# Supplementary material for: Metatranscriptome Analysis of Nasopharyngeal Swabs across the Varying Severity of COVID-19 Disease Demonstrated Unprecedented Species Diversity
Source: Microorganisms. 2023 Jul 14;11(7):1804. doi: 10.3390/microorganisms11071804 (PMC10384460; doi:10.3390/microorganisms11071804)
Supplement: Supplementary file 1 [file microorganisms-11-01804-s001.zip › Maaslin_results_ALL.zip!/Maaslin_results/Age.pdf]

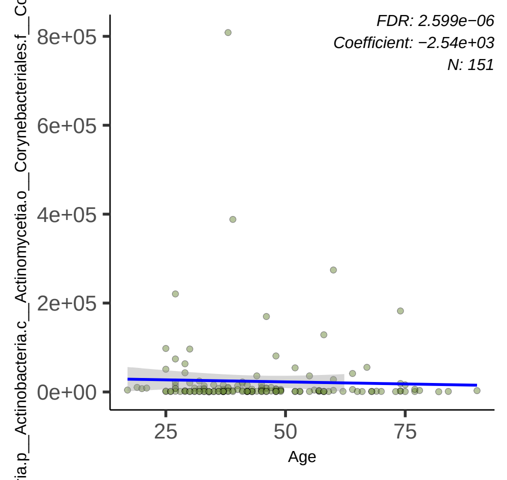
FDR: 2.599e−06
Coefficient: −2.54e+03
N: 151
8e+05
6e+05
4e+05
2e+05
0e+00
25
50
75
Age
ria.p__Actinobacteria.c__Actinomycetia.o__Corynebacteriales.f__Co

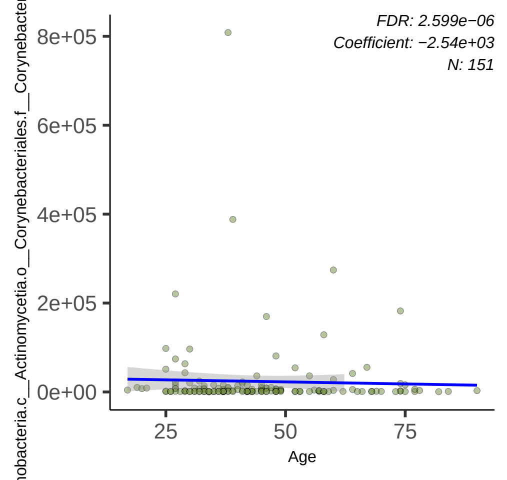

FDR: 2.599e−06
Coefficient: −2.54e+03
N: 151
8e+05
6e+05
4e+05
2e+05
0e+00
25
50
75
Age
nobacteria.c__Actinomycetia.o__Corynebacteriales.f__Corynebacter

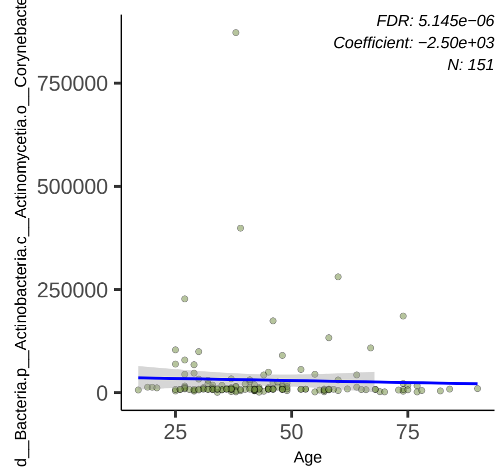
FDR: 5.145e−06
Coefficient: −2.50e+03
N: 151
d__Bacteria.p__Actinobacteria.c__Actinomycetia.o__Corynebacte
750000
500000
250000
0
25
50
75
Age

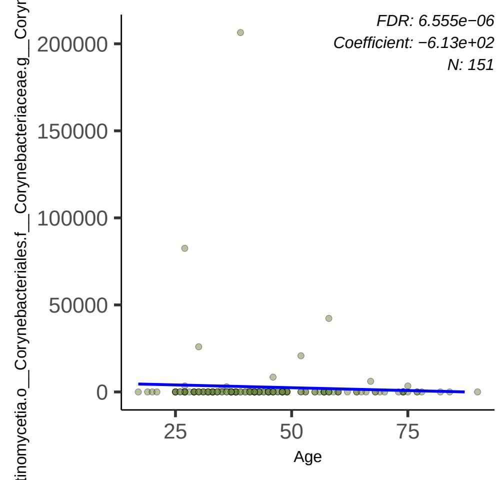

FDR: 6.555e−06
Coefficient: −6.13e+02
N: 151
200000
150000
100000
50000
0
25
50
75
Age
tinomycetia.o__Corynebacteriales.f__Corynebacteriaceae.g__Coryn

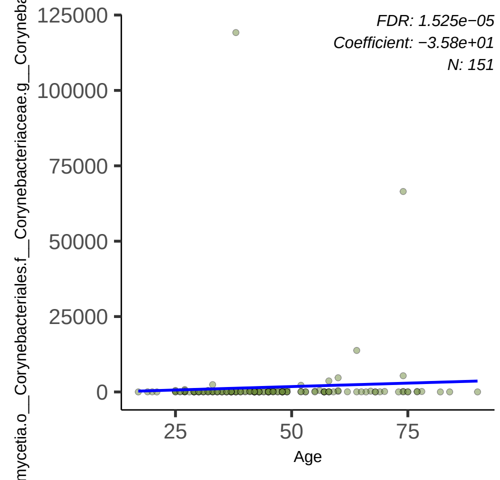

FDR: 1.525e−05
Coefficient: −3.58e+01
N: 151
125000
100000
75000
50000
25000
0
25
50
75
Age
mycetia.o__Corynebacteriales.f__Corynebacteriaceae.g__Coryneba

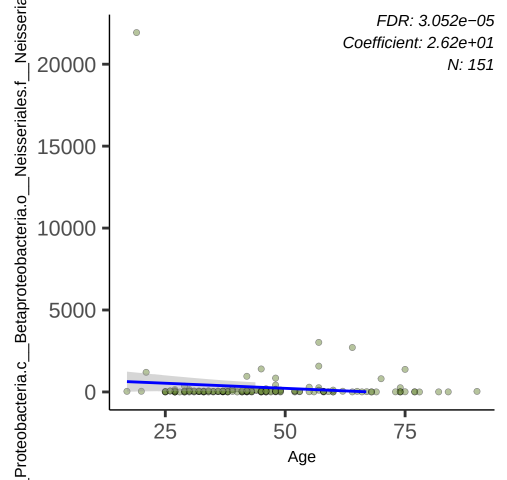
FDR: 3.052e−05
Coefficient: 2.62e+01
N: 151
Proteobacteria.c__Betaproteobacteria.o__Neisseriales.f__Neisseria
20000
15000
10000
5000
0
25
50
75
Age

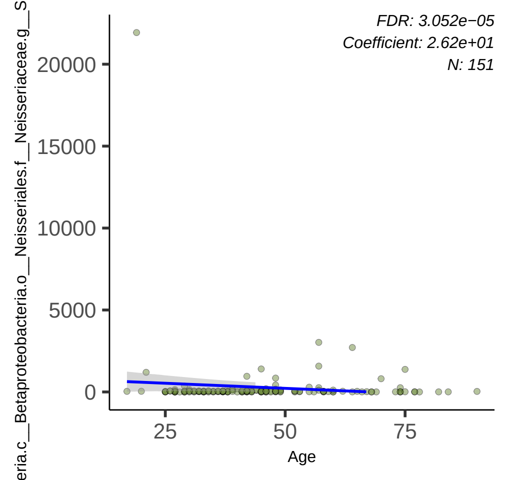

FDR: 3.052e−05
Coefficient: 2.62e+01
N: 151
20000
15000
10000
5000
0
25
50
75
Age
eria.c__Betaproteobacteria.o__Neisseriales.f__Neisseriaceae.g__S

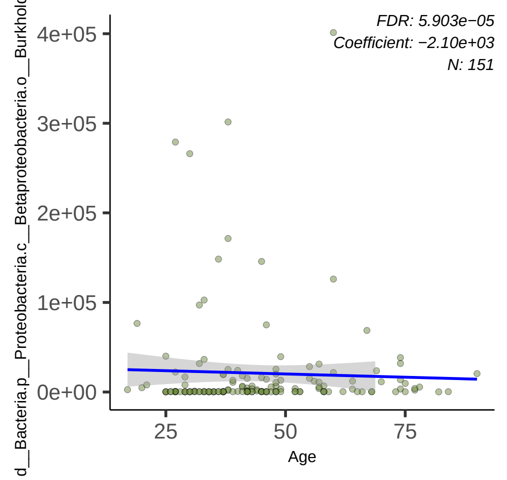

FDR: 5.903e−05
Coefficient: −2.10e+03
N: 151
4e+05
3e+05
2e+05
1e+05
0e+00
25
50
75
Age
d__Bacteria.p__Proteobacteria.c__Betaproteobacteria.o__Burkhold

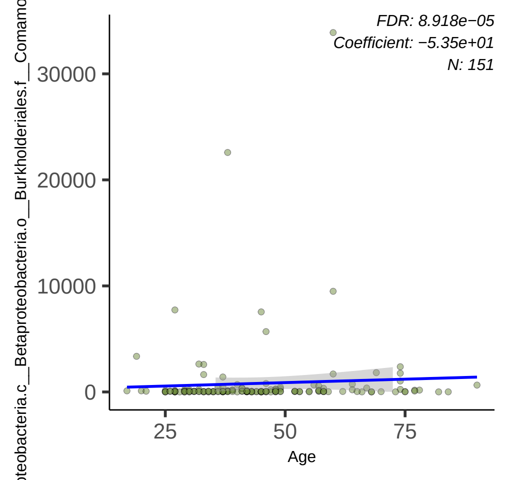
FDR: 8.918e−05
Coefficient: −5.35e+01
N: 151
oteobacteria.c__Betaproteobacteria.o__Burkholderiales.f__Comamo
30000
20000
10000
0
25
50
75
Age

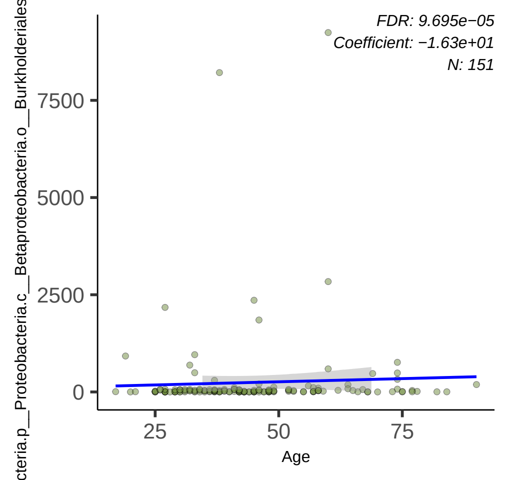
FDR: 9.695e−05
Coefficient: −1.63e+01
N: 151
cteria.p__Proteobacteria.c__Betaproteobacteria.o__Burkholderiales
7500
5000
2500
0
25
50
75
Age

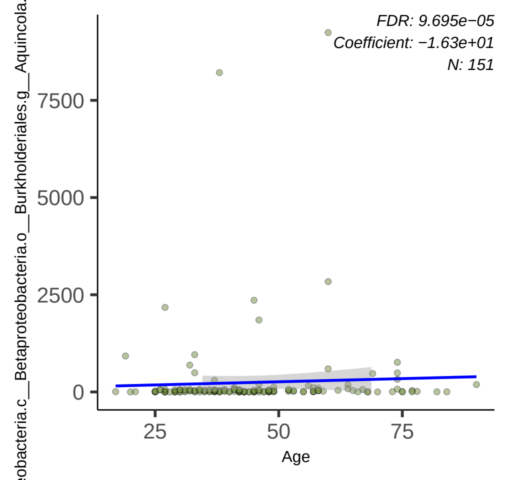
FDR: 9.695e−05
Coefficient: −1.63e+01
N: 151
eobacteria.c__Betaproteobacteria.o__Burkholderiales.g__Aquincola.
7500
5000
2500
0
25
50
75
Age

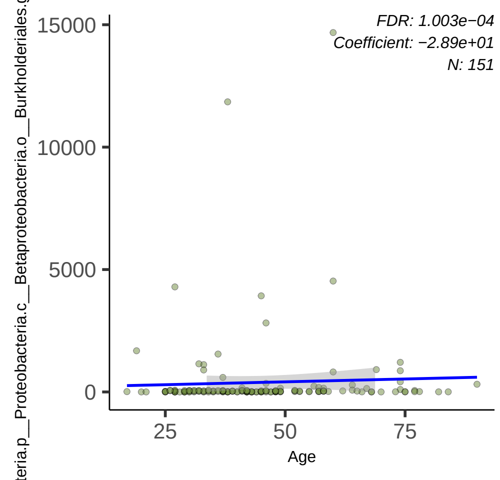
FDR: 1.003e−04
Coefficient: −2.89e+01
N: 151
15000
10000
5000
0
25
50
75
Age
teria.p__Proteobacteria.c__Betaproteobacteria.o__Burkholderiales.g

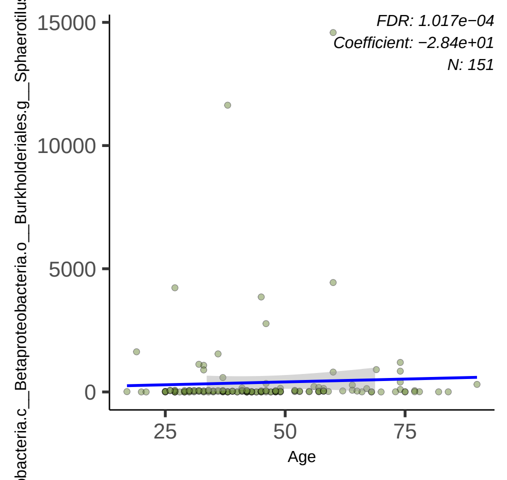

FDR: 1.017e−04
Coefficient: −2.84e+01
N: 151
15000
10000
5000
0
25
50
75
Age
obacteria.c__Betaproteobacteria.o__Burkholderiales.g__Sphaerotilus

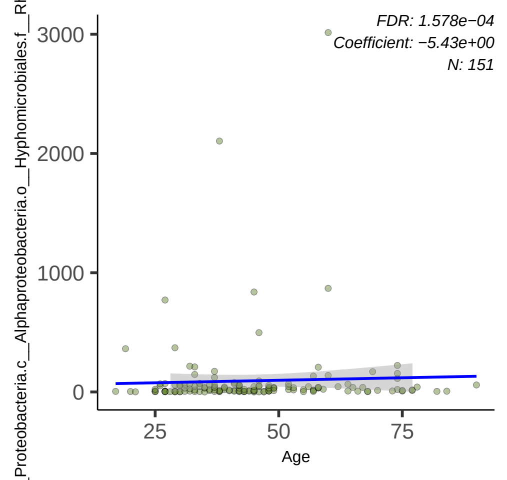
FDR: 1.578e−04
Coefficient: −5.43e+00
N: 151
3000
2000
1000
0
25
50
75
Age
Proteobacteria.c__Alphaproteobacteria.o__Hyphomicrobiales.f__Rh

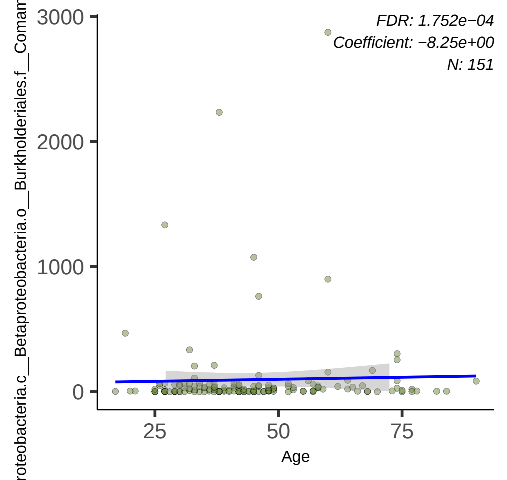

FDR: 1.752e−04
Coefficient: −8.25e+00
N: 151
3000
2000
1000
0
25
50
75
Age
roteobacteria.c__Betaproteobacteria.o__Burkholderiales.f__Comam

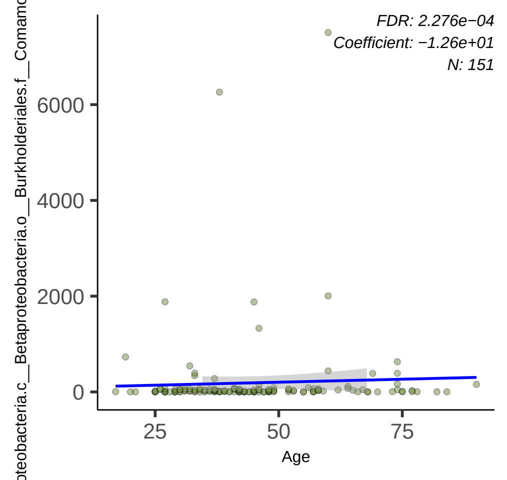

FDR: 2.276e−04
Coefficient: −1.26e+01
N: 151
0
2000
4000
6000
25
50
75
Age
oteobacteria.c__Betaproteobacteria.o__Burkholderiales.f__Comamo

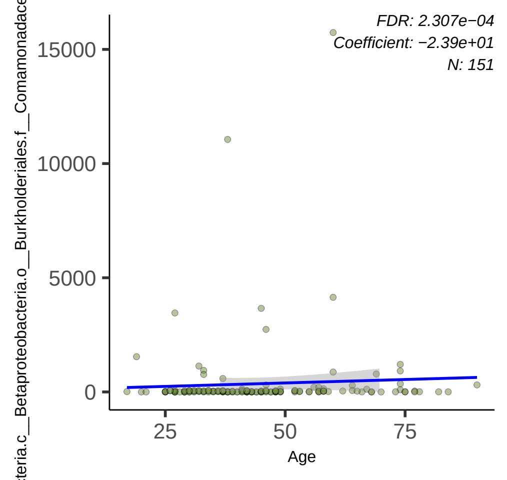

FDR: 2.307e−04
Coefficient: −2.39e+01
N: 151
15000
10000
5000
0
25
50
75
Age
cteria.c__Betaproteobacteria.o__Burkholderiales.f__Comamonadace

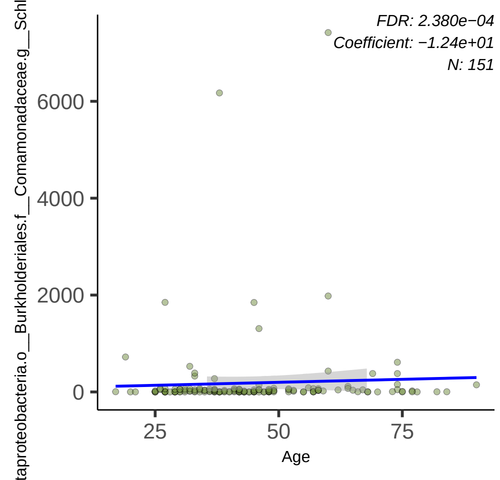

FDR: 2.380e−04
Coefficient: −1.24e+01
N: 151
6000
4000
2000
0
25
50
75
Age
taproteobacteria.o__Burkholderiales.f__Comamonadaceae.g__Schl

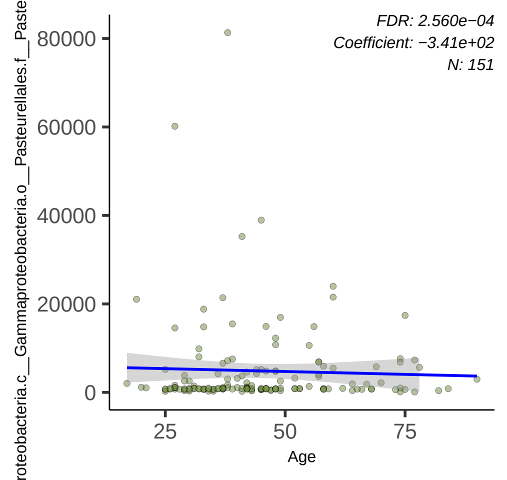
FDR: 2.560e−04
Coefficient: −3.41e+02
N: 151
80000
60000
40000
20000
0
25
50
75
Age
roteobacteria.c__Gammaproteobacteria.o__Pasteurellales.f__Paste

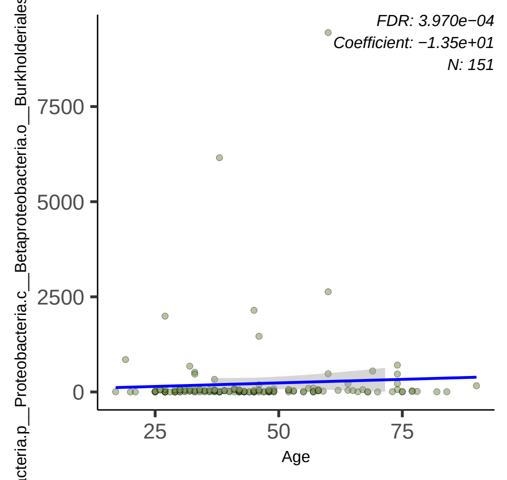

FDR: 3.970e−04
Coefficient: −1.35e+01
N: 151
acteria.p__Proteobacteria.c__Betaproteobacteria.o__Burkholderiales
7500
5000
2500
0
25
50
75
Age

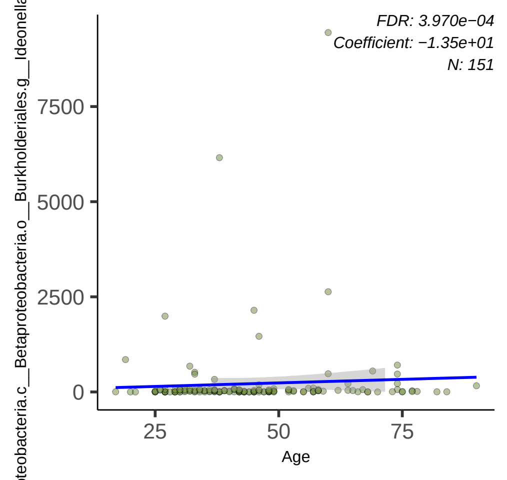
FDR: 3.970e−04
Coefficient: −1.35e+01
N: 151
7500
5000
2500
0
25
50
75
Age
oteobacteria.c__Betaproteobacteria.o__Burkholderiales.g__Ideonella

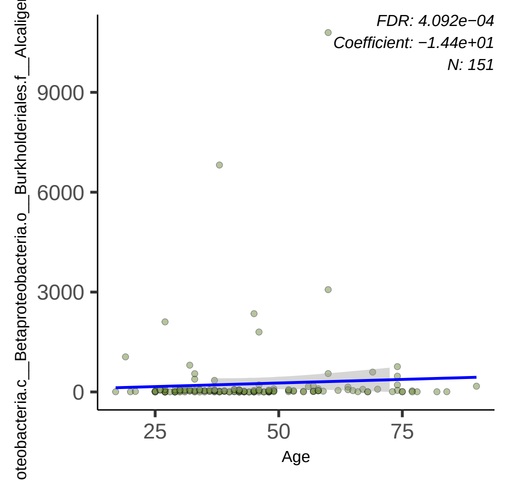

FDR: 4.092e−04
Coefficient: −1.44e+01
N: 151
oteobacteria.c__Betaproteobacteria.o__Burkholderiales.f__Alcaligen
9000
6000
3000
0
25
50
75
Age

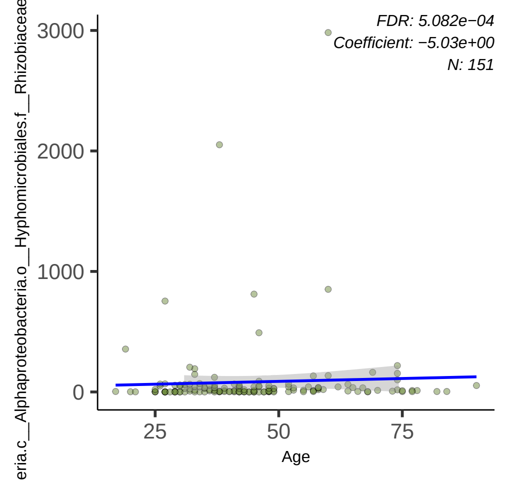

FDR: 5.082e−04
Coefficient: −5.03e+00
N: 151
3000
2000
1000
0
25
50
75
Age
eria.c__Alphaproteobacteria.o__Hyphomicrobiales.f__Rhizobiaceae

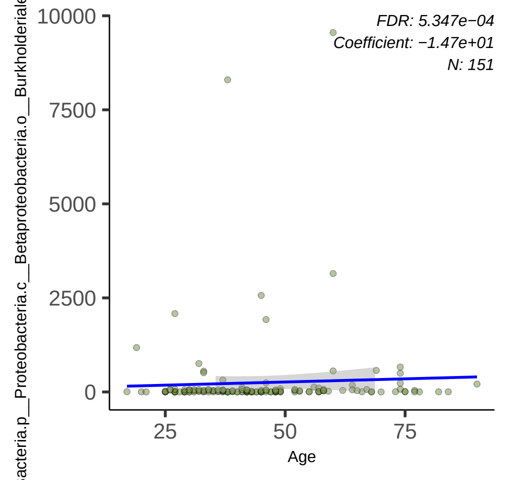
FDR: 5.347e−04
Coefficient: −1.47e+01
N: 151
10000
7500
5000
2500
0
25
50
75
Age
Proteobacteria.c__Betaproteobacteria.o__Burkholderiales

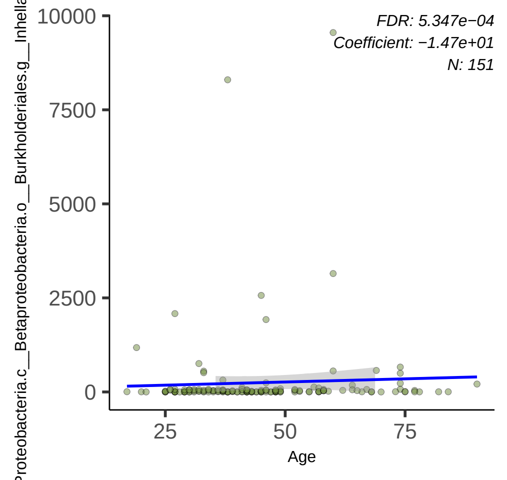
FDR: 5.347e−04
Coefficient: −1.47e+01
N: 151
10000
7500
5000
2500
0
25
50
75
Age
Proteobacteria.c__Betaproteobacteria.o__Burkholderiales.g__Inhella

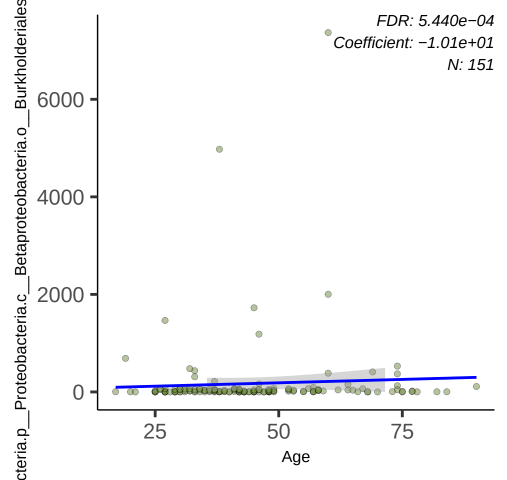

FDR: 5.440e−04
Coefficient: −1.01e+01
N: 151
0
2000
4000
6000
25
50
75
Age
cteria.p__Proteobacteria.c__Betaproteobacteria.o__Burkholderiales

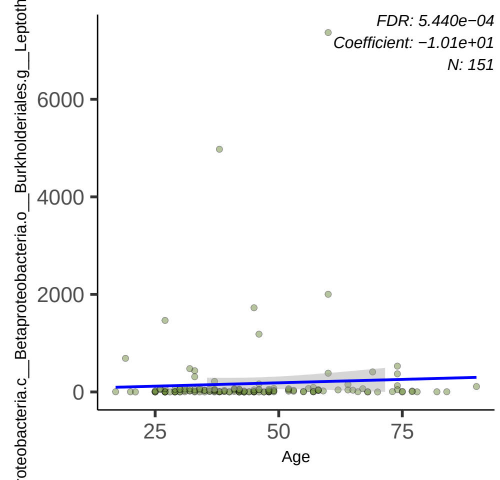
FDR: 5.440e−04
Coefficient: −1.01e+01
N: 151
6000
4000
2000
0
25
50
75
Age
roteobacteria.c__Betaproteobacteria.o__Burkholderiales.g__Leptoth

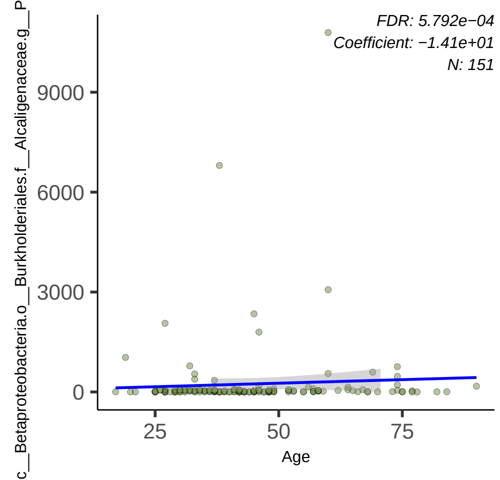

FDR: 5.792e−04
Coefficient: −1.41e+01
N: 151
c__Betaproteobacteria.o__Burkholderiales.f__Alcaligenaceae.g__P
9000
6000
3000
0
25
50
75
Age

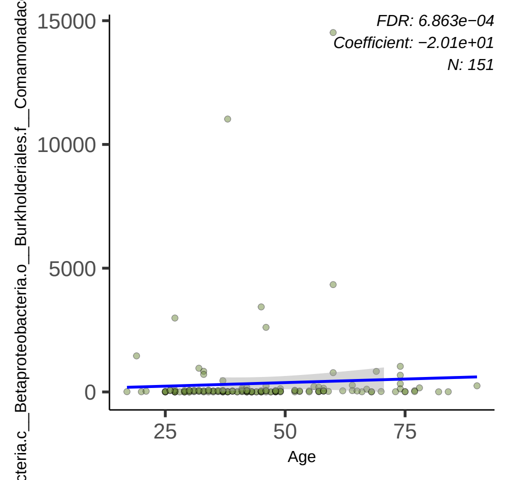

FDR: 6.863e−04
Coefficient: −2.01e+01
N: 151
15000
10000
5000
0
25
50
75
Age
cteria.c__Betaproteobacteria.o__Burkholderiales.f__Comamonadac

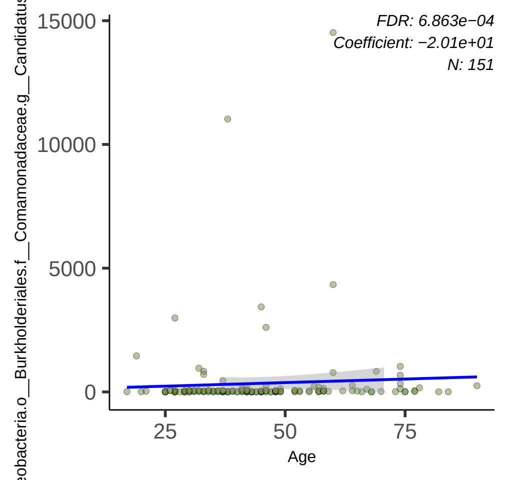

FDR: 6.863e−04
Coefficient: −2.01e+01
N: 151
15000
10000
5000
0
25
50
75
Age
eobacteria.o__Burkholderiales.f__Comamonadaceae.g__Candidatus

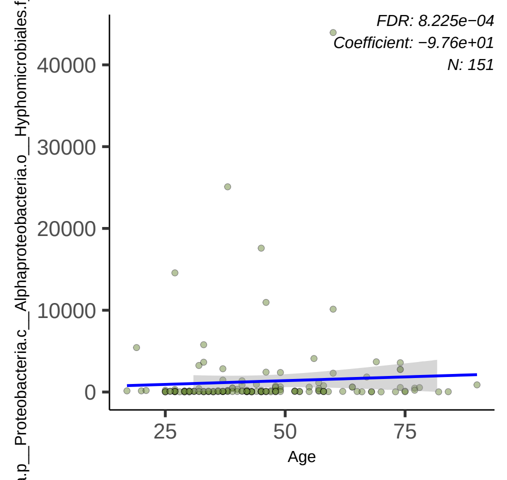
FDR: 8.225e−04
Coefficient: −9.76e+01
N: 151
a.p__Proteobacteria.c__Alphaproteobacteria.o__Hyphomicrobiales.f_
0
10000
20000
30000
40000
25
50
75
Age

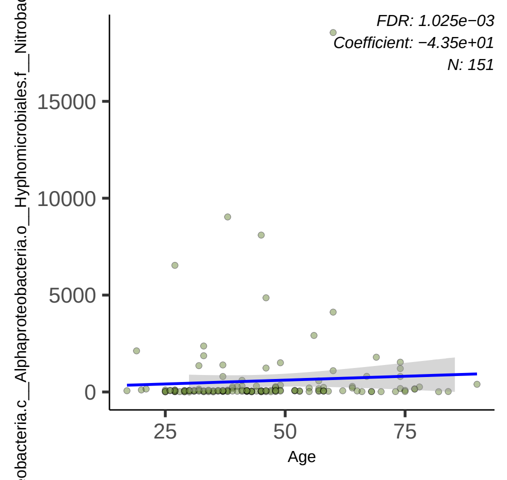

FDR: 1.025e−03
Coefficient: −4.35e+01
N: 151
0
5000
10000
15000
25
50
75
Age
eobacteria.c__Alphaproteobacteria.o__Hyphomicrobiales.f__Nitrobac

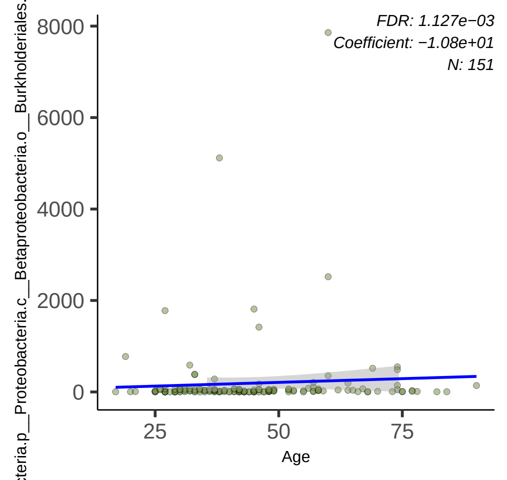
FDR: 1.127e−03
Coefficient: −1.08e+01
N: 151
8000
6000
4000
2000
0
25
50
75
Age
cteria.p__Proteobacteria.c__Betaproteobacteria.o__Burkholderiales.

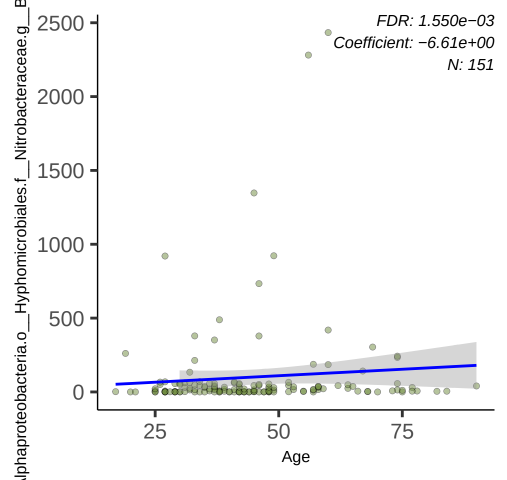
FDR: 1.550e−03
Coefficient: −6.61e+00
N: 151
lphaproteobacteria.o__Hyphomicrobiales.f__Nitrobacteraceae.g__B
2500
2000
1500
1000
500
0
25
50
75
Age

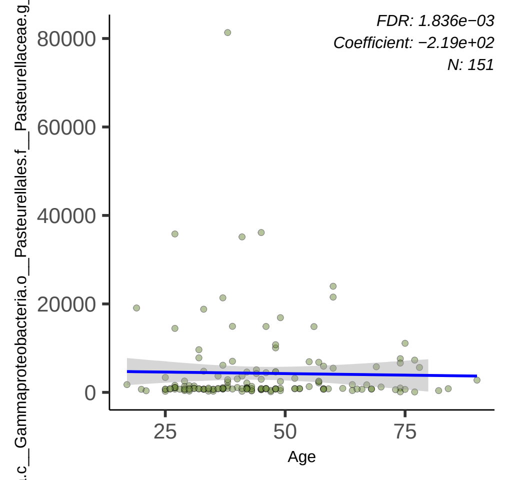
FDR: 1.836e−03
Coefficient: −2.19e+02
N: 151
80000
60000
40000
20000
0
25
50
75
Age
Gammaproteobacteria.o__Pasteurellales.f__Pasteurellaceae.g_

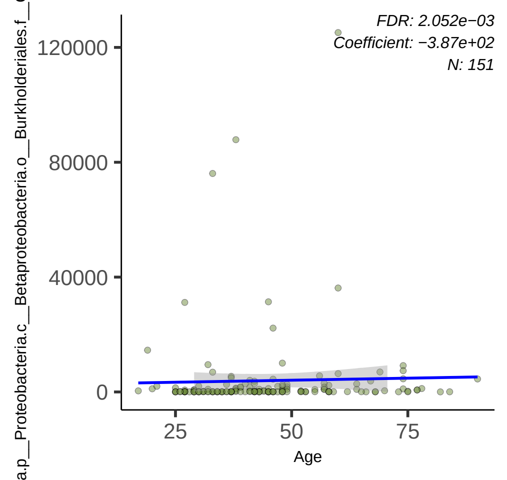
FDR: 2.052e−03
Coefficient: −3.87e+02
N: 151
120000
80000
40000
0
25
50
75
Age
a.p__Proteobacteria.c__Betaproteobacteria.o__Burkholderiales.f__

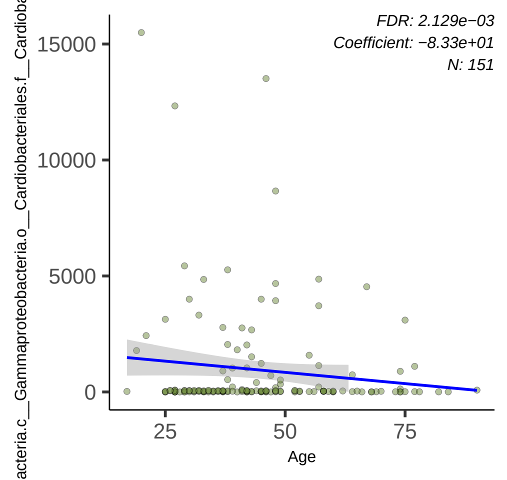

FDR: 2.129e−03
Coefficient: −8.33e+01
N: 151
acteria.c__Gammaproteobacteria.o__Cardiobacteriales.f__Cardioba
15000
10000
5000
0
25
50
75
Age

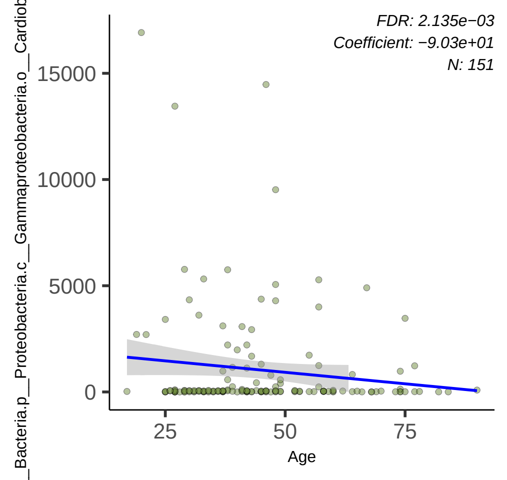
FDR: 2.135e−03
Coefficient: −9.03e+01
N: 151
Bacteria.p__Proteobacteria.c__Gammaproteobacteria.o__Cardiob
15000
10000
5000
0
25
50
75
Age

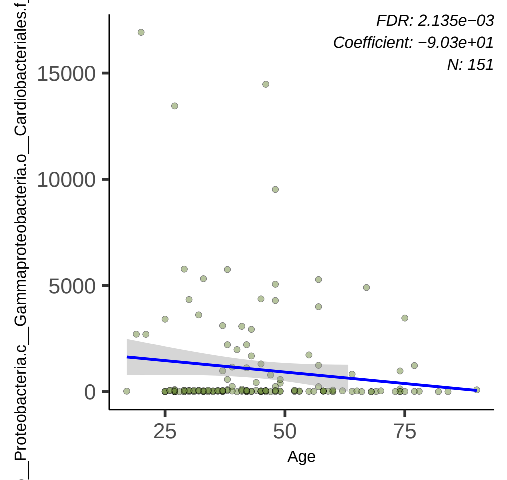

FDR: 2.135e−03
Coefficient: −9.03e+01
N: 151
Proteobacteria.c__Gammaproteobacteria.o__Cardiobacteriales.f_
15000
10000
5000
0
25
50
75
Age

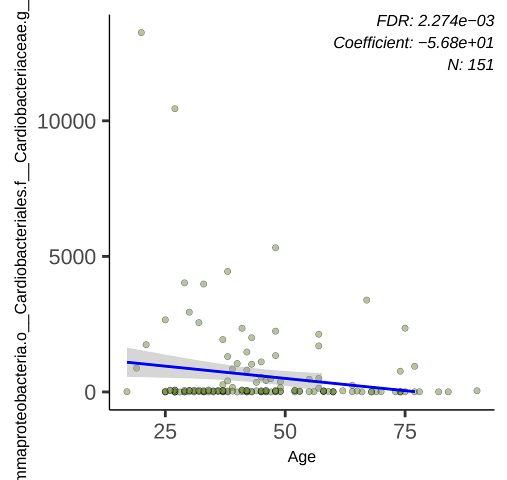

FDR: 2.274e−03
Coefficient: −5.68e+01
N: 151
mmaproteobacteria.o__Cardiobacteriales.f__Cardiobacteriaceae.g__
10000
5000
0
25
50
75
Age

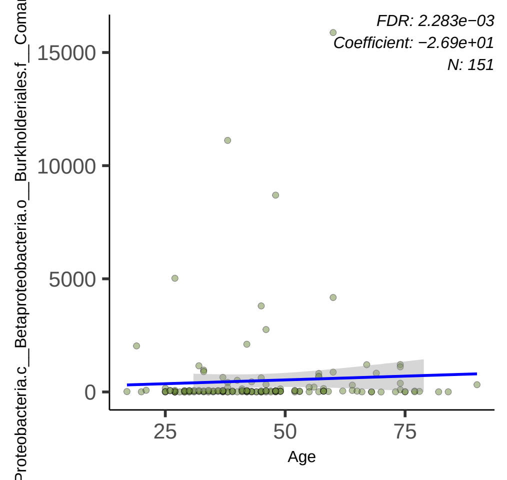
FDR: 2.283e−03
Coefficient: −2.69e+01
N: 151
15000
10000
5000
0
25
50
75
Age
Proteobacteria.c__Betaproteobacteria.o__Burkholderiales.f__Comar

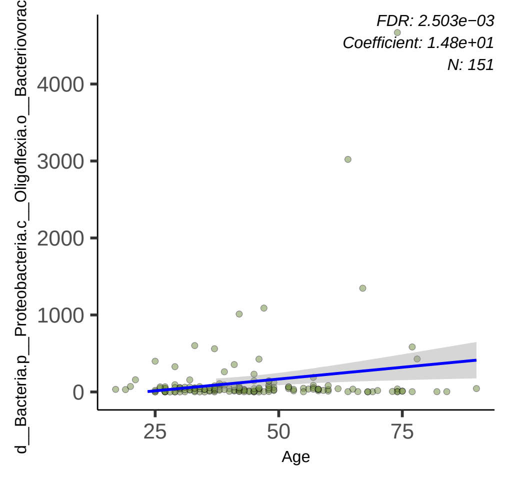
FDR: 2.503e−03
Coefficient: 1.48e+01
N: 151
d__Bacteria.p__Proteobacteria.c__Oligoflexia.o__Bacteriovorac
4000
3000
2000
1000
0
25
50
75
Age

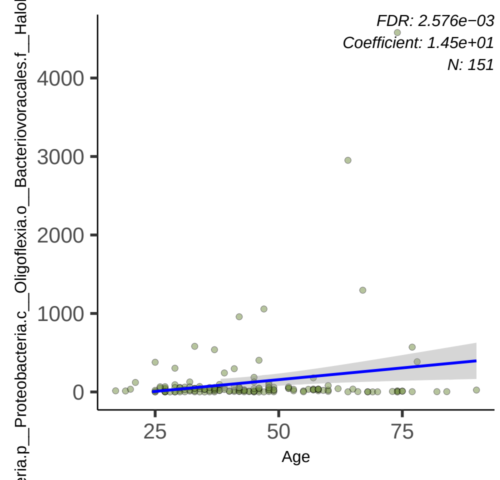

FDR: 2.576e−03
Coefficient: 1.45e+01
N: 151
4000
3000
2000
1000
0
25
50
75
Age
eria.p__Proteobacteria.c__Oligoflexia.o__Bacteriovoracales.f__Halol

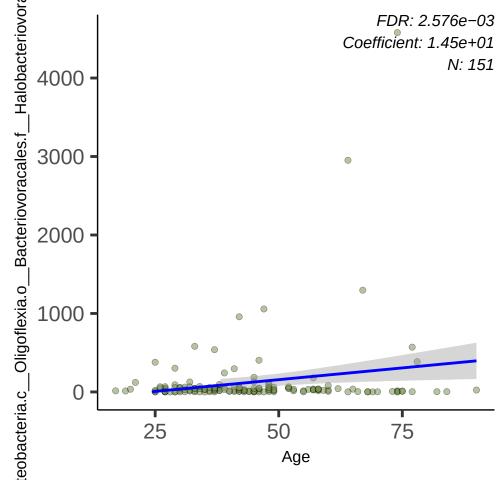

FDR: 2.576e−03
Coefficient: 1.45e+01
N: 151
4000
3000
2000
1000
0
25
50
75
Age
teobacteria.c__Oligoflexia.o__Bacteriovoracales.f__Halobacteriovora

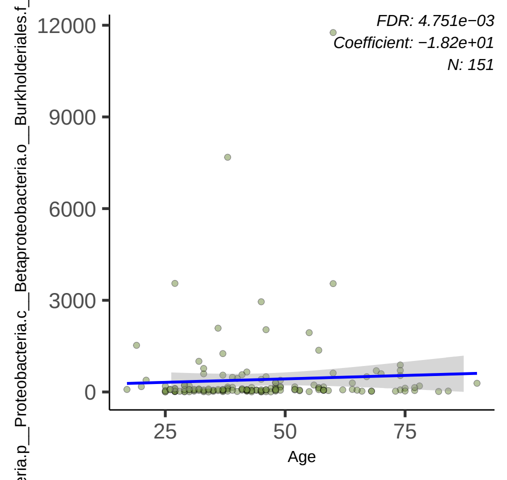

FDR: 4.751e−03
Coefficient: −1.82e+01
N: 151
12000
9000
6000
3000
0
25
50
75
Age
eria.p__Proteobacteria.c__Betaproteobacteria.o__Burkholderiales.f__

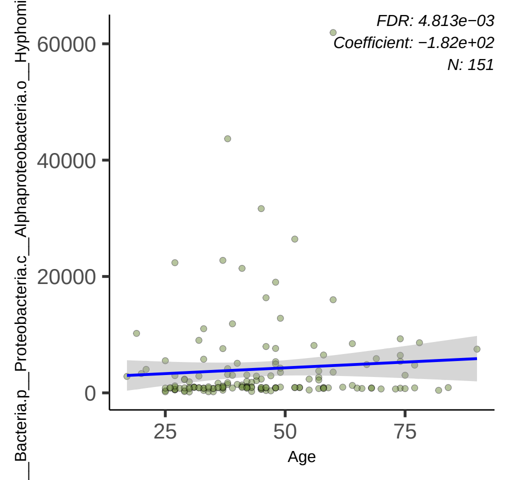
FDR: 4.813e−03
Coefficient: −1.82e+02
N: 151
0
20000
40000
60000
25
50
75
Age
__Bacteria.p__Proteobacteria.c__Alphaproteobacteria.o__Hyphomi

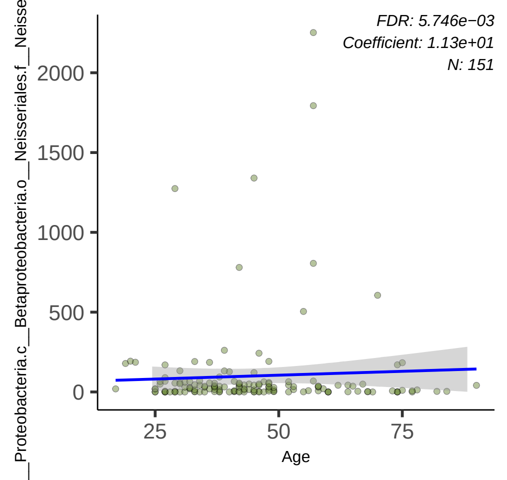

FDR: 5.746e−03
Coefficient: 1.13e+01
N: 151
2000
1500
1000
500
0
25
50
75
Age
_Proteobacteria.c__Betaproteobacteria.o__Neisseriales.f__Neisse

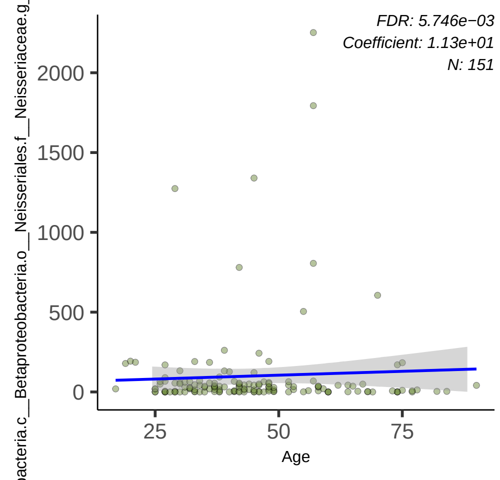

FDR: 5.746e−03
Coefficient: 1.13e+01
N: 151
bacteria.c__Betaproteobacteria.o__Neisseriales.f__Neisseriaceae.g__
2000
1500
1000
500
0
25
50
75
Age

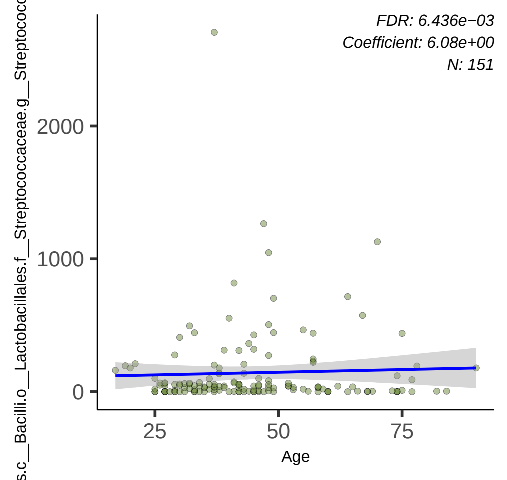

FDR: 6.436e−03
Coefficient: 6.08e+00
N: 151
s.c__Bacilli.o__Lactobacillales.f__Streptococcaceae.g__Streptococ
2000
1000
0
25
50
75
Age

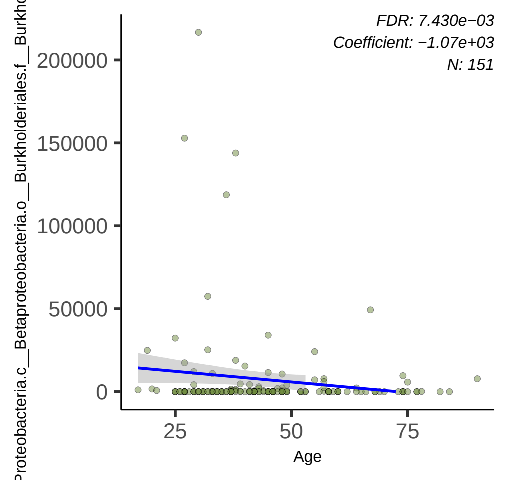

FDR: 7.430e−03
Coefficient: −1.07e+03
N: 151
200000
150000
100000
50000
0
25
50
75
Age
Proteobacteria.c__Betaproteobacteria.o__Burkholderiales.f__Burkho

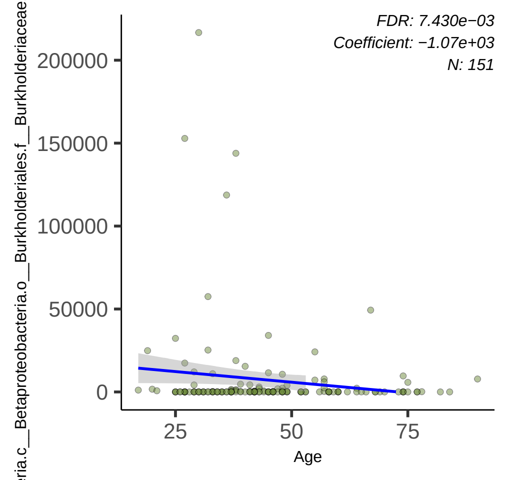
FDR: 7.430e−03
Coefficient: −1.07e+03
N: 151
200000
150000
100000
50000
0
25
50
75
Age
eria.c__Betaproteobacteria.o__Burkholderiales.f__Burkholderiaceae

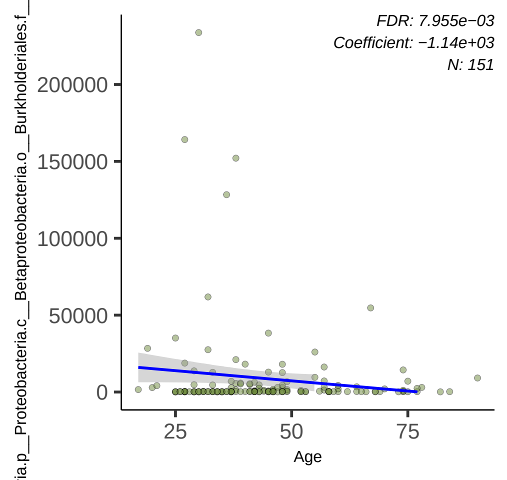

FDR: 7.955e−03
Coefficient: −1.14e+03
N: 151
ria.p__Proteobacteria.c__Betaproteobacteria.o__Burkholderiales.f_
200000
150000
100000
50000
0
25
50
75
Age

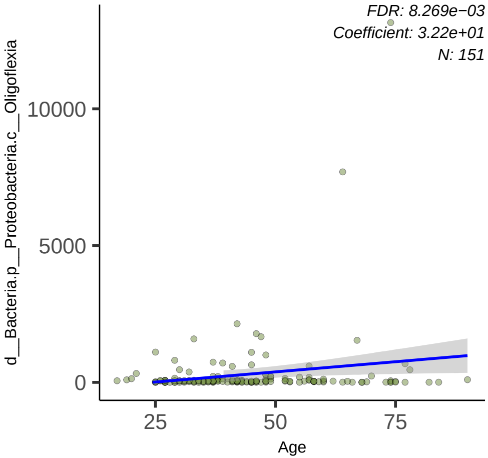
FDR: 8.269e−03
Coefficient: 3.22e+01
N: 151
d__Bacteria.p__Proteobacteria.c__Oligoflexia
10000
5000
0
25
50
75
Age

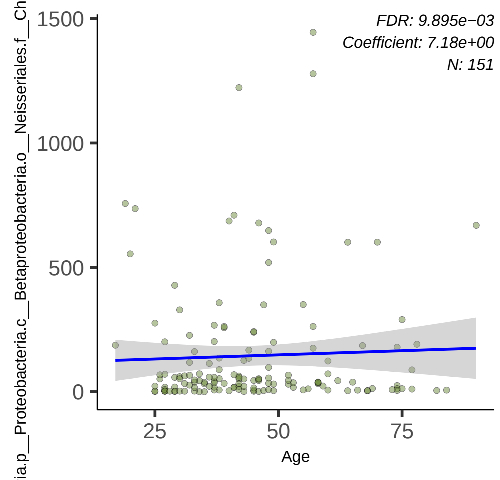
FDR: 9.895e−03
Coefficient: 7.18e+00
N: 151
1500
1000
500
0
25
50
75
Age
ia.p__Proteobacteria.c__Betaproteobacteria.o__Neisseriales.f__Ch

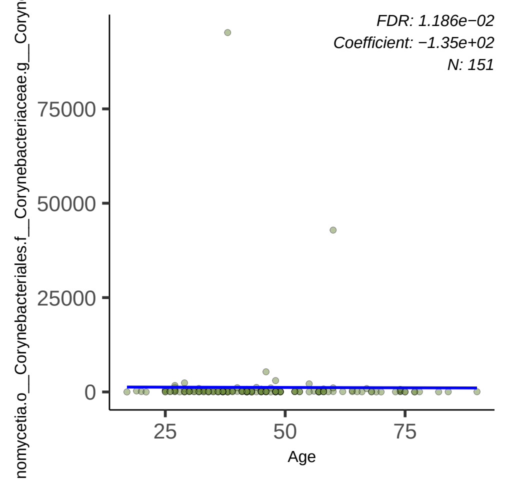

FDR: 1.186e−02
Coefficient: −1.35e+02
N: 151
nomycetia.o__Corynebacteriales.f__Corynebacteriaceae.g__Coryn
75000
50000
25000
0
25
50
75
Age

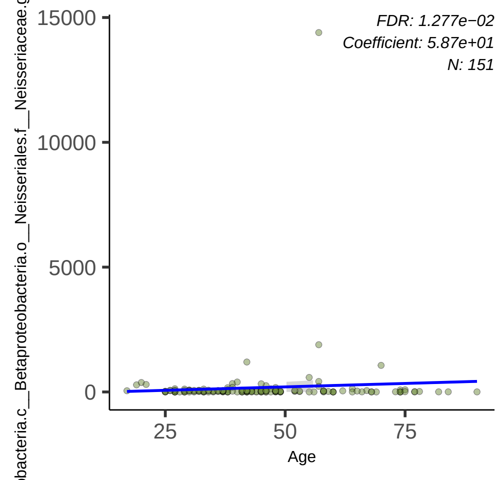

FDR: 1.277e−02
Coefficient: 5.87e+01
N: 151
15000
10000
5000
0
25
50
75
Age
obacteria.c__Betaproteobacteria.o__Neisseriales.f__Neisseriaceae.g

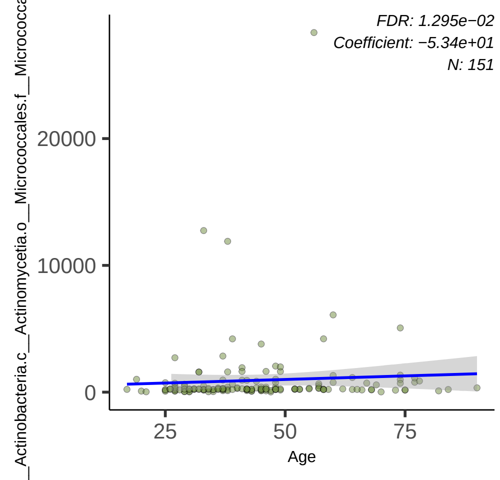

FDR: 1.295e−02
Coefficient: −5.34e+01
N: 151
_Actinobacteria.c__Actinomycetia.o__Micrococcales.f__Micrococca
20000
10000
0
25
50
75
Age

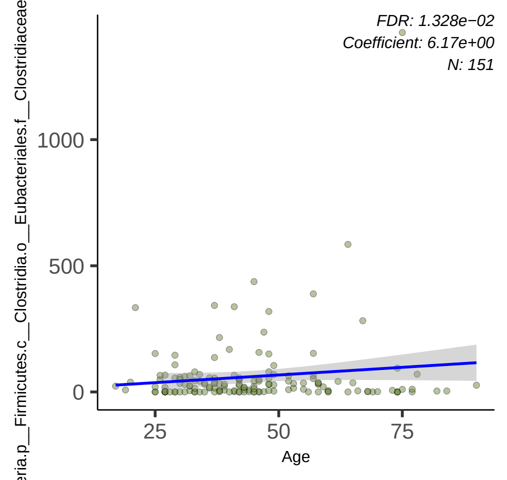
FDR: 1.328e−02
Coefficient: 6.17e+00
N: 151
1000
500
0
25
50
75
Age
eria.p__Firmicutes.c__Clostridia.o__Eubacteriales.f__Clostridiaceae

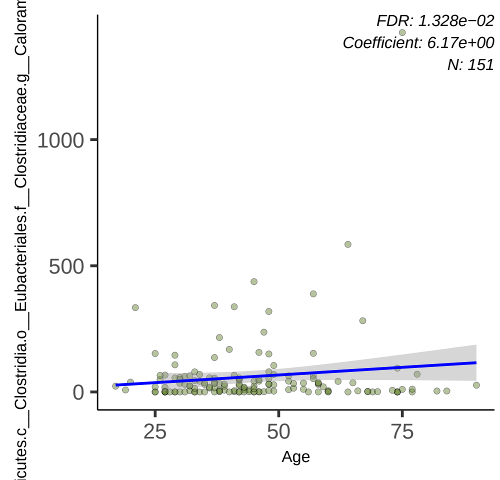

FDR: 1.328e−02
Coefficient: 6.17e+00
N: 151
1000
500
0
25
50
75
Age
icutes.c__Clostridia.o__Eubacteriales.f__Clostridiaceae.g__Caloram

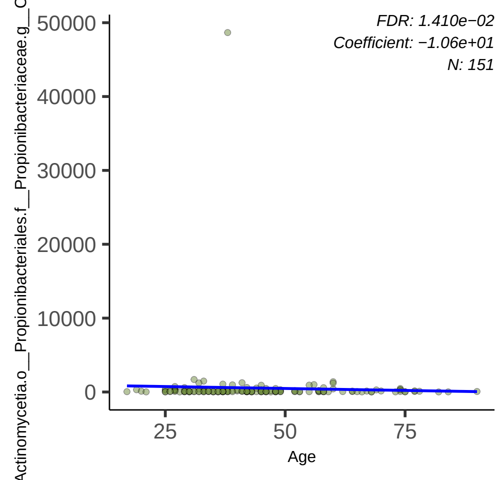

FDR: 1.410e−02
Coefficient: −1.06e+01
N: 151
Actinomycetia.o__Propionibacteriales.f__Propionibacteriaceae.g__C
50000
40000
30000
20000
10000
0
25
50
75
Age

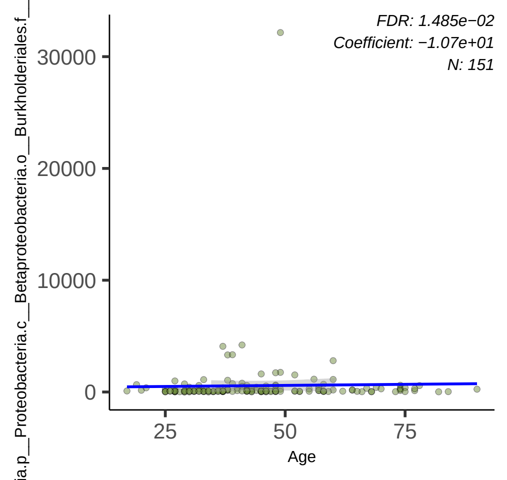

FDR: 1.485e−02
Coefficient: −1.07e+01
N: 151
ia.p__Proteobacteria.c__Betaproteobacteria.o__Burkholderiales.f_
30000
20000
10000
0
25
50
75
Age

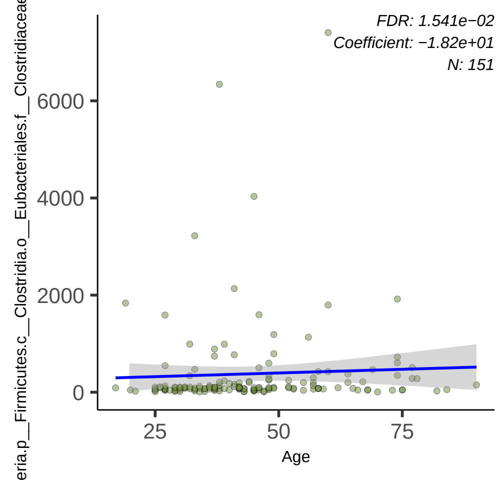

FDR: 1.541e−02
Coefficient: −1.82e+01
N: 151
eria.p__Firmicutes.c__Clostridia.o__Eubacteriales.f__Clostridiaceae
6000
4000
2000
0
25
50
75
Age

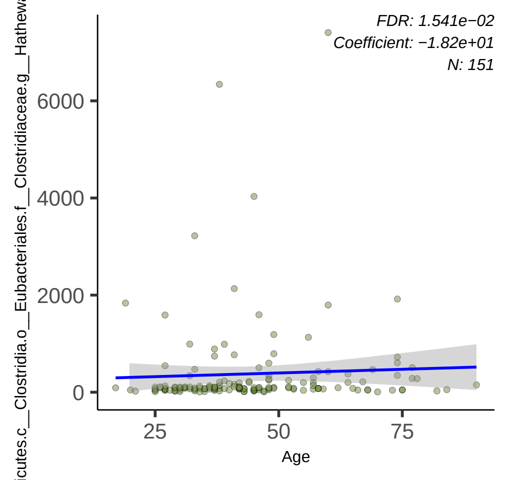
FDR: 1.541e−02
Coefficient: −1.82e+01
N: 151
icutes.c__Clostridia.o__Eubacteriales.f__Clostridiaceae.g__Hathewa
6000
4000
2000
0
25
50
75
Age

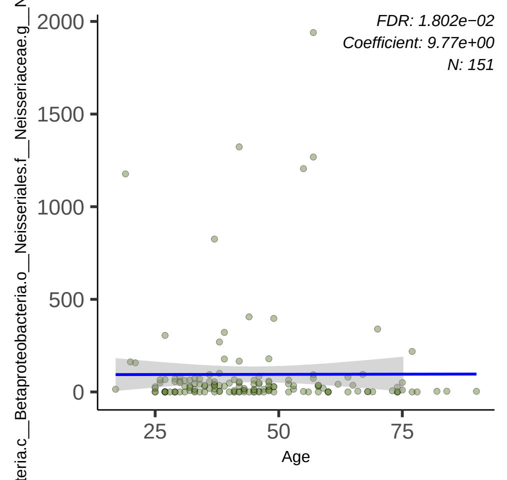

FDR: 1.802e−02
Coefficient: 9.77e+00
N: 151
2000
1500
1000
500
0
25
50
75
Age

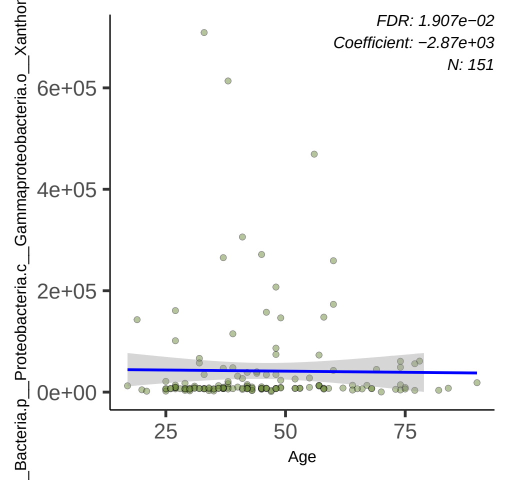

FDR: 1.907e−02
Coefficient: −2.87e+03
N: 151
6e+05
4e+05
2e+05
0e+00
25
50
75
Age
_Bacteria.p__Proteobacteria.c__Gammaproteobacteria.o__Xanthor

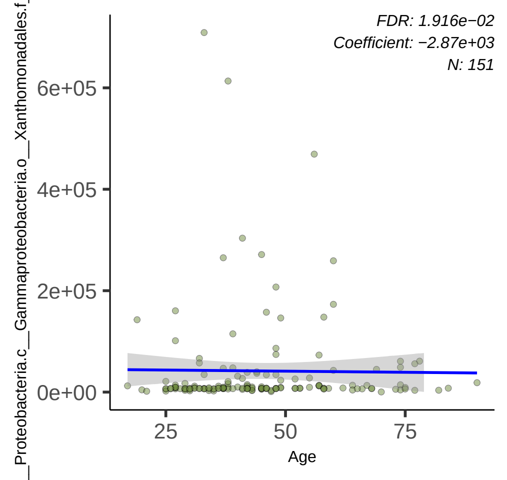
FDR: 1.916e−02
Coefficient: −2.87e+03
N: 151
Proteobacteria.c__Gammaproteobacteria.o__Xanthomonadales.f
6e+05
4e+05
2e+05
0e+00
25
50
75
Age

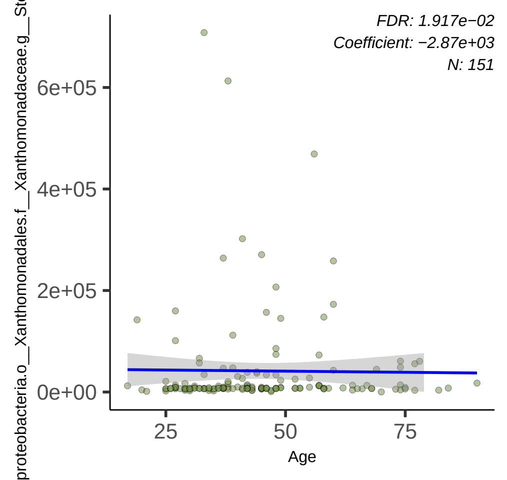

FDR: 1.917e−02
Coefficient: −2.87e+03
N: 151
6e+05
4e+05
2e+05
0e+00
25
50
75
Age
proteobacteria.o__Xanthomonadales.f__Xanthomonadaceae.g__St

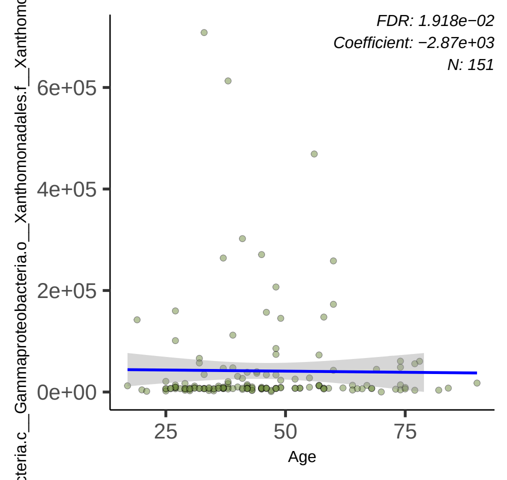

FDR: 1.918e−02
Coefficient: −2.87e+03
N: 151
cteria.c__Gammaproteobacteria.o__Xanthomonadales.f__Xanthomo
6e+05
4e+05
2e+05
0e+00
25
50
75
Age

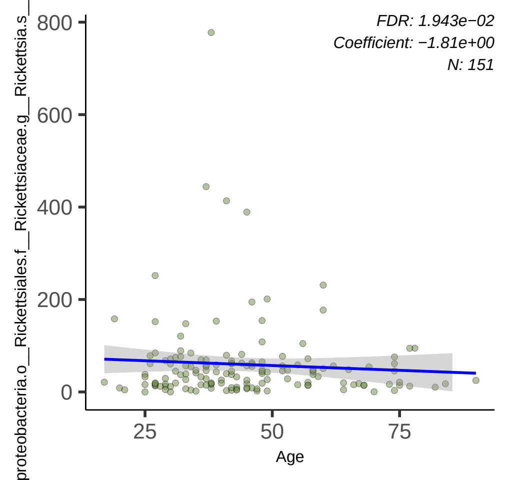
FDR: 1.943e−02
Coefficient: −1.81e+00
N: 151
proteobacteria.o__Rickettsiales.f__Rickettsiaceae.g__Rickettsia.s__
800
600
400
200
0
25
50
75
Age

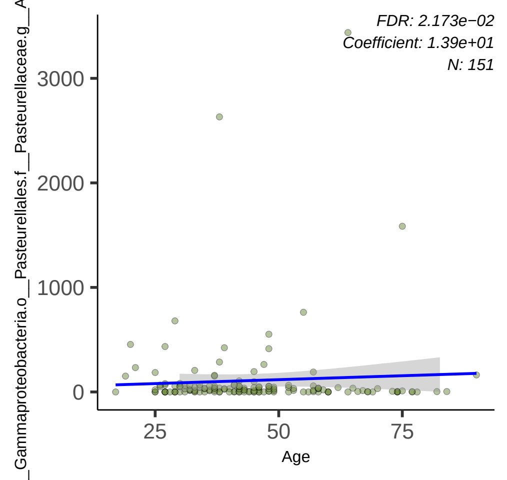

FDR: 2.173e−02
Coefficient: 1.39e+01
N: 151
3000
2000
1000
0
25
50
75
Age
_Gammaproteobacteria.o__Pasteurellales.f__Pasteurellaceae.g__A

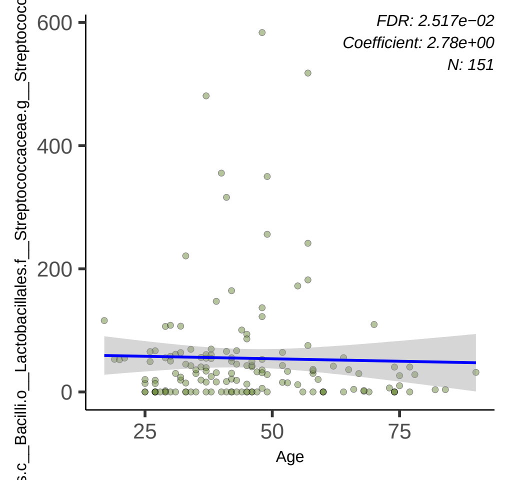

FDR: 2.517e−02
Coefficient: 2.78e+00
N: 151
s.c__Bacilli.o__Lactobacillales.f__Streptococcaceae.g__Streptococ
600
400
200
0
25
50
75
Age

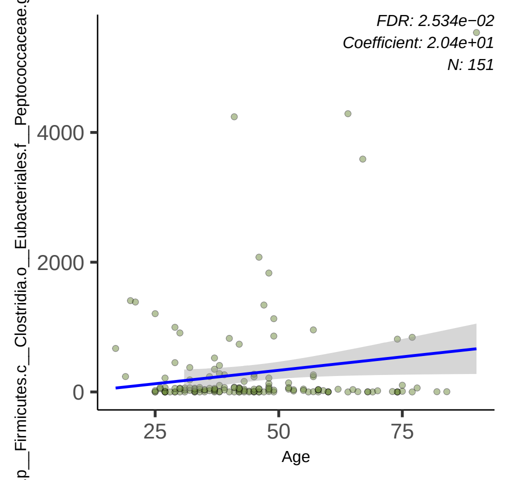

FDR: 2.534e−02
Coefficient: 2.04e+01
N: 151
p__Firmicutes.c__Clostridia.o__Eubacteriales.f__Peptococcaceae.g
4000
2000
0
25
50
75
Age

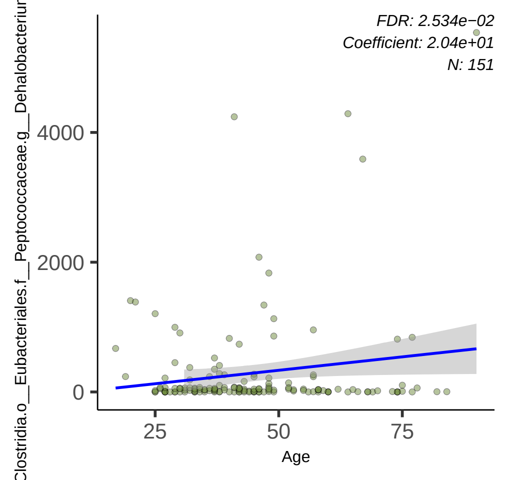

FDR: 2.534e−02
Coefficient: 2.04e+01
N: 151
Clostridia.o__Eubacteriales.f__Peptococcaceae.g__Dehalobacterium
4000
2000
0
25
50
75
Age

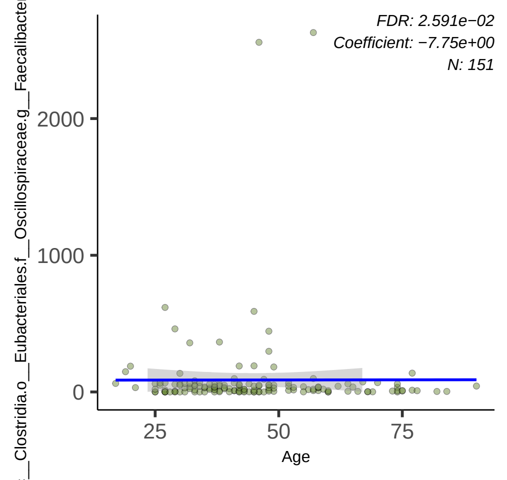

FDR: 2.591e−02
Coefficient: −7.75e+00
N: 151
2000
1000
0
25
50
75
Age
Clostridia.o__Eubacteriales.f__Oscillospiraceae.g__Faecalibacter

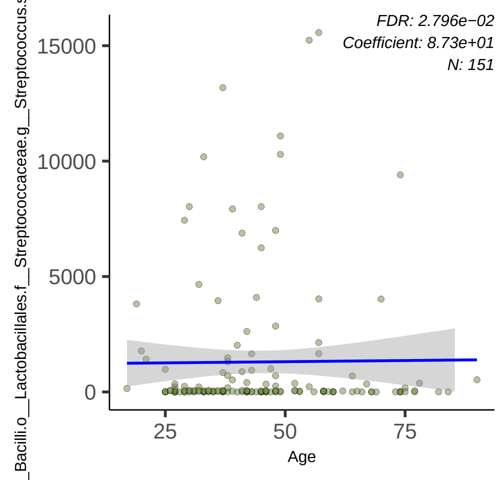

FDR: 2.796e−02
Coefficient: 8.73e+01
N: 151
Bacilli.o__Lactobacillales.f__Streptococcaceae.g__Streptococcus.s
0
5000
10000
15000
25
50
75
Age

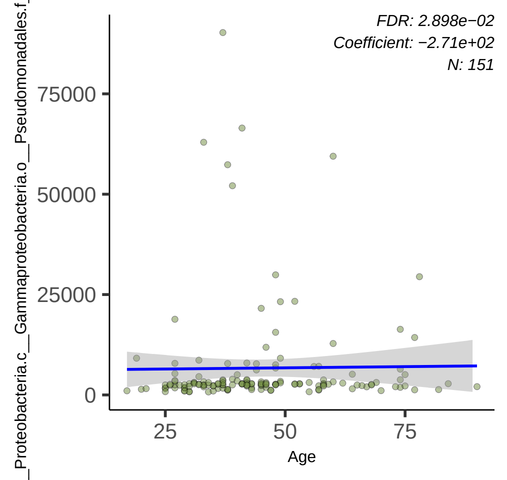
FDR: 2.898e−02
Coefficient: −2.71e+02
N: 151
_Proteobacteria.c__Gammaproteobacteria.o__Pseudomonadales.f_
75000
50000
25000
0
25
50
75
Age

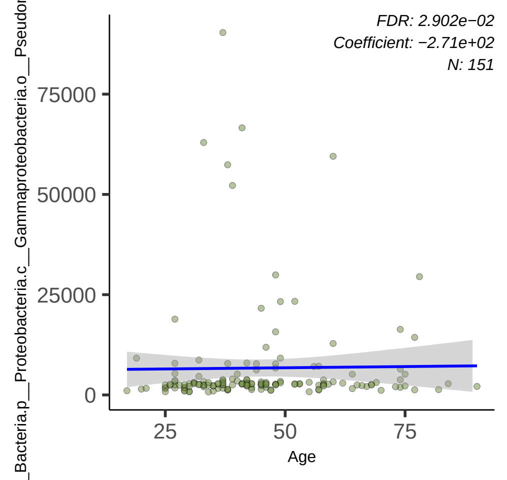
FDR: 2.902e−02
Coefficient: −2.71e+02
N: 151
75000
50000
25000
0
25
50
75
Age
Bacteria.p__Proteobacteria.c__Gammaproteobacteria.o__Pseudor

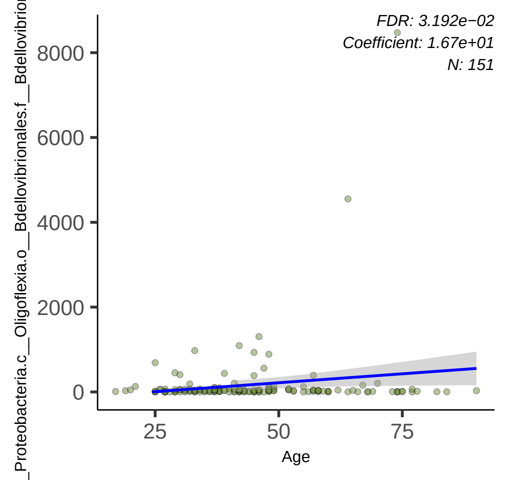

FDR: 3.192e−02
Coefficient: 1.67e+01
N: 151
8000
6000
4000
2000
0
25
50
75
Age
Proteobacteria.c__Oligoflexia.o__Bdellovibrionales.f__Bdellovibrion

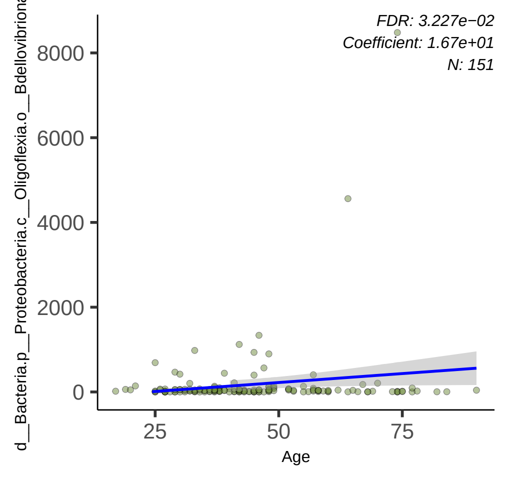

FDR: 3.227e−02
Coefficient: 1.67e+01
N: 151
d__Bacteria.p__Proteobacteria.c__Oligoflexia.o__Bdellovibriona
8000
6000
4000
2000
0
25
50
75
Age

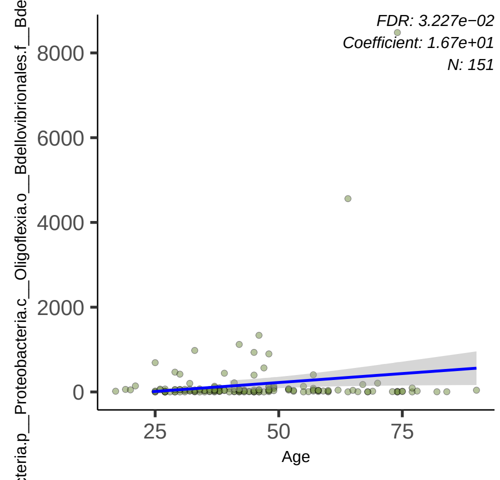
FDR: 3.227e−02
Coefficient: 1.67e+01
N: 151
cteria.p__Proteobacteria.c__Oligoflexia.o__Bdellovibrionales.f__Bde
8000
6000
4000
2000
0
25
50
75
Age

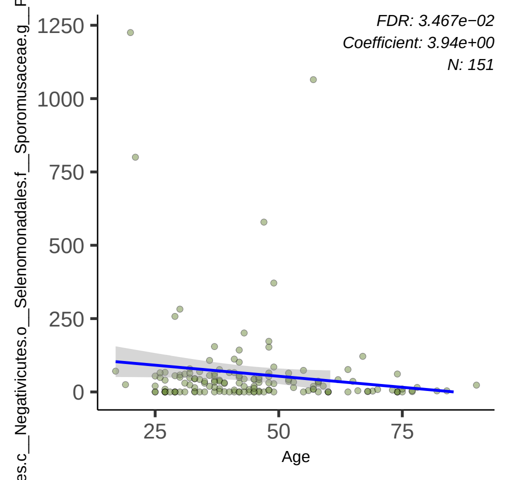

FDR: 3.467e−02
Coefficient: 3.94e+00
N: 151
1250
1000
750
500
250
0
25
50
75
Age
es.c__Negativicutes.o__Selenomonadales.f__Sporomusaceae.g__F

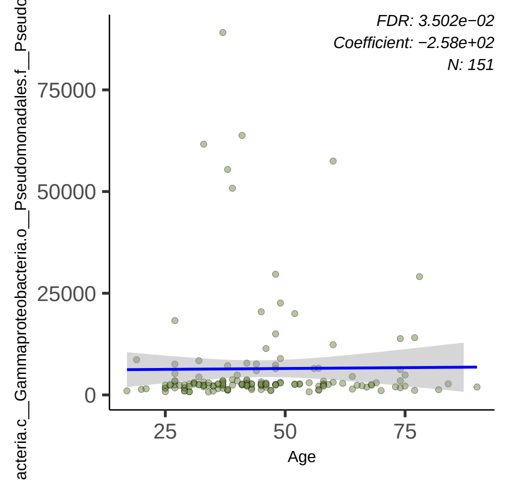

FDR: 3.502e−02
Coefficient: −2.58e+02
N: 151
acteria.c__Gammaproteobacteria.o__Pseudomonadales.f__Pseudo
75000
50000
25000
0
25
50
75
Age

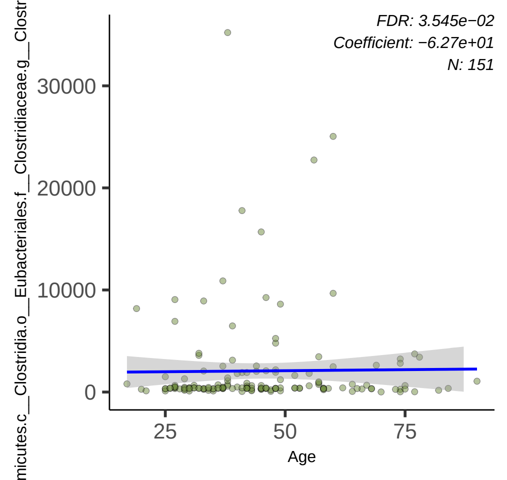

FDR: 3.545e−02
Coefficient: −6.27e+01
N: 151
micutes.c__Clostridia.o__Eubacteriales.f__Clostridiaceae.g__Clostr
0
10000
20000
30000
25
50
75
Age

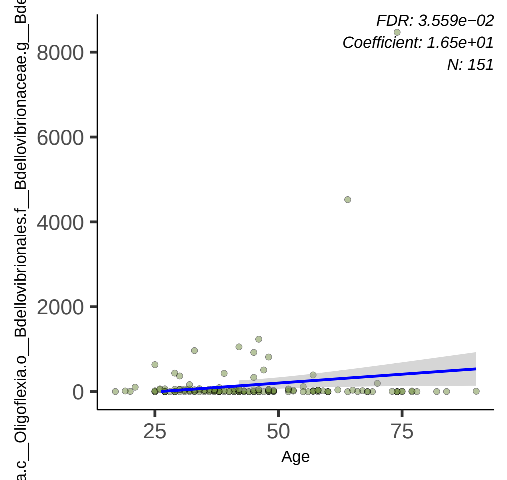

FDR: 3.559e−02
Coefficient: 1.65e+01
N: 151
8000
6000
4000
2000
0
25
50
75
Age
a.c__Oligoflexia.o__Bdellovibrionales.f__Bdellovibrionaceae.g__Bde

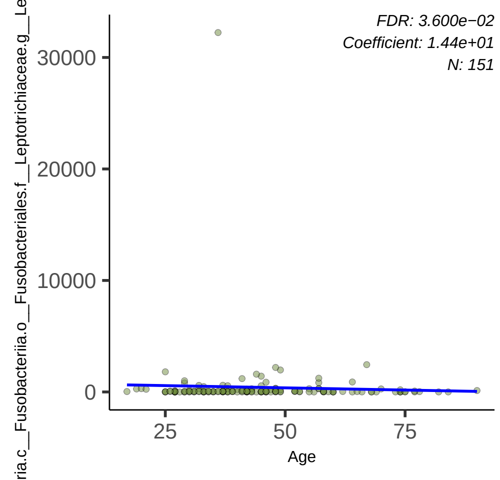

FDR: 3.600e−02
Coefficient: 1.44e+01
N: 151
30000
20000
10000
0
25
50
75
Age
ria.c__Fusobacteriia.o__Fusobacteriales.f__Leptotrichiaceae.g__Le

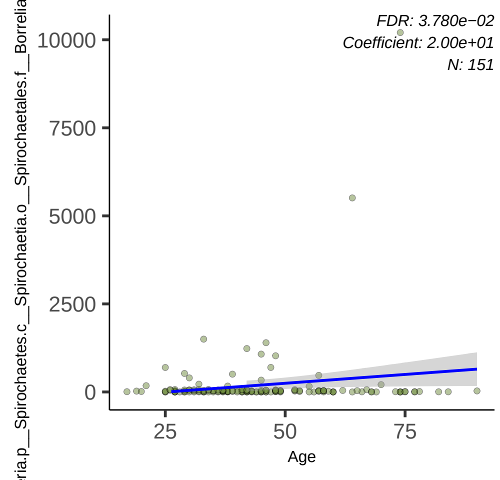

FDR: 3.780e−02
Coefficient: 2.00e+01
N: 151
10000
7500
5000
2500
0
25
50
75
Age
ria.p__Spirochaetes.c__Spirochaetia.o__Spirochaetales.f__Borrelia

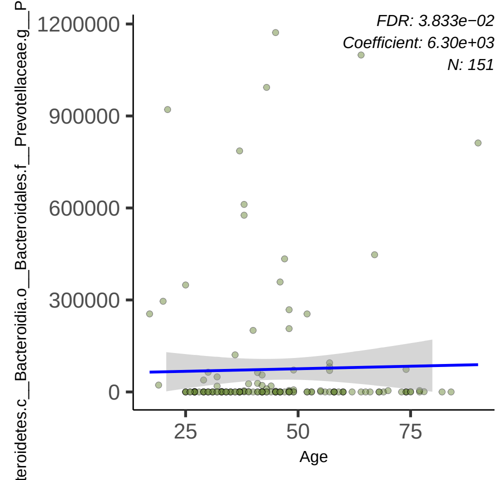

teroidetes.c__Bacteroidia.o__Bacteroidales.f__Prevotellaceae.g__P
FDR: 3.833e−02
Coefficient: 6.30e+03
N: 151
1200000
900000
600000
300000
0
25
50
75
Age

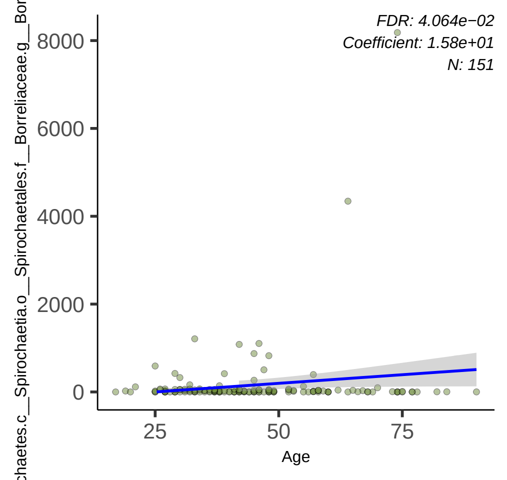

FDR: 4.064e−02
Coefficient: 1.58e+01
N: 151
8000
6000
4000
2000
0
25
50
75
Age
chaetes.c__Spirochaetia.o__Spirochaetales.f__Borreliaceae.g__Bor

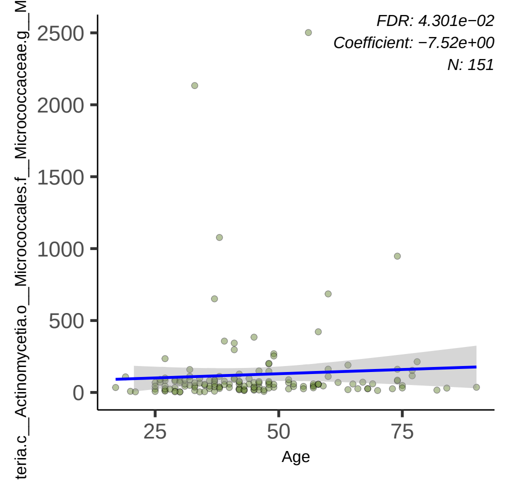

FDR: 4.301e−02
Coefficient: −7.52e+00
N: 151
2500
2000
1500
1000
500
0
25
50
75
Age
teria.c__Actinomycetia.o__Micrococcales.f__Micrococcaceae.g__M

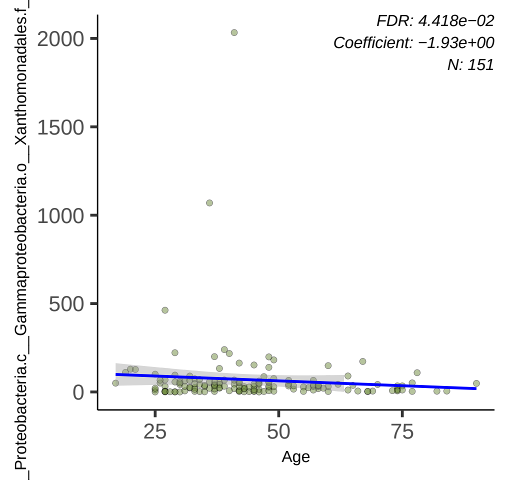
FDR: 4.418e−02
Coefficient: −1.93e+00
N: 151
2000
1500
1000
500
0
25
50
75
Age
Proteobacteria.c__Gammaproteobacteria.o__Xanthomonadales.f

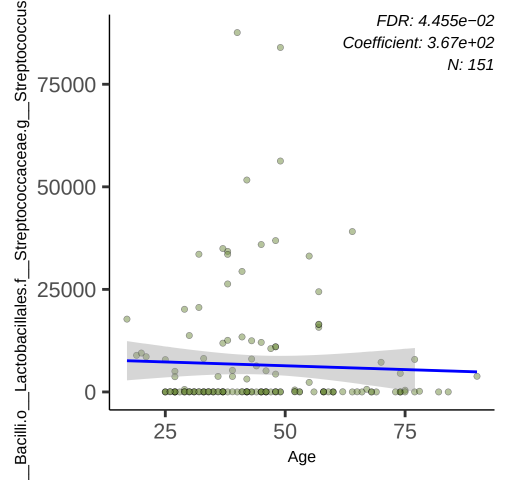
FDR: 4.455e−02
Coefficient: 3.67e+02
N: 151
Bacilli.o__Lactobacillales.f__Streptococcaceae.g__Streptococcus
75000
50000
25000
0
25
50
75
Age

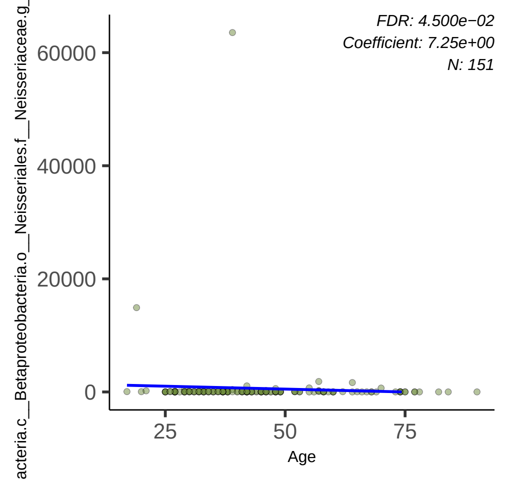

FDR: 4.500e−02
Coefficient: 7.25e+00
N: 151
60000
40000
20000
0
25
50
75
Age
acteria.c__Betaproteobacteria.o__Neisseriales.f__Neisseriaceae.g_

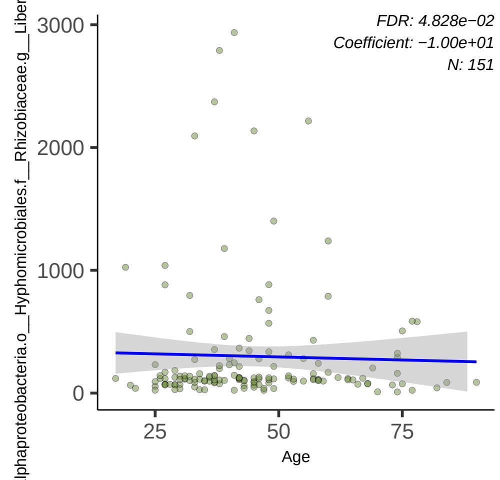

FDR: 4.828e−02
Coefficient: −1.00e+01
N: 151
lphaproteobacteria.o__Hyphomicrobiales.f__Rhizobiaceae.g__Liber
3000
2000
1000
0
25
50
75
Age

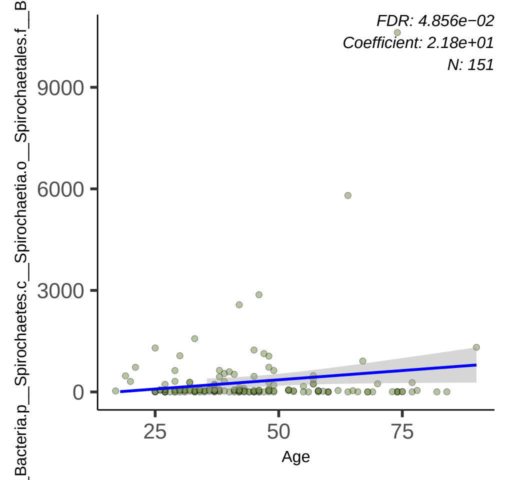

FDR: 4.856e−02
Coefficient: 2.18e+01
N: 151
Bacteria.p__Spirochaetes.c__Spirochaetia.o__Spirochaetales.f__B
9000
6000
3000
0
25
50
75
Age

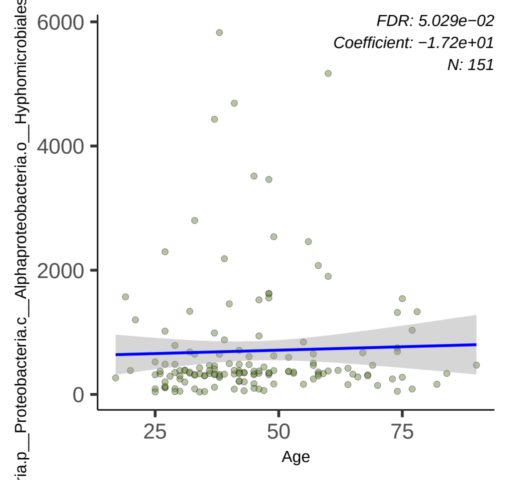

FDR: 5.029e−02
Coefficient: −1.72e+01
N: 151
ria.p__Proteobacteria.c__Alphaproteobacteria.o__Hyphomicrobiales
6000
4000
2000
0
25
50
75
Age

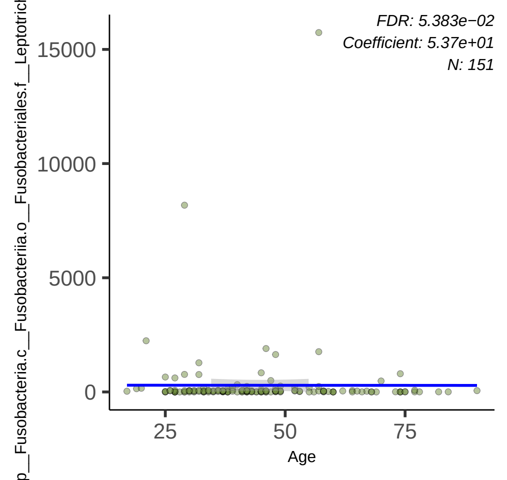
FDR: 5.383e−02
Coefficient: 5.37e+01
N: 151
15000
10000
5000
0
25
50
75
Age
p__Fusobacteria.c__Fusobacteriia.o__Fusobacteriales.f__Leptotrich

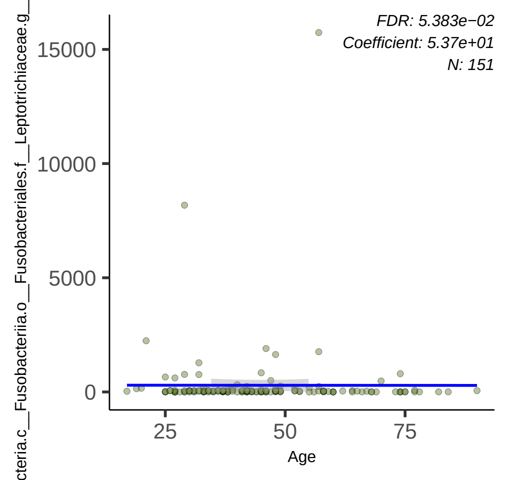
FDR: 5.383e−02
Coefficient: 5.37e+01
N: 151
15000
10000
5000
0
25
50
75
Age
cteria.c__Fusobacteriia.o__Fusobacteriales.f__Leptotrichiaceae.g__

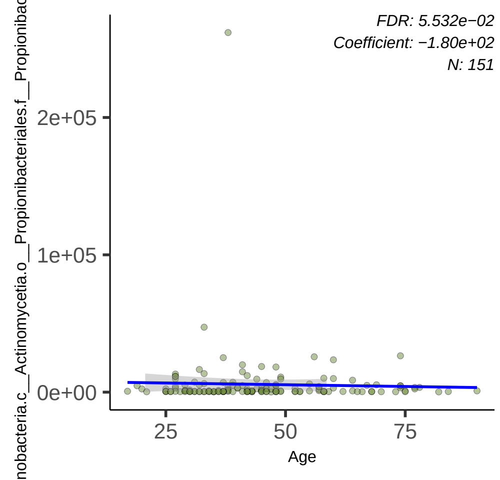

FDR: 5.532e−02
Coefficient: −1.80e+02
N: 151
nobacteria.c__Actinomycetia.o__Propionibacteriales.f__Propionibac
2e+05
1e+05
0e+00
25
50
75
Age

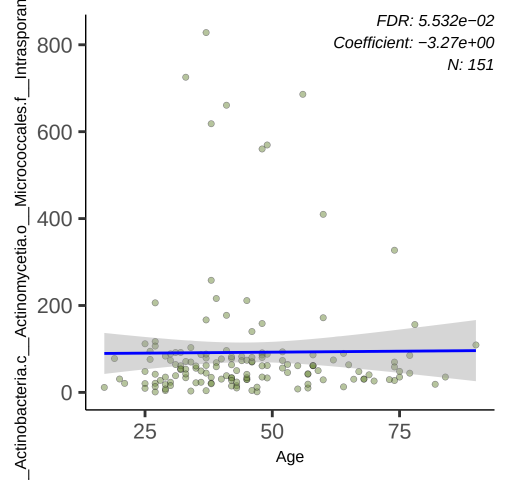
FDR: 5.532e−02
Coefficient: −3.27e+00
N: 151
_Actinobacteria.c__Actinomycetia.o__Micrococcales.f__Intrasporan
800
600
400
200
0
25
50
75
Age

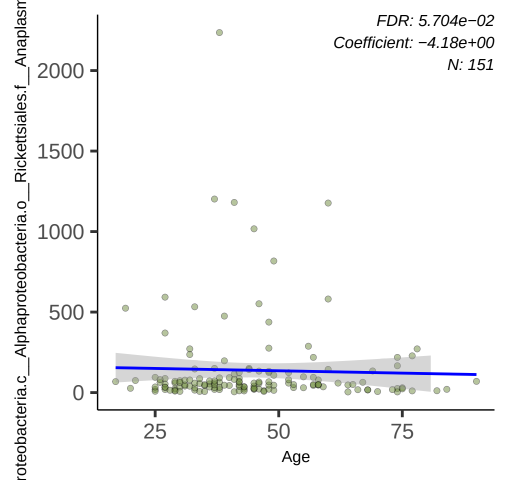

FDR: 5.704e−02
Coefficient: −4.18e+00
N: 151
roteobacteria.c__Alphaproteobacteria.o__Rickettsiales.f__Anaplasm
0
500
1000
1500
2000
25
50
75
Age

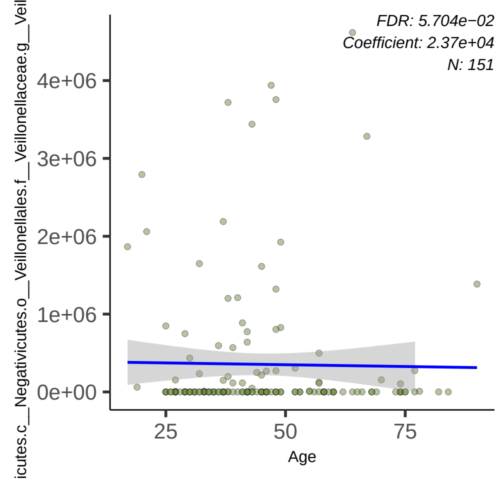

FDR: 5.704e−02
Coefficient: 2.37e+04
N: 151
icutes.c__Negativicutes.o__Veillonellales.f__Veillonellaceae.g__Veil
4e+06
3e+06
2e+06
1e+06
0e+00
25
50
75
Age

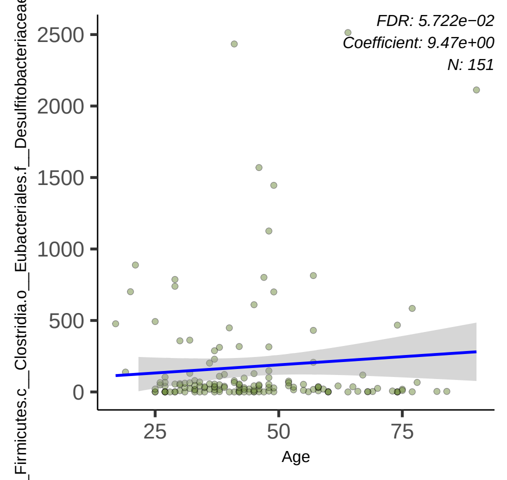
FDR: 5.722e−02
Coefficient: 9.47e+00
N: 151
Firmicutes.c__Clostridia.o__Eubacteriales.f__Desulfitobacteriaceae
2500
2000
1500
1000
500
0
25
50
75
Age

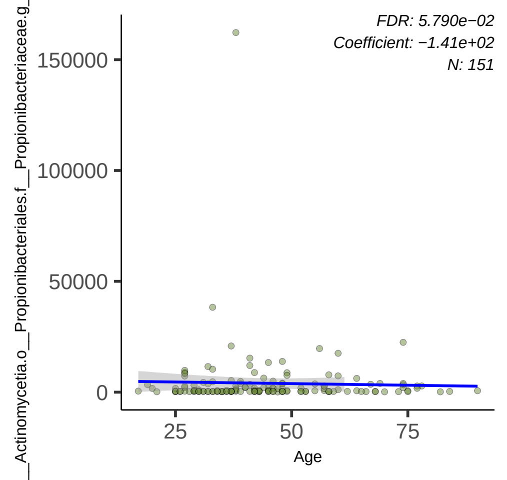

FDR: 5.790e−02
Coefficient: −1.41e+02
N: 151
150000
100000
50000
0
25
50
75
Age
Actinomycetia.o__Propionibacteriales.f__Propionibacteriaceae.g__

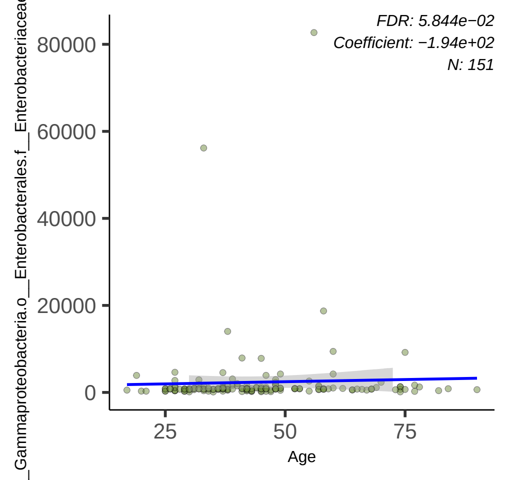

FDR: 5.844e−02
Coefficient: −1.94e+02
N: 151
80000
60000
40000
20000
0
25
50
75
Age
_Gammaproteobacteria.o__Enterobacterales.f__Enterobacteriaceae

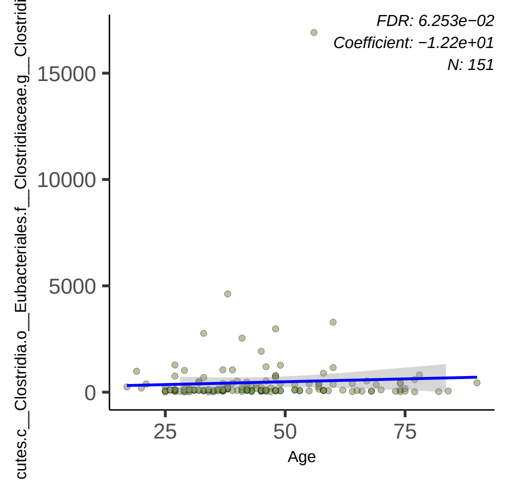

FDR: 6.253e−02
Coefficient: −1.22e+01
N: 151
cutes.c__Clostridia.o__Eubacteriales.f__Clostridiaceae.g__Clostridi
15000
10000
5000
0
25
50
75
Age

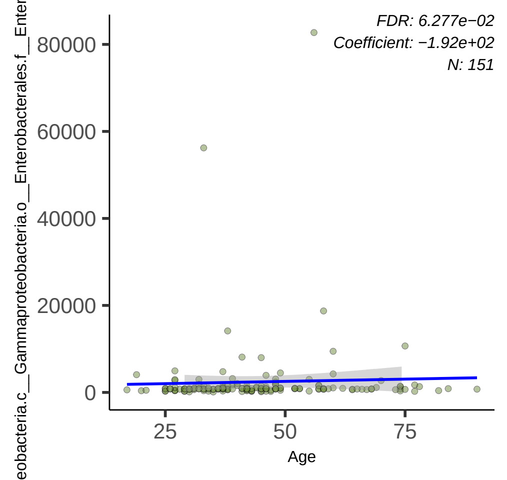

FDR: 6.277e−02
Coefficient: −1.92e+02
N: 151
80000
60000
40000
20000
0
25
50
75
Age
eobacteria.c__Gammaproteobacteria.o__Enterobacterales.f__Enter

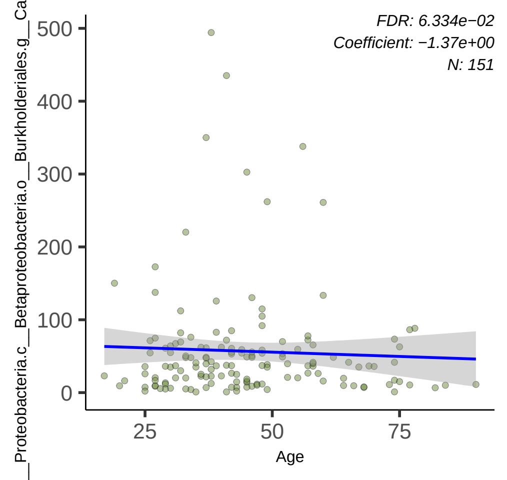
FDR: 6.334e−02
Coefficient: −1.37e+00
N: 151
Proteobacteria.c__Betaproteobacteria.o__Burkholderiales.g__Ca
Age
0
100
200
300
400
500
25
50
75

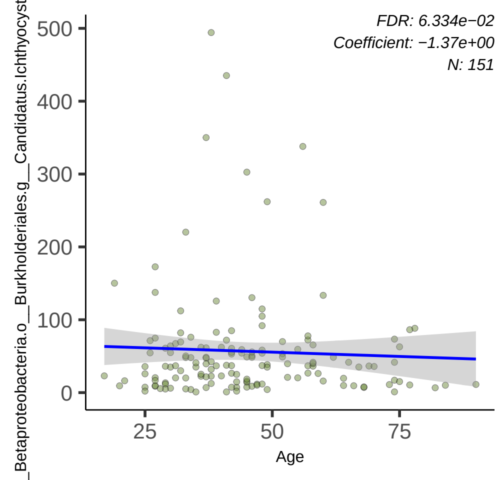
FDR: 6.334e−02
Coefficient: −1.37e+00
N: 151
Betaproteobacteria.o__Burkholderiales.g__Candidatus.Ichthyocyst
0
100
200
300
400
500
25
50
75
Age

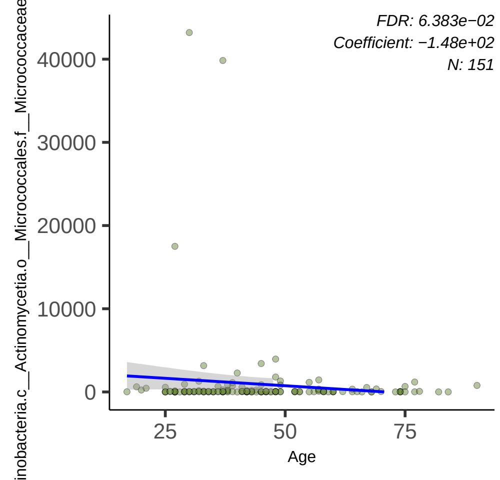
FDR: 6.383e−02
Coefficient: −1.48e+02
N: 151
inobacteria.c__Actinomycetia.o__Micrococcales.f__Micrococcaceae
40000
30000
20000
10000
0
25
50
75
Age

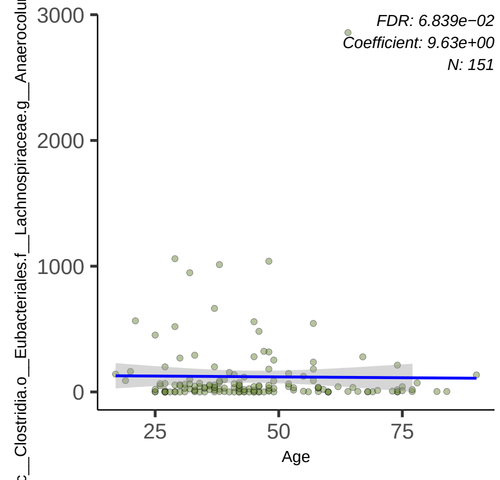

FDR: 6.839e−02
Coefficient: 9.63e+00
N: 151
3000
2000
1000
0
25
50
75
Age
c__Clostridia.o__Eubacteriales.f__Lachnospiraceae.g__Anaerocolu

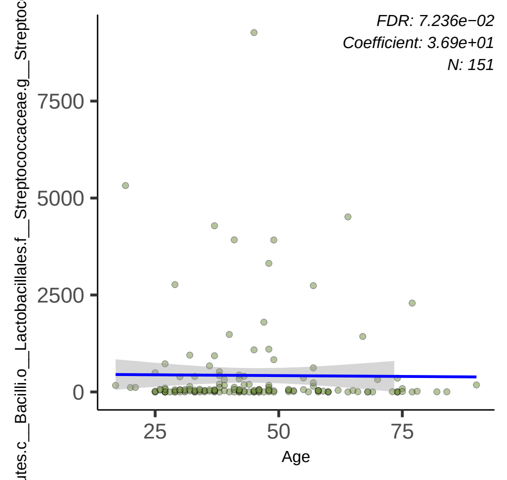
FDR: 7.236e−02
Coefficient: 3.69e+01
N: 151
7500
5000
2500
0
25
50
75
Age
utes.c__Bacilli.o__Lactobacillales.f__Streptococcaceae.g__Streptoc

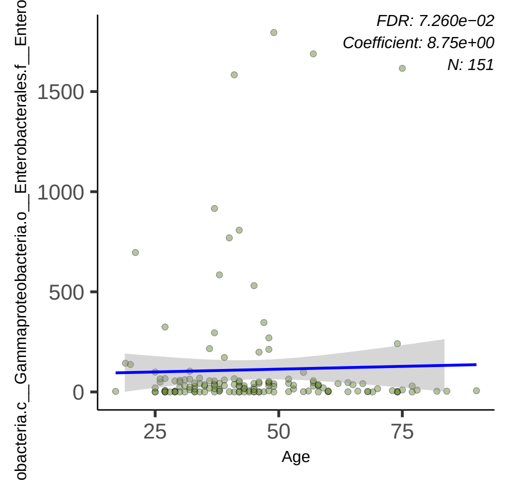

FDR: 7.260e−02
Coefficient: 8.75e+00
N: 151
obacteria.c__Gammaproteobacteria.o__Enterobacterales.f__Entero
1500
1000
500
0
25
50
75
Age

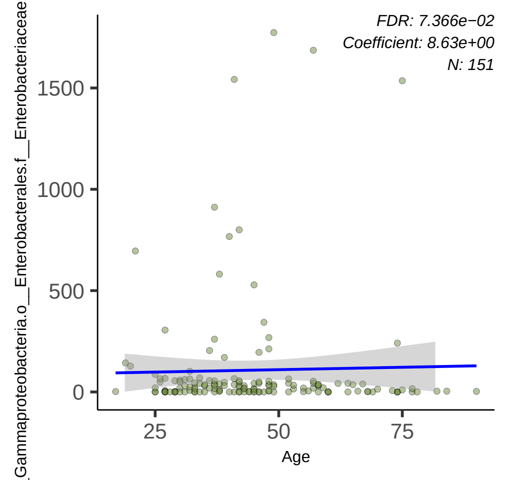

FDR: 7.366e−02
Coefficient: 8.63e+00
N: 151
1500
1000
500
0
25
50
75
Age
_Gammaproteobacteria.o__Enterobacterales.f__Enterobacteriaceae

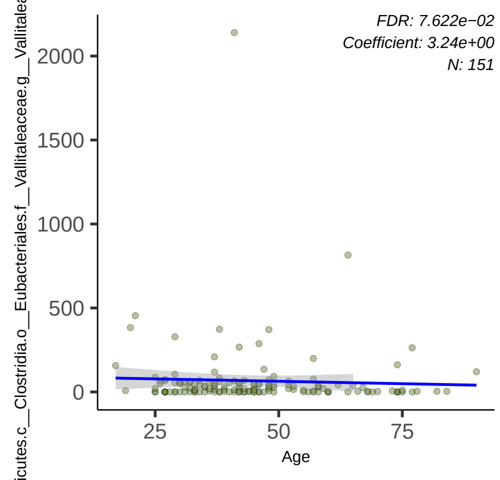
FDR: 7.622e−02
Coefficient: 3.24e+00
N: 151
2000
1500
1000
500
0
25
50
75
Age
icutes.c__Clostridia.o__Eubacteriales.f__Vallitaleaceae.g__Vallitalea

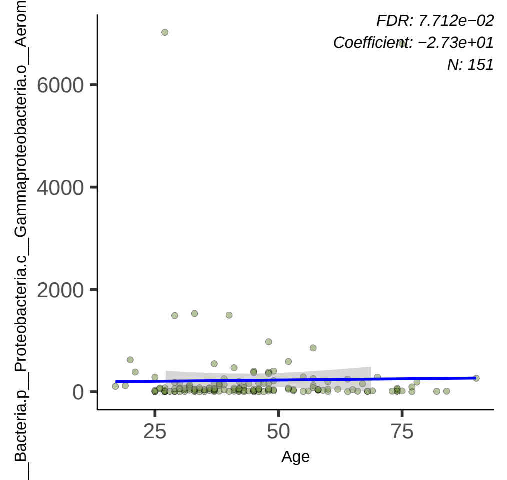

FDR: 7.712e−02
Coefficient: −2.73e+01
N: 151
6000
4000
2000
0
25
50
75
Age
Bacteria.p__Proteobacteria.c__Gammaproteobacteria.o__Aerom

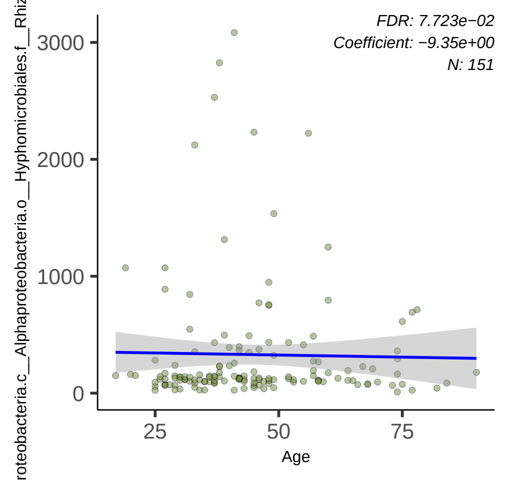

FDR: 7.723e−02
Coefficient: −9.35e+00
N: 151
3000
2000
1000
0
25
50
75
Age
roteobacteria.c__Alphaproteobacteria.o__Hyphomicrobiales.f__Rhiz

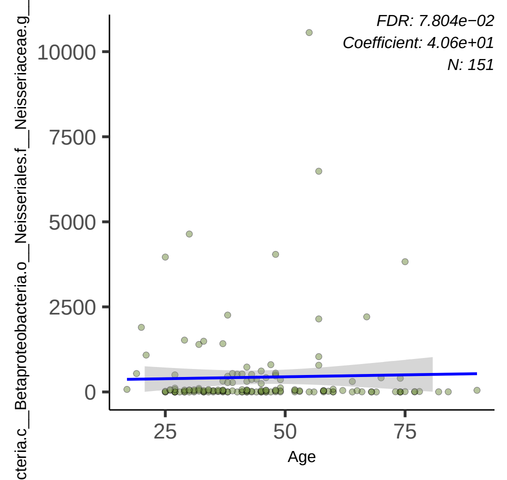
FDR: 7.804e−02
Coefficient: 4.06e+01
N: 151
10000
7500
5000
2500
0
25
50
75
Age
cteria.c__Betaproteobacteria.o__Neisseriales.f__Neisseriaceae.g__

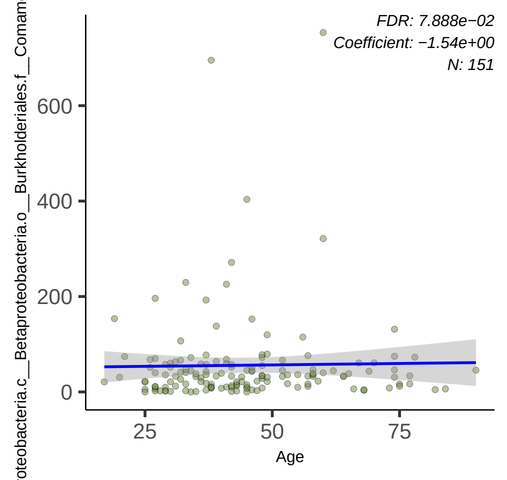
FDR: 7.888e−02
Coefficient: −1.54e+00
N: 151
0
200
400
600
25
50
75
Age
oteobacteria.c__Betaproteobacteria.o__Burkholderiales.f__Comam

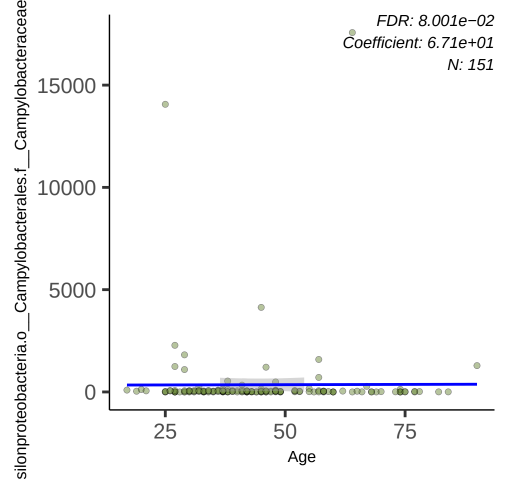

FDR: 8.001e−02
Coefficient: 6.71e+01
N: 151
15000
10000
5000
0
25
50
75
Age
silonproteobacteria.o__Campylobacterales.f__Campylobacteraceae

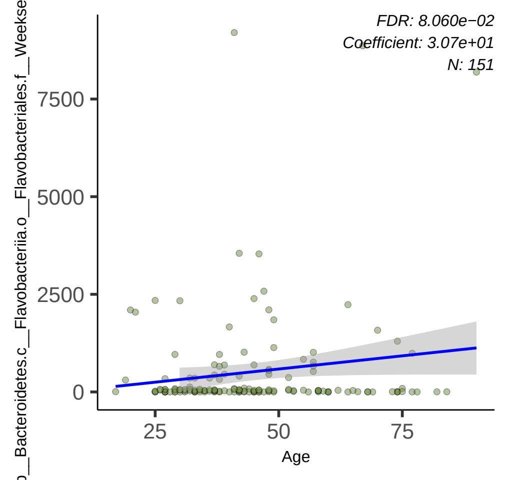

FDR: 8.060e−02
Coefficient: 3.07e+01
N: 151
0
2500
5000
7500
25
50
75
Age
o__Bacteroidetes.c__Flavobacteriia.o__Flavobacteriales.f__Weekse

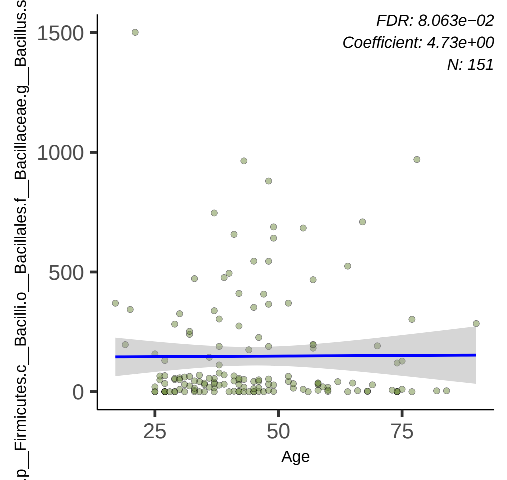

FDR: 8.063e−02
Coefficient: 4.73e+00
N: 151
p__Firmicutes.c__Bacilli.o__Bacillales.f__Bacillaceae.g__Bacillus.s
0
500
1000
1500
25
50
75
Age

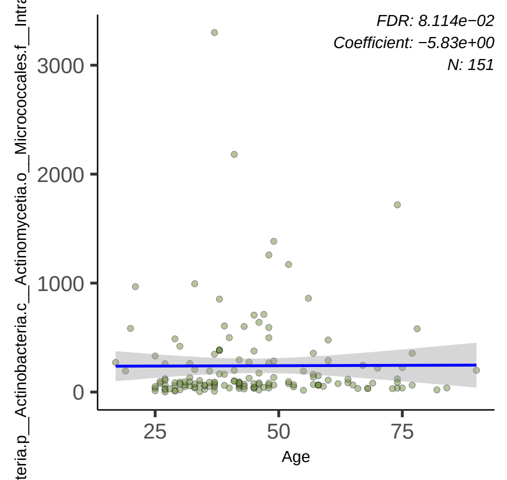

FDR: 8.114e−02
Coefficient: −5.83e+00
N: 151
3000
2000
1000
0
25
50
75
Age
teria.p__Actinobacteria.c__Actinomycetia.o__Micrococcales.f__Intra

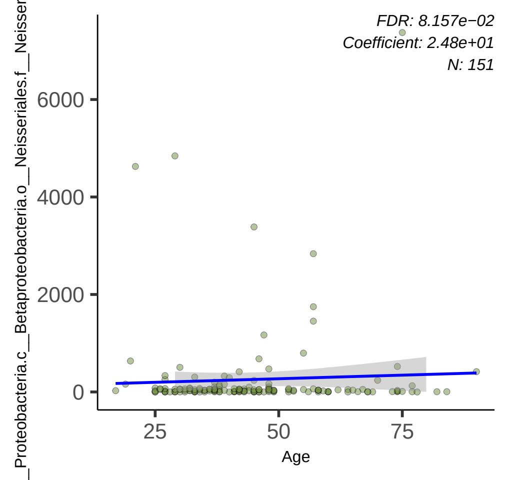
FDR: 8.157e−02
Coefficient: 2.48e+01
N: 151
6000
4000
2000
0
25
50
75
Age
_Proteobacteria.c__Betaproteobacteria.o__Neisseriales.f__Neisser

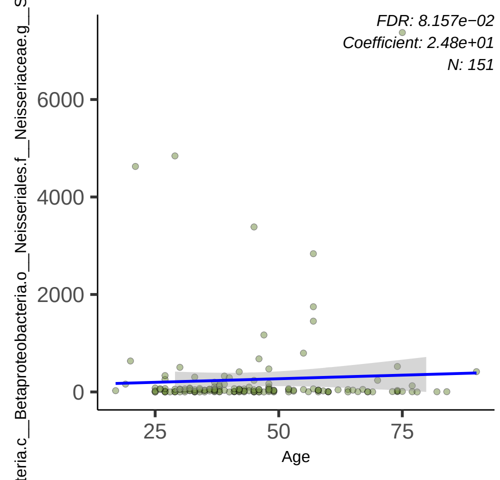

FDR: 8.157e−02
Coefficient: 2.48e+01
N: 151
6000
4000
2000
0
25
50
75
Age
teria.c__Betaproteobacteria.o__Neisseriales.f__Neisseriaceae.g__S

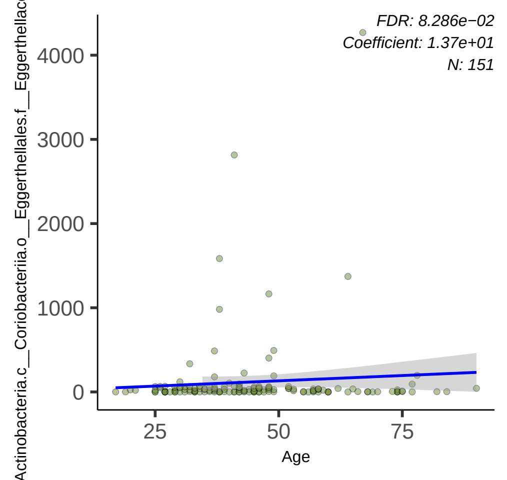
FDR: 8.286e−02
Coefficient: 1.37e+01
N: 151
4000
3000
2000
1000
0
25
50
75
Age
Actinobacteria.c__Coriobacteriia.o__Eggerthellales.f__Eggerthellac

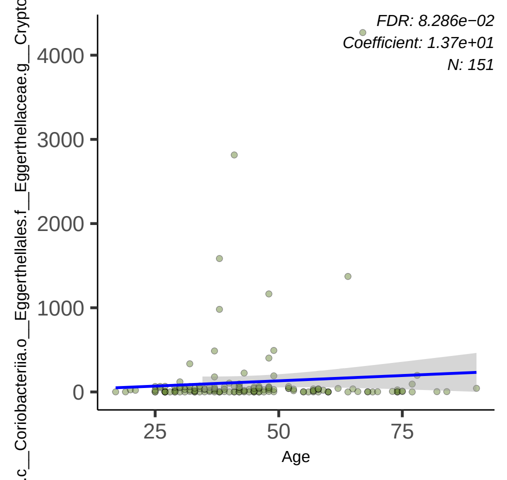

FDR: 8.286e−02
Coefficient: 1.37e+01
N: 151
4000
3000
2000
1000
0
25
50
75
Age
c__Coriobacteriia.o__Eggerthellales.f__Eggerthellaceae.g__Crypto

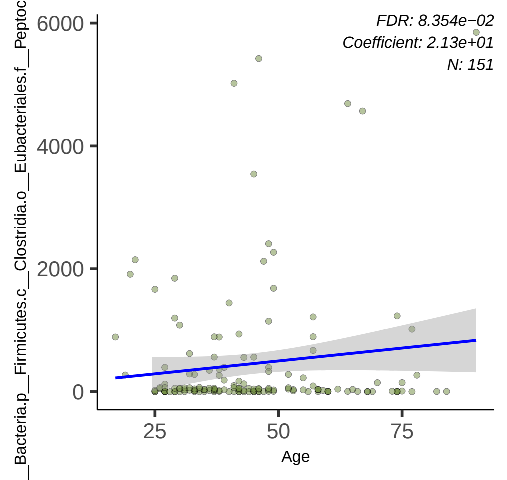
FDR: 8.354e−02
Coefficient: 2.13e+01
N: 151
Bacteria.p__Firmicutes.c__Clostridia.o__Eubacteriales.f__Peptoc
6000
4000
2000
0
25
50
75
Age

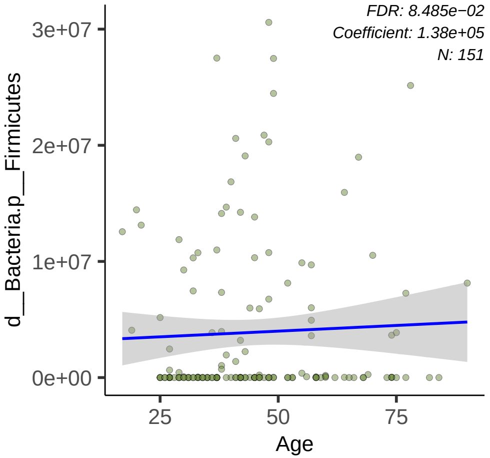

FDR: 8.485e−02
Coefficient: 1.38e+05
N: 151
3e+07
2e+07
1e+07
0e+00
d__Bacteria.p__Firmicutes
25
50
75
Age

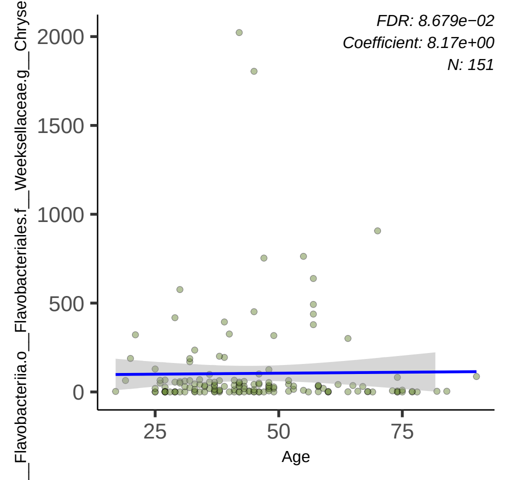

FDR: 8.679e−02
Coefficient: 8.17e+00
N: 151
Flavobacteriia.o__Flavobacteriales.f__Weeksellaceae.g__Chryse
2000
1500
1000
500
0
25
50
75
Age

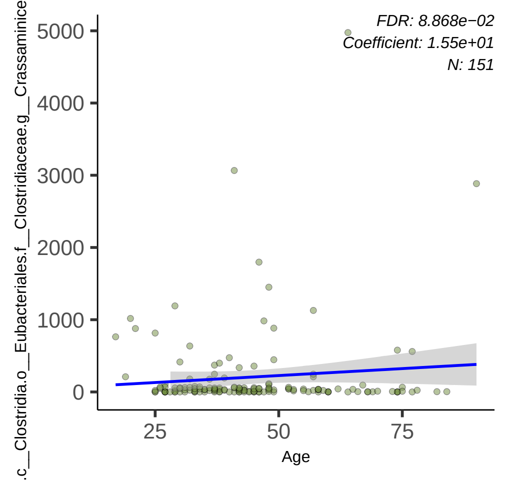
FDR: 8.868e−02
Coefficient: 1.55e+01
N: 151
c__Clostridia.o__Eubacteriales.f__Clostridiaceae.g__Crassaminice
5000
4000
3000
2000
1000
0
25
50
75
Age

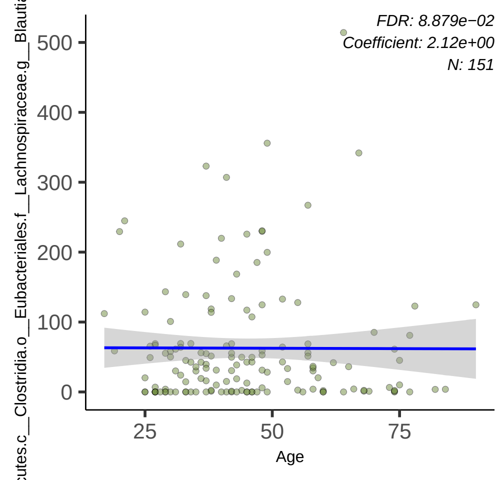
FDR: 8.879e−02
Coefficient: 2.12e+00
N: 151
cutes.c__Clostridia.o__Eubacteriales.f__Lachnospiraceae.g__Blauti
500
400
300
200
100
0
25
50
75
Age

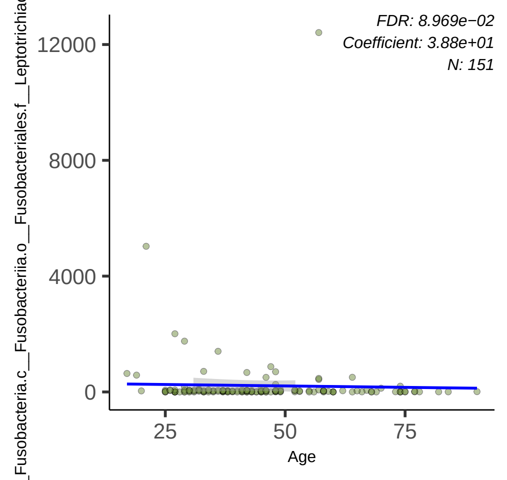
FDR: 8.969e−02
Coefficient: 3.88e+01
N: 151
12000
8000
4000
0
25
50
75
Age
Fusobacteria.c__Fusobacteriia.o__Fusobacteriales.f__Leptotrichia

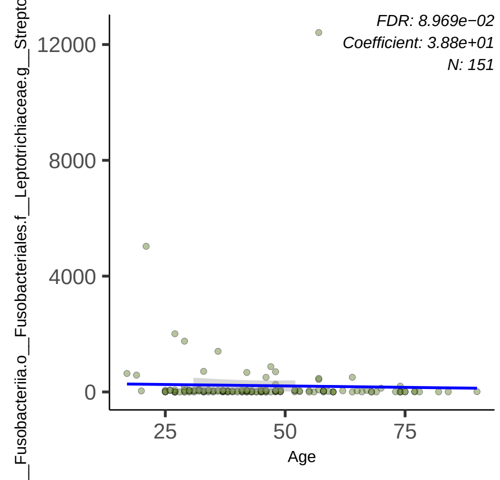

FDR: 8.969e−02
Coefficient: 3.88e+01
N: 151
12000
8000
4000
0
25
50
75
Age
Fusobacteriia.o__Fusobacteriales.f__Leptotrichiaceae.g__Strepto

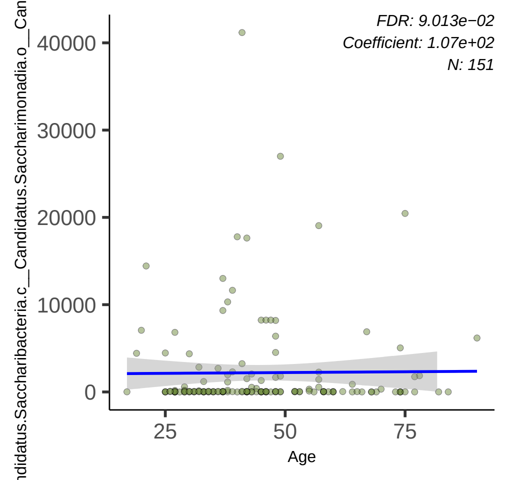
FDR: 9.013e−02
Coefficient: 1.07e+02
N: 151
ndidatus.Saccharibacteria.c__Candidatus.Saccharimonadia.o__Can
40000
30000
20000
10000
0
25
50
75
Age

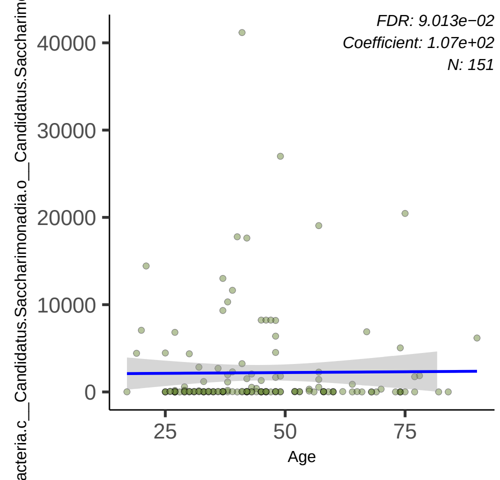
FDR: 9.013e−02
Coefficient: 1.07e+02
N: 151
acteria.c__Candidatus.Saccharimonadia.o__Candidatus.Saccharim
40000
30000
20000
10000
0
25
50
75
Age

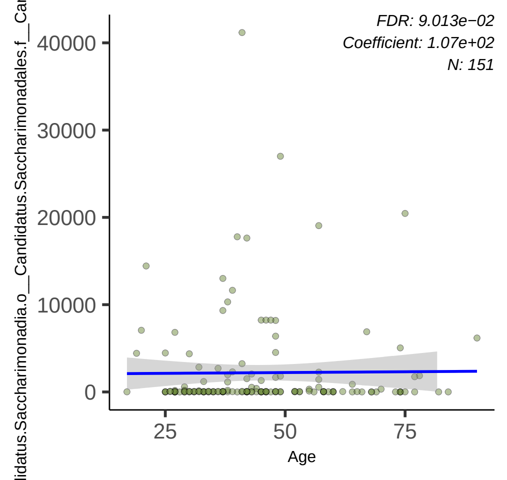
FDR: 9.013e−02
Coefficient: 1.07e+02
N: 151
40000
30000
20000
10000
0
25
50
75
Age
idatus.Saccharimonadia.o__Candidatus.Saccharimonadales.f__Ca

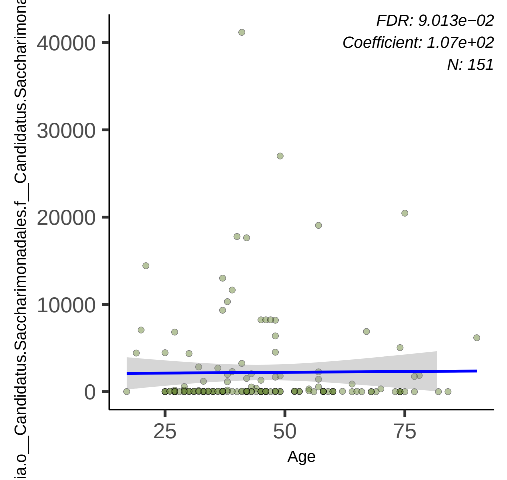
FDR: 9.013e−02
Coefficient: 1.07e+02
N: 151
ia.o__Candidatus.Saccharimonadales.f__Candidatus.Saccharimona
Age
0
10000
20000
30000
40000
25
50
75

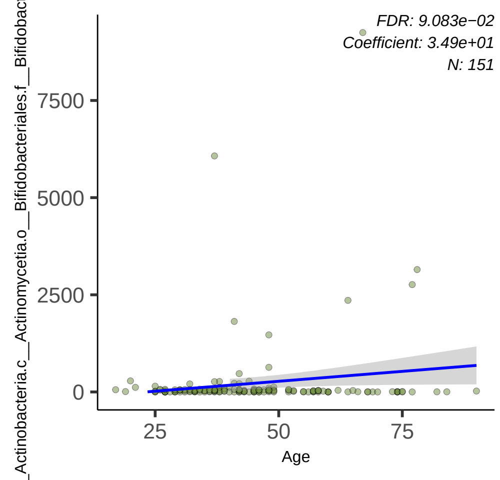

FDR: 9.083e−02
Coefficient: 3.49e+01
N: 151
7500
5000
2500
0
25
50
75
Age
Actinobacteria.c__Actinomycetia.o__Bifidobacteriales.f__Bifidobac

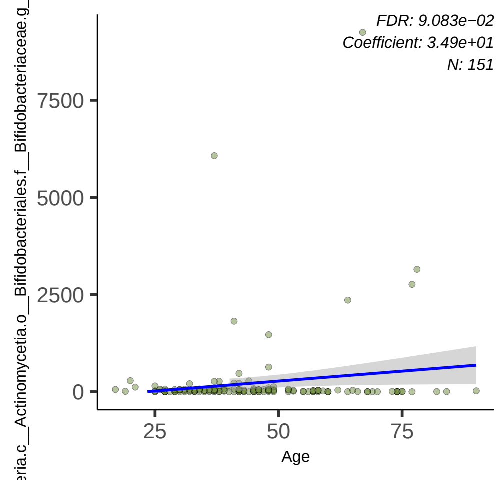
FDR: 9.083e−02
Coefficient: 3.49e+01
N: 151
7500
5000
2500
0
25
50
75
Age
eria.c__Actinomycetia.o__Bifidobacteriales.f__Bifidobacteriaceae.g__

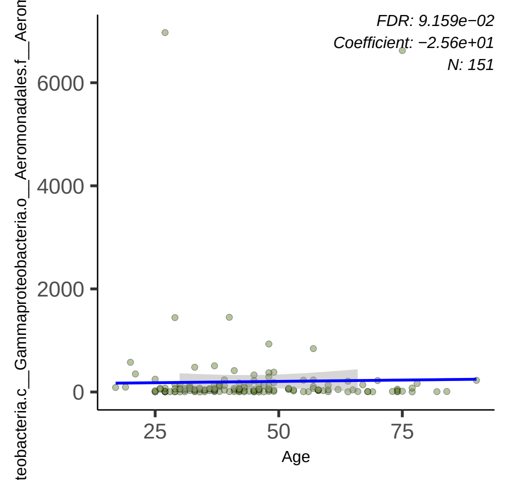
FDR: 9.159e−02
Coefficient: −2.56e+01
N: 151
teobacteria.c__Gammaproteobacteria.o__Aeromonadales.f__Aerom
6000
4000
2000
0
25
50
75
Age

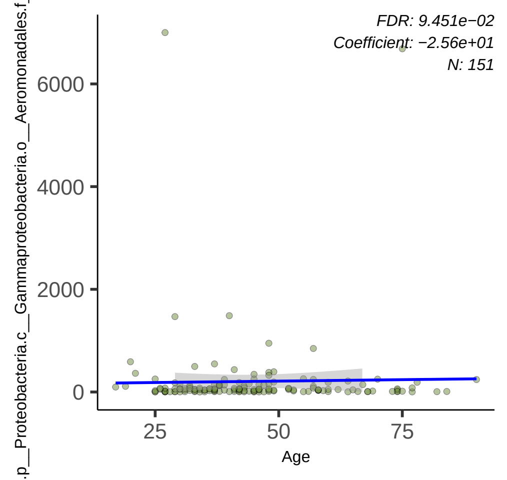

FDR: 9.451e−02
Coefficient: −2.56e+01
N: 151
6000
4000
2000
0
25
50
75
Age
p__Proteobacteria.c__Gammaproteobacteria.o__Aeromonadales.f_

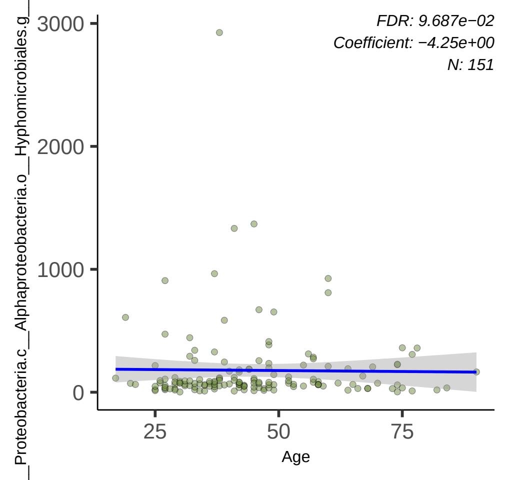

FDR: 9.687e−02
Coefficient: −4.25e+00
N: 151
Proteobacteria.c__Alphaproteobacteria.o__Hyphomicrobiales.g__
3000
2000
1000
0
25
50
75
Age

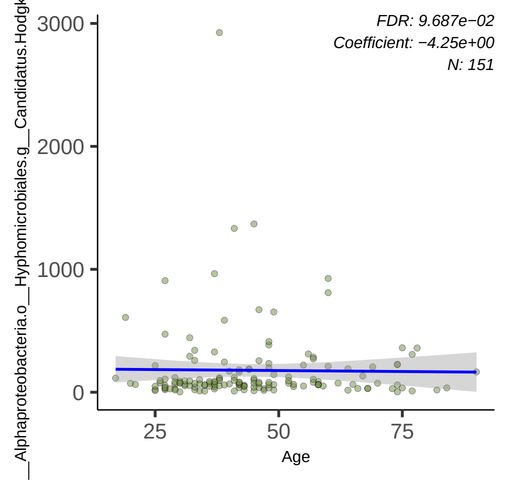

FDR: 9.687e−02
Coefficient: −4.25e+00
N: 151
3000
2000
1000
0
25
50
75
Age
_Alphaproteobacteria.o__Hyphomicrobiales.g__Candidatus.Hodgk

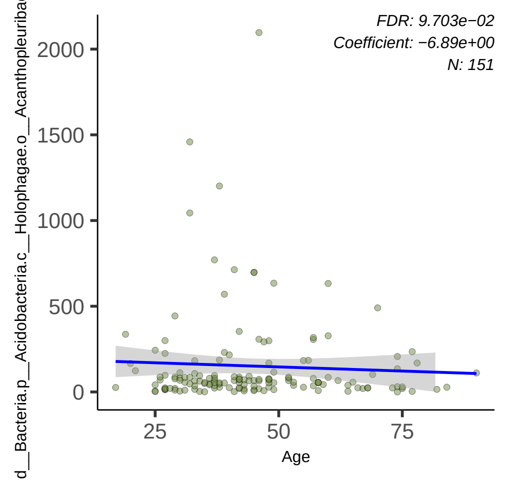

FDR: 9.703e−02
Coefficient: −6.89e+00
N: 151
d__Bacteria.p__Acidobacteria.c__Holophagae.o__Acanthopleuribac
0
500
1000
1500
2000
25
50
75
Age

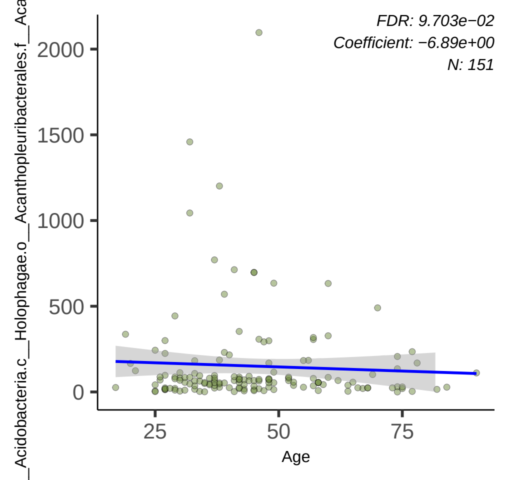

FDR: 9.703e−02
Coefficient: −6.89e+00
N: 151
Acidobacteria.c__Holophagae.o__Acanthopleuribacterales.f__Aca
0
500
1000
1500
2000
25
50
75
Age

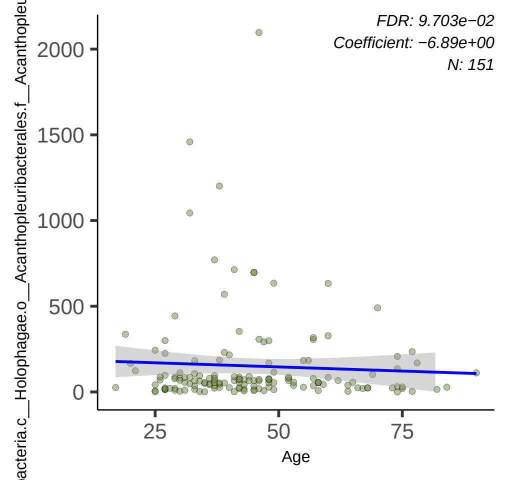

FDR: 9.703e−02
Coefficient: −6.89e+00
N: 151
acteria.c__Holophagae.o__Acanthopleuribacterales.f__Acanthopleu
0
500
1000
1500
2000
25
50
75
Age

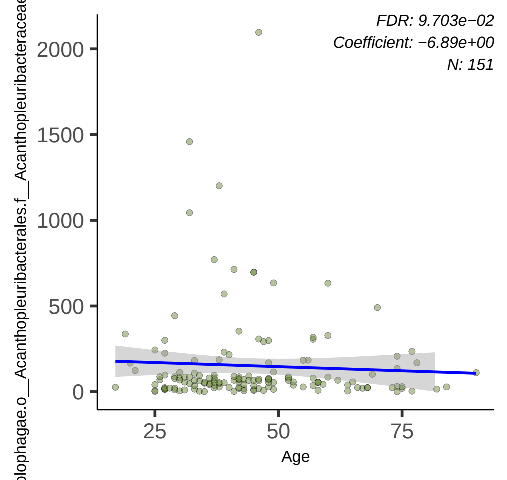
FDR: 9.703e−02
Coefficient: −6.89e+00
N: 151
0
500
1000
1500
2000
25
50
75
Age
lophagae.o__Acanthopleuribacterales.f__Acanthopleuribacteraceae

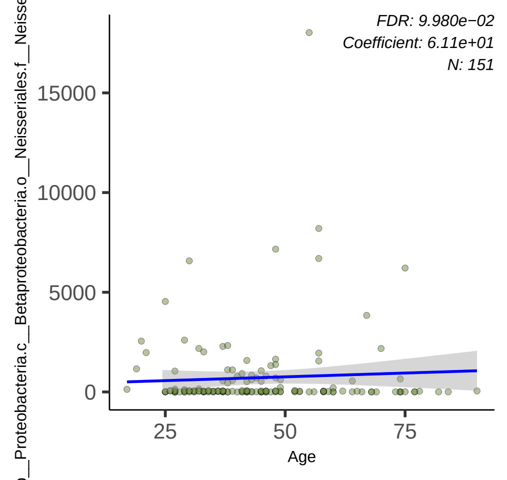

FDR: 9.980e−02
Coefficient: 6.11e+01
N: 151
15000
10000
5000
0
25
50
75
Age
o__Proteobacteria.c__Betaproteobacteria.o__Neisseriales.f__Neisse

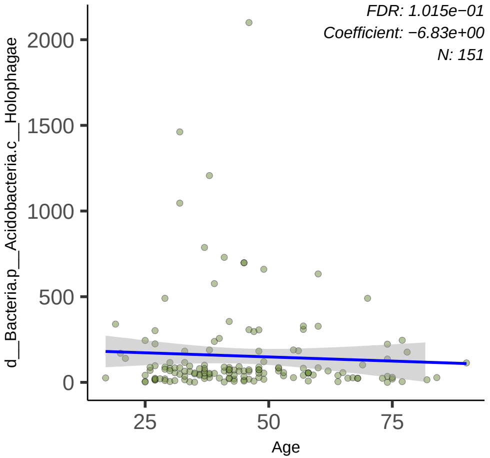
FDR: 1.015e−01
Coefficient: −6.83e+00
N: 151
d__Bacteria.p__Acidobacteria.c__Holophagae
0
500
1000
1500
2000
25
50
75
Age

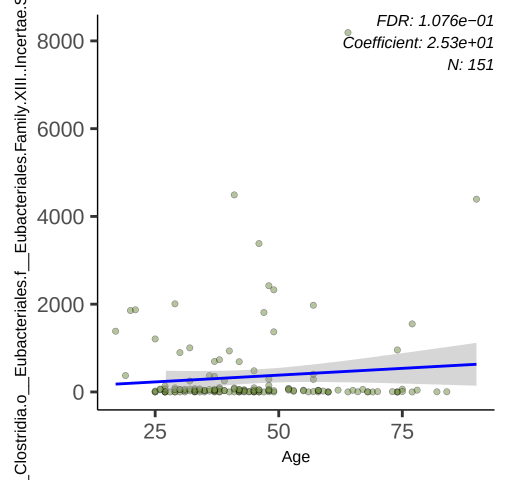
FDR: 1.076e−01
Coefficient: 2.53e+01
N: 151
8000
6000
4000
2000
0
25
50
75
Age
Clostridia.o__Eubacteriales.f__Eubacteriales.Family.XIII..Incertae.S

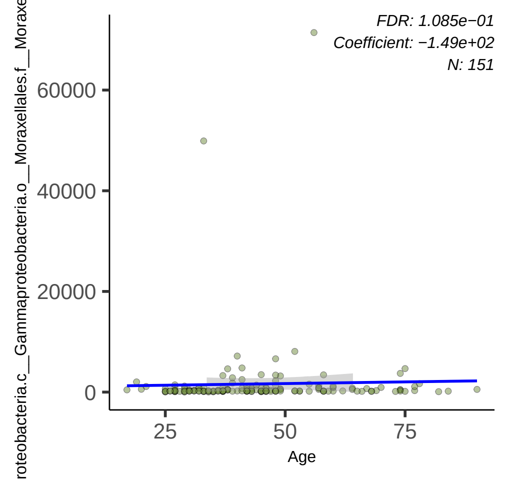
FDR: 1.085e−01
Coefficient: −1.49e+02
N: 151
roteobacteria.c__Gammaproteobacteria.o__Moraxellales.f__Moraxe
60000
40000
20000
0
25
50
75
Age

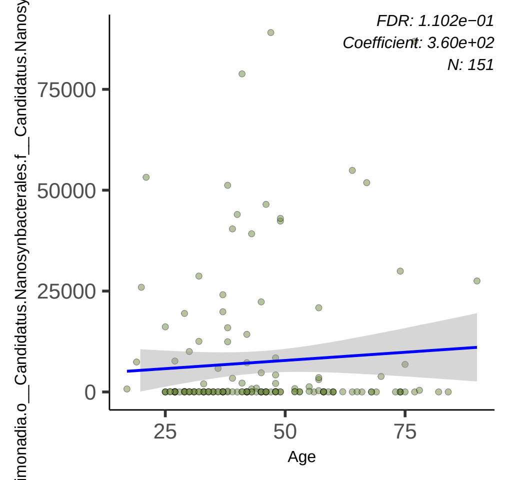
FDR: 1.102e−01
Coefficient: 3.60e+02
N: 151
75000
50000
25000
0
25
50
75
Age
imonadia.o__Candidatus.Nanosynbacterales.f__Candidatus.Nanosy

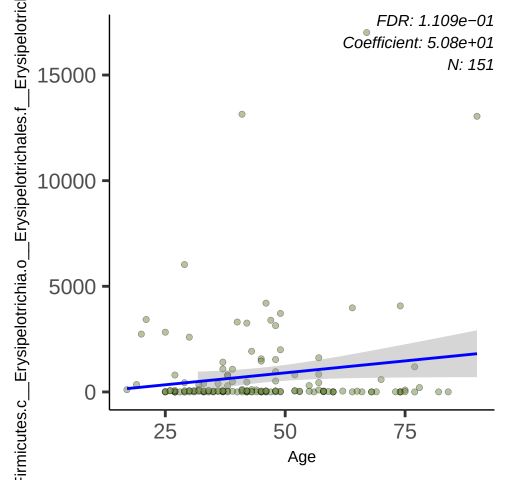

FDR: 1.109e−01
Coefficient: 5.08e+01
N: 151
Firmicutes.c__Erysipelotrichia.o__Erysipelotrichales.f__Erysipelotric
15000
10000
5000
0
25
50
75
Age

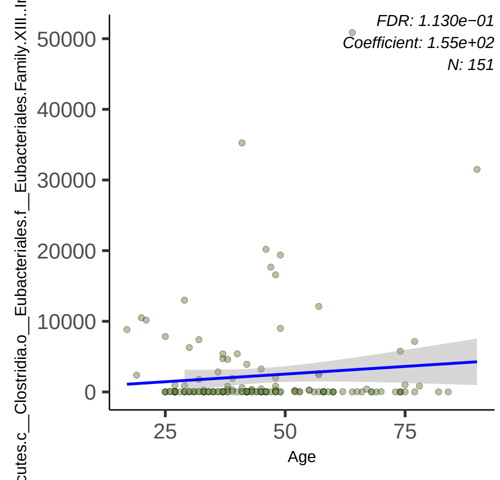

FDR: 1.130e−01
Coefficient: 1.55e+02
N: 151
cutes.c__Clostridia.o__Eubacteriales.f__Eubacteriales.Family.XIII..In
50000
40000
30000
20000
10000
0
25
50
75
Age

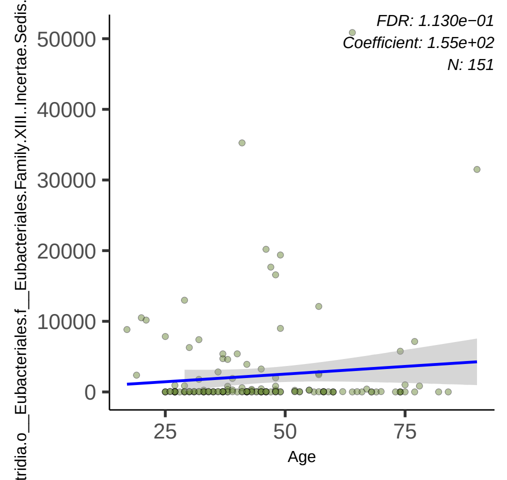

FDR: 1.130e−01
Coefficient: 1.55e+02
N: 151
50000
40000
30000
20000
10000
0
25
50
75
Age
tridia.o__Eubacteriales.f__Eubacteriales.Family.XIII..Incertae.Sedis.

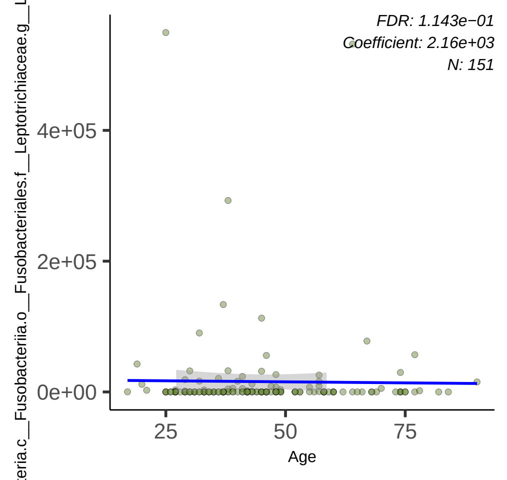

FDR: 1.143e−01
Coefficient: 2.16e+03
N: 151
4e+05
2e+05
0e+00
25
50
75
Age
teria.c__Fusobacteriia.o__Fusobacteriales.f__Leptotrichiaceae.g__L

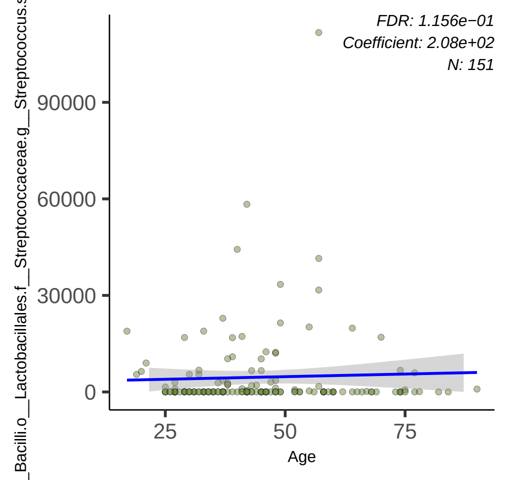
FDR: 1.156e−01
Coefficient: 2.08e+02
N: 151
Bacilli.o__Lactobacillales.f__Streptococcaceae.g__Streptococcus.s
90000
60000
30000
0
25
50
75
Age

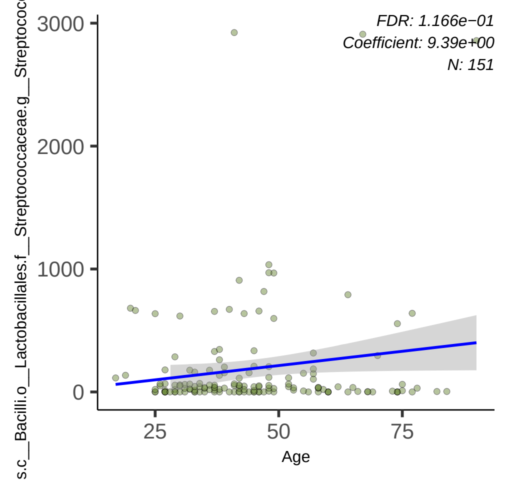

FDR: 1.166e−01
Coefficient: 9.39e+00
N: 151
s.c__Bacilli.o__Lactobacillales.f__Streptococcaceae.g__Streptococ
3000
2000
1000
0
25
50
75
Age

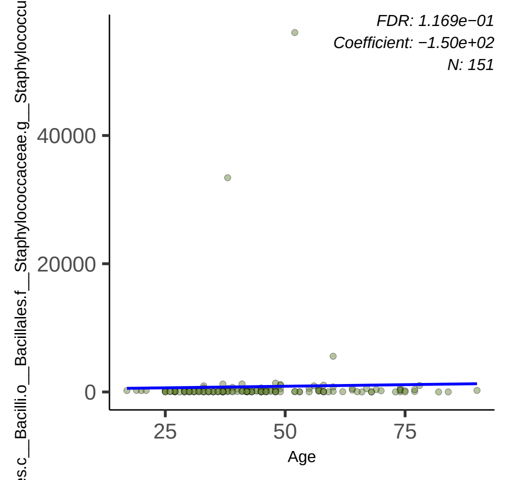

FDR: 1.169e−01
Coefficient: −1.50e+02
N: 151
40000
20000
0
25
50
75
Age
es.c__Bacilli.o__Bacillales.f__Staphylococcaceae.g__Staphylococcu

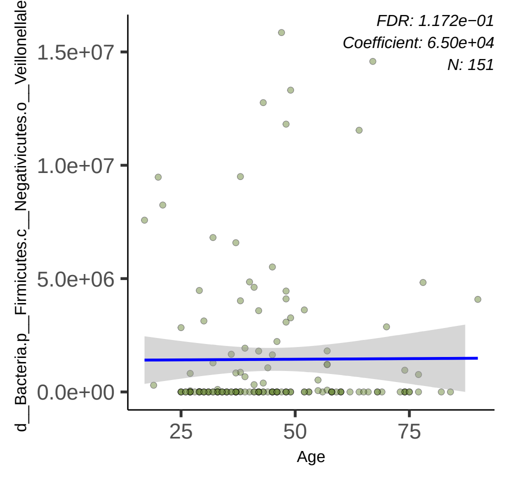
FDR: 1.172e−01
Coefficient: 6.50e+04
N: 151
d__Bacteria.p__Firmicutes.c__Negativicutes.o__Veillonellale
1.5e+07
1.0e+07
5.0e+06
0.0e+00
25
50
75
Age

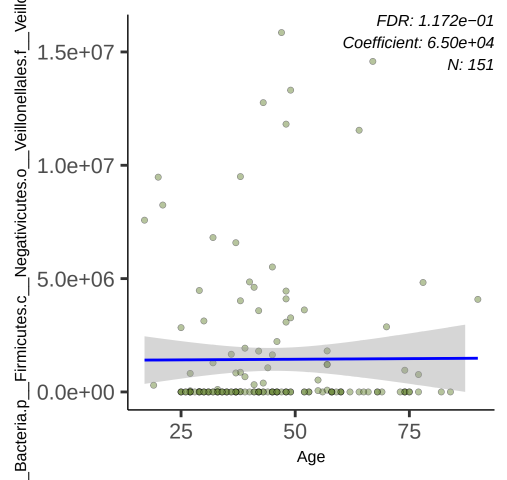
FDR: 1.172e−01
Coefficient: 6.50e+04
N: 151
1.5e+07
1.0e+07
5.0e+06
0.0e+00
25
50
75
Age
Bacteria.p__Firmicutes.c__Negativicutes.o__Veillonellales.f__Veillo

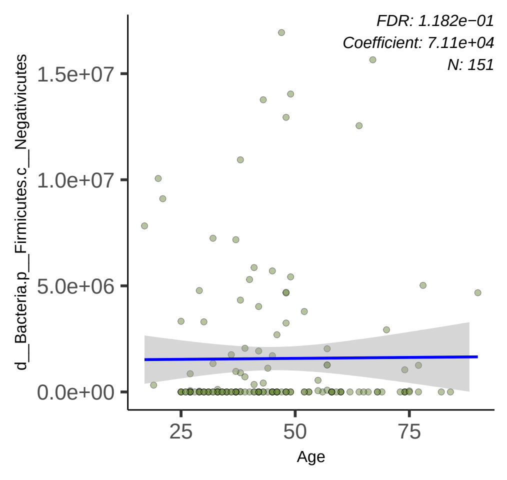
FDR: 1.182e−01
Coefficient: 7.11e+04
N: 151
d__Bacteria.p__Firmicutes.c__Negativicutes
1.5e+07
1.0e+07
5.0e+06
0.0e+00
25
50
75
Age

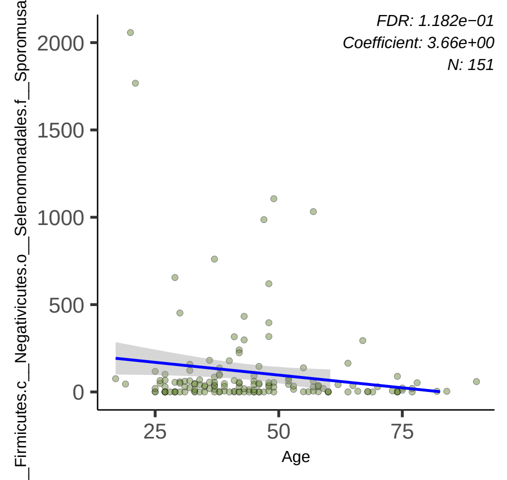

FDR: 1.182e−01
Coefficient: 3.66e+00
N: 151
Firmicutes.c__Negativicutes.o__Selenomonadales.f__Sporomusa
2000
1500
1000
500
0
25
50
75
Age

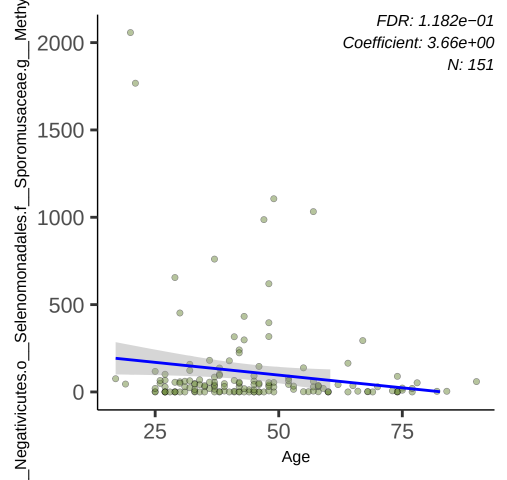

FDR: 1.182e−01
Coefficient: 3.66e+00
N: 151
_Negativicutes.o__Selenomonadales.f__Sporomusaceae.g__Methy
2000
1500
1000
500
0
25
50
75
Age

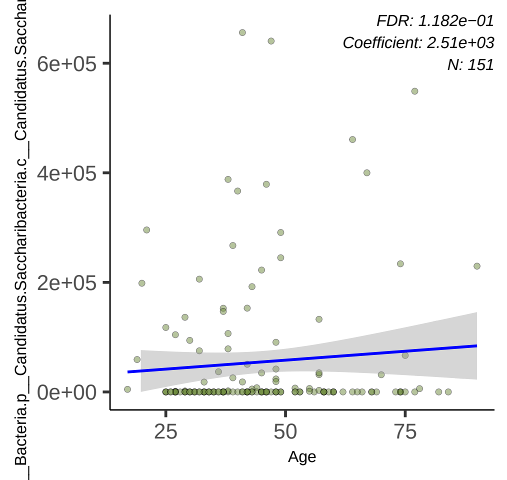

FDR: 1.182e−01
Coefficient: 2.51e+03
N: 151
6e+05
4e+05
2e+05
0e+00
25
50
75
Age
Bacteria.p__Candidatus.Saccharibacteria.c__Candidatus.Sacchar

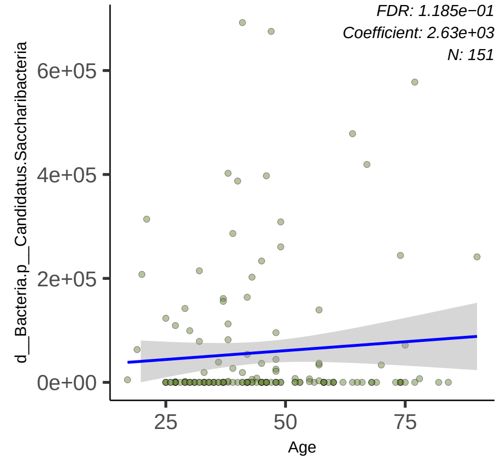
FDR: 1.185e−01
Coefficient: 2.63e+03
N: 151
d__Bacteria.p__Candidatus.Saccharibacteria
6e+05
4e+05
2e+05
0e+00
25
50
75
Age

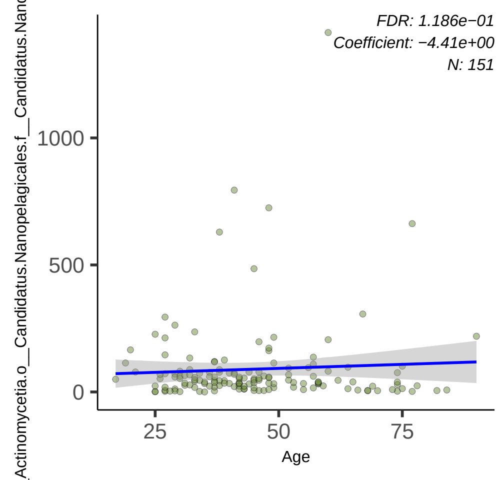

FDR: 1.186e−01
Coefficient: −4.41e+00
N: 151
1000
500
0
25
50
75
Age
Actinomycetia.o__Candidatus.Nanopelagicales.f__Candidatus.Nano

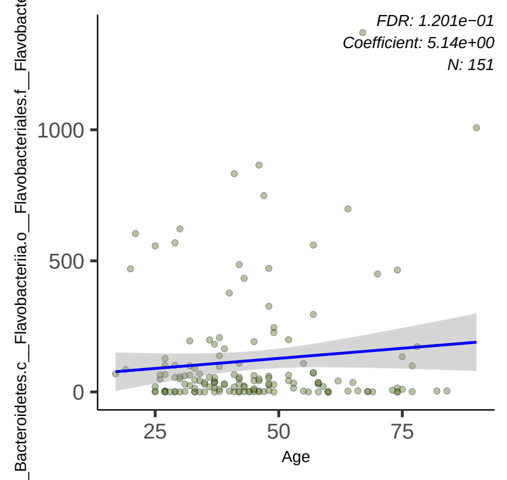

FDR: 1.201e−01
Coefficient: 5.14e+00
N: 151
1000
500
0
25
50
75
Age
_Bacteroidetes.c__Flavobacteriia.o__Flavobacteriales.f__Flavobacte

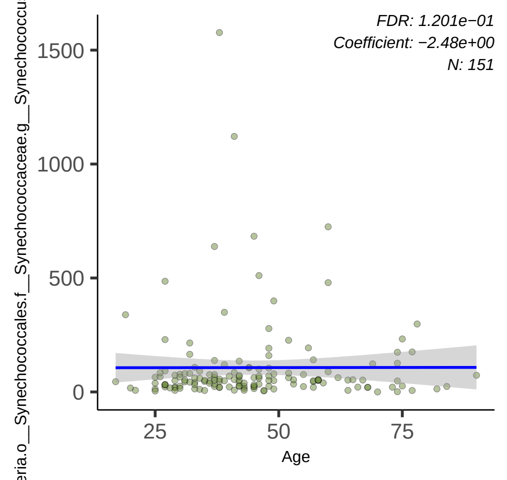
FDR: 1.201e−01
Coefficient: −2.48e+00
N: 151
1500
1000
500
0
25
50
75
Age
eria.o__Synechococcales.f__Synechococcaceae.g__Synechococcus

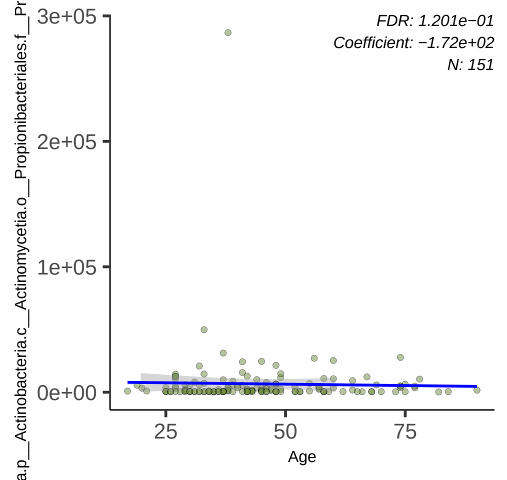

FDR: 1.201e−01
Coefficient: −1.72e+02
N: 151
3e+05
2e+05
1e+05
0e+00
25
50
75
Age
a.p__Actinobacteria.c__Actinomycetia.o__Propionibacteriales.f__Pr

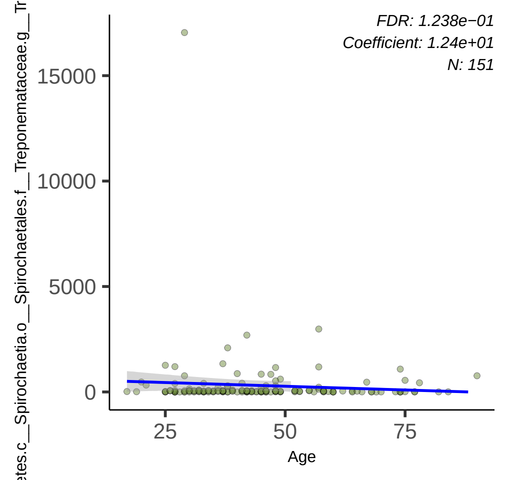

FDR: 1.238e−01
Coefficient: 1.24e+01
N: 151
15000
10000
5000
0
25
50
75
Age
etes.c__Spirochaetia.o__Spirochaetales.f__Treponemataceae.g__Tr

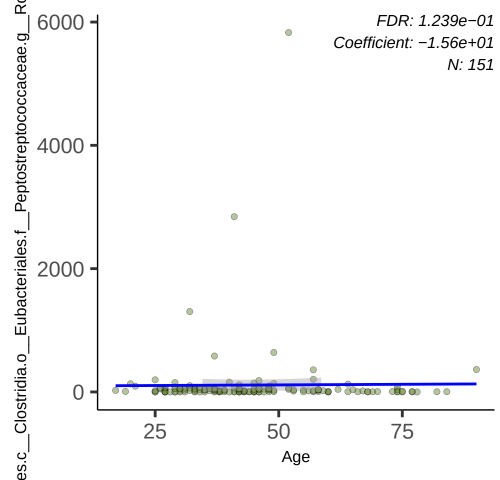

FDR: 1.239e−01
Coefficient: −1.56e+01
N: 151
6000
4000
2000
0
25
50
75
Age
es.c__Clostridia.o__Eubacteriales.f__Peptostreptococcaceae.g__Ro

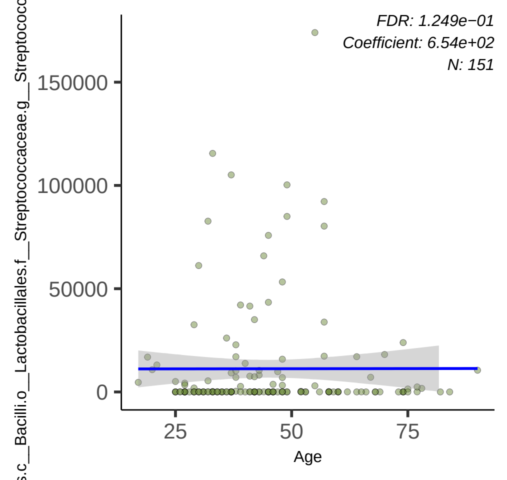

FDR: 1.249e−01
Coefficient: 6.54e+02
N: 151
150000
100000
50000
0
25
50
75
Age
s.c__Bacilli.o__Lactobacillales.f__Streptococcaceae.g__Streptococc

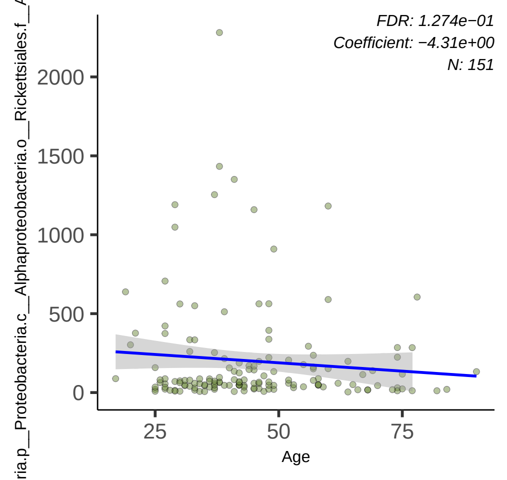
FDR: 1.274e−01
Coefficient: −4.31e+00
N: 151
ria.p__Proteobacteria.c__Alphaproteobacteria.o__Rickettsiales.f__
0
500
1000
1500
2000
25
50
75
Age

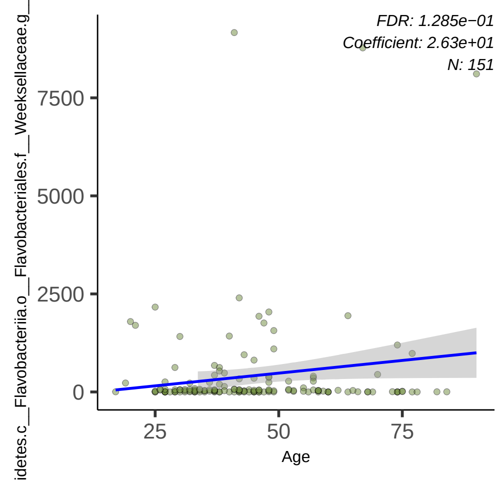
FDR: 1.285e−01
Coefficient: 2.63e+01
N: 151
idetes.c__Flavobacteriia.o__Flavobacteriales.f__Weeksellaceae.g__
7500
5000
2500
0
25
50
75
Age

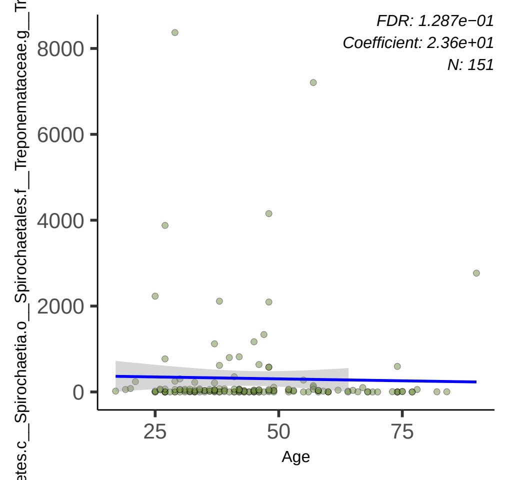
FDR: 1.287e−01
Coefficient: 2.36e+01
N: 151
8000
6000
4000
2000
0
25
50
75
Age

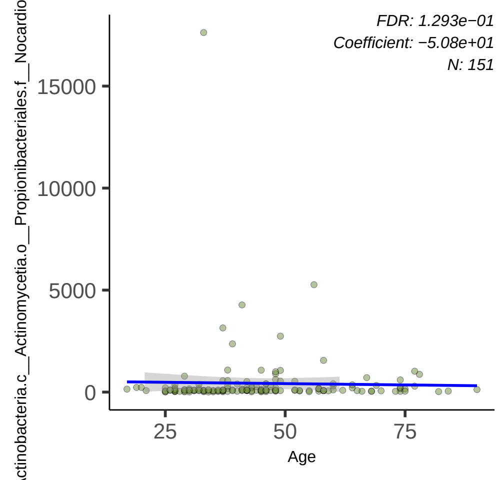

FDR: 1.293e−01
Coefficient: −5.08e+01
N: 151
15000
10000
5000
0
25
50
75
Age
Actinobacteria.c__Actinomycetia.o__Propionibacteriales.f__Nocardio

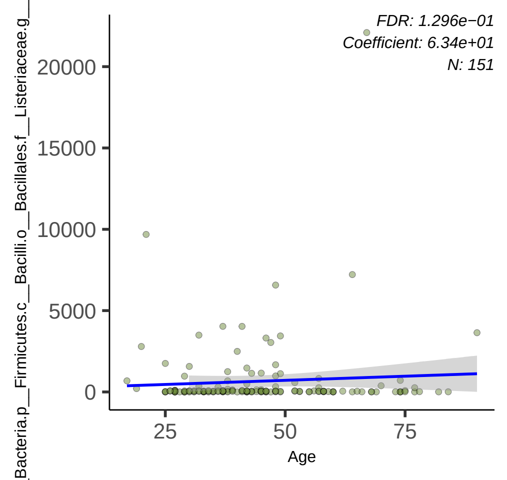
FDR: 1.296e−01
Coefficient: 6.34e+01
N: 151
Bacteria.p__Firmicutes.c__Bacilli.o__Bacillales.f__Listeriaceae.g__
20000
15000
10000
5000
0
25
50
75
Age

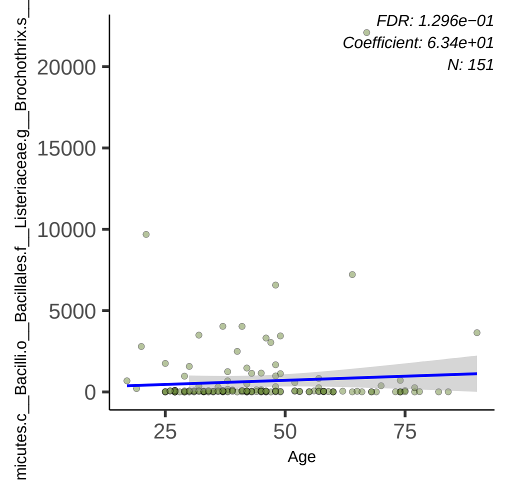

FDR: 1.296e−01
Coefficient: 6.34e+01
N: 151
micutes.c__Bacilli.o__Bacillales.f__Listeriaceae.g__Brochothrix.s__
20000
15000
10000
5000
0
25
50
75
Age

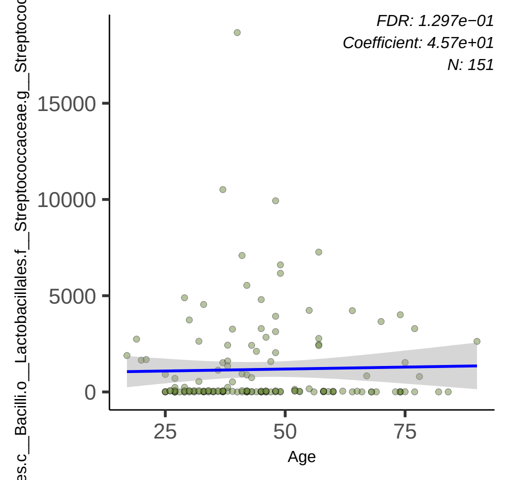

FDR: 1.297e−01
Coefficient: 4.57e+01
N: 151
15000
10000
5000
0
25
50
75
Age
es.c__Bacilli.o__Lactobacillales.f__Streptococcaceae.g__Streptococ

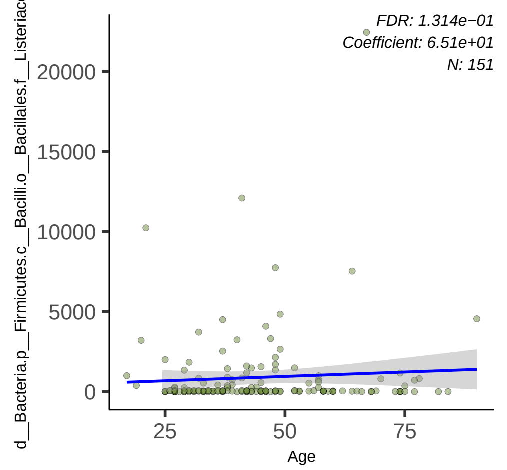
FDR: 1.314e−01
Coefficient: 6.51e+01
N: 151
d__Bacteria.p__Firmicutes.c__Bacilli.o__Bacillales.f__Listeriac
20000
15000
10000
5000
0
25
50
75
Age

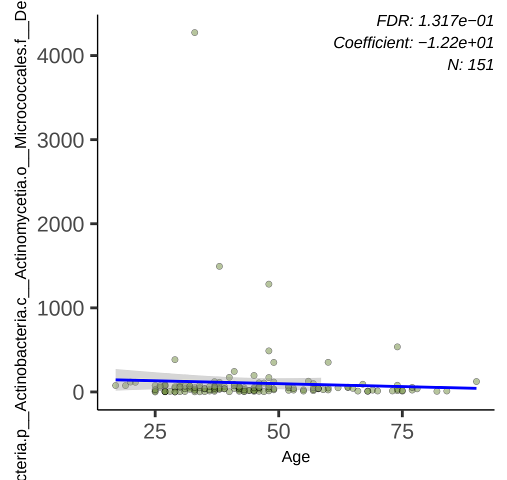

FDR: 1.317e−01
Coefficient: −1.22e+01
N: 151
cteria.p__Actinobacteria.c__Actinomycetia.o__Micrococcales.f__De
4000
3000
2000
1000
0
25
50
75
Age

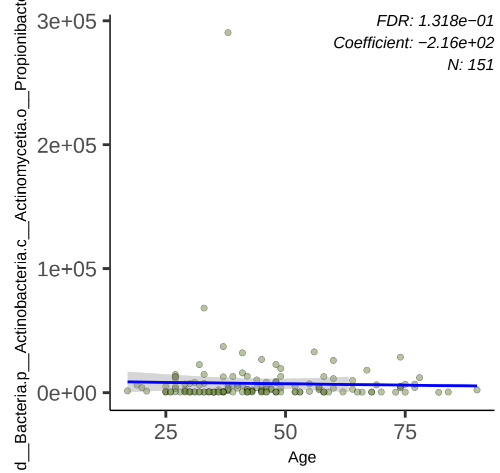

FDR: 1.318e−01
Coefficient: −2.16e+02
N: 151
3e+05
2e+05
1e+05
0e+00
25
50
75
Age
d__Bacteria.p__Actinobacteria.c__Actinomycetia.o__Propionibacte

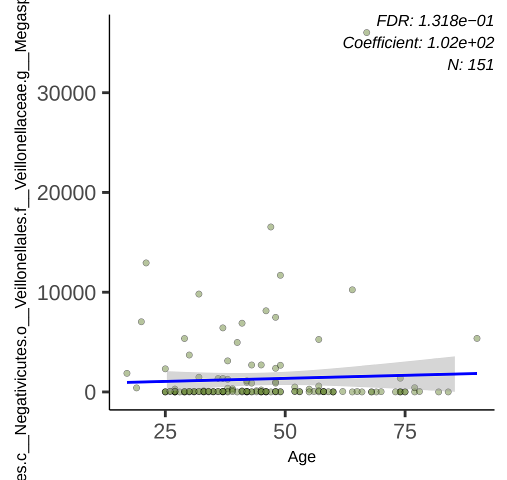

FDR: 1.318e−01
Coefficient: 1.02e+02
N: 151
es.c__Negativicutes.o__Veillonellales.f__Veillonellaceae.g__Megasp
30000
20000
10000
0
25
50
75
Age

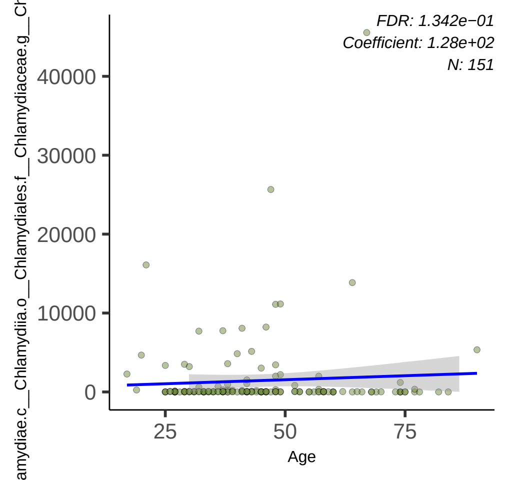

FDR: 1.342e−01
Coefficient: 1.28e+02
N: 151
amydiae.c__Chlamydiia.o__Chlamydiales.f__Chlamydiaceae.g__Ch
40000
30000
20000
10000
0
25
50
75
Age

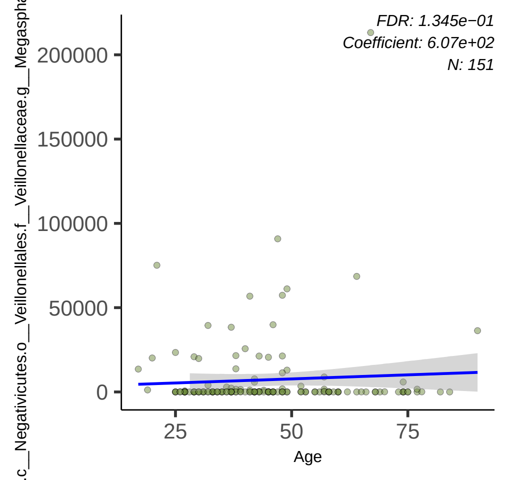

FDR: 1.345e−01
Coefficient: 6.07e+02
N: 151
200000
150000
100000
50000
0
25
50
75
Age
c__Negativicutes.o__Veillonellales.f__Veillonellaceae.g__Megaspha

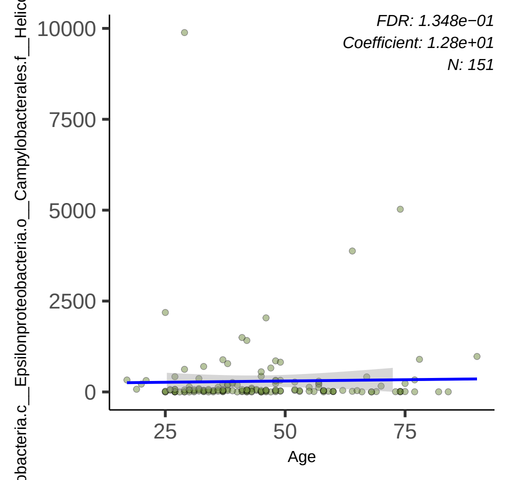

FDR: 1.348e−01
Coefficient: 1.28e+01
N: 151
10000
7500
5000
2500
0
25
50
75
Age
obacteria.c__Epsilonproteobacteria.o__Campylobacterales.f__Helico

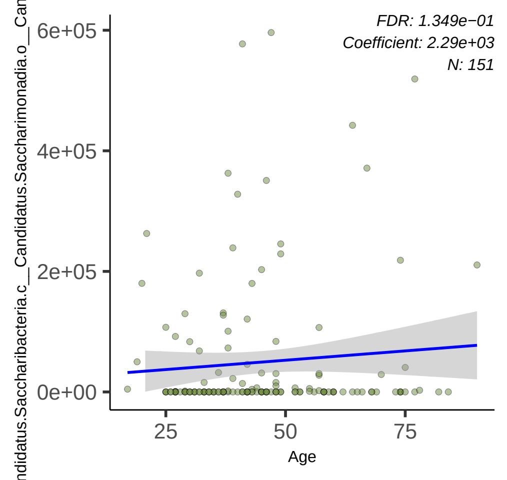

FDR: 1.349e−01
Coefficient: 2.29e+03
N: 151
ndidatus.Saccharibacteria.c__Candidatus.Saccharimonadia.o__Can
6e+05
4e+05
2e+05
0e+00
25
50
75
Age

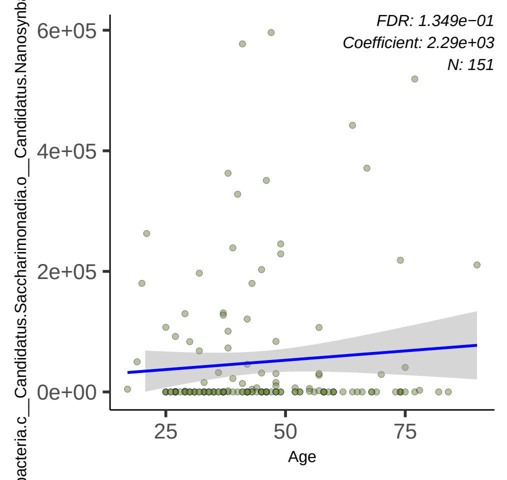

FDR: 1.349e−01
Coefficient: 2.29e+03
N: 151
6e+05
4e+05
2e+05
0e+00
25
50
75
Age
bacteria.c__Candidatus.Saccharimonadia.o__Candidatus.Nanosynb

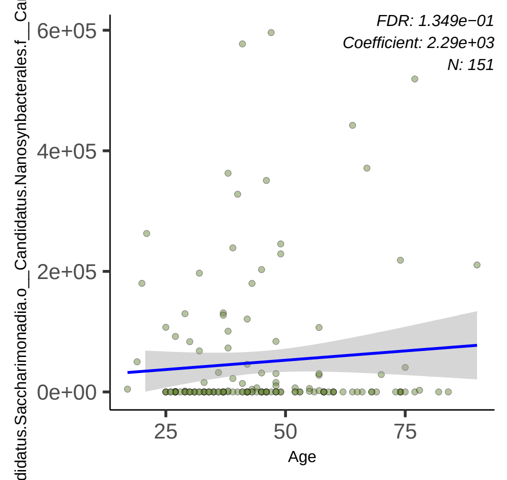

FDR: 1.349e−01
Coefficient: 2.29e+03
N: 151
didatus.Saccharimonadia.o__Candidatus.Nanosynbacterales.f__Ca
6e+05
4e+05
2e+05
0e+00
25
50
75
Age

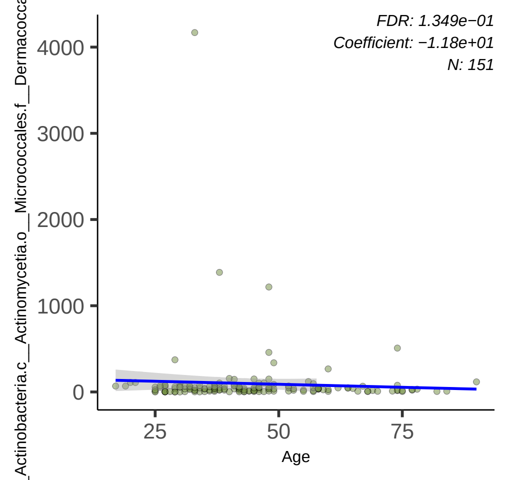

FDR: 1.349e−01
Coefficient: −1.18e+01
N: 151
Actinobacteria.c__Actinomycetia.o__Micrococcales.f__Dermacocca
4000
3000
2000
1000
0
25
50
75
Age

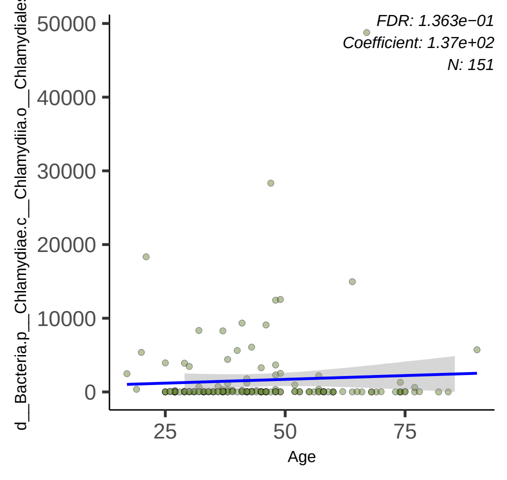
FDR: 1.363e−01
Coefficient: 1.37e+02
N: 151
d__Bacteria.p__Chlamydiae.c__Chlamydiia.o__Chlamydiales
50000
40000
30000
20000
10000
0
25
50
75
Age

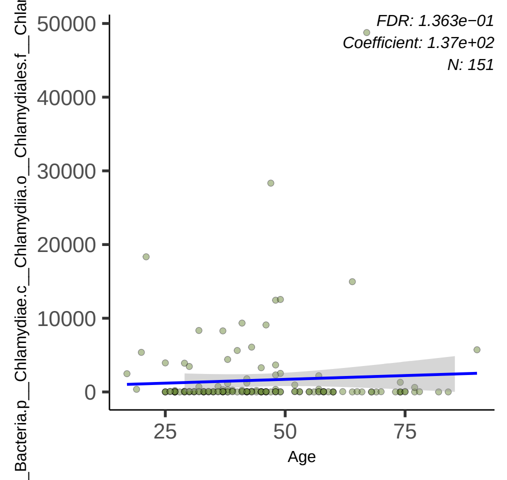

FDR: 1.363e−01
Coefficient: 1.37e+02
N: 151
_Bacteria.p__Chlamydiae.c__Chlamydiia.o__Chlamydiales.f__Chlar
50000
40000
30000
20000
10000
0
25
50
75
Age

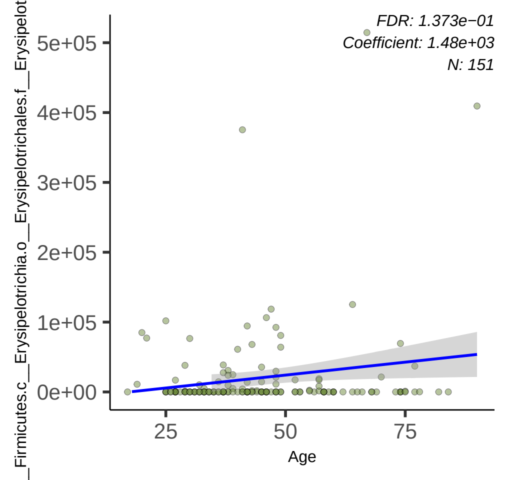
FDR: 1.373e−01
Coefficient: 1.48e+03
N: 151
Firmicutes.c__Erysipelotrichia.o__Erysipelotrichales.f__Erysipelot
5e+05
4e+05
3e+05
2e+05
1e+05
0e+00
25
50
75
Age

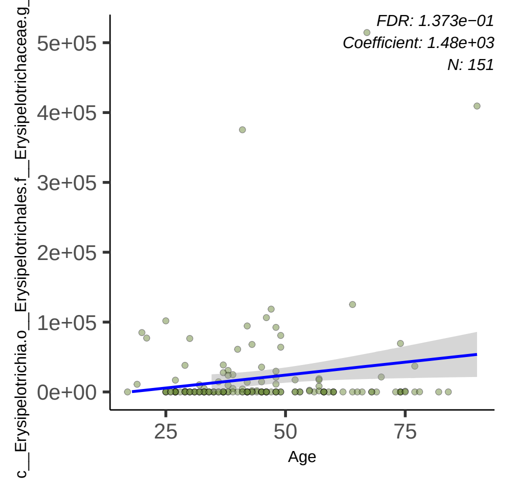

FDR: 1.373e−01
Coefficient: 1.48e+03
N: 151
c__Erysipelotrichia.o__Erysipelotrichales.f__Erysipelotrichaceae.g_
5e+05
4e+05
3e+05
2e+05
1e+05
0e+00
25
50
75
Age

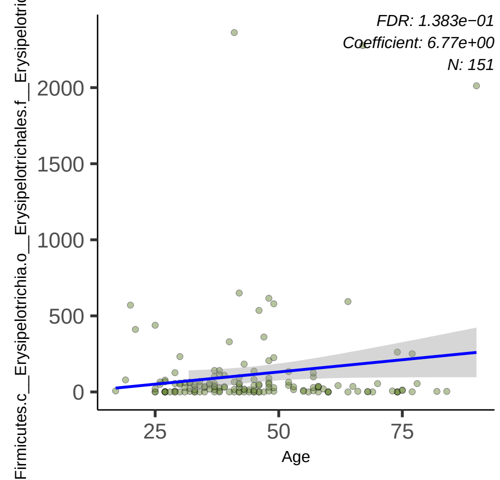

FDR: 1.383e−01
Coefficient: 6.77e+00
N: 151
Firmicutes.c__Erysipelotrichia.o__Erysipelotrichales.f__Erysipelotric
0
500
1000
1500
2000
25
50
75
Age

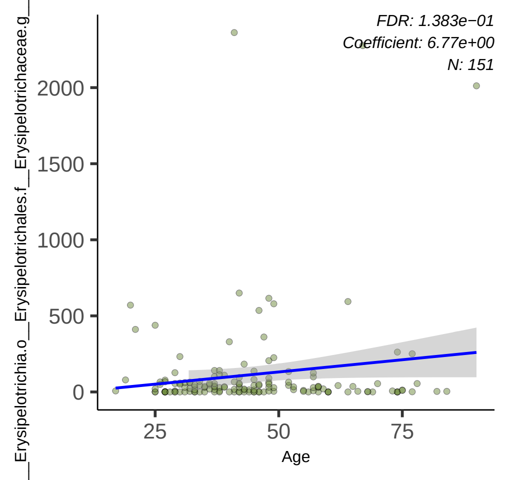
FDR: 1.383e−01
Coefficient: 6.77e+00
N: 151
Erysipelotrichia.o__Erysipelotrichales.f__Erysipelotrichaceae.g__
2000
1500
1000
500
0
25
50
75
Age

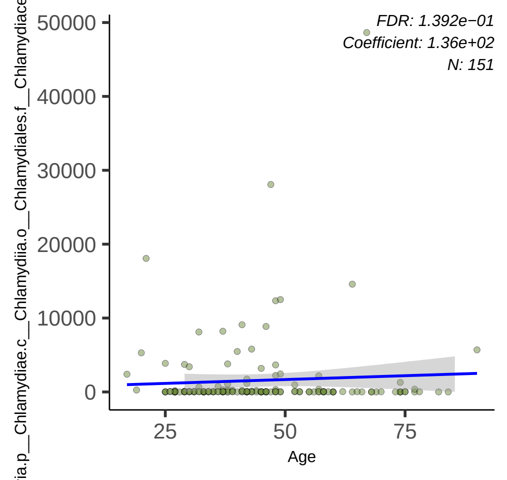
FDR: 1.392e−01
Coefficient: 1.36e+02
N: 151
ia.p__Chlamydiae.c__Chlamydiia.o__Chlamydiales.f__Chlamydiace
50000
40000
30000
20000
10000
0
25
50
75
Age

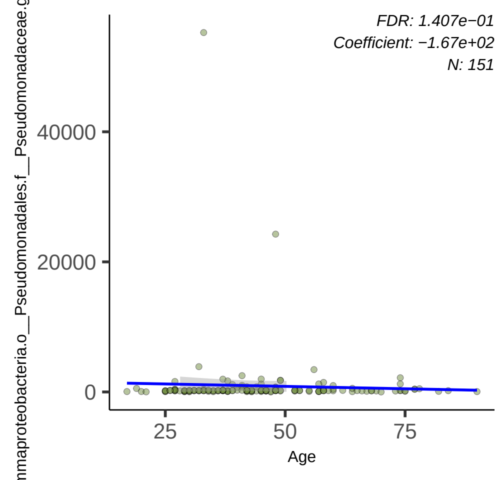
FDR: 1.407e−01
Coefficient: −1.67e+02
N: 151
mmaproteobacteria.o__Pseudomonadales.f__Pseudomonadaceae.g
40000
20000
0
25
50
75
Age

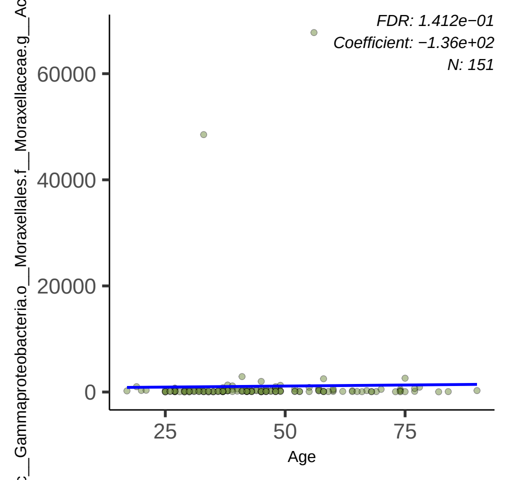
FDR: 1.412e−01
Coefficient: −1.36e+02
N: 151
60000
40000
20000
0
25
50
75
Age
c__Gammaproteobacteria.o__Moraxellales.f__Moraxellaceae.g__Ac

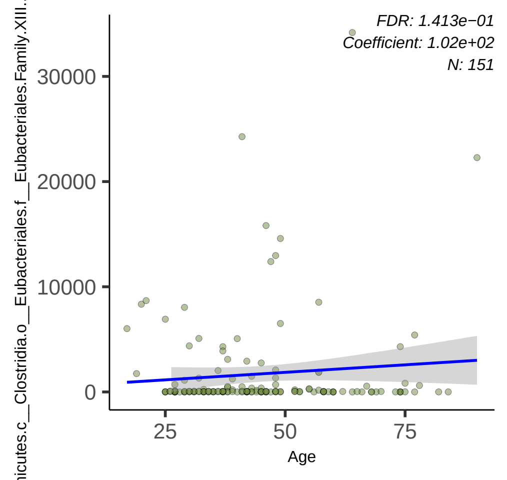

FDR: 1.413e−01
Coefficient: 1.02e+02
N: 151
30000
20000
10000
0
25
50
75
Age
nicutes.c__Clostridia.o__Eubacteriales.f__Eubacteriales.Family.XIII.

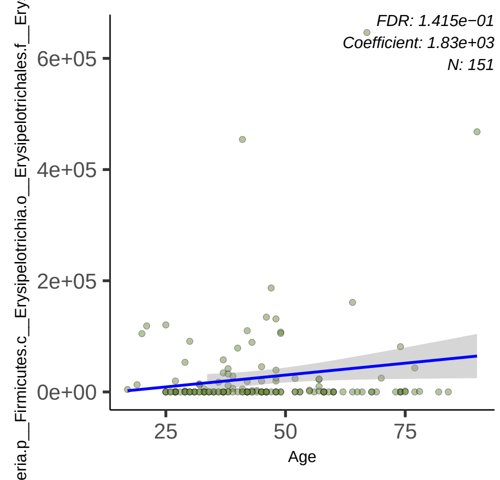

FDR: 1.415e−01
Coefficient: 1.83e+03
N: 151
6e+05
4e+05
2e+05
0e+00
25
50
75
Age
eria.p__Firmicutes.c__Erysipelotrichia.o__Erysipelotrichales.f__Ery

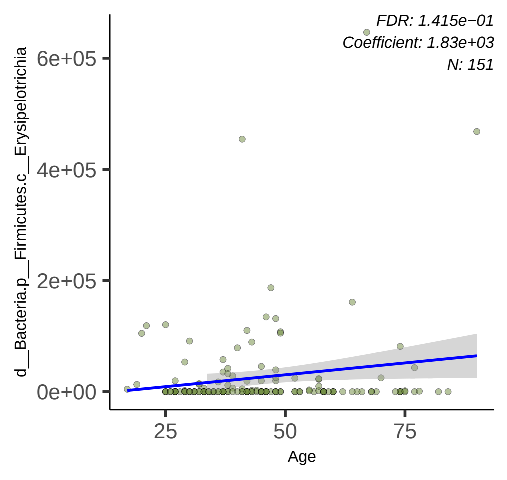

FDR: 1.415e−01
Coefficient: 1.83e+03
N: 151
d__Bacteria.p__Firmicutes.c__Erysipelotrichia
6e+05
4e+05
2e+05
0e+00
25
50
75
Age

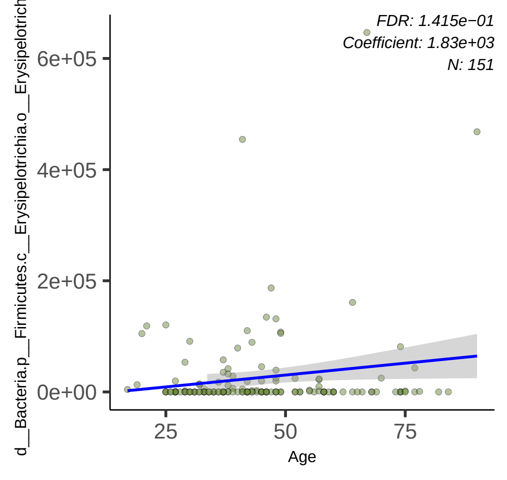
FDR: 1.415e−01
Coefficient: 1.83e+03
N: 151
6e+05
4e+05
2e+05
0e+00
25
50
75
Age
d__Bacteria.p__Firmicutes.c__Erysipelotrichia.o__Erysipelotrich

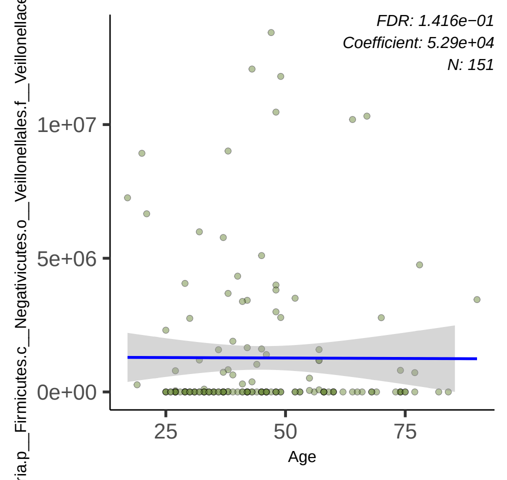

FDR: 1.416e−01
Coefficient: 5.29e+04
N: 151
1e+07
5e+06
0e+00
25
50
75
Age
ria.p__Firmicutes.c__Negativicutes.o__Veillonellales.f__Veillonellace

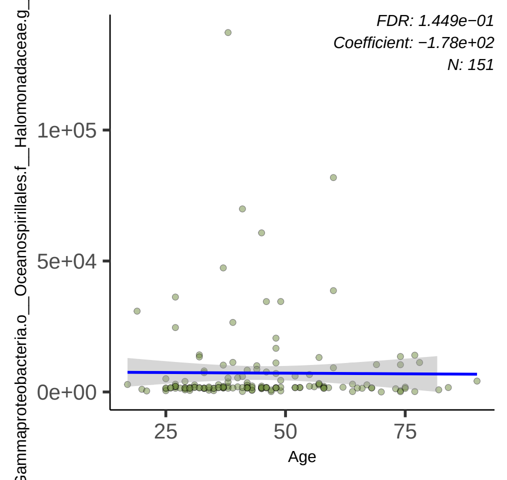

FDR: 1.449e−01
Coefficient: −1.78e+02
N: 151
Gammaproteobacteria.o__Oceanospirillales.f__Halomonadaceae.g_
1e+05
5e+04
0e+00
25
50
75
Age

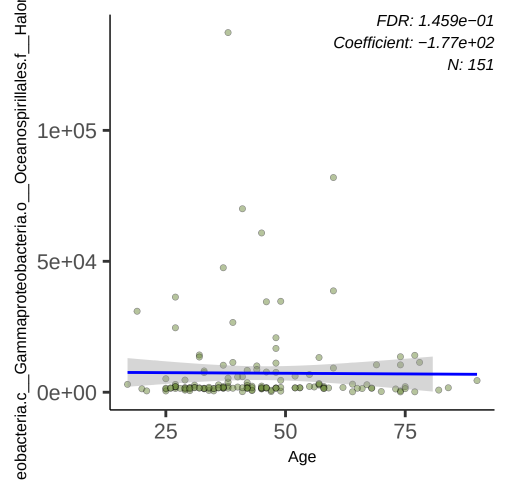

FDR: 1.459e−01
Coefficient: −1.77e+02
N: 151
1e+05
5e+04
0e+00
25
50
75
Age
eobacteria.c__Gammaproteobacteria.o__Oceanospirillales.f__Halom

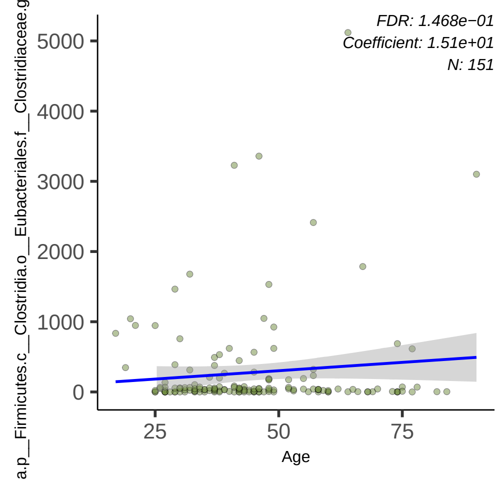
FDR: 1.468e−01
Coefficient: 1.51e+01
N: 151
a.p__Firmicutes.c__Clostridia.o__Eubacteriales.f__Clostridiaceae.g
5000
4000
3000
2000
1000
0
25
50
75
Age

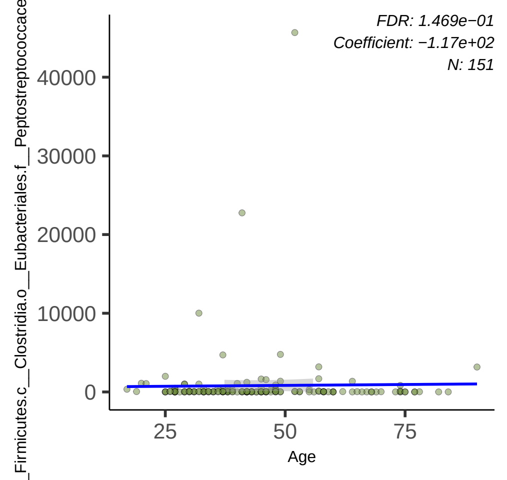

FDR: 1.469e−01
Coefficient: −1.17e+02
N: 151
Firmicutes.c__Clostridia.o__Eubacteriales.f__Peptostreptococcace
40000
30000
20000
10000
0
25
50
75
Age

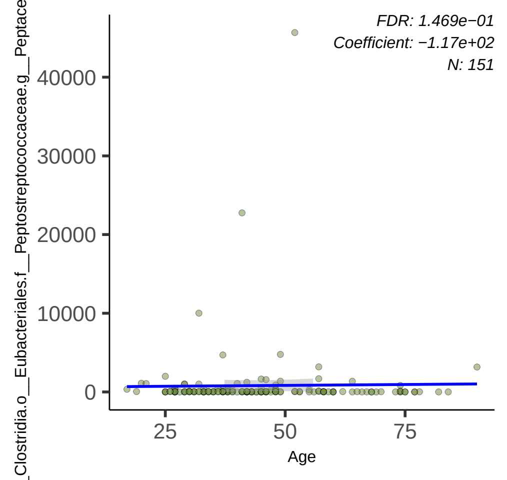

FDR: 1.469e−01
Coefficient: −1.17e+02
N: 151
40000
30000
20000
10000
0
25
50
75
Age
Clostridia.o__Eubacteriales.f__Peptostreptococcaceae.g__Peptace

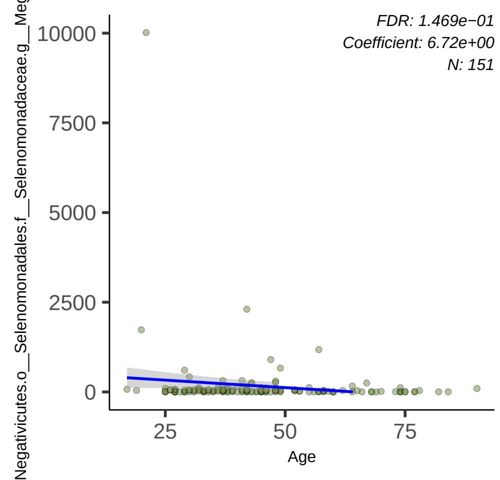

FDR: 1.469e−01
Coefficient: 6.72e+00
N: 151
10000
7500
5000
2500
0
25
50
75
Age
Negativicutes.o__Selenomonadales.f__Selenomonadaceae.g__Meg

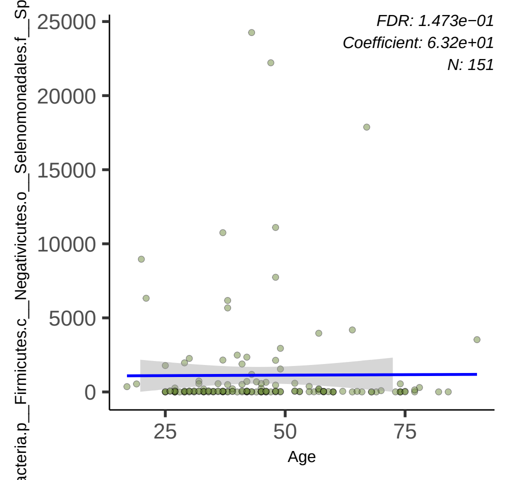

FDR: 1.473e−01
Coefficient: 6.32e+01
N: 151
acteria.p__Firmicutes.c__Negativicutes.o__Selenomonadales.f__Sp
25000
20000
15000
10000
5000
0
25
50
75
Age

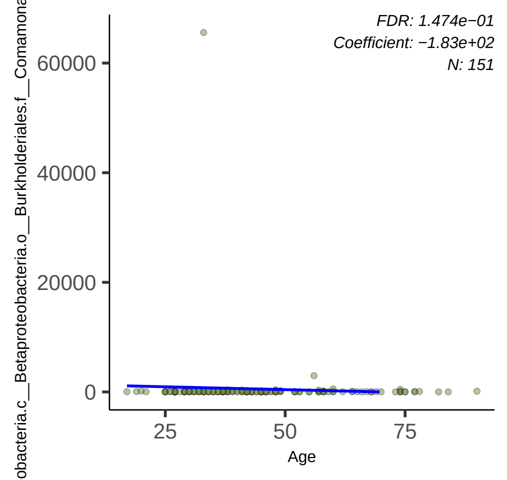

FDR: 1.474e−01
Coefficient: −1.83e+02
N: 151
60000
40000
20000
0
25
50
75
Age
obacteria.c__Betaproteobacteria.o__Burkholderiales.f__Comamona

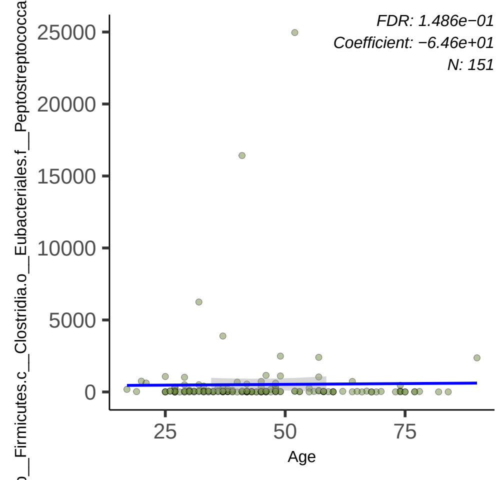

FDR: 1.486e−01
Coefficient: −6.46e+01
N: 151
o__Firmicutes.c__Clostridia.o__Eubacteriales.f__Peptostreptococca
25000
20000
15000
10000
5000
0
25
50
75
Age

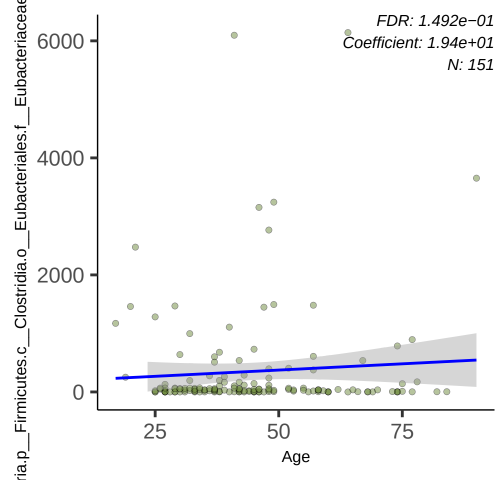

FDR: 1.492e−01
Coefficient: 1.94e+01
N: 151
6000
4000
2000
0
25
50
75
Age
ria.p__Firmicutes.c__Clostridia.o__Eubacteriales.f__Eubacteriaceae

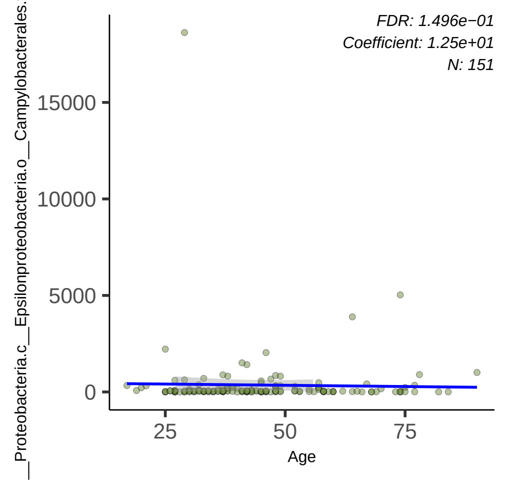
FDR: 1.496e−01
Coefficient: 1.25e+01
N: 151
15000
10000
5000
0
25
50
75
Age
_Proteobacteria.c__Epsilonproteobacteria.o__Campylobacterales.

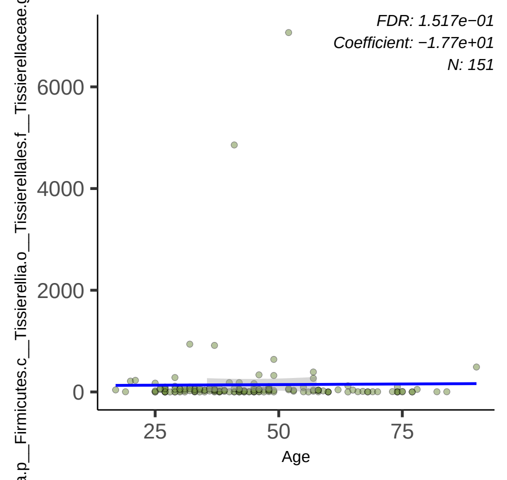
FDR: 1.517e−01
Coefficient: −1.77e+01
N: 151
6000
4000
2000
0
25
50
75
Age
a.p__Firmicutes.c__Tissierellia.o__Tissierellales.f__Tissierellaceae.g

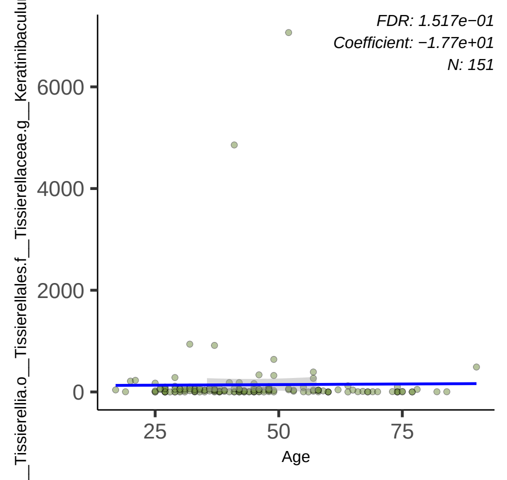
FDR: 1.517e−01
Coefficient: −1.77e+01
N: 151
6000
4000
2000
0
25
50
75
Age
Tissierellia.o__Tissierellales.f__Tissierellaceae.g__Keratinibaculum

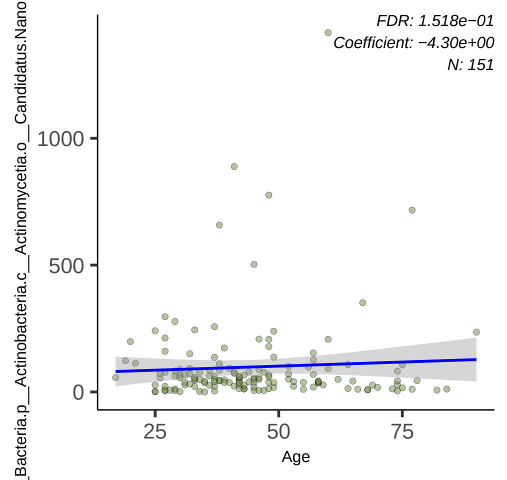

FDR: 1.518e−01
Coefficient: −4.30e+00
N: 151
Bacteria.p__Actinobacteria.c__Actinomycetia.o__Candidatus.Nano
1000
500
0
25
50
75
Age

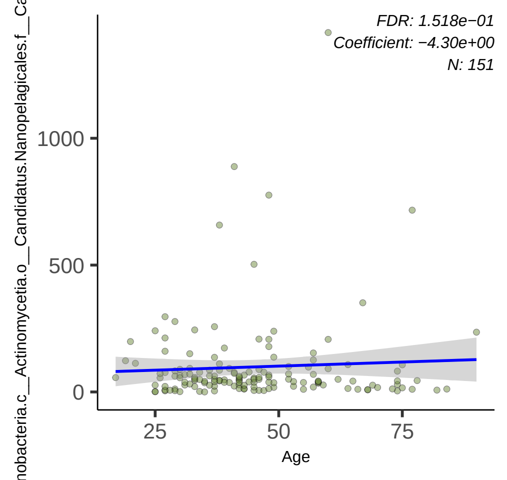
FDR: 1.518e−01
Coefficient: −4.30e+00
N: 151
1000
500
0
25
50
75
Age
nobacteria.c__Actinomycetia.o__Candidatus.Nanopelagicales.f__Ca

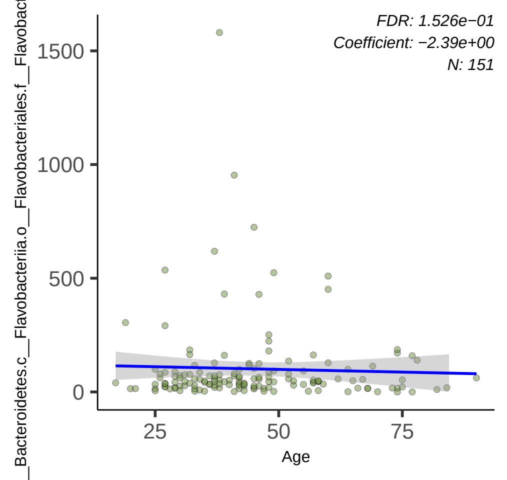
FDR: 1.526e−01
Coefficient: −2.39e+00
N: 151
1500
1000
500
0
25
50
75
Age
_Bacteroidetes.c__Flavobacteriia.o__Flavobacteriales.f__Flavobact

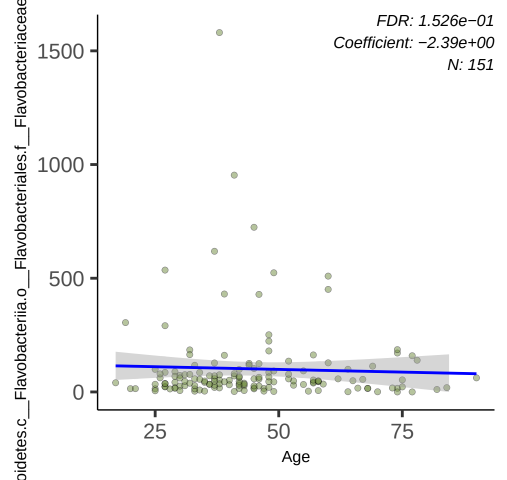

FDR: 1.526e−01
Coefficient: −2.39e+00
N: 151
oidetes.c__Flavobacteriia.o__Flavobacteriales.f__Flavobacteriaceae
1500
1000
500
0
25
50
75
Age

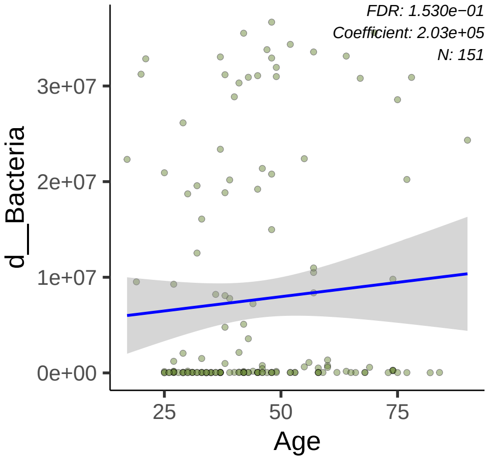
FDR: 1.530e−01
Coefficient: 2.03e+05
N: 151
3e+07
2e+07
1e+07
0e+00
d__Bacteria
25
50
75
Age

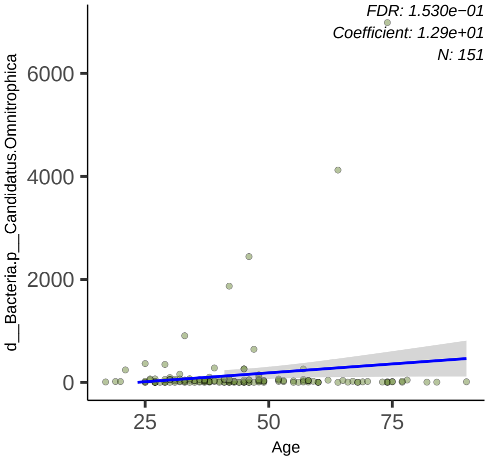

FDR: 1.530e−01
Coefficient: 1.29e+01
N: 151
d__Bacteria.p__Candidatus.Omnitrophica
6000
4000
2000
0
25
50
75
Age

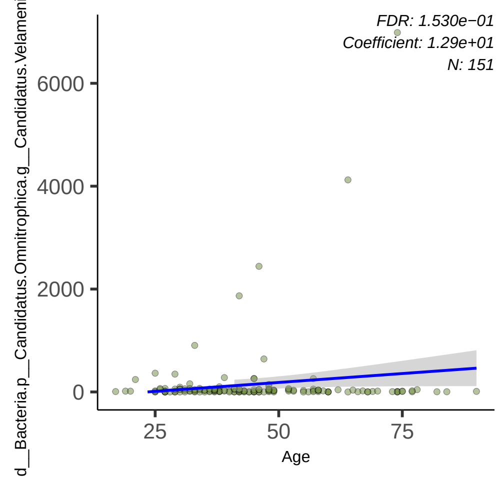

FDR: 1.530e−01
Coefficient: 1.29e+01
N: 151
6000
4000
2000
0
25
50
75
Age
d__Bacteria.p__Candidatus.Omnitrophica.g__Candidatus.Velameni

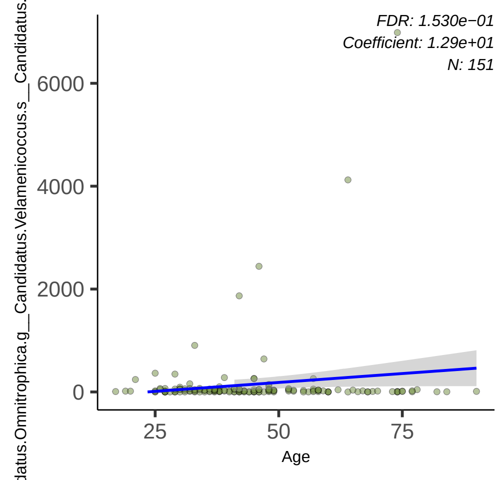

FDR: 1.530e−01
Coefficient: 1.29e+01
N: 151
6000
4000
2000
0
25
50
75
Age
datus.Omnitrophica.g__Candidatus.Velamenicoccus.s__Candidatus.

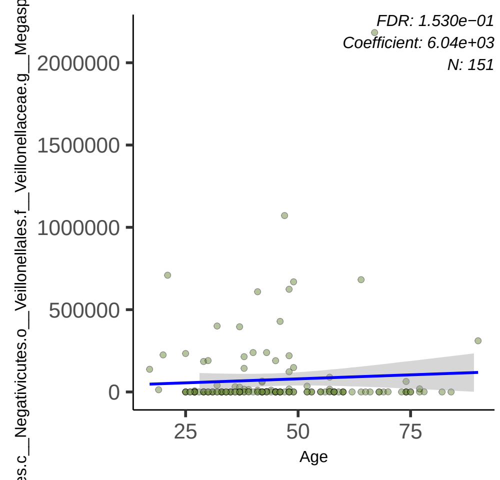

FDR: 1.530e−01
Coefficient: 6.04e+03
N: 151
2000000
1500000
1000000
500000
0
25
50
75
Age
es.c__Negativicutes.o__Veillonellales.f__Veillonellaceae.g__Megasp

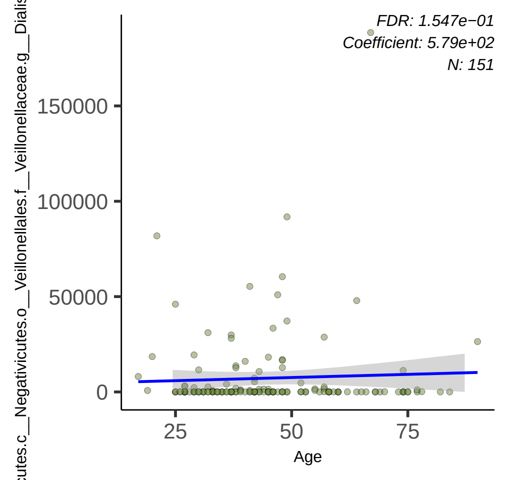

FDR: 1.547e−01
Coefficient: 5.79e+02
N: 151
150000
100000
50000
0
25
50
75
Age
cutes.c__Negativicutes.o__Veillonellales.f__Veillonellaceae.g__Dialis

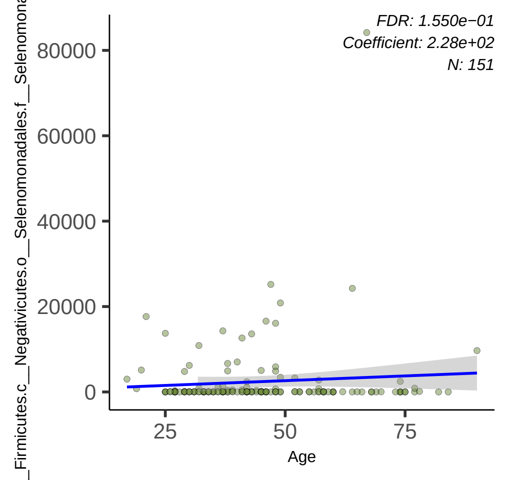
FDR: 1.550e−01
Coefficient: 2.28e+02
N: 151
80000
60000
40000
20000
0
25
50
75
Age
Firmicutes.c__Negativicutes.o__Selenomonadales.f__Selenomona

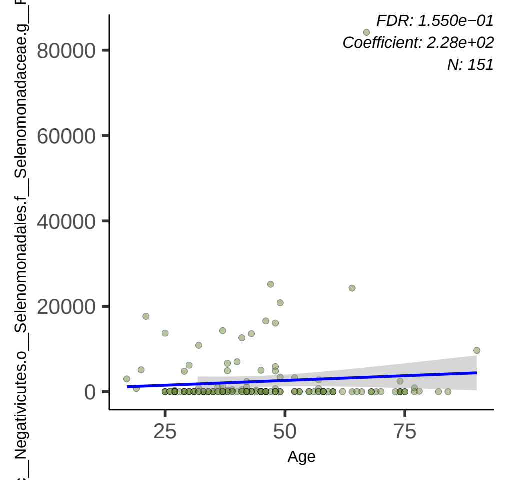

FDR: 1.550e−01
Coefficient: 2.28e+02
N: 151
80000
60000
40000
20000
0
25
50
75
Age
Negativicutes.o__Selenomonadales.f__Selenomonadaceae.g__

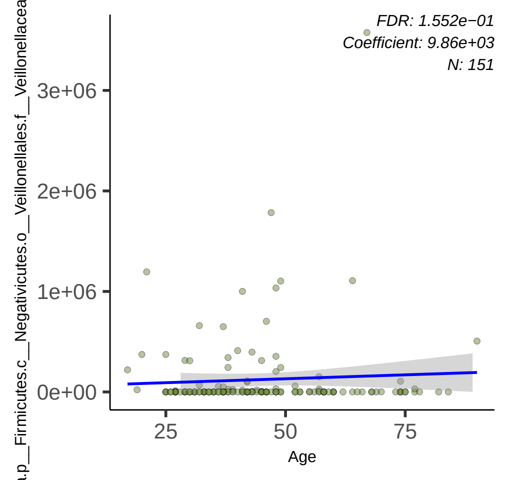

FDR: 1.552e−01
Coefficient: 9.86e+03
N: 151
Firmicutes.c__Negativicutes.o__Veillonellales.f__Veillonellacea
3e+06
2e+06
1e+06
0e+00
25
50
75
Age

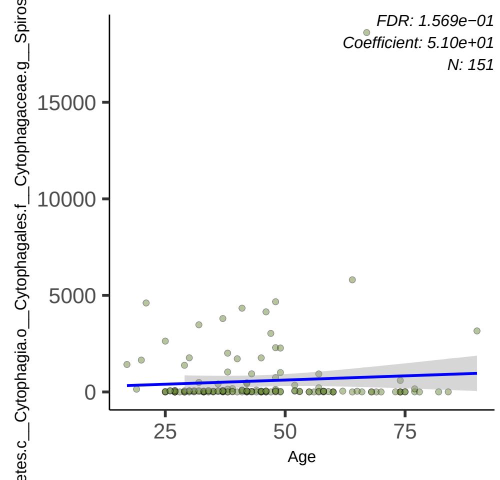

FDR: 1.569e−01
Coefficient: 5.10e+01
N: 151
15000
10000
5000
0
25
50
75
Age
etes.c__Cytophagia.o__Cytophagales.f__Cytophagaceae.g__Spiros

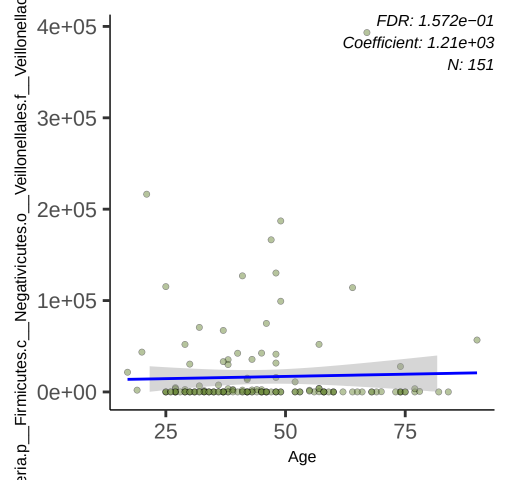

FDR: 1.572e−01
Coefficient: 1.21e+03
N: 151
4e+05
3e+05
2e+05
1e+05
0e+00
25
50
75
Age
eria.p__Firmicutes.c__Negativicutes.o__Veillonellales.f__Veillonellac

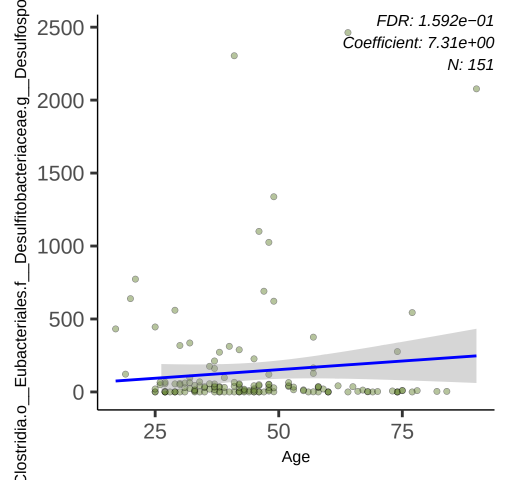

FDR: 1.592e−01
Coefficient: 7.31e+00
N: 151
Clostridia.o__Eubacteriales.f__Desulfitobacteriaceae.g__Desulfospo
2500
2000
1500
1000
500
0
25
50
75
Age

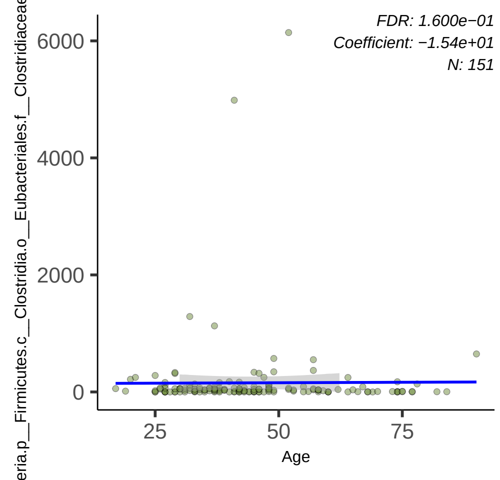

FDR: 1.600e−01
Coefficient: −1.54e+01
N: 151
6000
4000
2000
0
25
50
75
Age
eria.p__Firmicutes.c__Clostridia.o__Eubacteriales.f__Clostridiaceae

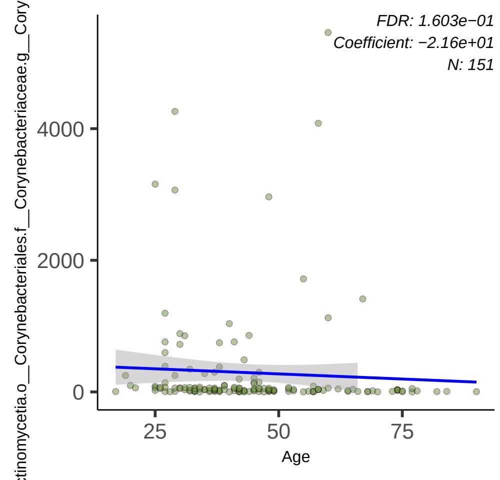
FDR: 1.603e−01
Coefficient: −2.16e+01
N: 151
4000
2000
0
25
50
75
Age
ctinomycetia.o__Corynebacteriales.f__Corynebacteriaceae.g__Cory

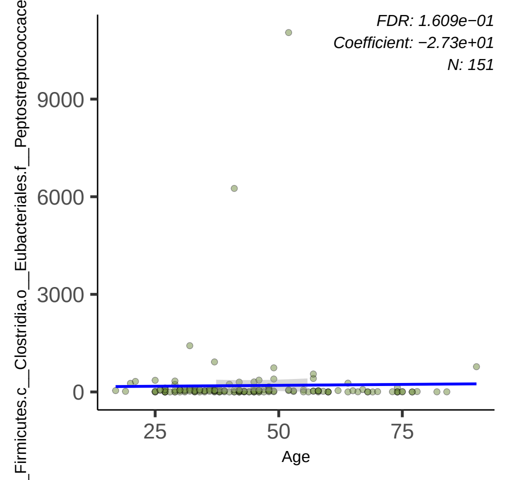

FDR: 1.609e−01
Coefficient: −2.73e+01
N: 151
9000
6000
3000
0
25
50
75
Age
Firmicutes.c__Clostridia.o__Eubacteriales.f__Peptostreptococcace

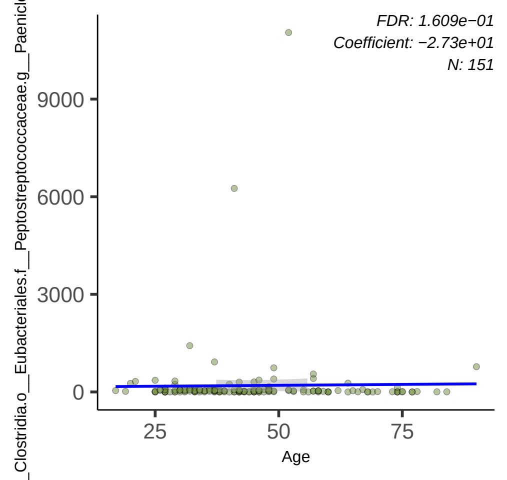
FDR: 1.609e−01
Coefficient: −2.73e+01
N: 151
9000
6000
3000
0
25
50
75
Age
_Clostridia.o__Eubacteriales.f__Peptostreptococcaceae.g__Paenicl

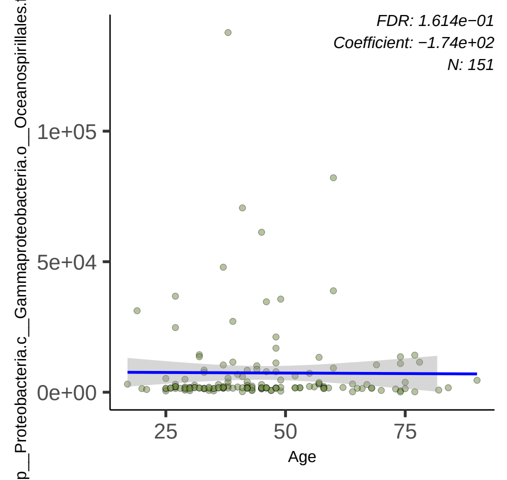
FDR: 1.614e−01
Coefficient: −1.74e+02
N: 151
p__Proteobacteria.c__Gammaproteobacteria.o__Oceanospirillales.f
1e+05
5e+04
0e+00
25
50
75
Age

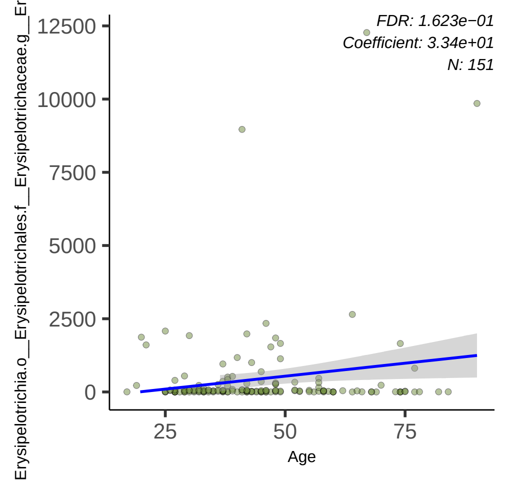

FDR: 1.623e−01
Coefficient: 3.34e+01
N: 151
Erysipelotrichia.o__Erysipelotrichales.f__Erysipelotrichaceae.g__Er
12500
10000
7500
5000
2500
0
25
50
75
Age

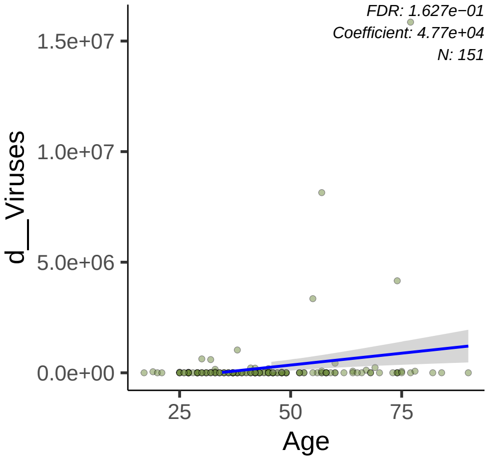

FDR: 1.627e−01
Coefficient: 4.77e+04
N: 151
d__Viruses
1.5e+07
1.0e+07
5.0e+06
0.0e+00
25
50
75
Age

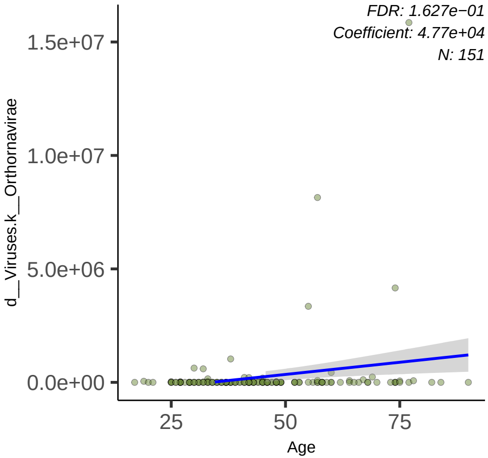
FDR: 1.627e−01
Coefficient: 4.77e+04
N: 151
1.5e+07
1.0e+07
5.0e+06
0.0e+00
d__Viruses.k__Orthornavirae
25
50
75
Age

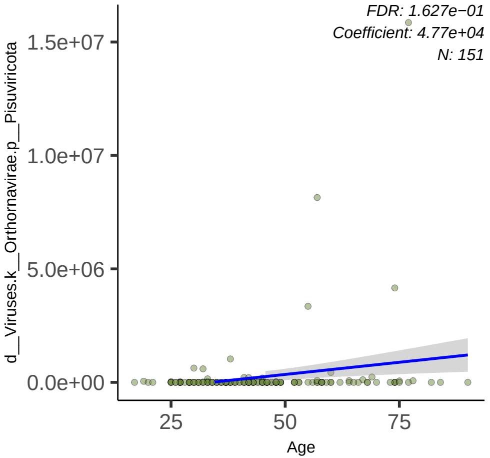

FDR: 1.627e−01
Coefficient: 4.77e+04
N: 151
d__Viruses.k__Orthornavirae.p__Pisuviricota
1.5e+07
1.0e+07
5.0e+06
0.0e+00
25
50
75
Age

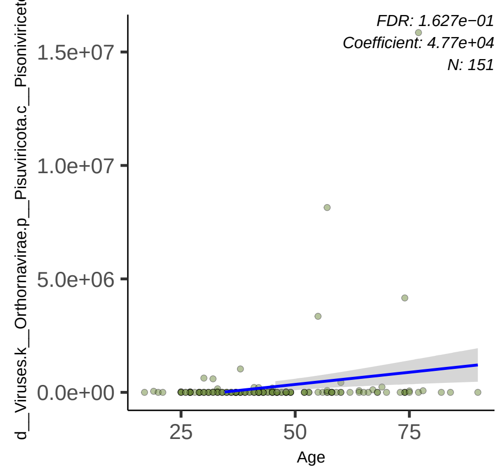
FDR: 1.627e−01
Coefficient: 4.77e+04
N: 151
1.5e+07
1.0e+07
5.0e+06
0.0e+00
d__Viruses.k__Orthornavirae.p__Pisuviricota.c__Pisoniviricet
25
50
75
Age

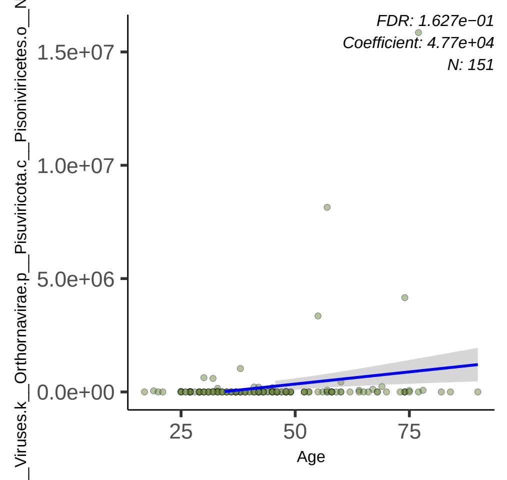
FDR: 1.627e−01
Coefficient: 4.77e+04
N: 151
1.5e+07
1.0e+07
5.0e+06
0.0e+00
25
50
75
Age
Viruses.k__Orthornavirae.p__Pisuviricota.c__Pisoniviricetes.o__N

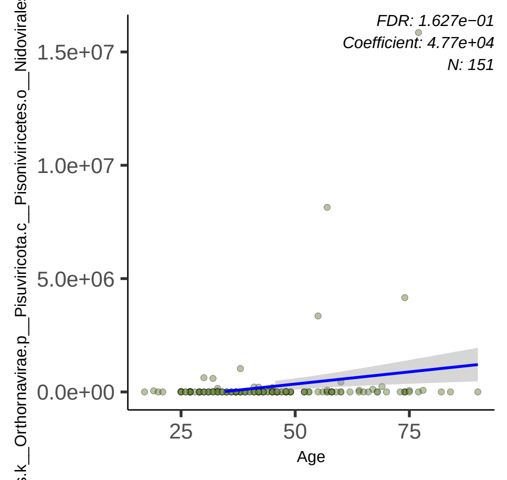

FDR: 1.627e−01
Coefficient: 4.77e+04
N: 151
1.5e+07
1.0e+07
5.0e+06
0.0e+00
25
50
75
Age
s.k__Orthornavirae.p__Pisuviricota.c__Pisoniviricetes.o__Nidovirales

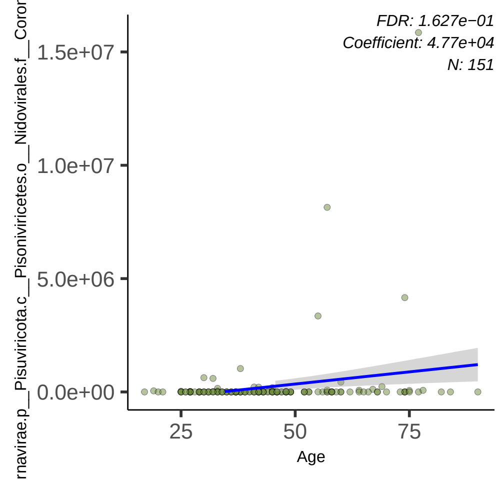
FDR: 1.627e−01
Coefficient: 4.77e+04
N: 151
rnavirae.p__Pisuviricota.c__Pisoniviricetes.o__Nidovirales.f__Coron
1.5e+07
1.0e+07
5.0e+06
0.0e+00
25
50
75
Age

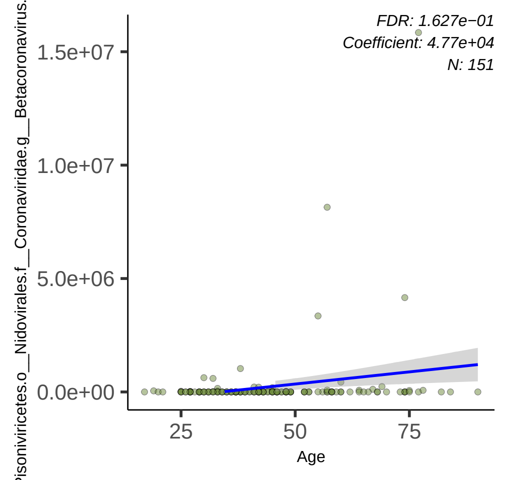

FDR: 1.627e−01
Coefficient: 4.77e+04
N: 151
Pisoniviricetes.o__Nidovirales.f__Coronaviridae.g__Betacoronavirus.
1.5e+07
1.0e+07
5.0e+06
0.0e+00
25
50
75
Age

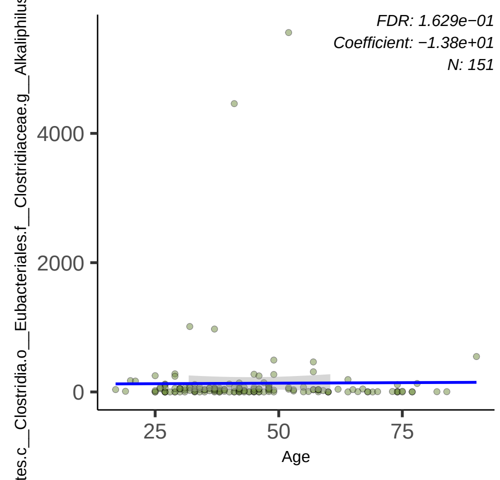

FDR: 1.629e−01
Coefficient: −1.38e+01
N: 151
tes.c__Clostridia.o__Eubacteriales.f__Clostridiaceae.g__Alkaliphilus
4000
2000
0
25
50
75
Age

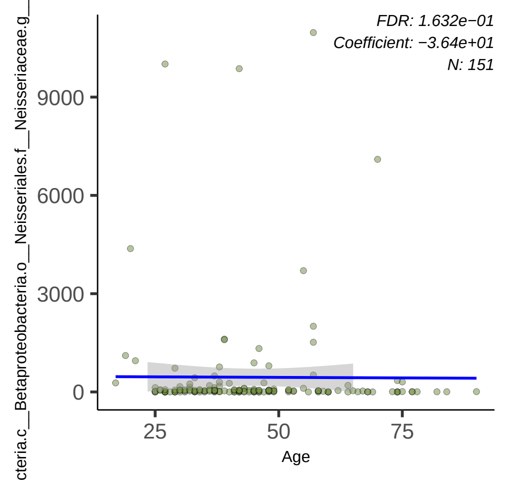

FDR: 1.632e−01
Coefficient: −3.64e+01
N: 151
9000
6000
3000
0
25
50
75
Age
cteria.c__Betaproteobacteria.o__Neisseriales.f__Neisseriaceae.g__

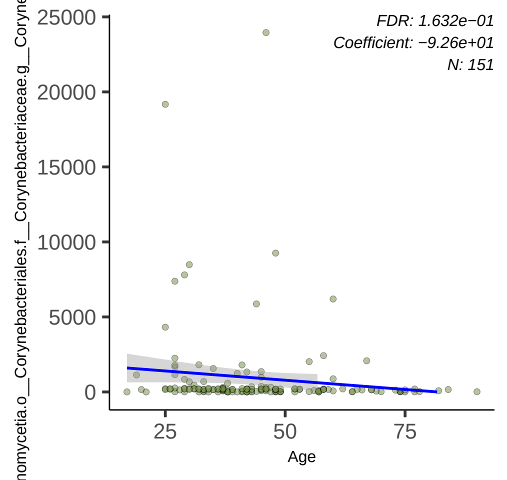
FDR: 1.632e−01
Coefficient: −9.26e+01
N: 151
25000
20000
15000
10000
5000
0
25
50
75
Age
nomycetia.o__Corynebacteriales.f__Corynebacteriaceae.g__Coryne

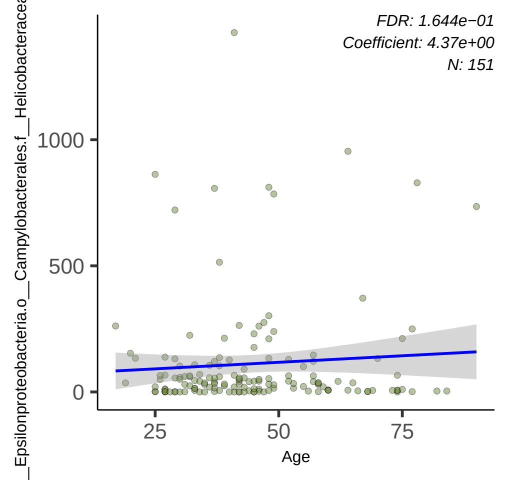
FDR: 1.644e−01
Coefficient: 4.37e+00
N: 151
Epsilonproteobacteria.o__Campylobacterales.f__Helicobacteracea
1000
500
0
25
50
75
Age

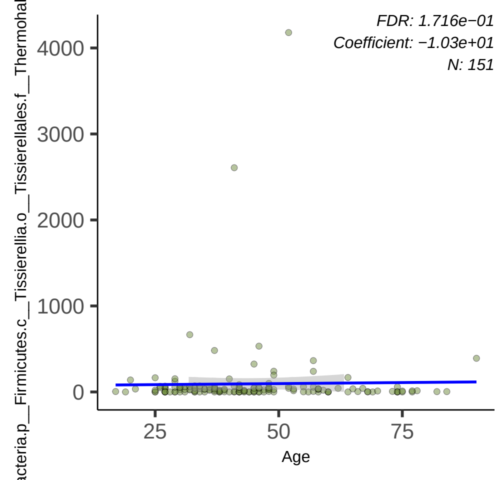

FDR: 1.716e−01
Coefficient: −1.03e+01
N: 151
4000
3000
2000
1000
0
25
50
75
Age
acteria.p__Firmicutes.c__Tissierellia.o__Tissierellales.f__Thermohal

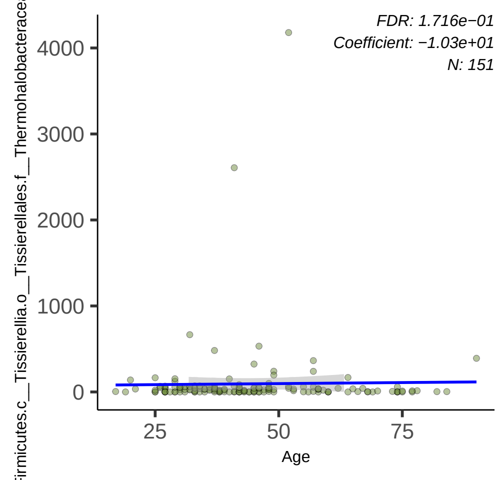
FDR: 1.716e−01
Coefficient: −1.03e+01
N: 151
Firmicutes.c__Tissierellia.o__Tissierellales.f__Thermohalobacterace
0
1000
2000
3000
4000
25
50
75
Age

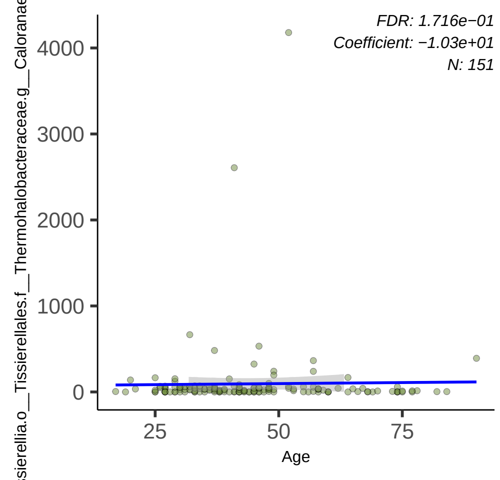

FDR: 1.716e−01
Coefficient: −1.03e+01
N: 151
sierellia.o__Tissierellales.f__Thermohalobacteraceae.g__Caloranae
4000
3000
2000
1000
0
25
50
75
Age

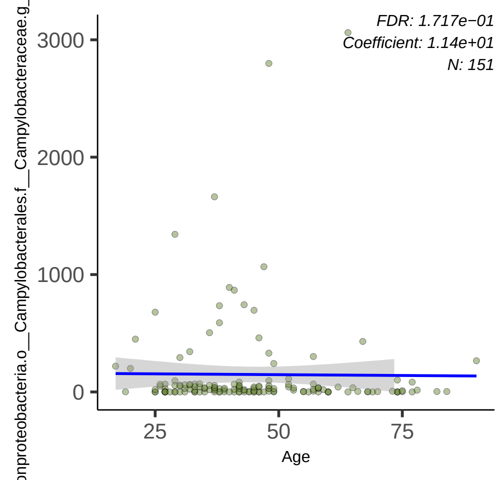

FDR: 1.717e−01
Coefficient: 1.14e+01
N: 151
3000
2000
1000
0
25
50
75
Age
onproteobacteria.o__Campylobacterales.f__Campylobacteraceae.g_

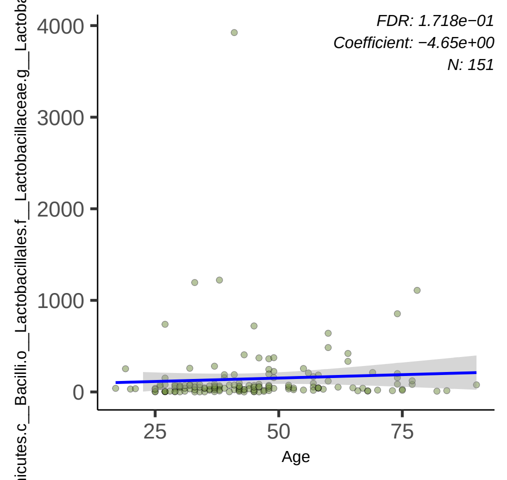
FDR: 1.718e−01
Coefficient: −4.65e+00
N: 151
icutes.c__Bacilli.o__Lactobacillales.f__Lactobacillaceae.g__Lactoba
4000
3000
2000
1000
0
25
50
75
Age

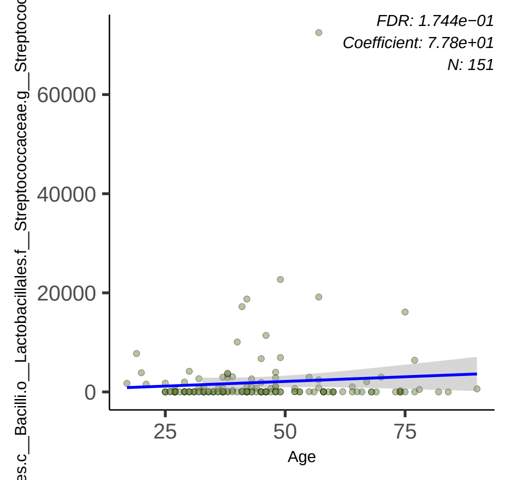
FDR: 1.744e−01
Coefficient: 7.78e+01
N: 151
60000
40000
20000
0
25
50
75
Age
es.c__Bacilli.o__Lactobacillales.f__Streptococcaceae.g__Streptococ

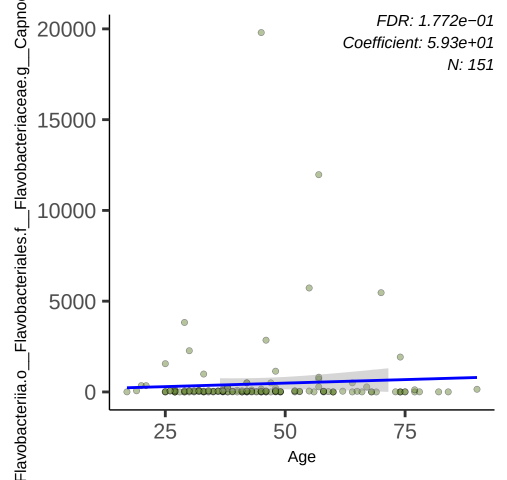

FDR: 1.772e−01
Coefficient: 5.93e+01
N: 151
Flavobacteriia.o__Flavobacteriales.f__Flavobacteriaceae.g__Capno
20000
15000
10000
5000
0
25
50
75
Age

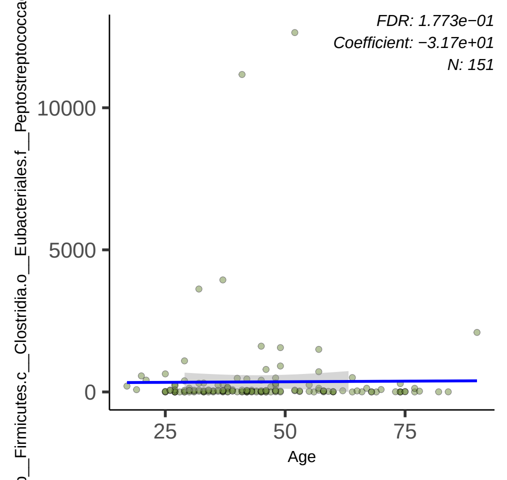
FDR: 1.773e−01
Coefficient: −3.17e+01
N: 151
10000
5000
0
25
50
75
Age
o__Firmicutes.c__Clostridia.o__Eubacteriales.f__Peptostreptococca

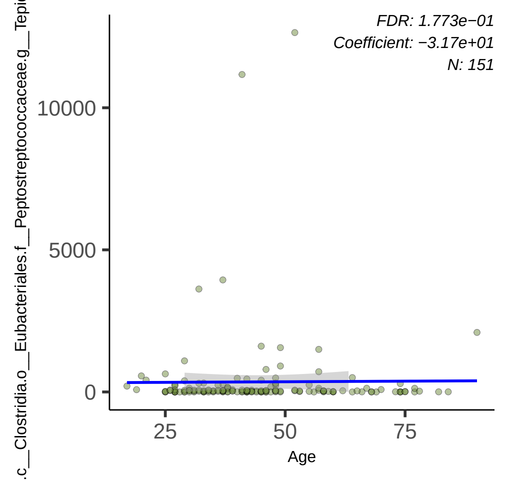

FDR: 1.773e−01
Coefficient: −3.17e+01
N: 151
10000
5000
0
25
50
75
Age

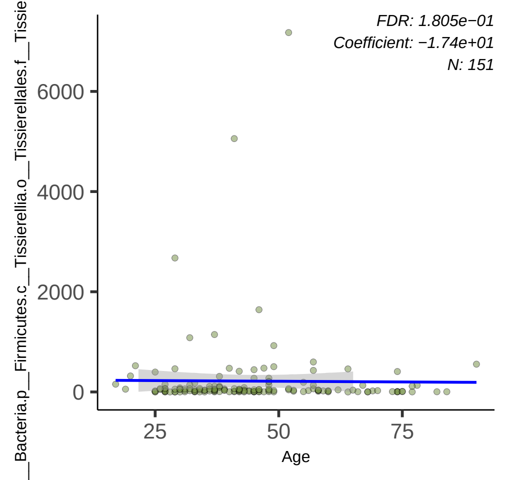
FDR: 1.805e−01
Coefficient: −1.74e+01
N: 151
Bacteria.p__Firmicutes.c__Tissierellia.o__Tissierellales.f__Tissie
6000
4000
2000
0
25
50
75
Age

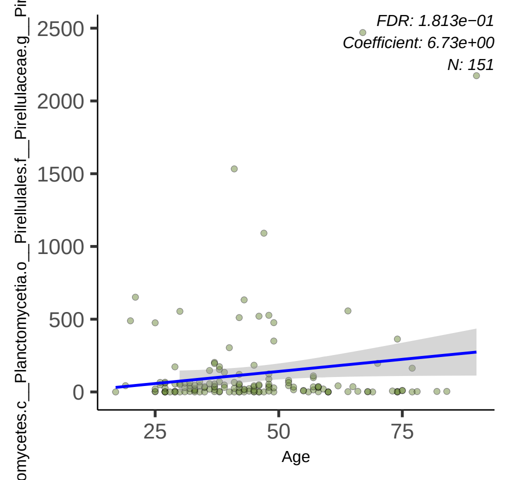

FDR: 1.813e−01
Coefficient: 6.73e+00
N: 151
2500
2000
1500
1000
500
0
25
50
75
Age
omycetes.c__Planctomycetia.o__Pirellulales.f__Pirellulaceae.g__Pir

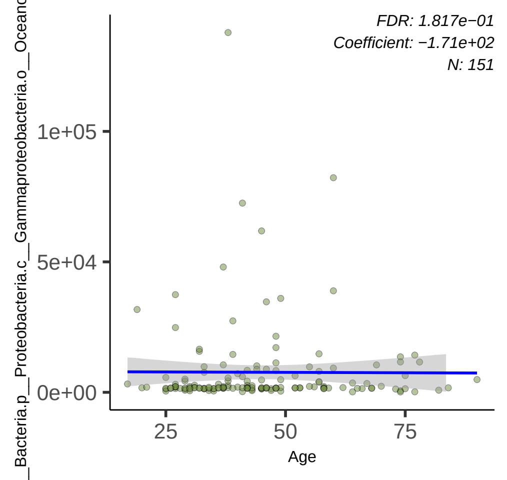

FDR: 1.817e−01
Coefficient: −1.71e+02
N: 151
1e+05
5e+04
0e+00
25
50
75
Age
Bacteria.p__Proteobacteria.c__Gammaproteobacteria.o__Oceano

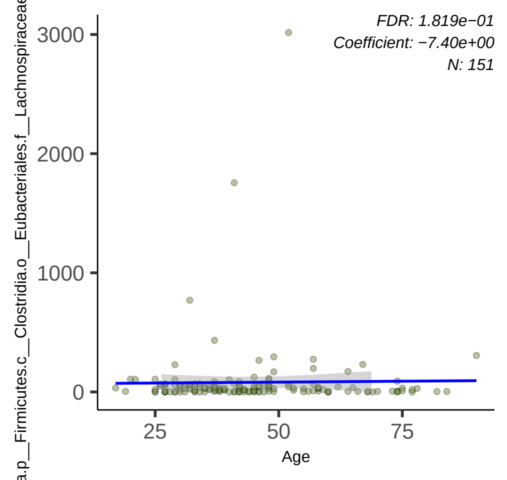

FDR: 1.819e−01
Coefficient: −7.40e+00
N: 151
a.p__Firmicutes.c__Clostridia.o__Eubacteriales.f__Lachnospiraceae
3000
2000
1000
0
25
50
75
Age

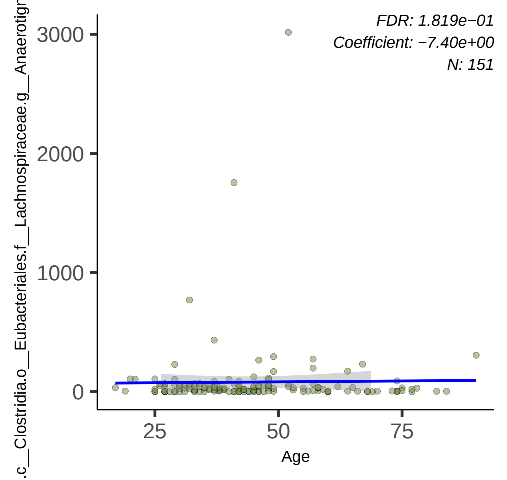

FDR: 1.819e−01
Coefficient: −7.40e+00
N: 151
3000
2000
1000
0
25
50
75
Age
.c__Clostridia.o__Eubacteriales.f__Lachnospiraceae.g__Anaerotign

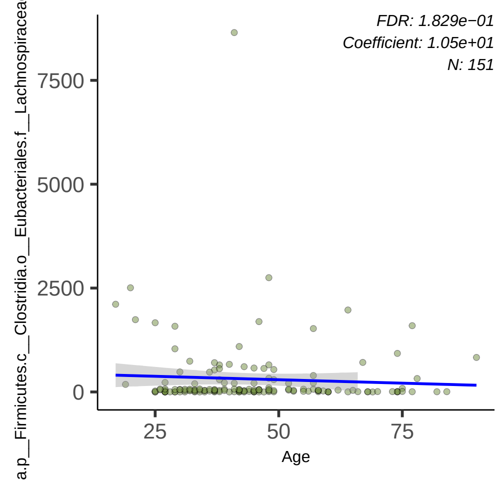

FDR: 1.829e−01
Coefficient: 1.05e+01
N: 151
a.p__Firmicutes.c__Clostridia.o__Eubacteriales.f__Lachnospiraceae
7500
5000
2500
0
25
50
75
Age

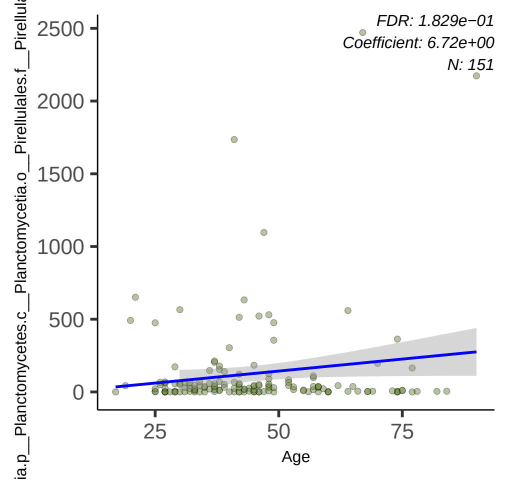

FDR: 1.829e−01
Coefficient: 6.72e+00
N: 151
2500
2000
1500
1000
500
0
25
50
75
Age
ia.p__Planctomycetes.c__Planctomycetia.o__Pirellulales.f__Pirellula

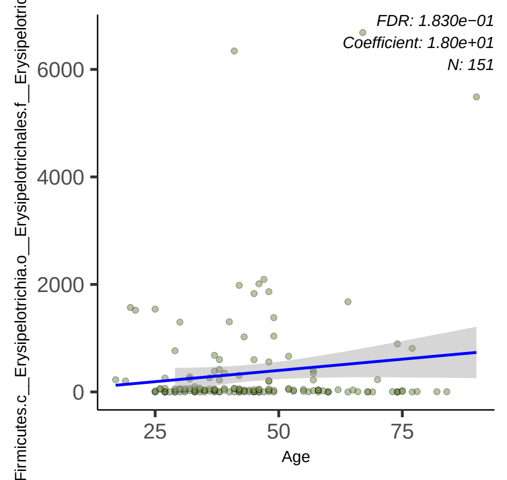

FDR: 1.830e−01
Coefficient: 1.80e+01
N: 151
Firmicutes.c__Erysipelotrichia.o__Erysipelotrichales.f__Erysipelotric
0
2000
4000
6000
25
50
75
Age

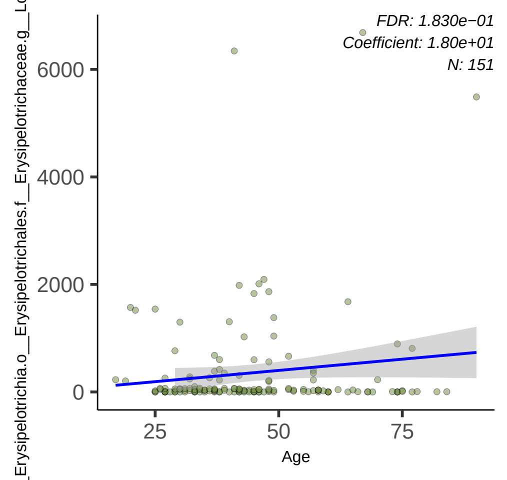

FDR: 1.830e−01
Coefficient: 1.80e+01
N: 151
6000
4000
2000
0
25
50
75
Age
Erysipelotrichia.o__Erysipelotrichales.f__Erysipelotrichaceae.g__Lo

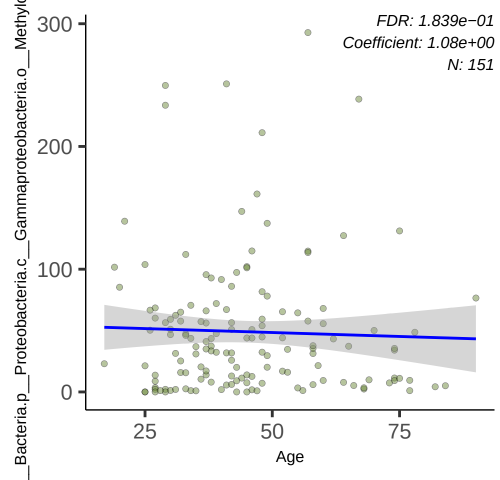

FDR: 1.839e−01
Coefficient: 1.08e+00
N: 151
300
200
100
0
25
50
75
Age
Bacteria.p__Proteobacteria.c__Gammaproteobacteria.o__Methylo

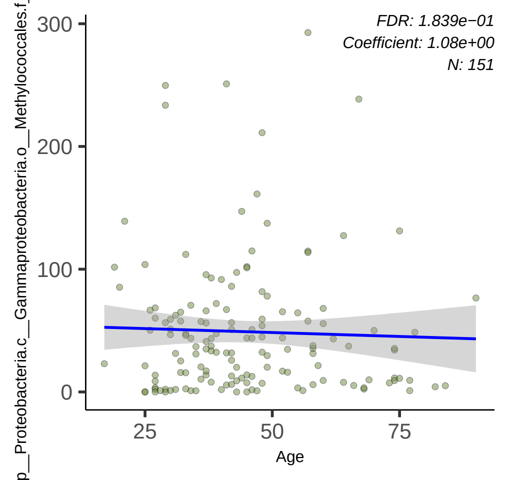

FDR: 1.839e−01
Coefficient: 1.08e+00
N: 151
p__Proteobacteria.c__Gammaproteobacteria.o__Methylococcales.f_
300
200
100
0
25
50
75
Age

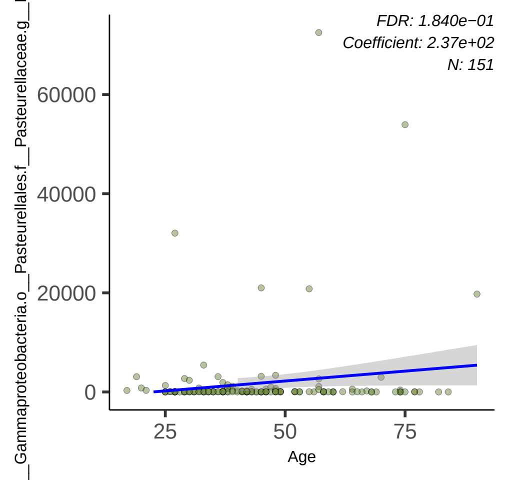

FDR: 1.840e−01
Coefficient: 2.37e+02
N: 151
Gammaproteobacteria.o__Pasteurellales.f__Pasteurellaceae.g__
60000
40000
20000
0
25
50
75
Age

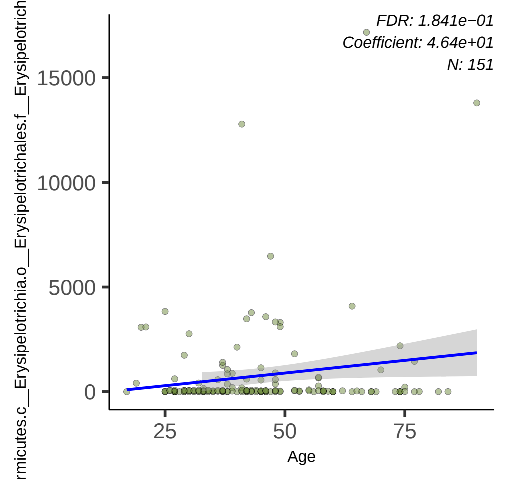

FDR: 1.841e−01
Coefficient: 4.64e+01
N: 151
rmicutes.c__Erysipelotrichia.o__Erysipelotrichales.f__Erysipelotrich
15000
10000
5000
0
25
50
75
Age

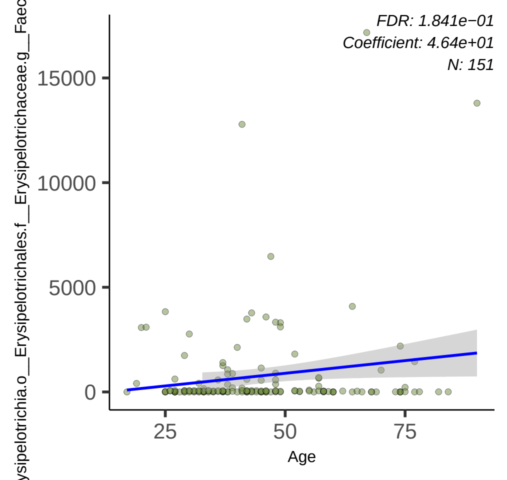
FDR: 1.841e−01
Coefficient: 4.64e+01
N: 151
15000
10000
5000
0
25
50
75
Age
rysipelotrichia.o__Erysipelotrichales.f__Erysipelotrichaceae.g__Faec

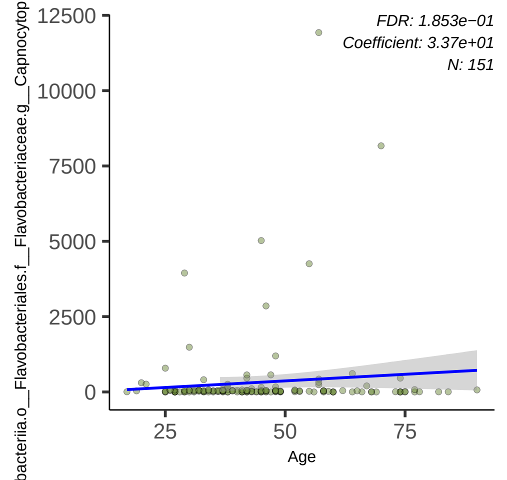

FDR: 1.853e−01
Coefficient: 3.37e+01
N: 151
bacteriia.o__Flavobacteriales.f__Flavobacteriaceae.g__Capnocytop
12500
10000
7500
5000
2500
0
25
50
75
Age

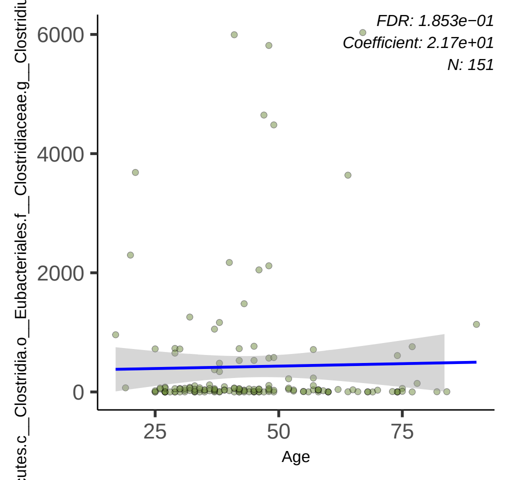
FDR: 1.853e−01
Coefficient: 2.17e+01
N: 151
6000
4000
2000
0
25
50
75
Age
cutes.c__Clostridia.o__Eubacteriales.f__Clostridiaceae.g__Clostridiu

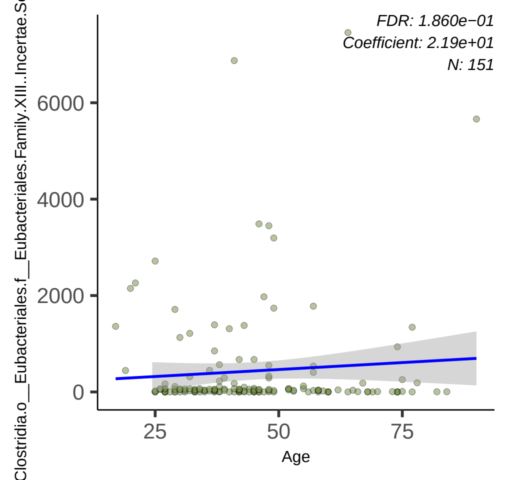
FDR: 1.860e−01
Coefficient: 2.19e+01
N: 151
6000
4000
2000
0
25
50
75
Age
Clostridia.o__Eubacteriales.f__Eubacteriales.Family.XIII..Incertae.S

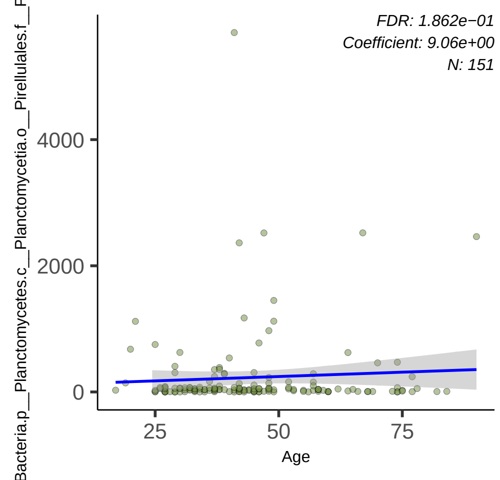

FDR: 1.862e−01
Coefficient: 9.06e+00
N: 151
Bacteria.p__Planctomycetes.c__Planctomycetia.o__Pirellulales.f__
4000
2000
0
25
50
75
Age

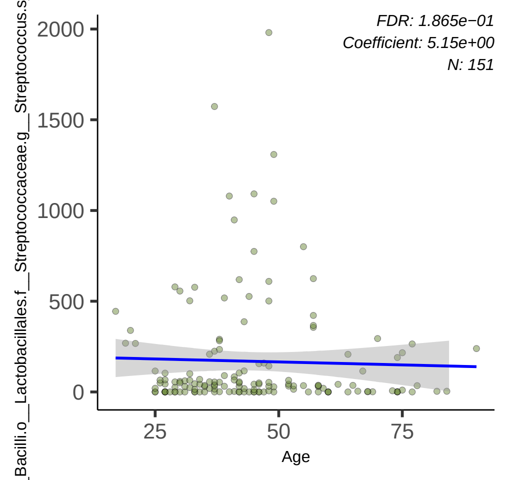

FDR: 1.865e−01
Coefficient: 5.15e+00
N: 151
2000
1500
1000
500
0
25
50
75
Age
Bacilli.o__Lactobacillales.f__Streptococcaceae.g__Streptococcus.s

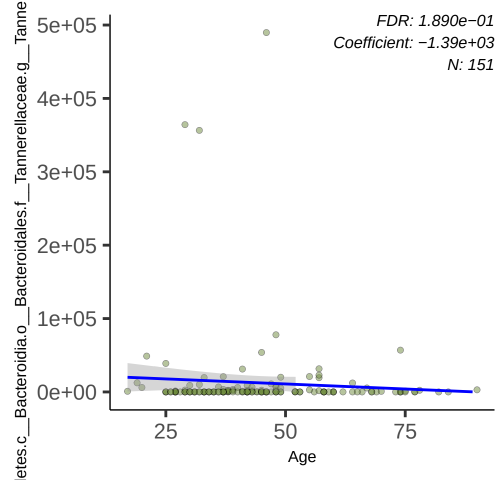

FDR: 1.890e−01
Coefficient: −1.39e+03
N: 151
5e+05
4e+05
3e+05
2e+05
1e+05
0e+00
25
50
75
Age
letes.c__Bacteroidia.o__Bacteroidales.f__Tannerellaceae.g__Tanne

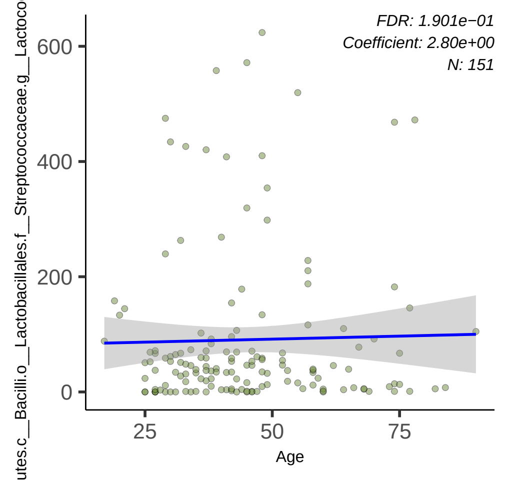
FDR: 1.901e−01
Coefficient: 2.80e+00
N: 151
utes.c__Bacilli.o__Lactobacillales.f__Streptococcaceae.g__Lactoco
600
400
200
0
25
50
75
Age

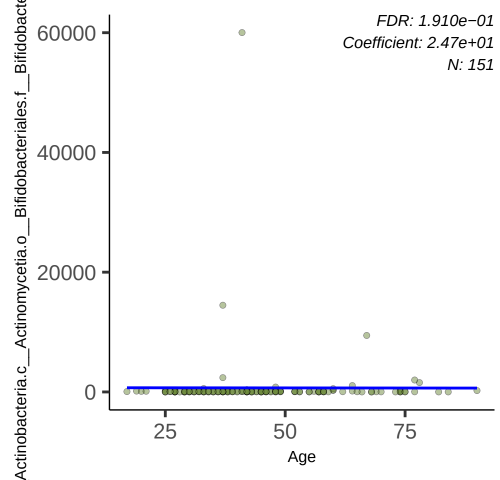
FDR: 1.910e−01
Coefficient: 2.47e+01
N: 151
60000
40000
20000
0
25
50
75
Age
Actinobacteria.c__Actinomycetia.o__Bifidobacteriales.f__Bifidobacte

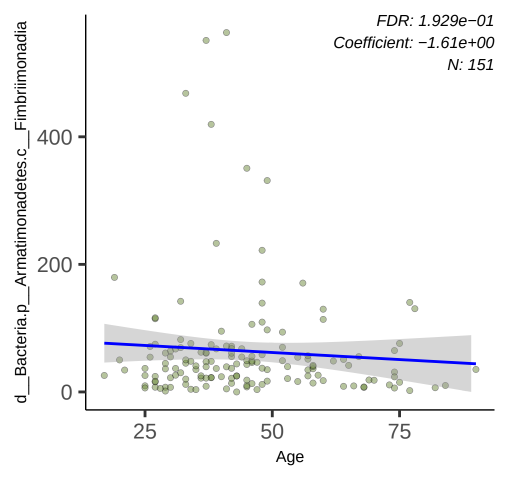
FDR: 1.929e−01
Coefficient: −1.61e+00
N: 151
d__Bacteria.p__Armatimonadetes.c__Fimbriimonadia
400
200
0
25
50
75
Age

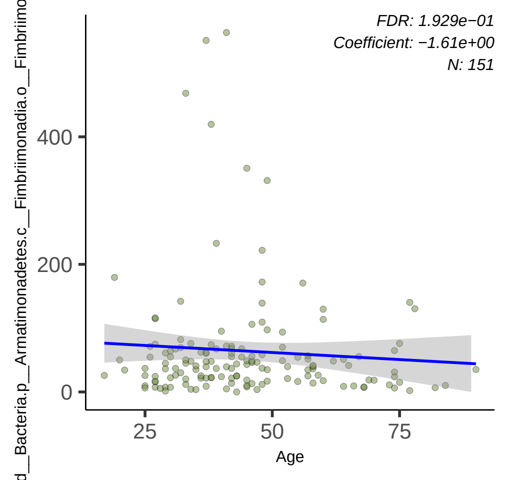
FDR: 1.929e−01
Coefficient: −1.61e+00
N: 151
d__Bacteria.p__Armatimonadetes.c__Fimbriimonadia.o__Fimbriimo
0
200
400
25
50
75
Age

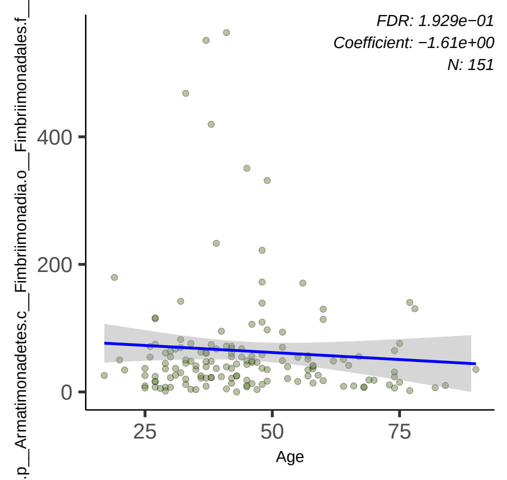
FDR: 1.929e−01
Coefficient: −1.61e+00
N: 151
p__Armatimonadetes.c__Fimbriimonadia.o__Fimbriimonadales.f__
0
200
400
25
50
75
Age

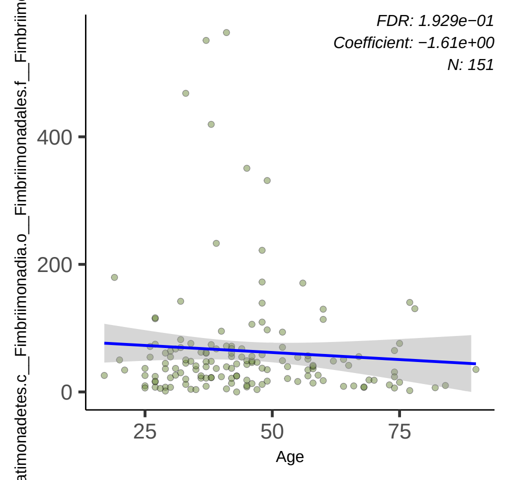

FDR: 1.929e−01
Coefficient: −1.61e+00
N: 151
atimonadetes.c__Fimbriimonadia.o__Fimbriimonadales.f__Fimbriimo
400
200
0
25
50
75
Age

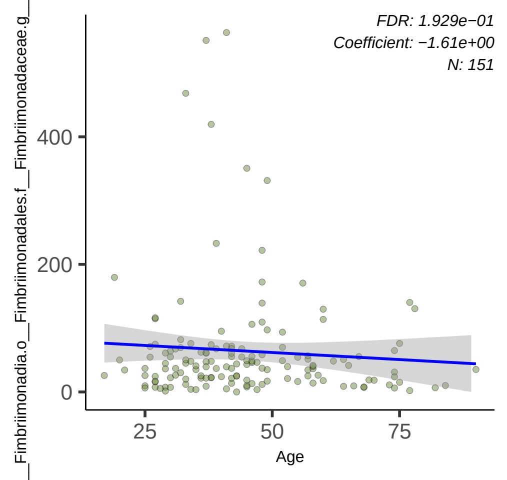

FDR: 1.929e−01
Coefficient: −1.61e+00
N: 151
Fimbriimonadia.o__Fimbriimonadales.f__Fimbriimonadaceae.g__
400
200
0
25
50
75
Age

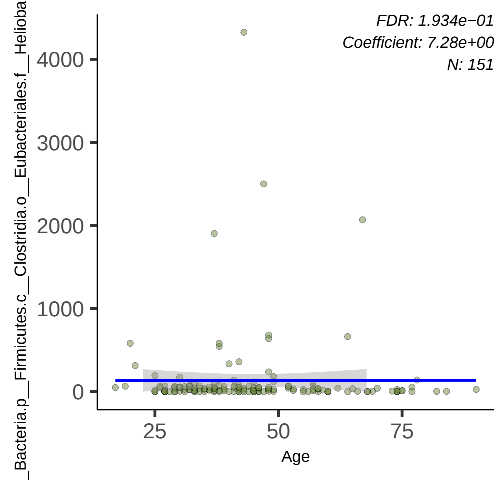
FDR: 1.934e−01
Coefficient: 7.28e+00
N: 151
Bacteria.p__Firmicutes.c__Clostridia.o__Eubacteriales.f__Heliobac
0
1000
2000
3000
4000
25
50
75
Age

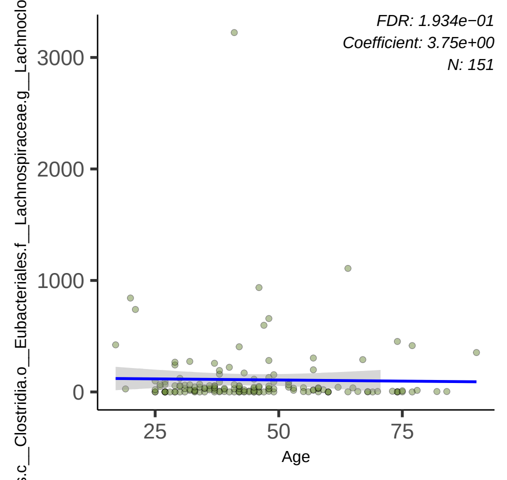

FDR: 1.934e−01
Coefficient: 3.75e+00
N: 151
3000
2000
1000
0
25
50
75
Age
s.c__Clostridia.o__Eubacteriales.f__Lachnospiraceae.g__Lachnoclo

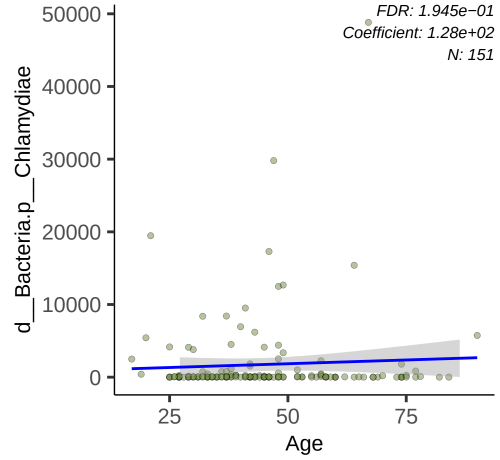

FDR: 1.945e−01
Coefficient: 1.28e+02
N: 151
d__Bacteria.p__Chlamydiae
50000
40000
30000
20000
10000
0
25
50
75
Age

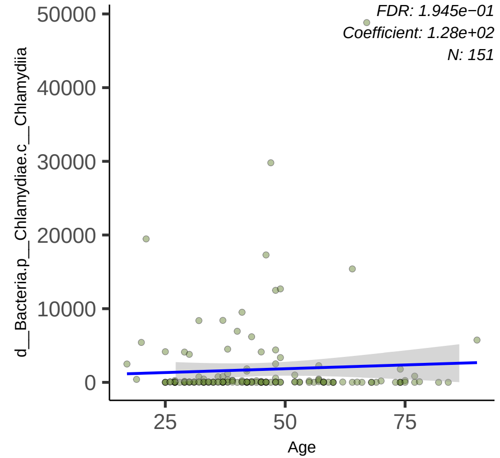
FDR: 1.945e−01
Coefficient: 1.28e+02
N: 151
d__Bacteria.p__Chlamydiae.c__Chlamydiia
50000
40000
30000
20000
10000
0
25
50
75
Age

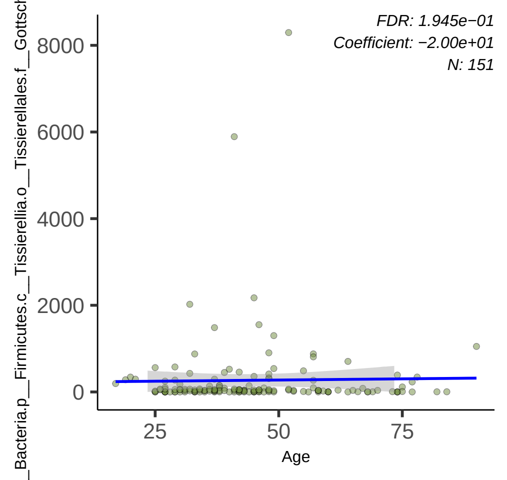

FDR: 1.945e−01
Coefficient: −2.00e+01
N: 151
Bacteria.p__Firmicutes.c__Tissierellia.o__Tissierellales.f__Gottsch
0
2000
4000
6000
8000
25
50
75
Age

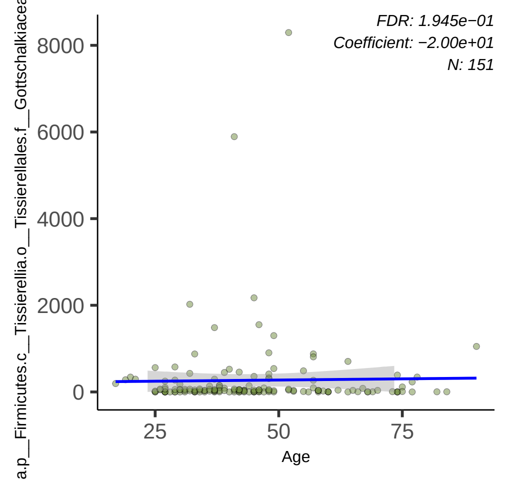

FDR: 1.945e−01
Coefficient: −2.00e+01
N: 151
a.p__Firmicutes.c__Tissierellia.o__Tissierellales.f__Gottschalkiacea
0
2000
4000
6000
8000
25
50
75
Age

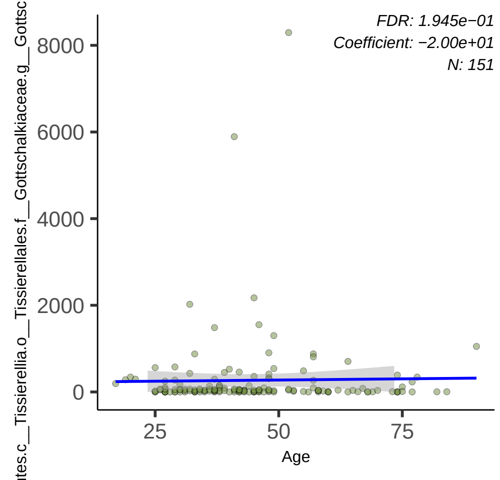
FDR: 1.945e−01
Coefficient: −2.00e+01
N: 151
8000
6000
4000
2000
0
25
50
75
Age
tes.c__Tissierellia.o__Tissierellales.f__Gottschalkiaceae.g__Gottsc

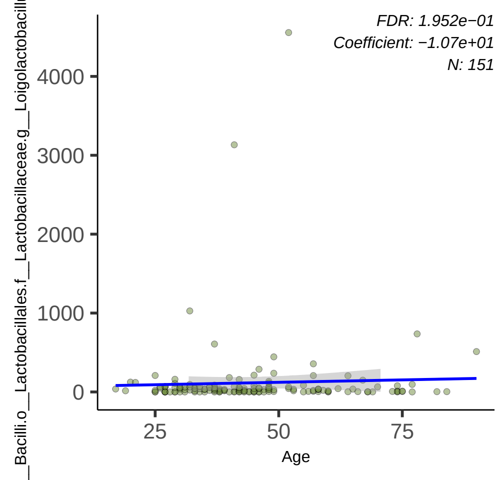

FDR: 1.952e−01
Coefficient: −1.07e+01
N: 151
Bacilli.o__Lactobacillales.f__Lactobacillaceae.g__Loigolactobacill
4000
3000
2000
1000
0
25
50
75
Age

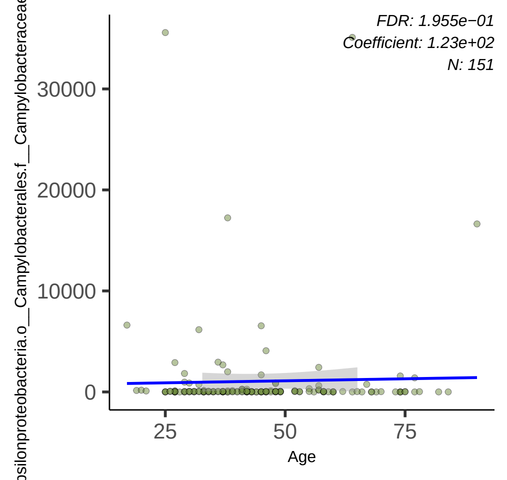

FDR: 1.955e−01
Coefficient: 1.23e+02
N: 151
psilonproteobacteria.o__Campylobacterales.f__Campylobacteraceae
0
10000
20000
30000
25
50
75
Age

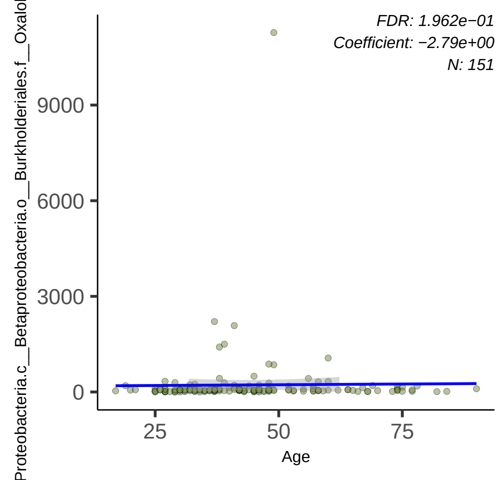

FDR: 1.962e−01
Coefficient: −2.79e+00
N: 151
Proteobacteria.c__Betaproteobacteria.o__Burkholderiales.f__Oxalob
9000
6000
3000
0
25
50
75
Age

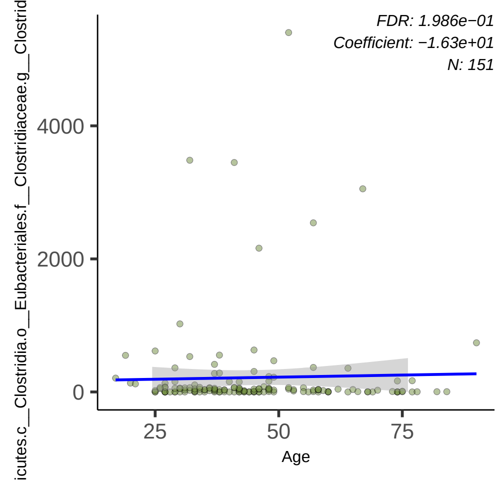
FDR: 1.986e−01
Coefficient: −1.63e+01
N: 151
icutes.c__Clostridia.o__Eubacteriales.f__Clostridiaceae.g__Clostrid
4000
2000
0
25
50
75
Age

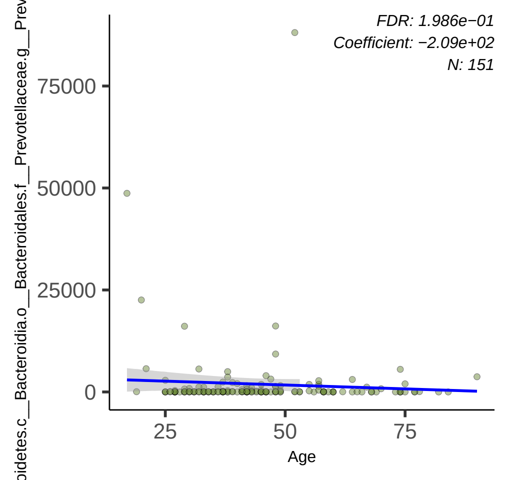
FDR: 1.986e−01
Coefficient: −2.09e+02
N: 151
75000
50000
25000
0
25
50
75
Age
oidetes.c__Bacteroidia.o__Bacteroidales.f__Prevotellaceae.g__Prev

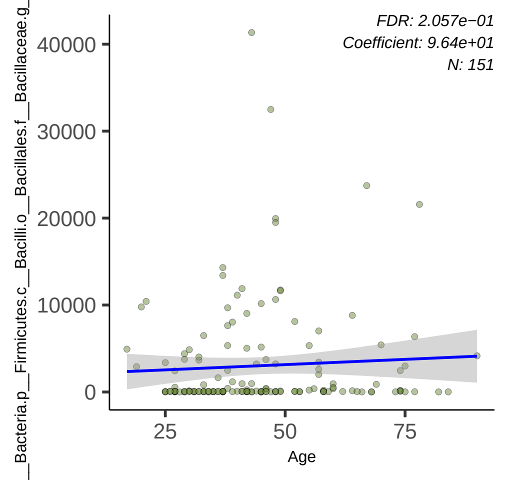

FDR: 2.057e−01
Coefficient: 9.64e+01
N: 151
Bacteria.p__Firmicutes.c__Bacilli.o__Bacillales.f__Bacillaceae.g__
0
10000
20000
30000
40000
25
50
75
Age

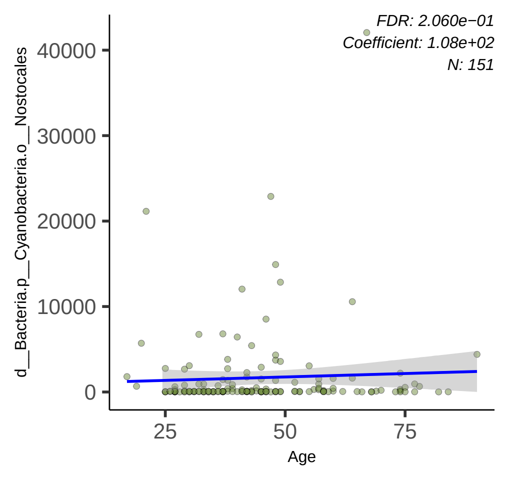

FDR: 2.060e−01
Coefficient: 1.08e+02
N: 151
d__Bacteria.p__Cyanobacteria.o__Nostocales
40000
30000
20000
10000
0
25
50
75
Age

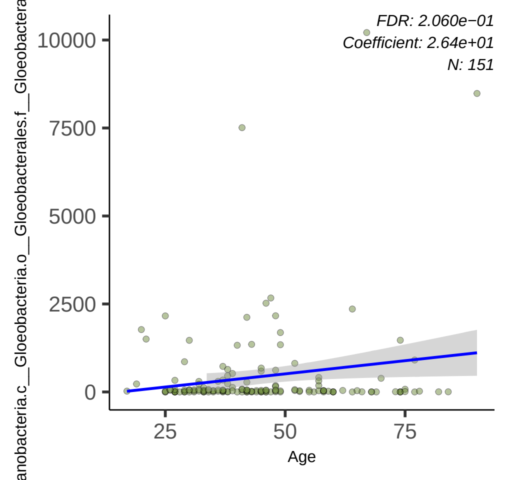
FDR: 2.060e−01
Coefficient: 2.64e+01
N: 151
10000
7500
5000
2500
0
25
50
75
Age
anobacteria.c__Gloeobacteria.o__Gloeobacterales.f__Gloeobactera

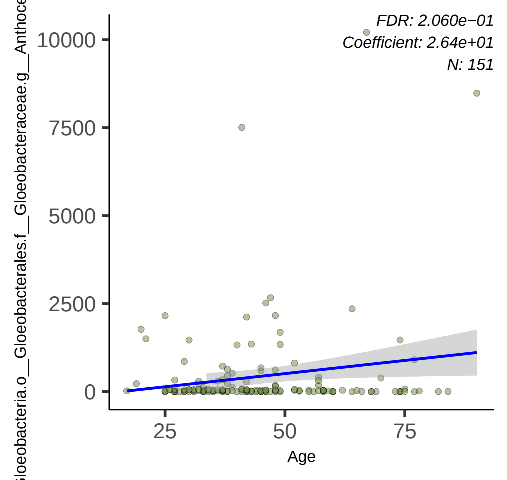
FDR: 2.060e−01
Coefficient: 2.64e+01
N: 151
10000
7500
5000
2500
0
25
50
75
Age
Gloeobacteria.o__Gloeobacterales.f__Gloeobacteraceae.g__Anthoce

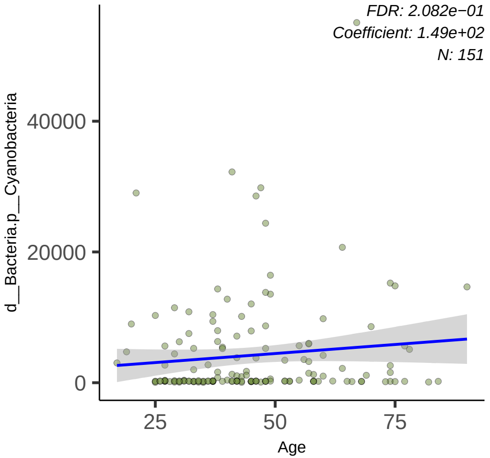

FDR: 2.082e−01
Coefficient: 1.49e+02
N: 151
d__Bacteria.p__Cyanobacteria
40000
20000
0
25
50
75
Age

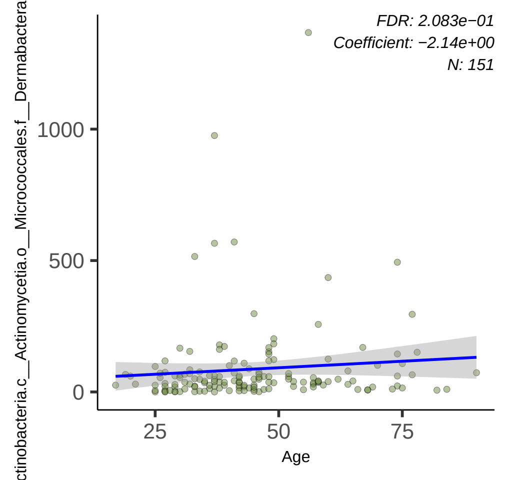

FDR: 2.083e−01
Coefficient: −2.14e+00
N: 151
1000
500
0
25
50
75
Age
ctinobacteria.c__Actinomycetia.o__Micrococcales.f__Dermabactera

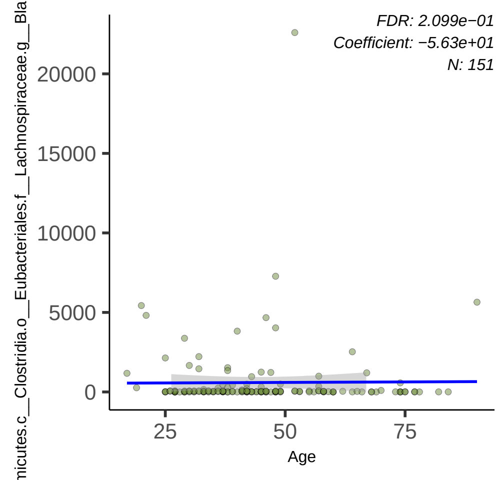

FDR: 2.099e−01
Coefficient: −5.63e+01
N: 151
micutes.c__Clostridia.o__Eubacteriales.f__Lachnospiraceae.g__Bla
0
5000
10000
15000
20000
25
50
75
Age

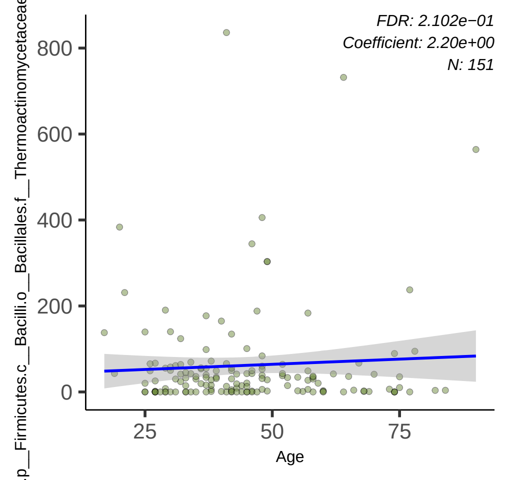
FDR: 2.102e−01
Coefficient: 2.20e+00
N: 151
800
600
400
200
0
25
50
75
Age
p__Firmicutes.c__Bacilli.o__Bacillales.f__Thermoactinomycetaceae

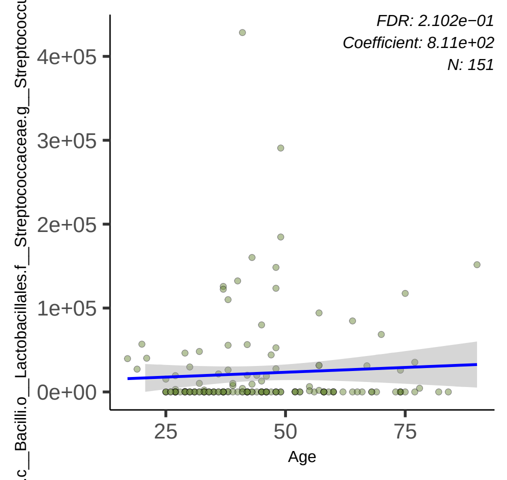

FDR: 2.102e−01
Coefficient: 8.11e+02
N: 151
c__Bacilli.o__Lactobacillales.f__Streptococcaceae.g__Streptococcu
4e+05
3e+05
2e+05
1e+05
0e+00
25
50
75
Age

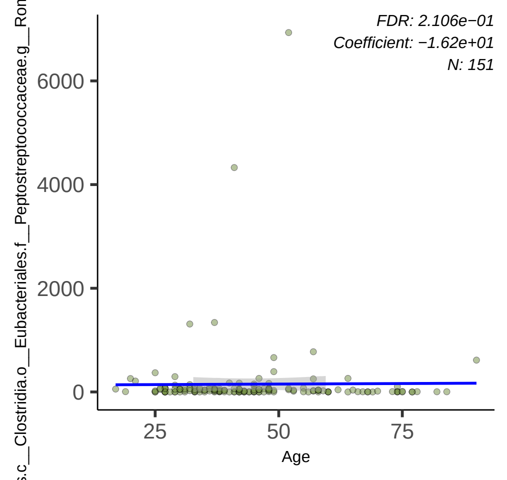
FDR: 2.106e−01
Coefficient: −1.62e+01
N: 151
s.c__Clostridia.o__Eubacteriales.f__Peptostreptococcaceae.g__Ron
6000
4000
2000
0
25
50
75
Age

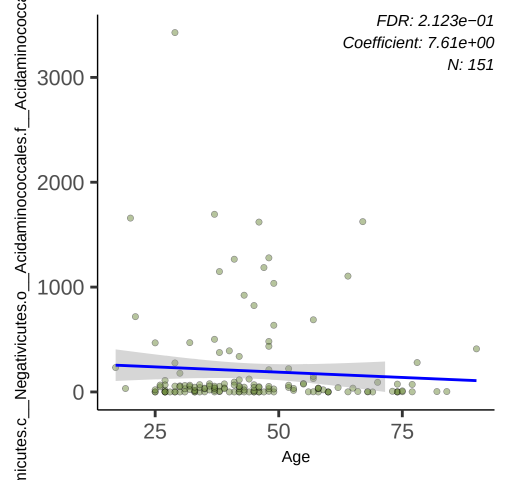
FDR: 2.123e−01
Coefficient: 7.61e+00
N: 151
3000
2000
1000
0
25
50
75
Age
nicutes.c__Negativicutes.o__Acidaminococcales.f__Acidaminococca

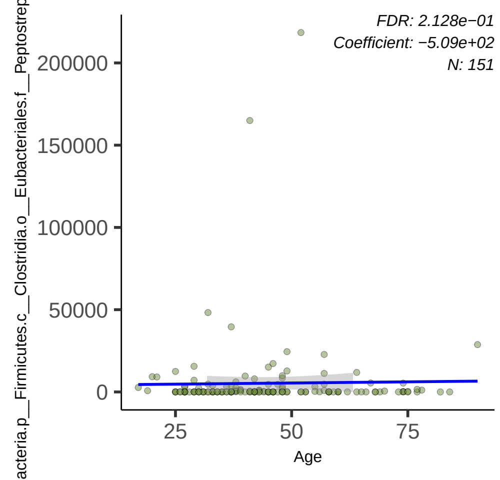

FDR: 2.128e−01
Coefficient: −5.09e+02
N: 151
acteria.p__Firmicutes.c__Clostridia.o__Eubacteriales.f__Peptostrep
200000
150000
100000
50000
0
25
50
75
Age

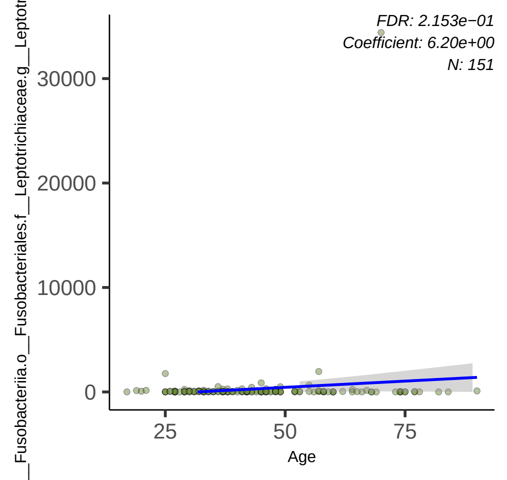
FDR: 2.153e−01
Coefficient: 6.20e+00
N: 151
Fusobacteriia.o__Fusobacteriales.f__Leptotrichiaceae.g__Leptotr
30000
20000
10000
0
25
50
75
Age

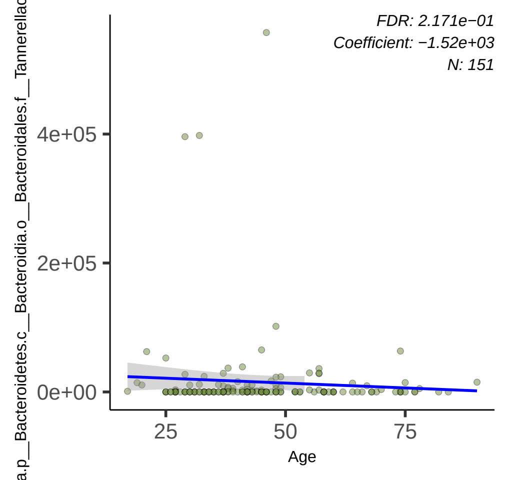

FDR: 2.171e−01
Coefficient: −1.52e+03
N: 151
4e+05
2e+05
0e+00
25
50
75
Age
a.p__Bacteroidetes.c__Bacteroidia.o__Bacteroidales.f__Tannerellac

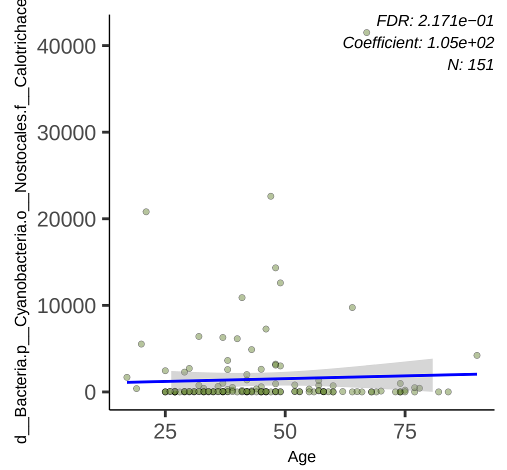

FDR: 2.171e−01
Coefficient: 1.05e+02
N: 151
d__Bacteria.p__Cyanobacteria.o__Nostocales.f__Calotrichace
40000
30000
20000
10000
0
25
50
75
Age

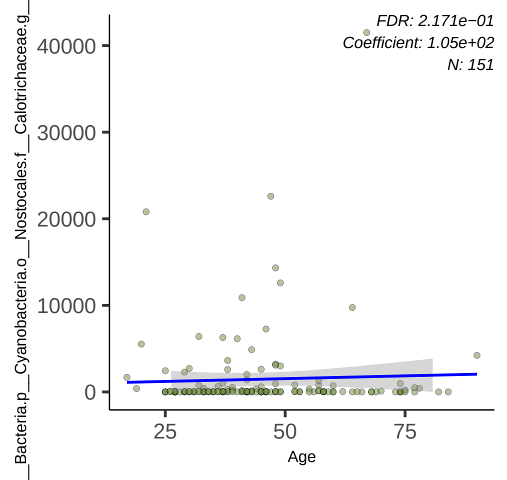

FDR: 2.171e−01
Coefficient: 1.05e+02
N: 151
Bacteria.p__Cyanobacteria.o__Nostocales.f__Calotrichaceae.g__
40000
30000
20000
10000
0
25
50
75
Age

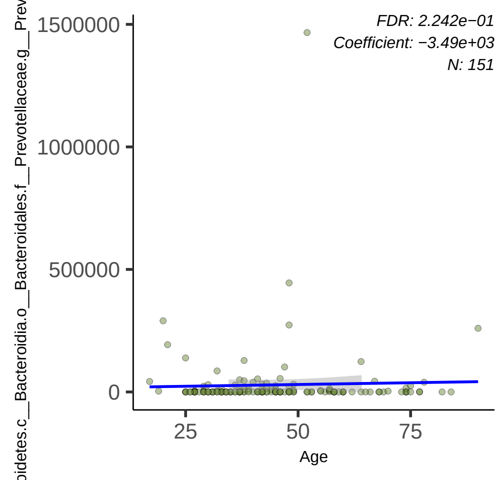
FDR: 2.242e−01
Coefficient: −3.49e+03
N: 151
1500000
1000000
500000
0
25
50
75
Age
oidetes.c__Bacteroidia.o__Bacteroidales.f__Prevotellaceae.g__Prev

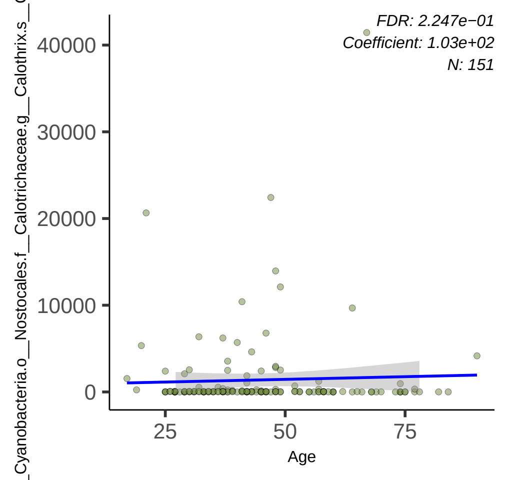
FDR: 2.247e−01
Coefficient: 1.03e+02
N: 151
Cyanobacteria.o__Nostocales.f__Calotrichaceae.g__Calothrix.s__
40000
30000
20000
10000
0
25
50
75
Age

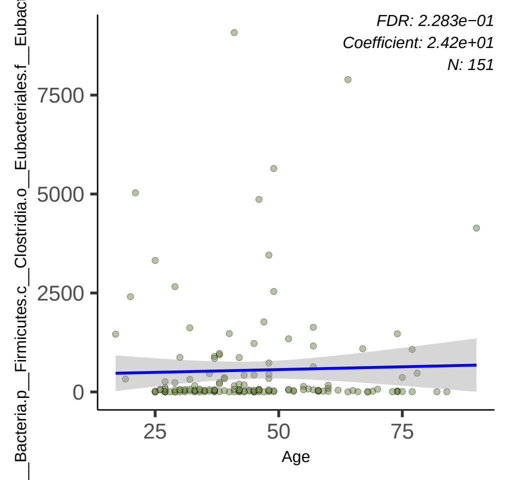
FDR: 2.283e−01
Coefficient: 2.42e+01
N: 151
Bacteria.p__Firmicutes.c__Clostridia.o__Eubacteriales.f__Eubact
7500
5000
2500
0
25
50
75
Age

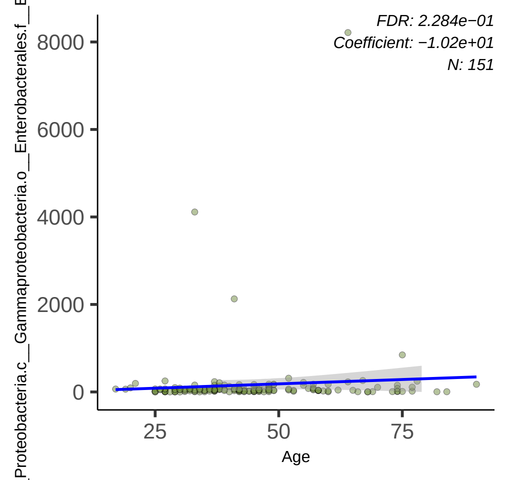

FDR: 2.284e−01
Coefficient: −1.02e+01
N: 151
8000
6000
4000
2000
0
25
50
75
Age
Proteobacteria.c__Gammaproteobacteria.o__Enterobacterales.f__E

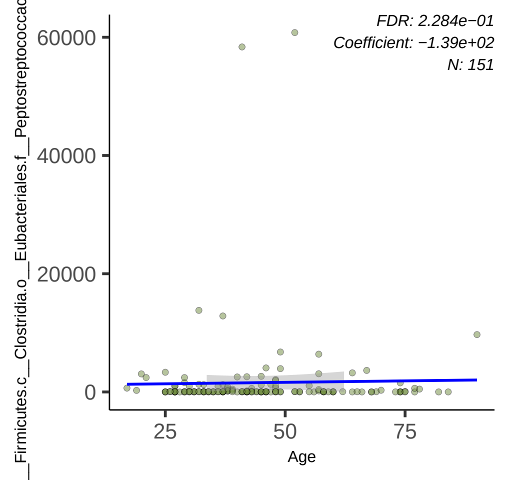

FDR: 2.284e−01
Coefficient: −1.39e+02
N: 151
60000
40000
20000
0
25
50
75
Age
__Firmicutes.c__Clostridia.o__Eubacteriales.f__Peptostreptococcac

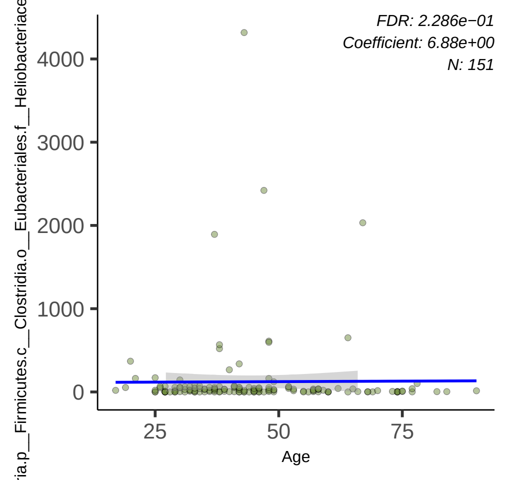
FDR: 2.286e−01
Coefficient: 6.88e+00
N: 151
ria.p__Firmicutes.c__Clostridia.o__Eubacteriales.f__Heliobacteriace
4000
3000
2000
1000
0
25
50
75
Age

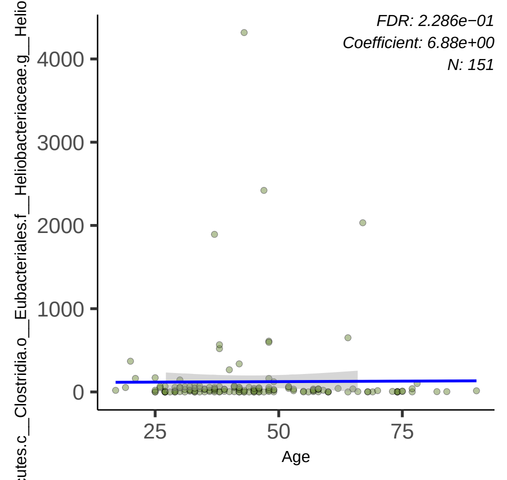
FDR: 2.286e−01
Coefficient: 6.88e+00
N: 151
4000
3000
2000
1000
0
25
50
75
Age
cutes.c__Clostridia.o__Eubacteriales.f__Heliobacteriaceae.g__Helio

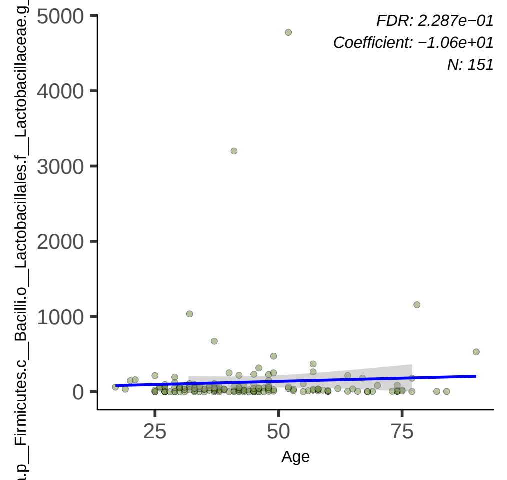
FDR: 2.287e−01
Coefficient: −1.06e+01
N: 151
Age
Firmicutes.c__Bacilli.o__Lactobacillales.f__Lactobacillaceae.g_
5000
4000
3000
2000
1000
0
25
50
75

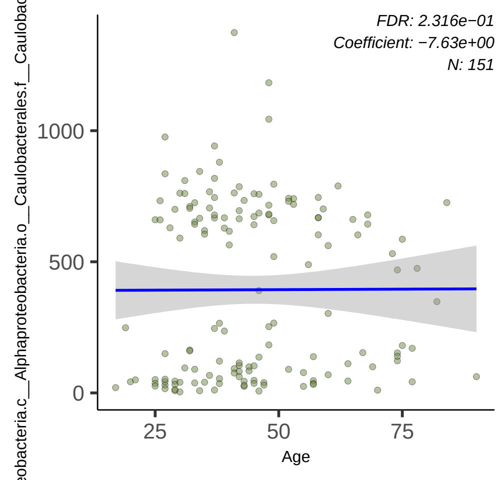
FDR: 2.316e−01
Coefficient: −7.63e+00
N: 151
eobacteria.c__Alphaproteobacteria.o__Caulobacterales.f__Caulobac
1000
500
0
25
50
75
Age

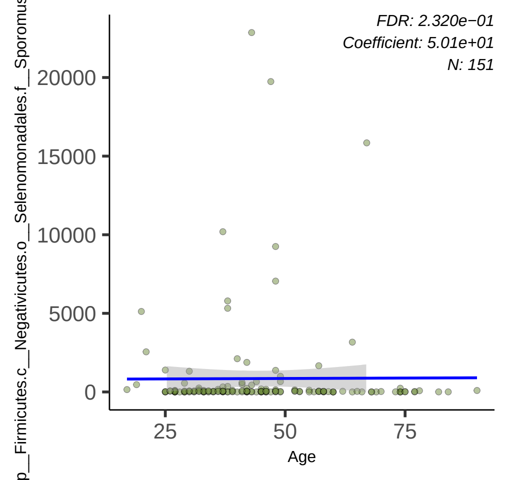
FDR: 2.320e−01
Coefficient: 5.01e+01
N: 151
p__Firmicutes.c__Negativicutes.o__Selenomonadales.f__Sporomus
20000
15000
10000
5000
0
25
50
75
Age

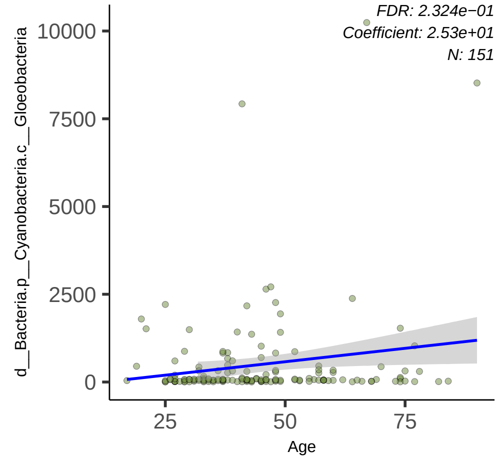
FDR: 2.324e−01
Coefficient: 2.53e+01
N: 151
d__Bacteria.p__Cyanobacteria.c__Gloeobacteria
10000
7500
5000
2500
0
25
50
75
Age

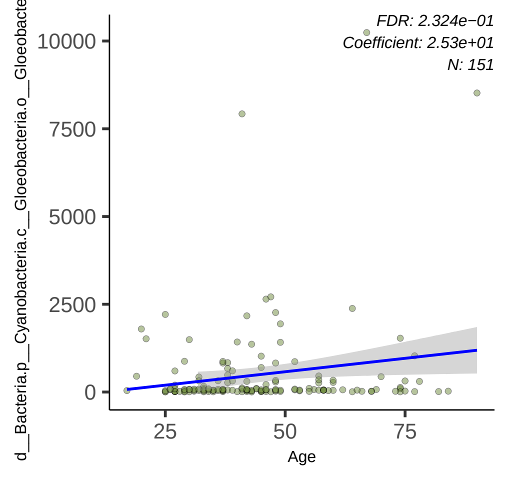

FDR: 2.324e−01
Coefficient: 2.53e+01
N: 151
d__Bacteria.p__Cyanobacteria.c__Gloeobacteria.o__Gloeobacte
10000
7500
5000
2500
0
25
50
75
Age

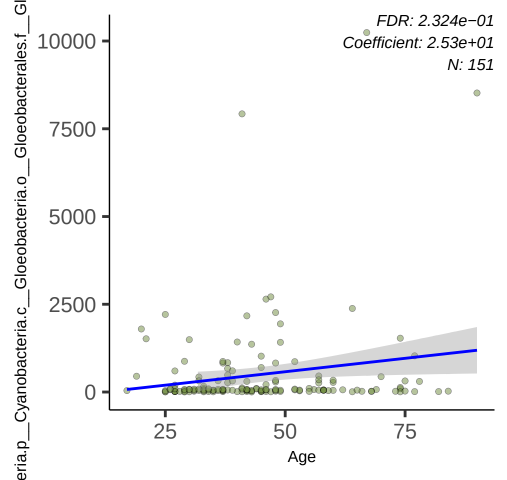

FDR: 2.324e−01
Coefficient: 2.53e+01
N: 151
10000
7500
5000
2500
0
25
50
75
Age
eria.p__Cyanobacteria.c__Gloeobacteria.o__Gloeobacterales.f__Gl

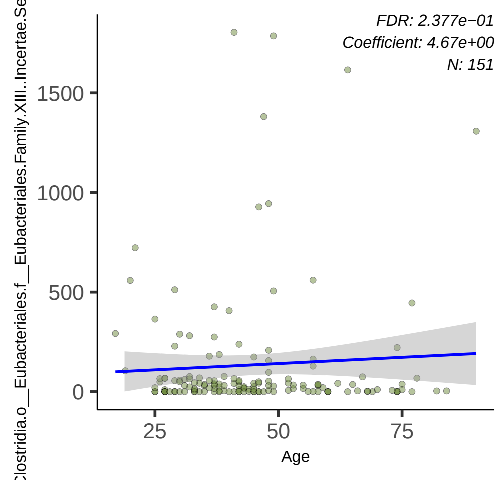
FDR: 2.377e−01
Coefficient: 4.67e+00
N: 151
1500
1000
500
0
25
50
75
Age
Clostridia.o__Eubacteriales.f__Eubacteriales.Family.XIII..Incertae.Se

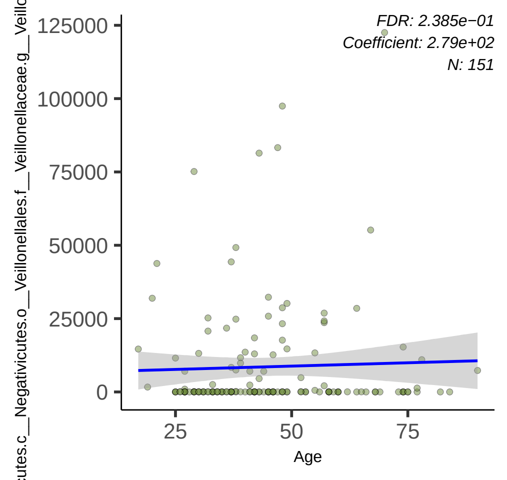
FDR: 2.385e−01
Coefficient: 2.79e+02
N: 151
125000
100000
75000
50000
25000
0
25
50
75
Age
cutes.c__Negativicutes.o__Veillonellales.f__Veillonellaceae.g__Veillo

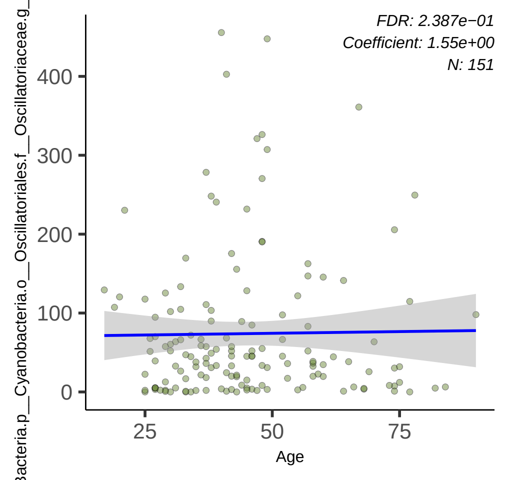
FDR: 2.387e−01
Coefficient: 1.55e+00
N: 151
Bacteria.p__Cyanobacteria.o__Oscillatoriales.f__Oscillatoriaceae.g__
400
300
200
100
0
25
50
75
Age

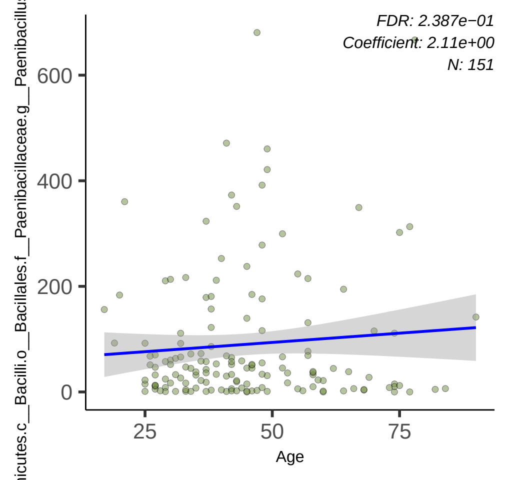
FDR: 2.387e−01
Coefficient: 2.11e+00
N: 151
icutes.c__Bacilli.o__Bacillales.f__Paenibacillaceae.g__Paenibacillus
0
200
400
600
25
50
75
Age

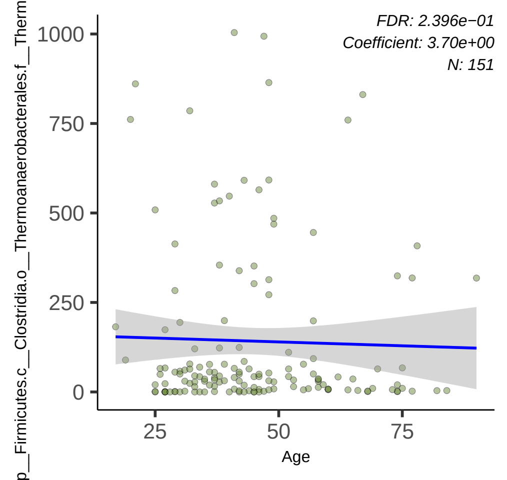

FDR: 2.396e−01
Coefficient: 3.70e+00
N: 151
p__Firmicutes.c__Clostridia.o__Thermoanaerobacterales.f__Therm
0
250
500
750
1000
25
50
75
Age

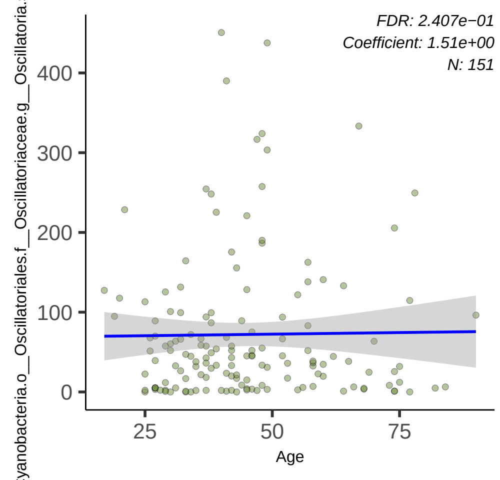

FDR: 2.407e−01
Coefficient: 1.51e+00
N: 151
0
100
200
300
400
25
50
75
Age
yanobacteria.o__Oscillatoriales.f__Oscillatoriaceae.g__Oscillatoria.

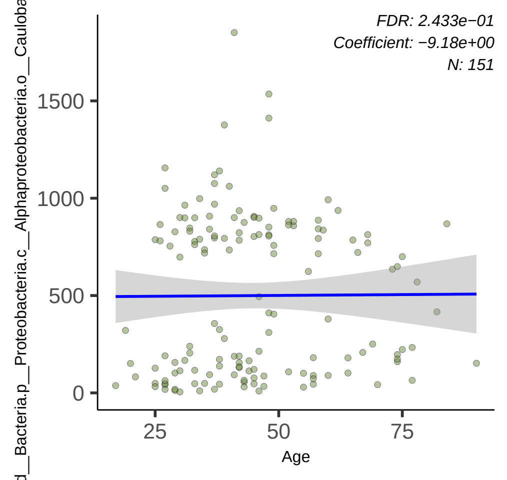

FDR: 2.433e−01
Coefficient: −9.18e+00
N: 151
d__Bacteria.p__Proteobacteria.c__Alphaproteobacteria.o__Caulobа
0
500
1000
1500
25
50
75
Age

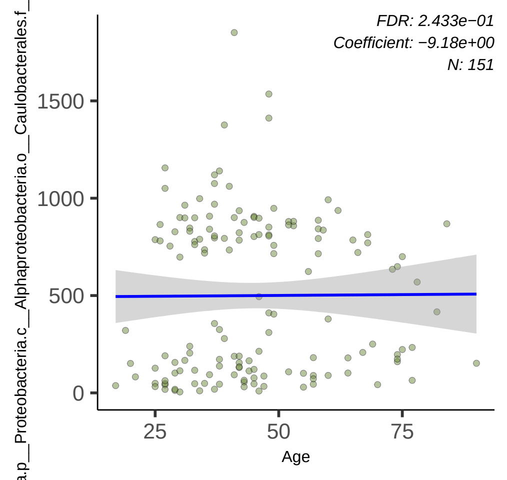

FDR: 2.433e−01
Coefficient: −9.18e+00
N: 151
a.p__Proteobacteria.c__Alphaproteobacteria.o__Caulobacterales.f__
1500
1000
500
0
25
50
75
Age

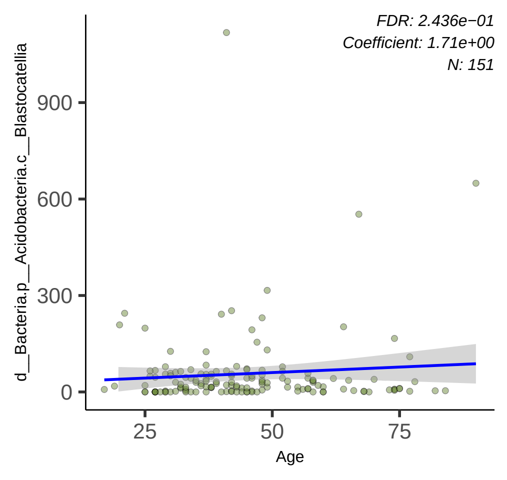

FDR: 2.436e−01
Coefficient: 1.71e+00
N: 151
d__Bacteria.p__Acidobacteria.c__Blastocatellia
900
600
300
0
25
50
75
Age

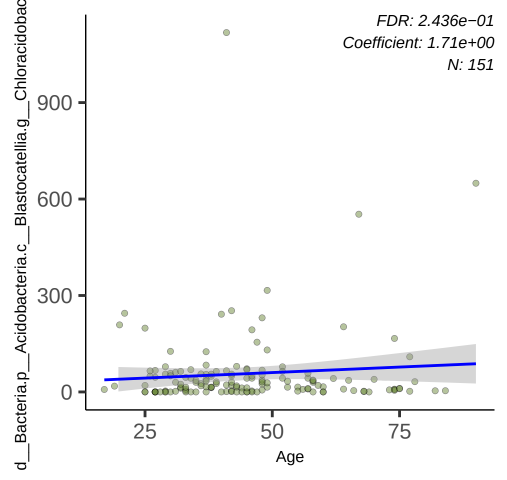
FDR: 2.436e−01
Coefficient: 1.71e+00
N: 151
d__Bacteria.p__Acidobacteria.c__Blastocatellia.g__Chloracidobac
900
600
300
0
25
50
75
Age

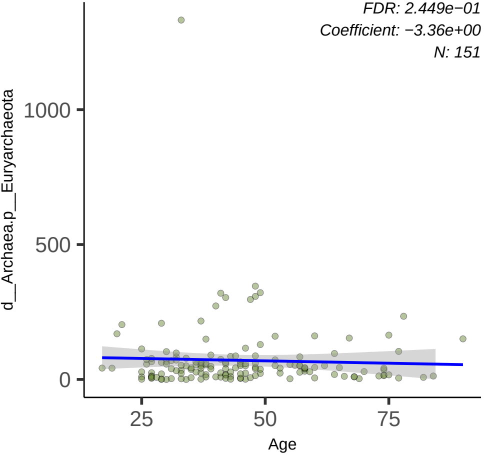

FDR: 2.449e−01
Coefficient: −3.36e+00
N: 151
d__Archaea.p__Euryarchaeota
1000
500
0
25
50
75
Age

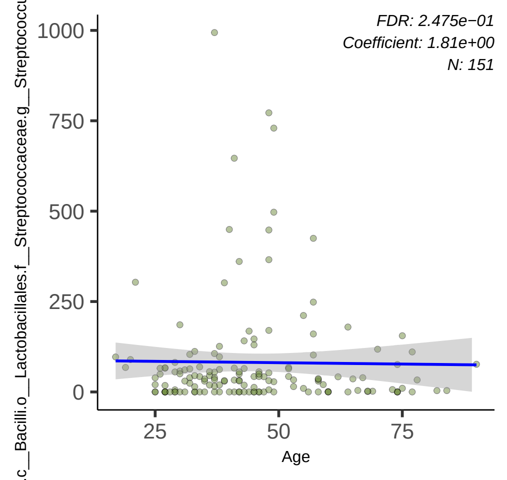
FDR: 2.475e−01
Coefficient: 1.81e+00
N: 151
c__Bacilli.o__Lactobacillales.f__Streptococcaceae.g__Streptococcu
1000
750
500
250
0
25
50
75
Age
