# Supplementary material for: Metatranscriptome Analysis of Nasopharyngeal Swabs across the Varying Severity of COVID-19 Disease Demonstrated Unprecedented Species Diversity
Source: Microorganisms. 2023 Jul 14;11(7):1804. doi: 10.3390/microorganisms11071804 (PMC10384460; doi:10.3390/microorganisms11071804)
Supplement: Supplementary file 1 [file microorganisms-11-01804-s001.zip › Maaslin_results_ALL.zip!/Maaslin_results/Age_category.pdf]

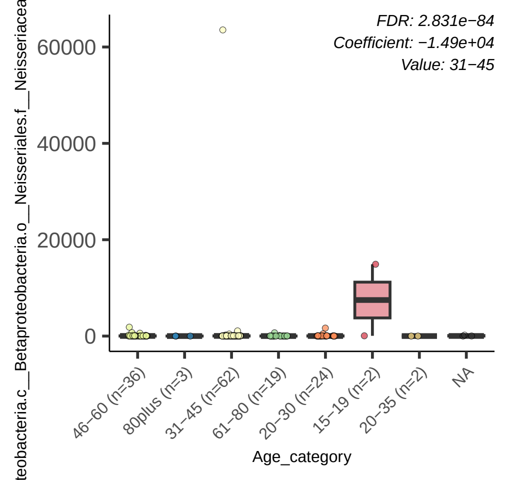

FDR: 2.831e−84
Coefficient: −1.49e+04
Value: 31−45
teobacteria.c__Betaproteobacteria.o__Neisseriales.f__Neisseriacea
60000
40000
20000
0
46−60 (n=36)
80plus (n=3)
31−45 (n=62)
61−80 (n=19)
20−30 (n=24)
15−19 (n=2)
20−35 (n=2)
NA
Age_category

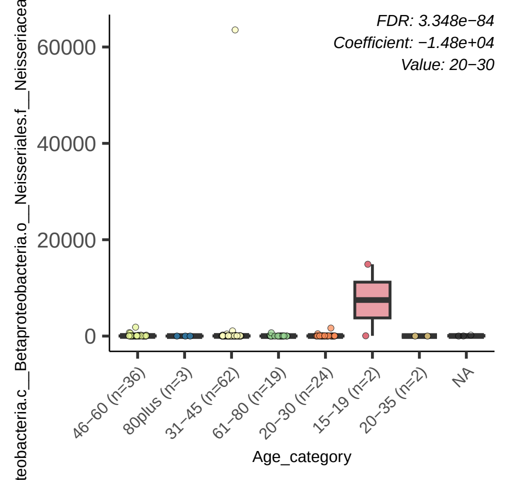

FDR: 3.348e−84
Coefficient: −1.48e+04
Value: 20−30
60000
40000
20000
0
teobacteria.c__Betaproteobacteria.o__Neisseriales.f__Neisseriacea
46−60 (n=36)
80plus (n=3)
31−45 (n=62)
61−80 (n=19)
20−30 (n=24)
15−19 (n=2)
20−35 (n=2)
NA
Age_category

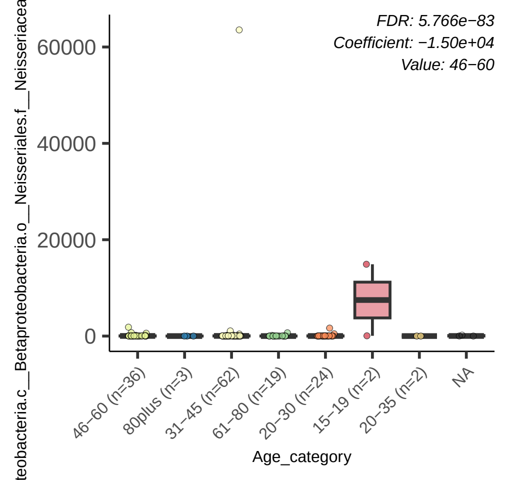

FDR: 5.766e−83
Coefficient: −1.50e+04
Value: 46−60
60000
40000
20000
0
teobacteria.c__Betaproteobacteria.o__Neisseriales.f__Neisseriacea
46−60 (n=36)
80plus (n=3)
31−45 (n=62)
61−80 (n=19)
20−30 (n=24)
15−19 (n=2)
20−35 (n=2)
NA
Age_category

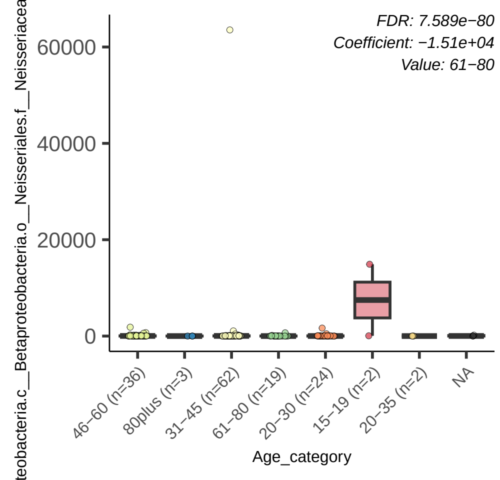

FDR: 7.589e−80
Coefficient: −1.51e+04
Value: 61−80
teobacteria.c__Betaproteobacteria.o__Neisseriales.f__Neisseriacea
60000
40000
20000
0
46−60 (n=36)
80plus (n=3)
31−45 (n=62)
61−80 (n=19)
20−30 (n=24)
15−19 (n=2)
20−35 (n=2)
NA
Age_category

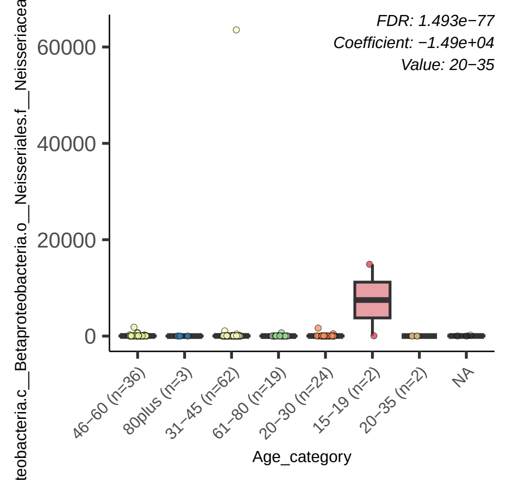
FDR: 1.493e−77
Coefficient: −1.49e+04
Value: 20−35
60000
40000
20000
0
teobacteria.c__Betaproteobacteria.o__Neisseriales.f__Neisseriacea
46−60 (n=36)
80plus (n=3)
31−45 (n=62)
61−80 (n=19)
20−30 (n=24)
15−19 (n=2)
20−35 (n=2)
NA
Age_category

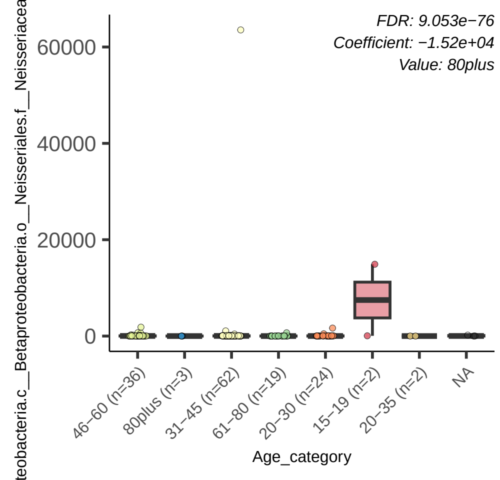

FDR: 9.053e−76
Coefficient: −1.52e+04
Value: 80plus
teobacteria.c__Betaproteobacteria.o__Neisseriales.f__Neisseriacea
60000
40000
20000
0
46−60 (n=36)
80plus (n=3)
31−45 (n=62)
61−80 (n=19)
20−30 (n=24)
15−19 (n=2)
20−35 (n=2)
NA
Age_category

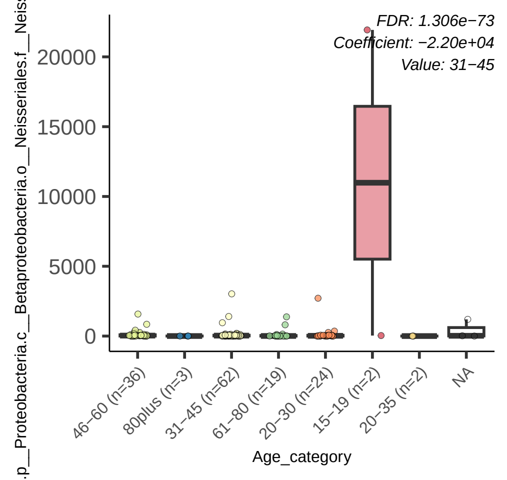
FDR: 1.306e−73
Coefficient: −2.20e+04
Value: 31−45
20000
15000
10000
5000
0
.p__Proteobacteria.c__Betaproteobacteria.o__Neisseriales.f__Neiss
46−60 (n=36)
80plus (n=3)
31−45 (n=62)
61−80 (n=19)
20−30 (n=24)
15−19 (n=2)
20−35 (n=2)
NA
Age_category

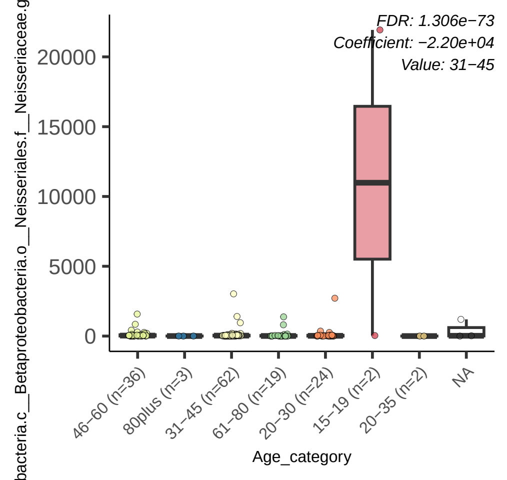
FDR: 1.306e−73
Coefficient: −2.20e+04
Value: 31−45
20000
15000
10000
5000
0
bacteria.c__Betaproteobacteria.o__Neisseriales.f__Neisseriaceae.g
46−60 (n=36)
80plus (n=3)
31−45 (n=62)
61−80 (n=19)
20−30 (n=24)
15−19 (n=2)
20−35 (n=2)
NA
Age_category

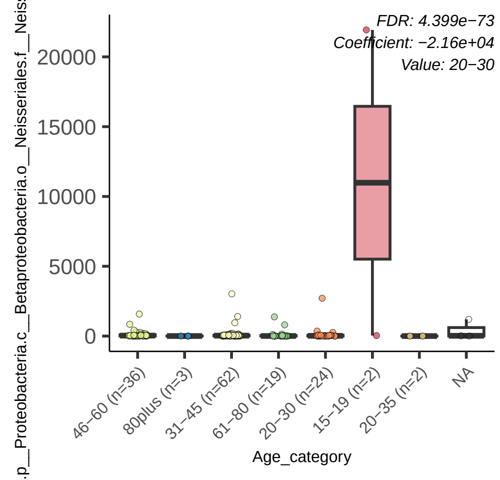

FDR: 4.399e−73
Coefficient: −2.16e+04
Value: 20−30
p__Proteobacteria.c__Betaproteobacteria.o__Neisseriales.f__Neiss
20000
15000
10000
5000
0
46−60 (n=36)
80plus (n=3)
31−45 (n=62)
61−80 (n=19)
20−30 (n=24)
15−19 (n=2)
20−35 (n=2)
NA
Age_category

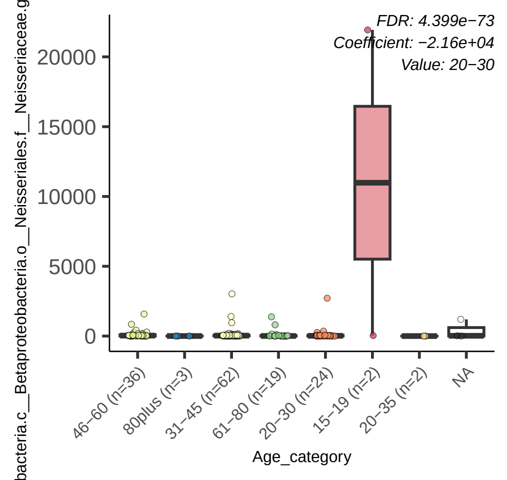

FDR: 4.399e−73
Coefficient: −2.16e+04
Value: 20−30
20000
15000
10000
5000
0
bacteria.c__Betaproteobacteria.o__Neisseriales.f__Neisseriaceae.g
46−60 (n=36)
80plus (n=3)
31−45 (n=62)
61−80 (n=19)
20−30 (n=24)
15−19 (n=2)
20−35 (n=2)
NA
Age_category

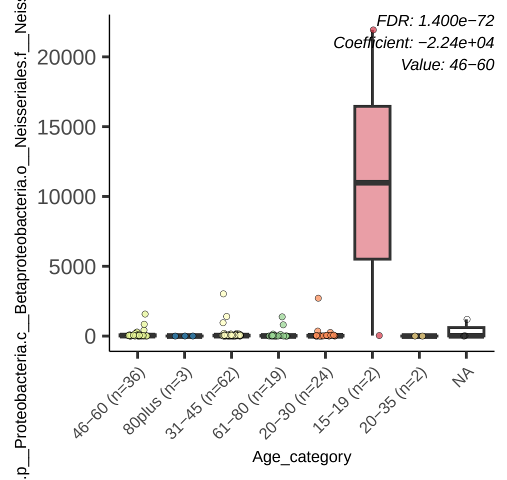

FDR: 1.400e−72
Coefficient: −2.24e+04
Value: 46−60
20000
15000
10000
5000
0
p__Proteobacteria.c__Betaproteobacteria.o__Neisseriales.f__Neiss
46−60 (n=36)
80plus (n=3)
31−45 (n=62)
61−80 (n=19)
20−30 (n=24)
15−19 (n=2)
20−35 (n=2)
NA
Age_category

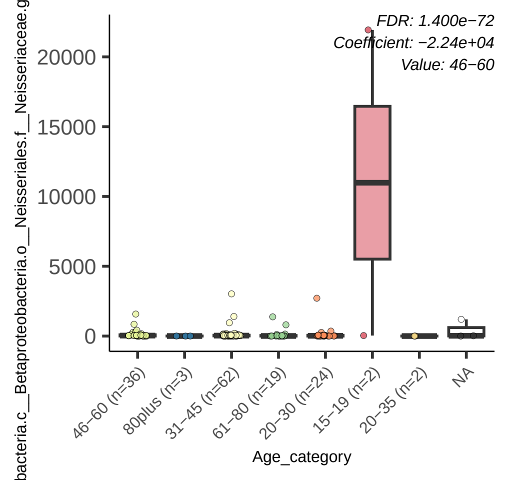
FDR: 1.400e−72
Coefficient: −2.24e+04
Value: 46−60
bacteria.c__Betaproteobacteria.o__Neisseriales.f__Neisseriaceae.g
20000
15000
10000
5000
0
46−60 (n=36)
80plus (n=3)
31−45 (n=62)
61−80 (n=19)
20−30 (n=24)
15−19 (n=2)
20−35 (n=2)
NA
Age_category

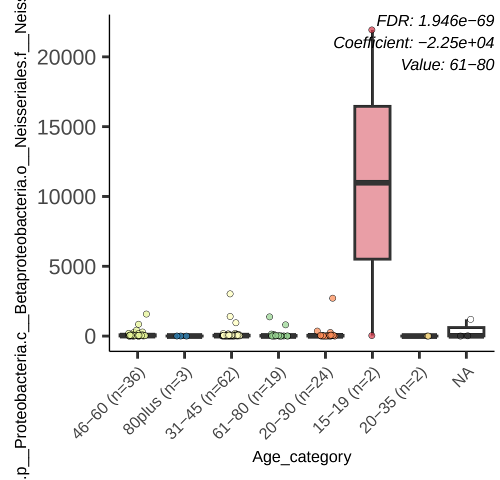

FDR: 1.946e−69
Coefficient: −2.25e+04
Value: 61−80
p__Proteobacteria.c__Betaproteobacteria.o__Neisseriales.f__Neiss
20000
15000
10000
5000
0
46−60 (n=36)
80plus (n=3)
31−45 (n=62)
61−80 (n=19)
20−30 (n=24)
15−19 (n=2)
20−35 (n=2)
NA
Age_category

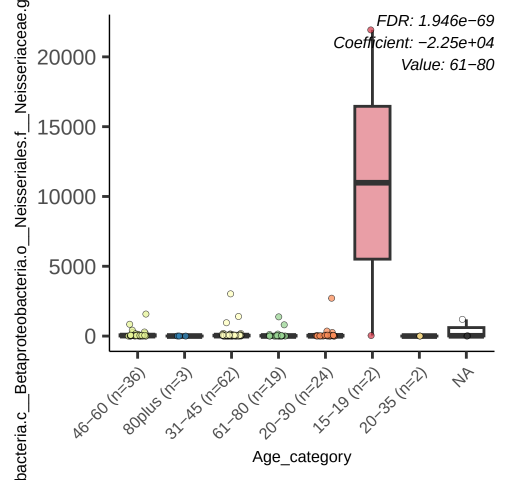

FDR: 1.946e−69
Coefficient: −2.25e+04
Value: 61−80
bacteria.c__Betaproteobacteria.o__Neisseriales.f__Neisseriaceae.g
20000
15000
10000
5000
0
46−60 (n=36)
80plus (n=3)
31−45 (n=62)
61−80 (n=19)
20−30 (n=24)
15−19 (n=2)
20−35 (n=2)
NA
Age_category

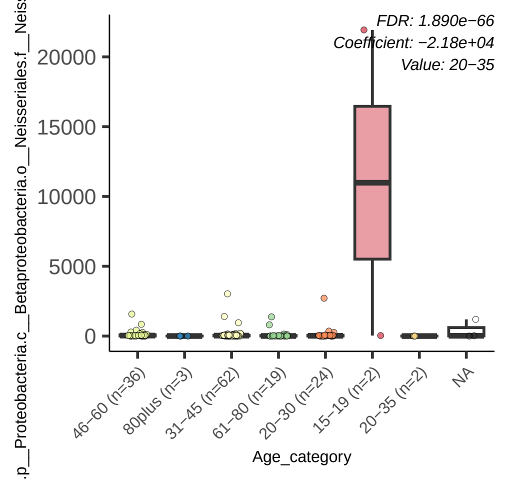
FDR: 1.890e−66
Coefficient: −2.18e+04
Value: 20−35
p__Proteobacteria.c__Betaproteobacteria.o__Neisseriales.f__Neiss
20000
15000
10000
5000
0
46−60 (n=36)
80plus (n=3)
31−45 (n=62)
61−80 (n=19)
20−30 (n=24)
15−19 (n=2)
20−35 (n=2)
NA
Age_category

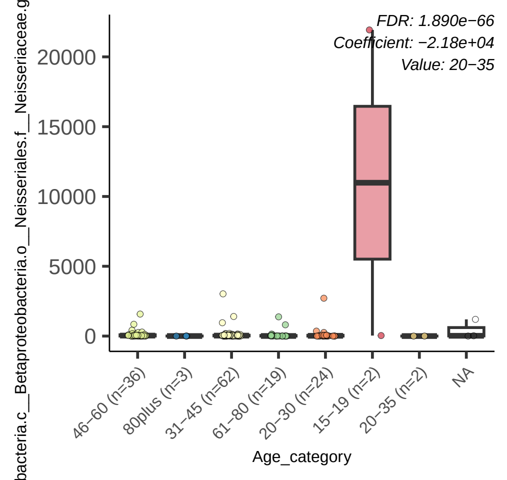

FDR: 1.890e−66
Coefficient: −2.18e+04
Value: 20−35
bacteria.c__Betaproteobacteria.o__Neisseriales.f__Neisseriaceae.g
20000
15000
10000
5000
0
46−60 (n=36)
80plus (n=3)
31−45 (n=62)
61−80 (n=19)
20−30 (n=24)
15−19 (n=2)
20−35 (n=2)
NA
Age_category

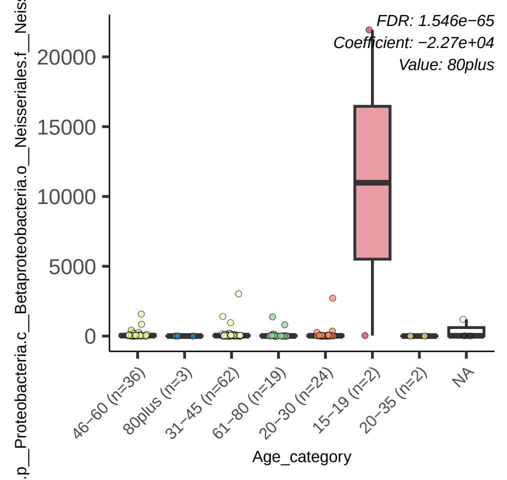

FDR: 1.546e−65
Coefficient: −2.27e+04
Value: 80plus
p__Proteobacteria.c__Betaproteobacteria.o__Neisseriales.f__Neiss
0
5000
10000
15000
20000
46−60 (n=36)
80plus (n=3)
31−45 (n=62)
61−80 (n=19)
20−30 (n=24)
15−19 (n=2)
20−35 (n=2)
NA
Age_category

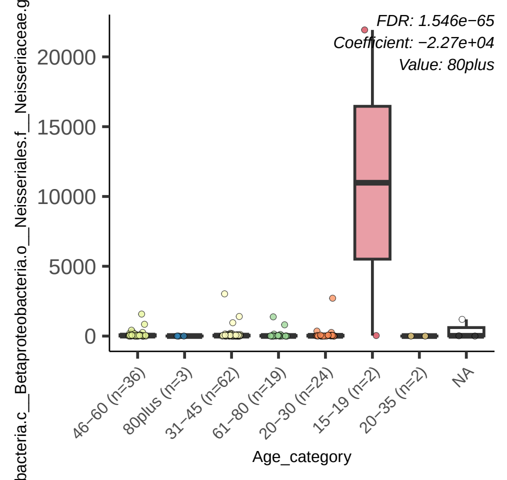
FDR: 1.546e−65
Coefficient: −2.27e+04
Value: 80plus
bacteria.c__Betaproteobacteria.o__Neisseriales.f__Neisseriaceae.g
20000
15000
10000
5000
0
46−60 (n=36)
80plus (n=3)
31−45 (n=62)
61−80 (n=19)
20−30 (n=24)
15−19 (n=2)
20−35 (n=2)
NA
Age_category

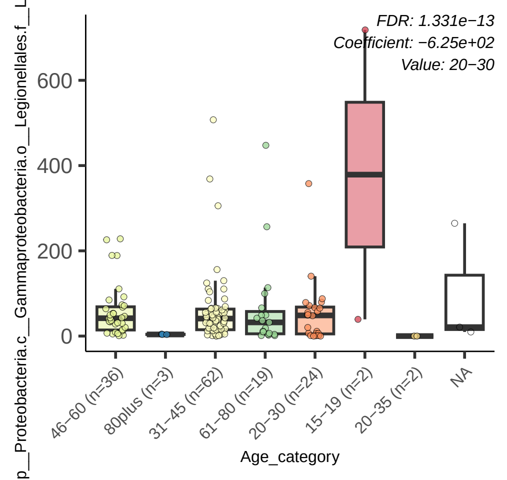
FDR: 1.331e−13
Coefficient: −6.25e+02
Value: 20−30
p__Proteobacteria.c__Gammaproteobacteria.o__Legionellales.f__L
600
400
200
0
46−60 (n=36)
80plus (n=3)
31−45 (n=62)
61−80 (n=19)
20−30 (n=24)
15−19 (n=2)
20−35 (n=2)
NA
Age_category

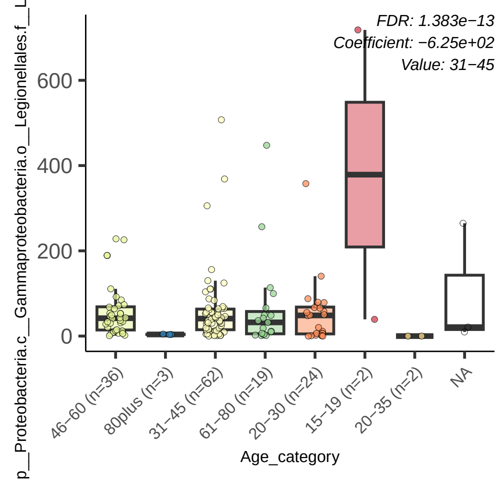

FDR: 1.383e−13
Coefficient: −6.25e+02
Value: 31−45
p__Proteobacteria.c__Gammaproteobacteria.o__Legionellales.f__L
600
400
200
0
46−60 (n=36)
80plus (n=3)
31−45 (n=62)
61−80 (n=19)
20−30 (n=24)
15−19 (n=2)
20−35 (n=2)
NA
Age_category

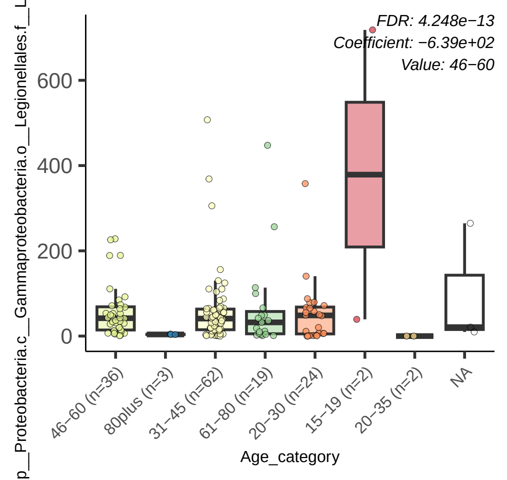

FDR: 4.248e−13
Coefficient: −6.39e+02
Value: 46−60
p__Proteobacteria.c__Gammaproteobacteria.o__Legionellales.f__L
0
200
400
600
46−60 (n=36)
80plus (n=3)
31−45 (n=62)
61−80 (n=19)
20−30 (n=24)
15−19 (n=2)
20−35 (n=2)
NA
Age_category

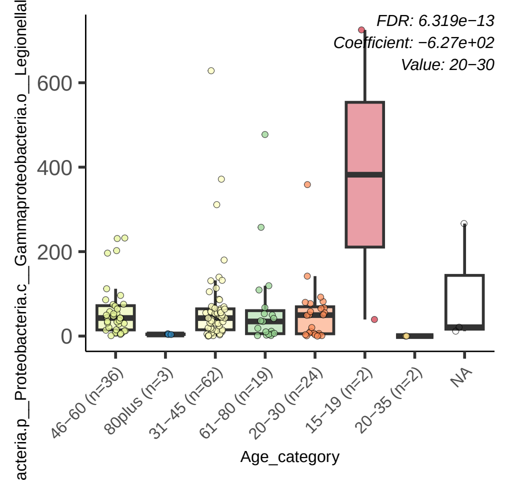
FDR: 6.319e−13
Coefficient: −6.27e+02
Value: 20−30
acteria.p__Proteobacteria.c__Gammaproteobacteria.o__Legionellal
600
400
200
0
46−60 (n=36)
80plus (n=3)
31−45 (n=62)
61−80 (n=19)
20−30 (n=24)
15−19 (n=2)
20−35 (n=2)
NA
Age_category

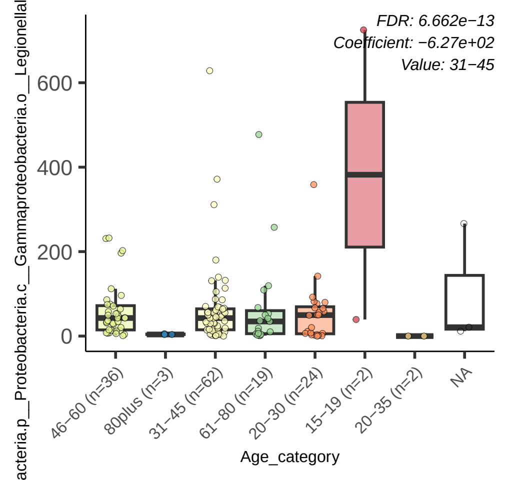

FDR: 6.662e−13
Coefficient: −6.27e+02
Value: 31−45
acteria.p__Proteobacteria.c__Gammaproteobacteria.o__Legionellal
0
200
400
600
46−60 (n=36)
80plus (n=3)
31−45 (n=62)
61−80 (n=19)
20−30 (n=24)
15−19 (n=2)
20−35 (n=2)
NA
Age_category

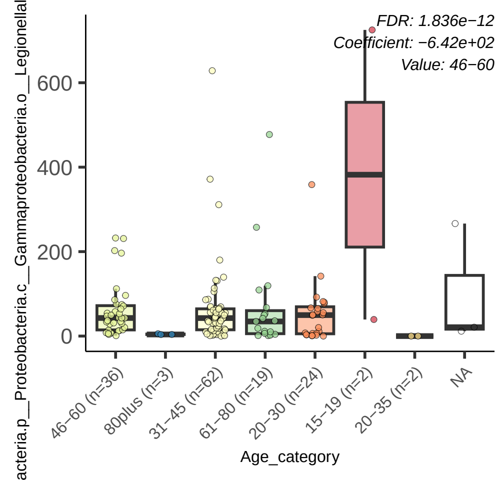

FDR: 1.836e−12
Coefficient: −6.42e+02
Value: 46−60
acteria.p__Proteobacteria.c__Gammaproteobacteria.o__Legionellal
0
200
400
600
46−60 (n=36)
80plus (n=3)
31−45 (n=62)
61−80 (n=19)
20−30 (n=24)
15−19 (n=2)
20−35 (n=2)
NA
Age_category

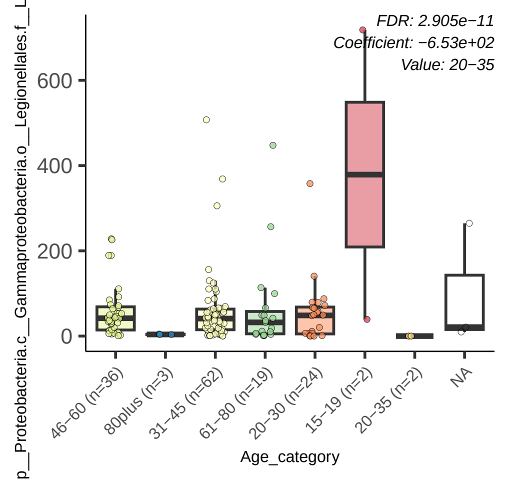

FDR: 2.905e−11
Coefficient: −6.53e+02
Value: 20−35
600
400
200
0
p__Proteobacteria.c__Gammaproteobacteria.o__Legionellales.f__L
46−60 (n=36)
80plus (n=3)
31−45 (n=62)
61−80 (n=19)
20−30 (n=24)
15−19 (n=2)
20−35 (n=2)
NA
Age_category

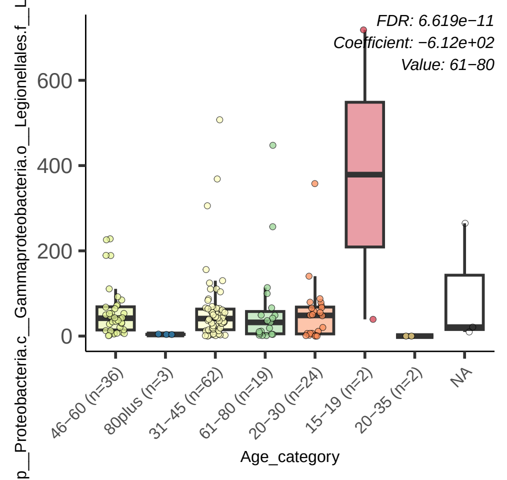

FDR: 6.619e−11
Coefficient: −6.12e+02
Value: 61−80
p__Proteobacteria.c__Gammaproteobacteria.o__Legionellales.f__L
600
400
200
0
46−60 (n=36)
80plus (n=3)
31−45 (n=62)
61−80 (n=19)
20−30 (n=24)
15−19 (n=2)
20−35 (n=2)
NA
Age_category

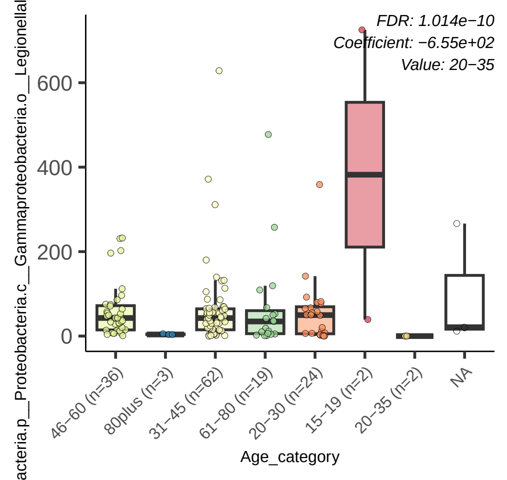

FDR: 1.014e−10
Coefficient: −6.55e+02
Value: 20−35
acteria.p__Proteobacteria.c__Gammaproteobacteria.o__Legionellal
0
200
400
600
46−60 (n=36)
80plus (n=3)
31−45 (n=62)
61−80 (n=19)
20−30 (n=24)
15−19 (n=2)
20−35 (n=2)
NA
Age_category

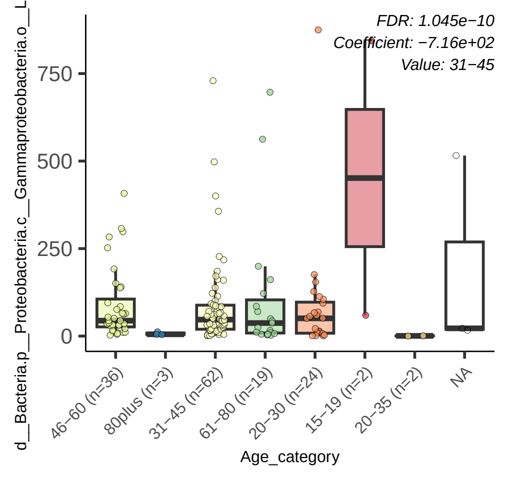

FDR: 1.045e−10
Coefficient: −7.16e+02
Value: 31−45
d__Bacteria.p__Proteobacteria.c__Gammaproteobacteria.o__L
750
500
250
0
46−60 (n=36)
80plus (n=3)
31−45 (n=62)
61−80 (n=19)
20−30 (n=24)
15−19 (n=2)
20−35 (n=2)
NA
Age_category

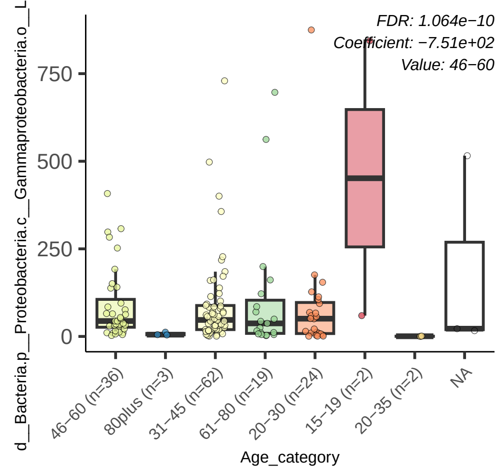
FDR: 1.064e−10
Coefficient: −7.51e+02
Value: 46−60
d__Bacteria.p__Proteobacteria.c__Gammaproteobacteria.o__L
750
500
250
0
46−60 (n=36)
80plus (n=3)
31−45 (n=62)
61−80 (n=19)
20−30 (n=24)
15−19 (n=2)
20−35 (n=2)
NA
Age_category

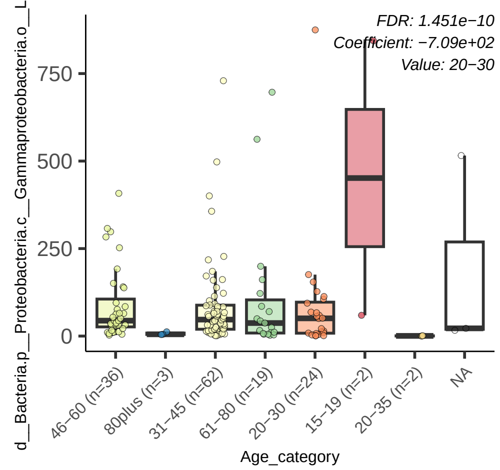

FDR: 1.451e−10
Coefficient: −7.09e+02
Value: 20−30
d__Bacteria.p__Proteobacteria.c__Gammaproteobacteria.o__L
750
500
250
0
46−60 (n=36)
80plus (n=3)
31−45 (n=62)
61−80 (n=19)
20−30 (n=24)
15−19 (n=2)
20−35 (n=2)
NA
Age_category

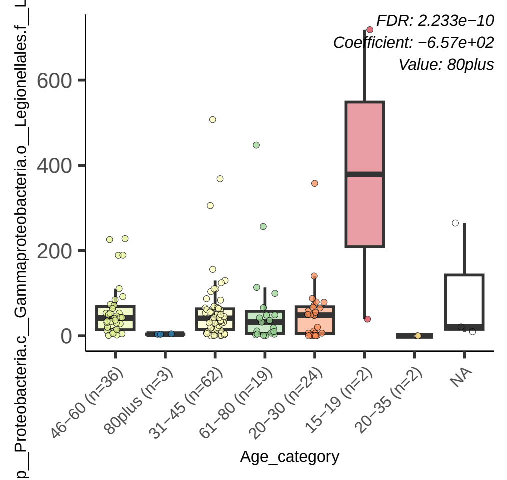

FDR: 2.233e−10
Coefficient: −6.57e+02
Value: 80plus
600
400
200
0
p__Proteobacteria.c__Gammaproteobacteria.o__Legionellales.f__L
46−60 (n=36)
80plus (n=3)
31−45 (n=62)
61−80 (n=19)
20−30 (n=24)
15−19 (n=2)
20−35 (n=2)
NA
Age_category

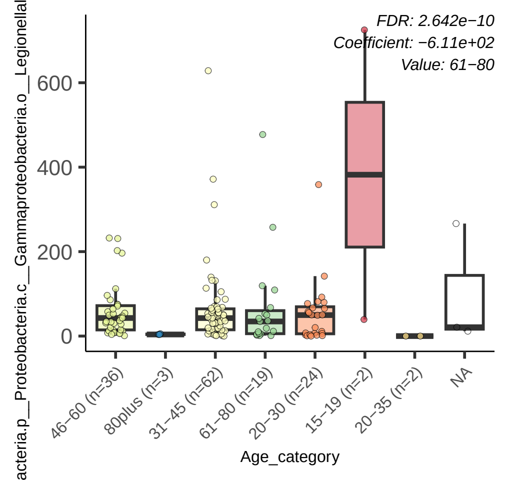
FDR: 2.642e−10
Coefficient: −6.11e+02
Value: 61−80
acteria.p__Proteobacteria.c__Gammaproteobacteria.o__Legionellal
600
400
200
0
46−60 (n=36)
80plus (n=3)
31−45 (n=62)
61−80 (n=19)
20−30 (n=24)
15−19 (n=2)
20−35 (n=2)
NA
Age_category

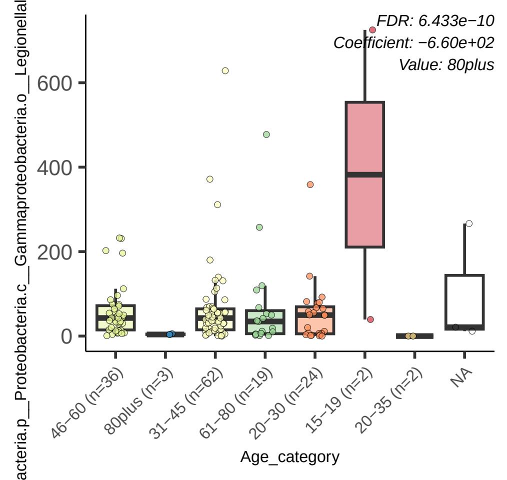
FDR: 6.433e−10
Coefficient: −6.60e+02
Value: 80plus
acteria.p__Proteobacteria.c__Gammaproteobacteria.o__Legionellal
0
200
400
600
46−60 (n=36)
80plus (n=3)
31−45 (n=62)
61−80 (n=19)
20−30 (n=24)
15−19 (n=2)
20−35 (n=2)
NA
Age_category

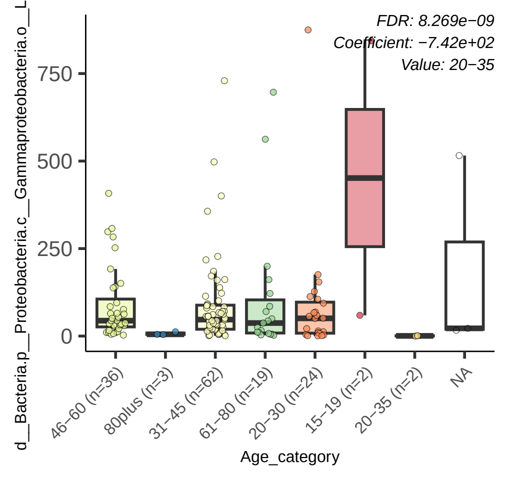

FDR: 8.269e−09
Coefficient: −7.42e+02
Value: 20−35
d__Bacteria.p__Proteobacteria.c__Gammaproteobacteria.o__L
750
500
250
0
46−60 (n=36)
80plus (n=3)
31−45 (n=62)
61−80 (n=19)
20−30 (n=24)
15−19 (n=2)
20−35 (n=2)
NA
Age_category

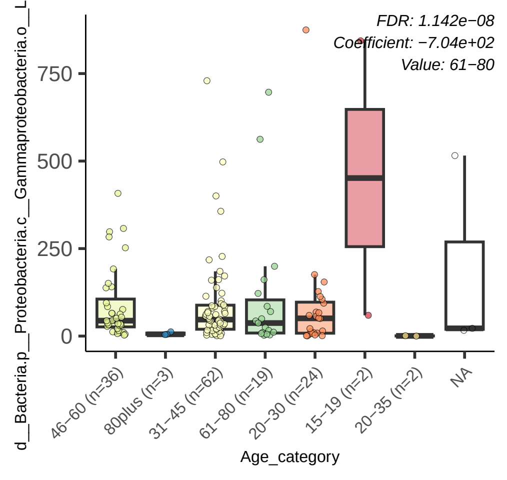

FDR: 1.142e−08
Coefficient: −7.04e+02
Value: 61−80
d__Bacteria.p__Proteobacteria.c__Gammaproteobacteria.o__L
750
500
250
0
46−60 (n=36)
80plus (n=3)
31−45 (n=62)
61−80 (n=19)
20−30 (n=24)
15−19 (n=2)
20−35 (n=2)
NA
Age_category

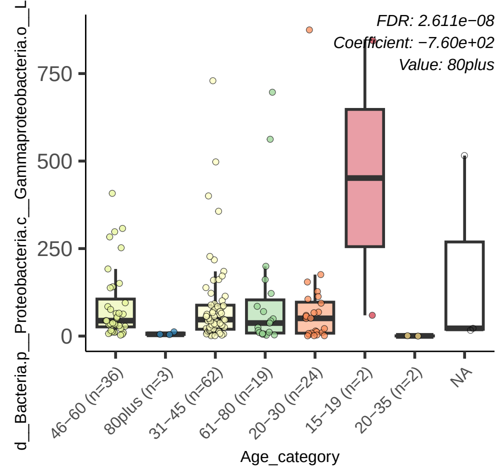

FDR: 2.611e−08
Coefficient: −7.60e+02
Value: 80plus
d__Bacteria.p__Proteobacteria.c__Gammaproteobacteria.o__L
750
500
250
0
46−60 (n=36)
80plus (n=3)
31−45 (n=62)
61−80 (n=19)
20−30 (n=24)
15−19 (n=2)
20−35 (n=2)
NA
Age_category

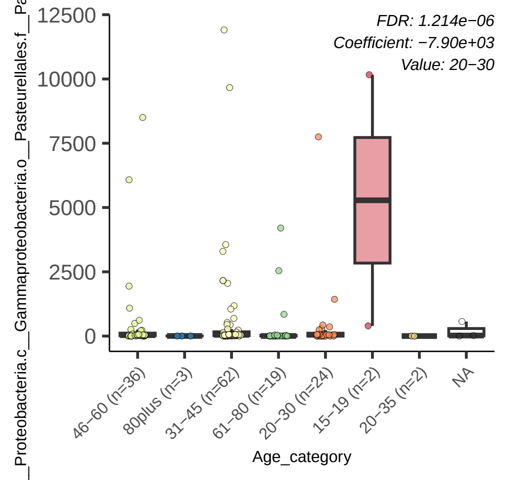

FDR: 1.214e−06
Coefficient: −7.90e+03
Value: 20−30
12500
10000
7500
5000
2500
0
_Proteobacteria.c__Gammaproteobacteria.o__Pasteurellales.f__Pa
46−60 (n=36)
80plus (n=3)
31−45 (n=62)
61−80 (n=19)
20−30 (n=24)
15−19 (n=2)
20−35 (n=2)
NA
Age_category

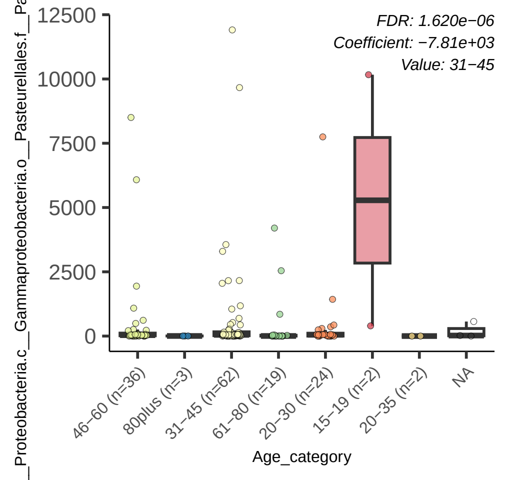
FDR: 1.620e−06
Coefficient: −7.81e+03
Value: 31−45
Proteobacteria.c__Gammaproteobacteria.o__Pasteurellales.f__Pa
12500
10000
7500
5000
2500
0
46−60 (n=36)
80plus (n=3)
31−45 (n=62)
61−80 (n=19)
20−30 (n=24)
15−19 (n=2)
20−35 (n=2)
NA
Age_category

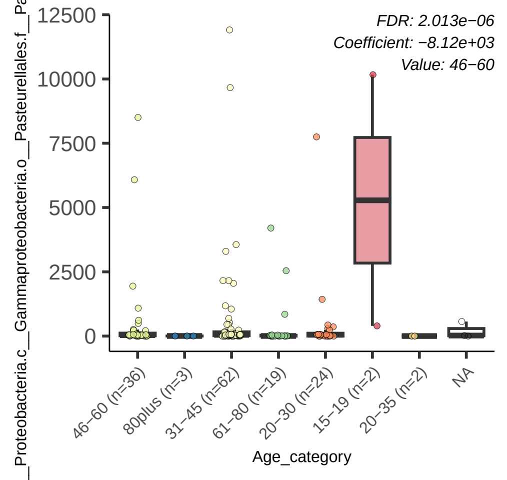

FDR: 2.013e−06
Coefficient: −8.12e+03
Value: 46−60
Proteobacteria.c__Gammaproteobacteria.o__Pasteurellales.f__Pa
12500
10000
7500
5000
2500
0
46−60 (n=36)
80plus (n=3)
31−45 (n=62)
61−80 (n=19)
20−30 (n=24)
15−19 (n=2)
20−35 (n=2)
NA
Age_category

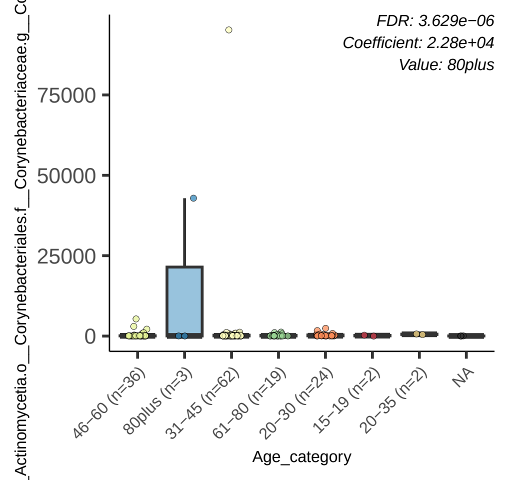
FDR: 3.629e−06
Coefficient: 2.28e+04
Value: 80plus
Actinomycetia.o__Corynebacteriales.f__Corynebacteriaceae.g__Co
75000
50000
25000
0
46−60 (n=36)
80plus (n=3)
31−45 (n=62)
61−80 (n=19)
20−30 (n=24)
15−19 (n=2)
20−35 (n=2)
NA
Age_category

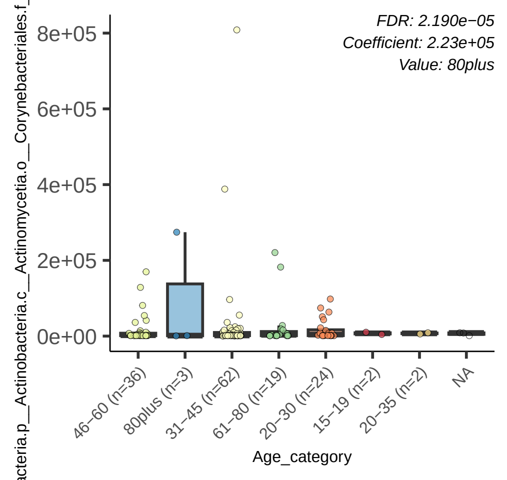

FDR: 2.190e−05
Coefficient: 2.23e+05
Value: 80plus
8e+05
6e+05
4e+05
2e+05
0e+00
46−60 (n=36)
80plus (n=3)
31−45 (n=62)
61−80 (n=19)
20−30 (n=24)
15−19 (n=2)
20−35 (n=2)
NA
Age_category
acteria.p__Actinobacteria.c__Actinomycetia.o__Corynebacteriales.f_

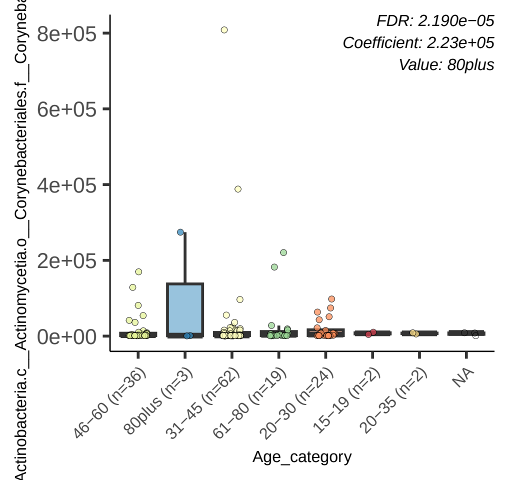
FDR: 2.190e−05
Coefficient: 2.23e+05
Value: 80plus
Actinobacteria.c__Actinomycetia.o__Corynebacteriales.f__Coryneba
8e+05
6e+05
4e+05
2e+05
0e+00
46−60 (n=36)
80plus (n=3)
31−45 (n=62)
61−80 (n=19)
20−30 (n=24)
15−19 (n=2)
20−35 (n=2)
NA
Age_category

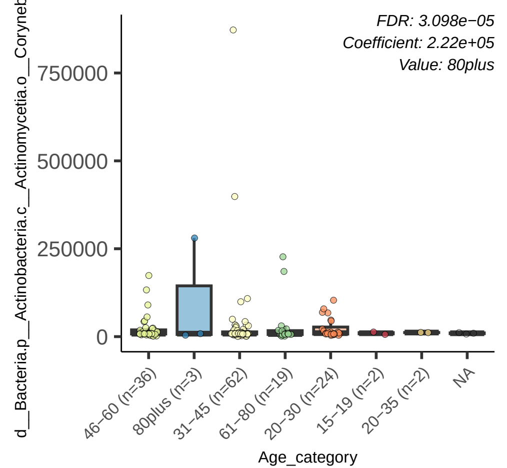

FDR: 3.098e−05
Coefficient: 2.22e+05
Value: 80plus
d__Bacteria.p__Actinobacteria.c__Actinomycetia.o__Coryneb
750000
500000
250000
0
46−60 (n=36)
80plus (n=3)
31−45 (n=62)
61−80 (n=19)
20−30 (n=24)
15−19 (n=2)
20−35 (n=2)
NA
Age_category

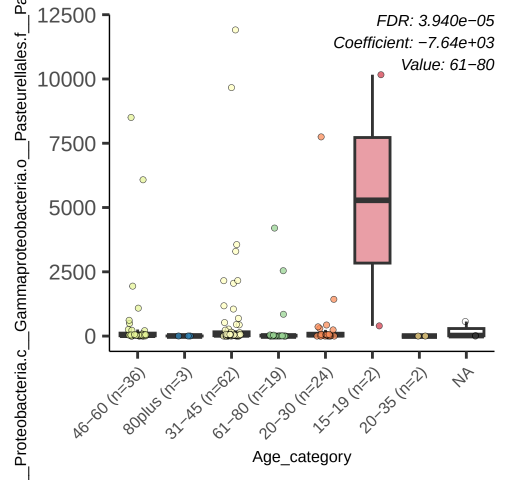

FDR: 3.940e−05
Coefficient: −7.64e+03
Value: 61−80
12500
10000
7500
5000
2500
0
46−60 (n=36)
80plus (n=3)
31−45 (n=62)
61−80 (n=19)
20−30 (n=24)
15−19 (n=2)
20−35 (n=2)
NA
Age_category
Proteobacteria.c__Gammaproteobacteria.o__Pasteurellales.f__Pa

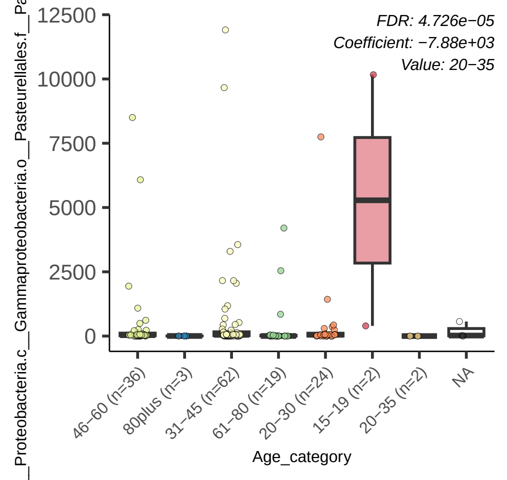

FDR: 4.726e−05
Coefficient: −7.88e+03
Value: 20−35
_Proteobacteria.c__Gammaproteobacteria.o__Pasteurellales.f__Pa
12500
10000
7500
5000
2500
0
46−60 (n=36)
80plus (n=3)
31−45 (n=62)
61−80 (n=19)
20−30 (n=24)
15−19 (n=2)
20−35 (n=2)
NA
Age_category

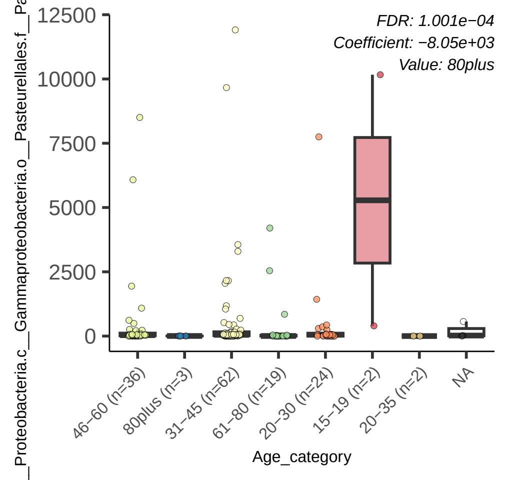

FDR: 1.001e−04
Coefficient: −8.05e+03
Value: 80plus
__Proteobacteria.c__Gammaproteobacteria.o__Pasteurellales.f__Pa
12500
10000
7500
5000
2500
0
46−60 (n=36)
80plus (n=3)
31−45 (n=62)
61−80 (n=19)
20−30 (n=24)
15−19 (n=2)
20−35 (n=2)
NA
Age_category

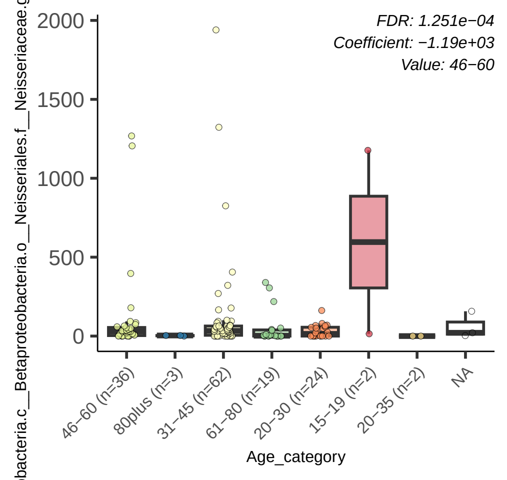

FDR: 1.251e−04
Coefficient: −1.19e+03
Value: 46−60
2000
1500
1000
500
0
obacteria.c__Betaproteobacteria.o__Neisseriales.f__Neisseriaceae.g
46−60 (n=36)
80plus (n=3)
31−45 (n=62)
61−80 (n=19)
20−30 (n=24)
15−19 (n=2)
20−35 (n=2)
NA
Age_category

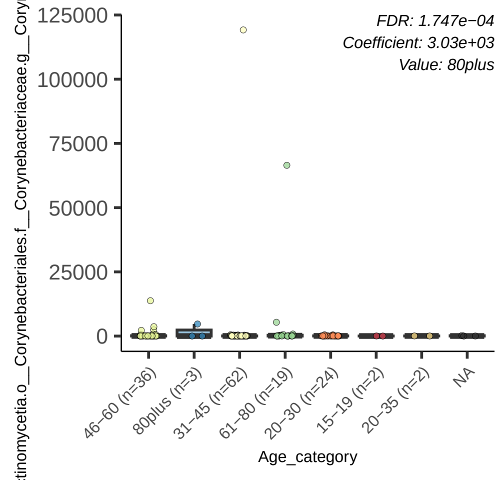
FDR: 1.747e−04
Coefficient: 3.03e+03
Value: 80plus
125000
100000
75000
50000
25000
0
46−60 (n=36)
80plus (n=3)
31−45 (n=62)
61−80 (n=19)
20−30 (n=24)
15−19 (n=2)
20−35 (n=2)
NA
Age_category

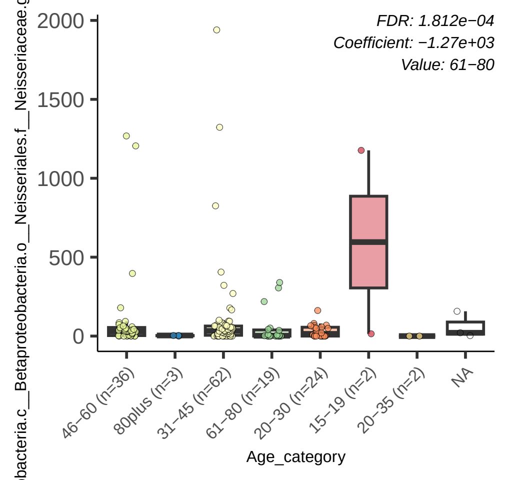

FDR: 1.812e−04
Coefficient: −1.27e+03
Value: 61−80
2000
1500
1000
500
0
obacteria.c__Betaproteobacteria.o__Neisseriales.f__Neisseriaceae.g
46−60 (n=36)
80plus (n=3)
31−45 (n=62)
61−80 (n=19)
20−30 (n=24)
15−19 (n=2)
20−35 (n=2)
NA
Age_category

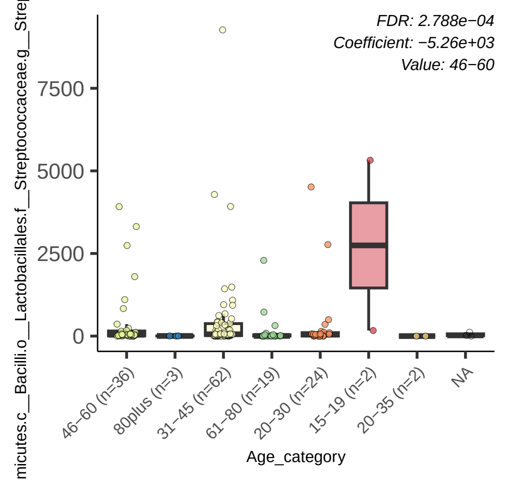

FDR: 2.788e−04
Coefficient: −5.26e+03
Value: 46−60
7500
5000
2500
0
micutes.c__Bacilli.o__Lactobacillales.f__Streptococcaceae.g__Strep
46−60 (n=36)
80plus (n=3)
31−45 (n=62)
61−80 (n=19)
20−30 (n=24)
15−19 (n=2)
20−35 (n=2)
NA
Age_category

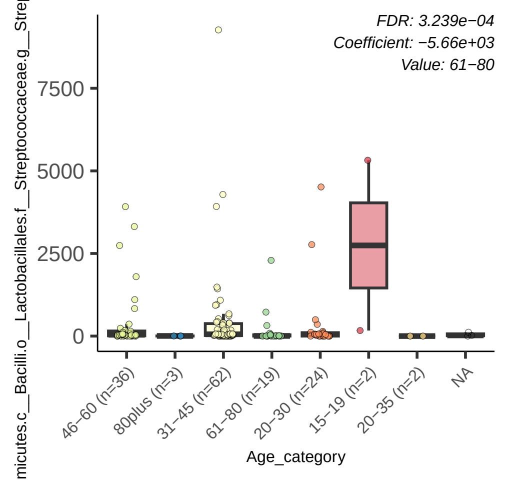
FDR: 3.239e−04
Coefficient: −5.66e+03
Value: 61−80
7500
5000
2500
0
46−60 (n=36)
80plus (n=3)
31−45 (n=62)
61−80 (n=19)
20−30 (n=24)
15−19 (n=2)
20−35 (n=2)
NA
Age_category
micutes.c__Bacilli.o__Lactobacillales.f__Streptococcaceae.g__Strep

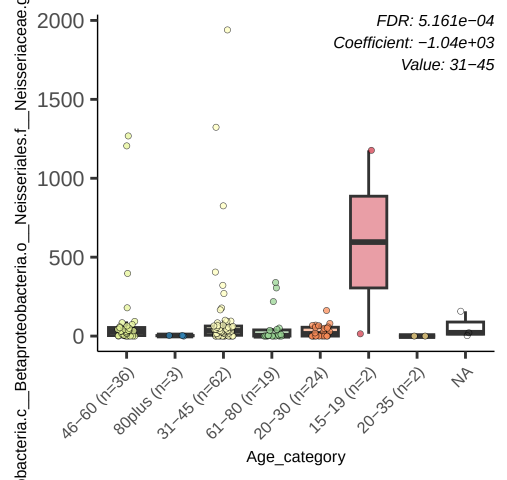
FDR: 5.161e−04
Coefficient: −1.04e+03
Value: 31−45
obacteria.c__Betaproteobacteria.o__Neisseriales.f__Neisseriaceae.g
2000
1500
1000
500
0
46−60 (n=36)
80plus (n=3)
31−45 (n=62)
61−80 (n=19)
20−30 (n=24)
15−19 (n=2)
20−35 (n=2)
NA
Age_category

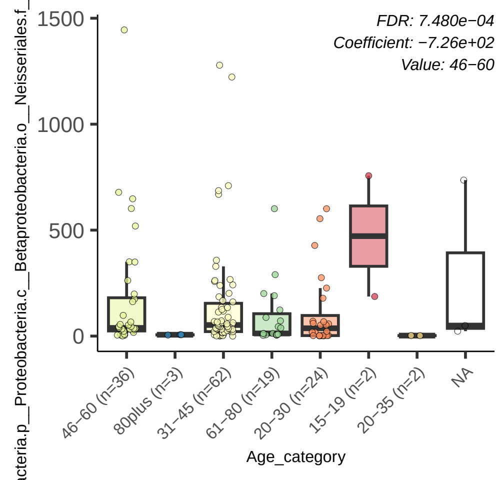

FDR: 7.480e−04
Coefficient: −7.26e+02
Value: 46−60
acteria.p__Proteobacteria.c__Betaproteobacteria.o__Neisseriales.f
1500
1000
500
0
46−60 (n=36)
80plus (n=3)
31−45 (n=62)
61−80 (n=19)
20−30 (n=24)
15−19 (n=2)
20−35 (n=2)
NA
Age_category

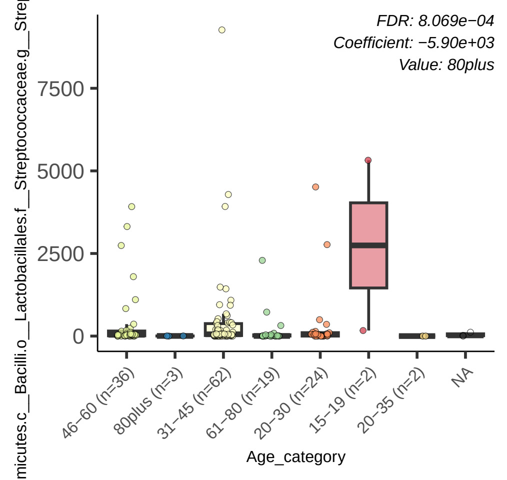

FDR: 8.069e−04
Coefficient: −5.90e+03
Value: 80plus
micutes.c__Bacilli.o__Lactobacillales.f__Streptococcaceae.g__Strep
7500
5000
2500
0
46−60 (n=36)
80plus (n=3)
31−45 (n=62)
61−80 (n=19)
20−30 (n=24)
15−19 (n=2)
20−35 (n=2)
NA
Age_category

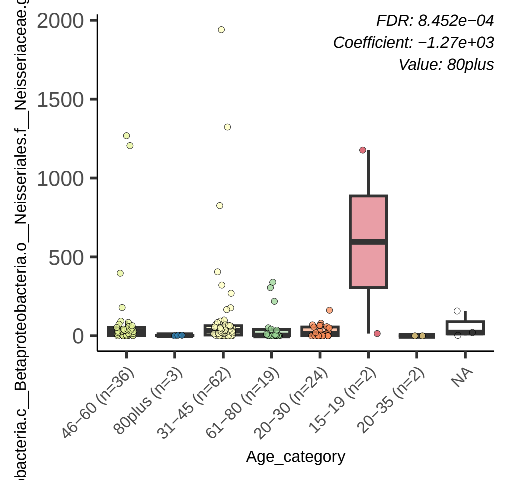
FDR: 8.452e−04
Coefficient: −1.27e+03
Value: 80plus
2000
1500
1000
500
0
46−60 (n=36)
80plus (n=3)
31−45 (n=62)
61−80 (n=19)
20−30 (n=24)
15−19 (n=2)
20−35 (n=2)
NA
Age_category

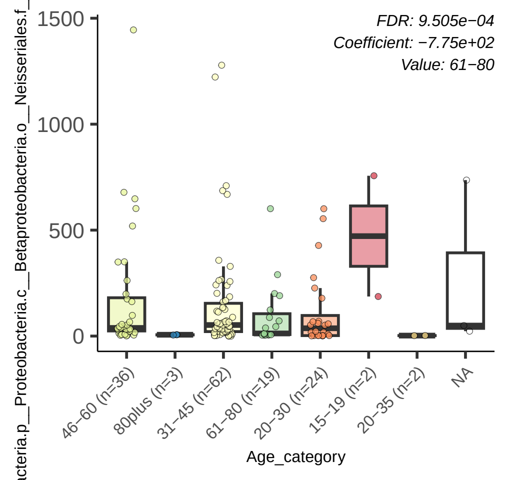

FDR: 9.505e−04
Coefficient: −7.75e+02
Value: 61−80
1500
1000
500
0
acteria.p__Proteobacteria.c__Betaproteobacteria.o__Neisseriales.f
46−60 (n=36)
80plus (n=3)
31−45 (n=62)
61−80 (n=19)
20−30 (n=24)
15−19 (n=2)
20−35 (n=2)
NA
Age_category

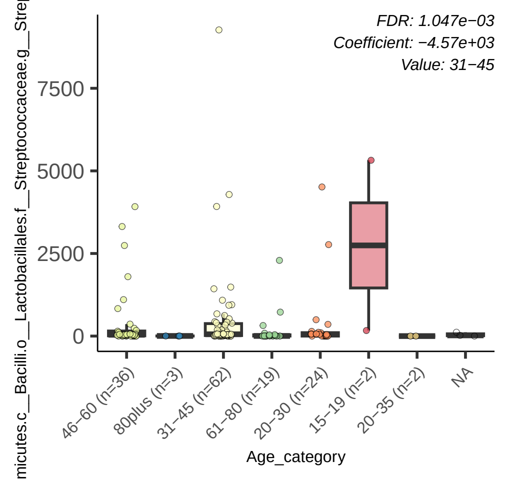
FDR: 1.047e−03
Coefficient: −4.57e+03
Value: 31−45
micutes.c__Bacilli.o__Lactobacillales.f__Streptococcaceae.g__Strep
7500
5000
2500
0
46−60 (n=36)
80plus (n=3)
31−45 (n=62)
61−80 (n=19)
20−30 (n=24)
15−19 (n=2)
20−35 (n=2)
NA
Age_category

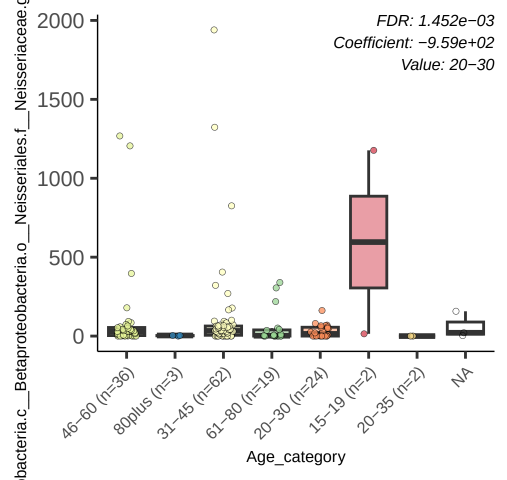

FDR: 1.452e−03
Coefficient: −9.59e+02
Value: 20−30
2000
1500
1000
500
0
obacteria.c__Betaproteobacteria.o__Neisseriales.f__Neisseriaceae.g
46−60 (n=36)
80plus (n=3)
31−45 (n=62)
61−80 (n=19)
20−30 (n=24)
15−19 (n=2)
20−35 (n=2)
NA
Age_category

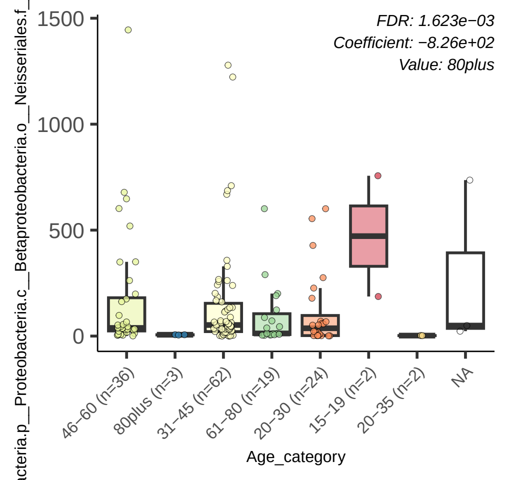

FDR: 1.623e−03
Coefficient: −8.26e+02
Value: 80plus
acteria.p__Proteobacteria.c__Betaproteobacteria.o__Neisseriales.f
1500
1000
500
0
46−60 (n=36)
80plus (n=3)
31−45 (n=62)
61−80 (n=19)
20−30 (n=24)
15−19 (n=2)
20−35 (n=2)
NA
Age_category

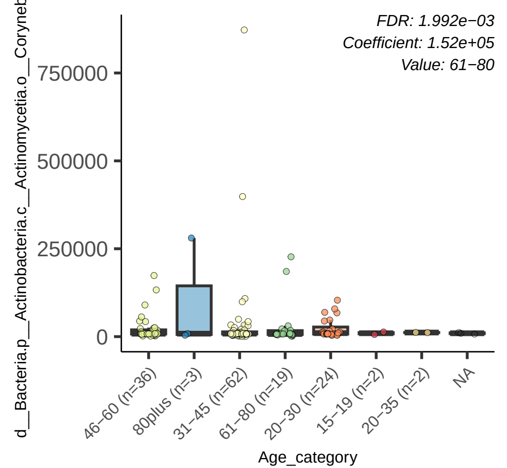
FDR: 1.992e−03
Coefficient: 1.52e+05
Value: 61−80
d__Bacteria.p__Actinobacteria.c__Actinomycetia.o__Coryneb
750000
500000
250000
0
46−60 (n=36)
80plus (n=3)
31−45 (n=62)
61−80 (n=19)
20−30 (n=24)
15−19 (n=2)
20−35 (n=2)
NA
Age_category

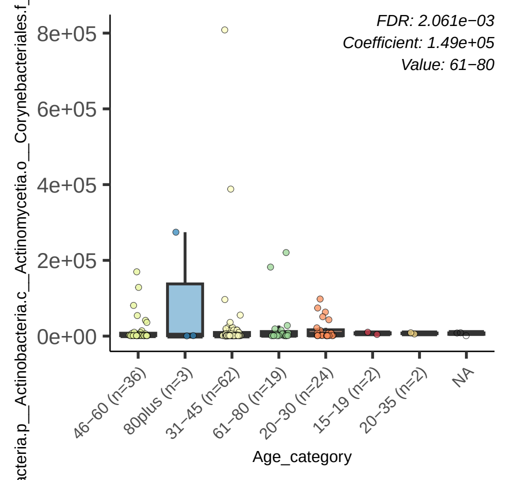

FDR: 2.061e−03
Coefficient: 1.49e+05
Value: 61−80
8e+05
6e+05
4e+05
2e+05
0e+00
acteria.p__Actinobacteria.c__Actinomycetia.o__Corynebacteriales.f_
46−60 (n=36)
80plus (n=3)
31−45 (n=62)
61−80 (n=19)
20−30 (n=24)
15−19 (n=2)
20−35 (n=2)
NA
Age_category

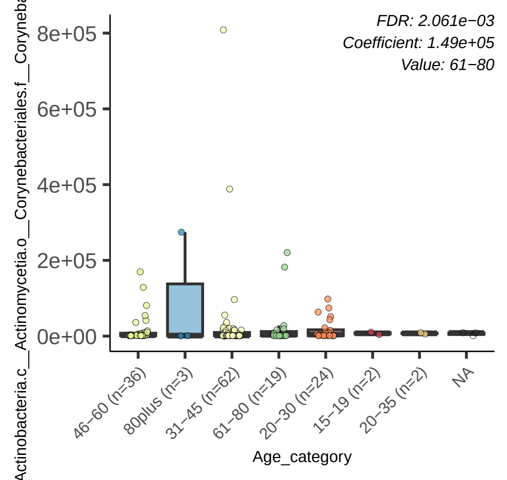
FDR: 2.061e−03
Coefficient: 1.49e+05
Value: 61−80
Actinobacteria.c__Actinomycetia.o__Corynebacteriales.f__Coryneba
8e+05
6e+05
4e+05
2e+05
0e+00
46−60 (n=36)
80plus (n=3)
31−45 (n=62)
61−80 (n=19)
20−30 (n=24)
15−19 (n=2)
20−35 (n=2)
NA
Age_category

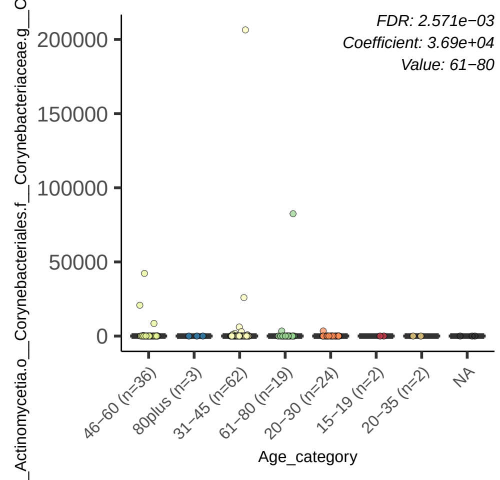

FDR: 2.571e−03
Coefficient: 3.69e+04
Value: 61−80
Actinomycetia.o__Corynebacteriales.f__Corynebacteriaceae.g__C
200000
150000
100000
50000
0
46−60 (n=36)
80plus (n=3)
31−45 (n=62)
61−80 (n=19)
20−30 (n=24)
15−19 (n=2)
20−35 (n=2)
NA
Age_category

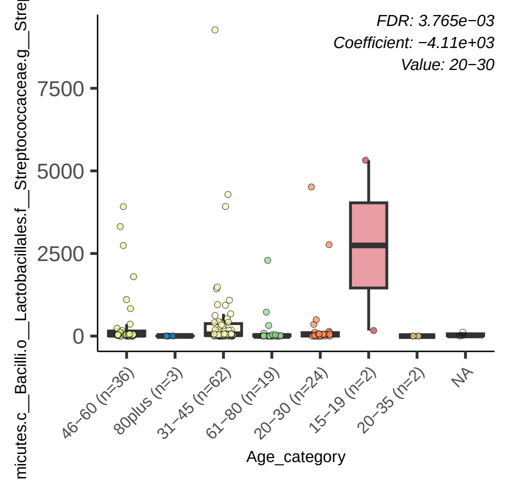
FDR: 3.765e−03
Coefficient: −4.11e+03
Value: 20−30
7500
5000
2500
0
micutes.c__Bacilli.o__Lactobacillales.f__Streptococcaceae.g__Strep
46−60 (n=36)
80plus (n=3)
31−45 (n=62)
61−80 (n=19)
20−30 (n=24)
15−19 (n=2)
20−35 (n=2)
NA
Age_category

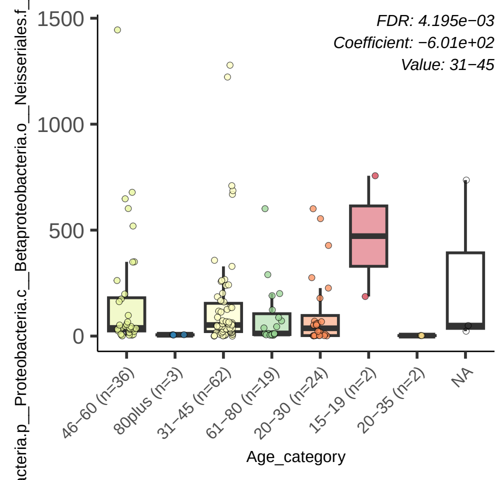

FDR: 4.195e−03
Coefficient: −6.01e+02
Value: 31−45
1500
1000
500
0
acteria.p__Proteobacteria.c__Betaproteobacteria.o__Neisseriales.f_
46−60 (n=36)
80plus (n=3)
31−45 (n=62)
61−80 (n=19)
20−30 (n=24)
15−19 (n=2)
20−35 (n=2)
NA
Age_category

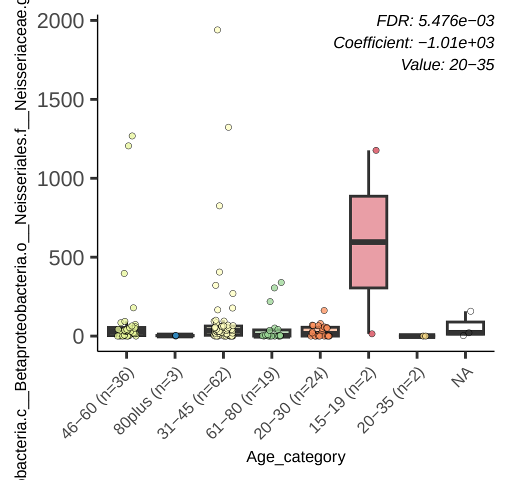

FDR: 5.476e−03
Coefficient: −1.01e+03
Value: 20−35
2000
1500
1000
500
0
46−60 (n=36)
80plus (n=3)
31−45 (n=62)
61−80 (n=19)
20−30 (n=24)
15−19 (n=2)
20−35 (n=2)
NA
Age_category

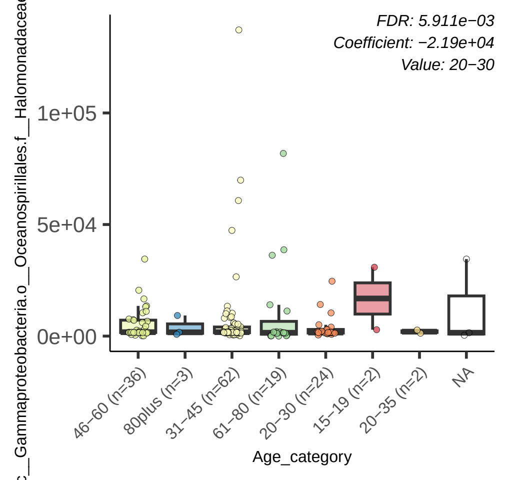

FDR: 5.911e−03
Coefficient: −2.19e+04
Value: 20−30
c__Gammaproteobacteria.o__Oceanospirillales.f__Halomonadaceae
1e+05
5e+04
0e+00
46−60 (n=36)
80plus (n=3)
31−45 (n=62)
61−80 (n=19)
20−30 (n=24)
15−19 (n=2)
20−35 (n=2)
NA
Age_category

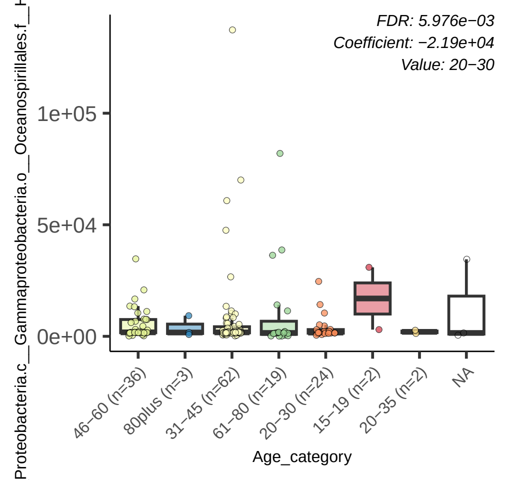
FDR: 5.976e−03
Coefficient: −2.19e+04
Value: 20−30
Proteobacteria.c__Gammaproteobacteria.o__Oceanospirillales.f__H
1e+05
5e+04
0e+00
46−60 (n=36)
80plus (n=3)
31−45 (n=62)
61−80 (n=19)
20−30 (n=24)
15−19 (n=2)
20−35 (n=2)
NA
Age_category

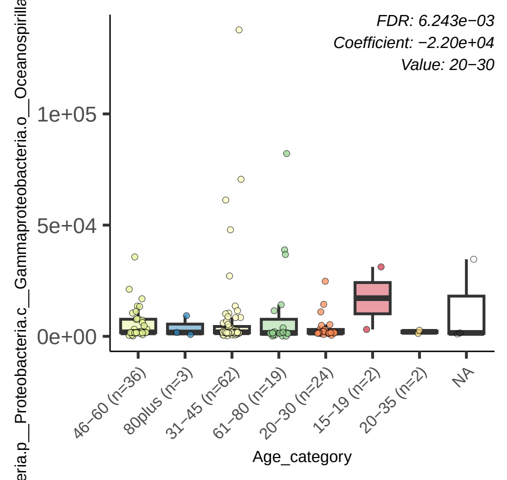
FDR: 6.243e−03
Coefficient: −2.20e+04
Value: 20−30
1e+05
5e+04
0e+00
ria.p__Proteobacteria.c__Gammaproteobacteria.o__Oceanospirilla
46−60 (n=36)
80plus (n=3)
31−45 (n=62)
61−80 (n=19)
20−30 (n=24)
15−19 (n=2)
20−35 (n=2)
NA
Age_category

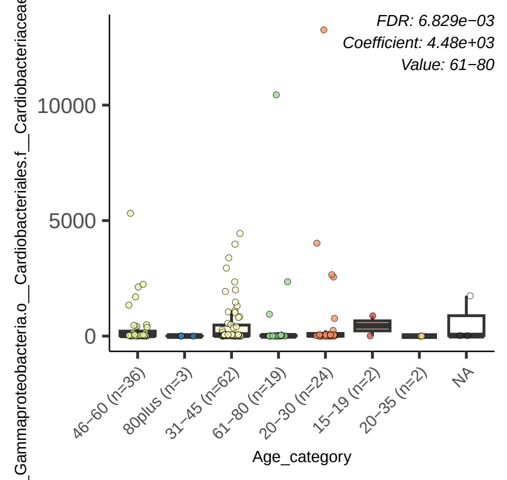

FDR: 6.829e−03
Coefficient: 4.48e+03
Value: 61−80
10000
5000
0
_Gammaproteobacteria.o__Cardiobacteriales.f__Cardiobacteriaceae
46−60 (n=36)
80plus (n=3)
31−45 (n=62)
61−80 (n=19)
20−30 (n=24)
15−19 (n=2)
20−35 (n=2)
NA
Age_category

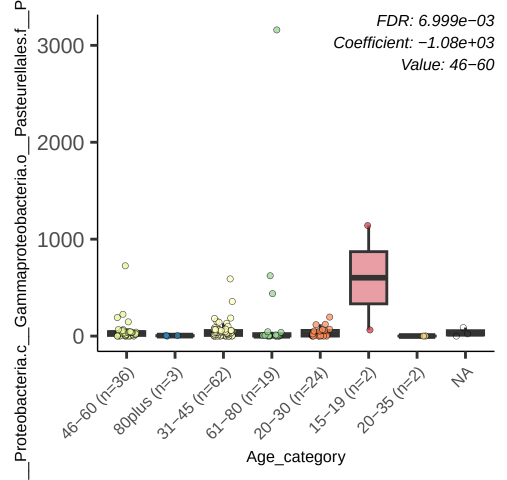

FDR: 6.999e−03
Coefficient: −1.08e+03
Value: 46−60
3000
2000
1000
0
_Proteobacteria.c__Gammaproteobacteria.o__Pasteurellales.f__P
46−60 (n=36)
80plus (n=3)
31−45 (n=62)
61−80 (n=19)
20−30 (n=24)
15−19 (n=2)
20−35 (n=2)
NA
Age_category

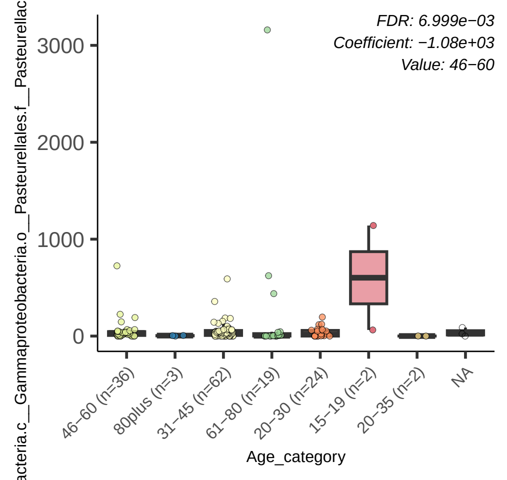

FDR: 6.999e−03
Coefficient: −1.08e+03
Value: 46−60
3000
2000
1000
0
acteria.c__Gammaproteobacteria.o__Pasteurellales.f__Pasteurellac
46−60 (n=36)
80plus (n=3)
31−45 (n=62)
61−80 (n=19)
20−30 (n=24)
15−19 (n=2)
20−35 (n=2)
NA
Age_category

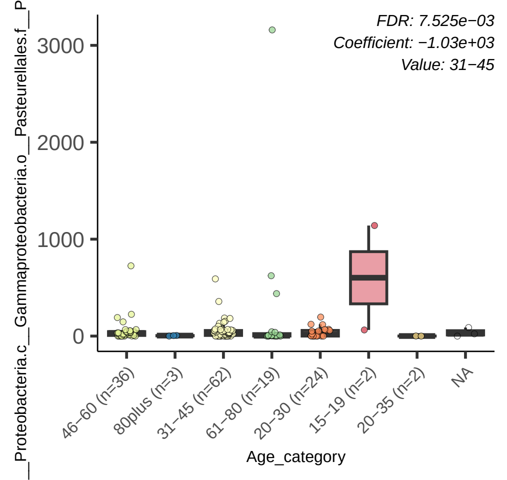
FDR: 7.525e−03
Coefficient: −1.03e+03
Value: 31−45
3000
2000
1000
0
Proteobacteria.c__Gammaproteobacteria.o__Pasteurellales.f__P
46−60 (n=36)
80plus (n=3)
31−45 (n=62)
61−80 (n=19)
20−30 (n=24)
15−19 (n=2)
20−35 (n=2)
NA
Age_category

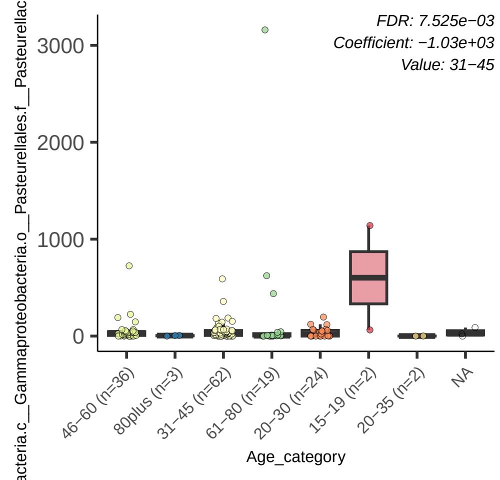

FDR: 7.525e−03
Coefficient: −1.03e+03
Value: 31−45
acteria.c__Gammaproteobacteria.o__Pasteurellales.f__Pasteurellac
3000
2000
1000
0
46−60 (n=36)
80plus (n=3)
31−45 (n=62)
61−80 (n=19)
20−30 (n=24)
15−19 (n=2)
20−35 (n=2)
NA
Age_category

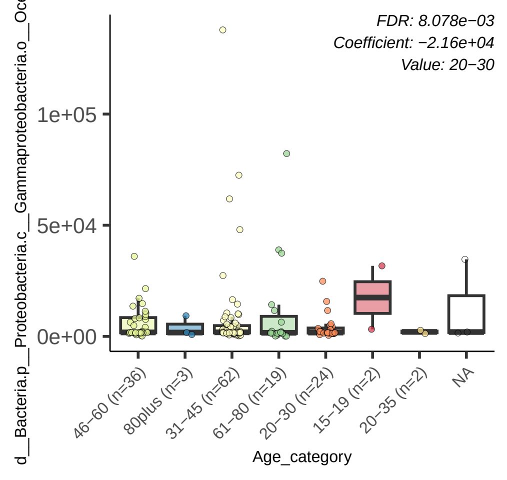
FDR: 8.078e−03
Coefficient: −2.16e+04
Value: 20−30
d__Bacteria.p__Proteobacteria.c__Gammaproteobacteria.o__Oce
1e+05
5e+04
0e+00
46−60 (n=36)
80plus (n=3)
31−45 (n=62)
61−80 (n=19)
20−30 (n=24)
15−19 (n=2)
20−35 (n=2)
NA
Age_category

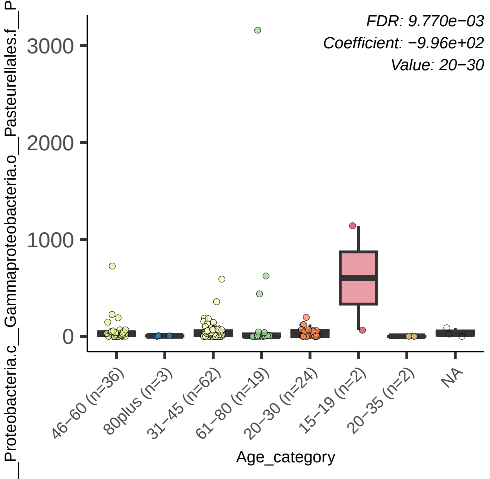
FDR: 9.770e−03
Coefficient: −9.96e+02
Value: 20−30
3000
2000
1000
0
_Proteobacteria.c__Gammaproteobacteria.o__Pasteurellales.f__P
46−60 (n=36)
80plus (n=3)
31−45 (n=62)
61−80 (n=19)
20−30 (n=24)
15−19 (n=2)
20−35 (n=2)
NA
Age_category

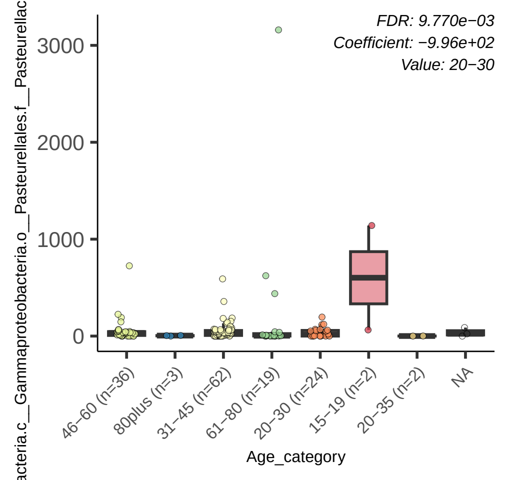

FDR: 9.770e−03
Coefficient: −9.96e+02
Value: 20−30
3000
2000
1000
0
46−60 (n=36)
80plus (n=3)
31−45 (n=62)
61−80 (n=19)
20−30 (n=24)
15−19 (n=2)
20−35 (n=2)
NA
Age_category
acteria.c__Gammaproteobacteria.o__Pasteurellales.f__Pasteurellac

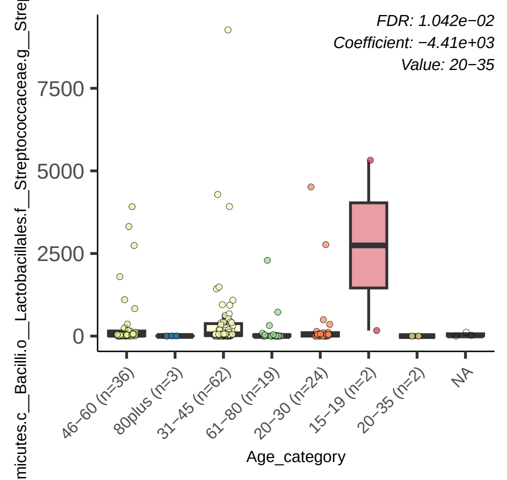

FDR: 1.042e−02
Coefficient: −4.41e+03
Value: 20−35
7500
5000
2500
0
micutes.c__Bacilli.o__Lactobacillales.f__Streptococcaceae.g__Strep
46−60 (n=36)
80plus (n=3)
31−45 (n=62)
61−80 (n=19)
20−30 (n=24)
15−19 (n=2)
20−35 (n=2)
NA
Age_category

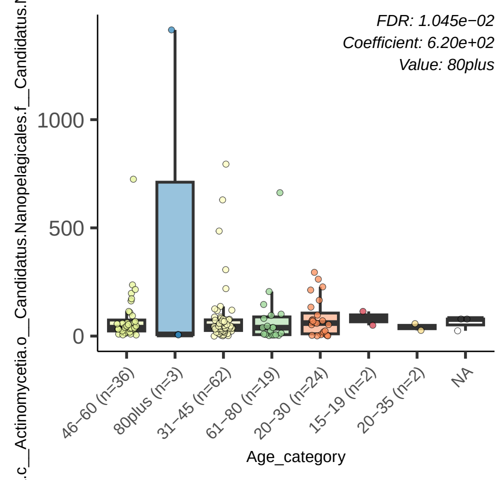

FDR: 1.045e−02
Coefficient: 6.20e+02
Value: 80plus
1000
500
0
46−60 (n=36)
80plus (n=3)
31−45 (n=62)
61−80 (n=19)
20−30 (n=24)
15−19 (n=2)
20−35 (n=2)
NA
Age_category
.c__Actinomycetia.o__Candidatus.Nanopelagicales.f__Candidatus.N

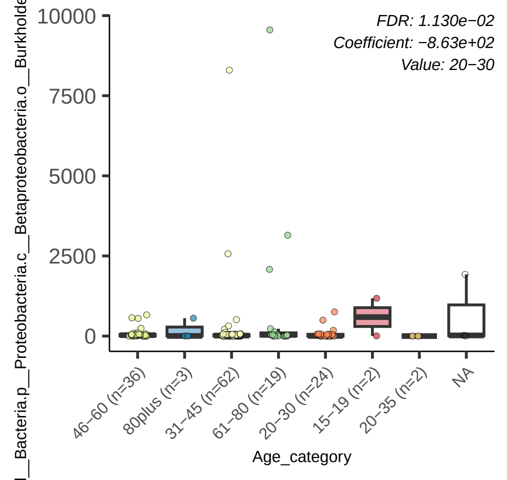
FDR: 1.130e−02
Coefficient: −8.63e+02
Value: 20−30
10000
7500
5000
2500
0
Bacteria.p__Proteobacteria.c__Betaproteobacteria.o__Burkholde
46−60 (n=36)
80plus (n=3)
31−45 (n=62)
61−80 (n=19)
20−30 (n=24)
15−19 (n=2)
20−35 (n=2)
NA
Age_category

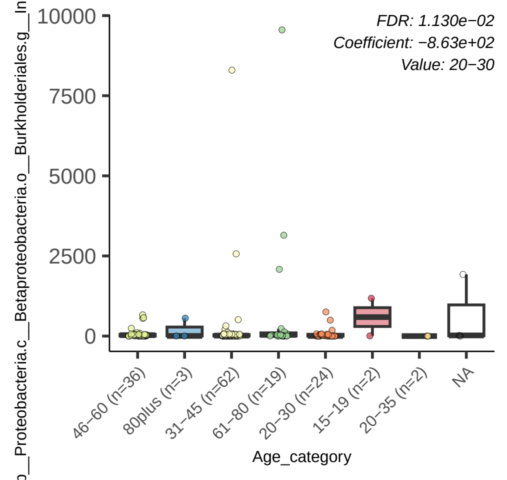

FDR: 1.130e−02
Coefficient: −8.63e+02
Value: 20−30
o__Proteobacteria.c__Betaproteobacteria.o__Burkholderiales.g__In
10000
7500
5000
2500
0
46−60 (n=36)
80plus (n=3)
31−45 (n=62)
61−80 (n=19)
20−30 (n=24)
15−19 (n=2)
20−35 (n=2)
NA
Age_category

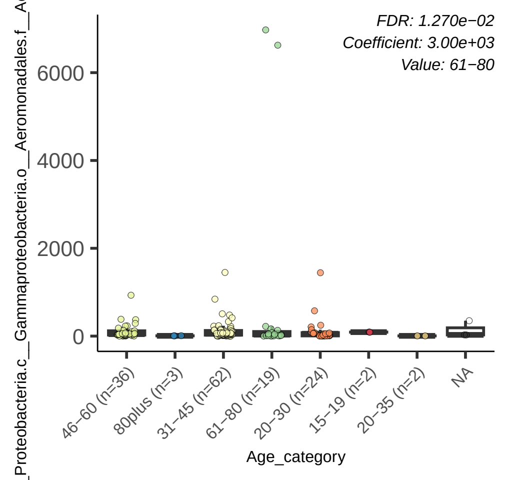

FDR: 1.270e−02
Coefficient: 3.00e+03
Value: 61−80
Proteobacteria.c__Gammaproteobacteria.o__Aeromonadales.f__Ae
0
2000
4000
6000
46−60 (n=36)
80plus (n=3)
31−45 (n=62)
61−80 (n=19)
20−30 (n=24)
15−19 (n=2)
20−35 (n=2)
NA
Age_category

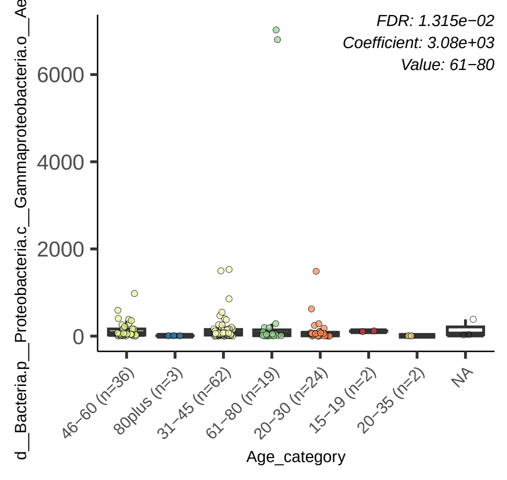
FDR: 1.315e−02
Coefficient: 3.08e+03
Value: 61−80
d__Bacteria.p__Proteobacteria.c__Gammaproteobacteria.o__Ae
6000
4000
2000
0
46−60 (n=36)
80plus (n=3)
31−45 (n=62)
61−80 (n=19)
20−30 (n=24)
15−19 (n=2)
20−35 (n=2)
NA
Age_category

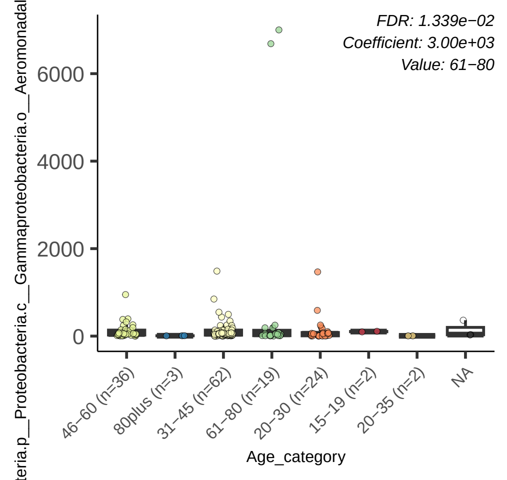
FDR: 1.339e−02
Coefficient: 3.00e+03
Value: 61−80
6000
4000
2000
0
teria.p__Proteobacteria.c__Gammaproteobacteria.o__Aeromonadal
46−60 (n=36)
80plus (n=3)
31−45 (n=62)
61−80 (n=19)
20−30 (n=24)
15−19 (n=2)
20−35 (n=2)
NA
Age_category

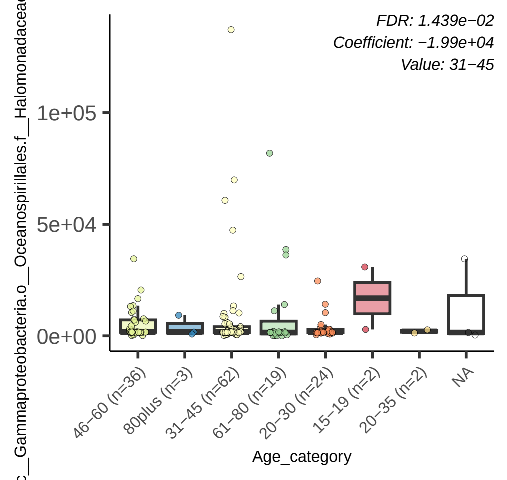
FDR: 1.439e−02
Coefficient: −1.99e+04
Value: 31−45
c__Gammaproteobacteria.o__Oceanospirillales.f__Halomonadaceae
1e+05
5e+04
0e+00
46−60 (n=36)
80plus (n=3)
31−45 (n=62)
61−80 (n=19)
20−30 (n=24)
15−19 (n=2)
20−35 (n=2)
NA
Age_category

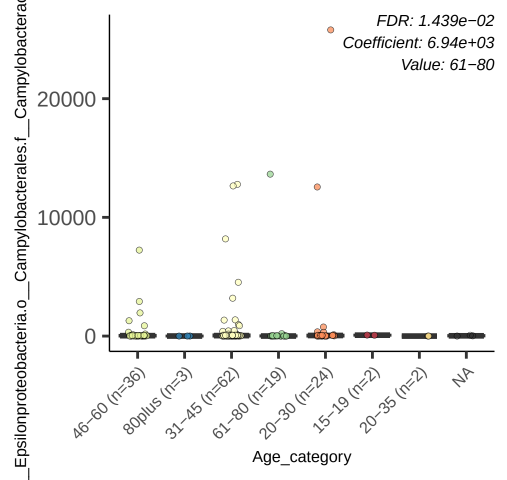

FDR: 1.439e−02
Coefficient: 6.94e+03
Value: 61−80
20000
10000
0
_Epsilonproteobacteria.o__Campylobacterales.f__Campylobacterac
46−60 (n=36)
80plus (n=3)
31−45 (n=62)
61−80 (n=19)
20−30 (n=24)
15−19 (n=2)
20−35 (n=2)
NA
Age_category

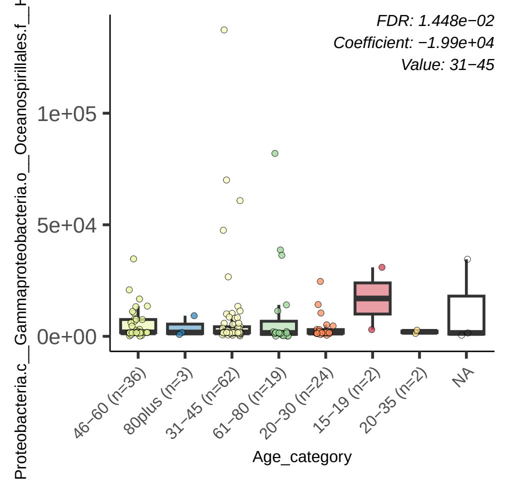

FDR: 1.448e−02
Coefficient: −1.99e+04
Value: 31−45
Proteobacteria.c__Gammaproteobacteria.o__Oceanospirillales.f__H
1e+05
5e+04
0e+00
46−60 (n=36)
80plus (n=3)
31−45 (n=62)
61−80 (n=19)
20−30 (n=24)
15−19 (n=2)
20−35 (n=2)
NA
Age_category

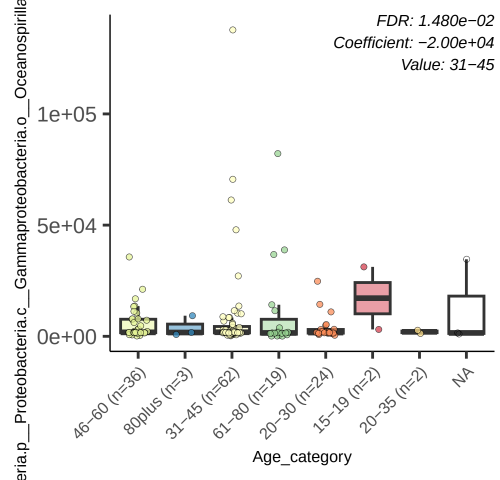

FDR: 1.480e−02
Coefficient: −2.00e+04
Value: 31−45
1e+05
5e+04
0e+00
46−60 (n=36)
80plus (n=3)
31−45 (n=62)
61−80 (n=19)
20−30 (n=24)
15−19 (n=2)
20−35 (n=2)
NA
Age_category

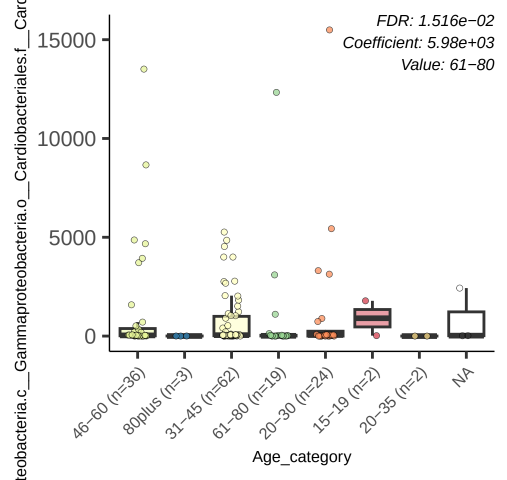

FDR: 1.516e−02
Coefficient: 5.98e+03
Value: 61−80
15000
10000
5000
0
teobacteria.c__Gammaproteobacteria.o__Cardiobacteriales.f__Card
46−60 (n=36)
80plus (n=3)
31−45 (n=62)
61−80 (n=19)
20−30 (n=24)
15−19 (n=2)
20−35 (n=2)
NA
Age_category

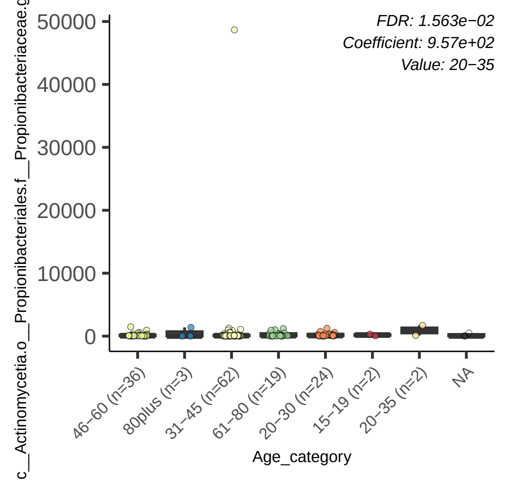
FDR: 1.563e−02
Coefficient: 9.57e+02
Value: 20−35
c__Actinomycetia.o__Propionibacteriales.f__Propionibacteriaceae.g
50000
40000
30000
20000
10000
0
46−60 (n=36)
80plus (n=3)
31−45 (n=62)
61−80 (n=19)
20−30 (n=24)
15−19 (n=2)
20−35 (n=2)
NA
Age_category

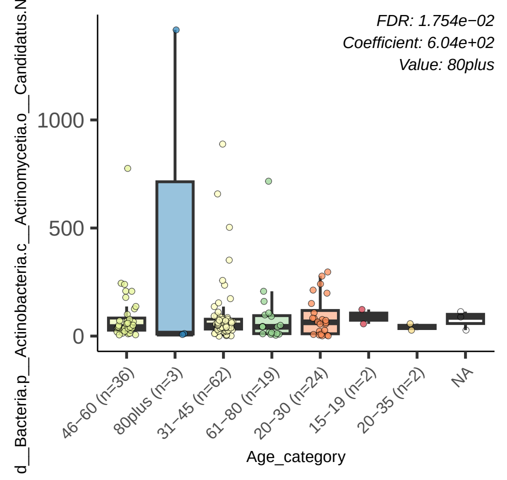

FDR: 1.754e−02
Coefficient: 6.04e+02
Value: 80plus
d__Bacteria.p__Actinobacteria.c__Actinomycetia.o__Candidatus.N
1000
500
0
46−60 (n=36)
80plus (n=3)
31−45 (n=62)
61−80 (n=19)
20−30 (n=24)
15−19 (n=2)
20−35 (n=2)
NA
Age_category

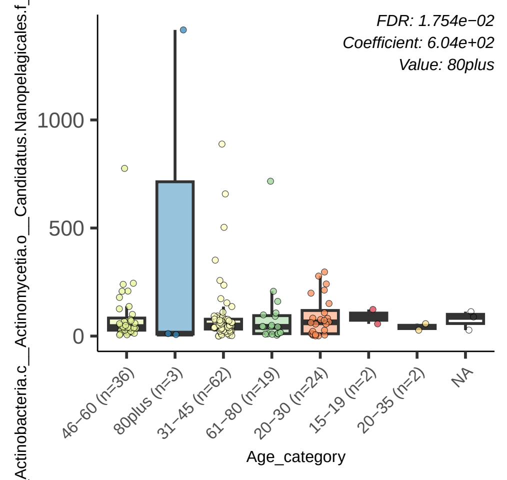

FDR: 1.754e−02
Coefficient: 6.04e+02
Value: 80plus
Actinobacteria.c__Actinomycetia.o__Candidatus.Nanopelagicales.f_
1000
500
0
46−60 (n=36)
80plus (n=3)
31−45 (n=62)
61−80 (n=19)
20−30 (n=24)
15−19 (n=2)
20−35 (n=2)
NA
Age_category

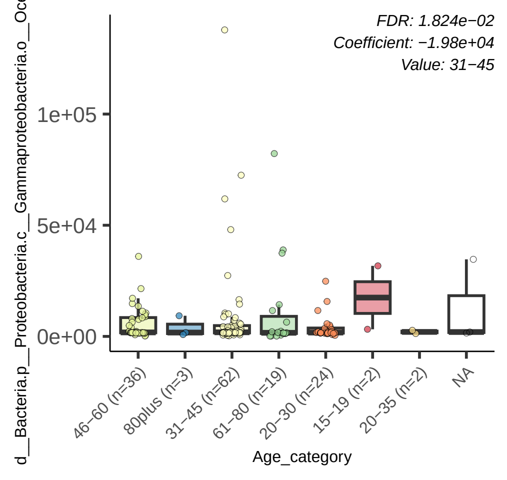
FDR: 1.824e−02
Coefficient: −1.98e+04
Value: 31−45
d__Bacteria.p__Proteobacteria.c__Gammaproteobacteria.o__Oce
1e+05
5e+04
0e+00
46−60 (n=36)
80plus (n=3)
31−45 (n=62)
61−80 (n=19)
20−30 (n=24)
15−19 (n=2)
20−35 (n=2)
NA
Age_category

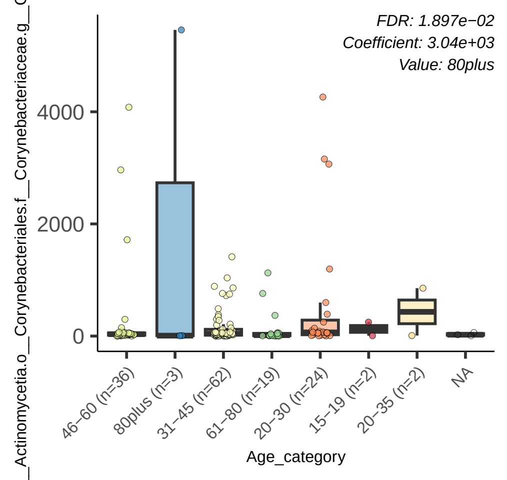

FDR: 1.897e−02
Coefficient: 3.04e+03
Value: 80plus
4000
2000
0
Actinomycetia.o__Corynebacteriales.f__Corynebacteriaceae.g__C
46−60 (n=36)
80plus (n=3)
31−45 (n=62)
61−80 (n=19)
20−30 (n=24)
15−19 (n=2)
20−35 (n=2)
NA
Age_category

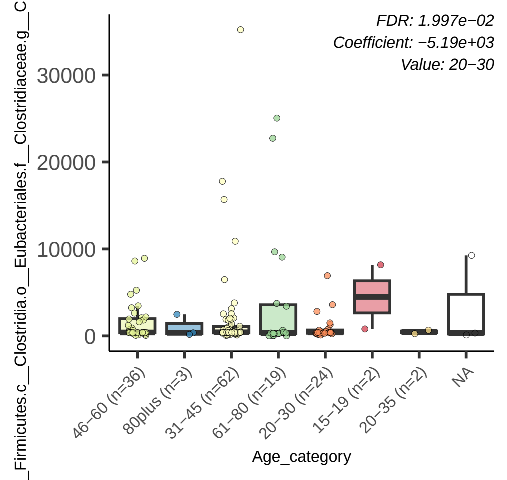

FDR: 1.997e−02
Coefficient: −5.19e+03
Value: 20−30
Firmicutes.c__Clostridia.o__Eubacteriales.f__Clostridiaceae.g__C
30000
20000
10000
0
46−60 (n=36)
80plus (n=3)
31−45 (n=62)
61−80 (n=19)
20−30 (n=24)
15−19 (n=2)
20−35 (n=2)
NA
Age_category

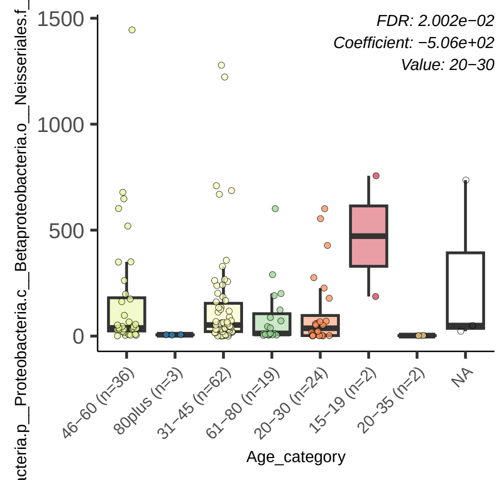

FDR: 2.002e−02
Coefficient: −5.06e+02
Value: 20−30
1500
1000
500
0
acteria.p__Proteobacteria.c__Betaproteobacteria.o__Neisseriales.f_
46−60 (n=36)
80plus (n=3)
31−45 (n=62)
61−80 (n=19)
20−30 (n=24)
15−19 (n=2)
20−35 (n=2)
NA
Age_category

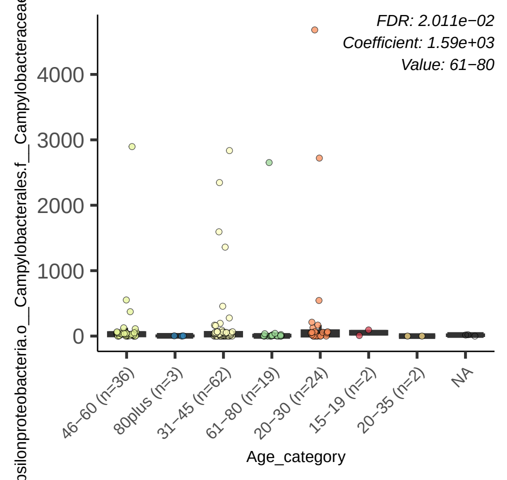
FDR: 2.011e−02
Coefficient: 1.59e+03
Value: 61−80
osilonproteobacteria.o__Campylobacterales.f__Campylobacterace
4000
3000
2000
1000
0
46−60 (n=36)
80plus (n=3)
31−45 (n=62)
61−80 (n=19)
20−30 (n=24)
15−19 (n=2)
20−35 (n=2)
NA
Age_category

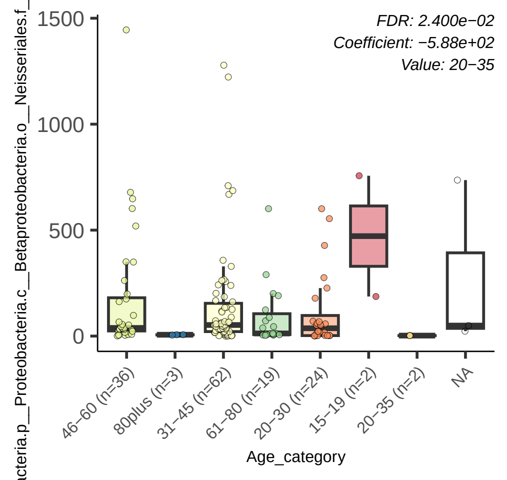

FDR: 2.400e−02
Coefficient: −5.88e+02
Value: 20−35
acteria.p__Proteobacteria.c__Betaproteobacteria.o__Neisseriales.f__
1500
1000
500
0
46−60 (n=36)
80plus (n=3)
31−45 (n=62)
61−80 (n=19)
20−30 (n=24)
15−19 (n=2)
20−35 (n=2)
NA
Age_category

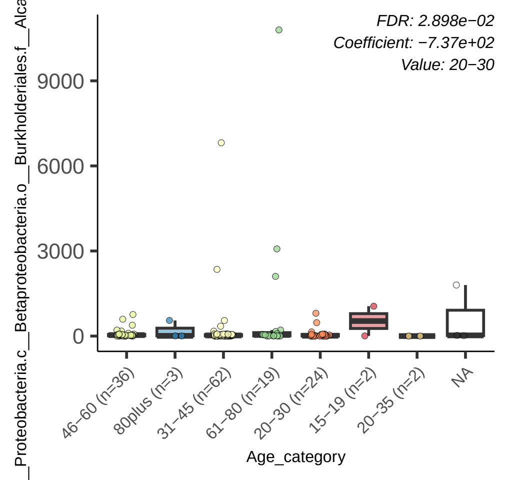

FDR: 2.898e−02
Coefficient: −7.37e+02
Value: 20−30
9000
6000
3000
0
Proteobacteria.c__Betaproteobacteria.o__Burkholderiales.f__Alca
46−60 (n=36)
80plus (n=3)
31−45 (n=62)
61−80 (n=19)
20−30 (n=24)
15−19 (n=2)
20−35 (n=2)
NA
Age_category

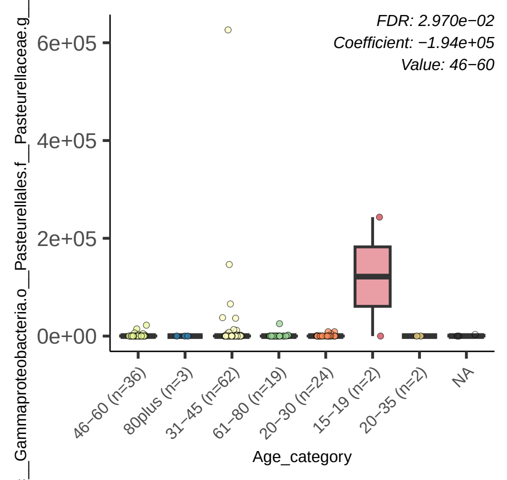
FDR: 2.970e−02
Coefficient: −1.94e+05
Value: 46−60
Gammaproteobacteria.o__Pasteurellales.f__Pasteurellaceae.g__
6e+05
4e+05
2e+05
0e+00
46−60 (n=36)
80plus (n=3)
31−45 (n=62)
61−80 (n=19)
20−30 (n=24)
15−19 (n=2)
20−35 (n=2)
NA
Age_category

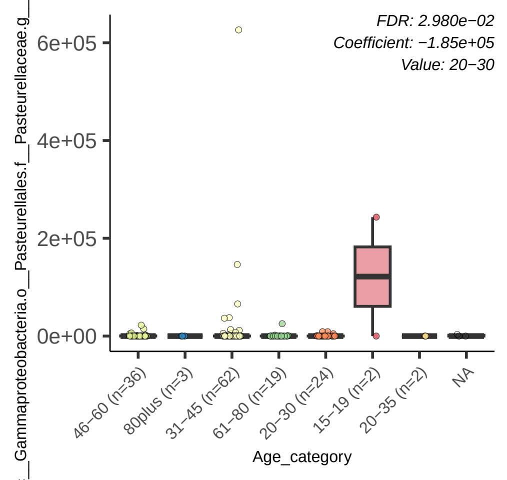

FDR: 2.980e−02
Coefficient: −1.85e+05
Value: 20−30
Gammaproteobacteria.o__Pasteurellales.f__Pasteurellaceae.g__
6e+05
4e+05
2e+05
0e+00
46−60 (n=36)
80plus (n=3)
31−45 (n=62)
61−80 (n=19)
20−30 (n=24)
15−19 (n=2)
20−35 (n=2)
NA
Age_category

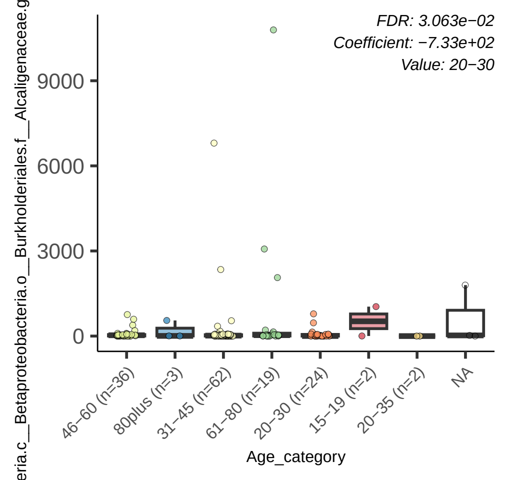

FDR: 3.063e−02
Coefficient: −7.33e+02
Value: 20−30
9000
6000
3000
0
46−60 (n=36)
80plus (n=3)
31−45 (n=62)
61−80 (n=19)
20−30 (n=24)
15−19 (n=2)
20−35 (n=2)
NA
Age_category
eria.c__Betaproteobacteria.o__Burkholderiales.f__Alcaligenaceae.g

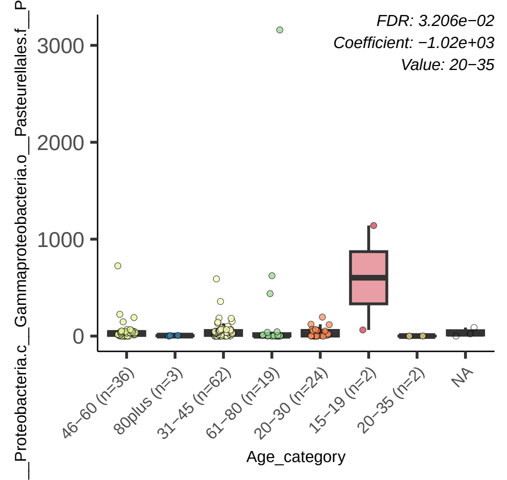

FDR: 3.206e−02
Coefficient: −1.02e+03
Value: 20−35
3000
2000
1000
0
Proteobacteria.c__Gammaproteobacteria.o__Pasteurellales.f__P
46−60 (n=36)
80plus (n=3)
31−45 (n=62)
61−80 (n=19)
20−30 (n=24)
15−19 (n=2)
20−35 (n=2)
NA
Age_category

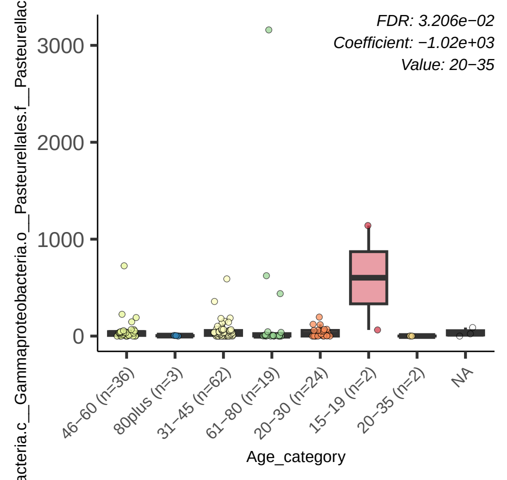

FDR: 3.206e−02
Coefficient: −1.02e+03
Value: 20−35
3000
2000
1000
0
acteria.c__Gammaproteobacteria.o__Pasteurellales.f__Pasteurellac
46−60 (n=36)
80plus (n=3)
31−45 (n=62)
61−80 (n=19)
20−30 (n=24)
15−19 (n=2)
20−35 (n=2)
NA
Age_category

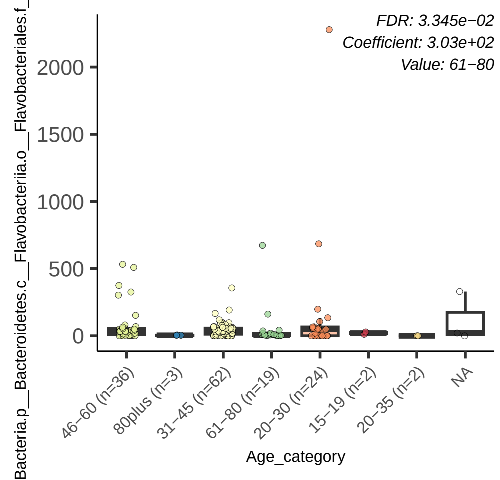
FDR: 3.345e−02
Coefficient: 3.03e+02
Value: 61−80
Bacteria.p__Bacteroidetes.c__Flavobacteriia.o__Flavobacteriales.f__
2000
1500
1000
500
0
46−60 (n=36)
80plus (n=3)
31−45 (n=62)
61−80 (n=19)
20−30 (n=24)
15−19 (n=2)
20−35 (n=2)
NA
Age_category

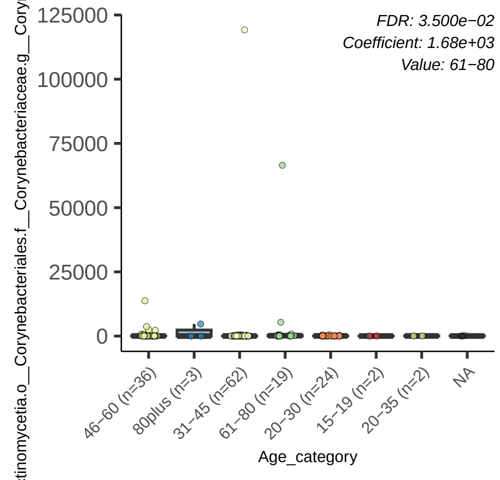
FDR: 3.500e−02
Coefficient: 1.68e+03
Value: 61−80
125000
100000
75000
50000
25000
0
46−60 (n=36)
80plus (n=3)
31−45 (n=62)
61−80 (n=19)
20−30 (n=24)
15−19 (n=2)
20−35 (n=2)
NA
Age_category
ctinomycetia.o__Corynebacteriales.f__Corynebacteriaceae.g__Cory

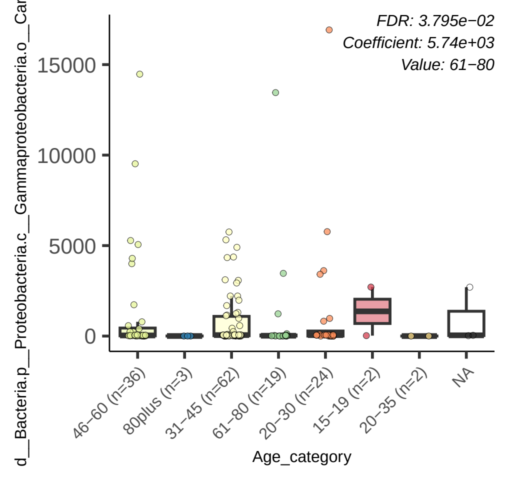

FDR: 3.795e−02
Coefficient: 5.74e+03
Value: 61−80
d__Bacteria.p__Proteobacteria.c__Gammaproteobacteria.o__Car
15000
10000
5000
0
46−60 (n=36)
80plus (n=3)
31−45 (n=62)
61−80 (n=19)
20−30 (n=24)
15−19 (n=2)
20−35 (n=2)
NA
Age_category

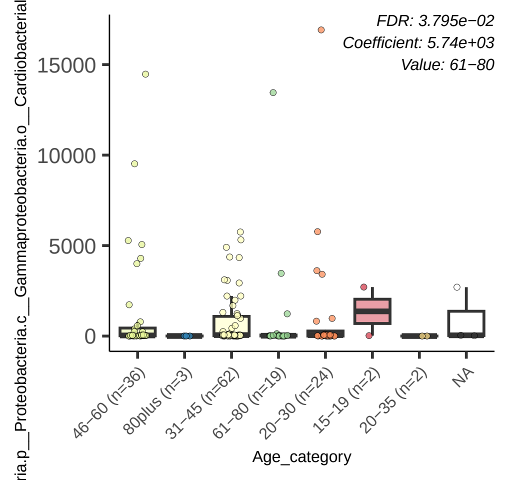

FDR: 3.795e−02
Coefficient: 5.74e+03
Value: 61−80
15000
10000
5000
0
ria.p__Proteobacteria.c__Gammaproteobacteria.o__Cardiobacterial
46−60 (n=36)
80plus (n=3)
31−45 (n=62)
61−80 (n=19)
20−30 (n=24)
15−19 (n=2)
20−35 (n=2)
NA
Age_category

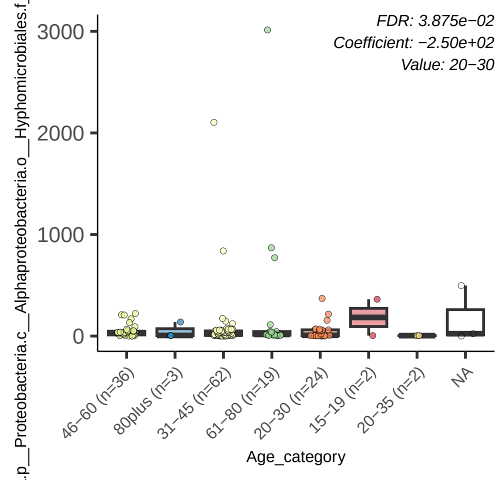

FDR: 3.875e−02
Coefficient: −2.50e+02
Value: 20−30
p__Proteobacteria.c__Alphaproteobacteria.o__Hyphomicrobiales.f__
3000
2000
1000
0
46−60 (n=36)
80plus (n=3)
31−45 (n=62)
61−80 (n=19)
20−30 (n=24)
15−19 (n=2)
20−35 (n=2)
NA
Age_category

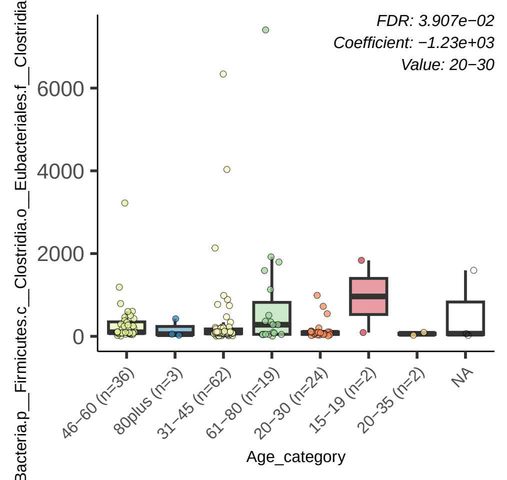

FDR: 3.907e−02
Coefficient: −1.23e+03
Value: 20−30
Bacteria.p__Firmicutes.c__Clostridia.o__Eubacteriales.f__Clostridia
6000
4000
2000
0
46−60 (n=36)
80plus (n=3)
31−45 (n=62)
61−80 (n=19)
20−30 (n=24)
15−19 (n=2)
20−35 (n=2)
NA
Age_category

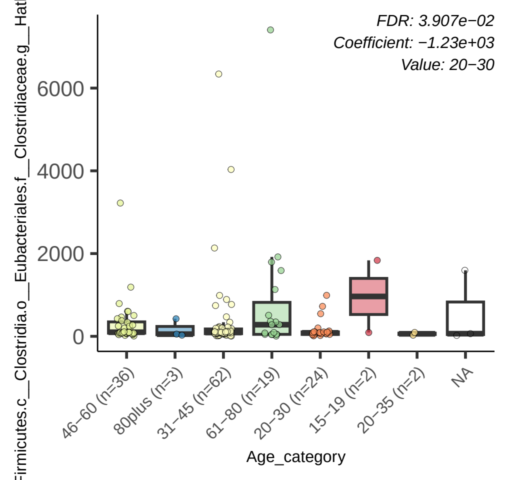

FDR: 3.907e−02
Coefficient: −1.23e+03
Value: 20−30
Firmicutes.c__Clostridia.o__Eubacteriales.f__Clostridiaceae.g__Hat
0
2000
4000
6000
46−60 (n=36)
80plus (n=3)
31−45 (n=62)
61−80 (n=19)
20−30 (n=24)
15−19 (n=2)
20−35 (n=2)
NA
Age_category

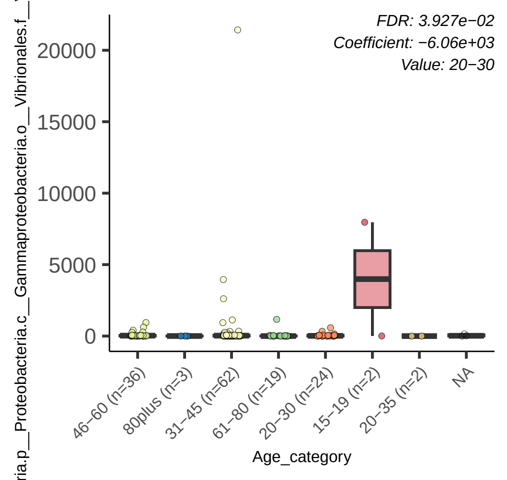

FDR: 3.927e−02
Coefficient: −6.06e+03
Value: 20−30
ria.p__Proteobacteria.c__Gammaproteobacteria.o__Vibrionales.f__
20000
15000
10000
5000
0
46−60 (n=36)
80plus (n=3)
31−45 (n=62)
61−80 (n=19)
20−30 (n=24)
15−19 (n=2)
20−35 (n=2)
NA
Age_category

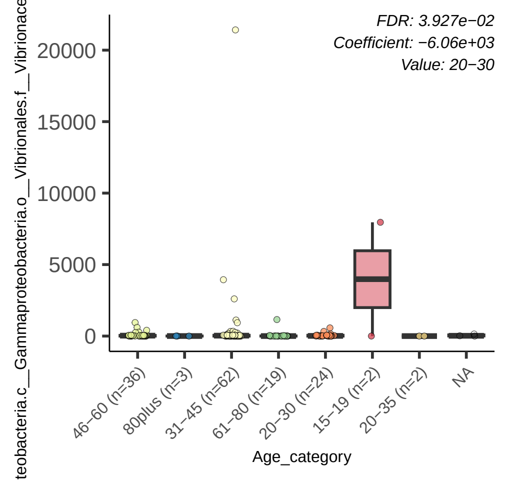
FDR: 3.927e−02
Coefficient: −6.06e+03
Value: 20−30
teobacteria.c__Gammaproteobacteria.o__Vibrionales.f__Vibrionace
20000
15000
10000
5000
0
46−60 (n=36)
80plus (n=3)
31−45 (n=62)
61−80 (n=19)
20−30 (n=24)
15−19 (n=2)
20−35 (n=2)
NA
Age_category

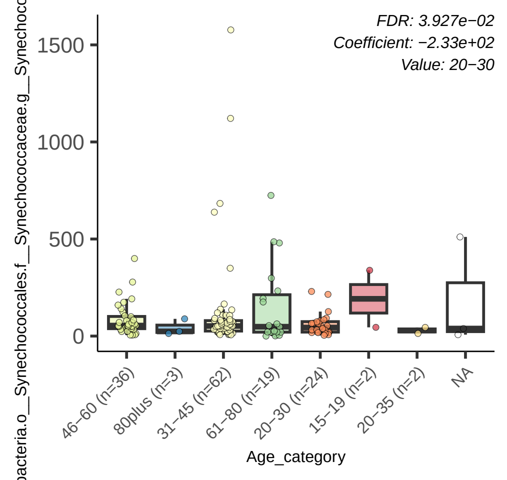

FDR: 3.927e−02
Coefficient: −2.33e+02
Value: 20−30
1500
1000
500
0
bacteria.o__Synechococcales.f__Synechococcaceae.g__Synechoco
46−60 (n=36)
80plus (n=3)
31−45 (n=62)
61−80 (n=19)
20−30 (n=24)
15−19 (n=2)
20−35 (n=2)
NA
Age_category

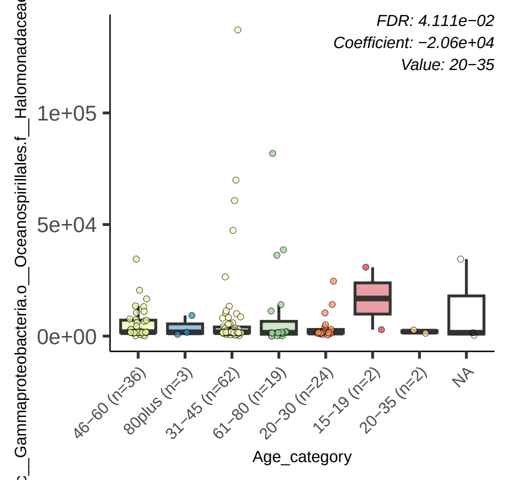

FDR: 4.111e−02
Coefficient: −2.06e+04
Value: 20−35
c__Gammaproteobacteria.o__Oceanospirillales.f__Halomonadaceae
1e+05
5e+04
0e+00
46−60 (n=36)
80plus (n=3)
31−45 (n=62)
61−80 (n=19)
20−30 (n=24)
15−19 (n=2)
20−35 (n=2)
NA
Age_category

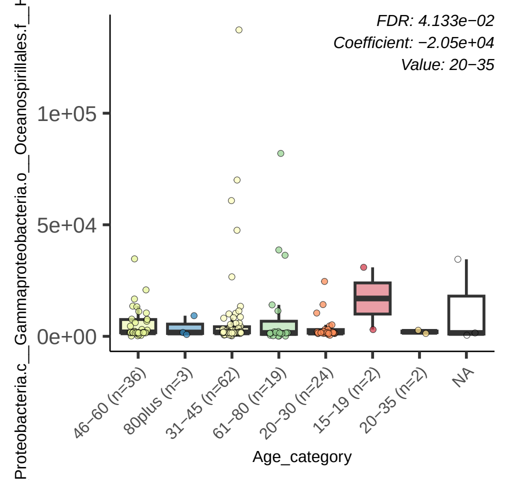

FDR: 4.133e−02
Coefficient: −2.05e+04
Value: 20−35
Proteobacteria.c__Gammaproteobacteria.o__Oceanospirillales.f__H
1e+05
5e+04
0e+00
46−60 (n=36)
80plus (n=3)
31−45 (n=62)
61−80 (n=19)
20−30 (n=24)
15−19 (n=2)
20−35 (n=2)
NA
Age_category

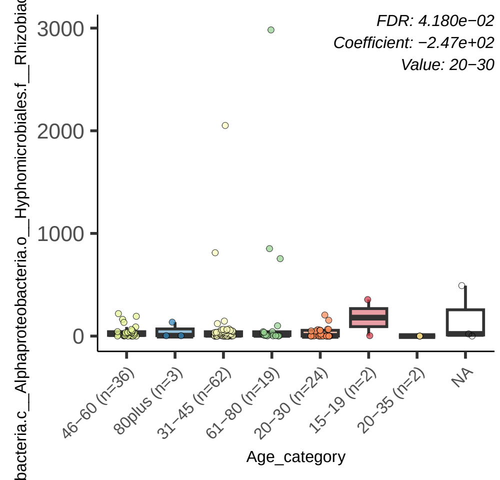

FDR: 4.180e−02
Coefficient: −2.47e+02
Value: 20−30
bacteria.c__Alphaproteobacteria.o__Hyphomicrobiales.f__Rhizobiac
3000
2000
1000
0
46−60 (n=36)
80plus (n=3)
31−45 (n=62)
61−80 (n=19)
20−30 (n=24)
15−19 (n=2)
20−35 (n=2)
NA
Age_category

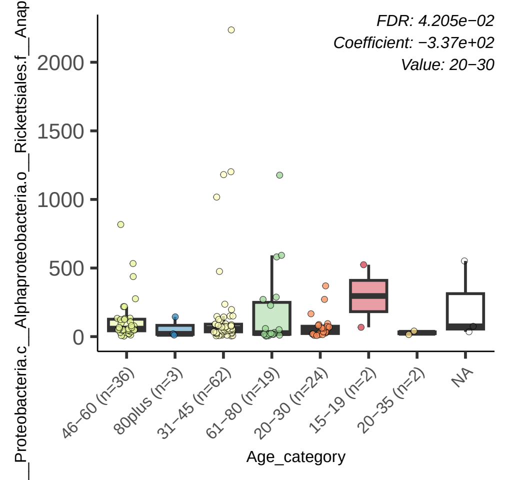

FDR: 4.205e−02
Coefficient: −3.37e+02
Value: 20−30
Proteobacteria.c__Alphaproteobacteria.o__Rickettsiales.f__Anap
0
500
1000
1500
2000
46−60 (n=36)
80plus (n=3)
31−45 (n=62)
61−80 (n=19)
20−30 (n=24)
15−19 (n=2)
20−35 (n=2)
NA
Age_category

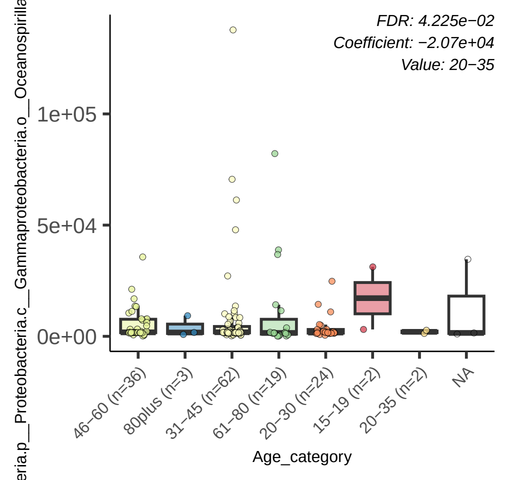

FDR: 4.225e−02
Coefficient: −2.07e+04
Value: 20−35
1e+05
5e+04
0e+00
eria.p__Proteobacteria.c__Gammaproteobacteria.o__Oceanospirilla
46−60 (n=36)
80plus (n=3)
31−45 (n=62)
61−80 (n=19)
20−30 (n=24)
15−19 (n=2)
20−35 (n=2)
NA
Age_category

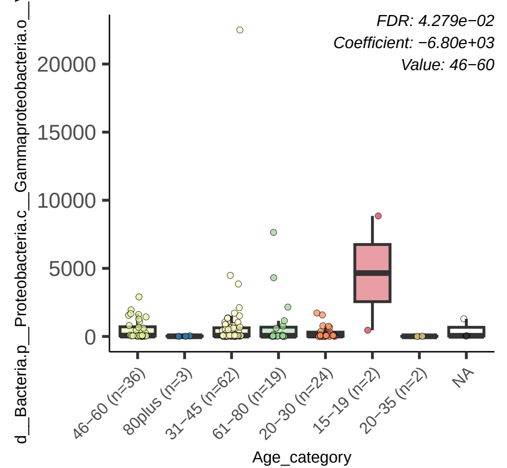
FDR: 4.279e−02
Coefficient: −6.80e+03
Value: 46−60
d__Bacteria.p__Proteobacteria.c__Gammaproteobacteria.o__
20000
15000
10000
5000
0
46−60 (n=36)
80plus (n=3)
31−45 (n=62)
61−80 (n=19)
20−30 (n=24)
15−19 (n=2)
20−35 (n=2)
NA
Age_category

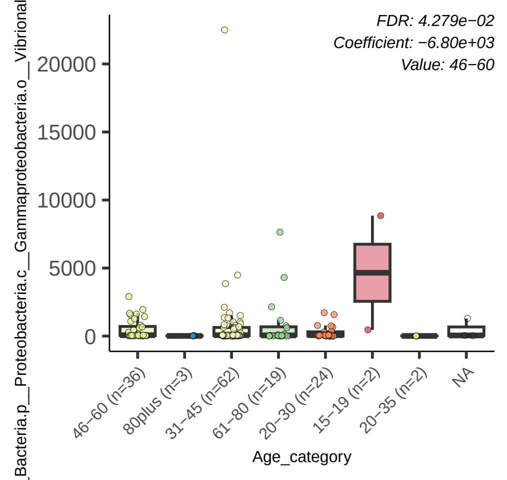

FDR: 4.279e−02
Coefficient: −6.80e+03
Value: 46−60
_Bacteria.p__Proteobacteria.c__Gammaproteobacteria.o__Vibrional
20000
15000
10000
5000
0
46−60 (n=36)
80plus (n=3)
31−45 (n=62)
61−80 (n=19)
20−30 (n=24)
15−19 (n=2)
20−35 (n=2)
NA
Age_category

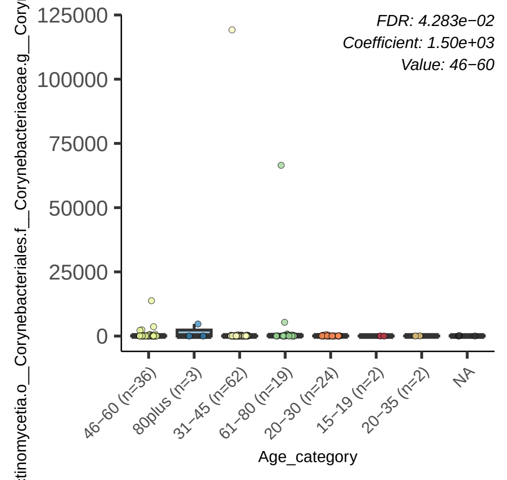

FDR: 4.283e−02
Coefficient: 1.50e+03
Value: 46−60
125000
100000
75000
50000
25000
0
46−60 (n=36)
80plus (n=3)
31−45 (n=62)
61−80 (n=19)
20−30 (n=24)
15−19 (n=2)
20−35 (n=2)
NA
Age_category

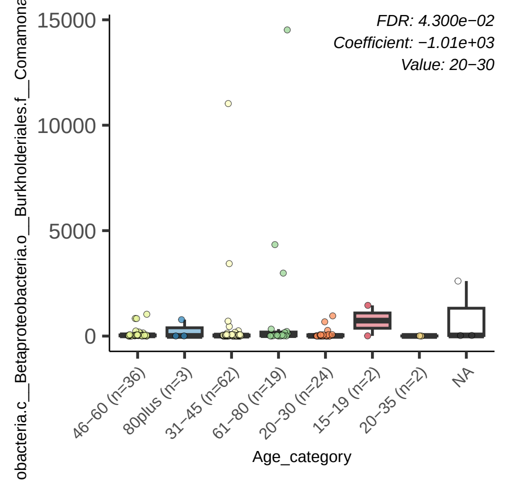

FDR: 4.300e−02
Coefficient: −1.01e+03
Value: 20−30
15000
10000
5000
0
obacteria.c__Betaproteobacteria.o__Burkholderiales.f__Comamona
46−60 (n=36)
80plus (n=3)
31−45 (n=62)
61−80 (n=19)
20−30 (n=24)
15−19 (n=2)
20−35 (n=2)
NA
Age_category

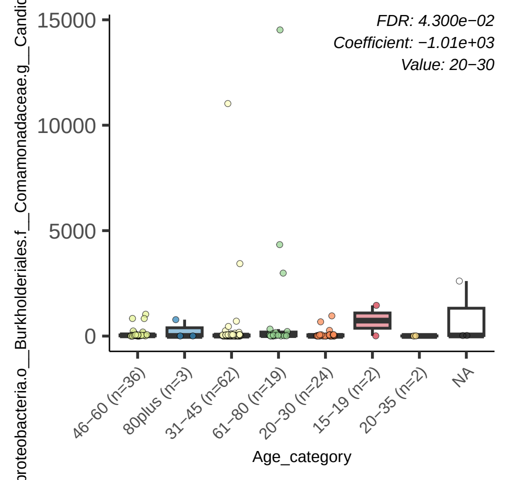
FDR: 4.300e−02
Coefficient: −1.01e+03
Value: 20−30
15000
10000
5000
0
roteobacteria.o__Burkholderiales.f__Comamonadaceae.g__Candid
46−60 (n=36)
80plus (n=3)
31−45 (n=62)
61−80 (n=19)
20−30 (n=24)
15−19 (n=2)
20−35 (n=2)
NA
Age_category

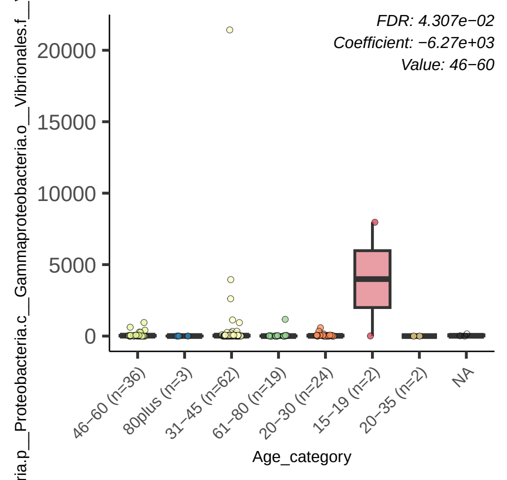

FDR: 4.307e−02
Coefficient: −6.27e+03
Value: 46−60
20000
15000
10000
5000
0
ria.p__Proteobacteria.c__Gammaproteobacteria.o__Vibrionales.f__
46−60 (n=36)
80plus (n=3)
31−45 (n=62)
61−80 (n=19)
20−30 (n=24)
15−19 (n=2)
20−35 (n=2)
NA
Age_category

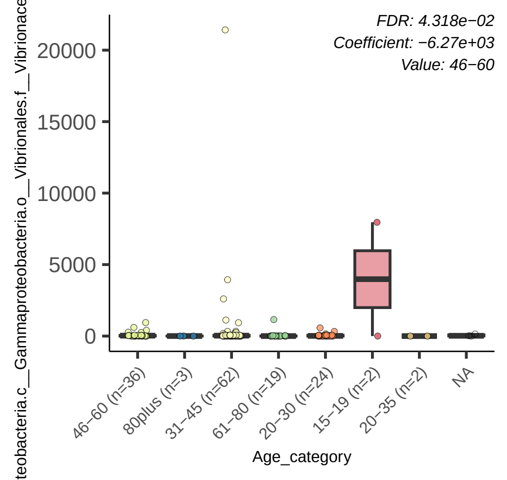
FDR: 4.318e−02
Coefficient: −6.27e+03
Value: 46−60
teobacteria.c__Gammaproteobacteria.o__Vibrionales.f__Vibrionace
20000
15000
10000
5000
0
46−60 (n=36)
80plus (n=3)
31−45 (n=62)
61−80 (n=19)
20−30 (n=24)
15−19 (n=2)
20−35 (n=2)
NA
Age_category

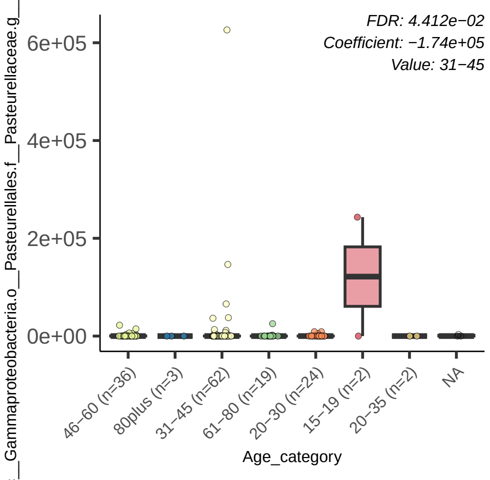

FDR: 4.412e−02
Coefficient: −1.74e+05
Value: 31−45
6e+05
4e+05
2e+05
0e+00
Gammaproteobacteria.o__Pasteurellales.f__Pasteurellaceae.g__
46−60 (n=36)
80plus (n=3)
31−45 (n=62)
61−80 (n=19)
20−30 (n=24)
15−19 (n=2)
20−35 (n=2)
NA
Age_category

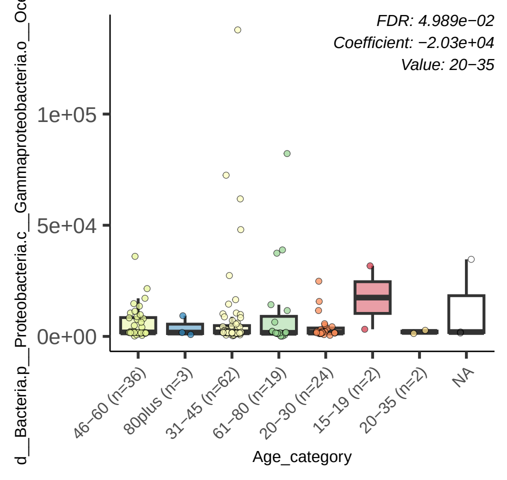

FDR: 4.989e−02
Coefficient: −2.03e+04
Value: 20−35
d__Bacteria.p__Proteobacteria.c__Gammaproteobacteria.o__Oce
1e+05
5e+04
0e+00
46−60 (n=36)
80plus (n=3)
31−45 (n=62)
61−80 (n=19)
20−30 (n=24)
15−19 (n=2)
20−35 (n=2)
NA
Age_category

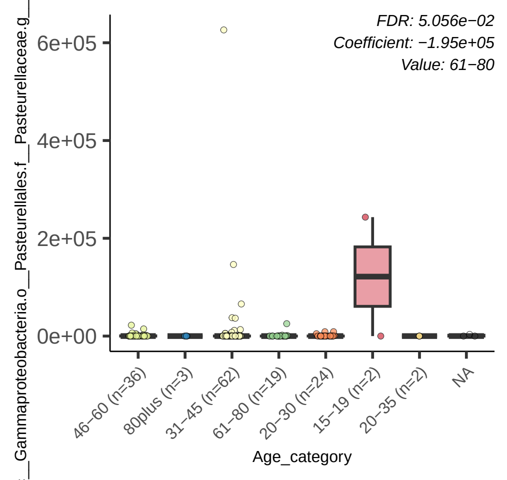
FDR: 5.056e−02
Coefficient: −1.95e+05
Value: 61−80
Gammaproteobacteria.o__Pasteurellales.f__Pasteurellaceae.g__
6e+05
4e+05
2e+05
0e+00
46−60 (n=36)
80plus (n=3)
31−45 (n=62)
61−80 (n=19)
20−30 (n=24)
15−19 (n=2)
20−35 (n=2)
NA
Age_category

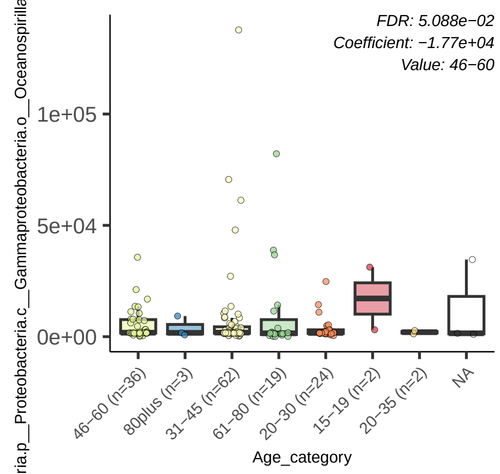

FDR: 5.088e−02
Coefficient: −1.77e+04
Value: 46−60
1e+05
5e+04
0e+00
eria.p__Proteobacteria.c__Gammaproteobacteria.o__Oceanospirilla
46−60 (n=36)
80plus (n=3)
31−45 (n=62)
61−80 (n=19)
20−30 (n=24)
15−19 (n=2)
20−35 (n=2)
NA
Age_category

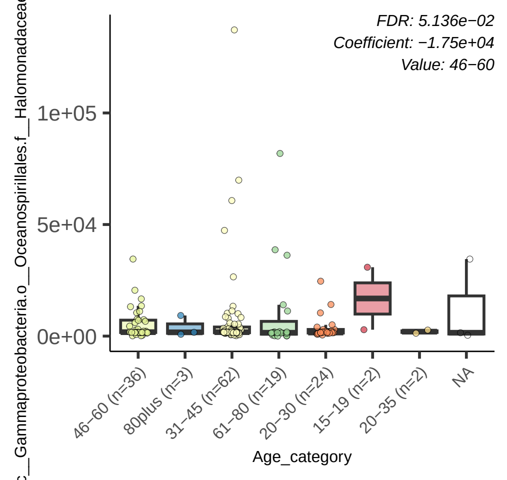
FDR: 5.136e−02
Coefficient: −1.75e+04
Value: 46−60
c__Gammaproteobacteria.o__Oceanospirillales.f__Halomonadaceae
1e+05
5e+04
0e+00
46−60 (n=36)
80plus (n=3)
31−45 (n=62)
61−80 (n=19)
20−30 (n=24)
15−19 (n=2)
20−35 (n=2)
NA
Age_category

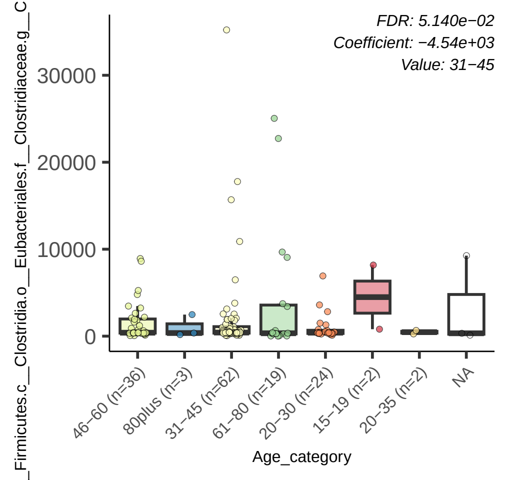
FDR: 5.140e−02
Coefficient: −4.54e+03
Value: 31−45
Firmicutes.c__Clostridia.o__Eubacteriales.f__Clostridiaceae.g__C
30000
20000
10000
0
46−60 (n=36)
80plus (n=3)
31−45 (n=62)
61−80 (n=19)
20−30 (n=24)
15−19 (n=2)
20−35 (n=2)
NA
Age_category

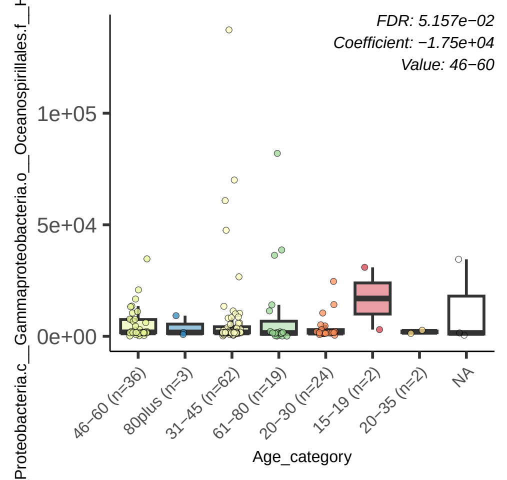
FDR: 5.157e−02
Coefficient: −1.75e+04
Value: 46−60
Proteobacteria.c__Gammaproteobacteria.o__Oceanospirillales.f__H
1e+05
5e+04
0e+00
46−60 (n=36)
80plus (n=3)
31−45 (n=62)
61−80 (n=19)
20−30 (n=24)
15−19 (n=2)
20−35 (n=2)
NA
Age_category

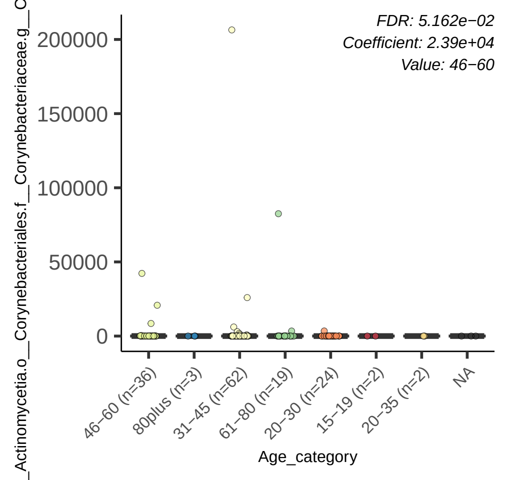

FDR: 5.162e−02
Coefficient: 2.39e+04
Value: 46−60
200000
150000
100000
50000
0
46−60 (n=36)
80plus (n=3)
31−45 (n=62)
61−80 (n=19)
20−30 (n=24)
15−19 (n=2)
20−35 (n=2)
NA
Age_category
Actinomycetia.o__Corynebacteriales.f__Corynebacteriaceae.g__C

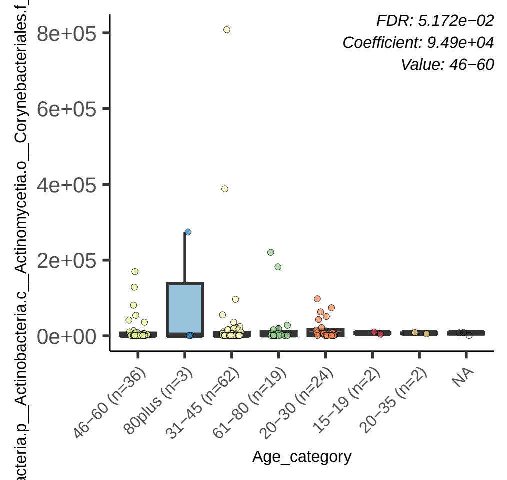

FDR: 5.172e−02
Coefficient: 9.49e+04
Value: 46−60
8e+05
6e+05
4e+05
2e+05
0e+00
acteria.p__Actinobacteria.c__Actinomycetia.o__Corynebacteriales.f_
46−60 (n=36)
80plus (n=3)
31−45 (n=62)
61−80 (n=19)
20−30 (n=24)
15−19 (n=2)
20−35 (n=2)
NA
Age_category

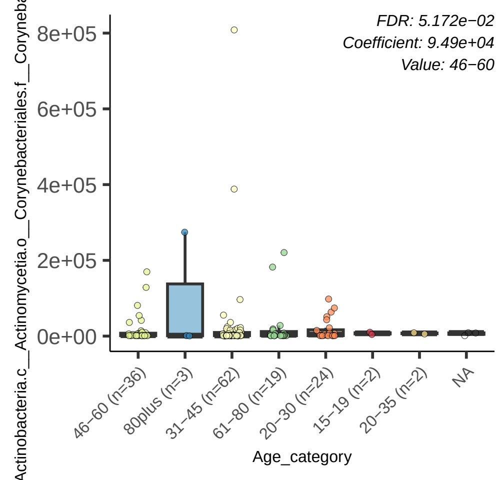

FDR: 5.172e−02
Coefficient: 9.49e+04
Value: 46−60
8e+05
6e+05
4e+05
2e+05
0e+00
Actinobacteria.c__Actinomycetia.o__Corynebacteriales.f__Coryneba
46−60 (n=36)
80plus (n=3)
31−45 (n=62)
61−80 (n=19)
20−30 (n=24)
15−19 (n=2)
20−35 (n=2)
NA
Age_category

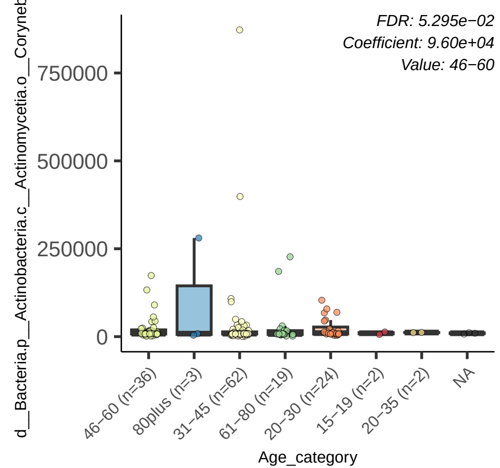

FDR: 5.295e−02
Coefficient: 9.60e+04
Value: 46−60
d__Bacteria.p__Actinobacteria.c__Actinomycetia.o__Coryneb
750000
500000
250000
0
46−60 (n=36)
80plus (n=3)
31−45 (n=62)
61−80 (n=19)
20−30 (n=24)
15−19 (n=2)
20−35 (n=2)
NA
Age_category

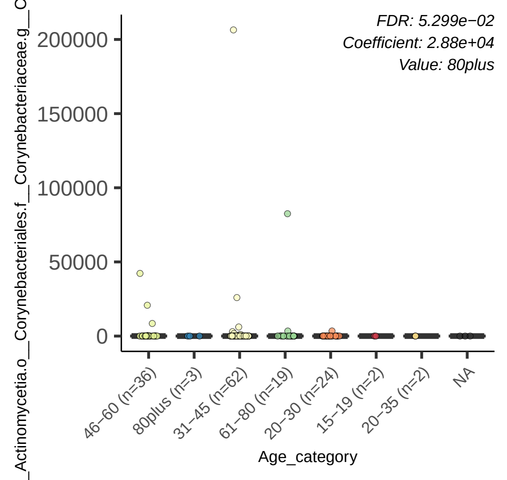
FDR: 5.299e−02
Coefficient: 2.88e+04
Value: 80plus
_Actinomycetia.o__Corynebacteriales.f__Corynebacteriaceae.g__C
200000
150000
100000
50000
0
46−60 (n=36)
80plus (n=3)
31−45 (n=62)
61−80 (n=19)
20−30 (n=24)
15−19 (n=2)
20−35 (n=2)
NA
Age_category

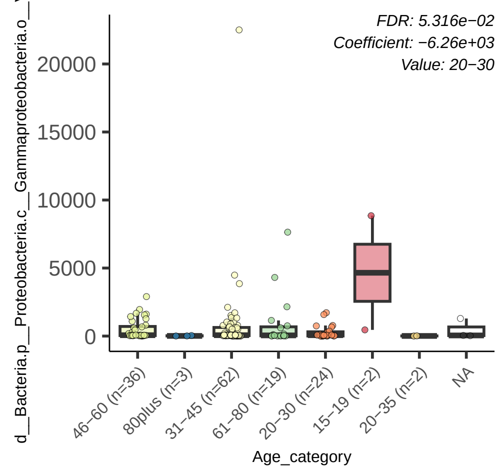

FDR: 5.316e−02
Coefficient: −6.26e+03
Value: 20−30
d__Bacteria.p__Proteobacteria.c__Gammaproteobacteria.o__
20000
15000
10000
5000
0
46−60 (n=36)
80plus (n=3)
31−45 (n=62)
61−80 (n=19)
20−30 (n=24)
15−19 (n=2)
20−35 (n=2)
NA
Age_category

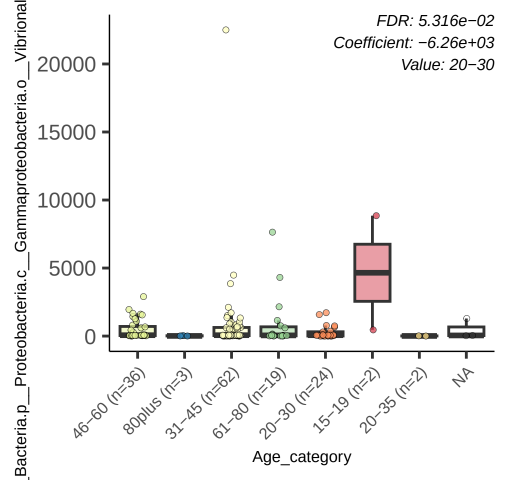

FDR: 5.316e−02
Coefficient: −6.26e+03
Value: 20−30
_Bacteria.p__Proteobacteria.c__Gammaproteobacteria.o__Vibrional
0
5000
10000
15000
20000
46−60 (n=36)
80plus (n=3)
31−45 (n=62)
61−80 (n=19)
20−30 (n=24)
15−19 (n=2)
20−35 (n=2)
NA
Age_category

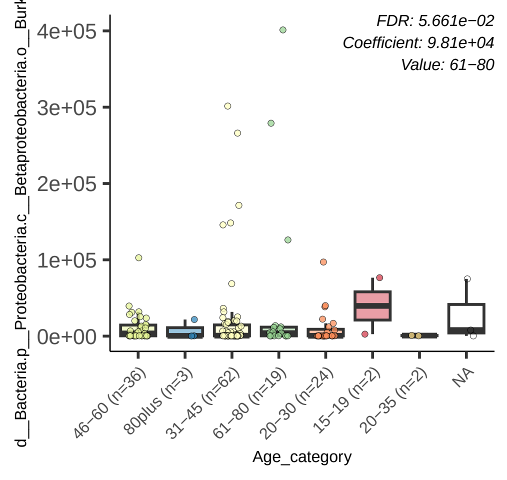

FDR: 5.661e−02
Coefficient: 9.81e+04
Value: 61−80
d__Bacteria.p__Proteobacteria.c__Betaproteobacteria.o__Burk
4e+05
3e+05
2e+05
1e+05
0e+00
46−60 (n=36)
80plus (n=3)
31−45 (n=62)
61−80 (n=19)
20−30 (n=24)
15−19 (n=2)
20−35 (n=2)
NA
Age_category

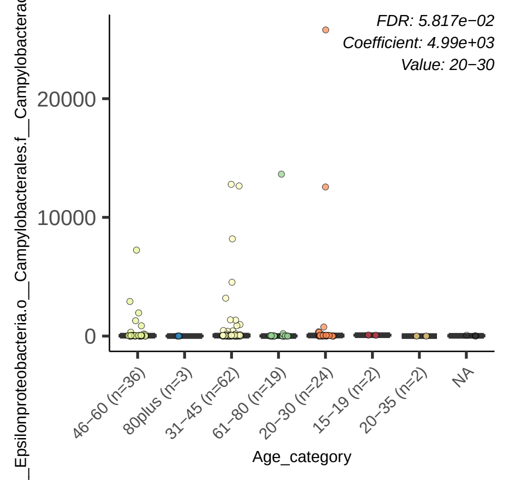
FDR: 5.817e−02
Coefficient: 4.99e+03
Value: 20−30
20000
10000
0
_Epsilonproteobacteria.o__Campylobacterales.f__Campylobacterac
46−60 (n=36)
80plus (n=3)
31−45 (n=62)
61−80 (n=19)
20−30 (n=24)
15−19 (n=2)
20−35 (n=2)
NA
Age_category

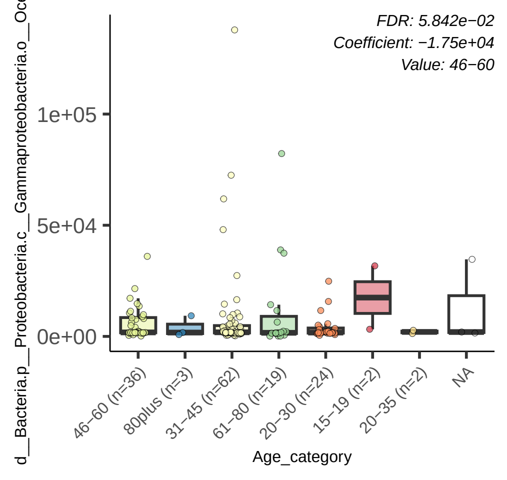

FDR: 5.842e−02
Coefficient: −1.75e+04
Value: 46−60
d__Bacteria.p__Proteobacteria.c__Gammaproteobacteria.o__Oce
1e+05
5e+04
0e+00
46−60 (n=36)
80plus (n=3)
31−45 (n=62)
61−80 (n=19)
20−30 (n=24)
15−19 (n=2)
20−35 (n=2)
NA
Age_category

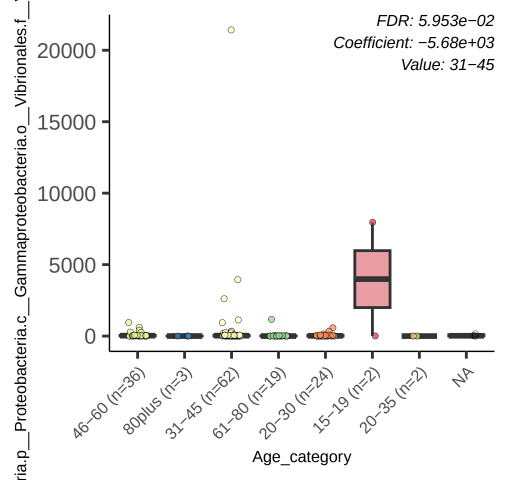
FDR: 5.953e−02
Coefficient: −5.68e+03
Value: 31−45
ria.p__Proteobacteria.c__Gammaproteobacteria.o__Vibrionales.f__
20000
15000
10000
5000
0
46−60 (n=36)
80plus (n=3)
31−45 (n=62)
61−80 (n=19)
20−30 (n=24)
15−19 (n=2)
20−35 (n=2)
NA
Age_category

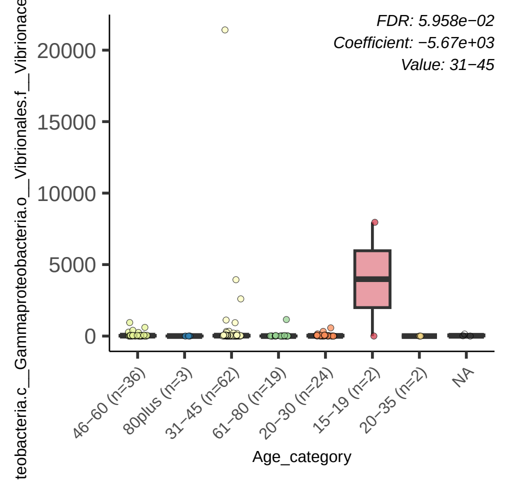

FDR: 5.958e−02
Coefficient: −5.67e+03
Value: 31−45
20000
15000
10000
5000
0
teobacteria.c__Gammaproteobacteria.o__Vibrionales.f__Vibrionace
46−60 (n=36)
80plus (n=3)
31−45 (n=62)
61−80 (n=19)
20−30 (n=24)
15−19 (n=2)
20−35 (n=2)
NA
Age_category

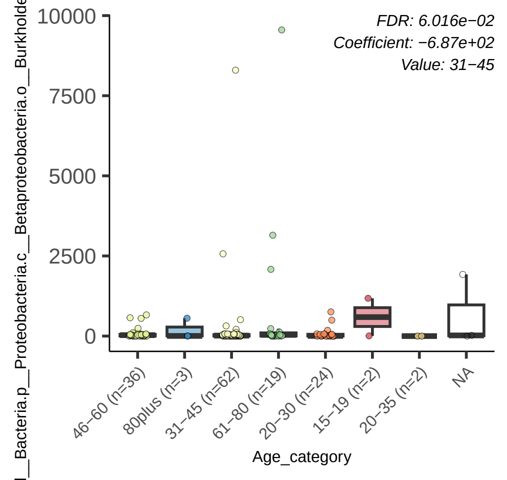

FDR: 6.016e−02
Coefficient: −6.87e+02
Value: 31−45
10000
7500
5000
2500
0
46−60 (n=36)
80plus (n=3)
31−45 (n=62)
61−80 (n=19)
20−30 (n=24)
15−19 (n=2)
20−35 (n=2)
NA
Age_category
Bacteria.p__Proteobacteria.c__Betaproteobacteria.o__Burkholde

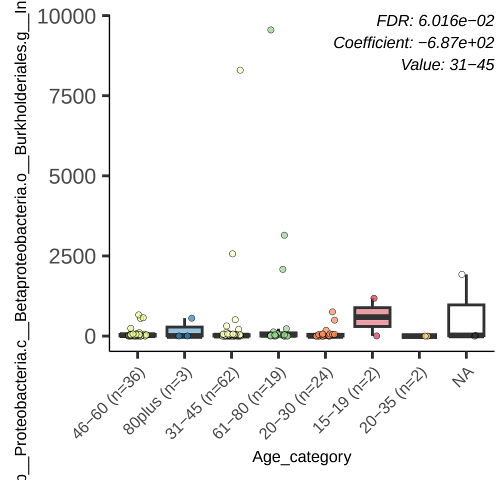
FDR: 6.016e−02
Coefficient: −6.87e+02
Value: 31−45
10000
7500
5000
2500
0
46−60 (n=36)
80plus (n=3)
31−45 (n=62)
61−80 (n=19)
20−30 (n=24)
15−19 (n=2)
20−35 (n=2)
NA
Age_category
o__Proteobacteria.c__Betaproteobacteria.o__Burkholderiales.g__In

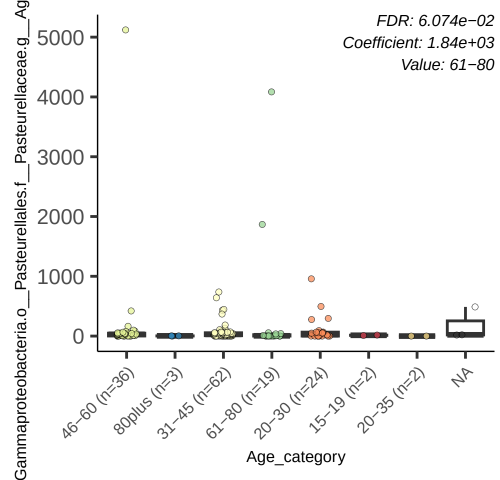
FDR: 6.074e−02
Coefficient: 1.84e+03
Value: 61−80
Gammaproteobacteria.o__Pasteurellales.f__Pasteurellaceae.g__Ag
5000
4000
3000
2000
1000
0
46−60 (n=36)
80plus (n=3)
31−45 (n=62)
61−80 (n=19)
20−30 (n=24)
15−19 (n=2)
20−35 (n=2)
NA
Age_category

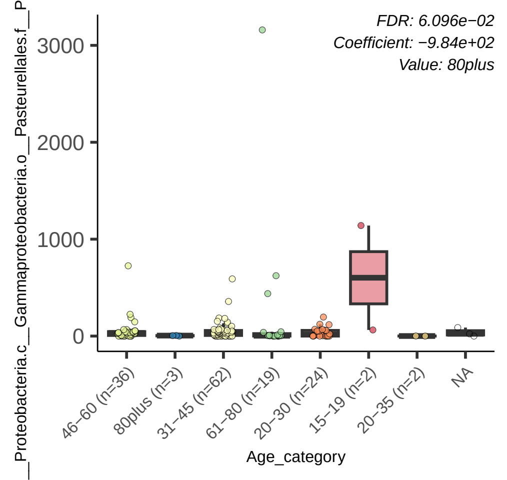

FDR: 6.096e−02
Coefficient: −9.84e+02
Value: 80plus
Proteobacteria.c__Gammaproteobacteria.o__Pasteurellales.f__P
3000
2000
1000
0
46−60 (n=36)
80plus (n=3)
31−45 (n=62)
61−80 (n=19)
20−30 (n=24)
15−19 (n=2)
20−35 (n=2)
NA
Age_category

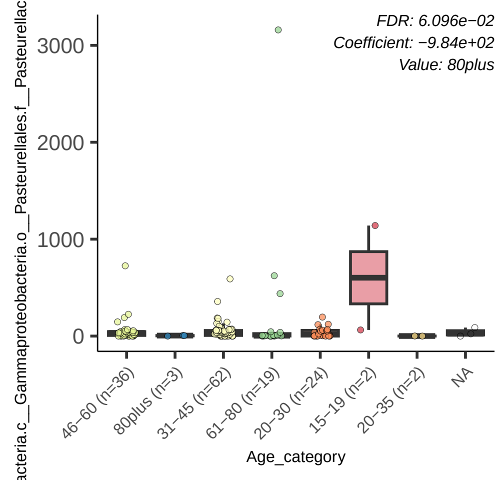

FDR: 6.096e−02
Coefficient: −9.84e+02
Value: 80plus
acteria.c__Gammaproteobacteria.o__Pasteurellales.f__Pasteurellac
3000
2000
1000
0
46−60 (n=36)
80plus (n=3)
31−45 (n=62)
61−80 (n=19)
20−30 (n=24)
15−19 (n=2)
20−35 (n=2)
NA
Age_category

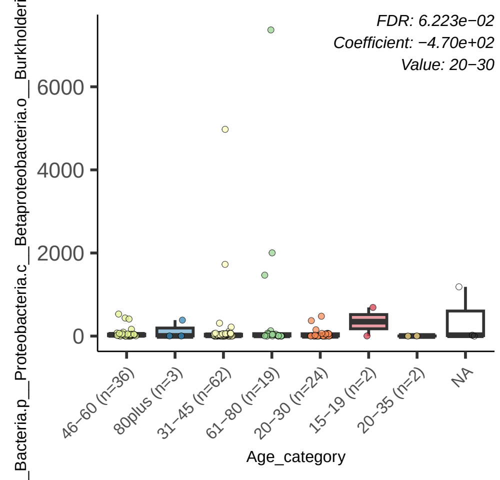

FDR: 6.223e−02
Coefficient: −4.70e+02
Value: 20−30
_Bacteria.p__Proteobacteria.c__Betaproteobacteria.o__Burkholderi
6000
4000
2000
0
46−60 (n=36)
80plus (n=3)
31−45 (n=62)
61−80 (n=19)
20−30 (n=24)
15−19 (n=2)
20−35 (n=2)
NA
Age_category

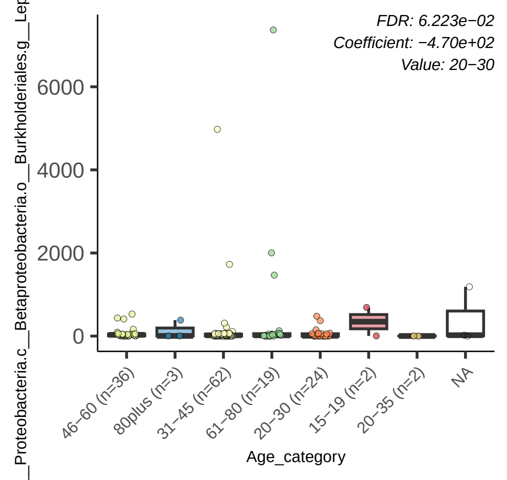

FDR: 6.223e−02
Coefficient: −4.70e+02
Value: 20−30
6000
4000
2000
0
46−60 (n=36)
80plus (n=3)
31−45 (n=62)
61−80 (n=19)
20−30 (n=24)
15−19 (n=2)
20−35 (n=2)
NA
Age_category
Proteobacteria.c__Betaproteobacteria.o__Burkholderiales.g__Lep

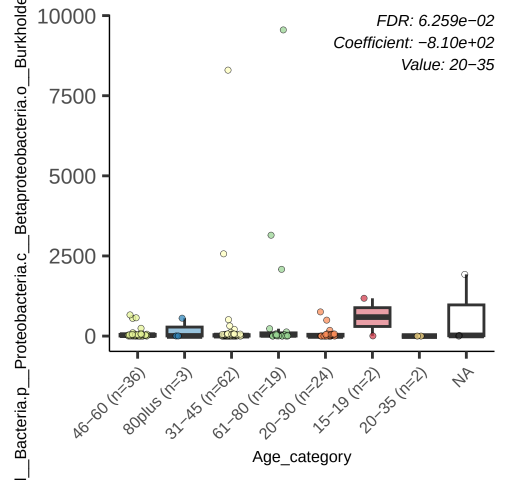

FDR: 6.259e−02
Coefficient: −8.10e+02
Value: 20−35
10000
7500
5000
2500
0
_Bacteria.p__Proteobacteria.c__Betaproteobacteria.o__Burkholde
46−60 (n=36)
80plus (n=3)
31−45 (n=62)
61−80 (n=19)
20−30 (n=24)
15−19 (n=2)
20−35 (n=2)
NA
Age_category

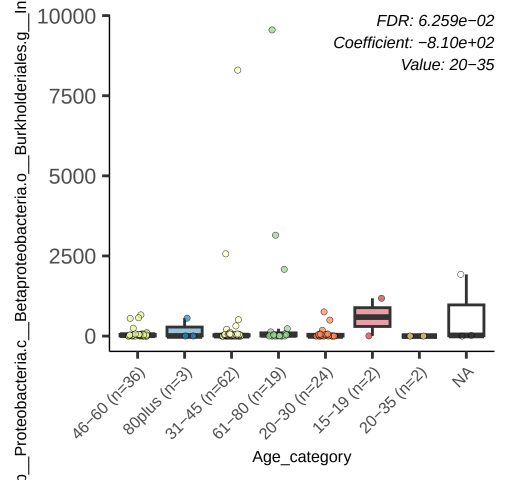
FDR: 6.259e−02
Coefficient: −8.10e+02
Value: 20−35
10000
7500
5000
2500
0
o__Proteobacteria.c__Betaproteobacteria.o__Burkholderiales.g__In
46−60 (n=36)
80plus (n=3)
31−45 (n=62)
61−80 (n=19)
20−30 (n=24)
15−19 (n=2)
20−35 (n=2)
NA
Age_category

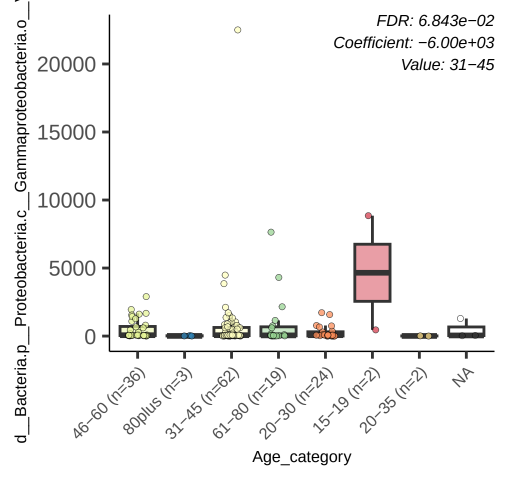

FDR: 6.843e−02
Coefficient: −6.00e+03
Value: 31−45
d__Bacteria.p__Proteobacteria.c__Gammaproteobacteria.o__
20000
15000
10000
5000
0
46−60 (n=36)
80plus (n=3)
31−45 (n=62)
61−80 (n=19)
20−30 (n=24)
15−19 (n=2)
20−35 (n=2)
NA
Age_category

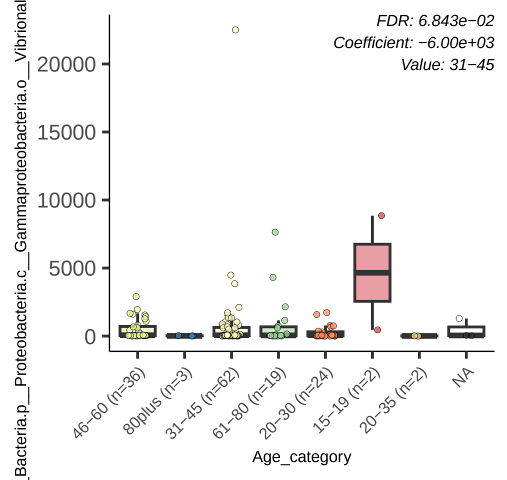
FDR: 6.843e−02
Coefficient: −6.00e+03
Value: 31−45
Bacteria.p__Proteobacteria.c__Gammaproteobacteria.o__Vibrional
20000
15000
10000
5000
0
46−60 (n=36)
80plus (n=3)
31−45 (n=62)
61−80 (n=19)
20−30 (n=24)
15−19 (n=2)
20−35 (n=2)
NA
Age_category

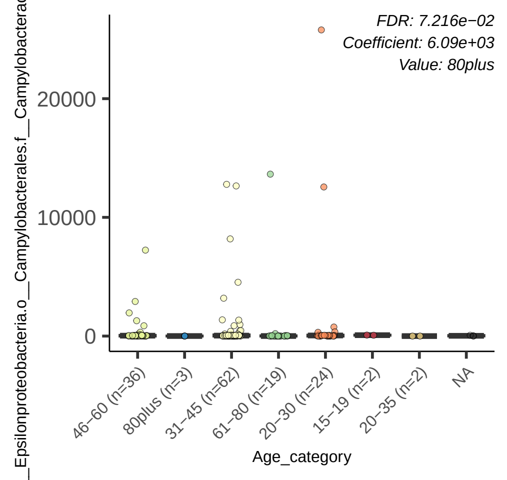
FDR: 7.216e−02
Coefficient: 6.09e+03
Value: 80plus
_Epsilonproteobacteria.o__Campylobacterales.f__Campylobacterac
20000
10000
0
46−60 (n=36)
80plus (n=3)
31−45 (n=62)
61−80 (n=19)
20−30 (n=24)
15−19 (n=2)
20−35 (n=2)
NA
Age_category

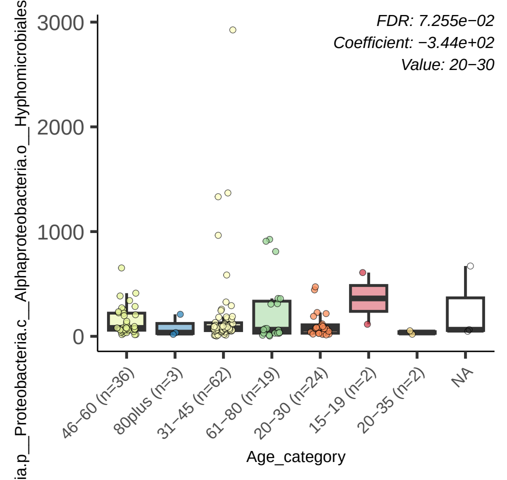

FDR: 7.255e−02
Coefficient: −3.44e+02
Value: 20−30
ia.p__Proteobacteria.c__Alphaproteobacteria.o__Hyphomicrobiales
3000
2000
1000
0
46−60 (n=36)
80plus (n=3)
31−45 (n=62)
61−80 (n=19)
20−30 (n=24)
15−19 (n=2)
20−35 (n=2)
NA
Age_category

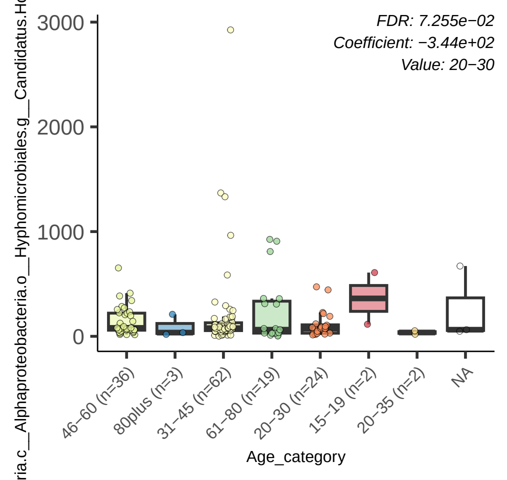

FDR: 7.255e−02
Coefficient: −3.44e+02
Value: 20−30
ria.c__Alphaproteobacteria.o__Hyphomicrobiales.g__Candidatus.Ho
3000
2000
1000
0
46−60 (n=36)
80plus (n=3)
31−45 (n=62)
61−80 (n=19)
20−30 (n=24)
15−19 (n=2)
20−35 (n=2)
NA
Age_category

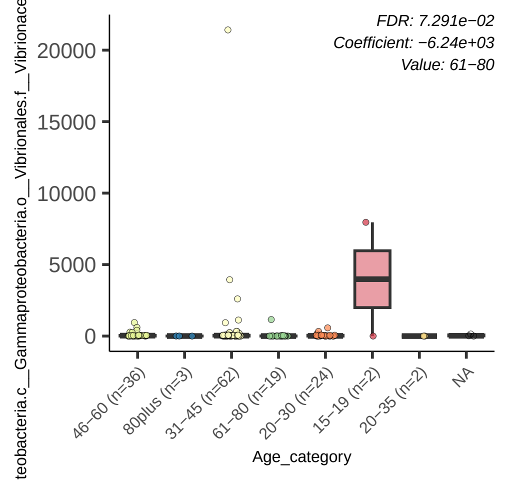

FDR: 7.291e−02
Coefficient: −6.24e+03
Value: 61−80
20000
15000
10000
5000
0
teobacteria.c__Gammaproteobacteria.o__Vibrionales.f__Vibrionace
46−60 (n=36)
80plus (n=3)
31−45 (n=62)
61−80 (n=19)
20−30 (n=24)
15−19 (n=2)
20−35 (n=2)
NA
Age_category

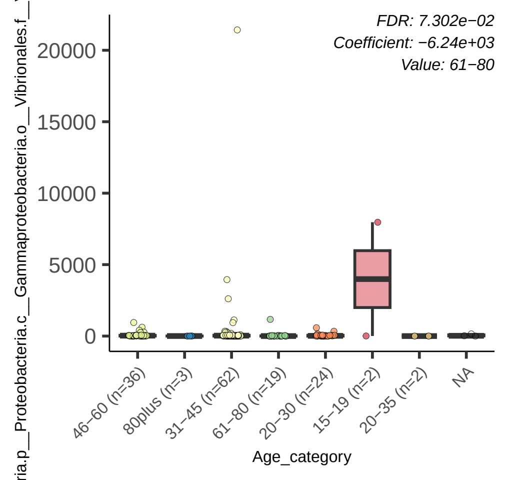
FDR: 7.302e−02
Coefficient: −6.24e+03
Value: 61−80
ria.p__Proteobacteria.c__Gammaproteobacteria.o__Vibrionales.f__
20000
15000
10000
5000
0
46−60 (n=36)
80plus (n=3)
31−45 (n=62)
61−80 (n=19)
20−30 (n=24)
15−19 (n=2)
20−35 (n=2)
NA
Age_category

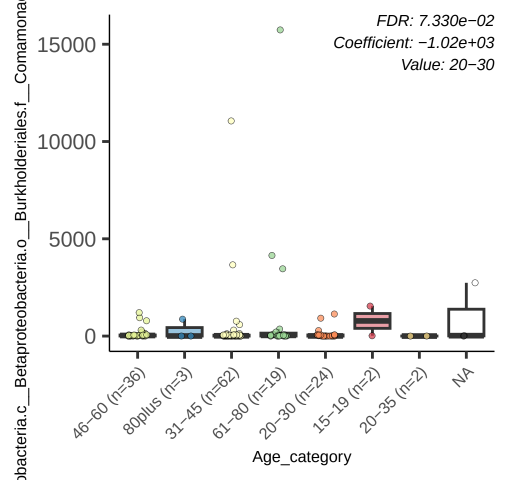
FDR: 7.330e−02
Coefficient: −1.02e+03
Value: 20−30
15000
10000
5000
0
46−60 (n=36)
80plus (n=3)
31−45 (n=62)
61−80 (n=19)
20−30 (n=24)
15−19 (n=2)
20−35 (n=2)
NA
Age_category
obacteria.c__Betaproteobacteria.o__Burkholderiales.f__Comamonad

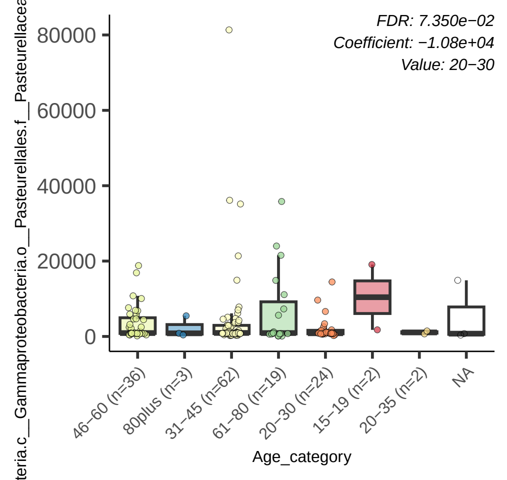

FDR: 7.350e−02
Coefficient: −1.08e+04
Value: 20−30
teria.c__Gammaproteobacteria.o__Pasteurellales.f__Pasteurellacea
80000
60000
40000
20000
0
46−60 (n=36)
80plus (n=3)
31−45 (n=62)
61−80 (n=19)
20−30 (n=24)
15−19 (n=2)
20−35 (n=2)
NA
Age_category

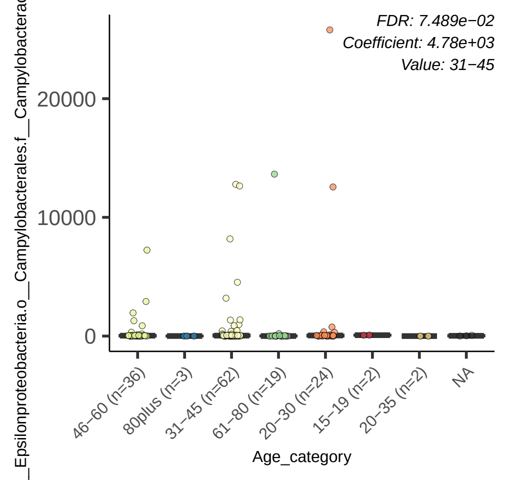

FDR: 7.489e−02
Coefficient: 4.78e+03
Value: 31−45
Epsilonproteobacteria.o__Campylobacterales.f__Campylobacterac
20000
10000
0
46−60 (n=36)
80plus (n=3)
31−45 (n=62)
61−80 (n=19)
20−30 (n=24)
15−19 (n=2)
20−35 (n=2)
NA
Age_category

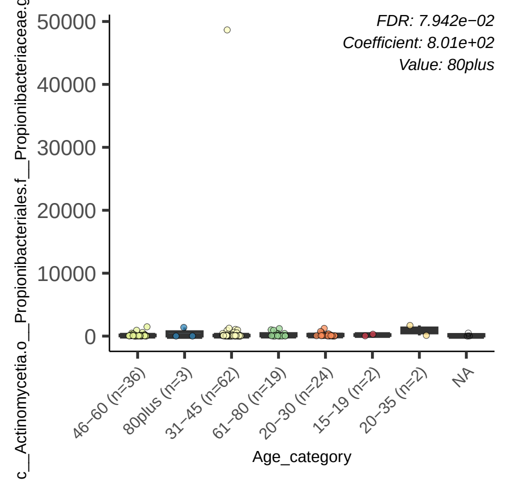

FDR: 7.942e−02
Coefficient: 8.01e+02
Value: 80plus
c__Actinomycetia.o__Propionibacteriales.f__Propionibacteriaceae.g
50000
40000
30000
20000
10000
0
46−60 (n=36)
80plus (n=3)
31−45 (n=62)
61−80 (n=19)
20−30 (n=24)
15−19 (n=2)
20−35 (n=2)
NA
Age_category

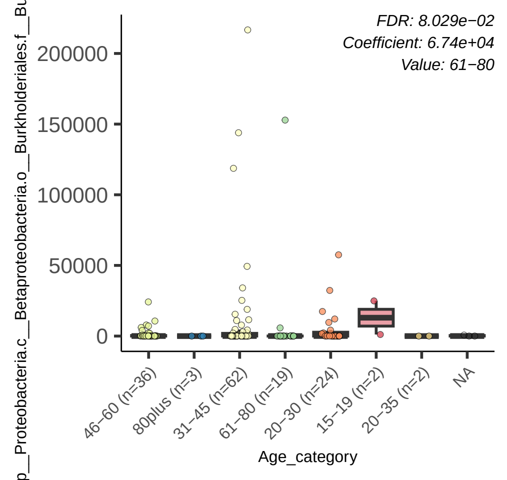

FDR: 8.029e−02
Coefficient: 6.74e+04
Value: 61−80
p__Proteobacteria.c__Betaproteobacteria.o__Burkholderiales.f__Bu
200000
150000
100000
50000
0
46−60 (n=36)
80plus (n=3)
31−45 (n=62)
61−80 (n=19)
20−30 (n=24)
15−19 (n=2)
20−35 (n=2)
NA
Age_category

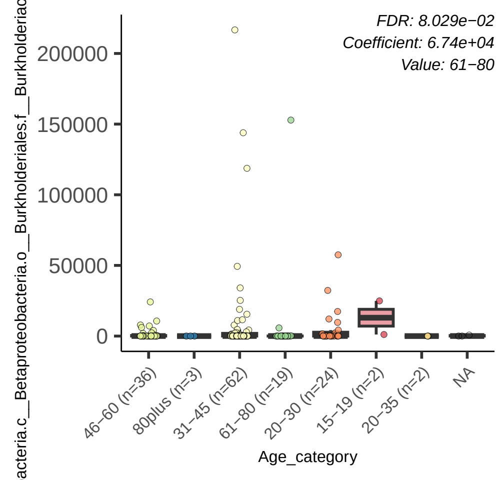

FDR: 8.029e−02
Coefficient: 6.74e+04
Value: 61−80
0
50000
100000
150000
200000
bacteria.c__Betaproteobacteria.o__Burkholderiales.f__Burkholderiac
46−60 (n=36)
80plus (n=3)
31−45 (n=62)
61−80 (n=19)
20−30 (n=24)
15−19 (n=2)
20−35 (n=2)
NA
Age_category

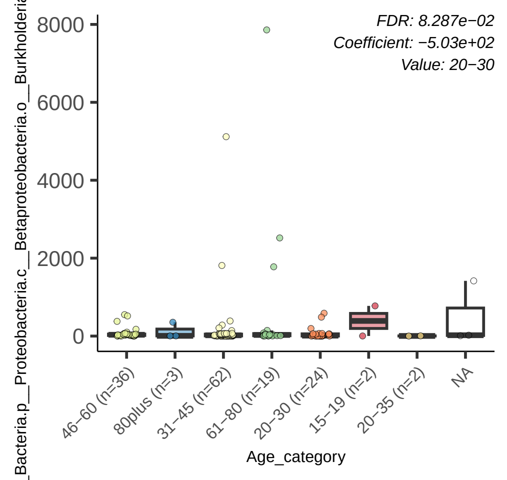
FDR: 8.287e−02
Coefficient: −5.03e+02
Value: 20−30
Bacteria.p__Proteobacteria.c__Betaproteobacteria.o__Burkholderia
8000
6000
4000
2000
0
46−60 (n=36)
80plus (n=3)
31−45 (n=62)
61−80 (n=19)
20−30 (n=24)
15−19 (n=2)
20−35 (n=2)
NA
Age_category

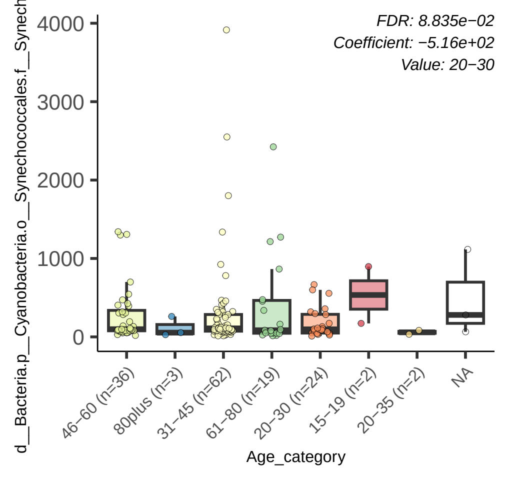
FDR: 8.835e−02
Coefficient: −5.16e+02
Value: 20−30
d__Bacteria.p__Cyanobacteria.o__Synechococcales.f__Synech
4000
3000
2000
1000
0
46−60 (n=36)
80plus (n=3)
31−45 (n=62)
61−80 (n=19)
20−30 (n=24)
15−19 (n=2)
20−35 (n=2)
NA
Age_category

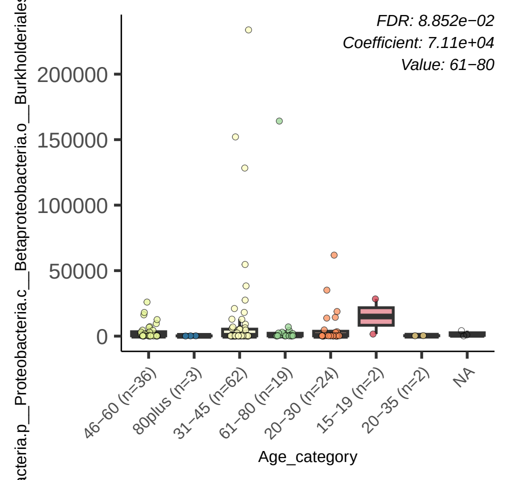
FDR: 8.852e−02
Coefficient: 7.11e+04
Value: 61−80
200000
150000
100000
50000
0
acteria.p__Proteobacteria.c__Betaproteobacteria.o__Burkholderiales
46−60 (n=36)
80plus (n=3)
31−45 (n=62)
61−80 (n=19)
20−30 (n=24)
15−19 (n=2)
20−35 (n=2)
NA
Age_category

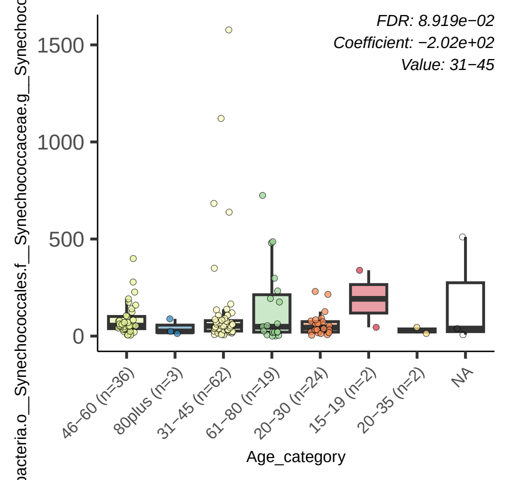

FDR: 8.919e−02
Coefficient: −2.02e+02
Value: 31−45
1500
1000
500
0
bacteria.o__Synechococcales.f__Synechococcaceae.g__Synechoco
46−60 (n=36)
80plus (n=3)
31−45 (n=62)
61−80 (n=19)
20−30 (n=24)
15−19 (n=2)
20−35 (n=2)
NA
Age_category

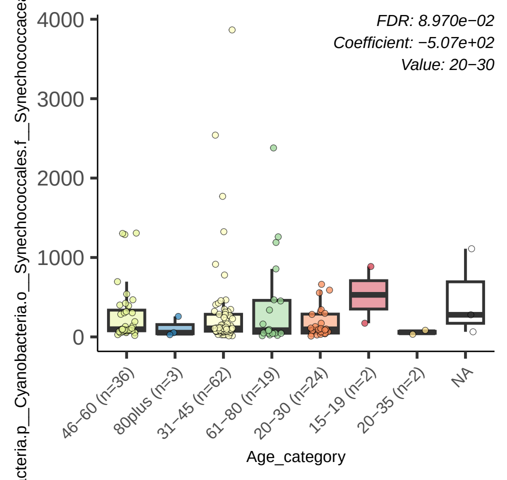

FDR: 8.970e−02
Coefficient: −5.07e+02
Value: 20−30
4000
3000
2000
1000
0
cteria.p__Cyanobacteria.o__Synechococcales.f__Synechococcace
46−60 (n=36)
80plus (n=3)
31−45 (n=62)
61−80 (n=19)
20−30 (n=24)
15−19 (n=2)
20−35 (n=2)
NA
Age_category

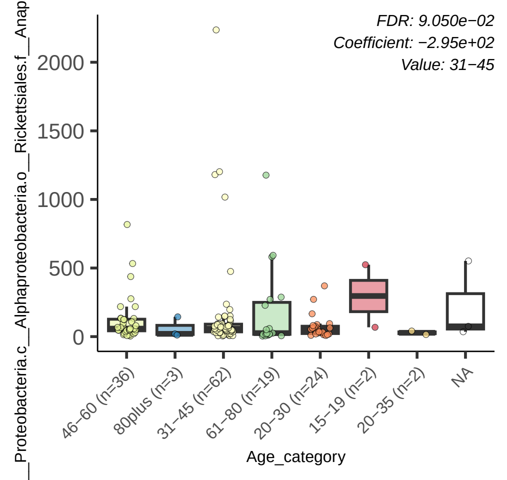

FDR: 9.050e−02
Coefficient: −2.95e+02
Value: 31−45
0
500
1000
1500
2000
_Proteobacteria.c__Alphaproteobacteria.o__Rickettsiales.f__Anap
46−60 (n=36)
80plus (n=3)
31−45 (n=62)
61−80 (n=19)
20−30 (n=24)
15−19 (n=2)
20−35 (n=2)
NA
Age_category

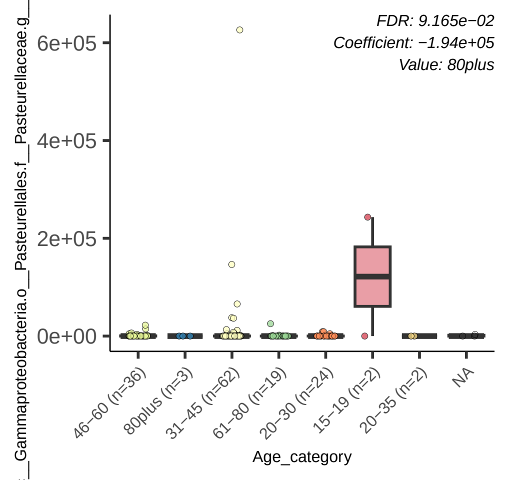

FDR: 9.165e−02
Coefficient: −1.94e+05
Value: 80plus
Gammaproteobacteria.o__Pasteurellales.f__Pasteurellaceae.g__
6e+05
4e+05
2e+05
0e+00
46−60 (n=36)
80plus (n=3)
31−45 (n=62)
61−80 (n=19)
20−30 (n=24)
15−19 (n=2)
20−35 (n=2)
NA
Age_category

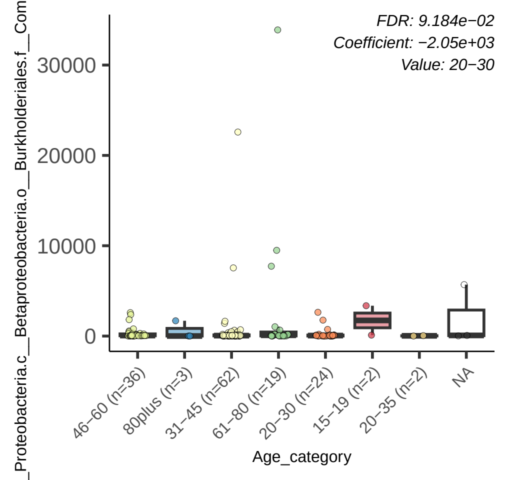

FDR: 9.184e−02
Coefficient: −2.05e+03
Value: 20−30
30000
20000
10000
0
_Proteobacteria.c__Betaproteobacteria.o__Burkholderiales.f__Com
46−60 (n=36)
80plus (n=3)
31−45 (n=62)
61−80 (n=19)
20−30 (n=24)
15−19 (n=2)
20−35 (n=2)
NA
Age_category

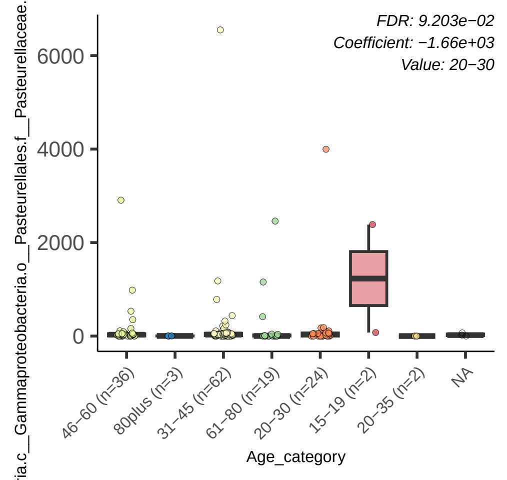

FDR: 9.203e−02
Coefficient: −1.66e+03
Value: 20−30
ria.c__Gammaproteobacteria.o__Pasteurellales.f__Pasteurellaceae.
0
2000
4000
6000
46−60 (n=36)
80plus (n=3)
31−45 (n=62)
61−80 (n=19)
20−30 (n=24)
15−19 (n=2)
20−35 (n=2)
NA
Age_category

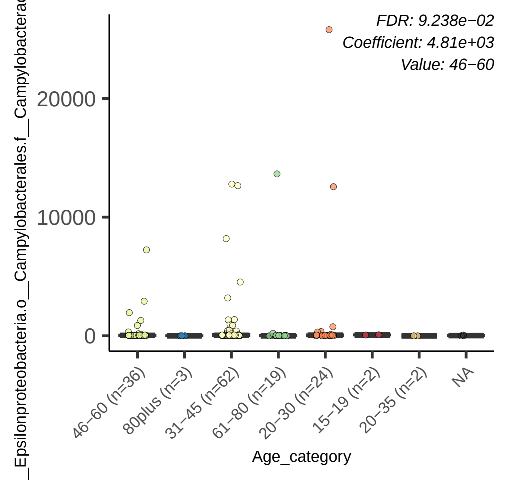
FDR: 9.238e−02
Coefficient: 4.81e+03
Value: 46−60
20000
10000
0
_Epsilonproteobacteria.o__Campylobacterales.f__Campylobacterac
46−60 (n=36)
80plus (n=3)
31−45 (n=62)
61−80 (n=19)
20−30 (n=24)
15−19 (n=2)
20−35 (n=2)
NA
Age_category

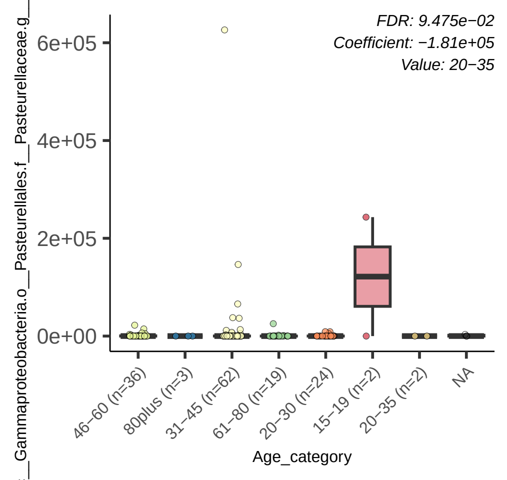

FDR: 9.475e−02
Coefficient: −1.81e+05
Value: 20−35
6e+05
4e+05
2e+05
0e+00
Gammaproteobacteria.o__Pasteurellales.f__Pasteurellaceae.g__
46−60 (n=36)
80plus (n=3)
31−45 (n=62)
61−80 (n=19)
20−30 (n=24)
15−19 (n=2)
20−35 (n=2)
NA
Age_category

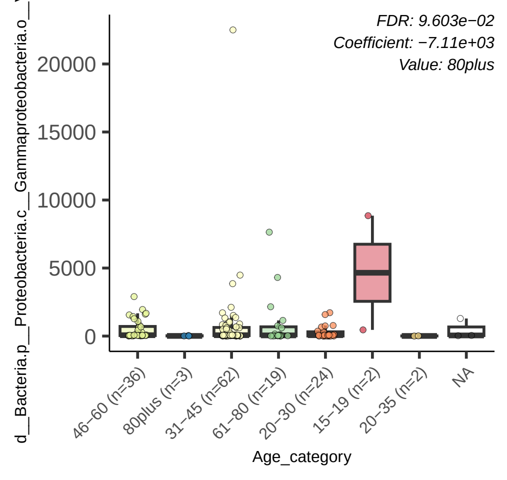
FDR: 9.603e−02
Coefficient: −7.11e+03
Value: 80plus
d__Bacteria.p__Proteobacteria.c__Gammaproteobacteria.o__
20000
15000
10000
5000
0
46−60 (n=36)
80plus (n=3)
31−45 (n=62)
61−80 (n=19)
20−30 (n=24)
15−19 (n=2)
20−35 (n=2)
NA
Age_category

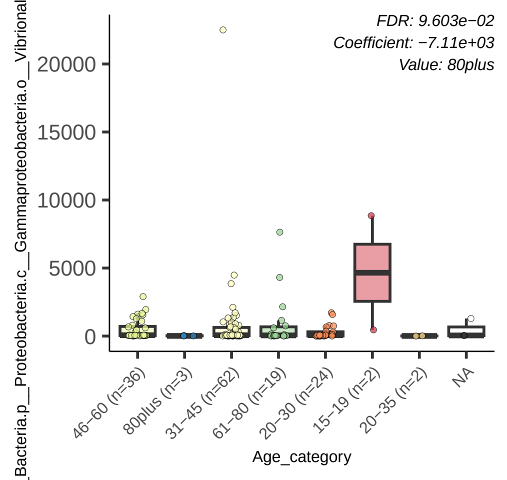

FDR: 9.603e−02
Coefficient: −7.11e+03
Value: 80plus
Bacteria.p__Proteobacteria.c__Gammaproteobacteria.o__Vibrional
20000
15000
10000
5000
0
46−60 (n=36)
80plus (n=3)
31−45 (n=62)
61−80 (n=19)
20−30 (n=24)
15−19 (n=2)
20−35 (n=2)
NA
Age_category

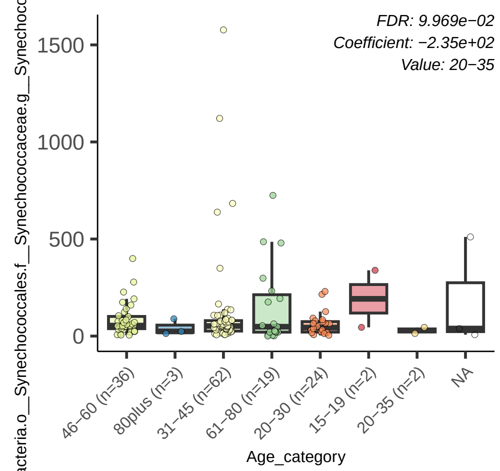

FDR: 9.969e−02
Coefficient: −2.35e+02
Value: 20−35
1500
1000
500
0
bacteria.o__Synechococcales.f__Synechococcaceae.g__Synechoco
46−60 (n=36)
80plus (n=3)
31−45 (n=62)
61−80 (n=19)
20−30 (n=24)
15−19 (n=2)
20−35 (n=2)
NA
Age_category

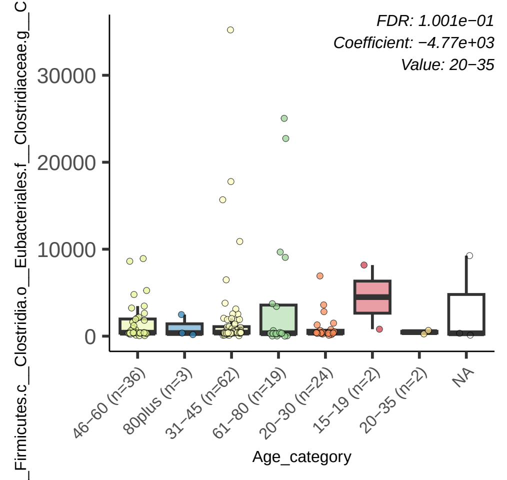

FDR: 1.001e−01
Coefficient: −4.77e+03
Value: 20−35
Firmicutes.c__Clostridia.o__Eubacteriales.f__Clostridiaceae.g__C
0
10000
20000
30000
46−60 (n=36)
80plus (n=3)
31−45 (n=62)
61−80 (n=19)
20−30 (n=24)
15−19 (n=2)
20−35 (n=2)
NA
Age_category

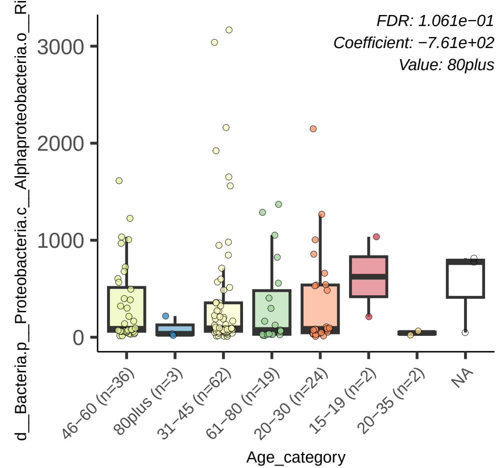

FDR: 1.061e−01
Coefficient: −7.61e+02
Value: 80plus
d__Bacteria.p__Proteobacteria.c__Alphaproteobacteria.o__Ri
3000
2000
1000
0
46−60 (n=36)
80plus (n=3)
31−45 (n=62)
61−80 (n=19)
20−30 (n=24)
15−19 (n=2)
20−35 (n=2)
NA
Age_category

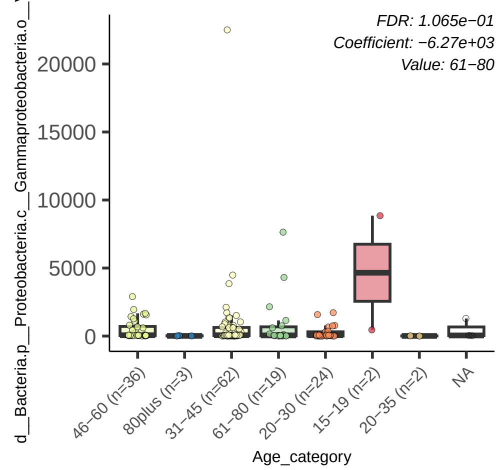

FDR: 1.065e−01
Coefficient: −6.27e+03
Value: 61−80
d__Bacteria.p__Proteobacteria.c__Gammaproteobacteria.o__
20000
15000
10000
5000
0
46−60 (n=36)
80plus (n=3)
31−45 (n=62)
61−80 (n=19)
20−30 (n=24)
15−19 (n=2)
20−35 (n=2)
NA
Age_category

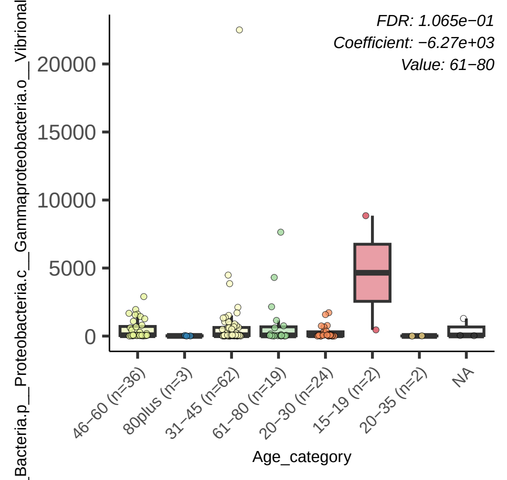
FDR: 1.065e−01
Coefficient: −6.27e+03
Value: 61−80
Bacteria.p__Proteobacteria.c__Gammaproteobacteria.o__Vibrional
20000
15000
10000
5000
0
46−60 (n=36)
80plus (n=3)
31−45 (n=62)
61−80 (n=19)
20−30 (n=24)
15−19 (n=2)
20−35 (n=2)
NA
Age_category

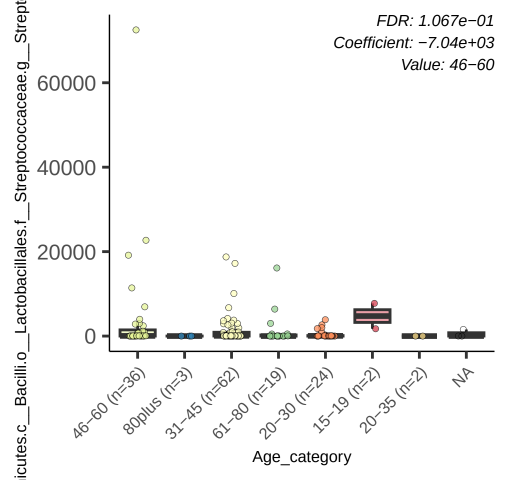

FDR: 1.067e−01
Coefficient: −7.04e+03
Value: 46−60
60000
40000
20000
0
icutes.c__Bacilli.o__Lactobacillales.f__Streptococcaceae.g__Strept
46−60 (n=36)
80plus (n=3)
31−45 (n=62)
61−80 (n=19)
20−30 (n=24)
15−19 (n=2)
20−35 (n=2)
NA
Age_category

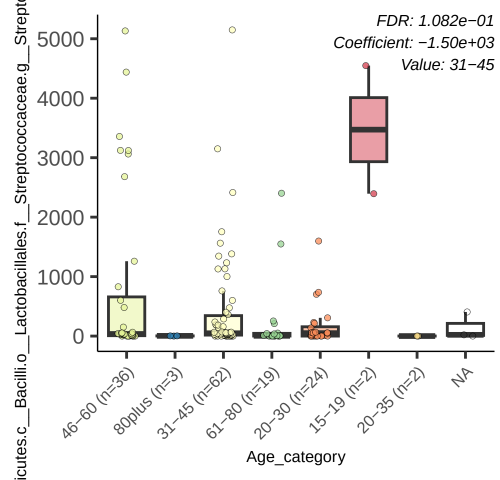

FDR: 1.082e−01
Coefficient: −1.50e+03
Value: 31−45
0
1000
2000
3000
4000
5000
46−60 (n=36)
80plus (n=3)
31−45 (n=62)
61−80 (n=19)
20−30 (n=24)
15−19 (n=2)
20−35 (n=2)
NA
Age_category

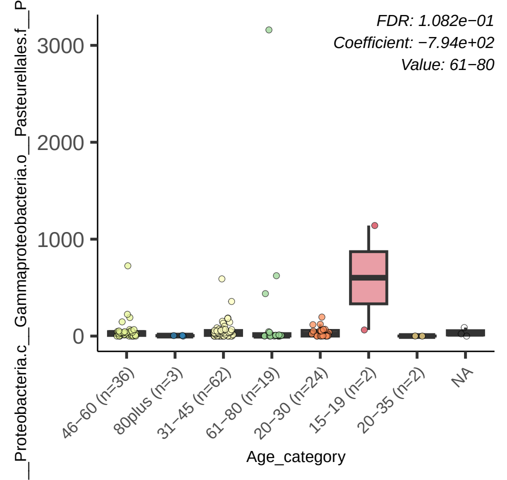

FDR: 1.082e−01
Coefficient: −7.94e+02
Value: 61−80
Proteobacteria.c__Gammaproteobacteria.o__Pasteurellales.f__P
0
1000
2000
3000
46−60 (n=36)
80plus (n=3)
31−45 (n=62)
61−80 (n=19)
20−30 (n=24)
15−19 (n=2)
20−35 (n=2)
NA
Age_category

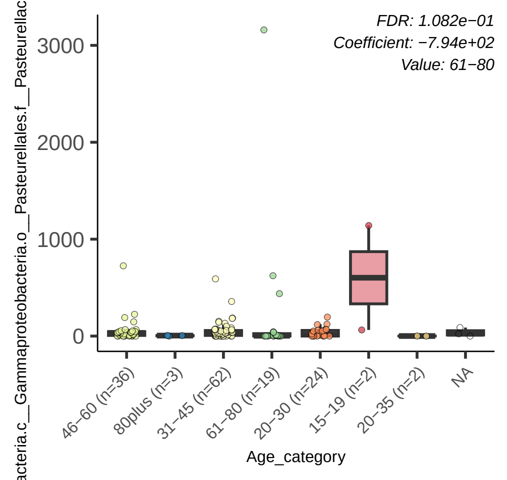
FDR: 1.082e−01
Coefficient: −7.94e+02
Value: 61−80
3000
2000
1000
0
acteria.c__Gammaproteobacteria.o__Pasteurellales.f__Pasteurellac
46−60 (n=36)
80plus (n=3)
31−45 (n=62)
61−80 (n=19)
20−30 (n=24)
15−19 (n=2)
20−35 (n=2)
NA
Age_category

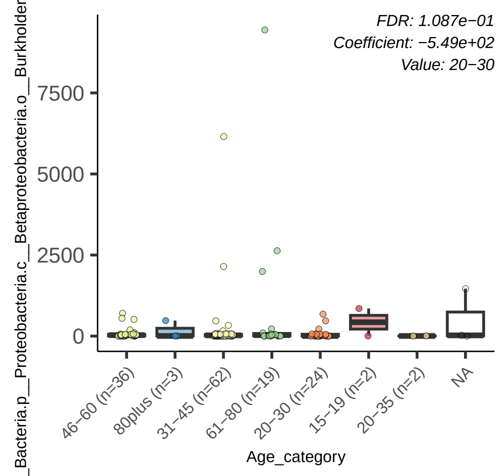

FDR: 1.087e−01
Coefficient: −5.49e+02
Value: 20−30
Bacteria.p__Proteobacteria.c__Betaproteobacteria.o__Burkholder
7500
5000
2500
0
46−60 (n=36)
80plus (n=3)
31−45 (n=62)
61−80 (n=19)
20−30 (n=24)
15−19 (n=2)
20−35 (n=2)
NA
Age_category

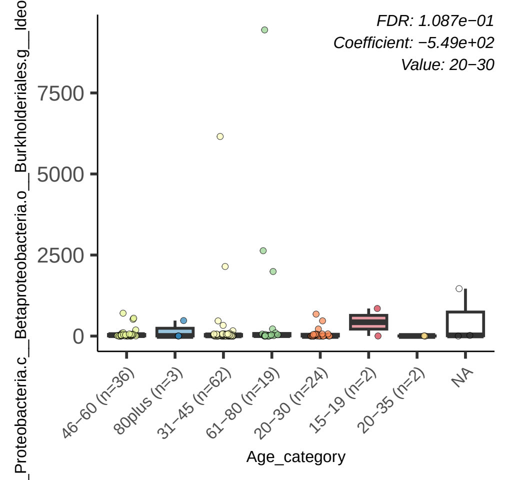

FDR: 1.087e−01
Coefficient: −5.49e+02
Value: 20−30
7500
5000
2500
0
Proteobacteria.c__Betaproteobacteria.o__Burkholderiales.g__Ideo
46−60 (n=36)
80plus (n=3)
31−45 (n=62)
61−80 (n=19)
20−30 (n=24)
15−19 (n=2)
20−35 (n=2)
NA
Age_category

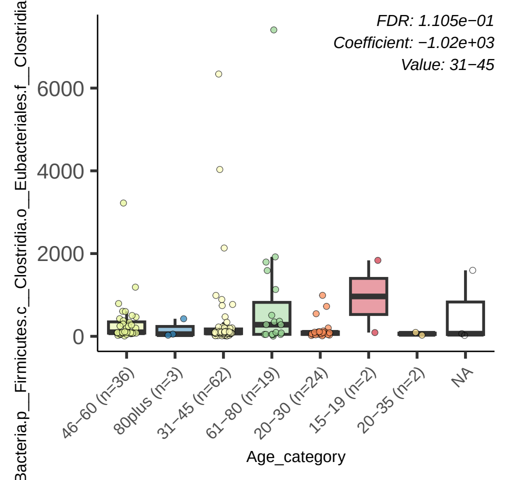

FDR: 1.105e−01
Coefficient: −1.02e+03
Value: 31−45
Bacteria.p__Firmicutes.c__Clostridia.o__Eubacteriales.f__Clostridia
0
2000
4000
6000
46−60 (n=36)
80plus (n=3)
31−45 (n=62)
61−80 (n=19)
20−30 (n=24)
15−19 (n=2)
20−35 (n=2)
NA
Age_category

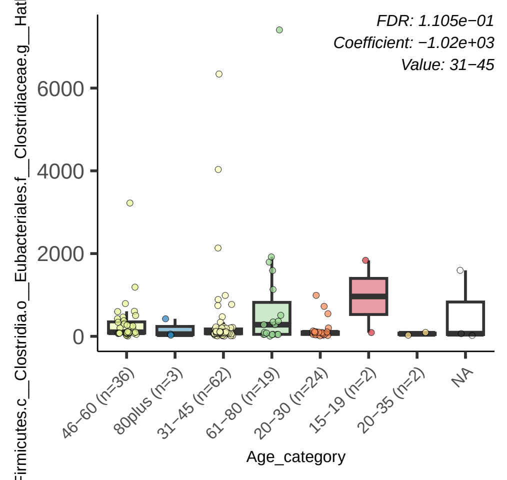
FDR: 1.105e−01
Coefficient: −1.02e+03
Value: 31−45
Firmicutes.c__Clostridia.o__Eubacteriales.f__Clostridiaceae.g__Hat
6000
4000
2000
0
46−60 (n=36)
80plus (n=3)
31−45 (n=62)
61−80 (n=19)
20−30 (n=24)
15−19 (n=2)
20−35 (n=2)
NA
Age_category

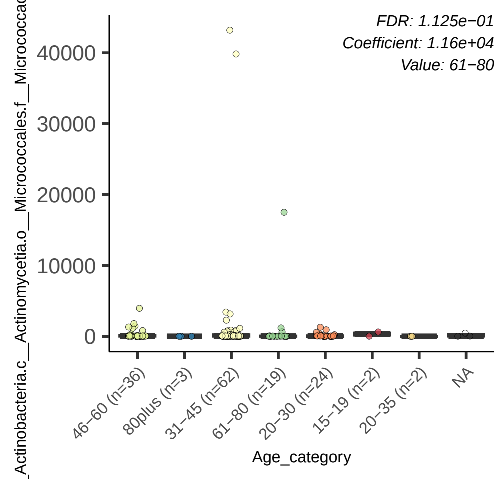

FDR: 1.125e−01
Coefficient: 1.16e+04
Value: 61−80
40000
30000
20000
10000
0
46−60 (n=36)
80plus (n=3)
31−45 (n=62)
61−80 (n=19)
20−30 (n=24)
15−19 (n=2)
20−35 (n=2)
NA
Age_category

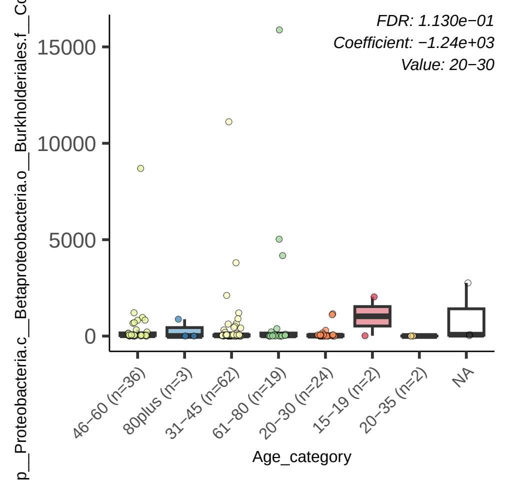

FDR: 1.130e−01
Coefficient: −1.24e+03
Value: 20−30
p__Proteobacteria.c__Betaproteobacteria.o__Burkholderiales.f__Co
15000
10000
5000
0
46−60 (n=36)
80plus (n=3)
31−45 (n=62)
61−80 (n=19)
20−30 (n=24)
15−19 (n=2)
20−35 (n=2)
NA
Age_category

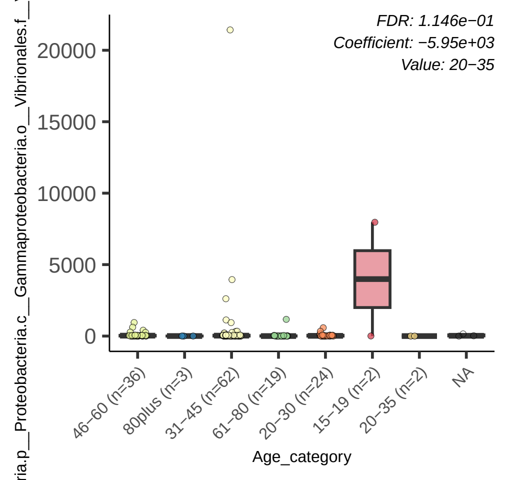

FDR: 1.146e−01
Coefficient: −5.95e+03
Value: 20−35
ria.p__Proteobacteria.c__Gammaproteobacteria.o__Vibrionales.f__
20000
15000
10000
5000
0
46−60 (n=36)
80plus (n=3)
31−45 (n=62)
61−80 (n=19)
20−30 (n=24)
15−19 (n=2)
20−35 (n=2)
NA
Age_category

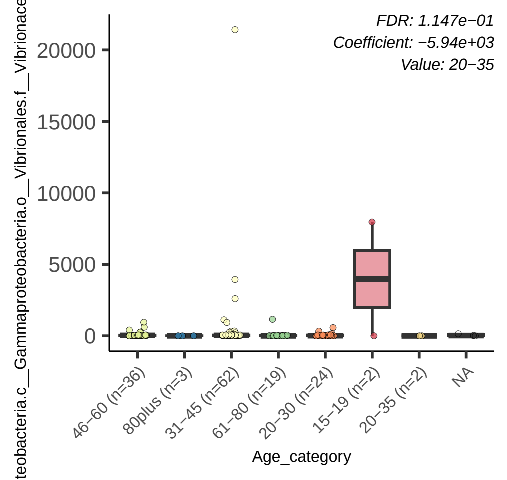
FDR: 1.147e−01
Coefficient: −5.94e+03
Value: 20−35
20000
15000
10000
5000
0
teobacteria.c__Gammaproteobacteria.o__Vibrionales.f__Vibrionace
46−60 (n=36)
80plus (n=3)
31−45 (n=62)
61−80 (n=19)
20−30 (n=24)
15−19 (n=2)
20−35 (n=2)
NA
Age_category

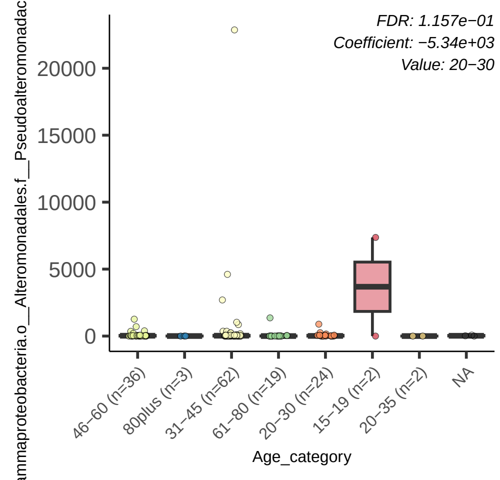
FDR: 1.157e−01
Coefficient: −5.34e+03
Value: 20−30
ammaproteobacteria.o__Alteromonadales.f__Pseudoalteromonadac
0
5000
10000
15000
20000
46−60 (n=36)
80plus (n=3)
31−45 (n=62)
61−80 (n=19)
20−30 (n=24)
15−19 (n=2)
20−35 (n=2)
NA
Age_category

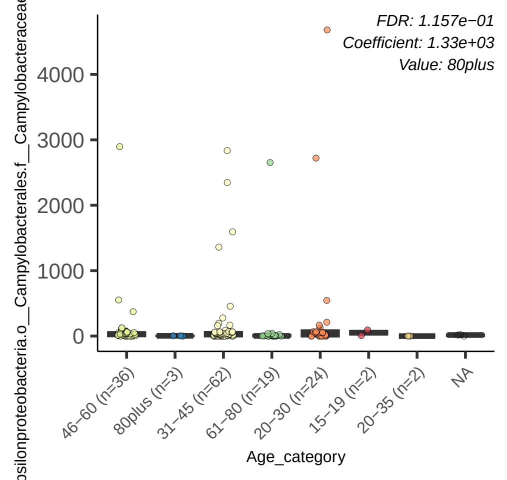

FDR: 1.157e−01
Coefficient: 1.33e+03
Value: 80plus
0
1000
2000
3000
4000
psilonproteobacteria.o__Campylobacterales.f__Campylobacteraceae
46−60 (n=36)
80plus (n=3)
31−45 (n=62)
61−80 (n=19)
20−30 (n=24)
15−19 (n=2)
20−35 (n=2)
NA
Age_category

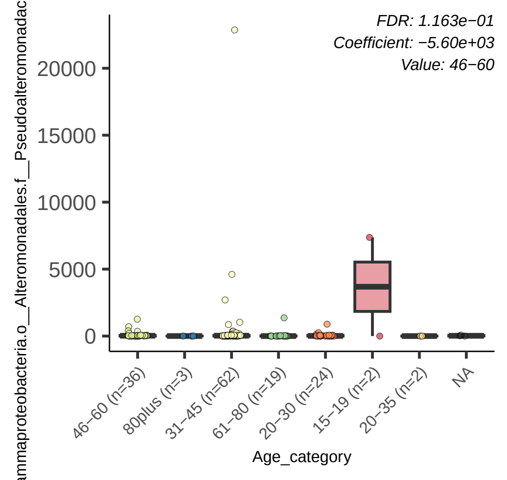
FDR: 1.163e−01
Coefficient: −5.60e+03
Value: 46−60
ammaproteobacteria.o__Alteromonadales.f__Pseudoalteromonadac
20000
15000
10000
5000
0
46−60 (n=36)
80plus (n=3)
31−45 (n=62)
61−80 (n=19)
20−30 (n=24)
15−19 (n=2)
20−35 (n=2)
NA
Age_category

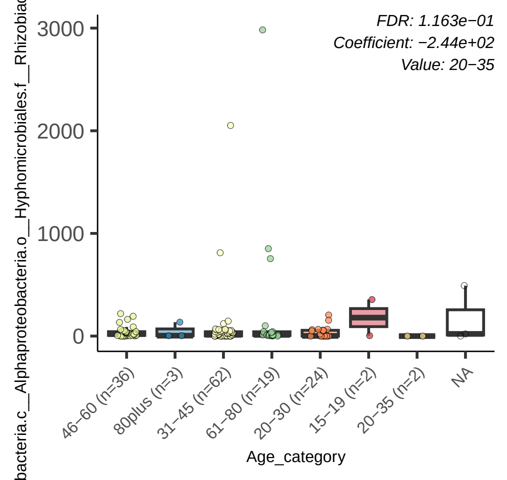

FDR: 1.163e−01
Coefficient: −2.44e+02
Value: 20−35
3000
2000
1000
0
bacteria.c__Alphaproteobacteria.o__Hyphomicrobiales.f__Rhizobiac
46−60 (n=36)
80plus (n=3)
31−45 (n=62)
61−80 (n=19)
20−30 (n=24)
15−19 (n=2)
20−35 (n=2)
NA
Age_category

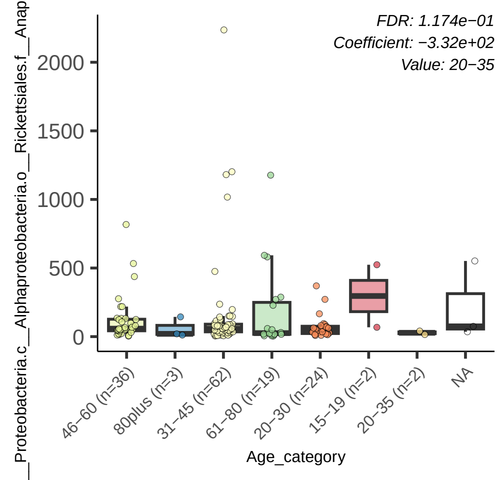

FDR: 1.174e−01
Coefficient: −3.32e+02
Value: 20−35
0
500
1000
1500
2000
Proteobacteria.c__Alphaproteobacteria.o__Rickettsiales.f__Anap
46−60 (n=36)
80plus (n=3)
31−45 (n=62)
61−80 (n=19)
20−30 (n=24)
15−19 (n=2)
20−35 (n=2)
NA
Age_category

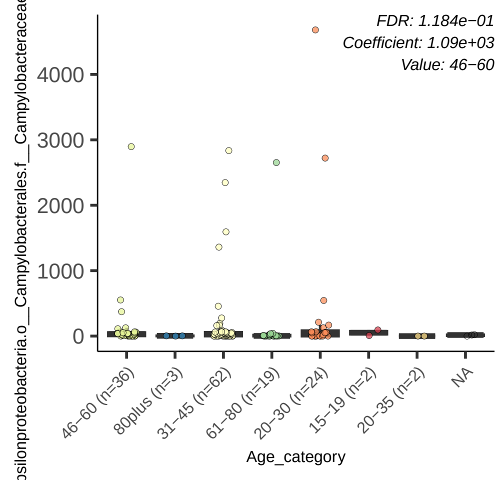

FDR: 1.184e−01
Coefficient: 1.09e+03
Value: 46−60
4000
3000
2000
1000
0
psilonproteobacteria.o__Campylobacterales.f__Campylobacterace
46−60 (n=36)
80plus (n=3)
31−45 (n=62)
61−80 (n=19)
20−30 (n=24)
15−19 (n=2)
20−35 (n=2)
NA
Age_category

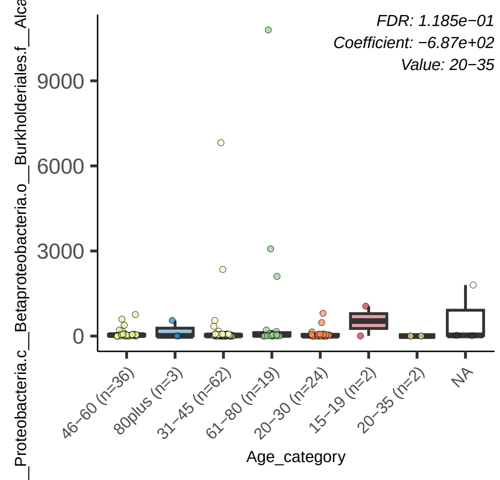
FDR: 1.185e−01
Coefficient: −6.87e+02
Value: 20−35
9000
6000
3000
0
Proteobacteria.c__Betaproteobacteria.o__Burkholderiales.f__Alca
46−60 (n=36)
80plus (n=3)
31−45 (n=62)
61−80 (n=19)
20−30 (n=24)
15−19 (n=2)
20−35 (n=2)
NA
Age_category

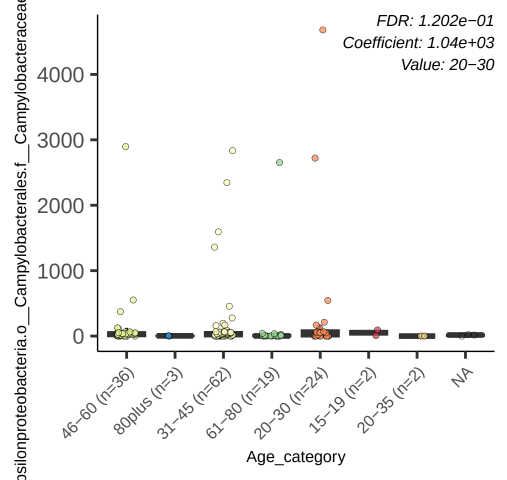
FDR: 1.202e−01
Coefficient: 1.04e+03
Value: 20−30
0
1000
2000
3000
4000
psilonproteobacteria.o__Campylobacterales.f__Campylobacterace
46−60 (n=36)
80plus (n=3)
31−45 (n=62)
61−80 (n=19)
20−30 (n=24)
15−19 (n=2)
20−35 (n=2)
NA
Age_category

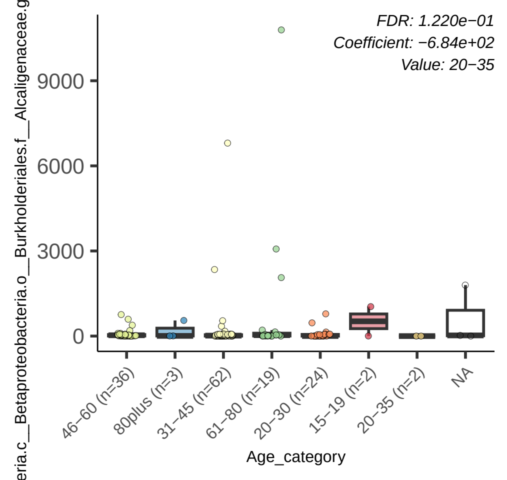

FDR: 1.220e−01
Coefficient: −6.84e+02
Value: 20−35
9000
6000
3000
0
46−60 (n=36)
80plus (n=3)
31−45 (n=62)
61−80 (n=19)
20−30 (n=24)
15−19 (n=2)
20−35 (n=2)
NA
Age_category

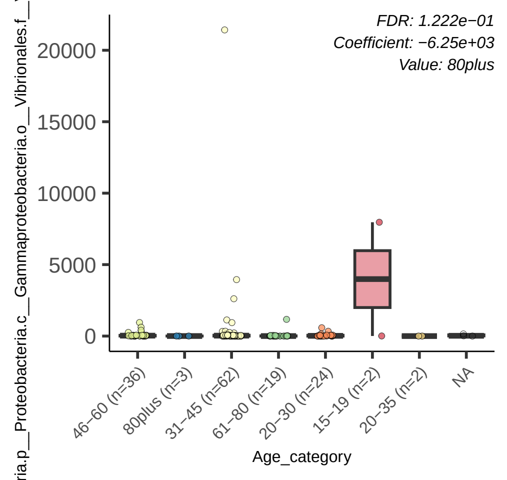

FDR: 1.222e−01
Coefficient: −6.25e+03
Value: 80plus
ria.p__Proteobacteria.c__Gammaproteobacteria.o__Vibrionales.f__
20000
15000
10000
5000
0
46−60 (n=36)
80plus (n=3)
31−45 (n=62)
61−80 (n=19)
20−30 (n=24)
15−19 (n=2)
20−35 (n=2)
NA
Age_category

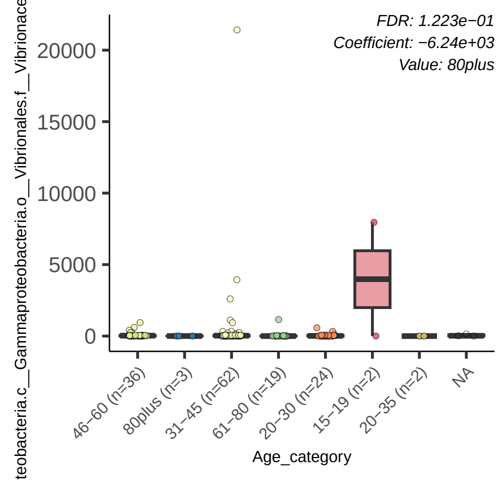

FDR: 1.223e−01
Coefficient: −6.24e+03
Value: 80plus
teobacteria.c__Gammaproteobacteria.o__Vibrionales.f__Vibrionace
20000
15000
10000
5000
0
46−60 (n=36)
80plus (n=3)
31−45 (n=62)
61−80 (n=19)
20−30 (n=24)
15−19 (n=2)
20−35 (n=2)
NA
Age_category

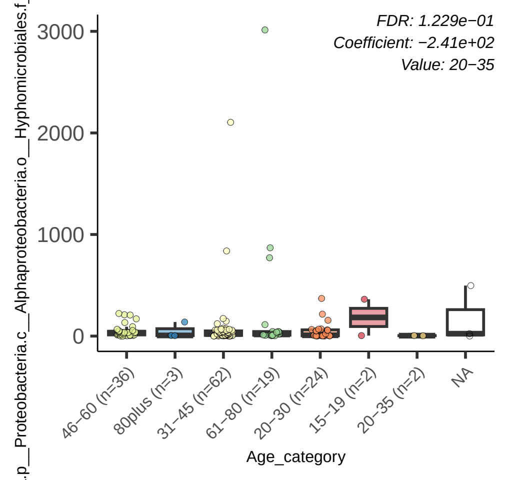
FDR: 1.229e−01
Coefficient: −2.41e+02
Value: 20−35
3000
2000
1000
0
p__Proteobacteria.c__Alphaproteobacteria.o__Hyphomicrobiales.f_
46−60 (n=36)
80plus (n=3)
31−45 (n=62)
61−80 (n=19)
20−30 (n=24)
15−19 (n=2)
20−35 (n=2)
NA
Age_category

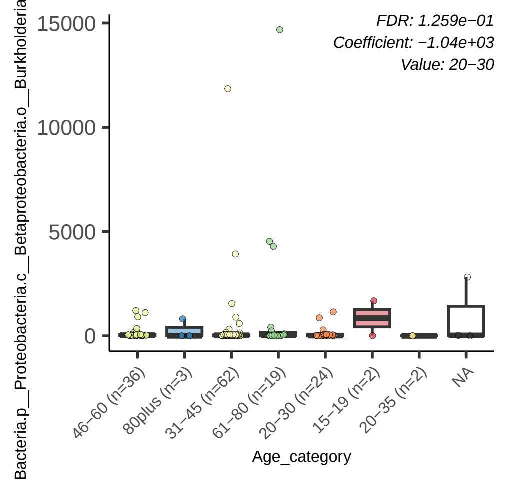

FDR: 1.259e−01
Coefficient: −1.04e+03
Value: 20−30
15000
10000
5000
0
Bacteria.p__Proteobacteria.c__Betaproteobacteria.o__Burkholderia
46−60 (n=36)
80plus (n=3)
31−45 (n=62)
61−80 (n=19)
20−30 (n=24)
15−19 (n=2)
20−35 (n=2)
NA
Age_category

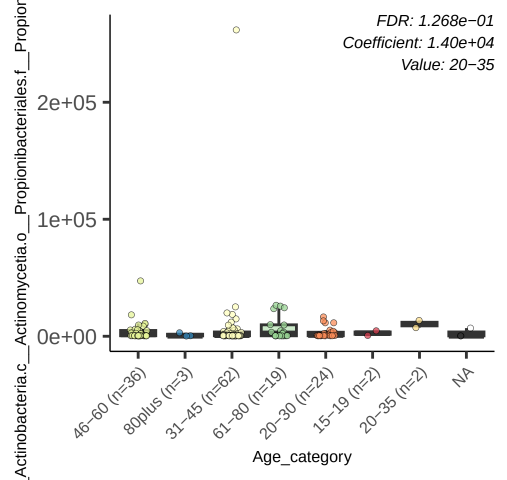
FDR: 1.268e−01
Coefficient: 1.40e+04
Value: 20−35
Actinobacteria.c__Actinomycetia.o__Propionibacteriales.f__Propion
2e+05
1e+05
0e+00
46−60 (n=36)
80plus (n=3)
31−45 (n=62)
61−80 (n=19)
20−30 (n=24)
15−19 (n=2)
20−35 (n=2)
NA
Age_category

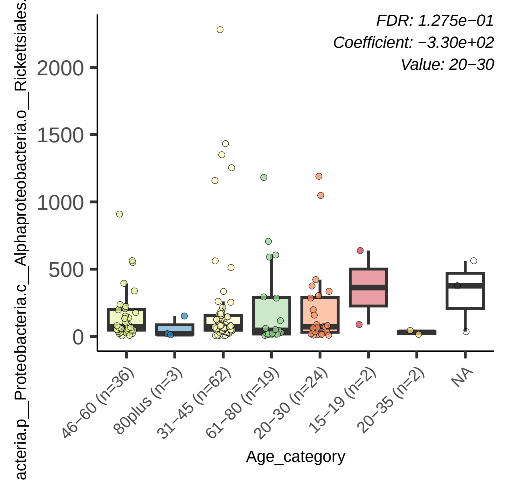

FDR: 1.275e−01
Coefficient: −3.30e+02
Value: 20−30
acteria.p__Proteobacteria.c__Alphaproteobacteria.o__Rickettsiales.
2000
1500
1000
500
0
46−60 (n=36)
80plus (n=3)
31−45 (n=62)
61−80 (n=19)
20−30 (n=24)
15−19 (n=2)
20−35 (n=2)
NA
Age_category

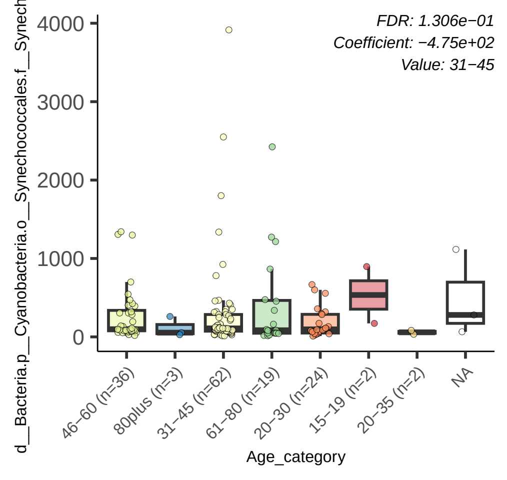
FDR: 1.306e−01
Coefficient: −4.75e+02
Value: 31−45
d__Bacteria.p__Cyanobacteria.o__Synechococcales.f__Synech
4000
3000
2000
1000
0
46−60 (n=36)
80plus (n=3)
31−45 (n=62)
61−80 (n=19)
20−30 (n=24)
15−19 (n=2)
20−35 (n=2)
NA
Age_category

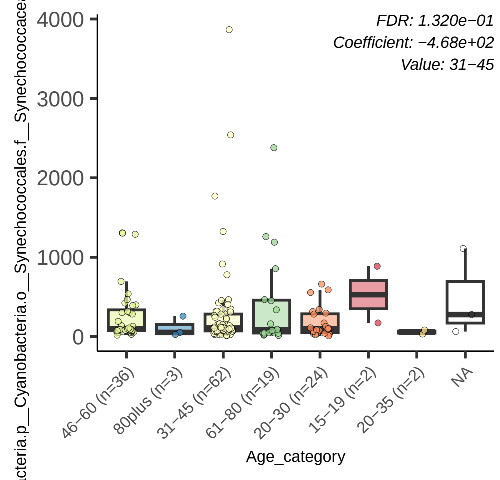

FDR: 1.320e−01
Coefficient: −4.68e+02
Value: 31−45
4000
3000
2000
1000
0
cteria.p__Cyanobacteria.o__Synechococcales.f__Synechococcace
46−60 (n=36)
80plus (n=3)
31−45 (n=62)
61−80 (n=19)
20−30 (n=24)
15−19 (n=2)
20−35 (n=2)
NA
Age_category

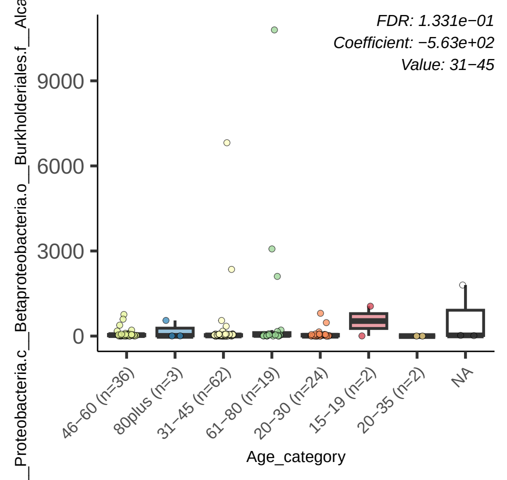

FDR: 1.331e−01
Coefficient: −5.63e+02
Value: 31−45
9000
6000
3000
0
Proteobacteria.c__Betaproteobacteria.o__Burkholderiales.f__Alca
46−60 (n=36)
80plus (n=3)
31−45 (n=62)
61−80 (n=19)
20−30 (n=24)
15−19 (n=2)
20−35 (n=2)
NA
Age_category

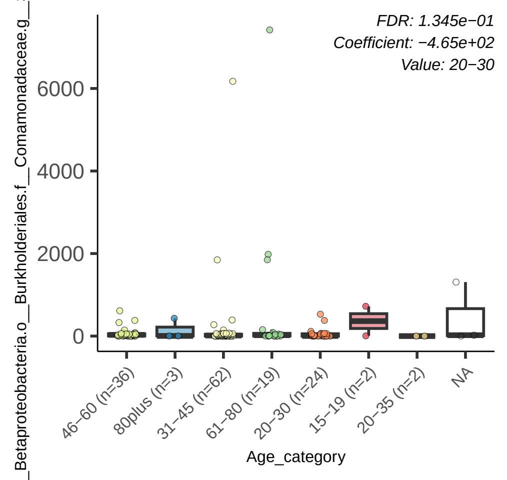
FDR: 1.345e−01
Coefficient: −4.65e+02
Value: 20−30
Betaproteobacteria.o__Burkholderiales.f__Comamonadaceae.g__
6000
4000
2000
0
46−60 (n=36)
80plus (n=3)
31−45 (n=62)
61−80 (n=19)
20−30 (n=24)
15−19 (n=2)
20−35 (n=2)
NA
Age_category

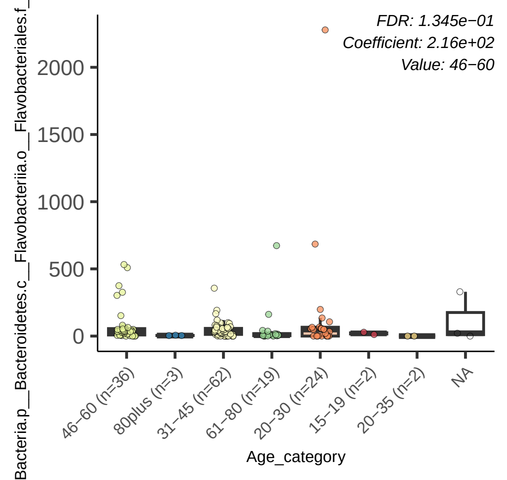
FDR: 1.345e−01
Coefficient: 2.16e+02
Value: 46−60
2000
1500
1000
500
0
Bacteria.p__Bacteroidetes.c__Flavobacteriia.o__Flavobacteriales.f__
46−60 (n=36)
80plus (n=3)
31−45 (n=62)
61−80 (n=19)
20−30 (n=24)
15−19 (n=2)
20−35 (n=2)
NA
Age_category

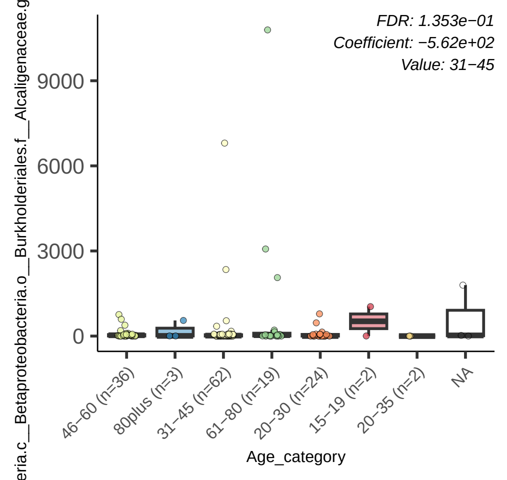

FDR: 1.353e−01
Coefficient: −5.62e+02
Value: 31−45
9000
6000
3000
0
46−60 (n=36)
80plus (n=3)
31−45 (n=62)
61−80 (n=19)
20−30 (n=24)
15−19 (n=2)
20−35 (n=2)
NA
Age_category
eria.c__Betaproteobacteria.o__Burkholderiales.f__Alcaligenaceae.g

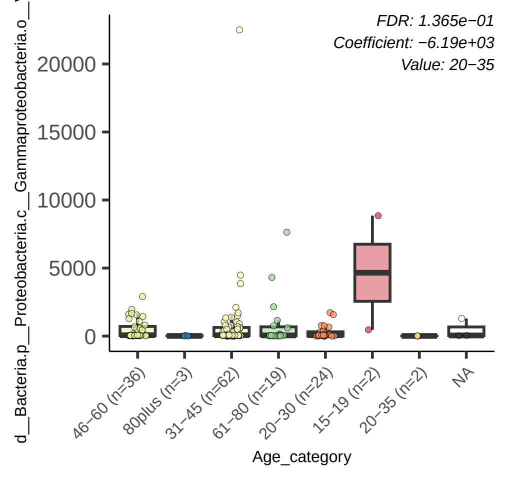
FDR: 1.365e−01
Coefficient: −6.19e+03
Value: 20−35
d__Bacteria.p__Proteobacteria.c__Gammaproteobacteria.o__
0
5000
10000
15000
20000
46−60 (n=36)
80plus (n=3)
31−45 (n=62)
61−80 (n=19)
20−30 (n=24)
15−19 (n=2)
20−35 (n=2)
NA
Age_category

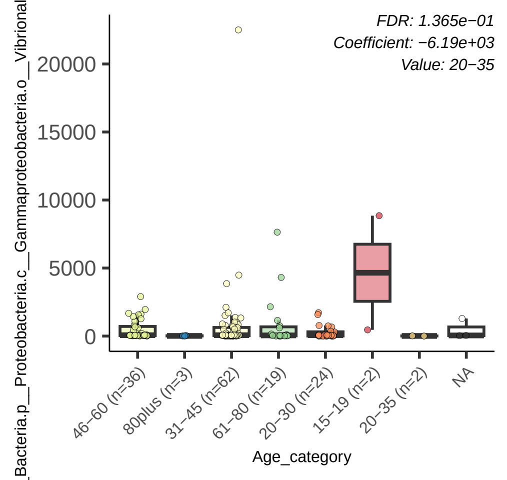

FDR: 1.365e−01
Coefficient: −6.19e+03
Value: 20−35
20000
15000
10000
5000
0
_Bacteria.p__Proteobacteria.c__Gammaproteobacteria.o__Vibrional
46−60 (n=36)
80plus (n=3)
31−45 (n=62)
61−80 (n=19)
20−30 (n=24)
15−19 (n=2)
20−35 (n=2)
NA
Age_category

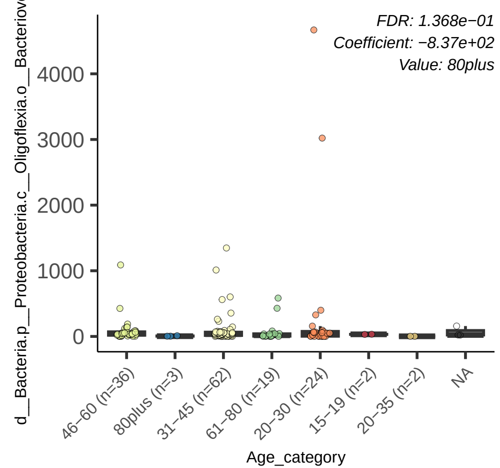

FDR: 1.368e−01
Coefficient: −8.37e+02
Value: 80plus
d__Bacteria.p__Proteobacteria.c__Oligoflexia.o__Bacteriov
4000
3000
2000
1000
0
46−60 (n=36)
80plus (n=3)
31−45 (n=62)
61−80 (n=19)
20−30 (n=24)
15−19 (n=2)
20−35 (n=2)
NA
Age_category

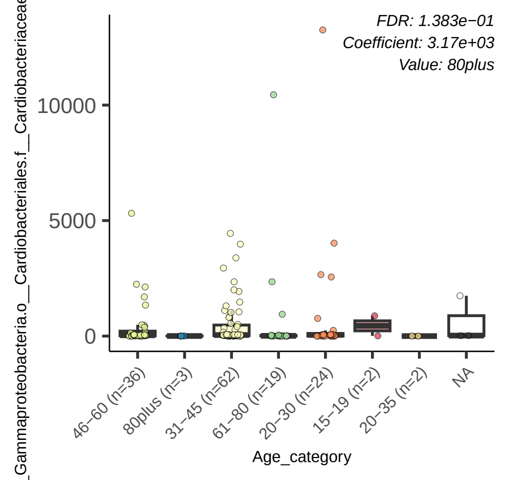

FDR: 1.383e−01
Coefficient: 3.17e+03
Value: 80plus
_Gammaproteobacteria.o__Cardiobacteriales.f__Cardiobacteriaceae
10000
5000
0
46−60 (n=36)
80plus (n=3)
31−45 (n=62)
61−80 (n=19)
20−30 (n=24)
15−19 (n=2)
20−35 (n=2)
NA
Age_category

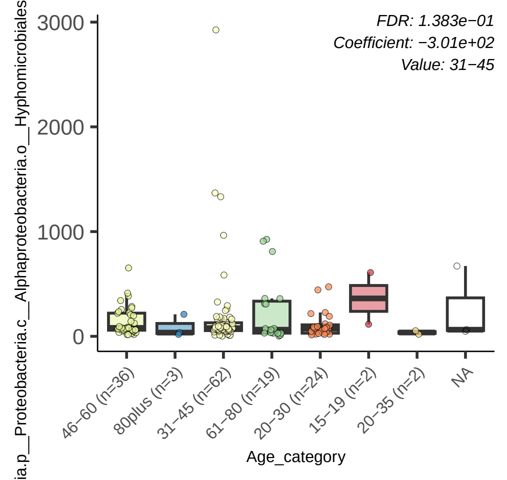

FDR: 1.383e−01
Coefficient: −3.01e+02
Value: 31−45
ia.p__Proteobacteria.c__Alphaproteobacteria.o__Hyphomicrobiales
3000
2000
1000
0
46−60 (n=36)
80plus (n=3)
31−45 (n=62)
61−80 (n=19)
20−30 (n=24)
15−19 (n=2)
20−35 (n=2)
NA
Age_category

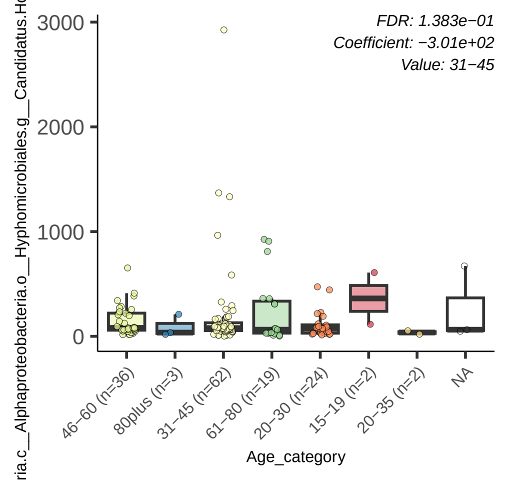

FDR: 1.383e−01
Coefficient: −3.01e+02
Value: 31−45
3000
2000
1000
0
ria.c__Alphaproteobacteria.o__Hyphomicrobiales.g__Candidatus.Ho
46−60 (n=36)
80plus (n=3)
31−45 (n=62)
61−80 (n=19)
20−30 (n=24)
15−19 (n=2)
20−35 (n=2)
NA
Age_category

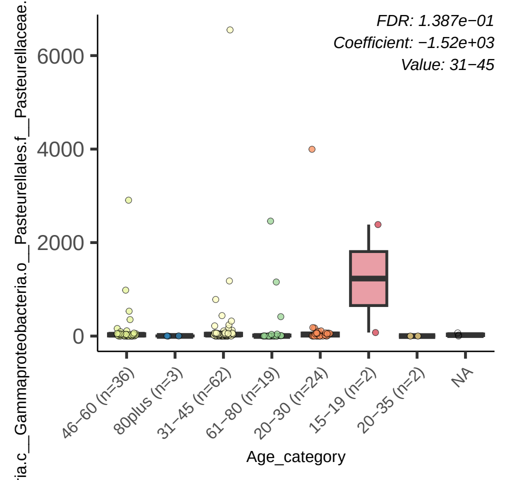

FDR: 1.387e−01
Coefficient: −1.52e+03
Value: 31−45
ria.c__Gammaproteobacteria.o__Pasteurellales.f__Pasteurellaceae.
6000
4000
2000
0
46−60 (n=36)
80plus (n=3)
31−45 (n=62)
61−80 (n=19)
20−30 (n=24)
15−19 (n=2)
20−35 (n=2)
NA
Age_category

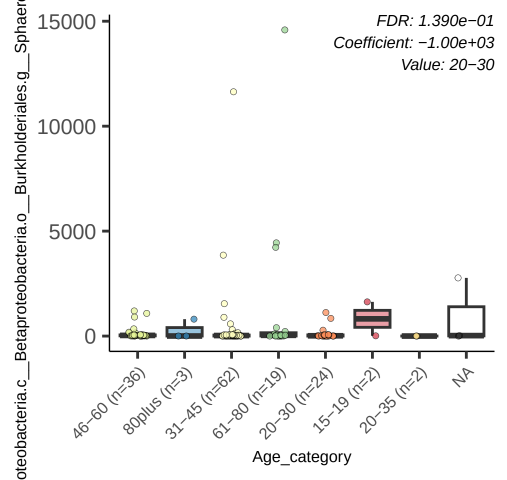

FDR: 1.390e−01
Coefficient: −1.00e+03
Value: 20−30
15000
10000
5000
0
oteobacteria.c__Betaproteobacteria.o__Burkholderiales.g__Sphaero
46−60 (n=36)
80plus (n=3)
31−45 (n=62)
61−80 (n=19)
20−30 (n=24)
15−19 (n=2)
20−35 (n=2)
NA
Age_category

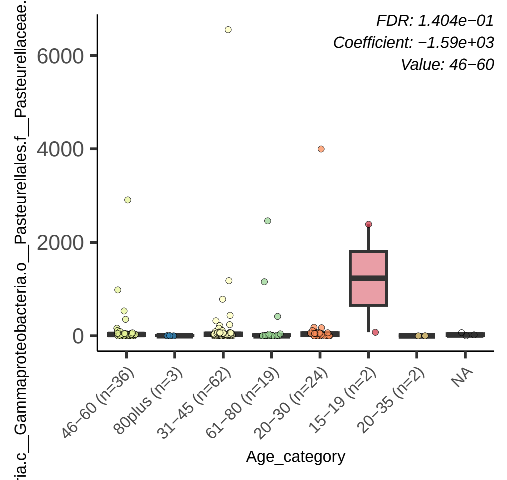
FDR: 1.404e−01
Coefficient: −1.59e+03
Value: 46−60
ria.c__Gammaproteobacteria.o__Pasteurellales.f__Pasteurellaceae.
6000
4000
2000
0
46−60 (n=36)
80plus (n=3)
31−45 (n=62)
61−80 (n=19)
20−30 (n=24)
15−19 (n=2)
20−35 (n=2)
NA
Age_category

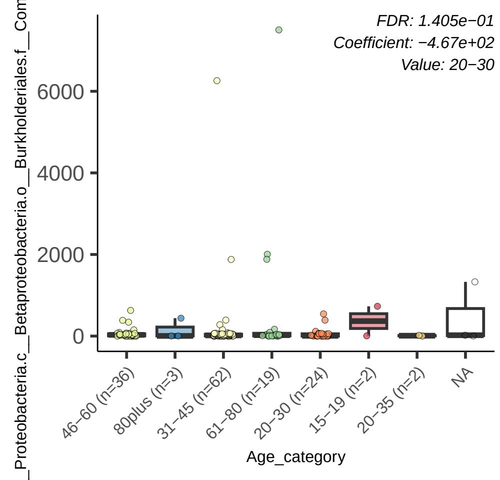

FDR: 1.405e−01
Coefficient: −4.67e+02
Value: 20−30
_Proteobacteria.c__Betaproteobacteria.o__Burkholderiales.f__Com
6000
4000
2000
0
46−60 (n=36)
80plus (n=3)
31−45 (n=62)
61−80 (n=19)
20−30 (n=24)
15−19 (n=2)
20−35 (n=2)
NA
Age_category

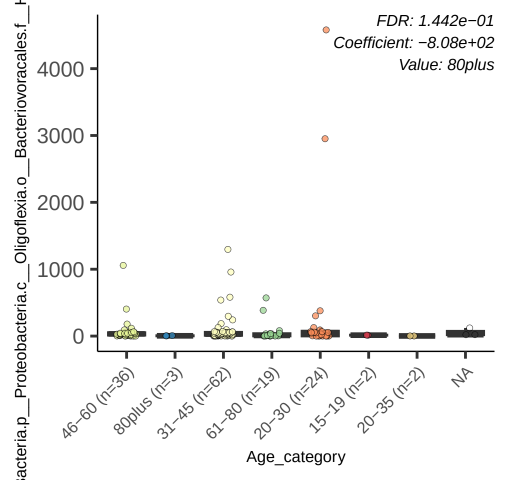

FDR: 1.442e−01
Coefficient: −8.08e+02
Value: 80plus
bacteria.p__Proteobacteria.c__Oligoflexia.o__Bacteriovoracales.f__H
4000
3000
2000
1000
0
46−60 (n=36)
80plus (n=3)
31−45 (n=62)
61−80 (n=19)
20−30 (n=24)
15−19 (n=2)
20−35 (n=2)
NA
Age_category

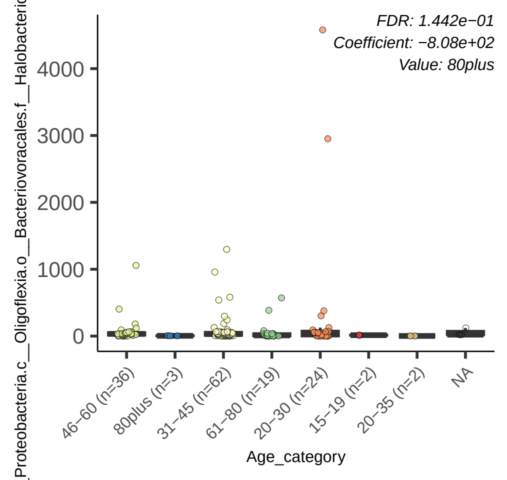

FDR: 1.442e−01
Coefficient: −8.08e+02
Value: 80plus
Proteobacteria.c__Oligoflexia.o__Bacteriovoracales.f__Halobacterio
4000
3000
2000
1000
0
46−60 (n=36)
80plus (n=3)
31−45 (n=62)
61−80 (n=19)
20−30 (n=24)
15−19 (n=2)
20−35 (n=2)
NA
Age_category

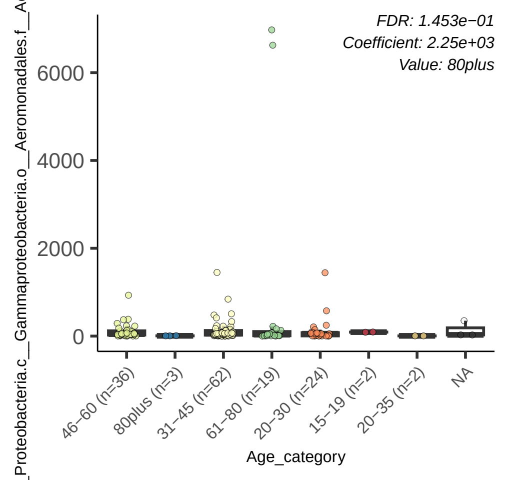

FDR: 1.453e−01
Coefficient: 2.25e+03
Value: 80plus
6000
4000
2000
0
Proteobacteria.c__Gammaproteobacteria.o__Aeromonadales.f__Ae
46−60 (n=36)
80plus (n=3)
31−45 (n=62)
61−80 (n=19)
20−30 (n=24)
15−19 (n=2)
20−35 (n=2)
NA
Age_category

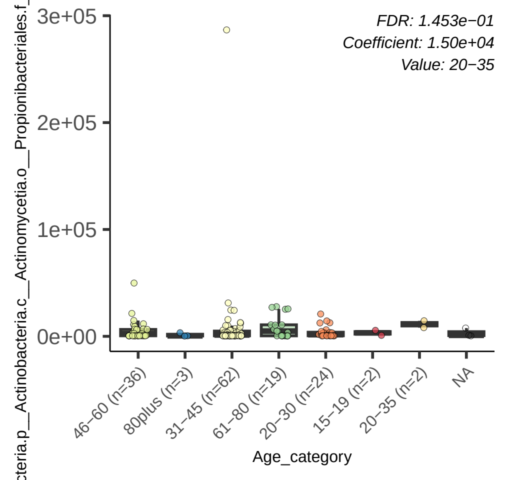

FDR: 1.453e−01
Coefficient: 1.50e+04
Value: 20−35
3e+05
2e+05
1e+05
0e+00
cteria.p__Actinobacteria.c__Actinomycetia.o__Propionibacteriales.f_
46−60 (n=36)
80plus (n=3)
31−45 (n=62)
61−80 (n=19)
20−30 (n=24)
15−19 (n=2)
20−35 (n=2)
NA
Age_category

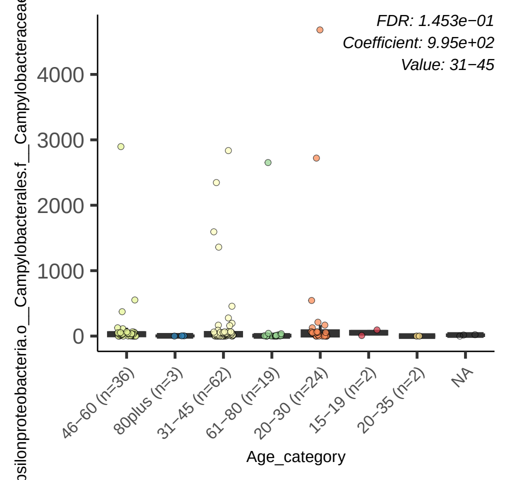
FDR: 1.453e−01
Coefficient: 9.95e+02
Value: 31−45
4000
3000
2000
1000
0
psilonproteobacteria.o__Campylobacterales.f__Campylobacteraceae
46−60 (n=36)
80plus (n=3)
31−45 (n=62)
61−80 (n=19)
20−30 (n=24)
15−19 (n=2)
20−35 (n=2)
NA
Age_category

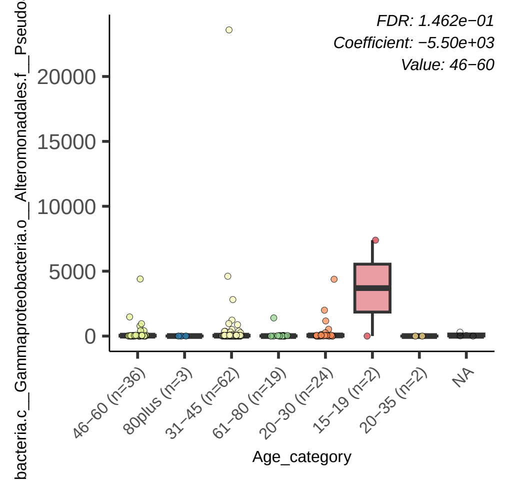

FDR: 1.462e−01
Coefficient: −5.50e+03
Value: 46−60
bacteria.c__Gammaproteobacteria.o__Alteromonadales.f__Pseudo
20000
15000
10000
5000
0
46−60 (n=36)
80plus (n=3)
31−45 (n=62)
61−80 (n=19)
20−30 (n=24)
15−19 (n=2)
20−35 (n=2)
NA
Age_category

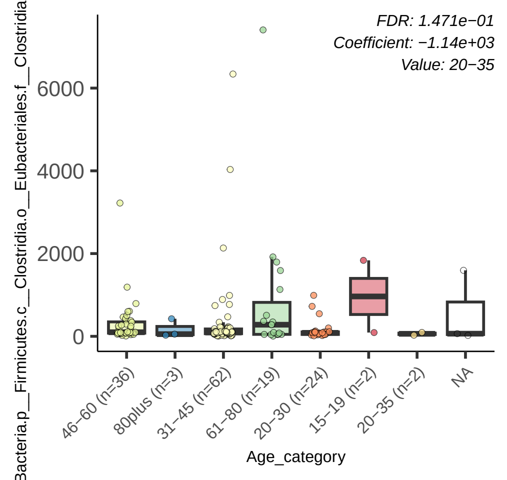

FDR: 1.471e−01
Coefficient: −1.14e+03
Value: 20−35
Bacteria.p__Firmicutes.c__Clostridia.o__Eubacteriales.f__Clostridia
0
2000
4000
6000
46−60 (n=36)
80plus (n=3)
31−45 (n=62)
61−80 (n=19)
20−30 (n=24)
15−19 (n=2)
20−35 (n=2)
NA
Age_category

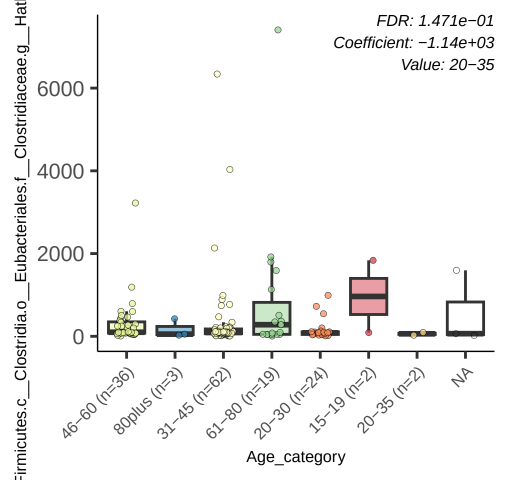

FDR: 1.471e−01
Coefficient: −1.14e+03
Value: 20−35
Firmicutes.c__Clostridia.o__Eubacteriales.f__Clostridiaceae.g__Hat
6000
4000
2000
0
46−60 (n=36)
80plus (n=3)
31−45 (n=62)
61−80 (n=19)
20−30 (n=24)
15−19 (n=2)
20−35 (n=2)
NA
Age_category

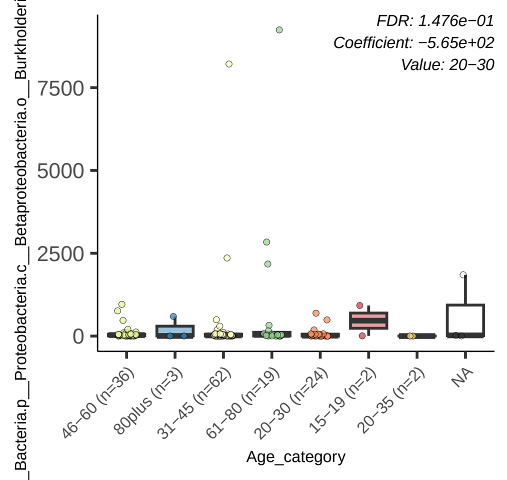

FDR: 1.476e−01
Coefficient: −5.65e+02
Value: 20−30
Bacteria.p__Proteobacteria.c__Betaproteobacteria.o__Burkholderi
7500
5000
2500
0
46−60 (n=36)
80plus (n=3)
31−45 (n=62)
61−80 (n=19)
20−30 (n=24)
15−19 (n=2)
20−35 (n=2)
NA
Age_category

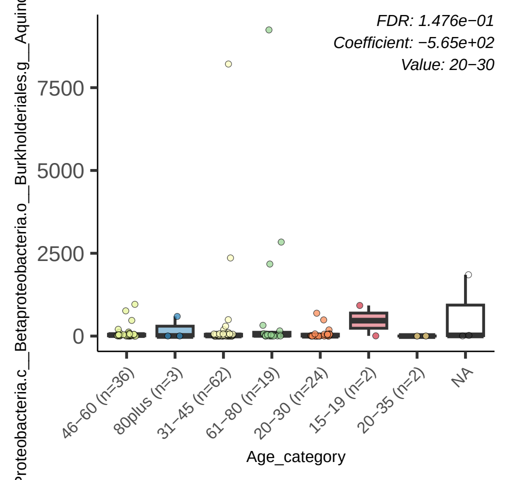

FDR: 1.476e−01
Coefficient: −5.65e+02
Value: 20−30
Proteobacteria.c__Betaproteobacteria.o__Burkholderiales.g__Aquin
7500
5000
2500
0
46−60 (n=36)
80plus (n=3)
31−45 (n=62)
61−80 (n=19)
20−30 (n=24)
15−19 (n=2)
20−35 (n=2)
NA
Age_category

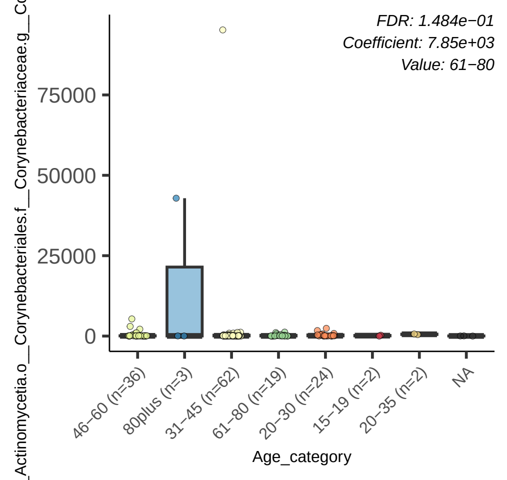

FDR: 1.484e−01
Coefficient: 7.85e+03
Value: 61−80
Actinomycetia.o__Corynebacteriales.f__Corynebacteriaceae.g__Co
75000
50000
25000
0
46−60 (n=36)
80plus (n=3)
31−45 (n=62)
61−80 (n=19)
20−30 (n=24)
15−19 (n=2)
20−35 (n=2)
NA
Age_category

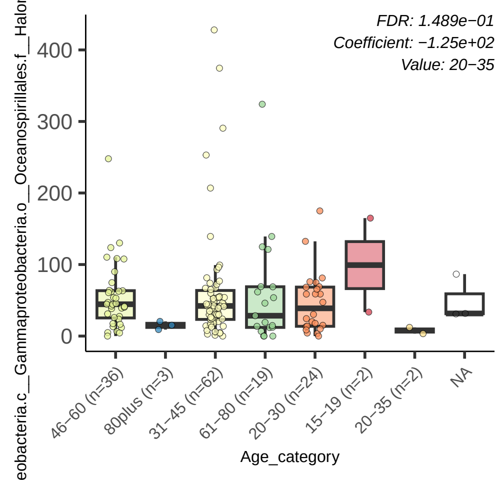

FDR: 1.489e−01
Coefficient: −1.25e+02
Value: 20−35
eobacteria.c__Gammaproteobacteria.o__Oceanospirillales.f__Halor
400
300
200
100
0
46−60 (n=36)
80plus (n=3)
31−45 (n=62)
61−80 (n=19)
20−30 (n=24)
15−19 (n=2)
20−35 (n=2)
NA
Age_category

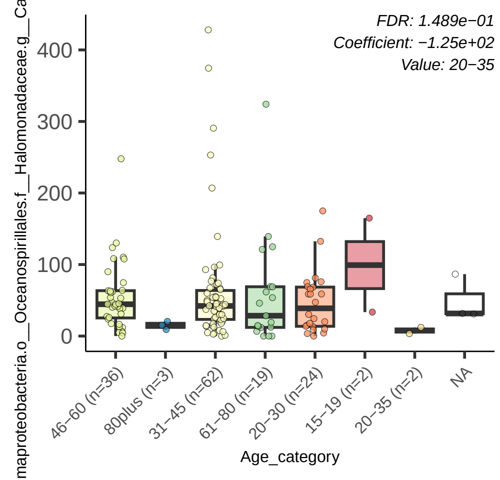

FDR: 1.489e−01
Coefficient: −1.25e+02
Value: 20−35
maproteobacteria.o__Oceanospirillales.f__Halomonadaceae.g__Ca
400
300
200
100
0
46−60 (n=36)
80plus (n=3)
31−45 (n=62)
61−80 (n=19)
20−30 (n=24)
15−19 (n=2)
20−35 (n=2)
NA
Age_category

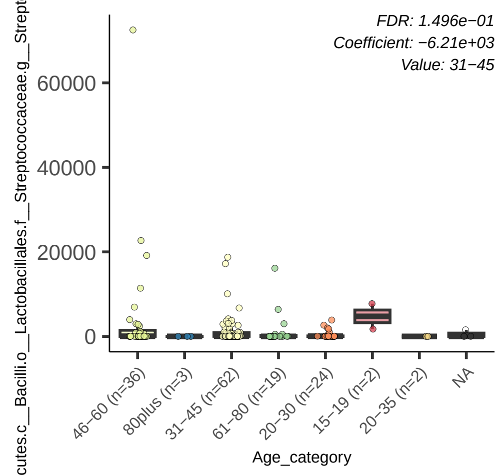

FDR: 1.496e−01
Coefficient: −6.21e+03
Value: 31−45
icutes.c__Bacilli.o__Lactobacillales.f__Streptococcaceae.g__Strept
60000
40000
20000
0
46−60 (n=36)
80plus (n=3)
31−45 (n=62)
61−80 (n=19)
20−30 (n=24)
15−19 (n=2)
20−35 (n=2)
NA
Age_category

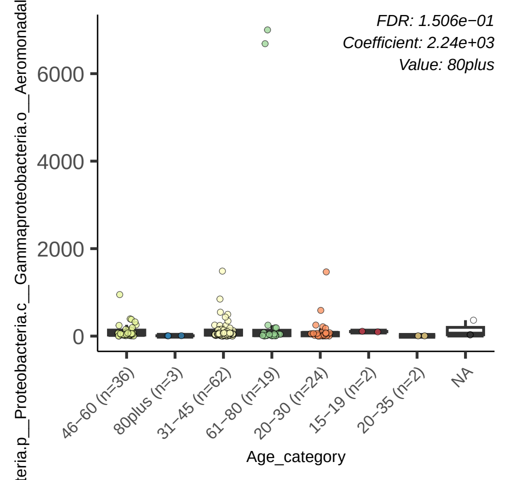

FDR: 1.506e−01
Coefficient: 2.24e+03
Value: 80plus
6000
4000
2000
0
teria.p__Proteobacteria.c__Gammaproteobacteria.o__Aeromonadal
46−60 (n=36)
80plus (n=3)
31−45 (n=62)
61−80 (n=19)
20−30 (n=24)
15−19 (n=2)
20−35 (n=2)
NA
Age_category

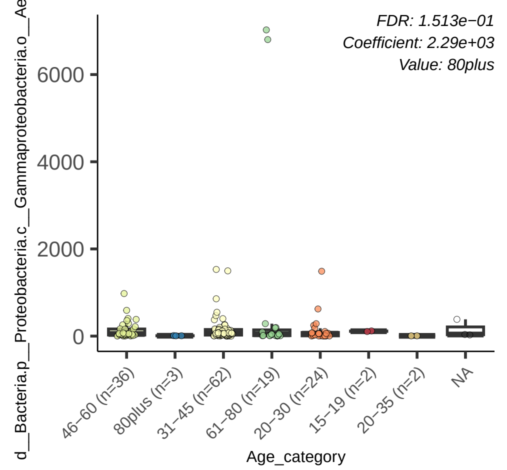
FDR: 1.513e−01
Coefficient: 2.29e+03
Value: 80plus
d__Bacteria.p__Proteobacteria.c__Gammaproteobacteria.o__Ae
6000
4000
2000
0
46−60 (n=36)
80plus (n=3)
31−45 (n=62)
61−80 (n=19)
20−30 (n=24)
15−19 (n=2)
20−35 (n=2)
NA
Age_category

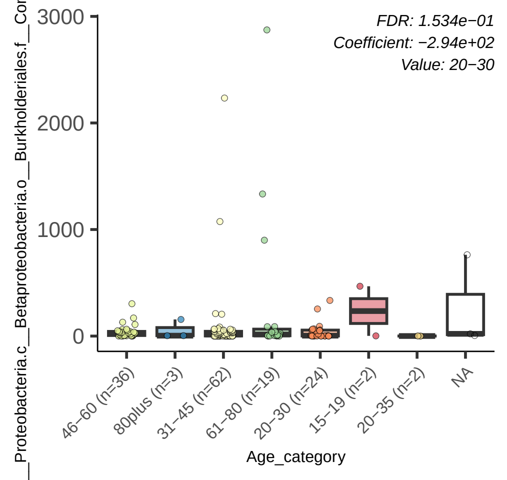

FDR: 1.534e−01
Coefficient: −2.94e+02
Value: 20−30
0
1000
2000
3000
46−60 (n=36)
80plus (n=3)
31−45 (n=62)
61−80 (n=19)
20−30 (n=24)
15−19 (n=2)
20−35 (n=2)
NA
Age_category

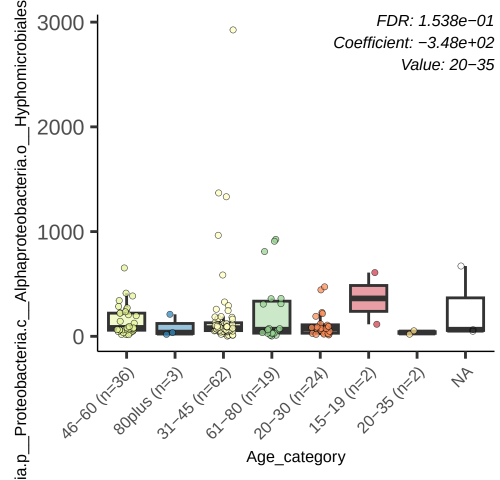

FDR: 1.538e−01
Coefficient: −3.48e+02
Value: 20−35
ia.p__Proteobacteria.c__Alphaproteobacteria.o__Hyphomicrobiales
3000
2000
1000
0
46−60 (n=36)
80plus (n=3)
31−45 (n=62)
61−80 (n=19)
20−30 (n=24)
15−19 (n=2)
20−35 (n=2)
NA
Age_category

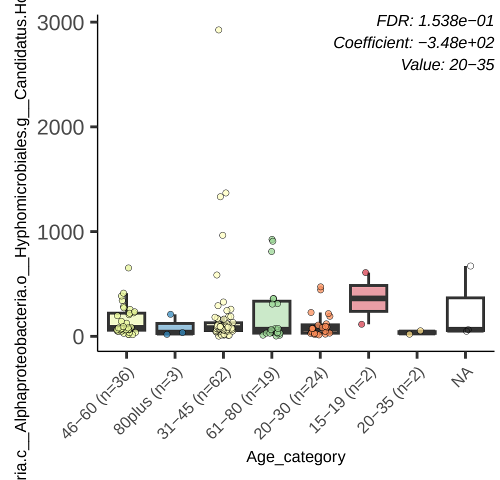

FDR: 1.538e−01
Coefficient: −3.48e+02
Value: 20−35
ria.c__Alphaproteobacteria.o__Hyphomicrobiales.g__Candidatus.Ho
3000
2000
1000
0
46−60 (n=36)
80plus (n=3)
31−45 (n=62)
61−80 (n=19)
20−30 (n=24)
15−19 (n=2)
20−35 (n=2)
NA
Age_category

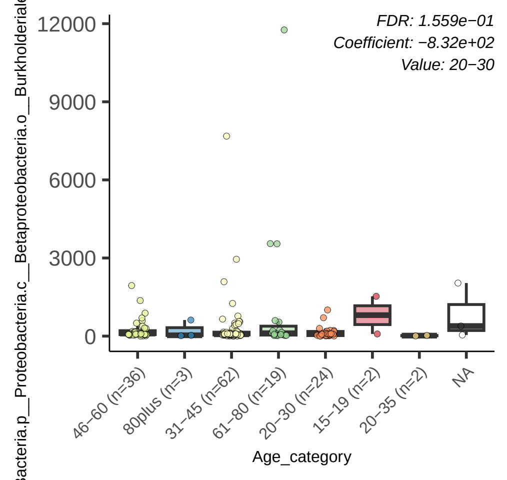

Bacteria.p__Proteobacteria.c__Betaproteobacteria.o__Burkholderiale
FDR: 1.559e−01
Coefficient: −8.32e+02
Value: 20−30
12000
9000
6000
3000
0
46−60 (n=36)
80plus (n=3)
31−45 (n=62)
61−80 (n=19)
20−30 (n=24)
15−19 (n=2)
20−35 (n=2)
NA
Age_category

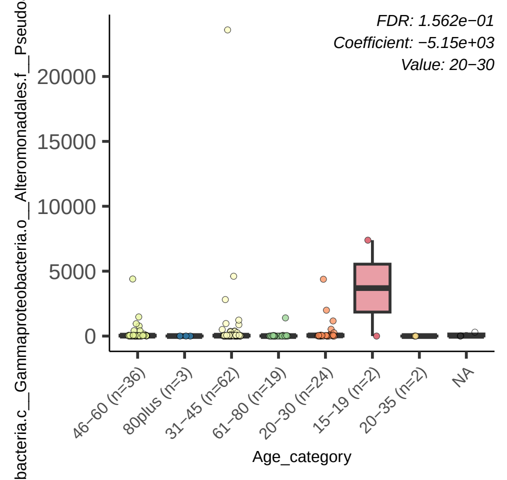

FDR: 1.562e−01
Coefficient: −5.15e+03
Value: 20−30
bacteria.c__Gammaproteobacteria.o__Alteromonadales.f__Pseudo
20000
15000
10000
5000
0
46−60 (n=36)
80plus (n=3)
31−45 (n=62)
61−80 (n=19)
20−30 (n=24)
15−19 (n=2)
20−35 (n=2)
NA
Age_category

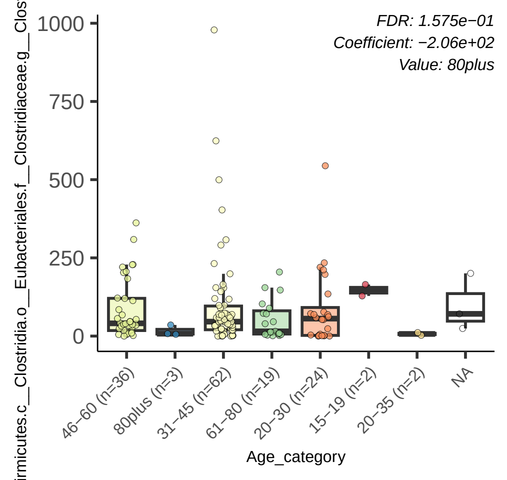
FDR: 1.575e−01
Coefficient: −2.06e+02
Value: 80plus
1000
750
500
250
0
irmicutes.c__Clostridia.o__Eubacteriales.f__Clostridiaceae.g__Clos
46−60 (n=36)
80plus (n=3)
31−45 (n=62)
61−80 (n=19)
20−30 (n=24)
15−19 (n=2)
20−35 (n=2)
NA
Age_category

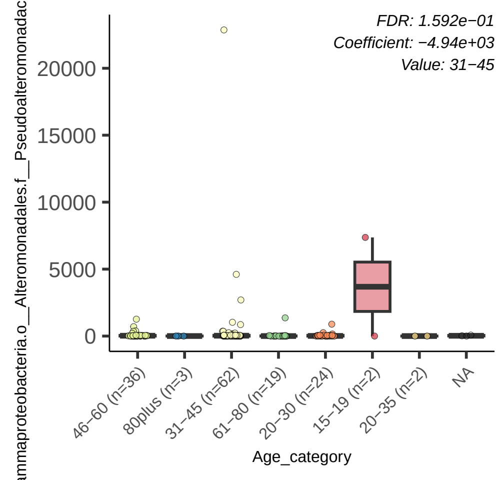

FDR: 1.592e−01
Coefficient: −4.94e+03
Value: 31−45
20000
15000
10000
5000
0
ammaproteobacteria.o__Alteromonadales.f__Pseudoalteromonadac
46−60 (n=36)
80plus (n=3)
31−45 (n=62)
61−80 (n=19)
20−30 (n=24)
15−19 (n=2)
20−35 (n=2)
NA
Age_category

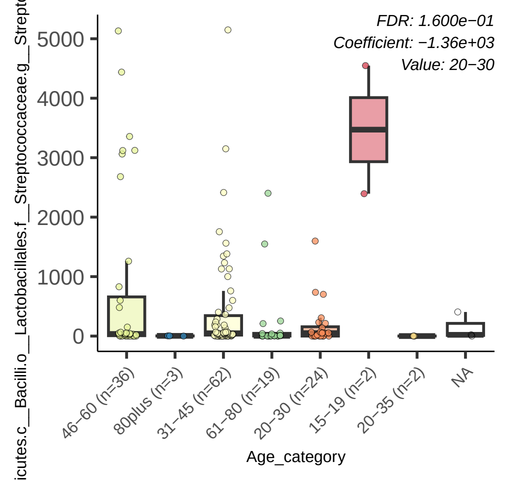

FDR: 1.600e−01
Coefficient: −1.36e+03
Value: 20−30
5000
4000
3000
2000
1000
0
46−60 (n=36)
80plus (n=3)
31−45 (n=62)
61−80 (n=19)
20−30 (n=24)
15−19 (n=2)
20−35 (n=2)
NA
Age_category
icutes.c__Bacilli.o__Lactobacillales.f__Streptococcaceae.g__Strepto

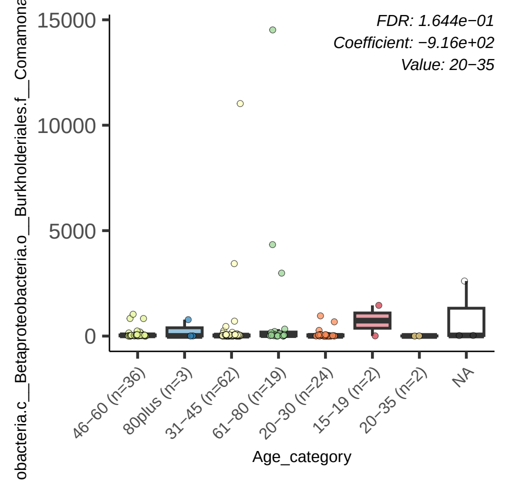

FDR: 1.644e−01
Coefficient: −9.16e+02
Value: 20−35
15000
10000
5000
0
obacteria.c__Betaproteobacteria.o__Burkholderiales.f__Comamona
46−60 (n=36)
80plus (n=3)
31−45 (n=62)
61−80 (n=19)
20−30 (n=24)
15−19 (n=2)
20−35 (n=2)
NA
Age_category

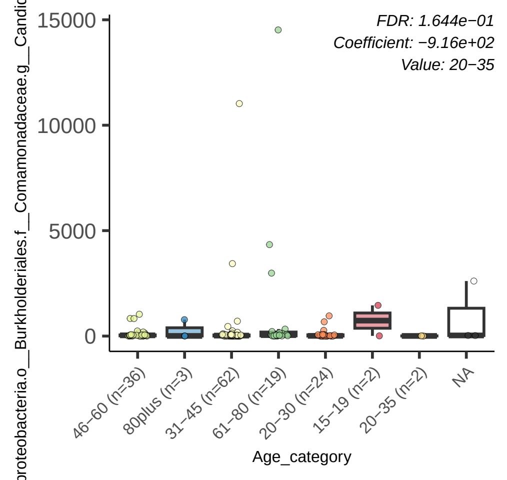

FDR: 1.644e−01
Coefficient: −9.16e+02
Value: 20−35
15000
10000
5000
0
roteobacteria.o__Burkholderiales.f__Comamonadaceae.g__Candic
46−60 (n=36)
80plus (n=3)
31−45 (n=62)
61−80 (n=19)
20−30 (n=24)
15−19 (n=2)
20−35 (n=2)
NA
Age_category

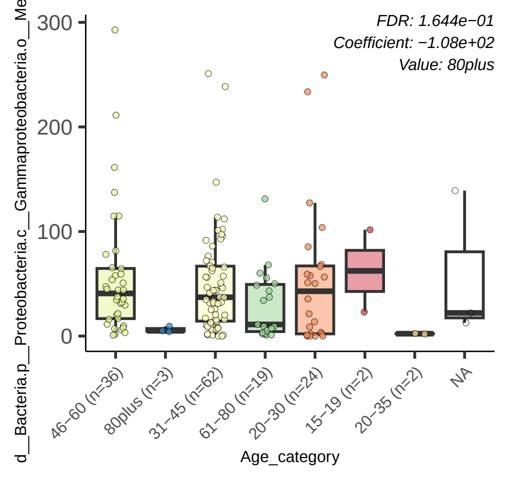

FDR: 1.644e−01
Coefficient: −1.08e+02
Value: 80plus
d__Bacteria.p__Proteobacteria.c__Gammaproteobacteria.o__Me
300
200
100
0
46−60 (n=36)
80plus (n=3)
31−45 (n=62)
61−80 (n=19)
20−30 (n=24)
15−19 (n=2)
20−35 (n=2)
NA
Age_category

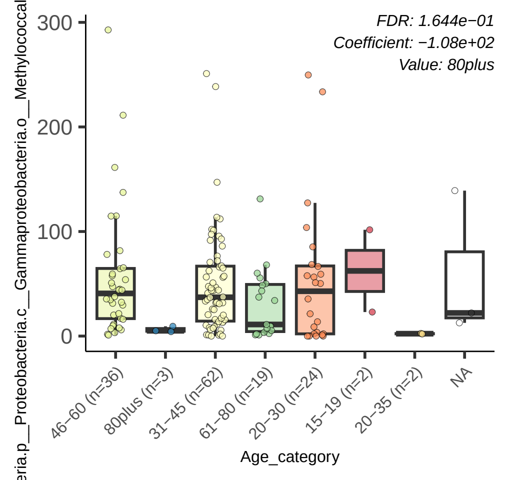

FDR: 1.644e−01
Coefficient: −1.08e+02
Value: 80plus
eria.p__Proteobacteria.c__Gammaproteobacteria.o__Methylococcal
300
200
100
0
46−60 (n=36)
80plus (n=3)
31−45 (n=62)
61−80 (n=19)
20−30 (n=24)
15−19 (n=2)
20−35 (n=2)
NA
Age_category

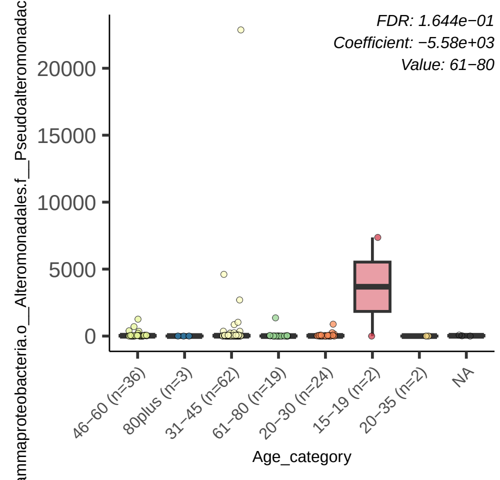

FDR: 1.644e−01
Coefficient: −5.58e+03
Value: 61−80
20000
15000
10000
5000
0
ammaproteobacteria.o__Alteromonadales.f__Pseudoalteromonadac
46−60 (n=36)
80plus (n=3)
31−45 (n=62)
61−80 (n=19)
20−30 (n=24)
15−19 (n=2)
20−35 (n=2)
NA
Age_category

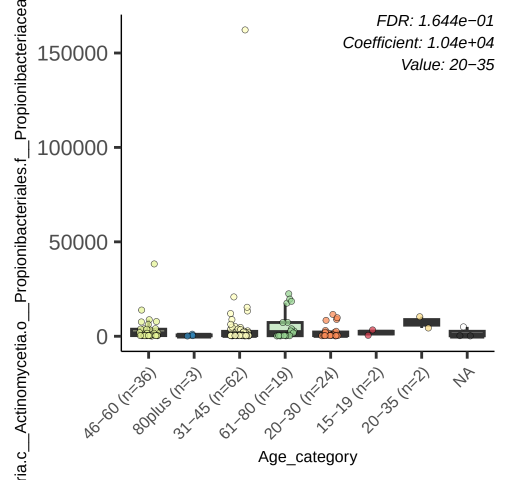

FDR: 1.644e−01
Coefficient: 1.04e+04
Value: 20−35
ria.c__Actinomycetia.o__Propionibacteriales.f__Propionibacteriacea
150000
100000
50000
0
46−60 (n=36)
80plus (n=3)
31−45 (n=62)
61−80 (n=19)
20−30 (n=24)
15−19 (n=2)
20−35 (n=2)
NA
Age_category

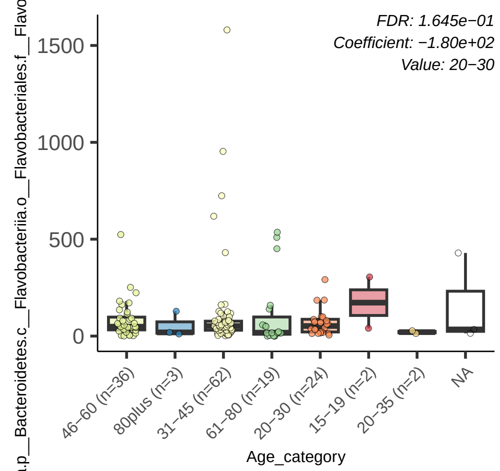

FDR: 1.645e−01
Coefficient: −1.80e+02
Value: 20−30
ia.p__Bacteroidetes.c__Flavobacteriia.o__Flavobacteriales.f__Flavo
1500
1000
500
0
46−60 (n=36)
80plus (n=3)
31−45 (n=62)
61−80 (n=19)
20−30 (n=24)
15−19 (n=2)
20−35 (n=2)
NA
Age_category

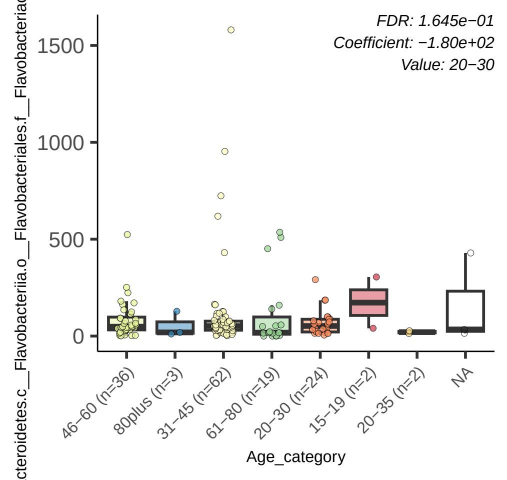

FDR: 1.645e−01
Coefficient: −1.80e+02
Value: 20−30
1500
1000
500
0
cteroidetes.c__Flavobacteriia.o__Flavobacteriales.f__Flavobacteriac
46−60 (n=36)
80plus (n=3)
31−45 (n=62)
61−80 (n=19)
20−30 (n=24)
15−19 (n=2)
20−35 (n=2)
NA
Age_category

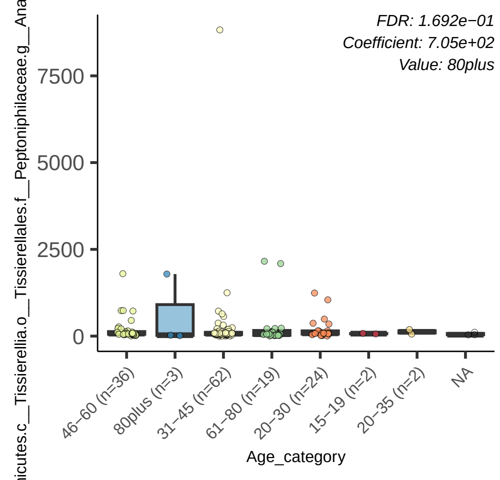

FDR: 1.692e−01
Coefficient: 7.05e+02
Value: 80plus
7500
5000
2500
0
46−60 (n=36)
80plus (n=3)
31−45 (n=62)
61−80 (n=19)
20−30 (n=24)
15−19 (n=2)
20−35 (n=2)
NA
Age_category

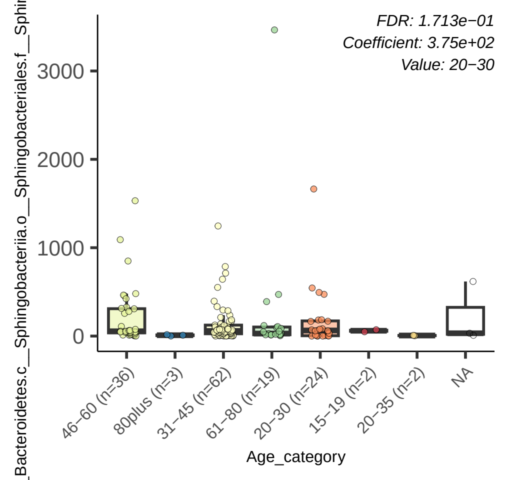

FDR: 1.713e−01
Coefficient: 3.75e+02
Value: 20−30
3000
2000
1000
0
Bacteroidetes.c__Sphingobacteriia.o__Sphingobacteriales.f__Sphi
46−60 (n=36)
80plus (n=3)
31−45 (n=62)
61−80 (n=19)
20−30 (n=24)
15−19 (n=2)
20−35 (n=2)
NA
Age_category

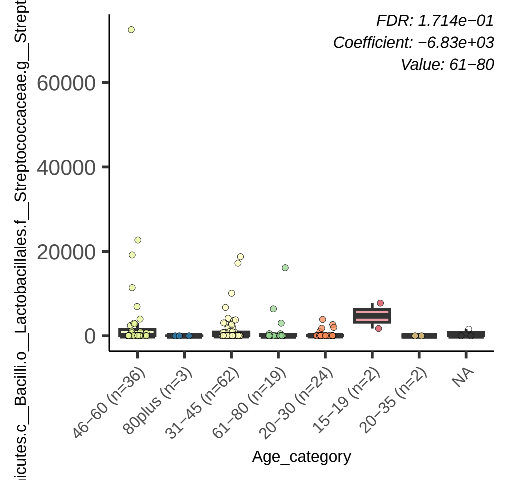

FDR: 1.714e−01
Coefficient: −6.83e+03
Value: 61−80
60000
40000
20000
0
46−60 (n=36)
80plus (n=3)
31−45 (n=62)
61−80 (n=19)
20−30 (n=24)
15−19 (n=2)
20−35 (n=2)
NA
Age_category
icutes.c__Bacilli.o__Lactobacillales.f__Streptococcaceae.g__Strept

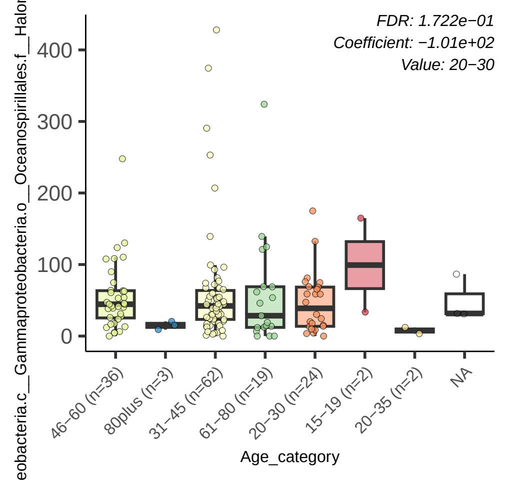

FDR: 1.722e−01
Coefficient: −1.01e+02
Value: 20−30
0
100
200
300
400
eobacteria.c__Gammaproteobacteria.o__Oceanospirillales.f__Halo
46−60 (n=36)
80plus (n=3)
31−45 (n=62)
61−80 (n=19)
20−30 (n=24)
15−19 (n=2)
20−35 (n=2)
NA
Age_category

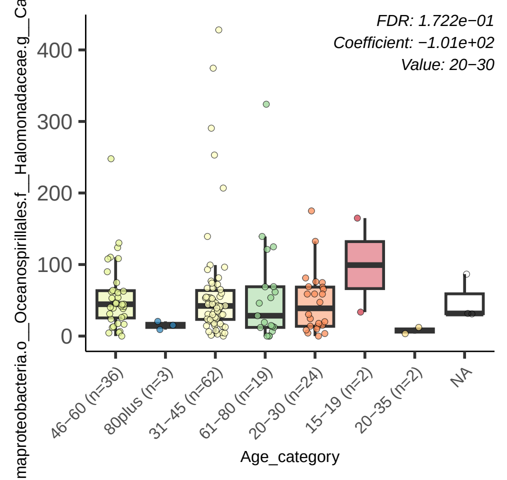

FDR: 1.722e−01
Coefficient: −1.01e+02
Value: 20−30
maproteobacteria.o__Oceanospirillales.f__Halomonadaceae.g__Ca
0
100
200
300
400
46−60 (n=36)
80plus (n=3)
31−45 (n=62)
61−80 (n=19)
20−30 (n=24)
15−19 (n=2)
20−35 (n=2)
NA
Age_category

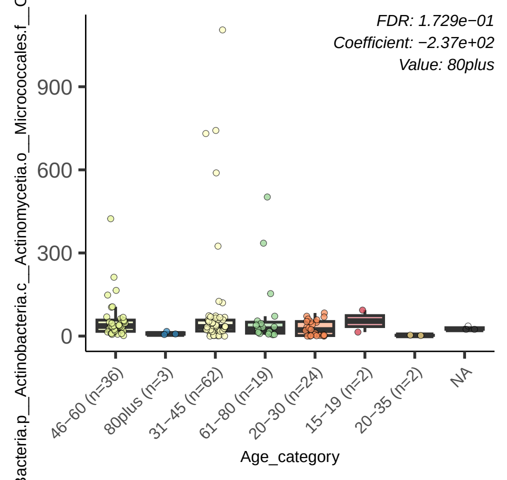

FDR: 1.729e−01
Coefficient: −2.37e+02
Value: 80plus
Bacteria.p__Actinobacteria.c__Actinomycetia.o__Micrococcales.f__C
900
600
300
0
46−60 (n=36)
80plus (n=3)
31−45 (n=62)
61−80 (n=19)
20−30 (n=24)
15−19 (n=2)
20−35 (n=2)
NA
Age_category

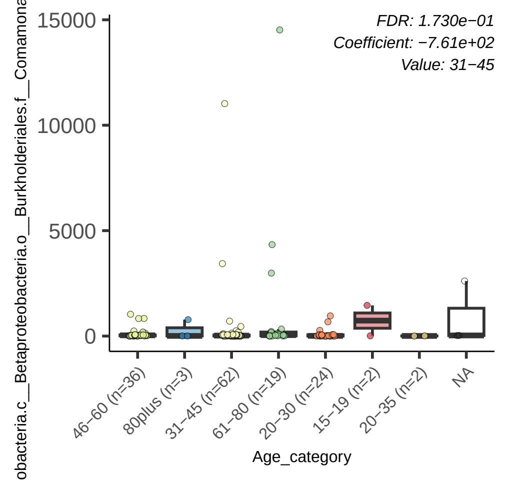

FDR: 1.730e−01
Coefficient: −7.61e+02
Value: 31−45
15000
10000
5000
0
obacteria.c__Betaproteobacteria.o__Burkholderiales.f__Comamona
46−60 (n=36)
80plus (n=3)
31−45 (n=62)
61−80 (n=19)
20−30 (n=24)
15−19 (n=2)
20−35 (n=2)
NA
Age_category

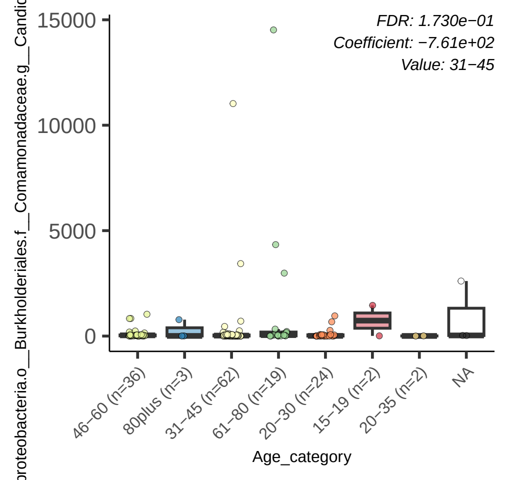
FDR: 1.730e−01
Coefficient: −7.61e+02
Value: 31−45
proteobacteria.o__Burkholderiales.f__Comamonadaceae.g__Candid
15000
10000
5000
0
46−60 (n=36)
80plus (n=3)
31−45 (n=62)
61−80 (n=19)
20−30 (n=24)
15−19 (n=2)
20−35 (n=2)
NA
Age_category

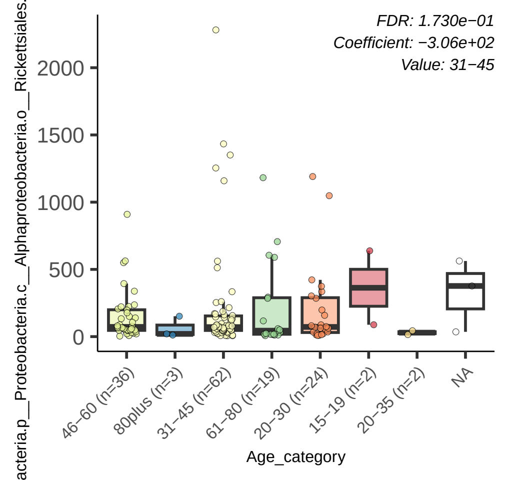
FDR: 1.730e−01
Coefficient: −3.06e+02
Value: 31−45
acteria.p__Proteobacteria.c__Alphaproteobacteria.o__Rickettsiales.
2000
1500
1000
500
0
46−60 (n=36)
80plus (n=3)
31−45 (n=62)
61−80 (n=19)
20−30 (n=24)
15−19 (n=2)
20−35 (n=2)
NA
Age_category

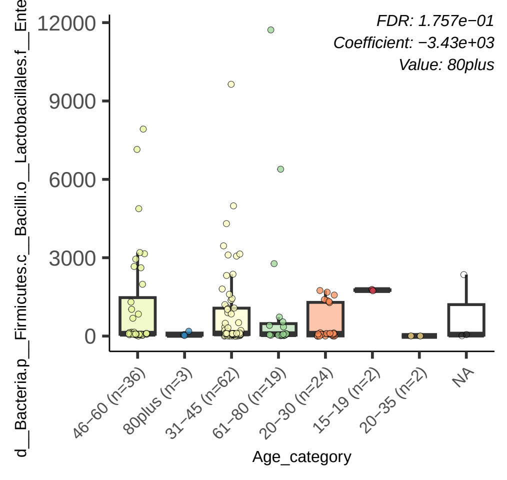

FDR: 1.757e−01
Coefficient: −3.43e+03
Value: 80plus
d__Bacteria.p__Firmicutes.c__Bacilli.o__Lactobacillales.f__Ente
12000
9000
6000
3000
0
46−60 (n=36)
80plus (n=3)
31−45 (n=62)
61−80 (n=19)
20−30 (n=24)
15−19 (n=2)
20−35 (n=2)
NA
Age_category

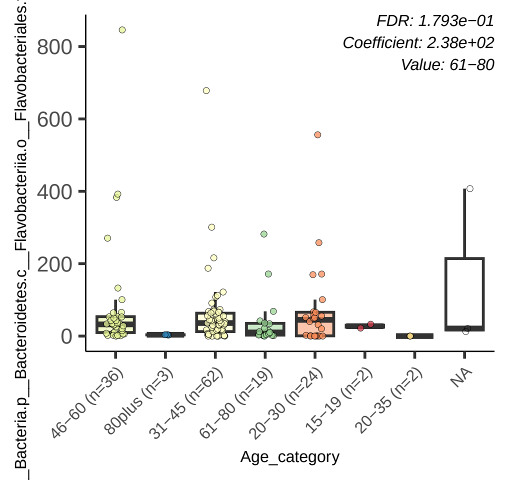

FDR: 1.793e−01
Coefficient: 2.38e+02
Value: 61−80
k__Bacteria.p__Bacteroidetes.c__Flavobacteriia.o__Flavobacteriales.f
0
200
400
600
800
46−60 (n=36)
80plus (n=3)
31−45 (n=62)
61−80 (n=19)
20−30 (n=24)
15−19 (n=2)
20−35 (n=2)
NA
Age_category

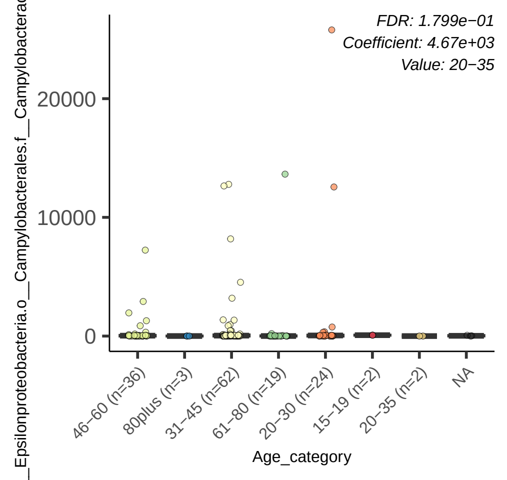

FDR: 1.799e−01
Coefficient: 4.67e+03
Value: 20−35
20000
10000
0
_Epsilonproteobacteria.o__Campylobacterales.f__Campylobacterac
46−60 (n=36)
80plus (n=3)
31−45 (n=62)
61−80 (n=19)
20−30 (n=24)
15−19 (n=2)
20−35 (n=2)
NA
Age_category

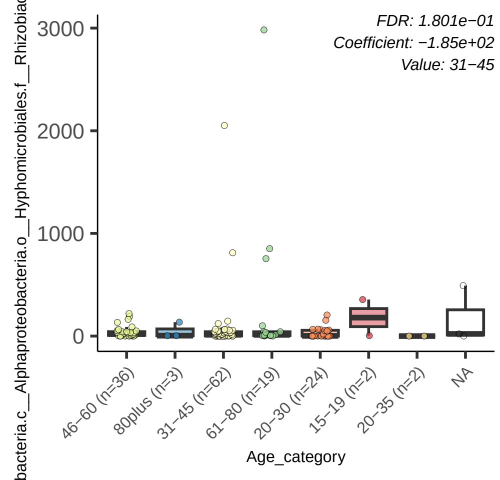

FDR: 1.801e−01
Coefficient: −1.85e+02
Value: 31−45
3000
2000
1000
0
bacteria.c__Alphaproteobacteria.o__Hyphomicrobiales.f__Rhizobiac
46−60 (n=36)
80plus (n=3)
31−45 (n=62)
61−80 (n=19)
20−30 (n=24)
15−19 (n=2)
20−35 (n=2)
NA
Age_category

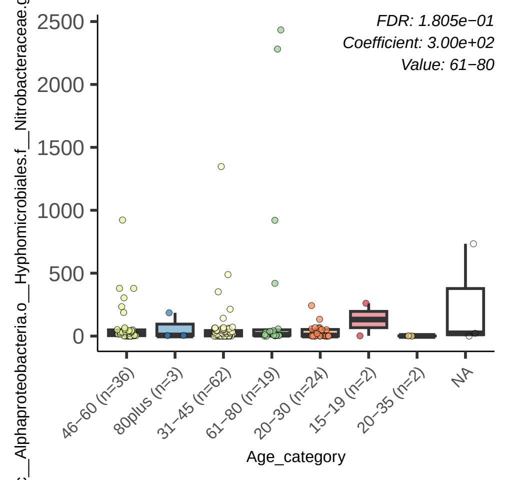

FDR: 1.805e−01
Coefficient: 3.00e+02
Value: 61−80
2500
2000
1500
1000
500
0
46−60 (n=36)
80plus (n=3)
31−45 (n=62)
61−80 (n=19)
20−30 (n=24)
15−19 (n=2)
20−35 (n=2)
NA
Age_category
Alphaproteobacteria.o__Hyphomicrobiales.f__Nitrobacteraceae.g

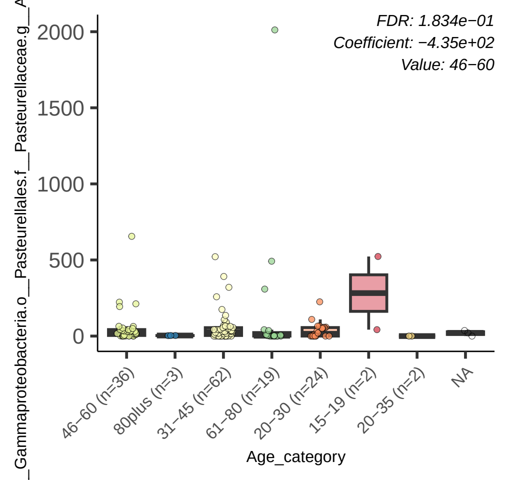

FDR: 1.834e−01
Coefficient: −4.35e+02
Value: 46−60
_Gammaproteobacteria.o__Pasteurellales.f__Pasteurellaceae.g__A
2000
1500
1000
500
0
46−60 (n=36)
80plus (n=3)
31−45 (n=62)
61−80 (n=19)
20−30 (n=24)
15−19 (n=2)
20−35 (n=2)
NA
Age_category

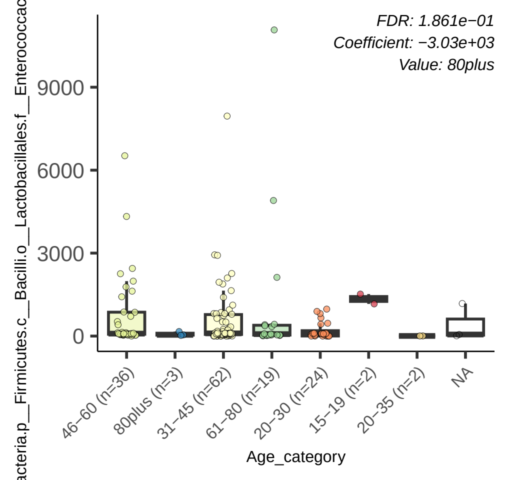
FDR: 1.861e−01
Coefficient: −3.03e+03
Value: 80plus
acteria.p__Firmicutes.c__Bacilli.o__Lactobacillales.f__Enterococcac
9000
6000
3000
0
46−60 (n=36)
80plus (n=3)
31−45 (n=62)
61−80 (n=19)
20−30 (n=24)
15−19 (n=2)
20−35 (n=2)
NA
Age_category

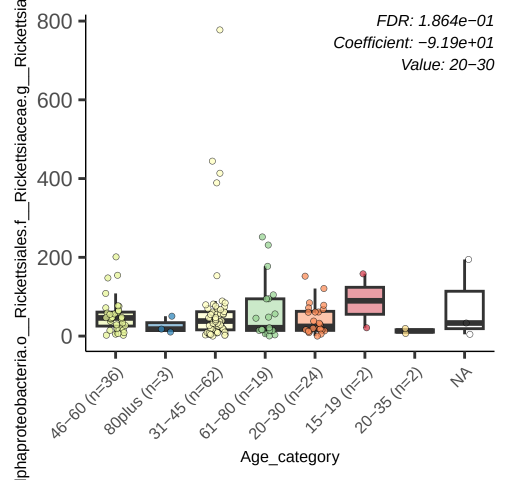
FDR: 1.864e−01
Coefficient: −9.19e+01
Value: 20−30
lphaproteobacteria.o__Rickettsiales.f__Rickettsiaceae.g__Rickettsia
800
600
400
200
0
46−60 (n=36)
80plus (n=3)
31−45 (n=62)
61−80 (n=19)
20−30 (n=24)
15−19 (n=2)
20−35 (n=2)
NA
Age_category

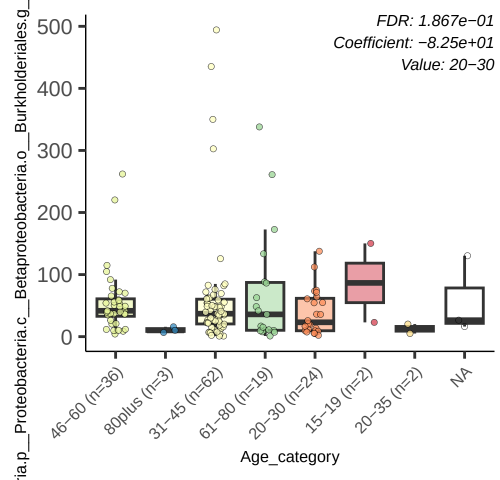

FDR: 1.867e−01
Coefficient: −8.25e+01
Value: 20−30
ria.p__Proteobacteria.c__Betaproteobacteria.o__Burkholderiales.g_
500
400
300
200
100
0
46−60 (n=36)
80plus (n=3)
31−45 (n=62)
61−80 (n=19)
20−30 (n=24)
15−19 (n=2)
20−35 (n=2)
NA
Age_category

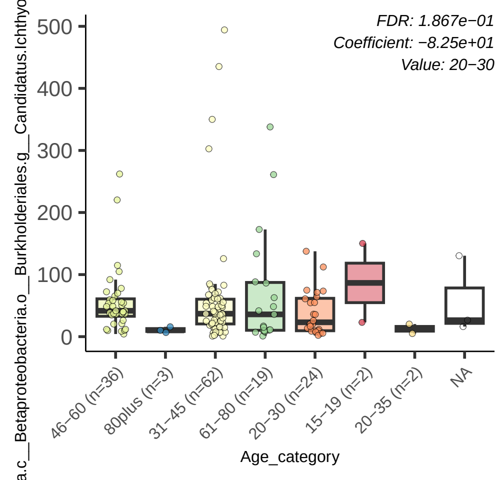
FDR: 1.867e−01
Coefficient: −8.25e+01
Value: 20−30
a.c__Betaproteobacteria.o__Burkholderiales.g__Candidatus.Ichthyo
500
400
300
200
100
0
46−60 (n=36)
80plus (n=3)
31−45 (n=62)
61−80 (n=19)
20−30 (n=24)
15−19 (n=2)
20−35 (n=2)
NA
Age_category

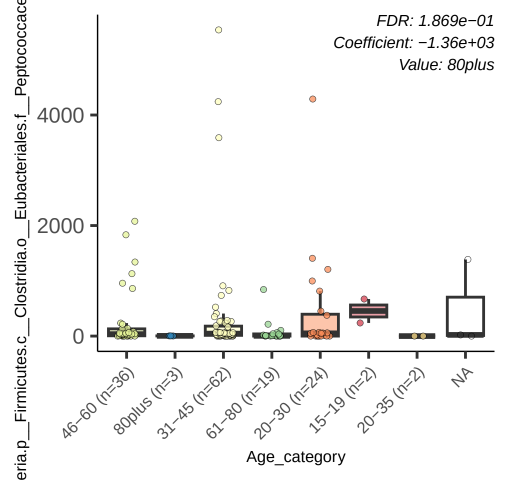
eria.p__Firmicutes.c__Clostridia.o__Eubacteriales.f__Peptococcace
FDR: 1.869e−01
Coefficient: −1.36e+03
Value: 80plus
4000
2000
0
46−60 (n=36)
80plus (n=3)
31−45 (n=62)
61−80 (n=19)
20−30 (n=24)
15−19 (n=2)
20−35 (n=2)
NA
Age_category

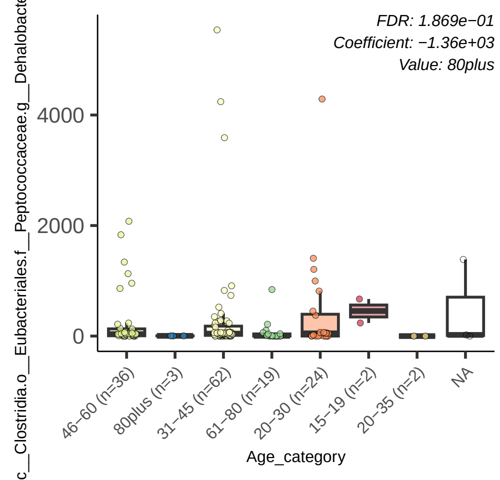

FDR: 1.869e−01
Coefficient: −1.36e+03
Value: 80plus
c__Clostridia.o__Eubacteriales.f__Peptococcaceae.g__Dehalobacte
4000
2000
0
46−60 (n=36)
80plus (n=3)
31−45 (n=62)
61−80 (n=19)
20−30 (n=24)
15−19 (n=2)
20−35 (n=2)
NA
Age_category

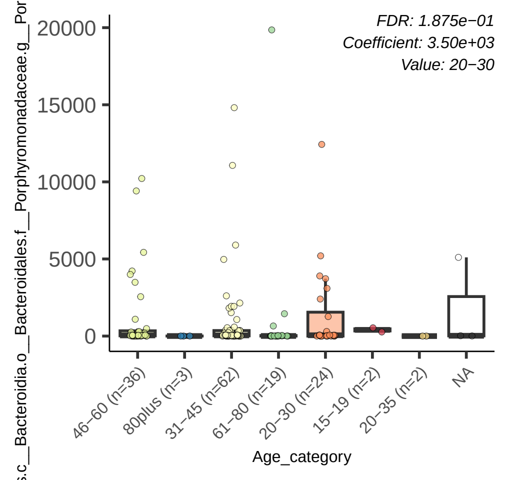

FDR: 1.875e−01
Coefficient: 3.50e+03
Value: 20−30
20000
15000
10000
5000
0
s.c__Bacteroidia.o__Bacteroidales.f__Porphyromonadaceae.g__Por
46−60 (n=36)
80plus (n=3)
31−45 (n=62)
61−80 (n=19)
20−30 (n=24)
15−19 (n=2)
20−35 (n=2)
NA
Age_category

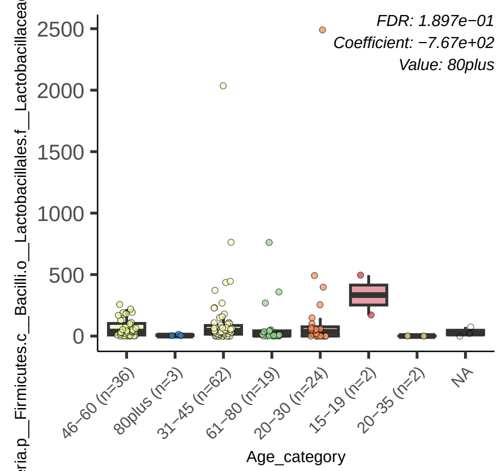

FDR: 1.897e−01
Coefficient: −7.67e+02
Value: 80plus
teria.p__Firmicutes.c__Bacilli.o__Lactobacillales.f__Lactobacillaceae
2500
2000
1500
1000
500
0
46−60 (n=36)
80plus (n=3)
31−45 (n=62)
61−80 (n=19)
20−30 (n=24)
15−19 (n=2)
20−35 (n=2)
NA
Age_category

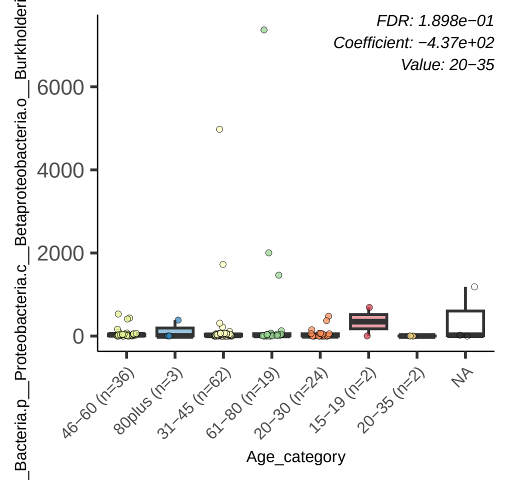

FDR: 1.898e−01
Coefficient: −4.37e+02
Value: 20−35
Bacteria.p__Proteobacteria.c__Betaproteobacteria.o__Burkholderi
0
2000
4000
6000
46−60 (n=36)
80plus (n=3)
31−45 (n=62)
61−80 (n=19)
20−30 (n=24)
15−19 (n=2)
20−35 (n=2)
NA
Age_category

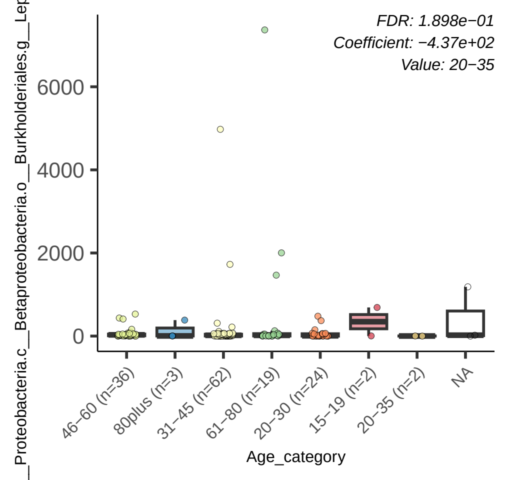

FDR: 1.898e−01
Coefficient: −4.37e+02
Value: 20−35
_Proteobacteria.c__Betaproteobacteria.o__Burkholderiales.g__Lep
6000
4000
2000
0
46−60 (n=36)
80plus (n=3)
31−45 (n=62)
61−80 (n=19)
20−30 (n=24)
15−19 (n=2)
20−35 (n=2)
NA
Age_category

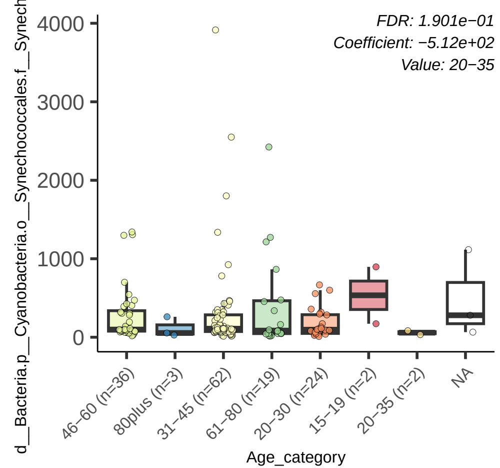

FDR: 1.901e−01
Coefficient: −5.12e+02
Value: 20−35
d__Bacteria.p__Cyanobacteria.o__Synechococcales.f__Synech
4000
3000
2000
1000
0
46−60 (n=36)
80plus (n=3)
31−45 (n=62)
61−80 (n=19)
20−30 (n=24)
15−19 (n=2)
20−35 (n=2)
NA
Age_category

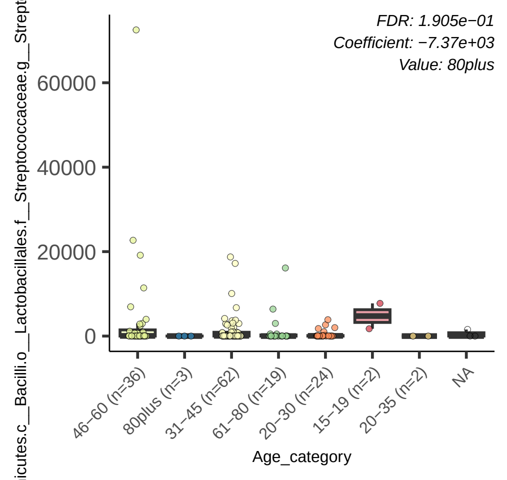

FDR: 1.905e−01
Coefficient: −7.37e+03
Value: 80plus
60000
40000
20000
0
46−60 (n=36)
80plus (n=3)
31−45 (n=62)
61−80 (n=19)
20−30 (n=24)
15−19 (n=2)
20−35 (n=2)
NA
Age_category

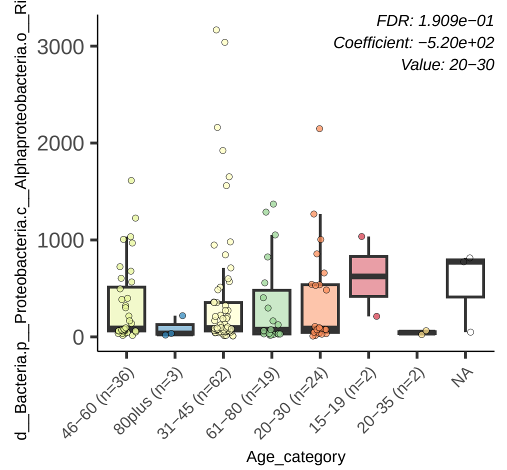

FDR: 1.909e−01
Coefficient: −5.20e+02
Value: 20−30
d__Bacteria.p__Proteobacteria.c__Alphaproteobacteria.o__Ri
3000
2000
1000
0
46−60 (n=36)
80plus (n=3)
31−45 (n=62)
61−80 (n=19)
20−30 (n=24)
15−19 (n=2)
20−35 (n=2)
NA
Age_category

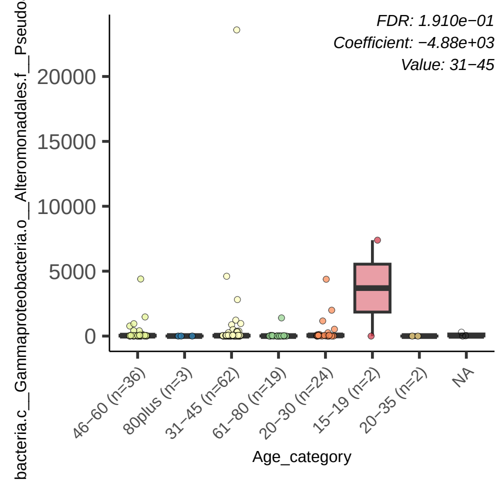

FDR: 1.910e−01
Coefficient: −4.88e+03
Value: 31−45
bacteria.c__Gammaproteobacteria.o__Alteromonadales.f__Pseudo
20000
15000
10000
5000
0
46−60 (n=36)
80plus (n=3)
31−45 (n=62)
61−80 (n=19)
20−30 (n=24)
15−19 (n=2)
20−35 (n=2)
NA
Age_category

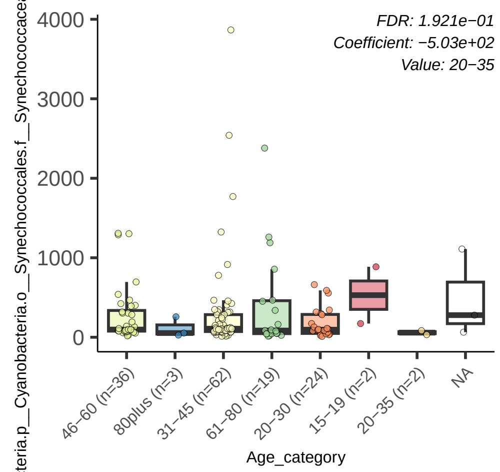

FDR: 1.921e−01
Coefficient: −5.03e+02
Value: 20−35
cteria.p__Cyanobacteria.o__Synechococcales.f__Synechococcace
0
1000
2000
3000
4000
46−60 (n=36)
80plus (n=3)
31−45 (n=62)
61−80 (n=19)
20−30 (n=24)
15−19 (n=2)
20−35 (n=2)
NA
Age_category

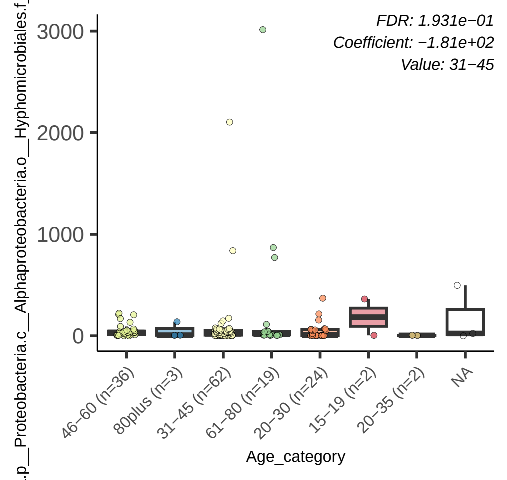

FDR: 1.931e−01
Coefficient: −1.81e+02
Value: 31−45
p__Proteobacteria.c__Alphaproteobacteria.o__Hyphomicrobiales.f_
3000
2000
1000
0
46−60 (n=36)
80plus (n=3)
31−45 (n=62)
61−80 (n=19)
20−30 (n=24)
15−19 (n=2)
20−35 (n=2)
NA
Age_category

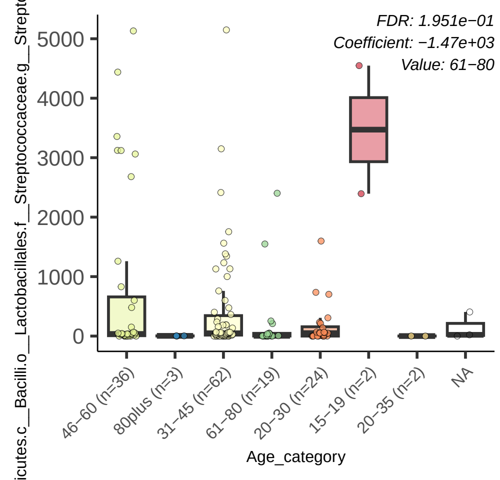

FDR: 1.951e−01
Coefficient: −1.47e+03
Value: 61−80
5000
4000
3000
2000
1000
0
icutes.c__Bacilli.o__Lactobacillales.f__Streptococcaceae.g__Strept
46−60 (n=36)
80plus (n=3)
31−45 (n=62)
61−80 (n=19)
20−30 (n=24)
15−19 (n=2)
20−35 (n=2)
NA
Age_category

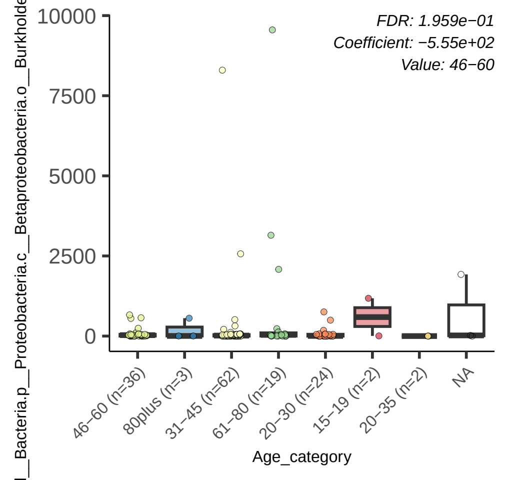

FDR: 1.959e−01
Coefficient: −5.55e+02
Value: 46−60
10000
7500
5000
2500
0
l__Bacteria.p__Proteobacteria.c__Betaproteobacteria.o__Burkholde
46−60 (n=36)
80plus (n=3)
31−45 (n=62)
61−80 (n=19)
20−30 (n=24)
15−19 (n=2)
20−35 (n=2)
NA
Age_category

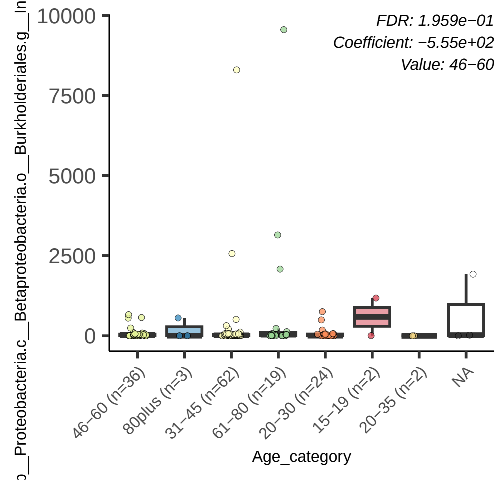

FDR: 1.959e−01
Coefficient: −5.55e+02
Value: 46−60
10000
7500
5000
2500
0
o__Proteobacteria.c__Betaproteobacteria.o__Burkholderiales.g__In
46−60 (n=36)
80plus (n=3)
31−45 (n=62)
61−80 (n=19)
20−30 (n=24)
15−19 (n=2)
20−35 (n=2)
NA
Age_category

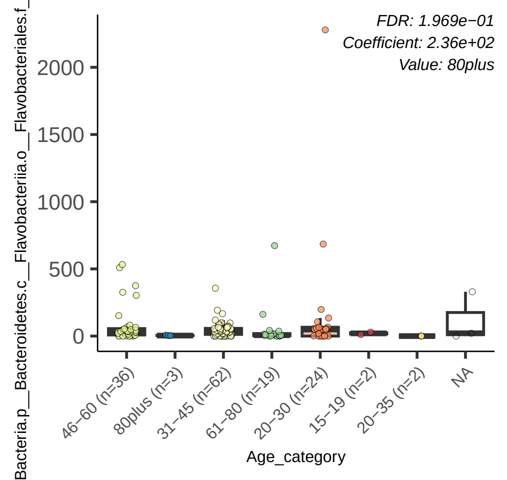

FDR: 1.969e−01
Coefficient: 2.36e+02
Value: 80plus
Bacteria.p__Bacteroidetes.c__Flavobacteriia.o__Flavobacteriales.f_
2000
1500
1000
500
0
46−60 (n=36)
80plus (n=3)
31−45 (n=62)
61−80 (n=19)
20−30 (n=24)
15−19 (n=2)
20−35 (n=2)
NA
Age_category

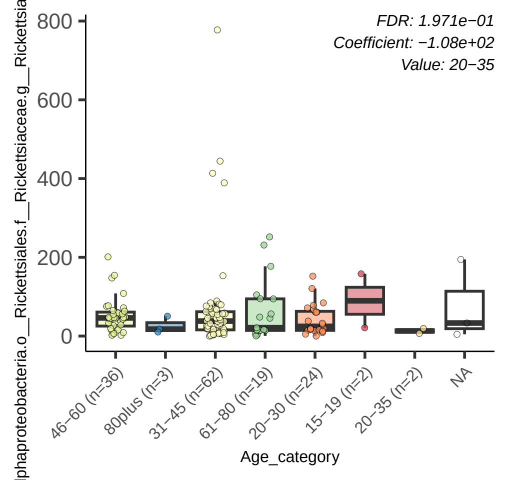
FDR: 1.971e−01
Coefficient: −1.08e+02
Value: 20−35
lphaproteobacteria.o__Rickettsiales.f__Rickettsiaceae.g__Rickettsia
800
600
400
200
0
46−60 (n=36)
80plus (n=3)
31−45 (n=62)
61−80 (n=19)
20−30 (n=24)
15−19 (n=2)
20−35 (n=2)
NA
Age_category

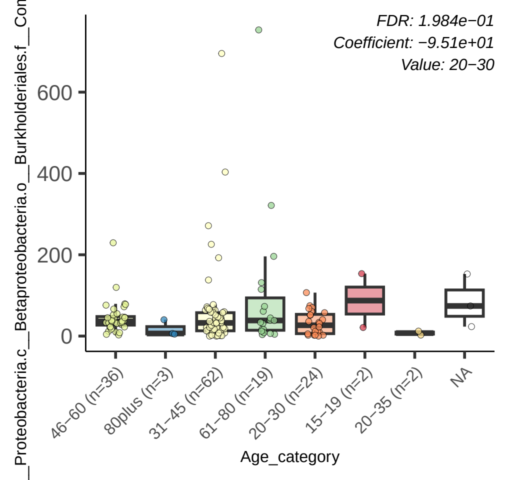

FDR: 1.984e−01
Coefficient: −9.51e+01
Value: 20−30
0
200
400
600
_Proteobacteria.c__Betaproteobacteria.o__Burkholderiales.f__Con
46−60 (n=36)
80plus (n=3)
31−45 (n=62)
61−80 (n=19)
20−30 (n=24)
15−19 (n=2)
20−35 (n=2)
NA
Age_category

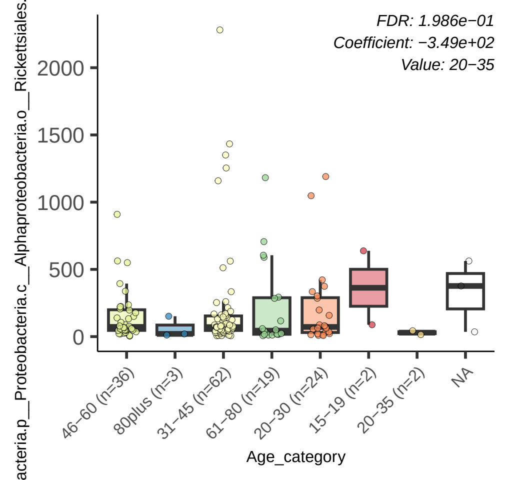

FDR: 1.986e−01
Coefficient: −3.49e+02
Value: 20−35
acteria.p__Proteobacteria.c__Alphaproteobacteria.o__Rickettsiales.
0
500
1000
1500
2000
46−60 (n=36)
80plus (n=3)
31−45 (n=62)
61−80 (n=19)
20−30 (n=24)
15−19 (n=2)
20−35 (n=2)
NA
Age_category

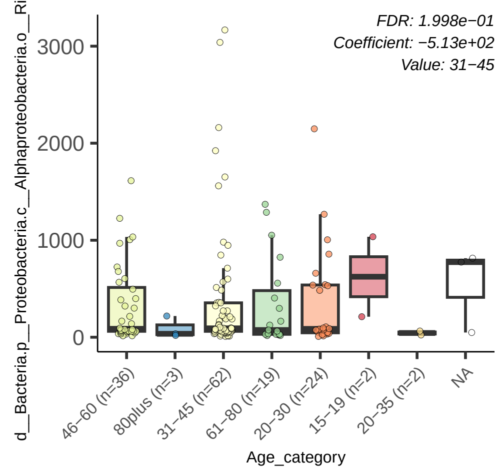

FDR: 1.998e−01
Coefficient: −5.13e+02
Value: 31−45
3000
2000
1000
0
d__Bacteria.p__Proteobacteria.c__Alphaproteobacteria.o__Ri
46−60 (n=36)
80plus (n=3)
31−45 (n=62)
61−80 (n=19)
20−30 (n=24)
15−19 (n=2)
20−35 (n=2)
NA
Age_category

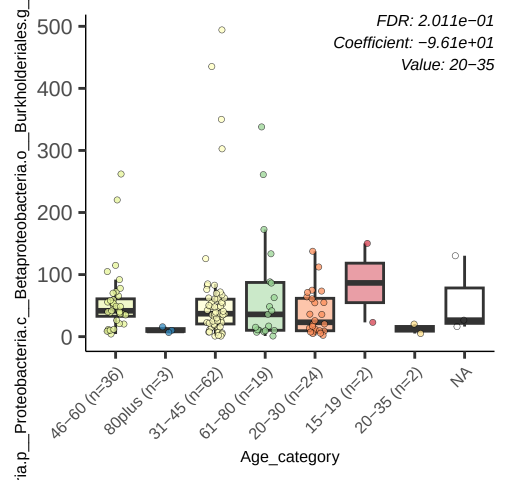

FDR: 2.011e−01
Coefficient: −9.61e+01
Value: 20−35
ria.p__Proteobacteria.c__Betaproteobacteria.o__Burkholderiales.g__
500
400
300
200
100
0
46−60 (n=36)
80plus (n=3)
31−45 (n=62)
61−80 (n=19)
20−30 (n=24)
15−19 (n=2)
20−35 (n=2)
NA
Age_category

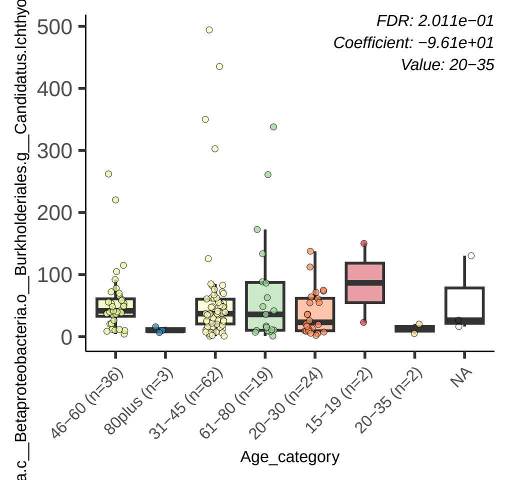

FDR: 2.011e−01
Coefficient: −9.61e+01
Value: 20−35
a.c__Betaproteobacteria.o__Burkholderiales.g__Candidatus.Ichthyo
0
100
200
300
400
500
46−60 (n=36)
80plus (n=3)
31−45 (n=62)
61−80 (n=19)
20−30 (n=24)
15−19 (n=2)
20−35 (n=2)
NA
Age_category

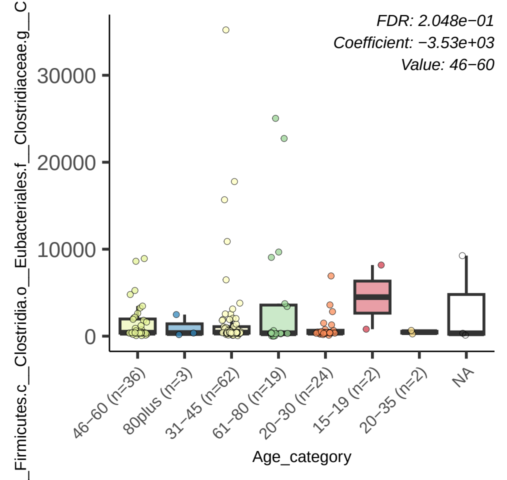

FDR: 2.048e−01
Coefficient: −3.53e+03
Value: 46−60
Firmicutes.c__Clostridia.o__Eubacteriales.f__Clostridiaceae.g__C
30000
20000
10000
0
46−60 (n=36)
80plus (n=3)
31−45 (n=62)
61−80 (n=19)
20−30 (n=24)
15−19 (n=2)
20−35 (n=2)
NA
Age_category

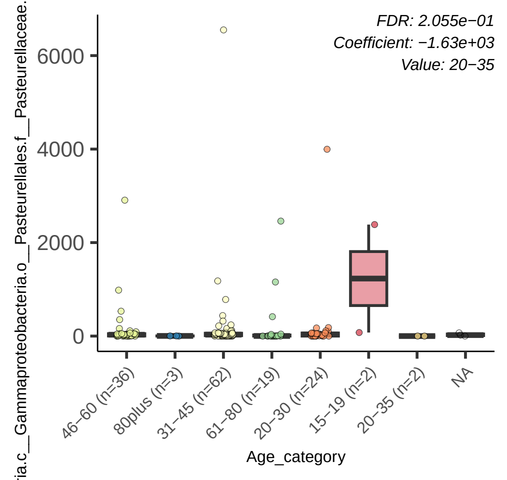
FDR: 2.055e−01
Coefficient: −1.63e+03
Value: 20−35
ria.c__Gammaproteobacteria.o__Pasteurellales.f__Pasteurellaceae.
6000
4000
2000
0
46−60 (n=36)
80plus (n=3)
31−45 (n=62)
61−80 (n=19)
20−30 (n=24)
15−19 (n=2)
20−35 (n=2)
NA
Age_category

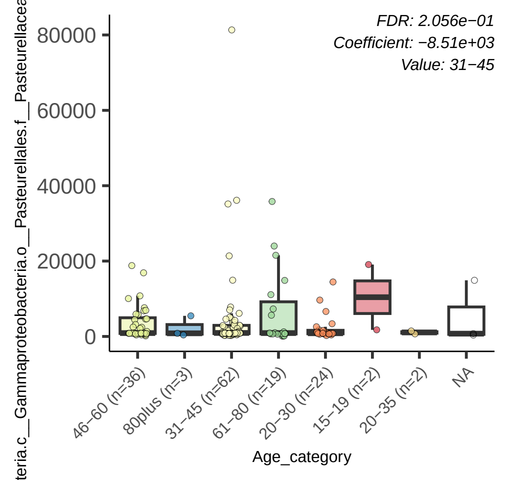

FDR: 2.056e−01
Coefficient: −8.51e+03
Value: 31−45
80000
60000
40000
20000
0
teria.c__Gammaproteobacteria.o__Pasteurellales.f__Pasteurellacea
46−60 (n=36)
80plus (n=3)
31−45 (n=62)
61−80 (n=19)
20−30 (n=24)
15−19 (n=2)
20−35 (n=2)
NA
Age_category

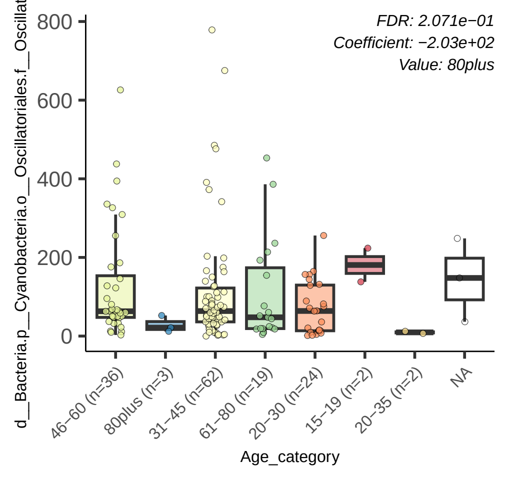

FDR: 2.071e−01
Coefficient: −2.03e+02
Value: 80plus
d__Bacteria.p__Cyanobacteria.o__Oscillatoriales.f__Oscillat
800
600
400
200
0
46−60 (n=36)
80plus (n=3)
31−45 (n=62)
61−80 (n=19)
20−30 (n=24)
15−19 (n=2)
20−35 (n=2)
NA
Age_category

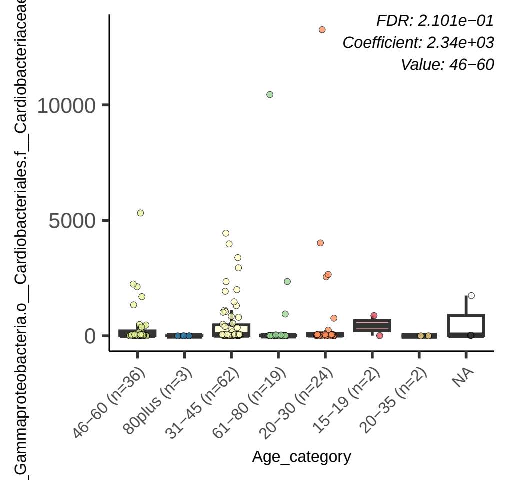

FDR: 2.101e−01
Coefficient: 2.34e+03
Value: 46−60
_Gammaproteobacteria.o__Cardiobacteriales.f__Cardiobacteriaceae
10000
5000
0
46−60 (n=36)
80plus (n=3)
31−45 (n=62)
61−80 (n=19)
20−30 (n=24)
15−19 (n=2)
20−35 (n=2)
NA
Age_category

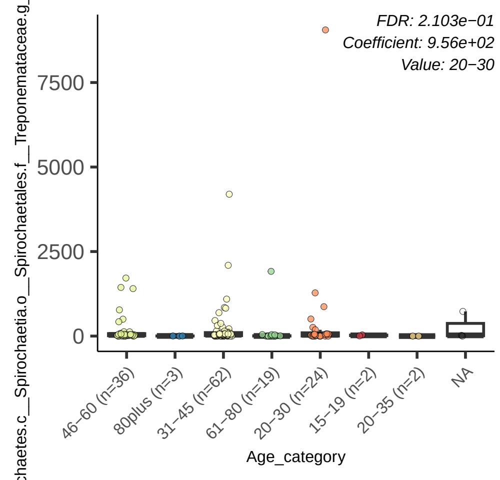

FDR: 2.103e−01
Coefficient: 9.56e+02
Value: 20−30
7500
5000
2500
0
46−60 (n=36)
80plus (n=3)
31−45 (n=62)
61−80 (n=19)
20−30 (n=24)
15−19 (n=2)
20−35 (n=2)
NA
Age_category

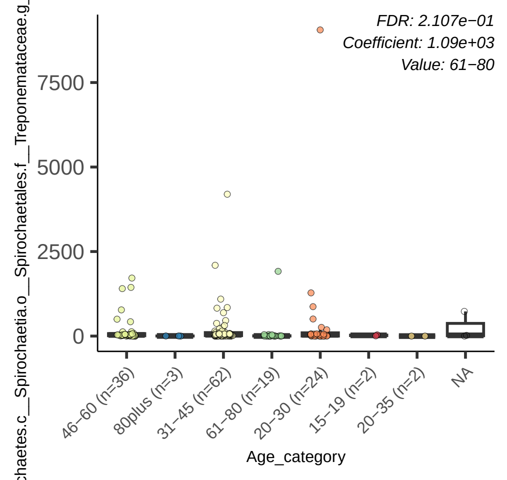

FDR: 2.107e−01
Coefficient: 1.09e+03
Value: 61−80
7500
5000
2500
0
46−60 (n=36)
80plus (n=3)
31−45 (n=62)
61−80 (n=19)
20−30 (n=24)
15−19 (n=2)
20−35 (n=2)
NA
Age_category

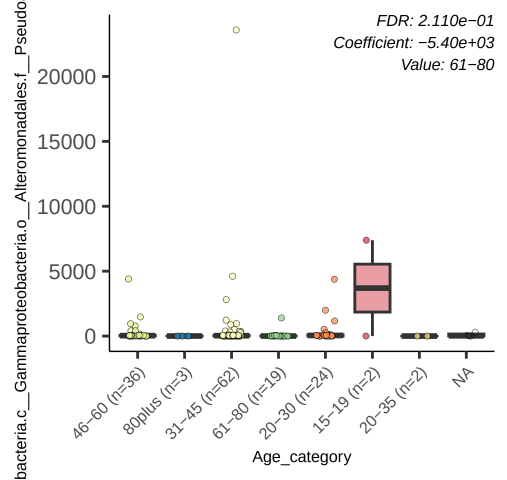
FDR: 2.110e−01
Coefficient: −5.40e+03
Value: 61−80
bacteria.c__Gammaproteobacteria.o__Alteromonadales.f__Pseudo
20000
15000
10000
5000
0
46−60 (n=36)
80plus (n=3)
31−45 (n=62)
61−80 (n=19)
20−30 (n=24)
15−19 (n=2)
20−35 (n=2)
NA
Age_category

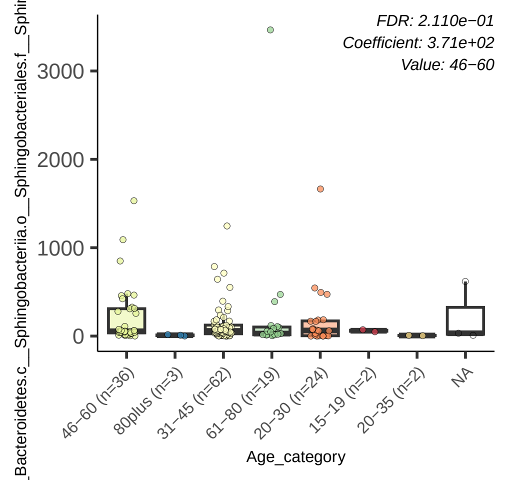

FDR: 2.110e−01
Coefficient: 3.71e+02
Value: 46−60
Bacteroidetes.c__Sphingobacteriia.o__Sphingobacteriales.f__Sphi
0
1000
2000
3000
46−60 (n=36)
80plus (n=3)
31−45 (n=62)
61−80 (n=19)
20−30 (n=24)
15−19 (n=2)
20−35 (n=2)
NA
Age_category

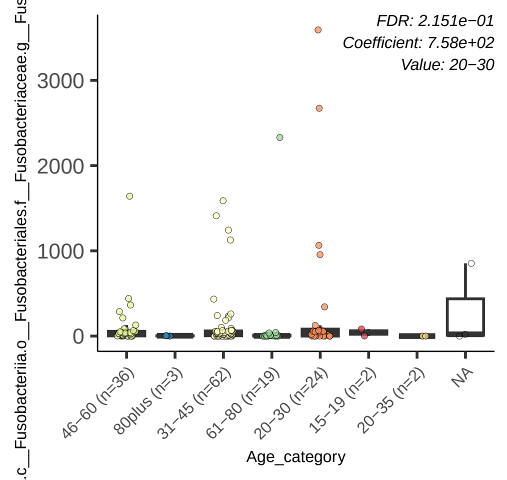

FDR: 2.151e−01
Coefficient: 7.58e+02
Value: 20−30
c__Fusobacteriia.o__Fusobacteriales.f__Fusobacteriaceae.g__Fus
3000
2000
1000
0
46−60 (n=36)
80plus (n=3)
31−45 (n=62)
61−80 (n=19)
20−30 (n=24)
15−19 (n=2)
20−35 (n=2)
NA
Age_category

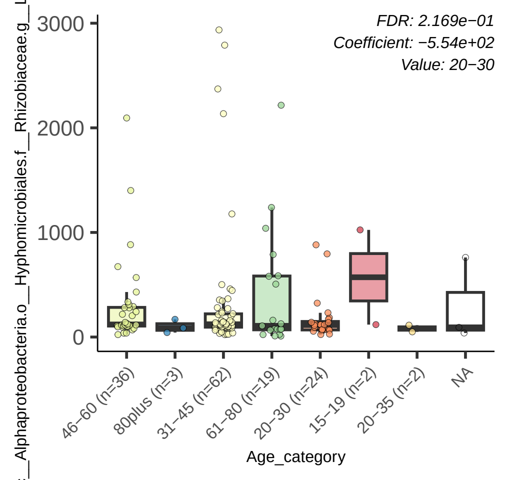

FDR: 2.169e−01
Coefficient: −5.54e+02
Value: 20−30
Alphaproteobacteria.o__Hyphomicrobiales.f__Rhizobiaceae.g__
3000
2000
1000
0
46−60 (n=36)
80plus (n=3)
31−45 (n=62)
61−80 (n=19)
20−30 (n=24)
15−19 (n=2)
20−35 (n=2)
NA
Age_category

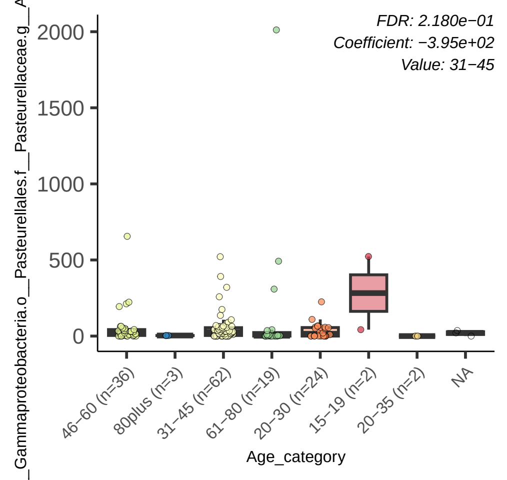

FDR: 2.180e−01
Coefficient: −3.95e+02
Value: 31−45
2000
1500
1000
500
0
_Gammaproteobacteria.o__Pasteurellales.f__Pasteurellaceae.g__A
46−60 (n=36)
80plus (n=3)
31−45 (n=62)
61−80 (n=19)
20−30 (n=24)
15−19 (n=2)
20−35 (n=2)
NA
Age_category

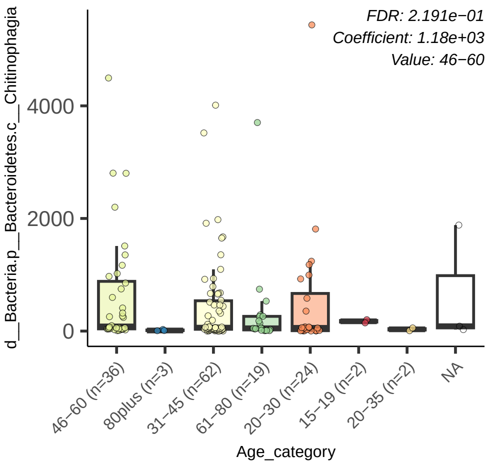
FDR: 2.191e−01
Coefficient: 1.18e+03
Value: 46−60
d__Bacteria.p__Bacteroidetes.c__Chitinophagia
0
2000
4000
46−60 (n=36)
80plus (n=3)
31−45 (n=62)
61−80 (n=19)
20−30 (n=24)
15−19 (n=2)
20−35 (n=2)
NA
Age_category

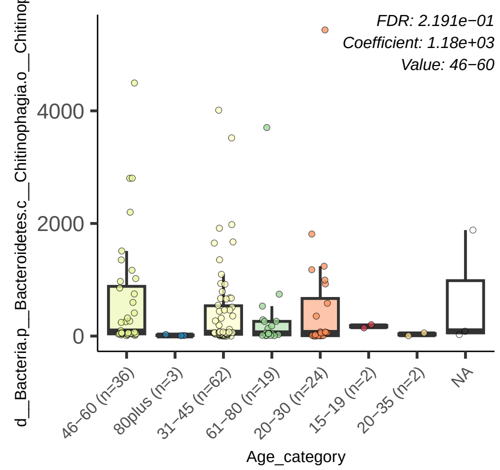

FDR: 2.191e−01
Coefficient: 1.18e+03
Value: 46−60
4000
2000
0
d__Bacteria.p__Bacteroidetes.c__Chitinophagia.o__Chitinop
46−60 (n=36)
80plus (n=3)
31−45 (n=62)
61−80 (n=19)
20−30 (n=24)
15−19 (n=2)
20−35 (n=2)
NA
Age_category

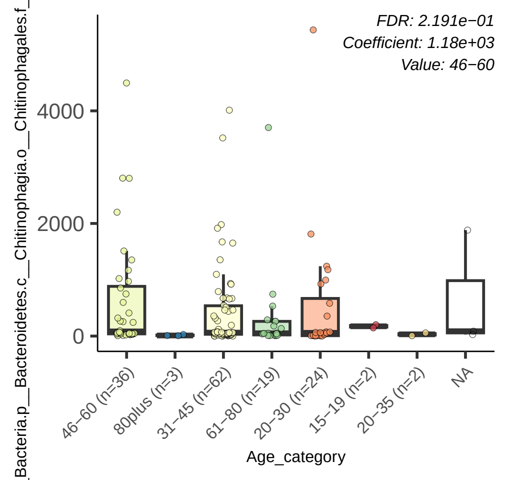

FDR: 2.191e−01
Coefficient: 1.18e+03
Value: 46−60
Bacteria.p__Bacteroidetes.c__Chitinophagia.o__Chitinophagales.f__
4000
2000
0
46−60 (n=36)
80plus (n=3)
31−45 (n=62)
61−80 (n=19)
20−30 (n=24)
15−19 (n=2)
20−35 (n=2)
NA
Age_category

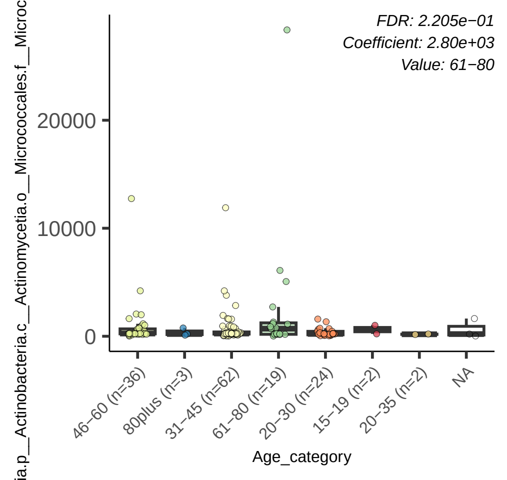

FDR: 2.205e−01
Coefficient: 2.80e+03
Value: 61−80
ia.p__Actinobacteria.c__Actinomycetia.o__Micrococcales.f__Microc
20000
10000
0
46−60 (n=36)
80plus (n=3)
31−45 (n=62)
61−80 (n=19)
20−30 (n=24)
15−19 (n=2)
20−35 (n=2)
NA
Age_category

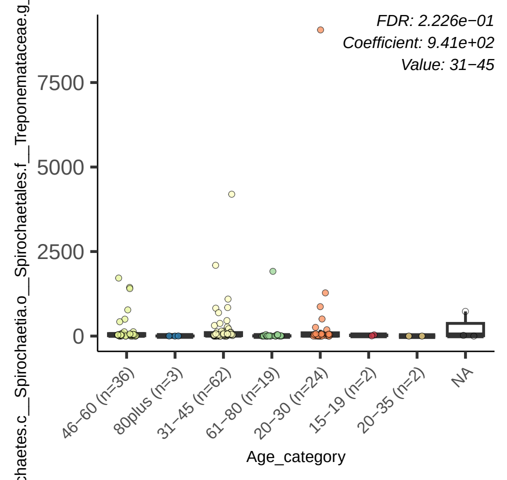

FDR: 2.226e−01
Coefficient: 9.41e+02
Value: 31−45
7500
5000
2500
0
chaetes.c__Spirochaetia.o__Spirochaetales.f__Treponemataceae.g_
46−60 (n=36)
80plus (n=3)
31−45 (n=62)
61−80 (n=19)
20−30 (n=24)
15−19 (n=2)
20−35 (n=2)
NA
Age_category

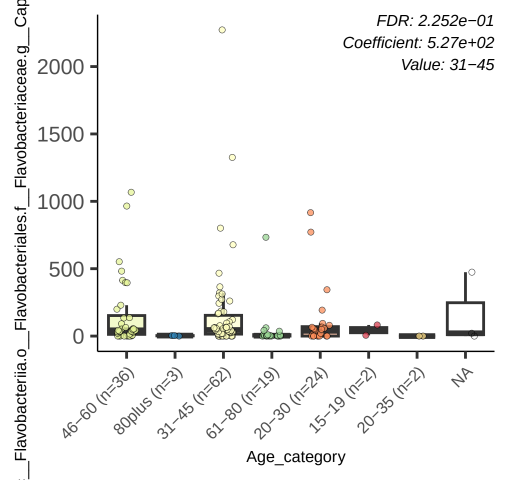

FDR: 2.252e−01
Coefficient: 5.27e+02
Value: 31−45
2000
1500
1000
500
0
46−60 (n=36)
80plus (n=3)
31−45 (n=62)
61−80 (n=19)
20−30 (n=24)
15−19 (n=2)
20−35 (n=2)
NA
Age_category
Flavobacteriia.o__Flavobacteriales.f__Flavobacteriaceae.g__Cap

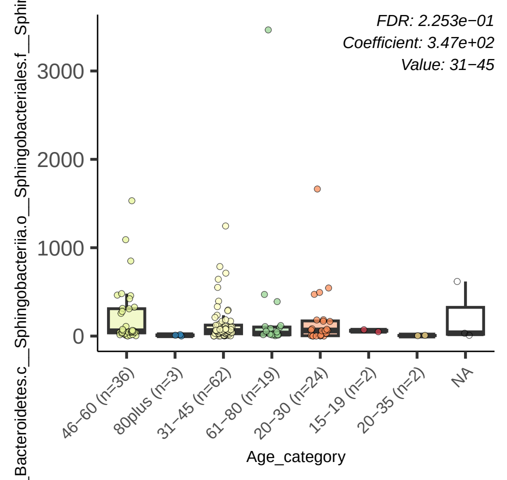
FDR: 2.253e−01
Coefficient: 3.47e+02
Value: 31−45
Bacteroidetes.c__Sphingobacteriia.o__Sphingobacteriales.f__Sphi
3000
2000
1000
0
46−60 (n=36)
80plus (n=3)
31−45 (n=62)
61−80 (n=19)
20−30 (n=24)
15−19 (n=2)
20−35 (n=2)
NA
Age_category

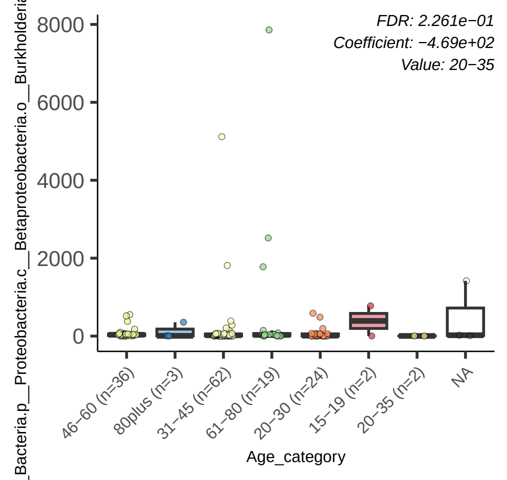

FDR: 2.261e−01
Coefficient: −4.69e+02
Value: 20−35
8000
6000
4000
2000
0
Bacteria.p__Proteobacteria.c__Betaproteobacteria.o__Burkholderia
46−60 (n=36)
80plus (n=3)
31−45 (n=62)
61−80 (n=19)
20−30 (n=24)
15−19 (n=2)
20−35 (n=2)
NA
Age_category

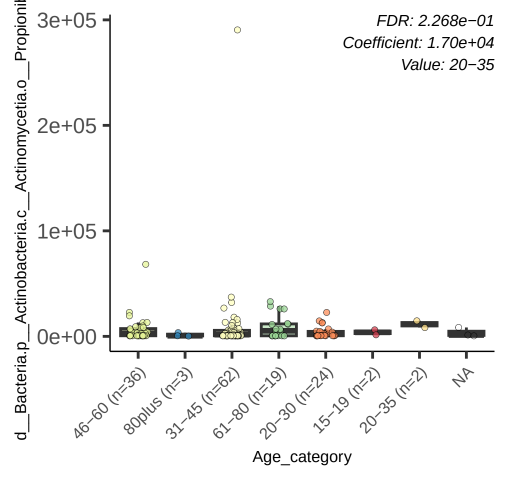

FDR: 2.268e−01
Coefficient: 1.70e+04
Value: 20−35
d__Bacteria.p__Actinobacteria.c__Actinomycetia.o__Propioni
3e+05
2e+05
1e+05
0e+00
46−60 (n=36)
80plus (n=3)
31−45 (n=62)
61−80 (n=19)
20−30 (n=24)
15−19 (n=2)
20−35 (n=2)
NA
Age_category

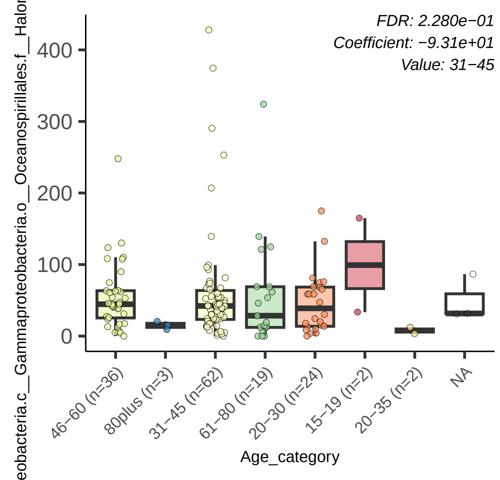

FDR: 2.280e−01
Coefficient: −9.31e+01
Value: 31−45
eobacteria.c__Gammaproteobacteria.o__Oceanospirillales.f__Halon
400
300
200
100
0
46−60 (n=36)
80plus (n=3)
31−45 (n=62)
61−80 (n=19)
20−30 (n=24)
15−19 (n=2)
20−35 (n=2)
NA
Age_category

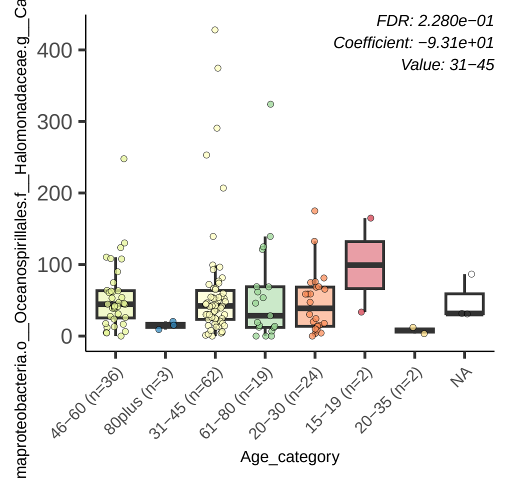
FDR: 2.280e−01
Coefficient: −9.31e+01
Value: 31−45
maproteobacteria.o__Oceanospirillales.f__Halomonadaceae.g__Ca
0
100
200
300
400
46−60 (n=36)
80plus (n=3)
31−45 (n=62)
61−80 (n=19)
20−30 (n=24)
15−19 (n=2)
20−35 (n=2)
NA
Age_category

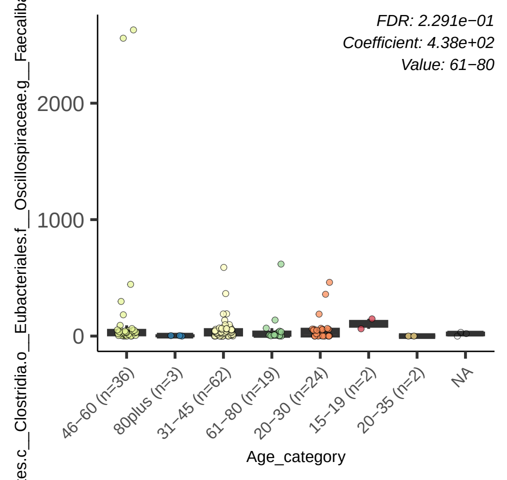

FDR: 2.291e−01
Coefficient: 4.38e+02
Value: 61−80
2000
1000
0
46−60 (n=36)
80plus (n=3)
31−45 (n=62)
61−80 (n=19)
20−30 (n=24)
15−19 (n=2)
20−35 (n=2)
NA
Age_category
tes.c__Clostridia.o__Eubacteriales.f__Oscillospiraceae.g__Faecaliba

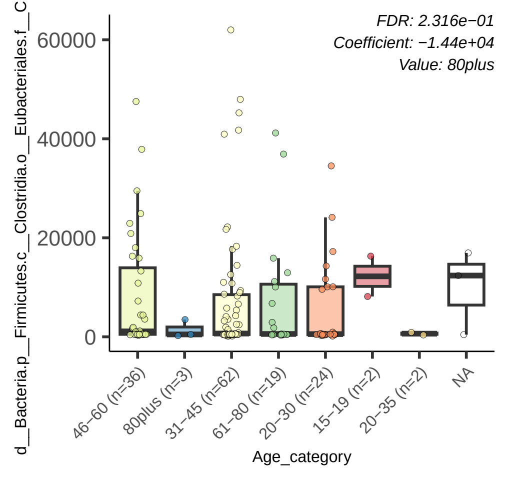

FDR: 2.316e−01
Coefficient: −1.44e+04
Value: 80plus
d__Bacteria.p__Firmicutes.c__Clostridia.o__Eubacteriales.f__C
60000
40000
20000
0
46−60 (n=36)
80plus (n=3)
31−45 (n=62)
61−80 (n=19)
20−30 (n=24)
15−19 (n=2)
20−35 (n=2)
NA
Age_category

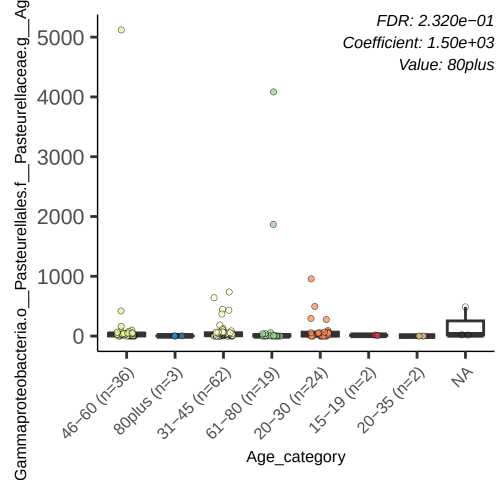

FDR: 2.320e−01
Coefficient: 1.50e+03
Value: 80plus
Gammaproteobacteria.o__Pasteurellales.f__Pasteurellaceae.g__Ag
5000
4000
3000
2000
1000
0
46−60 (n=36)
80plus (n=3)
31−45 (n=62)
61−80 (n=19)
20−30 (n=24)
15−19 (n=2)
20−35 (n=2)
NA
Age_category

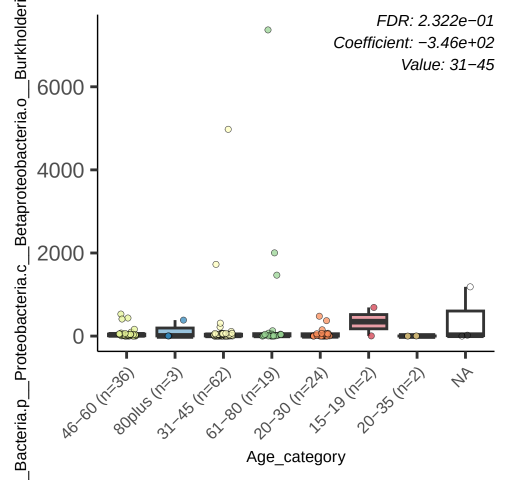

Bacteria.p__Proteobacteria.c__Betaproteobacteria.o__Burkholderi
FDR: 2.322e−01
Coefficient: −3.46e+02
Value: 31−45
6000
4000
2000
0
46−60 (n=36)
80plus (n=3)
31−45 (n=62)
61−80 (n=19)
20−30 (n=24)
15−19 (n=2)
20−35 (n=2)
NA
Age_category

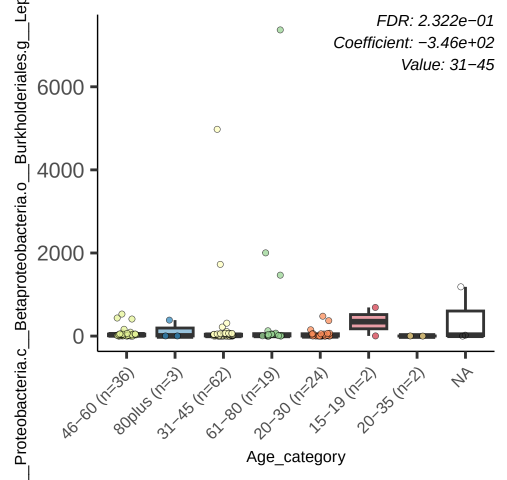
FDR: 2.322e−01
Coefficient: −3.46e+02
Value: 31−45
_Proteobacteria.c__Betaproteobacteria.o__Burkholderiales.g__Lep
6000
4000
2000
0
46−60 (n=36)
80plus (n=3)
31−45 (n=62)
61−80 (n=19)
20−30 (n=24)
15−19 (n=2)
20−35 (n=2)
NA
Age_category

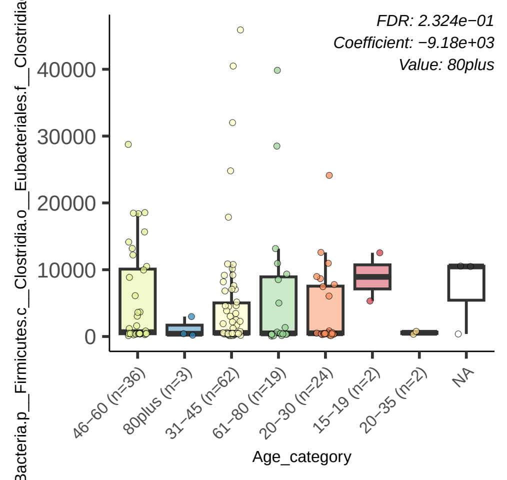

FDR: 2.324e−01
Coefficient: −9.18e+03
Value: 80plus
Bacteria.p__Firmicutes.c__Clostridia.o__Eubacteriales.f__Clostridia
40000
30000
20000
10000
0
46−60 (n=36)
80plus (n=3)
31−45 (n=62)
61−80 (n=19)
20−30 (n=24)
15−19 (n=2)
20−35 (n=2)
NA
Age_category

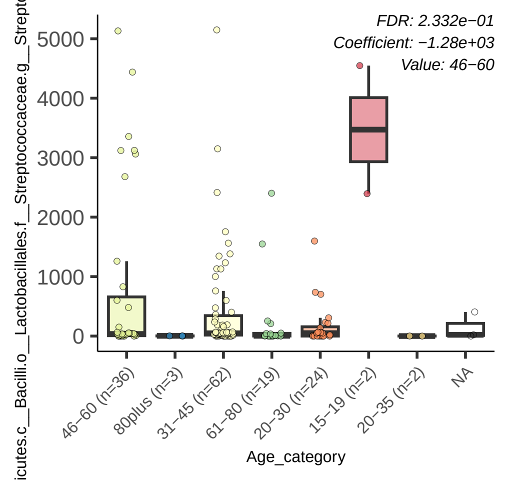

FDR: 2.332e−01
Coefficient: −1.28e+03
Value: 46−60
5000
4000
3000
2000
1000
0
46−60 (n=36)
80plus (n=3)
31−45 (n=62)
61−80 (n=19)
20−30 (n=24)
15−19 (n=2)
20−35 (n=2)
NA
Age_category

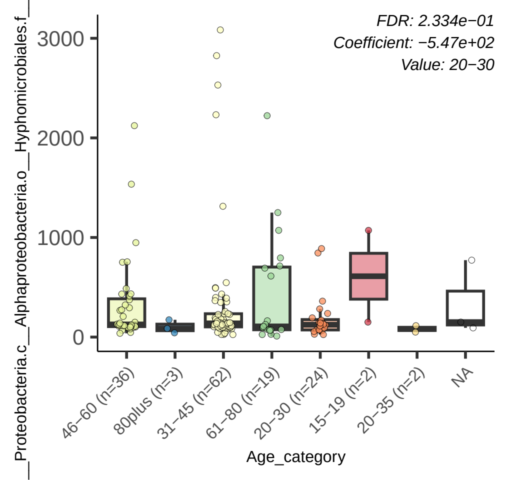

FDR: 2.334e−01
Coefficient: −5.47e+02
Value: 20−30
Proteobacteria.c__Alphaproteobacteria.o__Hyphomicrobiales.f
3000
2000
1000
0
46−60 (n=36)
80plus (n=3)
31−45 (n=62)
61−80 (n=19)
20−30 (n=24)
15−19 (n=2)
20−35 (n=2)
NA
Age_category

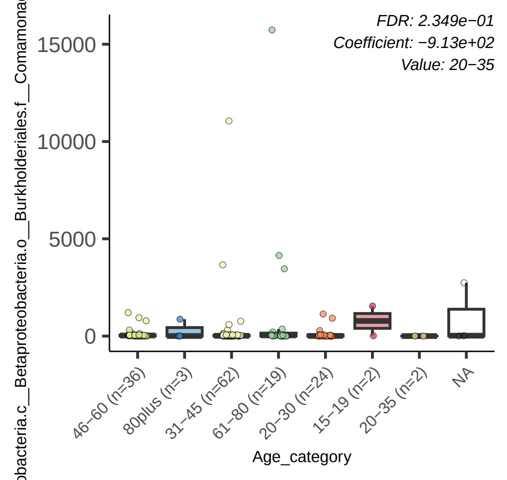

FDR: 2.349e−01
Coefficient: −9.13e+02
Value: 20−35
15000
10000
5000
0
obacteria.c__Betaproteobacteria.o__Burkholderiales.f__Comamonad
46−60 (n=36)
80plus (n=3)
31−45 (n=62)
61−80 (n=19)
20−30 (n=24)
15−19 (n=2)
20−35 (n=2)
NA
Age_category

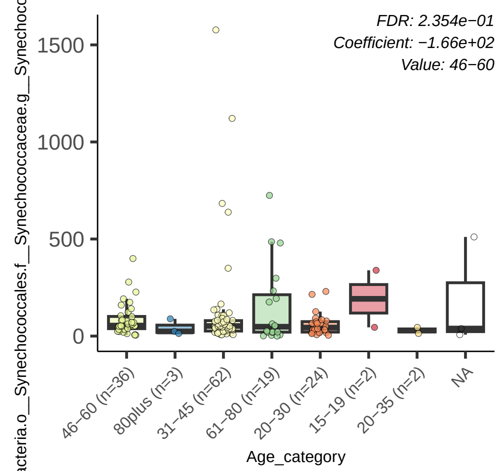

FDR: 2.354e−01
Coefficient: −1.66e+02
Value: 46−60
1500
1000
500
0
bacteria.o__Synechococcales.f__Synechococcaceae.g__Synechoco
46−60 (n=36)
80plus (n=3)
31−45 (n=62)
61−80 (n=19)
20−30 (n=24)
15−19 (n=2)
20−35 (n=2)
NA
Age_category

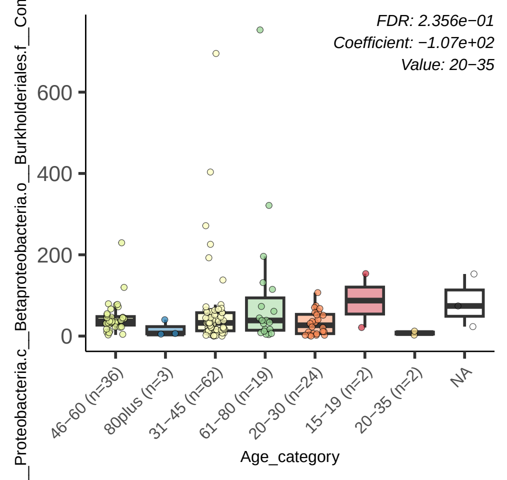
FDR: 2.356e−01
Coefficient: −1.07e+02
Value: 20−35
_Proteobacteria.c__Betaproteobacteria.o__Burkholderiales.f__Con
600
400
200
0
46−60 (n=36)
80plus (n=3)
31−45 (n=62)
61−80 (n=19)
20−30 (n=24)
15−19 (n=2)
20−35 (n=2)
NA
Age_category

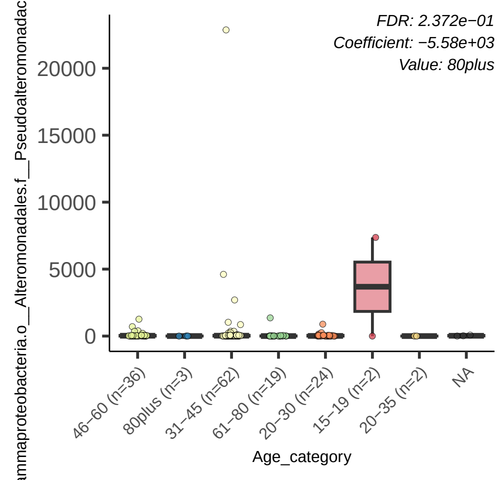

FDR: 2.372e−01
Coefficient: −5.58e+03
Value: 80plus
ammaproteobacteria.o__Alteromonadales.f__Pseudoalteromonadac
20000
15000
10000
5000
0
46−60 (n=36)
80plus (n=3)
31−45 (n=62)
61−80 (n=19)
20−30 (n=24)
15−19 (n=2)
20−35 (n=2)
NA
Age_category

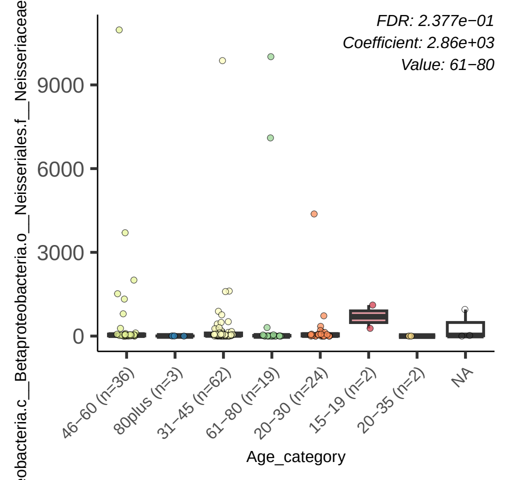
FDR: 2.377e−01
Coefficient: 2.86e+03
Value: 61−80
eobacteria.c__Betaproteobacteria.o__Neisseriales.f__Neisseriaceae
9000
6000
3000
0
46−60 (n=36)
80plus (n=3)
31−45 (n=62)
61−80 (n=19)
20−30 (n=24)
15−19 (n=2)
20−35 (n=2)
NA
Age_category

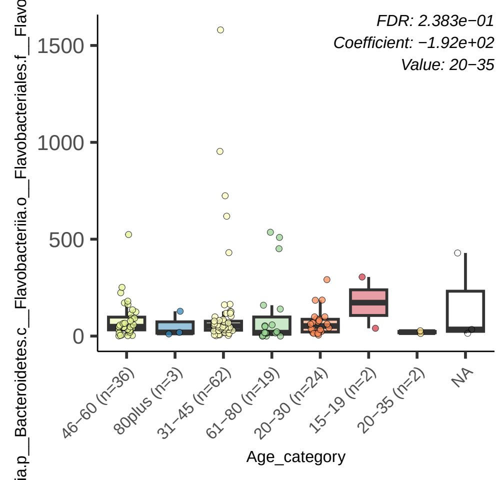

FDR: 2.383e−01
Coefficient: −1.92e+02
Value: 20−35
1500
1000
500
0
ia.p__Bacteroidetes.c__Flavobacteriia.o__Flavobacteriales.f__Flavo
46−60 (n=36)
80plus (n=3)
31−45 (n=62)
61−80 (n=19)
20−30 (n=24)
15−19 (n=2)
20−35 (n=2)
NA
Age_category

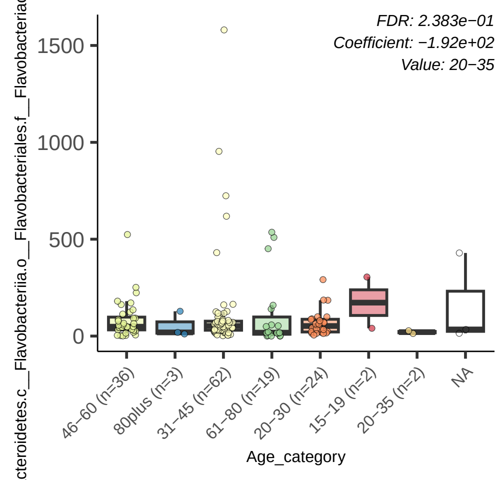
FDR: 2.383e−01
Coefficient: −1.92e+02
Value: 20−35
1500
1000
500
0
cteroidetes.c__Flavobacteriia.o__Flavobacteriales.f__Flavobacteria
46−60 (n=36)
80plus (n=3)
31−45 (n=62)
61−80 (n=19)
20−30 (n=24)
15−19 (n=2)
20−35 (n=2)
NA
Age_category

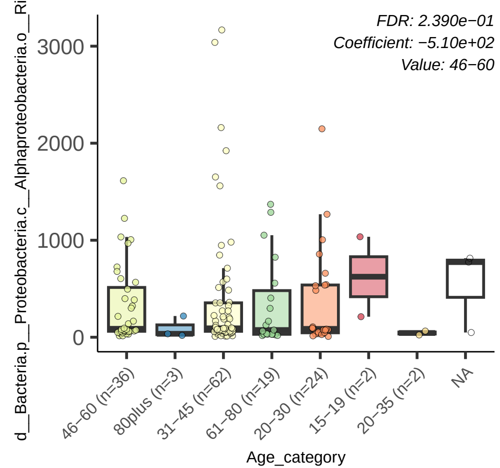

FDR: 2.390e−01
Coefficient: −5.10e+02
Value: 46−60
d__Bacteria.p__Proteobacteria.c__Alphaproteobacteria.o__Ri
3000
2000
1000
0
46−60 (n=36)
80plus (n=3)
31−45 (n=62)
61−80 (n=19)
20−30 (n=24)
15−19 (n=2)
20−35 (n=2)
NA
Age_category

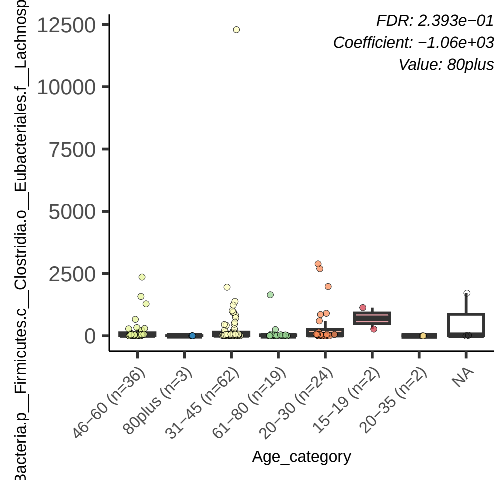

FDR: 2.393e−01
Coefficient: −1.06e+03
Value: 80plus
Bacteria.p__Firmicutes.c__Clostridia.o__Eubacteriales.f__Lachnosp
12500
10000
7500
5000
2500
0
46−60 (n=36)
80plus (n=3)
31−45 (n=62)
61−80 (n=19)
20−30 (n=24)
15−19 (n=2)
20−35 (n=2)
NA
Age_category

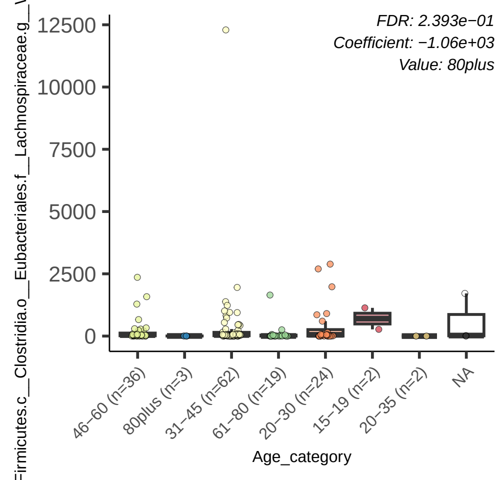

FDR: 2.393e−01
Coefficient: −1.06e+03
Value: 80plus
12500
10000
7500
5000
2500
0
46−60 (n=36)
80plus (n=3)
31−45 (n=62)
61−80 (n=19)
20−30 (n=24)
15−19 (n=2)
20−35 (n=2)
NA
Age_category
Firmicutes.c__Clostridia.o__Eubacteriales.f__Lachnospiraceae.g__

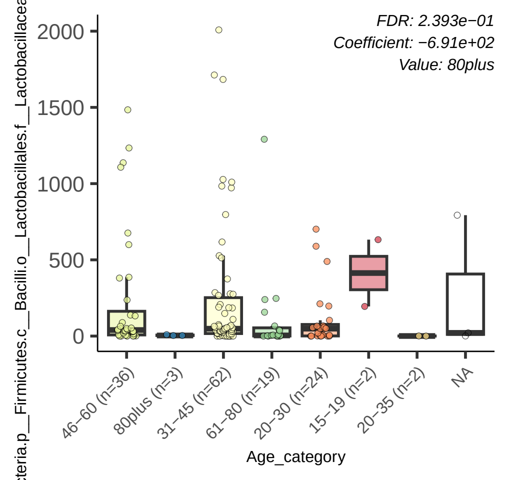

FDR: 2.393e−01
Coefficient: −6.91e+02
Value: 80plus
cteria.p__Firmicutes.c__Bacilli.o__Lactobacillales.f__Lactobacillacea
2000
1500
1000
500
0
46−60 (n=36)
80plus (n=3)
31−45 (n=62)
61−80 (n=19)
20−30 (n=24)
15−19 (n=2)
20−35 (n=2)
NA
Age_category

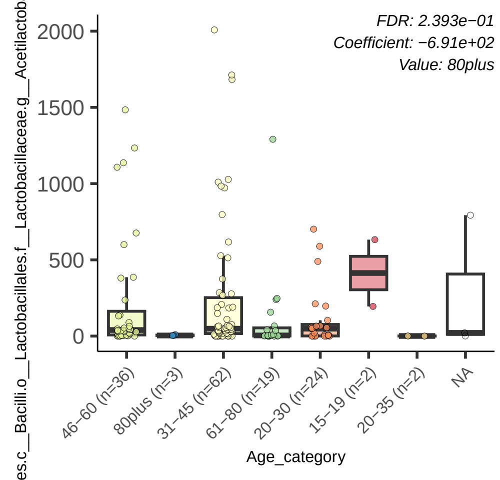
FDR: 2.393e−01
Coefficient: −6.91e+02
Value: 80plus
es.c__Bacilli.o__Lactobacillales.f__Lactobacillaceae.g__Acetilactob
0
500
1000
1500
2000
46−60 (n=36)
80plus (n=3)
31−45 (n=62)
61−80 (n=19)
20−30 (n=24)
15−19 (n=2)
20−35 (n=2)
NA
Age_category

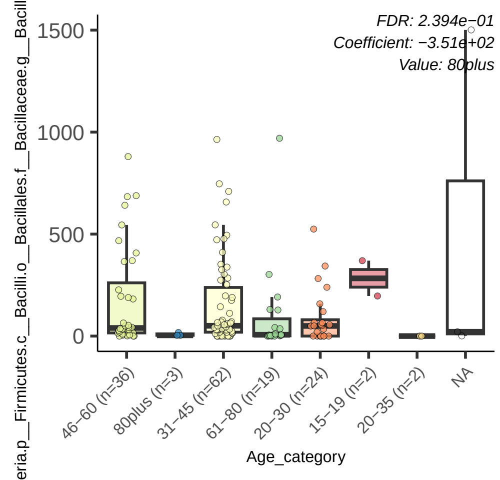

FDR: 2.394e−01
Coefficient: −3.51e+02
Value: 80plus
eria.p__Firmicutes.c__Bacilli.o__Bacillales.f__Bacillaceae.g__Bacill
1500
1000
500
0
46−60 (n=36)
80plus (n=3)
31−45 (n=62)
61−80 (n=19)
20−30 (n=24)
15−19 (n=2)
20−35 (n=2)
NA
Age_category

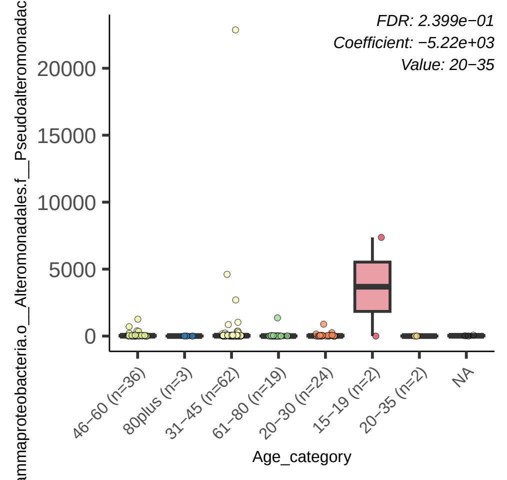
FDR: 2.399e−01
Coefficient: −5.22e+03
Value: 20−35
ammaproteobacteria.o__Alteromonadales.f__Pseudoalteromonadac
20000
15000
10000
5000
0
46−60 (n=36)
80plus (n=3)
31−45 (n=62)
61−80 (n=19)
20−30 (n=24)
15−19 (n=2)
20−35 (n=2)
NA
Age_category

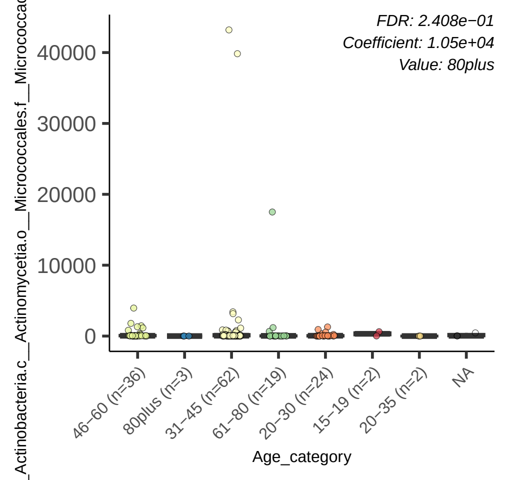
FDR: 2.408e−01
Coefficient: 1.05e+04
Value: 80plus
40000
30000
20000
10000
0
_Actinobacteria.c__Actinomycetia.o__Micrococcales.f__Micrococcac
46−60 (n=36)
80plus (n=3)
31−45 (n=62)
61−80 (n=19)
20−30 (n=24)
15−19 (n=2)
20−35 (n=2)
NA
Age_category

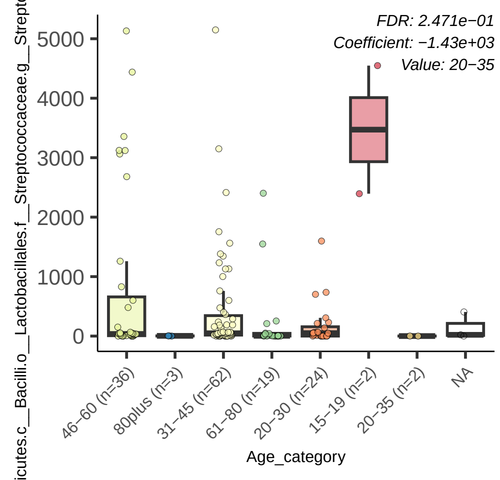
FDR: 2.471e−01
Coefficient: −1.43e+03
Value: 20−35
0
1000
2000
3000
4000
5000
Firmicutes.c__Bacilli.o__Lactobacillales.f__Streptococcaceae.g__Strepto
46−60 (n=36)
80plus (n=3)
31−45 (n=62)
61−80 (n=19)
20−30 (n=24)
15−19 (n=2)
20−35 (n=2)
NA
Age_category

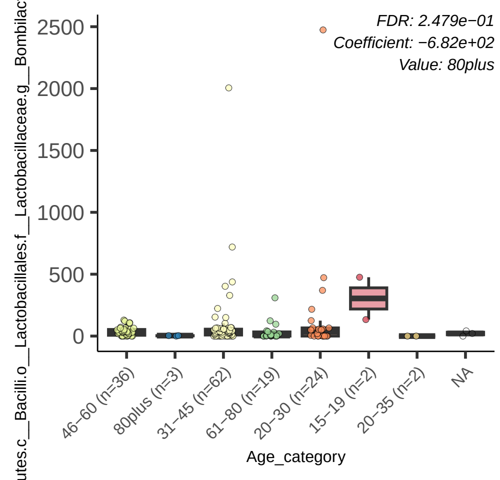

FDR: 2.479e−01
Coefficient: −6.82e+02
Value: 80plus
utes.c__Bacilli.o__Lactobacillales.f__Lactobacillaceae.g__Bombilac
2500
2000
1500
1000
500
0
46−60 (n=36)
80plus (n=3)
31−45 (n=62)
61−80 (n=19)
20−30 (n=24)
15−19 (n=2)
20−35 (n=2)
NA
Age_category

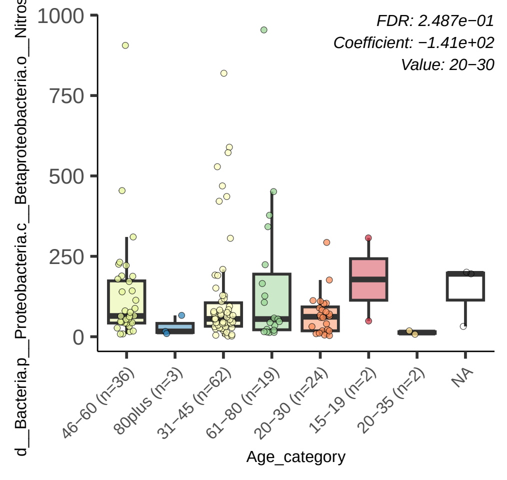

d__Bacteria.p__Proteobacteria.c__Betaproteobacteria.o__Nitros
1000
750
500
250
0
FDR: 2.487e−01
Coefficient: −1.41e+02
Value: 20−30
46−60 (n=36)
80plus (n=3)
31−45 (n=62)
61−80 (n=19)
20−30 (n=24)
15−19 (n=2)
20−35 (n=2)
NA
Age_category

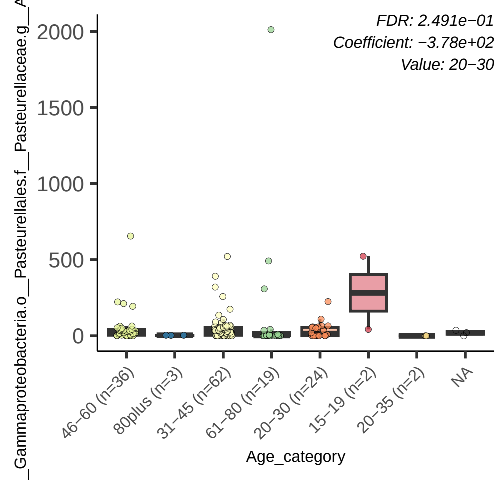

FDR: 2.491e−01
Coefficient: −3.78e+02
Value: 20−30
2000
1500
1000
500
0
_Gammaproteobacteria.o__Pasteurellales.f__Pasteurellaceae.g__A
46−60 (n=36)
80plus (n=3)
31−45 (n=62)
61−80 (n=19)
20−30 (n=24)
15−19 (n=2)
20−35 (n=2)
NA
Age_category
