# Supplementary material for: Metatranscriptome Analysis of Nasopharyngeal Swabs across the Varying Severity of COVID-19 Disease Demonstrated Unprecedented Species Diversity
Source: Microorganisms. 2023 Jul 14;11(7):1804. doi: 10.3390/microorganisms11071804 (PMC10384460; doi:10.3390/microorganisms11071804)
Supplement: Supplementary file 1 [file microorganisms-11-01804-s001.zip › Maaslin_results_ALL.zip!/Maaslin_results/ATBTreatment.pdf]

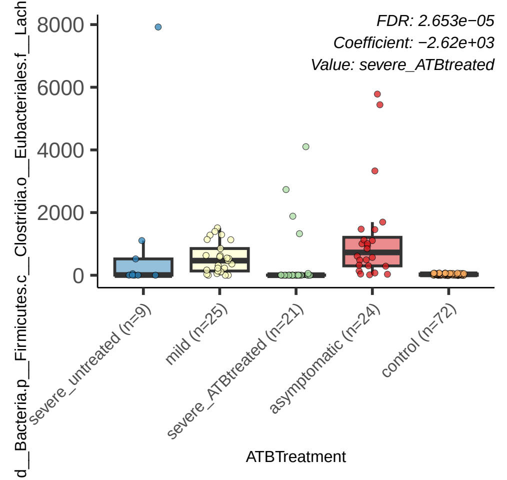
FDR: 2.653e−05
Coefficient: −2.62e+03
Value: severe_ATBtreated
d__Bacteria.p__Firmicutes.c__Clostridia.o__Eubacteriales.f__Lach
8000
6000
4000
2000
0
severe_untreated (n=9)
mild (n=25)
severe_ATBtreated (n=21)
asymptomatic (n=24)
control (n=72)
ATBTreatment

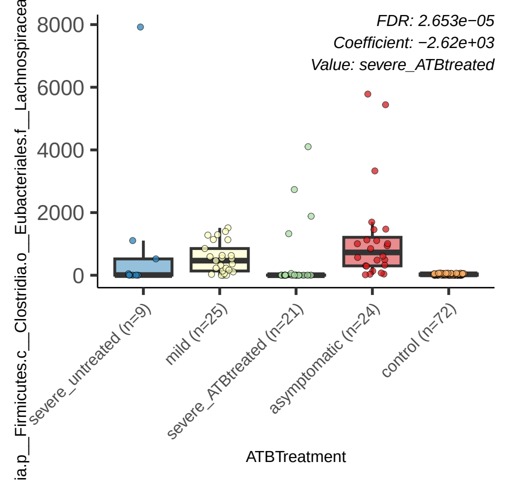

FDR: 2.653e−05
Coefficient: −2.62e+03
Value: severe_ATBtreated
8000
6000
4000
2000
0
ia.p__Firmicutes.c__Clostridia.o__Eubacteriales.f__Lachnospiracea
severe_untreated (n=9)
mild (n=25)
severe_ATBtreated (n=21)
asymptomatic (n=24)
control (n=72)
ATBTreatment

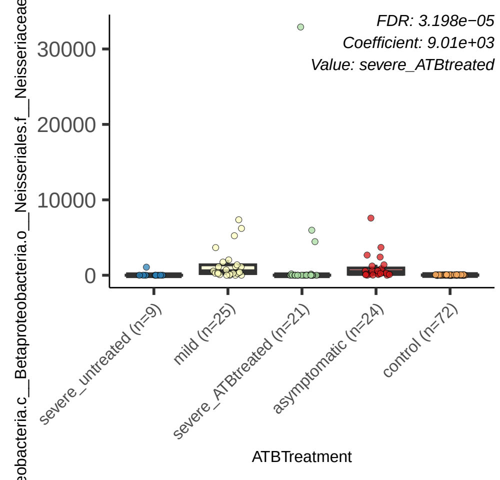

FDR: 3.198e−05
Coefficient: 9.01e+03
Value: severe_ATBtreated
30000
20000
10000
0
eobacteria.c__Betaproteobacteria.o__Neisseriales.f__Neisseriaceae
severe_untreated (n=9)
mild (n=25)
severe_ATBtreated (n=21)
asymptomatic (n=24)
control (n=72)
ATBTreatment

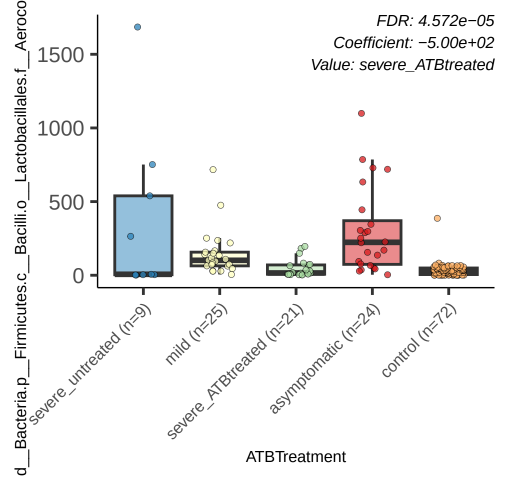

d__Bacteria.p__Firmicutes.c__Bacilli.o__Lactobacillales.f__Aeroco
FDR: 4.572e−05
Coefficient: −5.00e+02
Value: severe_ATBtreated
1500
1000
500
0
severe_untreated (n=9)
mild (n=25)
severe_ATBtreated (n=21)
asymptomatic (n=24)
control (n=72)
ATBTreatment

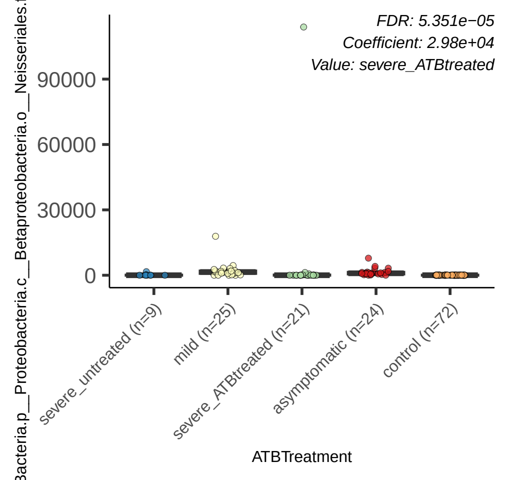
FDR: 5.351e−05
Coefficient: 2.98e+04
Value: severe_ATBtreated
Bacteria.p__Proteobacteria.c__Betaproteobacteria.o__Neisseriales.f
90000
60000
30000
0
severe_untreated (n=9)
mild (n=25)
severe_ATBtreated (n=21)
asymptomatic (n=24)
control (n=72)
ATBTreatment

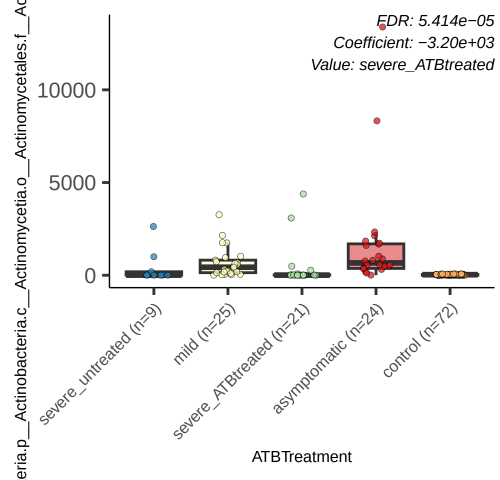
FDR: 5.414e−05
Coefficient: −3.20e+03
Value: severe_ATBtreated
10000
5000
0
Actinobacteria.p__Actinobacteria.c__Actinomycetia.o__Actinomycetales.f__Ac
severe_untreated (n=9)
mild (n=25)
severe_ATBtreated (n=21)
asymptomatic (n=24)
control (n=72)
ATBTreatment

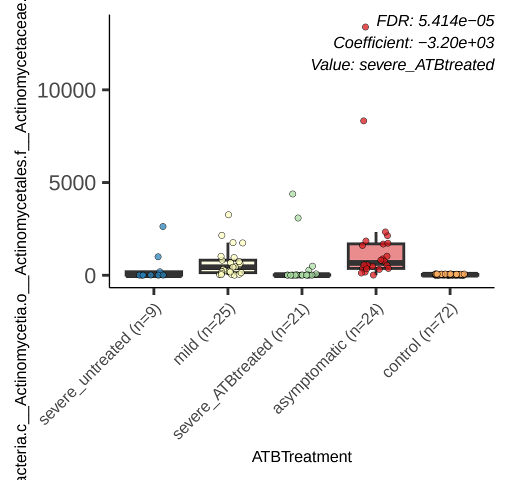

FDR: 5.414e−05
Coefficient: −3.20e+03
Value: severe_ATBtreated
10000
5000
0
severe_untreated (n=9)
mild (n=25)
severe_ATBtreated (n=21)
asymptomatic (n=24)
control (n=72)
ATBTreatment
acteria.c__Actinomycetia.o__Actinomycetales.f__Actinomycetaceae.

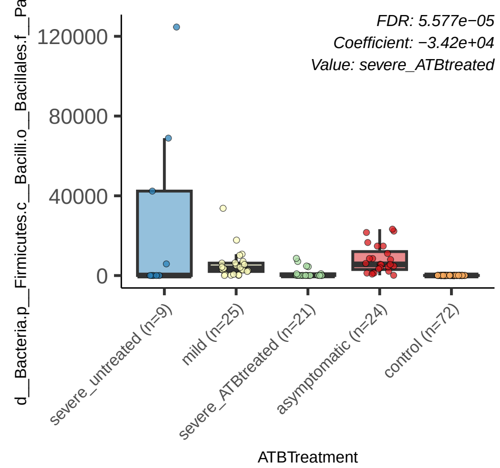
FDR: 5.577e−05
Coefficient: −3.42e+04
Value: severe_ATBtreated
d__Bacteria.p__Firmicutes.c__Bacilli.o__Bacillales.f__Pa
120000
80000
40000
0
severe_untreated (n=9)
mild (n=25)
severe_ATBtreated (n=21)
asymptomatic (n=24)
control (n=72)
ATBTreatment

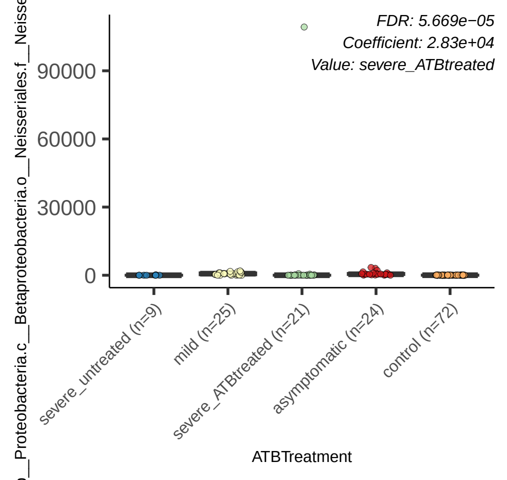
FDR: 5.669e−05
Coefficient: 2.83e+04
Value: severe_ATBtreated
90000
60000
30000
0
severe_untreated (n=9)
mild (n=25)
severe_ATBtreated (n=21)
asymptomatic (n=24)
control (n=72)
ATBTreatment
o__Proteobacteria.c__Betaproteobacteria.o__Neisseriales.f__Neisse

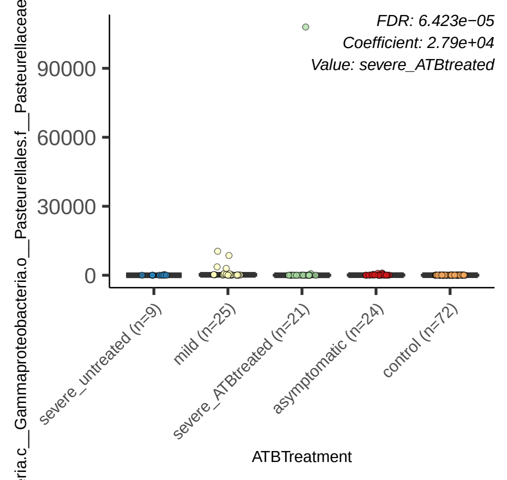

FDR: 6.423e−05
Coefficient: 2.79e+04
Value: severe_ATBtreated
90000
60000
30000
0
severe_untreated (n=9)
mild (n=25)
severe_ATBtreated (n=21)
asymptomatic (n=24)
control (n=72)
ATBTreatment
ria.c__Gammaproteobacteria.o__Pasteurellales.f__Pasteurellaceae

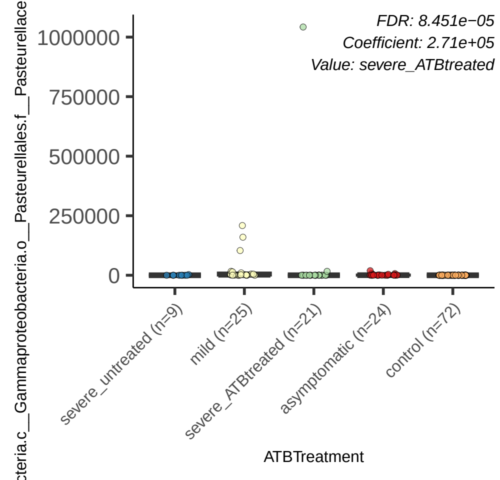

FDR: 8.451e−05
Coefficient: 2.71e+05
Value: severe_ATBtreated
1000000
750000
500000
250000
0
severe_untreated (n=9)
mild (n=25)
severe_ATBtreated (n=21)
asymptomatic (n=24)
control (n=72)
ATBTreatment
cteria.c__Gammaproteobacteria.o__Pasteurellales.f__Pasteurellace

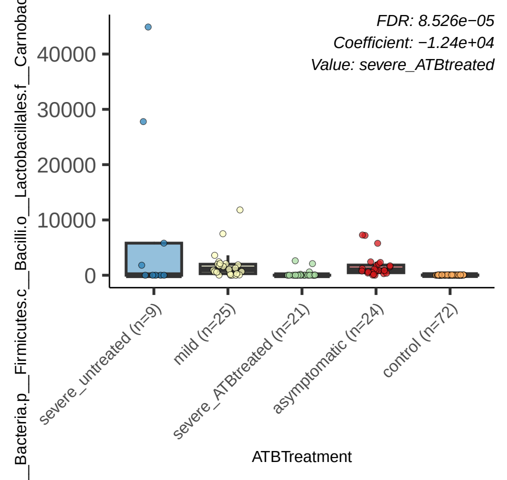
FDR: 8.526e−05
Coefficient: −1.24e+04
Value: severe_ATBtreated
40000
30000
20000
10000
0
severe_untreated (n=9)
mild (n=25)
severe_ATBtreated (n=21)
asymptomatic (n=24)
control (n=72)
ATBTreatment

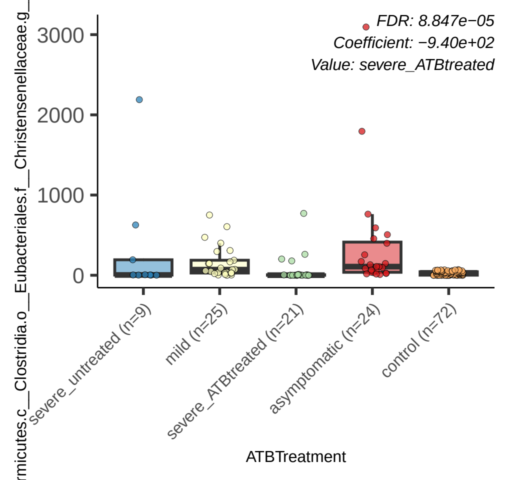
FDR: 8.847e−05
Coefficient: −9.40e+02
Value: severe_ATBtreated
3000
2000
1000
0
rmicutes.c__Clostridia.o__Eubacteriales.f__Christensenellaceae.g__
severe_untreated (n=9)
mild (n=25)
severe_ATBtreated (n=21)
asymptomatic (n=24)
control (n=72)
ATBTreatment

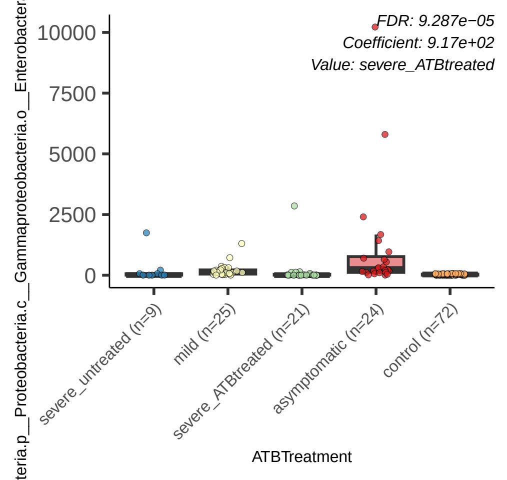

FDR: 9.287e−05
Coefficient: 9.17e+02
Value: severe_ATBtreated
10000
7500
5000
2500
0
teria.p__Proteobacteria.c__Gammaproteobacteria.o__Enterobacter
severe_untreated (n=9)
mild (n=25)
severe_ATBtreated (n=21)
asymptomatic (n=24)
control (n=72)
ATBTreatment

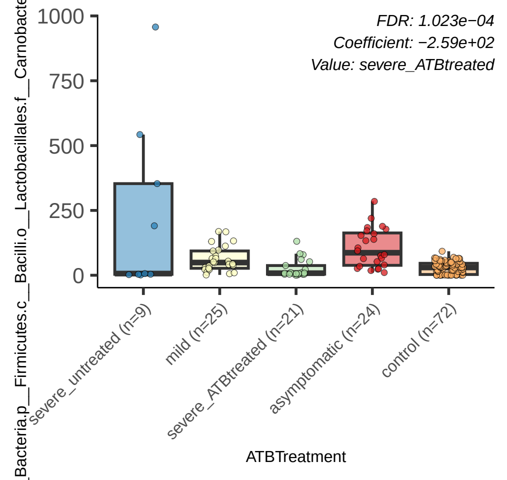

FDR: 1.023e−04
Coefficient: −2.59e+02
Value: severe_ATBtreated
Bacteria.p__Firmicutes.c__Bacilli.o__Lactobacillales.f__Carnobacte
1000
750
500
250
0
severe_untreated (n=9)
mild (n=25)
severe_ATBtreated (n=21)
asymptomatic (n=24)
control (n=72)
ATBTreatment

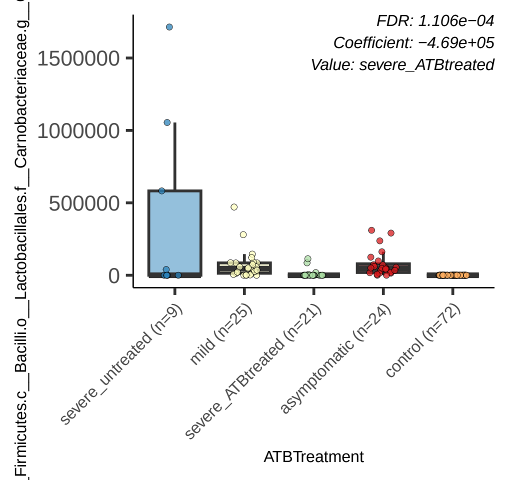

FDR: 1.106e−04
Coefficient: −4.69e+05
Value: severe_ATBtreated
Firmicutes.c__Bacilli.o__Lactobacillales.f__Carnobacteriaceae.g__
1500000
1000000
500000
0
severe_untreated (n=9)
mild (n=25)
severe_ATBtreated (n=21)
asymptomatic (n=24)
control (n=72)
ATBTreatment

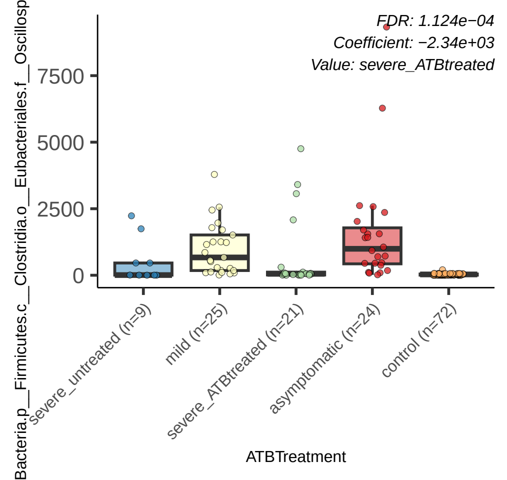

FDR: 1.124e−04
Coefficient: −2.34e+03
Value: severe_ATBtreated
Bacteria.p__Firmicutes.c__Clostridia.o__Eubacteriales.f__Oscillosp
0
2500
5000
7500
severe_untreated (n=9)
mild (n=25)
severe_ATBtreated (n=21)
asymptomatic (n=24)
control (n=72)
ATBTreatment

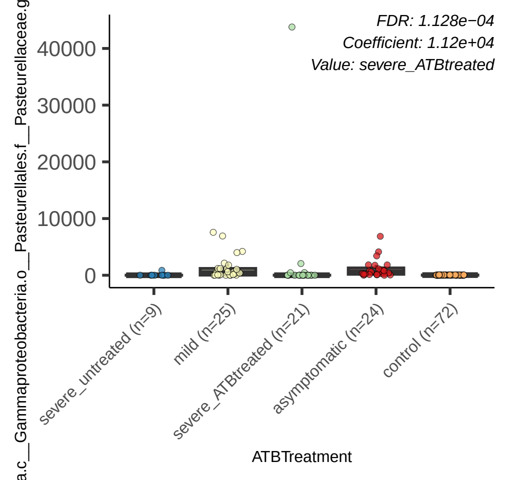
FDR: 1.128e−04
Coefficient: 1.12e+04
Value: severe_ATBtreated
a.c__Gammaproteobacteria.o__Pasteurellales.f__Pasteurellaceae.g
40000
30000
20000
10000
0
severe_untreated (n=9)
mild (n=25)
severe_ATBtreated (n=21)
asymptomatic (n=24)
control (n=72)
ATBTreatment

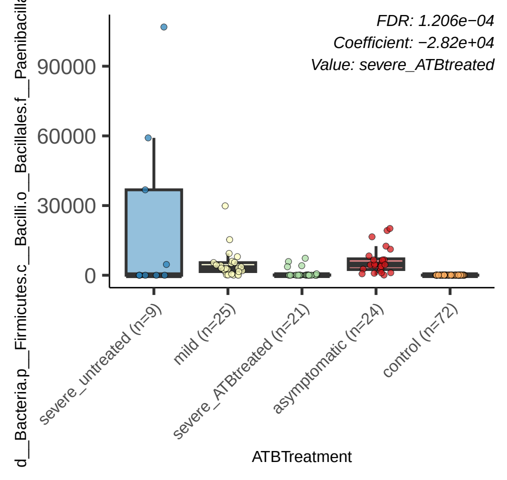

FDR: 1.206e−04
Coefficient: −2.82e+04
Value: severe_ATBtreated
d__Bacteria.p__Firmicutes.c__Bacilli.o__Bacillales.f__Paenibacilla
90000
60000
30000
0
severe_untreated (n=9)
mild (n=25)
severe_ATBtreated (n=21)
asymptomatic (n=24)
control (n=72)
ATBTreatment

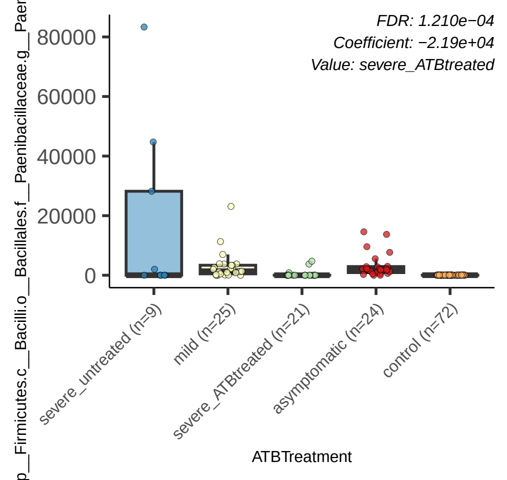
FDR: 1.210e−04
Coefficient: −2.19e+04
Value: severe_ATBtreated
p__Firmicutes.c__Bacilli.o__Bacillales.f__Paenibacillaceae.g__Paer
80000
60000
40000
20000
0
severe_untreated (n=9)
mild (n=25)
severe_ATBtreated (n=21)
asymptomatic (n=24)
control (n=72)
ATBTreatment

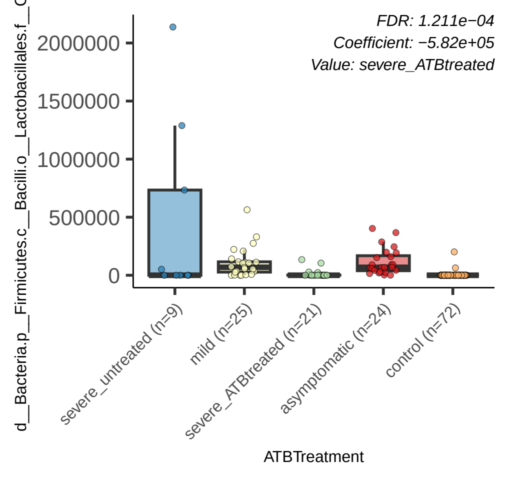

FDR: 1.211e−04
Coefficient: −5.82e+05
Value: severe_ATBtreated
2000000
1500000
1000000
500000
0
d__Bacteria.p__Firmicutes.c__Bacilli.o__Lactobacillales.f__C
severe_untreated (n=9)
mild (n=25)
severe_ATBtreated (n=21)
asymptomatic (n=24)
control (n=72)
ATBTreatment

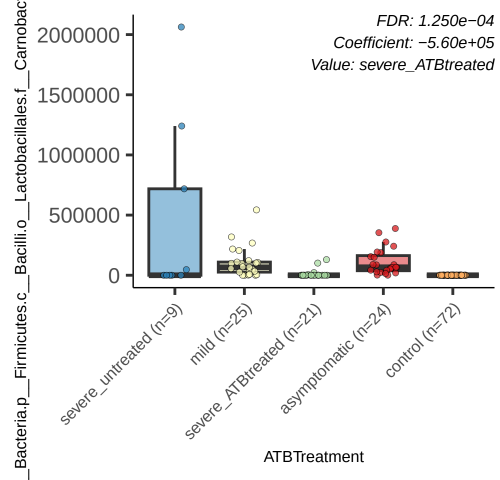

FDR: 1.250e−04
Coefficient: −5.60e+05
Value: severe_ATBtreated
Bacteria.p__Firmicutes.c__Bacilli.o__Lactobacillales.f__Carnobac
2000000
1500000
1000000
500000
0
severe_untreated (n=9)
mild (n=25)
severe_ATBtreated (n=21)
asymptomatic (n=24)
control (n=72)
ATBTreatment

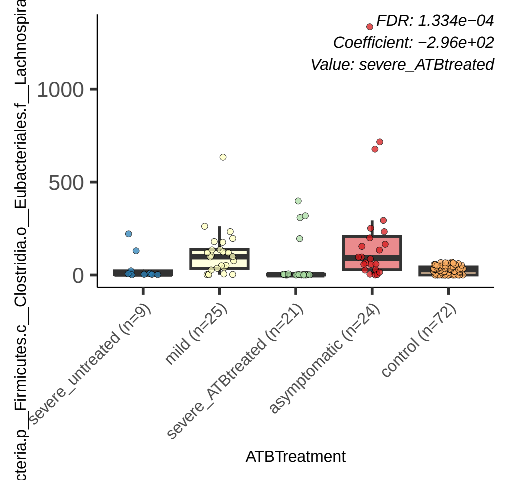

FDR: 1.334e−04
Coefficient: −2.96e+02
Value: severe_ATBtreated
1000
500
0
severe_untreated (n=9)
mild (n=25)
severe_ATBtreated (n=21)
asymptomatic (n=24)
control (n=72)
ATBTreatment
cteria.p__Firmicutes.c__Clostridia.o__Eubacteriales.f__Lachnospira

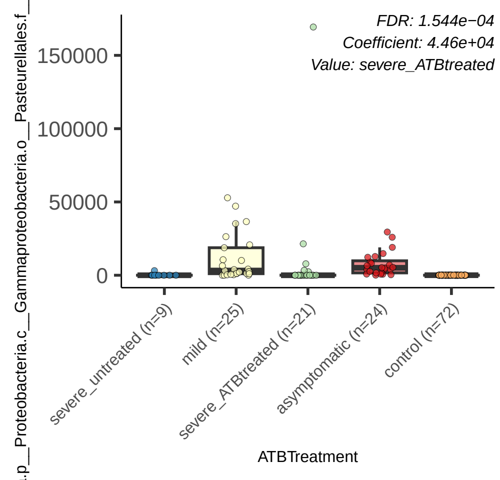

FDR: 1.544e−04
Coefficient: 4.46e+04
Value: severe_ATBtreated
150000
100000
50000
0
Proteobacteria.c__Gammaproteobacteria.o__Pasteurellales.f__
severe_untreated (n=9)
mild (n=25)
severe_ATBtreated (n=21)
asymptomatic (n=24)
control (n=72)
ATBTreatment

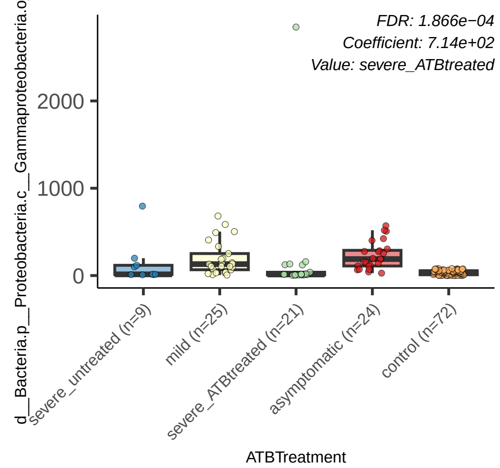

FDR: 1.866e−04
Coefficient: 7.14e+02
Value: severe_ATBtreated
d__Bacteria.p__Proteobacteria.c__Gammaproteobacteria.o
2000
1000
0
severe_untreated (n=9)
mild (n=25)
severe_ATBtreated (n=21)
asymptomatic (n=24)
control (n=72)
ATBTreatment

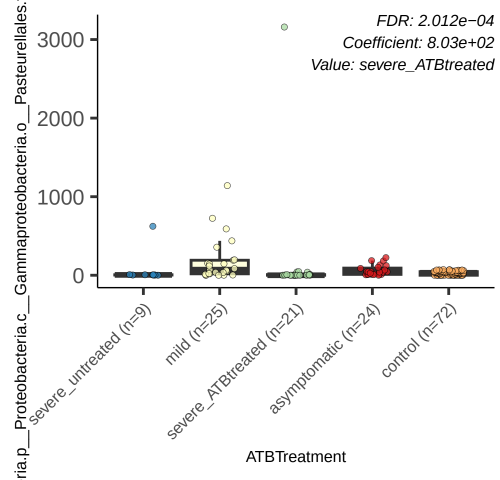

FDR: 2.012e−04
Coefficient: 8.03e+02
Value: severe_ATBtreated
3000
2000
1000
0
ria.p__Proteobacteria.c__Gammaproteobacteria.o__Pasteurellales.
severe_untreated (n=9)
mild (n=25)
severe_ATBtreated (n=21)
asymptomatic (n=24)
control (n=72)
ATBTreatment

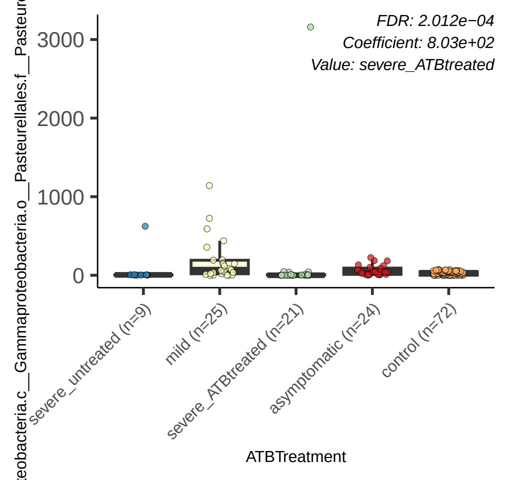

FDR: 2.012e−04
Coefficient: 8.03e+02
Value: severe_ATBtreated
0
1000
2000
3000
severe_untreated (n=9)
mild (n=25)
severe_ATBtreated (n=21)
asymptomatic (n=24)
control (n=72)
ATBTreatment

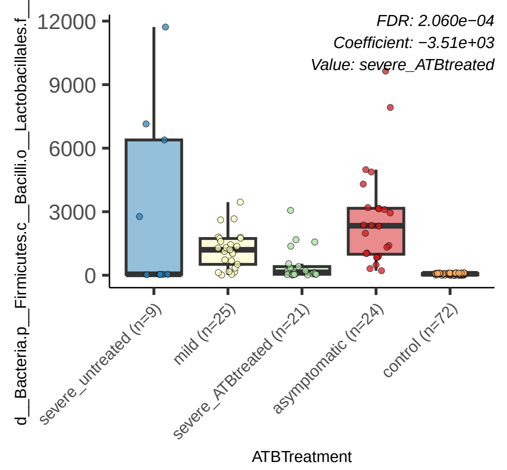

FDR: 2.060e−04
Coefficient: −3.51e+03
Value: severe_ATBtreated
d__Bacteria.p__Firmicutes.c__Bacilli.o__Lactobacillales.f__
12000
9000
6000
3000
0
severe_untreated (n=9)
mild (n=25)
severe_ATBtreated (n=21)
asymptomatic (n=24)
control (n=72)
ATBTreatment

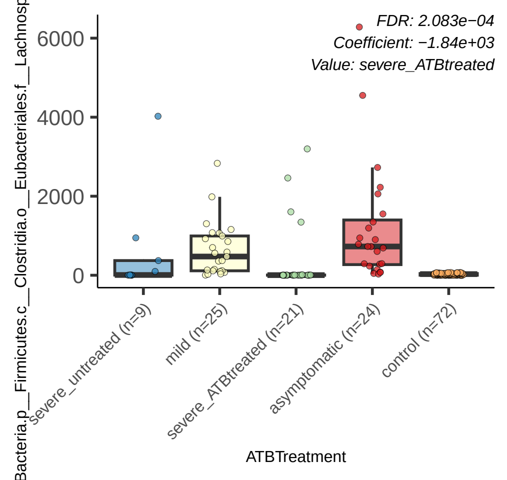
FDR: 2.083e−04
Coefficient: −1.84e+03
Value: severe_ATBtreated
Bacteria.p__Firmicutes.c__Clostridia.o__Eubacteriales.f__Lachnosp
6000
4000
2000
0
severe_untreated (n=9)
mild (n=25)
severe_ATBtreated (n=21)
asymptomatic (n=24)
control (n=72)
ATBTreatment

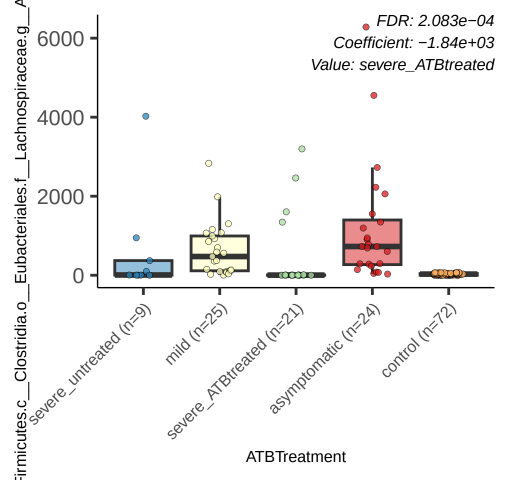

FDR: 2.083e−04
Coefficient: −1.84e+03
Value: severe_ATBtreated
Firmicutes.c__Clostridia.o__Eubacteriales.f__Lachnospiraceae.g__A
6000
4000
2000
0
severe_untreated (n=9)
mild (n=25)
severe_ATBtreated (n=21)
asymptomatic (n=24)
control (n=72)
ATBTreatment

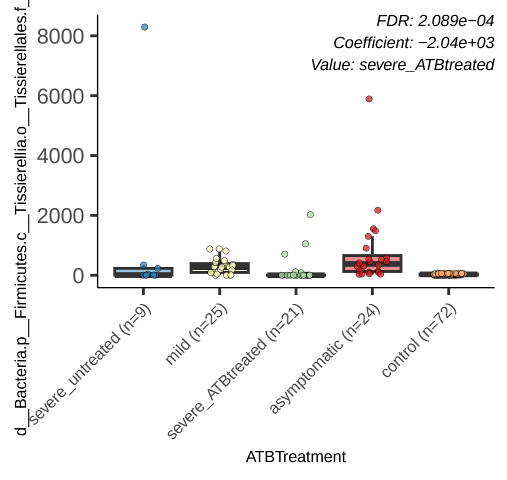
FDR: 2.089e−04
Coefficient: −2.04e+03
Value: severe_ATBtreated
d__Bacteria.p__Firmicutes.c__Tissierellia.o__Tissierellales.f_
8000
6000
4000
2000
0
severe_untreated (n=9)
mild (n=25)
severe_ATBtreated (n=21)
asymptomatic (n=24)
control (n=72)
ATBTreatment

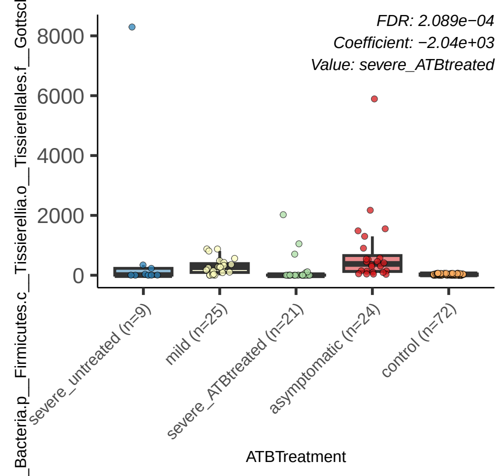
FDR: 2.089e−04
Coefficient: −2.04e+03
Value: severe_ATBtreated
8000
6000
4000
2000
0
Bacteria.p__Firmicutes.c__Tissierellia.o__Tissierellales.f__Gottsch
severe_untreated (n=9)
mild (n=25)
severe_ATBtreated (n=21)
asymptomatic (n=24)
control (n=72)
ATBTreatment

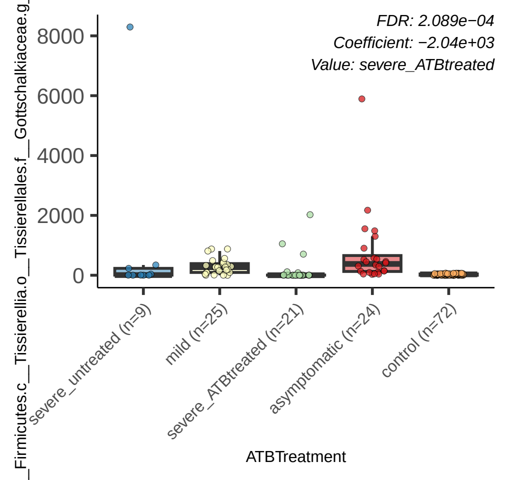

FDR: 2.089e−04
Coefficient: −2.04e+03
Value: severe_ATBtreated
_Firmicutes.c__Tissierellia.o__Tissierellales.f__Gottschalkiaceae.g_
8000
6000
4000
2000
0
severe_untreated (n=9)
mild (n=25)
severe_ATBtreated (n=21)
asymptomatic (n=24)
control (n=72)
ATBTreatment

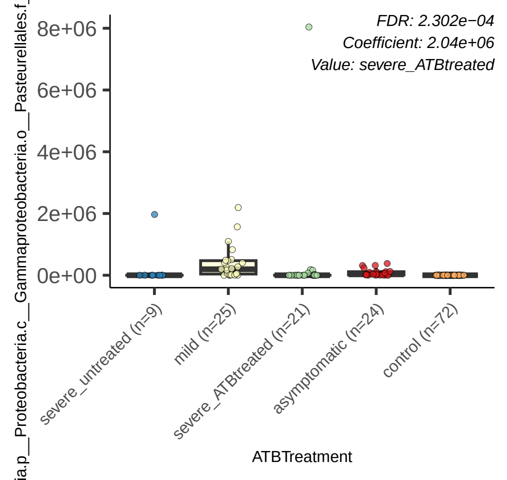

FDR: 2.302e−04
Coefficient: 2.04e+06
Value: severe_ATBtreated
8e+06
6e+06
4e+06
2e+06
0e+00
severe_untreated (n=9)
mild (n=25)
severe_ATBtreated (n=21)
asymptomatic (n=24)
control (n=72)
ATBTreatment
ia.p__Proteobacteria.c__Gammaproteobacteria.o__Pasteurellales.f__

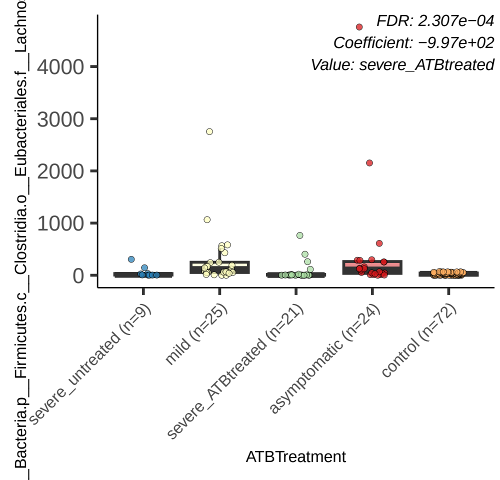

FDR: 2.307e−04
Coefficient: −9.97e+02
Value: severe_ATBtreated
__Bacteria.p__Firmicutes.c__Clostridia.o__Eubacteriales.f__Lachno
4000
3000
2000
1000
0
severe_untreated (n=9)
mild (n=25)
severe_ATBtreated (n=21)
asymptomatic (n=24)
control (n=72)
ATBTreatment

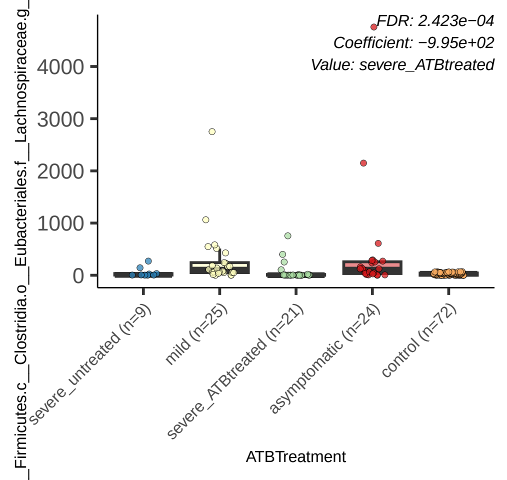

FDR: 2.423e−04
Coefficient: −9.95e+02
Value: severe_ATBtreated
4000
3000
2000
1000
0
Firmicutes.c__Clostridia.o__Eubacteriales.f__Lachnospiraceae.g__
severe_untreated (n=9)
mild (n=25)
severe_ATBtreated (n=21)
asymptomatic (n=24)
control (n=72)
ATBTreatment

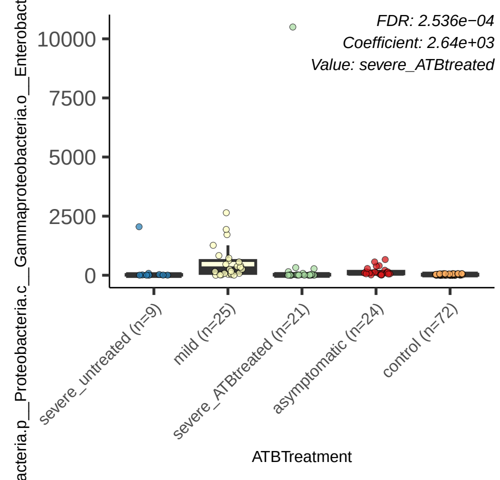
FDR: 2.536e−04
Coefficient: 2.64e+03
Value: severe_ATBtreated
acteria.p__Proteobacteria.c__Gammaproteobacteria.o__Enterobact
10000
7500
5000
2500
0
severe_untreated (n=9)
mild (n=25)
severe_ATBtreated (n=21)
asymptomatic (n=24)
control (n=72)
ATBTreatment

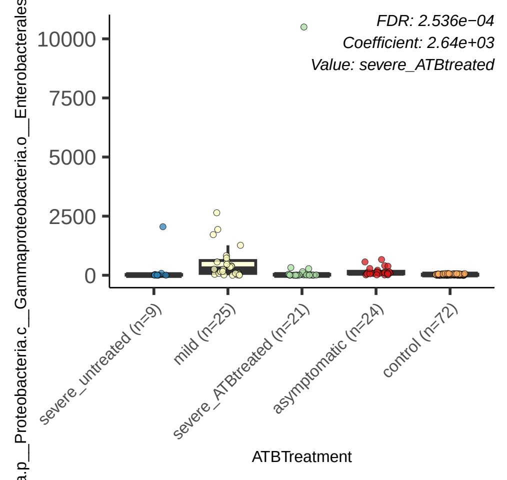

FDR: 2.536e−04
Coefficient: 2.64e+03
Value: severe_ATBtreated
a.p__Proteobacteria.c__Gammaproteobacteria.o__Enterobacterales
10000
7500
5000
2500
0
severe_untreated (n=9)
mild (n=25)
severe_ATBtreated (n=21)
asymptomatic (n=24)
control (n=72)
ATBTreatment

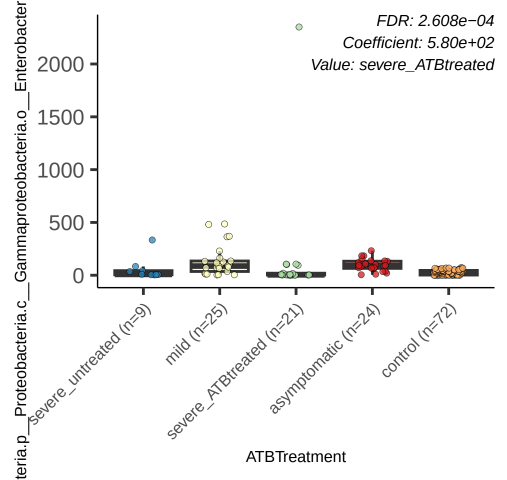
FDR: 2.608e−04
Coefficient: 5.80e+02
Value: severe_ATBtreated
teria.p__Proteobacteria.c__Gammaproteobacteria.o__Enterobacter
2000
1500
1000
500
0
severe_untreated (n=9)
mild (n=25)
severe_ATBtreated (n=21)
asymptomatic (n=24)
control (n=72)
ATBTreatment

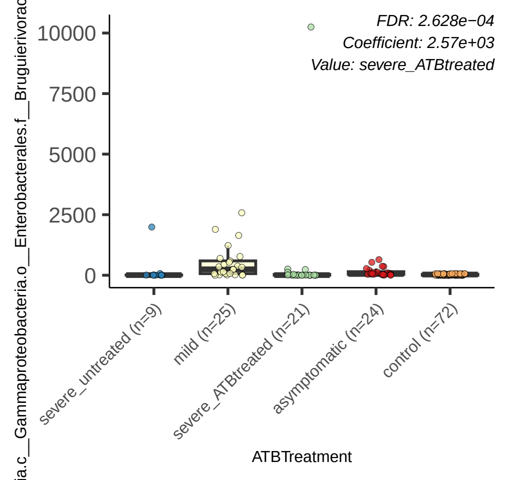

FDR: 2.628e−04
Coefficient: 2.57e+03
Value: severe_ATBtreated
10000
7500
5000
2500
0
ia.c__Gammaproteobacteria.o__Enterobacterales.f__Bruguierivorac
severe_untreated (n=9)
mild (n=25)
severe_ATBtreated (n=21)
asymptomatic (n=24)
control (n=72)
ATBTreatment

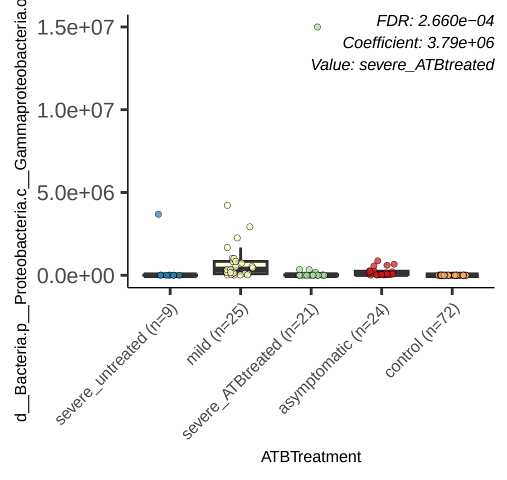
FDR: 2.660e−04
Coefficient: 3.79e+06
Value: severe_ATBtreated
d__Bacteria.p__Proteobacteria.c__Gammaproteobacteria.o
1.5e+07
1.0e+07
5.0e+06
0.0e+00
severe_untreated (n=9)
mild (n=25)
severe_ATBtreated (n=21)
asymptomatic (n=24)
control (n=72)
ATBTreatment

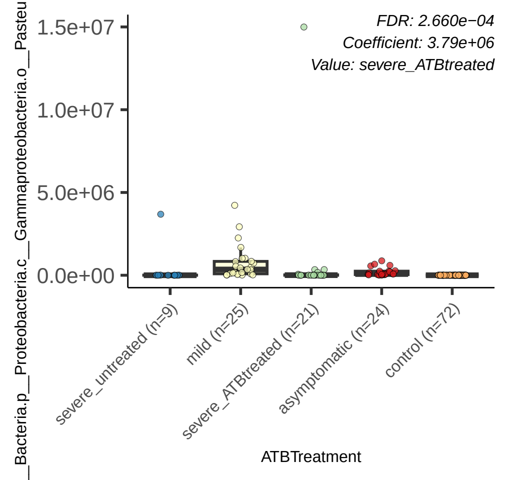

FDR: 2.660e−04
Coefficient: 3.79e+06
Value: severe_ATBtreated
1.5e+07
1.0e+07
5.0e+06
0.0e+00
Bacteria.p__Proteobacteria.c__Gammaproteobacteria.o__Pasteu
severe_untreated (n=9)
mild (n=25)
severe_ATBtreated (n=21)
asymptomatic (n=24)
control (n=72)
ATBTreatment

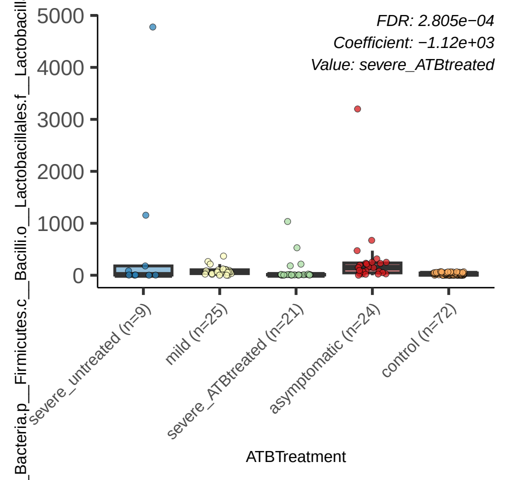

FDR: 2.805e−04
Coefficient: −1.12e+03
Value: severe_ATBtreated
Bacteria.p__Firmicutes.c__Bacilli.o__Lactobacillales.f__Lactobacill
5000
4000
3000
2000
1000
0
severe_untreated (n=9)
mild (n=25)
severe_ATBtreated (n=21)
asymptomatic (n=24)
control (n=72)
ATBTreatment

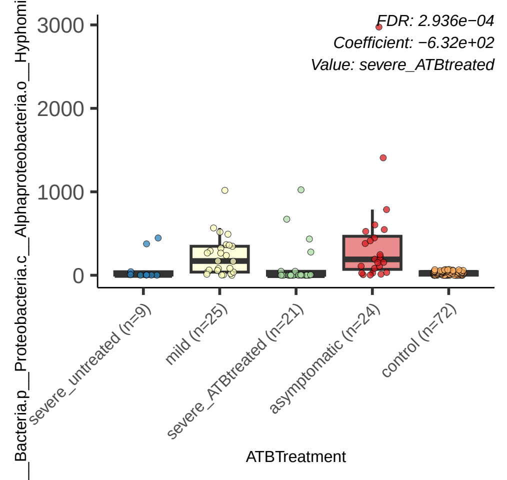

FDR: 2.936e−04
Coefficient: −6.32e+02
Value: severe_ATBtreated
Bacteria.p__Proteobacteria.c__Alphaproteobacteria.o__Hyphomi
3000
2000
1000
0
severe_untreated (n=9)
mild (n=25)
severe_ATBtreated (n=21)
asymptomatic (n=24)
control (n=72)
ATBTreatment

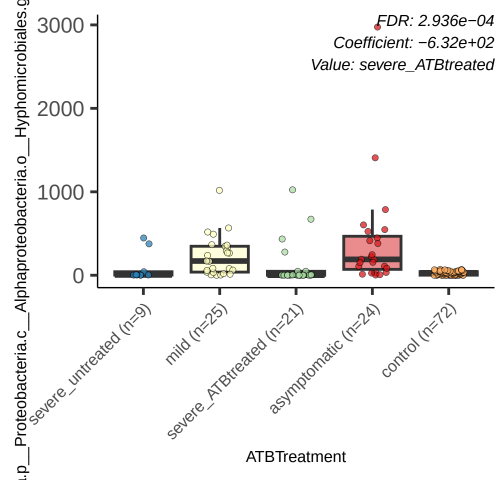

FDR: 2.936e−04
Coefficient: −6.32e+02
Value: severe_ATBtreated
3000
2000
1000
0
severe_untreated (n=9)
mild (n=25)
severe_ATBtreated (n=21)
asymptomatic (n=24)
control (n=72)
ATBTreatment
Proteobacteria.c__Alphaproteobacteria.o__Hyphomicrobiales.g

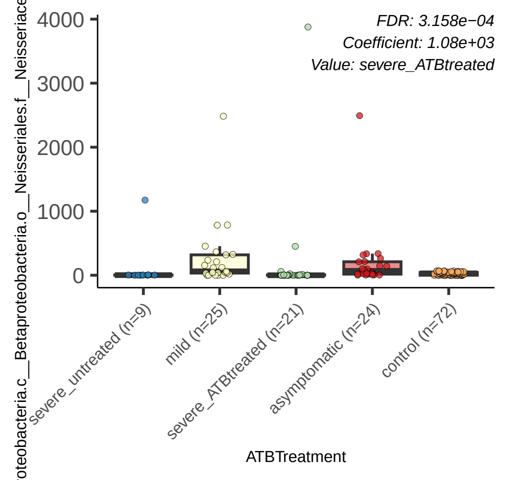
FDR: 3.158e−04
Coefficient: 1.08e+03
Value: severe_ATBtreated
roteobacteria.c__Betaproteobacteria.o__Neisseriales.f__Neisseriace
4000
3000
2000
1000
0
severe_untreated (n=9)
mild (n=25)
severe_ATBtreated (n=21)
asymptomatic (n=24)
control (n=72)
ATBTreatment

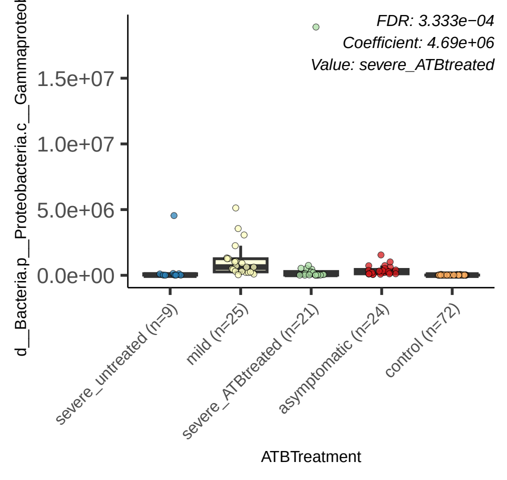

FDR: 3.333e−04
Coefficient: 4.69e+06
Value: severe_ATBtreated
d__Bacteria.p__Proteobacteria.c__Gammaproteob
1.5e+07
1.0e+07
5.0e+06
0.0e+00
severe_untreated (n=9)
mild (n=25)
severe_ATBtreated (n=21)
asymptomatic (n=24)
control (n=72)
ATBTreatment

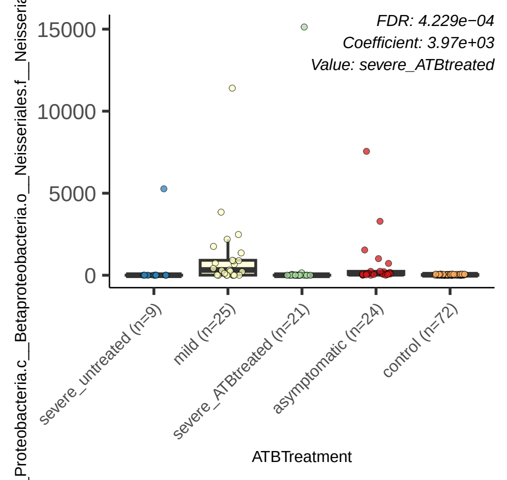

FDR: 4.229e−04
Coefficient: 3.97e+03
Value: severe_ATBtreated
15000
10000
5000
0
Proteobacteria.c__Betaproteobacteria.o__Neisseriales.f__Neisseria
severe_untreated (n=9)
mild (n=25)
severe_ATBtreated (n=21)
asymptomatic (n=24)
control (n=72)
ATBTreatment

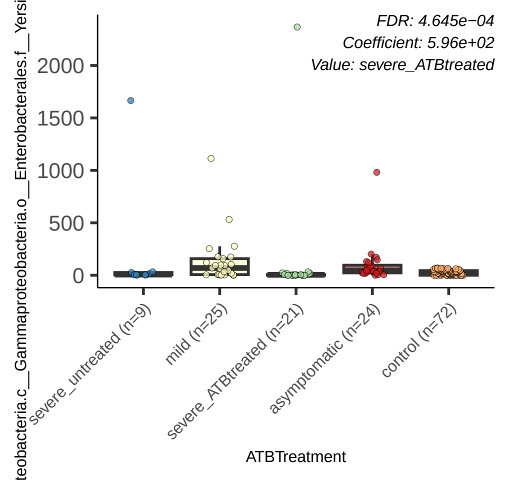

FDR: 4.645e−04
Coefficient: 5.96e+02
Value: severe_ATBtreated
2000
1500
1000
500
0
teobacteria.c__Gammaproteobacteria.o__Enterobacterales.f__Yersi
severe_untreated (n=9)
mild (n=25)
severe_ATBtreated (n=21)
asymptomatic (n=24)
control (n=72)
ATBTreatment

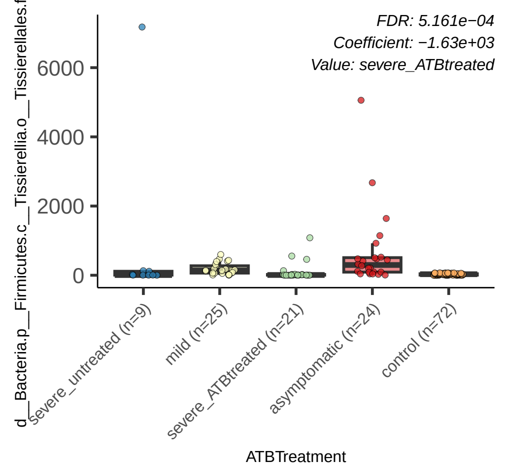
FDR: 5.161e−04
Coefficient: −1.63e+03
Value: severe_ATBtreated
d__Bacteria.p__Firmicutes.c__Tissierellia.o__Tissierellales.f
0
2000
4000
6000
severe_untreated (n=9)
mild (n=25)
severe_ATBtreated (n=21)
asymptomatic (n=24)
control (n=72)
ATBTreatment

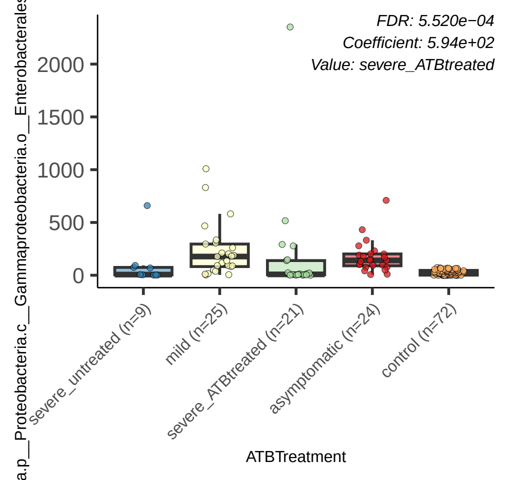

FDR: 5.520e−04
Coefficient: 5.94e+02
Value: severe_ATBtreated
a.p__Proteobacteria.c__Gammaproteobacteria.o__Enterobacterales
0
500
1000
1500
2000
severe_untreated (n=9)
mild (n=25)
severe_ATBtreated (n=21)
asymptomatic (n=24)
control (n=72)
ATBTreatment

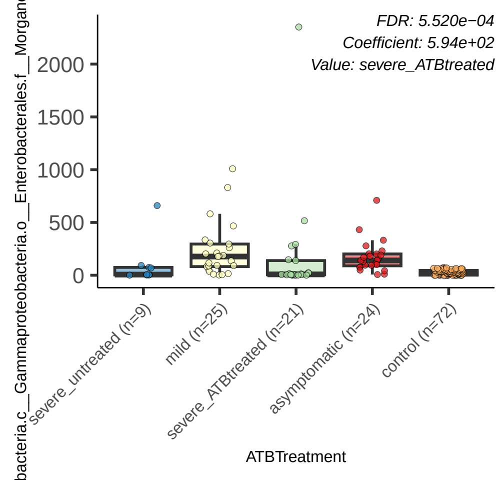
FDR: 5.520e−04
Coefficient: 5.94e+02
Value: severe_ATBtreated
0
500
1000
1500
2000
bacteria.c__Gammaproteobacteria.o__Enterobacterales.f__Morgan
severe_untreated (n=9)
mild (n=25)
severe_ATBtreated (n=21)
asymptomatic (n=24)
control (n=72)
ATBTreatment

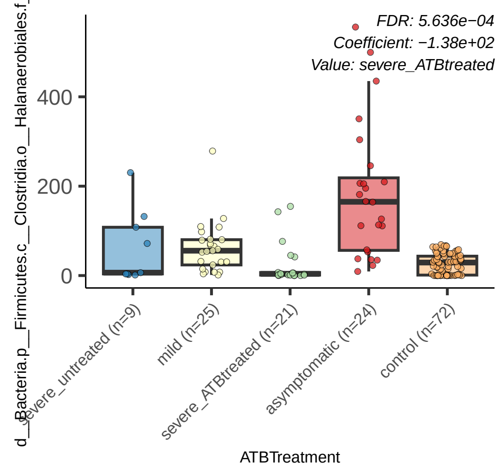

FDR: 5.636e−04
Coefficient: −1.38e+02
Value: severe_ATBtreated
d__Bacteria.p__Firmicutes.c__Clostridia.o__Halanaerobiales.f_
0
200
400
severe_untreated (n=9)
mild (n=25)
severe_ATBtreated (n=21)
asymptomatic (n=24)
control (n=72)
ATBTreatment

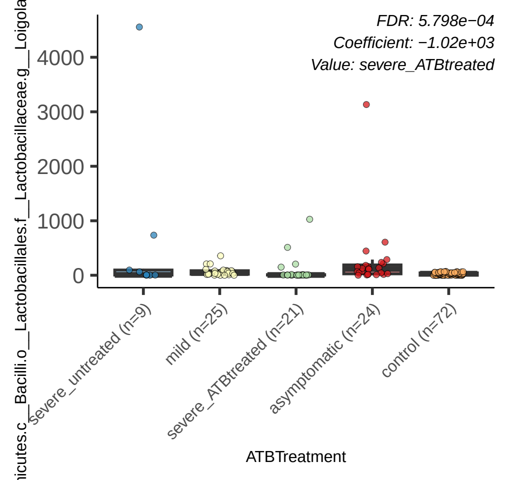

FDR: 5.798e−04
Coefficient: −1.02e+03
Value: severe_ATBtreated
4000
3000
2000
1000
0
severe_untreated (n=9)
mild (n=25)
severe_ATBtreated (n=21)
asymptomatic (n=24)
control (n=72)
ATBTreatment
nicutes.c__Bacilli.o__Lactobacillales.f__Lactobacillaceae.g__Loigola

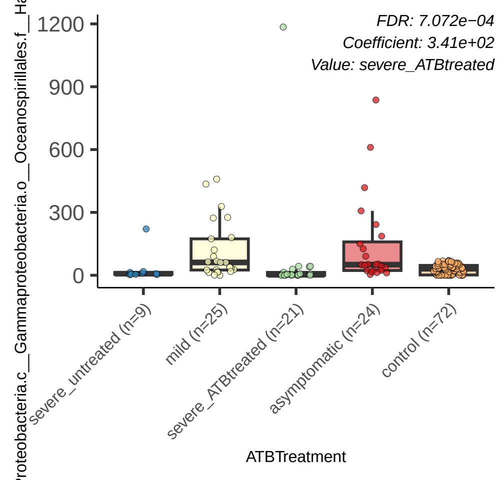

FDR: 7.072e−04
Coefficient: 3.41e+02
Value: severe_ATBtreated
1200
900
600
300
0
Proteobacteria.c__Gammaproteobacteria.o__Oceanospirillales.f__Ha
severe_untreated (n=9)
mild (n=25)
severe_ATBtreated (n=21)
asymptomatic (n=24)
control (n=72)
ATBTreatment

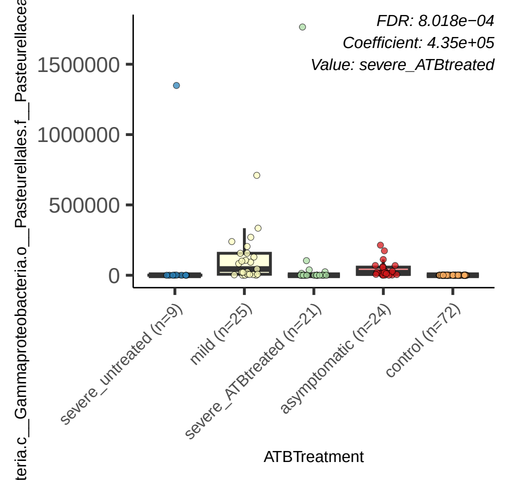
FDR: 8.018e−04
Coefficient: 4.35e+05
Value: severe_ATBtreated
1500000
1000000
500000
0
teria.c__Gammaproteobacteria.o__Pasteurellales.f__Pasteurellacea
severe_untreated (n=9)
mild (n=25)
severe_ATBtreated (n=21)
asymptomatic (n=24)
control (n=72)
ATBTreatment

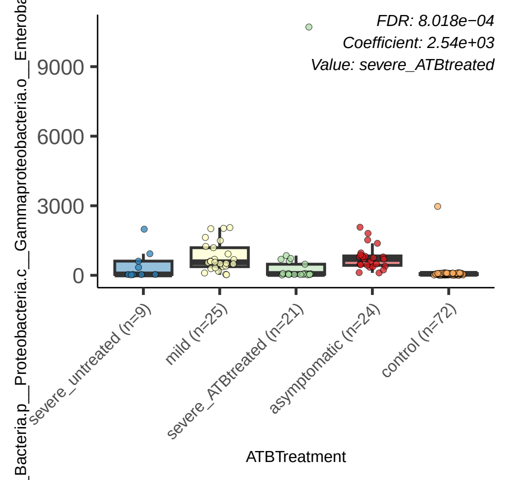
FDR: 8.018e−04
Coefficient: 2.54e+03
Value: severe_ATBtreated
Bacteria.p__Proteobacteria.c__Gammaproteobacteria.o__Enteroba
9000
6000
3000
0
severe_untreated (n=9)
mild (n=25)
severe_ATBtreated (n=21)
asymptomatic (n=24)
control (n=72)
ATBTreatment

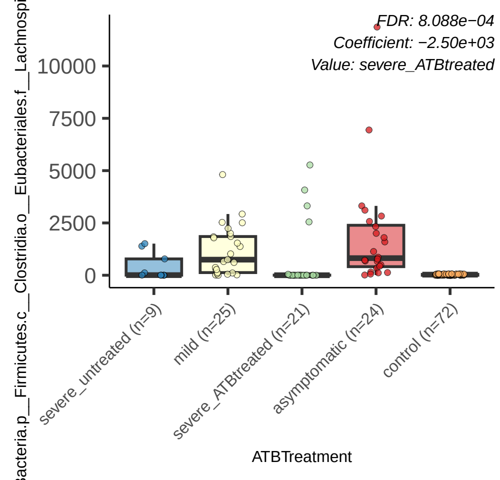

FDR: 8.088e−04
Coefficient: −2.50e+03
Value: severe_ATBtreated
Bacteria.p__Firmicutes.c__Clostridia.o__Eubacteriales.f__Lachnospi
10000
7500
5000
2500
0
severe_untreated (n=9)
mild (n=25)
severe_ATBtreated (n=21)
asymptomatic (n=24)
control (n=72)
ATBTreatment

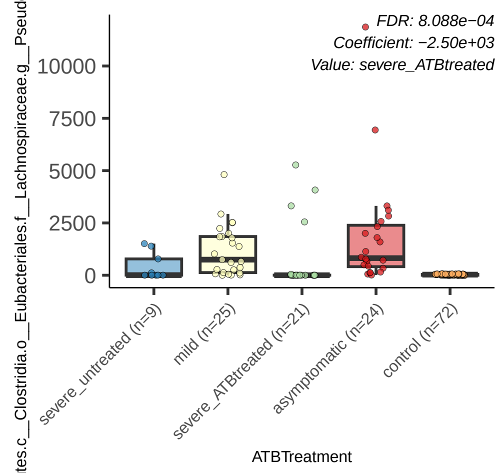

FDR: 8.088e−04
Coefficient: −2.50e+03
Value: severe_ATBtreated
utes.c__Clostridia.o__Eubacteriales.f__Lachnospiraceae.g__Pseud
10000
7500
5000
2500
0
severe_untreated (n=9)
mild (n=25)
severe_ATBtreated (n=21)
asymptomatic (n=24)
control (n=72)
ATBTreatment

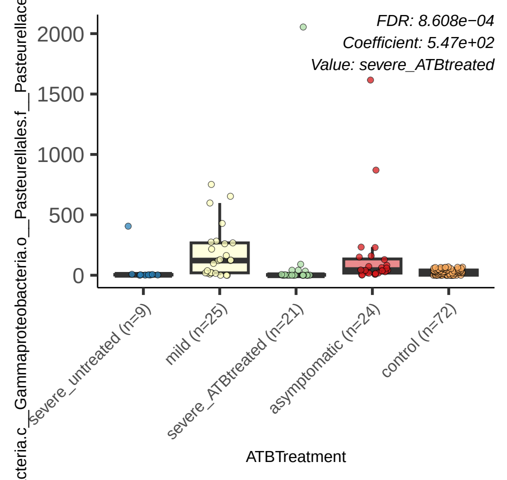

FDR: 8.608e−04
Coefficient: 5.47e+02
Value: severe_ATBtreated
2000
1500
1000
500
0
cteria.c__Gammaproteobacteria.o__Pasteurellales.f__Pasteurellace
severe_untreated (n=9)
mild (n=25)
severe_ATBtreated (n=21)
asymptomatic (n=24)
control (n=72)
ATBTreatment

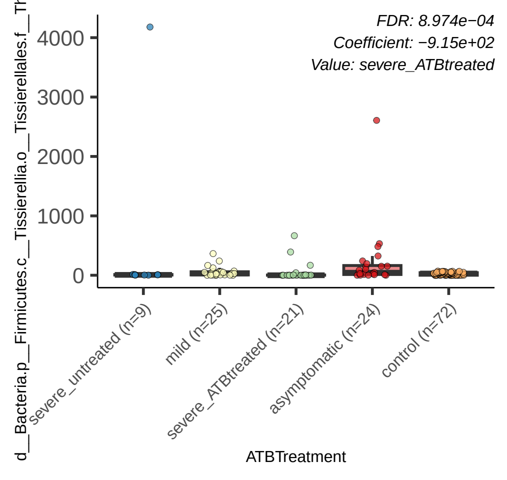

FDR: 8.974e−04
Coefficient: −9.15e+02
Value: severe_ATBtreated
4000
3000
2000
1000
0
d__Bacteria.p__Firmicutes.c__Tissierellia.o__Tissierellales.f__Tr
severe_untreated (n=9)
mild (n=25)
severe_ATBtreated (n=21)
asymptomatic (n=24)
control (n=72)
ATBTreatment

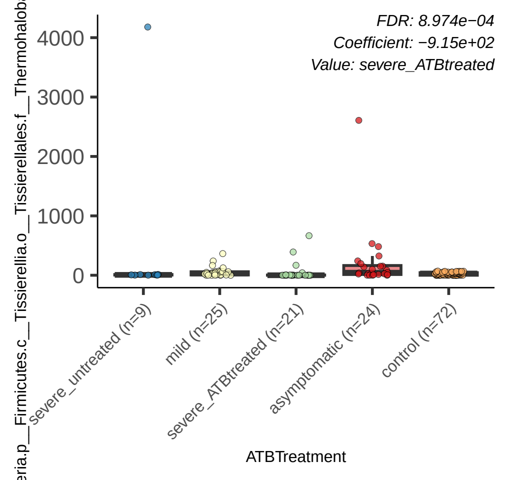
FDR: 8.974e−04
Coefficient: −9.15e+02
Value: severe_ATBtreated
0
1000
2000
3000
4000
severe_untreated (n=9)
mild (n=25)
severe_ATBtreated (n=21)
asymptomatic (n=24)
control (n=72)
ATBTreatment

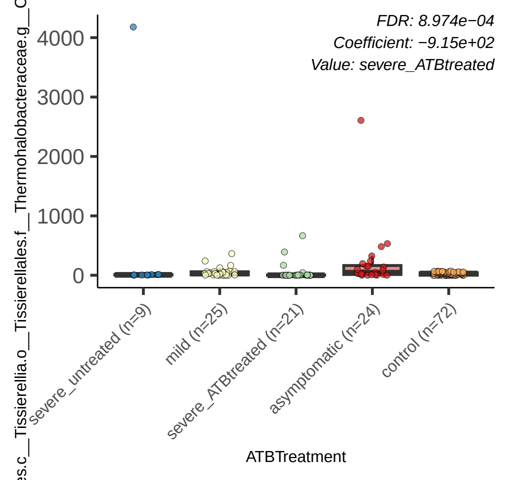

FDR: 8.974e−04
Coefficient: −9.15e+02
Value: severe_ATBtreated
4000
3000
2000
1000
0
severe_untreated (n=9)
mild (n=25)
severe_ATBtreated (n=21)
asymptomatic (n=24)
control (n=72)
ATBTreatment
es.c__Tissierellia.o__Tissierellales.f__Thermohalobacteraceae.g__C

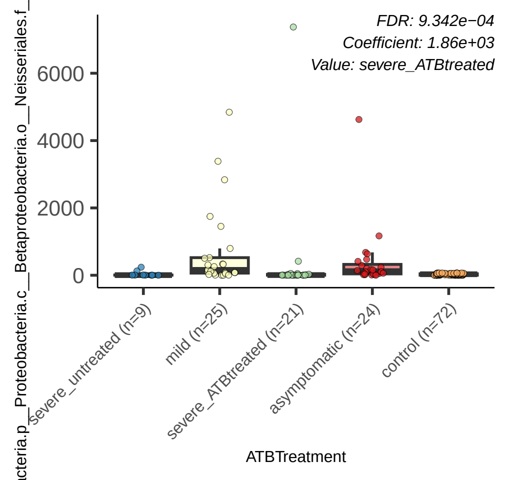

FDR: 9.342e−04
Coefficient: 1.86e+03
Value: severe_ATBtreated
6000
4000
2000
0
acteria.p__Proteobacteria.c__Betaproteobacteria.o__Neisseriales.f
severe_untreated (n=9)
mild (n=25)
severe_ATBtreated (n=21)
asymptomatic (n=24)
control (n=72)
ATBTreatment

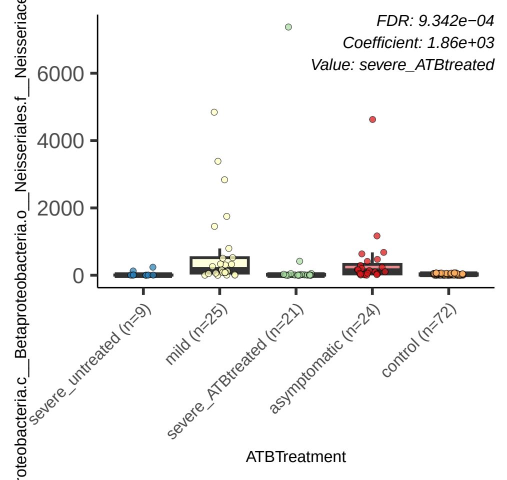

FDR: 9.342e−04
Coefficient: 1.86e+03
Value: severe_ATBtreated
6000
4000
2000
0
roteobacteria.c__Betaproteobacteria.o__Neisseriales.f__Neisseriace
severe_untreated (n=9)
mild (n=25)
severe_ATBtreated (n=21)
asymptomatic (n=24)
control (n=72)
ATBTreatment

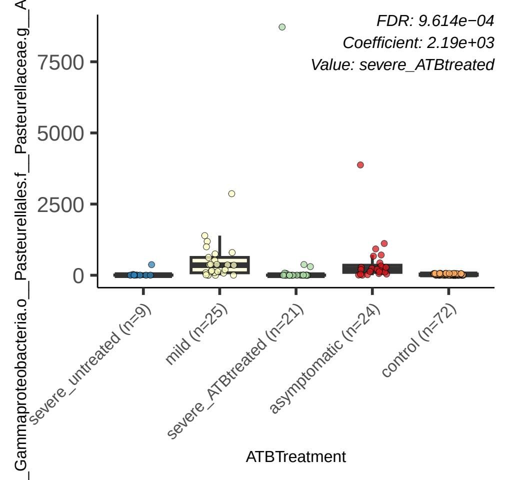
FDR: 9.614e−04
Coefficient: 2.19e+03
Value: severe_ATBtreated
7500
5000
2500
0
Gammaproteobacteria.o__Pasteurellales.f__Pasteurellaceae.g__A
severe_untreated (n=9)
mild (n=25)
severe_ATBtreated (n=21)
asymptomatic (n=24)
control (n=72)
ATBTreatment

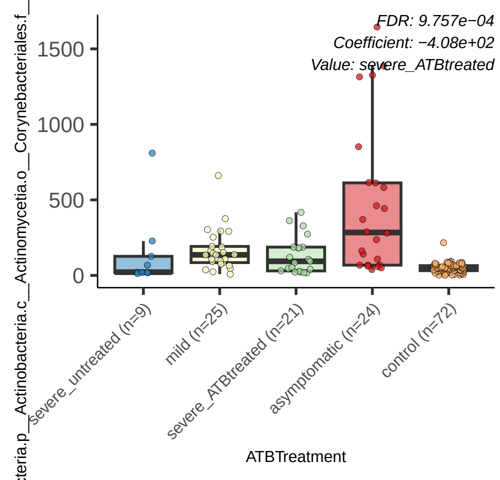

FDR: 9.757e−04
Coefficient: −4.08e+02
Value: severe_ATBtreated
1500
1000
500
0
cteria.p__Actinobacteria.c__Actinomycetia.o__Corynebacteriales.f__
severe_untreated (n=9)
mild (n=25)
severe_ATBtreated (n=21)
asymptomatic (n=24)
control (n=72)
ATBTreatment

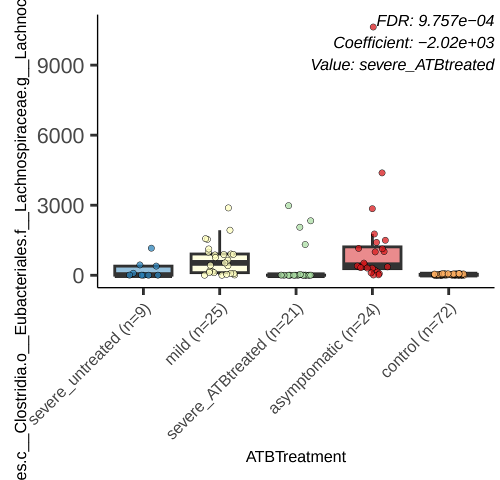

FDR: 9.757e−04
Coefficient: −2.02e+03
Value: severe_ATBtreated
9000
6000
3000
0
es.c__Clostridia.o__Eubacteriales.f__Lachnospiraceae.g__Lachnoc
severe_untreated (n=9)
mild (n=25)
severe_ATBtreated (n=21)
asymptomatic (n=24)
control (n=72)
ATBTreatment

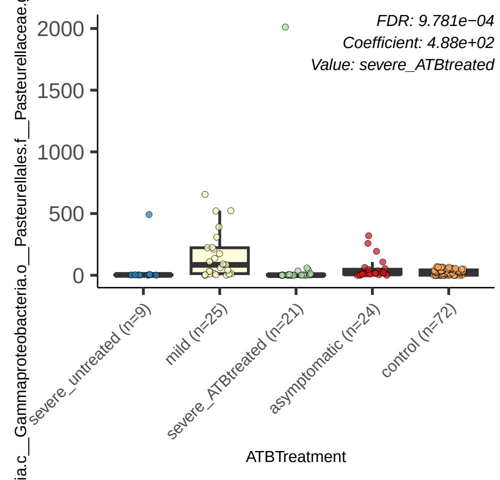

FDR: 9.781e−04
Coefficient: 4.88e+02
Value: severe_ATBtreated
2000
1500
1000
500
0
ia.c__Gammaproteobacteria.o__Pasteurellales.f__Pasteurellaceae.g
severe_untreated (n=9)
mild (n=25)
severe_ATBtreated (n=21)
asymptomatic (n=24)
control (n=72)
ATBTreatment

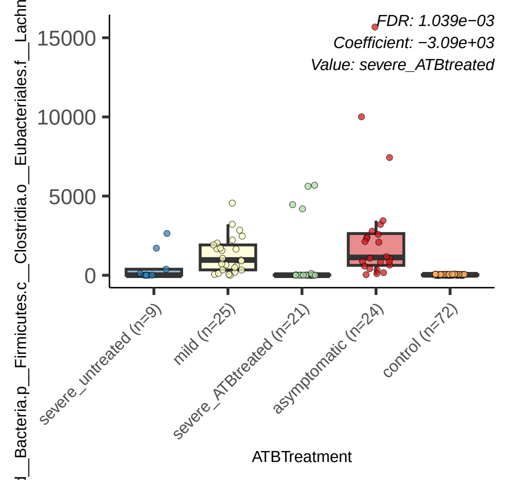

FDR: 1.039e−03
Coefficient: −3.09e+03
Value: severe_ATBtreated
15000
10000
5000
0
d__Bacteria.p__Firmicutes.c__Clostridia.o__Eubacteriales.f__Lachn
severe_untreated (n=9)
mild (n=25)
severe_ATBtreated (n=21)
asymptomatic (n=24)
control (n=72)
ATBTreatment

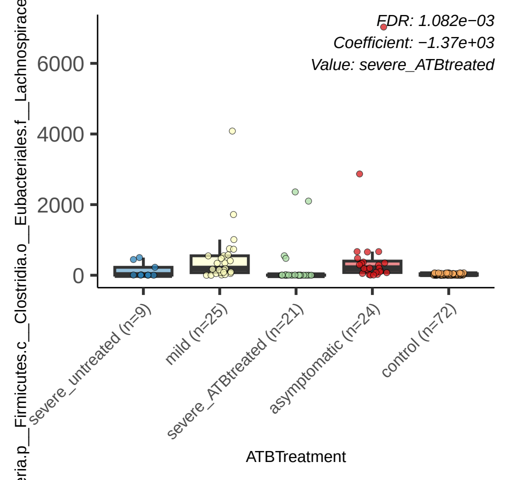

FDR: 1.082e−03
Coefficient: −1.37e+03
Value: severe_ATBtreated
6000
4000
2000
0
severe_untreated (n=9)
mild (n=25)
severe_ATBtreated (n=21)
asymptomatic (n=24)
control (n=72)
ATBTreatment

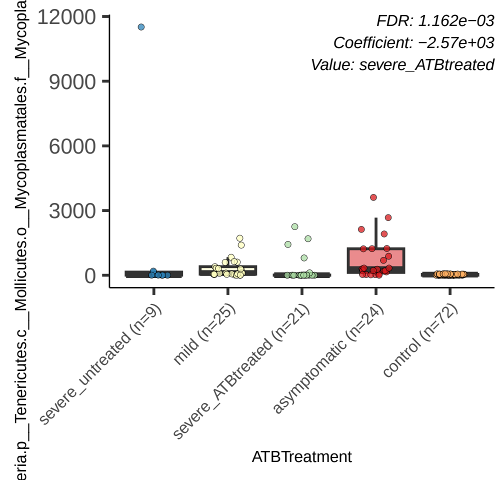
FDR: 1.162e−03
Coefficient: −2.57e+03
Value: severe_ATBtreated
12000
9000
6000
3000
0
eria.p__Tenericutes.c__Mollicutes.o__Mycoplasmatales.f__Mycopla
severe_untreated (n=9)
mild (n=25)
severe_ATBtreated (n=21)
asymptomatic (n=24)
control (n=72)
ATBTreatment

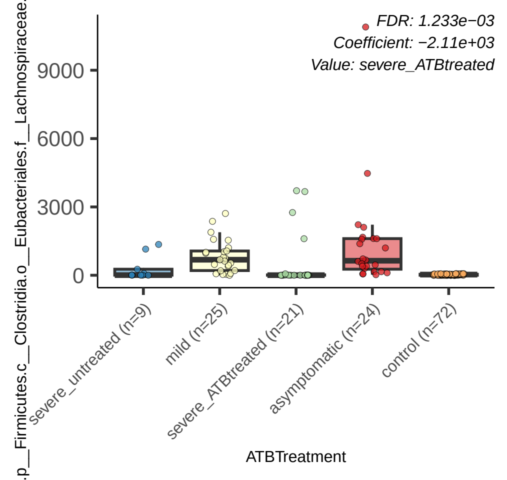
FDR: 1.233e−03
Coefficient: −2.11e+03
Value: severe_ATBtreated
p__Firmicutes.c__Clostridia.o__Eubacteriales.f__Lachnospiraceae.
9000
6000
3000
0
severe_untreated (n=9)
mild (n=25)
severe_ATBtreated (n=21)
asymptomatic (n=24)
control (n=72)
ATBTreatment

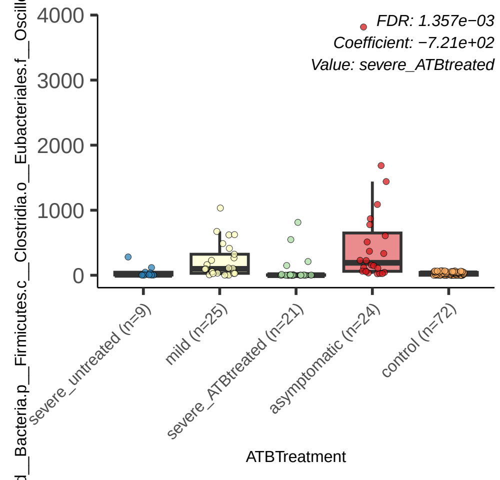
FDR: 1.357e−03
Coefficient: −7.21e+02
Value: severe_ATBtreated
4000
3000
2000
1000
0
d__Bacteria.p__Firmicutes.c__Clostridia.o__Eubacteriales.f__Oscill
severe_untreated (n=9)
mild (n=25)
severe_ATBtreated (n=21)
asymptomatic (n=24)
control (n=72)
ATBTreatment

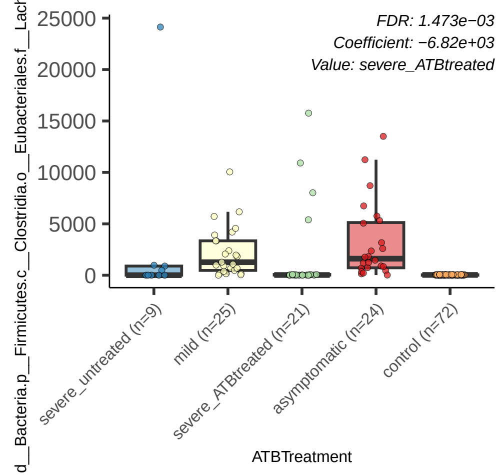

FDR: 1.473e−03
Coefficient: −6.82e+03
Value: severe_ATBtreated
25000
20000
15000
10000
5000
0
d__Bacteria.p__Firmicutes.c__Clostridia.o__Eubacteriales.f__Lach
severe_untreated (n=9)
mild (n=25)
severe_ATBtreated (n=21)
asymptomatic (n=24)
control (n=72)
ATBTreatment

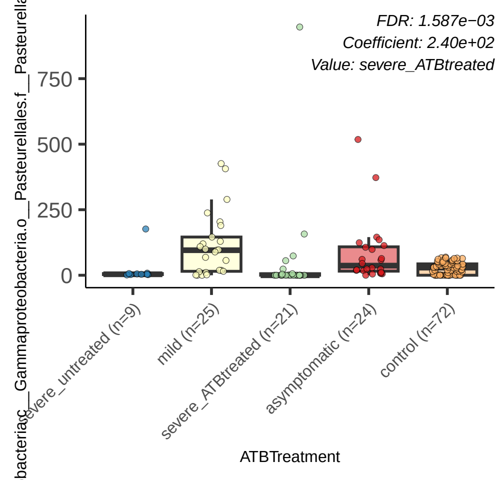

FDR: 1.587e−03
Coefficient: 2.40e+02
Value: severe_ATBtreated
750
500
250
0
bacteria.c__Gammaproteobacteria.o__Pasteurellales.f__Pasteurella
severe_untreated (n=9)
mild (n=25)
severe_ATBtreated (n=21)
asymptomatic (n=24)
control (n=72)
ATBTreatment

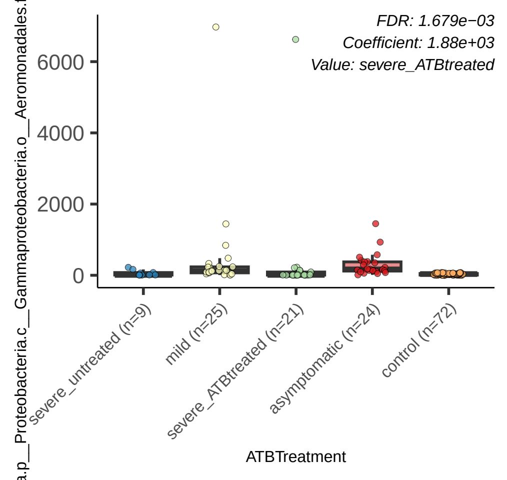

FDR: 1.679e−03
Coefficient: 1.88e+03
Value: severe_ATBtreated
6000
4000
2000
0
a.p__Proteobacteria.c__Gammaproteobacteria.o__Aeromonadales.f
severe_untreated (n=9)
mild (n=25)
severe_ATBtreated (n=21)
asymptomatic (n=24)
control (n=72)
ATBTreatment

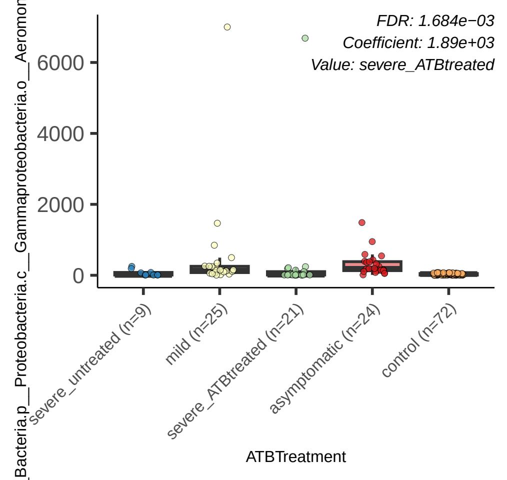
FDR: 1.684e−03
Coefficient: 1.89e+03
Value: severe_ATBtreated
Bacteria.p__Proteobacteria.c__Gammaproteobacteria.o__Aeromon
6000
4000
2000
0
severe_untreated (n=9)
mild (n=25)
severe_ATBtreated (n=21)
asymptomatic (n=24)
control (n=72)
ATBTreatment

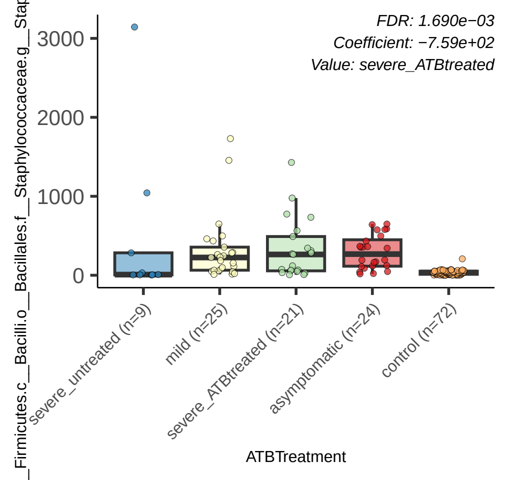

FDR: 1.690e−03
Coefficient: −7.59e+02
Value: severe_ATBtreated
3000
2000
1000
0
_Firmicutes.c__Bacilli.o__Bacillales.f__Staphylococcaceae.g__Stap
severe_untreated (n=9)
mild (n=25)
severe_ATBtreated (n=21)
asymptomatic (n=24)
control (n=72)
ATBTreatment

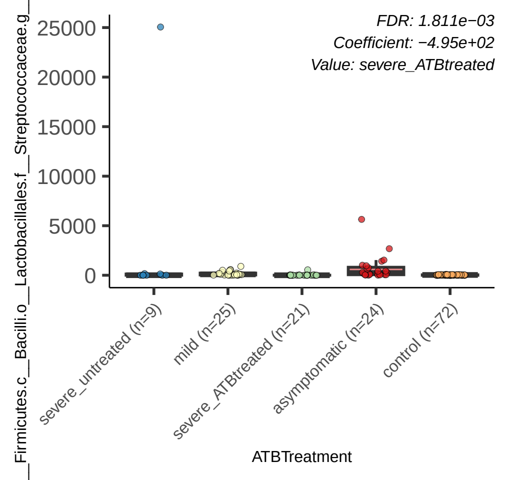

FDR: 1.811e−03
Coefficient: −4.95e+02
Value: severe_ATBtreated
Firmicutes.c__Bacilli.o__Lactobacillales.f__Streptococcaceae.g__
25000
20000
15000
10000
5000
0
severe_untreated (n=9)
mild (n=25)
severe_ATBtreated (n=21)
asymptomatic (n=24)
control (n=72)
ATBTreatment

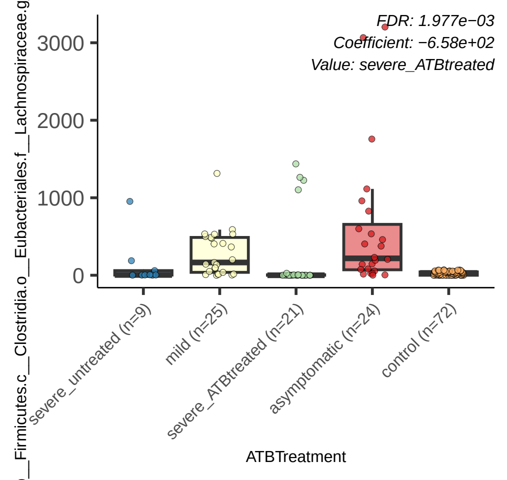
FDR: 1.977e−03
Coefficient: −6.58e+02
Value: severe_ATBtreated
3000
2000
1000
0
Firmicutes.c__Clostridia.o__Eubacteriales.f__Lachnospiraceae.g
severe_untreated (n=9)
mild (n=25)
severe_ATBtreated (n=21)
asymptomatic (n=24)
control (n=72)
ATBTreatment

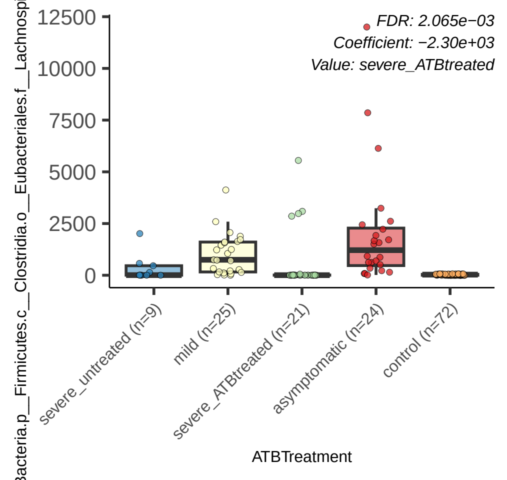

FDR: 2.065e−03
Coefficient: −2.30e+03
Value: severe_ATBtreated
12500
10000
7500
5000
2500
0
Bacteria.p__Firmicutes.c__Clostridia.o__Eubacteriales.f__Lachnospi
severe_untreated (n=9)
mild (n=25)
severe_ATBtreated (n=21)
asymptomatic (n=24)
control (n=72)
ATBTreatment

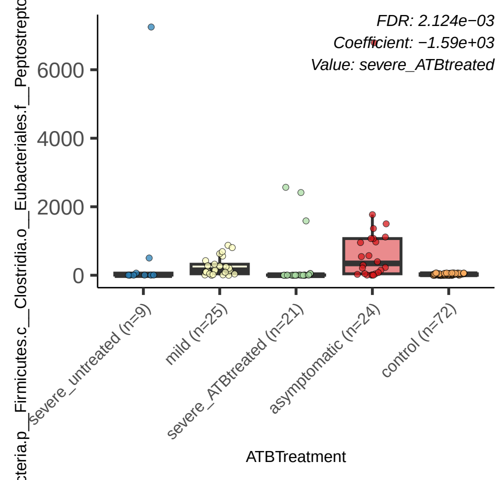

FDR: 2.124e−03
Coefficient: −1.59e+03
Value: severe_ATBtreated
6000
4000
2000
0
severe_untreated (n=9)
mild (n=25)
severe_ATBtreated (n=21)
asymptomatic (n=24)
control (n=72)
ATBTreatment

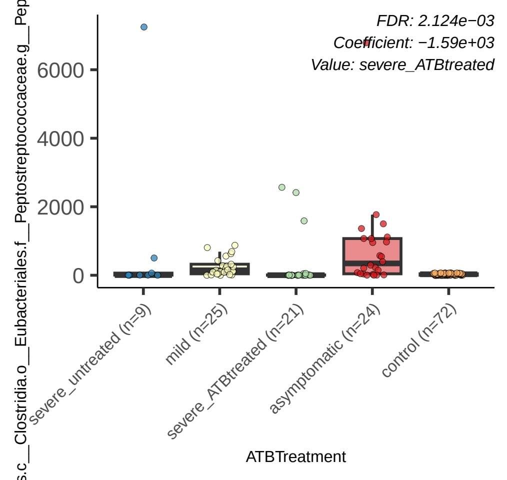

FDR: 2.124e−03
Coefficient: −1.59e+03
Value: severe_ATBtreated
s.c__Clostridia.o__Eubacteriales.f__Peptostreptococcaceae.g__Pep
6000
4000
2000
0
severe_untreated (n=9)
mild (n=25)
severe_ATBtreated (n=21)
asymptomatic (n=24)
control (n=72)
ATBTreatment

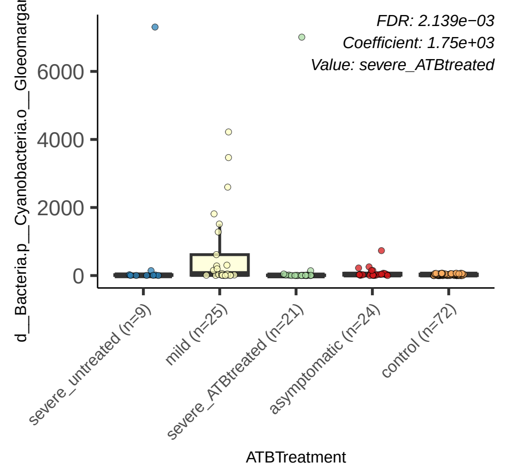

FDR: 2.139e−03
Coefficient: 1.75e+03
Value: severe_ATBtreated
d__Bacteria.p__Cyanobacteria.o__Gloeomargar
6000
4000
2000
0
severe_untreated (n=9)
mild (n=25)
severe_ATBtreated (n=21)
asymptomatic (n=24)
control (n=72)
ATBTreatment

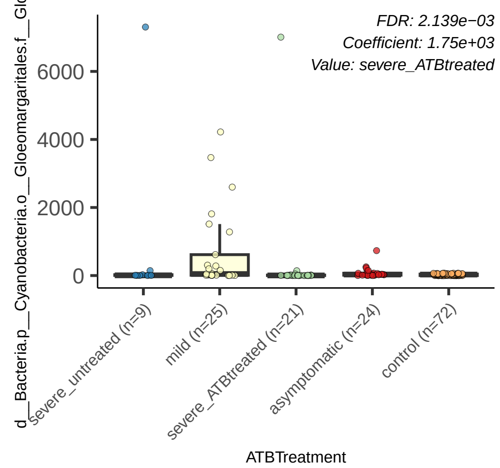

FDR: 2.139e−03
Coefficient: 1.75e+03
Value: severe_ATBtreated
d__Bacteria.p__Cyanobacteria.o__Gloeomargaritales.f__Glo
6000
4000
2000
0
severe_untreated (n=9)
mild (n=25)
severe_ATBtreated (n=21)
asymptomatic (n=24)
control (n=72)
ATBTreatment

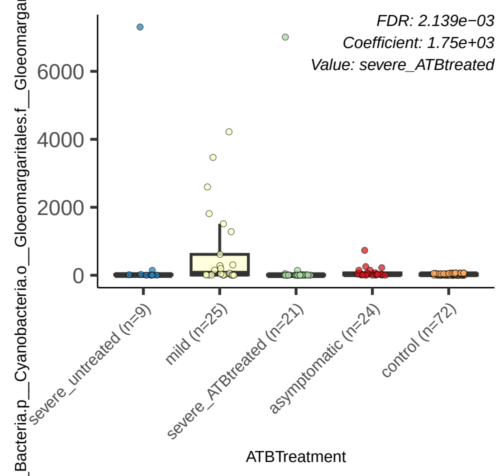

FDR: 2.139e−03
Coefficient: 1.75e+03
Value: severe_ATBtreated
_Bacteria.p__Cyanobacteria.o__Gloeomargaritales.f__Gloeomargar
6000
4000
2000
0
severe_untreated (n=9)
mild (n=25)
severe_ATBtreated (n=21)
asymptomatic (n=24)
control (n=72)
ATBTreatment

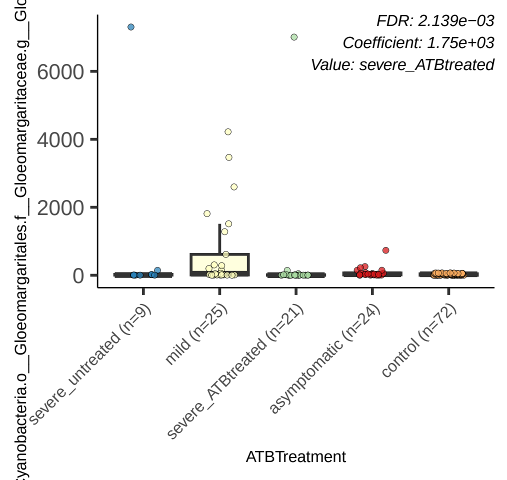
FDR: 2.139e−03
Coefficient: 1.75e+03
Value: severe_ATBtreated
6000
4000
2000
0
severe_untreated (n=9)
mild (n=25)
severe_ATBtreated (n=21)
asymptomatic (n=24)
control (n=72)
ATBTreatment

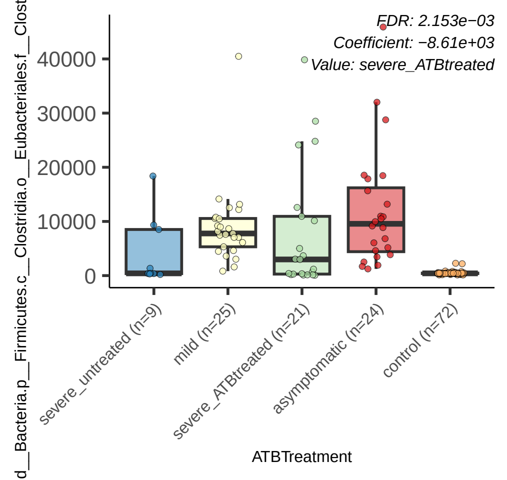
FDR: 2.153e−03
Coefficient: −8.61e+03
Value: severe_ATBtreated
d__Bacteria.p__Firmicutes.c__Clostridia.o__Eubacteriales.f__Clost
40000
30000
20000
10000
0
severe_untreated (n=9)
mild (n=25)
severe_ATBtreated (n=21)
asymptomatic (n=24)
control (n=72)
ATBTreatment

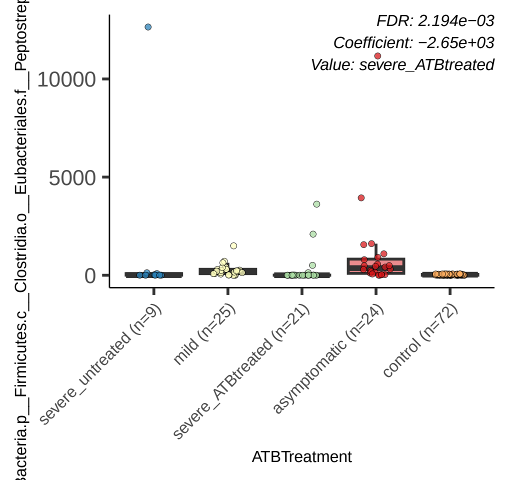

FDR: 2.194e−03
Coefficient: −2.65e+03
Value: severe_ATBtreated
Bacteria.p__Firmicutes.c__Clostridia.o__Eubacteriales.f__Peptostrep
10000
5000
0
severe_untreated (n=9)
mild (n=25)
severe_ATBtreated (n=21)
asymptomatic (n=24)
control (n=72)
ATBTreatment

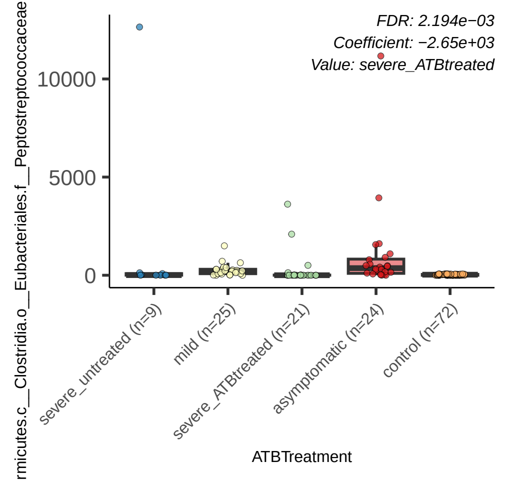

FDR: 2.194e−03
Coefficient: −2.65e+03
Value: severe_ATBtreated
rmicutes.c__Clostridia.o__Eubacteriales.f__Peptostreptococcaceae
10000
5000
0
severe_untreated (n=9)
mild (n=25)
severe_ATBtreated (n=21)
asymptomatic (n=24)
control (n=72)
ATBTreatment

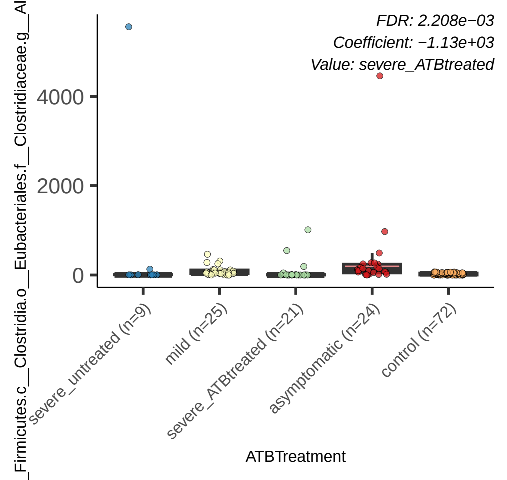

FDR: 2.208e−03
Coefficient: −1.13e+03
Value: severe_ATBtreated
4000
2000
0
severe_untreated (n=9)
mild (n=25)
severe_ATBtreated (n=21)
asymptomatic (n=24)
control (n=72)
ATBTreatment
Firmicutes.c__Clostridia.o__Eubacteriales.f__Clostridiaceae.g__Al

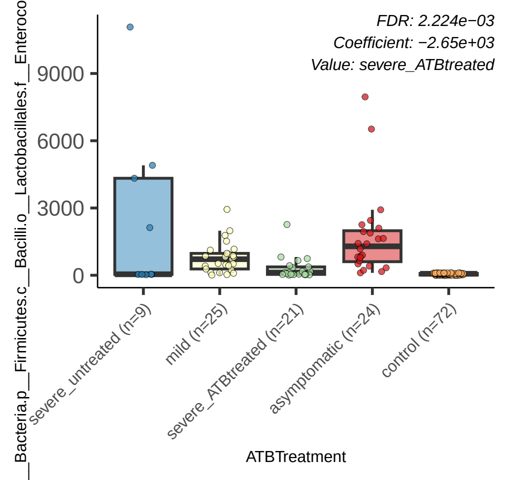

FDR: 2.224e−03
Coefficient: −2.65e+03
Value: severe_ATBtreated
9000
6000
3000
0
severe_untreated (n=9)
mild (n=25)
severe_ATBtreated (n=21)
asymptomatic (n=24)
control (n=72)
ATBTreatment
Bacteria.p__Firmicutes.c__Bacilli.o__Lactobacillales.f__Enteroco

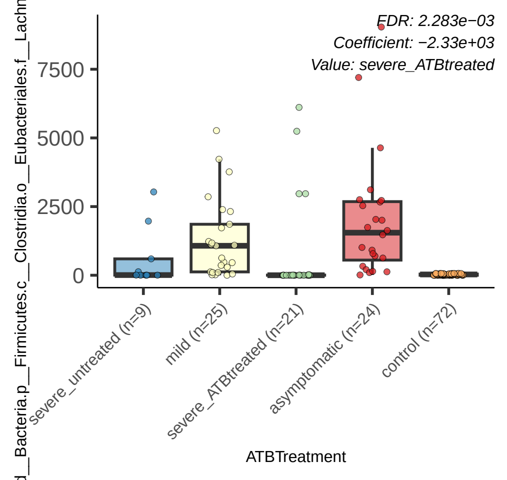
FDR: 2.283e−03
Coefficient: −2.33e+03
Value: severe_ATBtreated
7500
5000
2500
0
severe_untreated (n=9)
mild (n=25)
severe_ATBtreated (n=21)
asymptomatic (n=24)
control (n=72)
ATBTreatment
d__Bacteria.p__Firmicutes.c__Clostridia.o__Eubacteriales.f__Lachn

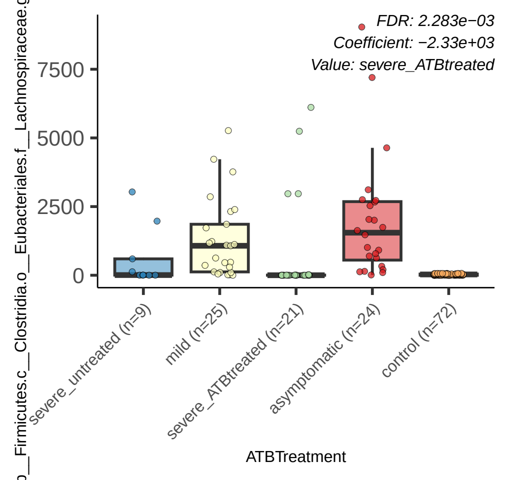

FDR: 2.283e−03
Coefficient: −2.33e+03
Value: severe_ATBtreated
o__Firmicutes.c__Clostridia.o__Eubacteriales.f__Lachnospiraceae.g
7500
5000
2500
0
severe_untreated (n=9)
mild (n=25)
severe_ATBtreated (n=21)
asymptomatic (n=24)
control (n=72)
ATBTreatment

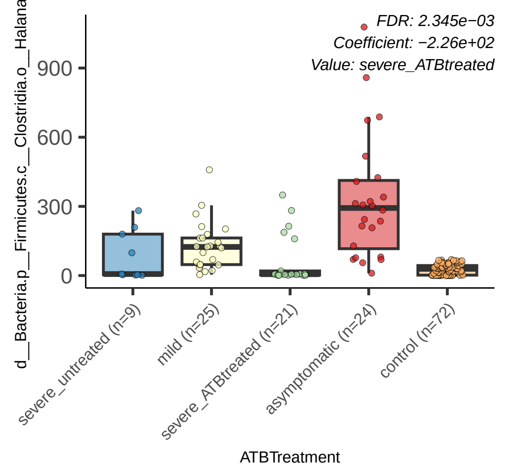

FDR: 2.345e−03
Coefficient: −2.26e+02
Value: severe_ATBtreated
d__Bacteria.p__Firmicutes.c__Clostridia.o__Halana
900
600
300
0
severe_untreated (n=9)
mild (n=25)
severe_ATBtreated (n=21)
asymptomatic (n=24)
control (n=72)
ATBTreatment

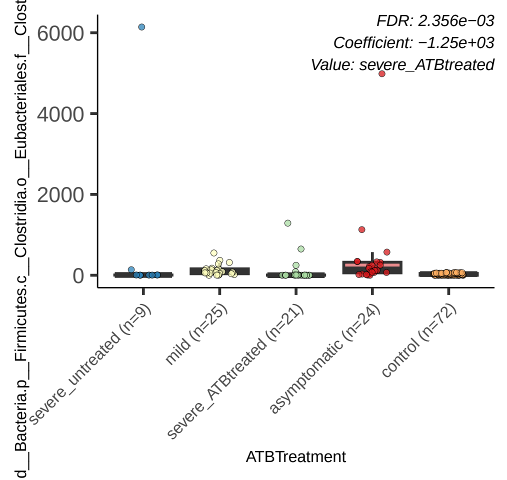
d__Bacteria.p__Firmicutes.c__Clostridia.o__Eubacteriales.f__Clost
FDR: 2.356e−03
Coefficient: −1.25e+03
Value: severe_ATBtreated
6000
4000
2000
0
severe_untreated (n=9)
mild (n=25)
severe_ATBtreated (n=21)
asymptomatic (n=24)
control (n=72)
ATBTreatment

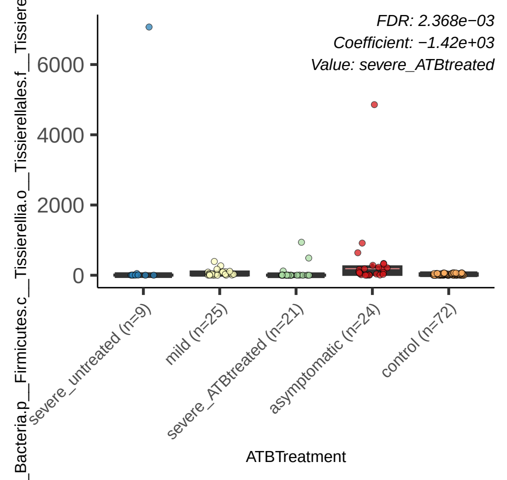

FDR: 2.368e−03
Coefficient: −1.42e+03
Value: severe_ATBtreated
6000
4000
2000
0
Bacteria.p__Firmicutes.c__Tissierellia.o__Tissierellales.f__Tissiere
severe_untreated (n=9)
mild (n=25)
severe_ATBtreated (n=21)
asymptomatic (n=24)
control (n=72)
ATBTreatment

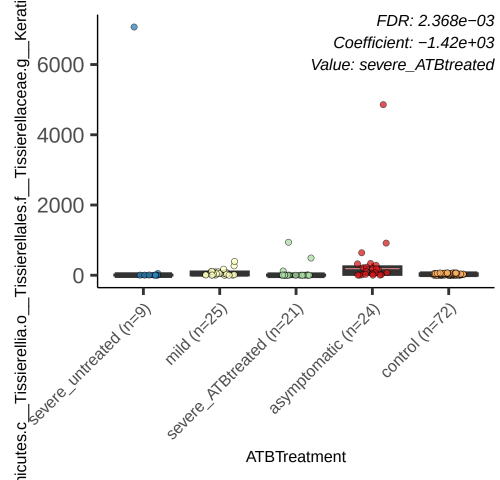

FDR: 2.368e−03
Coefficient: −1.42e+03
Value: severe_ATBtreated
6000
4000
2000
0
Tissierellales.f__Tissierellaceae.g__Kerati
nicutes.c__Tissierellia.o__
severe_untreated (n=9)
mild (n=25)
severe_ATBtreated (n=21)
asymptomatic (n=24)
control (n=72)
ATBTreatment

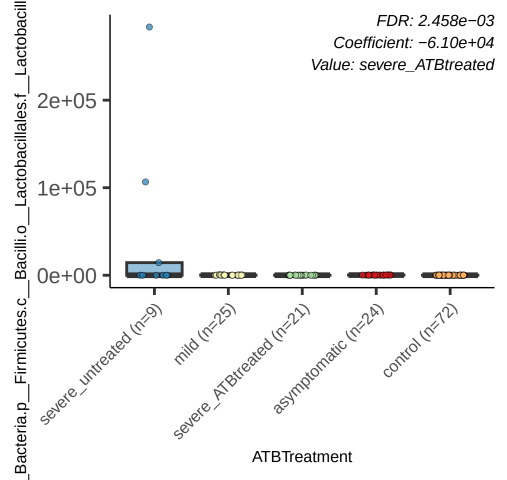

FDR: 2.458e−03
Coefficient: −6.10e+04
Value: severe_ATBtreated
2e+05
1e+05
0e+00
Bacteria.p__Firmicutes.c__Bacilli.o__Lactobacillales.f__Lactobacill
severe_untreated (n=9)
mild (n=25)
severe_ATBtreated (n=21)
asymptomatic (n=24)
control (n=72)
ATBTreatment

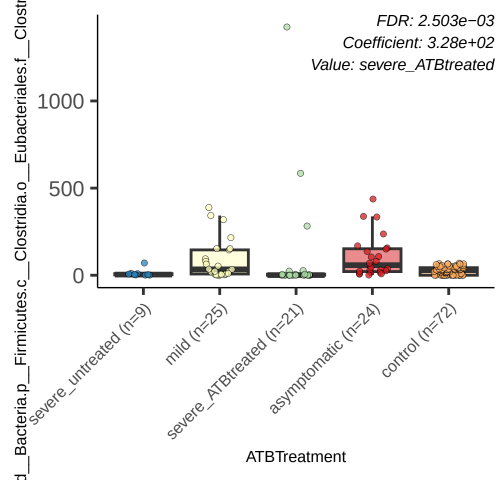

FDR: 2.503e−03
Coefficient: 3.28e+02
Value: severe_ATBtreated
d__Bacteria.p__Firmicutes.c__Clostridia.o__Eubacteriales.f__Clostr
1000
500
0
severe_untreated (n=9)
mild (n=25)
severe_ATBtreated (n=21)
asymptomatic (n=24)
control (n=72)
ATBTreatment

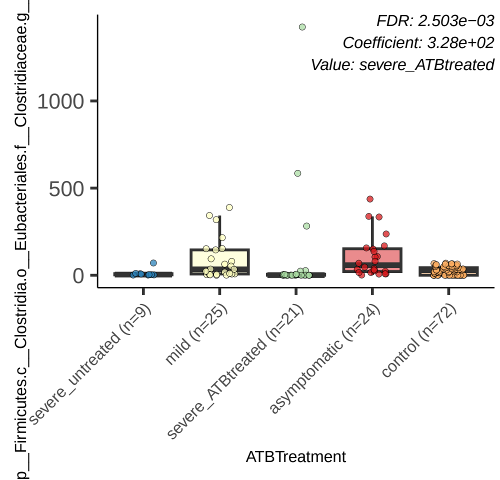

FDR: 2.503e−03
Coefficient: 3.28e+02
Value: severe_ATBtreated
p__Firmicutes.c__Clostridia.o__Eubacteriales.f__Clostridiaceae.g__
1000
500
0
severe_untreated (n=9)
mild (n=25)
severe_ATBtreated (n=21)
asymptomatic (n=24)
control (n=72)
ATBTreatment

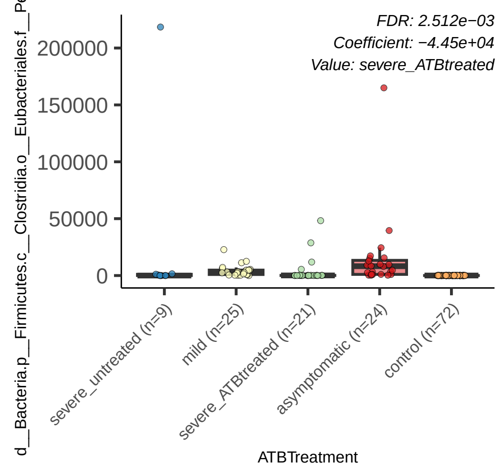
FDR: 2.512e−03
Coefficient: −4.45e+04
Value: severe_ATBtreated
d__Bacteria.p__Firmicutes.c__Clostridia.o__Eubacteriales.f__Pe
200000
150000
100000
50000
0
severe_untreated (n=9)
mild (n=25)
severe_ATBtreated (n=21)
asymptomatic (n=24)
control (n=72)
ATBTreatment

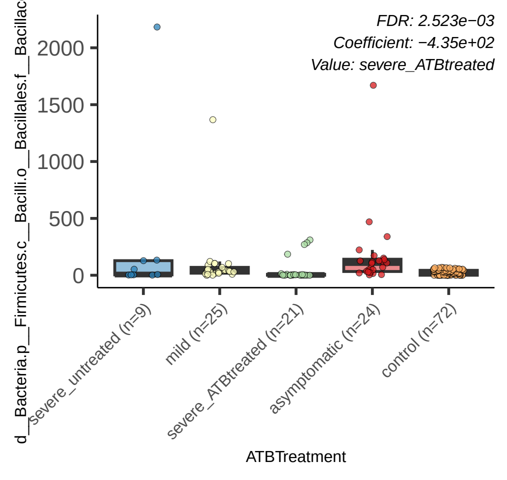

FDR: 2.523e−03
Coefficient: −4.35e+02
Value: severe_ATBtreated
d__Bacteria.p__Firmicutes.c__Bacilli.o__Bacillales.f__Bacillac
2000
1500
1000
500
0
severe_untreated (n=9)
mild (n=25)
severe_ATBtreated (n=21)
asymptomatic (n=24)
control (n=72)
ATBTreatment

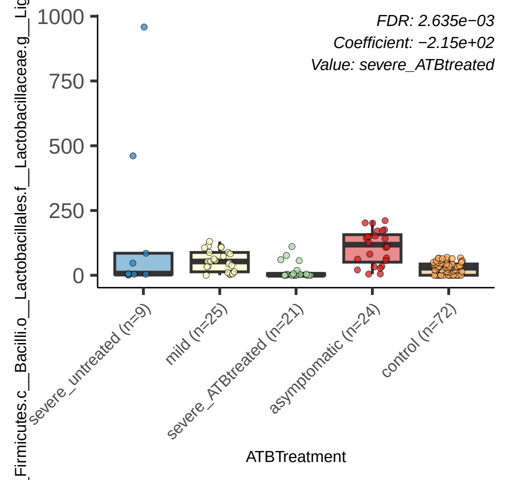

FDR: 2.635e−03
Coefficient: −2.15e+02
Value: severe_ATBtreated
1000
750
500
250
0
Firmicutes.c__Bacilli.o__Lactobacillales.f__Lactobacillaceae.g__Lig
severe_untreated (n=9)
mild (n=25)
severe_ATBtreated (n=21)
asymptomatic (n=24)
control (n=72)
ATBTreatment

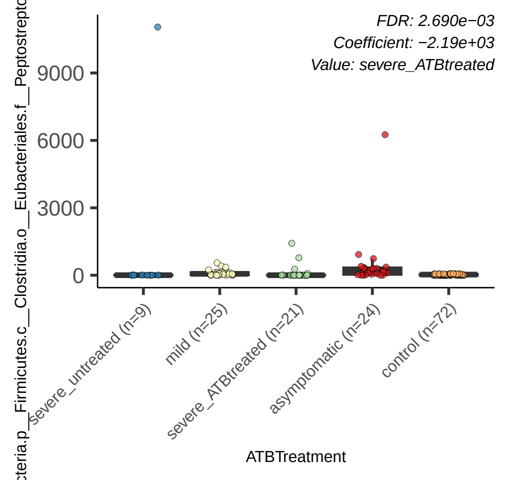

FDR: 2.690e−03
Coefficient: −2.19e+03
Value: severe_ATBtreated
9000
6000
3000
0
Firmicutes.c__Clostridia.o__Eubacteriales.f__Peptostrepto
cteria.p
severe_untreated (n=9)
mild (n=25)
severe_ATBtreated (n=21)
asymptomatic (n=24)
control (n=72)
ATBTreatment

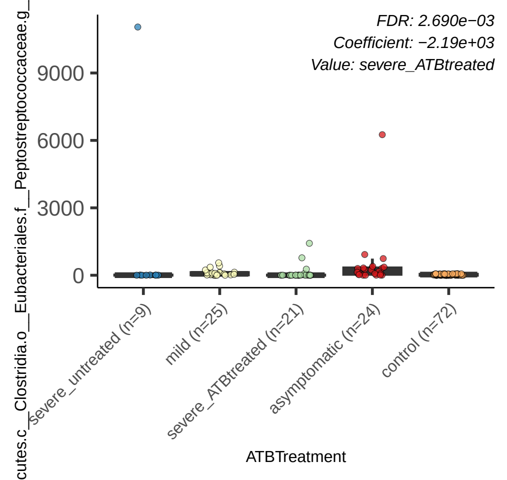

FDR: 2.690e−03
Coefficient: −2.19e+03
Value: severe_ATBtreated
cutes.c__Clostridia.o__Eubacteriales.f__Peptostreptococcaceae.g__
9000
6000
3000
0
severe_untreated (n=9)
mild (n=25)
severe_ATBtreated (n=21)
asymptomatic (n=24)
control (n=72)
ATBTreatment

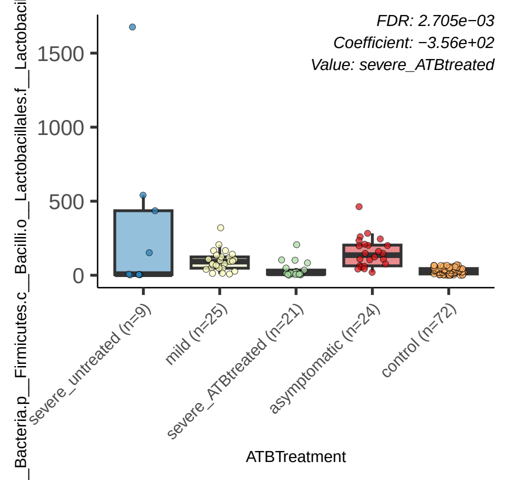
FDR: 2.705e−03
Coefficient: −3.56e+02
Value: severe_ATBtreated
1500
1000
500
0
Bacteria.p__Firmicutes.c__Bacilli.o__Lactobacillales.f__Lactobaci
severe_untreated (n=9)
mild (n=25)
severe_ATBtreated (n=21)
asymptomatic (n=24)
control (n=72)
ATBTreatment

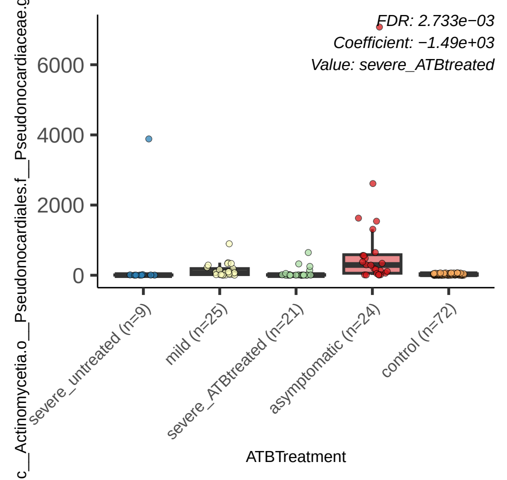
FDR: 2.733e−03
Coefficient: −1.49e+03
Value: severe_ATBtreated
c__Actinomycetia.o__Pseudonocardiales.f__Pseudonocardiaceae.g
6000
4000
2000
0
severe_untreated (n=9)
mild (n=25)
severe_ATBtreated (n=21)
asymptomatic (n=24)
control (n=72)
ATBTreatment

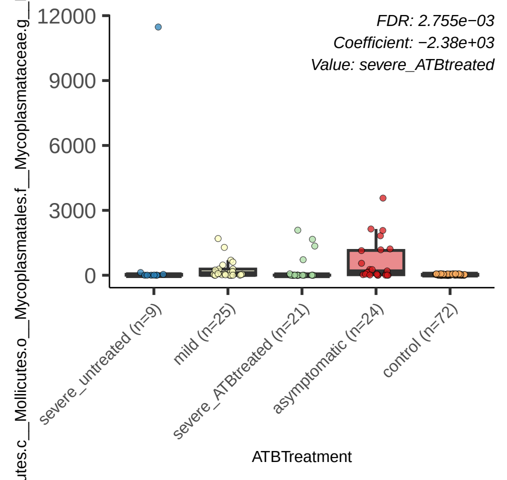

FDR: 2.755e−03
Coefficient: −2.38e+03
Value: severe_ATBtreated
12000
9000
6000
3000
0
utes.c__Mollicutes.o__Mycoplasmatales.f__Mycoplasmataceae.g_
severe_untreated (n=9)
mild (n=25)
severe_ATBtreated (n=21)
asymptomatic (n=24)
control (n=72)
ATBTreatment

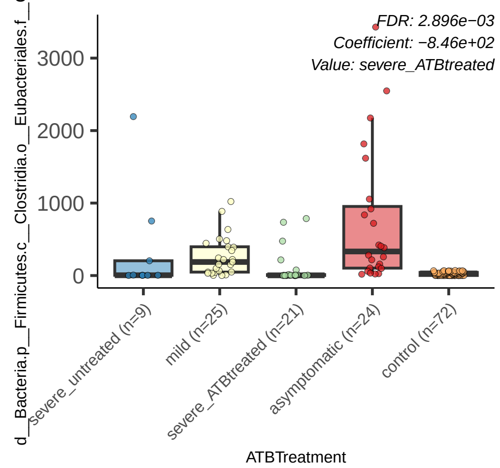

FDR: 2.896e−03
Coefficient: −8.46e+02
Value: severe_ATBtreated
d__Bacteria.p__Firmicutes.c__Clostridia.o__Eubacteriales.f__
3000
2000
1000
0
severe_untreated (n=9)
mild (n=25)
severe_ATBtreated (n=21)
asymptomatic (n=24)
control (n=72)
ATBTreatment

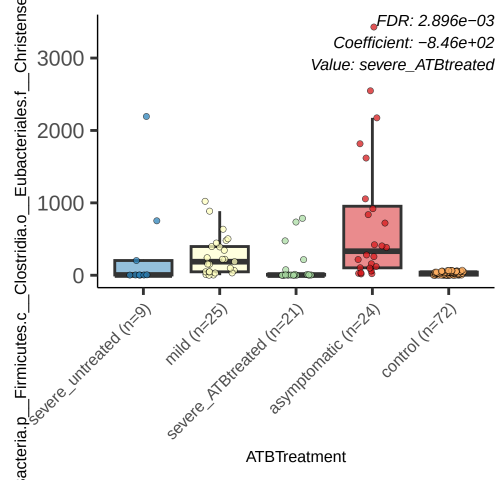

FDR: 2.896e−03
Coefficient: −8.46e+02
Value: severe_ATBtreated
3000
2000
1000
0
severe_untreated (n=9)
mild (n=25)
severe_ATBtreated (n=21)
asymptomatic (n=24)
control (n=72)
ATBTreatment

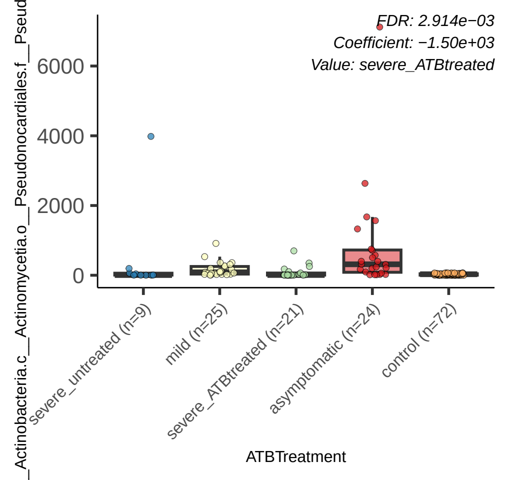

FDR: 2.914e−03
Coefficient: −1.50e+03
Value: severe_ATBtreated
6000
4000
2000
0
_Actinobacteria.c__Actinomycetia.o__Pseudonocardiales.f__Pseud
severe_untreated (n=9)
mild (n=25)
severe_ATBtreated (n=21)
asymptomatic (n=24)
control (n=72)
ATBTreatment

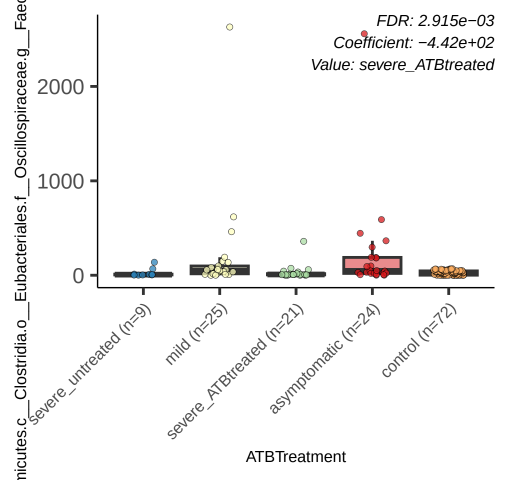
FDR: 2.915e−03
Coefficient: −4.42e+02
Value: severe_ATBtreated
2000
1000
0
severe_untreated (n=9)
mild (n=25)
severe_ATBtreated (n=21)
asymptomatic (n=24)
control (n=72)
ATBTreatment
micutes.c__Clostridia.o__Eubacteriales.f__Oscillospiraceae.g__Faec

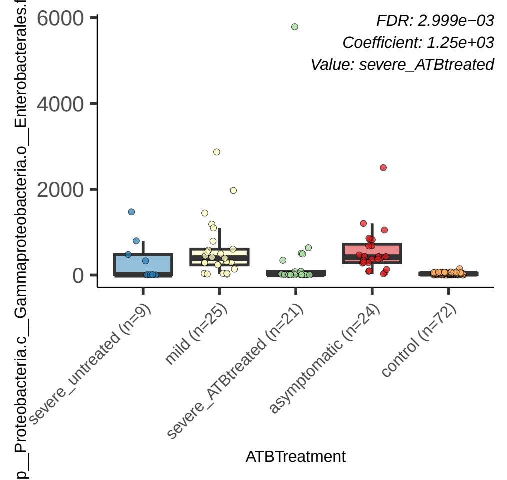

p__Proteobacteria.c__Gammaproteobacteria.o__Enterobacterales.f
6000
4000
2000
0
FDR: 2.999e−03
Coefficient: 1.25e+03
Value: severe_ATBtreated
severe_untreated (n=9)
mild (n=25)
severe_ATBtreated (n=21)
asymptomatic (n=24)
control (n=72)
ATBTreatment

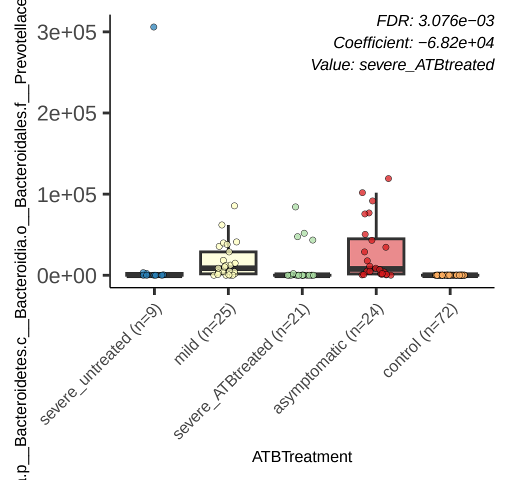

FDR: 3.076e−03
Coefficient: −6.82e+04
Value: severe_ATBtreated
k__Bacteria.p__Bacteroidetes.c__Bacteroidia.o__Bacteroidales.f__Prevotellaceae
3e+05
2e+05
1e+05
0e+00
severe_untreated (n=9)
mild (n=25)
severe_ATBtreated (n=21)
asymptomatic (n=24)
control (n=72)
ATBTreatment

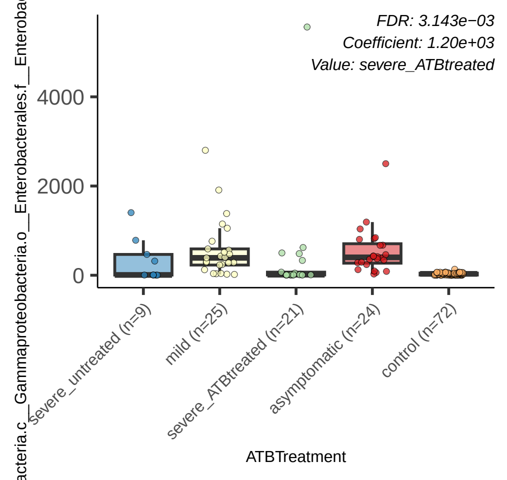

FDR: 3.143e−03
Coefficient: 1.20e+03
Value: severe_ATBtreated
0
2000
4000
acteria.c__Gammaproteobacteria.o__Enterobacterales.f__Enterobac
severe_untreated (n=9)
mild (n=25)
severe_ATBtreated (n=21)
asymptomatic (n=24)
control (n=72)
ATBTreatment

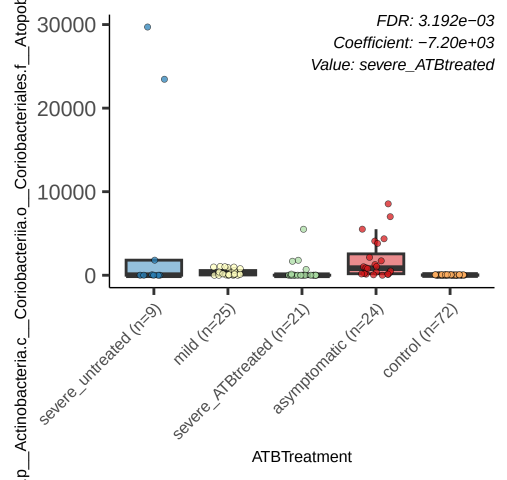

FDR: 3.192e−03
Coefficient: −7.20e+03
Value: severe_ATBtreated
30000
20000
10000
0
p__Actinobacteria.c__Coriobacteriia.o__Coriobacteriales.f__Atopob
severe_untreated (n=9)
mild (n=25)
severe_ATBtreated (n=21)
asymptomatic (n=24)
control (n=72)
ATBTreatment

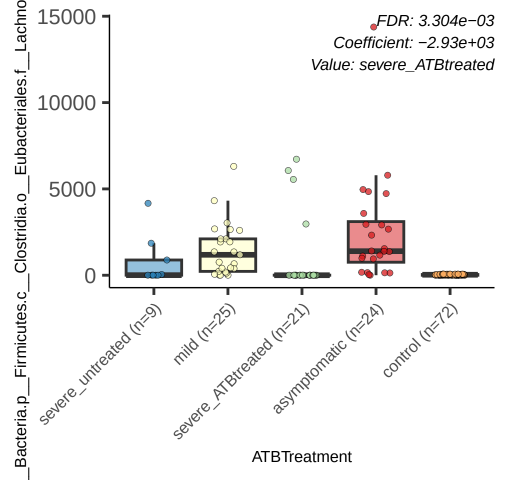

FDR: 3.304e−03
Coefficient: −2.93e+03
Value: severe_ATBtreated
Bacteria.p__Firmicutes.c__Clostridia.o__Eubacteriales.f__Lachno
15000
10000
5000
0
severe_untreated (n=9)
mild (n=25)
severe_ATBtreated (n=21)
asymptomatic (n=24)
control (n=72)
ATBTreatment

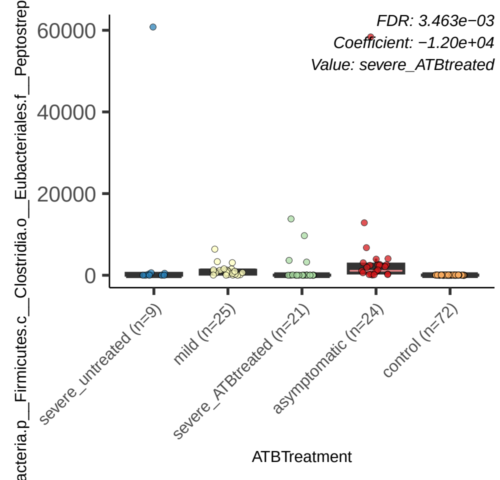

FDR: 3.463e−03
Coefficient: −1.20e+04
Value: severe_ATBtreated
60000
40000
20000
0
acteria.p__Firmicutes.c__Clostridia.o__Eubacteriales.f__Peptostrep
severe_untreated (n=9)
mild (n=25)
severe_ATBtreated (n=21)
asymptomatic (n=24)
control (n=72)
ATBTreatment

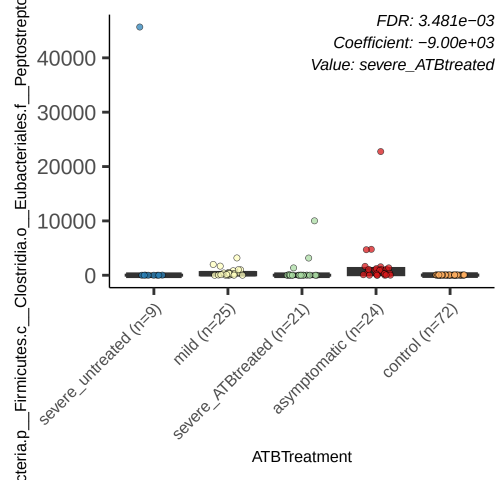

FDR: 3.481e−03
Coefficient: −9.00e+03
Value: severe_ATBtreated
40000
30000
20000
10000
0
severe_untreated (n=9)
mild (n=25)
severe_ATBtreated (n=21)
asymptomatic (n=24)
control (n=72)
ATBTreatment
cteria.p__Firmicutes.c__Clostridia.o__Eubacteriales.f__Peptostrepto

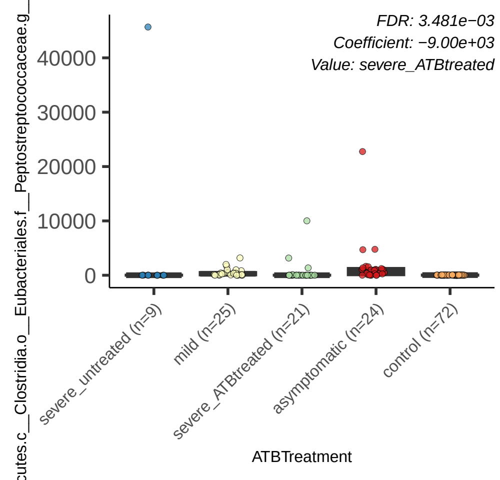

FDR: 3.481e−03
Coefficient: −9.00e+03
Value: severe_ATBtreated
40000
30000
20000
10000
0
cutes.c__Clostridia.o__Eubacteriales.f__Peptostreptococcaceae.g__
severe_untreated (n=9)
mild (n=25)
severe_ATBtreated (n=21)
asymptomatic (n=24)
control (n=72)
ATBTreatment

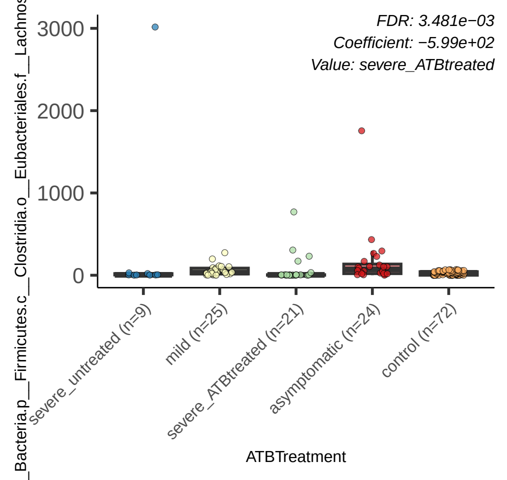

FDR: 3.481e−03
Coefficient: −5.99e+02
Value: severe_ATBtreated
Bacteria.p__Firmicutes.c__Clostridia.o__Eubacteriales.f__Lachnos
3000
2000
1000
0
severe_untreated (n=9)
mild (n=25)
severe_ATBtreated (n=21)
asymptomatic (n=24)
control (n=72)
ATBTreatment

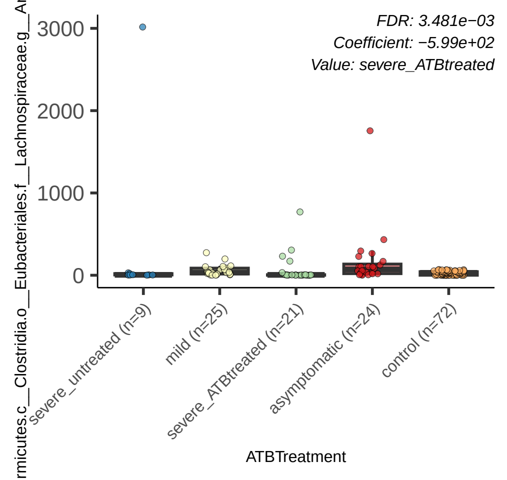

FDR: 3.481e−03
Coefficient: −5.99e+02
Value: severe_ATBtreated
3000
2000
1000
0
rmicutes.c__Clostridia.o__Eubacteriales.f__Lachnospiraceae.g__An
severe_untreated (n=9)
mild (n=25)
severe_ATBtreated (n=21)
asymptomatic (n=24)
control (n=72)
ATBTreatment

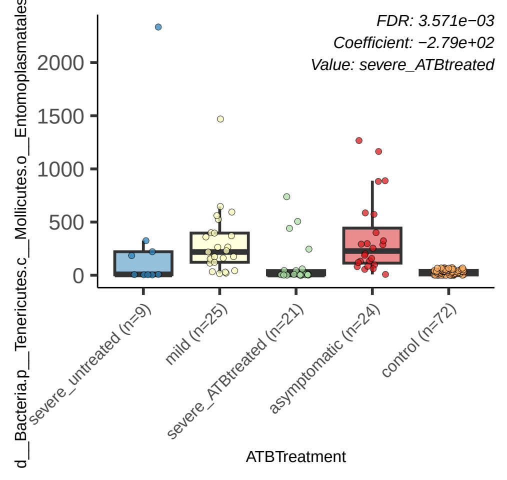

FDR: 3.571e−03
Coefficient: −2.79e+02
Value: severe_ATBtreated
d__Bacteria.p__Tenericutes.c__Mollicutes.o__Entomoplasmatales
2000
1500
1000
500
0
severe_untreated (n=9)
mild (n=25)
severe_ATBtreated (n=21)
asymptomatic (n=24)
control (n=72)
ATBTreatment

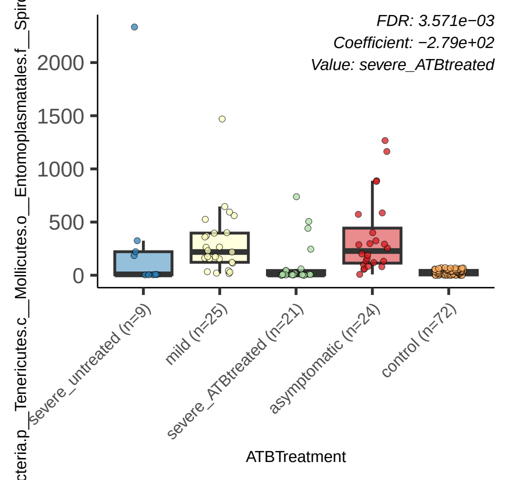

FDR: 3.571e−03
Coefficient: −2.79e+02
Value: severe_ATBtreated
cteria.p__Tenericutes.c__Mollicutes.o__Entomoplasmatales.f__Spiro
2000
1500
1000
500
0
severe_untreated (n=9)
mild (n=25)
severe_ATBtreated (n=21)
asymptomatic (n=24)
control (n=72)
ATBTreatment

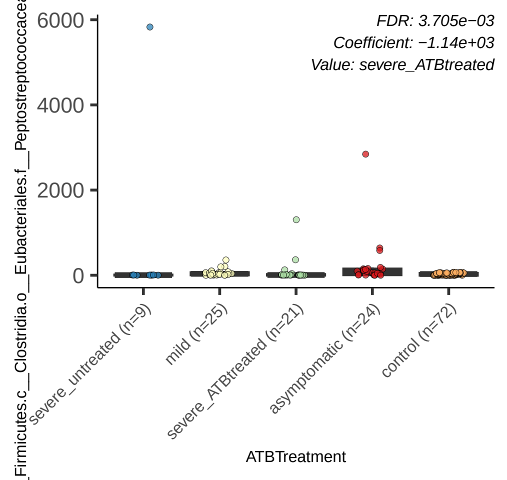

Firmicutes.c__Clostridia.o__Eubacteriales.f__Peptostreptococcaceae
6000
4000
2000
0
FDR: 3.705e−03
Coefficient: −1.14e+03
Value: severe_ATBtreated
severe_untreated (n=9)
mild (n=25)
severe_ATBtreated (n=21)
asymptomatic (n=24)
control (n=72)
ATBTreatment

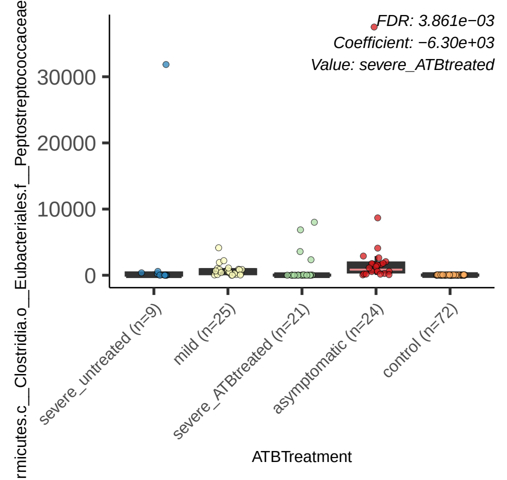

FDR: 3.861e−03
Coefficient: −6.30e+03
Value: severe_ATBtreated
rmicutes.c__Clostridia.o__Eubacteriales.f__Peptostreptococcaceae
30000
20000
10000
0
severe_untreated (n=9)
mild (n=25)
severe_ATBtreated (n=21)
asymptomatic (n=24)
control (n=72)
ATBTreatment

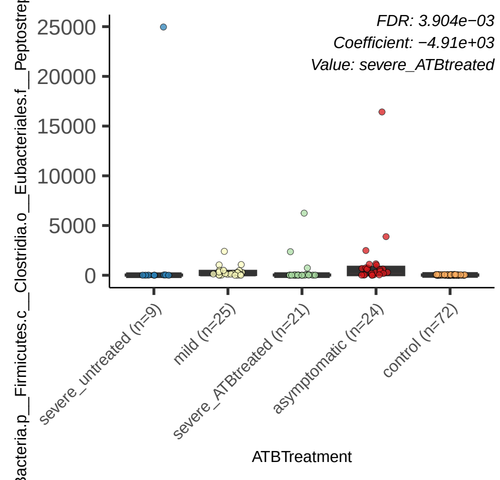

FDR: 3.904e−03
Coefficient: −4.91e+03
Value: severe_ATBtreated
Bacteria.p__Firmicutes.c__Clostridia.o__Eubacteriales.f__Peptostrep
25000
20000
15000
10000
5000
0
severe_untreated (n=9)
mild (n=25)
severe_ATBtreated (n=21)
asymptomatic (n=24)
control (n=72)
ATBTreatment

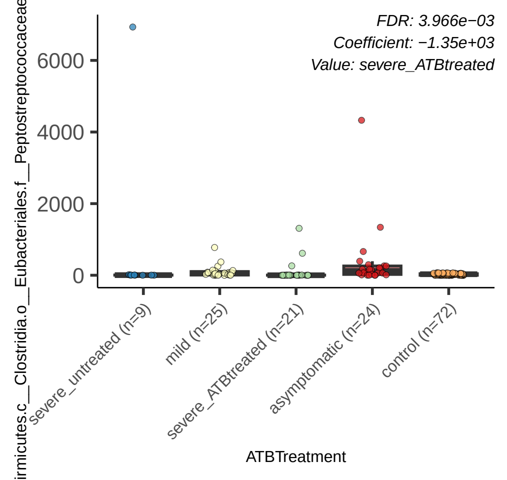
FDR: 3.966e−03
Coefficient: −1.35e+03
Value: severe_ATBtreated
irmicutes.c__Clostridia.o__Eubacteriales.f__Peptostreptococcaceae
6000
4000
2000
0
severe_untreated (n=9)
mild (n=25)
severe_ATBtreated (n=21)
asymptomatic (n=24)
control (n=72)
ATBTreatment

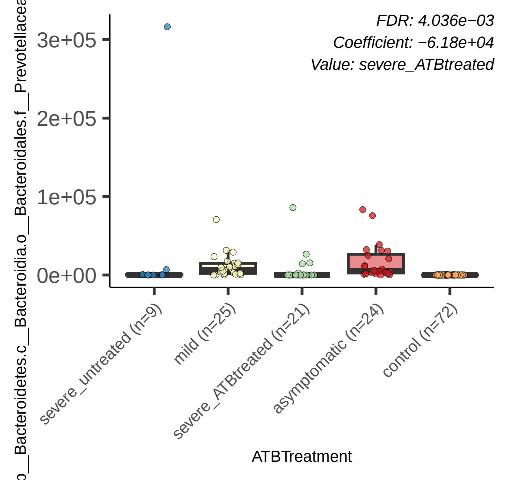
FDR: 4.036e−03
Coefficient: −6.18e+04
Value: severe_ATBtreated
3e+05
2e+05
1e+05
0e+00
o__Bacteroidetes.c__Bacteroidia.o__Bacteroidales.f__Prevotellacea
severe_untreated (n=9)
mild (n=25)
severe_ATBtreated (n=21)
asymptomatic (n=24)
control (n=72)
ATBTreatment

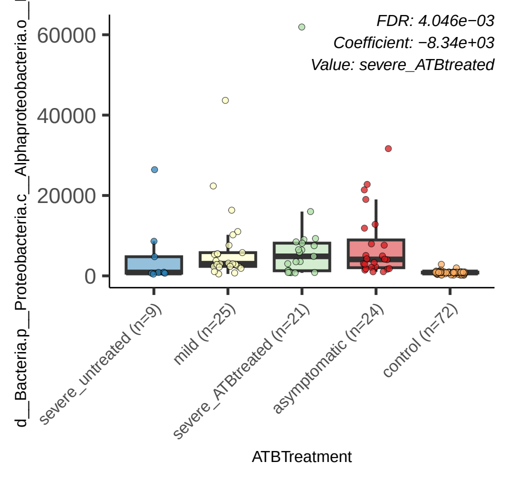
FDR: 4.046e−03
Coefficient: −8.34e+03
Value: severe_ATBtreated
d__Bacteria.p__Proteobacteria.c__Alphaproteobacteria.o__
60000
40000
20000
0
severe_untreated (n=9)
mild (n=25)
severe_ATBtreated (n=21)
asymptomatic (n=24)
control (n=72)
ATBTreatment

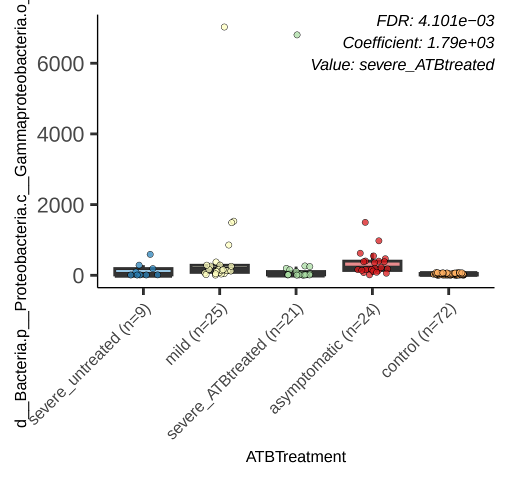

FDR: 4.101e−03
Coefficient: 1.79e+03
Value: severe_ATBtreated
6000
4000
2000
0
d__Bacteria.p__Proteobacteria.c__Gammaproteobacteria.o_
severe_untreated (n=9)
mild (n=25)
severe_ATBtreated (n=21)
asymptomatic (n=24)
control (n=72)
ATBTreatment

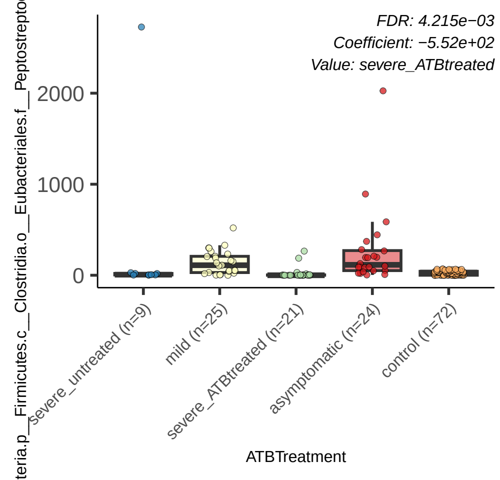

FDR: 4.215e−03
Coefficient: −5.52e+02
Value: severe_ATBtreated
2000
1000
0
teria.p__Firmicutes.c__Clostridia.o__Eubacteriales.f__Peptostrepto
severe_untreated (n=9)
mild (n=25)
severe_ATBtreated (n=21)
asymptomatic (n=24)
control (n=72)
ATBTreatment

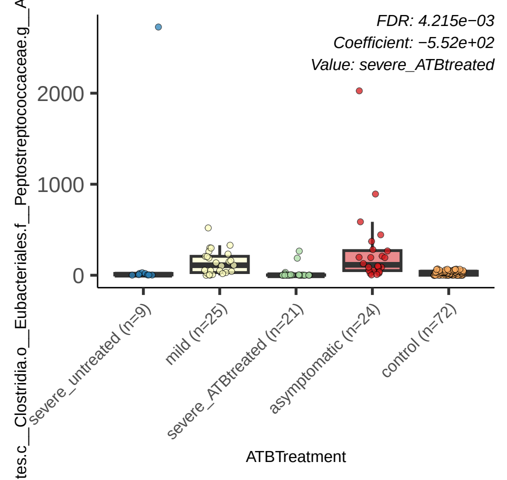
FDR: 4.215e−03
Coefficient: −5.52e+02
Value: severe_ATBtreated
2000
1000
0
tes.c__Clostridia.o__Eubacteriales.f__Peptostreptococcaceae.g__A
severe_untreated (n=9)
mild (n=25)
severe_ATBtreated (n=21)
asymptomatic (n=24)
control (n=72)
ATBTreatment

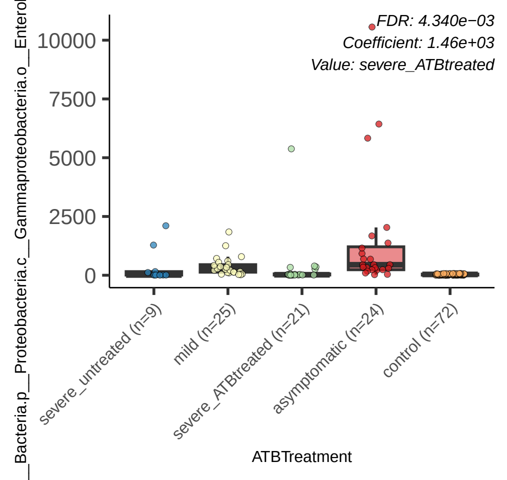
FDR: 4.340e−03
Coefficient: 1.46e+03
Value: severe_ATBtreated
10000
7500
5000
2500
0
severe_untreated (n=9)
mild (n=25)
severe_ATBtreated (n=21)
asymptomatic (n=24)
control (n=72)
ATBTreatment
_Bacteria.p__Proteobacteria.c__Gammaproteobacteria.o__Enterob

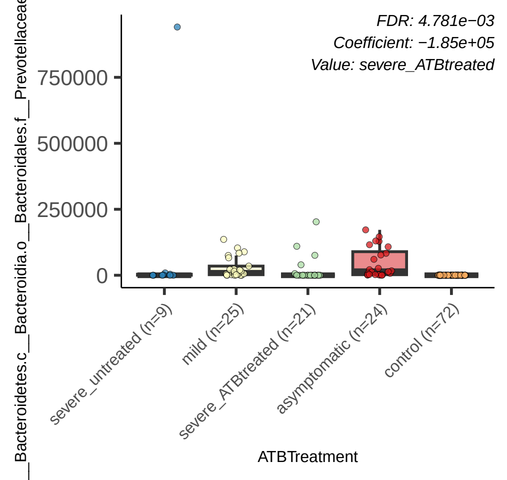

FDR: 4.781e−03
Coefficient: −1.85e+05
Value: severe_ATBtreated
Bacteroidetes.c__Bacteroidia.o__Bacteroidales.f__Prevotellaceae
750000
500000
250000
0
severe_untreated (n=9)
mild (n=25)
severe_ATBtreated (n=21)
asymptomatic (n=24)
control (n=72)
ATBTreatment

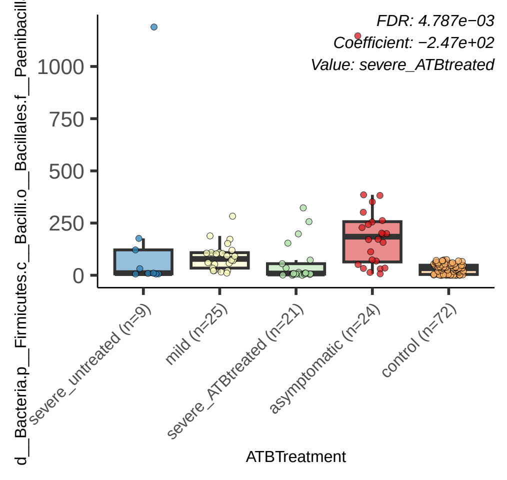
FDR: 4.787e−03
Coefficient: −2.47e+02
Value: severe_ATBtreated
d__Bacteria.p__Firmicutes.c__Bacilli.o__Bacillales.f__Paenibacill
1000
750
500
250
0
severe_untreated (n=9)
mild (n=25)
severe_ATBtreated (n=21)
asymptomatic (n=24)
control (n=72)
ATBTreatment

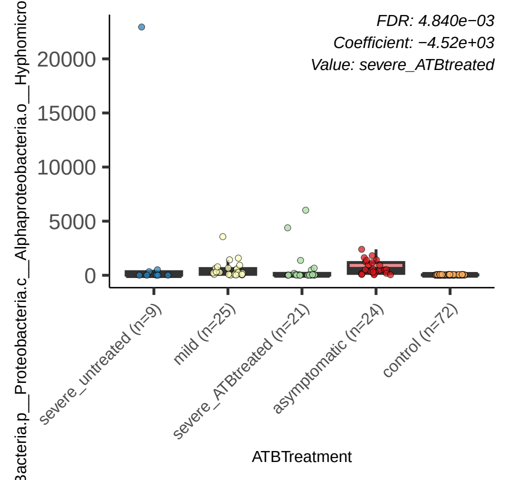

FDR: 4.840e−03
Coefficient: −4.52e+03
Value: severe_ATBtreated
20000
15000
10000
5000
0
Bacteria.p__Proteobacteria.c__Alphaproteobacteria.o__Hyphomicro
severe_untreated (n=9)
mild (n=25)
severe_ATBtreated (n=21)
asymptomatic (n=24)
control (n=72)
ATBTreatment

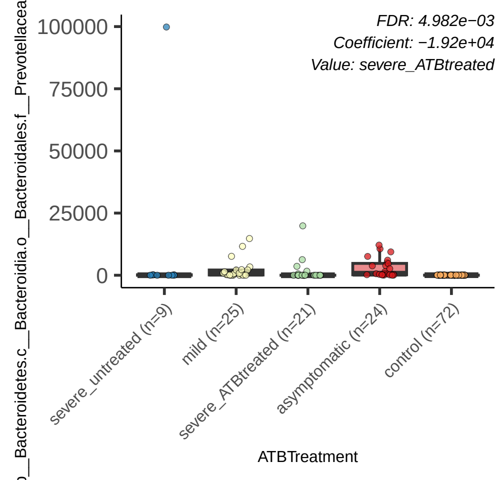
FDR: 4.982e−03
Coefficient: −1.92e+04
Value: severe_ATBtreated
o__Bacteroidetes.c__Bacteroidia.o__Bacteroidales.f__Prevotellacea
100000
75000
50000
25000
0
severe_untreated (n=9)
mild (n=25)
severe_ATBtreated (n=21)
asymptomatic (n=24)
control (n=72)
ATBTreatment

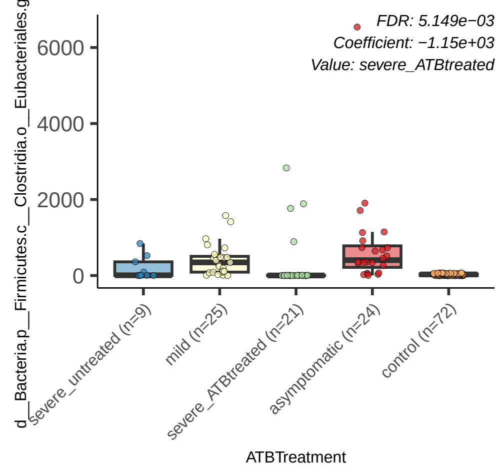
FDR: 5.149e−03
Coefficient: −1.15e+03
Value: severe_ATBtreated
d__Bacteria.p__Firmicutes.c__Clostridia.o__Eubacteriales.g
6000
4000
2000
0
severe_untreated (n=9)
mild (n=25)
severe_ATBtreated (n=21)
asymptomatic (n=24)
control (n=72)
ATBTreatment

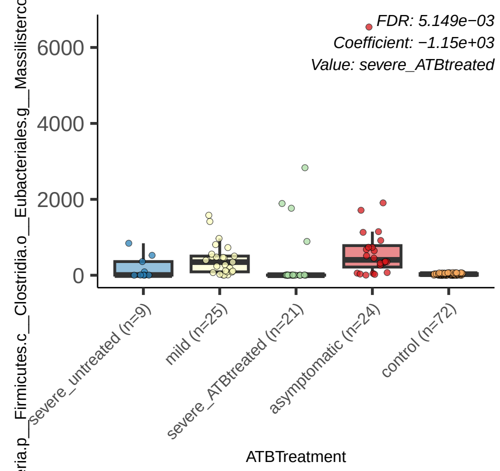

FDR: 5.149e−03
Coefficient: −1.15e+03
Value: severe_ATBtreated
6000
4000
2000
0
teria.p__Firmicutes.c__Clostridia.o__Eubacteriales.g__Massilisterco
severe_untreated (n=9)
mild (n=25)
severe_ATBtreated (n=21)
asymptomatic (n=24)
control (n=72)
ATBTreatment

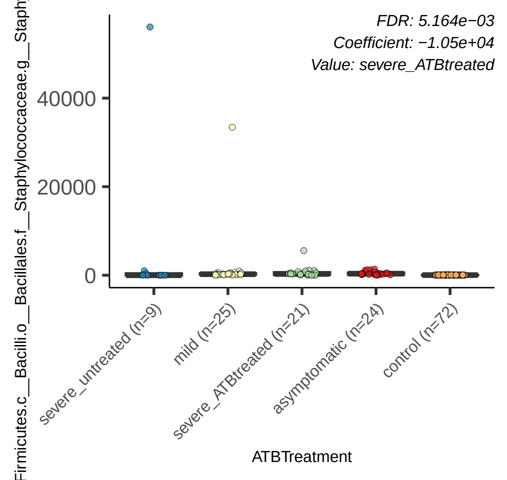

FDR: 5.164e−03
Coefficient: −1.05e+04
Value: severe_ATBtreated
40000
20000
0
severe_untreated (n=9)
mild (n=25)
severe_ATBtreated (n=21)
asymptomatic (n=24)
control (n=72)
ATBTreatment
Firmicutes.c__Bacilli.o__Bacillales.f__Staphylococcaceae.g__Staph

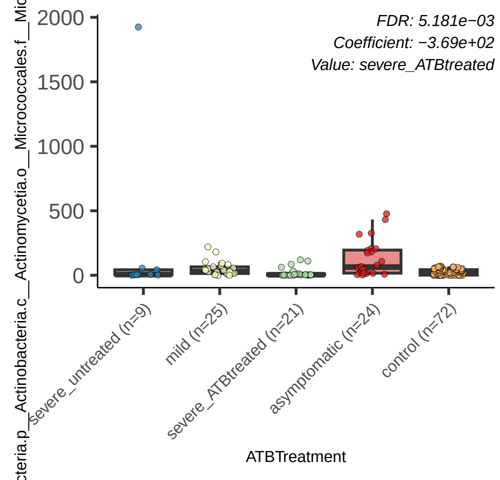

FDR: 5.181e−03
Coefficient: −3.69e+02
Value: severe_ATBtreated
2000
1500
1000
500
0
severe_untreated (n=9)
mild (n=25)
severe_ATBtreated (n=21)
asymptomatic (n=24)
control (n=72)
ATBTreatment

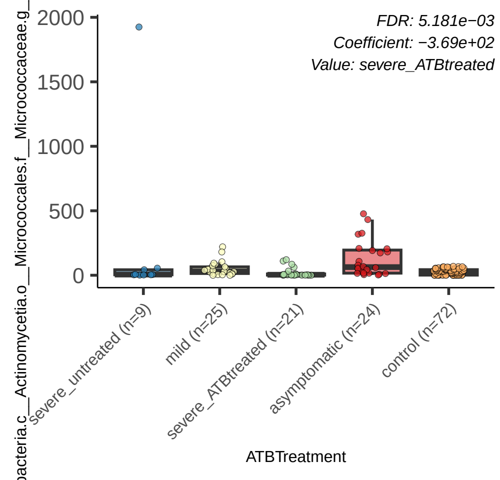

FDR: 5.181e−03
Coefficient: −3.69e+02
Value: severe_ATBtreated
2000
1500
1000
500
0
bacteria.c__Actinomycetia.o__Micrococcales.f__Micrococcaceae.g__
severe_untreated (n=9)
mild (n=25)
severe_ATBtreated (n=21)
asymptomatic (n=24)
control (n=72)
ATBTreatment

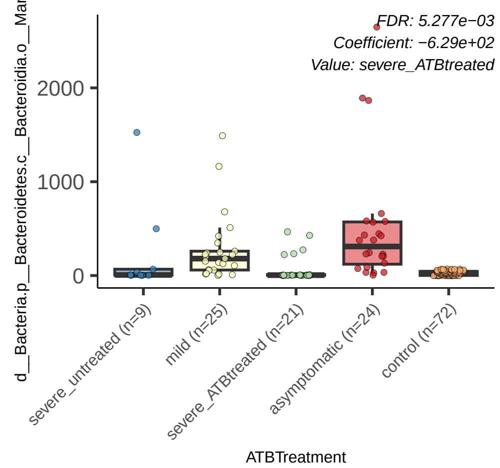

FDR: 5.277e−03
Coefficient: −6.29e+02
Value: severe_ATBtreated
d__Bacteria.p__Bacteroidetes.c__Bacteroidia.o__Mar
2000
1000
0
severe_untreated (n=9)
mild (n=25)
severe_ATBtreated (n=21)
asymptomatic (n=24)
control (n=72)
ATBTreatment

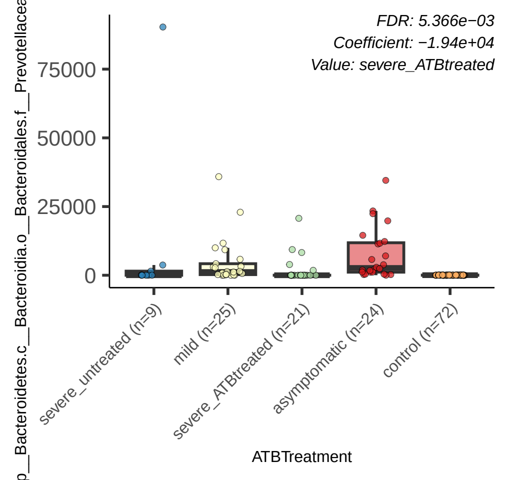

FDR: 5.366e−03
Coefficient: −1.94e+04
Value: severe_ATBtreated
75000
50000
25000
0
p__Bacteroidetes.c__Bacteroidia.o__Bacteroidales.f__Prevotellaceae
severe_untreated (n=9)
mild (n=25)
severe_ATBtreated (n=21)
asymptomatic (n=24)
control (n=72)
ATBTreatment

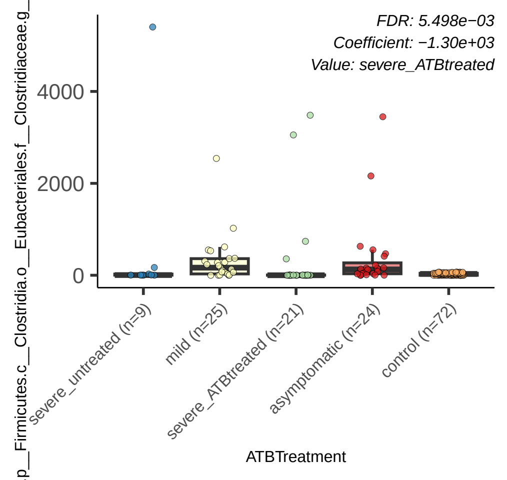
FDR: 5.498e−03
Coefficient: −1.30e+03
Value: severe_ATBtreated
4000
2000
0
p__Firmicutes.c__Clostridia.o__Eubacteriales.f__Clostridiaceae.g__
severe_untreated (n=9)
mild (n=25)
severe_ATBtreated (n=21)
asymptomatic (n=24)
control (n=72)
ATBTreatment

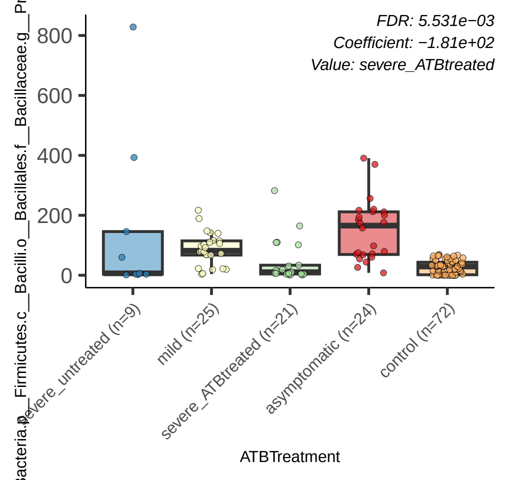
FDR: 5.531e−03
Coefficient: −1.81e+02
Value: severe_ATBtreated
Bacteria.p__Firmicutes.c__Bacilli.o__Bacillales.f__Bacillaceae.g__Pr
800
600
400
200
0
severe_untreated (n=9)
mild (n=25)
severe_ATBtreated (n=21)
asymptomatic (n=24)
control (n=72)
ATBTreatment

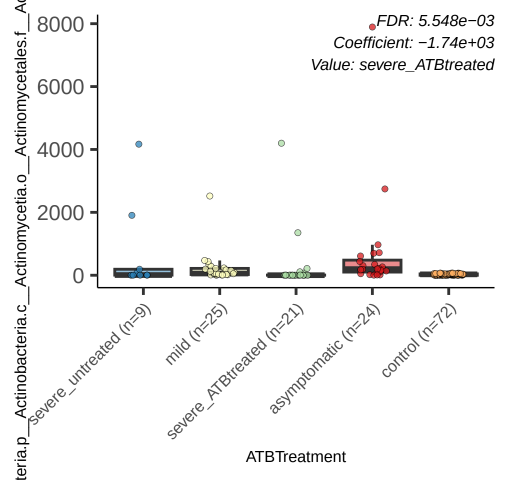
FDR: 5.548e−03
Coefficient: −1.74e+03
Value: severe_ATBtreated
0
2000
4000
6000
8000
teria.p__Actinobacteria.c__Actinomycetia.o__Actinomycetales.f__A
severe_untreated (n=9)
mild (n=25)
severe_ATBtreated (n=21)
asymptomatic (n=24)
control (n=72)
ATBTreatment

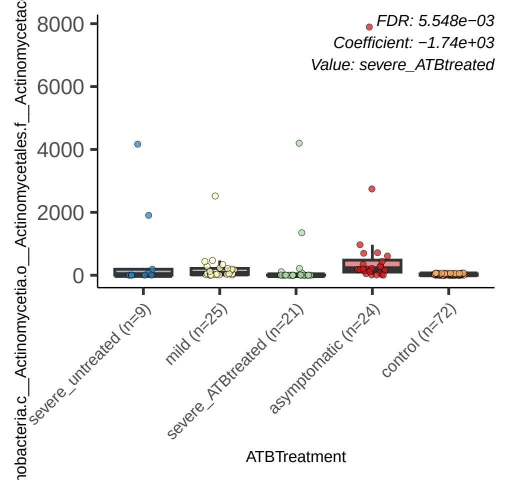

FDR: 5.548e−03
Coefficient: −1.74e+03
Value: severe_ATBtreated
8000
6000
4000
2000
0
nobacteria.c__Actinomycetia.o__Actinomycetales.f__Actinomycetac
severe_untreated (n=9)
mild (n=25)
severe_ATBtreated (n=21)
asymptomatic (n=24)
control (n=72)
ATBTreatment

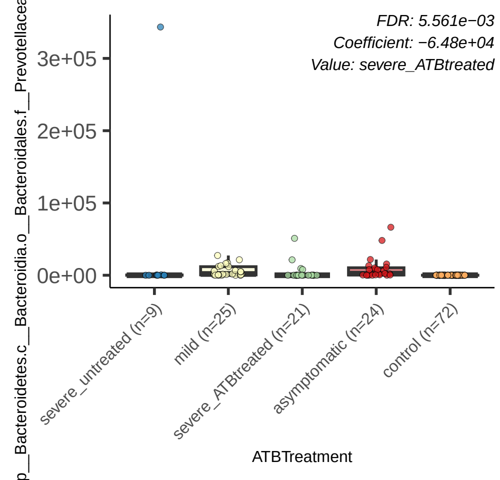

FDR: 5.561e−03
Coefficient: −6.48e+04
Value: severe_ATBtreated
p__Bacteroidetes.c__Bacteroidia.o__Bacteroidales.f__Prevotellacea
3e+05
2e+05
1e+05
0e+00
severe_untreated (n=9)
mild (n=25)
severe_ATBtreated (n=21)
asymptomatic (n=24)
control (n=72)
ATBTreatment

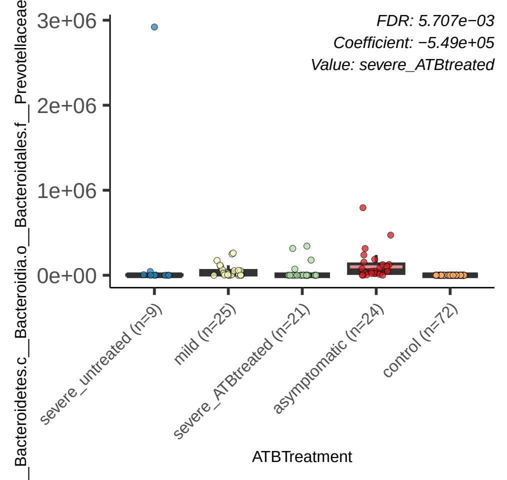
FDR: 5.707e−03
Coefficient: −5.49e+05
Value: severe_ATBtreated
Bacteroidetes.c__Bacteroidia.o__Bacteroidales.f__Prevotellaceae
3e+06
2e+06
1e+06
0e+00
severe_untreated (n=9)
mild (n=25)
severe_ATBtreated (n=21)
asymptomatic (n=24)
control (n=72)
ATBTreatment

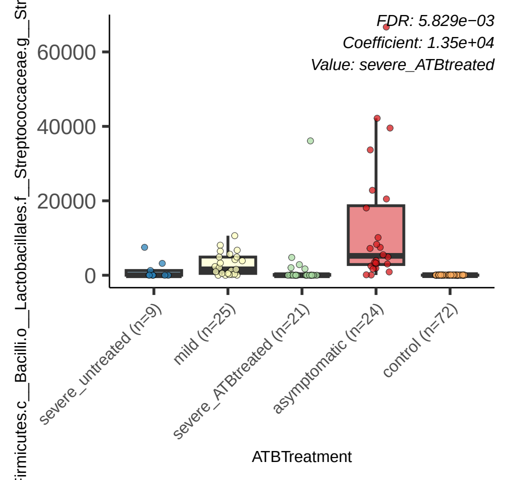

FDR: 5.829e−03
Coefficient: 1.35e+04
Value: severe_ATBtreated
60000
40000
20000
0
Firmicutes.c__Bacilli.o__Lactobacillales.f__Streptococcaceae.g__Str
severe_untreated (n=9)
mild (n=25)
severe_ATBtreated (n=21)
asymptomatic (n=24)
control (n=72)
ATBTreatment

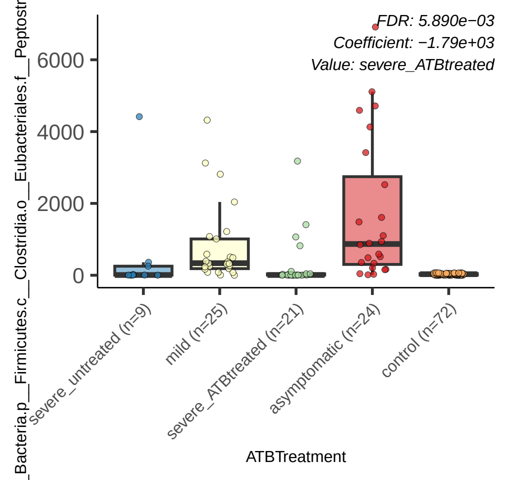

FDR: 5.890e−03
Coefficient: −1.79e+03
Value: severe_ATBtreated
Bacteria.p__Firmicutes.c__Clostridia.o__Eubacteriales.f__Peptostr
0
2000
4000
6000
severe_untreated (n=9)
mild (n=25)
severe_ATBtreated (n=21)
asymptomatic (n=24)
control (n=72)
ATBTreatment

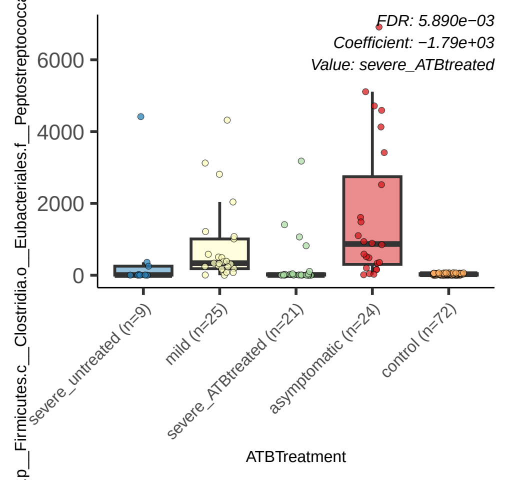
FDR: 5.890e−03
Coefficient: −1.79e+03
Value: severe_ATBtreated
6000
4000
2000
0
p__Firmicutes.c__Clostridia.o__Eubacteriales.f__Peptostreptococca
severe_untreated (n=9)
mild (n=25)
severe_ATBtreated (n=21)
asymptomatic (n=24)
control (n=72)
ATBTreatment

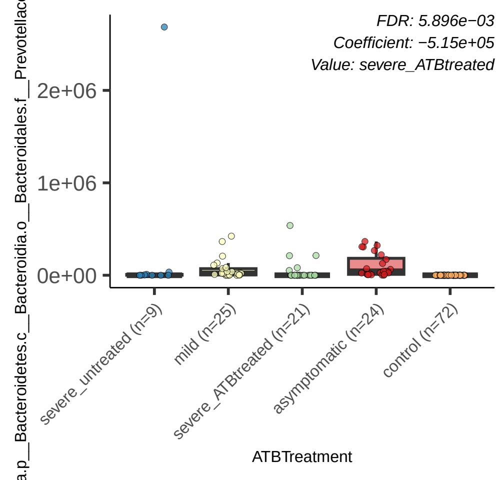

FDR: 5.896e−03
Coefficient: −5.15e+05
Value: severe_ATBtreated
2e+06
1e+06
0e+00
severe_untreated (n=9)
mild (n=25)
severe_ATBtreated (n=21)
asymptomatic (n=24)
control (n=72)
ATBTreatment

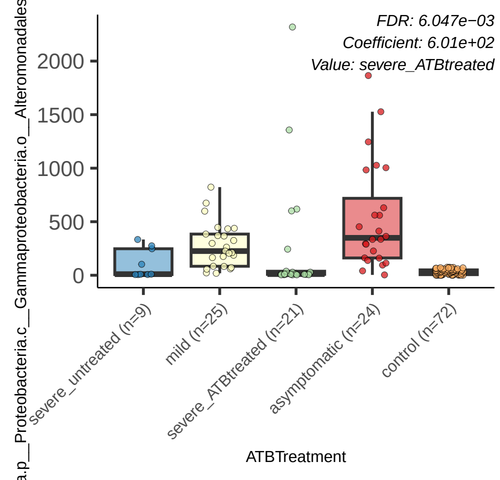

FDR: 6.047e−03
Coefficient: 6.01e+02
Value: severe_ATBtreated
a.p__Proteobacteria.c__Gammaproteobacteria.o__Alteromonadales
2000
1500
1000
500
0
severe_untreated (n=9)
mild (n=25)
severe_ATBtreated (n=21)
asymptomatic (n=24)
control (n=72)
ATBTreatment

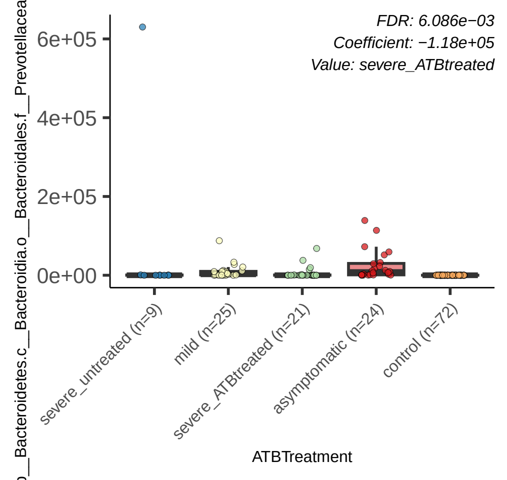
FDR: 6.086e−03
Coefficient: −1.18e+05
Value: severe_ATBtreated
6e+05
4e+05
2e+05
0e+00
o__Bacteroidetes.c__Bacteroidia.o__Bacteroidales.f__Prevotellacea
severe_untreated (n=9)
mild (n=25)
severe_ATBtreated (n=21)
asymptomatic (n=24)
control (n=72)
ATBTreatment

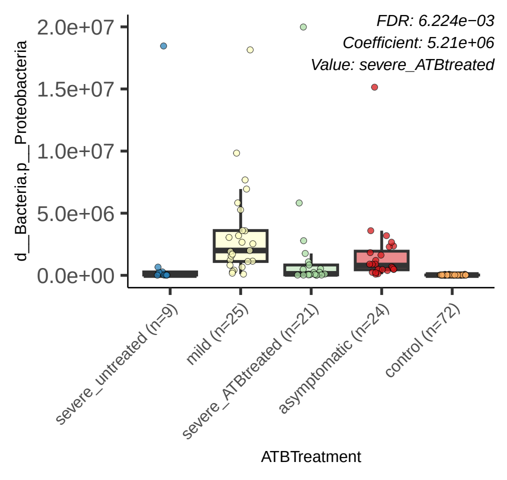

FDR: 6.224e−03
Coefficient: 5.21e+06
Value: severe_ATBtreated
d__Bacteria.p__Proteobacteria
2.0e+07
1.5e+07
1.0e+07
5.0e+06
0.0e+00
severe_untreated (n=9)
mild (n=25)
severe_ATBtreated (n=21)
asymptomatic (n=24)
control (n=72)
ATBTreatment

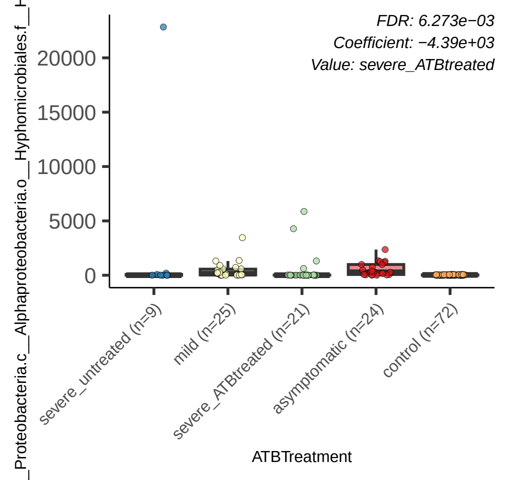

FDR: 6.273e−03
Coefficient: −4.39e+03
Value: severe_ATBtreated
_Proteobacteria.c__Alphaproteobacteria.o__Hyphomicrobiales.f__H
20000
15000
10000
5000
0
severe_untreated (n=9)
mild (n=25)
severe_ATBtreated (n=21)
asymptomatic (n=24)
control (n=72)
ATBTreatment

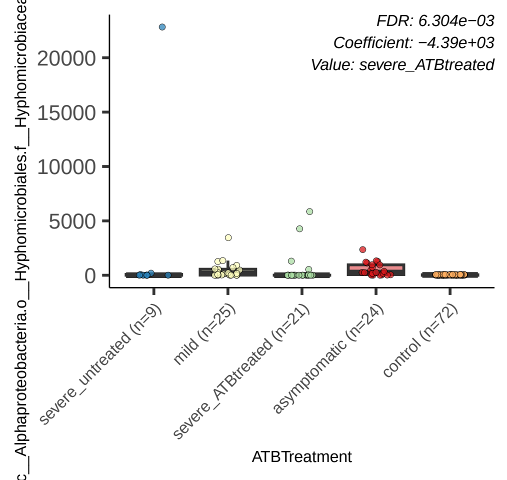
FDR: 6.304e−03
Coefficient: −4.39e+03
Value: severe_ATBtreated
c__Alphaproteobacteria.o__Hyphomicrobiales.f__Hyphomicrobiacea
20000
15000
10000
5000
0
severe_untreated (n=9)
mild (n=25)
severe_ATBtreated (n=21)
asymptomatic (n=24)
control (n=72)
ATBTreatment

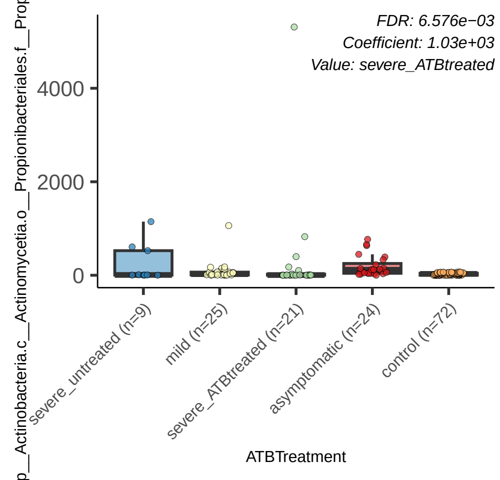

FDR: 6.576e−03
Coefficient: 1.03e+03
Value: severe_ATBtreated
4000
2000
0
p__Actinobacteria.c__Actinomycetia.o__Propionibacteriales.f__Prop
severe_untreated (n=9)
mild (n=25)
severe_ATBtreated (n=21)
asymptomatic (n=24)
control (n=72)
ATBTreatment

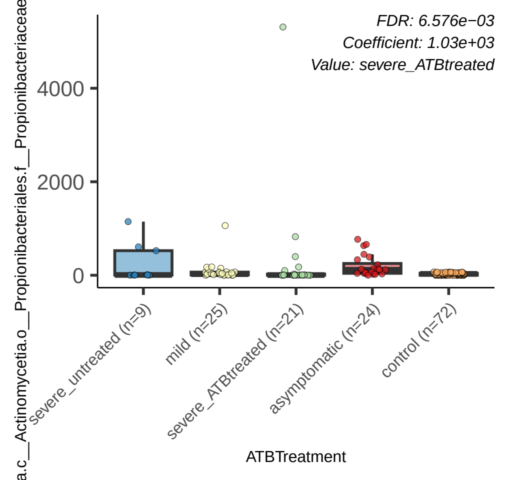

FDR: 6.576e−03
Coefficient: 1.03e+03
Value: severe_ATBtreated
4000
2000
0
a.c__Actinomycetia.o__Propionibacteriales.f__Propionibacteriaceae
severe_untreated (n=9)
mild (n=25)
severe_ATBtreated (n=21)
asymptomatic (n=24)
control (n=72)
ATBTreatment

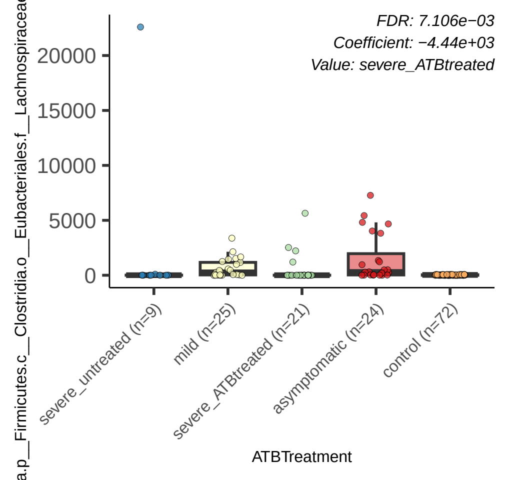

FDR: 7.106e−03
Coefficient: −4.44e+03
Value: severe_ATBtreated
a.p__Firmicutes.c__Clostridia.o__Eubacteriales.f__Lachnospiracea
20000
15000
10000
5000
0
severe_untreated (n=9)
mild (n=25)
severe_ATBtreated (n=21)
asymptomatic (n=24)
control (n=72)
ATBTreatment

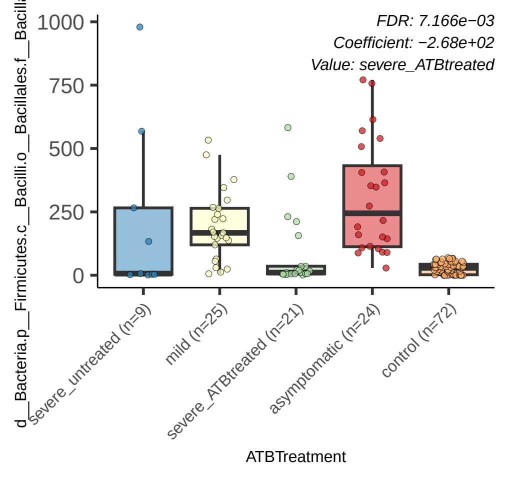
FDR: 7.166e−03
Coefficient: −2.68e+02
Value: severe_ATBtreated
1000
750
500
250
0
d__Bacteria.p__Firmicutes.c__Bacilli.o__Bacillales.f__Bacill
severe_untreated (n=9)
mild (n=25)
severe_ATBtreated (n=21)
asymptomatic (n=24)
control (n=72)
ATBTreatment

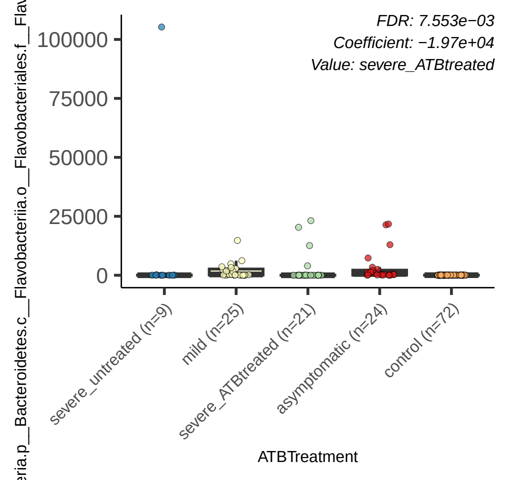

FDR: 7.553e−03
Coefficient: −1.97e+04
Value: severe_ATBtreated
100000
75000
50000
25000
0
severe_untreated (n=9)
mild (n=25)
severe_ATBtreated (n=21)
asymptomatic (n=24)
control (n=72)
ATBTreatment
eria.p__Bacteroidetes.c__Flavobacteriia.o__Flavobacteriales.f__Flav

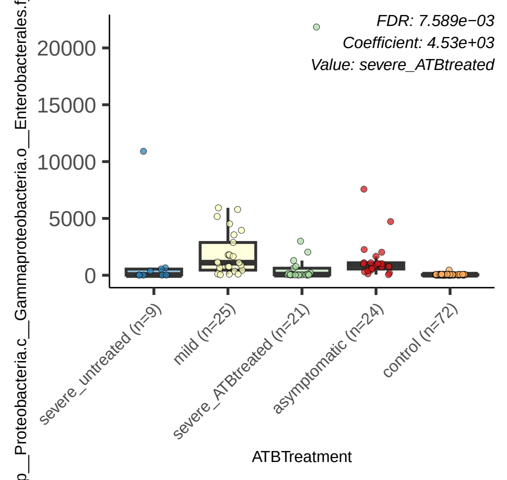

FDR: 7.589e−03
Coefficient: 4.53e+03
Value: severe_ATBtreated
20000
15000
10000
5000
0
p__Proteobacteria.c__Gammaproteobacteria.o__Enterobacterales.f
severe_untreated (n=9)
mild (n=25)
severe_ATBtreated (n=21)
asymptomatic (n=24)
control (n=72)
ATBTreatment

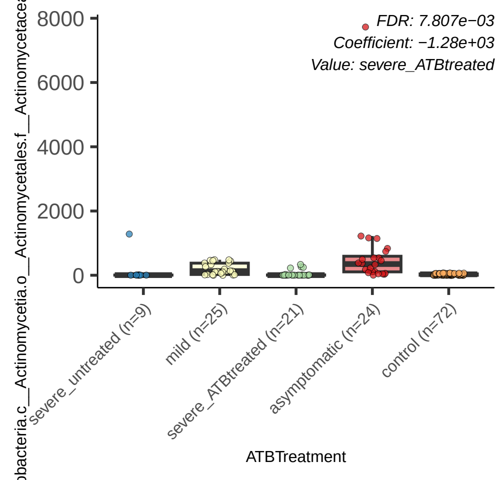

FDR: 7.807e−03
Coefficient: −1.28e+03
Value: severe_ATBtreated
8000
6000
4000
2000
0
severe_untreated (n=9)
mild (n=25)
severe_ATBtreated (n=21)
asymptomatic (n=24)
control (n=72)
ATBTreatment
obacteria.c__Actinomycetia.o__Actinomycetales.f__Actinomycetacea

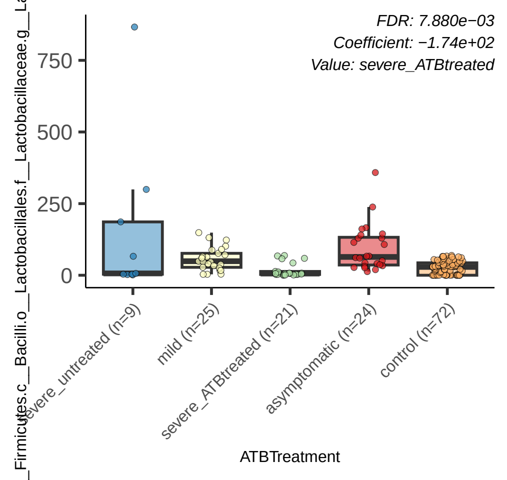

FDR: 7.880e−03
Coefficient: −1.74e+02
Value: severe_ATBtreated
Firmicutes.c__Bacilli.o__Lactobacillales.f__Lactobacillaceae.g__La
750
500
250
0
severe_untreated (n=9)
mild (n=25)
severe_ATBtreated (n=21)
asymptomatic (n=24)
control (n=72)
ATBTreatment

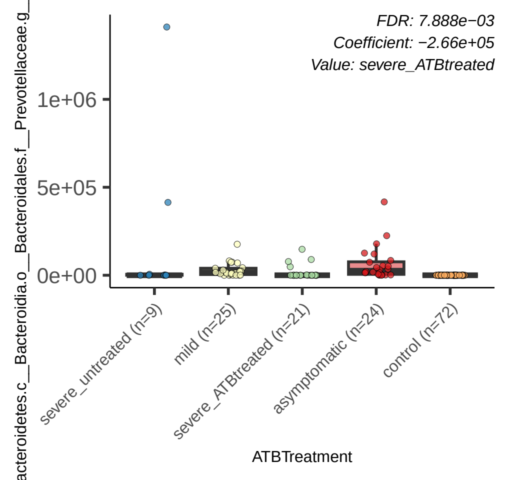

FDR: 7.888e−03
Coefficient: −2.66e+05
Value: severe_ATBtreated
1e+06
5e+05
0e+00
acteroidetes.c__Bacteroidia.o__Bacteroidales.f__Prevotellaceae.g__
severe_untreated (n=9)
mild (n=25)
severe_ATBtreated (n=21)
asymptomatic (n=24)
control (n=72)
ATBTreatment

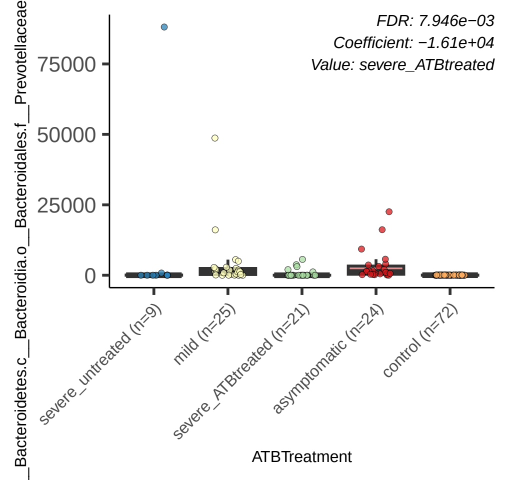

FDR: 7.946e−03
Coefficient: −1.61e+04
Value: severe_ATBtreated
Bacteroidetes.c__Bacteroidia.o__Bacteroidales.f__Prevotellaceae
75000
50000
25000
0
severe_untreated (n=9)
mild (n=25)
severe_ATBtreated (n=21)
asymptomatic (n=24)
control (n=72)
ATBTreatment

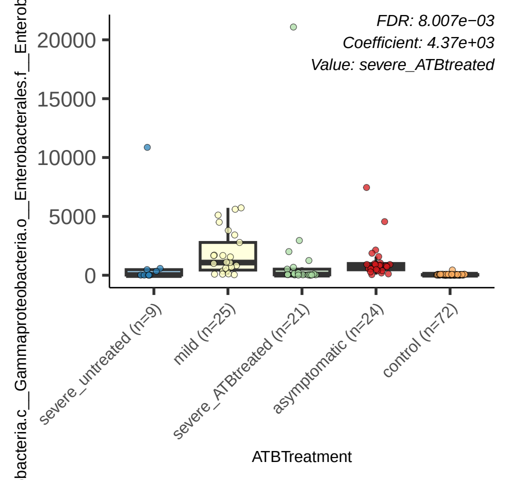

FDR: 8.007e−03
Coefficient: 4.37e+03
Value: severe_ATBtreated
20000
15000
10000
5000
0
bacteria.c__Gammaproteobacteria.o__Enterobacterales.f__Enterob
severe_untreated (n=9)
mild (n=25)
severe_ATBtreated (n=21)
asymptomatic (n=24)
control (n=72)
ATBTreatment

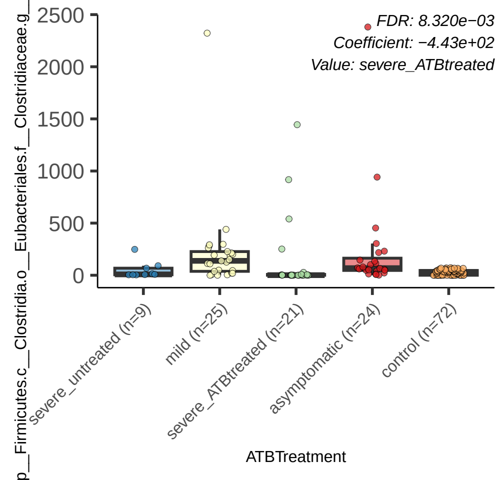

FDR: 8.320e−03
Coefficient: −4.43e+02
Value: severe_ATBtreated
p__Firmicutes.c__Clostridia.o__Eubacteriales.f__Clostridiaceae.g__
2500
2000
1500
1000
500
0
severe_untreated (n=9)
mild (n=25)
severe_ATBtreated (n=21)
asymptomatic (n=24)
control (n=72)
ATBTreatment

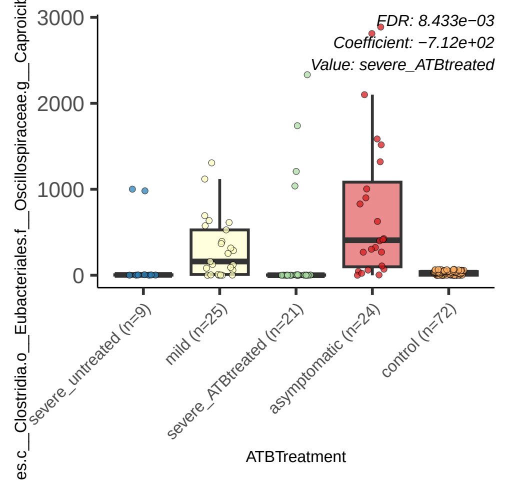

FDR: 8.433e−03
Coefficient: −7.12e+02
Value: severe_ATBtreated
3000
2000
1000
0
es.c__Clostridia.o__Eubacteriales.f__Oscillospiraceae.g__Caproicib
severe_untreated (n=9)
mild (n=25)
severe_ATBtreated (n=21)
asymptomatic (n=24)
control (n=72)
ATBTreatment

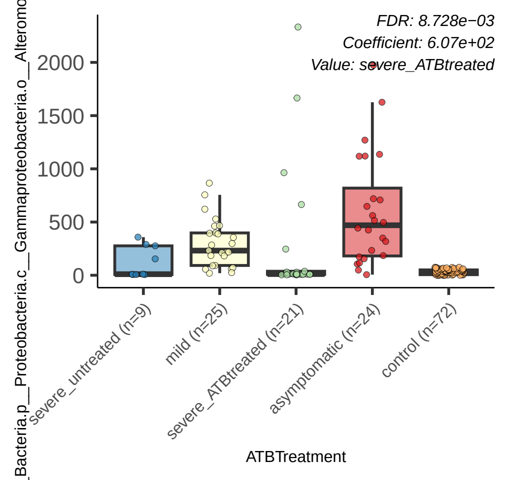

FDR: 8.728e−03
Coefficient: 6.07e+02
Value: severe_ATBtreated
Bacteria.p__Proteobacteria.c__Gammaproteobacteria.o__Alteromo
0
500
1000
1500
2000
severe_untreated (n=9)
mild (n=25)
severe_ATBtreated (n=21)
asymptomatic (n=24)
control (n=72)
ATBTreatment

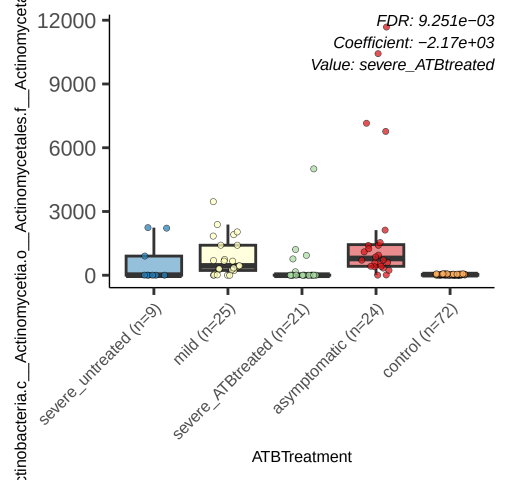

FDR: 9.251e−03
Coefficient: −2.17e+03
Value: severe_ATBtreated
12000
9000
6000
3000
0
Actinobacteria.c__Actinomycetia.o__Actinomycetales.f__Actinomyceta
severe_untreated (n=9)
mild (n=25)
severe_ATBtreated (n=21)
asymptomatic (n=24)
control (n=72)
ATBTreatment

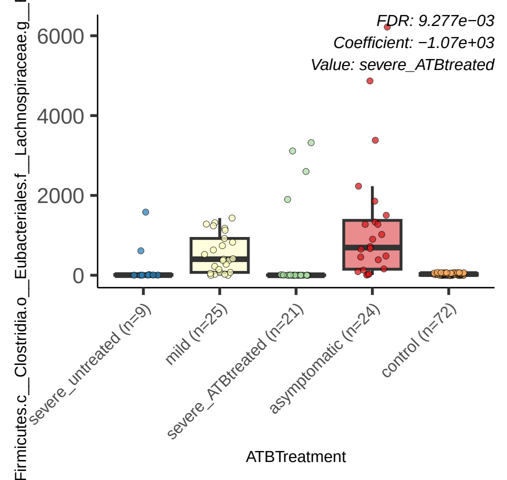
FDR: 9.277e−03
Coefficient: −1.07e+03
Value: severe_ATBtreated
Firmicutes.c__Clostridia.o__Eubacteriales.f__Lachnospiraceae.g__
6000
4000
2000
0
severe_untreated (n=9)
mild (n=25)
severe_ATBtreated (n=21)
asymptomatic (n=24)
control (n=72)
ATBTreatment

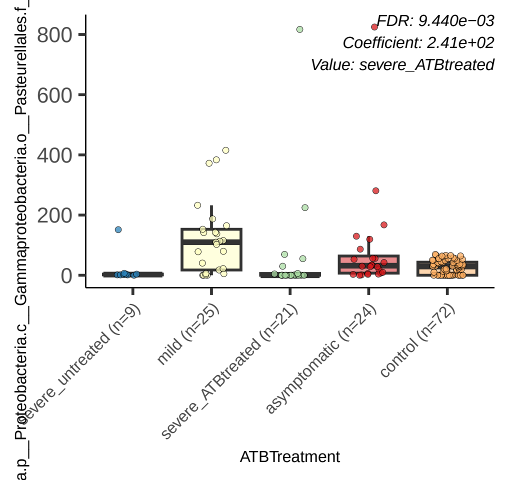

FDR: 9.440e−03
Coefficient: 2.41e+02
Value: severe_ATBtreated
a.p__Proteobacteria.c__Gammaproteobacteria.o__Pasteurellales.f
800
600
400
200
0
severe_untreated (n=9)
mild (n=25)
severe_ATBtreated (n=21)
asymptomatic (n=24)
control (n=72)
ATBTreatment

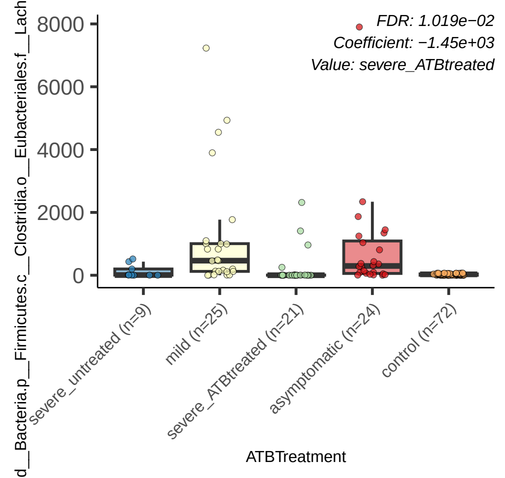
FDR: 1.019e−02
Coefficient: −1.45e+03
Value: severe_ATBtreated
8000
6000
4000
2000
0
d__Bacteria.p__Firmicutes.c__Clostridia.o__Eubacteriales.f__Lach
severe_untreated (n=9)
mild (n=25)
severe_ATBtreated (n=21)
asymptomatic (n=24)
control (n=72)
ATBTreatment

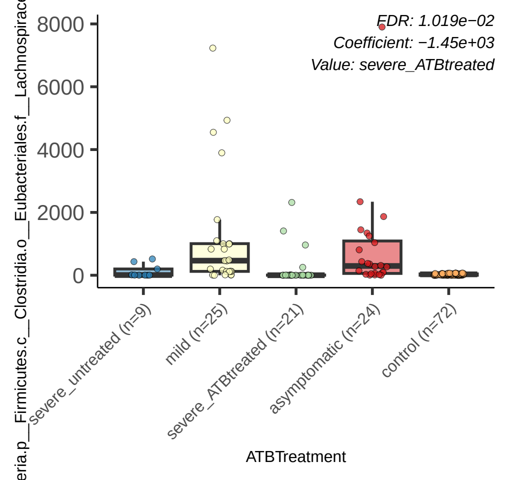

FDR: 1.019e−02
Coefficient: −1.45e+03
Value: severe_ATBtreated
8000
6000
4000
2000
0
severe_untreated (n=9)
mild (n=25)
severe_ATBtreated (n=21)
asymptomatic (n=24)
control (n=72)
ATBTreatment

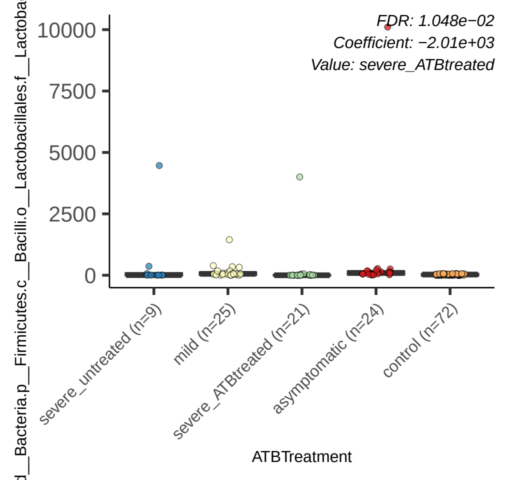
FDR: 1.048e−02
Coefficient: −2.01e+03
Value: severe_ATBtreated
10000
7500
5000
2500
0
severe_untreated (n=9)
mild (n=25)
severe_ATBtreated (n=21)
asymptomatic (n=24)
control (n=72)
ATBTreatment
d__Bacteria.p__Firmicutes.c__Bacilli.o__Lactobacillales.f__Lactoba

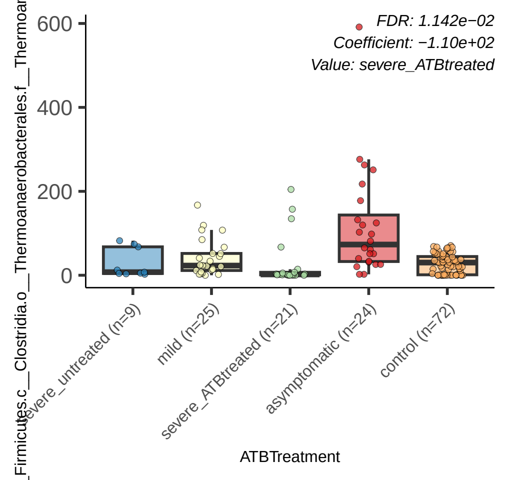
FDR: 1.142e−02
Coefficient: −1.10e+02
Value: severe_ATBtreated
600
400
200
0
Firmicutes.c__Clostridia.o__Thermoanaerobacterales.f__Thermoar
severe_untreated (n=9)
mild (n=25)
severe_ATBtreated (n=21)
asymptomatic (n=24)
control (n=72)
ATBTreatment

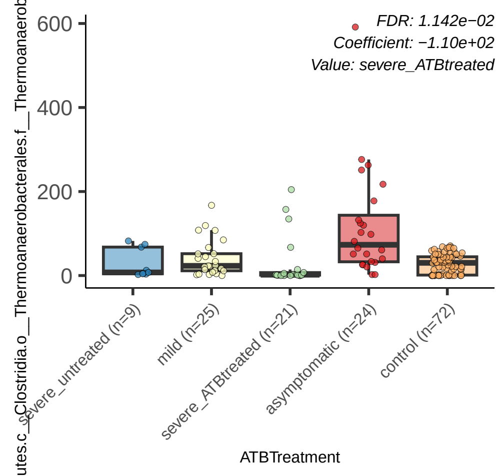
FDR: 1.142e−02
Coefficient: −1.10e+02
Value: severe_ATBtreated
600
400
200
0
cutes.c__Clostridia.o__Thermoanaerobacterales.f__Thermoanaerob
severe_untreated (n=9)
mild (n=25)
severe_ATBtreated (n=21)
asymptomatic (n=24)
control (n=72)
ATBTreatment

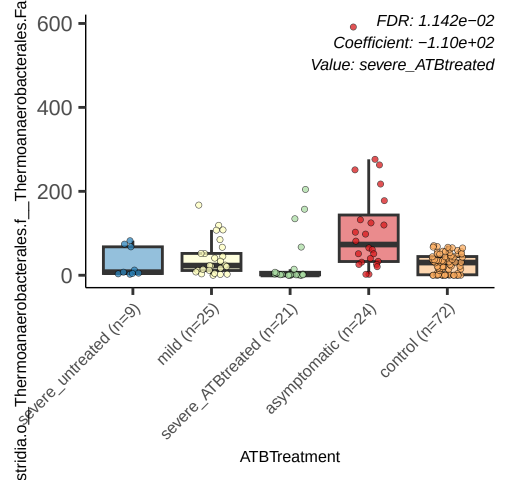

FDR: 1.142e−02
Coefficient: −1.10e+02
Value: severe_ATBtreated
600
400
200
0
stridia.o__Thermoanaerobacterales.f__Thermoanaerobacterales.Fa
severe_untreated (n=9)
mild (n=25)
severe_ATBtreated (n=21)
asymptomatic (n=24)
control (n=72)
ATBTreatment

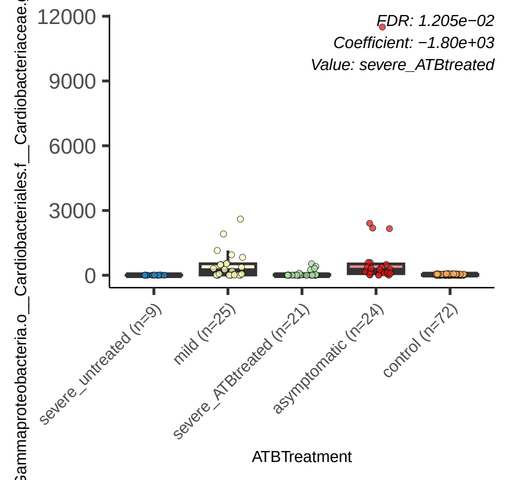

FDR: 1.205e−02
Coefficient: −1.80e+03
Value: severe_ATBtreated
12000
9000
6000
3000
0
Gammaproteobacteria.o__Cardiobacteriales.f__Cardiobacteriaceae.g
severe_untreated (n=9)
mild (n=25)
severe_ATBtreated (n=21)
asymptomatic (n=24)
control (n=72)
ATBTreatment

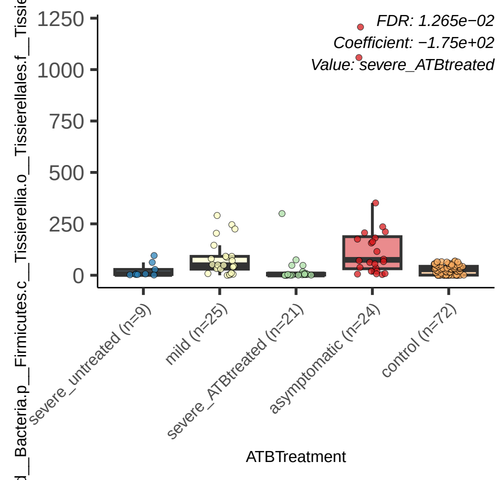

FDR: 1.265e−02
Coefficient: −1.75e+02
Value: severe_ATBtreated
1250
1000
750
500
250
0
d__Bacteria.p__Firmicutes.c__Tissierellia.o__Tissierellales.f__Tissie
severe_untreated (n=9)
mild (n=25)
severe_ATBtreated (n=21)
asymptomatic (n=24)
control (n=72)
ATBTreatment

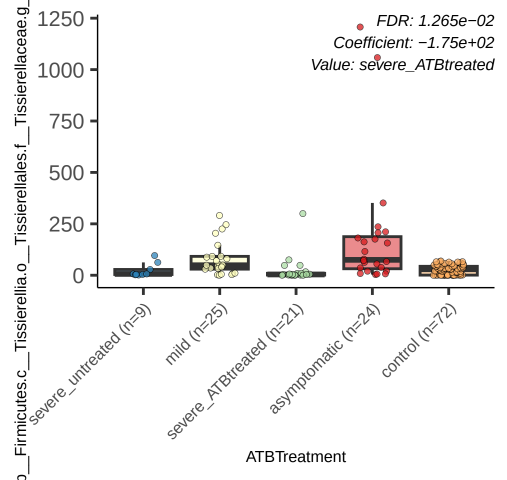

FDR: 1.265e−02
Coefficient: −1.75e+02
Value: severe_ATBtreated
o__Firmicutes.c__Tissierellia.o__Tissierellales.f__Tissierellaceae.g_
1250
1000
750
500
250
0
severe_untreated (n=9)
mild (n=25)
severe_ATBtreated (n=21)
asymptomatic (n=24)
control (n=72)
ATBTreatment

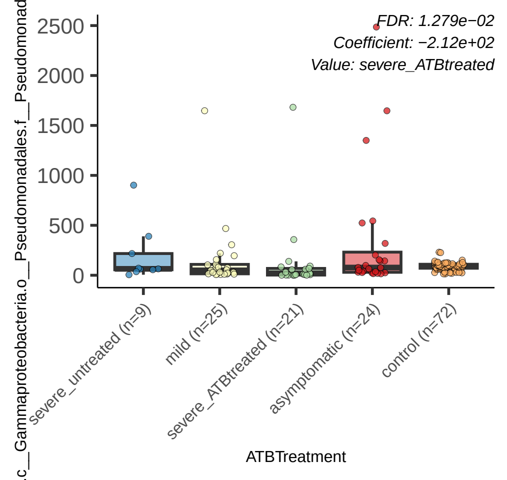

FDR: 1.279e−02
Coefficient: −2.12e+02
Value: severe_ATBtreated
c__Gammaproteobacteria.o__Pseudomonadales.f__Pseudomonad
2500
2000
1500
1000
500
0
severe_untreated (n=9)
mild (n=25)
severe_ATBtreated (n=21)
asymptomatic (n=24)
control (n=72)
ATBTreatment

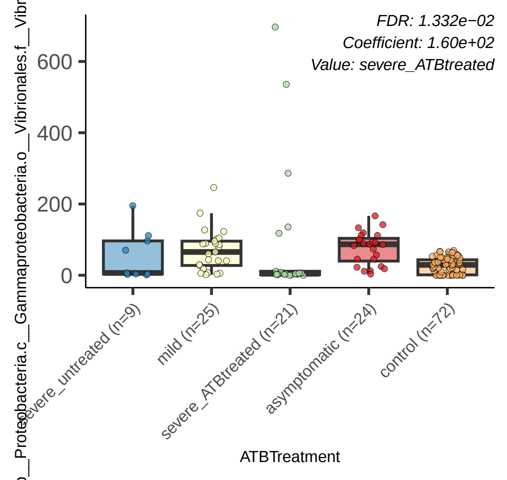

FDR: 1.332e−02
Coefficient: 1.60e+02
Value: severe_ATBtreated
600
400
200
0
o__Proteobacteria.c__Gammaproteobacteria.o__Vibrionales.f__Vibr
severe_untreated (n=9)
mild (n=25)
severe_ATBtreated (n=21)
asymptomatic (n=24)
control (n=72)
ATBTreatment

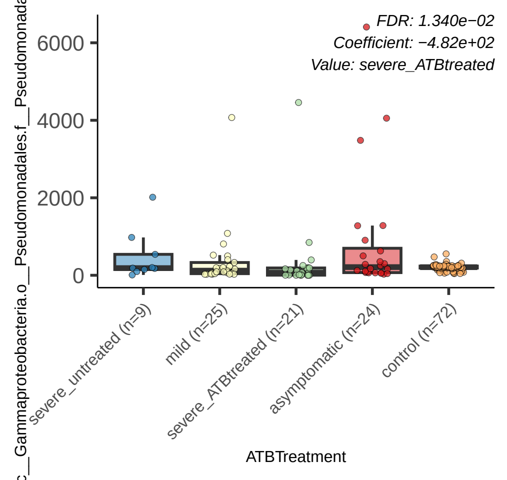
FDR: 1.340e−02
Coefficient: −4.82e+02
Value: severe_ATBtreated
c__Gammaproteobacteria.o__Pseudomonadales.f__Pseudomonada
6000
4000
2000
0
severe_untreated (n=9)
mild (n=25)
severe_ATBtreated (n=21)
asymptomatic (n=24)
control (n=72)
ATBTreatment

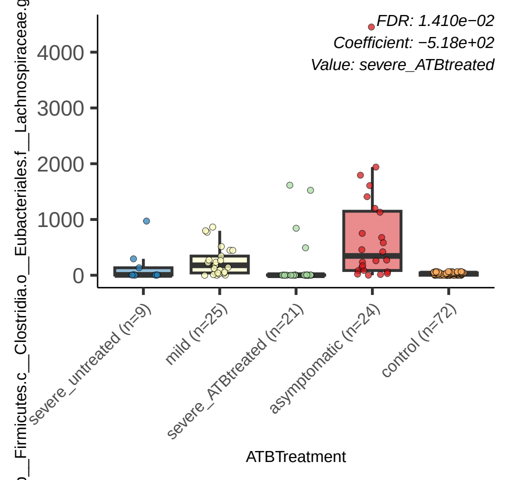
FDR: 1.410e−02
Coefficient: −5.18e+02
Value: severe_ATBtreated
0
1000
2000
3000
4000
o__Firmicutes.c__Clostridia.o__Eubacteriales.f__Lachnospiraceae.g
severe_untreated (n=9)
mild (n=25)
severe_ATBtreated (n=21)
asymptomatic (n=24)
control (n=72)
ATBTreatment

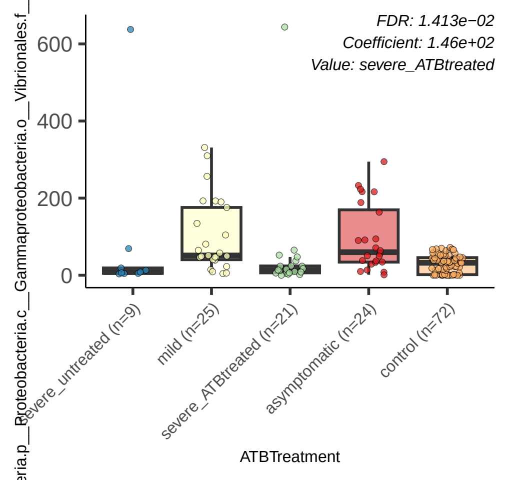
FDR: 1.413e−02
Coefficient: 1.46e+02
Value: severe_ATBtreated
eria.p__Proteobacteria.c__Gammaproteobacteria.o__Vibrionales.f__
600
400
200
0
severe_untreated (n=9)
mild (n=25)
severe_ATBtreated (n=21)
asymptomatic (n=24)
control (n=72)
ATBTreatment

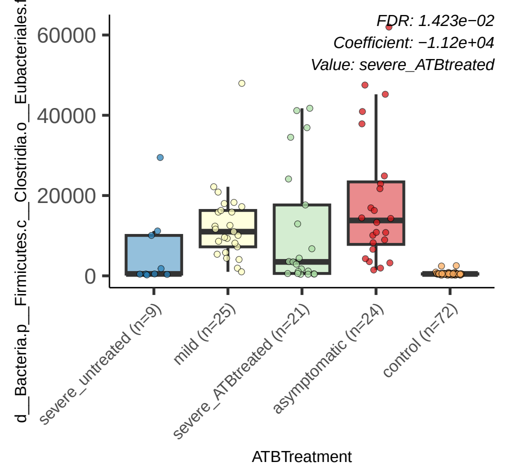
FDR: 1.423e−02
Coefficient: −1.12e+04
Value: severe_ATBtreated
d__Bacteria.p__Firmicutes.c__Clostridia.o__Eubacteriales.f
60000
40000
20000
0
severe_untreated (n=9)
mild (n=25)
severe_ATBtreated (n=21)
asymptomatic (n=24)
control (n=72)
ATBTreatment

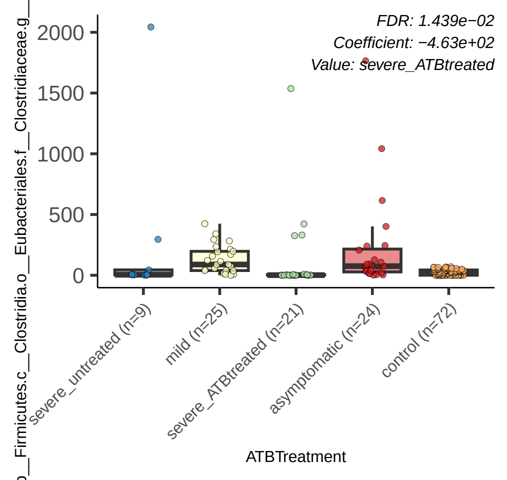

FDR: 1.439e−02
Coefficient: −4.63e+02
Value: severe_ATBtreated
Firmicutes.c__Clostridia.o__Eubacteriales.f__Clostridiaceae.g__
2000
1500
1000
500
0
severe_untreated (n=9)
mild (n=25)
severe_ATBtreated (n=21)
asymptomatic (n=24)
control (n=72)
ATBTreatment

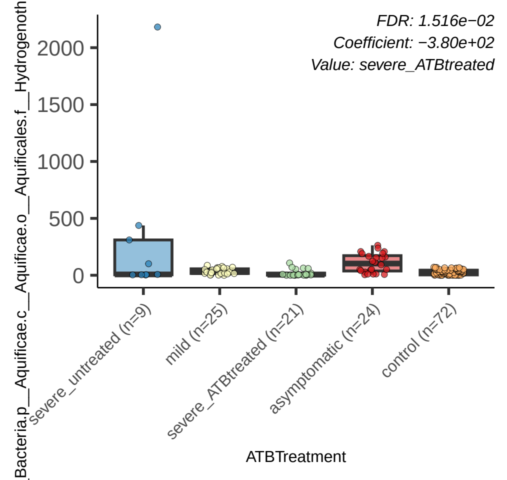
FDR: 1.516e−02
Coefficient: −3.80e+02
Value: severe_ATBtreated
Bacteria.p__Aquificae.c__Aquificae.o__Aquificales.f__Hydrogenoth
2000
1500
1000
500
0
severe_untreated (n=9)
mild (n=25)
severe_ATBtreated (n=21)
asymptomatic (n=24)
control (n=72)
ATBTreatment

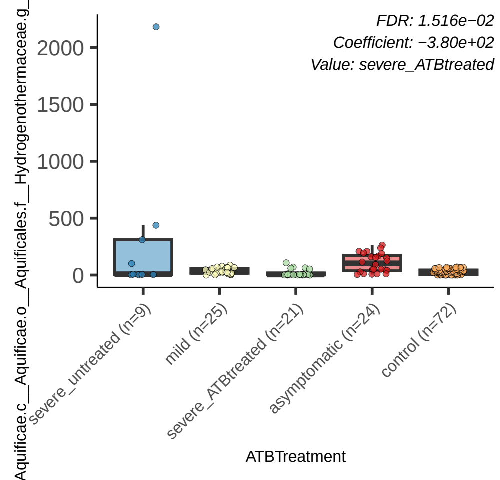

FDR: 1.516e−02
Coefficient: −3.80e+02
Value: severe_ATBtreated
Aquificae.c__Aquificae.o__Aquificales.f__Hydrogenothermaceae.g__
2000
1500
1000
500
0
severe_untreated (n=9)
mild (n=25)
severe_ATBtreated (n=21)
asymptomatic (n=24)
control (n=72)
ATBTreatment

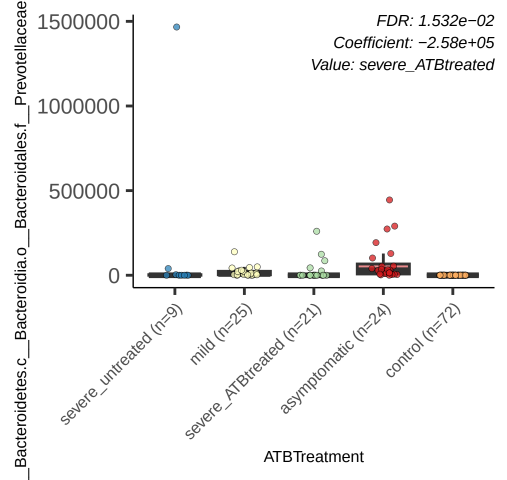

FDR: 1.532e−02
Coefficient: −2.58e+05
Value: severe_ATBtreated
1500000
1000000
500000
0
Bacteroidetes.c__Bacteroidia.o__Bacteroidales.f__Prevotellaceae
severe_untreated (n=9)
mild (n=25)
severe_ATBtreated (n=21)
asymptomatic (n=24)
control (n=72)
ATBTreatment

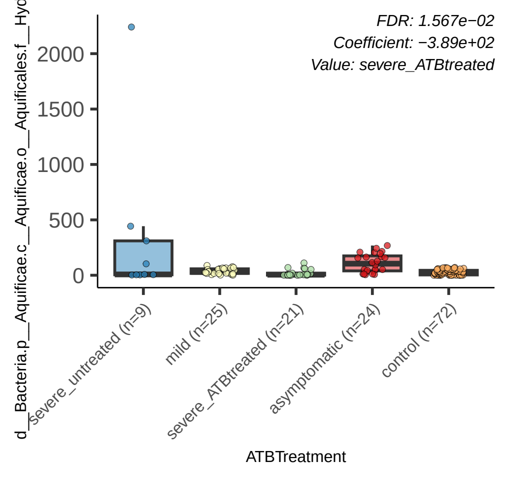

FDR: 1.567e−02
Coefficient: −3.89e+02
Value: severe_ATBtreated
d__Bacteria.p__Aquificae.c__Aquificae.o__Aquificales.f__Hyd
2000
1500
1000
500
0
severe_untreated (n=9)
mild (n=25)
severe_ATBtreated (n=21)
asymptomatic (n=24)
control (n=72)
ATBTreatment

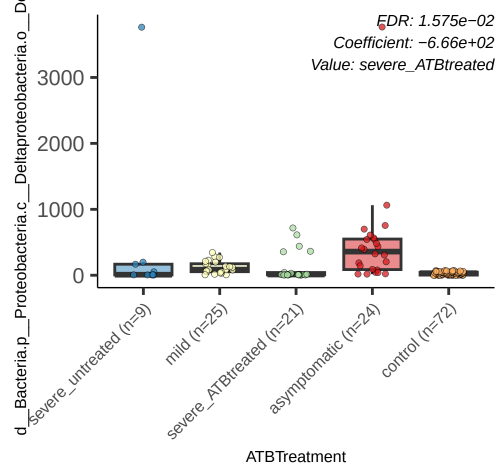

FDR: 1.575e−02
Coefficient: −6.66e+02
Value: severe_ATBtreated
d__Bacteria.p__Proteobacteria.c__Deltaproteobacteria.o__De
3000
2000
1000
0
severe_untreated (n=9)
mild (n=25)
severe_ATBtreated (n=21)
asymptomatic (n=24)
control (n=72)
ATBTreatment

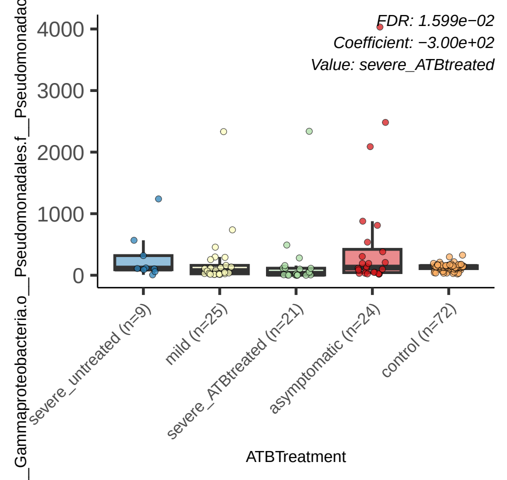

FDR: 1.599e−02
Coefficient: −3.00e+02
Value: severe_ATBtreated
Gammaproteobacteria.o__Pseudomonadales.f__Pseudomonadac
4000
3000
2000
1000
0
severe_untreated (n=9)
mild (n=25)
severe_ATBtreated (n=21)
asymptomatic (n=24)
control (n=72)
ATBTreatment

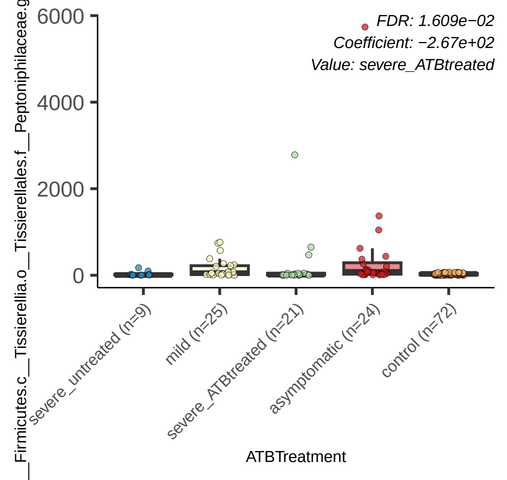

Firmicutes.c__Tissierellia.o__Tissierellales.f__Peptoniphilaceae.g
6000
4000
2000
0
FDR: 1.609e−02
Coefficient: −2.67e+02
Value: severe_ATBtreated
severe_untreated (n=9)
mild (n=25)
severe_ATBtreated (n=21)
asymptomatic (n=24)
control (n=72)
ATBTreatment

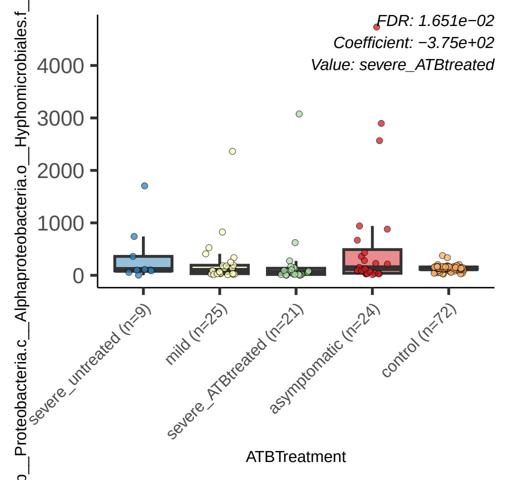

FDR: 1.651e−02
Coefficient: −3.75e+02
Value: severe_ATBtreated
o__Proteobacteria.c__Alphaproteobacteria.o__Hyphomicrobiales.f__
0
1000
2000
3000
4000
severe_untreated (n=9)
mild (n=25)
severe_ATBtreated (n=21)
asymptomatic (n=24)
control (n=72)
ATBTreatment

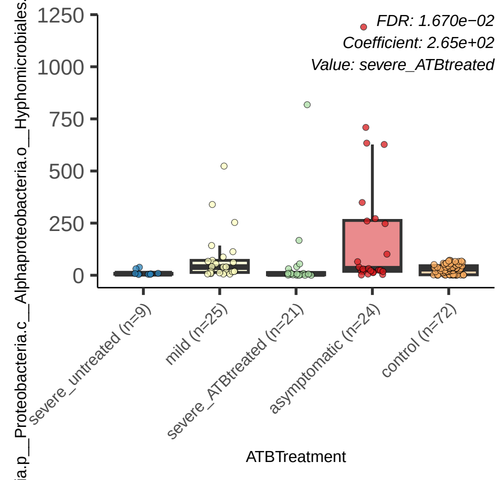

FDR: 1.670e−02
Coefficient: 2.65e+02
Value: severe_ATBtreated
ia.p__Proteobacteria.c__Alphaproteobacteria.o__Hyphomicrobiales.
1250
1000
750
500
250
0
severe_untreated (n=9)
mild (n=25)
severe_ATBtreated (n=21)
asymptomatic (n=24)
control (n=72)
ATBTreatment

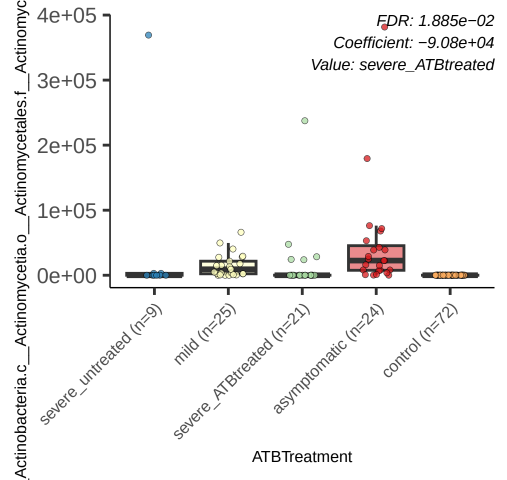
FDR: 1.885e−02
Coefficient: −9.08e+04
Value: severe_ATBtreated
Actinobacteria.c__Actinomycetia.o__Actinomycetales.f__Actinomyc
4e+05
3e+05
2e+05
1e+05
0e+00
severe_untreated (n=9)
mild (n=25)
severe_ATBtreated (n=21)
asymptomatic (n=24)
control (n=72)
ATBTreatment

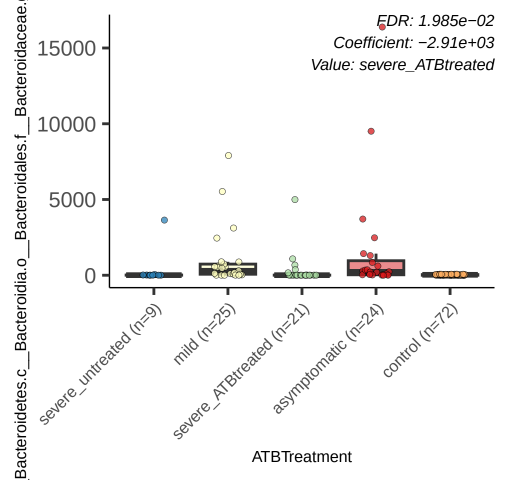

FDR: 1.985e−02
Coefficient: −2.91e+03
Value: severe_ATBtreated
15000
10000
5000
0
severe_untreated (n=9)
mild (n=25)
severe_ATBtreated (n=21)
asymptomatic (n=24)
control (n=72)
ATBTreatment
Bacteroidetes.c__Bacteroidia.o__Bacteroidales.f__Bacteroidaceae.g

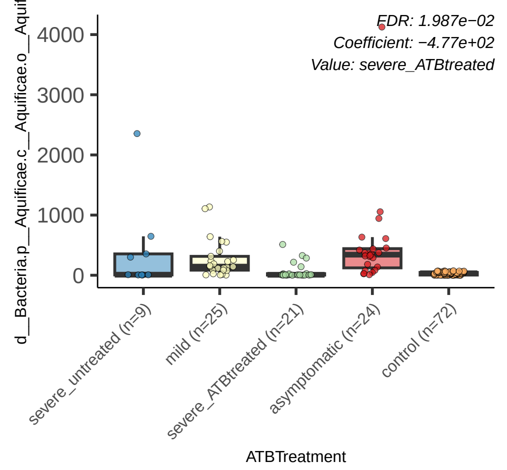
FDR: 1.987e−02
Coefficient: −4.77e+02
Value: severe_ATBtreated
d__Bacteria.p__Aquificae.c__Aquificae.o__Aquif
4000
3000
2000
1000
0
severe_untreated (n=9)
mild (n=25)
severe_ATBtreated (n=21)
asymptomatic (n=24)
control (n=72)
ATBTreatment

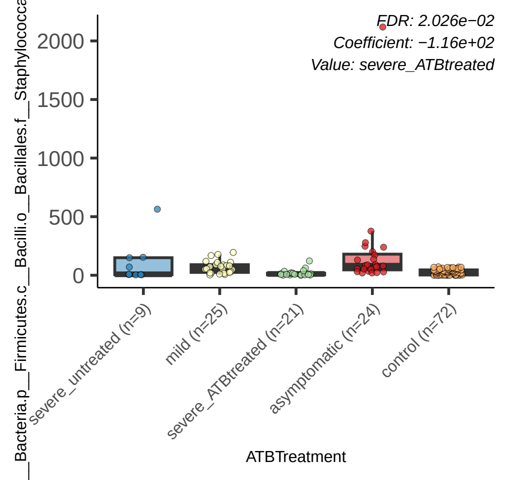
FDR: 2.026e−02
Coefficient: −1.16e+02
Value: severe_ATBtreated
2000
1500
1000
500
0
_Bacteria.p__Firmicutes.c__Bacilli.o__Bacillales.f__Staphylococca
severe_untreated (n=9)
mild (n=25)
severe_ATBtreated (n=21)
asymptomatic (n=24)
control (n=72)
ATBTreatment

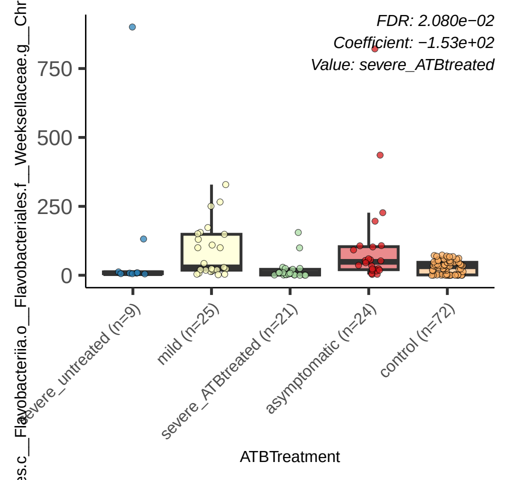

FDR: 2.080e−02
Coefficient: −1.53e+02
Value: severe_ATBtreated
750
500
250
0
es.c__Flavobacteriia.o__Flavobacteriales.f__Weeksellaceae.g__Chr
severe_untreated (n=9)
mild (n=25)
severe_ATBtreated (n=21)
asymptomatic (n=24)
control (n=72)
ATBTreatment

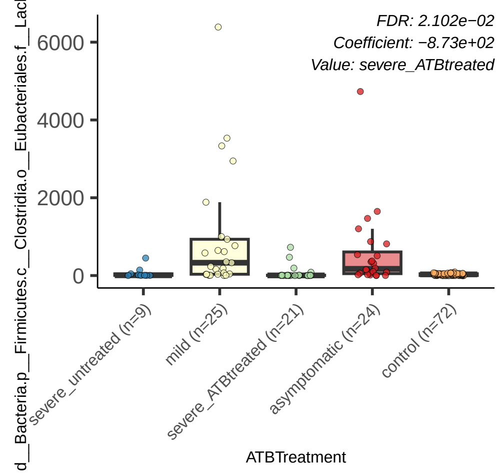

d__Bacteria.p__Firmicutes.c__Clostridia.o__Eubacteriales.f__Lac
FDR: 2.102e−02
Coefficient: −8.73e+02
Value: severe_ATBtreated
6000
4000
2000
0
severe_untreated (n=9)
mild (n=25)
severe_ATBtreated (n=21)
asymptomatic (n=24)
control (n=72)
ATBTreatment

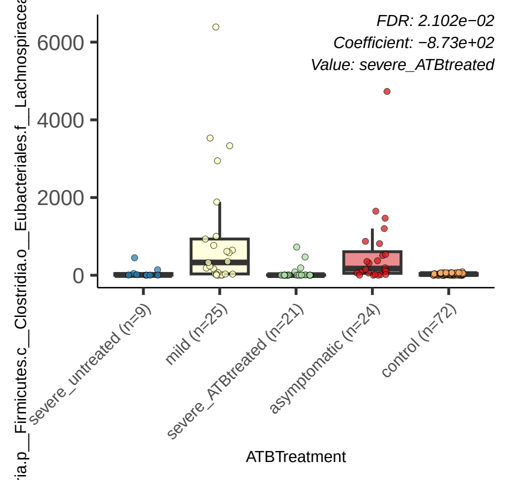

FDR: 2.102e−02
Coefficient: −8.73e+02
Value: severe_ATBtreated
ria.p__Firmicutes.c__Clostridia.o__Eubacteriales.f__Lachnospiracea
6000
4000
2000
0
severe_untreated (n=9)
mild (n=25)
severe_ATBtreated (n=21)
asymptomatic (n=24)
control (n=72)
ATBTreatment

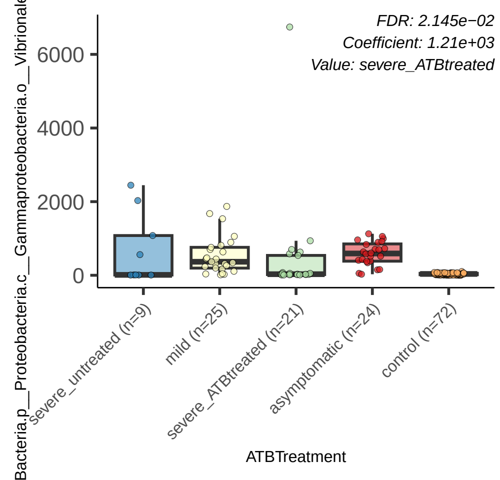

FDR: 2.145e−02
Coefficient: 1.21e+03
Value: severe_ATBtreated
Bacteria.p__Proteobacteria.c__Gammaproteobacteria.o__Vibrionale
6000
4000
2000
0
severe_untreated (n=9)
mild (n=25)
severe_ATBtreated (n=21)
asymptomatic (n=24)
control (n=72)
ATBTreatment

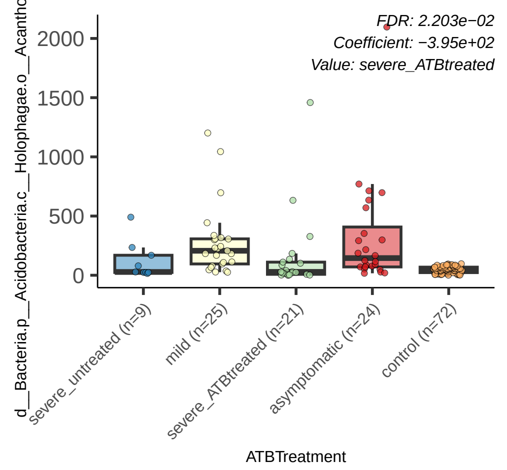

FDR: 2.203e−02
Coefficient: −3.95e+02
Value: severe_ATBtreated
2000
1500
1000
500
0
d__Bacteria.p__Acidobacteria.c__Holophagae.o__Acantho
severe_untreated (n=9)
mild (n=25)
severe_ATBtreated (n=21)
asymptomatic (n=24)
control (n=72)
ATBTreatment

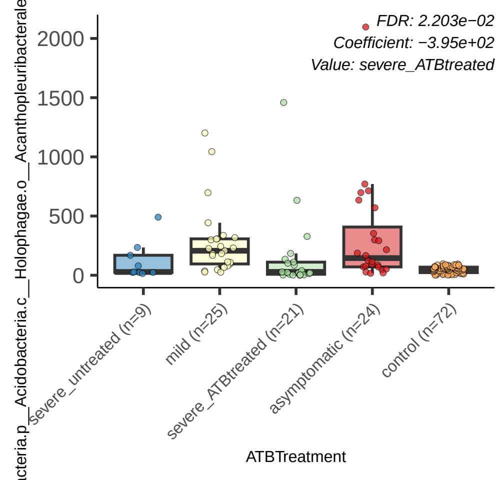
FDR: 2.203e−02
Coefficient: −3.95e+02
Value: severe_ATBtreated
2000
1500
1000
500
0
acteria.p__Acidobacteria.c__Holophagae.o__Acanthopleuribacterale
severe_untreated (n=9)
mild (n=25)
severe_ATBtreated (n=21)
asymptomatic (n=24)
control (n=72)
ATBTreatment

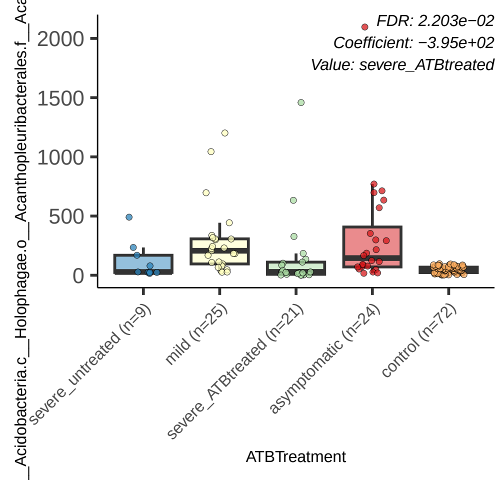

FDR: 2.203e−02
Coefficient: −3.95e+02
Value: severe_ATBtreated
2000
1500
1000
500
0
Acidobacteria.c__Holophagae.o__Acanthopleuribacterales.f__Aca
severe_untreated (n=9)
mild (n=25)
severe_ATBtreated (n=21)
asymptomatic (n=24)
control (n=72)
ATBTreatment

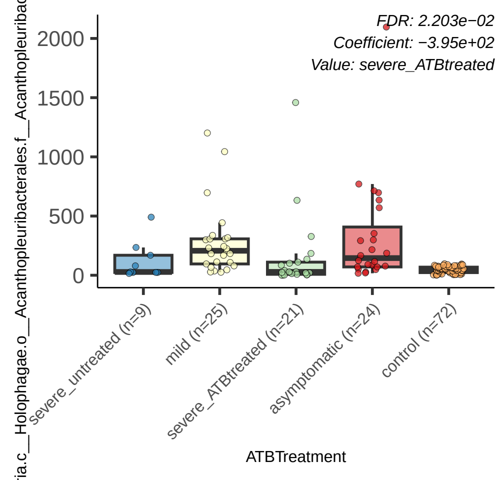
FDR: 2.203e−02
Coefficient: −3.95e+02
Value: severe_ATBtreated
2000
1500
1000
500
0
severe_untreated (n=9)
mild (n=25)
severe_ATBtreated (n=21)
asymptomatic (n=24)
control (n=72)
ATBTreatment
ria.c__Holophagae.o__Acanthopleuribacterales.f__Acanthopleuribac

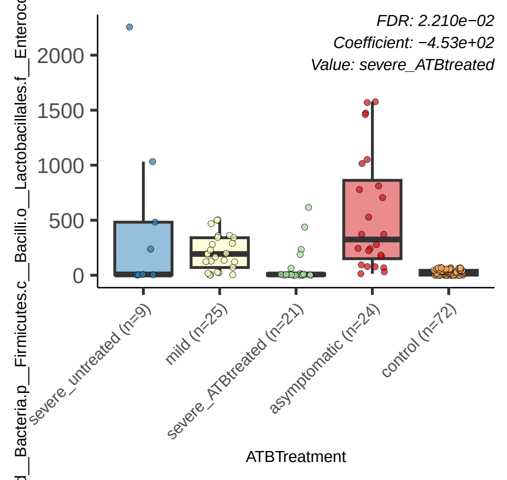
FDR: 2.210e−02
Coefficient: −4.53e+02
Value: severe_ATBtreated
d__Bacteria.p__Firmicutes.c__Bacilli.o__Lactobacillales.f__Enteroco
2000
1500
1000
500
0
severe_untreated (n=9)
mild (n=25)
severe_ATBtreated (n=21)
asymptomatic (n=24)
control (n=72)
ATBTreatment

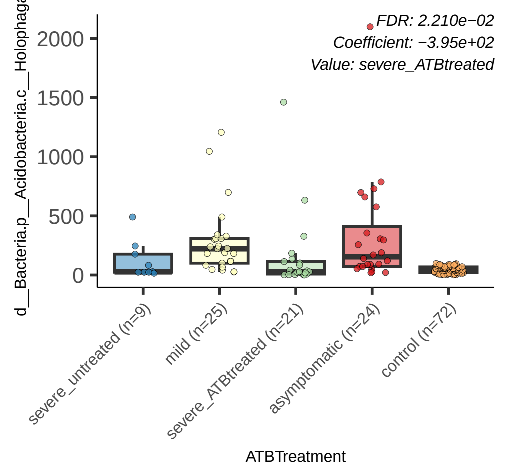

FDR: 2.210e−02
Coefficient: −3.95e+02
Value: severe_ATBtreated
d__Bacteria.p__Acidobacteria.c__Holophaga
2000
1500
1000
500
0
severe_untreated (n=9)
mild (n=25)
severe_ATBtreated (n=21)
asymptomatic (n=24)
control (n=72)
ATBTreatment

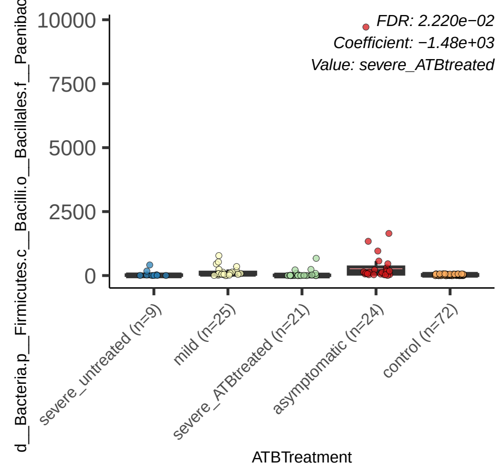

FDR: 2.220e−02
Coefficient: −1.48e+03
Value: severe_ATBtreated
d__Bacteria.p__Firmicutes.c__Bacilli.o__Bacillales.f__Paenibac
10000
7500
5000
2500
0
severe_untreated (n=9)
mild (n=25)
severe_ATBtreated (n=21)
asymptomatic (n=24)
control (n=72)
ATBTreatment

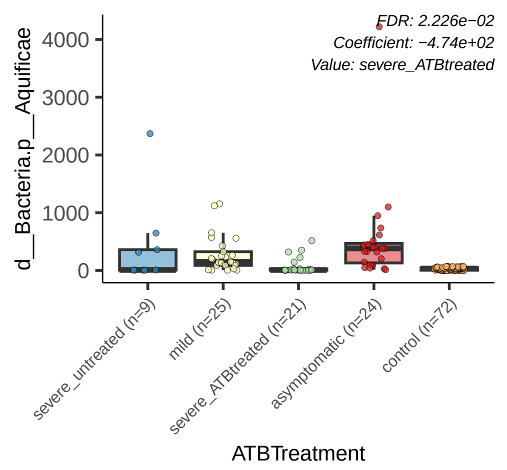

FDR: 2.226e−02
Coefficient: −4.74e+02
Value: severe_ATBtreated
d__Bacteria.p__Aquificae
4000
3000
2000
1000
0
severe_untreated (n=9)
mild (n=25)
severe_ATBtreated (n=21)
asymptomatic (n=24)
control (n=72)
ATBTreatment

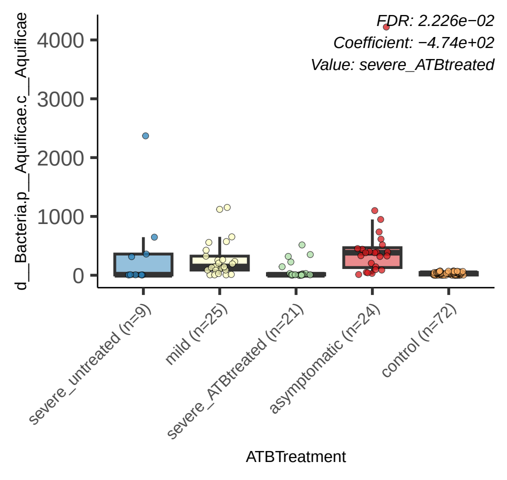

FDR: 2.226e−02
Coefficient: −4.74e+02
Value: severe_ATBtreated
d__Bacteria.p__Aquificae.c__Aquificae
4000
3000
2000
1000
0
severe_untreated (n=9)
mild (n=25)
severe_ATBtreated (n=21)
asymptomatic (n=24)
control (n=72)
ATBTreatment

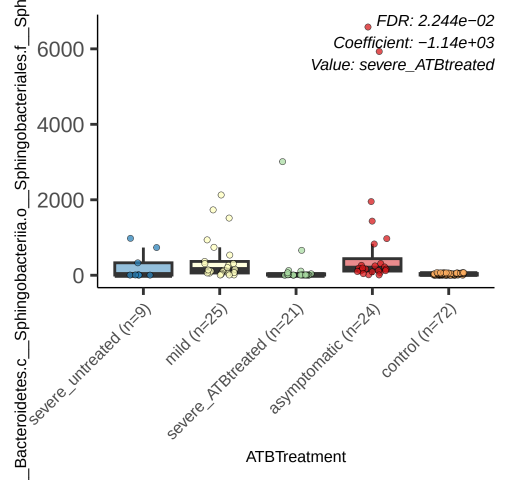
FDR: 2.244e−02
Coefficient: −1.14e+03
Value: severe_ATBtreated
0
2000
4000
6000
Bacteroidetes.c__Sphingobacteriia.o__Sphingobacteriales.f__Sph
severe_untreated (n=9)
mild (n=25)
severe_ATBtreated (n=21)
asymptomatic (n=24)
control (n=72)
ATBTreatment

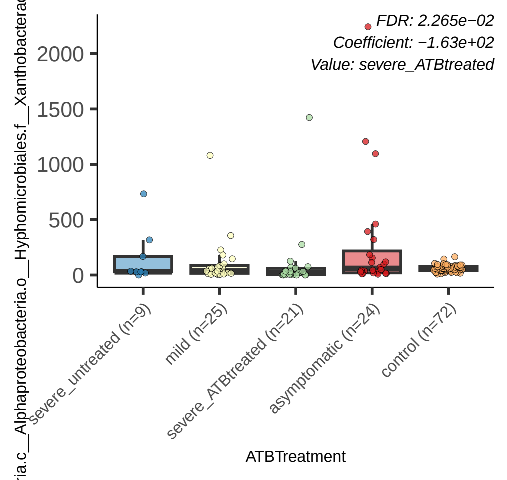
FDR: 2.265e−02
Coefficient: −1.63e+02
Value: severe_ATBtreated
2000
1500
1000
500
0
ria.c__Alphaproteobacteria.o__Hyphomicrobiales.f__Xanthobacterac
severe_untreated (n=9)
mild (n=25)
severe_ATBtreated (n=21)
asymptomatic (n=24)
control (n=72)
ATBTreatment

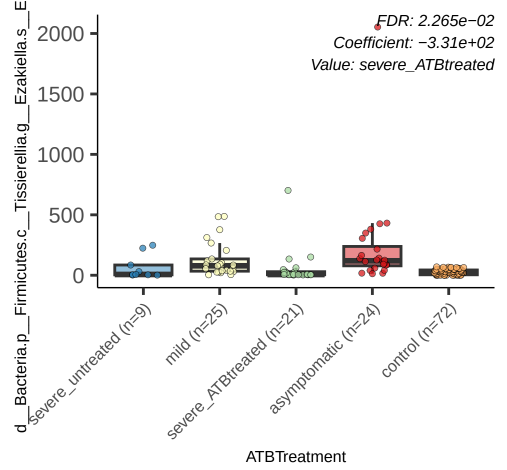
FDR: 2.265e−02
Coefficient: −3.31e+02
Value: severe_ATBtreated
d__Bacteria.p__Firmicutes.c__Tissierellia.g__Ezakiella.s__E
2000
1500
1000
500
0
severe_untreated (n=9)
mild (n=25)
severe_ATBtreated (n=21)
asymptomatic (n=24)
control (n=72)
ATBTreatment

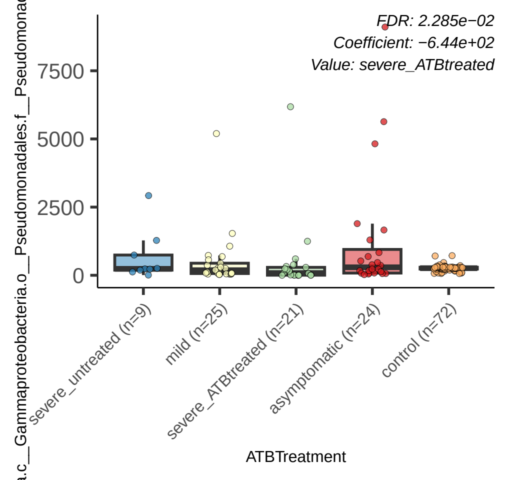

FDR: 2.285e−02
Coefficient: −6.44e+02
Value: severe_ATBtreated
7500
5000
2500
0
a.c__Gammaproteobacteria.o__Pseudomonadales.f__Pseudomonad
severe_untreated (n=9)
mild (n=25)
severe_ATBtreated (n=21)
asymptomatic (n=24)
control (n=72)
ATBTreatment

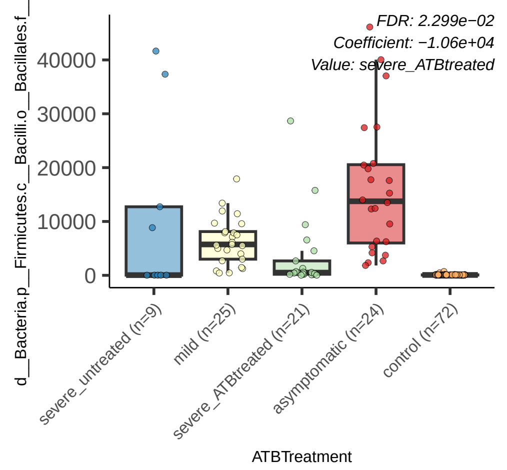

FDR: 2.299e−02
Coefficient: −1.06e+04
Value: severe_ATBtreated
d__Bacteria.p__Firmicutes.c__Bacilli.o__Bacillales.f__
40000
30000
20000
10000
0
severe_untreated (n=9)
mild (n=25)
severe_ATBtreated (n=21)
asymptomatic (n=24)
control (n=72)
ATBTreatment

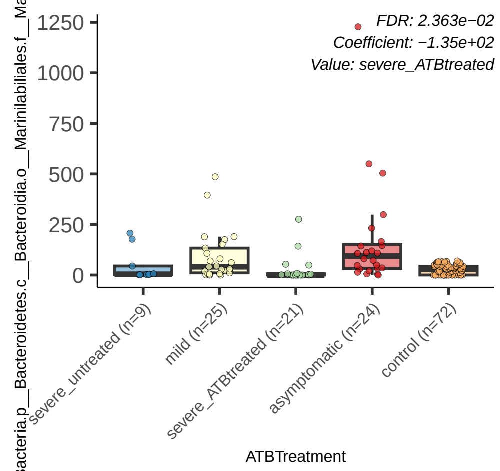

FDR: 2.363e−02
Coefficient: −1.35e+02
Value: severe_ATBtreated
Bacteria.p__Bacteroidetes.c__Bacteroidia.o__Marinilabiliales.f__Ma
1250
1000
750
500
250
0
severe_untreated (n=9)
mild (n=25)
severe_ATBtreated (n=21)
asymptomatic (n=24)
control (n=72)
ATBTreatment

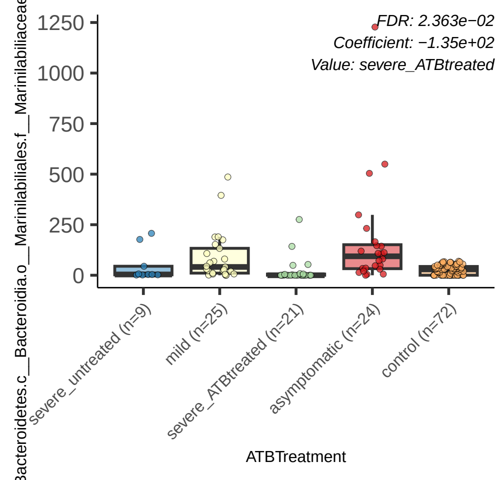

FDR: 2.363e−02
Coefficient: −1.35e+02
Value: severe_ATBtreated
1250
1000
750
500
250
0
Bacteroidetes.c__Bacteroidia.o__Marinilabiliales.f__Marinilabiliaceae
severe_untreated (n=9)
mild (n=25)
severe_ATBtreated (n=21)
asymptomatic (n=24)
control (n=72)
ATBTreatment

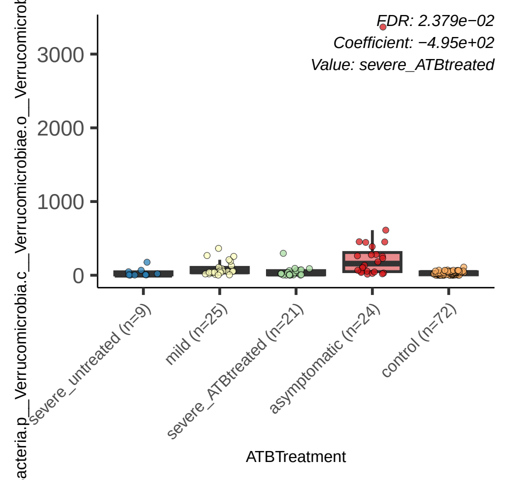

FDR: 2.379e−02
Coefficient: −4.95e+02
Value: severe_ATBtreated
3000
2000
1000
0
acteria.p__Verrucomicrobia.c__Verrucomicrobiae.o__Verrucomicrob
severe_untreated (n=9)
mild (n=25)
severe_ATBtreated (n=21)
asymptomatic (n=24)
control (n=72)
ATBTreatment

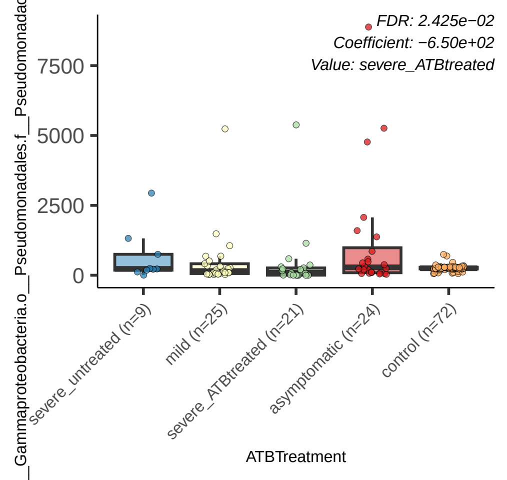
FDR: 2.425e−02
Coefficient: −6.50e+02
Value: severe_ATBtreated
_Gammaproteobacteria.o__Pseudomonadales.f__Pseudomonadac
7500
5000
2500
0
severe_untreated (n=9)
mild (n=25)
severe_ATBtreated (n=21)
asymptomatic (n=24)
control (n=72)
ATBTreatment

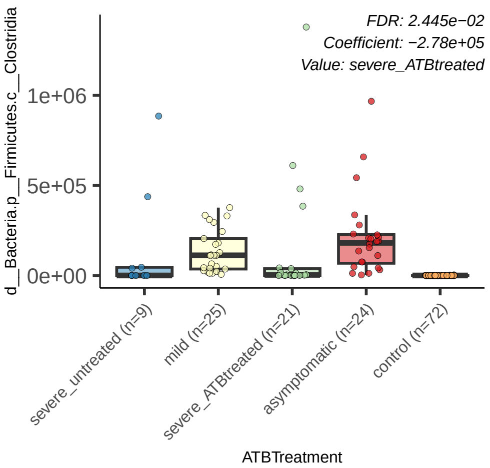

FDR: 2.445e−02
Coefficient: −2.78e+05
Value: severe_ATBtreated
d__Bacteria.p__Firmicutes.c__Clostridia
1e+06
5e+05
0e+00
severe_untreated (n=9)
mild (n=25)
severe_ATBtreated (n=21)
asymptomatic (n=24)
control (n=72)
ATBTreatment

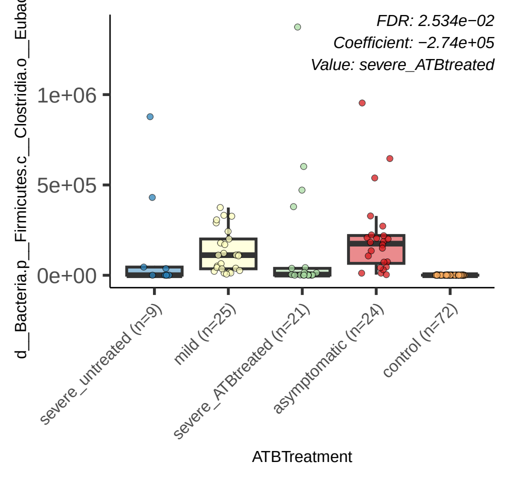

FDR: 2.534e−02
Coefficient: −2.74e+05
Value: severe_ATBtreated
1e+06
5e+05
0e+00
d__Bacteria.p__Firmicutes.c__Clostridia.o__Eubac
severe_untreated (n=9)
mild (n=25)
severe_ATBtreated (n=21)
asymptomatic (n=24)
control (n=72)
ATBTreatment

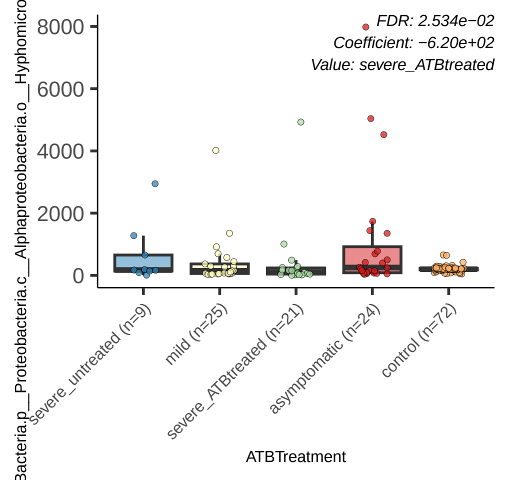

FDR: 2.534e−02
Coefficient: −6.20e+02
Value: severe_ATBtreated
8000
6000
4000
2000
0
Bacteria.p__Proteobacteria.c__Alphaproteobacteria.o__Hyphomicro
severe_untreated (n=9)
mild (n=25)
severe_ATBtreated (n=21)
asymptomatic (n=24)
control (n=72)
ATBTreatment

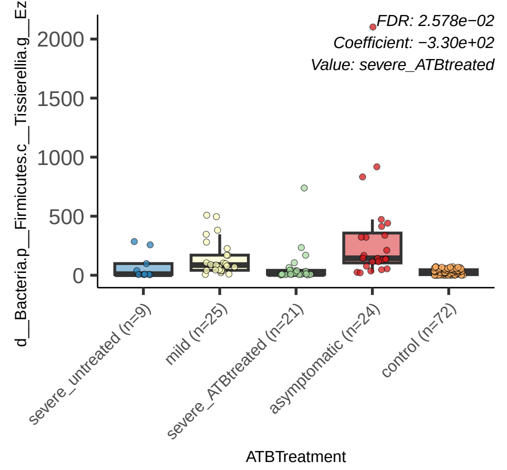
FDR: 2.578e−02
Coefficient: −3.30e+02
Value: severe_ATBtreated
d__Bacteria.p__Firmicutes.c__Tissierellia.g__Ez
2000
1500
1000
500
0
severe_untreated (n=9)
mild (n=25)
severe_ATBtreated (n=21)
asymptomatic (n=24)
control (n=72)
ATBTreatment

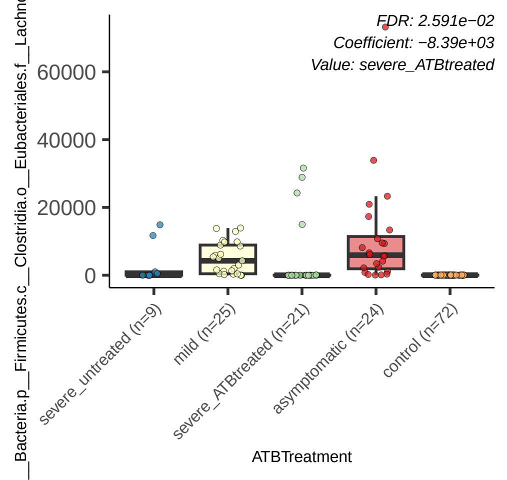

FDR: 2.591e−02
Coefficient: −8.39e+03
Value: severe_ATBtreated
Bacteria.p__Firmicutes.c__Clostridia.o__Eubacteriales.f__Lachno
60000
40000
20000
0
severe_untreated (n=9)
mild (n=25)
severe_ATBtreated (n=21)
asymptomatic (n=24)
control (n=72)
ATBTreatment

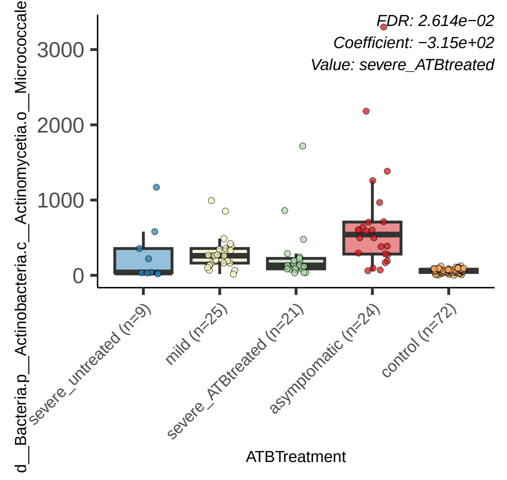

FDR: 2.614e−02
Coefficient: −3.15e+02
Value: severe_ATBtreated
3000
2000
1000
0
d__Bacteria.p__Actinobacteria.c__Actinomycetia.o__Micrococcale
severe_untreated (n=9)
mild (n=25)
severe_ATBtreated (n=21)
asymptomatic (n=24)
control (n=72)
ATBTreatment

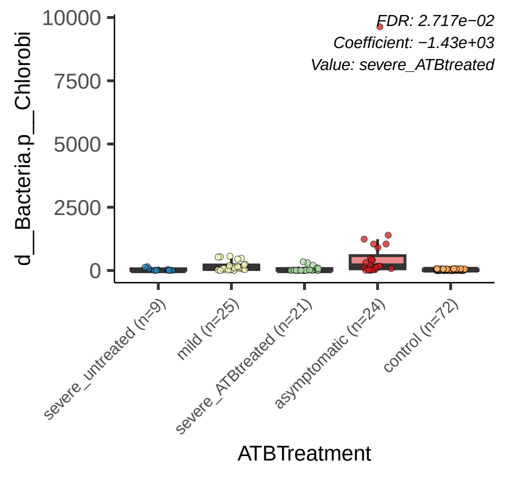

FDR: 2.717e−02
Coefficient: −1.43e+03
Value: severe_ATBtreated
d__Bacteria.p__Chlorobi
10000
7500
5000
2500
0
severe_untreated (n=9)
mild (n=25)
severe_ATBtreated (n=21)
asymptomatic (n=24)
control (n=72)
ATBTreatment

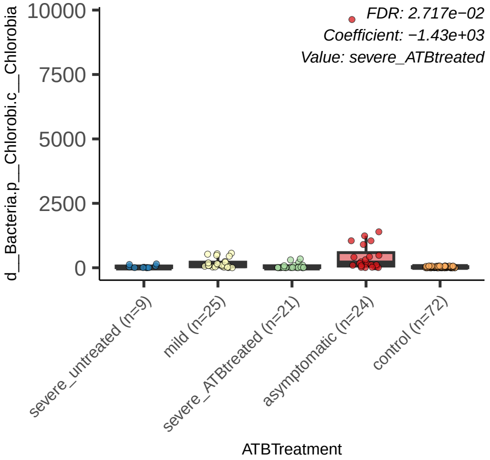
FDR: 2.717e−02
Coefficient: −1.43e+03
Value: severe_ATBtreated
d__Bacteria.p__Chlorobi.c__Chlorobia
10000
7500
5000
2500
0
severe_untreated (n=9)
mild (n=25)
severe_ATBtreated (n=21)
asymptomatic (n=24)
control (n=72)
ATBTreatment

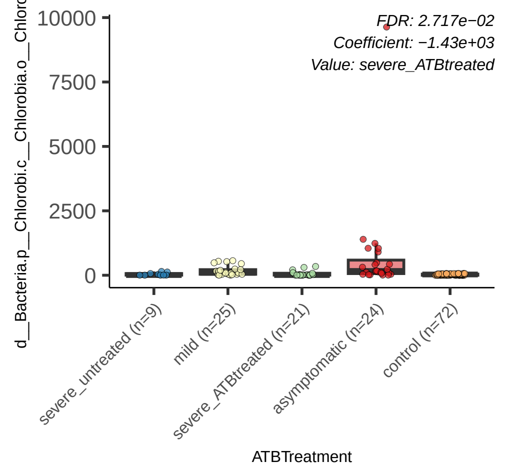

d__Bacteria.p__Chlorobi.c__Chlorobia.o__Chloro
10000
7500
5000
2500
0
FDR: 2.717e−02
Coefficient: −1.43e+03
Value: severe_ATBtreated
severe_untreated (n=9)
mild (n=25)
severe_ATBtreated (n=21)
asymptomatic (n=24)
control (n=72)
ATBTreatment

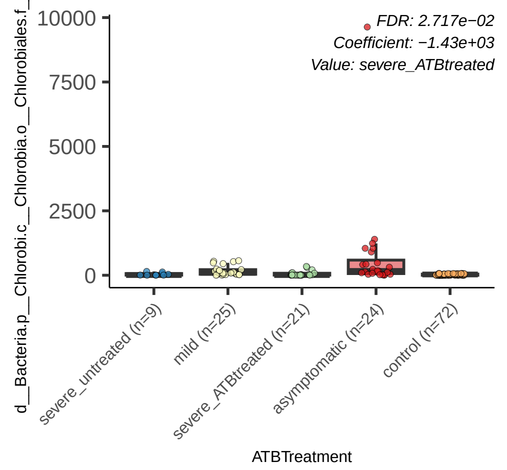

FDR: 2.717e−02
Coefficient: −1.43e+03
Value: severe_ATBtreated
10000
7500
5000
2500
0
d__Bacteria.p__Chlorobi.c__Chlorobia.o__Chlorobiales.f
severe_untreated (n=9)
mild (n=25)
severe_ATBtreated (n=21)
asymptomatic (n=24)
control (n=72)
ATBTreatment

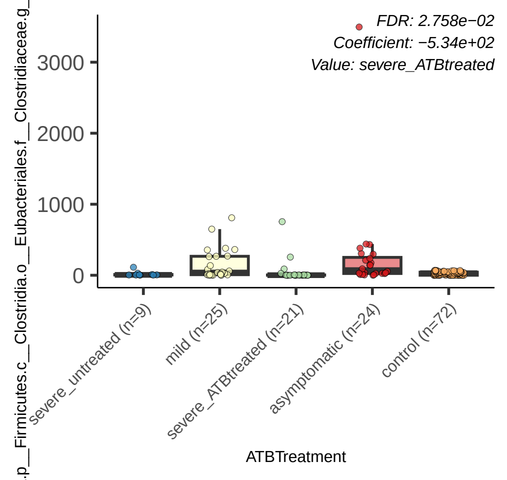

FDR: 2.758e−02
Coefficient: −5.34e+02
Value: severe_ATBtreated
3000
2000
1000
0
p__Firmicutes.c__Clostridia.o__Eubacteriales.f__Clostridiaceae.g__
severe_untreated (n=9)
mild (n=25)
severe_ATBtreated (n=21)
asymptomatic (n=24)
control (n=72)
ATBTreatment

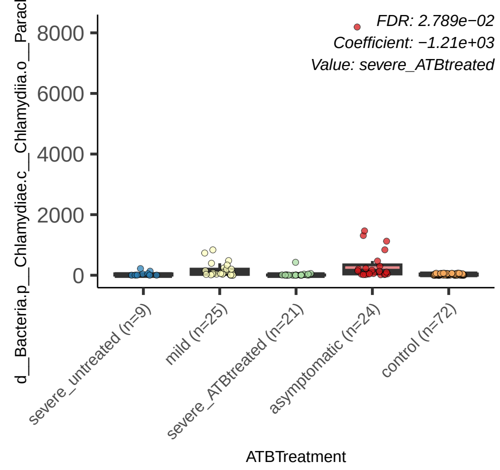

FDR: 2.789e−02
Coefficient: −1.21e+03
Value: severe_ATBtreated
d__Bacteria.p__Chlamydiae.c__Chlamydiia.o__Parach
8000
6000
4000
2000
0
severe_untreated (n=9)
mild (n=25)
severe_ATBtreated (n=21)
asymptomatic (n=24)
control (n=72)
ATBTreatment

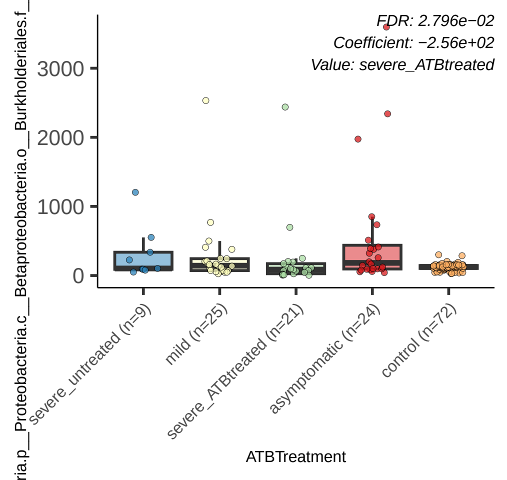

FDR: 2.796e−02
Coefficient: −2.56e+02
Value: severe_ATBtreated
3000
2000
1000
0
ria.p__Proteobacteria.c__Betaproteobacteria.o__Burkholderiales.f__
severe_untreated (n=9)
mild (n=25)
severe_ATBtreated (n=21)
asymptomatic (n=24)
control (n=72)
ATBTreatment

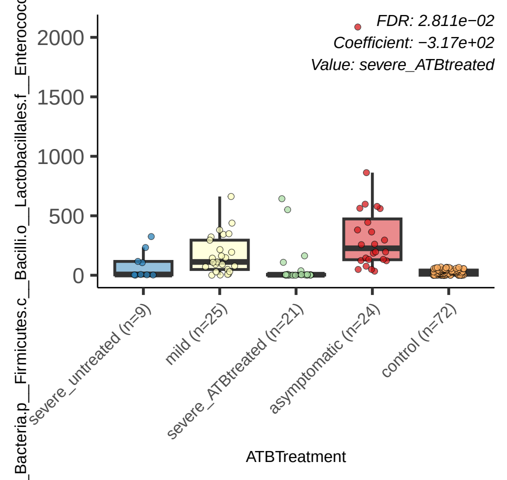

FDR: 2.811e−02
Coefficient: −3.17e+02
Value: severe_ATBtreated
2000
1500
1000
500
0
Bacteria.p__Firmicutes.c__Bacilli.o__Lactobacillales.f__Enterococc
severe_untreated (n=9)
mild (n=25)
severe_ATBtreated (n=21)
asymptomatic (n=24)
control (n=72)
ATBTreatment

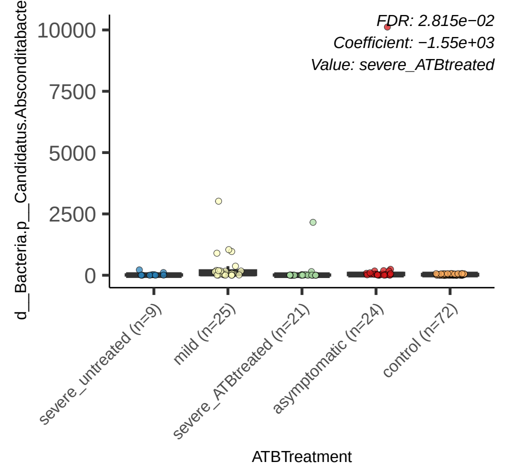

FDR: 2.815e−02
Coefficient: −1.55e+03
Value: severe_ATBtreated
d__Bacteria.p__Candidatus.Absconditabacte
10000
7500
5000
2500
0
severe_untreated (n=9)
mild (n=25)
severe_ATBtreated (n=21)
asymptomatic (n=24)
control (n=72)
ATBTreatment

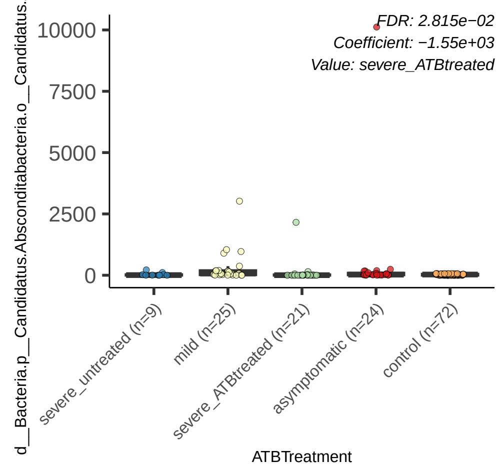
FDR: 2.815e−02
Coefficient: −1.55e+03
Value: severe_ATBtreated
10000
7500
5000
2500
0
d__Bacteria.p__Candidatus.Absconditabacteria.o__Candidatus.
severe_untreated (n=9)
mild (n=25)
severe_ATBtreated (n=21)
asymptomatic (n=24)
control (n=72)
ATBTreatment

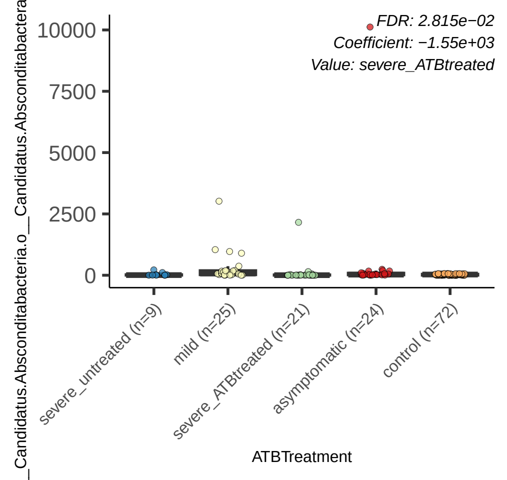

FDR: 2.815e−02
Coefficient: −1.55e+03
Value: severe_ATBtreated
10000
7500
5000
2500
0
__Candidatus.Absconditabacteria.o__Candidatus.Absconditabactera
severe_untreated (n=9)
mild (n=25)
severe_ATBtreated (n=21)
asymptomatic (n=24)
control (n=72)
ATBTreatment

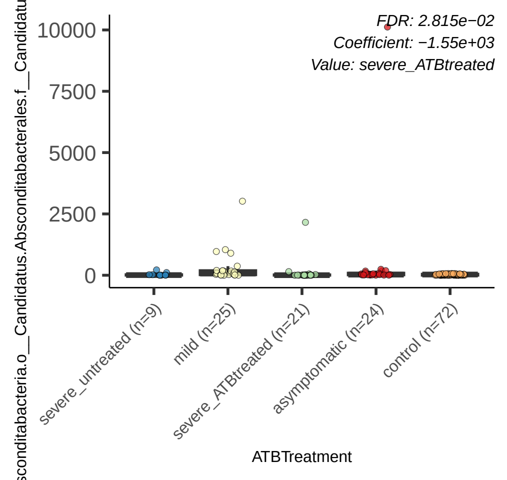

FDR: 2.815e−02
Coefficient: −1.55e+03
Value: severe_ATBtreated
10000
7500
5000
2500
0
Candidatus.Abscondita bacteria.o__Candidatus.Abscondita bacterales.f__Candidatu
severe_untreated (n=9)
mild (n=25)
severe_ATBtreated (n=21)
asymptomatic (n=24)
control (n=72)
ATBTreatment

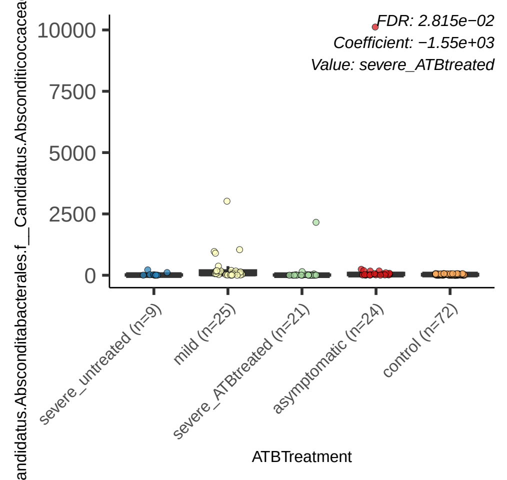

FDR: 2.815e−02
Coefficient: −1.55e+03
Value: severe_ATBtreated
10000
7500
5000
2500
0
andidatus.Absconditabacterales.f__Candidatus.Absconditicoccacea
severe_untreated (n=9)
mild (n=25)
severe_ATBtreated (n=21)
asymptomatic (n=24)
control (n=72)
ATBTreatment

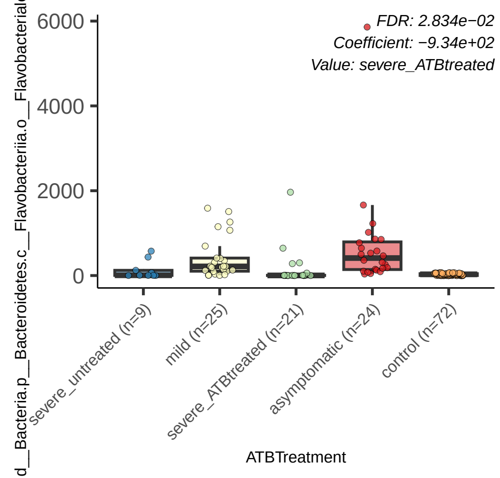

FDR: 2.834e−02
Coefficient: −9.34e+02
Value: severe_ATBtreated
6000
4000
2000
0
d__Bacteria.p__Bacteroidetes.c__Flavobacteriia.o__Flavobacterial
severe_untreated (n=9)
mild (n=25)
severe_ATBtreated (n=21)
asymptomatic (n=24)
control (n=72)
ATBTreatment

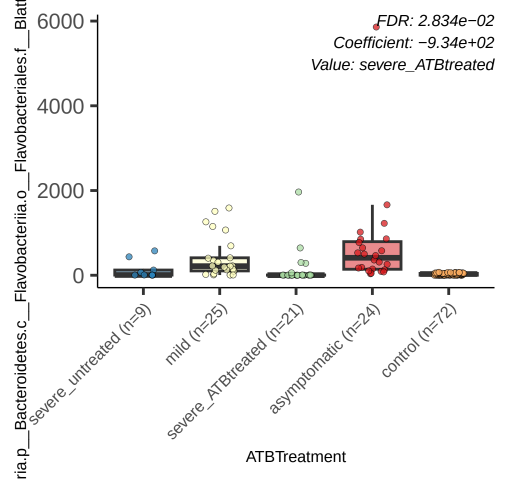

FDR: 2.834e−02
Coefficient: −9.34e+02
Value: severe_ATBtreated
6000
4000
2000
0
ria.p__Bacteroidetes.c__Flavobacteriia.o__Flavobacteriales.f__Blatt
severe_untreated (n=9)
mild (n=25)
severe_ATBtreated (n=21)
asymptomatic (n=24)
control (n=72)
ATBTreatment

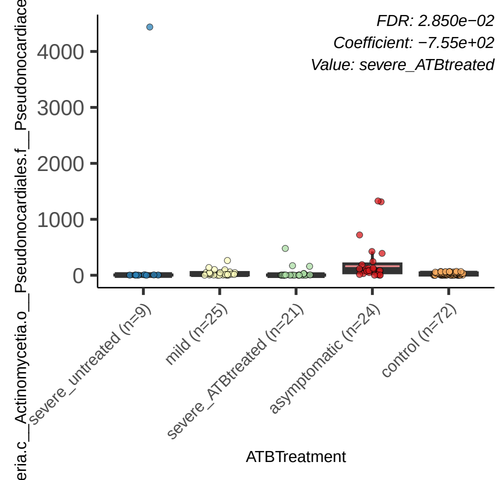

FDR: 2.850e−02
Coefficient: −7.55e+02
Value: severe_ATBtreated
0
1000
2000
3000
4000
eria.c__Actinomycetia.o__Pseudonocardiales.f__Pseudonocardiace
severe_untreated (n=9)
mild (n=25)
severe_ATBtreated (n=21)
asymptomatic (n=24)
control (n=72)
ATBTreatment

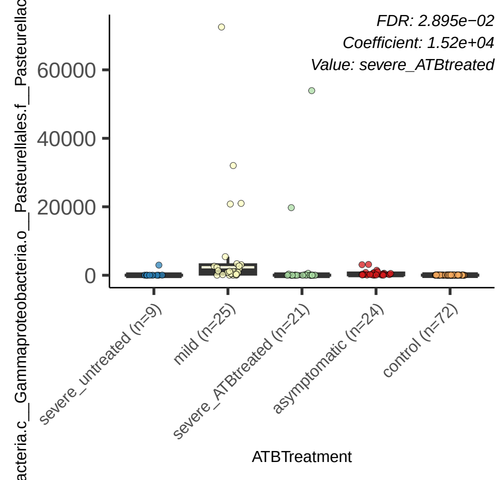

FDR: 2.895e−02
Coefficient: 1.52e+04
Value: severe_ATBtreated
60000
40000
20000
0
acteria.c__Gammaproteobacteria.o__Pasteurellales.f__Pasteurellac
severe_untreated (n=9)
mild (n=25)
severe_ATBtreated (n=21)
asymptomatic (n=24)
control (n=72)
ATBTreatment

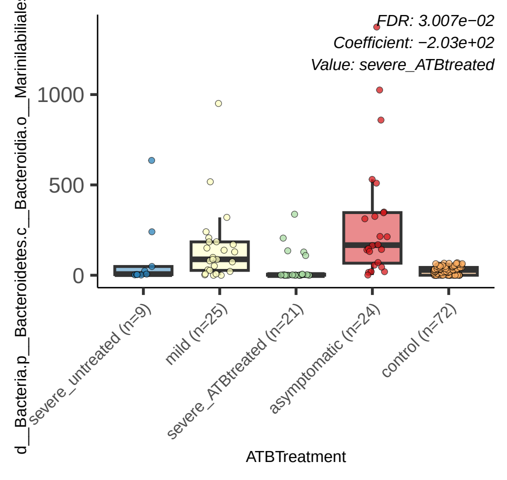

FDR: 3.007e−02
Coefficient: −2.03e+02
Value: severe_ATBtreated
d__Bacteria.p__Bacteroidetes.c__Bacteroidia.o__Marinilabiliales
1000
500
0
severe_untreated (n=9)
mild (n=25)
severe_ATBtreated (n=21)
asymptomatic (n=24)
control (n=72)
ATBTreatment

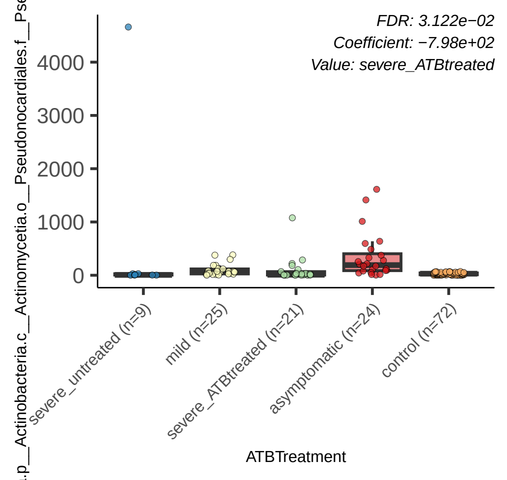

FDR: 3.122e−02
Coefficient: −7.98e+02
Value: severe_ATBtreated
p__Actinobacteria.c__Actinomycetia.o__Pseudonocardiales.f__Pse
4000
3000
2000
1000
0
severe_untreated (n=9)
mild (n=25)
severe_ATBtreated (n=21)
asymptomatic (n=24)
control (n=72)
ATBTreatment

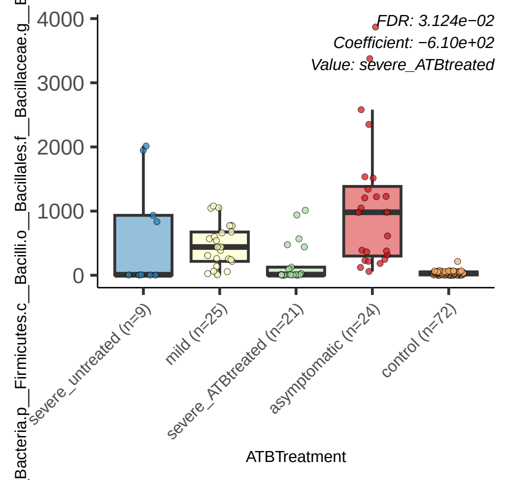

FDR: 3.124e−02
Coefficient: −6.10e+02
Value: severe_ATBtreated
4000
3000
2000
1000
0
Bacteria.p__Firmicutes.c__Bacilli.o__Bacillales.f__Bacillaceae.g__B
severe_untreated (n=9)
mild (n=25)
severe_ATBtreated (n=21)
asymptomatic (n=24)
control (n=72)
ATBTreatment

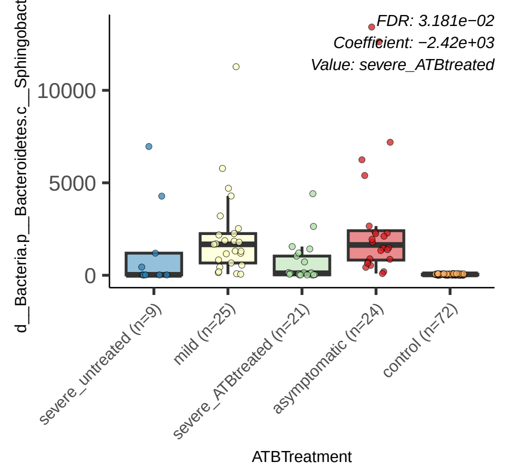
FDR: 3.181e−02
Coefficient: −2.42e+03
Value: severe_ATBtreated
d__Bacteria.p__Bacteroidetes.c__Sphingobact
10000
5000
0
severe_untreated (n=9)
mild (n=25)
severe_ATBtreated (n=21)
asymptomatic (n=24)
control (n=72)
ATBTreatment

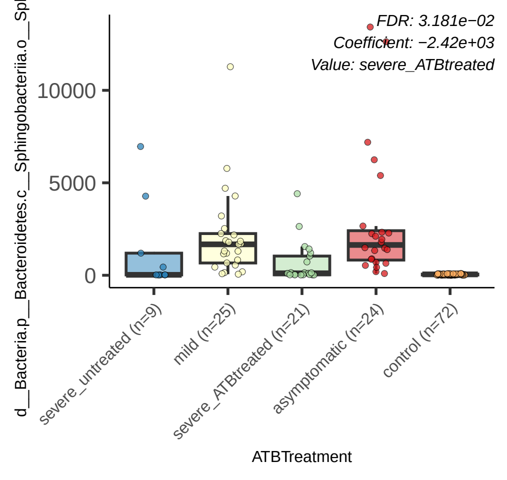
FDR: 3.181e−02
Coefficient: −2.42e+03
Value: severe_ATBtreated
d__Bacteria.p__Bacteroidetes.c__Sphingobacteriia.o__Sph
10000
5000
0
severe_untreated (n=9)
mild (n=25)
severe_ATBtreated (n=21)
asymptomatic (n=24)
control (n=72)
ATBTreatment

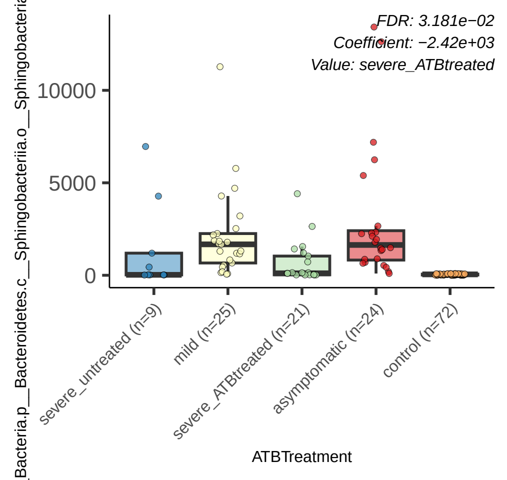

FDR: 3.181e−02
Coefficient: −2.42e+03
Value: severe_ATBtreated
10000
5000
0
Bacteria.p__Bacteroidetes.c__Sphingobacteriia.o__Sphingobacteria
severe_untreated (n=9)
mild (n=25)
severe_ATBtreated (n=21)
asymptomatic (n=24)
control (n=72)
ATBTreatment

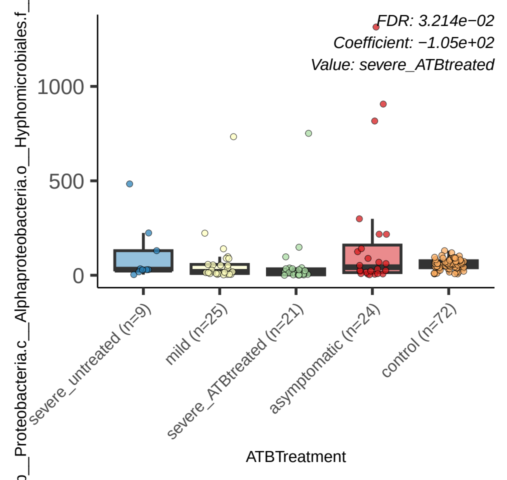

FDR: 3.214e−02
Coefficient: −1.05e+02
Value: severe_ATBtreated
o__Proteobacteria.c__Alphaproteobacteria.o__Hyphomicrobiales.f__
1000
500
0
severe_untreated (n=9)
mild (n=25)
severe_ATBtreated (n=21)
asymptomatic (n=24)
control (n=72)
ATBTreatment

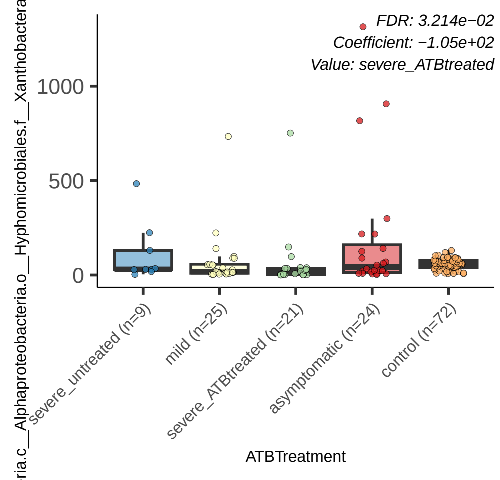

FDR: 3.214e−02
Coefficient: −1.05e+02
Value: severe_ATBtreated
1000
500
0
ria.c__Alphaproteobacteria.o__Hyphomicrobiales.f__Xanthobactera
severe_untreated (n=9)
mild (n=25)
severe_ATBtreated (n=21)
asymptomatic (n=24)
control (n=72)
ATBTreatment

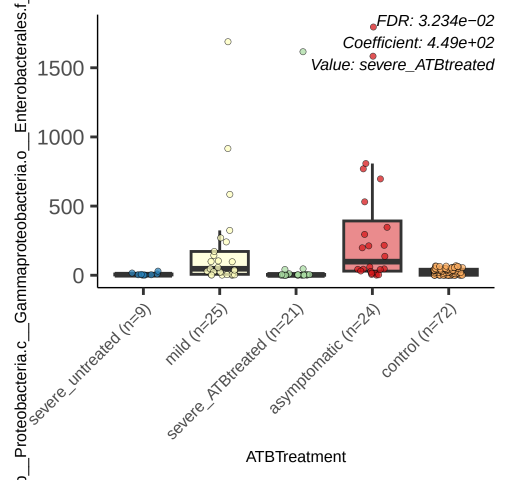

FDR: 3.234e−02
Coefficient: 4.49e+02
Value: severe_ATBtreated
o__Proteobacteria.c__Gammaproteobacteria.o__Enterobacterales.f_
0
500
1000
1500
severe_untreated (n=9)
mild (n=25)
severe_ATBtreated (n=21)
asymptomatic (n=24)
control (n=72)
ATBTreatment

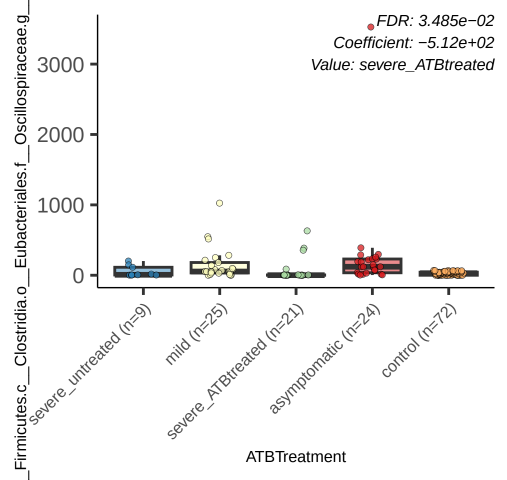

FDR: 3.485e−02
Coefficient: −5.12e+02
Value: severe_ATBtreated
Firmicutes.c__Clostridia.o__Eubacteriales.f__Oscillospiraceae.g__
3000
2000
1000
0
severe_untreated (n=9)
mild (n=25)
severe_ATBtreated (n=21)
asymptomatic (n=24)
control (n=72)
ATBTreatment

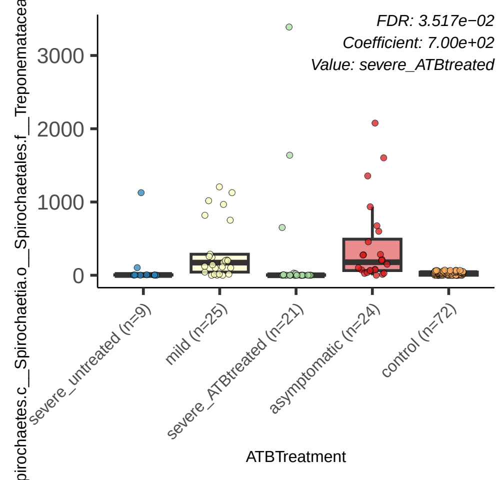

FDR: 3.517e−02
Coefficient: 7.00e+02
Value: severe_ATBtreated
3000
2000
1000
0
pirochaetes.c__Spirochaetia.o__Spirochaetales.f__Treponematacea
severe_untreated (n=9)
mild (n=25)
severe_ATBtreated (n=21)
asymptomatic (n=24)
control (n=72)
ATBTreatment

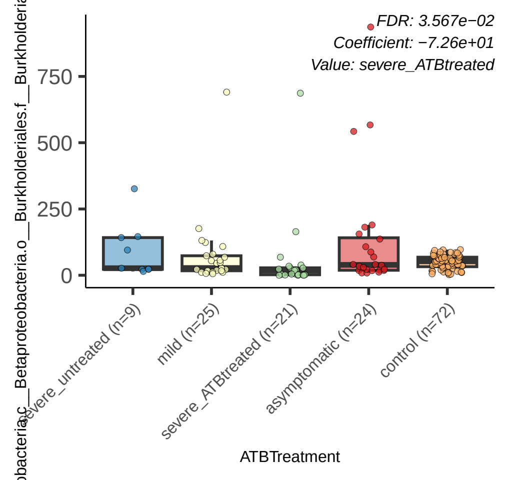

FDR: 3.567e−02
Coefficient: −7.26e+01
Value: severe_ATBtreated
obacteria.c__Betaproteobacteria.o__Burkholderiales.f__Burkholderia
750
500
250
0
severe_untreated (n=9)
mild (n=25)
severe_ATBtreated (n=21)
asymptomatic (n=24)
control (n=72)
ATBTreatment

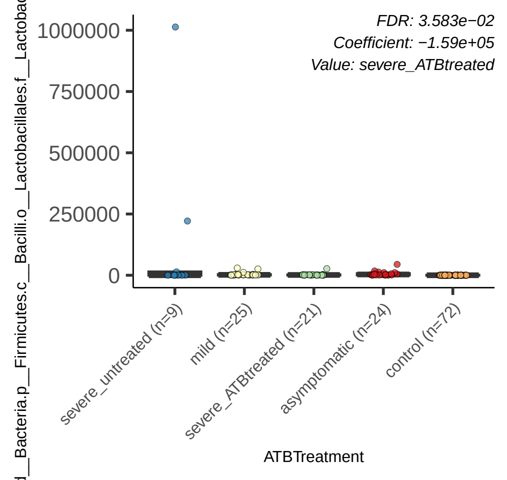

FDR: 3.583e−02
Coefficient: −1.59e+05
Value: severe_ATBtreated
1000000
750000
500000
250000
0
severe_untreated (n=9)
mild (n=25)
severe_ATBtreated (n=21)
asymptomatic (n=24)
control (n=72)
ATBTreatment
Bacteria.p__Firmicutes.c__Bacilli.o__Lactobacillales.f__Lactobac

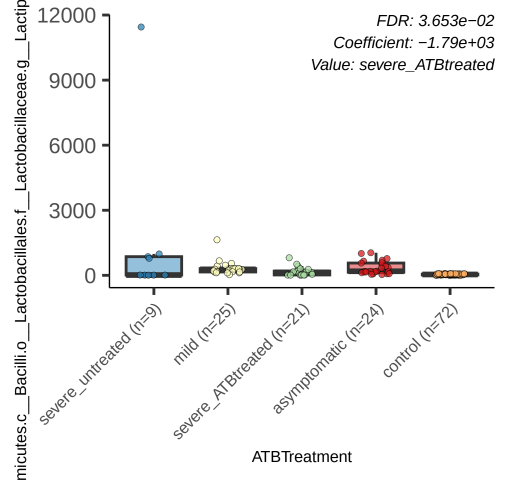

FDR: 3.653e−02
Coefficient: −1.79e+03
Value: severe_ATBtreated
12000
9000
6000
3000
0
severe_untreated (n=9)
mild (n=25)
severe_ATBtreated (n=21)
asymptomatic (n=24)
control (n=72)
ATBTreatment
micutes.c__Bacilli.o__Lactobacillales.f__Lactobacillaceae.g__Lactip

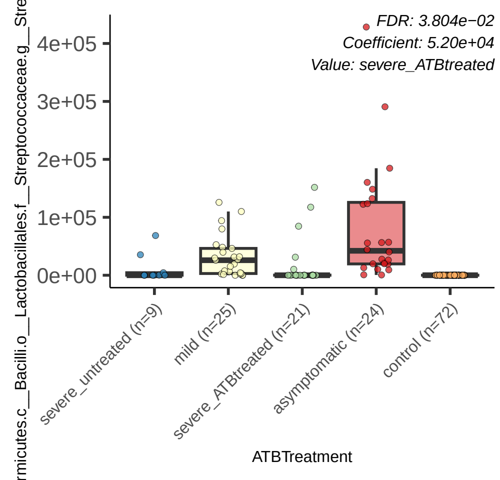

FDR: 3.804e−02
Coefficient: 5.20e+04
Value: severe_ATBtreated
4e+05
3e+05
2e+05
1e+05
0e+00
rmicutes.c__Bacilli.o__Lactobacillales.f__Streptococcaceae.g__Stre
severe_untreated (n=9)
mild (n=25)
severe_ATBtreated (n=21)
asymptomatic (n=24)
control (n=72)
ATBTreatment

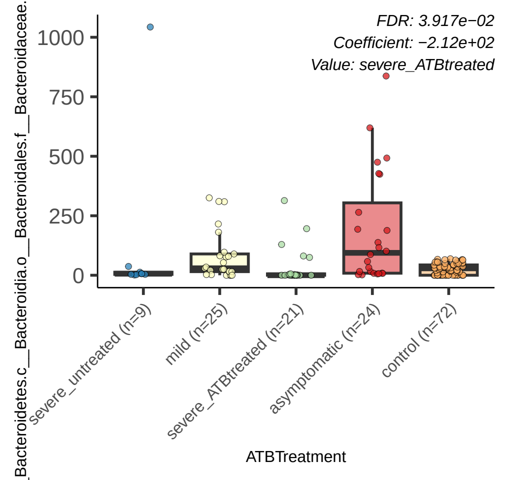

FDR: 3.917e−02
Coefficient: −2.12e+02
Value: severe_ATBtreated
Bacteroidetes.c__Bacteroidia.o__Bacteroidales.f__Bacteroidaceae.
1000
750
500
250
0
severe_untreated (n=9)
mild (n=25)
severe_ATBtreated (n=21)
asymptomatic (n=24)
control (n=72)
ATBTreatment

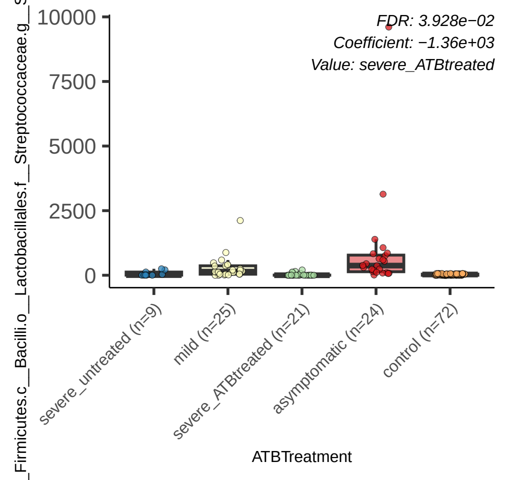

FDR: 3.928e−02
Coefficient: −1.36e+03
Value: severe_ATBtreated
10000
7500
5000
2500
0
severe_untreated (n=9)
mild (n=25)
severe_ATBtreated (n=21)
asymptomatic (n=24)
control (n=72)
ATBTreatment
Firmicutes.c__Bacilli.o__Lactobacillales.f__Streptococcaceae.g__

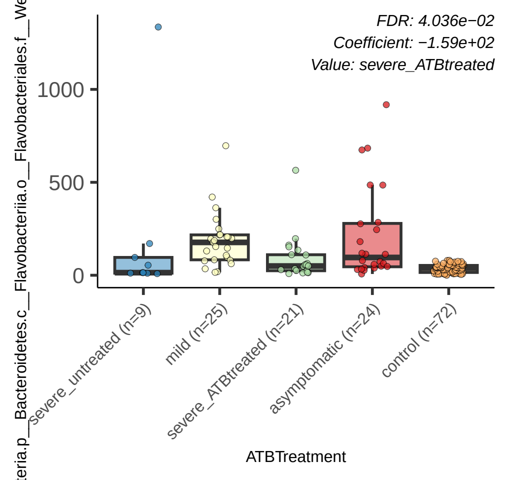

FDR: 4.036e−02
Coefficient: −1.59e+02
Value: severe_ATBtreated
1000
500
0
severe_untreated (n=9)
mild (n=25)
severe_ATBtreated (n=21)
asymptomatic (n=24)
control (n=72)
ATBTreatment
teria.p__Bacteroidetes.c__Flavobacteriia.o__Flavobacteriales.f__We

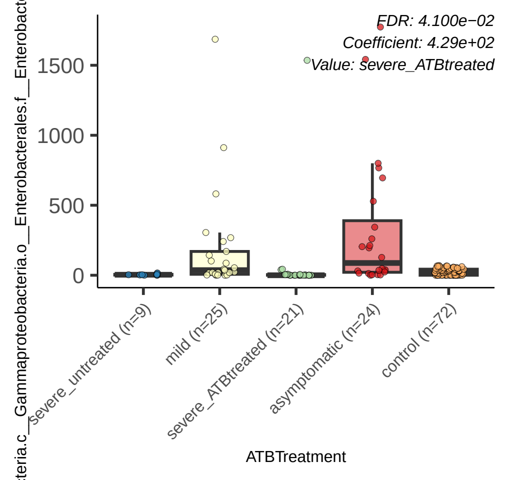

FDR: 4.100e−02
Coefficient: 4.29e+02
Value: severe_ATBtreated
teria.c__Gammaproteobacteria.o__Enterobacterales.f__Enterobact
1500
1000
500
0
severe_untreated (n=9)
mild (n=25)
severe_ATBtreated (n=21)
asymptomatic (n=24)
control (n=72)
ATBTreatment

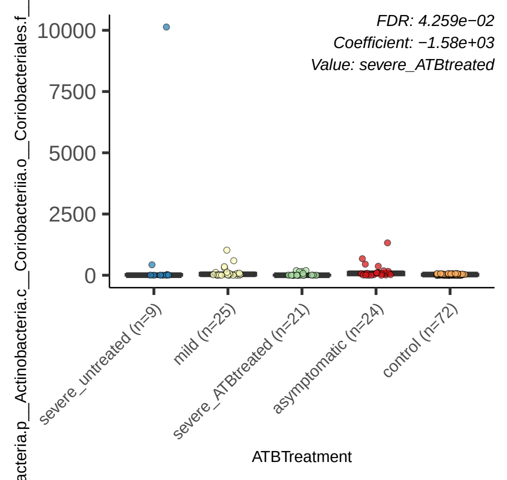
FDR: 4.259e−02
Coefficient: −1.58e+03
Value: severe_ATBtreated
acteria.p__Actinobacteria.c__Coriobacteriia.o__Coriobacteriales.f__
10000
7500
5000
2500
0
severe_untreated (n=9)
mild (n=25)
severe_ATBtreated (n=21)
asymptomatic (n=24)
control (n=72)
ATBTreatment

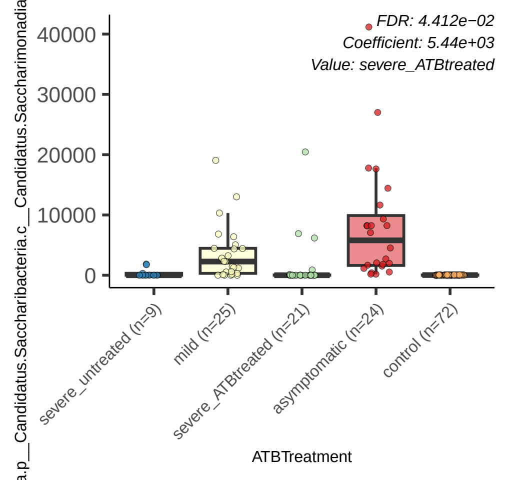
FDR: 4.412e−02
Coefficient: 5.44e+03
Value: severe_ATBtreated
40000
30000
20000
10000
0
a.p__Candidatus.Saccharibacteria.c__Candidatus.Saccharimonadia
severe_untreated (n=9)
mild (n=25)
severe_ATBtreated (n=21)
asymptomatic (n=24)
control (n=72)
ATBTreatment

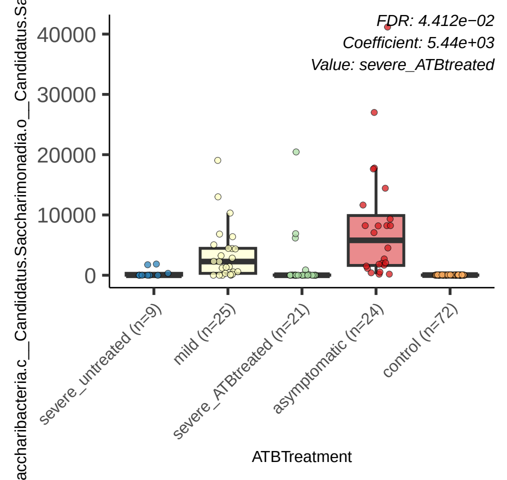

FDR: 4.412e−02
Coefficient: 5.44e+03
Value: severe_ATBtreated
40000
30000
20000
10000
0
accharibacteria.c__Candidatus.Saccharimonadia.o__Candidatus.Sa
severe_untreated (n=9)
mild (n=25)
severe_ATBtreated (n=21)
asymptomatic (n=24)
control (n=72)
ATBTreatment

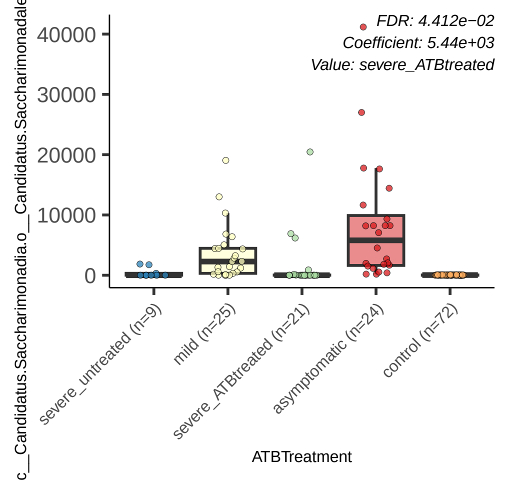

FDR: 4.412e−02
Coefficient: 5.44e+03
Value: severe_ATBtreated
c__Candidatus.Saccharimonadia.o__Candidatus.Saccharimonadale
40000
30000
20000
10000
0
severe_untreated (n=9)
mild (n=25)
severe_ATBtreated (n=21)
asymptomatic (n=24)
control (n=72)
ATBTreatment

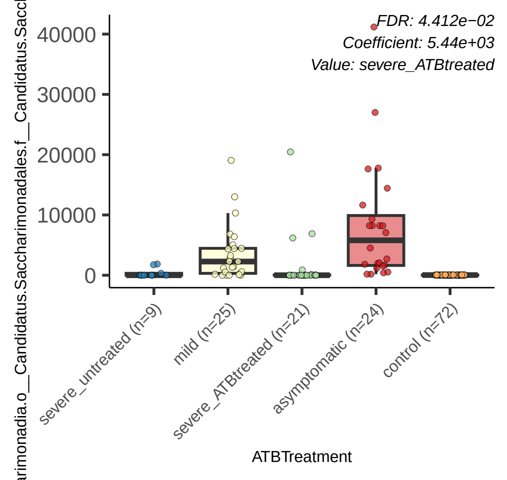

FDR: 4.412e−02
Coefficient: 5.44e+03
Value: severe_ATBtreated
40000
30000
20000
10000
0
arimonadia.o__Candidatus.Saccharimonadales.f__Candidatus.Sacc
severe_untreated (n=9)
mild (n=25)
severe_ATBtreated (n=21)
asymptomatic (n=24)
control (n=72)
ATBTreatment

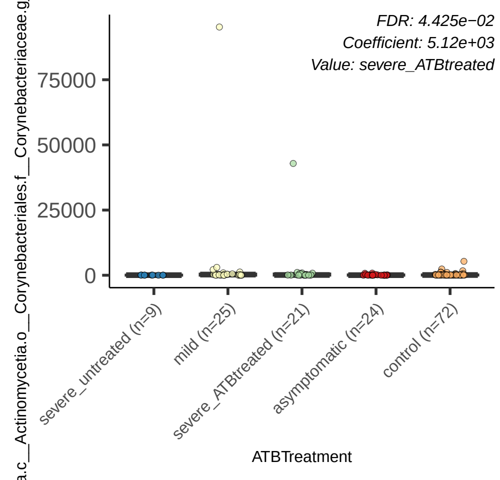

FDR: 4.425e−02
Coefficient: 5.12e+03
Value: severe_ATBtreated
75000
50000
25000
0
a.c__Actinomycetia.o__Corynebacteriales.f__Corynebacteriaceae.g.
severe_untreated (n=9)
mild (n=25)
severe_ATBtreated (n=21)
asymptomatic (n=24)
control (n=72)
ATBTreatment

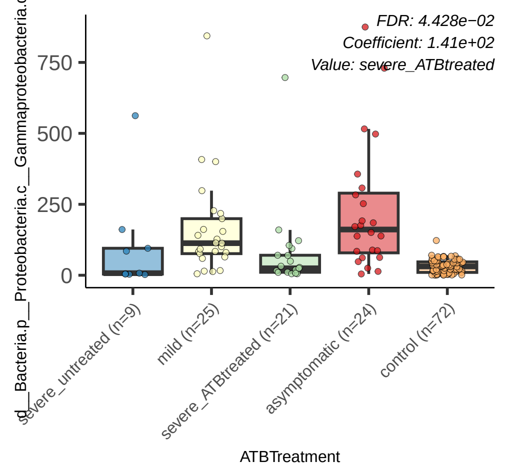

d__Bacteria.p__Proteobacteria.c__Gammaproteobacteria.c
FDR: 4.428e−02
Coefficient: 1.41e+02
Value: severe_ATBtreated
750
500
250
0
severe_untreated (n=9)
mild (n=25)
severe_ATBtreated (n=21)
asymptomatic (n=24)
control (n=72)
ATBTreatment

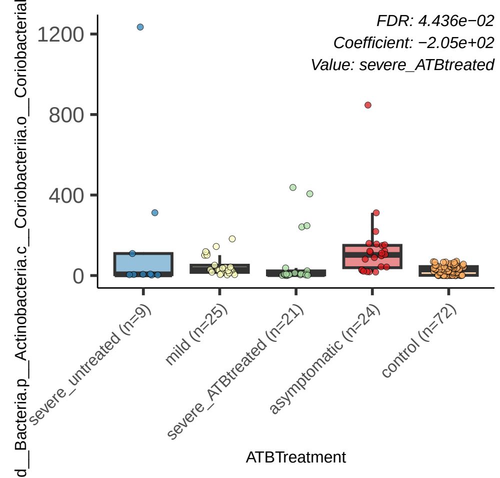
FDR: 4.436e−02
Coefficient: −2.05e+02
Value: severe_ATBtreated
d__Bacteria.p__Actinobacteria.c__Coriobacteriia.o__Coriobacteriales
1200
800
400
0
severe_untreated (n=9)
mild (n=25)
severe_ATBtreated (n=21)
asymptomatic (n=24)
control (n=72)
ATBTreatment

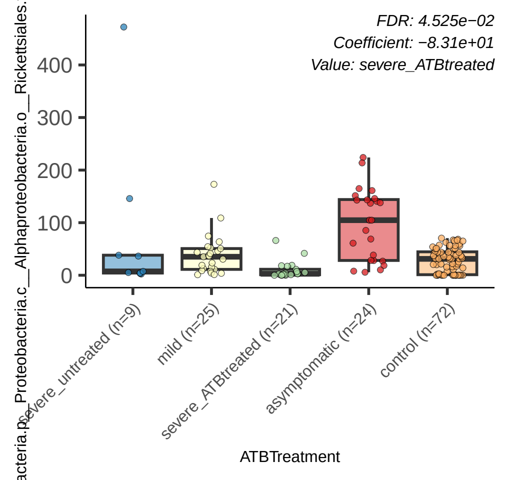

FDR: 4.525e−02
Coefficient: −8.31e+01
Value: severe_ATBtreated
acteria.p__Proteobacteria.c__Alphaproteobacteria.o__Rickettsiales.
400
300
200
100
0
severe_untreated (n=9)
mild (n=25)
severe_ATBtreated (n=21)
asymptomatic (n=24)
control (n=72)
ATBTreatment

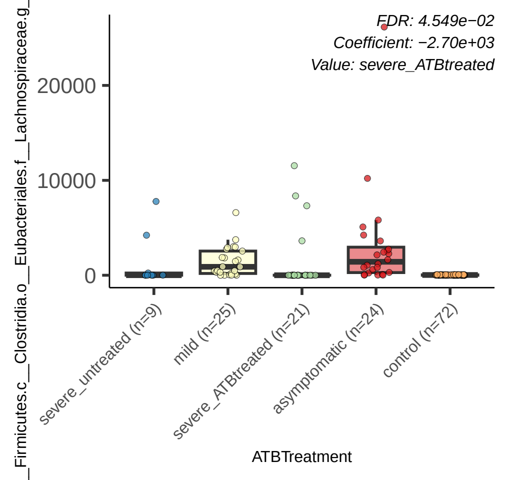

FDR: 4.549e−02
Coefficient: −2.70e+03
Value: severe_ATBtreated
Firmicutes.c__Clostridia.o__Eubacteriales.f__Lachnospiraceae.g_
20000
10000
0
severe_untreated (n=9)
mild (n=25)
severe_ATBtreated (n=21)
asymptomatic (n=24)
control (n=72)
ATBTreatment

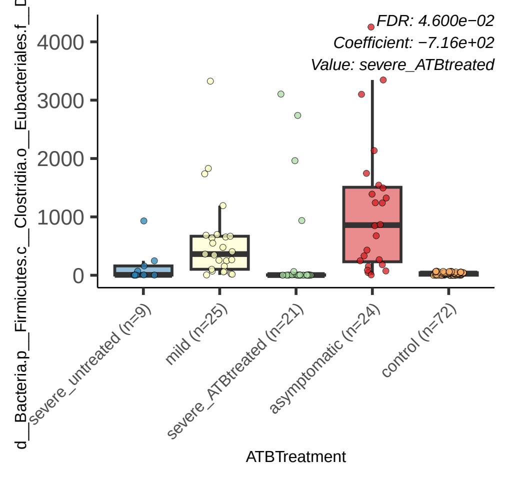
FDR: 4.600e−02
Coefficient: −7.16e+02
Value: severe_ATBtreated
d__Bacteria.p__Firmicutes.c__Clostridia.o__Eubacteriales.f__D
4000
3000
2000
1000
0
severe_untreated (n=9)
mild (n=25)
severe_ATBtreated (n=21)
asymptomatic (n=24)
control (n=72)
ATBTreatment

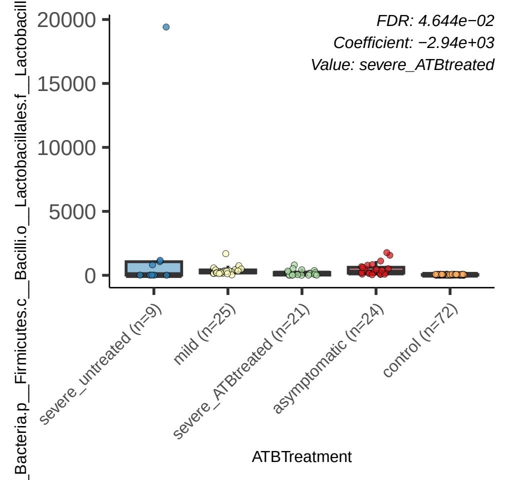
FDR: 4.644e−02
Coefficient: −2.94e+03
Value: severe_ATBtreated
Bacteria.p__Firmicutes.c__Bacilli.o__Lactobacillales.f__Lactobacill
20000
15000
10000
5000
0
severe_untreated (n=9)
mild (n=25)
severe_ATBtreated (n=21)
asymptomatic (n=24)
control (n=72)
ATBTreatment

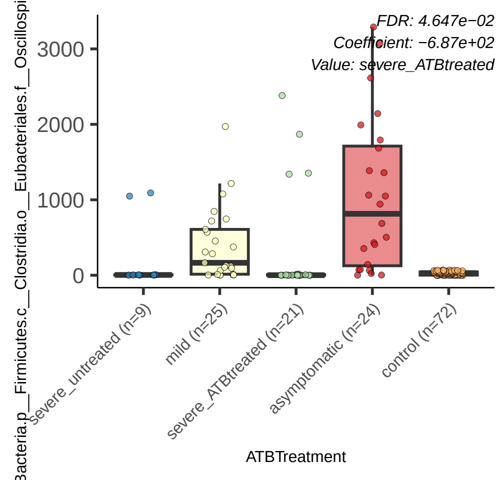

FDR: 4.647e−02
Coefficient: −6.87e+02
Value: severe_ATBtreated
Bacteria.p__Firmicutes.c__Clostridia.o__Eubacteriales.f__Oscillospi
3000
2000
1000
0
severe_untreated (n=9)
mild (n=25)
severe_ATBtreated (n=21)
asymptomatic (n=24)
control (n=72)
ATBTreatment

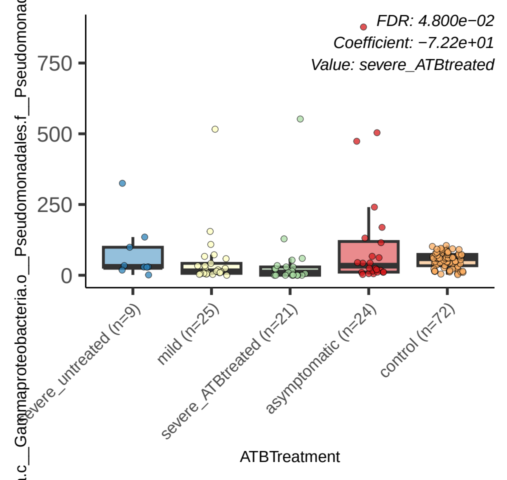

FDR: 4.800e−02
Coefficient: −7.22e+01
Value: severe_ATBtreated
750
500
250
0
Gammaproteobacteria.o__Pseudomonadales.f__Pseudomonad
severe_untreated (n=9)
mild (n=25)
severe_ATBtreated (n=21)
asymptomatic (n=24)
control (n=72)
ATBTreatment

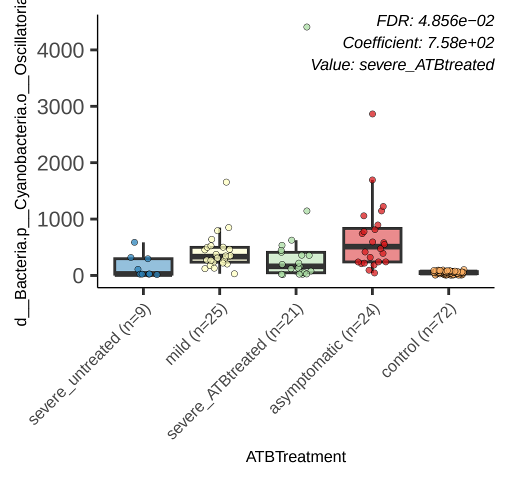
FDR: 4.856e−02
Coefficient: 7.58e+02
Value: severe_ATBtreated
d__Bacteria.p__Cyanobacteria.o__Oscillatoria
4000
3000
2000
1000
0
severe_untreated (n=9)
mild (n=25)
severe_ATBtreated (n=21)
asymptomatic (n=24)
control (n=72)
ATBTreatment

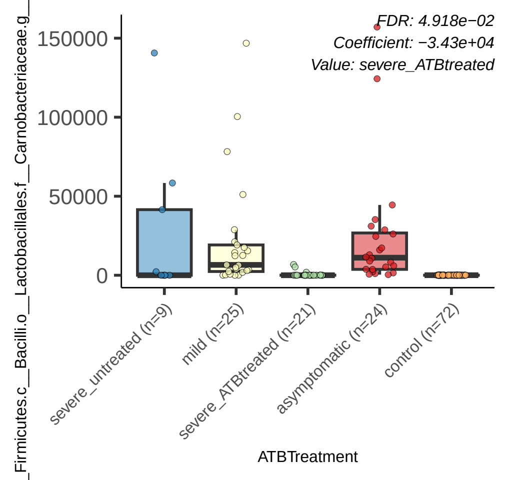

FDR: 4.918e−02
Coefficient: −3.43e+04
Value: severe_ATBtreated
Firmicutes.c__Bacilli.o__Lactobacillales.f__Carnobacteriaceae.g__
150000
100000
50000
0
severe_untreated (n=9)
mild (n=25)
severe_ATBtreated (n=21)
asymptomatic (n=24)
control (n=72)
ATBTreatment

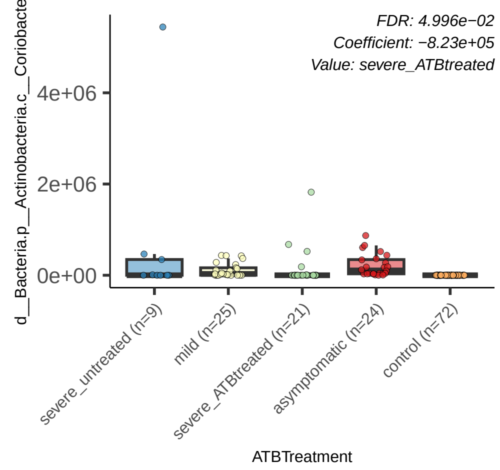

FDR: 4.996e−02
Coefficient: −8.23e+05
Value: severe_ATBtreated
d__Bacteria.p__Actinobacteria.c__Coriobacte
4e+06
2e+06
0e+00
severe_untreated (n=9)
mild (n=25)
severe_ATBtreated (n=21)
asymptomatic (n=24)
control (n=72)
ATBTreatment

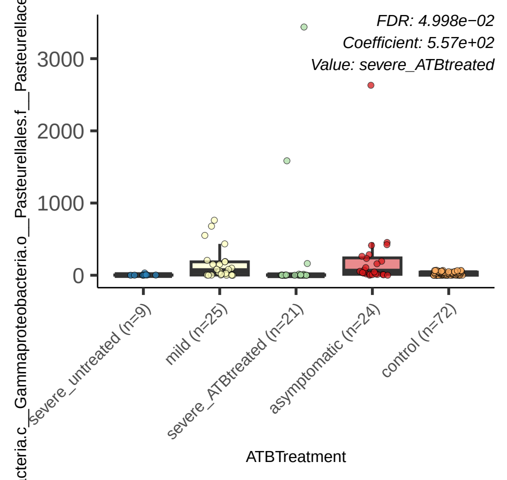

FDR: 4.998e−02
Coefficient: 5.57e+02
Value: severe_ATBtreated
3000
2000
1000
0
severe_untreated (n=9)
mild (n=25)
severe_ATBtreated (n=21)
asymptomatic (n=24)
control (n=72)
ATBTreatment
cteria.c__Gammaproteobacteria.o__Pasteurellales.f__Pasteurellace

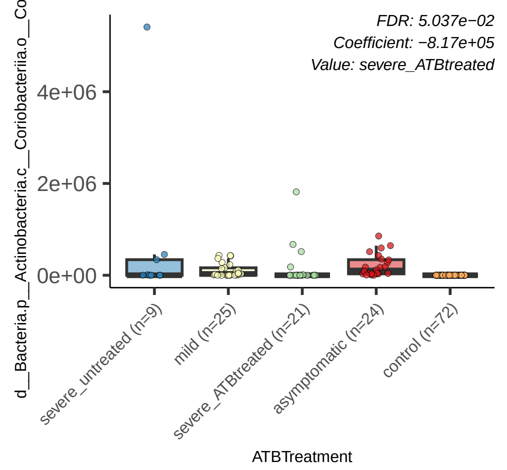
FDR: 5.037e−02
Coefficient: −8.17e+05
Value: severe_ATBtreated
4e+06
2e+06
0e+00
d__Bacteria.p__Actinobacteria.c__Coriobacteriia.o__Co
severe_untreated (n=9)
mild (n=25)
severe_ATBtreated (n=21)
asymptomatic (n=24)
control (n=72)
ATBTreatment

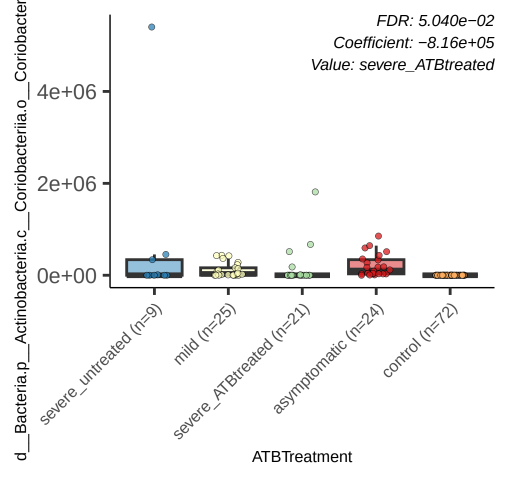

FDR: 5.040e−02
Coefficient: −8.16e+05
Value: severe_ATBtreated
4e+06
2e+06
0e+00
d__Bacteria.p__Actinobacteria.c__Coriobacteriia.o__Coriobacter
severe_untreated (n=9)
mild (n=25)
severe_ATBtreated (n=21)
asymptomatic (n=24)
control (n=72)
ATBTreatment

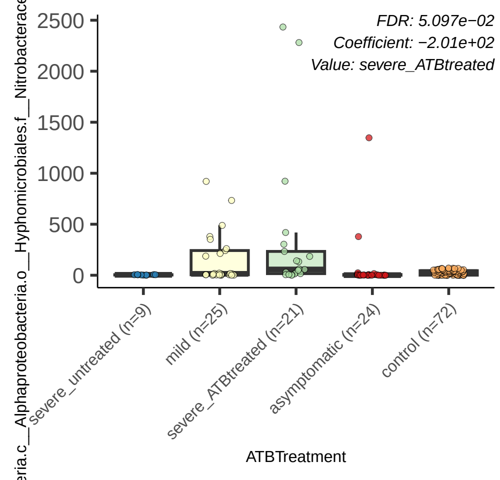

FDR: 5.097e−02
Coefficient: −2.01e+02
Value: severe_ATBtreated
2500
2000
1500
1000
500
0
Alphaproteobacteria.c__Alphaproteobacteria.o__Hyphomicrobiales.f__Nitrobacterace
severe_untreated (n=9)
mild (n=25)
severe_ATBtreated (n=21)
asymptomatic (n=24)
control (n=72)
ATBTreatment

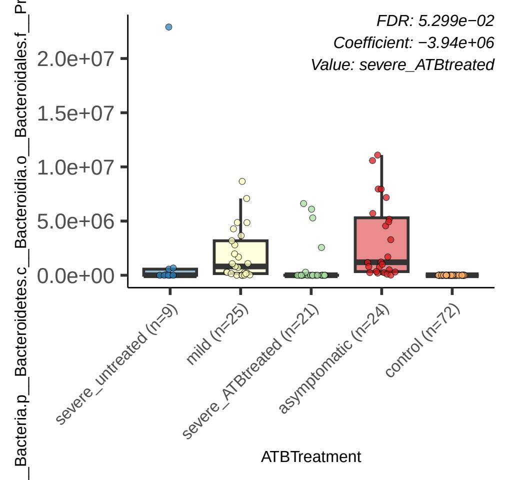

FDR: 5.299e−02
Coefficient: −3.94e+06
Value: severe_ATBtreated
2.0e+07
1.5e+07
1.0e+07
5.0e+06
0.0e+00
severe_untreated (n=9)
mild (n=25)
severe_ATBtreated (n=21)
asymptomatic (n=24)
control (n=72)
ATBTreatment

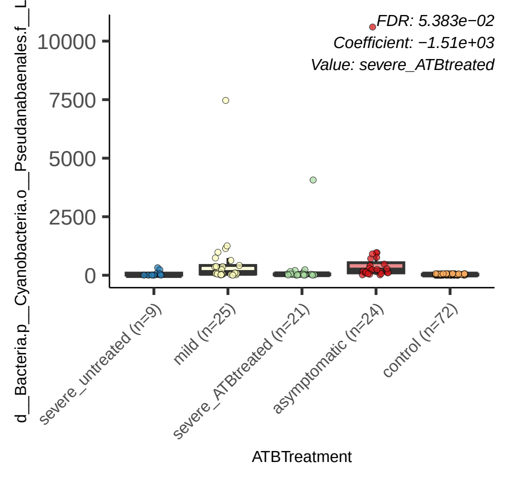

FDR: 5.383e−02
Coefficient: −1.51e+03
Value: severe_ATBtreated
d__Bacteria.p__Cyanobacteria.o__Pseudanabaenales.f__L
10000
7500
5000
2500
0
severe_untreated (n=9)
mild (n=25)
severe_ATBtreated (n=21)
asymptomatic (n=24)
control (n=72)
ATBTreatment

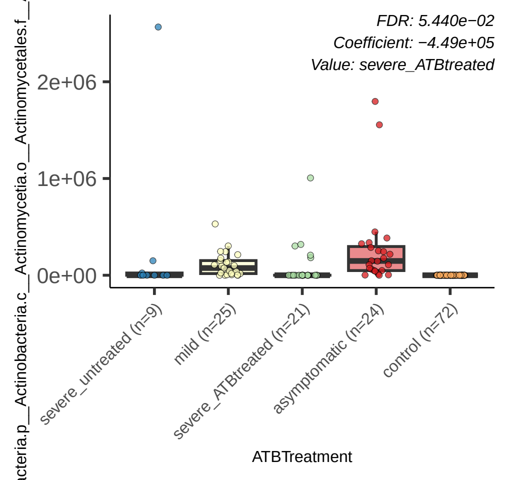
FDR: 5.440e−02
Coefficient: −4.49e+05
Value: severe_ATBtreated
acteria.p__Actinobacteria.c__Actinomycetia.o__Actinomycetales.f__
2e+06
1e+06
0e+00
severe_untreated (n=9)
mild (n=25)
severe_ATBtreated (n=21)
asymptomatic (n=24)
control (n=72)
ATBTreatment

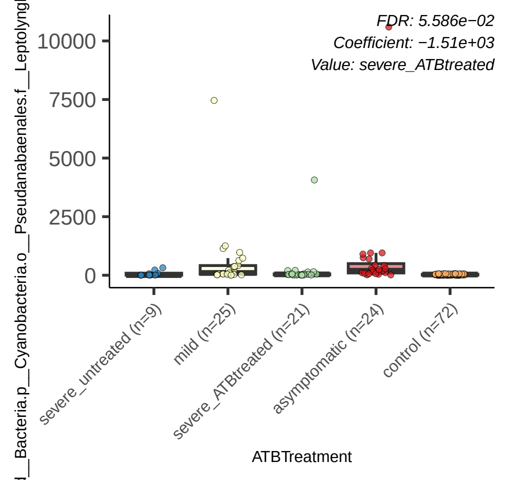
FDR: 5.586e−02
Coefficient: −1.51e+03
Value: severe_ATBtreated
10000
7500
5000
2500
0
d__Bacteria.p__Cyanobacteria.o__Pseudanabaenales.f__Leptolyngb
severe_untreated (n=9)
mild (n=25)
severe_ATBtreated (n=21)
asymptomatic (n=24)
control (n=72)
ATBTreatment

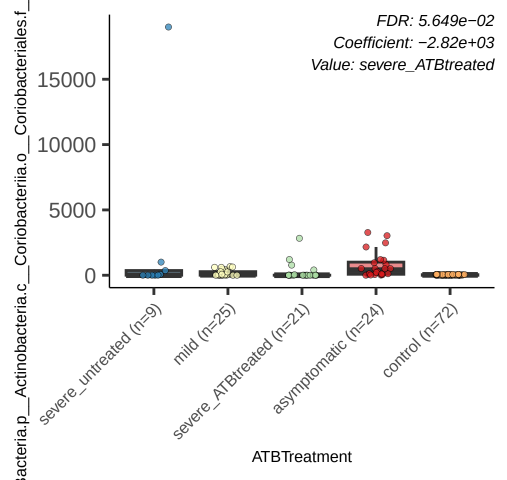
FDR: 5.649e−02
Coefficient: −2.82e+03
Value: severe_ATBtreated
Bacteria.p__Actinobacteria.c__Coriobacteriia.o__Coriobacteriales.f__
15000
10000
5000
0
severe_untreated (n=9)
mild (n=25)
severe_ATBtreated (n=21)
asymptomatic (n=24)
control (n=72)
ATBTreatment

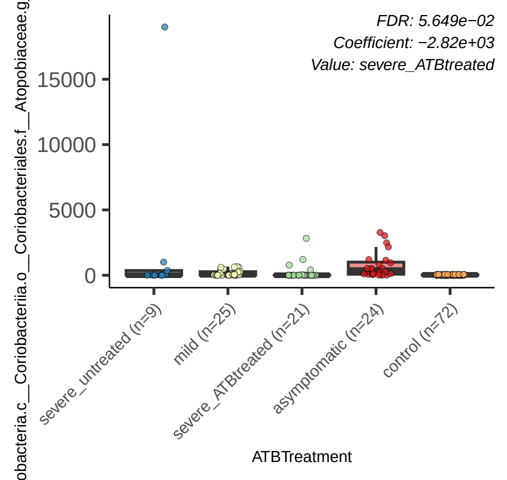
FDR: 5.649e−02
Coefficient: −2.82e+03
Value: severe_ATBtreated
obacteria.c__Coriobacteriia.o__Coriobacteriales.f__Atopobiaceae.g
15000
10000
5000
0
severe_untreated (n=9)
mild (n=25)
severe_ATBtreated (n=21)
asymptomatic (n=24)
control (n=72)
ATBTreatment

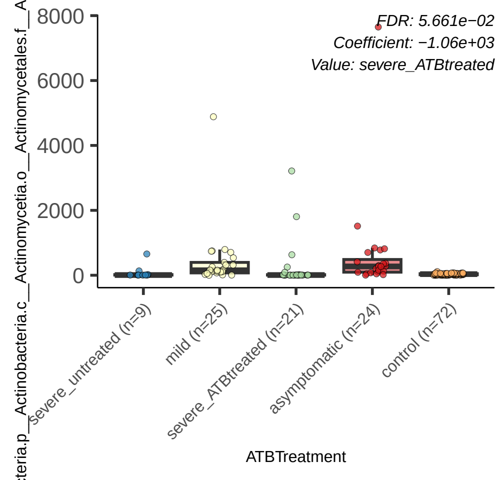

FDR: 5.661e−02
Coefficient: −1.06e+03
Value: severe_ATBtreated
8000
6000
4000
2000
0
severe_untreated (n=9)
mild (n=25)
severe_ATBtreated (n=21)
asymptomatic (n=24)
control (n=72)
ATBTreatment

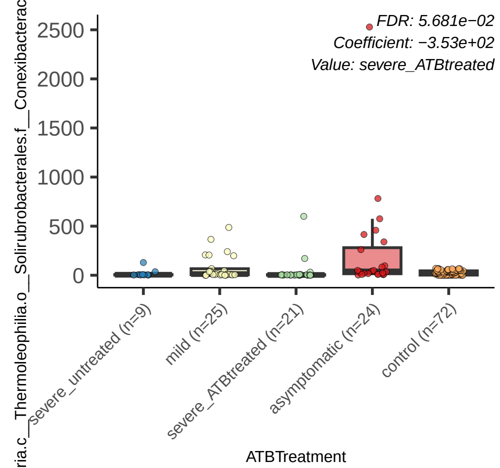

FDR: 5.681e−02
Coefficient: −3.53e+02
Value: severe_ATBtreated
2500
2000
1500
1000
500
0
teria.c__Thermoleophilia.o__Solirubrobacterales.f__Conexibacterac
severe_untreated (n=9)
mild (n=25)
severe_ATBtreated (n=21)
asymptomatic (n=24)
control (n=72)
ATBTreatment

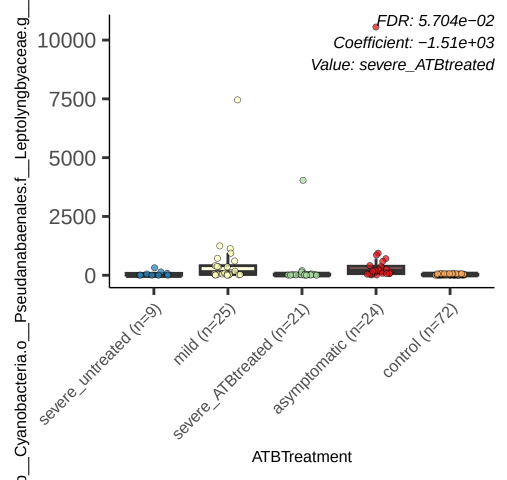

FDR: 5.704e−02
Coefficient: −1.51e+03
Value: severe_ATBtreated
10000
7500
5000
2500
0
o__Cyanobacteria.o__Pseudanabaenales.f__Leptolyngbyaceae.g__
severe_untreated (n=9)
mild (n=25)
severe_ATBtreated (n=21)
asymptomatic (n=24)
control (n=72)
ATBTreatment

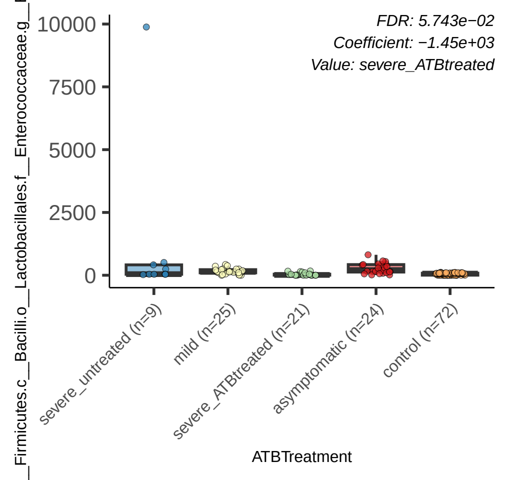

FDR: 5.743e−02
Coefficient: −1.45e+03
Value: severe_ATBtreated
Firmicutes.c__Bacilli.o__Lactobacillales.f__Enterococcaceae.g__
10000
7500
5000
2500
0
severe_untreated (n=9)
mild (n=25)
severe_ATBtreated (n=21)
asymptomatic (n=24)
control (n=72)
ATBTreatment

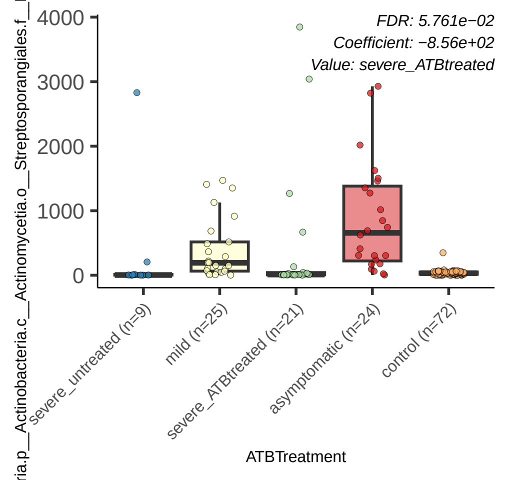

FDR: 5.761e−02
Coefficient: −8.56e+02
Value: severe_ATBtreated
ria.p__Actinobacteria.c__Actinomycetia.o__Streptosporangiales.f__
4000
3000
2000
1000
0
severe_untreated (n=9)
mild (n=25)
severe_ATBtreated (n=21)
asymptomatic (n=24)
control (n=72)
ATBTreatment

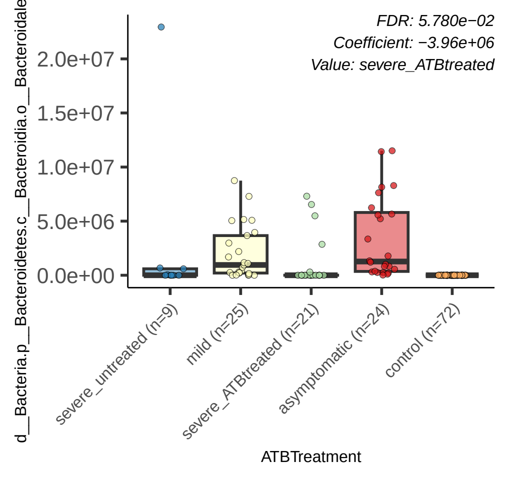

FDR: 5.780e−02
Coefficient: −3.96e+06
Value: severe_ATBtreated
d__Bacteria.p__Bacteroidetes.c__Bacteroidia.o__Bacteroidale
2.0e+07
1.5e+07
1.0e+07
5.0e+06
0.0e+00
severe_untreated (n=9)
mild (n=25)
severe_ATBtreated (n=21)
asymptomatic (n=24)
control (n=72)
ATBTreatment

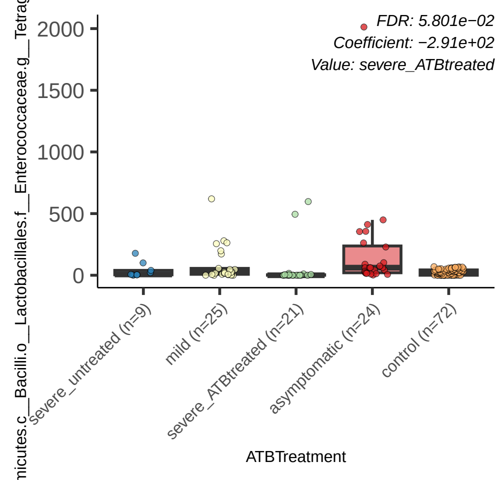
FDR: 5.801e−02
Coefficient: −2.91e+02
Value: severe_ATBtreated
micutes.c__Bacilli.o__Lactobacillales.f__Enterococcaceae.g__Tetrag
2000
1500
1000
500
0
severe_untreated (n=9)
mild (n=25)
severe_ATBtreated (n=21)
asymptomatic (n=24)
control (n=72)
ATBTreatment

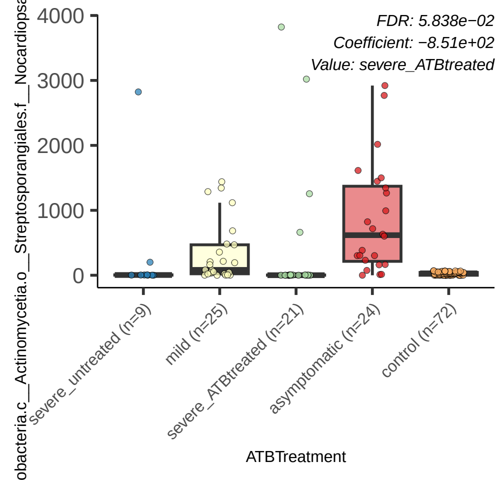

FDR: 5.838e−02
Coefficient: −8.51e+02
Value: severe_ATBtreated
obacteria.c__Actinomycetia.o__Streptosporangiales.f__Nocardiopsa
4000
3000
2000
1000
0
severe_untreated (n=9)
mild (n=25)
severe_ATBtreated (n=21)
asymptomatic (n=24)
control (n=72)
ATBTreatment

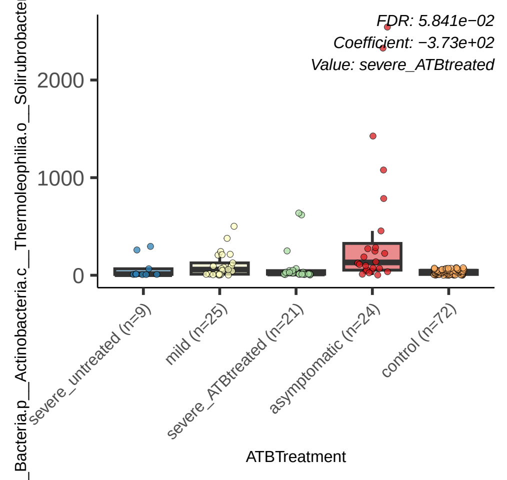

FDR: 5.841e−02
Coefficient: −3.73e+02
Value: severe_ATBtreated
2000
1000
0
_Bacteria.p__Actinobacteria.c__Thermoleophilia.o__Solirubrobacte
severe_untreated (n=9)
mild (n=25)
severe_ATBtreated (n=21)
asymptomatic (n=24)
control (n=72)
ATBTreatment

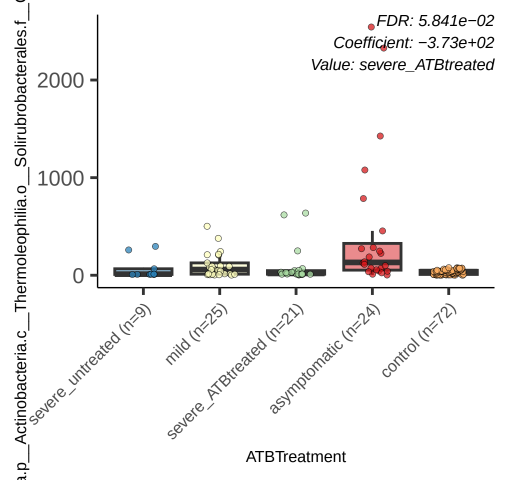
FDR: 5.841e−02
Coefficient: −3.73e+02
Value: severe_ATBtreated
2000
1000
0
severe_untreated (n=9)
mild (n=25)
severe_ATBtreated (n=21)
asymptomatic (n=24)
control (n=72)
ATBTreatment
a.p__Actinobacteria.c__Thermoleophilia.o__Solirubrobacterales.f__

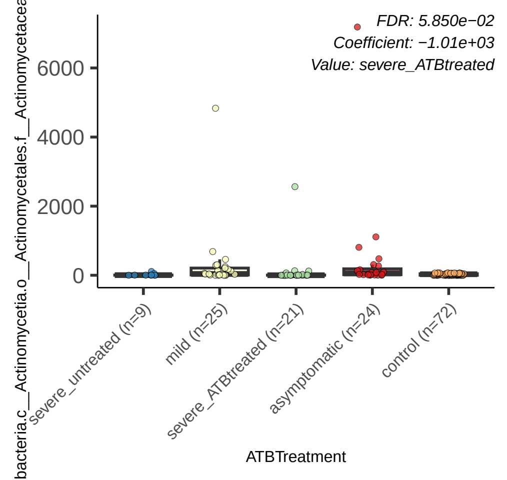

FDR: 5.850e−02
Coefficient: −1.01e+03
Value: severe_ATBtreated
6000
4000
2000
0
bacteria.c__Actinomycetia.o__Actinomycetales.f__Actinomycetacea
severe_untreated (n=9)
mild (n=25)
severe_ATBtreated (n=21)
asymptomatic (n=24)
control (n=72)
ATBTreatment

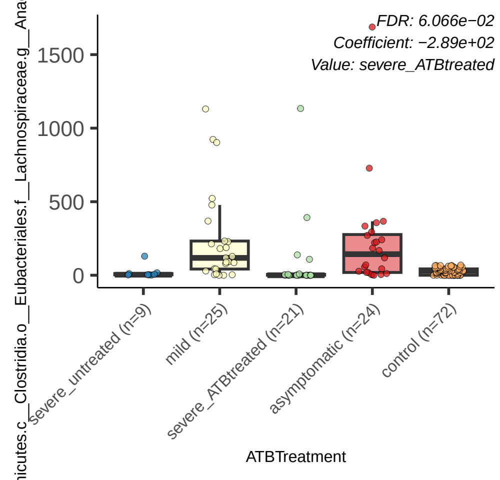

FDR: 6.066e−02
Coefficient: −2.89e+02
Value: severe_ATBtreated
1500
1000
500
0
nicutes.c__Clostridia.o__Eubacteriales.f__Lachnospiraceae.g__Ana
severe_untreated (n=9)
mild (n=25)
severe_ATBtreated (n=21)
asymptomatic (n=24)
control (n=72)
ATBTreatment

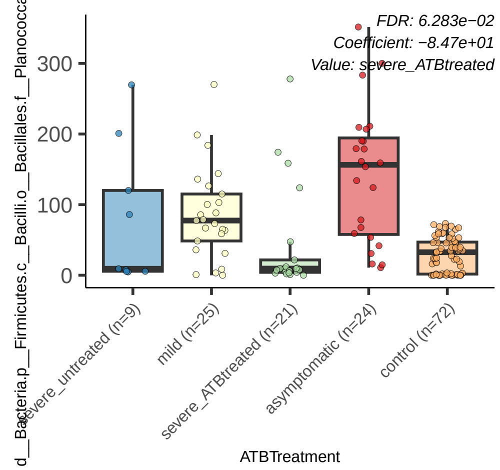

FDR: 6.283e−02
Coefficient: −8.47e+01
Value: severe_ATBtreated
d__Bacteria.p__Firmicutes.c__Bacilli.o__Bacillales.f__Planococca
300
200
100
0
severe_untreated (n=9)
mild (n=25)
severe_ATBtreated (n=21)
asymptomatic (n=24)
control (n=72)
ATBTreatment

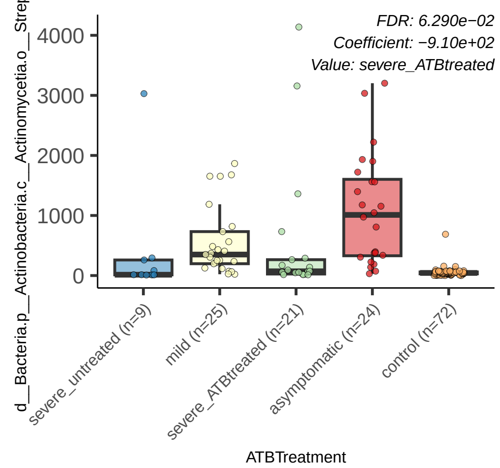

FDR: 6.290e−02
Coefficient: −9.10e+02
Value: severe_ATBtreated
d__Bacteria.p__Actinobacteria.c__Actinomycetia.o__Strep
4000
3000
2000
1000
0
severe_untreated (n=9)
mild (n=25)
severe_ATBtreated (n=21)
asymptomatic (n=24)
control (n=72)
ATBTreatment

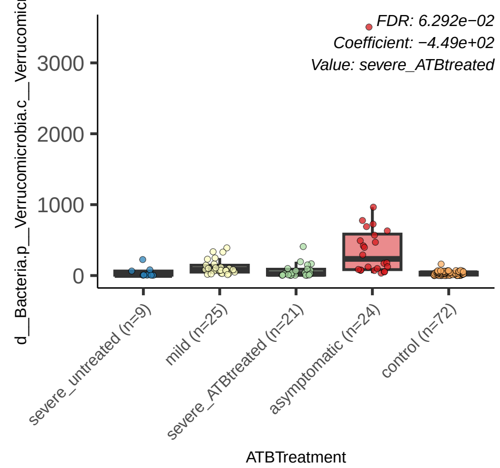

FDR: 6.292e−02
Coefficient: −4.49e+02
Value: severe_ATBtreated
d__Bacteria.p__Verrucomicrobia.c__Verrucomic
3000
2000
1000
0
severe_untreated (n=9)
mild (n=25)
severe_ATBtreated (n=21)
asymptomatic (n=24)
control (n=72)
ATBTreatment

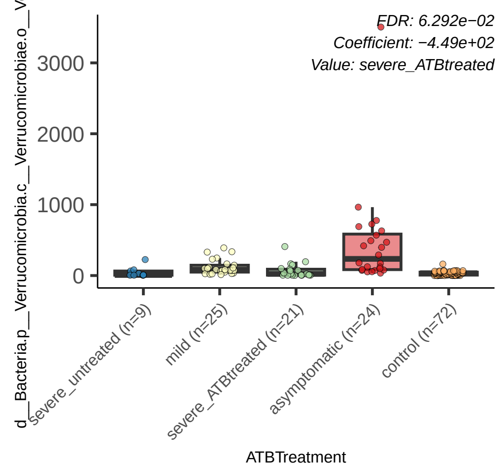
FDR: 6.292e−02
Coefficient: −4.49e+02
Value: severe_ATBtreated
d__Bacteria.p__Verrucomicrobia.c__Verrucomicrobiae.o__V
3000
2000
1000
0
severe_untreated (n=9)
mild (n=25)
severe_ATBtreated (n=21)
asymptomatic (n=24)
control (n=72)
ATBTreatment

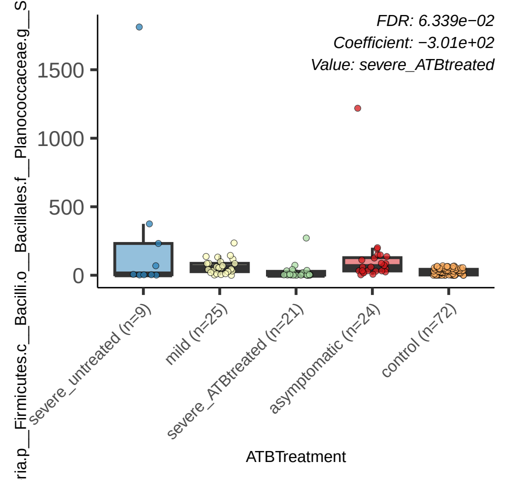
FDR: 6.339e−02
Coefficient: −3.01e+02
Value: severe_ATBtreated
ria.p__Firmicutes.c__Bacilli.o__Bacillales.f__Planococcaceae.g__S
1500
1000
500
0
severe_untreated (n=9)
mild (n=25)
severe_ATBtreated (n=21)
asymptomatic (n=24)
control (n=72)
ATBTreatment

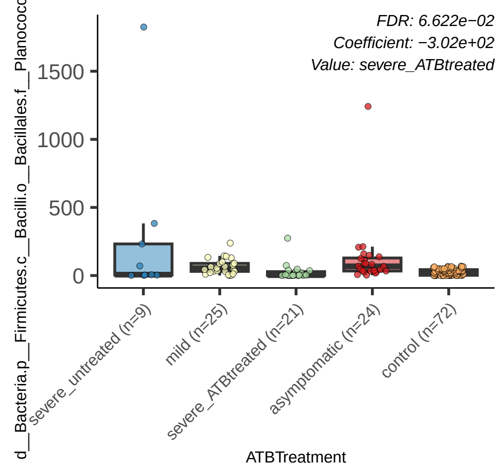

FDR: 6.622e−02
Coefficient: −3.02e+02
Value: severe_ATBtreated
d__Bacteria.p__Firmicutes.c__Bacilli.o__Bacillales.f__Planococc
1500
1000
500
0
severe_untreated (n=9)
mild (n=25)
severe_ATBtreated (n=21)
asymptomatic (n=24)
control (n=72)
ATBTreatment

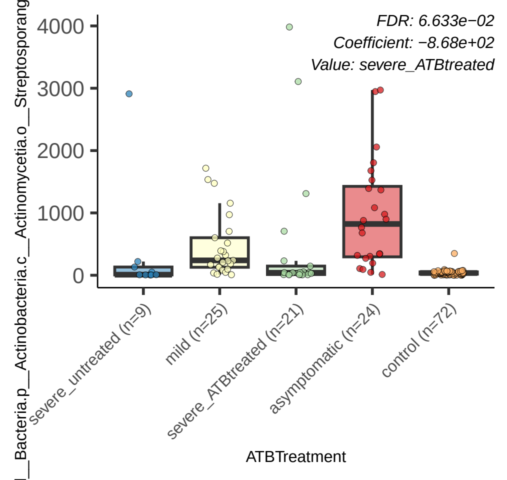

FDR: 6.633e−02
Coefficient: −8.68e+02
Value: severe_ATBtreated
__Bacteria.p__Actinobacteria.c__Actinomycetia.o__Streptosporang
4000
3000
2000
1000
0
severe_untreated (n=9)
mild (n=25)
severe_ATBtreated (n=21)
asymptomatic (n=24)
control (n=72)
ATBTreatment

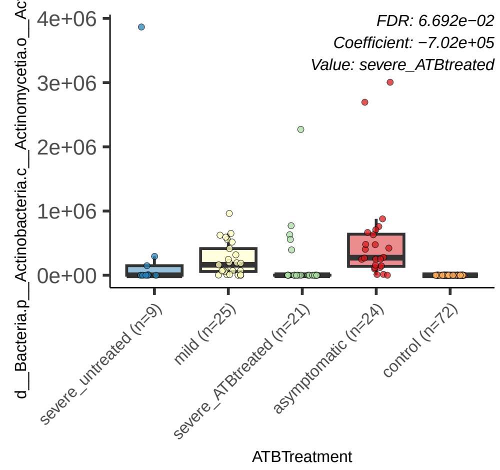

FDR: 6.692e−02
Coefficient: −7.02e+05
Value: severe_ATBtreated
d__Bacteria.p__Actinobacteria.c__Actinomycetia.o__Ac
4e+06
3e+06
2e+06
1e+06
0e+00
severe_untreated (n=9)
mild (n=25)
severe_ATBtreated (n=21)
asymptomatic (n=24)
control (n=72)
ATBTreatment

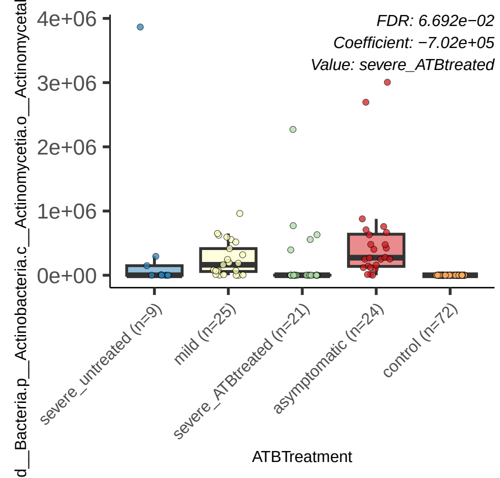

FDR: 6.692e−02
Coefficient: −7.02e+05
Value: severe_ATBtreated
d__Bacteria.p__Actinobacteria.c__Actinomycetia.o__Actinomycetal
4e+06
3e+06
2e+06
1e+06
0e+00
severe_untreated (n=9)
mild (n=25)
severe_ATBtreated (n=21)
asymptomatic (n=24)
control (n=72)
ATBTreatment

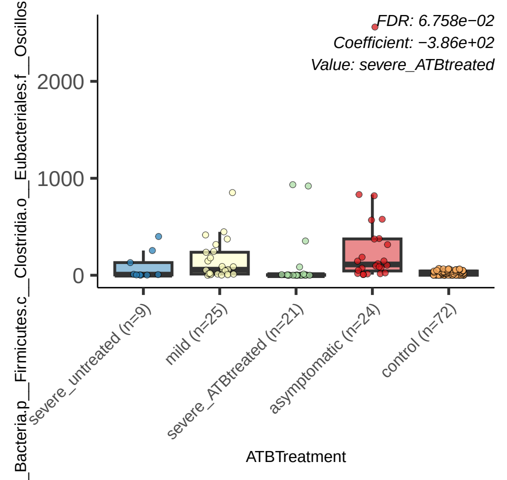

FDR: 6.758e−02
Coefficient: −3.86e+02
Value: severe_ATBtreated
Bacteria.p__Firmicutes.c__Clostridia.o__Eubacteriales.f__Oscillos
2000
1000
0
severe_untreated (n=9)
mild (n=25)
severe_ATBtreated (n=21)
asymptomatic (n=24)
control (n=72)
ATBTreatment

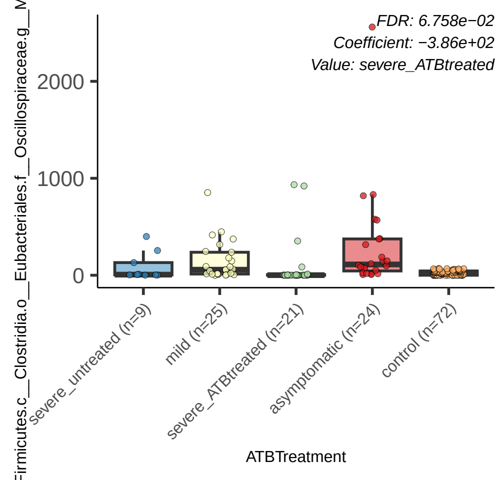

FDR: 6.758e−02
Coefficient: −3.86e+02
Value: severe_ATBtreated
Firmicutes.c__Clostridia.o__Eubacteriales.f__Oscillospiraceae.g__M
2000
1000
0
severe_untreated (n=9)
mild (n=25)
severe_ATBtreated (n=21)
asymptomatic (n=24)
control (n=72)
ATBTreatment

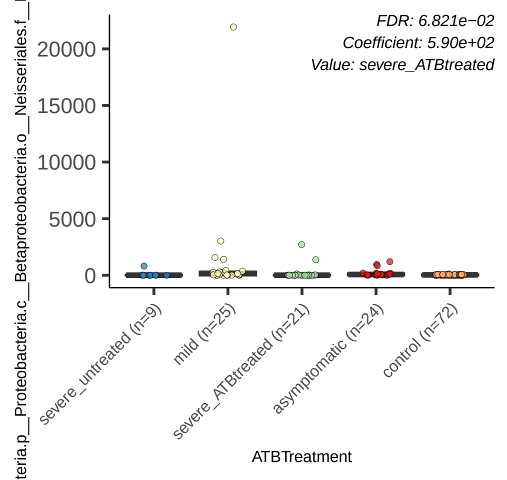

FDR: 6.821e−02
Coefficient: 5.90e+02
Value: severe_ATBtreated
teria.p__Proteobacteria.c__Betaproteobacteria.o__Neisseriales.f__
20000
15000
10000
5000
0
severe_untreated (n=9)
mild (n=25)
severe_ATBtreated (n=21)
asymptomatic (n=24)
control (n=72)
ATBTreatment

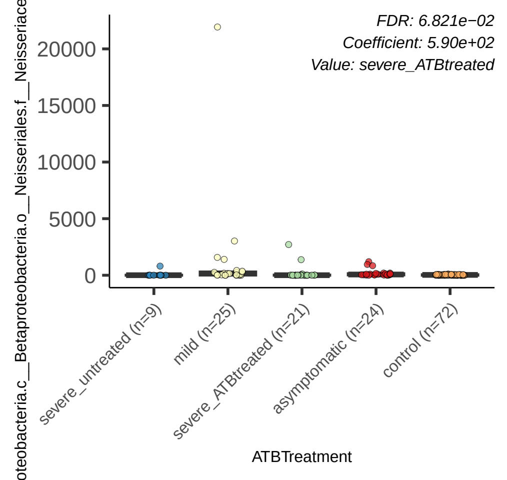

FDR: 6.821e−02
Coefficient: 5.90e+02
Value: severe_ATBtreated
20000
15000
10000
5000
0
oteobacteria.c__Betaproteobacteria.o__Neisseriales.f__Neisseriace
severe_untreated (n=9)
mild (n=25)
severe_ATBtreated (n=21)
asymptomatic (n=24)
control (n=72)
ATBTreatment

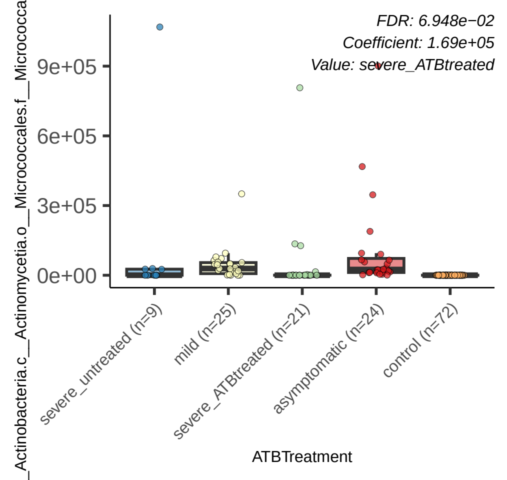

FDR: 6.948e−02
Coefficient: 1.69e+05
Value: severe_ATBtreated
9e+05
6e+05
3e+05
0e+00
severe_untreated (n=9)
mild (n=25)
severe_ATBtreated (n=21)
asymptomatic (n=24)
control (n=72)
ATBTreatment
_Actinobacteria.c__Actinomycetia.o__Micrococcales.f__Micrococca

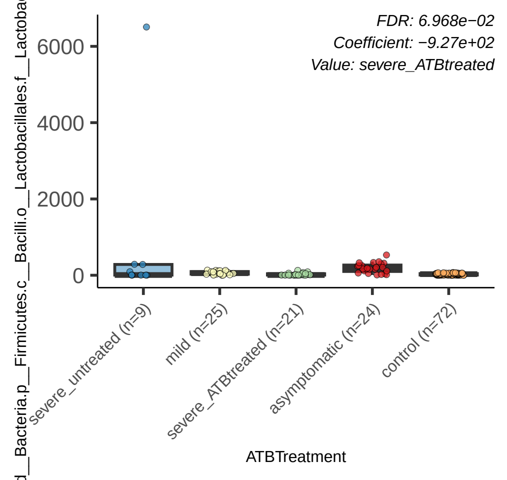

FDR: 6.968e−02
Coefficient: −9.27e+02
Value: severe_ATBtreated
d__Bacteria.p__Firmicutes.c__Bacilli.o__Lactobacillales.f__Lactobac
6000
4000
2000
0
severe_untreated (n=9)
mild (n=25)
severe_ATBtreated (n=21)
asymptomatic (n=24)
control (n=72)
ATBTreatment

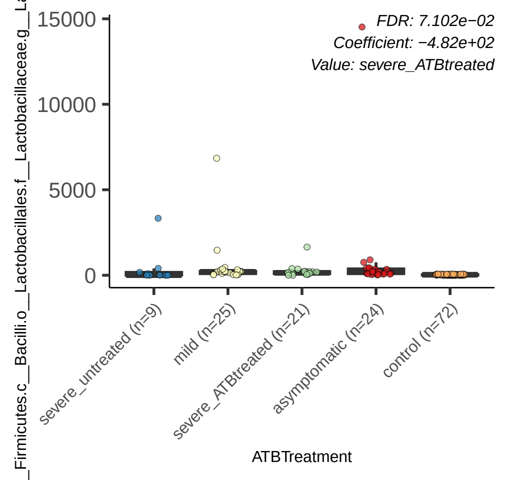
FDR: 7.102e−02
Coefficient: −4.82e+02
Value: severe_ATBtreated
15000
10000
5000
0
Firmicutes.c__Bacilli.o__Lactobacillales.f__Lactobacillaceae.g__La
severe_untreated (n=9)
mild (n=25)
severe_ATBtreated (n=21)
asymptomatic (n=24)
control (n=72)
ATBTreatment

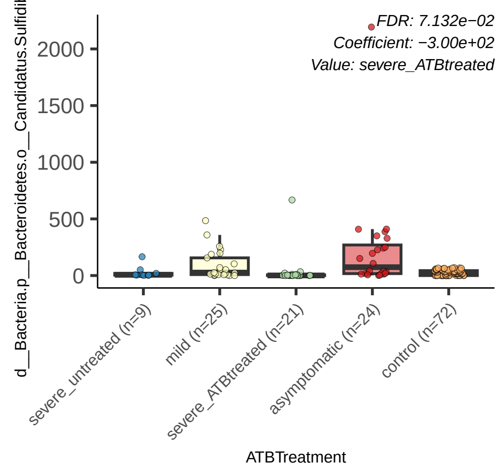
FDR: 7.132e−02
Coefficient: −3.00e+02
Value: severe_ATBtreated
d__Bacteria.p__Bacteroidetes.o__Candidatus.Sulfidib
2000
1500
1000
500
0
severe_untreated (n=9)
mild (n=25)
severe_ATBtreated (n=21)
asymptomatic (n=24)
control (n=72)
ATBTreatment

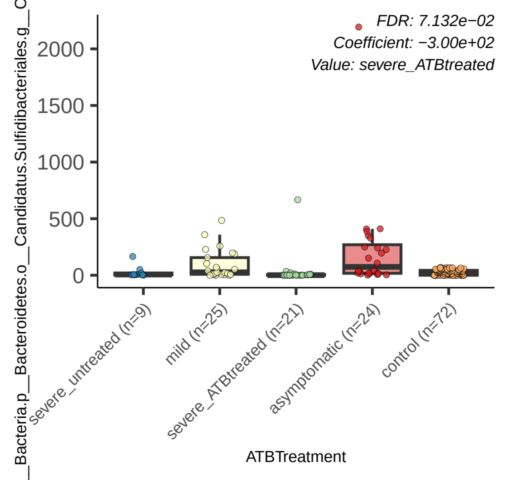

FDR: 7.132e−02
Coefficient: −3.00e+02
Value: severe_ATBtreated
2000
1500
1000
500
0
Bacteria.p__Bacteroidetes.o__Candidatus.Sulfidibacteriales.g__C
severe_untreated (n=9)
mild (n=25)
severe_ATBtreated (n=21)
asymptomatic (n=24)
control (n=72)
ATBTreatment

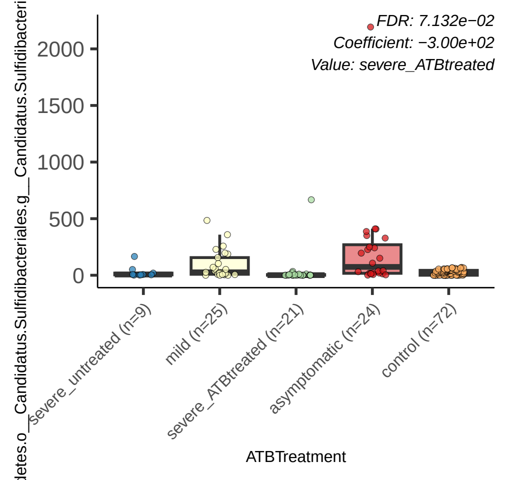

FDR: 7.132e−02
Coefficient: −3.00e+02
Value: severe_ATBtreated
2000
1500
1000
500
0
detes.o__Candidatus.Sulfidibacteriales.g__Candidatus.Sulfidibacteri
severe_untreated (n=9)
mild (n=25)
severe_ATBtreated (n=21)
asymptomatic (n=24)
control (n=72)
ATBTreatment

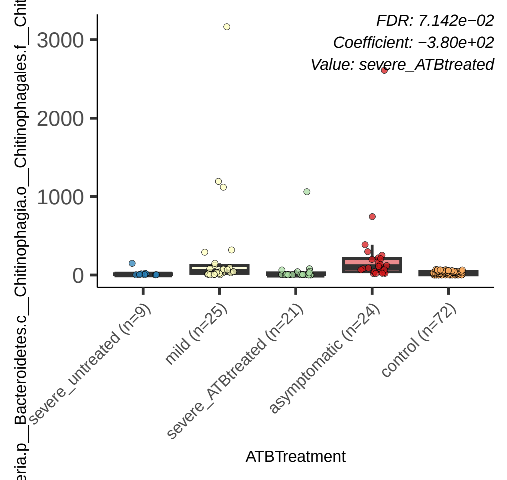

FDR: 7.142e−02
Coefficient: −3.80e+02
Value: severe_ATBtreated
3000
2000
1000
0
eria.p__Bacteroidetes.c__Chitinophagia.o__Chitinophagales.f__Chit
severe_untreated (n=9)
mild (n=25)
severe_ATBtreated (n=21)
asymptomatic (n=24)
control (n=72)
ATBTreatment

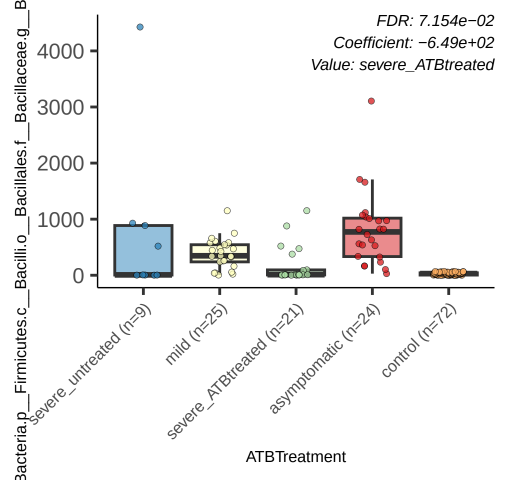
FDR: 7.154e−02
Coefficient: −6.49e+02
Value: severe_ATBtreated
Bacteria.p__Firmicutes.c__Bacilli.o__Bacillales.f__Bacillaceae.g__B
4000
3000
2000
1000
0
severe_untreated (n=9)
mild (n=25)
severe_ATBtreated (n=21)
asymptomatic (n=24)
control (n=72)
ATBTreatment

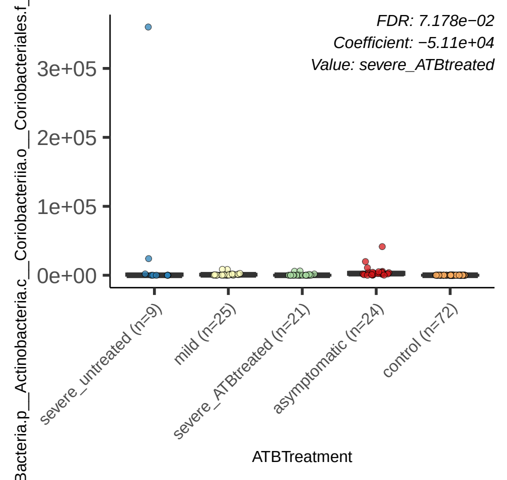

FDR: 7.178e−02
Coefficient: −5.11e+04
Value: severe_ATBtreated
3e+05
2e+05
1e+05
0e+00
Bacteria.p__Actinobacteria.c__Coriobacteriia.o__Coriobacteriales.f_
severe_untreated (n=9)
mild (n=25)
severe_ATBtreated (n=21)
asymptomatic (n=24)
control (n=72)
ATBTreatment

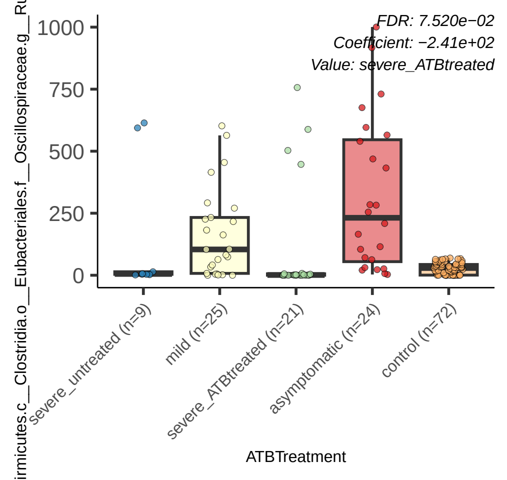

FDR: 7.520e−02
Coefficient: −2.41e+02
Value: severe_ATBtreated
1000
750
500
250
0
irmicutes.c__Clostridia.o__Eubacteriales.f__Oscillospiraceae.g__Ru
severe_untreated (n=9)
mild (n=25)
severe_ATBtreated (n=21)
asymptomatic (n=24)
control (n=72)
ATBTreatment

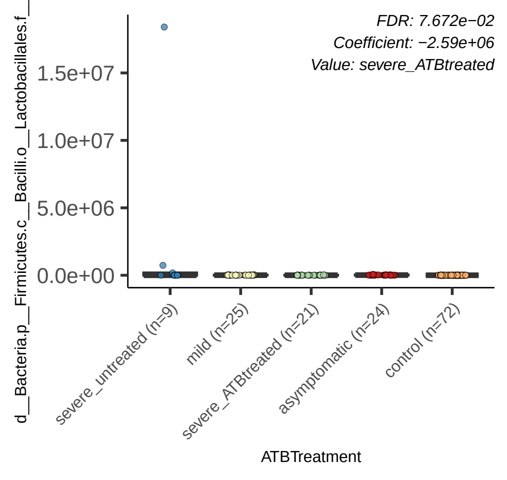

FDR: 7.672e−02
Coefficient: −2.59e+06
Value: severe_ATBtreated
d__Bacteria.p__Firmicutes.c__Bacilli.o__Lactobacillales.f__
1.5e+07
1.0e+07
5.0e+06
0.0e+00
severe_untreated (n=9)
mild (n=25)
severe_ATBtreated (n=21)
asymptomatic (n=24)
control (n=72)
ATBTreatment

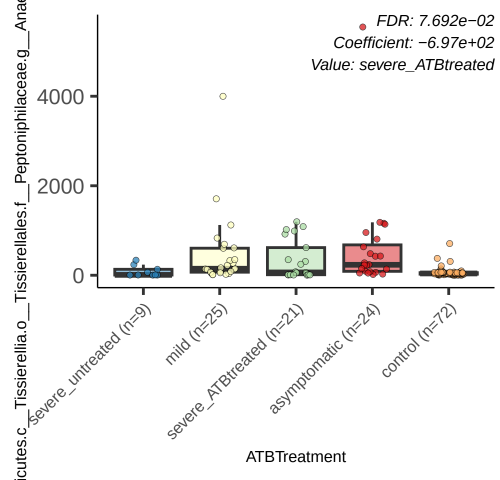

FDR: 7.692e−02
Coefficient: −6.97e+02
Value: severe_ATBtreated
4000
2000
0
severe_untreated (n=9)
mild (n=25)
severe_ATBtreated (n=21)
asymptomatic (n=24)
control (n=72)
ATBTreatment
icutes.c__Tissierellia.o__Tissierellales.f__Peptoniphilaceae.g__Anac

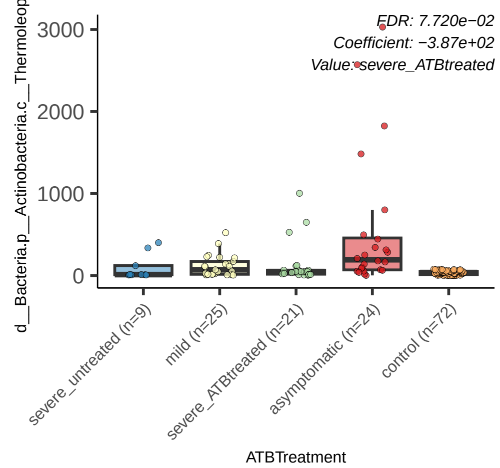

FDR: 7.720e−02
Coefficient: −3.87e+02
Value: severe_ATBtreated
d__Bacteria.p__Actinobacteria.c__Thermoleop
3000
2000
1000
0
severe_untreated (n=9)
mild (n=25)
severe_ATBtreated (n=21)
asymptomatic (n=24)
control (n=72)
ATBTreatment

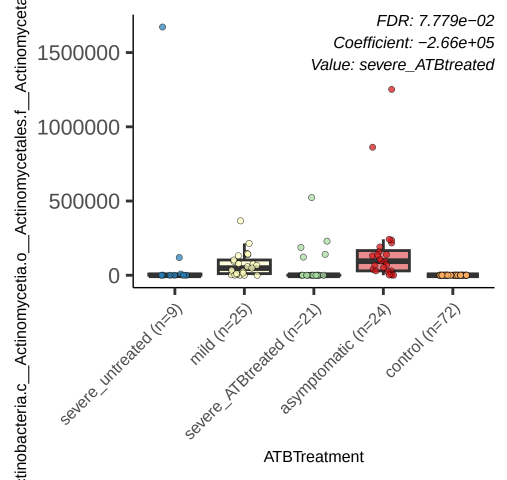

FDR: 7.779e−02
Coefficient: −2.66e+05
Value: severe_ATBtreated
1500000
1000000
500000
0
severe_untreated (n=9)
mild (n=25)
severe_ATBtreated (n=21)
asymptomatic (n=24)
control (n=72)
ATBTreatment

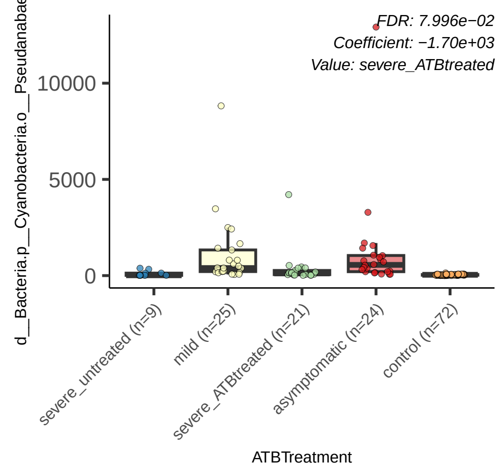

FDR: 7.996e−02
Coefficient: −1.70e+03
Value: severe_ATBtreated
d__Bacteria.p__Cyanobacteria.o__Pseudanabae
10000
5000
0
severe_untreated (n=9)
mild (n=25)
severe_ATBtreated (n=21)
asymptomatic (n=24)
control (n=72)
ATBTreatment

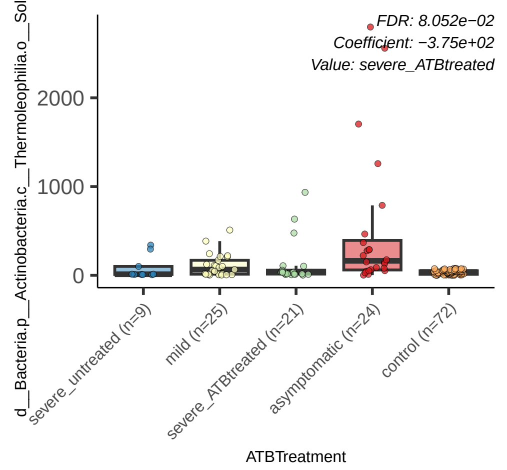
FDR: 8.052e−02
Coefficient: −3.75e+02
Value: severe_ATBtreated
d__Bacteria.p__Actinobacteria.c__Thermoleophilia.o__Sol
2000
1000
0
severe_untreated (n=9)
mild (n=25)
severe_ATBtreated (n=21)
asymptomatic (n=24)
control (n=72)
ATBTreatment

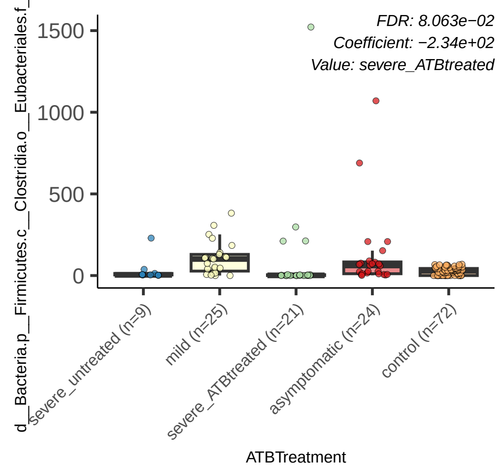

FDR: 8.063e−02
Coefficient: −2.34e+02
Value: severe_ATBtreated
d__Bacteria.p__Firmicutes.c__Clostridia.o__Eubacteriales.f__
1500
1000
500
0
severe_untreated (n=9)
mild (n=25)
severe_ATBtreated (n=21)
asymptomatic (n=24)
control (n=72)
ATBTreatment

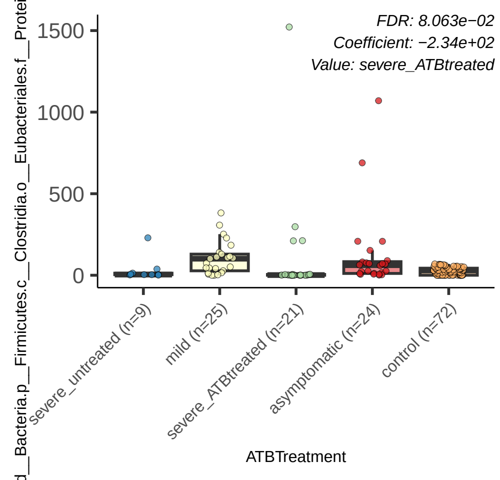

FDR: 8.063e−02
Coefficient: −2.34e+02
Value: severe_ATBtreated
1500
1000
500
0
severe_untreated (n=9)
mild (n=25)
severe_ATBtreated (n=21)
asymptomatic (n=24)
control (n=72)
ATBTreatment
d__Bacteria.p__Firmicutes.c__Clostridia.o__Eubacteriales.f__Protei

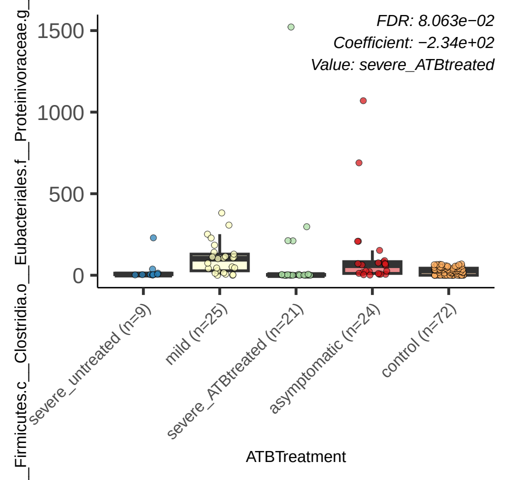

FDR: 8.063e−02
Coefficient: −2.34e+02
Value: severe_ATBtreated
Firmicutes.c__Clostridia.o__Eubacteriales.f__Proteinivoraceae.g__
0
500
1000
1500
severe_untreated (n=9)
mild (n=25)
severe_ATBtreated (n=21)
asymptomatic (n=24)
control (n=72)
ATBTreatment

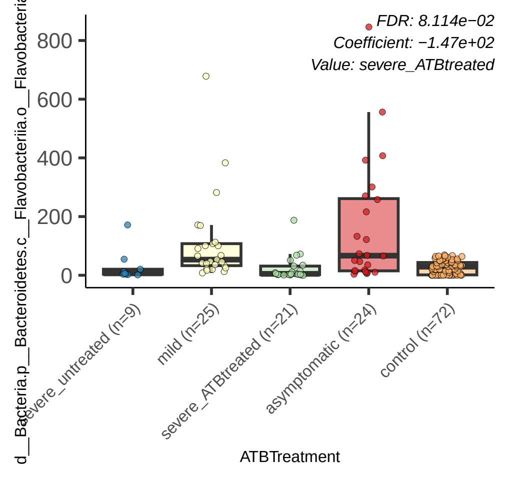

FDR: 8.114e−02
Coefficient: −1.47e+02
Value: severe_ATBtreated
d__Bacteria.p__Bacteroidetes.c__Flavobacteriia.o__Flavobacteria
800
600
400
200
0
severe_untreated (n=9)
mild (n=25)
severe_ATBtreated (n=21)
asymptomatic (n=24)
control (n=72)
ATBTreatment

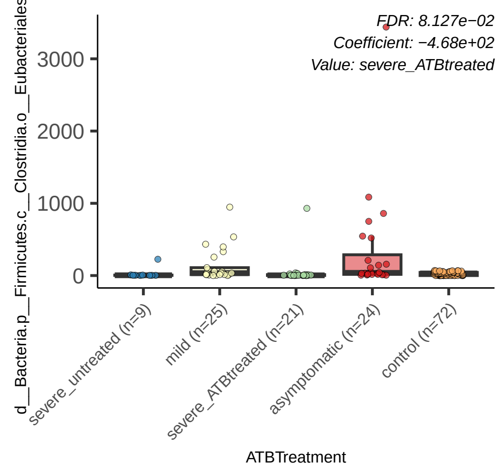

FDR: 8.127e−02
Coefficient: −4.68e+02
Value: severe_ATBtreated
3000
2000
1000
0
d__Bacteria.p__Firmicutes.c__Clostridia.o__Eubacteriales
severe_untreated (n=9)
mild (n=25)
severe_ATBtreated (n=21)
asymptomatic (n=24)
control (n=72)
ATBTreatment

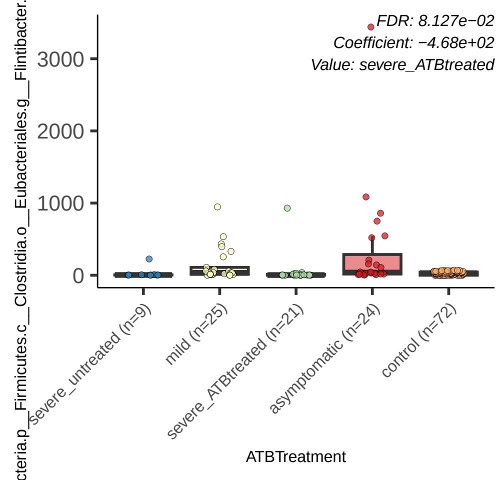

FDR: 8.127e−02
Coefficient: −4.68e+02
Value: severe_ATBtreated
cteria.p__Firmicutes.c__Clostridia.o__Eubacteriales.g__Flintibacter.
3000
2000
1000
0
severe_untreated (n=9)
mild (n=25)
severe_ATBtreated (n=21)
asymptomatic (n=24)
control (n=72)
ATBTreatment

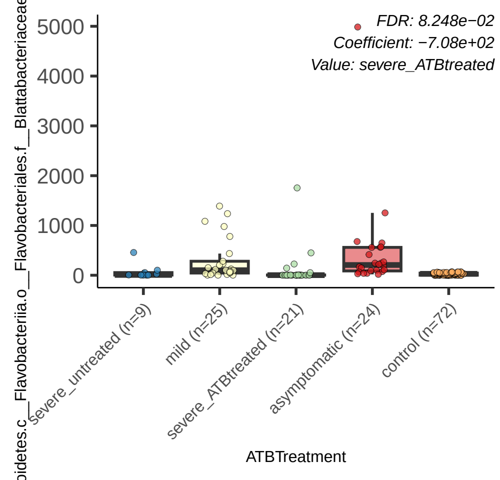

FDR: 8.248e−02
Coefficient: −7.08e+02
Value: severe_ATBtreated
5000
4000
3000
2000
1000
0
severe_untreated (n=9)
mild (n=25)
severe_ATBtreated (n=21)
asymptomatic (n=24)
control (n=72)
ATBTreatment

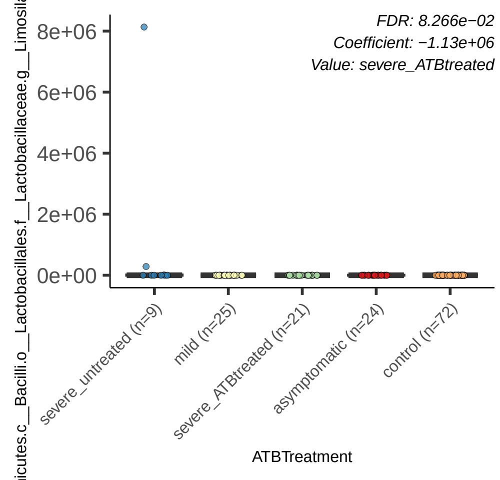
FDR: 8.266e−02
Coefficient: −1.13e+06
Value: severe_ATBtreated
8e+06
6e+06
4e+06
2e+06
0e+00
severe_untreated (n=9)
mild (n=25)
severe_ATBtreated (n=21)
asymptomatic (n=24)
control (n=72)
ATBTreatment

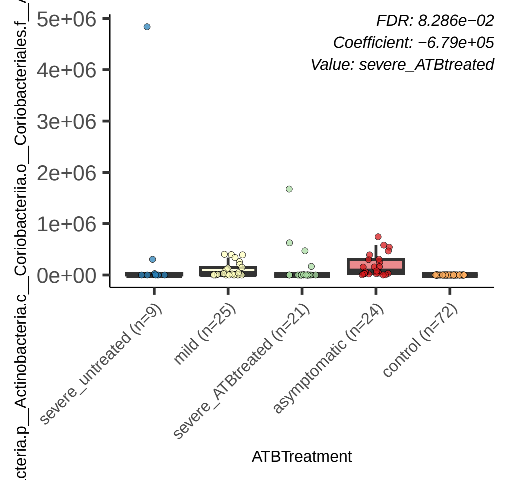
FDR: 8.286e−02
Coefficient: −6.79e+05
Value: severe_ATBtreated
5e+06
4e+06
3e+06
2e+06
1e+06
0e+00
Coriobacteriia.o__Coriobacteriales.f
Actinobacteria.c__
cteria.p__Actinobacteria.c__Coriobacteriia.o__Coriobacteriales.f
severe_untreated (n=9)
mild (n=25)
severe_ATBtreated (n=21)
asymptomatic (n=24)
control (n=72)
ATBTreatment

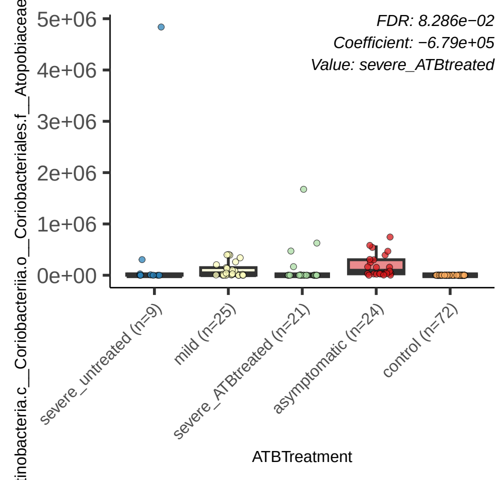
FDR: 8.286e−02
Coefficient: −6.79e+05
Value: severe_ATBtreated
5e+06
4e+06
3e+06
2e+06
1e+06
0e+00
tinobacteria.c__Coriobacteriia.o__Coriobacteriales.f__Atopobiaceae
severe_untreated (n=9)
mild (n=25)
severe_ATBtreated (n=21)
asymptomatic (n=24)
control (n=72)
ATBTreatment

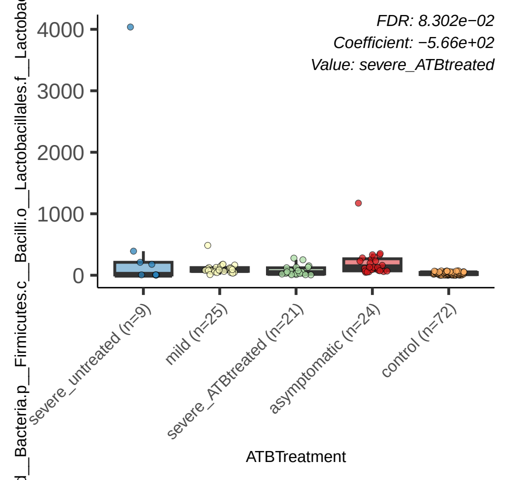
FDR: 8.302e−02
Coefficient: −5.66e+02
Value: severe_ATBtreated
d__Bacteria.p__Firmicutes.c__Bacilli.o__Lactobacillales.f__Lactobac
4000
3000
2000
1000
0
severe_untreated (n=9)
mild (n=25)
severe_ATBtreated (n=21)
asymptomatic (n=24)
control (n=72)
ATBTreatment

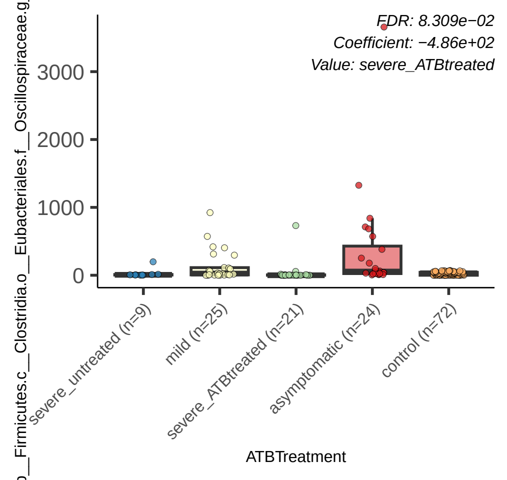
FDR: 8.309e−02
Coefficient: −4.86e+02
Value: severe_ATBtreated
3000
2000
1000
0
o__Firmicutes.c__Clostridia.o__Eubacteriales.f__Oscillospiraceae.g
severe_untreated (n=9)
mild (n=25)
severe_ATBtreated (n=21)
asymptomatic (n=24)
control (n=72)
ATBTreatment

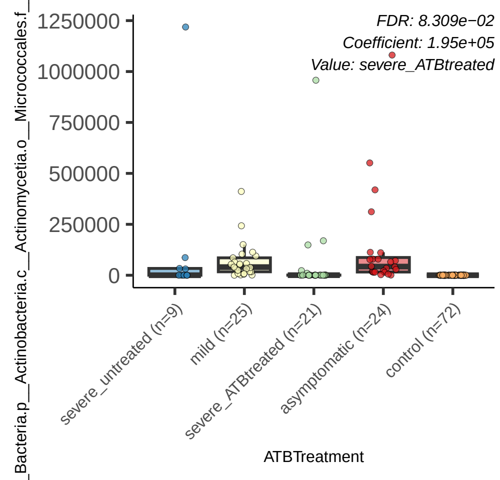

FDR: 8.309e−02
Coefficient: 1.95e+05
Value: severe_ATBtreated
Bacteria.p__Actinobacteria.c__Actinomycetia.o__Micrococcales.f
1250000
1000000
750000
500000
250000
0
severe_untreated (n=9)
mild (n=25)
severe_ATBtreated (n=21)
asymptomatic (n=24)
control (n=72)
ATBTreatment

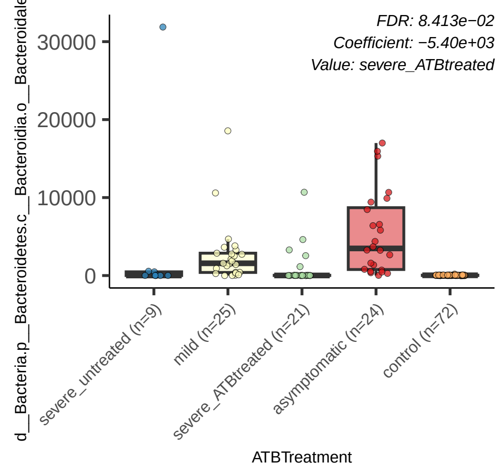

FDR: 8.413e−02
Coefficient: −5.40e+03
Value: severe_ATBtreated
30000
20000
10000
0
d__Bacteria.p__Bacteroidetes.c__Bacteroidia.o__Bacteroidale
severe_untreated (n=9)
mild (n=25)
severe_ATBtreated (n=21)
asymptomatic (n=24)
control (n=72)
ATBTreatment

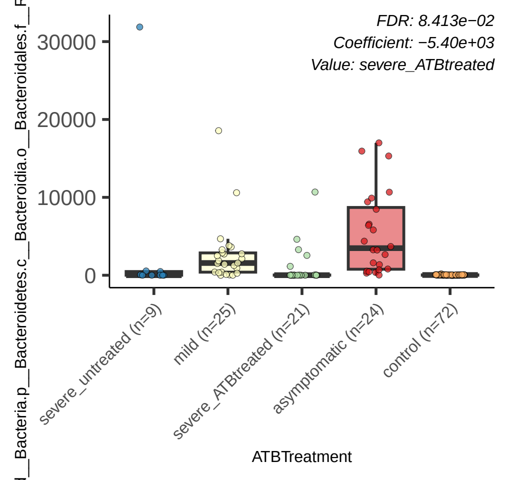

FDR: 8.413e−02
Coefficient: −5.40e+03
Value: severe_ATBtreated
30000
20000
10000
0
severe_untreated (n=9)
mild (n=25)
severe_ATBtreated (n=21)
asymptomatic (n=24)
control (n=72)
ATBTreatment

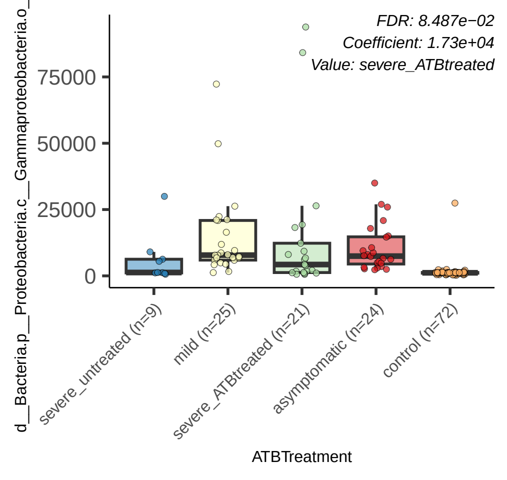

FDR: 8.487e−02
Coefficient: 1.73e+04
Value: severe_ATBtreated
d__Bacteria.p__Proteobacteria.c__Gammaproteobacteria.o__
75000
50000
25000
0
severe_untreated (n=9)
mild (n=25)
severe_ATBtreated (n=21)
asymptomatic (n=24)
control (n=72)
ATBTreatment

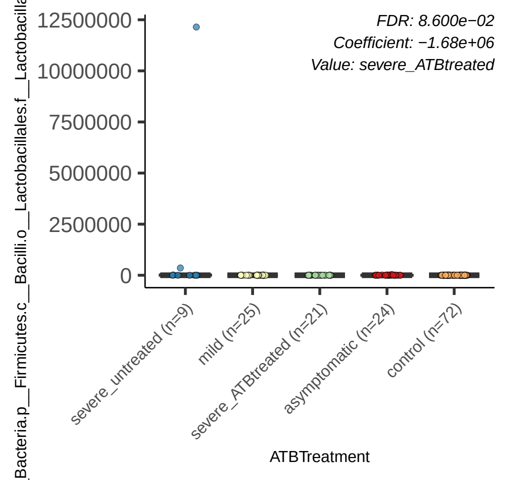

FDR: 8.600e−02
Coefficient: −1.68e+06
Value: severe_ATBtreated
Bacteria.p__Firmicutes.c__Bacilli.o__Lactobacillales.f__Lactobacilla
12500000
10000000
7500000
5000000
2500000
0
severe_untreated (n=9)
mild (n=25)
severe_ATBtreated (n=21)
asymptomatic (n=24)
control (n=72)
ATBTreatment

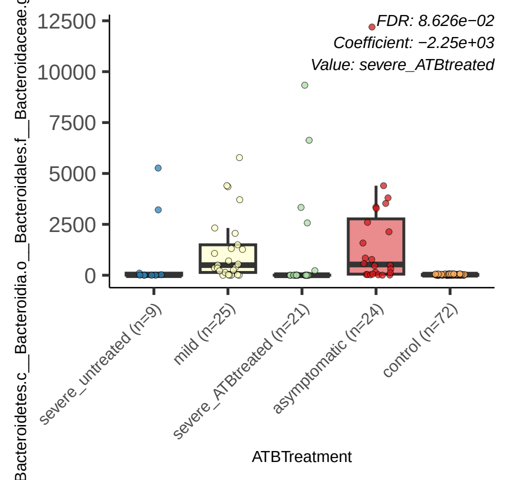

FDR: 8.626e−02
Coefficient: −2.25e+03
Value: severe_ATBtreated
12500
10000
7500
5000
2500
0
Bacteroidetes.c__Bacteroidia.o__Bacteroidales.f__Bacteroidaceae.g
severe_untreated (n=9)
mild (n=25)
severe_ATBtreated (n=21)
asymptomatic (n=24)
control (n=72)
ATBTreatment

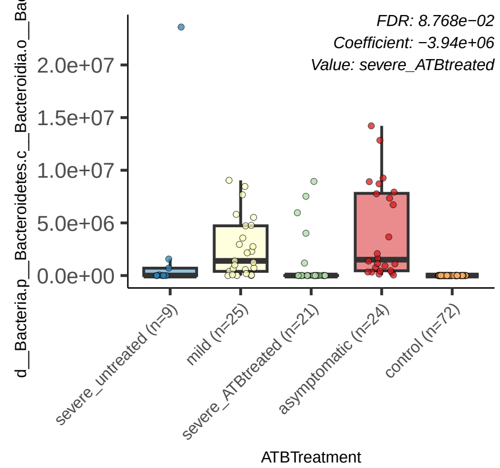

FDR: 8.768e−02
Coefficient: −3.94e+06
Value: severe_ATBtreated
d__Bacteria.p__Bacteroidetes.c__Bacteroidia.o__Bac
2.0e+07
1.5e+07
1.0e+07
5.0e+06
0.0e+00
severe_untreated (n=9)
mild (n=25)
severe_ATBtreated (n=21)
asymptomatic (n=24)
control (n=72)
ATBTreatment

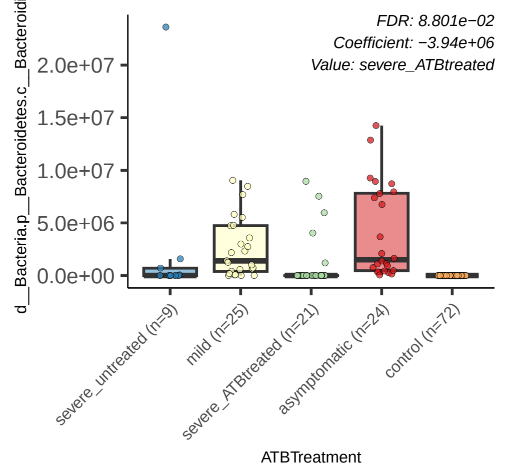

FDR: 8.801e−02
Coefficient: −3.94e+06
Value: severe_ATBtreated
d__Bacteria.p__Bacteroidetes.c__Bacteroidi
2.0e+07
1.5e+07
1.0e+07
5.0e+06
0.0e+00
severe_untreated (n=9)
mild (n=25)
severe_ATBtreated (n=21)
asymptomatic (n=24)
control (n=72)
ATBTreatment

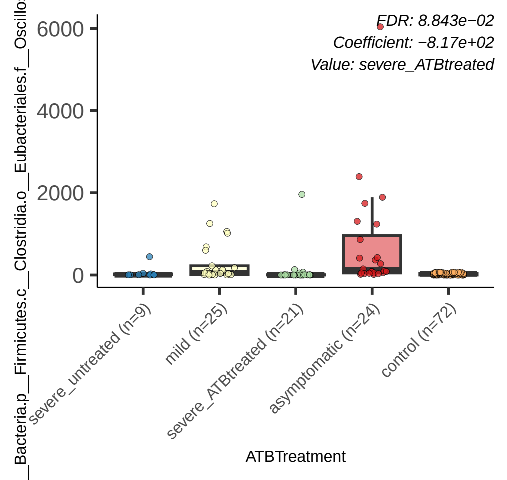

FDR: 8.843e−02
Coefficient: −8.17e+02
Value: severe_ATBtreated
6000
4000
2000
0
severe_untreated (n=9)
mild (n=25)
severe_ATBtreated (n=21)
asymptomatic (n=24)
control (n=72)
ATBTreatment

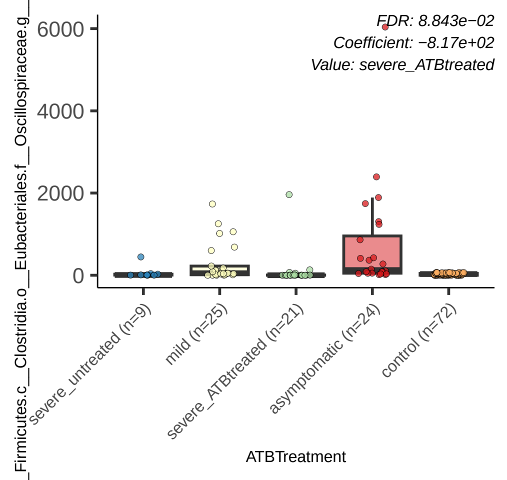

FDR: 8.843e−02
Coefficient: −8.17e+02
Value: severe_ATBtreated
Firmicutes.c__Clostridia.o__Eubacteriales.f__Oscillospiraceae.g__
6000
4000
2000
0
severe_untreated (n=9)
mild (n=25)
severe_ATBtreated (n=21)
asymptomatic (n=24)
control (n=72)
ATBTreatment

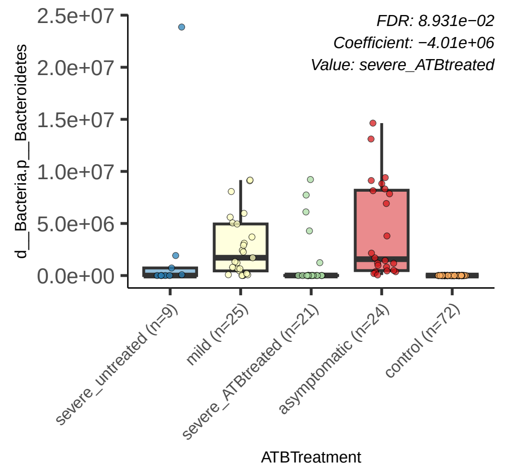
FDR: 8.931e−02
Coefficient: −4.01e+06
Value: severe_ATBtreated
d__Bacteria.p__Bacteroidetes
2.5e+07
2.0e+07
1.5e+07
1.0e+07
5.0e+06
0.0e+00
severe_untreated (n=9)
mild (n=25)
severe_ATBtreated (n=21)
asymptomatic (n=24)
control (n=72)
ATBTreatment

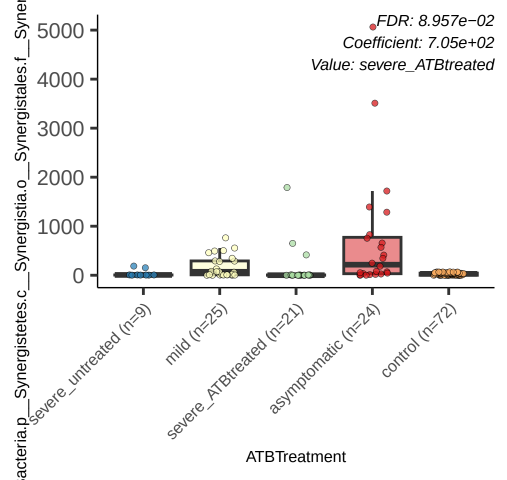
FDR: 8.957e−02
Coefficient: 7.05e+02
Value: severe_ATBtreated
5000
4000
3000
2000
1000
0
Bacteria.p__Synergistetes.c__Synergistia.o__Synergistales.f__Syner
severe_untreated (n=9)
mild (n=25)
severe_ATBtreated (n=21)
asymptomatic (n=24)
control (n=72)
ATBTreatment

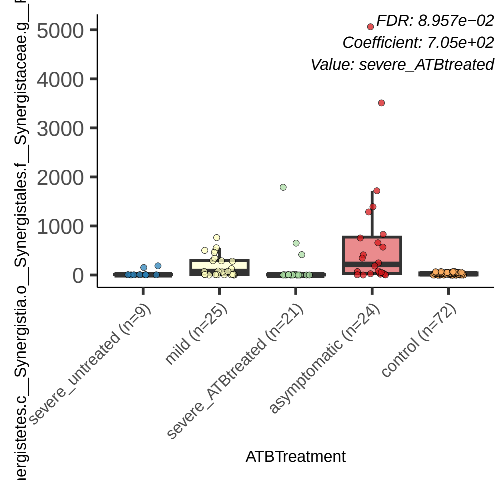

FDR: 8.957e−02
Coefficient: 7.05e+02
Value: severe_ATBtreated
5000
4000
3000
2000
1000
0
ergistetes.c__Synergistia.o__Synergistales.f__Synergistaceae.g__
severe_untreated (n=9)
mild (n=25)
severe_ATBtreated (n=21)
asymptomatic (n=24)
control (n=72)
ATBTreatment

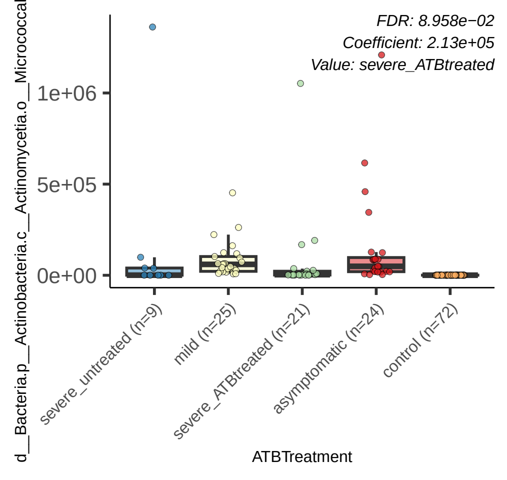
FDR: 8.958e−02
Coefficient: 2.13e+05
Value: severe_ATBtreated
d__Bacteria.p__Actinobacteria.c__Actinomycetia.o__Micrococcal
1e+06
5e+05
0e+00
severe_untreated (n=9)
mild (n=25)
severe_ATBtreated (n=21)
asymptomatic (n=24)
control (n=72)
ATBTreatment

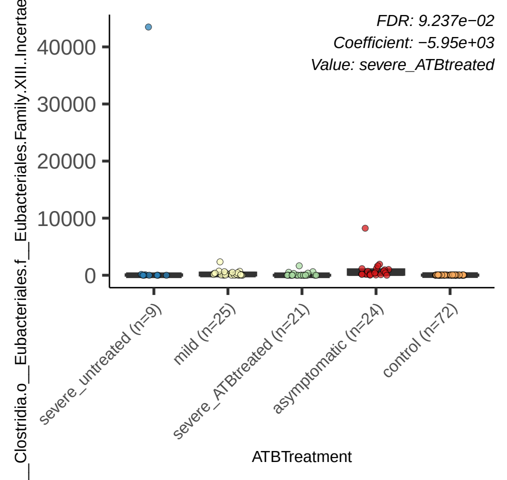

FDR: 9.237e−02
Coefficient: −5.95e+03
Value: severe_ATBtreated
40000
30000
20000
10000
0
__Clostridia.o__Eubacteriales.f__Eubacteriales.Family.XIII..Incertae
severe_untreated (n=9)
mild (n=25)
severe_ATBtreated (n=21)
asymptomatic (n=24)
control (n=72)
ATBTreatment

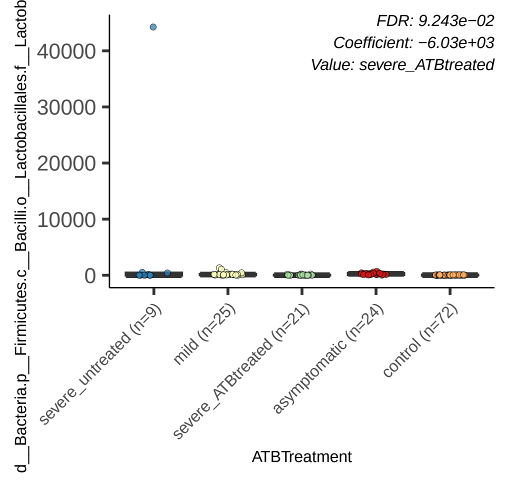
FDR: 9.243e−02
Coefficient: −6.03e+03
Value: severe_ATBtreated
d__Bacteria.p__Firmicutes.c__Bacilli.o__Lactobacillales.f__Lactob
40000
30000
20000
10000
0
severe_untreated (n=9)
mild (n=25)
severe_ATBtreated (n=21)
asymptomatic (n=24)
control (n=72)
ATBTreatment

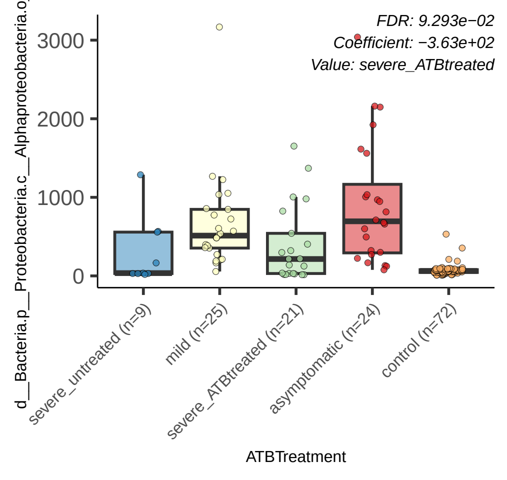
FDR: 9.293e−02
Coefficient: −3.63e+02
Value: severe_ATBtreated
d__Bacteria.p__Proteobacteria.c__Alphaproteobacteria.o_
3000
2000
1000
0
severe_untreated (n=9)
mild (n=25)
severe_ATBtreated (n=21)
asymptomatic (n=24)
control (n=72)
ATBTreatment

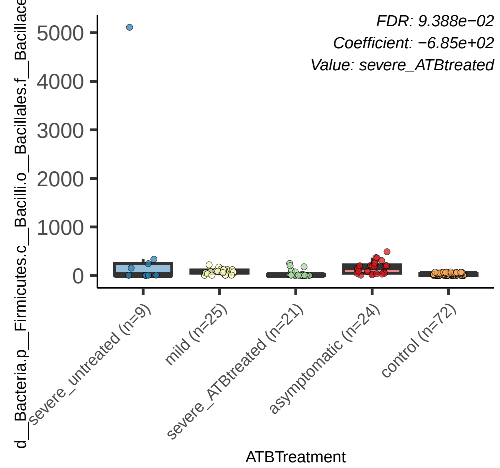
FDR: 9.388e−02
Coefficient: −6.85e+02
Value: severe_ATBtreated
d__Bacteria.p__Firmicutes.c__Bacilli.o__Bacillales.f__Bacillace
5000
4000
3000
2000
1000
0
severe_untreated (n=9)
mild (n=25)
severe_ATBtreated (n=21)
asymptomatic (n=24)
control (n=72)
ATBTreatment

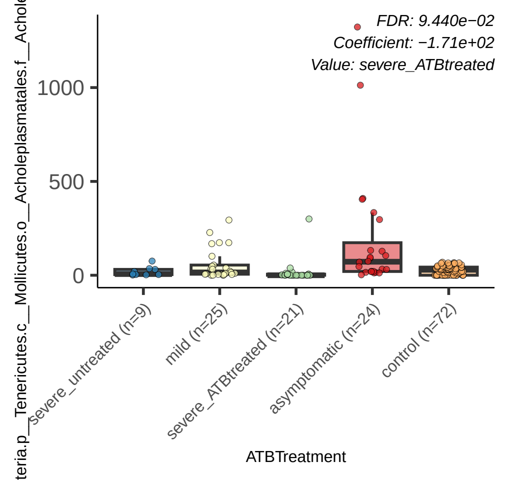
FDR: 9.440e−02
Coefficient: −1.71e+02
Value: severe_ATBtreated
1000
500
0
severe_untreated (n=9)
mild (n=25)
severe_ATBtreated (n=21)
asymptomatic (n=24)
control (n=72)
ATBTreatment
teria.p__Tenericutes.c__Mollicutes.o__Acholeplasmatales.f__Achole

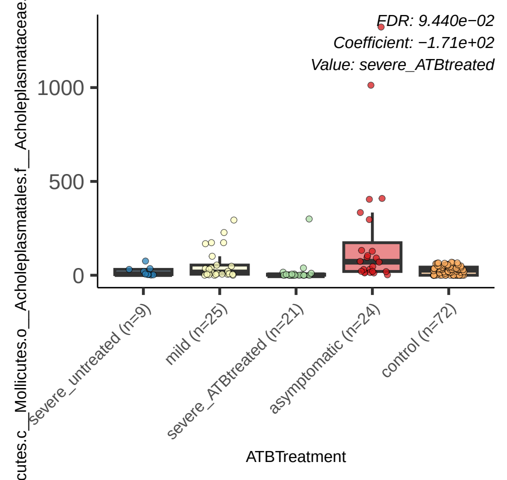
FDR: 9.440e−02
Coefficient: −1.71e+02
Value: severe_ATBtreated
1000
500
0
cutes.c__Mollicutes.o__Acholeplasmatales.f__Acholeplasmataceae.
severe_untreated (n=9)
mild (n=25)
severe_ATBtreated (n=21)
asymptomatic (n=24)
control (n=72)
ATBTreatment

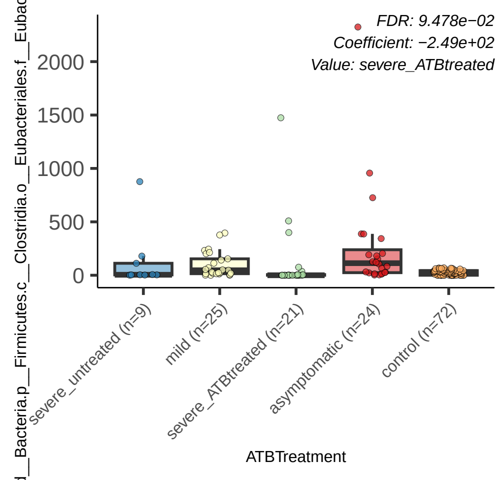

FDR: 9.478e−02
Coefficient: −2.49e+02
Value: severe_ATBtreated
2000
1500
1000
500
0
d__Bacteria.p__Firmicutes.c__Clostridia.o__Eubacteriales.f__Eubac
severe_untreated (n=9)
mild (n=25)
severe_ATBtreated (n=21)
asymptomatic (n=24)
control (n=72)
ATBTreatment

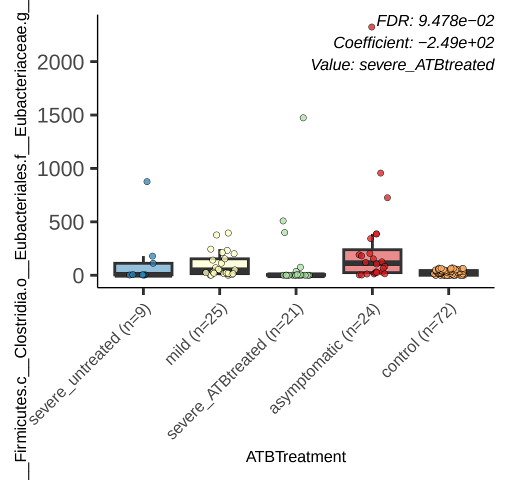
FDR: 9.478e−02
Coefficient: −2.49e+02
Value: severe_ATBtreated
Firmicutes.c__Clostridia.o__Eubacteriales.f__Eubacteriaceae.g__
2000
1500
1000
500
0
severe_untreated (n=9)
mild (n=25)
severe_ATBtreated (n=21)
asymptomatic (n=24)
control (n=72)
ATBTreatment

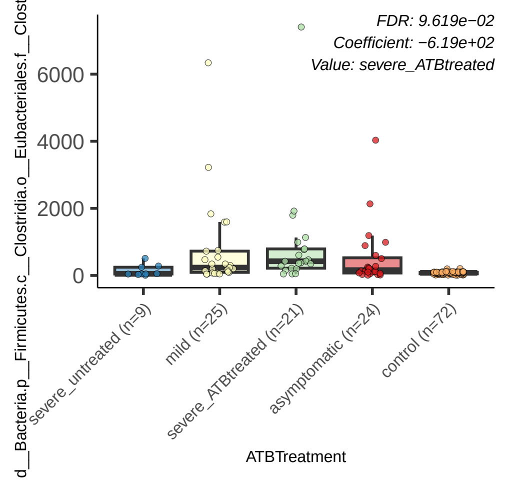

d__Bacteria.p__Firmicutes.c__Clostridia.o__Eubacteriales.f__Clost
FDR: 9.619e−02
Coefficient: −6.19e+02
Value: severe_ATBtreated
6000
4000
2000
0
severe_untreated (n=9)
mild (n=25)
severe_ATBtreated (n=21)
asymptomatic (n=24)
control (n=72)
ATBTreatment

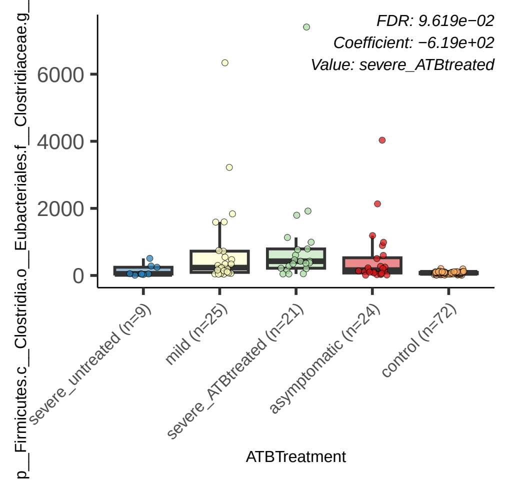
FDR: 9.619e−02
Coefficient: −6.19e+02
Value: severe_ATBtreated
p__Firmicutes.c__Clostridia.o__Eubacteriales.f__Clostridiaceae.g__
6000
4000
2000
0
severe_untreated (n=9)
mild (n=25)
severe_ATBtreated (n=21)
asymptomatic (n=24)
control (n=72)
ATBTreatment

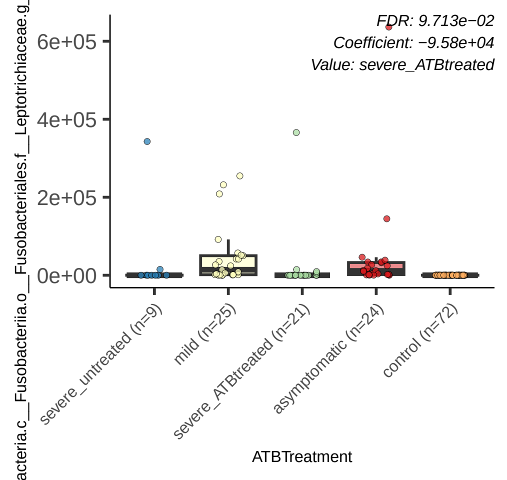

FDR: 9.713e−02
Coefficient: −9.58e+04
Value: severe_ATBtreated
acteria.c__Fusobacteriia.o__Fusobacteriales.f__Leptotrichiaceae.g_
6e+05
4e+05
2e+05
0e+00
severe_untreated (n=9)
mild (n=25)
severe_ATBtreated (n=21)
asymptomatic (n=24)
control (n=72)
ATBTreatment

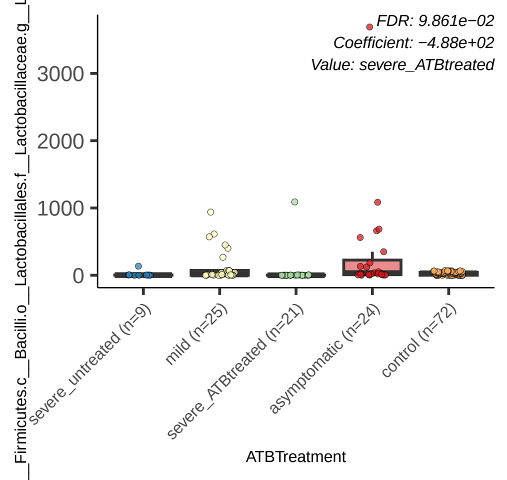
Firmicutes.c__Bacilli.o__Lactobacillales.f__Lactobacillaceae.g__L
FDR: 9.861e−02
Coefficient: −4.88e+02
Value: severe_ATBtreated
3000
2000
1000
0
severe_untreated (n=9)
mild (n=25)
severe_ATBtreated (n=21)
asymptomatic (n=24)
control (n=72)
ATBTreatment

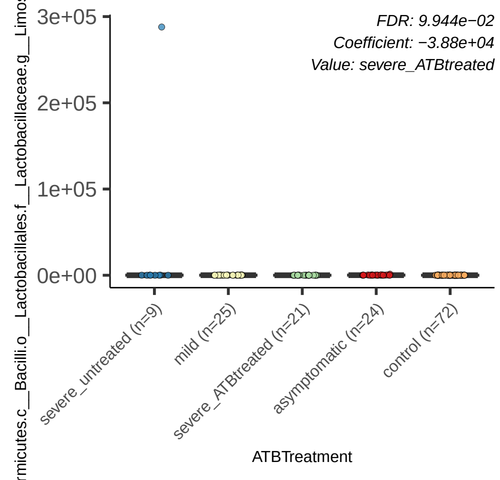
FDR: 9.944e−02
Coefficient: −3.88e+04
Value: severe_ATBtreated
3e+05
2e+05
1e+05
0e+00
rmicutes.c__Bacilli.o__Lactobacillales.f__Lactobacillaceae.g__Limos
severe_untreated (n=9)
mild (n=25)
severe_ATBtreated (n=21)
asymptomatic (n=24)
control (n=72)
ATBTreatment

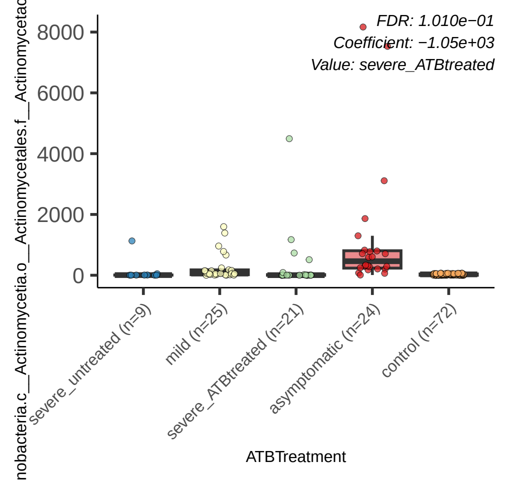

FDR: 1.010e−01
Coefficient: −1.05e+03
Value: severe_ATBtreated
8000
6000
4000
2000
0
nobacteria.c__Actinomycetia.o__Actinomycetales.f__Actinomycetac
severe_untreated (n=9)
mild (n=25)
severe_ATBtreated (n=21)
asymptomatic (n=24)
control (n=72)
ATBTreatment

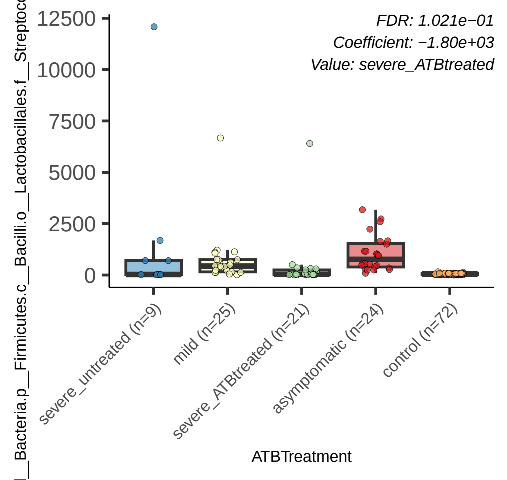

FDR: 1.021e−01
Coefficient: −1.80e+03
Value: severe_ATBtreated
__Bacteria.p__Firmicutes.c__Bacilli.o__Lactobacillales.f__Streptoco
12500
10000
7500
5000
2500
0
severe_untreated (n=9)
mild (n=25)
severe_ATBtreated (n=21)
asymptomatic (n=24)
control (n=72)
ATBTreatment

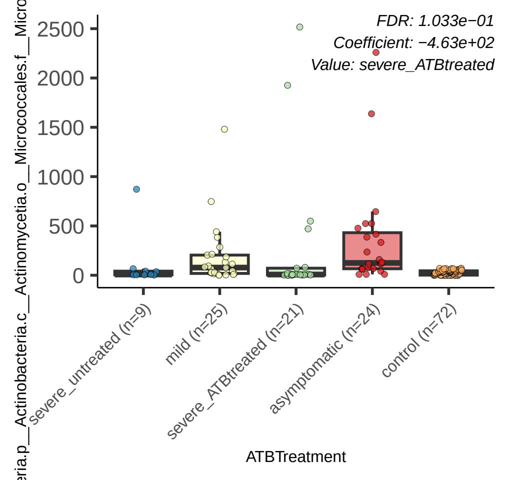

FDR: 1.033e−01
Coefficient: −4.63e+02
Value: severe_ATBtreated
2500
2000
1500
1000
500
0
severe_untreated (n=9)
mild (n=25)
severe_ATBtreated (n=21)
asymptomatic (n=24)
control (n=72)
ATBTreatment

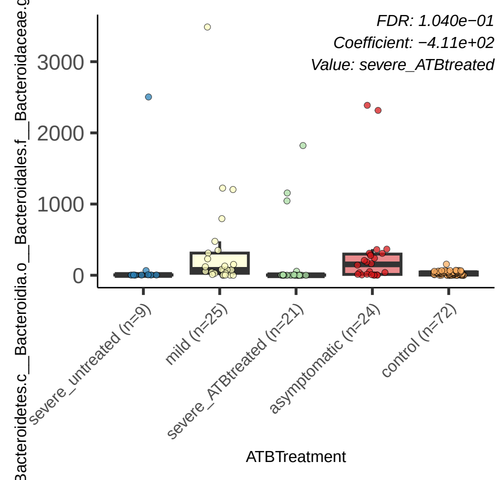

FDR: 1.040e−01
Coefficient: −4.11e+02
Value: severe_ATBtreated
0
1000
2000
3000
Bacteroidetes.c__Bacteroidia.o__Bacteroidales.f__Bacteroidaceae.g
severe_untreated (n=9)
mild (n=25)
severe_ATBtreated (n=21)
asymptomatic (n=24)
control (n=72)
ATBTreatment

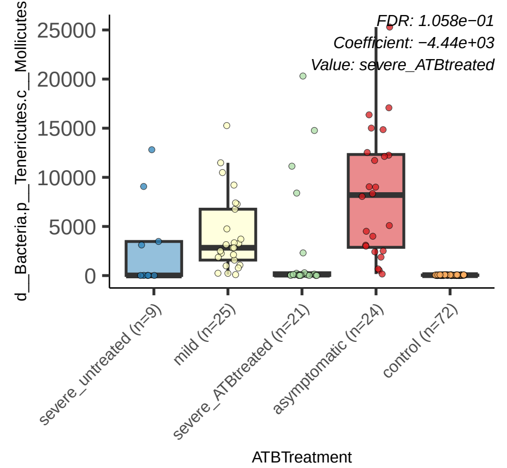
FDR: 1.058e−01
Coefficient: −4.44e+03
Value: severe_ATBtreated
d__Bacteria.p__Tenericutes.c__Mollicutes
25000
20000
15000
10000
5000
0
severe_untreated (n=9)
mild (n=25)
severe_ATBtreated (n=21)
asymptomatic (n=24)
control (n=72)
ATBTreatment

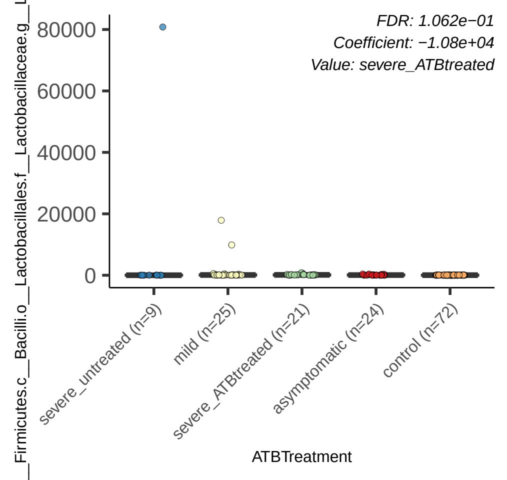
FDR: 1.062e−01
Coefficient: −1.08e+04
Value: severe_ATBtreated
Firmicutes.c__Bacilli.o__Lactobacillales.f__Lactobacillaceae.g__L
80000
60000
40000
20000
0
severe_untreated (n=9)
mild (n=25)
severe_ATBtreated (n=21)
asymptomatic (n=24)
control (n=72)
ATBTreatment

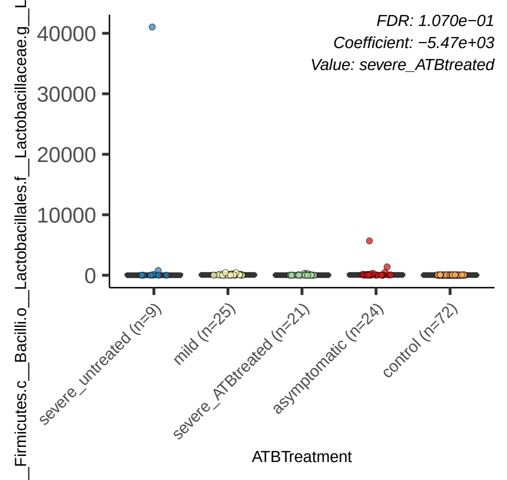

FDR: 1.070e−01
Coefficient: −5.47e+03
Value: severe_ATBtreated
Firmicutes.c__Bacilli.o__Lactobacillales.f__Lactobacillaceae.g__L
40000
30000
20000
10000
0
severe_untreated (n=9)
mild (n=25)
severe_ATBtreated (n=21)
asymptomatic (n=24)
control (n=72)
ATBTreatment

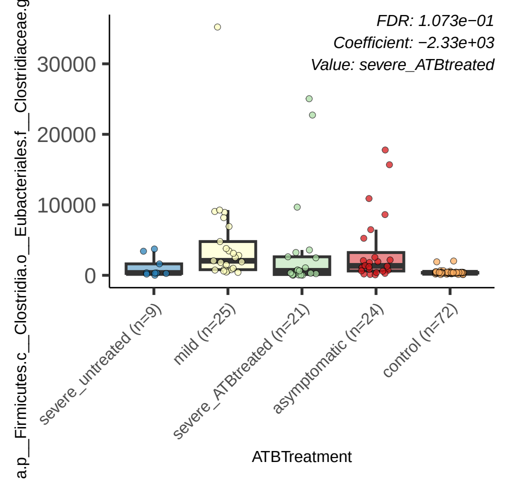

FDR: 1.073e−01
Coefficient: −2.33e+03
Value: severe_ATBtreated
30000
20000
10000
0
a.p__Firmicutes.c__Clostridia.o__Eubacteriales.f__Clostridiaceae.g
severe_untreated (n=9)
mild (n=25)
severe_ATBtreated (n=21)
asymptomatic (n=24)
control (n=72)
ATBTreatment

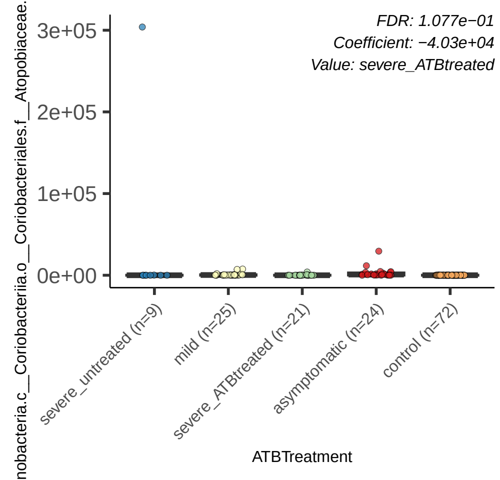

FDR: 1.077e−01
Coefficient: −4.03e+04
Value: severe_ATBtreated
3e+05
2e+05
1e+05
0e+00
nobacteria.c__Coriobacteriia.o__Coriobacteriales.f__Atopobiaceae.
severe_untreated (n=9)
mild (n=25)
severe_ATBtreated (n=21)
asymptomatic (n=24)
control (n=72)
ATBTreatment

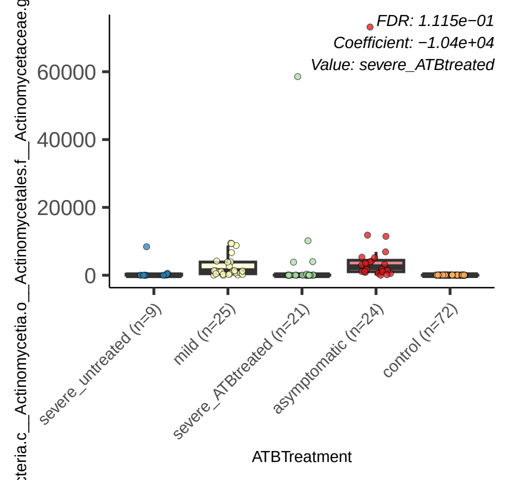

FDR: 1.115e−01
Coefficient: −1.04e+04
Value: severe_ATBtreated
60000
40000
20000
0
severe_untreated (n=9)
mild (n=25)
severe_ATBtreated (n=21)
asymptomatic (n=24)
control (n=72)
ATBTreatment
cteria.c__Actinomycetia.o__Actinomycetales.f__Actinomycetaceae.g

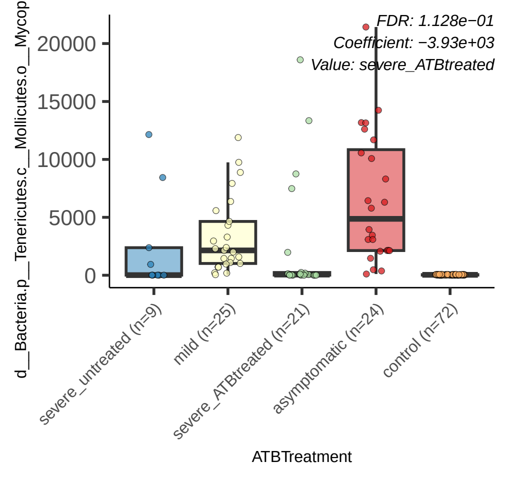

FDR: 1.128e−01
Coefficient: −3.93e+03
Value: severe_ATBtreated
d__Bacteria.p__Tenericutes.c__Mollicutes.o__Mycop
20000
15000
10000
5000
0
severe_untreated (n=9)
mild (n=25)
severe_ATBtreated (n=21)
asymptomatic (n=24)
control (n=72)
ATBTreatment

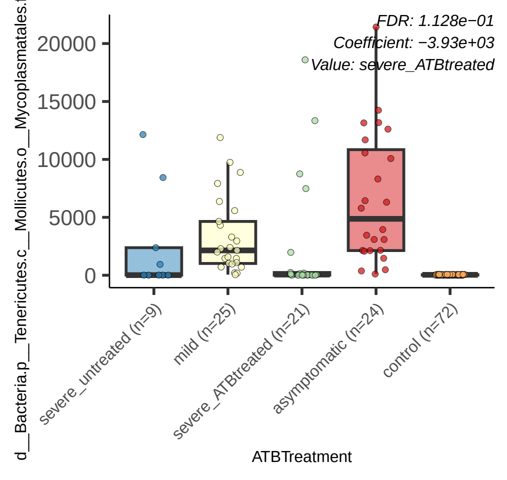
FDR: 1.128e−01
Coefficient: −3.93e+03
Value: severe_ATBtreated
d__Bacteria.p__Tenericutes.c__Mollicutes.o__Mycoplasmatales.f
20000
15000
10000
5000
0
severe_untreated (n=9)
mild (n=25)
severe_ATBtreated (n=21)
asymptomatic (n=24)
control (n=72)
ATBTreatment

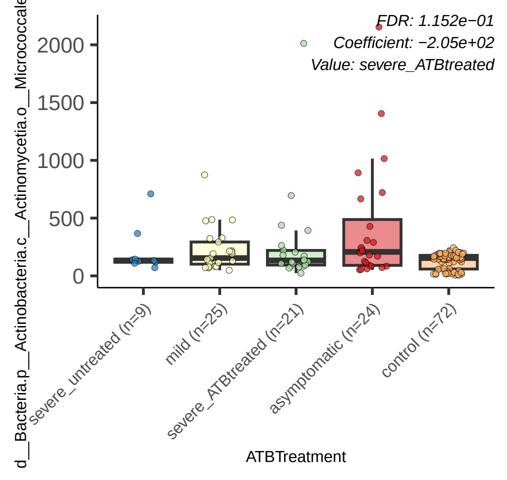

FDR: 1.152e−01
Coefficient: −2.05e+02
Value: severe_ATBtreated
d__Bacteria.p__Actinobacteria.c__Actinomycetia.o__Micrococcale
0
500
1000
1500
2000
severe_untreated (n=9)
mild (n=25)
severe_ATBtreated (n=21)
asymptomatic (n=24)
control (n=72)
ATBTreatment

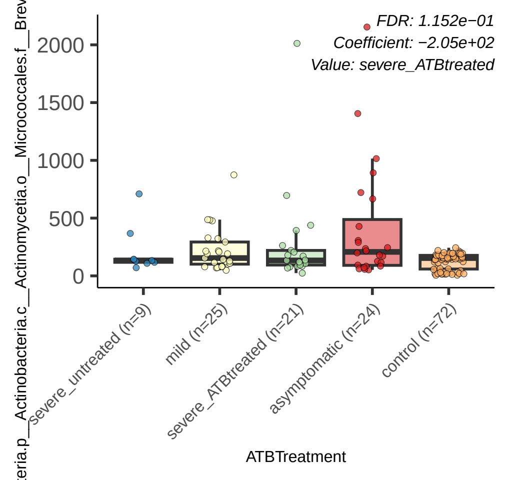

FDR: 1.152e−01
Coefficient: −2.05e+02
Value: severe_ATBtreated
2000
1500
1000
500
0
teria.p__Actinobacteria.c__Actinomycetia.o__Micrococcales.f__Brev
severe_untreated (n=9)
mild (n=25)
severe_ATBtreated (n=21)
asymptomatic (n=24)
control (n=72)
ATBTreatment

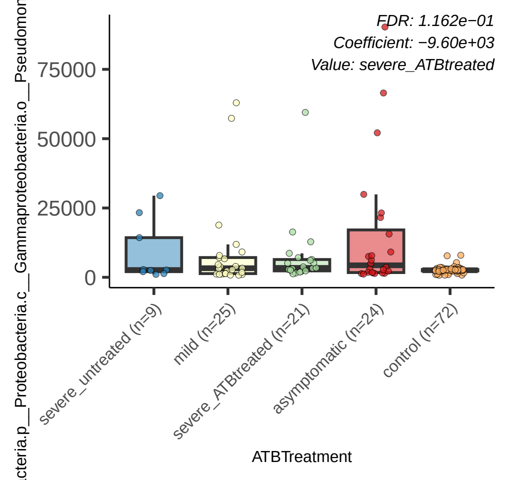

FDR: 1.162e−01
Coefficient: −9.60e+03
Value: severe_ATBtreated
75000
50000
25000
0
cteria.p__Proteobacteria.c__Gammaproteobacteria.o__Pseudomon
severe_untreated (n=9)
mild (n=25)
severe_ATBtreated (n=21)
asymptomatic (n=24)
control (n=72)
ATBTreatment

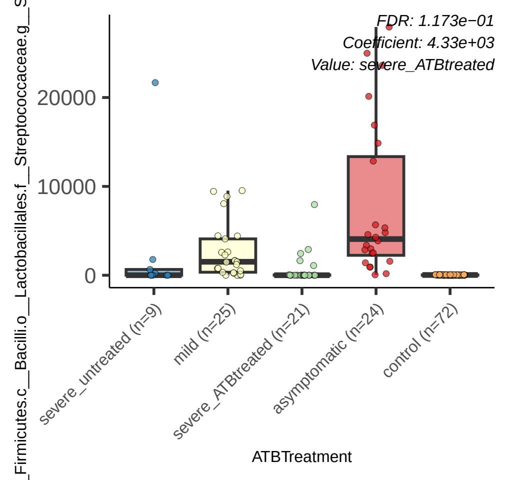

FDR: 1.173e−01
Coefficient: 4.33e+03
Value: severe_ATBtreated
20000
10000
0
Firmicutes.c__Bacilli.o__Lactobacillales.f__Streptococcaceae.g__S
severe_untreated (n=9)
mild (n=25)
severe_ATBtreated (n=21)
asymptomatic (n=24)
control (n=72)
ATBTreatment

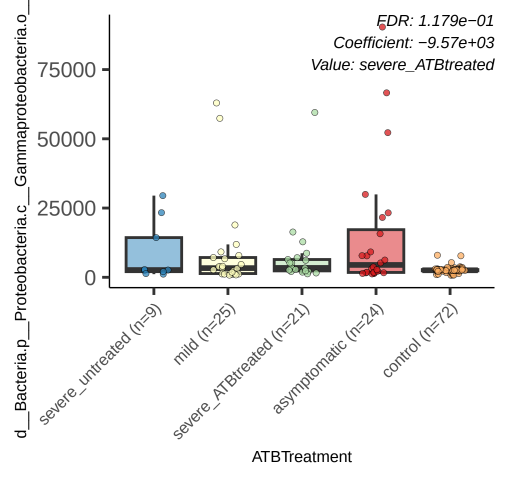

FDR: 1.179e−01
Coefficient: −9.57e+03
Value: severe_ATBtreated
d__Bacteria.p__Proteobacteria.c__Gammaproteobacteria.o__
75000
50000
25000
0
severe_untreated (n=9)
mild (n=25)
severe_ATBtreated (n=21)
asymptomatic (n=24)
control (n=72)
ATBTreatment

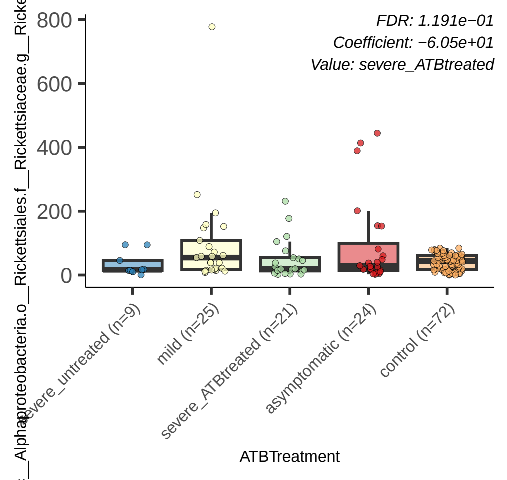

FDR: 1.191e−01
Coefficient: −6.05e+01
Value: severe_ATBtreated
800
600
400
200
0
Alphaproteobacteria.o__Rickettsiales.f__Rickettsiaceae.g__Ricke
severe_untreated (n=9)
mild (n=25)
severe_ATBtreated (n=21)
asymptomatic (n=24)
control (n=72)
ATBTreatment

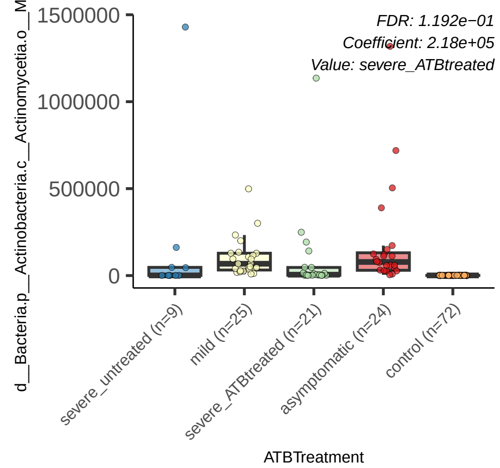

FDR: 1.192e−01
Coefficient: 2.18e+05
Value: severe_ATBtreated
d__Bacteria.p__Actinobacteria.c__Actinomycetia.o__M
1500000
1000000
500000
0
severe_untreated (n=9)
mild (n=25)
severe_ATBtreated (n=21)
asymptomatic (n=24)
control (n=72)
ATBTreatment

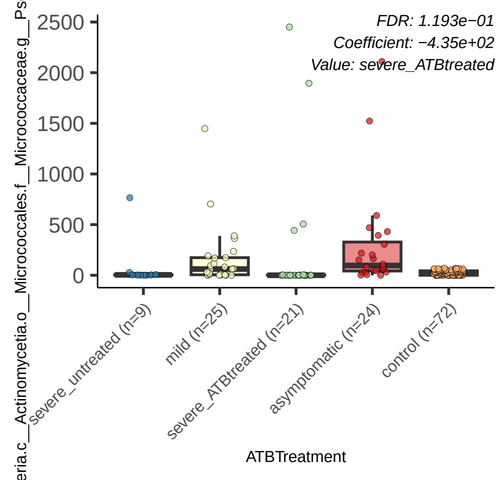

FDR: 1.193e−01
Coefficient: −4.35e+02
Value: severe_ATBtreated
2500
2000
1500
1000
500
0
eria.c__Actinomycetia.o__Micrococcales.f__Micrococcaceae.g__Ps
severe_untreated (n=9)
mild (n=25)
severe_ATBtreated (n=21)
asymptomatic (n=24)
control (n=72)
ATBTreatment

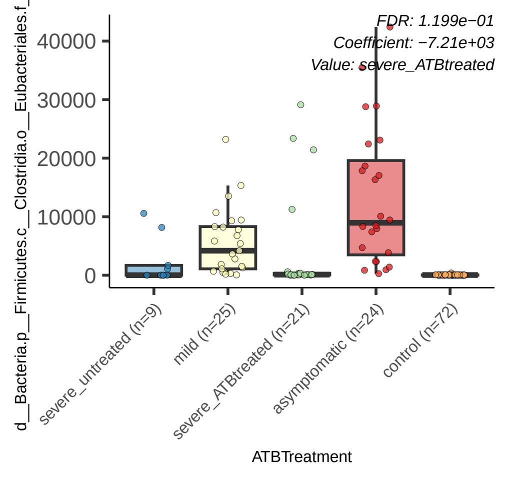

FDR: 1.199e−01
Coefficient: −7.21e+03
Value: severe_ATBtreated
d__Bacteria.p__Firmicutes.c__Clostridia.o__Eubacteriales.f_
40000
30000
20000
10000
0
severe_untreated (n=9)
mild (n=25)
severe_ATBtreated (n=21)
asymptomatic (n=24)
control (n=72)
ATBTreatment

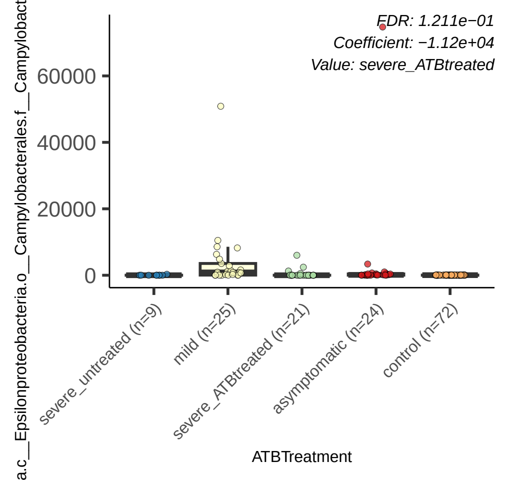

FDR: 1.211e−01
Coefficient: −1.12e+04
Value: severe_ATBtreated
60000
40000
20000
0
a.c__Epsilonproteobacteria.o__Campylobacterales.f__Campylobact
severe_untreated (n=9)
mild (n=25)
severe_ATBtreated (n=21)
asymptomatic (n=24)
control (n=72)
ATBTreatment

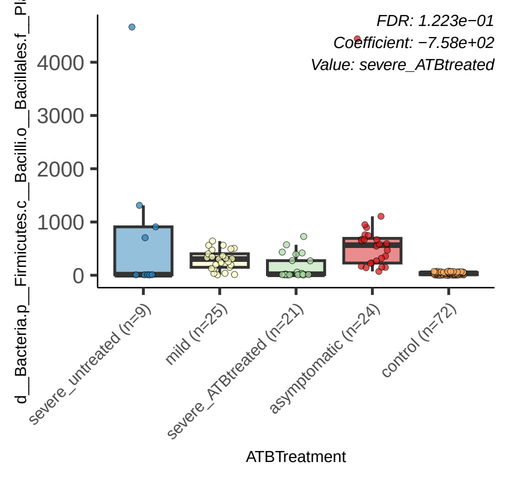

FDR: 1.223e−01
Coefficient: −7.58e+02
Value: severe_ATBtreated
d__Bacteria.p__Firmicutes.c__Bacilli.o__Bacillales.f__Pl
4000
3000
2000
1000
0
severe_untreated (n=9)
mild (n=25)
severe_ATBtreated (n=21)
asymptomatic (n=24)
control (n=72)
ATBTreatment

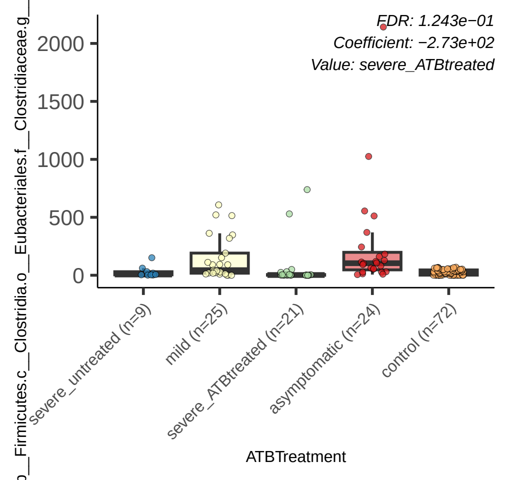
FDR: 1.243e−01
Coefficient: −2.73e+02
Value: severe_ATBtreated
2000
1500
1000
500
0
o__Clostridia.o__Eubacteriales.f__Clostridiaceae.g__
Firmicutes.c__
severe_untreated (n=9)
mild (n=25)
severe_ATBtreated (n=21)
asymptomatic (n=24)
control (n=72)
ATBTreatment

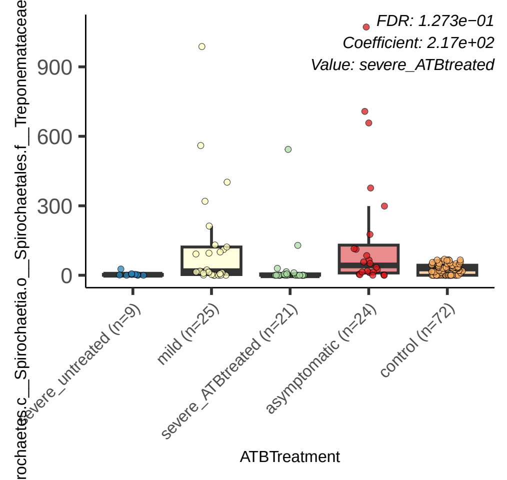

FDR: 1.273e−01
Coefficient: 2.17e+02
Value: severe_ATBtreated
rochaetes.c__Spirochaetia.o__Spirochaetales.f__Treponemataceae
900
600
300
0
severe_untreated (n=9)
mild (n=25)
severe_ATBtreated (n=21)
asymptomatic (n=24)
control (n=72)
ATBTreatment

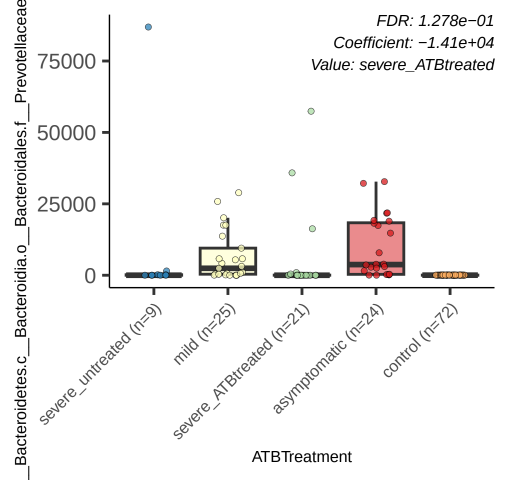
FDR: 1.278e−01
Coefficient: −1.41e+04
Value: severe_ATBtreated
Bacteroidetes.c__Bacteroidia.o__Bacteroidales.f__Prevotellaceae
75000
50000
25000
0
severe_untreated (n=9)
mild (n=25)
severe_ATBtreated (n=21)
asymptomatic (n=24)
control (n=72)
ATBTreatment

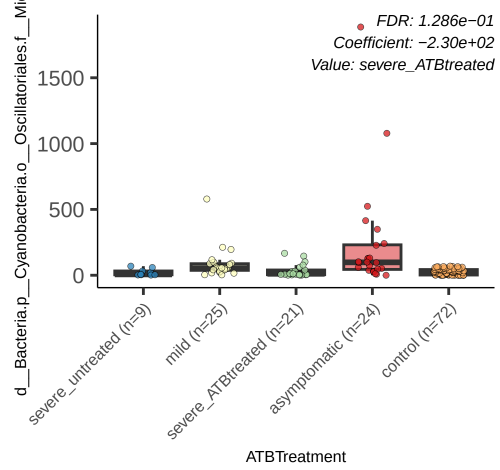

FDR: 1.286e−01
Coefficient: −2.30e+02
Value: severe_ATBtreated
d__Bacteria.p__Cyanobacteria.o__Oscillatoriales.f__Mi
1500
1000
500
0
severe_untreated (n=9)
mild (n=25)
severe_ATBtreated (n=21)
asymptomatic (n=24)
control (n=72)
ATBTreatment

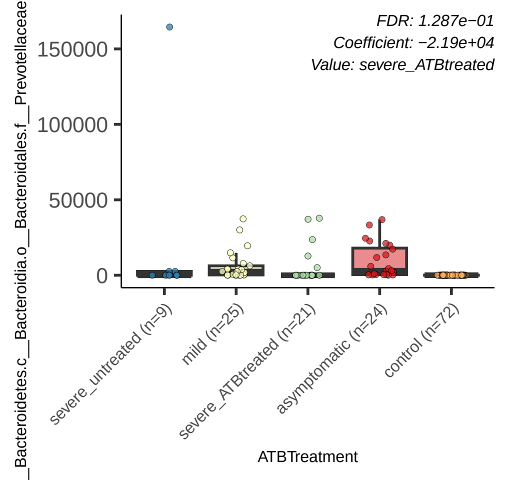

FDR: 1.287e−01
Coefficient: −2.19e+04
Value: severe_ATBtreated
150000
100000
50000
0
Bacteroidetes.c__Bacteroidia.o__Bacteroidales.f__Prevotellaceae
severe_untreated (n=9)
mild (n=25)
severe_ATBtreated (n=21)
asymptomatic (n=24)
control (n=72)
ATBTreatment

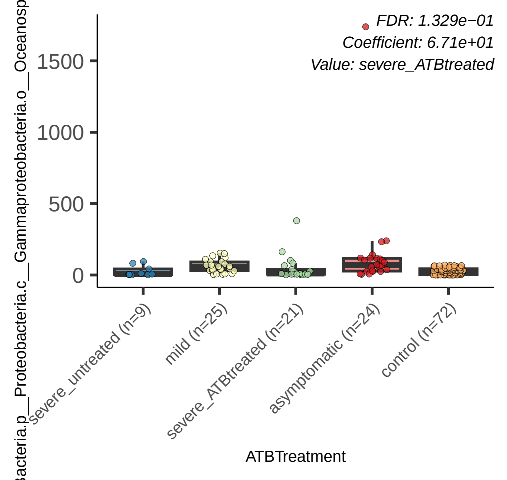
FDR: 1.329e−01
Coefficient: 6.71e+01
Value: severe_ATBtreated
Bacteria.p__Proteobacteria.c__Gammaproteobacteria.o__Oceanosp
1500
1000
500
0
severe_untreated (n=9)
mild (n=25)
severe_ATBtreated (n=21)
asymptomatic (n=24)
control (n=72)
ATBTreatment

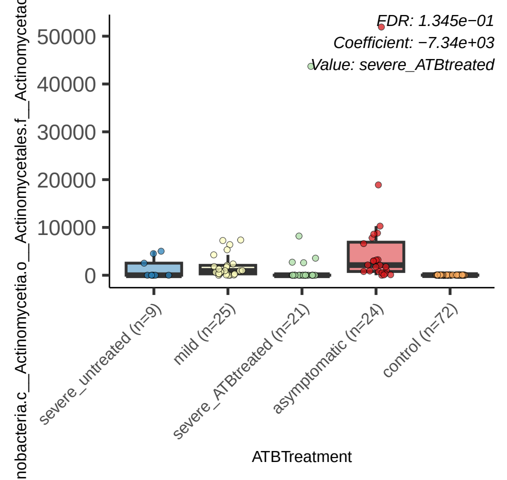

nobacteria.c__Actinomycetia.o__Actinomycetales.f__Actinomycetac
50000
40000
30000
20000
10000
0
FDR: 1.345e−01
Coefficient: −7.34e+03
Value: severe_ATBtreated
severe_untreated (n=9)
mild (n=25)
severe_ATBtreated (n=21)
asymptomatic (n=24)
control (n=72)
ATBTreatment

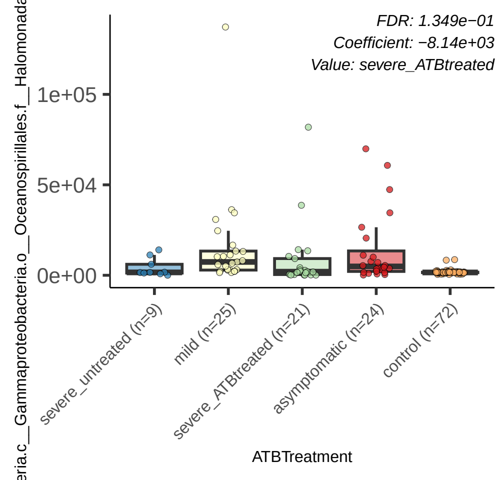
FDR: 1.349e−01
Coefficient: −8.14e+03
Value: severe_ATBtreated
1e+05
5e+04
0e+00
severe_untreated (n=9)
mild (n=25)
severe_ATBtreated (n=21)
asymptomatic (n=24)
control (n=72)
ATBTreatment

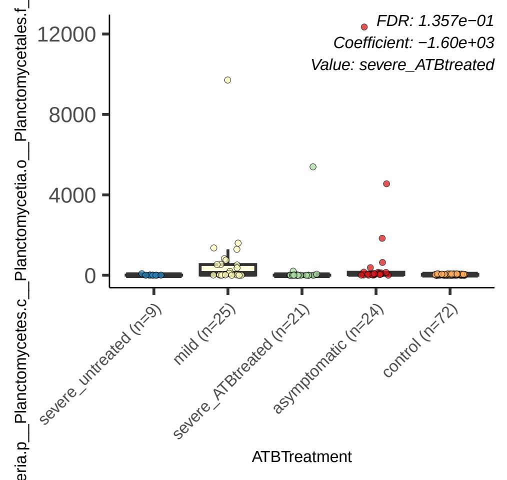

FDR: 1.357e−01
Coefficient: −1.60e+03
Value: severe_ATBtreated
12000
8000
4000
0
severe_untreated (n=9)
mild (n=25)
severe_ATBtreated (n=21)
asymptomatic (n=24)
control (n=72)
ATBTreatment
eria.p__Planctomycetes.c__Planctomycetia.o__Planctomycetales.f__

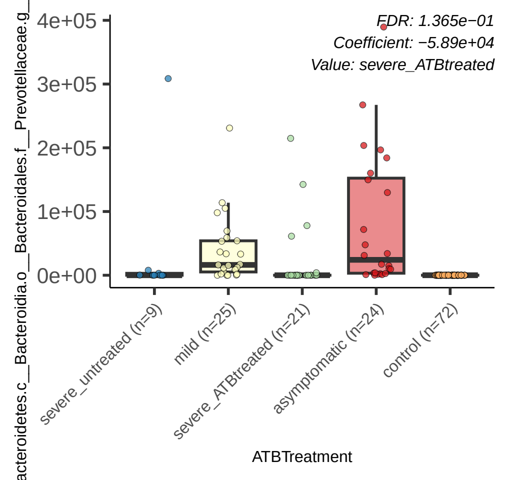

FDR: 1.365e−01
Coefficient: −5.89e+04
Value: severe_ATBtreated
4e+05
3e+05
2e+05
1e+05
0e+00
acteroidetes.c__Bacteroidia.o__Bacteroidales.f__Prevotellaceae.g__
severe_untreated (n=9)
mild (n=25)
severe_ATBtreated (n=21)
asymptomatic (n=24)
control (n=72)
ATBTreatment

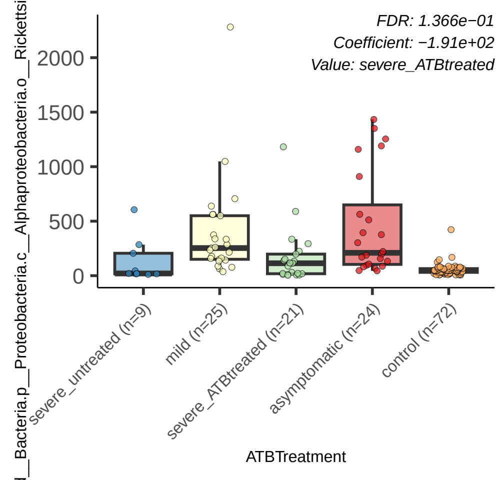
FDR: 1.366e−01
Coefficient: −1.91e+02
Value: severe_ATBtreated
0
500
1000
1500
2000
severe_untreated (n=9)
mild (n=25)
severe_ATBtreated (n=21)
asymptomatic (n=24)
control (n=72)
ATBTreatment
_Bacteria.p__Proteobacteria.c__Alphaproteobacteria.o__Rickettsi

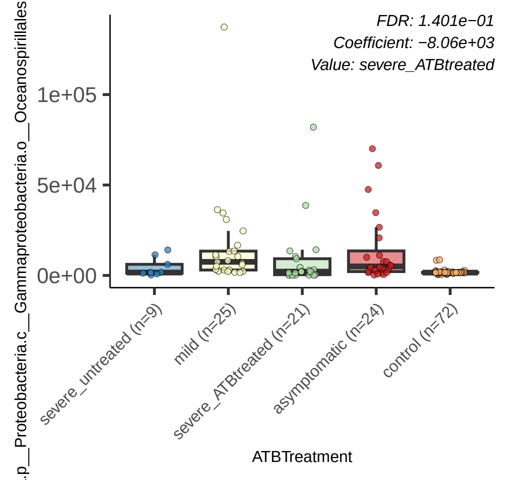
FDR: 1.401e−01
Coefficient: −8.06e+03
Value: severe_ATBtreated
p__Proteobacteria.c__Gammaproteobacteria.o__Oceanospirillales
1e+05
5e+04
0e+00
severe_untreated (n=9)
mild (n=25)
severe_ATBtreated (n=21)
asymptomatic (n=24)
control (n=72)
ATBTreatment

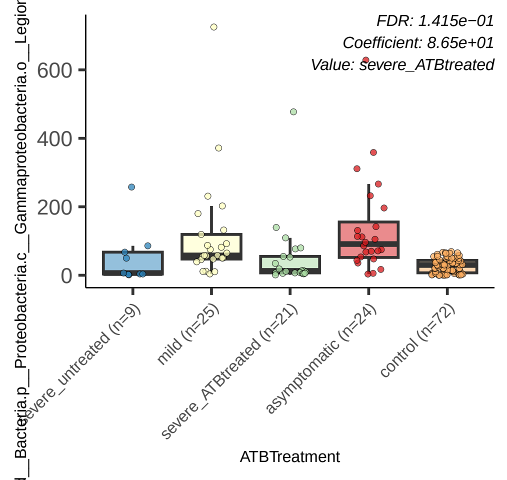

FDR: 1.415e−01
Coefficient: 8.65e+01
Value: severe_ATBtreated
600
400
200
0
Bacteria.p__Proteobacteria.c__Gammaproteobacteria.o__Legion
severe_untreated (n=9)
mild (n=25)
severe_ATBtreated (n=21)
asymptomatic (n=24)
control (n=72)
ATBTreatment

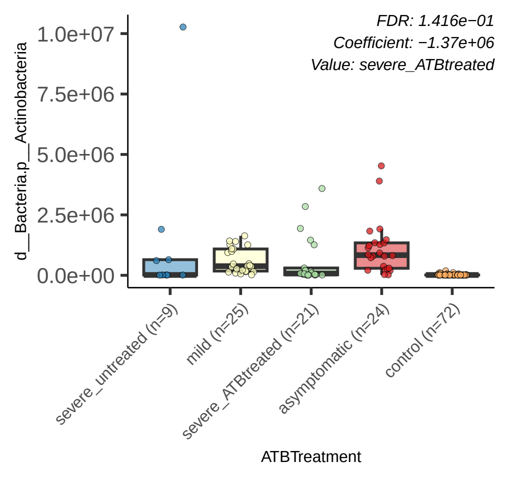
FDR: 1.416e−01
Coefficient: −1.37e+06
Value: severe_ATBtreated
d__Bacteria.p__Actinobacteria
1.0e+07
7.5e+06
5.0e+06
2.5e+06
0.0e+00
severe_untreated (n=9)
mild (n=25)
severe_ATBtreated (n=21)
asymptomatic (n=24)
control (n=72)
ATBTreatment

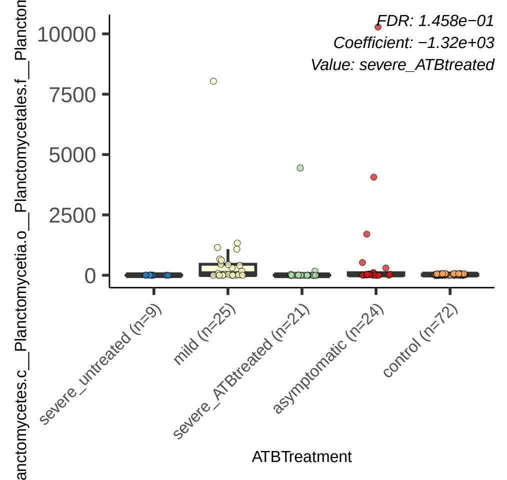

FDR: 1.458e−01
Coefficient: −1.32e+03
Value: severe_ATBtreated
10000
7500
5000
2500
0
anctomycetes.c__Planctomycetia.o__Planctomycetales.f__Planctom
severe_untreated (n=9)
mild (n=25)
severe_ATBtreated (n=21)
asymptomatic (n=24)
control (n=72)
ATBTreatment

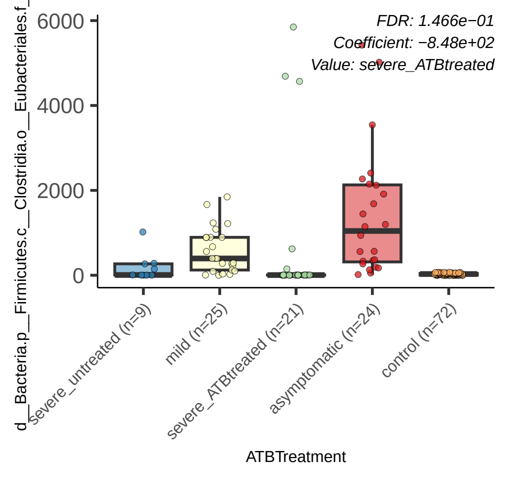

FDR: 1.466e−01
Coefficient: −8.48e+02
Value: severe_ATBtreated
d__Bacteria.p__Firmicutes.c__Clostridia.o__Eubacteriales.f_
6000
4000
2000
0
severe_untreated (n=9)
mild (n=25)
severe_ATBtreated (n=21)
asymptomatic (n=24)
control (n=72)
ATBTreatment

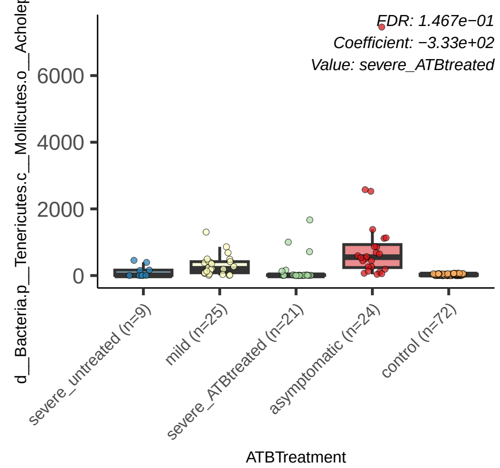

FDR: 1.467e−01
Coefficient: −3.33e+02
Value: severe_ATBtreated
d__Bacteria.p__Tenericutes.c__Mollicutes.o__Acholep
6000
4000
2000
0
severe_untreated (n=9)
mild (n=25)
severe_ATBtreated (n=21)
asymptomatic (n=24)
control (n=72)
ATBTreatment

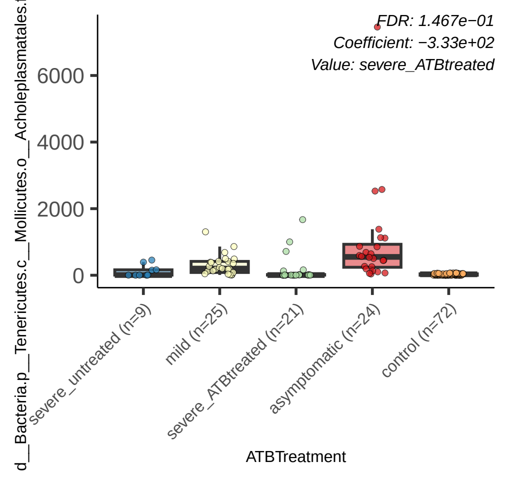
FDR: 1.467e−01
Coefficient: −3.33e+02
Value: severe_ATBtreated
d__Bacteria.p__Tenericutes.c__Mollicutes.o__Acholeplasmatales.f
6000
4000
2000
0
severe_untreated (n=9)
mild (n=25)
severe_ATBtreated (n=21)
asymptomatic (n=24)
control (n=72)
ATBTreatment

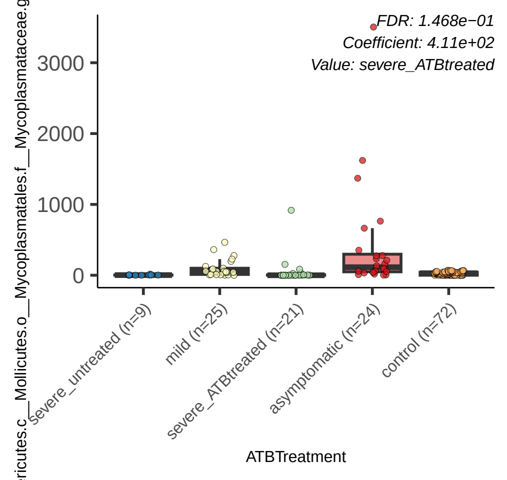
FDR: 1.468e−01
Coefficient: 4.11e+02
Value: severe_ATBtreated
3000
2000
1000
0
ricutes.c__Mollicutes.o__Mycoplasmatales.f__Mycoplasmataceae.g
severe_untreated (n=9)
mild (n=25)
severe_ATBtreated (n=21)
asymptomatic (n=24)
control (n=72)
ATBTreatment

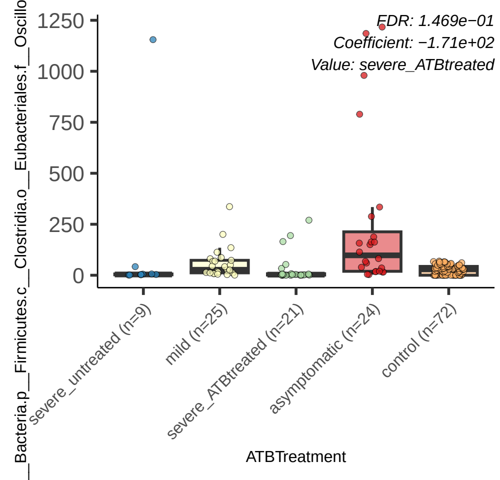
FDR: 1.469e−01
Coefficient: −1.71e+02
Value: severe_ATBtreated
Bacteria.p__Firmicutes.c__Clostridia.o__Eubacteriales.f__Oscillo
1250
1000
750
500
250
0
severe_untreated (n=9)
mild (n=25)
severe_ATBtreated (n=21)
asymptomatic (n=24)
control (n=72)
ATBTreatment

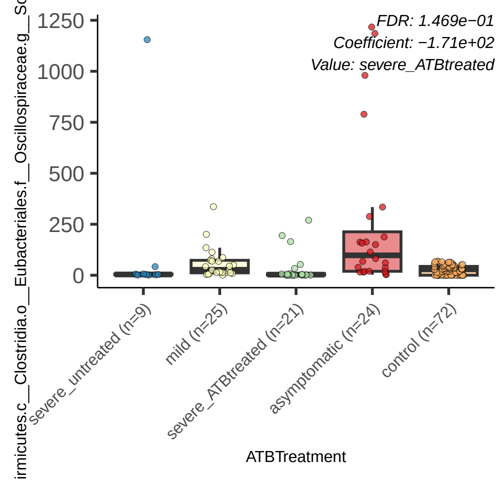

FDR: 1.469e−01
Coefficient: −1.71e+02
Value: severe_ATBtreated
1250
1000
750
500
250
0
irmicutes.c__Clostridia.o__Eubacteriales.f__Oscillospiraceae.g__Sc
severe_untreated (n=9)
mild (n=25)
severe_ATBtreated (n=21)
asymptomatic (n=24)
control (n=72)
ATBTreatment

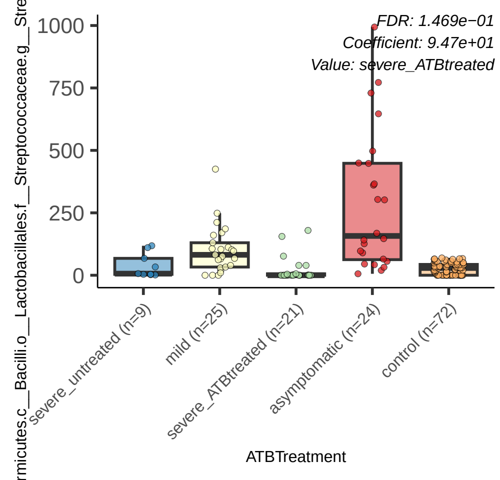

FDR: 1.469e−01
Coefficient: 9.47e+01
Value: severe_ATBtreated
1000
750
500
250
0
severe_untreated (n=9)
mild (n=25)
severe_ATBtreated (n=21)
asymptomatic (n=24)
control (n=72)
ATBTreatment
rmicutes.c__Bacilli.o__Lactobacillales.f__Streptococcaceae.g__Stre

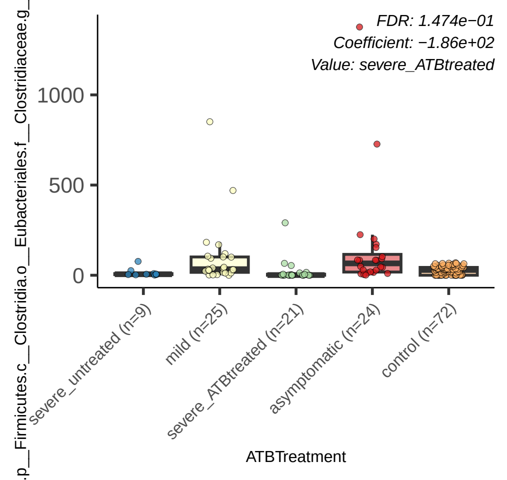
FDR: 1.474e−01
Coefficient: −1.86e+02
Value: severe_ATBtreated
1000
500
0
p__Firmicutes.c__Clostridia.o__Eubacteriales.f__Clostridiaceae.g__
severe_untreated (n=9)
mild (n=25)
severe_ATBtreated (n=21)
asymptomatic (n=24)
control (n=72)
ATBTreatment

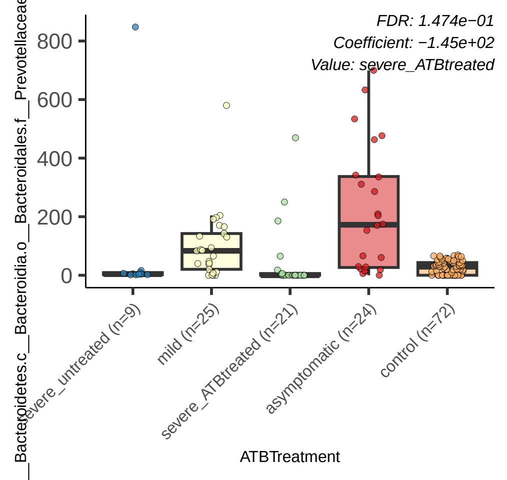

FDR: 1.474e−01
Coefficient: −1.45e+02
Value: severe_ATBtreated
800
600
400
200
0
Bacteroidetes.c__Bacteroidia.o__Bacteroidales.f__Prevotellaceae
severe_untreated (n=9)
mild (n=25)
severe_ATBtreated (n=21)
asymptomatic (n=24)
control (n=72)
ATBTreatment

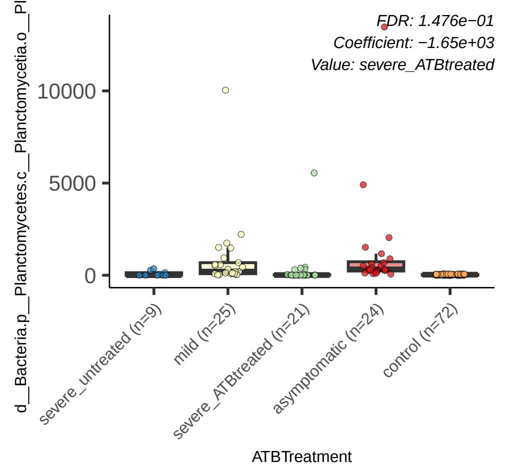
FDR: 1.476e−01
Coefficient: −1.65e+03
Value: severe_ATBtreated
d__Bacteria.p__Planctomycetes.c__Planctomycetia.o__Pl
10000
5000
0
severe_untreated (n=9)
mild (n=25)
severe_ATBtreated (n=21)
asymptomatic (n=24)
control (n=72)
ATBTreatment

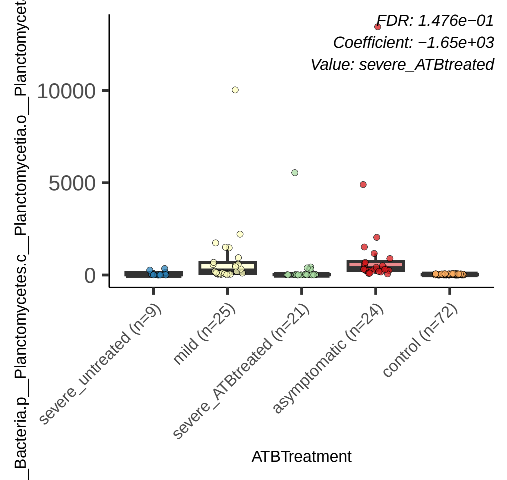
FDR: 1.476e−01
Coefficient: −1.65e+03
Value: severe_ATBtreated
10000
5000
0
severe_untreated (n=9)
mild (n=25)
severe_ATBtreated (n=21)
asymptomatic (n=24)
control (n=72)
ATBTreatment
Bacteria.p__Planctomycetes.c__Planctomycetia.o__Planctomycet

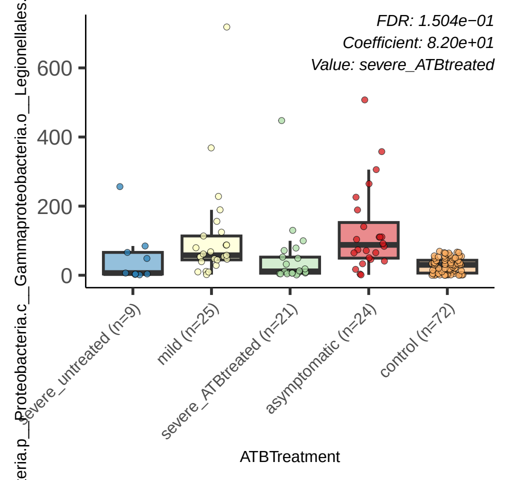

FDR: 1.504e−01
Coefficient: 8.20e+01
Value: severe_ATBtreated
0
200
400
600
severe_untreated (n=9)
mild (n=25)
severe_ATBtreated (n=21)
asymptomatic (n=24)
control (n=72)
ATBTreatment

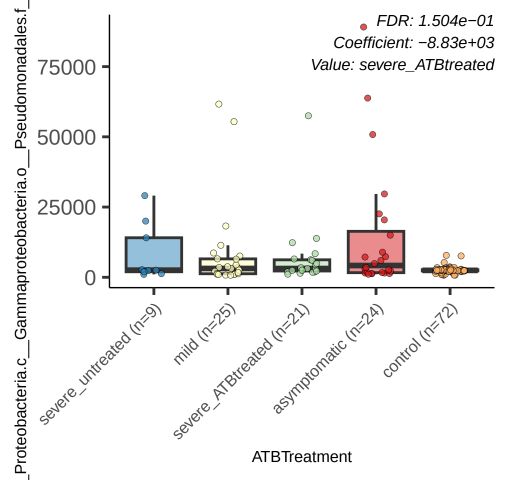
FDR: 1.504e−01
Coefficient: −8.83e+03
Value: severe_ATBtreated
Proteobacteria.c__Gammaproteobacteria.o__Pseudomonadales.f__
75000
50000
25000
0
severe_untreated (n=9)
mild (n=25)
severe_ATBtreated (n=21)
asymptomatic (n=24)
control (n=72)
ATBTreatment

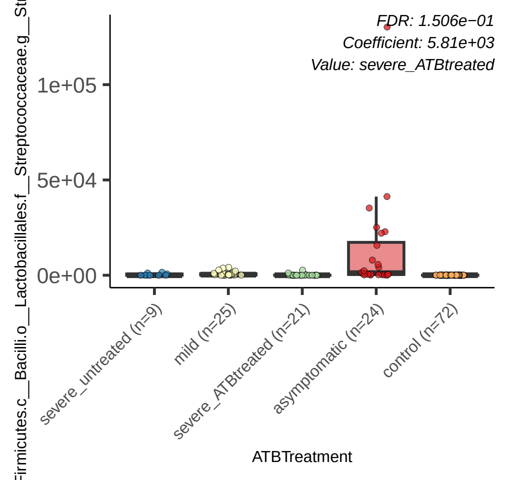
FDR: 1.506e−01
Coefficient: 5.81e+03
Value: severe_ATBtreated
1e+05
5e+04
0e+00
Firmicutes.c__Bacilli.o__Lactobacillales.f__Streptococcaceae.g__St
severe_untreated (n=9)
mild (n=25)
severe_ATBtreated (n=21)
asymptomatic (n=24)
control (n=72)
ATBTreatment

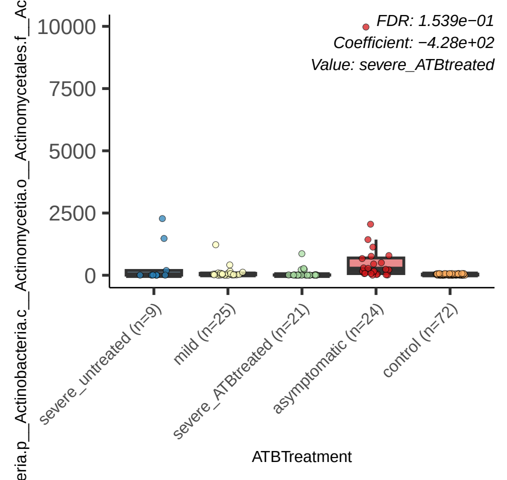

FDR: 1.539e−01
Coefficient: −4.28e+02
Value: severe_ATBtreated
10000
7500
5000
2500
0
severe_untreated (n=9)
mild (n=25)
severe_ATBtreated (n=21)
asymptomatic (n=24)
control (n=72)
ATBTreatment

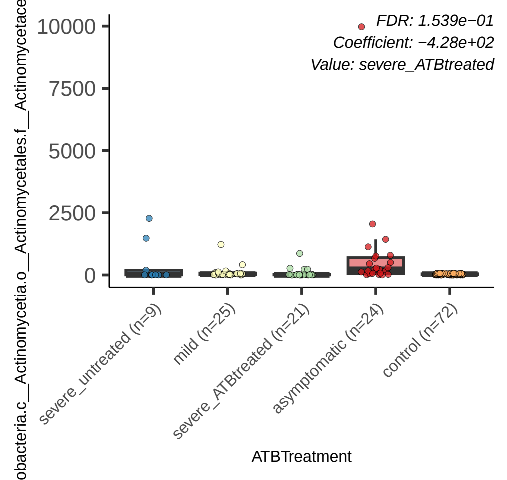

FDR: 1.539e−01
Coefficient: −4.28e+02
Value: severe_ATBtreated
10000
7500
5000
2500
0
obacteria.c__Actinomycetia.o__Actinomycetales.f__Actinomycetace
severe_untreated (n=9)
mild (n=25)
severe_ATBtreated (n=21)
asymptomatic (n=24)
control (n=72)
ATBTreatment

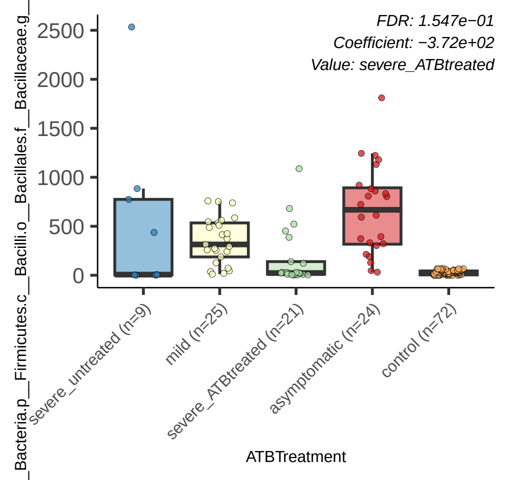

FDR: 1.547e−01
Coefficient: −3.72e+02
Value: severe_ATBtreated
Bacteria.p__Firmicutes.c__Bacilli.o__Bacillales.f__Bacillaceae.g__
2500
2000
1500
1000
500
0
severe_untreated (n=9)
mild (n=25)
severe_ATBtreated (n=21)
asymptomatic (n=24)
control (n=72)
ATBTreatment

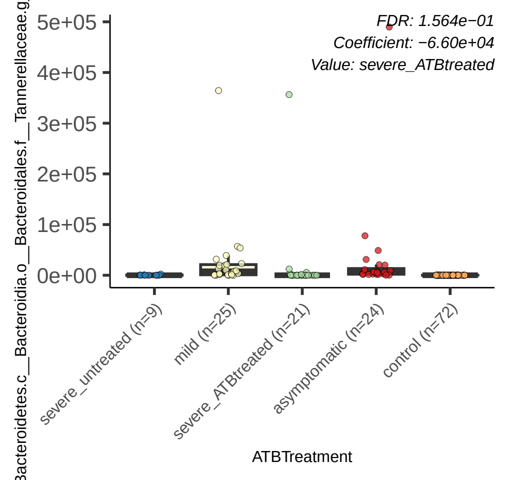

FDR: 1.564e−01
Coefficient: −6.60e+04
Value: severe_ATBtreated
Bacteroidetes.c__Bacteroidia.o__Bacteroidales.f__Tannerellaceae.g
5e+05
4e+05
3e+05
2e+05
1e+05
0e+00
severe_untreated (n=9)
mild (n=25)
severe_ATBtreated (n=21)
asymptomatic (n=24)
control (n=72)
ATBTreatment

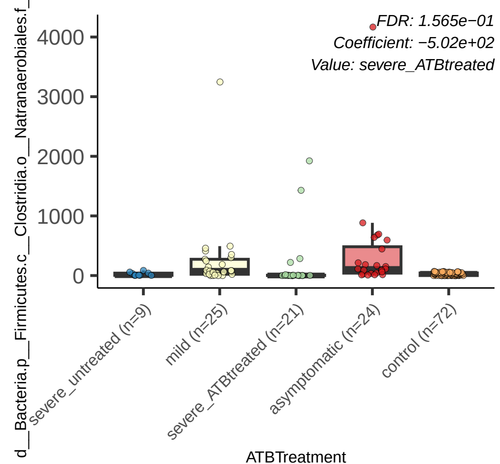
FDR: 1.565e−01
Coefficient: −5.02e+02
Value: severe_ATBtreated
d__Bacteria.p__Firmicutes.c__Clostridia.o__Natranaerobiales.f
4000
3000
2000
1000
0
severe_untreated (n=9)
mild (n=25)
severe_ATBtreated (n=21)
asymptomatic (n=24)
control (n=72)
ATBTreatment

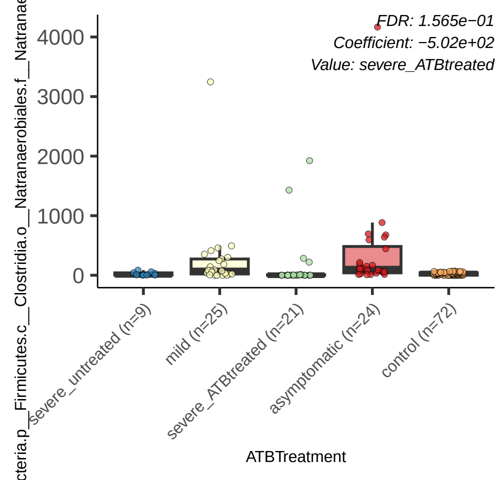

FDR: 1.565e−01
Coefficient: −5.02e+02
Value: severe_ATBtreated
4000
3000
2000
1000
0
severe_untreated (n=9)
mild (n=25)
severe_ATBtreated (n=21)
asymptomatic (n=24)
control (n=72)
ATBTreatment
cteria.p__Firmicutes.c__Clostridia.o__Natranaerobiales.f__Natranae

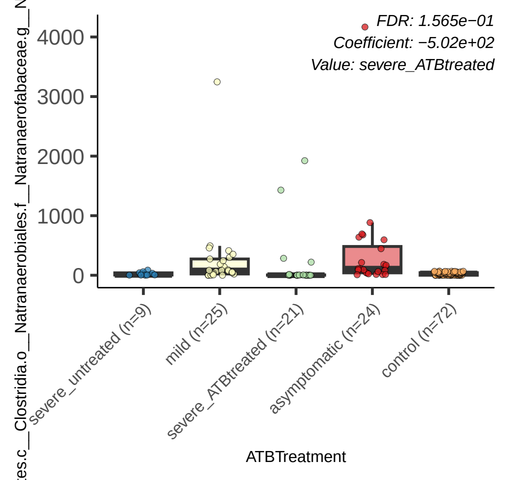
FDR: 1.565e−01
Coefficient: −5.02e+02
Value: severe_ATBtreated
4000
3000
2000
1000
0
severe_untreated (n=9)
mild (n=25)
severe_ATBtreated (n=21)
asymptomatic (n=24)
control (n=72)
ATBTreatment
Natranaerobiales.f__Natranaerofabaceae.g__
Clostridia.o__Natranaerobiales.f__Natranaerofabaceae.g__

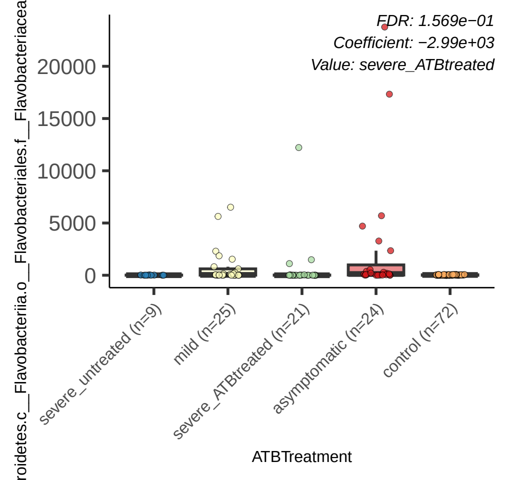

FDR: 1.569e−01
Coefficient: −2.99e+03
Value: severe_ATBtreated
20000
15000
10000
5000
0
roidetes.c__Flavobacteriia.o__Flavobacteriales.f__Flavobacteriacea
severe_untreated (n=9)
mild (n=25)
severe_ATBtreated (n=21)
asymptomatic (n=24)
control (n=72)
ATBTreatment

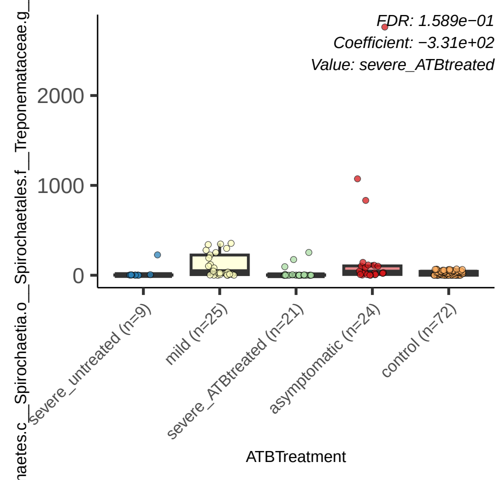
FDR: 1.589e−01
Coefficient: −3.31e+02
Value: severe_ATBtreated
2000
1000
0
naetes.c__Spirochaetia.o__Spirochaetales.f__Treponemataceae.g__
severe_untreated (n=9)
mild (n=25)
severe_ATBtreated (n=21)
asymptomatic (n=24)
control (n=72)
ATBTreatment

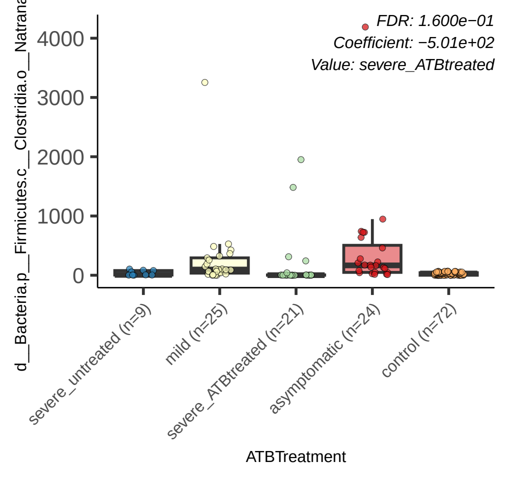

FDR: 1.600e−01
Coefficient: −5.01e+02
Value: severe_ATBtreated
d__Bacteria.p__Firmicutes.c__Clostridia.o__Natrana
4000
3000
2000
1000
0
severe_untreated (n=9)
mild (n=25)
severe_ATBtreated (n=21)
asymptomatic (n=24)
control (n=72)
ATBTreatment

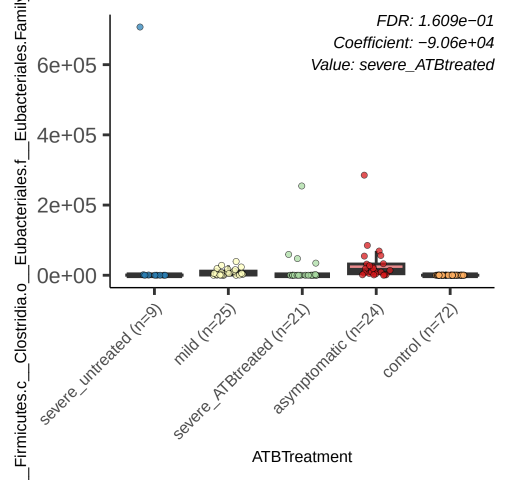
FDR: 1.609e−01
Coefficient: −9.06e+04
Value: severe_ATBtreated
6e+05
4e+05
2e+05
0e+00
_Firmicutes.c__Clostridia.o__Eubacteriales.f__Eubacteriales.Family
severe_untreated (n=9)
mild (n=25)
severe_ATBtreated (n=21)
asymptomatic (n=24)
control (n=72)
ATBTreatment

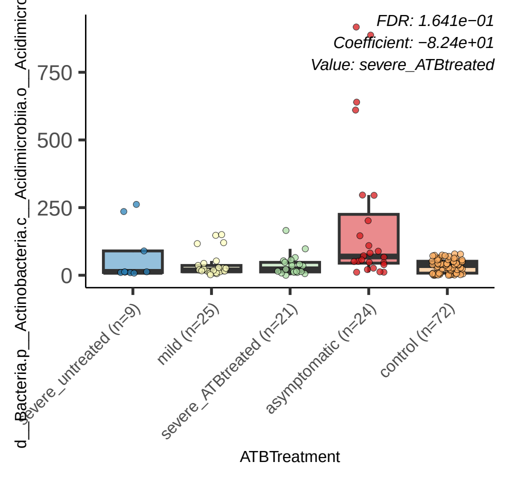

FDR: 1.641e−01
Coefficient: −8.24e+01
Value: severe_ATBtreated
d__Bacteria.p__Actinobacteria.c__Acidimicrobiia.o__Acidimicro
0
250
500
750
severe_untreated (n=9)
mild (n=25)
severe_ATBtreated (n=21)
asymptomatic (n=24)
control (n=72)
ATBTreatment

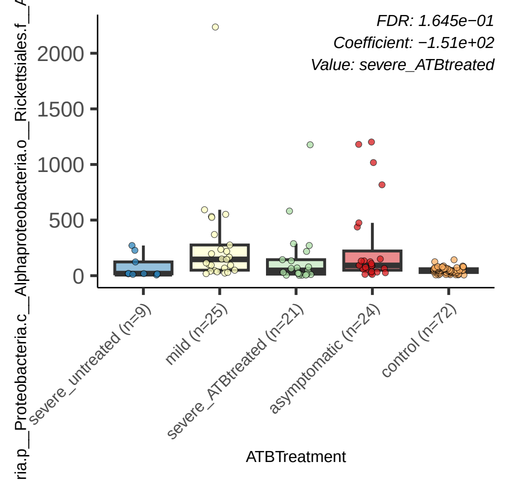
FDR: 1.645e−01
Coefficient: −1.51e+02
Value: severe_ATBtreated
ria.p__Proteobacteria.c__Alphaproteobacteria.o__Rickettsiales.f__A
2000
1500
1000
500
0
severe_untreated (n=9)
mild (n=25)
severe_ATBtreated (n=21)
asymptomatic (n=24)
control (n=72)
ATBTreatment

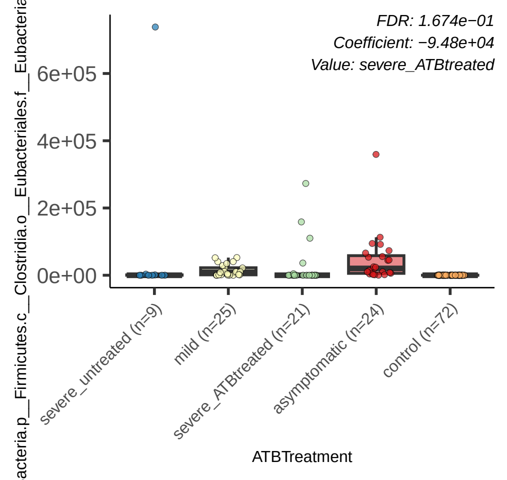
FDR: 1.674e−01
Coefficient: −9.48e+04
Value: severe_ATBtreated
0e+00
2e+05
4e+05
6e+05
acteria.p__Firmicutes.c__Clostridia.o__Eubacteriales.f__Eubacteria
severe_untreated (n=9)
mild (n=25)
severe_ATBtreated (n=21)
asymptomatic (n=24)
control (n=72)
ATBTreatment

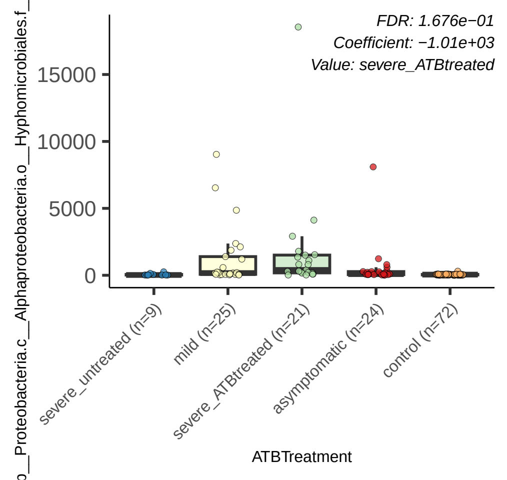

FDR: 1.676e−01
Coefficient: −1.01e+03
Value: severe_ATBtreated
o__Proteobacteria.c__Alphaproteobacteria.o__Hyphomicrobiales.f
15000
10000
5000
0
severe_untreated (n=9)
mild (n=25)
severe_ATBtreated (n=21)
asymptomatic (n=24)
control (n=72)
ATBTreatment

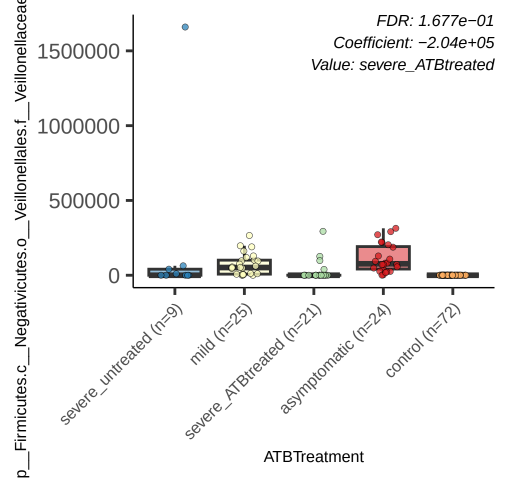

FDR: 1.677e−01
Coefficient: −2.04e+05
Value: severe_ATBtreated
p__Firmicutes.c__Negativicutes.o__Veillonellales.f__Veillonellaceae
1500000
1000000
500000
0
severe_untreated (n=9)
mild (n=25)
severe_ATBtreated (n=21)
asymptomatic (n=24)
control (n=72)
ATBTreatment

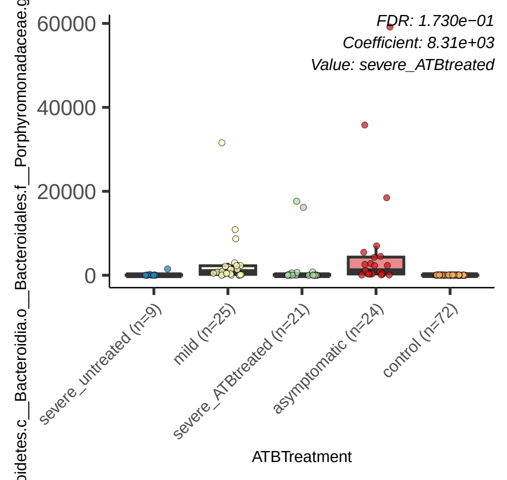

FDR: 1.730e−01
Coefficient: 8.31e+03
Value: severe_ATBtreated
60000
40000
20000
0
severe_untreated (n=9)
mild (n=25)
severe_ATBtreated (n=21)
asymptomatic (n=24)
control (n=72)
ATBTreatment

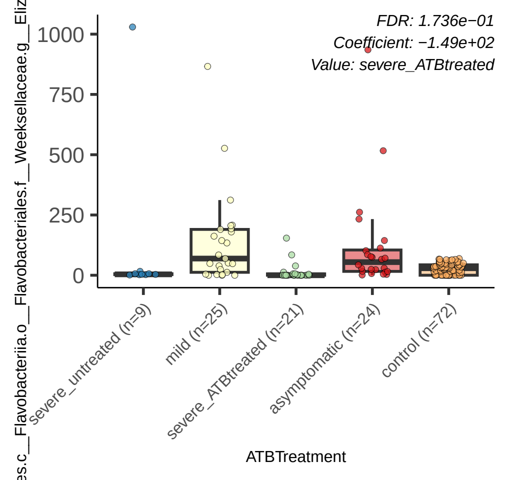

FDR: 1.736e−01
Coefficient: −1.49e+02
Value: severe_ATBtreated
1000
750
500
250
0
severe_untreated (n=9)
mild (n=25)
severe_ATBtreated (n=21)
asymptomatic (n=24)
control (n=72)
ATBTreatment
es.c__Flavobacteriia.o__Flavobacteriales.f__Weeksellaceae.g__Eliz

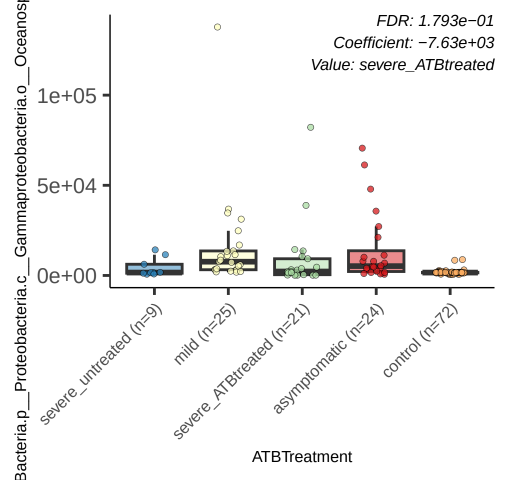
FDR: 1.793e−01
Coefficient: −7.63e+03
Value: severe_ATBtreated
1e+05
5e+04
0e+00
Bacteria.p__Proteobacteria.c__Gammaproteobacteria.o__Oceanosp
severe_untreated (n=9)
mild (n=25)
severe_ATBtreated (n=21)
asymptomatic (n=24)
control (n=72)
ATBTreatment

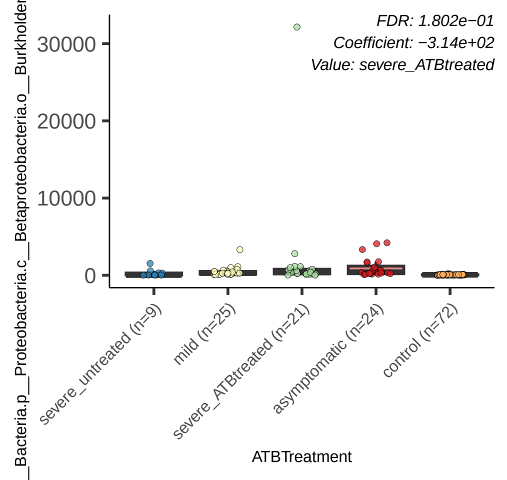
FDR: 1.802e−01
Coefficient: −3.14e+02
Value: severe_ATBtreated
30000
20000
10000
0
_Bacteria.p__Proteobacteria.c__Betaproteobacteria.o__Burkholder
severe_untreated (n=9)
mild (n=25)
severe_ATBtreated (n=21)
asymptomatic (n=24)
control (n=72)
ATBTreatment

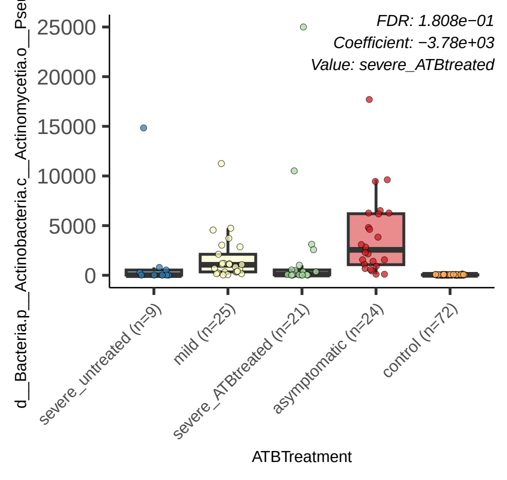

FDR: 1.808e−01
Coefficient: −3.78e+03
Value: severe_ATBtreated
d__Bacteria.p__Actinobacteria.c__Actinomycetia.o__Pse
25000
20000
15000
10000
5000
0
severe_untreated (n=9)
mild (n=25)
severe_ATBtreated (n=21)
asymptomatic (n=24)
control (n=72)
ATBTreatment

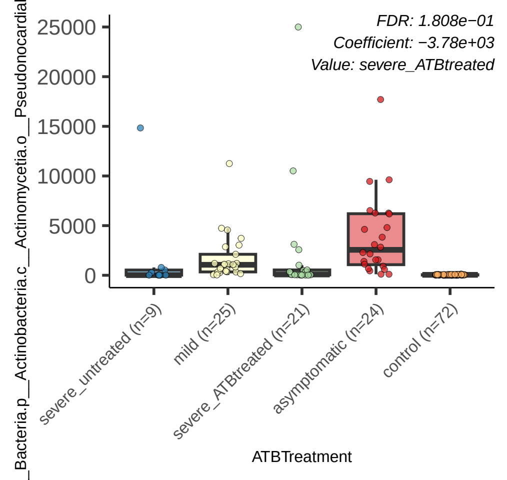
FDR: 1.808e−01
Coefficient: −3.78e+03
Value: severe_ATBtreated
Bacteria.p__Actinobacteria.c__Actinomycetia.o__Pseudonocardial
25000
20000
15000
10000
5000
0
severe_untreated (n=9)
mild (n=25)
severe_ATBtreated (n=21)
asymptomatic (n=24)
control (n=72)
ATBTreatment

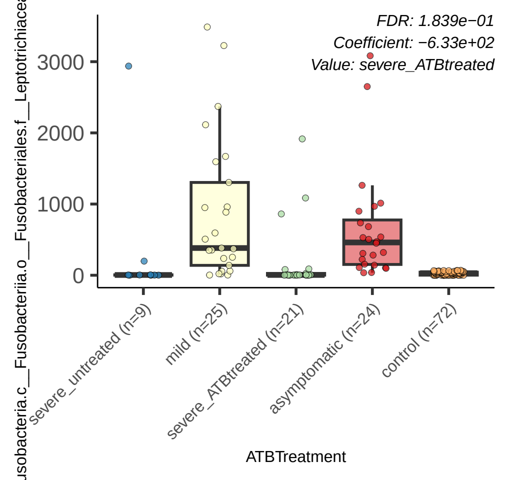

FDR: 1.839e−01
Coefficient: −6.33e+02
Value: severe_ATBtreated
3000
2000
1000
0
Fusobacteria.c__Fusobacteriia.o__Fusobacteriales.f__Leptotrichiacea
severe_untreated (n=9)
mild (n=25)
severe_ATBtreated (n=21)
asymptomatic (n=24)
control (n=72)
ATBTreatment

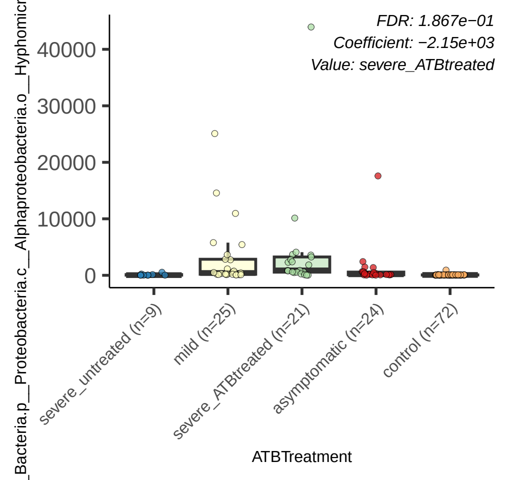

_Bacteria.p__Proteobacteria.c__Alphaproteobacteria.o__Hyphomicr
FDR: 1.867e−01
Coefficient: −2.15e+03
Value: severe_ATBtreated
40000
30000
20000
10000
0
severe_untreated (n=9)
mild (n=25)
severe_ATBtreated (n=21)
asymptomatic (n=24)
control (n=72)
ATBTreatment

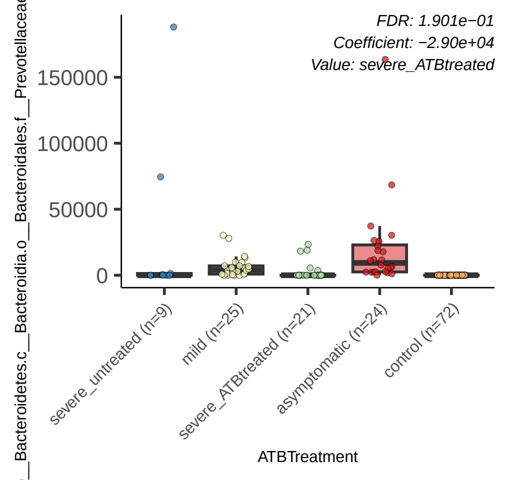
FDR: 1.901e−01
Coefficient: −2.90e+04
Value: severe_ATBtreated
150000
100000
50000
0
Bacteroidetes.c__Bacteroidia.o__Bacteroidales.f__Prevotellacea
severe_untreated (n=9)
mild (n=25)
severe_ATBtreated (n=21)
asymptomatic (n=24)
control (n=72)
ATBTreatment

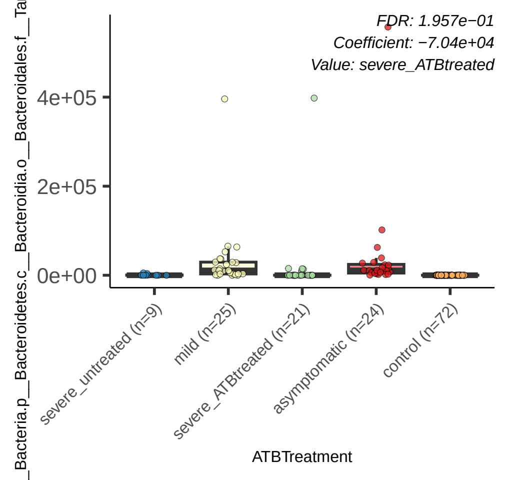

FDR: 1.957e−01
Coefficient: −7.04e+04
Value: severe_ATBtreated
4e+05
2e+05
0e+00
severe_untreated (n=9)
mild (n=25)
severe_ATBtreated (n=21)
asymptomatic (n=24)
control (n=72)
ATBTreatment
_Bacteria.p__Bacteroidetes.c__Bacteroidia.o__Bacteroidales.f__Ta

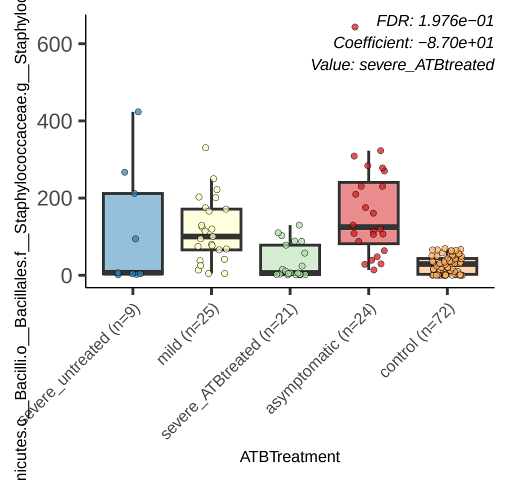

FDR: 1.976e−01
Coefficient: −8.70e+01
Value: severe_ATBtreated
600
400
200
0
severe_untreated (n=9)
mild (n=25)
severe_ATBtreated (n=21)
asymptomatic (n=24)
control (n=72)
ATBTreatment
nicutes.c__Bacilli.o__Bacillales.f__Staphylococcaceae.g__Staphyloc

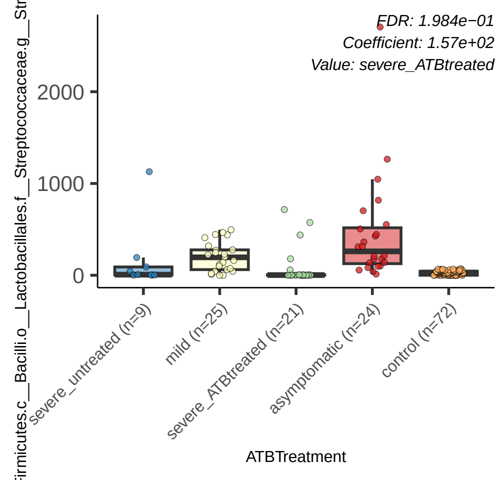

FDR: 1.984e−01
Coefficient: 1.57e+02
Value: severe_ATBtreated
2000
1000
0
Firmicutes.c__Bacilli.o__Lactobacillales.f__Streptococcaceae.g__Str
severe_untreated (n=9)
mild (n=25)
severe_ATBtreated (n=21)
asymptomatic (n=24)
control (n=72)
ATBTreatment

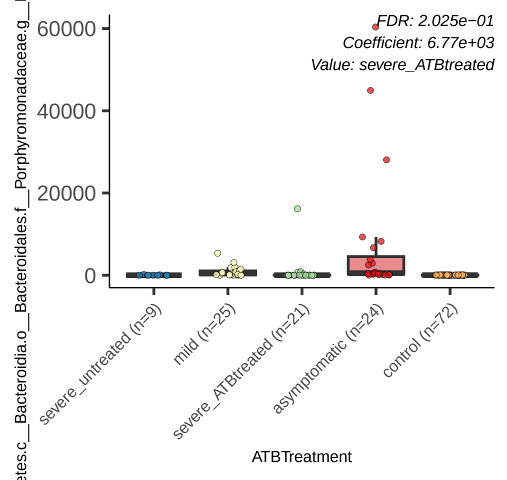

FDR: 2.025e−01
Coefficient: 6.77e+03
Value: severe_ATBtreated
60000
40000
20000
0
etes.c__Bacteroidia.o__Bacteroidales.f__Porphyromonadaceae.g__
severe_untreated (n=9)
mild (n=25)
severe_ATBtreated (n=21)
asymptomatic (n=24)
control (n=72)
ATBTreatment

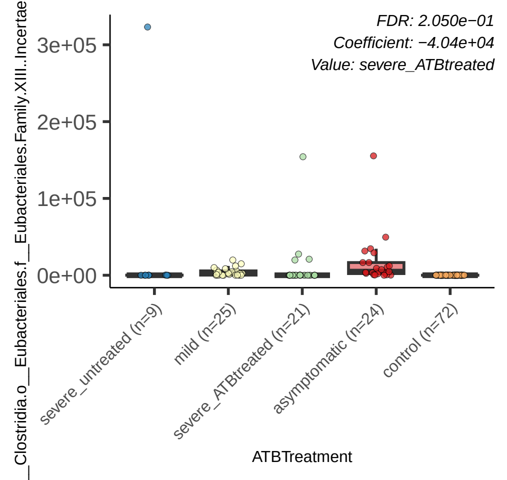
FDR: 2.050e−01
Coefficient: −4.04e+04
Value: severe_ATBtreated
3e+05
2e+05
1e+05
0e+00
__Clostridia.o__Eubacteriales.f__Eubacteriales.Family.XIII..Incertae
severe_untreated (n=9)
mild (n=25)
severe_ATBtreated (n=21)
asymptomatic (n=24)
control (n=72)
ATBTreatment

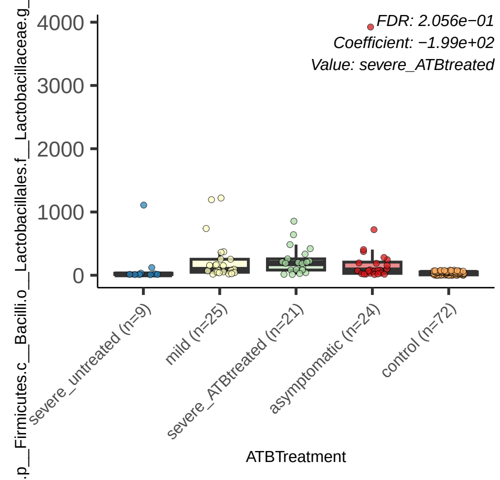

FDR: 2.056e−01
Coefficient: −1.99e+02
Value: severe_ATBtreated
p__Firmicutes.c__Bacilli.o__Lactobacillales.f__Lactobacillaceae.g__
4000
3000
2000
1000
0
severe_untreated (n=9)
mild (n=25)
severe_ATBtreated (n=21)
asymptomatic (n=24)
control (n=72)
ATBTreatment

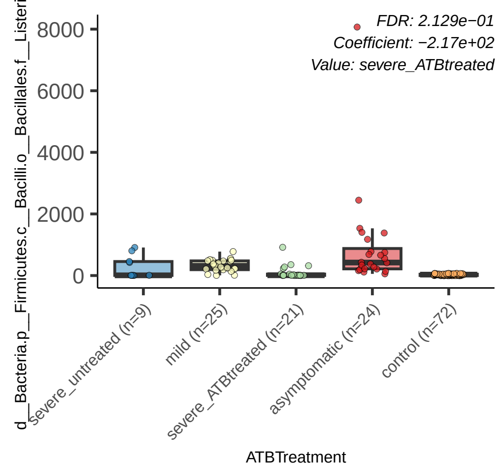

FDR: 2.129e−01
Coefficient: −2.17e+02
Value: severe_ATBtreated
d__Bacteria.p__Firmicutes.c__Bacilli.o__Bacillales.f__Listeri
8000
6000
4000
2000
0
severe_untreated (n=9)
mild (n=25)
severe_ATBtreated (n=21)
asymptomatic (n=24)
control (n=72)
ATBTreatment

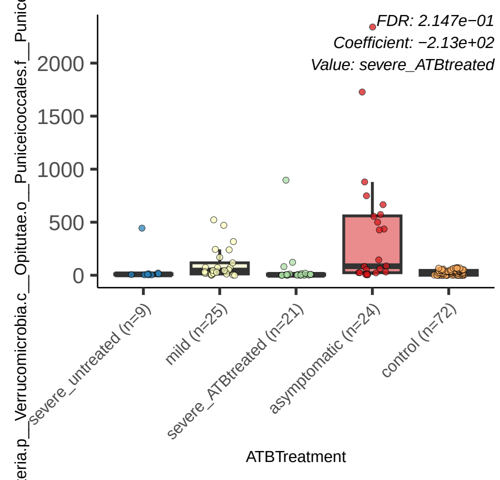

FDR: 2.147e−01
Coefficient: −2.13e+02
Value: severe_ATBtreated
2000
1500
1000
500
0
severe_untreated (n=9)
mild (n=25)
severe_ATBtreated (n=21)
asymptomatic (n=24)
control (n=72)
ATBTreatment

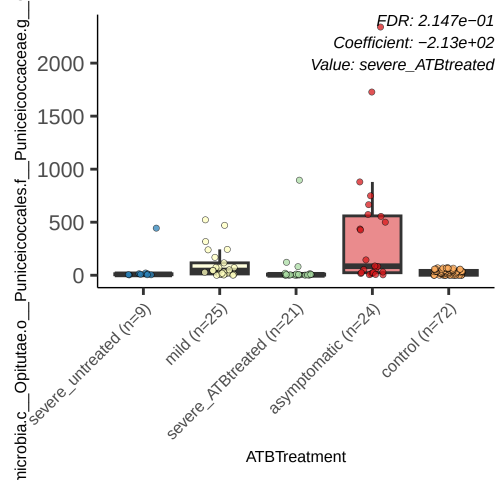

FDR: 2.147e−01
Coefficient: −2.13e+02
Value: severe_ATBtreated
microbia.c__Opitutae.o__Puniceicoccales.f__Puniceicoccaceae.g__
2000
1500
1000
500
0
severe_untreated (n=9)
mild (n=25)
severe_ATBtreated (n=21)
asymptomatic (n=24)
control (n=72)
ATBTreatment

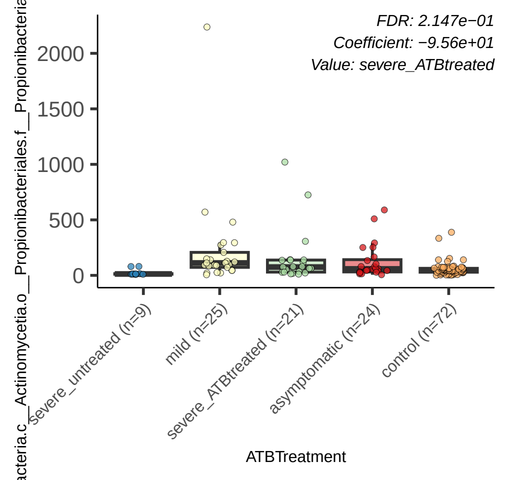

FDR: 2.147e−01
Coefficient: −9.56e+01
Value: severe_ATBtreated
acteria.c__Actinomycetia.o__Propionibacteriales.f__Propionibacteria
2000
1500
1000
500
0
severe_untreated (n=9)
mild (n=25)
severe_ATBtreated (n=21)
asymptomatic (n=24)
control (n=72)
ATBTreatment

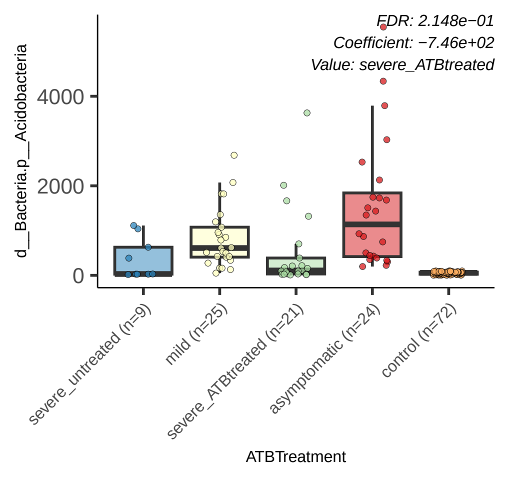

FDR: 2.148e−01
Coefficient: −7.46e+02
Value: severe_ATBtreated
d__Bacteria.p__Acidobacteria
4000
2000
0
severe_untreated (n=9)
mild (n=25)
severe_ATBtreated (n=21)
asymptomatic (n=24)
control (n=72)
ATBTreatment

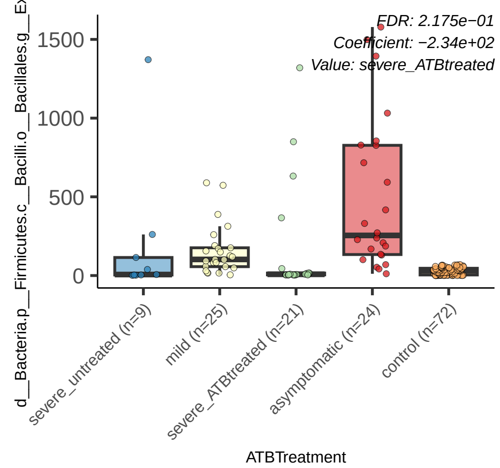
FDR: 2.175e−01
Coefficient: −2.34e+02
Value: severe_ATBtreated
1500
1000
500
0
d__Bacteria.p__Firmicutes.c__Bacilli.o__Bacillales.g__Ex
severe_untreated (n=9)
mild (n=25)
severe_ATBtreated (n=21)
asymptomatic (n=24)
control (n=72)
ATBTreatment

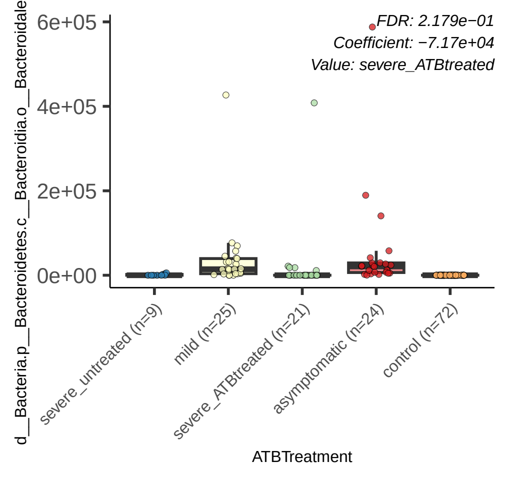

FDR: 2.179e−01
Coefficient: −7.17e+04
Value: severe_ATBtreated
6e+05
4e+05
2e+05
0e+00
d__Bacteria.p__Bacteroidetes.c__Bacteroidia.o__Bacteroidale
severe_untreated (n=9)
mild (n=25)
severe_ATBtreated (n=21)
asymptomatic (n=24)
control (n=72)
ATBTreatment

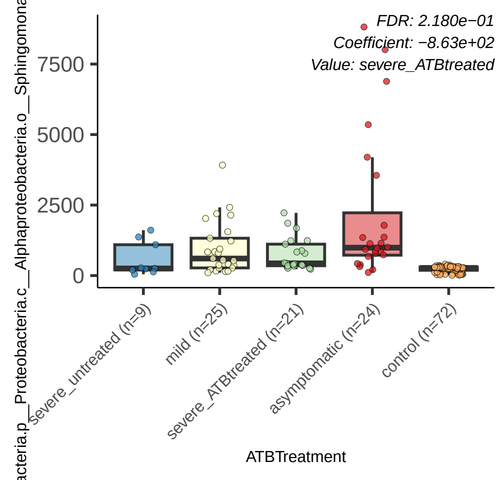

FDR: 2.180e−01
Coefficient: −8.63e+02
Value: severe_ATBtreated
acteria.p__Proteobacteria.c__Alphaproteobacteria.o__Sphingomona
7500
5000
2500
0
severe_untreated (n=9)
mild (n=25)
severe_ATBtreated (n=21)
asymptomatic (n=24)
control (n=72)
ATBTreatment

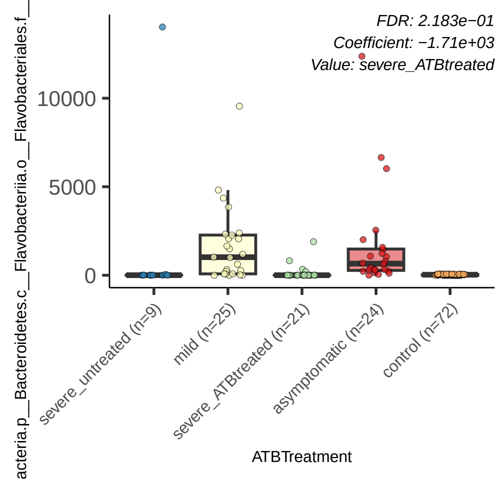

FDR: 2.183e−01
Coefficient: −1.71e+03
Value: severe_ATBtreated
acteria.p__Bacteroidetes.c__Flavobacteriia.o__Flavobacteriales.f__
10000
5000
0
severe_untreated (n=9)
mild (n=25)
severe_ATBtreated (n=21)
asymptomatic (n=24)
control (n=72)
ATBTreatment

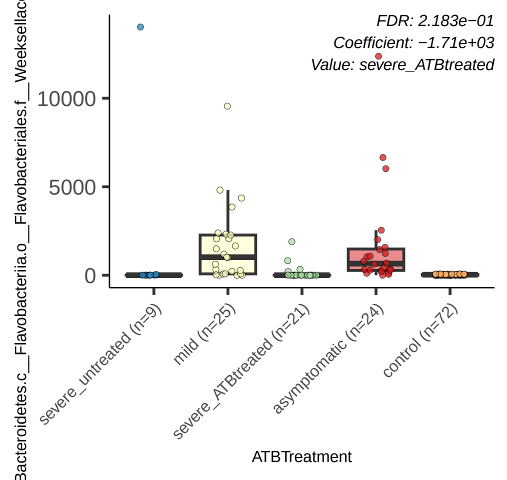

FDR: 2.183e−01
Coefficient: −1.71e+03
Value: severe_ATBtreated
10000
5000
0
severe_untreated (n=9)
mild (n=25)
severe_ATBtreated (n=21)
asymptomatic (n=24)
control (n=72)
ATBTreatment
Bacteroidetes.c__Flavobacteriia.o__Flavobacteriales.f__Weeksellac

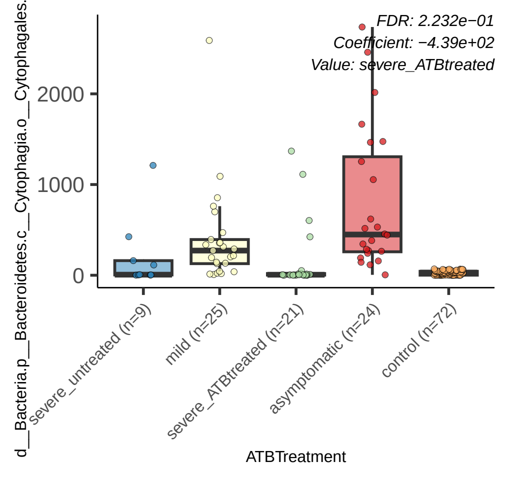
FDR: 2.232e−01
Coefficient: −4.39e+02
Value: severe_ATBtreated
d__Bacteria.p__Bacteroidetes.c__Cytophagia.o__Cytophagales.
0
1000
2000
severe_untreated (n=9)
mild (n=25)
severe_ATBtreated (n=21)
asymptomatic (n=24)
control (n=72)
ATBTreatment

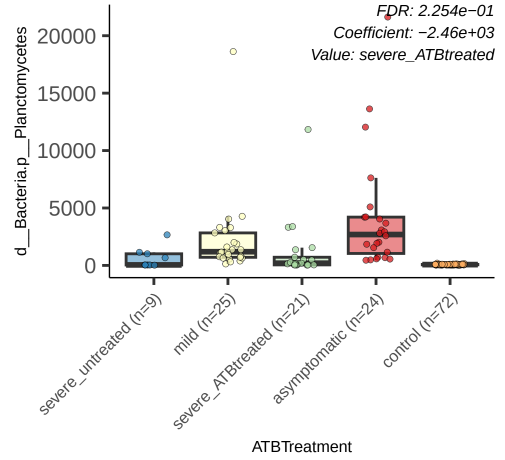

FDR: 2.254e−01
Coefficient: −2.46e+03
Value: severe_ATBtreated
d__Bacteria.p__Planctomycetes
20000
15000
10000
5000
0
severe_untreated (n=9)
mild (n=25)
severe_ATBtreated (n=21)
asymptomatic (n=24)
control (n=72)
ATBTreatment

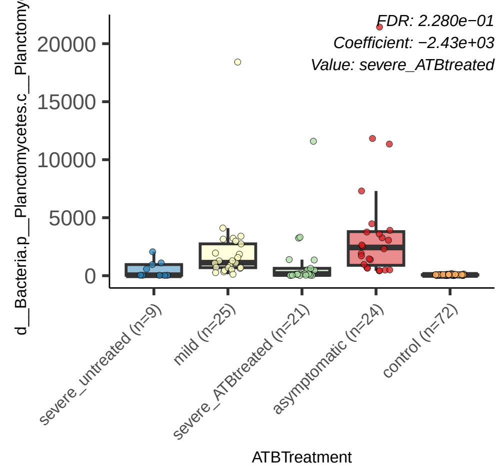
FDR: 2.280e−01
Coefficient: −2.43e+03
Value: severe_ATBtreated
d__Bacteria.p__Planctomycetes.c__Planctomy
20000
15000
10000
5000
0
severe_untreated (n=9)
mild (n=25)
severe_ATBtreated (n=21)
asymptomatic (n=24)
control (n=72)
ATBTreatment

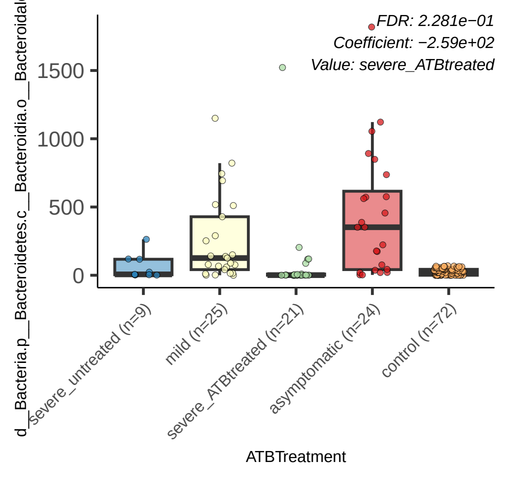
FDR: 2.281e−01
Coefficient: −2.59e+02
Value: severe_ATBtreated
d__Bacteria.p__Bacteroidetes.c__Bacteroidia.o__Bacteroidale
1500
1000
500
0
severe_untreated (n=9)
mild (n=25)
severe_ATBtreated (n=21)
asymptomatic (n=24)
control (n=72)
ATBTreatment

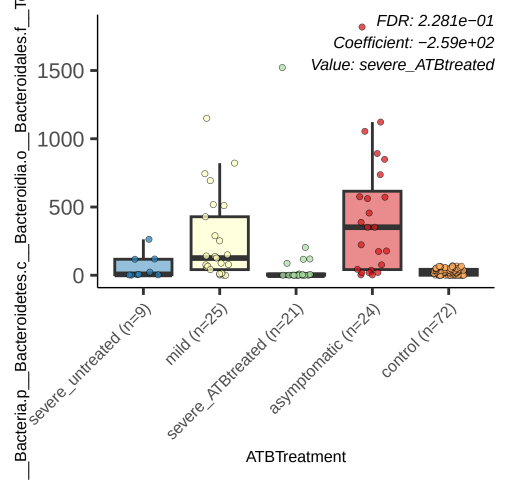
FDR: 2.281e−01
Coefficient: −2.59e+02
Value: severe_ATBtreated
1500
1000
500
0
severe_untreated (n=9)
mild (n=25)
severe_ATBtreated (n=21)
asymptomatic (n=24)
control (n=72)
ATBTreatment
_Bacteria.p__Bacteroidetes.c__Bacteroidia.o__Bacteroidales.f__T

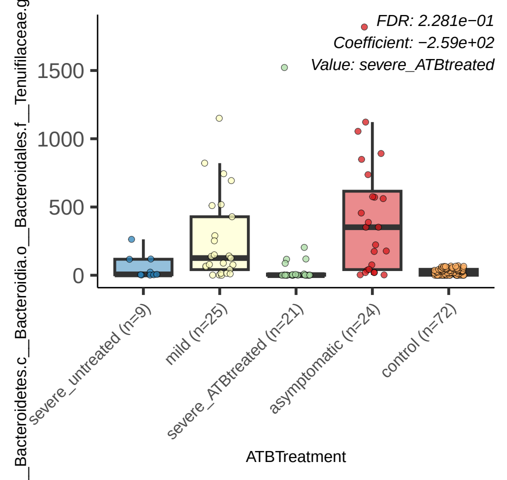

FDR: 2.281e−01
Coefficient: −2.59e+02
Value: severe_ATBtreated
1500
1000
500
0
severe_untreated (n=9)
mild (n=25)
severe_ATBtreated (n=21)
asymptomatic (n=24)
control (n=72)
ATBTreatment

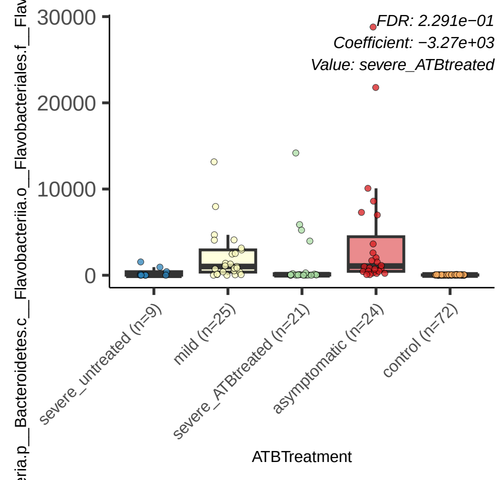

FDR: 2.291e−01
Coefficient: −3.27e+03
Value: severe_ATBtreated
30000
20000
10000
0
eria.p__Bacteroidetes.c__Flavobacteriia.o__Flavobacteriales.f__Flav
severe_untreated (n=9)
mild (n=25)
severe_ATBtreated (n=21)
asymptomatic (n=24)
control (n=72)
ATBTreatment

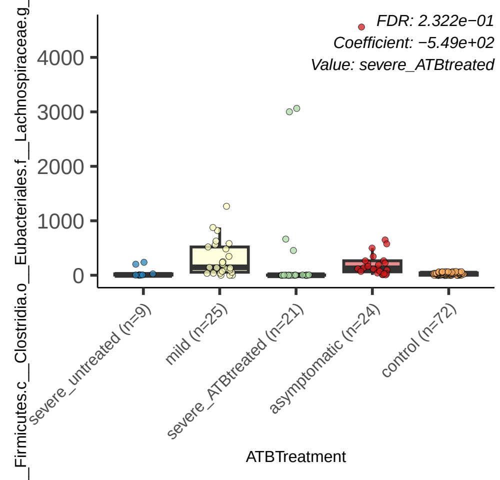
FDR: 2.322e−01
Coefficient: −5.49e+02
Value: severe_ATBtreated
Firmicutes.c__Clostridia.o__Eubacteriales.f__Lachnospiraceae.g__
4000
3000
2000
1000
0
severe_untreated (n=9)
mild (n=25)
severe_ATBtreated (n=21)
asymptomatic (n=24)
control (n=72)
ATBTreatment

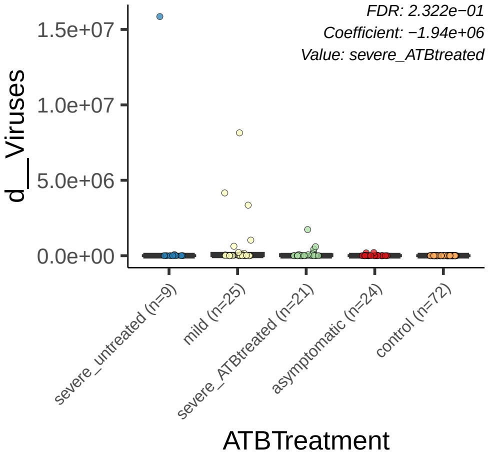

FDR: 2.322e−01
Coefficient: −1.94e+06
Value: severe_ATBtreated
d__Viruses
1.5e+07
1.0e+07
5.0e+06
0.0e+00
severe_untreated (n=9)
mild (n=25)
severe_ATBtreated (n=21)
asymptomatic (n=24)
control (n=72)
ATBTreatment

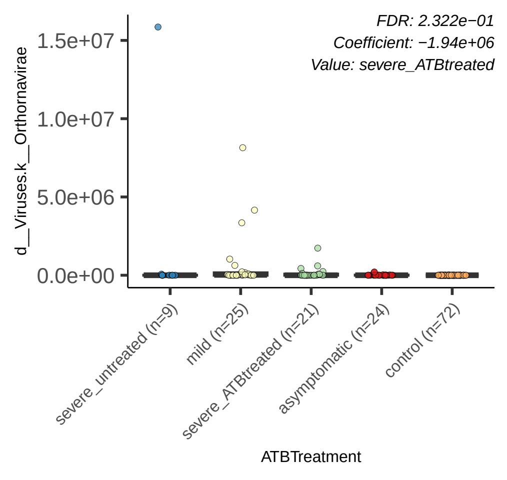
FDR: 2.322e−01
Coefficient: −1.94e+06
Value: severe_ATBtreated
d__Viruses.k__Orthornavirae
1.5e+07
1.0e+07
5.0e+06
0.0e+00
severe_untreated (n=9)
mild (n=25)
severe_ATBtreated (n=21)
asymptomatic (n=24)
control (n=72)
ATBTreatment

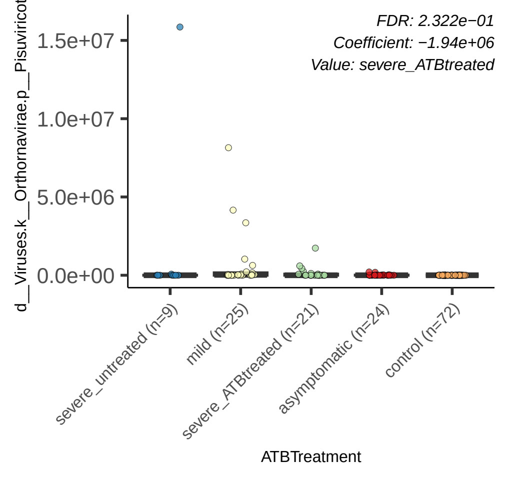

FDR: 2.322e−01
Coefficient: −1.94e+06
Value: severe_ATBtreated
d__Viruses.k__Orthornavirae.p__Pisuviricot
1.5e+07
1.0e+07
5.0e+06
0.0e+00
severe_untreated (n=9)
mild (n=25)
severe_ATBtreated (n=21)
asymptomatic (n=24)
control (n=72)
ATBTreatment

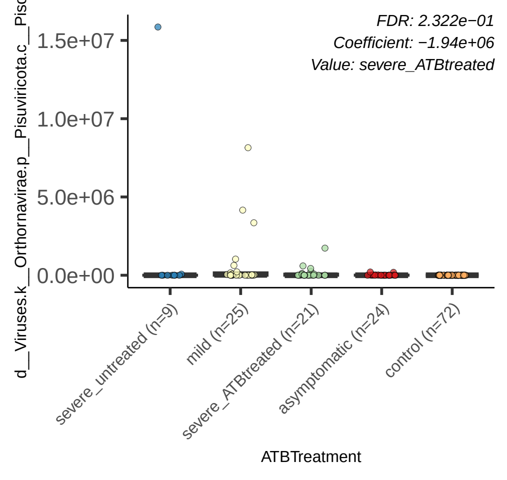

FDR: 2.322e−01
Coefficient: −1.94e+06
Value: severe_ATBtreated
d__Viruses.k__Orthornavirae.p__Pisuviricota.c__Pisc
1.5e+07
1.0e+07
5.0e+06
0.0e+00
severe_untreated (n=9)
mild (n=25)
severe_ATBtreated (n=21)
asymptomatic (n=24)
control (n=72)
ATBTreatment

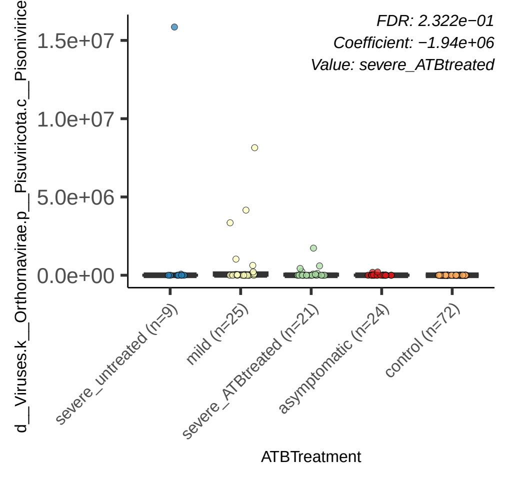

FDR: 2.322e−01
Coefficient: −1.94e+06
Value: severe_ATBtreated
d__Viruses.k__Orthornavirae.p__Pisuviricota.c__Pisonivirice
1.5e+07
1.0e+07
5.0e+06
0.0e+00
severe_untreated (n=9)
mild (n=25)
severe_ATBtreated (n=21)
asymptomatic (n=24)
control (n=72)
ATBTreatment

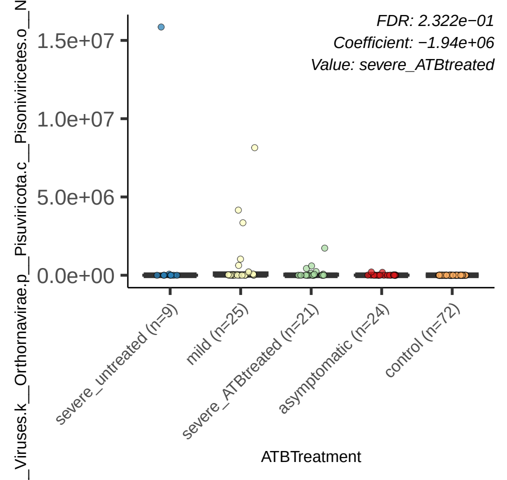

FDR: 2.322e−01
Coefficient: −1.94e+06
Value: severe_ATBtreated
1.5e+07
1.0e+07
5.0e+06
0.0e+00
Viruses.k__Orthornavirae.p__Pisuviricota.c__Pisoniviricetes.o__N
severe_untreated (n=9)
mild (n=25)
severe_ATBtreated (n=21)
asymptomatic (n=24)
control (n=72)
ATBTreatment

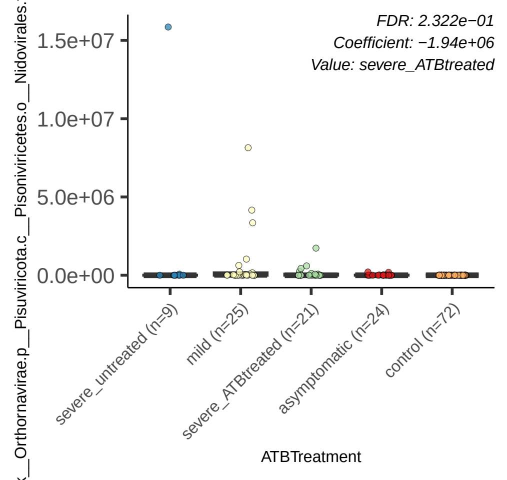

FDR: 2.322e−01
Coefficient: −1.94e+06
Value: severe_ATBtreated
1.5e+07
1.0e+07
5.0e+06
0.0e+00
severe_untreated (n=9)
mild (n=25)
severe_ATBtreated (n=21)
asymptomatic (n=24)
control (n=72)
ATBTreatment

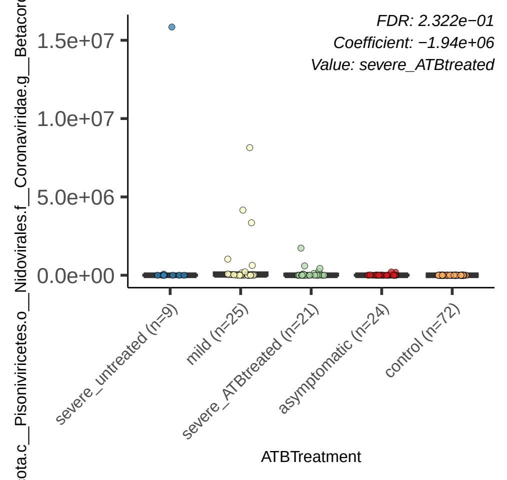

FDR: 2.322e−01
Coefficient: −1.94e+06
Value: severe_ATBtreated
1.5e+07
1.0e+07
5.0e+06
0.0e+00
ota.c__Pisoniviricetes.o__Nidovirales.f__Coronaviridae.g__Betacoro
severe_untreated (n=9)
mild (n=25)
severe_ATBtreated (n=21)
asymptomatic (n=24)
control (n=72)
ATBTreatment

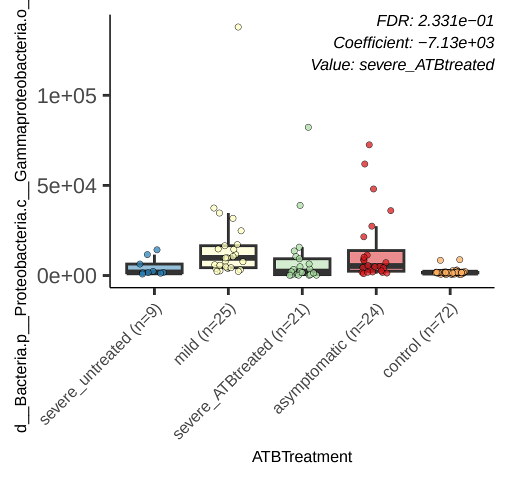

FDR: 2.331e−01
Coefficient: −7.13e+03
Value: severe_ATBtreated
d__Bacteria.p__Proteobacteria.c__Gammaproteobacteria.o__
1e+05
5e+04
0e+00
severe_untreated (n=9)
mild (n=25)
severe_ATBtreated (n=21)
asymptomatic (n=24)
control (n=72)
ATBTreatment

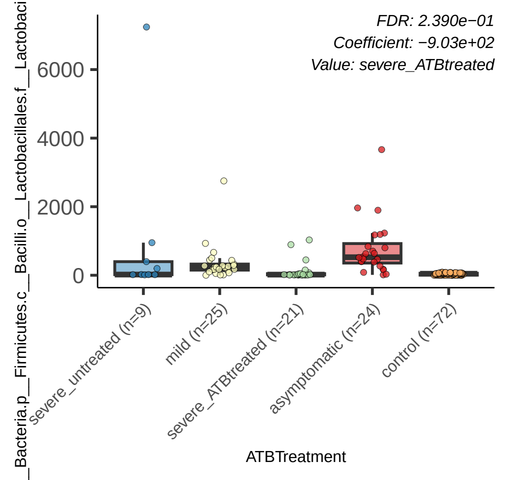

FDR: 2.390e−01
Coefficient: −9.03e+02
Value: severe_ATBtreated
_Bacteria.p__Firmicutes.c__Bacilli.o__Lactobacillales.f__Lactobaci
6000
4000
2000
0
severe_untreated (n=9)
mild (n=25)
severe_ATBtreated (n=21)
asymptomatic (n=24)
control (n=72)
ATBTreatment

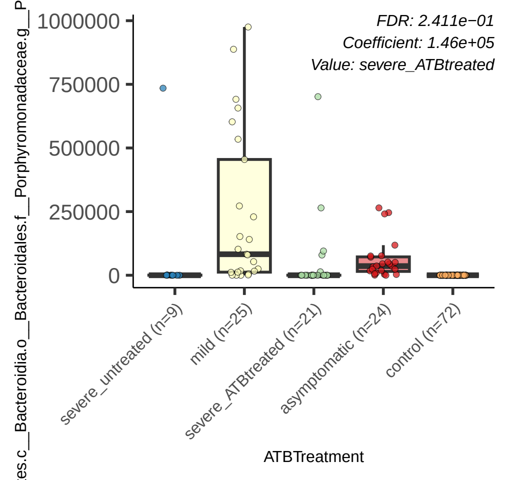

FDR: 2.411e−01
Coefficient: 1.46e+05
Value: severe_ATBtreated
1000000
750000
500000
250000
0
es.c__Bacteroidia.o__Bacteroidales.f__Porphyromonadaceae.g__P
severe_untreated (n=9)
mild (n=25)
severe_ATBtreated (n=21)
asymptomatic (n=24)
control (n=72)
ATBTreatment

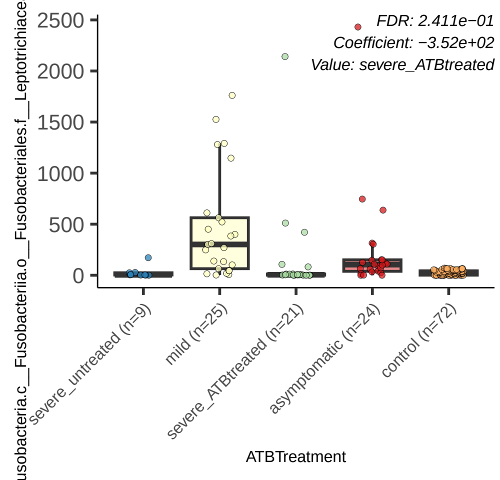

FDR: 2.411e−01
Coefficient: −3.52e+02
Value: severe_ATBtreated
usobacteria.c__Fusobacteriia.o__Fusobacteriales.f__Leptotrichiace
2500
2000
1500
1000
500
0
severe_untreated (n=9)
mild (n=25)
severe_ATBtreated (n=21)
asymptomatic (n=24)
control (n=72)
ATBTreatment

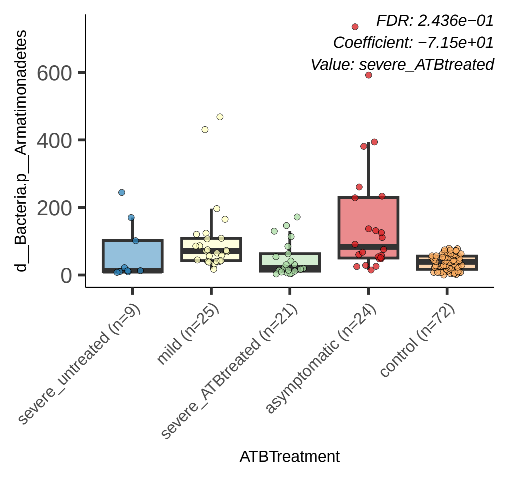
FDR: 2.436e−01
Coefficient: −7.15e+01
Value: severe_ATBtreated
d__Bacteria.p__Armatimonadetes
600
400
200
0
severe_untreated (n=9)
mild (n=25)
severe_ATBtreated (n=21)
asymptomatic (n=24)
control (n=72)
ATBTreatment

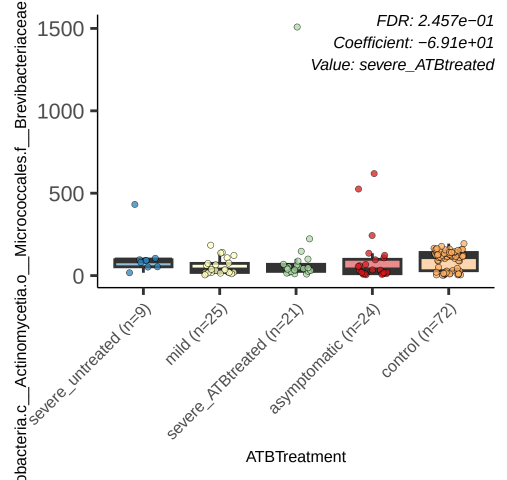
FDR: 2.457e−01
Coefficient: −6.91e+01
Value: severe_ATBtreated
0
500
1000
1500
obacteria.c__Actinomycetia.o__Micrococcales.f__Brevibacteriaceae
severe_untreated (n=9)
mild (n=25)
severe_ATBtreated (n=21)
asymptomatic (n=24)
control (n=72)
ATBTreatment

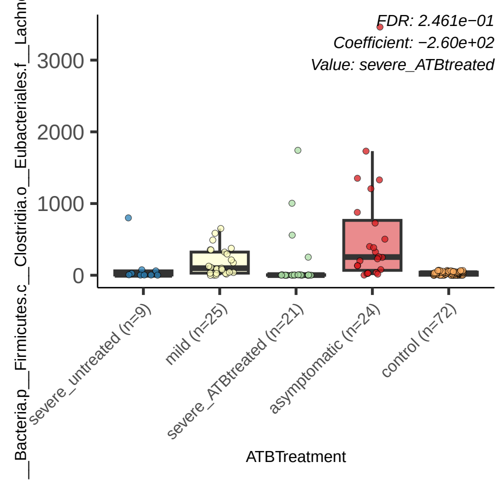
FDR: 2.461e−01
Coefficient: −2.60e+02
Value: severe_ATBtreated
_Bacteria.p__Firmicutes.c__Clostridia.o__Eubacteriales.f__Lachno
0
1000
2000
3000
severe_untreated (n=9)
mild (n=25)
severe_ATBtreated (n=21)
asymptomatic (n=24)
control (n=72)
ATBTreatment

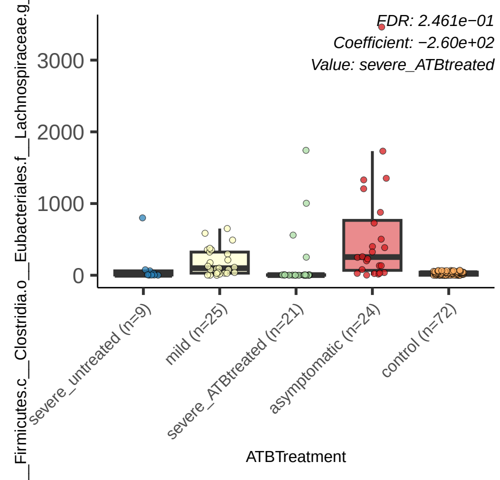
FDR: 2.461e−01
Coefficient: −2.60e+02
Value: severe_ATBtreated
Firmicutes.c__Clostridia.o__Eubacteriales.f__Lachnospiraceae.g__
3000
2000
1000
0
severe_untreated (n=9)
mild (n=25)
severe_ATBtreated (n=21)
asymptomatic (n=24)
control (n=72)
ATBTreatment
