# Supplementary material for: Metatranscriptome Analysis of Nasopharyngeal Swabs across the Varying Severity of COVID-19 Disease Demonstrated Unprecedented Species Diversity
Source: Microorganisms. 2023 Jul 14;11(7):1804. doi: 10.3390/microorganisms11071804 (PMC10384460; doi:10.3390/microorganisms11071804)
Supplement: Supplementary file 1 [file microorganisms-11-01804-s001.zip › Maaslin_results_ALL.zip!/Maaslin_results/heatmap.pdf]

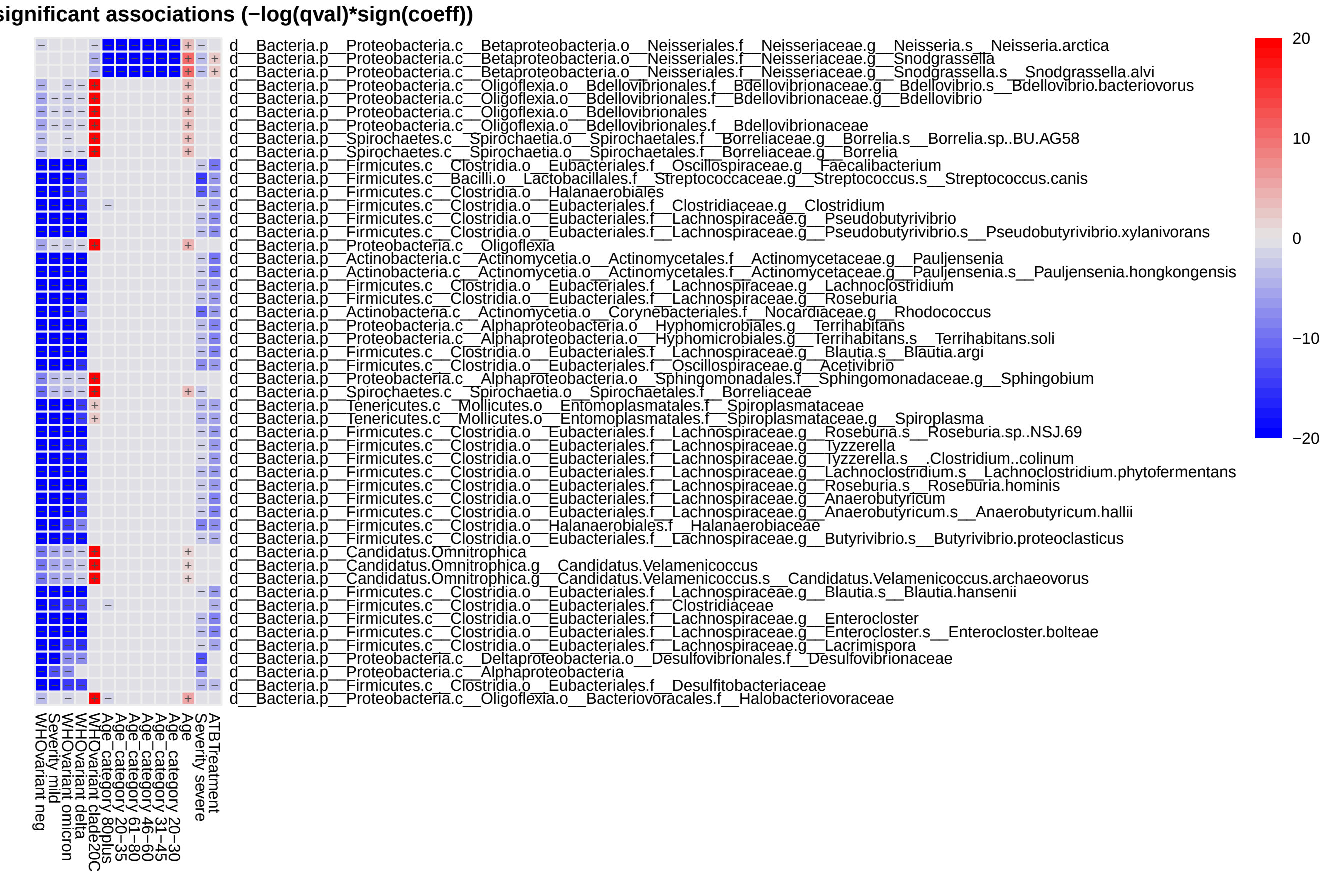
significant associations (−log(qval)*sign(coeff))
d__Bacteria.p__Proteobacteria.c__Betaproteobacteria.o__Neisseriales.f__Neisseriaceae.g__Neisseria.s__Neisseria.arctica
d__Bacteria.p__Proteobacteria.c__Betaproteobacteria.o__Neisseriales.f__Neisseriaceae.g__Snodgrassella
d__Bacteria.p__Proteobacteria.c__Betaproteobacteria.o__Neisseriales.f__Neisseriaceae.g__Snodgrassella.s__Snodgrassella.alvi
d__Bacteria.p__Proteobacteria.c__Oligoflexia.o__Bdellovibrionales.f__Bdellovibrionaceae.g__Bdellovibrio.s__Bdellovibrio.bacteriovorus
d__Bacteria.p__Proteobacteria.c__Oligoflexia.o__Bdellovibrionales.f__Bdellovibrionaceae.g__Bdellovibrio
d__Bacteria.p__Proteobacteria.c__Oligoflexia.o__Bdellovibrionales
d__Bacteria.p__Proteobacteria.c__Oligoflexia.o__Bdellovibrionales.f__Bdellovibrionaceae
d__Bacteria.p__Spirochaetes.c__Spirochaetia.o__Spirochaetales.f__Borreliaceae.g__Borrelia.s__Borrelia.sp..BU.AG58
d__Bacteria.p__Spirochaetes.c__Spirochaetia.o__Spirochaetales.f__Borreliaceae.g__Borrelia
d__Bacteria.p__Firmicutes.c__Clostridia.o__Eubacteriales.f__Oscillospiraceae.g__Faecalibacterium
d__Bacteria.p__Firmicutes.c__Bacilli.o__Lactobacillales.f__Streptococcaceae.g__Streptococcus.s__Streptococcus.canis
d__Bacteria.p__Firmicutes.c__Clostridia.o__Halanaerobiales
d__Bacteria.p__Firmicutes.c__Clostridia.o__Eubacteriales.f__Clostridiaceae.g__Clostridium
d__Bacteria.p__Firmicutes.c__Clostridia.o__Eubacteriales.f__Lachnospiraceae.g__Pseudobutyrivibrio
d__Bacteria.p__Firmicutes.c__Clostridia.o__Eubacteriales.f__Lachnospiraceae.g__Pseudobutyrivibrio.s__Pseudobutyrivibrio.xylanivorans
d__Bacteria.p__Proteobacteria.c__Oligoflexia
d__Bacteria.p__Actinobacteria.c__Actinomycetia.o__Actinomycetales.f__Actinomycetaceae.g__Pauljensenia
d__Bacteria.p__Actinobacteria.c__Actinomycetia.o__Actinomycetales.f__Actinomycetaceae.g__Pauljensenia.s__Pauljensenia.hongkongensis
d__Bacteria.p__Firmicutes.c__Clostridia.o__Eubacteriales.f__Lachnospiraceae.g__Lachnoclostridium
d__Bacteria.p__Firmicutes.c__Clostridia.o__Eubacteriales.f__Lachnospiraceae.g__Roseburia
d__Bacteria.p__Actinobacteria.c__Actinomycetia.o__Corynebacteriales.f__Nocardiaceae.g__Rhodococcus
d__Bacteria.p__Proteobacteria.c__Alphaproteobacteria.o__Hyphomicrobiales.g__Terrihabitans
d__Bacteria.p__Proteobacteria.c__Alphaproteobacteria.o__Hyphomicrobiales.g__Terrihabitans.s__Terrihabitans.soli
d__Bacteria.p__Firmicutes.c__Clostridia.o__Eubacteriales.f__Lachnospiraceae.g__Blautia.s__Blautia.argi
d__Bacteria.p__Firmicutes.c__Clostridia.o__Eubacteriales.f__Oscillospiraceae.g__Acetivibrio
d__Bacteria.p__Proteobacteria.c__Alphaproteobacteria.o__Sphingomonadales.f__Sphingomonadaceae.g__Sphingobium
d__Bacteria.p__Spirochaetes.c__Spirochaetia.o__Spirochaetales.f__Borreliaceae
d__Bacteria.p__Tenericutes.c__Mollicutes.o__Entomoplasmatales.f__Spiroplasmataceae
d__Bacteria.p__Tenericutes.c__Mollicutes.o__Entomoplasmatales.f__Spiroplasmataceae.g__Spiroplasma
d__Bacteria.p__Firmicutes.c__Clostridia.o__Eubacteriales.f__Lachnospiraceae.g__Roseburia.s__Roseburia.sp..NSJ.69
d__Bacteria.p__Firmicutes.c__Clostridia.o__Eubacteriales.f__Lachnospiraceae.g__Tyzzerella
d__Bacteria.p__Firmicutes.c__Clostridia.o__Eubacteriales.f__Lachnospiraceae.g__Tyzzerella.s__.Clostridium..colinum
d__Bacteria.p__Firmicutes.c__Clostridia.o__Eubacteriales.f__Lachnospiraceae.g__Lachnoclostridium.s__Lachnoclostridium.phytofermentans
d__Bacteria.p__Firmicutes.c__Clostridia.o__Eubacteriales.f__Lachnospiraceae.g__Roseburia.s__Roseburia.hominis
d__Bacteria.p__Firmicutes.c__Clostridia.o__Eubacteriales.f__Lachnospiraceae.g__Anaerobutyricum
d__Bacteria.p__Firmicutes.c__Clostridia.o__Eubacteriales.f__Lachnospiraceae.g__Anaerobutyricum.s__Anaerobutyricum.hallii
d__Bacteria.p__Firmicutes.c__Clostridia.o__Halanaerobiales.f__Halanaerobiaceae
d__Bacteria.p__Firmicutes.c__Clostridia.o__Eubacteriales.f__Lachnospiraceae.g__Butyrivibrio.s__Butyrivibrio.proteoclasticus
d__Bacteria.p__Candidatus.Omnitrophica
d__Bacteria.p__Candidatus.Omnitrophica.g__Candidatus.Velamenicoccus
d__Bacteria.p__Candidatus.Omnitrophica.g__Candidatus.Velamenicoccus.s__Candidatus.Velamenicoccus.archaeovorus
d__Bacteria.p__Firmicutes.c__Clostridia.o__Eubacteriales.f__Lachnospiraceae.g__Blautia.s__Blautia.hansenii
d__Bacteria.p__Firmicutes.c__Clostridia.o__Eubacteriales.f__Clostridiaceae
d__Bacteria.p__Firmicutes.c__Clostridia.o__Eubacteriales.f__Lachnospiraceae.g__Enterocloster
d__Bacteria.p__Firmicutes.c__Clostridia.o__Eubacteriales.f__Lachnospiraceae.g__Enterocloster.s__Enterocloster.bolteae
d__Bacteria.p__Firmicutes.c__Clostridia.o__Eubacteriales.f__Lachnospiraceae.g__Lacrimispora
d__Bacteria.p__Proteobacteria.c__Deltaproteobacteria.o__Desulfovibrionales.f__Desulfovibrionaceae
d__Bacteria.p__Proteobacteria.c__Alphaproteobacteria
d__Bacteria.p__Firmicutes.c__Clostridia.o__Eubacteriales.f__Desulfitobacteriaceae
d__Bacteria.p__Proteobacteria.c__Oligoflexia.o__Bacteriovoracales.f__Halobacteriovoraceae
WHOvariant neg
Severity mild
WHOvariant omicron
WHOvariant delta
WHOvariant clade20C
Age_category 80plus
Age_category 20−35
Age_category 61−80
Age_category 46−60
Age_category 31−45
Age_category 20−30
Age
Severity severe
ATBTreatment
20
10
0
−10
−20
