# Supplementary material for: Metatranscriptome Analysis of Nasopharyngeal Swabs across the Varying Severity of COVID-19 Disease Demonstrated Unprecedented Species Diversity
Source: Microorganisms. 2023 Jul 14;11(7):1804. doi: 10.3390/microorganisms11071804 (PMC10384460; doi:10.3390/microorganisms11071804)
Supplement: Supplementary file 1 [file microorganisms-11-01804-s001.zip › Maaslin_results_ALL.zip!/Maaslin_results/Severity.pdf]

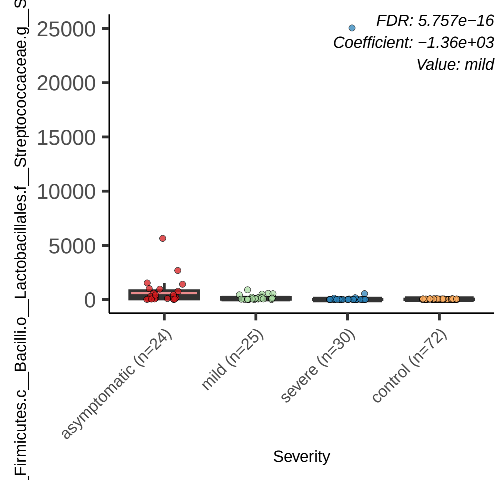

Firmicutes.c__Bacilli.o__Lactobacillales.f__Streptococcaceae.g__S
FDR: 5.757e−16
Coefficient: −1.36e+03
Value: mild
25000
20000
15000
10000
5000
0
asymptomatic (n=24)
mild (n=25)
severe (n=30)
control (n=72)
Severity

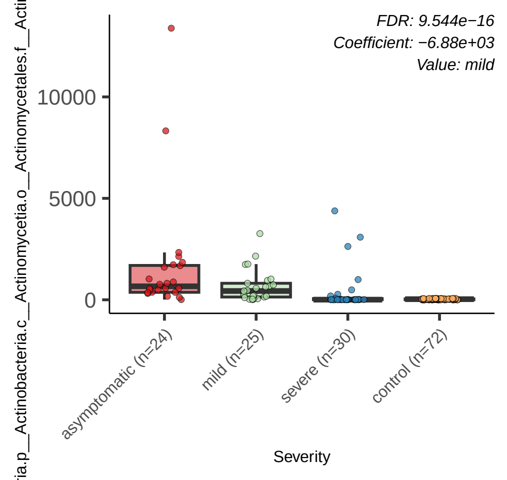

FDR: 9.544e−16
Coefficient: −6.88e+03
Value: mild
10000
5000
0
ria.p__Actinobacteria.c__Actinomycetia.o__Actinomycetales.f__Acti
asymptomatic (n=24)
mild (n=25)
severe (n=30)
control (n=72)
Severity

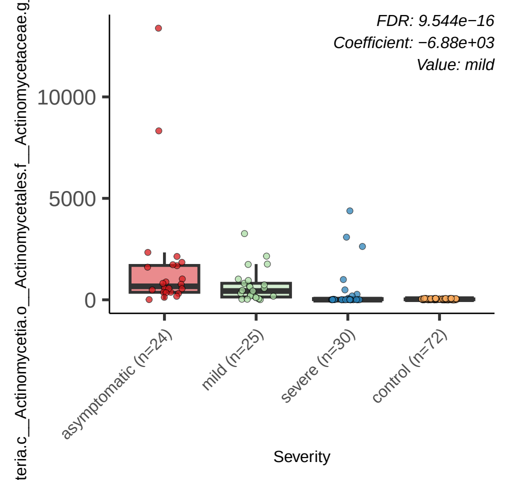

FDR: 9.544e−16
Coefficient: −6.88e+03
Value: mild
10000
5000
0
teria.c__Actinomycetia.o__Actinomycetales.f__Actinomycetaceae.g_
asymptomatic (n=24)
mild (n=25)
severe (n=30)
control (n=72)
Severity

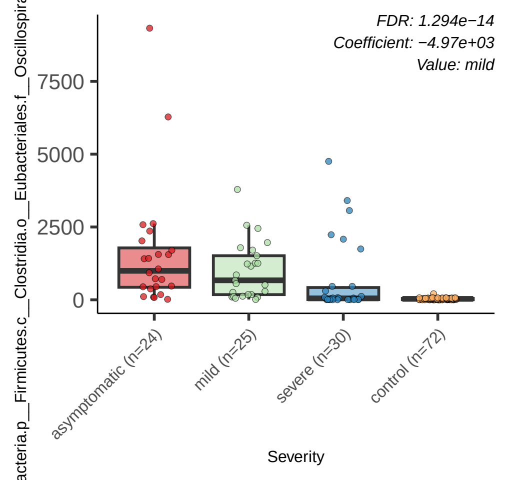
FDR: 1.294e−14
Coefficient: −4.97e+03
Value: mild
7500
5000
2500
0
acteria.p__Firmicutes.c__Clostridia.o__Eubacteriales.f__Oscillospira
asymptomatic (n=24)
mild (n=25)
severe (n=30)
control (n=72)
Severity

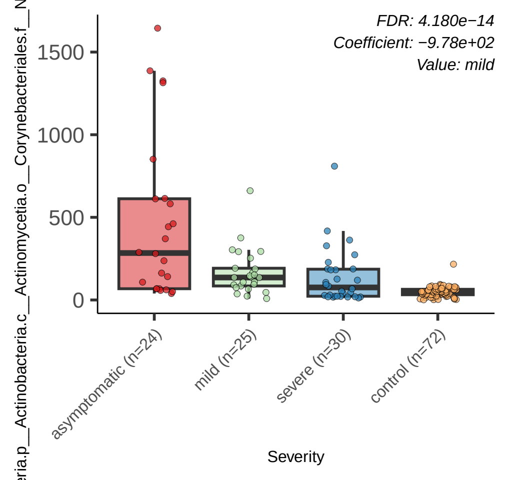

FDR: 4.180e−14
Coefficient: −9.78e+02
Value: mild
Actinobacteria.p__Actinobacteria.c__Actinomycetia.o__Corynebacteriales.f__N
1500
1000
500
0
asymptomatic (n=24)
mild (n=25)
severe (n=30)
control (n=72)
Severity

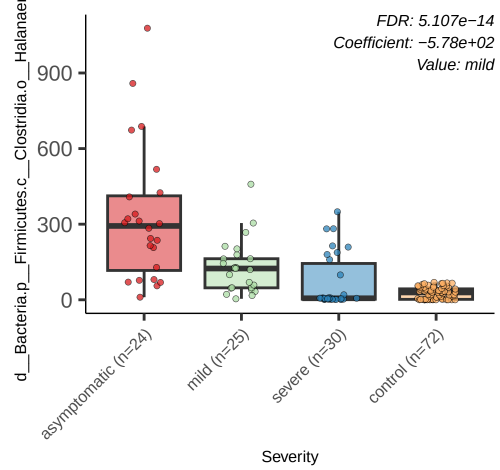

FDR: 5.107e−14
Coefficient: −5.78e+02
Value: mild
d__Bacteria.p__Firmicutes.c__Clostridia.o__Halanaer
900
600
300
0
asymptomatic (n=24)
mild (n=25)
severe (n=30)
control (n=72)
Severity

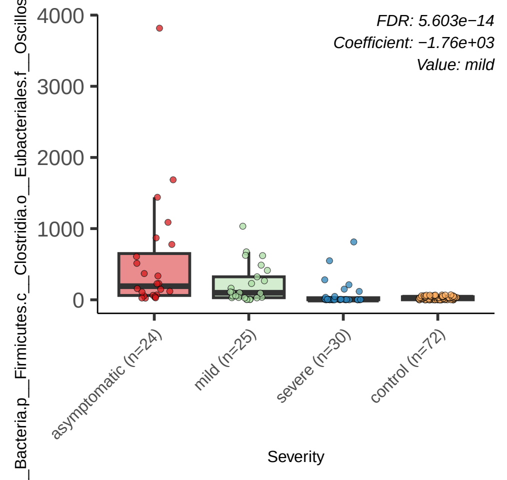

FDR: 5.603e−14
Coefficient: −1.76e+03
Value: mild
4000
3000
2000
1000
0
Bacteria.p__Firmicutes.c__Clostridia.o__Eubacteriales.f__Oscillos
asymptomatic (n=24)
mild (n=25)
severe (n=30)
control (n=72)
Severity

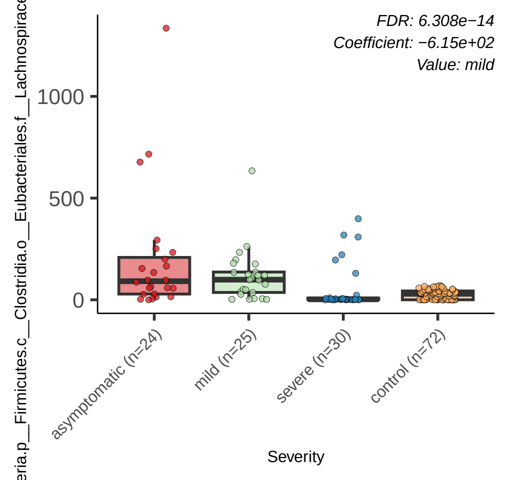

FDR: 6.308e−14
Coefficient: −6.15e+02
Value: mild
1000
500
0
asymptomatic (n=24)
mild (n=25)
severe (n=30)
control (n=72)
Severity
eria.p__Firmicutes.c__Clostridia.o__Eubacteriales.f__Lachnospirace

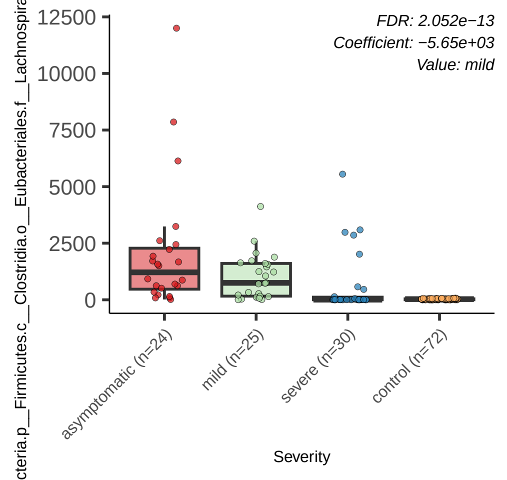

cteria.p__Firmicutes.c__Clostridia.o__Eubacteriales.f__Lachnospira
12500
10000
7500
5000
2500
0
FDR: 2.052e−13
Coefficient: −5.65e+03
Value: mild
asymptomatic (n=24)
mild (n=25)
severe (n=30)
control (n=72)
Severity

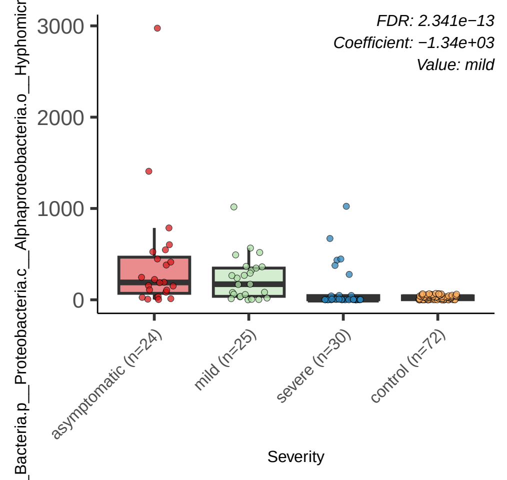

FDR: 2.341e−13
Coefficient: −1.34e+03
Value: mild
3000
2000
1000
0
Bacteria.p__Proteobacteria.c__Alphaproteobacteria.o__Hyphomicr
asymptomatic (n=24)
mild (n=25)
severe (n=30)
control (n=72)
Severity

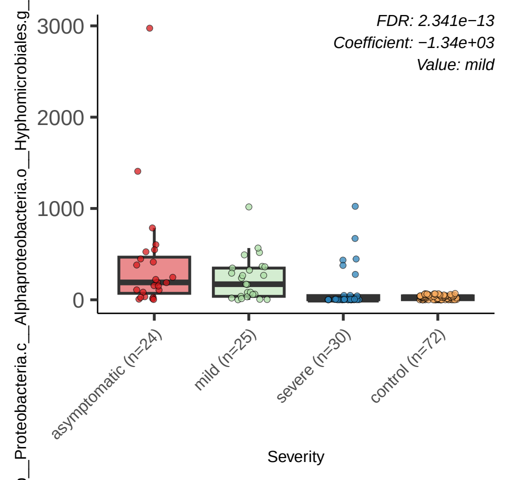
FDR: 2.341e−13
Coefficient: −1.34e+03
Value: mild
3000
2000
1000
0
o__Proteobacteria.c__Alphaproteobacteria.o__Hyphomicrobiales.g__
asymptomatic (n=24)
mild (n=25)
severe (n=30)
control (n=72)
Severity

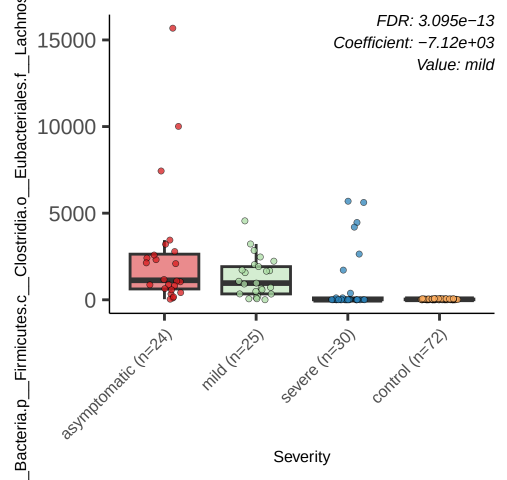

FDR: 3.095e−13
Coefficient: −7.12e+03
Value: mild
15000
10000
5000
0
Bacteria.p__Firmicutes.c__Clostridia.o__Eubacteriales.f__Lachnos
asymptomatic (n=24)
mild (n=25)
severe (n=30)
control (n=72)
Severity

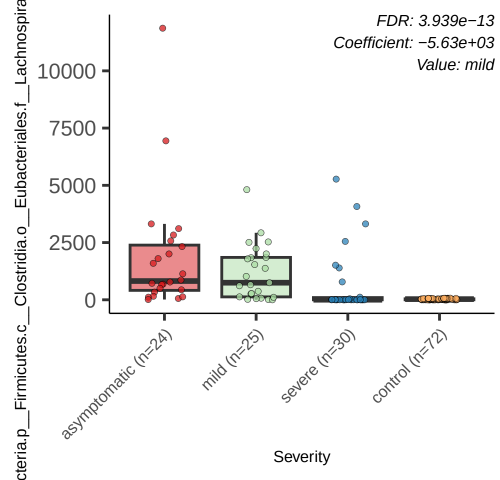

FDR: 3.939e−13
Coefficient: −5.63e+03
Value: mild
cteria.p__Firmicutes.c__Clostridia.o__Eubacteriales.f__Lachnospira
10000
7500
5000
2500
0
asymptomatic (n=24)
mild (n=25)
severe (n=30)
control (n=72)
Severity

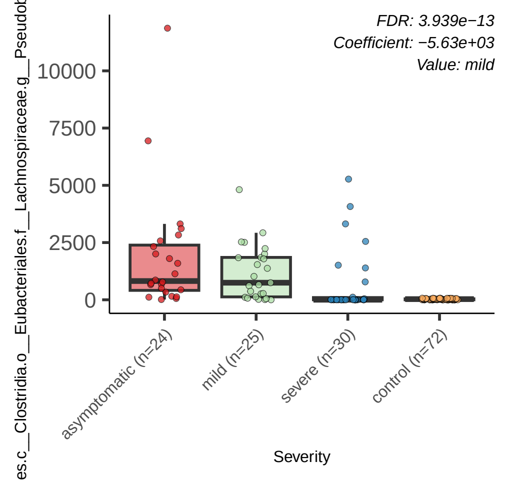

FDR: 3.939e−13
Coefficient: −5.63e+03
Value: mild
10000
7500
5000
2500
0
asymptomatic (n=24)
mild (n=25)
severe (n=30)
control (n=72)
Severity
es.c__Clostridia.o__Eubacteriales.f__Lachnospiraceae.g__Pseudob

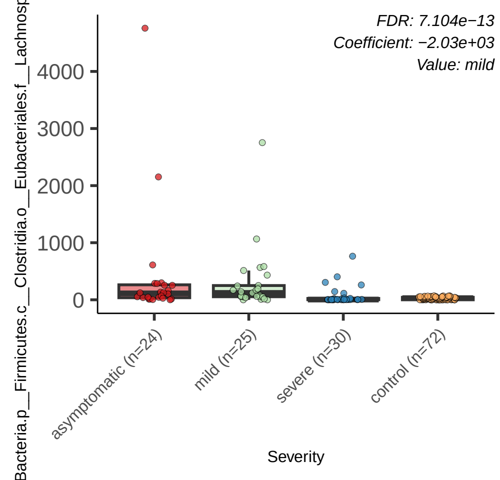
FDR: 7.104e−13
Coefficient: −2.03e+03
Value: mild
Bacteria.p__Firmicutes.c__Clostridia.o__Eubacteriales.f__Lachnosp
4000
3000
2000
1000
0
asymptomatic (n=24)
mild (n=25)
severe (n=30)
control (n=72)
Severity

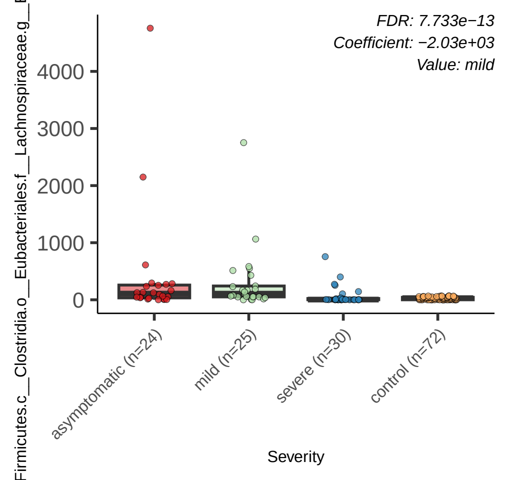

FDR: 7.733e−13
Coefficient: −2.03e+03
Value: mild
Firmicutes.c__Clostridia.o__Eubacteriales.f__Lachnospiraceae.g__
4000
3000
2000
1000
0
asymptomatic (n=24)
mild (n=25)
severe (n=30)
control (n=72)
Severity

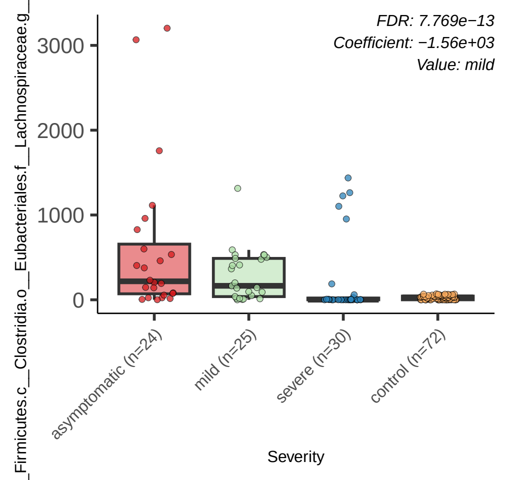

FDR: 7.769e−13
Coefficient: −1.56e+03
Value: mild
Firmicutes.c__Clostridia.o__Eubacteriales.f__Lachnospiraceae.g__
3000
2000
1000
0
asymptomatic (n=24)
mild (n=25)
severe (n=30)
control (n=72)
Severity

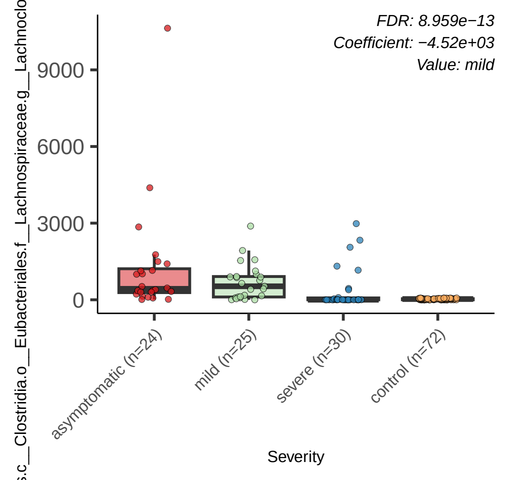
FDR: 8.959e−13
Coefficient: −4.52e+03
Value: mild
9000
6000
3000
0
asymptomatic (n=24)
mild (n=25)
severe (n=30)
control (n=72)
Severity
s.c__Clostridia.o__Eubacteriales.f__Lachnospiraceae.g__Lachnoclo

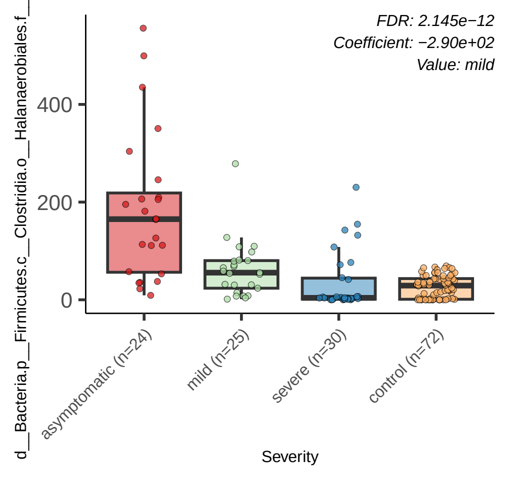

FDR: 2.145e−12
Coefficient: −2.90e+02
Value: mild
d__Bacteria.p__Firmicutes.c__Clostridia.o__Halanaerobiales.f__
400
200
0
asymptomatic (n=24)
mild (n=25)
severe (n=30)
control (n=72)
Severity

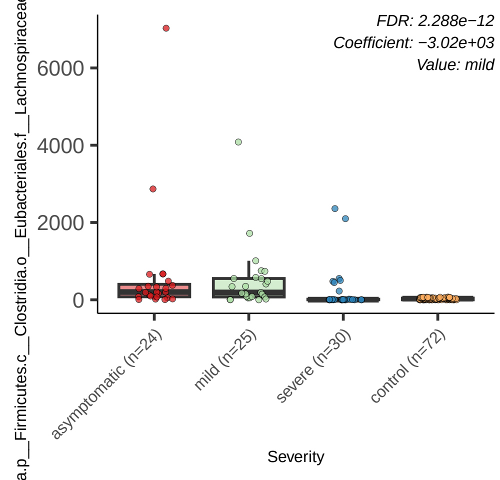
FDR: 2.288e−12
Coefficient: −3.02e+03
Value: mild
6000
4000
2000
0
a.p__Firmicutes.c__Clostridia.o__Eubacteriales.f__Lachnospiraceae
asymptomatic (n=24)
mild (n=25)
severe (n=30)
control (n=72)
Severity

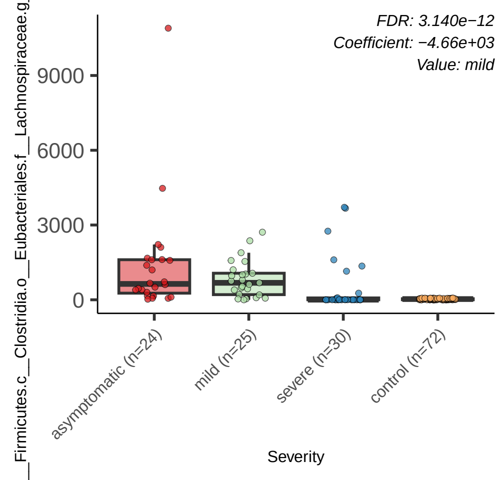
FDR: 3.140e−12
Coefficient: −4.66e+03
Value: mild
Firmicutes.c__Clostridia.o__Eubacteriales.f__Lachnospiraceae.g__
9000
6000
3000
0
asymptomatic (n=24)
mild (n=25)
severe (n=30)
control (n=72)
Severity

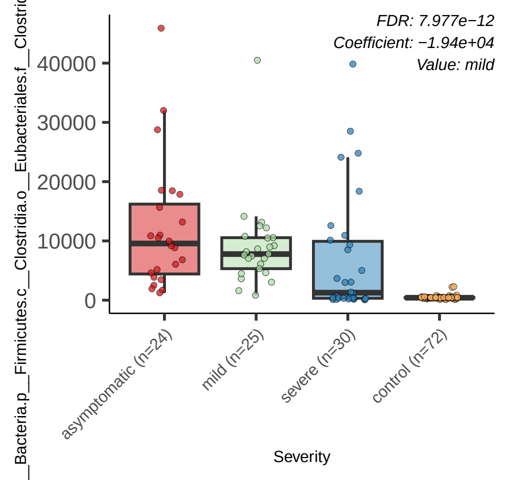
FDR: 7.977e−12
Coefficient: −1.94e+04
Value: mild
0
10000
20000
30000
40000
Bacteria.p__Firmicutes.c__Clostridia.o__Eubacteriales.f__Clostrid
asymptomatic (n=24)
mild (n=25)
severe (n=30)
control (n=72)
Severity

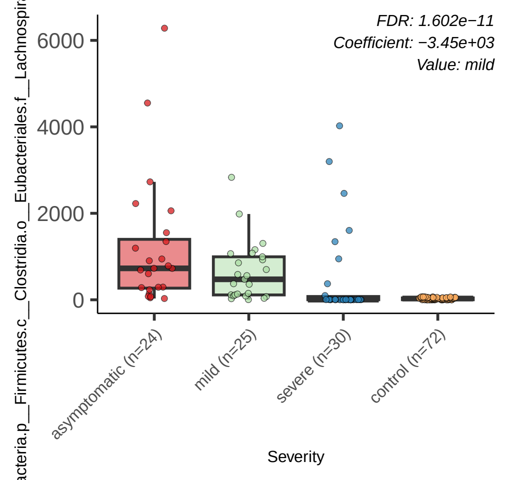
FDR: 1.602e−11
Coefficient: −3.45e+03
Value: mild
6000
4000
2000
0
acteria.p__Firmicutes.c__Clostridia.o__Eubacteriales.f__Lachnospira
asymptomatic (n=24)
mild (n=25)
severe (n=30)
control (n=72)
Severity

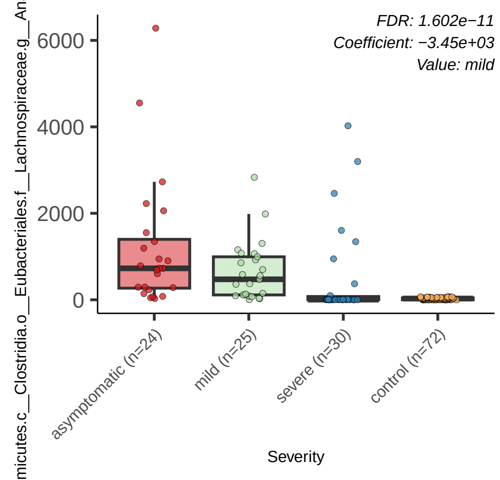

FDR: 1.602e−11
Coefficient: −3.45e+03
Value: mild
6000
4000
2000
0
micutes.c__Clostridia.o__Eubacteriales.f__Lachnospiraceae.g__An
asymptomatic (n=24)
mild (n=25)
severe (n=30)
control (n=72)
Severity

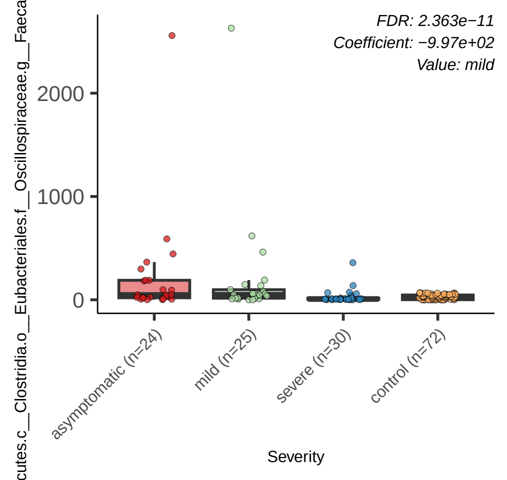

FDR: 2.363e−11
Coefficient: −9.97e+02
Value: mild
2000
1000
0
asymptomatic (n=24)
mild (n=25)
severe (n=30)
control (n=72)
Severity
cutes.c__Clostridia.o__Eubacteriales.f__Oscillospiraceae.g__Faeca

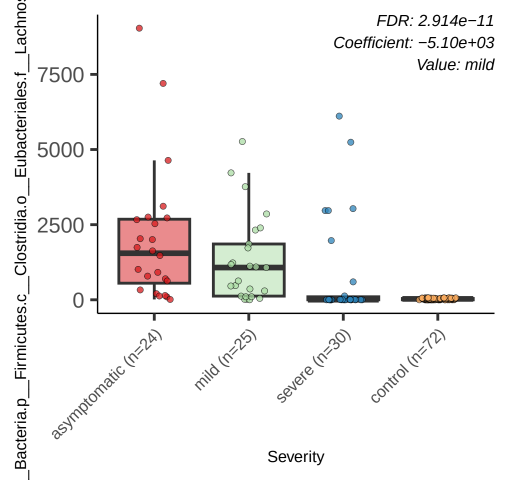

FDR: 2.914e−11
Coefficient: −5.10e+03
Value: mild
7500
5000
2500
0
_Bacteria.p__Firmicutes.c__Clostridia.o__Eubacteriales.f__Lachnos
asymptomatic (n=24)
mild (n=25)
severe (n=30)
control (n=72)
Severity

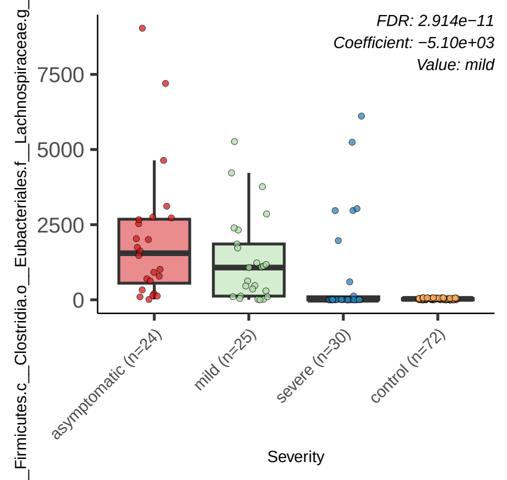
FDR: 2.914e−11
Coefficient: −5.10e+03
Value: mild
Firmicutes.c__Clostridia.o__Eubacteriales.f__Lachnospiraceae.g__
7500
5000
2500
0
asymptomatic (n=24)
mild (n=25)
severe (n=30)
control (n=72)
Severity

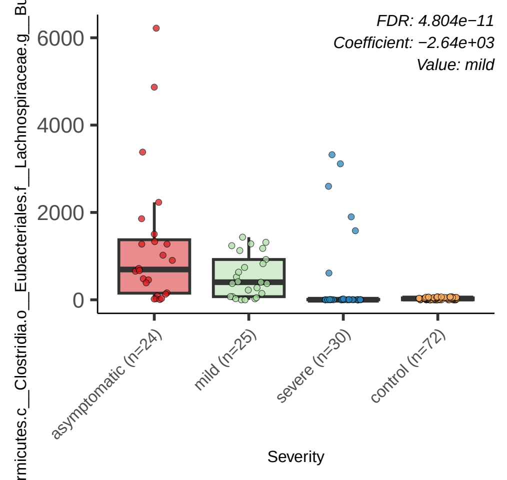

FDR: 4.804e−11
Coefficient: −2.64e+03
Value: mild
6000
4000
2000
0
rmicutes.c__Clostridia.o__Eubacteriales.f__Lachnospiraceae.g__Bu
asymptomatic (n=24)
mild (n=25)
severe (n=30)
control (n=72)
Severity

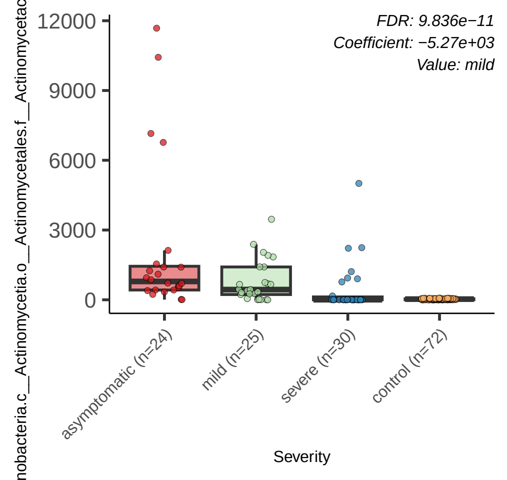

FDR: 9.836e−11
Coefficient: −5.27e+03
Value: mild
12000
9000
6000
3000
0
nobacteria.c__Actinomycetia.o__Actinomycetales.f__Actinomycetac
asymptomatic (n=24)
mild (n=25)
severe (n=30)
control (n=72)
Severity

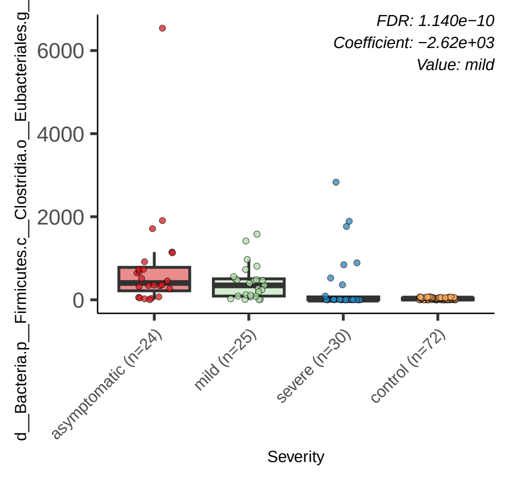

FDR: 1.140e−10
Coefficient: −2.62e+03
Value: mild
d__Bacteria.p__Firmicutes.c__Clostridia.o__Eubacteriales.g__
6000
4000
2000
0
asymptomatic (n=24)
mild (n=25)
severe (n=30)
control (n=72)
Severity

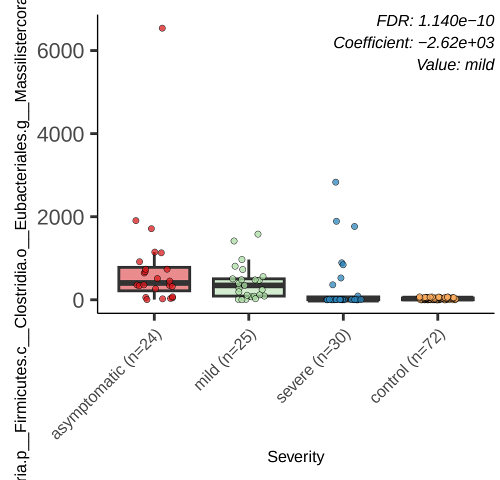

FDR: 1.140e−10
Coefficient: −2.62e+03
Value: mild
6000
4000
2000
0
ria.p__Firmicutes.c__Clostridia.o__Eubacteriales.g__Massilistercora
asymptomatic (n=24)
mild (n=25)
severe (n=30)
control (n=72)
Severity

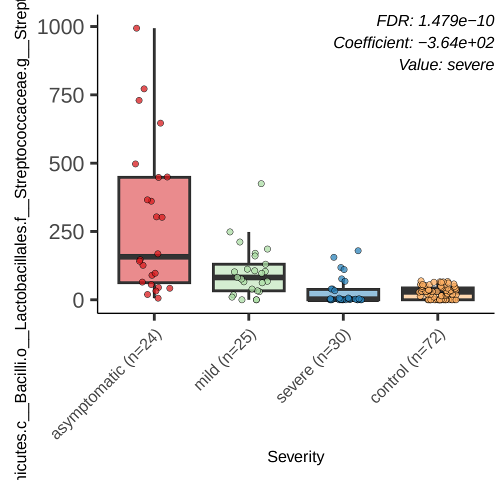

FDR: 1.479e−10
Coefficient: −3.64e+02
Value: severe
1000
750
500
250
0
asymptomatic (n=24)
mild (n=25)
severe (n=30)
control (n=72)
Severity

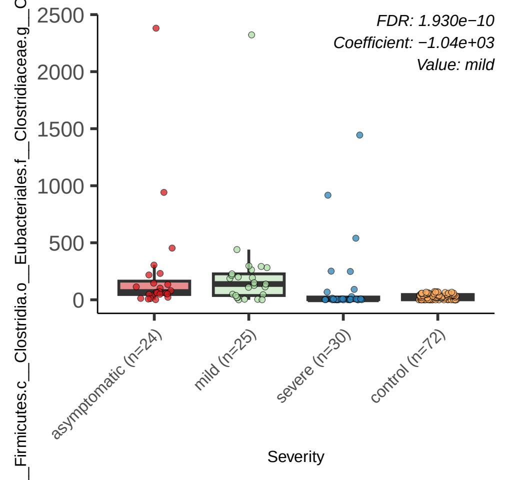
Firmicutes.c__Clostridia.o__Eubacteriales.f__Clostridiaceae.g__C
2500
2000
1500
1000
500
0
FDR: 1.930e−10
Coefficient: −1.04e+03
Value: mild
asymptomatic (n=24)
mild (n=25)
severe (n=30)
control (n=72)
Severity

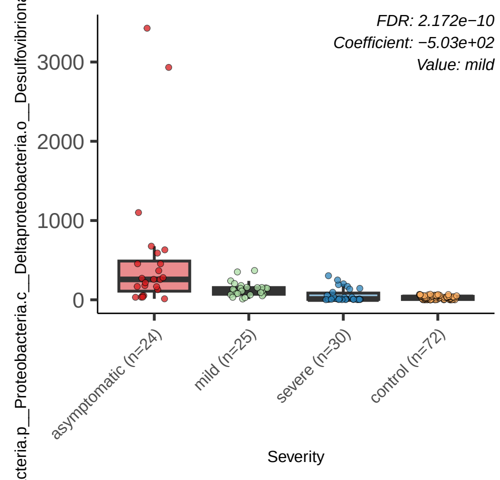

FDR: 2.172e−10
Coefficient: −5.03e+02
Value: mild
3000
2000
1000
0
cteria.p__Proteobacteria.c__Deltaproteobacteria.o__Desulfovibriona
asymptomatic (n=24)
mild (n=25)
severe (n=30)
control (n=72)
Severity

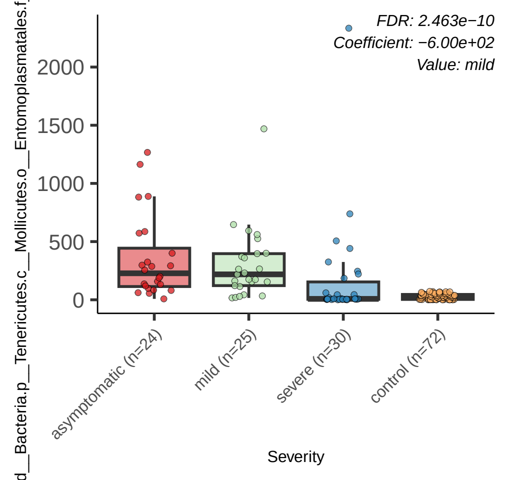

d__Bacteria.p__Tenericutes.c__Mollicutes.o__Entomoplasmatales.f__
FDR: 2.463e−10
Coefficient: −6.00e+02
Value: mild
2000
1500
1000
500
0
asymptomatic (n=24)
mild (n=25)
severe (n=30)
control (n=72)
Severity

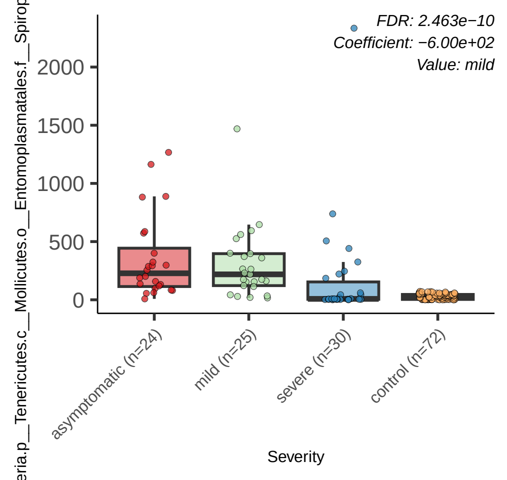

FDR: 2.463e−10
Coefficient: −6.00e+02
Value: mild
2000
1500
1000
500
0
eria.p__Tenericutes.c__Mollicutes.o__Entomoplasmatales.f__Spirop
asymptomatic (n=24)
mild (n=25)
severe (n=30)
control (n=72)
Severity

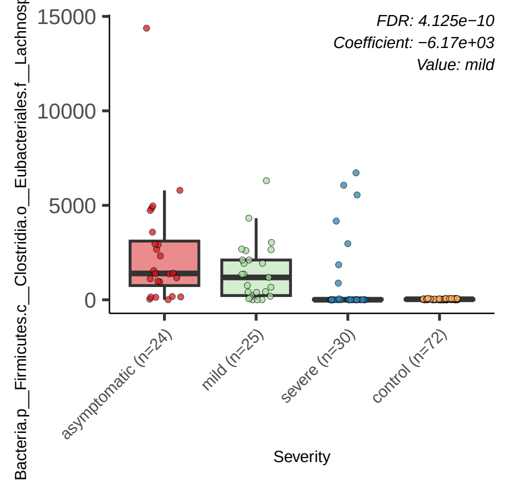

FDR: 4.125e−10
Coefficient: −6.17e+03
Value: mild
Bacteria.p__Firmicutes.c__Clostridia.o__Eubacteriales.f__Lachnosp
15000
10000
5000
0
asymptomatic (n=24)
mild (n=25)
severe (n=30)
control (n=72)
Severity

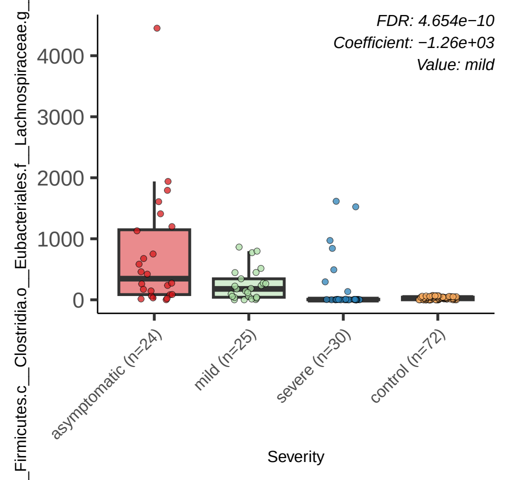

FDR: 4.654e−10
Coefficient: −1.26e+03
Value: mild
Firmicutes.c__Clostridia.o__Eubacteriales.f__Lachnospiraceae.g__
4000
3000
2000
1000
0
asymptomatic (n=24)
mild (n=25)
severe (n=30)
control (n=72)
Severity

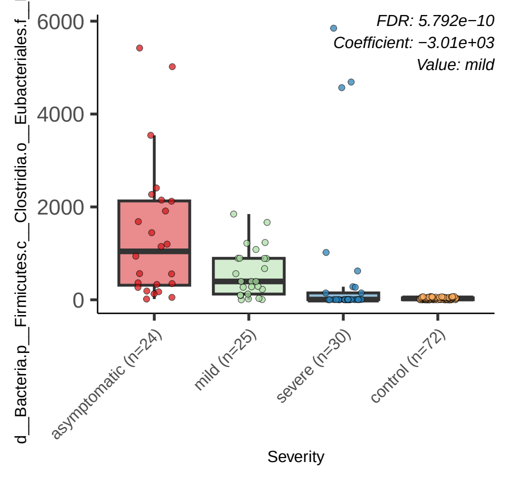

FDR: 5.792e−10
Coefficient: −3.01e+03
Value: mild
d__Bacteria.p__Firmicutes.c__Clostridia.o__Eubacteriales.f__
6000
4000
2000
0
asymptomatic (n=24)
mild (n=25)
severe (n=30)
control (n=72)
Severity

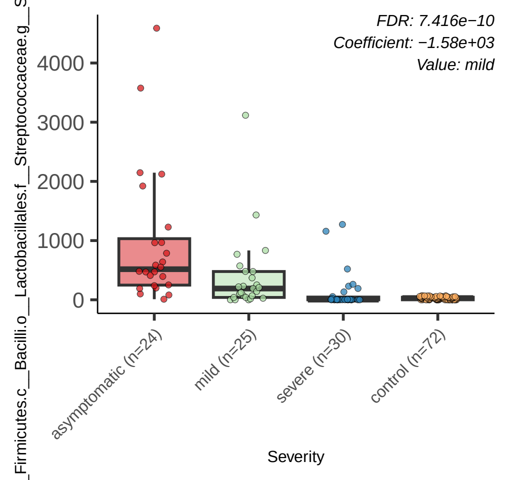

FDR: 7.416e−10
Coefficient: −1.58e+03
Value: mild
0
1000
2000
3000
4000
Firmicutes.c__Bacilli.o__Lactobacillales.f__Streptococcaceae.g__
asymptomatic (n=24)
mild (n=25)
severe (n=30)
control (n=72)
Severity

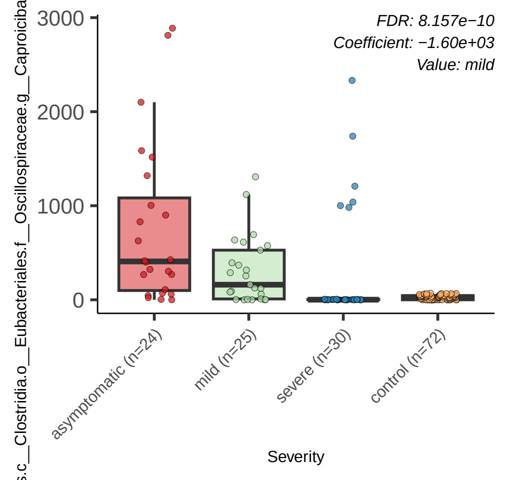

FDR: 8.157e−10
Coefficient: −1.60e+03
Value: mild
3000
2000
1000
0
s.c__Clostridia.o__Eubacteriales.f__Oscillospiraceae.g__Caproiciba
asymptomatic (n=24)
mild (n=25)
severe (n=30)
control (n=72)
Severity

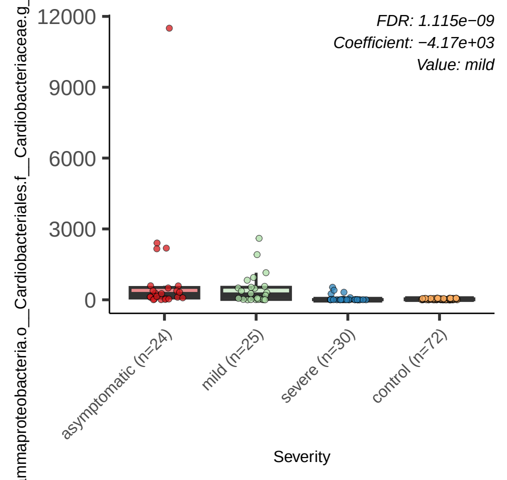

FDR: 1.115e−09
Coefficient: −4.17e+03
Value: mild
12000
9000
6000
3000
0
asymptomatic (n=24)
mild (n=25)
severe (n=30)
control (n=72)
Severity
mmaproteobacteria.o__Cardiobacteriales.f__Cardiobacteriaceae.g_

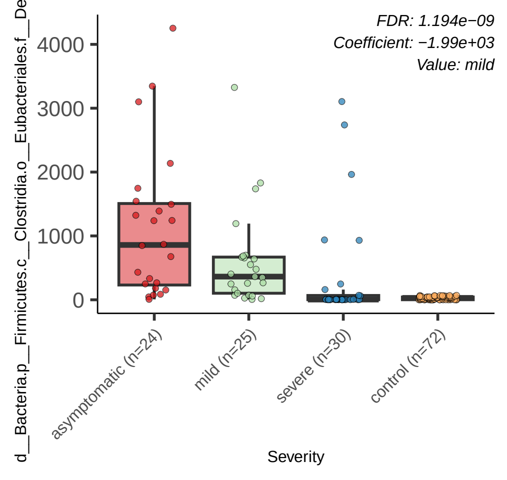

FDR: 1.194e−09
Coefficient: −1.99e+03
Value: mild
d__Bacteria.p__Firmicutes.c__Clostridia.o__Eubacteriales.f__De
0
1000
2000
3000
4000
asymptomatic (n=24)
mild (n=25)
severe (n=30)
control (n=72)
Severity

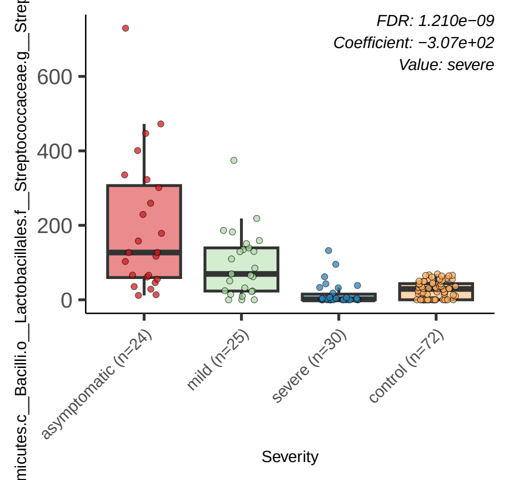
FDR: 1.210e−09
Coefficient: −3.07e+02
Value: severe
0
200
400
600
micutes.c__Bacilli.o__Lactobacillales.f__Streptococcaceae.g__Strep
asymptomatic (n=24)
mild (n=25)
severe (n=30)
control (n=72)
Severity

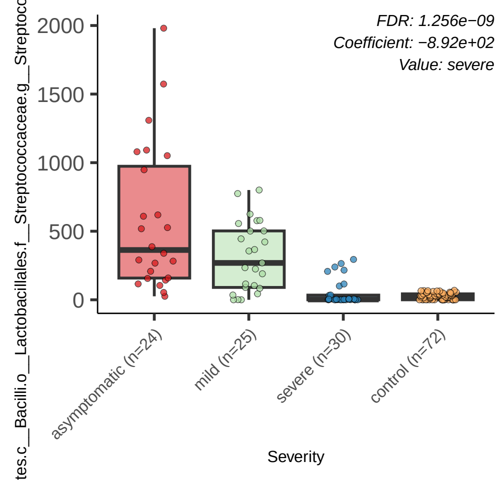
FDR: 1.256e−09
Coefficient: −8.92e+02
Value: severe
2000
1500
1000
500
0
tes.c__Bacilli.o__Lactobacillales.f__Streptococcaceae.g__Streptococc
asymptomatic (n=24)
mild (n=25)
severe (n=30)
control (n=72)
Severity

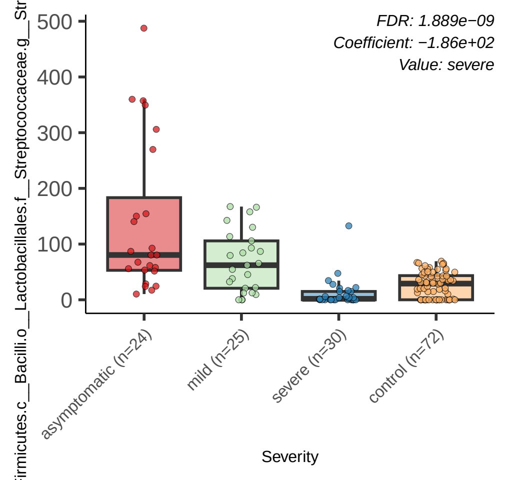

FDR: 1.889e−09
Coefficient: −1.86e+02
Value: severe
Firmicutes.c__Bacilli.o__Lactobacillales.f__Streptococcaceae.g__Str
500
400
300
200
100
0
asymptomatic (n=24)
mild (n=25)
severe (n=30)
control (n=72)
Severity

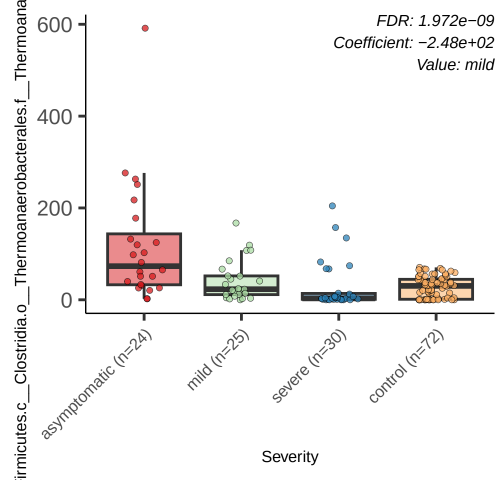

FDR: 1.972e−09
Coefficient: −2.48e+02
Value: mild
irmicutes.c__Clostridia.o__Thermoanaerobacterales.f__Thermoana
600
400
200
0
asymptomatic (n=24)
mild (n=25)
severe (n=30)
control (n=72)
Severity

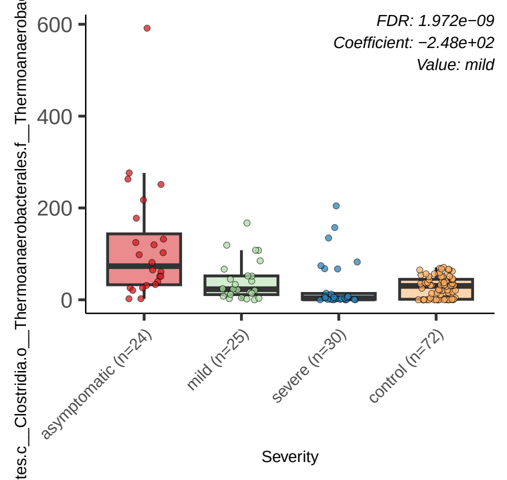

FDR: 1.972e−09
Coefficient: −2.48e+02
Value: mild
600
400
200
0
tes.c__Clostridia.o__Thermoanaerobacterales.f__Thermoanaerobac
asymptomatic (n=24)
mild (n=25)
severe (n=30)
control (n=72)
Severity

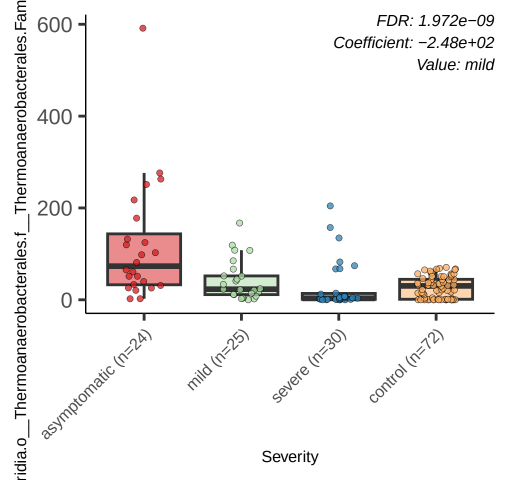
FDR: 1.972e−09
Coefficient: −2.48e+02
Value: mild
ridia.o__Thermoanaerobacterales.f__Thermoanaerobacterales.Fam
600
400
200
0
asymptomatic (n=24)
mild (n=25)
severe (n=30)
control (n=72)
Severity

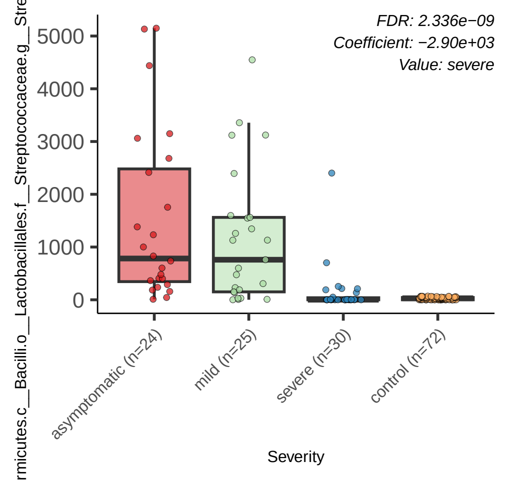

FDR: 2.336e−09
Coefficient: −2.90e+03
Value: severe
rmicutes.c__Bacilli.o__Lactobacillales.f__Streptococcaceae.g__Stre
5000
4000
3000
2000
1000
0
asymptomatic (n=24)
mild (n=25)
severe (n=30)
control (n=72)
Severity

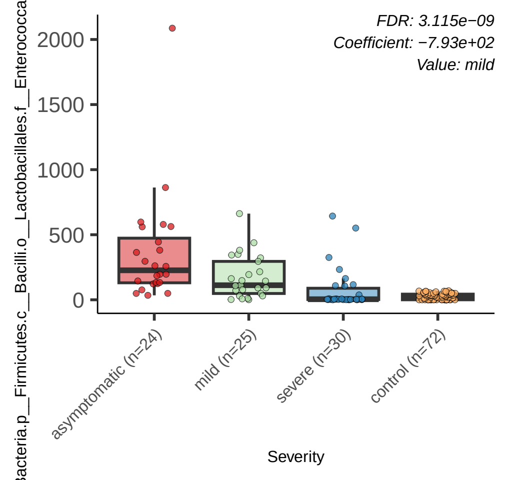

FDR: 3.115e−09
Coefficient: −7.93e+02
Value: mild
2000
1500
1000
500
0
Bacteria.p__Firmicutes.c__Bacilli.o__Lactobacillales.f__Enterococca
asymptomatic (n=24)
mild (n=25)
severe (n=30)
control (n=72)
Severity

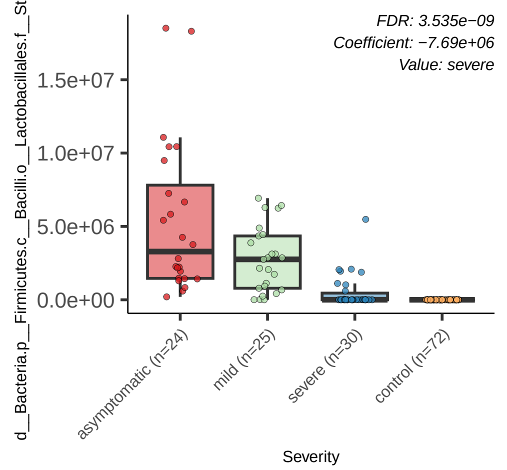

FDR: 3.535e−09
Coefficient: −7.69e+06
Value: severe
d__Bacteria.p__Firmicutes.c__Bacilli.o__Lactobacillales.f__St
0.0e+00
5.0e+06
1.0e+07
1.5e+07
asymptomatic (n=24)
mild (n=25)
severe (n=30)
control (n=72)
Severity

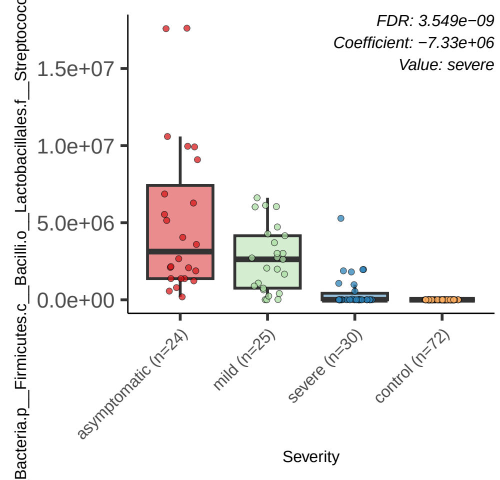
FDR: 3.549e−09
Coefficient: −7.33e+06
Value: severe
Bacteria.p__Firmicutes.c__Bacilli.o__Lactobacillales.f__Streptococc
1.5e+07
1.0e+07
5.0e+06
0.0e+00
asymptomatic (n=24)
mild (n=25)
severe (n=30)
control (n=72)
Severity

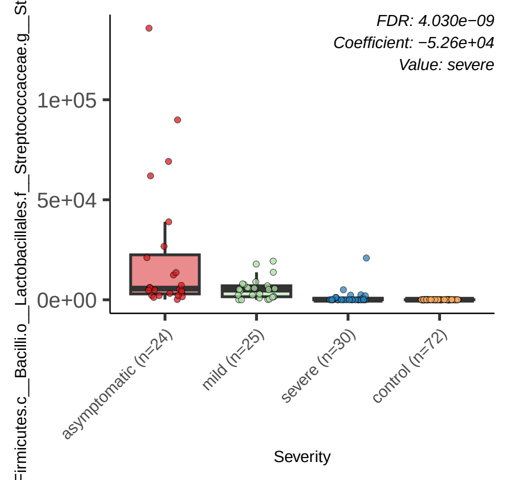

FDR: 4.030e−09
Coefficient: −5.26e+04
Value: severe
Firmicutes.c__Bacilli.o__Lactobacillales.f__Streptococcaceae.g__St
1e+05
5e+04
0e+00
asymptomatic (n=24)
mild (n=25)
severe (n=30)
control (n=72)
Severity

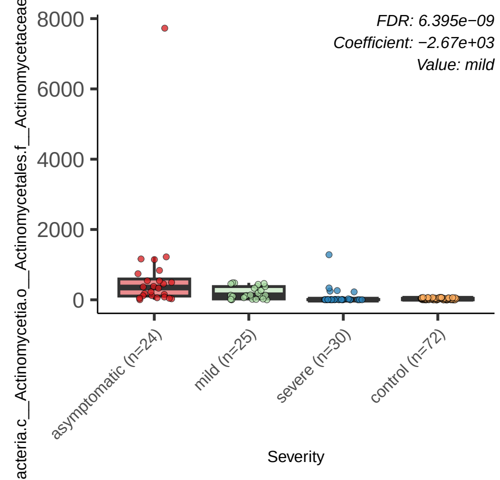

FDR: 6.395e−09
Coefficient: −2.67e+03
Value: mild
acteria.c__Actinomycetia.o__Actinomycetales.f__Actinomycetaceae
8000
6000
4000
2000
0
asymptomatic (n=24)
mild (n=25)
severe (n=30)
control (n=72)
Severity

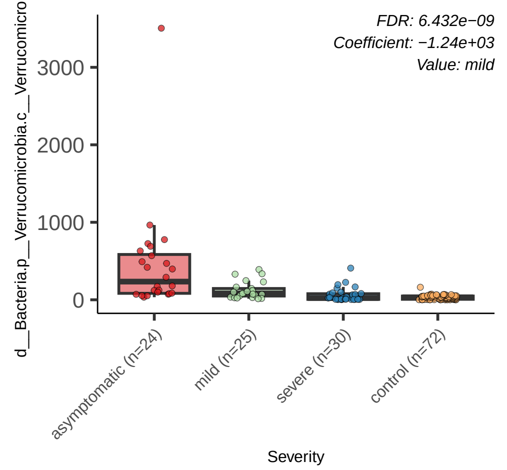

FDR: 6.432e−09
Coefficient: −1.24e+03
Value: mild
d__Bacteria.p__Verrucomicrobia.c__Verrucomicro
3000
2000
1000
0
asymptomatic (n=24)
mild (n=25)
severe (n=30)
control (n=72)
Severity

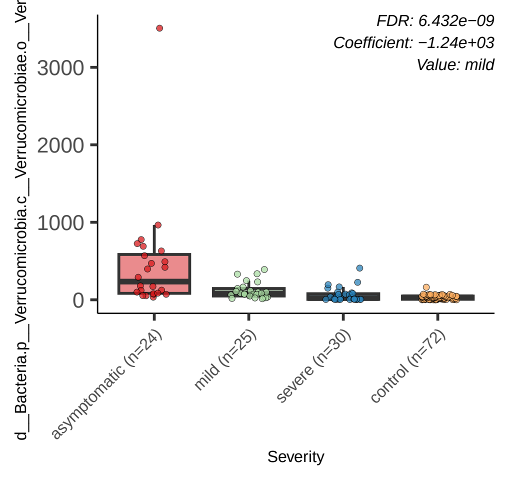
FDR: 6.432e−09
Coefficient: −1.24e+03
Value: mild
d__Bacteria.p__Verrucomicrobia.c__Verrucomicrobiae.o__Ver
3000
2000
1000
0
asymptomatic (n=24)
mild (n=25)
severe (n=30)
control (n=72)
Severity

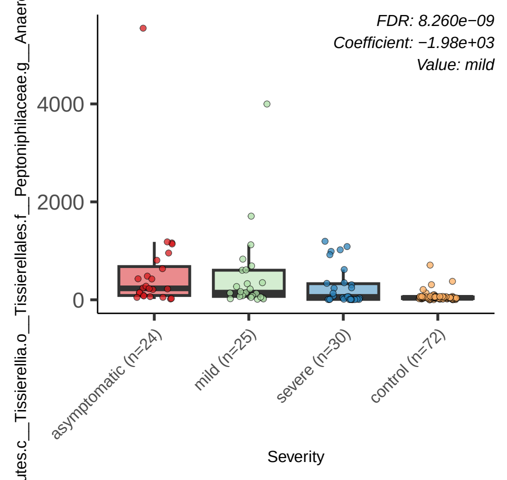

FDR: 8.260e−09
Coefficient: −1.98e+03
Value: mild
4000
2000
0
asymptomatic (n=24)
mild (n=25)
severe (n=30)
control (n=72)
Severity
utes.c__Tissierellia.o__Tissierellales.f__Peptoniphilaceae.g__Anaer

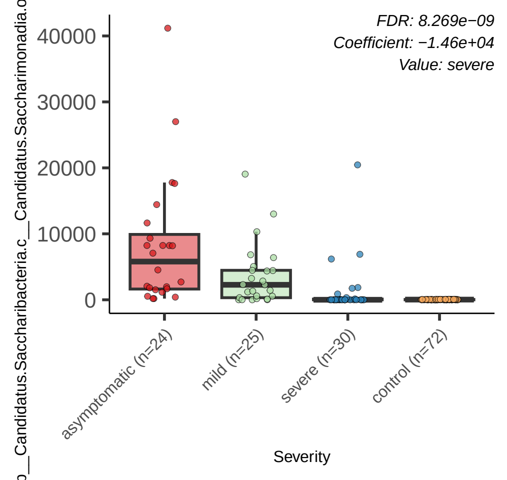
FDR: 8.269e−09
Coefficient: −1.46e+04
Value: severe
40000
30000
20000
10000
0
asymptomatic (n=24)
mild (n=25)
severe (n=30)
control (n=72)
Severity
Candidatus.Saccharibacteria.c__Candidatus.Saccharimonadia.o

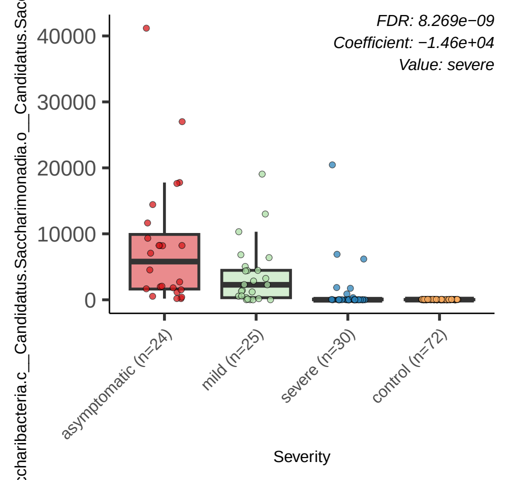

FDR: 8.269e−09
Coefficient: −1.46e+04
Value: severe
40000
30000
20000
10000
0
ccharibacteria.c__Candidatus.Saccharimonadia.o__Candidatus.Sacc
asymptomatic (n=24)
mild (n=25)
severe (n=30)
control (n=72)
Severity

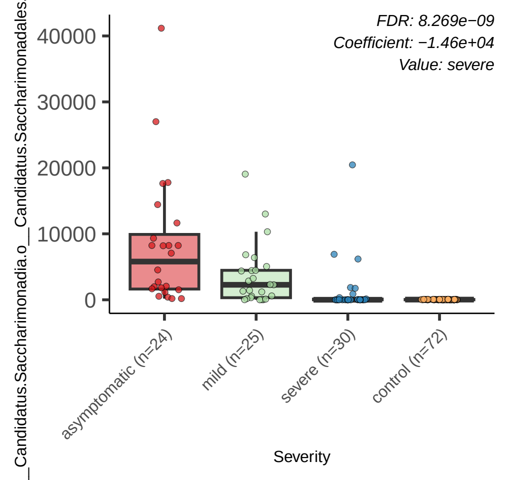
FDR: 8.269e−09
Coefficient: −1.46e+04
Value: severe
_Candidatus.Saccharimonadia.o__Candidatus.Saccharimonadales.
40000
30000
20000
10000
0
asymptomatic (n=24)
mild (n=25)
severe (n=30)
control (n=72)
Severity

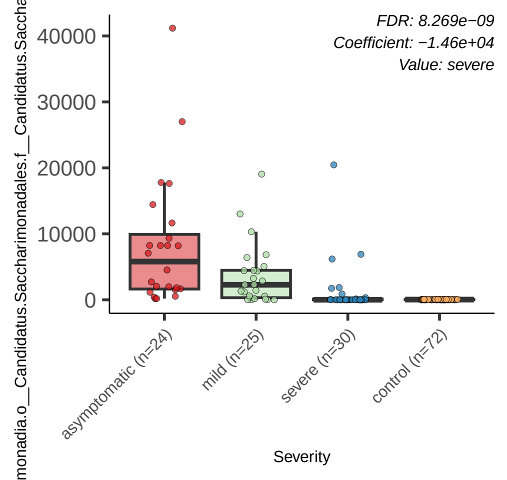

FDR: 8.269e−09
Coefficient: −1.46e+04
Value: severe
40000
30000
20000
10000
0
monadia.o__Candidatus.Saccharimonadales.f__Candidatus.Saccha
asymptomatic (n=24)
mild (n=25)
severe (n=30)
control (n=72)
Severity

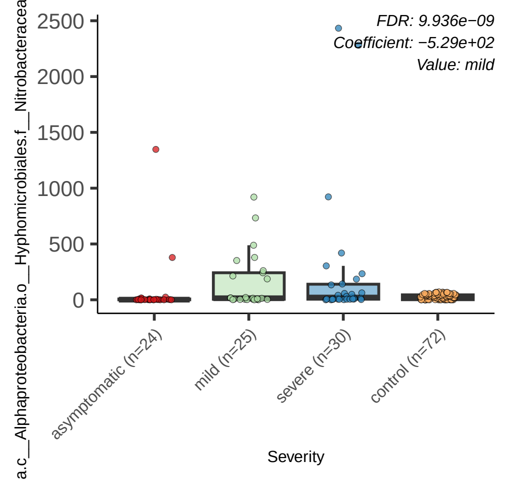

FDR: 9.936e−09
Coefficient: −5.29e+02
Value: mild
2500
2000
1500
1000
500
0
a.c__Alphaproteobacteria.o__Hyphomicrobiales.f__Nitrobacteracea
asymptomatic (n=24)
mild (n=25)
severe (n=30)
control (n=72)
Severity

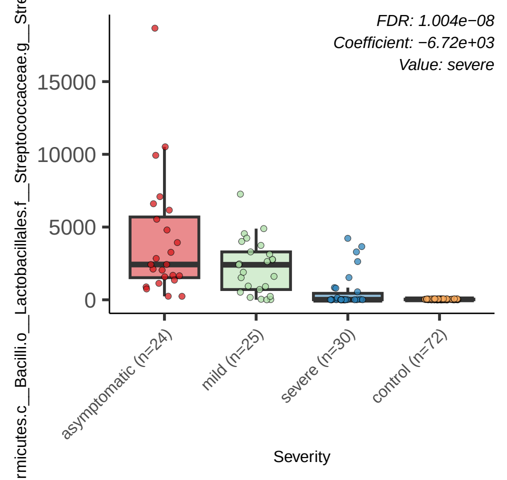

FDR: 1.004e−08
Coefficient: −6.72e+03
Value: severe
rmicutes.c__Bacilli.o__Lactobacillales.f__Streptococcaceae.g__Stre
15000
10000
5000
0
asymptomatic (n=24)
mild (n=25)
severe (n=30)
control (n=72)
Severity

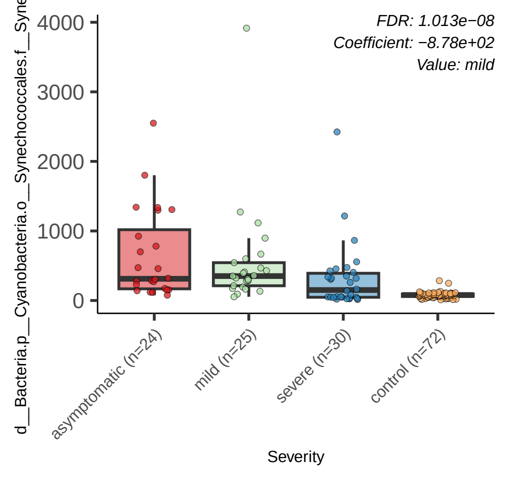

FDR: 1.013e−08
Coefficient: −8.78e+02
Value: mild
d__Bacteria.p__Cyanobacteria.o__Synechococcales.f__Syne
4000
3000
2000
1000
0
asymptomatic (n=24)
mild (n=25)
severe (n=30)
control (n=72)
Severity

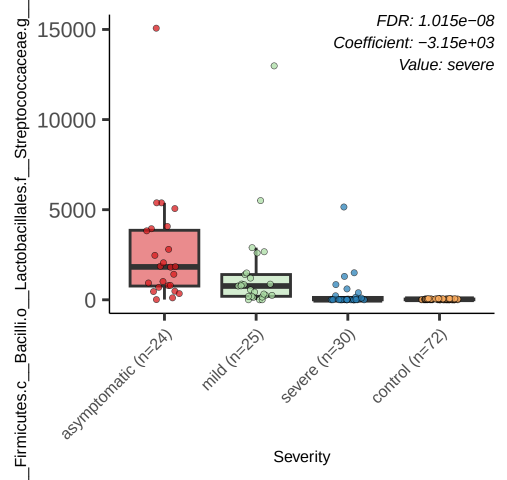
FDR: 1.015e−08
Coefficient: −3.15e+03
Value: severe
Firmicutes.c__Bacilli.o__Lactobacillales.f__Streptococcaceae.g__
15000
10000
5000
0
asymptomatic (n=24)
mild (n=25)
severe (n=30)
control (n=72)
Severity

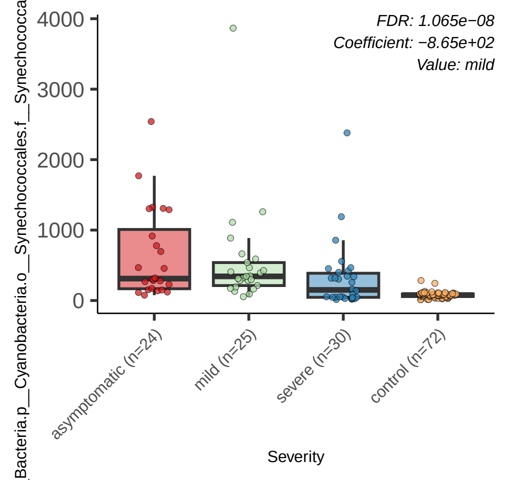
FDR: 1.065e−08
Coefficient: −8.65e+02
Value: mild
4000
3000
2000
1000
0
Bacteria.p__Cyanobacteria.o__Synechococcales.f__Synechococca
asymptomatic (n=24)
mild (n=25)
severe (n=30)
control (n=72)
Severity

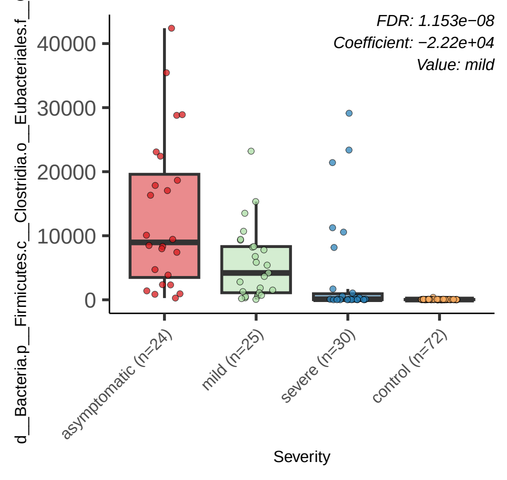

FDR: 1.153e−08
Coefficient: −2.22e+04
Value: mild
d__Bacteria.p__Firmicutes.c__Clostridia.o__Eubacteriales.f__
40000
30000
20000
10000
0
asymptomatic (n=24)
mild (n=25)
severe (n=30)
control (n=72)
Severity

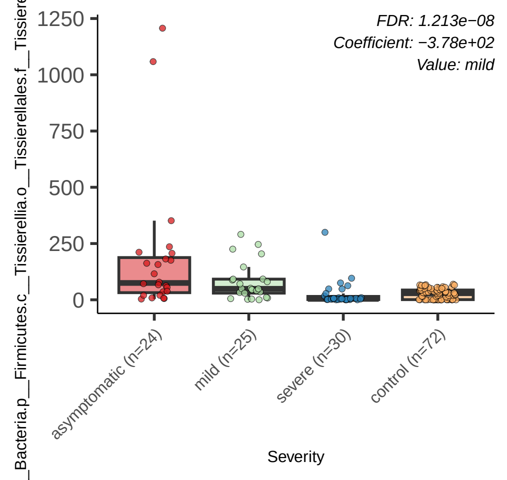
FDR: 1.213e−08
Coefficient: −3.78e+02
Value: mild
_Bacteria.p__Firmicutes.c__Tissierellia.o__Tissierellales.f__Tissiere
1250
1000
750
500
250
0
asymptomatic (n=24)
mild (n=25)
severe (n=30)
control (n=72)
Severity

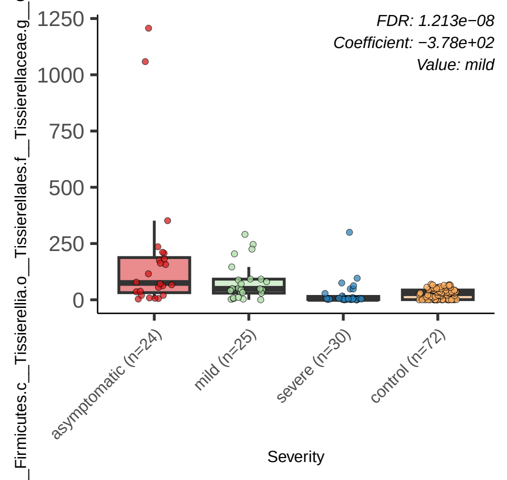
FDR: 1.213e−08
Coefficient: −3.78e+02
Value: mild
_Firmicutes.c__Tissierellia.o__Tissierellales.f__Tissierellaceae.g__
1250
1000
750
500
250
0
asymptomatic (n=24)
mild (n=25)
severe (n=30)
control (n=72)
Severity

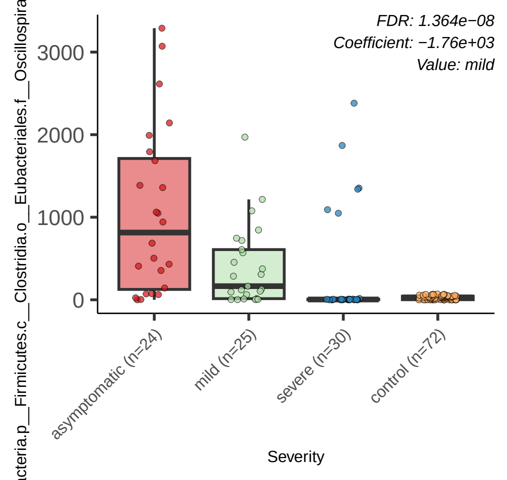

FDR: 1.364e−08
Coefficient: −1.76e+03
Value: mild
3000
2000
1000
0
acteria.p__Firmicutes.c__Clostridia.o__Eubacteriales.f__Oscillospira
asymptomatic (n=24)
mild (n=25)
severe (n=30)
control (n=72)
Severity

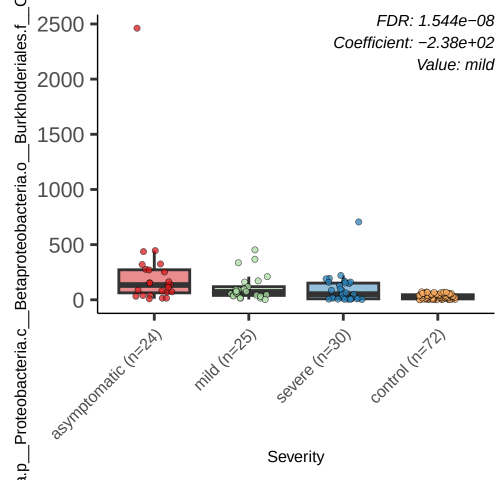

FDR: 1.544e−08
Coefficient: −2.38e+02
Value: mild
a.p__Proteobacteria.c__Betaproteobacteria.o__Burkholderiales.f__C
2500
2000
1500
1000
500
0
asymptomatic (n=24)
mild (n=25)
severe (n=30)
control (n=72)
Severity

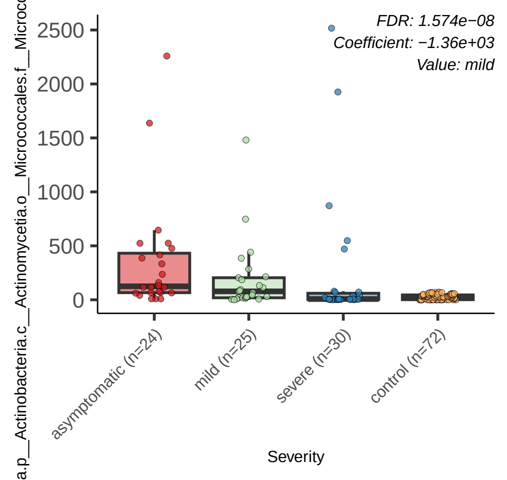
FDR: 1.574e−08
Coefficient: −1.36e+03
Value: mild
a.p__Actinobacteria.c__Actinomycetia.o__Micrococcales.f__Microco
2500
2000
1500
1000
500
0
asymptomatic (n=24)
mild (n=25)
severe (n=30)
control (n=72)
Severity

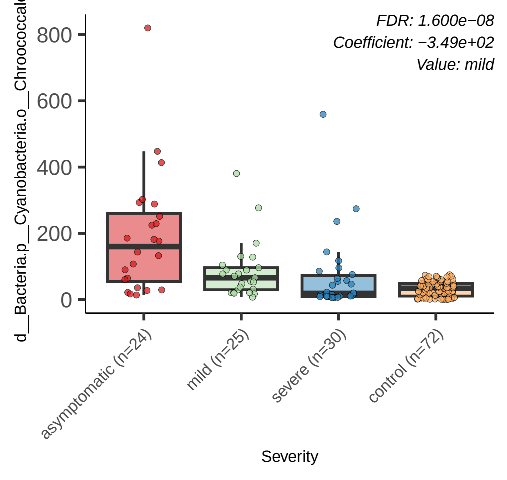

FDR: 1.600e−08
Coefficient: −3.49e+02
Value: mild
d__Bacteria.p__Cyanobacteria.o__Chroococcale
800
600
400
200
0
asymptomatic (n=24)
mild (n=25)
severe (n=30)
control (n=72)
Severity

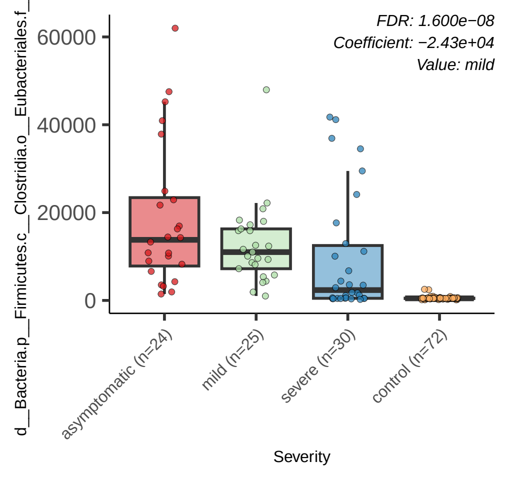

FDR: 1.600e−08
Coefficient: −2.43e+04
Value: mild
d__Bacteria.p__Firmicutes.c__Clostridia.o__Eubacteriales.f__
60000
40000
20000
0
asymptomatic (n=24)
mild (n=25)
severe (n=30)
control (n=72)
Severity

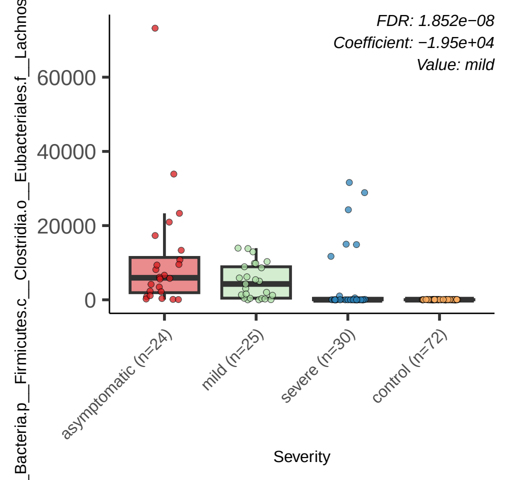

FDR: 1.852e−08
Coefficient: −1.95e+04
Value: mild
_Bacteria.p__Firmicutes.c__Clostridia.o__Eubacteriales.f__Lachnos
60000
40000
20000
0
asymptomatic (n=24)
mild (n=25)
severe (n=30)
control (n=72)
Severity

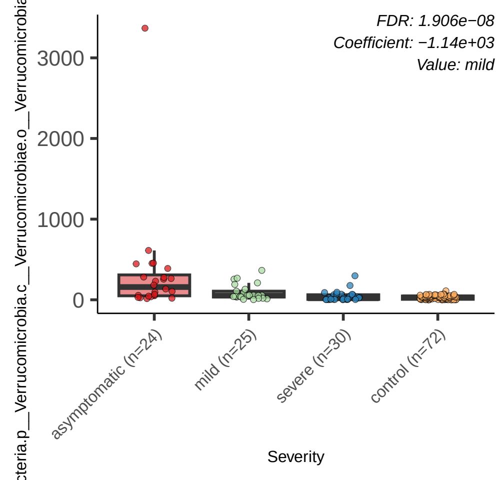

FDR: 1.906e−08
Coefficient: −1.14e+03
Value: mild
3000
2000
1000
0
cteria.p__Verrucomicrobia.c__Verrucomicrobiae.o__Verrucomicrobia
asymptomatic (n=24)
mild (n=25)
severe (n=30)
control (n=72)
Severity

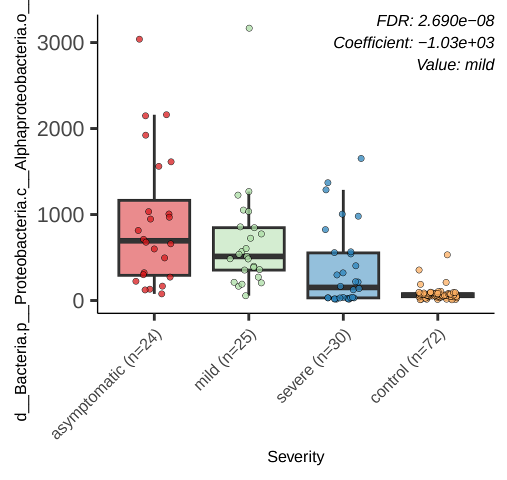

FDR: 2.690e−08
Coefficient: −1.03e+03
Value: mild
d__Bacteria.p__Proteobacteria.c__Alphaproteobacteria.o__
3000
2000
1000
0
asymptomatic (n=24)
mild (n=25)
severe (n=30)
control (n=72)
Severity

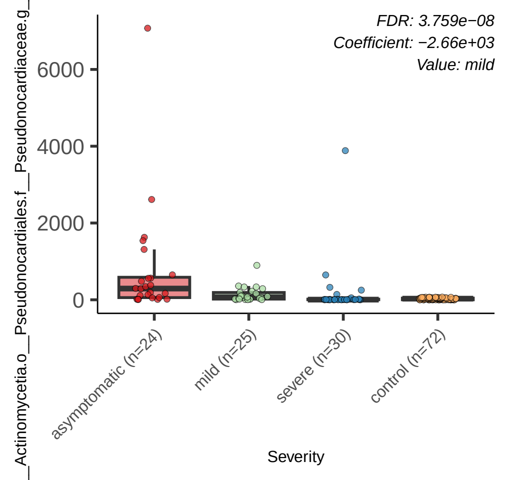

FDR: 3.759e−08
Coefficient: −2.66e+03
Value: mild
6000
4000
2000
0
Actinomycetia.o__Pseudonocardiales.f__Pseudonocardiaceae.g__
asymptomatic (n=24)
mild (n=25)
severe (n=30)
control (n=72)
Severity

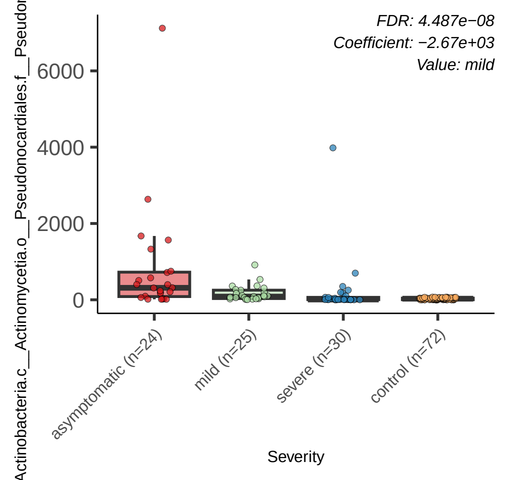

FDR: 4.487e−08
Coefficient: −2.67e+03
Value: mild
Actinobacteria.c__Actinomycetia.o__Pseudonocardiales.f__Pseudon
6000
4000
2000
0
asymptomatic (n=24)
mild (n=25)
severe (n=30)
control (n=72)
Severity

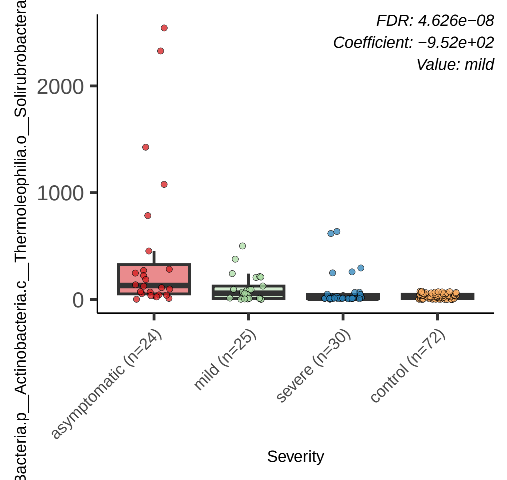

FDR: 4.626e−08
Coefficient: −9.52e+02
Value: mild
Bacteria.p__Actinobacteria.c__Thermoleophilia.o__Solirubrobactera
0
1000
2000
asymptomatic (n=24)
mild (n=25)
severe (n=30)
control (n=72)
Severity

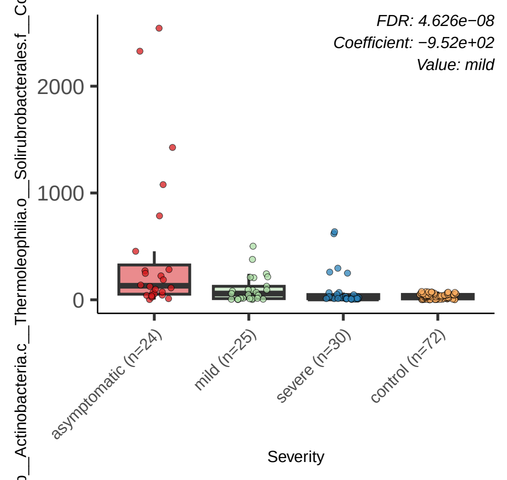
FDR: 4.626e−08
Coefficient: −9.52e+02
Value: mild
2000
1000
0
asymptomatic (n=24)
mild (n=25)
severe (n=30)
control (n=72)
Severity
o__Actinobacteria.c__Thermoleophilia.o__Solirubrobacterales.f__Co

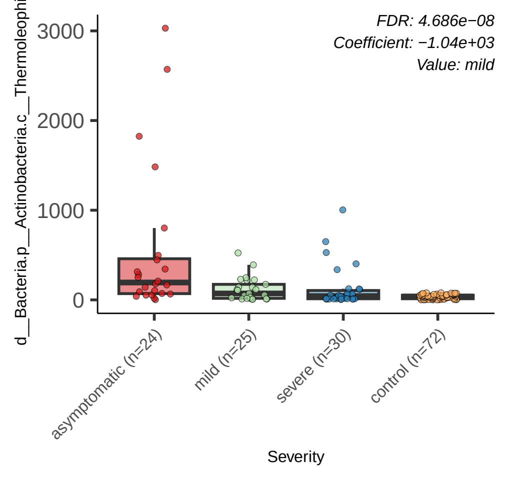
FDR: 4.686e−08
Coefficient: −1.04e+03
Value: mild
d__Bacteria.p__Actinobacteria.c__Thermoleophi
3000
2000
1000
0
asymptomatic (n=24)
mild (n=25)
severe (n=30)
control (n=72)
Severity

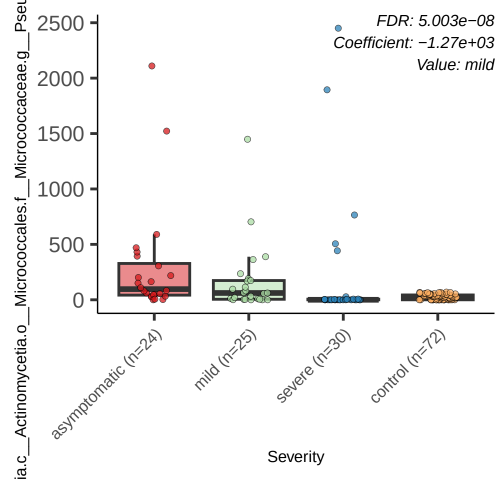

FDR: 5.003e−08
Coefficient: −1.27e+03
Value: mild
ia.c__Actinomycetia.o__Micrococcales.f__Micrococcaceae.g__Pseu
0
500
1000
1500
2000
2500
asymptomatic (n=24)
mild (n=25)
severe (n=30)
control (n=72)
Severity

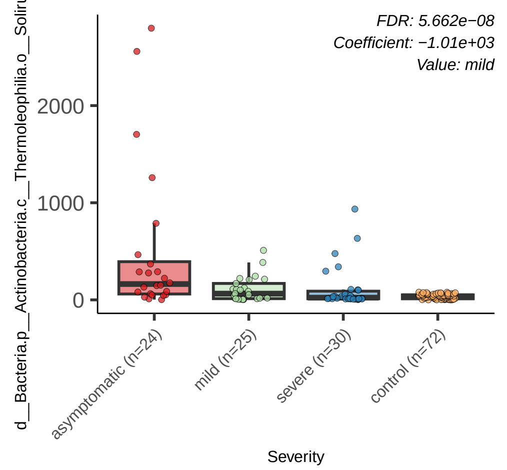

FDR: 5.662e−08
Coefficient: −1.01e+03
Value: mild
d__Bacteria.p__Actinobacteria.c__Thermoleophilia.o__Soliru
2000
1000
0
asymptomatic (n=24)
mild (n=25)
severe (n=30)
control (n=72)
Severity

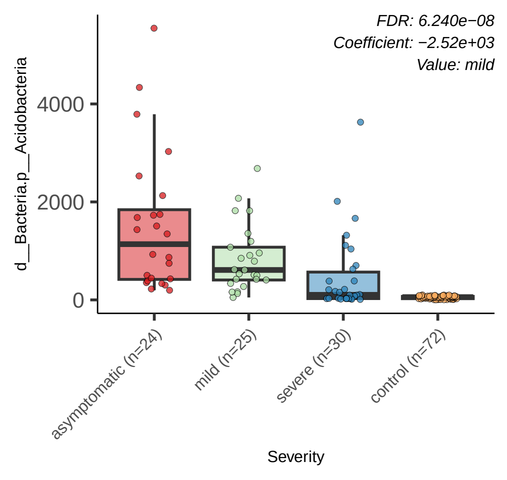
FDR: 6.240e−08
Coefficient: −2.52e+03
Value: mild
d__Bacteria.p__Acidobacteria
4000
2000
0
asymptomatic (n=24)
mild (n=25)
severe (n=30)
control (n=72)
Severity

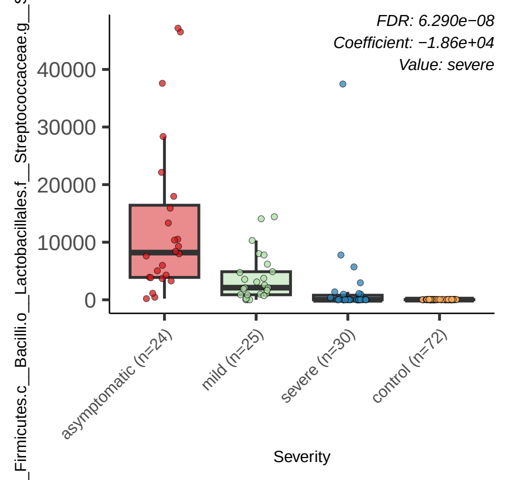

FDR: 6.290e−08
Coefficient: −1.86e+04
Value: severe
Firmicutes.c__Bacilli.o__Lactobacillales.f__Streptococcaceae.g__
40000
30000
20000
10000
0
asymptomatic (n=24)
mild (n=25)
severe (n=30)
control (n=72)
Severity

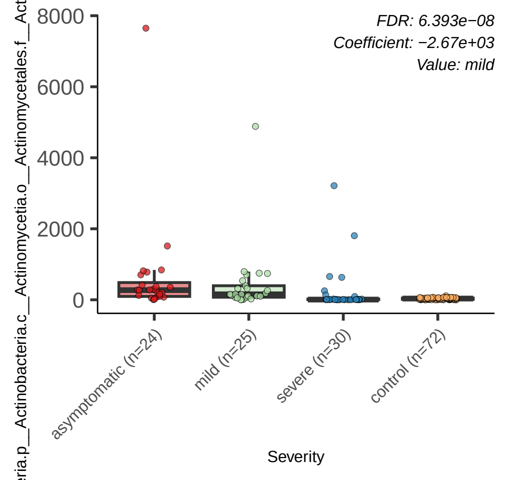
FDR: 6.393e−08
Coefficient: −2.67e+03
Value: mild
8000
6000
4000
2000
0
asymptomatic (n=24)
mild (n=25)
severe (n=30)
control (n=72)
Severity
Actinobacteria.c__Actinomycetia.o__Actinomycetales.f__Act

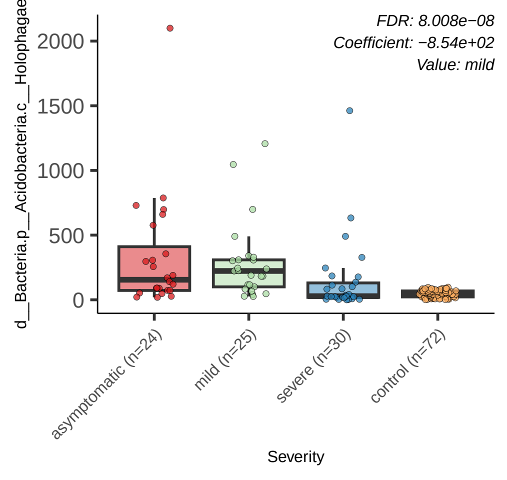

FDR: 8.008e−08
Coefficient: −8.54e+02
Value: mild
d__Bacteria.p__Acidobacteria.c__Holophagae
2000
1500
1000
500
0
asymptomatic (n=24)
mild (n=25)
severe (n=30)
control (n=72)
Severity

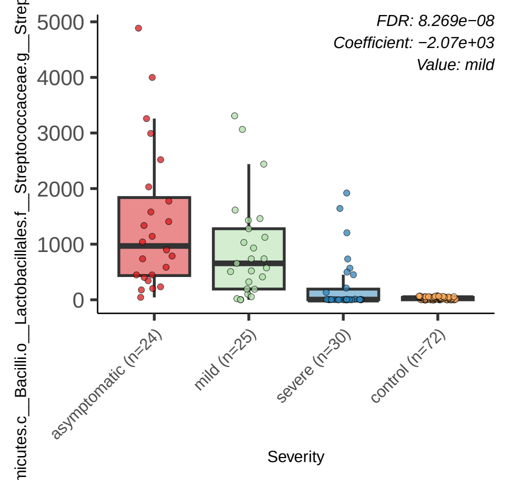

FDR: 8.269e−08
Coefficient: −2.07e+03
Value: mild
5000
4000
3000
2000
1000
0
micutes.c__Bacilli.o__Lactobacillales.f__Streptococcaceae.g__Strep
asymptomatic (n=24)
mild (n=25)
severe (n=30)
control (n=72)
Severity

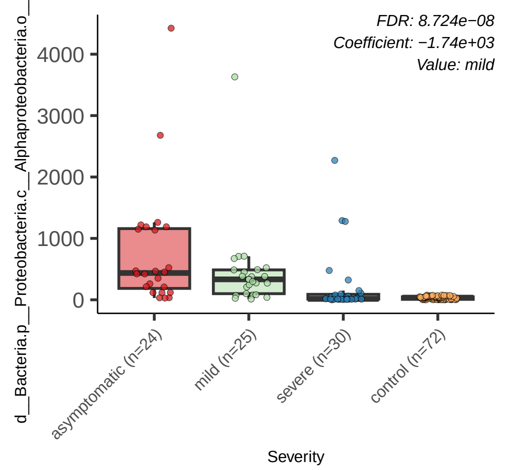
FDR: 8.724e−08
Coefficient: −1.74e+03
Value: mild
d__Bacteria.p__Proteobacteria.c__Alphaproteobacteria.o__
4000
3000
2000
1000
0
asymptomatic (n=24)
mild (n=25)
severe (n=30)
control (n=72)
Severity

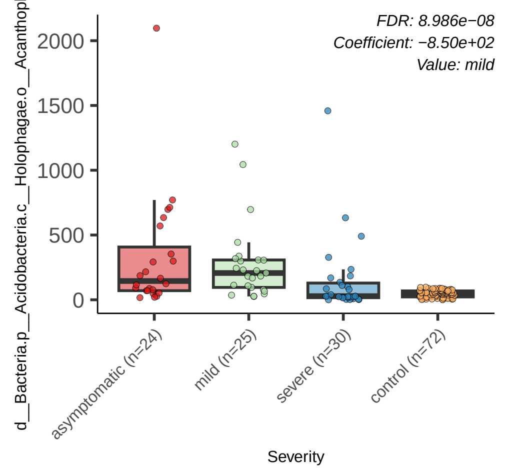

FDR: 8.986e−08
Coefficient: −8.50e+02
Value: mild
d__Bacteria.p__Acidobacteria.c__Holophagae.o__Acanthopl
2000
1500
1000
500
0
asymptomatic (n=24)
mild (n=25)
severe (n=30)
control (n=72)
Severity

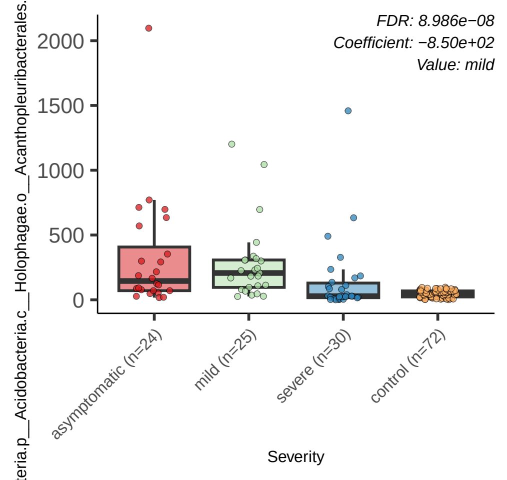

FDR: 8.986e−08
Coefficient: −8.50e+02
Value: mild
2000
1500
1000
500
0
teria.p__Acidobacteria.c__Holophagae.o__Acanthopleuribacterales.
asymptomatic (n=24)
mild (n=25)
severe (n=30)
control (n=72)
Severity

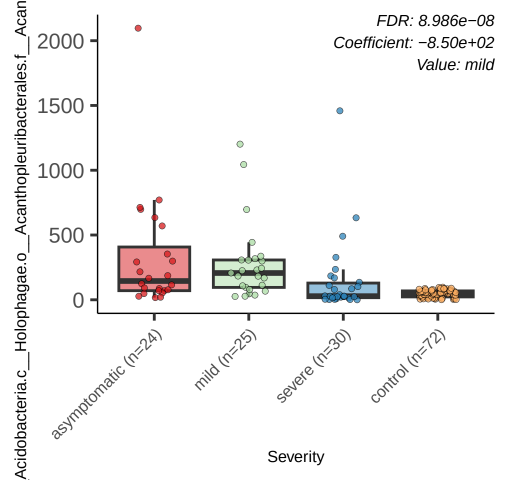

FDR: 8.986e−08
Coefficient: −8.50e+02
Value: mild
2000
1500
1000
500
0
Acidobacteria.c__Holophagae.o__Acanthopleuribacterales.f__Acan
asymptomatic (n=24)
mild (n=25)
severe (n=30)
control (n=72)
Severity

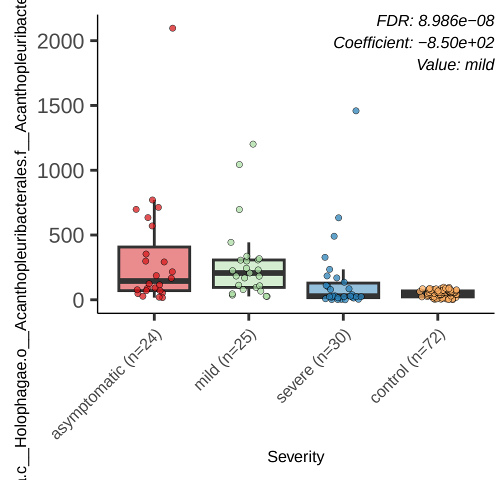

FDR: 8.986e−08
Coefficient: −8.50e+02
Value: mild
2000
1500
1000
500
0
.c__Holophagae.o__Acanthopleuribacterales.f__Acanthopleuribacte
asymptomatic (n=24)
mild (n=25)
severe (n=30)
control (n=72)
Severity

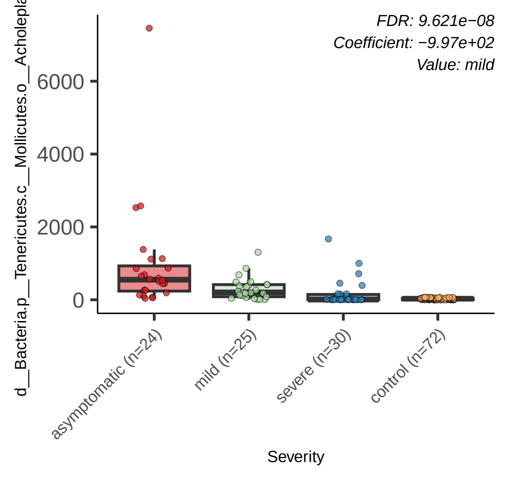
FDR: 9.621e−08
Coefficient: −9.97e+02
Value: mild
d__Bacteria.p__Tenericutes.c__Mollicutes.o__Achol
0
2000
4000
6000
asymptomatic (n=24)
mild (n=25)
severe (n=30)
control (n=72)
Severity

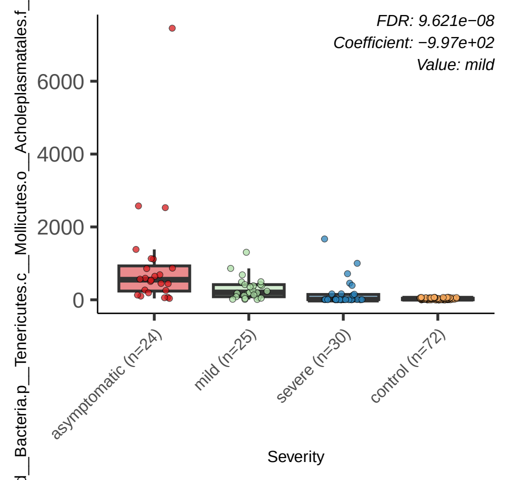

FDR: 9.621e−08
Coefficient: −9.97e+02
Value: mild
d__Bacteria.p__Tenericutes.c__Mollicutes.o__Acholeplasmatales.f
6000
4000
2000
0
asymptomatic (n=24)
mild (n=25)
severe (n=30)
control (n=72)
Severity

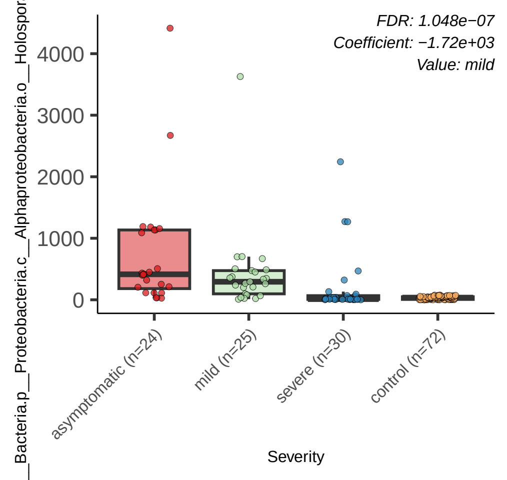
FDR: 1.048e−07
Coefficient: −1.72e+03
Value: mild
_Bacteria.p__Proteobacteria.c__Alphaproteobacteria.o__Holospor
4000
3000
2000
1000
0
asymptomatic (n=24)
mild (n=25)
severe (n=30)
control (n=72)
Severity

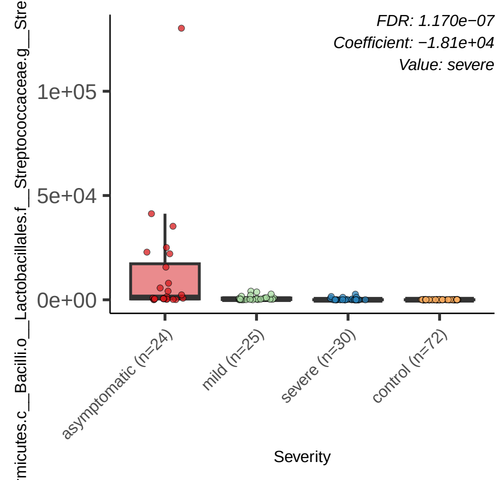

FDR: 1.170e−07
Coefficient: −1.81e+04
Value: severe
1e+05
5e+04
0e+00
rmicutes.c__Bacilli.o__Lactobacillales.f__Streptococcaceae.g__Stre
asymptomatic (n=24)
mild (n=25)
severe (n=30)
control (n=72)
Severity

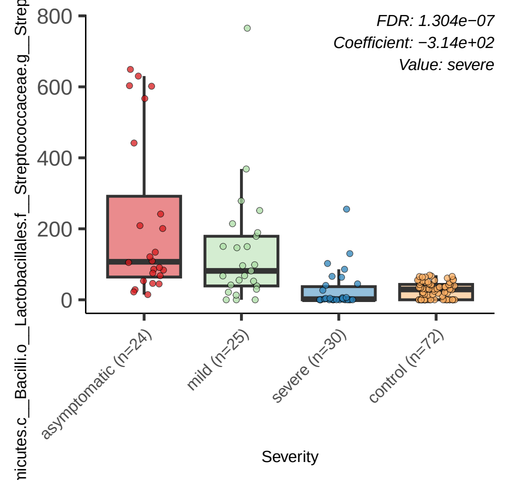
FDR: 1.304e−07
Coefficient: −3.14e+02
Value: severe
800
600
400
200
0
micutes.c__Bacilli.o__Lactobacillales.f__Streptococcaceae.g__Strep
asymptomatic (n=24)
mild (n=25)
severe (n=30)
control (n=72)
Severity

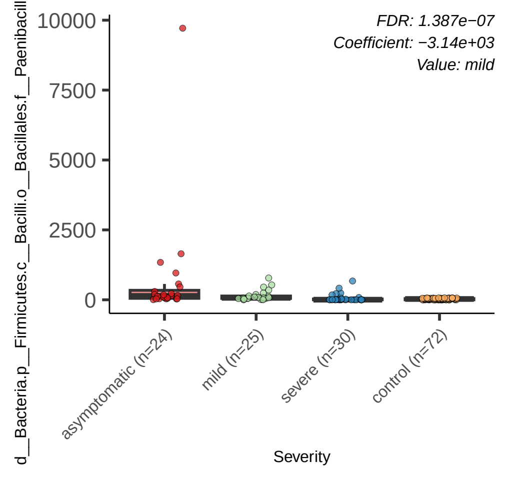
FDR: 1.387e−07
Coefficient: −3.14e+03
Value: mild
d__Bacteria.p__Firmicutes.c__Bacilli.o__Bacillales.f__Paenibacill
10000
7500
5000
2500
0
asymptomatic (n=24)
mild (n=25)
severe (n=30)
control (n=72)
Severity

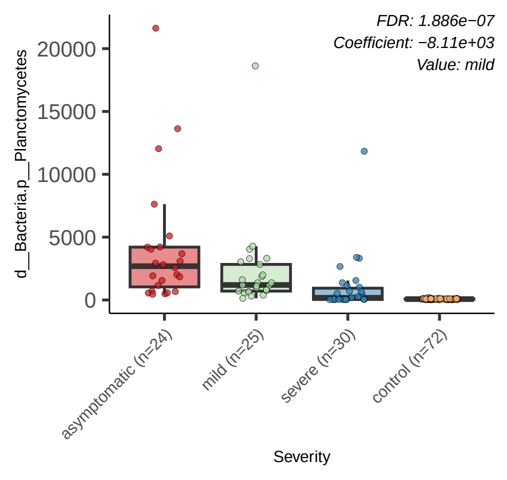

FDR: 1.886e−07
Coefficient: −8.11e+03
Value: mild
d__Bacteria.p__Planctomycetes
20000
15000
10000
5000
0
asymptomatic (n=24)
mild (n=25)
severe (n=30)
control (n=72)
Severity

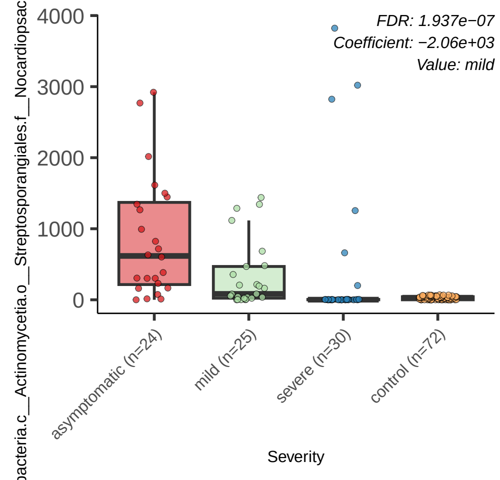
FDR: 1.937e−07
Coefficient: −2.06e+03
Value: mild
4000
3000
2000
1000
0
bacteria.c__Actinomycetia.o__Streptosporangiales.f__Nocardiopsac
asymptomatic (n=24)
mild (n=25)
severe (n=30)
control (n=72)
Severity

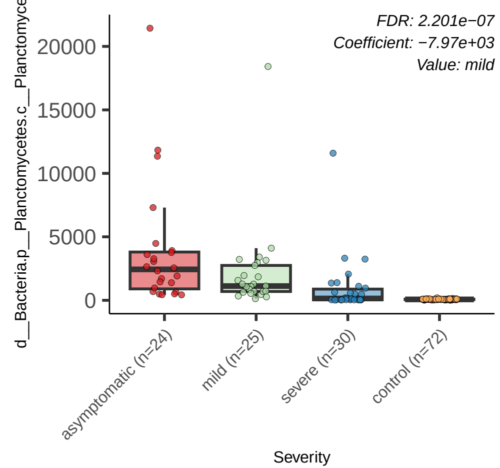

FDR: 2.201e−07
Coefficient: −7.97e+03
Value: mild
d__Bacteria.p__Planctomycetes.c__Planctomyce
20000
15000
10000
5000
0
asymptomatic (n=24)
mild (n=25)
severe (n=30)
control (n=72)
Severity

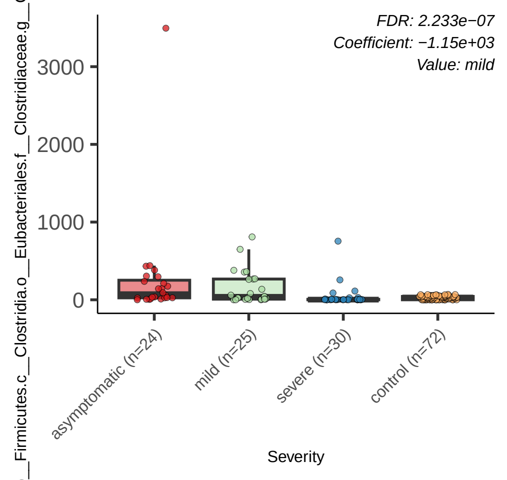

FDR: 2.233e−07
Coefficient: −1.15e+03
Value: mild
Firmicutes.c__Clostridia.o__Eubacteriales.f__Clostridiaceae.g__
3000
2000
1000
0
asymptomatic (n=24)
mild (n=25)
severe (n=30)
control (n=72)
Severity

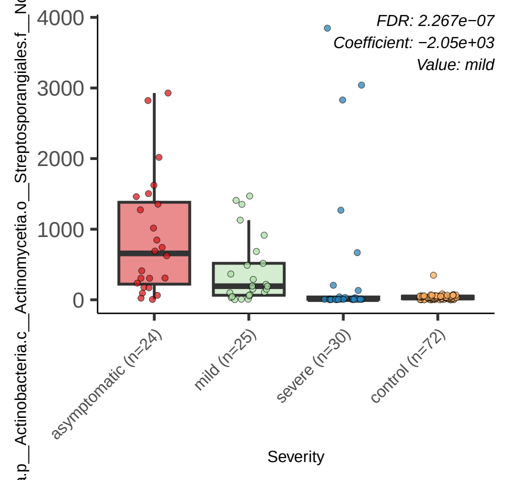

FDR: 2.267e−07
Coefficient: −2.05e+03
Value: mild
4000
3000
2000
1000
0
asymptomatic (n=24)
mild (n=25)
severe (n=30)
control (n=72)
Severity
a.p__Actinobacteria.c__Actinomycetia.o__Streptosporangiales.f__No

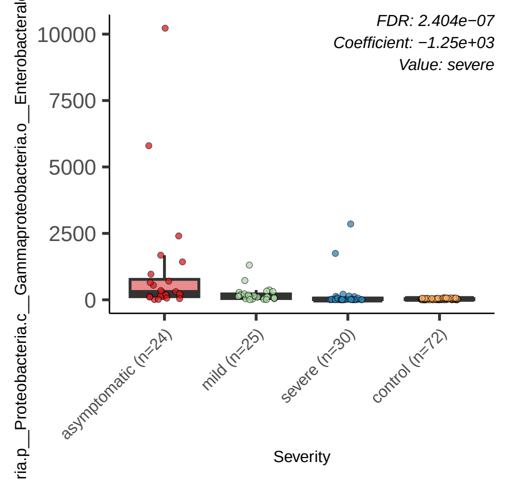

FDR: 2.404e−07
Coefficient: −1.25e+03
Value: severe
10000
7500
5000
2500
0
ria.p__Proteobacteria.c__Gammaproteobacteria.o__Enterobacterale
asymptomatic (n=24)
mild (n=25)
severe (n=30)
control (n=72)
Severity

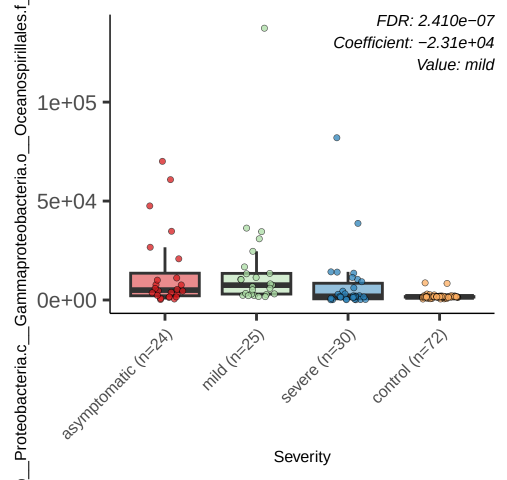
FDR: 2.410e−07
Coefficient: −2.31e+04
Value: mild
1e+05
5e+04
0e+00
Proteobacteria.c__Gammaproteobacteria.o__Oceanospirillales.f_
asymptomatic (n=24)
mild (n=25)
severe (n=30)
control (n=72)
Severity

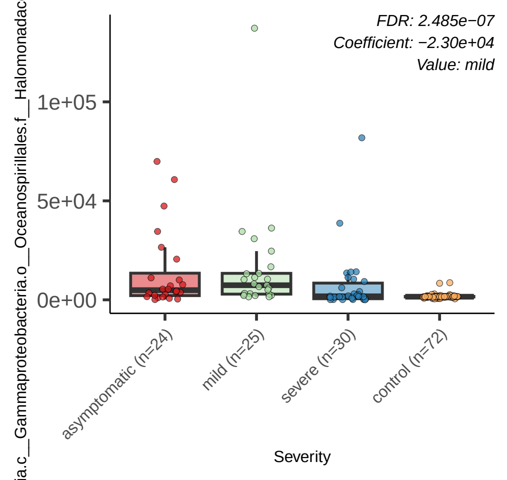
FDR: 2.485e−07
Coefficient: −2.30e+04
Value: mild
ia.c__Gammaproteobacteria.o__Oceanospirillales.f__Halomonadac
1e+05
5e+04
0e+00
asymptomatic (n=24)
mild (n=25)
severe (n=30)
control (n=72)
Severity

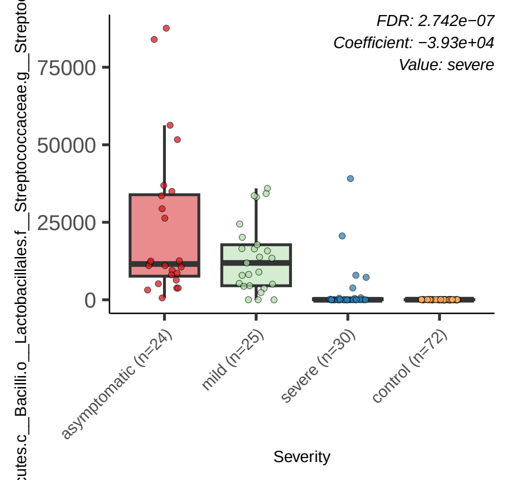

FDR: 2.742e−07
Coefficient: −3.93e+04
Value: severe
75000
50000
25000
0
asymptomatic (n=24)
mild (n=25)
severe (n=30)
control (n=72)
Severity
cutes.c__Bacilli.o__Lactobacillales.f__Streptococcaceae.g__Strepto

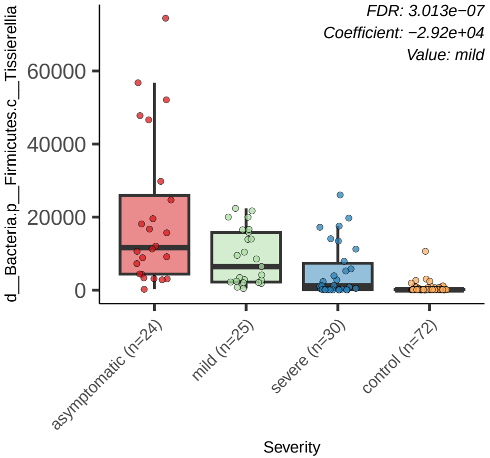
FDR: 3.013e−07
Coefficient: −2.92e+04
Value: mild
d__Bacteria.p__Firmicutes.c__Tissierellia
60000
40000
20000
0
asymptomatic (n=24)
mild (n=25)
severe (n=30)
control (n=72)
Severity

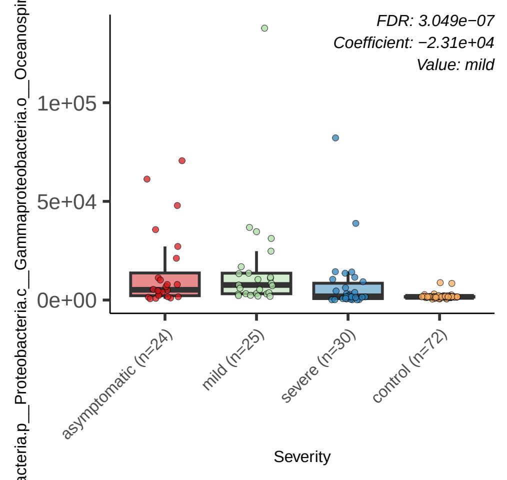
FDR: 3.049e−07
Coefficient: −2.31e+04
Value: mild
1e+05
5e+04
0e+00
acteria.p__Proteobacteria.c__Gammaproteobacteria.o__Oceanospi
asymptomatic (n=24)
mild (n=25)
severe (n=30)
control (n=72)
Severity

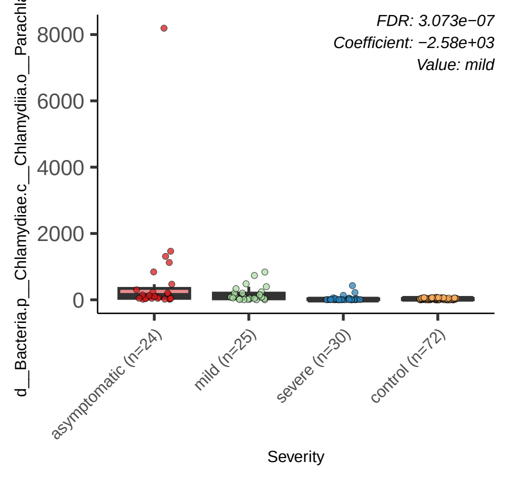

FDR: 3.073e−07
Coefficient: −2.58e+03
Value: mild
d__Bacteria.p__Chlamydiae.c__Chlamydiia.o__Parachla
8000
6000
4000
2000
0
asymptomatic (n=24)
mild (n=25)
severe (n=30)
control (n=72)
Severity

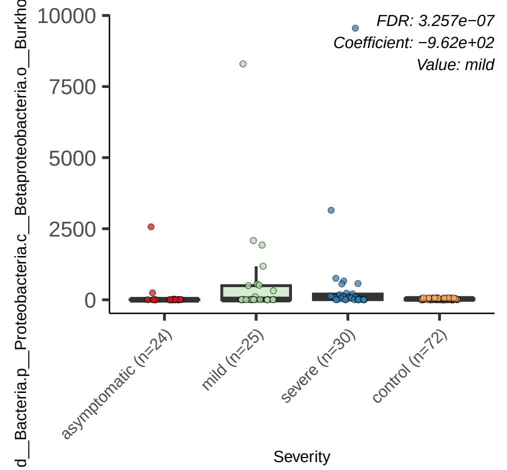
FDR: 3.257e−07
Coefficient: −9.62e+02
Value: mild
d__Bacteria.p__Proteobacteria.c__Betaproteobacteria.o__Burkho
10000
7500
5000
2500
0
asymptomatic (n=24)
mild (n=25)
severe (n=30)
control (n=72)
Severity

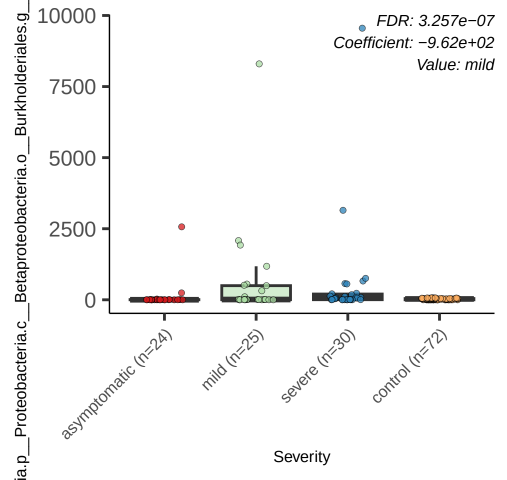

FDR: 3.257e−07
Coefficient: −9.62e+02
Value: mild
10000
7500
5000
2500
0
ia.p__Proteobacteria.c__Betaproteobacteria.o__Burkholderiales.g__
asymptomatic (n=24)
mild (n=25)
severe (n=30)
control (n=72)
Severity

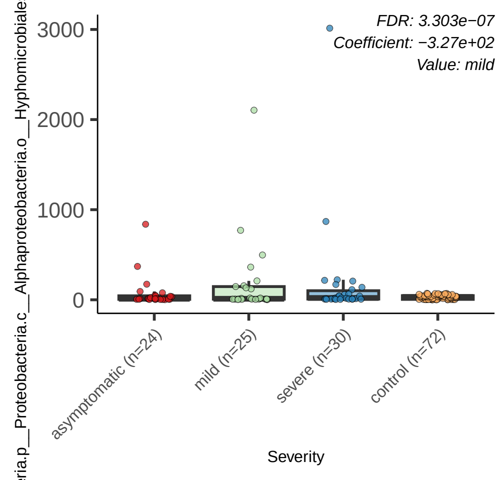
FDR: 3.303e−07
Coefficient: −3.27e+02
Value: mild
3000
2000
1000
0
asymptomatic (n=24)
mild (n=25)
severe (n=30)
control (n=72)
Severity
eria.p__Proteobacteria.c__Alphaproteobacteria.o__Hyphomicrobiale

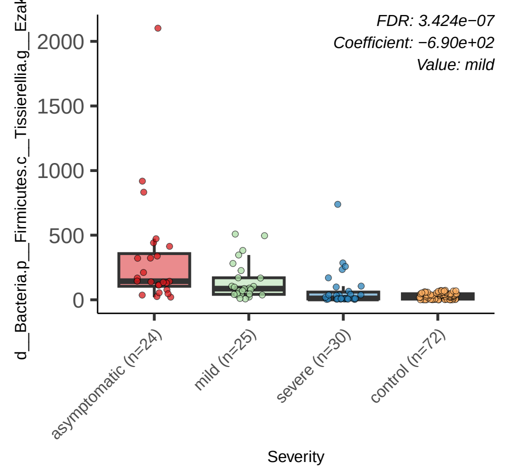
FDR: 3.424e−07
Coefficient: −6.90e+02
Value: mild
d__Bacteria.p__Firmicutes.c__Tissierellia.g__Ezak
2000
1500
1000
500
0
asymptomatic (n=24)
mild (n=25)
severe (n=30)
control (n=72)
Severity

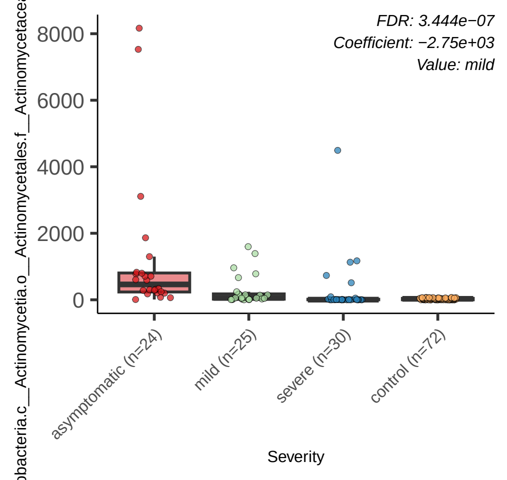
FDR: 3.444e−07
Coefficient: −2.75e+03
Value: mild
8000
6000
4000
2000
0
bacteria.c__Actinomycetia.o__Actinomycetales.f__Actinomycetace
asymptomatic (n=24)
mild (n=25)
severe (n=30)
control (n=72)
Severity

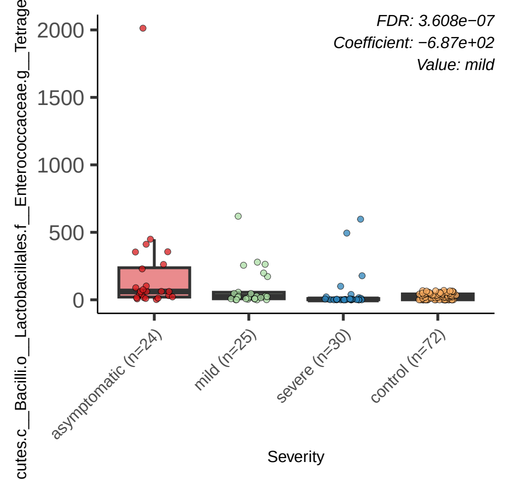

FDR: 3.608e−07
Coefficient: −6.87e+02
Value: mild
2000
1500
1000
500
0
cutes.c__Bacilli.o__Lactobacillales.f__Enterococcaceae.g__Tetrage
asymptomatic (n=24)
mild (n=25)
severe (n=30)
control (n=72)
Severity

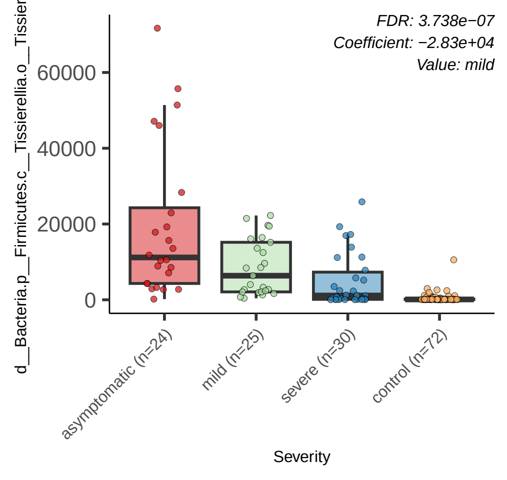

FDR: 3.738e−07
Coefficient: −2.83e+04
Value: mild
d__Bacteria.p__Firmicutes.c__Tissierellia.o__Tissier
60000
40000
20000
0
asymptomatic (n=24)
mild (n=25)
severe (n=30)
control (n=72)
Severity

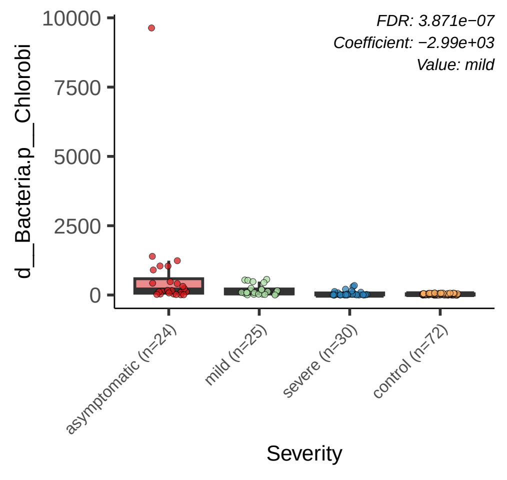
FDR: 3.871e−07
Coefficient: −2.99e+03
Value: mild
d__Bacteria.p__Chlorobi
10000
7500
5000
2500
0
asymptomatic (n=24)
mild (n=25)
severe (n=30)
control (n=72)
Severity

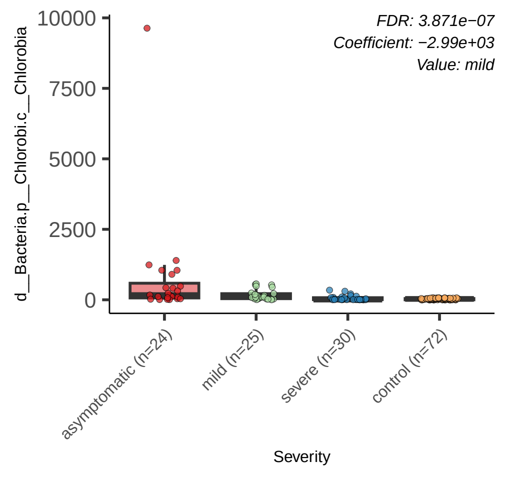
FDR: 3.871e−07
Coefficient: −2.99e+03
Value: mild
d__Bacteria.p__Chlorobi.c__Chlorobia
10000
7500
5000
2500
0
asymptomatic (n=24)
mild (n=25)
severe (n=30)
control (n=72)
Severity

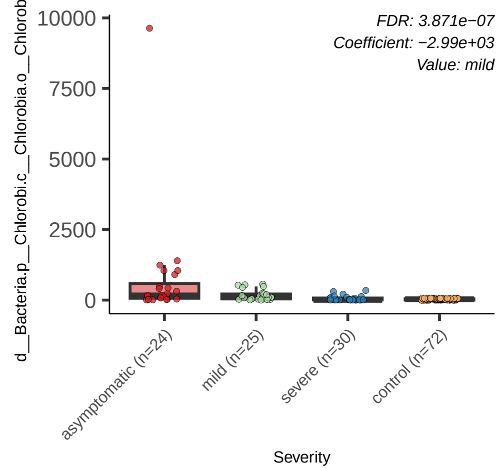
FDR: 3.871e−07
Coefficient: −2.99e+03
Value: mild
d__Bacteria.p__Chlorobi.c__Chlorobia.o__Chlorob
10000
7500
5000
2500
0
asymptomatic (n=24)
mild (n=25)
severe (n=30)
control (n=72)
Severity

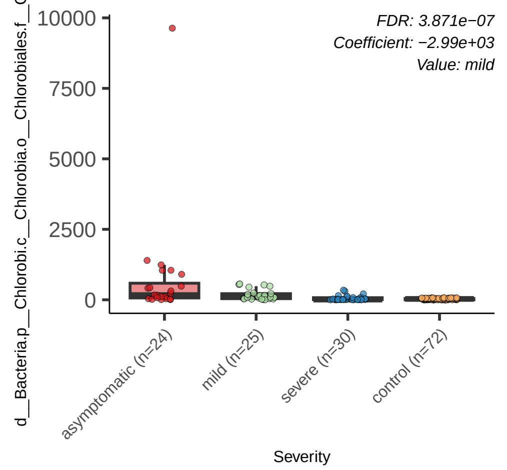

FDR: 3.871e−07
Coefficient: −2.99e+03
Value: mild
d__Bacteria.p__Chlorobi.c__Chlorobia.o__Chlorobiales.f__C
10000
7500
5000
2500
0
asymptomatic (n=24)
mild (n=25)
severe (n=30)
control (n=72)
Severity

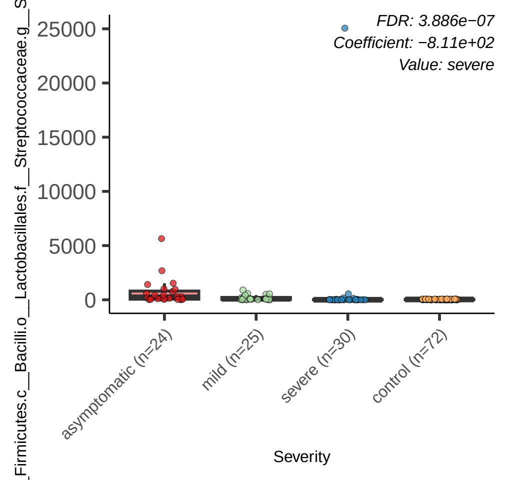

FDR: 3.886e−07
Coefficient: −8.11e+02
Value: severe
Firmicutes.c__Bacilli.o__Lactobacillales.f__Streptococcaceae.g__S
25000
20000
15000
10000
5000
0
asymptomatic (n=24)
mild (n=25)
severe (n=30)
control (n=72)
Severity

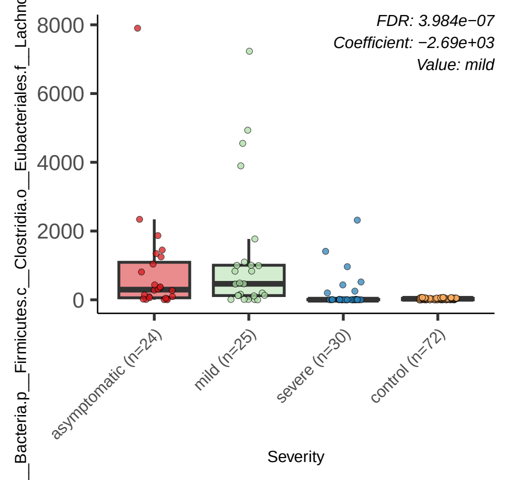

FDR: 3.984e−07
Coefficient: −2.69e+03
Value: mild
8000
6000
4000
2000
0
asymptomatic (n=24)
mild (n=25)
severe (n=30)
control (n=72)
Severity
Bacteria.p__Firmicutes.c__Clostridia.o__Eubacteriales.f__Lachno

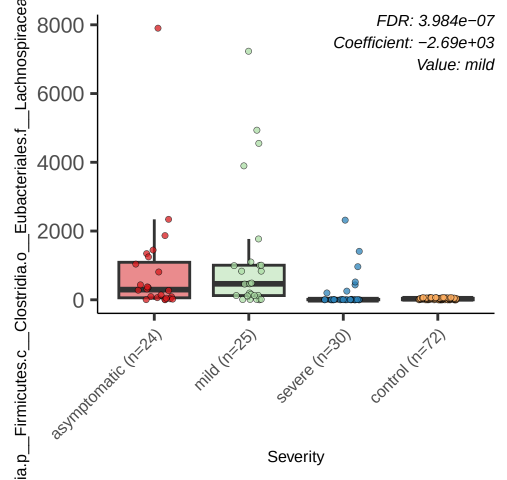
FDR: 3.984e−07
Coefficient: −2.69e+03
Value: mild
8000
6000
4000
2000
0
ia.p__Firmicutes.c__Clostridia.o__Eubacteriales.f__Lachnospiracea
asymptomatic (n=24)
mild (n=25)
severe (n=30)
control (n=72)
Severity

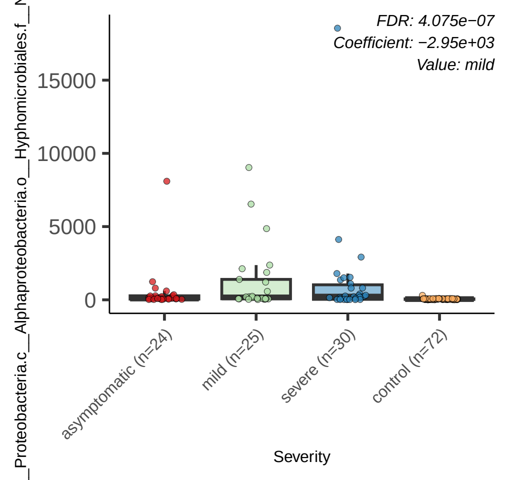

FDR: 4.075e−07
Coefficient: −2.95e+03
Value: mild
Proteobacteria.c__Alphaproteobacteria.o__Hyphomicrobiales.f__
15000
10000
5000
0
asymptomatic (n=24)
mild (n=25)
severe (n=30)
control (n=72)
Severity

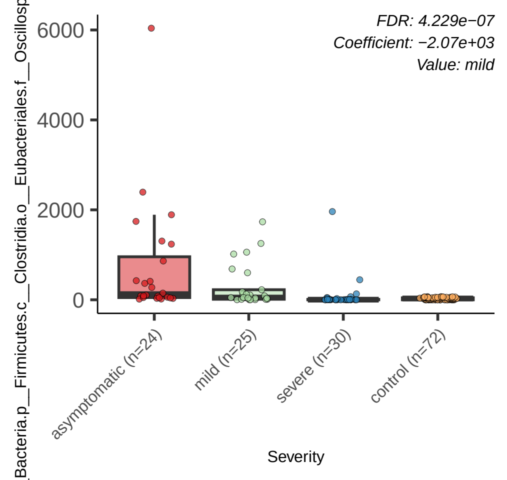

FDR: 4.229e−07
Coefficient: −2.07e+03
Value: mild
6000
4000
2000
0
asymptomatic (n=24)
mild (n=25)
severe (n=30)
control (n=72)
Severity
Bacteria.p__Firmicutes.c__Clostridia.o__Eubacteriales.f__Oscillosp

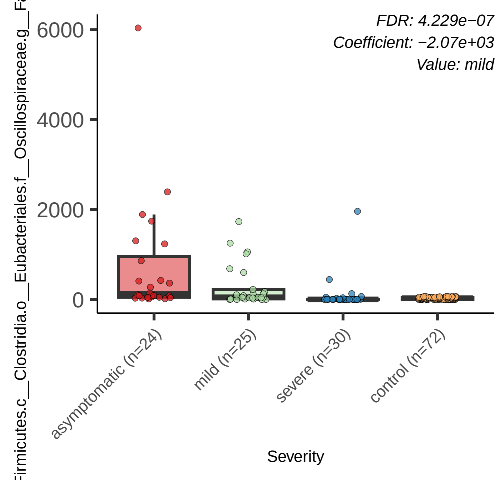

FDR: 4.229e−07
Coefficient: −2.07e+03
Value: mild
6000
4000
2000
0
Firmicutes.c__Clostridia.o__Eubacteriales.f__Oscillospiraceae.g__Fa
asymptomatic (n=24)
mild (n=25)
severe (n=30)
control (n=72)
Severity

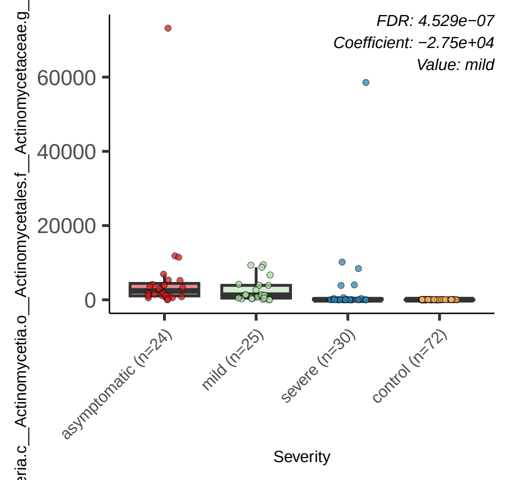
FDR: 4.529e−07
Coefficient: −2.75e+04
Value: mild
eria.c__Actinomycetia.o__Actinomycetales.f__Actinomycetaceae.g__
60000
40000
20000
0
asymptomatic (n=24)
mild (n=25)
severe (n=30)
control (n=72)
Severity

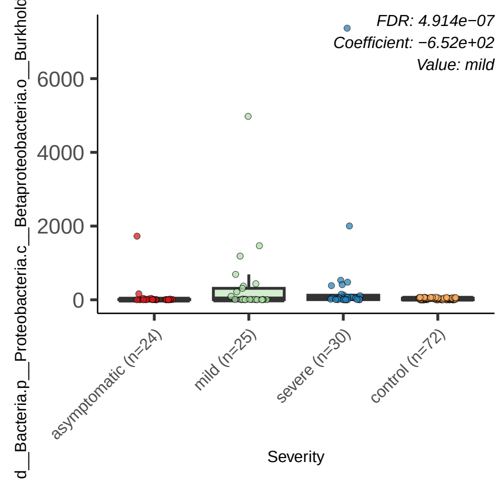

FDR: 4.914e−07
Coefficient: −6.52e+02
Value: mild
d__Bacteria.p__Proteobacteria.c__Betaproteobacteria.o__Burkhold
6000
4000
2000
0
asymptomatic (n=24)
mild (n=25)
severe (n=30)
control (n=72)
Severity

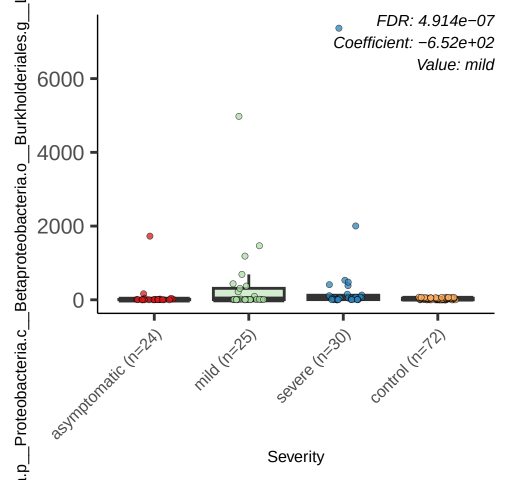
FDR: 4.914e−07
Coefficient: −6.52e+02
Value: mild
6000
4000
2000
0
asymptomatic (n=24)
mild (n=25)
severe (n=30)
control (n=72)
Severity
a.p__Proteobacteria.c__Betaproteobacteria.o__Burkholderiales.g__

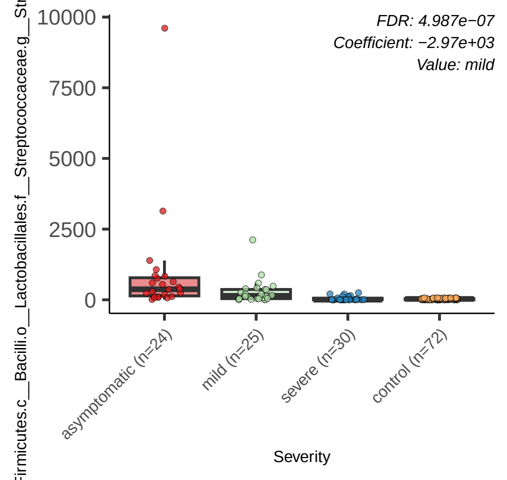

FDR: 4.987e−07
Coefficient: −2.97e+03
Value: mild
10000
7500
5000
2500
0
Firmicutes.c__Bacilli.o__Lactobacillales.f__Streptococcaceae.g__Str
asymptomatic (n=24)
mild (n=25)
severe (n=30)
control (n=72)
Severity

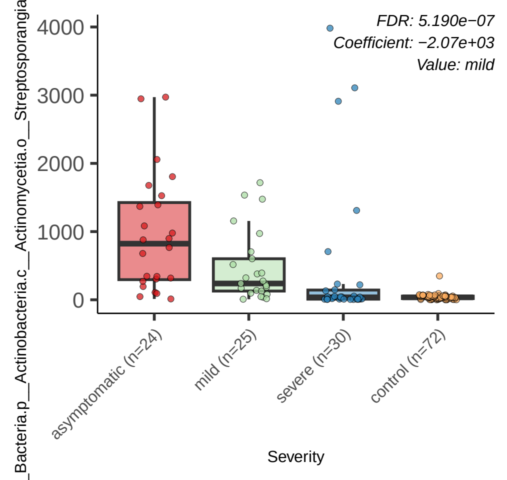

FDR: 5.190e−07
Coefficient: −2.07e+03
Value: mild
4000
3000
2000
1000
0
Bacteria.p__Actinobacteria.c__Actinomycetia.o__Streptosporangiales
asymptomatic (n=24)
mild (n=25)
severe (n=30)
control (n=72)
Severity

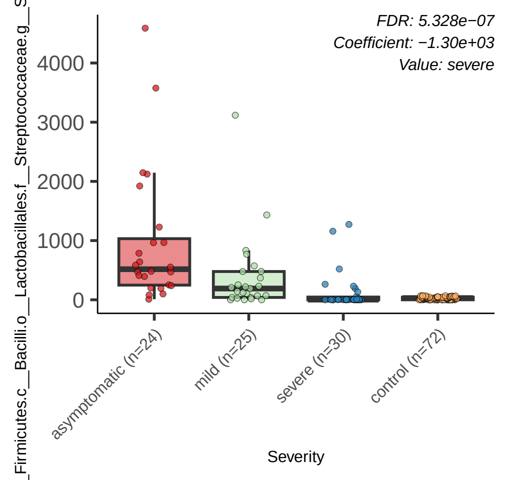

FDR: 5.328e−07
Coefficient: −1.30e+03
Value: severe
4000
3000
2000
1000
0
Firmicutes.c__Bacilli.o__Lactobacillales.f__Streptococcaceae.g__S
asymptomatic (n=24)
mild (n=25)
severe (n=30)
control (n=72)
Severity

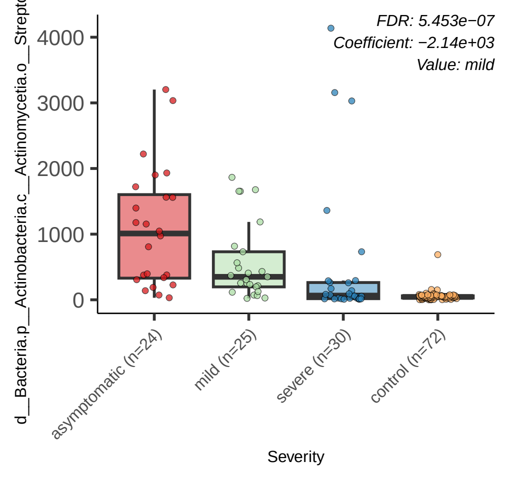

FDR: 5.453e−07
Coefficient: −2.14e+03
Value: mild
d__Bacteria.p__Actinobacteria.c__Actinomycetia.o__Strepto
4000
3000
2000
1000
0
asymptomatic (n=24)
mild (n=25)
severe (n=30)
control (n=72)
Severity

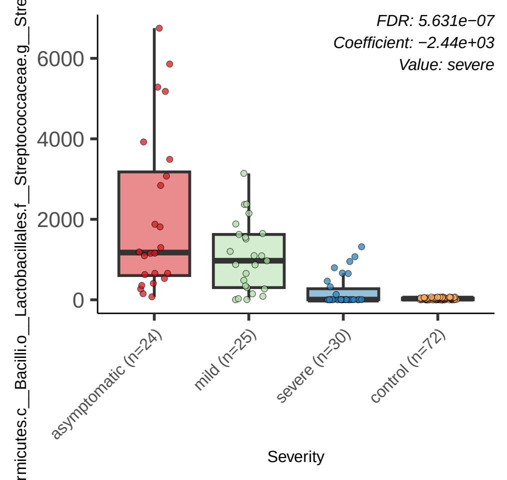
FDR: 5.631e−07
Coefficient: −2.44e+03
Value: severe
rmicutes.c__Bacilli.o__Lactobacillales.f__Streptococcaceae.g__Stre
6000
4000
2000
0
asymptomatic (n=24)
mild (n=25)
severe (n=30)
control (n=72)
Severity

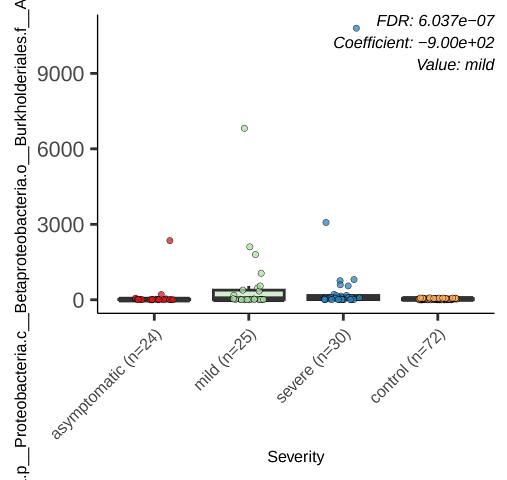

FDR: 6.037e−07
Coefficient: −9.00e+02
Value: mild
9000
6000
3000
0
asymptomatic (n=24)
mild (n=25)
severe (n=30)
control (n=72)
Severity
p__Proteobacteria.c__Betaproteobacteria.o__Burkholderiales.f__A

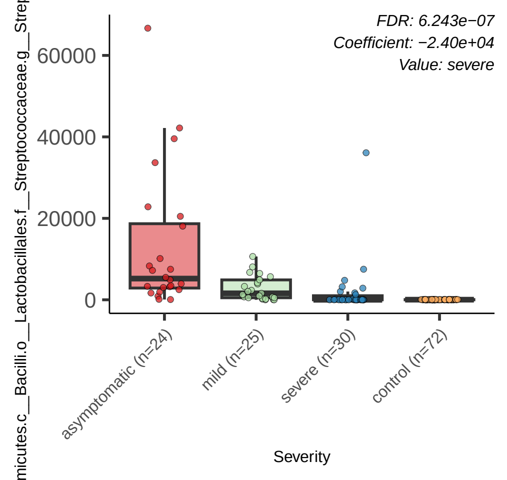

FDR: 6.243e−07
Coefficient: −2.40e+04
Value: severe
60000
40000
20000
0
micutes.c__Bacilli.o__Lactobacillales.f__Streptococcaceae.g__Strep
asymptomatic (n=24)
mild (n=25)
severe (n=30)
control (n=72)
Severity

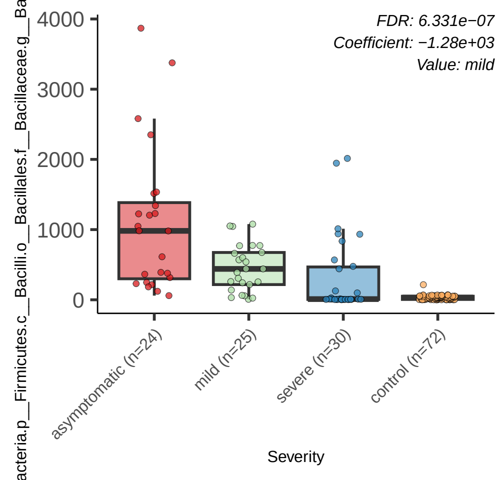

FDR: 6.331e−07
Coefficient: −1.28e+03
Value: mild
4000
3000
2000
1000
0
asymptomatic (n=24)
mild (n=25)
severe (n=30)
control (n=72)
Severity

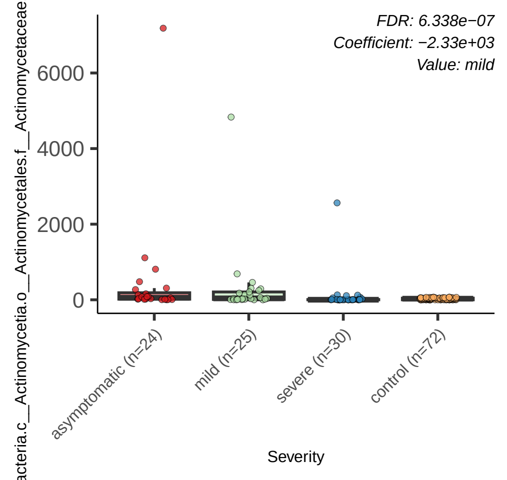

FDR: 6.338e−07
Coefficient: −2.33e+03
Value: mild
6000
4000
2000
0
acteria.c__Actinomycetia.o__Actinomycetales.f__Actinomycetaceae
asymptomatic (n=24)
mild (n=25)
severe (n=30)
control (n=72)
Severity

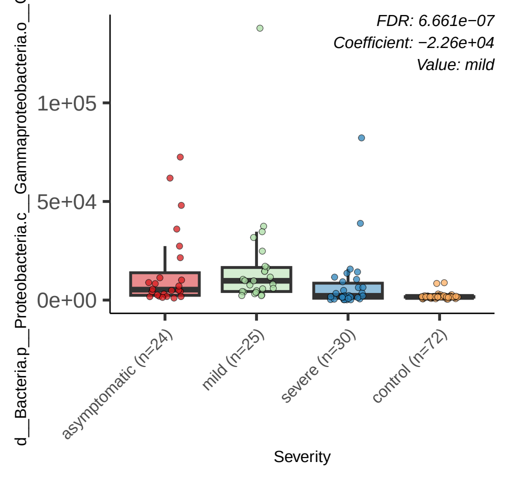
FDR: 6.661e−07
Coefficient: −2.26e+04
Value: mild
d__Bacteria.p__Proteobacteria.c__Gammaproteobacteria.o__
1e+05
5e+04
0e+00
asymptomatic (n=24)
mild (n=25)
severe (n=30)
control (n=72)
Severity

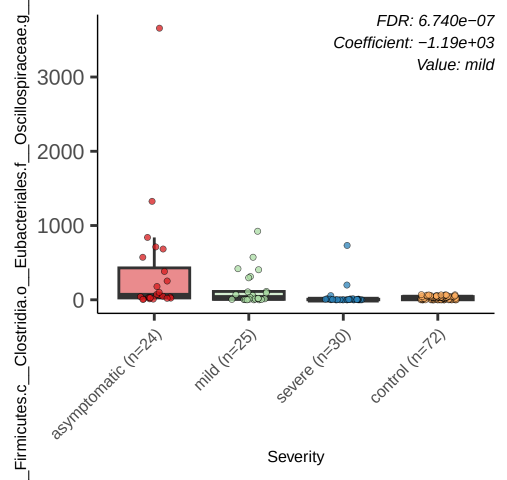

FDR: 6.740e−07
Coefficient: −1.19e+03
Value: mild
Firmicutes.c__Clostridia.o__Eubacteriales.f__Oscillospiraceae.g__
3000
2000
1000
0
asymptomatic (n=24)
mild (n=25)
severe (n=30)
control (n=72)
Severity

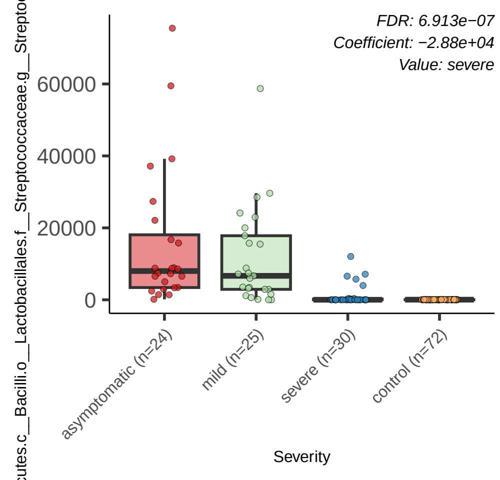
FDR: 6.913e−07
Coefficient: −2.88e+04
Value: severe
cutes.c__Bacilli.o__Lactobacillales.f__Streptococcaceae.g__Strepto
60000
40000
20000
0
asymptomatic (n=24)
mild (n=25)
severe (n=30)
control (n=72)
Severity

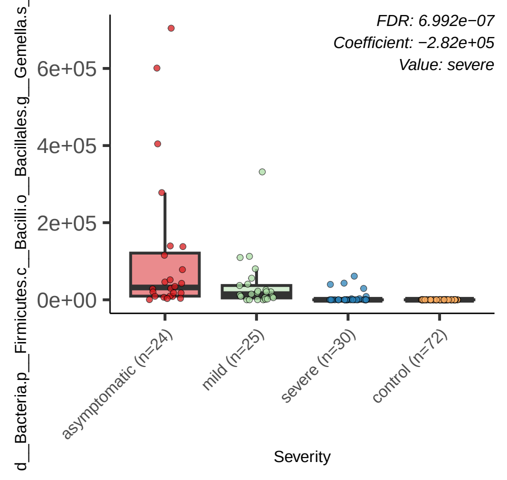

FDR: 6.992e−07
Coefficient: −2.82e+05
Value: severe
d__Bacteria.p__Firmicutes.c__Bacilli.o__Bacillales.g__Gemella.s__
6e+05
4e+05
2e+05
0e+00
asymptomatic (n=24)
mild (n=25)
severe (n=30)
control (n=72)
Severity

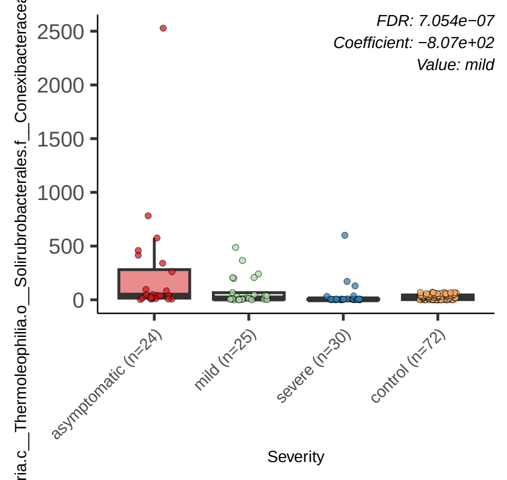

FDR: 7.054e−07
Coefficient: −8.07e+02
Value: mild
0
500
1000
1500
2000
2500
ria.c__Thermoleophilia.o__Solirubrobacterales.f__Conexibacteracea
asymptomatic (n=24)
mild (n=25)
severe (n=30)
control (n=72)
Severity

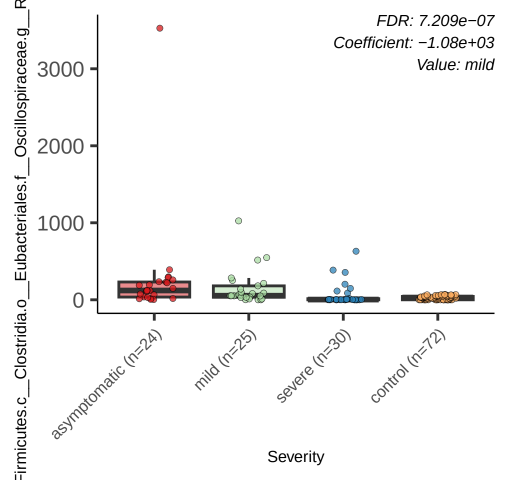

FDR: 7.209e−07
Coefficient: −1.08e+03
Value: mild
Firmicutes.c__Clostridia.o__Eubacteriales.f__Oscillospiraceae.g__R
3000
2000
1000
0
asymptomatic (n=24)
mild (n=25)
severe (n=30)
control (n=72)
Severity

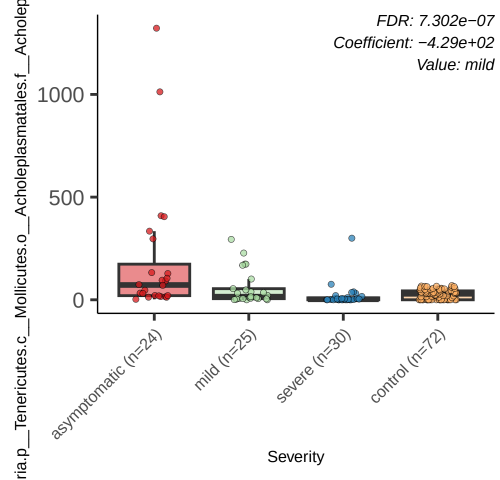
FDR: 7.302e−07
Coefficient: −4.29e+02
Value: mild
ria.p__Tenericutes.c__Mollicutes.o__Acholeplasmatales.f__Acholep
1000
500
0
asymptomatic (n=24)
mild (n=25)
severe (n=30)
control (n=72)
Severity

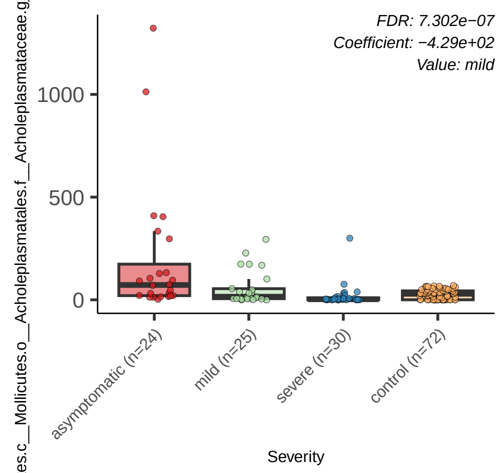

FDR: 7.302e−07
Coefficient: −4.29e+02
Value: mild
1000
500
0
utes.c__Mollicutes.o__Acholeplasmatales.f__Acholeplasmataceae.g
asymptomatic (n=24)
mild (n=25)
severe (n=30)
control (n=72)
Severity

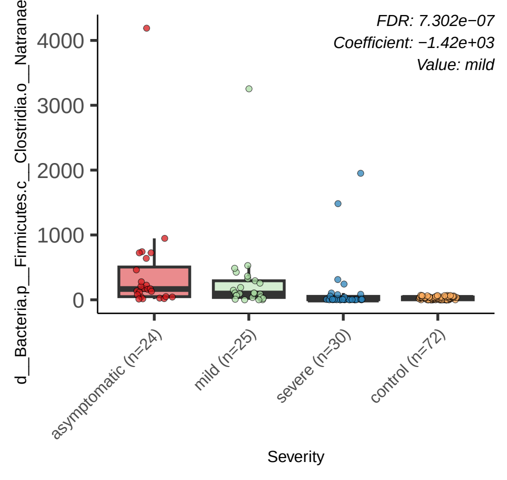

FDR: 7.302e−07
Coefficient: −1.42e+03
Value: mild
d__Bacteria.p__Firmicutes.c__Clostridia.o__Natranae
4000
3000
2000
1000
0
asymptomatic (n=24)
mild (n=25)
severe (n=30)
control (n=72)
Severity

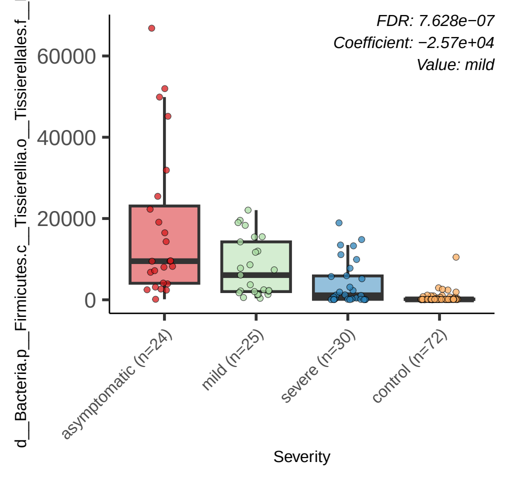
FDR: 7.628e−07
Coefficient: −2.57e+04
Value: mild
d__Bacteria.p__Firmicutes.c__Tissierellia.o__Tissierellales.f__
60000
40000
20000
0
asymptomatic (n=24)
mild (n=25)
severe (n=30)
control (n=72)
Severity

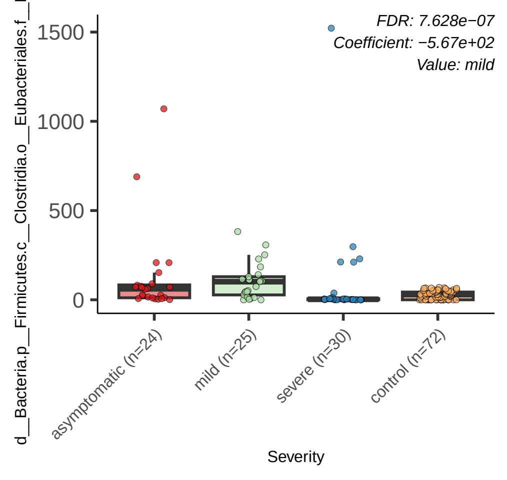

FDR: 7.628e−07
Coefficient: −5.67e+02
Value: mild
d__Bacteria.p__Firmicutes.c__Clostridia.o__Eubacteriales.f__
1500
1000
500
0
asymptomatic (n=24)
mild (n=25)
severe (n=30)
control (n=72)
Severity

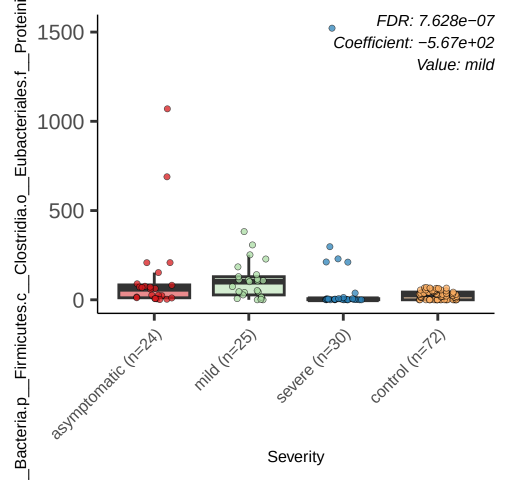
FDR: 7.628e−07
Coefficient: −5.67e+02
Value: mild
1500
1000
500
0
_Bacteria.p__Firmicutes.c__Clostridia.o__Eubacteriales.f__Proteini
asymptomatic (n=24)
mild (n=25)
severe (n=30)
control (n=72)
Severity

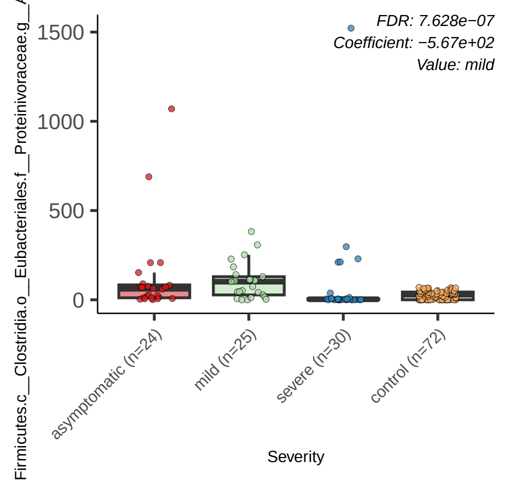

FDR: 7.628e−07
Coefficient: −5.67e+02
Value: mild
1500
1000
500
0
Firmicutes.c__Clostridia.o__Eubacteriales.f__Proteinivoraceae.g__A
asymptomatic (n=24)
mild (n=25)
severe (n=30)
control (n=72)
Severity

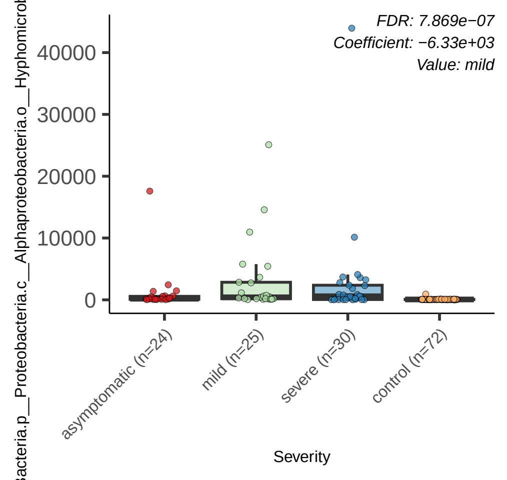

FDR: 7.869e−07
Coefficient: −6.33e+03
Value: mild
40000
30000
20000
10000
0
Bacteria.p__Proteobacteria.c__Alphaproteobacteria.o__Hyphomicrob
asymptomatic (n=24)
mild (n=25)
severe (n=30)
control (n=72)
Severity

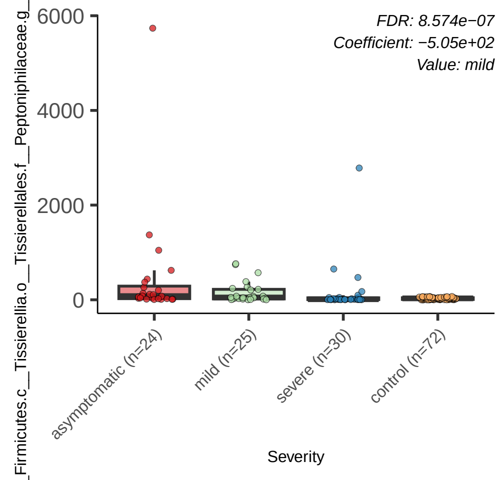

FDR: 8.574e−07
Coefficient: −5.05e+02
Value: mild
Firmicutes.c__Tissierellia.o__Tissierellales.f__Peptoniphilaceae.g__
6000
4000
2000
0
asymptomatic (n=24)
mild (n=25)
severe (n=30)
control (n=72)
Severity

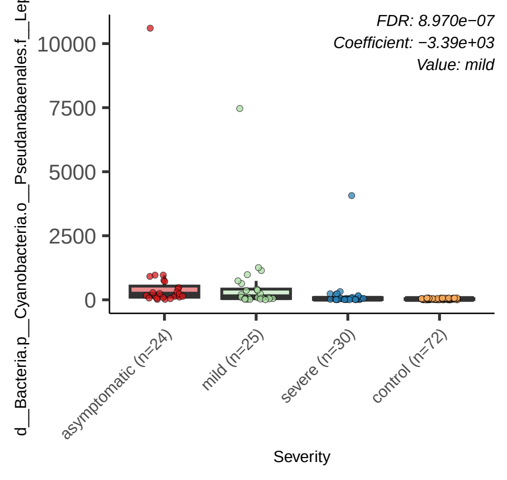

FDR: 8.970e−07
Coefficient: −3.39e+03
Value: mild
d__Bacteria.p__Cyanobacteria.o__Pseudanabaenales.f__Lep
10000
7500
5000
2500
0
asymptomatic (n=24)
mild (n=25)
severe (n=30)
control (n=72)
Severity

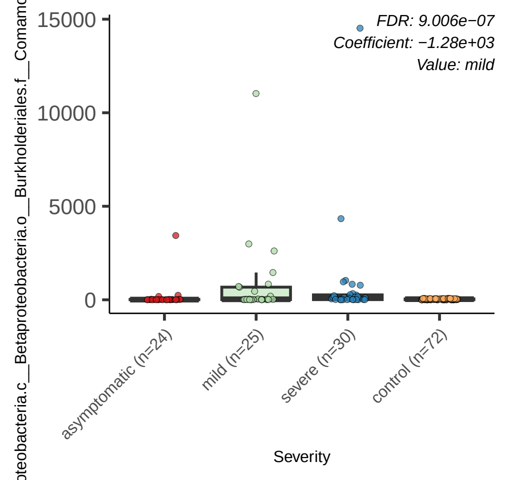

FDR: 9.006e−07
Coefficient: −1.28e+03
Value: mild
15000
10000
5000
0
oteobacteria.c__Betaproteobacteria.o__Burkholderiales.f__Comamo
asymptomatic (n=24)
mild (n=25)
severe (n=30)
control (n=72)
Severity

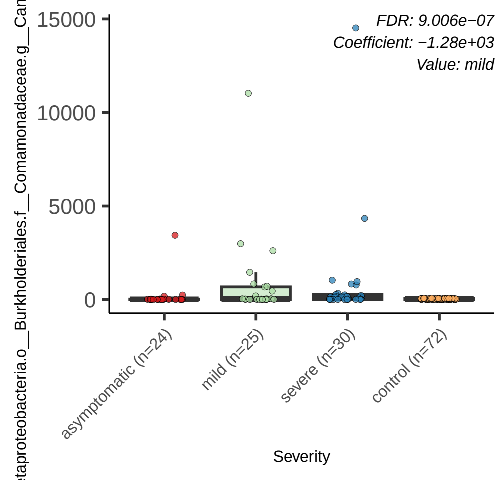

etaproteobacteria.o__Burkholderiales.f__Comamonadaceae.g__Can
15000
10000
5000
0
FDR: 9.006e−07
Coefficient: −1.28e+03
Value: mild
asymptomatic (n=24)
mild (n=25)
severe (n=30)
control (n=72)
Severity

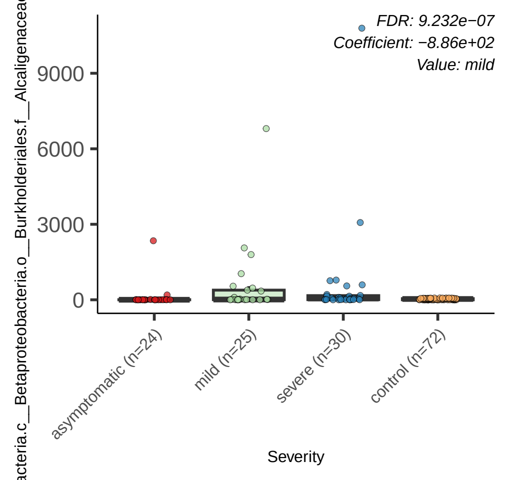
FDR: 9.232e−07
Coefficient: −8.86e+02
Value: mild
9000
6000
3000
0
acteria.c__Betaproteobacteria.o__Burkholderiales.f__Alcaligenaceae
asymptomatic (n=24)
mild (n=25)
severe (n=30)
control (n=72)
Severity

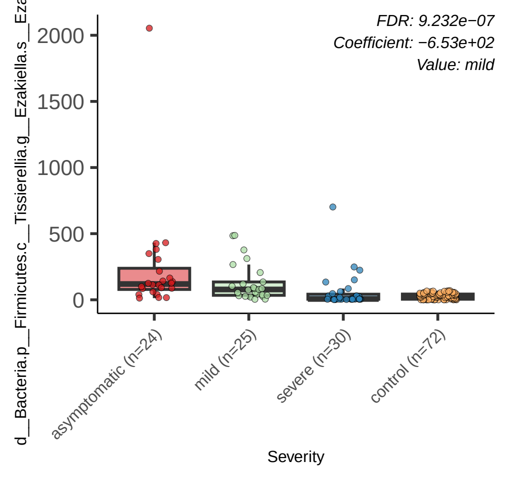

FDR: 9.232e−07
Coefficient: −6.53e+02
Value: mild
d__Bacteria.p__Firmicutes.c__Tissierellia.g__Ezakiella.s__Eza
2000
1500
1000
500
0
asymptomatic (n=24)
mild (n=25)
severe (n=30)
control (n=72)
Severity

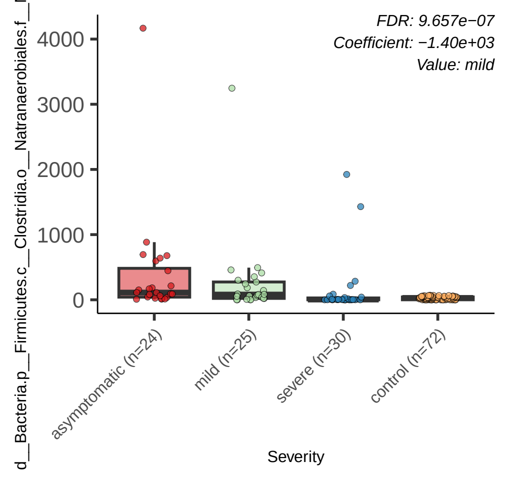

FDR: 9.657e−07
Coefficient: −1.40e+03
Value: mild
d__Bacteria.p__Firmicutes.c__Clostridia.o__Natranaerobiales.f__
0
1000
2000
3000
4000
asymptomatic (n=24)
mild (n=25)
severe (n=30)
control (n=72)
Severity

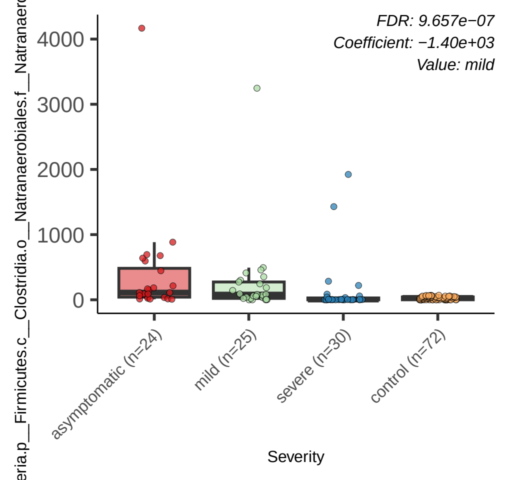
FDR: 9.657e−07
Coefficient: −1.40e+03
Value: mild
4000
3000
2000
1000
0
asymptomatic (n=24)
mild (n=25)
severe (n=30)
control (n=72)
Severity
eria.p__Firmicutes.c__Clostridia.o__Natranaerobiales.f__Natranaero

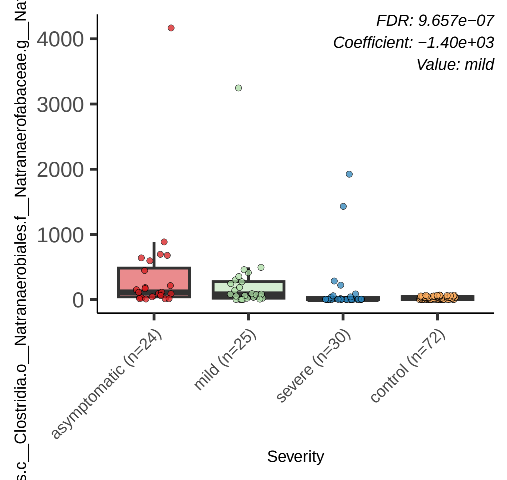

FDR: 9.657e−07
Coefficient: −1.40e+03
Value: mild
4000
3000
2000
1000
0
s.c__Clostridia.o__Natranaerobiales.f__Natranaerofabaceae.g__Na
asymptomatic (n=24)
mild (n=25)
severe (n=30)
control (n=72)
Severity

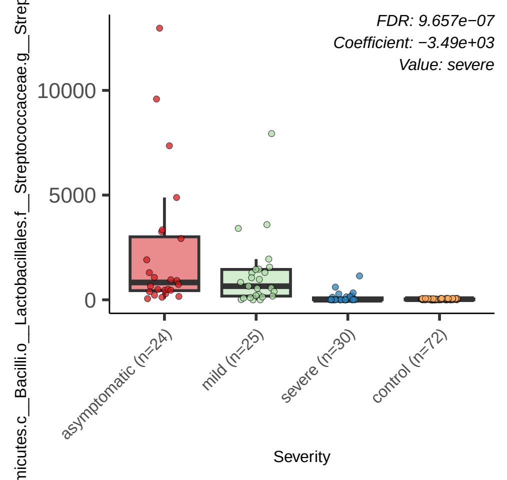
FDR: 9.657e−07
Coefficient: −3.49e+03
Value: severe
10000
5000
0
asymptomatic (n=24)
mild (n=25)
severe (n=30)
control (n=72)
Severity
micutes.c__Bacilli.o__Lactobacillales.f__Streptococcaceae.g__Strep

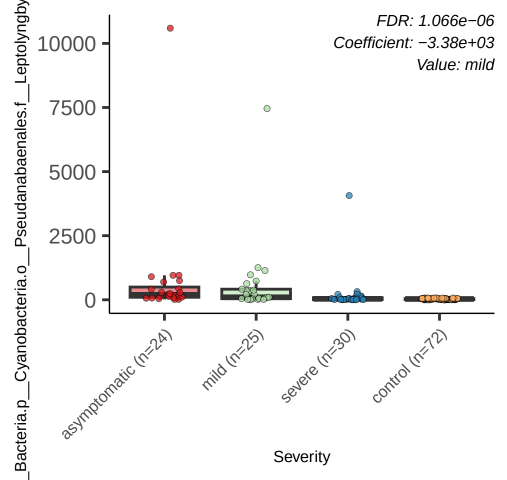

FDR: 1.066e−06
Coefficient: −3.38e+03
Value: mild
10000
7500
5000
2500
0
_Bacteria.p__Cyanobacteria.o__Pseudanabaenales.f__Leptolyngby
asymptomatic (n=24)
mild (n=25)
severe (n=30)
control (n=72)
Severity

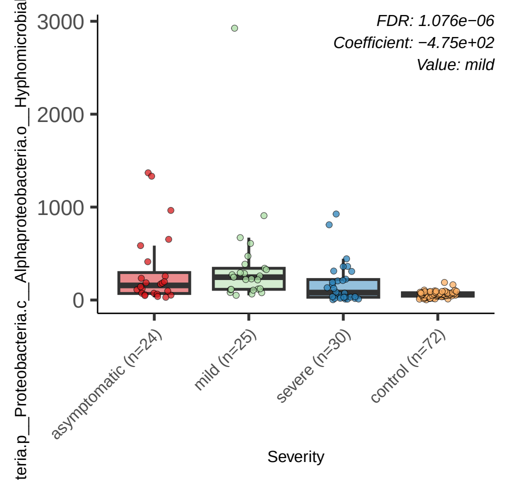
FDR: 1.076e−06
Coefficient: −4.75e+02
Value: mild
3000
2000
1000
0
teria.p__Proteobacteria.c__Alphaproteobacteria.o__Hyphomicrobial
asymptomatic (n=24)
mild (n=25)
severe (n=30)
control (n=72)
Severity

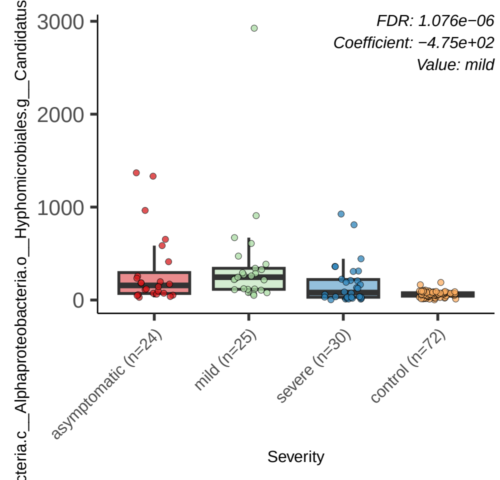

FDR: 1.076e−06
Coefficient: −4.75e+02
Value: mild
3000
2000
1000
0
cteria.c__Alphaproteobacteria.o__Hyphomicrobiales.g__Candidatus
asymptomatic (n=24)
mild (n=25)
severe (n=30)
control (n=72)
Severity

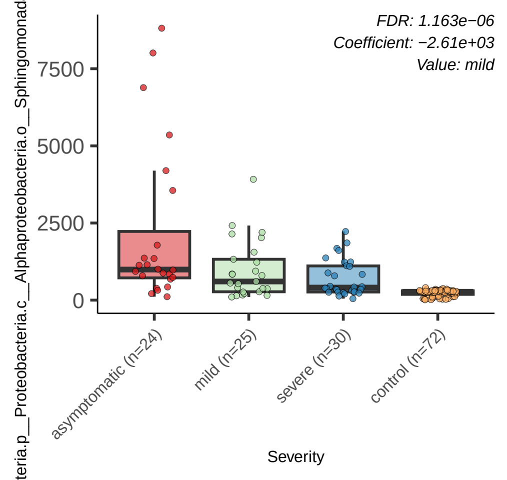
FDR: 1.163e−06
Coefficient: −2.61e+03
Value: mild
teria.p__Proteobacteria.c__Alphaproteobacteria.o__Sphingomonad
7500
5000
2500
0
asymptomatic (n=24)
mild (n=25)
severe (n=30)
control (n=72)
Severity

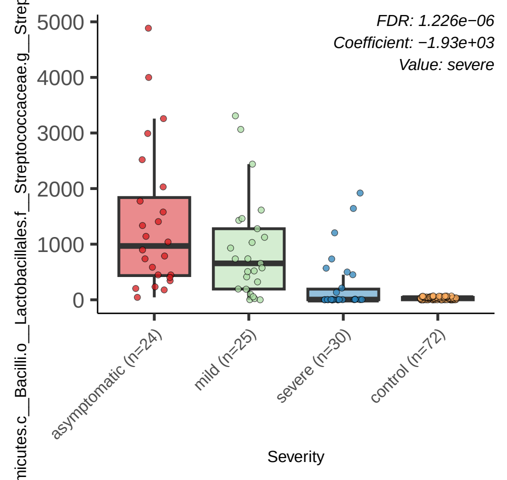

FDR: 1.226e−06
Coefficient: −1.93e+03
Value: severe
micutes.c__Bacilli.o__Lactobacillales.f__Streptococcaceae.g__Strep
5000
4000
3000
2000
1000
0
asymptomatic (n=24)
mild (n=25)
severe (n=30)
control (n=72)
Severity

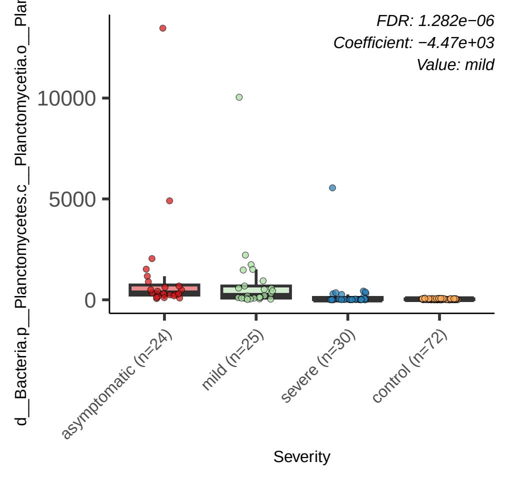

FDR: 1.282e−06
Coefficient: −4.47e+03
Value: mild
d__Bacteria.p__Planctomycetes.c__Planctomycetia.o__Plar
10000
5000
0
asymptomatic (n=24)
mild (n=25)
severe (n=30)
control (n=72)
Severity

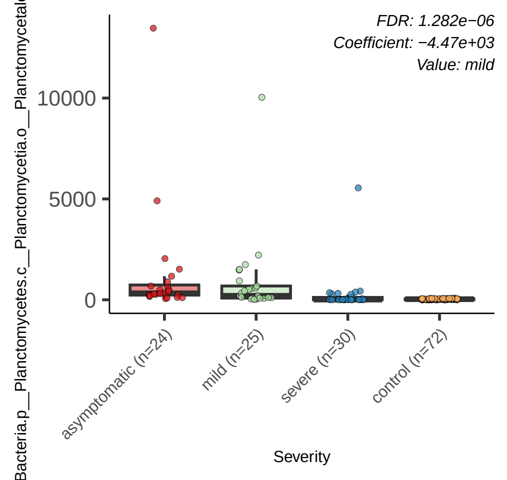

FDR: 1.282e−06
Coefficient: −4.47e+03
Value: mild
10000
5000
0
asymptomatic (n=24)
mild (n=25)
severe (n=30)
control (n=72)
Severity
Bacteria.p__Planctomycetes.c__Planctomycetia.o__Planctomycetal

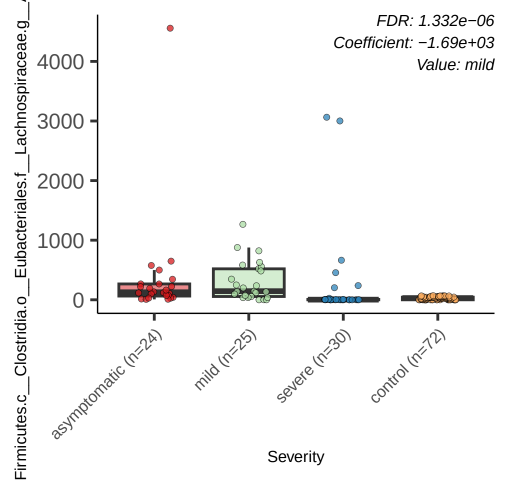

FDR: 1.332e−06
Coefficient: −1.69e+03
Value: mild
0
1000
2000
3000
4000
Firmicutes.c__Clostridia.o__Eubacteriales.f__Lachnospiraceae.g__
asymptomatic (n=24)
mild (n=25)
severe (n=30)
control (n=72)
Severity

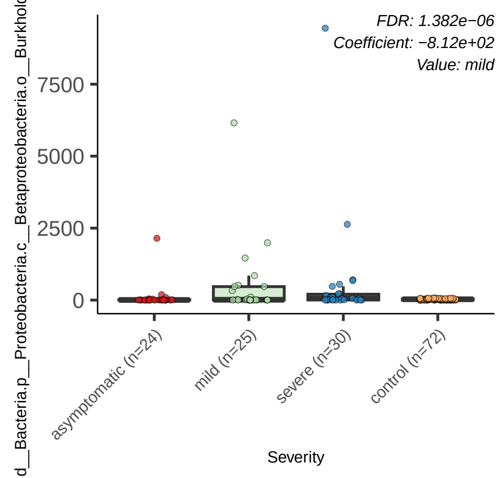
FDR: 1.382e−06
Coefficient: −8.12e+02
Value: mild
7500
5000
2500
0
asymptomatic (n=24)
mild (n=25)
severe (n=30)
control (n=72)
Severity
d__Bacteria.p__Proteobacteria.c__Betaproteobacteria.o__Burkhold

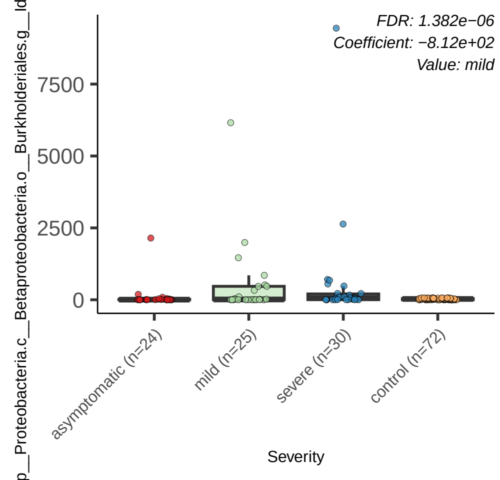

FDR: 1.382e−06
Coefficient: −8.12e+02
Value: mild
7500
5000
2500
0
p__Proteobacteria.c__Betaproteobacteria.o__Burkholderiales.g__Id
asymptomatic (n=24)
mild (n=25)
severe (n=30)
control (n=72)
Severity

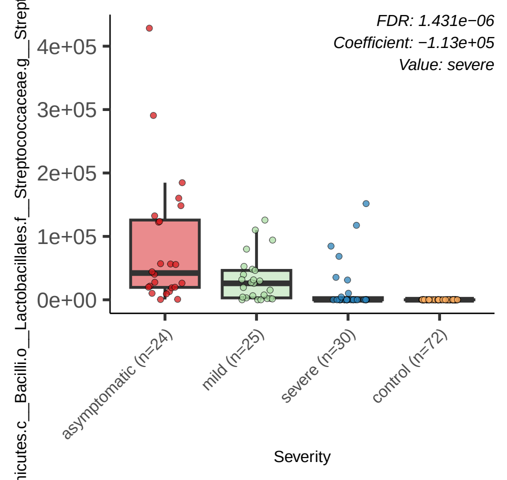
FDR: 1.431e−06
Coefficient: −1.13e+05
Value: severe
4e+05
3e+05
2e+05
1e+05
0e+00
asymptomatic (n=24)
mild (n=25)
severe (n=30)
control (n=72)
Severity
nicutes.c__Bacilli.o__Lactobacillales.f__Streptococcaceae.g__Strep

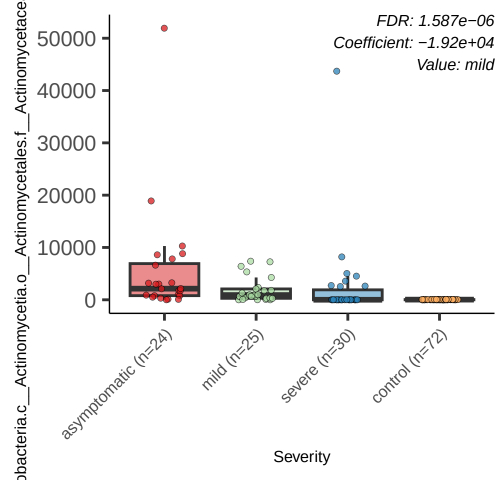
FDR: 1.587e−06
Coefficient: −1.92e+04
Value: mild
obacteria.c__Actinomycetia.o__Actinomycetales.f__Actinomycetace
50000
40000
30000
20000
10000
0
asymptomatic (n=24)
mild (n=25)
severe (n=30)
control (n=72)
Severity

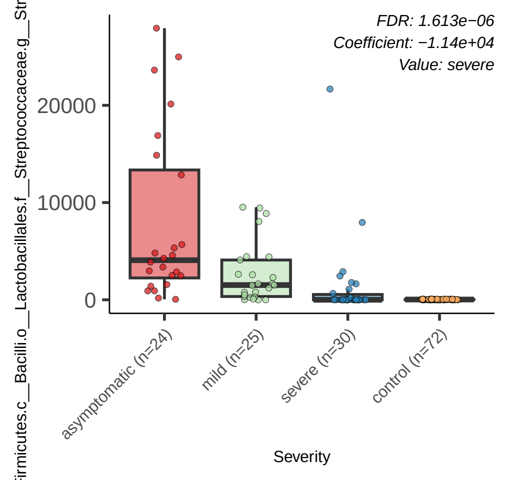

FDR: 1.613e−06
Coefficient: −1.14e+04
Value: severe
0
10000
20000
Firmicutes.c__Bacilli.o__Lactobacillales.f__Streptococcaceae.g__Str
asymptomatic (n=24)
mild (n=25)
severe (n=30)
control (n=72)
Severity

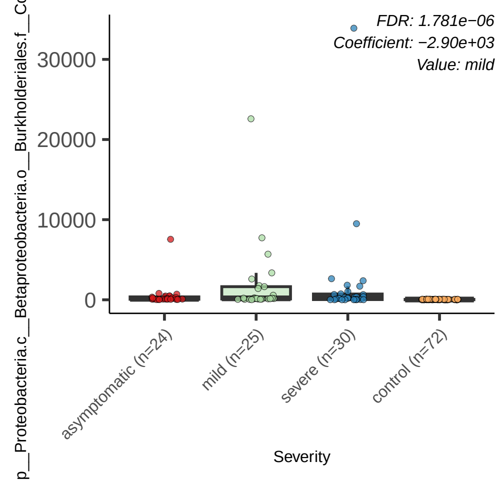
FDR: 1.781e−06
Coefficient: −2.90e+03
Value: mild
30000
20000
10000
0
asymptomatic (n=24)
mild (n=25)
severe (n=30)
control (n=72)
Severity
p__Proteobacteria.c__Betaproteobacteria.o__Burkholderiales.f__Co

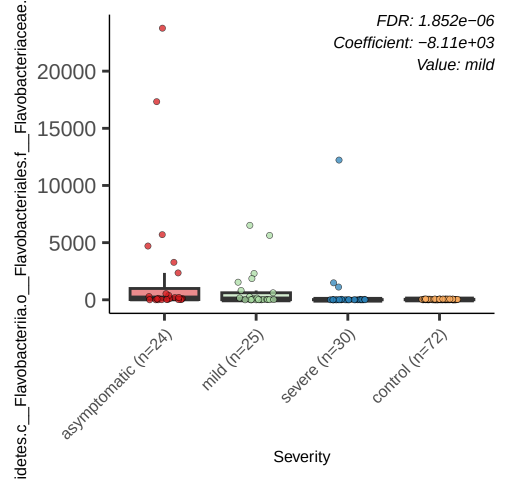

FDR: 1.852e−06
Coefficient: −8.11e+03
Value: mild
idetes.c__Flavobacteriia.o__Flavobacteriales.f__Flavobacteriaceae.
20000
15000
10000
5000
0
asymptomatic (n=24)
mild (n=25)
severe (n=30)
control (n=72)
Severity

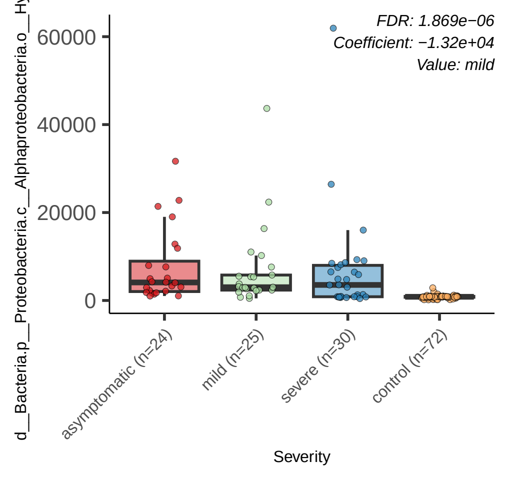

FDR: 1.869e−06
Coefficient: −1.32e+04
Value: mild
d__Bacteria.p__Proteobacteria.c__Alphaproteobacteria.o__Hy
60000
40000
20000
0
asymptomatic (n=24)
mild (n=25)
severe (n=30)
control (n=72)
Severity

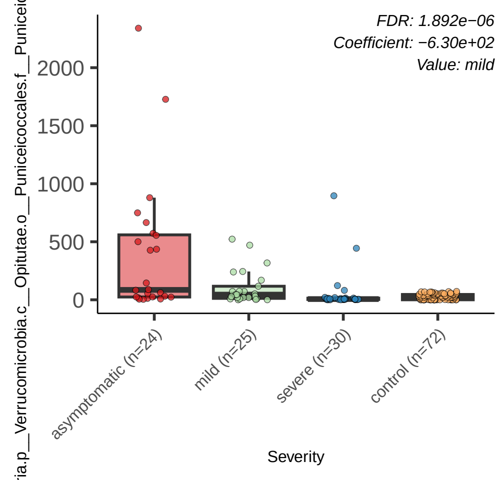

FDR: 1.892e−06
Coefficient: −6.30e+02
Value: mild
ria.p__Verrucomicrobia.c__Opitutae.o__Puniceicoccales.f__Puniceic
0
500
1000
1500
2000
asymptomatic (n=24)
mild (n=25)
severe (n=30)
control (n=72)
Severity

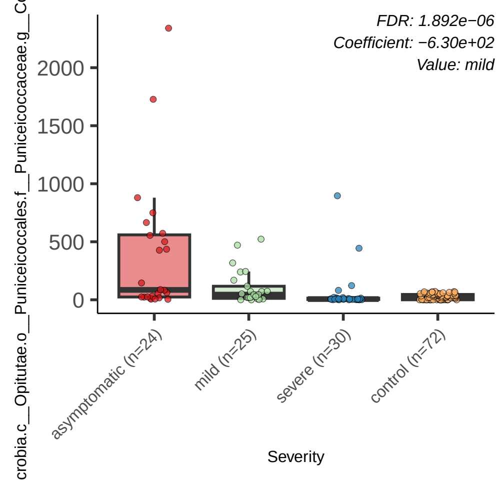

FDR: 1.892e−06
Coefficient: −6.30e+02
Value: mild
crobia.c__Opitutae.o__Puniceicoccales.f__Puniceicoccaceae.g__Co
2000
1500
1000
500
0
asymptomatic (n=24)
mild (n=25)
severe (n=30)
control (n=72)
Severity

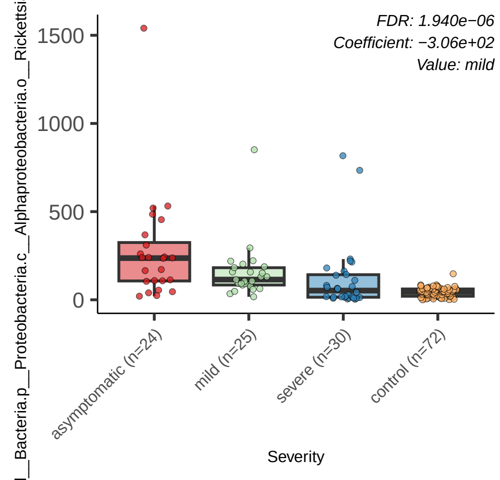
FDR: 1.940e−06
Coefficient: −3.06e+02
Value: mild
1500
1000
500
0
Bacteria.p__Proteobacteria.c__Alphaproteobacteria.o__Ricketts
asymptomatic (n=24)
mild (n=25)
severe (n=30)
control (n=72)
Severity

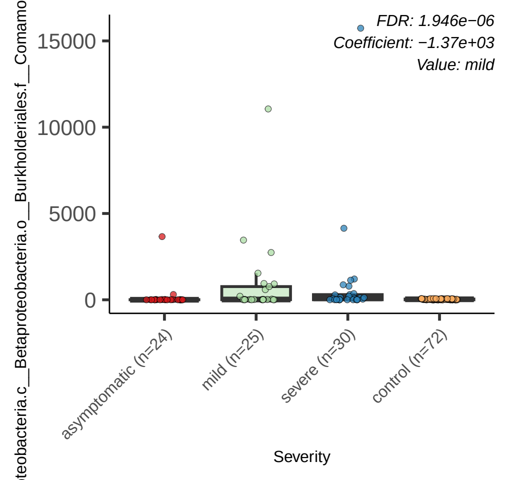

FDR: 1.946e−06
Coefficient: −1.37e+03
Value: mild
15000
10000
5000
0
asymptomatic (n=24)
mild (n=25)
severe (n=30)
control (n=72)
Severity
eobacteria.c__Betaproteobacteria.o__Burkholderiales.f__Comamo

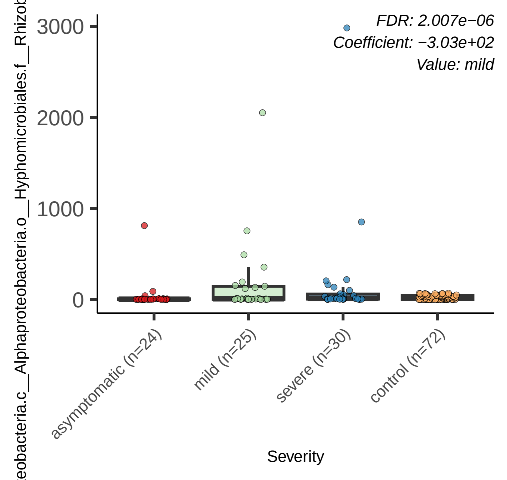

FDR: 2.007e−06
Coefficient: −3.03e+02
Value: mild
3000
2000
1000
0
eobacteria.c__Alphaproteobacteria.o__Hyphomicrobiales.f__Rhizob
asymptomatic (n=24)
mild (n=25)
severe (n=30)
control (n=72)
Severity

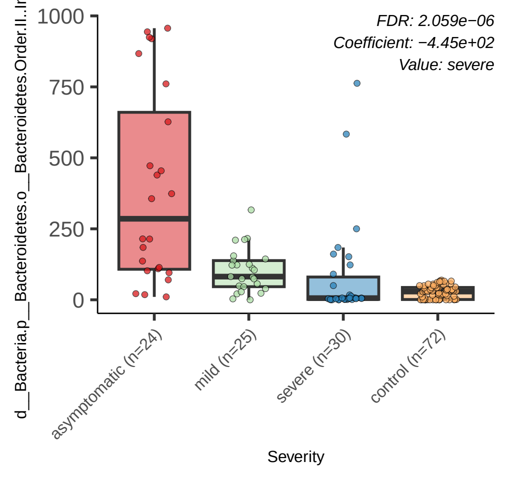

FDR: 2.059e−06
Coefficient: −4.45e+02
Value: severe
d__Bacteria.p__Bacteroidetes.o__Bacteroidetes.Order.II..In
1000
750
500
250
0
asymptomatic (n=24)
mild (n=25)
severe (n=30)
control (n=72)
Severity

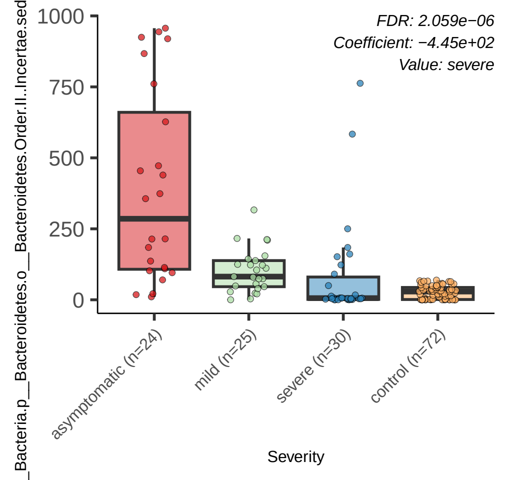

FDR: 2.059e−06
Coefficient: −4.45e+02
Value: severe
1000
750
500
250
0
Bacteria.p__Bacteroidetes.o__Bacteroidetes.Order.II..Incertae.sed
asymptomatic (n=24)
mild (n=25)
severe (n=30)
control (n=72)
Severity

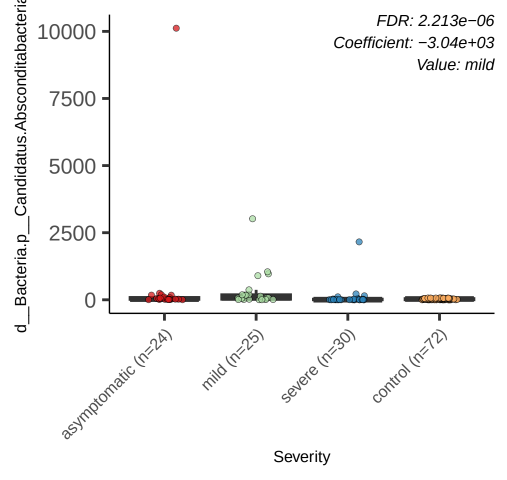
FDR: 2.213e−06
Coefficient: −3.04e+03
Value: mild
d__Bacteria.p__Candidatus.Absconditabacteria
10000
7500
5000
2500
0
asymptomatic (n=24)
mild (n=25)
severe (n=30)
control (n=72)
Severity

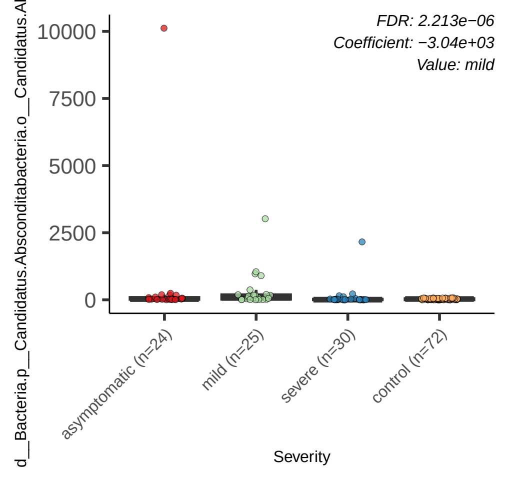

FDR: 2.213e−06
Coefficient: −3.04e+03
Value: mild
10000
7500
5000
2500
0
d__Bacteria.p__Candidatus.Absconditabacteria.o__Candidatus.Al
asymptomatic (n=24)
mild (n=25)
severe (n=30)
control (n=72)
Severity

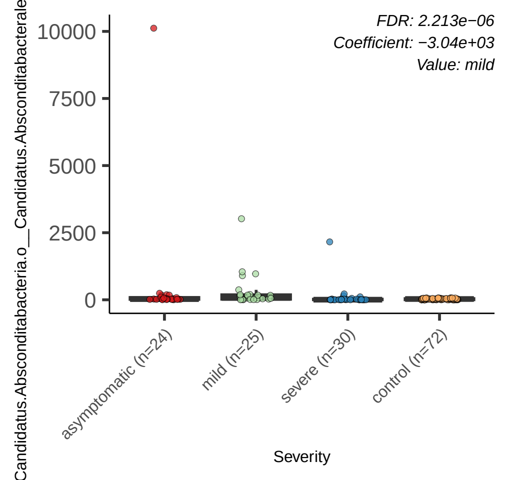

FDR: 2.213e−06
Coefficient: −3.04e+03
Value: mild
Candidatus.Absconditabacteria.o__Candidatus.Absconditabacterale
10000
7500
5000
2500
0
asymptomatic (n=24)
mild (n=25)
severe (n=30)
control (n=72)
Severity

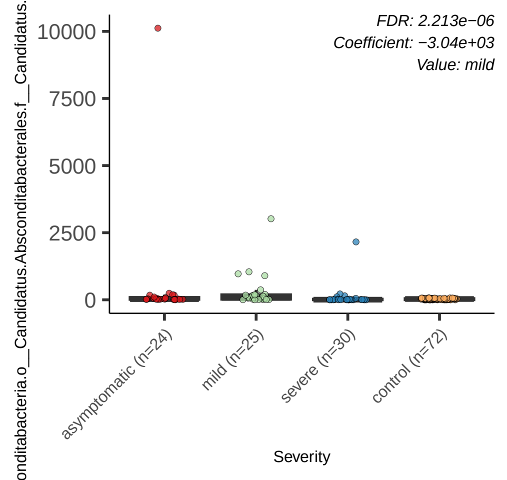

FDR: 2.213e−06
Coefficient: −3.04e+03
Value: mild
10000
7500
5000
2500
0
asymptomatic (n=24)
mild (n=25)
severe (n=30)
control (n=72)
Severity
onditabacteria.o__Candidatus.Absconditabacterales.f__Candidatus.

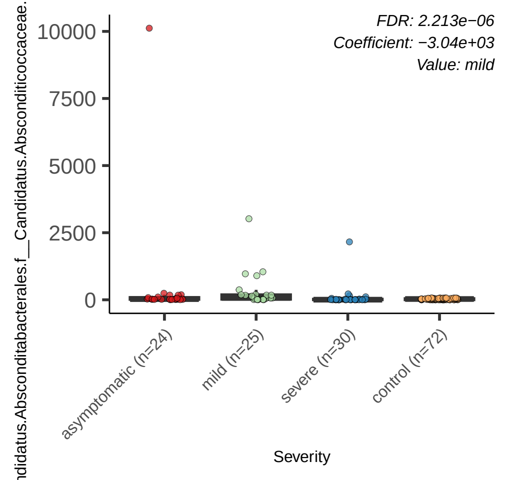

FDR: 2.213e−06
Coefficient: −3.04e+03
Value: mild
10000
7500
5000
2500
0
ndidatus.Absconditabacterales.f__Candidatus.Absconditicoccaceae.
asymptomatic (n=24)
mild (n=25)
severe (n=30)
control (n=72)
Severity

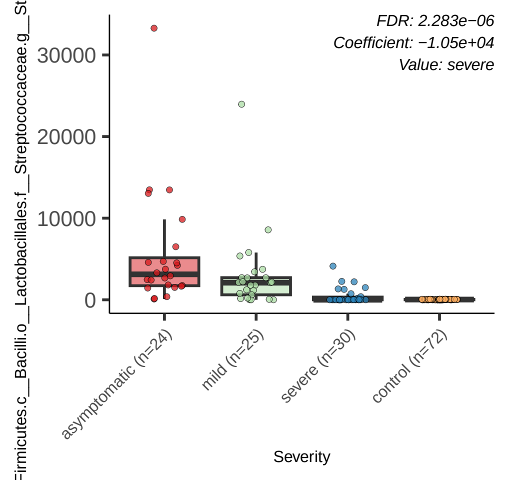

FDR: 2.283e−06
Coefficient: −1.05e+04
Value: severe
30000
20000
10000
0
Firmicutes.c__Bacilli.o__Lactobacillales.f__Streptococcaceae.g__St
asymptomatic (n=24)
mild (n=25)
severe (n=30)
control (n=72)
Severity

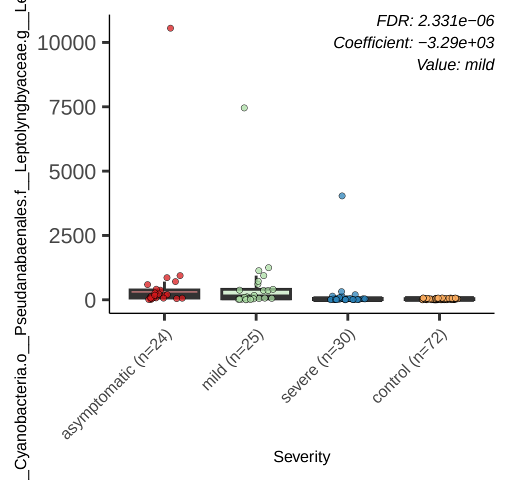
FDR: 2.331e−06
Coefficient: −3.29e+03
Value: mild
_Cyanobacteria.o__Pseudanabaenales.f__Leptolyngbyaceae.g__Le
10000
7500
5000
2500
0
asymptomatic (n=24)
mild (n=25)
severe (n=30)
control (n=72)
Severity

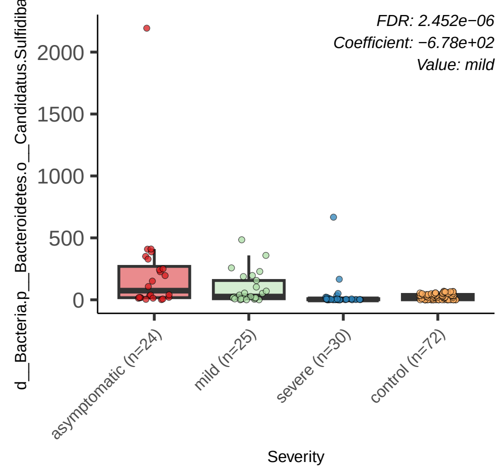

FDR: 2.452e−06
Coefficient: −6.78e+02
Value: mild
d__Bacteria.p__Bacteroidetes.o__Candidatus.Sulfidiba
2000
1500
1000
500
0
asymptomatic (n=24)
mild (n=25)
severe (n=30)
control (n=72)
Severity

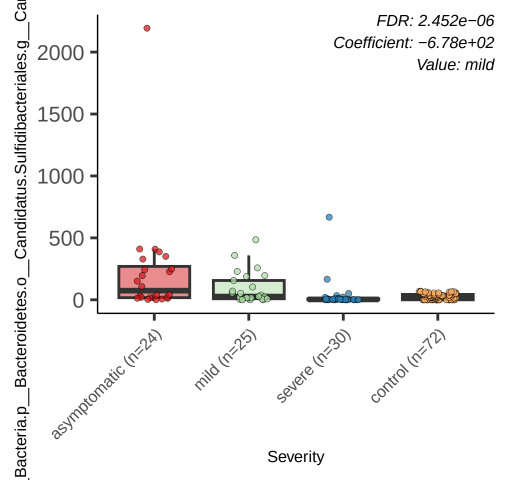

FDR: 2.452e−06
Coefficient: −6.78e+02
Value: mild
2000
1500
1000
500
0
Bacteria.p__Bacteroidetes.o__Candidatus.Sulfidibacteriales.g__Ca
asymptomatic (n=24)
mild (n=25)
severe (n=30)
control (n=72)
Severity

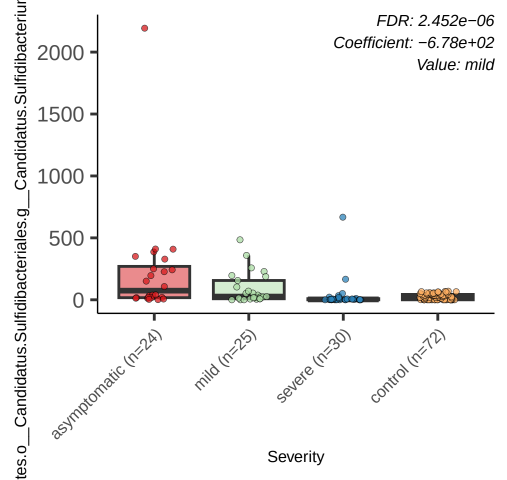
FDR: 2.452e−06
Coefficient: −6.78e+02
Value: mild
2000
1500
1000
500
0
asymptomatic (n=24)
mild (n=25)
severe (n=30)
control (n=72)
Severity
tes.o__Candidatus.Sulfidibacteriales.g__Candidatus.Sulfidibacteriu

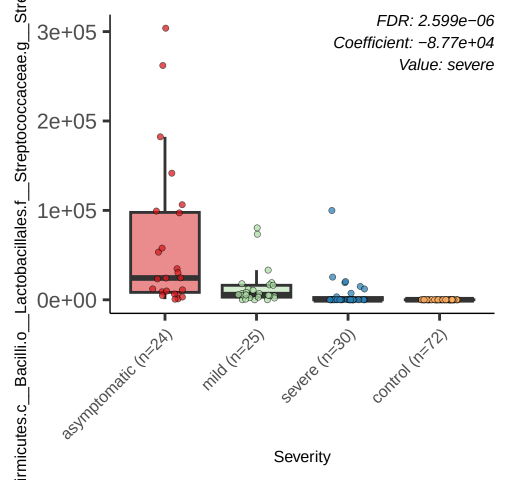

FDR: 2.599e−06
Coefficient: −8.77e+04
Value: severe
3e+05
2e+05
1e+05
0e+00
irmicutes.c__Bacilli.o__Lactobacillales.f__Streptococcaceae.g__Stre
asymptomatic (n=24)
mild (n=25)
severe (n=30)
control (n=72)
Severity

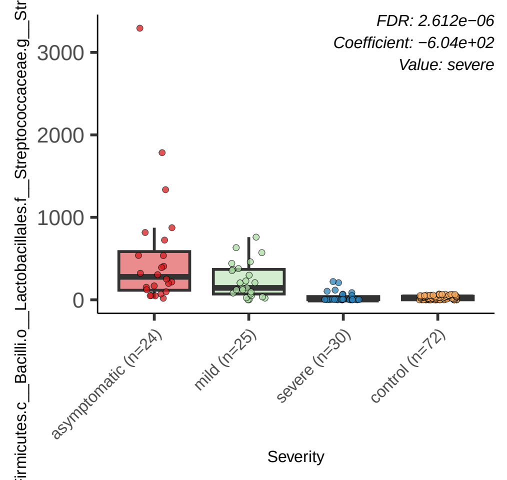

FDR: 2.612e−06
Coefficient: −6.04e+02
Value: severe
3000
2000
1000
0
asymptomatic (n=24)
mild (n=25)
severe (n=30)
control (n=72)
Severity
irmicutes.c__Bacilli.o__Lactobacillales.f__Streptococcaceae.g__Str

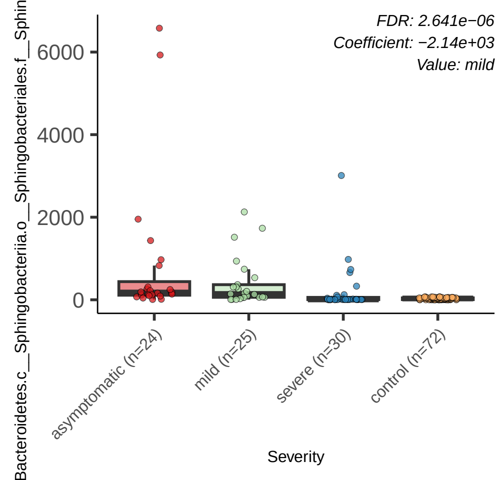

FDR: 2.641e−06
Coefficient: −2.14e+03
Value: mild
6000
4000
2000
0
Bacteroidetes.c__Sphingobacteriia.o__Sphingobacteriales.f__Sphin
asymptomatic (n=24)
mild (n=25)
severe (n=30)
control (n=72)
Severity

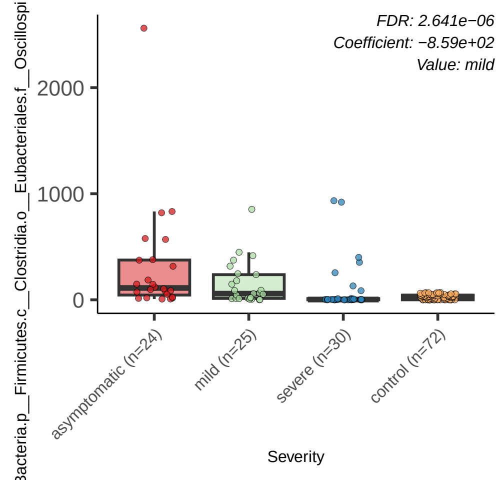

FDR: 2.641e−06
Coefficient: −8.59e+02
Value: mild
Bacteria.p__Firmicutes.c__Clostridia.o__Eubacteriales.f__Oscillospi
2000
1000
0
asymptomatic (n=24)
mild (n=25)
severe (n=30)
control (n=72)
Severity

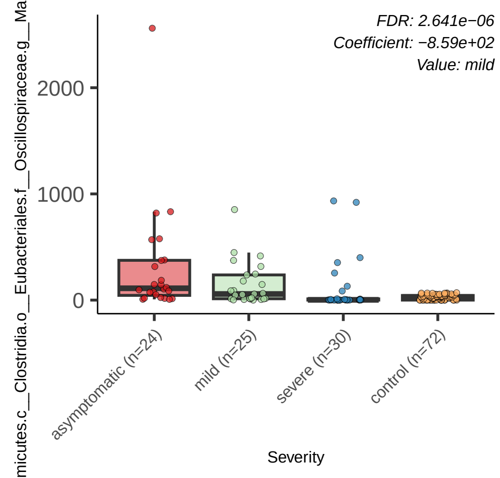

FDR: 2.641e−06
Coefficient: −8.59e+02
Value: mild
2000
1000
0
asymptomatic (n=24)
mild (n=25)
severe (n=30)
control (n=72)
Severity

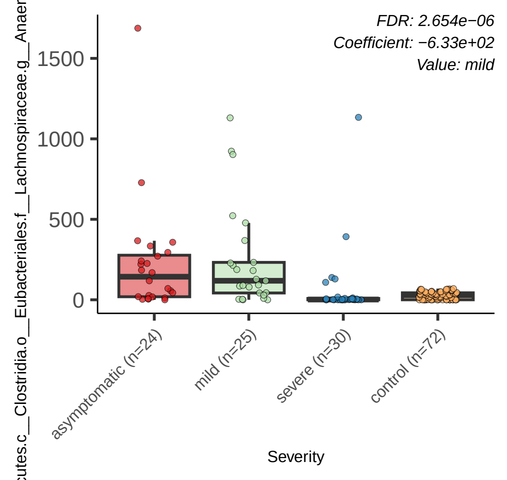

FDR: 2.654e−06
Coefficient: −6.33e+02
Value: mild
cutes.c__Clostridia.o__Eubacteriales.f__Lachnospiraceae.g__Anaer
0
500
1000
1500
asymptomatic (n=24)
mild (n=25)
severe (n=30)
control (n=72)
Severity

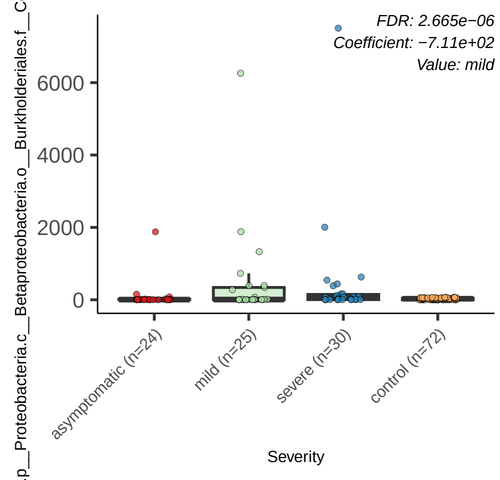
FDR: 2.665e−06
Coefficient: −7.11e+02
Value: mild
6000
4000
2000
0
p__Proteobacteria.c__Betaproteobacteria.o__Burkholderiales.f__C
asymptomatic (n=24)
mild (n=25)
severe (n=30)
control (n=72)
Severity

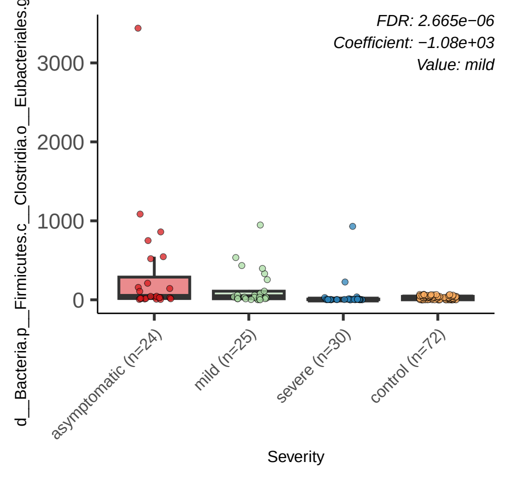

FDR: 2.665e−06
Coefficient: −1.08e+03
Value: mild
d__Bacteria.p__Firmicutes.c__Clostridia.o__Eubacteriales.g
3000
2000
1000
0
asymptomatic (n=24)
mild (n=25)
severe (n=30)
control (n=72)
Severity

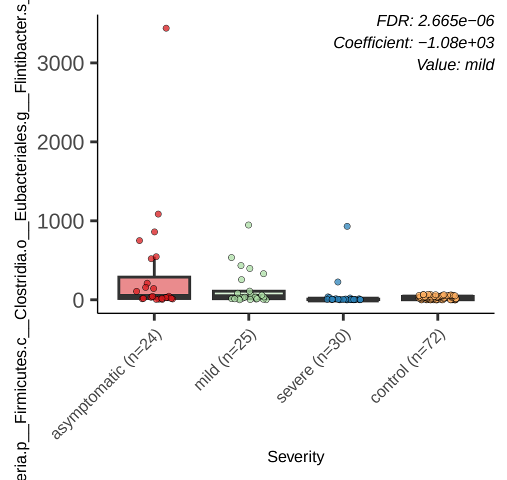

FDR: 2.665e−06
Coefficient: −1.08e+03
Value: mild
0
1000
2000
3000
eria.p__Firmicutes.c__Clostridia.o__Eubacteriales.g__Flintibacter.s_
asymptomatic (n=24)
mild (n=25)
severe (n=30)
control (n=72)
Severity

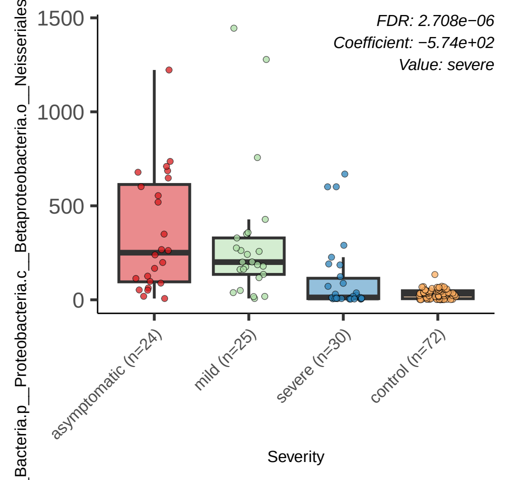

FDR: 2.708e−06
Coefficient: −5.74e+02
Value: severe
Bacteria.p__Proteobacteria.c__Betaproteobacteria.o__Neisseriales
1500
1000
500
0
asymptomatic (n=24)
mild (n=25)
severe (n=30)
control (n=72)
Severity

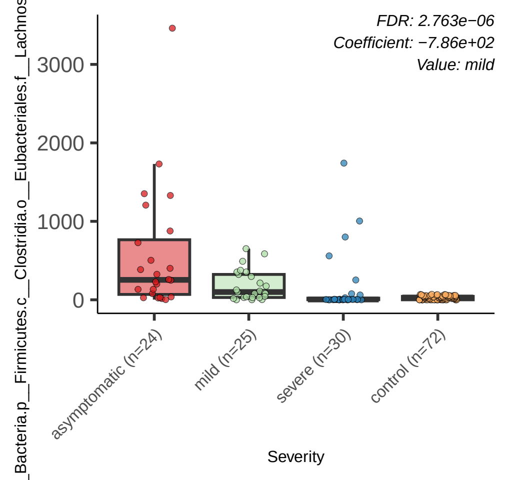

FDR: 2.763e−06
Coefficient: −7.86e+02
Value: mild
Bacteria.p__Firmicutes.c__Clostridia.o__Eubacteriales.f__Lachnos
3000
2000
1000
0
asymptomatic (n=24)
mild (n=25)
severe (n=30)
control (n=72)
Severity

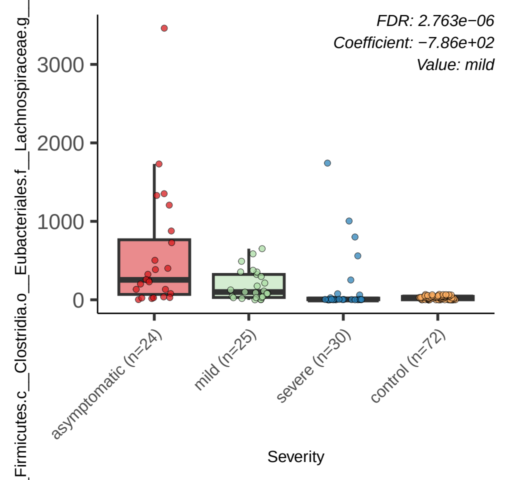

FDR: 2.763e−06
Coefficient: −7.86e+02
Value: mild
Firmicutes.c__Clostridia.o__Eubacteriales.f__Lachnospiraceae.g__
3000
2000
1000
0
asymptomatic (n=24)
mild (n=25)
severe (n=30)
control (n=72)
Severity

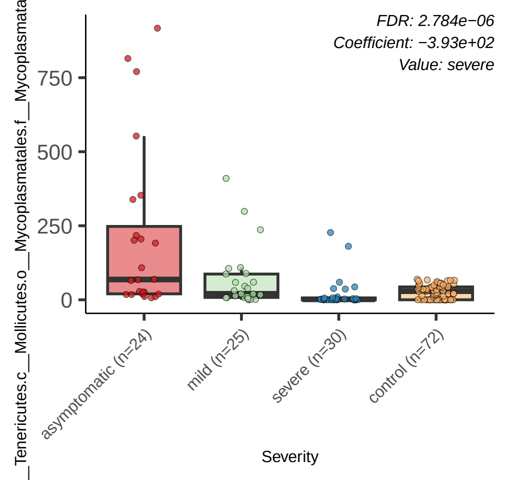
FDR: 2.784e−06
Coefficient: −3.93e+02
Value: severe
Tenericutes.c__Mollicutes.o__Mycoplasmatales.f__Mycoplasmata
0
250
500
750
asymptomatic (n=24)
mild (n=25)
severe (n=30)
control (n=72)
Severity

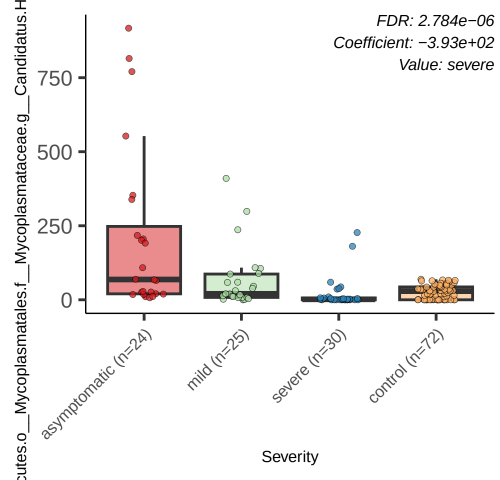

FDR: 2.784e−06
Coefficient: −3.93e+02
Value: severe
cutes.o__Mycoplasmatales.f__Mycoplasmataceae.g__Candidatus.H
750
500
250
0
asymptomatic (n=24)
mild (n=25)
severe (n=30)
control (n=72)
Severity

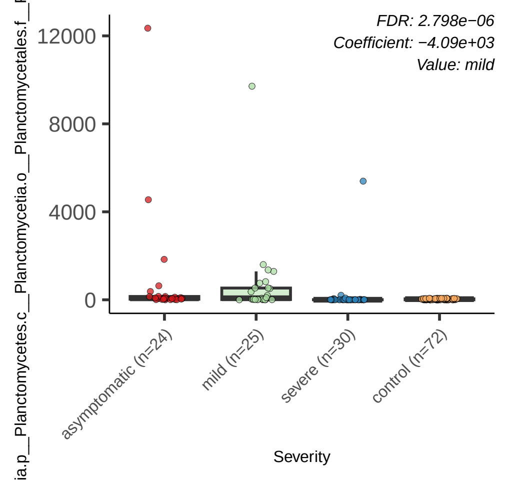
FDR: 2.798e−06
Coefficient: −4.09e+03
Value: mild
12000
8000
4000
0
ia.p__Planctomycetes.c__Planctomycetia.o__Planctomycetales.f__P
asymptomatic (n=24)
mild (n=25)
severe (n=30)
control (n=72)
Severity

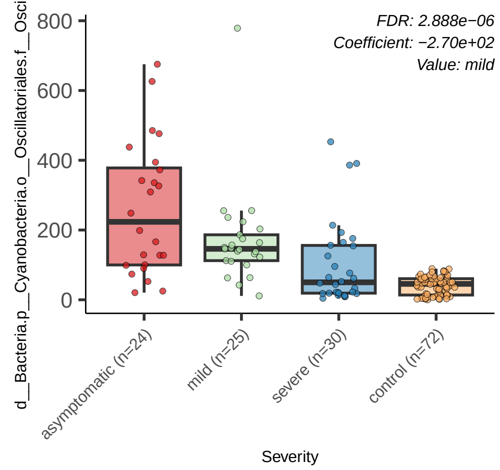
FDR: 2.888e−06
Coefficient: −2.70e+02
Value: mild
d__Bacteria.p__Cyanobacteria.o__Oscillatoriales.f__Osci
800
600
400
200
0
asymptomatic (n=24)
mild (n=25)
severe (n=30)
control (n=72)
Severity

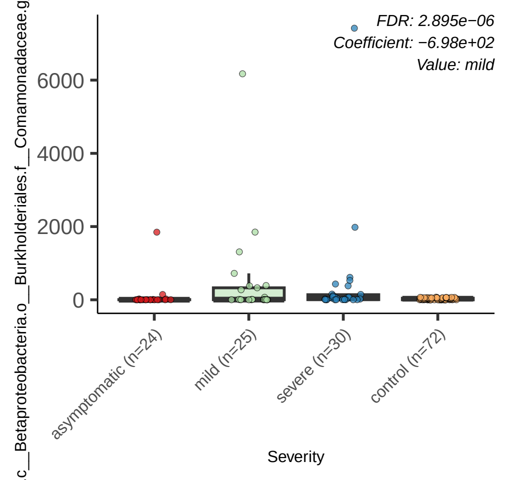

FDR: 2.895e−06
Coefficient: −6.98e+02
Value: mild
6000
4000
2000
0
c__Betaproteobacteria.o__Burkholderiales.f__Comamonadaceae.g
asymptomatic (n=24)
mild (n=25)
severe (n=30)
control (n=72)
Severity

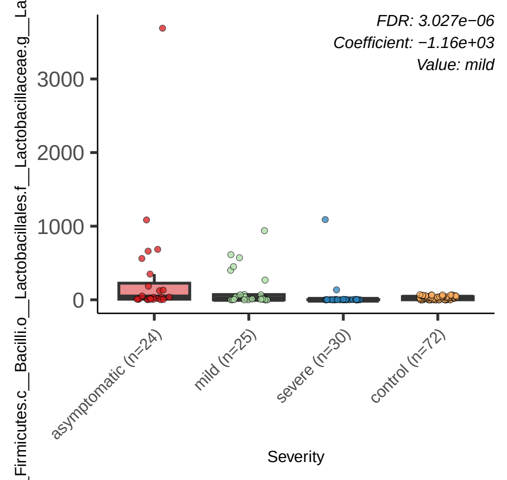
FDR: 3.027e−06
Coefficient: −1.16e+03
Value: mild
Firmicutes.c__Bacilli.o__Lactobacillales.f__Lactobacillaceae.g__La
3000
2000
1000
0
asymptomatic (n=24)
mild (n=25)
severe (n=30)
control (n=72)
Severity

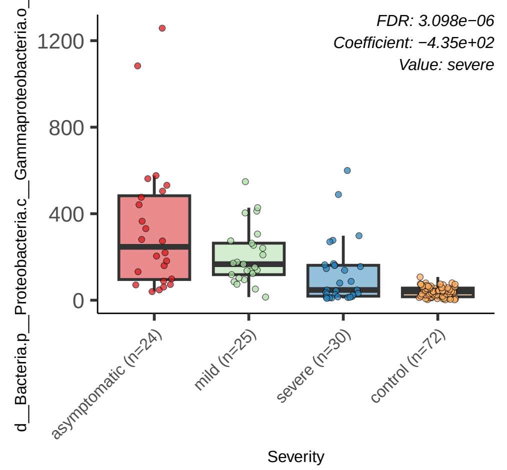

FDR: 3.098e−06
Coefficient: −4.35e+02
Value: severe
d__Bacteria.p__Proteobacteria.c__Gammaproteobacteria.o__
1200
800
400
0
asymptomatic (n=24)
mild (n=25)
severe (n=30)
control (n=72)
Severity

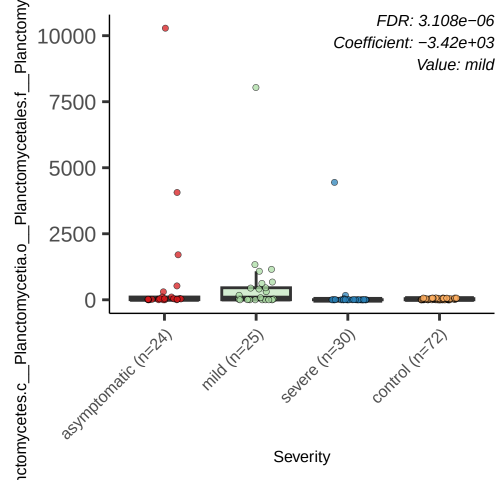

FDR: 3.108e−06
Coefficient: −3.42e+03
Value: mild
10000
7500
5000
2500
0
asymptomatic (n=24)
mild (n=25)
severe (n=30)
control (n=72)
Severity
nctomycetes.c__Planctomycetia.o__Planctomycetales.f__Planctomy

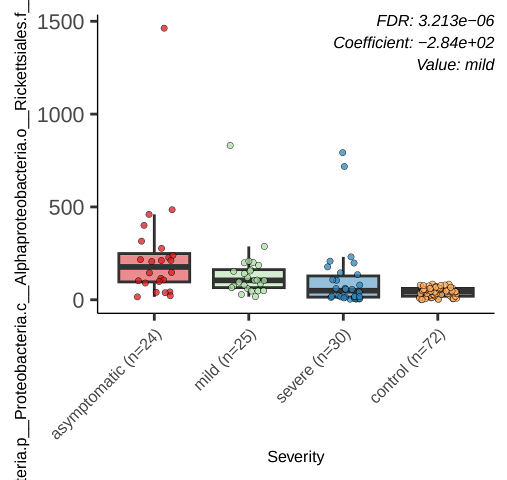

FDR: 3.213e−06
Coefficient: −2.84e+02
Value: mild
1500
1000
500
0
teria.p__Proteobacteria.c__Alphaproteobacteria.o__Rickettsiales.f__
asymptomatic (n=24)
mild (n=25)
severe (n=30)
control (n=72)
Severity

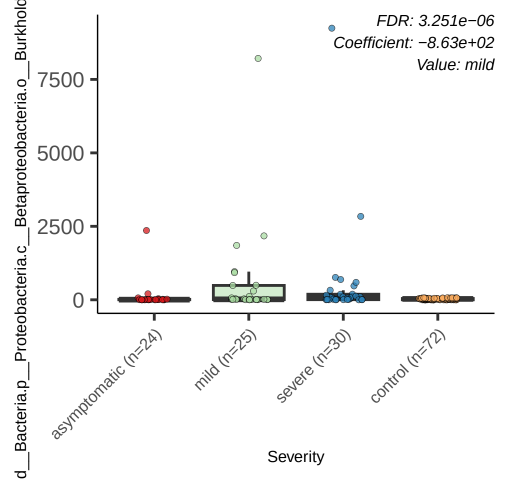

FDR: 3.251e−06
Coefficient: −8.63e+02
Value: mild
d__Bacteria.p__Proteobacteria.c__Betaproteobacteria.o__Burkhold
7500
5000
2500
0
asymptomatic (n=24)
mild (n=25)
severe (n=30)
control (n=72)
Severity

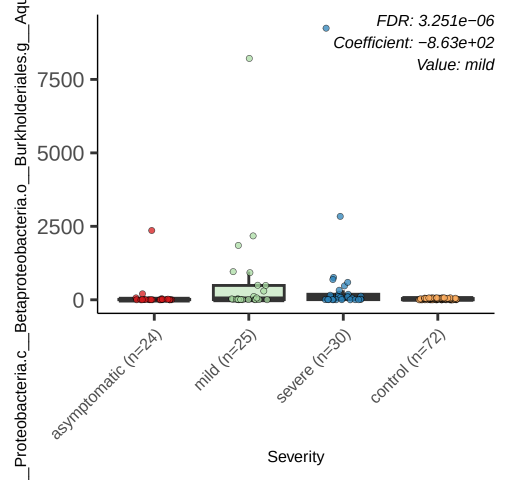
FDR: 3.251e−06
Coefficient: −8.63e+02
Value: mild
7500
5000
2500
0
asymptomatic (n=24)
mild (n=25)
severe (n=30)
control (n=72)
Severity
_Proteobacteria.c__Betaproteobacteria.o__Burkholderiales.g__Aqu

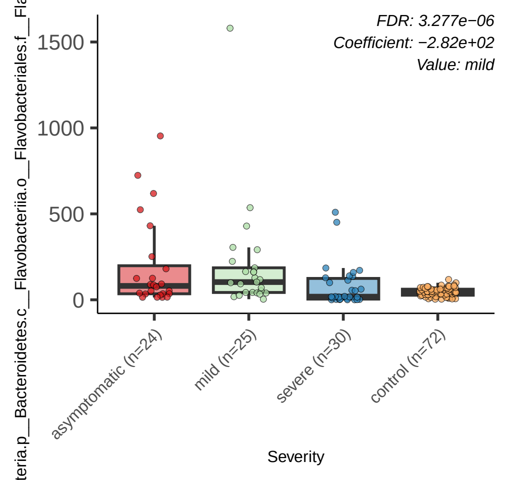

FDR: 3.277e−06
Coefficient: −2.82e+02
Value: mild
1500
1000
500
0
teria.p__Bacteroidetes.c__Flavobacteriia.o__Flavobacteriales.f__Fla
asymptomatic (n=24)
mild (n=25)
severe (n=30)
control (n=72)
Severity

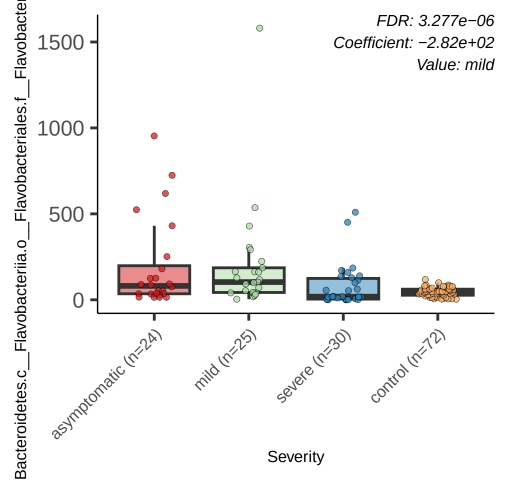
FDR: 3.277e−06
Coefficient: −2.82e+02
Value: mild
Bacteroidetes.c__Flavobacteriia.o__Flavobacteriales.f__Flavobacter
1500
1000
500
0
asymptomatic (n=24)
mild (n=25)
severe (n=30)
control (n=72)
Severity

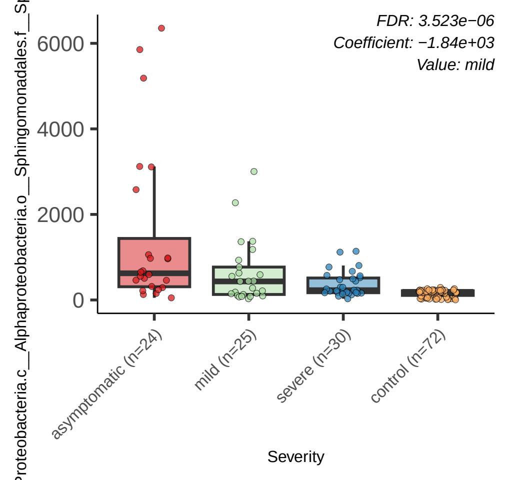

FDR: 3.523e−06
Coefficient: −1.84e+03
Value: mild
6000
4000
2000
0
Proteobacteria.c__Alphaproteobacteria.o__Sphingomonadales.f__Sp
asymptomatic (n=24)
mild (n=25)
severe (n=30)
control (n=72)
Severity

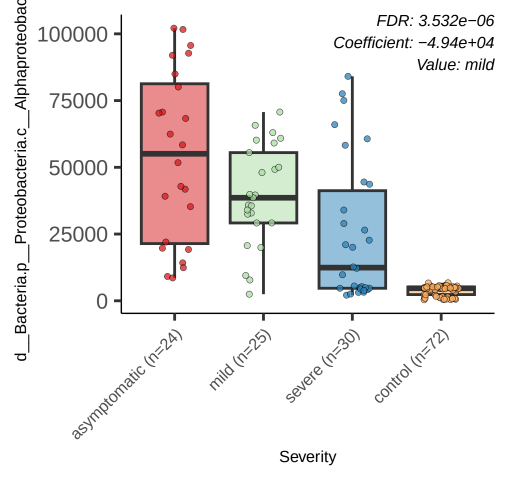
FDR: 3.532e−06
Coefficient: −4.94e+04
Value: mild
d__Bacteria.p__Proteobacteria.c__Alphaproteobac
100000
75000
50000
25000
0
asymptomatic (n=24)
mild (n=25)
severe (n=30)
control (n=72)
Severity

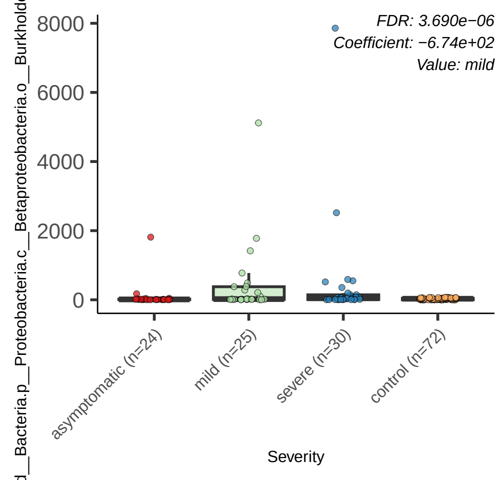

FDR: 3.690e−06
Coefficient: −6.74e+02
Value: mild
d__Bacteria.p__Proteobacteria.c__Betaproteobacteria.o__Burkhold
8000
6000
4000
2000
0
asymptomatic (n=24)
mild (n=25)
severe (n=30)
control (n=72)
Severity

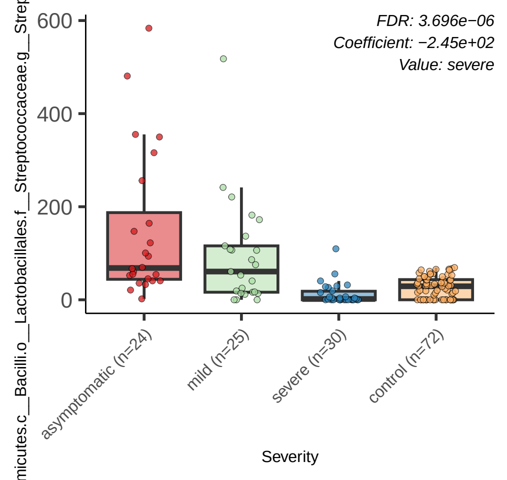
FDR: 3.696e−06
Coefficient: −2.45e+02
Value: severe
micutes.c__Bacilli.o__Lactobacillales.f__Streptococcaceae.g__Strep
600
400
200
0
asymptomatic (n=24)
mild (n=25)
severe (n=30)
control (n=72)
Severity

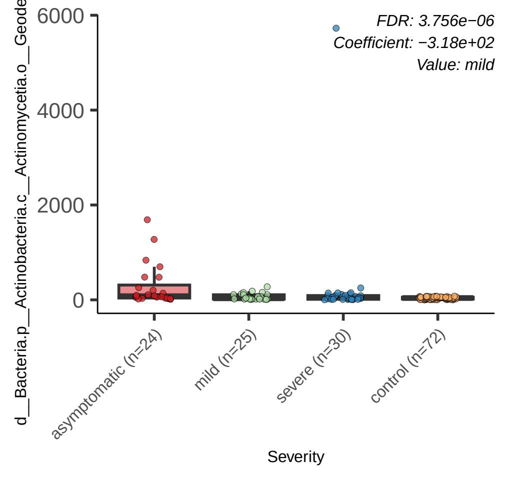
FDR: 3.756e−06
Coefficient: −3.18e+02
Value: mild
6000
4000
2000
0
d__Bacteria.p__Actinobacteria.c__Actinomycetia.o__Geode
asymptomatic (n=24)
mild (n=25)
severe (n=30)
control (n=72)
Severity

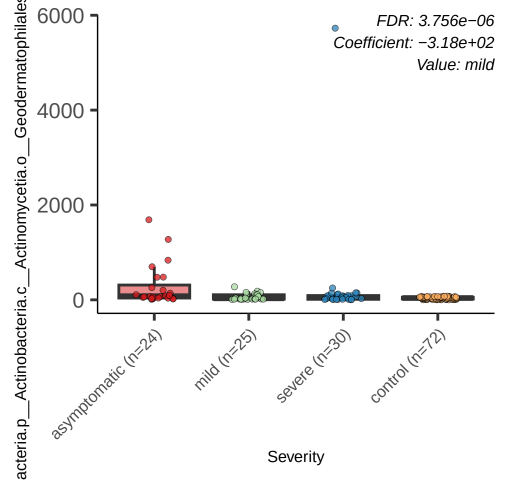

FDR: 3.756e−06
Coefficient: −3.18e+02
Value: mild
6000
4000
2000
0
acteria.p__Actinobacteria.c__Actinomycetia.o__Geodermatophilales
asymptomatic (n=24)
mild (n=25)
severe (n=30)
control (n=72)
Severity

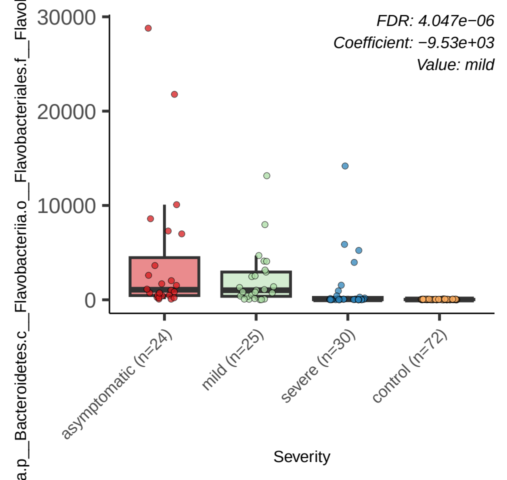

FDR: 4.047e−06
Coefficient: −9.53e+03
Value: mild
30000
20000
10000
0
a.p__Bacteroidetes.c__Flavobacteriia.o__Flavobacteriales.f__Flavo
asymptomatic (n=24)
mild (n=25)
severe (n=30)
control (n=72)
Severity

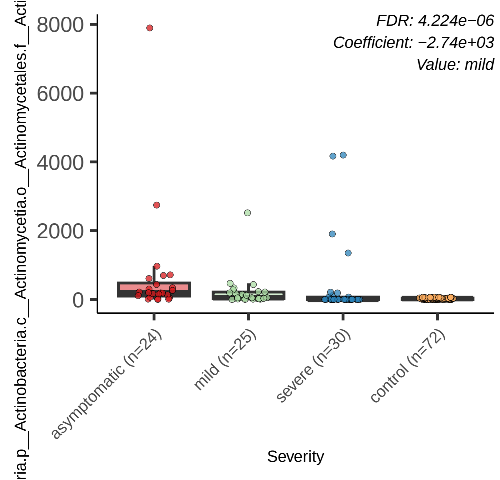

FDR: 4.224e−06
Coefficient: −2.74e+03
Value: mild
8000
6000
4000
2000
0
asymptomatic (n=24)
mild (n=25)
severe (n=30)
control (n=72)
Severity

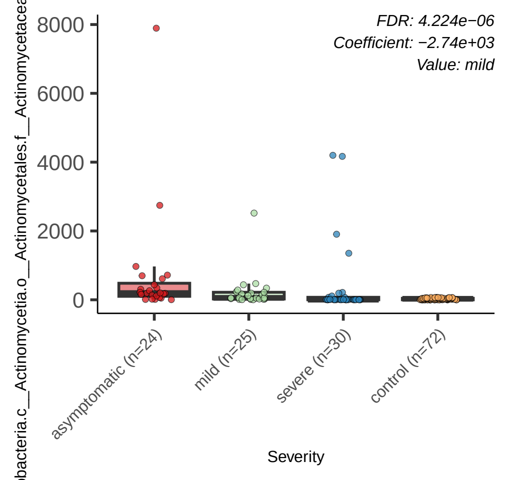
FDR: 4.224e−06
Coefficient: −2.74e+03
Value: mild
8000
6000
4000
2000
0
obacteria.c__Actinomycetia.o__Actinomycetales.f__Actinomycetacea
asymptomatic (n=24)
mild (n=25)
severe (n=30)
control (n=72)
Severity

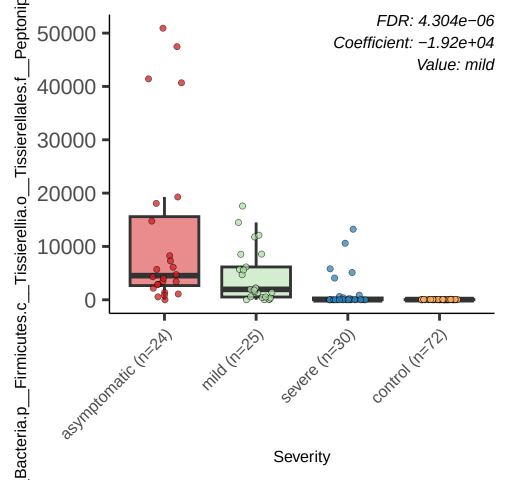

FDR: 4.304e−06
Coefficient: −1.92e+04
Value: mild
Bacteria.p__Firmicutes.c__Tissierellia.o__Tissierellales.f__Peptonip
50000
40000
30000
20000
10000
0
asymptomatic (n=24)
mild (n=25)
severe (n=30)
control (n=72)
Severity

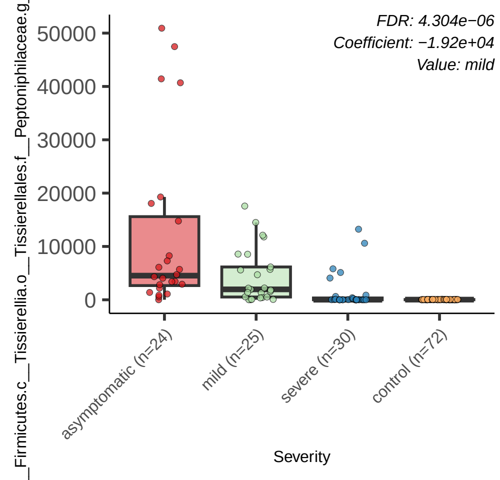

FDR: 4.304e−06
Coefficient: −1.92e+04
Value: mild
_Firmicutes.c__Tissierellia.o__Tissierellales.f__Peptoniphilaceae.g_
50000
40000
30000
20000
10000
0
asymptomatic (n=24)
mild (n=25)
severe (n=30)
control (n=72)
Severity

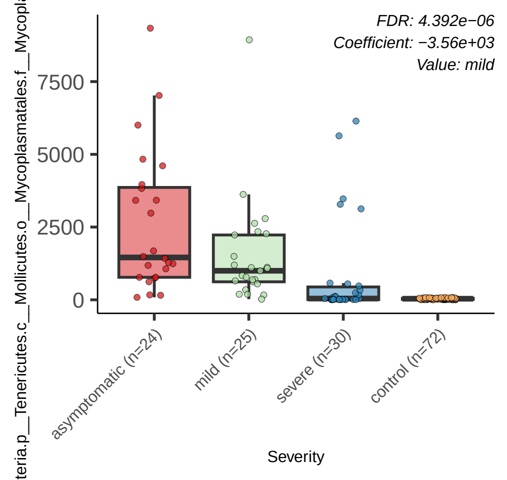

FDR: 4.392e−06
Coefficient: −3.56e+03
Value: mild
teria.p__Tenericutes.c__Mollicutes.o__Mycoplasmatales.f__Mycopla
7500
5000
2500
0
asymptomatic (n=24)
mild (n=25)
severe (n=30)
control (n=72)
Severity

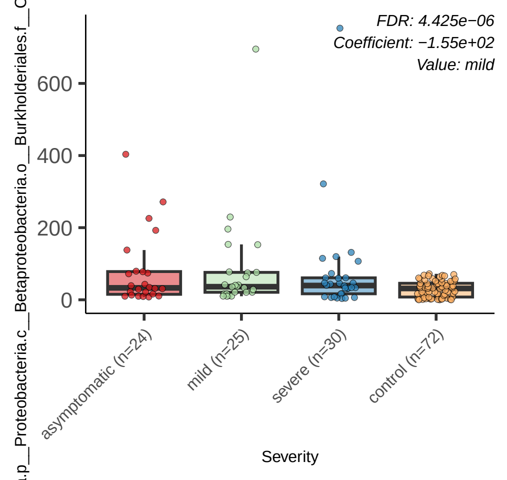

FDR: 4.425e−06
Coefficient: −1.55e+02
Value: mild
p__Proteobacteria.c__Betaproteobacteria.o__Burkholderiales.f__C
600
400
200
0
asymptomatic (n=24)
mild (n=25)
severe (n=30)
control (n=72)
Severity

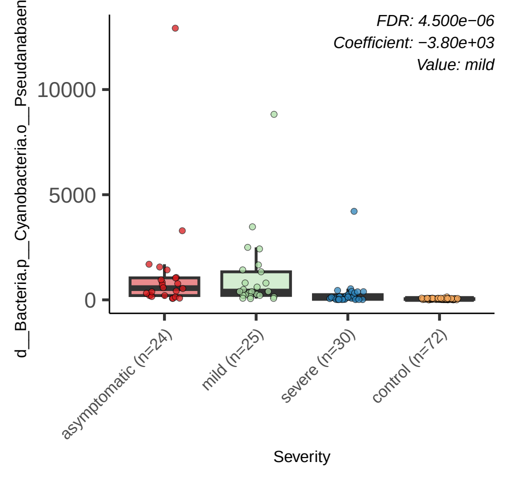

FDR: 4.500e−06
Coefficient: −3.80e+03
Value: mild
d__Bacteria.p__Cyanobacteria.o__Pseudanabaen
10000
5000
0
asymptomatic (n=24)
mild (n=25)
severe (n=30)
control (n=72)
Severity

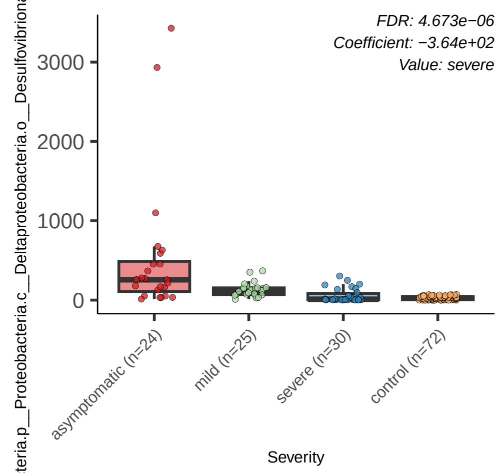

FDR: 4.673e−06
Coefficient: −3.64e+02
Value: severe
3000
2000
1000
0
cteria.p__Proteobacteria.c__Deltaproteobacteria.o__Desulfovibriona
asymptomatic (n=24)
mild (n=25)
severe (n=30)
control (n=72)
Severity

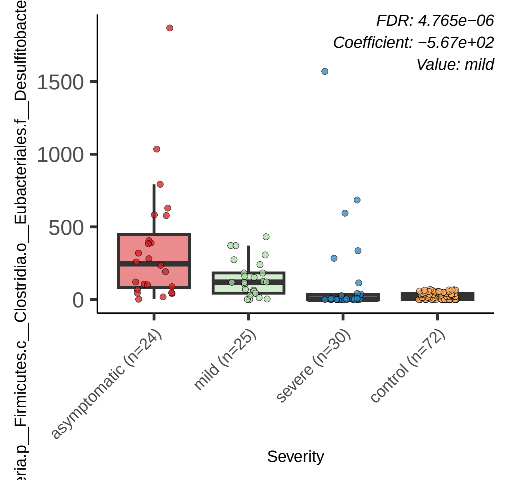

FDR: 4.765e−06
Coefficient: −5.67e+02
Value: mild
1500
1000
500
0
eria.p__Firmicutes.c__Clostridia.o__Eubacteriales.f__Desulfitobacte
asymptomatic (n=24)
mild (n=25)
severe (n=30)
control (n=72)
Severity

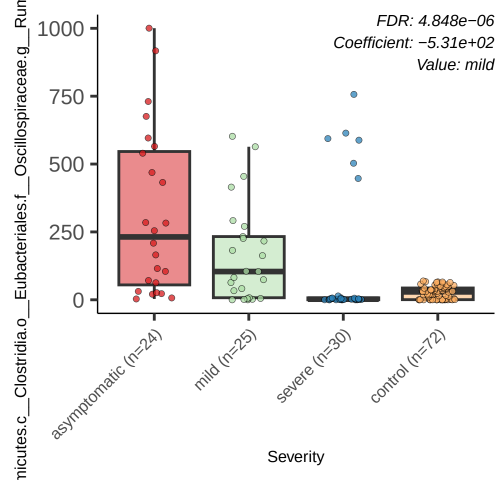

FDR: 4.848e−06
Coefficient: −5.31e+02
Value: mild
1000
750
500
250
0
micutes.c__Clostridia.o__Eubacteriales.f__Oscillospiraceae.g__Rum
asymptomatic (n=24)
mild (n=25)
severe (n=30)
control (n=72)
Severity

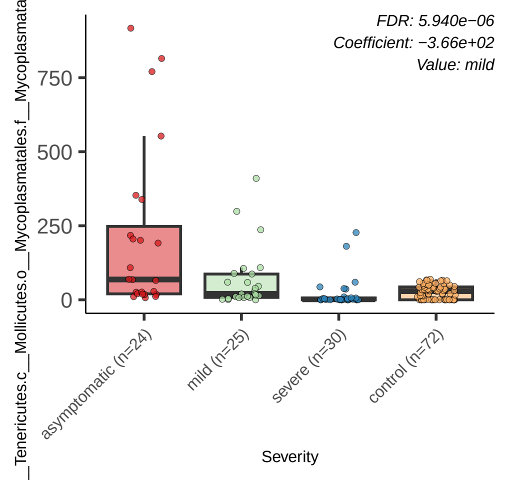
FDR: 5.940e−06
Coefficient: −3.66e+02
Value: mild
Tenericutes.c__Mollicutes.o__Mycoplasmatales.f__Mycoplasmata
0
250
500
750
asymptomatic (n=24)
mild (n=25)
severe (n=30)
control (n=72)
Severity

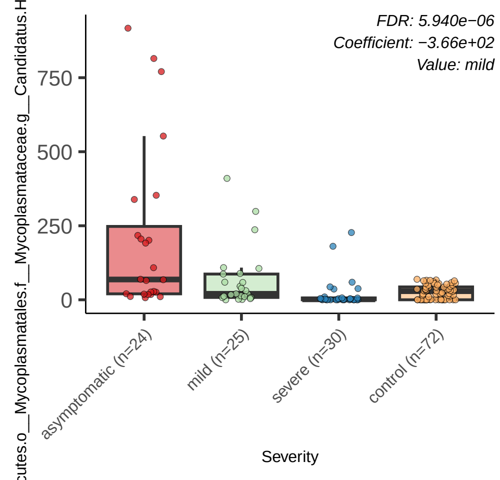

FDR: 5.940e−06
Coefficient: −3.66e+02
Value: mild
cutes.o__Mycoplasmatales.f__Mycoplasmataceae.g__Candidatus.H
750
500
250
0
asymptomatic (n=24)
mild (n=25)
severe (n=30)
control (n=72)
Severity

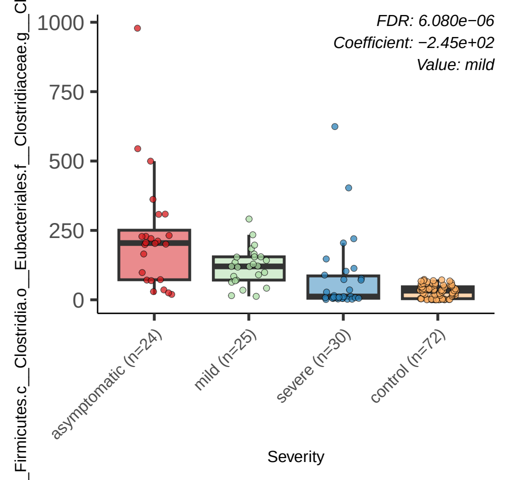

FDR: 6.080e−06
Coefficient: −2.45e+02
Value: mild
1000
750
500
250
0
Firmicutes.c__Clostridia.o__Eubacteriales.f__Clostridiaceae.g__Cl
asymptomatic (n=24)
mild (n=25)
severe (n=30)
control (n=72)
Severity

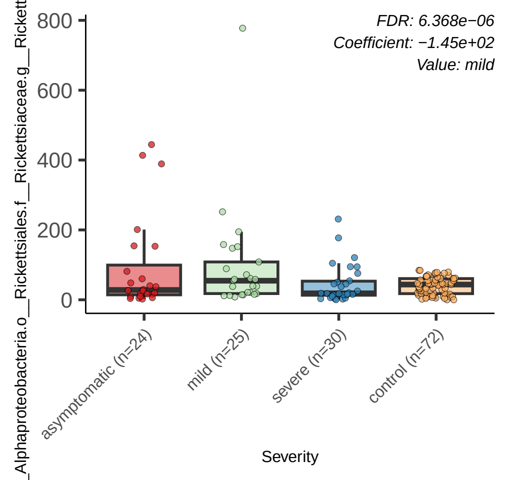

FDR: 6.368e−06
Coefficient: −1.45e+02
Value: mild
Alphaproteobacteria.o__Rickettsiales.f__Rickettsiaceae.g__Rickett
800
600
400
200
0
asymptomatic (n=24)
mild (n=25)
severe (n=30)
control (n=72)
Severity

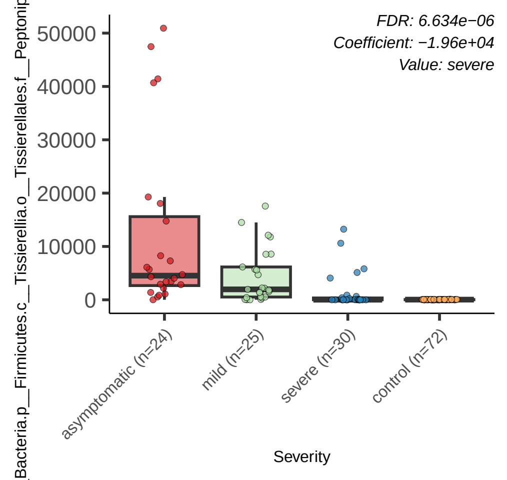

FDR: 6.634e−06
Coefficient: −1.96e+04
Value: severe
50000
40000
30000
20000
10000
0
Bacteria.p__Firmicutes.c__Tissierellia.o__Tissierellales.f__Peptonip
asymptomatic (n=24)
mild (n=25)
severe (n=30)
control (n=72)
Severity

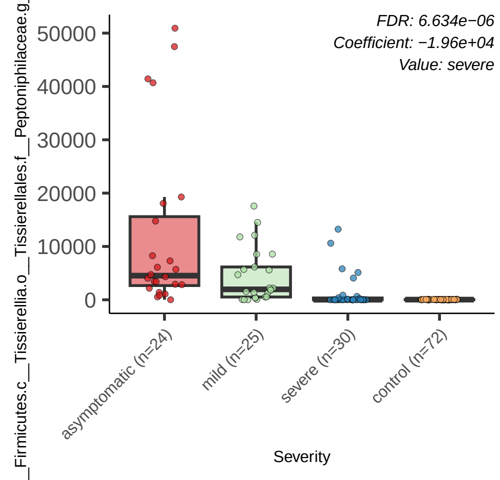
FDR: 6.634e−06
Coefficient: −1.96e+04
Value: severe
Firmicutes.c__Tissierellia.o__Tissierellales.f__Peptoniphilaceae.g_
50000
40000
30000
20000
10000
0
asymptomatic (n=24)
mild (n=25)
severe (n=30)
control (n=72)
Severity

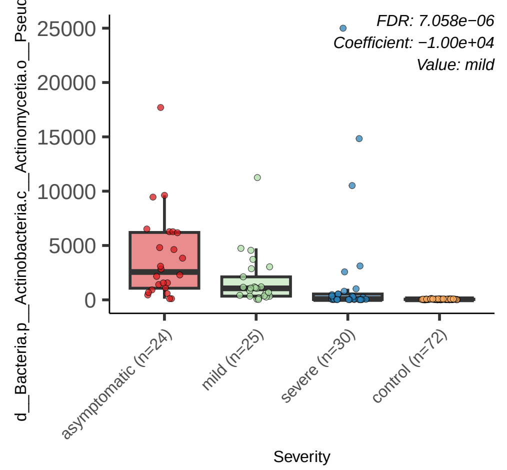
FDR: 7.058e−06
Coefficient: −1.00e+04
Value: mild
25000
20000
15000
10000
5000
0
d__Bacteria.p__Actinobacteria.c__Actinomycetia.o__Pseud
asymptomatic (n=24)
mild (n=25)
severe (n=30)
control (n=72)
Severity

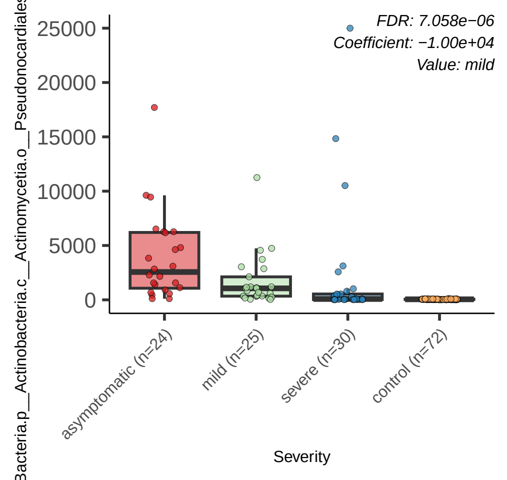

FDR: 7.058e−06
Coefficient: −1.00e+04
Value: mild
Bacteria.p__Actinobacteria.c__Actinomycetia.o__Pseudonocardiales
25000
20000
15000
10000
5000
0
asymptomatic (n=24)
mild (n=25)
severe (n=30)
control (n=72)
Severity

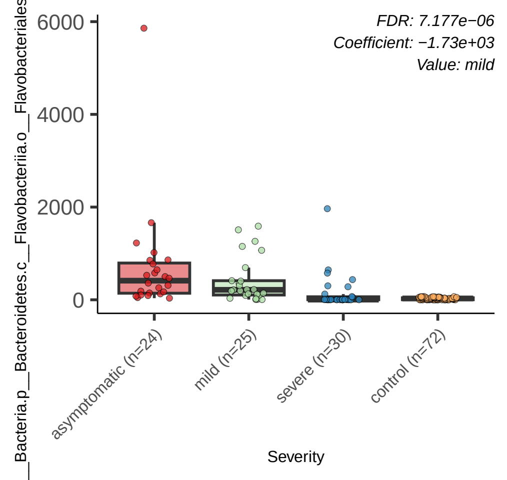
FDR: 7.177e−06
Coefficient: −1.73e+03
Value: mild
Bacteria.p__Bacteroidetes.c__Flavobacteriia.o__Flavobacteriales
6000
4000
2000
0
asymptomatic (n=24)
mild (n=25)
severe (n=30)
control (n=72)
Severity

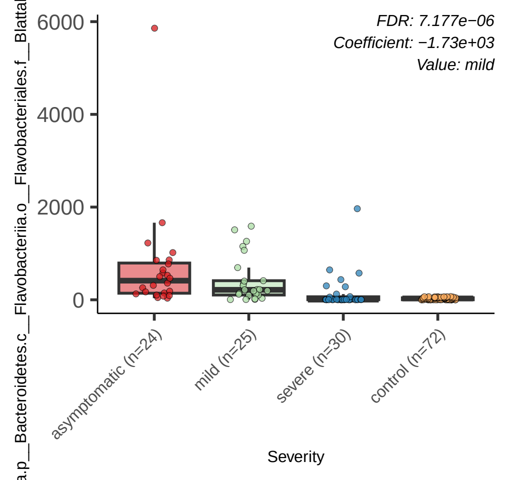

FDR: 7.177e−06
Coefficient: −1.73e+03
Value: mild
6000
4000
2000
0
a.p__Bacteroidetes.c__Flavobacteriia.o__Flavobacteriales.f__Blattab
asymptomatic (n=24)
mild (n=25)
severe (n=30)
control (n=72)
Severity

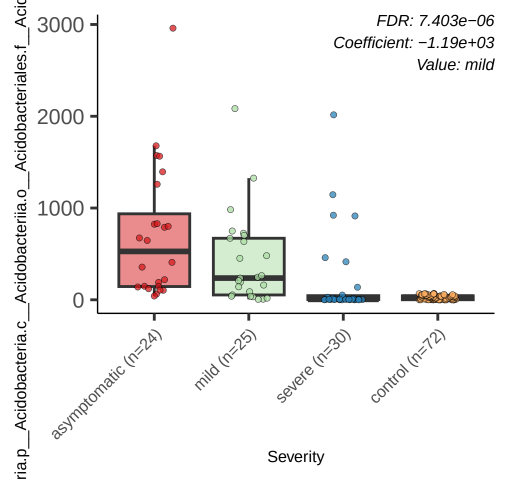

FDR: 7.403e−06
Coefficient: −1.19e+03
Value: mild
ria.p__Acidobacteria.c__Acidobacteria.o__Acidobacteriales.f__Acid
3000
2000
1000
0
asymptomatic (n=24)
mild (n=25)
severe (n=30)
control (n=72)
Severity

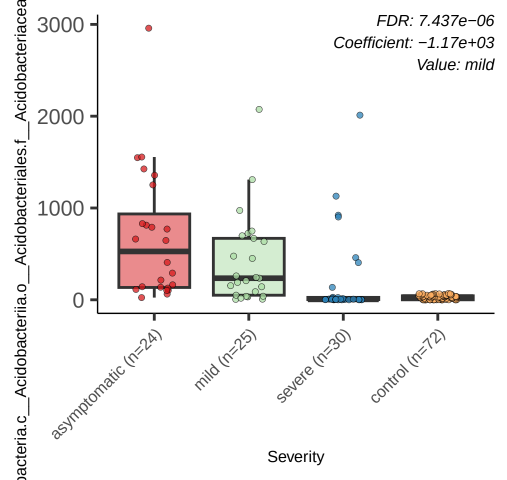
FDR: 7.437e−06
Coefficient: −1.17e+03
Value: mild
3000
2000
1000
0
bacteria.c__Acidobacteriia.o__Acidobacteriales.f__Acidobacteriacea
asymptomatic (n=24)
mild (n=25)
severe (n=30)
control (n=72)
Severity

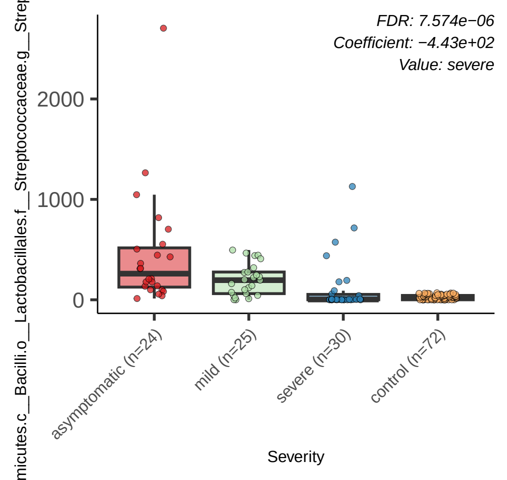

FDR: 7.574e−06
Coefficient: −4.43e+02
Value: severe
micutes.c__Bacilli.o__Lactobacillales.f__Streptococcaceae.g__Strep
2000
1000
0
asymptomatic (n=24)
mild (n=25)
severe (n=30)
control (n=72)
Severity

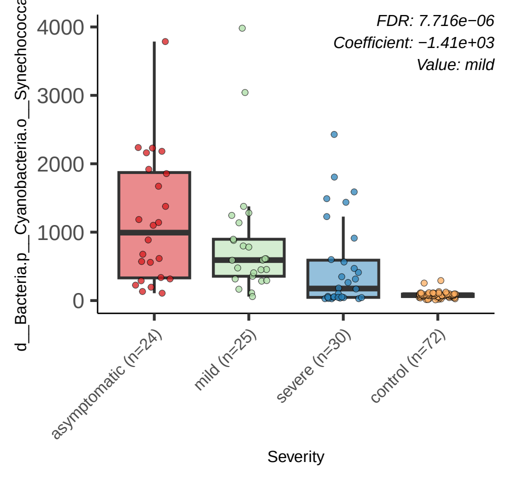

FDR: 7.716e−06
Coefficient: −1.41e+03
Value: mild
d__Bacteria.p__Cyanobacteria.o__Synechococca
4000
3000
2000
1000
0
asymptomatic (n=24)
mild (n=25)
severe (n=30)
control (n=72)
Severity

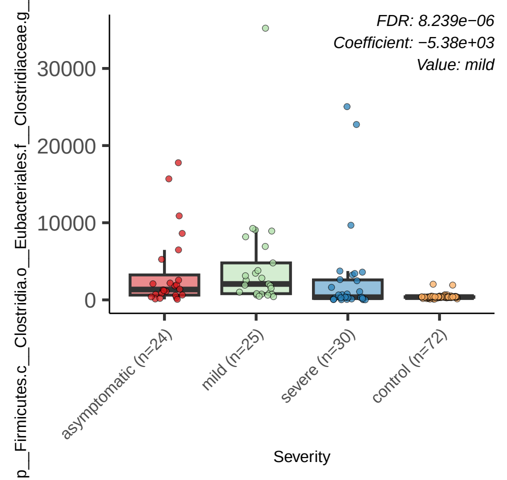

FDR: 8.239e−06
Coefficient: −5.38e+03
Value: mild
p__Firmicutes.c__Clostridia.o__Eubacteriales.f__Clostridiaceae.g__
30000
20000
10000
0
asymptomatic (n=24)
mild (n=25)
severe (n=30)
control (n=72)
Severity

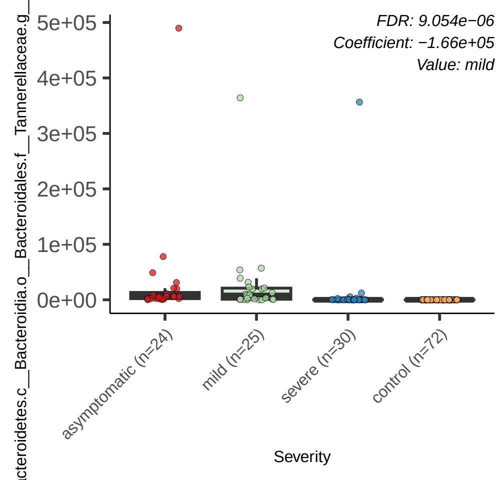
FDR: 9.054e−06
Coefficient: −1.66e+05
Value: mild
5e+05
4e+05
3e+05
2e+05
1e+05
0e+00
Bacteroidetes.c__Bacteroidia.o__Bacteroidales.f__Tannerellaceae.g__
asymptomatic (n=24)
mild (n=25)
severe (n=30)
control (n=72)
Severity

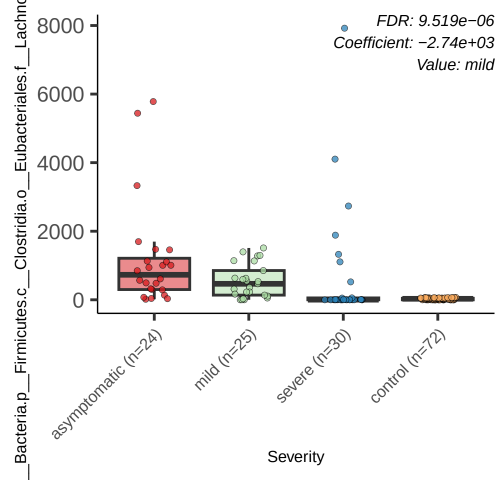

FDR: 9.519e−06
Coefficient: −2.74e+03
Value: mild
8000
6000
4000
2000
0
Bacteria.p__Firmicutes.c__Clostridia.o__Eubacteriales.f__Lachno
asymptomatic (n=24)
mild (n=25)
severe (n=30)
control (n=72)
Severity

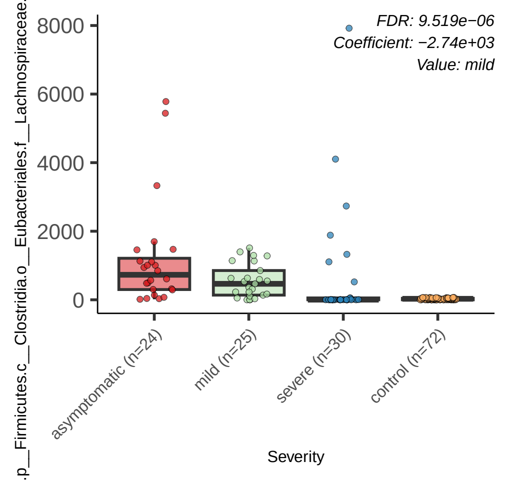

FDR: 9.519e−06
Coefficient: −2.74e+03
Value: mild
p__Firmicutes.c__Clostridia.o__Eubacteriales.f__Lachnospiraceae.
8000
6000
4000
2000
0
asymptomatic (n=24)
mild (n=25)
severe (n=30)
control (n=72)
Severity

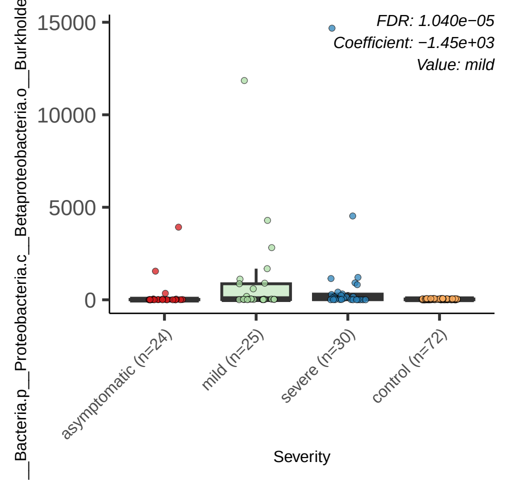
FDR: 1.040e−05
Coefficient: −1.45e+03
Value: mild
15000
10000
5000
0
_Bacteria.p__Proteobacteria.c__Betaproteobacteria.o__Burkholde
asymptomatic (n=24)
mild (n=25)
severe (n=30)
control (n=72)
Severity

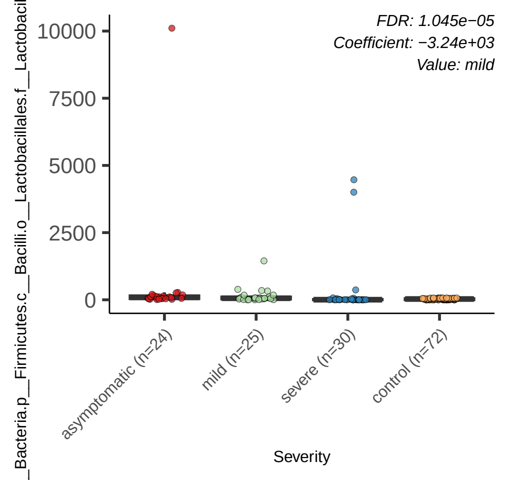

FDR: 1.045e−05
Coefficient: −3.24e+03
Value: mild
10000
7500
5000
2500
0
asymptomatic (n=24)
mild (n=25)
severe (n=30)
control (n=72)
Severity
Bacteria.p__Firmicutes.c__Bacilli.o__Lactobacillales.f__Lactobacil

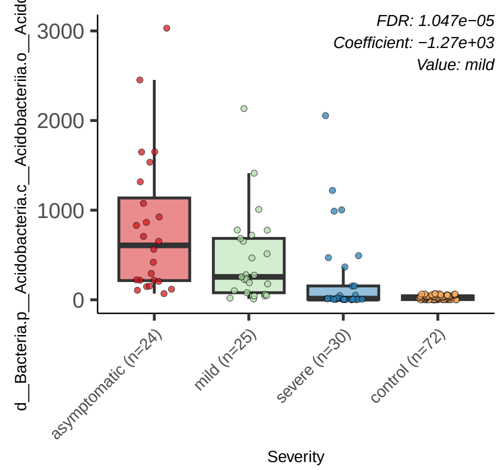
FDR: 1.047e−05
Coefficient: −1.27e+03
Value: mild
d__Bacteria.p__Acidobacteria.c__Acidobacteria.o__Acido
3000
2000
1000
0
asymptomatic (n=24)
mild (n=25)
severe (n=30)
control (n=72)
Severity

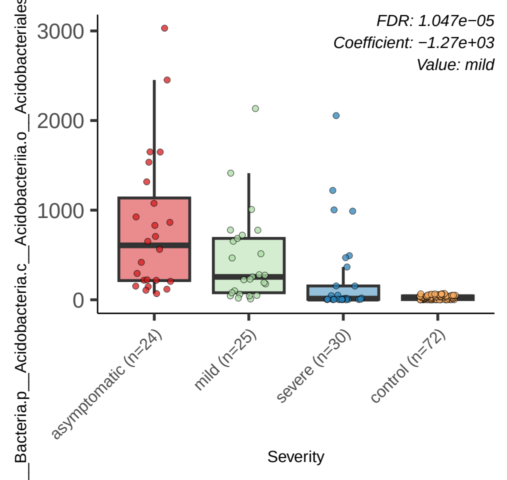

FDR: 1.047e−05
Coefficient: −1.27e+03
Value: mild
3000
2000
1000
0
Bacteria.p__Acidobacteria.c__Acidobacteriia.o__Acidobacteriales
asymptomatic (n=24)
mild (n=25)
severe (n=30)
control (n=72)
Severity

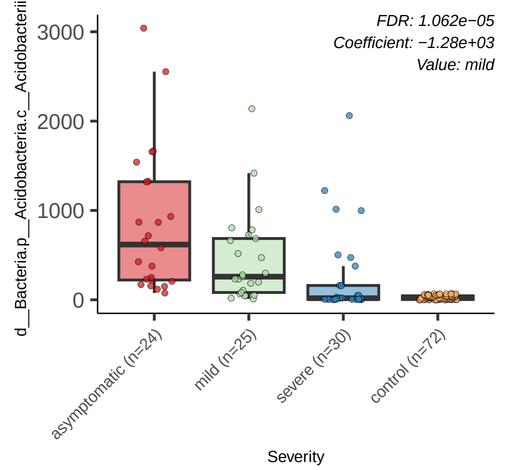
FDR: 1.062e−05
Coefficient: −1.28e+03
Value: mild
d__Bacteria.p__Acidobacteria.c__Acidobacteriia
3000
2000
1000
0
asymptomatic (n=24)
mild (n=25)
severe (n=30)
control (n=72)
Severity

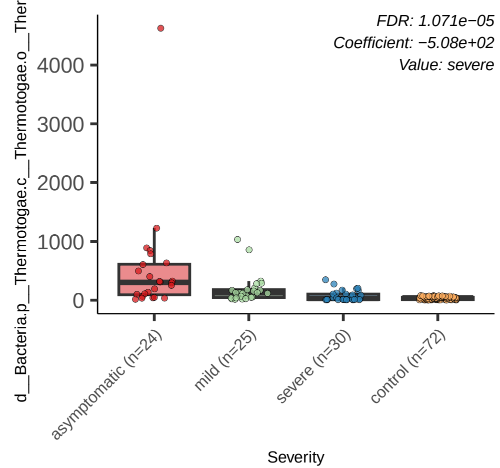

FDR: 1.071e−05
Coefficient: −5.08e+02
Value: severe
d__Bacteria.p__Thermotogae.c__Thermotogae.o__Ther
4000
3000
2000
1000
0
asymptomatic (n=24)
mild (n=25)
severe (n=30)
control (n=72)
Severity

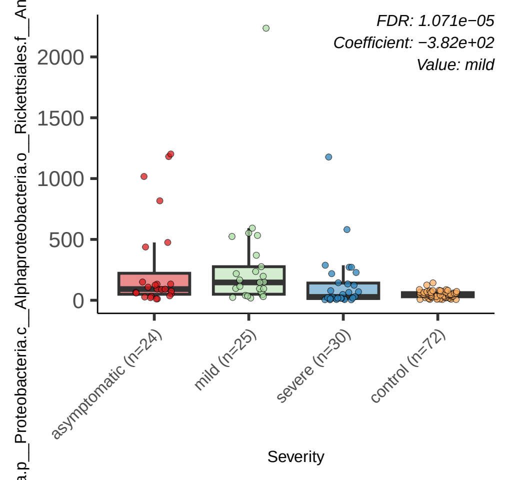

FDR: 1.071e−05
Coefficient: −3.82e+02
Value: mild
a.p__Proteobacteria.c__Alphaproteobacteria.o__Rickettsiales.f__An
2000
1500
1000
500
0
asymptomatic (n=24)
mild (n=25)
severe (n=30)
control (n=72)
Severity

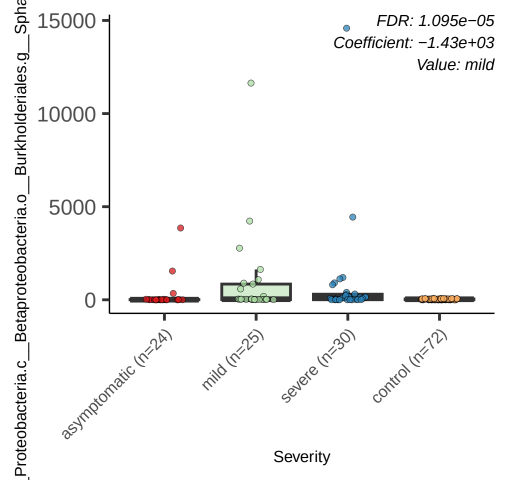

FDR: 1.095e−05
Coefficient: −1.43e+03
Value: mild
15000
10000
5000
0
Proteobacteria.c__Betaproteobacteria.o__Burkholderiales.g__Spha
asymptomatic (n=24)
mild (n=25)
severe (n=30)
control (n=72)
Severity

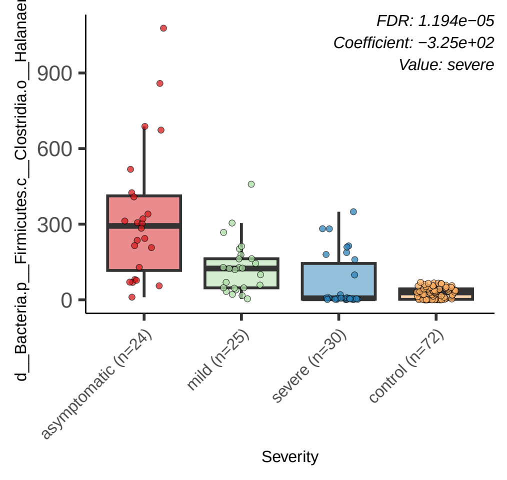

FDR: 1.194e−05
Coefficient: −3.25e+02
Value: severe
d__Bacteria.p__Firmicutes.c__Clostridia.o__Halanaer
900
600
300
0
asymptomatic (n=24)
mild (n=25)
severe (n=30)
control (n=72)
Severity

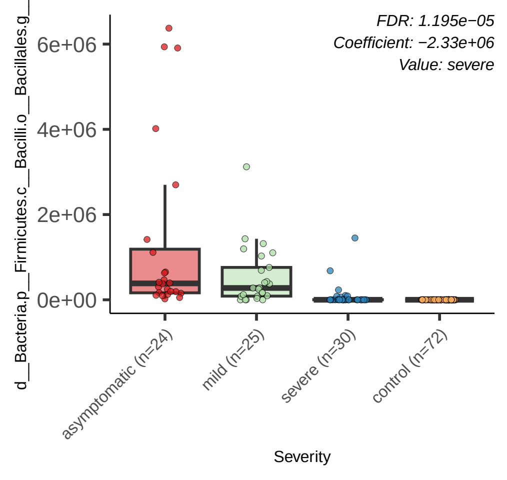

FDR: 1.195e−05
Coefficient: −2.33e+06
Value: severe
d__Bacteria.p__Firmicutes.c__Bacilli.o__Bacillales.g__
6e+06
4e+06
2e+06
0e+00
asymptomatic (n=24)
mild (n=25)
severe (n=30)
control (n=72)
Severity

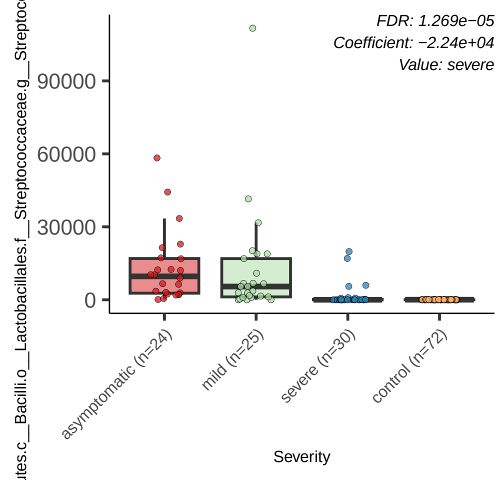

FDR: 1.269e−05
Coefficient: −2.24e+04
Value: severe
90000
60000
30000
0
asymptomatic (n=24)
mild (n=25)
severe (n=30)
control (n=72)
Severity
utes.c__Bacilli.o__Lactobacillales.f__Streptococcaceae.g__Streptoc

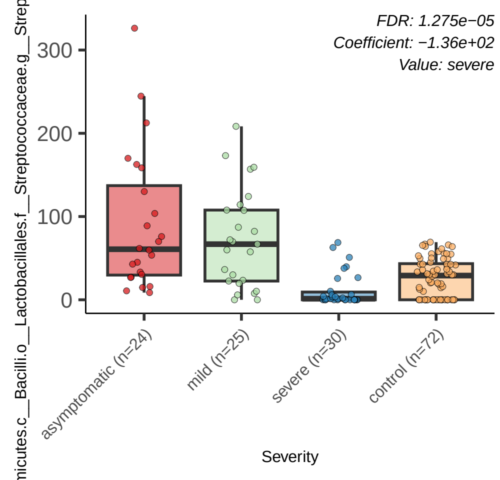

FDR: 1.275e−05
Coefficient: −1.36e+02
Value: severe
300
200
100
0
micutes.c__Bacilli.o__Lactobacillales.f__Streptococcaceae.g__Strep
asymptomatic (n=24)
mild (n=25)
severe (n=30)
control (n=72)
Severity

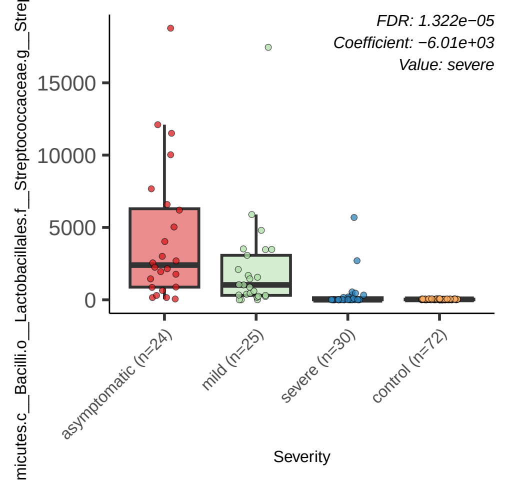
FDR: 1.322e−05
Coefficient: −6.01e+03
Value: severe
micutes.c__Bacilli.o__Lactobacillales.f__Streptococcaceae.g__Strep
15000
10000
5000
0
asymptomatic (n=24)
mild (n=25)
severe (n=30)
control (n=72)
Severity

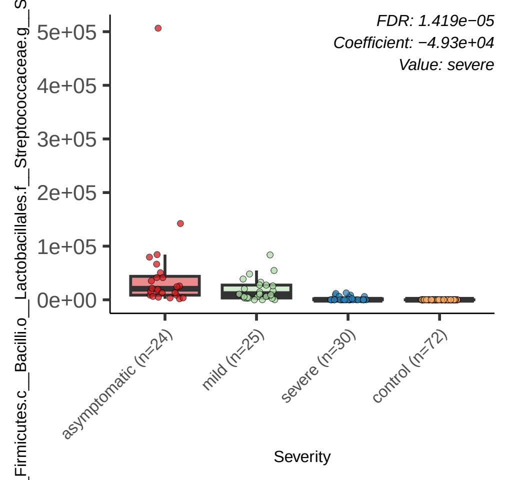

FDR: 1.419e−05
Coefficient: −4.93e+04
Value: severe
Firmicutes.c__Bacilli.o__Lactobacillales.f__Streptococcaceae.g__S
5e+05
4e+05
3e+05
2e+05
1e+05
0e+00
asymptomatic (n=24)
mild (n=25)
severe (n=30)
control (n=72)
Severity

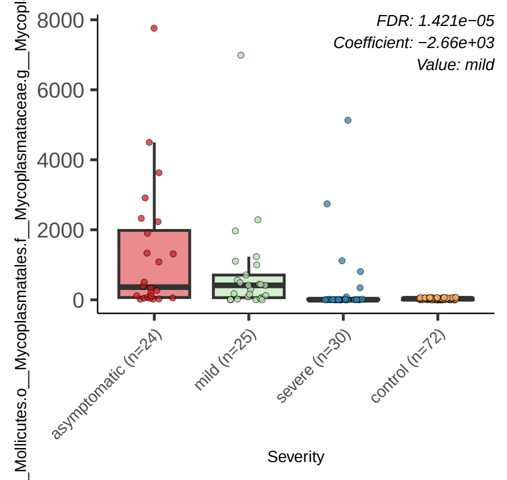

FDR: 1.421e−05
Coefficient: −2.66e+03
Value: mild
8000
6000
4000
2000
0
_Mollicutes.o__Mycoplasmatales.f__Mycoplasmataceae.g__Mycopl
asymptomatic (n=24)
mild (n=25)
severe (n=30)
control (n=72)
Severity

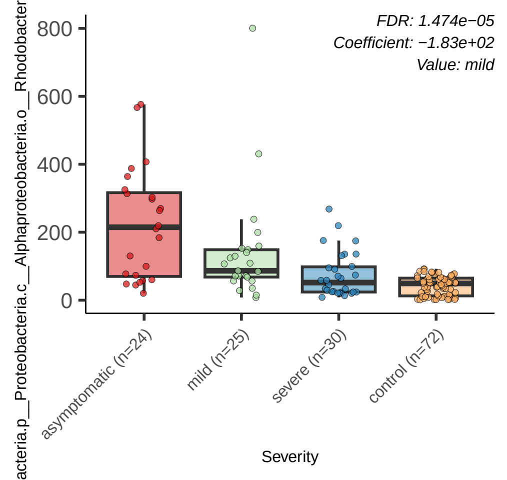

FDR: 1.474e−05
Coefficient: −1.83e+02
Value: mild
800
600
400
200
0
acteria.p__Proteobacteria.c__Alphaproteobacteria.o__Rhodobacter
asymptomatic (n=24)
mild (n=25)
severe (n=30)
control (n=72)
Severity

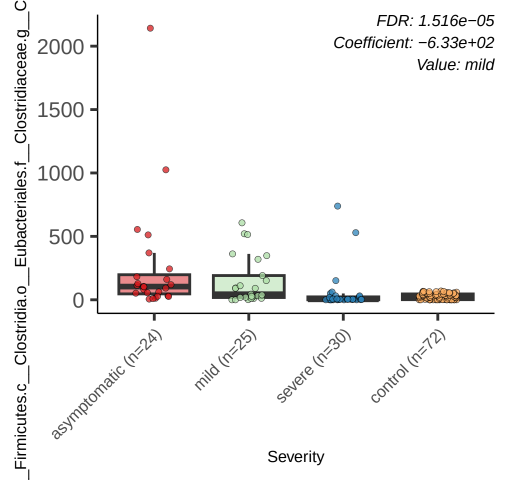
FDR: 1.516e−05
Coefficient: −6.33e+02
Value: mild
Firmicutes.c__Clostridia.o__Eubacteriales.f__Clostridiaceae.g__C
2000
1500
1000
500
0
asymptomatic (n=24)
mild (n=25)
severe (n=30)
control (n=72)
Severity

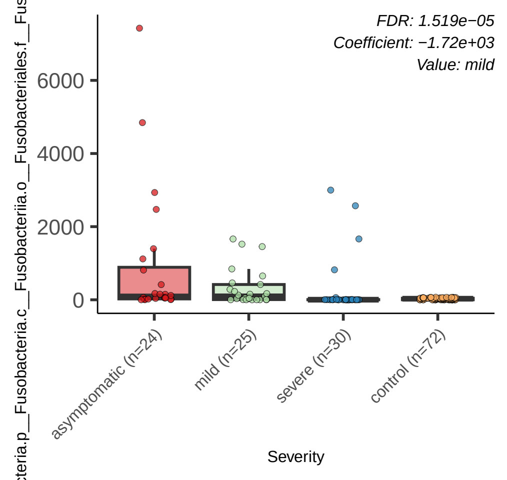

FDR: 1.519e−05
Coefficient: −1.72e+03
Value: mild
6000
4000
2000
0
teria.p__Fusobacteria.c__Fusobacteriia.o__Fusobacteriales.f__Fus
asymptomatic (n=24)
mild (n=25)
severe (n=30)
control (n=72)
Severity

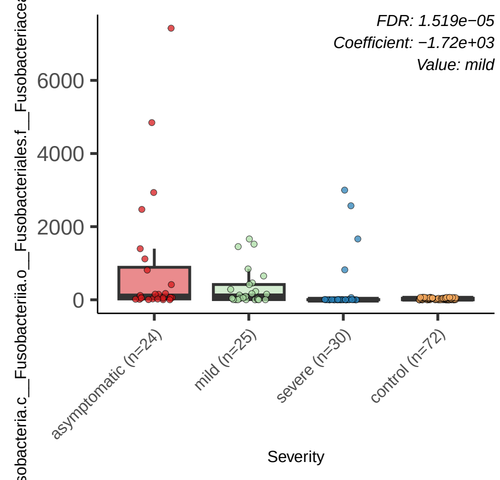

FDR: 1.519e−05
Coefficient: −1.72e+03
Value: mild
6000
4000
2000
0
asymptomatic (n=24)
mild (n=25)
severe (n=30)
control (n=72)
Severity
sobacteria.c__Fusobacteriia.o__Fusobacteriales.f__Fusobacteriacea

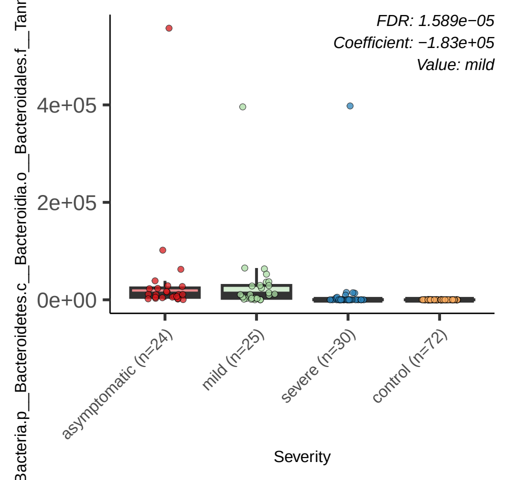
FDR: 1.589e−05
Coefficient: −1.83e+05
Value: mild
4e+05
2e+05
0e+00
Bacteria.p__Bacteroidetes.c__Bacteroidia.o__Bacteroidales.f__Tann
asymptomatic (n=24)
mild (n=25)
severe (n=30)
control (n=72)
Severity

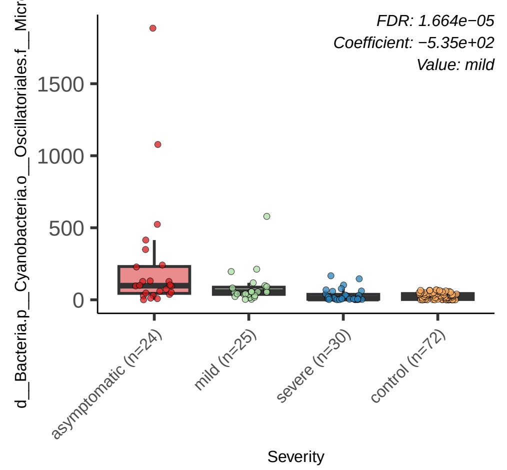

FDR: 1.664e−05
Coefficient: −5.35e+02
Value: mild
d__Bacteria.p__Cyanobacteria.o__Oscillatoriales.f__Micr
0
500
1000
1500
asymptomatic (n=24)
mild (n=25)
severe (n=30)
control (n=72)
Severity

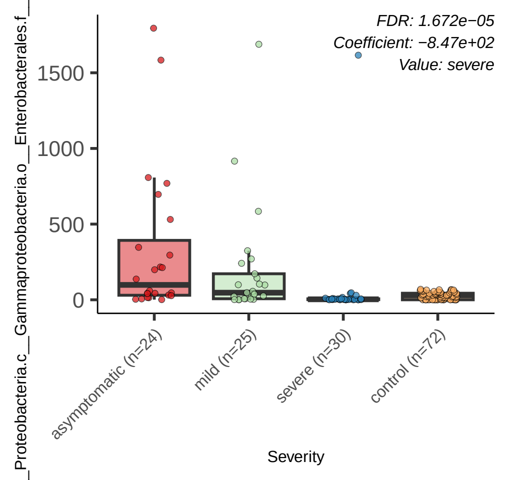

FDR: 1.672e−05
Coefficient: −8.47e+02
Value: severe
Proteobacteria.c__Gammaproteobacteria.o__Enterobacterales.f
1500
1000
500
0
asymptomatic (n=24)
mild (n=25)
severe (n=30)
control (n=72)
Severity

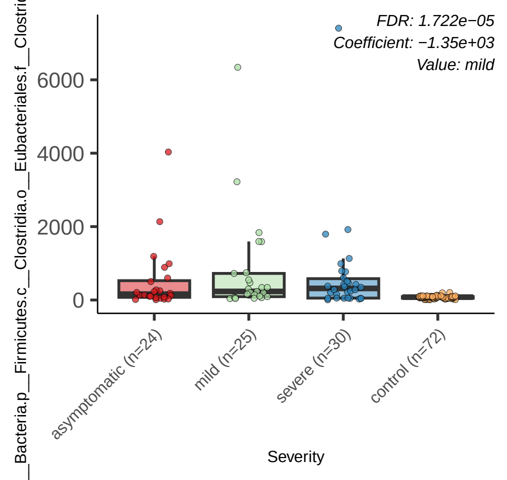

FDR: 1.722e−05
Coefficient: −1.35e+03
Value: mild
6000
4000
2000
0
Bacteria.p__Firmicutes.c__Clostridia.o__Eubacteriales.f__Clostrid
asymptomatic (n=24)
mild (n=25)
severe (n=30)
control (n=72)
Severity

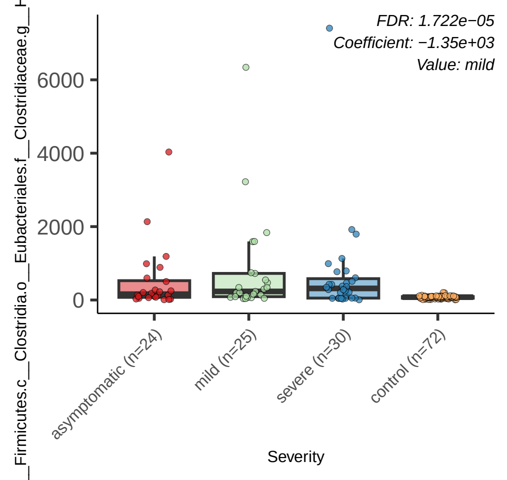
FDR: 1.722e−05
Coefficient: −1.35e+03
Value: mild
6000
4000
2000
0
asymptomatic (n=24)
mild (n=25)
severe (n=30)
control (n=72)
Severity
Firmicutes.c__Clostridia.o__Eubacteriales.f__Clostridiaceae.g__

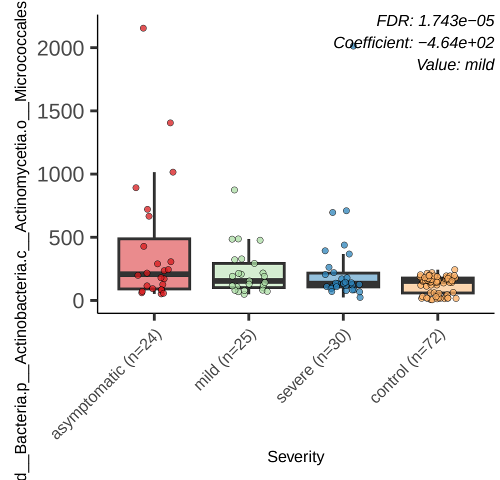

FDR: 1.743e−05
Coefficient: −4.64e+02
Value: mild
d__Bacteria.p__Actinobacteria.c__Actinomycetia.o__Micrococcales
2000
1500
1000
500
0
asymptomatic (n=24)
mild (n=25)
severe (n=30)
control (n=72)
Severity

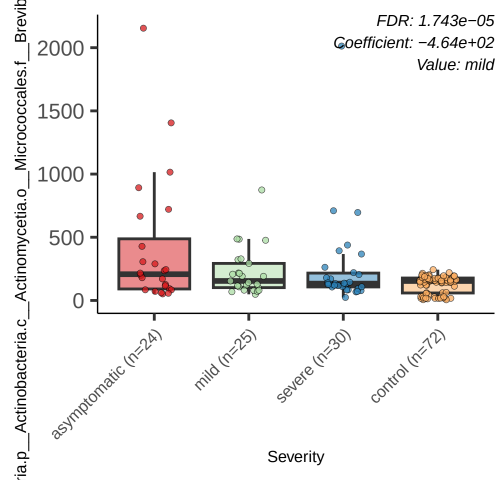
FDR: 1.743e−05
Coefficient: −4.64e+02
Value: mild
2000
1500
1000
500
0
asymptomatic (n=24)
mild (n=25)
severe (n=30)
control (n=72)
Severity

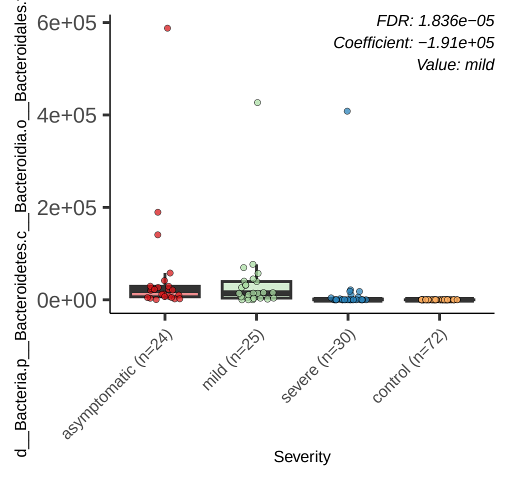

FDR: 1.836e−05
Coefficient: −1.91e+05
Value: mild
6e+05
4e+05
2e+05
0e+00
d__Bacteria.p__Bacteroidetes.c__Bacteroidia.o__Bacteroidales.f
asymptomatic (n=24)
mild (n=25)
severe (n=30)
control (n=72)
Severity

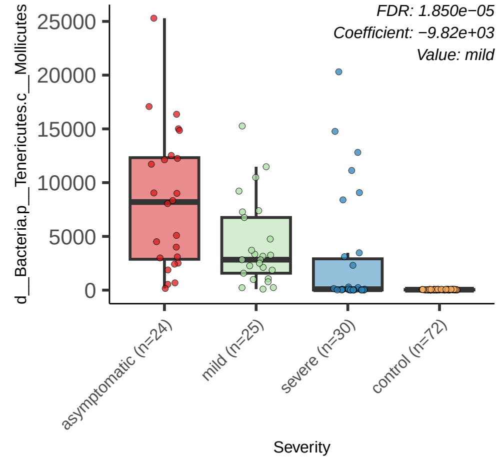
FDR: 1.850e−05
Coefficient: −9.82e+03
Value: mild
d__Bacteria.p__Tenericutes.c__Mollicutes
25000
20000
15000
10000
5000
0
asymptomatic (n=24)
mild (n=25)
severe (n=30)
control (n=72)
Severity

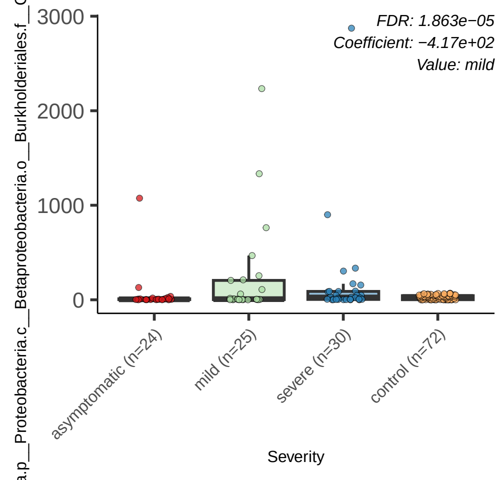

FDR: 1.863e−05
Coefficient: −4.17e+02
Value: mild
3000
2000
1000
0
a.p__Proteobacteria.c__Betaproteobacteria.o__Burkholderiales.f__C
asymptomatic (n=24)
mild (n=25)
severe (n=30)
control (n=72)
Severity

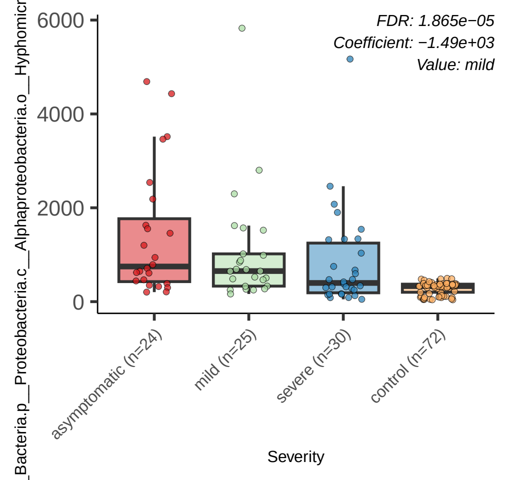
FDR: 1.865e−05
Coefficient: −1.49e+03
Value: mild
6000
4000
2000
0
Bacteria.p__Proteobacteria.c__Alphaproteobacteria.o__Hyphomicr
asymptomatic (n=24)
mild (n=25)
severe (n=30)
control (n=72)
Severity

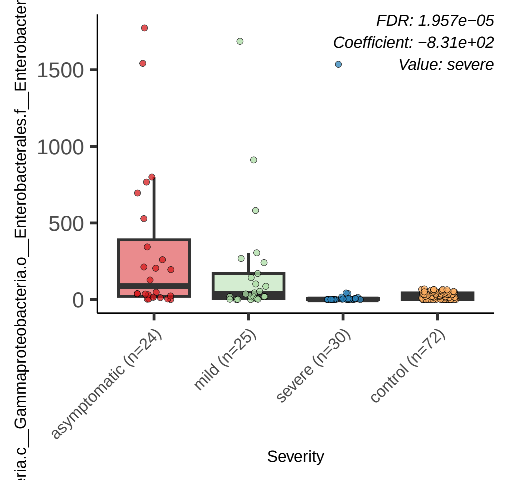

FDR: 1.957e−05
Coefficient: −8.31e+02
Value: severe
1500
1000
500
0
ria.c__Gammaproteobacteria.o__Enterobacterales.f__Enterobacter
asymptomatic (n=24)
mild (n=25)
severe (n=30)
control (n=72)
Severity

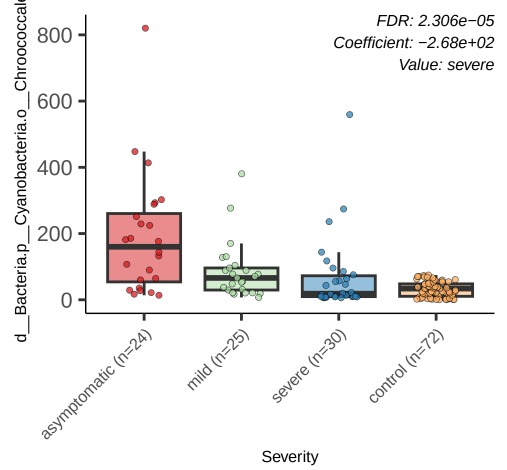

FDR: 2.306e−05
Coefficient: −2.68e+02
Value: severe
d__Bacteria.p__Cyanobacteria.o__Chroococcale
800
600
400
200
0
asymptomatic (n=24)
mild (n=25)
severe (n=30)
control (n=72)
Severity

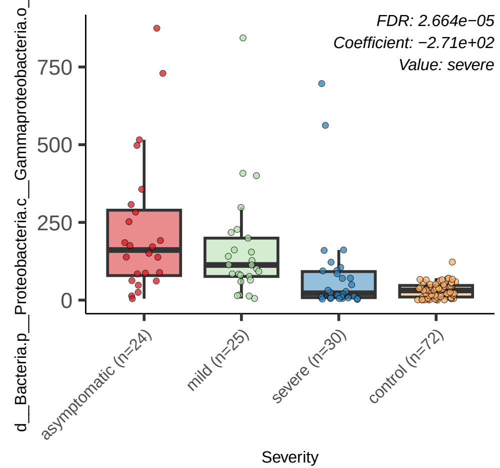
FDR: 2.664e−05
Coefficient: −2.71e+02
Value: severe
d__Bacteria.p__Proteobacteria.c__Gammaproteobacteria.o__
0
250
500
750
asymptomatic (n=24)
mild (n=25)
severe (n=30)
control (n=72)
Severity

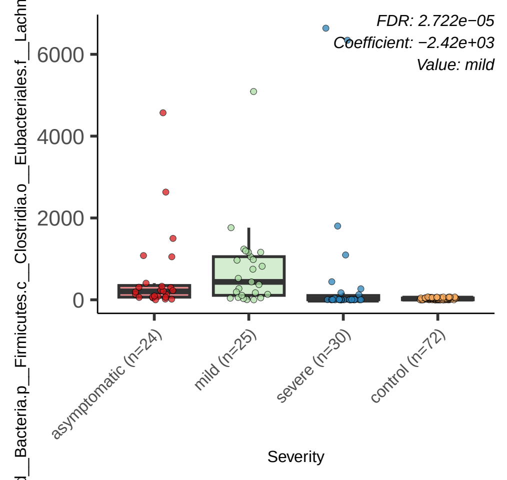

FDR: 2.722e−05
Coefficient: −2.42e+03
Value: mild
6000
4000
2000
0
d__Bacteria.p__Firmicutes.c__Clostridia.o__Eubacteriales.f__Lachn
asymptomatic (n=24)
mild (n=25)
severe (n=30)
control (n=72)
Severity

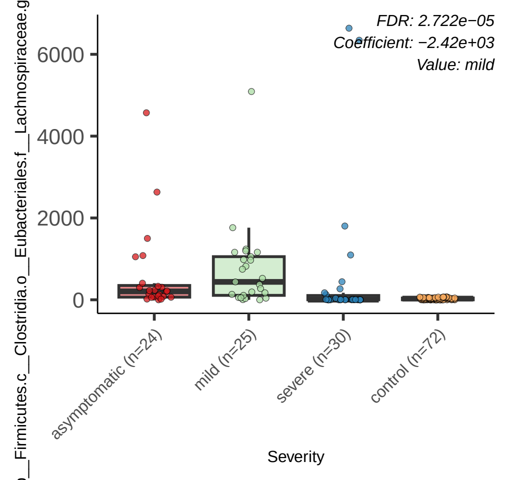
FDR: 2.722e−05
Coefficient: −2.42e+03
Value: mild
6000
4000
2000
0
asymptomatic (n=24)
mild (n=25)
severe (n=30)
control (n=72)
Severity
Firmicutes.c__Clostridia.o__Eubacteriales.f__Lachnospiraceae.g

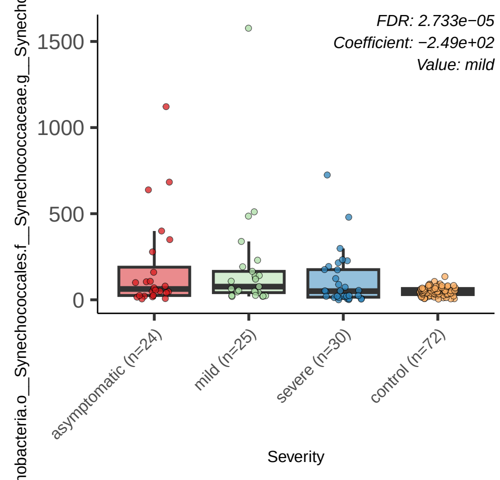

FDR: 2.733e−05
Coefficient: −2.49e+02
Value: mild
1500
1000
500
0
asymptomatic (n=24)
mild (n=25)
severe (n=30)
control (n=72)
Severity
nobacteria.o__Synechococcales.f__Synechococcaceae.g__Synecho

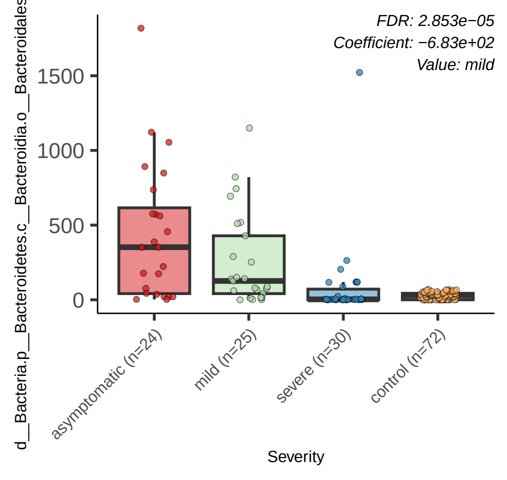
FDR: 2.853e−05
Coefficient: −6.83e+02
Value: mild
d__Bacteria.p__Bacteroidetes.c__Bacteroidia.o__Bacteroidales
1500
1000
500
0
asymptomatic (n=24)
mild (n=25)
severe (n=30)
control (n=72)
Severity

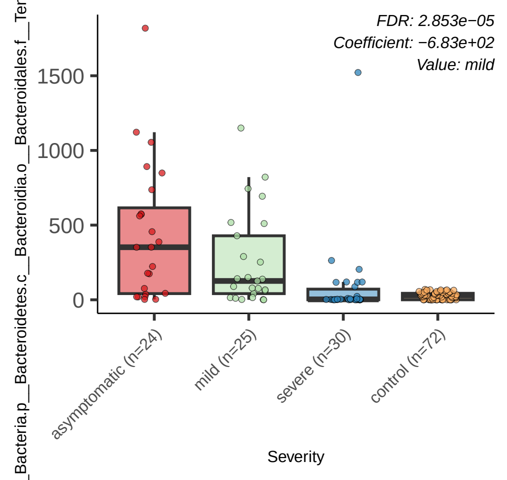
FDR: 2.853e−05
Coefficient: −6.83e+02
Value: mild
0
500
1000
1500
asymptomatic (n=24)
mild (n=25)
severe (n=30)
control (n=72)
Severity
Bacteria.p__Bacteroidetes.c__Bacteroidia.o__Bacteroidales.f__Ter

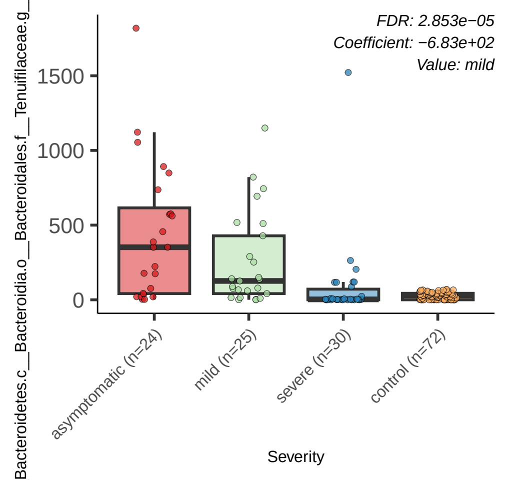
FDR: 2.853e−05
Coefficient: −6.83e+02
Value: mild
Bacteroidetes.c__Bacteroidia.o__Bacteroidales.f__Tenuifilaceae.g__
1500
1000
500
0
asymptomatic (n=24)
mild (n=25)
severe (n=30)
control (n=72)
Severity

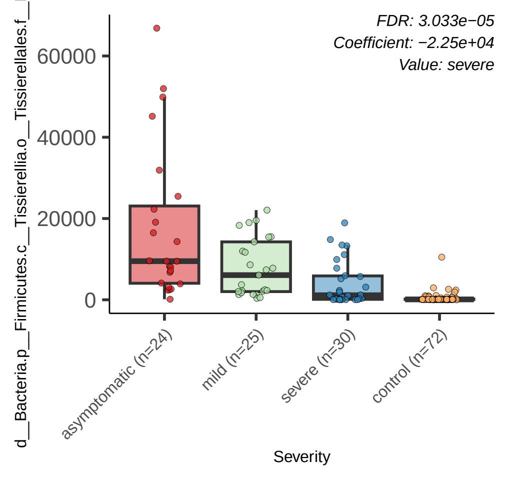

FDR: 3.033e−05
Coefficient: −2.25e+04
Value: severe
d__Bacteria.p__Firmicutes.c__Tissierellia.o__Tissierellales.f__
0
20000
40000
60000
asymptomatic (n=24)
mild (n=25)
severe (n=30)
control (n=72)
Severity

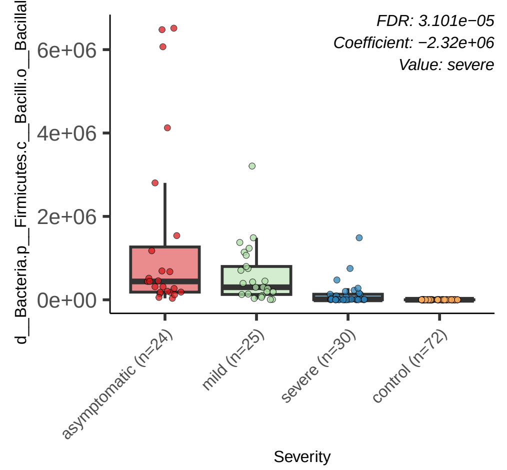
FDR: 3.101e−05
Coefficient: −2.32e+06
Value: severe
d__Bacteria.p__Firmicutes.c__Bacilli.o__Bacillal
6e+06
4e+06
2e+06
0e+00
asymptomatic (n=24)
mild (n=25)
severe (n=30)
control (n=72)
Severity

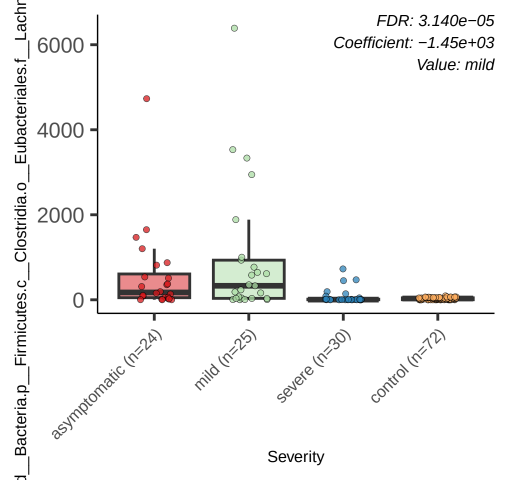

FDR: 3.140e−05
Coefficient: −1.45e+03
Value: mild
6000
4000
2000
0
d__Bacteria.p__Firmicutes.c__Clostridia.o__Eubacteriales.f__Lachn
asymptomatic (n=24)
mild (n=25)
severe (n=30)
control (n=72)
Severity

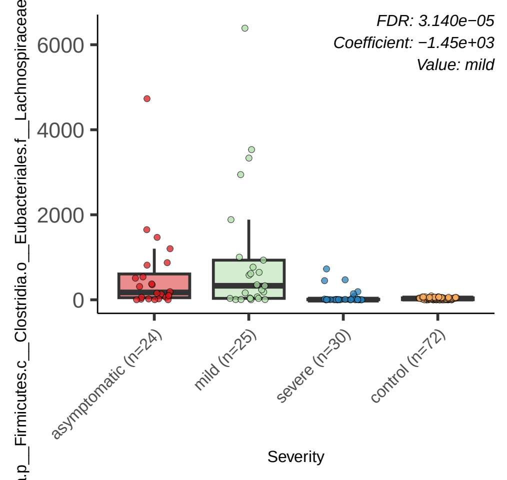

FDR: 3.140e−05
Coefficient: −1.45e+03
Value: mild
6000
4000
2000
0
a.p__Firmicutes.c__Clostridia.o__Eubacteriales.f__Lachnospiraceae
asymptomatic (n=24)
mild (n=25)
severe (n=30)
control (n=72)
Severity

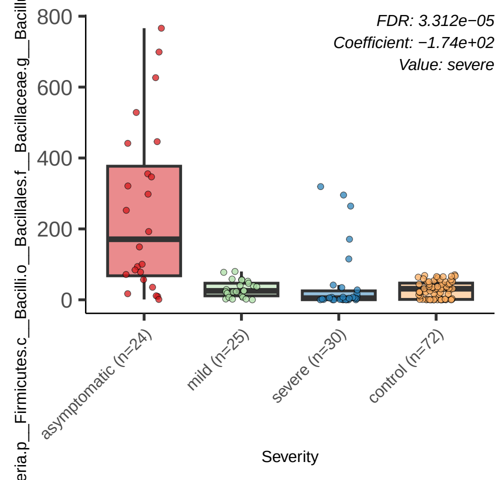

FDR: 3.312e−05
Coefficient: −1.74e+02
Value: severe
800
600
400
200
0
asymptomatic (n=24)
mild (n=25)
severe (n=30)
control (n=72)
Severity
eria.p__Firmicutes.c__Bacilli.o__Bacillales.f__Bacillaceae.g__Bacill

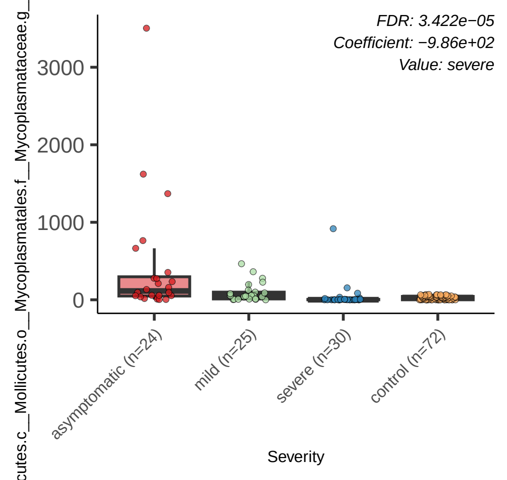

FDR: 3.422e−05
Coefficient: −9.86e+02
Value: severe
0
1000
2000
3000
cutes.c__Mollicutes.o__Mycoplasmatales.f__Mycoplasmataceae.g__
asymptomatic (n=24)
mild (n=25)
severe (n=30)
control (n=72)
Severity

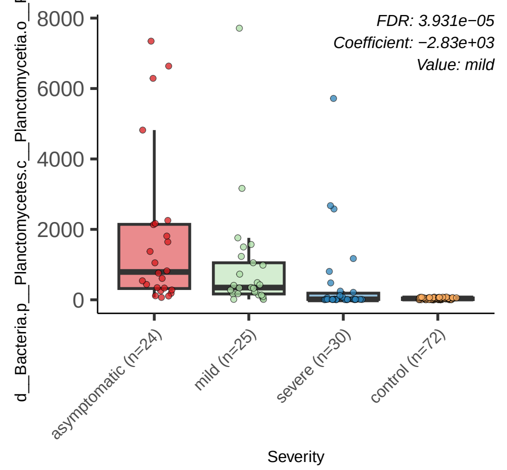

FDR: 3.931e−05
Coefficient: −2.83e+03
Value: mild
d__Bacteria.p__Planctomycetes.c__Planctomycetia.o__P
8000
6000
4000
2000
0
asymptomatic (n=24)
mild (n=25)
severe (n=30)
control (n=72)
Severity

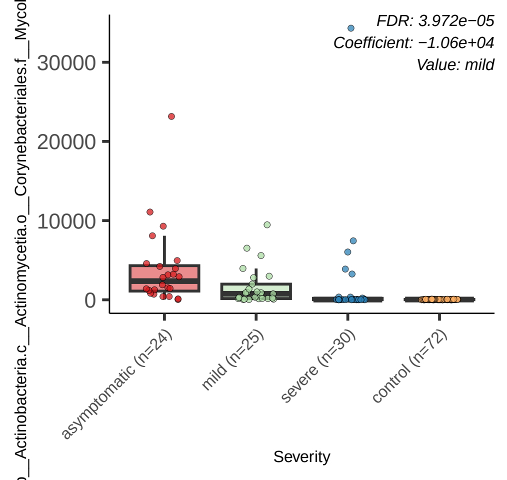

FDR: 3.972e−05
Coefficient: −1.06e+04
Value: mild
30000
20000
10000
0
asymptomatic (n=24)
mild (n=25)
severe (n=30)
control (n=72)
Severity
o__Actinobacteria.c__Actinomycetia.o__Corynebacteriales.f__Mycol

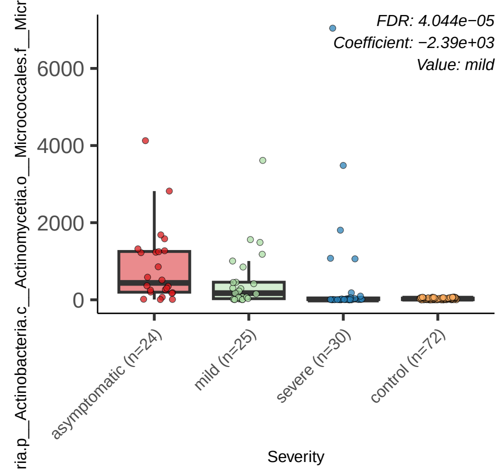

FDR: 4.044e−05
Coefficient: −2.39e+03
Value: mild
6000
4000
2000
0
asymptomatic (n=24)
mild (n=25)
severe (n=30)
control (n=72)
Severity
teria.p__Actinobacteria.c__Actinomycetia.o__Micrococcales.f__Micr

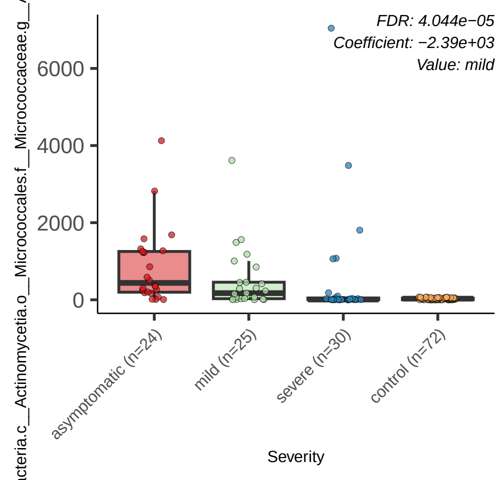

FDR: 4.044e−05
Coefficient: −2.39e+03
Value: mild
6000
4000
2000
0
acteria.c__Actinomycetia.o__Micrococcales.f__Micrococcaceae.g__
asymptomatic (n=24)
mild (n=25)
severe (n=30)
control (n=72)
Severity

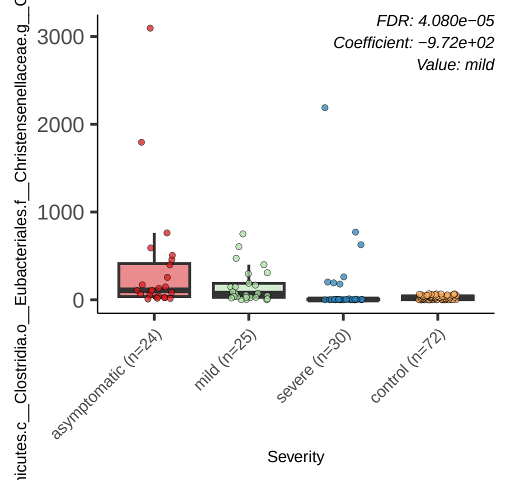
FDR: 4.080e−05
Coefficient: −9.72e+02
Value: mild
3000
2000
1000
0
asymptomatic (n=24)
mild (n=25)
severe (n=30)
control (n=72)
Severity
nicutes.c__Clostridia.o__Eubacteriales.f__Christensenellaceae.g__C

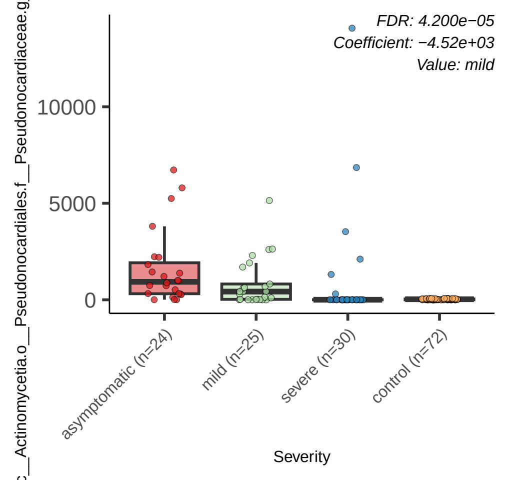

FDR: 4.200e−05
Coefficient: −4.52e+03
Value: mild
10000
5000
0
c__Actinomycetia.o__Pseudonocardiales.f__Pseudonocardiaceae.g
asymptomatic (n=24)
mild (n=25)
severe (n=30)
control (n=72)
Severity

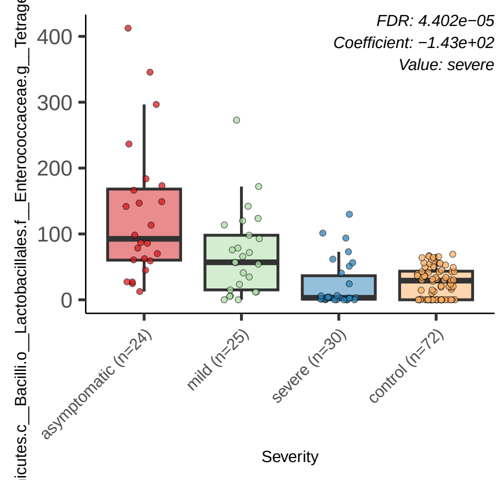

FDR: 4.402e−05
Coefficient: −1.43e+02
Value: severe
400
300
200
100
0
icutes.c__Bacilli.o__Lactobacillales.f__Enterococcaceae.g__Tetrage
asymptomatic (n=24)
mild (n=25)
severe (n=30)
control (n=72)
Severity

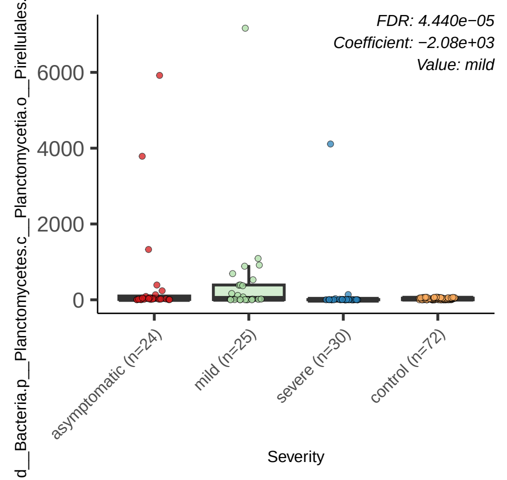

FDR: 4.440e−05
Coefficient: −2.08e+03
Value: mild
d__Bacteria.p__Planctomycetes.c__Planctomycetia.o__Pirellulales.
6000
4000
2000
0
asymptomatic (n=24)
mild (n=25)
severe (n=30)
control (n=72)
Severity

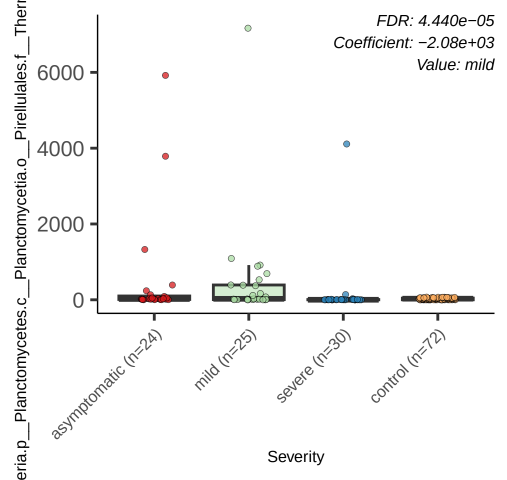

FDR: 4.440e−05
Coefficient: −2.08e+03
Value: mild
6000
4000
2000
0
asymptomatic (n=24)
mild (n=25)
severe (n=30)
control (n=72)
Severity
eria.p__Planctomycetes.c__Planctomycetia.o__Pirellulales.f__Therr

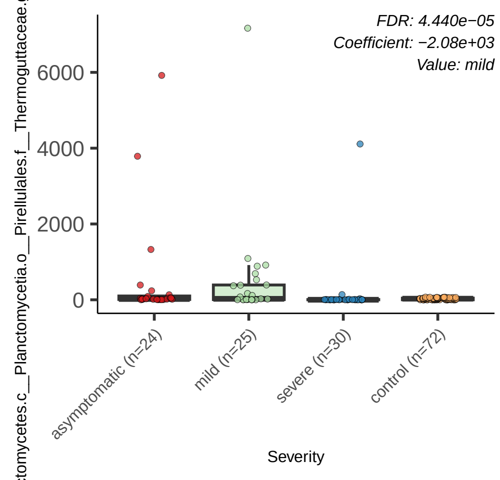

FDR: 4.440e−05
Coefficient: −2.08e+03
Value: mild
0
2000
4000
6000
ctomycetes.c__Planctomycetia.o__Pirellulales.f__Thermoguttaceae.g
asymptomatic (n=24)
mild (n=25)
severe (n=30)
control (n=72)
Severity

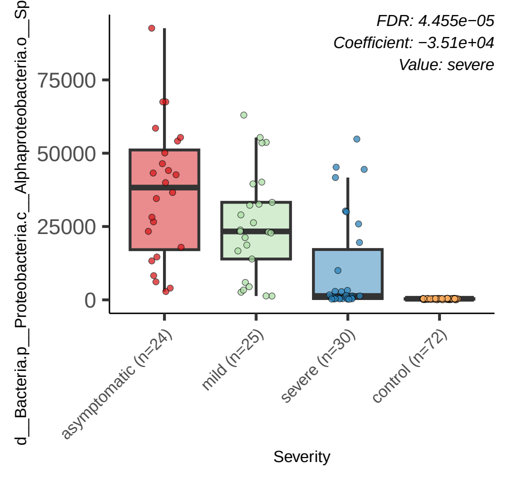

FDR: 4.455e−05
Coefficient: −3.51e+04
Value: severe
d__Bacteria.p__Proteobacteria.c__Alphaproteobacteria.o__Sp
75000
50000
25000
0
asymptomatic (n=24)
mild (n=25)
severe (n=30)
control (n=72)
Severity

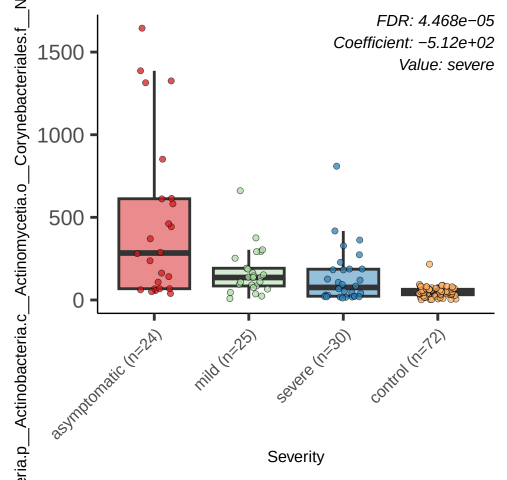

FDR: 4.468e−05
Coefficient: −5.12e+02
Value: severe
1500
1000
500
0
asymptomatic (n=24)
mild (n=25)
severe (n=30)
control (n=72)
Severity

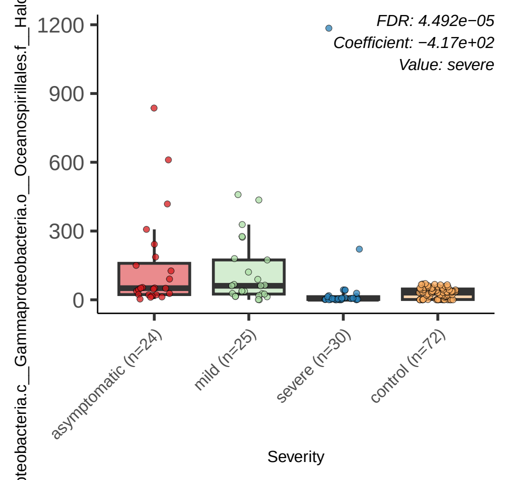

FDR: 4.492e−05
Coefficient: −4.17e+02
Value: severe
1200
900
600
300
0
asymptomatic (n=24)
mild (n=25)
severe (n=30)
control (n=72)
Severity
oteobacteria.c__Gammaproteobacteria.o__Oceanospirillales.f__Halo

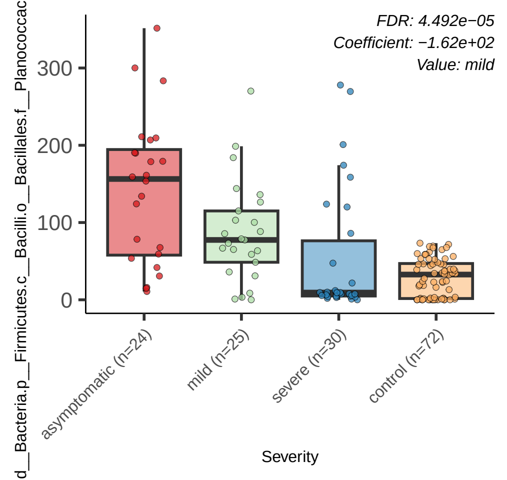

FDR: 4.492e−05
Coefficient: −1.62e+02
Value: mild
d__Bacteria.p__Firmicutes.c__Bacilli.o__Bacillales.f__Planococcac
300
200
100
0
asymptomatic (n=24)
mild (n=25)
severe (n=30)
control (n=72)
Severity

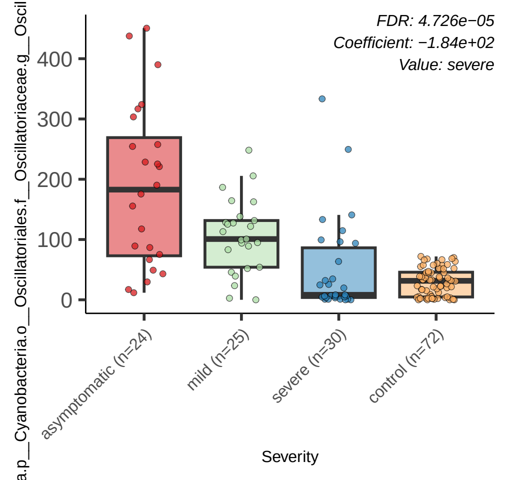

FDR: 4.726e−05
Coefficient: −1.84e+02
Value: severe
a.p__Cyanobacteria.o__Oscillatoriales.f__Oscillatoriaceae.g__Oscil
0
100
200
300
400
asymptomatic (n=24)
mild (n=25)
severe (n=30)
control (n=72)
Severity

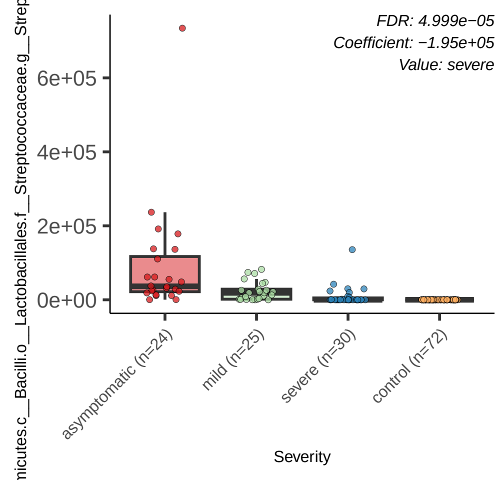

FDR: 4.999e−05
Coefficient: −1.95e+05
Value: severe
6e+05
4e+05
2e+05
0e+00
micutes.c__Bacilli.o__Lactobacillales.f__Streptococcaceae.g__Strep
asymptomatic (n=24)
mild (n=25)
severe (n=30)
control (n=72)
Severity

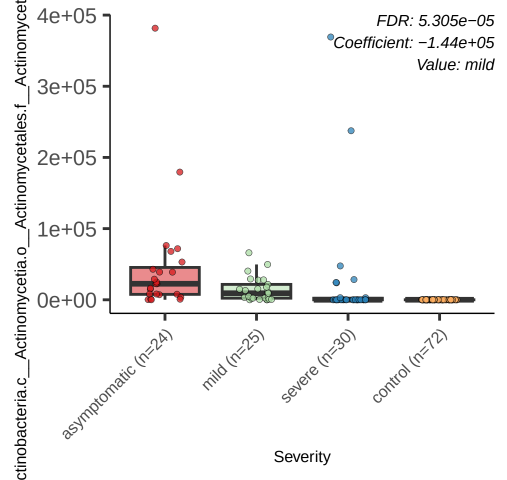

FDR: 5.305e−05
Coefficient: −1.44e+05
Value: mild
4e+05
3e+05
2e+05
1e+05
0e+00
ctinobacteria.c__Actinomycetia.o__Actinomycetales.f__Actinomycet
asymptomatic (n=24)
mild (n=25)
severe (n=30)
control (n=72)
Severity

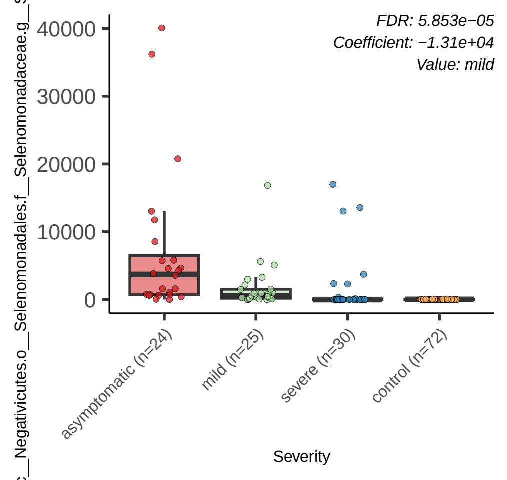
FDR: 5.853e−05
Coefficient: −1.31e+04
Value: mild
40000
30000
20000
10000
0
Negativicutes.o__Selenomonadales.f__Selenomonadaceae.g__
asymptomatic (n=24)
mild (n=25)
severe (n=30)
control (n=72)
Severity

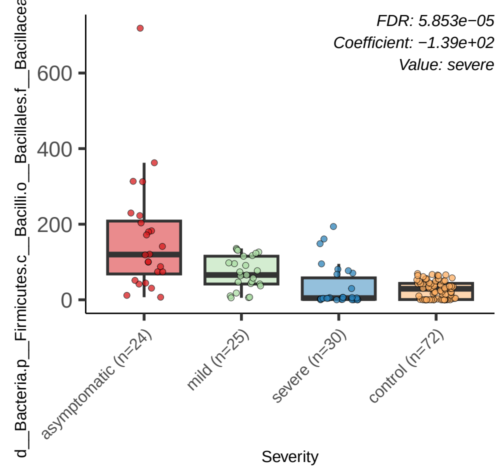

FDR: 5.853e−05
Coefficient: −1.39e+02
Value: severe
d__Bacteria.p__Firmicutes.c__Bacilli.o__Bacillales.f__Bacillacea
0
200
400
600
asymptomatic (n=24)
mild (n=25)
severe (n=30)
control (n=72)
Severity

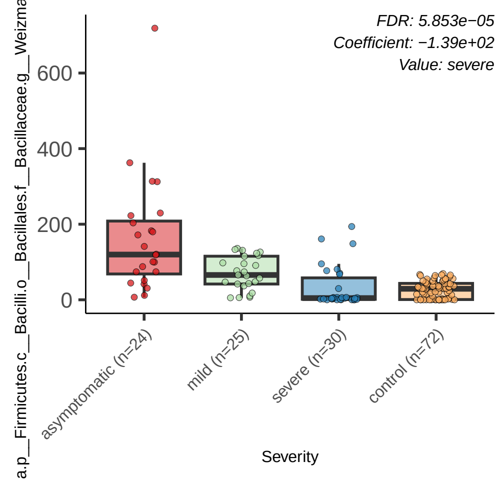

FDR: 5.853e−05
Coefficient: −1.39e+02
Value: severe
a.p__Firmicutes.c__Bacilli.o__Bacillales.f__Bacillaceae.g__Weizma
0
200
400
600
asymptomatic (n=24)
mild (n=25)
severe (n=30)
control (n=72)
Severity

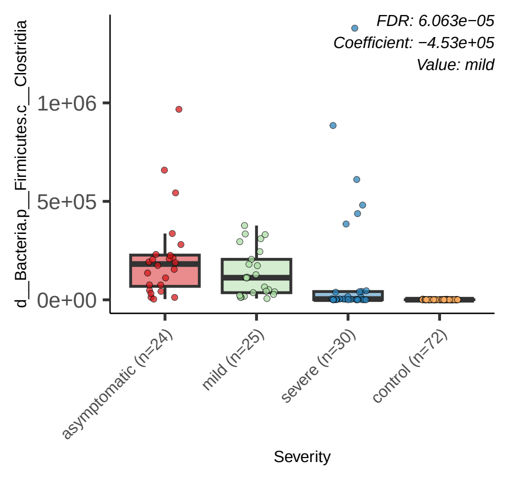

FDR: 6.063e−05
Coefficient: −4.53e+05
Value: mild
d__Bacteria.p__Firmicutes.c__Clostridia
1e+06
5e+05
0e+00
asymptomatic (n=24)
mild (n=25)
severe (n=30)
control (n=72)
Severity

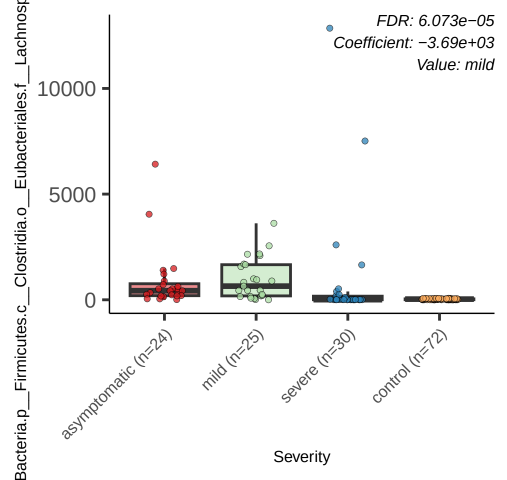
FDR: 6.073e−05
Coefficient: −3.69e+03
Value: mild
Bacteria.p__Firmicutes.c__Clostridia.o__Eubacteriales.f__Lachnosp
10000
5000
0
asymptomatic (n=24)
mild (n=25)
severe (n=30)
control (n=72)
Severity

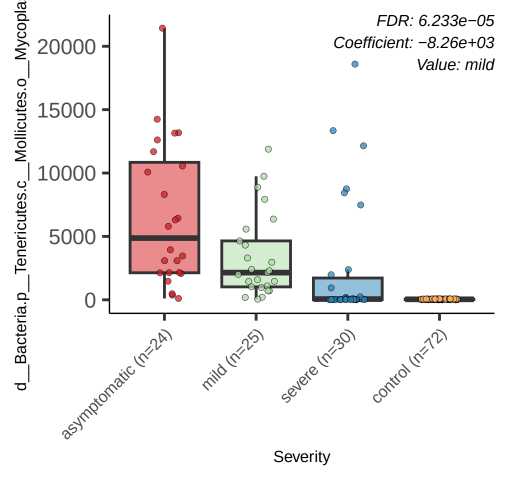
FDR: 6.233e−05
Coefficient: −8.26e+03
Value: mild
d__Bacteria.p__Tenericutes.c__Mollicutes.o__Mycopla
20000
15000
10000
5000
0
asymptomatic (n=24)
mild (n=25)
severe (n=30)
control (n=72)
Severity

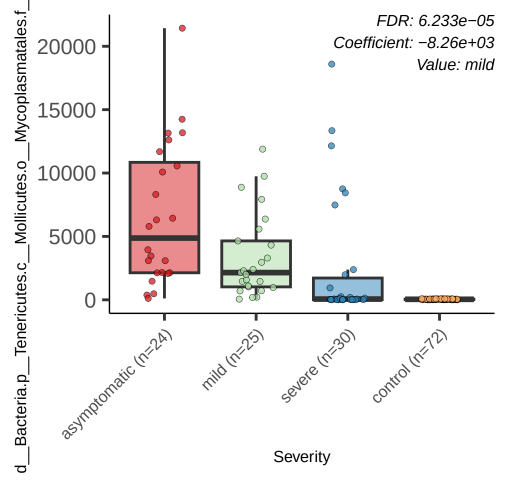

FDR: 6.233e−05
Coefficient: −8.26e+03
Value: mild
d__Bacteria.p__Tenericutes.c__Mollicutes.o__Mycoplasmatales.f__
20000
15000
10000
5000
0
asymptomatic (n=24)
mild (n=25)
severe (n=30)
control (n=72)
Severity

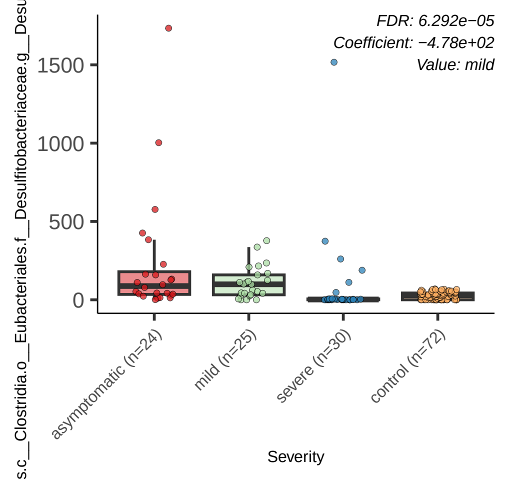

FDR: 6.292e−05
Coefficient: −4.78e+02
Value: mild
s.c__Clostridia.o__Eubacteriales.f__Desulfitobacteriaceae.g__Desu
1500
1000
500
0
asymptomatic (n=24)
mild (n=25)
severe (n=30)
control (n=72)
Severity

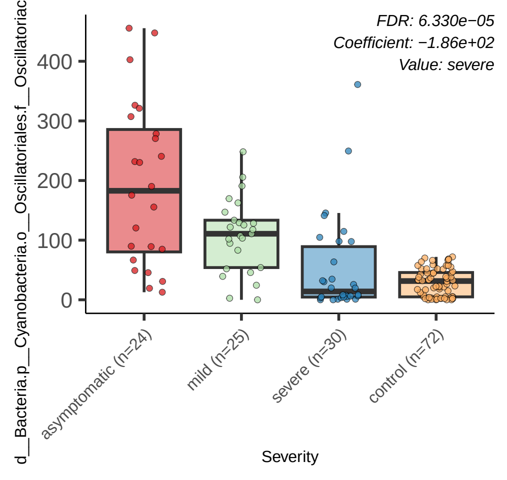

FDR: 6.330e−05
Coefficient: −1.86e+02
Value: severe
d__Bacteria.p__Cyanobacteria.o__Oscillatoriales.f__Oscillatoriac
0
100
200
300
400
asymptomatic (n=24)
mild (n=25)
severe (n=30)
control (n=72)
Severity

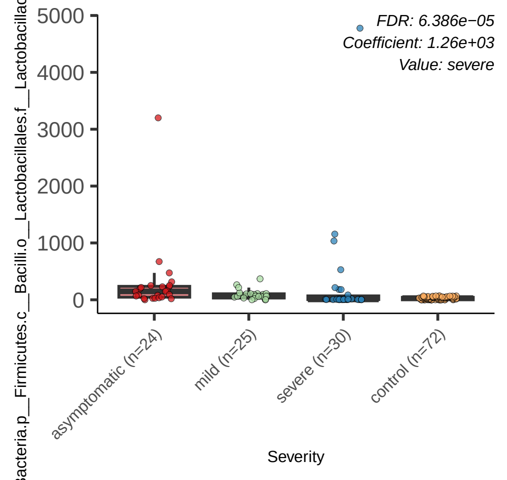

FDR: 6.386e−05
Coefficient: 1.26e+03
Value: severe
5000
4000
3000
2000
1000
0
asymptomatic (n=24)
mild (n=25)
severe (n=30)
control (n=72)
Severity
Bacteria.p__Firmicutes.c__Bacilli.o__Lactobacillales.f__Lactobacillac

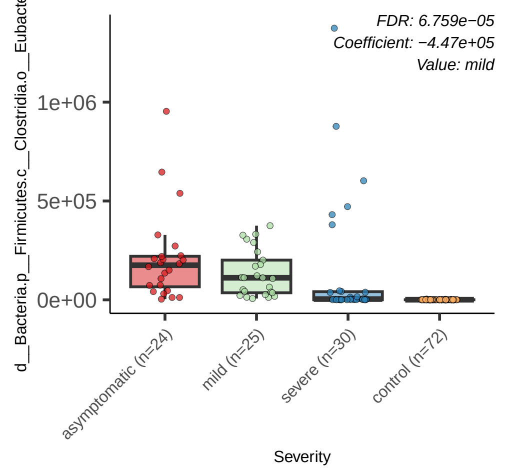

FDR: 6.759e−05
Coefficient: −4.47e+05
Value: mild
d__Bacteria.p__Firmicutes.c__Clostridia.o__Eubacte
1e+06
5e+05
0e+00
asymptomatic (n=24)
mild (n=25)
severe (n=30)
control (n=72)
Severity

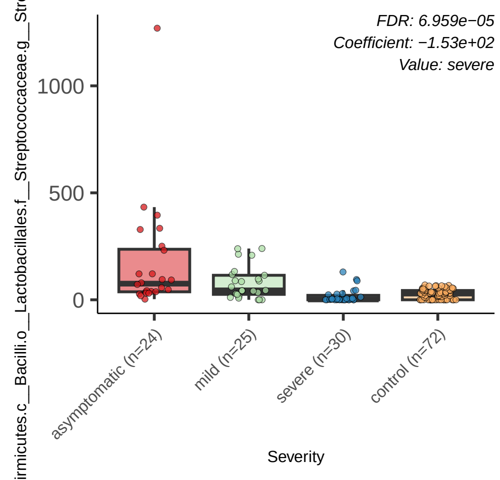

FDR: 6.959e−05
Coefficient: −1.53e+02
Value: severe
1000
500
0
asymptomatic (n=24)
mild (n=25)
severe (n=30)
control (n=72)
Severity
irmicutes.c__Bacilli.o__Lactobacillales.f__Streptococcaceae.g__Str

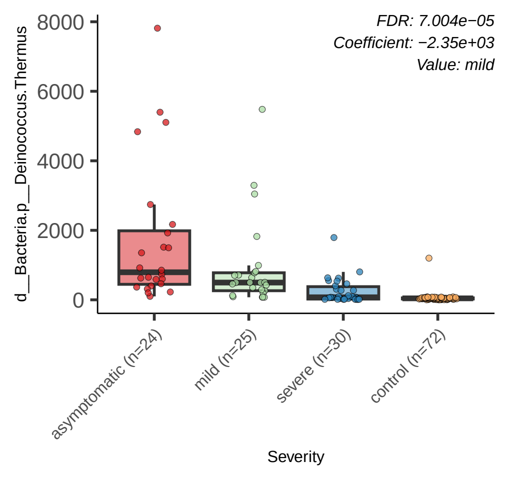

FDR: 7.004e−05
Coefficient: −2.35e+03
Value: mild
d__Bacteria.p__Deinococcus.Thermus
8000
6000
4000
2000
0
asymptomatic (n=24)
mild (n=25)
severe (n=30)
control (n=72)
Severity

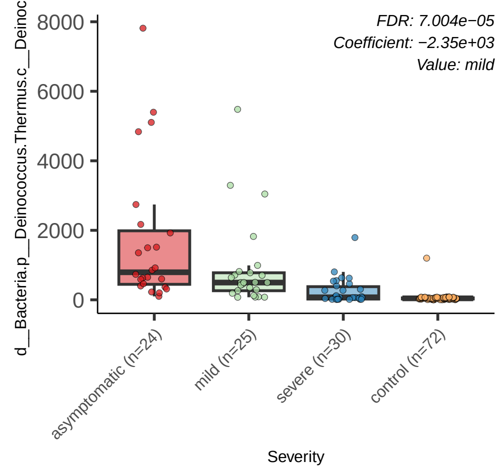
FDR: 7.004e−05
Coefficient: −2.35e+03
Value: mild
d__Bacteria.p__Deinococcus.Thermus.c__Deinoc
8000
6000
4000
2000
0
asymptomatic (n=24)
mild (n=25)
severe (n=30)
control (n=72)
Severity

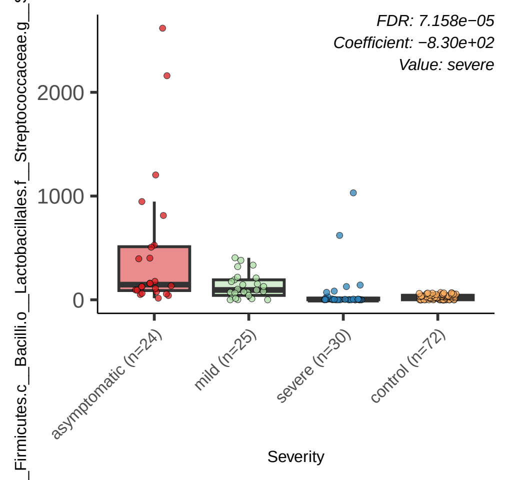
FDR: 7.158e−05
Coefficient: −8.30e+02
Value: severe
Firmicutes.c__Bacilli.o__Lactobacillales.f__Streptococcaceae.g__
2000
1000
0
asymptomatic (n=24)
mild (n=25)
severe (n=30)
control (n=72)
Severity

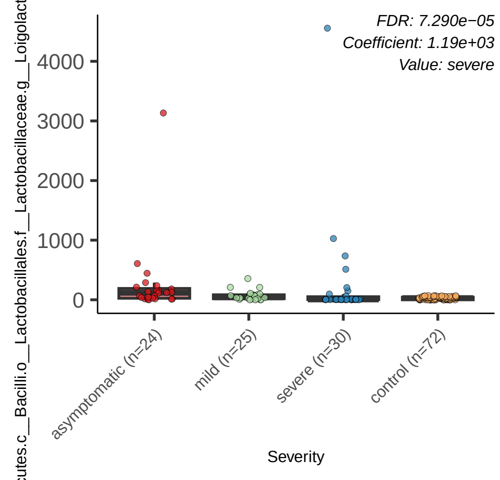
FDR: 7.290e−05
Coefficient: 1.19e+03
Value: severe
4000
3000
2000
1000
0
asymptomatic (n=24)
mild (n=25)
severe (n=30)
control (n=72)
Severity

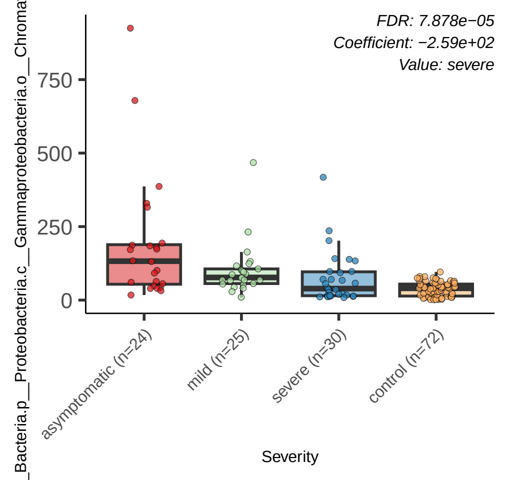
FDR: 7.878e−05
Coefficient: −2.59e+02
Value: severe
0
250
500
750
asymptomatic (n=24)
mild (n=25)
severe (n=30)
control (n=72)
Severity
Bacteria.p__Proteobacteria.c__Gammaproteobacteria.o__Chroma

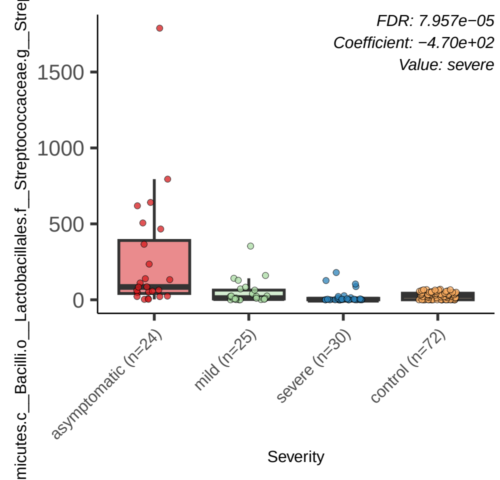
FDR: 7.957e−05
Coefficient: −4.70e+02
Value: severe
1500
1000
500
0
micutes.c__Bacilli.o__Lactobacillales.f__Streptococcaceae.g__Strep
asymptomatic (n=24)
mild (n=25)
severe (n=30)
control (n=72)
Severity

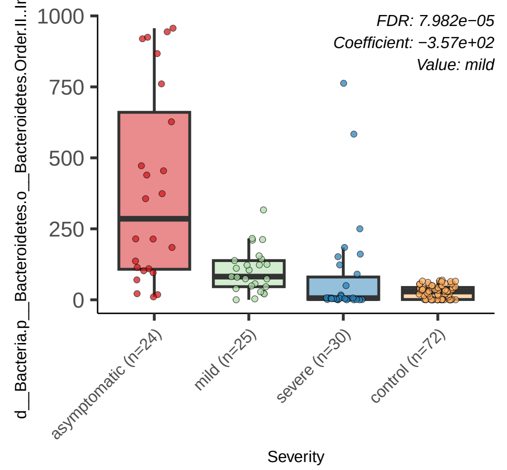

FDR: 7.982e−05
Coefficient: −3.57e+02
Value: mild
d__Bacteria.p__Bacteroidetes.o__Bacteroidetes.Order.II..In
1000
750
500
250
0
asymptomatic (n=24)
mild (n=25)
severe (n=30)
control (n=72)
Severity

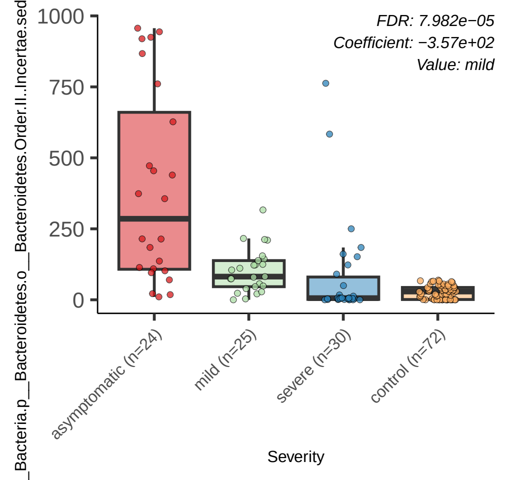
FDR: 7.982e−05
Coefficient: −3.57e+02
Value: mild
1000
750
500
250
0
Bacteria.p__Bacteroidetes.c__Bacteroidetes.o__Bacteroidetes.Order.II..Incertae.sed
asymptomatic (n=24)
mild (n=25)
severe (n=30)
control (n=72)
Severity

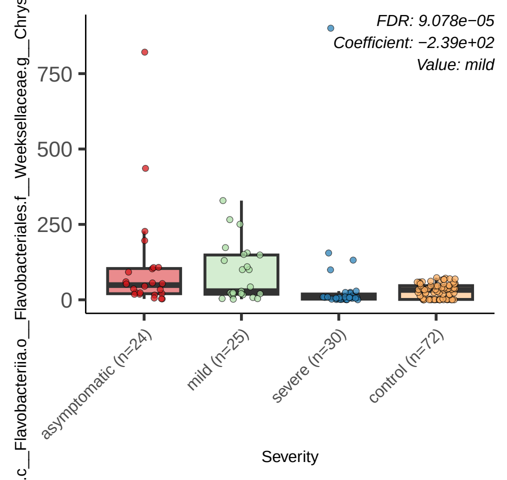

FDR: 9.078e−05
Coefficient: −2.39e+02
Value: mild
750
500
250
0
.c__Flavobacteriia.o__Flavobacteriales.f__Weeksellaceae.g__Chrys
asymptomatic (n=24)
mild (n=25)
severe (n=30)
control (n=72)
Severity

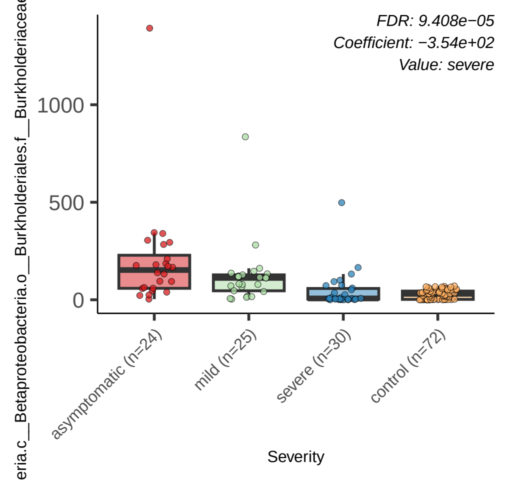

FDR: 9.408e−05
Coefficient: −3.54e+02
Value: severe
eria.c__Betaproteobacteria.o__Burkholderiales.f__Burkholderiaceae
1000
500
0
asymptomatic (n=24)
mild (n=25)
severe (n=30)
control (n=72)
Severity

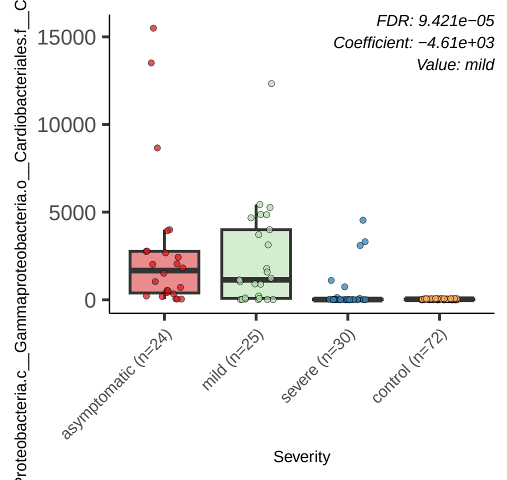
FDR: 9.421e−05
Coefficient: −4.61e+03
Value: mild
Proteobacteria.c__Gammaproteobacteria.o__Cardiobacteriales.f__C
15000
10000
5000
0
asymptomatic (n=24)
mild (n=25)
severe (n=30)
control (n=72)
Severity

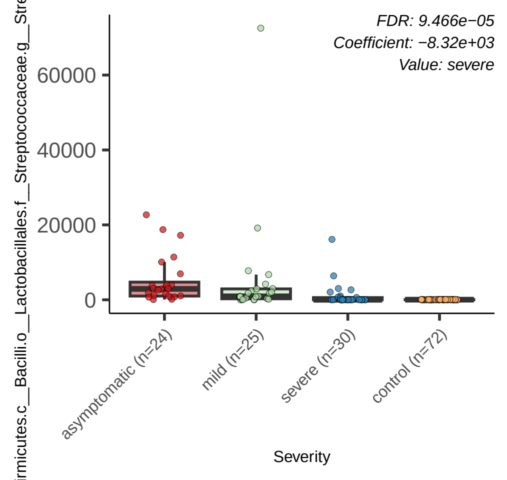

FDR: 9.466e−05
Coefficient: −8.32e+03
Value: severe
60000
40000
20000
0
irmicutes.c__Bacilli.o__Lactobacillales.f__Streptococcaceae.g__Stre
asymptomatic (n=24)
mild (n=25)
severe (n=30)
control (n=72)
Severity

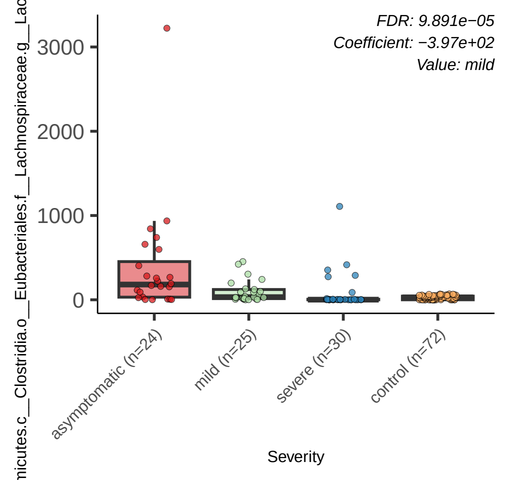
FDR: 9.891e−05
Coefficient: −3.97e+02
Value: mild
3000
2000
1000
0
micutes.c__Clostridia.o__Eubacteriales.f__Lachnospiraceae.g__Lac
asymptomatic (n=24)
mild (n=25)
severe (n=30)
control (n=72)
Severity

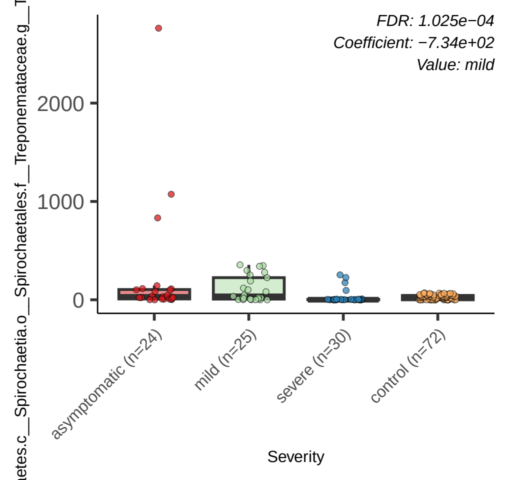

FDR: 1.025e−04
Coefficient: −7.34e+02
Value: mild
2000
1000
0
asymptomatic (n=24)
mild (n=25)
severe (n=30)
control (n=72)
Severity

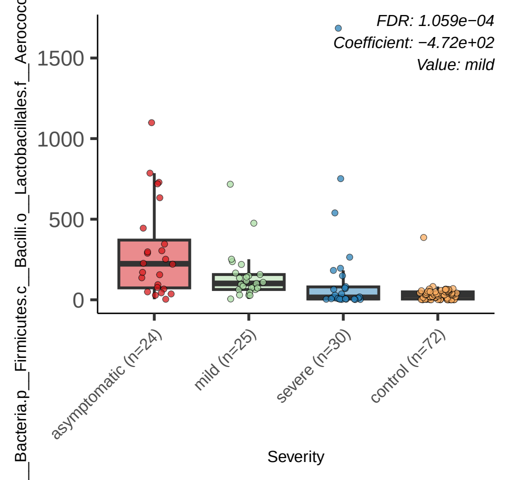

FDR: 1.059e−04
Coefficient: −4.72e+02
Value: mild
1500
1000
500
0
Bacteria.p__Firmicutes.c__Bacilli.o__Lactobacillales.f__Aerococc
asymptomatic (n=24)
mild (n=25)
severe (n=30)
control (n=72)
Severity

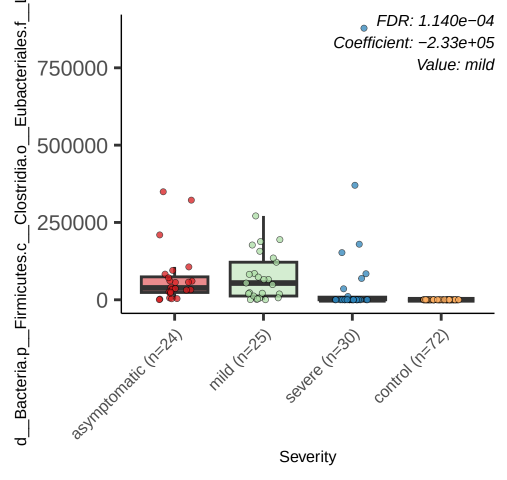

FDR: 1.140e−04
Coefficient: −2.33e+05
Value: mild
750000
500000
250000
0
d__Bacteria.p__Firmicutes.c__Clostridia.o__Eubacteriales.f__
asymptomatic (n=24)
mild (n=25)
severe (n=30)
control (n=72)
Severity

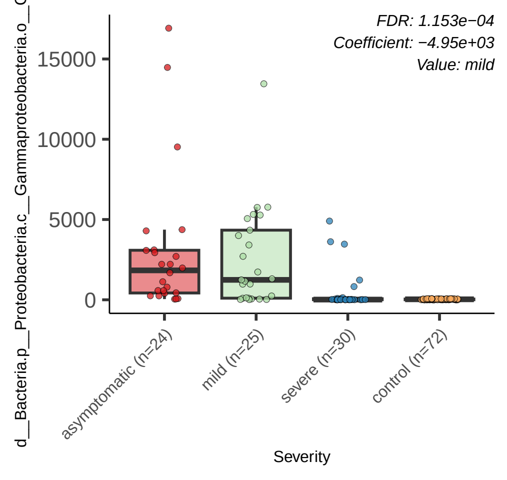

FDR: 1.153e−04
Coefficient: −4.95e+03
Value: mild
d__Bacteria.p__Proteobacteria.c__Gammaproteobacteria.o__C
15000
10000
5000
0
asymptomatic (n=24)
mild (n=25)
severe (n=30)
control (n=72)
Severity

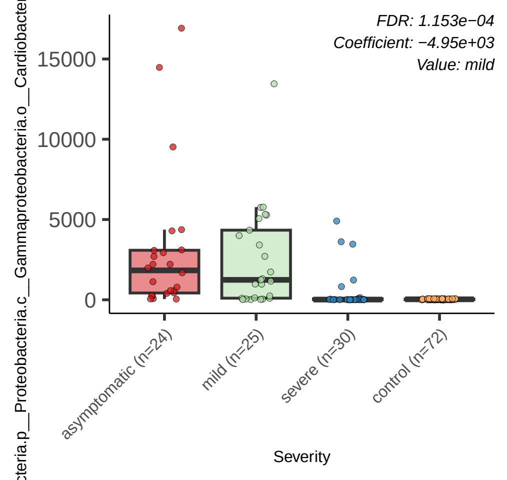

FDR: 1.153e−04
Coefficient: −4.95e+03
Value: mild
15000
10000
5000
0
cteria.p__Proteobacteria.c__Gammaproteobacteria.o__Cardiobacte
asymptomatic (n=24)
mild (n=25)
severe (n=30)
control (n=72)
Severity

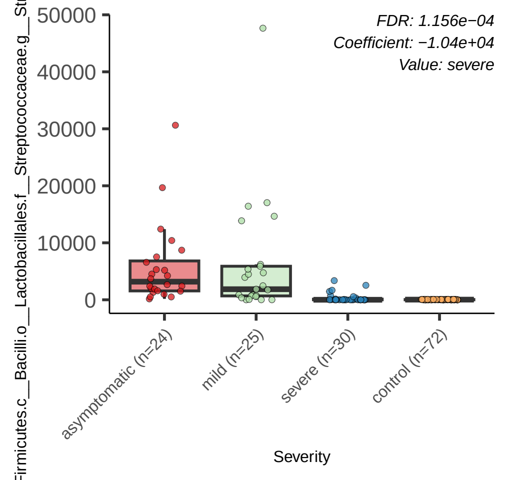
FDR: 1.156e−04
Coefficient: −1.04e+04
Value: severe
50000
40000
30000
20000
10000
0
Firmicutes.c__Bacilli.o__Lactobacillales.f__Streptococcaceae.g__St
asymptomatic (n=24)
mild (n=25)
severe (n=30)
control (n=72)
Severity

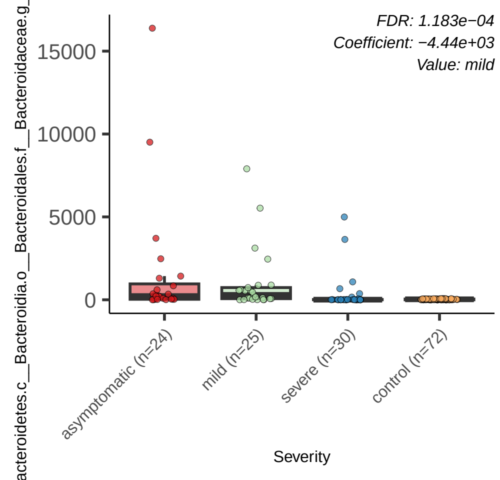

FDR: 1.183e−04
Coefficient: −4.44e+03
Value: mild
acteroidetes.c__Bacteroidia.o__Bacteroidales.f__Bacteroidaceae.g_
15000
10000
5000
0
asymptomatic (n=24)
mild (n=25)
severe (n=30)
control (n=72)
Severity

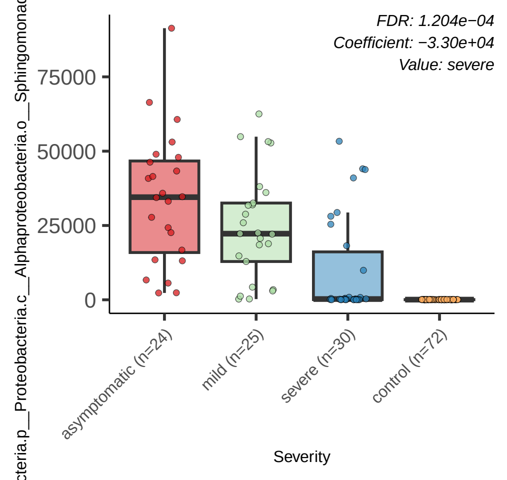

FDR: 1.204e−04
Coefficient: −3.30e+04
Value: severe
75000
50000
25000
0
cteria.p__Proteobacteria.c__Alphaproteobacteria.o__Sphingomonad
asymptomatic (n=24)
mild (n=25)
severe (n=30)
control (n=72)
Severity

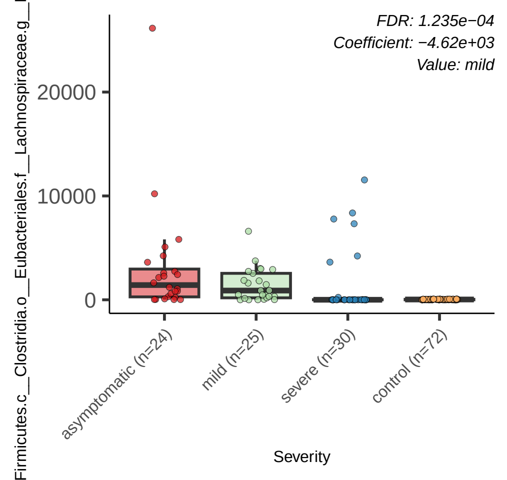
FDR: 1.235e−04
Coefficient: −4.62e+03
Value: mild
Firmicutes.c__Clostridia.o__Eubacteriales.f__Lachnospiraceae.g__
20000
10000
0
asymptomatic (n=24)
mild (n=25)
severe (n=30)
control (n=72)
Severity

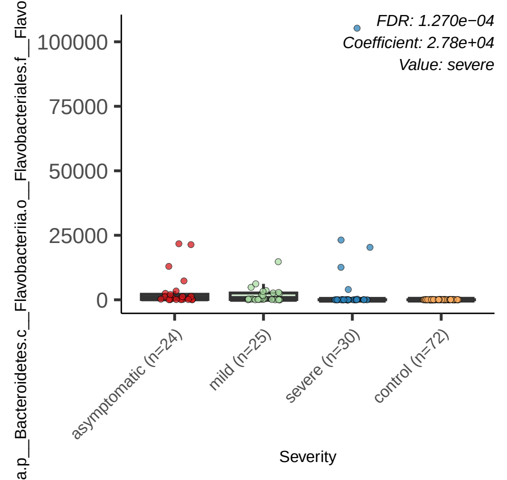

FDR: 1.270e−04
Coefficient: 2.78e+04
Value: severe
100000
75000
50000
25000
0
asymptomatic (n=24)
mild (n=25)
severe (n=30)
control (n=72)
Severity
a.p__Bacteroidetes.c__Flavobacteriia.o__Flavobacteriales.f__Flavo

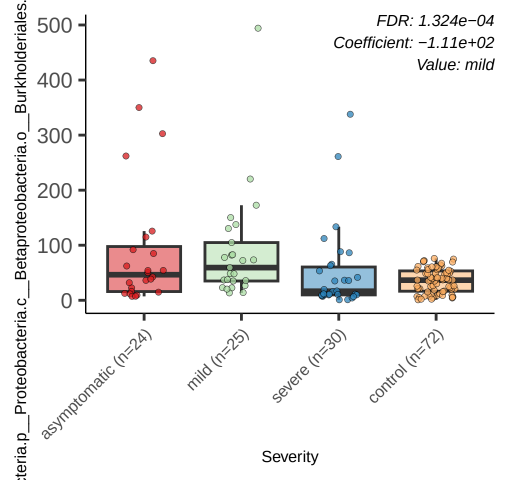
FDR: 1.324e−04
Coefficient: −1.11e+02
Value: mild
cteria.p__Proteobacteria.c__Betaproteobacteria.o__Burkholderiales.
500
400
300
200
100
0
asymptomatic (n=24)
mild (n=25)
severe (n=30)
control (n=72)
Severity

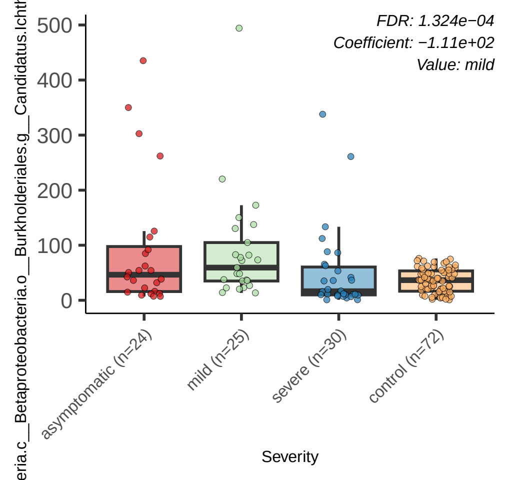

FDR: 1.324e−04
Coefficient: −1.11e+02
Value: mild
eria.c__Betaproteobacteria.o__Burkholderiales.g__Candidatus.Ichth
500
400
300
200
100
0
asymptomatic (n=24)
mild (n=25)
severe (n=30)
control (n=72)
Severity

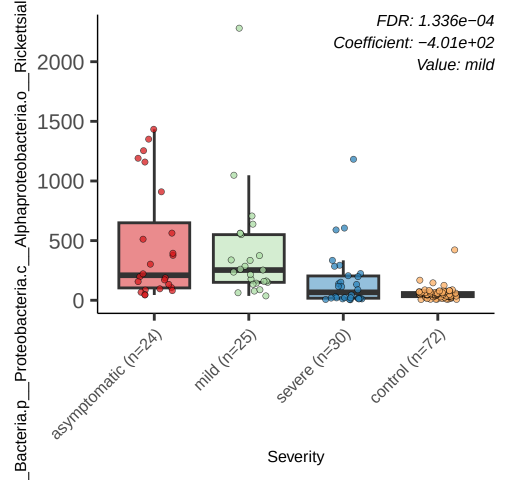

FDR: 1.336e−04
Coefficient: −4.01e+02
Value: mild
Bacteria.p__Proteobacteria.c__Alphaproteobacteria.o__Rickettsial
0
500
1000
1500
2000
asymptomatic (n=24)
mild (n=25)
severe (n=30)
control (n=72)
Severity

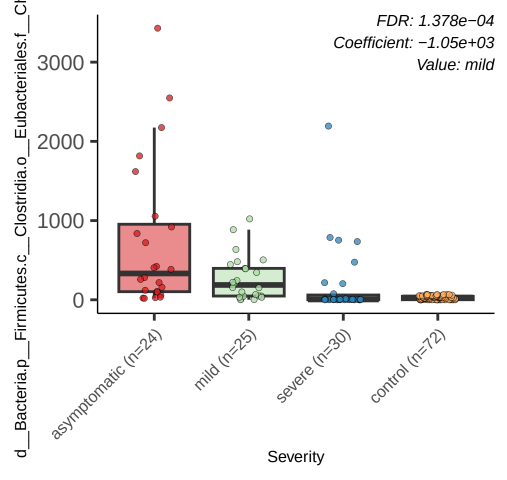

FDR: 1.378e−04
Coefficient: −1.05e+03
Value: mild
d__Bacteria.p__Firmicutes.c__Clostridia.o__Eubacteriales.f__Cl
3000
2000
1000
0
asymptomatic (n=24)
mild (n=25)
severe (n=30)
control (n=72)
Severity

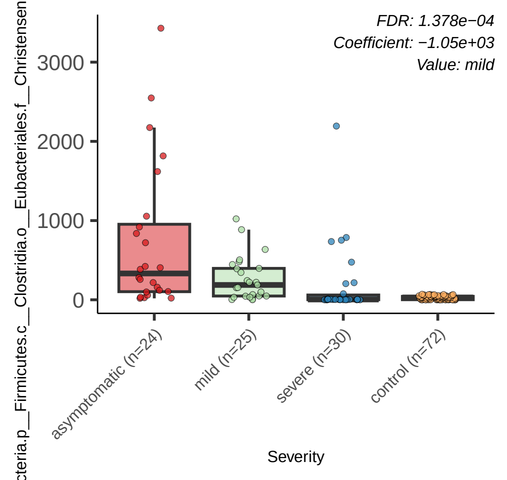

FDR: 1.378e−04
Coefficient: −1.05e+03
Value: mild
3000
2000
1000
0
cteria.p__Firmicutes.c__Clostridia.o__Eubacteriales.f__Christensen
asymptomatic (n=24)
mild (n=25)
severe (n=30)
control (n=72)
Severity

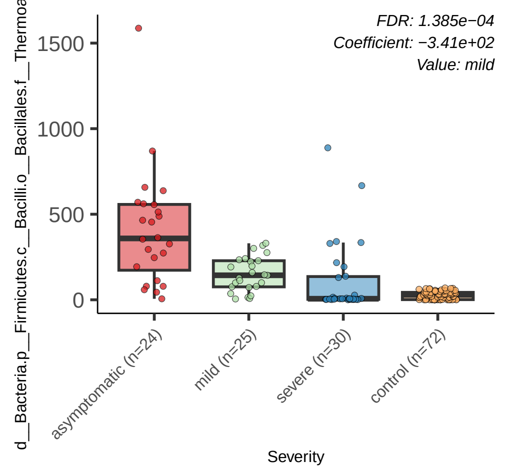
FDR: 1.385e−04
Coefficient: −3.41e+02
Value: mild
d__Bacteria.p__Firmicutes.c__Bacilli.o__Bacillales.f__Thermoa
1500
1000
500
0
asymptomatic (n=24)
mild (n=25)
severe (n=30)
control (n=72)
Severity

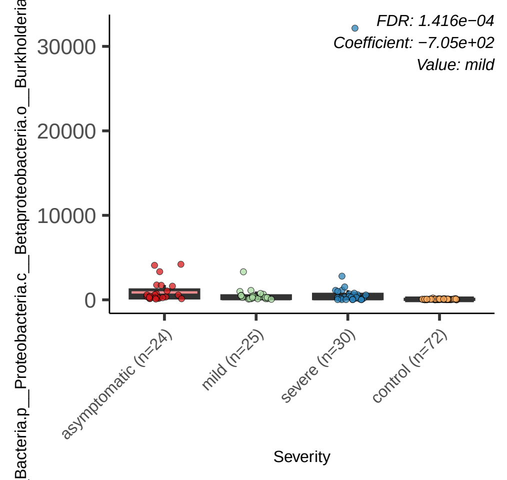

FDR: 1.416e−04
Coefficient: −7.05e+02
Value: mild
30000
20000
10000
0
Bacteria.p__Proteobacteria.c__Betaproteobacteria.o__Burkholderia
asymptomatic (n=24)
mild (n=25)
severe (n=30)
control (n=72)
Severity

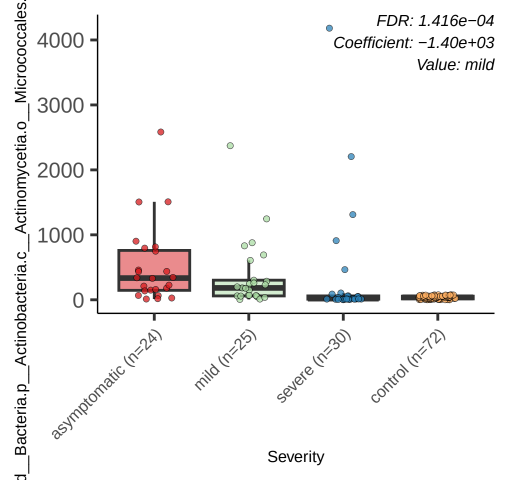

FDR: 1.416e−04
Coefficient: −1.40e+03
Value: mild
d__Bacteria.p__Actinobacteria.c__Actinomycetia.o__Micrococcales.
4000
3000
2000
1000
0
asymptomatic (n=24)
mild (n=25)
severe (n=30)
control (n=72)
Severity

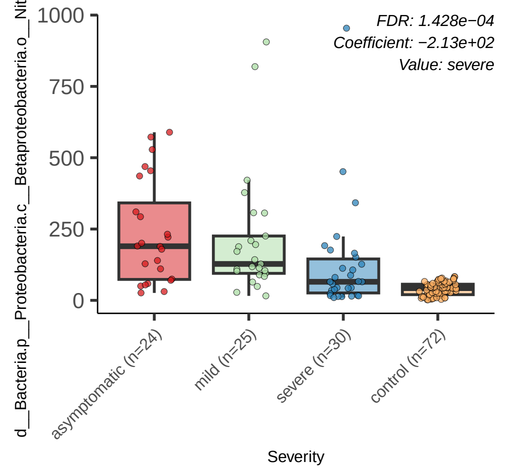

d__Bacteria.p__Proteobacteria.c__Betaproteobacteria.o__Nit
1000
750
500
250
0
FDR: 1.428e−04
Coefficient: −2.13e+02
Value: severe
asymptomatic (n=24)
mild (n=25)
severe (n=30)
control (n=72)
Severity

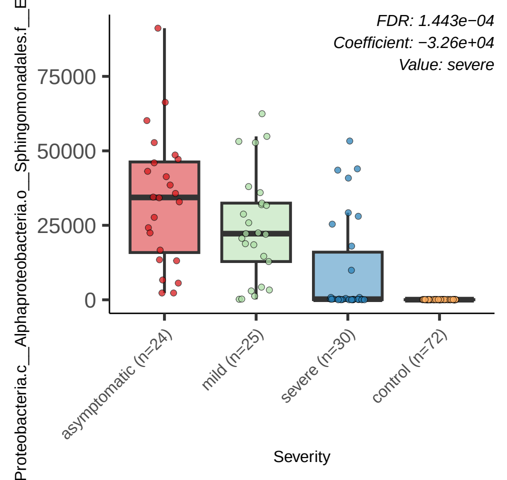

FDR: 1.443e−04
Coefficient: −3.26e+04
Value: severe
Proteobacteria.c__Alphaproteobacteria.o__Sphingomonadales.f__E
75000
50000
25000
0
asymptomatic (n=24)
mild (n=25)
severe (n=30)
control (n=72)
Severity

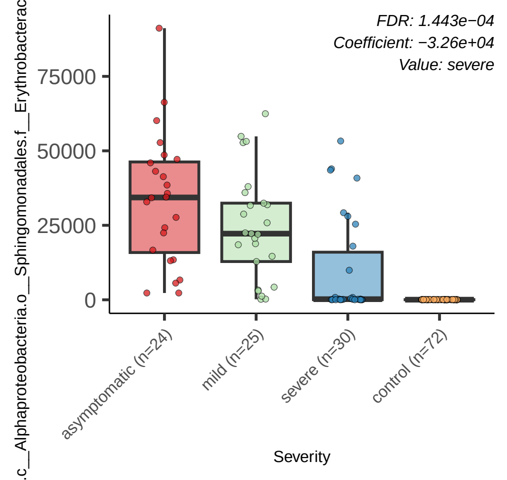

FDR: 1.443e−04
Coefficient: −3.26e+04
Value: severe
75000
50000
25000
0
c__Alphaproteobacteria.o__Sphingomonadales.f__Erythrobacterac
asymptomatic (n=24)
mild (n=25)
severe (n=30)
control (n=72)
Severity

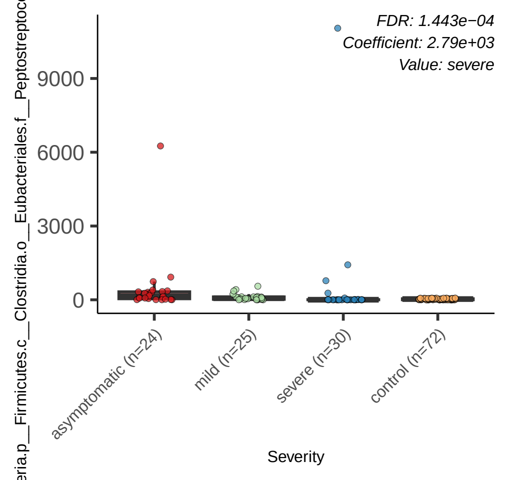
FDR: 1.443e−04
Coefficient: 2.79e+03
Value: severe
9000
6000
3000
0
asymptomatic (n=24)
mild (n=25)
severe (n=30)
control (n=72)
Severity
eria.p__Firmicutes.c__Clostridia.o__Eubacteriales.f__Peptostreptoc

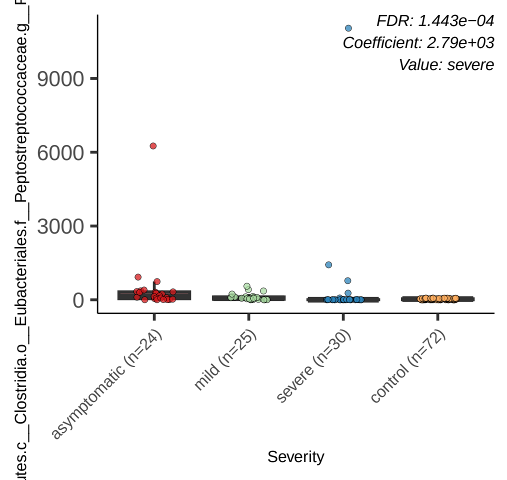

FDR: 1.443e−04
Coefficient: 2.79e+03
Value: severe
9000
6000
3000
0
asymptomatic (n=24)
mild (n=25)
severe (n=30)
control (n=72)
Severity
utes.c__Clostridia.o__Eubacteriales.f__Peptostreptococcaceae.g__F

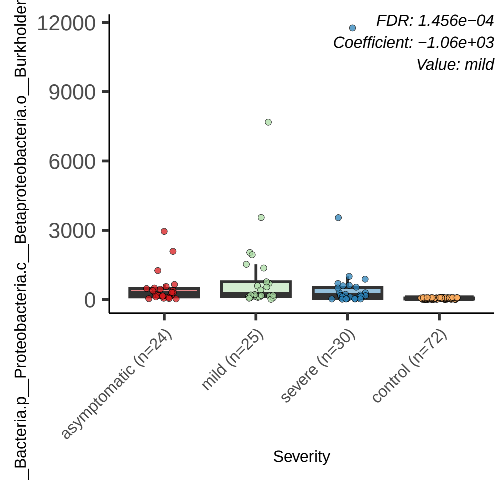

FDR: 1.456e−04
Coefficient: −1.06e+03
Value: mild
12000
9000
6000
3000
0
asymptomatic (n=24)
mild (n=25)
severe (n=30)
control (n=72)
Severity
_Bacteria.p__Proteobacteria.c__Betaproteobacteria.o__Burkholder

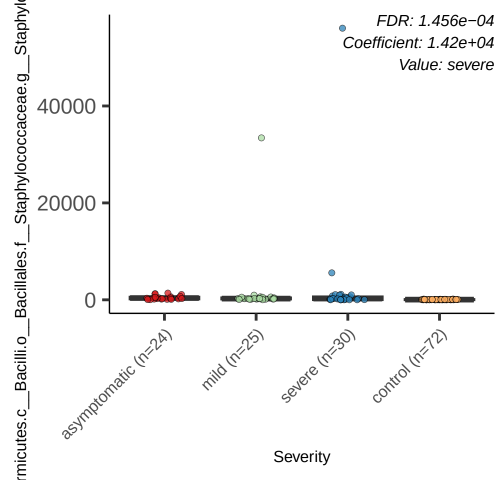

FDR: 1.456e−04
Coefficient: 1.42e+04
Value: severe
40000
20000
0
asymptomatic (n=24)
mild (n=25)
severe (n=30)
control (n=72)
Severity
rmicutes.c__Bacilli.o__Bacillales.f__Staphylococcaceae.g__Staphylo

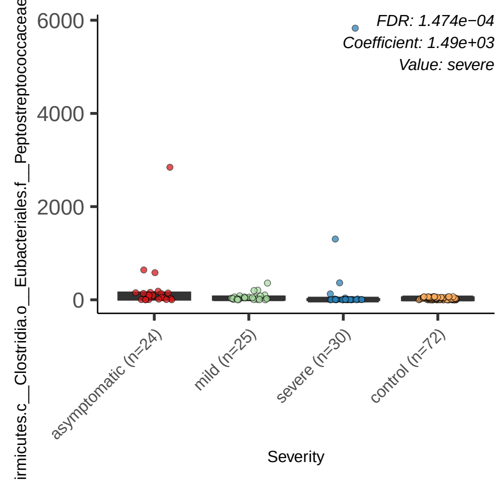

FDR: 1.474e−04
Coefficient: 1.49e+03
Value: severe
6000
4000
2000
0
irmicutes.c__Clostridia.o__Eubacteriales.f__Peptostreptococcaceae
asymptomatic (n=24)
mild (n=25)
severe (n=30)
control (n=72)
Severity

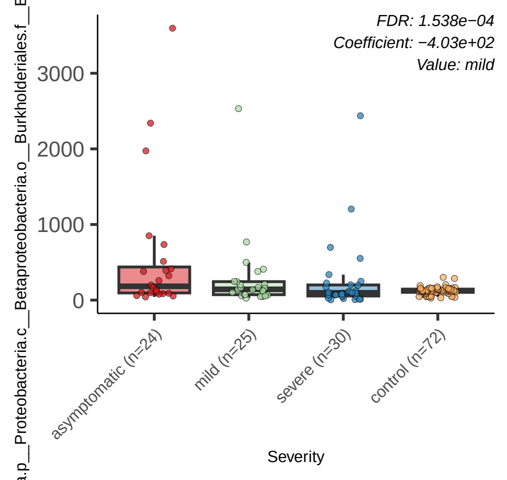
FDR: 1.538e−04
Coefficient: −4.03e+02
Value: mild
3000
2000
1000
0
Proteobacteria.c__Betaproteobacteria.o__Burkholderiales.f__
asymptomatic (n=24)
mild (n=25)
severe (n=30)
control (n=72)
Severity

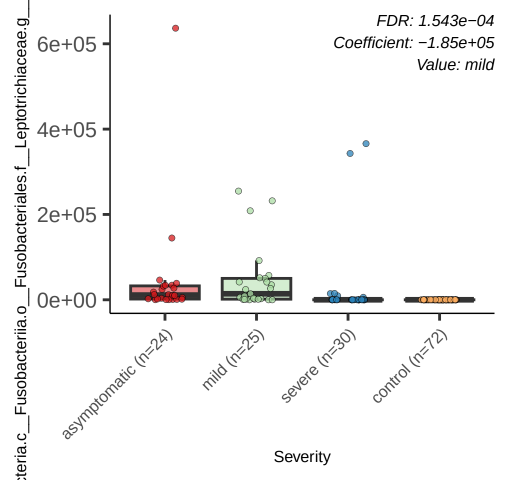
FDR: 1.543e−04
Coefficient: −1.85e+05
Value: mild
6e+05
4e+05
2e+05
0e+00
cteria.c__Fusobacteriia.o__Fusobacteriales.f__Leptotrichiaceae.g__
asymptomatic (n=24)
mild (n=25)
severe (n=30)
control (n=72)
Severity

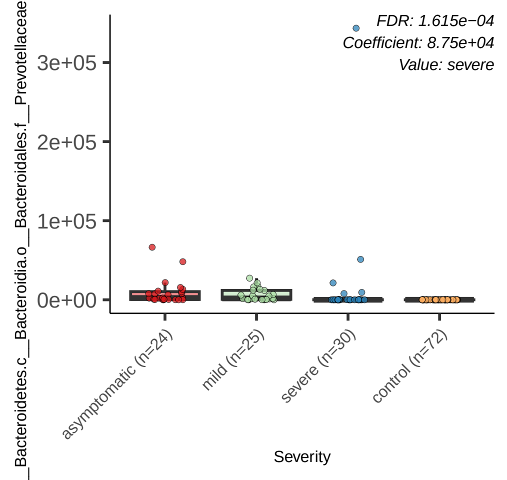

FDR: 1.615e−04
Coefficient: 8.75e+04
Value: severe
3e+05
2e+05
1e+05
0e+00
Bacteroidetes.c__Bacteroidia.o__Bacteroidales.f__Prevotellaceae
asymptomatic (n=24)
mild (n=25)
severe (n=30)
control (n=72)
Severity

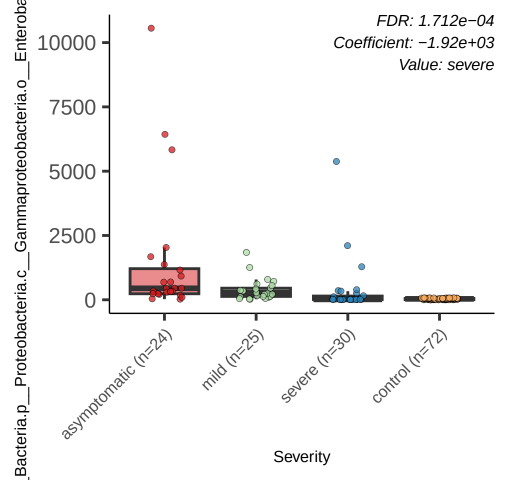

FDR: 1.712e−04
Coefficient: −1.92e+03
Value: severe
10000
7500
5000
2500
0
_Bacteria.p__Proteobacteria.c__Gammaproteobacteria.o__Enteroba
asymptomatic (n=24)
mild (n=25)
severe (n=30)
control (n=72)
Severity

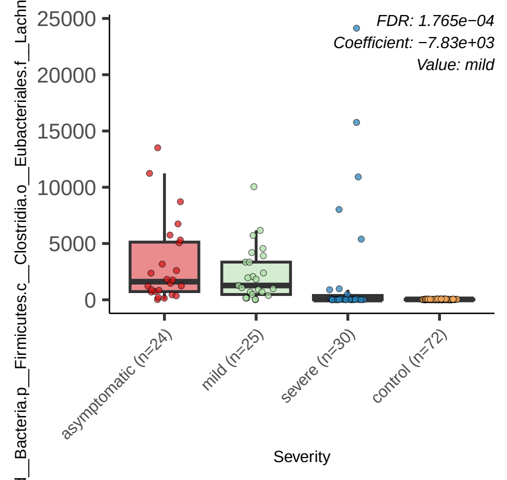
FDR: 1.765e−04
Coefficient: −7.83e+03
Value: mild
25000
20000
15000
10000
5000
0
d__Bacteria.p__Firmicutes.c__Clostridia.o__Eubacteriales.f__Lachn
asymptomatic (n=24)
mild (n=25)
severe (n=30)
control (n=72)
Severity

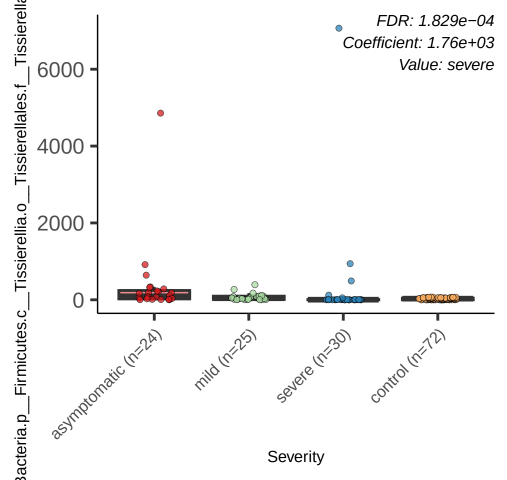

FDR: 1.829e−04
Coefficient: 1.76e+03
Value: severe
6000
4000
2000
0
asymptomatic (n=24)
mild (n=25)
severe (n=30)
control (n=72)
Severity
Bacteria.p__Firmicutes.c__Tissierellia.o__Tissierellales.f__Tissierella

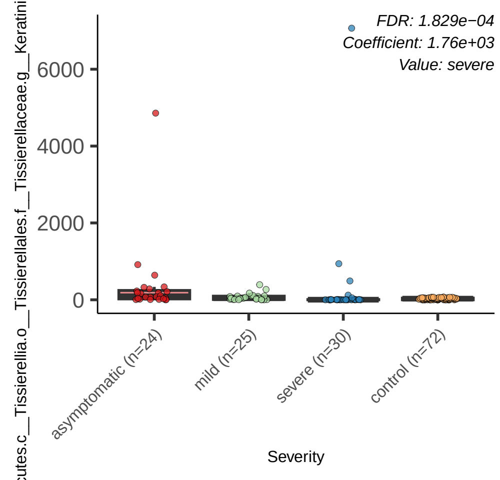

FDR: 1.829e−04
Coefficient: 1.76e+03
Value: severe
6000
4000
2000
0
cutes.c__Tissierellia.o__Tissierellales.f__Tissierellaceae.g__Keratini
asymptomatic (n=24)
mild (n=25)
severe (n=30)
control (n=72)
Severity

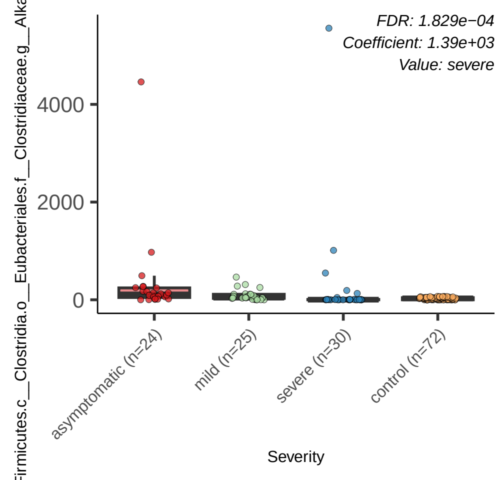
FDR: 1.829e−04
Coefficient: 1.39e+03
Value: severe
Firmicutes.c__Clostridia.o__Eubacteriales.f__Clostridiaceae.g__Alka
4000
2000
0
asymptomatic (n=24)
mild (n=25)
severe (n=30)
control (n=72)
Severity

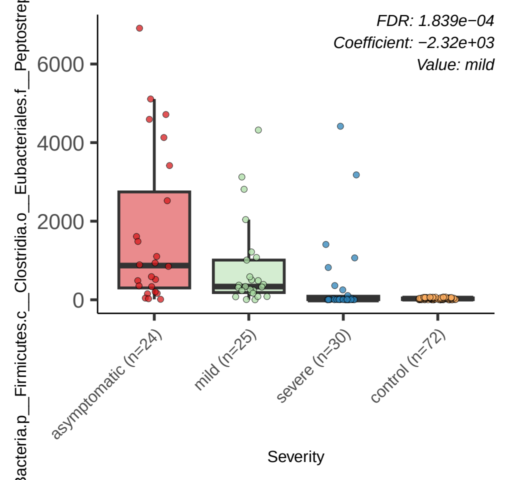

FDR: 1.839e−04
Coefficient: −2.32e+03
Value: mild
6000
4000
2000
0
Bacteria.p__Firmicutes.c__Clostridia.o__Eubacteriales.f__Peptostrep
asymptomatic (n=24)
mild (n=25)
severe (n=30)
control (n=72)
Severity

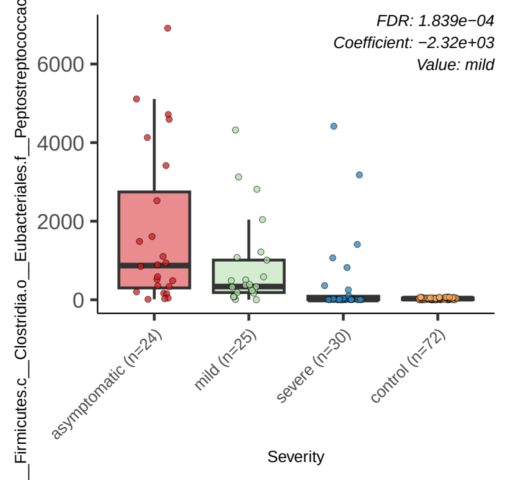
FDR: 1.839e−04
Coefficient: −2.32e+03
Value: mild
Firmicutes.c__Clostridia.o__Eubacteriales.f__Peptostreptococcac
6000
4000
2000
0
asymptomatic (n=24)
mild (n=25)
severe (n=30)
control (n=72)
Severity

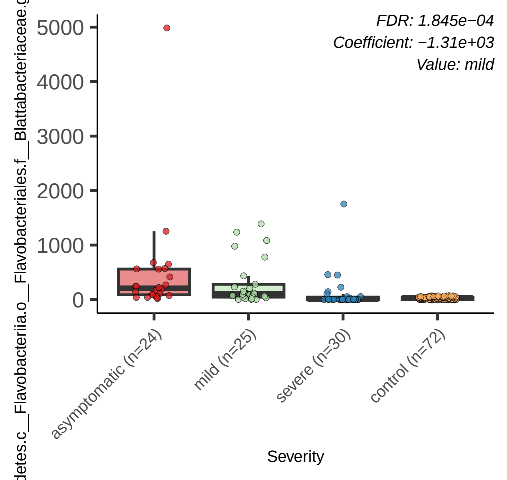
FDR: 1.845e−04
Coefficient: −1.31e+03
Value: mild
5000
4000
3000
2000
1000
0
asymptomatic (n=24)
mild (n=25)
severe (n=30)
control (n=72)
Severity
detes.c__Flavobacteriia.o__Flavobacteriales.f__Blattabacteriaceae.g

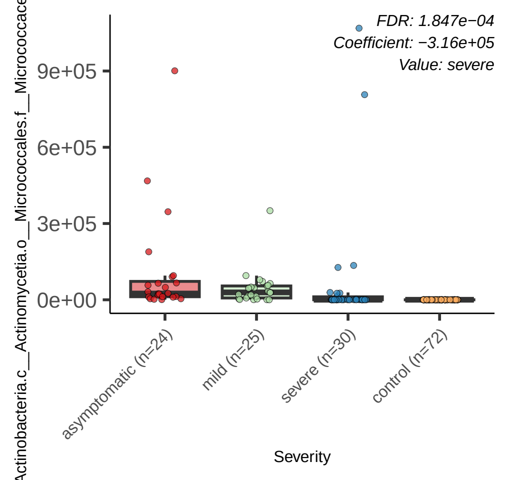

FDR: 1.847e−04
Coefficient: −3.16e+05
Value: severe
9e+05
6e+05
3e+05
0e+00
Actinobacteria.c__Actinomycetia.o__Micrococcales.f__Micrococcace
asymptomatic (n=24)
mild (n=25)
severe (n=30)
control (n=72)
Severity

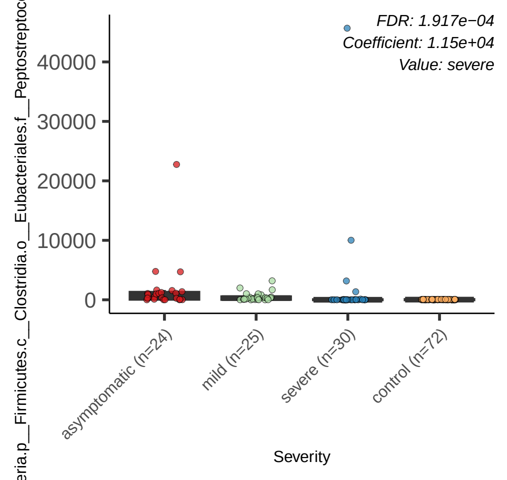

FDR: 1.917e−04
Coefficient: 1.15e+04
Value: severe
40000
30000
20000
10000
0
asymptomatic (n=24)
mild (n=25)
severe (n=30)
control (n=72)
Severity
eria.p__Firmicutes.c__Clostridia.o__Eubacteriales.f__Peptostreptoc

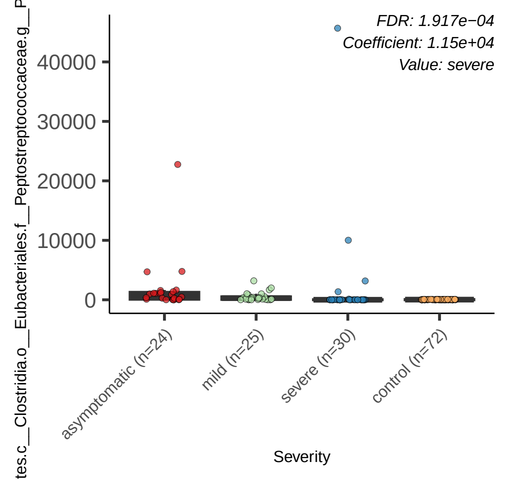

FDR: 1.917e−04
Coefficient: 1.15e+04
Value: severe
tes.c__Clostridia.o__Eubacteriales.f__Peptostreptococcaceae.g__P
40000
30000
20000
10000
0
asymptomatic (n=24)
mild (n=25)
severe (n=30)
control (n=72)
Severity

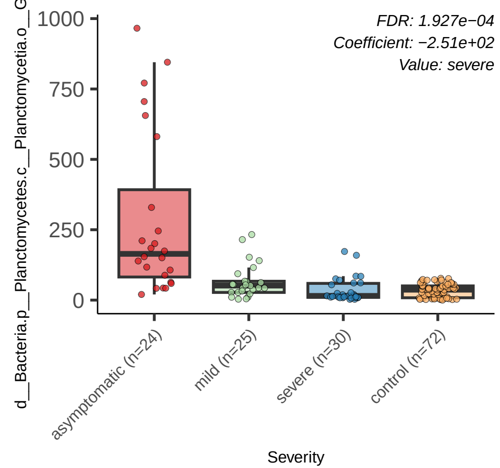

FDR: 1.927e−04
Coefficient: −2.51e+02
Value: severe
d__Bacteria.p__Planctomycetes.c__Planctomycetia.o__G
1000
750
500
250
0
asymptomatic (n=24)
mild (n=25)
severe (n=30)
control (n=72)
Severity

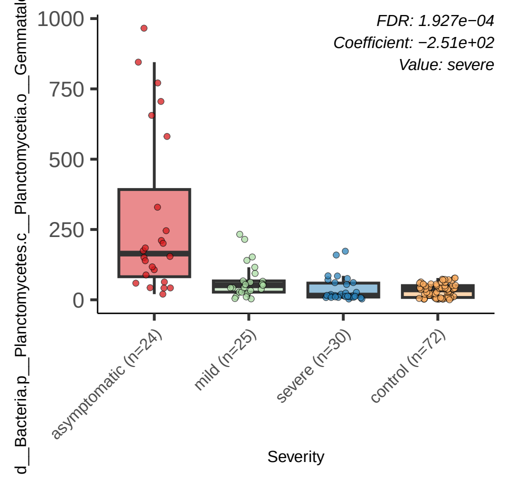
FDR: 1.927e−04
Coefficient: −2.51e+02
Value: severe
d__Bacteria.p__Planctomycetes.c__Planctomycetia.o__Gemmatalo
1000
750
500
250
0
asymptomatic (n=24)
mild (n=25)
severe (n=30)
control (n=72)
Severity

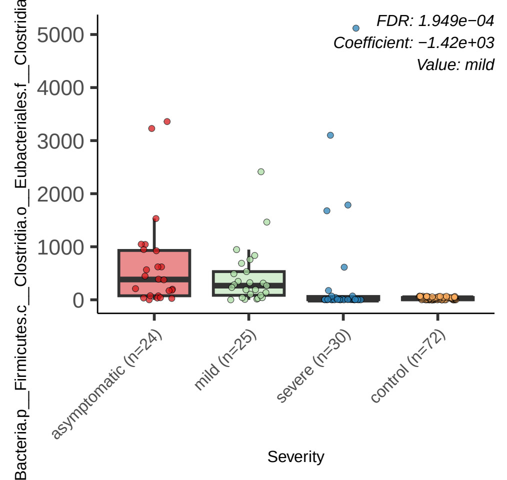

FDR: 1.949e−04
Coefficient: −1.42e+03
Value: mild
Bacteria.p__Firmicutes.c__Clostridia.o__Eubacteriales.f__Clostridia
0
1000
2000
3000
4000
5000
asymptomatic (n=24)
mild (n=25)
severe (n=30)
control (n=72)
Severity

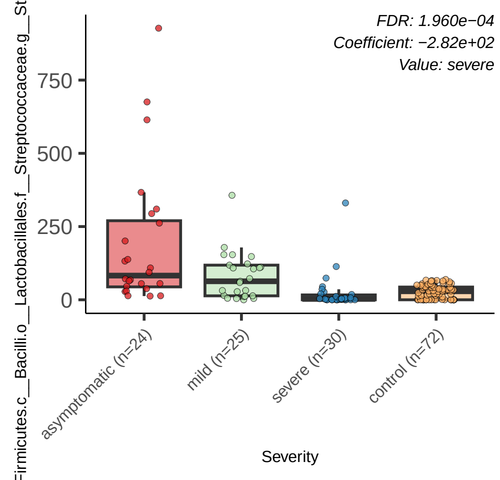

FDR: 1.960e−04
Coefficient: −2.82e+02
Value: severe
Firmicutes.c__Bacilli.o__Lactobacillales.f__Streptococcaceae.g__St
0
250
500
750
asymptomatic (n=24)
mild (n=25)
severe (n=30)
control (n=72)
Severity

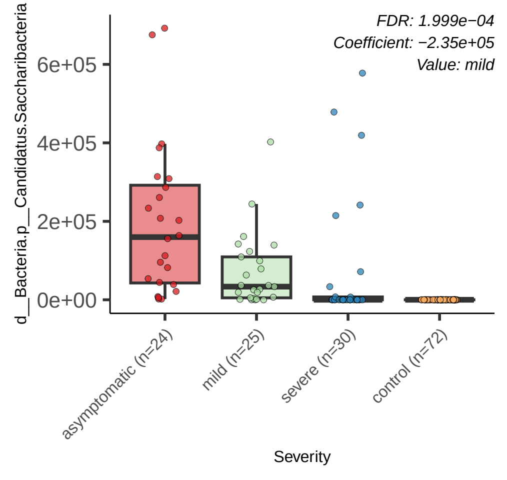

FDR: 1.999e−04
Coefficient: −2.35e+05
Value: mild
d__Bacteria.p__Candidatus.Saccharibacteria
6e+05
4e+05
2e+05
0e+00
asymptomatic (n=24)
mild (n=25)
severe (n=30)
control (n=72)
Severity

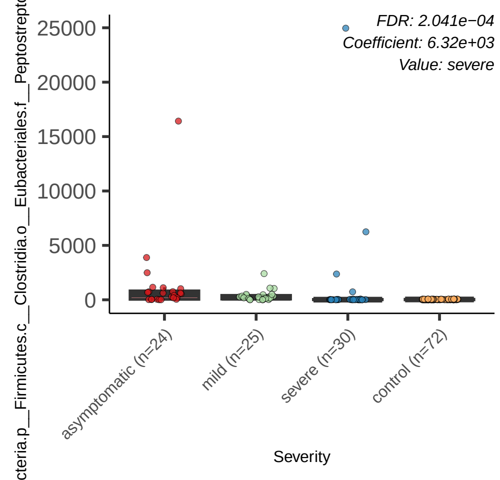

FDR: 2.041e−04
Coefficient: 6.32e+03
Value: severe
25000
20000
15000
10000
5000
0
asymptomatic (n=24)
mild (n=25)
severe (n=30)
control (n=72)
Severity
cteria.p__Firmicutes.c__Clostridia.o__Eubacteriales.f__Peptostrepto

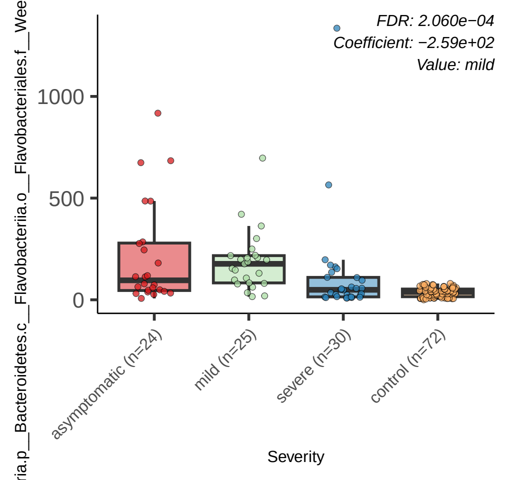

FDR: 2.060e−04
Coefficient: −2.59e+02
Value: mild
1000
500
0
asymptomatic (n=24)
mild (n=25)
severe (n=30)
control (n=72)
Severity
ria.p__Bacteroidetes.c__Flavobacteriia.o__Flavobacteriales.f__Wee

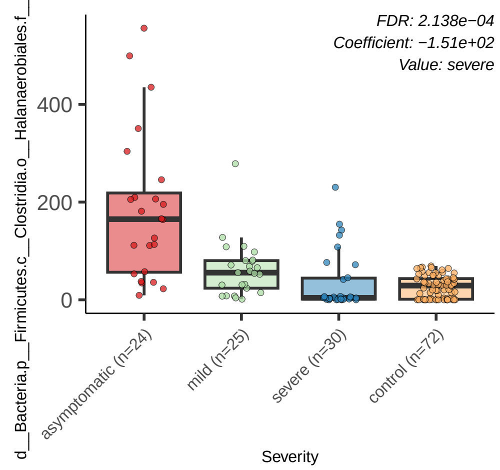

FDR: 2.138e−04
Coefficient: −1.51e+02
Value: severe
d__Bacteria.p__Firmicutes.c__Clostridia.o__Halanaerobiales.f__
400
200
0
asymptomatic (n=24)
mild (n=25)
severe (n=30)
control (n=72)
Severity

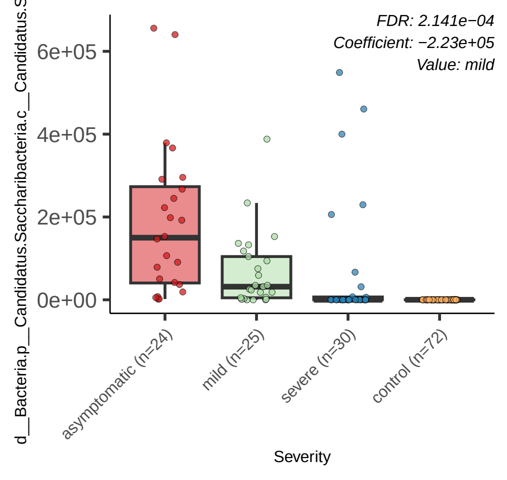

FDR: 2.141e−04
Coefficient: −2.23e+05
Value: mild
6e+05
4e+05
2e+05
0e+00
d__Bacteria.p__Candidatus.Saccharibacteria.c__Candidatus.S
asymptomatic (n=24)
mild (n=25)
severe (n=30)
control (n=72)
Severity

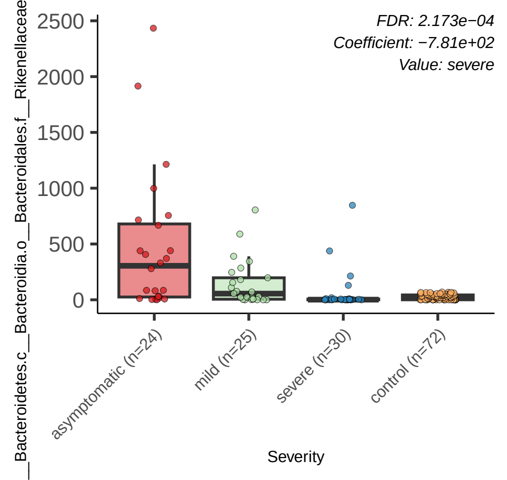
FDR: 2.173e−04
Coefficient: −7.81e+02
Value: severe
_Bacteroidetes.c__Bacteroidia.o__Bacteroidales.f__Rikenellaceae
2500
2000
1500
1000
500
0
asymptomatic (n=24)
mild (n=25)
severe (n=30)
control (n=72)
Severity

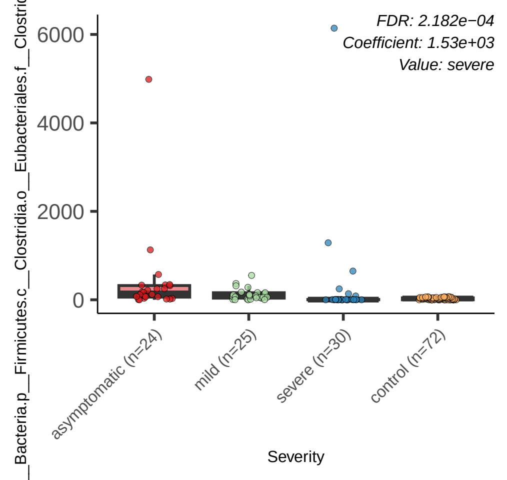

Bacteria.p__Firmicutes.c__Clostridia.o__Eubacteriales.f__Clostrid
FDR: 2.182e−04
Coefficient: 1.53e+03
Value: severe
6000
4000
2000
0
asymptomatic (n=24)
mild (n=25)
severe (n=30)
control (n=72)
Severity

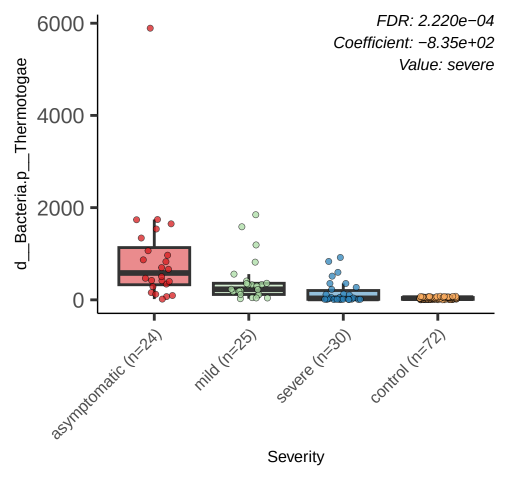

FDR: 2.220e−04
Coefficient: −8.35e+02
Value: severe
d__Bacteria.p__Thermotogae
6000
4000
2000
0
asymptomatic (n=24)
mild (n=25)
severe (n=30)
control (n=72)
Severity

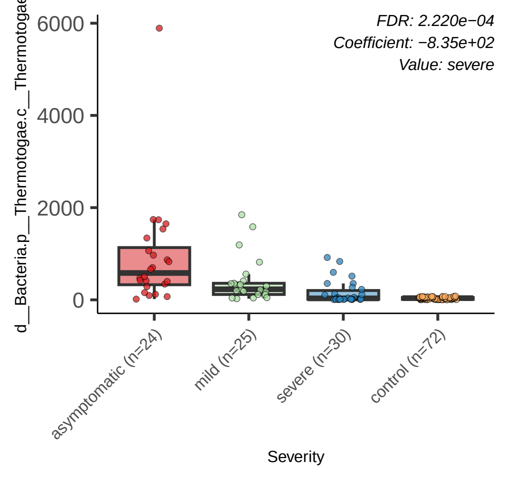

FDR: 2.220e−04
Coefficient: −8.35e+02
Value: severe
d__Bacteria.p__Thermotogae.c__Thermotogae
6000
4000
2000
0
asymptomatic (n=24)
mild (n=25)
severe (n=30)
control (n=72)
Severity

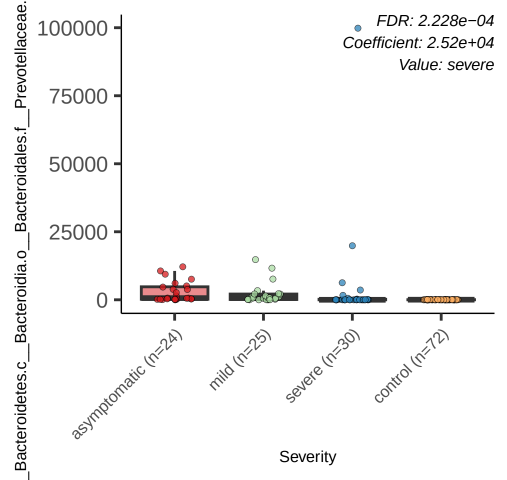
FDR: 2.228e−04
Coefficient: 2.52e+04
Value: severe
100000
75000
50000
25000
0
asymptomatic (n=24)
mild (n=25)
severe (n=30)
control (n=72)
Severity
_Bacteroidetes.c__Bacteroidia.o__Bacteroidales.f__Prevotellaceae.

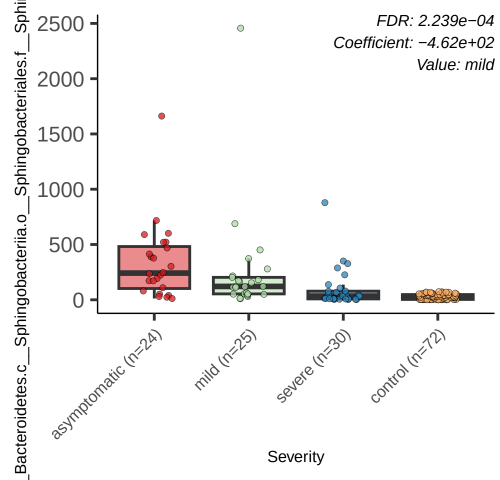

FDR: 2.239e−04
Coefficient: −4.62e+02
Value: mild
0
500
1000
1500
2000
2500
asymptomatic (n=24)
mild (n=25)
severe (n=30)
control (n=72)
Severity
Bacteroidetes.c__Sphingobacteriia.o__Sphingobacteriales.f__Sphi

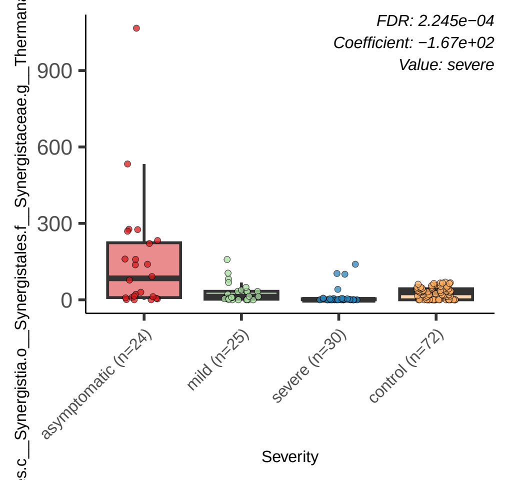

FDR: 2.245e−04
Coefficient: −1.67e+02
Value: severe
s.c__Synergistia.o__Synergistales.f__Synergistaceae.g__Therman
900
600
300
0
asymptomatic (n=24)
mild (n=25)
severe (n=30)
control (n=72)
Severity

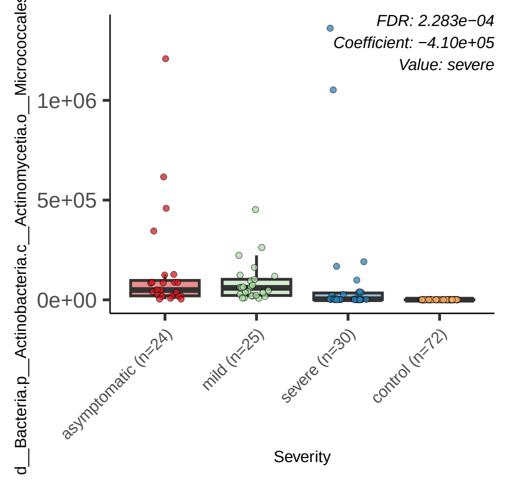

FDR: 2.283e−04
Coefficient: −4.10e+05
Value: severe
d__Bacteria.p__Actinobacteria.c__Actinomycetia.o__Micrococcales
1e+06
5e+05
0e+00
asymptomatic (n=24)
mild (n=25)
severe (n=30)
control (n=72)
Severity

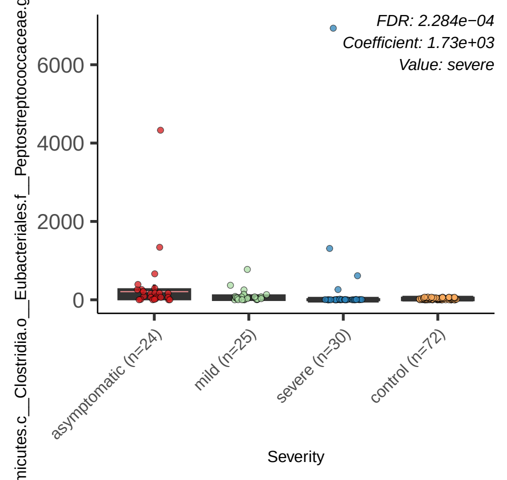

FDR: 2.284e−04
Coefficient: 1.73e+03
Value: severe
6000
4000
2000
0
micutes.c__Clostridia.o__Eubacteriales.f__Peptostreptococcaceae.g
asymptomatic (n=24)
mild (n=25)
severe (n=30)
control (n=72)
Severity

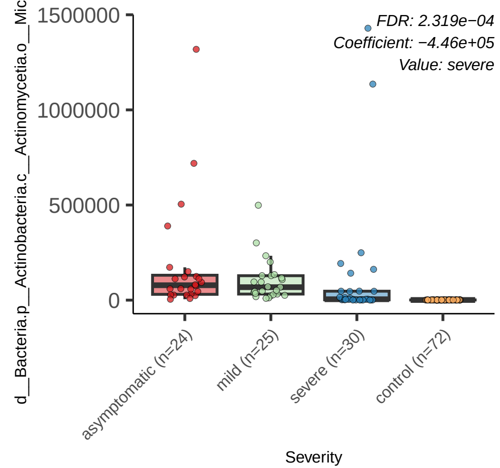

d__Bacteria.p__Actinobacteria.c__Actinomycetia.o__Mic
1500000
1000000
500000
0
FDR: 2.319e−04
Coefficient: −4.46e+05
Value: severe
asymptomatic (n=24)
mild (n=25)
severe (n=30)
control (n=72)
Severity

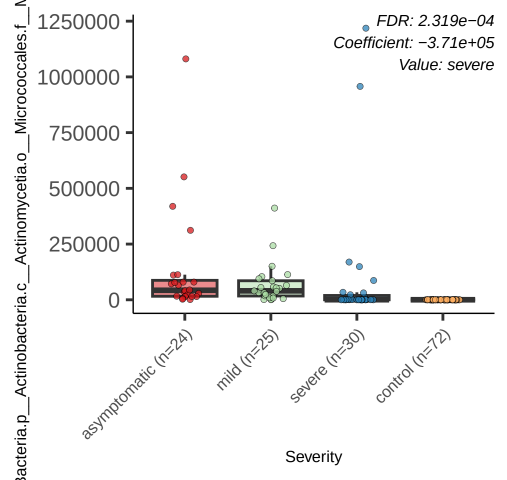

Bacteria.p__Actinobacteria.c__Actinomycetia.o__Micrococcales.f__M
1250000
1000000
750000
500000
250000
0
FDR: 2.319e−04
Coefficient: −3.71e+05
Value: severe
asymptomatic (n=24)
mild (n=25)
severe (n=30)
control (n=72)
Severity

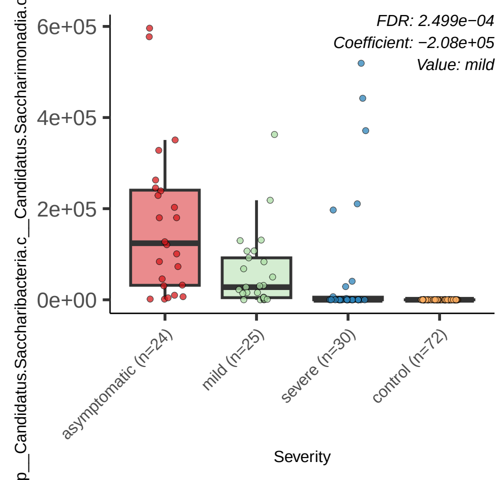

FDR: 2.499e−04
Coefficient: −2.08e+05
Value: mild
6e+05
4e+05
2e+05
0e+00
p__Candidatus.Saccharibacteria.c__Candidatus.Saccharimonadia.c
asymptomatic (n=24)
mild (n=25)
severe (n=30)
control (n=72)
Severity

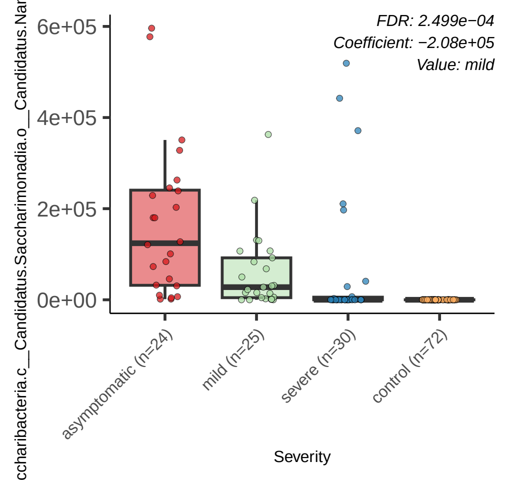

FDR: 2.499e−04
Coefficient: −2.08e+05
Value: mild
6e+05
4e+05
2e+05
0e+00
ccharibacteria.c__Candidatus.Saccharimonadia.o__Candidatus.Nar
asymptomatic (n=24)
mild (n=25)
severe (n=30)
control (n=72)
Severity

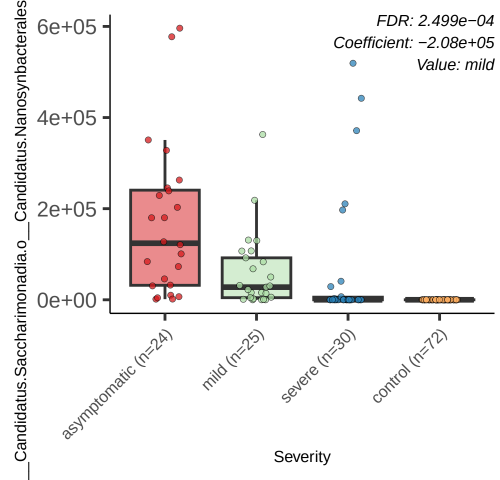

FDR: 2.499e−04
Coefficient: −2.08e+05
Value: mild
6e+05
4e+05
2e+05
0e+00
_Candidatus.Saccharimonadia.o__Candidatus.Nanosynbacterales
asymptomatic (n=24)
mild (n=25)
severe (n=30)
control (n=72)
Severity

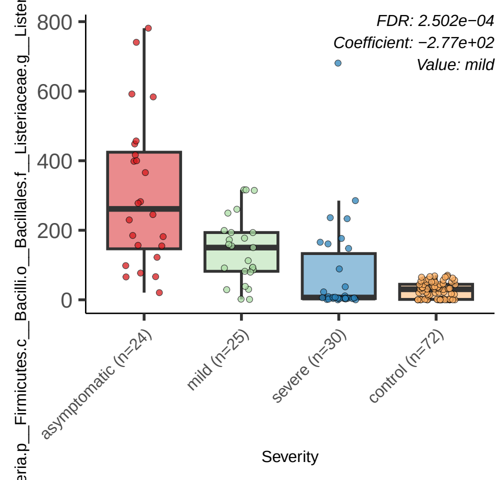

FDR: 2.502e−04
Coefficient: −2.77e+02
Value: mild
0
200
400
600
800
asymptomatic (n=24)
mild (n=25)
severe (n=30)
control (n=72)
Severity

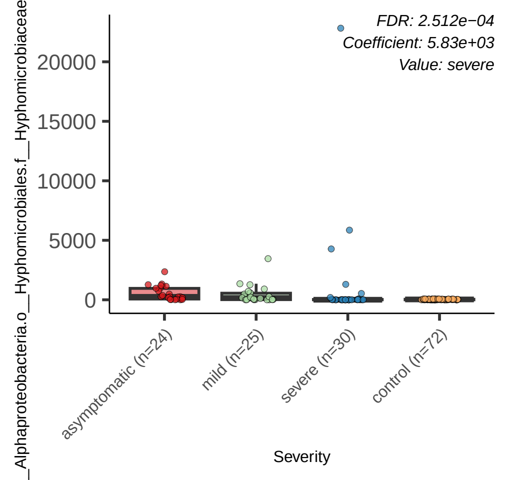

FDR: 2.512e−04
Coefficient: 5.83e+03
Value: severe
20000
15000
10000
5000
0
Alphaproteobacteria.o__Hyphomicrobiales.f__Hyphomicrobiaceae
asymptomatic (n=24)
mild (n=25)
severe (n=30)
control (n=72)
Severity

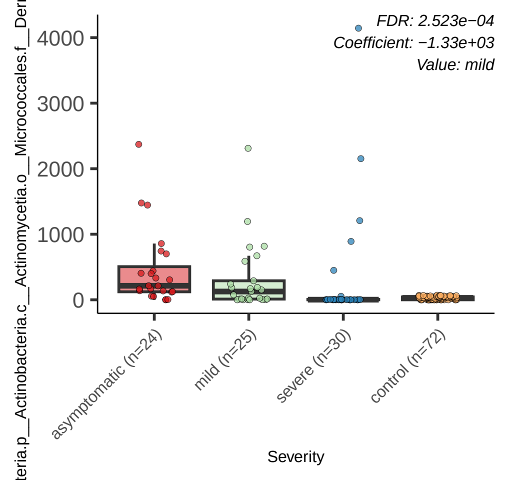

FDR: 2.523e−04
Coefficient: −1.33e+03
Value: mild
teria.p__Actinobacteria.c__Actinomycetia.o__Micrococcales.f__Derr
4000
3000
2000
1000
0
asymptomatic (n=24)
mild (n=25)
severe (n=30)
control (n=72)
Severity

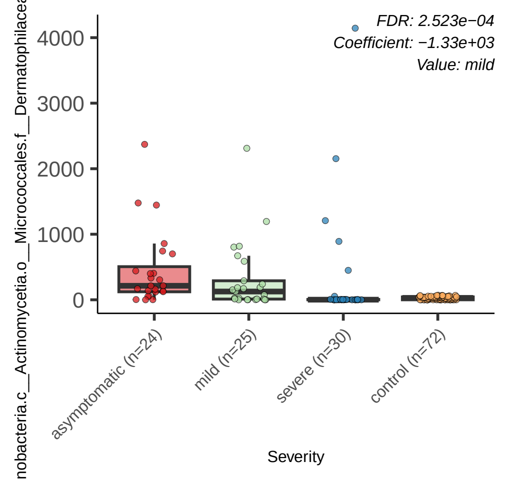

FDR: 2.523e−04
Coefficient: −1.33e+03
Value: mild
4000
3000
2000
1000
0
nobacteria.c__Actinomycetia.o__Micrococcales.f__Dermatophilacea
asymptomatic (n=24)
mild (n=25)
severe (n=30)
control (n=72)
Severity

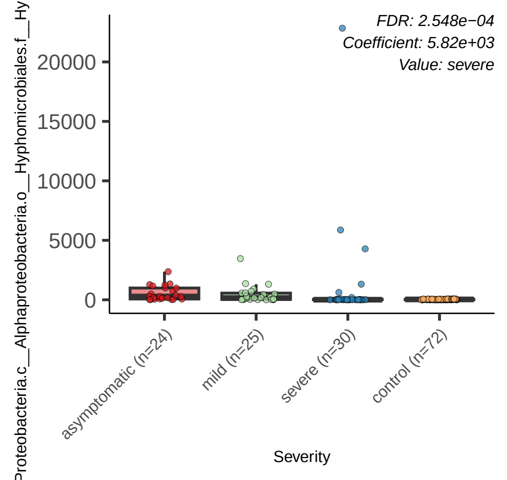

FDR: 2.548e−04
Coefficient: 5.82e+03
Value: severe
Proteobacteria.c__Alphaproteobacteria.o__Hyphomicrobiales.f__Hy
20000
15000
10000
5000
0
asymptomatic (n=24)
mild (n=25)
severe (n=30)
control (n=72)
Severity

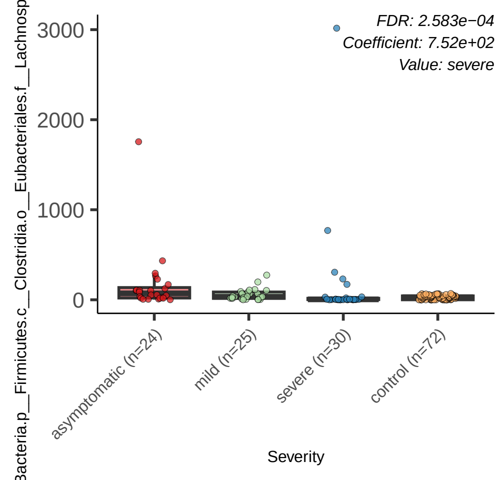
FDR: 2.583e−04
Coefficient: 7.52e+02
Value: severe
3000
2000
1000
0
Bacteria.p__Firmicutes.c__Clostridia.o__Eubacteriales.f__Lachnosp
asymptomatic (n=24)
mild (n=25)
severe (n=30)
control (n=72)
Severity

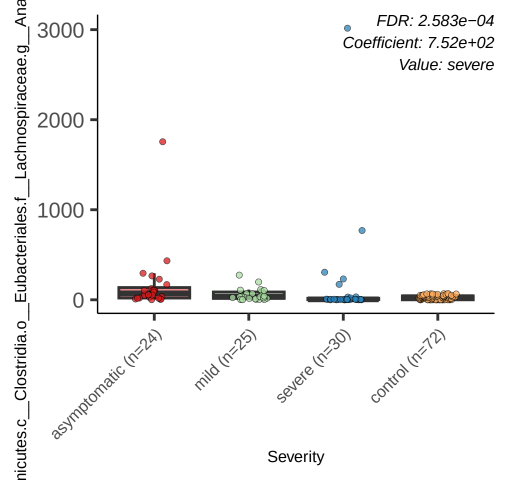

FDR: 2.583e−04
Coefficient: 7.52e+02
Value: severe
3000
2000
1000
0
asymptomatic (n=24)
mild (n=25)
severe (n=30)
control (n=72)
Severity
nicutes.c__Clostridia.o__Eubacteriales.f__Lachnospiraceae.g__Ana

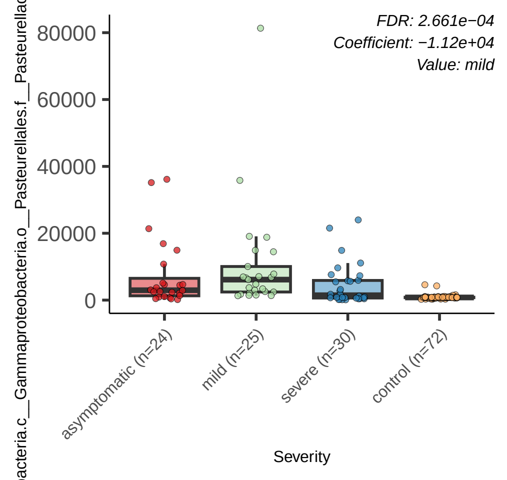

FDR: 2.661e−04
Coefficient: −1.12e+04
Value: mild
80000
60000
40000
20000
0
acteria.c__Gammaproteobacteria.o__Pasteurellales.f__Pasteurellac
asymptomatic (n=24)
mild (n=25)
severe (n=30)
control (n=72)
Severity

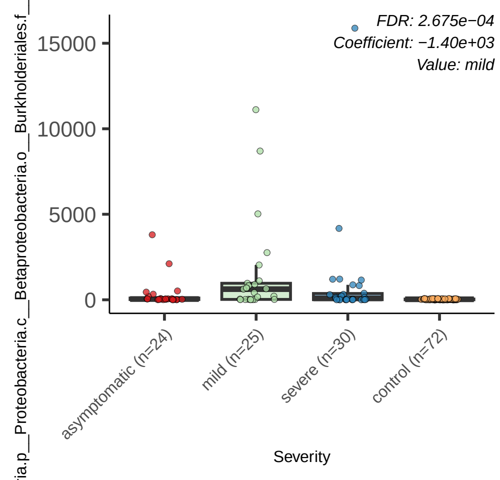

FDR: 2.675e−04
Coefficient: −1.40e+03
Value: mild
ria.p__Proteobacteria.c__Betaproteobacteria.o__Burkholderiales.f_
15000
10000
5000
0
asymptomatic (n=24)
mild (n=25)
severe (n=30)
control (n=72)
Severity

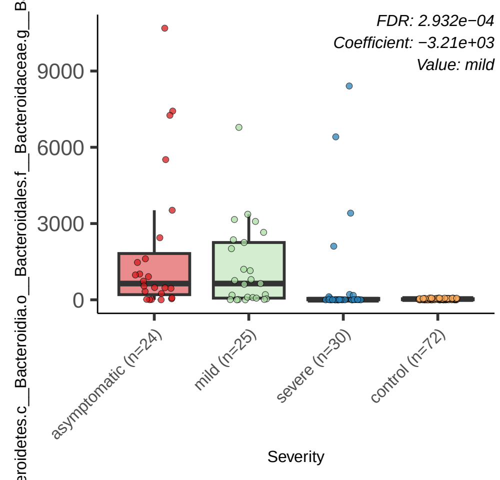
FDR: 2.932e−04
Coefficient: −3.21e+03
Value: mild
9000
6000
3000
0
asymptomatic (n=24)
mild (n=25)
severe (n=30)
control (n=72)
Severity
eroidetes.c__Bacteroidia.o__Bacteroidales.f__Bacteroidaceae.g__B

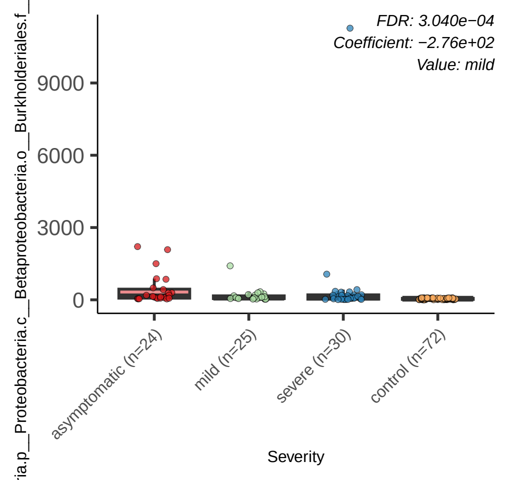

FDR: 3.040e−04
Coefficient: −2.76e+02
Value: mild
9000
6000
3000
0
ria.p__Proteobacteria.c__Betaproteobacteria.o__Burkholderiales.f__
asymptomatic (n=24)
mild (n=25)
severe (n=30)
control (n=72)
Severity

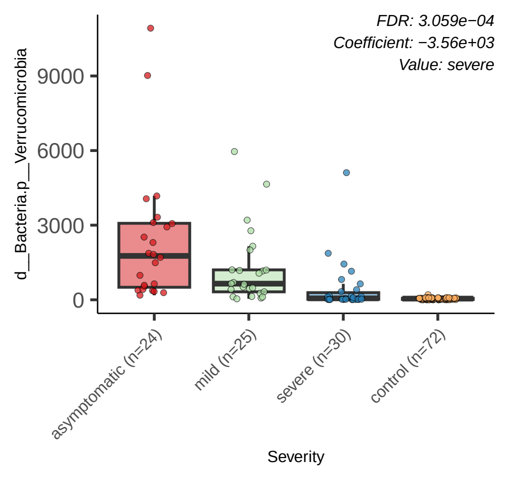
FDR: 3.059e−04
Coefficient: −3.56e+03
Value: severe
d__Bacteria.p__Verrucomicrobia
9000
6000
3000
0
asymptomatic (n=24)
mild (n=25)
severe (n=30)
control (n=72)
Severity

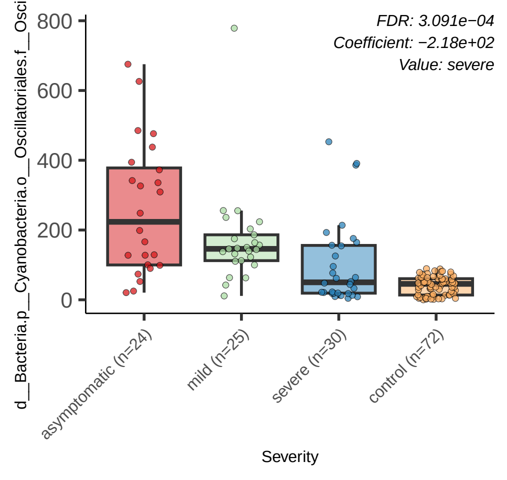
FDR: 3.091e−04
Coefficient: −2.18e+02
Value: severe
d__Bacteria.p__Cyanobacteria.o__Oscillatoriales.f__Osci
800
600
400
200
0
asymptomatic (n=24)
mild (n=25)
severe (n=30)
control (n=72)
Severity

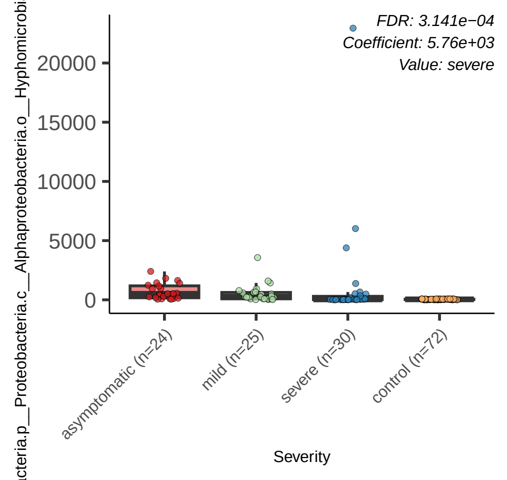

FDR: 3.141e−04
Coefficient: 5.76e+03
Value: severe
20000
15000
10000
5000
0
acteria.p__Proteobacteria.c__Alphaproteobacteria.o__Hyphomicrobi
asymptomatic (n=24)
mild (n=25)
severe (n=30)
control (n=72)
Severity

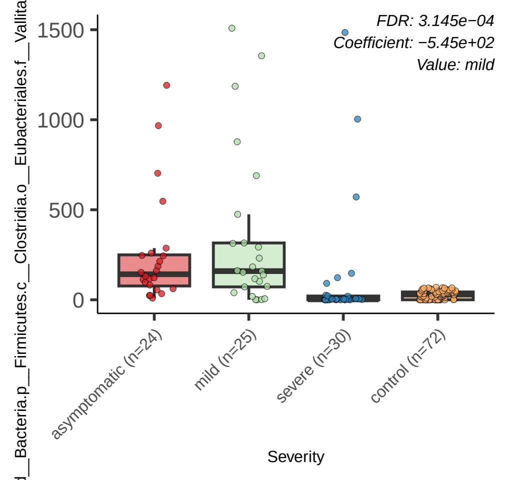
d__Bacteria.p__Firmicutes.c__Clostridia.o__Eubacteriales.f__Vallita
FDR: 3.145e−04
Coefficient: −5.45e+02
Value: mild
1500
1000
500
0
asymptomatic (n=24)
mild (n=25)
severe (n=30)
control (n=72)
Severity

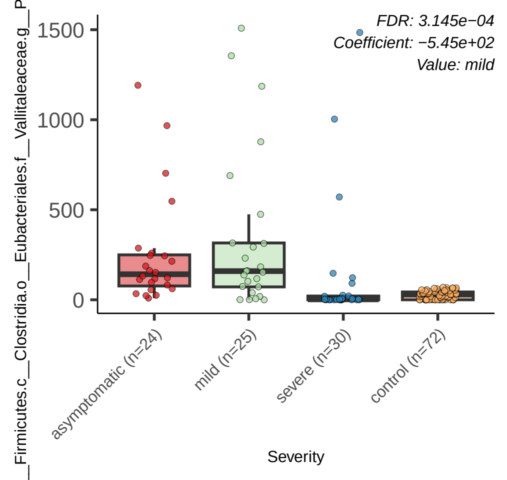

FDR: 3.145e−04
Coefficient: −5.45e+02
Value: mild
Firmicutes.c__Clostridia.o__Eubacteriales.f__Vallitaleaceae.g__P
1500
1000
500
0
asymptomatic (n=24)
mild (n=25)
severe (n=30)
control (n=72)
Severity

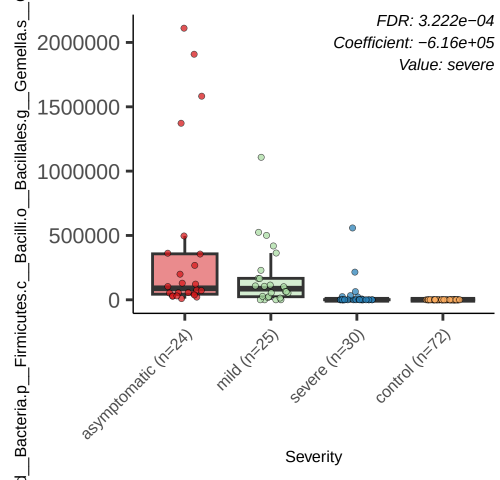

FDR: 3.222e−04
Coefficient: −6.16e+05
Value: severe
2000000
1500000
1000000
500000
0
asymptomatic (n=24)
mild (n=25)
severe (n=30)
control (n=72)
Severity
Bacteria.p__Firmicutes.c__Bacilli.o__Bacillales.g__Gemella.s__

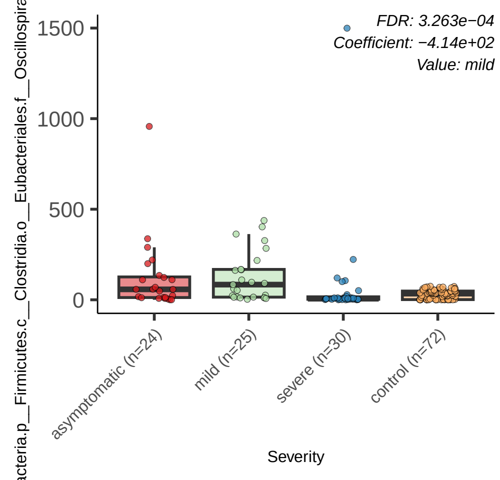
FDR: 3.263e−04
Coefficient: −4.14e+02
Value: mild
1500
1000
500
0
acteria.p__Firmicutes.c__Clostridia.o__Eubacteriales.f__Oscillospira
asymptomatic (n=24)
mild (n=25)
severe (n=30)
control (n=72)
Severity

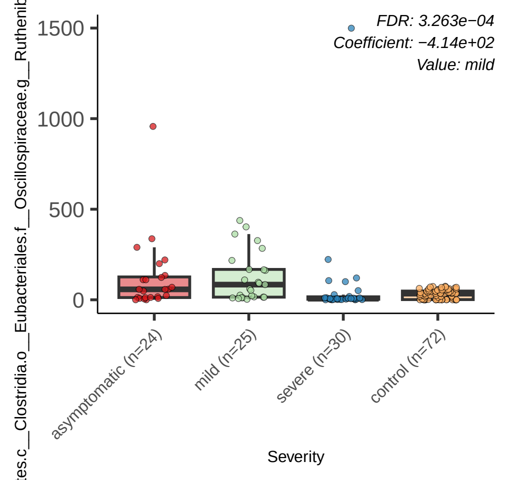

FDR: 3.263e−04
Coefficient: −4.14e+02
Value: mild
1500
1000
500
0
tes.c__Clostridia.o__Eubacteriales.f__Oscillospiraceae.g__Ruthenib
asymptomatic (n=24)
mild (n=25)
severe (n=30)
control (n=72)
Severity

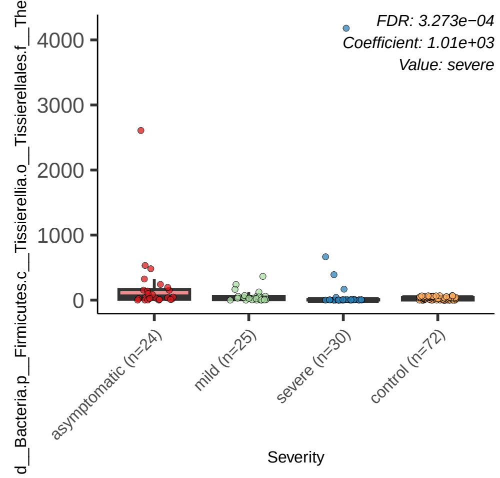

FDR: 3.273e−04
Coefficient: 1.01e+03
Value: severe
d__Bacteria.p__Firmicutes.c__Tissierellia.o__Tissierellales.f__The
4000
3000
2000
1000
0
asymptomatic (n=24)
mild (n=25)
severe (n=30)
control (n=72)
Severity

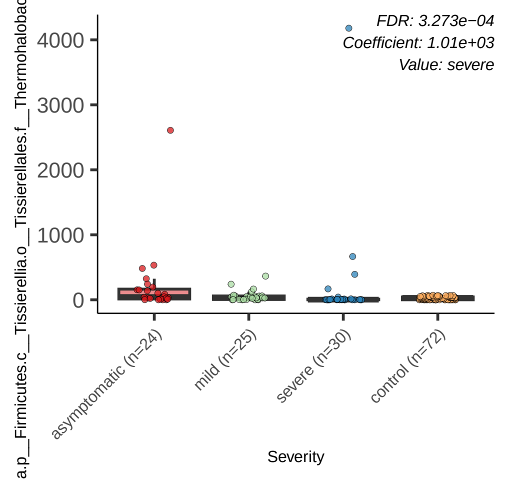
FDR: 3.273e−04
Coefficient: 1.01e+03
Value: severe
a.p__Firmicutes.c__Tissierellia.o__Tissierellales.f__Thermohalobac
4000
3000
2000
1000
0
asymptomatic (n=24)
mild (n=25)
severe (n=30)
control (n=72)
Severity

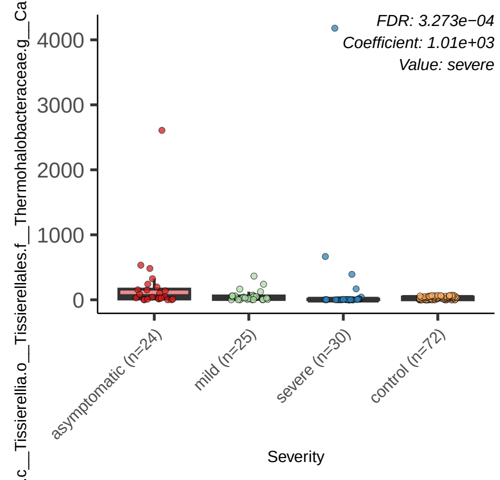

FDR: 3.273e−04
Coefficient: 1.01e+03
Value: severe
c__Tissierellia.o__Tissierellales.f__Thermohalobacteraceae.g__Ca
4000
3000
2000
1000
0
asymptomatic (n=24)
mild (n=25)
severe (n=30)
control (n=72)
Severity

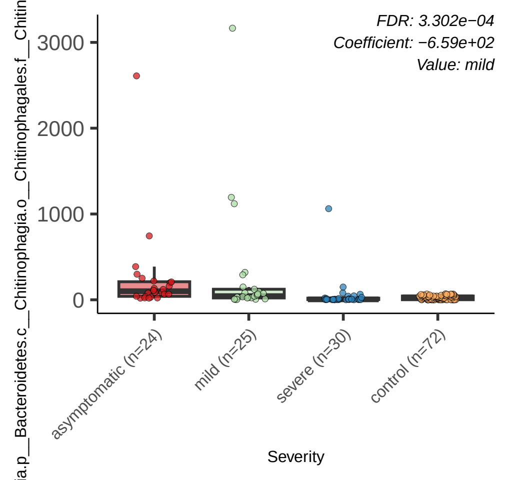

FDR: 3.302e−04
Coefficient: −6.59e+02
Value: mild
ia.p__Bacteroidetes.c__Chitinophagia.o__Chitinophagales.f__Chitin
3000
2000
1000
0
asymptomatic (n=24)
mild (n=25)
severe (n=30)
control (n=72)
Severity

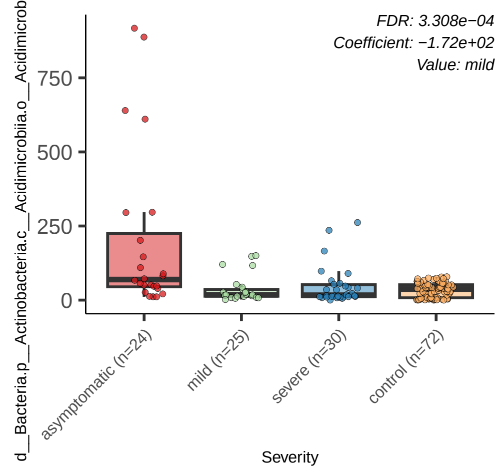

FDR: 3.308e−04
Coefficient: −1.72e+02
Value: mild
d__Bacteria.p__Actinobacteria.c__Acidimicrobiia.o__Acidimicrob
750
500
250
0
asymptomatic (n=24)
mild (n=25)
severe (n=30)
control (n=72)
Severity

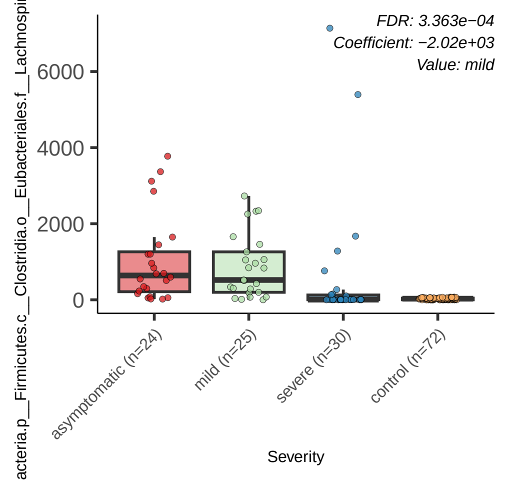

FDR: 3.363e−04
Coefficient: −2.02e+03
Value: mild
acteria.p__Firmicutes.c__Clostridia.o__Eubacteriales.f__Lachnospi
6000
4000
2000
0
asymptomatic (n=24)
mild (n=25)
severe (n=30)
control (n=72)
Severity

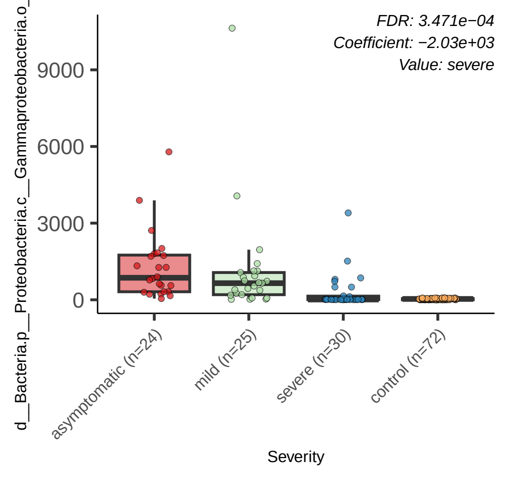
FDR: 3.471e−04
Coefficient: −2.03e+03
Value: severe
d__Bacteria.p__Proteobacteria.c__Gammaproteobacteria.o__
9000
6000
3000
0
asymptomatic (n=24)
mild (n=25)
severe (n=30)
control (n=72)
Severity

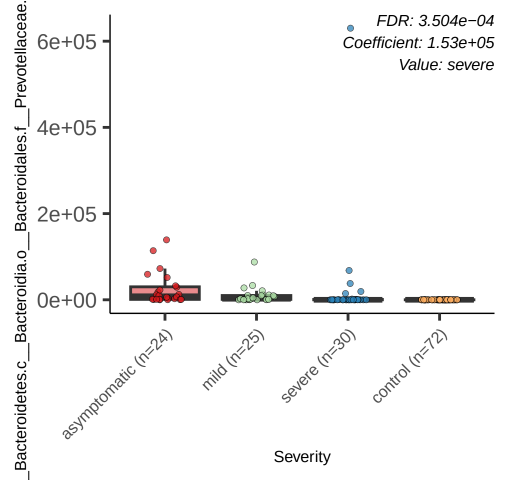

FDR: 3.504e−04
Coefficient: 1.53e+05
Value: severe
6e+05
4e+05
2e+05
0e+00
_Bacteroidetes.c__Bacteroidia.o__Bacteroidales.f__Prevotellaceae.
asymptomatic (n=24)
mild (n=25)
severe (n=30)
control (n=72)
Severity

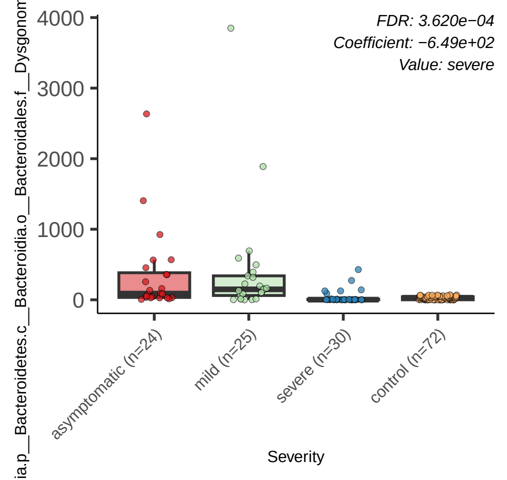
FDR: 3.620e−04
Coefficient: −6.49e+02
Value: severe
ia.p__Bacteroidetes.c__Bacteroidia.o__Bacteroidales.f__Dysgonom
4000
3000
2000
1000
0
asymptomatic (n=24)
mild (n=25)
severe (n=30)
control (n=72)
Severity

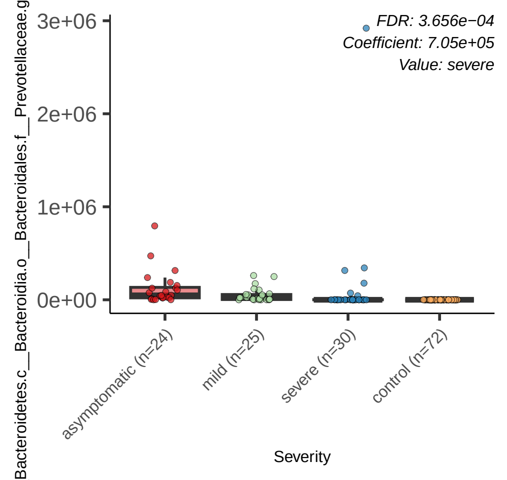

FDR: 3.656e−04
Coefficient: 7.05e+05
Value: severe
3e+06
2e+06
1e+06
0e+00
Bacteroidetes.c__Bacteroidia.o__Bacteroidales.f__Prevotellaceae.g
asymptomatic (n=24)
mild (n=25)
severe (n=30)
control (n=72)
Severity

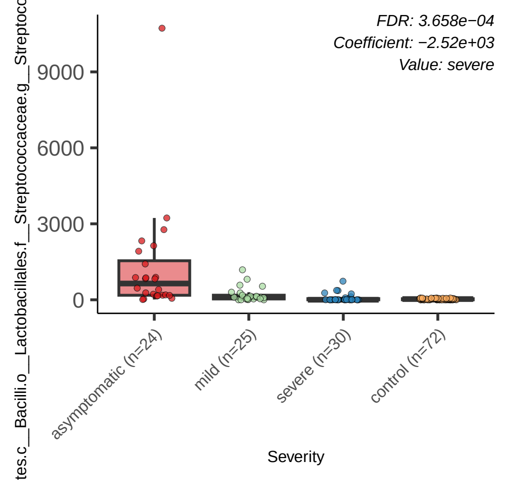

FDR: 3.658e−04
Coefficient: −2.52e+03
Value: severe
9000
6000
3000
0
tes.c__Bacilli.o__Lactobacillales.f__Streptococcaceae.g__Streptoco
asymptomatic (n=24)
mild (n=25)
severe (n=30)
control (n=72)
Severity

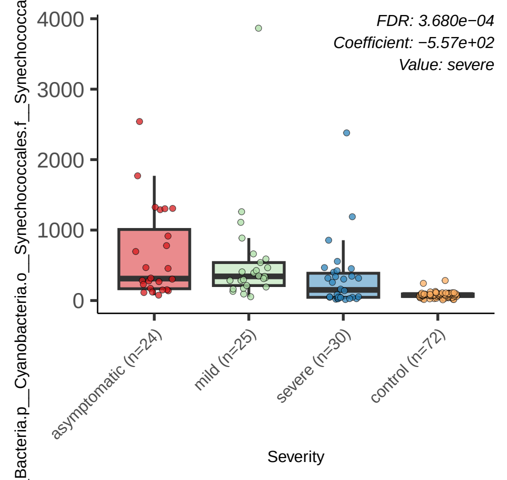

FDR: 3.680e−04
Coefficient: −5.57e+02
Value: severe
Bacteria.p__Cyanobacteria.o__Synechococcales.f__Synechococca
4000
3000
2000
1000
0
asymptomatic (n=24)
mild (n=25)
severe (n=30)
control (n=72)
Severity

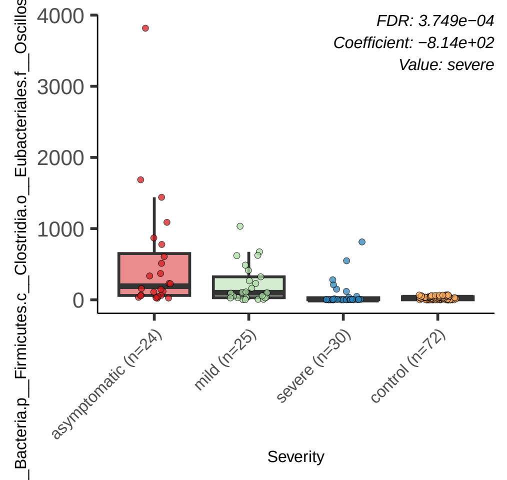

Bacteria.p__Firmicutes.c__Clostridia.o__Eubacteriales.f__Oscillos
4000
3000
2000
1000
0
FDR: 3.749e−04
Coefficient: −8.14e+02
Value: severe
asymptomatic (n=24)
mild (n=25)
severe (n=30)
control (n=72)
Severity

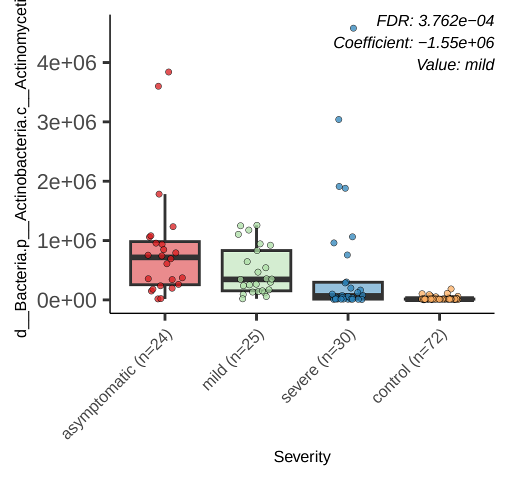
FDR: 3.762e−04
Coefficient: −1.55e+06
Value: mild
d__Bacteria.p__Actinobacteria.c__Actinomyceti
4e+06
3e+06
2e+06
1e+06
0e+00
asymptomatic (n=24)
mild (n=25)
severe (n=30)
control (n=72)
Severity

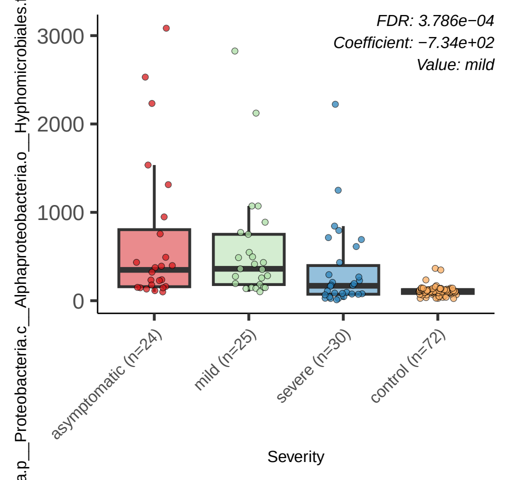

FDR: 3.786e−04
Coefficient: −7.34e+02
Value: mild
a.p__Proteobacteria.c__Alphaproteobacteria.o__Hyphomicrobiales.f
3000
2000
1000
0
asymptomatic (n=24)
mild (n=25)
severe (n=30)
control (n=72)
Severity

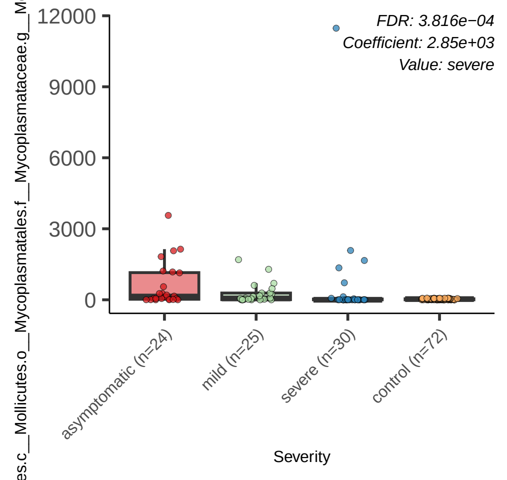

FDR: 3.816e−04
Coefficient: 2.85e+03
Value: severe
12000
9000
6000
3000
0
asymptomatic (n=24)
mild (n=25)
severe (n=30)
control (n=72)
Severity
es.c__Mollicutes.o__Mycoplasmatales.f__Mycoplasmataceae.g__M

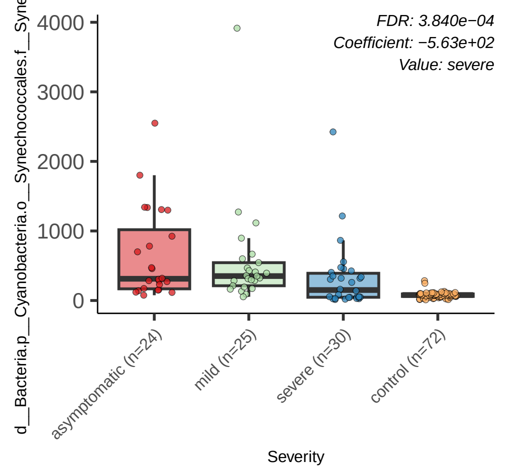

FDR: 3.840e−04
Coefficient: −5.63e+02
Value: severe
d__Bacteria.p__Cyanobacteria.o__Synechococcales.f__Syne
4000
3000
2000
1000
0
asymptomatic (n=24)
mild (n=25)
severe (n=30)
control (n=72)
Severity

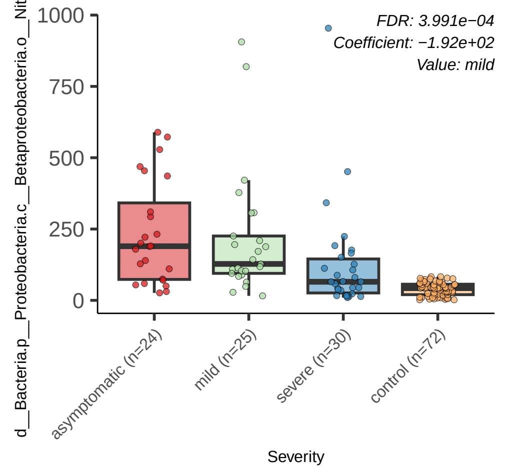

FDR: 3.991e−04
Coefficient: −1.92e+02
Value: mild
d__Bacteria.p__Proteobacteria.c__Betaproteobacteria.o__Nit
1000
750
500
250
0
asymptomatic (n=24)
mild (n=25)
severe (n=30)
control (n=72)
Severity

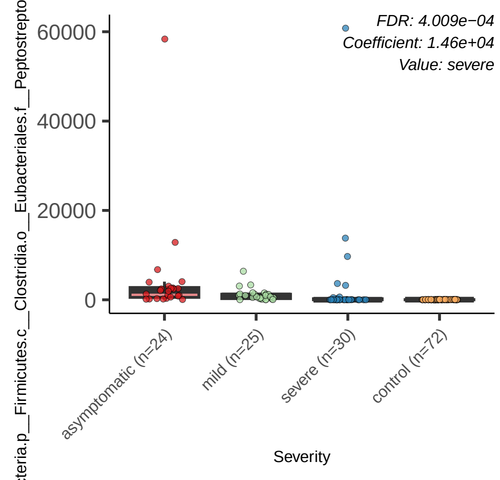
FDR: 4.009e−04
Coefficient: 1.46e+04
Value: severe
60000
40000
20000
0
asymptomatic (n=24)
mild (n=25)
severe (n=30)
control (n=72)
Severity
cteria.p__Firmicutes.c__Clostridia.o__Eubacteriales.f__Peptostrepto

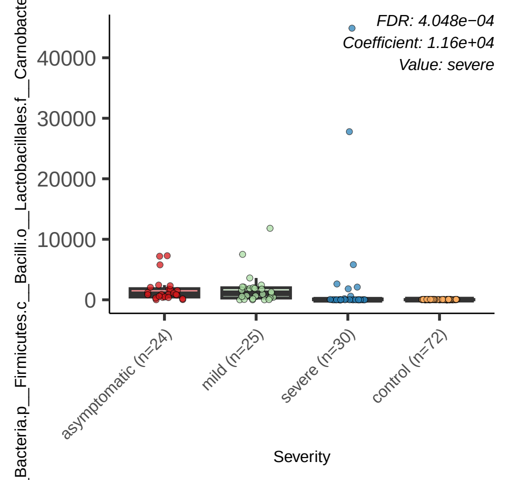

FDR: 4.048e−04
Coefficient: 1.16e+04
Value: severe
Bacteria.p__Firmicutes.c__Bacilli.o__Lactobacillales.f__Carnobacte
40000
30000
20000
10000
0
asymptomatic (n=24)
mild (n=25)
severe (n=30)
control (n=72)
Severity

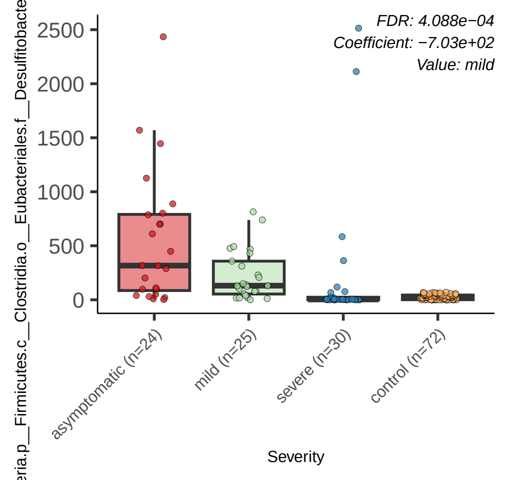

FDR: 4.088e−04
Coefficient: −7.03e+02
Value: mild
eria.p__Firmicutes.c__Clostridia.o__Eubacteriales.f__Desulfitobacte
2500
2000
1500
1000
500
0
asymptomatic (n=24)
mild (n=25)
severe (n=30)
control (n=72)
Severity

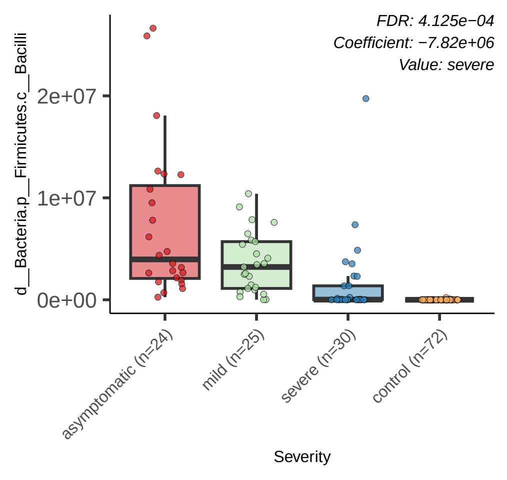
FDR: 4.125e−04
Coefficient: −7.82e+06
Value: severe
d__Bacteria.p__Firmicutes.c__Bacilli
2e+07
1e+07
0e+00
asymptomatic (n=24)
mild (n=25)
severe (n=30)
control (n=72)
Severity

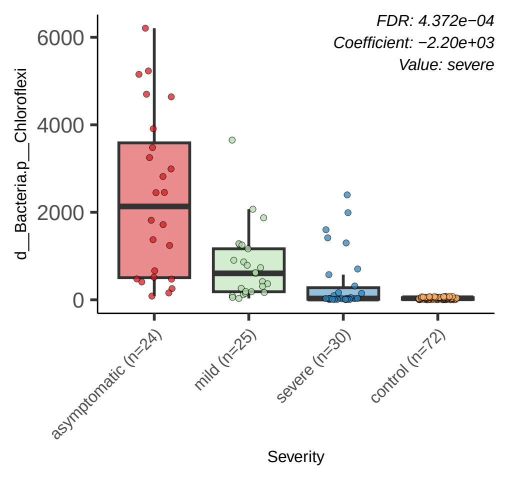
FDR: 4.372e−04
Coefficient: −2.20e+03
Value: severe
d__Bacteria.p__Chloroflexi
6000
4000
2000
0
asymptomatic (n=24)
mild (n=25)
severe (n=30)
control (n=72)
Severity

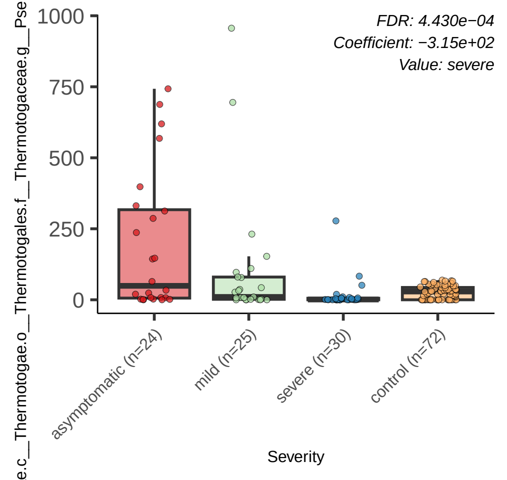

FDR: 4.430e−04
Coefficient: −3.15e+02
Value: severe
1000
750
500
250
0
e.c__Thermotogae.o__Thermotogales.f__Thermotogaceae.g__Pse
asymptomatic (n=24)
mild (n=25)
severe (n=30)
control (n=72)
Severity

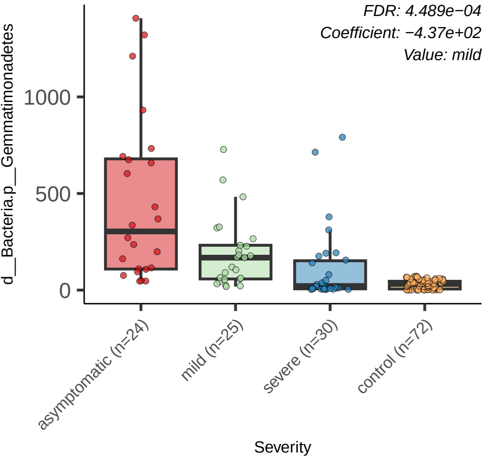

FDR: 4.489e−04
Coefficient: −4.37e+02
Value: mild
d__Bacteria.p__Gemmatimonadetes
1000
500
0
asymptomatic (n=24)
mild (n=25)
severe (n=30)
control (n=72)
Severity

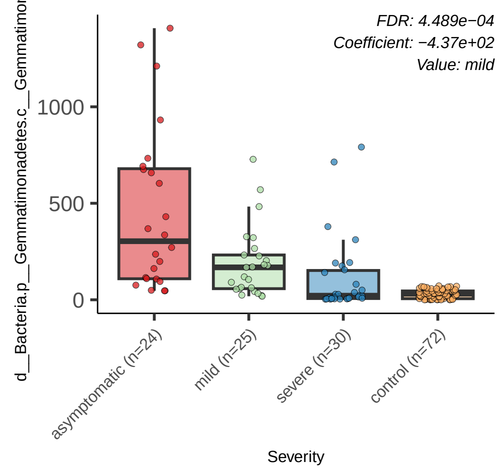
FDR: 4.489e−04
Coefficient: −4.37e+02
Value: mild
d__Bacteria.p__Gemmatimonadetes.c__Gemmatimon
1000
500
0
asymptomatic (n=24)
mild (n=25)
severe (n=30)
control (n=72)
Severity

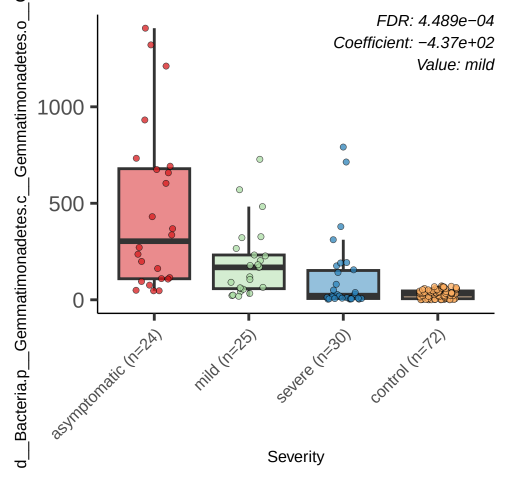

FDR: 4.489e−04
Coefficient: −4.37e+02
Value: mild
d__Bacteria.p__Gemmatimonadetes.c__Gemmatimonadetes.o__G
1000
500
0
asymptomatic (n=24)
mild (n=25)
severe (n=30)
control (n=72)
Severity

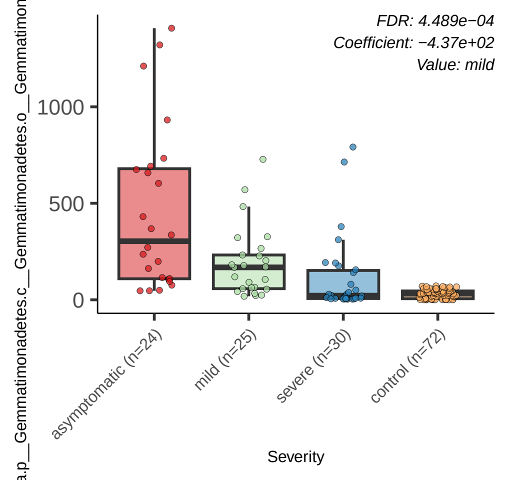
FDR: 4.489e−04
Coefficient: −4.37e+02
Value: mild
1000
500
0
a.p__Gemmatimonadetes.c__Gemmatimonadetes.o__Gemmatimon
asymptomatic (n=24)
mild (n=25)
severe (n=30)
control (n=72)
Severity

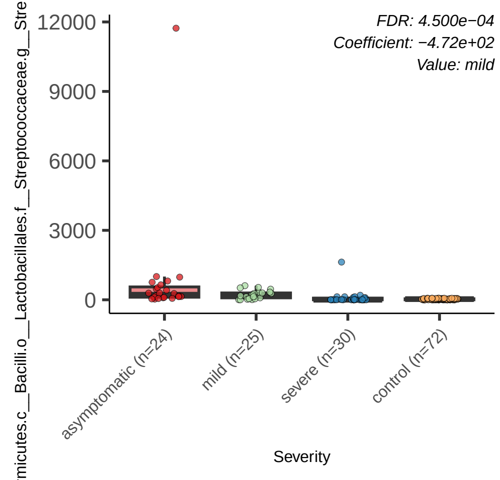
FDR: 4.500e−04
Coefficient: −4.72e+02
Value: mild
12000
9000
6000
3000
0
rmicutes.c__Bacilli.o__Lactobacillales.f__Streptococcaceae.g__Stre
asymptomatic (n=24)
mild (n=25)
severe (n=30)
control (n=72)
Severity

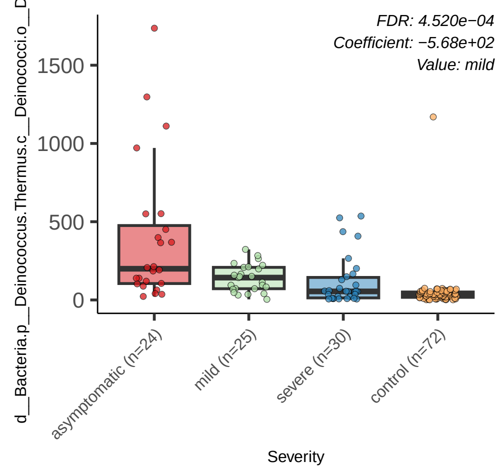

FDR: 4.520e−04
Coefficient: −5.68e+02
Value: mild
d__Bacteria.p__Deinococcus.Thermus.c__Deinococci.o__D
0
500
1000
1500
asymptomatic (n=24)
mild (n=25)
severe (n=30)
control (n=72)
Severity

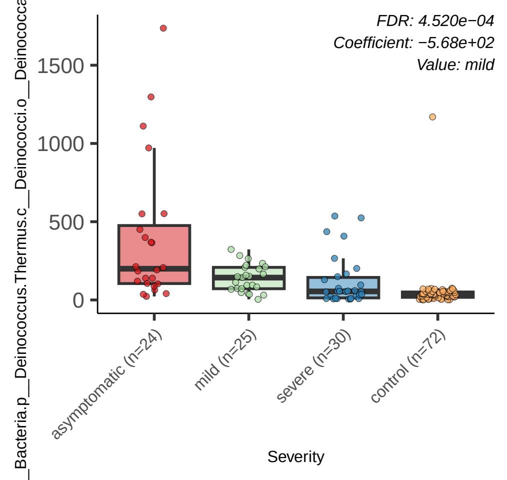
FDR: 4.520e−04
Coefficient: −5.68e+02
Value: mild
1500
1000
500
0
asymptomatic (n=24)
mild (n=25)
severe (n=30)
control (n=72)
Severity
Bacteria.p__Deinococcus.Thermus.c__Deinococci.o__Deinococca

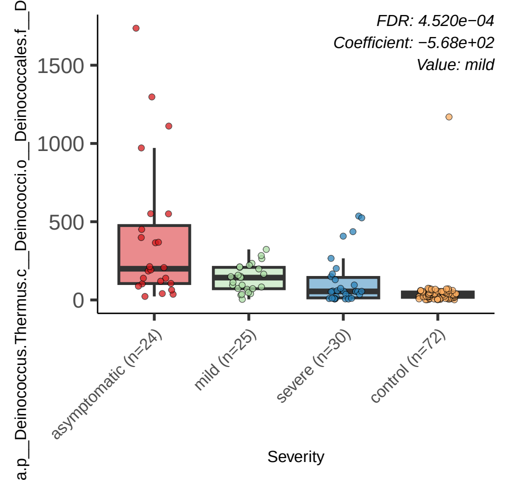
FDR: 4.520e−04
Coefficient: −5.68e+02
Value: mild
1500
1000
500
0
a.p__Deinococcus.Thermus.c__Deinococci.o__Deinococcales.f__D
asymptomatic (n=24)
mild (n=25)
severe (n=30)
control (n=72)
Severity

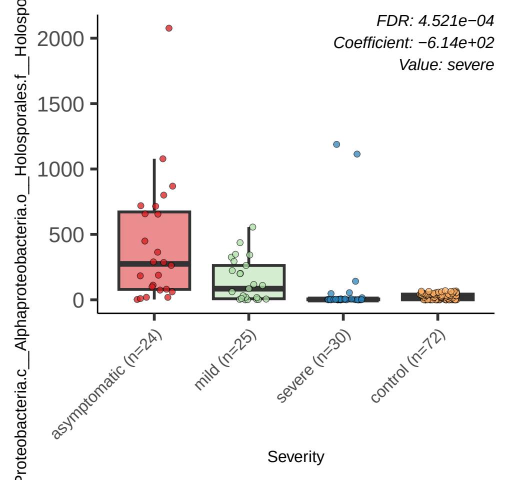
FDR: 4.521e−04
Coefficient: −6.14e+02
Value: severe
Proteobacteria.c__Alphaproteobacteria.o__Holosporales.f__Holospo
2000
1500
1000
500
0
asymptomatic (n=24)
mild (n=25)
severe (n=30)
control (n=72)
Severity

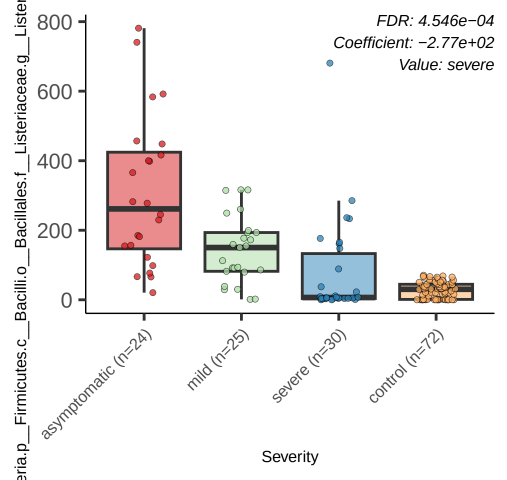

FDR: 4.546e−04
Coefficient: −2.77e+02
Value: severe
800
600
400
200
0
eria.p__Firmicutes.c__Bacilli.o__Bacillales.f__Listeriaceae.g__Lister
asymptomatic (n=24)
mild (n=25)
severe (n=30)
control (n=72)
Severity

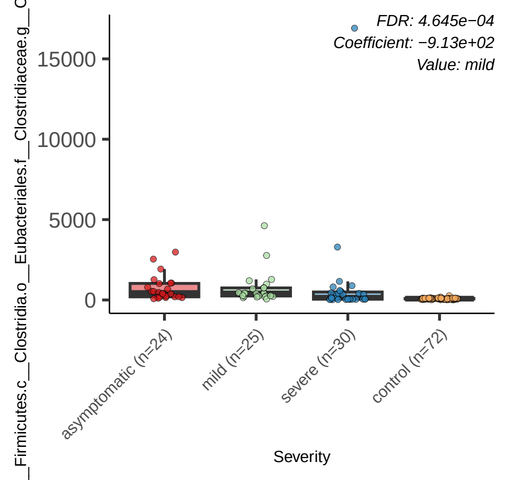

FDR: 4.645e−04
Coefficient: −9.13e+02
Value: mild
15000
10000
5000
0
Firmicutes.c__Clostridia.o__Eubacteriales.f__Clostridiaceae.g__C
asymptomatic (n=24)
mild (n=25)
severe (n=30)
control (n=72)
Severity

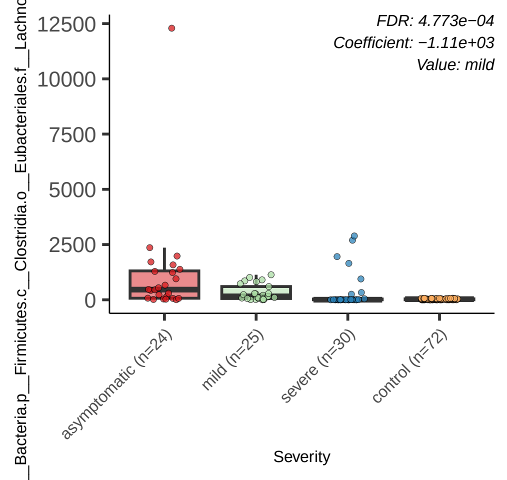

FDR: 4.773e−04
Coefficient: −1.11e+03
Value: mild
12500
10000
7500
5000
2500
0
Bacteria.p__Firmicutes.c__Clostridia.o__Eubacteriales.f__Lachno
asymptomatic (n=24)
mild (n=25)
severe (n=30)
control (n=72)
Severity

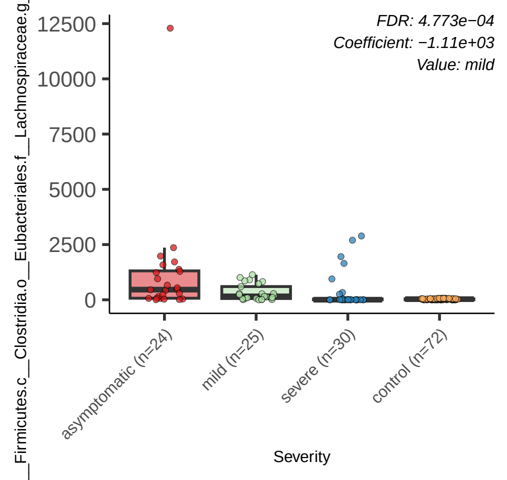

Firmicutes.c__Clostridia.o__Eubacteriales.f__Lachnospiraceae.g_
FDR: 4.773e−04
Coefficient: −1.11e+03
Value: mild
12500
10000
7500
5000
2500
0
asymptomatic (n=24)
mild (n=25)
severe (n=30)
control (n=72)
Severity

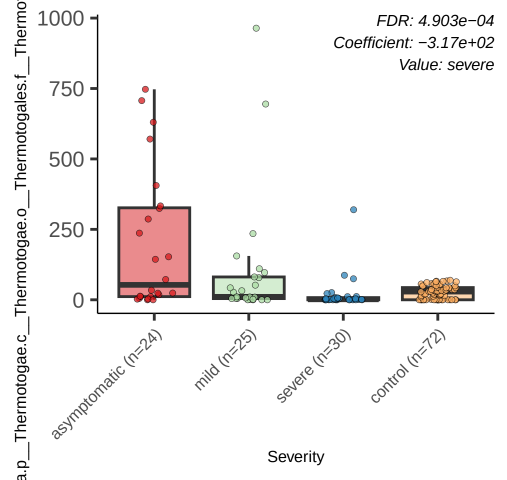

FDR: 4.903e−04
Coefficient: −3.17e+02
Value: severe
1000
750
500
250
0
a.p__Thermotogae.c__Thermotogae.o__Thermotogales.f__Thermo
asymptomatic (n=24)
mild (n=25)
severe (n=30)
control (n=72)
Severity

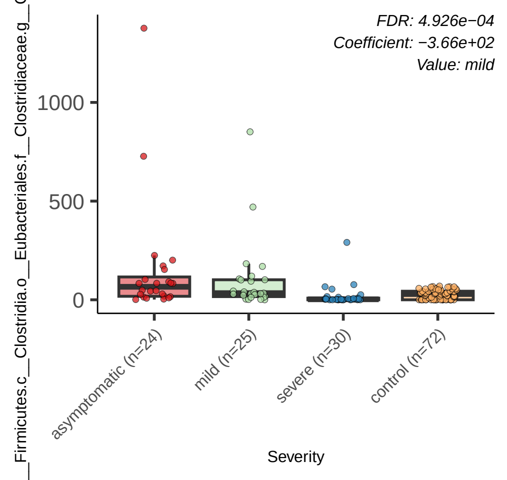

FDR: 4.926e−04
Coefficient: −3.66e+02
Value: mild
1000
500
0
Firmicutes.c__Clostridia.o__Eubacteriales.f__Clostridiaceae.g__C
asymptomatic (n=24)
mild (n=25)
severe (n=30)
control (n=72)
Severity

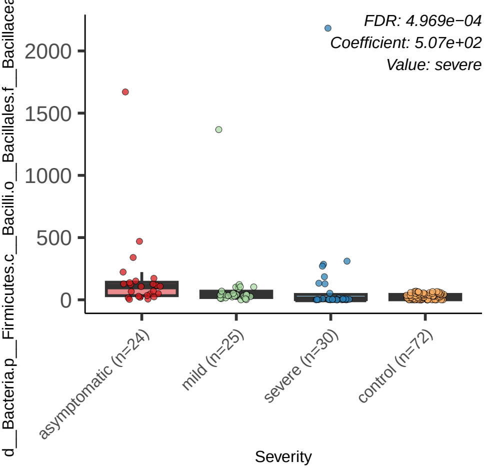

FDR: 4.969e−04
Coefficient: 5.07e+02
Value: severe
d__Bacteria.p__Firmicutes.c__Bacilli.o__Bacillales.f__Bacillacea
0
500
1000
1500
2000
asymptomatic (n=24)
mild (n=25)
severe (n=30)
control (n=72)
Severity

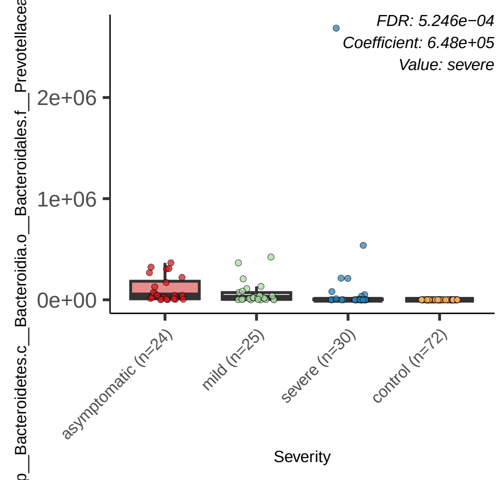
FDR: 5.246e−04
Coefficient: 6.48e+05
Value: severe
2e+06
1e+06
0e+00
asymptomatic (n=24)
mild (n=25)
severe (n=30)
control (n=72)
Severity
p__Bacteroidetes.c__Bacteroidia.o__Bacteroidales.f__Prevotellacea

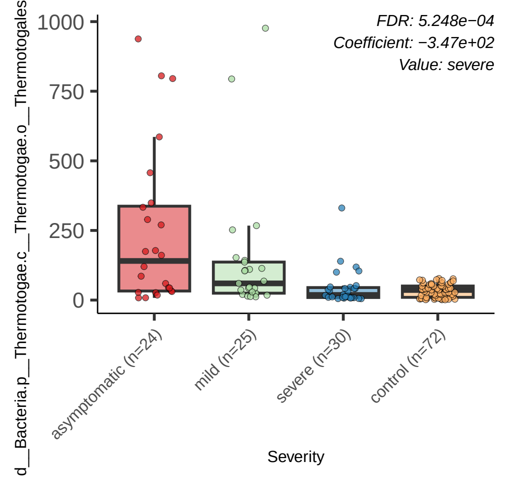

FDR: 5.248e−04
Coefficient: −3.47e+02
Value: severe
d__Bacteria.p__Thermotogae.c__Thermotogae.o__Thermotogales
1000
750
500
250
0
asymptomatic (n=24)
mild (n=25)
severe (n=30)
control (n=72)
Severity

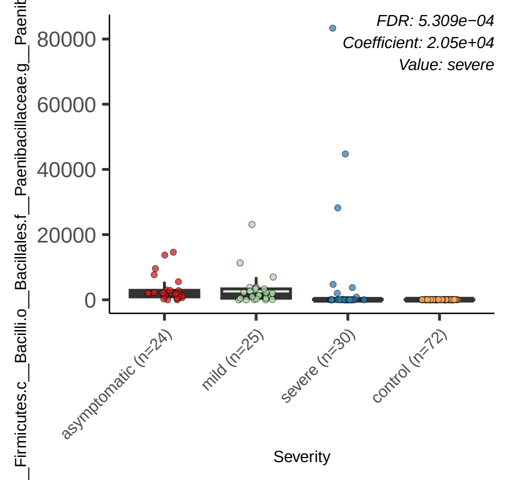

FDR: 5.309e−04
Coefficient: 2.05e+04
Value: severe
80000
60000
40000
20000
0
asymptomatic (n=24)
mild (n=25)
severe (n=30)
control (n=72)
Severity
Firmicutes.c__Bacilli.o__Bacillales.f__Paenibacillaceae.g__Paenib

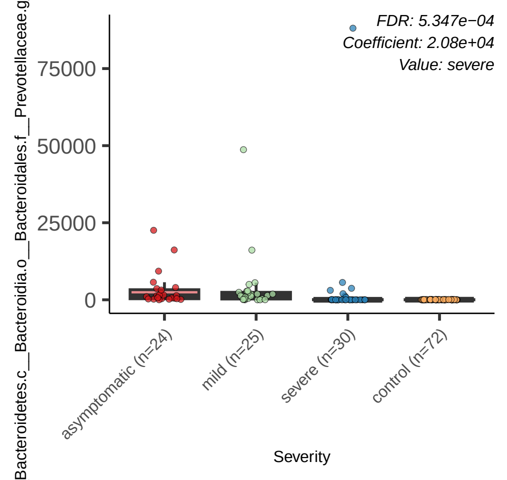

FDR: 5.347e−04
Coefficient: 2.08e+04
Value: severe
Bacteroidetes.c__Bacteroidia.o__Bacteroidales.f__Prevotellaceae.g
75000
50000
25000
0
asymptomatic (n=24)
mild (n=25)
severe (n=30)
control (n=72)
Severity

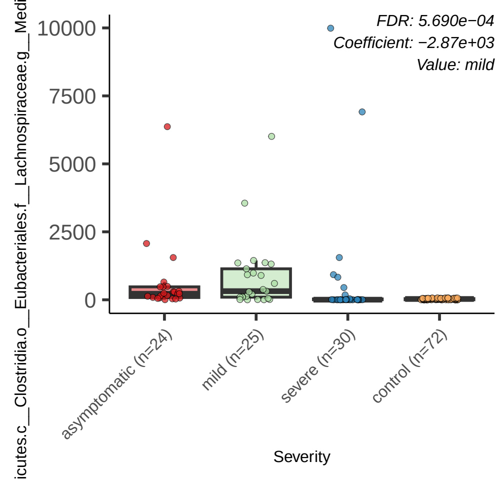
FDR: 5.690e−04
Coefficient: −2.87e+03
Value: mild
10000
7500
5000
2500
0
icutes.c__Clostridia.o__Eubacteriales.f__Lachnospiraceae.g__Medi
asymptomatic (n=24)
mild (n=25)
severe (n=30)
control (n=72)
Severity

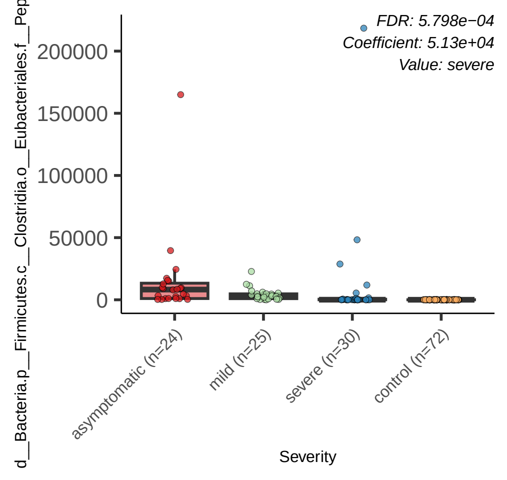

FDR: 5.798e−04
Coefficient: 5.13e+04
Value: severe
200000
150000
100000
50000
0
d__Bacteria.p__Firmicutes.c__Clostridia.o__Eubacteriales.f__Pep
asymptomatic (n=24)
mild (n=25)
severe (n=30)
control (n=72)
Severity

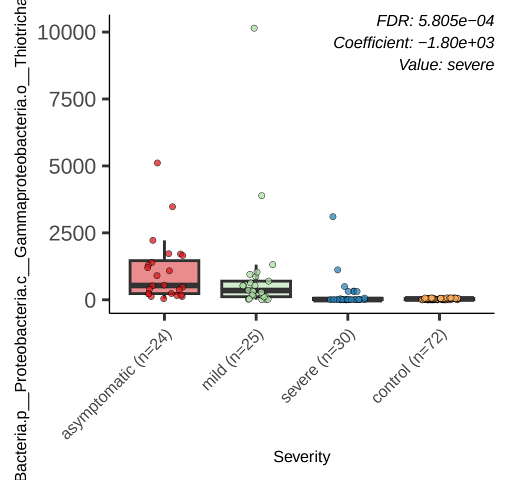

FDR: 5.805e−04
Coefficient: −1.80e+03
Value: severe
10000
7500
5000
2500
0
Bacteria.p__Proteobacteria.c__Gammaproteobacteria.o__Thiotricha
asymptomatic (n=24)
mild (n=25)
severe (n=30)
control (n=72)
Severity

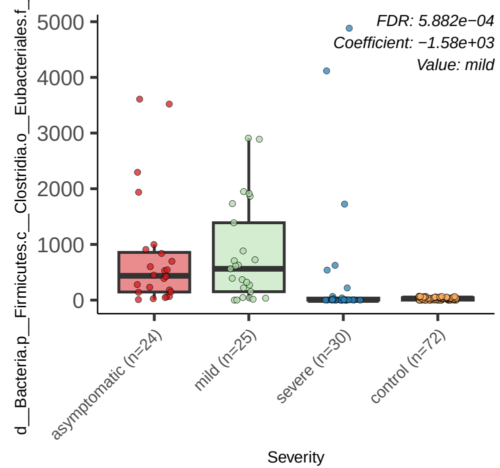

d__Bacteria.p__Firmicutes.c__Clostridia.o__Eubacteriales.f
5000
4000
3000
2000
1000
0
FDR: 5.882e−04
Coefficient: −1.58e+03
Value: mild
asymptomatic (n=24)
mild (n=25)
severe (n=30)
control (n=72)
Severity

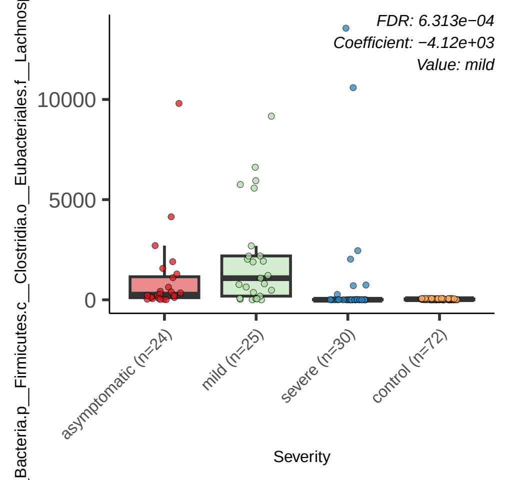

FDR: 6.313e−04
Coefficient: −4.12e+03
Value: mild
10000
5000
0
Bacteria.p__Firmicutes.c__Clostridia.o__Eubacteriales.f__Lachnos
asymptomatic (n=24)
mild (n=25)
severe (n=30)
control (n=72)
Severity

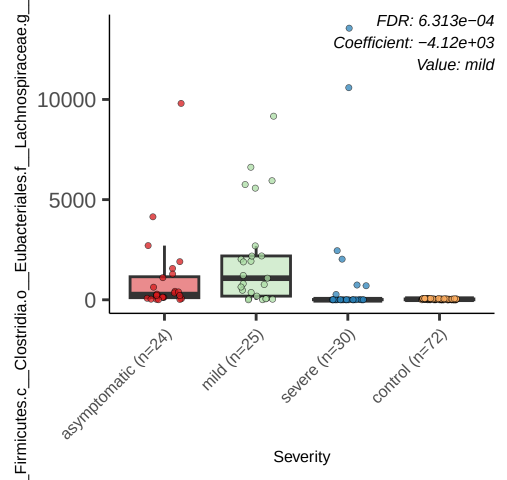
FDR: 6.313e−04
Coefficient: −4.12e+03
Value: mild
Firmicutes.c__Clostridia.o__Eubacteriales.f__Lachnospiraceae.g__
10000
5000
0
asymptomatic (n=24)
mild (n=25)
severe (n=30)
control (n=72)
Severity

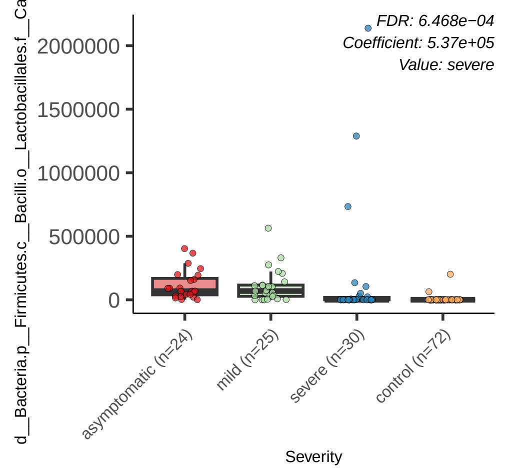

FDR: 6.468e−04
Coefficient: 5.37e+05
Value: severe
d__Bacteria.p__Firmicutes.c__Bacilli.o__Lactobacillales.f__Ca
2000000
1500000
1000000
500000
0
asymptomatic (n=24)
mild (n=25)
severe (n=30)
control (n=72)
Severity

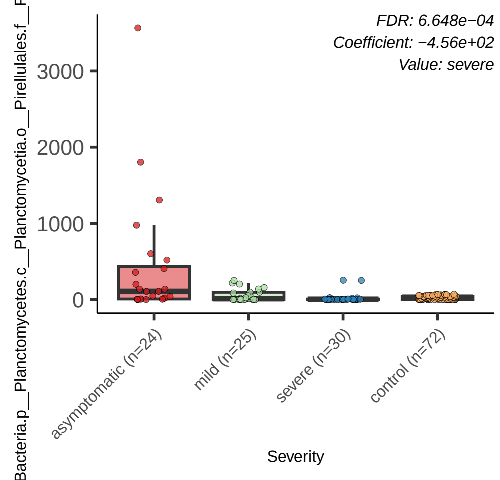
FDR: 6.648e−04
Coefficient: −4.56e+02
Value: severe
Bacteria.p__Planctomycetes.c__Planctomycetia.o__Pirellulales.f__P
3000
2000
1000
0
asymptomatic (n=24)
mild (n=25)
severe (n=30)
control (n=72)
Severity

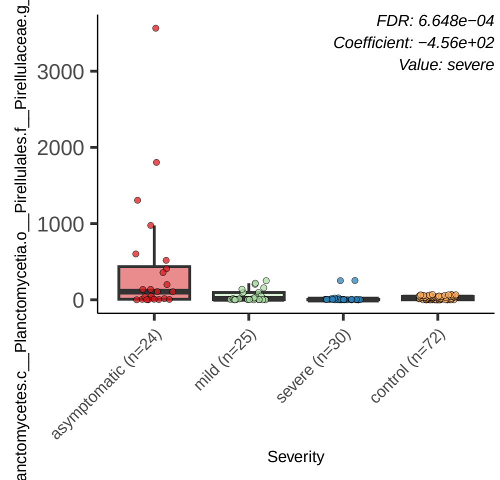

FDR: 6.648e−04
Coefficient: −4.56e+02
Value: severe
3000
2000
1000
0
lanctomycetes.c__Planctomycetia.o__Pirellulales.f__Pirelluliaceae.g_
asymptomatic (n=24)
mild (n=25)
severe (n=30)
control (n=72)
Severity

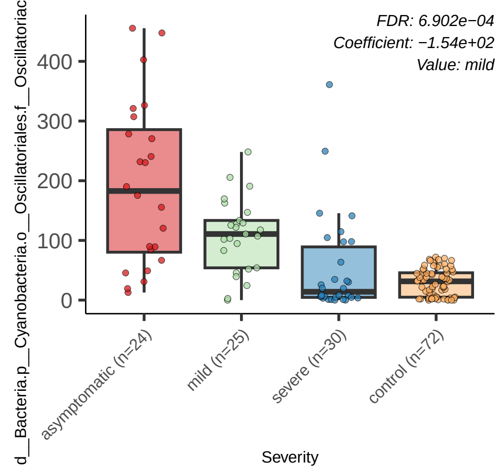

FDR: 6.902e−04
Coefficient: −1.54e+02
Value: mild
0
100
200
300
400
d__Bacteria.p__Cyanobacteria.o__Oscillatoriales.f__Oscillatoriac
asymptomatic (n=24)
mild (n=25)
severe (n=30)
control (n=72)
Severity

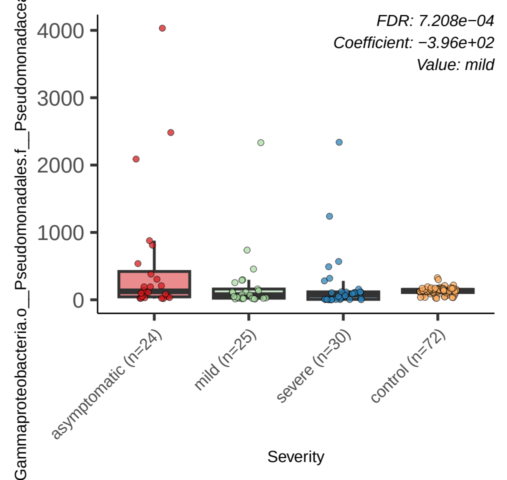

FDR: 7.208e−04
Coefficient: −3.96e+02
Value: mild
Gammaproteobacteria.o__Pseudomonadales.f__Pseudomonadacea
4000
3000
2000
1000
0
asymptomatic (n=24)
mild (n=25)
severe (n=30)
control (n=72)
Severity

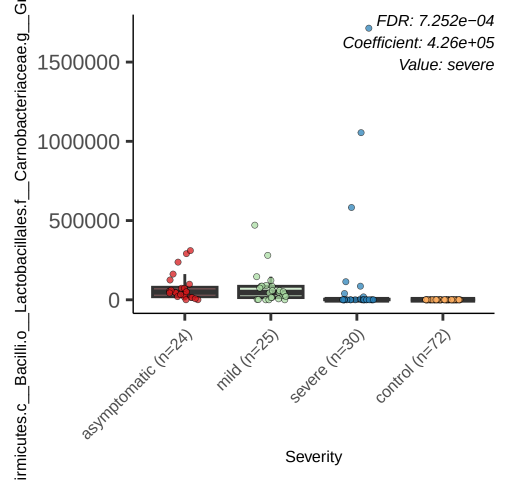

FDR: 7.252e−04
Coefficient: 4.26e+05
Value: severe
1500000
1000000
500000
0
irmicutes.c__Bacilli.o__Lactobacillales.f__Carnobacteriaceae.g__G
asymptomatic (n=24)
mild (n=25)
severe (n=30)
control (n=72)
Severity

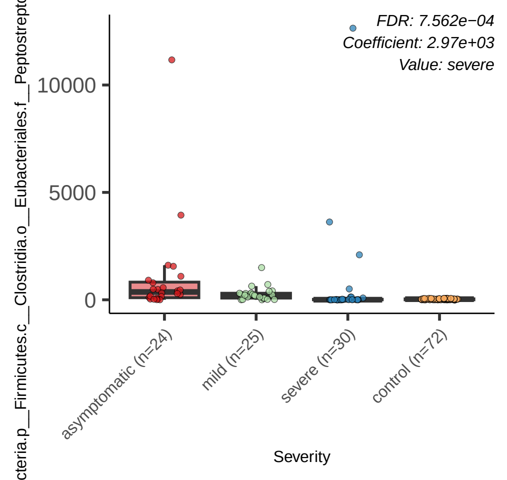

FDR: 7.562e−04
Coefficient: 2.97e+03
Value: severe
10000
5000
0
asymptomatic (n=24)
mild (n=25)
severe (n=30)
control (n=72)
Severity
cteria.p__Firmicutes.c__Clostridia.o__Eubacteriales.f__Peptostrepto

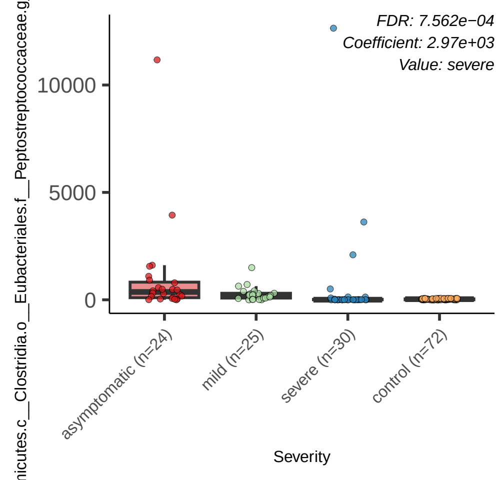
FDR: 7.562e−04
Coefficient: 2.97e+03
Value: severe
10000
5000
0
asymptomatic (n=24)
mild (n=25)
severe (n=30)
control (n=72)
Severity
nicutes.c__Clostridia.o__Eubacteriales.f__Peptostreptococcaceae.g

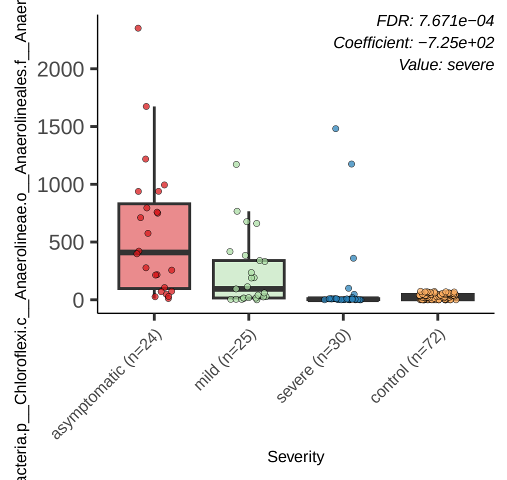

FDR: 7.671e−04
Coefficient: −7.25e+02
Value: severe
acteria.p__Chloroflexi.c__Anaerolineae.o__Anaerolineales.f__Anaer
2000
1500
1000
500
0
asymptomatic (n=24)
mild (n=25)
severe (n=30)
control (n=72)
Severity

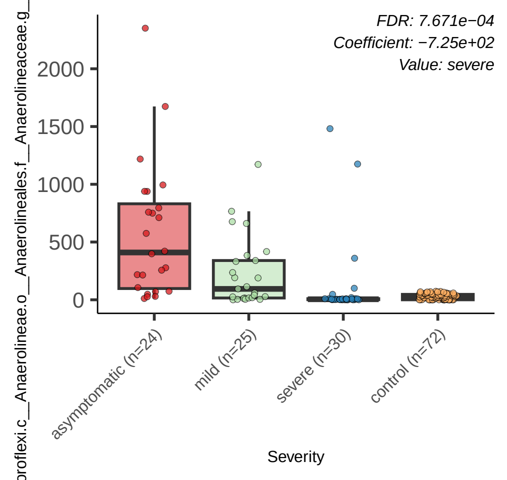

FDR: 7.671e−04
Coefficient: −7.25e+02
Value: severe
0
500
1000
1500
2000
roflexi.c__Anaerolineae.o__Anaerolineales.f__Anaerolineaceae.g__
asymptomatic (n=24)
mild (n=25)
severe (n=30)
control (n=72)
Severity

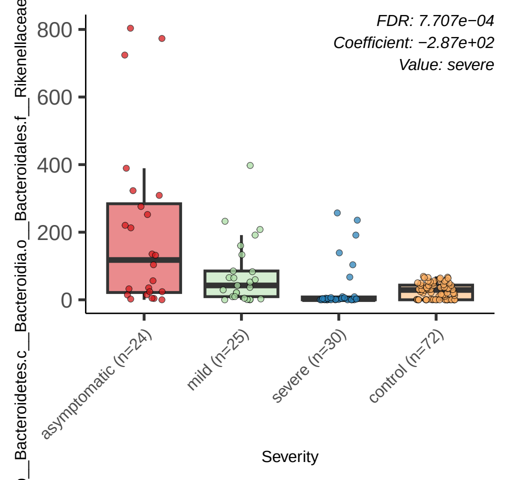

FDR: 7.707e−04
Coefficient: −2.87e+02
Value: severe
o__Bacteroidetes.c__Bacteroidia.o__Bacteroidales.f__Rikenellaceae
800
600
400
200
0
asymptomatic (n=24)
mild (n=25)
severe (n=30)
control (n=72)
Severity

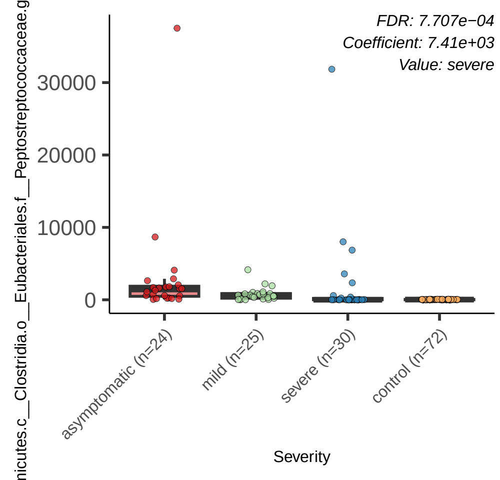

FDR: 7.707e−04
Coefficient: 7.41e+03
Value: severe
30000
20000
10000
0
nicutes.c__Clostridia.o__Eubacteriales.f__Peptostreptococcaceae.g
asymptomatic (n=24)
mild (n=25)
severe (n=30)
control (n=72)
Severity

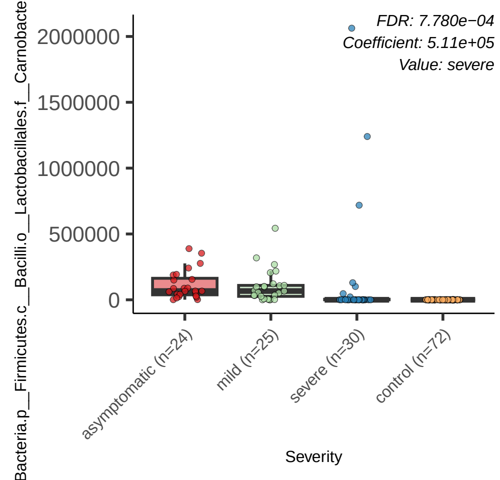
FDR: 7.780e−04
Coefficient: 5.11e+05
Value: severe
Bacteria.p__Firmicutes.c__Bacilli.o__Lactobacillales.f__Carnobacte
2000000
1500000
1000000
500000
0
asymptomatic (n=24)
mild (n=25)
severe (n=30)
control (n=72)
Severity

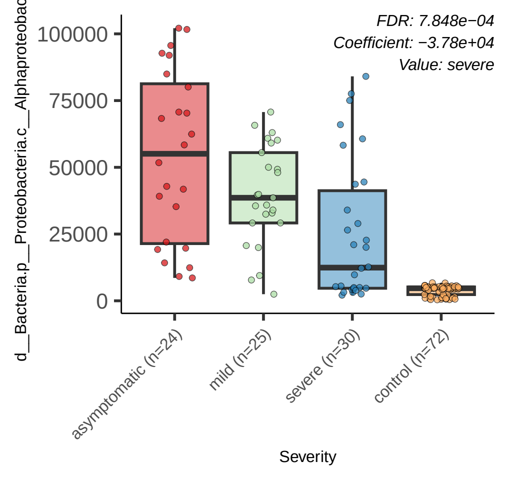

FDR: 7.848e−04
Coefficient: −3.78e+04
Value: severe
d__Bacteria.p__Proteobacteria.c__Alphaproteobac
100000
75000
50000
25000
0
asymptomatic (n=24)
mild (n=25)
severe (n=30)
control (n=72)
Severity

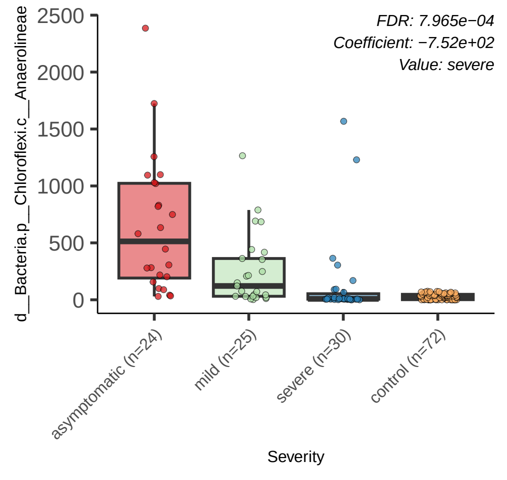

FDR: 7.965e−04
Coefficient: −7.52e+02
Value: severe
d__Bacteria.p__Chloroflexi.c__Anaerolineae
2500
2000
1500
1000
500
0
asymptomatic (n=24)
mild (n=25)
severe (n=30)
control (n=72)
Severity

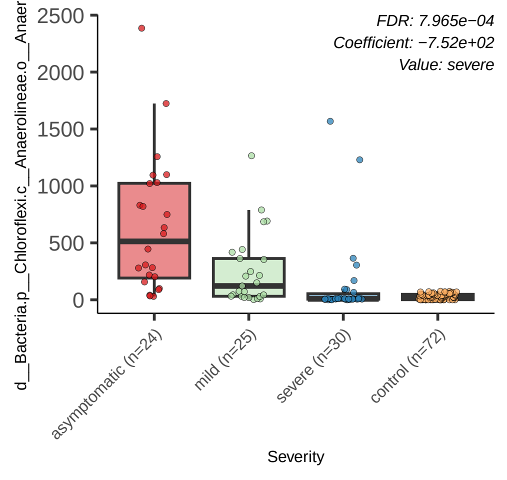

FDR: 7.965e−04
Coefficient: −7.52e+02
Value: severe
d__Bacteria.p__Chloroflexi.c__Anaerolineae.o__Anaer
2500
2000
1500
1000
500
0
asymptomatic (n=24)
mild (n=25)
severe (n=30)
control (n=72)
Severity

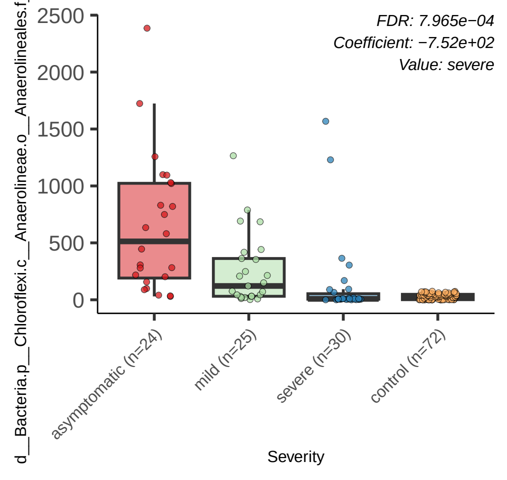

FDR: 7.965e−04
Coefficient: −7.52e+02
Value: severe
d__Bacteria.p__Chloroflexi.c__Anaerolineae.o__Anaerolineales.f
2500
2000
1500
1000
500
0
asymptomatic (n=24)
mild (n=25)
severe (n=30)
control (n=72)
Severity

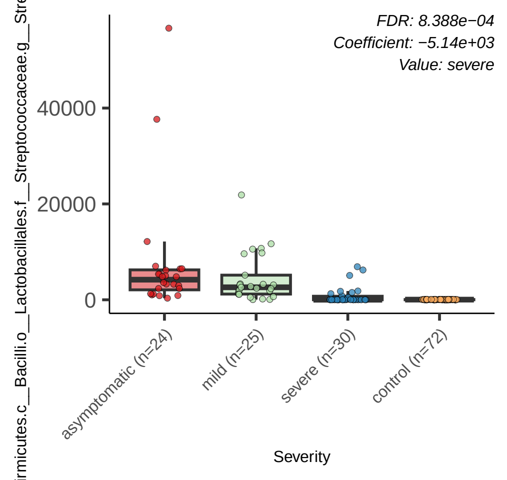

FDR: 8.388e−04
Coefficient: −5.14e+03
Value: severe
40000
20000
0
irmicutes.c__Bacilli.o__Lactobacillales.f__Streptococcaceae.g__Stre
asymptomatic (n=24)
mild (n=25)
severe (n=30)
control (n=72)
Severity

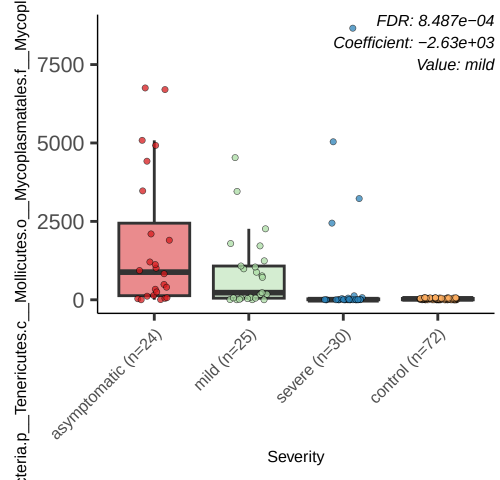

FDR: 8.487e−04
Coefficient: −2.63e+03
Value: mild
7500
5000
2500
0
cteria.p__Tenericutes.c__Mollicutes.o__Mycoplasmatales.f__Mycopl
asymptomatic (n=24)
mild (n=25)
severe (n=30)
control (n=72)
Severity

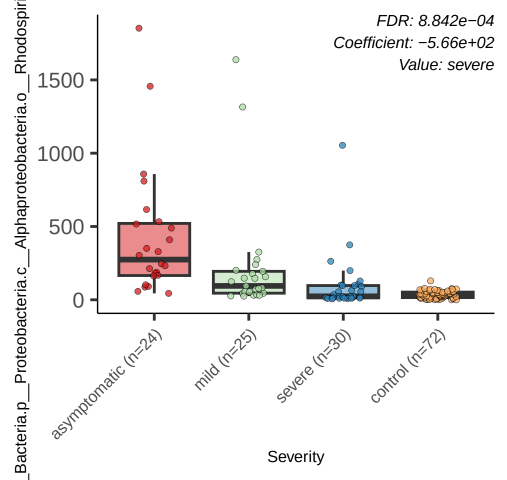

FDR: 8.842e−04
Coefficient: −5.66e+02
Value: severe
Bacteria.p__Proteobacteria.c__Alphaproteobacteria.o__Rhodospiri
0
500
1000
1500
asymptomatic (n=24)
mild (n=25)
severe (n=30)
control (n=72)
Severity

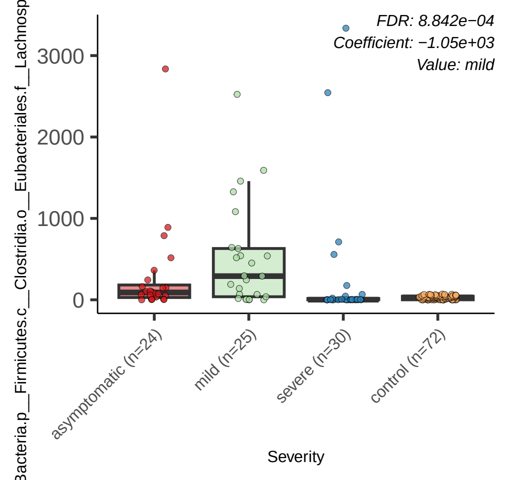

FDR: 8.842e−04
Coefficient: −1.05e+03
Value: mild
3000
2000
1000
0
Bacteria.p__Firmicutes.c__Clostridia.o__Eubacteriales.f__Lachnosp
asymptomatic (n=24)
mild (n=25)
severe (n=30)
control (n=72)
Severity

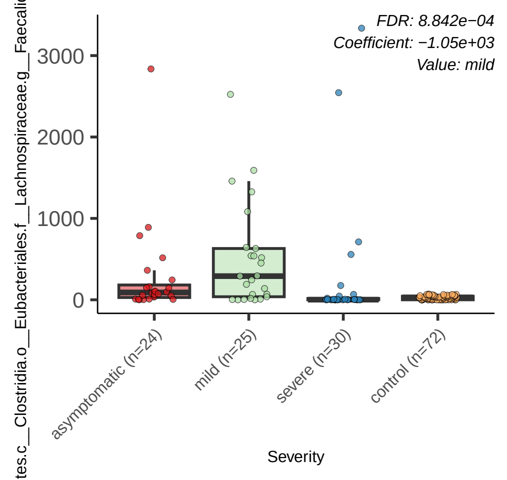

FDR: 8.842e−04
Coefficient: −1.05e+03
Value: mild
3000
2000
1000
0
tes.c__Clostridia.o__Eubacteriales.f__Lachnospiraceae.g__Faecalic
asymptomatic (n=24)
mild (n=25)
severe (n=30)
control (n=72)
Severity

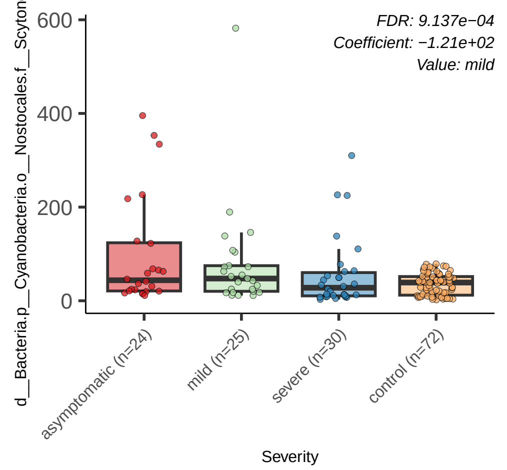

FDR: 9.137e−04
Coefficient: −1.21e+02
Value: mild
600
400
200
0
d__Bacteria.p__Cyanobacteria.o__Nostocales.f__Scyton
asymptomatic (n=24)
mild (n=25)
severe (n=30)
control (n=72)
Severity

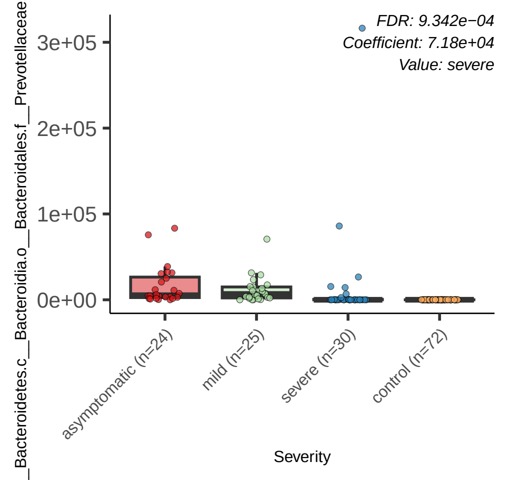

FDR: 9.342e−04
Coefficient: 7.18e+04
Value: severe
3e+05
2e+05
1e+05
0e+00
Bacteroidetes.c__Bacteroidia.o__Bacteroidales.f__Prevotellaceae
asymptomatic (n=24)
mild (n=25)
severe (n=30)
control (n=72)
Severity

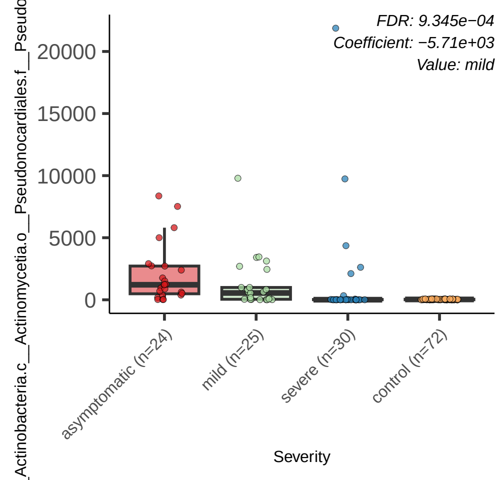

FDR: 9.345e−04
Coefficient: −5.71e+03
Value: mild
20000
15000
10000
5000
0
Actinobacteria.c__Actinomycetia.o__Pseudonocardiales.f__Pseudo
asymptomatic (n=24)
mild (n=25)
severe (n=30)
control (n=72)
Severity

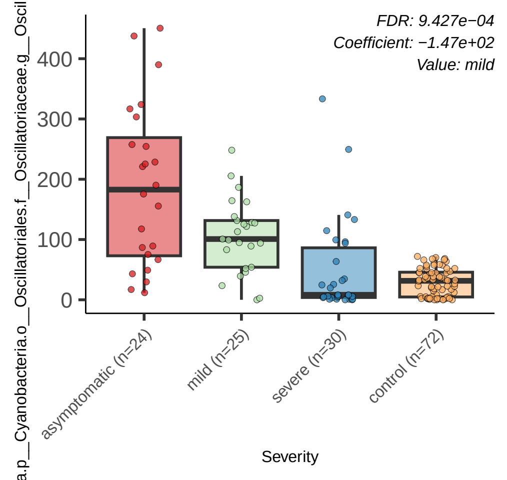
FDR: 9.427e−04
Coefficient: −1.47e+02
Value: mild
a.p__Cyanobacteria.o__Oscillatoriales.f__Oscillatoriaceae.g__Oscil
400
300
200
100
0
asymptomatic (n=24)
mild (n=25)
severe (n=30)
control (n=72)
Severity

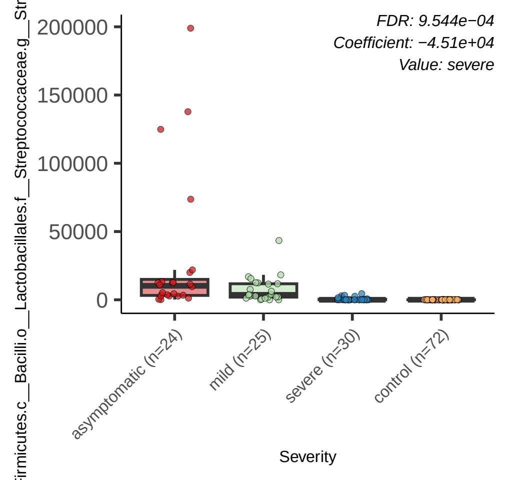

FDR: 9.544e−04
Coefficient: −4.51e+04
Value: severe
200000
150000
100000
50000
0
asymptomatic (n=24)
mild (n=25)
severe (n=30)
control (n=72)
Severity
irmicutes.c__Bacilli.o__Lactobacillales.f__Streptococcaceae.g__Str

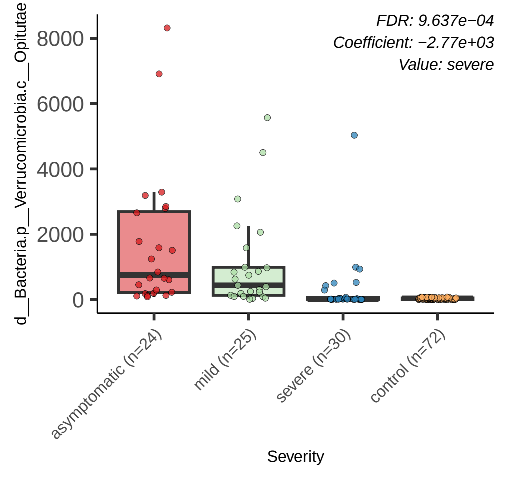
FDR: 9.637e−04
Coefficient: −2.77e+03
Value: severe
d__Bacteria.p__Verrucomicrobia.c__Opitutae
0
2000
4000
6000
8000
asymptomatic (n=24)
mild (n=25)
severe (n=30)
control (n=72)
Severity

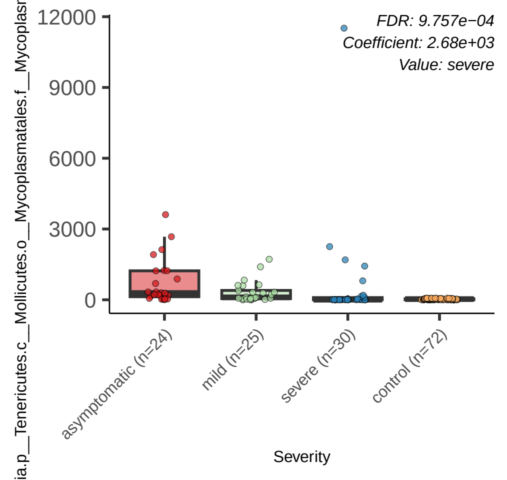

FDR: 9.757e−04
Coefficient: 2.68e+03
Value: severe
12000
9000
6000
3000
0
ia.p__Tenericutes.c__Mollicutes.o__Mycoplasmatales.f__Mycoplasm
asymptomatic (n=24)
mild (n=25)
severe (n=30)
control (n=72)
Severity

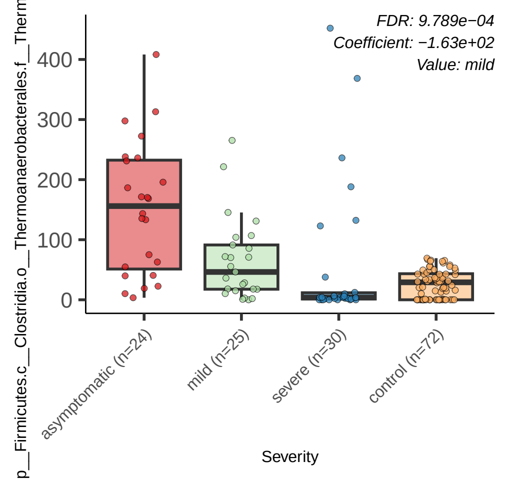

FDR: 9.789e−04
Coefficient: −1.63e+02
Value: mild
p__Firmicutes.c__Clostridia.o__Thermoanaerobacterales.f__Therm
0
100
200
300
400
asymptomatic (n=24)
mild (n=25)
severe (n=30)
control (n=72)
Severity

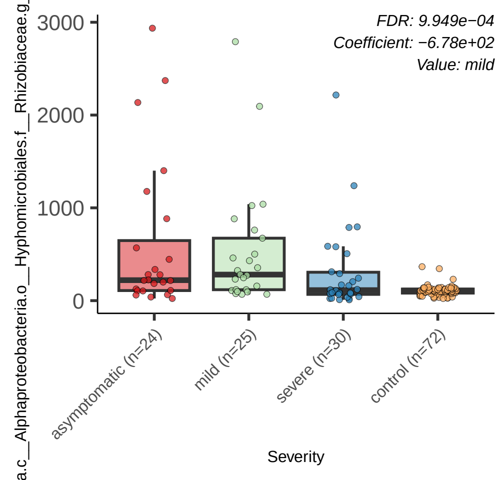
FDR: 9.949e−04
Coefficient: −6.78e+02
Value: mild
3000
2000
1000
0
a.c__Alphaproteobacteria.o__Hyphomicrobiales.f__Rhizobiaceae.g__
asymptomatic (n=24)
mild (n=25)
severe (n=30)
control (n=72)
Severity

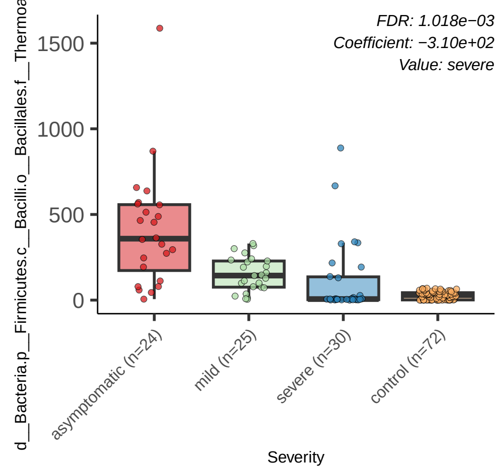

FDR: 1.018e−03
Coefficient: −3.10e+02
Value: severe
d__Bacteria.p__Firmicutes.c__Bacilli.o__Bacillales.f__Thermoa
1500
1000
500
0
asymptomatic (n=24)
mild (n=25)
severe (n=30)
control (n=72)
Severity

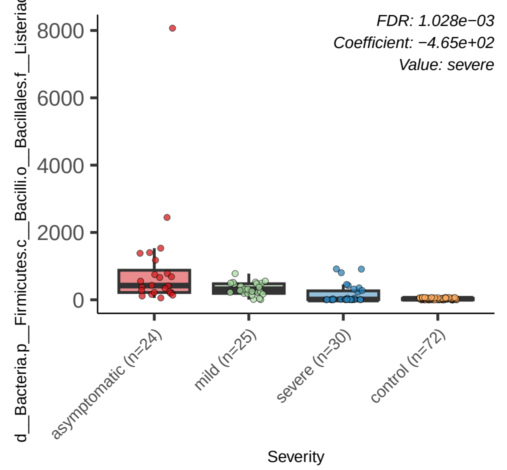

FDR: 1.028e−03
Coefficient: −4.65e+02
Value: severe
d__Bacteria.p__Firmicutes.c__Bacilli.o__Bacillales.f__Listeriac
8000
6000
4000
2000
0
asymptomatic (n=24)
mild (n=25)
severe (n=30)
control (n=72)
Severity

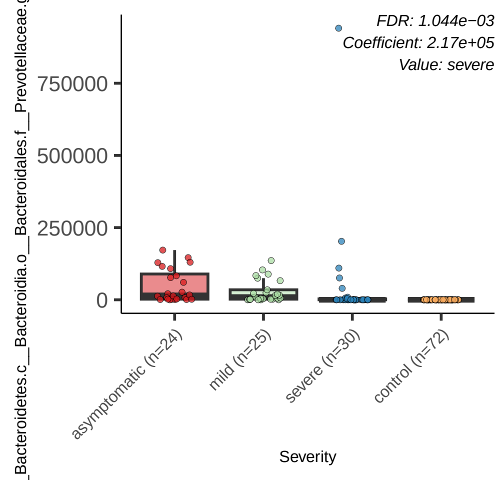

FDR: 1.044e−03
Coefficient: 2.17e+05
Value: severe
750000
500000
250000
0
Bacteroidetes.c__Bacteroidia.o__Bacteroidales.f__Prevotellaceae.g
asymptomatic (n=24)
mild (n=25)
severe (n=30)
control (n=72)
Severity

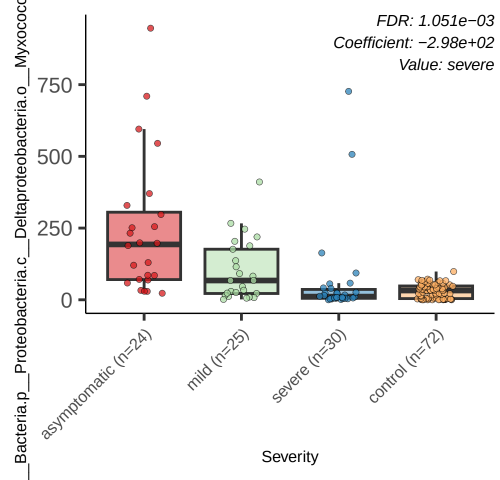

FDR: 1.051e−03
Coefficient: −2.98e+02
Value: severe
__Bacteria.p__Proteobacteria.c__Deltaproteobacteria.o__Myxococc
750
500
250
0
asymptomatic (n=24)
mild (n=25)
severe (n=30)
control (n=72)
Severity

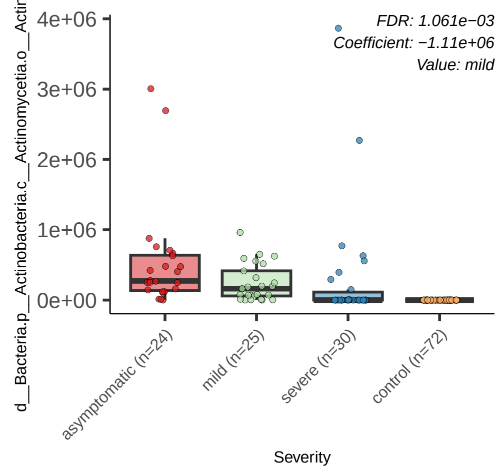

FDR: 1.061e−03
Coefficient: −1.11e+06
Value: mild
d__Bacteria.p__Actinobacteria.c__Actinomycetia.o__Actin
4e+06
3e+06
2e+06
1e+06
0e+00
asymptomatic (n=24)
mild (n=25)
severe (n=30)
control (n=72)
Severity

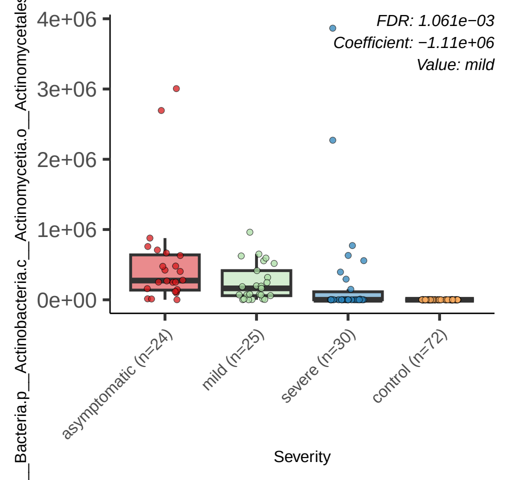
FDR: 1.061e−03
Coefficient: −1.11e+06
Value: mild
4e+06
3e+06
2e+06
1e+06
0e+00
Bacteria.p__Actinobacteria.c__Actinomycetia.o__Actinomycetales
asymptomatic (n=24)
mild (n=25)
severe (n=30)
control (n=72)
Severity

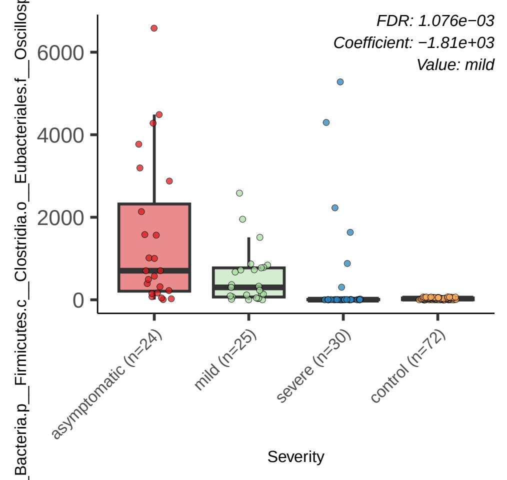

FDR: 1.076e−03
Coefficient: −1.81e+03
Value: mild
Bacteria.p__Firmicutes.c__Clostridia.o__Eubacteriales.f__Oscillosp
6000
4000
2000
0
asymptomatic (n=24)
mild (n=25)
severe (n=30)
control (n=72)
Severity

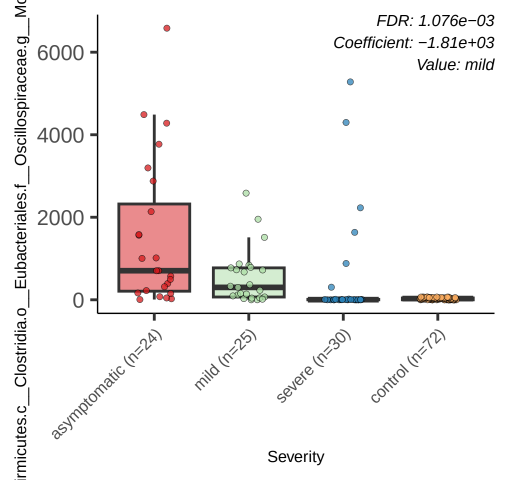

FDR: 1.076e−03
Coefficient: −1.81e+03
Value: mild
0
2000
4000
6000
asymptomatic (n=24)
mild (n=25)
severe (n=30)
control (n=72)
Severity
irmicutes.c__Clostridia.o__Eubacteriales.f__Oscillospiraceae.g__Mo

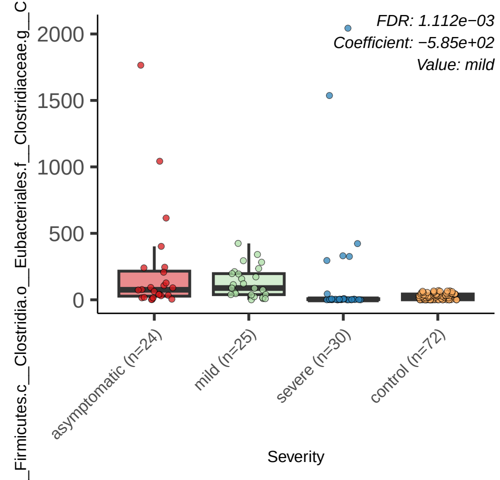

FDR: 1.112e−03
Coefficient: −5.85e+02
Value: mild
Firmicutes.c__Clostridia.o__Eubacteriales.f__Clostridiaceae.g__C
2000
1500
1000
500
0
asymptomatic (n=24)
mild (n=25)
severe (n=30)
control (n=72)
Severity

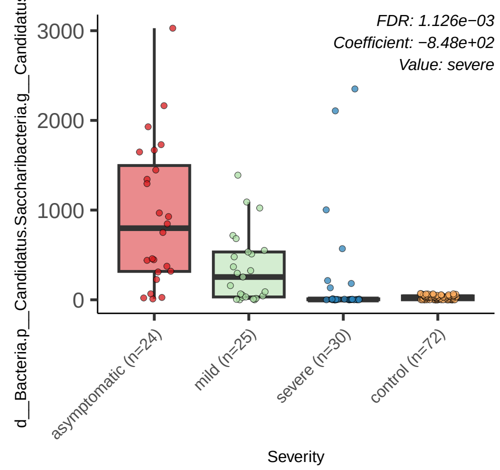

FDR: 1.126e−03
Coefficient: −8.48e+02
Value: severe
d__Bacteria.p__Candidatus.Saccharibacteria.g__Candidatus
3000
2000
1000
0
asymptomatic (n=24)
mild (n=25)
severe (n=30)
control (n=72)
Severity

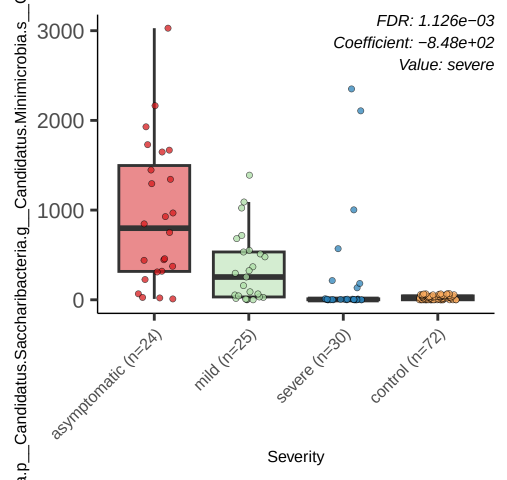

FDR: 1.126e−03
Coefficient: −8.48e+02
Value: severe
3000
2000
1000
0
a.p__Candidatus.Saccharibacteria.g__Candidatus.Minimicrobia.s__C
asymptomatic (n=24)
mild (n=25)
severe (n=30)
control (n=72)
Severity

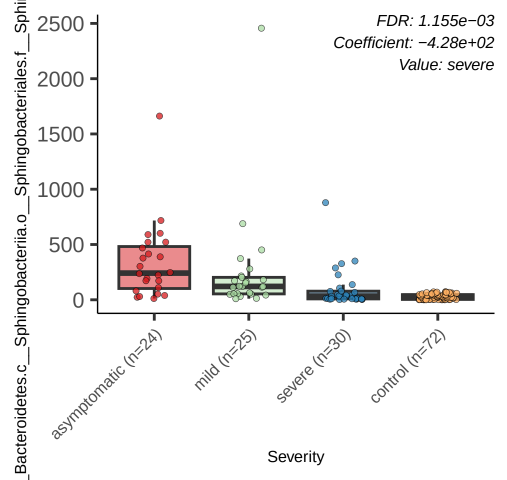

FDR: 1.155e−03
Coefficient: −4.28e+02
Value: severe
_Bacteroidetes.c__Sphingobacteriia.o__Sphingobacteriales.f__Sphi
2500
2000
1500
1000
500
0
asymptomatic (n=24)
mild (n=25)
severe (n=30)
control (n=72)
Severity

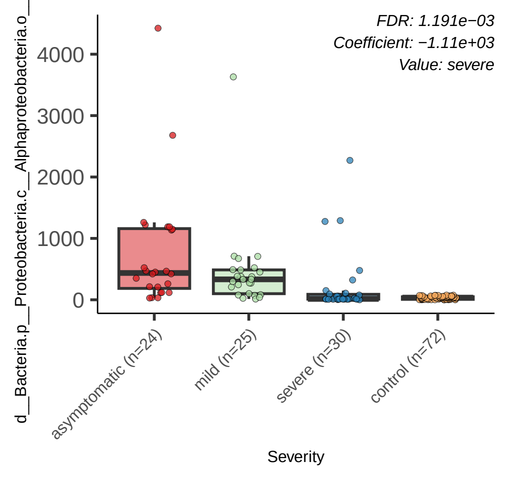

FDR: 1.191e−03
Coefficient: −1.11e+03
Value: severe
d__Bacteria.p__Proteobacteria.c__Alphaproteobacteria.o__
4000
3000
2000
1000
0
asymptomatic (n=24)
mild (n=25)
severe (n=30)
control (n=72)
Severity

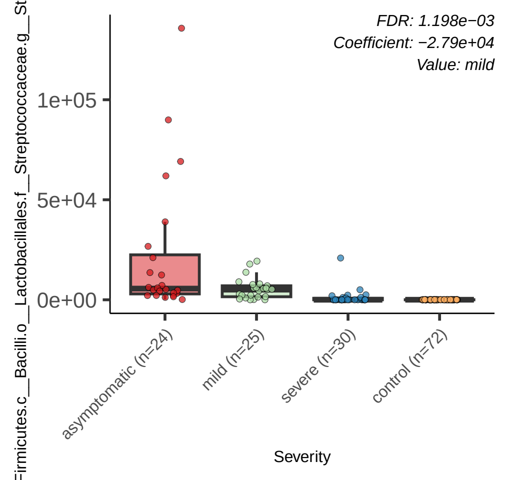
FDR: 1.198e−03
Coefficient: −2.79e+04
Value: mild
Firmicutes.c__Bacilli.o__Lactobacillales.f__Streptococcaceae.g__St
1e+05
5e+04
0e+00
asymptomatic (n=24)
mild (n=25)
severe (n=30)
control (n=72)
Severity

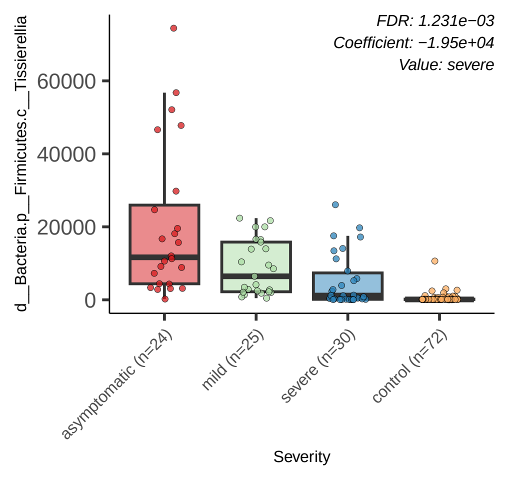

FDR: 1.231e−03
Coefficient: −1.95e+04
Value: severe
d__Bacteria.p__Firmicutes.c__Tissierellia
60000
40000
20000
0
asymptomatic (n=24)
mild (n=25)
severe (n=30)
control (n=72)
Severity

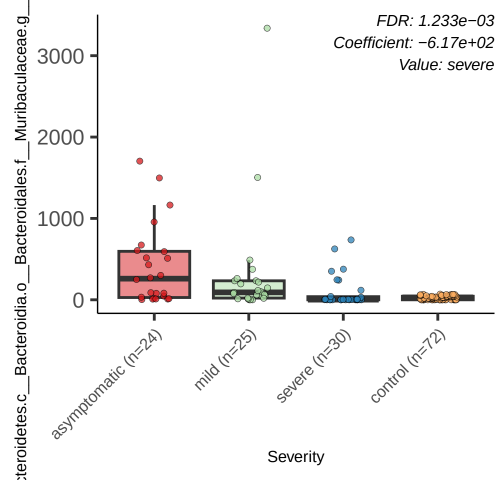

FDR: 1.233e−03
Coefficient: −6.17e+02
Value: severe
3000
2000
1000
0
asymptomatic (n=24)
mild (n=25)
severe (n=30)
control (n=72)
Severity
cteroidetes.c__Bacteroidia.o__Bacteroidales.f__Muribaculaceae.g__

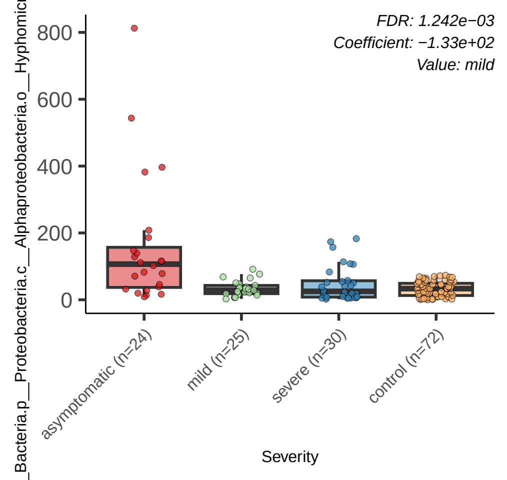

FDR: 1.242e−03
Coefficient: −1.33e+02
Value: mild
Bacteria.p__Proteobacteria.c__Alphaproteobacteria.o__Hyphomic
800
600
400
200
0
asymptomatic (n=24)
mild (n=25)
severe (n=30)
control (n=72)
Severity

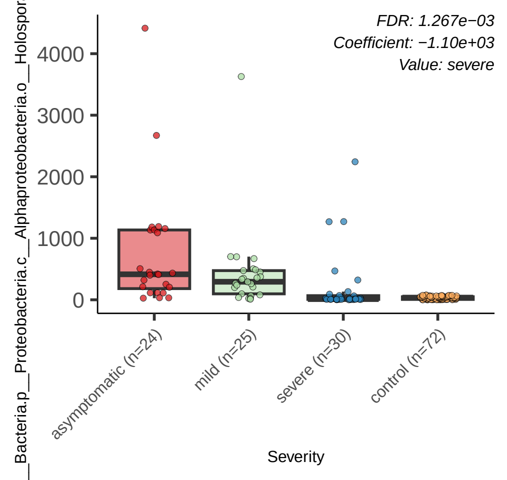

FDR: 1.267e−03
Coefficient: −1.10e+03
Value: severe
4000
3000
2000
1000
0
Bacteria.p__Proteobacteria.c__Alphaproteobacteria.o__Holospor
asymptomatic (n=24)
mild (n=25)
severe (n=30)
control (n=72)
Severity

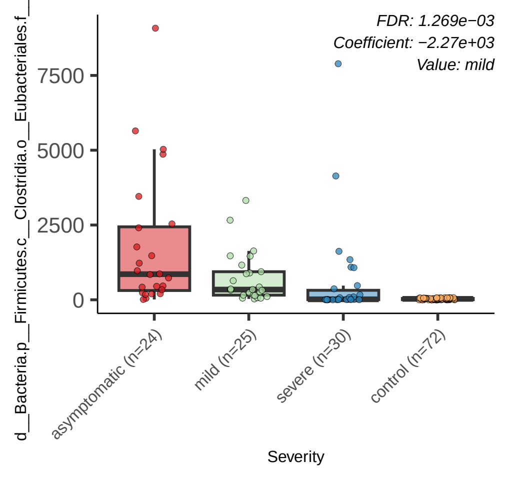
FDR: 1.269e−03
Coefficient: −2.27e+03
Value: mild
d__Bacteria.p__Firmicutes.c__Clostridia.o__Eubacteriales.f
7500
5000
2500
0
asymptomatic (n=24)
mild (n=25)
severe (n=30)
control (n=72)
Severity

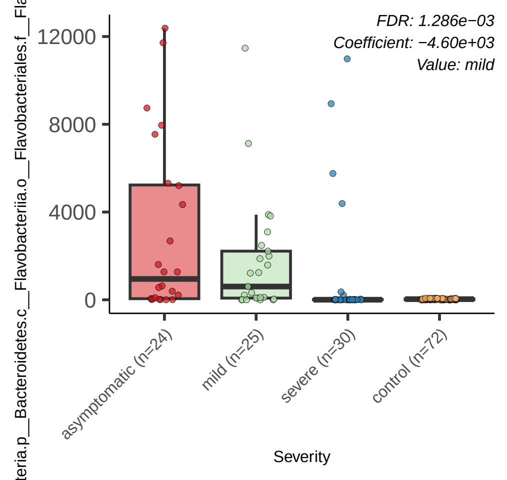

FDR: 1.286e−03
Coefficient: −4.60e+03
Value: mild
12000
8000
4000
0
teria.p__Bacteroidetes.c__Flavobacteriia.o__Flavobacteriales.f__Fla
asymptomatic (n=24)
mild (n=25)
severe (n=30)
control (n=72)
Severity

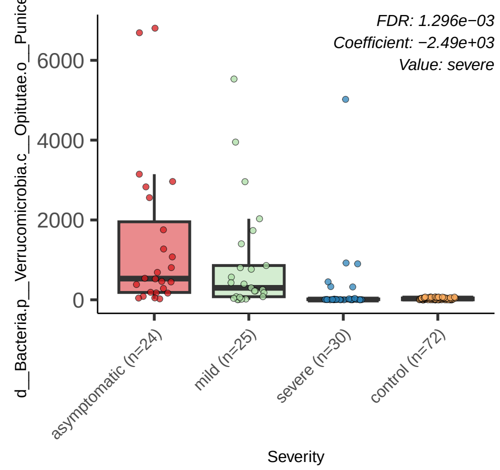
FDR: 1.296e−03
Coefficient: −2.49e+03
Value: severe
d__Bacteria.p__Verrucomicrobia.c__Opitutae.o__Punice
6000
4000
2000
0
asymptomatic (n=24)
mild (n=25)
severe (n=30)
control (n=72)
Severity

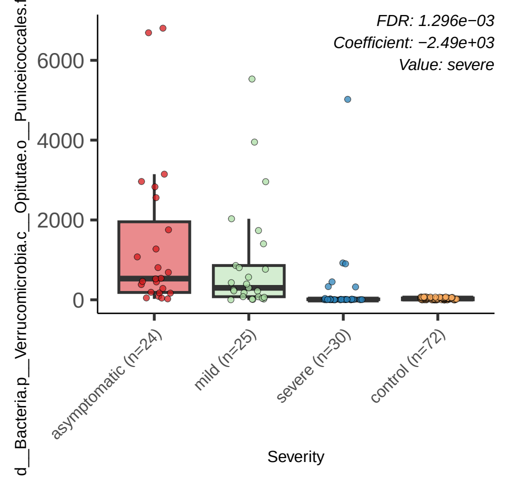
FDR: 1.296e−03
Coefficient: −2.49e+03
Value: severe
d__Bacteria.p__Verrucomicrobia.c__Opitutae.o__Puniceicoccales.f
6000
4000
2000
0
asymptomatic (n=24)
mild (n=25)
severe (n=30)
control (n=72)
Severity

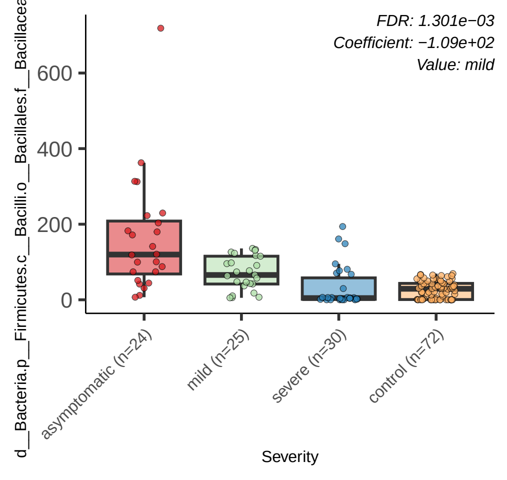

FDR: 1.301e−03
Coefficient: −1.09e+02
Value: mild
d__Bacteria.p__Firmicutes.c__Bacilli.o__Bacillales.f__Bacillacea
0
200
400
600
asymptomatic (n=24)
mild (n=25)
severe (n=30)
control (n=72)
Severity

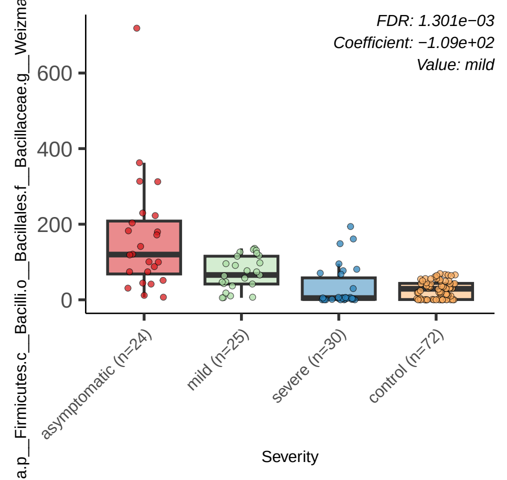
FDR: 1.301e−03
Coefficient: −1.09e+02
Value: mild
a.p__Firmicutes.c__Bacilli.o__Bacillales.f__Bacillaceae.g__Weizma
0
200
400
600
asymptomatic (n=24)
mild (n=25)
severe (n=30)
control (n=72)
Severity

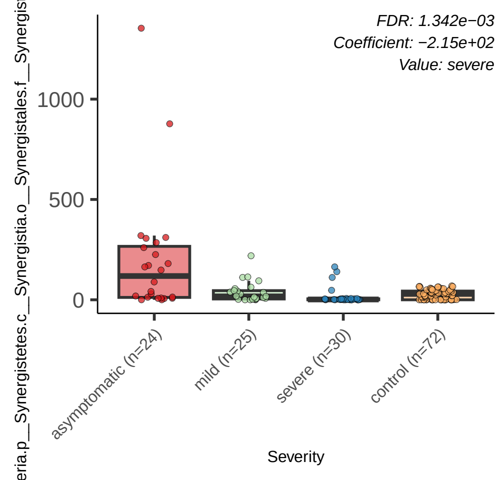

FDR: 1.342e−03
Coefficient: −2.15e+02
Value: severe
1000
500
0
asymptomatic (n=24)
mild (n=25)
severe (n=30)
control (n=72)
Severity
eria.p__Synergistetes.c__Synergistia.o__Synergistales.f__Synergis

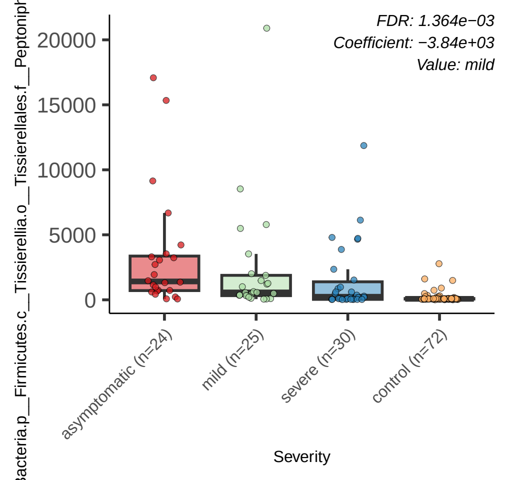
FDR: 1.364e−03
Coefficient: −3.84e+03
Value: mild
20000
15000
10000
5000
0
Bacteria.p__Firmicutes.c__Tissierellia.o__Tissierellales.f__Peptoniph
asymptomatic (n=24)
mild (n=25)
severe (n=30)
control (n=72)
Severity

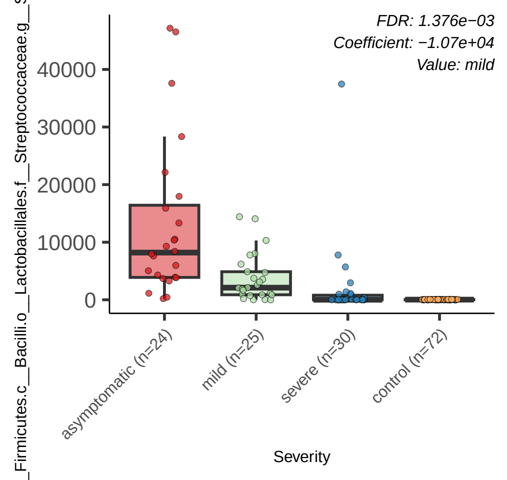

FDR: 1.376e−03
Coefficient: −1.07e+04
Value: mild
Firmicutes.c__Bacilli.o__Lactobacillales.f__Streptococcaceae.g__
40000
30000
20000
10000
0
asymptomatic (n=24)
mild (n=25)
severe (n=30)
control (n=72)
Severity

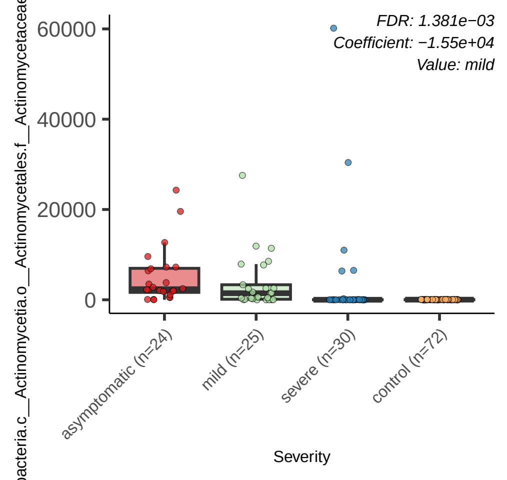

FDR: 1.381e−03
Coefficient: −1.55e+04
Value: mild
60000
40000
20000
0
bacteria.c__Actinomycetia.o__Actinomycetales.f__Actinomycetaceae
asymptomatic (n=24)
mild (n=25)
severe (n=30)
control (n=72)
Severity

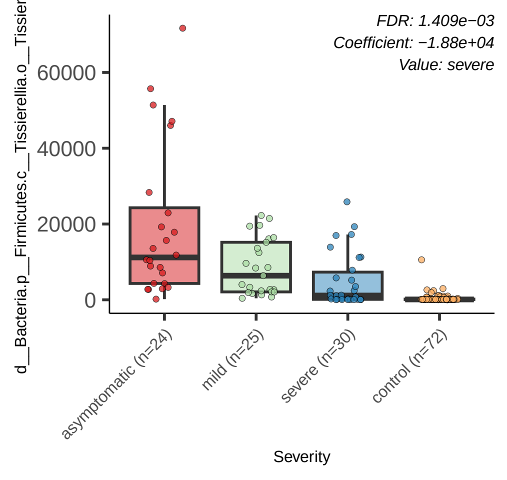

FDR: 1.409e−03
Coefficient: −1.88e+04
Value: severe
d__Bacteria.p__Firmicutes.c__Tissierellia.o__Tissier
0
20000
40000
60000
asymptomatic (n=24)
mild (n=25)
severe (n=30)
control (n=72)
Severity

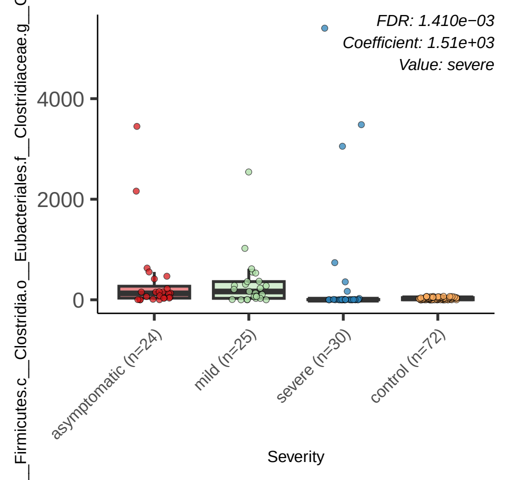

FDR: 1.410e−03
Coefficient: 1.51e+03
Value: severe
4000
2000
0
asymptomatic (n=24)
mild (n=25)
severe (n=30)
control (n=72)
Severity
Firmicutes.c__Clostridia.o__Eubacteriales.f__Clostridiaceae.g__C

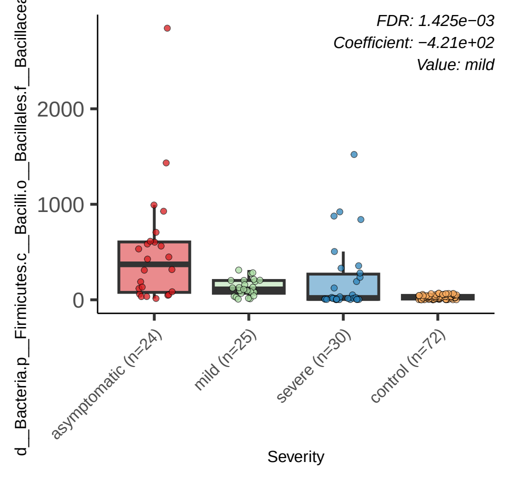

FDR: 1.425e−03
Coefficient: −4.21e+02
Value: mild
2000
1000
0
d__Bacteria.p__Firmicutes.c__Bacilli.o__Bacillales.f__Bacillacea
asymptomatic (n=24)
mild (n=25)
severe (n=30)
control (n=72)
Severity

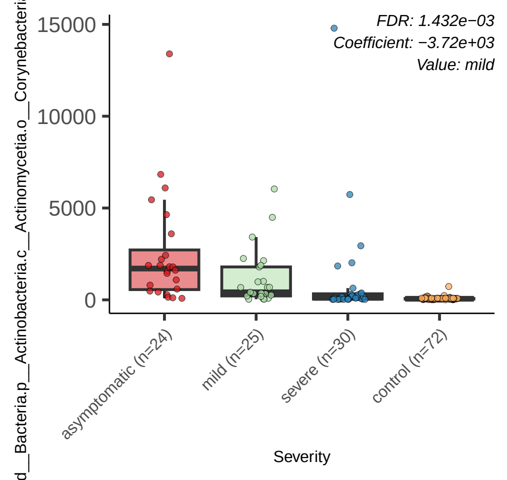

FDR: 1.432e−03
Coefficient: −3.72e+03
Value: mild
15000
10000
5000
0
d__Bacteria.p__Actinobacteria.c__Actinomycetia.o__Corynebacteri
asymptomatic (n=24)
mild (n=25)
severe (n=30)
control (n=72)
Severity

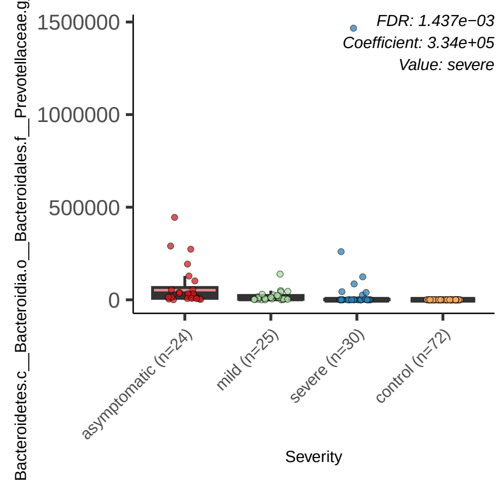

FDR: 1.437e−03
Coefficient: 3.34e+05
Value: severe
Bacteroidetes.c__Bacteroidia.o__Bacteroidales.f__Prevotellaceae.g
1500000
1000000
500000
0
asymptomatic (n=24)
mild (n=25)
severe (n=30)
control (n=72)
Severity

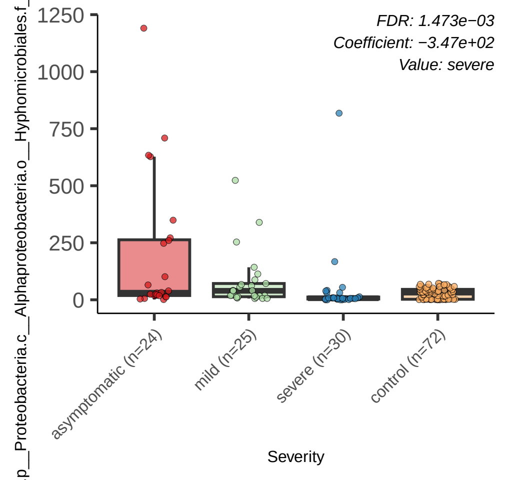

p__Proteobacteria.c__Alphaproteobacteria.o__Hyphomicrobiales.f__
FDR: 1.473e−03
Coefficient: −3.47e+02
Value: severe
1250
1000
750
500
250
0
asymptomatic (n=24)
mild (n=25)
severe (n=30)
control (n=72)
Severity

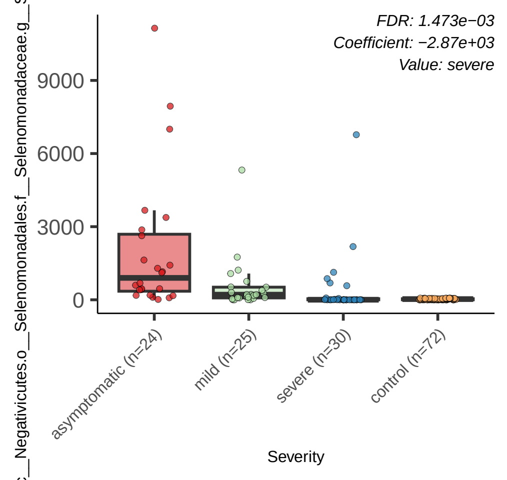

FDR: 1.473e−03
Coefficient: −2.87e+03
Value: severe
9000
6000
3000
0
Negativicutes.o__Selenomonadales.f__Selenomonadaceae.g__
asymptomatic (n=24)
mild (n=25)
severe (n=30)
control (n=72)
Severity

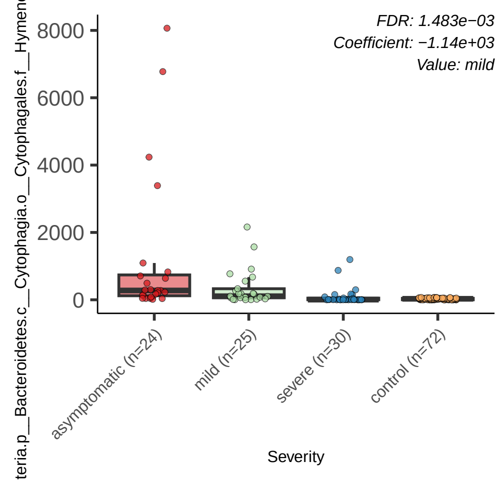
FDR: 1.483e−03
Coefficient: −1.14e+03
Value: mild
8000
6000
4000
2000
0
teria.p__Bacteroidetes.c__Cytophagia.o__Cytophagales.f__Hymeno
asymptomatic (n=24)
mild (n=25)
severe (n=30)
control (n=72)
Severity

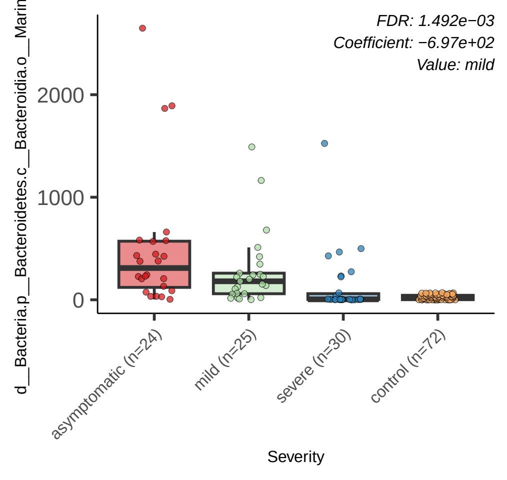
FDR: 1.492e−03
Coefficient: −6.97e+02
Value: mild
d__Bacteria.p__Bacteroidetes.c__Bacteroidia.o__Marin
2000
1000
0
asymptomatic (n=24)
mild (n=25)
severe (n=30)
control (n=72)
Severity

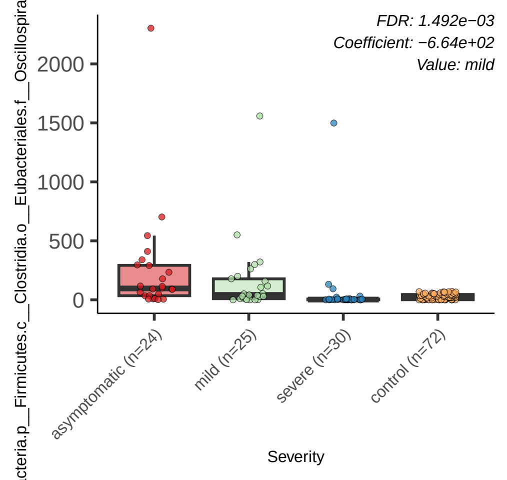

FDR: 1.492e−03
Coefficient: −6.64e+02
Value: mild
2000
1500
1000
500
0
asymptomatic (n=24)
mild (n=25)
severe (n=30)
control (n=72)
Severity
cteria.p__Firmicutes.c__Clostridia.o__Eubacteriales.f__Oscillospira

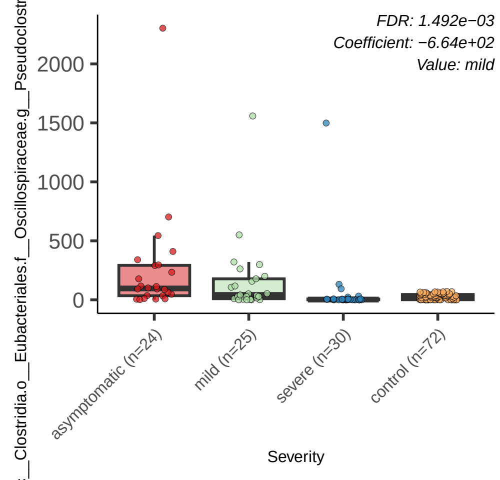

FDR: 1.492e−03
Coefficient: −6.64e+02
Value: mild
Clostridia.o__Eubacteriales.f__Oscillospiraceae.g__Pseudoclostr
2000
1500
1000
500
0
asymptomatic (n=24)
mild (n=25)
severe (n=30)
control (n=72)
Severity

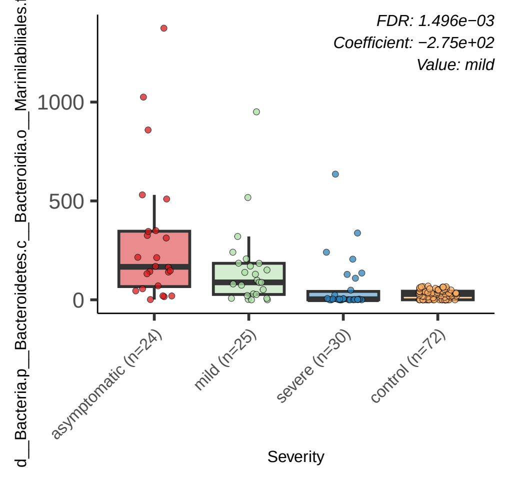
FDR: 1.496e−03
Coefficient: −2.75e+02
Value: mild
d__Bacteria.p__Bacteroidetes.c__Bacteroidia.o__Marinilabiliales.f
1000
500
0
asymptomatic (n=24)
mild (n=25)
severe (n=30)
control (n=72)
Severity

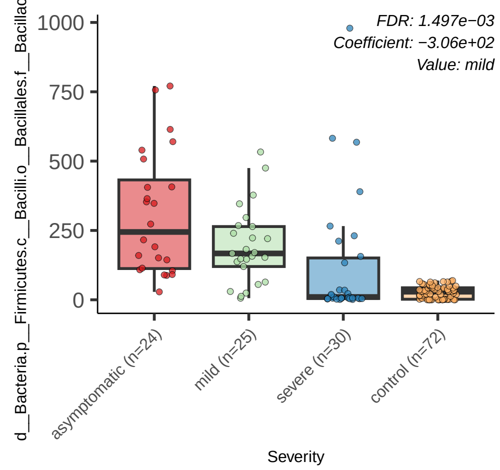

d__Bacteria.p__Firmicutes.c__Bacilli.o__Bacillales.f__Bacillac
FDR: 1.497e−03
Coefficient: −3.06e+02
Value: mild
1000
750
500
250
0
asymptomatic (n=24)
mild (n=25)
severe (n=30)
control (n=72)
Severity

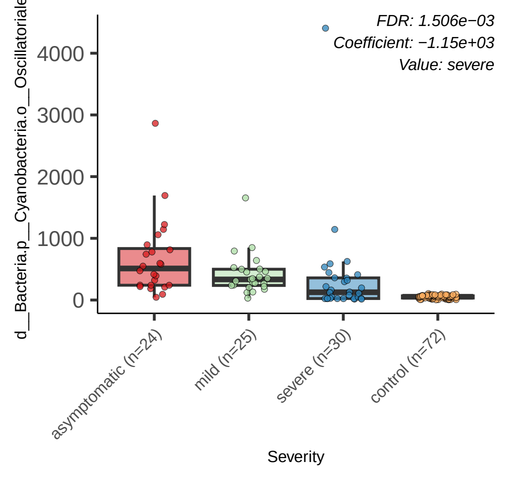

FDR: 1.506e−03
Coefficient: −1.15e+03
Value: severe
d__Bacteria.p__Cyanobacteria.o__Oscillatoriale
4000
3000
2000
1000
0
asymptomatic (n=24)
mild (n=25)
severe (n=30)
control (n=72)
Severity

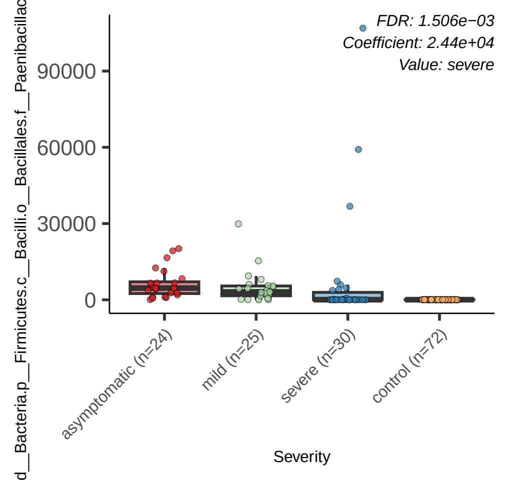
FDR: 1.506e−03
Coefficient: 2.44e+04
Value: severe
90000
60000
30000
0
d__Bacteria.p__Firmicutes.c__Bacilli.o__Bacillales.f__Paenibacillac
asymptomatic (n=24)
mild (n=25)
severe (n=30)
control (n=72)
Severity

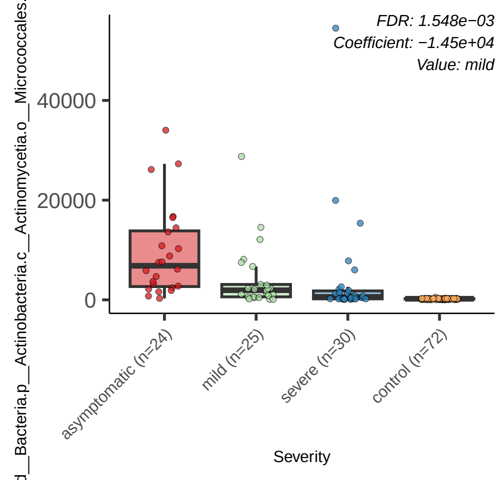
FDR: 1.548e−03
Coefficient: −1.45e+04
Value: mild
d__Bacteria.p__Actinobacteria.c__Actinomycetia.o__Micrococcales.
40000
20000
0
asymptomatic (n=24)
mild (n=25)
severe (n=30)
control (n=72)
Severity

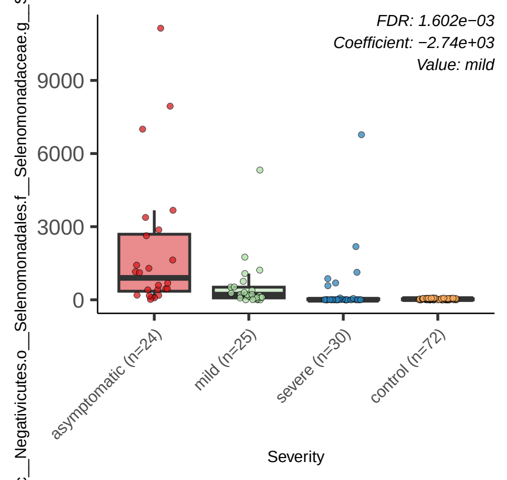

FDR: 1.602e−03
Coefficient: −2.74e+03
Value: mild
9000
6000
3000
0
Negativicutes.o__Selenomonadales.f__Selenomonadaceae.g__
asymptomatic (n=24)
mild (n=25)
severe (n=30)
control (n=72)
Severity

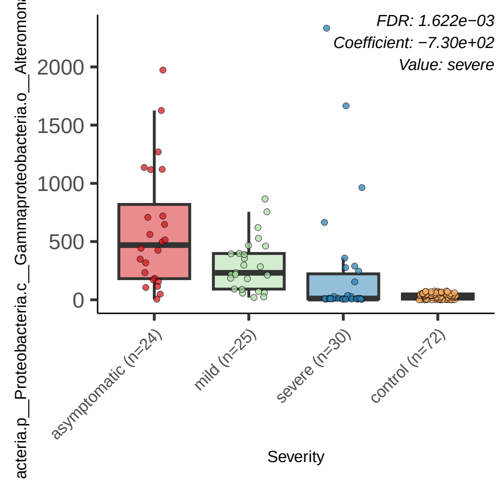

FDR: 1.622e−03
Coefficient: −7.30e+02
Value: severe
acteria.p__Proteobacteria.c__Gammaproteobacteria.o__Alteromona
2000
1500
1000
500
0
asymptomatic (n=24)
mild (n=25)
severe (n=30)
control (n=72)
Severity

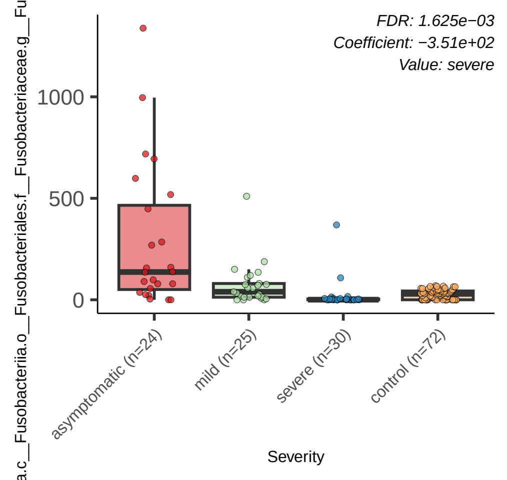
FDR: 1.625e−03
Coefficient: −3.51e+02
Value: severe
a.c__Fusobacteriia.o__Fusobacteriales.f__Fusobacteriaceae.g__Fu
1000
500
0
asymptomatic (n=24)
mild (n=25)
severe (n=30)
control (n=72)
Severity

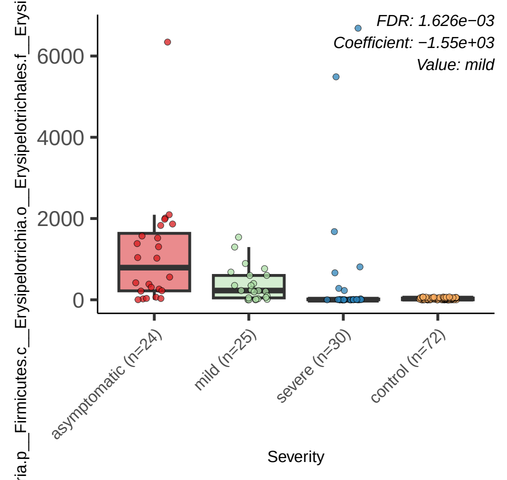
FDR: 1.626e−03
Coefficient: −1.55e+03
Value: mild
6000
4000
2000
0
ria.p__Firmicutes.c__Erysipelotrichia.o__Erysipelotrichales.f__Erysi
asymptomatic (n=24)
mild (n=25)
severe (n=30)
control (n=72)
Severity

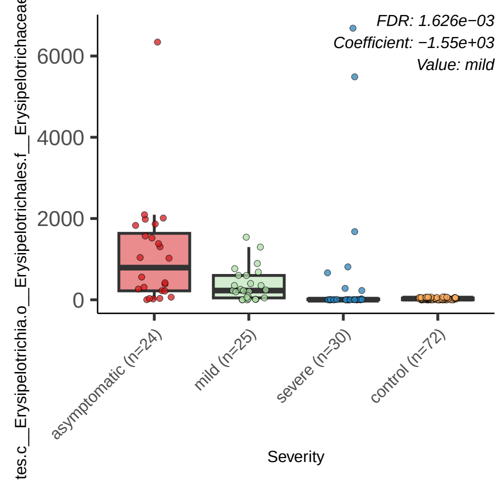

FDR: 1.626e−03
Coefficient: −1.55e+03
Value: mild
6000
4000
2000
0
tes.c__Erysipelotrichia.o__Erysipelotrichales.f__Erysipelotrichaceae
asymptomatic (n=24)
mild (n=25)
severe (n=30)
control (n=72)
Severity

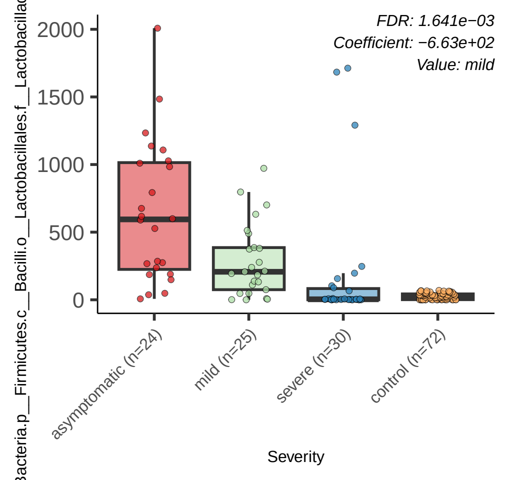

FDR: 1.641e−03
Coefficient: −6.63e+02
Value: mild
Bacteria.p__Firmicutes.c__Bacilli.o__Lactobacillales.f__Lactobacillac
2000
1500
1000
500
0
asymptomatic (n=24)
mild (n=25)
severe (n=30)
control (n=72)
Severity

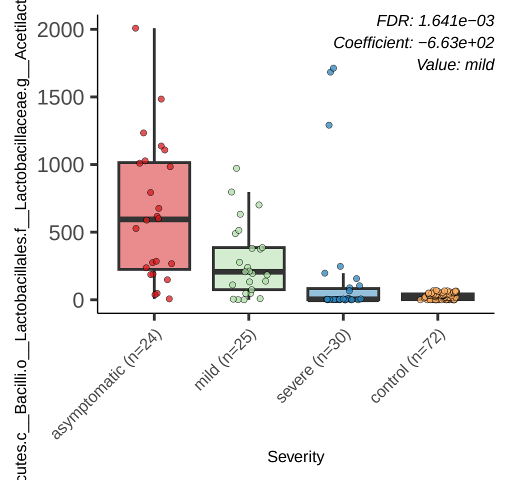
FDR: 1.641e−03
Coefficient: −6.63e+02
Value: mild
cutes.c__Bacilli.o__Lactobacillales.f__Lactobacillaceae.g__Acetilact
2000
1500
1000
500
0
asymptomatic (n=24)
mild (n=25)
severe (n=30)
control (n=72)
Severity

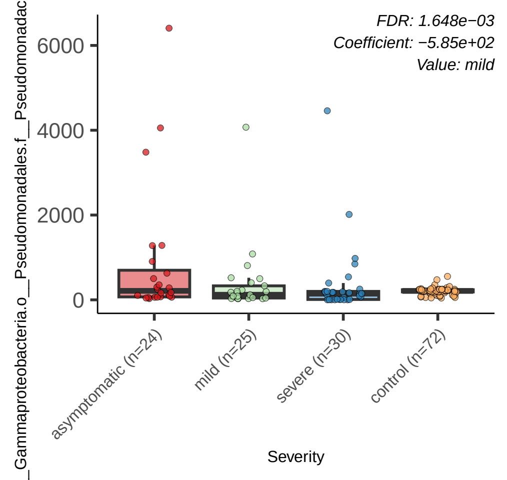

FDR: 1.648e−03
Coefficient: −5.85e+02
Value: mild
6000
4000
2000
0
_Gammaproteobacteria.o__Pseudomonadales.f__Pseudomonadac
asymptomatic (n=24)
mild (n=25)
severe (n=30)
control (n=72)
Severity

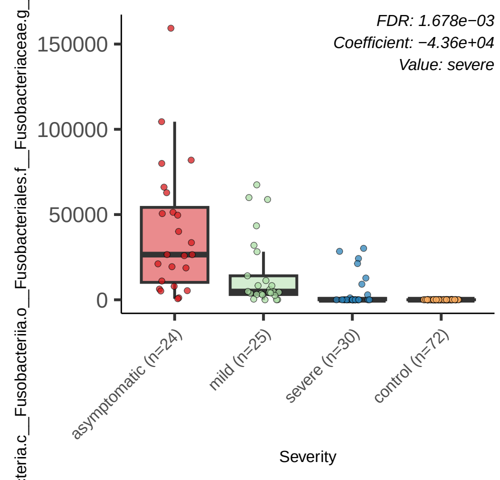
FDR: 1.678e−03
Coefficient: −4.36e+04
Value: severe
150000
100000
50000
0
asymptomatic (n=24)
mild (n=25)
severe (n=30)
control (n=72)
Severity
cteria.c__Fusobacteriia.o__Fusobacteriales.f__Fusobacteriaceae.g__

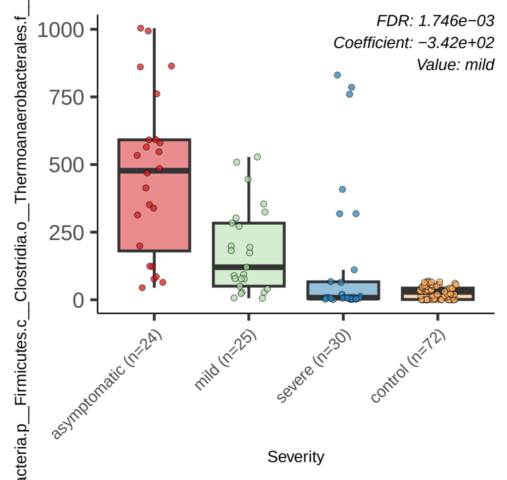

FDR: 1.746e−03
Coefficient: −3.42e+02
Value: mild
acteria.p__Firmicutes.c__Clostridia.o__Thermoanaerobacterales.f
1000
750
500
250
0
asymptomatic (n=24)
mild (n=25)
severe (n=30)
control (n=72)
Severity

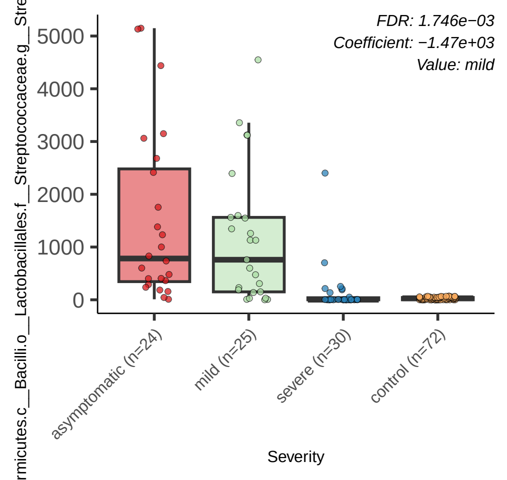

FDR: 1.746e−03
Coefficient: −1.47e+03
Value: mild
rmicutes.c__Bacilli.o__Lactobacillales.f__Streptococcaceae.g__Stre
5000
4000
3000
2000
1000
0
asymptomatic (n=24)
mild (n=25)
severe (n=30)
control (n=72)
Severity

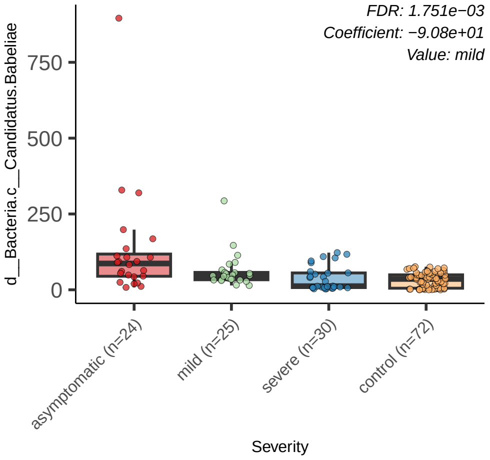

FDR: 1.751e−03
Coefficient: −9.08e+01
Value: mild
d__Bacteria.c__Candidatus.Babeliae
0
250
500
750
asymptomatic (n=24)
mild (n=25)
severe (n=30)
control (n=72)
Severity

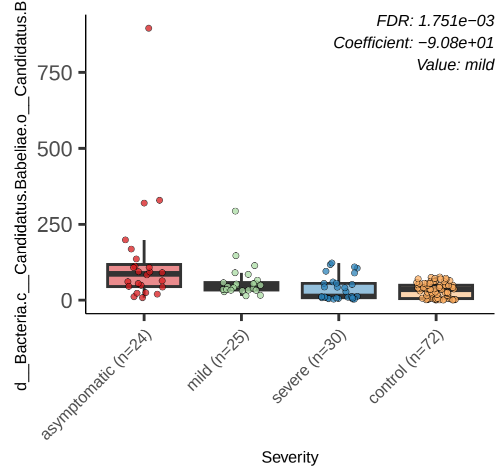

FDR: 1.751e−03
Coefficient: −9.08e+01
Value: mild
750
500
250
0
d__Bacteria.c__Candidatus.Babeliae.o__Candidatus.B
asymptomatic (n=24)
mild (n=25)
severe (n=30)
control (n=72)
Severity

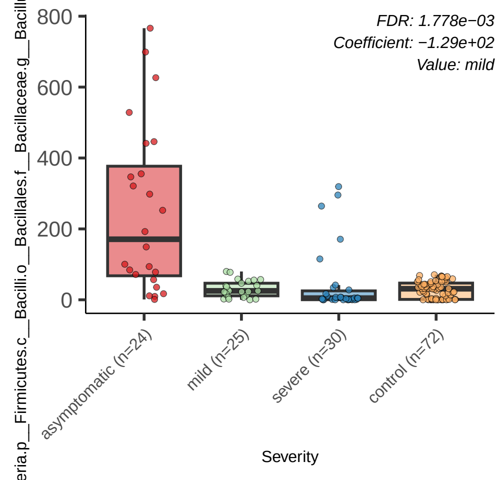

FDR: 1.778e−03
Coefficient: −1.29e+02
Value: mild
800
600
400
200
0
asymptomatic (n=24)
mild (n=25)
severe (n=30)
control (n=72)
Severity

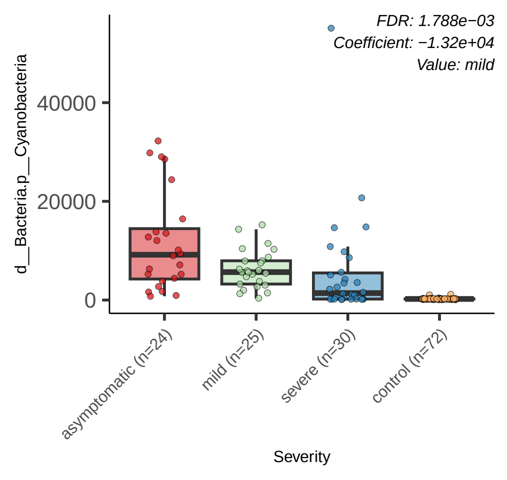
FDR: 1.788e−03
Coefficient: −1.32e+04
Value: mild
d__Bacteria.p__Cyanobacteria
40000
20000
0
asymptomatic (n=24)
mild (n=25)
severe (n=30)
control (n=72)
Severity

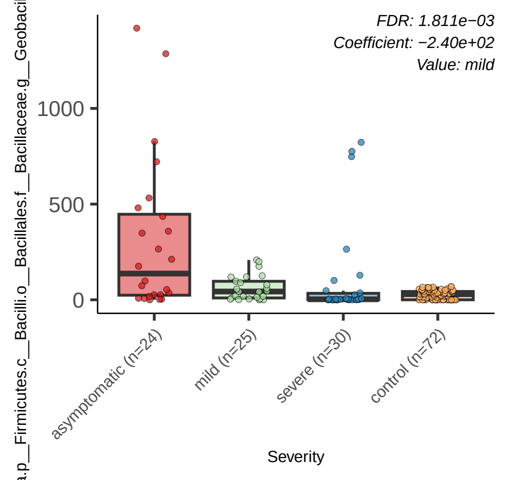

FDR: 1.811e−03
Coefficient: −2.40e+02
Value: mild
1000
500
0
asymptomatic (n=24)
mild (n=25)
severe (n=30)
control (n=72)
Severity
a.p__Firmicutes.c__Bacilli.o__Bacillales.f__Bacillaceae.g__Geobaci

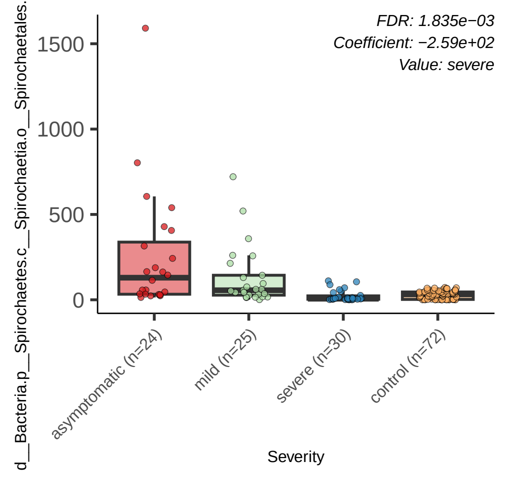

FDR: 1.835e−03
Coefficient: −2.59e+02
Value: severe
d__Bacteria.p__Spirochaetes.c__Spirochaetia.o__Spirochaetales.
1500
1000
500
0
asymptomatic (n=24)
mild (n=25)
severe (n=30)
control (n=72)
Severity

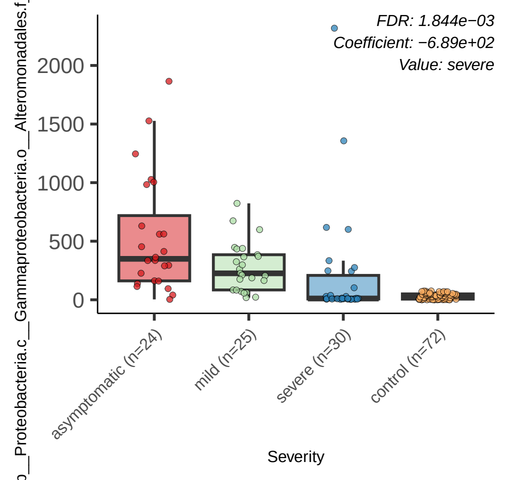

FDR: 1.844e−03
Coefficient: −6.89e+02
Value: severe
2000
1500
1000
500
0
o__Proteobacteria.c__Gammaproteobacteria.o__Alteromonadales.f_
asymptomatic (n=24)
mild (n=25)
severe (n=30)
control (n=72)
Severity

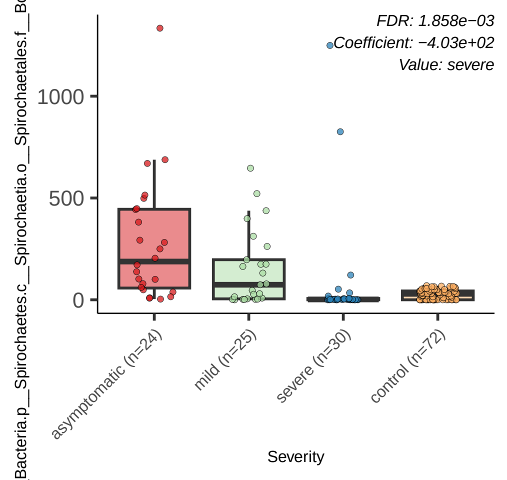

FDR: 1.858e−03
Coefficient: −4.03e+02
Value: severe
1000
500
0
Bacteria.p__Spirochaetes.c__Spirochaetia.o__Spirochaetales.f__Bo
asymptomatic (n=24)
mild (n=25)
severe (n=30)
control (n=72)
Severity

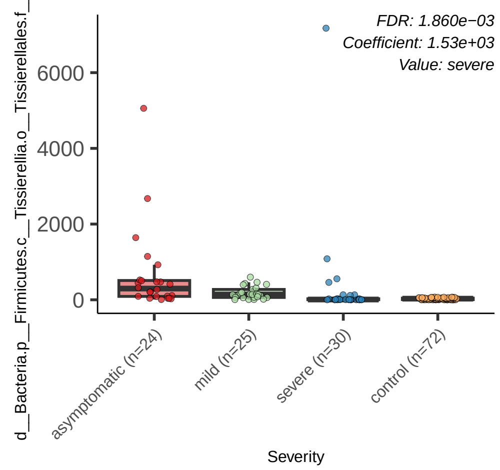

FDR: 1.860e−03
Coefficient: 1.53e+03
Value: severe
d__Bacteria.p__Firmicutes.c__Tissierellia.o__Tissierellales.f__
6000
4000
2000
0
asymptomatic (n=24)
mild (n=25)
severe (n=30)
control (n=72)
Severity

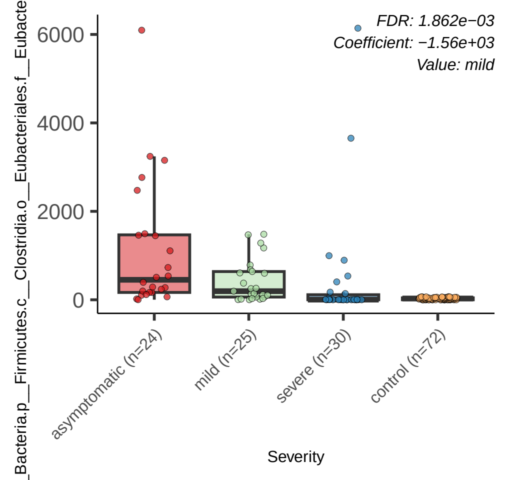

FDR: 1.862e−03
Coefficient: −1.56e+03
Value: mild
Bacteria.p__Firmicutes.c__Clostridia.o__Eubacteriales.f__Eubacte
6000
4000
2000
0
asymptomatic (n=24)
mild (n=25)
severe (n=30)
control (n=72)
Severity

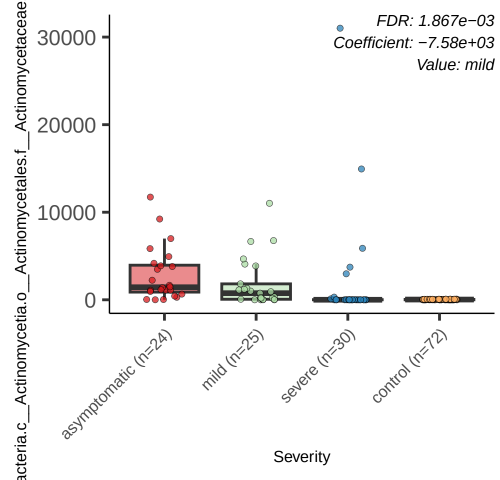

FDR: 1.867e−03
Coefficient: −7.58e+03
Value: mild
acteria.c__Actinomycetia.o__Actinomycetales.f__Actinomycetaceae
30000
20000
10000
0
asymptomatic (n=24)
mild (n=25)
severe (n=30)
control (n=72)
Severity

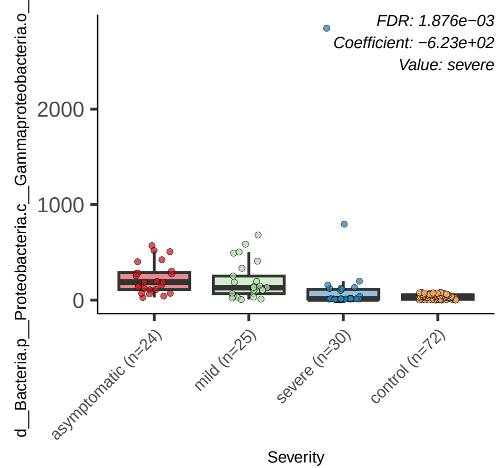

FDR: 1.876e−03
Coefficient: −6.23e+02
Value: severe
d__Bacteria.p__Proteobacteria.c__Gammaproteobacteria.o__
0
1000
2000
asymptomatic (n=24)
mild (n=25)
severe (n=30)
control (n=72)
Severity

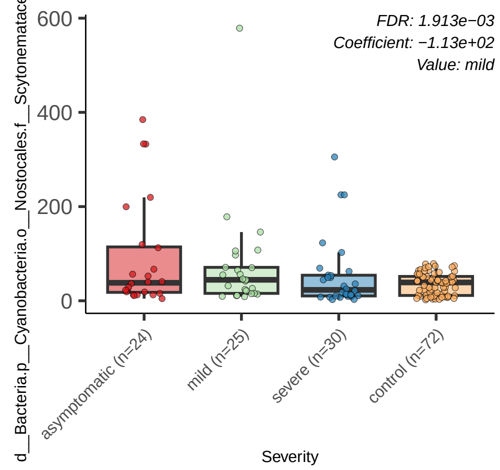

d__Bacteria.p__Cyanobacteria.o__Nostocales.f__Scytonematace
FDR: 1.913e−03
Coefficient: −1.13e+02
Value: mild
600
400
200
0
asymptomatic (n=24)
mild (n=25)
severe (n=30)
control (n=72)
Severity

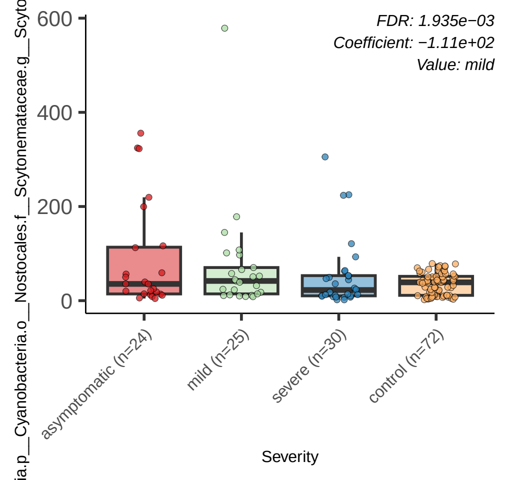

FDR: 1.935e−03
Coefficient: −1.11e+02
Value: mild
ia.p__Cyanobacteria.o__Nostocales.f__Scytonemataceae.g__Scyto
600
400
200
0
asymptomatic (n=24)
mild (n=25)
severe (n=30)
control (n=72)
Severity

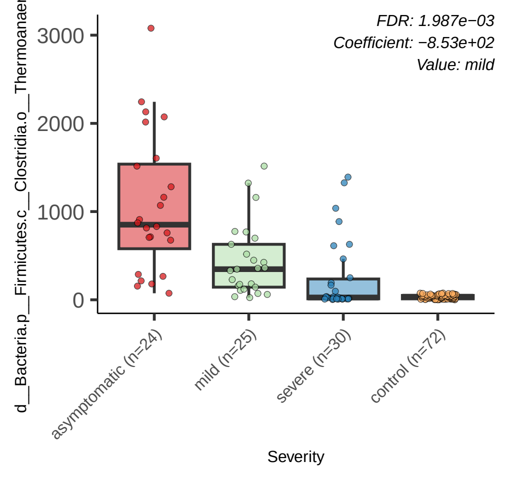

FDR: 1.987e−03
Coefficient: −8.53e+02
Value: mild
d__Bacteria.p__Firmicutes.c__Clostridia.o__Thermoanae
3000
2000
1000
0
asymptomatic (n=24)
mild (n=25)
severe (n=30)
control (n=72)
Severity

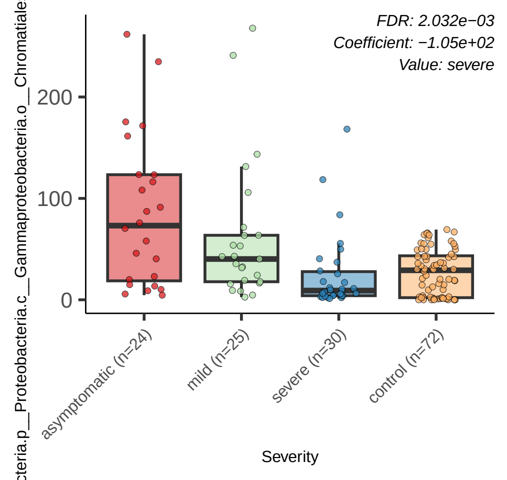
FDR: 2.032e−03
Coefficient: −1.05e+02
Value: severe
cteria.p__Proteobacteria.c__Gammaproteobacteria.o__Chromatiales
200
100
0
asymptomatic (n=24)
mild (n=25)
severe (n=30)
control (n=72)
Severity

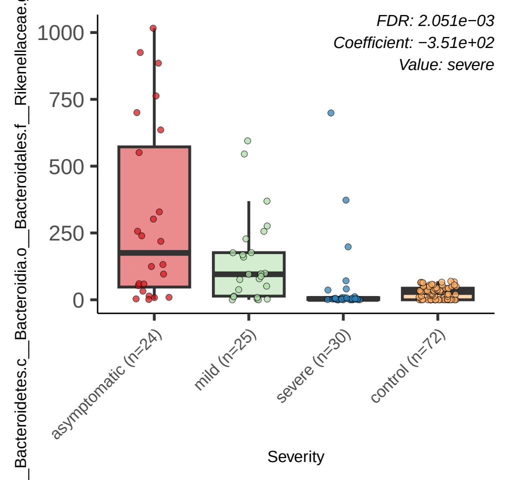

FDR: 2.051e−03
Coefficient: −3.51e+02
Value: severe
Bacteroidetes.c__Bacteroidia.o__Bacteroidales.f__Rikenellaceae.g
1000
750
500
250
0
asymptomatic (n=24)
mild (n=25)
severe (n=30)
control (n=72)
Severity

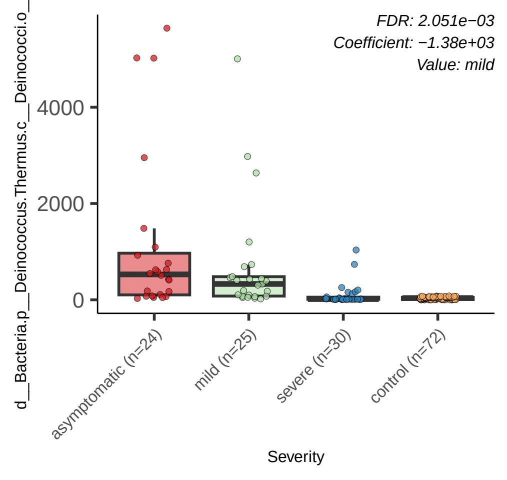

FDR: 2.051e−03
Coefficient: −1.38e+03
Value: mild
d__Bacteria.p__Deinococcus.Thermus.c__Deinococci.o__
4000
2000
0
asymptomatic (n=24)
mild (n=25)
severe (n=30)
control (n=72)
Severity

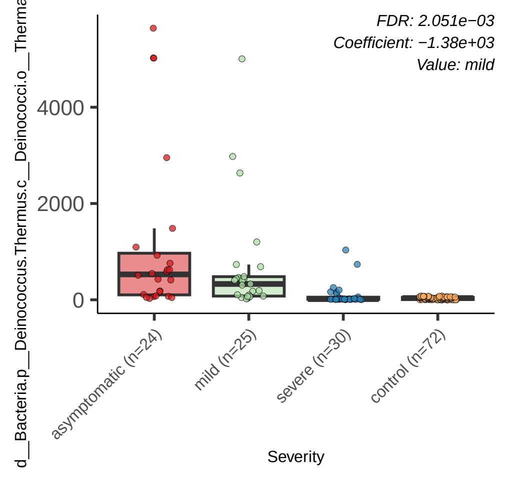
FDR: 2.051e−03
Coefficient: −1.38e+03
Value: mild
4000
2000
0
asymptomatic (n=24)
mild (n=25)
severe (n=30)
control (n=72)
Severity
d__Bacteria.p__Deinococcus.Thermus.c__Deinococci.o__Therma

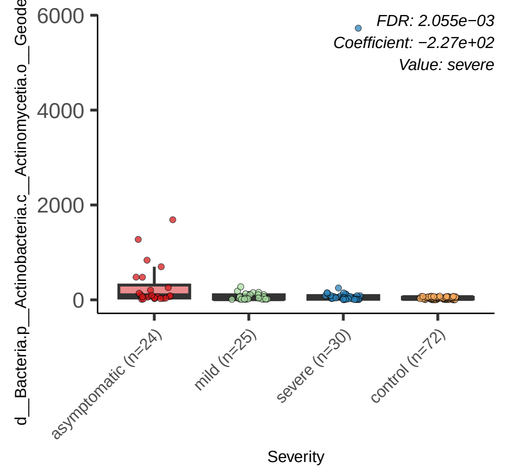

FDR: 2.055e−03
Coefficient: −2.27e+02
Value: severe
d__Bacteria.p__Actinobacteria.c__Actinomycetia.o__Geode
6000
4000
2000
0
asymptomatic (n=24)
mild (n=25)
severe (n=30)
control (n=72)
Severity

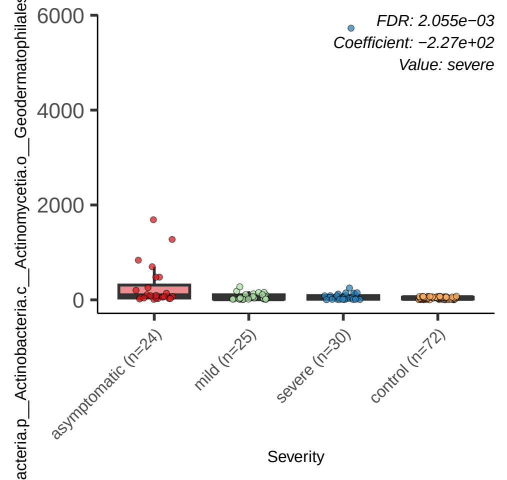
FDR: 2.055e−03
Coefficient: −2.27e+02
Value: severe
6000
4000
2000
0
acteria.p__Actinobacteria.c__Actinomycetia.o__Geodermatophilales
asymptomatic (n=24)
mild (n=25)
severe (n=30)
control (n=72)
Severity

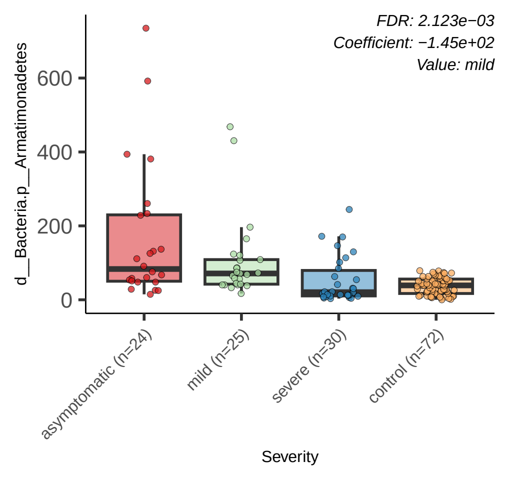
FDR: 2.123e−03
Coefficient: −1.45e+02
Value: mild
d__Bacteria.p__Armatimonadetes
0
200
400
600
asymptomatic (n=24)
mild (n=25)
severe (n=30)
control (n=72)
Severity

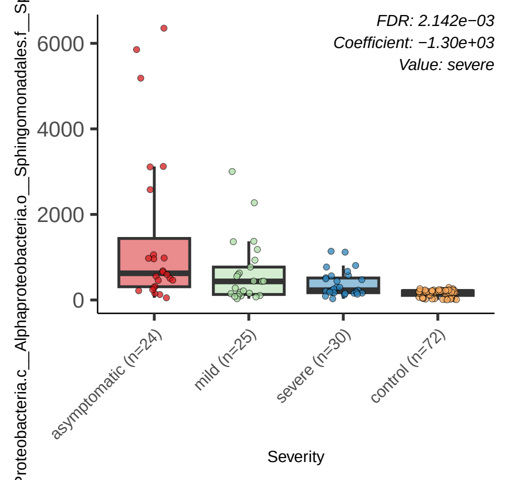

FDR: 2.142e−03
Coefficient: −1.30e+03
Value: severe
6000
4000
2000
0
Proteobacteria.c__Alphaproteobacteria.o__Sphingomonadales.f__Sp
asymptomatic (n=24)
mild (n=25)
severe (n=30)
control (n=72)
Severity

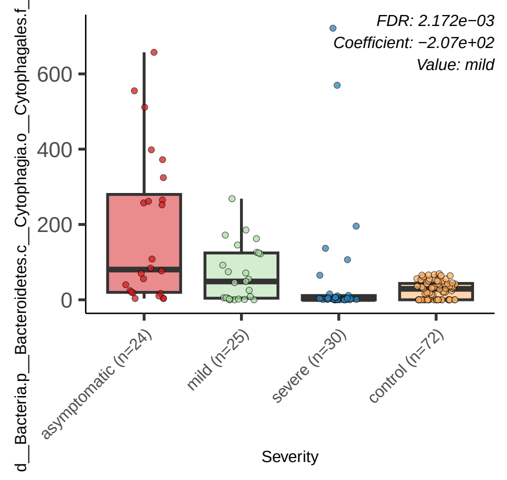

FDR: 2.172e−03
Coefficient: −2.07e+02
Value: mild
d__Bacteria.p__Bacteroidetes.c__Cytophagia.o__Cytophagales.f__
0
200
400
600
asymptomatic (n=24)
mild (n=25)
severe (n=30)
control (n=72)
Severity

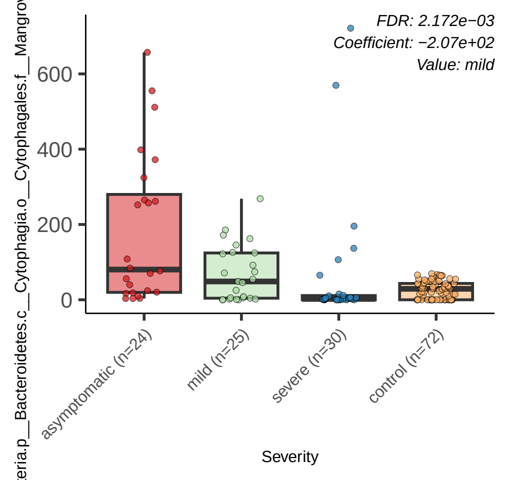

FDR: 2.172e−03
Coefficient: −2.07e+02
Value: mild
0
200
400
600
asymptomatic (n=24)
mild (n=25)
severe (n=30)
control (n=72)
Severity
teria.p__Bacteroidetes.c__Cytophagia.o__Cytophagales.f__Mangrov

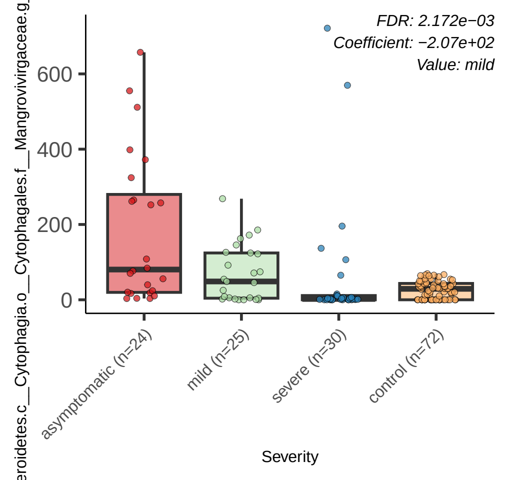
FDR: 2.172e−03
Coefficient: −2.07e+02
Value: mild
eroidetes.c__Cytophagia.o__Cytophagales.f__Mangrovivirgaceae.g_
600
400
200
0
asymptomatic (n=24)
mild (n=25)
severe (n=30)
control (n=72)
Severity

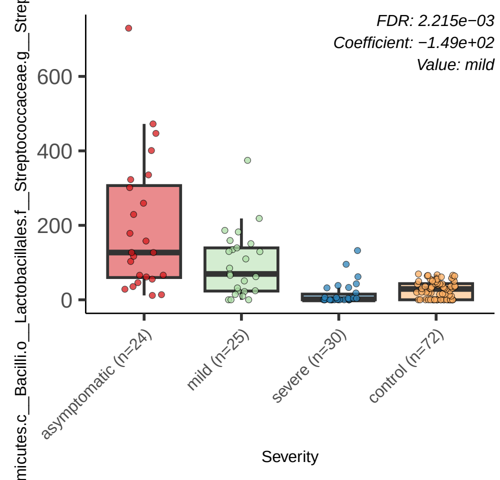

FDR: 2.215e−03
Coefficient: −1.49e+02
Value: mild
micutes.c__Bacilli.o__Lactobacillales.f__Streptococcaceae.g__Strep
600
400
200
0
asymptomatic (n=24)
mild (n=25)
severe (n=30)
control (n=72)
Severity

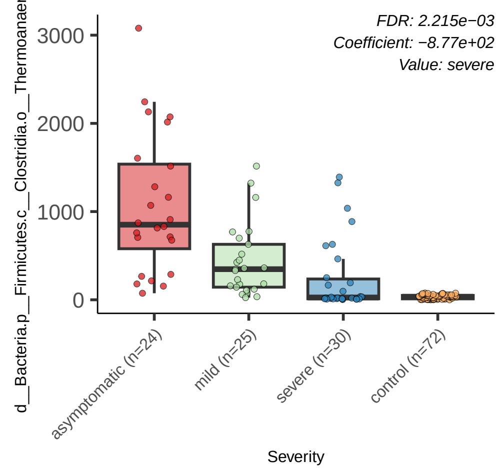

FDR: 2.215e−03
Coefficient: −8.77e+02
Value: severe
d__Bacteria.p__Firmicutes.c__Clostridia.o__Thermoanaer
3000
2000
1000
0
asymptomatic (n=24)
mild (n=25)
severe (n=30)
control (n=72)
Severity

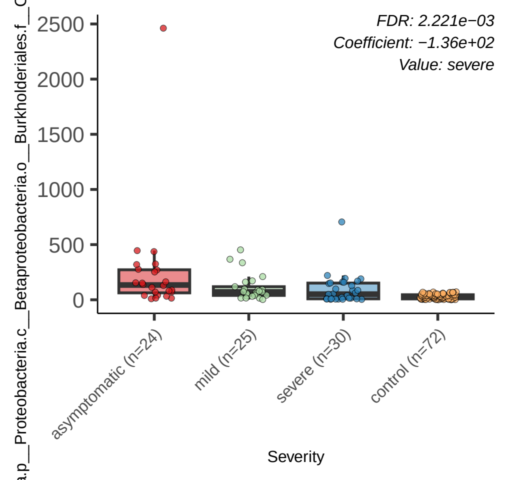
FDR: 2.221e−03
Coefficient: −1.36e+02
Value: severe
a.p__Proteobacteria.c__Betaproteobacteria.o__Burkholderiales.f__C
2500
2000
1500
1000
500
0
asymptomatic (n=24)
mild (n=25)
severe (n=30)
control (n=72)
Severity

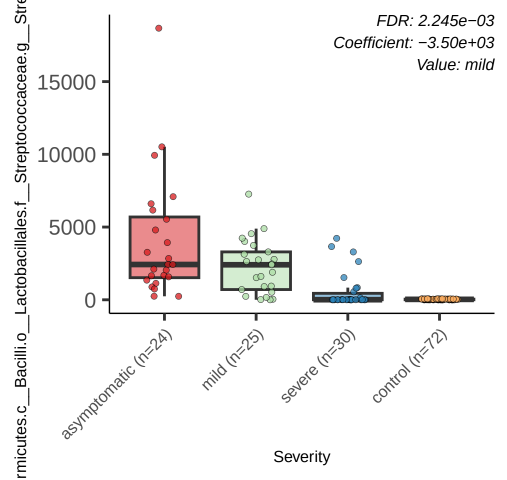

FDR: 2.245e−03
Coefficient: −3.50e+03
Value: mild
15000
10000
5000
0
rmicutes.c__Bacilli.o__Lactobacillales.f__Streptococcaceae.g__Stre
asymptomatic (n=24)
mild (n=25)
severe (n=30)
control (n=72)
Severity

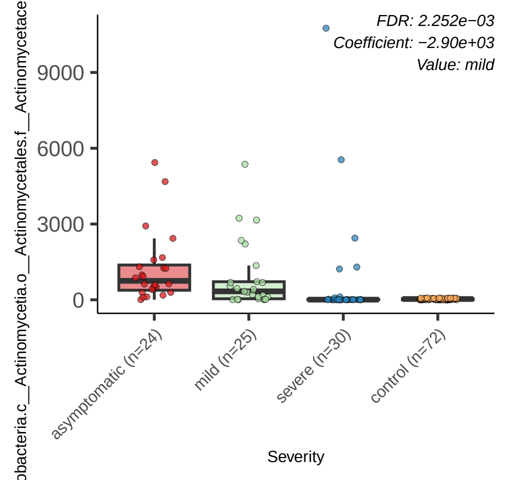

FDR: 2.252e−03
Coefficient: −2.90e+03
Value: mild
9000
6000
3000
0
obacteria.c__Actinomycetia.o__Actinomycetales.f__Actinomycetace
asymptomatic (n=24)
mild (n=25)
severe (n=30)
control (n=72)
Severity

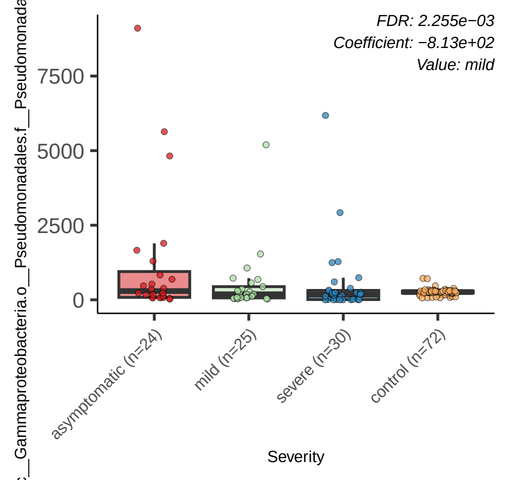

FDR: 2.255e−03
Coefficient: −8.13e+02
Value: mild
7500
5000
2500
0
c__Gammaproteobacteria.o__Pseudomonadales.f__Pseudomonada
asymptomatic (n=24)
mild (n=25)
severe (n=30)
control (n=72)
Severity

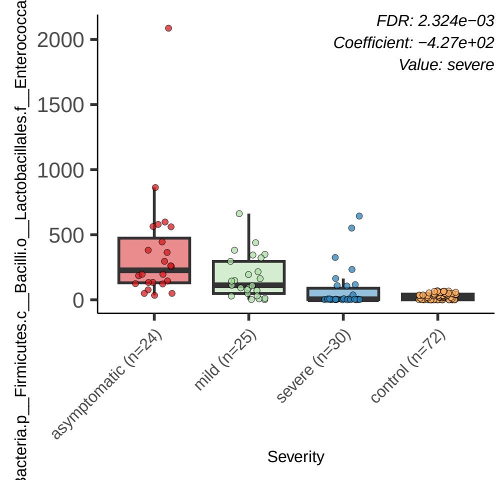

FDR: 2.324e−03
Coefficient: −4.27e+02
Value: severe
Bacteria.p__Firmicutes.c__Bacilli.o__Lactobacillales.f__Enterococca
2000
1500
1000
500
0
asymptomatic (n=24)
mild (n=25)
severe (n=30)
control (n=72)
Severity

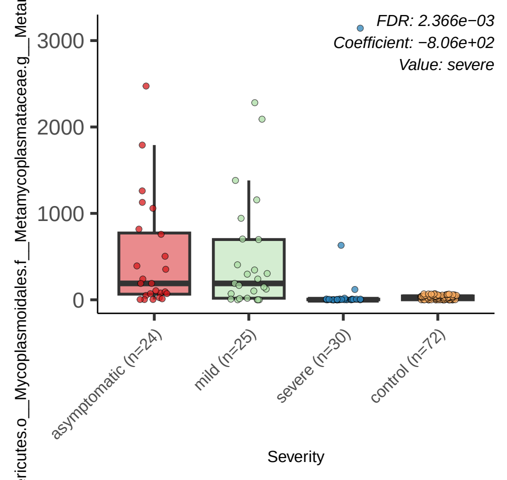

FDR: 2.366e−03
Coefficient: −8.06e+02
Value: severe
3000
2000
1000
0
ricutes.o__Mycoplasmoidales.f__Metamycoplasmataceae.g__Metam
asymptomatic (n=24)
mild (n=25)
severe (n=30)
control (n=72)
Severity

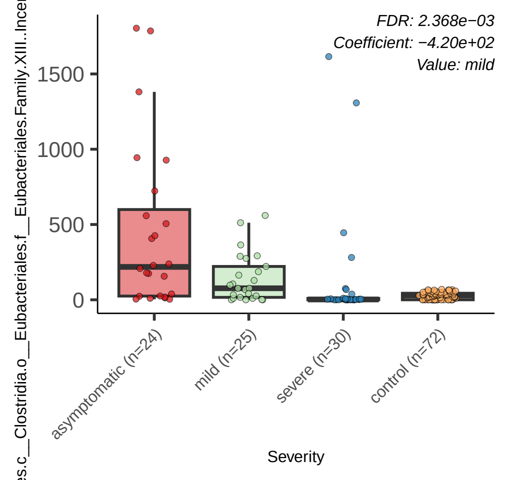

FDR: 2.368e−03
Coefficient: −4.20e+02
Value: mild
es.c__Clostridia.o__Eubacteriales.f__Eubacteriales.Family.XIII..Incer
1500
1000
500
0
asymptomatic (n=24)
mild (n=25)
severe (n=30)
control (n=72)
Severity

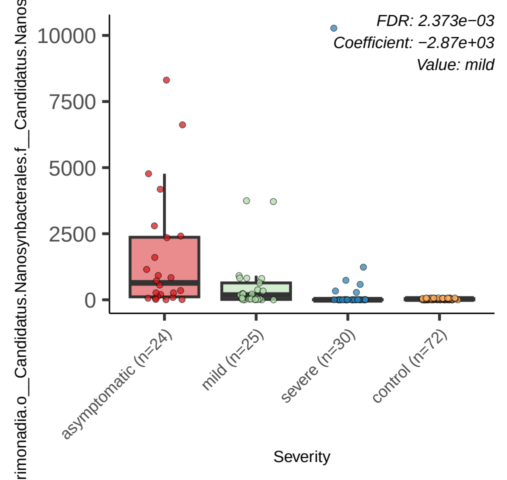
FDR: 2.373e−03
Coefficient: −2.87e+03
Value: mild
10000
7500
5000
2500
0
rimonadia.o__Candidatus.Nanosynbacterales.f__Candidatus.Nanos
asymptomatic (n=24)
mild (n=25)
severe (n=30)
control (n=72)
Severity

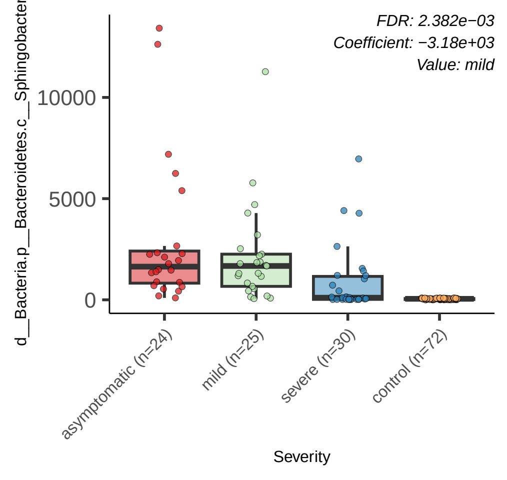

FDR: 2.382e−03
Coefficient: −3.18e+03
Value: mild
d__Bacteria.p__Bacteroidetes.c__Sphingobacter
10000
5000
0
asymptomatic (n=24)
mild (n=25)
severe (n=30)
control (n=72)
Severity

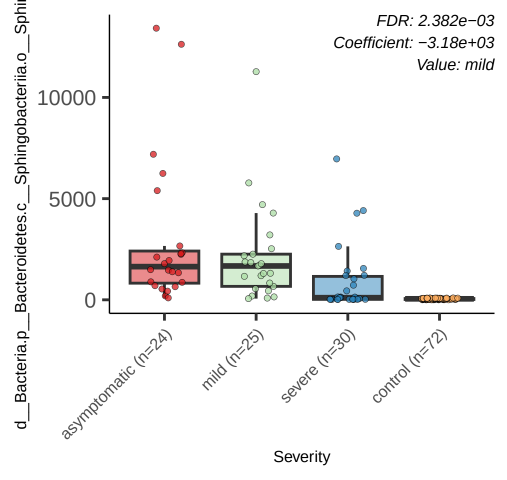
FDR: 2.382e−03
Coefficient: −3.18e+03
Value: mild
d__Bacteria.p__Bacteroidetes.c__Sphingobacteriia.o__Sphi
10000
5000
0
asymptomatic (n=24)
mild (n=25)
severe (n=30)
control (n=72)
Severity

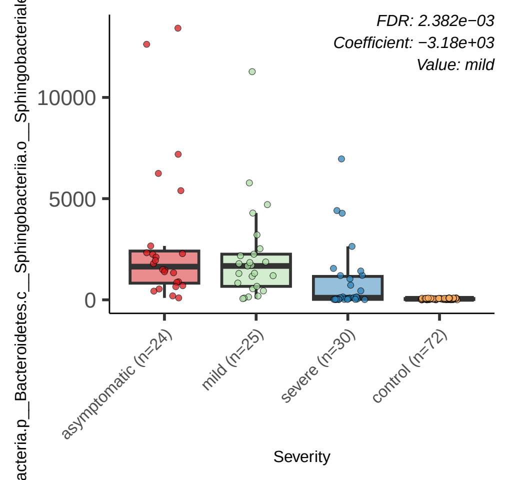
FDR: 2.382e−03
Coefficient: −3.18e+03
Value: mild
acteria.p__Bacteroidetes.c__Sphingobacteriia.o__Sphingobacteriale
10000
5000
0
asymptomatic (n=24)
mild (n=25)
severe (n=30)
control (n=72)
Severity

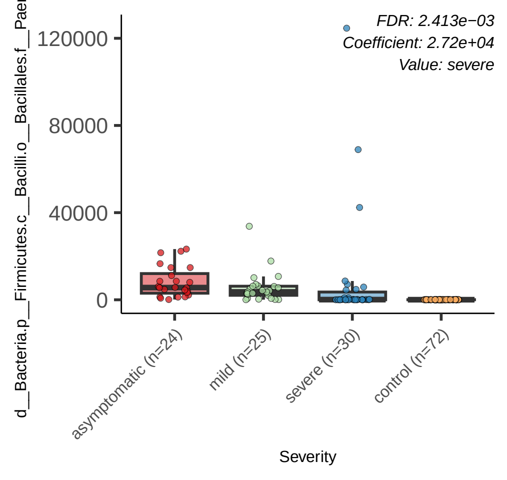

FDR: 2.413e−03
Coefficient: 2.72e+04
Value: severe
120000
80000
40000
0
d__Bacteria.p__Firmicutes.c__Bacilli.o__Bacillales.f__Pae
asymptomatic (n=24)
mild (n=25)
severe (n=30)
control (n=72)
Severity

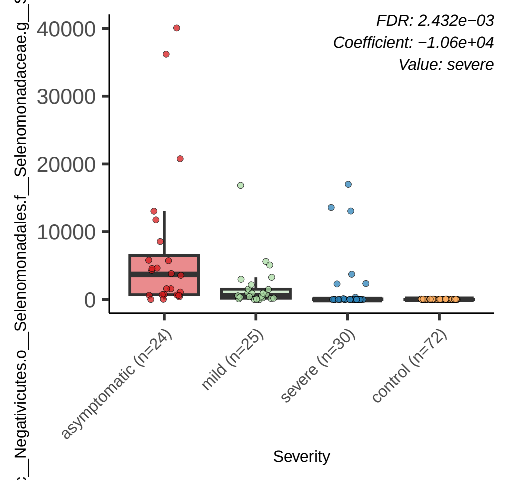

FDR: 2.432e−03
Coefficient: −1.06e+04
Value: severe
40000
30000
20000
10000
0
asymptomatic (n=24)
mild (n=25)
severe (n=30)
control (n=72)
Severity
Negativicutes.o__Selenomonadales.f__Selenomonadaceae.g__

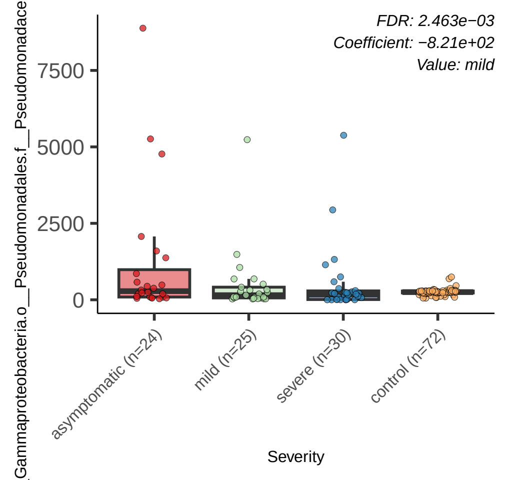

FDR: 2.463e−03
Coefficient: −8.21e+02
Value: mild
Gammaproteobacteria.o__Pseudomonadales.f__Pseudomonadace
7500
5000
2500
0
asymptomatic (n=24)
mild (n=25)
severe (n=30)
control (n=72)
Severity

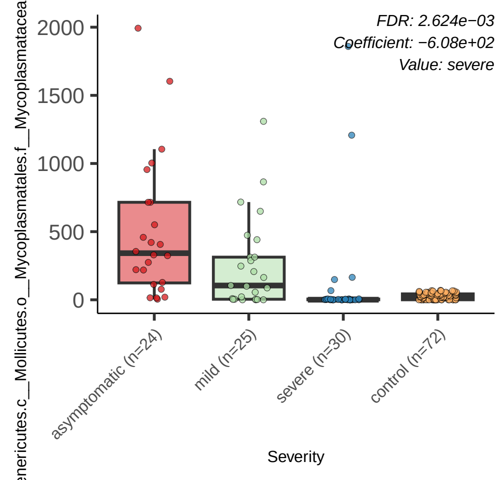
FDR: 2.624e−03
Coefficient: −6.08e+02
Value: severe
enericutes.c__Mollicutes.o__Mycoplasmatales.f__Mycoplasmatacea
2000
1500
1000
500
0
asymptomatic (n=24)
mild (n=25)
severe (n=30)
control (n=72)
Severity

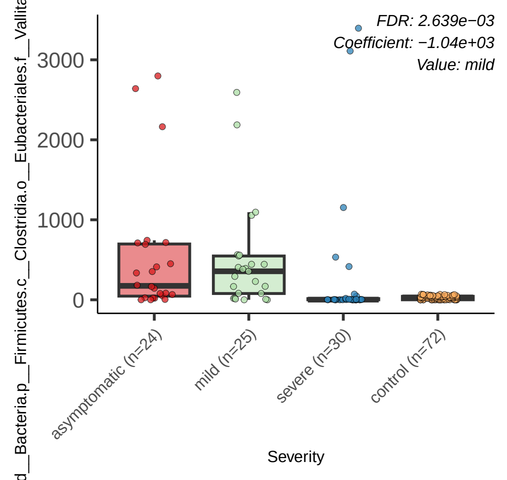
FDR: 2.639e−03
Coefficient: −1.04e+03
Value: mild
d__Bacteria.p__Firmicutes.c__Clostridia.o__Eubacteriales.f__Vallita
3000
2000
1000
0
asymptomatic (n=24)
mild (n=25)
severe (n=30)
control (n=72)
Severity

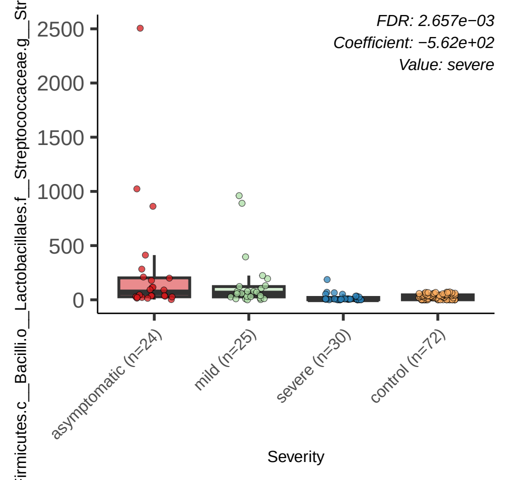

FDR: 2.657e−03
Coefficient: −5.62e+02
Value: severe
2500
2000
1500
1000
500
0
Firmicutes.c__Bacilli.o__Lactobacillales.f__Streptococcaceae.g__Str
asymptomatic (n=24)
mild (n=25)
severe (n=30)
control (n=72)
Severity

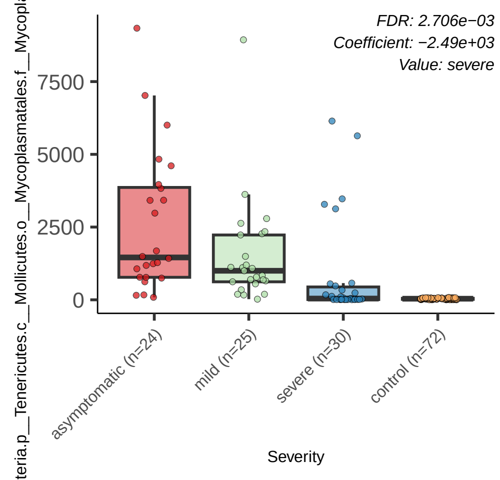

FDR: 2.706e−03
Coefficient: −2.49e+03
Value: severe
teria.p__Tenericutes.c__Mollicutes.o__Mycoplasmatales.f__Mycopla
7500
5000
2500
0
asymptomatic (n=24)
mild (n=25)
severe (n=30)
control (n=72)
Severity

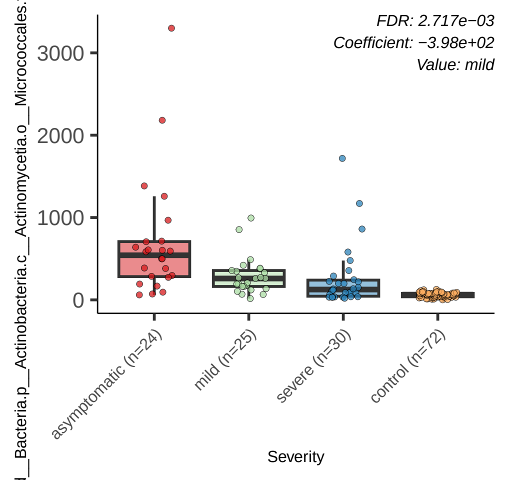

FDR: 2.717e−03
Coefficient: −3.98e+02
Value: mild
3000
2000
1000
0
d__Bacteria.p__Actinobacteria.c__Actinomycetia.o__Micrococcales.
asymptomatic (n=24)
mild (n=25)
severe (n=30)
control (n=72)
Severity

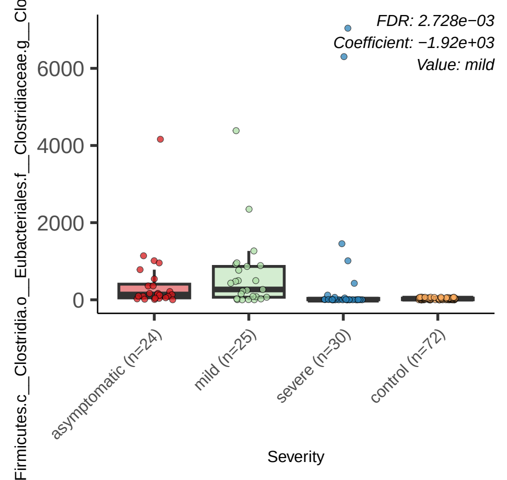

FDR: 2.728e−03
Coefficient: −1.92e+03
Value: mild
6000
4000
2000
0
asymptomatic (n=24)
mild (n=25)
severe (n=30)
control (n=72)
Severity
Firmicutes.c__Clostridia.o__Eubacteriales.f__Clostridiaceae.g__Clo

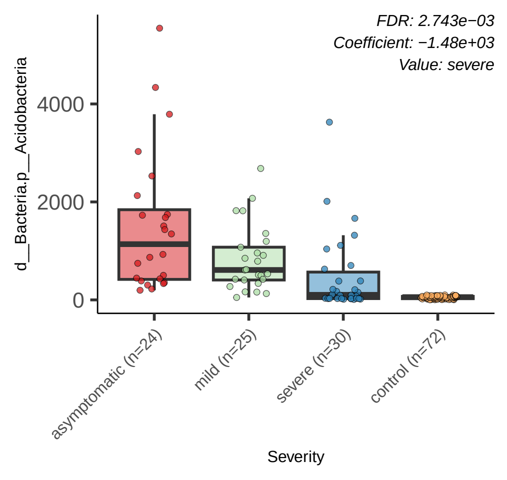

FDR: 2.743e−03
Coefficient: −1.48e+03
Value: severe
d__Bacteria.p__Acidobacteria
4000
2000
0
asymptomatic (n=24)
mild (n=25)
severe (n=30)
control (n=72)
Severity

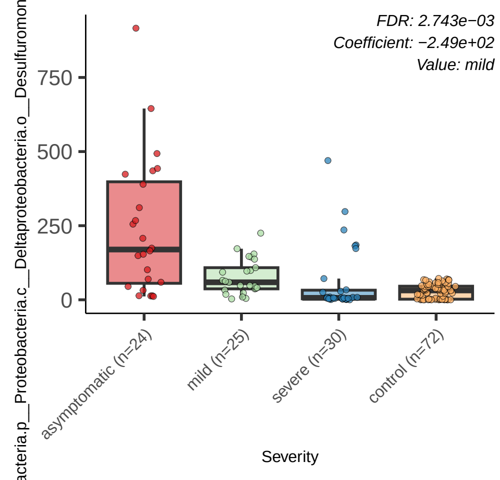

FDR: 2.743e−03
Coefficient: −2.49e+02
Value: mild
0
250
500
750
acteria.p__Proteobacteria.c__Deltaproteobacteria.o__Desulfuromon
asymptomatic (n=24)
mild (n=25)
severe (n=30)
control (n=72)
Severity

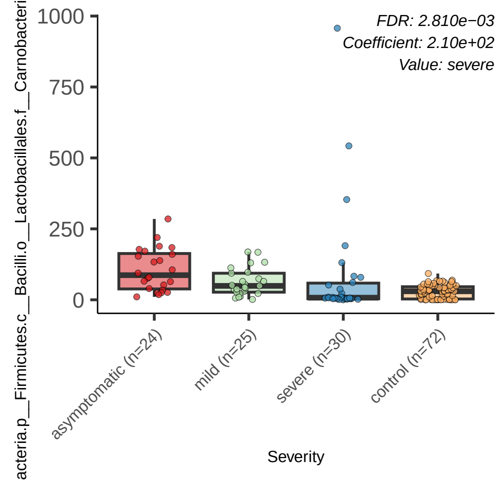

FDR: 2.810e−03
Coefficient: 2.10e+02
Value: severe
acteria.p__Firmicutes.c__Bacilli.o__Lactobacillales.f__Carnobacteri
1000
750
500
250
0
asymptomatic (n=24)
mild (n=25)
severe (n=30)
control (n=72)
Severity

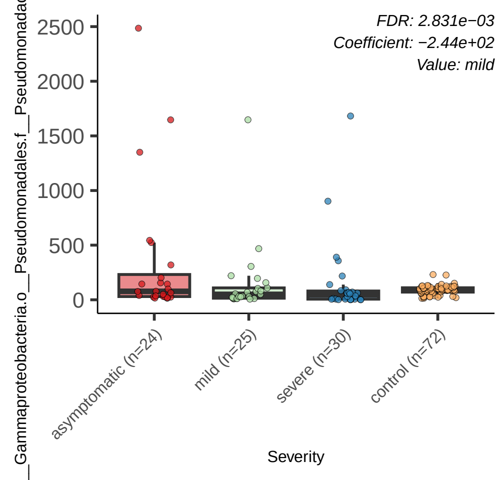

FDR: 2.831e−03
Coefficient: −2.44e+02
Value: mild
_Gammaproteobacteria.o__Pseudomonadales.f__Pseudomonadac
2500
2000
1500
1000
500
0
asymptomatic (n=24)
mild (n=25)
severe (n=30)
control (n=72)
Severity

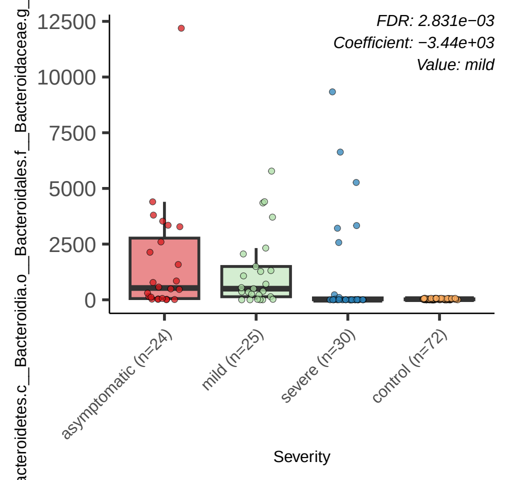

FDR: 2.831e−03
Coefficient: −3.44e+03
Value: mild
12500
10000
7500
5000
2500
0
acteroidetes.c__Bacteroidia.o__Bacteroidales.f__Bacteroidaceae.g__
asymptomatic (n=24)
mild (n=25)
severe (n=30)
control (n=72)
Severity

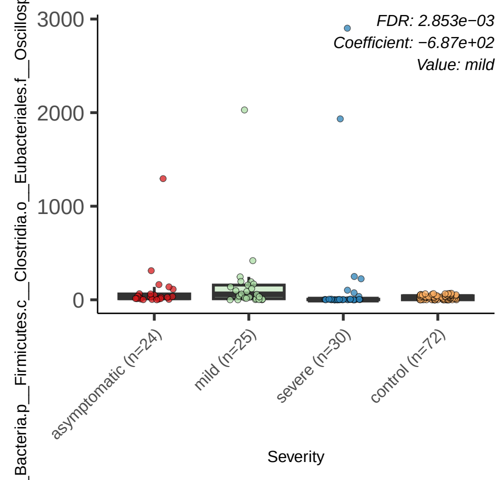
Bacteria.p__Firmicutes.c__Clostridia.o__Eubacteriales.f__Oscillosp
FDR: 2.853e−03
Coefficient: −6.87e+02
Value: mild
3000
2000
1000
0
asymptomatic (n=24)
mild (n=25)
severe (n=30)
control (n=72)
Severity

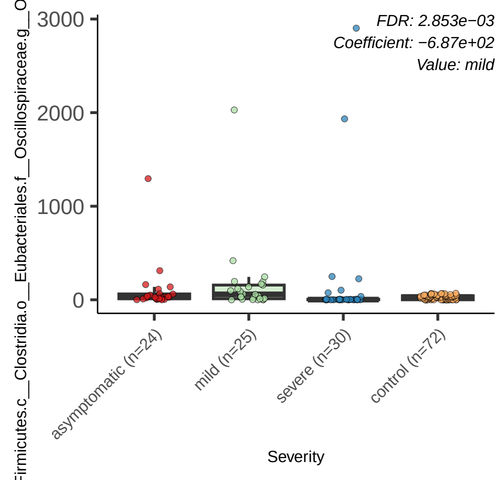

Firmicutes.c__Clostridia.o__Eubacteriales.f__Oscillospiraceae.g__O
3000
2000
1000
0
FDR: 2.853e−03
Coefficient: −6.87e+02
Value: mild
asymptomatic (n=24)
mild (n=25)
severe (n=30)
control (n=72)
Severity

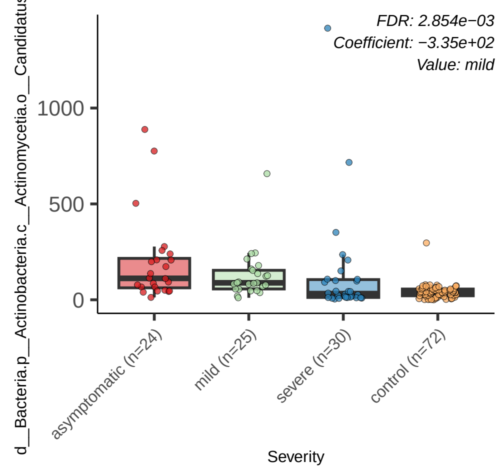

FDR: 2.854e−03
Coefficient: −3.35e+02
Value: mild
1000
500
0
d__Bacteria.p__Actinobacteria.c__Actinomycetia.o__Candidatus
asymptomatic (n=24)
mild (n=25)
severe (n=30)
control (n=72)
Severity

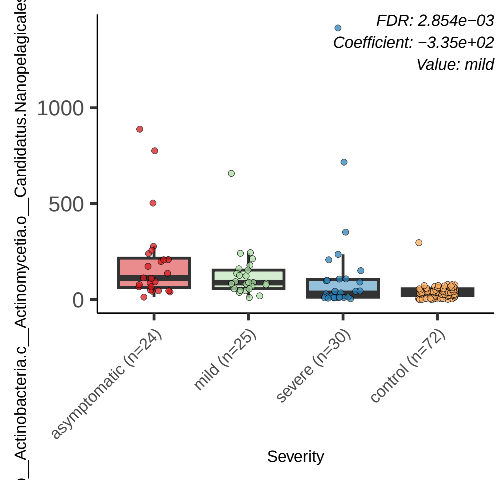
FDR: 2.854e−03
Coefficient: −3.35e+02
Value: mild
o__Actinobacteria.c__Actinomycetia.o__Candidatus.Nanopelagicales
1000
500
0
asymptomatic (n=24)
mild (n=25)
severe (n=30)
control (n=72)
Severity

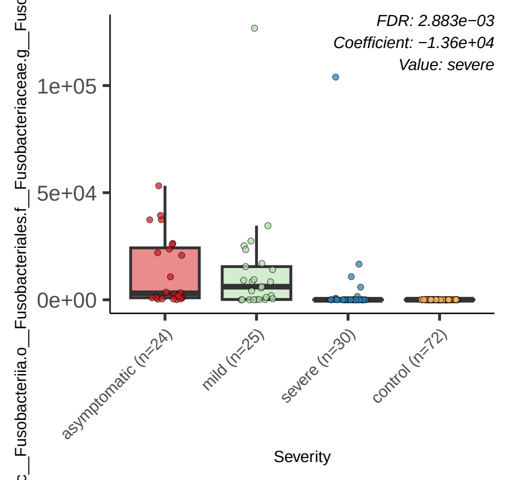

FDR: 2.883e−03
Coefficient: −1.36e+04
Value: severe
1e+05
5e+04
0e+00
c__Fusobacteriia.o__Fusobacteriales.f__Fusobacteriaceae.g__Fuso
asymptomatic (n=24)
mild (n=25)
severe (n=30)
control (n=72)
Severity

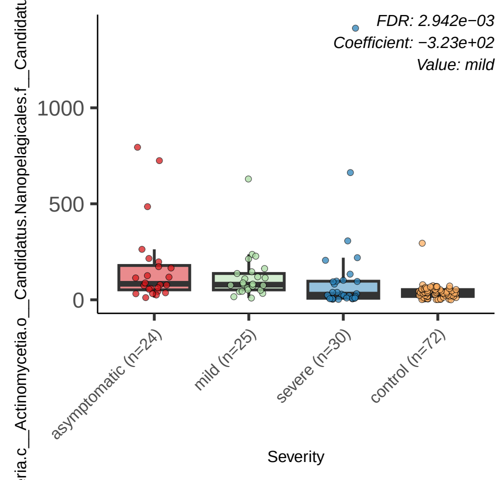

FDR: 2.942e−03
Coefficient: −3.23e+02
Value: mild
1000
500
0
asymptomatic (n=24)
mild (n=25)
severe (n=30)
control (n=72)
Severity

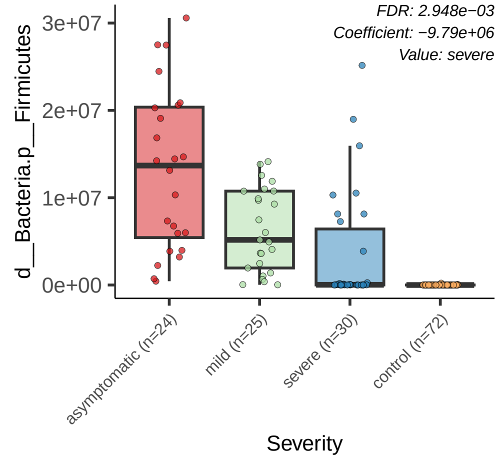

FDR: 2.948e−03
Coefficient: −9.79e+06
Value: severe
d__Bacteria.p__Firmicutes
3e+07
2e+07
1e+07
0e+00
asymptomatic (n=24)
mild (n=25)
severe (n=30)
control (n=72)
Severity

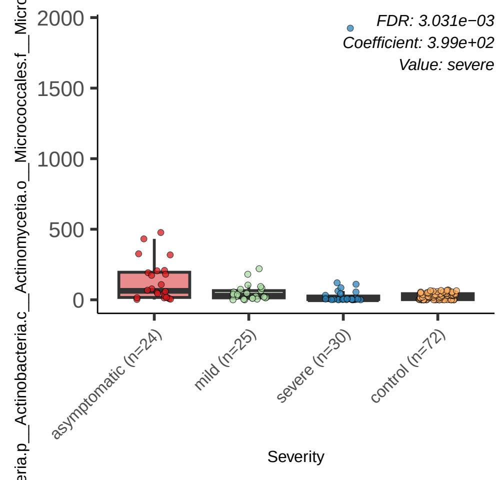

FDR: 3.031e−03
Coefficient: 3.99e+02
Value: severe
2000
1500
1000
500
0
eria.p__Actinobacteria.c__Actinomycetia.o__Micrococcales.f__Micro
asymptomatic (n=24)
mild (n=25)
severe (n=30)
control (n=72)
Severity

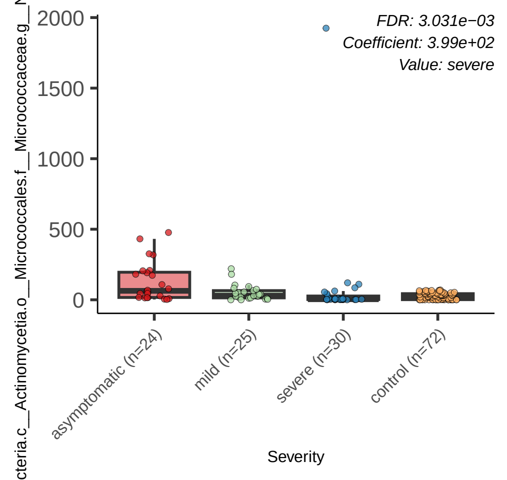

FDR: 3.031e−03
Coefficient: 3.99e+02
Value: severe
2000
1500
1000
500
0
cteria.c__Actinomycetia.o__Micrococcales.f__Micrococcaceae.g__
asymptomatic (n=24)
mild (n=25)
severe (n=30)
control (n=72)
Severity

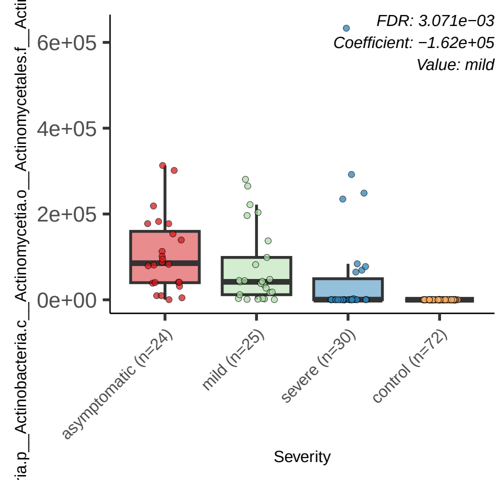

Actinobacteria.p__Actinobacteria.c__Actinomycetia.o__Actinomycetales.f__Acti
FDR: 3.071e−03
Coefficient: −1.62e+05
Value: mild
6e+05
4e+05
2e+05
0e+00
asymptomatic (n=24)
mild (n=25)
severe (n=30)
control (n=72)
Severity

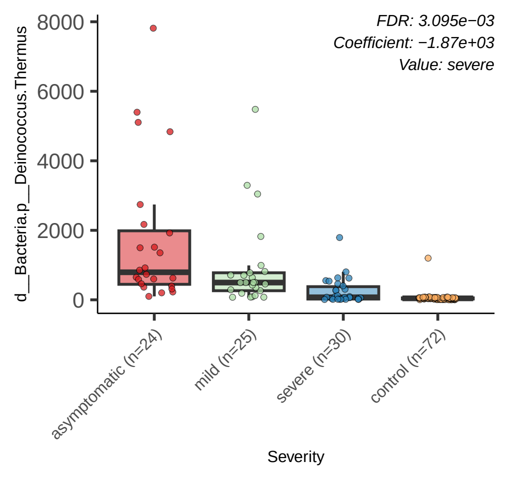

FDR: 3.095e−03
Coefficient: −1.87e+03
Value: severe
d__Bacteria.p__Deinococcus.Thermus
8000
6000
4000
2000
0
asymptomatic (n=24)
mild (n=25)
severe (n=30)
control (n=72)
Severity

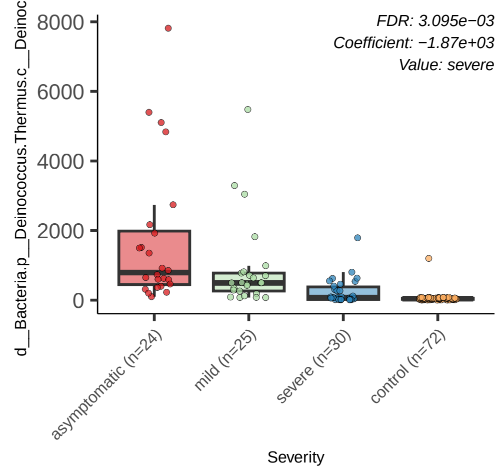

FDR: 3.095e−03
Coefficient: −1.87e+03
Value: severe
d__Bacteria.p__Deinococcus.Thermus.c__Deinoc
8000
6000
4000
2000
0
asymptomatic (n=24)
mild (n=25)
severe (n=30)
control (n=72)
Severity

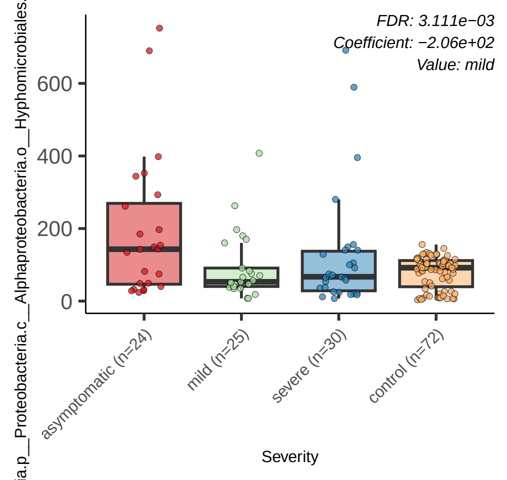

FDR: 3.111e−03
Coefficient: −2.06e+02
Value: mild
ia.p__Proteobacteria.c__Alphaproteobacteria.o__Hyphomicrobiales.
0
200
400
600
asymptomatic (n=24)
mild (n=25)
severe (n=30)
control (n=72)
Severity

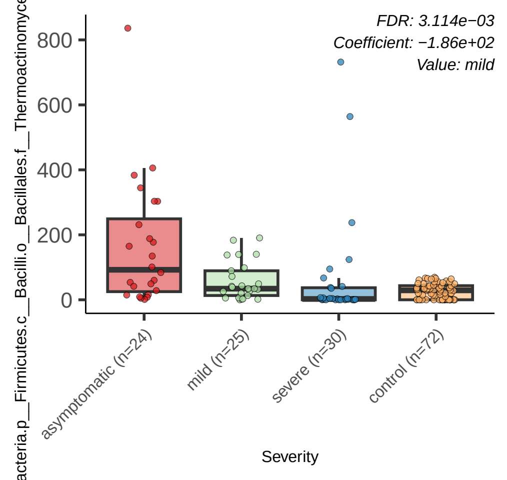

FDR: 3.114e−03
Coefficient: −1.86e+02
Value: mild
acteria.p__Firmicutes.c__Bacilli.o__Bacillales.f__Thermoactinomyce
800
600
400
200
0
asymptomatic (n=24)
mild (n=25)
severe (n=30)
control (n=72)
Severity

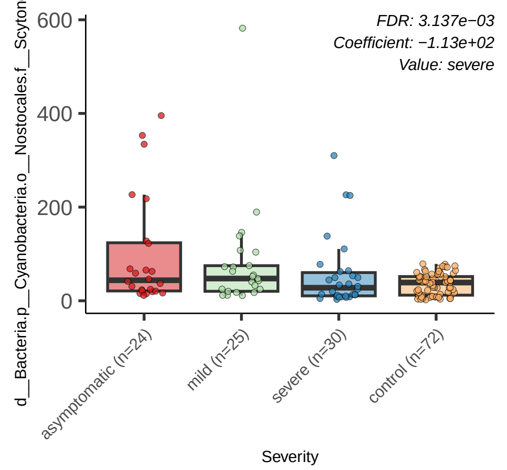

FDR: 3.137e−03
Coefficient: −1.13e+02
Value: severe
600
400
200
0
d__Bacteria.p__Cyanobacteria.o__Nostocales.f__Scyton
asymptomatic (n=24)
mild (n=25)
severe (n=30)
control (n=72)
Severity

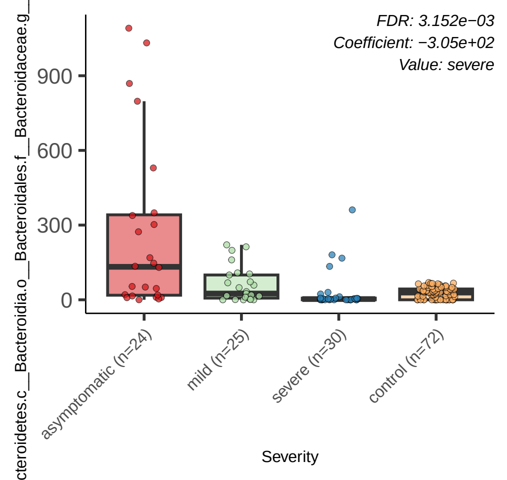

FDR: 3.152e−03
Coefficient: −3.05e+02
Value: severe
900
600
300
0
cteroidetes.c__Bacteroidia.o__Bacteroidales.f__Bacteroidaceae.g__
asymptomatic (n=24)
mild (n=25)
severe (n=30)
control (n=72)
Severity

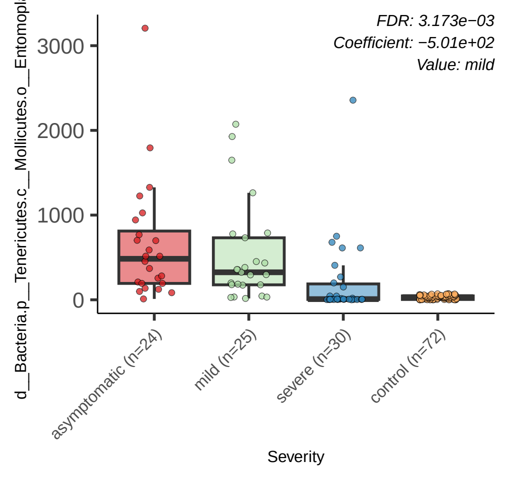

FDR: 3.173e−03
Coefficient: −5.01e+02
Value: mild
d__Bacteria.p__Tenericutes.c__Mollicutes.o__Entomopla
3000
2000
1000
0
asymptomatic (n=24)
mild (n=25)
severe (n=30)
control (n=72)
Severity

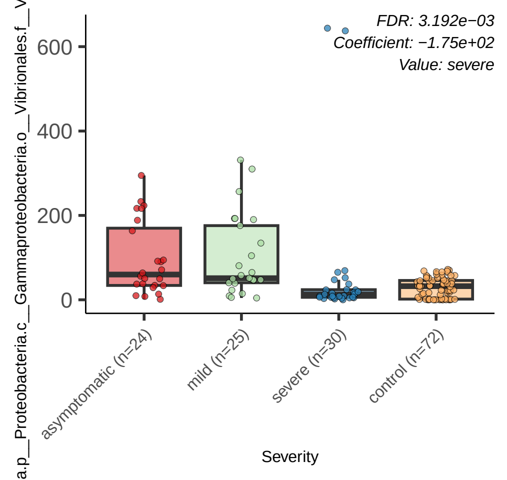

FDR: 3.192e−03
Coefficient: −1.75e+02
Value: severe
a.p__Proteobacteria.c__Gammaproteobacteria.o__Vibrionales.f__V
0
200
400
600
asymptomatic (n=24)
mild (n=25)
severe (n=30)
control (n=72)
Severity

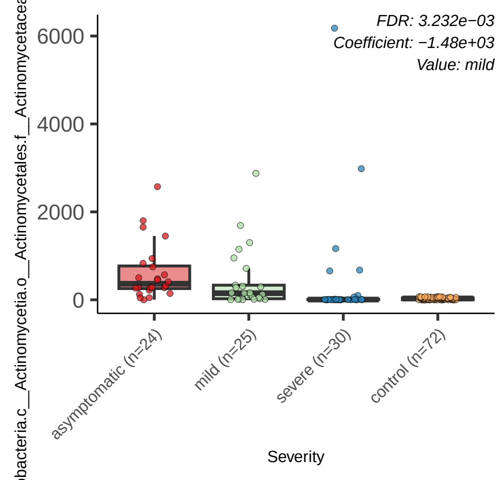

FDR: 3.232e−03
Coefficient: −1.48e+03
Value: mild
6000
4000
2000
0
bacteria.c__Actinomycetia.o__Actinomycetales.f__Actinomycetacea
asymptomatic (n=24)
mild (n=25)
severe (n=30)
control (n=72)
Severity

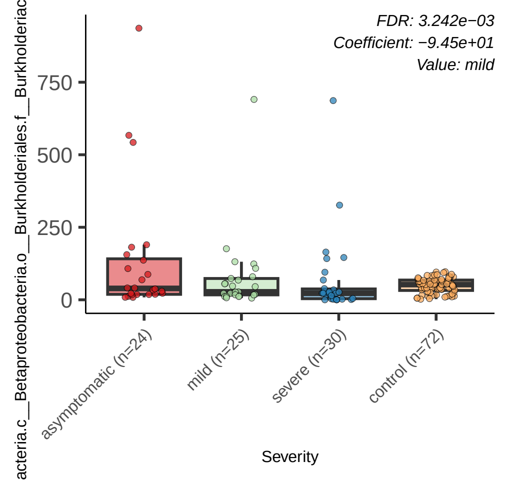

FDR: 3.242e−03
Coefficient: −9.45e+01
Value: mild
acteria.c__Betaproteobacteria.o__Burkholderiales.f__Burkholderiac
0
250
500
750
asymptomatic (n=24)
mild (n=25)
severe (n=30)
control (n=72)
Severity

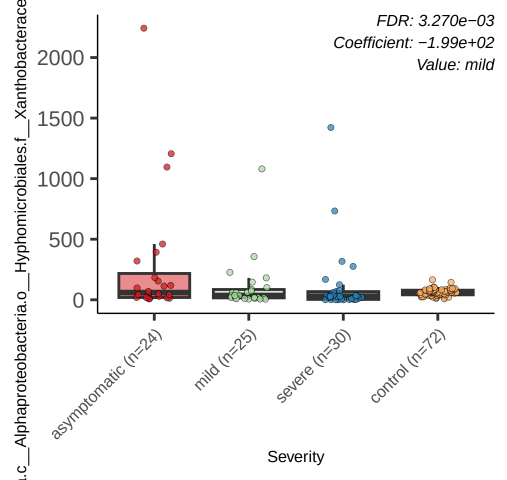
FDR: 3.270e−03
Coefficient: −1.99e+02
Value: mild
2000
1500
1000
500
0
asymptomatic (n=24)
mild (n=25)
severe (n=30)
control (n=72)
Severity
a.c__Alphaproteobacteria.o__Hyphomicrobiales.f__Xanthobacterace

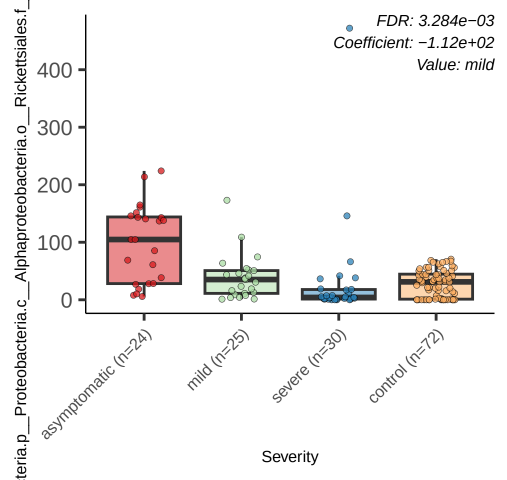

FDR: 3.284e−03
Coefficient: −1.12e+02
Value: mild
400
300
200
100
0
asymptomatic (n=24)
mild (n=25)
severe (n=30)
control (n=72)
Severity
cteria.p__Proteobacteria.c__Alphaproteobacteria.o__Rickettsiales.f__

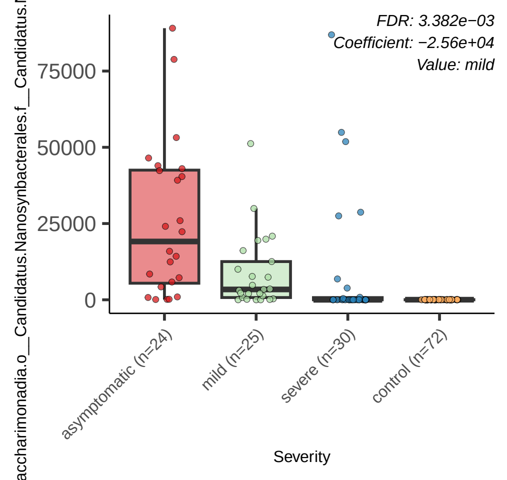

FDR: 3.382e−03
Coefficient: −2.56e+04
Value: mild
75000
50000
25000
0
accharimonadia.o__Candidatus.Nanosynbacterales.f__Candidatus.N
asymptomatic (n=24)
mild (n=25)
severe (n=30)
control (n=72)
Severity

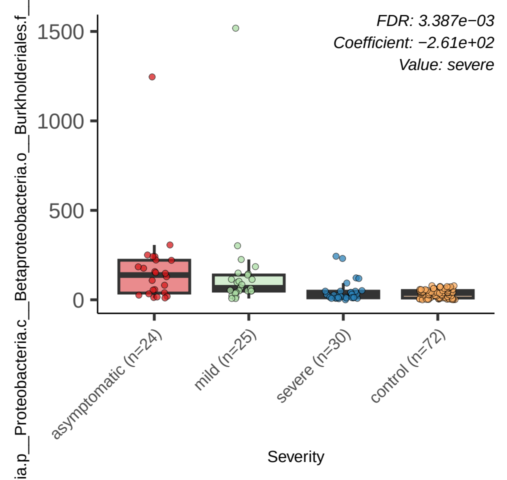
FDR: 3.387e−03
Coefficient: −2.61e+02
Value: severe
ia.p__Proteobacteria.c__Betaproteobacteria.o__Burkholderiales.f__
1500
1000
500
0
asymptomatic (n=24)
mild (n=25)
severe (n=30)
control (n=72)
Severity

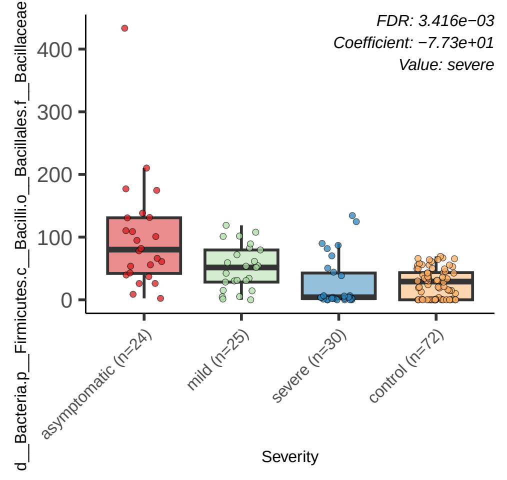
FDR: 3.416e−03
Coefficient: −7.73e+01
Value: severe
d__Bacteria.p__Firmicutes.c__Bacilli.o__Bacillales.f__Bacillaceae
0
100
200
300
400
asymptomatic (n=24)
mild (n=25)
severe (n=30)
control (n=72)
Severity

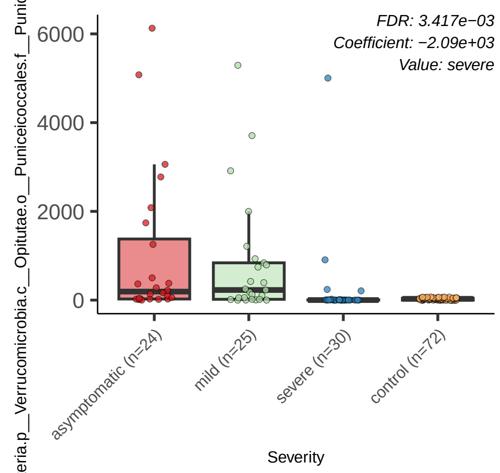

FDR: 3.417e−03
Coefficient: −2.09e+03
Value: severe
6000
4000
2000
0
asymptomatic (n=24)
mild (n=25)
severe (n=30)
control (n=72)
Severity
cteria.p__Verrucomicrobia.c__Opitutae.o__Puniceicoccales.f__Punic

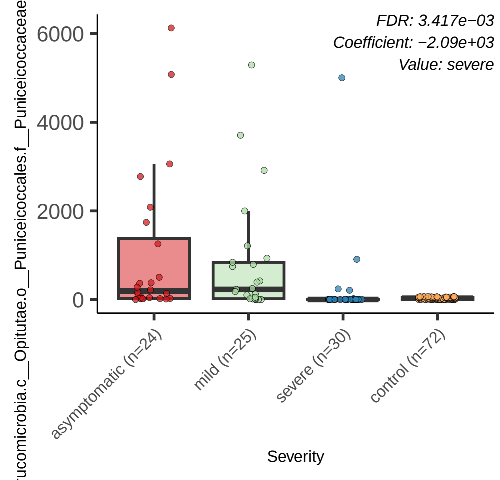
FDR: 3.417e−03
Coefficient: −2.09e+03
Value: severe
6000
4000
2000
0
rucomicrobia.c__Opitutae.o__Puniceicoccales.f__Puniceicoccaceae
asymptomatic (n=24)
mild (n=25)
severe (n=30)
control (n=72)
Severity

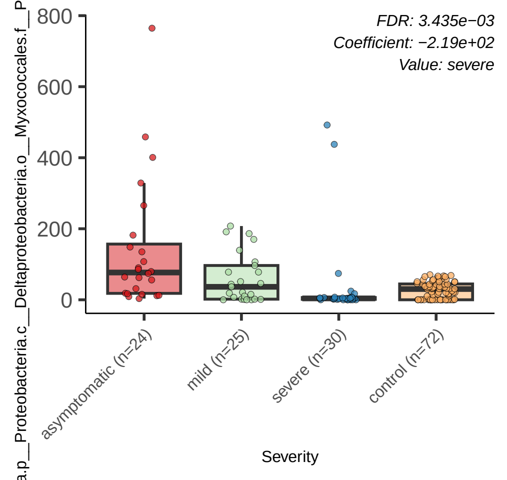

FDR: 3.435e−03
Coefficient: −2.19e+02
Value: severe
a.p__Proteobacteria.c__Deltaproteobacteria.o__Myxococcales.f__P
800
600
400
200
0
asymptomatic (n=24)
mild (n=25)
severe (n=30)
control (n=72)
Severity

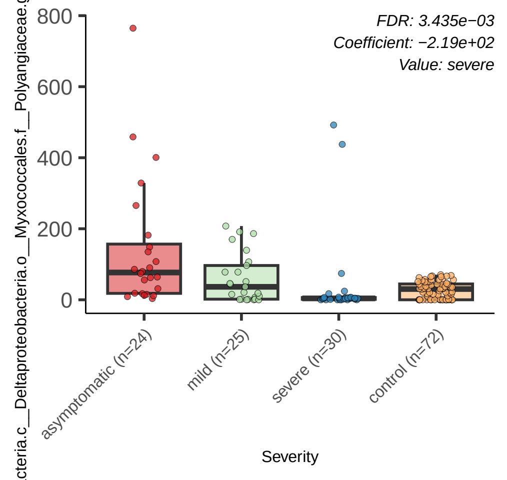

FDR: 3.435e−03
Coefficient: −2.19e+02
Value: severe
800
600
400
200
0
cteria.c__Deltaproteobacteria.o__Myxococcales.f__Polyangiaceae.g
asymptomatic (n=24)
mild (n=25)
severe (n=30)
control (n=72)
Severity

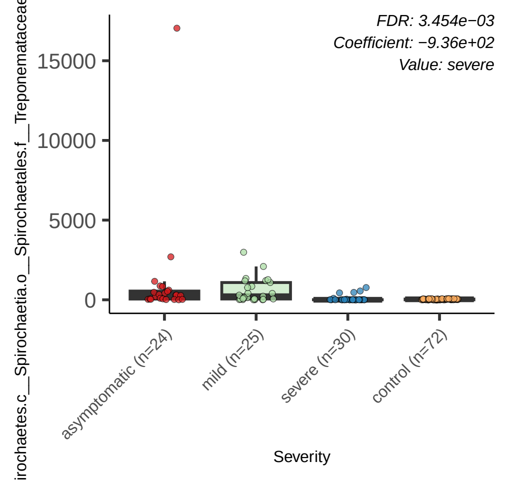
FDR: 3.454e−03
Coefficient: −9.36e+02
Value: severe
15000
10000
5000
0
irochaetes.c__Spirochaetia.o__Spirochaetales.f__Treponemataceae
asymptomatic (n=24)
mild (n=25)
severe (n=30)
control (n=72)
Severity

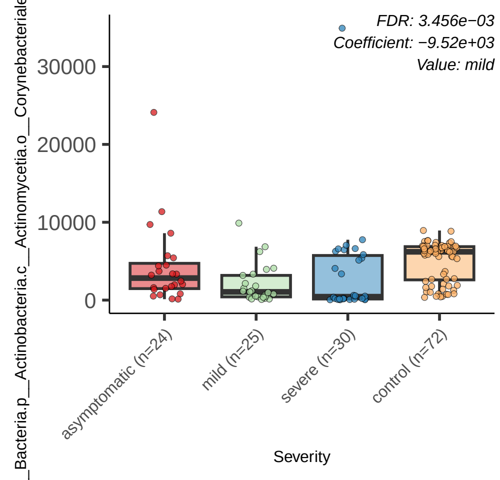

FDR: 3.456e−03
Coefficient: −9.52e+03
Value: mild
30000
20000
10000
0
_Bacteria.p__Actinobacteria.c__Actinomycetia.o__Corynebacteriale
asymptomatic (n=24)
mild (n=25)
severe (n=30)
control (n=72)
Severity

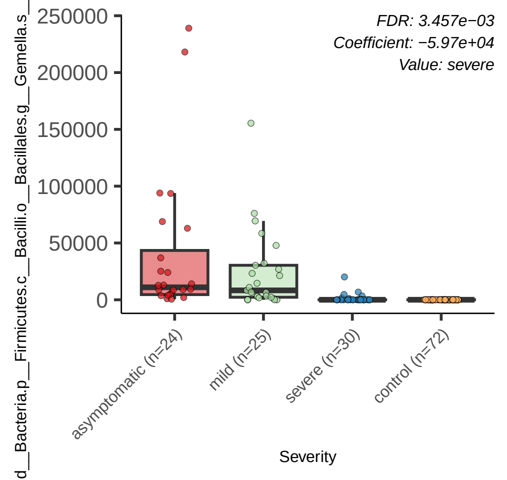
FDR: 3.457e−03
Coefficient: −5.97e+04
Value: severe
d__Bacteria.p__Firmicutes.c__Bacilli.o__Bacillales.g__Gemella.s
250000
200000
150000
100000
50000
0
asymptomatic (n=24)
mild (n=25)
severe (n=30)
control (n=72)
Severity

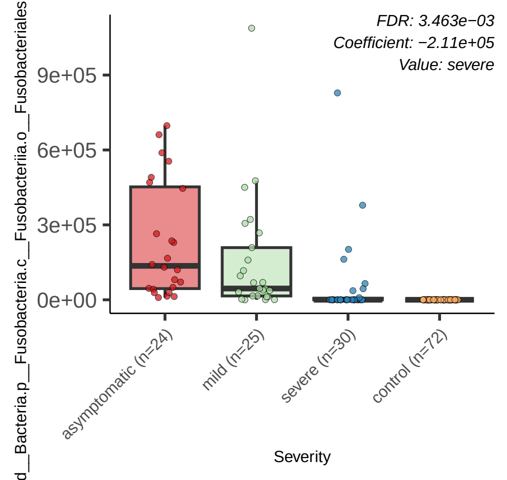

FDR: 3.463e−03
Coefficient: −2.11e+05
Value: severe
9e+05
6e+05
3e+05
0e+00
d__Bacteria.p__Fusobacteria.c__Fusobacteriia.o__Fusobacteriales
asymptomatic (n=24)
mild (n=25)
severe (n=30)
control (n=72)
Severity

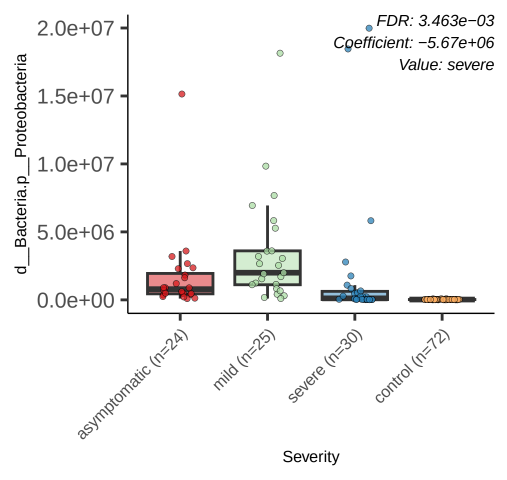
FDR: 3.463e−03
Coefficient: −5.67e+06
Value: severe
d__Bacteria.p__Proteobacteria
2.0e+07
1.5e+07
1.0e+07
5.0e+06
0.0e+00
asymptomatic (n=24)
mild (n=25)
severe (n=30)
control (n=72)
Severity

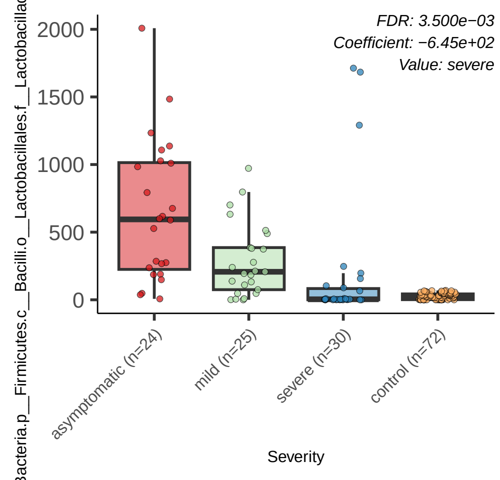

FDR: 3.500e−03
Coefficient: −6.45e+02
Value: severe
2000
1500
1000
500
0
Bacteria.p__Firmicutes.c__Bacilli.o__Lactobacillales.f__Lactobacillac
asymptomatic (n=24)
mild (n=25)
severe (n=30)
control (n=72)
Severity

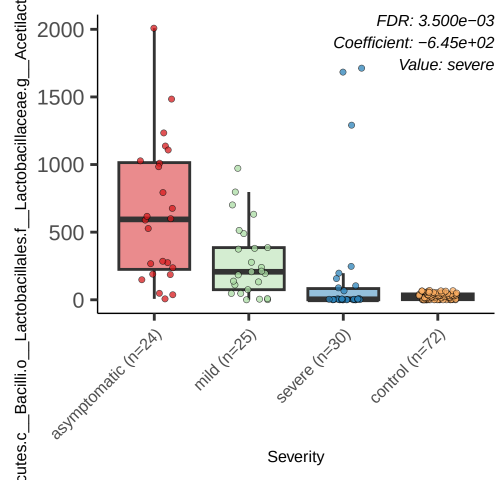

FDR: 3.500e−03
Coefficient: −6.45e+02
Value: severe
2000
1500
1000
500
0
cutes.c__Bacilli.o__Lactobacillales.f__Lactobacillaceae.g__Acetilact
asymptomatic (n=24)
mild (n=25)
severe (n=30)
control (n=72)
Severity

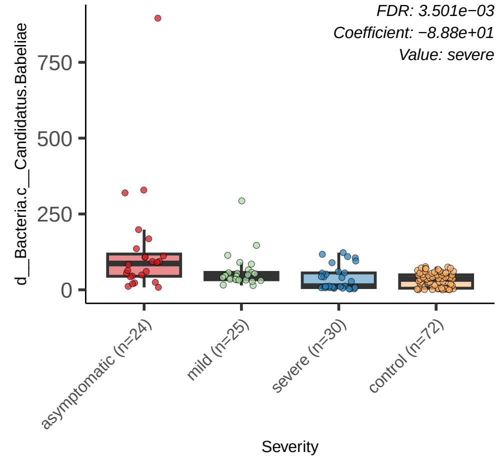

FDR: 3.501e−03
Coefficient: −8.88e+01
Value: severe
d__Bacteria.c__Candidatus.Babeliae
750
500
250
0
asymptomatic (n=24)
mild (n=25)
severe (n=30)
control (n=72)
Severity

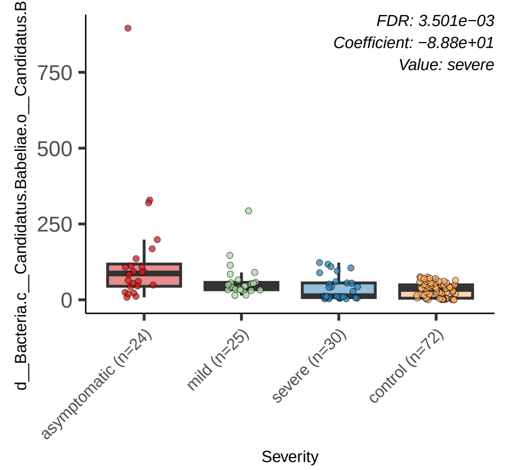

FDR: 3.501e−03
Coefficient: −8.88e+01
Value: severe
d__Bacteria.c__Candidatus.Babeliae.o__Candidatus.B
750
500
250
0
asymptomatic (n=24)
mild (n=25)
severe (n=30)
control (n=72)
Severity

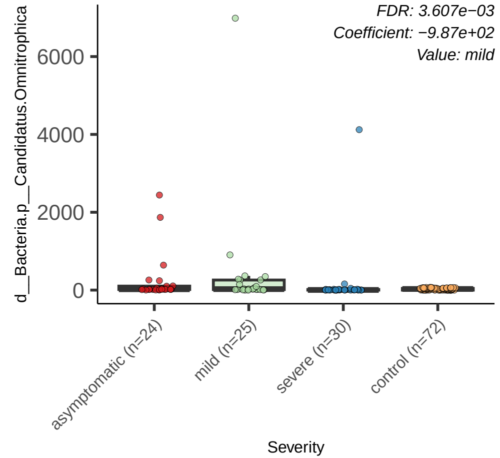

FDR: 3.607e−03
Coefficient: −9.87e+02
Value: mild
d__Bacteria.p__Candidatus.Omnitrophica
6000
4000
2000
0
asymptomatic (n=24)
mild (n=25)
severe (n=30)
control (n=72)
Severity

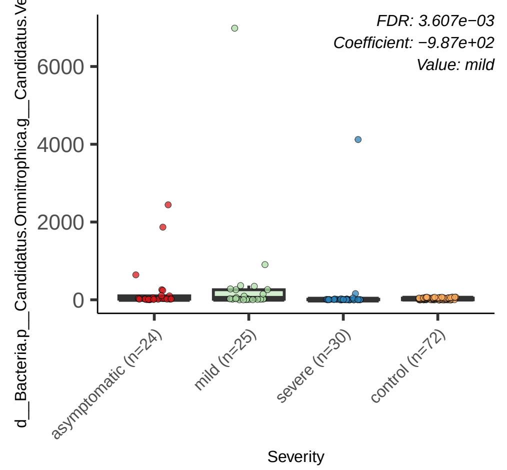

FDR: 3.607e−03
Coefficient: −9.87e+02
Value: mild
d__Bacteria.p__Candidatus.Omnitrophica.g__Candidatus.Ve
6000
4000
2000
0
asymptomatic (n=24)
mild (n=25)
severe (n=30)
control (n=72)
Severity

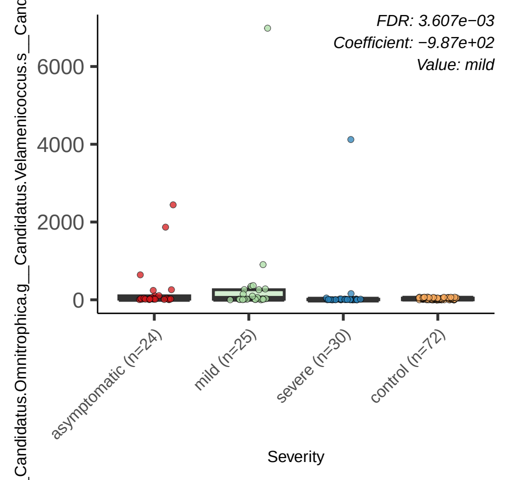

FDR: 3.607e−03
Coefficient: −9.87e+02
Value: mild
6000
4000
2000
0
_Candidatus.Omnitrophica.g__Candidatus.Velamenicoccus.s__Canc
asymptomatic (n=24)
mild (n=25)
severe (n=30)
control (n=72)
Severity

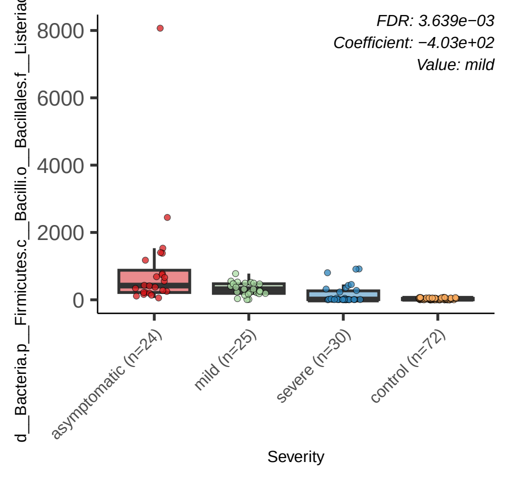

FDR: 3.639e−03
Coefficient: −4.03e+02
Value: mild
d__Bacteria.p__Firmicutes.c__Bacilli.o__Bacillales.f__Listeria
8000
6000
4000
2000
0
asymptomatic (n=24)
mild (n=25)
severe (n=30)
control (n=72)
Severity

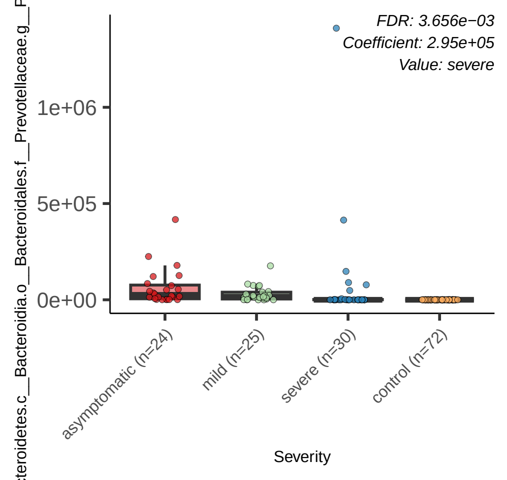

FDR: 3.656e−03
Coefficient: 2.95e+05
Value: severe
1e+06
5e+05
0e+00
asymptomatic (n=24)
mild (n=25)
severe (n=30)
control (n=72)
Severity

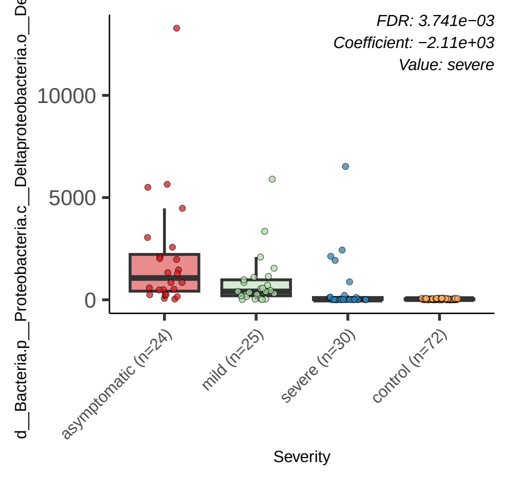

FDR: 3.741e−03
Coefficient: −2.11e+03
Value: severe
d__Bacteria.p__Proteobacteria.c__Deltaproteobacteria.o__De
10000
5000
0
asymptomatic (n=24)
mild (n=25)
severe (n=30)
control (n=72)
Severity

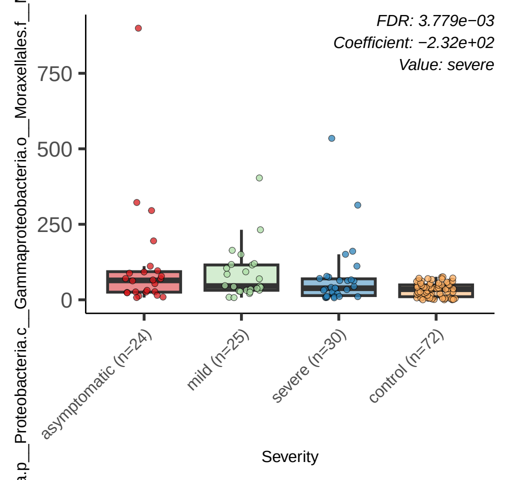

FDR: 3.779e−03
Coefficient: −2.32e+02
Value: severe
a.p__Proteobacteria.c__Gammaproteobacteria.o__Moraxellales.f__M
0
250
500
750
asymptomatic (n=24)
mild (n=25)
severe (n=30)
control (n=72)
Severity

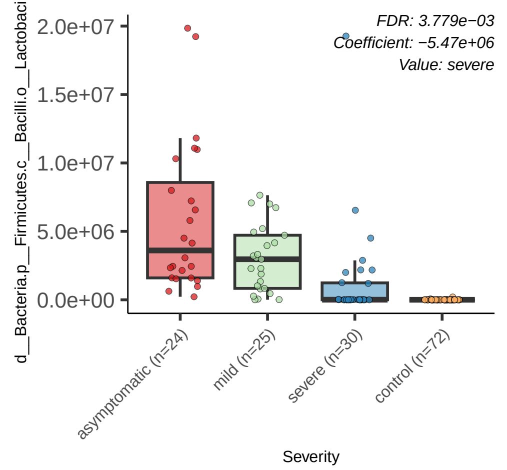

FDR: 3.779e−03
Coefficient: −5.47e+06
Value: severe
d__Bacteria.p__Firmicutes.c__Bacilli.o__Lactobaci
2.0e+07
1.5e+07
1.0e+07
5.0e+06
0.0e+00
asymptomatic (n=24)
mild (n=25)
severe (n=30)
control (n=72)
Severity

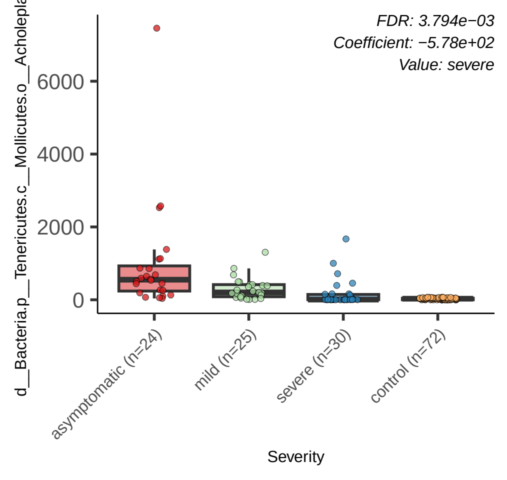

FDR: 3.794e−03
Coefficient: −5.78e+02
Value: severe
d__Bacteria.p__Tenericutes.c__Mollicutes.o__Achole
6000
4000
2000
0
asymptomatic (n=24)
mild (n=25)
severe (n=30)
control (n=72)
Severity

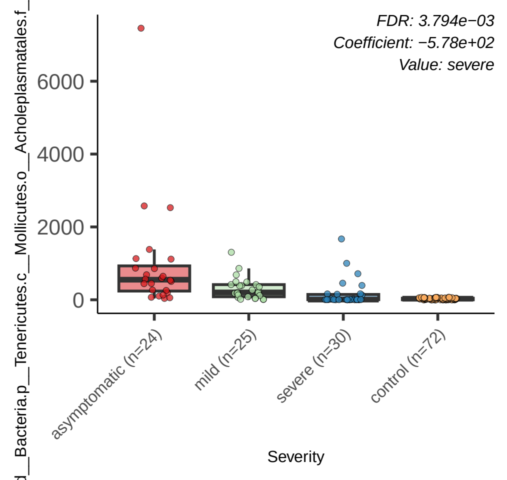

FDR: 3.794e−03
Coefficient: −5.78e+02
Value: severe
d__Bacteria.p__Tenericutes.c__Mollicutes.o__Acholeplasmatales.f__
0
2000
4000
6000
asymptomatic (n=24)
mild (n=25)
severe (n=30)
control (n=72)
Severity

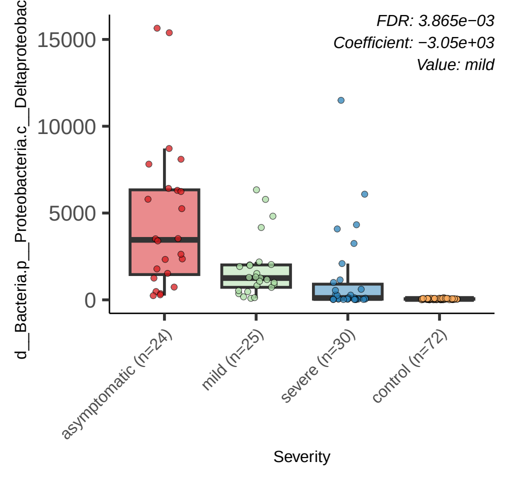
FDR: 3.865e−03
Coefficient: −3.05e+03
Value: mild
15000
10000
5000
0
d__Bacteria.p__Proteobacteria.c__Deltaproteobac
asymptomatic (n=24)
mild (n=25)
severe (n=30)
control (n=72)
Severity

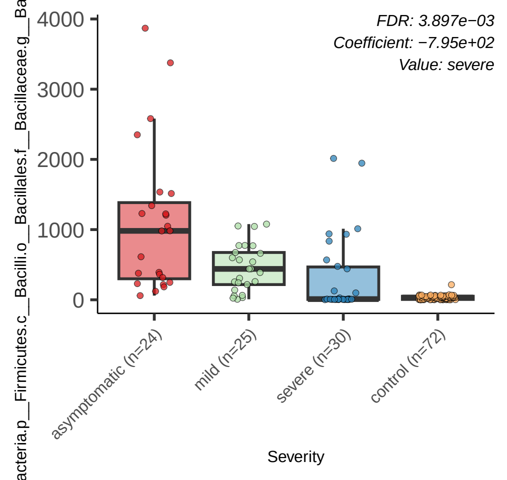

FDR: 3.897e−03
Coefficient: −7.95e+02
Value: severe
0
1000
2000
3000
4000
asymptomatic (n=24)
mild (n=25)
severe (n=30)
control (n=72)
Severity

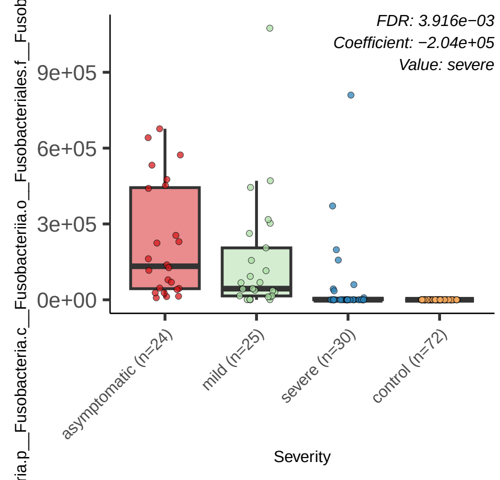

FDR: 3.916e−03
Coefficient: −2.04e+05
Value: severe
9e+05
6e+05
3e+05
0e+00
asymptomatic (n=24)
mild (n=25)
severe (n=30)
control (n=72)
Severity
ria.p__Fusobacteria.c__Fusobacteriia.o__Fusobacteriales.f__Fusob

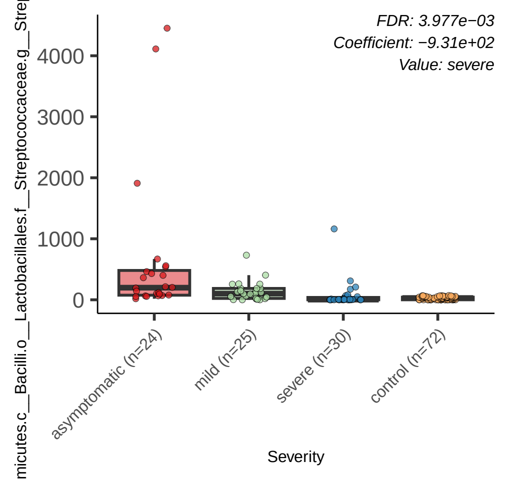

FDR: 3.977e−03
Coefficient: −9.31e+02
Value: severe
micutes.c__Bacilli.o__Lactobacillales.f__Streptococcaceae.g__Strep
4000
3000
2000
1000
0
asymptomatic (n=24)
mild (n=25)
severe (n=30)
control (n=72)
Severity

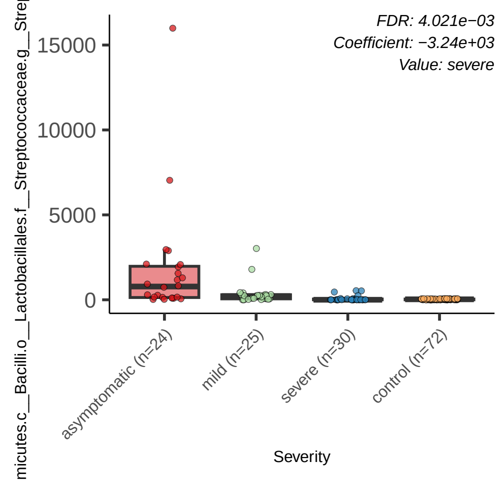
micutes.c__Bacilli.o__Lactobacillales.f__Streptococcaceae.g__Strep
15000
10000
5000
0
FDR: 4.021e−03
Coefficient: −3.24e+03
Value: severe
asymptomatic (n=24)
mild (n=25)
severe (n=30)
control (n=72)
Severity

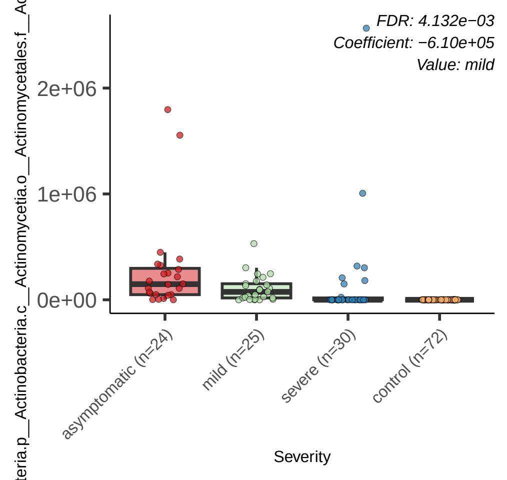

FDR: 4.132e−03
Coefficient: −6.10e+05
Value: mild
2e+06
1e+06
0e+00
asymptomatic (n=24)
mild (n=25)
severe (n=30)
control (n=72)
Severity

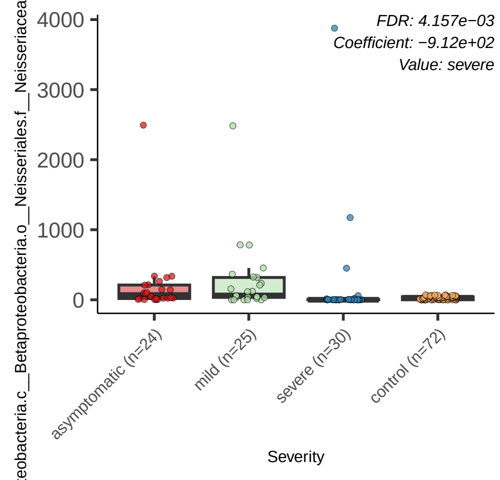

FDR: 4.157e−03
Coefficient: −9.12e+02
Value: severe
4000
3000
2000
1000
0
eobacteria.c__Betaproteobacteria.o__Neisseriales.f__Neisseriacea
asymptomatic (n=24)
mild (n=25)
severe (n=30)
control (n=72)
Severity

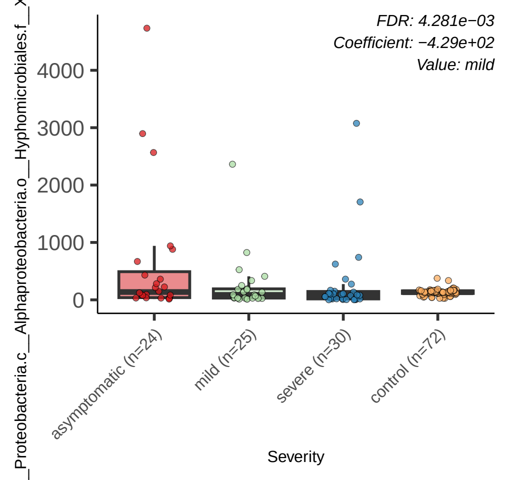

FDR: 4.281e−03
Coefficient: −4.29e+02
Value: mild
Proteobacteria.c__Alphaproteobacteria.o__Hyphomicrobiales.f__X
4000
3000
2000
1000
0
asymptomatic (n=24)
mild (n=25)
severe (n=30)
control (n=72)
Severity

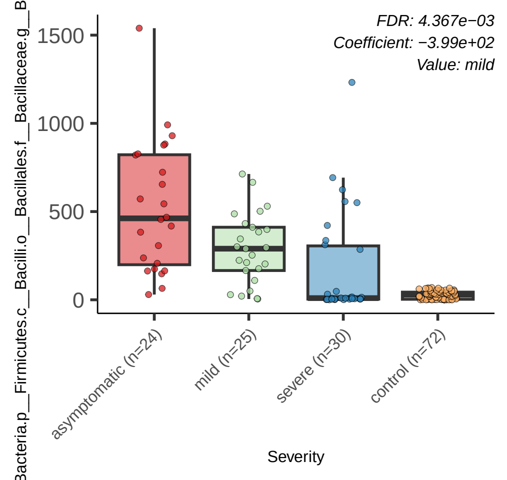

FDR: 4.367e−03
Coefficient: −3.99e+02
Value: mild
Bacteria.p__Firmicutes.c__Bacilli.o__Bacillales.f__Bacillaceae.g__B
0
500
1000
1500
asymptomatic (n=24)
mild (n=25)
severe (n=30)
control (n=72)
Severity

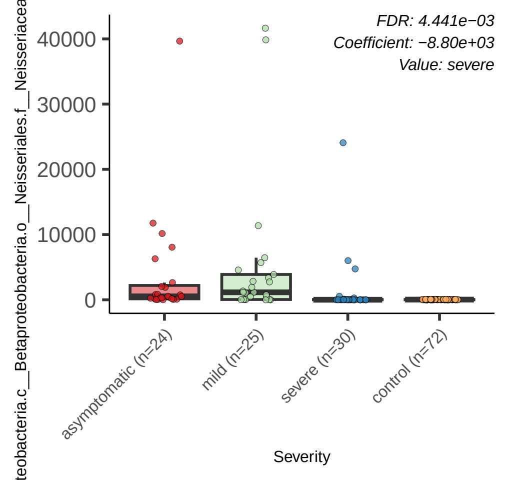

FDR: 4.441e−03
Coefficient: −8.80e+03
Value: severe
40000
30000
20000
10000
0
teobacteria.c__Betaproteobacteria.o__Neisseriales.f__Neisseriacea
asymptomatic (n=24)
mild (n=25)
severe (n=30)
control (n=72)
Severity

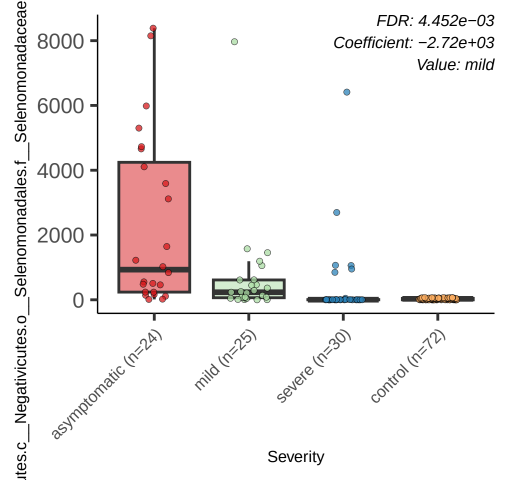
FDR: 4.452e−03
Coefficient: −2.72e+03
Value: mild
8000
6000
4000
2000
0
utes.c__Negativicutes.o__Selenomonadales.f__Selenomonadaceae
asymptomatic (n=24)
mild (n=25)
severe (n=30)
control (n=72)
Severity

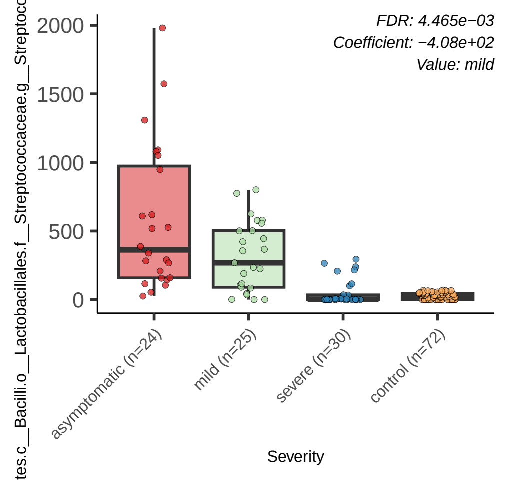

FDR: 4.465e−03
Coefficient: −4.08e+02
Value: mild
2000
1500
1000
500
0
tes.c__Bacilli.o__Lactobacillales.f__Streptococcaceae.g__Streptocc
asymptomatic (n=24)
mild (n=25)
severe (n=30)
control (n=72)
Severity

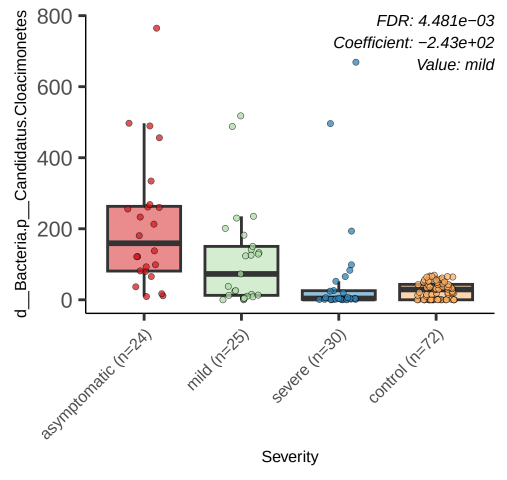

FDR: 4.481e−03
Coefficient: −2.43e+02
Value: mild
d__Bacteria.p__Candidatus.Cloacimonetes
800
600
400
200
0
asymptomatic (n=24)
mild (n=25)
severe (n=30)
control (n=72)
Severity

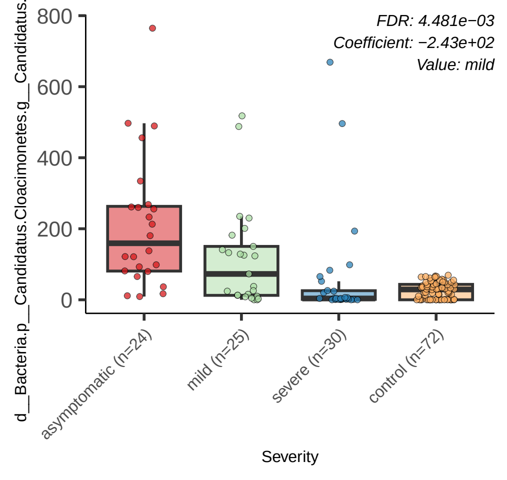
FDR: 4.481e−03
Coefficient: −2.43e+02
Value: mild
d__Bacteria.p__Candidatus.Cloacimonetes.g__Candidatus.
800
600
400
200
0
asymptomatic (n=24)
mild (n=25)
severe (n=30)
control (n=72)
Severity

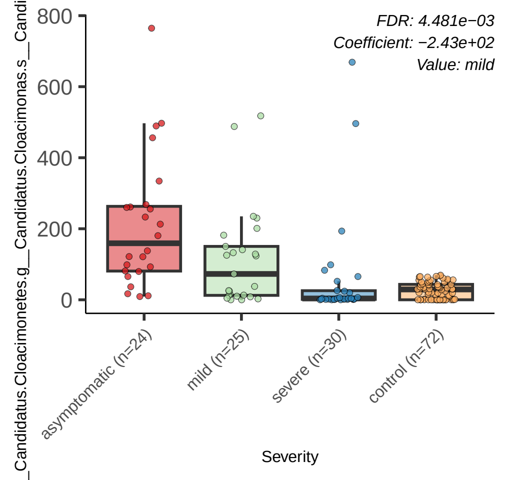

FDR: 4.481e−03
Coefficient: −2.43e+02
Value: mild
_Candidatus.Cloacimonetes.g__Candidatus.Cloacimonas.s__Candi
800
600
400
200
0
asymptomatic (n=24)
mild (n=25)
severe (n=30)
control (n=72)
Severity

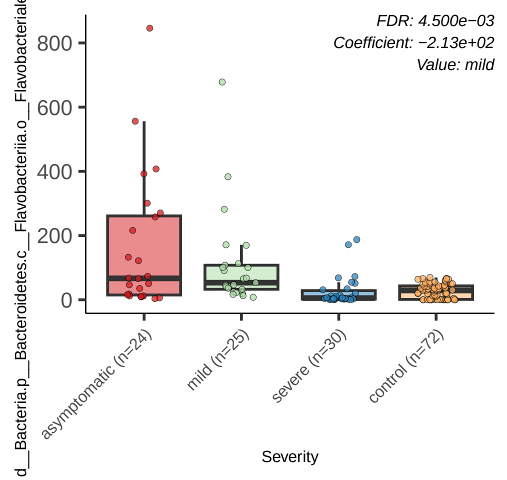
FDR: 4.500e−03
Coefficient: −2.13e+02
Value: mild
d__Bacteria.p__Bacteroidetes.c__Flavobacteriia.o__Flavobacteriale
0
200
400
600
800
asymptomatic (n=24)
mild (n=25)
severe (n=30)
control (n=72)
Severity

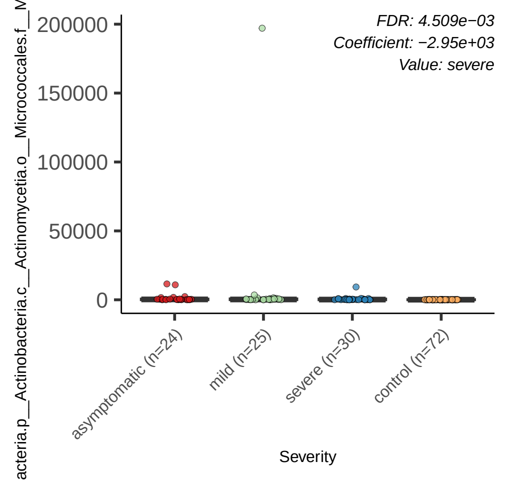

acteria.p__Actinobacteria.c__Actinomycetia.o__Micrococcales.f__M
FDR: 4.509e−03
Coefficient: −2.95e+03
Value: severe
200000
150000
100000
50000
0
asymptomatic (n=24)
mild (n=25)
severe (n=30)
control (n=72)
Severity

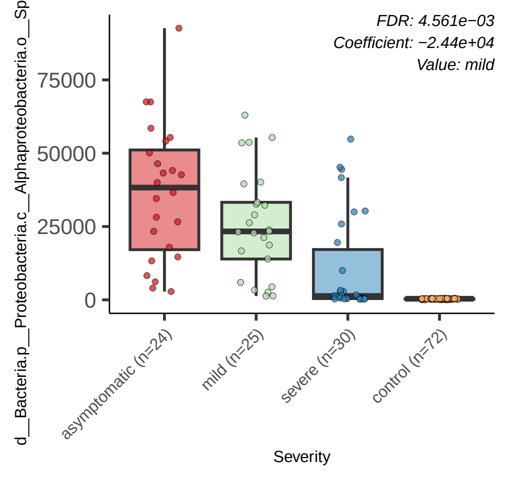
FDR: 4.561e−03
Coefficient: −2.44e+04
Value: mild
d__Bacteria.p__Proteobacteria.c__Alphaproteobacteria.o__Sp
75000
50000
25000
0
asymptomatic (n=24)
mild (n=25)
severe (n=30)
control (n=72)
Severity

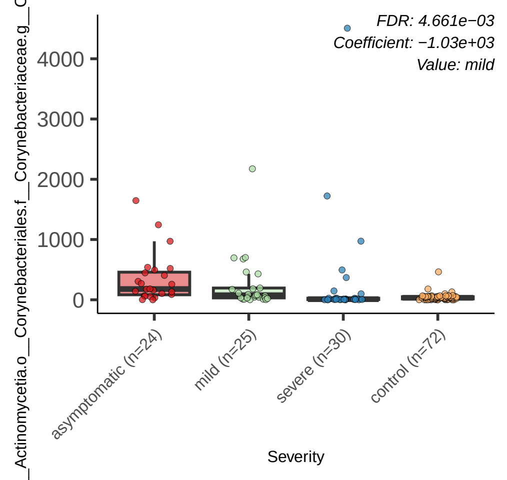

FDR: 4.661e−03
Coefficient: −1.03e+03
Value: mild
4000
3000
2000
1000
0
_Actinomycetia.o__Corynebacteriales.f__Corynebacteriaceae.g__C
asymptomatic (n=24)
mild (n=25)
severe (n=30)
control (n=72)
Severity

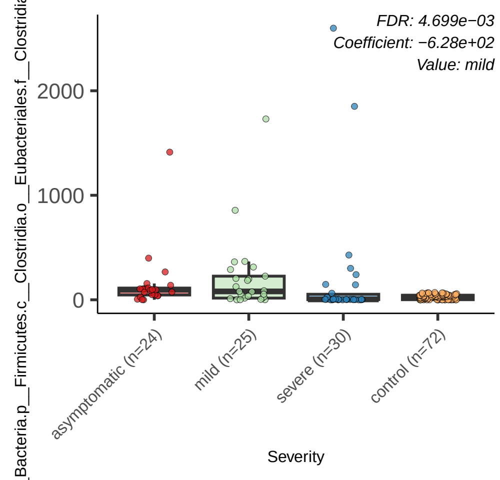

FDR: 4.699e−03
Coefficient: −6.28e+02
Value: mild
2000
1000
0
Bacteria.p__Firmicutes.c__Clostridia.o__Eubacteriales.f__Clostridia
asymptomatic (n=24)
mild (n=25)
severe (n=30)
control (n=72)
Severity

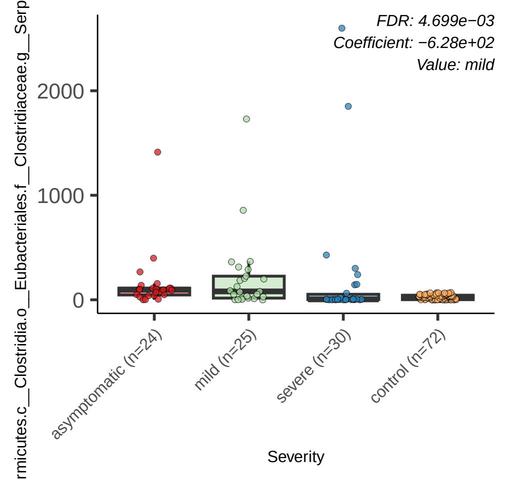

FDR: 4.699e−03
Coefficient: −6.28e+02
Value: mild
2000
1000
0
rmicutes.c__Clostridia.o__Eubacteriales.f__Clostridiaceae.g__Serp
asymptomatic (n=24)
mild (n=25)
severe (n=30)
control (n=72)
Severity

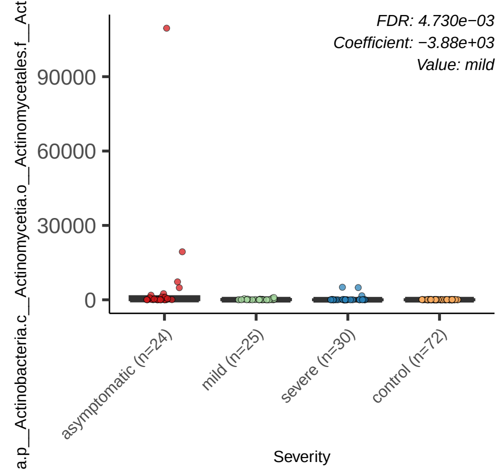

FDR: 4.730e−03
Coefficient: −3.88e+03
Value: mild
90000
60000
30000
0
asymptomatic (n=24)
mild (n=25)
severe (n=30)
control (n=72)
Severity

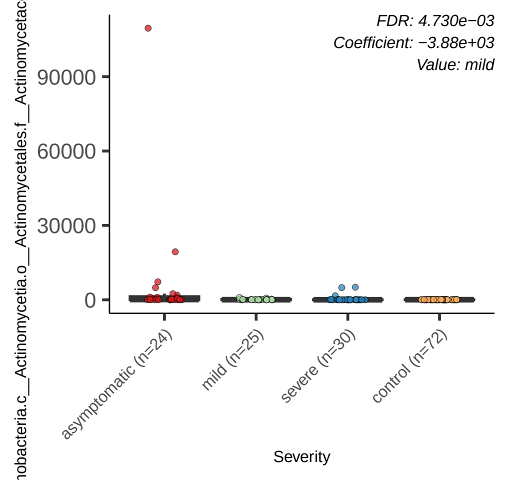

FDR: 4.730e−03
Coefficient: −3.88e+03
Value: mild
90000
60000
30000
0
nobacteria.c__Actinomycetia.o__Actinomycetales.f__Actinomycetac
asymptomatic (n=24)
mild (n=25)
severe (n=30)
control (n=72)
Severity

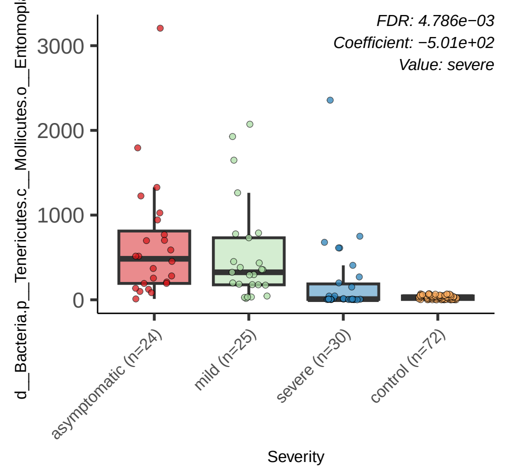

FDR: 4.786e−03
Coefficient: −5.01e+02
Value: severe
d__Bacteria.p__Tenericutes.c__Mollicutes.o__Entomopla
3000
2000
1000
0
asymptomatic (n=24)
mild (n=25)
severe (n=30)
control (n=72)
Severity

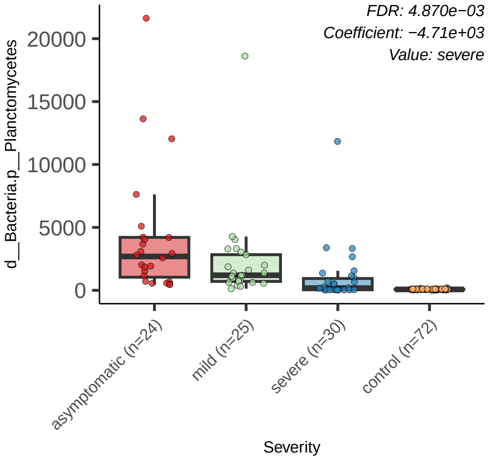
FDR: 4.870e−03
Coefficient: −4.71e+03
Value: severe
d__Bacteria.p__Planctomycetes
20000
15000
10000
5000
0
asymptomatic (n=24)
mild (n=25)
severe (n=30)
control (n=72)
Severity

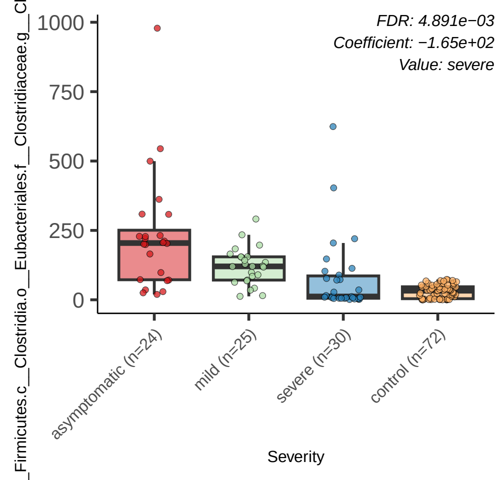

FDR: 4.891e−03
Coefficient: −1.65e+02
Value: severe
Firmicutes.c__Clostridia.o__Eubacteriales.f__Clostridiaceae.g__Cl
1000
750
500
250
0
asymptomatic (n=24)
mild (n=25)
severe (n=30)
control (n=72)
Severity

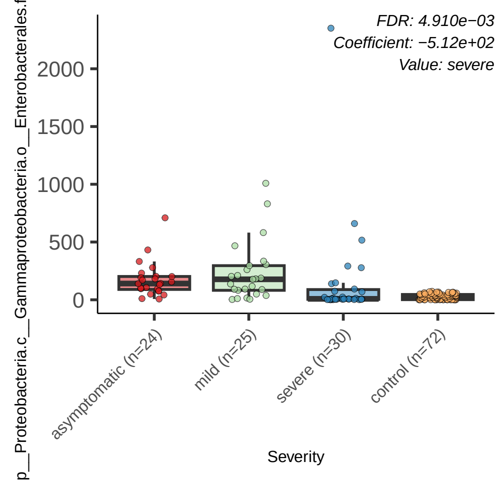

FDR: 4.910e−03
Coefficient: −5.12e+02
Value: severe
p__Proteobacteria.c__Gammaproteobacteria.o__Enterobacterales.f
0
500
1000
1500
2000
asymptomatic (n=24)
mild (n=25)
severe (n=30)
control (n=72)
Severity

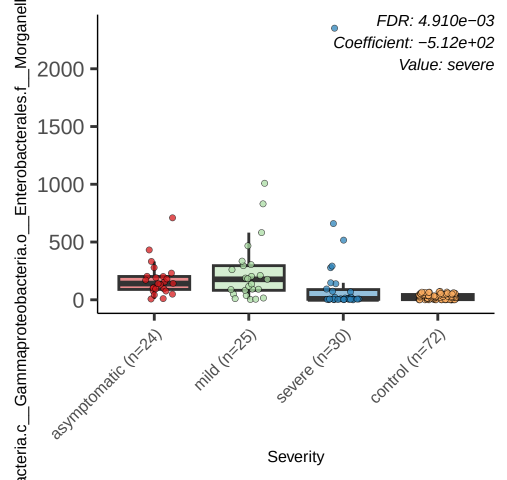
FDR: 4.910e−03
Coefficient: −5.12e+02
Value: severe
acteria.c__Gammaproteobacteria.o__Enterobacterales.f__Morganell
2000
1500
1000
500
0
asymptomatic (n=24)
mild (n=25)
severe (n=30)
control (n=72)
Severity

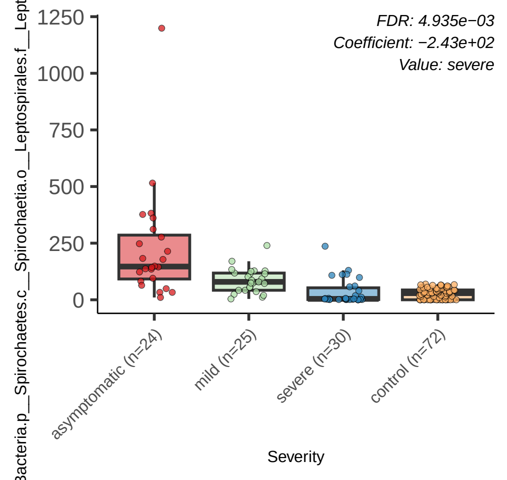

FDR: 4.935e−03
Coefficient: −2.43e+02
Value: severe
1250
1000
750
500
250
0
Bacteria.p__Spirochaetes.c__Spirochaetia.o__Leptospirales.f__Lept
asymptomatic (n=24)
mild (n=25)
severe (n=30)
control (n=72)
Severity

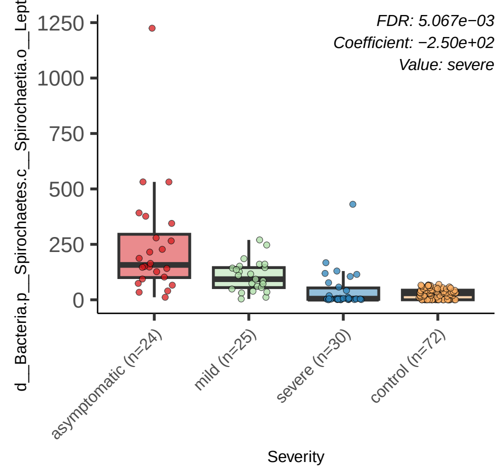

FDR: 5.067e−03
Coefficient: −2.50e+02
Value: severe
d__Bacteria.p__Spirochaetes.c__Spirochaetia.o__Lept
1250
1000
750
500
250
0
asymptomatic (n=24)
mild (n=25)
severe (n=30)
control (n=72)
Severity

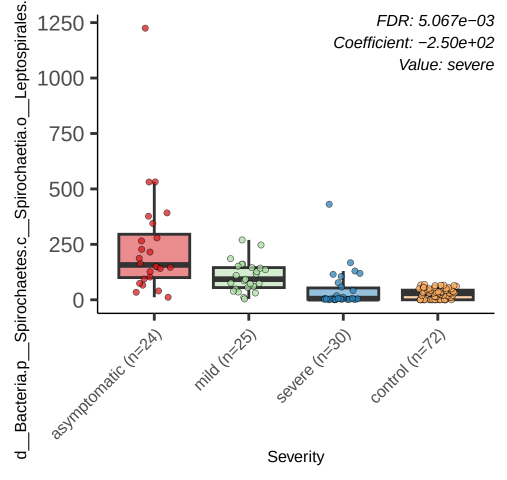
FDR: 5.067e−03
Coefficient: −2.50e+02
Value: severe
d__Bacteria.p__Spirochaetes.c__Spirochaetia.o__Leptospirales.
1250
1000
750
500
250
0
asymptomatic (n=24)
mild (n=25)
severe (n=30)
control (n=72)
Severity

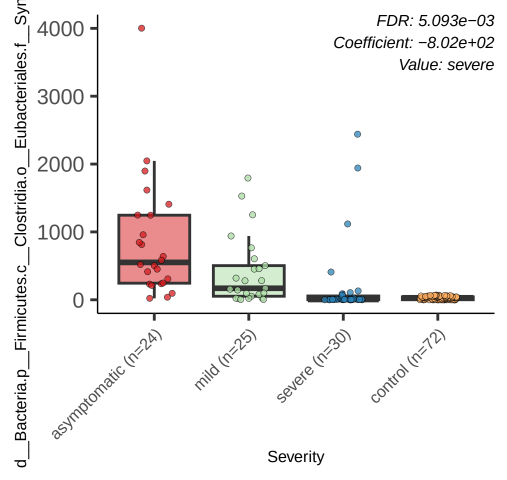
FDR: 5.093e−03
Coefficient: −8.02e+02
Value: severe
d__Bacteria.p__Firmicutes.c__Clostridia.o__Eubacteriales.f__Syn
4000
3000
2000
1000
0
asymptomatic (n=24)
mild (n=25)
severe (n=30)
control (n=72)
Severity

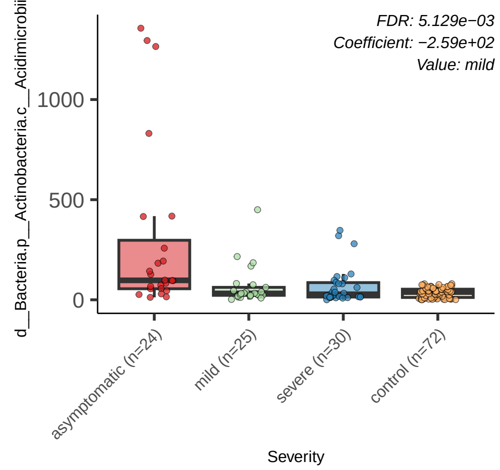

FDR: 5.129e−03
Coefficient: −2.59e+02
Value: mild
d__Bacteria.p__Actinobacteria.c__Acidimicrobii
1000
500
0
asymptomatic (n=24)
mild (n=25)
severe (n=30)
control (n=72)
Severity

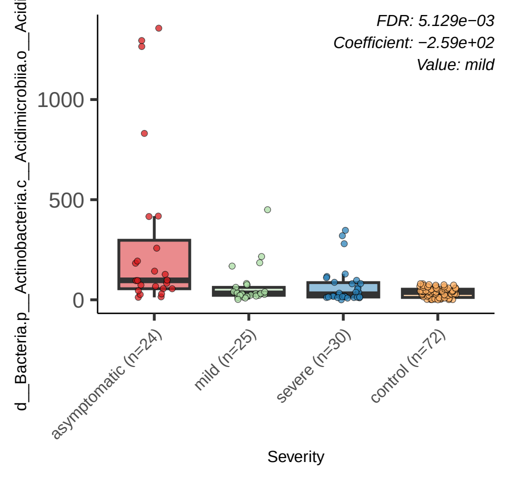

FDR: 5.129e−03
Coefficient: −2.59e+02
Value: mild
d__Bacteria.p__Actinobacteria.c__Acidimicrobiia.o__Acidi
1000
500
0
asymptomatic (n=24)
mild (n=25)
severe (n=30)
control (n=72)
Severity

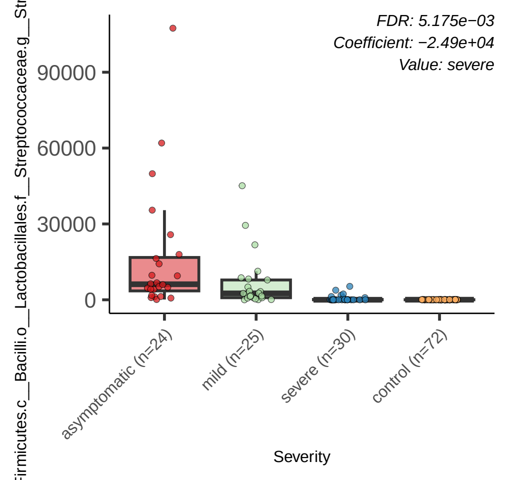
FDR: 5.175e−03
Coefficient: −2.49e+04
Value: severe
90000
60000
30000
0
Firmicutes.c__Bacilli.o__Lactobacillales.f__Streptococcaceae.g__Str
asymptomatic (n=24)
mild (n=25)
severe (n=30)
control (n=72)
Severity

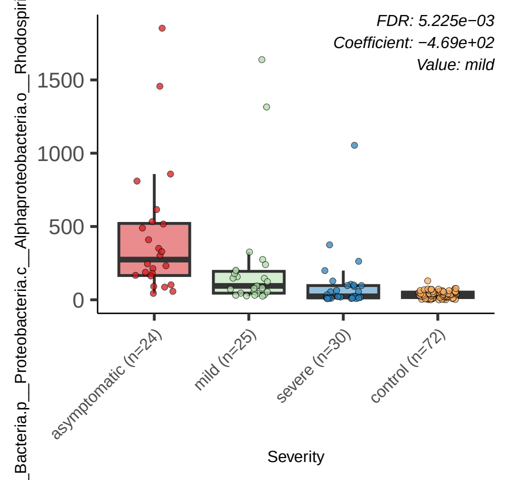
FDR: 5.225e−03
Coefficient: −4.69e+02
Value: mild
_Bacteria.p__Proteobacteria.c__Alphaproteobacteria.o__Rhodospiri
1500
1000
500
0
asymptomatic (n=24)
mild (n=25)
severe (n=30)
control (n=72)
Severity

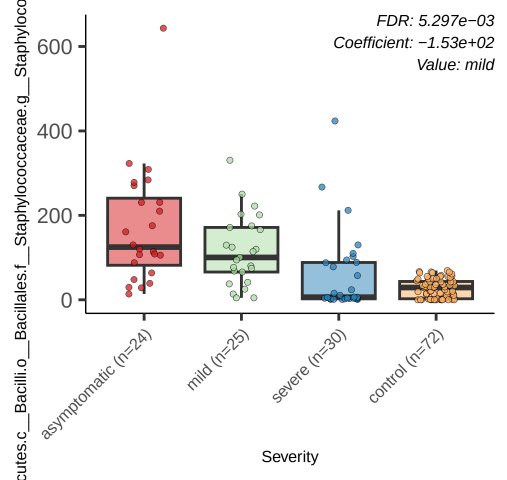

FDR: 5.297e−03
Coefficient: −1.53e+02
Value: mild
0
200
400
600
cutes.c__Bacilli.o__Bacillales.f__Staphylococcaceae.g__Staphyloco
asymptomatic (n=24)
mild (n=25)
severe (n=30)
control (n=72)
Severity

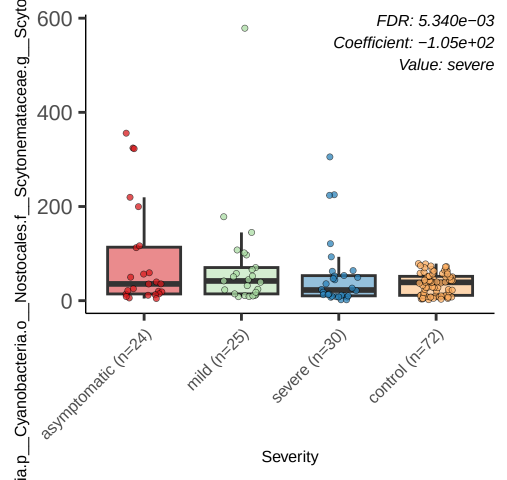

FDR: 5.340e−03
Coefficient: −1.05e+02
Value: severe
600
400
200
0
ia.p__Cyanobacteria.o__Nostocales.f__Scytonemataceae.g__Scyto
asymptomatic (n=24)
mild (n=25)
severe (n=30)
control (n=72)
Severity

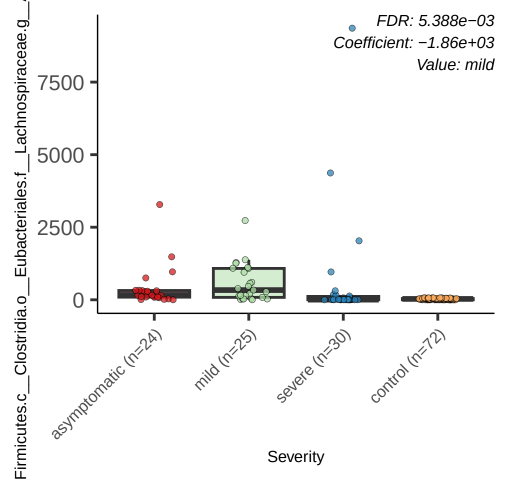

FDR: 5.388e−03
Coefficient: −1.86e+03
Value: mild
Firmicutes.c__Clostridia.o__Eubacteriales.f__Lachnospiraceae.g__
7500
5000
2500
0
asymptomatic (n=24)
mild (n=25)
severe (n=30)
control (n=72)
Severity

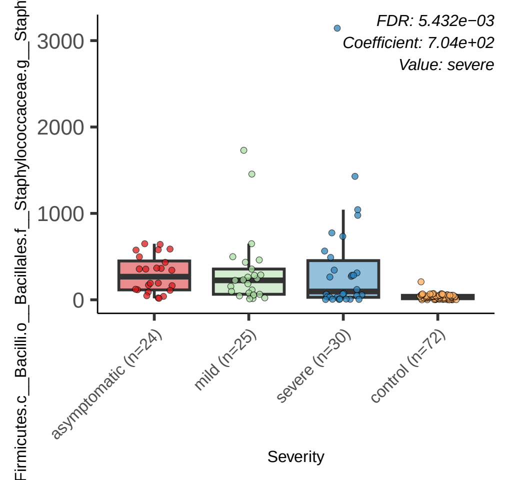

FDR: 5.432e−03
Coefficient: 7.04e+02
Value: severe
Firmicutes.c__Bacilli.o__Bacillales.f__Staphylococcaceae.g__Staph
3000
2000
1000
0
asymptomatic (n=24)
mild (n=25)
severe (n=30)
control (n=72)
Severity

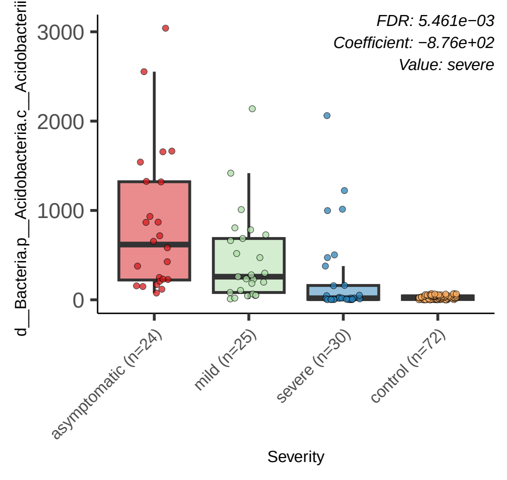

FDR: 5.461e−03
Coefficient: −8.76e+02
Value: severe
d__Bacteria.p__Acidobacteria.c__Acidobacterii
3000
2000
1000
0
asymptomatic (n=24)
mild (n=25)
severe (n=30)
control (n=72)
Severity

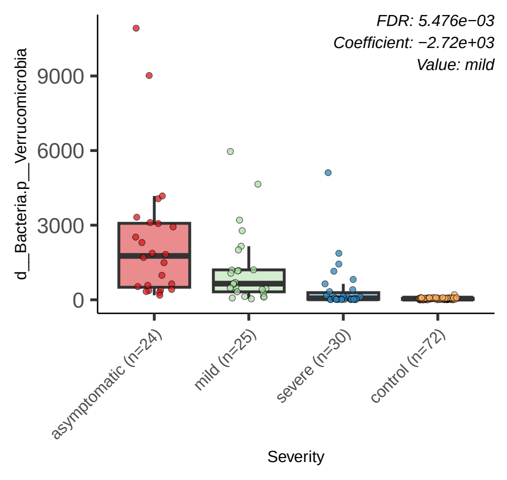
FDR: 5.476e−03
Coefficient: −2.72e+03
Value: mild
d__Bacteria.p__Verrucomicrobia
9000
6000
3000
0
asymptomatic (n=24)
mild (n=25)
severe (n=30)
control (n=72)
Severity

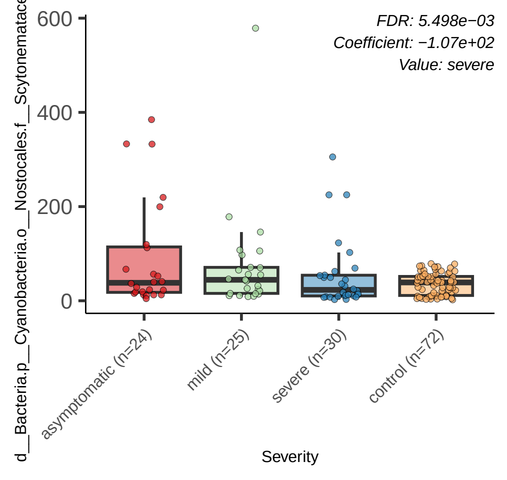
FDR: 5.498e−03
Coefficient: −1.07e+02
Value: severe
d__Bacteria.p__Cyanobacteria.o__Nostocales.f__Scytonematace
600
400
200
0
asymptomatic (n=24)
mild (n=25)
severe (n=30)
control (n=72)
Severity

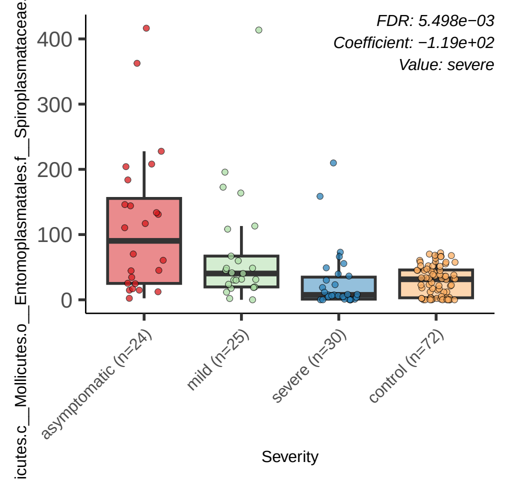

FDR: 5.498e−03
Coefficient: −1.19e+02
Value: severe
cutes.c__Mollicutes.o__Entomoplasmatales.f__Spiroplasmataceae.
0
100
200
300
400
asymptomatic (n=24)
mild (n=25)
severe (n=30)
control (n=72)
Severity

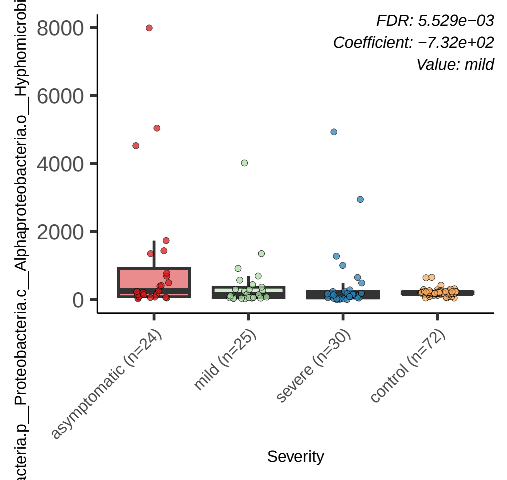
FDR: 5.529e−03
Coefficient: −7.32e+02
Value: mild
8000
6000
4000
2000
0
acteria.p__Proteobacteria.c__Alphaproteobacteria.o__Hyphomicrobi
asymptomatic (n=24)
mild (n=25)
severe (n=30)
control (n=72)
Severity

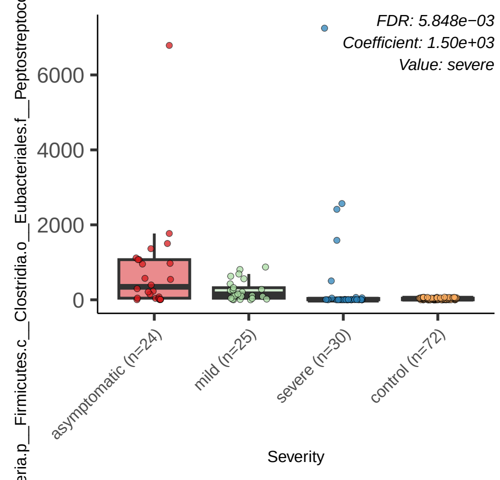

FDR: 5.848e−03
Coefficient: 1.50e+03
Value: severe
6000
4000
2000
0
asymptomatic (n=24)
mild (n=25)
severe (n=30)
control (n=72)
Severity
eria.p__Firmicutes.c__Clostridia.o__Eubacteriales.f__Peptostreptoco

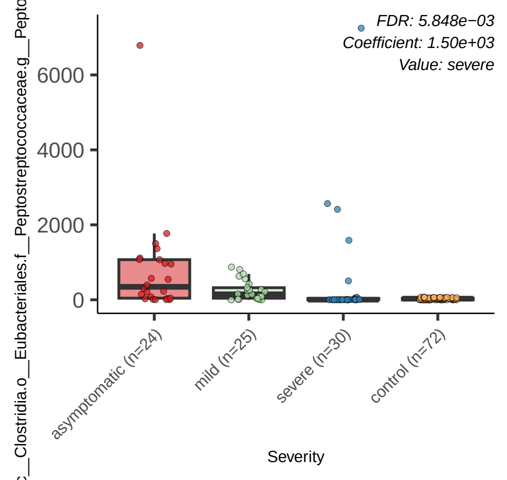
FDR: 5.848e−03
Coefficient: 1.50e+03
Value: severe
6000
4000
2000
0
c__Clostridia.o__Eubacteriales.f__Peptostreptococcaceae.g__Pepto
asymptomatic (n=24)
mild (n=25)
severe (n=30)
control (n=72)
Severity

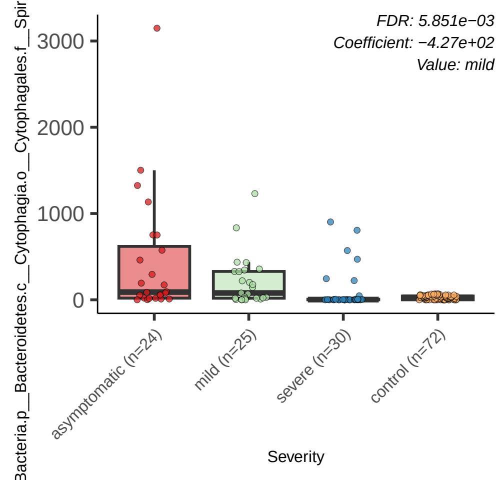

FDR: 5.851e−03
Coefficient: −4.27e+02
Value: mild
3000
2000
1000
0
Bacteria.p__Bacteroidetes.c__Cytophagia.o__Cytophagales.f__Spir
asymptomatic (n=24)
mild (n=25)
severe (n=30)
control (n=72)
Severity

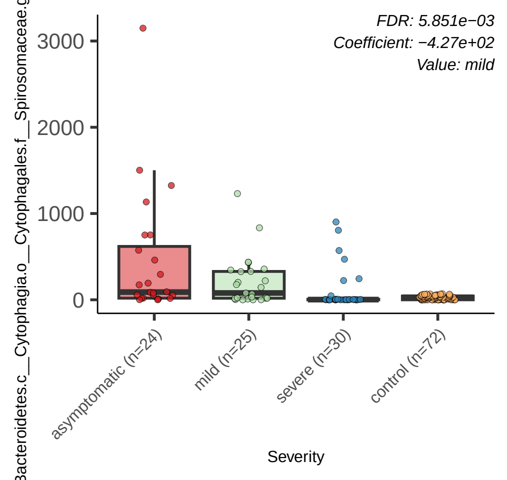

FDR: 5.851e−03
Coefficient: −4.27e+02
Value: mild
3000
2000
1000
0
Bacteroidetes.c__Cytophagia.o__Cytophagales.f__Spirosomaceae.g
asymptomatic (n=24)
mild (n=25)
severe (n=30)
control (n=72)
Severity

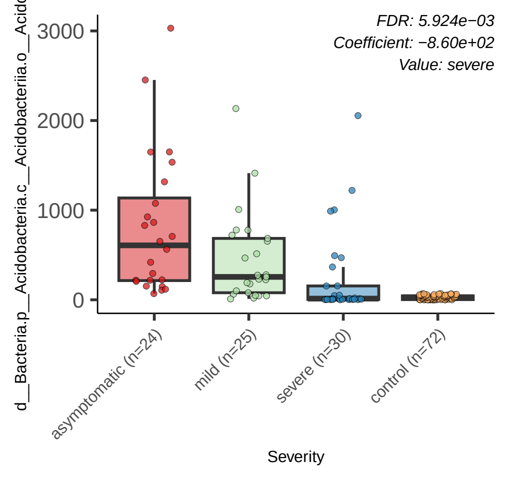

FDR: 5.924e−03
Coefficient: −8.60e+02
Value: severe
d__Bacteria.p__Acidobacteria.c__Acidobacteria.o__Acido
3000
2000
1000
0
asymptomatic (n=24)
mild (n=25)
severe (n=30)
control (n=72)
Severity

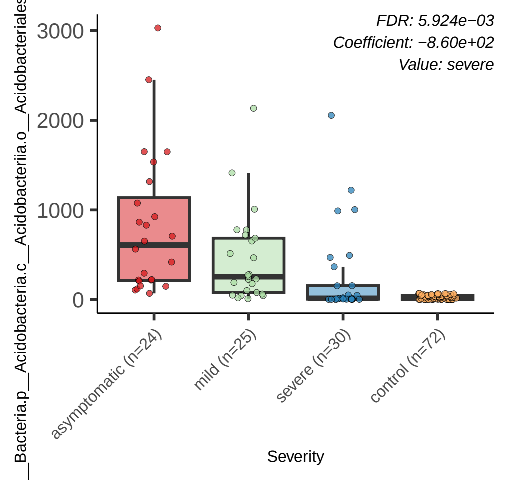

FDR: 5.924e−03
Coefficient: −8.60e+02
Value: severe
Bacteria.p__Acidobacteria.c__Acidobacteriia.o__Acidobacteriales
3000
2000
1000
0
asymptomatic (n=24)
mild (n=25)
severe (n=30)
control (n=72)
Severity

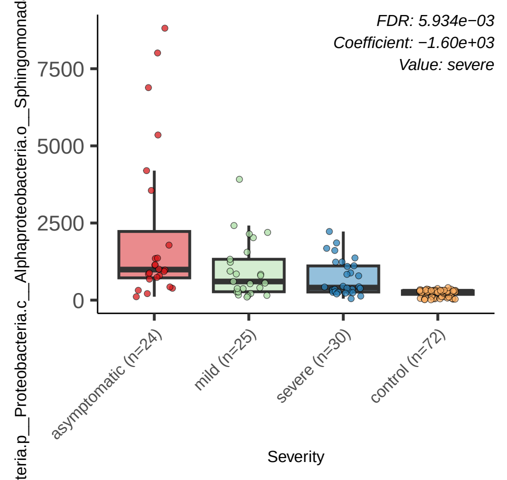

FDR: 5.934e−03
Coefficient: −1.60e+03
Value: severe
teria.p__Proteobacteria.c__Alphaproteobacteria.o__Sphingomonad
7500
5000
2500
0
asymptomatic (n=24)
mild (n=25)
severe (n=30)
control (n=72)
Severity

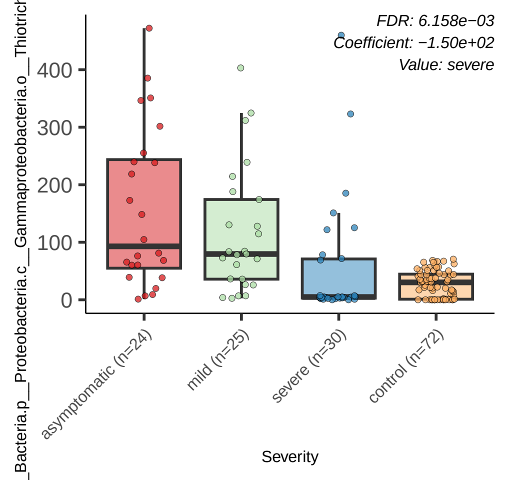
FDR: 6.158e−03
Coefficient: −1.50e+02
Value: severe
0
100
200
300
400
_Bacteria.p__Proteobacteria.c__Gammaproteobacteria.o__Thiotrich
asymptomatic (n=24)
mild (n=25)
severe (n=30)
control (n=72)
Severity

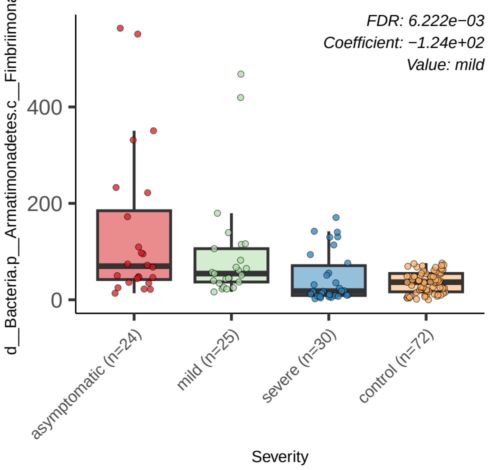

FDR: 6.222e−03
Coefficient: −1.24e+02
Value: mild
d__Bacteria.p__Armatimonadetes.c__Fimbriimona
400
200
0
asymptomatic (n=24)
mild (n=25)
severe (n=30)
control (n=72)
Severity

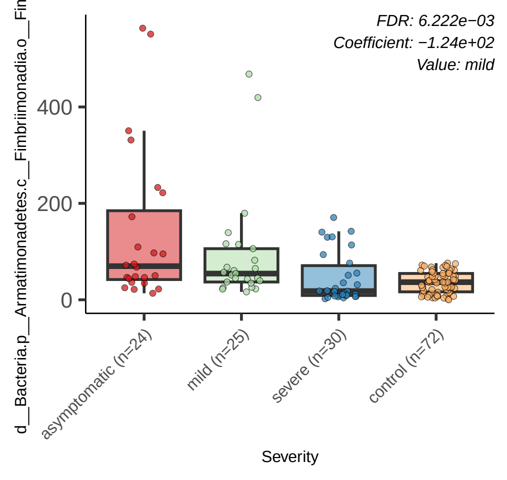
FDR: 6.222e−03
Coefficient: −1.24e+02
Value: mild
d__Bacteria.p__Armatimonadetes.c__Fimbriimonadia.o__Fim
400
200
0
asymptomatic (n=24)
mild (n=25)
severe (n=30)
control (n=72)
Severity

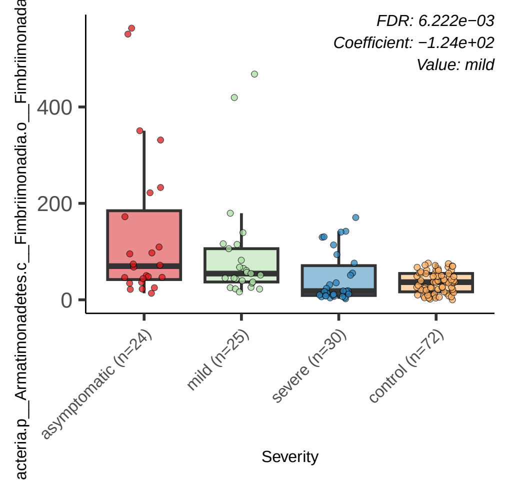

FDR: 6.222e−03
Coefficient: −1.24e+02
Value: mild
acteria.p__Armatimonadetes.c__Fimbriimonadia.o__Fimbriimonada
0
200
400
asymptomatic (n=24)
mild (n=25)
severe (n=30)
control (n=72)
Severity

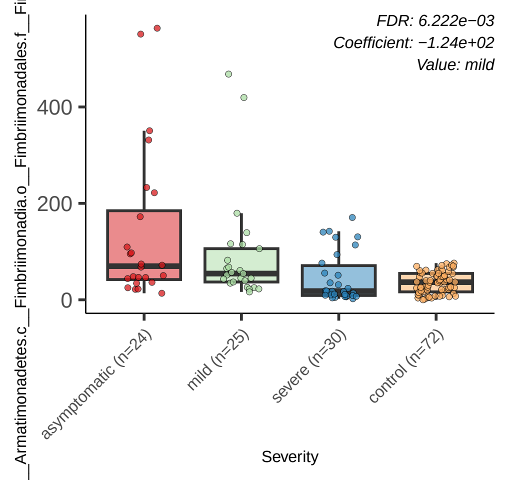

FDR: 6.222e−03
Coefficient: −1.24e+02
Value: mild
0
200
400
Armatimonadetes.c__Fimbriimonadia.o__Fimbriimonadales.f__Fi
asymptomatic (n=24)
mild (n=25)
severe (n=30)
control (n=72)
Severity

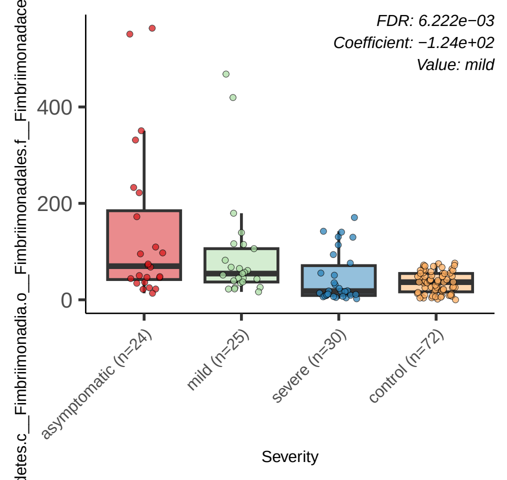
FDR: 6.222e−03
Coefficient: −1.24e+02
Value: mild
400
200
0
asymptomatic (n=24)
mild (n=25)
severe (n=30)
control (n=72)
Severity
detes.c__Fimbriimonadia.o__Fimbriimonadales.f__Fimbriimonadace

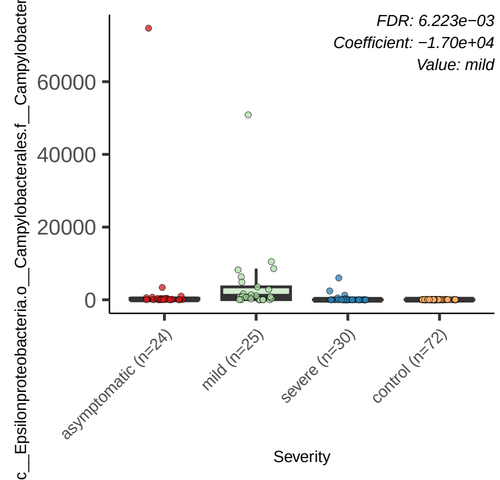

FDR: 6.223e−03
Coefficient: −1.70e+04
Value: mild
c__Epsilonproteobacteria.o__Campylobacterales.f__Campylobacter
60000
40000
20000
0
asymptomatic (n=24)
mild (n=25)
severe (n=30)
control (n=72)
Severity

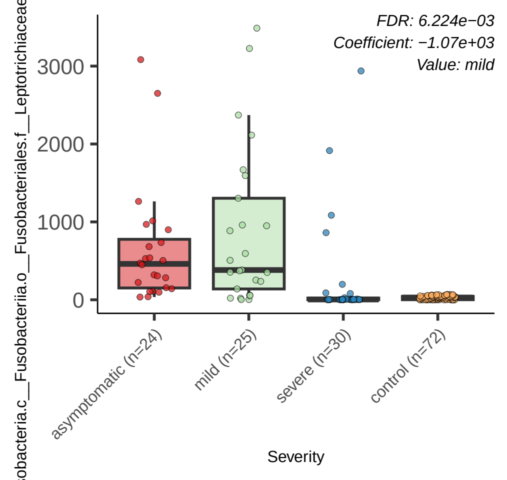

FDR: 6.224e−03
Coefficient: −1.07e+03
Value: mild
sobacteria.c__Fusobacteriia.o__Fusobacteriales.f__Leptotrichiaceae
3000
2000
1000
0
asymptomatic (n=24)
mild (n=25)
severe (n=30)
control (n=72)
Severity

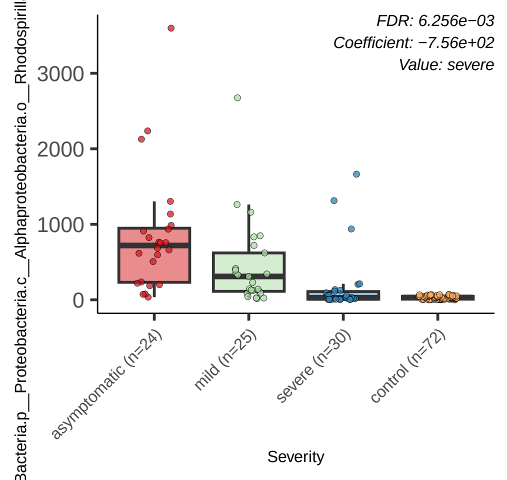

FDR: 6.256e−03
Coefficient: −7.56e+02
Value: severe
3000
2000
1000
0
asymptomatic (n=24)
mild (n=25)
severe (n=30)
control (n=72)
Severity
Bacteria.p__Proteobacteria.c__Alphaproteobacteria.o__Rhodospirill

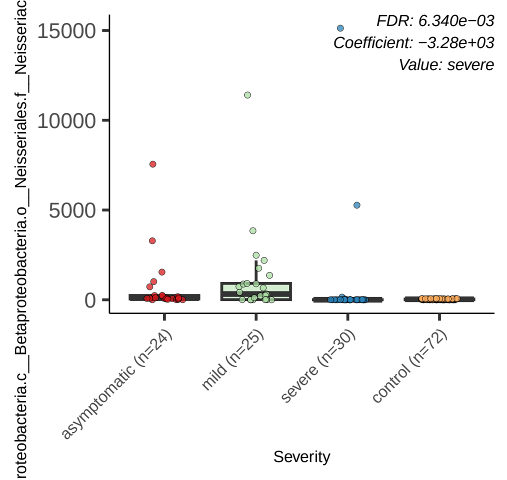
FDR: 6.340e−03
Coefficient: −3.28e+03
Value: severe
15000
10000
5000
0
roteobacteria.c__Betaproteobacteria.o__Neisseriales.f__Neisseriac
asymptomatic (n=24)
mild (n=25)
severe (n=30)
control (n=72)
Severity

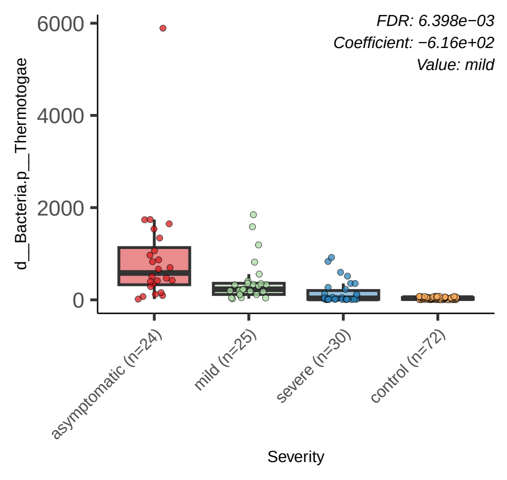

FDR: 6.398e−03
Coefficient: −6.16e+02
Value: mild
6000
4000
2000
0
d__Bacteria.p__Thermotogae
asymptomatic (n=24)
mild (n=25)
severe (n=30)
control (n=72)
Severity

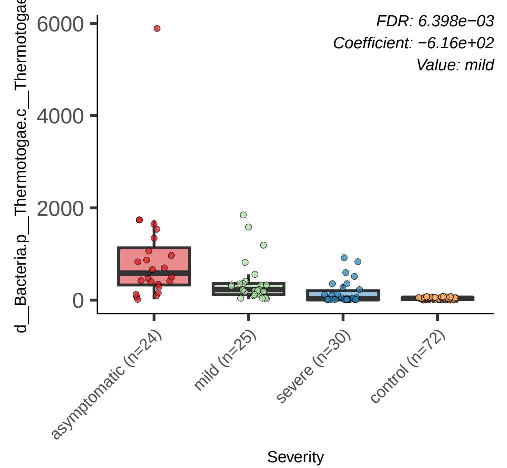

FDR: 6.398e−03
Coefficient: −6.16e+02
Value: mild
d__Bacteria.p__Thermotogae.c__Thermotogae
6000
4000
2000
0
asymptomatic (n=24)
mild (n=25)
severe (n=30)
control (n=72)
Severity

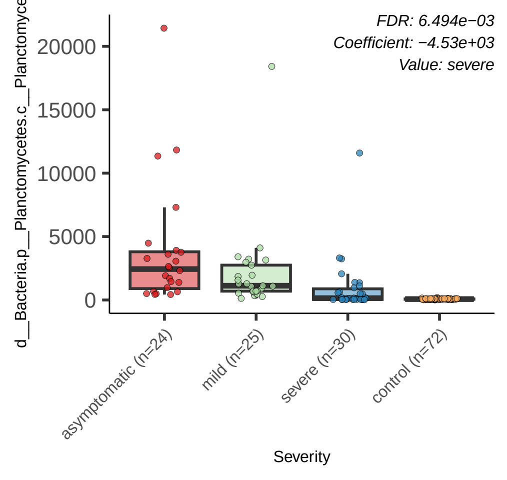
FDR: 6.494e−03
Coefficient: −4.53e+03
Value: severe
d__Bacteria.p__Planctomycetes.c__Planctomyce
20000
15000
10000
5000
0
asymptomatic (n=24)
mild (n=25)
severe (n=30)
control (n=72)
Severity

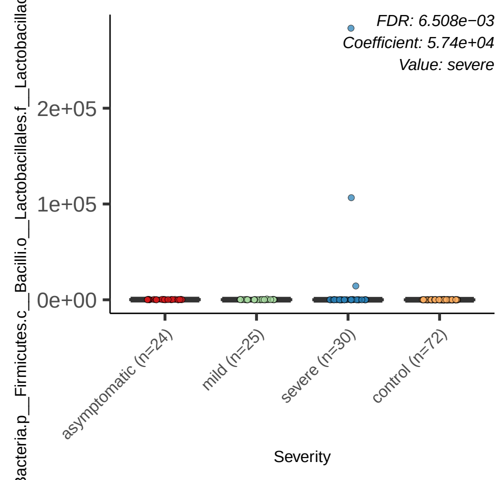

FDR: 6.508e−03
Coefficient: 5.74e+04
Value: severe
2e+05
1e+05
0e+00
asymptomatic (n=24)
mild (n=25)
severe (n=30)
control (n=72)
Severity
Bacteria.p__Firmicutes.c__Bacilli.o__Lactobacillales.f__Lactobacillac

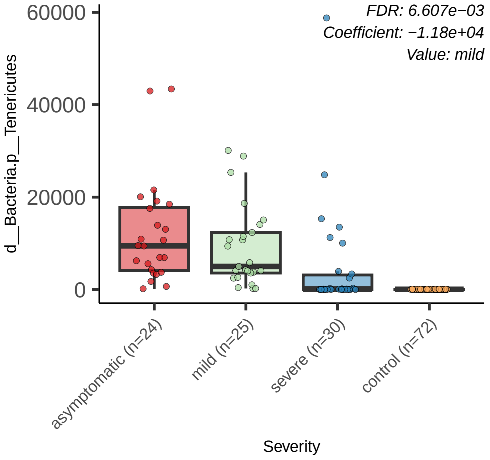

FDR: 6.607e−03
Coefficient: −1.18e+04
Value: mild
d__Bacteria.p__Tenericutes
60000
40000
20000
0
asymptomatic (n=24)
mild (n=25)
severe (n=30)
control (n=72)
Severity

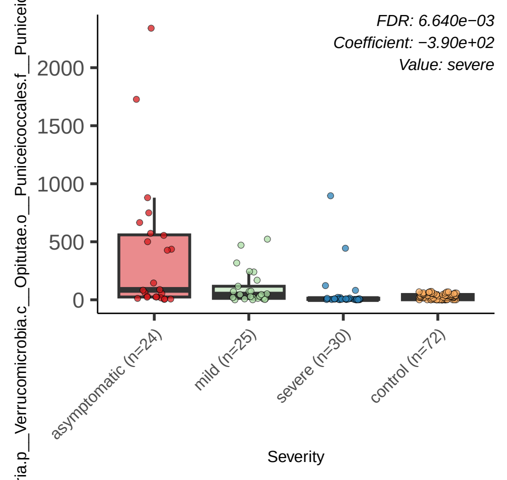

FDR: 6.640e−03
Coefficient: −3.90e+02
Value: severe
ria.p__Verrucomicrobia.c__Opitutae.o__Puniceicoccales.f__Puniceic
2000
1500
1000
500
0
asymptomatic (n=24)
mild (n=25)
severe (n=30)
control (n=72)
Severity

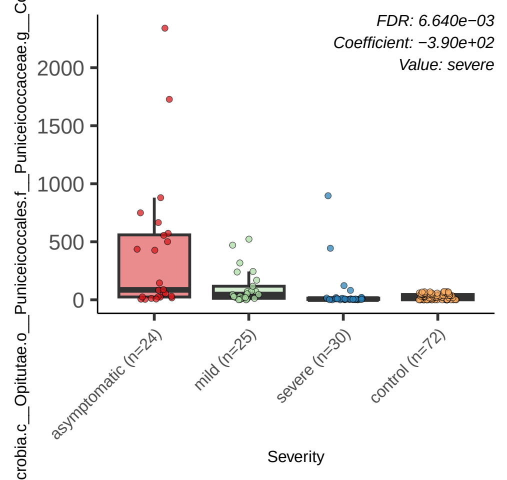
FDR: 6.640e−03
Coefficient: −3.90e+02
Value: severe
crobia.c__Opitutae.o__Puniceicoccales.f__Puniceicoccaceae.g__Co
2000
1500
1000
500
0
asymptomatic (n=24)
mild (n=25)
severe (n=30)
control (n=72)
Severity

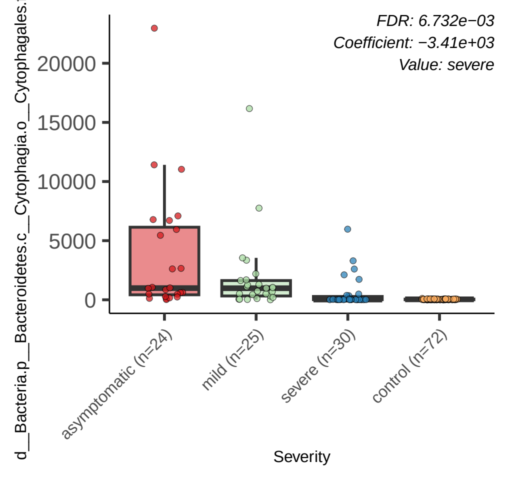

FDR: 6.732e−03
Coefficient: −3.41e+03
Value: severe
d__Bacteria.p__Bacteroidetes.c__Cytophagia.o__Cytophagales.f
20000
15000
10000
5000
0
asymptomatic (n=24)
mild (n=25)
severe (n=30)
control (n=72)
Severity

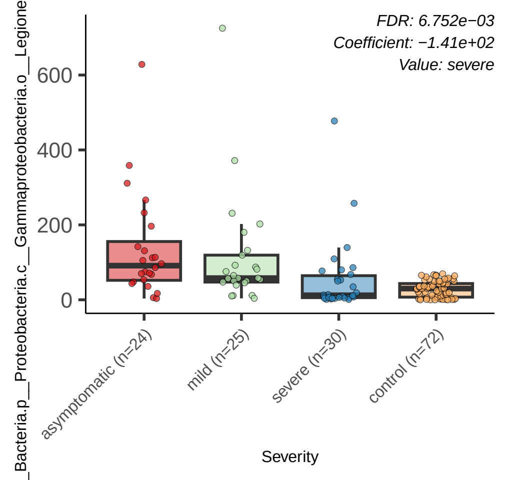

FDR: 6.752e−03
Coefficient: −1.41e+02
Value: severe
Bacteria.p__Proteobacteria.c__Gammaproteobacteria.o__Legione
0
200
400
600
asymptomatic (n=24)
mild (n=25)
severe (n=30)
control (n=72)
Severity

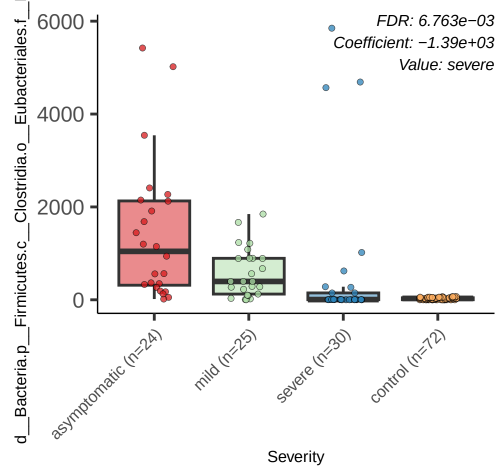

FDR: 6.763e−03
Coefficient: −1.39e+03
Value: severe
d__Bacteria.p__Firmicutes.c__Clostridia.o__Eubacteriales.f__
6000
4000
2000
0
asymptomatic (n=24)
mild (n=25)
severe (n=30)
control (n=72)
Severity

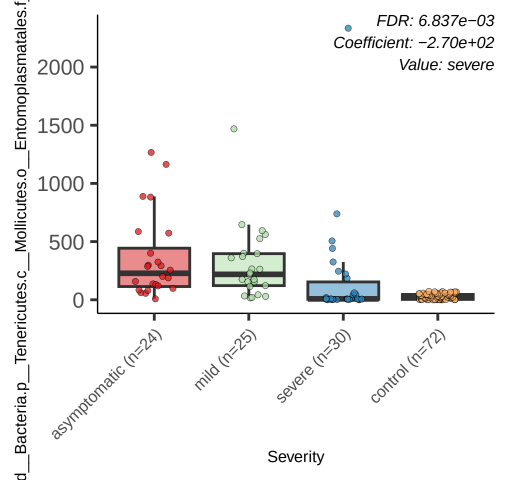

FDR: 6.837e−03
Coefficient: −2.70e+02
Value: severe
2000
1500
1000
500
0
d__Bacteria.p__Tenericutes.c__Mollicutes.o__Entomoplasmatales.f
asymptomatic (n=24)
mild (n=25)
severe (n=30)
control (n=72)
Severity

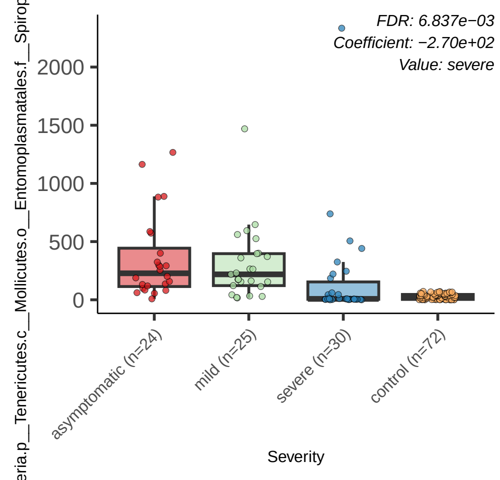

FDR: 6.837e−03
Coefficient: −2.70e+02
Value: severe
eria.p__Tenericutes.c__Mollicutes.o__Entomoplasmatales.f__Spirop
2000
1500
1000
500
0
asymptomatic (n=24)
mild (n=25)
severe (n=30)
control (n=72)
Severity

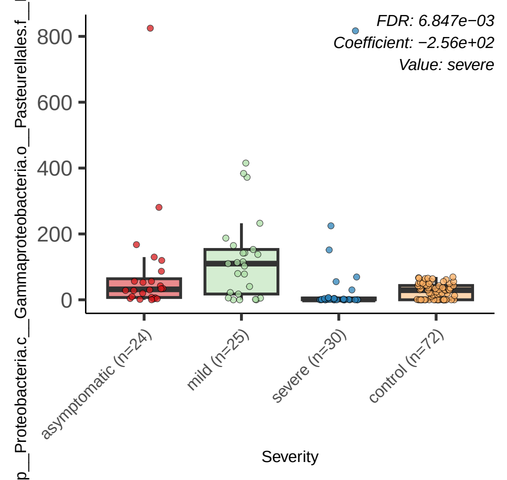
FDR: 6.847e−03
Coefficient: −2.56e+02
Value: severe
p__Proteobacteria.c__Gammaproteobacteria.o__Pasteurellales.f__
800
600
400
200
0
asymptomatic (n=24)
mild (n=25)
severe (n=30)
control (n=72)
Severity

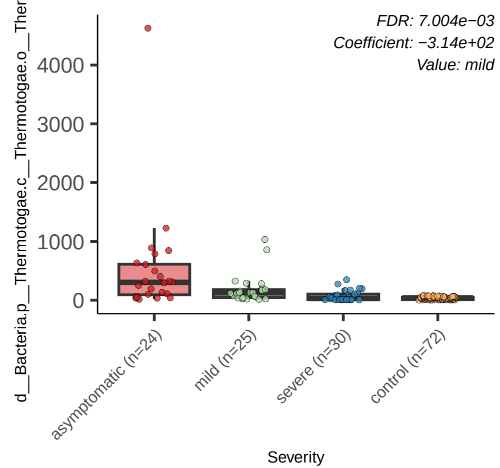

FDR: 7.004e−03
Coefficient: −3.14e+02
Value: mild
d__Bacteria.p__Thermotogae.c__Thermotogae.o__Ther
0
1000
2000
3000
4000
asymptomatic (n=24)
mild (n=25)
severe (n=30)
control (n=72)
Severity

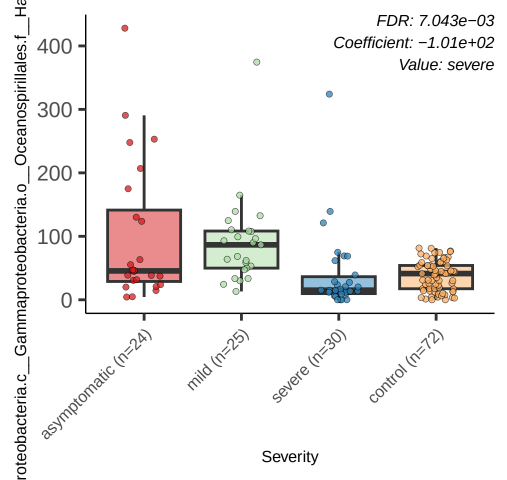

FDR: 7.043e−03
Coefficient: −1.01e+02
Value: severe
roteobacteria.c__Gammaproteobacteria.o__Oceanospirillales.f__Ha
400
300
200
100
0
asymptomatic (n=24)
mild (n=25)
severe (n=30)
control (n=72)
Severity

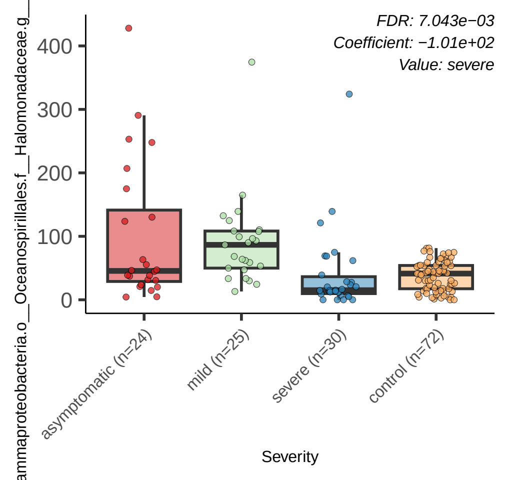

FDR: 7.043e−03
Coefficient: −1.01e+02
Value: severe
400
300
200
100
0
ammaproteobacteria.o__Oceanospirillales.f__Halomonadaceae.g__
asymptomatic (n=24)
mild (n=25)
severe (n=30)
control (n=72)
Severity

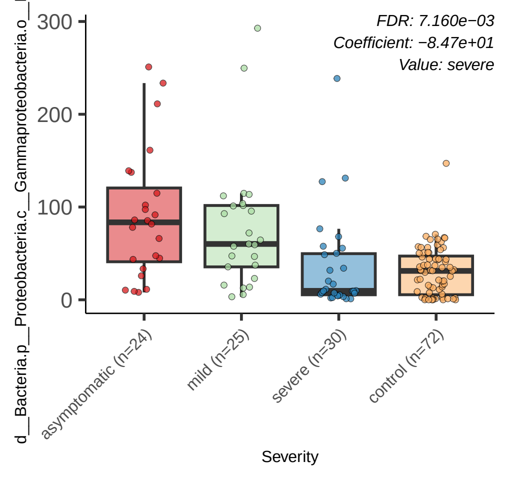

FDR: 7.160e−03
Coefficient: −8.47e+01
Value: severe
d__Bacteria.p__Proteobacteria.c__Gammaproteobacteria.o__
300
200
100
0
asymptomatic (n=24)
mild (n=25)
severe (n=30)
control (n=72)
Severity

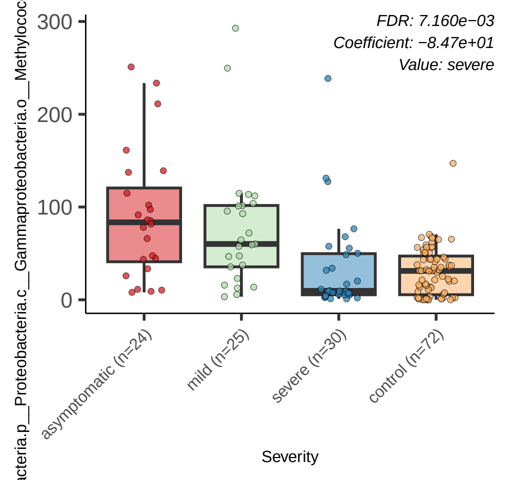

FDR: 7.160e−03
Coefficient: −8.47e+01
Value: severe
300
200
100
0
acteria.p__Proteobacteria.c__Gammaproteobacteria.o__Methylococ
asymptomatic (n=24)
mild (n=25)
severe (n=30)
control (n=72)
Severity

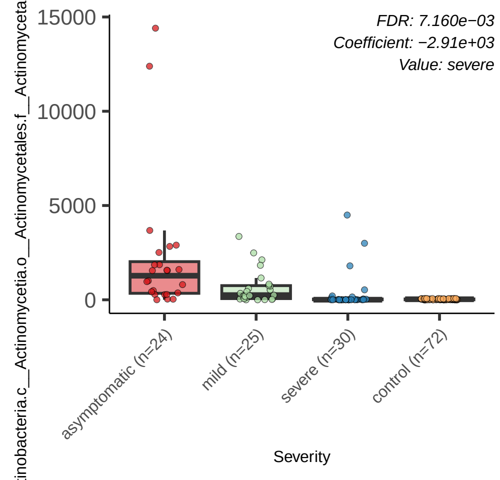

FDR: 7.160e−03
Coefficient: −2.91e+03
Value: severe
15000
10000
5000
0
inobacteria.c__Actinomycetia.o__Actinomycetales.f__Actinomyceta
asymptomatic (n=24)
mild (n=25)
severe (n=30)
control (n=72)
Severity

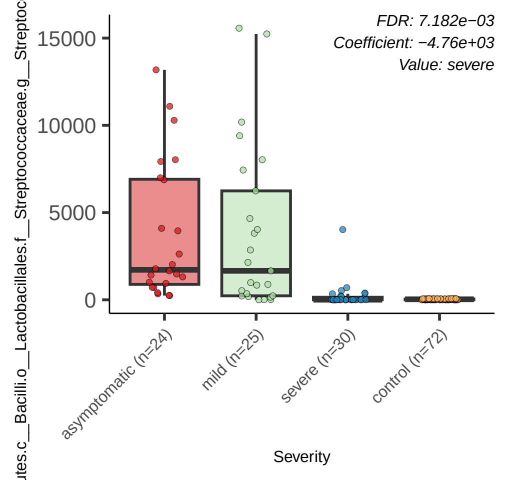

FDR: 7.182e−03
Coefficient: −4.76e+03
Value: severe
15000
10000
5000
0
utes.c__Bacilli.o__Lactobacillales.f__Streptococcaceae.g__Streptoc
asymptomatic (n=24)
mild (n=25)
severe (n=30)
control (n=72)
Severity

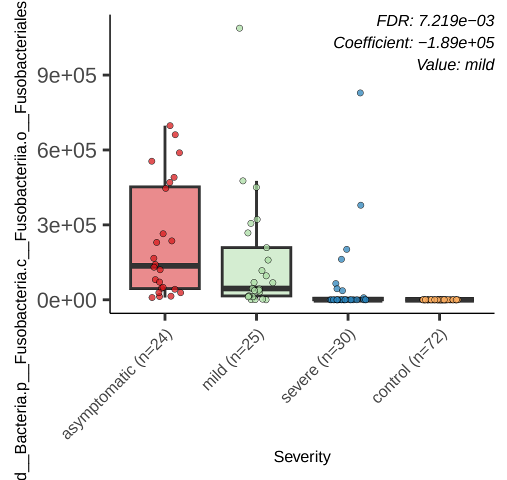

FDR: 7.219e−03
Coefficient: −1.89e+05
Value: mild
d__Bacteria.p__Fusobacteria.c__Fusobacteriia.o__Fusobacteriales
9e+05
6e+05
3e+05
0e+00
asymptomatic (n=24)
mild (n=25)
severe (n=30)
control (n=72)
Severity

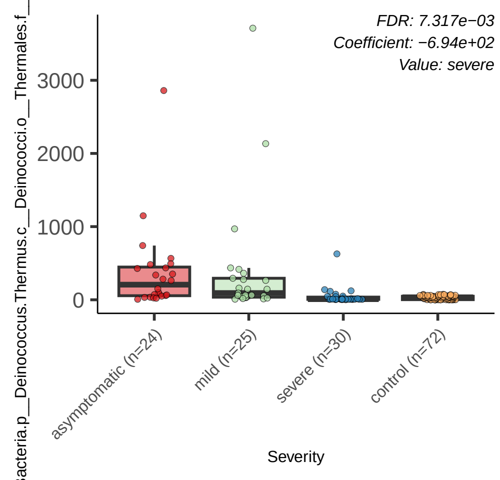
FDR: 7.317e−03
Coefficient: −6.94e+02
Value: severe
Bacteria.p__Deinococcus.Thermus.c__Deinococci.o__Thermales.f__
3000
2000
1000
0
asymptomatic (n=24)
mild (n=25)
severe (n=30)
control (n=72)
Severity

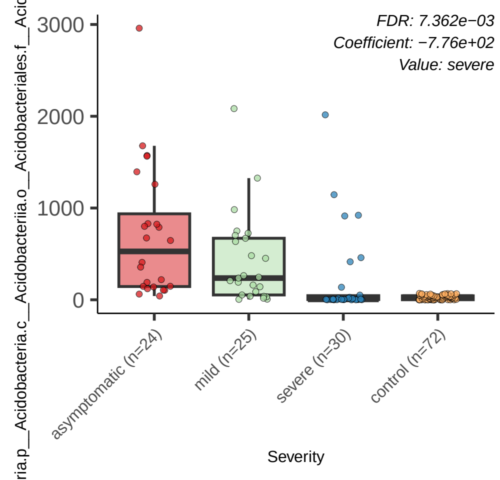

Acidobacteria.p__Acidobacteria.c__Acidobacteria.o__Acidobacteriales.f__Acid
FDR: 7.362e−03
Coefficient: −7.76e+02
Value: severe
3000
2000
1000
0
asymptomatic (n=24)
mild (n=25)
severe (n=30)
control (n=72)
Severity

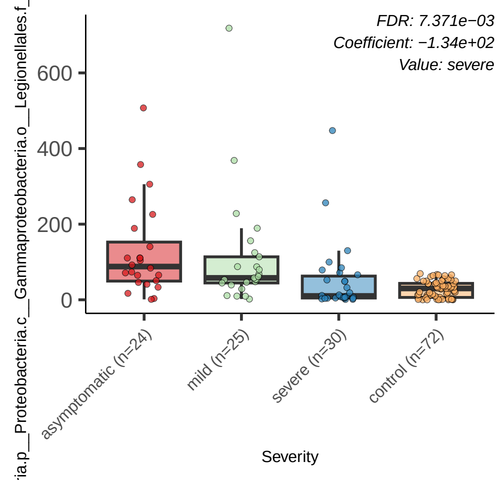
FDR: 7.371e−03
Coefficient: −1.34e+02
Value: severe
ria.p__Proteobacteria.c__Gammaproteobacteria.o__Legionellales.f_
600
400
200
0
asymptomatic (n=24)
mild (n=25)
severe (n=30)
control (n=72)
Severity

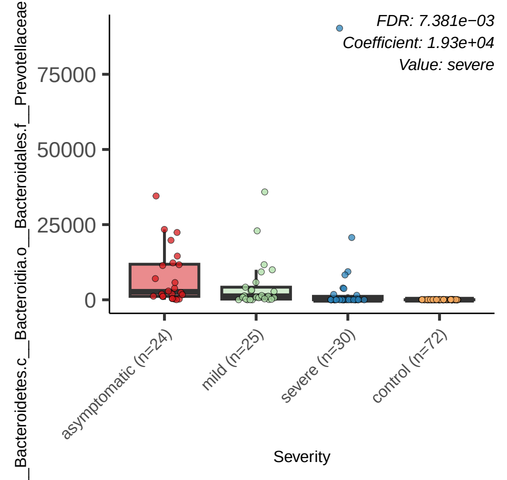

FDR: 7.381e−03
Coefficient: 1.93e+04
Value: severe
_Bacteroidetes.c__Bacteroidia.o__Bacteroidales.f__Prevotellaceae
75000
50000
25000
0
asymptomatic (n=24)
mild (n=25)
severe (n=30)
control (n=72)
Severity

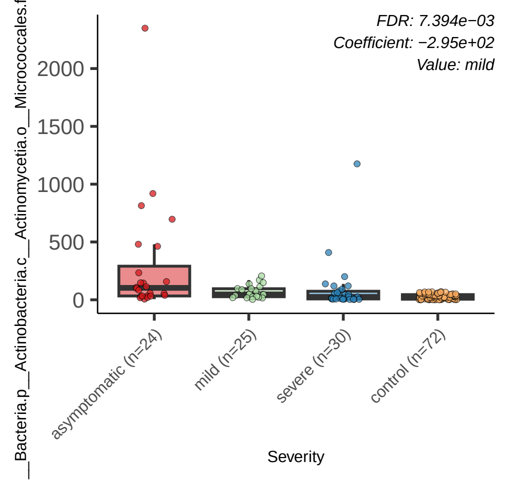

FDR: 7.394e−03
Coefficient: −2.95e+02
Value: mild
_Bacteria.p__Actinobacteria.c__Actinomycetia.o__Micrococcales.f
2000
1500
1000
500
0
asymptomatic (n=24)
mild (n=25)
severe (n=30)
control (n=72)
Severity

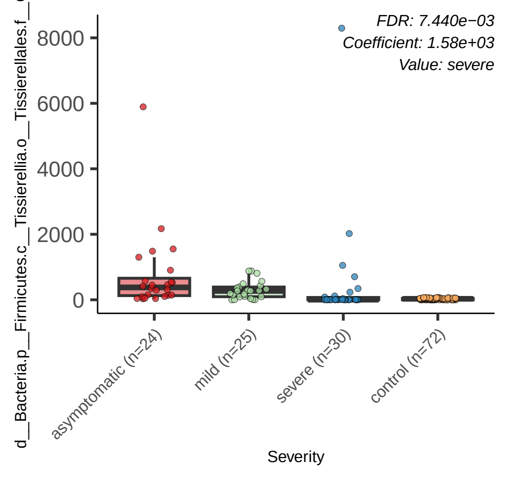

d__Bacteria.p__Firmicutes.c__Tissierellia.o__Tissierellales.f__
FDR: 7.440e−03
Coefficient: 1.58e+03
Value: severe
8000
6000
4000
2000
0
asymptomatic (n=24)
mild (n=25)
severe (n=30)
control (n=72)
Severity

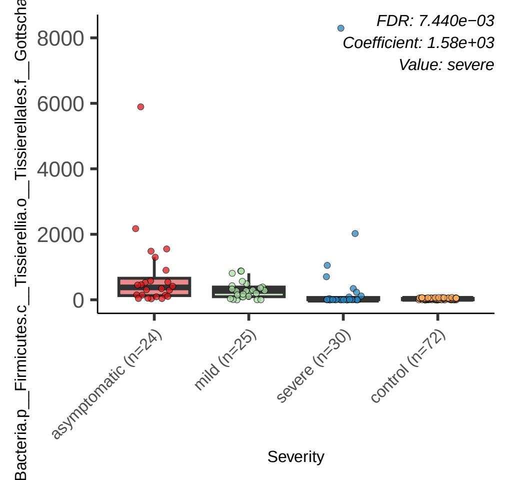
FDR: 7.440e−03
Coefficient: 1.58e+03
Value: severe
Bacteria.p__Firmicutes.c__Tissierellia.o__Tissierellales.f__Gottscha
8000
6000
4000
2000
0
asymptomatic (n=24)
mild (n=25)
severe (n=30)
control (n=72)
Severity

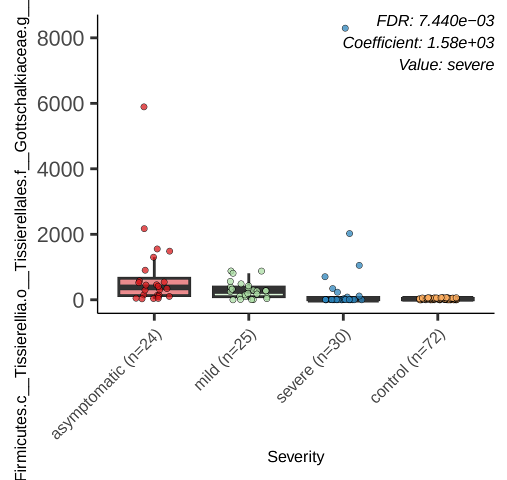
FDR: 7.440e−03
Coefficient: 1.58e+03
Value: severe
Firmicutes.c__Tissierellia.o__Tissierellales.f__Gottschalkiaceae.g__
8000
6000
4000
2000
0
asymptomatic (n=24)
mild (n=25)
severe (n=30)
control (n=72)
Severity

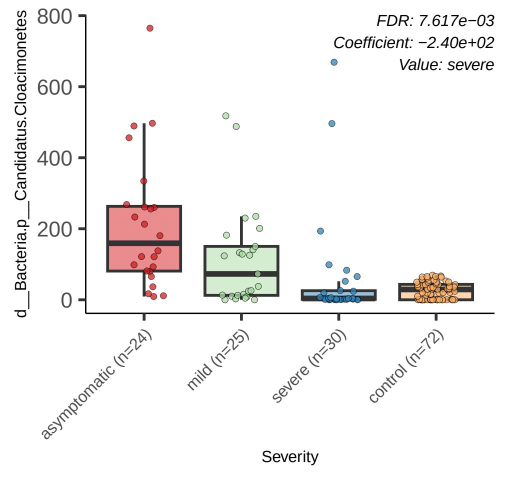

FDR: 7.617e−03
Coefficient: −2.40e+02
Value: severe
d__Bacteria.p__Candidatus.Cloacimonetes
800
600
400
200
0
asymptomatic (n=24)
mild (n=25)
severe (n=30)
control (n=72)
Severity

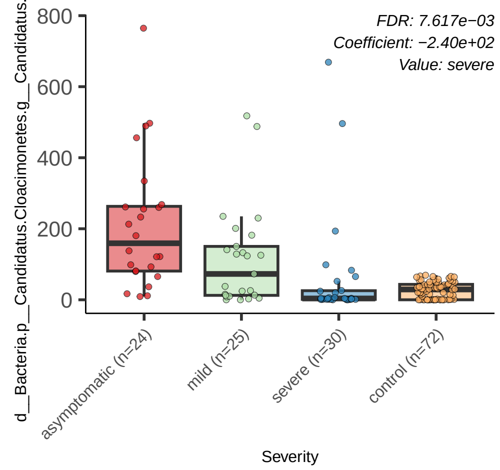

FDR: 7.617e−03
Coefficient: −2.40e+02
Value: severe
800
600
400
200
0
d__Bacteria.p__Candidatus.Cloacimonetes.g__Candidatus.
asymptomatic (n=24)
mild (n=25)
severe (n=30)
control (n=72)
Severity

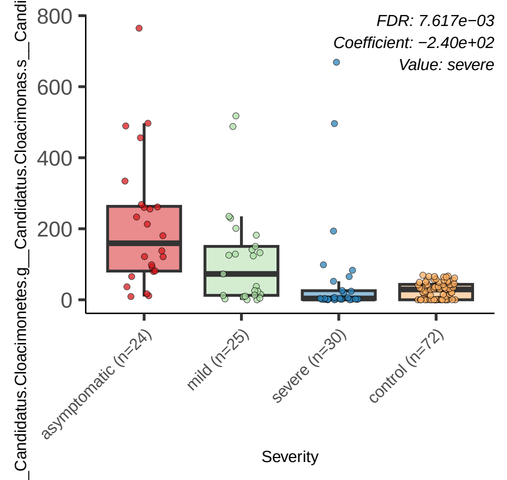
FDR: 7.617e−03
Coefficient: −2.40e+02
Value: severe
800
600
400
200
0
_Candidatus.Cloacimonetes.g__Candidatus.Cloacimonas.s__Candi
asymptomatic (n=24)
mild (n=25)
severe (n=30)
control (n=72)
Severity

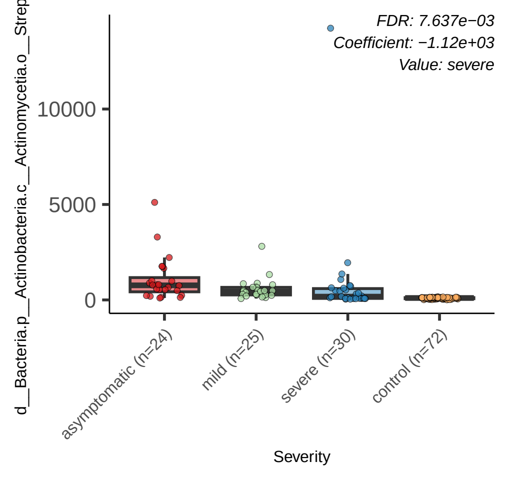

FDR: 7.637e−03
Coefficient: −1.12e+03
Value: severe
d__Bacteria.p__Actinobacteria.c__Actinomycetia.o__Strep
10000
5000
0
asymptomatic (n=24)
mild (n=25)
severe (n=30)
control (n=72)
Severity

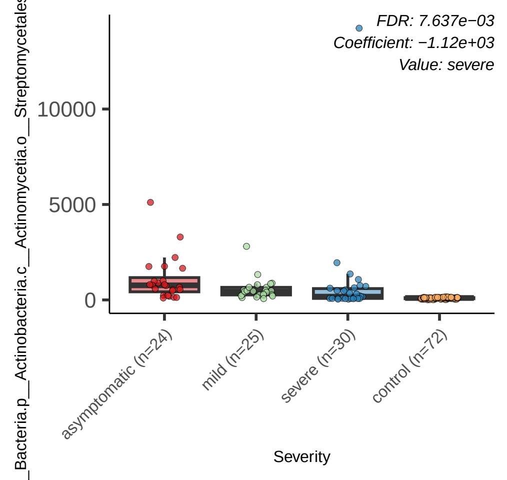
FDR: 7.637e−03
Coefficient: −1.12e+03
Value: severe
10000
5000
0
asymptomatic (n=24)
mild (n=25)
severe (n=30)
control (n=72)
Severity
_Bacteria.p__Actinobacteria.c__Actinomycetia.o__Streptomycetales

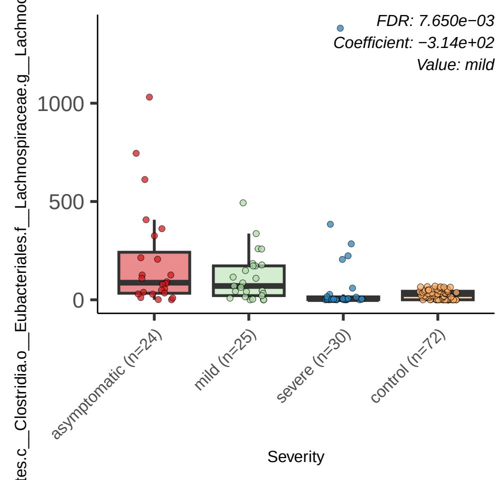

FDR: 7.650e−03
Coefficient: −3.14e+02
Value: mild
1000
500
0
asymptomatic (n=24)
mild (n=25)
severe (n=30)
control (n=72)
Severity
tes.c__Clostridia.o__Eubacteriales.f__Lachnospiraceae.g__Lachnoc

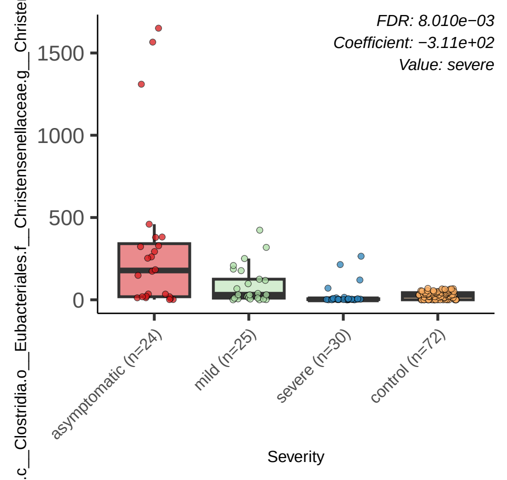
FDR: 8.010e−03
Coefficient: −3.11e+02
Value: severe
1500
1000
500
0
c__Clostridia.o__Eubacteriales.f__Christensenellaceae.g__Christe
asymptomatic (n=24)
mild (n=25)
severe (n=30)
control (n=72)
Severity

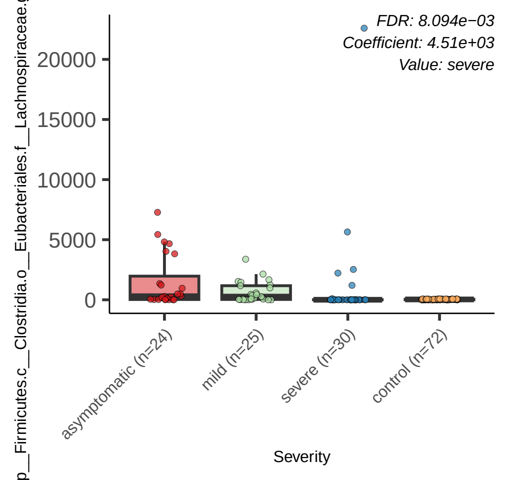

FDR: 8.094e−03
Coefficient: 4.51e+03
Value: severe
p__Firmicutes.c__Clostridia.o__Eubacteriales.f__Lachnospiraceae.g
0
5000
10000
15000
20000
asymptomatic (n=24)
mild (n=25)
severe (n=30)
control (n=72)
Severity

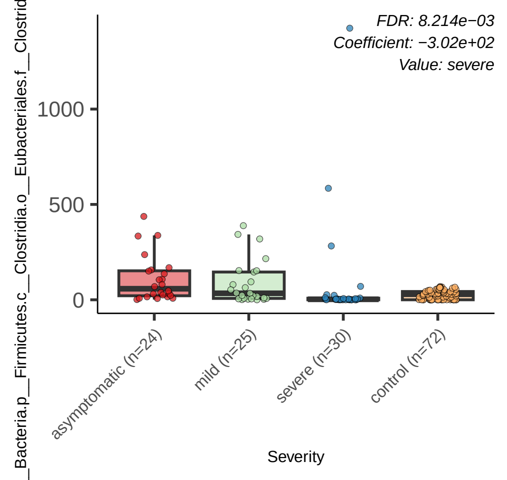

FDR: 8.214e−03
Coefficient: −3.02e+02
Value: severe
Bacteria.p__Firmicutes.c__Clostridia.o__Eubacteriales.f__Clostrid
1000
500
0
asymptomatic (n=24)
mild (n=25)
severe (n=30)
control (n=72)
Severity

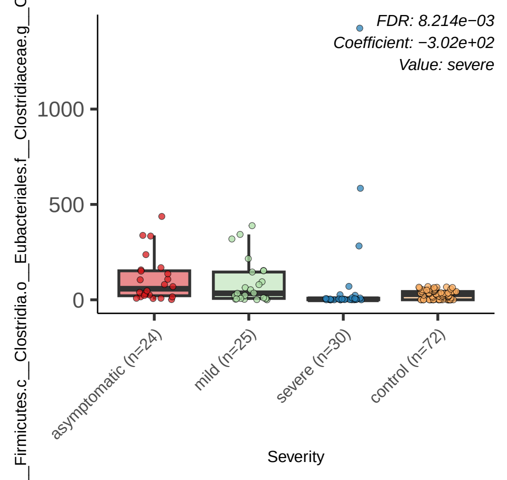
FDR: 8.214e−03
Coefficient: −3.02e+02
Value: severe
_Firmicutes.c__Clostridia.o__Eubacteriales.f__Clostridiaceae.g__C
1000
500
0
asymptomatic (n=24)
mild (n=25)
severe (n=30)
control (n=72)
Severity

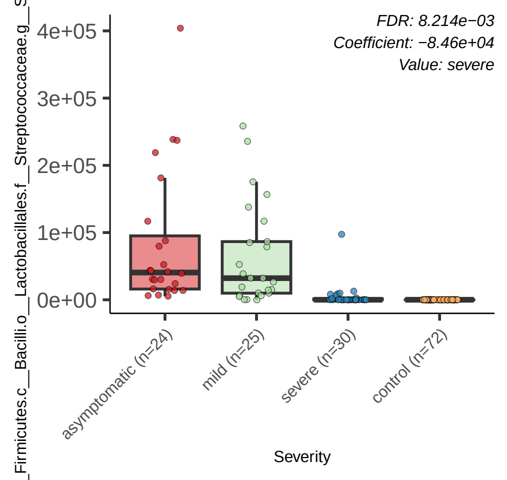

FDR: 8.214e−03
Coefficient: −8.46e+04
Value: severe
Firmicutes.c__Bacilli.o__Lactobacillales.f__Streptococcaceae.g__S
4e+05
3e+05
2e+05
1e+05
0e+00
asymptomatic (n=24)
mild (n=25)
severe (n=30)
control (n=72)
Severity

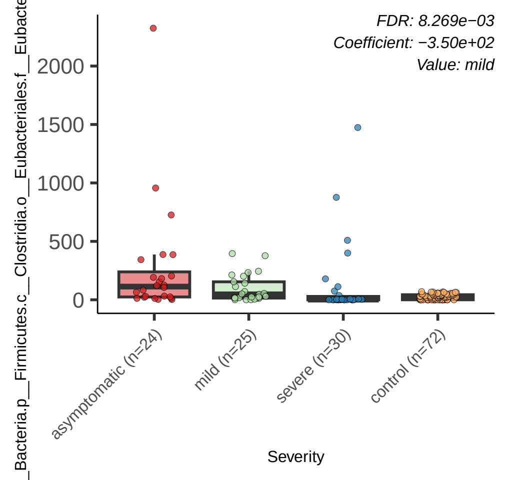
FDR: 8.269e−03
Coefficient: −3.50e+02
Value: mild
2000
1500
1000
500
0
asymptomatic (n=24)
mild (n=25)
severe (n=30)
control (n=72)
Severity
_Bacteria.p__Firmicutes.c__Clostridia.o__Eubacteriales.f__Eubacte

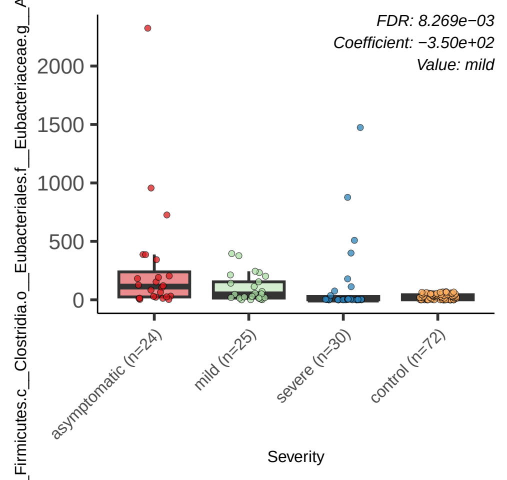

FDR: 8.269e−03
Coefficient: −3.50e+02
Value: mild
2000
1500
1000
500
0
asymptomatic (n=24)
mild (n=25)
severe (n=30)
control (n=72)
Severity
Firmicutes.c__Clostridia.o__Eubacteriales.f__Eubacteriaceae.g__

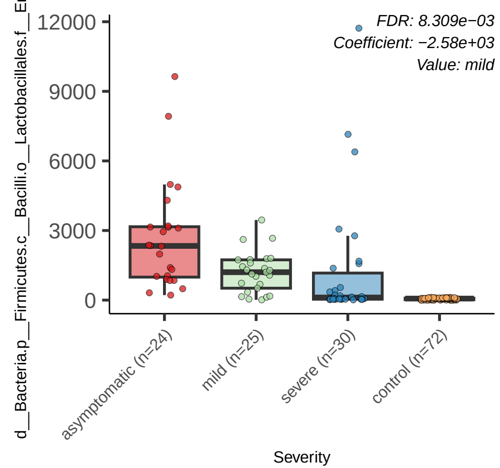
d__Bacteria.p__Firmicutes.c__Bacilli.o__Lactobacillales.f__E
FDR: 8.309e−03
Coefficient: −2.58e+03
Value: mild
12000
9000
6000
3000
0
asymptomatic (n=24)
mild (n=25)
severe (n=30)
control (n=72)
Severity

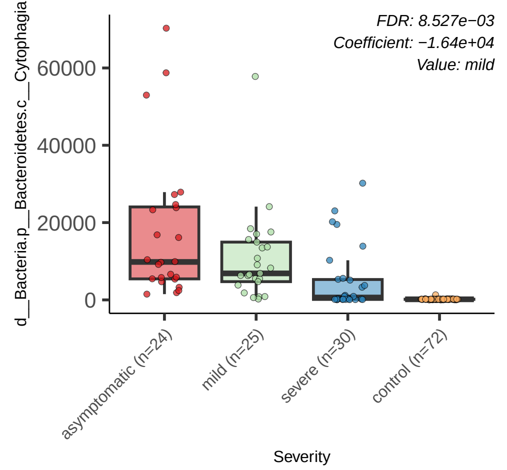
FDR: 8.527e−03
Coefficient: −1.64e+04
Value: mild
d__Bacteria.p__Bacteroidetes.c__Cytophagia
60000
40000
20000
0
asymptomatic (n=24)
mild (n=25)
severe (n=30)
control (n=72)
Severity

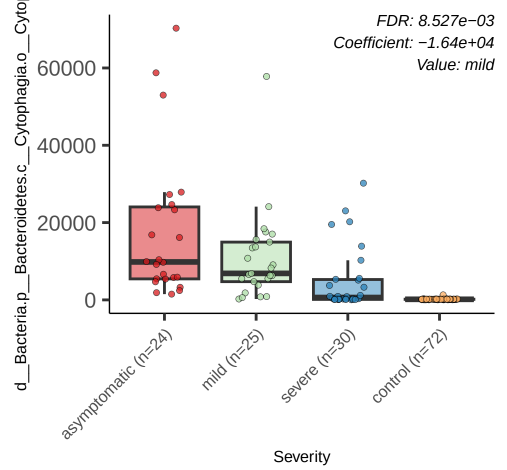

FDR: 8.527e−03
Coefficient: −1.64e+04
Value: mild
d__Bacteria.p__Bacteroidetes.c__Cytophagia.o__Cytop
60000
40000
20000
0
asymptomatic (n=24)
mild (n=25)
severe (n=30)
control (n=72)
Severity

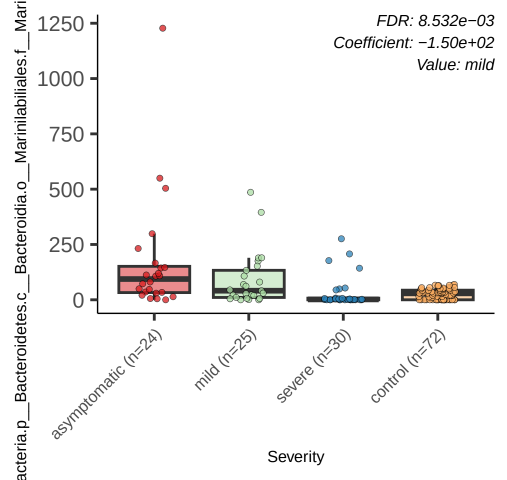
FDR: 8.532e−03
Coefficient: −1.50e+02
Value: mild
acteria.p__Bacteroidetes.c__Bacteroidia.o__Marinilabiliales.f__Mari
1250
1000
750
500
250
0
asymptomatic (n=24)
mild (n=25)
severe (n=30)
control (n=72)
Severity

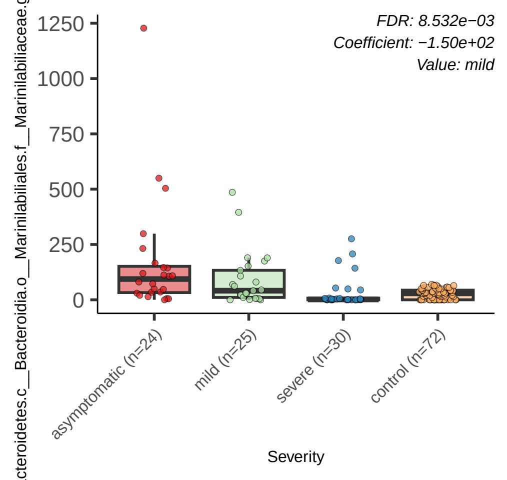

FDR: 8.532e−03
Coefficient: −1.50e+02
Value: mild
1250
1000
750
500
250
0
asymptomatic (n=24)
mild (n=25)
severe (n=30)
control (n=72)
Severity
cteroidetes.c__Bacteroidia.o__Marinilabiliales.f__Marinilabiliaceae.g

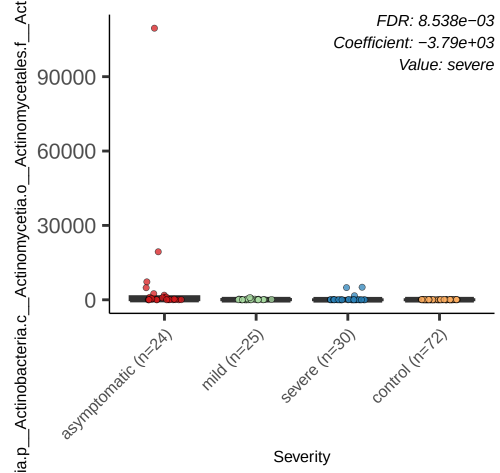

FDR: 8.538e−03
Coefficient: −3.79e+03
Value: severe
90000
60000
30000
0
asymptomatic (n=24)
mild (n=25)
severe (n=30)
control (n=72)
Severity

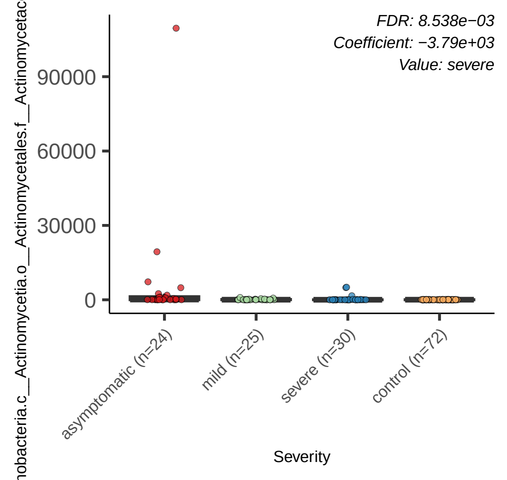

FDR: 8.538e−03
Coefficient: −3.79e+03
Value: severe
90000
60000
30000
0
nobacteria.c__Actinomycetia.o__Actinomycetales.f__Actinomycetac
asymptomatic (n=24)
mild (n=25)
severe (n=30)
control (n=72)
Severity

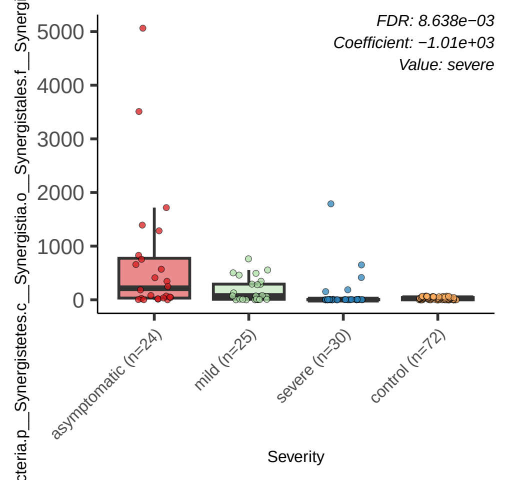

FDR: 8.638e−03
Coefficient: −1.01e+03
Value: severe
5000
4000
3000
2000
1000
0
cteria.p__Synergistetes.c__Synergistia.o__Synergistales.f__Synergi
asymptomatic (n=24)
mild (n=25)
severe (n=30)
control (n=72)
Severity

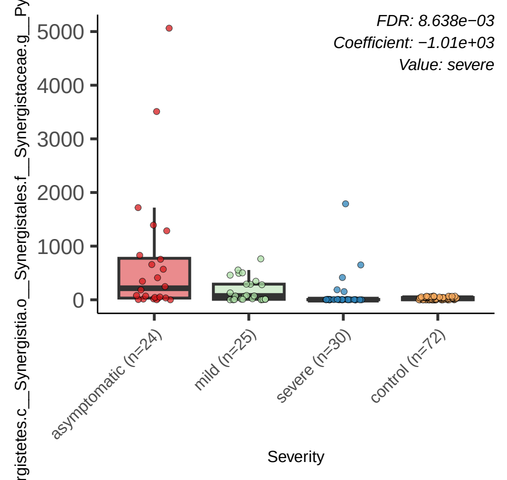

FDR: 8.638e−03
Coefficient: −1.01e+03
Value: severe
rgistetes.c__Synergistia.o__Synergistales.f__Synergistaceae.g__Py
5000
4000
3000
2000
1000
0
asymptomatic (n=24)
mild (n=25)
severe (n=30)
control (n=72)
Severity

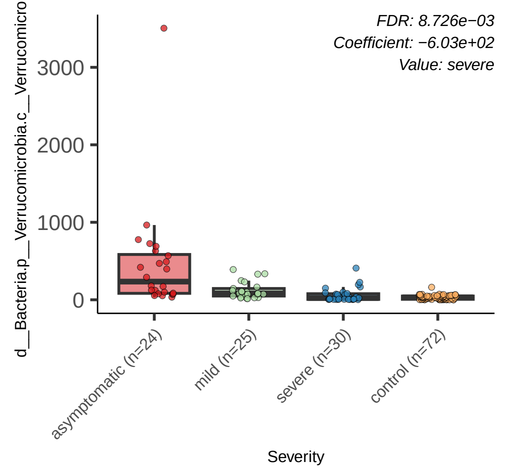

FDR: 8.726e−03
Coefficient: −6.03e+02
Value: severe
d__Bacteria.p__Verrucomicrobia.c__Verrucomicro
3000
2000
1000
0
asymptomatic (n=24)
mild (n=25)
severe (n=30)
control (n=72)
Severity

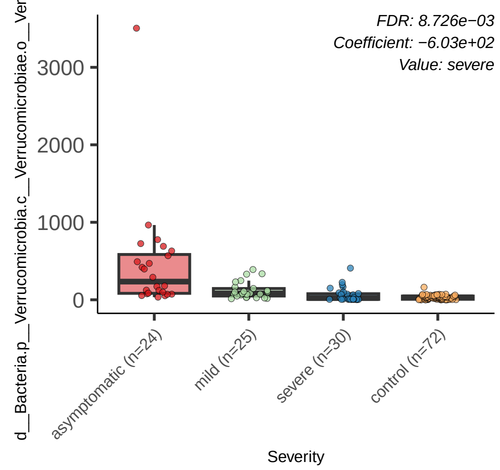
FDR: 8.726e−03
Coefficient: −6.03e+02
Value: severe
d__Bacteria.p__Verrucomicrobia.c__Verrucomicrobiae.o__Ver
3000
2000
1000
0
asymptomatic (n=24)
mild (n=25)
severe (n=30)
control (n=72)
Severity

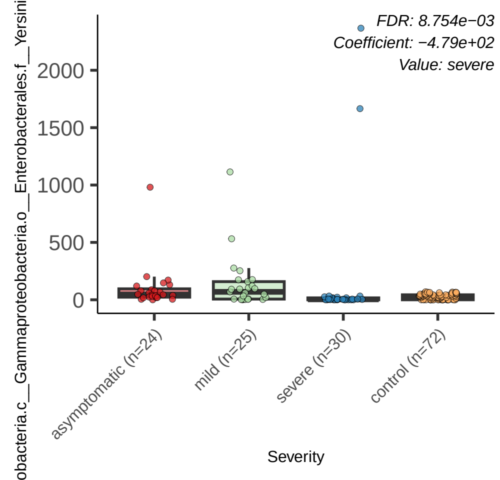

FDR: 8.754e−03
Coefficient: −4.79e+02
Value: severe
obacteria.c__Gammaproteobacteria.o__Enterobacterales.f__Yersini
2000
1500
1000
500
0
asymptomatic (n=24)
mild (n=25)
severe (n=30)
control (n=72)
Severity

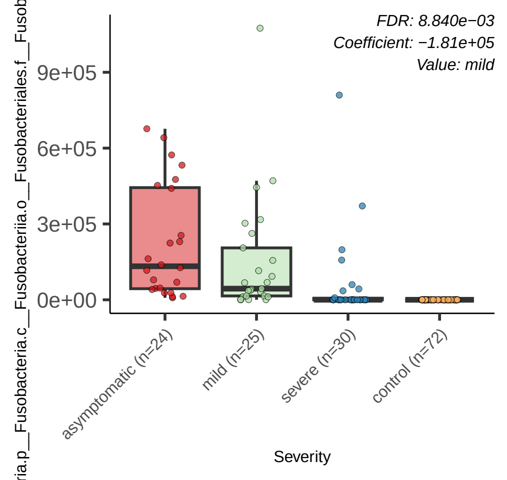
FDR: 8.840e−03
Coefficient: −1.81e+05
Value: mild
9e+05
6e+05
3e+05
0e+00
ria.p__Fusobacteria.c__Fusobacteriia.o__Fusobacteriales.f__Fusob
asymptomatic (n=24)
mild (n=25)
severe (n=30)
control (n=72)
Severity

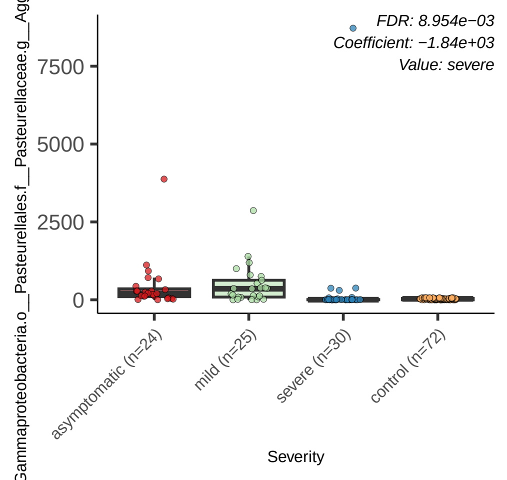

FDR: 8.954e−03
Coefficient: −1.84e+03
Value: severe
7500
5000
2500
0
Gammaproteobacteria.o__Pasteurellales.f__Pasteurellaceae.g__Agg
asymptomatic (n=24)
mild (n=25)
severe (n=30)
control (n=72)
Severity

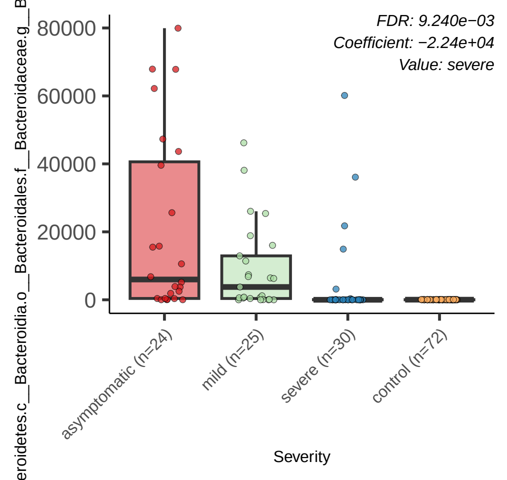
FDR: 9.240e−03
Coefficient: −2.24e+04
Value: severe
eroidetes.c__Bacteroidia.o__Bacteroidales.f__Bacteroidaceae.g__B
80000
60000
40000
20000
0
asymptomatic (n=24)
mild (n=25)
severe (n=30)
control (n=72)
Severity

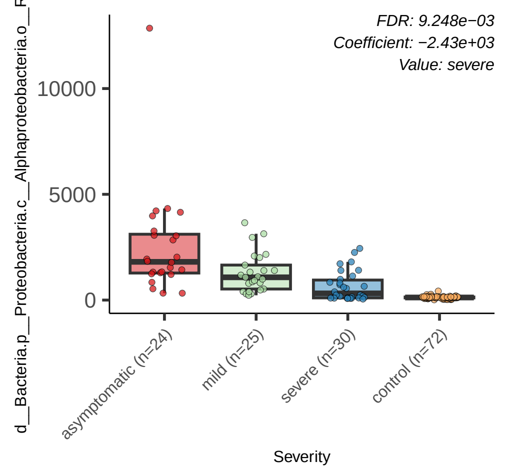

FDR: 9.248e−03
Coefficient: −2.43e+03
Value: severe
d__Bacteria.p__Proteobacteria.c__Alphaproteobacteria.o__R
10000
5000
0
asymptomatic (n=24)
mild (n=25)
severe (n=30)
control (n=72)
Severity

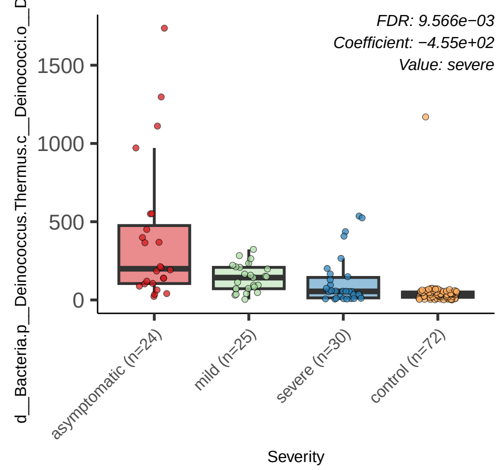

FDR: 9.566e−03
Coefficient: −4.55e+02
Value: severe
d__Bacteria.p__Deinococcus.Thermus.c__Deinococci.o__D
0
500
1000
1500
asymptomatic (n=24)
mild (n=25)
severe (n=30)
control (n=72)
Severity

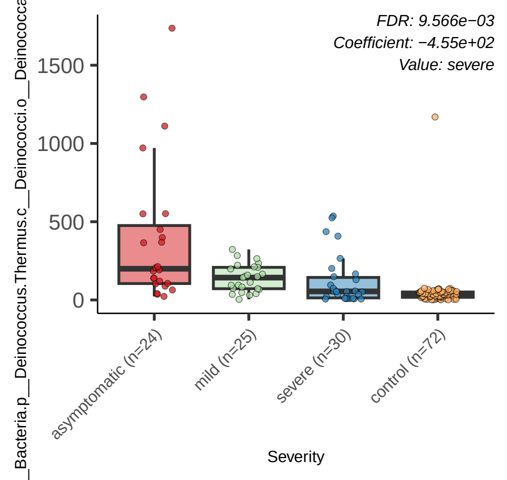

FDR: 9.566e−03
Coefficient: −4.55e+02
Value: severe
Bacteria.p__Deinococcus.Thermus.c__Deinococci.o__Deinococca
0
500
1000
1500
asymptomatic (n=24)
mild (n=25)
severe (n=30)
control (n=72)
Severity

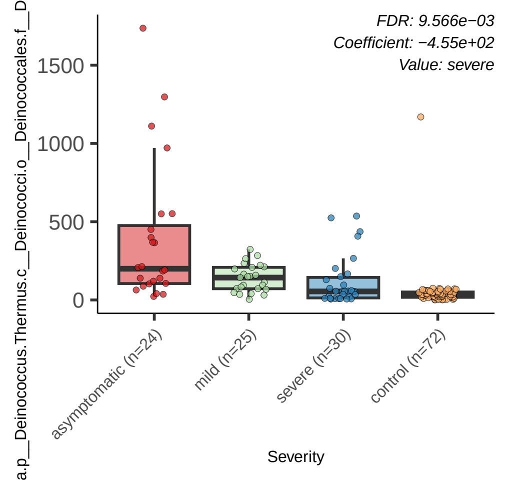

FDR: 9.566e−03
Coefficient: −4.55e+02
Value: severe
0
500
1000
1500
a.p__Deinococcus.Thermus.c__Deinococci.o__Deinococcales.f__D
asymptomatic (n=24)
mild (n=25)
severe (n=30)
control (n=72)
Severity

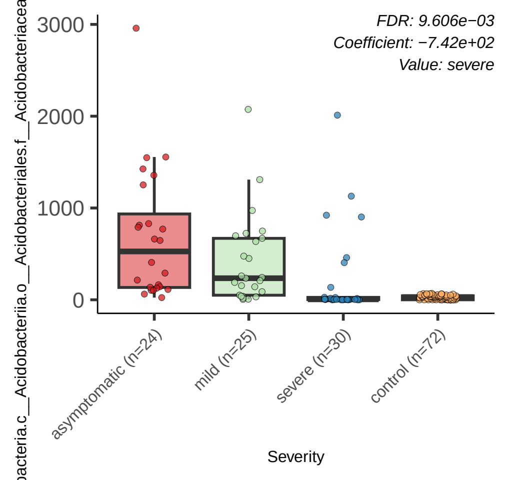

FDR: 9.606e−03
Coefficient: −7.42e+02
Value: severe
3000
2000
1000
0
bacteria.c__Acidobacteriia.o__Acidobacteriales.f__Acidobacteriacea
asymptomatic (n=24)
mild (n=25)
severe (n=30)
control (n=72)
Severity

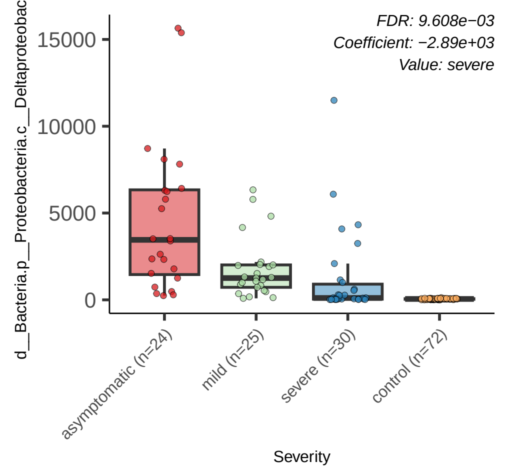

FDR: 9.608e−03
Coefficient: −2.89e+03
Value: severe
d__Bacteria.p__Proteobacteria.c__Deltaproteobac
15000
10000
5000
0
asymptomatic (n=24)
mild (n=25)
severe (n=30)
control (n=72)
Severity

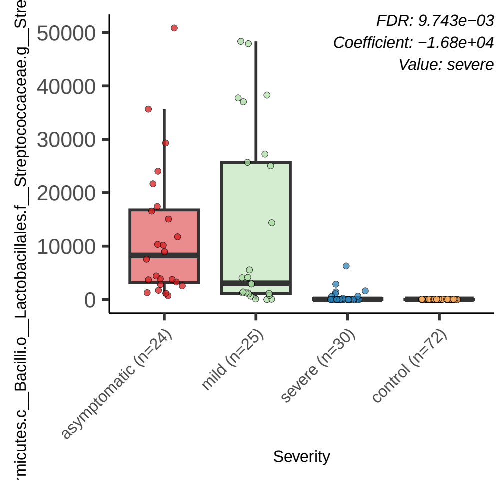

FDR: 9.743e−03
Coefficient: −1.68e+04
Value: severe
50000
40000
30000
20000
10000
0
rmicutes.c__Bacilli.o__Lactobacillales.f__Streptococcaceae.g__Stre
asymptomatic (n=24)
mild (n=25)
severe (n=30)
control (n=72)
Severity

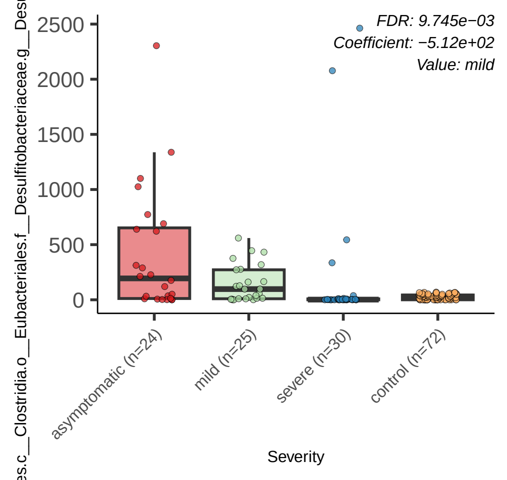

FDR: 9.745e−03
Coefficient: −5.12e+02
Value: mild
2500
2000
1500
1000
500
0
es.c__Clostridia.o__Eubacteriales.f__Desulfitobacteriaceae.g__Des
asymptomatic (n=24)
mild (n=25)
severe (n=30)
control (n=72)
Severity

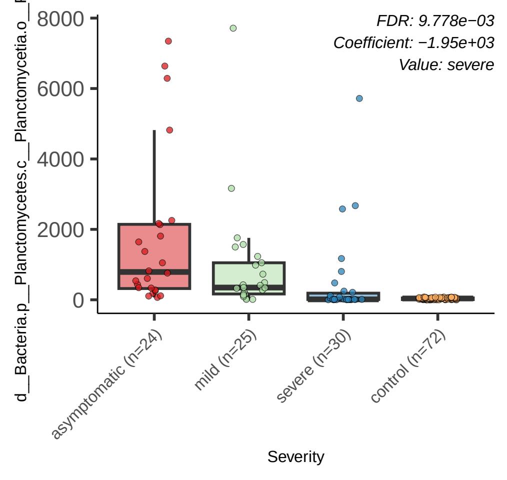
FDR: 9.778e−03
Coefficient: −1.95e+03
Value: severe
8000
6000
4000
2000
0
asymptomatic (n=24)
mild (n=25)
severe (n=30)
control (n=72)
Severity

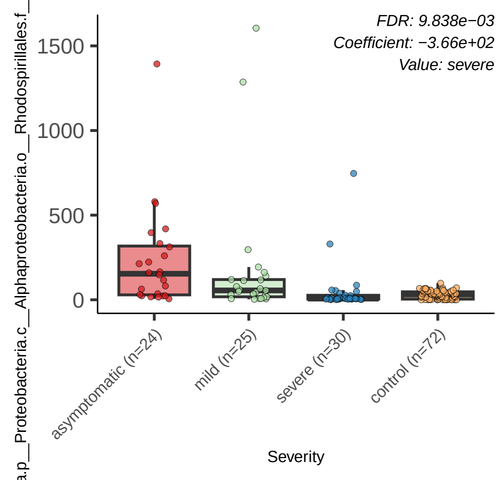
FDR: 9.838e−03
Coefficient: −3.66e+02
Value: severe
1500
1000
500
0
a.p__Proteobacteria.c__Alphaproteobacteria.o__Rhodospirillales.f__
asymptomatic (n=24)
mild (n=25)
severe (n=30)
control (n=72)
Severity

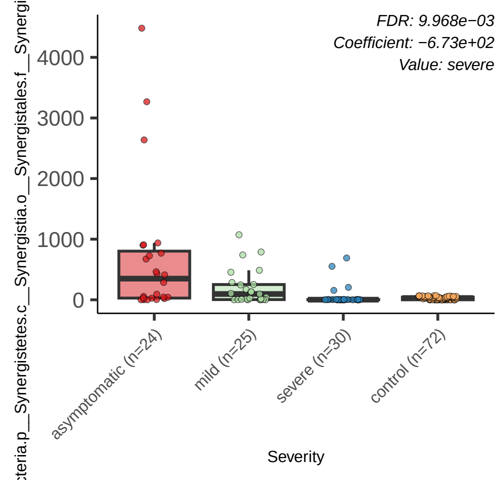

FDR: 9.968e−03
Coefficient: −6.73e+02
Value: severe
4000
3000
2000
1000
0
cteria.p__Synergistetes.c__Synergistia.o__Synergistales.f__Synergi
asymptomatic (n=24)
mild (n=25)
severe (n=30)
control (n=72)
Severity

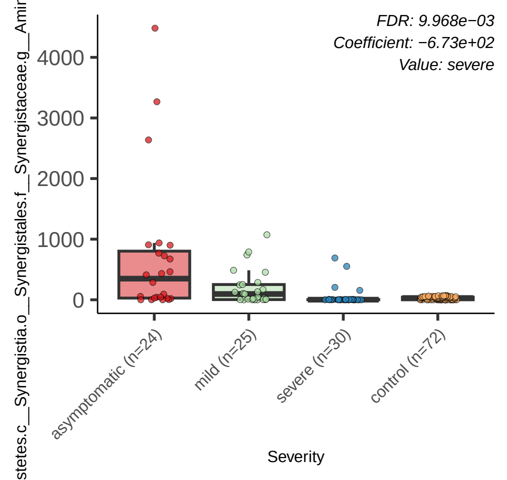

FDR: 9.968e−03
Coefficient: −6.73e+02
Value: severe
statetes.c__Synergistia.o__Synergistales.f__Synergistaceae.g__Amin
4000
3000
2000
1000
0
asymptomatic (n=24)
mild (n=25)
severe (n=30)
control (n=72)
Severity

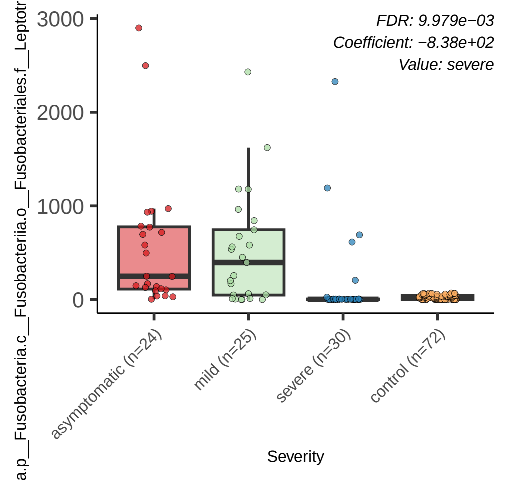

FDR: 9.979e−03
Coefficient: −8.38e+02
Value: severe
a.p__Fusobacteria.c__Fusobacteriia.o__Fusobacteriales.f__Leptotr
3000
2000
1000
0
asymptomatic (n=24)
mild (n=25)
severe (n=30)
control (n=72)
Severity

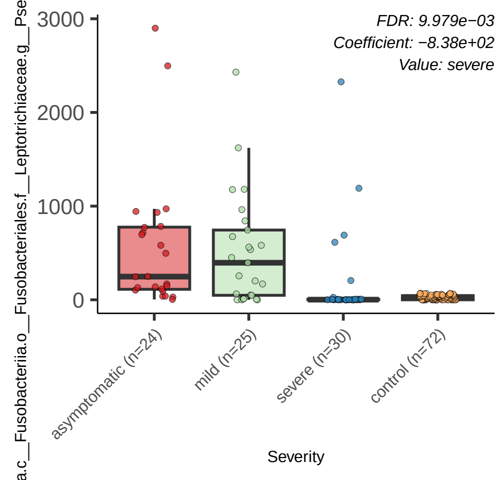

a.c__Fusobacteriia.o__Fusobacteriales.f__Leptotrichiaceae.g__Pse
3000
2000
1000
0
FDR: 9.979e−03
Coefficient: −8.38e+02
Value: severe
asymptomatic (n=24)
mild (n=25)
severe (n=30)
control (n=72)
Severity

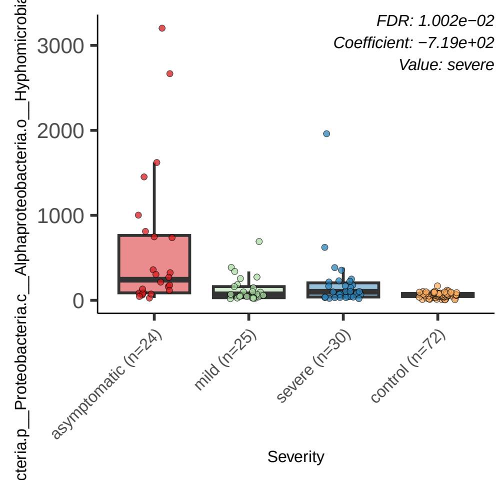
FDR: 1.002e−02
Coefficient: −7.19e+02
Value: severe
3000
2000
1000
0
cteria.p__Proteobacteria.c__Alphaproteobacteria.o__Hyphomicrobia
asymptomatic (n=24)
mild (n=25)
severe (n=30)
control (n=72)
Severity

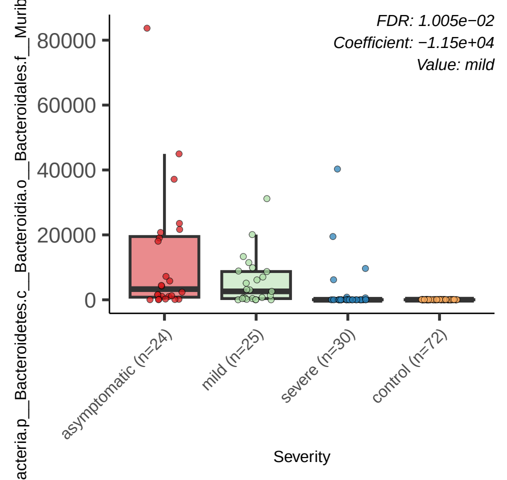

FDR: 1.005e−02
Coefficient: −1.15e+04
Value: mild
acteria.p__Bacteroidetes.c__Bacteroidia.o__Bacteroidales.f__Murib
80000
60000
40000
20000
0
asymptomatic (n=24)
mild (n=25)
severe (n=30)
control (n=72)
Severity

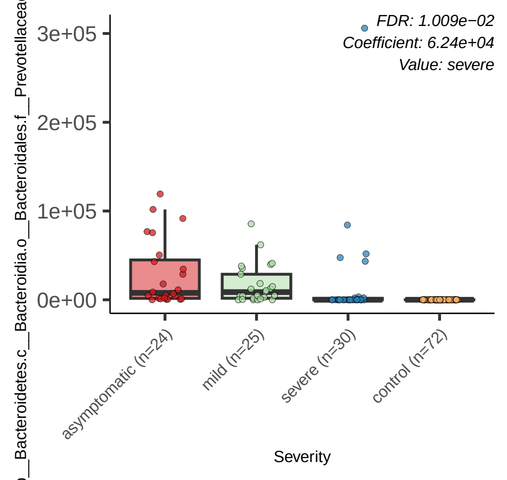

FDR: 1.009e−02
Coefficient: 6.24e+04
Value: severe
o__Bacteroidetes.c__Bacteroidia.o__Bacteroidales.f__Prevotellaceae
3e+05
2e+05
1e+05
0e+00
asymptomatic (n=24)
mild (n=25)
severe (n=30)
control (n=72)
Severity

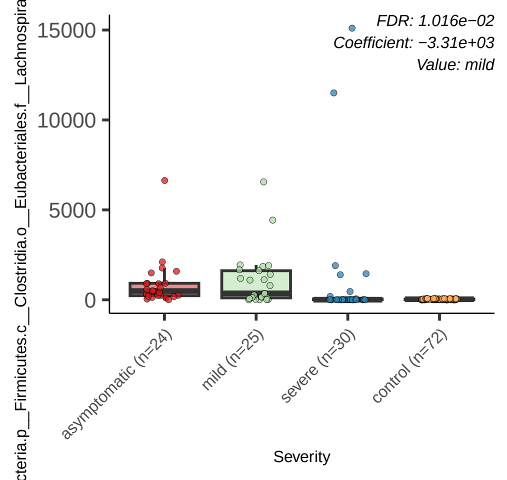

FDR: 1.016e−02
Coefficient: −3.31e+03
Value: mild
15000
10000
5000
0
cteria.p__Firmicutes.c__Clostridia.o__Eubacteriales.f__Lachnospira
asymptomatic (n=24)
mild (n=25)
severe (n=30)
control (n=72)
Severity

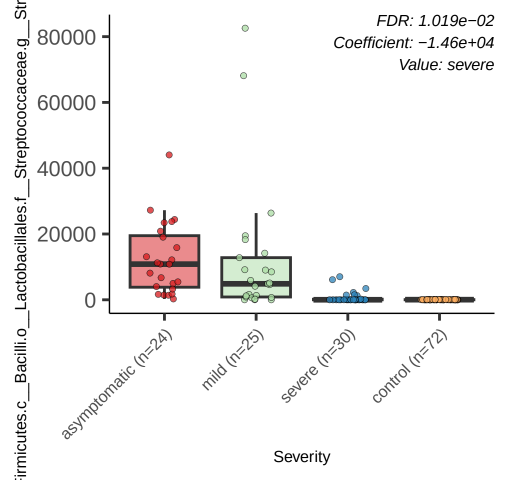
FDR: 1.019e−02
Coefficient: −1.46e+04
Value: severe
Firmicutes.c__Bacilli.o__Lactobacillales.f__Streptococcaceae.g__Str
80000
60000
40000
20000
0
asymptomatic (n=24)
mild (n=25)
severe (n=30)
control (n=72)
Severity

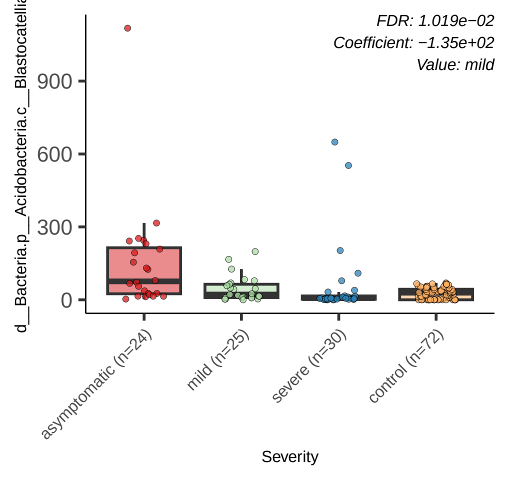

FDR: 1.019e−02
Coefficient: −1.35e+02
Value: mild
d__Bacteria.p__Acidobacteria.c__Blastocatellia
900
600
300
0
asymptomatic (n=24)
mild (n=25)
severe (n=30)
control (n=72)
Severity

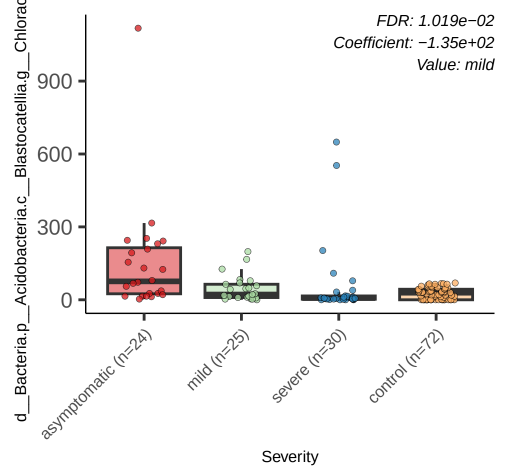

FDR: 1.019e−02
Coefficient: −1.35e+02
Value: mild
d__Bacteria.p__Acidobacteria.c__Blastocatellia.g__Chlorac
900
600
300
0
asymptomatic (n=24)
mild (n=25)
severe (n=30)
control (n=72)
Severity

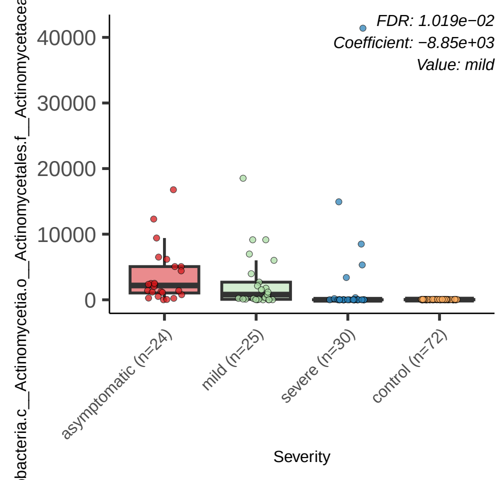

FDR: 1.019e−02
Coefficient: −8.85e+03
Value: mild
40000
30000
20000
10000
0
bacteria.c__Actinomycetia.o__Actinomycetales.f__Actinomycetacea
asymptomatic (n=24)
mild (n=25)
severe (n=30)
control (n=72)
Severity

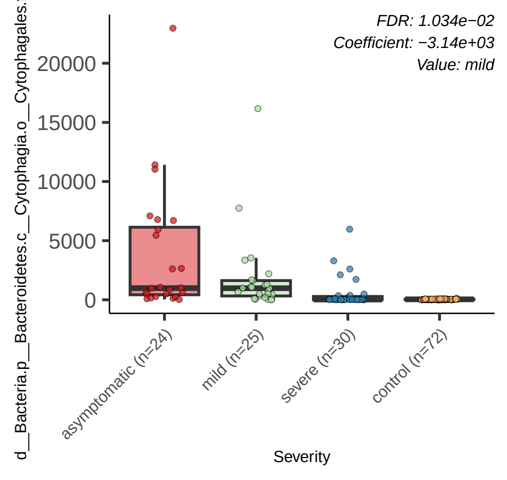
FDR: 1.034e−02
Coefficient: −3.14e+03
Value: mild
d__Bacteria.p__Bacteroidetes.c__Cytophagia.o__Cytophagales.
20000
15000
10000
5000
0
asymptomatic (n=24)
mild (n=25)
severe (n=30)
control (n=72)
Severity

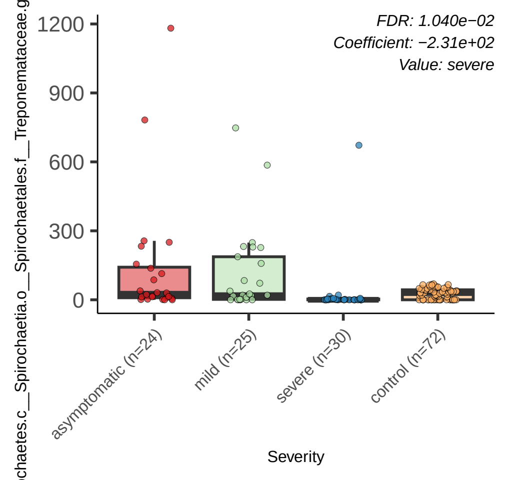

FDR: 1.040e−02
Coefficient: −2.31e+02
Value: severe
1200
900
600
300
0
chaetes.c__Spirochaetia.o__Spirochaetales.f__Treponemataceae.g
asymptomatic (n=24)
mild (n=25)
severe (n=30)
control (n=72)
Severity

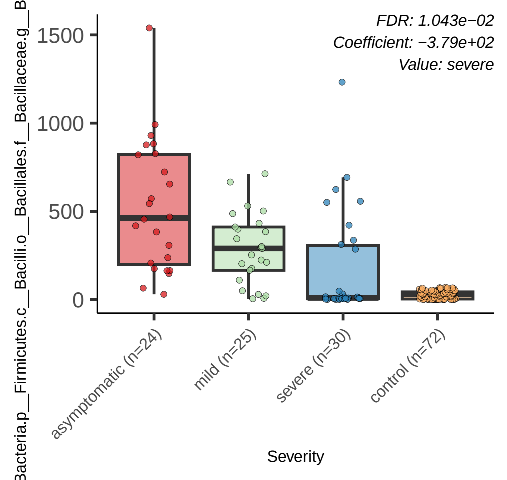

FDR: 1.043e−02
Coefficient: −3.79e+02
Value: severe
Bacteria.p__Firmicutes.c__Bacilli.o__Bacillales.f__Bacillaceae.g__B
1500
1000
500
0
asymptomatic (n=24)
mild (n=25)
severe (n=30)
control (n=72)
Severity

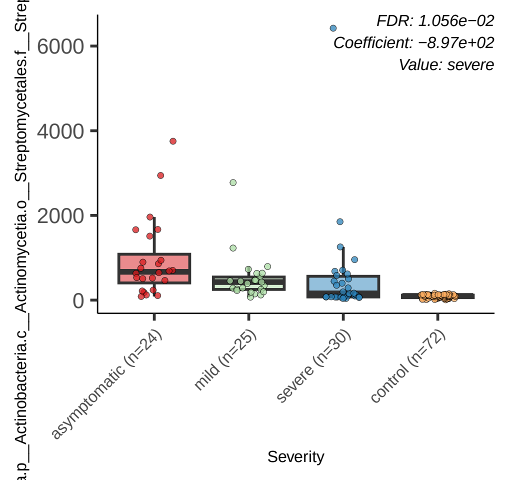

FDR: 1.056e−02
Coefficient: −8.97e+02
Value: severe
a.p__Actinobacteria.c__Actinomycetia.o__Streptomycetales.f__Strep
6000
4000
2000
0
asymptomatic (n=24)
mild (n=25)
severe (n=30)
control (n=72)
Severity

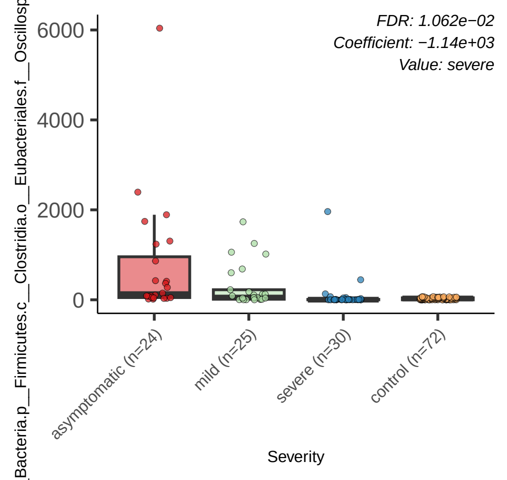
FDR: 1.062e−02
Coefficient: −1.14e+03
Value: severe
Bacteria.p__Firmicutes.c__Clostridia.o__Eubacteriales.f__Oscillosp
6000
4000
2000
0
asymptomatic (n=24)
mild (n=25)
severe (n=30)
control (n=72)
Severity

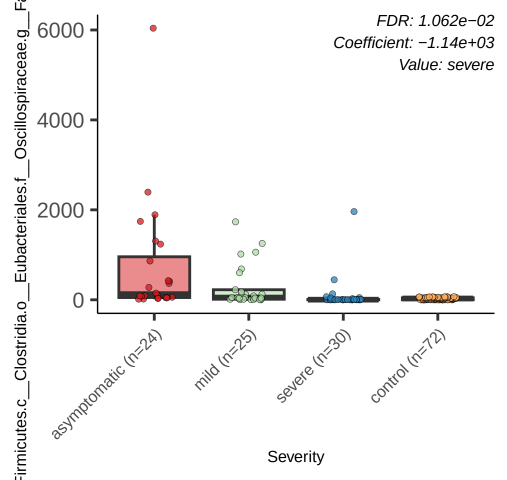

Firmicutes.c__Clostridia.o__Eubacteriales.f__Oscillospiraceae.g__Fa
FDR: 1.062e−02
Coefficient: −1.14e+03
Value: severe
6000
4000
2000
0
asymptomatic (n=24)
mild (n=25)
severe (n=30)
control (n=72)
Severity

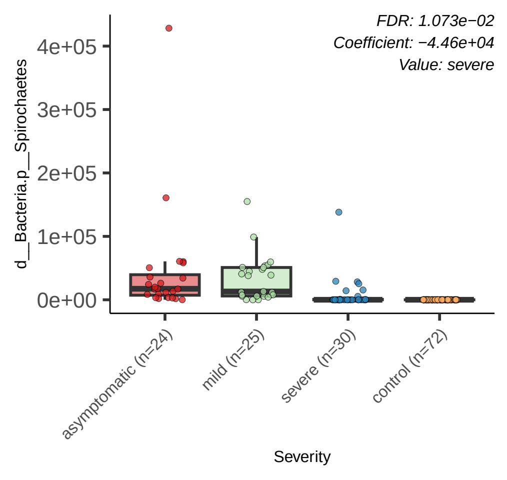

FDR: 1.073e−02
Coefficient: −4.46e+04
Value: severe
d__Bacteria.p__Spirochaetes
4e+05
3e+05
2e+05
1e+05
0e+00
asymptomatic (n=24)
mild (n=25)
severe (n=30)
control (n=72)
Severity

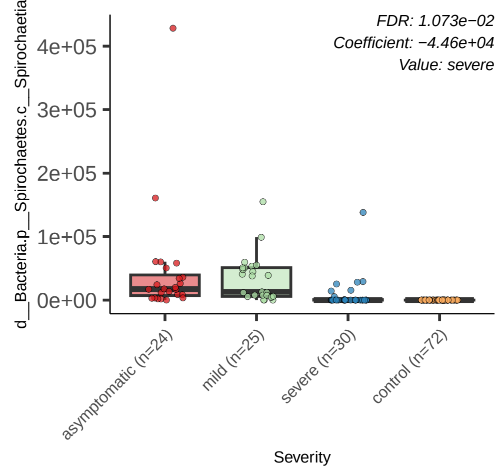
FDR: 1.073e−02
Coefficient: −4.46e+04
Value: severe
d__Bacteria.p__Spirochaetes.c__Spirochaetia
4e+05
3e+05
2e+05
1e+05
0e+00
asymptomatic (n=24)
mild (n=25)
severe (n=30)
control (n=72)
Severity

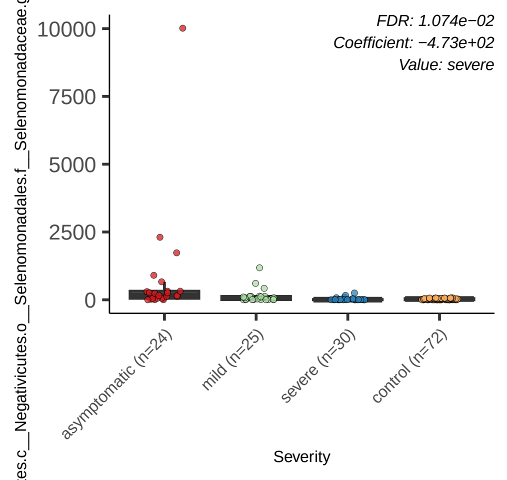

FDR: 1.074e−02
Coefficient: −4.73e+02
Value: severe
10000
7500
5000
2500
0
asymptomatic (n=24)
mild (n=25)
severe (n=30)
control (n=72)
Severity
tes.c__Negativicutes.o__Selenomonadales.f__Selenomonadaceae.g

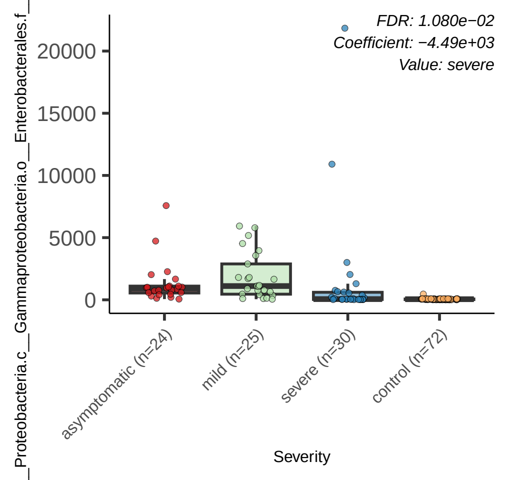

FDR: 1.080e−02
Coefficient: −4.49e+03
Value: severe
20000
15000
10000
5000
0
Proteobacteria.c__Gammaproteobacteria.o__Enterobacterales.f
asymptomatic (n=24)
mild (n=25)
severe (n=30)
control (n=72)
Severity

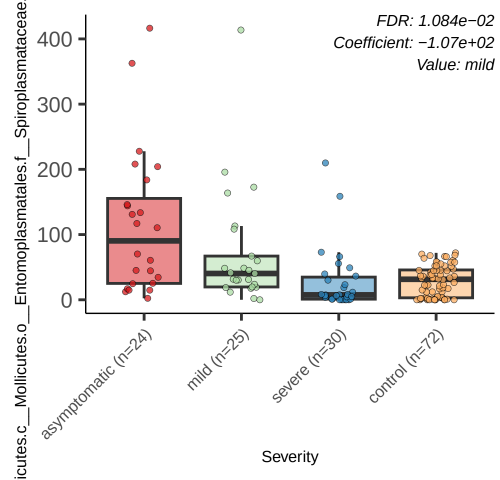

FDR: 1.084e−02
Coefficient: −1.07e+02
Value: mild
400
300
200
100
0
icutes.c__Mollicutes.o__Entomoplasmatales.f__Spiroplasmataceae.
asymptomatic (n=24)
mild (n=25)
severe (n=30)
control (n=72)
Severity

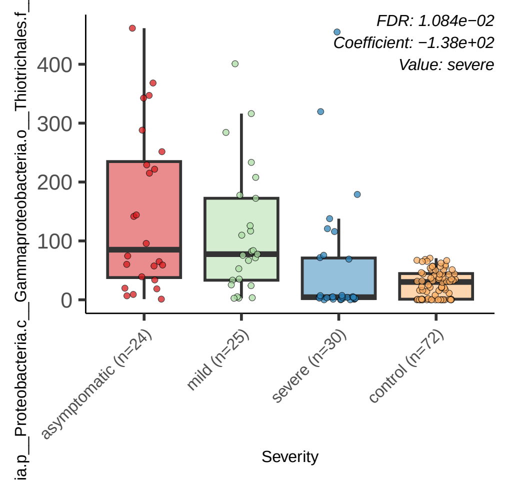
FDR: 1.084e−02
Coefficient: −1.38e+02
Value: severe
ia.p__Proteobacteria.c__Gammaproteobacteria.o__Thiotrichales.f__
0
100
200
300
400
asymptomatic (n=24)
mild (n=25)
severe (n=30)
control (n=72)
Severity

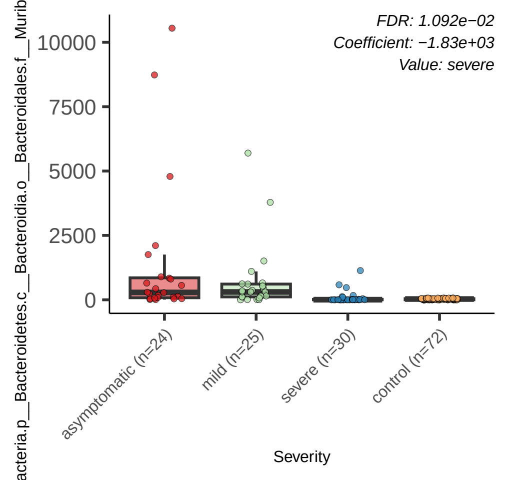

FDR: 1.092e−02
Coefficient: −1.83e+03
Value: severe
10000
7500
5000
2500
0
asymptomatic (n=24)
mild (n=25)
severe (n=30)
control (n=72)
Severity
acteria.p__Bacteroidetes.c__Bacteroidia.o__Bacteroidales.f__Murib

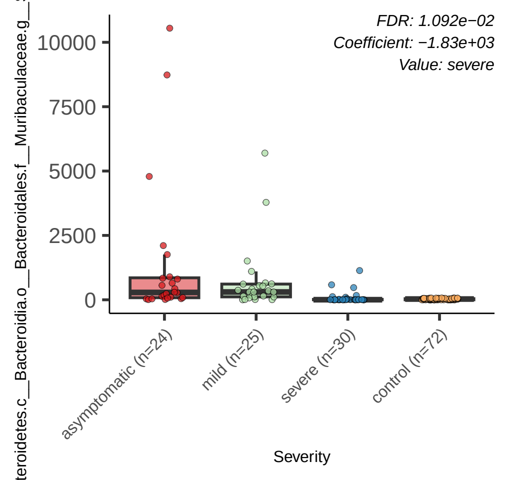
FDR: 1.092e−02
Coefficient: −1.83e+03
Value: severe
10000
7500
5000
2500
0
teroidetes.c__Bacteroidia.o__Bacteroidales.f__Muribaculaceae.g__
asymptomatic (n=24)
mild (n=25)
severe (n=30)
control (n=72)
Severity

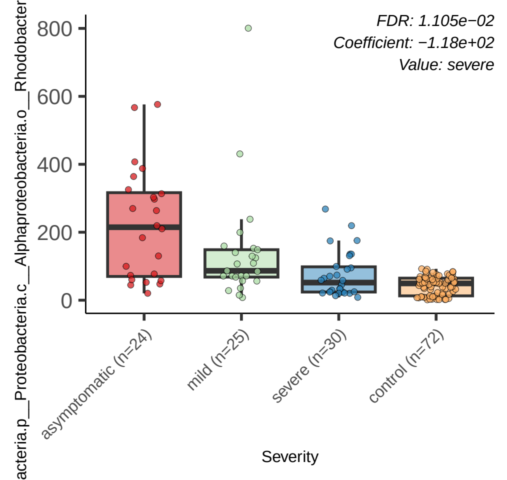

FDR: 1.105e−02
Coefficient: −1.18e+02
Value: severe
acteria.p__Proteobacteria.c__Alphaproteobacteria.o__Rhodobacter
800
600
400
200
0
asymptomatic (n=24)
mild (n=25)
severe (n=30)
control (n=72)
Severity

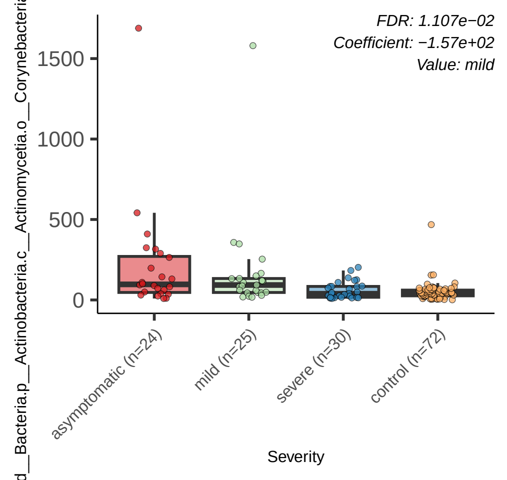

FDR: 1.107e−02
Coefficient: −1.57e+02
Value: mild
d__Bacteria.p__Actinobacteria.c__Actinomycetia.o__Corynebacteria
1500
1000
500
0
asymptomatic (n=24)
mild (n=25)
severe (n=30)
control (n=72)
Severity

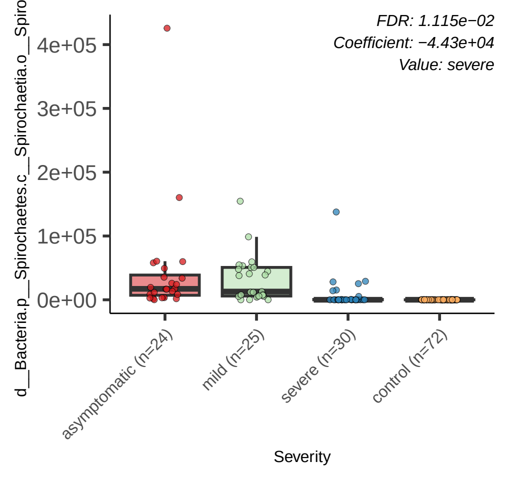

FDR: 1.115e−02
Coefficient: −4.43e+04
Value: severe
d__Bacteria.p__Spirochaetes.c__Spirochaetia.o__Spiro
4e+05
3e+05
2e+05
1e+05
0e+00
asymptomatic (n=24)
mild (n=25)
severe (n=30)
control (n=72)
Severity

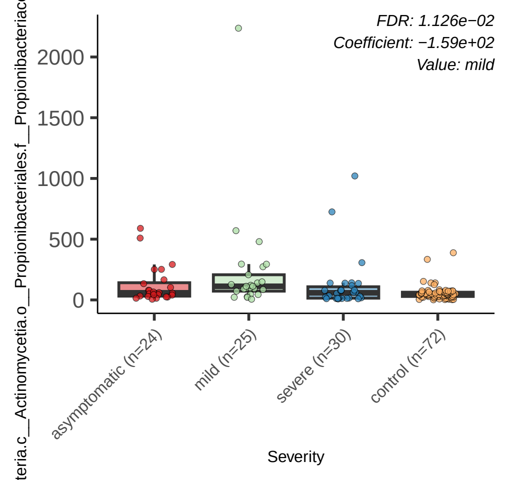

FDR: 1.126e−02
Coefficient: −1.59e+02
Value: mild
2000
1500
1000
500
0
asymptomatic (n=24)
mild (n=25)
severe (n=30)
control (n=72)
Severity

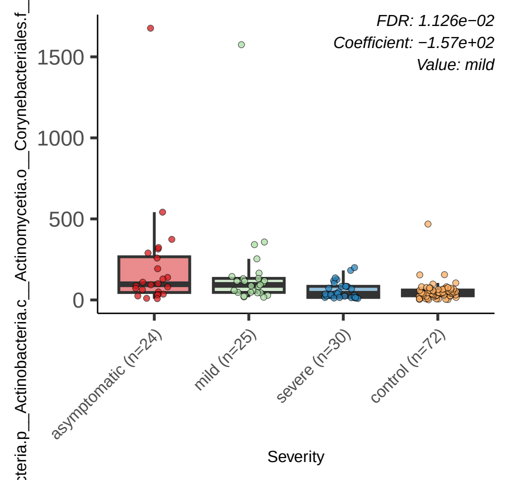

FDR: 1.126e−02
Coefficient: −1.57e+02
Value: mild
cteria.p__Actinobacteria.c__Actinomycetia.o__Corynebacteriales.f__
1500
1000
500
0
asymptomatic (n=24)
mild (n=25)
severe (n=30)
control (n=72)
Severity

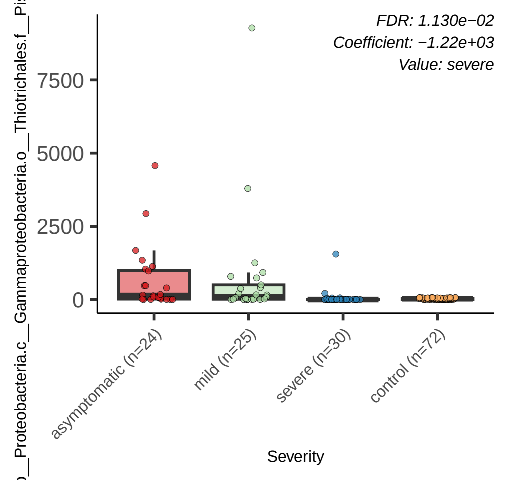

FDR: 1.130e−02
Coefficient: −1.22e+03
Value: severe
o__Proteobacteria.c__Gammaproteobacteria.o__Thiotrichales.f__Pis
7500
5000
2500
0
asymptomatic (n=24)
mild (n=25)
severe (n=30)
control (n=72)
Severity

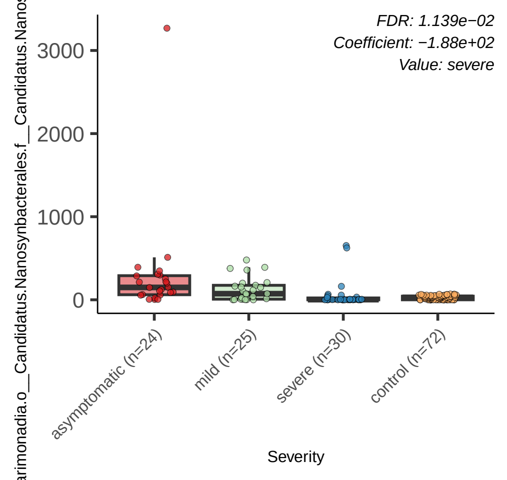
FDR: 1.139e−02
Coefficient: −1.88e+02
Value: severe
3000
2000
1000
0
arimonadia.o__Candidatus.Nanosynbacterales.f__Candidatus.Nanos
asymptomatic (n=24)
mild (n=25)
severe (n=30)
control (n=72)
Severity

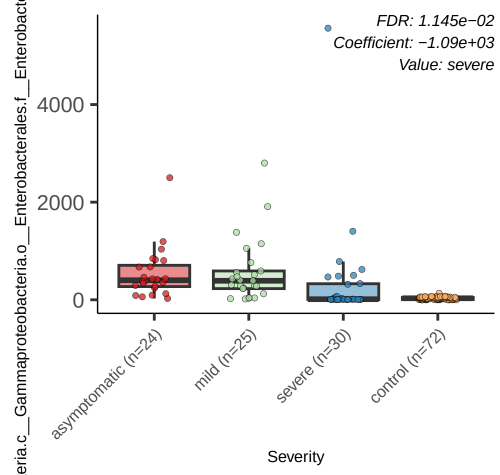

FDR: 1.145e−02
Coefficient: −1.09e+03
Value: severe
4000
2000
0
asymptomatic (n=24)
mild (n=25)
severe (n=30)
control (n=72)
Severity

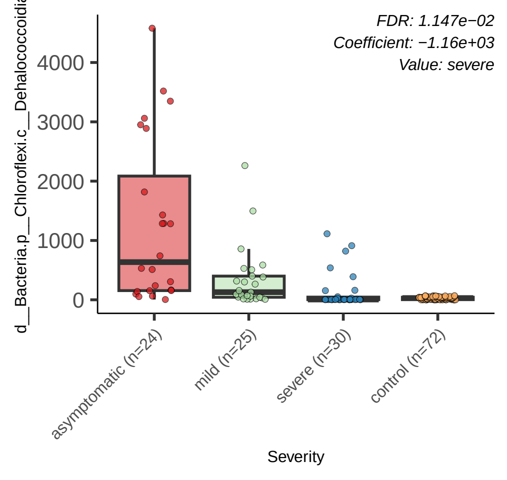

FDR: 1.147e−02
Coefficient: −1.16e+03
Value: severe
d__Bacteria.p__Chloroflexi.c__Dehalococcoidia
4000
3000
2000
1000
0
asymptomatic (n=24)
mild (n=25)
severe (n=30)
control (n=72)
Severity

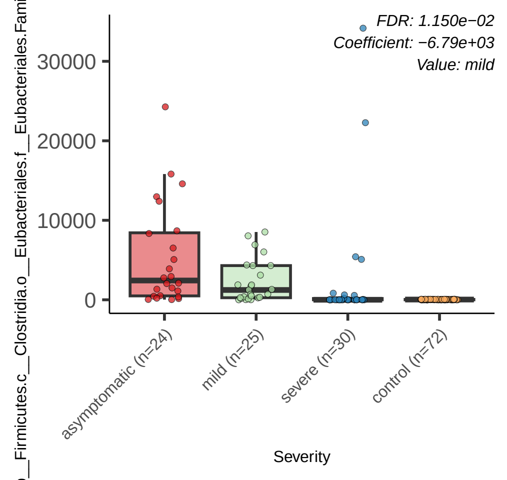

FDR: 1.150e−02
Coefficient: −6.79e+03
Value: mild
30000
20000
10000
0
o__Firmicutes.c__Clostridia.o__Eubacteriales.f__Eubacteriales.Fami
asymptomatic (n=24)
mild (n=25)
severe (n=30)
control (n=72)
Severity

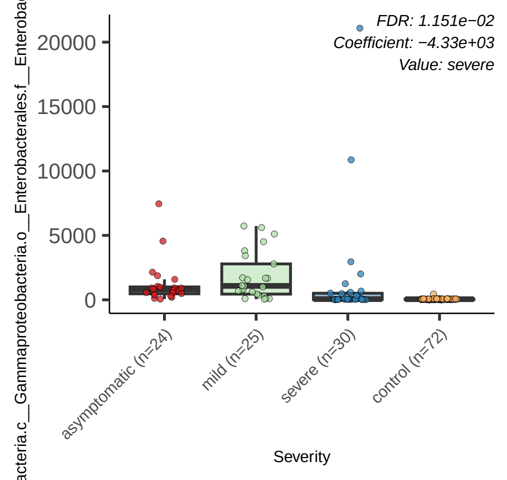

FDR: 1.151e−02
Coefficient: −4.33e+03
Value: severe
20000
15000
10000
5000
0
acteria.c__Gammaproteobacteria.o__Enterobacterales.f__Enterobac
asymptomatic (n=24)
mild (n=25)
severe (n=30)
control (n=72)
Severity

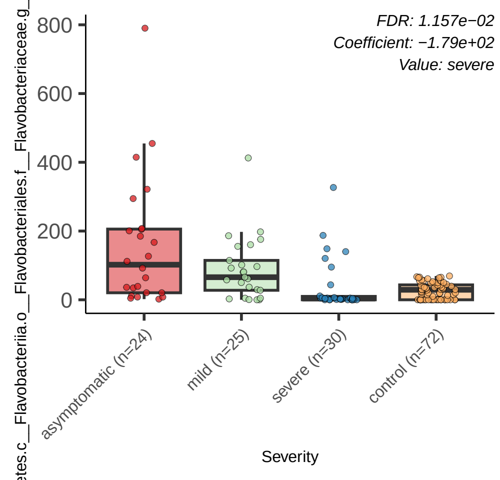

FDR: 1.157e−02
Coefficient: −1.79e+02
Value: severe
etes.c__Flavobacteriia.o__Flavobacteriales.f__Flavobacteriaceae.g__
800
600
400
200
0
asymptomatic (n=24)
mild (n=25)
severe (n=30)
control (n=72)
Severity

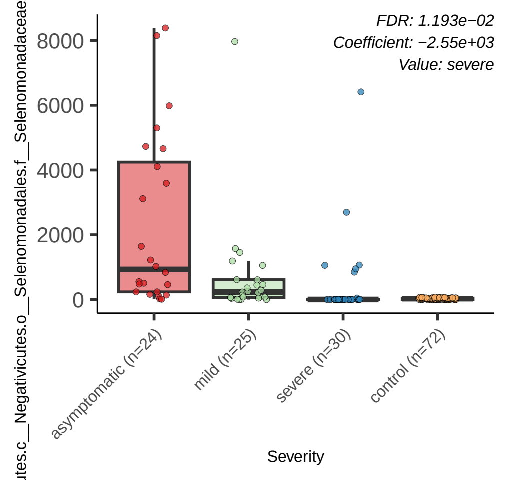
FDR: 1.193e−02
Coefficient: −2.55e+03
Value: severe
8000
6000
4000
2000
0
utes.c__Negativicutes.o__Selenomonadales.f__Selenomonadaceae
asymptomatic (n=24)
mild (n=25)
severe (n=30)
control (n=72)
Severity

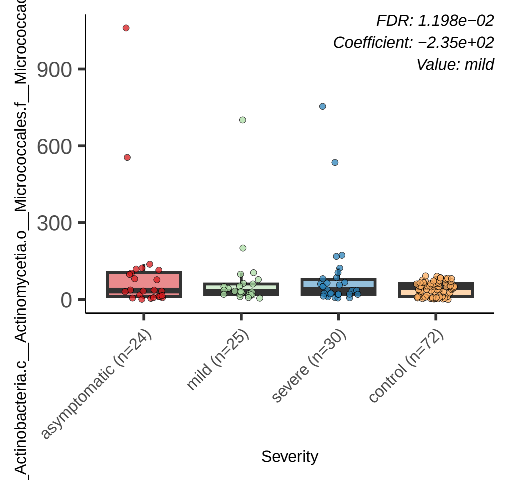
FDR: 1.198e−02
Coefficient: −2.35e+02
Value: mild
900
600
300
0
Actinobacteria.c__Actinomycetia.o__Micrococcales.f__Micrococcac
asymptomatic (n=24)
mild (n=25)
severe (n=30)
control (n=72)
Severity

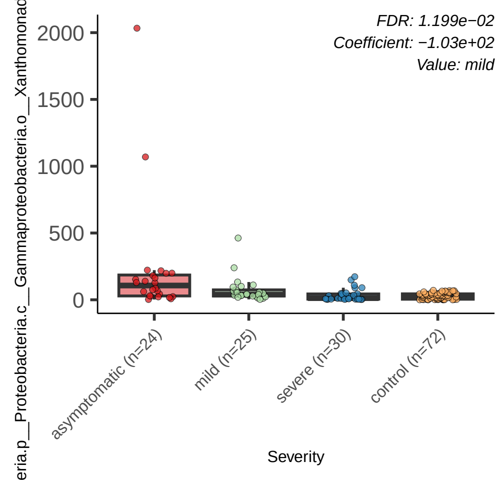

FDR: 1.199e−02
Coefficient: −1.03e+02
Value: mild
2000
1500
1000
500
0
eria.p__Proteobacteria.c__Gammaproteobacteria.o__Xanthomonad
asymptomatic (n=24)
mild (n=25)
severe (n=30)
control (n=72)
Severity

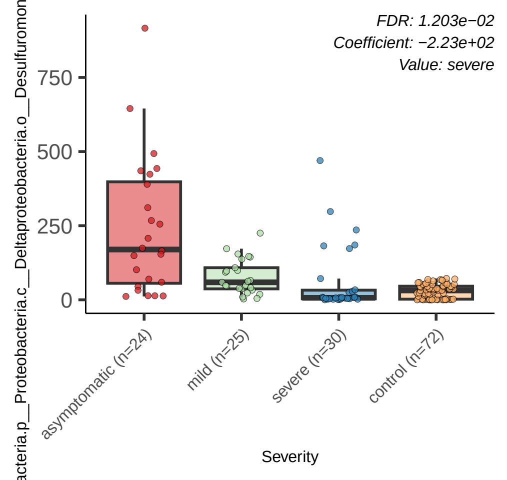

FDR: 1.203e−02
Coefficient: −2.23e+02
Value: severe
acteria.p__Proteobacteria.c__Deltaproteobacteria.o__Desulfuromon
750
500
250
0
asymptomatic (n=24)
mild (n=25)
severe (n=30)
control (n=72)
Severity

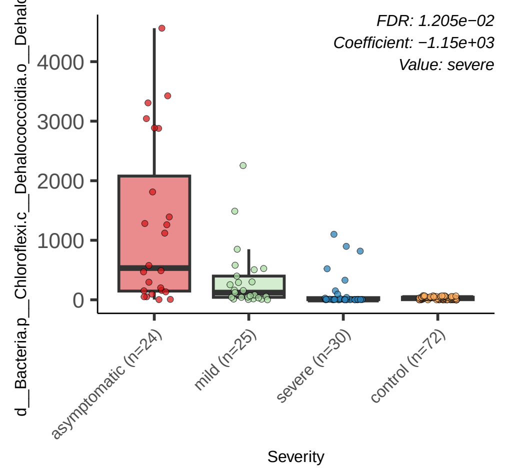

FDR: 1.205e−02
Coefficient: −1.15e+03
Value: severe
d__Bacteria.p__Chloroflexi.c__Dehalococcoidia.o__Dehalo
0
1000
2000
3000
4000
asymptomatic (n=24)
mild (n=25)
severe (n=30)
control (n=72)
Severity

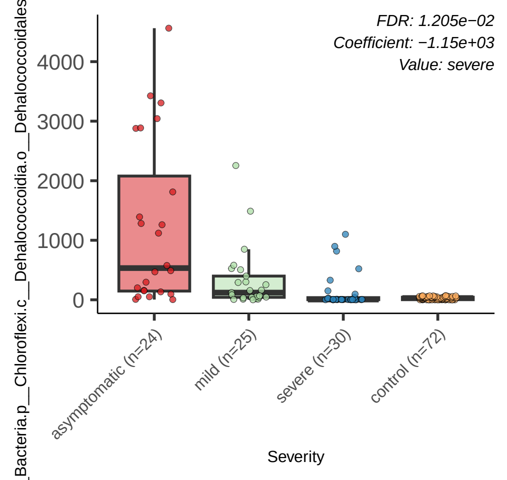

FDR: 1.205e−02
Coefficient: −1.15e+03
Value: severe
4000
3000
2000
1000
0
Bacteria.p__Chloroflexi.c__Dehalococcoidia.o__Dehalococcoidales
asymptomatic (n=24)
mild (n=25)
severe (n=30)
control (n=72)
Severity

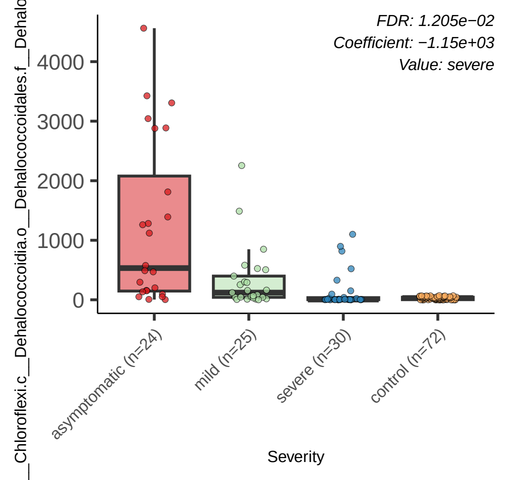
FDR: 1.205e−02
Coefficient: −1.15e+03
Value: severe
4000
3000
2000
1000
0
Chloroflexi.c__Dehalococcoidia.o__Dehalococcoidales.f__Dehalo
asymptomatic (n=24)
mild (n=25)
severe (n=30)
control (n=72)
Severity

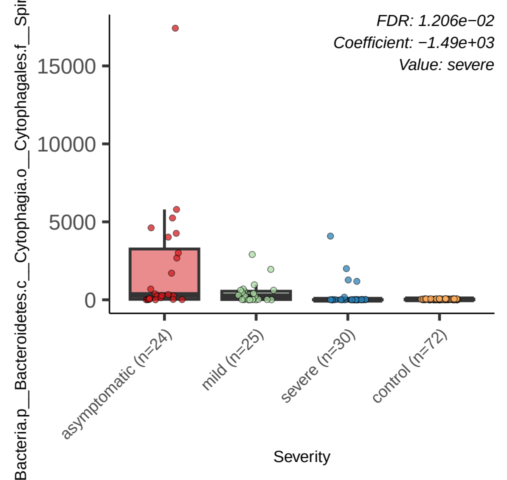

FDR: 1.206e−02
Coefficient: −1.49e+03
Value: severe
Bacteria.p__Bacteroidetes.c__Cytophagia.o__Cytophagales.f__Spi
15000
10000
5000
0
asymptomatic (n=24)
mild (n=25)
severe (n=30)
control (n=72)
Severity

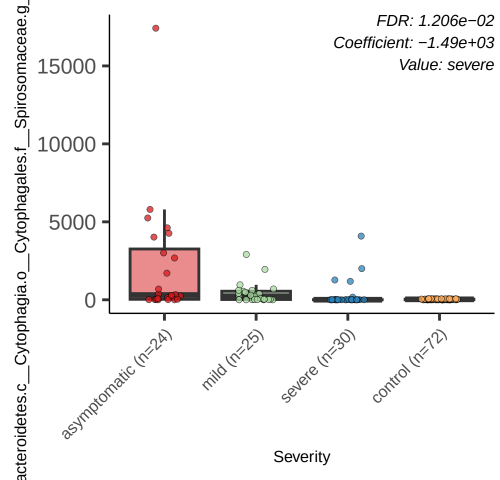

FDR: 1.206e−02
Coefficient: −1.49e+03
Value: severe
15000
10000
5000
0
acteroidetes.c__Cytophagia.o__Cytophagales.f__Spirosomaceae.g_
asymptomatic (n=24)
mild (n=25)
severe (n=30)
control (n=72)
Severity

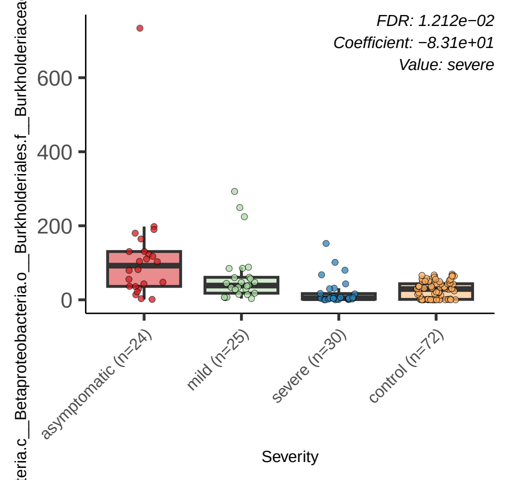

FDR: 1.212e−02
Coefficient: −8.31e+01
Value: severe
teria.c__Betaproteobacteria.o__Burkholderiales.f__Burkholderiacea
600
400
200
0
asymptomatic (n=24)
mild (n=25)
severe (n=30)
control (n=72)
Severity

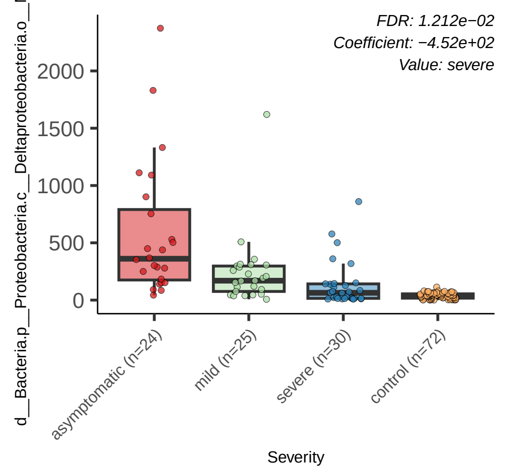
FDR: 1.212e−02
Coefficient: −4.52e+02
Value: severe
d__Bacteria.p__Proteobacteria.c__Deltaproteobacteria.o__
0
500
1000
1500
2000
asymptomatic (n=24)
mild (n=25)
severe (n=30)
control (n=72)
Severity

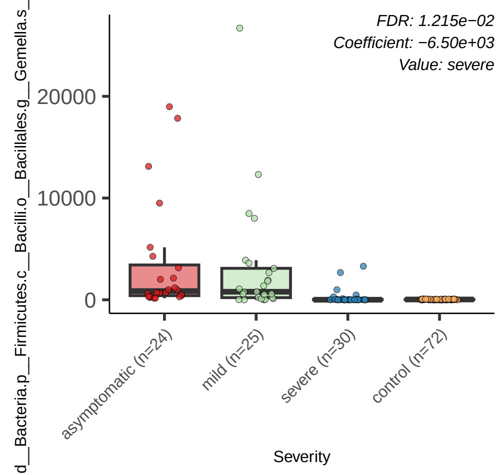

FDR: 1.215e−02
Coefficient: −6.50e+03
Value: severe
d__Bacteria.p__Firmicutes.c__Bacilli.o__Bacillales.g__Gemella.s__
20000
10000
0
asymptomatic (n=24)
mild (n=25)
severe (n=30)
control (n=72)
Severity

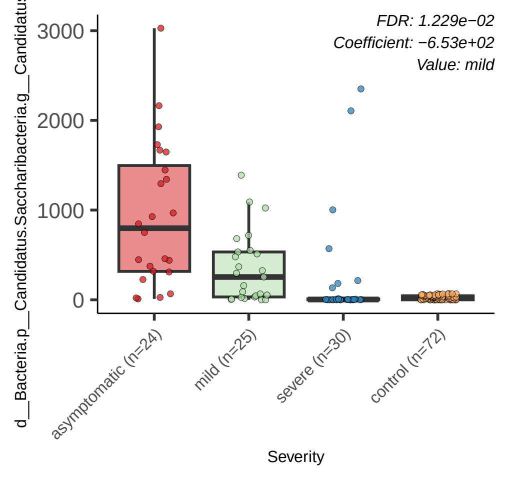

FDR: 1.229e−02
Coefficient: −6.53e+02
Value: mild
d__Bacteria.p__Candidatus.Saccharibacteria.g__Candidatus
3000
2000
1000
0
asymptomatic (n=24)
mild (n=25)
severe (n=30)
control (n=72)
Severity

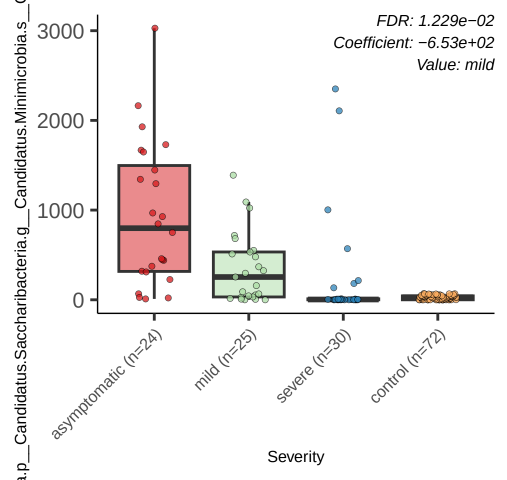

FDR: 1.229e−02
Coefficient: −6.53e+02
Value: mild
3000
2000
1000
0
a.p__Candidatus.Saccharibacteria.g__Candidatus.Minimicrobia.s__C
asymptomatic (n=24)
mild (n=25)
severe (n=30)
control (n=72)
Severity

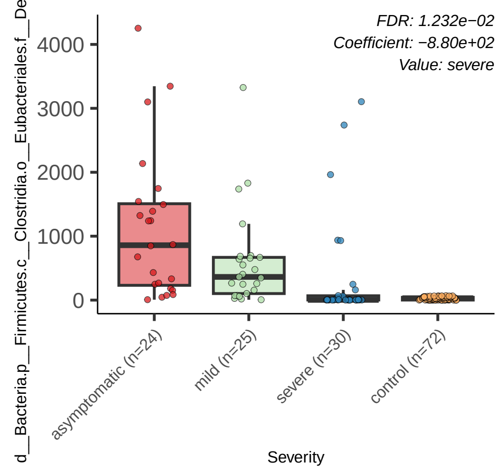
FDR: 1.232e−02
Coefficient: −8.80e+02
Value: severe
d__Bacteria.p__Firmicutes.c__Clostridia.o__Eubacteriales.f__De
4000
3000
2000
1000
0
asymptomatic (n=24)
mild (n=25)
severe (n=30)
control (n=72)
Severity

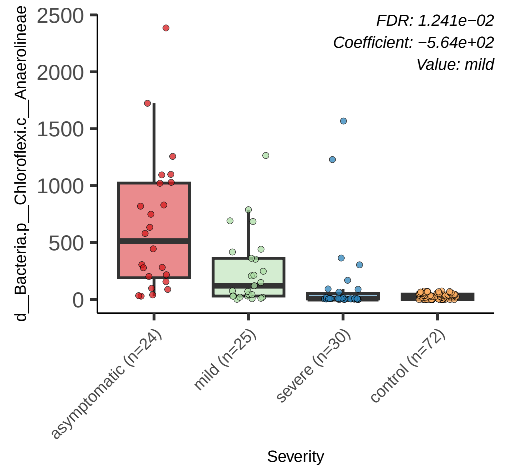

FDR: 1.241e−02
Coefficient: −5.64e+02
Value: mild
d__Bacteria.p__Chloroflexi.c__Anaerolineae
0
500
1000
1500
2000
2500
asymptomatic (n=24)
mild (n=25)
severe (n=30)
control (n=72)
Severity

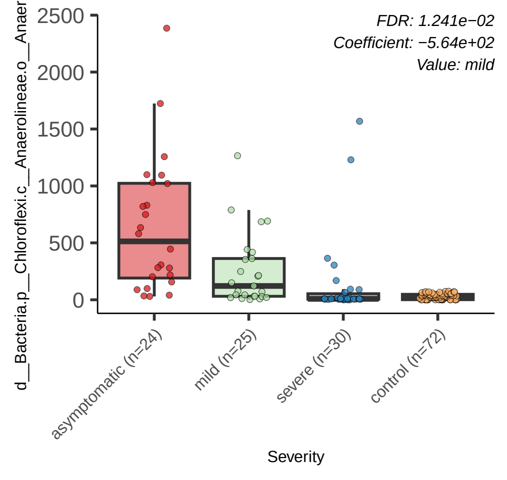

FDR: 1.241e−02
Coefficient: −5.64e+02
Value: mild
d__Bacteria.p__Chloroflexi.c__Anaerolineae.o__Anaer
2500
2000
1500
1000
500
0
asymptomatic (n=24)
mild (n=25)
severe (n=30)
control (n=72)
Severity

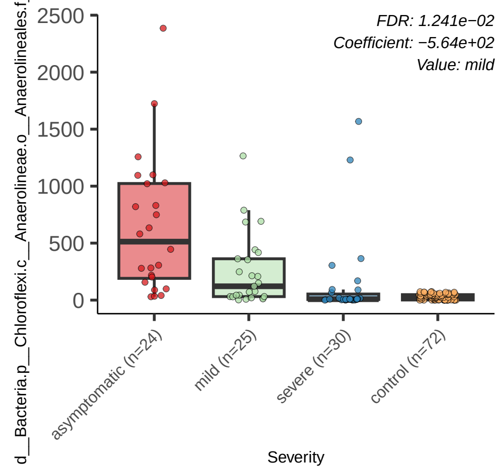

FDR: 1.241e−02
Coefficient: −5.64e+02
Value: mild
d__Bacteria.p__Chloroflexi.c__Anaerolineae.o__Anaerolineales.f
2500
2000
1500
1000
500
0
asymptomatic (n=24)
mild (n=25)
severe (n=30)
control (n=72)
Severity

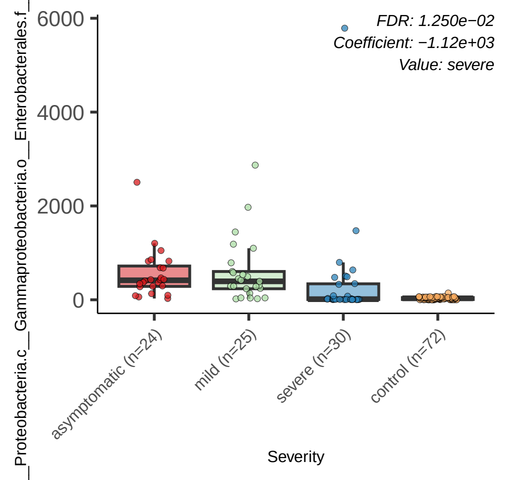

FDR: 1.250e−02
Coefficient: −1.12e+03
Value: severe
6000
4000
2000
0
Proteobacteria.c__Gammaproteobacteria.o__Enterobacterales.f
asymptomatic (n=24)
mild (n=25)
severe (n=30)
control (n=72)
Severity

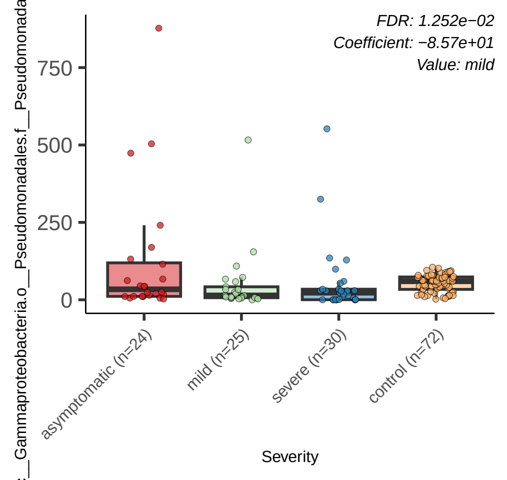
FDR: 1.252e−02
Coefficient: −8.57e+01
Value: mild
750
500
250
0
Gammaproteobacteria.o__Pseudomonadales.f__Pseudomonada
asymptomatic (n=24)
mild (n=25)
severe (n=30)
control (n=72)
Severity

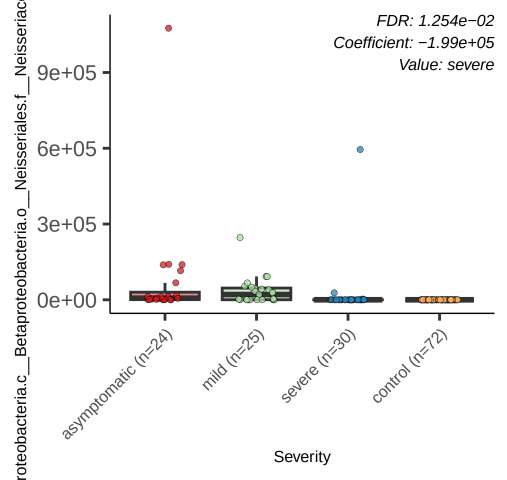
FDR: 1.254e−02
Coefficient: −1.99e+05
Value: severe
9e+05
6e+05
3e+05
0e+00
asymptomatic (n=24)
mild (n=25)
severe (n=30)
control (n=72)
Severity
roteobacteria.c__Betaproteobacteria.o__Neisseriales.f__Neisseriac

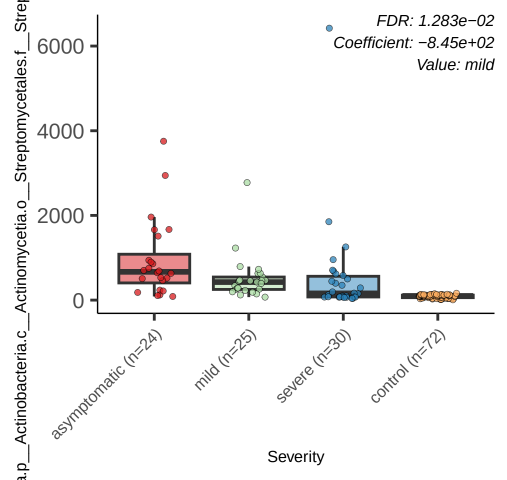
FDR: 1.283e−02
Coefficient: −8.45e+02
Value: mild
a.p__Actinobacteria.c__Actinomycetia.o__Streptomycetales.f__Strep
6000
4000
2000
0
asymptomatic (n=24)
mild (n=25)
severe (n=30)
control (n=72)
Severity

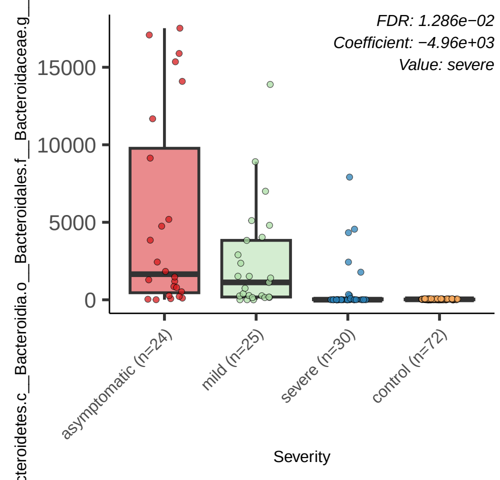

FDR: 1.286e−02
Coefficient: −4.96e+03
Value: severe
15000
10000
5000
0
cteroidetes.c__Bacteroidia.o__Bacteroidales.f__Bacteroidaceae.g__
asymptomatic (n=24)
mild (n=25)
severe (n=30)
control (n=72)
Severity

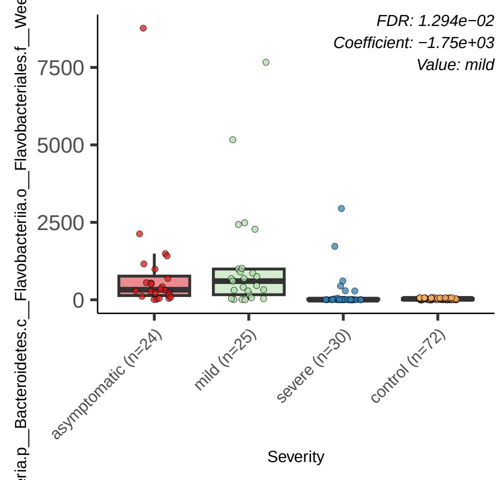

FDR: 1.294e−02
Coefficient: −1.75e+03
Value: mild
7500
5000
2500
0
asymptomatic (n=24)
mild (n=25)
severe (n=30)
control (n=72)
Severity

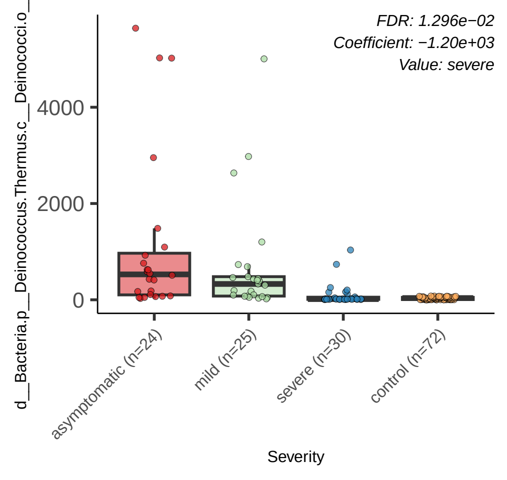

FDR: 1.296e−02
Coefficient: −1.20e+03
Value: severe
d__Bacteria.p__Deinococcus.Thermus.c__Deinococci.o__
0
2000
4000
asymptomatic (n=24)
mild (n=25)
severe (n=30)
control (n=72)
Severity

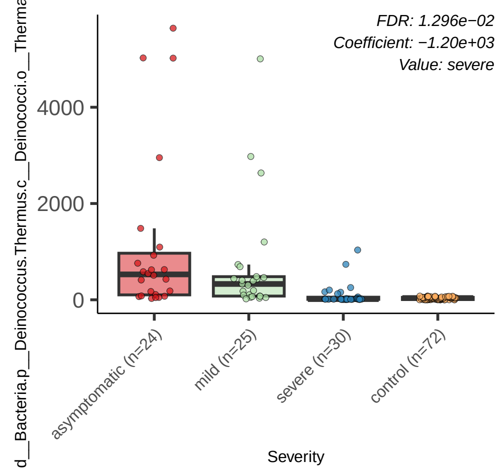

FDR: 1.296e−02
Coefficient: −1.20e+03
Value: severe
4000
2000
0
d__Bacteria.p__Deinococcus.Thermus.c__Deinococci.o__Therma
asymptomatic (n=24)
mild (n=25)
severe (n=30)
control (n=72)
Severity

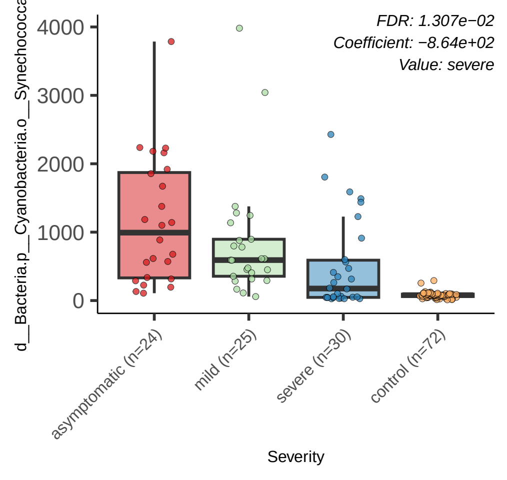
FDR: 1.307e−02
Coefficient: −8.64e+02
Value: severe
d__Bacteria.p__Cyanobacteria.o__Synechococca
4000
3000
2000
1000
0
asymptomatic (n=24)
mild (n=25)
severe (n=30)
control (n=72)
Severity

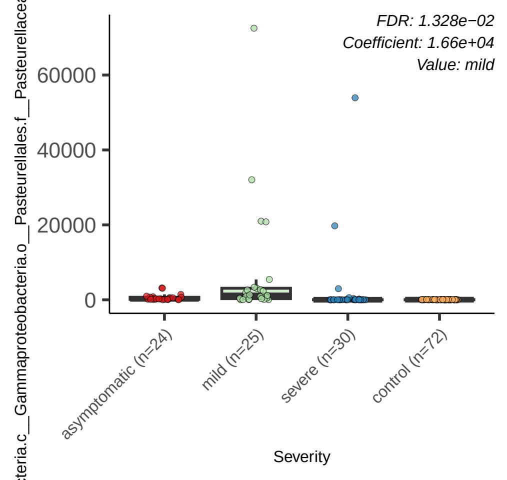

FDR: 1.328e−02
Coefficient: 1.66e+04
Value: mild
60000
40000
20000
0
cteria.c__Gammaproteobacteria.o__Pasteurellales.f__Pasteurellacea
asymptomatic (n=24)
mild (n=25)
severe (n=30)
control (n=72)
Severity

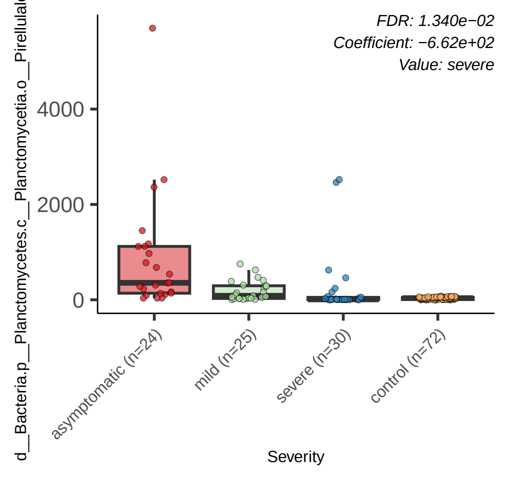

FDR: 1.340e−02
Coefficient: −6.62e+02
Value: severe
d__Bacteria.p__Planctomycetes.c__Planctomycetia.o__Pirellulal
0
2000
4000
asymptomatic (n=24)
mild (n=25)
severe (n=30)
control (n=72)
Severity

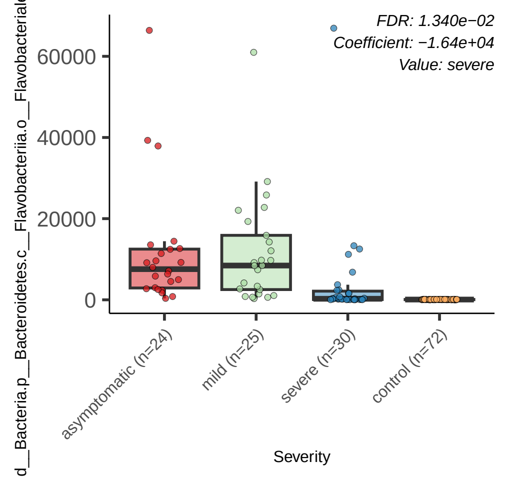
FDR: 1.340e−02
Coefficient: −1.64e+04
Value: severe
60000
40000
20000
0
asymptomatic (n=24)
mild (n=25)
severe (n=30)
control (n=72)
Severity
d__Bacteria.p__Bacteroidetes.c__Flavobacteriia.o__Flavobacteriale

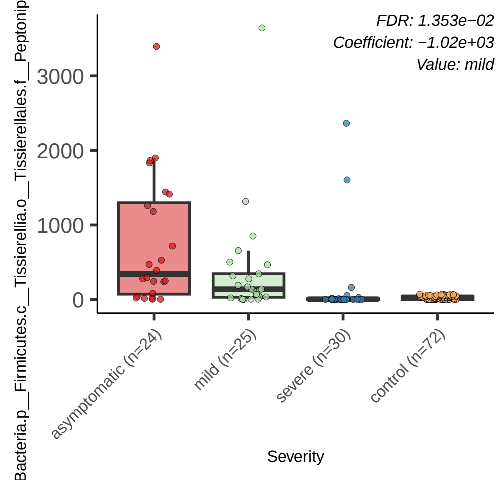

FDR: 1.353e−02
Coefficient: −1.02e+03
Value: mild
Bacteria.p__Firmicutes.c__Tissierellia.o__Tissierellales.f__Peptonip
3000
2000
1000
0
asymptomatic (n=24)
mild (n=25)
severe (n=30)
control (n=72)
Severity

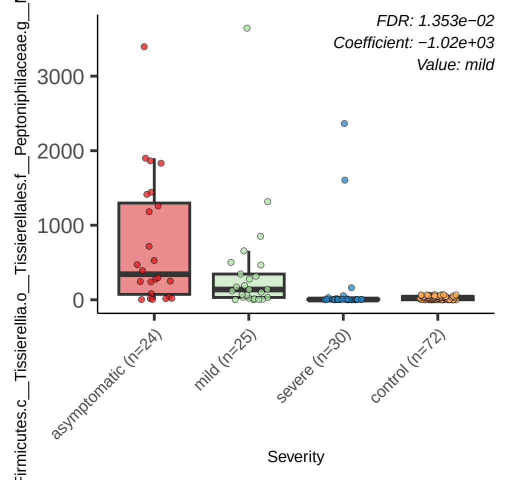

FDR: 1.353e−02
Coefficient: −1.02e+03
Value: mild
Firmicutes.c__Tissierellia.o__Tissierellales.f__Peptoniphilaceae.g__
3000
2000
1000
0
asymptomatic (n=24)
mild (n=25)
severe (n=30)
control (n=72)
Severity

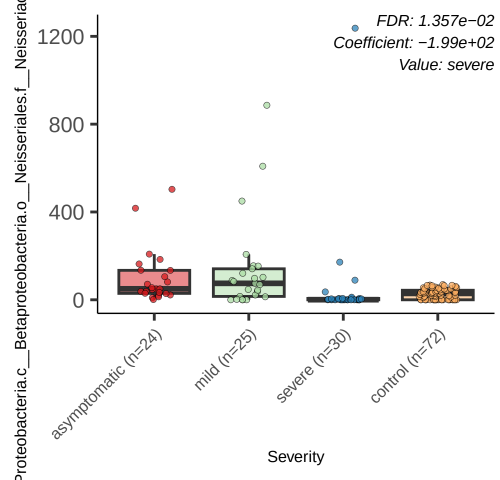

FDR: 1.357e−02
Coefficient: −1.99e+02
Value: severe
1200
800
400
0
Proteobacteria.c__Betaproteobacteria.o__Neisseriales.f__Neisseriac
asymptomatic (n=24)
mild (n=25)
severe (n=30)
control (n=72)
Severity

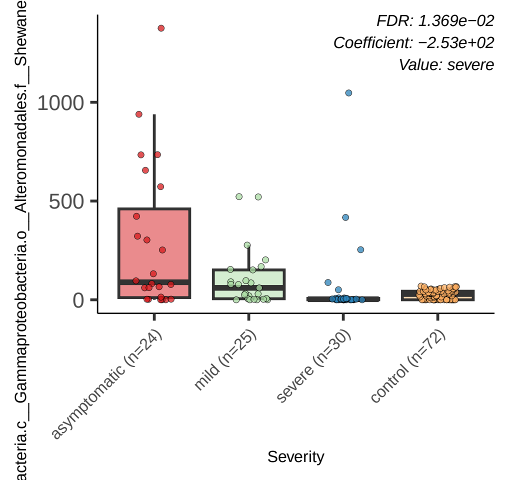

FDR: 1.369e−02
Coefficient: −2.53e+02
Value: severe
1000
500
0
acteria.c__Gammaproteobacteria.o__Alteromonadales.f__Shewane
asymptomatic (n=24)
mild (n=25)
severe (n=30)
control (n=72)
Severity

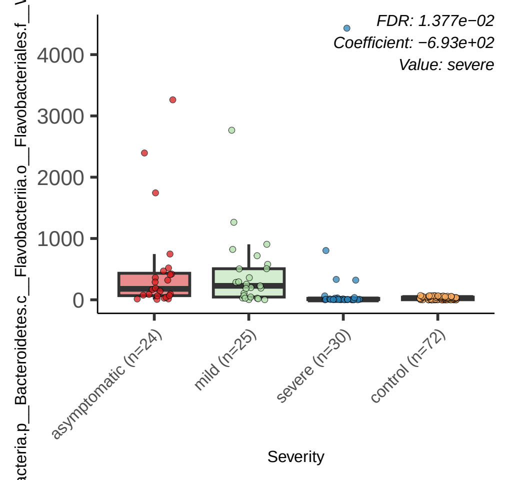

FDR: 1.377e−02
Coefficient: −6.93e+02
Value: severe
0
1000
2000
3000
4000
acteria.p__Bacteroidetes.c__Flavobacteriia.o__Flavobacteriales.f__W
asymptomatic (n=24)
mild (n=25)
severe (n=30)
control (n=72)
Severity

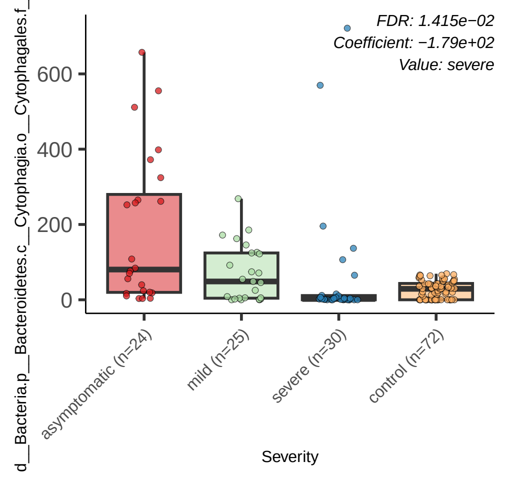

FDR: 1.415e−02
Coefficient: −1.79e+02
Value: severe
d__Bacteria.p__Bacteroidetes.c__Cytophagia.o__Cytophagales.f__
0
200
400
600
asymptomatic (n=24)
mild (n=25)
severe (n=30)
control (n=72)
Severity

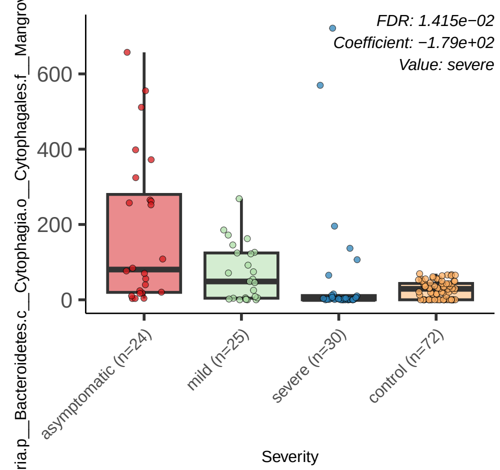
FDR: 1.415e−02
Coefficient: −1.79e+02
Value: severe
0
200
400
600
asymptomatic (n=24)
mild (n=25)
severe (n=30)
control (n=72)
Severity
teria.p__Bacteroidetes.c__Cytophagia.o__Cytophagales.f__Mangrov

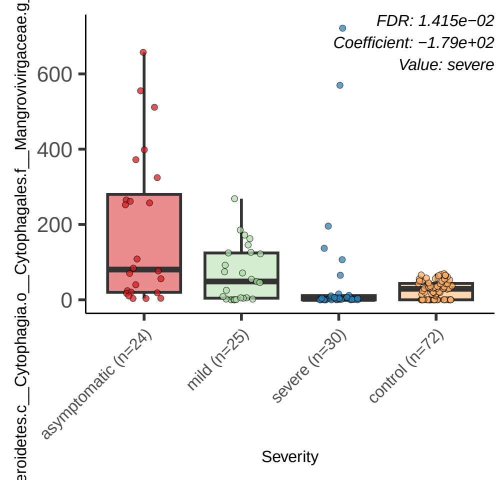
FDR: 1.415e−02
Coefficient: −1.79e+02
Value: severe
600
400
200
0
eroidetes.c__Cytophagia.o__Cytophagales.f__Mangrovivirgaceae.g
asymptomatic (n=24)
mild (n=25)
severe (n=30)
control (n=72)
Severity

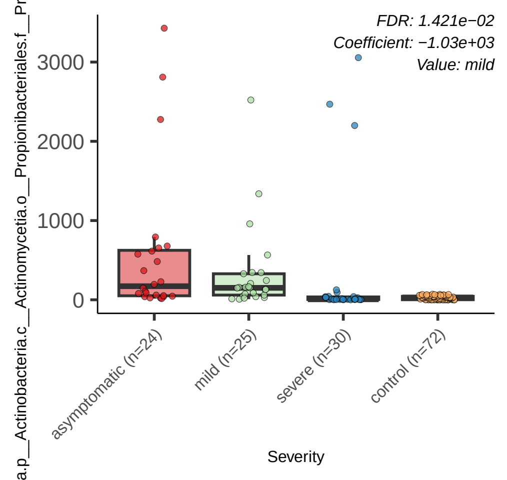

FDR: 1.421e−02
Coefficient: −1.03e+03
Value: mild
a.p__Actinobacteria.c__Actinomycetia.o__Propionibacteriales.f__Pr
3000
2000
1000
0
asymptomatic (n=24)
mild (n=25)
severe (n=30)
control (n=72)
Severity

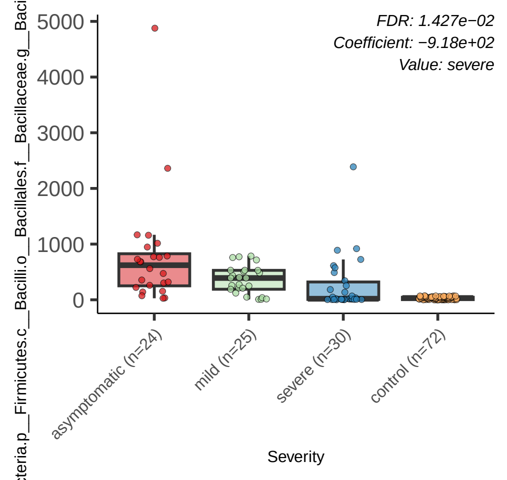

FDR: 1.427e−02
Coefficient: −9.18e+02
Value: severe
5000
4000
3000
2000
1000
0
asymptomatic (n=24)
mild (n=25)
severe (n=30)
control (n=72)
Severity
cteria.p__Firmicutes.c__Bacilli.o__Bacillales.f__Bacillaceae.g__Baci

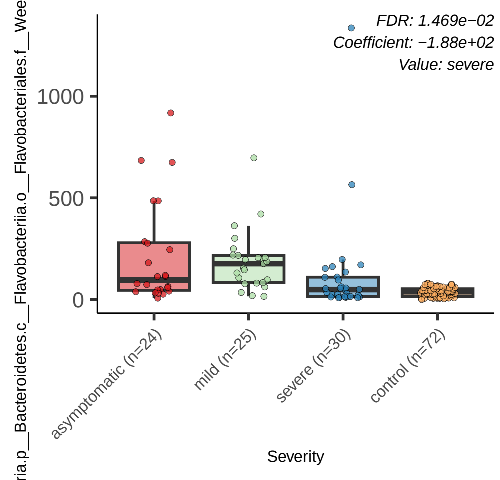

FDR: 1.469e−02
Coefficient: −1.88e+02
Value: severe
1000
500
0
ria.p__Bacteroidetes.c__Flavobacteriia.o__Flavobacteriales.f__Wee
asymptomatic (n=24)
mild (n=25)
severe (n=30)
control (n=72)
Severity

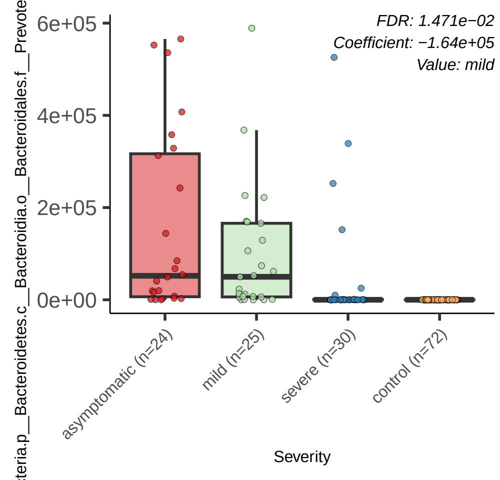
FDR: 1.471e−02
Coefficient: −1.64e+05
Value: mild
teria.p__Bacteroidetes.c__Bacteroidia.o__Bacteroidales.f__Prevote
6e+05
4e+05
2e+05
0e+00
asymptomatic (n=24)
mild (n=25)
severe (n=30)
control (n=72)
Severity

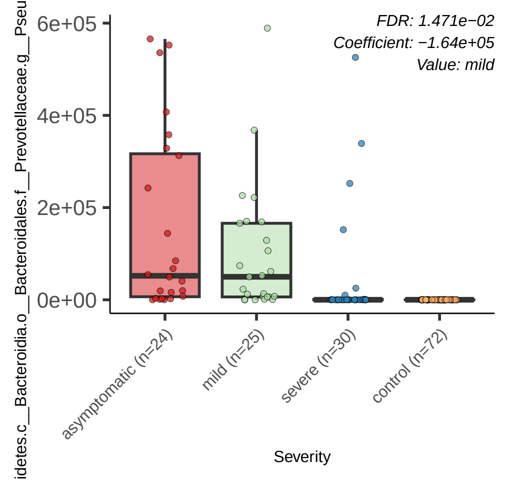
FDR: 1.471e−02
Coefficient: −1.64e+05
Value: mild
Bacteroidetes.c__Bacteroidia.o__Bacteroidales.f__Prevotellaceae.g__Pseu
6e+05
4e+05
2e+05
0e+00
asymptomatic (n=24)
mild (n=25)
severe (n=30)
control (n=72)
Severity

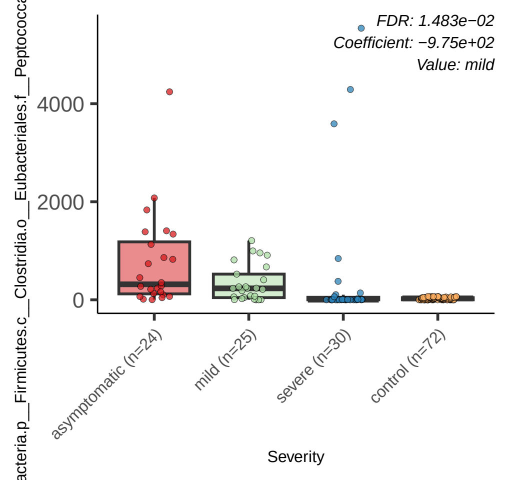

FDR: 1.483e−02
Coefficient: −9.75e+02
Value: mild
4000
2000
0
acteria.p__Firmicutes.c__Clostridia.o__Eubacteriales.f__Peptococca
asymptomatic (n=24)
mild (n=25)
severe (n=30)
control (n=72)
Severity

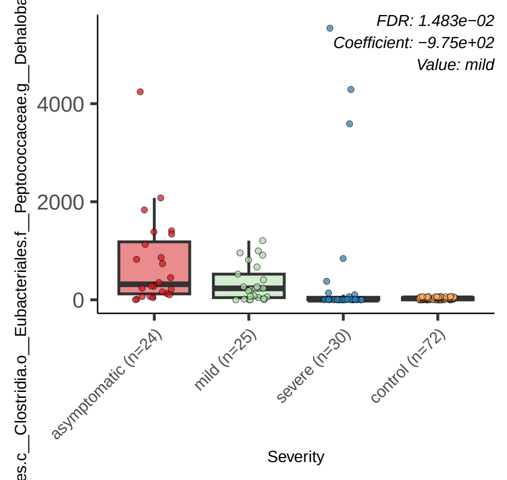

FDR: 1.483e−02
Coefficient: −9.75e+02
Value: mild
4000
2000
0
asymptomatic (n=24)
mild (n=25)
severe (n=30)
control (n=72)
Severity
es.c__Clostridia.o__Eubacteriales.f__Peptococcaceae.g__Dehaloba

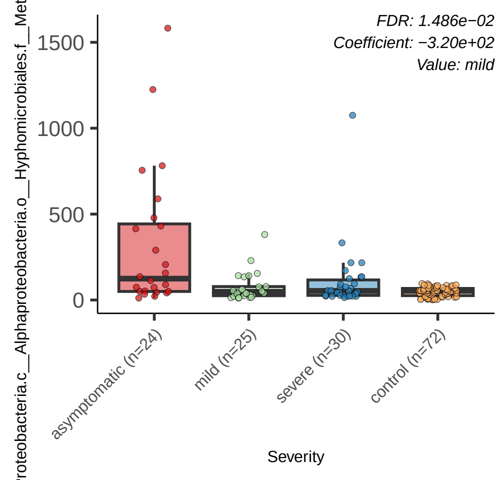
FDR: 1.486e−02
Coefficient: −3.20e+02
Value: mild
Proteobacteria.c__Alphaproteobacteria.o__Hyphomicrobiales.f__Met
1500
1000
500
0
asymptomatic (n=24)
mild (n=25)
severe (n=30)
control (n=72)
Severity

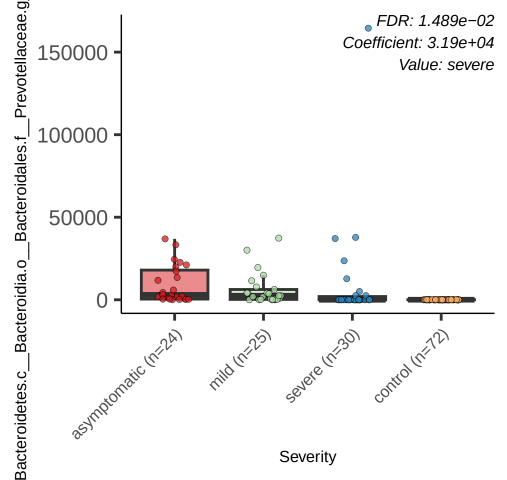

FDR: 1.489e−02
Coefficient: 3.19e+04
Value: severe
150000
100000
50000
0
asymptomatic (n=24)
mild (n=25)
severe (n=30)
control (n=72)
Severity
Bacteroidetes.c__Bacteroidia.o__Bacteroidales.f__Prevotellaceae.g

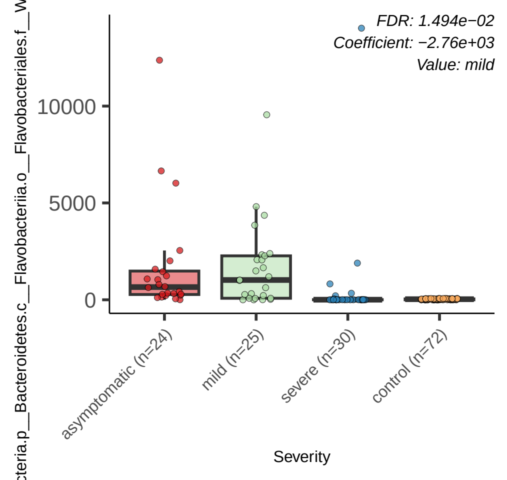

FDR: 1.494e−02
Coefficient: −2.76e+03
Value: mild
10000
5000
0
asymptomatic (n=24)
mild (n=25)
severe (n=30)
control (n=72)
Severity
cteria.p__Bacteroidetes.c__Flavobacteriia.o__Flavobacteriales.f__W

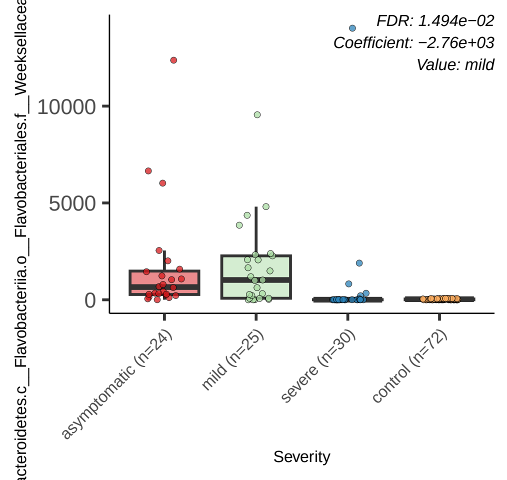
FDR: 1.494e−02
Coefficient: −2.76e+03
Value: mild
10000
5000
0
acteroidetes.c__Flavobacteriia.o__Flavobacteriales.f__Weeksellacea
asymptomatic (n=24)
mild (n=25)
severe (n=30)
control (n=72)
Severity

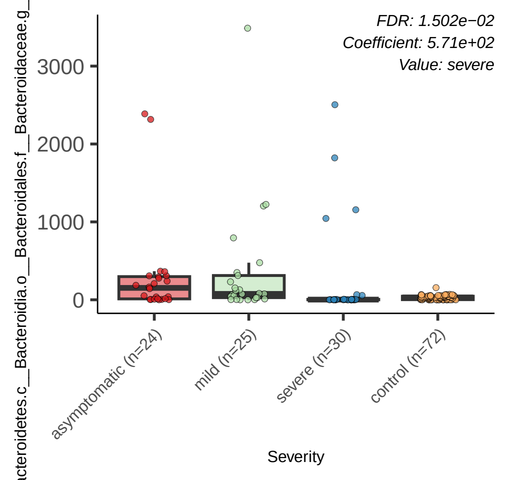
FDR: 1.502e−02
Coefficient: 5.71e+02
Value: severe
3000
2000
1000
0
acteroidetes.c__Bacteroidia.o__Bacteroidales.f__Bacteroidaceae.g__
asymptomatic (n=24)
mild (n=25)
severe (n=30)
control (n=72)
Severity

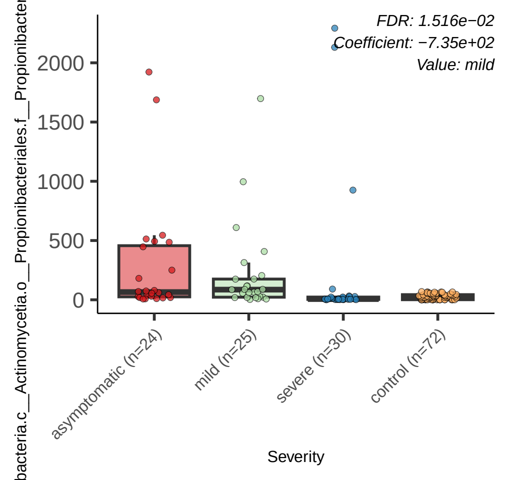

FDR: 1.516e−02
Coefficient: −7.35e+02
Value: mild
2000
1500
1000
500
0
bacteria.c__Actinomycetia.o__Propionibacteriales.f__Propionibacter
asymptomatic (n=24)
mild (n=25)
severe (n=30)
control (n=72)
Severity

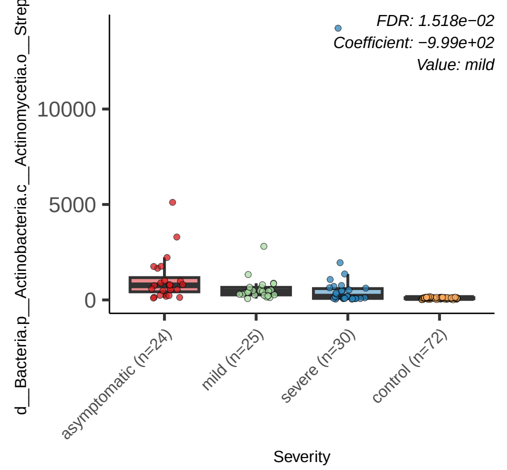
FDR: 1.518e−02
Coefficient: −9.99e+02
Value: mild
10000
5000
0
d__Bacteria.p__Actinobacteria.c__Actinomycetia.o__Strep
asymptomatic (n=24)
mild (n=25)
severe (n=30)
control (n=72)
Severity

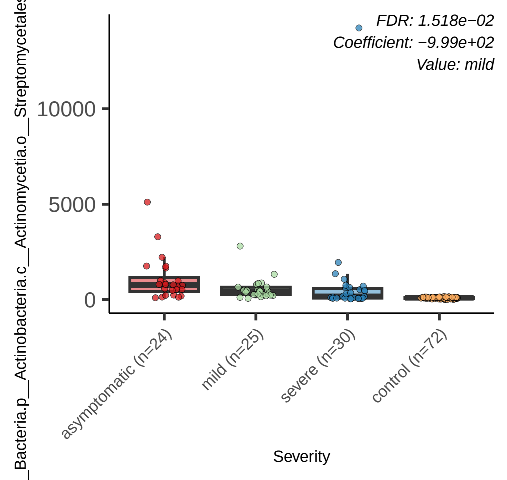

FDR: 1.518e−02
Coefficient: −9.99e+02
Value: mild
10000
5000
0
Bacteria.p__Actinobacteria.c__Actinomycetia.o__Streptomycetales
asymptomatic (n=24)
mild (n=25)
severe (n=30)
control (n=72)
Severity

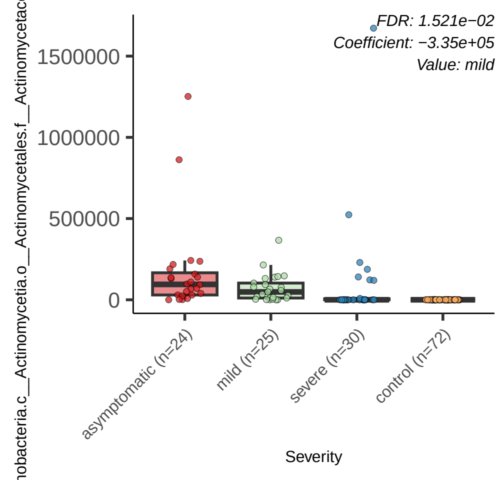

nobacteria.c__Actinomycetia.o__Actinomycetales.f__Actinomycetac
FDR: 1.521e−02
Coefficient: −3.35e+05
Value: mild
1500000
1000000
500000
0
asymptomatic (n=24)
mild (n=25)
severe (n=30)
control (n=72)
Severity

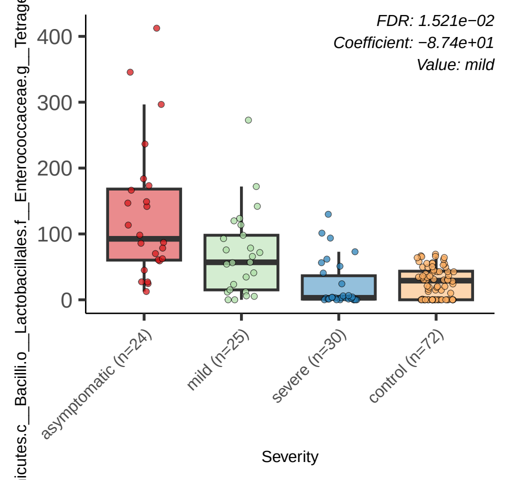

FDR: 1.521e−02
Coefficient: −8.74e+01
Value: mild
400
300
200
100
0
icutes.c__Bacilli.o__Lactobacillales.f__Enterococcaceae.g__Tetrage
asymptomatic (n=24)
mild (n=25)
severe (n=30)
control (n=72)
Severity

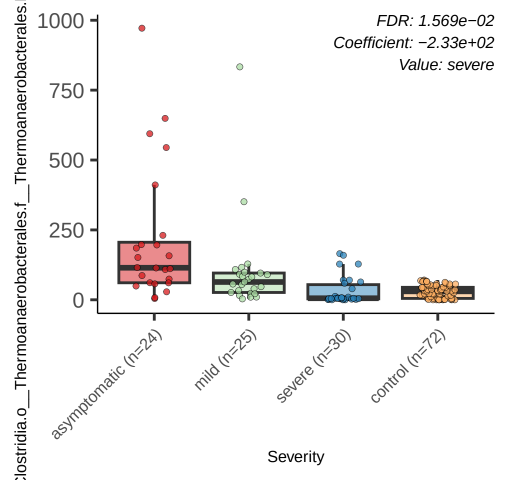

FDR: 1.569e−02
Coefficient: −2.33e+02
Value: severe
1000
750
500
250
0
Clostridia.o__Thermoanaerobacterales.f__Thermoanaerobacterales.
asymptomatic (n=24)
mild (n=25)
severe (n=30)
control (n=72)
Severity

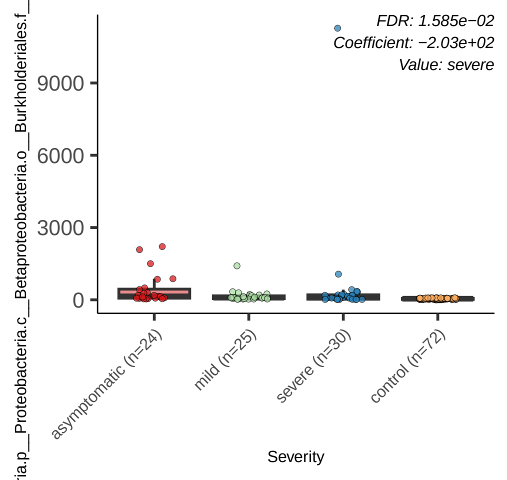
FDR: 1.585e−02
Coefficient: −2.03e+02
Value: severe
9000
6000
3000
0
ria.p__Proteobacteria.c__Betaproteobacteria.o__Burkholderiales.f__
asymptomatic (n=24)
mild (n=25)
severe (n=30)
control (n=72)
Severity

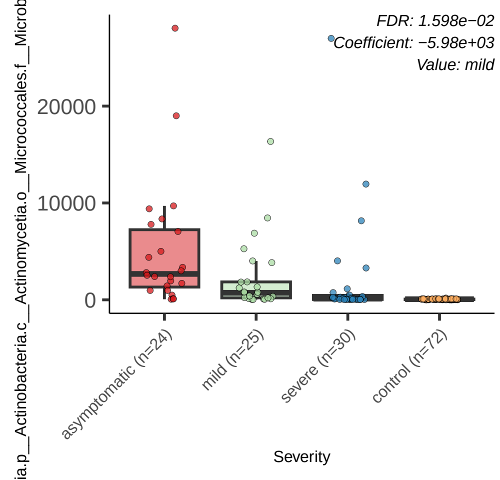

FDR: 1.598e−02
Coefficient: −5.98e+03
Value: mild
20000
10000
0
ia.p__Actinobacteria.c__Actinomycetia.o__Micrococcales.f__Microb
asymptomatic (n=24)
mild (n=25)
severe (n=30)
control (n=72)
Severity

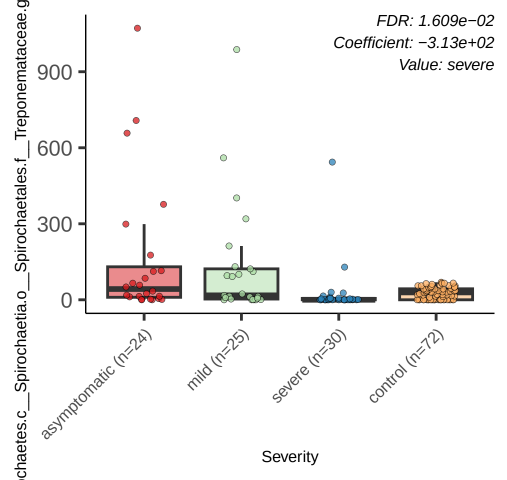

FDR: 1.609e−02
Coefficient: −3.13e+02
Value: severe
900
600
300
0
asymptomatic (n=24)
mild (n=25)
severe (n=30)
control (n=72)
Severity
chaetes.c__Spirochaetia.o__Spirochaetales.f__Treponemataceae.g

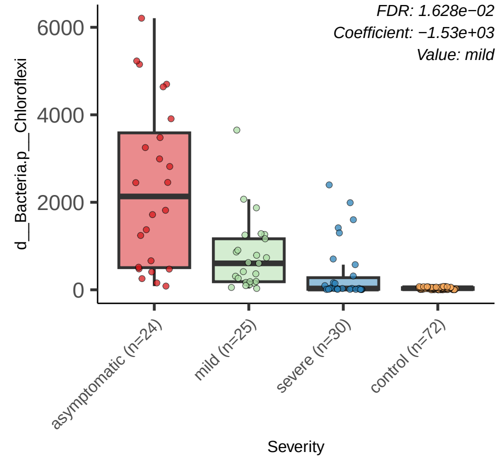
FDR: 1.628e−02
Coefficient: −1.53e+03
Value: mild
6000
4000
2000
0
d__Bacteria.p__Chloroflexi
asymptomatic (n=24)
mild (n=25)
severe (n=30)
control (n=72)
Severity

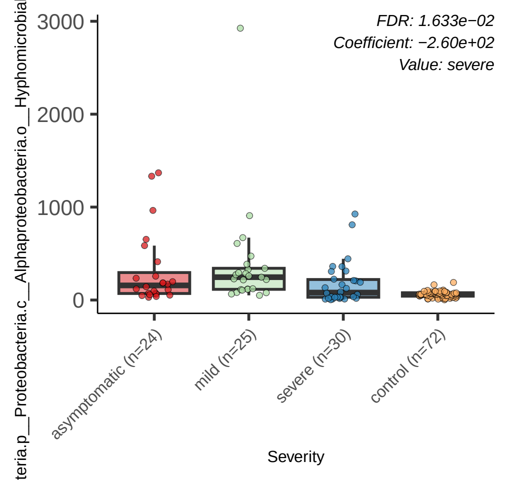

FDR: 1.633e−02
Coefficient: −2.60e+02
Value: severe
3000
2000
1000
0
teria.p__Proteobacteria.c__Alphaproteobacteria.o__Hyphomicrobial
asymptomatic (n=24)
mild (n=25)
severe (n=30)
control (n=72)
Severity

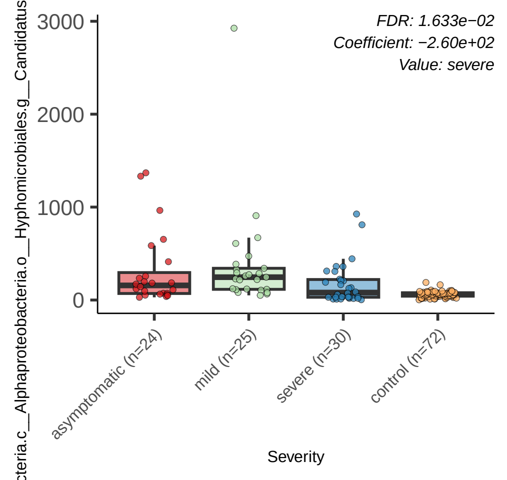
FDR: 1.633e−02
Coefficient: −2.60e+02
Value: severe
3000
2000
1000
0
cteria.c__Alphaproteobacteria.o__Hyphomicrobiales.g__Candidatus
asymptomatic (n=24)
mild (n=25)
severe (n=30)
control (n=72)
Severity

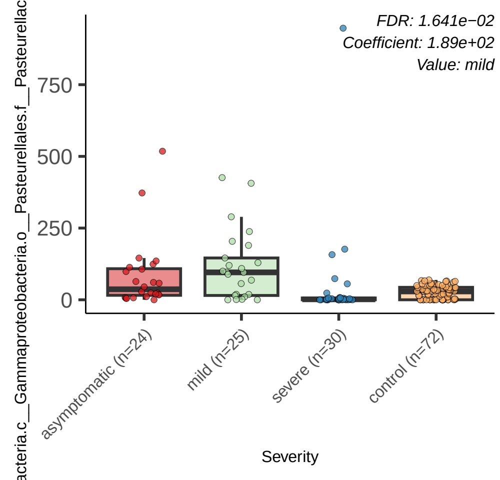

FDR: 1.641e−02
Coefficient: 1.89e+02
Value: mild
0
250
500
750
acteria.c__Gammaproteobacteria.o__Pasteurellales.f__Pasteurellac
asymptomatic (n=24)
mild (n=25)
severe (n=30)
control (n=72)
Severity

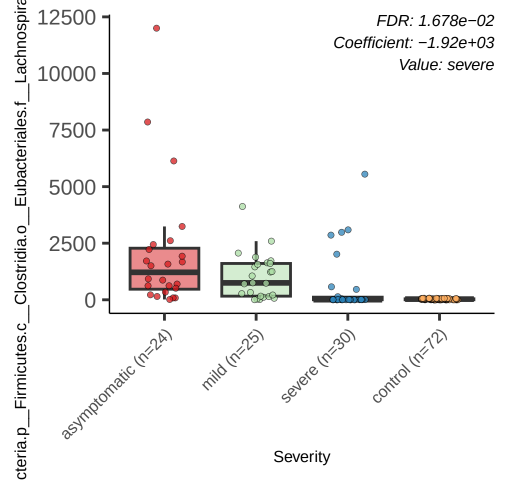

FDR: 1.678e−02
Coefficient: −1.92e+03
Value: severe
cteria.p__Firmicutes.c__Clostridia.o__Eubacteriales.f__Lachnospira
12500
10000
7500
5000
2500
0
asymptomatic (n=24)
mild (n=25)
severe (n=30)
control (n=72)
Severity

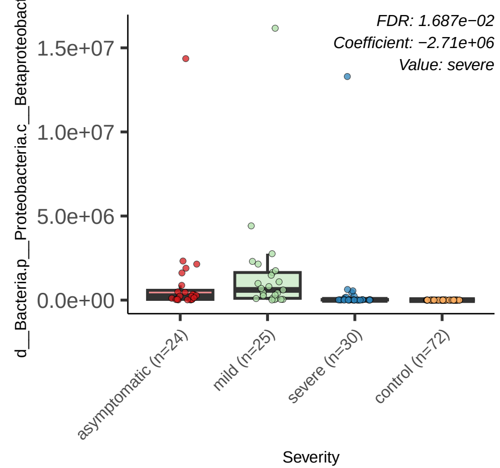

FDR: 1.687e−02
Coefficient: −2.71e+06
Value: severe
d__Bacteria.p__Proteobacteria.c__Betaproteobact
1.5e+07
1.0e+07
5.0e+06
0.0e+00
asymptomatic (n=24)
mild (n=25)
severe (n=30)
control (n=72)
Severity

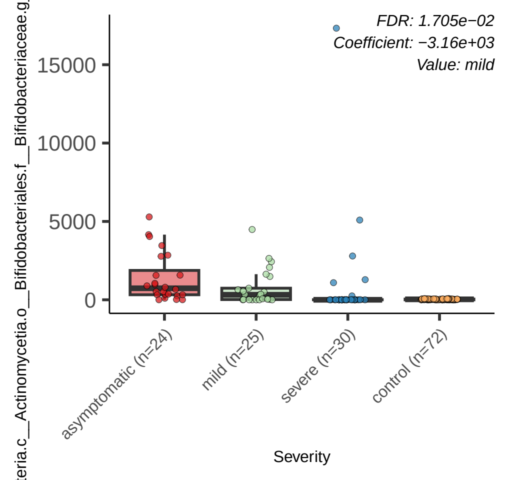

FDR: 1.705e−02
Coefficient: −3.16e+03
Value: mild
15000
10000
5000
0
teria.c__Actinomycetia.o__Bifidobacteriales.f__Bifidobacteriaceae.g
asymptomatic (n=24)
mild (n=25)
severe (n=30)
control (n=72)
Severity

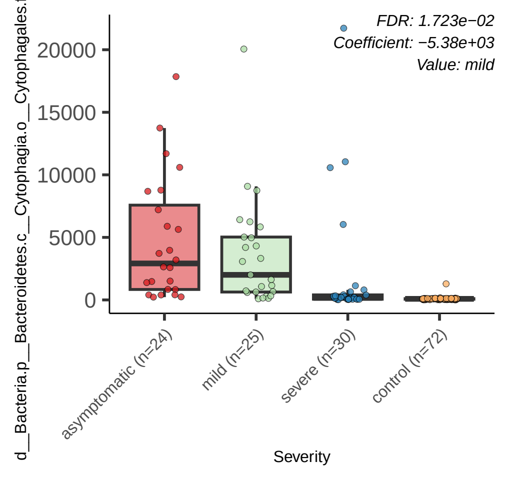

FDR: 1.723e−02
Coefficient: −5.38e+03
Value: mild
20000
15000
10000
5000
0
d__Bacteria.p__Bacteroidetes.c__Cytophagia.o__Cytophagales.f
asymptomatic (n=24)
mild (n=25)
severe (n=30)
control (n=72)
Severity

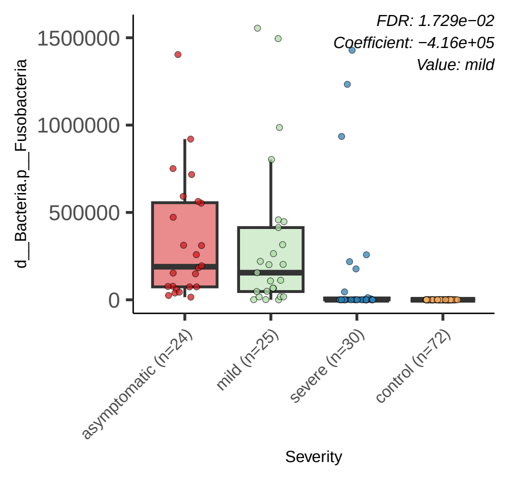

FDR: 1.729e−02
Coefficient: −4.16e+05
Value: mild
d__Bacteria.p__Fusobacteria
1500000
1000000
500000
0
asymptomatic (n=24)
mild (n=25)
severe (n=30)
control (n=72)
Severity

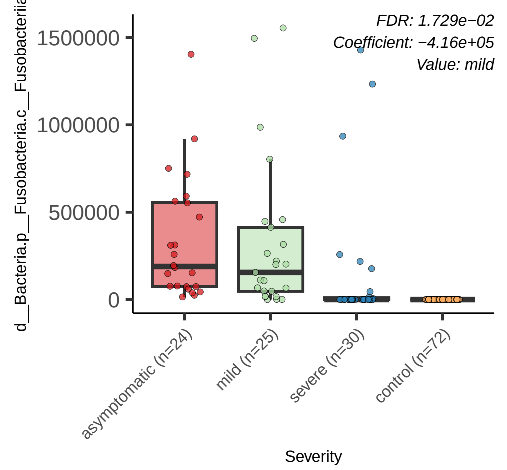

FDR: 1.729e−02
Coefficient: −4.16e+05
Value: mild
d__Bacteria.p__Fusobacteria.c__Fusobacteriia
1500000
1000000
500000
0
asymptomatic (n=24)
mild (n=25)
severe (n=30)
control (n=72)
Severity

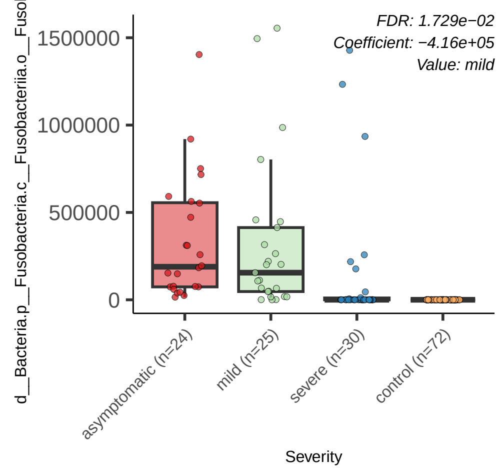

FDR: 1.729e−02
Coefficient: −4.16e+05
Value: mild
d__Bacteria.p__Fusobacteria.c__Fusobacteriia.o__Fuso
1500000
1000000
500000
0
asymptomatic (n=24)
mild (n=25)
severe (n=30)
control (n=72)
Severity

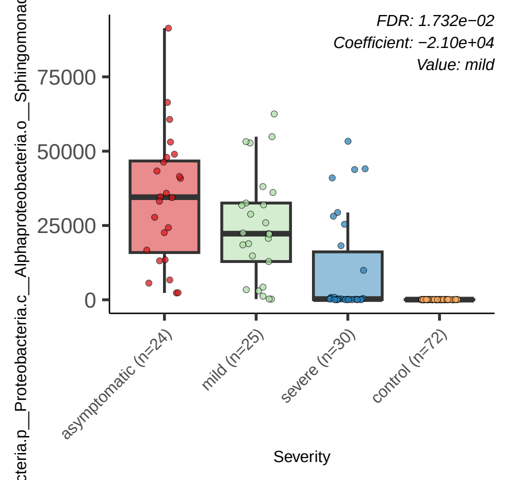

FDR: 1.732e−02
Coefficient: −2.10e+04
Value: mild
75000
50000
25000
0
cteria.p__Proteobacteria.c__Alphaproteobacteria.o__Sphingomonad
asymptomatic (n=24)
mild (n=25)
severe (n=30)
control (n=72)
Severity

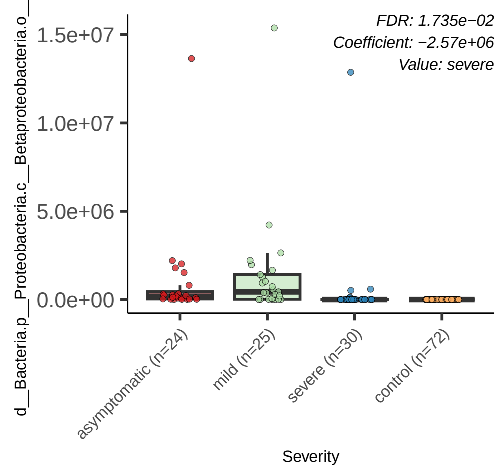

FDR: 1.735e−02
Coefficient: −2.57e+06
Value: severe
d__Bacteria.p__Proteobacteria.c__Betaproteobacteria.o__
1.5e+07
1.0e+07
5.0e+06
0.0e+00
asymptomatic (n=24)
mild (n=25)
severe (n=30)
control (n=72)
Severity

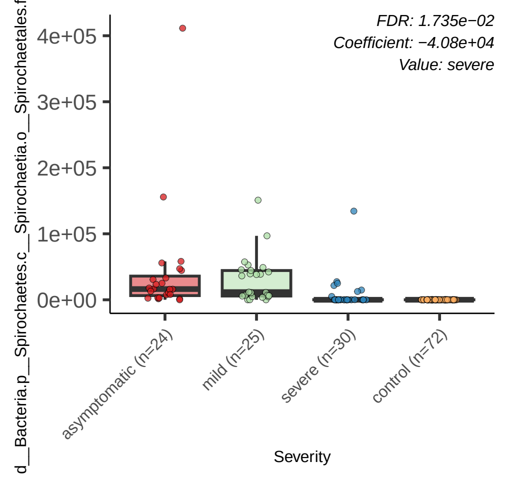

FDR: 1.735e−02
Coefficient: −4.08e+04
Value: severe
d__Bacteria.p__Spirochaetes.c__Spirochaetia.o__Spirochaetales.f
4e+05
3e+05
2e+05
1e+05
0e+00
asymptomatic (n=24)
mild (n=25)
severe (n=30)
control (n=72)
Severity

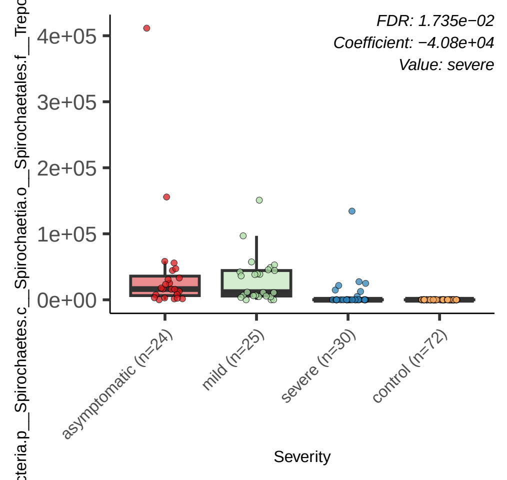

FDR: 1.735e−02
Coefficient: −4.08e+04
Value: severe
4e+05
3e+05
2e+05
1e+05
0e+00
asymptomatic (n=24)
mild (n=25)
severe (n=30)
control (n=72)
Severity
cteria.p__Spirochaetes.c__Spirochaetia.o__Spirochaetales.f__Trepo

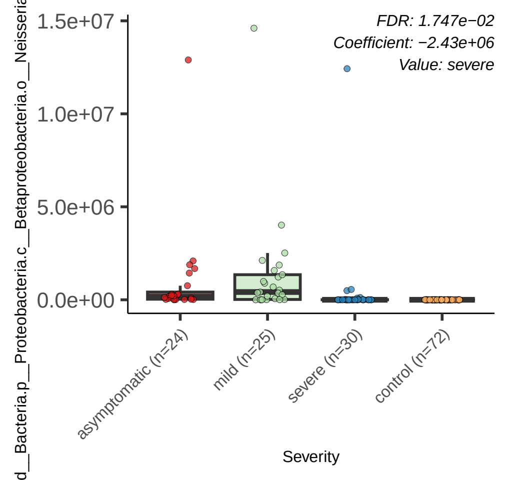

FDR: 1.747e−02
Coefficient: −2.43e+06
Value: severe
d__Bacteria.p__Proteobacteria.c__Betaproteobacteria.o__Neisseria
1.5e+07
1.0e+07
5.0e+06
0.0e+00
asymptomatic (n=24)
mild (n=25)
severe (n=30)
control (n=72)
Severity

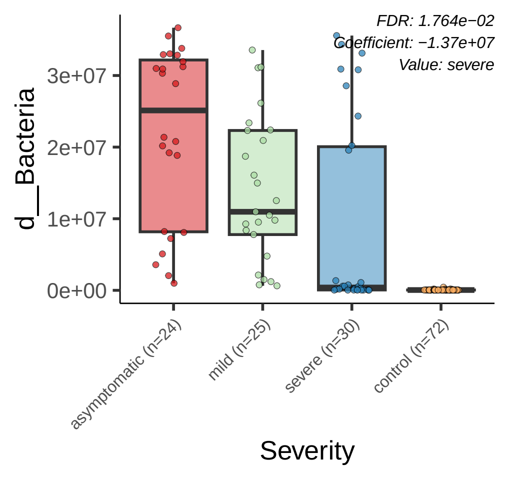
FDR: 1.764e−02
Coefficient: −1.37e+07
Value: severe
d__Bacteria
3e+07
2e+07
1e+07
0e+00
asymptomatic (n=24)
mild (n=25)
severe (n=30)
control (n=72)
Severity

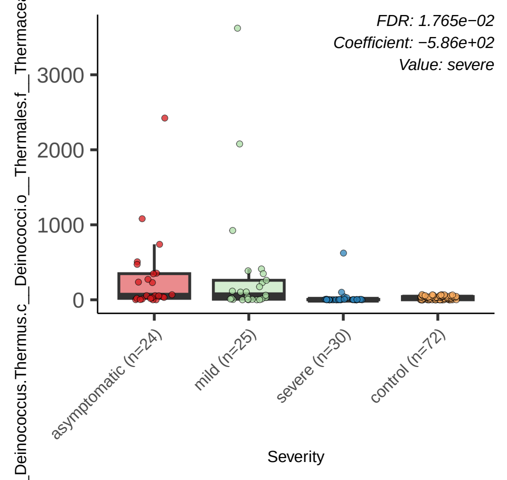

FDR: 1.765e−02
Coefficient: −5.86e+02
Value: severe
3000
2000
1000
0
__Deinococcus.Thermus.c__Deinococci.o__Thermales.f__Thermace
asymptomatic (n=24)
mild (n=25)
severe (n=30)
control (n=72)
Severity

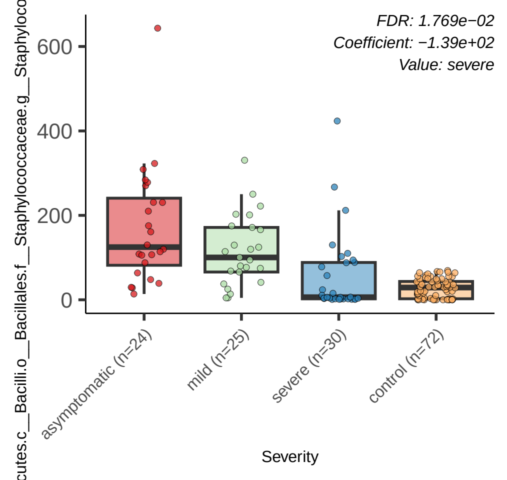

FDR: 1.769e−02
Coefficient: −1.39e+02
Value: severe
cutes.c__Bacilli.o__Bacillales.f__Staphylococcaceae.g__Staphyloco
600
400
200
0
asymptomatic (n=24)
mild (n=25)
severe (n=30)
control (n=72)
Severity

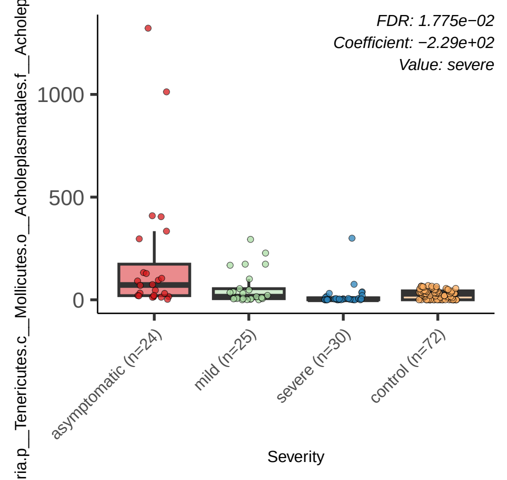

FDR: 1.775e−02
Coefficient: −2.29e+02
Value: severe
ria.p__Tenericutes.c__Mollicutes.o__Acholeplasmatales.f__Acholep
1000
500
0
asymptomatic (n=24)
mild (n=25)
severe (n=30)
control (n=72)
Severity

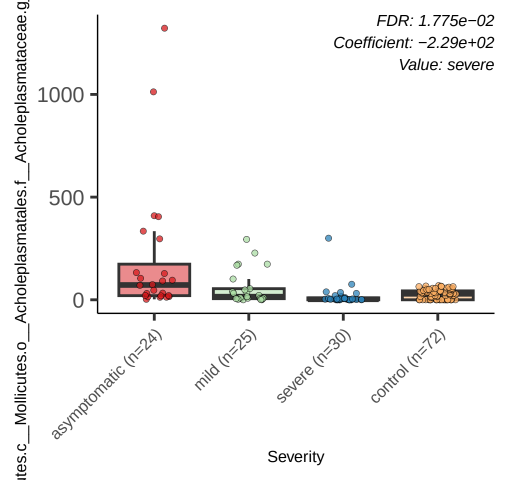

FDR: 1.775e−02
Coefficient: −2.29e+02
Value: severe
1000
500
0
ites.c__Mollicutes.o__Acholeplasmatales.f__Acholeplasmataceae.g.
asymptomatic (n=24)
mild (n=25)
severe (n=30)
control (n=72)
Severity

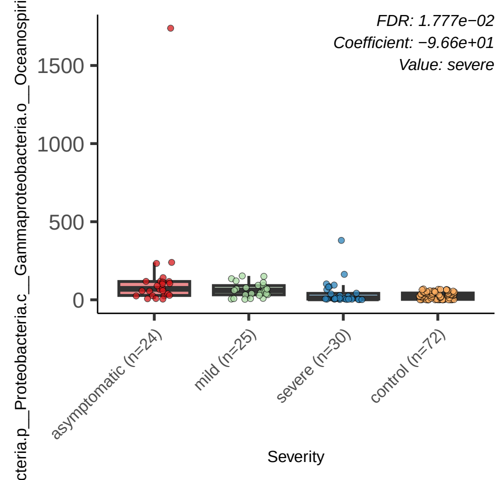

FDR: 1.777e−02
Coefficient: −9.66e+01
Value: severe
1500
1000
500
0
cteria.p__Proteobacteria.c__Gammaproteobacteria.o__Oceanospiri
asymptomatic (n=24)
mild (n=25)
severe (n=30)
control (n=72)
Severity

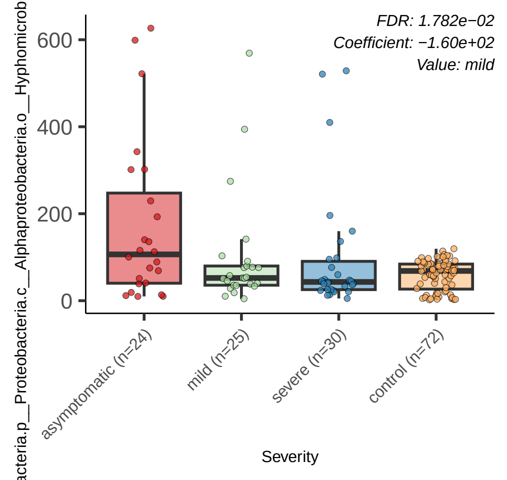

FDR: 1.782e−02
Coefficient: −1.60e+02
Value: mild
0
200
400
600
acteria.p__Proteobacteria.c__Alphaproteobacteria.o__Hyphomicrob
asymptomatic (n=24)
mild (n=25)
severe (n=30)
control (n=72)
Severity

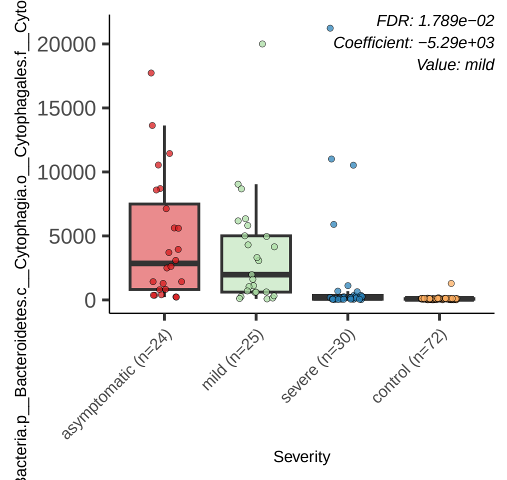
FDR: 1.789e−02
Coefficient: −5.29e+03
Value: mild
Bacteria.p__Bacteroidetes.c__Cytophagia.o__Cytophagales.f__Cyto
20000
15000
10000
5000
0
asymptomatic (n=24)
mild (n=25)
severe (n=30)
control (n=72)
Severity

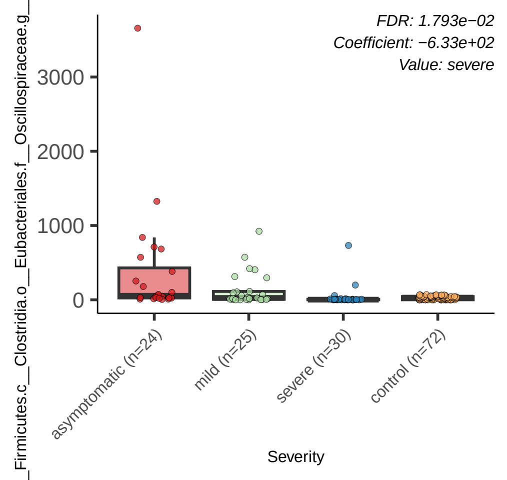

FDR: 1.793e−02
Coefficient: −6.33e+02
Value: severe
Firmicutes.c__Clostridia.o__Eubacteriales.f__Oscillospiraceae.g__
0
1000
2000
3000
asymptomatic (n=24)
mild (n=25)
severe (n=30)
control (n=72)
Severity

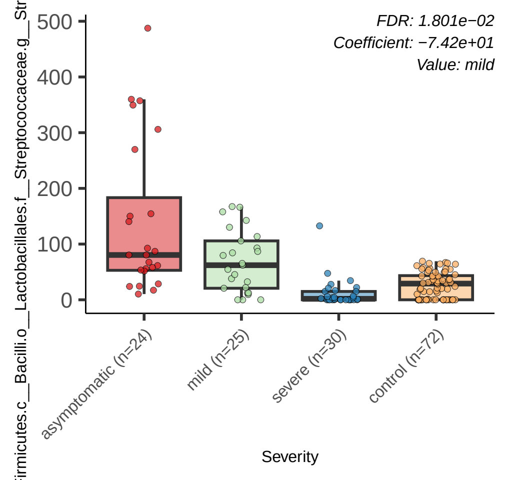

FDR: 1.801e−02
Coefficient: −7.42e+01
Value: mild
irmicutes.c__Bacilli.o__Lactobacillales.f__Streptococcaceae.g__Str
500
400
300
200
100
0
asymptomatic (n=24)
mild (n=25)
severe (n=30)
control (n=72)
Severity

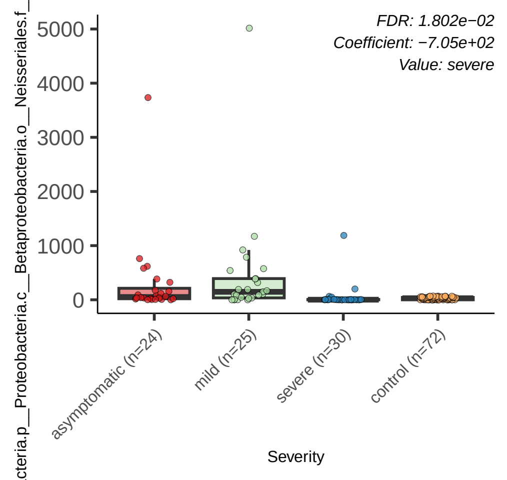

FDR: 1.802e−02
Coefficient: −7.05e+02
Value: severe
cteria.p__Proteobacteria.c__Betaproteobacteria.o__Neisseriales.f
5000
4000
3000
2000
1000
0
asymptomatic (n=24)
mild (n=25)
severe (n=30)
control (n=72)
Severity

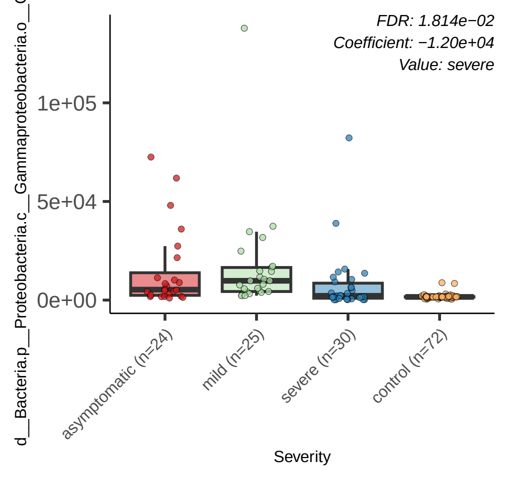

FDR: 1.814e−02
Coefficient: −1.20e+04
Value: severe
d__Bacteria.p__Proteobacteria.c__Gammaproteobacteria.o__
1e+05
5e+04
0e+00
asymptomatic (n=24)
mild (n=25)
severe (n=30)
control (n=72)
Severity

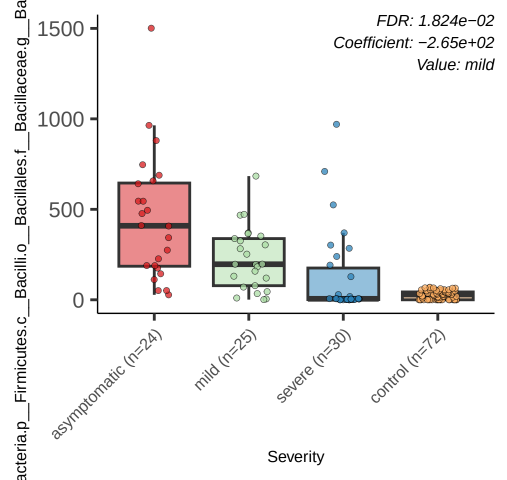

acteria.p__Firmicutes.c__Bacilli.o__Bacillales.f__Bacillaceae.g__Ba
FDR: 1.824e−02
Coefficient: −2.65e+02
Value: mild
1500
1000
500
0
asymptomatic (n=24)
mild (n=25)
severe (n=30)
control (n=72)
Severity

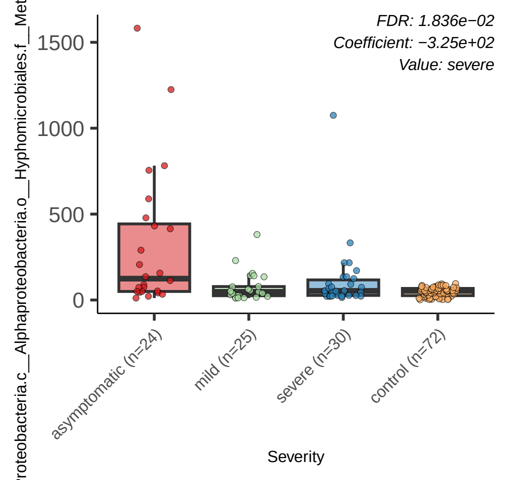

FDR: 1.836e−02
Coefficient: −3.25e+02
Value: severe
1500
1000
500
0
Proteobacteria.c__Alphaproteobacteria.o__Hyphomicrobiales.f__Met
asymptomatic (n=24)
mild (n=25)
severe (n=30)
control (n=72)
Severity

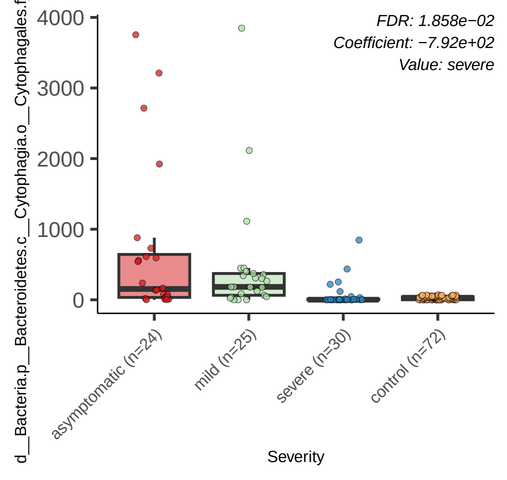

d__Bacteria.p__Bacteroidetes.c__Cytophagia.o__Cytophagales.f
4000
3000
2000
1000
0
FDR: 1.858e−02
Coefficient: −7.92e+02
Value: severe
asymptomatic (n=24)
mild (n=25)
severe (n=30)
control (n=72)
Severity

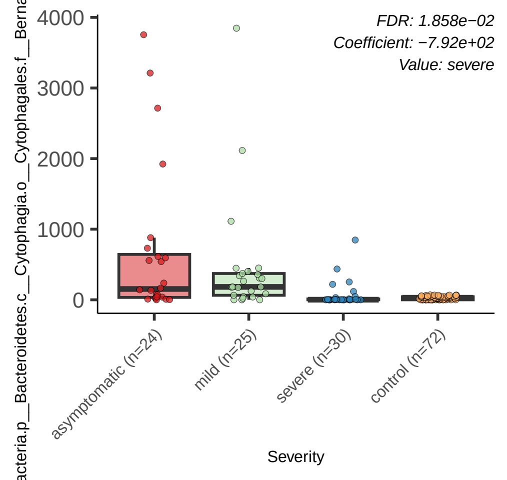

FDR: 1.858e−02
Coefficient: −7.92e+02
Value: severe
4000
3000
2000
1000
0
acteria.p__Bacteroidetes.c__Cytophagia.o__Cytophagales.f__Berna
asymptomatic (n=24)
mild (n=25)
severe (n=30)
control (n=72)
Severity

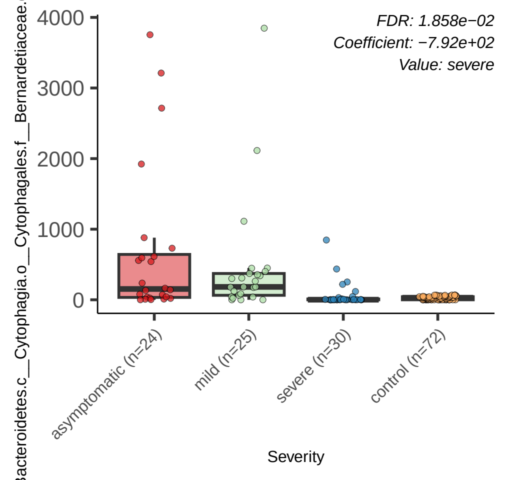

FDR: 1.858e−02
Coefficient: −7.92e+02
Value: severe
Bacteroidetes.c__Cytophagia.o__Cytophagales.f__Bernardetiaceae.
4000
3000
2000
1000
0
asymptomatic (n=24)
mild (n=25)
severe (n=30)
control (n=72)
Severity

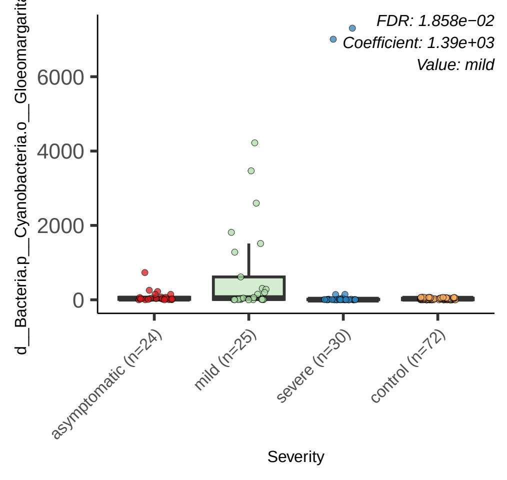
FDR: 1.858e−02
Coefficient: 1.39e+03
Value: mild
d__Bacteria.p__Cyanobacteria.o__Gloeomargarita
6000
4000
2000
0
asymptomatic (n=24)
mild (n=25)
severe (n=30)
control (n=72)
Severity

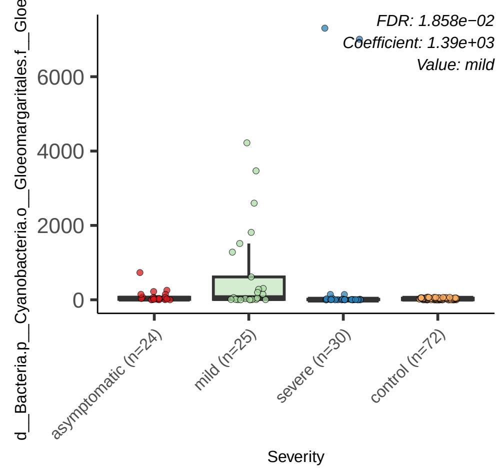
FDR: 1.858e−02
Coefficient: 1.39e+03
Value: mild
d__Bacteria.p__Cyanobacteria.o__Gloeomargaritales.f__Gloe
6000
4000
2000
0
asymptomatic (n=24)
mild (n=25)
severe (n=30)
control (n=72)
Severity

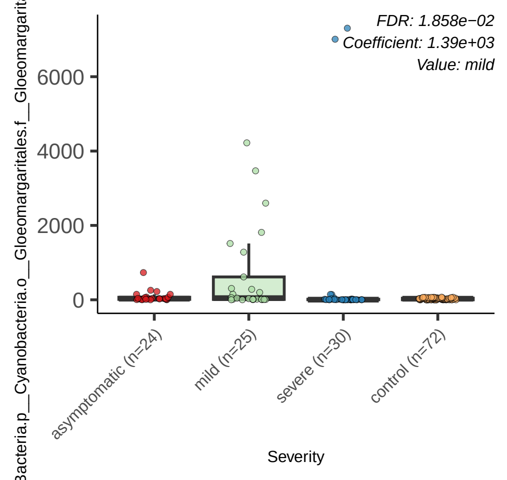
FDR: 1.858e−02
Coefficient: 1.39e+03
Value: mild
Bacteria.p__Cyanobacteria.o__Gloeomargaritales.f__Gloeomargarit
6000
4000
2000
0
asymptomatic (n=24)
mild (n=25)
severe (n=30)
control (n=72)
Severity

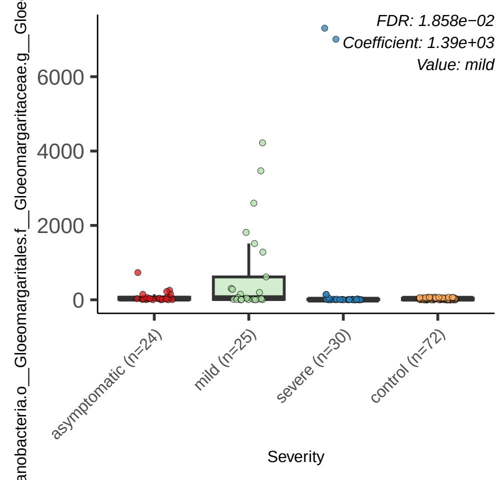
FDR: 1.858e−02
Coefficient: 1.39e+03
Value: mild
6000
4000
2000
0
asymptomatic (n=24)
mild (n=25)
severe (n=30)
control (n=72)
Severity
anobacteria.o__Gloeomargaritales.f__Gloeomargaritaceae.g__Gloe

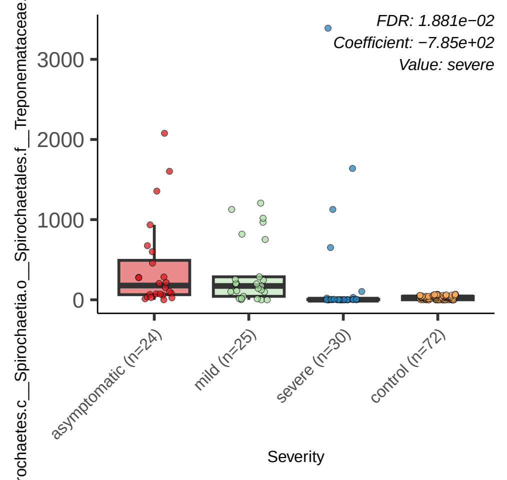
FDR: 1.881e−02
Coefficient: −7.85e+02
Value: severe
rochaetes.c__Spirochaetia.o__Spirochaetales.f__Treponemataceae.
3000
2000
1000
0
asymptomatic (n=24)
mild (n=25)
severe (n=30)
control (n=72)
Severity

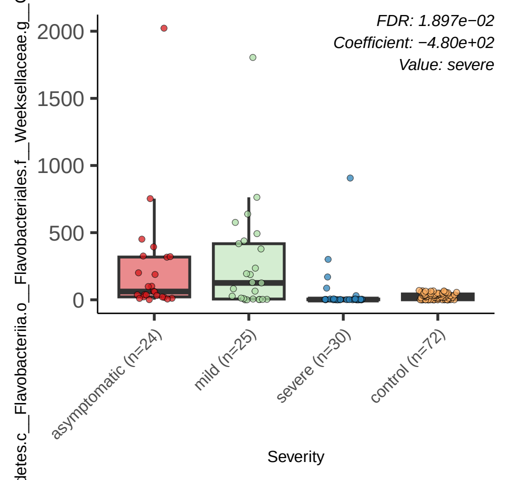
FDR: 1.897e−02
Coefficient: −4.80e+02
Value: severe
0
500
1000
1500
2000
detes.c__Flavobacteriia.o__Flavobacteriales.f__Weeksellaceae.g__
asymptomatic (n=24)
mild (n=25)
severe (n=30)
control (n=72)
Severity

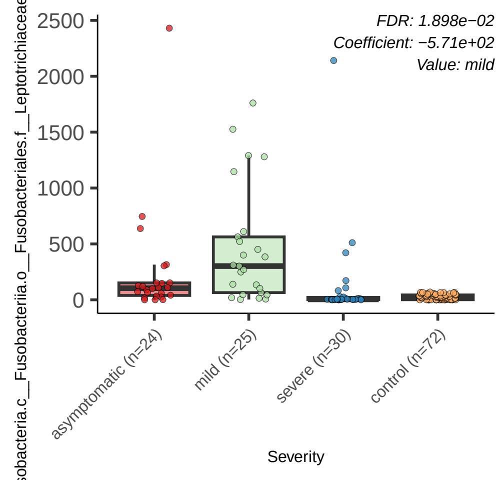
FDR: 1.898e−02
Coefficient: −5.71e+02
Value: mild
2500
2000
1500
1000
500
0
asymptomatic (n=24)
mild (n=25)
severe (n=30)
control (n=72)
Severity
sobacteria.c__Fusobacteriia.o__Fusobacteriales.f__Leptotrichiaceae

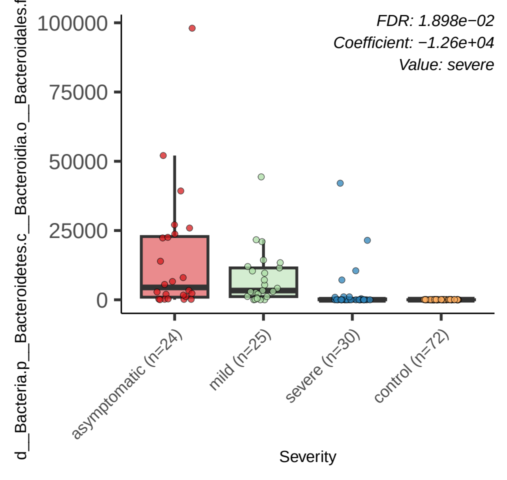

FDR: 1.898e−02
Coefficient: −1.26e+04
Value: severe
100000
75000
50000
25000
0
asymptomatic (n=24)
mild (n=25)
severe (n=30)
control (n=72)
Severity
d__Bacteria.p__Bacteroidetes.c__Bacteroidia.o__Bacteroidales.f

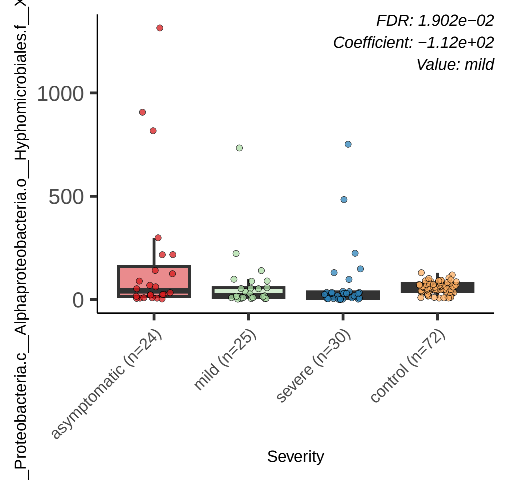
FDR: 1.902e−02
Coefficient: −1.12e+02
Value: mild
1000
500
0
asymptomatic (n=24)
mild (n=25)
severe (n=30)
control (n=72)
Severity
_Proteobacteria.c__Alphaproteobacteria.o__Hyphomicrobiales.f__X

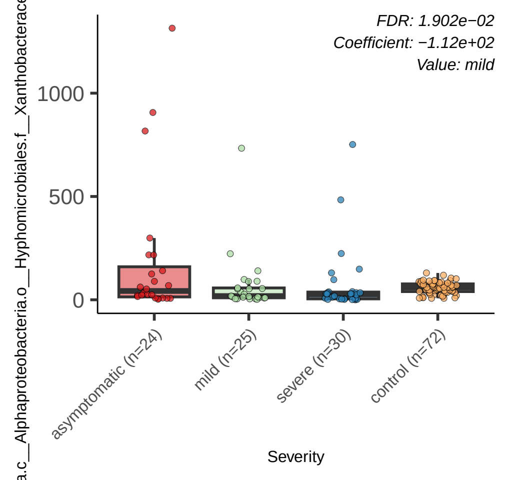

FDR: 1.902e−02
Coefficient: −1.12e+02
Value: mild
1000
500
0
a.c__Alphaproteobacteria.o__Hyphomicrobiales.f__Xanthobacterace
asymptomatic (n=24)
mild (n=25)
severe (n=30)
control (n=72)
Severity

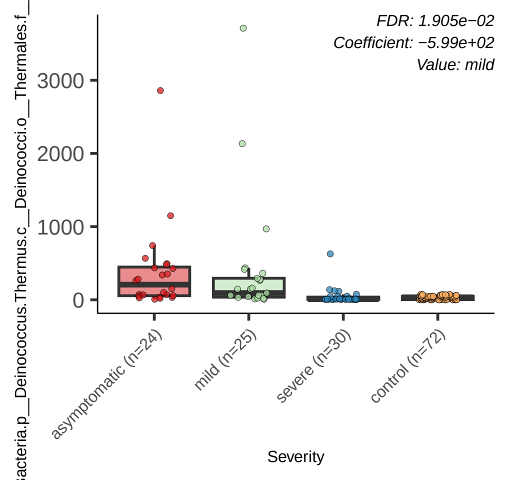
FDR: 1.905e−02
Coefficient: −5.99e+02
Value: mild
Bacteria.p__Deinococcus.Thermus.c__Deinococci.o__Thermales.f__
3000
2000
1000
0
asymptomatic (n=24)
mild (n=25)
severe (n=30)
control (n=72)
Severity

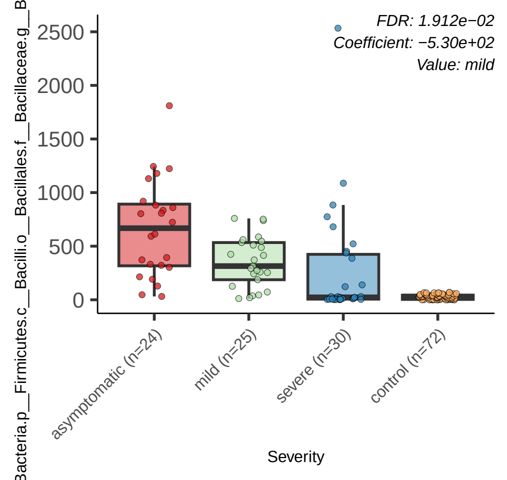

FDR: 1.912e−02
Coefficient: −5.30e+02
Value: mild
Bacteria.p__Firmicutes.c__Bacilli.o__Bacillales.f__Bacillaceae.g__B
2500
2000
1500
1000
500
0
asymptomatic (n=24)
mild (n=25)
severe (n=30)
control (n=72)
Severity

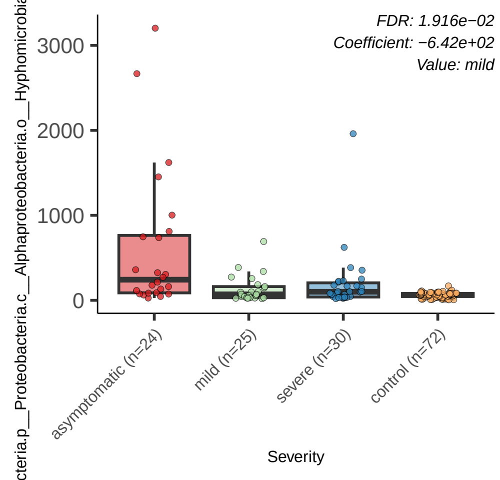

FDR: 1.916e−02
Coefficient: −6.42e+02
Value: mild
3000
2000
1000
0
cteria.p__Proteobacteria.c__Alphaproteobacteria.o__Hyphomicrobia
asymptomatic (n=24)
mild (n=25)
severe (n=30)
control (n=72)
Severity

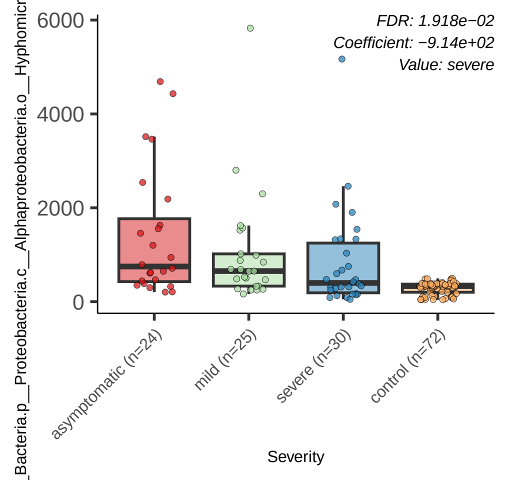

FDR: 1.918e−02
Coefficient: −9.14e+02
Value: severe
6000
4000
2000
0
Bacteria.p__Proteobacteria.c__Alphaproteobacteria.o__Hyphomicr
asymptomatic (n=24)
mild (n=25)
severe (n=30)
control (n=72)
Severity

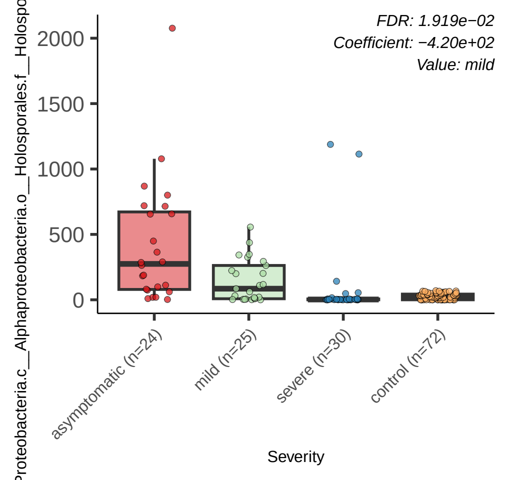

FDR: 1.919e−02
Coefficient: −4.20e+02
Value: mild
Proteobacteria.c__Alphaproteobacteria.o__Holosporales.f__Holospo
2000
1500
1000
500
0
asymptomatic (n=24)
mild (n=25)
severe (n=30)
control (n=72)
Severity

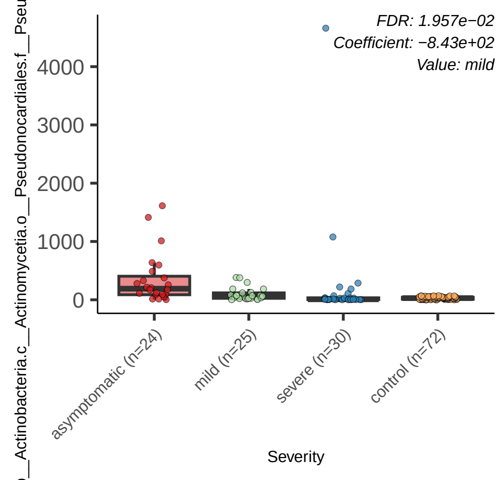

FDR: 1.957e−02
Coefficient: −8.43e+02
Value: mild
4000
3000
2000
1000
0
asymptomatic (n=24)
mild (n=25)
severe (n=30)
control (n=72)
Severity
o__Actinobacteria.c__Actinomycetia.o__Pseudonocardiales.f__Pseu

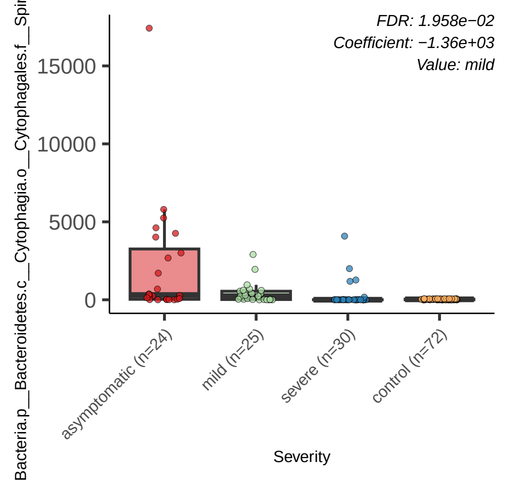

FDR: 1.958e−02
Coefficient: −1.36e+03
Value: mild
Bacteria.p__Bacteroidetes.c__Cytophagia.o__Cytophagales.f__Spir
15000
10000
5000
0
asymptomatic (n=24)
mild (n=25)
severe (n=30)
control (n=72)
Severity

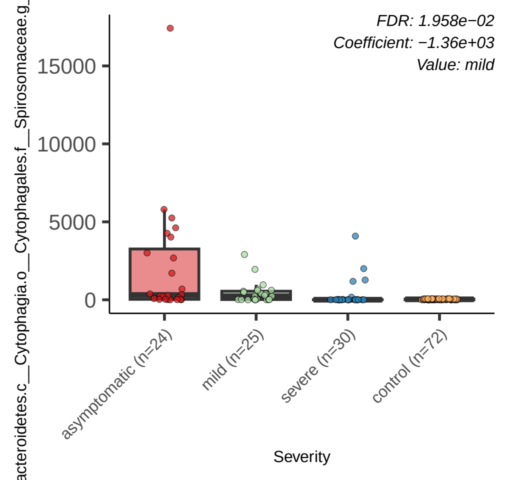
FDR: 1.958e−02
Coefficient: −1.36e+03
Value: mild
acteroidetes.c__Cytophagia.o__Cytophagales.f__Spirosomaceae.g_
15000
10000
5000
0
asymptomatic (n=24)
mild (n=25)
severe (n=30)
control (n=72)
Severity

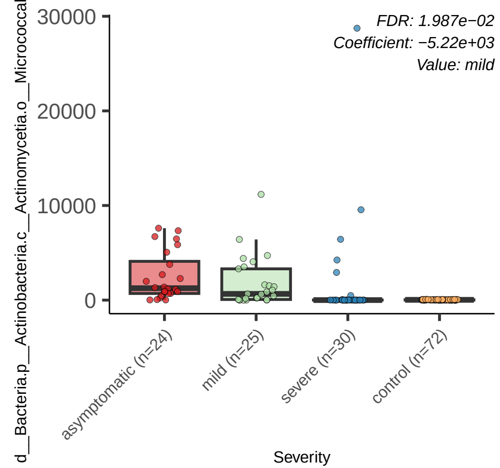

d__Bacteria.p__Actinobacteria.c__Actinomycetia.o__Micrococcal
30000
20000
10000
0
FDR: 1.987e−02
Coefficient: −5.22e+03
Value: mild
asymptomatic (n=24)
mild (n=25)
severe (n=30)
control (n=72)
Severity

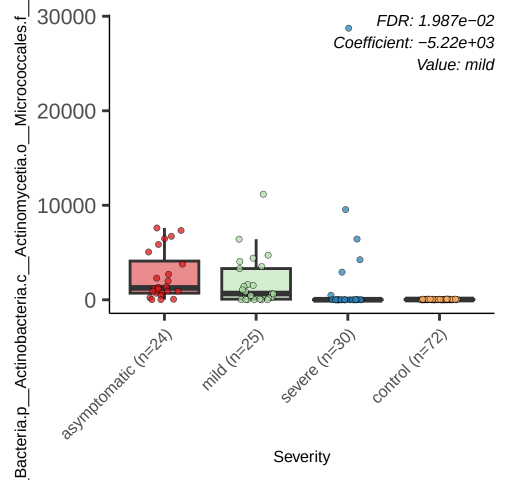
FDR: 1.987e−02
Coefficient: −5.22e+03
Value: mild
Bacteria.p__Actinobacteria.c__Actinomycetia.o__Micrococcales.f
30000
20000
10000
0
asymptomatic (n=24)
mild (n=25)
severe (n=30)
control (n=72)
Severity

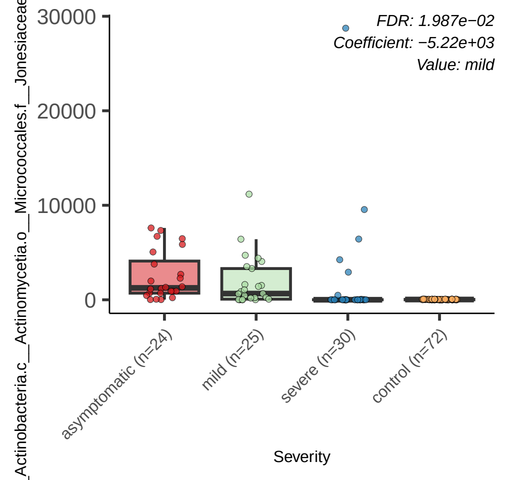

FDR: 1.987e−02
Coefficient: −5.22e+03
Value: mild
30000
20000
10000
0
asymptomatic (n=24)
mild (n=25)
severe (n=30)
control (n=72)
Severity
Actinobacteria.c__Actinomycetia.o__Micrococcales.f__Jonesiaceae

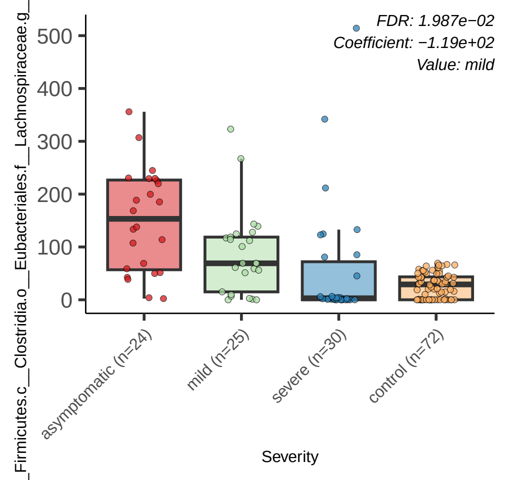

FDR: 1.987e−02
Coefficient: −1.19e+02
Value: mild
_Firmicutes.c__Clostridia.o__Eubacteriales.f__Lachnospiraceae.g__
500
400
300
200
100
0
asymptomatic (n=24)
mild (n=25)
severe (n=30)
control (n=72)
Severity

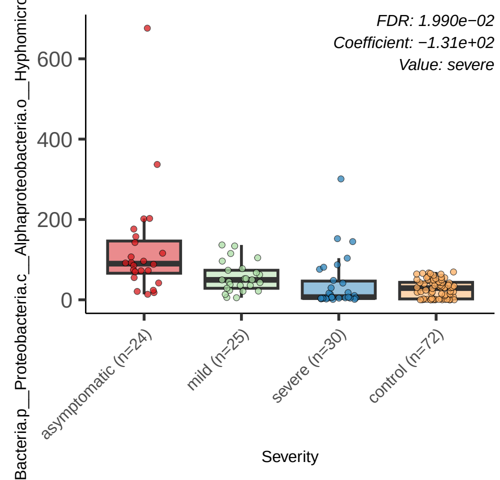
FDR: 1.990e−02
Coefficient: −1.31e+02
Value: severe
Bacteria.p__Proteobacteria.c__Alphaproteobacteria.o__Hyphomicro
0
200
400
600
asymptomatic (n=24)
mild (n=25)
severe (n=30)
control (n=72)
Severity

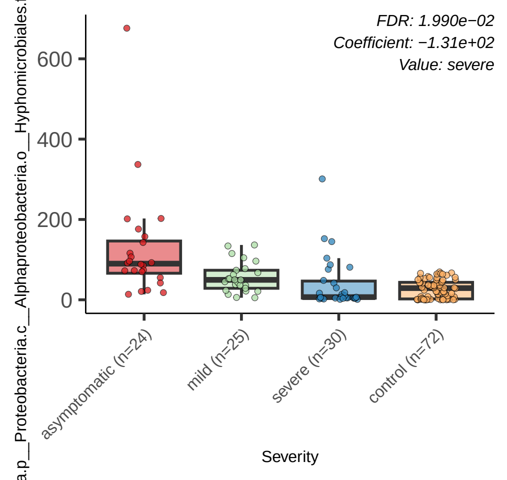
FDR: 1.990e−02
Coefficient: −1.31e+02
Value: severe
a.p__Proteobacteria.c__Alphaproteobacteria.o__Hyphomicrobiales.f
600
400
200
0
asymptomatic (n=24)
mild (n=25)
severe (n=30)
control (n=72)
Severity

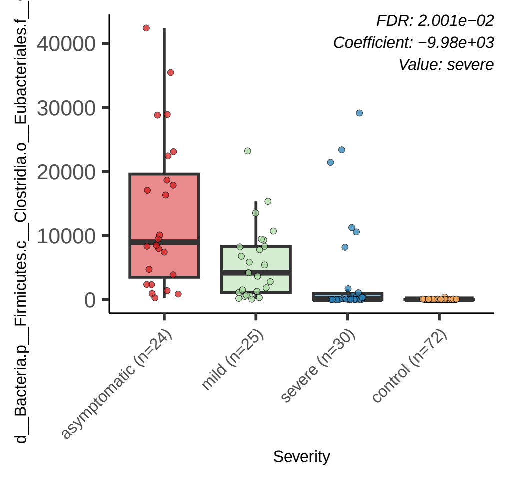

FDR: 2.001e−02
Coefficient: −9.98e+03
Value: severe
d__Bacteria.p__Firmicutes.c__Clostridia.o__Eubacteriales.f__
40000
30000
20000
10000
0
asymptomatic (n=24)
mild (n=25)
severe (n=30)
control (n=72)
Severity

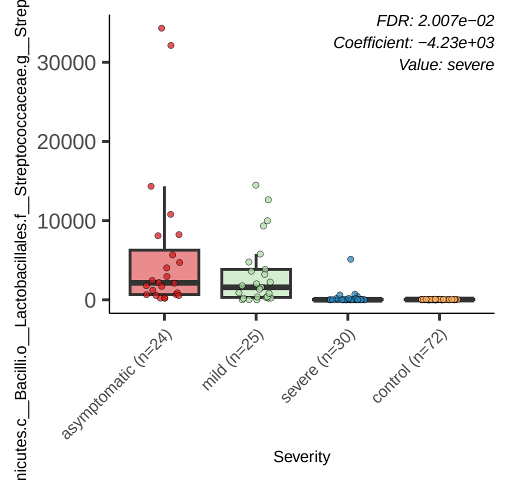

FDR: 2.007e−02
Coefficient: −4.23e+03
Value: severe
30000
20000
10000
0
nicutes.c__Bacilli.o__Lactobacillales.f__Streptococcaceae.g__Strep
asymptomatic (n=24)
mild (n=25)
severe (n=30)
control (n=72)
Severity

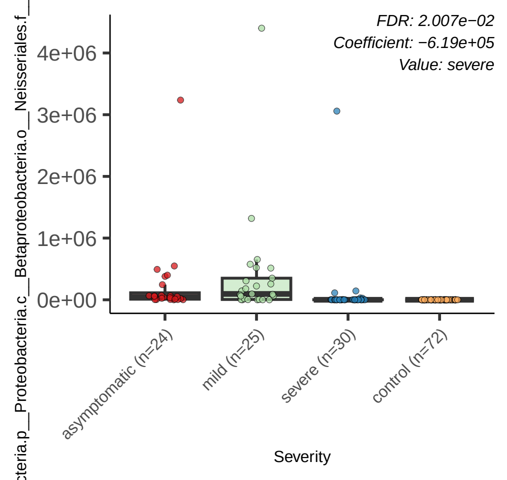
FDR: 2.007e−02
Coefficient: −6.19e+05
Value: severe
cteria.p__Proteobacteria.c__Betaproteobacteria.o__Neisseriales.f_
4e+06
3e+06
2e+06
1e+06
0e+00
asymptomatic (n=24)
mild (n=25)
severe (n=30)
control (n=72)
Severity

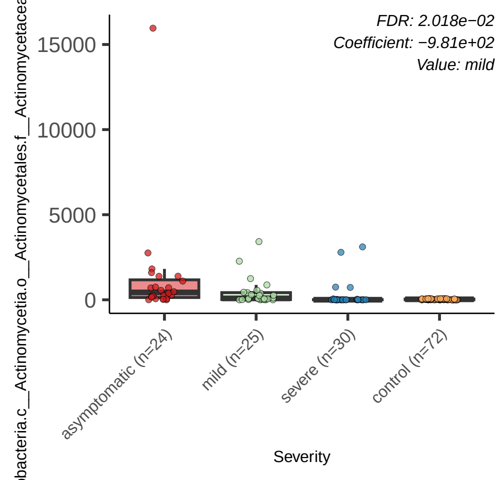
FDR: 2.018e−02
Coefficient: −9.81e+02
Value: mild
15000
10000
5000
0
asymptomatic (n=24)
mild (n=25)
severe (n=30)
control (n=72)
Severity
obacteria.c__Actinomycetia.o__Actinomycetales.f__Actinomycetacea

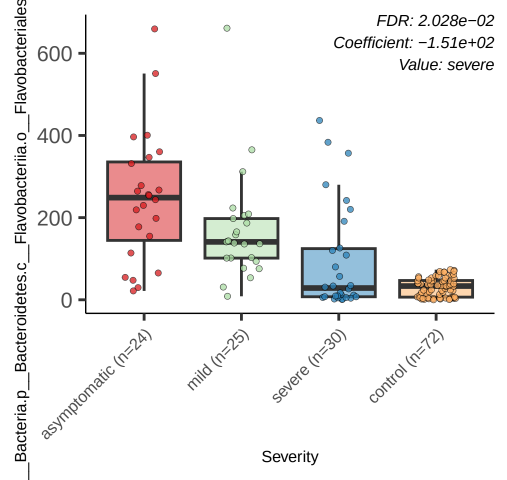
FDR: 2.028e−02
Coefficient: −1.51e+02
Value: severe
__Bacteria.p__Bacteroidetes.c__Flavobacteriia.o__Flavobacteriales
0
200
400
600
asymptomatic (n=24)
mild (n=25)
severe (n=30)
control (n=72)
Severity

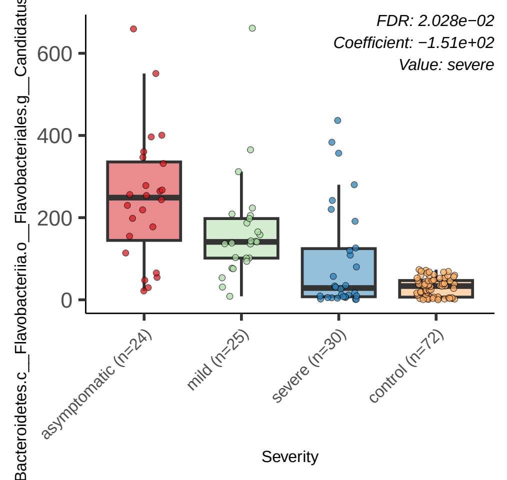

FDR: 2.028e−02
Coefficient: −1.51e+02
Value: severe
Bacteroidetes.c__Flavobacteriia.o__Flavobacteriales.g__Candidatus
600
400
200
0
asymptomatic (n=24)
mild (n=25)
severe (n=30)
control (n=72)
Severity

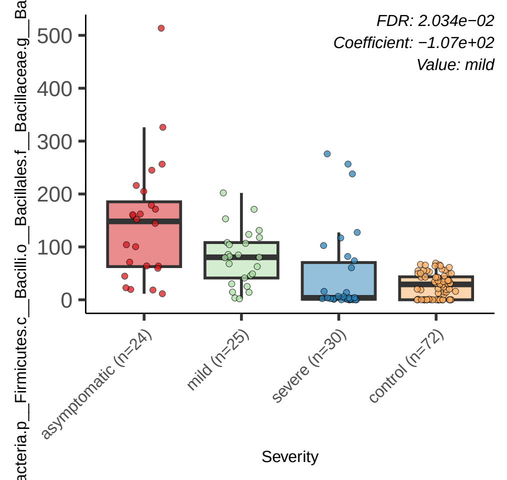

FDR: 2.034e−02
Coefficient: −1.07e+02
Value: mild
Firmicutes.c__Bacilli.o__Bacillales.f__Bacillaceae.g__Ba
acteria.p__
0
100
200
300
400
500
asymptomatic (n=24)
mild (n=25)
severe (n=30)
control (n=72)
Severity

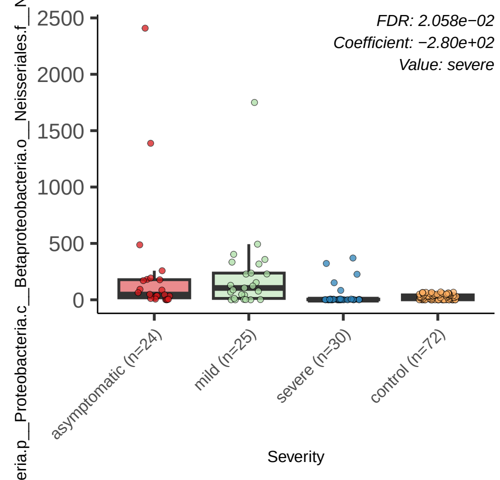
FDR: 2.058e−02
Coefficient: −2.80e+02
Value: severe
2500
2000
1500
1000
500
0
eria.p__Proteobacteria.c__Betaproteobacteria.o__Neisseriales.f__N
asymptomatic (n=24)
mild (n=25)
severe (n=30)
control (n=72)
Severity

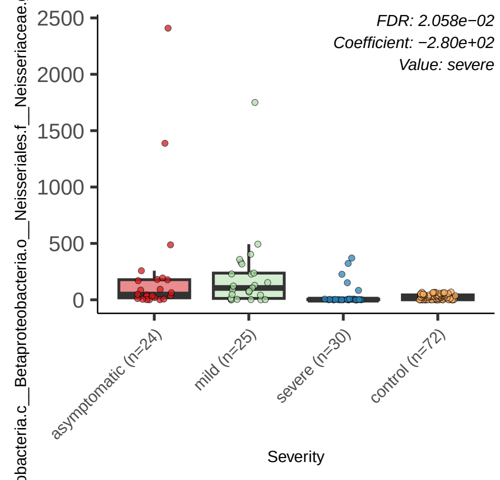

FDR: 2.058e−02
Coefficient: −2.80e+02
Value: severe
2500
2000
1500
1000
500
0
obacteria.c__Betaproteobacteria.o__Neisseriales.f__Neisseriaceae.
asymptomatic (n=24)
mild (n=25)
severe (n=30)
control (n=72)
Severity

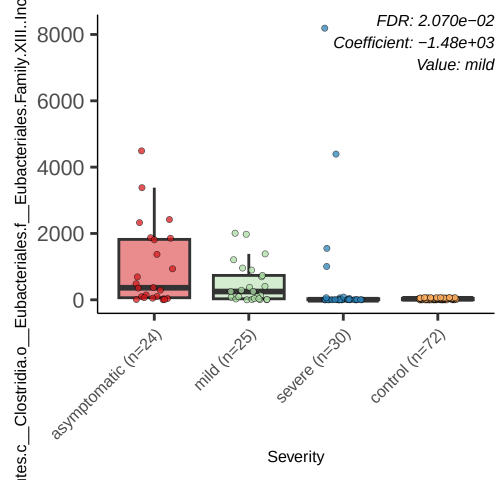

FDR: 2.070e−02
Coefficient: −1.48e+03
Value: mild
8000
6000
4000
2000
0
asymptomatic (n=24)
mild (n=25)
severe (n=30)
control (n=72)
Severity

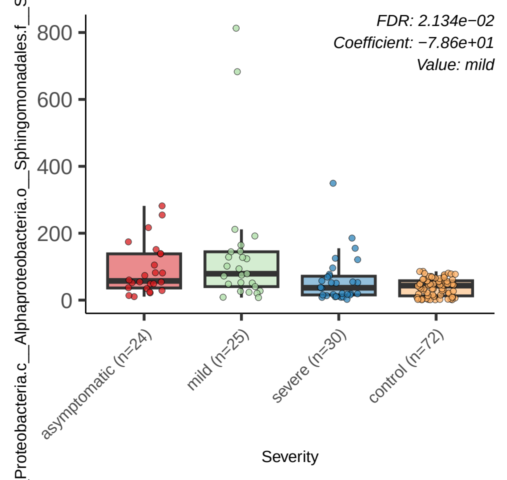

FDR: 2.134e−02
Coefficient: −7.86e+01
Value: mild
800
600
400
200
0
Proteobacteria.c__Alphaproteobacteria.o__Sphingomonadales.f__S
asymptomatic (n=24)
mild (n=25)
severe (n=30)
control (n=72)
Severity

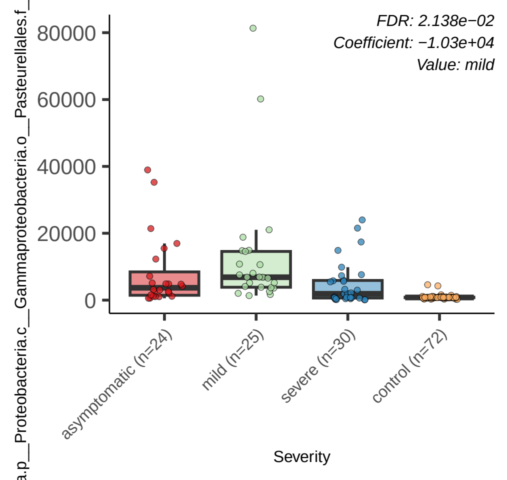
FDR: 2.138e−02
Coefficient: −1.03e+04
Value: mild
a.p__Proteobacteria.c__Gammaproteobacteria.o__Pasteurellales.f__
80000
60000
40000
20000
0
asymptomatic (n=24)
mild (n=25)
severe (n=30)
control (n=72)
Severity

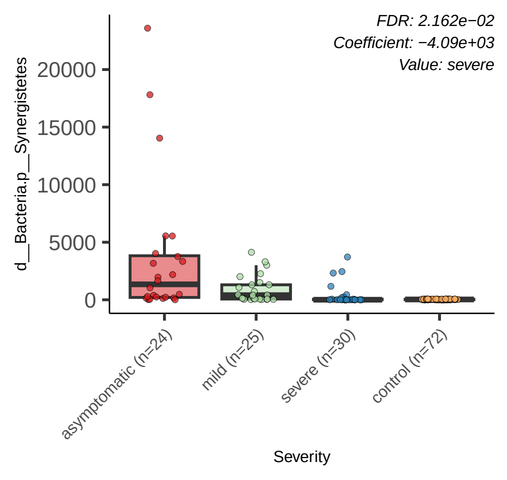

FDR: 2.162e−02
Coefficient: −4.09e+03
Value: severe
d__Bacteria.p__Synergistetes
20000
15000
10000
5000
0
asymptomatic (n=24)
mild (n=25)
severe (n=30)
control (n=72)
Severity

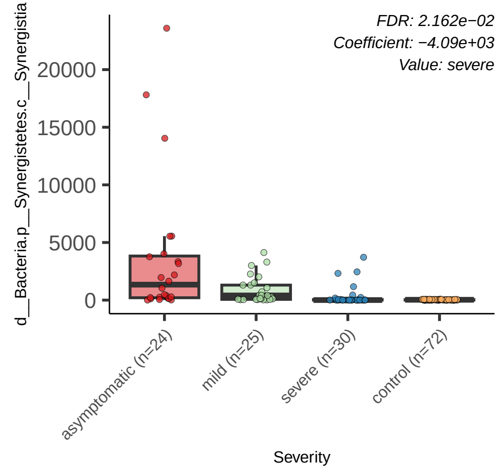

FDR: 2.162e−02
Coefficient: −4.09e+03
Value: severe
d__Bacteria.p__Synergistetes.c__Synergistia
20000
15000
10000
5000
0
asymptomatic (n=24)
mild (n=25)
severe (n=30)
control (n=72)
Severity

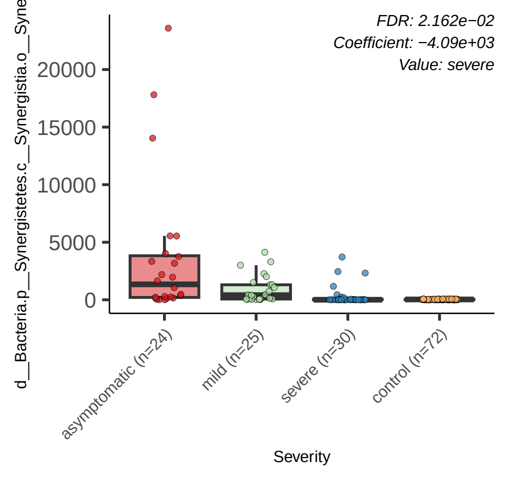

FDR: 2.162e−02
Coefficient: −4.09e+03
Value: severe
d__Bacteria.p__Synergistetes.c__Synergistia.o__Syne
20000
15000
10000
5000
0
asymptomatic (n=24)
mild (n=25)
severe (n=30)
control (n=72)
Severity

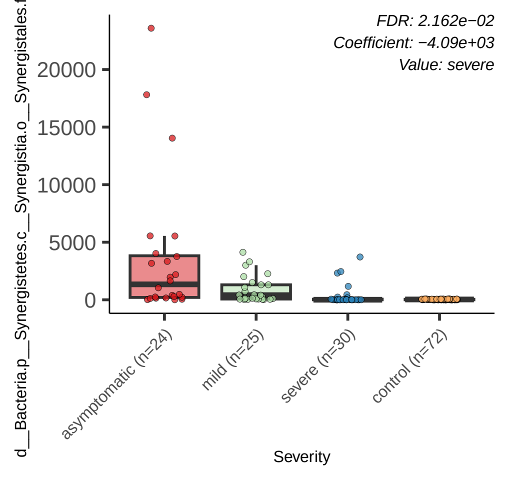

FDR: 2.162e−02
Coefficient: −4.09e+03
Value: severe
d__Bacteria.p__Synergistetes.c__Synergistia.o__Synergistales.f
20000
15000
10000
5000
0
asymptomatic (n=24)
mild (n=25)
severe (n=30)
control (n=72)
Severity

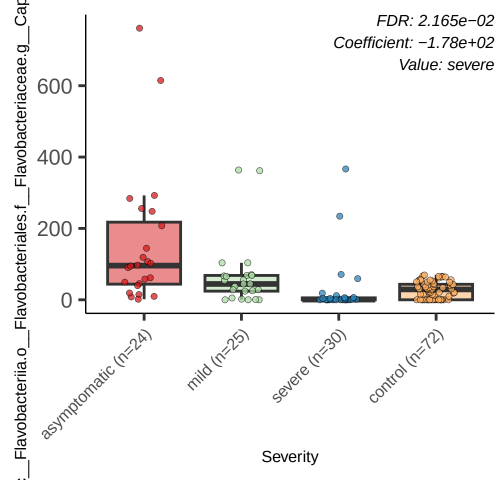

FDR: 2.165e−02
Coefficient: −1.78e+02
Value: severe
600
400
200
0
asymptomatic (n=24)
mild (n=25)
severe (n=30)
control (n=72)
Severity
Flavobacteriia.o__Flavobacteriales.f__Flavobacteriaceae.g__Cap

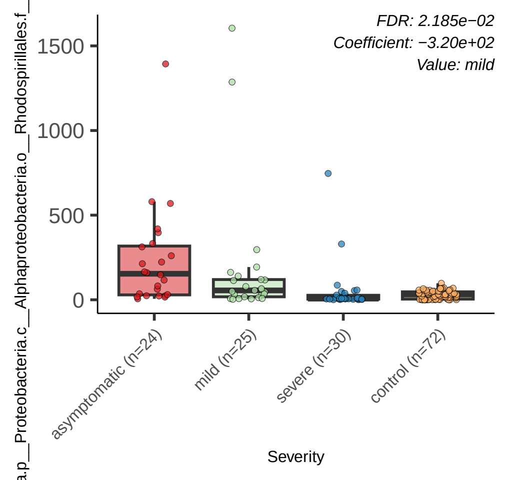

FDR: 2.185e−02
Coefficient: −3.20e+02
Value: mild
a.p__Proteobacteria.c__Alphaproteobacteria.o__Rhodospirillales.f__
1500
1000
500
0
asymptomatic (n=24)
mild (n=25)
severe (n=30)
control (n=72)
Severity

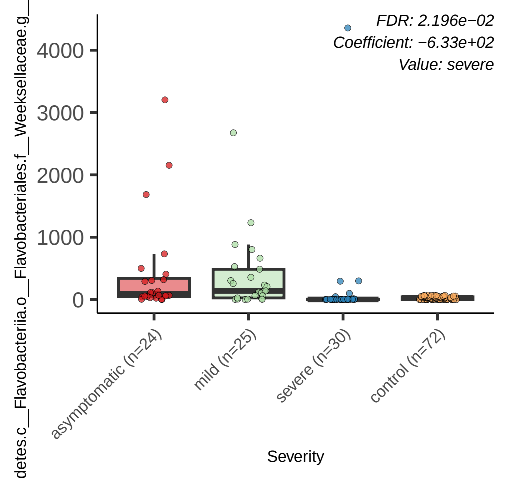

FDR: 2.196e−02
Coefficient: −6.33e+02
Value: severe
detes.c__Flavobacteriia.o__Flavobacteriales.f__Weeksellaceae.g__
4000
3000
2000
1000
0
asymptomatic (n=24)
mild (n=25)
severe (n=30)
control (n=72)
Severity

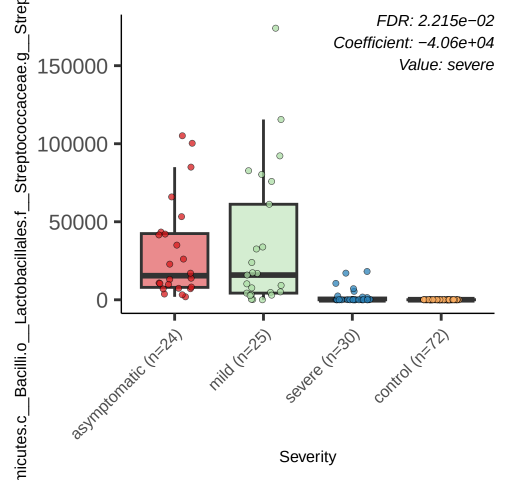

FDR: 2.215e−02
Coefficient: −4.06e+04
Value: severe
150000
100000
50000
0
micutes.c__Bacilli.o__Lactobacillales.f__Streptococcaceae.g__Strep
asymptomatic (n=24)
mild (n=25)
severe (n=30)
control (n=72)
Severity

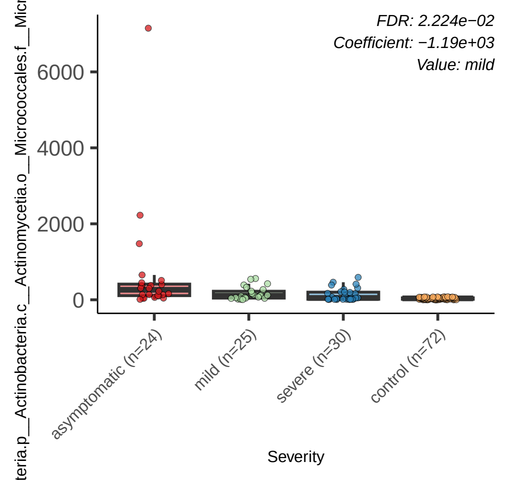

FDR: 2.224e−02
Coefficient: −1.19e+03
Value: mild
teria.p__Actinobacteria.c__Actinomycetia.o__Micrococcales.f__Micr
0
2000
4000
6000
asymptomatic (n=24)
mild (n=25)
severe (n=30)
control (n=72)
Severity

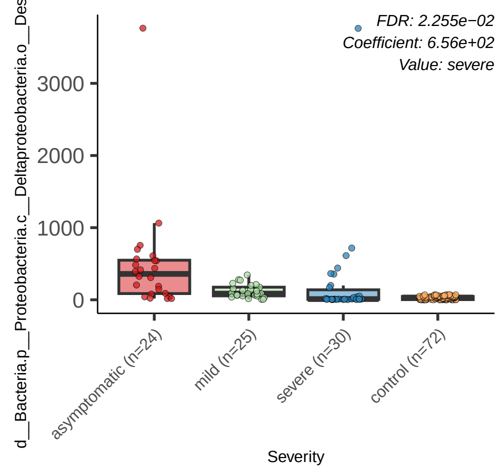

FDR: 2.255e−02
Coefficient: 6.56e+02
Value: severe
d__Bacteria.p__Proteobacteria.c__Deltaproteobacteria.o__Des
3000
2000
1000
0
asymptomatic (n=24)
mild (n=25)
severe (n=30)
control (n=72)
Severity

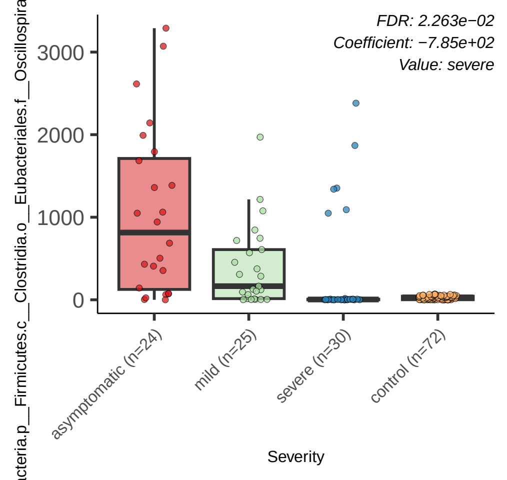

FDR: 2.263e−02
Coefficient: −7.85e+02
Value: severe
acteria.p__Firmicutes.c__Clostridia.o__Eubacteriales.f__Oscillospira
0
1000
2000
3000
asymptomatic (n=24)
mild (n=25)
severe (n=30)
control (n=72)
Severity

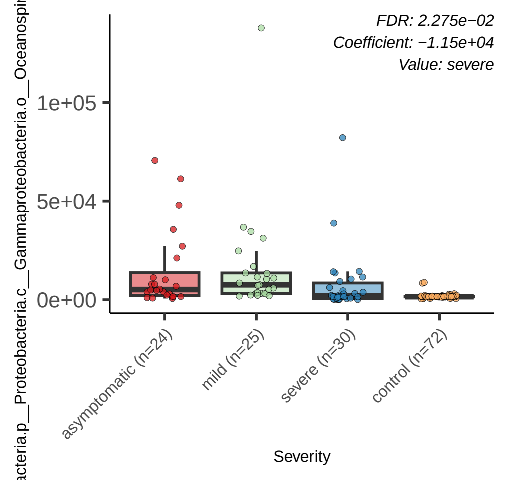

FDR: 2.275e−02
Coefficient: −1.15e+04
Value: severe
1e+05
5e+04
0e+00
acteria.p__Proteobacteria.c__Gammaproteobacteria.o__Oceanospi
asymptomatic (n=24)
mild (n=25)
severe (n=30)
control (n=72)
Severity

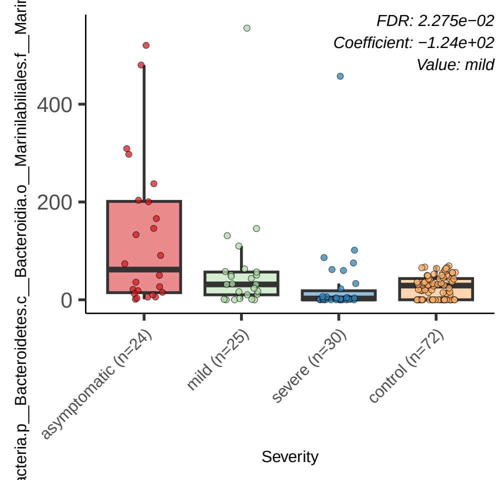

FDR: 2.275e−02
Coefficient: −1.24e+02
Value: mild
400
200
0
asymptomatic (n=24)
mild (n=25)
severe (n=30)
control (n=72)
Severity
acteria.p__Bacteroidetes.c__Bacteroidia.o__Marinilabiliales.f__Mari

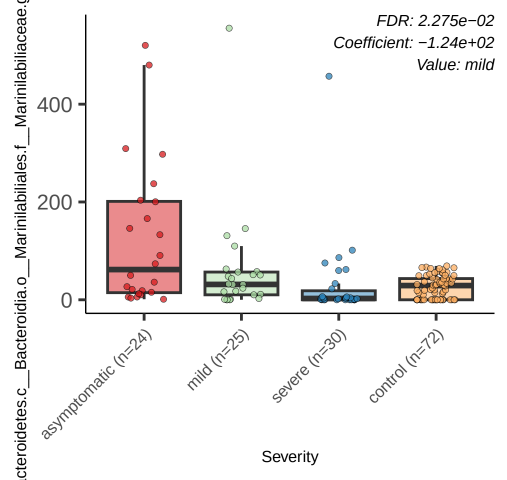

FDR: 2.275e−02
Coefficient: −1.24e+02
Value: mild
400
200
0
acteroidetes.c__Bacteroidia.o__Marinilabiliales.f__Marinilabiliaceae.g
asymptomatic (n=24)
mild (n=25)
severe (n=30)
control (n=72)
Severity

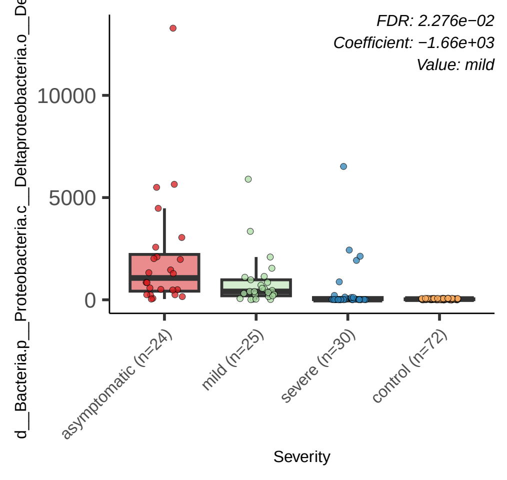
FDR: 2.276e−02
Coefficient: −1.66e+03
Value: mild
10000
5000
0
d__Bacteria.p__Proteobacteria.c__Deltaproteobacteria.o__De
asymptomatic (n=24)
mild (n=25)
severe (n=30)
control (n=72)
Severity

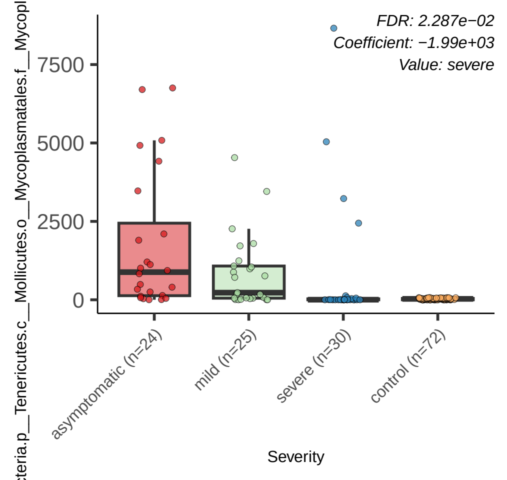

FDR: 2.287e−02
Coefficient: −1.99e+03
Value: severe
7500
5000
2500
0
teria.p__Tenericutes.c__Mollicutes.o__Mycoplasmatales.f__Mycopl
asymptomatic (n=24)
mild (n=25)
severe (n=30)
control (n=72)
Severity

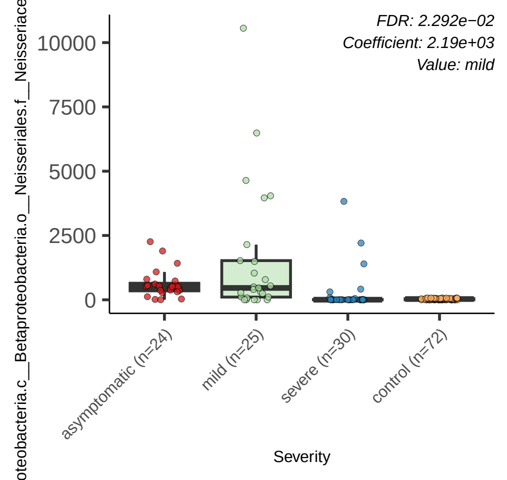

FDR: 2.292e−02
Coefficient: 2.19e+03
Value: mild
10000
7500
5000
2500
0
oteobacteria.c__Betaproteobacteria.o__Neisseriales.f__Neisseriace
asymptomatic (n=24)
mild (n=25)
severe (n=30)
control (n=72)
Severity

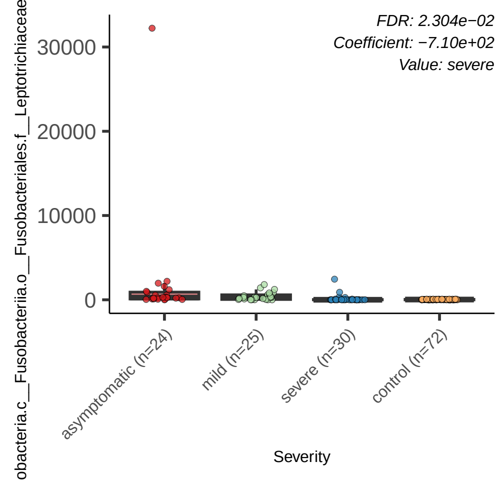

FDR: 2.304e−02
Coefficient: −7.10e+02
Value: severe
30000
20000
10000
0
obacteria.c__Fusobacteriia.o__Fusobacteriales.f__Leptotrichiaceae
asymptomatic (n=24)
mild (n=25)
severe (n=30)
control (n=72)
Severity

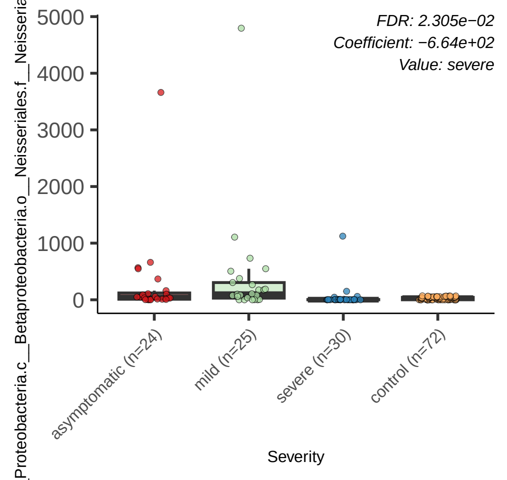

FDR: 2.305e−02
Coefficient: −6.64e+02
Value: severe
Proteobacteria.c__Betaproteobacteria.o__Neisseriales.f__Neisseria
5000
4000
3000
2000
1000
0
asymptomatic (n=24)
mild (n=25)
severe (n=30)
control (n=72)
Severity

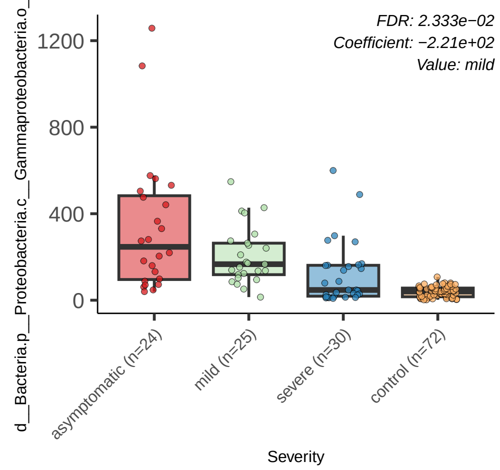

FDR: 2.333e−02
Coefficient: −2.21e+02
Value: mild
d__Bacteria.p__Proteobacteria.c__Gammaproteobacteria.o__
1200
800
400
0
asymptomatic (n=24)
mild (n=25)
severe (n=30)
control (n=72)
Severity

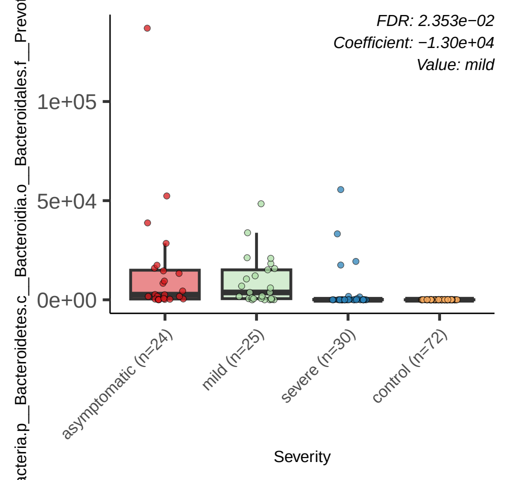

FDR: 2.353e−02
Coefficient: −1.30e+04
Value: mild
1e+05
5e+04
0e+00
acteria.p__Bacteroidetes.c__Bacteroidia.o__Bacteroidales.f__Prevot
asymptomatic (n=24)
mild (n=25)
severe (n=30)
control (n=72)
Severity

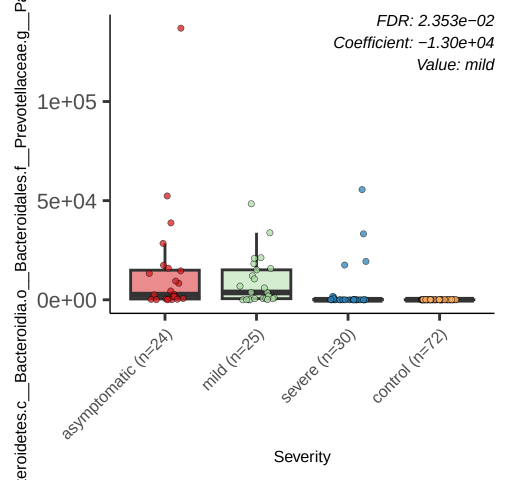

FDR: 2.353e−02
Coefficient: −1.30e+04
Value: mild
teroidetes.c__Bacteroidia.o__Bacteroidales.f__Prevotellaceae.g__Pa
1e+05
5e+04
0e+00
asymptomatic (n=24)
mild (n=25)
severe (n=30)
control (n=72)
Severity

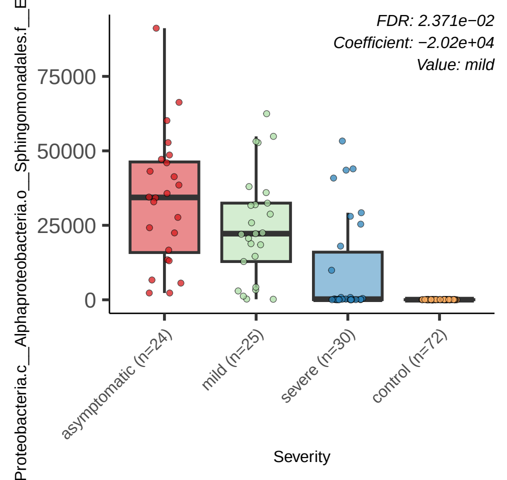
FDR: 2.371e−02
Coefficient: −2.02e+04
Value: mild
75000
50000
25000
0
asymptomatic (n=24)
mild (n=25)
severe (n=30)
control (n=72)
Severity
Proteobacteria.c__Alphaproteobacteria.o__Sphingomonadales.f__E

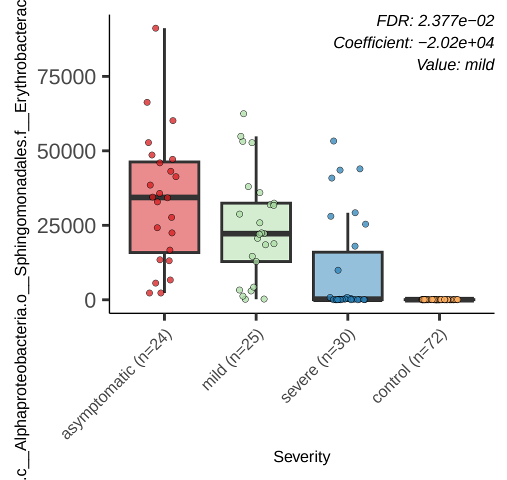

FDR: 2.377e−02
Coefficient: −2.02e+04
Value: mild
75000
50000
25000
0
.c__Alphaproteobacteria.o__Sphingomonadales.f__Erythrobacterac
asymptomatic (n=24)
mild (n=25)
severe (n=30)
control (n=72)
Severity

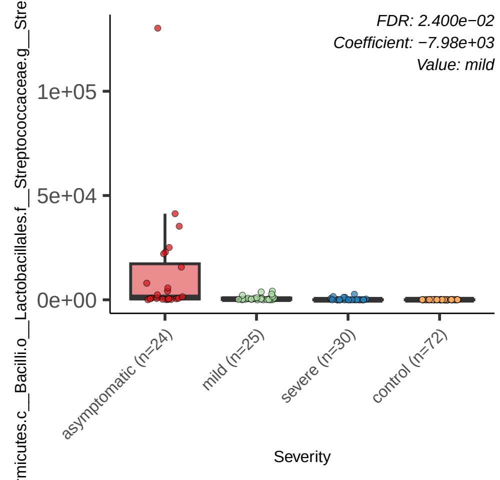
FDR: 2.400e−02
Coefficient: −7.98e+03
Value: mild
micutes.c__Bacilli.o__Lactobacillales.f__Streptococcaceae.g__Stre
1e+05
5e+04
0e+00
asymptomatic (n=24)
mild (n=25)
severe (n=30)
control (n=72)
Severity

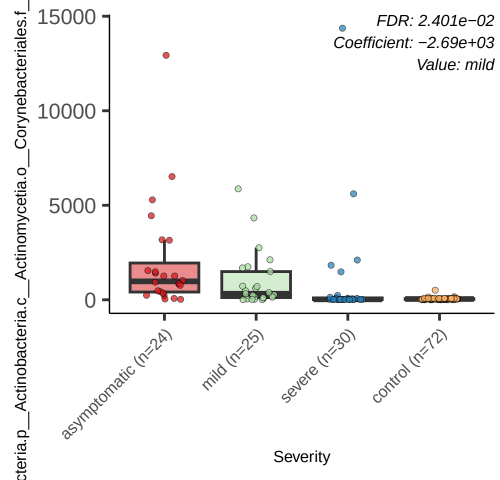

FDR: 2.401e−02
Coefficient: −2.69e+03
Value: mild
15000
10000
5000
0
cteria.p__Actinobacteria.c__Actinomycetia.o__Corynebacteriales.f__
asymptomatic (n=24)
mild (n=25)
severe (n=30)
control (n=72)
Severity

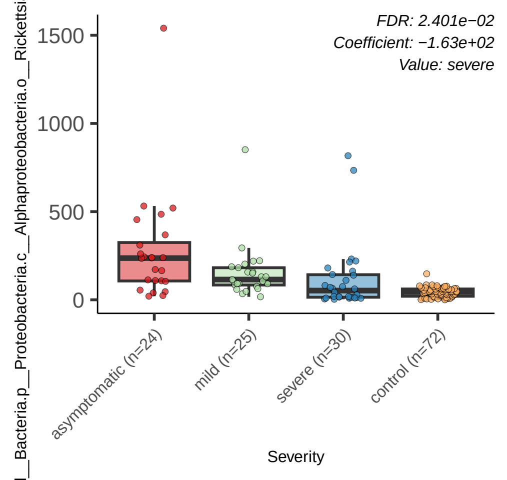
FDR: 2.401e−02
Coefficient: −1.63e+02
Value: severe
Bacteria.p__Proteobacteria.c__Alphaproteobacteria.o__Rickettsi
1500
1000
500
0
asymptomatic (n=24)
mild (n=25)
severe (n=30)
control (n=72)
Severity

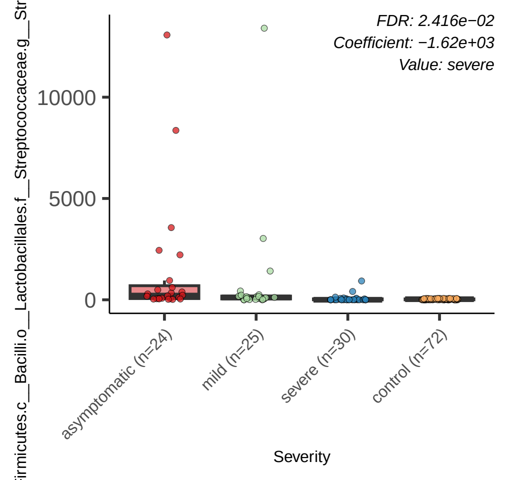

FDR: 2.416e−02
Coefficient: −1.62e+03
Value: severe
Firmicutes.c__Bacilli.o__Lactobacillales.f__Streptococcaceae.g__Str
10000
5000
0
asymptomatic (n=24)
mild (n=25)
severe (n=30)
control (n=72)
Severity

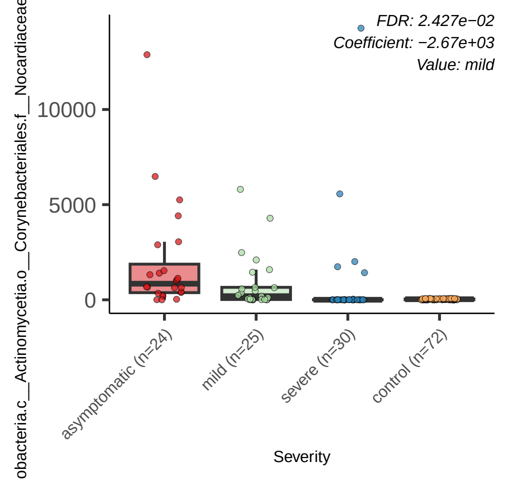
FDR: 2.427e−02
Coefficient: −2.67e+03
Value: mild
obacteria.c__Actinomycetia.o__Corynebacteriales.f__Nocardiaceae
10000
5000
0
asymptomatic (n=24)
mild (n=25)
severe (n=30)
control (n=72)
Severity

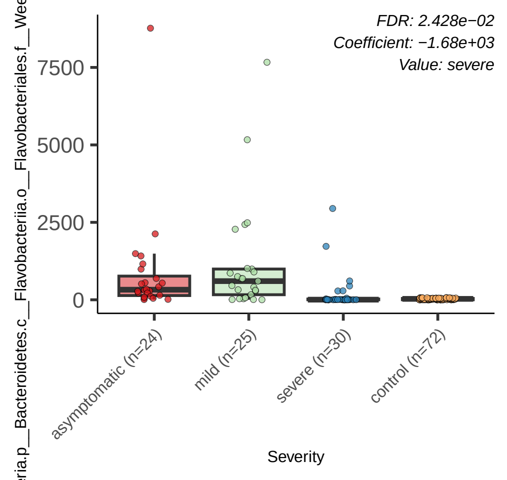

FDR: 2.428e−02
Coefficient: −1.68e+03
Value: severe
7500
5000
2500
0
asymptomatic (n=24)
mild (n=25)
severe (n=30)
control (n=72)
Severity
eria.p__Bacteroidetes.c__Flavobacteriia.o__Flavobacteriales.f__Wee

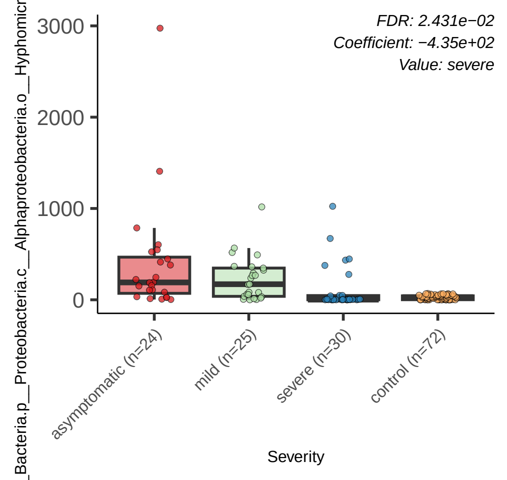
FDR: 2.431e−02
Coefficient: −4.35e+02
Value: severe
3000
2000
1000
0
_Bacteria.p__Proteobacteria.c__Alphaproteobacteria.o__Hyphomicr
asymptomatic (n=24)
mild (n=25)
severe (n=30)
control (n=72)
Severity

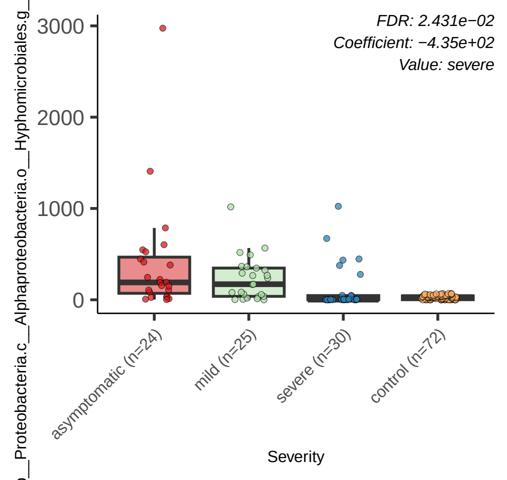

FDR: 2.431e−02
Coefficient: −4.35e+02
Value: severe
3000
2000
1000
0
Proteobacteria.c__Alphaproteobacteria.o__Hyphomicrobiales.g__
asymptomatic (n=24)
mild (n=25)
severe (n=30)
control (n=72)
Severity

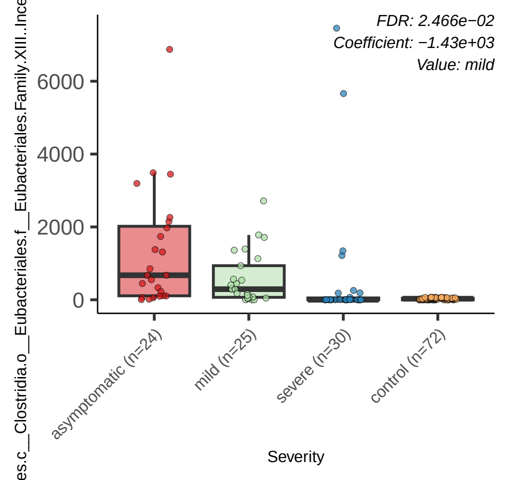

FDR: 2.466e−02
Coefficient: −1.43e+03
Value: mild
6000
4000
2000
0
es.c__Clostridia.o__Eubacteriales.f__Eubacteriales.Family.XIII..Ince
asymptomatic (n=24)
mild (n=25)
severe (n=30)
control (n=72)
Severity

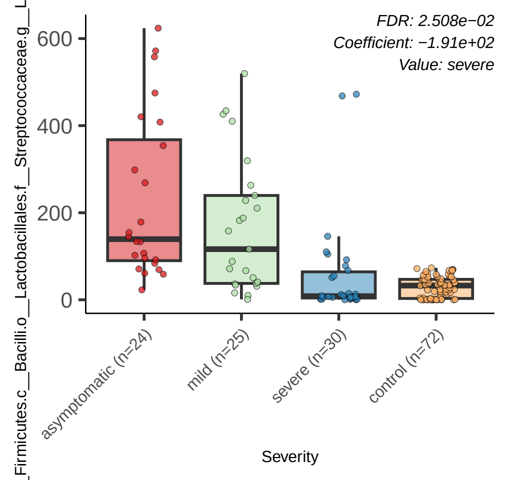
FDR: 2.508e−02
Coefficient: −1.91e+02
Value: severe
Firmicutes.c__Bacilli.o__Lactobacillales.f__Streptococcaceae.g__L
600
400
200
0
asymptomatic (n=24)
mild (n=25)
severe (n=30)
control (n=72)
Severity

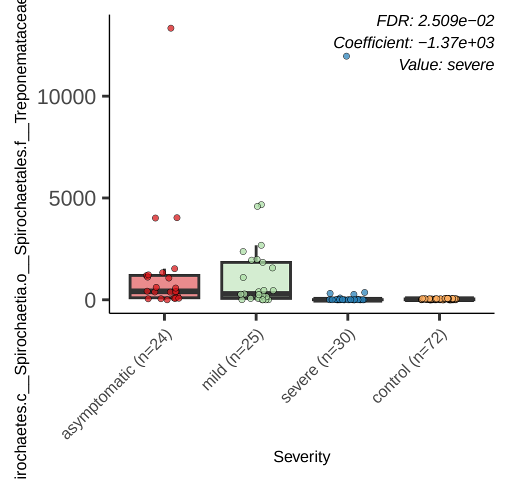

FDR: 2.509e−02
Coefficient: −1.37e+03
Value: severe
irochaetes.c__Spirochaetia.o__Spirochaetales.f__Treponemataceae
10000
5000
0
asymptomatic (n=24)
mild (n=25)
severe (n=30)
control (n=72)
Severity

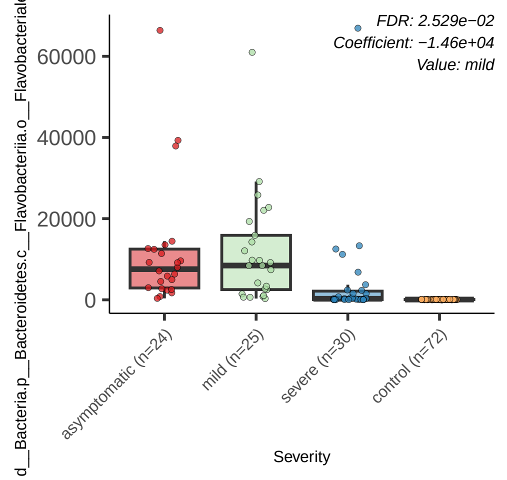

FDR: 2.529e−02
Coefficient: −1.46e+04
Value: mild
d__Bacteria.p__Bacteroidetes.c__Flavobacteriia.o__Flavobacteriale
60000
40000
20000
0
asymptomatic (n=24)
mild (n=25)
severe (n=30)
control (n=72)
Severity

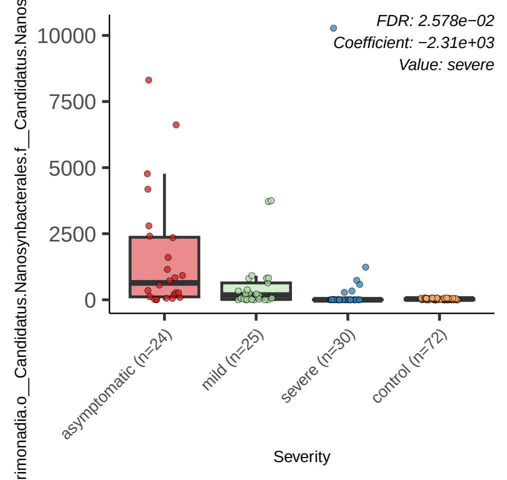

FDR: 2.578e−02
Coefficient: −2.31e+03
Value: severe
10000
7500
5000
2500
0
rimonadia.o__Candidatus.Nanosynbacterales.f__Candidatus.Nanos
asymptomatic (n=24)
mild (n=25)
severe (n=30)
control (n=72)
Severity

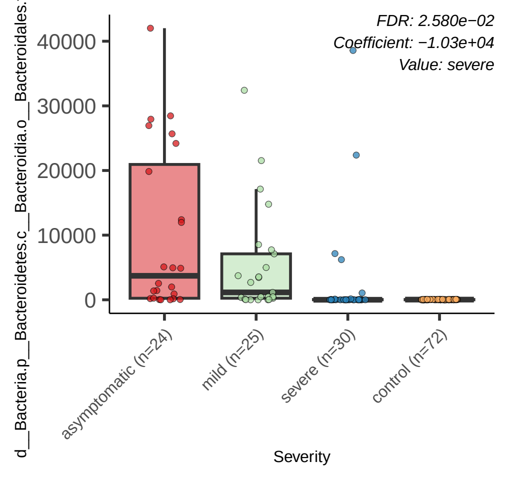

FDR: 2.580e−02
Coefficient: −1.03e+04
Value: severe
d__Bacteria.p__Bacteroidetes.c__Bacteroidia.o__Bacteroidales.f
40000
30000
20000
10000
0
asymptomatic (n=24)
mild (n=25)
severe (n=30)
control (n=72)
Severity

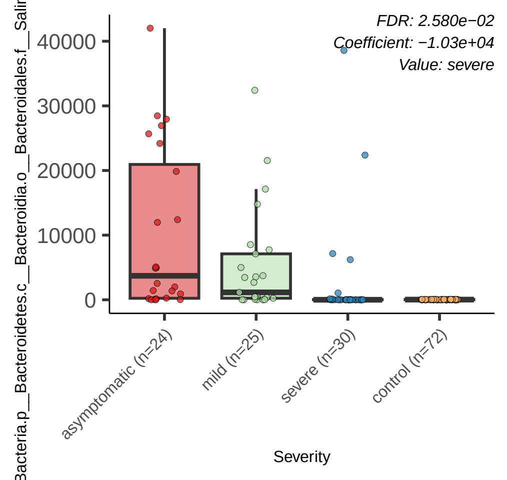
FDR: 2.580e−02
Coefficient: −1.03e+04
Value: severe
Bacteria.p__Bacteroidetes.c__Bacteroidia.o__Bacteroidales.f__Sali
40000
30000
20000
10000
0
asymptomatic (n=24)
mild (n=25)
severe (n=30)
control (n=72)
Severity

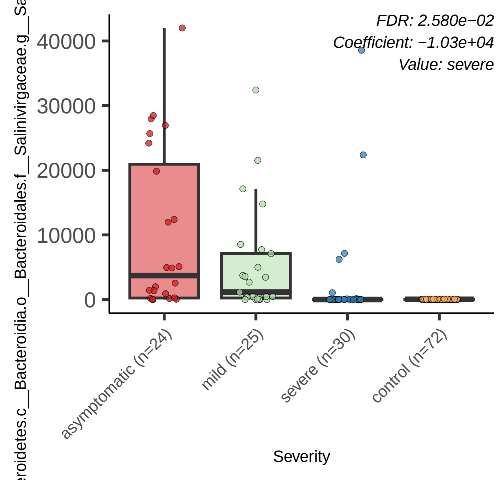

FDR: 2.580e−02
Coefficient: −1.03e+04
Value: severe
40000
30000
20000
10000
0
asymptomatic (n=24)
mild (n=25)
severe (n=30)
control (n=72)
Severity

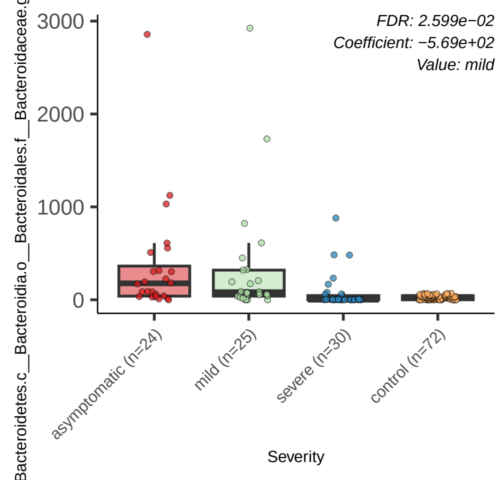
FDR: 2.599e−02
Coefficient: −5.69e+02
Value: mild
3000
2000
1000
0
Bacteroidetes.c__Bacteroidia.o__Bacteroidales.f__Bacteroidaceae.g
asymptomatic (n=24)
mild (n=25)
severe (n=30)
control (n=72)
Severity

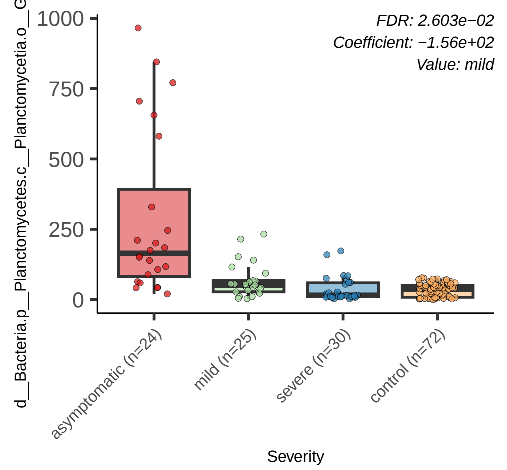
FDR: 2.603e−02
Coefficient: −1.56e+02
Value: mild
d__Bacteria.p__Planctomycetes.c__Planctomycetia.o__G
1000
750
500
250
0
asymptomatic (n=24)
mild (n=25)
severe (n=30)
control (n=72)
Severity

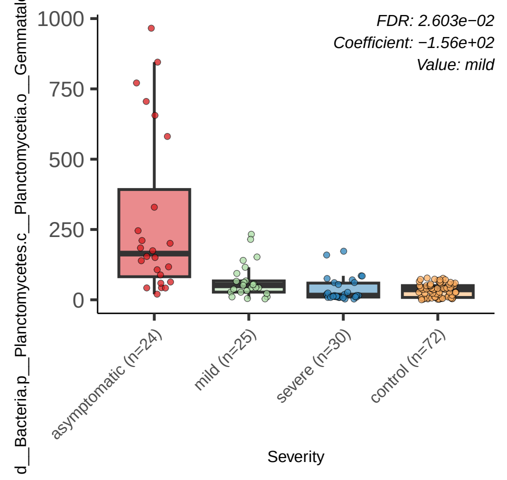

FDR: 2.603e−02
Coefficient: −1.56e+02
Value: mild
d__Bacteria.p__Planctomycetes.c__Planctomycetia.o__Gemmatalo
1000
750
500
250
0
asymptomatic (n=24)
mild (n=25)
severe (n=30)
control (n=72)
Severity

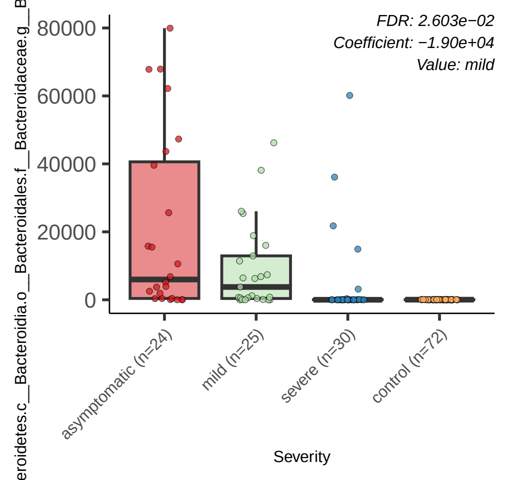

FDR: 2.603e−02
Coefficient: −1.90e+04
Value: mild
80000
60000
40000
20000
0
eroidetes.c__Bacteroidia.o__Bacteroidales.f__Bacteroidaceae.g__B
asymptomatic (n=24)
mild (n=25)
severe (n=30)
control (n=72)
Severity

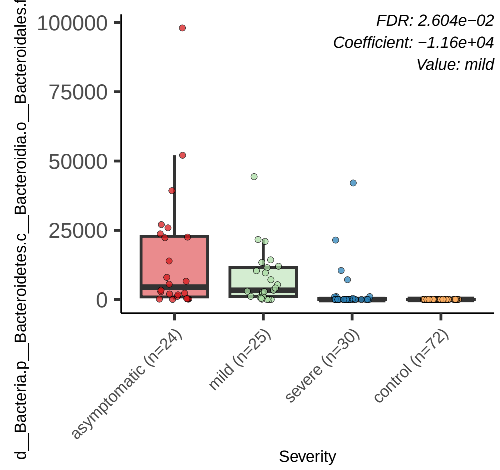

FDR: 2.604e−02
Coefficient: −1.16e+04
Value: mild
d__Bacteria.p__Bacteroidetes.c__Bacteroidia.o__Bacteroidales.f
100000
75000
50000
25000
0
asymptomatic (n=24)
mild (n=25)
severe (n=30)
control (n=72)
Severity

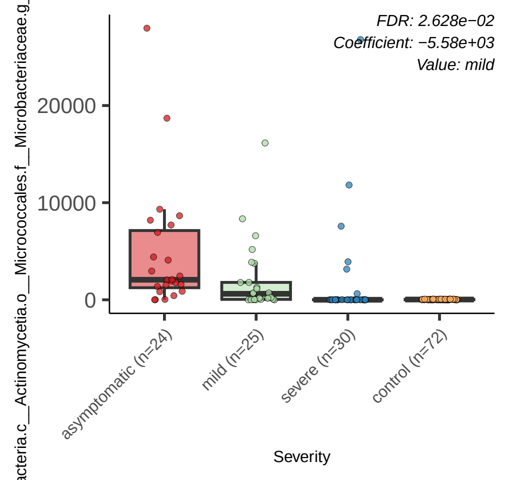

FDR: 2.628e−02
Coefficient: −5.58e+03
Value: mild
20000
10000
0
asymptomatic (n=24)
mild (n=25)
severe (n=30)
control (n=72)
Severity
acteria.c__Actinomycetia.o__Micrococcales.f__Microbacteriaceae.g_

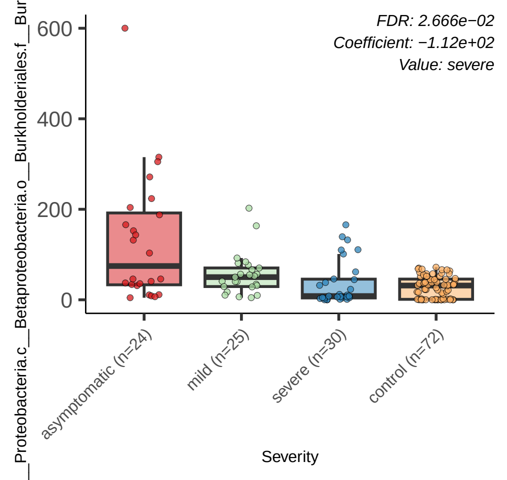
FDR: 2.666e−02
Coefficient: −1.12e+02
Value: severe
_Proteobacteria.c__Betaproteobacteria.o__Burkholderiales.f__Bur
600
400
200
0
asymptomatic (n=24)
mild (n=25)
severe (n=30)
control (n=72)
Severity

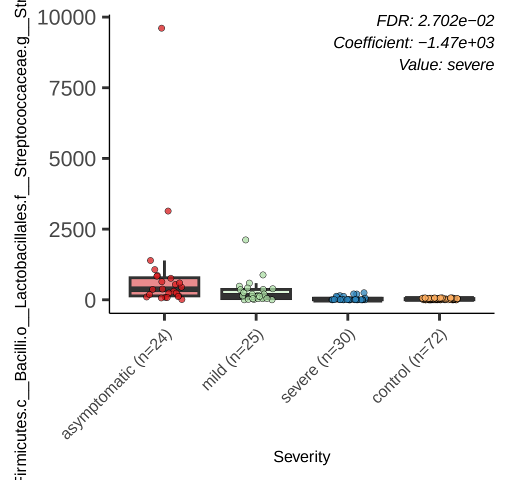

Firmicutes.c__Bacilli.o__Lactobacillales.f__Streptococcaceae.g__Str
FDR: 2.702e−02
Coefficient: −1.47e+03
Value: severe
10000
7500
5000
2500
0
asymptomatic (n=24)
mild (n=25)
severe (n=30)
control (n=72)
Severity

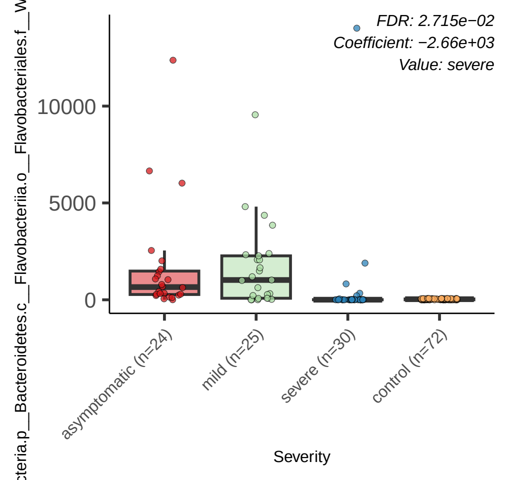

FDR: 2.715e−02
Coefficient: −2.66e+03
Value: severe
10000
5000
0
cteria.p__Bacteroidetes.c__Flavobacteriia.o__Flavobacteriales.f__W
asymptomatic (n=24)
mild (n=25)
severe (n=30)
control (n=72)
Severity

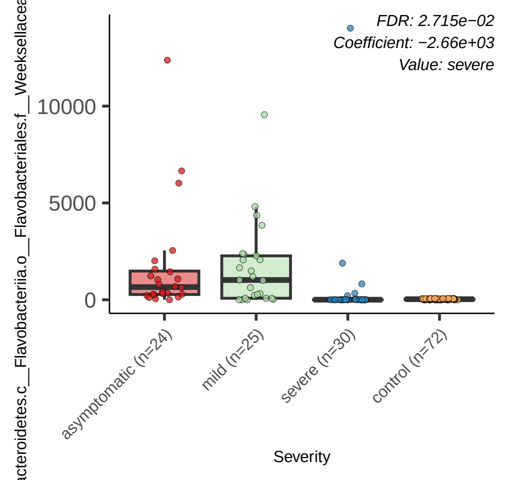
FDR: 2.715e−02
Coefficient: −2.66e+03
Value: severe
acteroidetes.c__Flavobacteriia.o__Flavobacteriales.f__Weeksellacea
10000
5000
0
asymptomatic (n=24)
mild (n=25)
severe (n=30)
control (n=72)
Severity

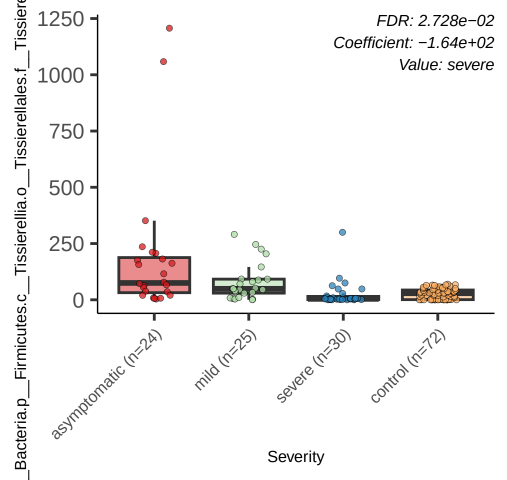

FDR: 2.728e−02
Coefficient: −1.64e+02
Value: severe
_Bacteria.p__Firmicutes.c__Tissierellia.o__Tissierellales.f__Tissiere
1250
1000
750
500
250
0
asymptomatic (n=24)
mild (n=25)
severe (n=30)
control (n=72)
Severity

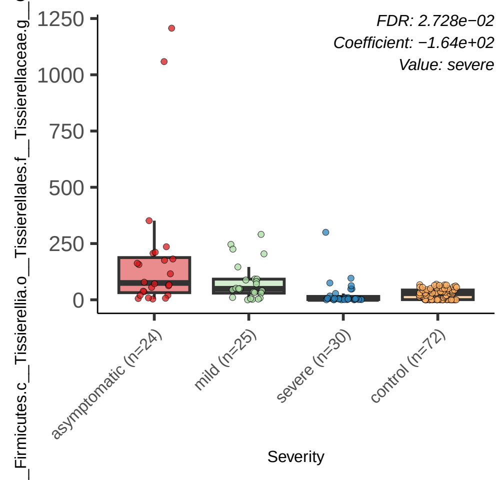

FDR: 2.728e−02
Coefficient: −1.64e+02
Value: severe
Firmicutes.c__Tissierellia.o__Tissierellales.f__Tissierellaceae.g__
1250
1000
750
500
250
0
asymptomatic (n=24)
mild (n=25)
severe (n=30)
control (n=72)
Severity

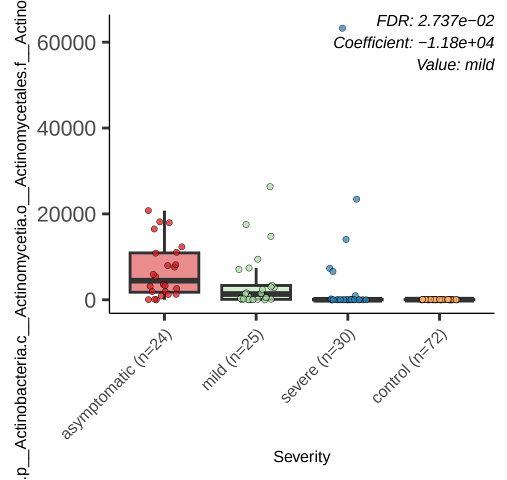
FDR: 2.737e−02
Coefficient: −1.18e+04
Value: mild
p__Actinobacteria.c__Actinomycetia.o__Actinomycetales.f__Actino
60000
40000
20000
0
asymptomatic (n=24)
mild (n=25)
severe (n=30)
control (n=72)
Severity

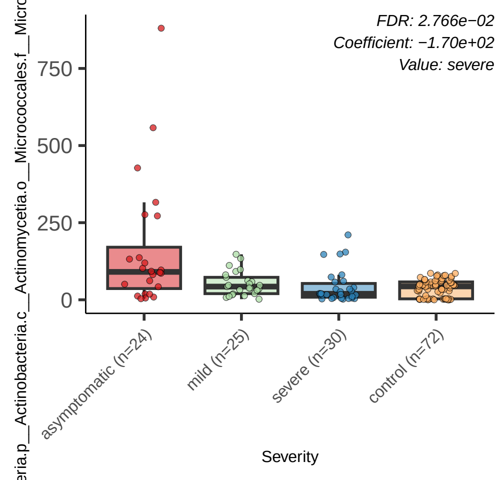

FDR: 2.766e−02
Coefficient: −1.70e+02
Value: severe
750
500
250
0
Actinobacteria.c__Actinomycetia.o__Micrococcales.f__Micro
asymptomatic (n=24)
mild (n=25)
severe (n=30)
control (n=72)
Severity

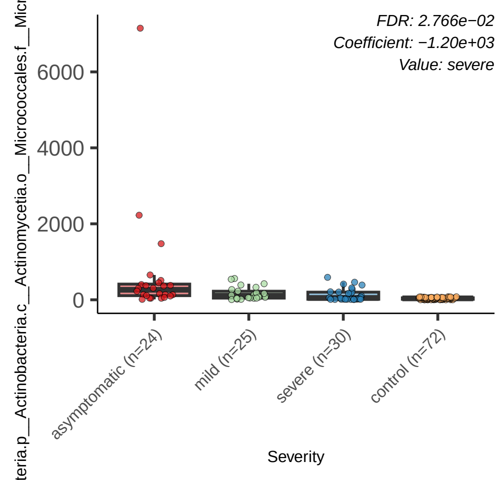

FDR: 2.766e−02
Coefficient: −1.20e+03
Value: severe
0
2000
4000
6000
teria.p__Actinobacteria.c__Actinomycetia.o__Micrococcales.f__Micr
asymptomatic (n=24)
mild (n=25)
severe (n=30)
control (n=72)
Severity

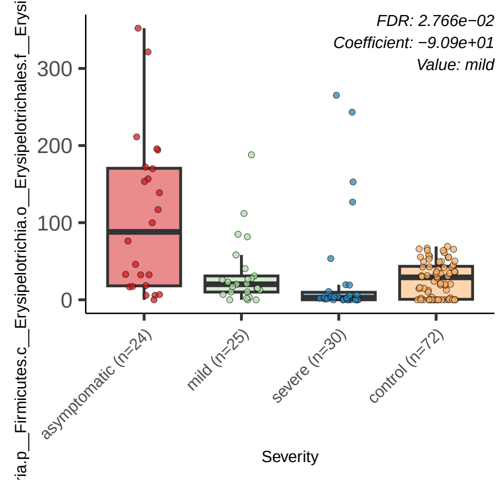
FDR: 2.766e−02
Coefficient: −9.09e+01
Value: mild
300
200
100
0
asymptomatic (n=24)
mild (n=25)
severe (n=30)
control (n=72)
Severity

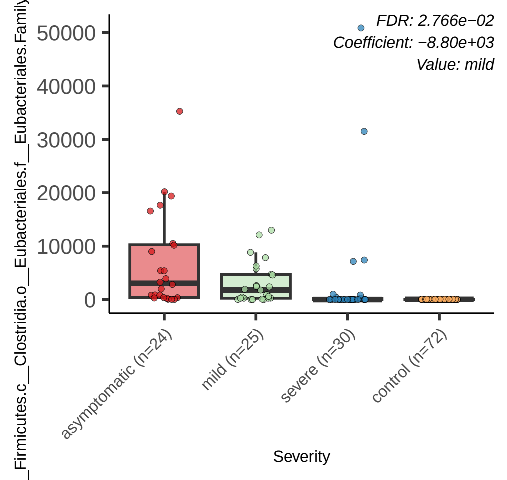
FDR: 2.766e−02
Coefficient: −8.80e+03
Value: mild
Firmicutes.c__Clostridia.o__Eubacteriales.f__Eubacteriales.Family
50000
40000
30000
20000
10000
0
asymptomatic (n=24)
mild (n=25)
severe (n=30)
control (n=72)
Severity

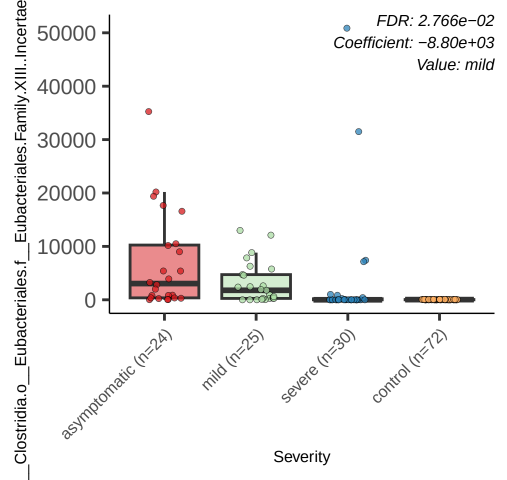

FDR: 2.766e−02
Coefficient: −8.80e+03
Value: mild
50000
40000
30000
20000
10000
0
Clostridia.o__Eubacteriales.f__Eubacteriales.Family.XIII..Incertae
asymptomatic (n=24)
mild (n=25)
severe (n=30)
control (n=72)
Severity

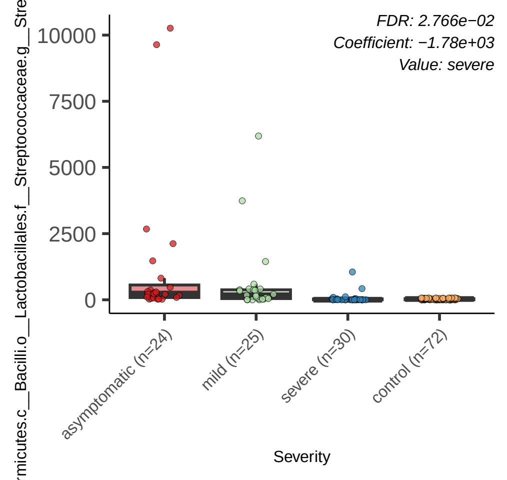

FDR: 2.766e−02
Coefficient: −1.78e+03
Value: severe
10000
7500
5000
2500
0
rmicutes.c__Bacilli.o__Lactobacillales.f__Streptococcaceae.g__Stre
asymptomatic (n=24)
mild (n=25)
severe (n=30)
control (n=72)
Severity

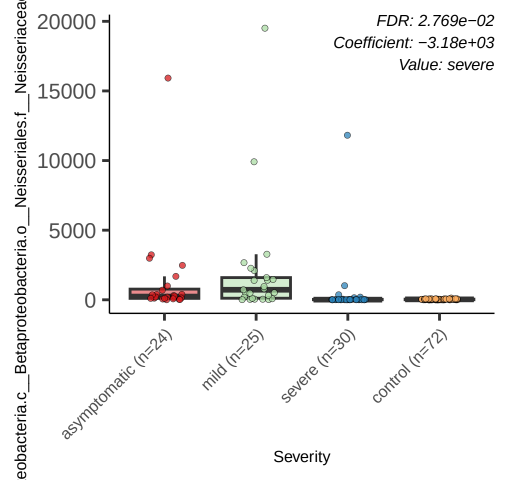

FDR: 2.769e−02
Coefficient: −3.18e+03
Value: severe
20000
15000
10000
5000
0
eobacteria.c__Betaproteobacteria.o__Neisseriales.f__Neisseriacea
asymptomatic (n=24)
mild (n=25)
severe (n=30)
control (n=72)
Severity

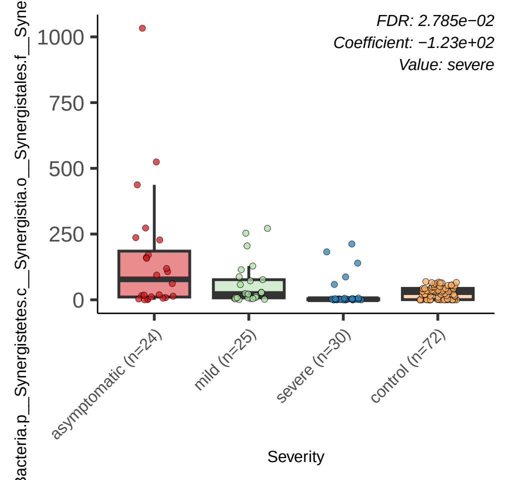

FDR: 2.785e−02
Coefficient: −1.23e+02
Value: severe
Bacteria.p__Synergistetes.c__Synergistia.o__Synergistales.f__Syne
1000
750
500
250
0
asymptomatic (n=24)
mild (n=25)
severe (n=30)
control (n=72)
Severity

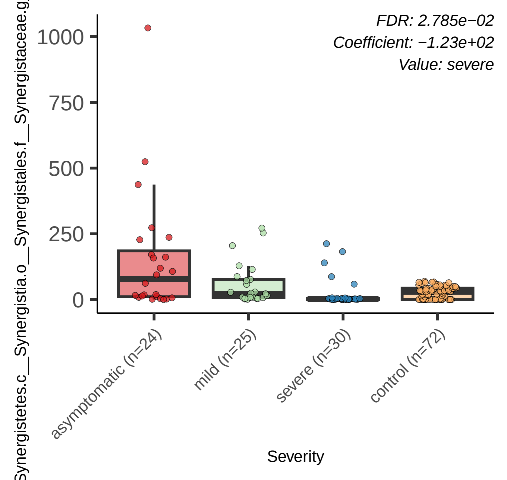

FDR: 2.785e−02
Coefficient: −1.23e+02
Value: severe
1000
750
500
250
0
Synergistetes.c__Synergistia.o__Synergistales.f__Synergistaceae.g
asymptomatic (n=24)
mild (n=25)
severe (n=30)
control (n=72)
Severity

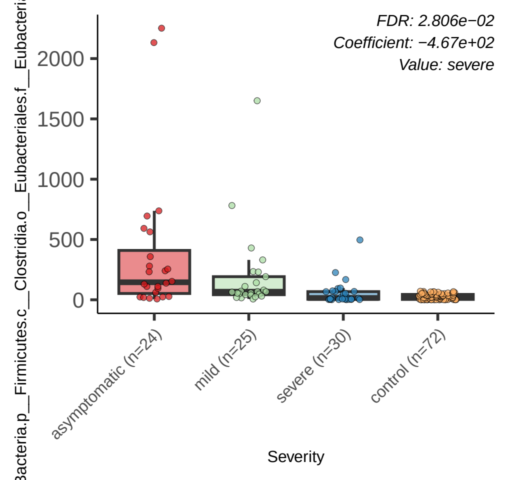

FDR: 2.806e−02
Coefficient: −4.67e+02
Value: severe
Bacteria.p__Firmicutes.c__Clostridia.o__Eubacteriales.f__Eubacteri
0
500
1000
1500
2000
asymptomatic (n=24)
mild (n=25)
severe (n=30)
control (n=72)
Severity

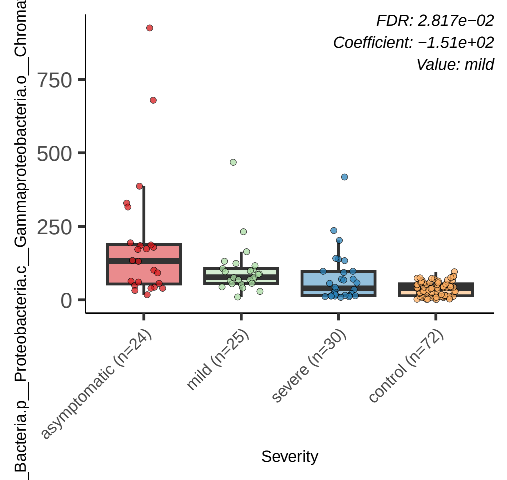

FDR: 2.817e−02
Coefficient: −1.51e+02
Value: mild
_Bacteria.p__Proteobacteria.c__Gammaproteobacteria.o__Chroma
750
500
250
0
asymptomatic (n=24)
mild (n=25)
severe (n=30)
control (n=72)
Severity

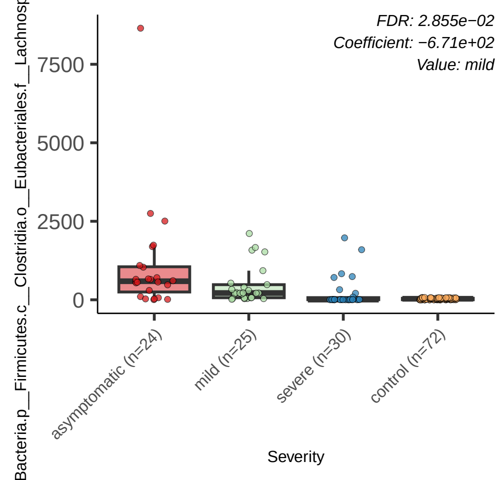

FDR: 2.855e−02
Coefficient: −6.71e+02
Value: mild
Bacteria.p__Firmicutes.c__Clostridia.o__Eubacteriales.f__Lachnosp
7500
5000
2500
0
asymptomatic (n=24)
mild (n=25)
severe (n=30)
control (n=72)
Severity

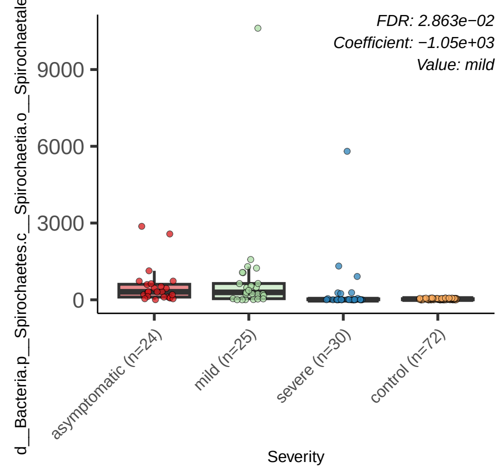

FDR: 2.863e−02
Coefficient: −1.05e+03
Value: mild
9000
6000
3000
0
d__Bacteria.p__Spirochaetes.c__Spirochaetia.o__Spirochaetale
asymptomatic (n=24)
mild (n=25)
severe (n=30)
control (n=72)
Severity

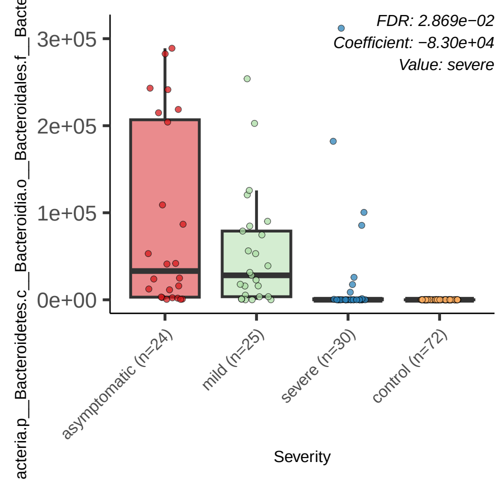

acteria.p__Bacteroidetes.c__Bacteroidia.o__Bacteroidales.f__Bacte
FDR: 2.869e−02
Coefficient: −8.30e+04
Value: severe
3e+05
2e+05
1e+05
0e+00
asymptomatic (n=24)
mild (n=25)
severe (n=30)
control (n=72)
Severity

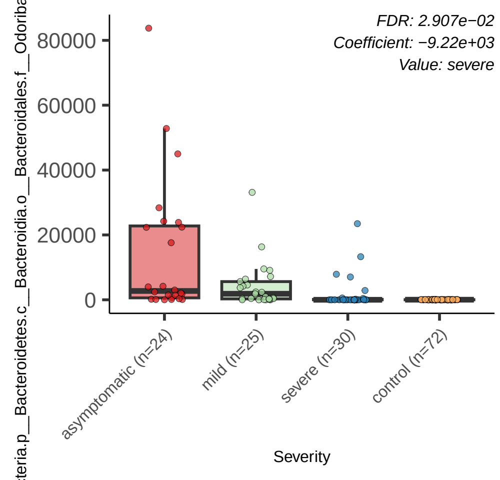

FDR: 2.907e−02
Coefficient: −9.22e+03
Value: severe
80000
60000
40000
20000
0
asymptomatic (n=24)
mild (n=25)
severe (n=30)
control (n=72)
Severity

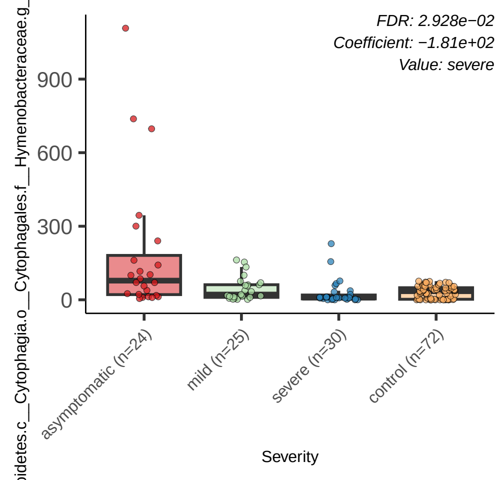

FDR: 2.928e−02
Coefficient: −1.81e+02
Value: severe
900
600
300
0
bidetes.c__Cytophagia.o__Cytophagales.f__Hymenobacteraceae.g_
asymptomatic (n=24)
mild (n=25)
severe (n=30)
control (n=72)
Severity

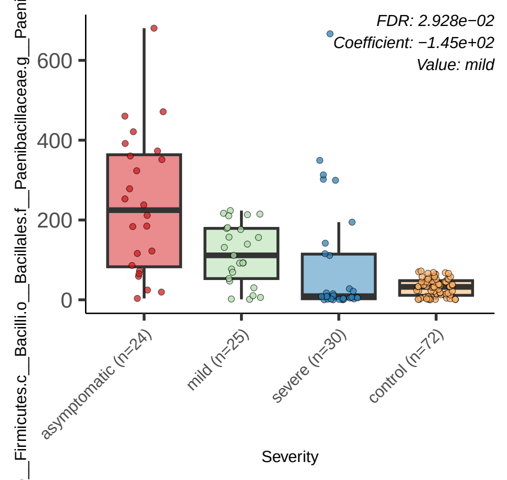

FDR: 2.928e−02
Coefficient: −1.45e+02
Value: mild
Firmicutes.c__Bacilli.o__Bacillales.f__Paenibacillaceae.g__Paeni
0
200
400
600
asymptomatic (n=24)
mild (n=25)
severe (n=30)
control (n=72)
Severity

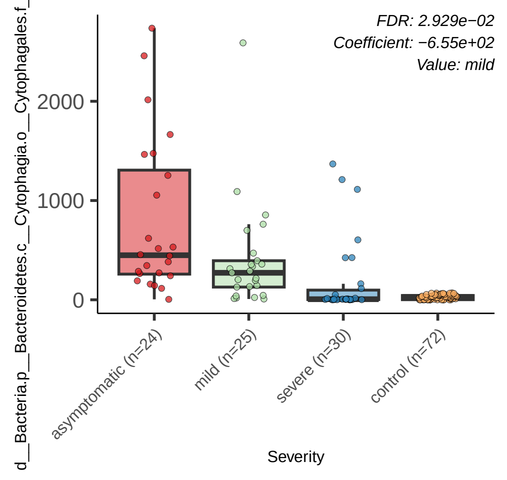

FDR: 2.929e−02
Coefficient: −6.55e+02
Value: mild
d__Bacteria.p__Bacteroidetes.c__Cytophagia.o__Cytophagales.f__
0
1000
2000
asymptomatic (n=24)
mild (n=25)
severe (n=30)
control (n=72)
Severity

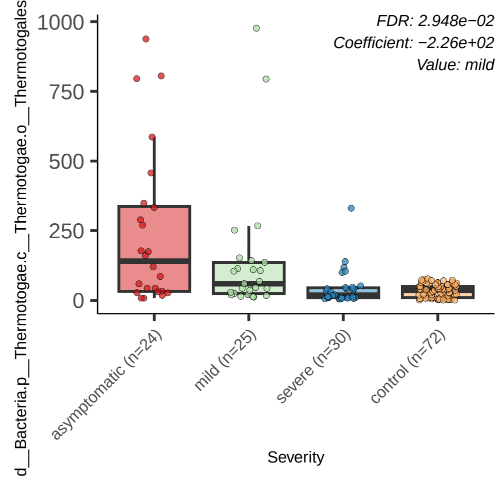

FDR: 2.948e−02
Coefficient: −2.26e+02
Value: mild
d__Bacteria.p__Thermotogae.c__Thermotogae.o__Thermotogales
1000
750
500
250
0
asymptomatic (n=24)
mild (n=25)
severe (n=30)
control (n=72)
Severity

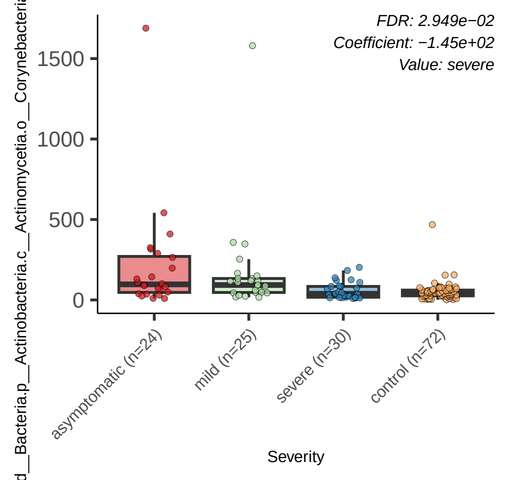
FDR: 2.949e−02
Coefficient: −1.45e+02
Value: severe
d__Bacteria.p__Actinobacteria.c__Actinomycetia.o__Corynebacteria
1500
1000
500
0
asymptomatic (n=24)
mild (n=25)
severe (n=30)
control (n=72)
Severity

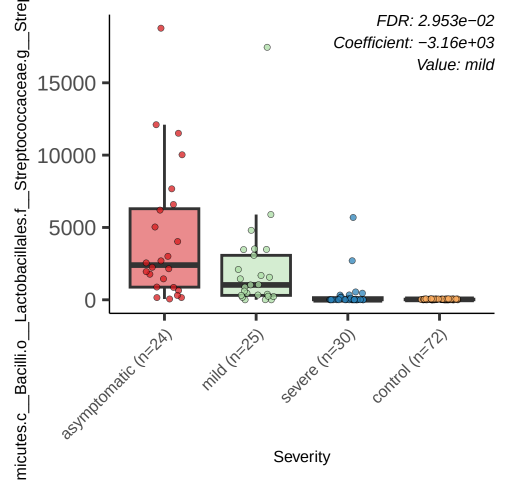

FDR: 2.953e−02
Coefficient: −3.16e+03
Value: mild
micutes.c__Bacilli.o__Lactobacillales.f__Streptococcaceae.g__Strep
15000
10000
5000
0
asymptomatic (n=24)
mild (n=25)
severe (n=30)
control (n=72)
Severity

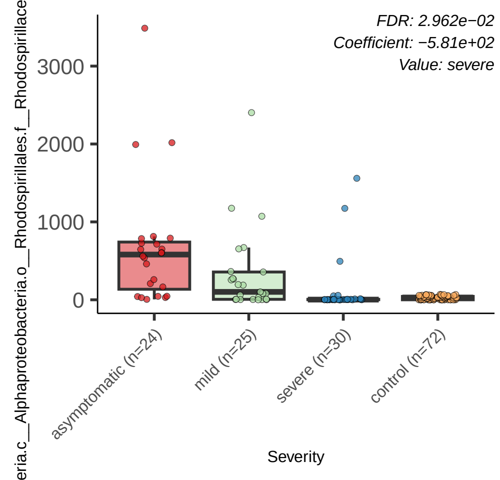

FDR: 2.962e−02
Coefficient: −5.81e+02
Value: severe
3000
2000
1000
0
eria.c__Alphaproteobacteria.o__Rhodospirillales.f__Rhodospirillace
asymptomatic (n=24)
mild (n=25)
severe (n=30)
control (n=72)
Severity

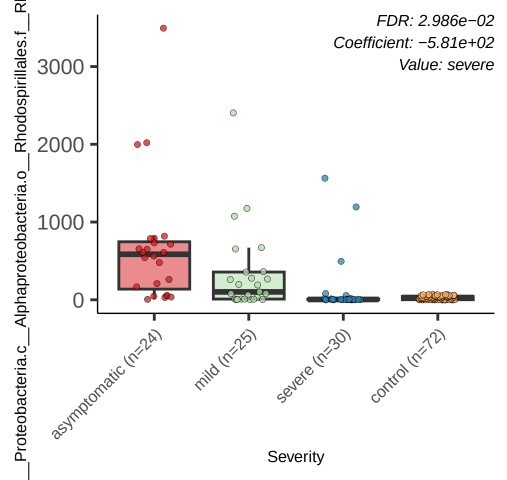
FDR: 2.986e−02
Coefficient: −5.81e+02
Value: severe
_Proteobacteria.c__Alphaproteobacteria.o__Rhodospirillales.f__Rh
3000
2000
1000
0
asymptomatic (n=24)
mild (n=25)
severe (n=30)
control (n=72)
Severity

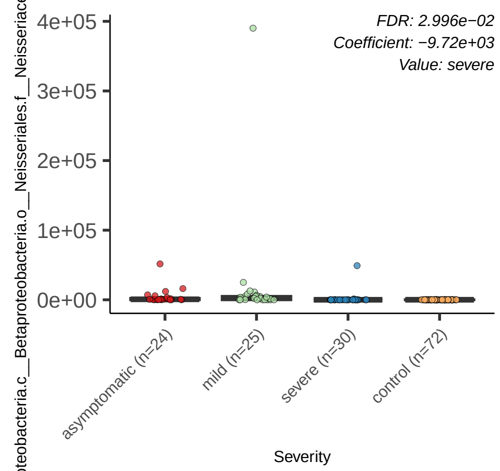
FDR: 2.996e−02
Coefficient: −9.72e+03
Value: severe
4e+05
3e+05
2e+05
1e+05
0e+00
asymptomatic (n=24)
mild (n=25)
severe (n=30)
control (n=72)
Severity

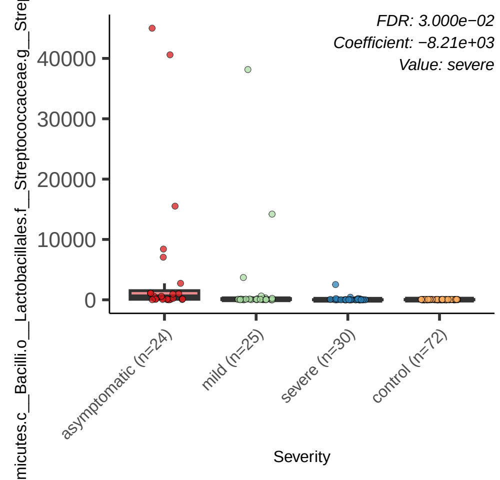

FDR: 3.000e−02
Coefficient: −8.21e+03
Value: severe
micutes.c__Bacilli.o__Lactobacillales.f__Streptococcaceae.g__Strep
0
10000
20000
30000
40000
asymptomatic (n=24)
mild (n=25)
severe (n=30)
control (n=72)
Severity

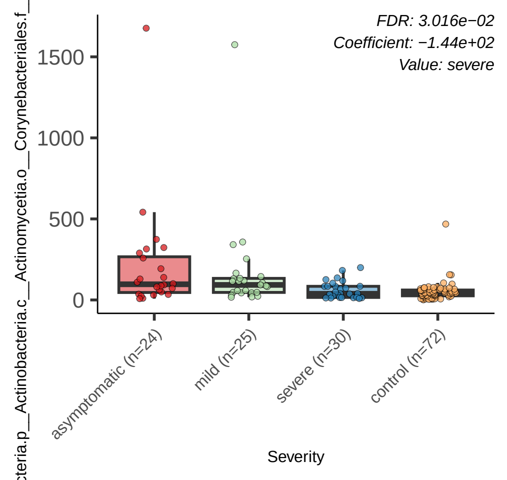
FDR: 3.016e−02
Coefficient: −1.44e+02
Value: severe
cteria.p__Actinobacteria.c__Actinomycetia.o__Corynebacteriales.f__
1500
1000
500
0
asymptomatic (n=24)
mild (n=25)
severe (n=30)
control (n=72)
Severity

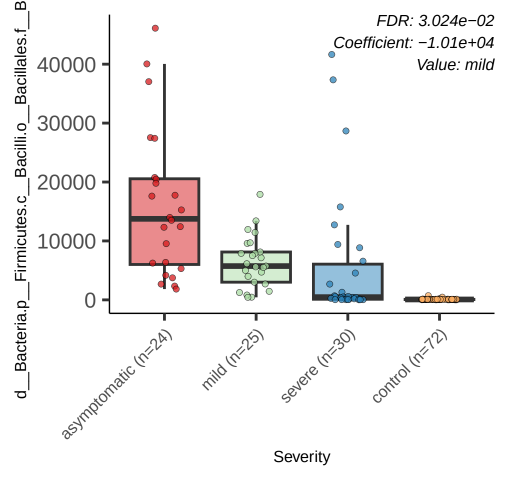

FDR: 3.024e−02
Coefficient: −1.01e+04
Value: mild
d__Bacteria.p__Firmicutes.c__Bacilli.o__Bacillales.f__B
40000
30000
20000
10000
0
asymptomatic (n=24)
mild (n=25)
severe (n=30)
control (n=72)
Severity

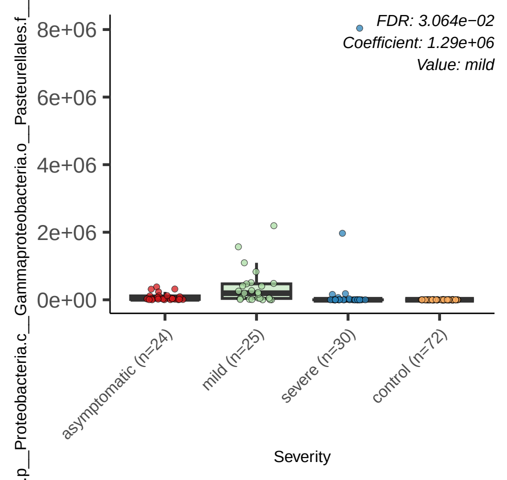
FDR: 3.064e−02
Coefficient: 1.29e+06
Value: mild
p__Proteobacteria.c__Gammaproteobacteria.o__Pasteurellales.f
8e+06
6e+06
4e+06
2e+06
0e+00
asymptomatic (n=24)
mild (n=25)
severe (n=30)
control (n=72)
Severity

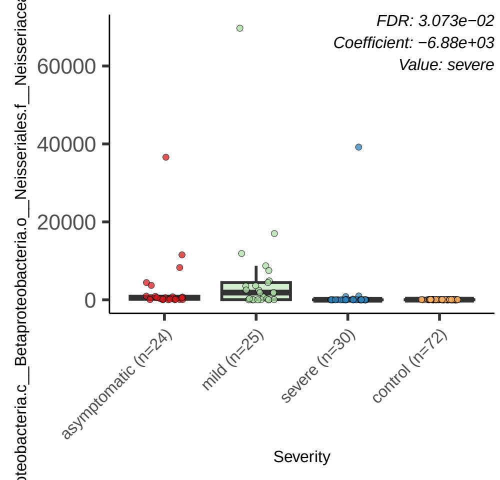

FDR: 3.073e−02
Coefficient: −6.88e+03
Value: severe
60000
40000
20000
0
asymptomatic (n=24)
mild (n=25)
severe (n=30)
control (n=72)
Severity
oteobacteria.c__Betaproteobacteria.o__Neisseriales.f__Neisseriace

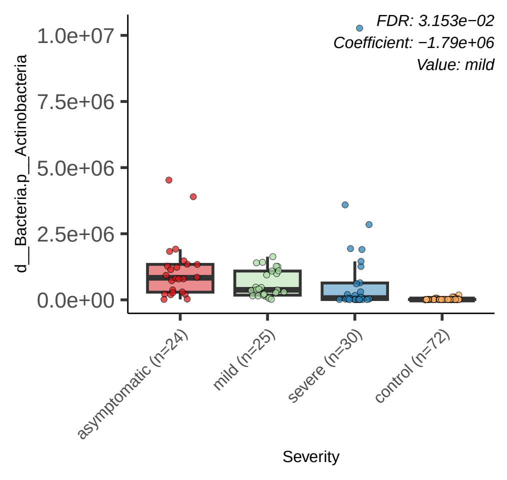
FDR: 3.153e−02
Coefficient: −1.79e+06
Value: mild
d__Bacteria.p__Actinobacteria
1.0e+07
7.5e+06
5.0e+06
2.5e+06
0.0e+00
asymptomatic (n=24)
mild (n=25)
severe (n=30)
control (n=72)
Severity

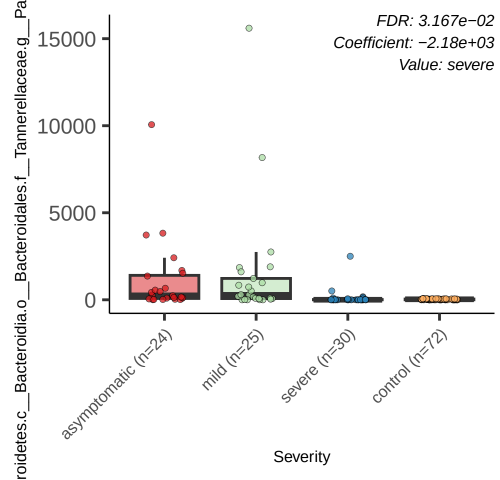

FDR: 3.167e−02
Coefficient: −2.18e+03
Value: severe
15000
10000
5000
0
asymptomatic (n=24)
mild (n=25)
severe (n=30)
control (n=72)
Severity
roidetes.c__Bacteroidia.o__Bacteroidales.f__Tannerellaceae.g__Pa

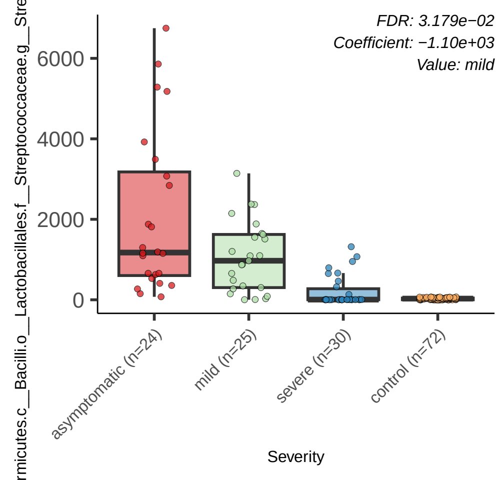

FDR: 3.179e−02
Coefficient: −1.10e+03
Value: mild
Firmicutes.c__Bacilli.o__Lactobacillales.f__Streptococcaceae.g__Stre
0
2000
4000
6000
asymptomatic (n=24)
mild (n=25)
severe (n=30)
control (n=72)
Severity

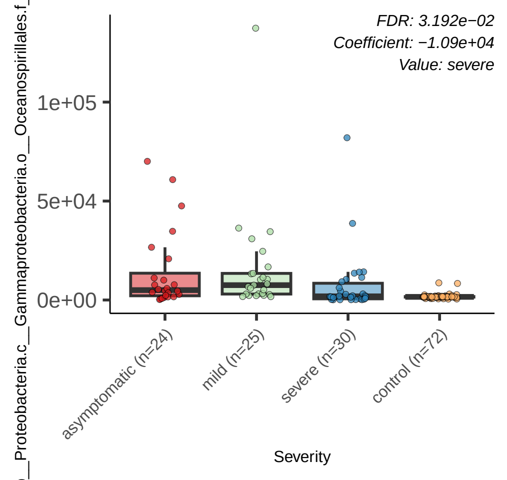
FDR: 3.192e−02
Coefficient: −1.09e+04
Value: severe
1e+05
5e+04
0e+00
o__Proteobacteria.c__Gammaproteobacteria.o__Oceanospirillales.f_
asymptomatic (n=24)
mild (n=25)
severe (n=30)
control (n=72)
Severity

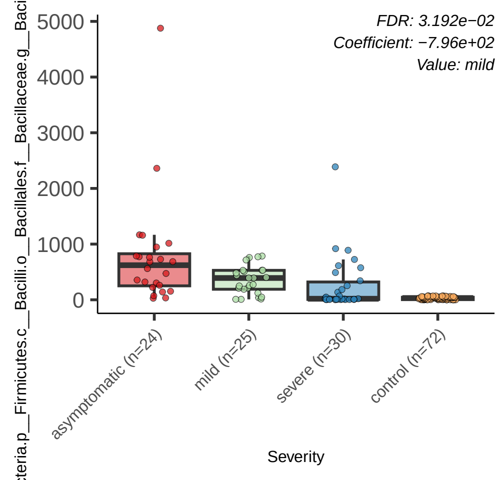

FDR: 3.192e−02
Coefficient: −7.96e+02
Value: mild
5000
4000
3000
2000
1000
0
asymptomatic (n=24)
mild (n=25)
severe (n=30)
control (n=72)
Severity
cteria.p__Firmicutes.c__Bacilli.o__Bacillales.f__Bacillaceae.g__Baci

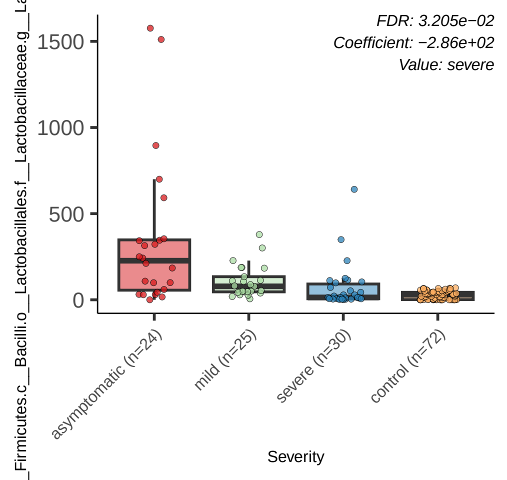

FDR: 3.205e−02
Coefficient: −2.86e+02
Value: severe
1500
1000
500
0
Firmicutes.c__Bacilli.o__Lactobacillales.f__Lactobacillaceae.g__La
asymptomatic (n=24)
mild (n=25)
severe (n=30)
control (n=72)
Severity

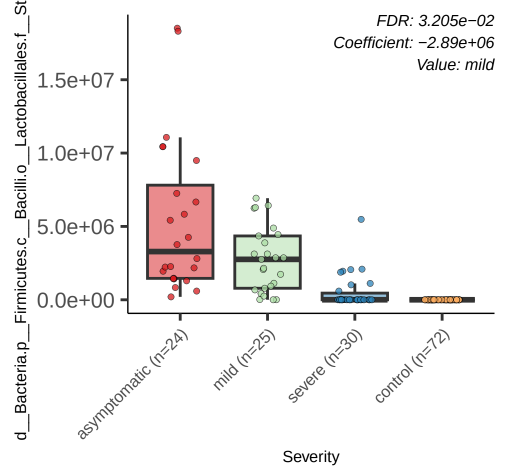
FDR: 3.205e−02
Coefficient: −2.89e+06
Value: mild
d__Bacteria.p__Firmicutes.c__Bacilli.o__Lactobacillales.f__St
1.5e+07
1.0e+07
5.0e+06
0.0e+00
asymptomatic (n=24)
mild (n=25)
severe (n=30)
control (n=72)
Severity

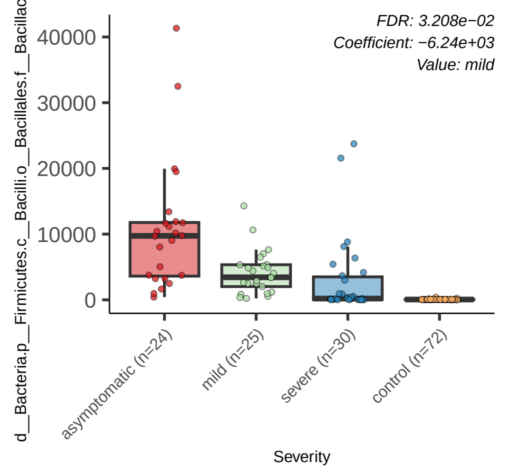
FDR: 3.208e−02
Coefficient: −6.24e+03
Value: mild
d__Bacteria.p__Firmicutes.c__Bacilli.o__Bacillales.f__Bacillac
40000
30000
20000
10000
0
asymptomatic (n=24)
mild (n=25)
severe (n=30)
control (n=72)
Severity

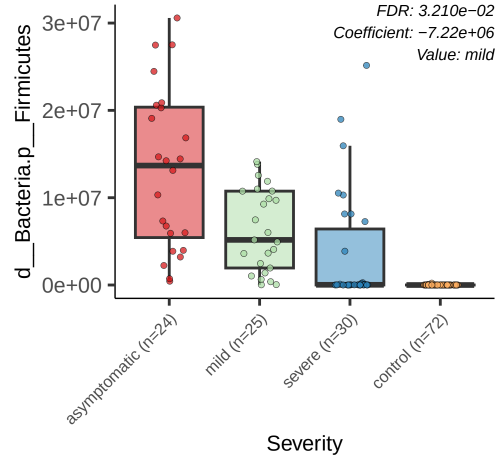

FDR: 3.210e−02
Coefficient: −7.22e+06
Value: mild
d__Bacteria.p__Firmicutes
3e+07
2e+07
1e+07
0e+00
asymptomatic (n=24)
mild (n=25)
severe (n=30)
control (n=72)
Severity

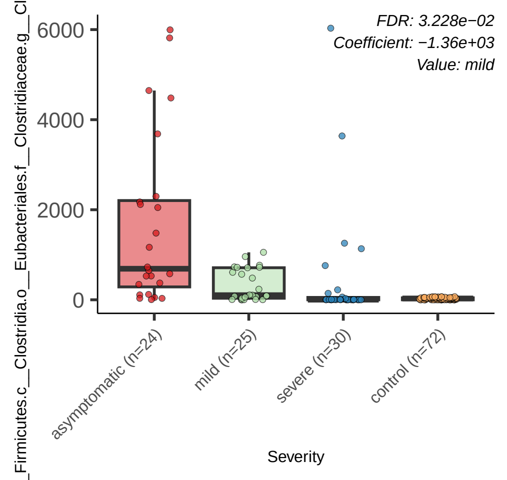

_Firmicutes.c__Clostridia.o__Eubacteriales.f__Clostridiaceae.g__Cl
FDR: 3.228e−02
Coefficient: −1.36e+03
Value: mild
6000
4000
2000
0
asymptomatic (n=24)
mild (n=25)
severe (n=30)
control (n=72)
Severity

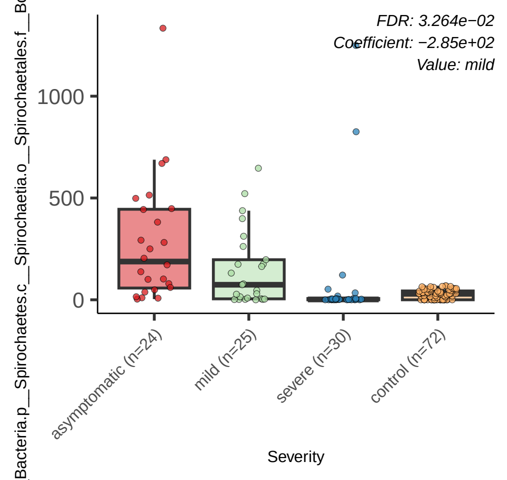

FDR: 3.264e−02
Coefficient: −2.85e+02
Value: mild
Bacteria.p__Spirochaetes.c__Spirochaetia.o__Spirochaetales.f__Bo
1000
500
0
asymptomatic (n=24)
mild (n=25)
severe (n=30)
control (n=72)
Severity

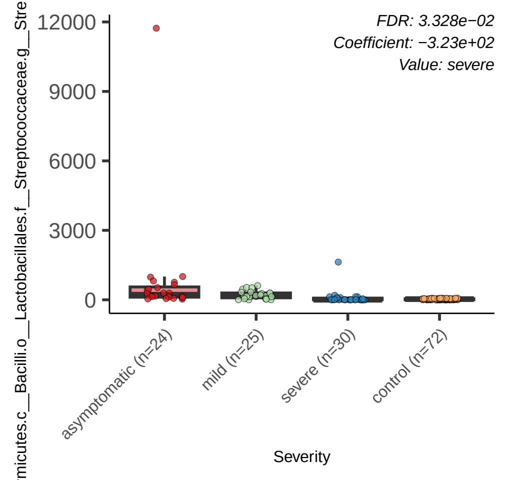
FDR: 3.328e−02
Coefficient: −3.23e+02
Value: severe
12000
9000
6000
3000
0
asymptomatic (n=24)
mild (n=25)
severe (n=30)
control (n=72)
Severity
rmicutes.c__Bacilli.o__Lactobacillales.f__Streptococcaceae.g__Stre

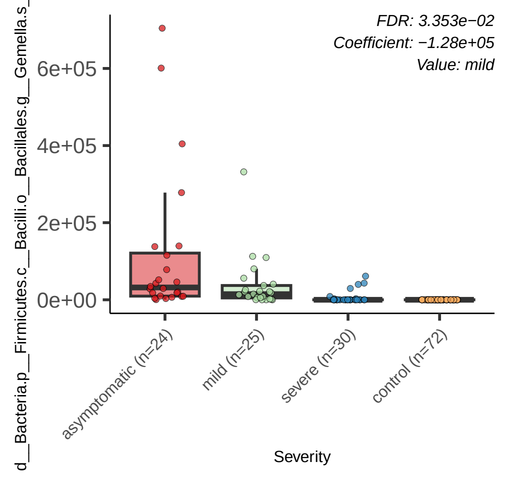

FDR: 3.353e−02
Coefficient: −1.28e+05
Value: mild
d__Bacteria.p__Firmicutes.c__Bacilli.o__Bacillales.g__Gemella.s_
6e+05
4e+05
2e+05
0e+00
asymptomatic (n=24)
mild (n=25)
severe (n=30)
control (n=72)
Severity

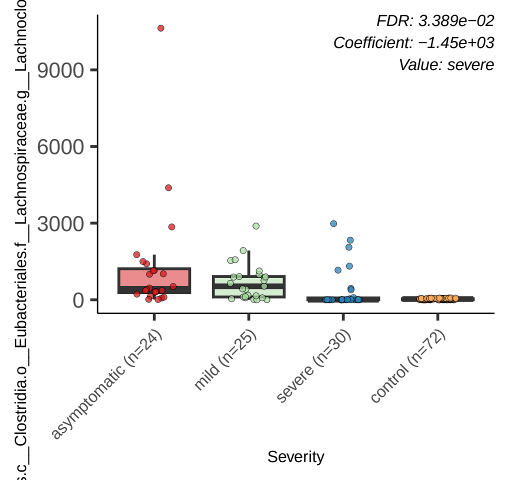
FDR: 3.389e−02
Coefficient: −1.45e+03
Value: severe
9000
6000
3000
0
s.c__Clostridia.o__Eubacteriales.f__Lachnospiraceae.g__Lachnoclo
asymptomatic (n=24)
mild (n=25)
severe (n=30)
control (n=72)
Severity

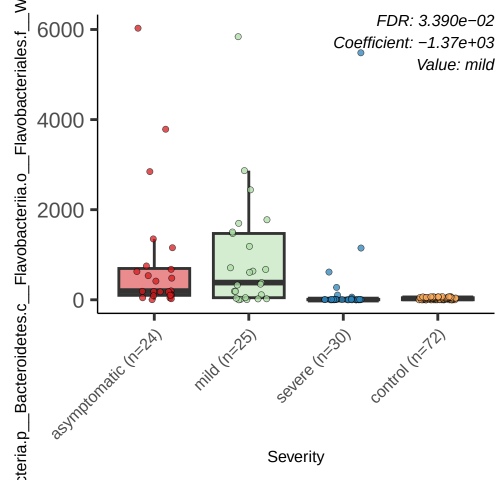
FDR: 3.390e−02
Coefficient: −1.37e+03
Value: mild
6000
4000
2000
0
asymptomatic (n=24)
mild (n=25)
severe (n=30)
control (n=72)
Severity
cteria.p__Bacteroidetes.c__Flavobacteriia.o__Flavobacteriales.f__W

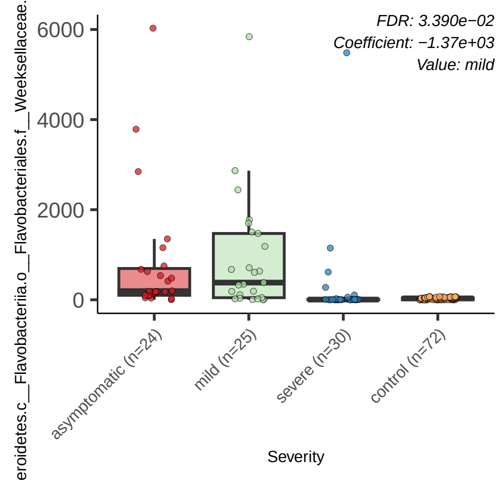

FDR: 3.390e−02
Coefficient: −1.37e+03
Value: mild
eroidetes.c__Flavobacteriia.o__Flavobacteriales.f__Weeksellaceae.
6000
4000
2000
0
asymptomatic (n=24)
mild (n=25)
severe (n=30)
control (n=72)
Severity

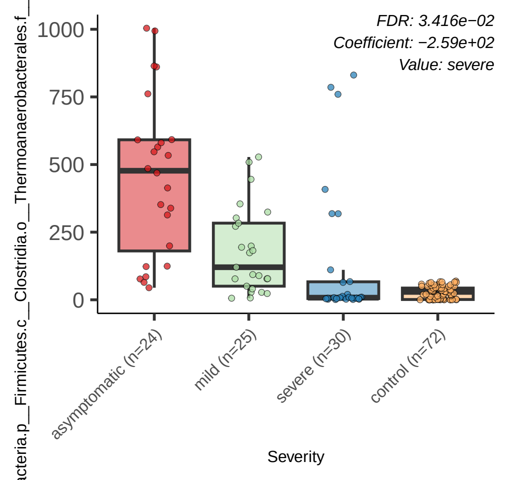

FDR: 3.416e−02
Coefficient: −2.59e+02
Value: severe
1000
750
500
250
0
acteria.p__Firmicutes.c__Clostridia.o__Thermoanaerobacterales.f__
asymptomatic (n=24)
mild (n=25)
severe (n=30)
control (n=72)
Severity

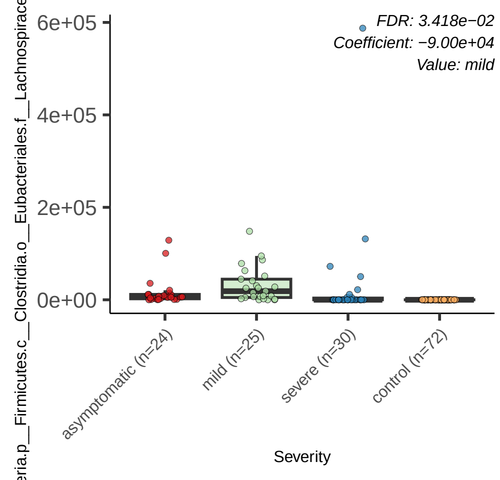
FDR: 3.418e−02
Coefficient: −9.00e+04
Value: mild
6e+05
4e+05
2e+05
0e+00
eria.p__Firmicutes.c__Clostridia.o__Eubacteriales.f__Lachnospirace
asymptomatic (n=24)
mild (n=25)
severe (n=30)
control (n=72)
Severity

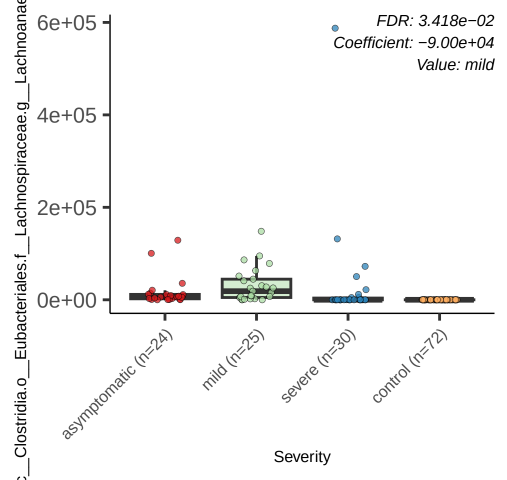

FDR: 3.418e−02
Coefficient: −9.00e+04
Value: mild
6e+05
4e+05
2e+05
0e+00
c__Clostridia.o__Eubacteriales.f__Lachnospiraceae.g__Lachnoanae
asymptomatic (n=24)
mild (n=25)
severe (n=30)
control (n=72)
Severity

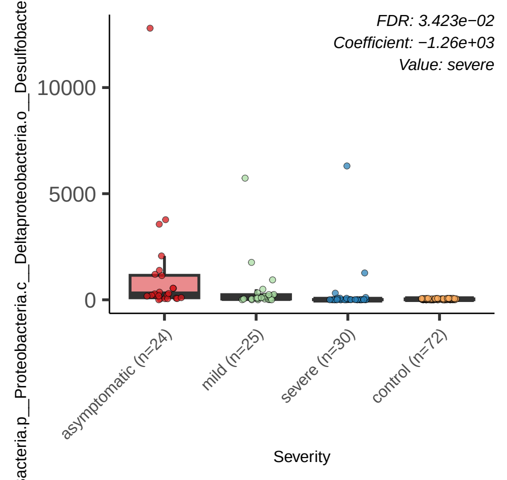

FDR: 3.423e−02
Coefficient: −1.26e+03
Value: severe
10000
5000
0
acteria.p__Proteobacteria.c__Deltaproteobacteria.o__Desulfobacte
asymptomatic (n=24)
mild (n=25)
severe (n=30)
control (n=72)
Severity

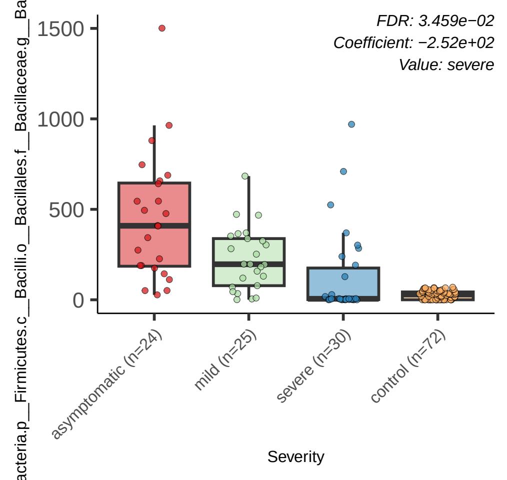
FDR: 3.459e−02
Coefficient: −2.52e+02
Value: severe
1500
1000
500
0
acteria.p__Firmicutes.c__Bacilli.o__Bacillales.f__Bacillaceae.g__Ba
asymptomatic (n=24)
mild (n=25)
severe (n=30)
control (n=72)
Severity

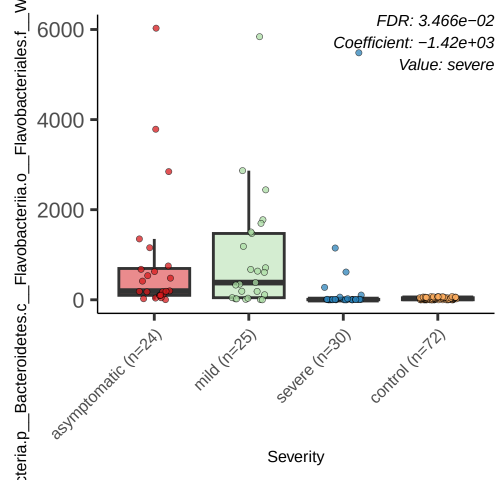

FDR: 3.466e−02
Coefficient: −1.42e+03
Value: severe
6000
4000
2000
0
asymptomatic (n=24)
mild (n=25)
severe (n=30)
control (n=72)
Severity
cteria.p__Bacteroidetes.c__Flavobacteriia.o__Flavobacteriales.f__W

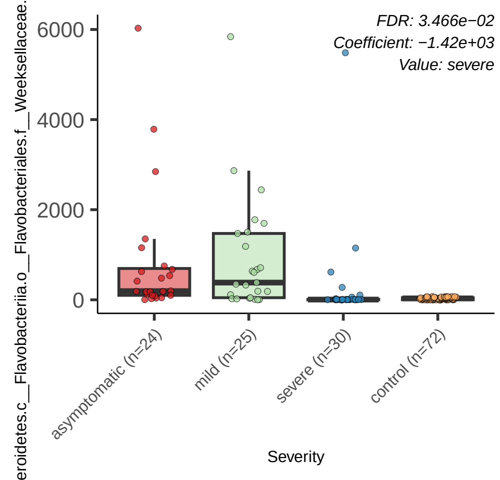

FDR: 3.466e−02
Coefficient: −1.42e+03
Value: severe
eroidetes.c__Flavobacteriia.o__Flavobacteriales.f__Weeksellaceae.
6000
4000
2000
0
asymptomatic (n=24)
mild (n=25)
severe (n=30)
control (n=72)
Severity

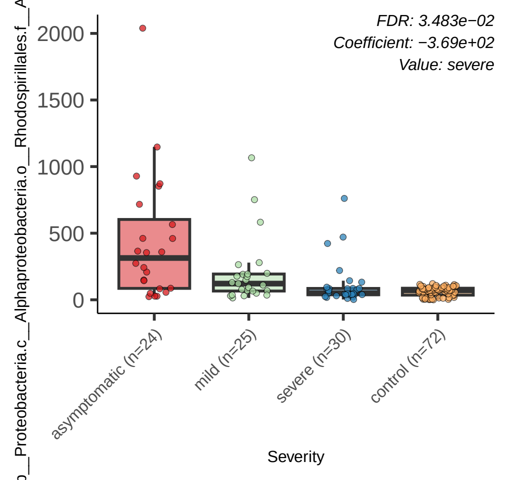
FDR: 3.483e−02
Coefficient: −3.69e+02
Value: severe
o__Proteobacteria.c__Alphaproteobacteria.o__Rhodospirillales.f__A
2000
1500
1000
500
0
asymptomatic (n=24)
mild (n=25)
severe (n=30)
control (n=72)
Severity

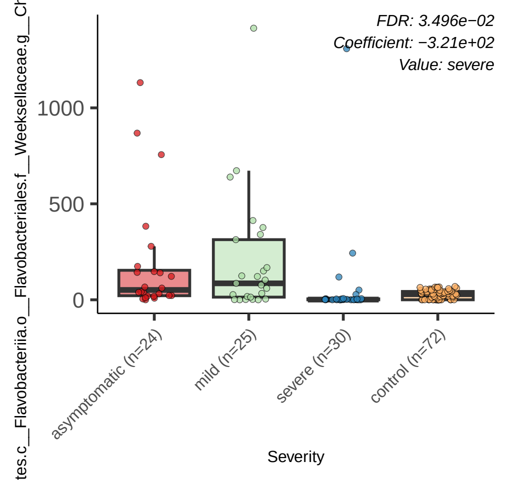

FDR: 3.496e−02
Coefficient: −3.21e+02
Value: severe
tes.c__Flavobacteriia.o__Flavobacteriales.f__Weeksellaceae.g__Ch
1000
500
0
asymptomatic (n=24)
mild (n=25)
severe (n=30)
control (n=72)
Severity

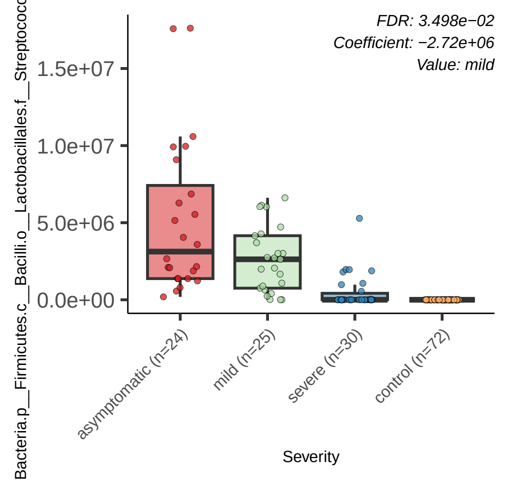
FDR: 3.498e−02
Coefficient: −2.72e+06
Value: mild
Bacteria.p__Firmicutes.c__Bacilli.o__Lactobacillales.f__Streptococc
0.0e+00
5.0e+06
1.0e+07
1.5e+07
asymptomatic (n=24)
mild (n=25)
severe (n=30)
control (n=72)
Severity

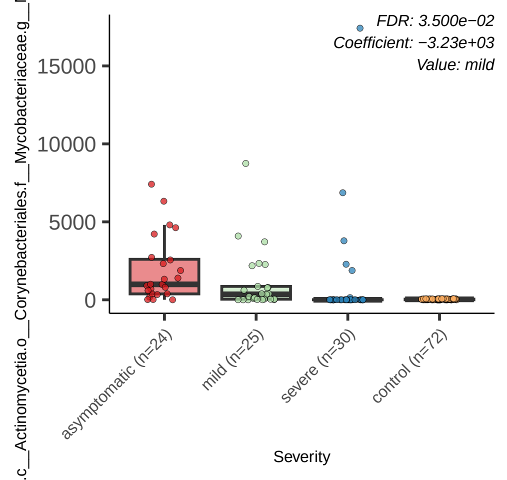

FDR: 3.500e−02
Coefficient: −3.23e+03
Value: mild
15000
10000
5000
0
c__Actinomycetia.o__Corynebacteriales.f__Mycobacteriaceae.g__
asymptomatic (n=24)
mild (n=25)
severe (n=30)
control (n=72)
Severity

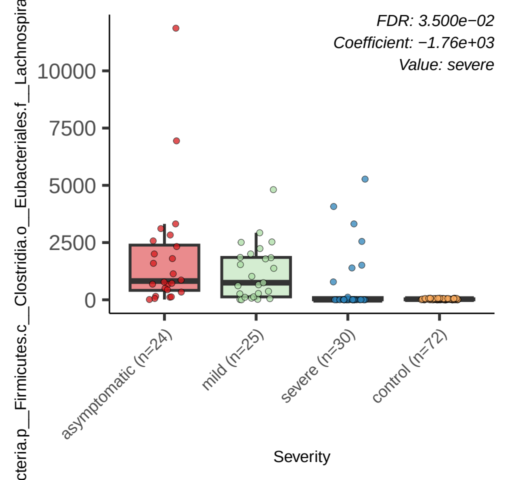

FDR: 3.500e−02
Coefficient: −1.76e+03
Value: severe
cteria.p__Firmicutes.c__Clostridia.o__Eubacteriales.f__Lachnospira
10000
7500
5000
2500
0
asymptomatic (n=24)
mild (n=25)
severe (n=30)
control (n=72)
Severity

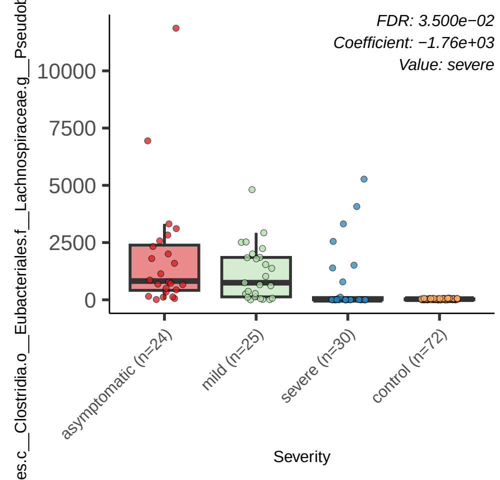

es.c__Clostridia.o__Eubacteriales.f__Lachnospiraceae.g__Pseudob
FDR: 3.500e−02
Coefficient: −1.76e+03
Value: severe
10000
7500
5000
2500
0
asymptomatic (n=24)
mild (n=25)
severe (n=30)
control (n=72)
Severity

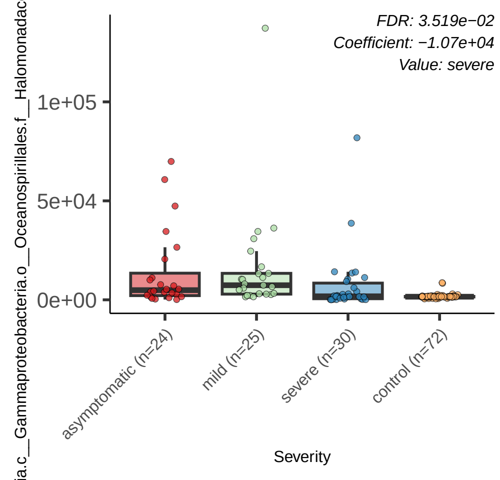

FDR: 3.519e−02
Coefficient: −1.07e+04
Value: severe
ia.c__Gammaproteobacteria.o__Oceanospirillales.f__Halomonadac
1e+05
5e+04
0e+00
asymptomatic (n=24)
mild (n=25)
severe (n=30)
control (n=72)
Severity

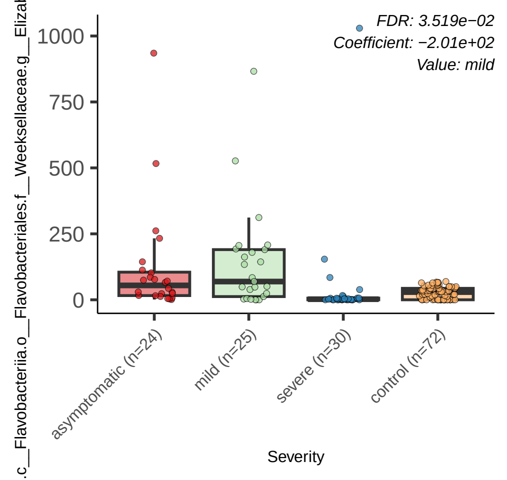

FDR: 3.519e−02
Coefficient: −2.01e+02
Value: mild
1000
750
500
250
0
c__Flavobacteriia.o__Flavobacteriales.f__Weeksellaceae.g__Elizab
asymptomatic (n=24)
mild (n=25)
severe (n=30)
control (n=72)
Severity

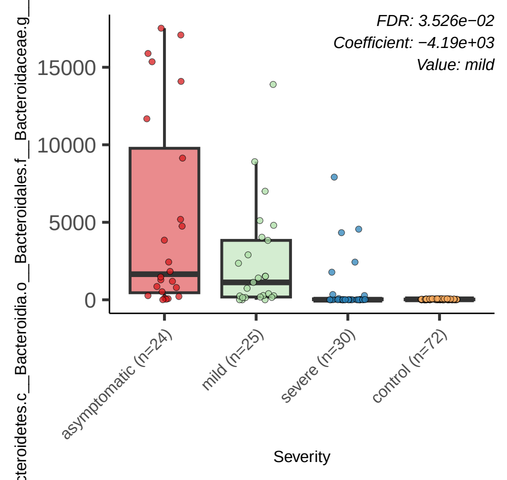

FDR: 3.526e−02
Coefficient: −4.19e+03
Value: mild
cteroidetes.c__Bacteroidia.o__Bacteroidales.f__Bacteroidaceae.g__
15000
10000
5000
0
asymptomatic (n=24)
mild (n=25)
severe (n=30)
control (n=72)
Severity

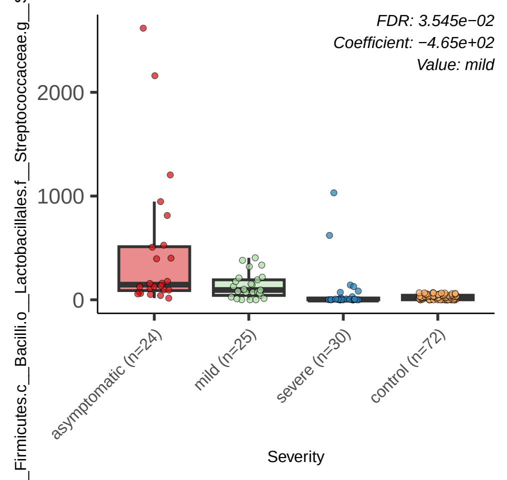

FDR: 3.545e−02
Coefficient: −4.65e+02
Value: mild
Firmicutes.c__Bacilli.o__Lactobacillales.f__Streptococcaceae.g__
2000
1000
0
asymptomatic (n=24)
mild (n=25)
severe (n=30)
control (n=72)
Severity

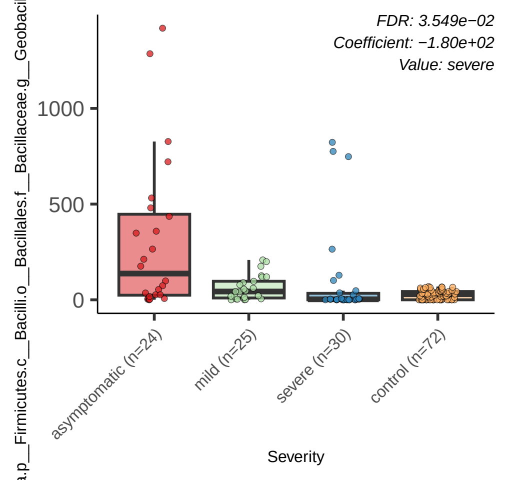
FDR: 3.549e−02
Coefficient: −1.80e+02
Value: severe
1000
500
0
asymptomatic (n=24)
mild (n=25)
severe (n=30)
control (n=72)
Severity
a.p__Firmicutes.c__Bacilli.o__Bacillales.f__Bacillaceae.g__Geobaci

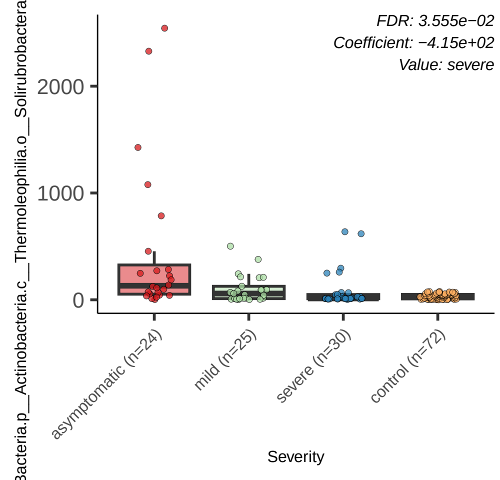

FDR: 3.555e−02
Coefficient: −4.15e+02
Value: severe
Bacteria.p__Actinobacteria.c__Thermoleophilia.o__Solirubrobacterales
2000
1000
0
asymptomatic (n=24)
mild (n=25)
severe (n=30)
control (n=72)
Severity

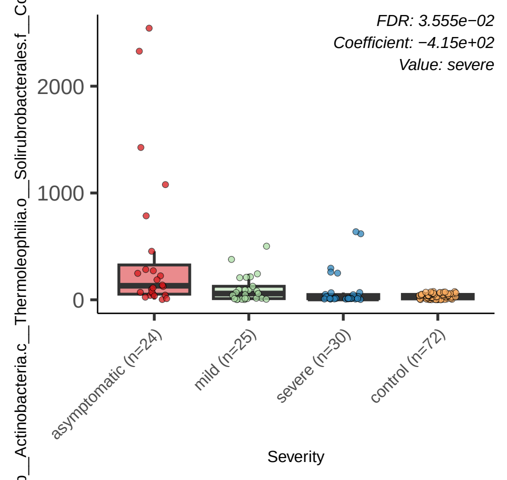
FDR: 3.555e−02
Coefficient: −4.15e+02
Value: severe
2000
1000
0
asymptomatic (n=24)
mild (n=25)
severe (n=30)
control (n=72)
Severity
o__Actinobacteria.c__Thermoleophilia.o__Solirubrobacterales.f__Co

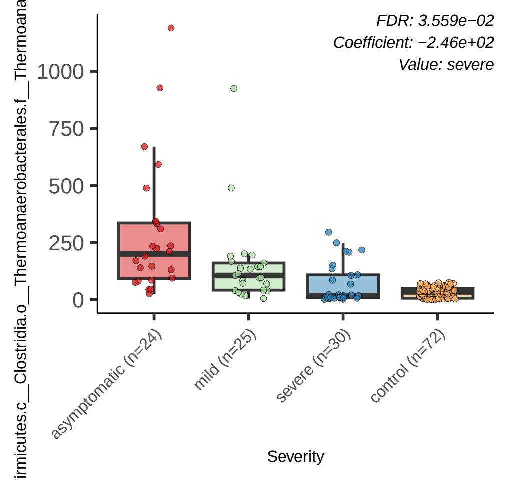

FDR: 3.559e−02
Coefficient: −2.46e+02
Value: severe
irmicutes.c__Clostridia.o__Thermoanaerobacterales.f__Thermoana
1000
750
500
250
0
asymptomatic (n=24)
mild (n=25)
severe (n=30)
control (n=72)
Severity

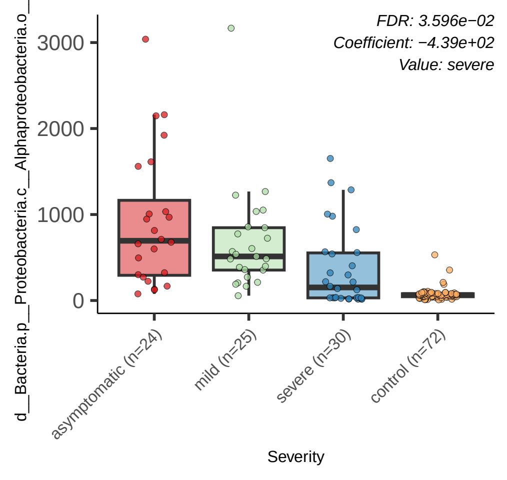

FDR: 3.596e−02
Coefficient: −4.39e+02
Value: severe
d__Bacteria.p__Proteobacteria.c__Alphaproteobacteria.o__
3000
2000
1000
0
asymptomatic (n=24)
mild (n=25)
severe (n=30)
control (n=72)
Severity

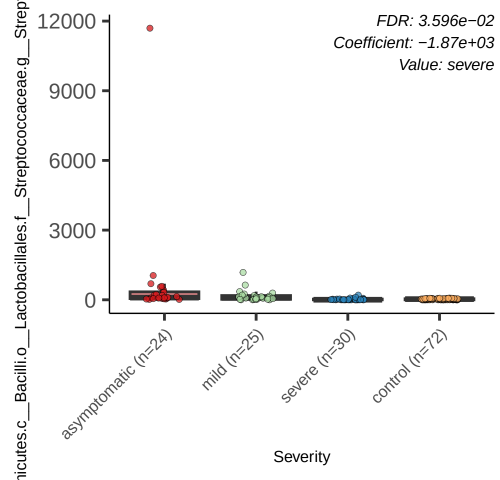

FDR: 3.596e−02
Coefficient: −1.87e+03
Value: severe
12000
9000
6000
3000
0
asymptomatic (n=24)
mild (n=25)
severe (n=30)
control (n=72)
Severity
nicutes.c__Bacilli.o__Lactobacillales.f__Streptococcaceae.g__Strep

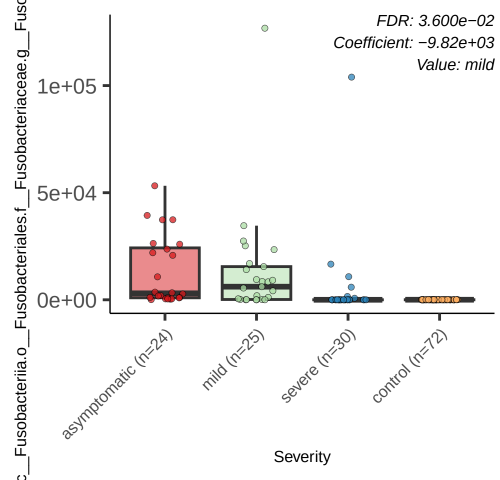

FDR: 3.600e−02
Coefficient: −9.82e+03
Value: mild
1e+05
5e+04
0e+00
c__Fusobacteriia.o__Fusobacteriales.f__Fusobacteriaceae.g__Fuso
asymptomatic (n=24)
mild (n=25)
severe (n=30)
control (n=72)
Severity

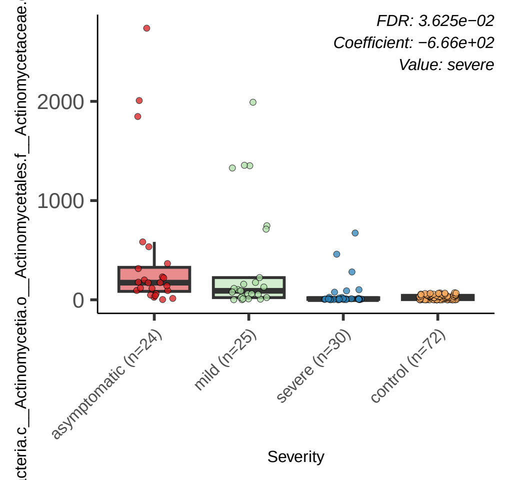

FDR: 3.625e−02
Coefficient: −6.66e+02
Value: severe
2000
1000
0
cteria.c__Actinomycetia.o__Actinomycetales.f__Actinomycetaceae.
asymptomatic (n=24)
mild (n=25)
severe (n=30)
control (n=72)
Severity

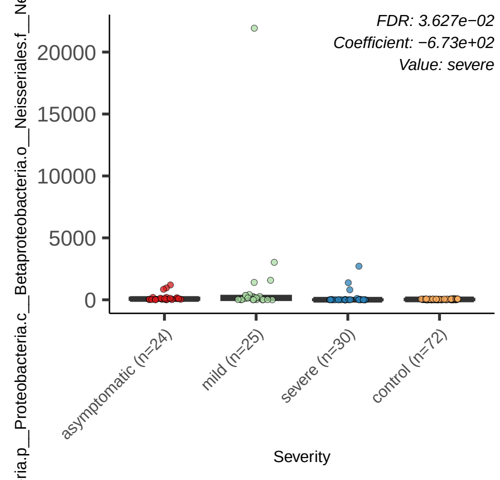

FDR: 3.627e−02
Coefficient: −6.73e+02
Value: severe
20000
15000
10000
5000
0
asymptomatic (n=24)
mild (n=25)
severe (n=30)
control (n=72)
Severity

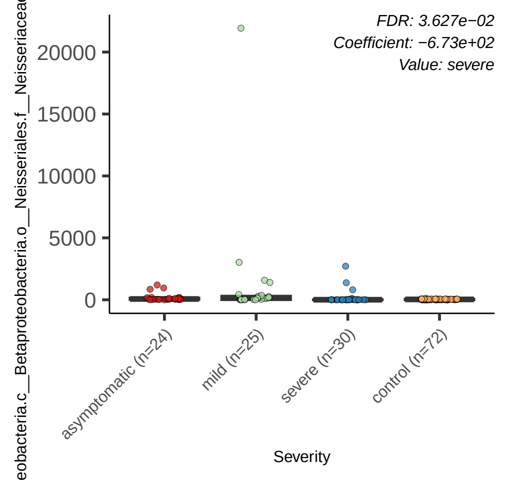
FDR: 3.627e−02
Coefficient: −6.73e+02
Value: severe
20000
15000
10000
5000
0
eobacteria.c__Betaproteobacteria.o__Neisseriales.f__Neisseriacea
asymptomatic (n=24)
mild (n=25)
severe (n=30)
control (n=72)
Severity

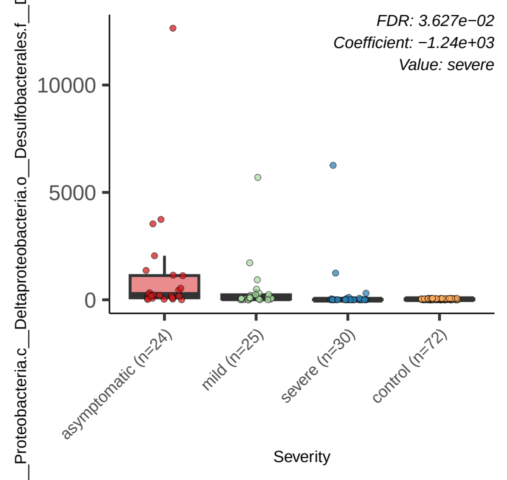
FDR: 3.627e−02
Coefficient: −1.24e+03
Value: severe
__Proteobacteria.c__Deltaproteobacteria.o__Desulfobacterales.f__D
10000
5000
0
asymptomatic (n=24)
mild (n=25)
severe (n=30)
control (n=72)
Severity

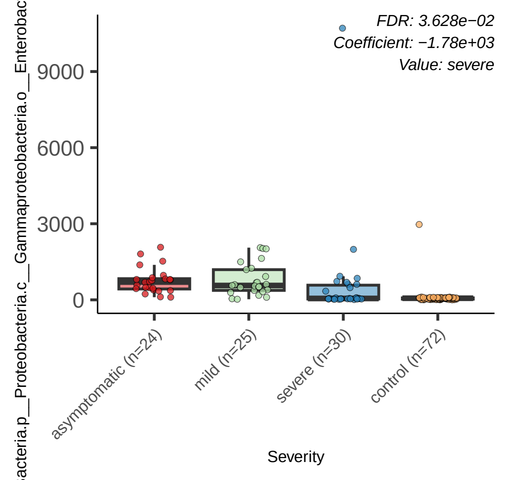
FDR: 3.628e−02
Coefficient: −1.78e+03
Value: severe
9000
6000
3000
0
acteria.p__Proteobacteria.c__Gammaproteobacteria.o__Enterobac
asymptomatic (n=24)
mild (n=25)
severe (n=30)
control (n=72)
Severity

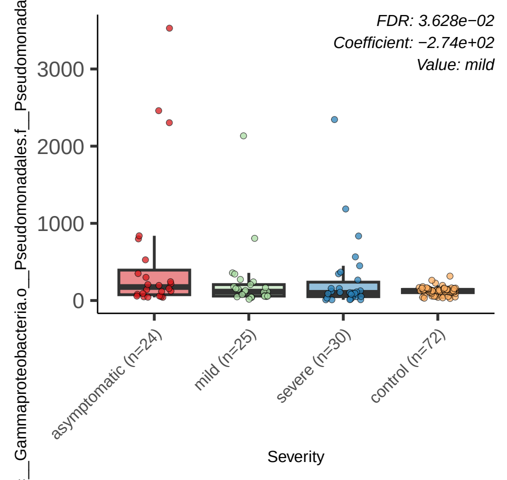
FDR: 3.628e−02
Coefficient: −2.74e+02
Value: mild
3000
2000
1000
0
Gammaproteobacteria.o__Pseudomonadales.f__Pseudomonada
asymptomatic (n=24)
mild (n=25)
severe (n=30)
control (n=72)
Severity

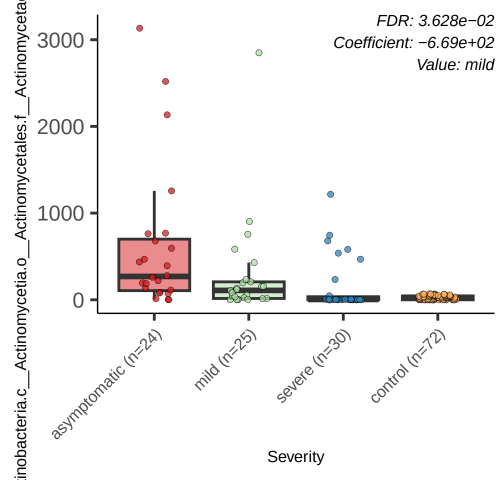

FDR: 3.628e−02
Coefficient: −6.69e+02
Value: mild
3000
2000
1000
0
asymptomatic (n=24)
mild (n=25)
severe (n=30)
control (n=72)
Severity

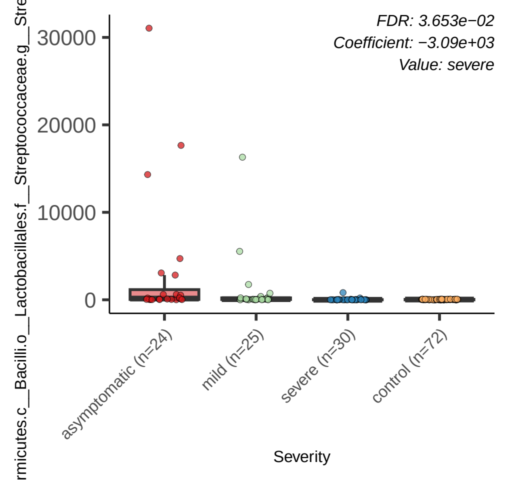

FDR: 3.653e−02
Coefficient: −3.09e+03
Value: severe
rmicutes.c__Bacilli.o__Lactobacillales.f__Streptococcaceae.g__Stre
30000
20000
10000
0
asymptomatic (n=24)
mild (n=25)
severe (n=30)
control (n=72)
Severity

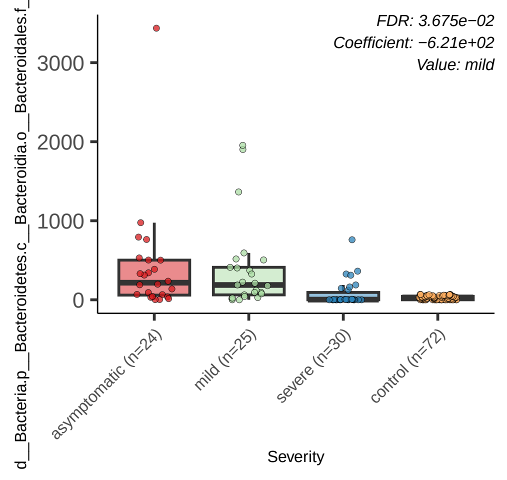

FDR: 3.675e−02
Coefficient: −6.21e+02
Value: mild
d__Bacteria.p__Bacteroidetes.c__Bacteroidia.o__Bacteroidales.f__
3000
2000
1000
0
asymptomatic (n=24)
mild (n=25)
severe (n=30)
control (n=72)
Severity

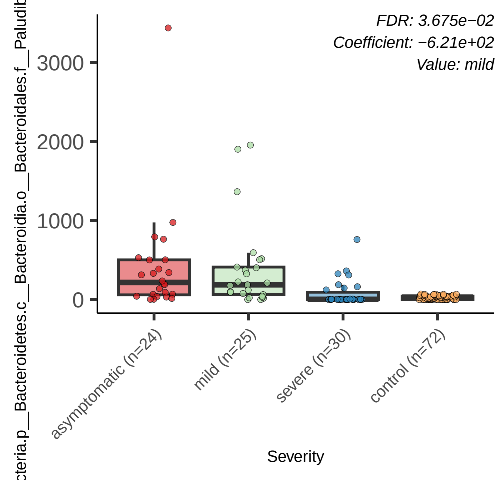
FDR: 3.675e−02
Coefficient: −6.21e+02
Value: mild
cteria.p__Bacteroidetes.c__Bacteroidia.o__Bacteroidales.f__Paludib
3000
2000
1000
0
asymptomatic (n=24)
mild (n=25)
severe (n=30)
control (n=72)
Severity

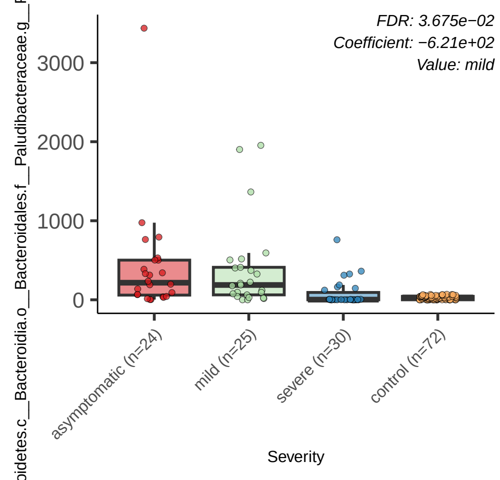

FDR: 3.675e−02
Coefficient: −6.21e+02
Value: mild
3000
2000
1000
0
oidetes.c__Bacteroidia.o__Bacteroidales.f__Paludibacteraceae.g__P
asymptomatic (n=24)
mild (n=25)
severe (n=30)
control (n=72)
Severity

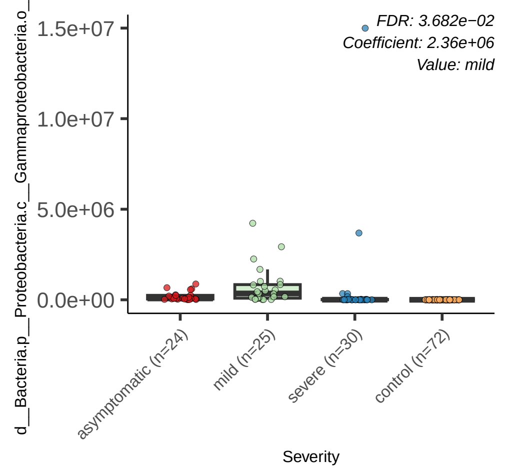

FDR: 3.682e−02
Coefficient: 2.36e+06
Value: mild
d__Bacteria.p__Proteobacteria.c__Gammaproteobacteria.o__
1.5e+07
1.0e+07
5.0e+06
0.0e+00
asymptomatic (n=24)
mild (n=25)
severe (n=30)
control (n=72)
Severity

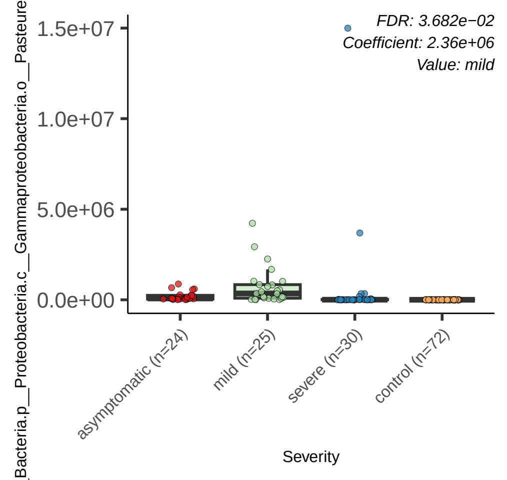

FDR: 3.682e−02
Coefficient: 2.36e+06
Value: mild
1.5e+07
1.0e+07
5.0e+06
0.0e+00
Bacteria.p__Proteobacteria.c__Gammaproteobacteria.o__Pasteure
asymptomatic (n=24)
mild (n=25)
severe (n=30)
control (n=72)
Severity

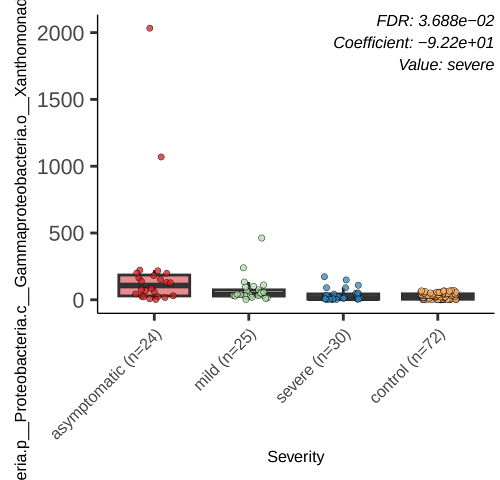

FDR: 3.688e−02
Coefficient: −9.22e+01
Value: severe
2000
1500
1000
500
0
eria.p__Proteobacteria.c__Gammaproteobacteria.o__Xanthomonad
asymptomatic (n=24)
mild (n=25)
severe (n=30)
control (n=72)
Severity

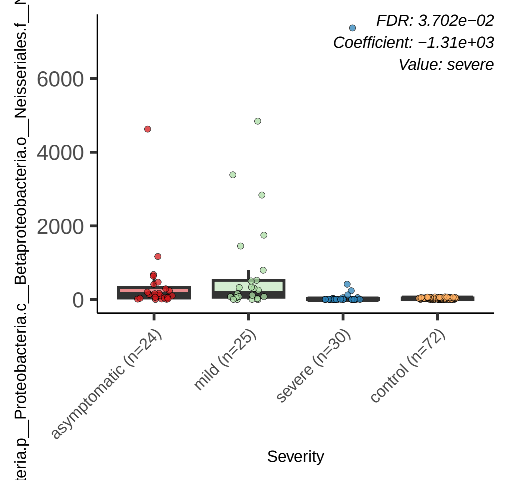

FDR: 3.702e−02
Coefficient: −1.31e+03
Value: severe
6000
4000
2000
0
teria.p__Proteobacteria.c__Betaproteobacteria.o__Neisseriales.f__N
asymptomatic (n=24)
mild (n=25)
severe (n=30)
control (n=72)
Severity

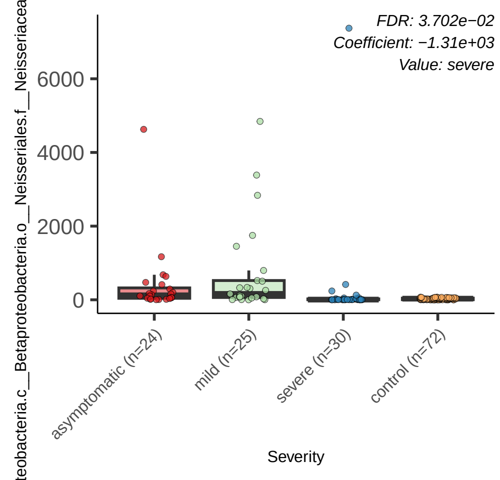
FDR: 3.702e−02
Coefficient: −1.31e+03
Value: severe
teobacteria.c__Betaproteobacteria.o__Neisseriales.f__Neisseriacea
6000
4000
2000
0
asymptomatic (n=24)
mild (n=25)
severe (n=30)
control (n=72)
Severity

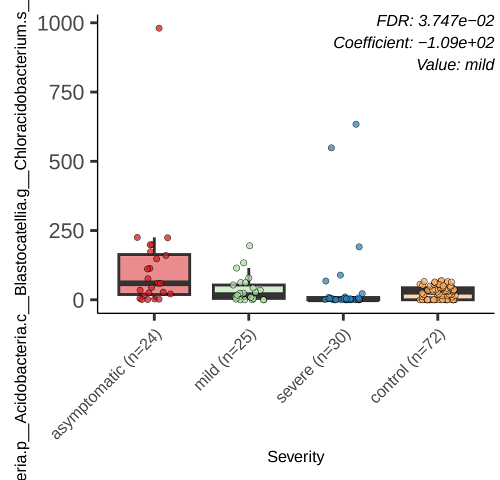

FDR: 3.747e−02
Coefficient: −1.09e+02
Value: mild
1000
750
500
250
0
eria.p__Acidobacteria.c__Blastocatellia.g__Chloracidobacterium.s_
asymptomatic (n=24)
mild (n=25)
severe (n=30)
control (n=72)
Severity

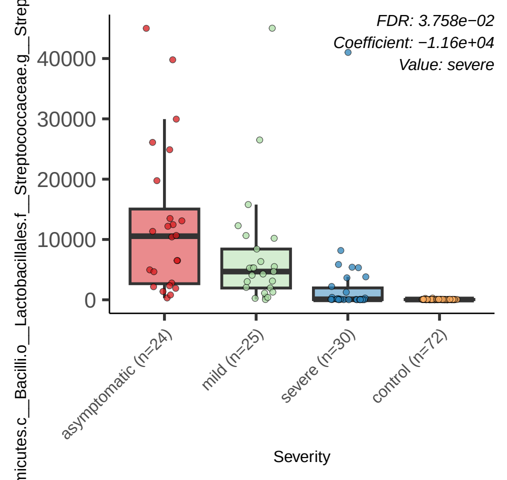

FDR: 3.758e−02
Coefficient: −1.16e+04
Value: severe
40000
30000
20000
10000
0
micutes.c__Bacilli.o__Lactobacillales.f__Streptococcaceae.g__Strep
asymptomatic (n=24)
mild (n=25)
severe (n=30)
control (n=72)
Severity

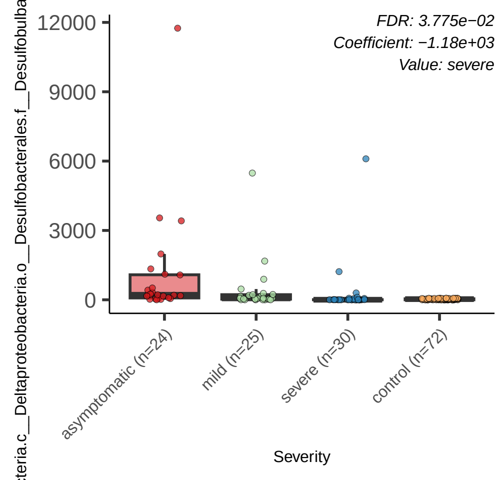
FDR: 3.775e−02
Coefficient: −1.18e+03
Value: severe
12000
9000
6000
3000
0
cteria.c__Deltaproteobacteria.o__Desulfobacterales.f__Desulfobulba
asymptomatic (n=24)
mild (n=25)
severe (n=30)
control (n=72)
Severity

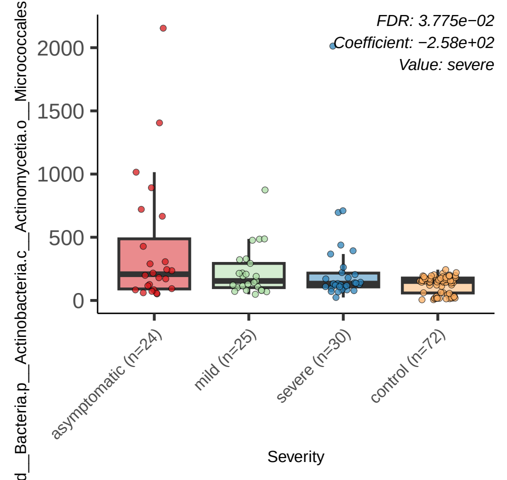

FDR: 3.775e−02
Coefficient: −2.58e+02
Value: severe
d__Bacteria.p__Actinobacteria.c__Actinomycetia.o__Micrococcales
2000
1500
1000
500
0
asymptomatic (n=24)
mild (n=25)
severe (n=30)
control (n=72)
Severity

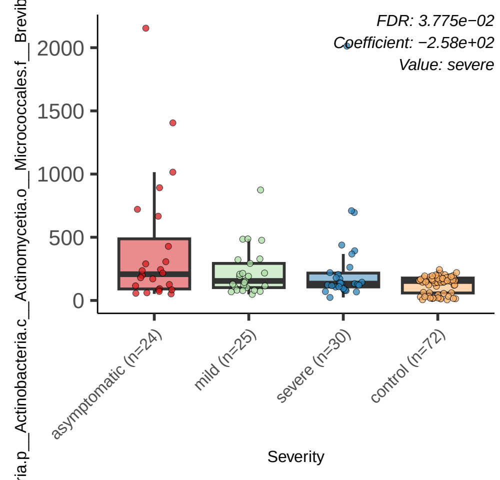

FDR: 3.775e−02
Coefficient: −2.58e+02
Value: severe
ria.p__Actinobacteria.c__Actinomycetia.o__Micrococcales.f__Brevib
2000
1500
1000
500
0
asymptomatic (n=24)
mild (n=25)
severe (n=30)
control (n=72)
Severity

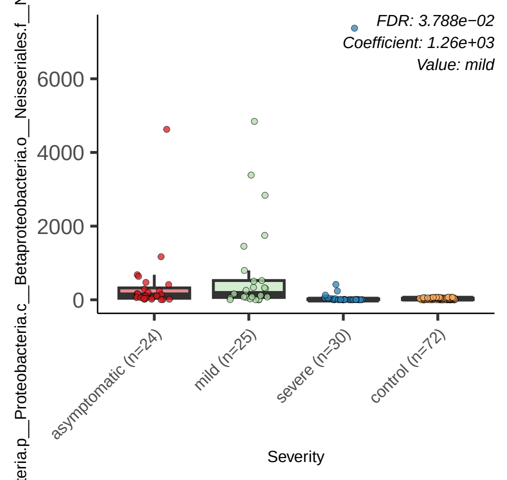
FDR: 3.788e−02
Coefficient: 1.26e+03
Value: mild
6000
4000
2000
0
teria.p__Proteobacteria.c__Betaproteobacteria.o__Neisseriales.f__N
asymptomatic (n=24)
mild (n=25)
severe (n=30)
control (n=72)
Severity

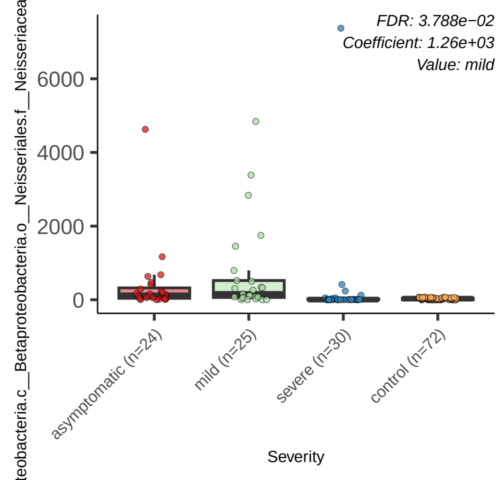

FDR: 3.788e−02
Coefficient: 1.26e+03
Value: mild
6000
4000
2000
0
teobacteria.c__Betaproteobacteria.o__Neisseriales.f__Neisseriacea
asymptomatic (n=24)
mild (n=25)
severe (n=30)
control (n=72)
Severity

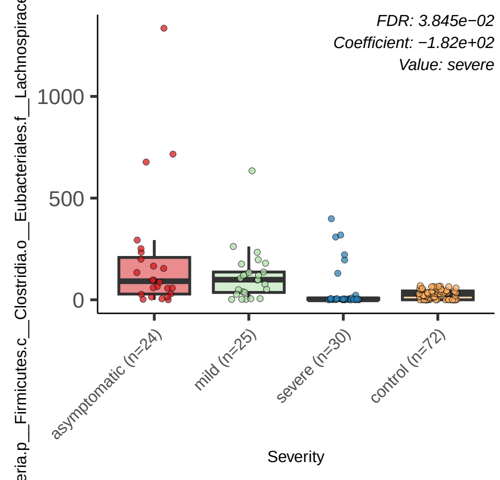
FDR: 3.845e−02
Coefficient: −1.82e+02
Value: severe
1000
500
0
asymptomatic (n=24)
mild (n=25)
severe (n=30)
control (n=72)
Severity
eria.p__Firmicutes.c__Clostridia.o__Eubacteriales.f__Lachnospirace

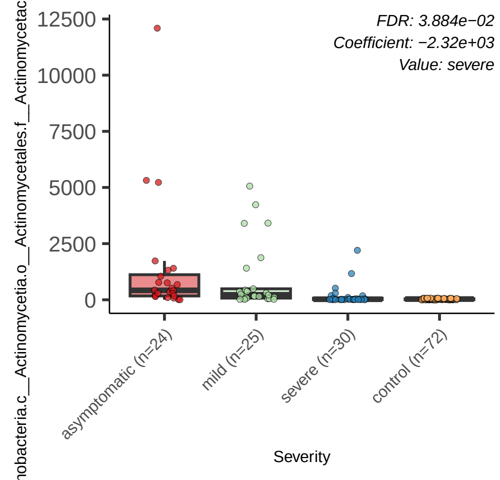
FDR: 3.884e−02
Coefficient: −2.32e+03
Value: severe
nobacteria.c__Actinomycetia.o__Actinomycetales.f__Actinomycetac
12500
10000
7500
5000
2500
0
asymptomatic (n=24)
mild (n=25)
severe (n=30)
control (n=72)
Severity

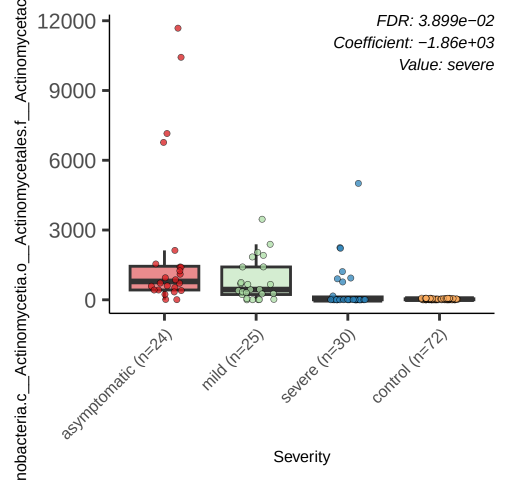

FDR: 3.899e−02
Coefficient: −1.86e+03
Value: severe
12000
9000
6000
3000
0
nobacteria.c__Actinomycetia.o__Actinomycetales.f__Actinomycetac
asymptomatic (n=24)
mild (n=25)
severe (n=30)
control (n=72)
Severity

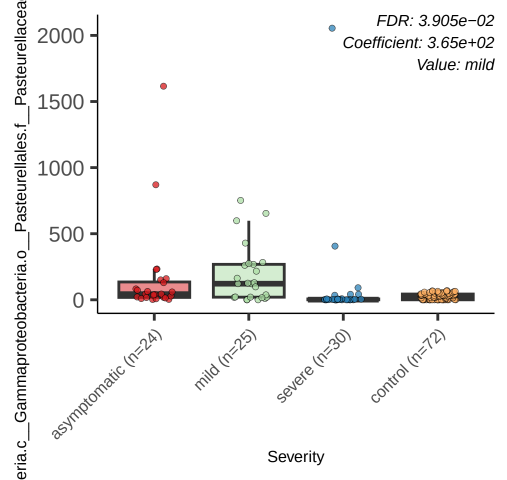

FDR: 3.905e−02
Coefficient: 3.65e+02
Value: mild
2000
1500
1000
500
0
eria.c__Gammaproteobacteria.o__Pasteurellales.f__Pasteurellacea
asymptomatic (n=24)
mild (n=25)
severe (n=30)
control (n=72)
Severity

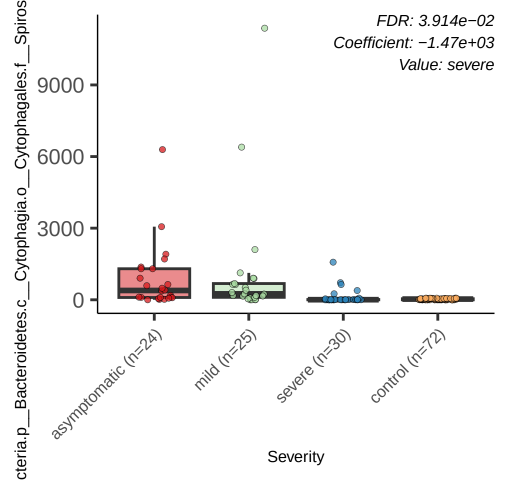

FDR: 3.914e−02
Coefficient: −1.47e+03
Value: severe
9000
6000
3000
0
cteria.p__Bacteroidetes.c__Cytophagia.o__Cytophagales.f__Spiros
asymptomatic (n=24)
mild (n=25)
severe (n=30)
control (n=72)
Severity

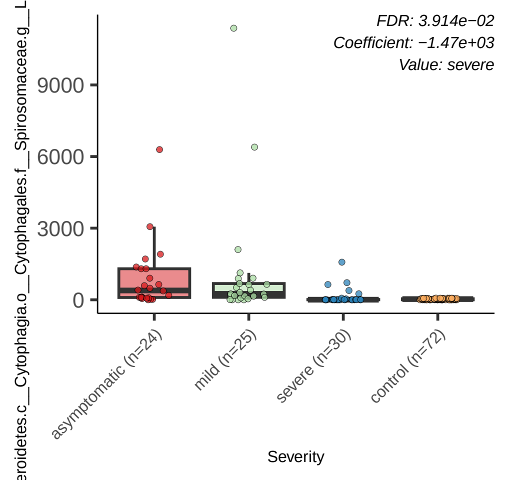

FDR: 3.914e−02
Coefficient: −1.47e+03
Value: severe
9000
6000
3000
0
eroidetes.c__Cytophagia.o__Cytophagales.f__Spirosomaceae.g__L
asymptomatic (n=24)
mild (n=25)
severe (n=30)
control (n=72)
Severity

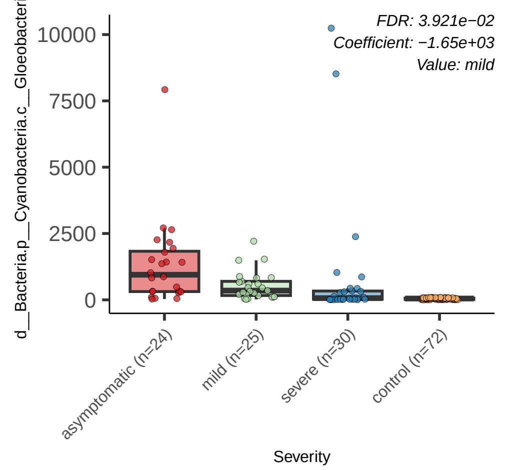
FDR: 3.921e−02
Coefficient: −1.65e+03
Value: mild
10000
7500
5000
2500
0
d__Bacteria.p__Cyanobacteria.c__Gloeobacteri
asymptomatic (n=24)
mild (n=25)
severe (n=30)
control (n=72)
Severity

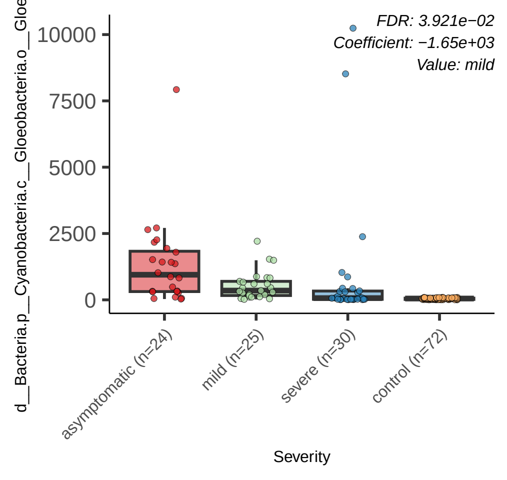

FDR: 3.921e−02
Coefficient: −1.65e+03
Value: mild
d__Bacteria.p__Cyanobacteria.c__Gloeobacteria.o__Gloe
10000
7500
5000
2500
0
asymptomatic (n=24)
mild (n=25)
severe (n=30)
control (n=72)
Severity

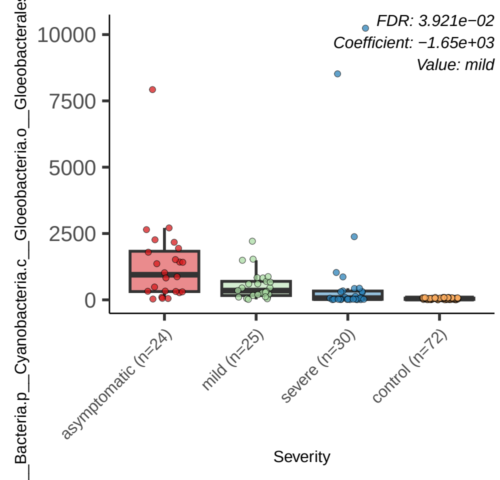

FDR: 3.921e−02
Coefficient: −1.65e+03
Value: mild
10000
7500
5000
2500
0
_Bacteria.p__Cyanobacteria.c__Gloeobacteria.o__Gloeobacterales
asymptomatic (n=24)
mild (n=25)
severe (n=30)
control (n=72)
Severity

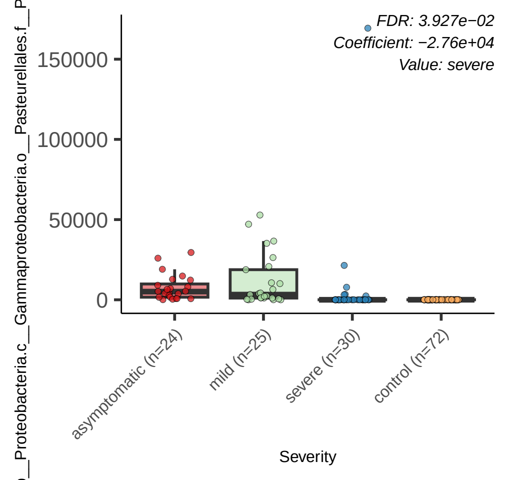
FDR: 3.927e−02
Coefficient: −2.76e+04
Value: severe
150000
100000
50000
0
asymptomatic (n=24)
mild (n=25)
severe (n=30)
control (n=72)
Severity
Proteobacteria.c__Gammaproteobacteria.o__Pasteurellales.f__P

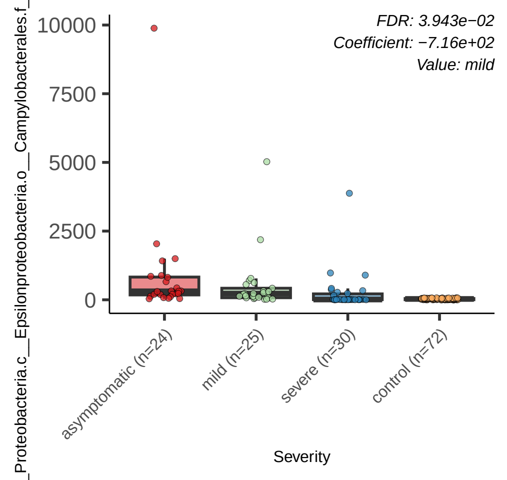
FDR: 3.943e−02
Coefficient: −7.16e+02
Value: mild
_Proteobacteria.c__Epsilonproteobacteria.o__Campylobacterales.f_
10000
7500
5000
2500
0
asymptomatic (n=24)
mild (n=25)
severe (n=30)
control (n=72)
Severity

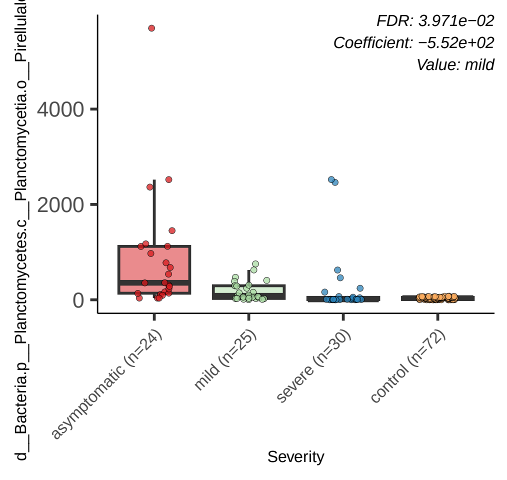

FDR: 3.971e−02
Coefficient: −5.52e+02
Value: mild
d__Bacteria.p__Planctomycetes.c__Planctomycetia.o__Pirellulale
0
2000
4000
asymptomatic (n=24)
mild (n=25)
severe (n=30)
control (n=72)
Severity

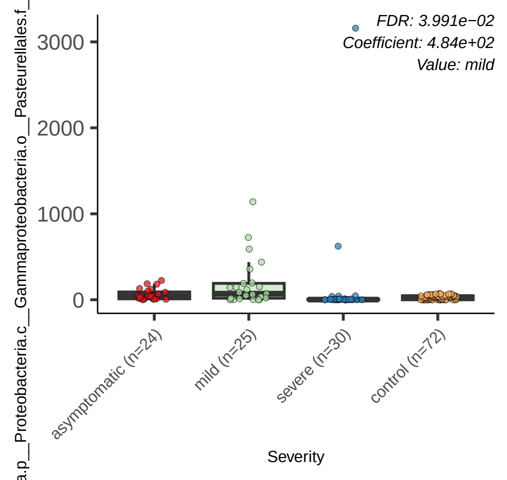

FDR: 3.991e−02
Coefficient: 4.84e+02
Value: mild
a.p__Proteobacteria.c__Gammaproteobacteria.o__Pasteurellales.f
3000
2000
1000
0
asymptomatic (n=24)
mild (n=25)
severe (n=30)
control (n=72)
Severity

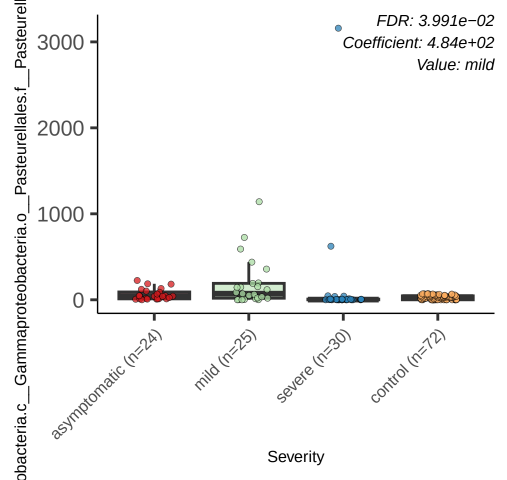

FDR: 3.991e−02
Coefficient: 4.84e+02
Value: mild
3000
2000
1000
0
obacteria.c__Gammaproteobacteria.o__Pasteurellales.f__Pasteurell
asymptomatic (n=24)
mild (n=25)
severe (n=30)
control (n=72)
Severity

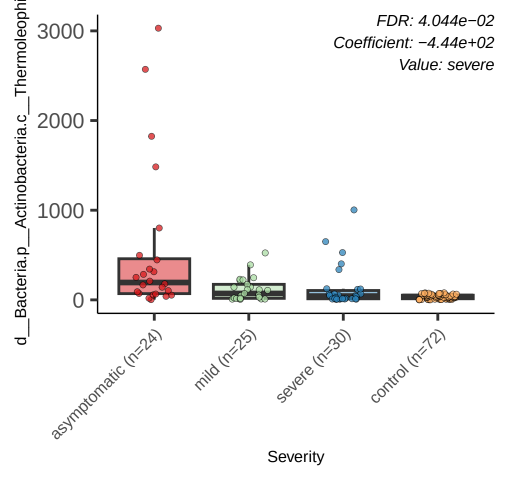
FDR: 4.044e−02
Coefficient: −4.44e+02
Value: severe
d__Bacteria.p__Actinobacteria.c__Thermoleophi
3000
2000
1000
0
asymptomatic (n=24)
mild (n=25)
severe (n=30)
control (n=72)
Severity

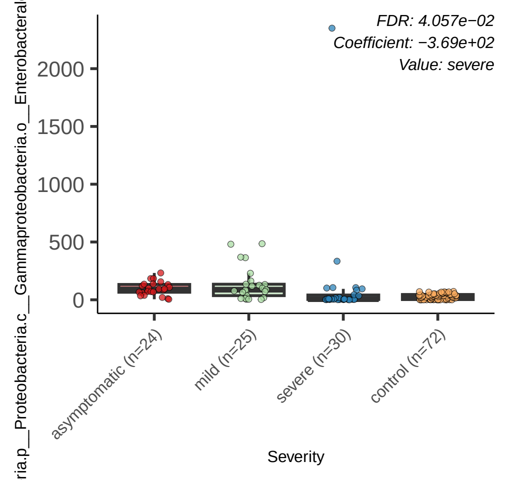

FDR: 4.057e−02
Coefficient: −3.69e+02
Value: severe
2000
1500
1000
500
0
ria.p__Proteobacteria.c__Gammaproteobacteria.o__Enterobacterale
asymptomatic (n=24)
mild (n=25)
severe (n=30)
control (n=72)
Severity

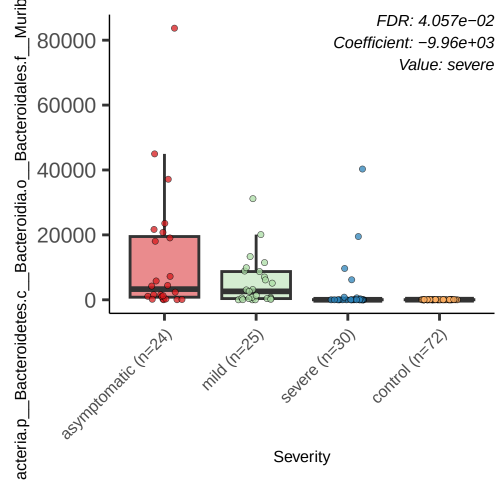
FDR: 4.057e−02
Coefficient: −9.96e+03
Value: severe
80000
60000
40000
20000
0
asymptomatic (n=24)
mild (n=25)
severe (n=30)
control (n=72)
Severity
acteria.p__Bacteroidetes.c__Bacteroidia.o__Bacteroidales.f__Murib

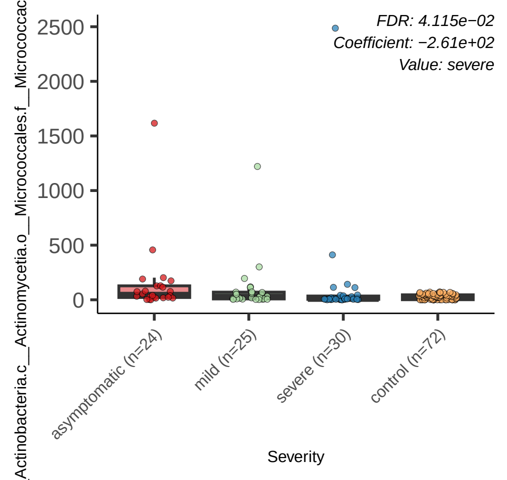
FDR: 4.115e−02
Coefficient: −2.61e+02
Value: severe
Actinobacteria.c__Actinomycetia.o__Micrococcales.f__Micrococcac
2500
2000
1500
1000
500
0
asymptomatic (n=24)
mild (n=25)
severe (n=30)
control (n=72)
Severity

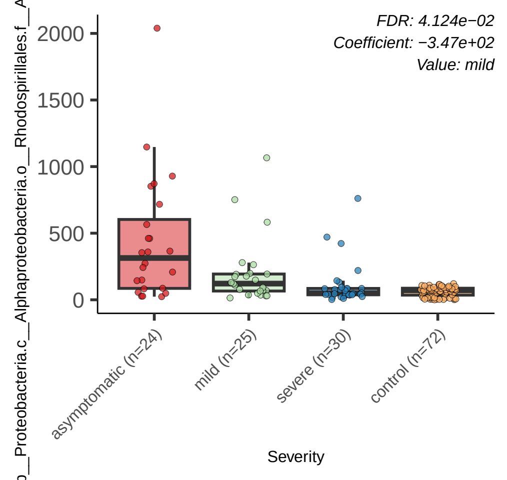
FDR: 4.124e−02
Coefficient: −3.47e+02
Value: mild
o__Proteobacteria.c__Alphaproteobacteria.o__Rhodospirillales.f__A
2000
1500
1000
500
0
asymptomatic (n=24)
mild (n=25)
severe (n=30)
control (n=72)
Severity

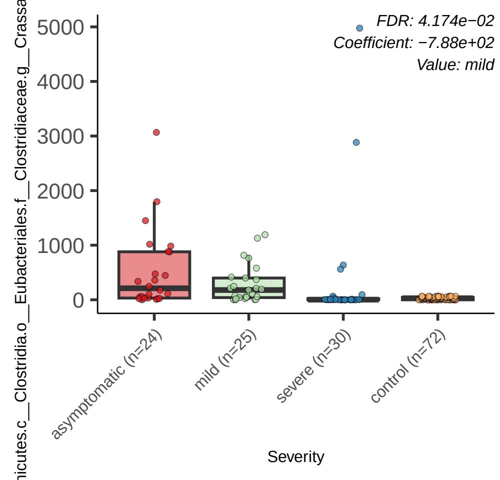

FDR: 4.174e−02
Coefficient: −7.88e+02
Value: mild
5000
4000
3000
2000
1000
0
nicutes.c__Clostridia.o__Eubacteriales.f__Clostridiaceae.g__Crassa
asymptomatic (n=24)
mild (n=25)
severe (n=30)
control (n=72)
Severity

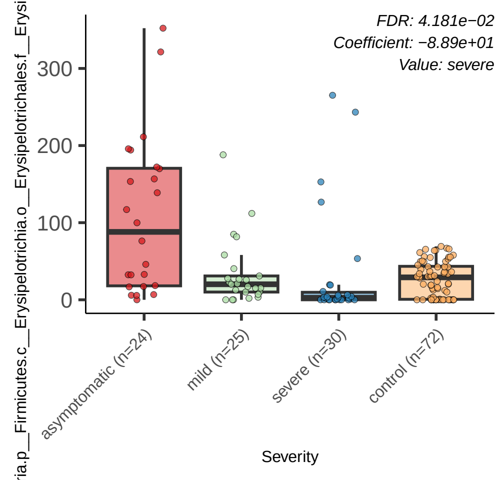

FDR: 4.181e−02
Coefficient: −8.89e+01
Value: severe
ria.p__Firmicutes.c__Erysipelotrichia.o__Erysipelotrichales.f__Erysi
300
200
100
0
asymptomatic (n=24)
mild (n=25)
severe (n=30)
control (n=72)
Severity

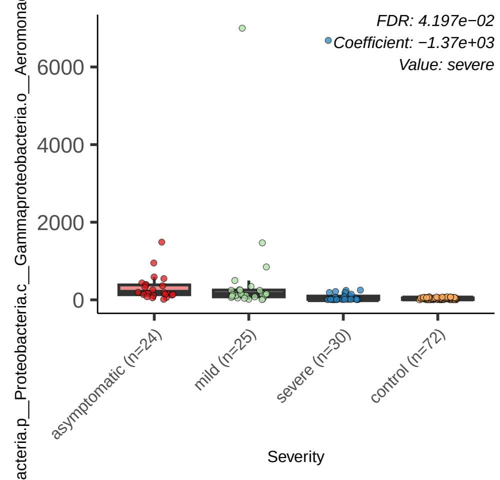

acteria.p__Proteobacteria.c__Gammaproteobacteria.o__Aeromonad
6000
4000
2000
0
FDR: 4.197e−02
Coefficient: −1.37e+03
Value: severe
asymptomatic (n=24)
mild (n=25)
severe (n=30)
control (n=72)
Severity

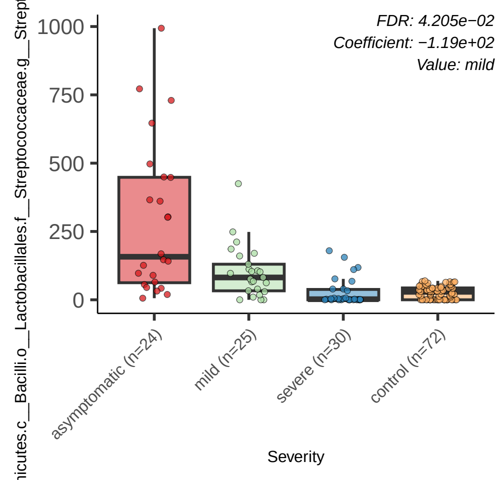

FDR: 4.205e−02
Coefficient: −1.19e+02
Value: mild
1000
750
500
250
0
nicutes.c__Bacilli.o__Lactobacillales.f__Streptococcaceae.g__Strept
asymptomatic (n=24)
mild (n=25)
severe (n=30)
control (n=72)
Severity

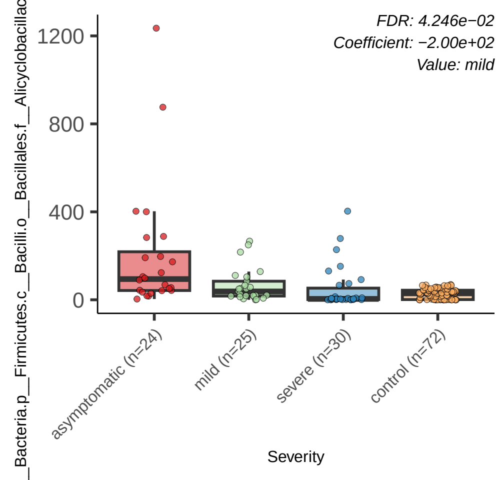
FDR: 4.246e−02
Coefficient: −2.00e+02
Value: mild
1200
800
400
0
asymptomatic (n=24)
mild (n=25)
severe (n=30)
control (n=72)
Severity

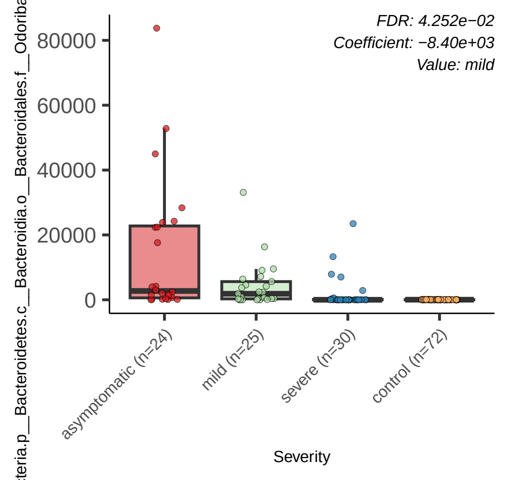

FDR: 4.252e−02
Coefficient: −8.40e+03
Value: mild
80000
60000
40000
20000
0
teria.p__Bacteroidetes.c__Bacteroidia.o__Bacteroidales.f__Odoriba
asymptomatic (n=24)
mild (n=25)
severe (n=30)
control (n=72)
Severity

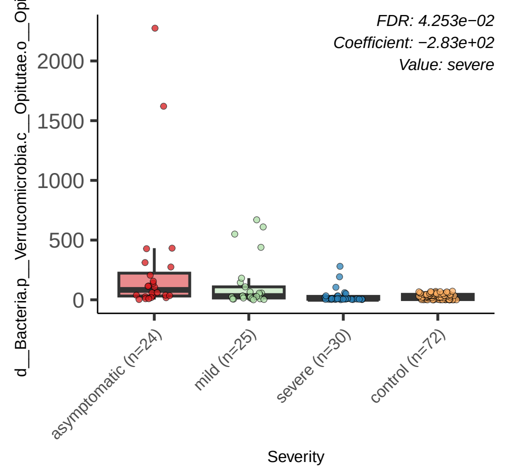

FDR: 4.253e−02
Coefficient: −2.83e+02
Value: severe
2000
1500
1000
500
0
d__Bacteria.p__Verrucomicrobia.c__Opitutae.o__Opi
asymptomatic (n=24)
mild (n=25)
severe (n=30)
control (n=72)
Severity

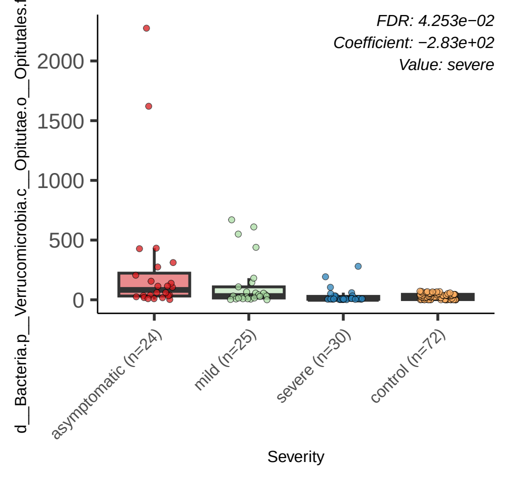
FDR: 4.253e−02
Coefficient: −2.83e+02
Value: severe
d__Bacteria.p__Verrucomicrobia.c__Opitutae.o__Opitutales.f
2000
1500
1000
500
0
asymptomatic (n=24)
mild (n=25)
severe (n=30)
control (n=72)
Severity

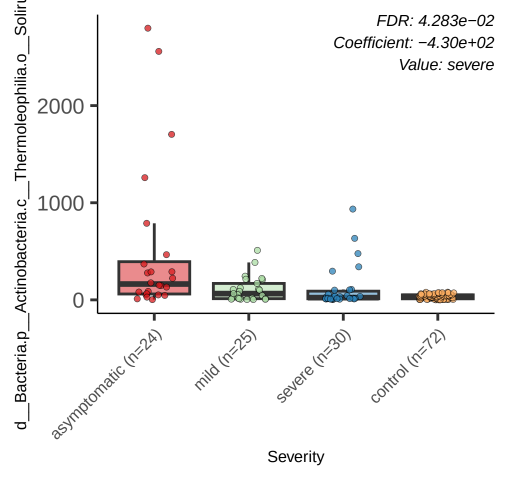

FDR: 4.283e−02
Coefficient: −4.30e+02
Value: severe
d__Bacteria.p__Actinobacteria.c__Thermoleophilia.o__Soliru
0
1000
2000
asymptomatic (n=24)
mild (n=25)
severe (n=30)
control (n=72)
Severity

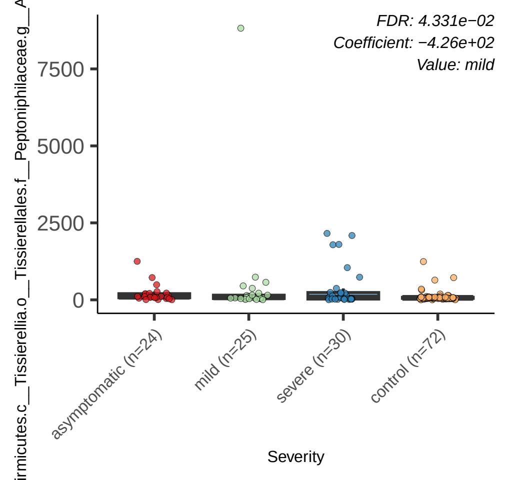

FDR: 4.331e−02
Coefficient: −4.26e+02
Value: mild
7500
5000
2500
0
irmicutes.c__Tissierellia.o__Tissierellales.f__Peptoniphilaceae.g__A
asymptomatic (n=24)
mild (n=25)
severe (n=30)
control (n=72)
Severity

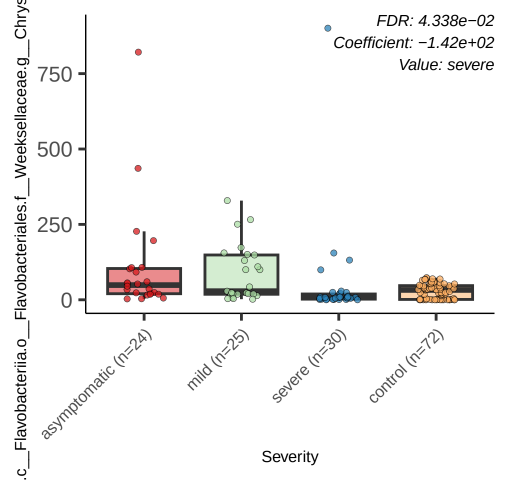

FDR: 4.338e−02
Coefficient: −1.42e+02
Value: severe
750
500
250
0
c__Flavobacteriia.o__Flavobacteriales.f__Weeksellaceae.g__Chrys
asymptomatic (n=24)
mild (n=25)
severe (n=30)
control (n=72)
Severity

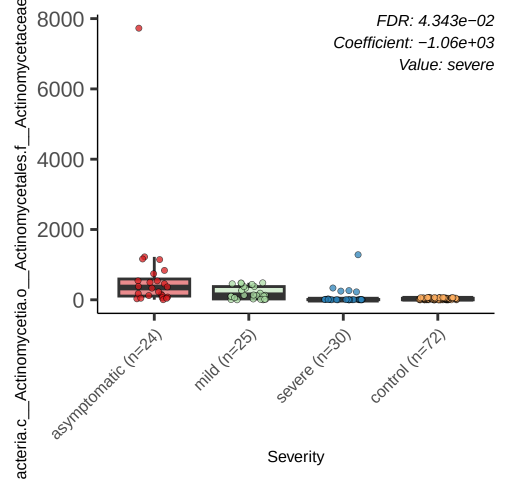
FDR: 4.343e−02
Coefficient: −1.06e+03
Value: severe
8000
6000
4000
2000
0
acteria.c__Actinomycetia.o__Actinomycetales.f__Actinomycetaceae
asymptomatic (n=24)
mild (n=25)
severe (n=30)
control (n=72)
Severity

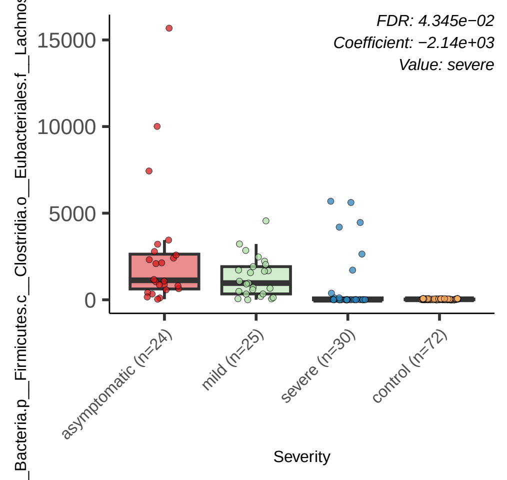
FDR: 4.345e−02
Coefficient: −2.14e+03
Value: severe
_Bacteria.p__Firmicutes.c__Clostridia.o__Eubacteriales.f__Lachnos
15000
10000
5000
0
asymptomatic (n=24)
mild (n=25)
severe (n=30)
control (n=72)
Severity

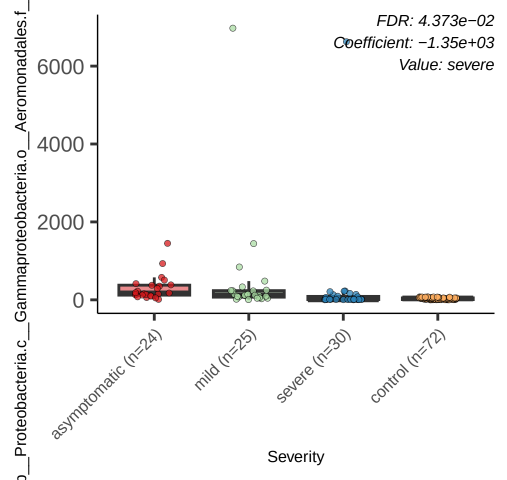

FDR: 4.373e−02
Coefficient: −1.35e+03
Value: severe
p__Proteobacteria.c__Gammaproteobacteria.o__Aeromonadales.f
0
2000
4000
6000
asymptomatic (n=24)
mild (n=25)
severe (n=30)
control (n=72)
Severity

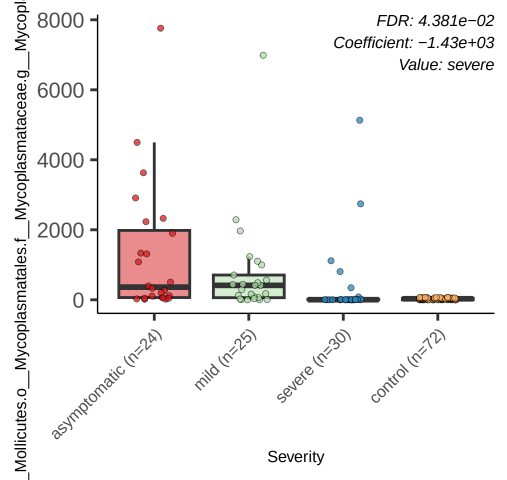
FDR: 4.381e−02
Coefficient: −1.43e+03
Value: severe
8000
6000
4000
2000
0
_Mollicutes.o__Mycoplasmatales.f__Mycoplasmataceae.g__Mycopl
asymptomatic (n=24)
mild (n=25)
severe (n=30)
control (n=72)
Severity

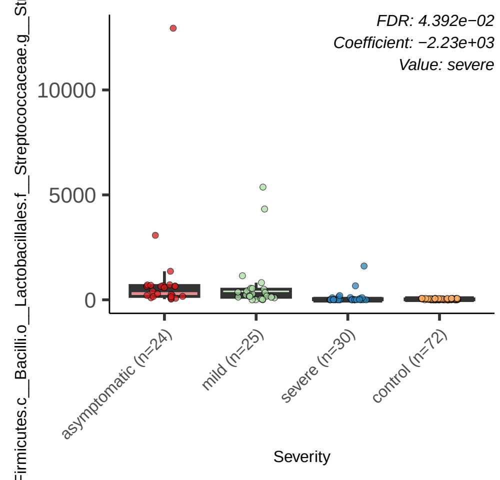
FDR: 4.392e−02
Coefficient: −2.23e+03
Value: severe
10000
5000
0
asymptomatic (n=24)
mild (n=25)
severe (n=30)
control (n=72)
Severity
Firmicutes.c__Bacilli.o__Lactobacillales.f__Streptococcaceae.g__St

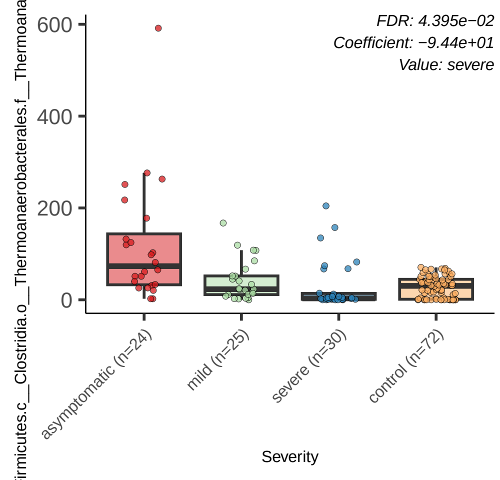

FDR: 4.395e−02
Coefficient: −9.44e+01
Value: severe
irmicutes.c__Clostridia.o__Thermoanaerobacterales.f__Thermoana
600
400
200
0
asymptomatic (n=24)
mild (n=25)
severe (n=30)
control (n=72)
Severity

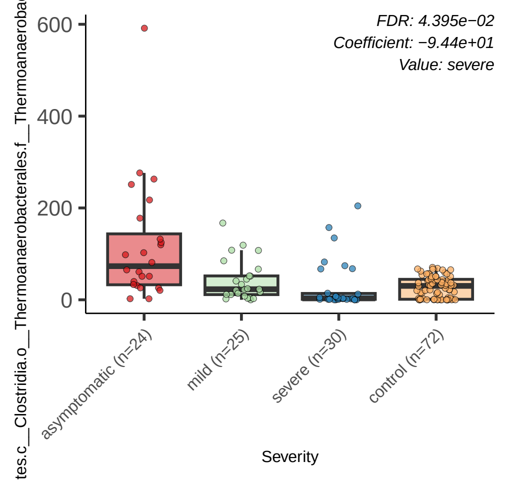

FDR: 4.395e−02
Coefficient: −9.44e+01
Value: severe
0
200
400
600
tes.c__Clostridia.o__Thermoanaerobacterales.f__Thermoanaerobac
asymptomatic (n=24)
mild (n=25)
severe (n=30)
control (n=72)
Severity

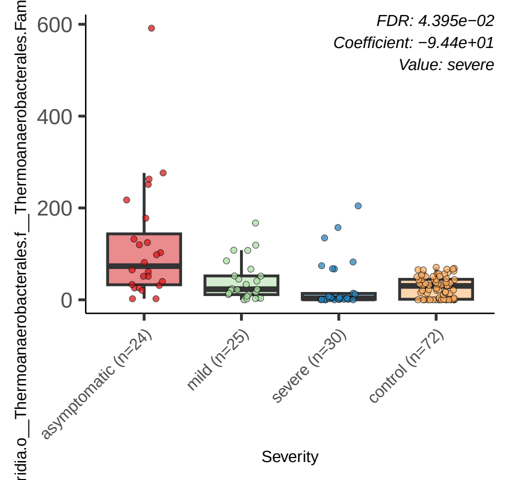

FDR: 4.395e−02
Coefficient: −9.44e+01
Value: severe
600
400
200
0
ridia.o__Thermoanaerobacterales.f__Thermoanaerobacterales.Fam
asymptomatic (n=24)
mild (n=25)
severe (n=30)
control (n=72)
Severity

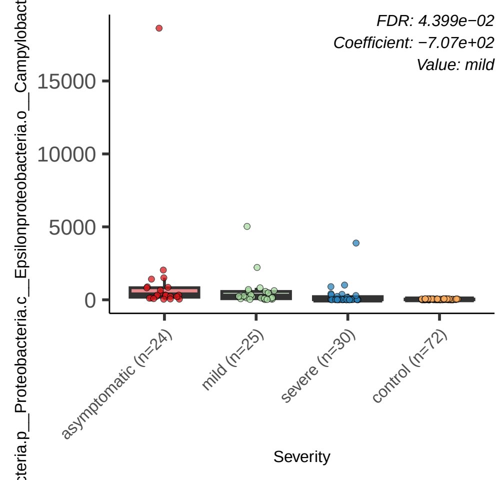

FDR: 4.399e−02
Coefficient: −7.07e+02
Value: mild
15000
10000
5000
0
acteria.p__Proteobacteria.c__Epsilonproteobacteria.o__Campylobact
asymptomatic (n=24)
mild (n=25)
severe (n=30)
control (n=72)
Severity

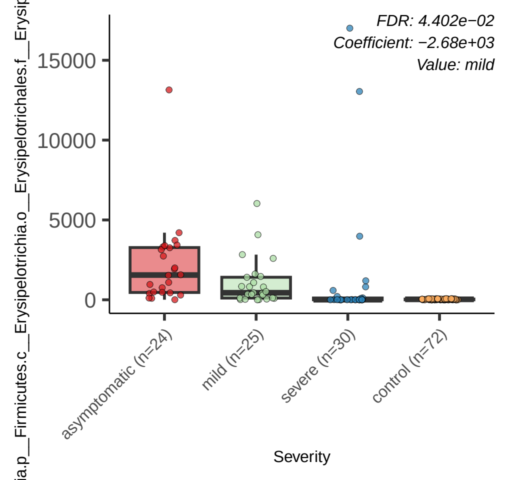

FDR: 4.402e−02
Coefficient: −2.68e+03
Value: mild
15000
10000
5000
0
asymptomatic (n=24)
mild (n=25)
severe (n=30)
control (n=72)
Severity
ia.p__Firmicutes.c__Erysipelotrichia.o__Erysipelotrichales.f__Erysip

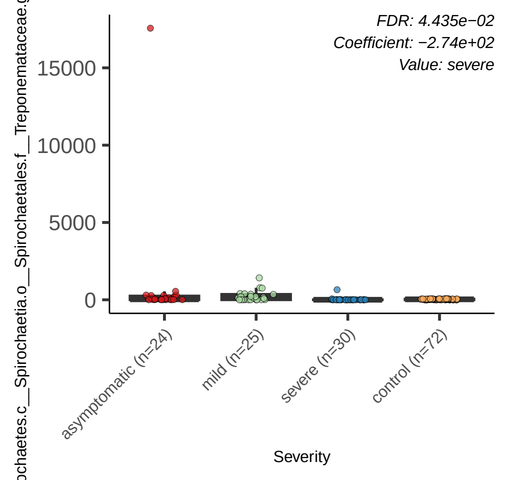

FDR: 4.435e−02
Coefficient: −2.74e+02
Value: severe
15000
10000
5000
0
ochaetes.c__Spirochaetia.o__Spirochaetales.f__Treponemataceae.g
asymptomatic (n=24)
mild (n=25)
severe (n=30)
control (n=72)
Severity

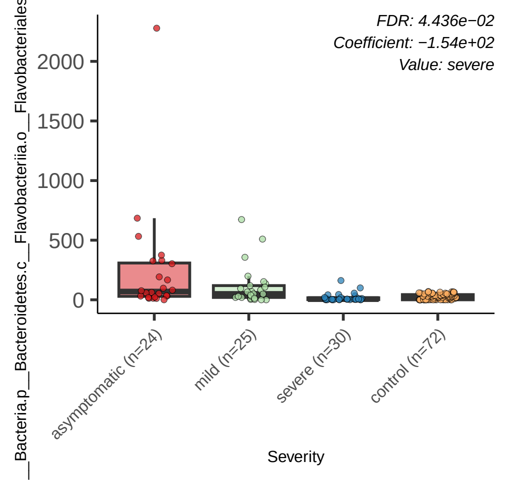
FDR: 4.436e−02
Coefficient: −1.54e+02
Value: severe
2000
1500
1000
500
0
_Bacteria.p__Bacteroidetes.c__Flavobacteriia.o__Flavobacteriales
asymptomatic (n=24)
mild (n=25)
severe (n=30)
control (n=72)
Severity

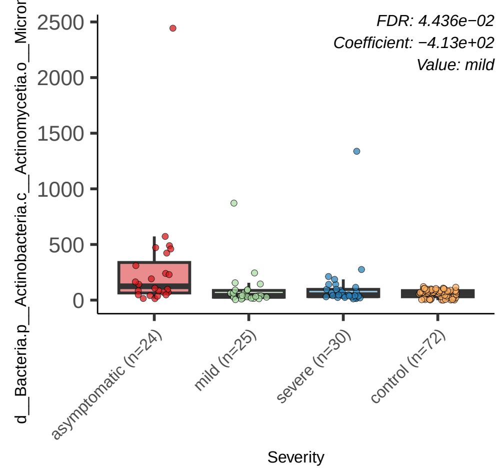

FDR: 4.436e−02
Coefficient: −4.13e+02
Value: mild
d__Bacteria.p__Actinobacteria.c__Actinomycetia.o__Micron
2500
2000
1500
1000
500
0
asymptomatic (n=24)
mild (n=25)
severe (n=30)
control (n=72)
Severity

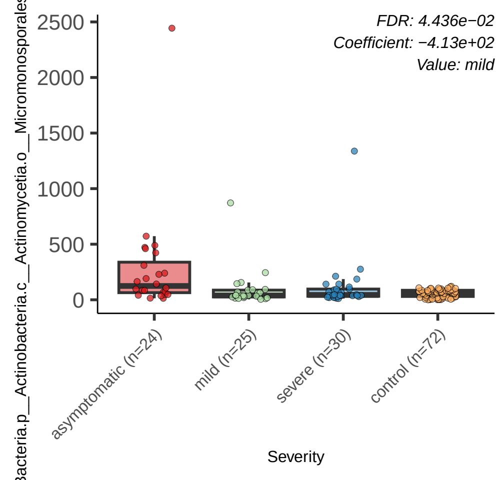

FDR: 4.436e−02
Coefficient: −4.13e+02
Value: mild
Bacteria.p__Actinobacteria.c__Actinomycetia.o__Micromonosporales
2500
2000
1500
1000
500
0
asymptomatic (n=24)
mild (n=25)
severe (n=30)
control (n=72)
Severity

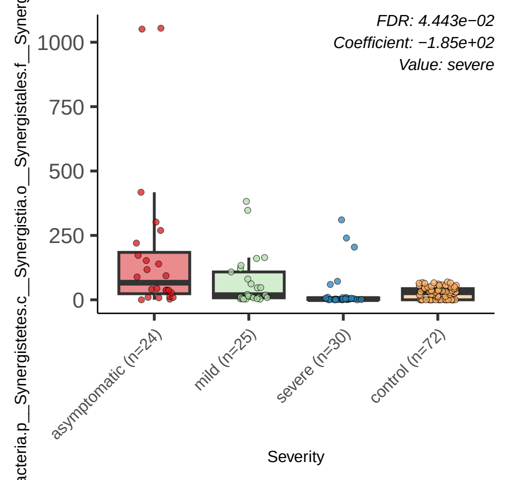
FDR: 4.443e−02
Coefficient: −1.85e+02
Value: severe
acteria.p__Synergistetes.c__Synergistia.o__Synergistales.f__Synerg
1000
750
500
250
0
asymptomatic (n=24)
mild (n=25)
severe (n=30)
control (n=72)
Severity

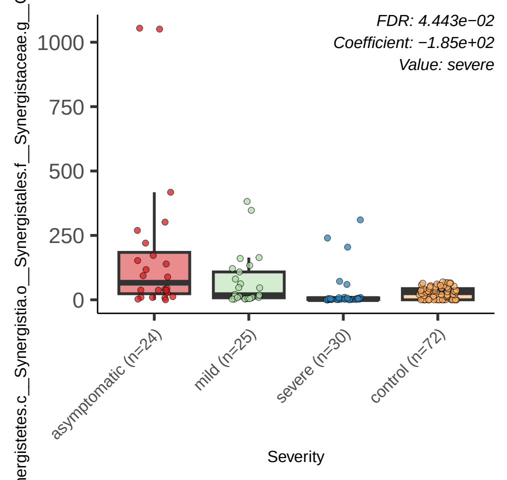

FDR: 4.443e−02
Coefficient: −1.85e+02
Value: severe
1000
750
500
250
0
ergistetes.c__Synergistia.o__Synergistales.f__Synergistaceae.g__C
asymptomatic (n=24)
mild (n=25)
severe (n=30)
control (n=72)
Severity

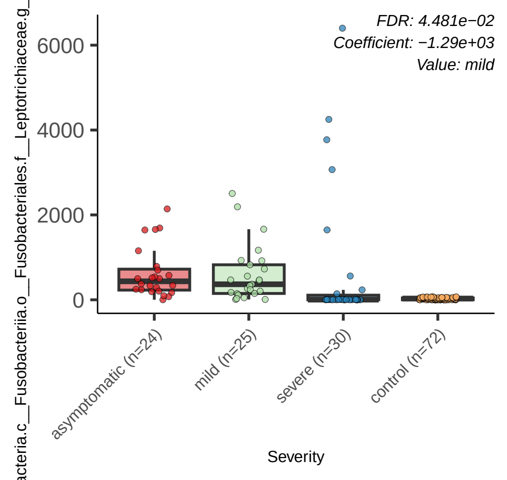

FDR: 4.481e−02
Coefficient: −1.29e+03
Value: mild
6000
4000
2000
0
acteria.c__Fusobacteriia.o__Fusobacteriales.f__Leptotrichiaceae.g_
asymptomatic (n=24)
mild (n=25)
severe (n=30)
control (n=72)
Severity

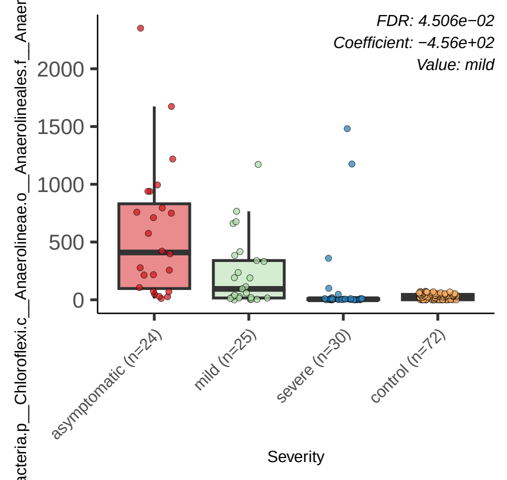

FDR: 4.506e−02
Coefficient: −4.56e+02
Value: mild
acteria.p__Chloroflexi.c__Anaerolineae.o__Anaerolineales.f__Anaer
2000
1500
1000
500
0
asymptomatic (n=24)
mild (n=25)
severe (n=30)
control (n=72)
Severity

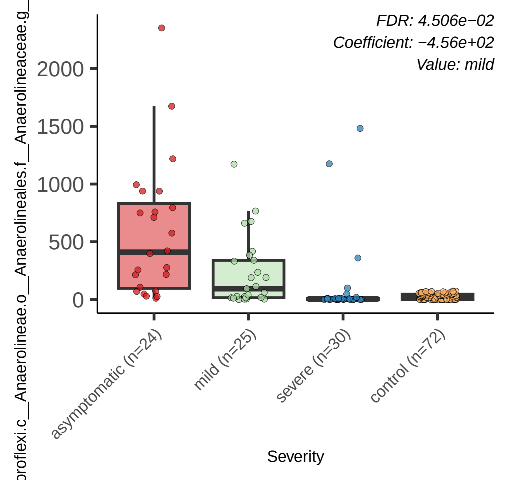

FDR: 4.506e−02
Coefficient: −4.56e+02
Value: mild
roflexi.c__Anaerolineae.o__Anaerolineales.f__Anaerolineaceae.g__
0
500
1000
1500
2000
asymptomatic (n=24)
mild (n=25)
severe (n=30)
control (n=72)
Severity

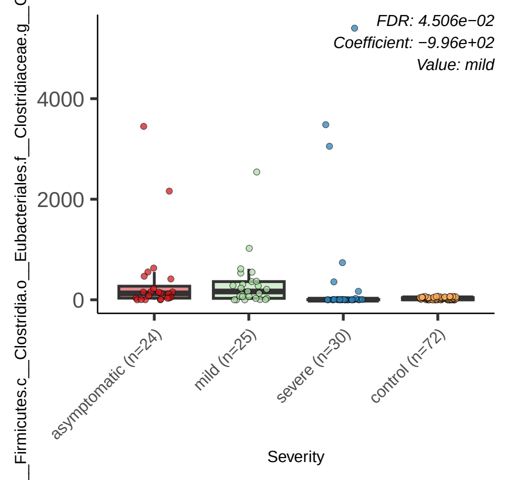
FDR: 4.506e−02
Coefficient: −9.96e+02
Value: mild
4000
2000
0
asymptomatic (n=24)
mild (n=25)
severe (n=30)
control (n=72)
Severity

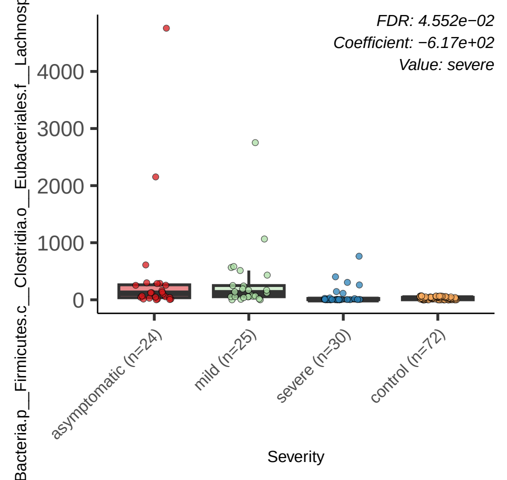
FDR: 4.552e−02
Coefficient: −6.17e+02
Value: severe
Bacteria.p__Firmicutes.c__Clostridia.o__Eubacteriales.f__Lachnosp
4000
3000
2000
1000
0
asymptomatic (n=24)
mild (n=25)
severe (n=30)
control (n=72)
Severity

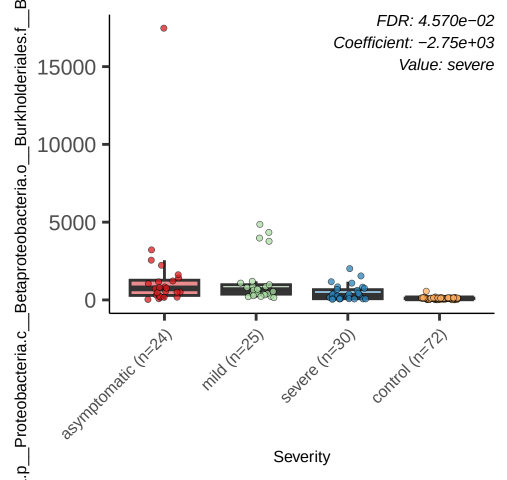

FDR: 4.570e−02
Coefficient: −2.75e+03
Value: severe
15000
10000
5000
0
p__Proteobacteria.c__Betaproteobacteria.o__Burkholderiales.f__B
asymptomatic (n=24)
mild (n=25)
severe (n=30)
control (n=72)
Severity

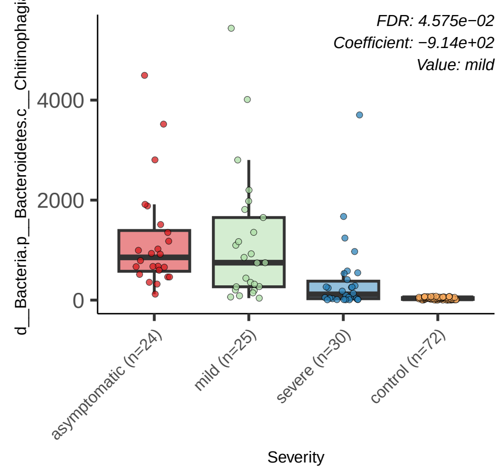
FDR: 4.575e−02
Coefficient: −9.14e+02
Value: mild
d__Bacteria.p__Bacteroidetes.c__Chitinophagia
4000
2000
0
asymptomatic (n=24)
mild (n=25)
severe (n=30)
control (n=72)
Severity

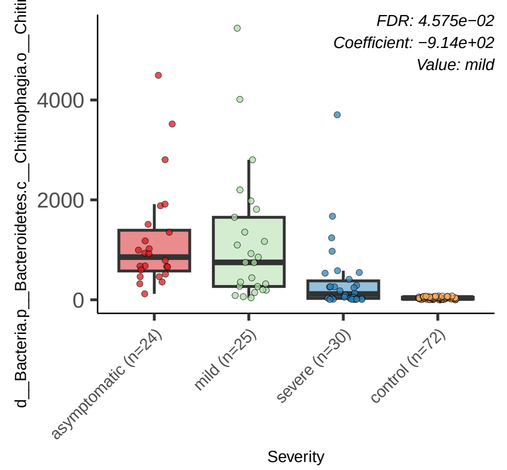

FDR: 4.575e−02
Coefficient: −9.14e+02
Value: mild
d__Bacteria.p__Bacteroidetes.c__Chitinophagia.o__Chiti
4000
2000
0
asymptomatic (n=24)
mild (n=25)
severe (n=30)
control (n=72)
Severity

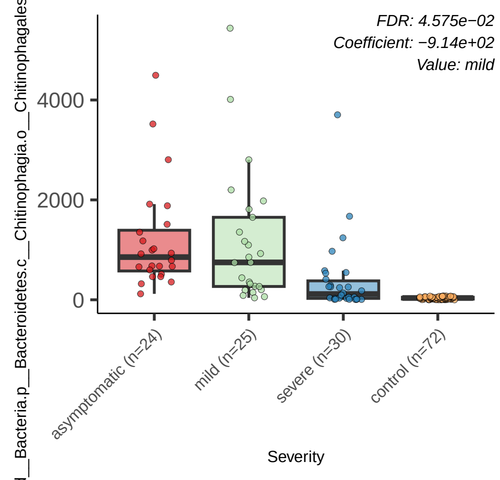

FDR: 4.575e−02
Coefficient: −9.14e+02
Value: mild
d__Bacteria.p__Bacteroidetes.c__Chitinophagia.o__Chitinophagales
0
2000
4000
asymptomatic (n=24)
mild (n=25)
severe (n=30)
control (n=72)
Severity

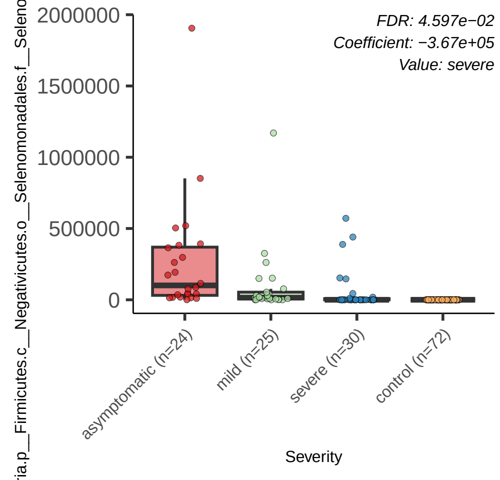

FDR: 4.597e−02
Coefficient: −3.67e+05
Value: severe
2000000
1500000
1000000
500000
0
ria.p__Firmicutes.c__Negativicutes.o__Selenomonadales.f__Seleno
asymptomatic (n=24)
mild (n=25)
severe (n=30)
control (n=72)
Severity

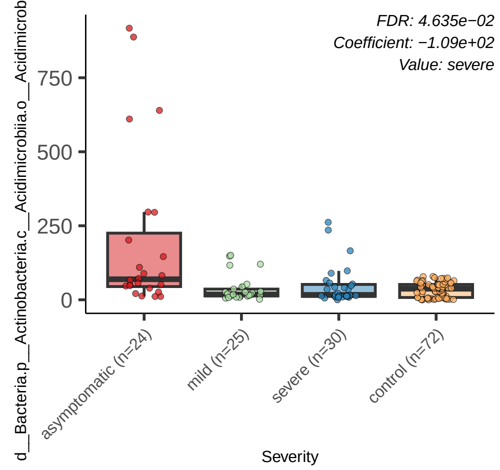

FDR: 4.635e−02
Coefficient: −1.09e+02
Value: severe
d__Bacteria.p__Actinobacteria.c__Acidimicrobiia.o__Acidimicrob
750
500
250
0
asymptomatic (n=24)
mild (n=25)
severe (n=30)
control (n=72)
Severity

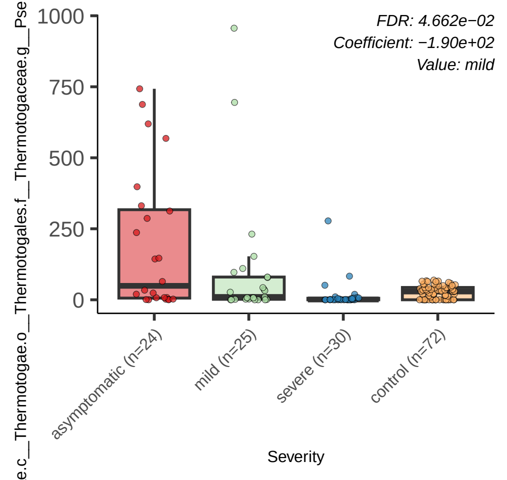

e.c__Thermotogae.o__Thermotogales.f__Thermotogaceae.g__Pse
1000
750
500
250
0
FDR: 4.662e−02
Coefficient: −1.90e+02
Value: mild
asymptomatic (n=24)
mild (n=25)
severe (n=30)
control (n=72)
Severity

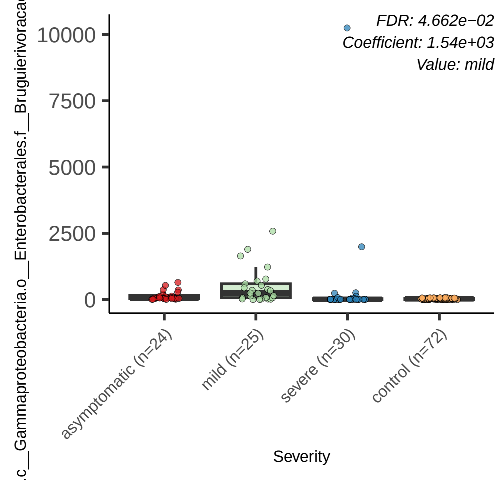

FDR: 4.662e−02
Coefficient: 1.54e+03
Value: mild
10000
7500
5000
2500
0
c__Gammaproteobacteria.o__Enterobacterales.f__Bruguierivoracae
asymptomatic (n=24)
mild (n=25)
severe (n=30)
control (n=72)
Severity

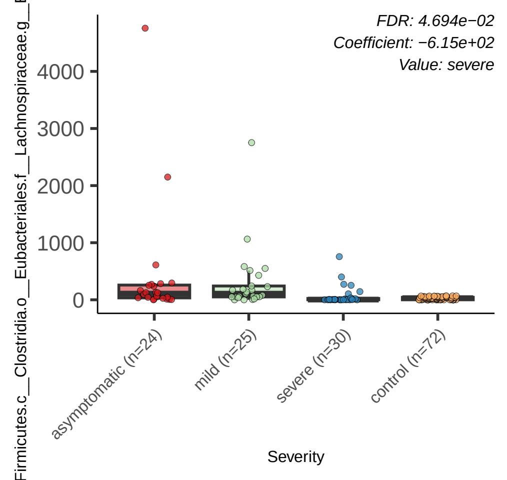

FDR: 4.694e−02
Coefficient: −6.15e+02
Value: severe
Firmicutes.c__Clostridia.o__Eubacteriales.f__Lachnospiraceae.g__
4000
3000
2000
1000
0
asymptomatic (n=24)
mild (n=25)
severe (n=30)
control (n=72)
Severity

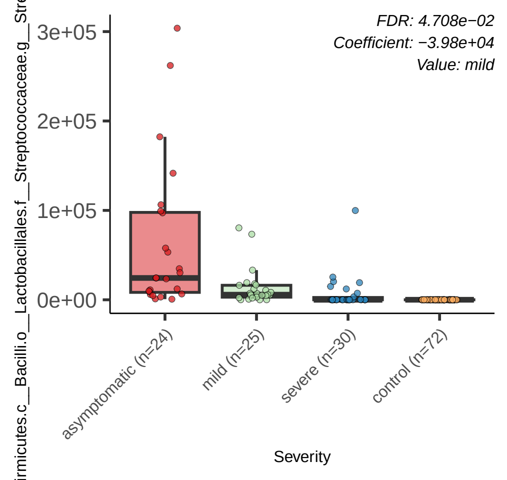
FDR: 4.708e−02
Coefficient: −3.98e+04
Value: mild
3e+05
2e+05
1e+05
0e+00
asymptomatic (n=24)
mild (n=25)
severe (n=30)
control (n=72)
Severity

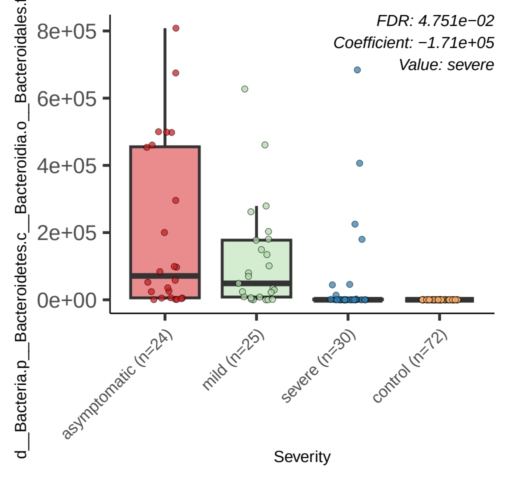

d__Bacteria.p__Bacteroidetes.c__Bacteroidia.o__Bacteroidales.f
FDR: 4.751e−02
Coefficient: −1.71e+05
Value: severe
8e+05
6e+05
4e+05
2e+05
0e+00
asymptomatic (n=24)
mild (n=25)
severe (n=30)
control (n=72)
Severity

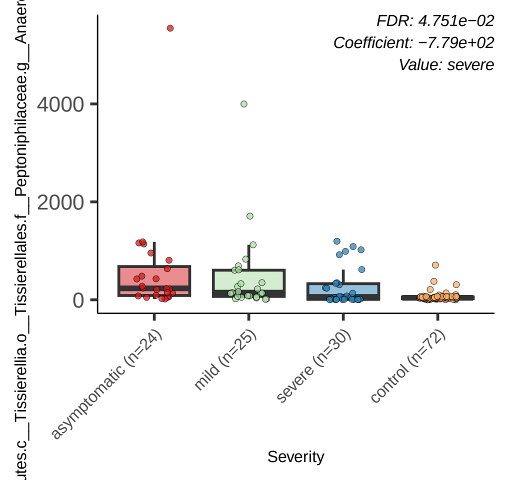

FDR: 4.751e−02
Coefficient: −7.79e+02
Value: severe
0
2000
4000
utes.c__Tissierellia.o__Tissierellales.f__Peptoniphilaceae.g__Anaer
asymptomatic (n=24)
mild (n=25)
severe (n=30)
control (n=72)
Severity

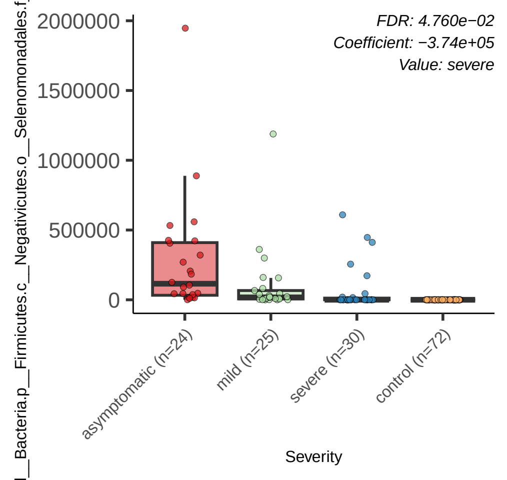

FDR: 4.760e−02
Coefficient: −3.74e+05
Value: severe
2000000
1500000
1000000
500000
0
Bacteria.p__Firmicutes.c__Negativicutes.o__Selenomonadales.f
asymptomatic (n=24)
mild (n=25)
severe (n=30)
control (n=72)
Severity

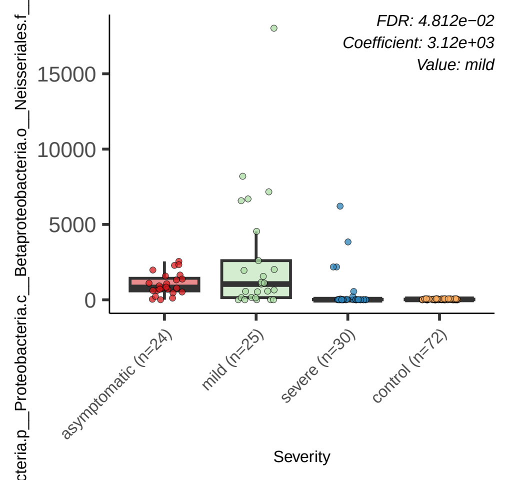
FDR: 4.812e−02
Coefficient: 3.12e+03
Value: mild
cteria.p__Proteobacteria.c__Betaproteobacteria.o__Neisseriales.f_
15000
10000
5000
0
asymptomatic (n=24)
mild (n=25)
severe (n=30)
control (n=72)
Severity

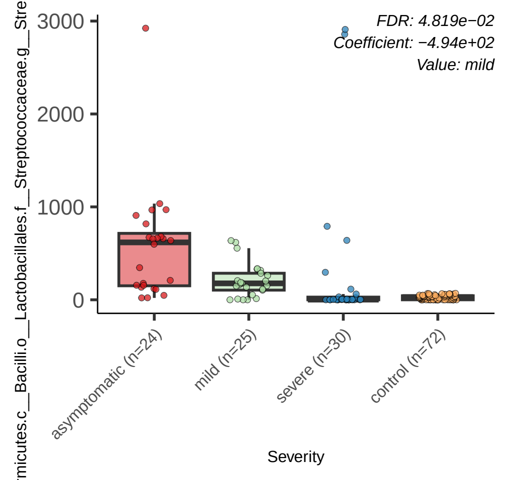

FDR: 4.819e−02
Coefficient: −4.94e+02
Value: mild
3000
2000
1000
0
micutes.c__Bacilli.o__Lactobacillales.f__Streptococcaceae.g__Stre
asymptomatic (n=24)
mild (n=25)
severe (n=30)
control (n=72)
Severity

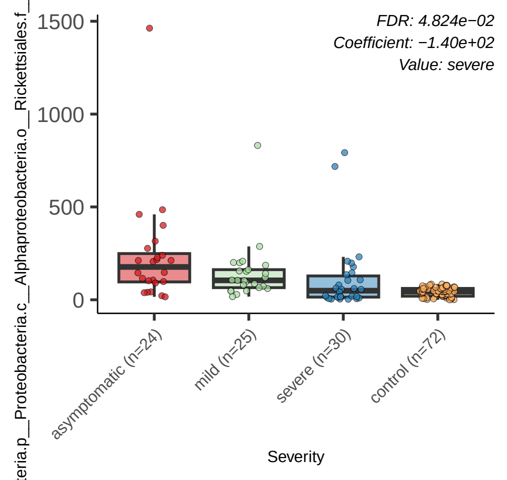
FDR: 4.824e−02
Coefficient: −1.40e+02
Value: severe
1500
1000
500
0
teria.p__Proteobacteria.c__Alphaproteobacteria.o__Rickettsiales.f__
asymptomatic (n=24)
mild (n=25)
severe (n=30)
control (n=72)
Severity

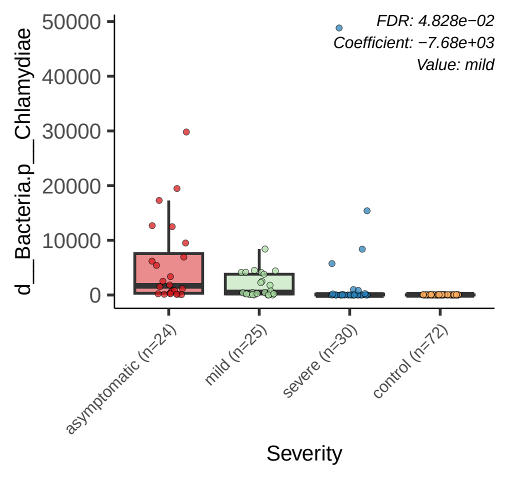

FDR: 4.828e−02
Coefficient: −7.68e+03
Value: mild
d__Bacteria.p__Chlamydiae
50000
40000
30000
20000
10000
0
asymptomatic (n=24)
mild (n=25)
severe (n=30)
control (n=72)
Severity

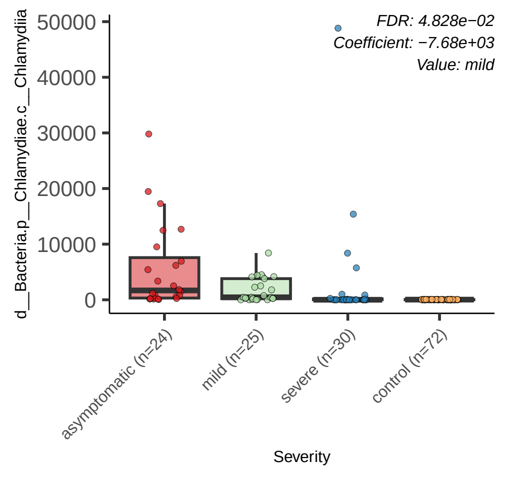
FDR: 4.828e−02
Coefficient: −7.68e+03
Value: mild
d__Bacteria.p__Chlamydiae.c__Chlamydiia
50000
40000
30000
20000
10000
0
asymptomatic (n=24)
mild (n=25)
severe (n=30)
control (n=72)
Severity

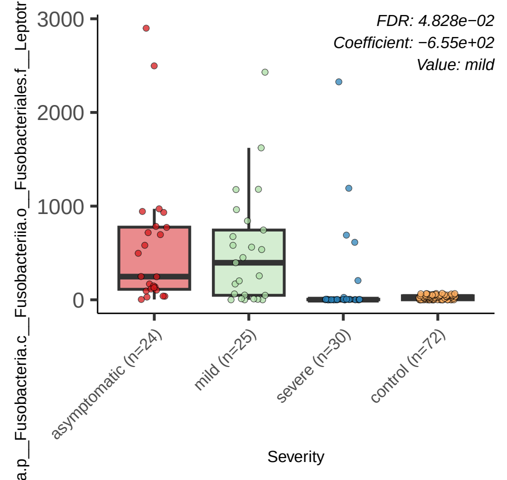

FDR: 4.828e−02
Coefficient: −6.55e+02
Value: mild
3000
2000
1000
0
a.p__Fusobacteria.c__Fusobacteriia.o__Fusobacteriales.f__Leptotr
asymptomatic (n=24)
mild (n=25)
severe (n=30)
control (n=72)
Severity

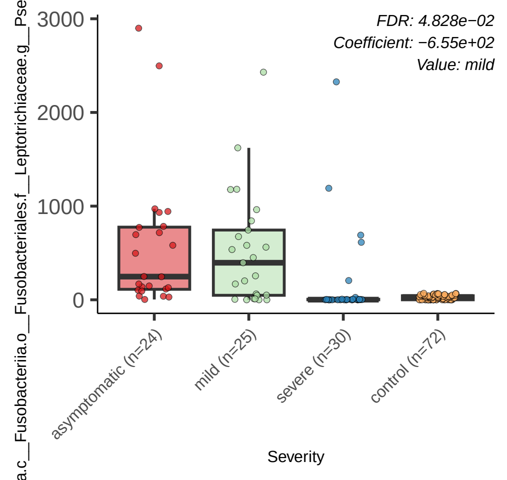

FDR: 4.828e−02
Coefficient: −6.55e+02
Value: mild
0
1000
2000
3000
a.c__Fusobacteriia.o__Fusobacteriales.f__Leptotrichiaceae.g__Pse
asymptomatic (n=24)
mild (n=25)
severe (n=30)
control (n=72)
Severity

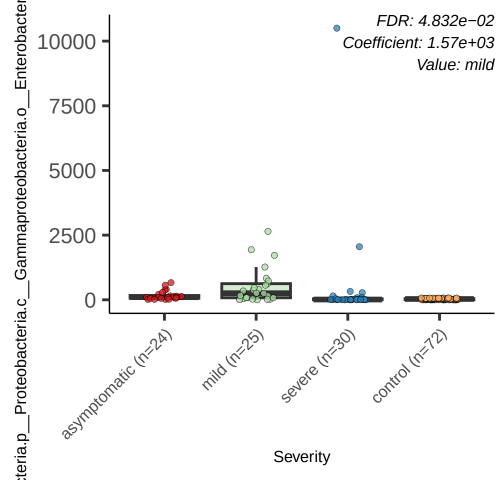
FDR: 4.832e−02
Coefficient: 1.57e+03
Value: mild
10000
7500
5000
2500
0
asymptomatic (n=24)
mild (n=25)
severe (n=30)
control (n=72)
Severity
cteria.p__Proteobacteria.c__Gammaproteobacteria.o__Enterobacter

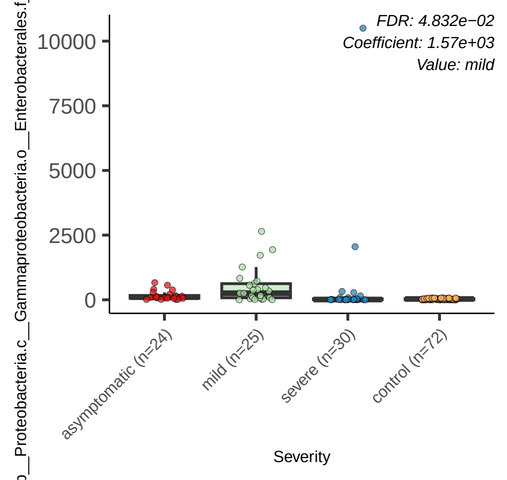

FDR: 4.832e−02
Coefficient: 1.57e+03
Value: mild
10000
7500
5000
2500
0
asymptomatic (n=24)
mild (n=25)
severe (n=30)
control (n=72)
Severity
p__Proteobacteria.c__Gammaproteobacteria.o__Enterobacterales.f_

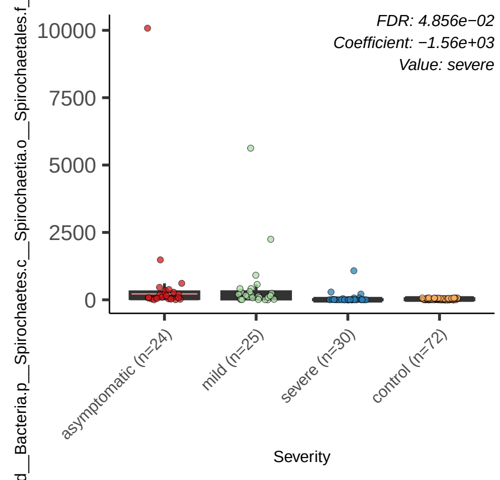
FDR: 4.856e−02
Coefficient: −1.56e+03
Value: severe
d__Bacteria.p__Spirochaetes.c__Spirochaetia.o__Spirochaetales.f_
10000
7500
5000
2500
0
asymptomatic (n=24)
mild (n=25)
severe (n=30)
control (n=72)
Severity

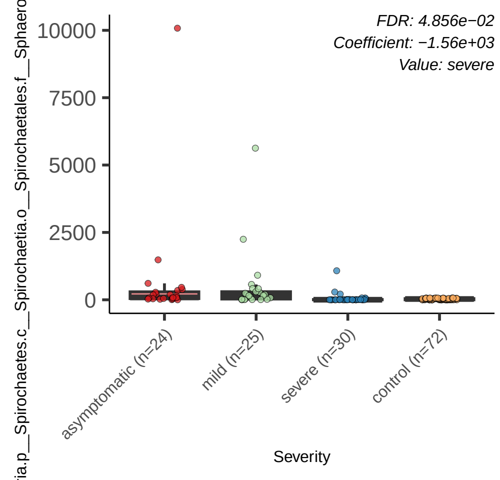
FDR: 4.856e−02
Coefficient: −1.56e+03
Value: severe
10000
7500
5000
2500
0
ia.p__Spirochaetes.c__Spirochaetia.o__Spirochaetales.f__Sphaero
asymptomatic (n=24)
mild (n=25)
severe (n=30)
control (n=72)
Severity

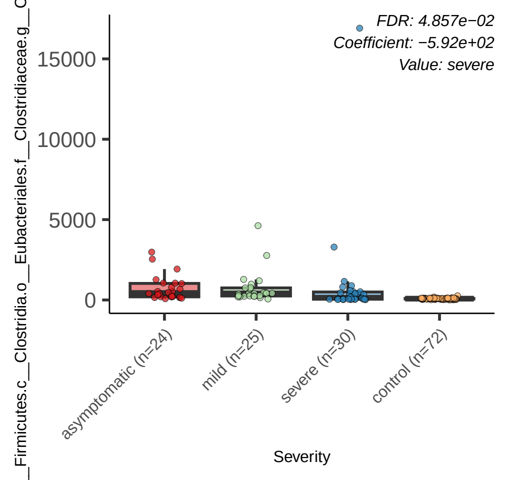

FDR: 4.857e−02
Coefficient: −5.92e+02
Value: severe
15000
10000
5000
0
Firmicutes.c__Clostridia.o__Eubacteriales.f__Clostridiaceae.g__C
asymptomatic (n=24)
mild (n=25)
severe (n=30)
control (n=72)
Severity

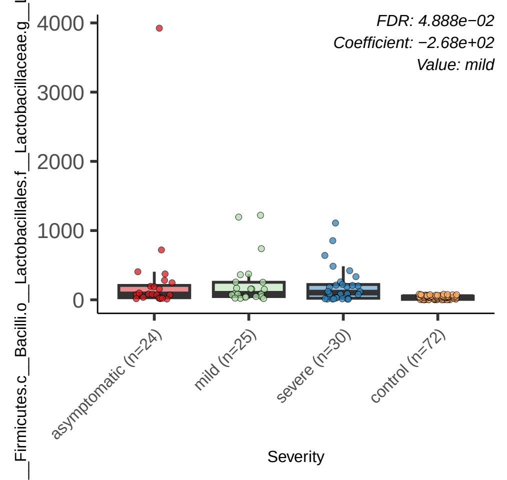
FDR: 4.888e−02
Coefficient: −2.68e+02
Value: mild
Firmicutes.c__Bacilli.o__Lactobacillales.f__Lactobacillaceae.g__L
4000
3000
2000
1000
0
asymptomatic (n=24)
mild (n=25)
severe (n=30)
control (n=72)
Severity

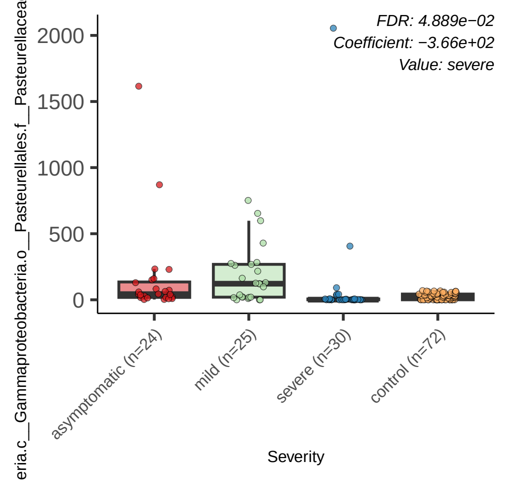

FDR: 4.889e−02
Coefficient: −3.66e+02
Value: severe
2000
1500
1000
500
0
eria.c__Gammaproteobacteria.o__Pasteurellales.f__Pasteurellacea
asymptomatic (n=24)
mild (n=25)
severe (n=30)
control (n=72)
Severity

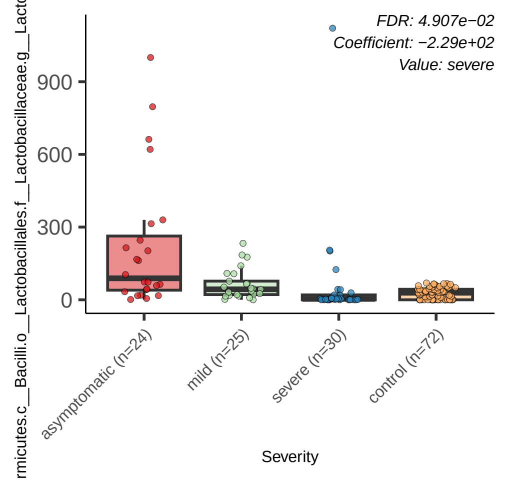

FDR: 4.907e−02
Coefficient: −2.29e+02
Value: severe
900
600
300
0
rmicutes.c__Bacilli.o__Lactobacillales.f__Lactobacillaceae.g__Lacto
asymptomatic (n=24)
mild (n=25)
severe (n=30)
control (n=72)
Severity

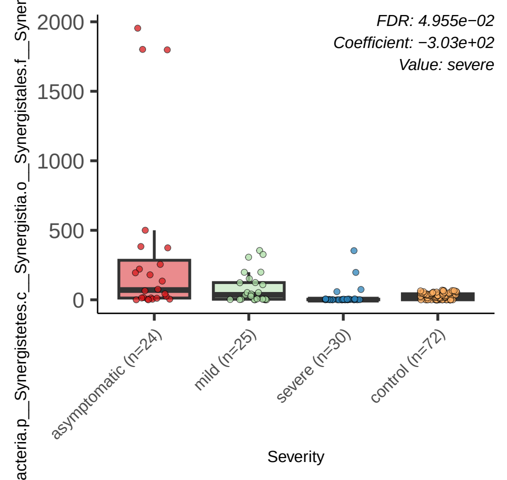
FDR: 4.955e−02
Coefficient: −3.03e+02
Value: severe
acteria.p__Synergistetes.c__Synergistia.o__Synergistales.f__Syner
2000
1500
1000
500
0
asymptomatic (n=24)
mild (n=25)
severe (n=30)
control (n=72)
Severity

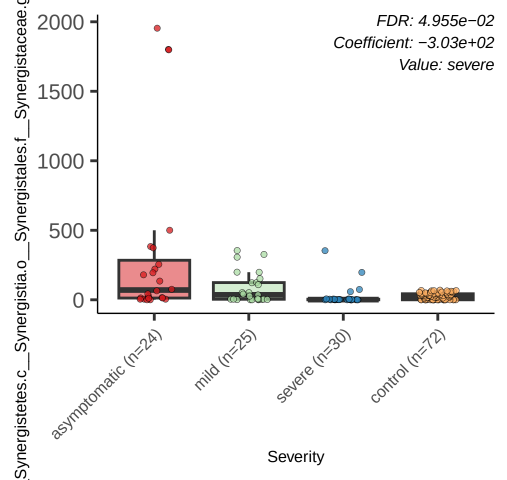

FDR: 4.955e−02
Coefficient: −3.03e+02
Value: severe
Synergistetes.c__Synergistia.o__Synergistales.f__Synergistaceae.g
2000
1500
1000
500
0
asymptomatic (n=24)
mild (n=25)
severe (n=30)
control (n=72)
Severity

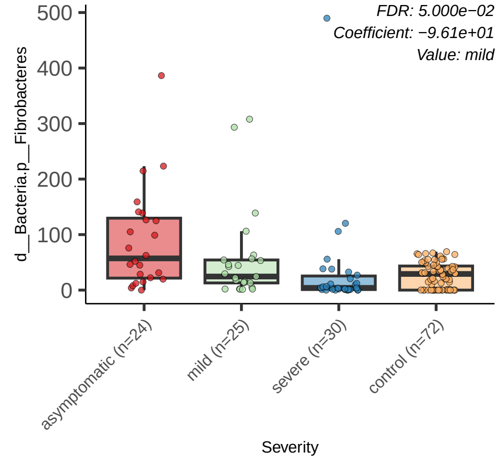

FDR: 5.000e−02
Coefficient: −9.61e+01
Value: mild
d__Bacteria.p__Fibrobacteres
500
400
300
200
100
0
asymptomatic (n=24)
mild (n=25)
severe (n=30)
control (n=72)
Severity

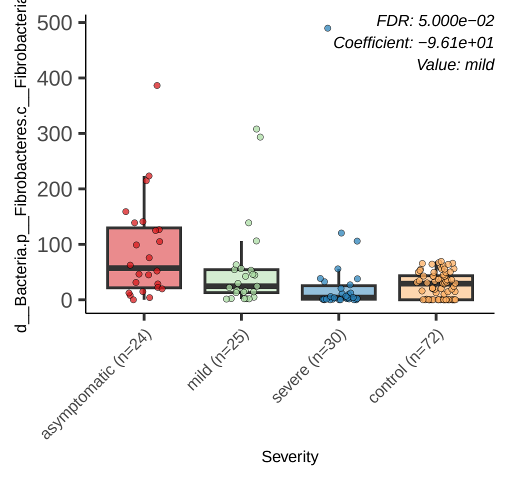

FDR: 5.000e−02
Coefficient: −9.61e+01
Value: mild
d__Bacteria.p__Fibrobacteres.c__Fibrobacteria
500
400
300
200
100
0
asymptomatic (n=24)
mild (n=25)
severe (n=30)
control (n=72)
Severity

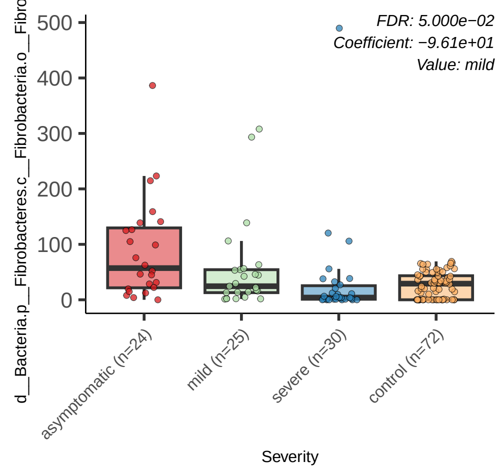

FDR: 5.000e−02
Coefficient: −9.61e+01
Value: mild
d__Bacteria.p__Fibrobacteres.c__Fibrobacteria.o__Fibro
500
400
300
200
100
0
asymptomatic (n=24)
mild (n=25)
severe (n=30)
control (n=72)
Severity

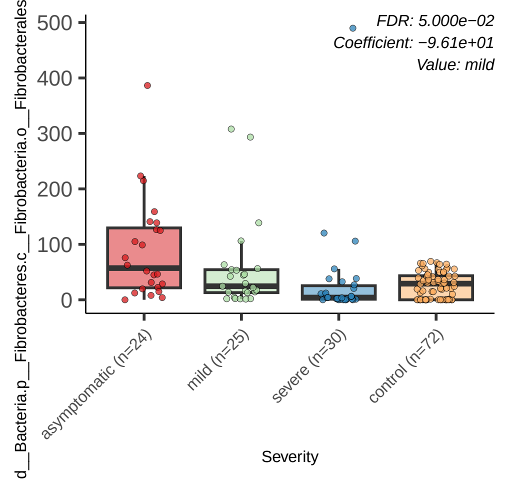

FDR: 5.000e−02
Coefficient: −9.61e+01
Value: mild
d__Bacteria.p__Fibrobacteres.c__Fibrobacteria.o__Fibrobacterales
0
100
200
300
400
500
asymptomatic (n=24)
mild (n=25)
severe (n=30)
control (n=72)
Severity

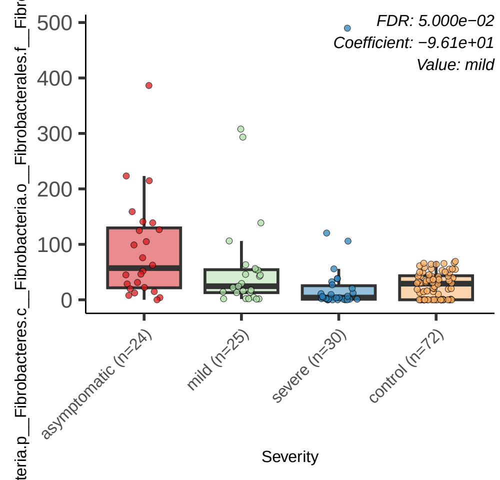
FDR: 5.000e−02
Coefficient: −9.61e+01
Value: mild
teria.p__Fibrobacteres.c__Fibrobacteria.o__Fibrobacterales.f__Fibro
500
400
300
200
100
0
asymptomatic (n=24)
mild (n=25)
severe (n=30)
control (n=72)
Severity

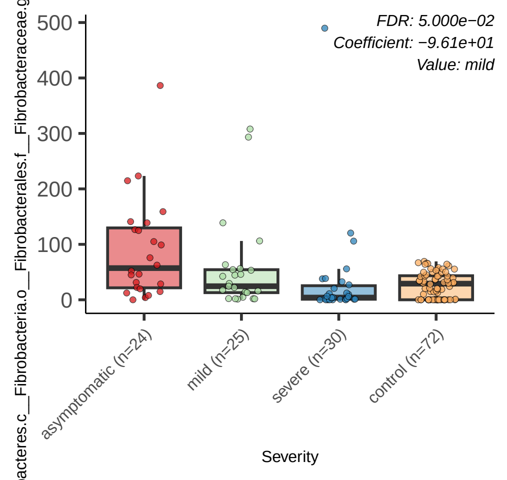

FDR: 5.000e−02
Coefficient: −9.61e+01
Value: mild
500
400
300
200
100
0
acteres.c__Fibrobacteria.o__Fibrobacterales.f__Fibrobacteraceae.g
asymptomatic (n=24)
mild (n=25)
severe (n=30)
control (n=72)
Severity

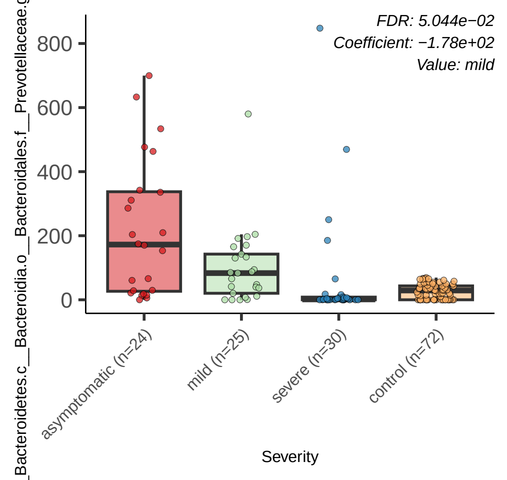
FDR: 5.044e−02
Coefficient: −1.78e+02
Value: mild
Bacteroidetes.c__Bacteroidia.o__Bacteroidales.f__Prevotellaceae.g
800
600
400
200
0
asymptomatic (n=24)
mild (n=25)
severe (n=30)
control (n=72)
Severity

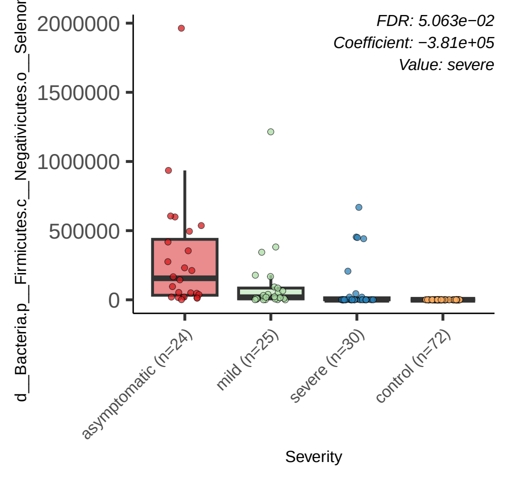

FDR: 5.063e−02
Coefficient: −3.81e+05
Value: severe
d__Bacteria.p__Firmicutes.c__Negativicutes.o__Selenor
2000000
1500000
1000000
500000
0
asymptomatic (n=24)
mild (n=25)
severe (n=30)
control (n=72)
Severity

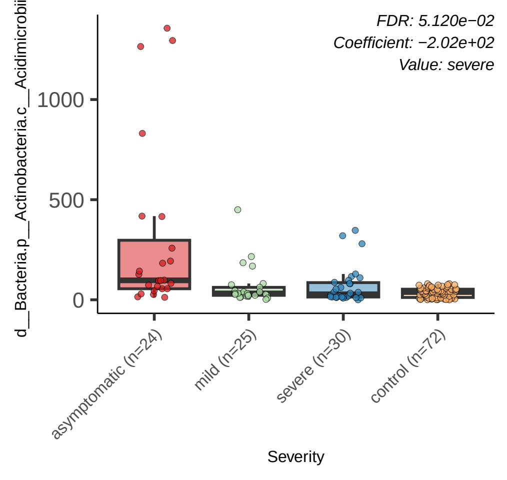

FDR: 5.120e−02
Coefficient: −2.02e+02
Value: severe
d__Bacteria.p__Actinobacteria.c__Acidimicrobii
0
500
1000
asymptomatic (n=24)
mild (n=25)
severe (n=30)
control (n=72)
Severity

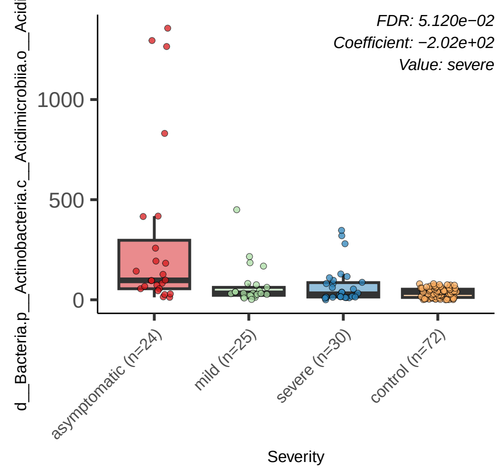

FDR: 5.120e−02
Coefficient: −2.02e+02
Value: severe
d__Bacteria.p__Actinobacteria.c__Acidimicrobiia.o__Acidi
0
500
1000
asymptomatic (n=24)
mild (n=25)
severe (n=30)
control (n=72)
Severity

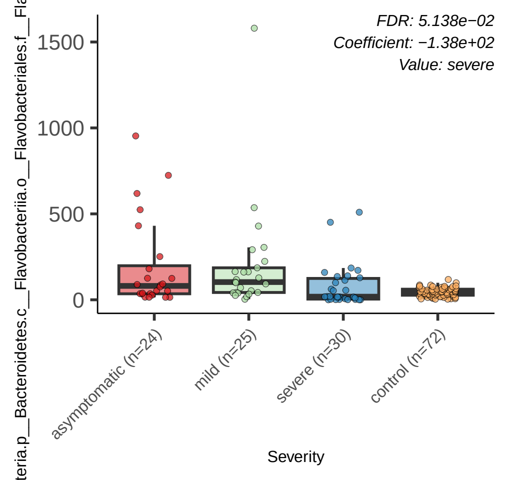

FDR: 5.138e−02
Coefficient: −1.38e+02
Value: severe
1500
1000
500
0
teria.p__Bacteroidetes.c__Flavobacteriia.o__Flavobacteriales.f__Fla
asymptomatic (n=24)
mild (n=25)
severe (n=30)
control (n=72)
Severity

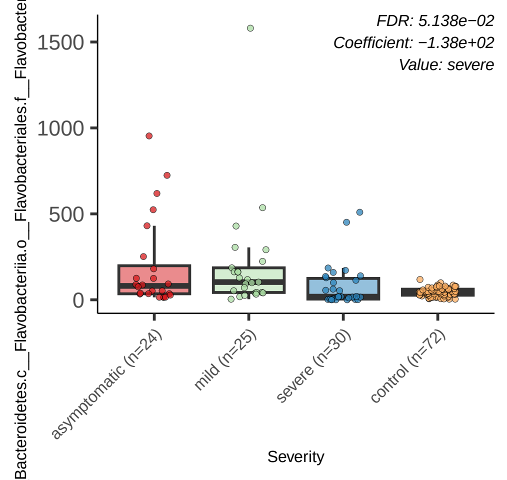
FDR: 5.138e−02
Coefficient: −1.38e+02
Value: severe
1500
1000
500
0
Bacteroidetes.c__Flavobacteriia.o__Flavobacteriales.f__Flavobacter
asymptomatic (n=24)
mild (n=25)
severe (n=30)
control (n=72)
Severity

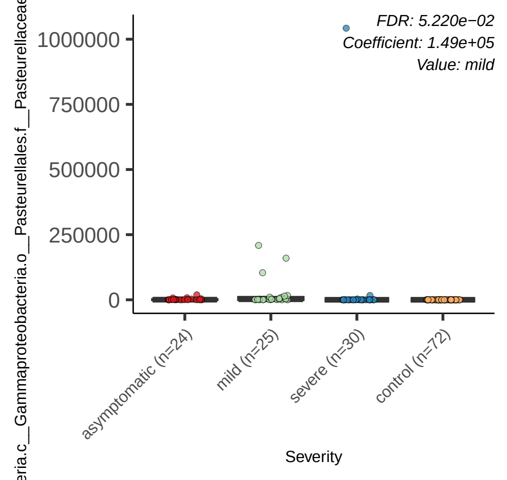

FDR: 5.220e−02
Coefficient: 1.49e+05
Value: mild
1000000
750000
500000
250000
0
asymptomatic (n=24)
mild (n=25)
severe (n=30)
control (n=72)
Severity
eria.c__Gammaproteobacteria.o__Pasteurellales.f__Pasteurellaceae

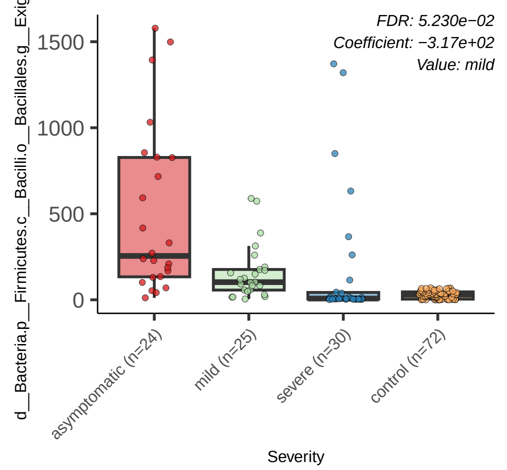
FDR: 5.230e−02
Coefficient: −3.17e+02
Value: mild
d__Bacteria.p__Firmicutes.c__Bacilli.o__Bacillales.g__Exig
1500
1000
500
0
asymptomatic (n=24)
mild (n=25)
severe (n=30)
control (n=72)
Severity

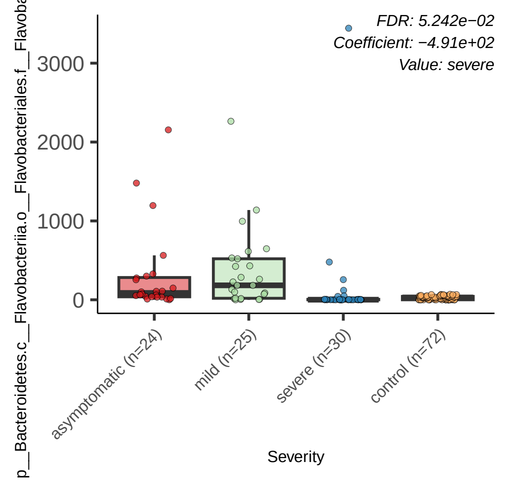
FDR: 5.242e−02
Coefficient: −4.91e+02
Value: severe
p__Bacteroidetes.c__Flavobacteriia.o__Flavobacteriales.f__Flavoba
3000
2000
1000
0
asymptomatic (n=24)
mild (n=25)
severe (n=30)
control (n=72)
Severity

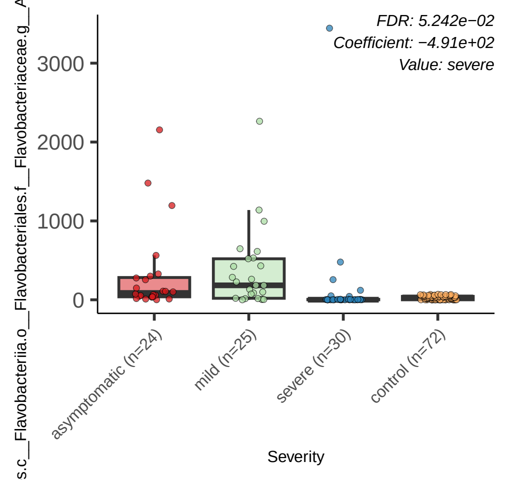

FDR: 5.242e−02
Coefficient: −4.91e+02
Value: severe
3000
2000
1000
0
s.c__Flavobacteriia.o__Flavobacteriales.f__Flavobacteriaceae.g__A
asymptomatic (n=24)
mild (n=25)
severe (n=30)
control (n=72)
Severity

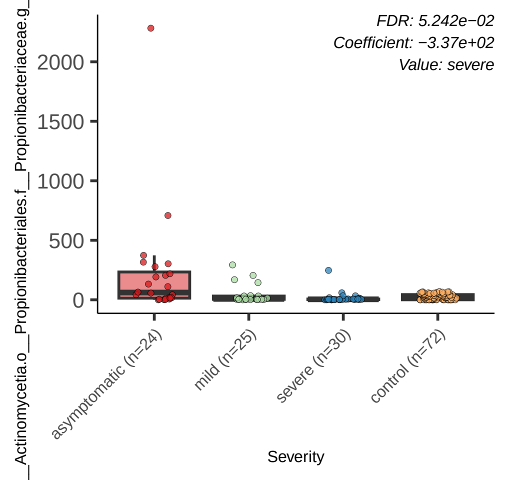

FDR: 5.242e−02
Coefficient: −3.37e+02
Value: severe
_Actinomycetia.o__Propionibacteriales.f__Propionibacteriaceae.g_
2000
1500
1000
500
0
asymptomatic (n=24)
mild (n=25)
severe (n=30)
control (n=72)
Severity

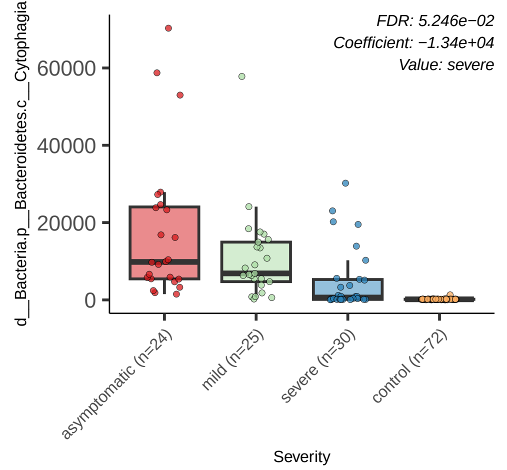
FDR: 5.246e−02
Coefficient: −1.34e+04
Value: severe
d__Bacteria.p__Bacteroidetes.c__Cytophagia
60000
40000
20000
0
asymptomatic (n=24)
mild (n=25)
severe (n=30)
control (n=72)
Severity

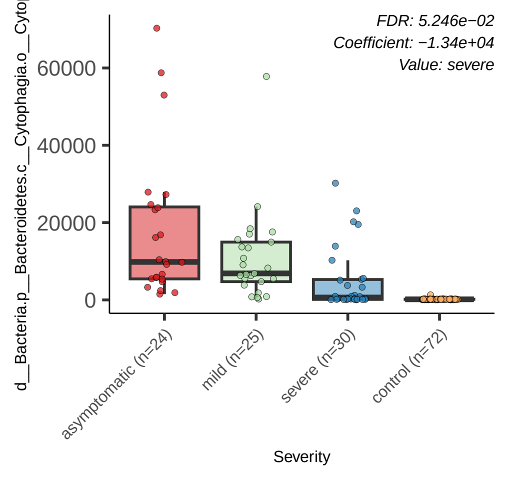

FDR: 5.246e−02
Coefficient: −1.34e+04
Value: severe
d__Bacteria.p__Bacteroidetes.c__Cytophagia.o__Cytop
0
20000
40000
60000
asymptomatic (n=24)
mild (n=25)
severe (n=30)
control (n=72)
Severity

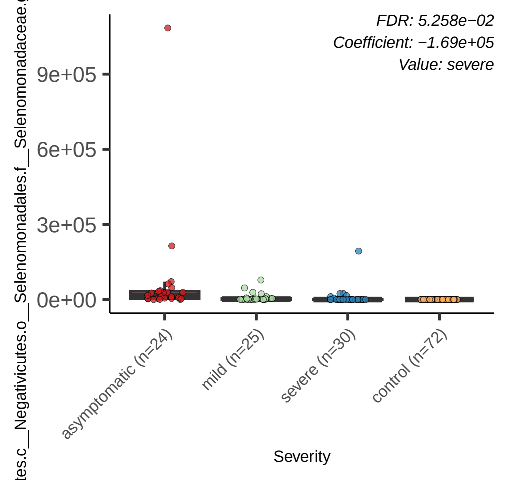
FDR: 5.258e−02
Coefficient: −1.69e+05
Value: severe
9e+05
6e+05
3e+05
0e+00
asymptomatic (n=24)
mild (n=25)
severe (n=30)
control (n=72)
Severity
tes.c__Negativicutes.o__Selenomonadales.f__Selenomonadaceae.g

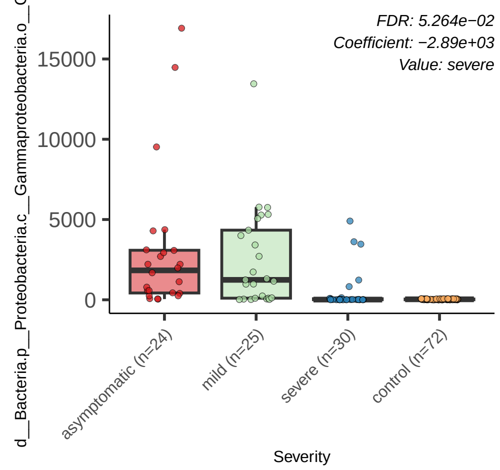

FDR: 5.264e−02
Coefficient: −2.89e+03
Value: severe
15000
10000
5000
0
asymptomatic (n=24)
mild (n=25)
severe (n=30)
control (n=72)
Severity
d__Bacteria.p__Proteobacteria.c__Gammaproteobacteria.o__C

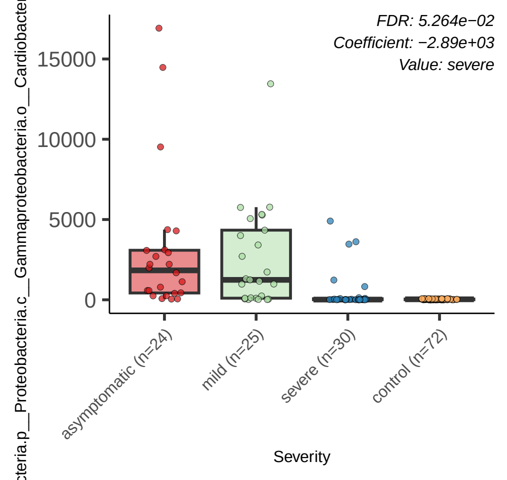

FDR: 5.264e−02
Coefficient: −2.89e+03
Value: severe
15000
10000
5000
0
cteria.p__Proteobacteria.c__Gammaproteobacteria.o__Cardiobacte
asymptomatic (n=24)
mild (n=25)
severe (n=30)
control (n=72)
Severity

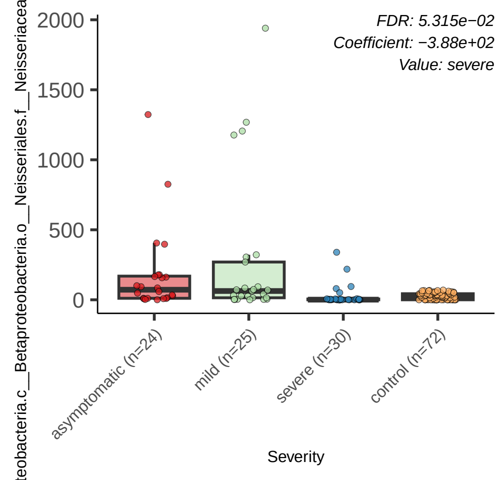
FDR: 5.315e−02
Coefficient: −3.88e+02
Value: severe
2000
1500
1000
500
0
teobacteria.c__Betaproteobacteria.o__Neisseriales.f__Neisseriacea
asymptomatic (n=24)
mild (n=25)
severe (n=30)
control (n=72)
Severity

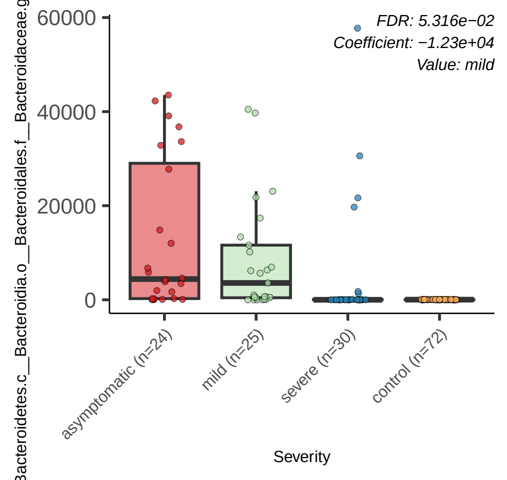

FDR: 5.316e−02
Coefficient: −1.23e+04
Value: mild
60000
40000
20000
0
Bacteroidetes.c__Bacteroidia.o__Bacteroidales.f__Bacteroidaceae.g
asymptomatic (n=24)
mild (n=25)
severe (n=30)
control (n=72)
Severity

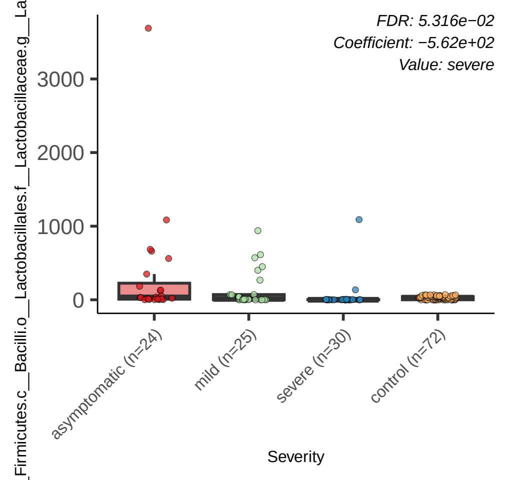

FDR: 5.316e−02
Coefficient: −5.62e+02
Value: severe
Firmicutes.c__Bacilli.o__Lactobacillales.f__Lactobacillaceae.g__La
3000
2000
1000
0
asymptomatic (n=24)
mild (n=25)
severe (n=30)
control (n=72)
Severity

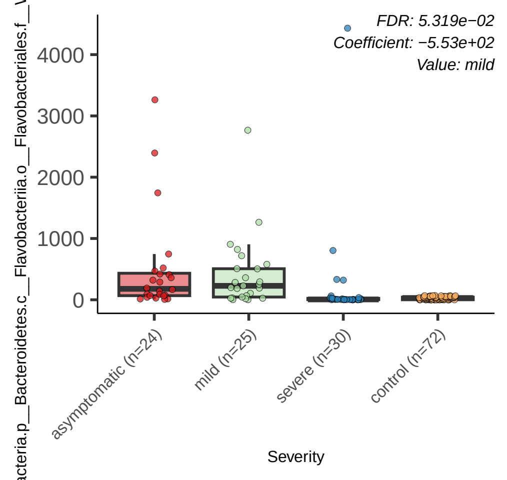

FDR: 5.319e−02
Coefficient: −5.53e+02
Value: mild
0
1000
2000
3000
4000
acteria.p__Bacteroidetes.c__Flavobacteriia.o__Flavobacteriales.f__W
asymptomatic (n=24)
mild (n=25)
severe (n=30)
control (n=72)
Severity

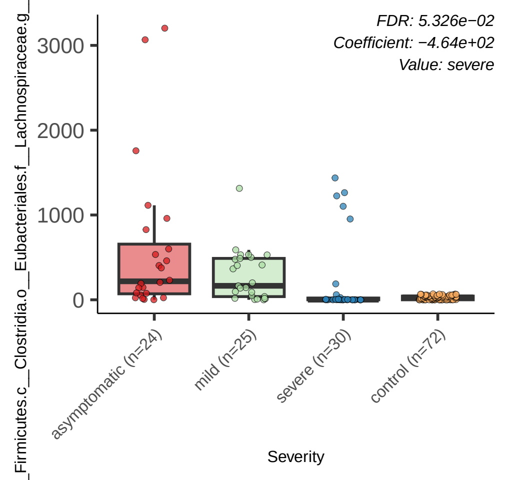
FDR: 5.326e−02
Coefficient: −4.64e+02
Value: severe
Firmicutes.c__Clostridia.o__Eubacteriales.f__Lachnospiraceae.g__
3000
2000
1000
0
asymptomatic (n=24)
mild (n=25)
severe (n=30)
control (n=72)
Severity

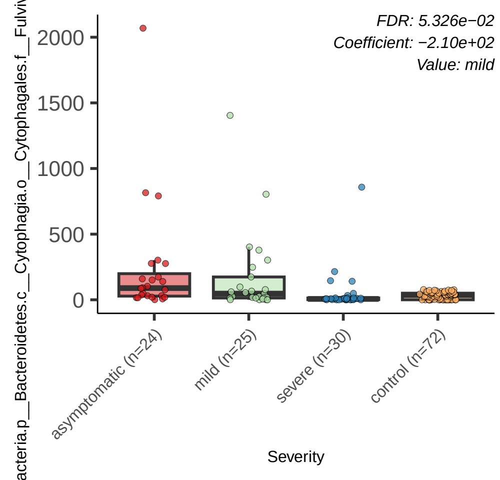

FDR: 5.326e−02
Coefficient: −2.10e+02
Value: mild
2000
1500
1000
500
0
acteria.p__Bacteroidetes.c__Cytophagia.o__Cytophagales.f__Fulviv
asymptomatic (n=24)
mild (n=25)
severe (n=30)
control (n=72)
Severity

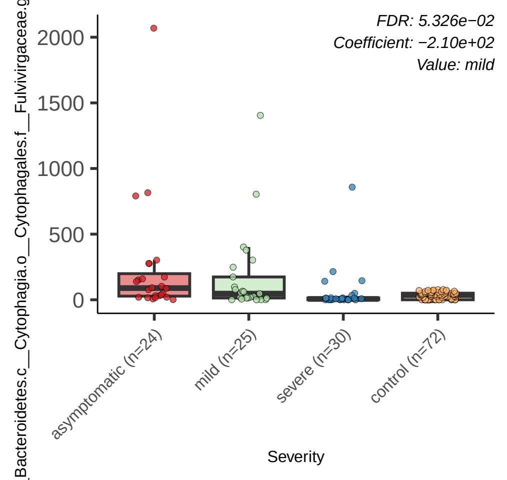
FDR: 5.326e−02
Coefficient: −2.10e+02
Value: mild
Bacteroidetes.c__Cytophagia.o__Cytophagales.f__Fulvivirgaceae.g
2000
1500
1000
500
0
asymptomatic (n=24)
mild (n=25)
severe (n=30)
control (n=72)
Severity

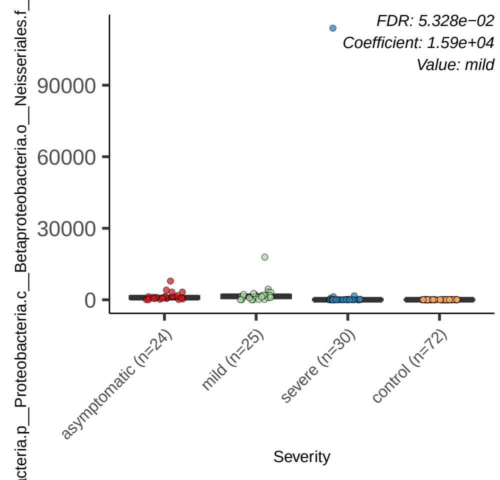

FDR: 5.328e−02
Coefficient: 1.59e+04
Value: mild
90000
60000
30000
0
cteria.p__Proteobacteria.c__Betaproteobacteria.o__Neisseriales.f__
asymptomatic (n=24)
mild (n=25)
severe (n=30)
control (n=72)
Severity

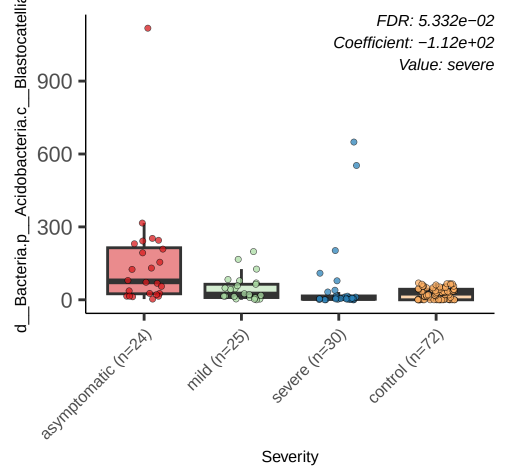

FDR: 5.332e−02
Coefficient: −1.12e+02
Value: severe
d__Bacteria.p__Acidobacteria.c__Blastocatellia
900
600
300
0
asymptomatic (n=24)
mild (n=25)
severe (n=30)
control (n=72)
Severity

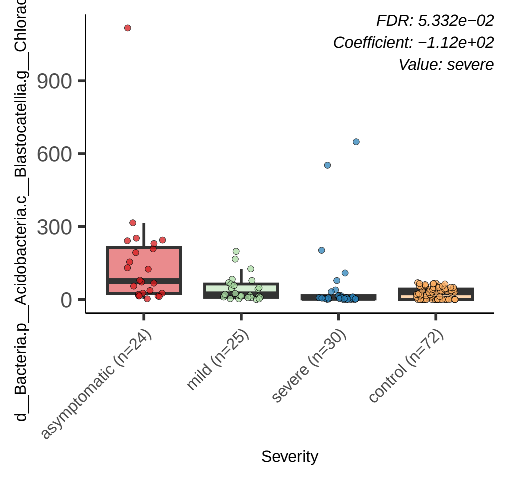

FDR: 5.332e−02
Coefficient: −1.12e+02
Value: severe
0
300
600
900
d__Bacteria.p__Acidobacteria.c__Blastocatellia.g__Chloroac
asymptomatic (n=24)
mild (n=25)
severe (n=30)
control (n=72)
Severity

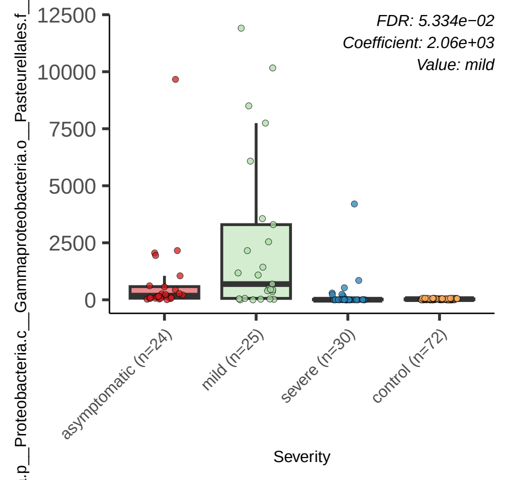

FDR: 5.334e−02
Coefficient: 2.06e+03
Value: mild
12500
10000
7500
5000
2500
0
asymptomatic (n=24)
mild (n=25)
severe (n=30)
control (n=72)
Severity

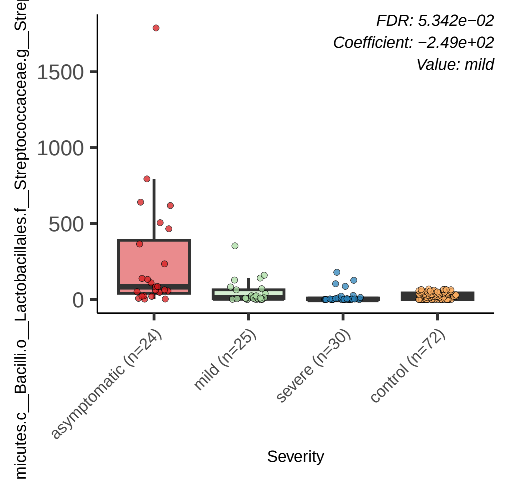
FDR: 5.342e−02
Coefficient: −2.49e+02
Value: mild
micutes.c__Bacilli.o__Lactobacillales.f__Streptococcaceae.g__Strep
1500
1000
500
0
asymptomatic (n=24)
mild (n=25)
severe (n=30)
control (n=72)
Severity

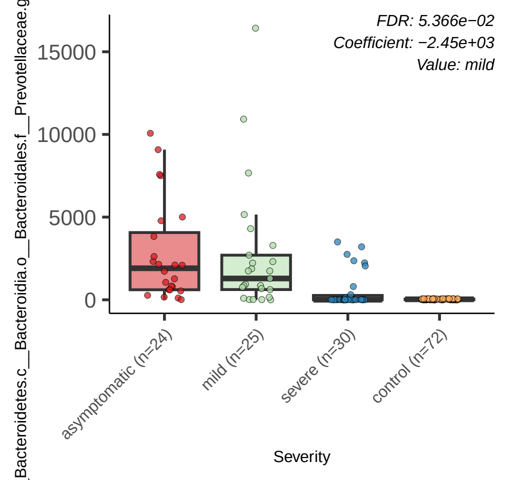

FDR: 5.366e−02
Coefficient: −2.45e+03
Value: mild
Bacteroidetes.c__Bacteroidia.o__Bacteroidales.f__Prevotellaceae.g
15000
10000
5000
0
asymptomatic (n=24)
mild (n=25)
severe (n=30)
control (n=72)
Severity

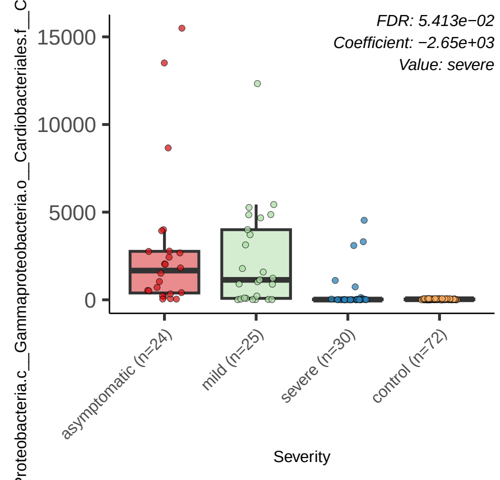
FDR: 5.413e−02
Coefficient: −2.65e+03
Value: severe
15000
10000
5000
0
Proteobacteria.c__Gammaproteobacteria.o__Cardiobacteriales.f__C
asymptomatic (n=24)
mild (n=25)
severe (n=30)
control (n=72)
Severity

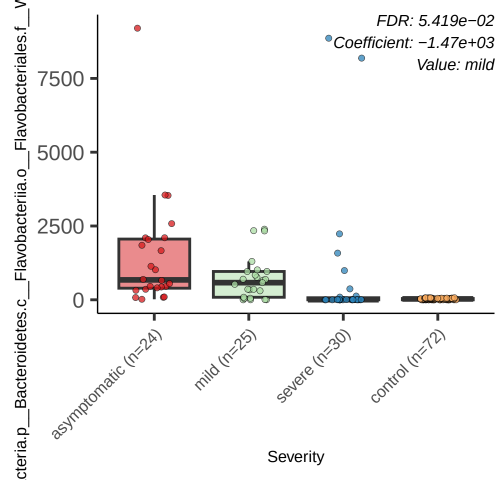

FDR: 5.419e−02
Coefficient: −1.47e+03
Value: mild
7500
5000
2500
0
asymptomatic (n=24)
mild (n=25)
severe (n=30)
control (n=72)
Severity
cteria.p__Bacteroidetes.c__Flavobacteriia.o__Flavobacteriales.f__W

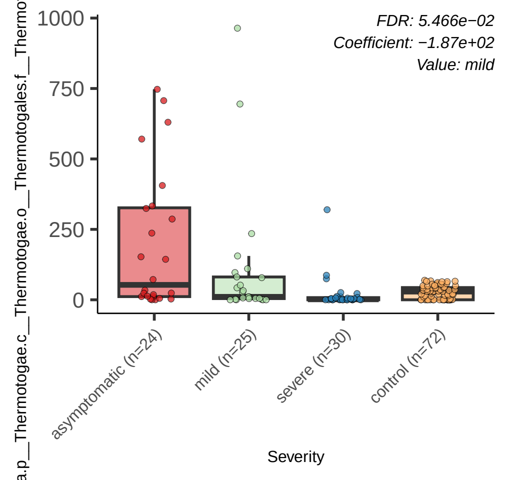

FDR: 5.466e−02
Coefficient: −1.87e+02
Value: mild
1000
750
500
250
0
a.p__Thermotogae.c__Thermotogae.o__Thermotogales.f__Thermo
asymptomatic (n=24)
mild (n=25)
severe (n=30)
control (n=72)
Severity

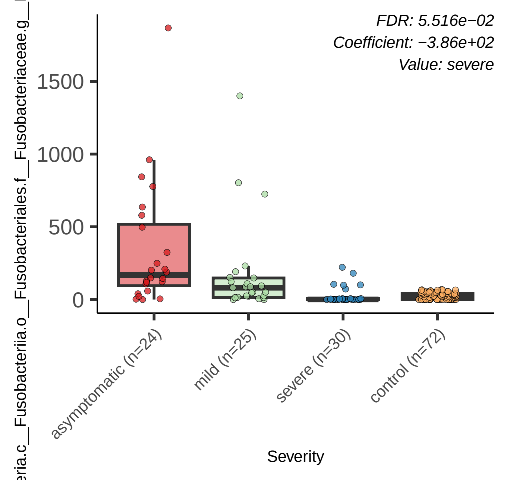

FDR: 5.516e−02
Coefficient: −3.86e+02
Value: severe
Fusobacteriia.c__Fusobacteriia.o__Fusobacteriales.f__Fusobacteriaceae.g__
1500
1000
500
0
asymptomatic (n=24)
mild (n=25)
severe (n=30)
control (n=72)
Severity

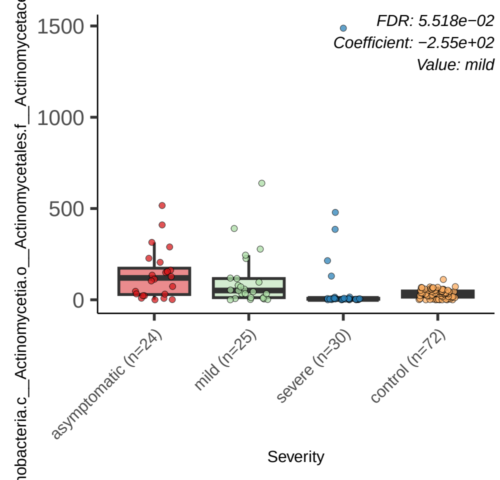

FDR: 5.518e−02
Coefficient: −2.55e+02
Value: mild
1500
1000
500
0
nobacteria.c__Actinomycetia.o__Actinomycetales.f__Actinomycetac
asymptomatic (n=24)
mild (n=25)
severe (n=30)
control (n=72)
Severity

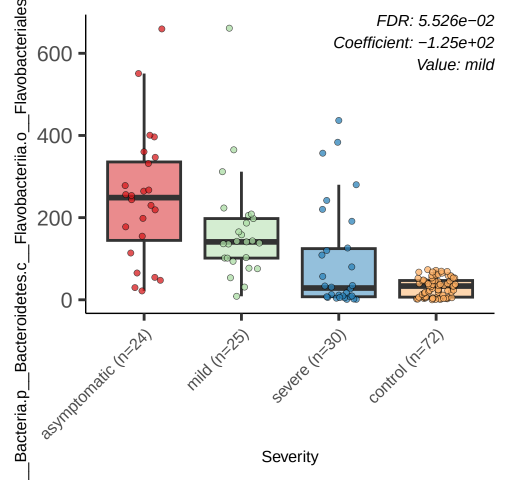
FDR: 5.526e−02
Coefficient: −1.25e+02
Value: mild
k__Bacteria.p__Bacteroidetes.c__Flavobacteriia.o__Flavobacteriales
0
200
400
600
asymptomatic (n=24)
mild (n=25)
severe (n=30)
control (n=72)
Severity

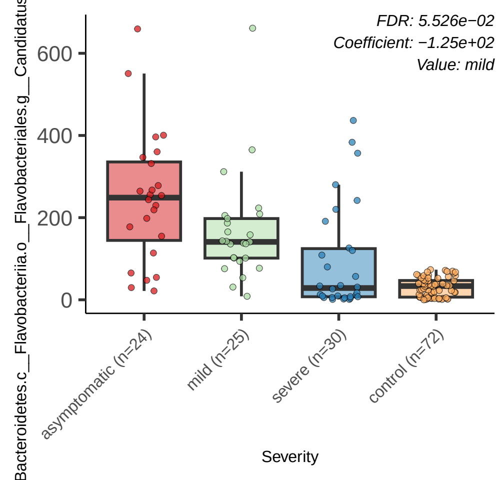

FDR: 5.526e−02
Coefficient: −1.25e+02
Value: mild
Bacteroidetes.c__Flavobacteriia.o__Flavobacteriales.g__Candidatus
600
400
200
0
asymptomatic (n=24)
mild (n=25)
severe (n=30)
control (n=72)
Severity

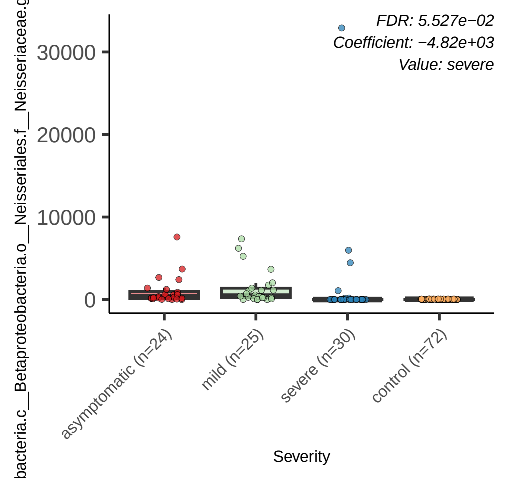
FDR: 5.527e−02
Coefficient: −4.82e+03
Value: severe
30000
20000
10000
0
bacteria.c__Betaproteobacteria.o__Neisseriales.f__Neisseriaceae.g
asymptomatic (n=24)
mild (n=25)
severe (n=30)
control (n=72)
Severity

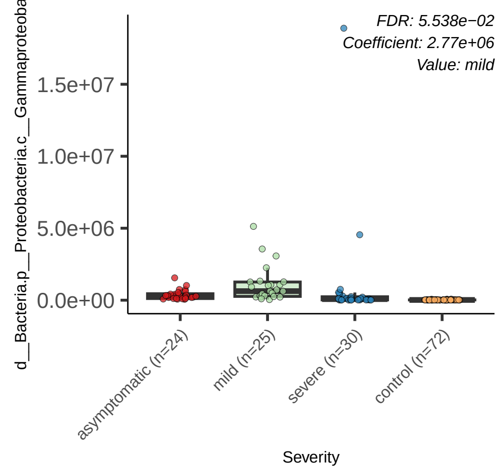

FDR: 5.538e−02
Coefficient: 2.77e+06
Value: mild
d__Bacteria.p__Proteobacteria.c__Gammaproteoba
1.5e+07
1.0e+07
5.0e+06
0.0e+00
asymptomatic (n=24)
mild (n=25)
severe (n=30)
control (n=72)
Severity

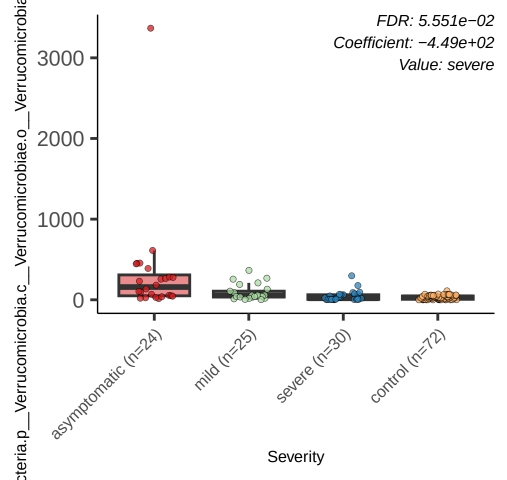

FDR: 5.551e−02
Coefficient: −4.49e+02
Value: severe
cteria.p__Verrucomicrobia.c__Verrucomicrobiae.o__Verrucomicrobia
3000
2000
1000
0
asymptomatic (n=24)
mild (n=25)
severe (n=30)
control (n=72)
Severity

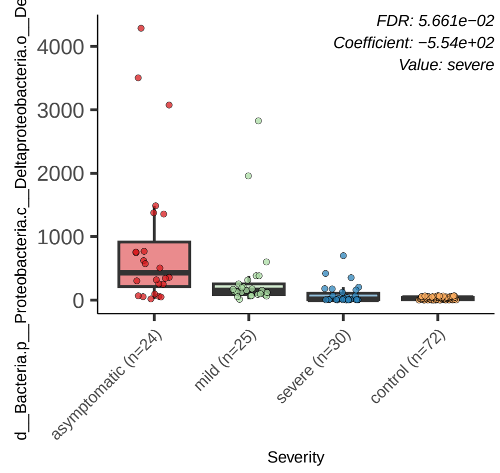

FDR: 5.661e−02
Coefficient: −5.54e+02
Value: severe
d__Bacteria.p__Proteobacteria.c__Deltaproteobacteria.o__De
4000
3000
2000
1000
0
asymptomatic (n=24)
mild (n=25)
severe (n=30)
control (n=72)
Severity

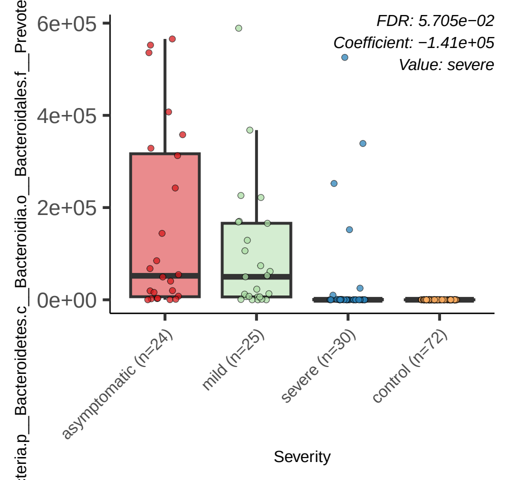

FDR: 5.705e−02
Coefficient: −1.41e+05
Value: severe
6e+05
4e+05
2e+05
0e+00
asymptomatic (n=24)
mild (n=25)
severe (n=30)
control (n=72)
Severity
teria.p__Bacteroidetes.c__Bacteroidia.o__Bacteroidales.f__Prevote

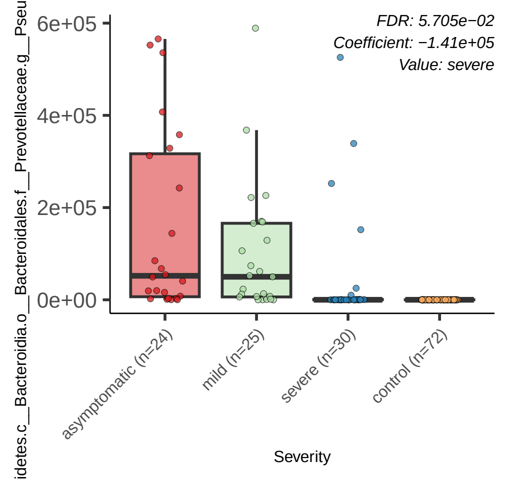

FDR: 5.705e−02
Coefficient: −1.41e+05
Value: severe
6e+05
4e+05
2e+05
0e+00
idetes.c__Bacteroidia.o__Bacteroidales.f__Prevotellaceae.g__Pseu
asymptomatic (n=24)
mild (n=25)
severe (n=30)
control (n=72)
Severity

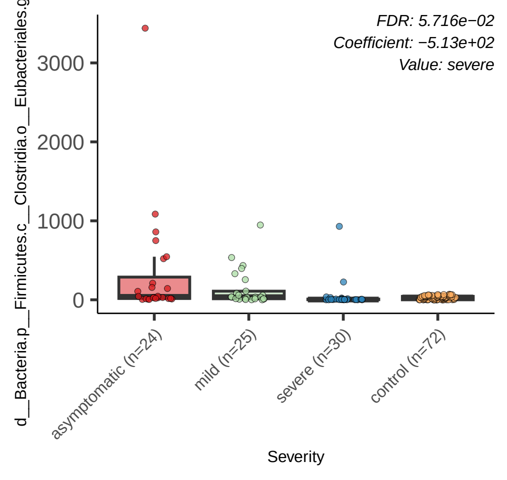

FDR: 5.716e−02
Coefficient: −5.13e+02
Value: severe
d__Bacteria.p__Firmicutes.c__Clostridia.o__Eubacteriales.g
0
1000
2000
3000
asymptomatic (n=24)
mild (n=25)
severe (n=30)
control (n=72)
Severity

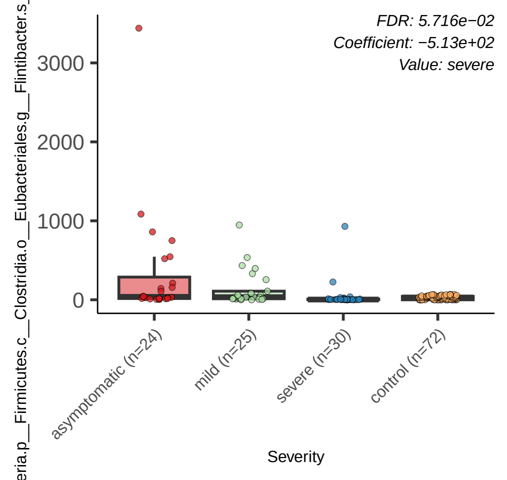

FDR: 5.716e−02
Coefficient: −5.13e+02
Value: severe
3000
2000
1000
0
asymptomatic (n=24)
mild (n=25)
severe (n=30)
control (n=72)
Severity
eria.p__Firmicutes.c__Clostridia.o__Eubacteriales.g__Flintibacter.s_

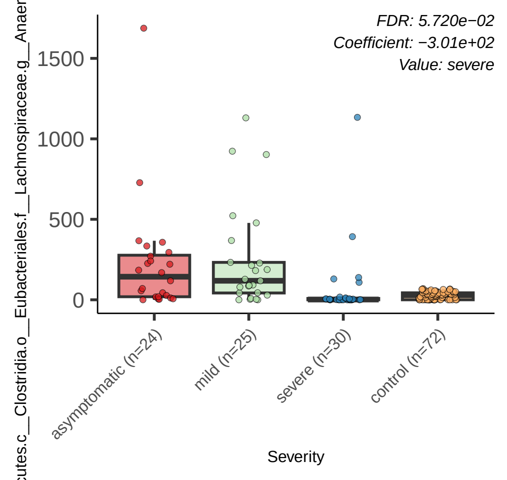
FDR: 5.720e−02
Coefficient: −3.01e+02
Value: severe
cutes.c__Clostridia.o__Eubacteriales.f__Lachnospiraceae.g__Anaer
1500
1000
500
0
asymptomatic (n=24)
mild (n=25)
severe (n=30)
control (n=72)
Severity

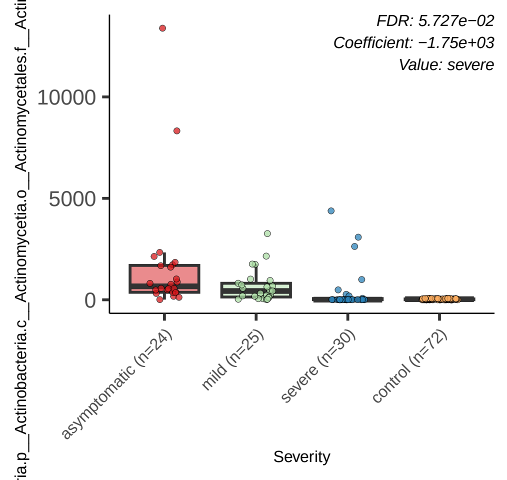
FDR: 5.727e−02
Coefficient: −1.75e+03
Value: severe
10000
5000
0
asymptomatic (n=24)
mild (n=25)
severe (n=30)
control (n=72)
Severity
ria.p__Actinobacteria.c__Actinomycetia.o__Actinomycetales.f__Acti

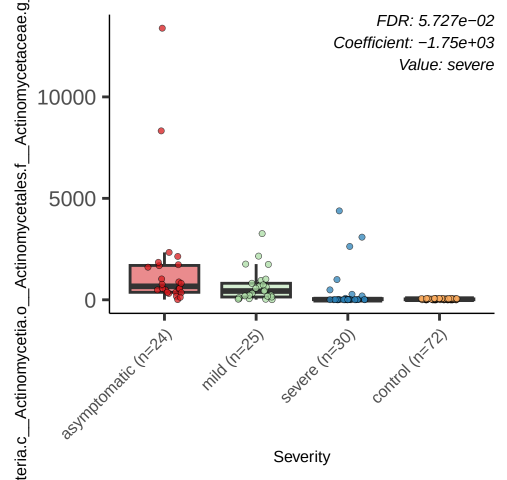

FDR: 5.727e−02
Coefficient: −1.75e+03
Value: severe
10000
5000
0
teria.c__Actinomycetia.o__Actinomycetales.f__Actinomycetaceae.g
asymptomatic (n=24)
mild (n=25)
severe (n=30)
control (n=72)
Severity

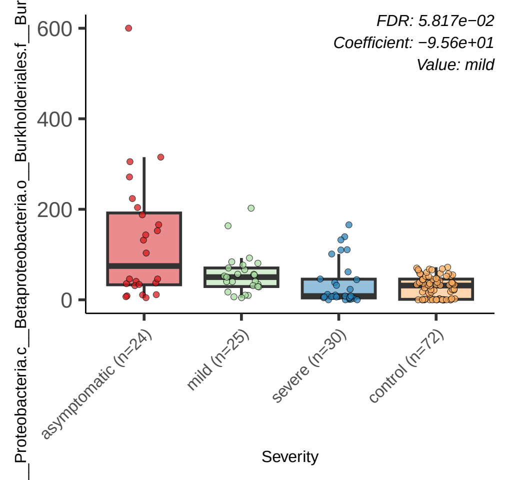

FDR: 5.817e−02
Coefficient: −9.56e+01
Value: mild
_Proteobacteria.c__Betaproteobacteria.o__Burkholderiales.f__Bur
600
400
200
0
asymptomatic (n=24)
mild (n=25)
severe (n=30)
control (n=72)
Severity

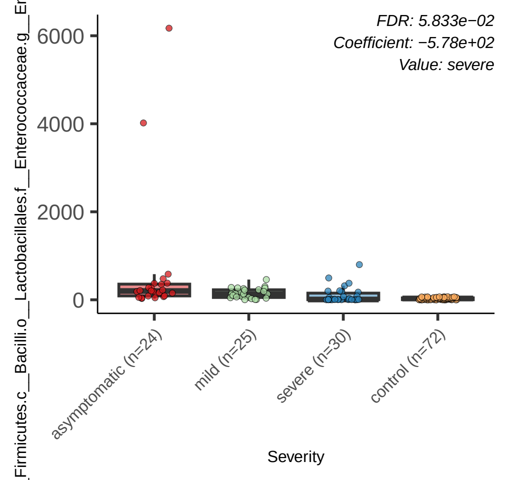

FDR: 5.833e−02
Coefficient: −5.78e+02
Value: severe
Firmicutes.c__Bacilli.o__Lactobacillales.f__Enterococcaceae.g__En
6000
4000
2000
0
asymptomatic (n=24)
mild (n=25)
severe (n=30)
control (n=72)
Severity

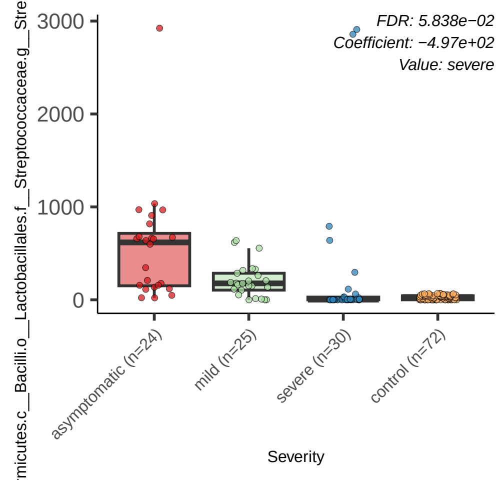
FDR: 5.838e−02
Coefficient: −4.97e+02
Value: severe
3000
2000
1000
0
micutes.c__Bacilli.o__Lactobacillales.f__Streptococcaceae.g__Stre
asymptomatic (n=24)
mild (n=25)
severe (n=30)
control (n=72)
Severity

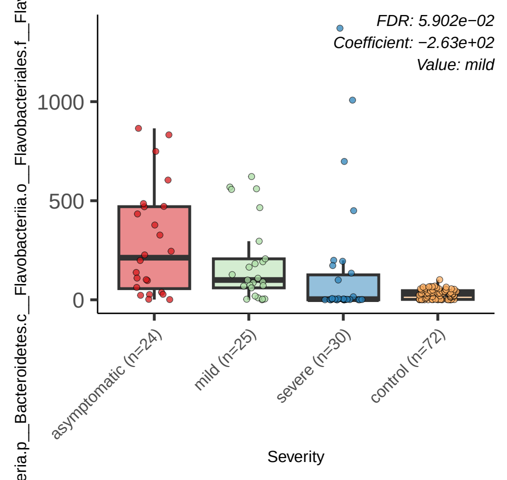
FDR: 5.902e−02
Coefficient: −2.63e+02
Value: mild
1000
500
0
asymptomatic (n=24)
mild (n=25)
severe (n=30)
control (n=72)
Severity
Bacteroidetes.c__Flavobacteriia.o__Flavobacteriales.f__Flav

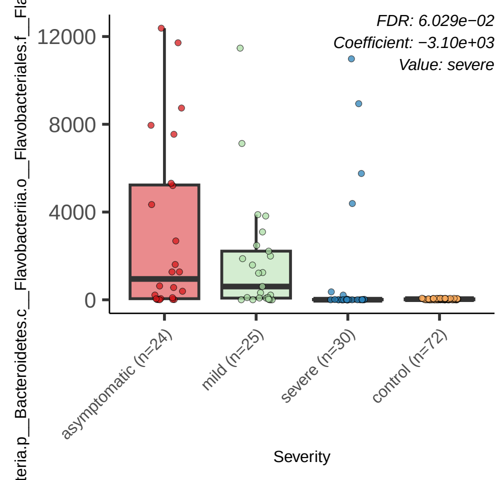

FDR: 6.029e−02
Coefficient: −3.10e+03
Value: severe
12000
8000
4000
0
asymptomatic (n=24)
mild (n=25)
severe (n=30)
control (n=72)
Severity
teria.p__Bacteroidetes.c__Flavobacteriia.o__Flavobacteriales.f__Fla

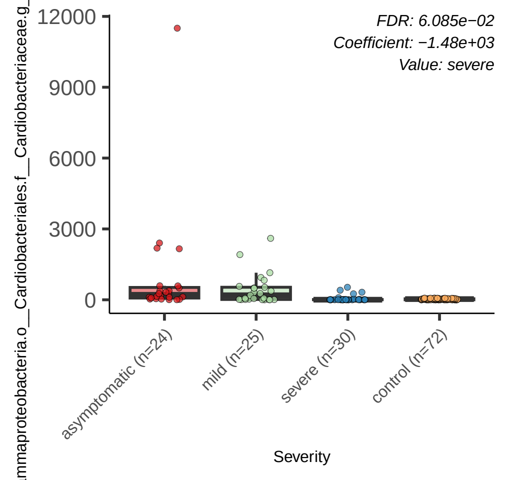
FDR: 6.085e−02
Coefficient: −1.48e+03
Value: severe
12000
9000
6000
3000
0
mmaproteobacteria.o__Cardiobacteriales.f__Cardiobacteriaceae.g_
asymptomatic (n=24)
mild (n=25)
severe (n=30)
control (n=72)
Severity

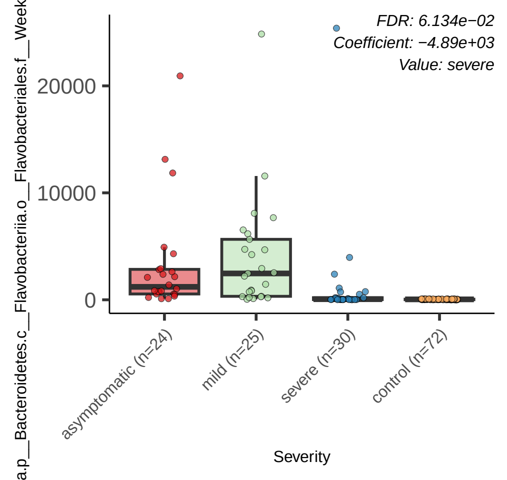

FDR: 6.134e−02
Coefficient: −4.89e+03
Value: severe
20000
10000
0
a.p__Bacteroidetes.c__Flavobacteriia.o__Flavobacteriales.f__Week
asymptomatic (n=24)
mild (n=25)
severe (n=30)
control (n=72)
Severity

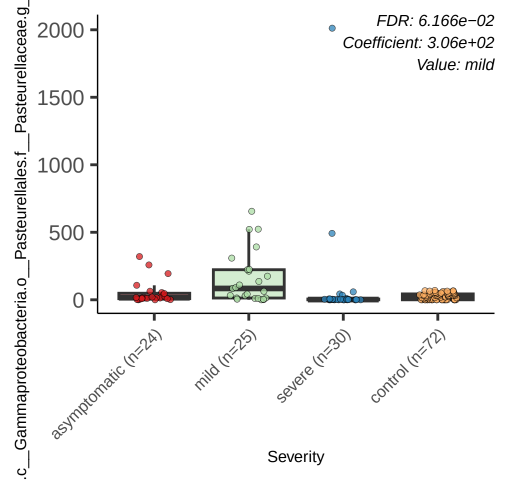

FDR: 6.166e−02
Coefficient: 3.06e+02
Value: mild
c__Gammaproteobacteria.o__Pasteurellales.f__Pasteurellaceae.g__
2000
1500
1000
500
0
asymptomatic (n=24)
mild (n=25)
severe (n=30)
control (n=72)
Severity

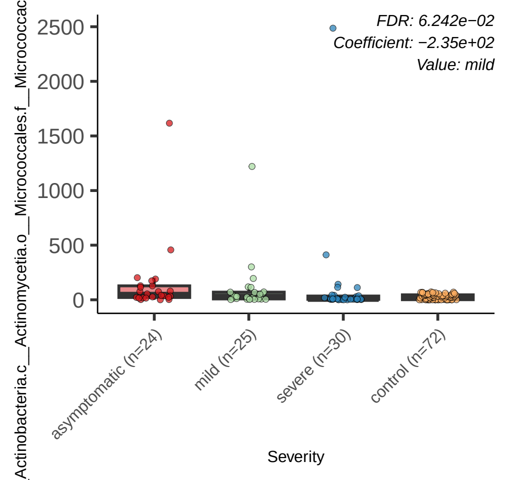

Actinobacteria.c__Actinomycetia.o__Micrococcales.f__Micrococcac
FDR: 6.242e−02
Coefficient: −2.35e+02
Value: mild
2500
2000
1500
1000
500
0
asymptomatic (n=24)
mild (n=25)
severe (n=30)
control (n=72)
Severity

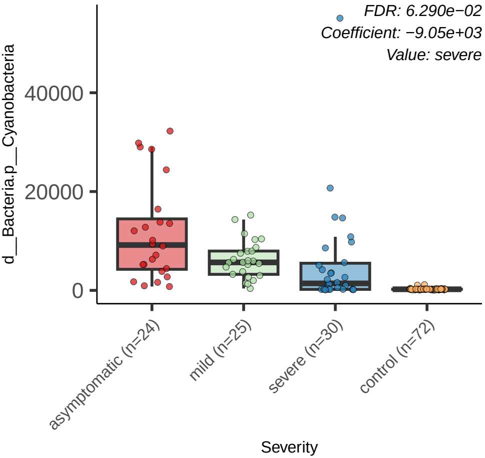

FDR: 6.290e−02
Coefficient: −9.05e+03
Value: severe
d__Bacteria.p__Cyanobacteria
40000
20000
0
asymptomatic (n=24)
mild (n=25)
severe (n=30)
control (n=72)
Severity

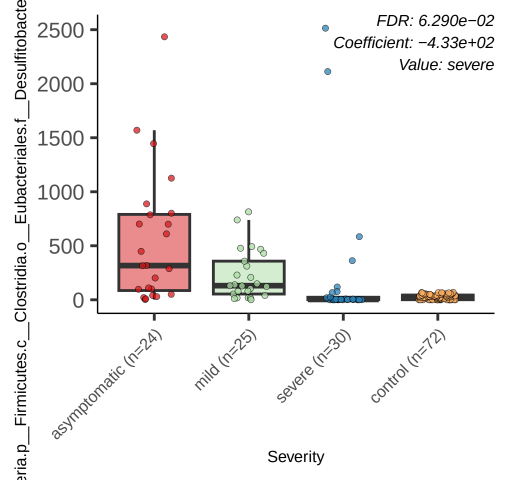

FDR: 6.290e−02
Coefficient: −4.33e+02
Value: severe
eria.p__Firmicutes.c__Clostridia.o__Eubacteriales.f__Desulfitobacte
2500
2000
1500
1000
500
0
asymptomatic (n=24)
mild (n=25)
severe (n=30)
control (n=72)
Severity

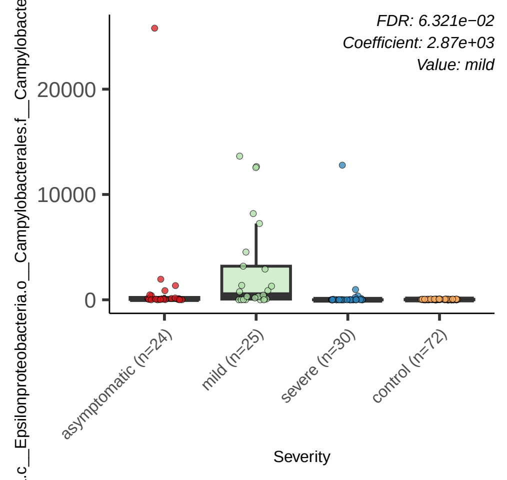

FDR: 6.321e−02
Coefficient: 2.87e+03
Value: mild
20000
10000
0
.c__Epsilonproteobacteria.o__Campylobacterales.f__Campylobacte
asymptomatic (n=24)
mild (n=25)
severe (n=30)
control (n=72)
Severity

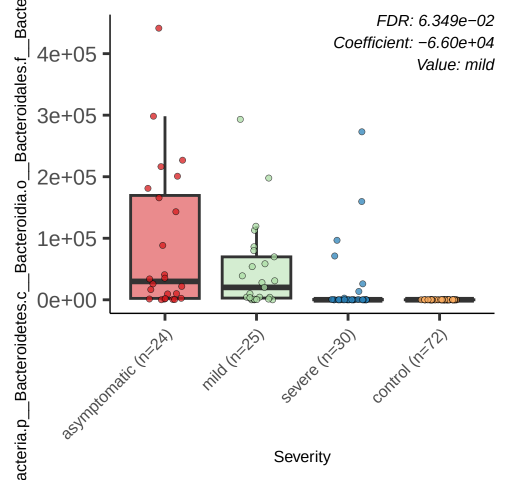

FDR: 6.349e−02
Coefficient: −6.60e+04
Value: mild
acteria.p__Bacteroidetes.c__Bacteroidia.o__Bacteroidales.f__Bacte
4e+05
3e+05
2e+05
1e+05
0e+00
asymptomatic (n=24)
mild (n=25)
severe (n=30)
control (n=72)
Severity

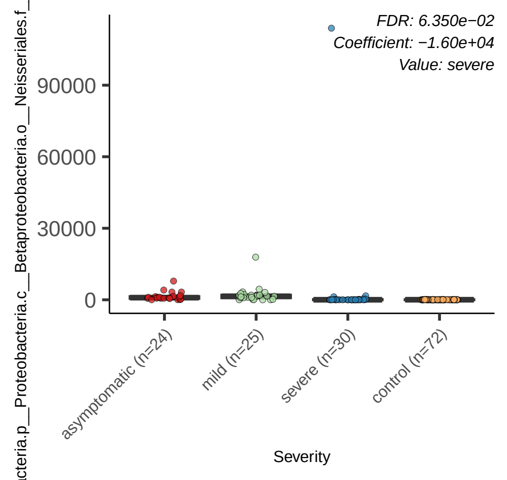

FDR: 6.350e−02
Coefficient: −1.60e+04
Value: severe
90000
60000
30000
0
acteria.p__Proteobacteria.c__Betaproteobacteria.o__Neisseriales.f__
asymptomatic (n=24)
mild (n=25)
severe (n=30)
control (n=72)
Severity

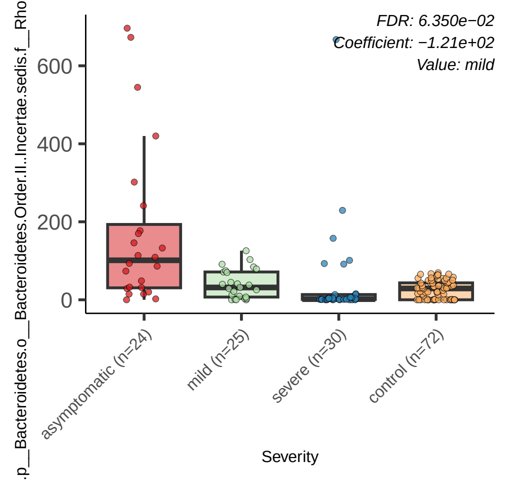

FDR: 6.350e−02
Coefficient: −1.21e+02
Value: mild
p__Bacteroidetes.o__Bacteroidetes.Order.II..Incertae.sedis.f__Rho
600
400
200
0
asymptomatic (n=24)
mild (n=25)
severe (n=30)
control (n=72)
Severity

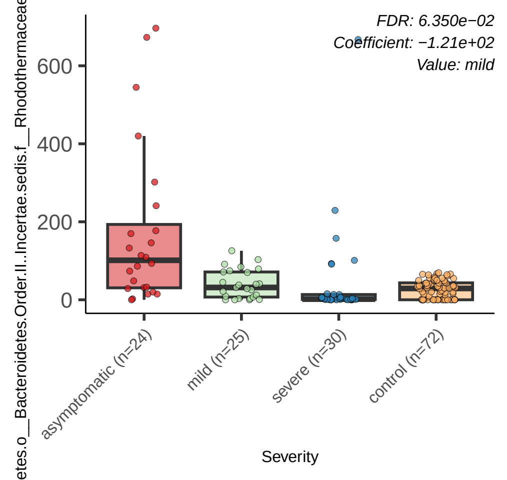

FDR: 6.350e−02
Coefficient: −1.21e+02
Value: mild
600
400
200
0
etes.o__Bacteroidetes.Order.II..Incertae.sedis.f__Rhodothermaceae
asymptomatic (n=24)
mild (n=25)
severe (n=30)
control (n=72)
Severity

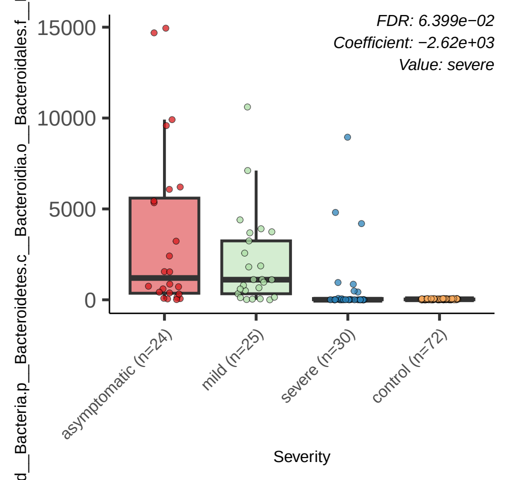

FDR: 6.399e−02
Coefficient: −2.62e+03
Value: severe
d__Bacteria.p__Bacteroidetes.c__Bacteroidia.o__Bacteroidales.f__
15000
10000
5000
0
asymptomatic (n=24)
mild (n=25)
severe (n=30)
control (n=72)
Severity

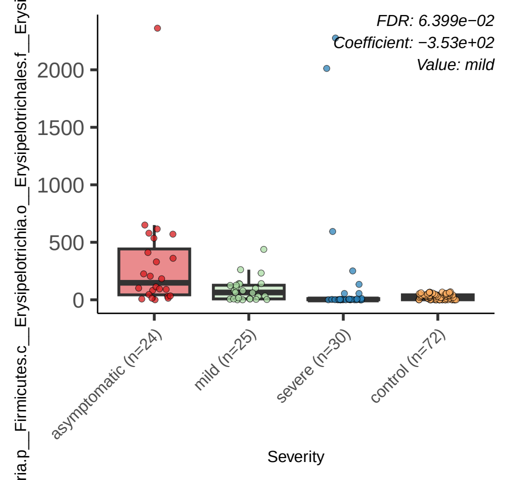
FDR: 6.399e−02
Coefficient: −3.53e+02
Value: mild
ria.p__Firmicutes.c__Erysipelotrichia.o__Erysipelotrichales.f__Erysi
0
500
1000
1500
2000
asymptomatic (n=24)
mild (n=25)
severe (n=30)
control (n=72)
Severity

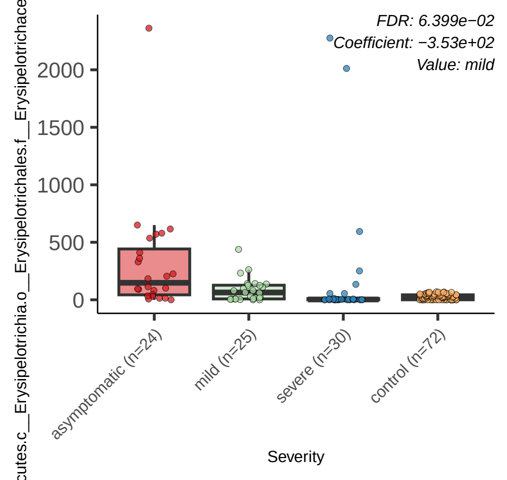

FDR: 6.399e−02
Coefficient: −3.53e+02
Value: mild
2000
1500
1000
500
0
cutes.c__Erysipelotrichia.o__Erysipelotrichales.f__Erysipelotrichace
asymptomatic (n=24)
mild (n=25)
severe (n=30)
control (n=72)
Severity

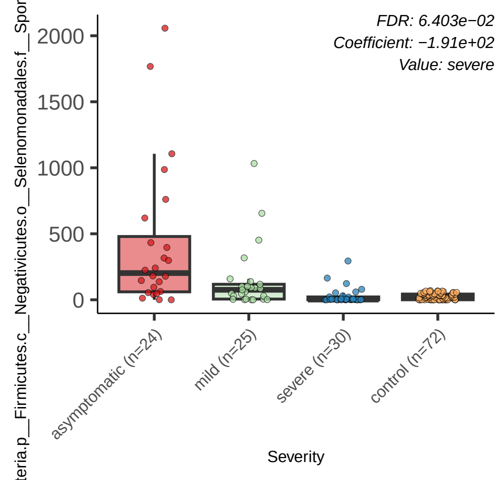

FDR: 6.403e−02
Coefficient: −1.91e+02
Value: severe
teria.p__Firmicutes.c__Negativicutes.o__Selenomonadales.f__Spor
0
500
1000
1500
2000
asymptomatic (n=24)
mild (n=25)
severe (n=30)
control (n=72)
Severity

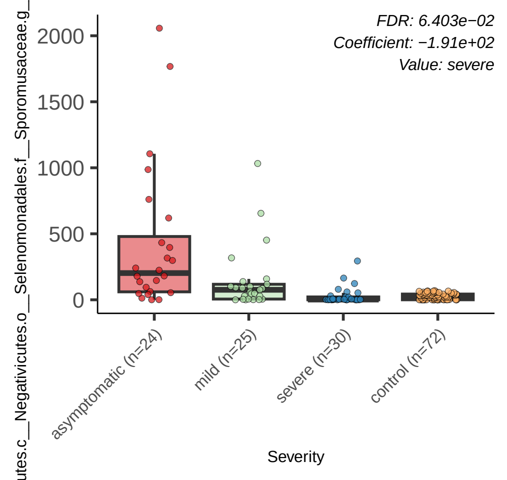

FDR: 6.403e−02
Coefficient: −1.91e+02
Value: severe
2000
1500
1000
500
0
utes.c__Negativicutes.o__Selenomonadales.f__Sporomusaceae.g__
asymptomatic (n=24)
mild (n=25)
severe (n=30)
control (n=72)
Severity

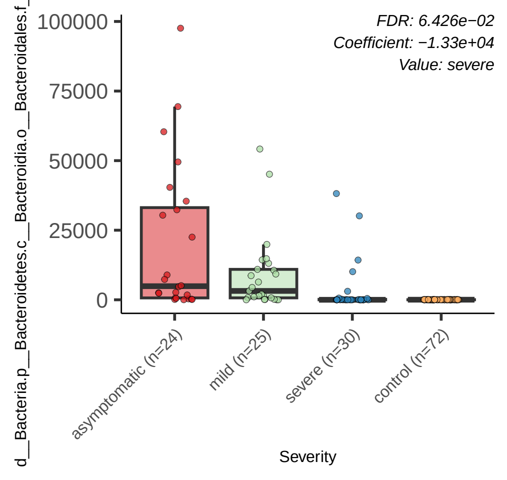

FDR: 6.426e−02
Coefficient: −1.33e+04
Value: severe
d__Bacteria.p__Bacteroidetes.c__Bacteroidia.o__Bacteroidales.f_
100000
75000
50000
25000
0
asymptomatic (n=24)
mild (n=25)
severe (n=30)
control (n=72)
Severity

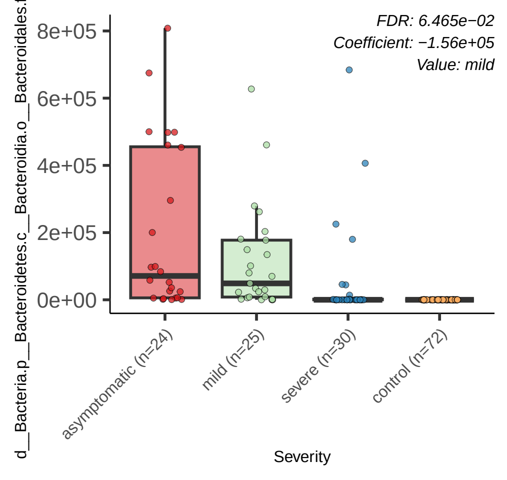

FDR: 6.465e−02
Coefficient: −1.56e+05
Value: mild
d__Bacteria.p__Bacteroidetes.c__Bacteroidia.o__Bacteroidales.f
8e+05
6e+05
4e+05
2e+05
0e+00
asymptomatic (n=24)
mild (n=25)
severe (n=30)
control (n=72)
Severity

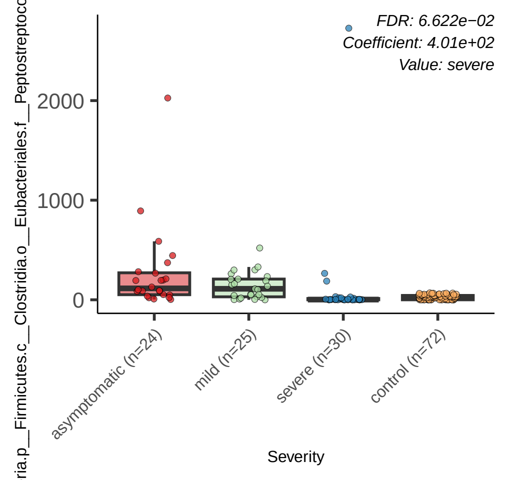

FDR: 6.622e−02
Coefficient: 4.01e+02
Value: severe
ria.p__Firmicutes.c__Clostridia.o__Eubacteriales.f__Peptostreptoco
2000
1000
0
asymptomatic (n=24)
mild (n=25)
severe (n=30)
control (n=72)
Severity

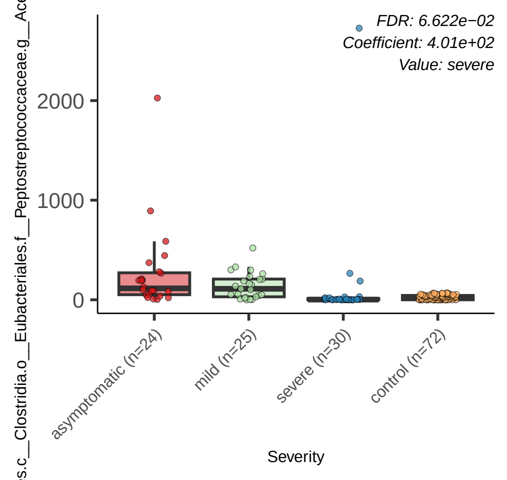

FDR: 6.622e−02
Coefficient: 4.01e+02
Value: severe
2000
1000
0
asymptomatic (n=24)
mild (n=25)
severe (n=30)
control (n=72)
Severity
s.c__Clostridia.o__Eubacteriales.f__Peptostreptococcaceae.g__Ace

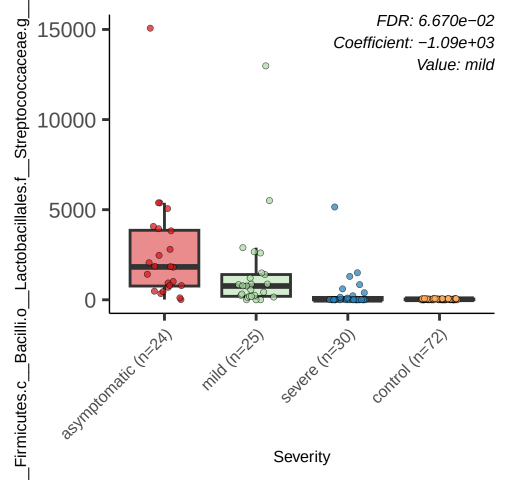
FDR: 6.670e−02
Coefficient: −1.09e+03
Value: mild
Firmicutes.c__Bacilli.o__Lactobacillales.f__Streptococcaceae.g__
15000
10000
5000
0
asymptomatic (n=24)
mild (n=25)
severe (n=30)
control (n=72)
Severity

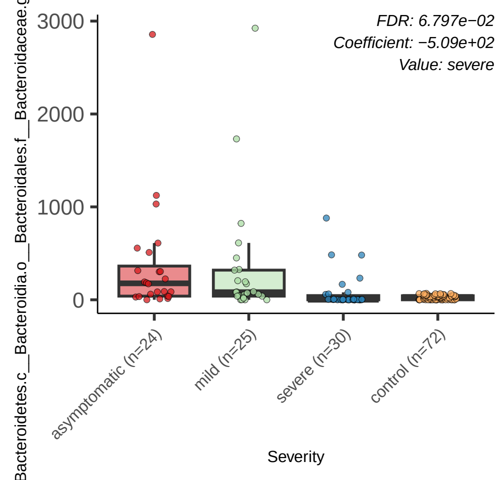

FDR: 6.797e−02
Coefficient: −5.09e+02
Value: severe
Bacteroidetes.c__Bacteroidia.o__Bacteroidales.f__Bacteroidaceae.g
3000
2000
1000
0
asymptomatic (n=24)
mild (n=25)
severe (n=30)
control (n=72)
Severity

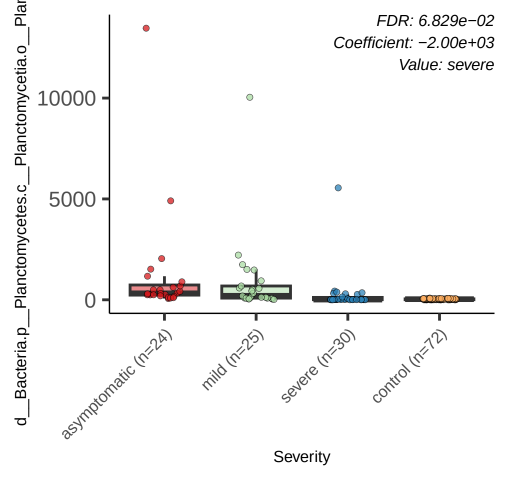

FDR: 6.829e−02
Coefficient: −2.00e+03
Value: severe
d__Bacteria.p__Planctomycetes.c__Planctomycetia.o__Plan
10000
5000
0
asymptomatic (n=24)
mild (n=25)
severe (n=30)
control (n=72)
Severity

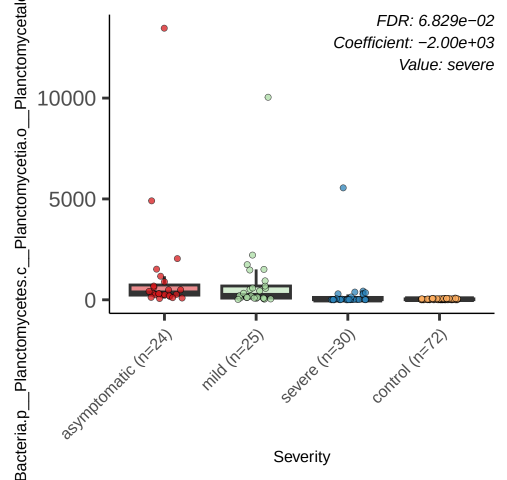
FDR: 6.829e−02
Coefficient: −2.00e+03
Value: severe
10000
5000
0
Bacteria.p__Planctomycetes.c__Planctomycetia.o__Planctomycetal
asymptomatic (n=24)
mild (n=25)
severe (n=30)
control (n=72)
Severity

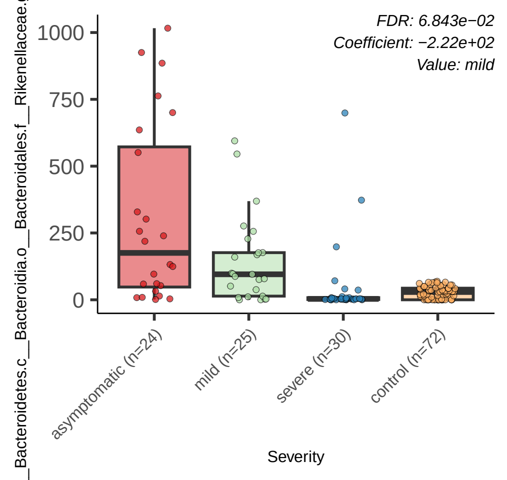

FDR: 6.843e−02
Coefficient: −2.22e+02
Value: mild
_Bacteroidetes.c__Bacteroidia.o__Bacteroidales.f__Rikenellaceae.g
1000
750
500
250
0
asymptomatic (n=24)
mild (n=25)
severe (n=30)
control (n=72)
Severity

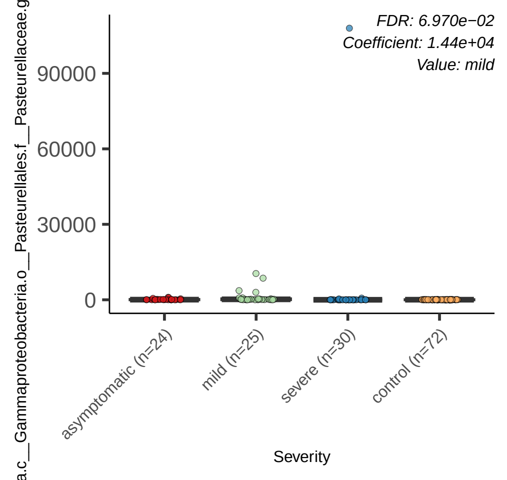
FDR: 6.970e−02
Coefficient: 1.44e+04
Value: mild
a.c__Gammaproteobacteria.o__Pasteurellales.f__Pasteurellaceae.g
90000
60000
30000
0
asymptomatic (n=24)
mild (n=25)
severe (n=30)
control (n=72)
Severity

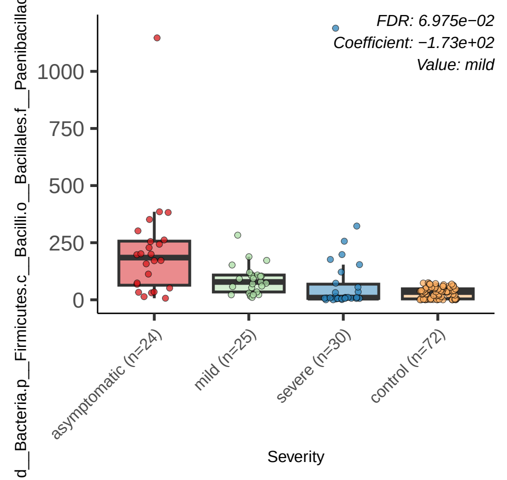

FDR: 6.975e−02
Coefficient: −1.73e+02
Value: mild
d__Bacteria.p__Firmicutes.c__Bacilli.o__Bacillales.f__Paenibacillac
1000
750
500
250
0
asymptomatic (n=24)
mild (n=25)
severe (n=30)
control (n=72)
Severity

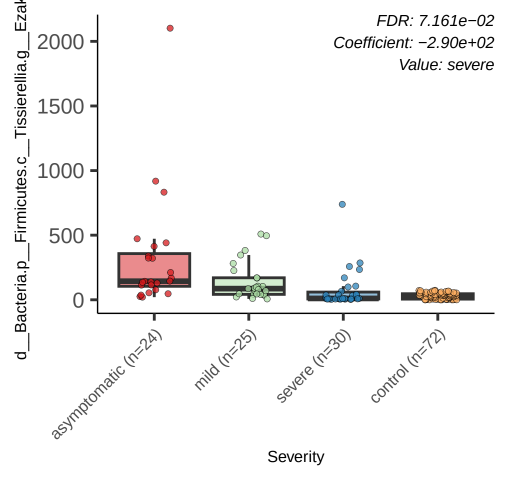

FDR: 7.161e−02
Coefficient: −2.90e+02
Value: severe
d__Bacteria.p__Firmicutes.c__Tissierellia.g__Ezak
0
500
1000
1500
2000
asymptomatic (n=24)
mild (n=25)
severe (n=30)
control (n=72)
Severity

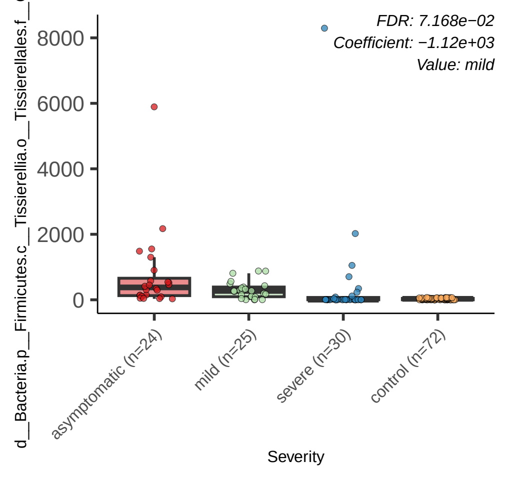

FDR: 7.168e−02
Coefficient: −1.12e+03
Value: mild
d__Bacteria.p__Firmicutes.c__Tissierellia.o__Tissierellales.f__
8000
6000
4000
2000
0
asymptomatic (n=24)
mild (n=25)
severe (n=30)
control (n=72)
Severity

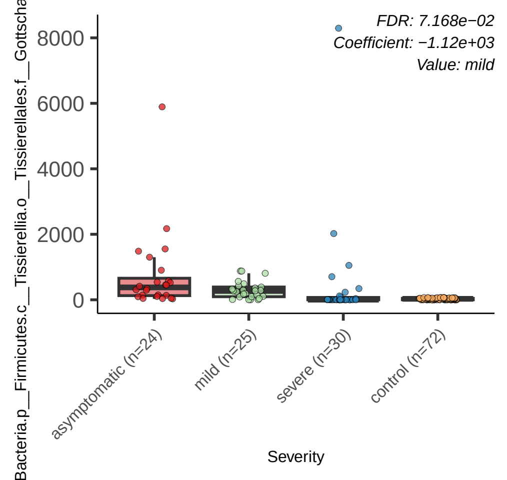

FDR: 7.168e−02
Coefficient: −1.12e+03
Value: mild
8000
6000
4000
2000
0
Bacteria.p__Firmicutes.c__Tissierellia.o__Tissierellales.f__Gottscha
asymptomatic (n=24)
mild (n=25)
severe (n=30)
control (n=72)
Severity

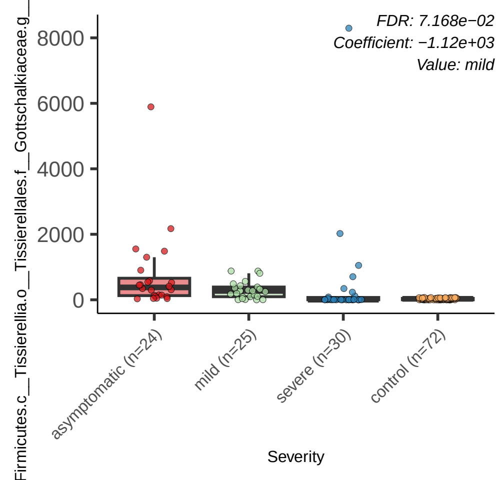
FDR: 7.168e−02
Coefficient: −1.12e+03
Value: mild
8000
6000
4000
2000
0
Firmicutes.c__Tissierellia.o__Tissierellales.f__Gottschalkiaceae.g__
asymptomatic (n=24)
mild (n=25)
severe (n=30)
control (n=72)
Severity

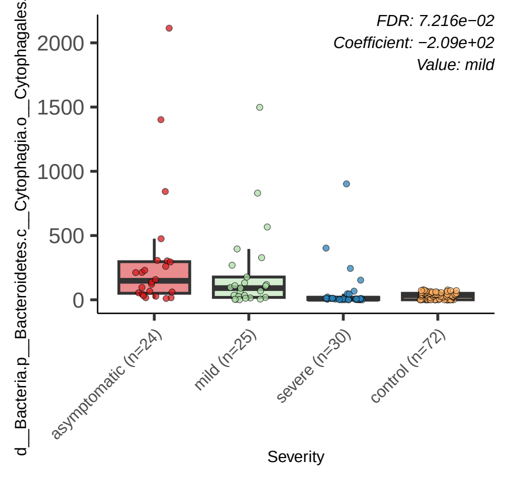

FDR: 7.216e−02
Coefficient: −2.09e+02
Value: mild
d__Bacteria.p__Bacteroidetes.c__Cytophagia.o__Cytophagales.
2000
1500
1000
500
0
asymptomatic (n=24)
mild (n=25)
severe (n=30)
control (n=72)
Severity

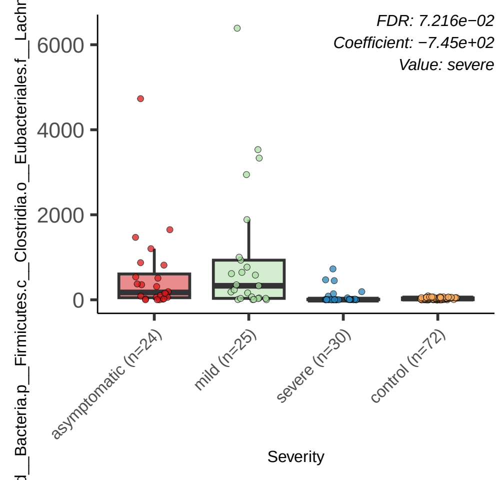

FDR: 7.216e−02
Coefficient: −7.45e+02
Value: severe
6000
4000
2000
0
d__Bacteria.p__Firmicutes.c__Clostridia.o__Eubacteriales.f__Lachn
asymptomatic (n=24)
mild (n=25)
severe (n=30)
control (n=72)
Severity

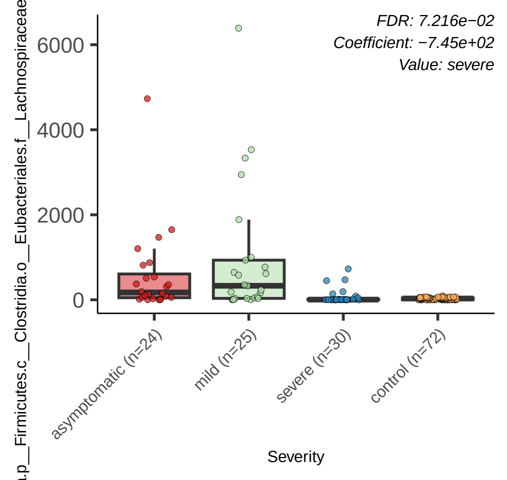
FDR: 7.216e−02
Coefficient: −7.45e+02
Value: severe
6000
4000
2000
0
asymptomatic (n=24)
mild (n=25)
severe (n=30)
control (n=72)
Severity
a.p__Firmicutes.c__Clostridia.o__Eubacteriales.f__Lachnospiraceae

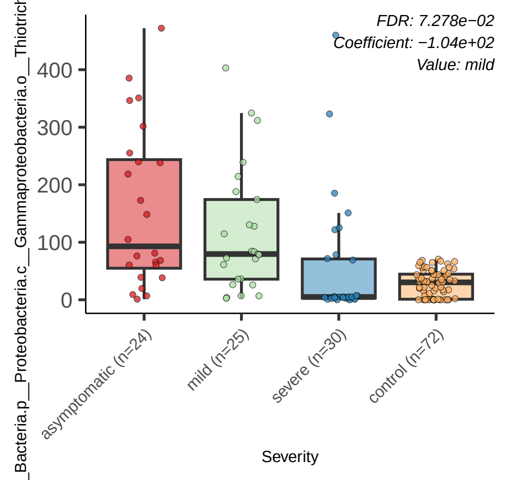

FDR: 7.278e−02
Coefficient: −1.04e+02
Value: mild
Bacteria.p__Proteobacteria.c__Gammaproteobacteria.o__Thiotrich
0
100
200
300
400
asymptomatic (n=24)
mild (n=25)
severe (n=30)
control (n=72)
Severity

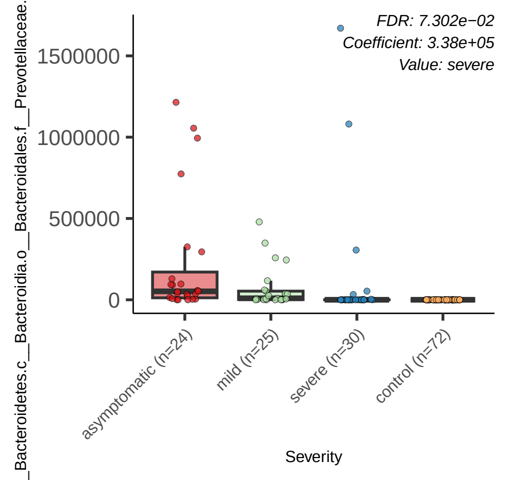

FDR: 7.302e−02
Coefficient: 3.38e+05
Value: severe
_Bacteroidetes.c__Bacteroidia.o__Bacteroidales.f__Prevotellaceae.
1500000
1000000
500000
0
asymptomatic (n=24)
mild (n=25)
severe (n=30)
control (n=72)
Severity

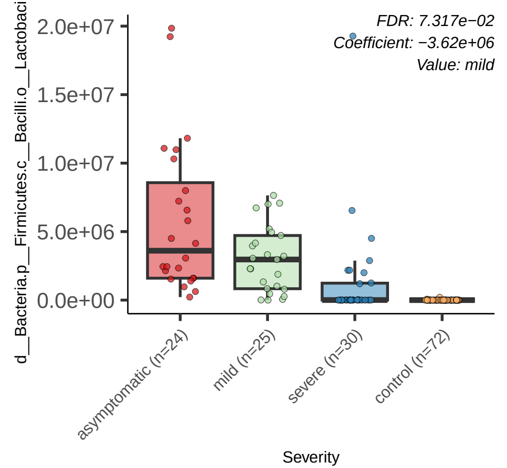

FDR: 7.317e−02
Coefficient: −3.62e+06
Value: mild
d__Bacteria.p__Firmicutes.c__Bacilli.o__Lactobaci
2.0e+07
1.5e+07
1.0e+07
5.0e+06
0.0e+00
asymptomatic (n=24)
mild (n=25)
severe (n=30)
control (n=72)
Severity

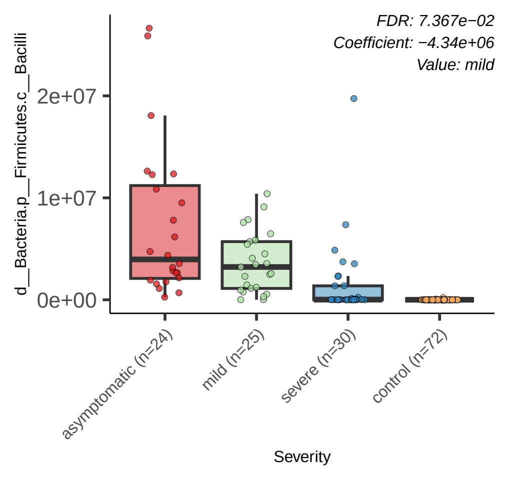
FDR: 7.367e−02
Coefficient: −4.34e+06
Value: mild
d__Bacteria.p__Firmicutes.c__Bacilli
2e+07
1e+07
0e+00
asymptomatic (n=24)
mild (n=25)
severe (n=30)
control (n=72)
Severity

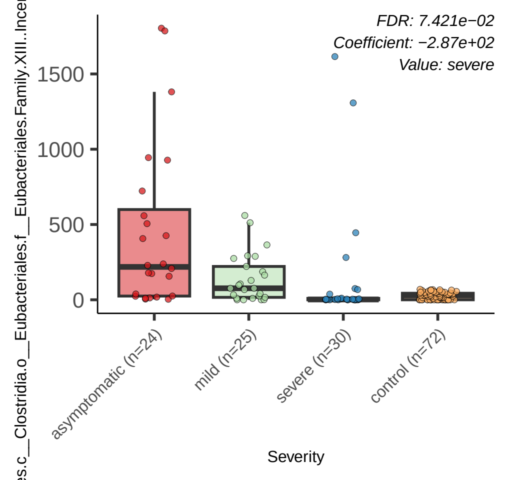

FDR: 7.421e−02
Coefficient: −2.87e+02
Value: severe
es.c__Clostridia.o__Eubacteriales.f__Eubacteriales.Family.XIII..Ince
0
500
1000
1500
asymptomatic (n=24)
mild (n=25)
severe (n=30)
control (n=72)
Severity

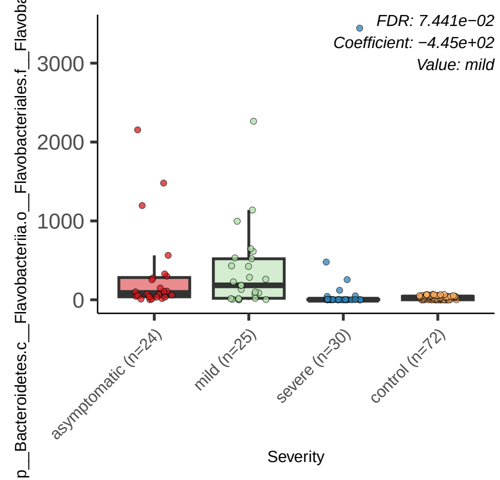
FDR: 7.441e−02
Coefficient: −4.45e+02
Value: mild
p__Bacteroidetes.c__Flavobacteriia.o__Flavobacteriales.f__Flavoba
3000
2000
1000
0
asymptomatic (n=24)
mild (n=25)
severe (n=30)
control (n=72)
Severity

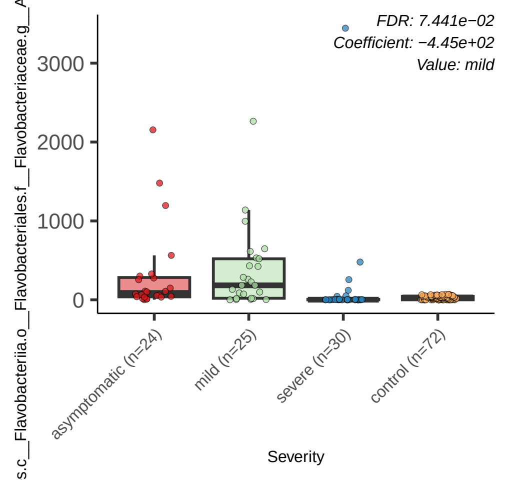

FDR: 7.441e−02
Coefficient: −4.45e+02
Value: mild
3000
2000
1000
0
s.c__Flavobacteriia.o__Flavobacteriales.f__Flavobacteriaceae.g__A
asymptomatic (n=24)
mild (n=25)
severe (n=30)
control (n=72)
Severity

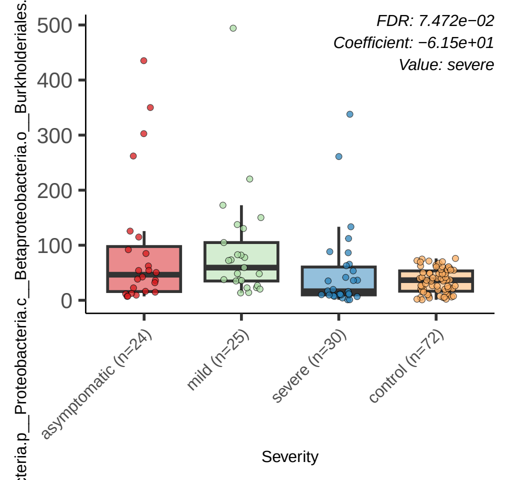

FDR: 7.472e−02
Coefficient: −6.15e+01
Value: severe
0
100
200
300
400
500
cteria.p__Proteobacteria.c__Betaproteobacteria.o__Burkholderiales.
asymptomatic (n=24)
mild (n=25)
severe (n=30)
control (n=72)
Severity

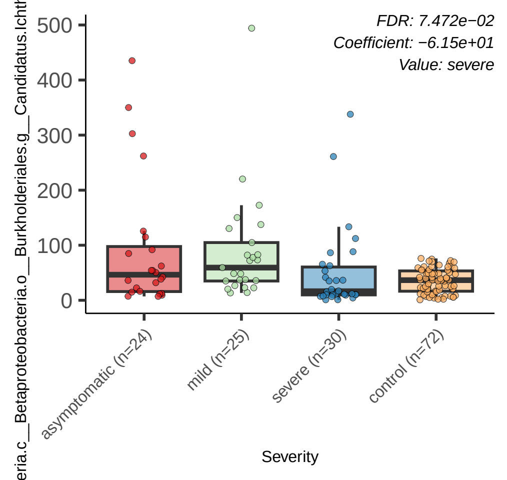

FDR: 7.472e−02
Coefficient: −6.15e+01
Value: severe
eria.c__Betaproteobacteria.o__Burkholderiales.g__Candidatus.Ichth
500
400
300
200
100
0
asymptomatic (n=24)
mild (n=25)
severe (n=30)
control (n=72)
Severity

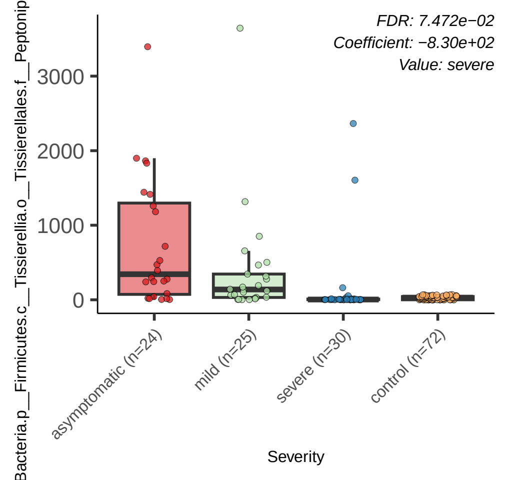

FDR: 7.472e−02
Coefficient: −8.30e+02
Value: severe
Bacteria.p__Firmicutes.c__Tissierellia.o__Tissierellales.f__Peptonip
3000
2000
1000
0
asymptomatic (n=24)
mild (n=25)
severe (n=30)
control (n=72)
Severity

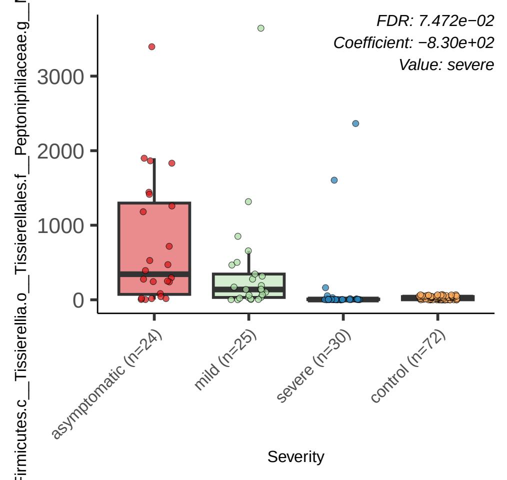
FDR: 7.472e−02
Coefficient: −8.30e+02
Value: severe
Firmicutes.c__Tissierellia.o__Tissierellales.f__Peptoniphilaceae.g__
0
1000
2000
3000
asymptomatic (n=24)
mild (n=25)
severe (n=30)
control (n=72)
Severity

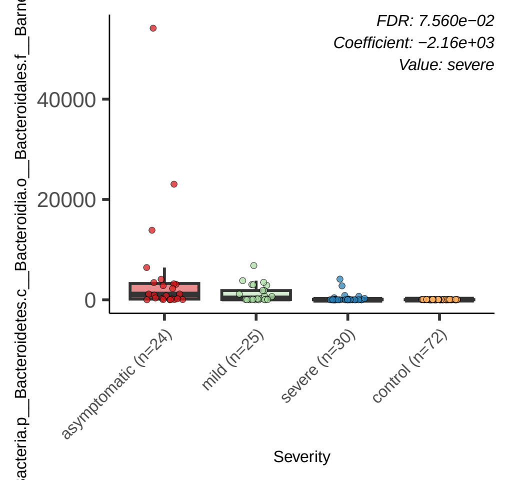

FDR: 7.560e−02
Coefficient: −2.16e+03
Value: severe
40000
20000
0
acteria.p__Bacteroidetes.c__Bacteroidia.o__Bacteroidales.f__Barn
asymptomatic (n=24)
mild (n=25)
severe (n=30)
control (n=72)
Severity

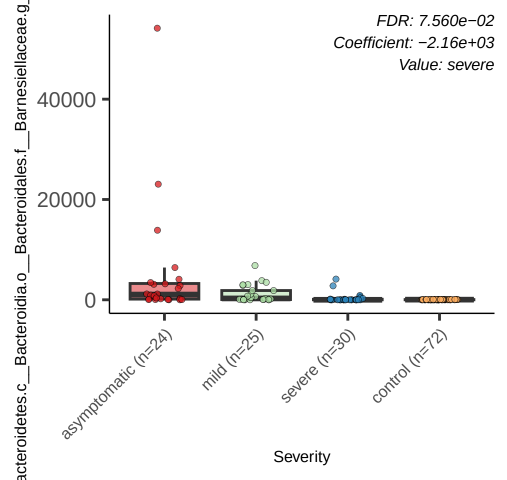

FDR: 7.560e−02
Coefficient: −2.16e+03
Value: severe
acteroidetes.c__Bacteroidia.o__Bacteroidales.f__Barnesiellaceae.g_
40000
20000
0
asymptomatic (n=24)
mild (n=25)
severe (n=30)
control (n=72)
Severity

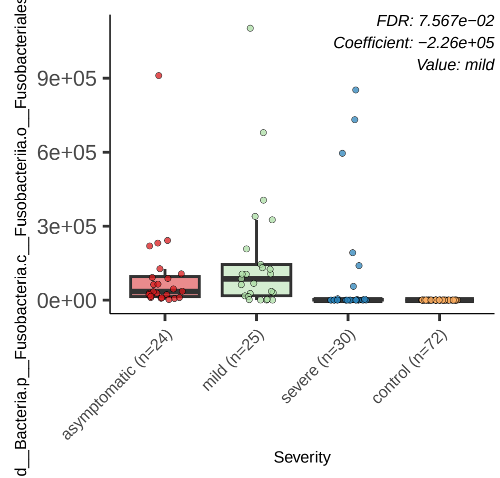

FDR: 7.567e−02
Coefficient: −2.26e+05
Value: mild
d__Bacteria.p__Fusobacteria.c__Fusobacteriia.o__Fusobacteriales
9e+05
6e+05
3e+05
0e+00
asymptomatic (n=24)
mild (n=25)
severe (n=30)
control (n=72)
Severity

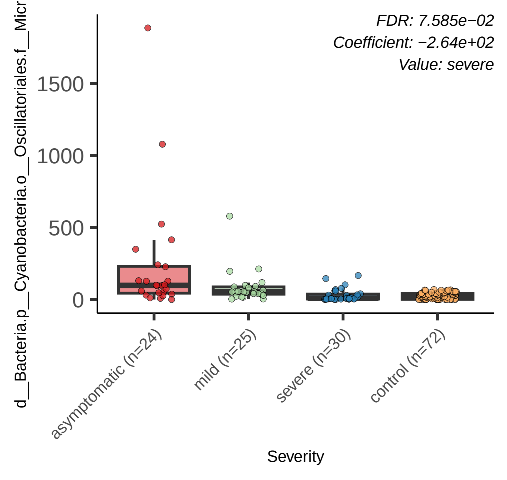

FDR: 7.585e−02
Coefficient: −2.64e+02
Value: severe
d__Bacteria.p__Cyanobacteria.o__Oscillatoriales.f__Micr
0
500
1000
1500
asymptomatic (n=24)
mild (n=25)
severe (n=30)
control (n=72)
Severity

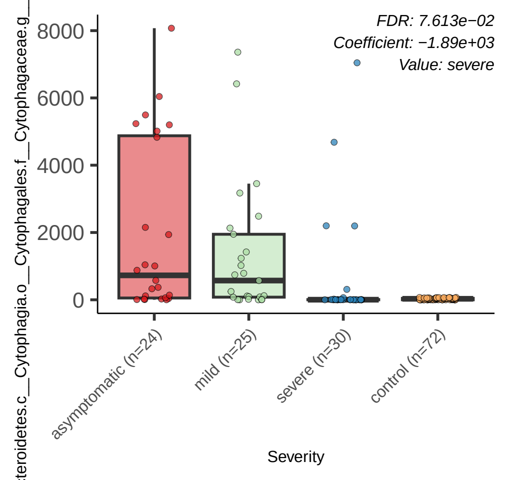

FDR: 7.613e−02
Coefficient: −1.89e+03
Value: severe
8000
6000
4000
2000
0
teroidetes.c__Cytophagia.o__Cytophagales.f__Cytophagaceae.g__
asymptomatic (n=24)
mild (n=25)
severe (n=30)
control (n=72)
Severity

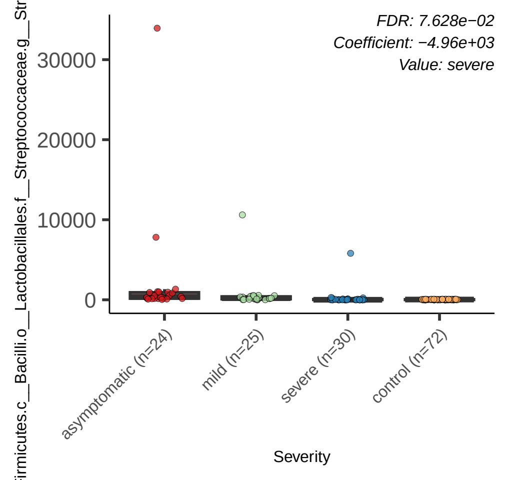
FDR: 7.628e−02
Coefficient: −4.96e+03
Value: severe
30000
20000
10000
0
irmicutes.c__Bacilli.o__Lactobacillales.f__Streptococcaceae.g__Str
asymptomatic (n=24)
mild (n=25)
severe (n=30)
control (n=72)
Severity

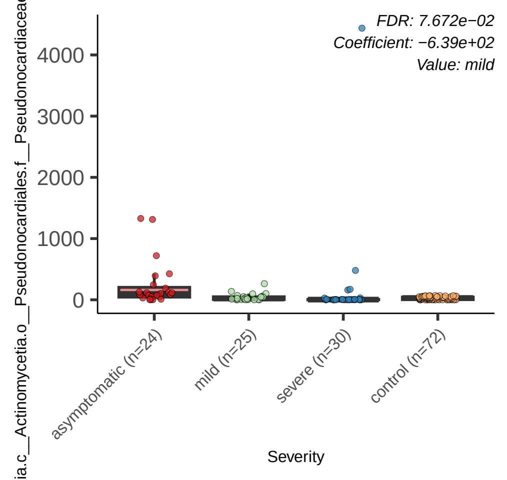

FDR: 7.672e−02
Coefficient: −6.39e+02
Value: mild
4000
3000
2000
1000
0
ia.c__Actinomycetia.o__Pseudonocardiales.f__Pseudonocardiaceae
asymptomatic (n=24)
mild (n=25)
severe (n=30)
control (n=72)
Severity

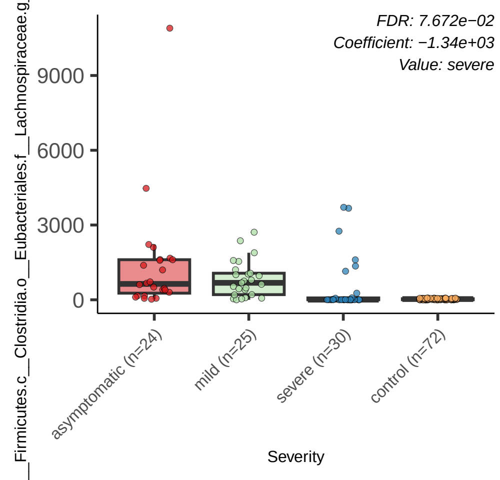
FDR: 7.672e−02
Coefficient: −1.34e+03
Value: severe
9000
6000
3000
0
Firmicutes.c__Clostridia.o__Eubacteriales.f__Lachnospiraceae.g
asymptomatic (n=24)
mild (n=25)
severe (n=30)
control (n=72)
Severity

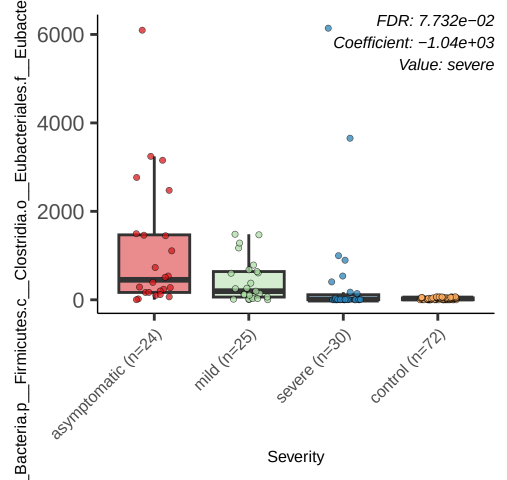

Bacteria.p__Firmicutes.c__Clostridia.o__Eubacteriales.f__Eubacte
FDR: 7.732e−02
Coefficient: −1.04e+03
Value: severe
6000
4000
2000
0
asymptomatic (n=24)
mild (n=25)
severe (n=30)
control (n=72)
Severity

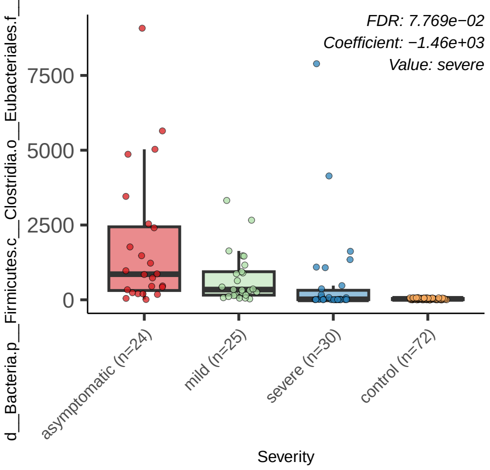

FDR: 7.769e−02
Coefficient: −1.46e+03
Value: severe
d__Bacteria.p__Firmicutes.c__Clostridia.o__Eubacteriales.f
7500
5000
2500
0
asymptomatic (n=24)
mild (n=25)
severe (n=30)
control (n=72)
Severity

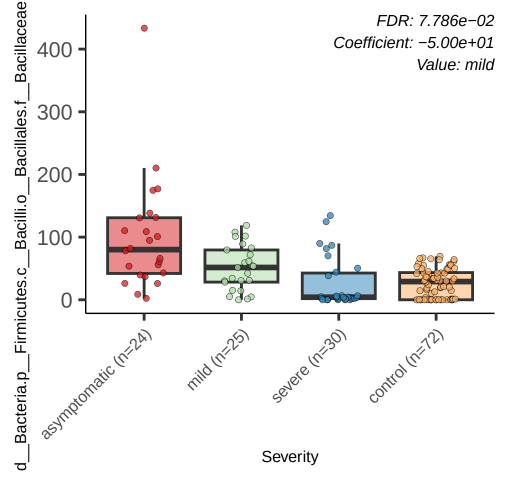
FDR: 7.786e−02
Coefficient: −5.00e+01
Value: mild
d__Bacteria.p__Firmicutes.c__Bacilli.o__Bacillales.f__Bacillaceae
400
300
200
100
0
asymptomatic (n=24)
mild (n=25)
severe (n=30)
control (n=72)
Severity

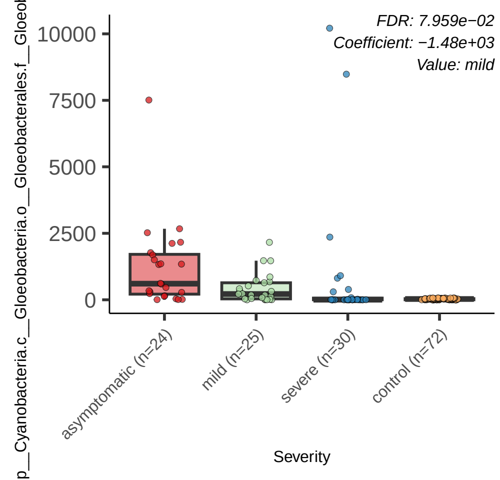

FDR: 7.959e−02
Coefficient: −1.48e+03
Value: mild
10000
7500
5000
2500
0
p__Cyanobacteria.c__Gloeobacteria.o__Gloeobacterales.f__Gloeob
asymptomatic (n=24)
mild (n=25)
severe (n=30)
control (n=72)
Severity

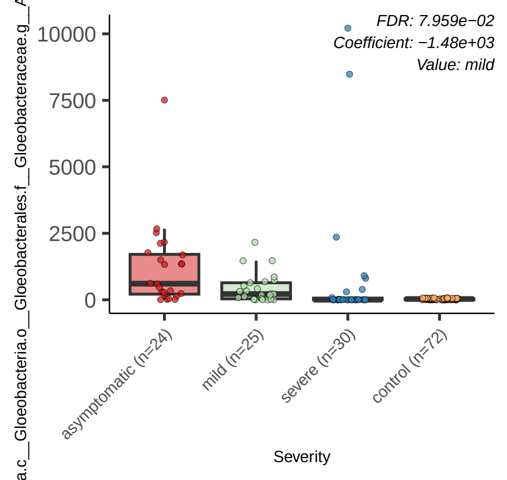

a.c__Gloeobacteria.o__Gloeobacterales.f__Gloeobacteraceae.g__A
FDR: 7.959e−02
Coefficient: −1.48e+03
Value: mild
10000
7500
5000
2500
0
asymptomatic (n=24)
mild (n=25)
severe (n=30)
control (n=72)
Severity

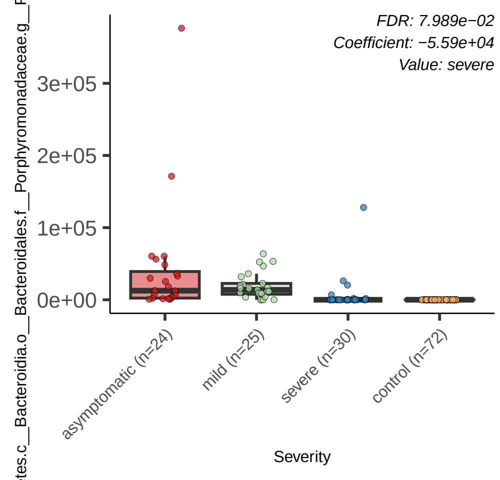
FDR: 7.989e−02
Coefficient: −5.59e+04
Value: severe
3e+05
2e+05
1e+05
0e+00
asymptomatic (n=24)
mild (n=25)
severe (n=30)
control (n=72)
Severity
Bacteroidia.o__Bacteroidales.f__Porphyromonadaceae.g__P

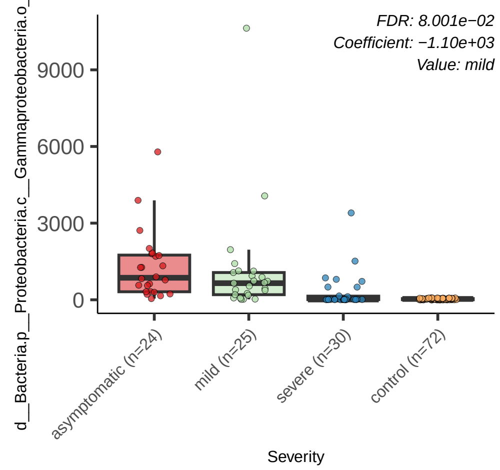

FDR: 8.001e−02
Coefficient: −1.10e+03
Value: mild
d__Bacteria.p__Proteobacteria.c__Gammaproteobacteria.o__
9000
6000
3000
0
asymptomatic (n=24)
mild (n=25)
severe (n=30)
control (n=72)
Severity

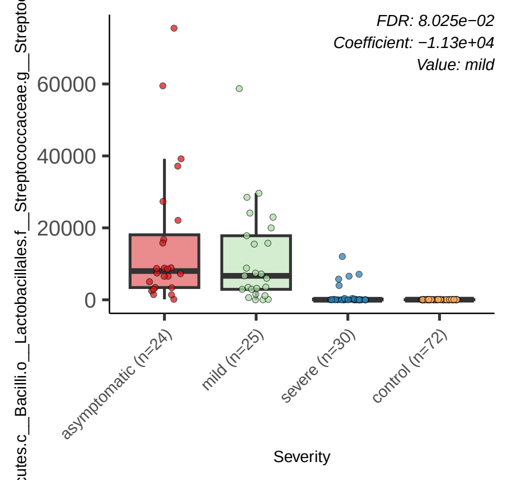
FDR: 8.025e−02
Coefficient: −1.13e+04
Value: mild
60000
40000
20000
0
asymptomatic (n=24)
mild (n=25)
severe (n=30)
control (n=72)
Severity
cutes.c__Bacilli.o__Lactobacillales.f__Streptococcaceae.g__Streptoc

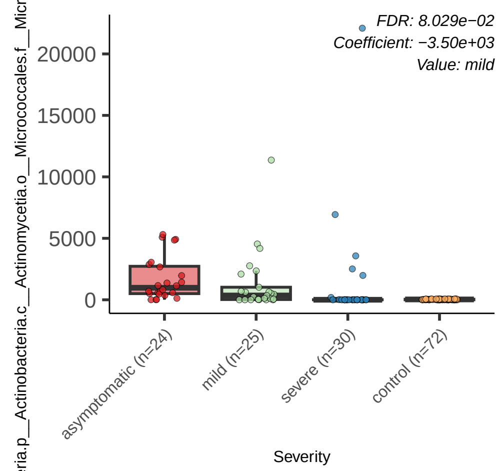
FDR: 8.029e−02
Coefficient: −3.50e+03
Value: mild
20000
15000
10000
5000
0
asymptomatic (n=24)
mild (n=25)
severe (n=30)
control (n=72)
Severity
teria.p__Actinobacteria.c__Actinomycetia.o__Micrococcales.f__Micr

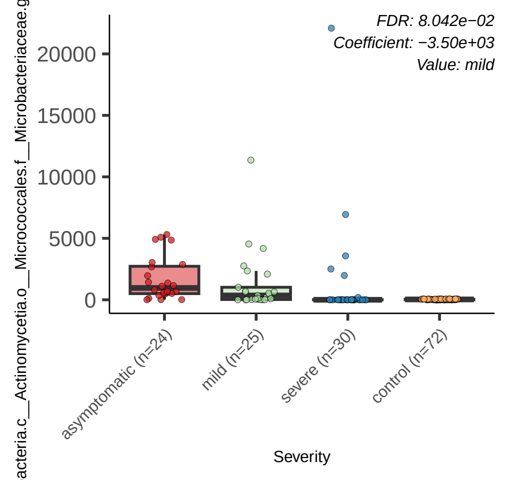
FDR: 8.042e−02
Coefficient: −3.50e+03
Value: mild
20000
15000
10000
5000
0
acteria.c__Actinomycetia.o__Micrococcales.f__Microbacteriaceae.g
asymptomatic (n=24)
mild (n=25)
severe (n=30)
control (n=72)
Severity

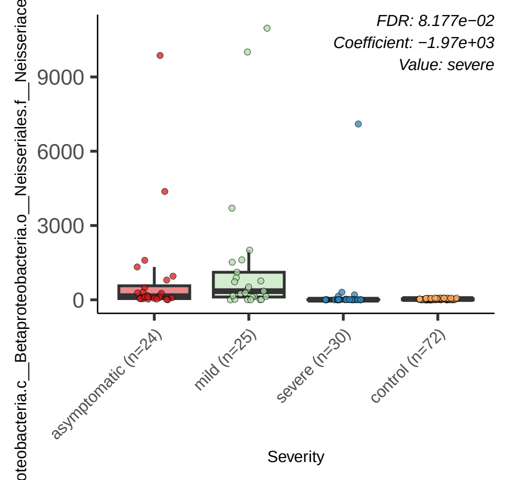

FDR: 8.177e−02
Coefficient: −1.97e+03
Value: severe
9000
6000
3000
0
oteobacteria.c__Betaproteobacteria.o__Neisseriales.f__Neisseriace
asymptomatic (n=24)
mild (n=25)
severe (n=30)
control (n=72)
Severity

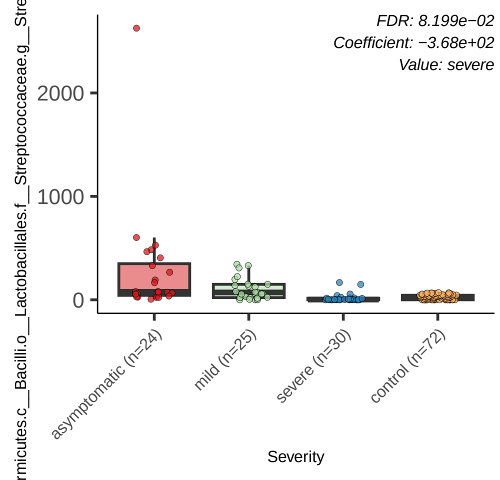

FDR: 8.199e−02
Coefficient: −3.68e+02
Value: severe
2000
1000
0
rmicutes.c__Bacilli.o__Lactobacillales.f__Streptococcaceae.g__Stre
asymptomatic (n=24)
mild (n=25)
severe (n=30)
control (n=72)
Severity

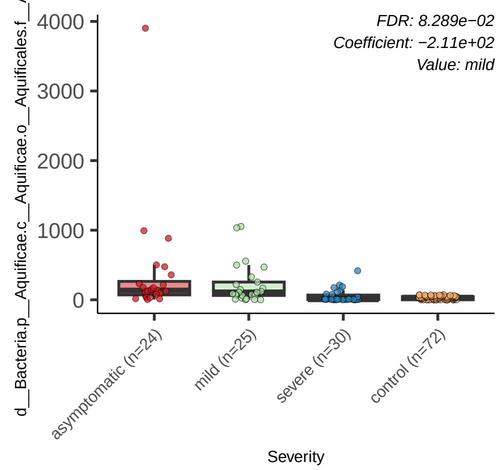

d__Bacteria.p__Aquificae.c__Aquificae.o__Aquificales.f__A
FDR: 8.289e−02
Coefficient: −2.11e+02
Value: mild
4000
3000
2000
1000
0
asymptomatic (n=24)
mild (n=25)
severe (n=30)
control (n=72)
Severity

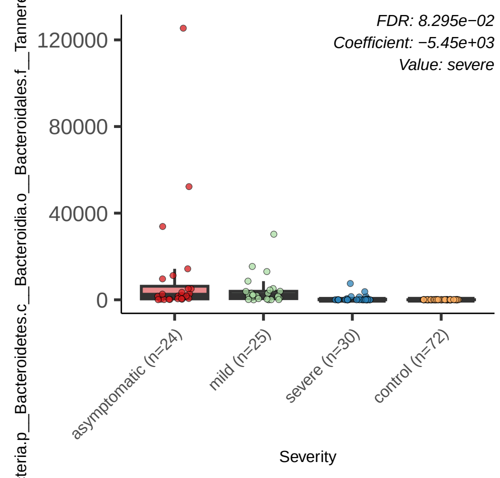

FDR: 8.295e−02
Coefficient: −5.45e+03
Value: severe
120000
80000
40000
0
asymptomatic (n=24)
mild (n=25)
severe (n=30)
control (n=72)
Severity
cteria.p__Bacteroidetes.c__Bacteroidia.o__Bacteroidales.f__Tanner

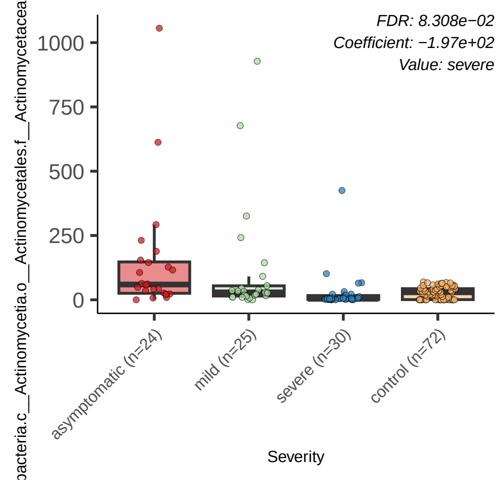

FDR: 8.308e−02
Coefficient: −1.97e+02
Value: severe
1000
750
500
250
0
acteria.c__Actinomycetia.o__Actinomycetales.f__Actinomycetacea
asymptomatic (n=24)
mild (n=25)
severe (n=30)
control (n=72)
Severity

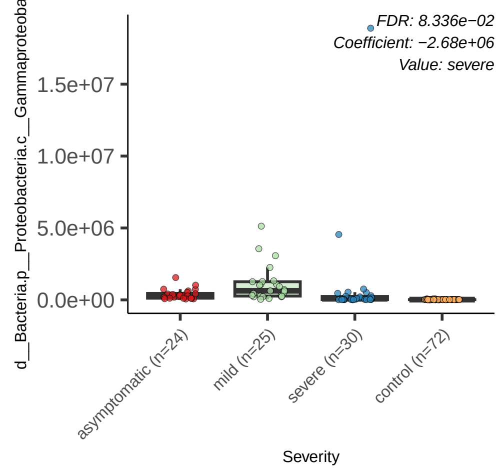

FDR: 8.336e−02
Coefficient: −2.68e+06
Value: severe
1.5e+07
1.0e+07
5.0e+06
0.0e+00
d__Bacteria.p__Proteobacteria.c__Gammaproteoba
asymptomatic (n=24)
mild (n=25)
severe (n=30)
control (n=72)
Severity

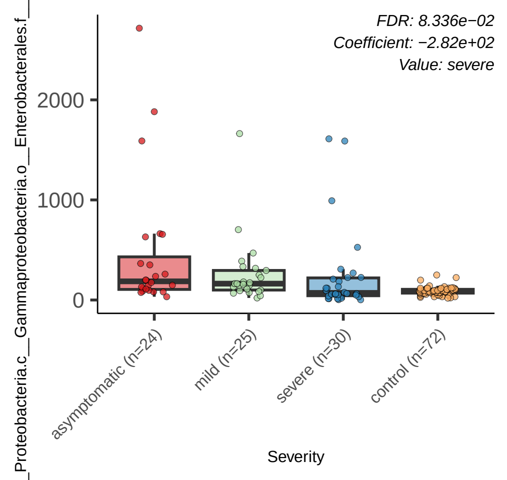

FDR: 8.336e−02
Coefficient: −2.82e+02
Value: severe
2000
1000
0
Proteobacteria.c__Gammaproteobacteria.o__Enterobacterales.f
asymptomatic (n=24)
mild (n=25)
severe (n=30)
control (n=72)
Severity

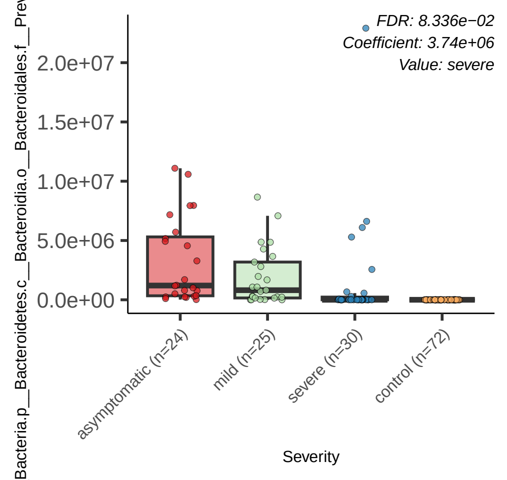

FDR: 8.336e−02
Coefficient: 3.74e+06
Value: severe
2.0e+07
1.5e+07
1.0e+07
5.0e+06
0.0e+00
Bacteria.p__Bacteroidetes.c__Bacteroidia.o__Bacteroidales.f__Prev
asymptomatic (n=24)
mild (n=25)
severe (n=30)
control (n=72)
Severity

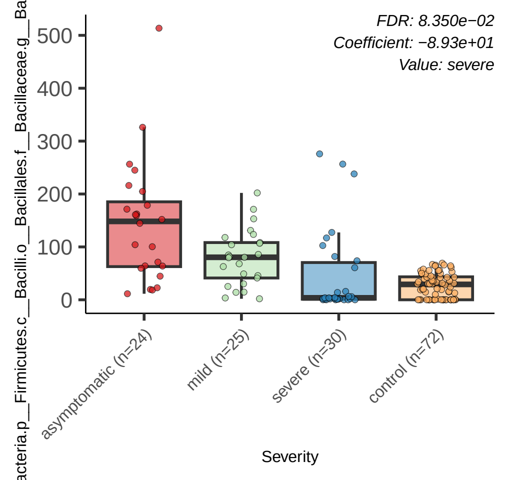

FDR: 8.350e−02
Coefficient: −8.93e+01
Value: severe
acteria.p__Firmicutes.c__Bacilli.o__Bacillales.f__Bacillaceae.g__Ba
0
100
200
300
400
500
asymptomatic (n=24)
mild (n=25)
severe (n=30)
control (n=72)
Severity

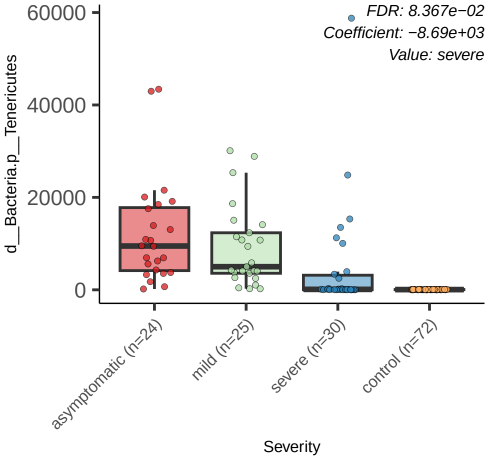
FDR: 8.367e−02
Coefficient: −8.69e+03
Value: severe
d__Bacteria.p__Tenericutes
60000
40000
20000
0
asymptomatic (n=24)
mild (n=25)
severe (n=30)
control (n=72)
Severity

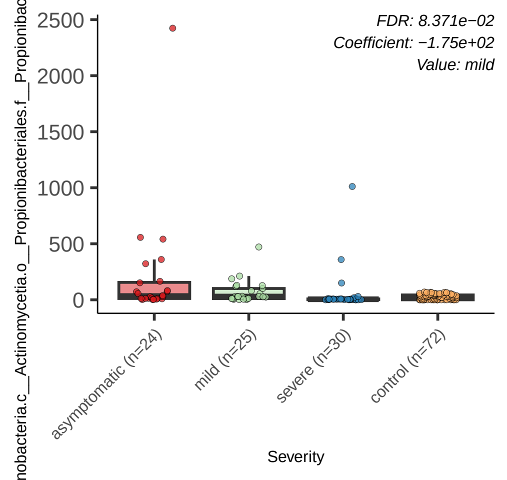

FDR: 8.371e−02
Coefficient: −1.75e+02
Value: mild
nobacteria.c__Actinomycetia.o__Propionibacteriales.f__Propionibac
2500
2000
1500
1000
500
0
asymptomatic (n=24)
mild (n=25)
severe (n=30)
control (n=72)
Severity

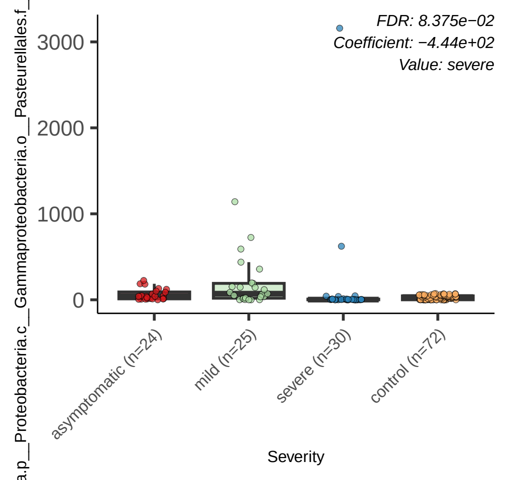
FDR: 8.375e−02
Coefficient: −4.44e+02
Value: severe
a.p__Proteobacteria.c__Gammaproteobacteria.o__Pasteurellales.f__
3000
2000
1000
0
asymptomatic (n=24)
mild (n=25)
severe (n=30)
control (n=72)
Severity

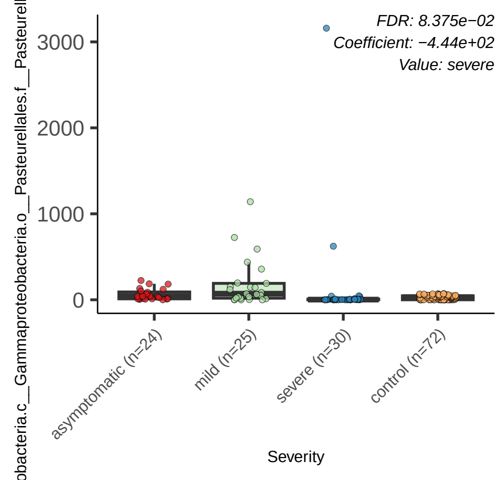

FDR: 8.375e−02
Coefficient: −4.44e+02
Value: severe
3000
2000
1000
0
obacteria.c__Gammaproteobacteria.o__Pasteurellales.f__Pasteurell
asymptomatic (n=24)
mild (n=25)
severe (n=30)
control (n=72)
Severity

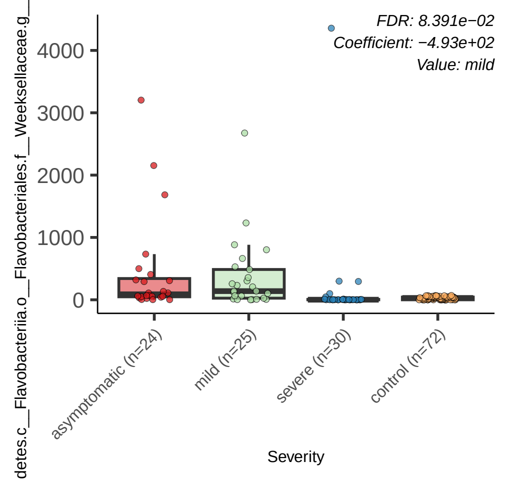
FDR: 8.391e−02
Coefficient: −4.93e+02
Value: mild
4000
3000
2000
1000
0
detes.c__Flavobacteriia.o__Flavobacteriales.f__Weeksellaceae.g__
asymptomatic (n=24)
mild (n=25)
severe (n=30)
control (n=72)
Severity

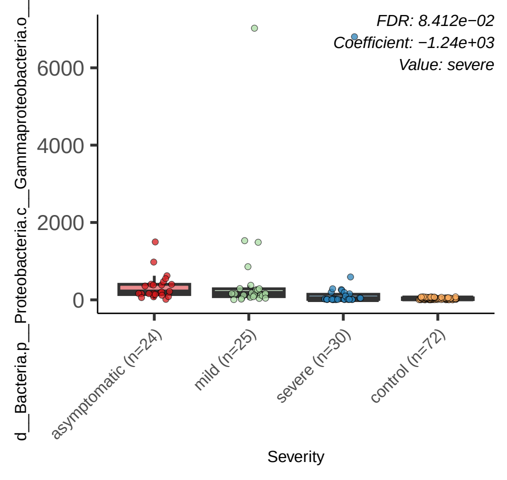

FDR: 8.412e−02
Coefficient: −1.24e+03
Value: severe
d__Bacteria.p__Proteobacteria.c__Gammaproteobacteria.o__
6000
4000
2000
0
asymptomatic (n=24)
mild (n=25)
severe (n=30)
control (n=72)
Severity

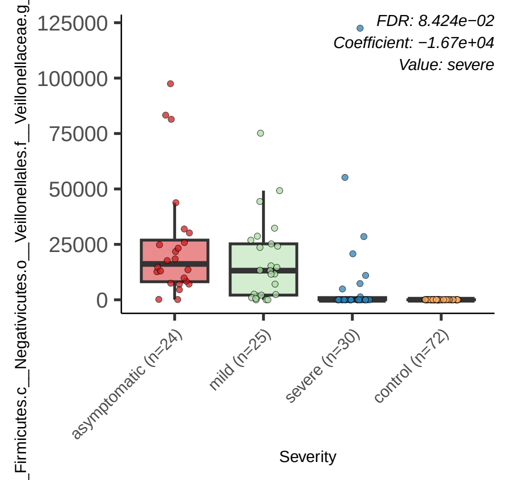

FDR: 8.424e−02
Coefficient: −1.67e+04
Value: severe
Firmicutes.c__Negativicutes.o__Veillonellales.f__Veillonellaceae.g_
125000
100000
75000
50000
25000
0
asymptomatic (n=24)
mild (n=25)
severe (n=30)
control (n=72)
Severity

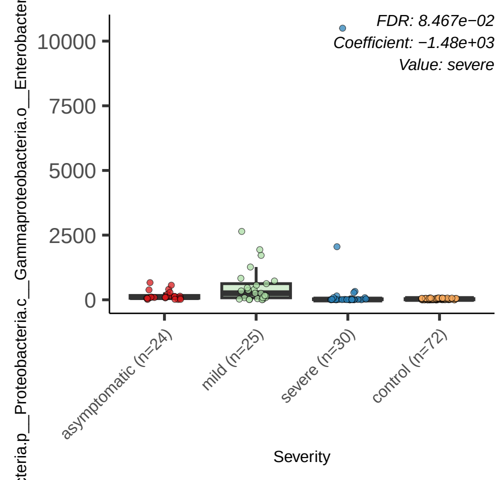
FDR: 8.467e−02
Coefficient: −1.48e+03
Value: severe
10000
7500
5000
2500
0
asymptomatic (n=24)
mild (n=25)
severe (n=30)
control (n=72)
Severity
cteria.p__Proteobacteria.c__Gammaproteobacteria.o__Enterobacter

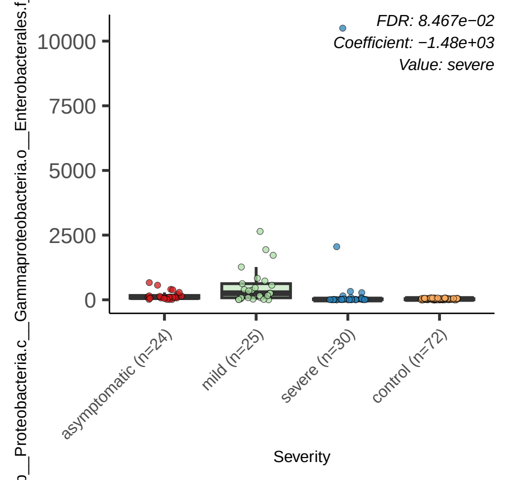

FDR: 8.467e−02
Coefficient: −1.48e+03
Value: severe
10000
7500
5000
2500
0
o__Proteobacteria.c__Gammaproteobacteria.o__Enterobacterales.f_
asymptomatic (n=24)
mild (n=25)
severe (n=30)
control (n=72)
Severity

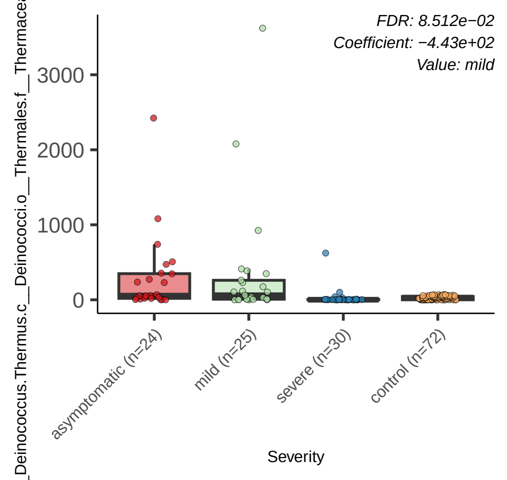

FDR: 8.512e−02
Coefficient: −4.43e+02
Value: mild
3000
2000
1000
0
_Deinococcus.Thermus.c__Deinococci.o__Thermales.f__Thermace
asymptomatic (n=24)
mild (n=25)
severe (n=30)
control (n=72)
Severity

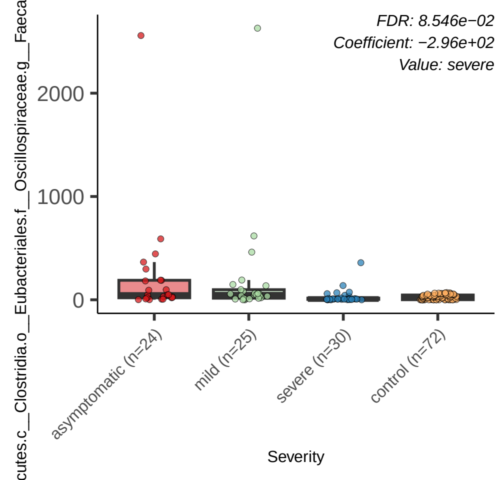
FDR: 8.546e−02
Coefficient: −2.96e+02
Value: severe
cutes.c__Clostridia.o__Eubacteriales.f__Oscillospiraceae.g__Faeca
2000
1000
0
asymptomatic (n=24)
mild (n=25)
severe (n=30)
control (n=72)
Severity

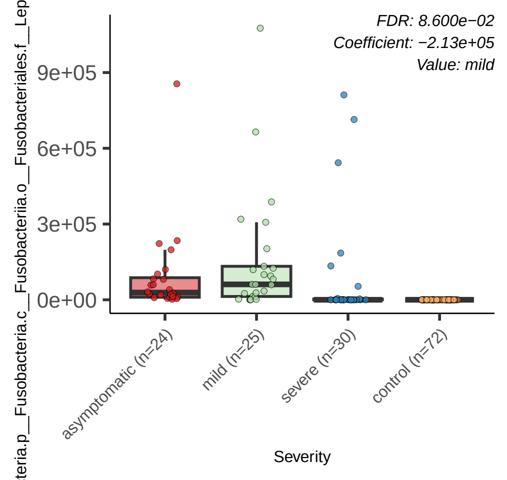

FDR: 8.600e−02
Coefficient: −2.13e+05
Value: mild
9e+05
6e+05
3e+05
0e+00
teria.p__Fusobacteria.c__Fusobacteriia.o__Fusobacteriales.f__Lep
asymptomatic (n=24)
mild (n=25)
severe (n=30)
control (n=72)
Severity

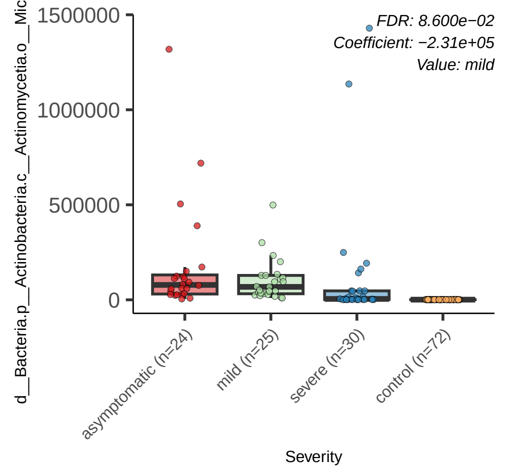

d__Bacteria.p__Actinobacteria.c__Actinobacteria.c__Actinomycetia.o__Mic
1500000
1000000
500000
0
FDR: 8.600e−02
Coefficient: −2.31e+05
Value: mild
asymptomatic (n=24)
mild (n=25)
severe (n=30)
control (n=72)
Severity

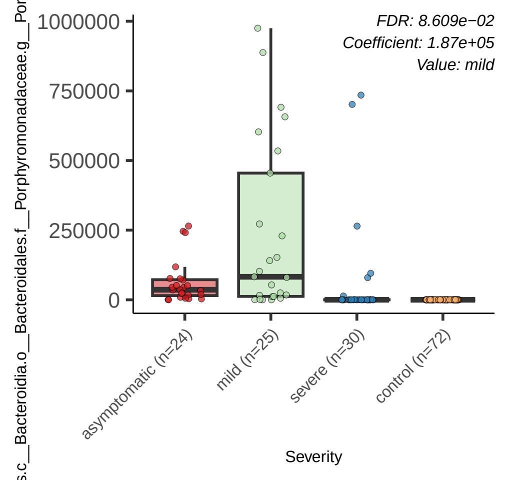

FDR: 8.609e−02
Coefficient: 1.87e+05
Value: mild
1000000
750000
500000
250000
0
s.c__Bacteroidia.o__Bacteroidales.f__Porphyromonadaceae.g__Por
asymptomatic (n=24)
mild (n=25)
severe (n=30)
control (n=72)
Severity

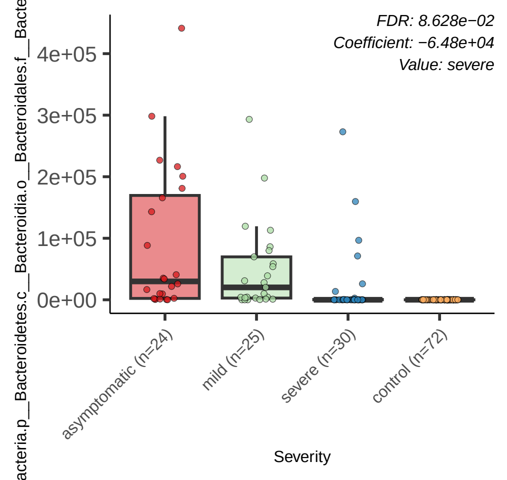

FDR: 8.628e−02
Coefficient: −6.48e+04
Value: severe
acteria.p__Bacteroidetes.c__Bacteroidia.o__Bacteroidales.f__Bacte
4e+05
3e+05
2e+05
1e+05
0e+00
asymptomatic (n=24)
mild (n=25)
severe (n=30)
control (n=72)
Severity

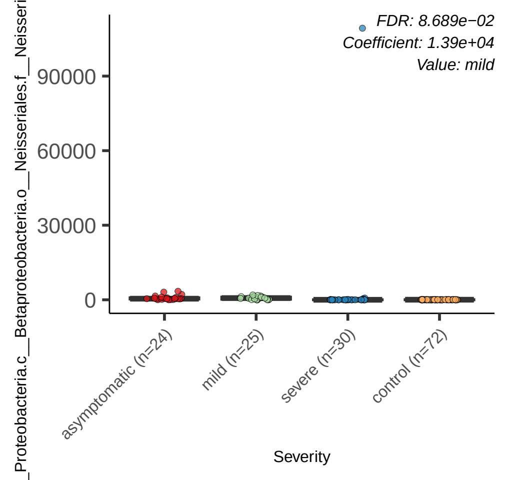

FDR: 8.689e−02
Coefficient: 1.39e+04
Value: mild
90000
60000
30000
0
_Proteobacteria.c__Betaproteobacteria.o__Neisseriales.f__Neisseri
asymptomatic (n=24)
mild (n=25)
severe (n=30)
control (n=72)
Severity

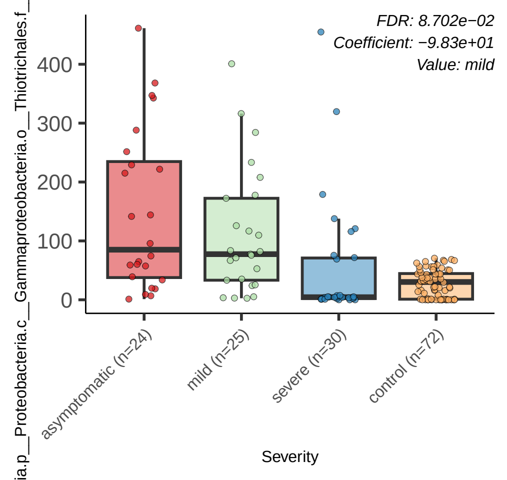

FDR: 8.702e−02
Coefficient: −9.83e+01
Value: mild
ia.p__Proteobacteria.c__Gammaproteobacteria.o__Thiotrichales.f__
0
100
200
300
400
asymptomatic (n=24)
mild (n=25)
severe (n=30)
control (n=72)
Severity

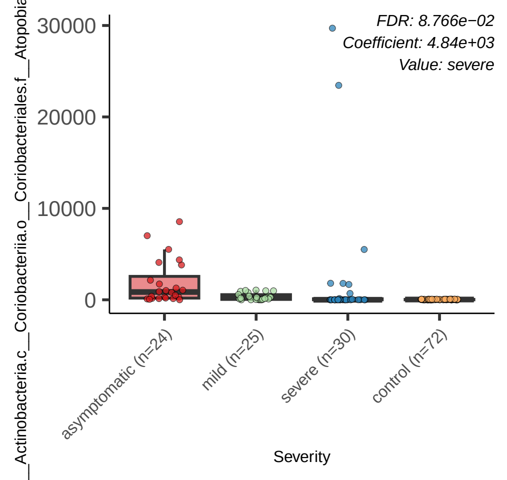
FDR: 8.766e−02
Coefficient: 4.84e+03
Value: severe
__Actinobacteria.c__Coriobacteriia.o__Coriobacteriales.f__Atopobia
30000
20000
10000
0
asymptomatic (n=24)
mild (n=25)
severe (n=30)
control (n=72)
Severity

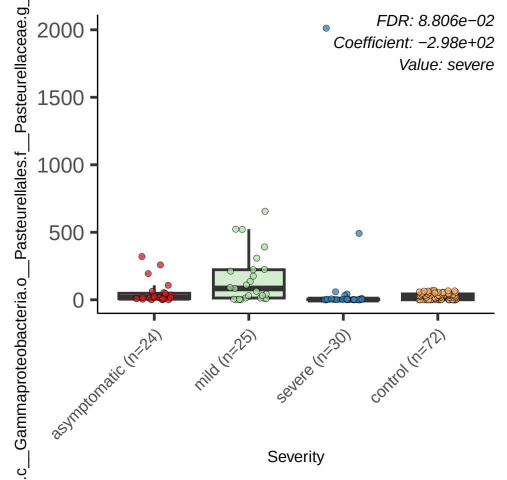
FDR: 8.806e−02
Coefficient: −2.98e+02
Value: severe
2000
1500
1000
500
0
c__Gammaproteobacteria.o__Pasteurellales.f__Pasteurellaceae.g__
asymptomatic (n=24)
mild (n=25)
severe (n=30)
control (n=72)
Severity

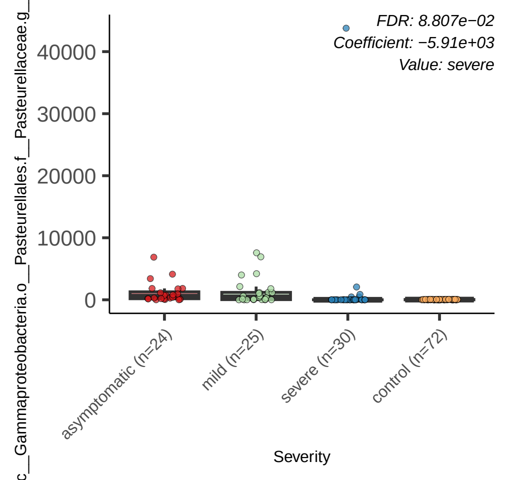

FDR: 8.807e−02
Coefficient: −5.91e+03
Value: severe
c__Gammaproteobacteria.o__Pasteurellales.f__Pasteurellaceae.g__
40000
30000
20000
10000
0
asymptomatic (n=24)
mild (n=25)
severe (n=30)
control (n=72)
Severity

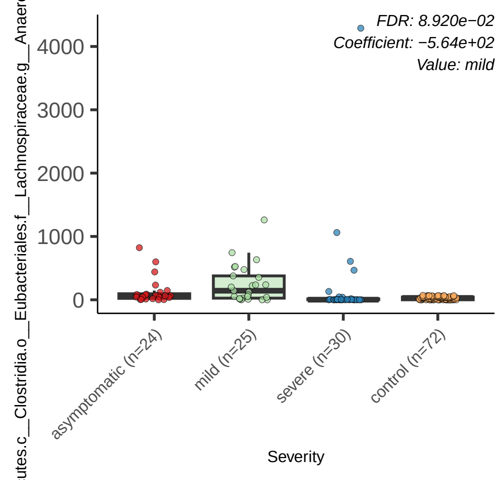

FDR: 8.920e−02
Coefficient: −5.64e+02
Value: mild
0
1000
2000
3000
4000
utes.c__Clostridia.o__Eubacteriales.f__Lachnospiraceae.g__Anaero
asymptomatic (n=24)
mild (n=25)
severe (n=30)
control (n=72)
Severity

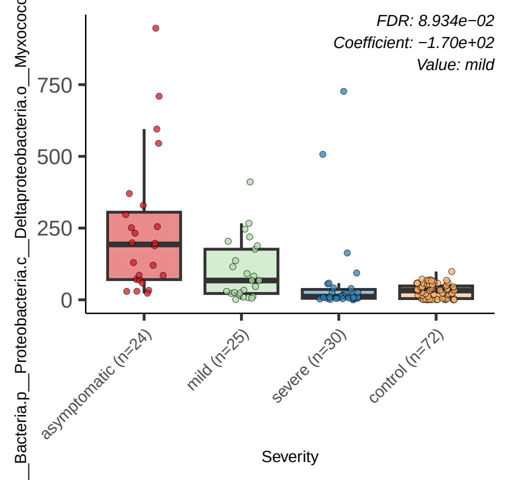
FDR: 8.934e−02
Coefficient: −1.70e+02
Value: mild
Bacteria.p__Proteobacteria.c__Deltaproteobacteria.o__Myxococc
750
500
250
0
asymptomatic (n=24)
mild (n=25)
severe (n=30)
control (n=72)
Severity

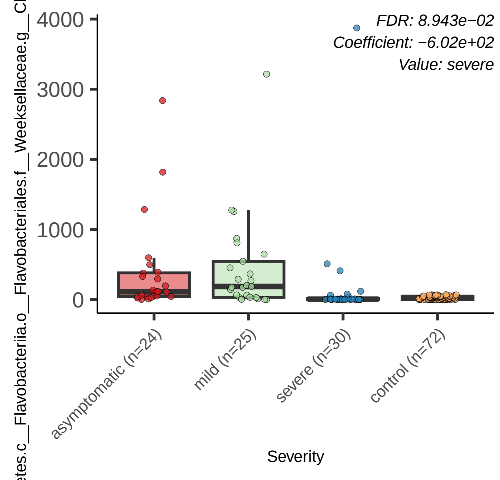

FDR: 8.943e−02
Coefficient: −6.02e+02
Value: severe
4000
3000
2000
1000
0
asymptomatic (n=24)
mild (n=25)
severe (n=30)
control (n=72)
Severity
etes.c__Flavobacteriia.o__Flavobacteriales.f__Weeksellaceae.g__Cl

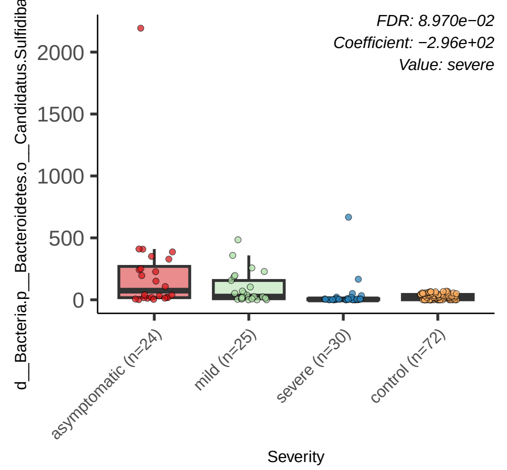

FDR: 8.970e−02
Coefficient: −2.96e+02
Value: severe
d__Bacteria.p__Bacteroidetes.o__Candidatus.Sulfidiba
2000
1500
1000
500
0
asymptomatic (n=24)
mild (n=25)
severe (n=30)
control (n=72)
Severity

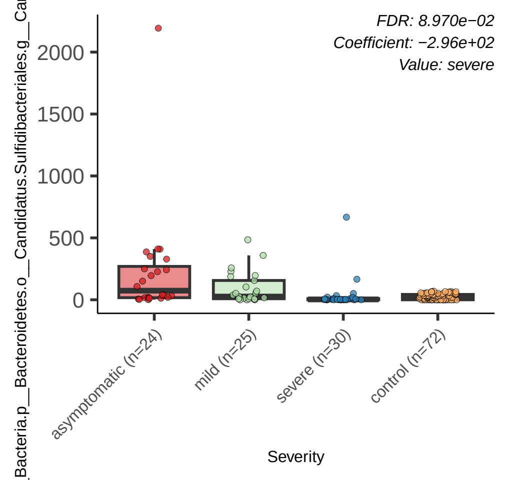

FDR: 8.970e−02
Coefficient: −2.96e+02
Value: severe
0
500
1000
1500
2000
Bacteria.p__Bacteroidetes.o__Candidatus.Sulfidibacteriales.g__Ca
asymptomatic (n=24)
mild (n=25)
severe (n=30)
control (n=72)
Severity

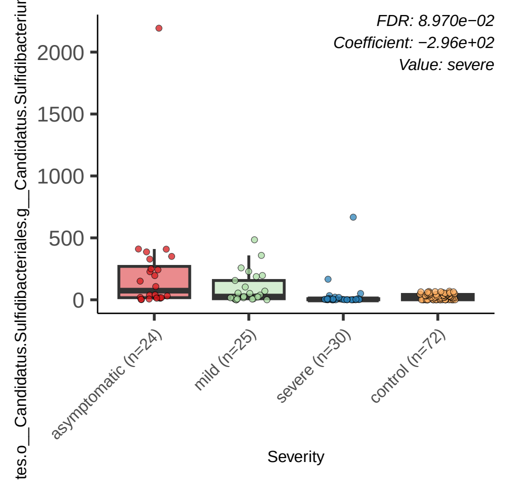

FDR: 8.970e−02
Coefficient: −2.96e+02
Value: severe
2000
1500
1000
500
0
tes.o__Candidatus.Sulfidibacteriales.g__Candidatus.Sulfidibacteriur
asymptomatic (n=24)
mild (n=25)
severe (n=30)
control (n=72)
Severity

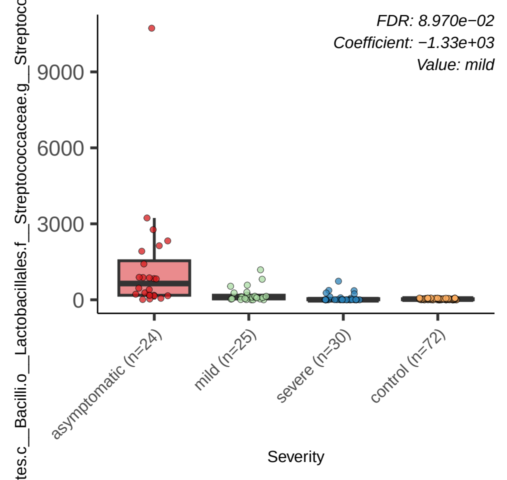

tes.c__Bacilli.o__Lactobacillales.f__Streptococcaceae.g__Streptococ
FDR: 8.970e−02
Coefficient: −1.33e+03
Value: mild
9000
6000
3000
0
asymptomatic (n=24)
mild (n=25)
severe (n=30)
control (n=72)
Severity

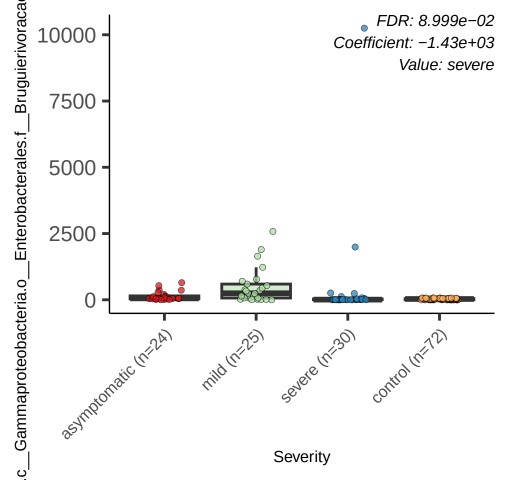
FDR: 8.999e−02
Coefficient: −1.43e+03
Value: severe
c__Gammaproteobacteria.o__Enterobacterales.f__Bruguierivoracae
10000
7500
5000
2500
0
asymptomatic (n=24)
mild (n=25)
severe (n=30)
control (n=72)
Severity

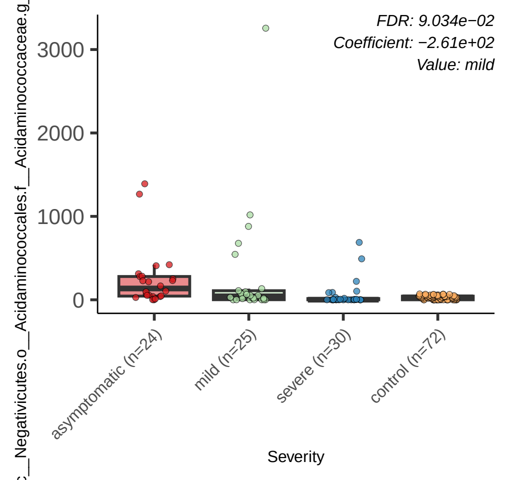

FDR: 9.034e−02
Coefficient: −2.61e+02
Value: mild
c__Negativicutes.o__Acidaminococcales.f__Acidaminococcaceae.g__
3000
2000
1000
0
asymptomatic (n=24)
mild (n=25)
severe (n=30)
control (n=72)
Severity

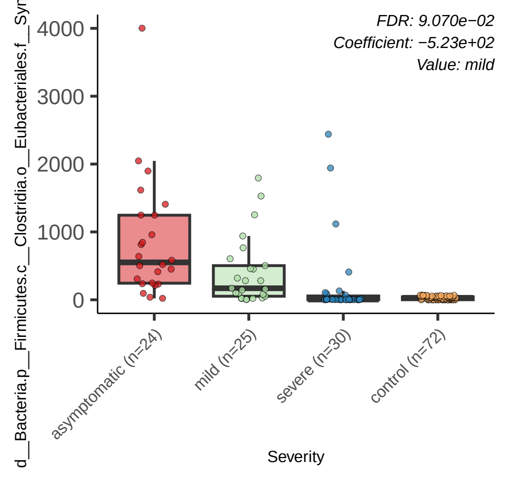

FDR: 9.070e−02
Coefficient: −5.23e+02
Value: mild
d__Bacteria.p__Firmicutes.c__Clostridia.o__Eubacteriales.f__Syn
4000
3000
2000
1000
0
asymptomatic (n=24)
mild (n=25)
severe (n=30)
control (n=72)
Severity

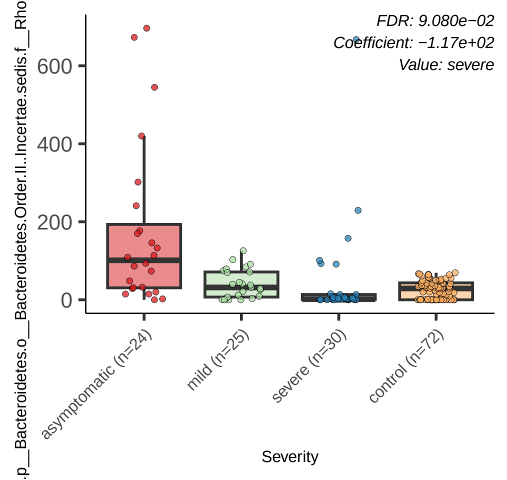

FDR: 9.080e−02
Coefficient: −1.17e+02
Value: severe
p__Bacteroidetes.o__Bacteroidetes.Order.II..Incertae.sedis.f__Rho
600
400
200
0
asymptomatic (n=24)
mild (n=25)
severe (n=30)
control (n=72)
Severity

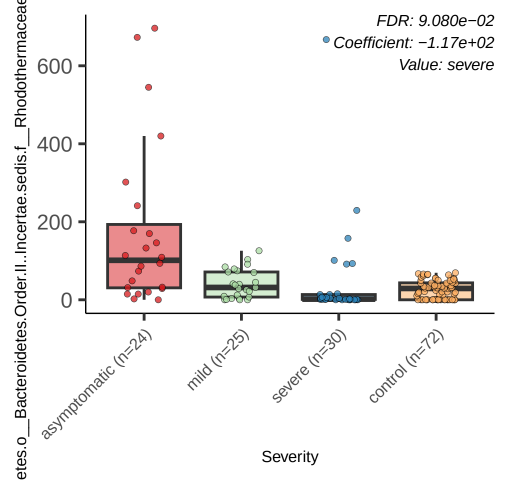

FDR: 9.080e−02
Coefficient: −1.17e+02
Value: severe
0
200
400
600
etes.o__Bacteroidetes.Order.II..Incertae.sedis.f__Rhodothermaceae
asymptomatic (n=24)
mild (n=25)
severe (n=30)
control (n=72)
Severity

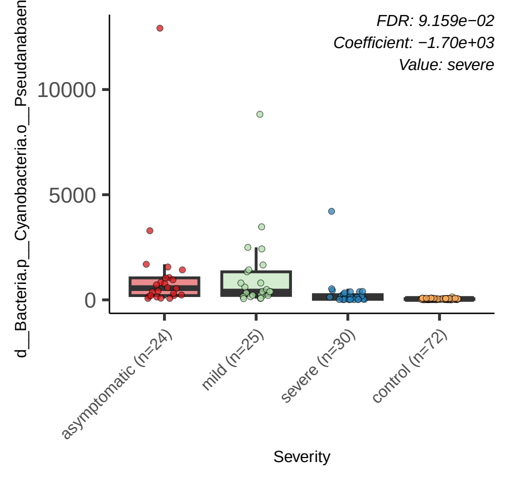

FDR: 9.159e−02
Coefficient: −1.70e+03
Value: severe
d__Bacteria.p__Cyanobacteria.o__Pseudanabaen
10000
5000
0
asymptomatic (n=24)
mild (n=25)
severe (n=30)
control (n=72)
Severity

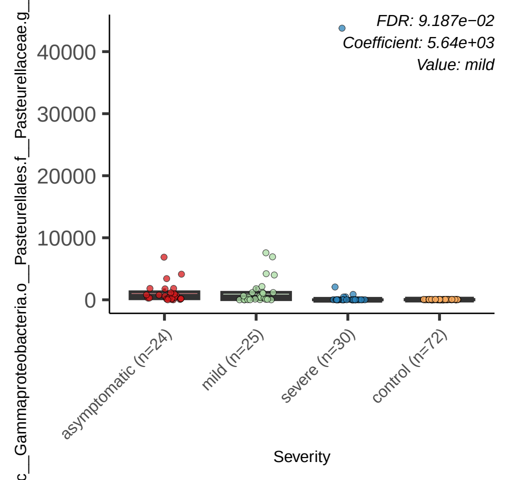

FDR: 9.187e−02
Coefficient: 5.64e+03
Value: mild
c__Gammaproteobacteria.o__Pasteurellales.f__Pasteurellaceae.g__
40000
30000
20000
10000
0
asymptomatic (n=24)
mild (n=25)
severe (n=30)
control (n=72)
Severity

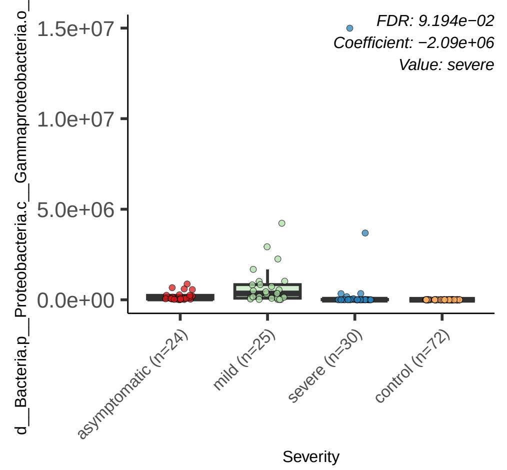

FDR: 9.194e−02
Coefficient: −2.09e+06
Value: severe
d__Bacteria.p__Proteobacteria.c__Gammaproteobacteria.o__
1.5e+07
1.0e+07
5.0e+06
0.0e+00
asymptomatic (n=24)
mild (n=25)
severe (n=30)
control (n=72)
Severity

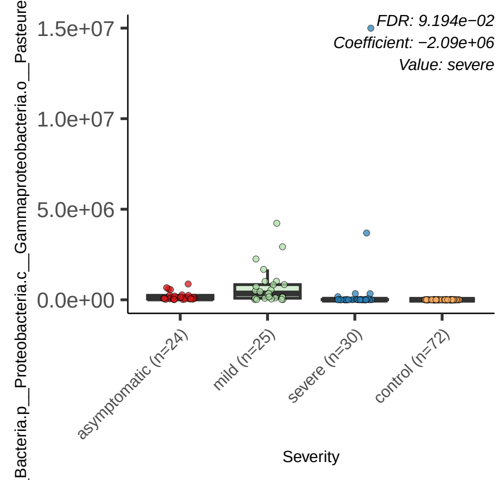
FDR: 9.194e−02
Coefficient: −2.09e+06
Value: severe
1.5e+07
1.0e+07
5.0e+06
0.0e+00
Bacteria.p__Proteobacteria.c__Gammaproteobacteria.o__Pasteure
asymptomatic (n=24)
mild (n=25)
severe (n=30)
control (n=72)
Severity

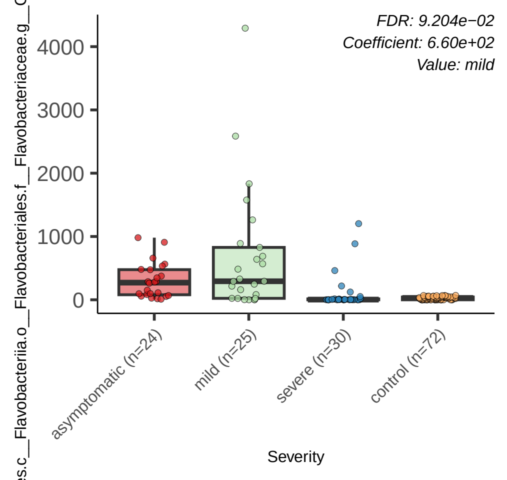
FDR: 9.204e−02
Coefficient: 6.60e+02
Value: mild
4000
3000
2000
1000
0
es.c__Flavobacteriia.o__Flavobacteriales.f__Flavobacteriaceae.g__C
asymptomatic (n=24)
mild (n=25)
severe (n=30)
control (n=72)
Severity

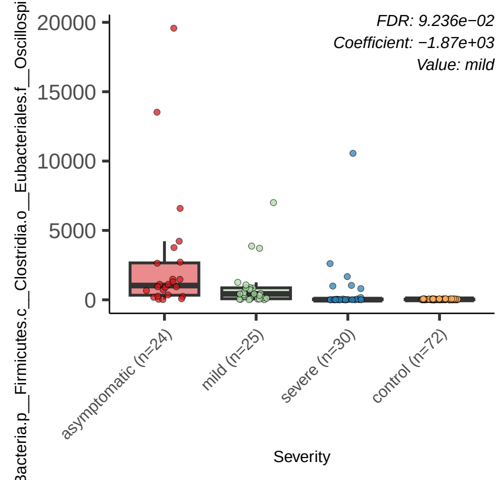

FDR: 9.236e−02
Coefficient: −1.87e+03
Value: mild
20000
15000
10000
5000
0
Bacteria.p__Firmicutes.c__Clostridia.o__Eubacteriales.f__Oscillospi
asymptomatic (n=24)
mild (n=25)
severe (n=30)
control (n=72)
Severity

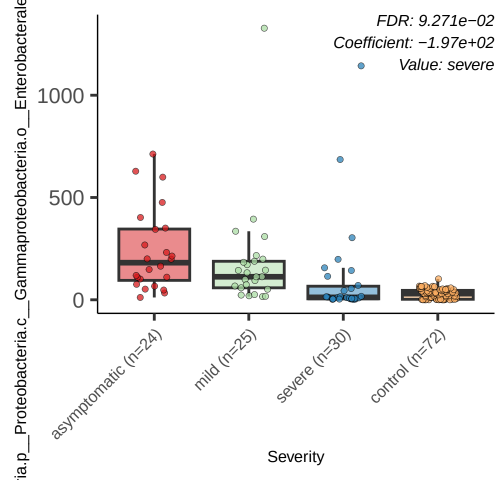
FDR: 9.271e−02
Coefficient: −1.97e+02
Value: severe
1000
500
0
ria.p__Proteobacteria.c__Gammaproteobacteria.o__Enterobacterale
asymptomatic (n=24)
mild (n=25)
severe (n=30)
control (n=72)
Severity

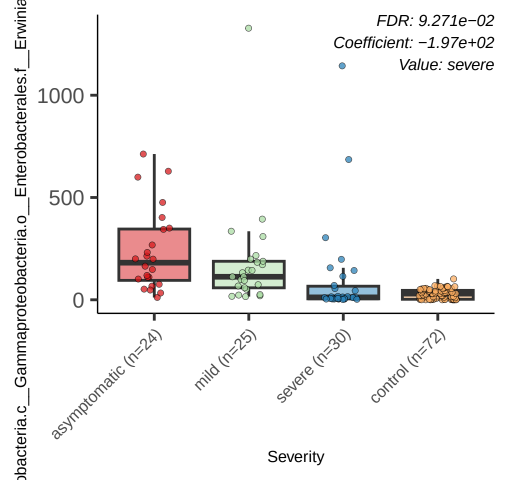

FDR: 9.271e−02
Coefficient: −1.97e+02
Value: severe
1000
500
0
obacteria.c__Gammaproteobacteria.o__Enterobacterales.f__Erwinia
asymptomatic (n=24)
mild (n=25)
severe (n=30)
control (n=72)
Severity

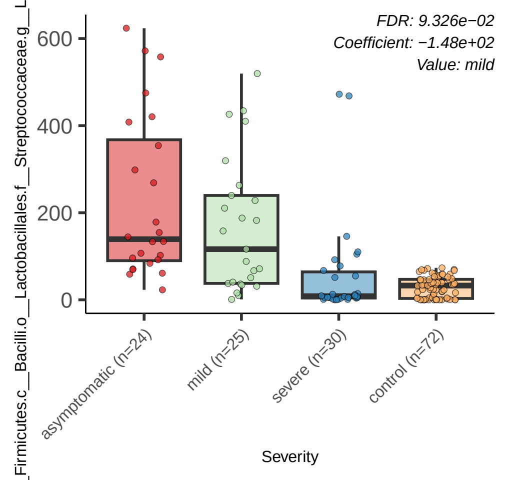

FDR: 9.326e−02
Coefficient: −1.48e+02
Value: mild
Firmicutes.c__Bacilli.o__Lactobacillales.f__Streptococcaceae.g__L
600
400
200
0
asymptomatic (n=24)
mild (n=25)
severe (n=30)
control (n=72)
Severity

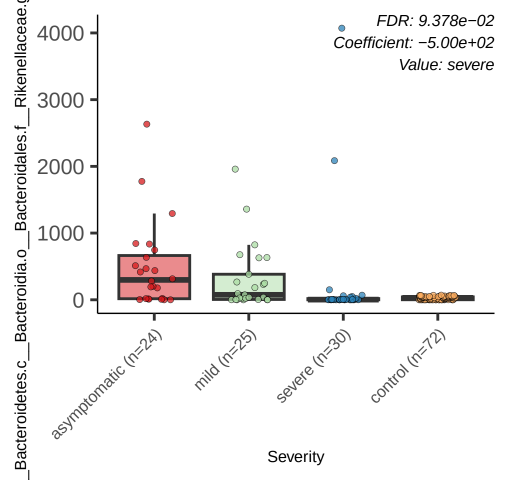

FDR: 9.378e−02
Coefficient: −5.00e+02
Value: severe
4000
3000
2000
1000
0
Bacteroidetes.c__Bacteroidia.o__Bacteroidales.f__Rikenellaceae.g
asymptomatic (n=24)
mild (n=25)
severe (n=30)
control (n=72)
Severity

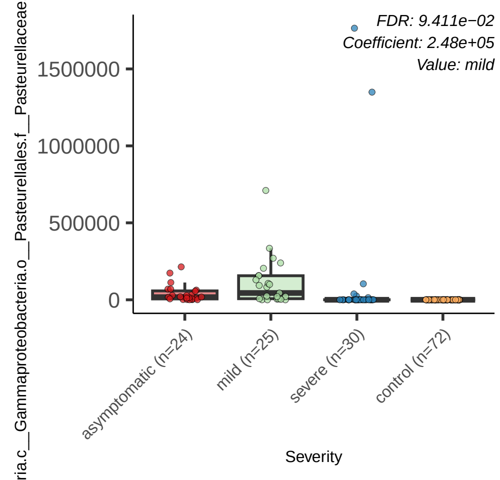
FDR: 9.411e−02
Coefficient: 2.48e+05
Value: mild
ria.c__Gammaproteobacteria.o__Pasteurellales.f__Pasteurellaceae
1500000
1000000
500000
0
asymptomatic (n=24)
mild (n=25)
severe (n=30)
control (n=72)
Severity

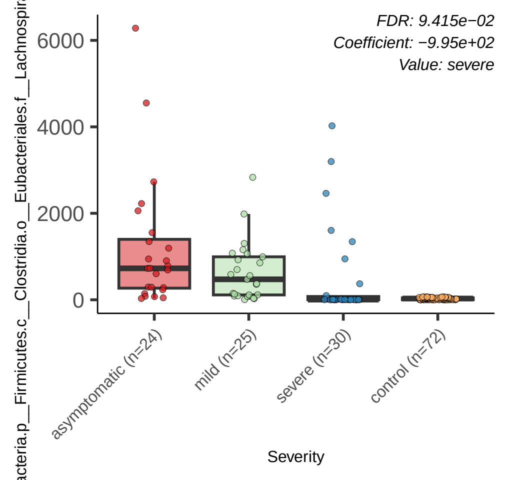

FDR: 9.415e−02
Coefficient: −9.95e+02
Value: severe
acteria.p__Firmicutes.c__Clostridia.o__Eubacteriales.f__Lachnospira
6000
4000
2000
0
asymptomatic (n=24)
mild (n=25)
severe (n=30)
control (n=72)
Severity

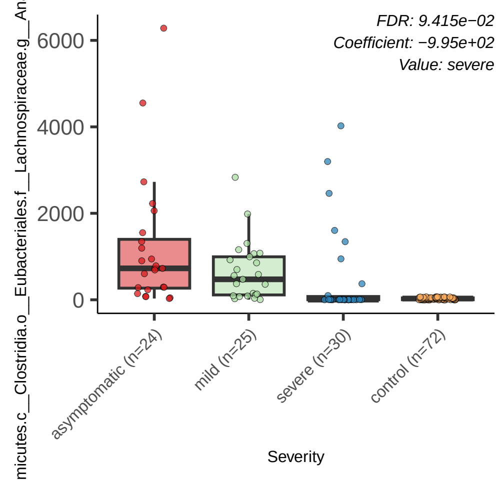

FDR: 9.415e−02
Coefficient: −9.95e+02
Value: severe
6000
4000
2000
0
micutes.c__Clostridia.o__Eubacteriales.f__Lachnospiraceae.g__An
asymptomatic (n=24)
mild (n=25)
severe (n=30)
control (n=72)
Severity

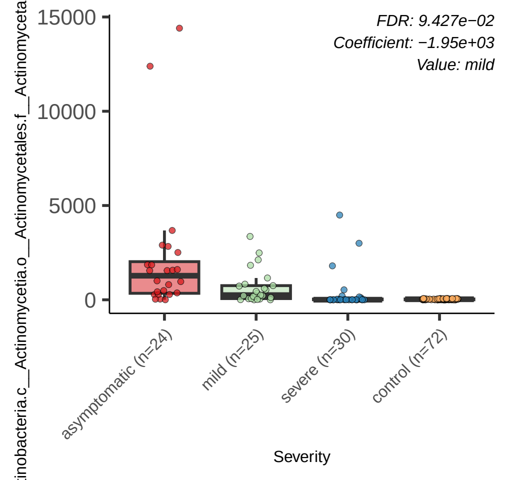

FDR: 9.427e−02
Coefficient: −1.95e+03
Value: mild
15000
10000
5000
0
inobacteria.c__Actinomycetia.o__Actinomycetales.f__Actinomyceta
asymptomatic (n=24)
mild (n=25)
severe (n=30)
control (n=72)
Severity

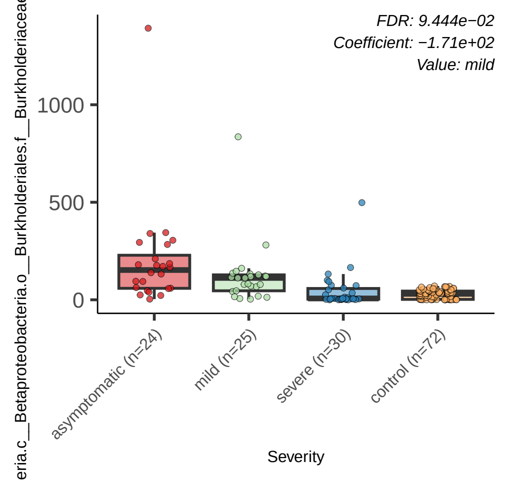
FDR: 9.444e−02
Coefficient: −1.71e+02
Value: mild
1000
500
0
eria.c__Betaproteobacteria.o__Burkholderiales.f__Burkholderiacea
asymptomatic (n=24)
mild (n=25)
severe (n=30)
control (n=72)
Severity

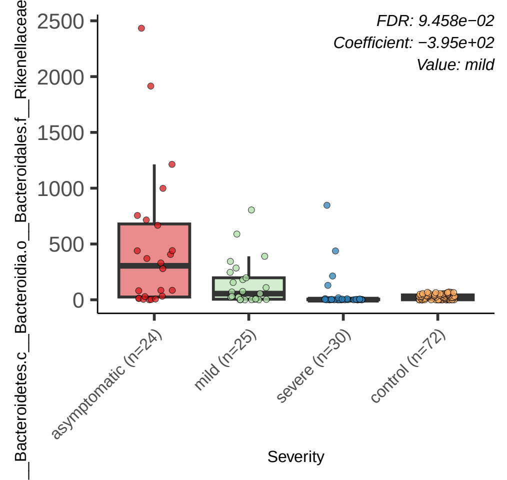
FDR: 9.458e−02
Coefficient: −3.95e+02
Value: mild
_Bacteroidetes.c__Bacteroidia.o__Bacteroidales.f__Rikenellaceae
2500
2000
1500
1000
500
0
asymptomatic (n=24)
mild (n=25)
severe (n=30)
control (n=72)
Severity

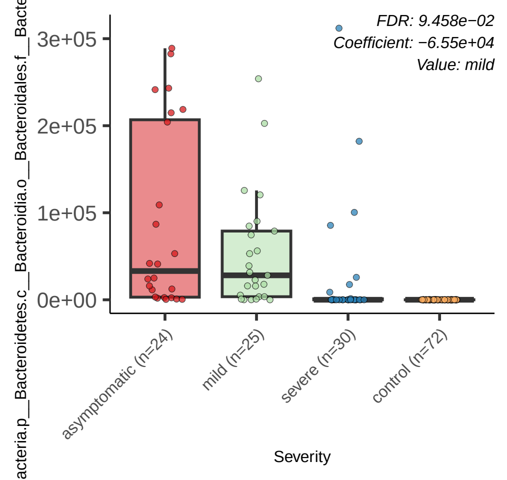
FDR: 9.458e−02
Coefficient: −6.55e+04
Value: mild
acteria.p__Bacteroidetes.c__Bacteroidia.o__Bacteroidales.f__Bacte
3e+05
2e+05
1e+05
0e+00
asymptomatic (n=24)
mild (n=25)
severe (n=30)
control (n=72)
Severity

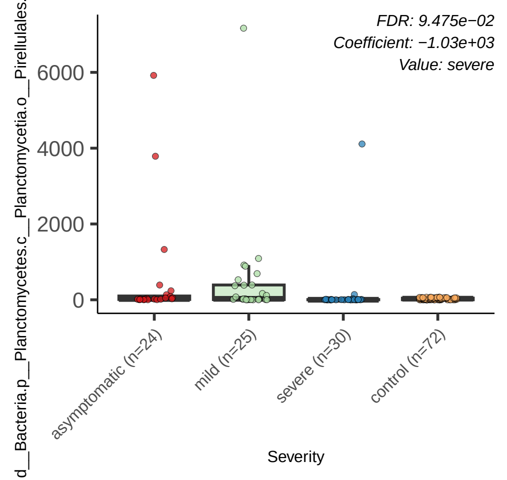

FDR: 9.475e−02
Coefficient: −1.03e+03
Value: severe
d__Bacteria.p__Planctomycetes.c__Planctomycetia.o__Pirellulales.
6000
4000
2000
0
asymptomatic (n=24)
mild (n=25)
severe (n=30)
control (n=72)
Severity

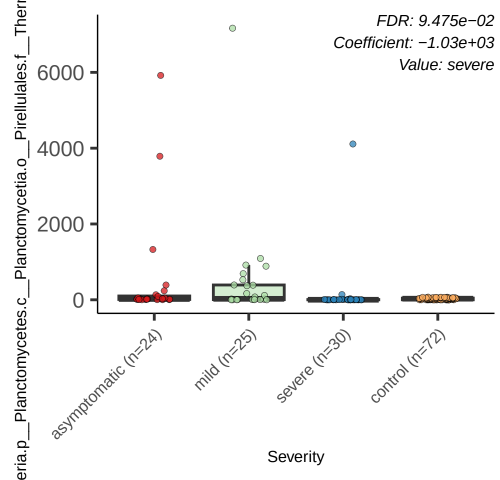

FDR: 9.475e−02
Coefficient: −1.03e+03
Value: severe
6000
4000
2000
0
asymptomatic (n=24)
mild (n=25)
severe (n=30)
control (n=72)
Severity
eria.p__Planctomycetes.c__Planctomycetia.o__Pirellulales.f__Therr

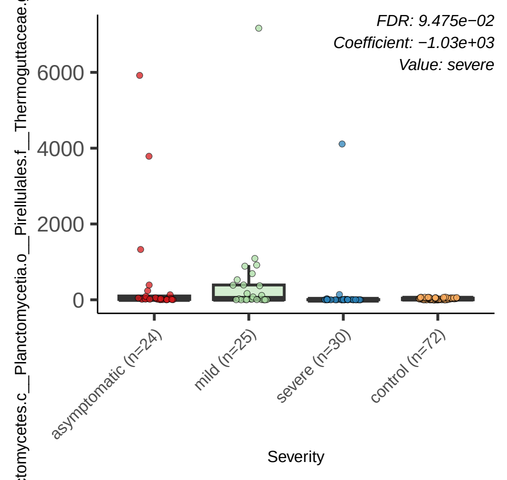

FDR: 9.475e−02
Coefficient: −1.03e+03
Value: severe
6000
4000
2000
0
ctomycetes.c__Planctomycetia.o__Pirellulales.f__Thermoguttaceae.g
asymptomatic (n=24)
mild (n=25)
severe (n=30)
control (n=72)
Severity

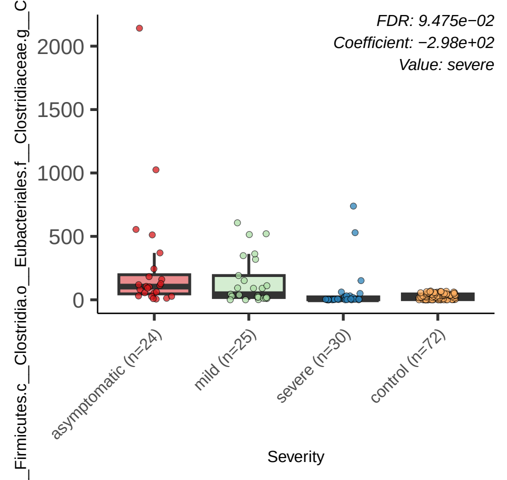
FDR: 9.475e−02
Coefficient: −2.98e+02
Value: severe
Firmicutes.c__Clostridia.o__Eubacteriales.f__Clostridiaceae.g__C
0
500
1000
1500
2000
asymptomatic (n=24)
mild (n=25)
severe (n=30)
control (n=72)
Severity

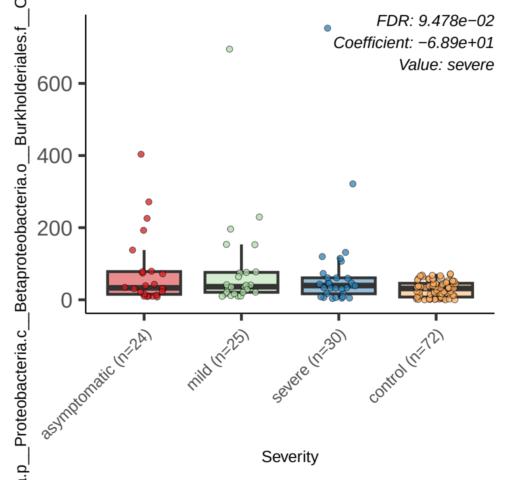

FDR: 9.478e−02
Coefficient: −6.89e+01
Value: severe
0
200
400
600
asymptomatic (n=24)
mild (n=25)
severe (n=30)
control (n=72)
Severity
Proteobacteria.c__Betaproteobacteria.o__Burkholderiales.f__

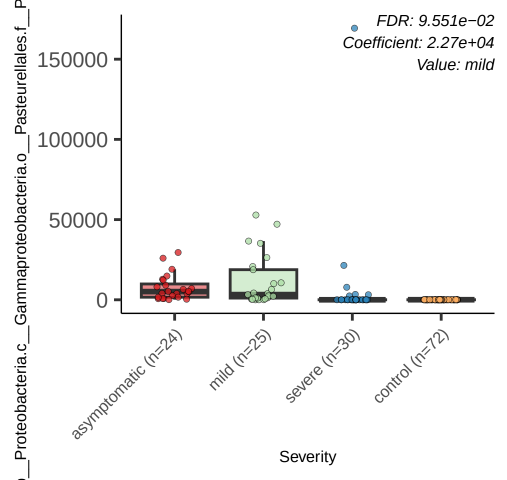
FDR: 9.551e−02
Coefficient: 2.27e+04
Value: mild
150000
100000
50000
0
asymptomatic (n=24)
mild (n=25)
severe (n=30)
control (n=72)
Severity
Proteobacteria.c__Gammaproteobacteria.o__Pasteurellales.f__P

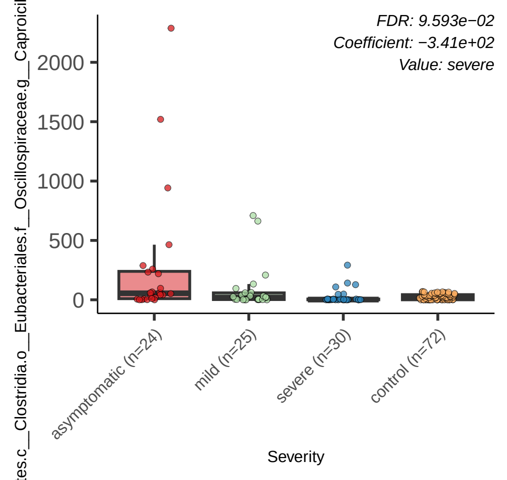

FDR: 9.593e−02
Coefficient: −3.41e+02
Value: severe
2000
1500
1000
500
0
asymptomatic (n=24)
mild (n=25)
severe (n=30)
control (n=72)
Severity
tes.c__Clostridia.o__Eubacteriales.f__Oscillospiraceae.g__Caproicib

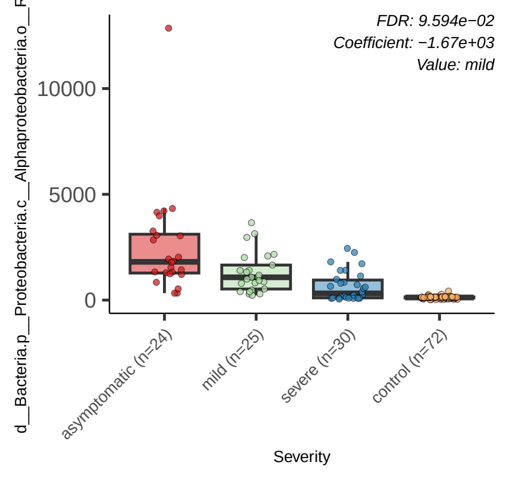
FDR: 9.594e−02
Coefficient: −1.67e+03
Value: mild
d__Bacteria.p__Proteobacteria.c__Alphaproteobacteria.o__R
10000
5000
0
asymptomatic (n=24)
mild (n=25)
severe (n=30)
control (n=72)
Severity

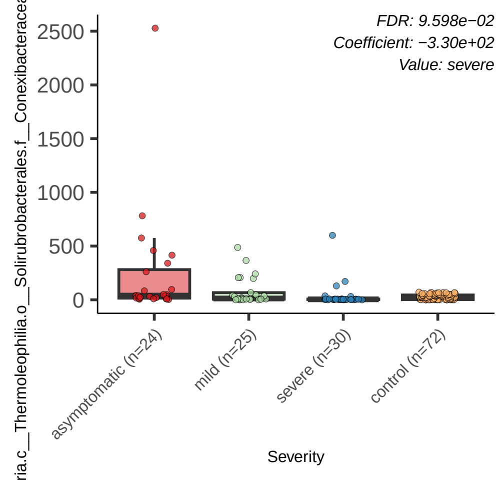

FDR: 9.598e−02
Coefficient: −3.30e+02
Value: severe
ria.c__Thermoleophilia.o__Solirubrobacterales.f__Conexibacteracea
2500
2000
1500
1000
500
0
asymptomatic (n=24)
mild (n=25)
severe (n=30)
control (n=72)
Severity

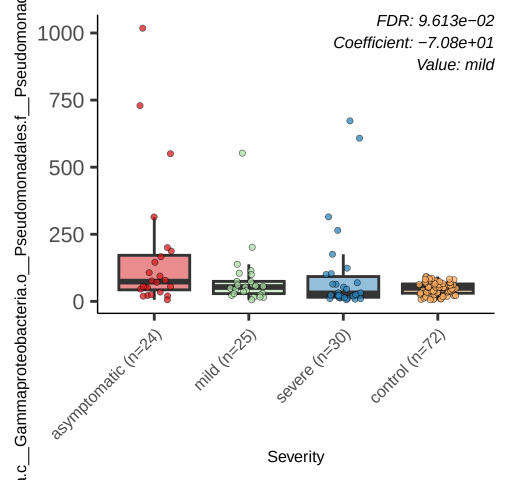

FDR: 9.613e−02
Coefficient: −7.08e+01
Value: mild
1000
750
500
250
0
asymptomatic (n=24)
mild (n=25)
severe (n=30)
control (n=72)
Severity

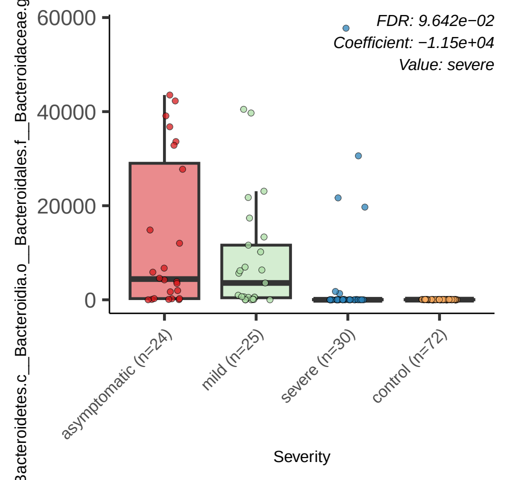
FDR: 9.642e−02
Coefficient: −1.15e+04
Value: severe
60000
40000
20000
0
asymptomatic (n=24)
mild (n=25)
severe (n=30)
control (n=72)
Severity
Bacteroidetes.c__Bacteroidia.o__Bacteroidales.f__Bacteroidaceae.g

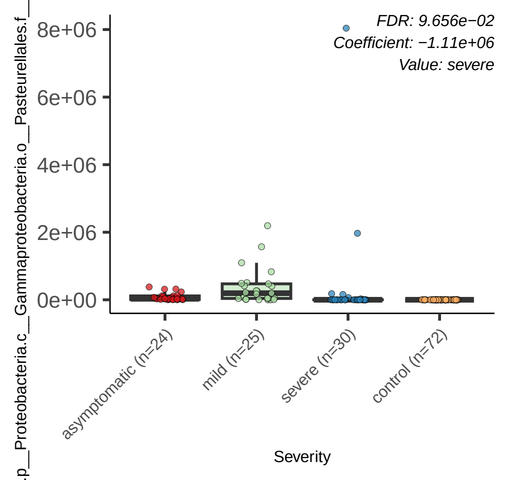
FDR: 9.656e−02
Coefficient: −1.11e+06
Value: severe
8e+06
6e+06
4e+06
2e+06
0e+00
p__Proteobacteria.c__Gammaproteobacteria.o__Pasteurellales.f
asymptomatic (n=24)
mild (n=25)
severe (n=30)
control (n=72)
Severity

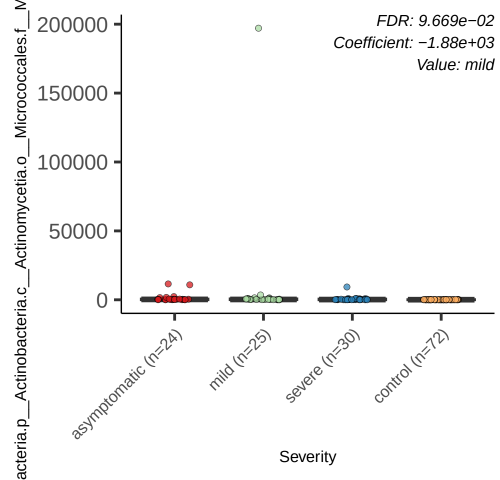

FDR: 9.669e−02
Coefficient: −1.88e+03
Value: mild
200000
150000
100000
50000
0
asymptomatic (n=24)
mild (n=25)
severe (n=30)
control (n=72)
Severity
acteria.p__Actinobacteria.c__Actinomycetia.o__Micrococcales.f__M

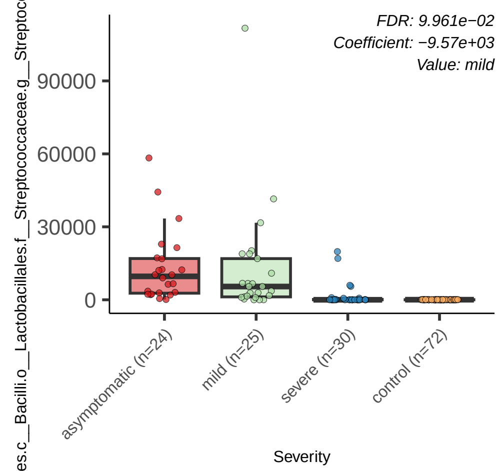

FDR: 9.961e−02
Coefficient: −9.57e+03
Value: mild
Streptococcaceae.g__Streptococ
utes.c__Bacilli.o__Lactobacillales.f__Streptococcaceae.g__Streptoc
90000
60000
30000
0
asymptomatic (n=24)
mild (n=25)
severe (n=30)
control (n=72)
Severity

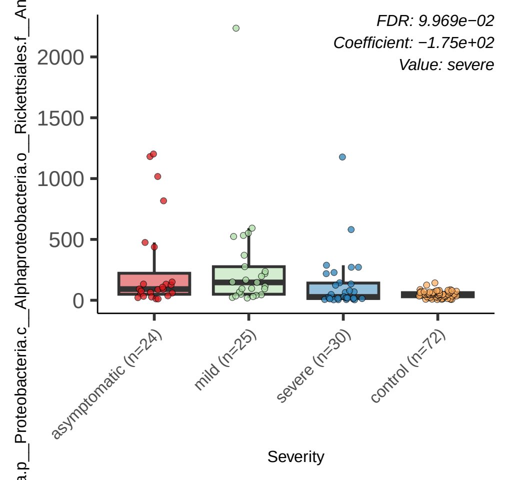
FDR: 9.969e−02
Coefficient: −1.75e+02
Value: severe
a.p__Proteobacteria.c__Alphaproteobacteria.o__Rickettsiales.f__An
2000
1500
1000
500
0
asymptomatic (n=24)
mild (n=25)
severe (n=30)
control (n=72)
Severity

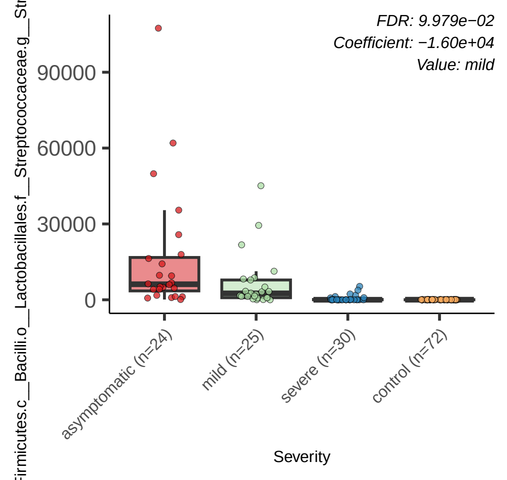

FDR: 9.979e−02
Coefficient: −1.60e+04
Value: mild
Firmicutes.c__Bacilli.o__Lactobacillales.f__Streptococcaceae.g__Str
90000
60000
30000
0
asymptomatic (n=24)
mild (n=25)
severe (n=30)
control (n=72)
Severity

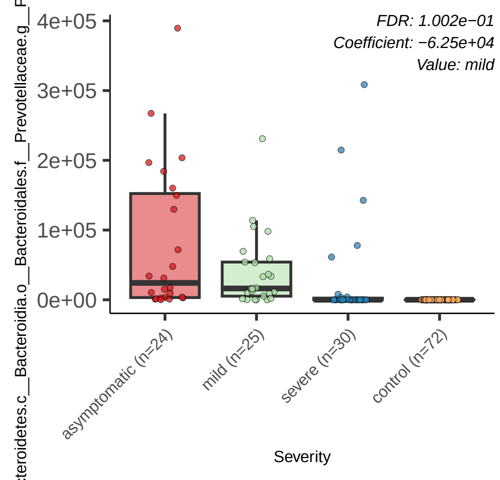

FDR: 1.002e−01
Coefficient: −6.25e+04
Value: mild
4e+05
3e+05
2e+05
1e+05
0e+00
asymptomatic (n=24)
mild (n=25)
severe (n=30)
control (n=72)
Severity

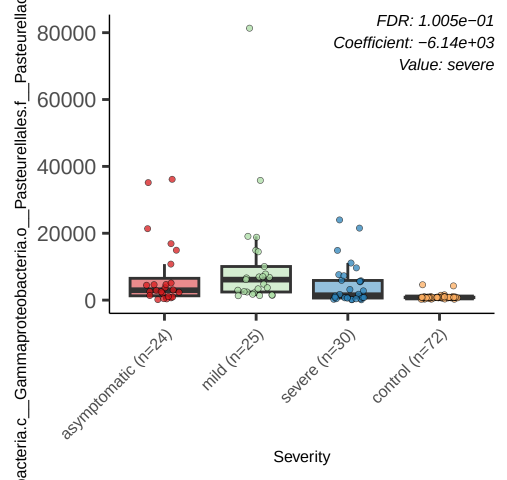

FDR: 1.005e−01
Coefficient: −6.14e+03
Value: severe
80000
60000
40000
20000
0
asymptomatic (n=24)
mild (n=25)
severe (n=30)
control (n=72)
Severity
acteria.c__Gammaproteobacteria.o__Pasteurellales.f__Pasteurellac

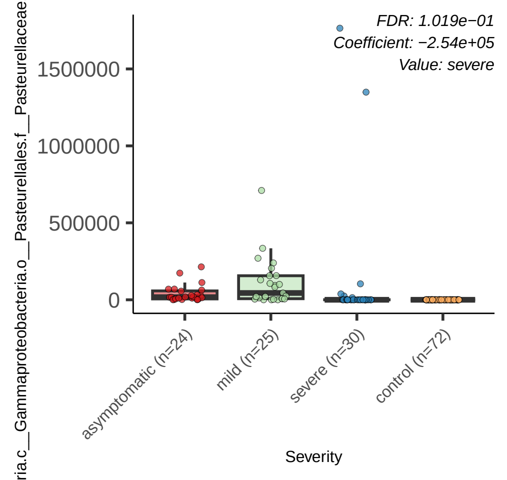
FDR: 1.019e−01
Coefficient: −2.54e+05
Value: severe
1500000
1000000
500000
0
ria.c__Gammaproteobacteria.o__Pasteurellales.f__Pasteurellaceae
asymptomatic (n=24)
mild (n=25)
severe (n=30)
control (n=72)
Severity

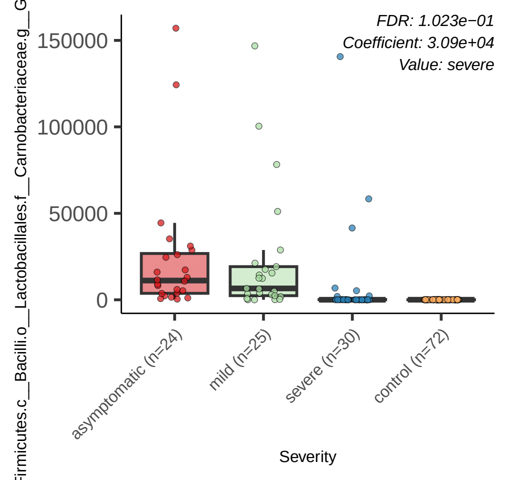
FDR: 1.023e−01
Coefficient: 3.09e+04
Value: severe
Firmicutes.c__Bacilli.o__Lactobacillales.f__Carnobacteriaceae.g__G
150000
100000
50000
0
asymptomatic (n=24)
mild (n=25)
severe (n=30)
control (n=72)
Severity

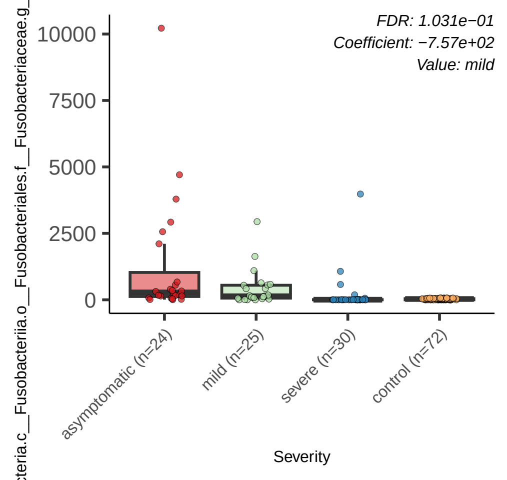

FDR: 1.031e−01
Coefficient: −7.57e+02
Value: mild
10000
7500
5000
2500
0
cteria.c__Fusobacteriia.o__Fusobacteriales.f__Fusobacteriaceae.g__
asymptomatic (n=24)
mild (n=25)
severe (n=30)
control (n=72)
Severity

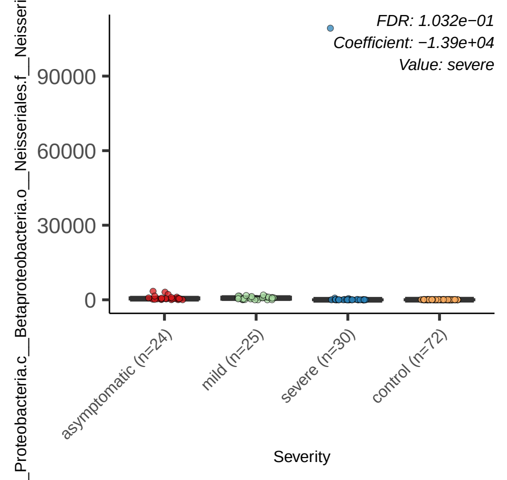
FDR: 1.032e−01
Coefficient: −1.39e+04
Value: severe
Proteobacteria.c__Betaproteobacteria.o__Neisseriales.f__Neisseri
90000
60000
30000
0
asymptomatic (n=24)
mild (n=25)
severe (n=30)
control (n=72)
Severity

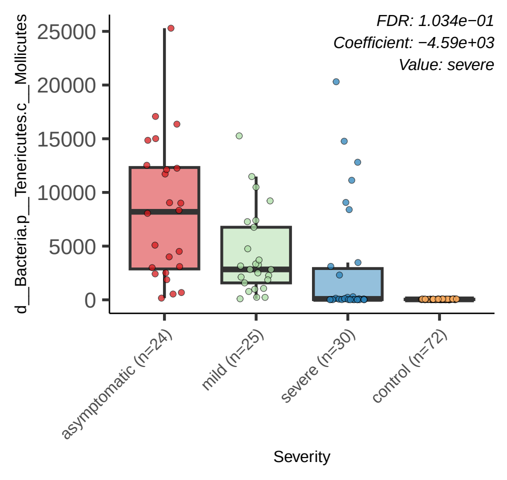

FDR: 1.034e−01
Coefficient: −4.59e+03
Value: severe
d__Bacteria.p__Tenericutes.c__Mollicutes
25000
20000
15000
10000
5000
0
asymptomatic (n=24)
mild (n=25)
severe (n=30)
control (n=72)
Severity

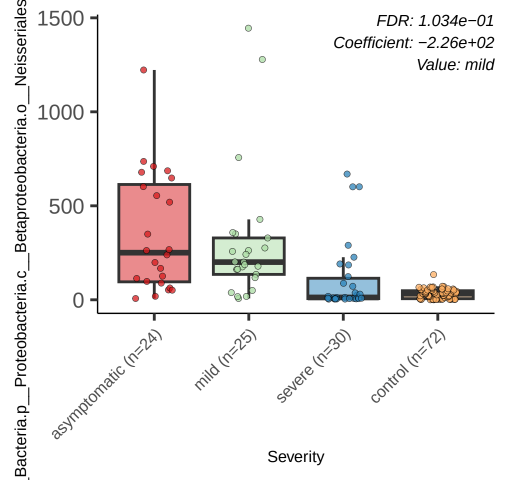

FDR: 1.034e−01
Coefficient: −2.26e+02
Value: mild
1500
1000
500
0
Bacteria.p__Proteobacteria.c__Betaproteobacteria.o__Neisseriales
asymptomatic (n=24)
mild (n=25)
severe (n=30)
control (n=72)
Severity

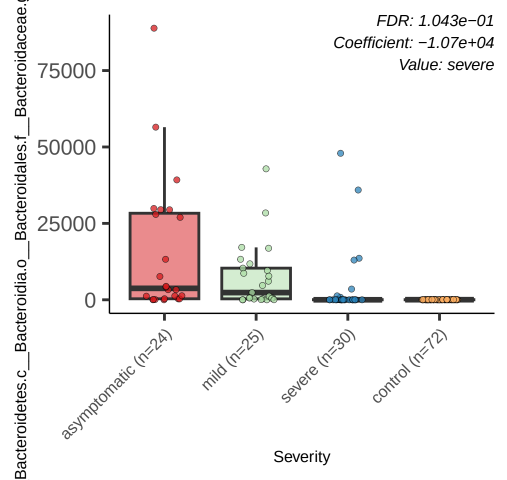
FDR: 1.043e−01
Coefficient: −1.07e+04
Value: severe
75000
50000
25000
0
Bacteroidetes.c__Bacteroidia.o__Bacteroidales.f__Bacteroidaceae.g
asymptomatic (n=24)
mild (n=25)
severe (n=30)
control (n=72)
Severity

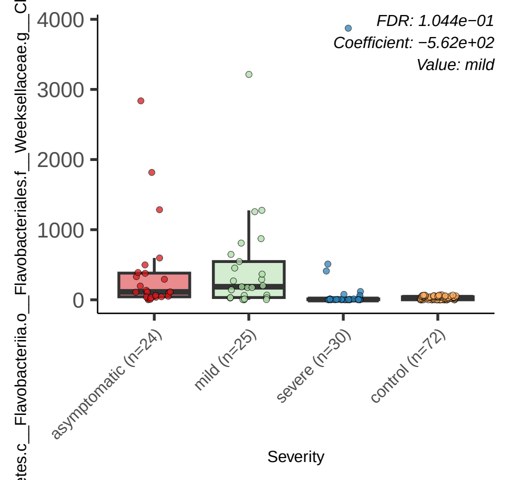

FDR: 1.044e−01
Coefficient: −5.62e+02
Value: mild
4000
3000
2000
1000
0
asymptomatic (n=24)
mild (n=25)
severe (n=30)
control (n=72)
Severity
etes.c__Flavobacteriia.o__Flavobacteriales.f__Weeksellaceae.g__Cl

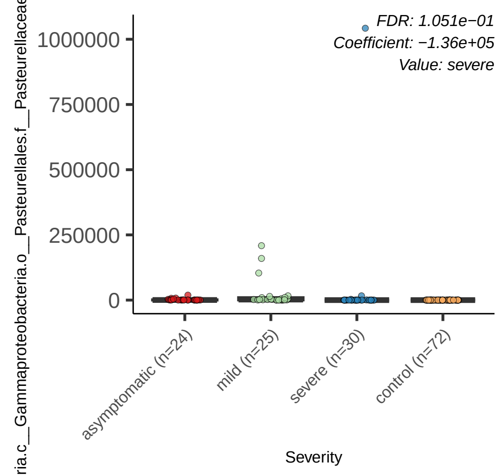

FDR: 1.051e−01
Coefficient: −1.36e+05
Value: severe
1000000
750000
500000
250000
0
asymptomatic (n=24)
mild (n=25)
severe (n=30)
control (n=72)
Severity
eria.c__Gammaproteobacteria.o__Pasteurellales.f__Pasteurellaceae

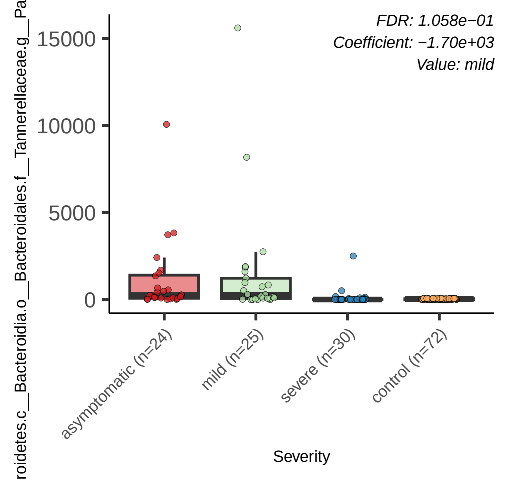

FDR: 1.058e−01
Coefficient: −1.70e+03
Value: mild
15000
10000
5000
0
roidetes.c__Bacteroidia.o__Bacteroidales.f__Tannerellaceae.g__Pa
asymptomatic (n=24)
mild (n=25)
severe (n=30)
control (n=72)
Severity

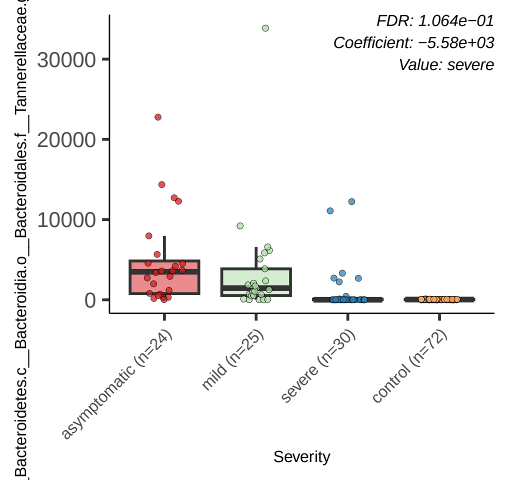

FDR: 1.064e−01
Coefficient: −5.58e+03
Value: severe
Bacteroidetes.c__Bacteroidia.o__Bacteroidales.f__Tannerellaceae.g
30000
20000
10000
0
asymptomatic (n=24)
mild (n=25)
severe (n=30)
control (n=72)
Severity

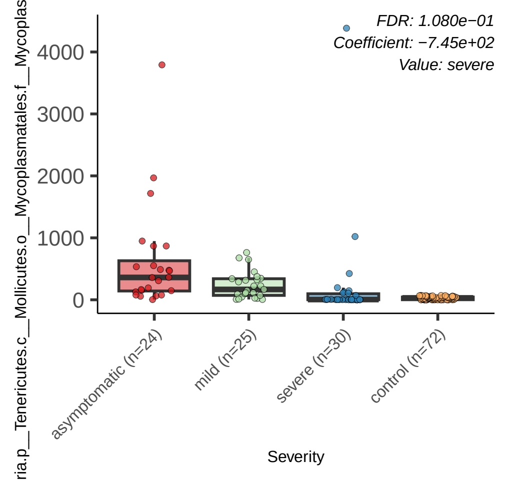

FDR: 1.080e−01
Coefficient: −7.45e+02
Value: severe
ria.p__Tenericutes.c__Mollicutes.o__Mycoplasmatales.f__Mycoplas
4000
3000
2000
1000
0
asymptomatic (n=24)
mild (n=25)
severe (n=30)
control (n=72)
Severity

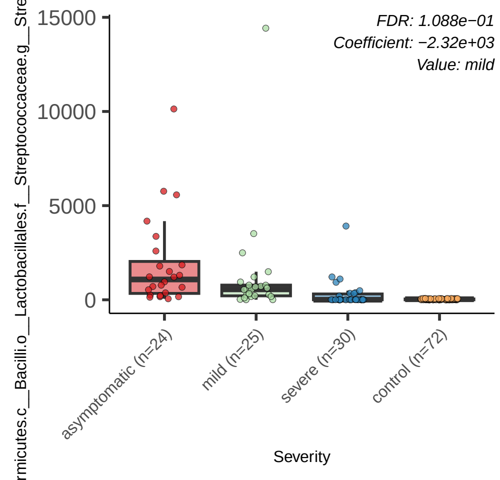

FDR: 1.088e−01
Coefficient: −2.32e+03
Value: mild
15000
10000
5000
0
rmicutes.c__Bacilli.o__Lactobacillales.f__Streptococcaceae.g__Stre
asymptomatic (n=24)
mild (n=25)
severe (n=30)
control (n=72)
Severity

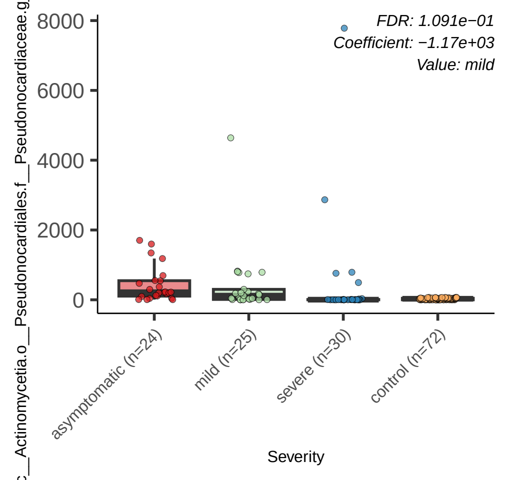

FDR: 1.091e−01
Coefficient: −1.17e+03
Value: mild
c__Actinomycetia.o__Pseudonocardiales.f__Pseudonocardiaceae.g
8000
6000
4000
2000
0
asymptomatic (n=24)
mild (n=25)
severe (n=30)
control (n=72)
Severity

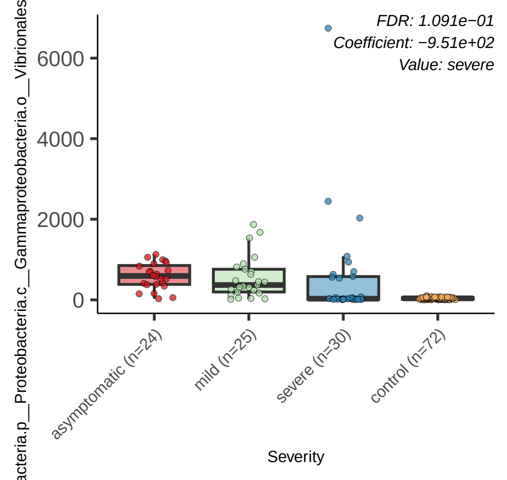
FDR: 1.091e−01
Coefficient: −9.51e+02
Value: severe
6000
4000
2000
0
acteria.p__Proteobacteria.c__Gammaproteobacteria.o__Vibrionales
asymptomatic (n=24)
mild (n=25)
severe (n=30)
control (n=72)
Severity

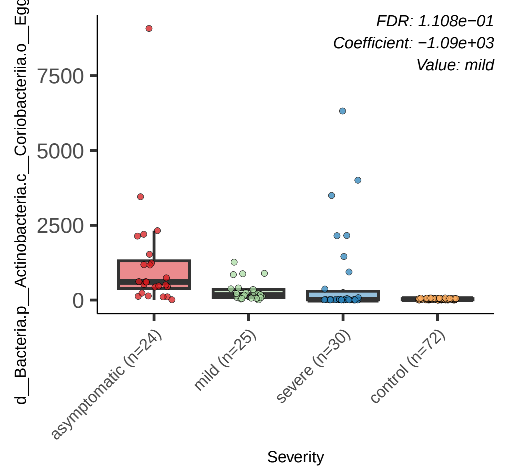
FDR: 1.108e−01
Coefficient: −1.09e+03
Value: mild
d__Bacteria.p__Actinobacteria.c__Coriobacteriia.o__Egg
7500
5000
2500
0
asymptomatic (n=24)
mild (n=25)
severe (n=30)
control (n=72)
Severity

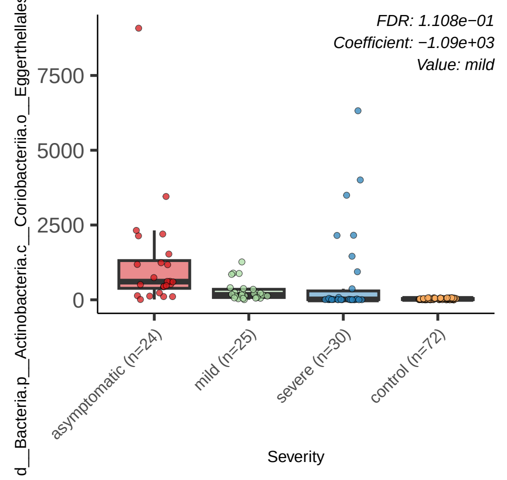
FDR: 1.108e−01
Coefficient: −1.09e+03
Value: mild
d__Bacteria.p__Actinobacteria.c__Coriobacteriia.o__Eggerthellales
7500
5000
2500
0
asymptomatic (n=24)
mild (n=25)
severe (n=30)
control (n=72)
Severity

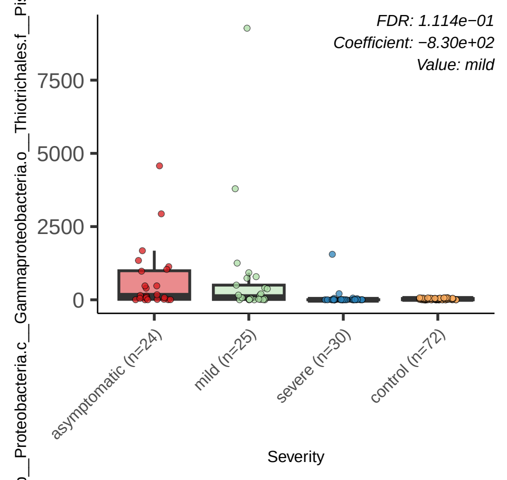

FDR: 1.114e−01
Coefficient: −8.30e+02
Value: mild
0
2500
5000
7500
o__Proteobacteria.c__Gammaproteobacteria.o__Thiotrichales.f__Pis
asymptomatic (n=24)
mild (n=25)
severe (n=30)
control (n=72)
Severity

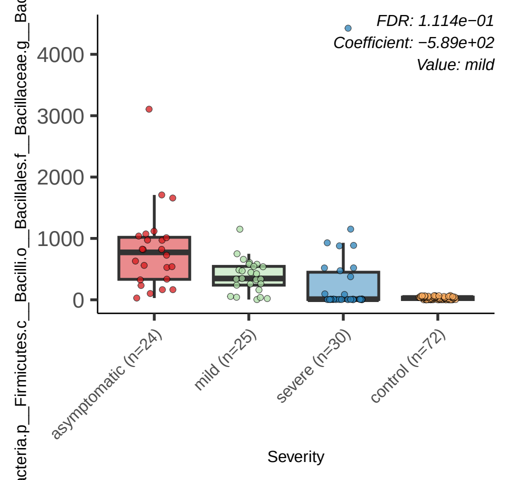
FDR: 1.114e−01
Coefficient: −5.89e+02
Value: mild
0
1000
2000
3000
4000
asymptomatic (n=24)
mild (n=25)
severe (n=30)
control (n=72)
Severity

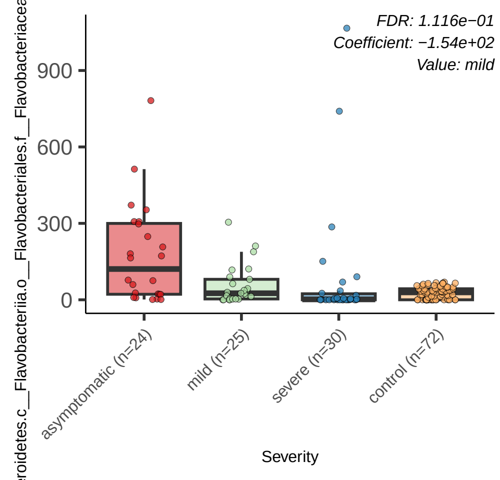
FDR: 1.116e−01
Coefficient: −1.54e+02
Value: mild
900
600
300
0
eroidetes.c__Flavobacteriia.o__Flavobacteriales.f__Flavobacteriacea
asymptomatic (n=24)
mild (n=25)
severe (n=30)
control (n=72)
Severity

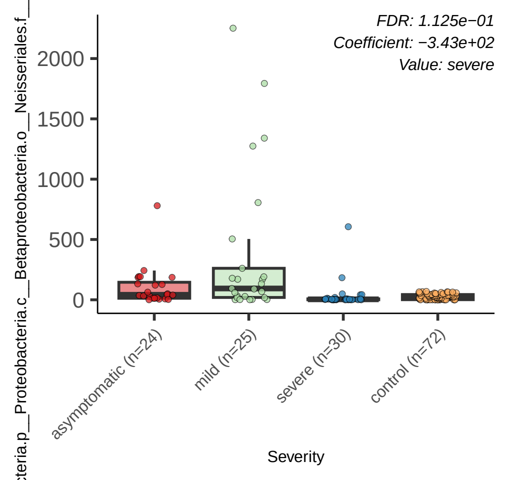

FDR: 1.125e−01
Coefficient: −3.43e+02
Value: severe
0
500
1000
1500
2000
cteria.p__Proteobacteria.c__Betaproteobacteria.o__Neisseriales.f__
asymptomatic (n=24)
mild (n=25)
severe (n=30)
control (n=72)
Severity

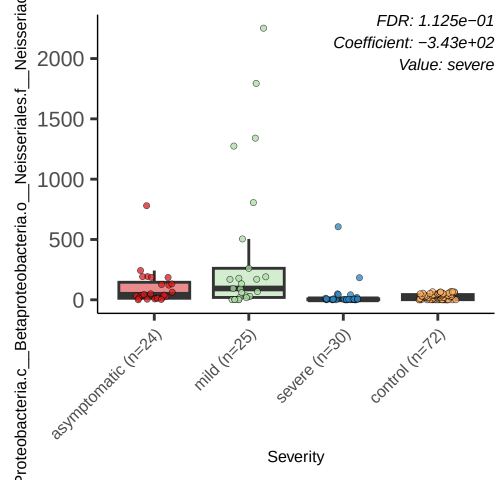
FDR: 1.125e−01
Coefficient: −3.43e+02
Value: severe
Proteobacteria.c__Betaproteobacteria.o__Neisseriales.f__Neisseriac
2000
1500
1000
500
0
asymptomatic (n=24)
mild (n=25)
severe (n=30)
control (n=72)
Severity

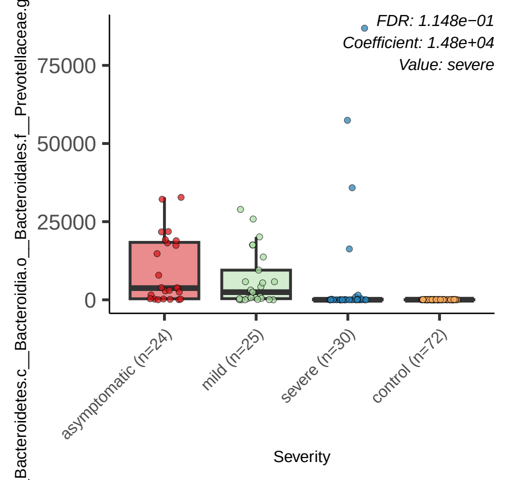

FDR: 1.148e−01
Coefficient: 1.48e+04
Value: severe
Bacteroidetes.c__Bacteroidia.o__Bacteroidales.f__Prevotellaceae.g
75000
50000
25000
0
asymptomatic (n=24)
mild (n=25)
severe (n=30)
control (n=72)
Severity

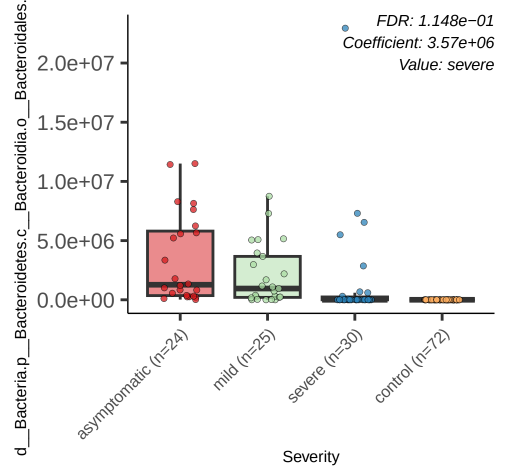

d__Bacteria.p__Bacteroidetes.c__Bacteroidia.o__Bacteroidales.
FDR: 1.148e−01
Coefficient: 3.57e+06
Value: severe
2.0e+07
1.5e+07
1.0e+07
5.0e+06
0.0e+00
asymptomatic (n=24)
mild (n=25)
severe (n=30)
control (n=72)
Severity

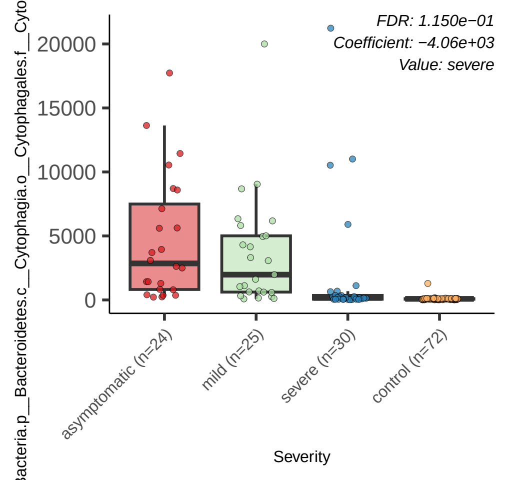
FDR: 1.150e−01
Coefficient: −4.06e+03
Value: severe
Bacteria.p__Bacteroidetes.c__Cytophagia.o__Cytophagales.f__Cyto
20000
15000
10000
5000
0
asymptomatic (n=24)
mild (n=25)
severe (n=30)
control (n=72)
Severity

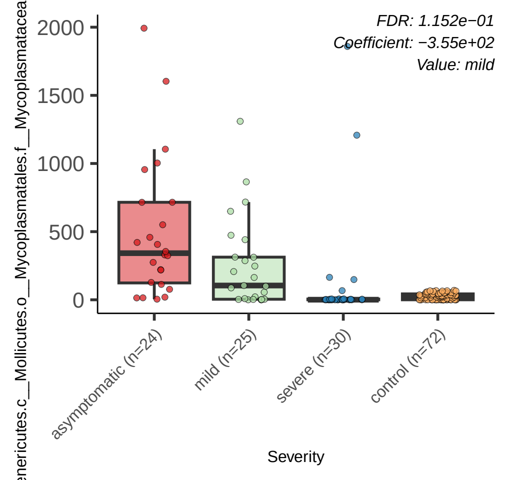
FDR: 1.152e−01
Coefficient: −3.55e+02
Value: mild
enericutes.c__Mollicutes.o__Mycoplasmatales.f__Mycoplasmatacea
2000
1500
1000
500
0
asymptomatic (n=24)
mild (n=25)
severe (n=30)
control (n=72)
Severity

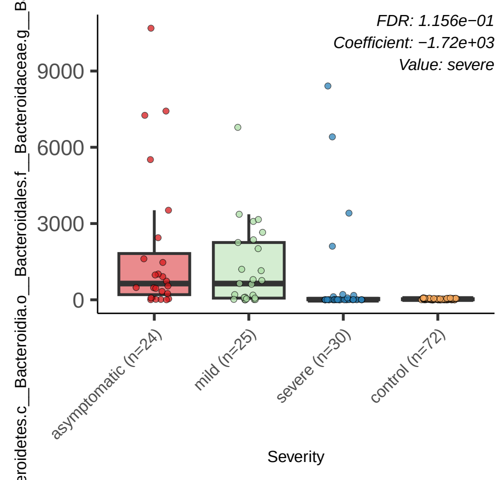

FDR: 1.156e−01
Coefficient: −1.72e+03
Value: severe
9000
6000
3000
0
eroidetes.c__Bacteroidia.o__Bacteroidales.f__Bacteroidaceae.g__B
asymptomatic (n=24)
mild (n=25)
severe (n=30)
control (n=72)
Severity

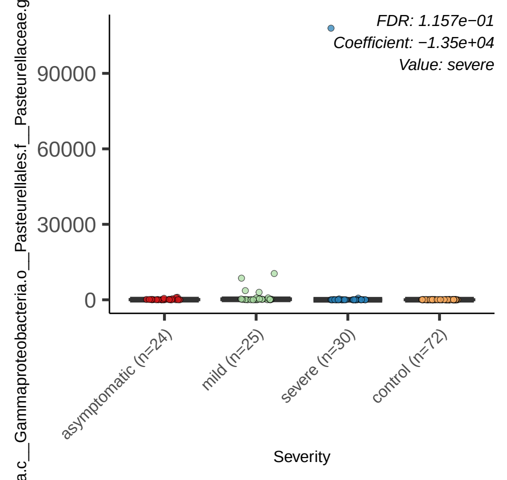

FDR: 1.157e−01
Coefficient: −1.35e+04
Value: severe
90000
60000
30000
0
a.c__Gammaproteobacteria.o__Pasteurellales.f__Pasteurellaceae.g
asymptomatic (n=24)
mild (n=25)
severe (n=30)
control (n=72)
Severity

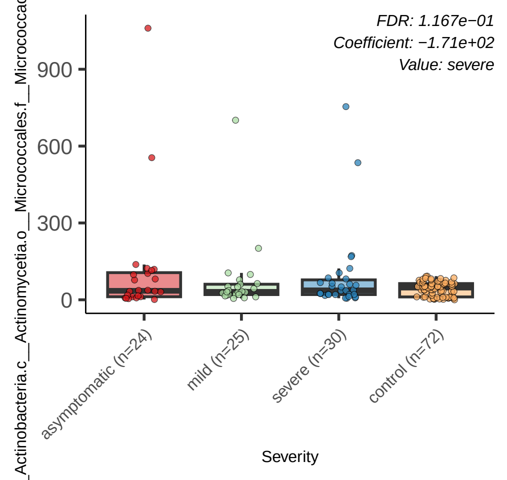

FDR: 1.167e−01
Coefficient: −1.71e+02
Value: severe
Actinobacteria.c__Actinomycetia.o__Micrococcales.f__Micrococcac
900
600
300
0
asymptomatic (n=24)
mild (n=25)
severe (n=30)
control (n=72)
Severity

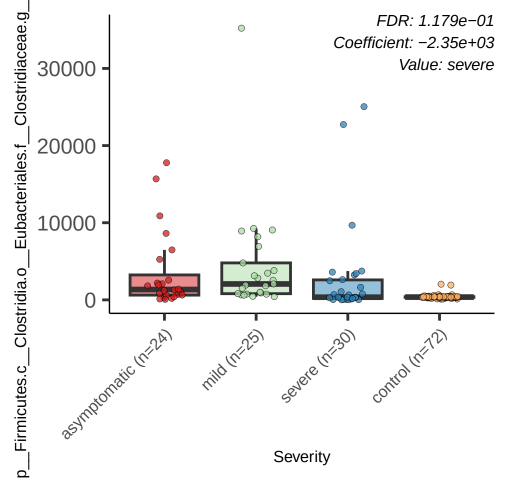

FDR: 1.179e−01
Coefficient: −2.35e+03
Value: severe
p__Firmicutes.c__Clostridia.o__Eubacteriales.f__Clostridiaceae.g__
30000
20000
10000
0
asymptomatic (n=24)
mild (n=25)
severe (n=30)
control (n=72)
Severity

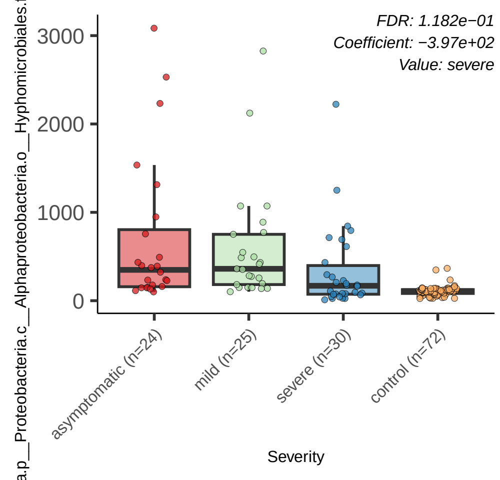

FDR: 1.182e−01
Coefficient: −3.97e+02
Value: severe
3000
2000
1000
0
a.p__Proteobacteria.c__Alphaproteobacteria.o__Hyphomicrobiales.f
asymptomatic (n=24)
mild (n=25)
severe (n=30)
control (n=72)
Severity

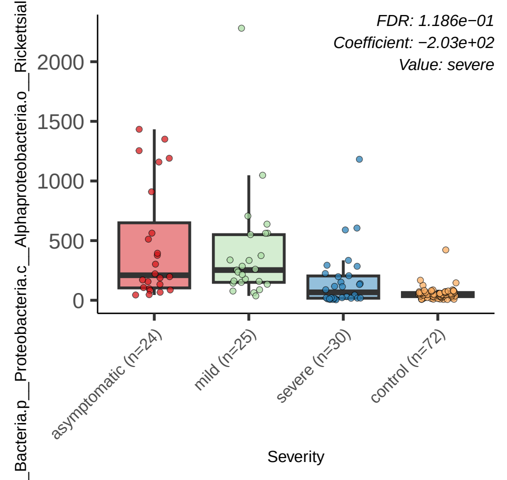

FDR: 1.186e−01
Coefficient: −2.03e+02
Value: severe
2000
1500
1000
500
0
_Bacteria.p__Proteobacteria.c__Alphaproteobacteria.o__Rickettsial
asymptomatic (n=24)
mild (n=25)
severe (n=30)
control (n=72)
Severity

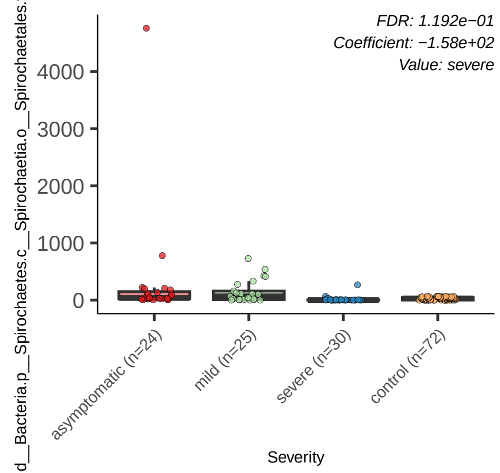

FDR: 1.192e−01
Coefficient: −1.58e+02
Value: severe
d__Bacteria.p__Spirochaetes.c__Spirochaetia.o__Spirochaetales.
4000
3000
2000
1000
0
asymptomatic (n=24)
mild (n=25)
severe (n=30)
control (n=72)
Severity

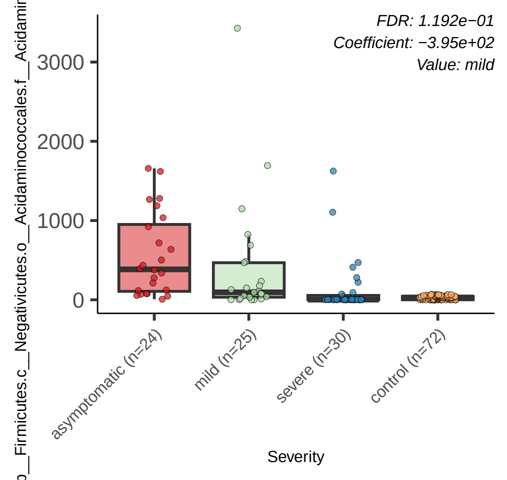
FDR: 1.192e−01
Coefficient: −3.95e+02
Value: mild
o__Firmicutes.c__Negativicutes.o__Acidaminococcales.f__Acidami
0
1000
2000
3000
asymptomatic (n=24)
mild (n=25)
severe (n=30)
control (n=72)
Severity

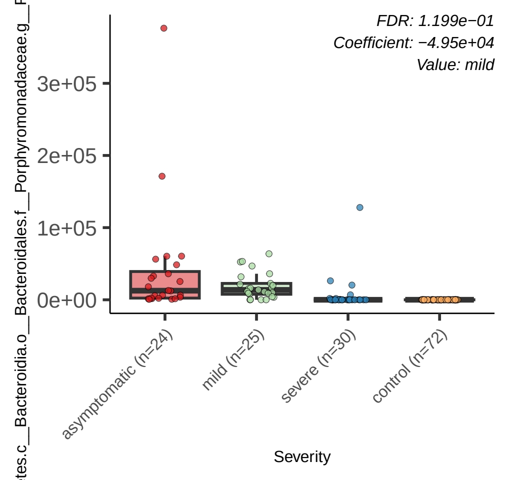
FDR: 1.199e−01
Coefficient: −4.95e+04
Value: mild
3e+05
2e+05
1e+05
0e+00
asymptomatic (n=24)
mild (n=25)
severe (n=30)
control (n=72)
Severity
tes.c__Bacteroidia.o__Bacteroidales.f__Porphyromonadaceae.g__P

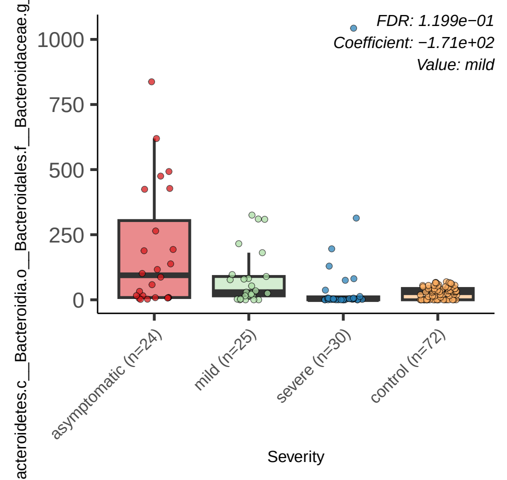

FDR: 1.199e−01
Coefficient: −1.71e+02
Value: mild
1000
750
500
250
0
acteroidetes.c__Bacteroidia.o__Bacteroidales.f__Bacteroidaceae.g_
asymptomatic (n=24)
mild (n=25)
severe (n=30)
control (n=72)
Severity

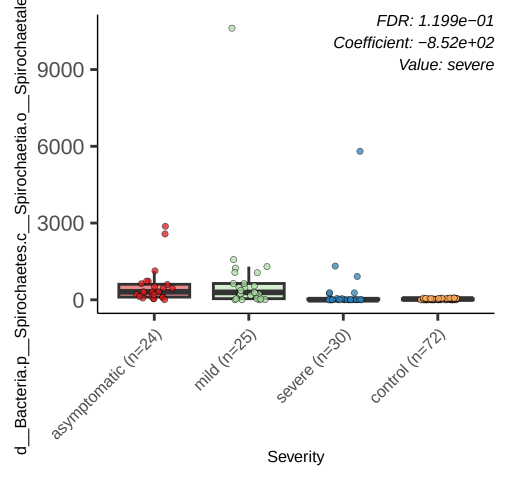

FDR: 1.199e−01
Coefficient: −8.52e+02
Value: severe
9000
6000
3000
0
d__Bacteria.p__Spirochaetes.c__Spirochaetia.o__Spirochaetale
asymptomatic (n=24)
mild (n=25)
severe (n=30)
control (n=72)
Severity

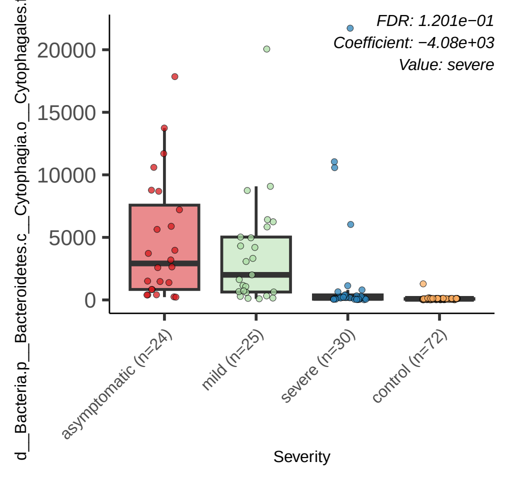
FDR: 1.201e−01
Coefficient: −4.08e+03
Value: severe
d__Bacteria.p__Bacteroidetes.c__Cytophagia.o__Cytophagales.f
20000
15000
10000
5000
0
asymptomatic (n=24)
mild (n=25)
severe (n=30)
control (n=72)
Severity

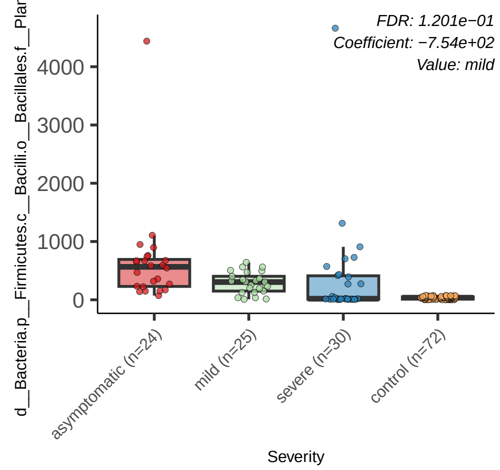

FDR: 1.201e−01
Coefficient: −7.54e+02
Value: mild
d__Bacteria.p__Firmicutes.c__Bacilli.o__Bacillales.f__Plar
0
1000
2000
3000
4000
asymptomatic (n=24)
mild (n=25)
severe (n=30)
control (n=72)
Severity

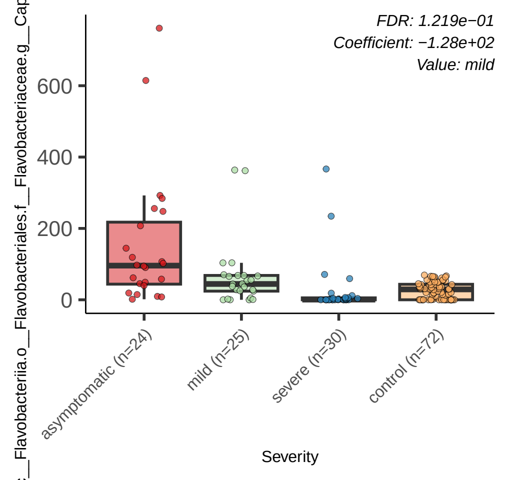

FDR: 1.219e−01
Coefficient: −1.28e+02
Value: mild
600
400
200
0
Flavobacteriia.o__Flavobacteriales.f__Flavobacteriaceae.g__Cap
asymptomatic (n=24)
mild (n=25)
severe (n=30)
control (n=72)
Severity

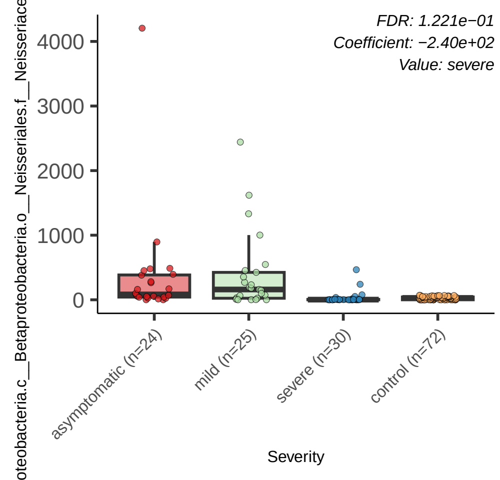

FDR: 1.221e−01
Coefficient: −2.40e+02
Value: severe
4000
3000
2000
1000
0
oteobacteria.c__Betaproteobacteria.o__Neisseriales.f__Neisseriace
asymptomatic (n=24)
mild (n=25)
severe (n=30)
control (n=72)
Severity

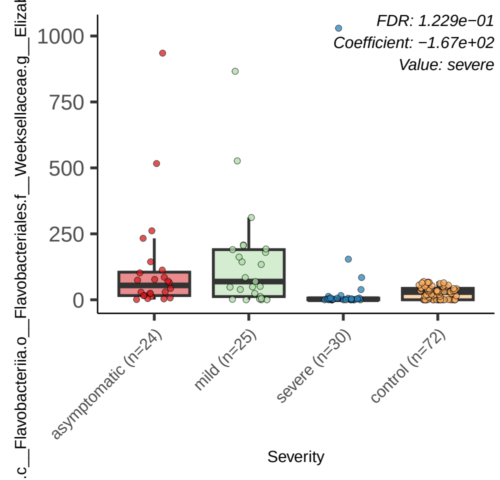
FDR: 1.229e−01
Coefficient: −1.67e+02
Value: severe
.c__Flavobacteriia.o__Flavobacteriales.f__Weeksellaceae.g__Elizab
1000
750
500
250
0
asymptomatic (n=24)
mild (n=25)
severe (n=30)
control (n=72)
Severity

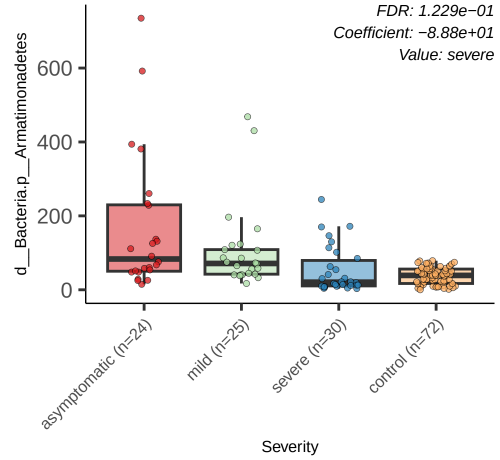

FDR: 1.229e−01
Coefficient: −8.88e+01
Value: severe
d__Bacteria.p__Armatimonadetes
0
200
400
600
asymptomatic (n=24)
mild (n=25)
severe (n=30)
control (n=72)
Severity

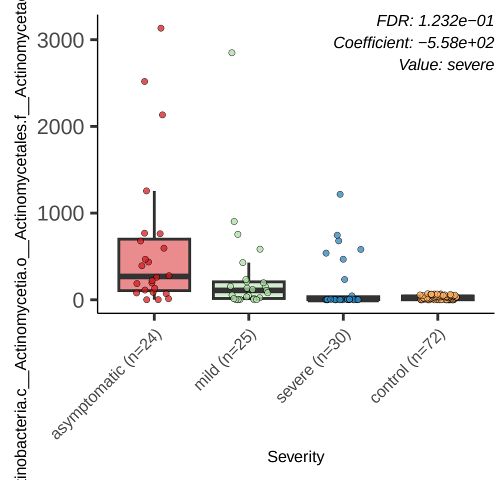

FDR: 1.232e−01
Coefficient: −5.58e+02
Value: severe
3000
2000
1000
0
asymptomatic (n=24)
mild (n=25)
severe (n=30)
control (n=72)
Severity

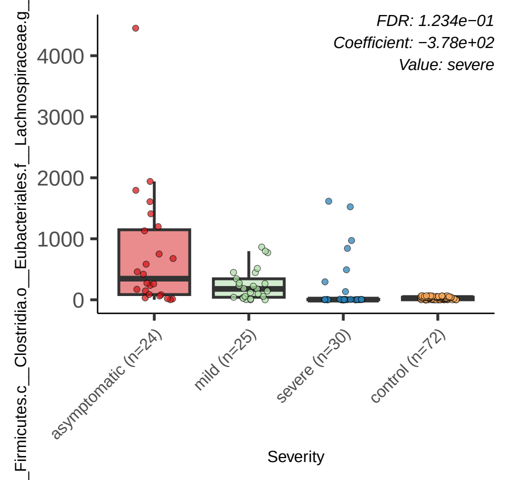

FDR: 1.234e−01
Coefficient: −3.78e+02
Value: severe
Firmicutes.c__Clostridia.o__Eubacteriales.f__Lachnospiraceae.g__
0
1000
2000
3000
4000
asymptomatic (n=24)
mild (n=25)
severe (n=30)
control (n=72)
Severity

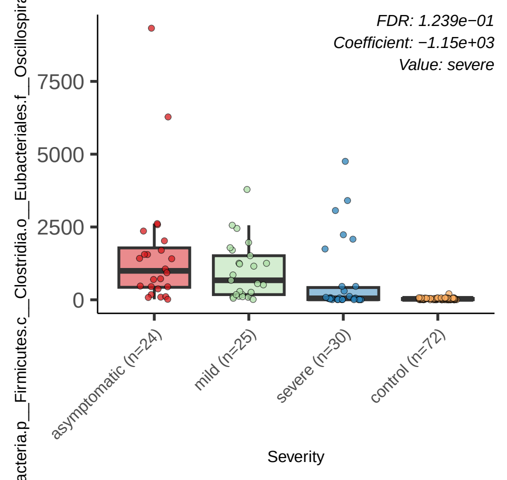

FDR: 1.239e−01
Coefficient: −1.15e+03
Value: severe
acteria.p__Firmicutes.c__Clostridia.o__Eubacteriales.f__Oscillospira
7500
5000
2500
0
asymptomatic (n=24)
mild (n=25)
severe (n=30)
control (n=72)
Severity

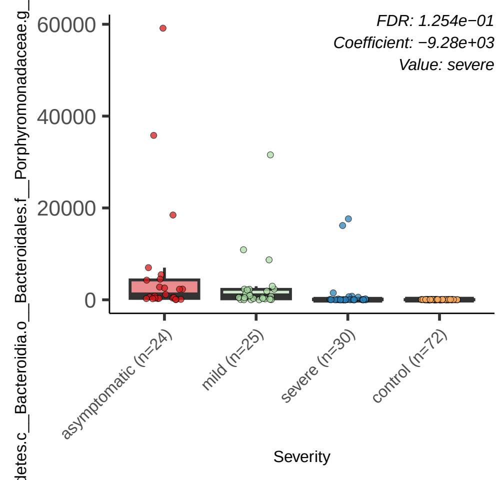
FDR: 1.254e−01
Coefficient: −9.28e+03
Value: severe
detes.c__Bacteroidia.o__Bacteroidales.f__Porphyromonadaceae.g__
60000
40000
20000
0
asymptomatic (n=24)
mild (n=25)
severe (n=30)
control (n=72)
Severity

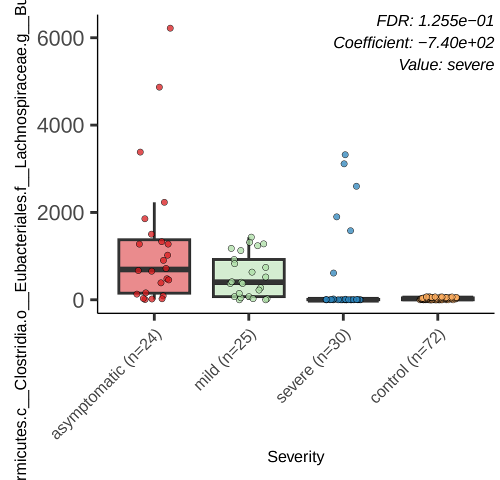

FDR: 1.255e−01
Coefficient: −7.40e+02
Value: severe
rmicutes.c__Clostridia.o__Eubacteriales.f__Lachnospiraceae.g__Bu
6000
4000
2000
0
asymptomatic (n=24)
mild (n=25)
severe (n=30)
control (n=72)
Severity

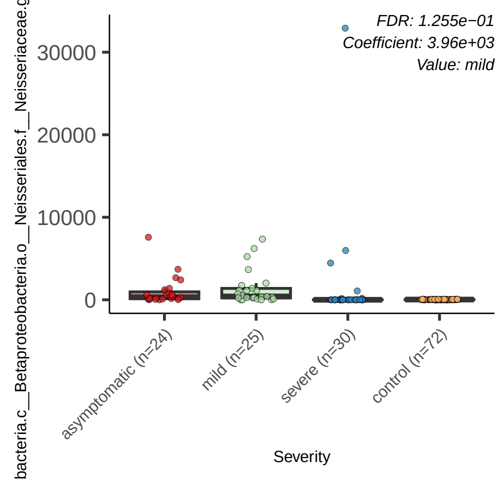

FDR: 1.255e−01
Coefficient: 3.96e+03
Value: mild
30000
20000
10000
0
bacteria.c__Betaproteobacteria.o__Neisseriales.f__Neisseriaceae.g
asymptomatic (n=24)
mild (n=25)
severe (n=30)
control (n=72)
Severity

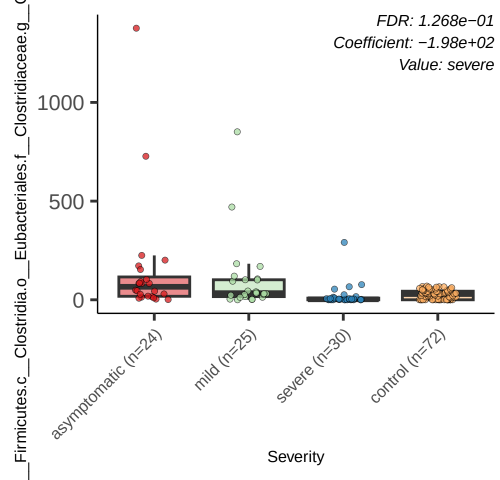

FDR: 1.268e−01
Coefficient: −1.98e+02
Value: severe
Firmicutes.c__Clostridia.o__Eubacteriales.f__Clostridiaceae.g__C
1000
500
0
asymptomatic (n=24)
mild (n=25)
severe (n=30)
control (n=72)
Severity

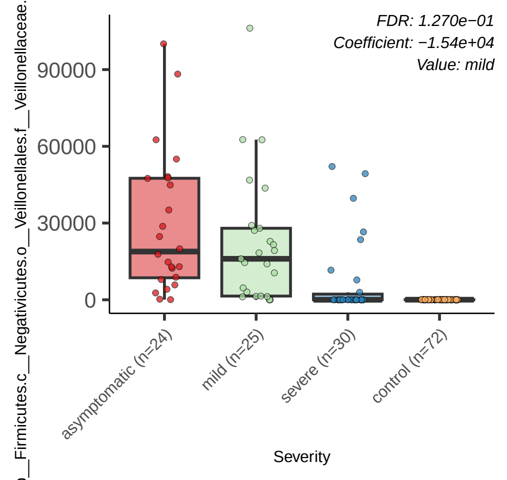
FDR: 1.270e−01
Coefficient: −1.54e+04
Value: mild
Firmicutes.c__Negativicutes.o__Veillonellales.f__Veillonellaceae.
90000
60000
30000
0
asymptomatic (n=24)
mild (n=25)
severe (n=30)
control (n=72)
Severity

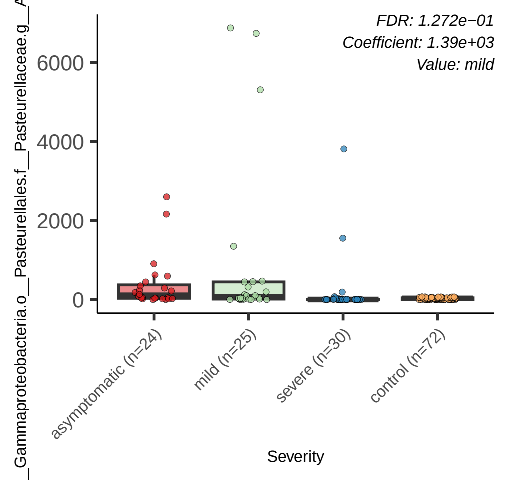
FDR: 1.272e−01
Coefficient: 1.39e+03
Value: mild
6000
4000
2000
0
asymptomatic (n=24)
mild (n=25)
severe (n=30)
control (n=72)
Severity
_Gammaproteobacteria.o__Pasteurellales.f__Pasteurellaceae.g__A

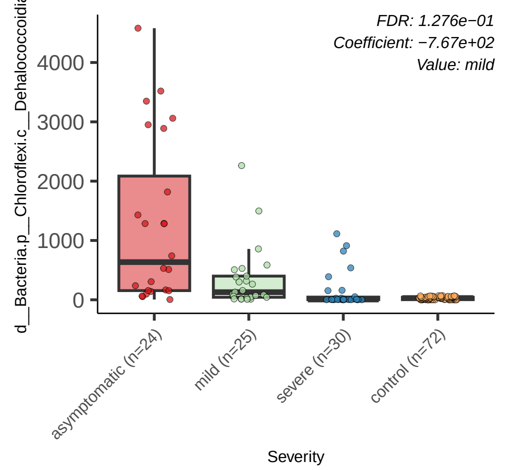
FDR: 1.276e−01
Coefficient: −7.67e+02
Value: mild
d__Bacteria.p__Chloroflexi.c__Dehalococcoidia
4000
3000
2000
1000
0
asymptomatic (n=24)
mild (n=25)
severe (n=30)
control (n=72)
Severity

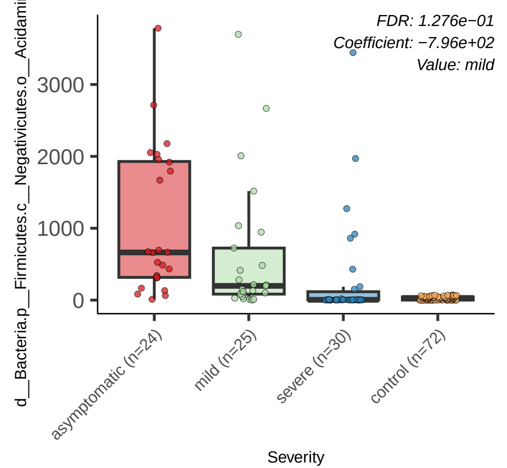

FDR: 1.276e−01
Coefficient: −7.96e+02
Value: mild
d__Bacteria.p__Firmicutes.c__Negativicutes.o__Acidami
3000
2000
1000
0
asymptomatic (n=24)
mild (n=25)
severe (n=30)
control (n=72)
Severity

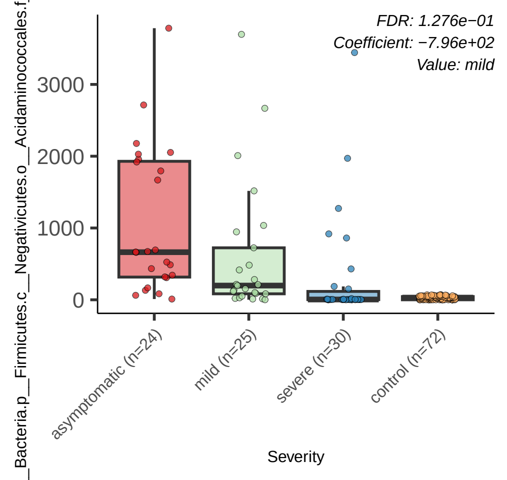

FDR: 1.276e−01
Coefficient: −7.96e+02
Value: mild
Bacteria.p__Firmicutes.c__Negativicutes.o__Acidaminococcales.f_
3000
2000
1000
0
asymptomatic (n=24)
mild (n=25)
severe (n=30)
control (n=72)
Severity

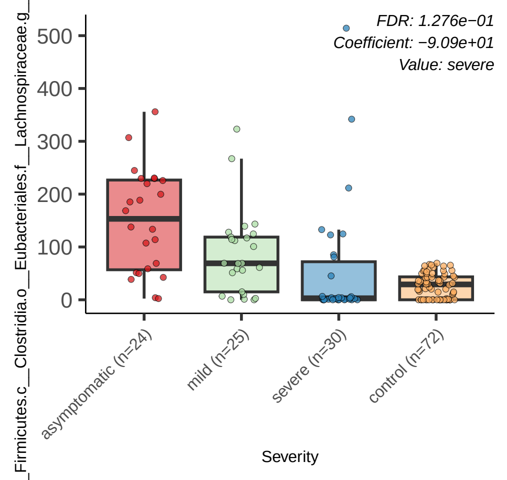
FDR: 1.276e−01
Coefficient: −9.09e+01
Value: severe
Firmicutes.c__Clostridia.o__Eubacteriales.f__Lachnospiraceae.g__
0
100
200
300
400
500
asymptomatic (n=24)
mild (n=25)
severe (n=30)
control (n=72)
Severity

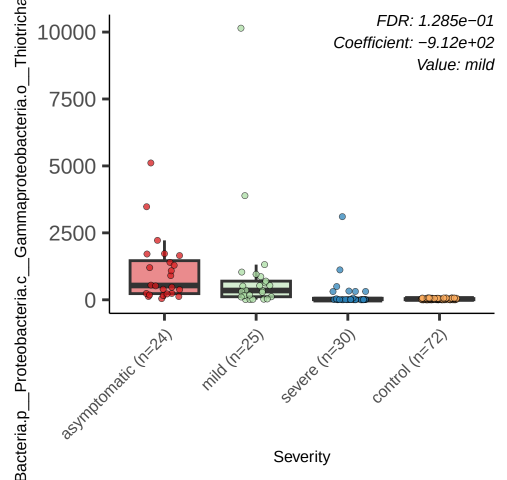
FDR: 1.285e−01
Coefficient: −9.12e+02
Value: mild
10000
7500
5000
2500
0
Bacteria.p__Proteobacteria.c__Gammaproteobacteria.o__Thiotricha
asymptomatic (n=24)
mild (n=25)
severe (n=30)
control (n=72)
Severity

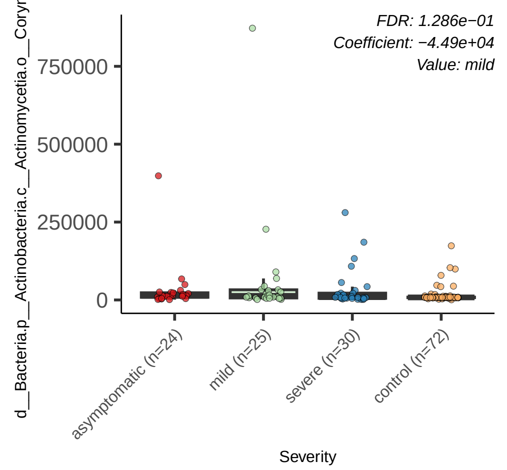

FDR: 1.286e−01
Coefficient: −4.49e+04
Value: mild
d__Bacteria.p__Actinobacteria.c__Actinomycetia.o__Coryn
750000
500000
250000
0
asymptomatic (n=24)
mild (n=25)
severe (n=30)
control (n=72)
Severity

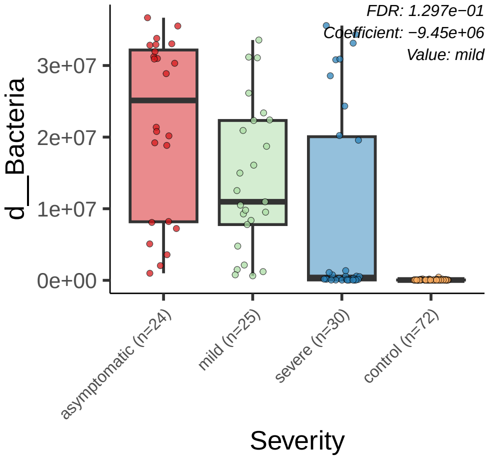
FDR: 1.297e−01
Coefficient: −9.45e+06
Value: mild
d__Bacteria
3e+07
2e+07
1e+07
0e+00
asymptomatic (n=24)
mild (n=25)
severe (n=30)
control (n=72)
Severity

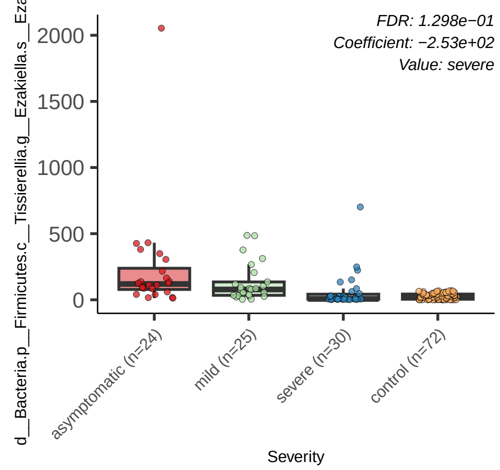

FDR: 1.298e−01
Coefficient: −2.53e+02
Value: severe
d__Bacteria.p__Firmicutes.c__Tissierellia.g__Ezakiella.s__Eza
2000
1500
1000
500
0
asymptomatic (n=24)
mild (n=25)
severe (n=30)
control (n=72)
Severity

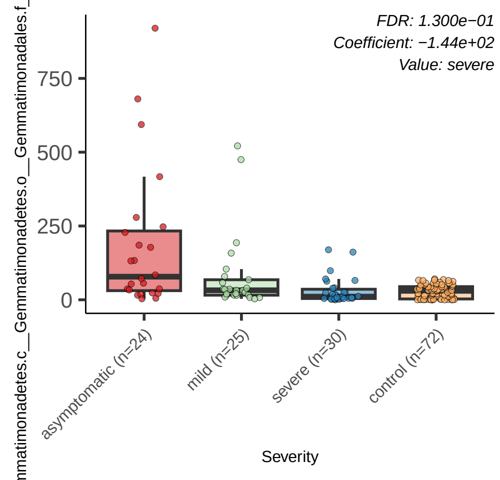

FDR: 1.300e−01
Coefficient: −1.44e+02
Value: severe
750
500
250
0
asymptomatic (n=24)
mild (n=25)
severe (n=30)
control (n=72)
Severity
nmatimonadetes.c__Gemmatimonadetes.o__Gemmatimonadales.f_

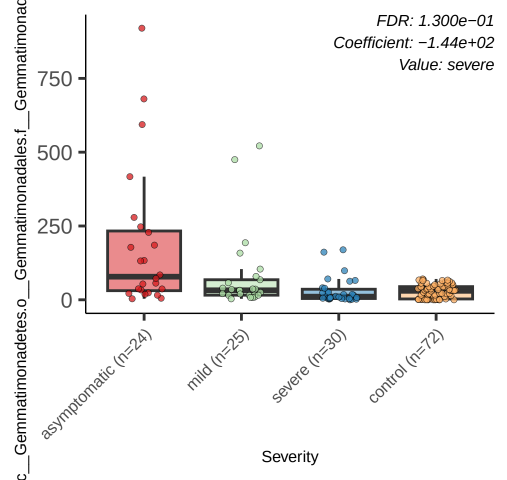

FDR: 1.300e−01
Coefficient: −1.44e+02
Value: severe
c__Gemmatimonadetes.o__Gemmatimonadales.f__Gemmatimonac
750
500
250
0
asymptomatic (n=24)
mild (n=25)
severe (n=30)
control (n=72)
Severity

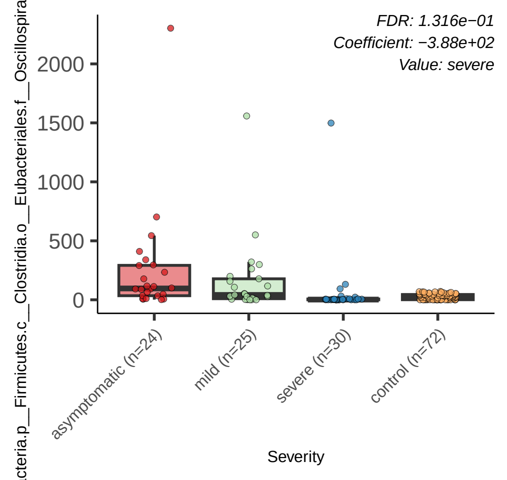
FDR: 1.316e−01
Coefficient: −3.88e+02
Value: severe
0
500
1000
1500
2000
cteria.p__Firmicutes.c__Clostridia.o__Eubacteriales.f__Oscillospira
asymptomatic (n=24)
mild (n=25)
severe (n=30)
control (n=72)
Severity

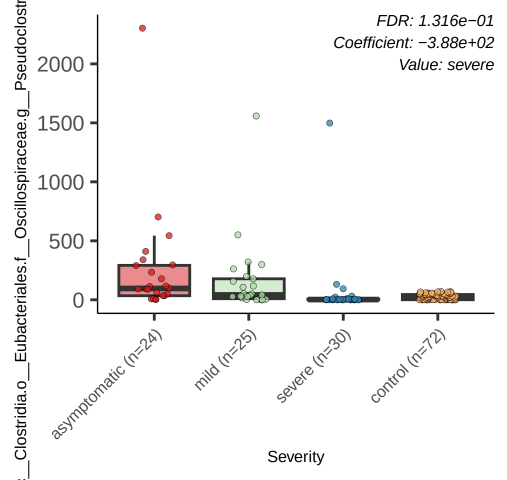

FDR: 1.316e−01
Coefficient: −3.88e+02
Value: severe
Clostridia.o__Eubacteriales.f__Oscillospiraceae.g__Pseudoclostr
2000
1500
1000
500
0
asymptomatic (n=24)
mild (n=25)
severe (n=30)
control (n=72)
Severity

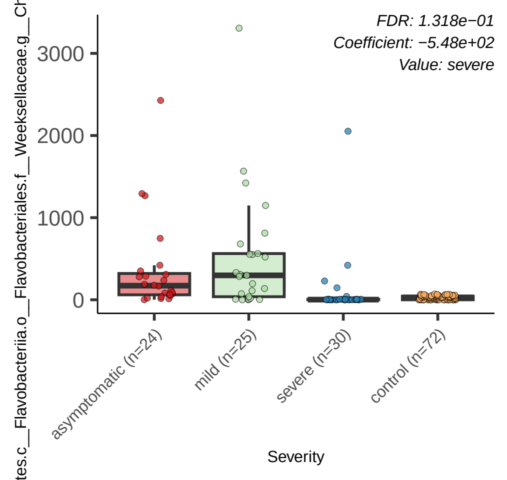

FDR: 1.318e−01
Coefficient: −5.48e+02
Value: severe
3000
2000
1000
0
asymptomatic (n=24)
mild (n=25)
severe (n=30)
control (n=72)
Severity

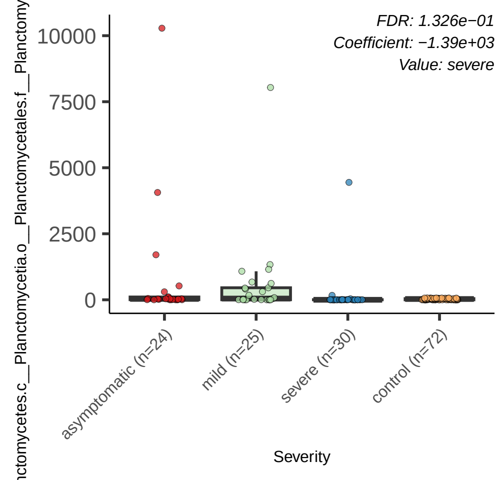
FDR: 1.326e−01
Coefficient: −1.39e+03
Value: severe
10000
7500
5000
2500
0
asymptomatic (n=24)
mild (n=25)
severe (n=30)
control (n=72)
Severity
nctomycetes.c__Planctomycetia.o__Planctomycetales.f__Planctomy

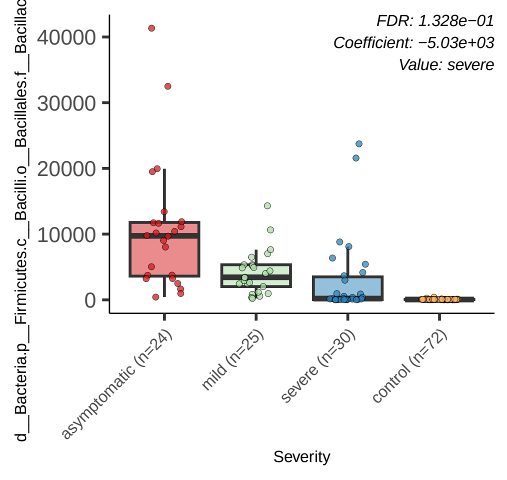
FDR: 1.328e−01
Coefficient: −5.03e+03
Value: severe
d__Bacteria.p__Firmicutes.c__Bacilli.o__Bacillales.f__Bacillac
40000
30000
20000
10000
0
asymptomatic (n=24)
mild (n=25)
severe (n=30)
control (n=72)
Severity

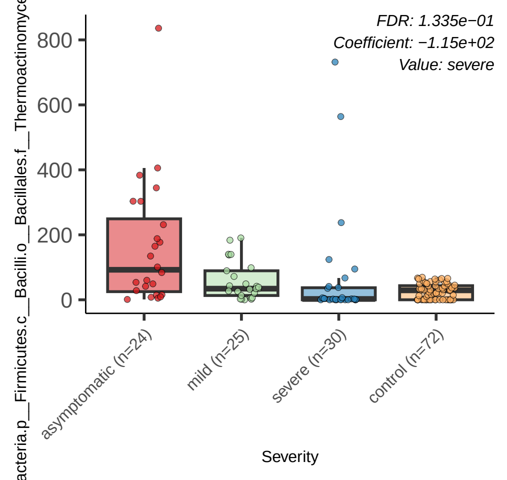
FDR: 1.335e−01
Coefficient: −1.15e+02
Value: severe
acteria.p__Firmicutes.c__Bacilli.o__Bacillales.f__Thermoactinomyce
0
200
400
600
800
asymptomatic (n=24)
mild (n=25)
severe (n=30)
control (n=72)
Severity

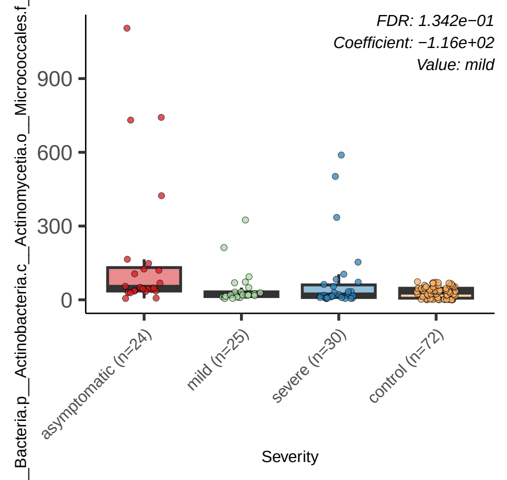
FDR: 1.342e−01
Coefficient: −1.16e+02
Value: mild
Bacteria.p__Actinobacteria.c__Actinomycetia.o__Micrococcales.f__
900
600
300
0
asymptomatic (n=24)
mild (n=25)
severe (n=30)
control (n=72)
Severity

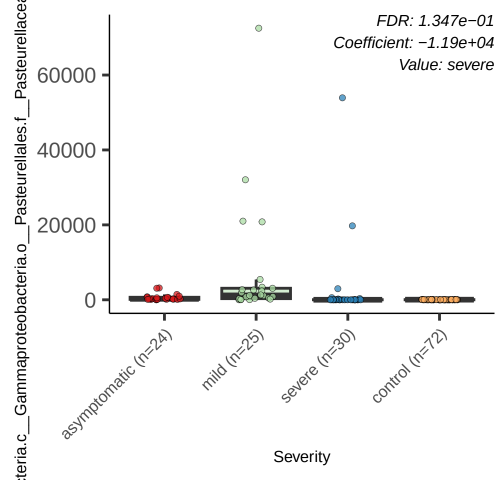

FDR: 1.347e−01
Coefficient: −1.19e+04
Value: severe
60000
40000
20000
0
teria.c__Gammaproteobacteria.o__Pasteurellales.f__Pasteurellacea
asymptomatic (n=24)
mild (n=25)
severe (n=30)
control (n=72)
Severity

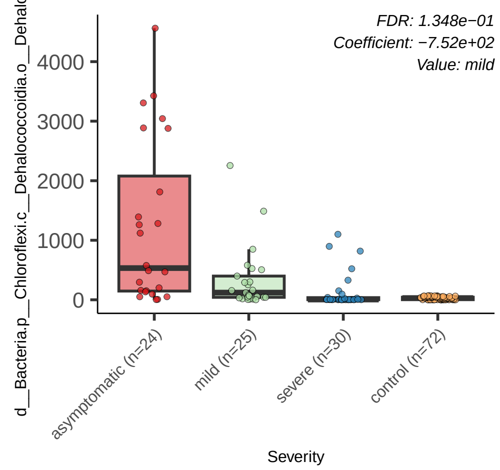
FDR: 1.348e−01
Coefficient: −7.52e+02
Value: mild
d__Bacteria.p__Chloroflexi.c__Dehalococcoidia.o__Dehalo
4000
3000
2000
1000
0
asymptomatic (n=24)
mild (n=25)
severe (n=30)
control (n=72)
Severity

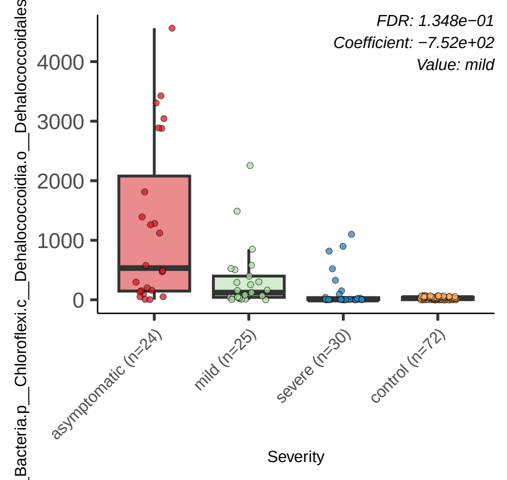
FDR: 1.348e−01
Coefficient: −7.52e+02
Value: mild
Bacteria.p__Chloroflexi.c__Dehalococcoidia.o__Dehalococcoidales
4000
3000
2000
1000
0
asymptomatic (n=24)
mild (n=25)
severe (n=30)
control (n=72)
Severity

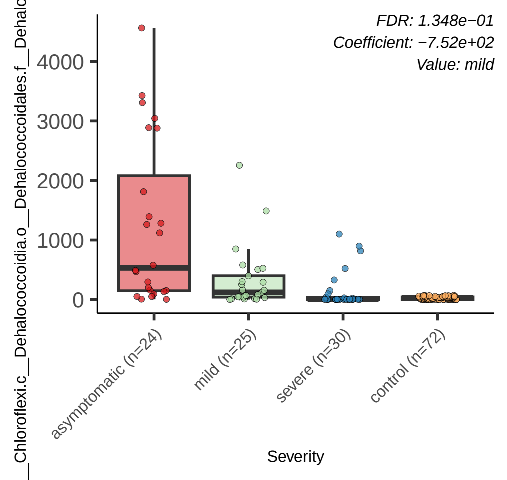

FDR: 1.348e−01
Coefficient: −7.52e+02
Value: mild
4000
3000
2000
1000
0
Chloroflexi.c__Dehalococcoidia.o__Dehalococcoidales.f__Dehalo
asymptomatic (n=24)
mild (n=25)
severe (n=30)
control (n=72)
Severity

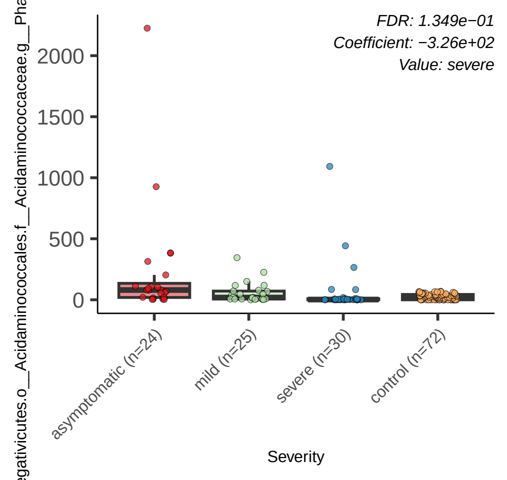

FDR: 1.349e−01
Coefficient: −3.26e+02
Value: severe
2000
1500
1000
500
0
egativicutes.o__Acidaminococcales.f__Acidaminococcaceae.g__Pha
asymptomatic (n=24)
mild (n=25)
severe (n=30)
control (n=72)
Severity

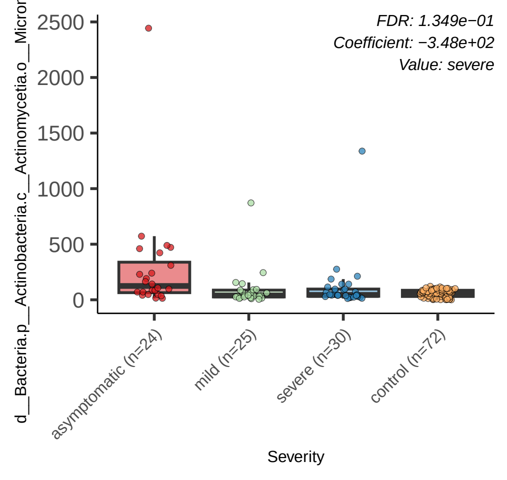

FDR: 1.349e−01
Coefficient: −3.48e+02
Value: severe
d__Bacteria.p__Actinobacteria.c__Actinomycetia.o__Microm
2500
2000
1500
1000
500
0
asymptomatic (n=24)
mild (n=25)
severe (n=30)
control (n=72)
Severity

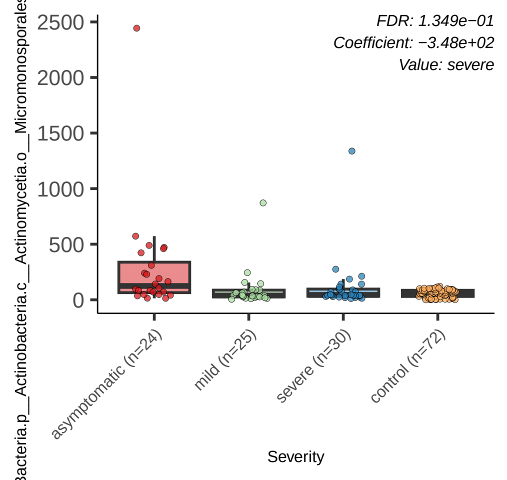
FDR: 1.349e−01
Coefficient: −3.48e+02
Value: severe
Bacteria.p__Actinobacteria.c__Actinomycetia.o__Micromonosporales
2500
2000
1500
1000
500
0
asymptomatic (n=24)
mild (n=25)
severe (n=30)
control (n=72)
Severity

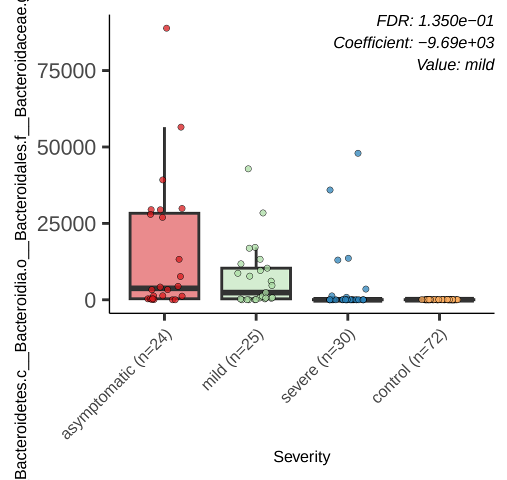

FDR: 1.350e−01
Coefficient: −9.69e+03
Value: mild
75000
50000
25000
0
asymptomatic (n=24)
mild (n=25)
severe (n=30)
control (n=72)
Severity
Bacteroidetes.c__Bacteroidia.o__Bacteroidales.f__Bacteroidaceae.g

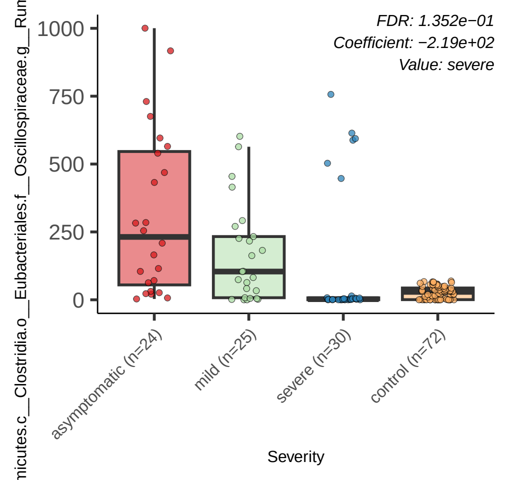
FDR: 1.352e−01
Coefficient: −2.19e+02
Value: severe
micutes.c__Clostridia.o__Eubacteriales.f__Oscillospiraceae.g__Rum
1000
750
500
250
0
asymptomatic (n=24)
mild (n=25)
severe (n=30)
control (n=72)
Severity

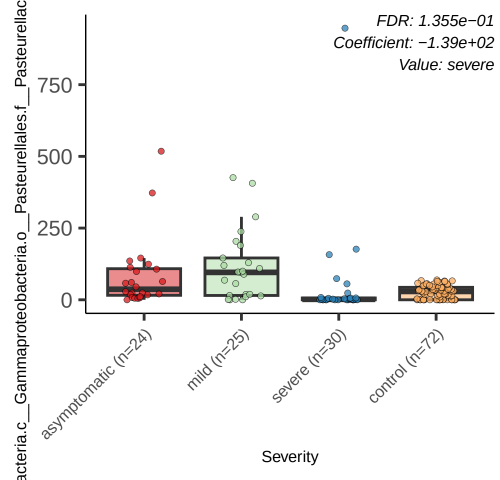
FDR: 1.355e−01
Coefficient: −1.39e+02
Value: severe
acteria.c__Gammaproteobacteria.o__Pasteurellales.f__Pasteurellac
750
500
250
0
asymptomatic (n=24)
mild (n=25)
severe (n=30)
control (n=72)
Severity

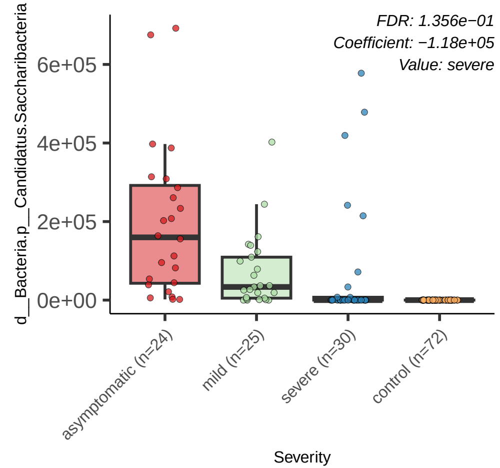

FDR: 1.356e−01
Coefficient: −1.18e+05
Value: severe
d__Bacteria.p__Candidatus.Saccharibacteria
6e+05
4e+05
2e+05
0e+00
asymptomatic (n=24)
mild (n=25)
severe (n=30)
control (n=72)
Severity

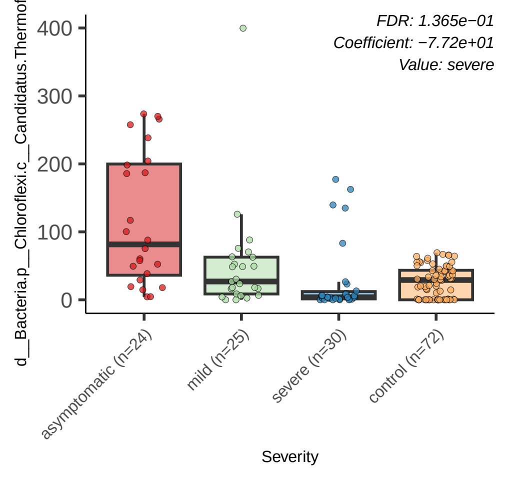
FDR: 1.365e−01
Coefficient: −7.72e+01
Value: severe
d__Bacteria.p__Chloroflexi.c__Candidatus.Thermof
0
100
200
300
400
asymptomatic (n=24)
mild (n=25)
severe (n=30)
control (n=72)
Severity

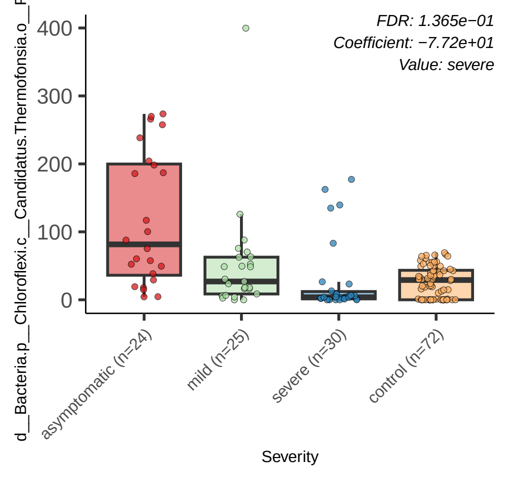

FDR: 1.365e−01
Coefficient: −7.72e+01
Value: severe
d__Bacteria.p__Chloroflexi.c__Candidatus.Thermofonsia.o__F
0
100
200
300
400
asymptomatic (n=24)
mild (n=25)
severe (n=30)
control (n=72)
Severity

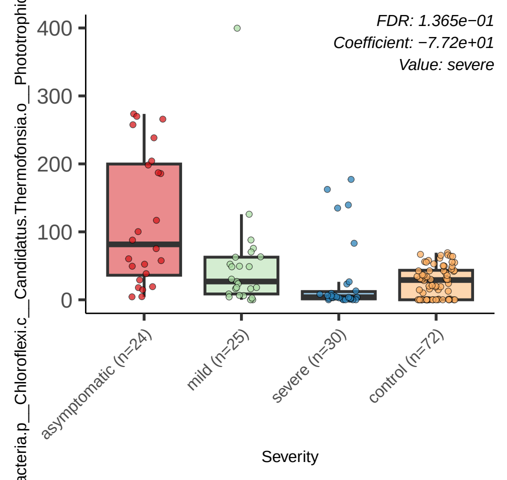

FDR: 1.365e−01
Coefficient: −7.72e+01
Value: severe
acteria.p__Chloroflexi.c__Candidatus.Thermofonsia.o__Phototrophic
0
100
200
300
400
asymptomatic (n=24)
mild (n=25)
severe (n=30)
control (n=72)
Severity

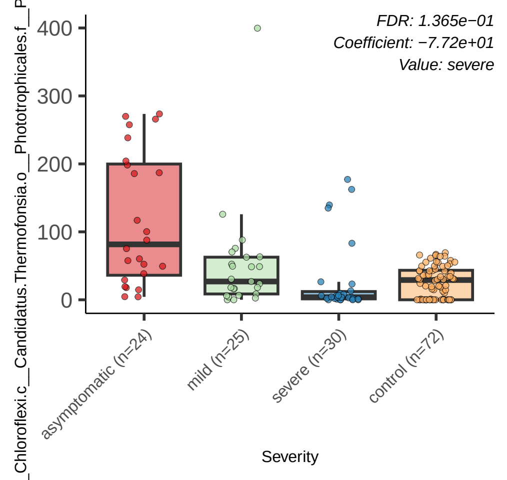
FDR: 1.365e−01
Coefficient: −7.72e+01
Value: severe
_Chloroflexi.c__Candidatus.Thermofonsia.o__Phototrophicales.f__P
0
100
200
300
400
asymptomatic (n=24)
mild (n=25)
severe (n=30)
control (n=72)
Severity

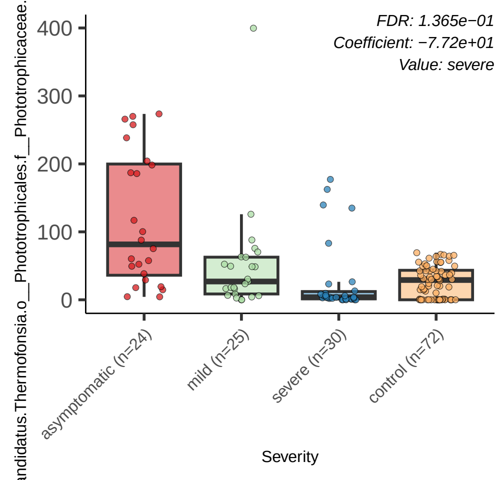

FDR: 1.365e−01
Coefficient: −7.72e+01
Value: severe
400
300
200
100
0
andidatus.Thermofonsia.o__Photototrophicales.f__Phototrophicaceae.
asymptomatic (n=24)
mild (n=25)
severe (n=30)
control (n=72)
Severity

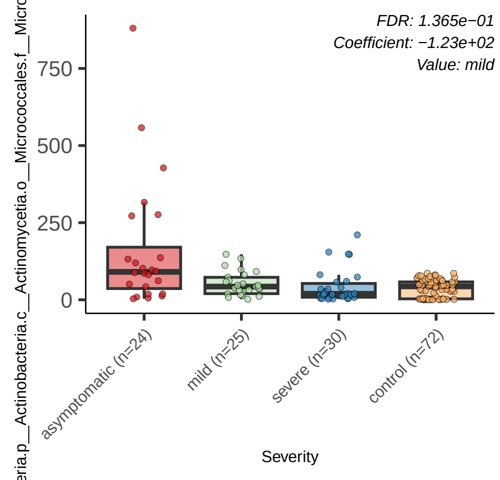
FDR: 1.365e−01
Coefficient: −1.23e+02
Value: mild
750
500
250
0
asymptomatic (n=24)
mild (n=25)
severe (n=30)
control (n=72)
Severity
eria.p__Actinobacteria.c__Actinomycetia.o__Micrococcales.f__Micro

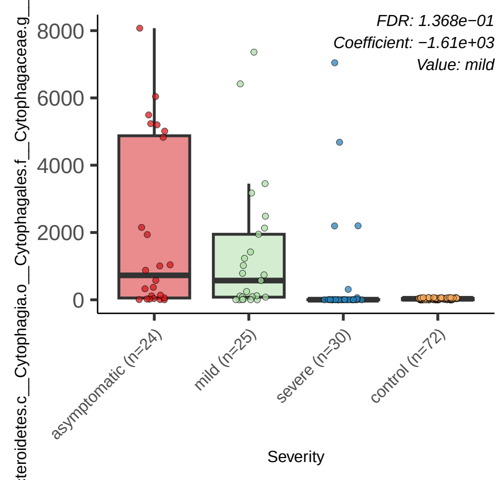

FDR: 1.368e−01
Coefficient: −1.61e+03
Value: mild
8000
6000
4000
2000
0
asymptomatic (n=24)
mild (n=25)
severe (n=30)
control (n=72)
Severity
cteroidetes.c__Cytophagia.o__Cytophagales.f__Cytophagaceae.g__

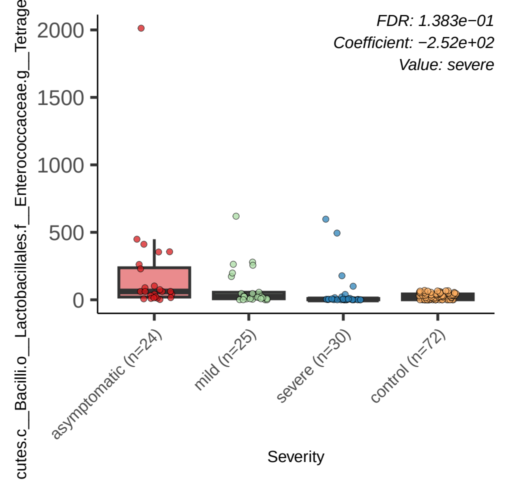

FDR: 1.383e−01
Coefficient: −2.52e+02
Value: severe
cutes.c__Bacilli.o__Lactobacillales.f__Enterococcaceae.g__Tetrage
2000
1500
1000
500
0
asymptomatic (n=24)
mild (n=25)
severe (n=30)
control (n=72)
Severity

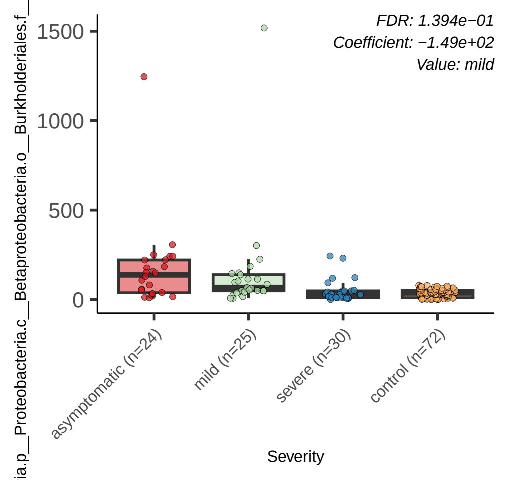
FDR: 1.394e−01
Coefficient: −1.49e+02
Value: mild
ia.p__Proteobacteria.c__Betaproteobacteria.o__Burkholderiales.f__
1500
1000
500
0
asymptomatic (n=24)
mild (n=25)
severe (n=30)
control (n=72)
Severity

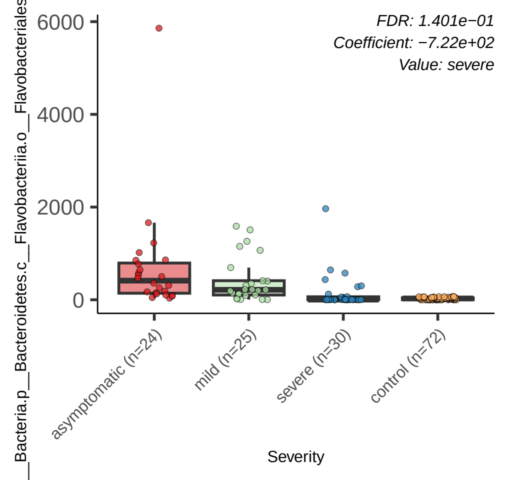
FDR: 1.401e−01
Coefficient: −7.22e+02
Value: severe
6000
4000
2000
0
Bacteria.p__Bacteroidetes.c__Flavobacteriia.o__Flavobacteriales
asymptomatic (n=24)
mild (n=25)
severe (n=30)
control (n=72)
Severity

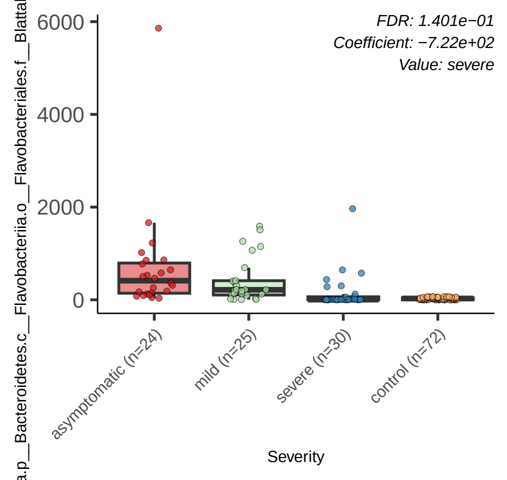
FDR: 1.401e−01
Coefficient: −7.22e+02
Value: severe
6000
4000
2000
0
asymptomatic (n=24)
mild (n=25)
severe (n=30)
control (n=72)
Severity
a.p__Bacteroidetes.c__Flavobacteriia.o__Flavobacteriales.f__Blattab

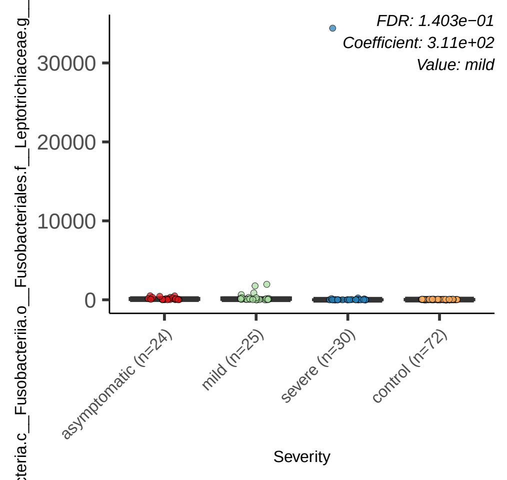

FDR: 1.403e−01
Coefficient: 3.11e+02
Value: mild
Fusobacteria.c__Fusobacteriia.o__Fusobacteriales.f__Leptotrichiaceae.g__
30000
20000
10000
0
asymptomatic (n=24)
mild (n=25)
severe (n=30)
control (n=72)
Severity

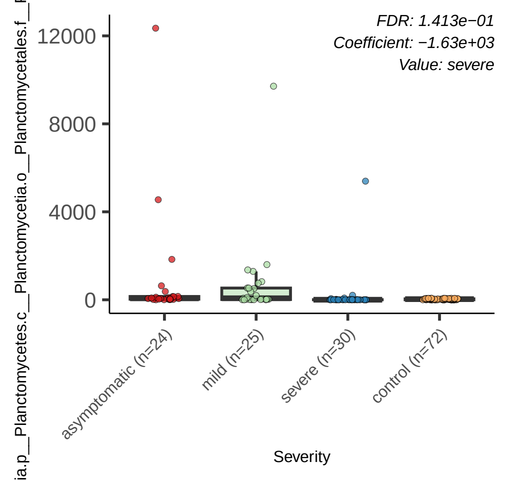

FDR: 1.413e−01
Coefficient: −1.63e+03
Value: severe
12000
8000
4000
0
ia.p__Planctomycetes.c__Planctomycetia.o__Planctomycetales.f__P
asymptomatic (n=24)
mild (n=25)
severe (n=30)
control (n=72)
Severity

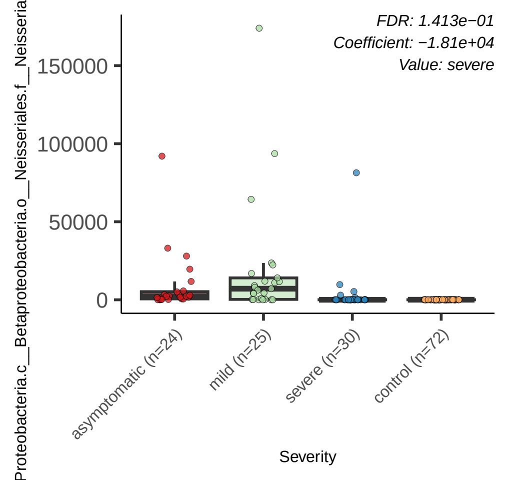
FDR: 1.413e−01
Coefficient: −1.81e+04
Value: severe
Proteobacteria.c__Betaproteobacteria.o__Neisseriales.f__Neisseria
150000
100000
50000
0
asymptomatic (n=24)
mild (n=25)
severe (n=30)
control (n=72)
Severity

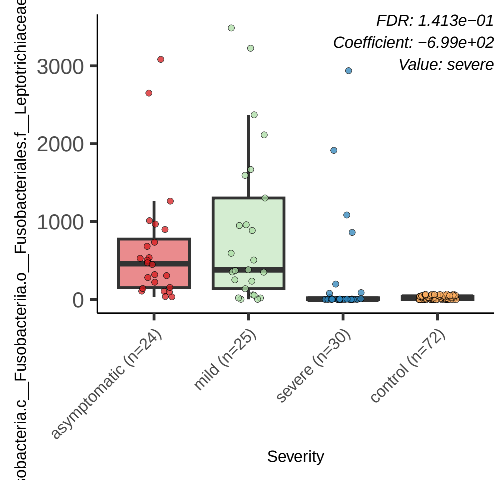

FDR: 1.413e−01
Coefficient: −6.99e+02
Value: severe
3000
2000
1000
0
asymptomatic (n=24)
mild (n=25)
severe (n=30)
control (n=72)
Severity
obacteria.c__Fusobacteriia.o__Fusobacteriales.f__Leptotrichiaceae

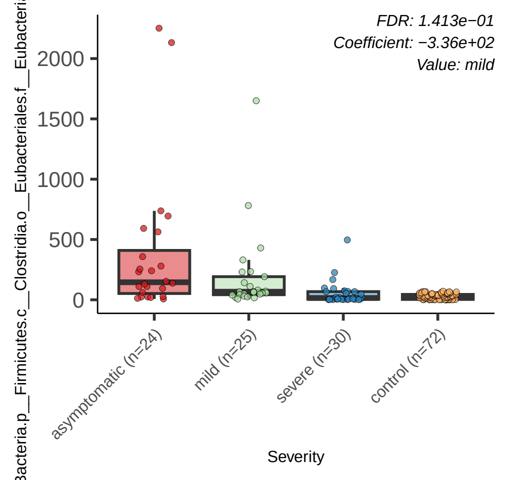

FDR: 1.413e−01
Coefficient: −3.36e+02
Value: mild
Bacteria.p__Firmicutes.c__Clostridia.o__Eubacteriales.f__Eubacteri
0
500
1000
1500
2000
asymptomatic (n=24)
mild (n=25)
severe (n=30)
control (n=72)
Severity

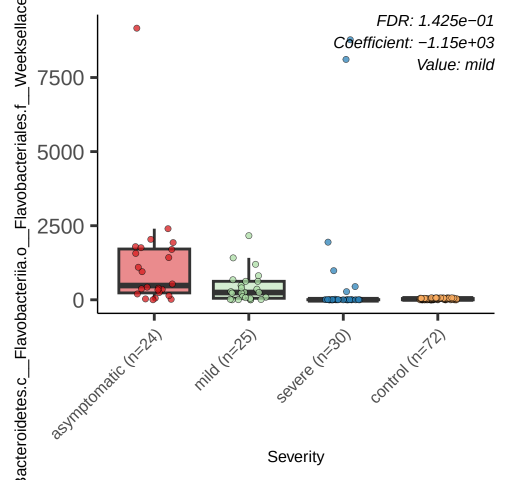

FDR: 1.425e−01
Coefficient: −1.15e+03
Value: mild
7500
5000
2500
0
Bacteroidetes.c__Flavobacteriia.o__Flavobacteriales.f__Weeksellace
asymptomatic (n=24)
mild (n=25)
severe (n=30)
control (n=72)
Severity

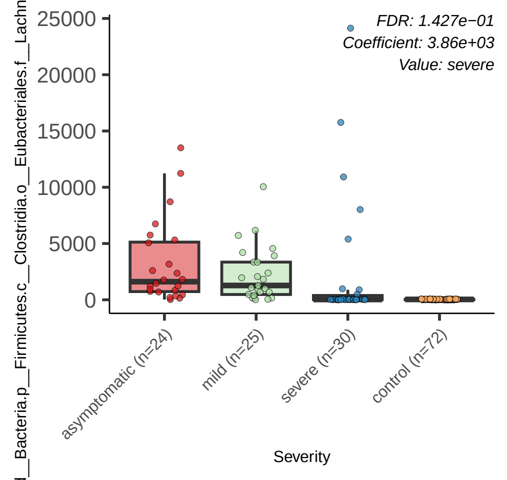

Bacteria.p__Firmicutes.c__Clostridia.o__Eubacteriales.f__Lachn
25000
20000
15000
10000
5000
0
FDR: 1.427e−01
Coefficient: 3.86e+03
Value: severe
asymptomatic (n=24)
mild (n=25)
severe (n=30)
control (n=72)
Severity

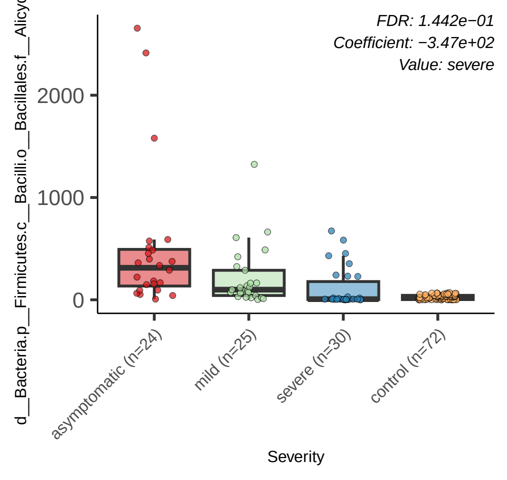

FDR: 1.442e−01
Coefficient: −3.47e+02
Value: severe
d__Bacteria.p__Firmicutes.c__Bacilli.o__Bacillales.f__Alicyc
2000
1000
0
asymptomatic (n=24)
mild (n=25)
severe (n=30)
control (n=72)
Severity

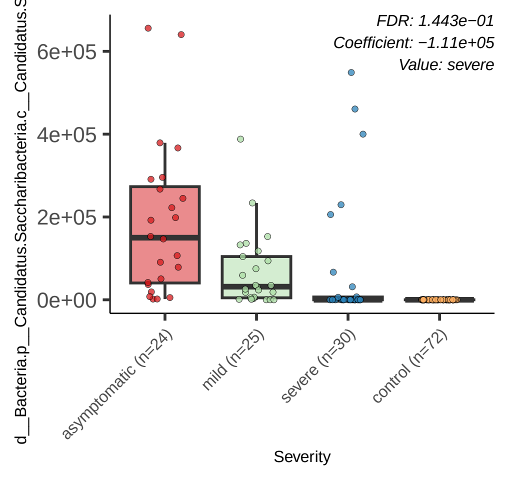

FDR: 1.443e−01
Coefficient: −1.11e+05
Value: severe
d__Bacteria.p__Candidatus.Saccharibacteria.c__Candidatus.S
6e+05
4e+05
2e+05
0e+00
asymptomatic (n=24)
mild (n=25)
severe (n=30)
control (n=72)
Severity

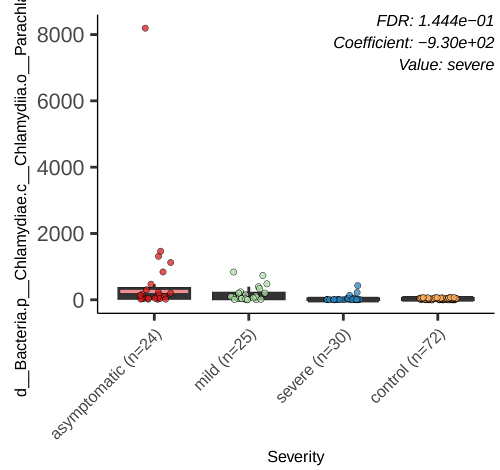

FDR: 1.444e−01
Coefficient: −9.30e+02
Value: severe
d__Bacteria.p__Chlamydiae.c__Chlamydiia.o__Parachla
8000
6000
4000
2000
0
asymptomatic (n=24)
mild (n=25)
severe (n=30)
control (n=72)
Severity

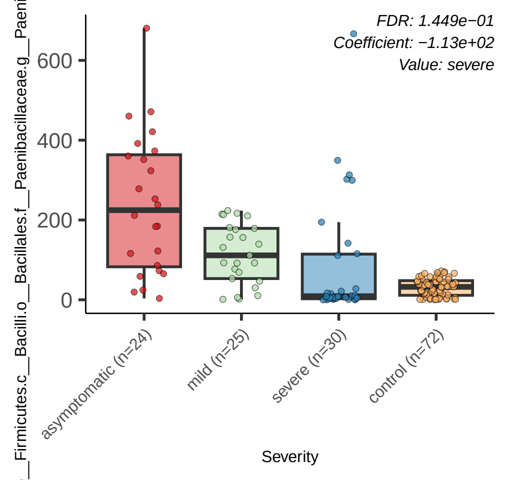
FDR: 1.449e−01
Coefficient: −1.13e+02
Value: severe
Firmicutes.c__Bacilli.o__Bacillales.f__Paenibacillaceae.g__Paeni
600
400
200
0
asymptomatic (n=24)
mild (n=25)
severe (n=30)
control (n=72)
Severity

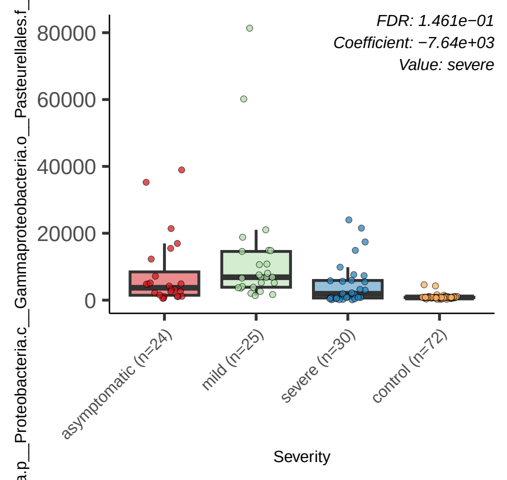
FDR: 1.461e−01
Coefficient: −7.64e+03
Value: severe
a.p__Proteobacteria.c__Gammaproteobacteria.o__Pasteurellales.f__
80000
60000
40000
20000
0
asymptomatic (n=24)
mild (n=25)
severe (n=30)
control (n=72)
Severity

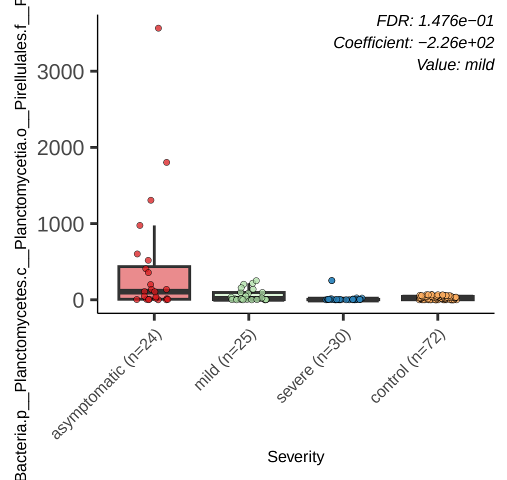

FDR: 1.476e−01
Coefficient: −2.26e+02
Value: mild
Bacteria.p__Planctomycetes.c__Planctomycetia.o__Pirellulales.f__P
3000
2000
1000
0
asymptomatic (n=24)
mild (n=25)
severe (n=30)
control (n=72)
Severity

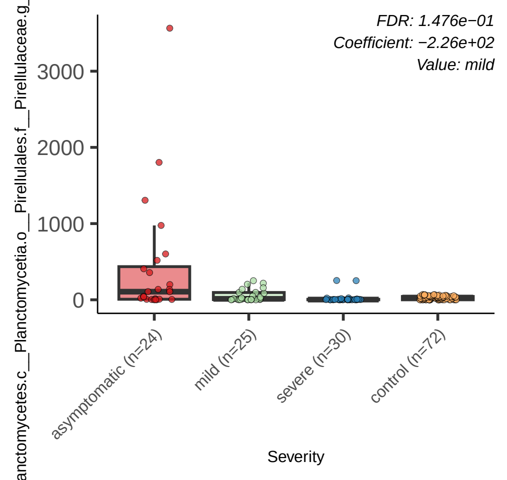

FDR: 1.476e−01
Coefficient: −2.26e+02
Value: mild
3000
2000
1000
0
asymptomatic (n=24)
mild (n=25)
severe (n=30)
control (n=72)
Severity
lanctomycetes.c__Planctomycetia.o__Pirellulales.f__Pirellulaceae.g_

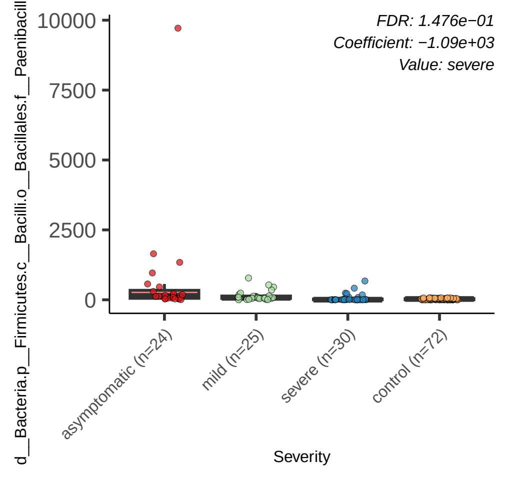
d__Bacteria.p__Firmicutes.c__Bacilli.o__Bacillales.f__Paenibacill
FDR: 1.476e−01
Coefficient: −1.09e+03
Value: severe
10000
7500
5000
2500
0
asymptomatic (n=24)
mild (n=25)
severe (n=30)
control (n=72)
Severity

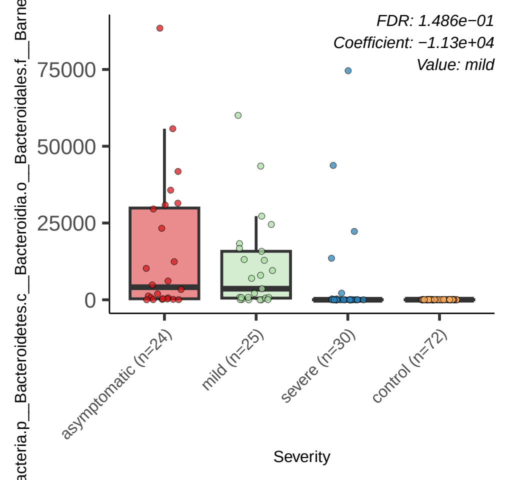
FDR: 1.486e−01
Coefficient: −1.13e+04
Value: mild
75000
50000
25000
0
acteria.p__Bacteroidetes.c__Bacteroidia.o__Bacteroidales.f__Barne
asymptomatic (n=24)
mild (n=25)
severe (n=30)
control (n=72)
Severity

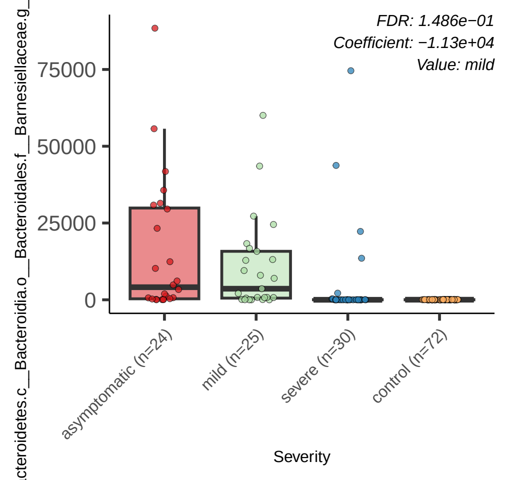

FDR: 1.486e−01
Coefficient: −1.13e+04
Value: mild
75000
50000
25000
0
acteroidetes.c__Bacteroidia.o__Bacteroidales.f__Barnesiellaceae.g__
asymptomatic (n=24)
mild (n=25)
severe (n=30)
control (n=72)
Severity

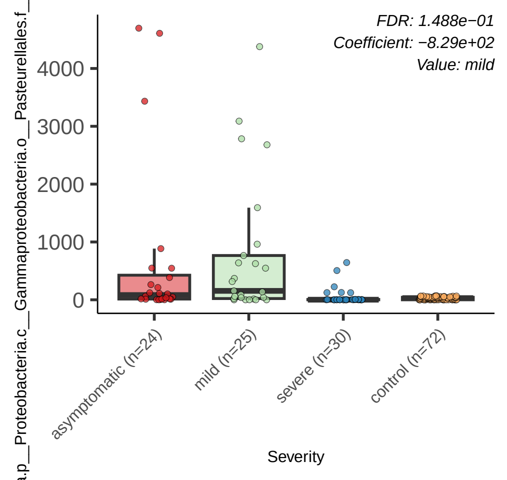

FDR: 1.488e−01
Coefficient: −8.29e+02
Value: mild
a.p__Proteobacteria.c__Gammaproteobacteria.o__Pasteurellales.f__
0
1000
2000
3000
4000
asymptomatic (n=24)
mild (n=25)
severe (n=30)
control (n=72)
Severity

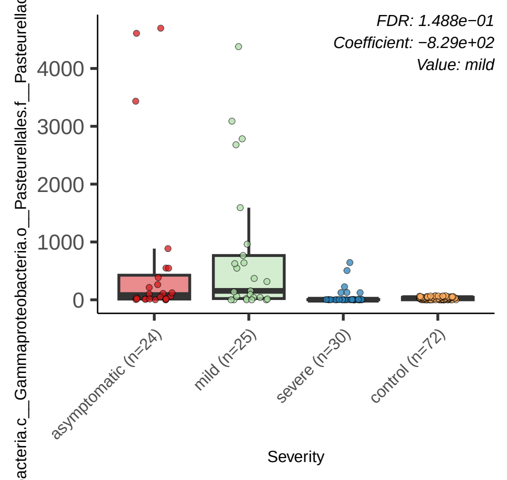

FDR: 1.488e−01
Coefficient: −8.29e+02
Value: mild
4000
3000
2000
1000
0
acteria.c__Gammaproteobacteria.o__Pasteurellales.f__Pasteurellac
asymptomatic (n=24)
mild (n=25)
severe (n=30)
control (n=72)
Severity

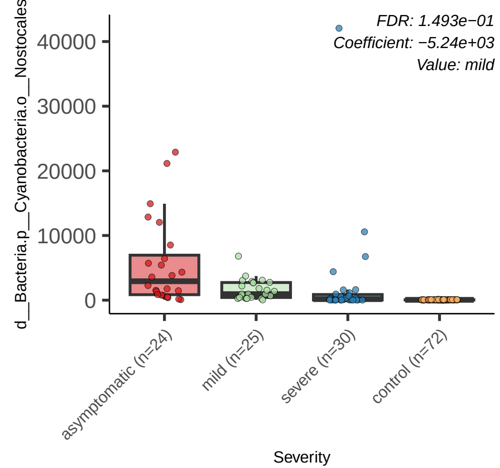

FDR: 1.493e−01
Coefficient: −5.24e+03
Value: mild
d__Bacteria.p__Cyanobacteria.o__Nostocales
40000
30000
20000
10000
0
asymptomatic (n=24)
mild (n=25)
severe (n=30)
control (n=72)
Severity

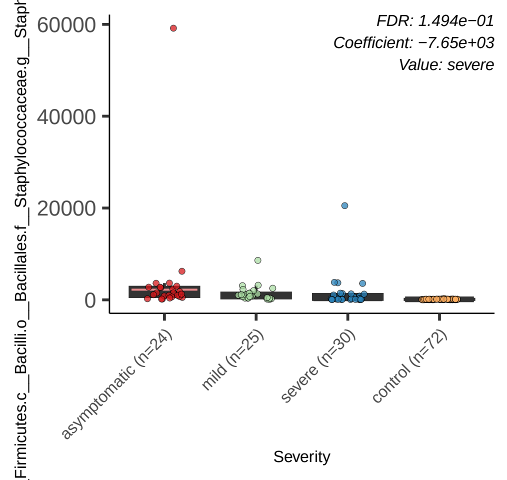

FDR: 1.494e−01
Coefficient: −7.65e+03
Value: severe
60000
40000
20000
0
Firmicutes.c__Bacilli.o__Bacillales.f__Staphylococcaceae.g__Staph
asymptomatic (n=24)
mild (n=25)
severe (n=30)
control (n=72)
Severity

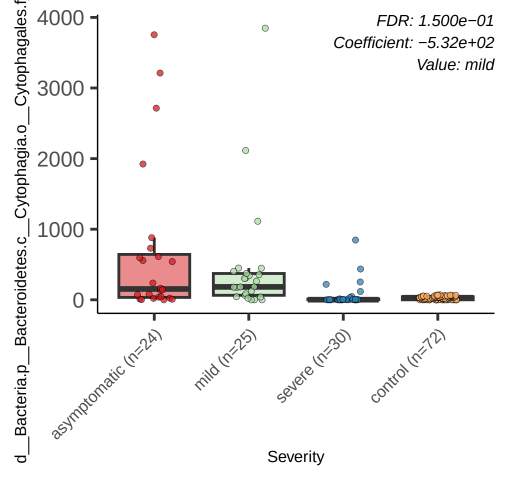

FDR: 1.500e−01
Coefficient: −5.32e+02
Value: mild
d__Bacteria.p__Bacteroidetes.c__Cytophagia.o__Cytophagales.f
4000
3000
2000
1000
0
asymptomatic (n=24)
mild (n=25)
severe (n=30)
control (n=72)
Severity

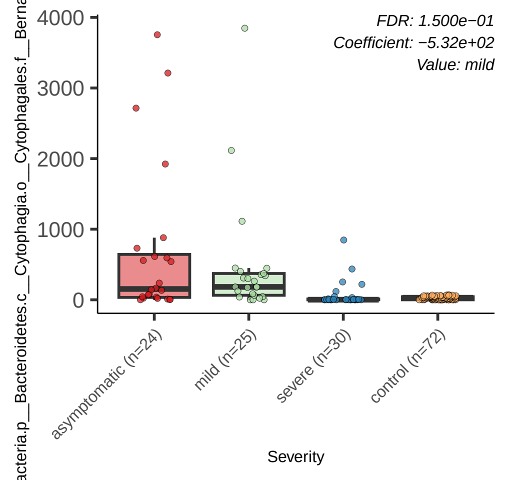

FDR: 1.500e−01
Coefficient: −5.32e+02
Value: mild
4000
3000
2000
1000
0
acteria.p__Bacteroidetes.c__Cytophagia.o__Cytophagales.f__Berna
asymptomatic (n=24)
mild (n=25)
severe (n=30)
control (n=72)
Severity

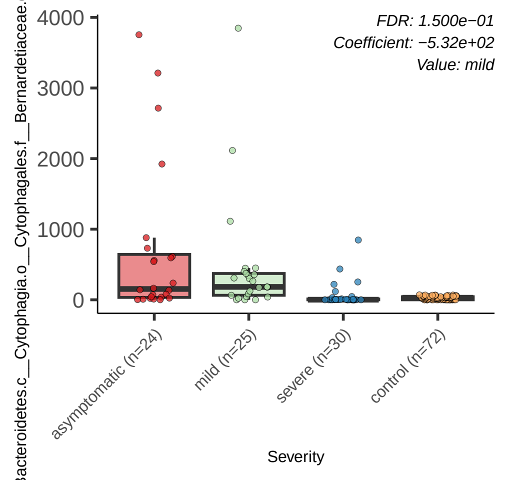

FDR: 1.500e−01
Coefficient: −5.32e+02
Value: mild
4000
3000
2000
1000
0
Bacteroidetes.c__Cytophagia.o__Cytophagales.f__Bernardetiaceae.
asymptomatic (n=24)
mild (n=25)
severe (n=30)
control (n=72)
Severity

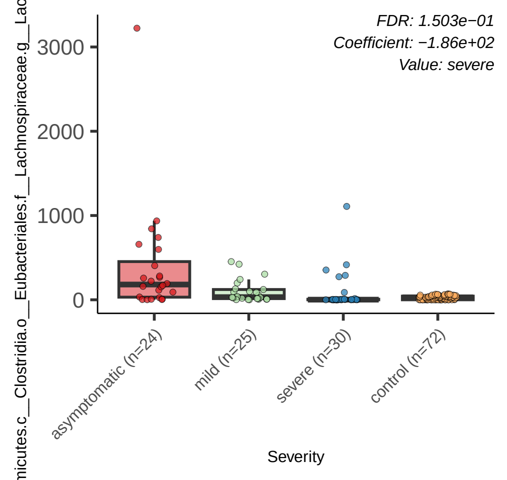
FDR: 1.503e−01
Coefficient: −1.86e+02
Value: severe
3000
2000
1000
0
micutes.c__Clostridia.o__Eubacteriales.f__Lachnospiraceae.g__Lac
asymptomatic (n=24)
mild (n=25)
severe (n=30)
control (n=72)
Severity

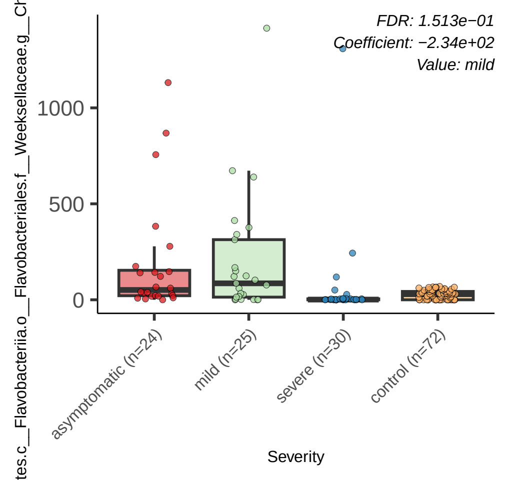

FDR: 1.513e−01
Coefficient: −2.34e+02
Value: mild
1000
500
0
tes.c__Flavobacteriia.o__Flavobacteriales.f__Weeksellaceae.g__Ch
asymptomatic (n=24)
mild (n=25)
severe (n=30)
control (n=72)
Severity

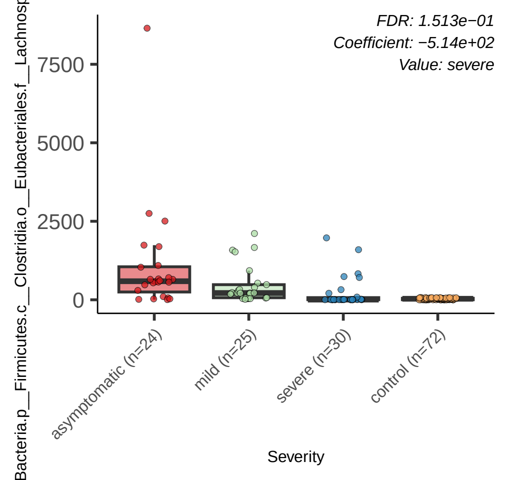

FDR: 1.513e−01
Coefficient: −5.14e+02
Value: severe
Bacteria.p__Firmicutes.c__Clostridia.o__Eubacteriales.f__Lachnosp
7500
5000
2500
0
asymptomatic (n=24)
mild (n=25)
severe (n=30)
control (n=72)
Severity

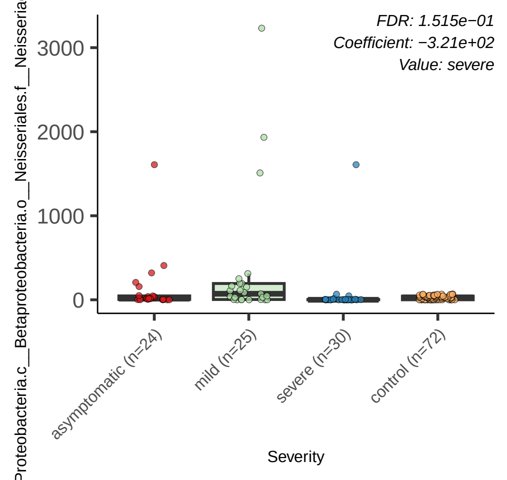

FDR: 1.515e−01
Coefficient: −3.21e+02
Value: severe
3000
2000
1000
0
Proteobacteria.c__Betaproteobacteria.o__Neisseriales.f__Neisseria
asymptomatic (n=24)
mild (n=25)
severe (n=30)
control (n=72)
Severity

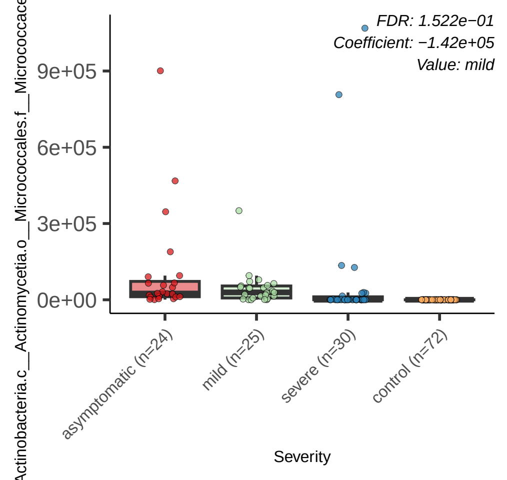

FDR: 1.522e−01
Coefficient: −1.42e+05
Value: mild
9e+05
6e+05
3e+05
0e+00
Actinobacteria.c__Actinomycetia.o__Micrococcales.f__Micrococcace
asymptomatic (n=24)
mild (n=25)
severe (n=30)
control (n=72)
Severity

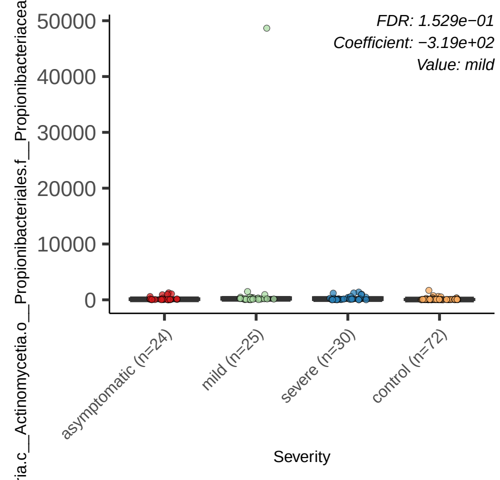

FDR: 1.529e−01
Coefficient: −3.19e+02
Value: mild
50000
40000
30000
20000
10000
0
ia.c__Actinomycetia.o__Propionibacteriales.f__Propionibacteriacea
asymptomatic (n=24)
mild (n=25)
severe (n=30)
control (n=72)
Severity

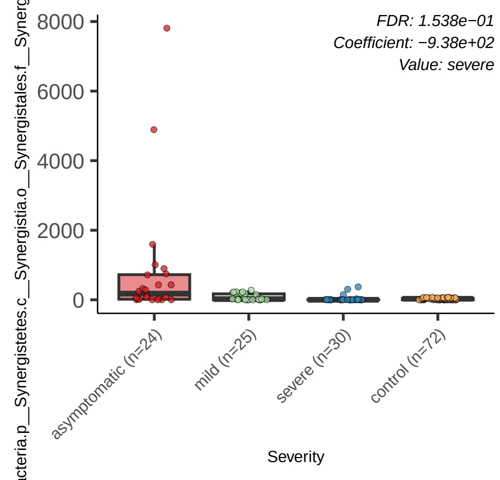

FDR: 1.538e−01
Coefficient: −9.38e+02
Value: severe
8000
6000
4000
2000
0
acteria.p__Synergistetes.c__Synergistia.o__Synergistales.f__Synerg
asymptomatic (n=24)
mild (n=25)
severe (n=30)
control (n=72)
Severity

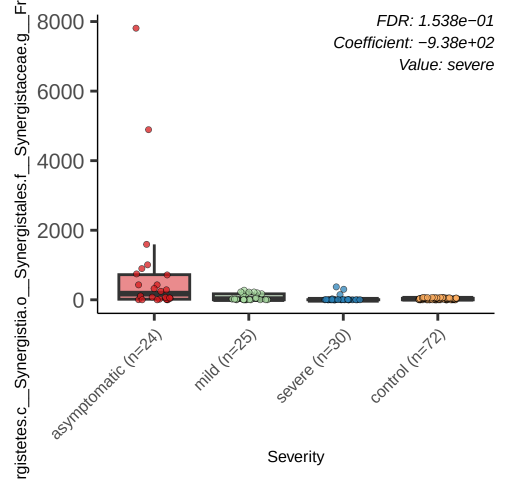
FDR: 1.538e−01
Coefficient: −9.38e+02
Value: severe
8000
6000
4000
2000
0
rgistetes.c__Synergistia.o__Synergistales.f__Synergistaceae.g__Fr
asymptomatic (n=24)
mild (n=25)
severe (n=30)
control (n=72)
Severity

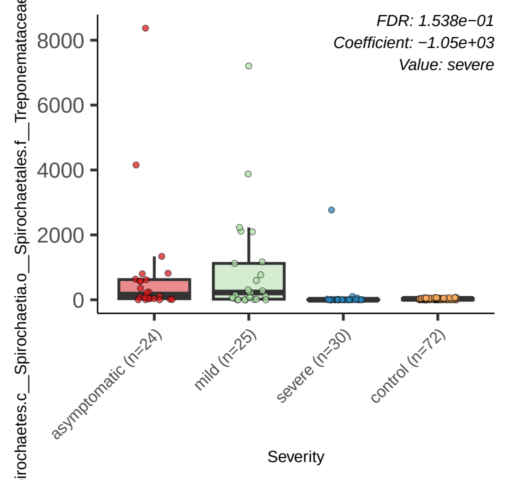

FDR: 1.538e−01
Coefficient: −1.05e+03
Value: severe
8000
6000
4000
2000
0
irochaetes.c__Spirochaetia.o__Spirochaetales.f__Treponemataceae
asymptomatic (n=24)
mild (n=25)
severe (n=30)
control (n=72)
Severity

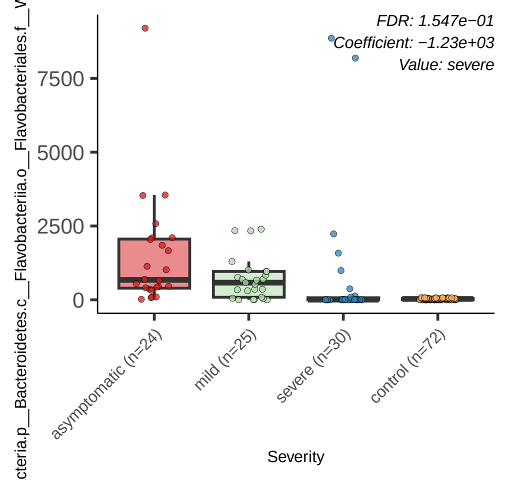

FDR: 1.547e−01
Coefficient: −1.23e+03
Value: severe
7500
5000
2500
0
asymptomatic (n=24)
mild (n=25)
severe (n=30)
control (n=72)
Severity
cteria.p__Bacteroidetes.c__Flavobacteriia.o__Flavobacteriales.f__W

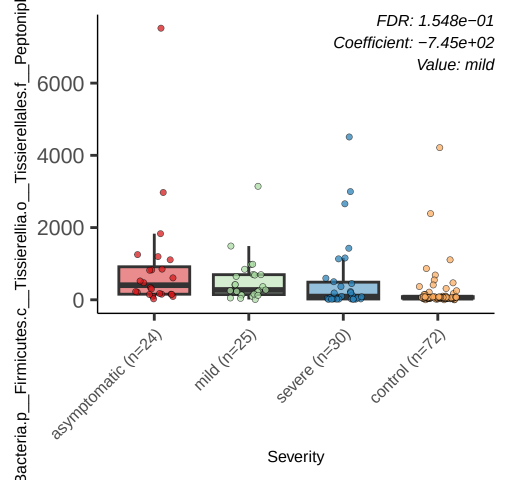

FDR: 1.548e−01
Coefficient: −7.45e+02
Value: mild
Bacteria.p__Firmicutes.c__Tissierellia.o__Tissierellales.f__Peptonipl
6000
4000
2000
0
asymptomatic (n=24)
mild (n=25)
severe (n=30)
control (n=72)
Severity

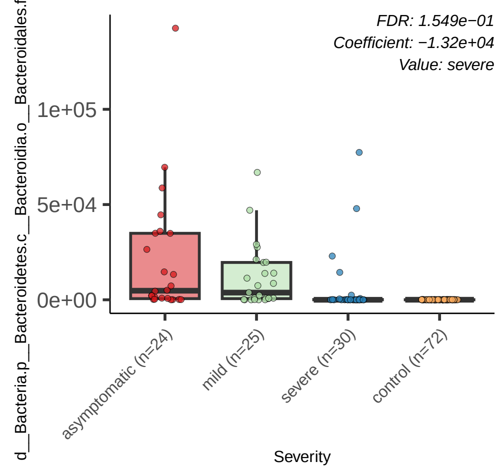
FDR: 1.549e−01
Coefficient: −1.32e+04
Value: severe
d__Bacteria.p__Bacteroidetes.c__Bacteroidia.o__Bacteroidales.f
1e+05
5e+04
0e+00
asymptomatic (n=24)
mild (n=25)
severe (n=30)
control (n=72)
Severity

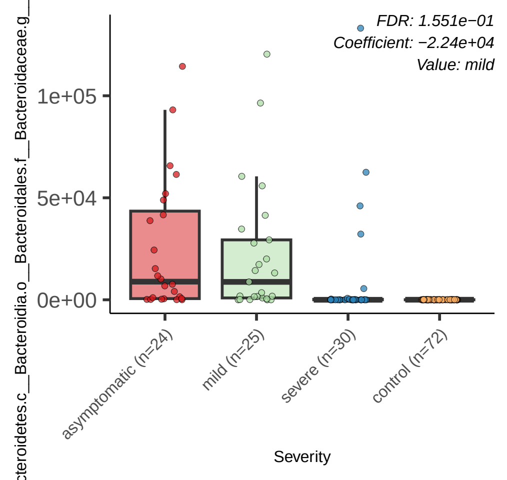
FDR: 1.551e−01
Coefficient: −2.24e+04
Value: mild
1e+05
5e+04
0e+00
cteroidetes.c__Bacteroidia.o__Bacteroidales.f__Bacteroidaceae.g__
asymptomatic (n=24)
mild (n=25)
severe (n=30)
control (n=72)
Severity

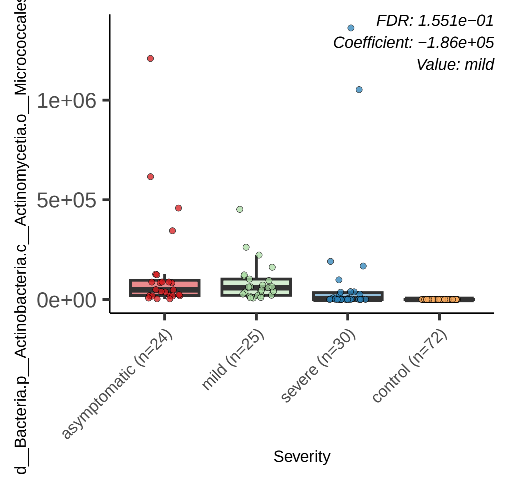

FDR: 1.551e−01
Coefficient: −1.86e+05
Value: mild
d__Bacteria.p__Actinobacteria.c__Actinomycetia.o__Micrococcales
1e+06
5e+05
0e+00
asymptomatic (n=24)
mild (n=25)
severe (n=30)
control (n=72)
Severity

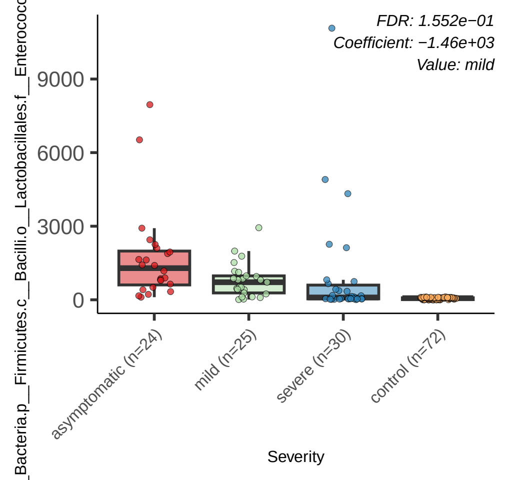

FDR: 1.552e−01
Coefficient: −1.46e+03
Value: mild
Bacteria.p__Firmicutes.c__Bacilli.o__Lactobacillales.f__Enterococc
9000
6000
3000
0
asymptomatic (n=24)
mild (n=25)
severe (n=30)
control (n=72)
Severity

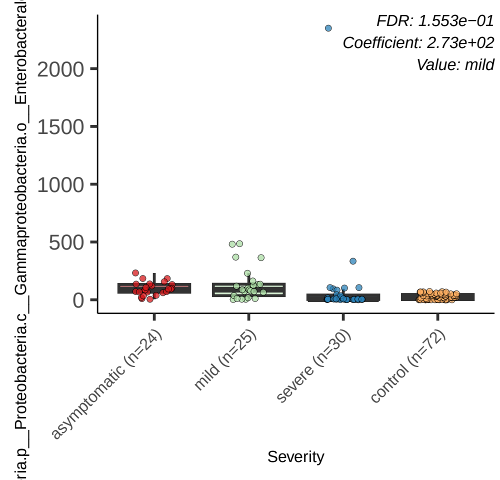
FDR: 1.553e−01
Coefficient: 2.73e+02
Value: mild
2000
1500
1000
500
0
ria.p__Proteobacteria.c__Gammaproteobacteria.o__Enterobacteral
asymptomatic (n=24)
mild (n=25)
severe (n=30)
control (n=72)
Severity

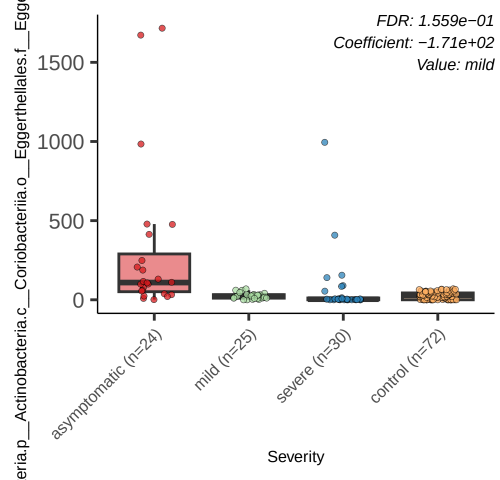
FDR: 1.559e−01
Coefficient: −1.71e+02
Value: mild
1500
1000
500
0
teria.p__Actinobacteria.c__Coriobacteriia.o__Eggerthellales.f__Egg
asymptomatic (n=24)
mild (n=25)
severe (n=30)
control (n=72)
Severity

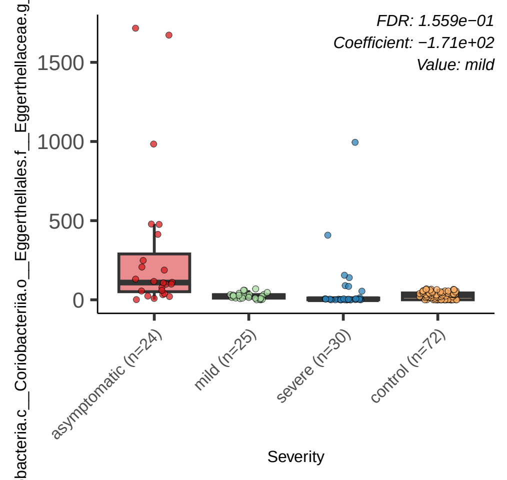

FDR: 1.559e−01
Coefficient: −1.71e+02
Value: mild
1500
1000
500
0
bacteria.c__Coriobacteriia.o__Eggerthellales.f__Eggerthellaceae.g_
asymptomatic (n=24)
mild (n=25)
severe (n=30)
control (n=72)
Severity

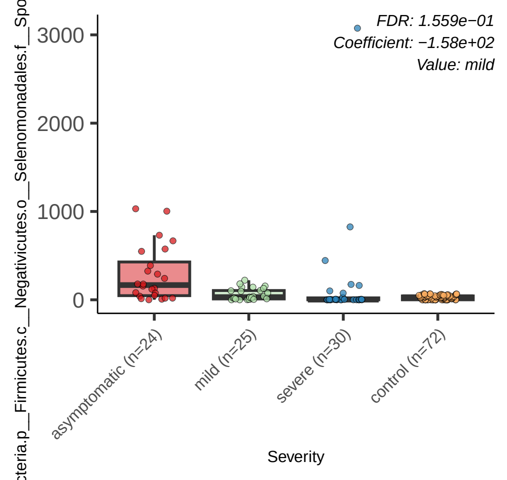
FDR: 1.559e−01
Coefficient: −1.58e+02
Value: mild
3000
2000
1000
0
asymptomatic (n=24)
mild (n=25)
severe (n=30)
control (n=72)
Severity
cteria.p__Firmicutes.c__Negativicutes.o__Selenomonadales.f__Spo

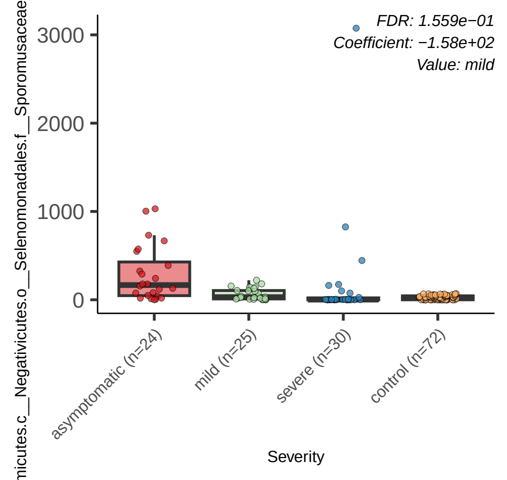

FDR: 1.559e−01
Coefficient: −1.58e+02
Value: mild
micutes.c__Negativicutes.o__Selenomonadales.f__Sporomusaceae
3000
2000
1000
0
asymptomatic (n=24)
mild (n=25)
severe (n=30)
control (n=72)
Severity

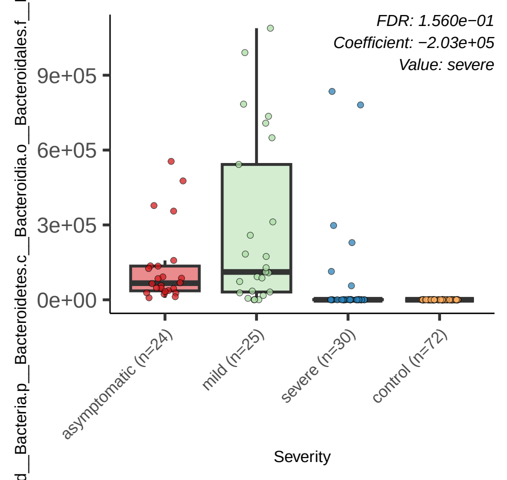

FDR: 1.560e−01
Coefficient: −2.03e+05
Value: severe
d__Bacteria.p__Bacteroidetes.c__Bacteroidia.o__Bacteroidales.f__
9e+05
6e+05
3e+05
0e+00
asymptomatic (n=24)
mild (n=25)
severe (n=30)
control (n=72)
Severity

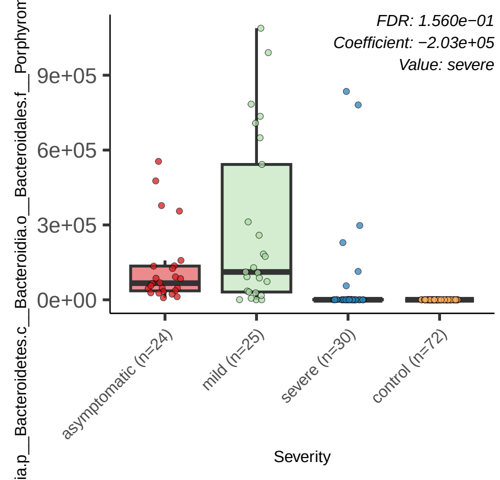

FDR: 1.560e−01
Coefficient: −2.03e+05
Value: severe
ia.p__Bacteroidetes.c__Bacteroidia.o__Bacteroidales.f__Porphyrom
9e+05
6e+05
3e+05
0e+00
asymptomatic (n=24)
mild (n=25)
severe (n=30)
control (n=72)
Severity

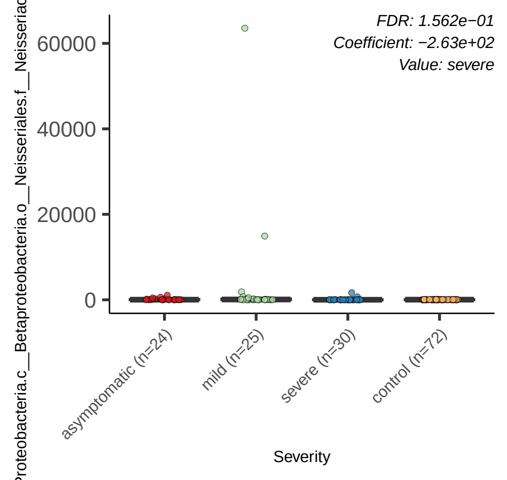
FDR: 1.562e−01
Coefficient: −2.63e+02
Value: severe
60000
40000
20000
0
Proteobacteria.c__Betaproteobacteria.o__Neisseriales.f__Neisseriac
asymptomatic (n=24)
mild (n=25)
severe (n=30)
control (n=72)
Severity

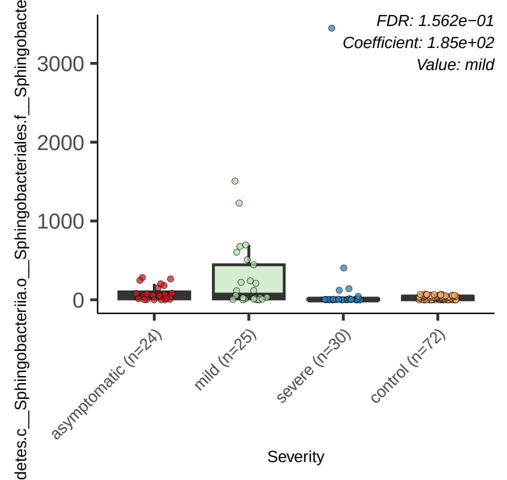

FDR: 1.562e−01
Coefficient: 1.85e+02
Value: mild
3000
2000
1000
0
detes.c__Sphingobacteriia.o__Sphingobacteriales.f__Sphingobacte
asymptomatic (n=24)
mild (n=25)
severe (n=30)
control (n=72)
Severity

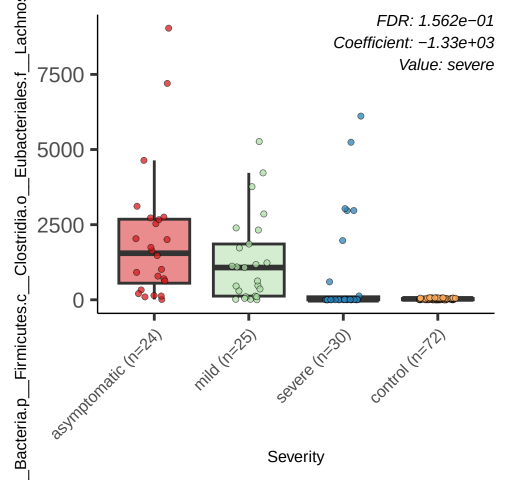

FDR: 1.562e−01
Coefficient: −1.33e+03
Value: severe
_Bacteria.p__Firmicutes.c__Clostridia.o__Eubacteriales.f__Lachnos
7500
5000
2500
0
asymptomatic (n=24)
mild (n=25)
severe (n=30)
control (n=72)
Severity

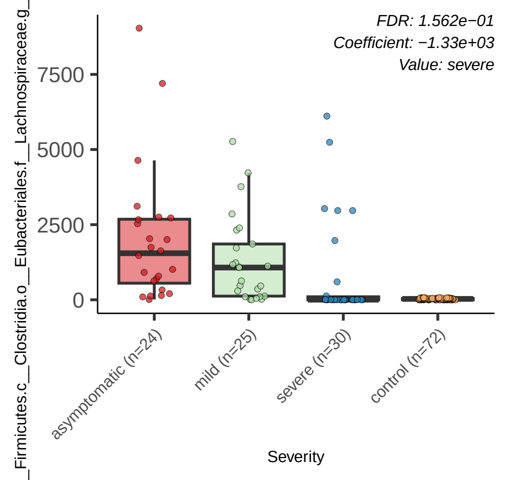
FDR: 1.562e−01
Coefficient: −1.33e+03
Value: severe
Firmicutes.c__Clostridia.o__Eubacteriales.f__Lachnospiraceae.g__
7500
5000
2500
0
asymptomatic (n=24)
mild (n=25)
severe (n=30)
control (n=72)
Severity

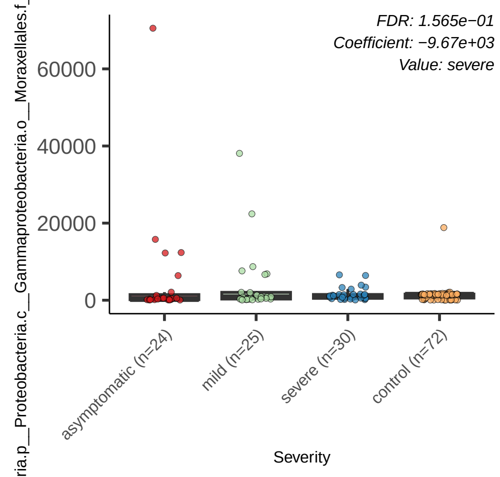
FDR: 1.565e−01
Coefficient: −9.67e+03
Value: severe
60000
40000
20000
0
eria.p__Proteobacteria.c__Gammaproteobacteria.o__Moraxellales.f_
asymptomatic (n=24)
mild (n=25)
severe (n=30)
control (n=72)
Severity

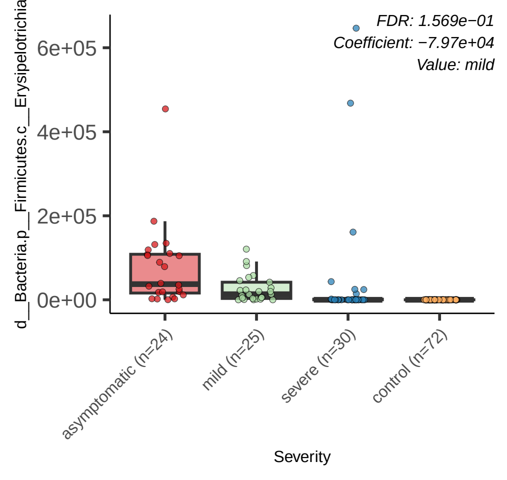

FDR: 1.569e−01
Coefficient: −7.97e+04
Value: mild
d__Bacteria.p__Firmicutes.c__Erysipelotrichia
6e+05
4e+05
2e+05
0e+00
asymptomatic (n=24)
mild (n=25)
severe (n=30)
control (n=72)
Severity

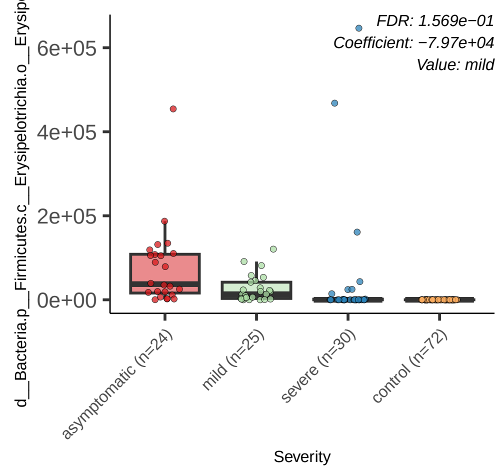

FDR: 1.569e−01
Coefficient: −7.97e+04
Value: mild
6e+05
4e+05
2e+05
0e+00
d__Bacteria.p__Firmicutes.c__Erysipelotrichia.o__Erysipe
asymptomatic (n=24)
mild (n=25)
severe (n=30)
control (n=72)
Severity

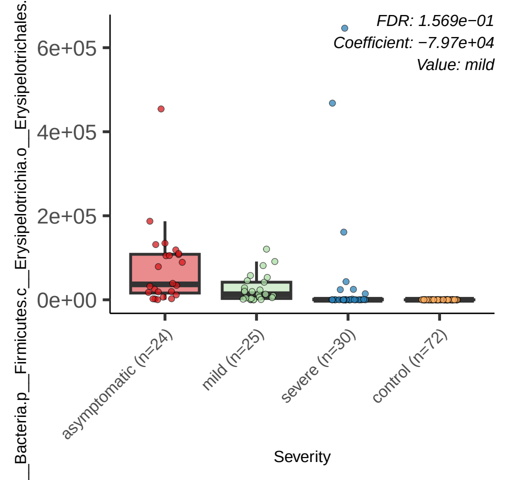
FDR: 1.569e−01
Coefficient: −7.97e+04
Value: mild
6e+05
4e+05
2e+05
0e+00
Bacteria.p__Firmicutes.c__Erysipelotrichia.o__Erysipelotrichales.
asymptomatic (n=24)
mild (n=25)
severe (n=30)
control (n=72)
Severity

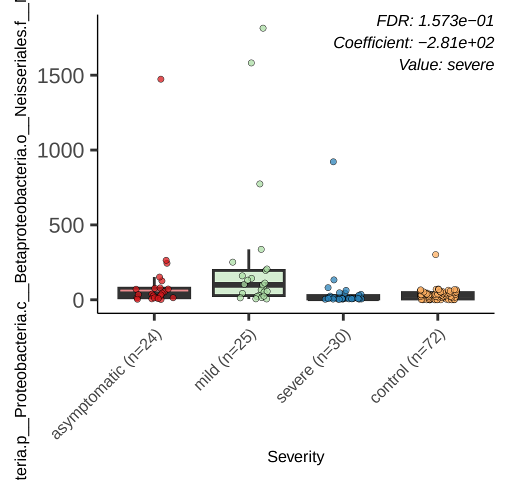
FDR: 1.573e−01
Coefficient: −2.81e+02
Value: severe
1500
1000
500
0
teria.p__Proteobacteria.c__Betaproteobacteria.o__Neisseriales.f__
asymptomatic (n=24)
mild (n=25)
severe (n=30)
control (n=72)
Severity

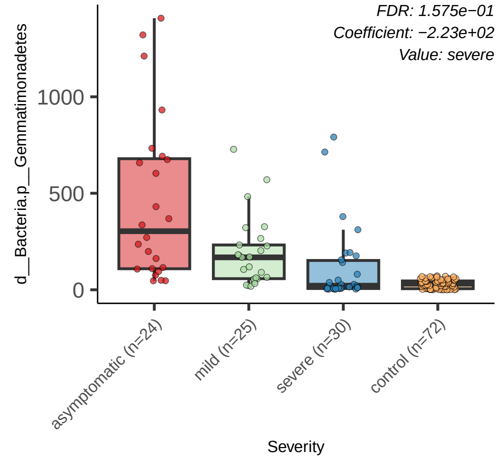

FDR: 1.575e−01
Coefficient: −2.23e+02
Value: severe
d__Bacteria.p__Gemmatimonadetes
1000
500
0
asymptomatic (n=24)
mild (n=25)
severe (n=30)
control (n=72)
Severity

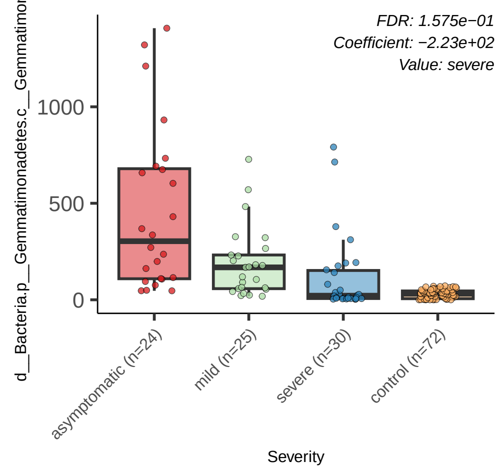

FDR: 1.575e−01
Coefficient: −2.23e+02
Value: severe
d__Bacteria.p__Gemmatimonadetes.c__Gemmatimon
1000
500
0
asymptomatic (n=24)
mild (n=25)
severe (n=30)
control (n=72)
Severity

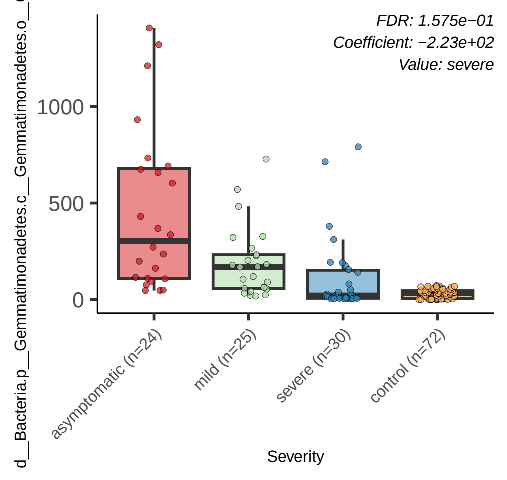
FDR: 1.575e−01
Coefficient: −2.23e+02
Value: severe
d__Bacteria.p__Gemmatimonadetes.c__Gemmatimonadetes.o__G
1000
500
0
asymptomatic (n=24)
mild (n=25)
severe (n=30)
control (n=72)
Severity

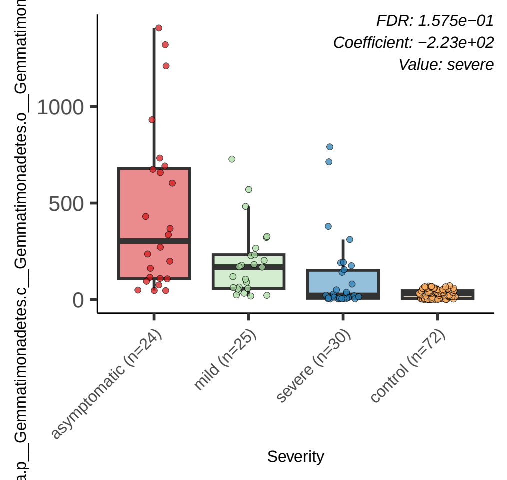

FDR: 1.575e−01
Coefficient: −2.23e+02
Value: severe
1000
500
0
a.p__Gemmatimonadetes.c__Gemmatimonadetes.o__Gemmatimon
asymptomatic (n=24)
mild (n=25)
severe (n=30)
control (n=72)
Severity

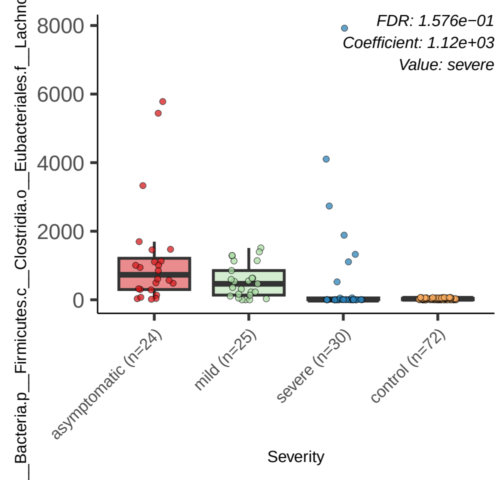

FDR: 1.576e−01
Coefficient: 1.12e+03
Value: severe
8000
6000
4000
2000
0
Bacteria.p__Firmicutes.c__Clostridia.o__Eubacteriales.f__Lachno
asymptomatic (n=24)
mild (n=25)
severe (n=30)
control (n=72)
Severity

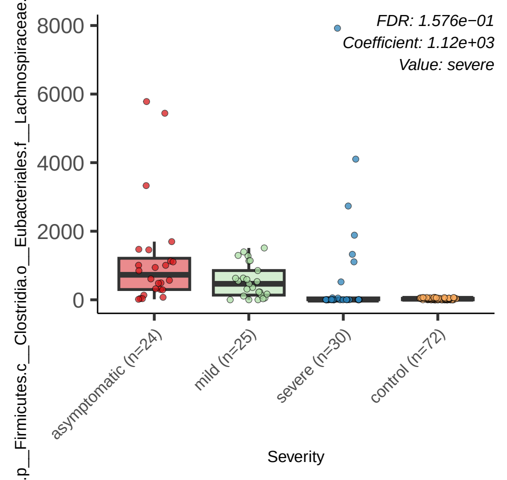

FDR: 1.576e−01
Coefficient: 1.12e+03
Value: severe
p__Firmicutes.c__Clostridia.o__Eubacteriales.f__Lachnospiraceae.
8000
6000
4000
2000
0
asymptomatic (n=24)
mild (n=25)
severe (n=30)
control (n=72)
Severity

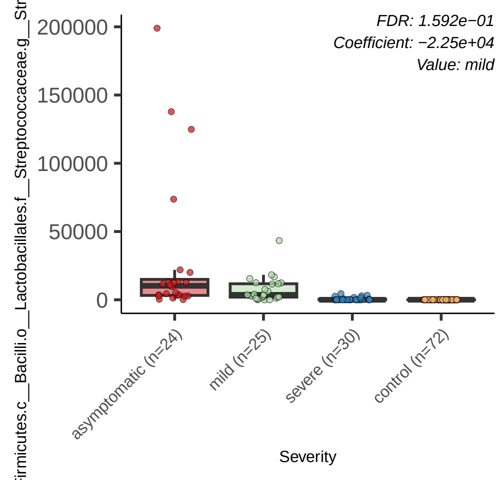

FDR: 1.592e−01
Coefficient: −2.25e+04
Value: mild
200000
150000
100000
50000
0
asymptomatic (n=24)
mild (n=25)
severe (n=30)
control (n=72)
Severity

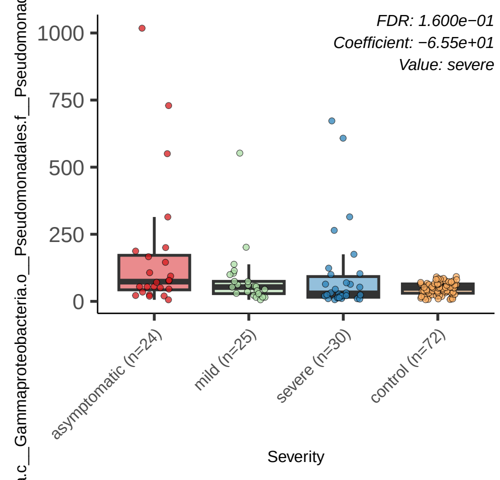
FDR: 1.600e−01
Coefficient: −6.55e+01
Value: severe
1000
750
500
250
0
asymptomatic (n=24)
mild (n=25)
severe (n=30)
control (n=72)
Severity

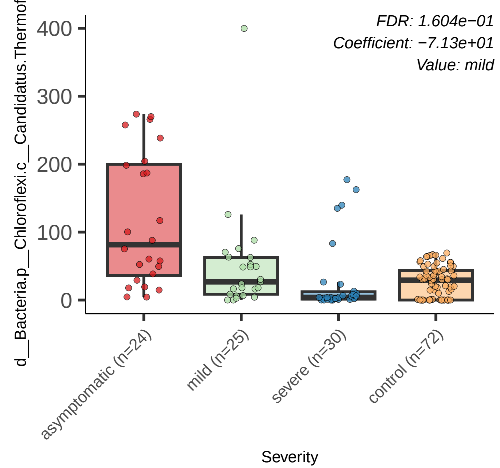

FDR: 1.604e−01
Coefficient: −7.13e+01
Value: mild
d__Bacteria.p__Chloroflexi.c__Candidatus.Thermof
0
100
200
300
400
asymptomatic (n=24)
mild (n=25)
severe (n=30)
control (n=72)
Severity

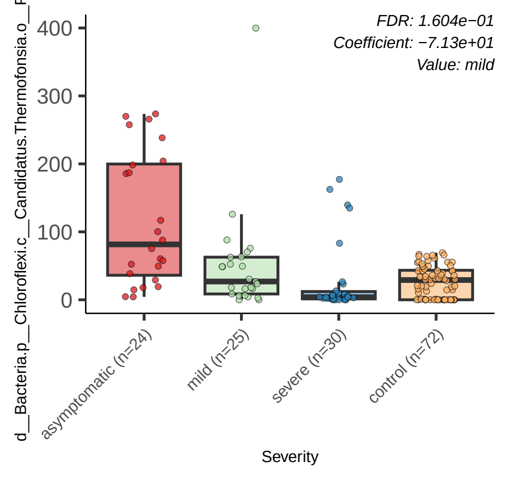

FDR: 1.604e−01
Coefficient: −7.13e+01
Value: mild
d__Bacteria.p__Chloroflexi.c__Candidatus.Thermofonsia.o__F
0
100
200
300
400
asymptomatic (n=24)
mild (n=25)
severe (n=30)
control (n=72)
Severity

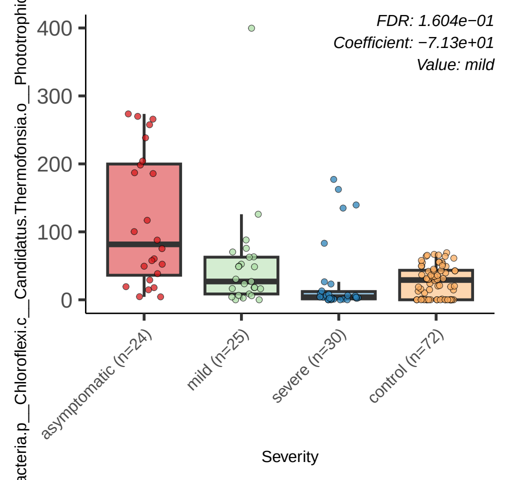
FDR: 1.604e−01
Coefficient: −7.13e+01
Value: mild
400
300
200
100
0
acteria.p__Chloroflexi.c__Candidatus.Thermofonsia.o__Phototrophic
asymptomatic (n=24)
mild (n=25)
severe (n=30)
control (n=72)
Severity

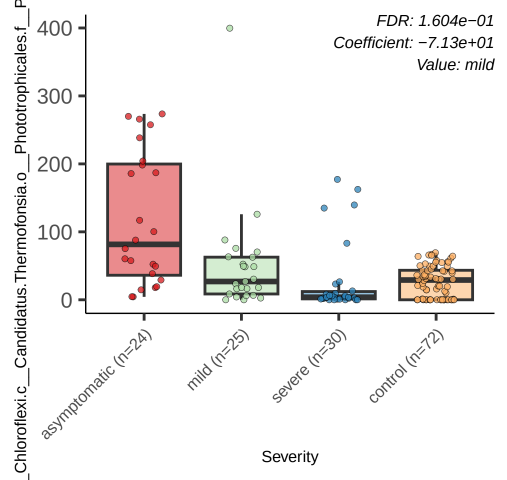

FDR: 1.604e−01
Coefficient: −7.13e+01
Value: mild
400
300
200
100
0
asymptomatic (n=24)
mild (n=25)
severe (n=30)
control (n=72)
Severity
Chloroflexi.c__Candidatus.Thermofonsia.o__Phototrophicales.f__P

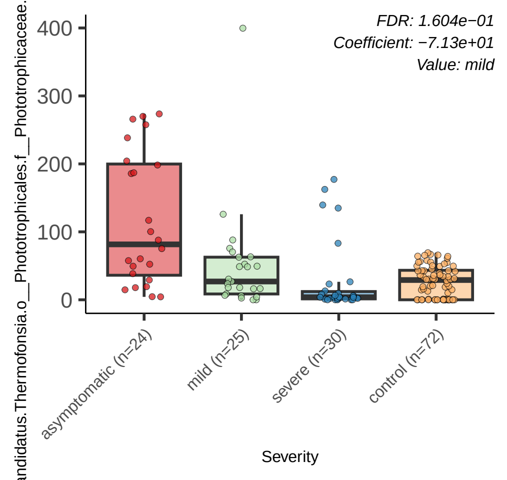

FDR: 1.604e−01
Coefficient: −7.13e+01
Value: mild
400
300
200
100
0
andidatus.Thermofonsia.o__Phototrophicales.f__Phototrophicaceae.
asymptomatic (n=24)
mild (n=25)
severe (n=30)
control (n=72)
Severity

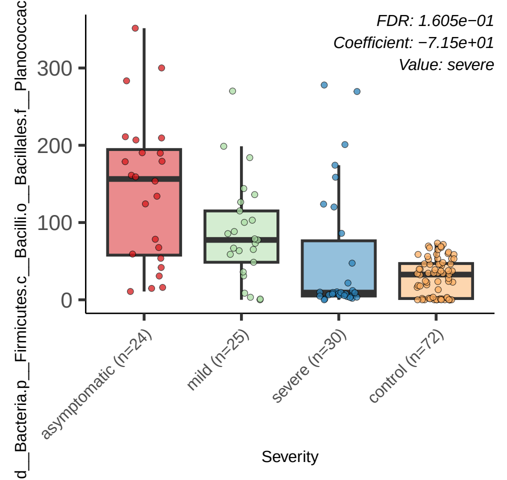

FDR: 1.605e−01
Coefficient: −7.15e+01
Value: severe
d__Bacteria.p__Firmicutes.c__Bacilli.o__Bacillales.f__Planococcac
0
100
200
300
asymptomatic (n=24)
mild (n=25)
severe (n=30)
control (n=72)
Severity

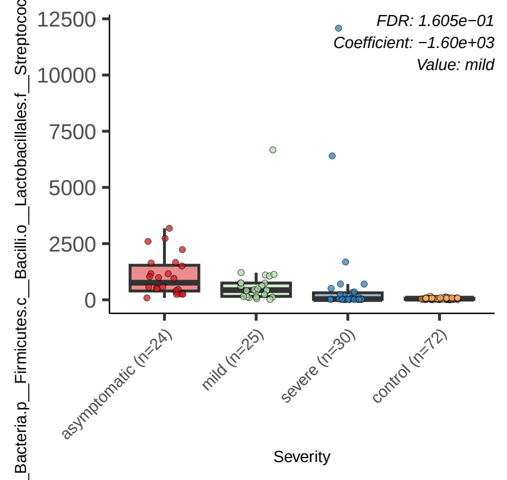

FDR: 1.605e−01
Coefficient: −1.60e+03
Value: mild
12500
10000
7500
5000
2500
0
Bacteria.p__Firmicutes.c__Bacilli.o__Lactobacillales.f__Streptococ
asymptomatic (n=24)
mild (n=25)
severe (n=30)
control (n=72)
Severity

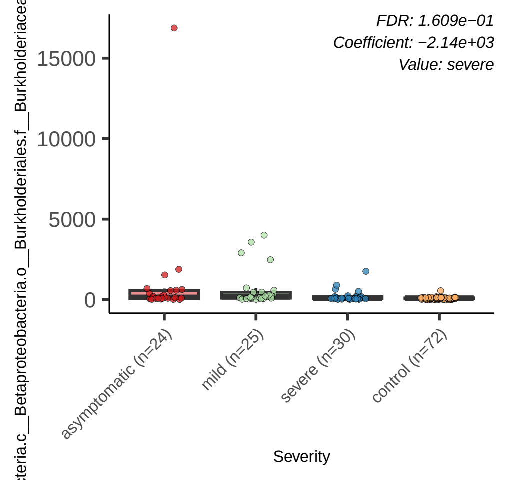

FDR: 1.609e−01
Coefficient: −2.14e+03
Value: severe
15000
10000
5000
0
cteria.c__Betaproteobacteria.o__Burkholderiales.f__Burkholderiacea
asymptomatic (n=24)
mild (n=25)
severe (n=30)
control (n=72)
Severity

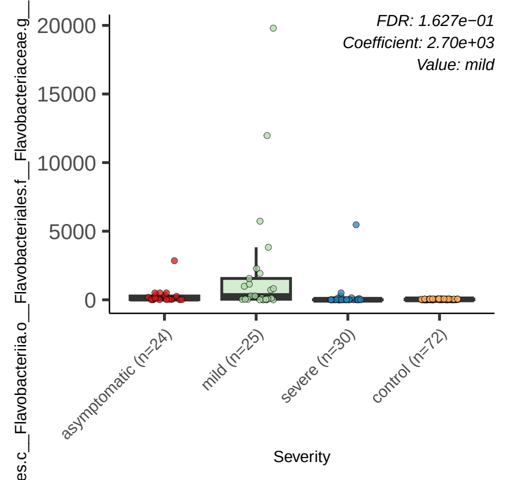
FDR: 1.627e−01
Coefficient: 2.70e+03
Value: mild
es.c__Flavobacteriia.o__Flavobacteriales.f__Flavobacteriaceae.g__
20000
15000
10000
5000
0
asymptomatic (n=24)
mild (n=25)
severe (n=30)
control (n=72)
Severity

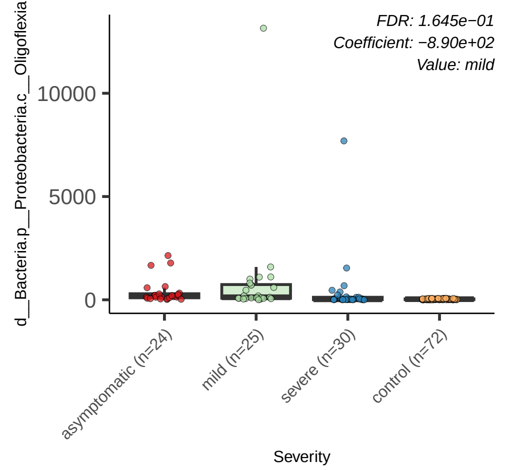

FDR: 1.645e−01
Coefficient: −8.90e+02
Value: mild
d__Bacteria.p__Proteobacteria.c__Oligoflexia
10000
5000
0
asymptomatic (n=24)
mild (n=25)
severe (n=30)
control (n=72)
Severity

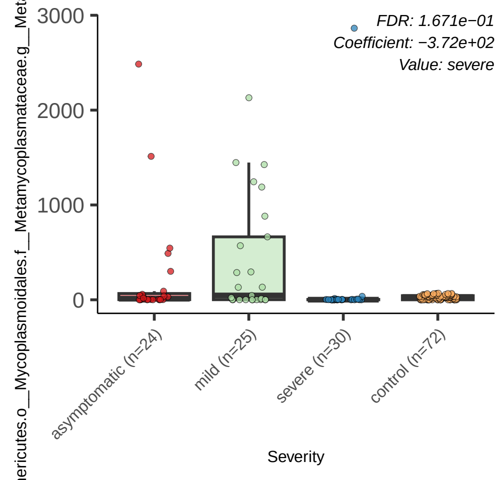

FDR: 1.671e−01
Coefficient: −3.72e+02
Value: severe
3000
2000
1000
0
asymptomatic (n=24)
mild (n=25)
severe (n=30)
control (n=72)
Severity

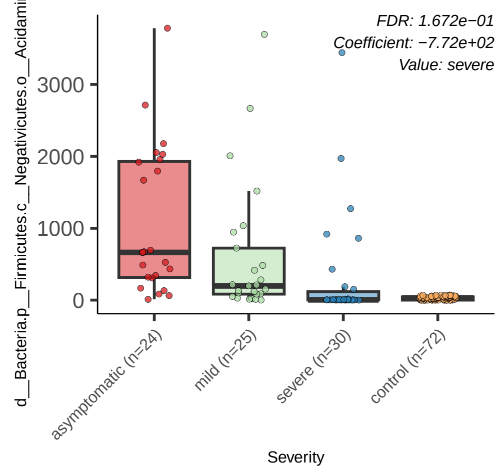
FDR: 1.672e−01
Coefficient: −7.72e+02
Value: severe
d__Bacteria.p__Firmicutes.c__Negativicutes.o__Acidami
3000
2000
1000
0
asymptomatic (n=24)
mild (n=25)
severe (n=30)
control (n=72)
Severity

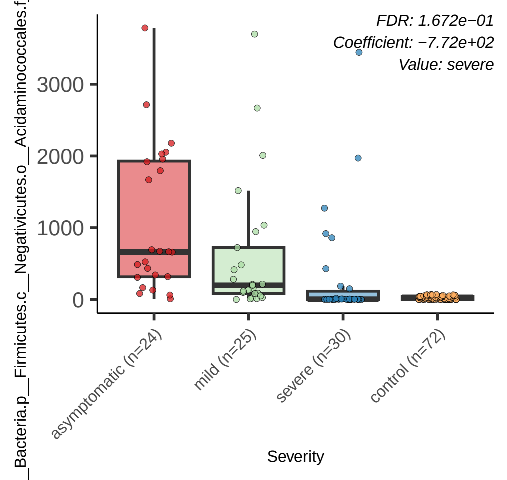
FDR: 1.672e−01
Coefficient: −7.72e+02
Value: severe
Bacteria.p__Firmicutes.c__Negativicutes.o__Acidaminococcales.f__
3000
2000
1000
0
asymptomatic (n=24)
mild (n=25)
severe (n=30)
control (n=72)
Severity

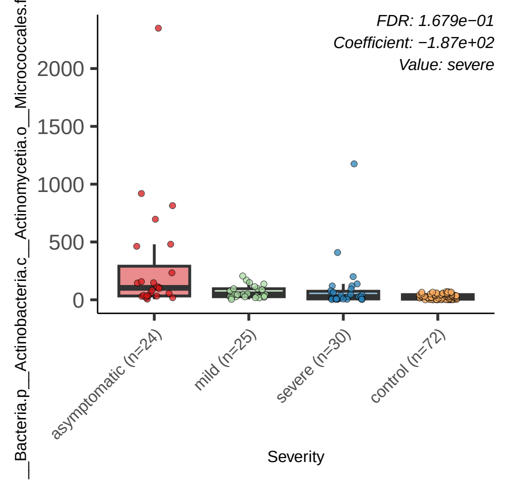

FDR: 1.679e−01
Coefficient: −1.87e+02
Value: severe
_Bacteria.p__Actinobacteria.c__Actinomycetia.o__Micrococcales.f
0
500
1000
1500
2000
asymptomatic (n=24)
mild (n=25)
severe (n=30)
control (n=72)
Severity

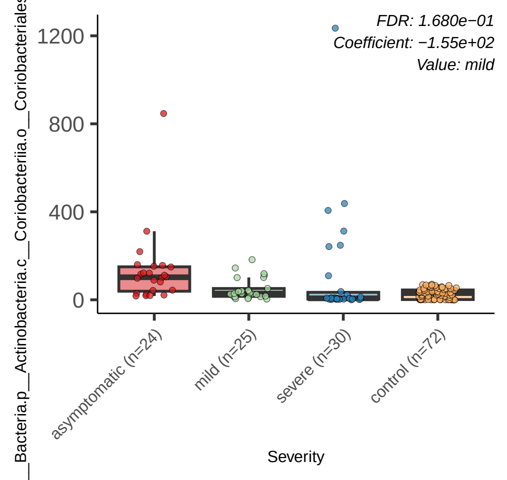
FDR: 1.680e−01
Coefficient: −1.55e+02
Value: mild
1200
800
400
0
_Bacteria.p__Actinobacteria.c__Coriobacteriia.o__Coriobacteriales
asymptomatic (n=24)
mild (n=25)
severe (n=30)
control (n=72)
Severity

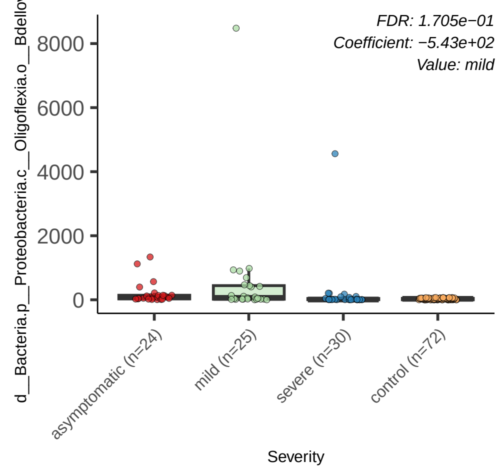
FDR: 1.705e−01
Coefficient: −5.43e+02
Value: mild
d__Bacteria.p__Proteobacteria.c__Oligoflexia.o__Bdellov
8000
6000
4000
2000
0
asymptomatic (n=24)
mild (n=25)
severe (n=30)
control (n=72)
Severity

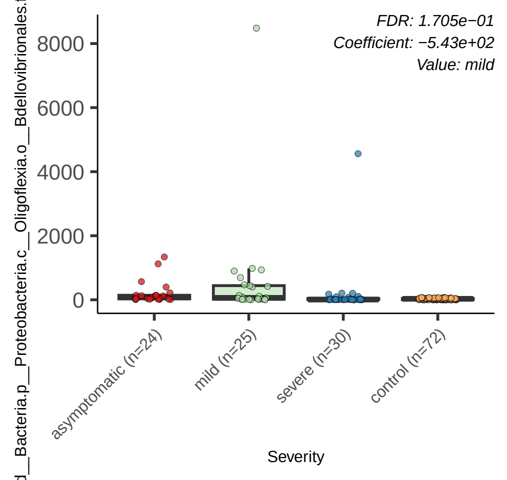

FDR: 1.705e−01
Coefficient: −5.43e+02
Value: mild
d__Bacteria.p__Proteobacteria.c__Oligoflexia.o__Bdellovibrionales.f
8000
6000
4000
2000
0
asymptomatic (n=24)
mild (n=25)
severe (n=30)
control (n=72)
Severity

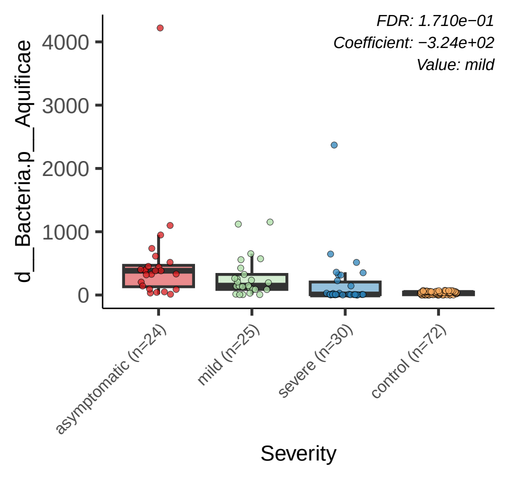

FDR: 1.710e−01
Coefficient: −3.24e+02
Value: mild
d__Bacteria.p__Aquificae
4000
3000
2000
1000
0
asymptomatic (n=24)
mild (n=25)
severe (n=30)
control (n=72)
Severity

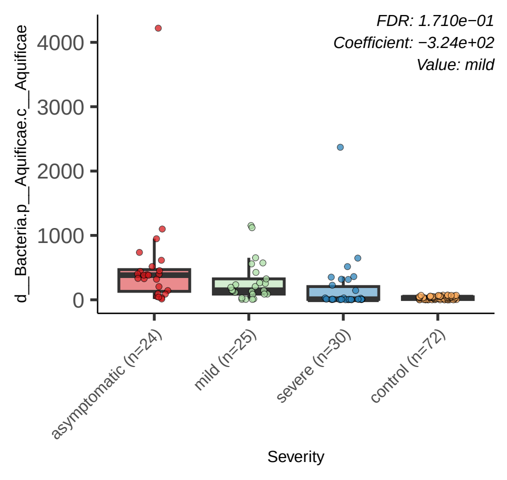
FDR: 1.710e−01
Coefficient: −3.24e+02
Value: mild
d__Bacteria.p__Aquificae.c__Aquificae
4000
3000
2000
1000
0
asymptomatic (n=24)
mild (n=25)
severe (n=30)
control (n=72)
Severity

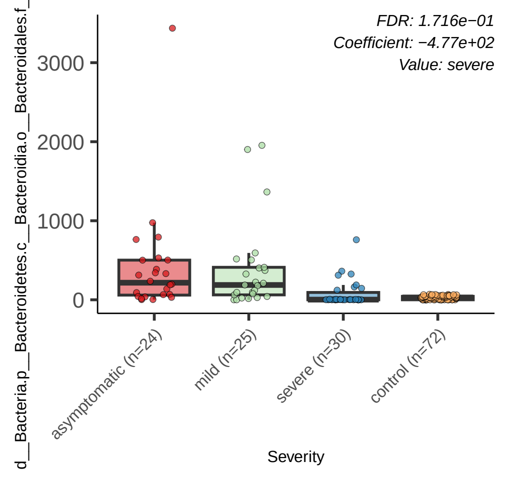

FDR: 1.716e−01
Coefficient: −4.77e+02
Value: severe
d__Bacteria.p__Bacteroidetes.c__Bacteroidia.o__Bacteroidales.f__
3000
2000
1000
0
asymptomatic (n=24)
mild (n=25)
severe (n=30)
control (n=72)
Severity

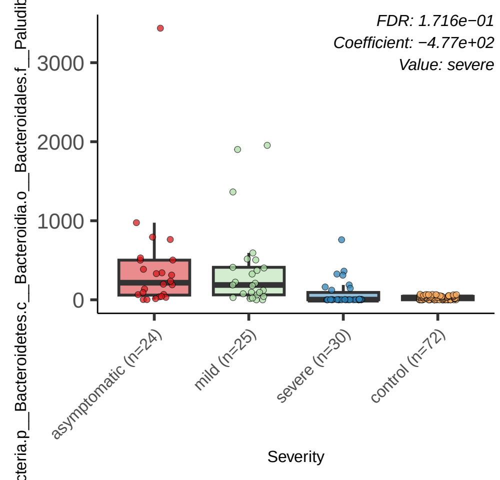

FDR: 1.716e−01
Coefficient: −4.77e+02
Value: severe
3000
2000
1000
0
cteria.p__Bacteroidetes.c__Bacteroidia.o__Bacteroidales.f__Paludib
asymptomatic (n=24)
mild (n=25)
severe (n=30)
control (n=72)
Severity

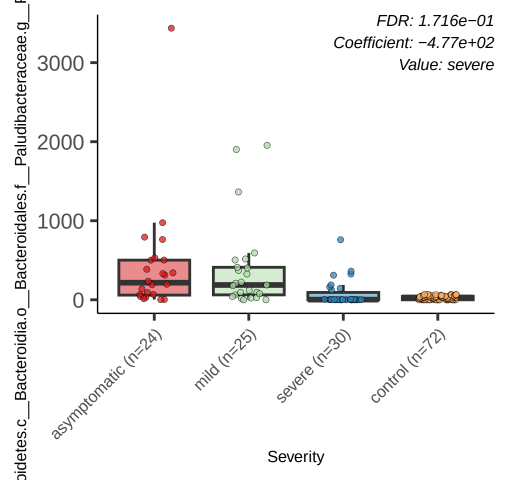

FDR: 1.716e−01
Coefficient: −4.77e+02
Value: severe
3000
2000
1000
0
oidetes.c__Bacteroidia.o__Bacteroidales.f__Paludibacteraceae.g__P
asymptomatic (n=24)
mild (n=25)
severe (n=30)
control (n=72)
Severity

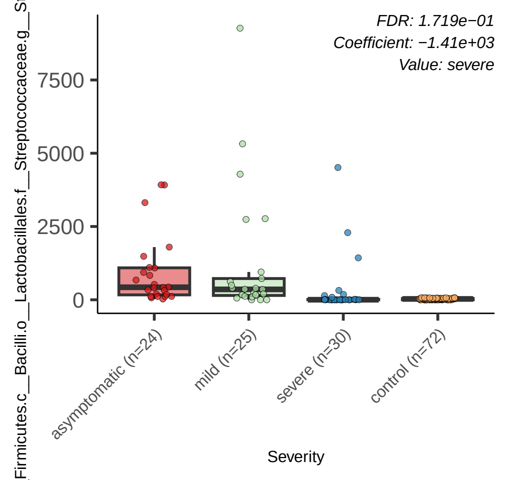

FDR: 1.719e−01
Coefficient: −1.41e+03
Value: severe
Firmicutes.c__Bacilli.o__Lactobacillales.f__Streptococcaceae.g__St
7500
5000
2500
0
asymptomatic (n=24)
mild (n=25)
severe (n=30)
control (n=72)
Severity

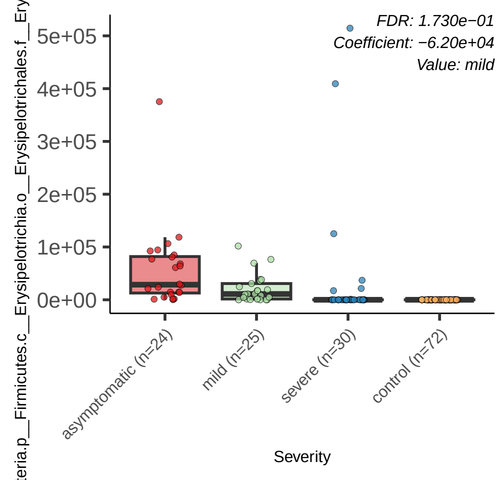

teria.p__Firmicutes.c__Erysipelotrichia.o__Erysipelotrichales.f__Ery
FDR: 1.730e−01
Coefficient: −6.20e+04
Value: mild
5e+05
4e+05
3e+05
2e+05
1e+05
0e+00
asymptomatic (n=24)
mild (n=25)
severe (n=30)
control (n=72)
Severity

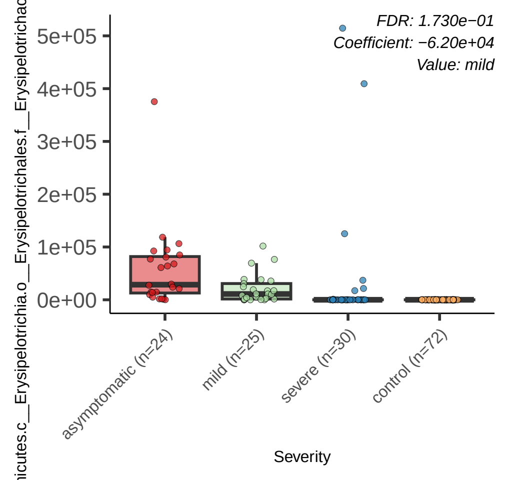

FDR: 1.730e−01
Coefficient: −6.20e+04
Value: mild
5e+05
4e+05
3e+05
2e+05
1e+05
0e+00
nicutes.c__Erysipelotrichia.o__Erysipelotrichales.f__Erysipelotrichac
asymptomatic (n=24)
mild (n=25)
severe (n=30)
control (n=72)
Severity

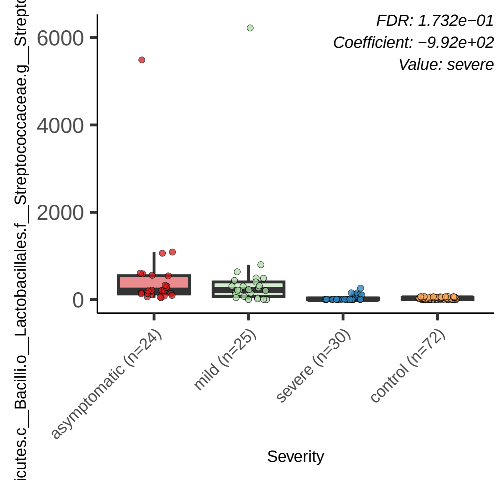

icutes.c__Bacilli.o__Lactobacillales.f__Streptococcaceae.g__Strepto
FDR: 1.732e−01
Coefficient: −9.92e+02
Value: severe
6000
4000
2000
0
asymptomatic (n=24)
mild (n=25)
severe (n=30)
control (n=72)
Severity

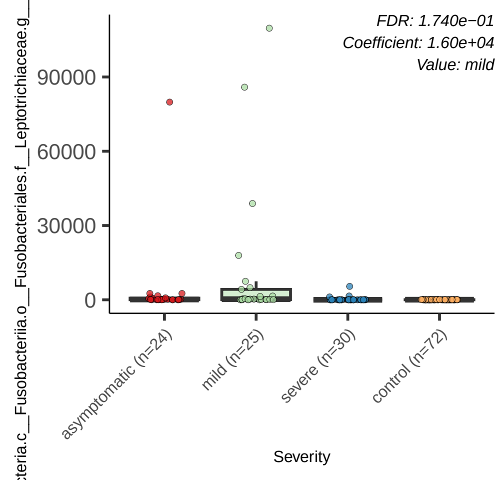

FDR: 1.740e−01
Coefficient: 1.60e+04
Value: mild
90000
60000
30000
0
Fusobacteria.c__Fusobacteriia.o__Fusobacteriales.f__Leptotrichiaceae.g__
asymptomatic (n=24)
mild (n=25)
severe (n=30)
control (n=72)
Severity

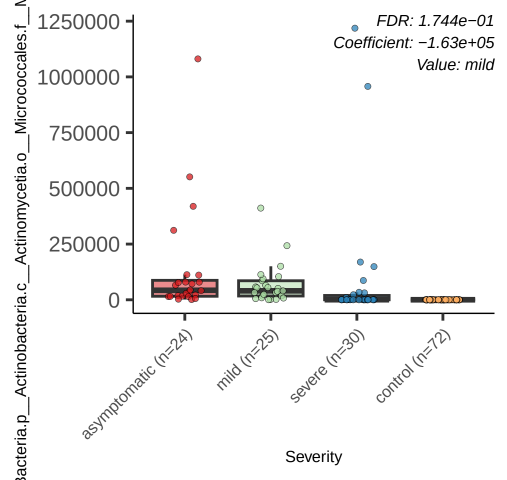
FDR: 1.744e−01
Coefficient: −1.63e+05
Value: mild
Bacteria.p__Actinobacteria.c__Actinomycetia.o__Micrococcales.f__M
1250000
1000000
750000
500000
250000
0
asymptomatic (n=24)
mild (n=25)
severe (n=30)
control (n=72)
Severity

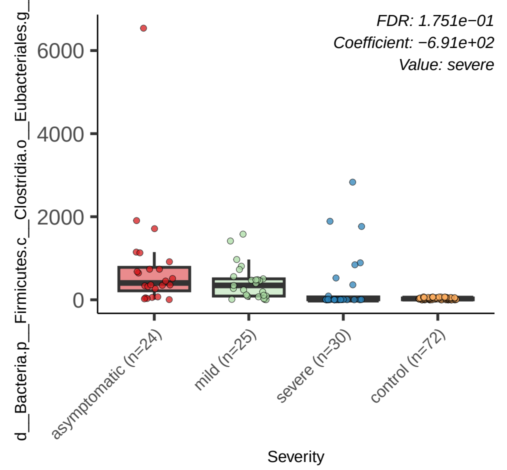

FDR: 1.751e−01
Coefficient: −6.91e+02
Value: severe
d__Bacteria.p__Firmicutes.c__Clostridia.o__Eubacteriales.g__
6000
4000
2000
0
asymptomatic (n=24)
mild (n=25)
severe (n=30)
control (n=72)
Severity

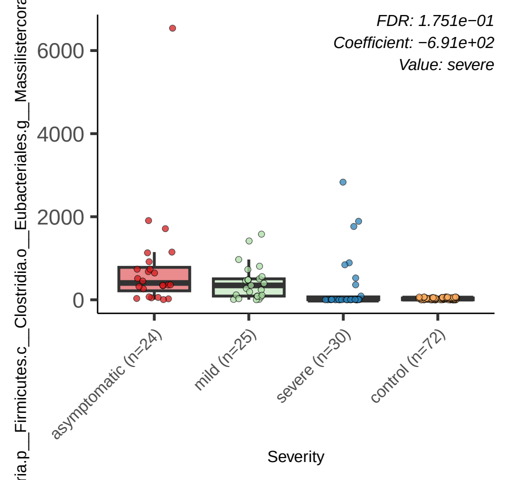

FDR: 1.751e−01
Coefficient: −6.91e+02
Value: severe
6000
4000
2000
0
ria.p__Firmicutes.c__Clostridia.o__Eubacteriales.g__Massilistercora
asymptomatic (n=24)
mild (n=25)
severe (n=30)
control (n=72)
Severity

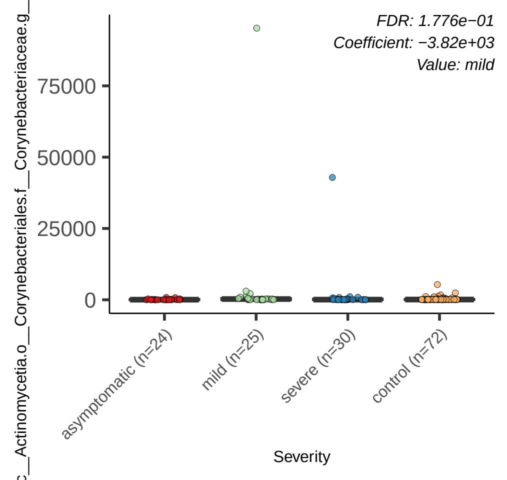
FDR: 1.776e−01
Coefficient: −3.82e+03
Value: mild
75000
50000
25000
0
c__Actinomycetia.o__Corynebacteriales.f__Corynebacteriaceae.g__
asymptomatic (n=24)
mild (n=25)
severe (n=30)
control (n=72)
Severity

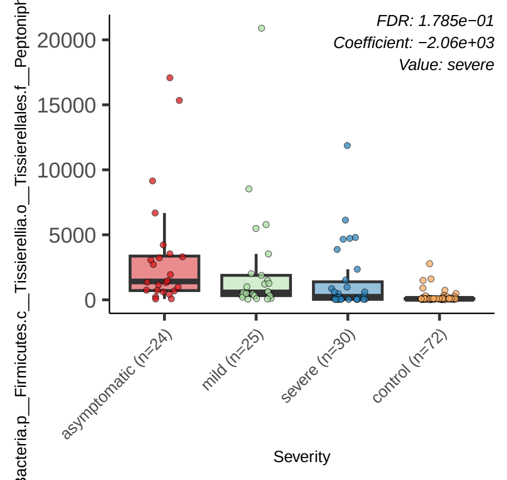

FDR: 1.785e−01
Coefficient: −2.06e+03
Value: severe
Bacteria.p__Firmicutes.c__Tissierellia.o__Tissierellales.f__Peptoniph
20000
15000
10000
5000
0
asymptomatic (n=24)
mild (n=25)
severe (n=30)
control (n=72)
Severity

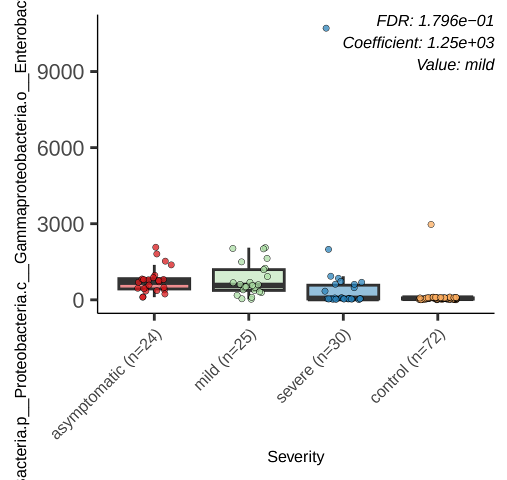

FDR: 1.796e−01
Coefficient: 1.25e+03
Value: mild
9000
6000
3000
0
acteria.p__Proteobacteria.c__Gammaproteobacteria.o__Enterobac
asymptomatic (n=24)
mild (n=25)
severe (n=30)
control (n=72)
Severity

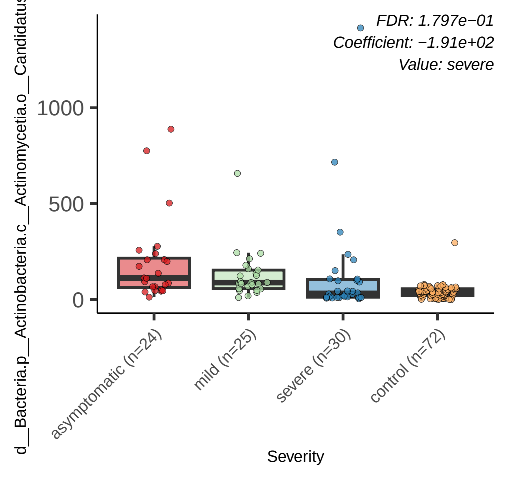
FDR: 1.797e−01
Coefficient: −1.91e+02
Value: severe
d__Bacteria.p__Actinobacteria.c__Actinomycetia.o__Candidatus
1000
500
0
asymptomatic (n=24)
mild (n=25)
severe (n=30)
control (n=72)
Severity

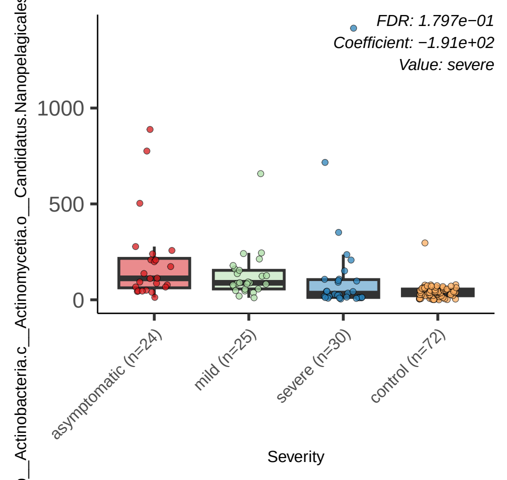

FDR: 1.797e−01
Coefficient: −1.91e+02
Value: severe
o__Actinobacteria.c__Actinomycetia.o__Candidatus.Nanopelagicales
1000
500
0
asymptomatic (n=24)
mild (n=25)
severe (n=30)
control (n=72)
Severity

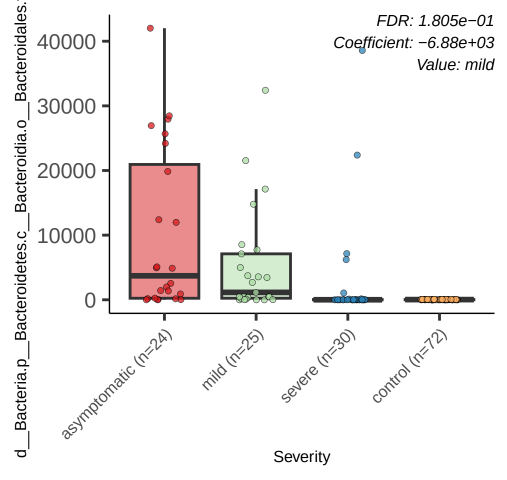

FDR: 1.805e−01
Coefficient: −6.88e+03
Value: mild
40000
30000
20000
10000
0
d__Bacteria.p__Bacteroidetes.c__Bacteroidia.o__Bacteroidales.f
asymptomatic (n=24)
mild (n=25)
severe (n=30)
control (n=72)
Severity

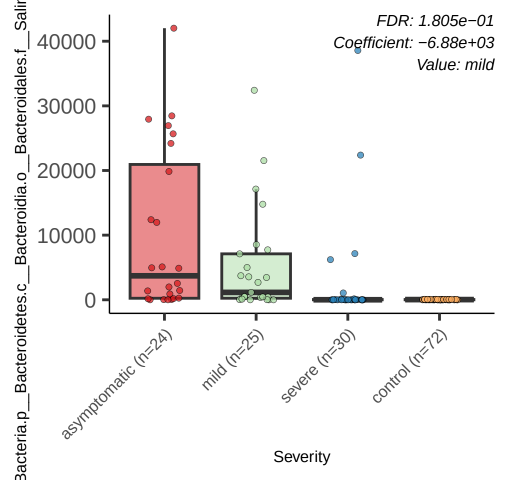

FDR: 1.805e−01
Coefficient: −6.88e+03
Value: mild
40000
30000
20000
10000
0
Bacteria.p__Bacteroidetes.c__Bacteroidia.o__Bacteroidales.f__Sali
asymptomatic (n=24)
mild (n=25)
severe (n=30)
control (n=72)
Severity

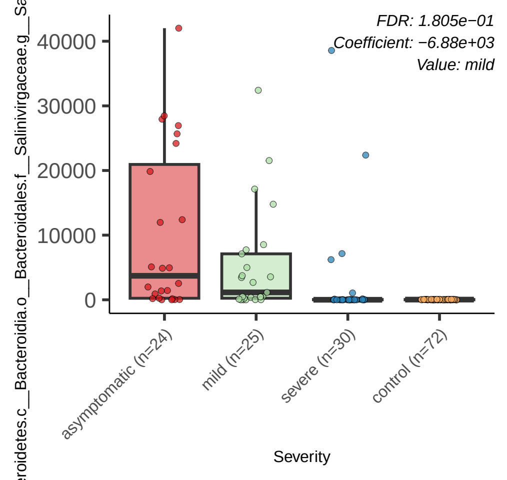

FDR: 1.805e−01
Coefficient: −6.88e+03
Value: mild
40000
30000
20000
10000
0
eroidetes.c__Bacteroidia.o__Bacteroidales.f__Salinivirgaceae.g__Sa
asymptomatic (n=24)
mild (n=25)
severe (n=30)
control (n=72)
Severity

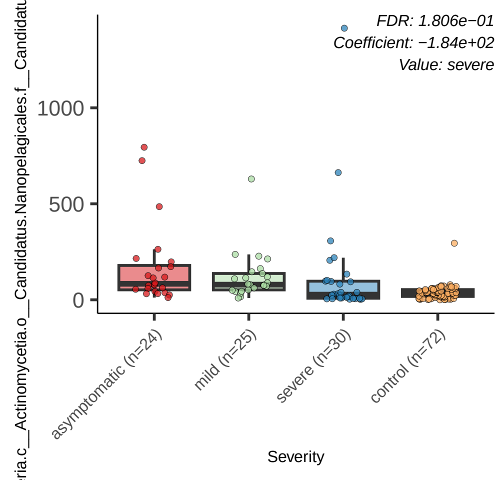

FDR: 1.806e−01
Coefficient: −1.84e+02
Value: severe
1000
500
0
asymptomatic (n=24)
mild (n=25)
severe (n=30)
control (n=72)
Severity

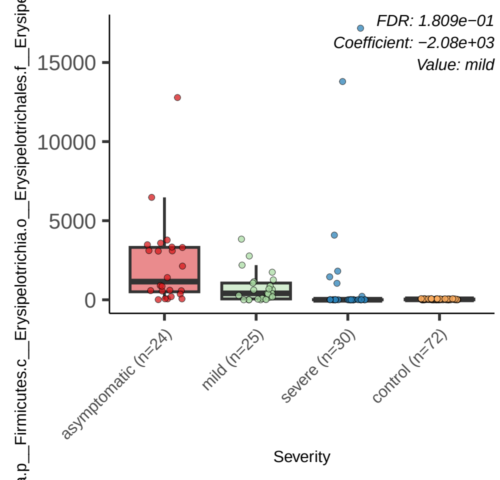

FDR: 1.809e−01
Coefficient: −2.08e+03
Value: mild
Firmicutes.c__Erysipelotrichia.o__Erysipelotrichales.f__Erysipe
15000
10000
5000
0
asymptomatic (n=24)
mild (n=25)
severe (n=30)
control (n=72)
Severity

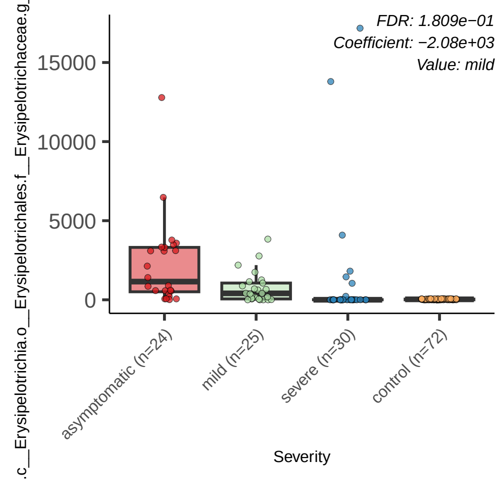
FDR: 1.809e−01
Coefficient: −2.08e+03
Value: mild
15000
10000
5000
0
c__Erysipelotrichia.o__Erysipelotrichales.f__Erysipelotrichaceae.g_
asymptomatic (n=24)
mild (n=25)
severe (n=30)
control (n=72)
Severity

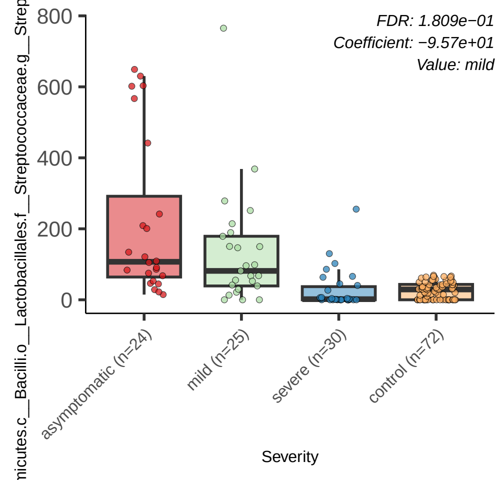
FDR: 1.809e−01
Coefficient: −9.57e+01
Value: mild
800
600
400
200
0
micutes.c__Bacilli.o__Lactobacillales.f__Streptococcaceae.g__Strep
asymptomatic (n=24)
mild (n=25)
severe (n=30)
control (n=72)
Severity

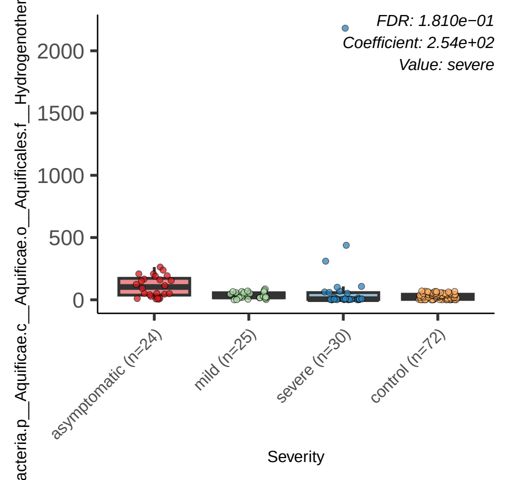

FDR: 1.810e−01
Coefficient: 2.54e+02
Value: severe
acteria.p__Aquificae.c__Aquificae.o__Aquificales.f__Hydrogenother
2000
1500
1000
500
0
asymptomatic (n=24)
mild (n=25)
severe (n=30)
control (n=72)
Severity

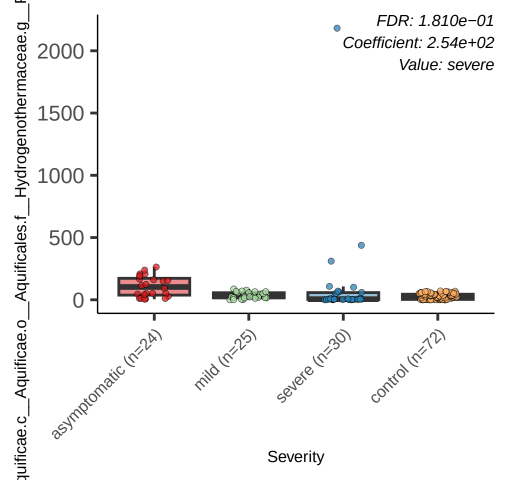

Aquificae.c__Aquificae.o__Aquificales.f__Hydrogenothermaceae.g__
FDR: 1.810e−01
Coefficient: 2.54e+02
Value: severe
2000
1500
1000
500
0
asymptomatic (n=24)
mild (n=25)
severe (n=30)
control (n=72)
Severity

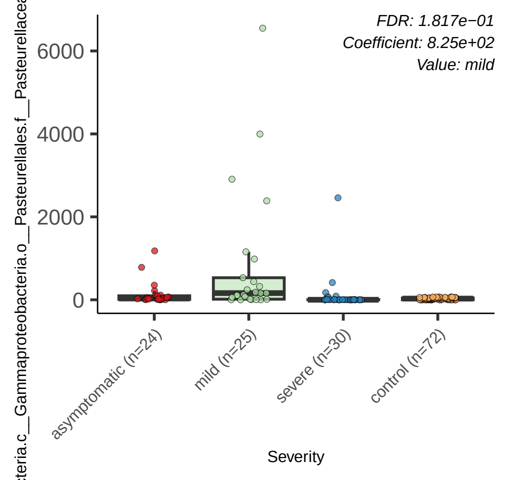

FDR: 1.817e−01
Coefficient: 8.25e+02
Value: mild
6000
4000
2000
0
teria.c__Gammaproteobacteria.o__Pasteurellales.f__Pasteurellacea
asymptomatic (n=24)
mild (n=25)
severe (n=30)
control (n=72)
Severity

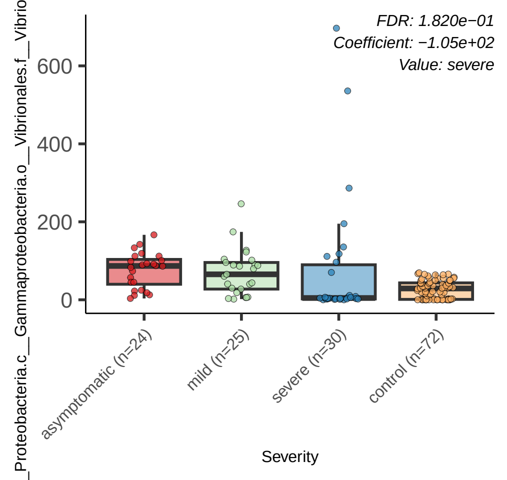

FDR: 1.820e−01
Coefficient: −1.05e+02
Value: severe
0
200
400
600
_Proteobacteria.c__Gammaproteobacteria.o__Vibrionales.f__Vibrio
asymptomatic (n=24)
mild (n=25)
severe (n=30)
control (n=72)
Severity

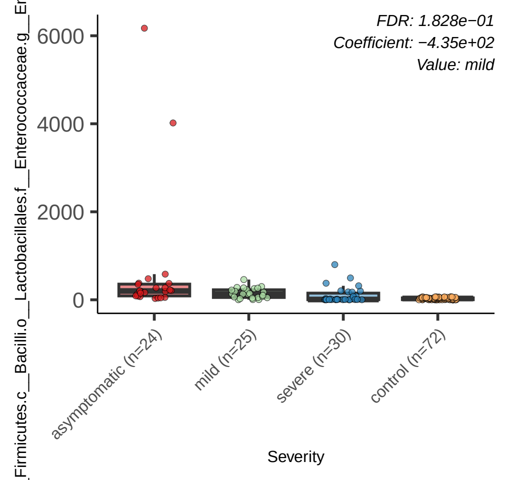
FDR: 1.828e−01
Coefficient: −4.35e+02
Value: mild
6000
4000
2000
0
Firmicutes.c__Bacilli.o__Lactobacillales.f__Enterococcaceae.g__En
asymptomatic (n=24)
mild (n=25)
severe (n=30)
control (n=72)
Severity

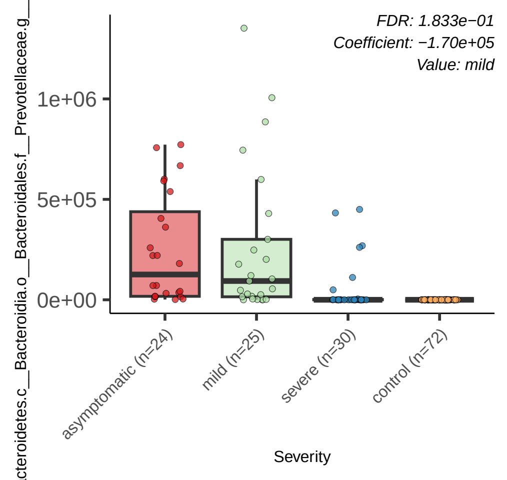

FDR: 1.833e−01
Coefficient: −1.70e+05
Value: mild
1e+06
5e+05
0e+00
asymptomatic (n=24)
mild (n=25)
severe (n=30)
control (n=72)
Severity
cteroidetes.c__Bacteroidia.o__Bacteroidales.f__Prevotellaceae.g__

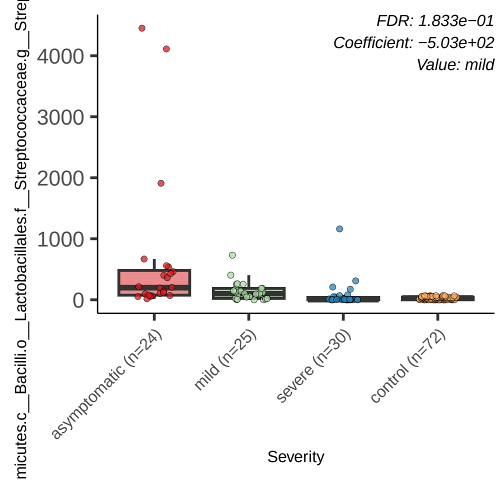

FDR: 1.833e−01
Coefficient: −5.03e+02
Value: mild
4000
3000
2000
1000
0
micutes.c__Bacilli.o__Lactobacillales.f__Streptococcaceae.g__Strep
asymptomatic (n=24)
mild (n=25)
severe (n=30)
control (n=72)
Severity

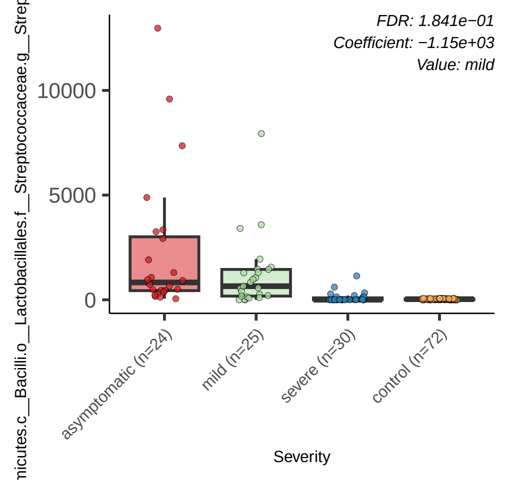

FDR: 1.841e−01
Coefficient: −1.15e+03
Value: mild
micutes.c__Bacilli.o__Lactobacillales.f__Streptococcaceae.g__Strep
10000
5000
0
asymptomatic (n=24)
mild (n=25)
severe (n=30)
control (n=72)
Severity

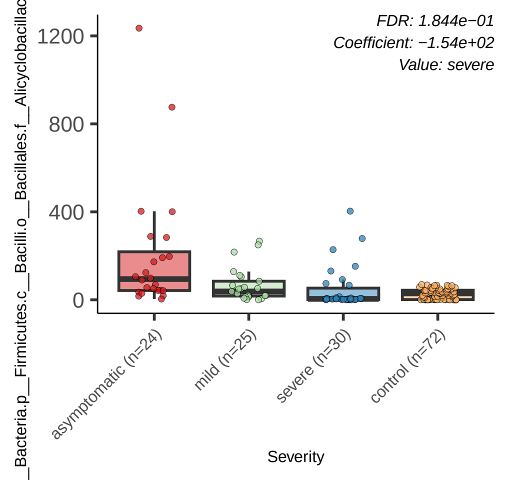
FDR: 1.844e−01
Coefficient: −1.54e+02
Value: severe
1200
800
400
0
asymptomatic (n=24)
mild (n=25)
severe (n=30)
control (n=72)
Severity
Bacteria.p__Firmicutes.c__Bacilli.o__Bacillales.f__Alicyclobacillac

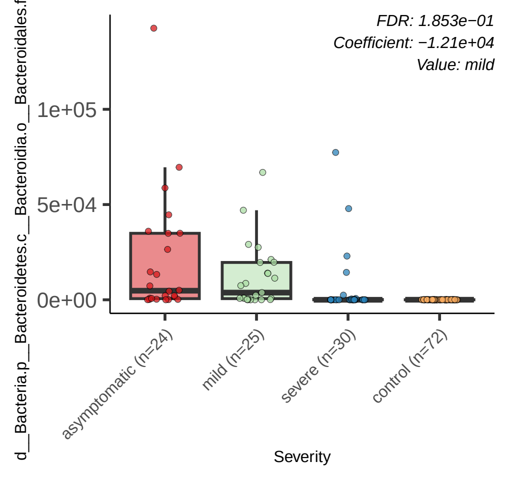
FDR: 1.853e−01
Coefficient: −1.21e+04
Value: mild
d__Bacteria.p__Bacteroidetes.c__Bacteroidia.o__Bacteroidales.f
1e+05
5e+04
0e+00
asymptomatic (n=24)
mild (n=25)
severe (n=30)
control (n=72)
Severity

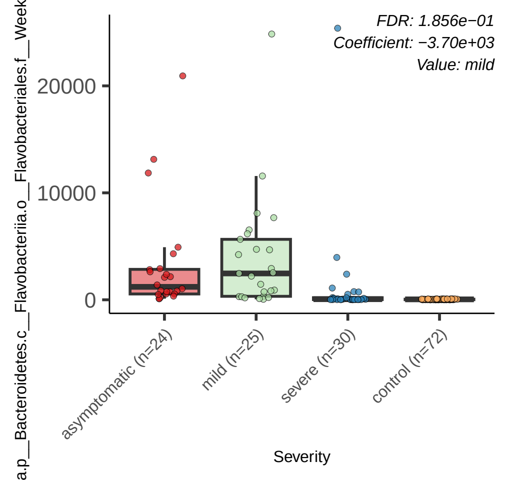

FDR: 1.856e−01
Coefficient: −3.70e+03
Value: mild
20000
10000
0
a.p__Bacteroidetes.c__Flavobacteriia.o__Flavobacteriales.f__Week
asymptomatic (n=24)
mild (n=25)
severe (n=30)
control (n=72)
Severity

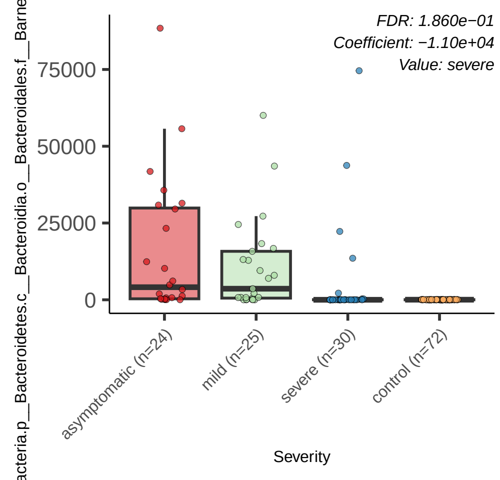

FDR: 1.860e−01
Coefficient: −1.10e+04
Value: severe
75000
50000
25000
0
acteria.p__Bacteroidetes.c__Bacteroidia.o__Bacteroidales.f__Barne
asymptomatic (n=24)
mild (n=25)
severe (n=30)
control (n=72)
Severity

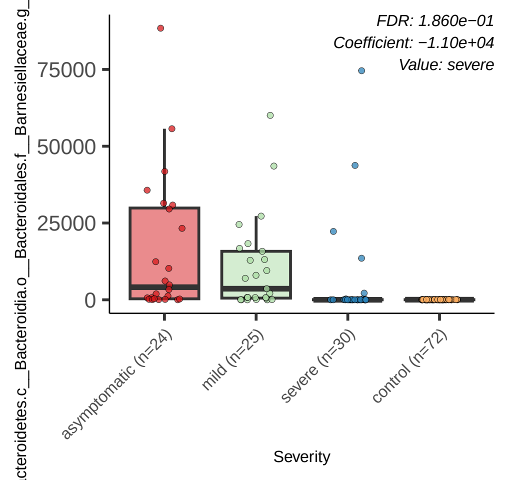

FDR: 1.860e−01
Coefficient: −1.10e+04
Value: severe
75000
50000
25000
0
acteroidetes.c__Bacteroidia.o__Bacteroidales.f__Barnesiellaceae.g__
asymptomatic (n=24)
mild (n=25)
severe (n=30)
control (n=72)
Severity

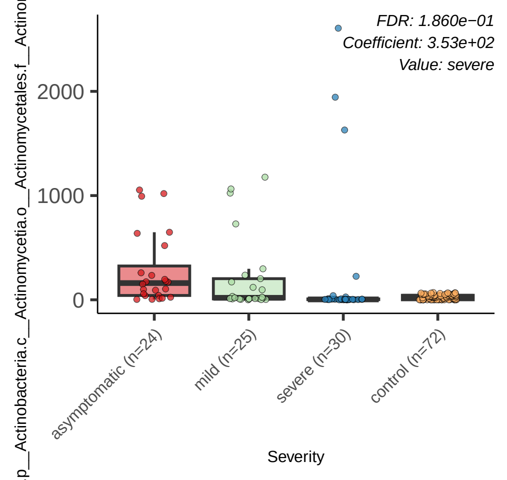

FDR: 1.860e−01
Coefficient: 3.53e+02
Value: severe
p__Actinobacteria.c__Actinomycetia.o__Actinomycetales.f__Actino
2000
1000
0
asymptomatic (n=24)
mild (n=25)
severe (n=30)
control (n=72)
Severity

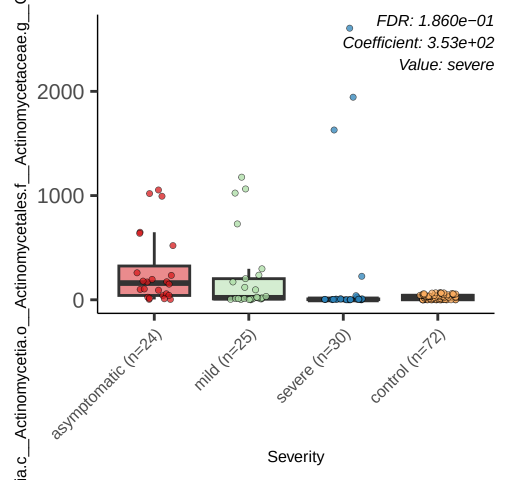

ia.c__Actinomycetia.o__Actinomycetales.f__Actinomycetaceae.g__C
FDR: 1.860e−01
Coefficient: 3.53e+02
Value: severe
2000
1000
0
asymptomatic (n=24)
mild (n=25)
severe (n=30)
control (n=72)
Severity

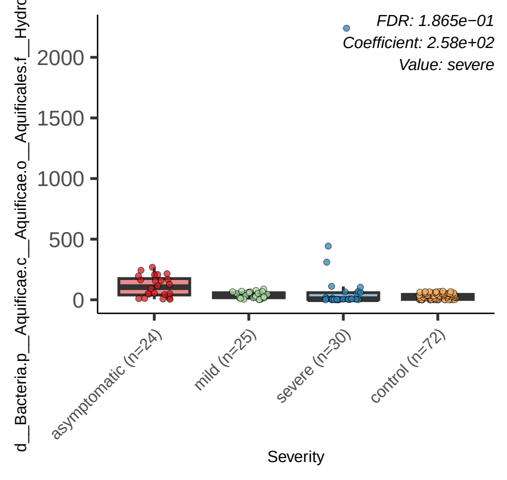

FDR: 1.865e−01
Coefficient: 2.58e+02
Value: severe
d__Bacteria.p__Aquificae.c__Aquificae.o__Aquificales.f__Hydro
2000
1500
1000
500
0
asymptomatic (n=24)
mild (n=25)
severe (n=30)
control (n=72)
Severity

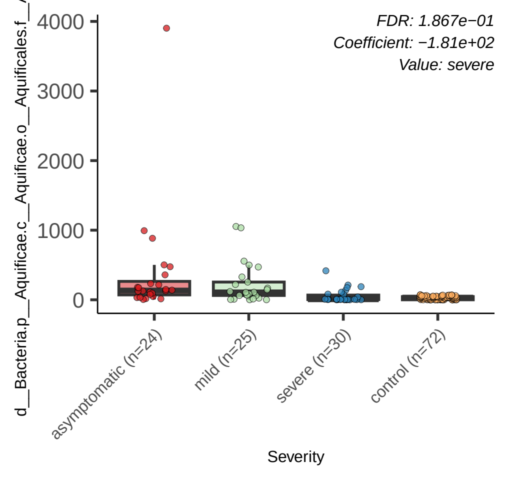

FDR: 1.867e−01
Coefficient: −1.81e+02
Value: severe
d__Bacteria.p__Aquificae.c__Aquificae.o__Aquificales.f__
4000
3000
2000
1000
0
asymptomatic (n=24)
mild (n=25)
severe (n=30)
control (n=72)
Severity

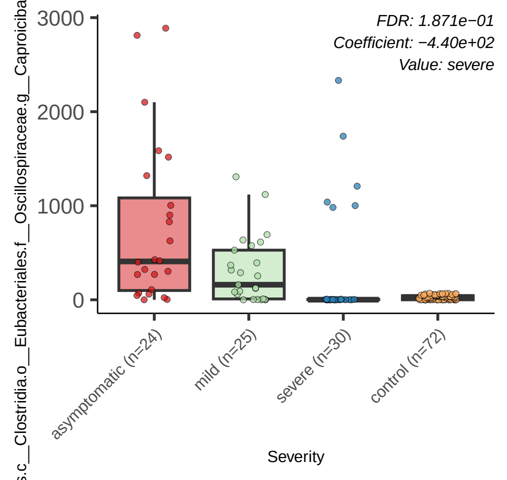

FDR: 1.871e−01
Coefficient: −4.40e+02
Value: severe
s.c__Clostridia.o__Eubacteriales.f__Oscillospiraceae.g__Caproiciba
3000
2000
1000
0
asymptomatic (n=24)
mild (n=25)
severe (n=30)
control (n=72)
Severity

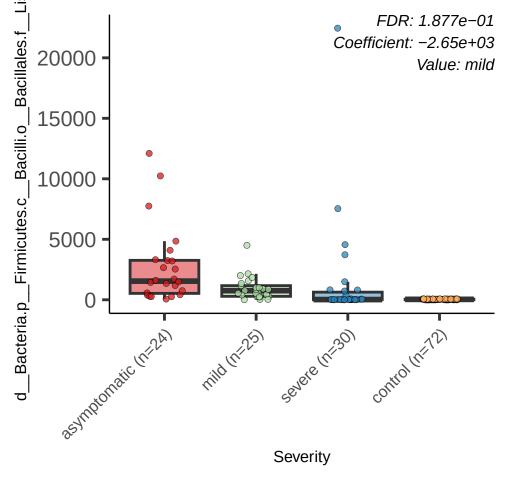

FDR: 1.877e−01
Coefficient: −2.65e+03
Value: mild
d__Bacteria.p__Firmicutes.c__Bacilli.o__Bacillales.f__Li
20000
15000
10000
5000
0
asymptomatic (n=24)
mild (n=25)
severe (n=30)
control (n=72)
Severity

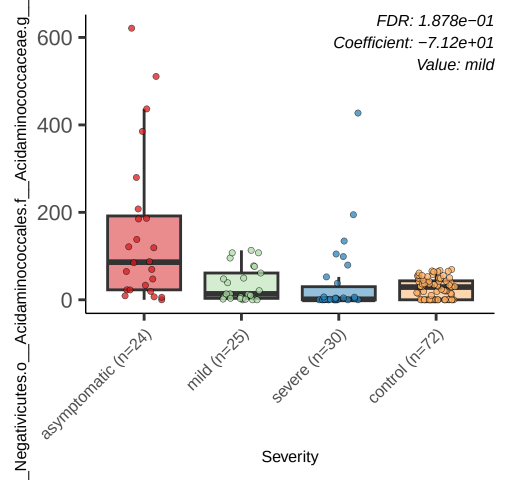
FDR: 1.878e−01
Coefficient: −7.12e+01
Value: mild
Negativicutes.o__Acidaminococcales.f__Acidaminococcaceae.g__
600
400
200
0
asymptomatic (n=24)
mild (n=25)
severe (n=30)
control (n=72)
Severity

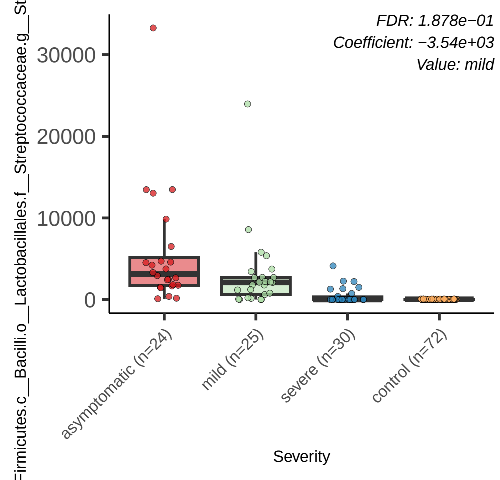
FDR: 1.878e−01
Coefficient: −3.54e+03
Value: mild
Firmicutes.c__Bacilli.o__Lactobacillales.f__Streptococcaceae.g__St
30000
20000
10000
0
asymptomatic (n=24)
mild (n=25)
severe (n=30)
control (n=72)
Severity

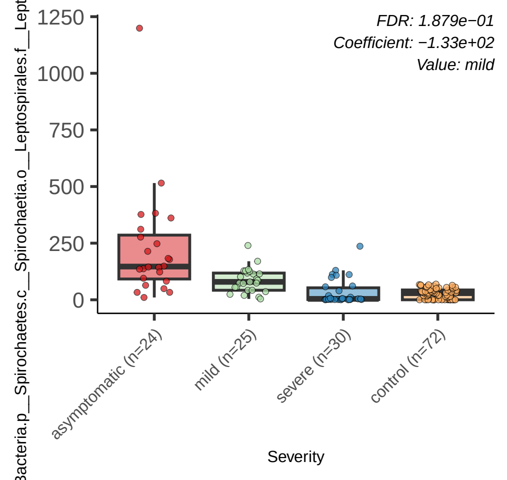

FDR: 1.879e−01
Coefficient: −1.33e+02
Value: mild
1250
1000
750
500
250
0
Bacteria.p__Spirochaetes.c__Spirochaetia.o__Leptospirales.f__Lept
asymptomatic (n=24)
mild (n=25)
severe (n=30)
control (n=72)
Severity

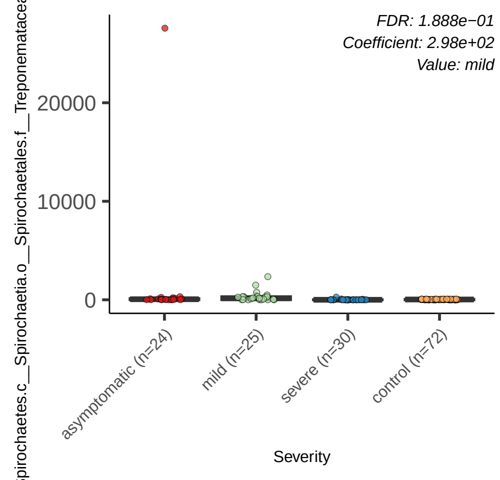
FDR: 1.888e−01
Coefficient: 2.98e+02
Value: mild
20000
10000
0
Spirochaetes.c__Spirochaetia.o__Spirochaetales.f__Treponemataceae
asymptomatic (n=24)
mild (n=25)
severe (n=30)
control (n=72)
Severity

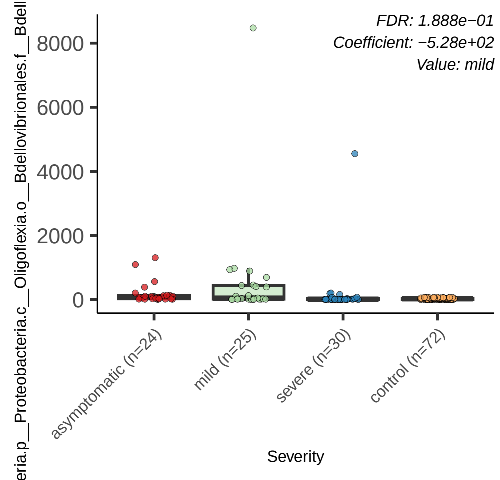

FDR: 1.888e−01
Coefficient: −5.28e+02
Value: mild
8000
6000
4000
2000
0
eria.p__Proteobacteria.c__Oligoflexia.o__Bdellovibrionales.f__Bdell
asymptomatic (n=24)
mild (n=25)
severe (n=30)
control (n=72)
Severity

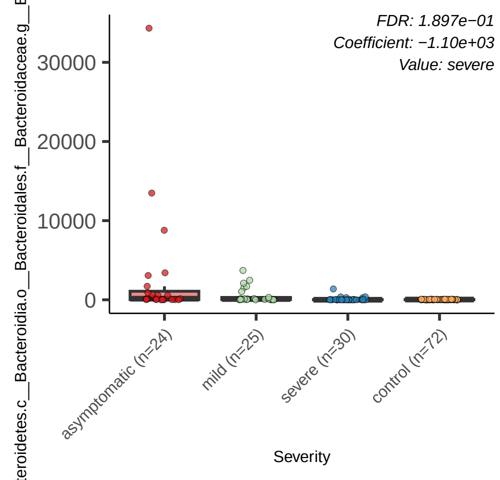
FDR: 1.897e−01
Coefficient: −1.10e+03
Value: severe
30000
20000
10000
0
asymptomatic (n=24)
mild (n=25)
severe (n=30)
control (n=72)
Severity

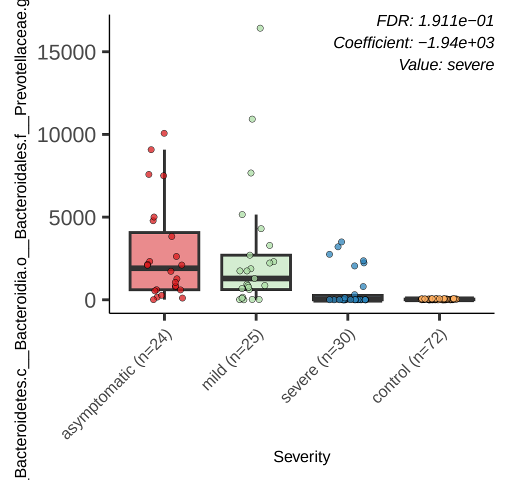

FDR: 1.911e−01
Coefficient: −1.94e+03
Value: severe
Bacteroidetes.c__Bacteroidia.o__Bacteroidales.f__Prevotellaceae.g
15000
10000
5000
0
asymptomatic (n=24)
mild (n=25)
severe (n=30)
control (n=72)
Severity

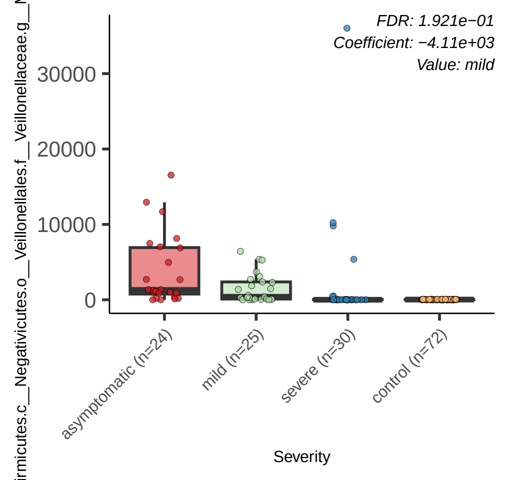

FDR: 1.921e−01
Coefficient: −4.11e+03
Value: mild
30000
20000
10000
0
asymptomatic (n=24)
mild (n=25)
severe (n=30)
control (n=72)
Severity
irmicutes.c__Negativicutes.o__Veillonellales.f__Veillonellaceae.g__

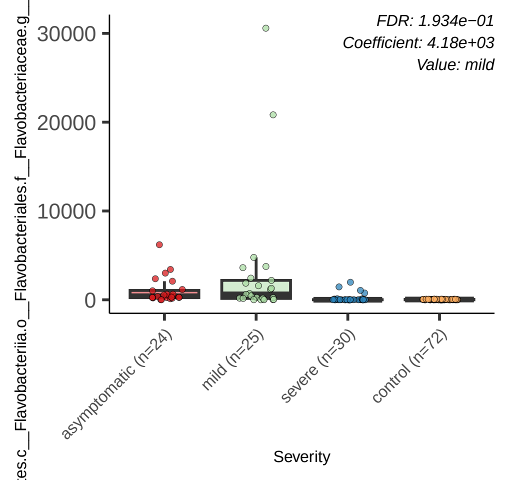

FDR: 1.934e−01
Coefficient: 4.18e+03
Value: mild
30000
20000
10000
0
asymptomatic (n=24)
mild (n=25)
severe (n=30)
control (n=72)
Severity
Flavobacteriia.o__Flavobacteriales.f__Flavobacteriaceae.g__

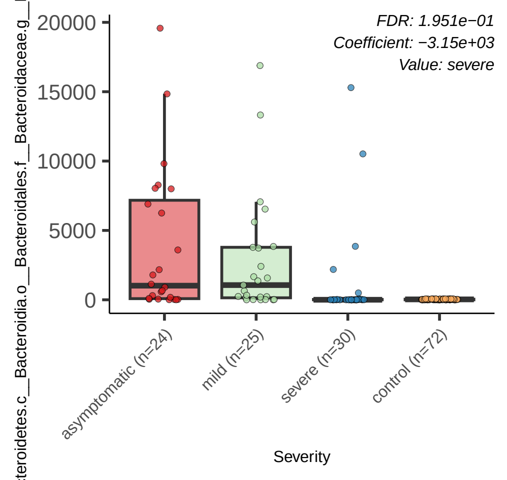

FDR: 1.951e−01
Coefficient: −3.15e+03
Value: severe
20000
15000
10000
5000
0
asymptomatic (n=24)
mild (n=25)
severe (n=30)
control (n=72)
Severity

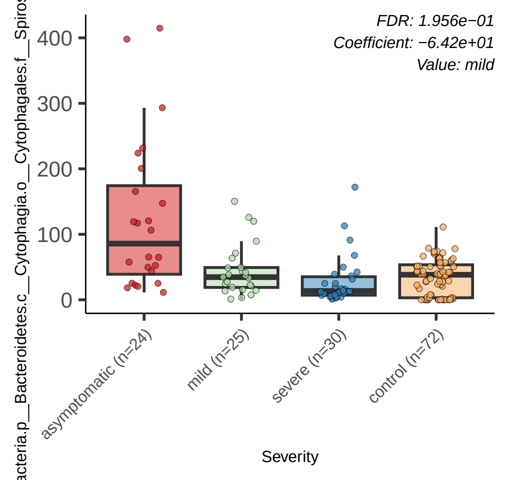

FDR: 1.956e−01
Coefficient: −6.42e+01
Value: mild
acteria.p__Bacteroidetes.c__Cytophagia.o__Cytophagales.f__Spiros
0
100
200
300
400
asymptomatic (n=24)
mild (n=25)
severe (n=30)
control (n=72)
Severity

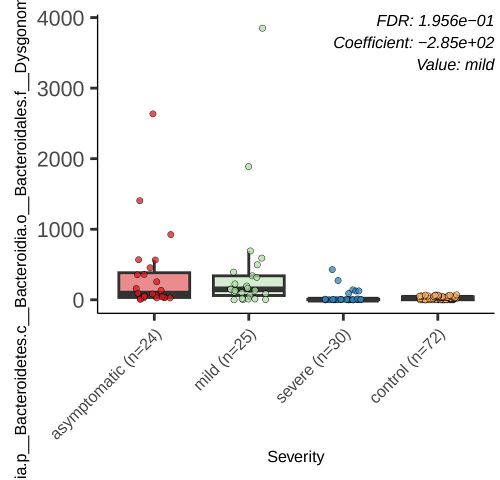

FDR: 1.956e−01
Coefficient: −2.85e+02
Value: mild
4000
3000
2000
1000
0
ia.p__Bacteroidetes.c__Bacteroidia.o__Bacteroidales.f__Dysgonom
asymptomatic (n=24)
mild (n=25)
severe (n=30)
control (n=72)
Severity

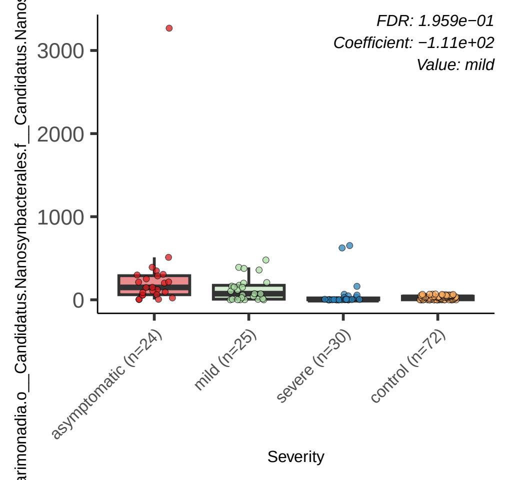

FDR: 1.959e−01
Coefficient: −1.11e+02
Value: mild
3000
2000
1000
0
arimonadia.o__Candidatus.Nanosynbacterales.f__Candidatus.Nanos
asymptomatic (n=24)
mild (n=25)
severe (n=30)
control (n=72)
Severity

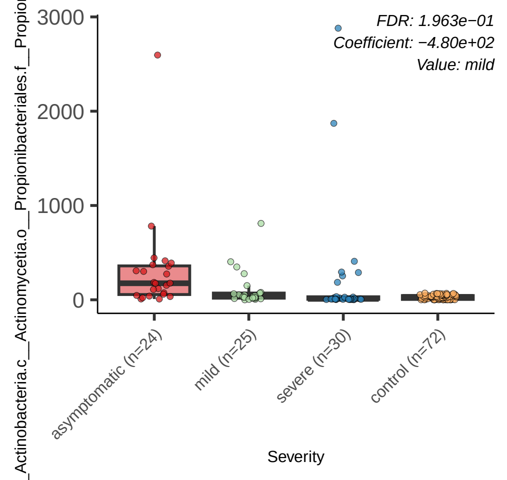

FDR: 1.963e−01
Coefficient: −4.80e+02
Value: mild
3000
2000
1000
0
_Actinobacteria.c__Actinomycetia.o__Propionibacteriales.f__Propion
asymptomatic (n=24)
mild (n=25)
severe (n=30)
control (n=72)
Severity

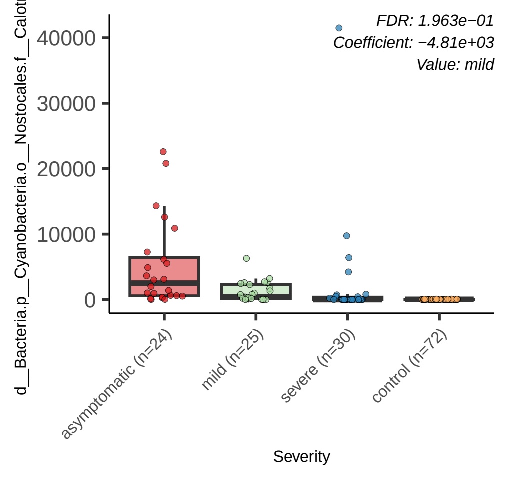

FDR: 1.963e−01
Coefficient: −4.81e+03
Value: mild
d__Bacteria.p__Cyanobacteria.o__Nostocales.f__Caloth
40000
30000
20000
10000
0
asymptomatic (n=24)
mild (n=25)
severe (n=30)
control (n=72)
Severity

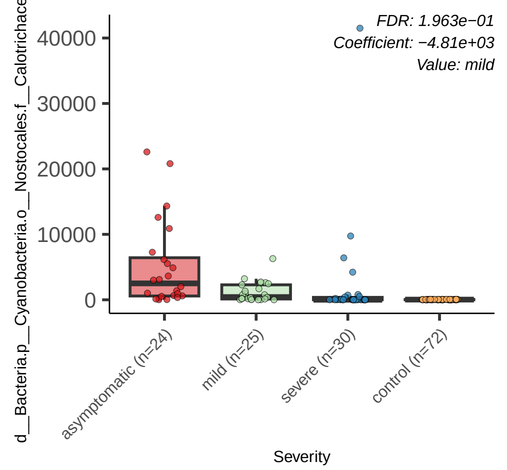

d__Bacteria.p__Cyanobacteria.o__Nostocales.f__Calotrichace
FDR: 1.963e−01
Coefficient: −4.81e+03
Value: mild
40000
30000
20000
10000
0
asymptomatic (n=24)
mild (n=25)
severe (n=30)
control (n=72)
Severity

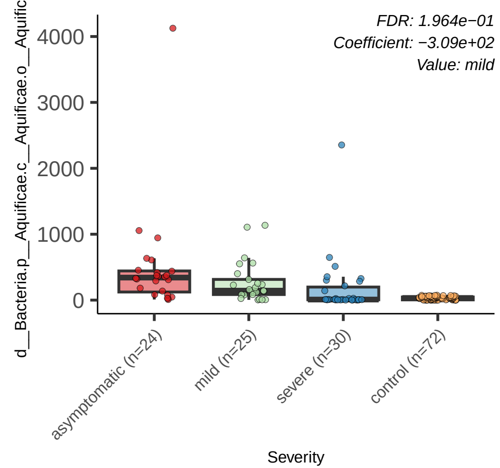
FDR: 1.964e−01
Coefficient: −3.09e+02
Value: mild
d__Bacteria.p__Aquificae.c__Aquificae.o__Aquific
4000
3000
2000
1000
0
asymptomatic (n=24)
mild (n=25)
severe (n=30)
control (n=72)
Severity

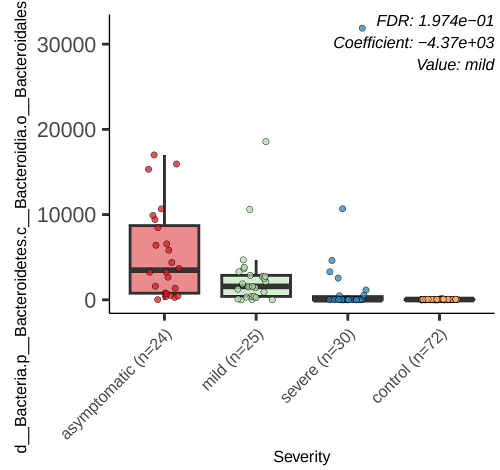

FDR: 1.974e−01
Coefficient: −4.37e+03
Value: mild
d__Bacteria.p__Bacteroidetes.c__Bacteroidia.o__Bacteroidales
30000
20000
10000
0
asymptomatic (n=24)
mild (n=25)
severe (n=30)
control (n=72)
Severity

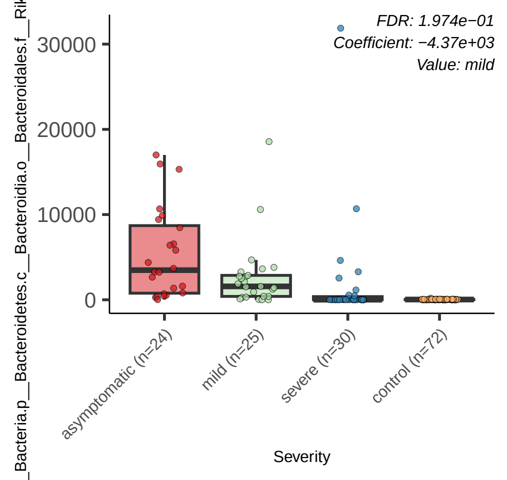

FDR: 1.974e−01
Coefficient: −4.37e+03
Value: mild
30000
20000
10000
0
__Bacteria.p__Bacteroidetes.c__Bacteroidia.o__Bacteroidales.f__Rik
asymptomatic (n=24)
mild (n=25)
severe (n=30)
control (n=72)
Severity

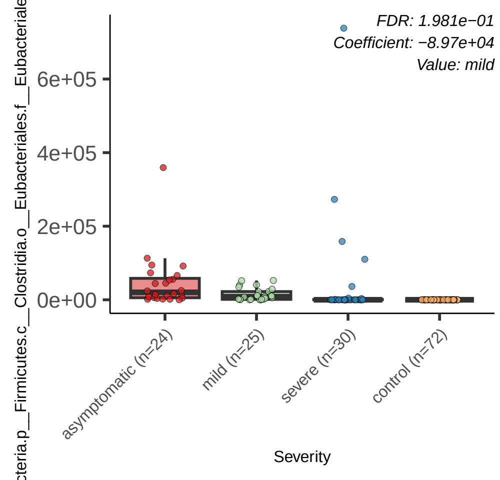
FDR: 1.981e−01
Coefficient: −8.97e+04
Value: mild
6e+05
4e+05
2e+05
0e+00
cteria.p__Firmicutes.c__Clostridia.o__Eubacteriales.f__Eubacteriale
asymptomatic (n=24)
mild (n=25)
severe (n=30)
control (n=72)
Severity

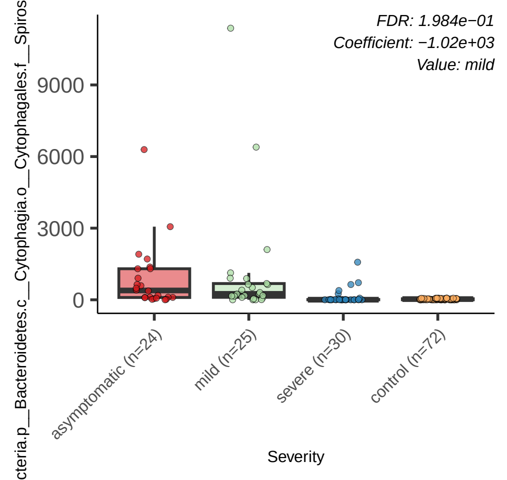

FDR: 1.984e−01
Coefficient: −1.02e+03
Value: mild
9000
6000
3000
0
cteria.p__Bacteroidetes.c__Cytophagia.o__Cytophagales.f__Spiros
asymptomatic (n=24)
mild (n=25)
severe (n=30)
control (n=72)
Severity

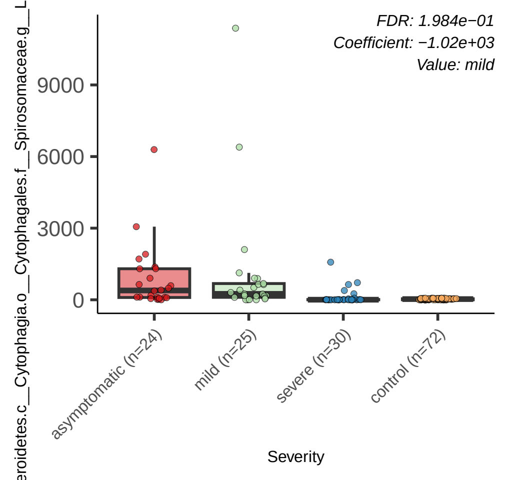

FDR: 1.984e−01
Coefficient: −1.02e+03
Value: mild
9000
6000
3000
0
eroidetes.c__Cytophagia.o__Cytophagales.f__Spirosomaceae.g__L
asymptomatic (n=24)
mild (n=25)
severe (n=30)
control (n=72)
Severity

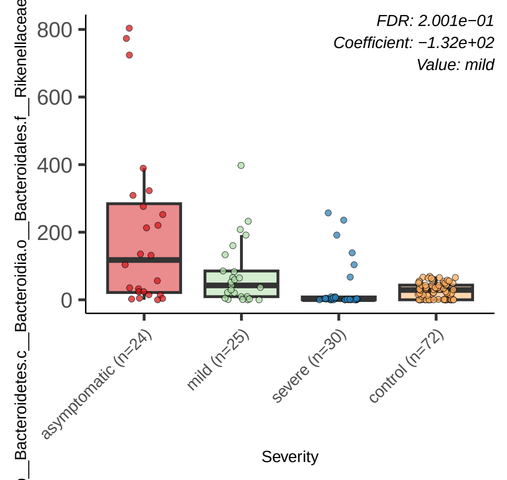

FDR: 2.001e−01
Coefficient: −1.32e+02
Value: mild
o__Bacteroidetes.c__Bacteroidia.o__Bacteroidales.f__Rikenellaceae
0
200
400
600
800
asymptomatic (n=24)
mild (n=25)
severe (n=30)
control (n=72)
Severity

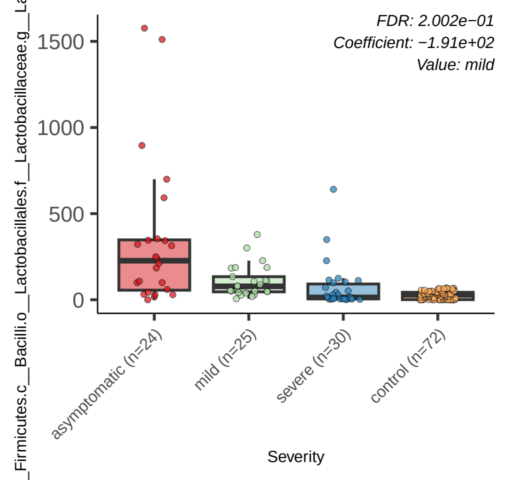
FDR: 2.002e−01
Coefficient: −1.91e+02
Value: mild
1500
1000
500
0
Firmicutes.c__Bacilli.o__Lactobacillales.f__Lactobacillaceae.g__La
asymptomatic (n=24)
mild (n=25)
severe (n=30)
control (n=72)
Severity

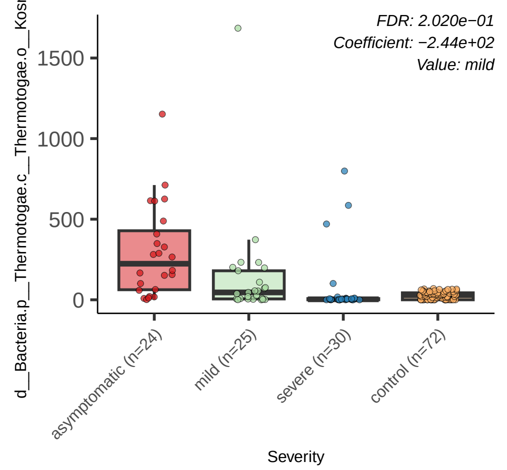

FDR: 2.020e−01
Coefficient: −2.44e+02
Value: mild
d__Bacteria.p__Thermotogae.c__Thermotogae.o__Kos
0
500
1000
1500
asymptomatic (n=24)
mild (n=25)
severe (n=30)
control (n=72)
Severity

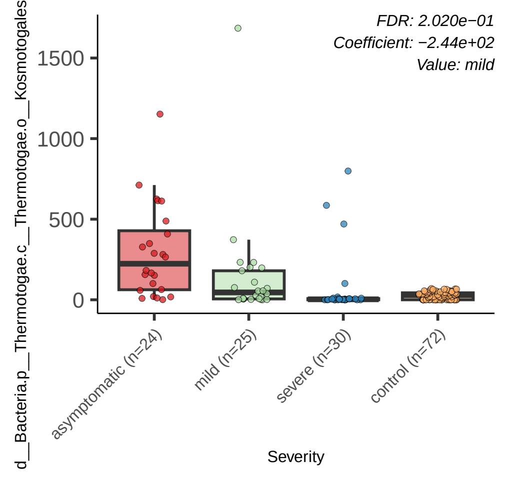
FDR: 2.020e−01
Coefficient: −2.44e+02
Value: mild
d__Bacteria.p__Thermotogae.c__Thermotogae.o__Kosmotogales
1500
1000
500
0
asymptomatic (n=24)
mild (n=25)
severe (n=30)
control (n=72)
Severity

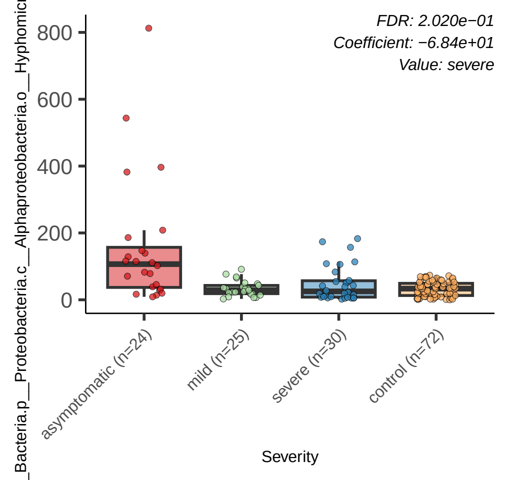

FDR: 2.020e−01
Coefficient: −6.84e+01
Value: severe
800
600
400
200
0
Bacteria.p__Proteobacteria.c__Alphaproteobacteria.o__Hyphomic
asymptomatic (n=24)
mild (n=25)
severe (n=30)
control (n=72)
Severity

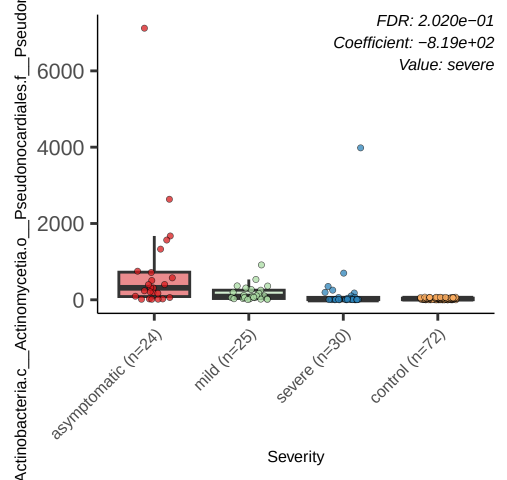

FDR: 2.020e−01
Coefficient: −8.19e+02
Value: severe
Actinobacteria.c__Actinomycetia.o__Pseudonocardiales.f__Pseudor
6000
4000
2000
0
asymptomatic (n=24)
mild (n=25)
severe (n=30)
control (n=72)
Severity

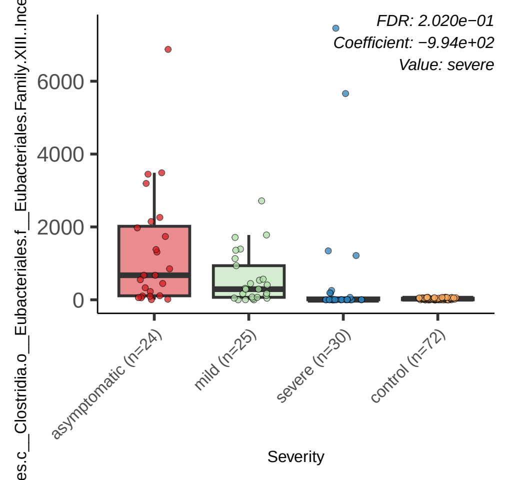

FDR: 2.020e−01
Coefficient: −9.94e+02
Value: severe
6000
4000
2000
0
es.c__Clostridia.o__Eubacteriales.f__Eubacteriales.Family.XIII..Ince
asymptomatic (n=24)
mild (n=25)
severe (n=30)
control (n=72)
Severity

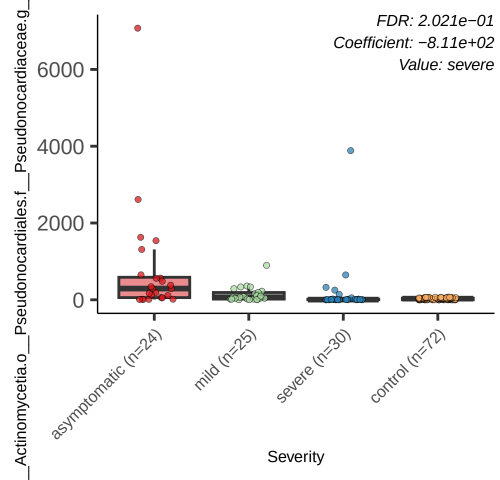
FDR: 2.021e−01
Coefficient: −8.11e+02
Value: severe
Actinomycetia.o__Pseudonocardiales.f__Pseudonocardiaceae.g__
0
2000
4000
6000
asymptomatic (n=24)
mild (n=25)
severe (n=30)
control (n=72)
Severity

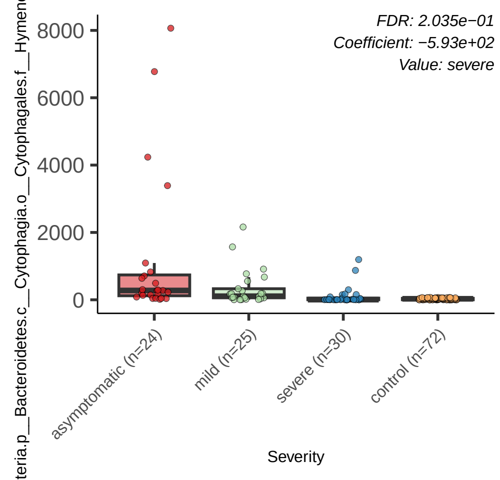
FDR: 2.035e−01
Coefficient: −5.93e+02
Value: severe
8000
6000
4000
2000
0
teria.p__Bacteroidetes.c__Cytophagia.o__Cytophagales.f__Hymeno
asymptomatic (n=24)
mild (n=25)
severe (n=30)
control (n=72)
Severity

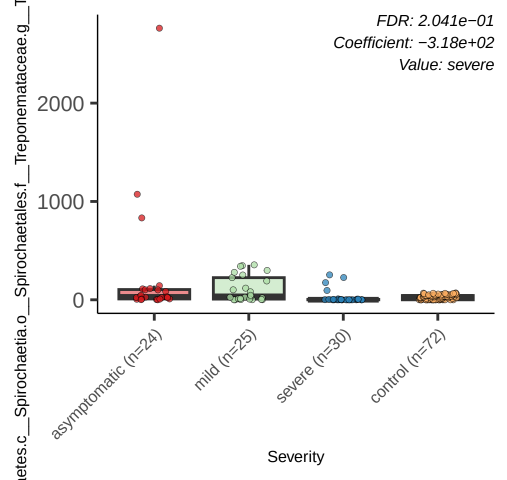

FDR: 2.041e−01
Coefficient: −3.18e+02
Value: severe
2000
1000
0
asymptomatic (n=24)
mild (n=25)
severe (n=30)
control (n=72)
Severity
etes.c__Spirochaetia.o__Spirochaetales.f__Treponemataceae.g__T

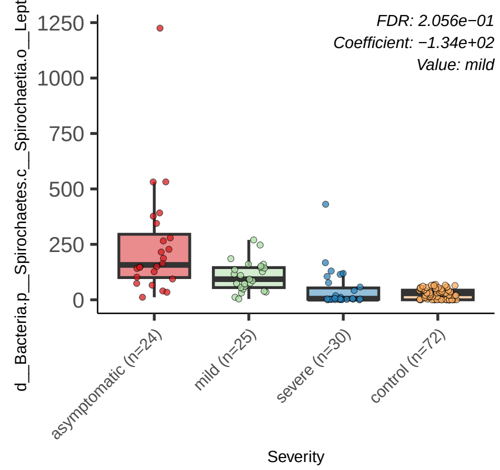

FDR: 2.056e−01
Coefficient: −1.34e+02
Value: mild
d__Bacteria.p__Spirochaetes.c__Spirochaetia.o__Lept
1250
1000
750
500
250
0
asymptomatic (n=24)
mild (n=25)
severe (n=30)
control (n=72)
Severity

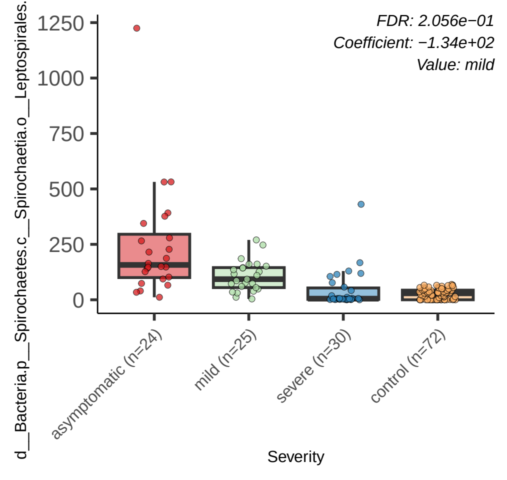
FDR: 2.056e−01
Coefficient: −1.34e+02
Value: mild
d__Bacteria.p__Spirochaetes.c__Spirochaetia.o__Leptospirales.
1250
1000
750
500
250
0
asymptomatic (n=24)
mild (n=25)
severe (n=30)
control (n=72)
Severity

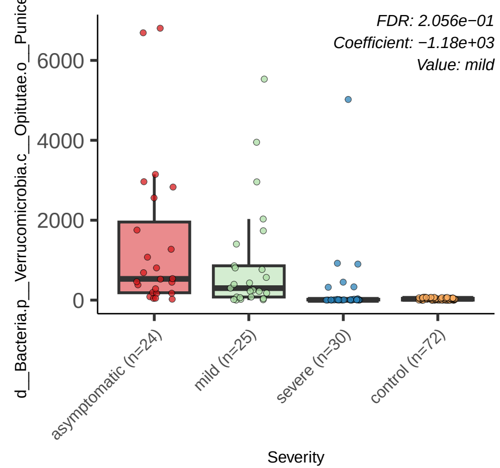

FDR: 2.056e−01
Coefficient: −1.18e+03
Value: mild
d__Bacteria.p__Verrucomicrobia.c__Opitutae.o__Punice
6000
4000
2000
0
asymptomatic (n=24)
mild (n=25)
severe (n=30)
control (n=72)
Severity

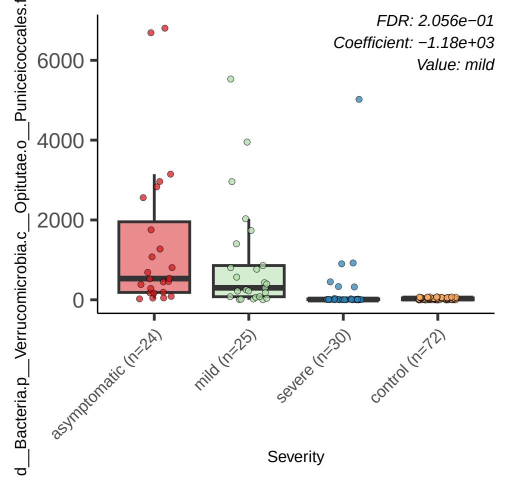

FDR: 2.056e−01
Coefficient: −1.18e+03
Value: mild
d__Bacteria.p__Verrucomicrobia.c__Opitutae.o__Puniceicoccales.f
6000
4000
2000
0
asymptomatic (n=24)
mild (n=25)
severe (n=30)
control (n=72)
Severity

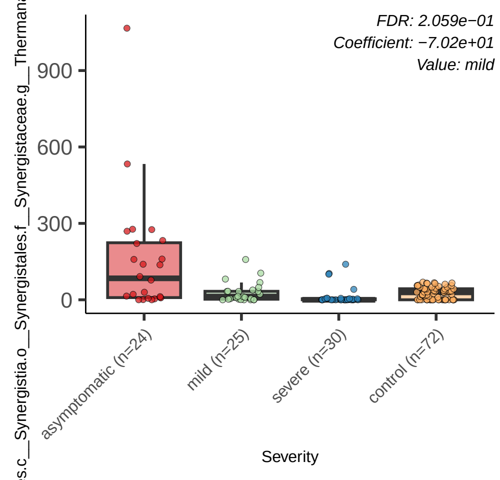

FDR: 2.059e−01
Coefficient: −7.02e+01
Value: mild
900
600
300
0
asymptomatic (n=24)
mild (n=25)
severe (n=30)
control (n=72)
Severity
s.c__Synergistia.o__Synergistales.f__Synergistaceae.g__Therman

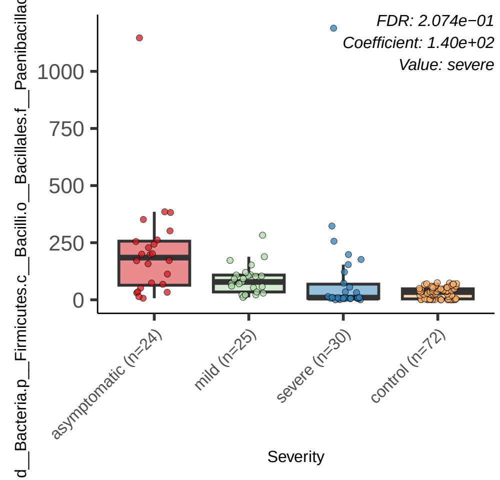

d__Bacteria.p__Firmicutes.c__Bacilli.o__Bacillales.f__Paenibacillac
FDR: 2.074e−01
Coefficient: 1.40e+02
Value: severe
1000
750
500
250
0
asymptomatic (n=24)
mild (n=25)
severe (n=30)
control (n=72)
Severity

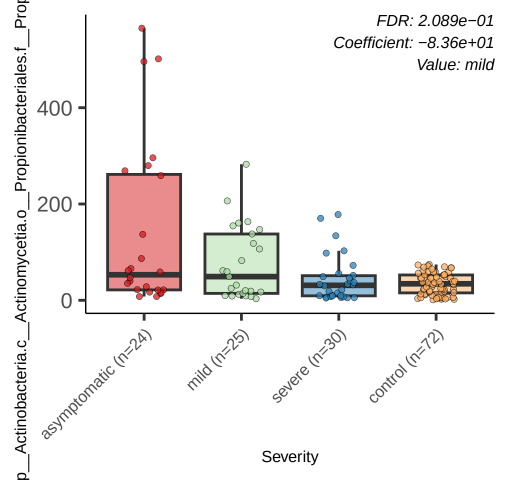

FDR: 2.089e−01
Coefficient: −8.36e+01
Value: mild
p__Actinobacteria.c__Actinomycetia.o__Propionibacteriales.f__Prop
0
200
400
asymptomatic (n=24)
mild (n=25)
severe (n=30)
control (n=72)
Severity

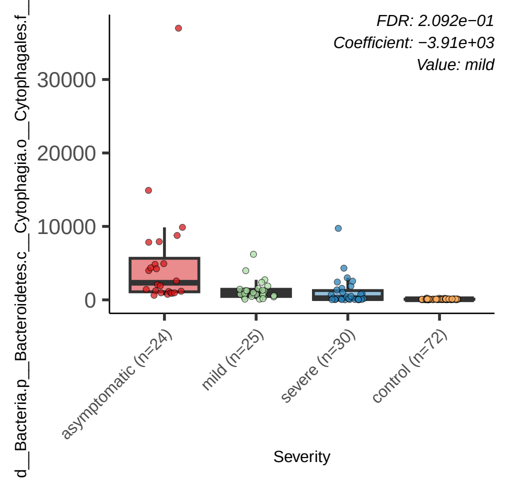

FDR: 2.092e−01
Coefficient: −3.91e+03
Value: mild
d__Bacteria.p__Bacteroidetes.c__Cytophagia.o__Cytophagales.f__
30000
20000
10000
0
asymptomatic (n=24)
mild (n=25)
severe (n=30)
control (n=72)
Severity

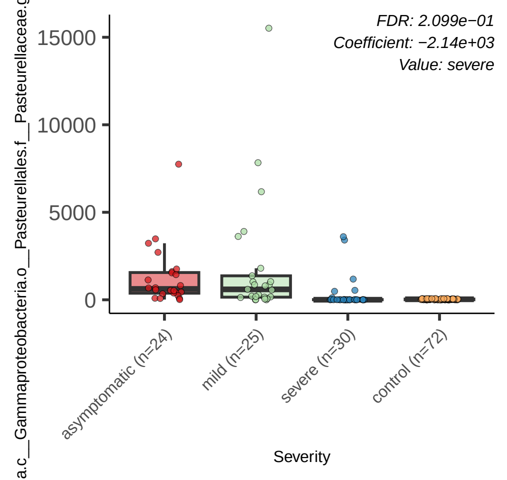
FDR: 2.099e−01
Coefficient: −2.14e+03
Value: severe
15000
10000
5000
0
a.c__Gammaproteobacteria.o__Pasteurellales.f__Pasteurellaceae.g
asymptomatic (n=24)
mild (n=25)
severe (n=30)
control (n=72)
Severity

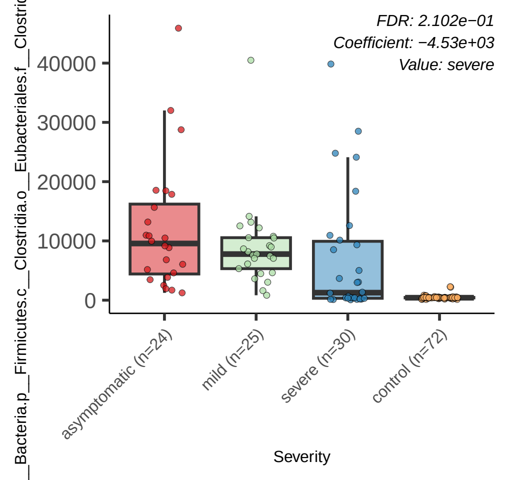
FDR: 2.102e−01
Coefficient: −4.53e+03
Value: severe
Bacteria.p__Firmicutes.c__Clostridia.o__Eubacteriales.f__Clostrid
40000
30000
20000
10000
0
asymptomatic (n=24)
mild (n=25)
severe (n=30)
control (n=72)
Severity

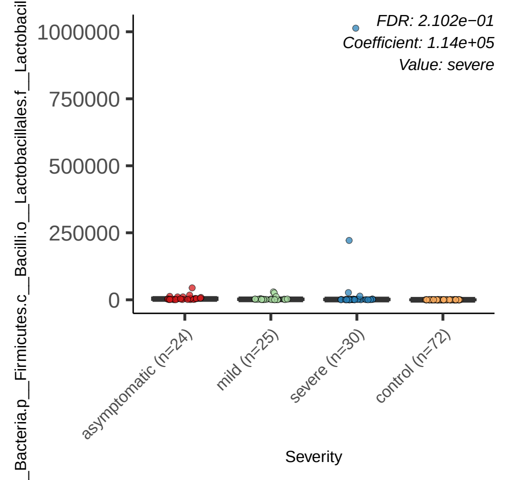

FDR: 2.102e−01
Coefficient: 1.14e+05
Value: severe
1000000
750000
500000
250000
0
Bacteria.p__Firmicutes.c__Bacilli.o__Lactobacillales.f__Lactobacil
asymptomatic (n=24)
mild (n=25)
severe (n=30)
control (n=72)
Severity

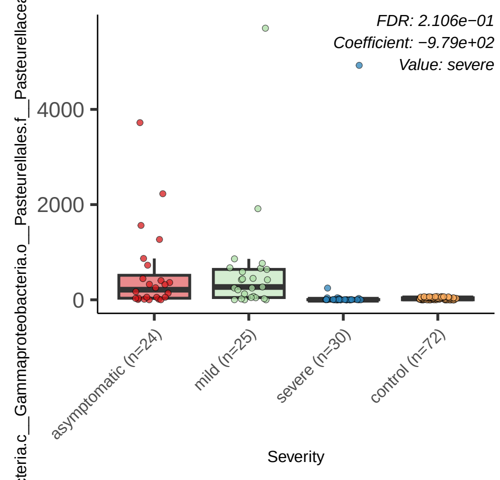
FDR: 2.106e−01
Coefficient: −9.79e+02
Value: severe
4000
2000
0
asymptomatic (n=24)
mild (n=25)
severe (n=30)
control (n=72)
Severity
cteria.c__Gammaproteobacteria.o__Pasteurellales.f__Pasteurellacea

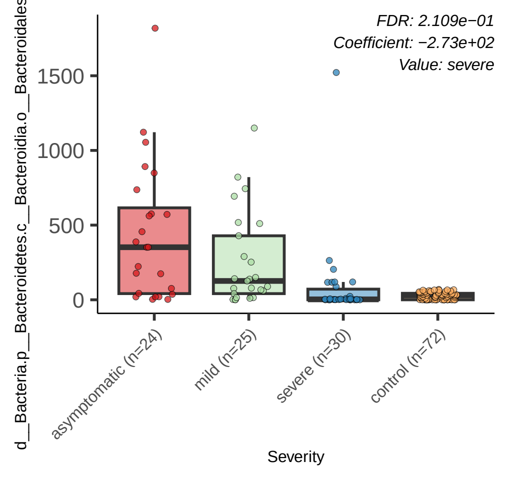
FDR: 2.109e−01
Coefficient: −2.73e+02
Value: severe
d__Bacteria.p__Bacteroidetes.c__Bacteroidia.o__Bacteroidales
1500
1000
500
0
asymptomatic (n=24)
mild (n=25)
severe (n=30)
control (n=72)
Severity

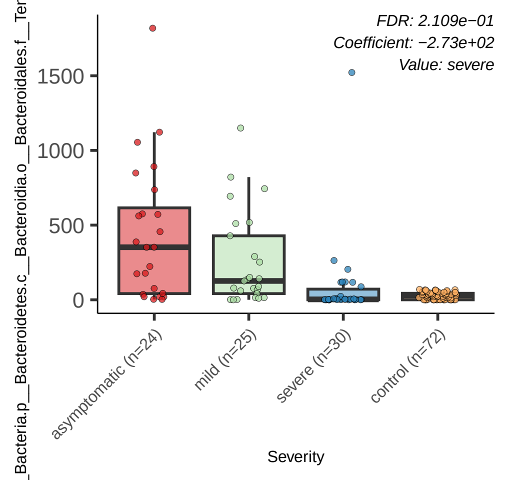

FDR: 2.109e−01
Coefficient: −2.73e+02
Value: severe
0
500
1000
1500
asymptomatic (n=24)
mild (n=25)
severe (n=30)
control (n=72)
Severity
_Bacteria.p__Bacteroidetes.c__Bacteroidia.o__Bacteroidales.f__Ter

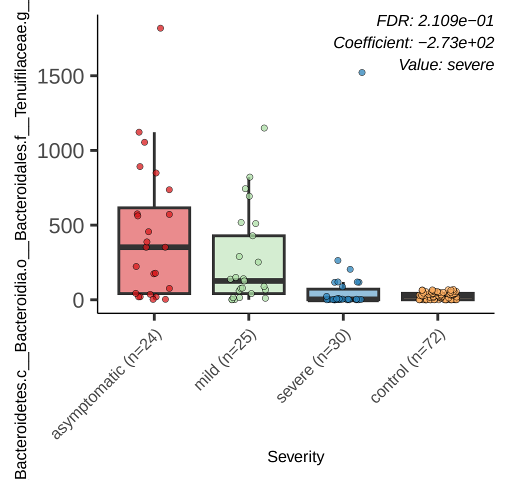
FDR: 2.109e−01
Coefficient: −2.73e+02
Value: severe
Bacteroidetes.c__Bacteroidia.o__Bacteroidales.f__Tenuifilaceae.g__
1500
1000
500
0
asymptomatic (n=24)
mild (n=25)
severe (n=30)
control (n=72)
Severity

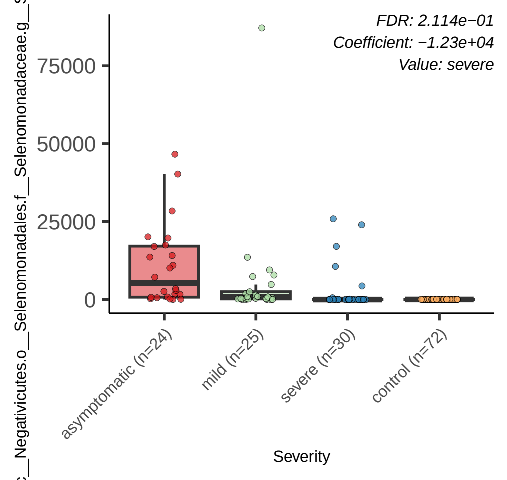
FDR: 2.114e−01
Coefficient: −1.23e+04
Value: severe
Negativicutes.o__Selenomonadales.f__Selenomonadaceae.g__
75000
50000
25000
0
asymptomatic (n=24)
mild (n=25)
severe (n=30)
control (n=72)
Severity

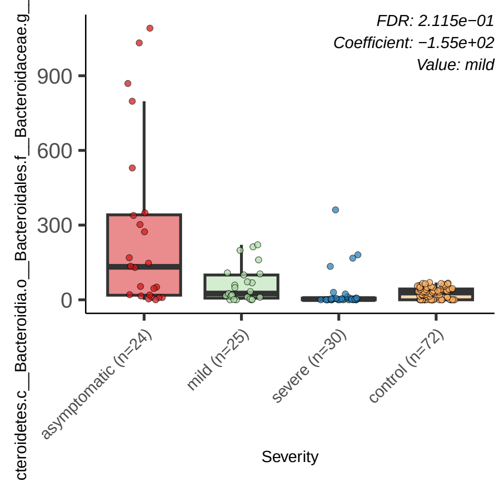

FDR: 2.115e−01
Coefficient: −1.55e+02
Value: mild
cteroidetes.c__Bacteroidia.o__Bacteroidales.f__Bacteroidaceae.g__
900
600
300
0
asymptomatic (n=24)
mild (n=25)
severe (n=30)
control (n=72)
Severity

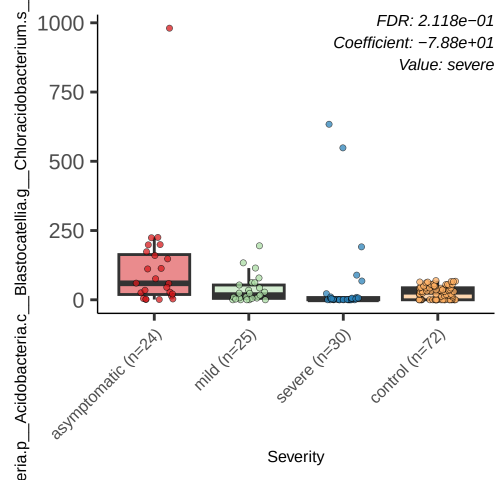

FDR: 2.118e−01
Coefficient: −7.88e+01
Value: severe
eria.p__Acidobacteria.c__Blastocatellia.g__Chloracidobacterium.s_
1000
750
500
250
0
asymptomatic (n=24)
mild (n=25)
severe (n=30)
control (n=72)
Severity

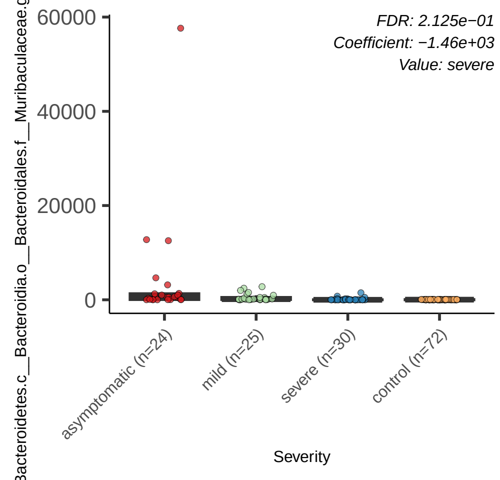

FDR: 2.125e−01
Coefficient: −1.46e+03
Value: severe
60000
40000
20000
0
asymptomatic (n=24)
mild (n=25)
severe (n=30)
control (n=72)
Severity

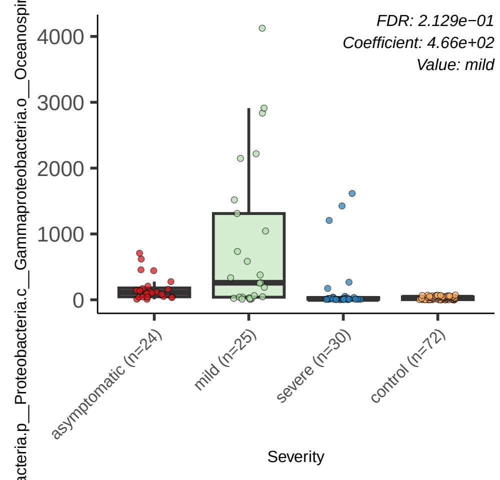
FDR: 2.129e−01
Coefficient: 4.66e+02
Value: mild
acteria.p__Proteobacteria.c__Gammaproteobacteria.o__Oceanospi
4000
3000
2000
1000
0
asymptomatic (n=24)
mild (n=25)
severe (n=30)
control (n=72)
Severity

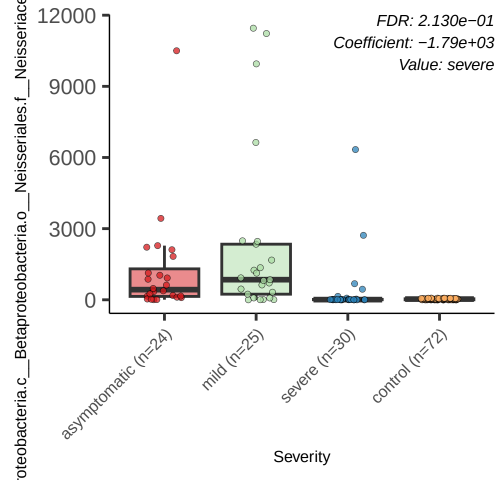

FDR: 2.130e−01
Coefficient: −1.79e+03
Value: severe
12000
9000
6000
3000
0
roteobacteria.c__Betaproteobacteria.o__Neisseriales.f__Neisseriace
asymptomatic (n=24)
mild (n=25)
severe (n=30)
control (n=72)
Severity

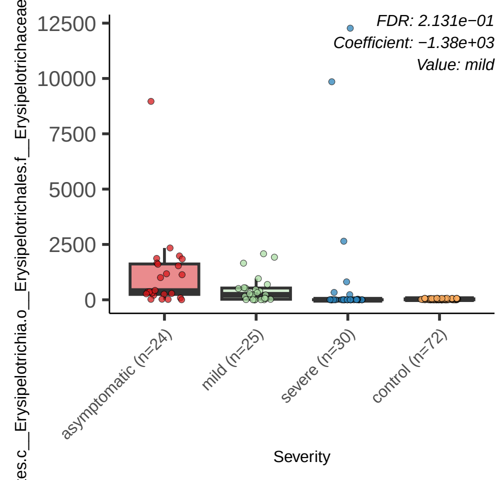

FDR: 2.131e−01
Coefficient: −1.38e+03
Value: mild
12500
10000
7500
5000
2500
0
asymptomatic (n=24)
mild (n=25)
severe (n=30)
control (n=72)
Severity
es.c__Erysipelotrichia.o__Erysipelotrichales.f__Erysipelotrichaceae

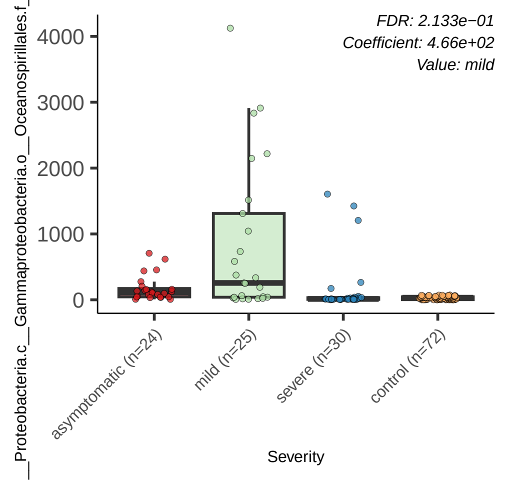
FDR: 2.133e−01
Coefficient: 4.66e+02
Value: mild
_Proteobacteria.c__Gammaproteobacteria.o__Oceanospirillales.f_
4000
3000
2000
1000
0
asymptomatic (n=24)
mild (n=25)
severe (n=30)
control (n=72)
Severity

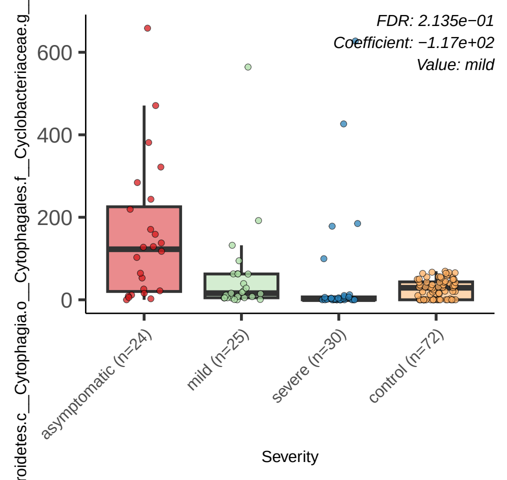
FDR: 2.135e−01
Coefficient: −1.17e+02
Value: mild
roidetes.c__Cytophagia.o__Cytophagales.f__Cyclobacteriaceae.g__
600
400
200
0
asymptomatic (n=24)
mild (n=25)
severe (n=30)
control (n=72)
Severity

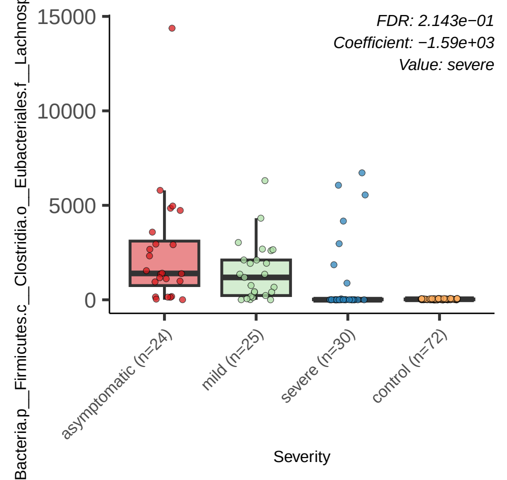
FDR: 2.143e−01
Coefficient: −1.59e+03
Value: severe
15000
10000
5000
0
Bacteria.p__Firmicutes.c__Clostridia.o__Eubacteriales.f__Lachnosp
asymptomatic (n=24)
mild (n=25)
severe (n=30)
control (n=72)
Severity

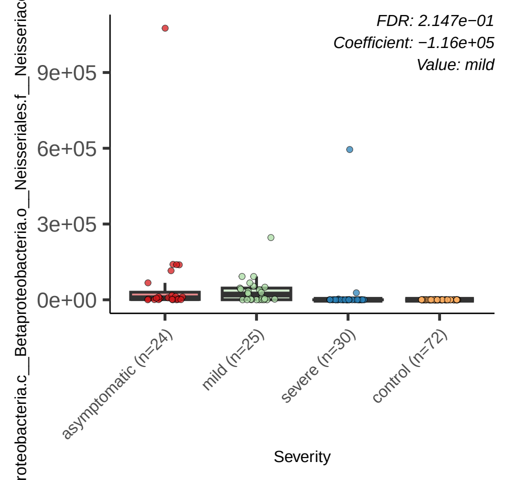

FDR: 2.147e−01
Coefficient: −1.16e+05
Value: mild
9e+05
6e+05
3e+05
0e+00
asymptomatic (n=24)
mild (n=25)
severe (n=30)
control (n=72)
Severity
roteobacteria.c__Betaproteobacteria.o__Neisseriales.f__Neisseriac

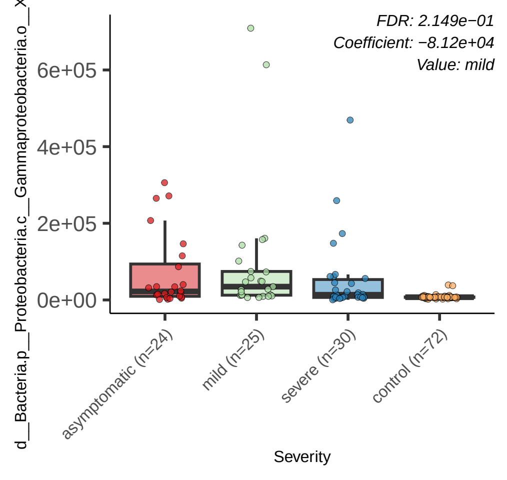

FDR: 2.149e−01
Coefficient: −8.12e+04
Value: mild
d__Bacteria.p__Proteobacteria.c__Gammaproteobacteria.o__X
6e+05
4e+05
2e+05
0e+00
asymptomatic (n=24)
mild (n=25)
severe (n=30)
control (n=72)
Severity

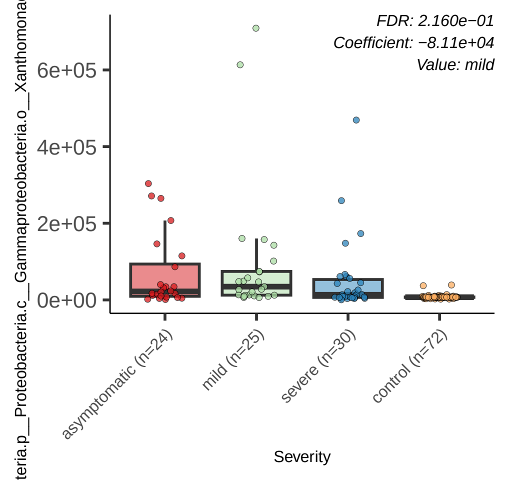
FDR: 2.160e−01
Coefficient: −8.11e+04
Value: mild
6e+05
4e+05
2e+05
0e+00
teria.p__Proteobacteria.c__Gammaproteobacteria.o__Xanthomonac
asymptomatic (n=24)
mild (n=25)
severe (n=30)
control (n=72)
Severity

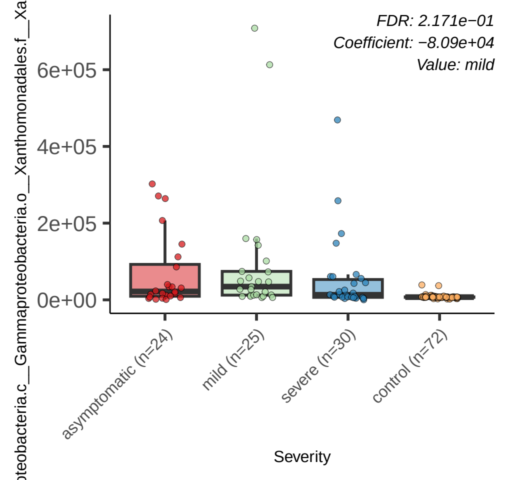

FDR: 2.171e−01
Coefficient: −8.09e+04
Value: mild
6e+05
4e+05
2e+05
0e+00
oteobacteria.c__Gammaproteobacteria.o__Xanthomonadales.f__Xa
asymptomatic (n=24)
mild (n=25)
severe (n=30)
control (n=72)
Severity

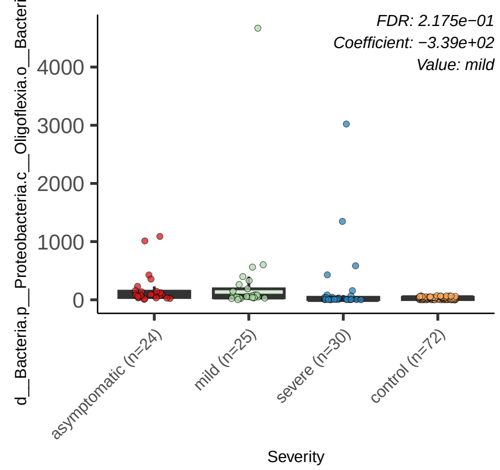

FDR: 2.175e−01
Coefficient: −3.39e+02
Value: mild
d__Bacteria.p__Proteobacteria.c__Oligoflexia.o__Bacteri
0
1000
2000
3000
4000
asymptomatic (n=24)
mild (n=25)
severe (n=30)
control (n=72)
Severity

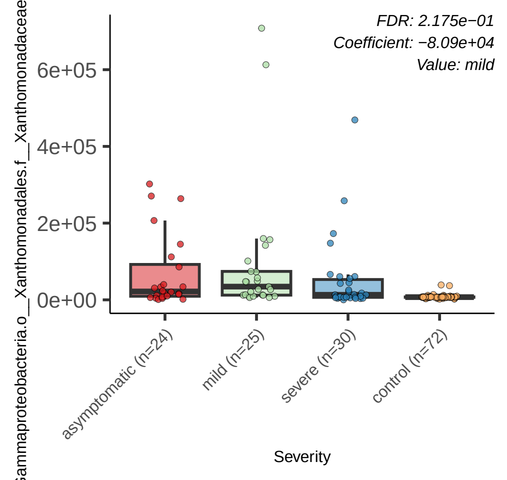
FDR: 2.175e−01
Coefficient: −8.09e+04
Value: mild
Gammaproteobacteria.o__Xanthomonadales.f__Xanthomonadaceae
6e+05
4e+05
2e+05
0e+00
asymptomatic (n=24)
mild (n=25)
severe (n=30)
control (n=72)
Severity

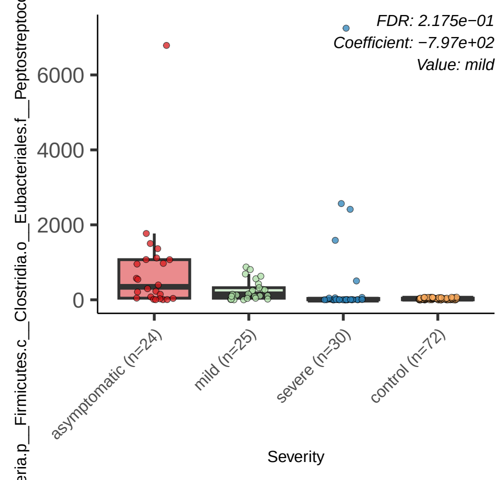

FDR: 2.175e−01
Coefficient: −7.97e+02
Value: mild
6000
4000
2000
0
eria.p__Firmicutes.c__Clostridia.o__Eubacteriales.f__Peptostreptoco
asymptomatic (n=24)
mild (n=25)
severe (n=30)
control (n=72)
Severity

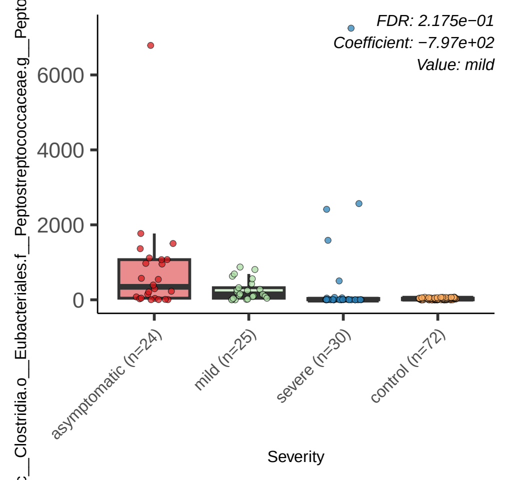
FDR: 2.175e−01
Coefficient: −7.97e+02
Value: mild
c__Clostridia.o__Eubacteriales.f__Peptostreptococcaceae.g__Pepto
6000
4000
2000
0
asymptomatic (n=24)
mild (n=25)
severe (n=30)
control (n=72)
Severity

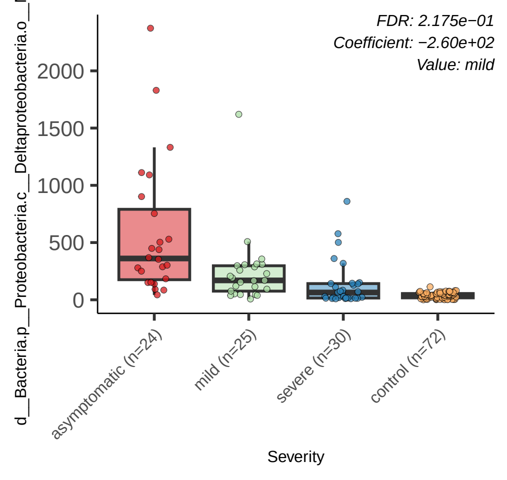
FDR: 2.175e−01
Coefficient: −2.60e+02
Value: mild
d__Bacteria.p__Proteobacteria.c__Deltaproteobacteria.o__
2000
1500
1000
500
0
asymptomatic (n=24)
mild (n=25)
severe (n=30)
control (n=72)
Severity

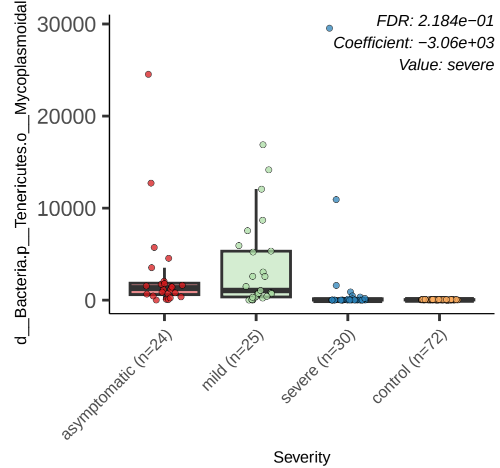

FDR: 2.184e−01
Coefficient: −3.06e+03
Value: severe
d__Bacteria.p__Tenericutes.o__Mycoplasmoidal
30000
20000
10000
0
asymptomatic (n=24)
mild (n=25)
severe (n=30)
control (n=72)
Severity

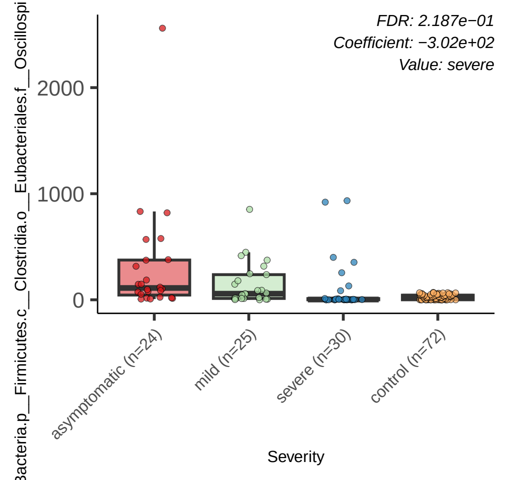

FDR: 2.187e−01
Coefficient: −3.02e+02
Value: severe
Bacteria.p__Firmicutes.c__Clostridia.o__Eubacteriales.f__Oscillospi
2000
1000
0
asymptomatic (n=24)
mild (n=25)
severe (n=30)
control (n=72)
Severity

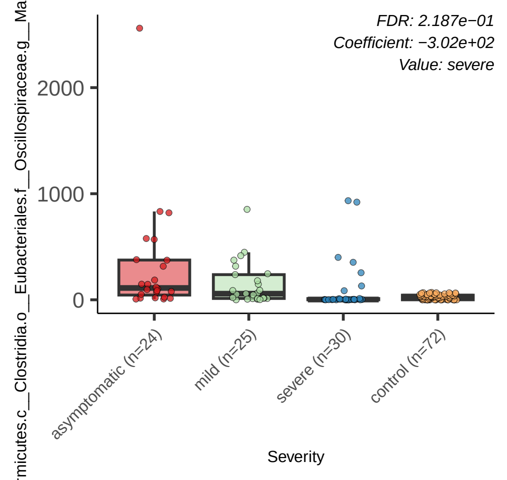

FDR: 2.187e−01
Coefficient: −3.02e+02
Value: severe
Firmicutes.c__Clostridia.o__Eubacteriales.f__Oscillospiraceae.g__Ma
2000
1000
0
asymptomatic (n=24)
mild (n=25)
severe (n=30)
control (n=72)
Severity

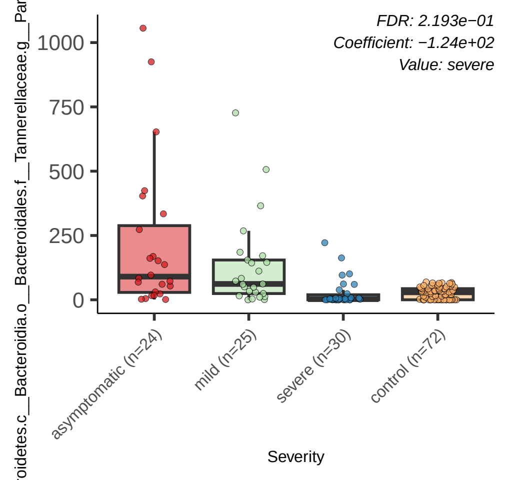

FDR: 2.193e−01
Coefficient: −1.24e+02
Value: severe
1000
750
500
250
0
Bacteroidetes.c__Bacteroidia.o__Bacteroidales.f__Tannerellaceae.g__Par
asymptomatic (n=24)
mild (n=25)
severe (n=30)
control (n=72)
Severity

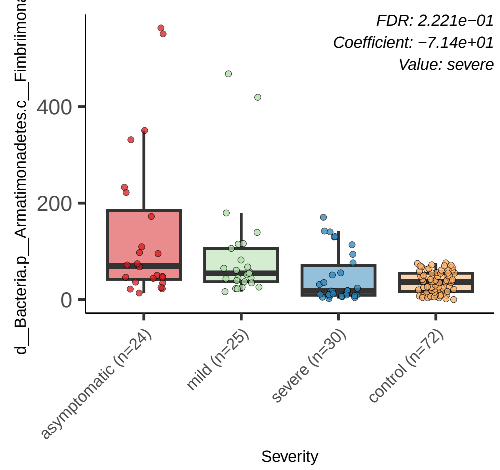

FDR: 2.221e−01
Coefficient: −7.14e+01
Value: severe
d__Bacteria.p__Armatimonadetes.c__Fimbriimona
400
200
0
asymptomatic (n=24)
mild (n=25)
severe (n=30)
control (n=72)
Severity

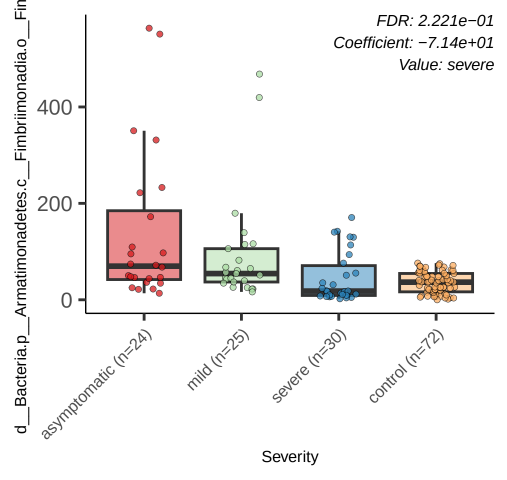

FDR: 2.221e−01
Coefficient: −7.14e+01
Value: severe
d__Bacteria.p__Armatimonadetes.c__Fimbriimonadia.o__Fim
0
200
400
asymptomatic (n=24)
mild (n=25)
severe (n=30)
control (n=72)
Severity

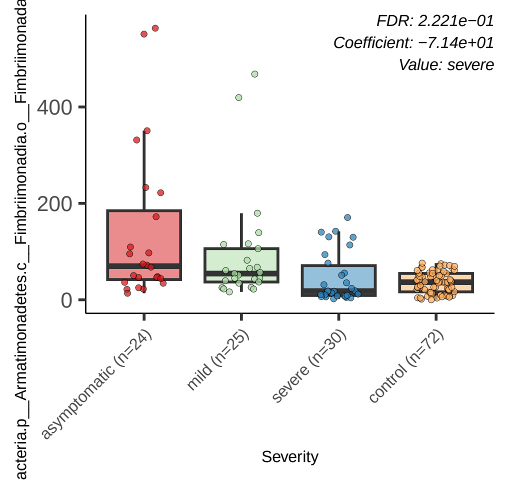

FDR: 2.221e−01
Coefficient: −7.14e+01
Value: severe
acteria.p__Armatimonadetes.c__Fimbriimonadia.o__Fimbriimonada
0
200
400
asymptomatic (n=24)
mild (n=25)
severe (n=30)
control (n=72)
Severity

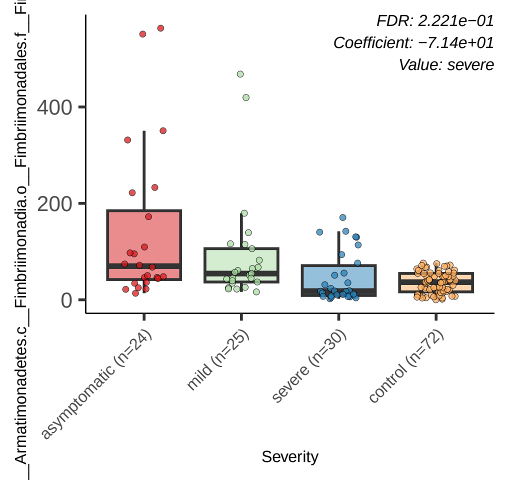
FDR: 2.221e−01
Coefficient: −7.14e+01
Value: severe
Armatimonadetes.c__Fimbriimonadia.o__Fimbriimonadales.f__Fim
0
200
400
asymptomatic (n=24)
mild (n=25)
severe (n=30)
control (n=72)
Severity

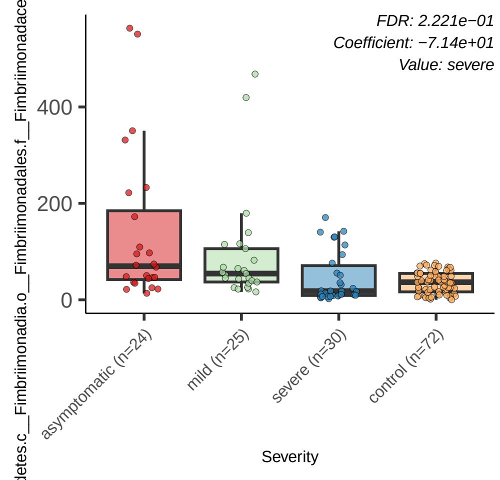

FDR: 2.221e−01
Coefficient: −7.14e+01
Value: severe
400
200
0
detes.c__Fimbriimonadia.o__Fimbriimonadales.f__Fimbriimonadace
asymptomatic (n=24)
mild (n=25)
severe (n=30)
control (n=72)
Severity

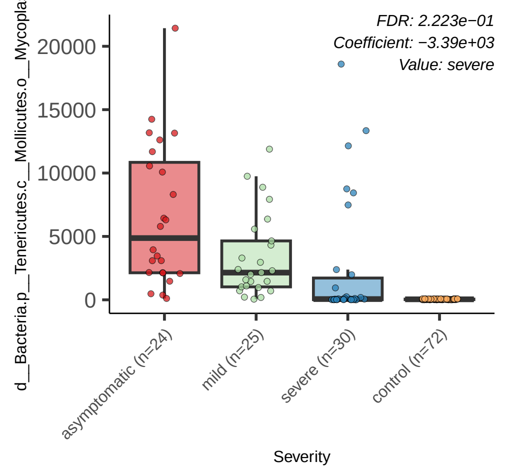

FDR: 2.223e−01
Coefficient: −3.39e+03
Value: severe
d__Bacteria.p__Tenericutes.c__Mollicutes.o__Mycopla
20000
15000
10000
5000
0
asymptomatic (n=24)
mild (n=25)
severe (n=30)
control (n=72)
Severity

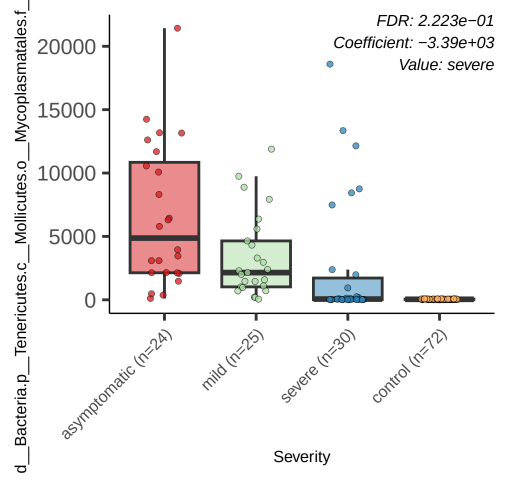
FDR: 2.223e−01
Coefficient: −3.39e+03
Value: severe
d__Bacteria.p__Tenericutes.c__Mollicutes.o__Mycoplasmatales.f__
20000
15000
10000
5000
0
asymptomatic (n=24)
mild (n=25)
severe (n=30)
control (n=72)
Severity

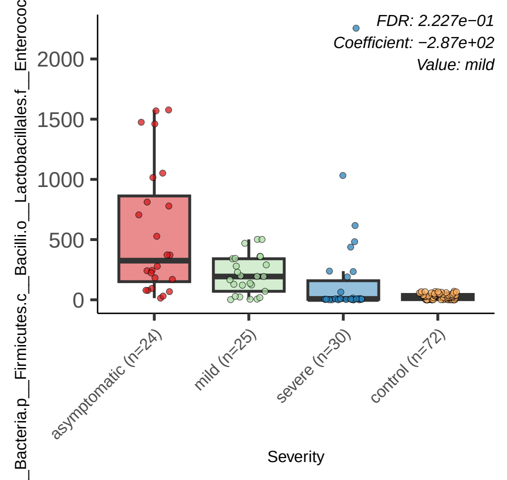

FDR: 2.227e−01
Coefficient: −2.87e+02
Value: mild
2000
1500
1000
500
0
Bacteria.p__Firmicutes.c__Bacilli.o__Lactobacillales.f__Enterococ
asymptomatic (n=24)
mild (n=25)
severe (n=30)
control (n=72)
Severity

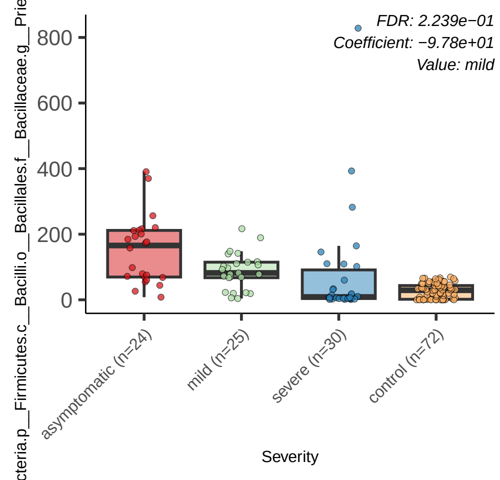

FDR: 2.239e−01
Coefficient: −9.78e+01
Value: mild
800
600
400
200
0
cteria.p__Firmicutes.c__Bacilli.o__Bacillales.f__Bacillaceae.g__Prie
asymptomatic (n=24)
mild (n=25)
severe (n=30)
control (n=72)
Severity

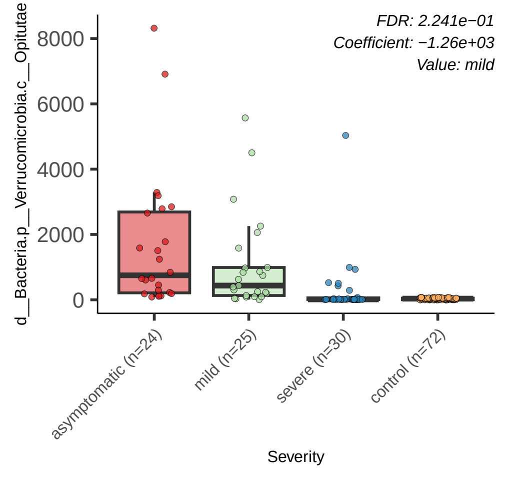

FDR: 2.241e−01
Coefficient: −1.26e+03
Value: mild
d__Bacteria.p__Verrucomicrobia.c__Opitutae
8000
6000
4000
2000
0
asymptomatic (n=24)
mild (n=25)
severe (n=30)
control (n=72)
Severity

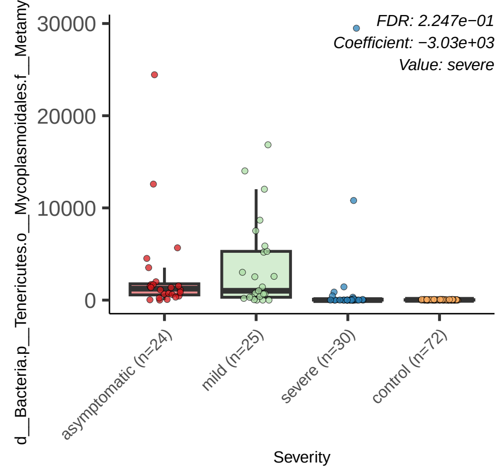

FDR: 2.247e−01
Coefficient: −3.03e+03
Value: severe
d__Bacteria.p__Tenericutes.o__Mycoplasmoidales.f__Metamy
30000
20000
10000
0
asymptomatic (n=24)
mild (n=25)
severe (n=30)
control (n=72)
Severity

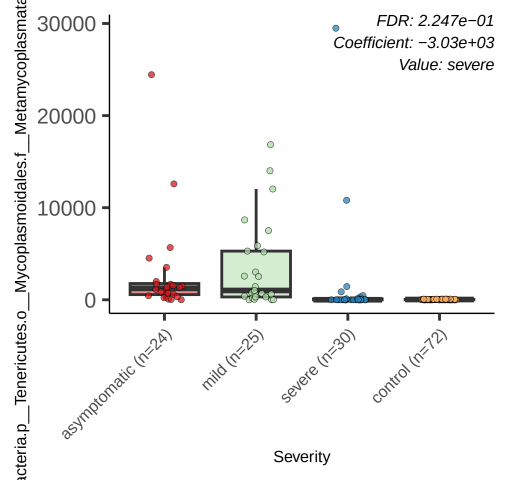

FDR: 2.247e−01
Coefficient: −3.03e+03
Value: severe
30000
20000
10000
0
acteria.p__Tenericutes.o__Mycoplasmoidales.f__Metamycoplasmata
asymptomatic (n=24)
mild (n=25)
severe (n=30)
control (n=72)
Severity

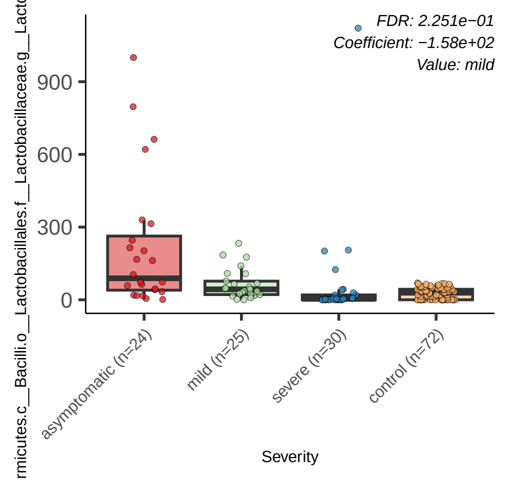

FDR: 2.251e−01
Coefficient: −1.58e+02
Value: mild
rmicutes.c__Bacilli.o__Lactobacillales.f__Lactobacillaceae.g__Lacto
900
600
300
0
asymptomatic (n=24)
mild (n=25)
severe (n=30)
control (n=72)
Severity

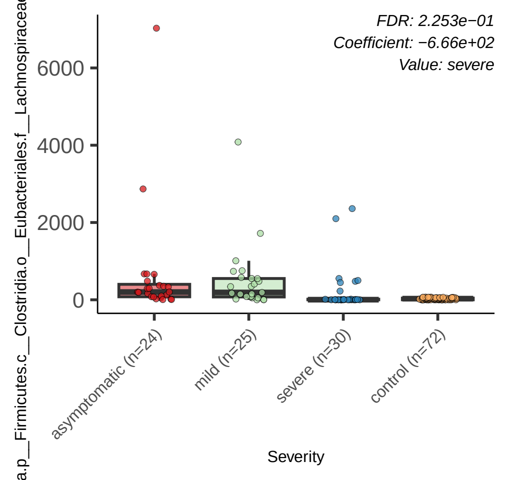
FDR: 2.253e−01
Coefficient: −6.66e+02
Value: severe
6000
4000
2000
0
a.p__Firmicutes.c__Clostridia.o__Eubacteriales.f__Lachnospiraceae
asymptomatic (n=24)
mild (n=25)
severe (n=30)
control (n=72)
Severity

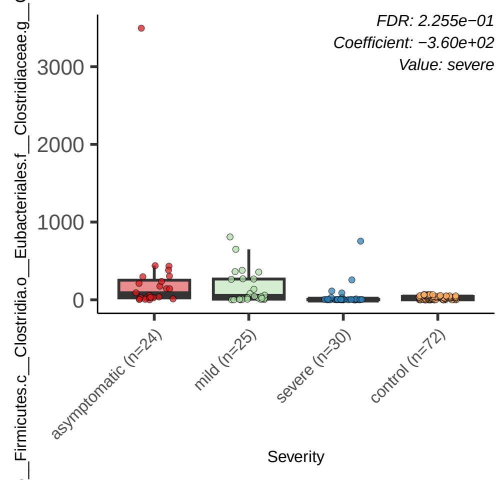
FDR: 2.255e−01
Coefficient: −3.60e+02
Value: severe
Firmicutes.c__Clostridia.o__Eubacteriales.f__Clostridiaceae.g__
3000
2000
1000
0
asymptomatic (n=24)
mild (n=25)
severe (n=30)
control (n=72)
Severity

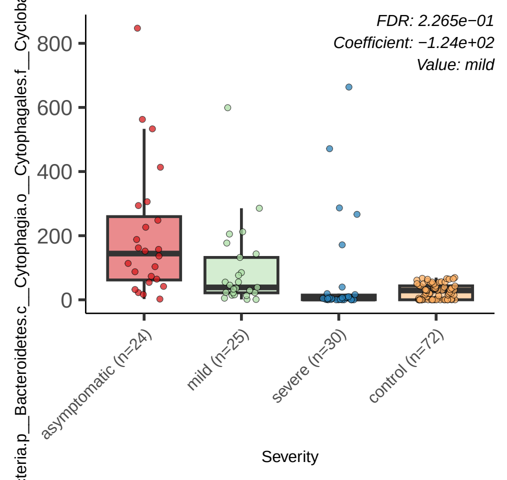

FDR: 2.265e−01
Coefficient: −1.24e+02
Value: mild
teria.p__Bacteroidetes.c__Cytophagia.o__Cytophagales.f__Cycloba
800
600
400
200
0
asymptomatic (n=24)
mild (n=25)
severe (n=30)
control (n=72)
Severity

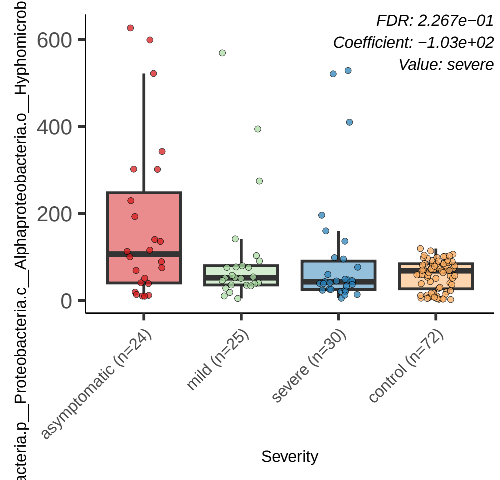
FDR: 2.267e−01
Coefficient: −1.03e+02
Value: severe
acteria.p__Proteobacteria.c__Alphaproteobacteria.o__Hyphomicrob
600
400
200
0
asymptomatic (n=24)
mild (n=25)
severe (n=30)
control (n=72)
Severity

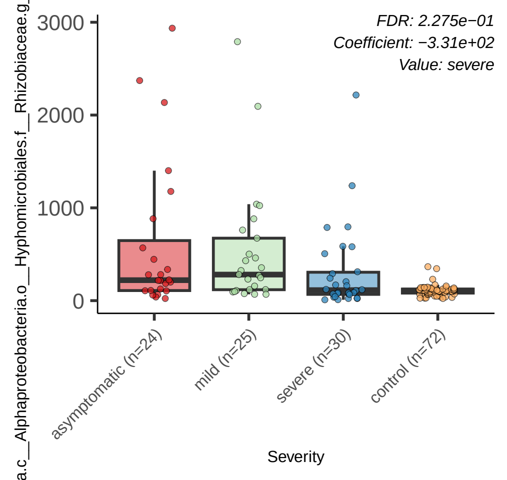

FDR: 2.275e−01
Coefficient: −3.31e+02
Value: severe
a.c__Alphaproteobacteria.o__Hyphomicrobiales.f__Rhizobiaceae.g_
3000
2000
1000
0
asymptomatic (n=24)
mild (n=25)
severe (n=30)
control (n=72)
Severity

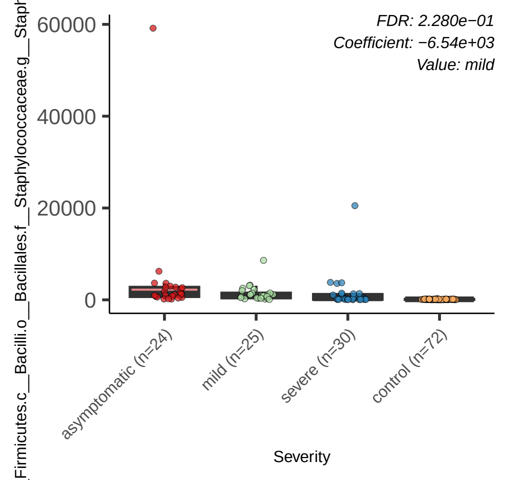

FDR: 2.280e−01
Coefficient: −6.54e+03
Value: mild
Firmicutes.c__Bacilli.o__Bacillales.f__Staphylococcaceae.g__Staph
60000
40000
20000
0
asymptomatic (n=24)
mild (n=25)
severe (n=30)
control (n=72)
Severity

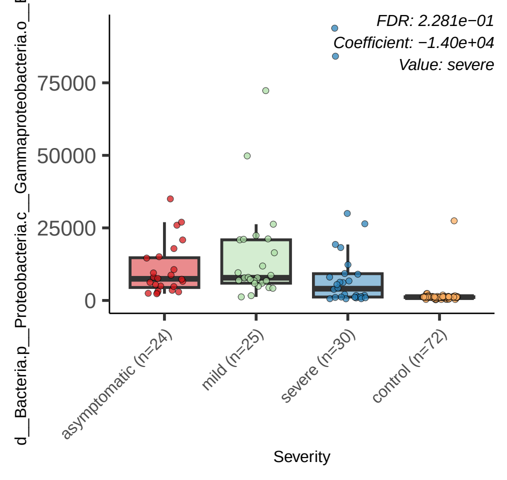
FDR: 2.281e−01
Coefficient: −1.40e+04
Value: severe
d__Bacteria.p__Proteobacteria.c__Gammaproteobacteria.o__B
75000
50000
25000
0
asymptomatic (n=24)
mild (n=25)
severe (n=30)
control (n=72)
Severity

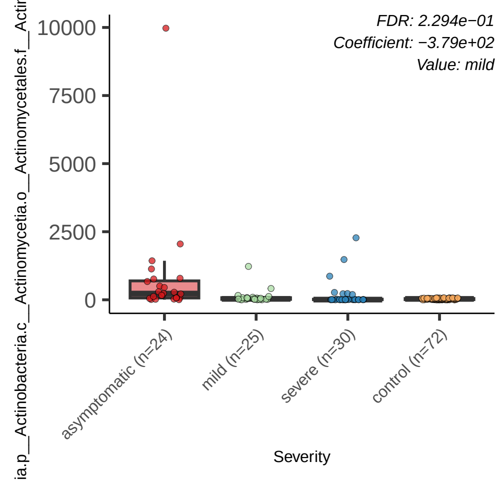

FDR: 2.294e−01
Coefficient: −3.79e+02
Value: mild
10000
7500
5000
2500
0
ia.p__Actinobacteria.c__Actinomycetia.o__Actinomycetales.f__Actin
asymptomatic (n=24)
mild (n=25)
severe (n=30)
control (n=72)
Severity

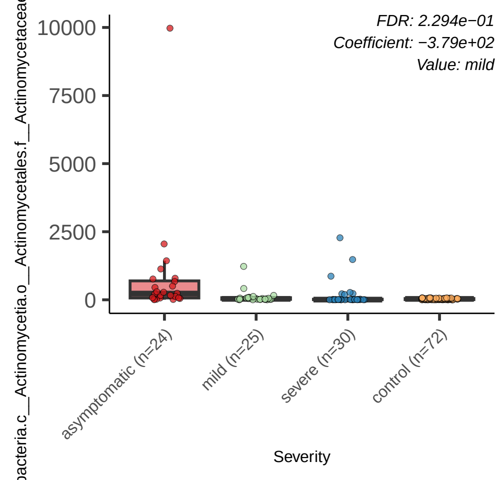

FDR: 2.294e−01
Coefficient: −3.79e+02
Value: mild
10000
7500
5000
2500
0
asymptomatic (n=24)
mild (n=25)
severe (n=30)
control (n=72)
Severity

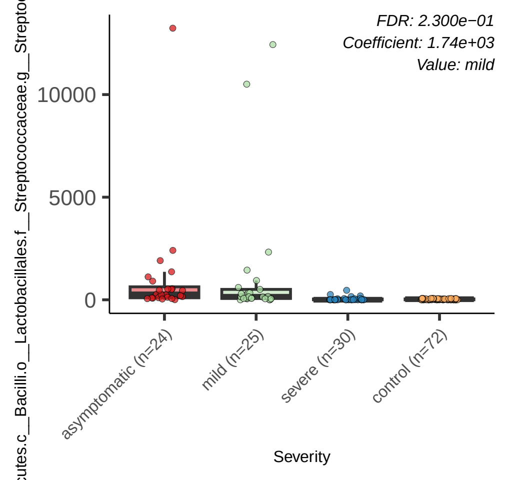

FDR: 2.300e−01
Coefficient: 1.74e+03
Value: mild
10000
5000
0
cutes.c__Bacilli.o__Lactobacillales.f__Streptococcaceae.g__Streptoc
asymptomatic (n=24)
mild (n=25)
severe (n=30)
control (n=72)
Severity

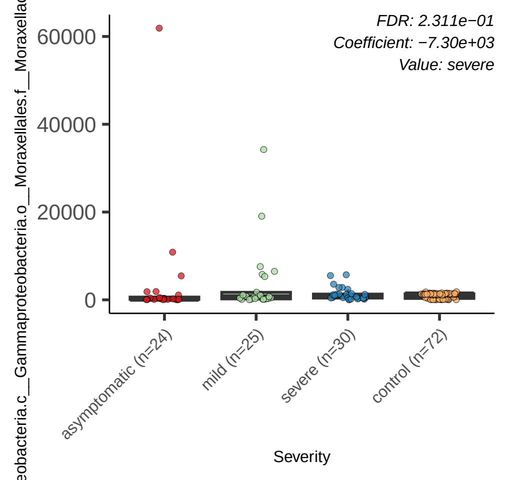
FDR: 2.311e−01
Coefficient: −7.30e+03
Value: severe
eobacteria.c__Gammaproteobacteria.o__Moraxellales.f__Moraxellac
60000
40000
20000
0
asymptomatic (n=24)
mild (n=25)
severe (n=30)
control (n=72)
Severity

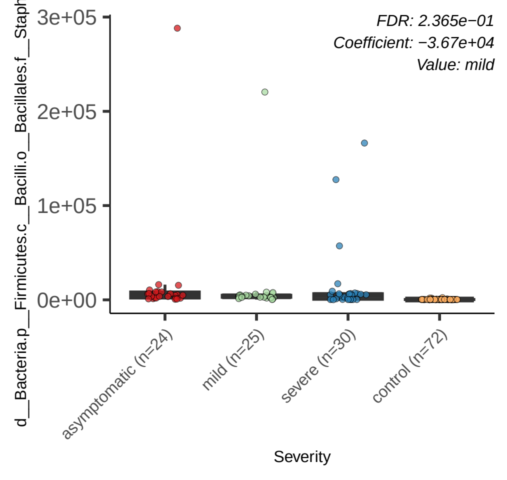

d__Bacteria.p__Firmicutes.c__Bacilli.o__Bacillales.f__Staph
3e+05
2e+05
1e+05
0e+00
FDR: 2.365e−01
Coefficient: −3.67e+04
Value: mild
asymptomatic (n=24)
mild (n=25)
severe (n=30)
control (n=72)
Severity

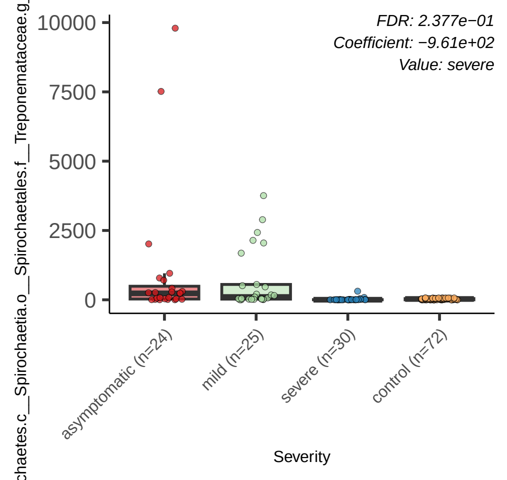

FDR: 2.377e−01
Coefficient: −9.61e+02
Value: severe
10000
7500
5000
2500
0
chaetes.c__Spirochaetia.o__Spirochaetales.f__Treponemataceae.g
asymptomatic (n=24)
mild (n=25)
severe (n=30)
control (n=72)
Severity

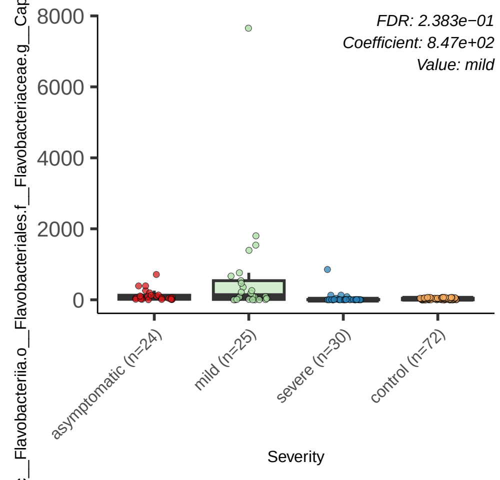
FDR: 2.383e−01
Coefficient: 8.47e+02
Value: mild
8000
6000
4000
2000
0
asymptomatic (n=24)
mild (n=25)
severe (n=30)
control (n=72)
Severity
Flavobacteriia.o__Flavobacteriales.f__Flavobacteriaceae.g__Cap

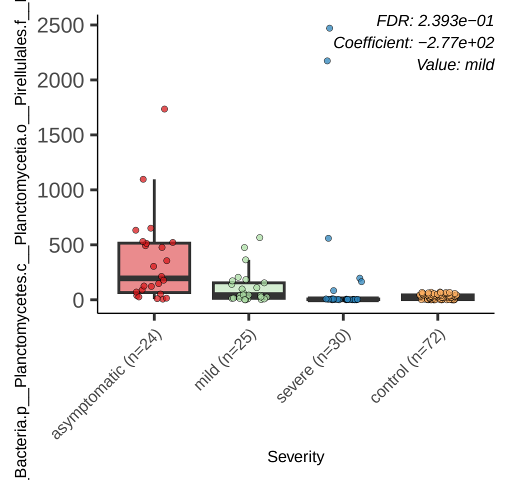

FDR: 2.393e−01
Coefficient: −2.77e+02
Value: mild
2500
2000
1500
1000
500
0
Bacteria.p__Planctomycetes.c__Planctomycetia.o__Pirellulales.f__
asymptomatic (n=24)
mild (n=25)
severe (n=30)
control (n=72)
Severity

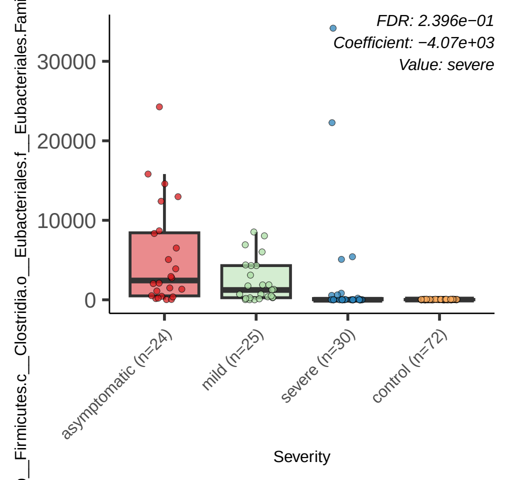

FDR: 2.396e−01
Coefficient: −4.07e+03
Value: severe
30000
20000
10000
0
o__Firmicutes.c__Clostridia.o__Eubacteriales.f__Eubacteriales.Fami
asymptomatic (n=24)
mild (n=25)
severe (n=30)
control (n=72)
Severity

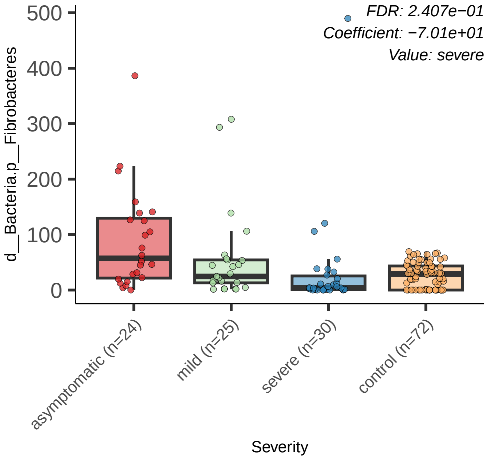
FDR: 2.407e−01
Coefficient: −7.01e+01
Value: severe
d__Bacteria.p__Fibrobacteres
500
400
300
200
100
0
asymptomatic (n=24)
mild (n=25)
severe (n=30)
control (n=72)
Severity

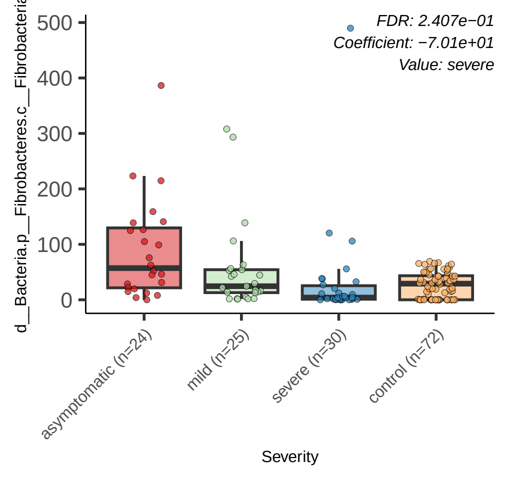
FDR: 2.407e−01
Coefficient: −7.01e+01
Value: severe
d__Bacteria.p__Fibrobacteres.c__Fibrobacteria
0
100
200
300
400
500
asymptomatic (n=24)
mild (n=25)
severe (n=30)
control (n=72)
Severity

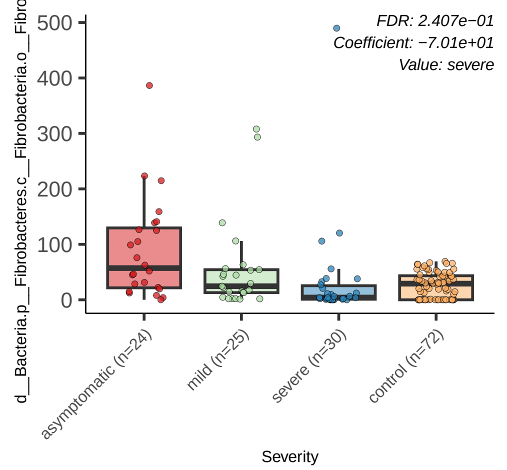

FDR: 2.407e−01
Coefficient: −7.01e+01
Value: severe
d__Bacteria.p__Fibrobacteres.c__Fibrobacteria.o__Fibro
500
400
300
200
100
0
asymptomatic (n=24)
mild (n=25)
severe (n=30)
control (n=72)
Severity

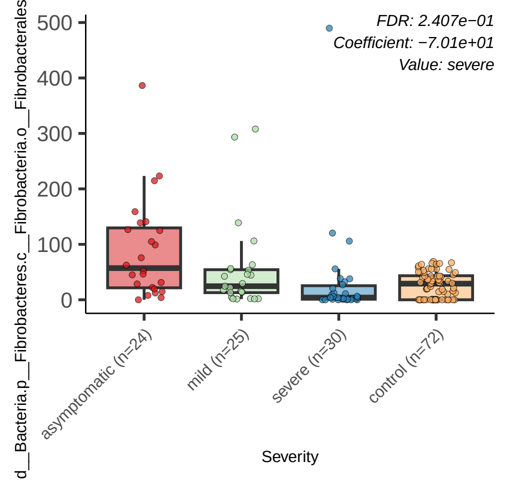

FDR: 2.407e−01
Coefficient: −7.01e+01
Value: severe
d__Bacteria.p__Fibrobacteres.c__Fibrobacteria.o__Fibrobacterales
500
400
300
200
100
0
asymptomatic (n=24)
mild (n=25)
severe (n=30)
control (n=72)
Severity

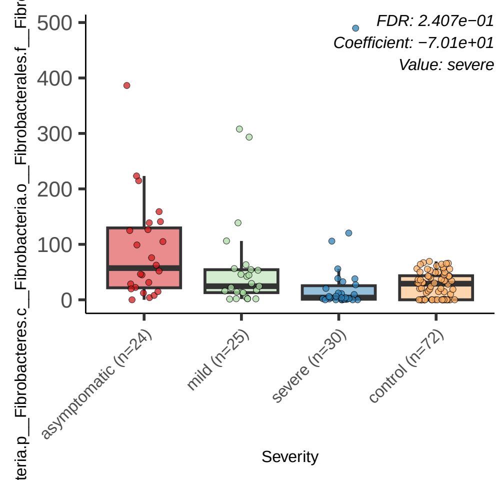

FDR: 2.407e−01
Coefficient: −7.01e+01
Value: severe
500
400
300
200
100
0
teria.p__Fibrobacteres.c__Fibrobacteria.o__Fibrobacterales.f__Fibro
asymptomatic (n=24)
mild (n=25)
severe (n=30)
control (n=72)
Severity

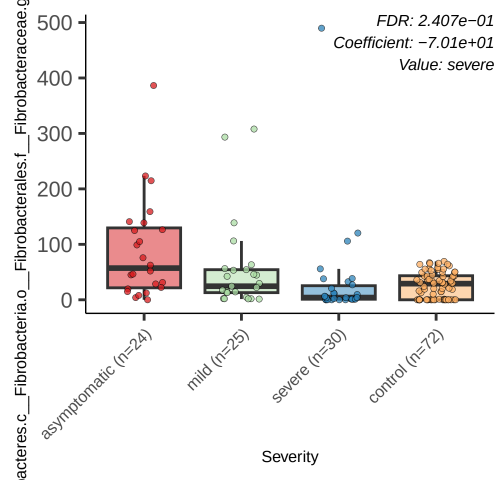
FDR: 2.407e−01
Coefficient: −7.01e+01
Value: severe
500
400
300
200
100
0
asymptomatic (n=24)
mild (n=25)
severe (n=30)
control (n=72)
Severity

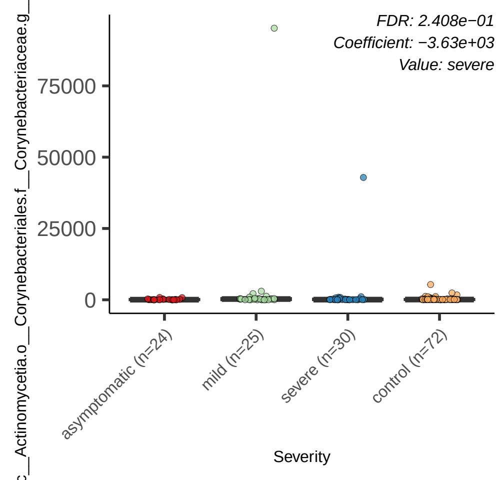
FDR: 2.408e−01
Coefficient: −3.63e+03
Value: severe
75000
50000
25000
0
c__Actinomycetia.o__Corynebacteriales.f__Corynebacteriaceae.g__
asymptomatic (n=24)
mild (n=25)
severe (n=30)
control (n=72)
Severity

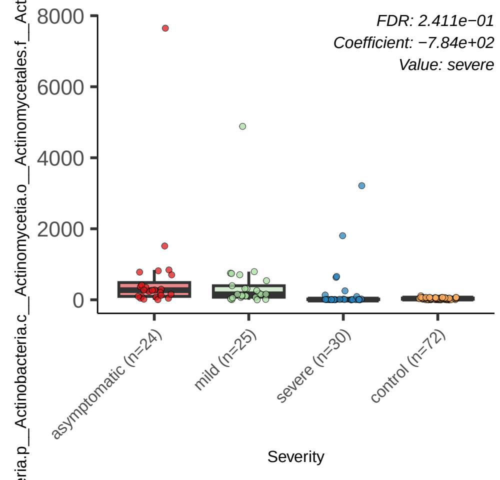

FDR: 2.411e−01
Coefficient: −7.84e+02
Value: severe
8000
6000
4000
2000
0
asymptomatic (n=24)
mild (n=25)
severe (n=30)
control (n=72)
Severity

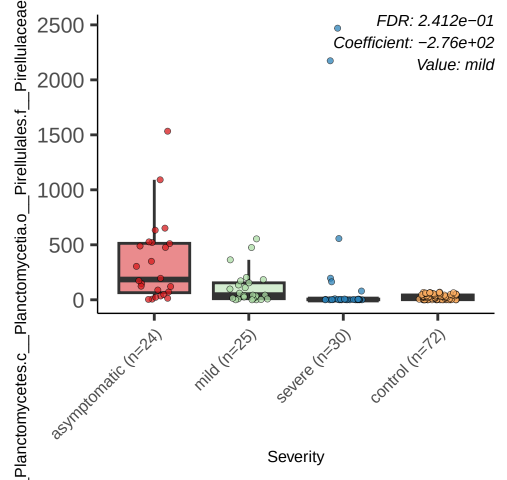
FDR: 2.412e−01
Coefficient: −2.76e+02
Value: mild
2500
2000
1500
1000
500
0
Planctomycetes.c__Planctomycetia.o__Pirellulales.f__Pirellulaceae
asymptomatic (n=24)
mild (n=25)
severe (n=30)
control (n=72)
Severity

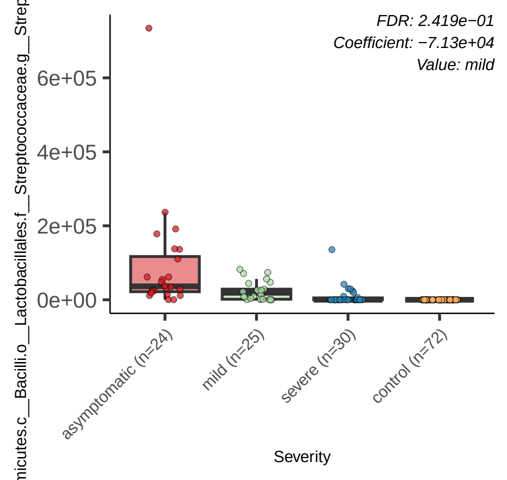

FDR: 2.419e−01
Coefficient: −7.13e+04
Value: mild
micutes.c__Bacilli.o__Lactobacillales.f__Streptococcaceae.g__Strep
6e+05
4e+05
2e+05
0e+00
asymptomatic (n=24)
mild (n=25)
severe (n=30)
control (n=72)
Severity

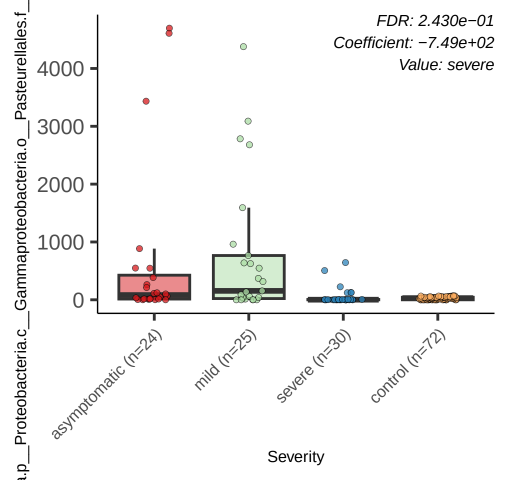

FDR: 2.430e−01
Coefficient: −7.49e+02
Value: severe
a.p__Proteobacteria.c__Gammaproteobacteria.o__Pasteurellales.f__
0
1000
2000
3000
4000
asymptomatic (n=24)
mild (n=25)
severe (n=30)
control (n=72)
Severity

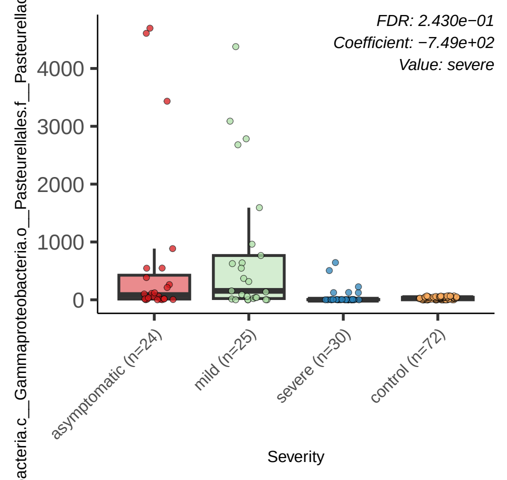
FDR: 2.430e−01
Coefficient: −7.49e+02
Value: severe
4000
3000
2000
1000
0
acteria.c__Gammaproteobacteria.o__Pasteurellales.f__Pasteurellac
asymptomatic (n=24)
mild (n=25)
severe (n=30)
control (n=72)
Severity

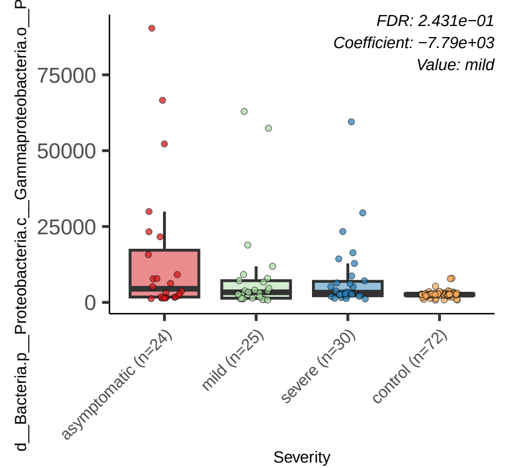

FDR: 2.431e−01
Coefficient: −7.79e+03
Value: mild
d__Bacteria.p__Proteobacteria.c__Gammaproteobacteria.o__P
75000
50000
25000
0
asymptomatic (n=24)
mild (n=25)
severe (n=30)
control (n=72)
Severity

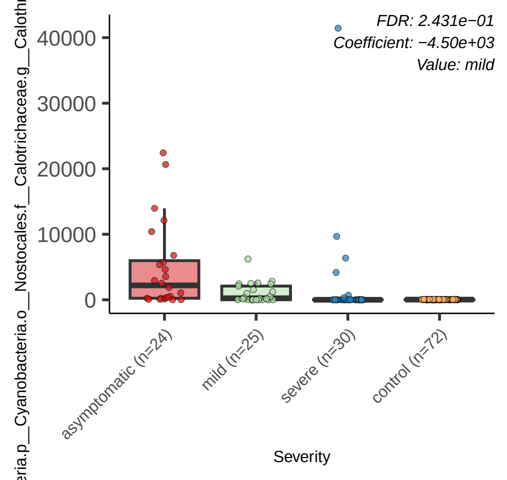

FDR: 2.431e−01
Coefficient: −4.50e+03
Value: mild
40000
30000
20000
10000
0
asymptomatic (n=24)
mild (n=25)
severe (n=30)
control (n=72)
Severity
eria.p__Cyanobacteria.o__Nostocales.f__Calotrichaceae.g__Calothr

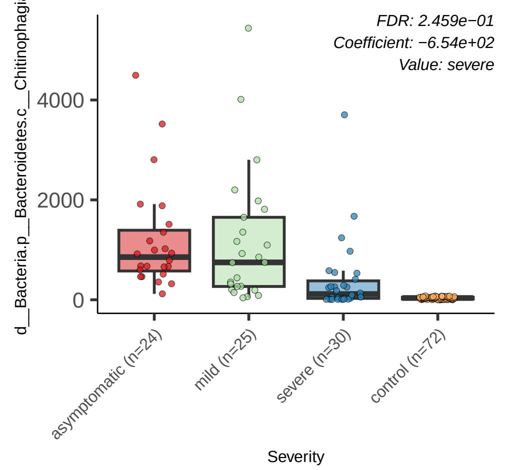

FDR: 2.459e−01
Coefficient: −6.54e+02
Value: severe
d__Bacteria.p__Bacteroidetes.c__Chitinophagia
0
2000
4000
asymptomatic (n=24)
mild (n=25)
severe (n=30)
control (n=72)
Severity

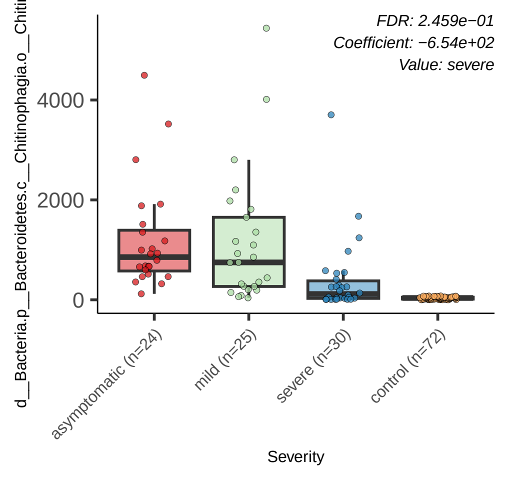

FDR: 2.459e−01
Coefficient: −6.54e+02
Value: severe
d__Bacteria.p__Bacteroidetes.c__Chitinophagia.o__Chiti
0
2000
4000
asymptomatic (n=24)
mild (n=25)
severe (n=30)
control (n=72)
Severity

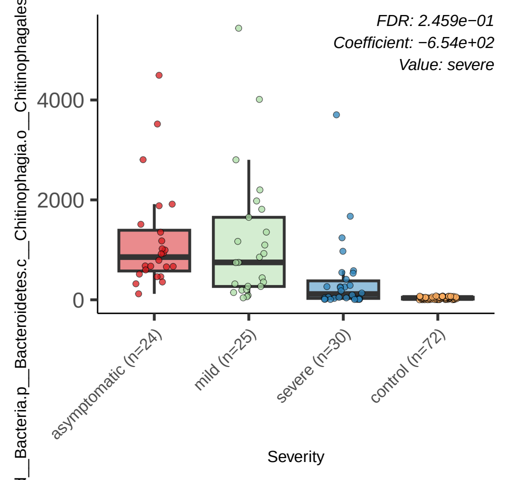
FDR: 2.459e−01
Coefficient: −6.54e+02
Value: severe
d__Bacteria.p__Bacteroidetes.c__Chitinophagia.o__Chitinophagales
4000
2000
0
asymptomatic (n=24)
mild (n=25)
severe (n=30)
control (n=72)
Severity

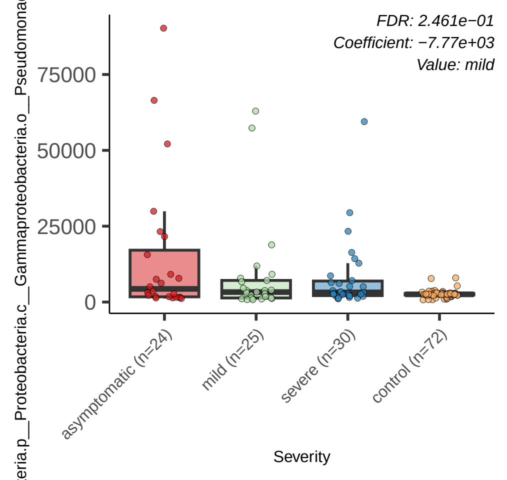
FDR: 2.461e−01
Coefficient: −7.77e+03
Value: mild
0
25000
50000
75000
teria.p__Proteobacteria.c__Gammaproteobacteria.o__Pseudomonad
asymptomatic (n=24)
mild (n=25)
severe (n=30)
control (n=72)
Severity
