# Supplementary material for: Metatranscriptome Analysis of Nasopharyngeal Swabs across the Varying Severity of COVID-19 Disease Demonstrated Unprecedented Species Diversity
Source: Microorganisms. 2023 Jul 14;11(7):1804. doi: 10.3390/microorganisms11071804 (PMC10384460; doi:10.3390/microorganisms11071804)
Supplement: Supplementary file 1 [file microorganisms-11-01804-s001.zip › Maaslin_results_ALL.zip!/Maaslin_results/WHOvariant.pdf]

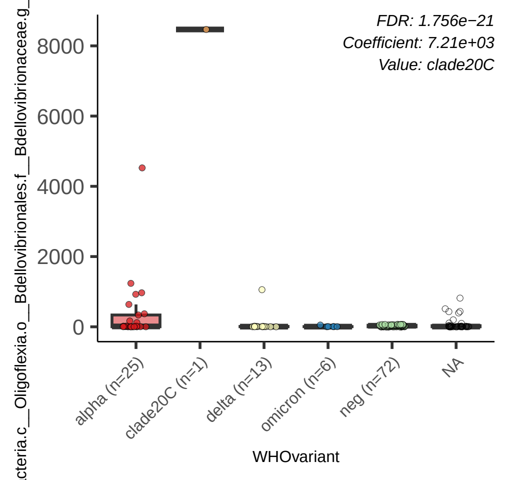

FDR: 1.756e−21
Coefficient: 7.21e+03
Value: clade20C
8000
6000
4000
2000
0
cteria.c__Oligoflexia.o__Bdellovibrionales.f__Bdellovibrionaceae.g__
alpha (n=25)
clade20C (n=1)
delta (n=13)
omicron (n=6)
neg (n=72)
NA
WHOvariant

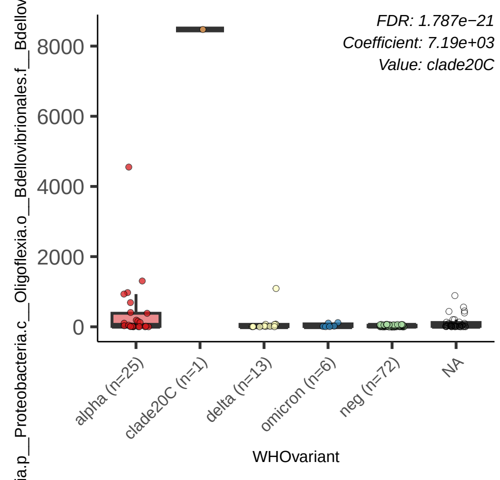

FDR: 1.787e−21
Coefficient: 7.19e+03
Value: clade20C
ia.p__Proteobacteria.c__Oligoflexia.o__Bdellovibrionales.f__Bdellov
8000
6000
4000
2000
0
alpha (n=25)
clade20C (n=1)
delta (n=13)
omicron (n=6)
neg (n=72)
NA
WHOvariant

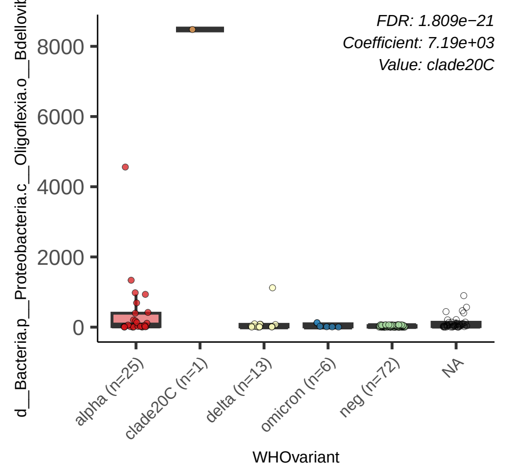
FDR: 1.809e−21
Coefficient: 7.19e+03
Value: clade20C
d__Bacteria.p__Proteobacteria.c__Oligoflexia.o__Bdellovib
8000
6000
4000
2000
0
alpha (n=25)
clade20C (n=1)
delta (n=13)
omicron (n=6)
neg (n=72)
NA
WHOvariant

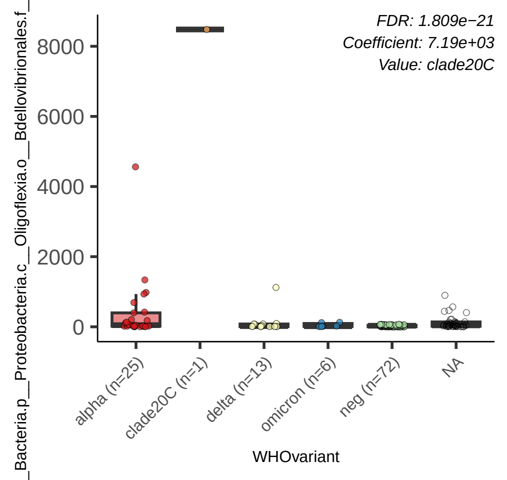

FDR: 1.809e−21
Coefficient: 7.19e+03
Value: clade20C
Bacteria.p__Proteobacteria.c__Oligoflexia.o__Bdellovibrionales.f
8000
6000
4000
2000
0
alpha (n=25)
clade20C (n=1)
delta (n=13)
omicron (n=6)
neg (n=72)
NA
WHOvariant

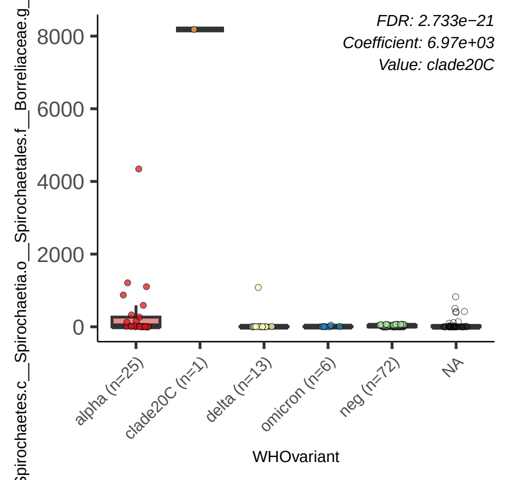

FDR: 2.733e−21
Coefficient: 6.97e+03
Value: clade20C
8000
6000
4000
2000
0
Spirochaetes.c__Spirochaetia.o__Spirochaetales.f__Borreliaceae.g__
alpha (n=25)
clade20C (n=1)
delta (n=13)
omicron (n=6)
neg (n=72)
NA
WHOvariant

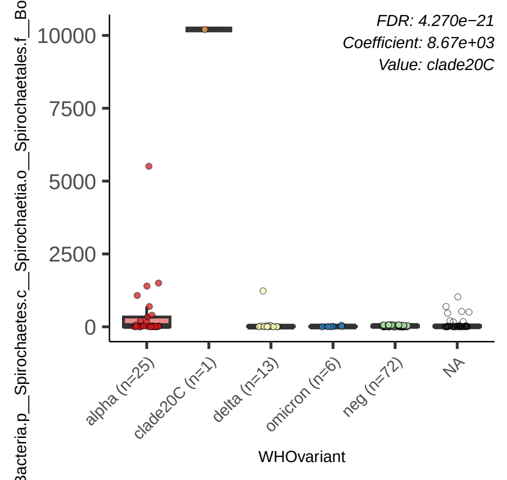
FDR: 4.270e−21
Coefficient: 8.67e+03
Value: clade20C
10000
7500
5000
2500
0
alpha (n=25)
clade20C (n=1)
delta (n=13)
omicron (n=6)
neg (n=72)
NA
WHOvariant
Bacteria.p__Spirochaetes.c__Spirochaetia.o__Spirochaetales.f__Bo

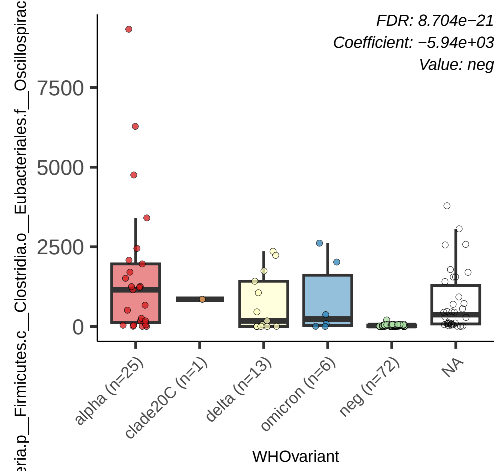

FDR: 8.704e−21
Coefficient: −5.94e+03
Value: neg
7500
5000
2500
0
teria.p__Firmicutes.c__Clostridia.o__Eubacteriales.f__Oscillospirac
alpha (n=25)
clade20C (n=1)
delta (n=13)
omicron (n=6)
neg (n=72)
NA
WHOvariant

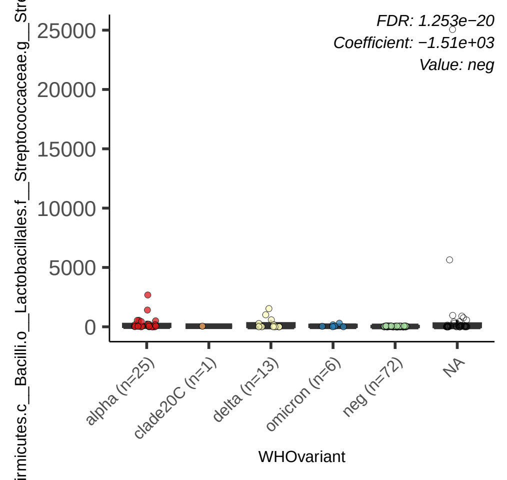
FDR: 1.253e−20
Coefficient: −1.51e+03
Value: neg
irmicutes.c__Bacilli.o__Lactobacillales.f__Streptococcaceae.g__Stre
25000
20000
15000
10000
5000
0
alpha (n=25)
clade20C (n=1)
delta (n=13)
omicron (n=6)
neg (n=72)
NA
WHOvariant

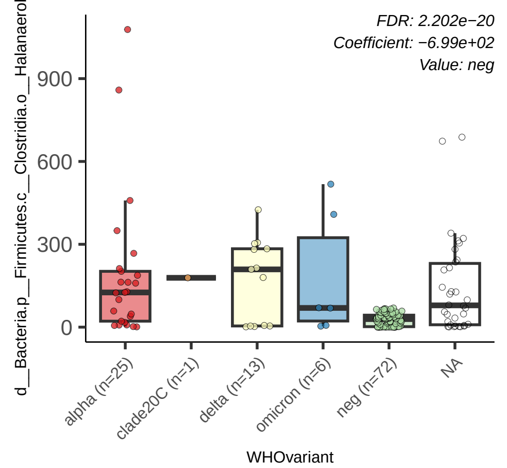

FDR: 2.202e−20
Coefficient: −6.99e+02
Value: neg
d__Bacteria.p__Firmicutes.c__Clostridia.o__Halanaerob
0
300
600
900
alpha (n=25)
clade20C (n=1)
delta (n=13)
omicron (n=6)
neg (n=72)
NA
WHOvariant

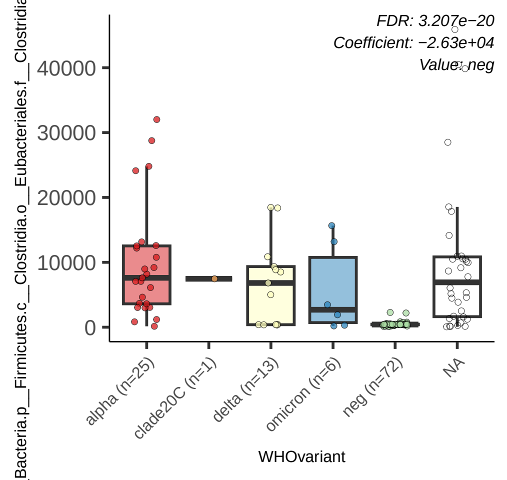
FDR: 3.207e−20
Coefficient: −2.63e+04
Value: neg
Bacteria.p__Firmicutes.c__Clostridia.o__Eubacteriales.f__Clostridia
0
10000
20000
30000
40000
alpha (n=25)
clade20C (n=1)
delta (n=13)
omicron (n=6)
neg (n=72)
NA
WHOvariant

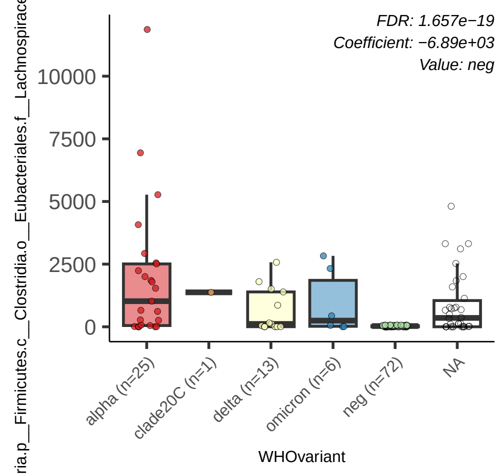
FDR: 1.657e−19
Coefficient: −6.89e+03
Value: neg
10000
7500
5000
2500
0
alpha (n=25)
clade20C (n=1)
delta (n=13)
omicron (n=6)
neg (n=72)
NA
WHOvariant

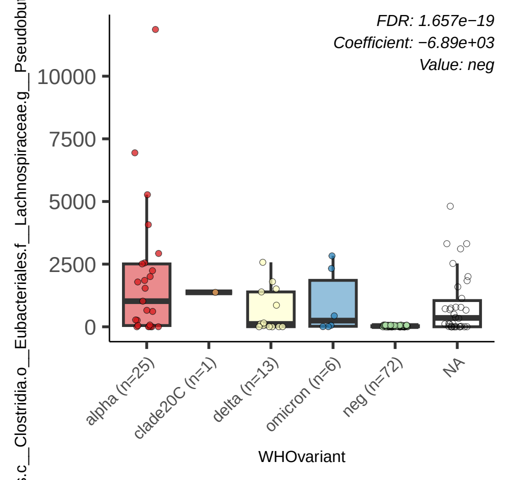
FDR: 1.657e−19
Coefficient: −6.89e+03
Value: neg
s.c__Clostridia.o__Eubacteriales.f__Lachnospiraceae.g__Pseudobu
10000
7500
5000
2500
0
alpha (n=25)
clade20C (n=1)
delta (n=13)
omicron (n=6)
neg (n=72)
NA
WHOvariant

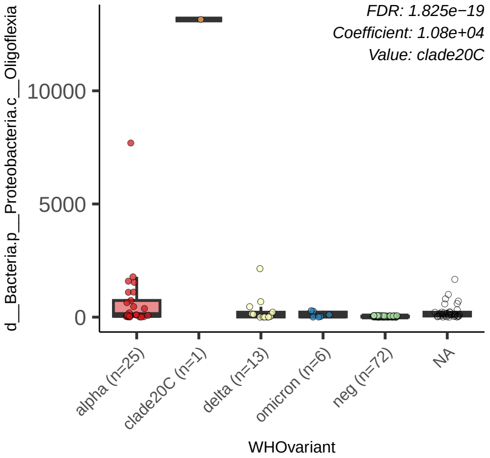

FDR: 1.825e−19
Coefficient: 1.08e+04
Value: clade20C
d__Bacteria.p__Proteobacteria.c__Oligoflexia
10000
5000
0
alpha (n=25)
clade20C (n=1)
delta (n=13)
omicron (n=6)
neg (n=72)
NA
WHOvariant

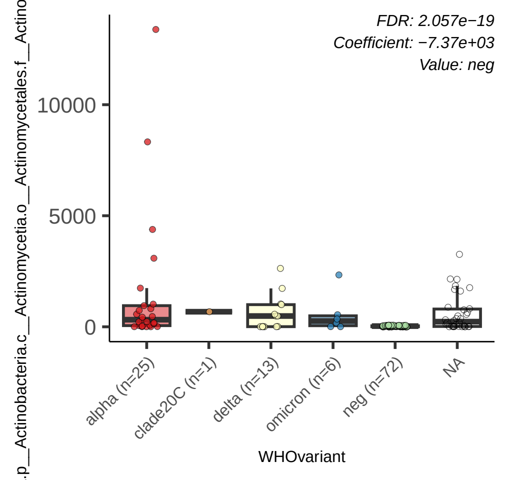

FDR: 2.057e−19
Coefficient: −7.37e+03
Value: neg
p__Actinobacteria.c__Actinomycetia.o__Actinomycetales.f__Actino
10000
5000
0
alpha (n=25)
clade20C (n=1)
delta (n=13)
omicron (n=6)
neg (n=72)
NA
WHOvariant

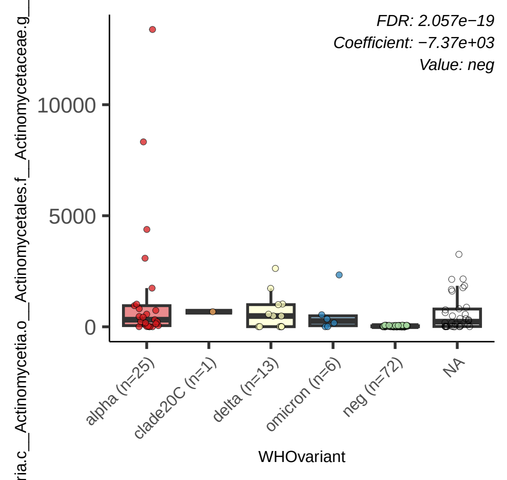
FDR: 2.057e−19
Coefficient: −7.37e+03
Value: neg
ria.c__Actinomycetia.o__Actinomycetales.f__Actinomycetaceae.g__
10000
5000
0
alpha (n=25)
clade20C (n=1)
delta (n=13)
omicron (n=6)
neg (n=72)
NA
WHOvariant

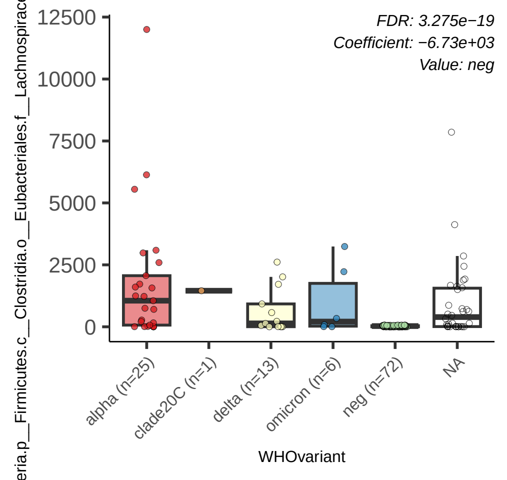

FDR: 3.275e−19
Coefficient: −6.73e+03
Value: neg
12500
10000
7500
5000
2500
0
alpha (n=25)
clade20C (n=1)
delta (n=13)
omicron (n=6)
neg (n=72)
NA
WHOvariant

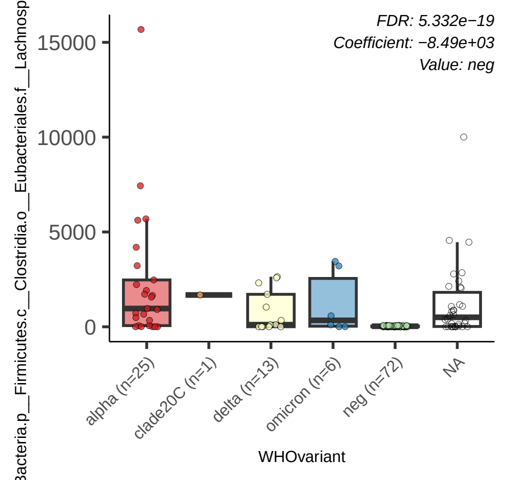
FDR: 5.332e−19
Coefficient: −8.49e+03
Value: neg
Bacteria.p__Firmicutes.c__Clostridia.o__Eubacteriales.f__Lachnosp
15000
10000
5000
0
alpha (n=25)
clade20C (n=1)
delta (n=13)
omicron (n=6)
neg (n=72)
NA
WHOvariant

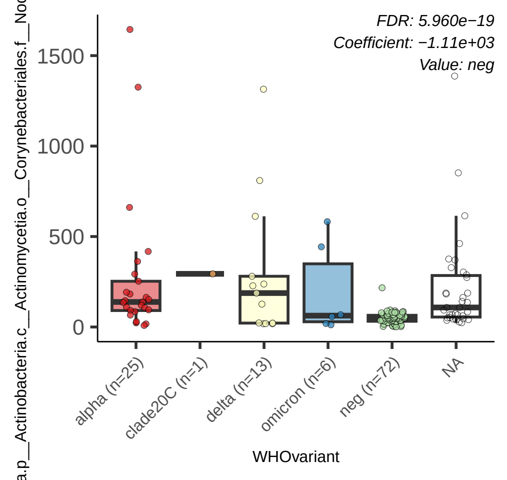

FDR: 5.960e−19
Coefficient: −1.11e+03
Value: neg
1500
1000
500
0
a.p__Actinobacteria.c__Actinomycetia.o__Corynebacteriales.f__Noc
alpha (n=25)
clade20C (n=1)
delta (n=13)
omicron (n=6)
neg (n=72)
NA
WHOvariant

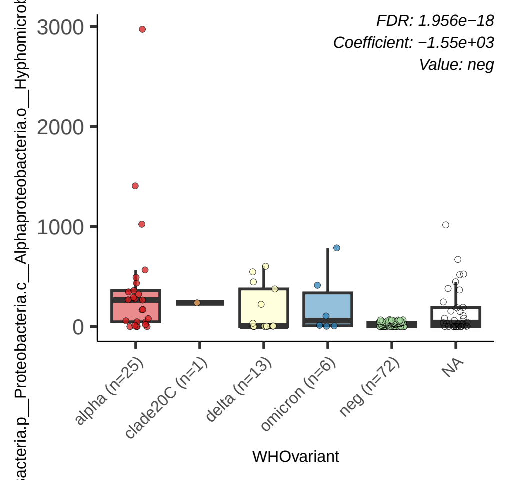

FDR: 1.956e−18
Coefficient: −1.55e+03
Value: neg
3000
2000
1000
0
Bacteria.p__Proteobacteria.c__Alphaproteobacteria.o__Hyphomicrob
alpha (n=25)
clade20C (n=1)
delta (n=13)
omicron (n=6)
neg (n=72)
NA
WHOvariant

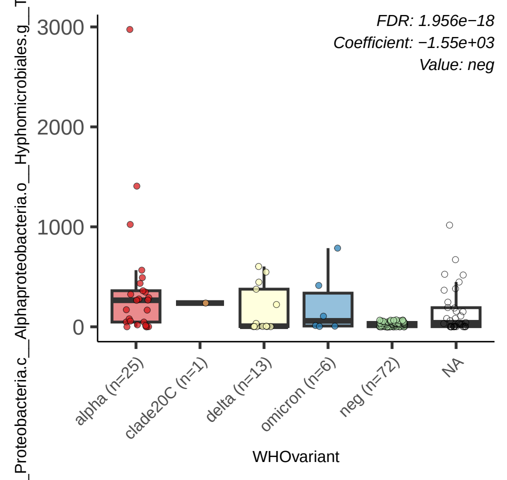
Proteobacteria.c__Alphaproteobacteria.o__Hyphomicrobiales.g__T
FDR: 1.956e−18
Coefficient: −1.55e+03
Value: neg
3000
2000
1000
0
alpha (n=25)
clade20C (n=1)
delta (n=13)
omicron (n=6)
neg (n=72)
NA
WHOvariant

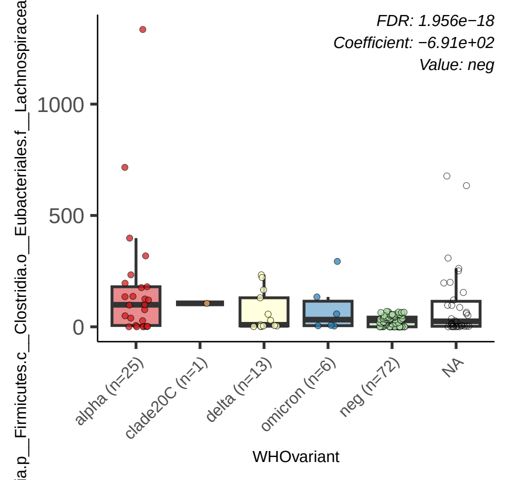

FDR: 1.956e−18
Coefficient: −6.91e+02
Value: neg
1000
500
0
ia.p__Firmicutes.c__Clostridia.o__Eubacteriales.f__Lachnospiracea
alpha (n=25)
clade20C (n=1)
delta (n=13)
omicron (n=6)
neg (n=72)
NA
WHOvariant

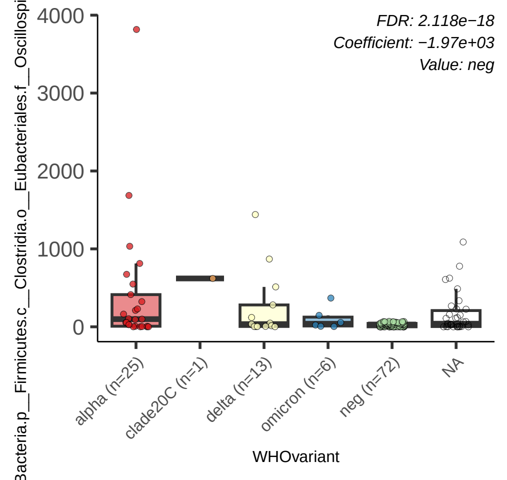

FDR: 2.118e−18
Coefficient: −1.97e+03
Value: neg
4000
3000
2000
1000
0
Bacteria.p__Firmicutes.c__Clostridia.o__Eubacteriales.f__Oscillospi
alpha (n=25)
clade20C (n=1)
delta (n=13)
omicron (n=6)
neg (n=72)
NA
WHOvariant

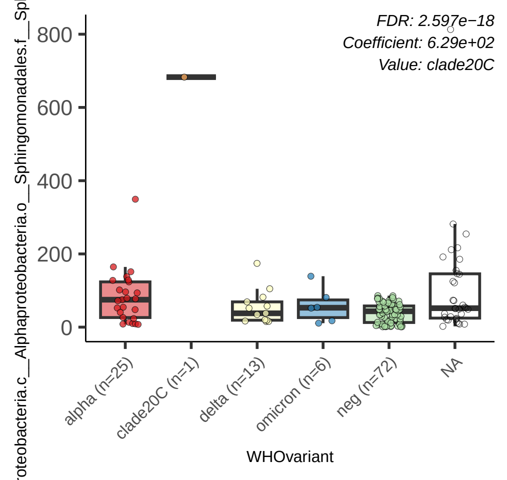

FDR: 2.597e−18
Coefficient: 6.29e+02
Value: clade20C
roteobacteria.c__Alphaproteobacteria.o__Sphingomonadales.f__Sph
800
600
400
200
0
alpha (n=25)
clade20C (n=1)
delta (n=13)
omicron (n=6)
neg (n=72)
NA
WHOvariant

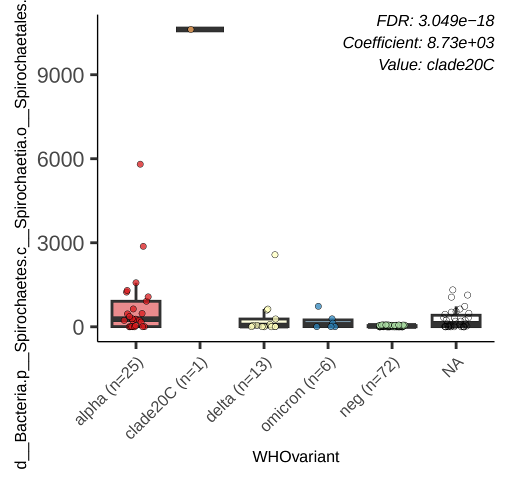
FDR: 3.049e−18
Coefficient: 8.73e+03
Value: clade20C
d__Bacteria.p__Spirochaetes.c__Spirochaetia.o__Spirochaetales.
0
3000
6000
9000
alpha (n=25)
clade20C (n=1)
delta (n=13)
omicron (n=6)
neg (n=72)
NA
WHOvariant

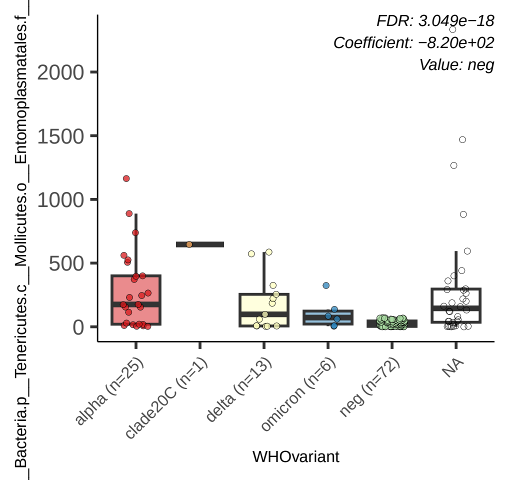
FDR: 3.049e−18
Coefficient: −8.20e+02
Value: neg
Bacteria.p__Tenericutes.c__Mollicutes.o__Entomoplasmatales.f__
2000
1500
1000
500
0
alpha (n=25)
clade20C (n=1)
delta (n=13)
omicron (n=6)
neg (n=72)
NA
WHOvariant

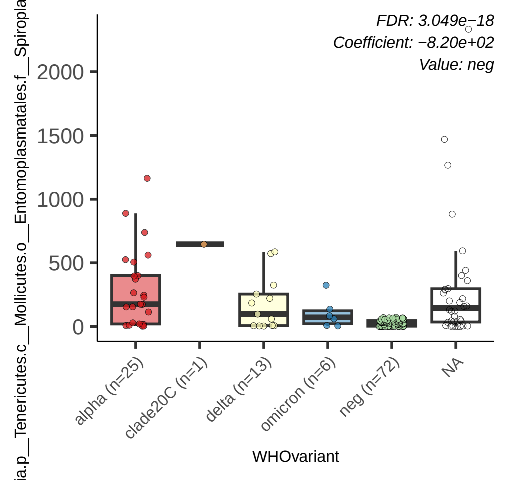

FDR: 3.049e−18
Coefficient: −8.20e+02
Value: neg
ia.p__Tenericutes.c__Mollicutes.o__Entomoplasmatales.f__Spiropla
0
500
1000
1500
2000
alpha (n=25)
clade20C (n=1)
delta (n=13)
omicron (n=6)
neg (n=72)
NA
WHOvariant

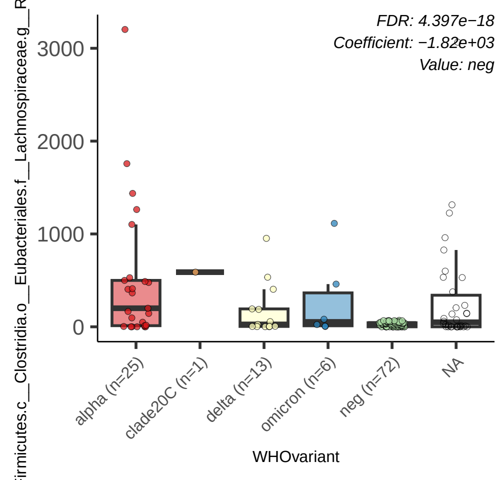
FDR: 4.397e−18
Coefficient: −1.82e+03
Value: neg
3000
2000
1000
0
Firmicutes.c__Clostridia.o__Eubacteriales.f__Lachnospiraceae.g__R
alpha (n=25)
clade20C (n=1)
delta (n=13)
omicron (n=6)
neg (n=72)
NA
WHOvariant

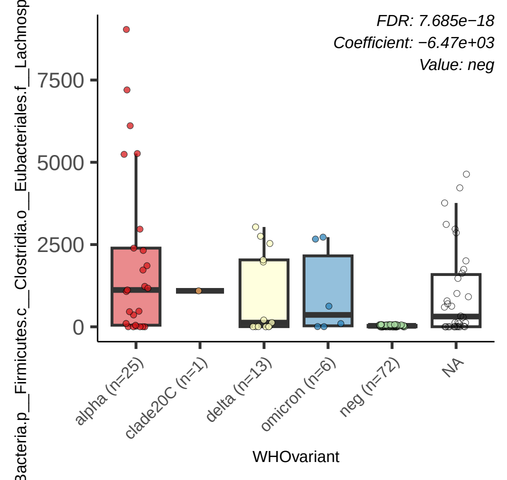
FDR: 7.685e−18
Coefficient: −6.47e+03
Value: neg
Bacteria.p__Firmicutes.c__Clostridia.o__Eubacteriales.f__Lachnosp
7500
5000
2500
0
alpha (n=25)
clade20C (n=1)
delta (n=13)
omicron (n=6)
neg (n=72)
NA
WHOvariant

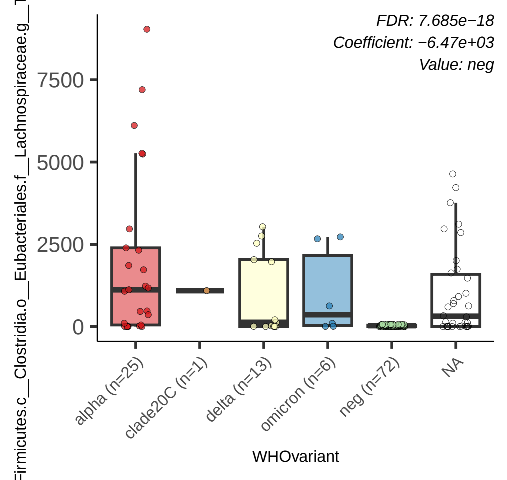
FDR: 7.685e−18
Coefficient: −6.47e+03
Value: neg
Firmicutes.c__Clostridia.o__Eubacteriales.f__Lachnospiraceae.g__T
7500
5000
2500
0
alpha (n=25)
clade20C (n=1)
delta (n=13)
omicron (n=6)
neg (n=72)
NA
WHOvariant

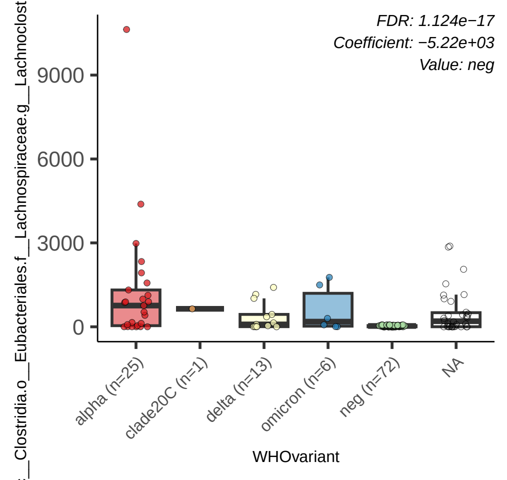

FDR: 1.124e−17
Coefficient: −5.22e+03
Value: neg
__Clostridia.o__Eubacteriales.f__Lachnospiraceae.g__Lachnoclost
9000
6000
3000
0
alpha (n=25)
clade20C (n=1)
delta (n=13)
omicron (n=6)
neg (n=72)
NA
WHOvariant

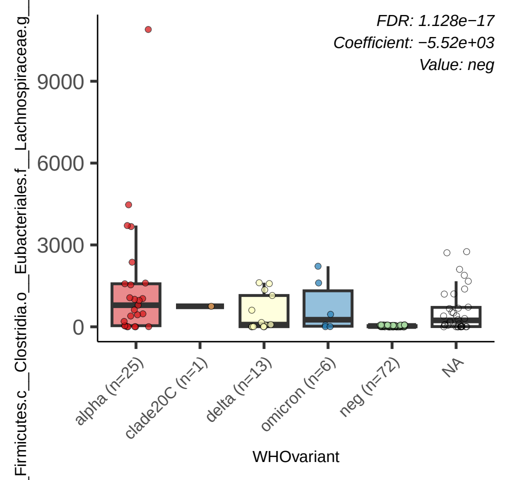
FDR: 1.128e−17
Coefficient: −5.52e+03
Value: neg
Firmicutes.c__Clostridia.o__Eubacteriales.f__Lachnospiraceae.g__
9000
6000
3000
0
alpha (n=25)
clade20C (n=1)
delta (n=13)
omicron (n=6)
neg (n=72)
NA
WHOvariant

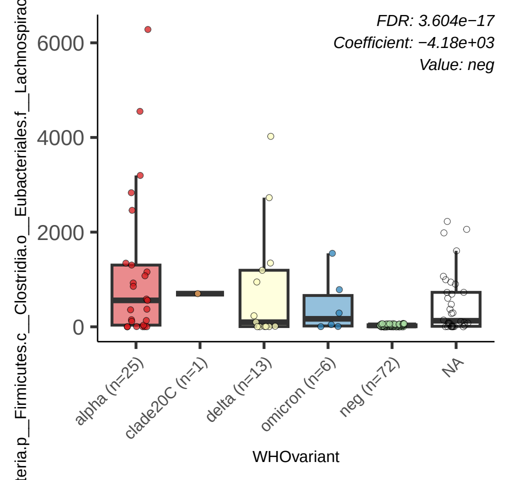

FDR: 3.604e−17
Coefficient: −4.18e+03
Value: neg
6000
4000
2000
0
teria.p__Firmicutes.c__Clostridia.o__Eubacteriales.f__Lachnospirac
alpha (n=25)
clade20C (n=1)
delta (n=13)
omicron (n=6)
neg (n=72)
NA
WHOvariant

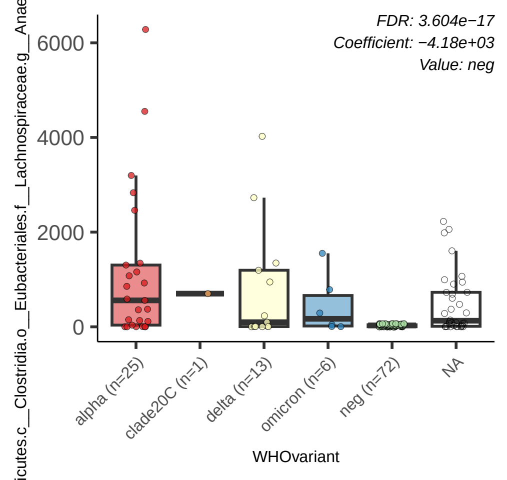

FDR: 3.604e−17
Coefficient: −4.18e+03
Value: neg
icutes.c__Clostridia.o__Eubacteriales.f__Lachnospiraceae.g__Anae
6000
4000
2000
0
alpha (n=25)
clade20C (n=1)
delta (n=13)
omicron (n=6)
neg (n=72)
NA
WHOvariant

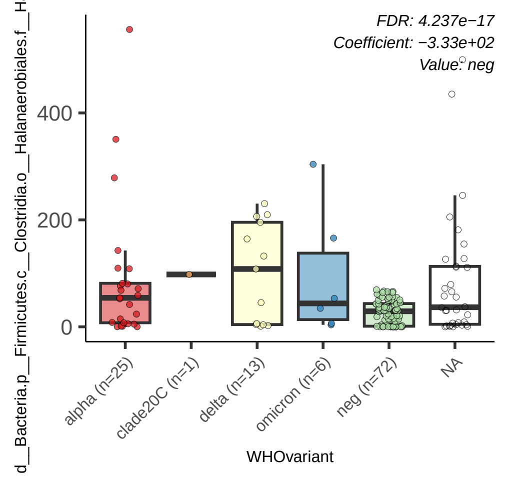

FDR: 4.237e−17
Coefficient: −3.33e+02
Value: neg
d__Bacteria.p__Firmicutes.c__Clostridia.o__Halanaerobiales.f__H
400
200
0
alpha (n=25)
clade20C (n=1)
delta (n=13)
omicron (n=6)
neg (n=72)
NA
WHOvariant

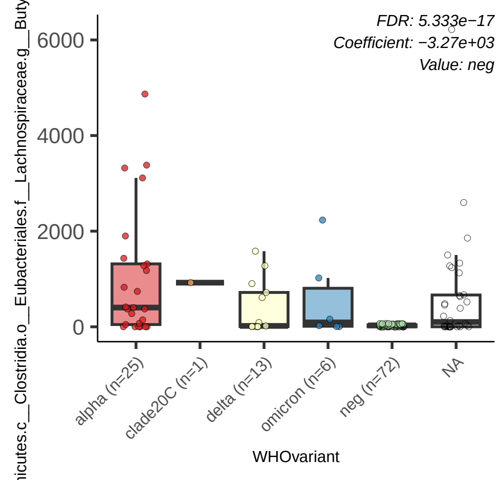

FDR: 5.333e−17
Coefficient: −3.27e+03
Value: neg
6000
4000
2000
0
alpha (n=25)
clade20C (n=1)
delta (n=13)
omicron (n=6)
neg (n=72)
NA
WHOvariant

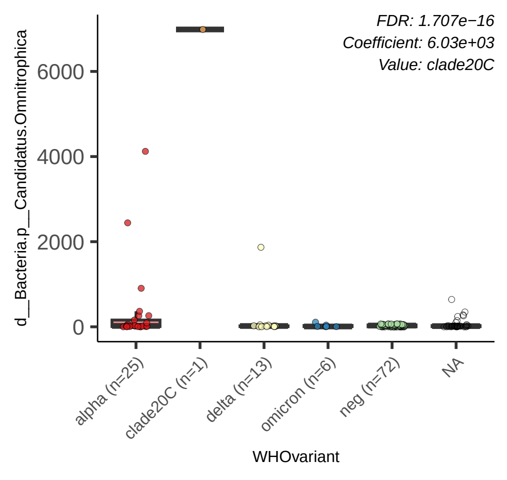

FDR: 1.707e−16
Coefficient: 6.03e+03
Value: clade20C
d__Bacteria.p__Candidatus.Omnitrophica
6000
4000
2000
0
alpha (n=25)
clade20C (n=1)
delta (n=13)
omicron (n=6)
neg (n=72)
NA
WHOvariant

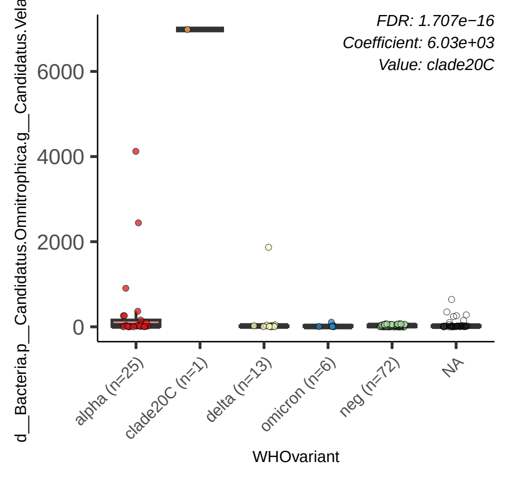

FDR: 1.707e−16
Coefficient: 6.03e+03
Value: clade20C
d__Bacteria.p__Candidatus.Omnitrophica.g__Candidatus.Vela
6000
4000
2000
0
alpha (n=25)
clade20C (n=1)
delta (n=13)
omicron (n=6)
neg (n=72)
NA
WHOvariant

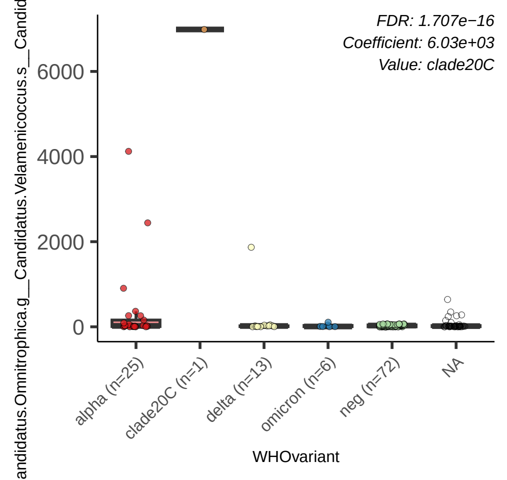
FDR: 1.707e−16
Coefficient: 6.03e+03
Value: clade20C
andidatus.Omnitrophica.g__Candidatus.Velamenicoccus.s__Candid
6000
4000
2000
0
alpha (n=25)
clade20C (n=1)
delta (n=13)
omicron (n=6)
neg (n=72)
NA
WHOvariant

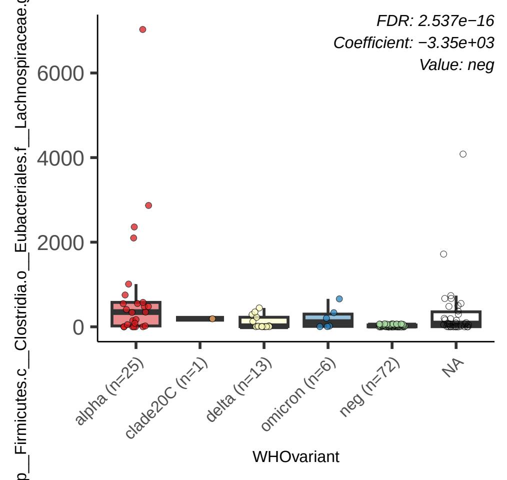
FDR: 2.537e−16
Coefficient: −3.35e+03
Value: neg
p__Firmicutes.c__Clostridia.o__Eubacteriales.f__Lachnospiraceae.g
6000
4000
2000
0
alpha (n=25)
clade20C (n=1)
delta (n=13)
omicron (n=6)
neg (n=72)
NA
WHOvariant

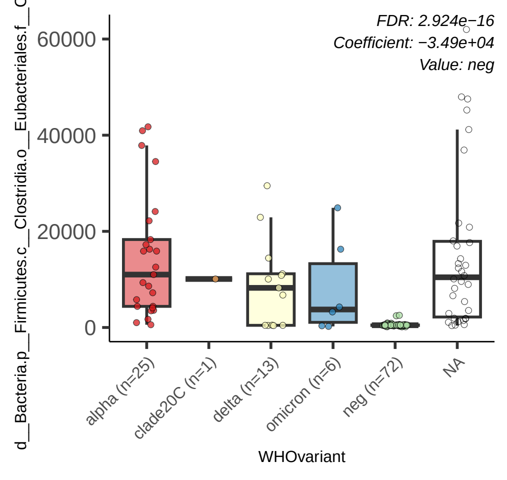

d__Bacteria.p__Firmicutes.c__Clostridia.o__Eubacteriales.f__C
FDR: 2.924e−16
Coefficient: −3.49e+04
Value: neg
60000
40000
20000
0
alpha (n=25)
clade20C (n=1)
delta (n=13)
omicron (n=6)
neg (n=72)
NA
WHOvariant

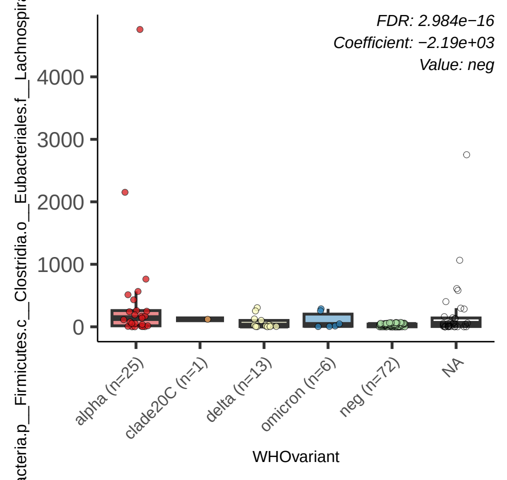

FDR: 2.984e−16
Coefficient: −2.19e+03
Value: neg
acteria.p__Firmicutes.c__Clostridia.o__Eubacteriales.f__Lachnospira
4000
3000
2000
1000
0
alpha (n=25)
clade20C (n=1)
delta (n=13)
omicron (n=6)
neg (n=72)
NA
WHOvariant

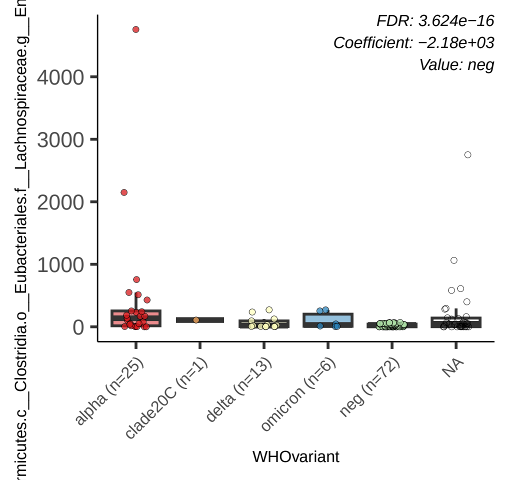

FDR: 3.624e−16
Coefficient: −2.18e+03
Value: neg
4000
3000
2000
1000
0
alpha (n=25)
clade20C (n=1)
delta (n=13)
omicron (n=6)
neg (n=72)
NA
WHOvariant

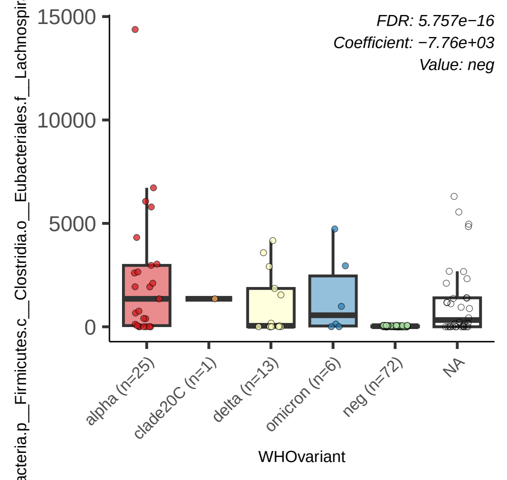

FDR: 5.757e−16
Coefficient: −7.76e+03
Value: neg
15000
10000
5000
0
acteria.p__Firmicutes.c__Clostridia.o__Eubacteriales.f__Lachnospi
alpha (n=25)
clade20C (n=1)
delta (n=13)
omicron (n=6)
neg (n=72)
NA
WHOvariant

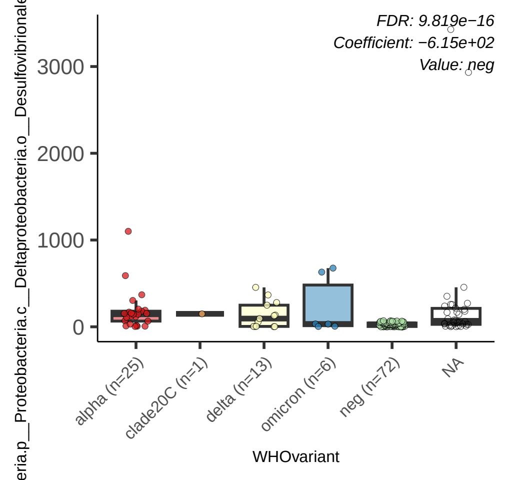

FDR: 9.819e−16
Coefficient: −6.15e+02
Value: neg
3000
2000
1000
0
eria.p__Proteobacteria.c__Deltaproteobacteria.o__Desulfovibrionale
alpha (n=25)
clade20C (n=1)
delta (n=13)
omicron (n=6)
neg (n=72)
NA
WHOvariant

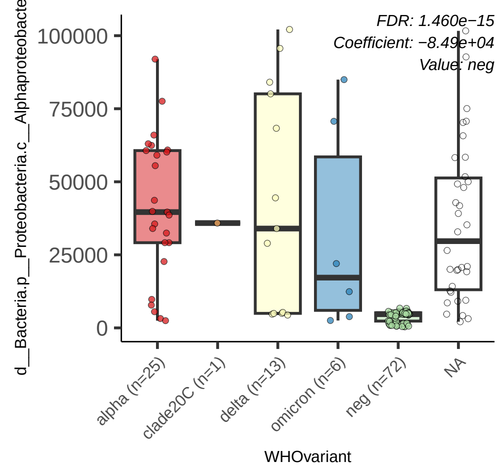
FDR: 1.460e−15
Coefficient: −8.49e+04
Value: neg
d__Bacteria.p__Proteobacteria.c__Alphaproteobacte
100000
75000
50000
25000
0
alpha (n=25)
clade20C (n=1)
delta (n=13)
omicron (n=6)
neg (n=72)
NA
WHOvariant

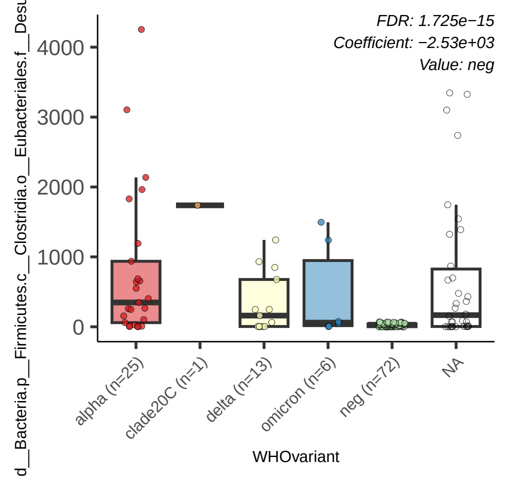

FDR: 1.725e−15
Coefficient: −2.53e+03
Value: neg
d__Bacteria.p__Firmicutes.c__Clostridia.o__Eubacteriales.f__Desu
4000
3000
2000
1000
0
alpha (n=25)
clade20C (n=1)
delta (n=13)
omicron (n=6)
neg (n=72)
NA
WHOvariant

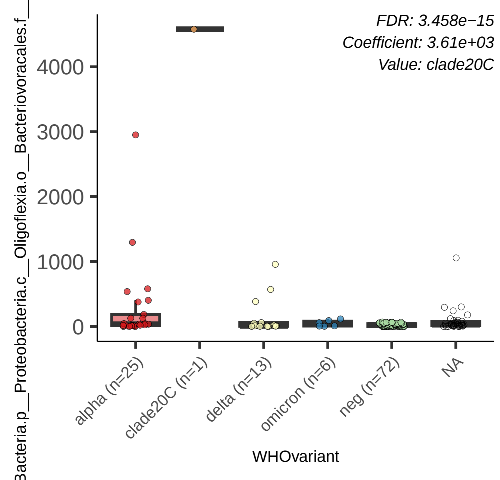

FDR: 3.458e−15
Coefficient: 3.61e+03
Value: clade20C
Bacteria.p__Proteobacteria.c__Oligoflexia.o__Bacteriovoracales.f__
4000
3000
2000
1000
0
alpha (n=25)
clade20C (n=1)
delta (n=13)
omicron (n=6)
neg (n=72)
NA
WHOvariant

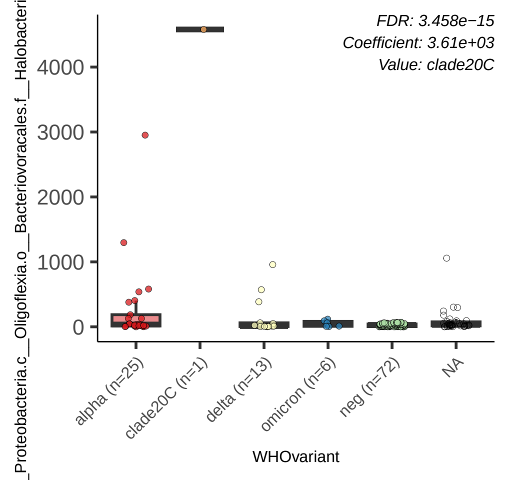
FDR: 3.458e−15
Coefficient: 3.61e+03
Value: clade20C
_Proteobacteria.c__Oligoflexia.o__Bacteriovoracales.f__Halobacteri
4000
3000
2000
1000
0
alpha (n=25)
clade20C (n=1)
delta (n=13)
omicron (n=6)
neg (n=72)
NA
WHOvariant

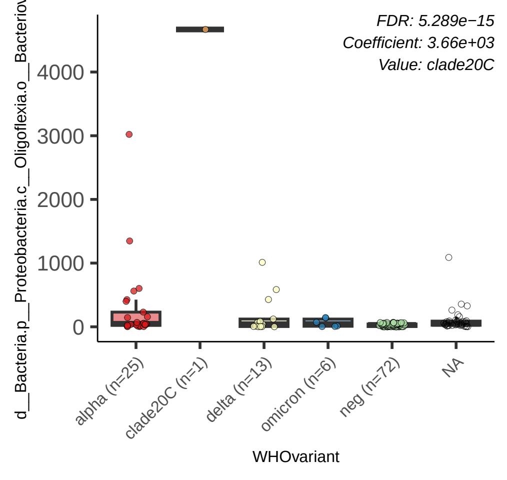

FDR: 5.289e−15
Coefficient: 3.66e+03
Value: clade20C
d__Bacteria.p__Proteobacteria.c__Oligoflexia.o__Bacteriov
4000
3000
2000
1000
0
alpha (n=25)
clade20C (n=1)
delta (n=13)
omicron (n=6)
neg (n=72)
NA
WHOvariant

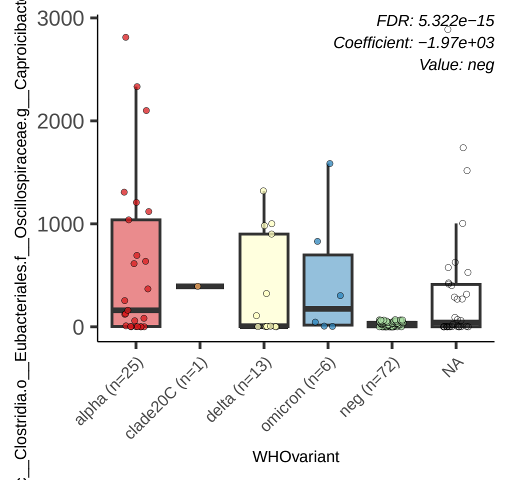
FDR: 5.322e−15
Coefficient: −1.97e+03
Value: neg
3000
2000
1000
0
Clostridia.o__Eubacteriales.f__Oscillospiraceae.g__Caproicibacte
alpha (n=25)
clade20C (n=1)
delta (n=13)
omicron (n=6)
neg (n=72)
NA
WHOvariant

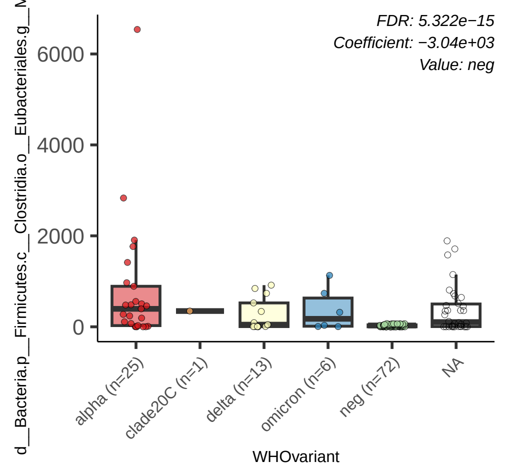

FDR: 5.322e−15
Coefficient: −3.04e+03
Value: neg
d__Bacteria.p__Firmicutes.c__Clostridia.o__Eubacteriales.g__M
6000
4000
2000
0
alpha (n=25)
clade20C (n=1)
delta (n=13)
omicron (n=6)
neg (n=72)
NA
WHOvariant

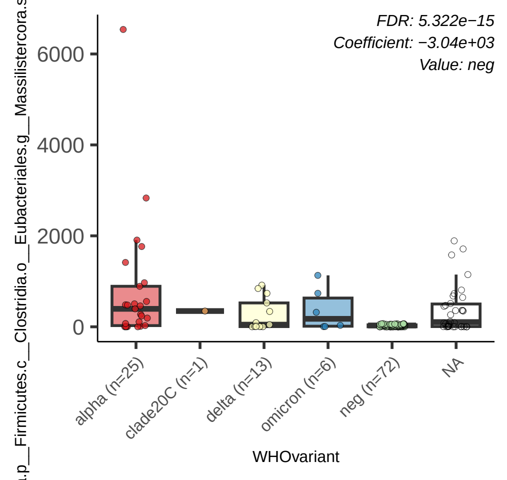

FDR: 5.322e−15
Coefficient: −3.04e+03
Value: neg
6000
4000
2000
0
alpha (n=25)
clade20C (n=1)
delta (n=13)
omicron (n=6)
neg (n=72)
NA
WHOvariant

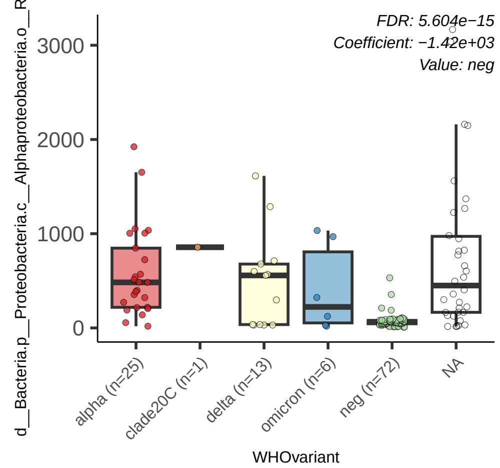

FDR: 5.604e−15
Coefficient: −1.42e+03
Value: neg
d__Bacteria.p__Proteobacteria.c__Alphaproteobacteria.o__R
3000
2000
1000
0
alpha (n=25)
clade20C (n=1)
delta (n=13)
omicron (n=6)
neg (n=72)
NA
WHOvariant

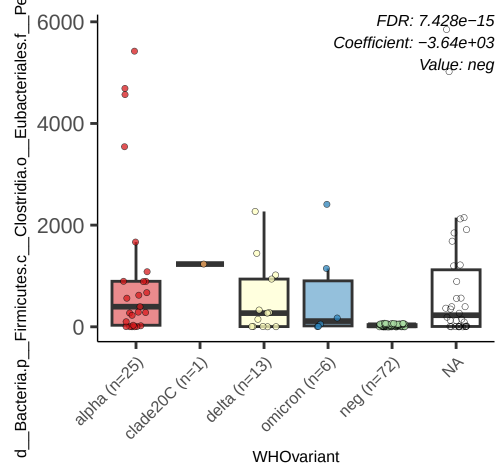

FDR: 7.428e−15
Coefficient: −3.64e+03
Value: neg
d__Bacteria.p__Firmicutes.c__Clostridia.o__Eubacteriales.f__Pe
6000
4000
2000
0
alpha (n=25)
clade20C (n=1)
delta (n=13)
omicron (n=6)
neg (n=72)
NA
WHOvariant

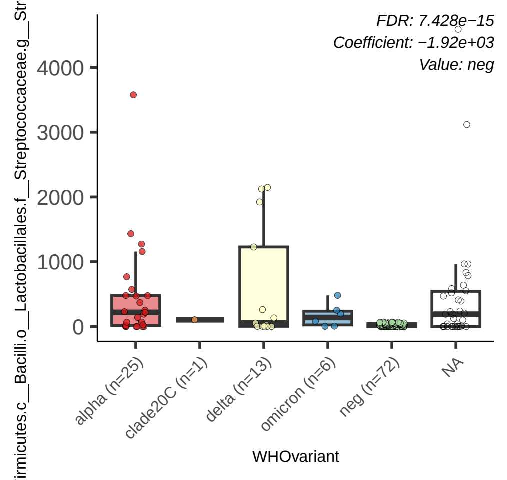

FDR: 7.428e−15
Coefficient: −1.92e+03
Value: neg
4000
3000
2000
1000
0
irmicutes.c__Bacilli.o__Lactobacillales.f__Streptococcaceae.g__Str
alpha (n=25)
clade20C (n=1)
delta (n=13)
omicron (n=6)
neg (n=72)
NA
WHOvariant

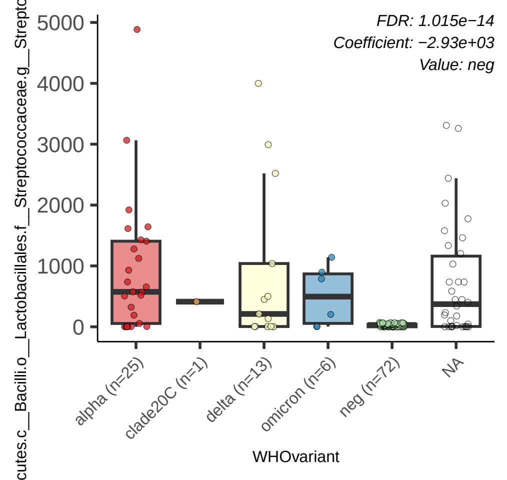
FDR: 1.015e−14
Coefficient: −2.93e+03
Value: neg
cutes.c__Bacilli.o__Lactobacillales.f__Streptococcaceae.g__Strepto
0
1000
2000
3000
4000
5000
alpha (n=25)
clade20C (n=1)
delta (n=13)
omicron (n=6)
neg (n=72)
NA
WHOvariant

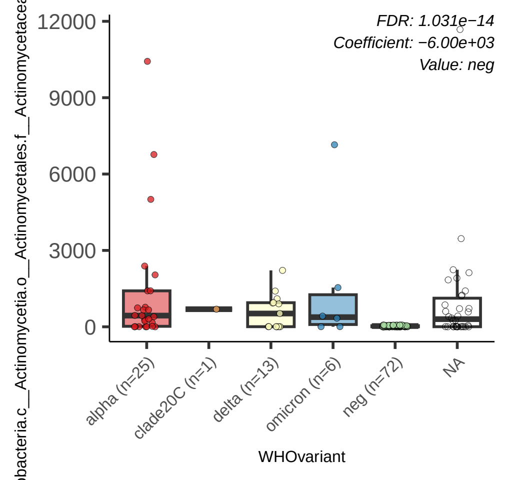
FDR: 1.031e−14
Coefficient: −6.00e+03
Value: neg
12000
9000
6000
3000
0
obacteria.c__Actinomycetia.o__Actinomycetales.f__Actinomycetacea
alpha (n=25)
clade20C (n=1)
delta (n=13)
omicron (n=6)
neg (n=72)
NA
WHOvariant

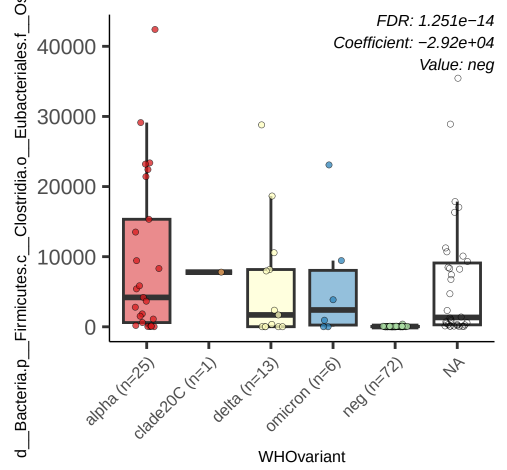

FDR: 1.251e−14
Coefficient: −2.92e+04
Value: neg
d__Bacteria.p__Firmicutes.c__Clostridia.o__Eubacteriales.f__Os
40000
30000
20000
10000
0
alpha (n=25)
clade20C (n=1)
delta (n=13)
omicron (n=6)
neg (n=72)
NA
WHOvariant

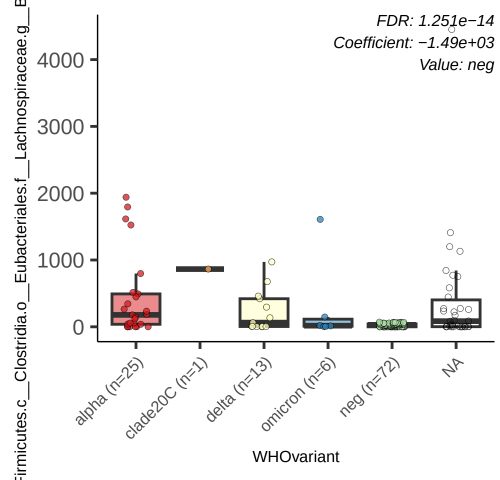

FDR: 1.251e−14
Coefficient: −1.49e+03
Value: neg
Firmicutes.c__Clostridia.o__Eubacteriales.f__Lachnospiraceae.g__B
4000
3000
2000
1000
0
alpha (n=25)
clade20C (n=1)
delta (n=13)
omicron (n=6)
neg (n=72)
NA
WHOvariant

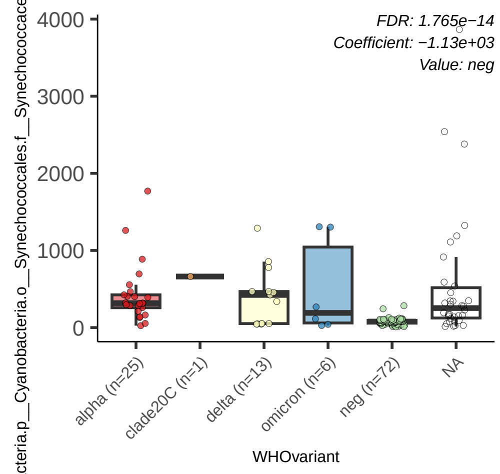

FDR: 1.765e−14
Coefficient: −1.13e+03
Value: neg
4000
3000
2000
1000
0
acteria.p__Cyanobacteria.o__Synechococcales.f__Synechococcace
alpha (n=25)
clade20C (n=1)
delta (n=13)
omicron (n=6)
neg (n=72)
NA
WHOvariant

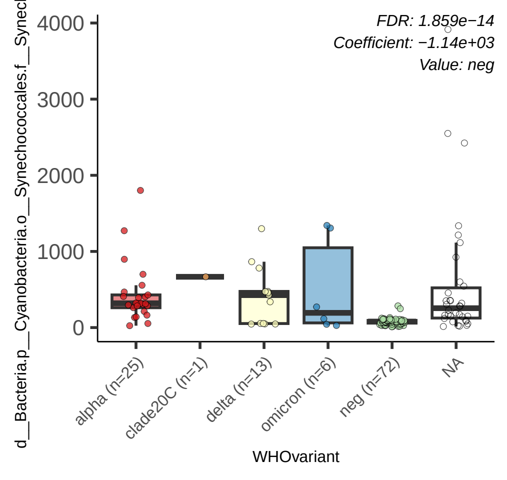

FDR: 1.859e−14
Coefficient: −1.14e+03
Value: neg
d__Bacteria.p__Cyanobacteria.o__Synechococcales.f__Synec
4000
3000
2000
1000
0
alpha (n=25)
clade20C (n=1)
delta (n=13)
omicron (n=6)
neg (n=72)
NA
WHOvariant

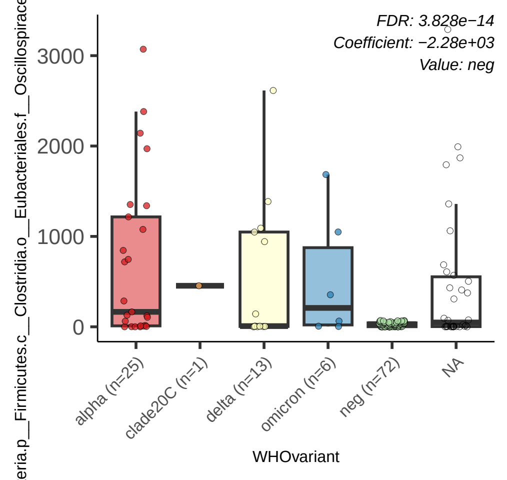

FDR: 3.828e−14
Coefficient: −2.28e+03
Value: neg
3000
2000
1000
0
alpha (n=25)
clade20C (n=1)
delta (n=13)
omicron (n=6)
neg (n=72)
NA
WHOvariant

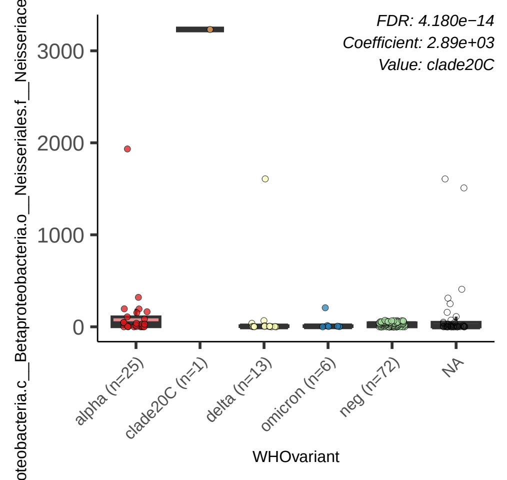

FDR: 4.180e−14
Coefficient: 2.89e+03
Value: clade20C
3000
2000
1000
0
oteobacteria.c__Betaproteobacteria.o__Neisseriales.f__Neisseriace
alpha (n=25)
clade20C (n=1)
delta (n=13)
omicron (n=6)
neg (n=72)
NA
WHOvariant

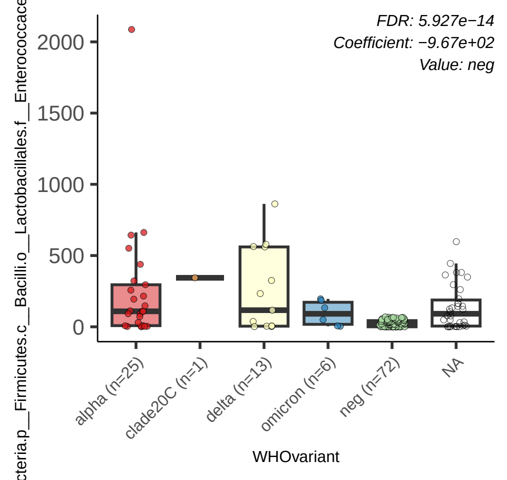

FDR: 5.927e−14
Coefficient: −9.67e+02
Value: neg
2000
1500
1000
500
0
cteria.p__Firmicutes.c__Bacilli.o__Lactobacillales.f__Enterococcace
alpha (n=25)
clade20C (n=1)
delta (n=13)
omicron (n=6)
neg (n=72)
NA
WHOvariant

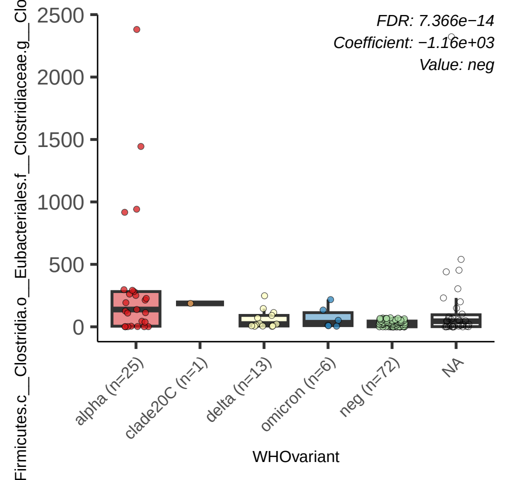

FDR: 7.366e−14
Coefficient: −1.16e+03
Value: neg
Firmicutes.c__Clostridia.o__Eubacteriales.f__Clostridiaceae.g__Clo
2500
2000
1500
1000
500
0
alpha (n=25)
clade20C (n=1)
delta (n=13)
omicron (n=6)
neg (n=72)
NA
WHOvariant

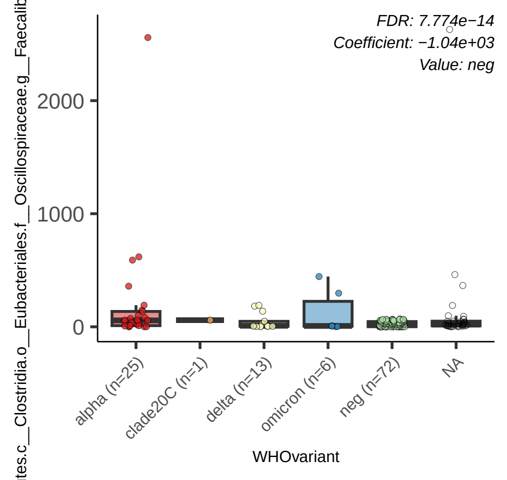

FDR: 7.774e−14
Coefficient: −1.04e+03
Value: neg
2000
1000
0
alpha (n=25)
clade20C (n=1)
delta (n=13)
omicron (n=6)
neg (n=72)
NA
WHOvariant

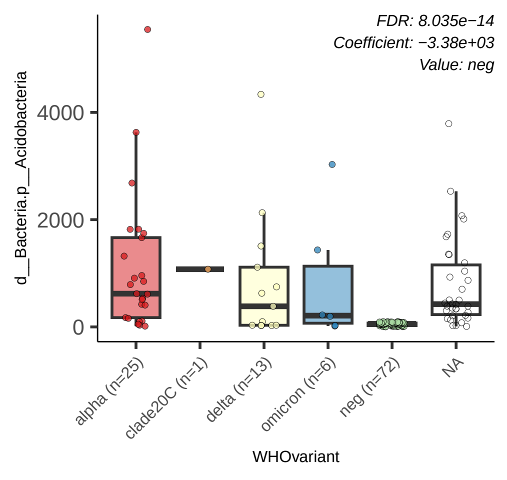
FDR: 8.035e−14
Coefficient: −3.38e+03
Value: neg
d__Bacteria.p__Acidobacteria
4000
2000
0
alpha (n=25)
clade20C (n=1)
delta (n=13)
omicron (n=6)
neg (n=72)
NA
WHOvariant

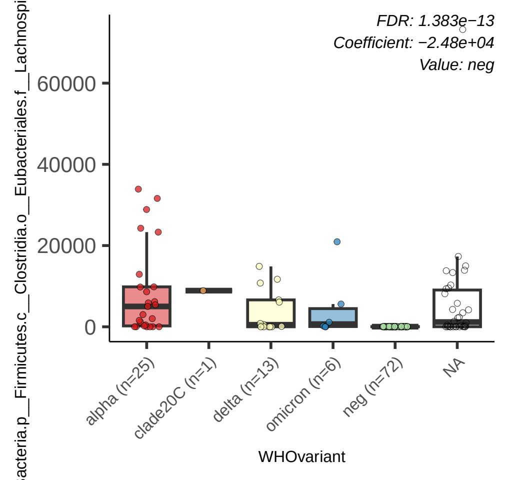

FDR: 1.383e−13
Coefficient: −2.48e+04
Value: neg
60000
40000
20000
0
acteria.p__Firmicutes.c__Clostridia.o__Eubacteriales.f__Lachnospi
alpha (n=25)
clade20C (n=1)
delta (n=13)
omicron (n=6)
neg (n=72)
NA
WHOvariant

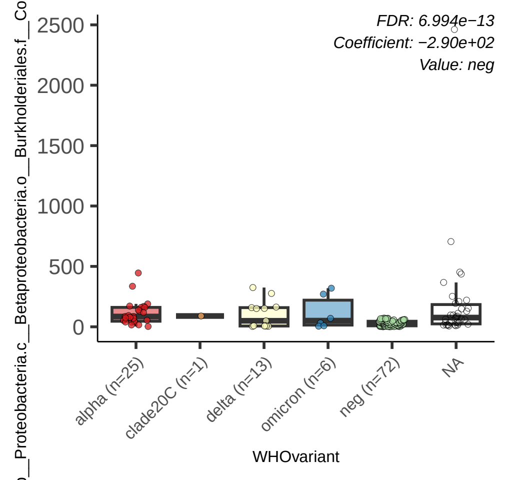

FDR: 6.994e−13
Coefficient: −2.90e+02
Value: neg
0
500
1000
1500
2000
2500
Proteobacteria.c__Betaproteobacteria.o__Burkholderiales.f__Co
alpha (n=25)
clade20C (n=1)
delta (n=13)
omicron (n=6)
neg (n=72)
NA
WHOvariant

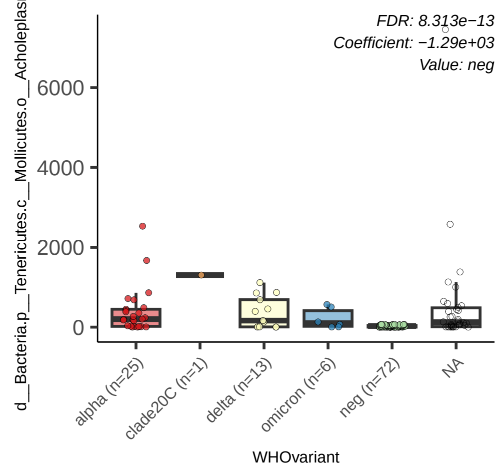

FDR: 8.313e−13
Coefficient: −1.29e+03
Value: neg
d__Bacteria.p__Tenericutes.c__Mollicutes.o__Acholeplas
6000
4000
2000
0
alpha (n=25)
clade20C (n=1)
delta (n=13)
omicron (n=6)
neg (n=72)
NA
WHOvariant

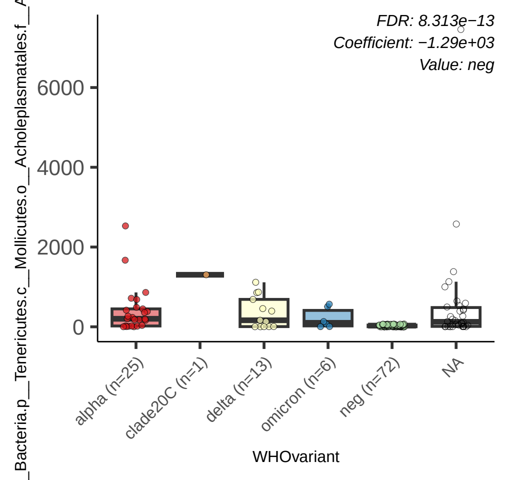

FDR: 8.313e−13
Coefficient: −1.29e+03
Value: neg
6000
4000
2000
0
alpha (n=25)
clade20C (n=1)
delta (n=13)
omicron (n=6)
neg (n=72)
NA
WHOvariant
Bacteria.p__Tenericutes.c__Mollicutes.o__Acholeplasmatales.f__A

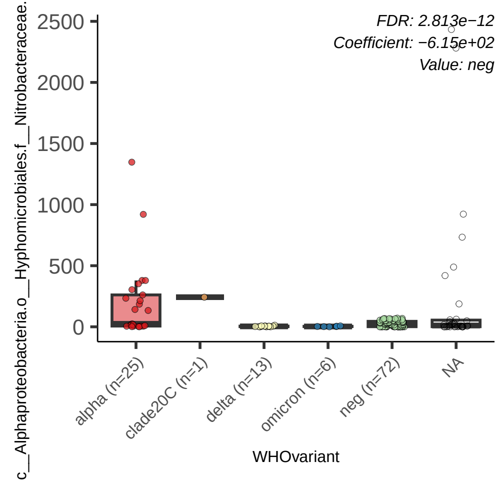

FDR: 2.813e−12
Coefficient: −6.15e+02
Value: neg
c__Alphaproteobacteria.o__Hyphomicrobiales.f__Nitrobacteraceae.
2500
2000
1500
1000
500
0
alpha (n=25)
clade20C (n=1)
delta (n=13)
omicron (n=6)
neg (n=72)
NA
WHOvariant

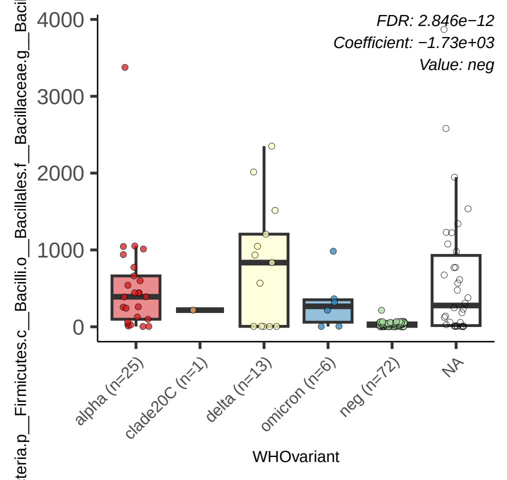

FDR: 2.846e−12
Coefficient: −1.73e+03
Value: neg
4000
3000
2000
1000
0
alpha (n=25)
clade20C (n=1)
delta (n=13)
omicron (n=6)
neg (n=72)
NA
WHOvariant

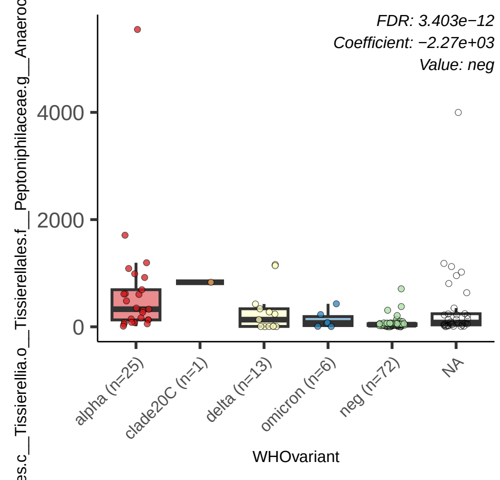

FDR: 3.403e−12
Coefficient: −2.27e+03
Value: neg
es.c__Tissierellia.o__Tissierellales.f__Peptoniphilaceae.g__Anaeroc
4000
2000
0
alpha (n=25)
clade20C (n=1)
delta (n=13)
omicron (n=6)
neg (n=72)
NA
WHOvariant

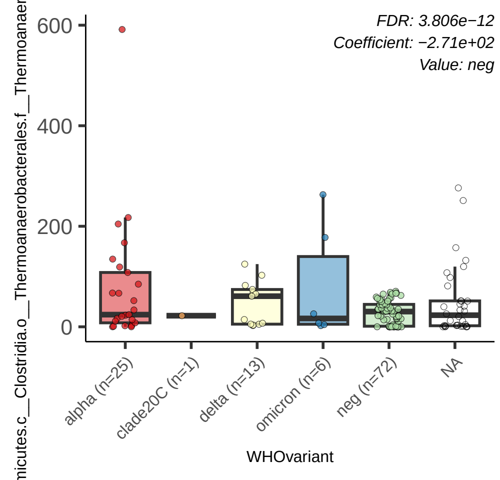

FDR: 3.806e−12
Coefficient: −2.71e+02
Value: neg
micutes.c__Clostridia.o__Thermoanaerobacterales.f__Thermoanaer
600
400
200
0
alpha (n=25)
clade20C (n=1)
delta (n=13)
omicron (n=6)
neg (n=72)
NA
WHOvariant

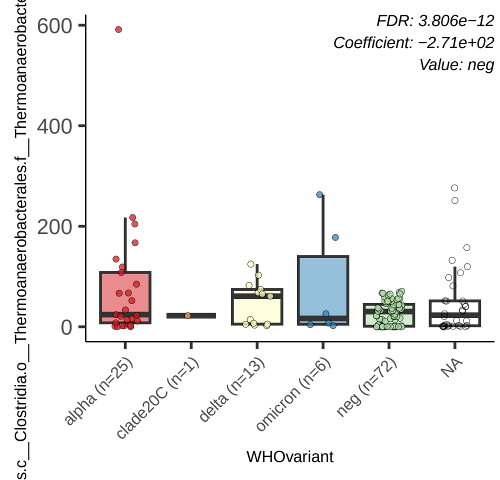

s.c__Clostridia.o__Thermoanaerobacterales.f__Thermoanaerobacte
600
400
200
0
FDR: 3.806e−12
Coefficient: −2.71e+02
Value: neg
alpha (n=25)
clade20C (n=1)
delta (n=13)
omicron (n=6)
neg (n=72)
NA
WHOvariant

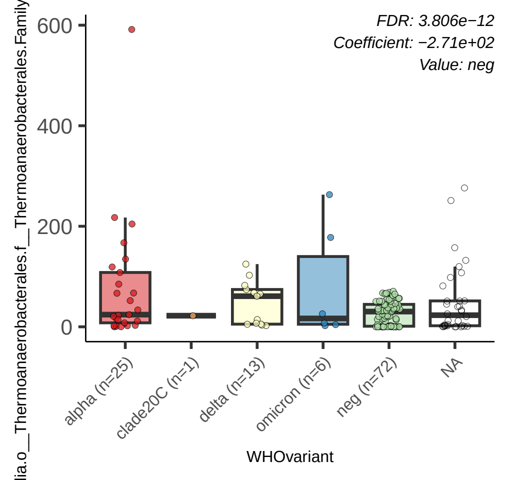

FDR: 3.806e−12
Coefficient: −2.71e+02
Value: neg
600
400
200
0
lia.o__Thermoanaerobacterales.f__Thermoanaerobacterales.Family
alpha (n=25)
clade20C (n=1)
delta (n=13)
omicron (n=6)
neg (n=72)
NA
WHOvariant

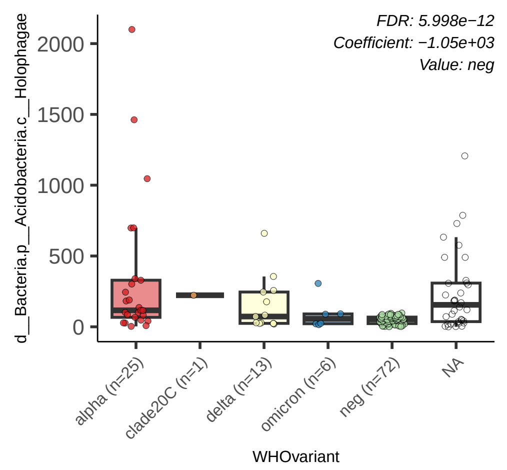

FDR: 5.998e−12
Coefficient: −1.05e+03
Value: neg
d__Bacteria.p__Acidobacteria.c__Holophagae
2000
1500
1000
500
0
alpha (n=25)
clade20C (n=1)
delta (n=13)
omicron (n=6)
neg (n=72)
NA
WHOvariant

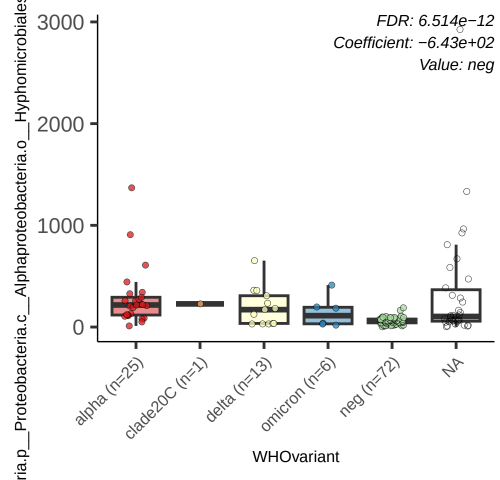

FDR: 6.514e−12
Coefficient: −6.43e+02
Value: neg
3000
2000
1000
0
ria.p__Proteobacteria.c__Alphaproteobacteria.o__Hyphomicrobiales
alpha (n=25)
clade20C (n=1)
delta (n=13)
omicron (n=6)
neg (n=72)
NA
WHOvariant

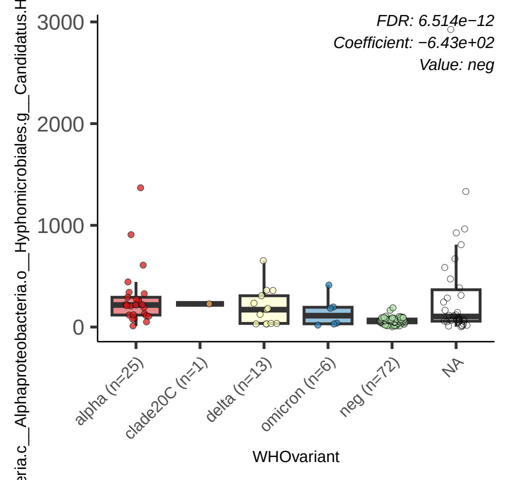

FDR: 6.514e−12
Coefficient: −6.43e+02
Value: neg
3000
2000
1000
0
alpha (n=25)
clade20C (n=1)
delta (n=13)
omicron (n=6)
neg (n=72)
NA
WHOvariant

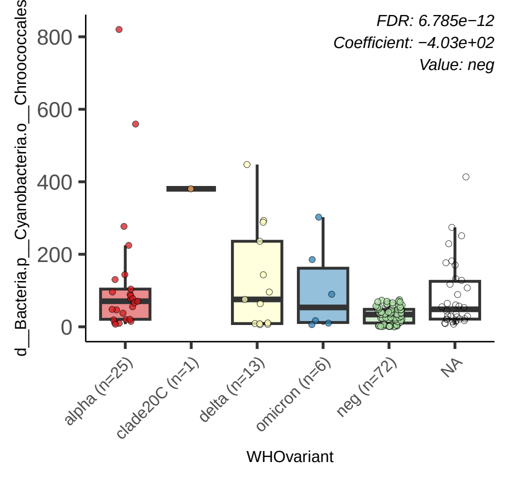
FDR: 6.785e−12
Coefficient: −4.03e+02
Value: neg
d__Bacteria.p__Cyanobacteria.o__Chroococcales
800
600
400
200
0
alpha (n=25)
clade20C (n=1)
delta (n=13)
omicron (n=6)
neg (n=72)
NA
WHOvariant

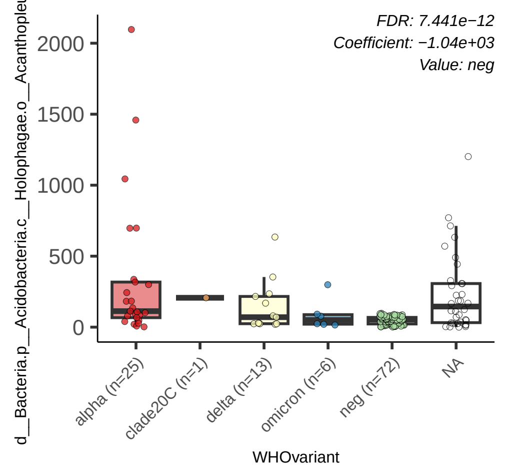
FDR: 7.441e−12
Coefficient: −1.04e+03
Value: neg
d__Bacteria.p__Acidobacteria.c__Holophagae.o__Acanthopleu
2000
1500
1000
500
0
alpha (n=25)
clade20C (n=1)
delta (n=13)
omicron (n=6)
neg (n=72)
NA
WHOvariant

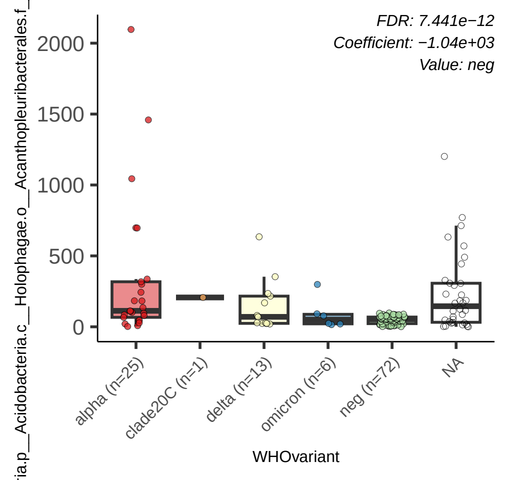

FDR: 7.441e−12
Coefficient: −1.04e+03
Value: neg
ria.p__Acidobacteria.c__Holophagae.o__Acanthopleuribacterales.f__
2000
1500
1000
500
0
alpha (n=25)
clade20C (n=1)
delta (n=13)
omicron (n=6)
neg (n=72)
NA
WHOvariant

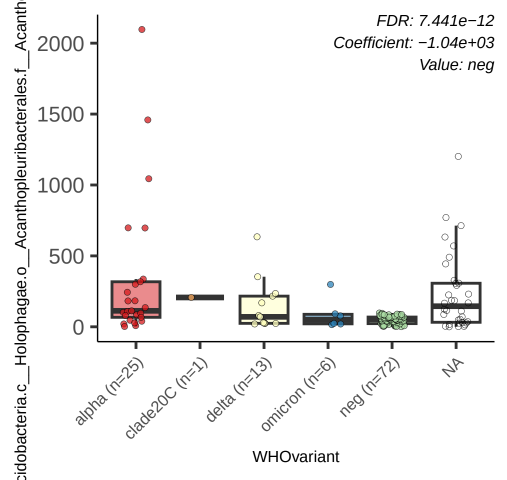

FDR: 7.441e−12
Coefficient: −1.04e+03
Value: neg
cidobacteria.c__Holophagae.o__Acanthopleuribacterales.f__Acanth
2000
1500
1000
500
0
alpha (n=25)
clade20C (n=1)
delta (n=13)
omicron (n=6)
neg (n=72)
NA
WHOvariant

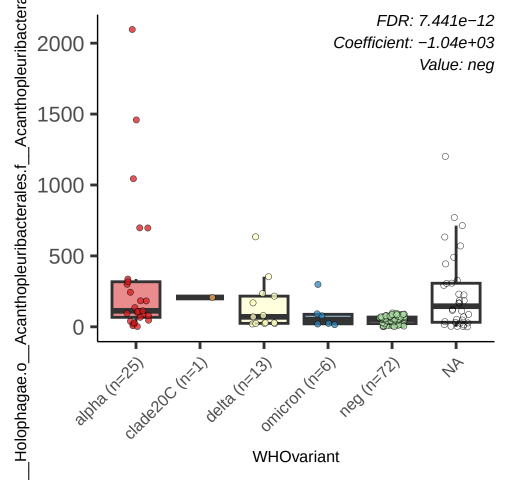

FDR: 7.441e−12
Coefficient: −1.04e+03
Value: neg
Holophagae.o__Acanthopleuribacterales.f__Acanthopleuribactera
2000
1500
1000
500
0
alpha (n=25)
clade20C (n=1)
delta (n=13)
omicron (n=6)
neg (n=72)
NA
WHOvariant

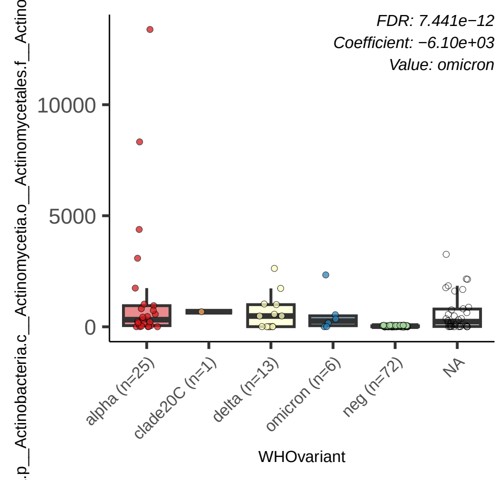

FDR: 7.441e−12
Coefficient: −6.10e+03
Value: omicron
10000
5000
0
alpha (n=25)
clade20C (n=1)
delta (n=13)
omicron (n=6)
neg (n=72)
NA
WHOvariant
p__Actinobacteria.c__Actinomycetia.o__Actinomycetales.f__Actino

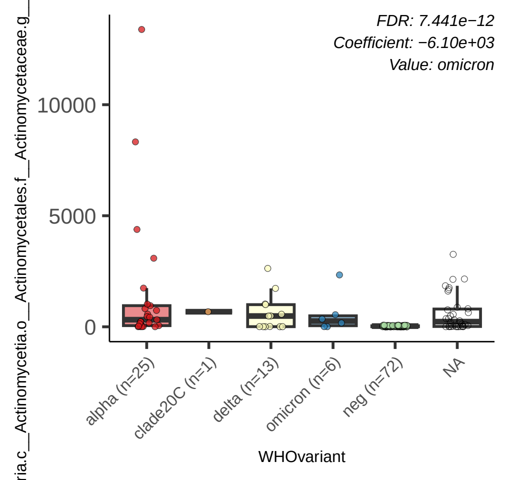

FDR: 7.441e−12
Coefficient: −6.10e+03
Value: omicron
10000
5000
0
ria.c__Actinomycetia.o__Actinomycetales.f__Actinomycetaceae.g__
alpha (n=25)
clade20C (n=1)
delta (n=13)
omicron (n=6)
neg (n=72)
NA
WHOvariant

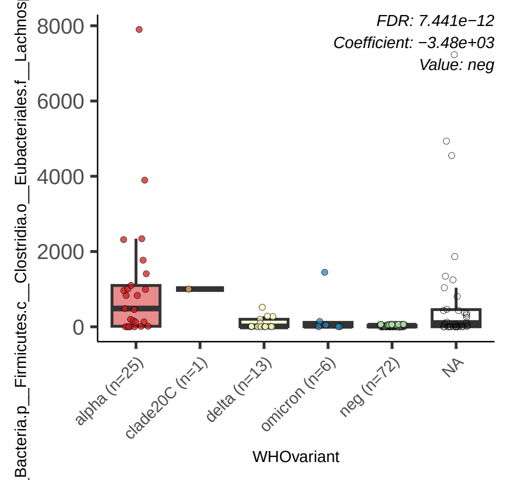
FDR: 7.441e−12
Coefficient: −3.48e+03
Value: neg
8000
6000
4000
2000
0
Bacteria.p__Firmicutes.c__Clostridia.o__Eubacteriales.f__Lachnos
alpha (n=25)
clade20C (n=1)
delta (n=13)
omicron (n=6)
neg (n=72)
NA
WHOvariant

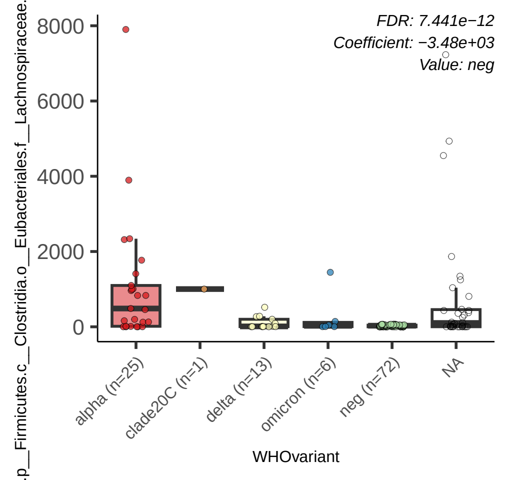

FDR: 7.441e−12
Coefficient: −3.48e+03
Value: neg
p__Firmicutes.c__Clostridia.o__Eubacteriales.f__Lachnospiraceae.
8000
6000
4000
2000
0
alpha (n=25)
clade20C (n=1)
delta (n=13)
omicron (n=6)
neg (n=72)
NA
WHOvariant

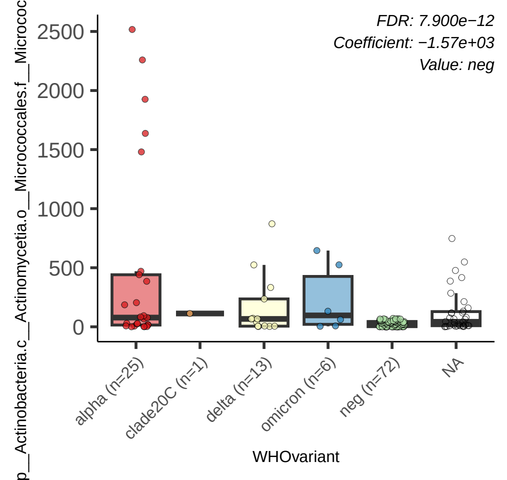

FDR: 7.900e−12
Coefficient: −1.57e+03
Value: neg
p__Actinobacteria.c__Actinomycetia.o__Micrococcales.f__Micrococ
0
500
1000
1500
2000
2500
alpha (n=25)
clade20C (n=1)
delta (n=13)
omicron (n=6)
neg (n=72)
NA
WHOvariant

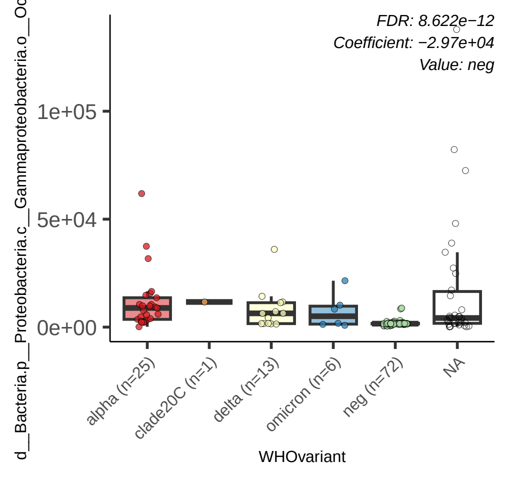
FDR: 8.622e−12
Coefficient: −2.97e+04
Value: neg
d__Bacteria.p__Proteobacteria.c__Gammaproteobacteria.o__Oc
1e+05
5e+04
0e+00
alpha (n=25)
clade20C (n=1)
delta (n=13)
omicron (n=6)
neg (n=72)
NA
WHOvariant

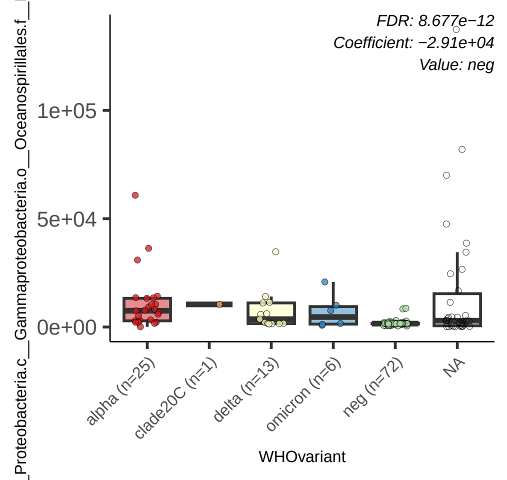
FDR: 8.677e−12
Coefficient: −2.91e+04
Value: neg
Proteobacteria.c__Gammaproteobacteria.o__Oceanospirillales.f__
1e+05
5e+04
0e+00
alpha (n=25)
clade20C (n=1)
delta (n=13)
omicron (n=6)
neg (n=72)
NA
WHOvariant

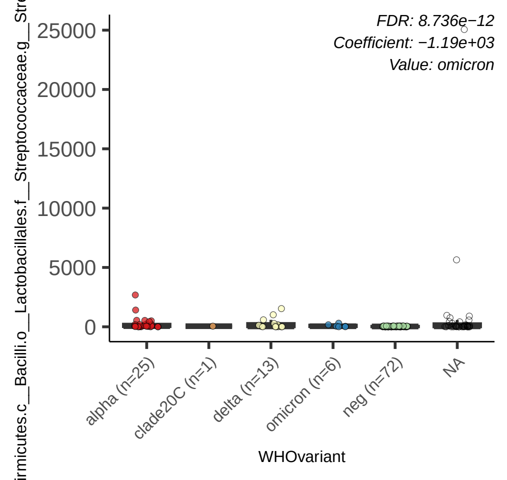
FDR: 8.736e−12
Coefficient: −1.19e+03
Value: omicron
25000
20000
15000
10000
5000
0
irmicutes.c__Bacilli.o__Lactobacillales.f__Streptococcaceae.g__Str
alpha (n=25)
clade20C (n=1)
delta (n=13)
omicron (n=6)
neg (n=72)
NA
WHOvariant

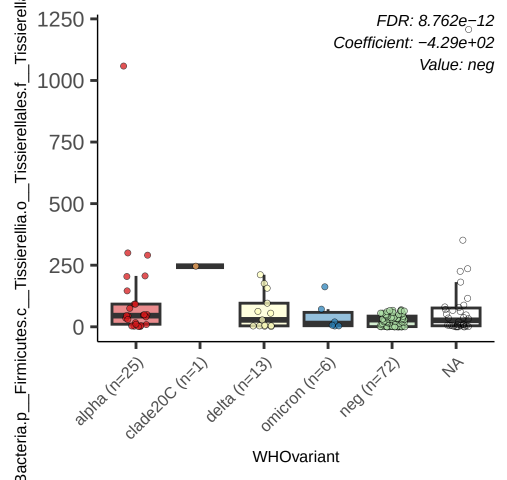
FDR: 8.762e−12
Coefficient: −4.29e+02
Value: neg
Bacteria.p__Firmicutes.c__Tissierellia.o__Tissierellales.f__Tissierella
1250
1000
750
500
250
0
alpha (n=25)
clade20C (n=1)
delta (n=13)
omicron (n=6)
neg (n=72)
NA
WHOvariant

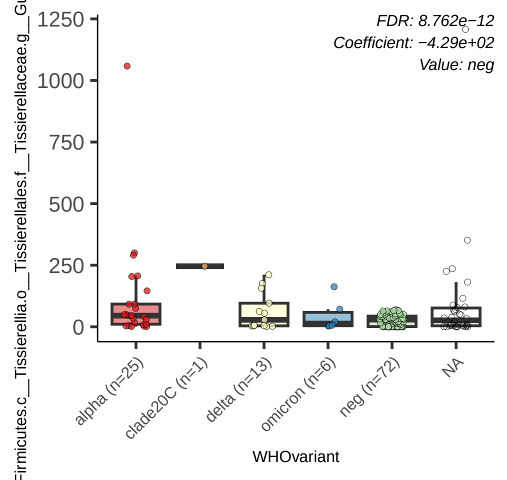

FDR: 8.762e−12
Coefficient: −4.29e+02
Value: neg
1250
1000
750
500
250
0
Firmicutes.c__Tissierellia.o__Tissierellales.f__Tissierellaceae.g__Gu
alpha (n=25)
clade20C (n=1)
delta (n=13)
omicron (n=6)
neg (n=72)
NA
WHOvariant

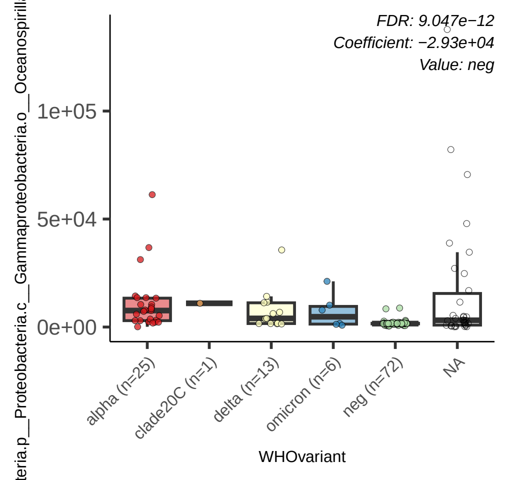
FDR: 9.047e−12
Coefficient: −2.93e+04
Value: neg
1e+05
5e+04
0e+00
alpha (n=25)
clade20C (n=1)
delta (n=13)
omicron (n=6)
neg (n=72)
NA
WHOvariant
teria.p__Proteobacteria.c__Gammaproteobacteria.o__Oceanospirill

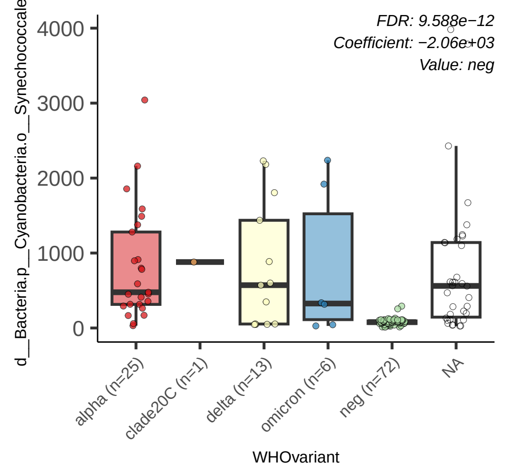

FDR: 9.588e−12
Coefficient: −2.06e+03
Value: neg
d__Bacteria.p__Cyanobacteria.o__Synechococcale
4000
3000
2000
1000
0
alpha (n=25)
clade20C (n=1)
delta (n=13)
omicron (n=6)
neg (n=72)
NA
WHOvariant

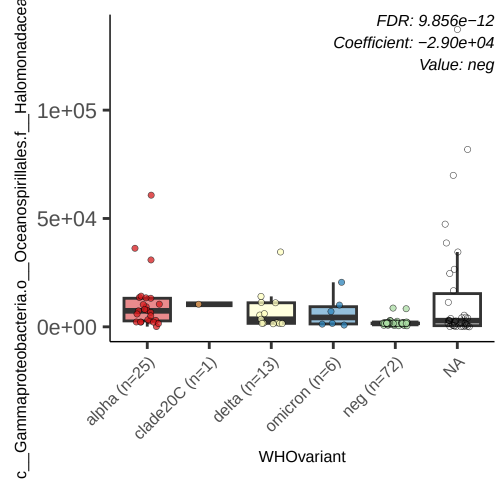

FDR: 9.856e−12
Coefficient: −2.90e+04
Value: neg
c__Gammaproteobacteria.o__Oceanospirillales.f__Halomonadaceae
1e+05
5e+04
0e+00
alpha (n=25)
clade20C (n=1)
delta (n=13)
omicron (n=6)
neg (n=72)
NA
WHOvariant

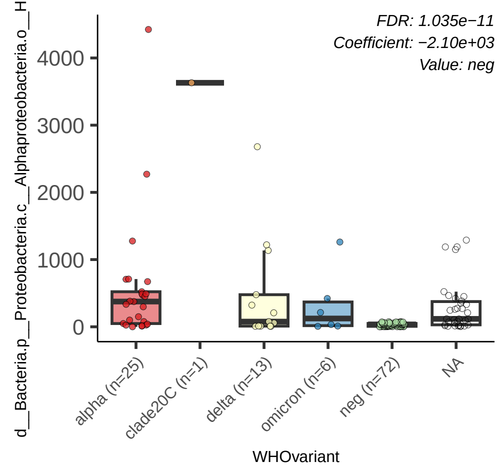

FDR: 1.035e−11
Coefficient: −2.10e+03
Value: neg
d__Bacteria.p__Proteobacteria.c__Alphaproteobacteria.o__H
4000
3000
2000
1000
0
alpha (n=25)
clade20C (n=1)
delta (n=13)
omicron (n=6)
neg (n=72)
NA
WHOvariant

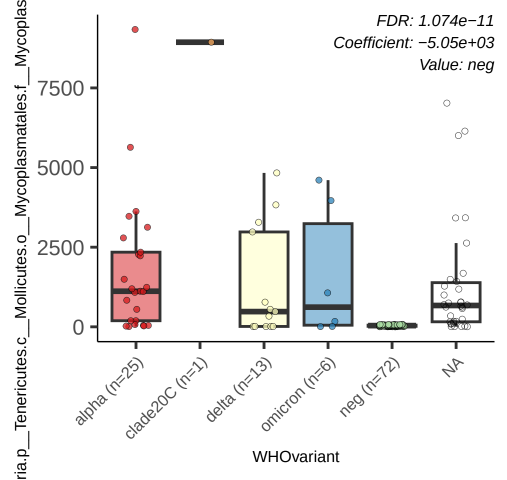

FDR: 1.074e−11
Coefficient: −5.05e+03
Value: neg
ria.p__Tenericutes.c__Mollicutes.o__Mycoplasmatales.f__Mycoplas
0
2500
5000
7500
alpha (n=25)
clade20C (n=1)
delta (n=13)
omicron (n=6)
neg (n=72)
NA
WHOvariant

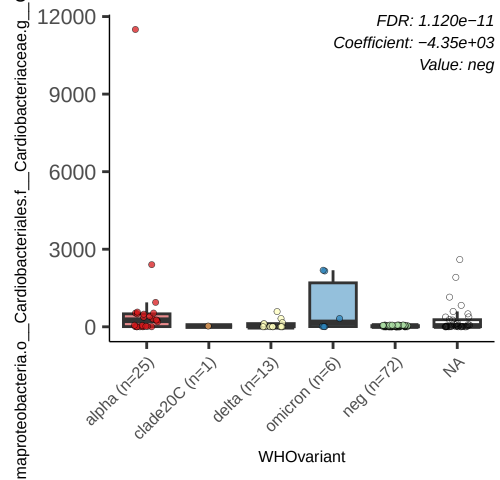

maproteobacteria.o__Cardiobacteriales.f__Cardiobacteriaceae.g__
FDR: 1.120e−11
Coefficient: −4.35e+03
Value: neg
12000
9000
6000
3000
0
alpha (n=25)
clade20C (n=1)
delta (n=13)
omicron (n=6)
neg (n=72)
NA
WHOvariant

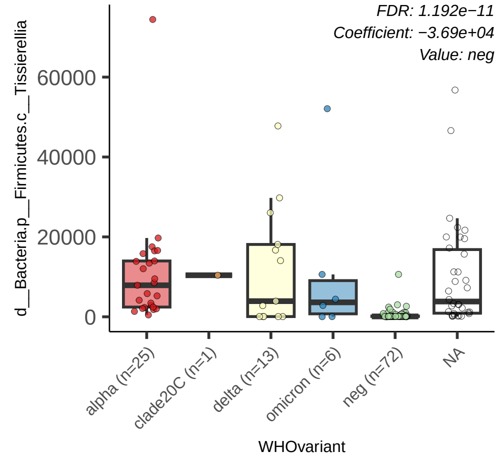
FDR: 1.192e−11
Coefficient: −3.69e+04
Value: neg
d__Bacteria.p__Firmicutes.c__Tissierellia
60000
40000
20000
0
alpha (n=25)
clade20C (n=1)
delta (n=13)
omicron (n=6)
neg (n=72)
NA
WHOvariant

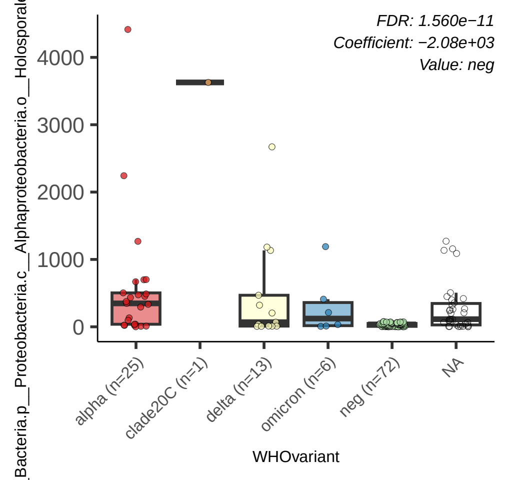

FDR: 1.560e−11
Coefficient: −2.08e+03
Value: neg
Bacteria.p__Proteobacteria.c__Alphaproteobacteria.o__Holosporale
4000
3000
2000
1000
0
alpha (n=25)
clade20C (n=1)
delta (n=13)
omicron (n=6)
neg (n=72)
NA
WHOvariant

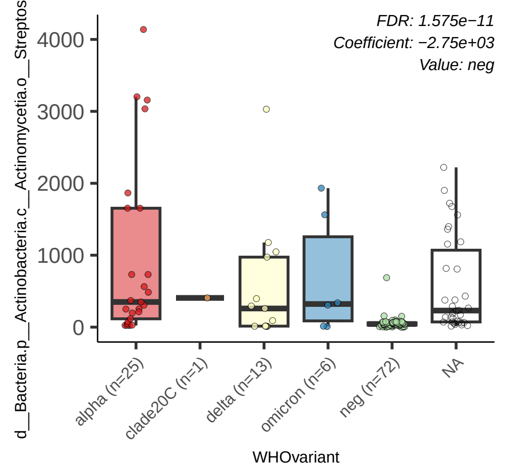
FDR: 1.575e−11
Coefficient: −2.75e+03
Value: neg
d__Bacteria.p__Actinobacteria.c__Actinomycetia.o__Streptos
4000
3000
2000
1000
0
alpha (n=25)
clade20C (n=1)
delta (n=13)
omicron (n=6)
neg (n=72)
NA
WHOvariant

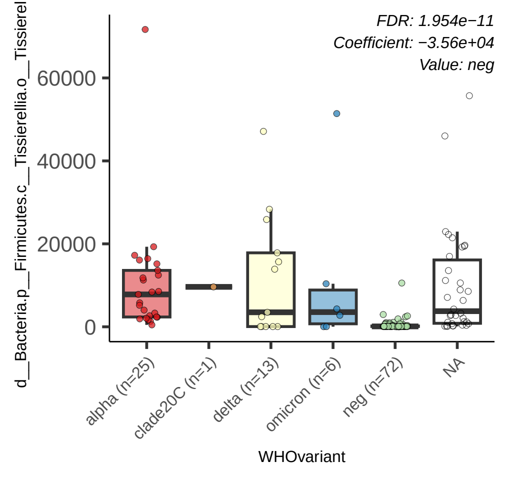
FDR: 1.954e−11
Coefficient: −3.56e+04
Value: neg
d__Bacteria.p__Firmicutes.c__Tissierellia.o__Tissierel
60000
40000
20000
0
alpha (n=25)
clade20C (n=1)
delta (n=13)
omicron (n=6)
neg (n=72)
NA
WHOvariant

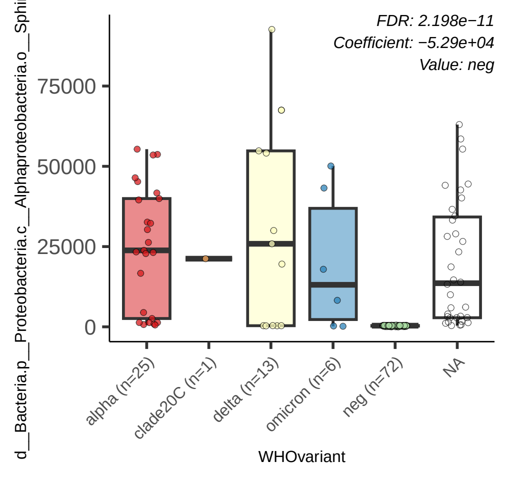

FDR: 2.198e−11
Coefficient: −5.29e+04
Value: neg
d__Bacteria.p__Proteobacteria.c__Alphaproteobacteria.o__Sphi
75000
50000
25000
0
alpha (n=25)
clade20C (n=1)
delta (n=13)
omicron (n=6)
neg (n=72)
NA
WHOvariant

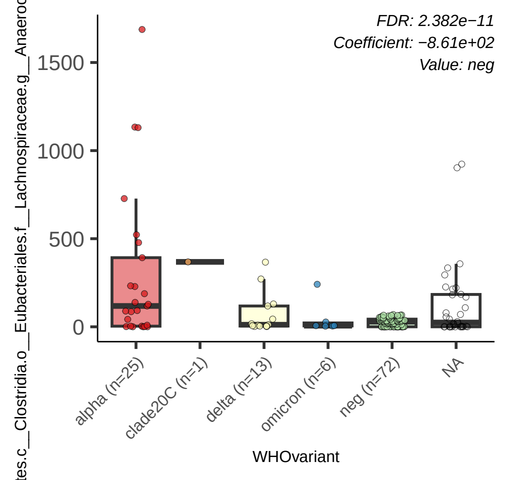

FDR: 2.382e−11
Coefficient: −8.61e+02
Value: neg
tes.c__Clostridia.o__Eubacteriales.f__Lachnospiraceae.g__Anaeroc
0
500
1000
1500
alpha (n=25)
clade20C (n=1)
delta (n=13)
omicron (n=6)
neg (n=72)
NA
WHOvariant

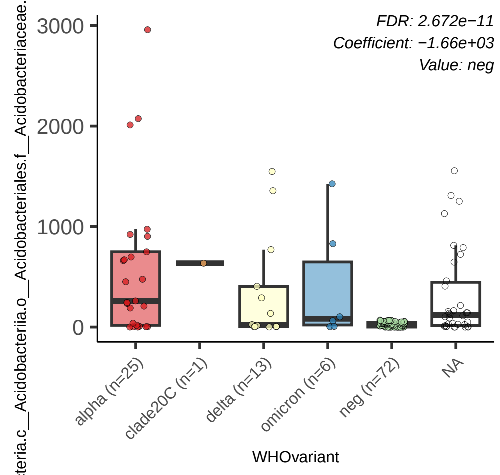

FDR: 2.672e−11
Coefficient: −1.66e+03
Value: neg
cteria.c__Acidobacteriia.o__Acidobacteriales.f__Acidobacteriaceae.
3000
2000
1000
0
alpha (n=25)
clade20C (n=1)
delta (n=13)
omicron (n=6)
neg (n=72)
NA
WHOvariant

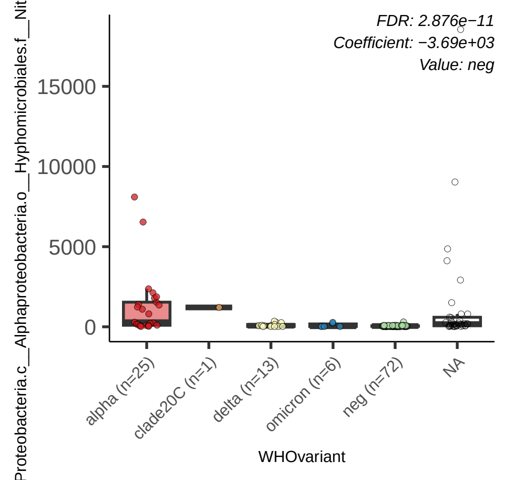
FDR: 2.876e−11
Coefficient: −3.69e+03
Value: neg
Proteobacteria.c__Alphaproteobacteria.o__Hyphomicrobiales.f__Nit
15000
10000
5000
0
alpha (n=25)
clade20C (n=1)
delta (n=13)
omicron (n=6)
neg (n=72)
NA
WHOvariant

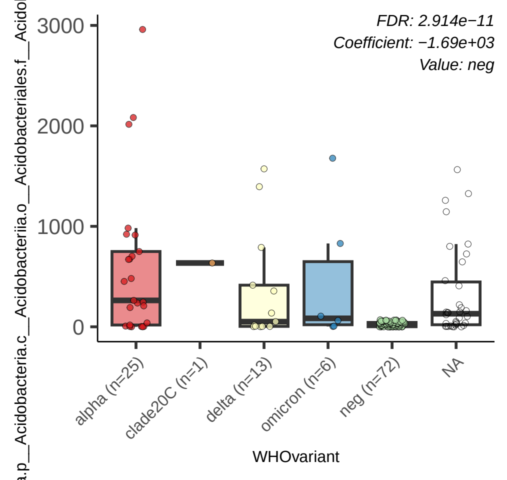

FDR: 2.914e−11
Coefficient: −1.69e+03
Value: neg
3000
2000
1000
0
alpha (n=25)
clade20C (n=1)
delta (n=13)
omicron (n=6)
neg (n=72)
NA
WHOvariant
a.p__Acidobacteria.c__Acidobacteriia.o__Acidobacteriales.f__Acidob

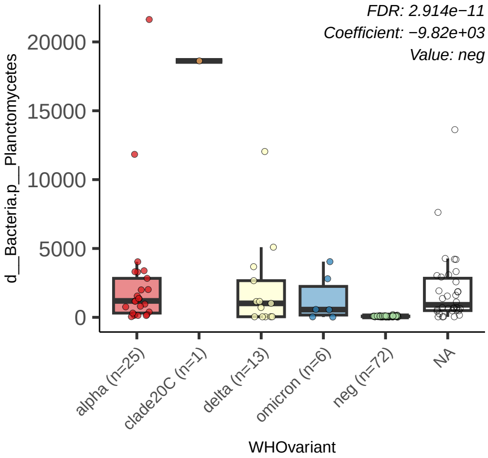
FDR: 2.914e−11
Coefficient: −9.82e+03
Value: neg
d__Bacteria.p__Planctomycetes
20000
15000
10000
5000
0
alpha (n=25)
clade20C (n=1)
delta (n=13)
omicron (n=6)
neg (n=72)
NA
WHOvariant

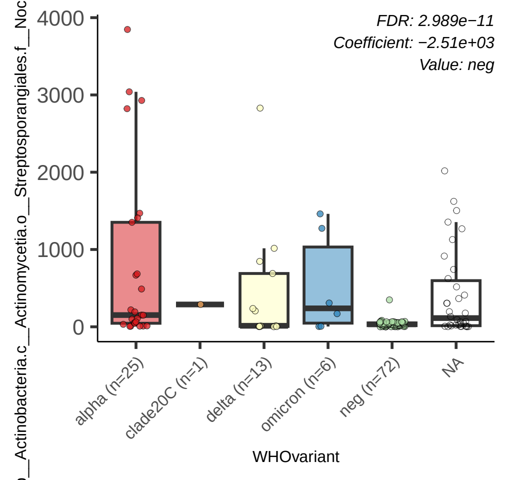

FDR: 2.989e−11
Coefficient: −2.51e+03
Value: neg
4000
3000
2000
1000
0
o__Actinobacteria.c__Actinomycetia.o__Streptosporangiales.f__Noc
alpha (n=25)
clade20C (n=1)
delta (n=13)
omicron (n=6)
neg (n=72)
NA
WHOvariant

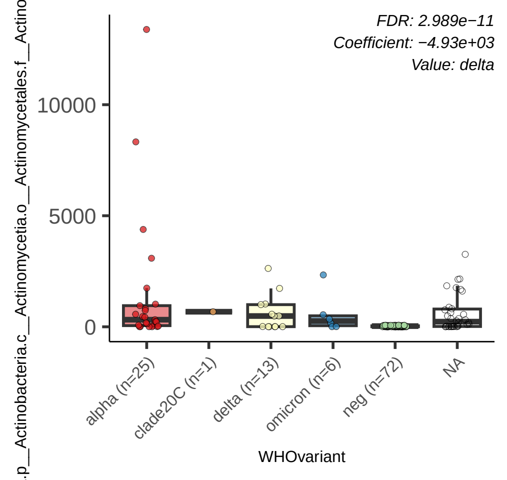

FDR: 2.989e−11
Coefficient: −4.93e+03
Value: delta
10000
5000
0
p__Actinobacteria.c__Actinomycetia.o__Actinomycetales.f__Actino
alpha (n=25)
clade20C (n=1)
delta (n=13)
omicron (n=6)
neg (n=72)
NA
WHOvariant

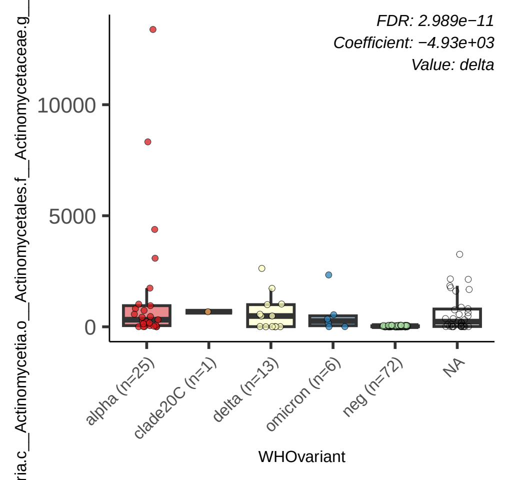

FDR: 2.989e−11
Coefficient: −4.93e+03
Value: delta
ria.c__Actinomycetia.o__Actinomycetales.f__Actinomycetaceae.g__
10000
5000
0
alpha (n=25)
clade20C (n=1)
delta (n=13)
omicron (n=6)
neg (n=72)
NA
WHOvariant

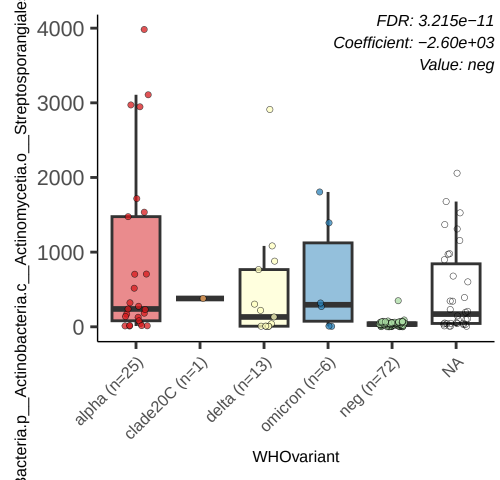

FDR: 3.215e−11
Coefficient: −2.60e+03
Value: neg
Bacteria.p__Actinobacteria.c__Actinomycetia.o__Streptosporangiales
4000
3000
2000
1000
0
alpha (n=25)
clade20C (n=1)
delta (n=13)
omicron (n=6)
neg (n=72)
NA
WHOvariant

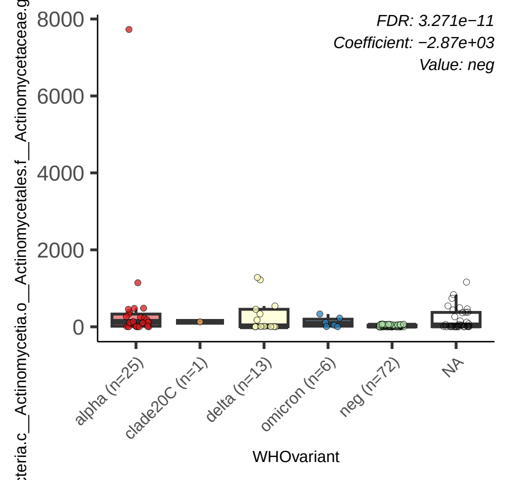

FDR: 3.271e−11
Coefficient: −2.87e+03
Value: neg
8000
6000
4000
2000
0
cteria.c__Actinomycetia.o__Actinomycetales.f__Actinomycetaceae.g
alpha (n=25)
clade20C (n=1)
delta (n=13)
omicron (n=6)
neg (n=72)
NA
WHOvariant

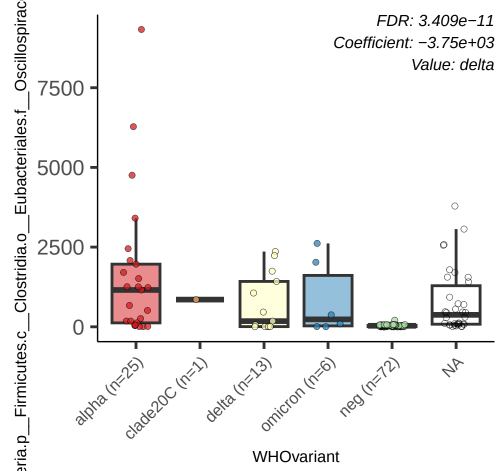

FDR: 3.409e−11
Coefficient: −3.75e+03
Value: delta
teria.p__Firmicutes.c__Clostridia.o__Eubacteriales.f__Oscillospirac
7500
5000
2500
0
alpha (n=25)
clade20C (n=1)
delta (n=13)
omicron (n=6)
neg (n=72)
NA
WHOvariant

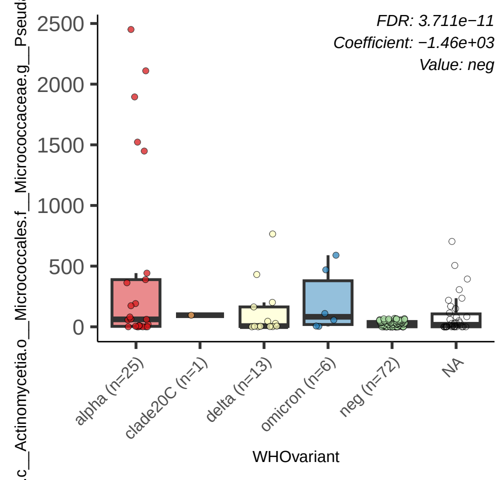

FDR: 3.711e−11
Coefficient: −1.46e+03
Value: neg
2500
2000
1500
1000
500
0
c__Actinomycetia.o__Micrococcales.f__Micrococcaceae.g__Pseud
alpha (n=25)
clade20C (n=1)
delta (n=13)
omicron (n=6)
neg (n=72)
NA
WHOvariant

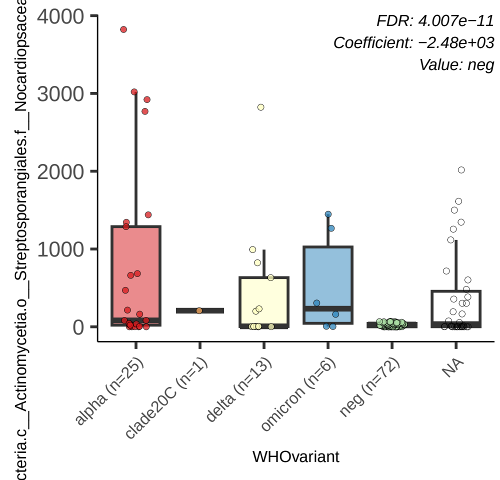

FDR: 4.007e−11
Coefficient: −2.48e+03
Value: neg
cteria.c__Actinomycetia.o__Streptosporangiales.f__Nocardiopsacea
4000
3000
2000
1000
0
alpha (n=25)
clade20C (n=1)
delta (n=13)
omicron (n=6)
neg (n=72)
NA
WHOvariant

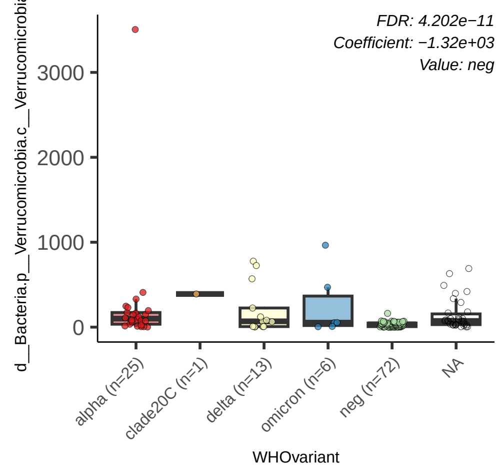
FDR: 4.202e−11
Coefficient: −1.32e+03
Value: neg
d__Bacteria.p__Verrucomicrobia.c__Verrucomicrobia
3000
2000
1000
0
alpha (n=25)
clade20C (n=1)
delta (n=13)
omicron (n=6)
neg (n=72)
NA
WHOvariant

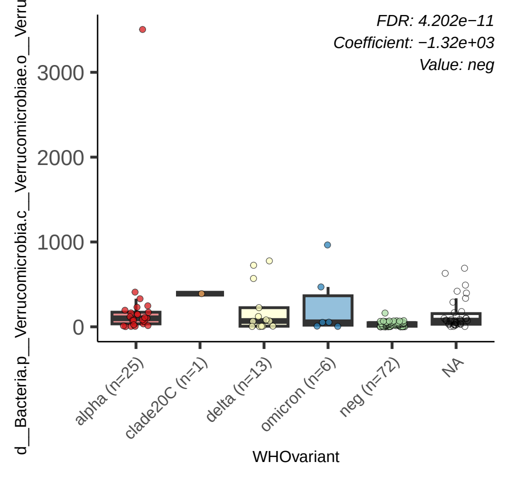

FDR: 4.202e−11
Coefficient: −1.32e+03
Value: neg
d__Bacteria.p__Verrucomicrobia.c__Verrucomicrobiae.o__Verru
0
1000
2000
3000
alpha (n=25)
clade20C (n=1)
delta (n=13)
omicron (n=6)
neg (n=72)
NA
WHOvariant

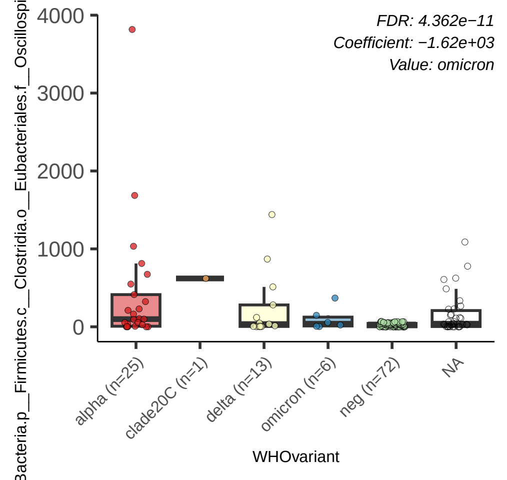

FDR: 4.362e−11
Coefficient: −1.62e+03
Value: omicron
Bacteria.p__Firmicutes.c__Clostridia.o__Eubacteriales.f__Oscillospi
4000
3000
2000
1000
0
alpha (n=25)
clade20C (n=1)
delta (n=13)
omicron (n=6)
neg (n=72)
NA
WHOvariant

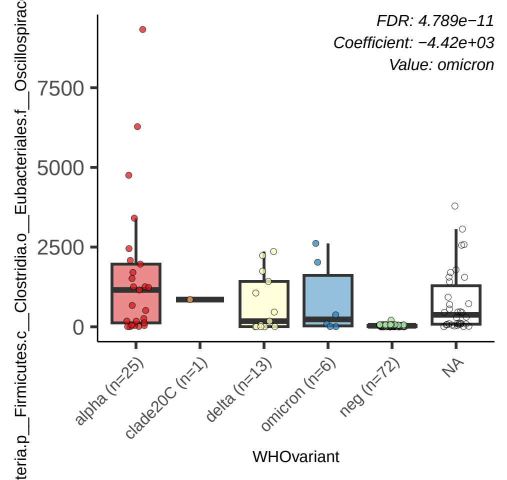

FDR: 4.789e−11
Coefficient: −4.42e+03
Value: omicron
7500
5000
2500
0
teria.p__Firmicutes.c__Clostridia.o__Eubacteriales.f__Oscillospirac
alpha (n=25)
clade20C (n=1)
delta (n=13)
omicron (n=6)
neg (n=72)
NA
WHOvariant

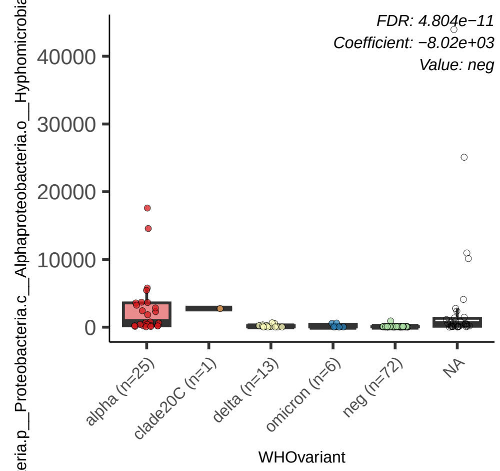

FDR: 4.804e−11
Coefficient: −8.02e+03
Value: neg
40000
30000
20000
10000
0
cteria.p__Proteobacteria.c__Alphaproteobacteria.o__Hyphomicrobia
alpha (n=25)
clade20C (n=1)
delta (n=13)
omicron (n=6)
neg (n=72)
NA
WHOvariant

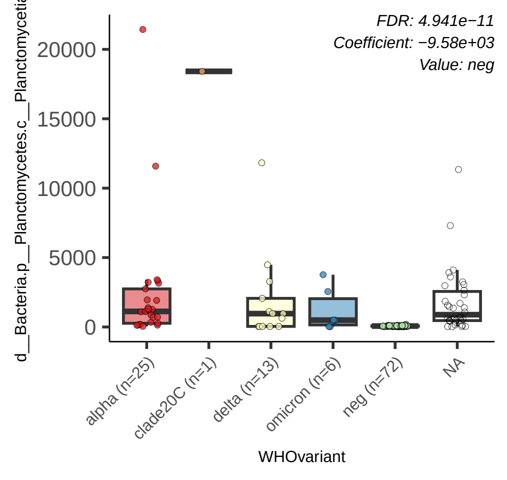
FDR: 4.941e−11
Coefficient: −9.58e+03
Value: neg
d__Bacteria.p__Planctomycetes.c__Planctomyceti
20000
15000
10000
5000
0
alpha (n=25)
clade20C (n=1)
delta (n=13)
omicron (n=6)
neg (n=72)
NA
WHOvariant

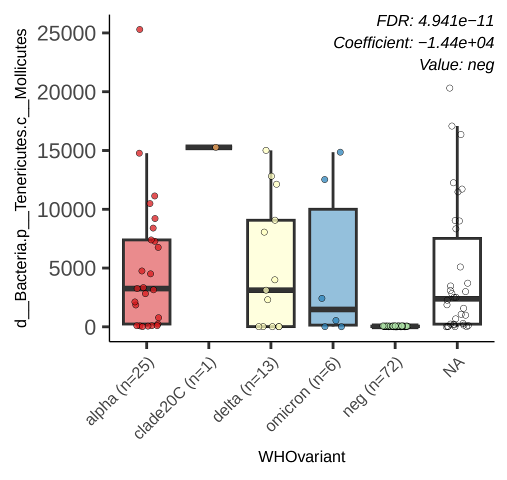

FDR: 4.941e−11
Coefficient: −1.44e+04
Value: neg
d__Bacteria.p__Tenericutes.c__Mollicutes
25000
20000
15000
10000
5000
0
alpha (n=25)
clade20C (n=1)
delta (n=13)
omicron (n=6)
neg (n=72)
NA
WHOvariant

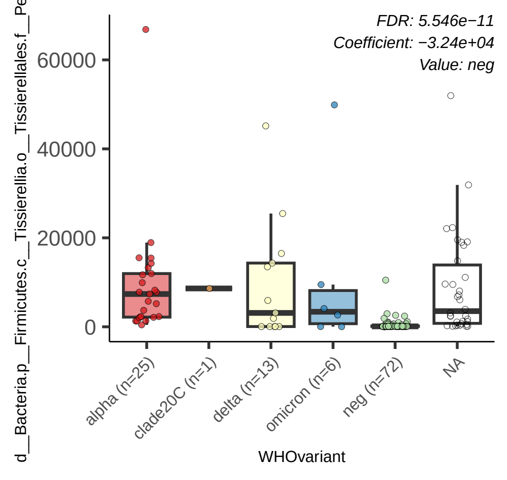
d__Bacteria.p__Firmicutes.c__Tissierellia.o__Tissierellales.f__Pe
FDR: 5.546e−11
Coefficient: −3.24e+04
Value: neg
60000
40000
20000
0
alpha (n=25)
clade20C (n=1)
delta (n=13)
omicron (n=6)
neg (n=72)
NA
WHOvariant

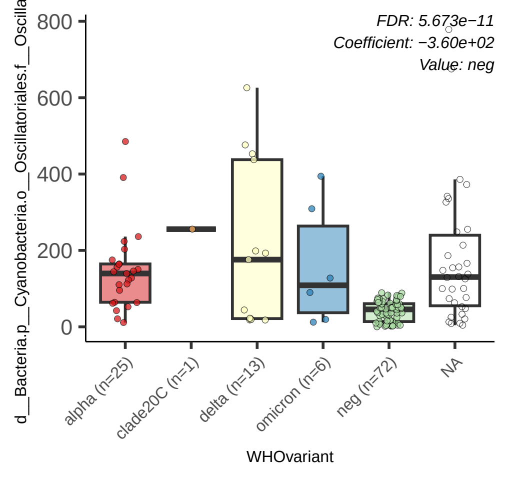

FDR: 5.673e−11
Coefficient: −3.60e+02
Value: neg
d__Bacteria.p__Cyanobacteria.o__Oscillatoriales.f__Oscilla
0
200
400
600
800
alpha (n=25)
clade20C (n=1)
delta (n=13)
omicron (n=6)
neg (n=72)
NA
WHOvariant

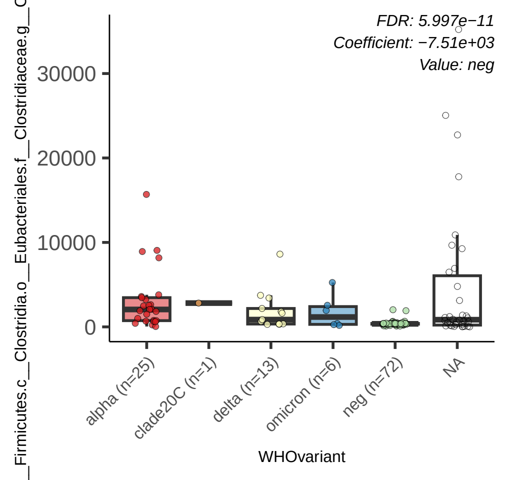
FDR: 5.997e−11
Coefficient: −7.51e+03
Value: neg
30000
20000
10000
0
Firmicutes.c__Clostridia.o__Eubacteriales.f__Clostridiaceae.g__C
alpha (n=25)
clade20C (n=1)
delta (n=13)
omicron (n=6)
neg (n=72)
NA
WHOvariant

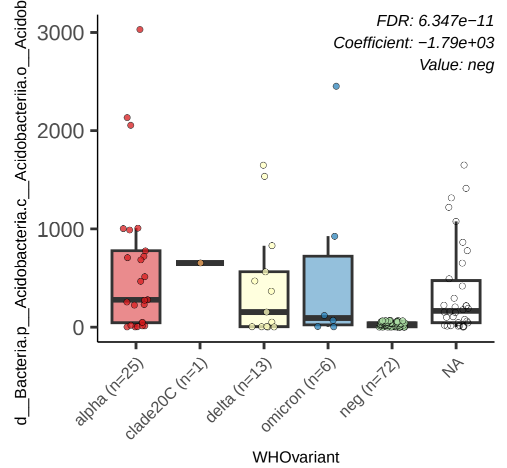

FDR: 6.347e−11
Coefficient: −1.79e+03
Value: neg
d__Bacteria.p__Acidobacteria.c__Acidobacteria.o__Acidob
3000
2000
1000
0
alpha (n=25)
clade20C (n=1)
delta (n=13)
omicron (n=6)
neg (n=72)
NA
WHOvariant

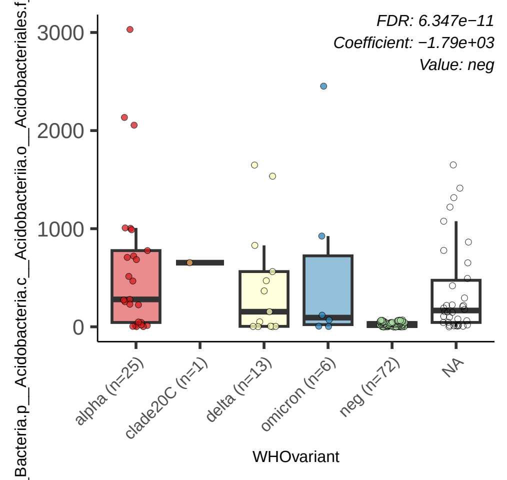

FDR: 6.347e−11
Coefficient: −1.79e+03
Value: neg
3000
2000
1000
0
Bacteria.p__Acidobacteria.c__Acidobacteriia.o__Acidobacteriales.f_
alpha (n=25)
clade20C (n=1)
delta (n=13)
omicron (n=6)
neg (n=72)
NA
WHOvariant

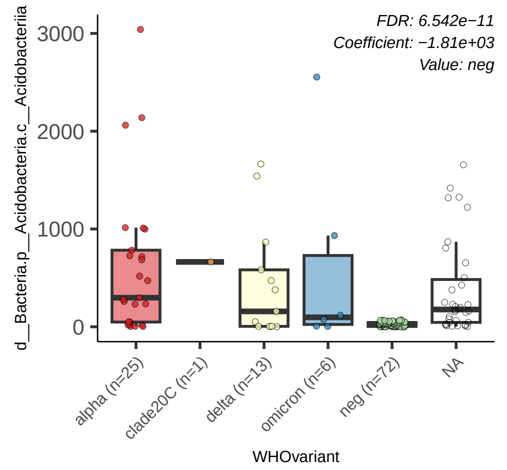

FDR: 6.542e−11
Coefficient: −1.81e+03
Value: neg
d__Bacteria.p__Acidobacteria.c__Acidobacteriia
3000
2000
1000
0
alpha (n=25)
clade20C (n=1)
delta (n=13)
omicron (n=6)
neg (n=72)
NA
WHOvariant

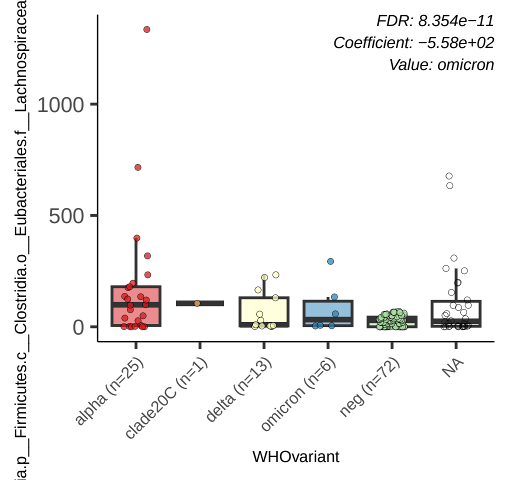
FDR: 8.354e−11
Coefficient: −5.58e+02
Value: omicron
ia.p__Firmicutes.c__Clostridia.o__Eubacteriales.f__Lachnospiracea
1000
500
0
alpha (n=25)
clade20C (n=1)
delta (n=13)
omicron (n=6)
neg (n=72)
NA
WHOvariant

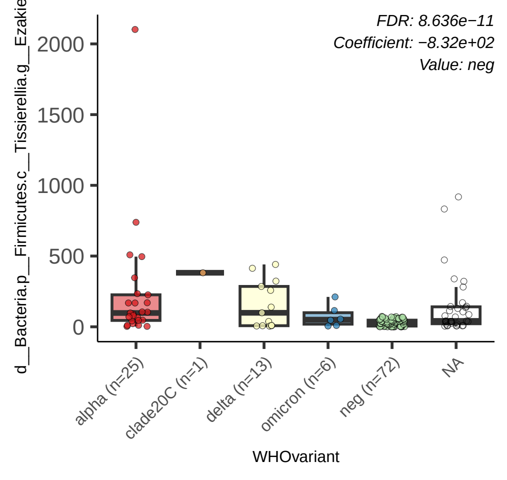

FDR: 8.636e−11
Coefficient: −8.32e+02
Value: neg
d__Bacteria.p__Firmicutes.c__Tissierellia.g__Ezakie
2000
1500
1000
500
0
alpha (n=25)
clade20C (n=1)
delta (n=13)
omicron (n=6)
neg (n=72)
NA
WHOvariant

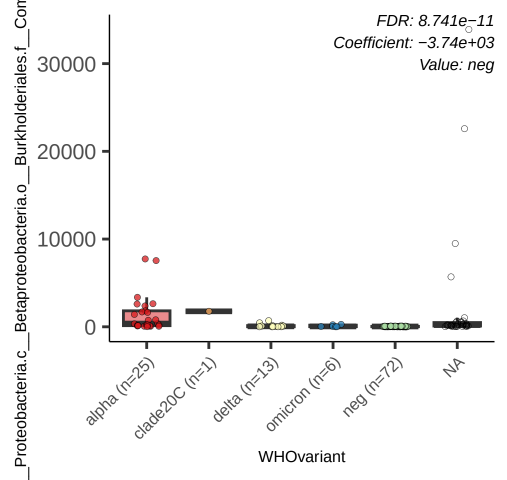
FDR: 8.741e−11
Coefficient: −3.74e+03
Value: neg
30000
20000
10000
0
alpha (n=25)
clade20C (n=1)
delta (n=13)
omicron (n=6)
neg (n=72)
NA
WHOvariant
_Proteobacteria.c__Betaproteobacteria.o__Burkholderiales.f__Com

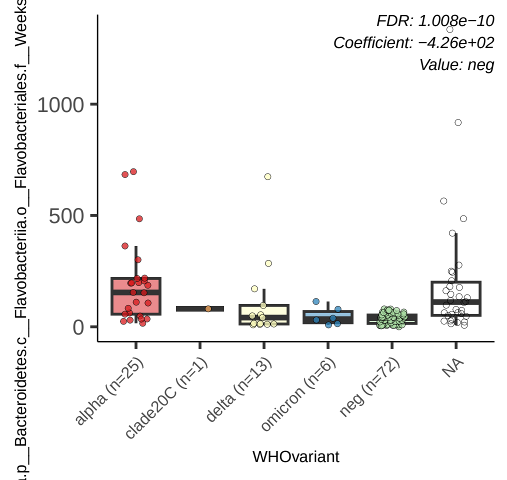
FDR: 1.008e−10
Coefficient: −4.26e+02
Value: neg
1000
500
0
a.p__Bacteroidetes.c__Flavobacteriia.o__Flavobacteriales.f__Weeks
alpha (n=25)
clade20C (n=1)
delta (n=13)
omicron (n=6)
neg (n=72)
NA
WHOvariant

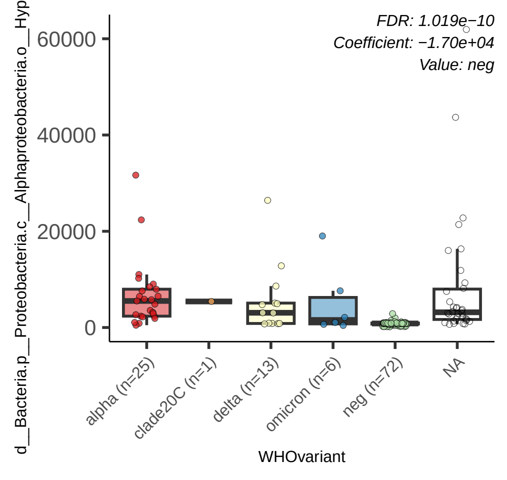
FDR: 1.019e−10
Coefficient: −1.70e+04
Value: neg
d__Bacteria.p__Proteobacteria.c__Alphaproteobacteria.o__Hyp
60000
40000
20000
0
alpha (n=25)
clade20C (n=1)
delta (n=13)
omicron (n=6)
neg (n=72)
NA
WHOvariant

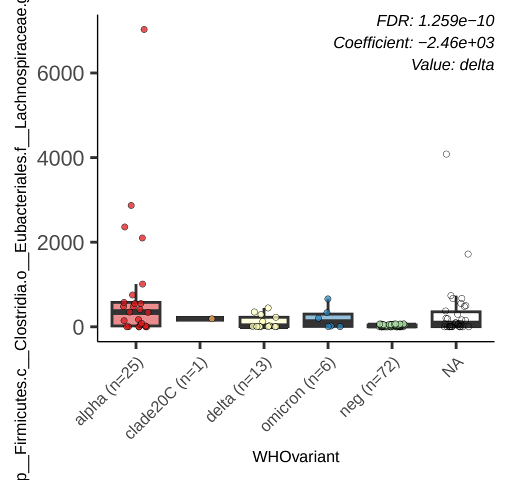

FDR: 1.259e−10
Coefficient: −2.46e+03
Value: delta
6000
4000
2000
0
p__Firmicutes.c__Clostridia.o__Eubacteriales.f__Lachnospiraceae.g
alpha (n=25)
clade20C (n=1)
delta (n=13)
omicron (n=6)
neg (n=72)
NA
WHOvariant

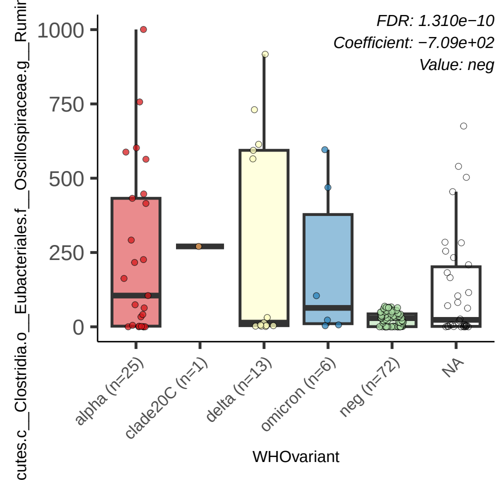
FDR: 1.310e−10
Coefficient: −7.09e+02
Value: neg
cutes.c__Clostridia.o__Eubacteriales.f__Oscillospiraceae.g__Rumi
1000
750
500
250
0
alpha (n=25)
clade20C (n=1)
delta (n=13)
omicron (n=6)
neg (n=72)
NA
WHOvariant

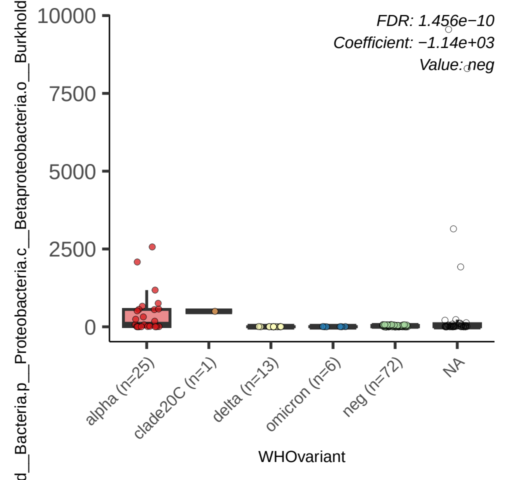

FDR: 1.456e−10
Coefficient: −1.14e+03
Value: neg
10000
7500
5000
2500
0
d__Bacteria.p__Proteobacteria.c__Betaproteobacteria.o__Burkhold
alpha (n=25)
clade20C (n=1)
delta (n=13)
omicron (n=6)
neg (n=72)
NA
WHOvariant

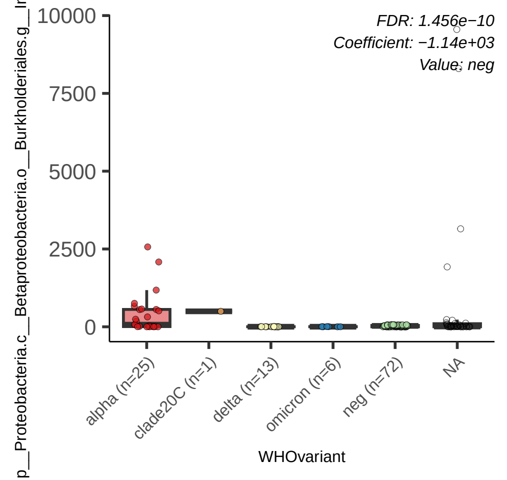
p__Proteobacteria.c__Betaproteobacteria.o__Burkholderiales.g__In
FDR: 1.456e−10
Coefficient: −1.14e+03
Value: neg
10000
7500
5000
2500
0
alpha (n=25)
clade20C (n=1)
delta (n=13)
omicron (n=6)
neg (n=72)
NA
WHOvariant

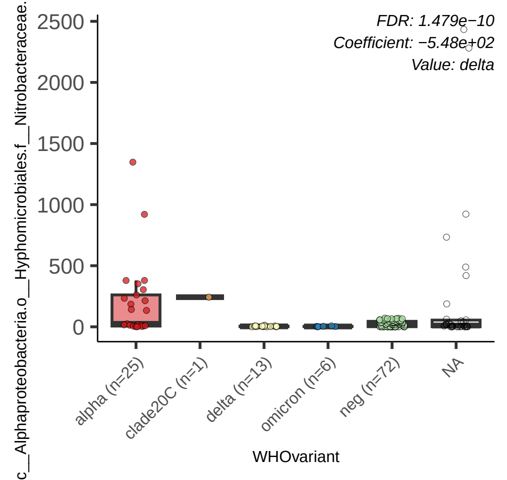

FDR: 1.479e−10
Coefficient: −5.48e+02
Value: delta
c__Alphaproteobacteria.o__Hyphomicrobiales.f__Nitrobacteraceae.
2500
2000
1500
1000
500
0
alpha (n=25)
clade20C (n=1)
delta (n=13)
omicron (n=6)
neg (n=72)
NA
WHOvariant

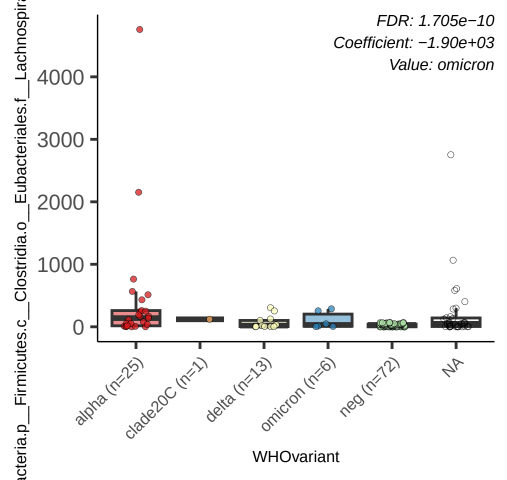

FDR: 1.705e−10
Coefficient: −1.90e+03
Value: omicron
4000
3000
2000
1000
0
acteria.p__Firmicutes.c__Clostridia.o__Eubacteriales.f__Lachnospira
alpha (n=25)
clade20C (n=1)
delta (n=13)
omicron (n=6)
neg (n=72)
NA
WHOvariant

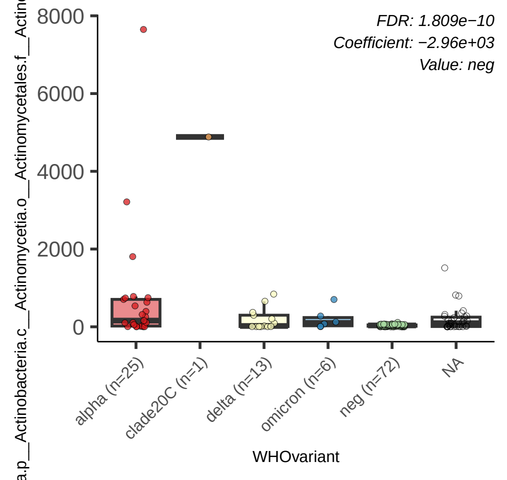

FDR: 1.809e−10
Coefficient: −2.96e+03
Value: neg
8000
6000
4000
2000
0
a.p__Actinobacteria.c__Actinomycetia.o__Actinomycetales.f__Actino
alpha (n=25)
clade20C (n=1)
delta (n=13)
omicron (n=6)
neg (n=72)
NA
WHOvariant

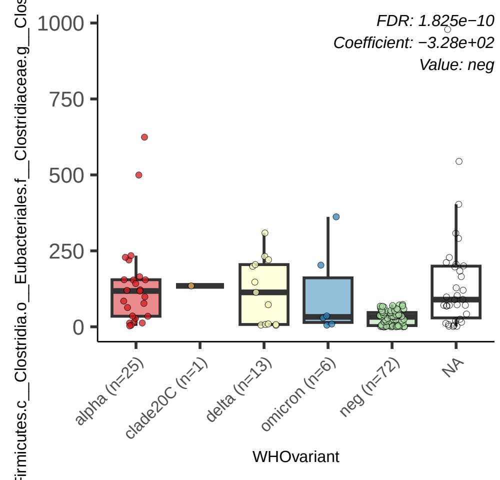

FDR: 1.825e−10
Coefficient: −3.28e+02
Value: neg
1000
750
500
250
0
Firmicutes.c__Clostridia.o__Eubacteriales.f__Clostridiaceae.g__Clos
alpha (n=25)
clade20C (n=1)
delta (n=13)
omicron (n=6)
neg (n=72)
NA
WHOvariant

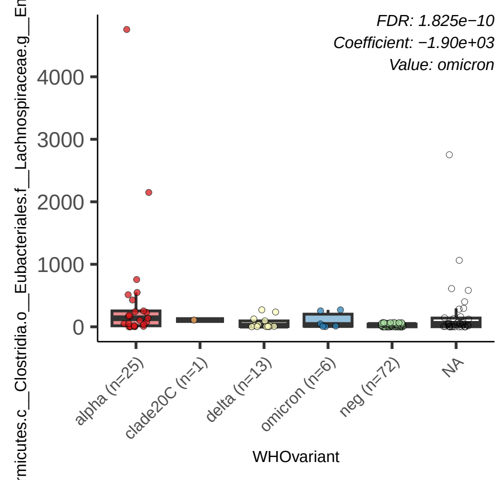

FDR: 1.825e−10
Coefficient: −1.90e+03
Value: omicron
4000
3000
2000
1000
0
alpha (n=25)
clade20C (n=1)
delta (n=13)
omicron (n=6)
neg (n=72)
NA
WHOvariant

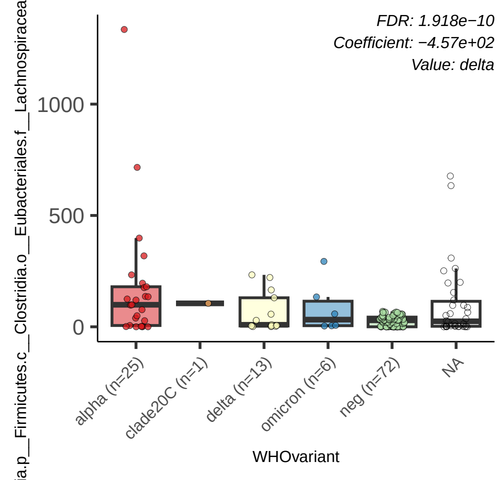

FDR: 1.918e−10
Coefficient: −4.57e+02
Value: delta
1000
500
0
alpha (n=25)
clade20C (n=1)
delta (n=13)
omicron (n=6)
neg (n=72)
NA
WHOvariant

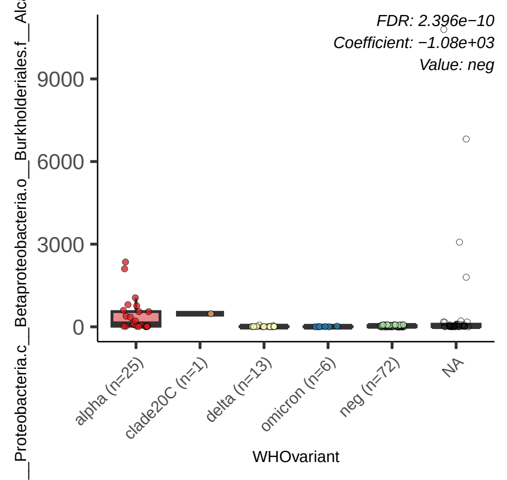

FDR: 2.396e−10
Coefficient: −1.08e+03
Value: neg
_Proteobacteria.c__Betaproteobacteria.o__Burkholderiales.f__Alc
9000
6000
3000
0
alpha (n=25)
clade20C (n=1)
delta (n=13)
omicron (n=6)
neg (n=72)
NA
WHOvariant

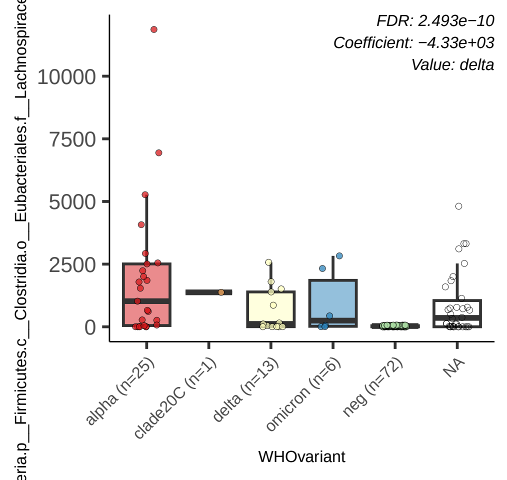
FDR: 2.493e−10
Coefficient: −4.33e+03
Value: delta
10000
7500
5000
2500
0
alpha (n=25)
clade20C (n=1)
delta (n=13)
omicron (n=6)
neg (n=72)
NA
WHOvariant
eria.p__Firmicutes.c__Clostridia.o__Eubacteriales.f__Lachnospirace

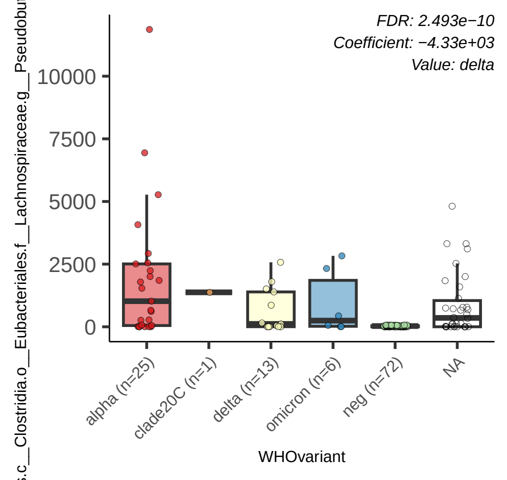

FDR: 2.493e−10
Coefficient: −4.33e+03
Value: delta
10000
7500
5000
2500
0
s.c__Clostridia.o__Eubacteriales.f__Lachnospiraceae.g__Pseudobu
alpha (n=25)
clade20C (n=1)
delta (n=13)
omicron (n=6)
neg (n=72)
NA
WHOvariant

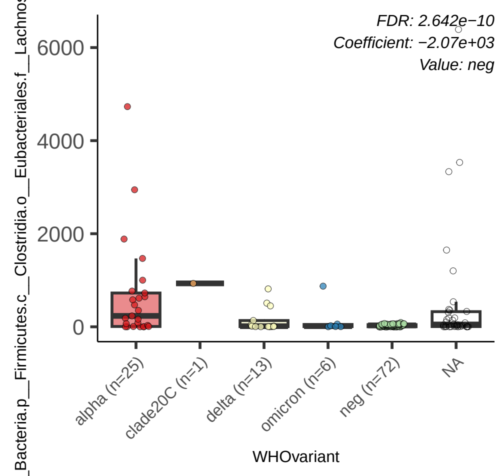

FDR: 2.642e−10
Coefficient: −2.07e+03
Value: neg
6000
4000
2000
0
_Bacteria.p__Firmicutes.c__Clostridia.o__Eubacteriales.f__Lachnos
alpha (n=25)
clade20C (n=1)
delta (n=13)
omicron (n=6)
neg (n=72)
NA
WHOvariant

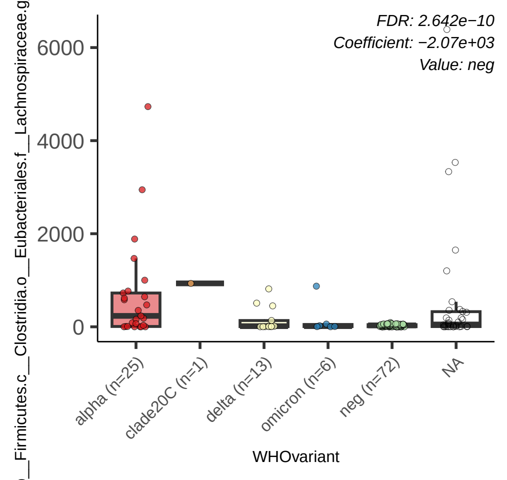
FDR: 2.642e−10
Coefficient: −2.07e+03
Value: neg
Firmicutes.c__Clostridia.o__Eubacteriales.f__Lachnospiraceae.g
6000
4000
2000
0
alpha (n=25)
clade20C (n=1)
delta (n=13)
omicron (n=6)
neg (n=72)
NA
WHOvariant

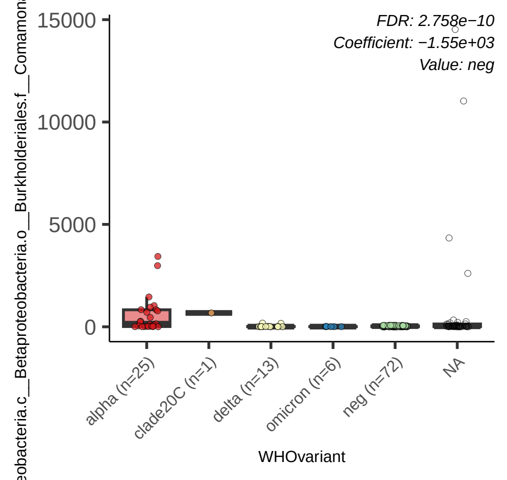

eobacteria.c__Betaproteobacteria.o__Burkholderiales.f__Comamona
15000
10000
5000
0
FDR: 2.758e−10
Coefficient: −1.55e+03
Value: neg
alpha (n=25)
clade20C (n=1)
delta (n=13)
omicron (n=6)
neg (n=72)
NA
WHOvariant

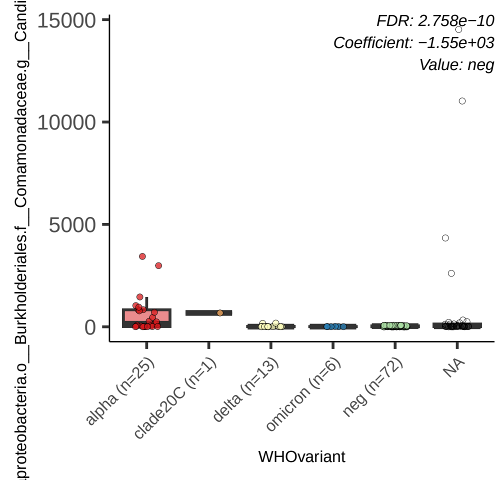
FDR: 2.758e−10
Coefficient: −1.55e+03
Value: neg
15000
10000
5000
0
proteobacteria.o__Burkholderiales.f__Comamonadaceae.g__Candi
alpha (n=25)
clade20C (n=1)
delta (n=13)
omicron (n=6)
neg (n=72)
NA
WHOvariant

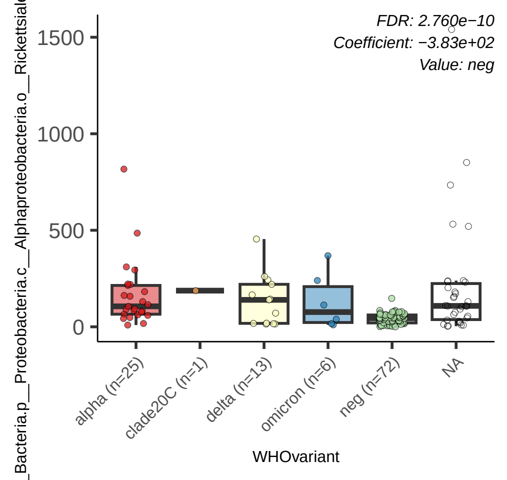

FDR: 2.760e−10
Coefficient: −3.83e+02
Value: neg
1500
1000
500
0
Bacteria.p__Proteobacteria.c__Alphaproteobacteria.o__Rickettsiale
alpha (n=25)
clade20C (n=1)
delta (n=13)
omicron (n=6)
neg (n=72)
NA
WHOvariant

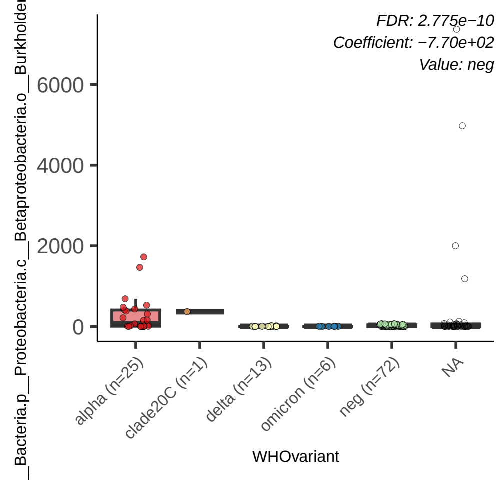
FDR: 2.775e−10
Coefficient: −7.70e+02
Value: neg
Bacteria.p__Proteobacteria.c__Betaproteobacteria.o__Burkholder
6000
4000
2000
0
alpha (n=25)
clade20C (n=1)
delta (n=13)
omicron (n=6)
neg (n=72)
NA
WHOvariant

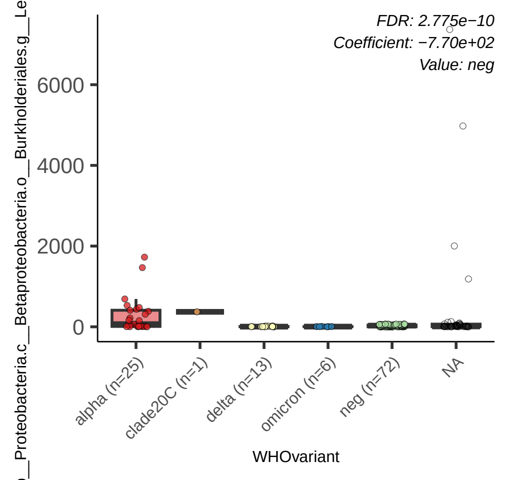
FDR: 2.775e−10
Coefficient: −7.70e+02
Value: neg
6000
4000
2000
0
alpha (n=25)
clade20C (n=1)
delta (n=13)
omicron (n=6)
neg (n=72)
NA
WHOvariant
o__Proteobacteria.c__Betaproteobacteria.o__Burkholderiales.g__Le

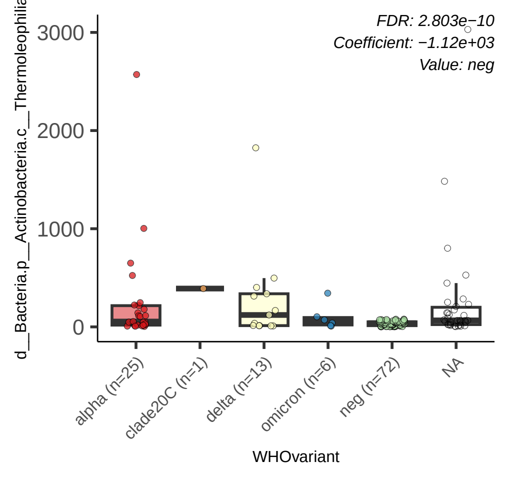

FDR: 2.803e−10
Coefficient: −1.12e+03
Value: neg
d__Bacteria.p__Actinobacteria.c__Thermoleophilia
3000
2000
1000
0
alpha (n=25)
clade20C (n=1)
delta (n=13)
omicron (n=6)
neg (n=72)
NA
WHOvariant

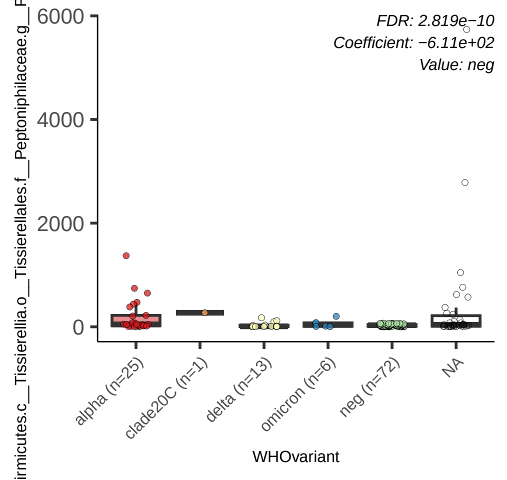

FDR: 2.819e−10
Coefficient: −6.11e+02
Value: neg
6000
4000
2000
0
irmicutes.c__Tissierellia.o__Tissierellales.f__Peptoniphilaceae.g__F
alpha (n=25)
clade20C (n=1)
delta (n=13)
omicron (n=6)
neg (n=72)
NA
WHOvariant

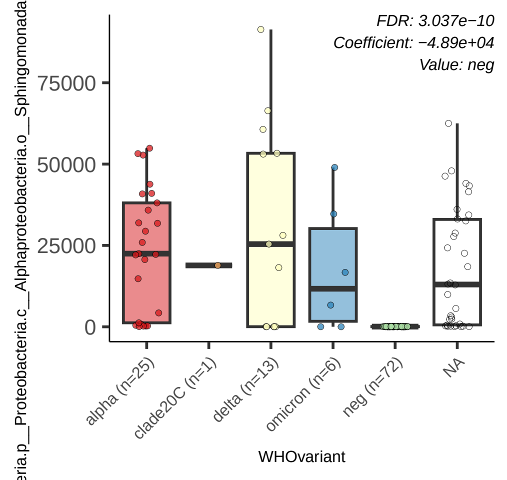

FDR: 3.037e−10
Coefficient: −4.89e+04
Value: neg
75000
50000
25000
0
eria.p__Proteobacteria.c__Alphaproteobacteria.o__Sphingomonada
alpha (n=25)
clade20C (n=1)
delta (n=13)
omicron (n=6)
neg (n=72)
NA
WHOvariant

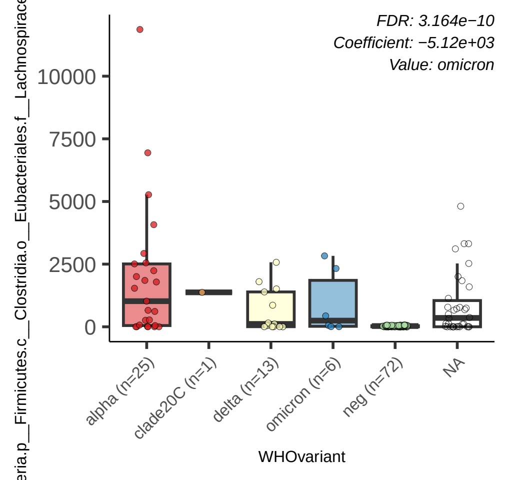

FDR: 3.164e−10
Coefficient: −5.12e+03
Value: omicron
10000
7500
5000
2500
0
alpha (n=25)
clade20C (n=1)
delta (n=13)
omicron (n=6)
neg (n=72)
NA
WHOvariant
eria.p__Firmicutes.c__Clostridia.o__Eubacteriales.f__Lachnospirace

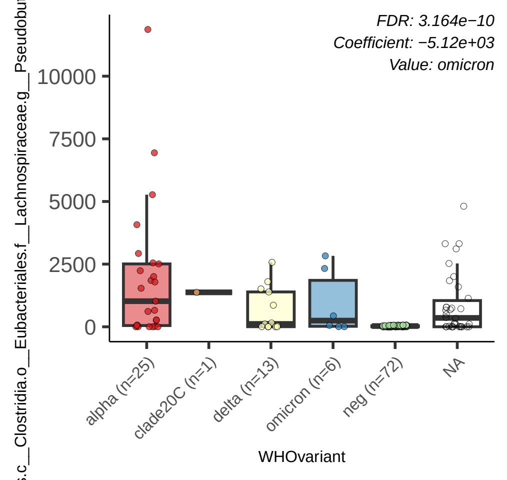
FDR: 3.164e−10
Coefficient: −5.12e+03
Value: omicron
s.c__Clostridia.o__Eubacteriales.f__Lachnospiraceae.g__Pseudobu
10000
7500
5000
2500
0
alpha (n=25)
clade20C (n=1)
delta (n=13)
omicron (n=6)
neg (n=72)
NA
WHOvariant

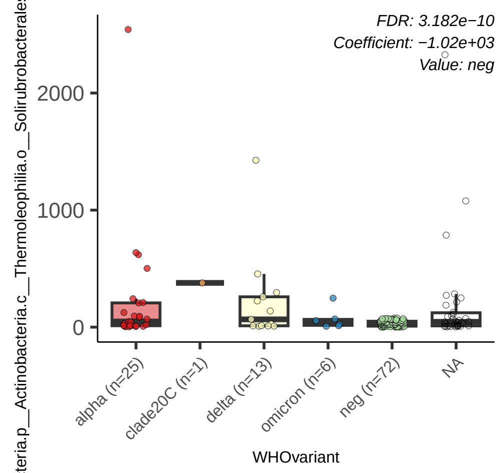
FDR: 3.182e−10
Coefficient: −1.02e+03
Value: neg
2000
1000
0
cteria.p__Actinobacteria.c__Thermoleophilia.o__Solirubrobacterales
alpha (n=25)
clade20C (n=1)
delta (n=13)
omicron (n=6)
neg (n=72)
NA
WHOvariant

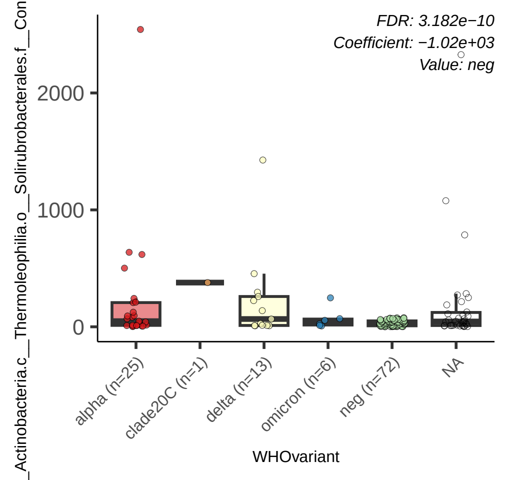

FDR: 3.182e−10
Coefficient: −1.02e+03
Value: neg
_Actinobacteria.c__Thermoleophilia.o__Solirubrobacterales.f__Con
2000
1000
0
alpha (n=25)
clade20C (n=1)
delta (n=13)
omicron (n=6)
neg (n=72)
NA
WHOvariant

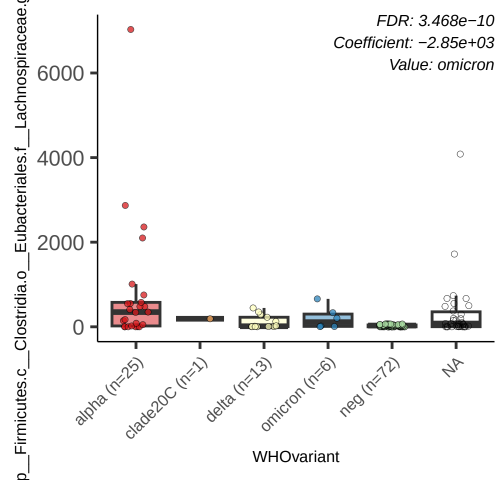
FDR: 3.468e−10
Coefficient: −2.85e+03
Value: omicron
p__Firmicutes.c__Clostridia.o__Eubacteriales.f__Lachnospiraceae.g
0
2000
4000
6000
alpha (n=25)
clade20C (n=1)
delta (n=13)
omicron (n=6)
neg (n=72)
NA
WHOvariant

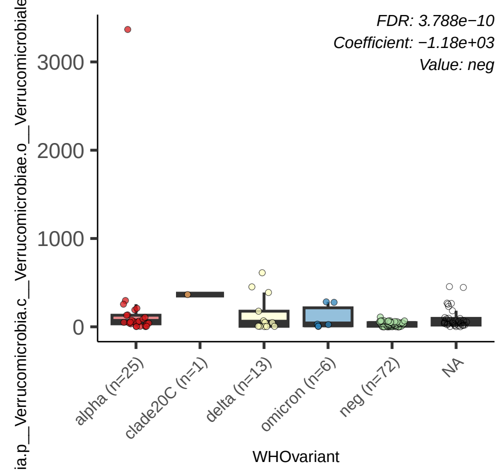

FDR: 3.788e−10
Coefficient: −1.18e+03
Value: neg
3000
2000
1000
0
eria.p__Verrucomicrobia.c__Verrucomicrobiae.o__Verrucomicrobiale
alpha (n=25)
clade20C (n=1)
delta (n=13)
omicron (n=6)
neg (n=72)
NA
WHOvariant

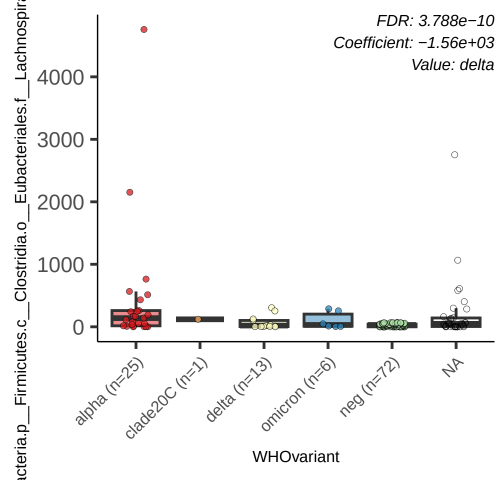
FDR: 3.788e−10
Coefficient: −1.56e+03
Value: delta
4000
3000
2000
1000
0
alpha (n=25)
clade20C (n=1)
delta (n=13)
omicron (n=6)
neg (n=72)
NA
WHOvariant

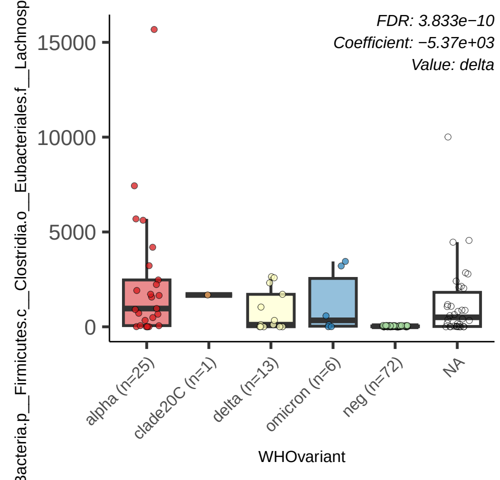
FDR: 3.833e−10
Coefficient: −5.37e+03
Value: delta
15000
10000
5000
0
Bacteria.p__Firmicutes.c__Clostridia.o__Eubacteriales.f__Lachnosp
alpha (n=25)
clade20C (n=1)
delta (n=13)
omicron (n=6)
neg (n=72)
NA
WHOvariant

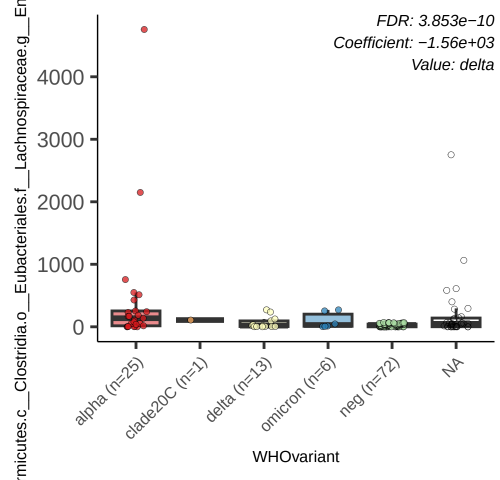
FDR: 3.853e−10
Coefficient: −1.56e+03
Value: delta
4000
3000
2000
1000
0
rmicutes.c__Clostridia.o__Eubacteriales.f__Lachnospiraceae.g__En
alpha (n=25)
clade20C (n=1)
delta (n=13)
omicron (n=6)
neg (n=72)
NA
WHOvariant

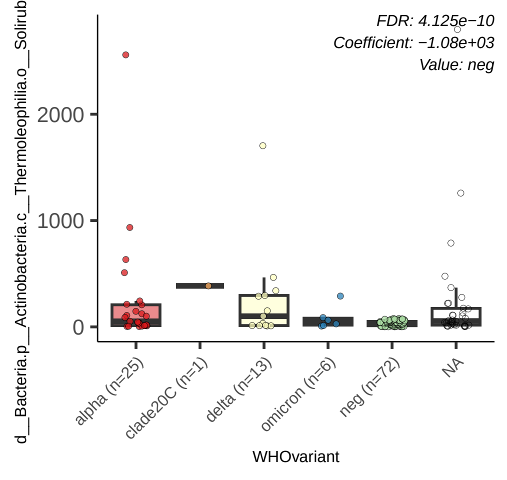

FDR: 4.125e−10
Coefficient: −1.08e+03
Value: neg
d__Bacteria.p__Actinobacteria.c__Thermoleophilia.o__Solirub
2000
1000
0
alpha (n=25)
clade20C (n=1)
delta (n=13)
omicron (n=6)
neg (n=72)
NA
WHOvariant

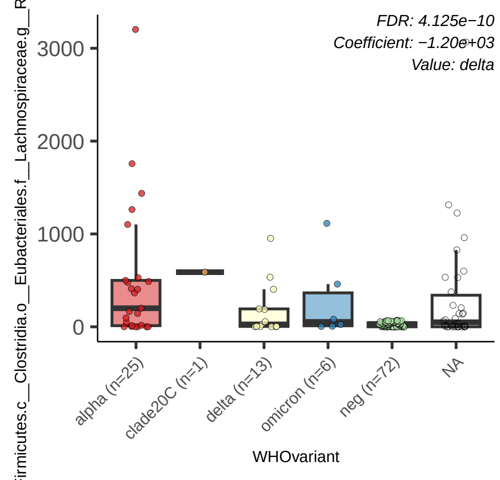

FDR: 4.125e−10
Coefficient: −1.20e+03
Value: delta
3000
2000
1000
0
irmicutes.c__Clostridia.o__Eubacteriales.f__Lachnospiraceae.g__R
alpha (n=25)
clade20C (n=1)
delta (n=13)
omicron (n=6)
neg (n=72)
NA
WHOvariant

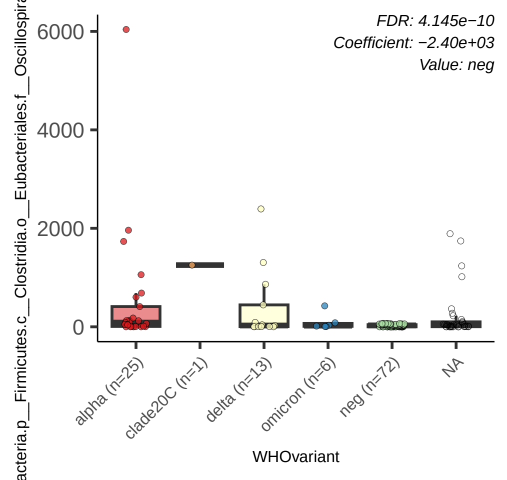
FDR: 4.145e−10
Coefficient: −2.40e+03
Value: neg
6000
4000
2000
0
acteria.p__Firmicutes.c__Clostridia.o__Eubacteriales.f__Oscillospira
alpha (n=25)
clade20C (n=1)
delta (n=13)
omicron (n=6)
neg (n=72)
NA
WHOvariant

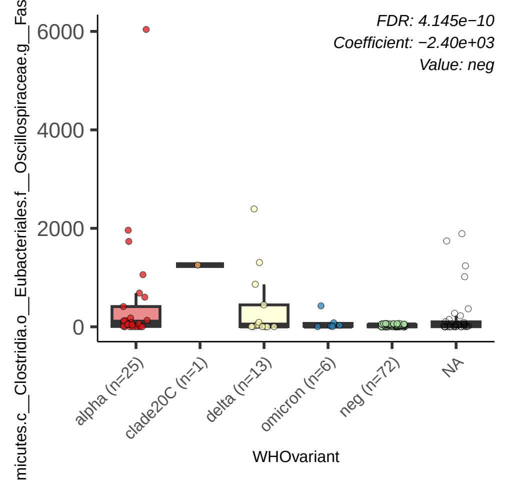

micutes.c__Clostridia.o__Eubacteriales.f__Oscillospiraceae.g__Fas
FDR: 4.145e−10
Coefficient: −2.40e+03
Value: neg
6000
4000
2000
0
alpha (n=25)
clade20C (n=1)
delta (n=13)
omicron (n=6)
neg (n=72)
NA
WHOvariant

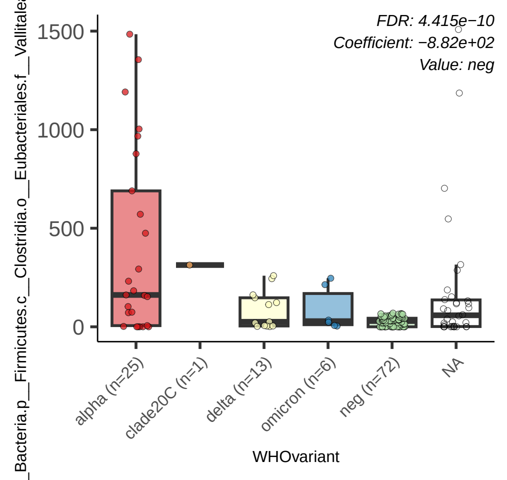

FDR: 4.415e−10
Coefficient: −8.82e+02
Value: neg
_Bacteria.p__Firmicutes.c__Clostridia.o__Eubacteriales.f__Vallitalea
1500
1000
500
0
alpha (n=25)
clade20C (n=1)
delta (n=13)
omicron (n=6)
neg (n=72)
NA
WHOvariant

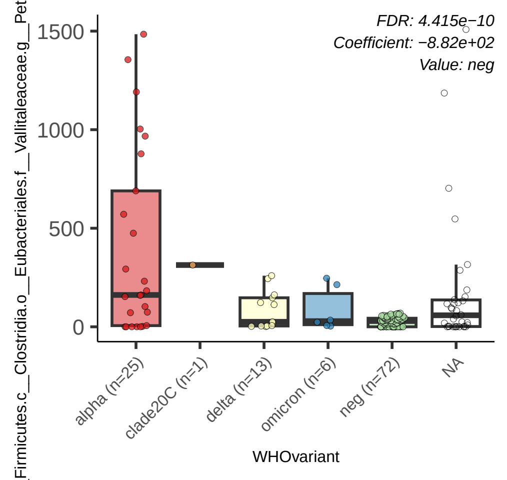

FDR: 4.415e−10
Coefficient: −8.82e+02
Value: neg
Firmicutes.c__Clostridia.o__Eubacteriales.f__Vallitaleaceae.g__Pet
1500
1000
500
0
alpha (n=25)
clade20C (n=1)
delta (n=13)
omicron (n=6)
neg (n=72)
NA
WHOvariant

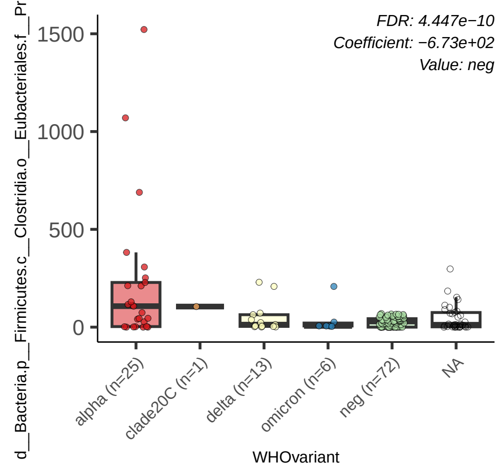

FDR: 4.447e−10
Coefficient: −6.73e+02
Value: neg
d__Bacteria.p__Firmicutes.c__Clostridia.o__Eubacteriales.f__Pr
1500
1000
500
0
alpha (n=25)
clade20C (n=1)
delta (n=13)
omicron (n=6)
neg (n=72)
NA
WHOvariant

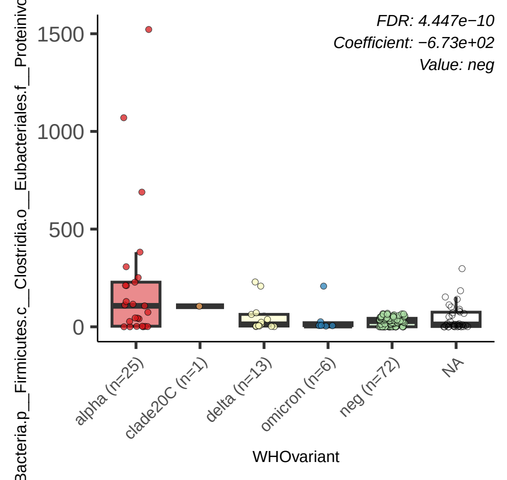

FDR: 4.447e−10
Coefficient: −6.73e+02
Value: neg
Bacteria.p__Firmicutes.c__Clostridia.o__Eubacteriales.f__Proteinivo
1500
1000
500
0
alpha (n=25)
clade20C (n=1)
delta (n=13)
omicron (n=6)
neg (n=72)
NA
WHOvariant

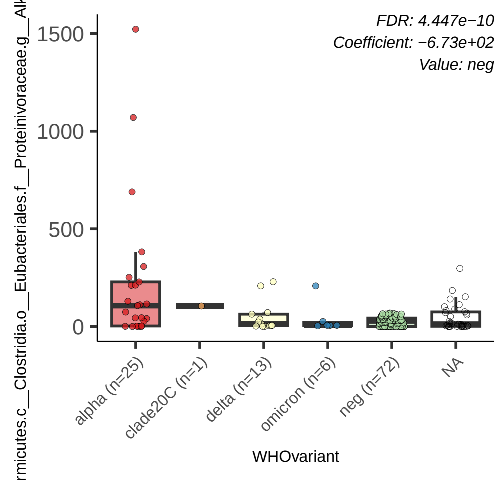
FDR: 4.447e−10
Coefficient: −6.73e+02
Value: neg
1500
1000
500
0
alpha (n=25)
clade20C (n=1)
delta (n=13)
omicron (n=6)
neg (n=72)
NA
WHOvariant

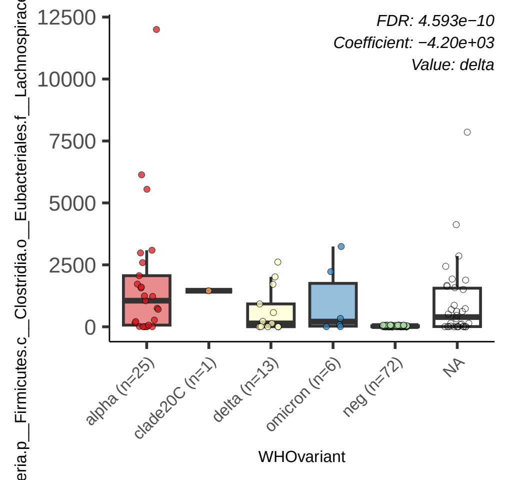

FDR: 4.593e−10
Coefficient: −4.20e+03
Value: delta
12500
10000
7500
5000
2500
0
alpha (n=25)
clade20C (n=1)
delta (n=13)
omicron (n=6)
neg (n=72)
NA
WHOvariant

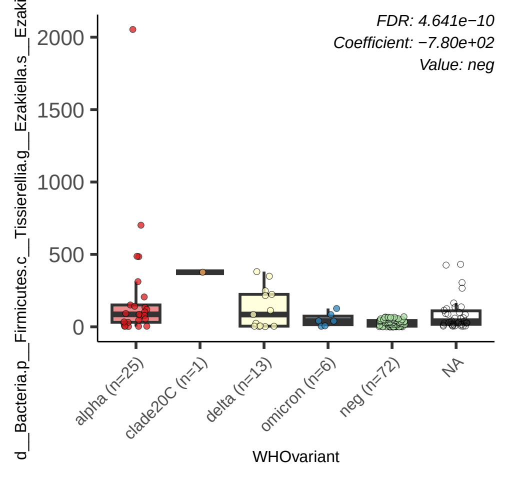

FDR: 4.641e−10
Coefficient: −7.80e+02
Value: neg
d__Bacteria.p__Firmicutes.c__Tissierellia.g__Ezakiella.s__Ezaki
2000
1500
1000
500
0
alpha (n=25)
clade20C (n=1)
delta (n=13)
omicron (n=6)
neg (n=72)
NA
WHOvariant

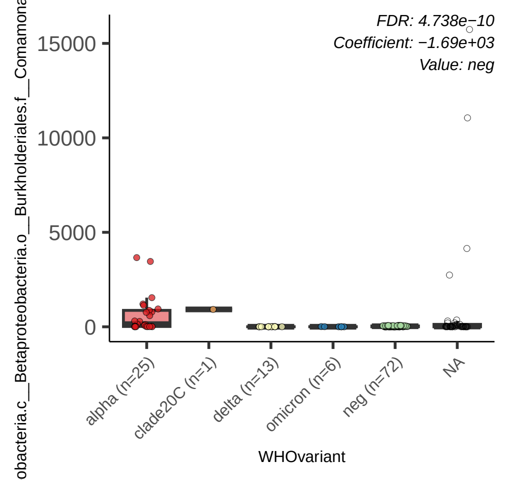

FDR: 4.738e−10
Coefficient: −1.69e+03
Value: neg
15000
10000
5000
0
obacteria.c__Betaproteobacteria.o__Burkholderiales.f__Comamona
alpha (n=25)
clade20C (n=1)
delta (n=13)
omicron (n=6)
neg (n=72)
NA
WHOvariant

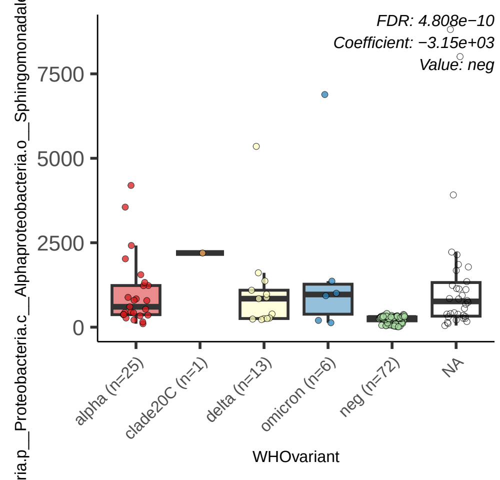

FDR: 4.808e−10
Coefficient: −3.15e+03
Value: neg
7500
5000
2500
0
ria.p__Proteobacteria.c__Alphaproteobacteria.o__Sphingomonadale
alpha (n=25)
clade20C (n=1)
delta (n=13)
omicron (n=6)
neg (n=72)
NA
WHOvariant

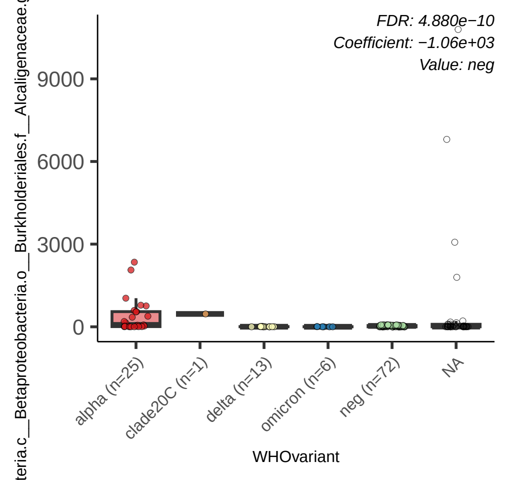

FDR: 4.880e−10
Coefficient: −1.06e+03
Value: neg
9000
6000
3000
0
teria.c__Betaproteobacteria.o__Burkholderiales.f__Alcaligenaceae.g
alpha (n=25)
clade20C (n=1)
delta (n=13)
omicron (n=6)
neg (n=72)
NA
WHOvariant

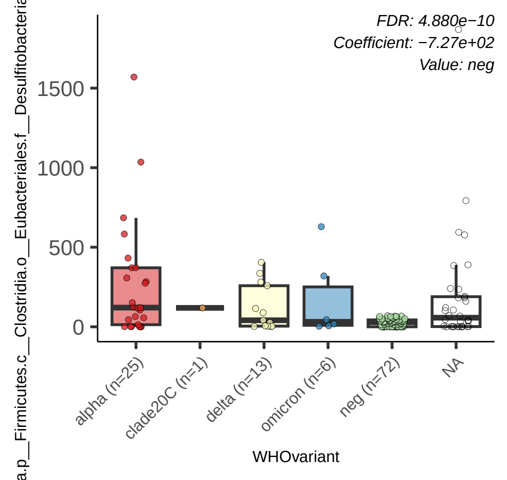
FDR: 4.880e−10
Coefficient: −7.27e+02
Value: neg
a.p__Firmicutes.c__Clostridia.o__Eubacteriales.f__Desulfitobacteria
1500
1000
500
0
alpha (n=25)
clade20C (n=1)
delta (n=13)
omicron (n=6)
neg (n=72)
NA
WHOvariant

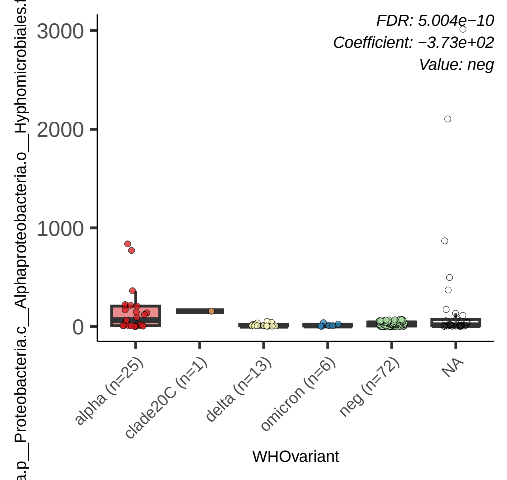

FDR: 5.004e−10
Coefficient: −3.73e+02
Value: neg
3000
2000
1000
0
alpha (n=25)
clade20C (n=1)
delta (n=13)
omicron (n=6)
neg (n=72)
NA
WHOvariant
a.p__Proteobacteria.c__Alphaproteobacteria.o__Hyphomicrobiales.f

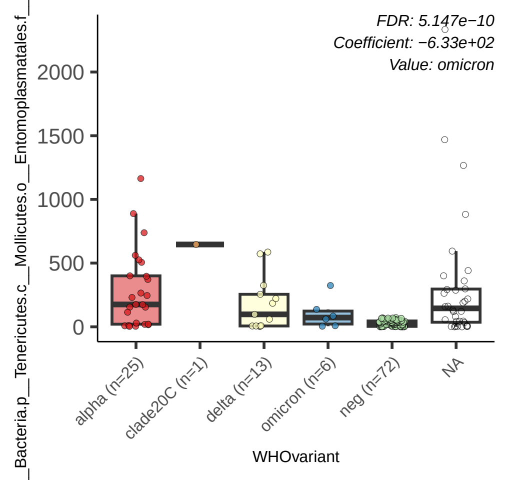
FDR: 5.147e−10
Coefficient: −6.33e+02
Value: omicron
Bacteria.p__Tenericutes.c__Mollicutes.o__Entomoplasmatales.f__
2000
1500
1000
500
0
alpha (n=25)
clade20C (n=1)
delta (n=13)
omicron (n=6)
neg (n=72)
NA
WHOvariant

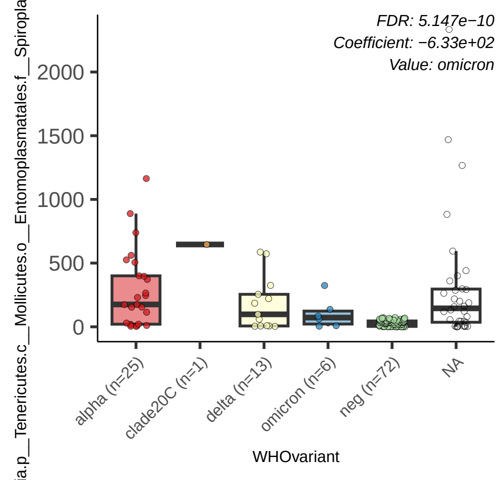

FDR: 5.147e−10
Coefficient: −6.33e+02
Value: omicron
ia.p__Tenericutes.c__Mollicutes.o__Entomoplasmatales.f__Spiropla
2000
1500
1000
500
0
alpha (n=25)
clade20C (n=1)
delta (n=13)
omicron (n=6)
neg (n=72)
NA
WHOvariant

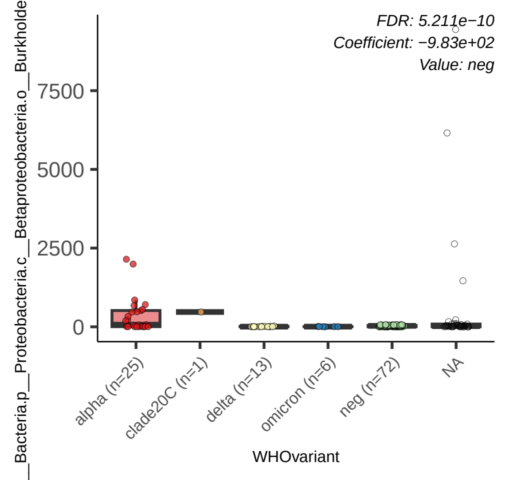

FDR: 5.211e−10
Coefficient: −9.83e+02
Value: neg
0
2500
5000
7500
_Bacteria.p__Proteobacteria.c__Betaproteobacteria.o__Burkholde
alpha (n=25)
clade20C (n=1)
delta (n=13)
omicron (n=6)
neg (n=72)
NA
WHOvariant

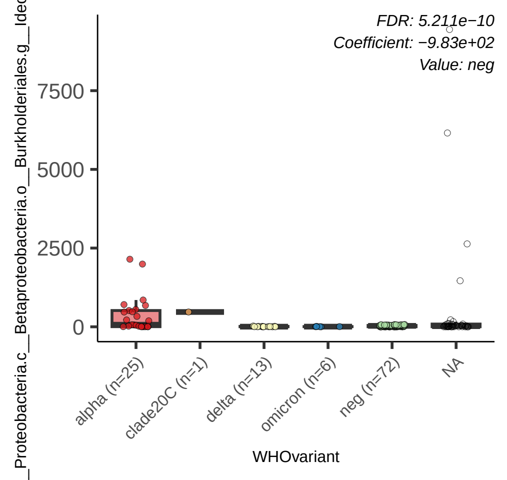

FDR: 5.211e−10
Coefficient: −9.83e+02
Value: neg
_Proteobacteria.c__Betaproteobacteria.o__Burkholderiales.g__Ide
7500
5000
2500
0
alpha (n=25)
clade20C (n=1)
delta (n=13)
omicron (n=6)
neg (n=72)
NA
WHOvariant

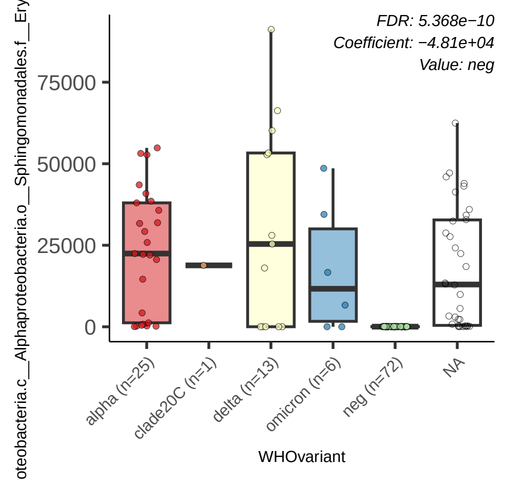

FDR: 5.368e−10
Coefficient: −4.81e+04
Value: neg
oteobacteria.c__Alphaproteobacteria.o__Sphingomonadales.f__Ery
75000
50000
25000
0
alpha (n=25)
clade20C (n=1)
delta (n=13)
omicron (n=6)
neg (n=72)
NA
WHOvariant

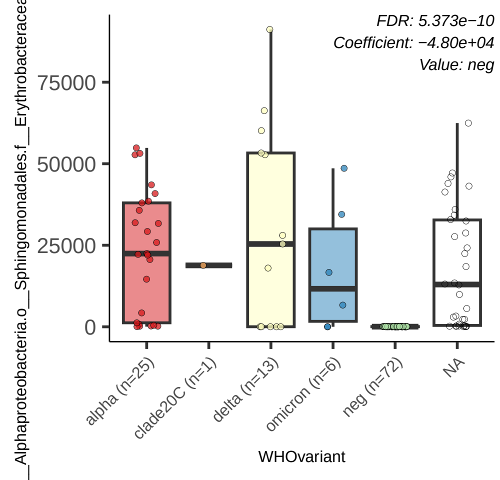
FDR: 5.373e−10
Coefficient: −4.80e+04
Value: neg
__Alphaproteobacteria.o__Sphingomonadales.f__Erythrobacteraceae
75000
50000
25000
0
alpha (n=25)
clade20C (n=1)
delta (n=13)
omicron (n=6)
neg (n=72)
NA
WHOvariant

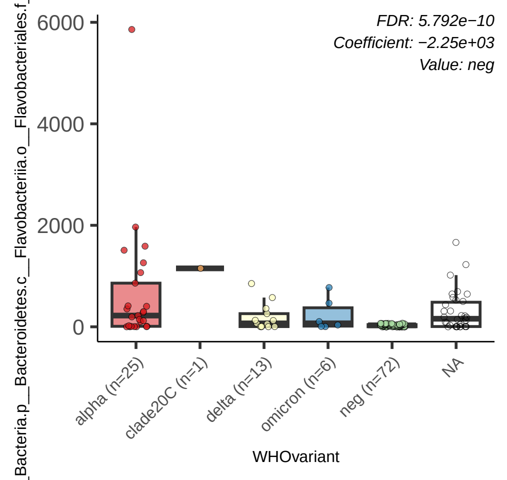

FDR: 5.792e−10
Coefficient: −2.25e+03
Value: neg
6000
4000
2000
0
Bacteria.p__Bacteroidetes.c__Flavobacteriia.o__Flavobacteriales.f_
alpha (n=25)
clade20C (n=1)
delta (n=13)
omicron (n=6)
neg (n=72)
NA
WHOvariant

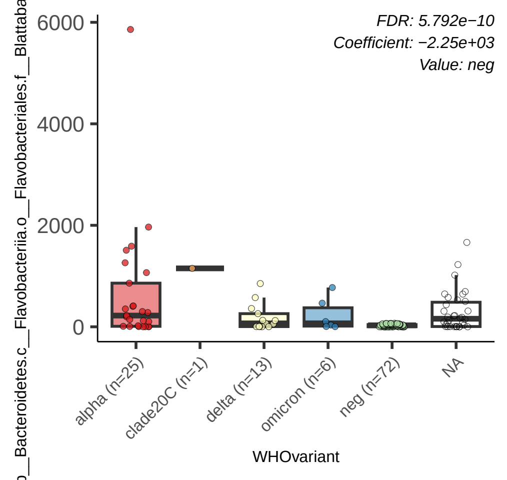

FDR: 5.792e−10
Coefficient: −2.25e+03
Value: neg
6000
4000
2000
0
o__Bacteroidetes.c__Flavobacteriia.o__Flavobacteriales.f__Blattaba
alpha (n=25)
clade20C (n=1)
delta (n=13)
omicron (n=6)
neg (n=72)
NA
WHOvariant

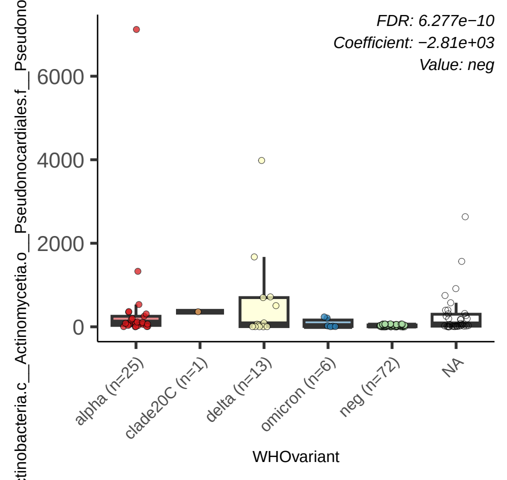

FDR: 6.277e−10
Coefficient: −2.81e+03
Value: neg
6000
4000
2000
0
ctinobacteria.c__Actinomycetia.o__Pseudonocardiales.f__Pseudono
alpha (n=25)
clade20C (n=1)
delta (n=13)
omicron (n=6)
neg (n=72)
NA
WHOvariant

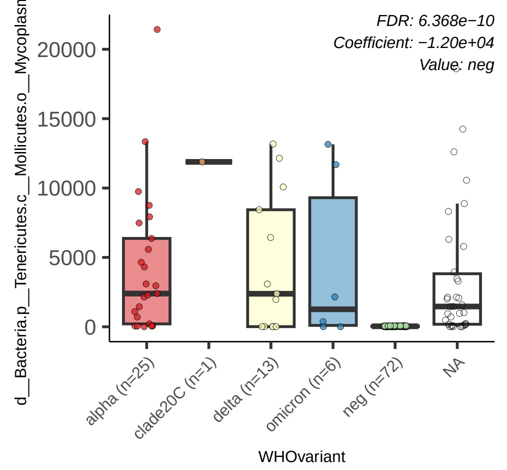

FDR: 6.368e−10
Coefficient: −1.20e+04
Value: neg
d__Bacteria.p__Tenericutes.c__Mollicutes.o__Mycoplasm
20000
15000
10000
5000
0
alpha (n=25)
clade20C (n=1)
delta (n=13)
omicron (n=6)
neg (n=72)
NA
WHOvariant

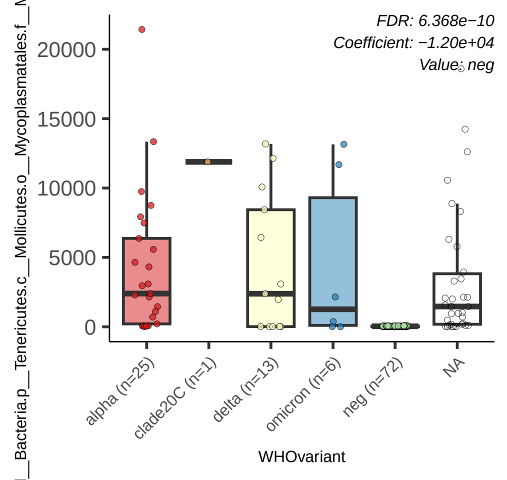

FDR: 6.368e−10
Coefficient: −1.20e+04
Value: neg
__Bacteria.p__Tenericutes.c__Mollicutes.o__Mycoplasmatales.f__M
20000
15000
10000
5000
0
alpha (n=25)
clade20C (n=1)
delta (n=13)
omicron (n=6)
neg (n=72)
NA
WHOvariant

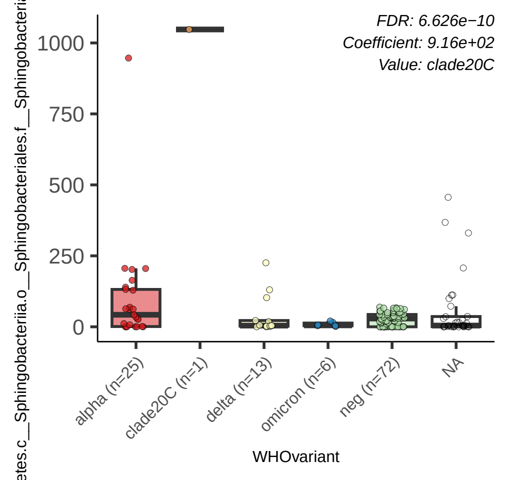

FDR: 6.626e−10
Coefficient: 9.16e+02
Value: clade20C
1000
750
500
250
0
etes.c__Sphingobacteriia.o__Sphingobacteriales.f__Sphingobacteri
alpha (n=25)
clade20C (n=1)
delta (n=13)
omicron (n=6)
neg (n=72)
NA
WHOvariant

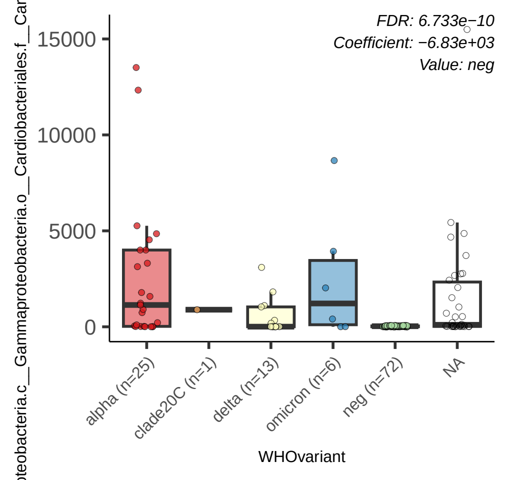
FDR: 6.733e−10
Coefficient: −6.83e+03
Value: neg
15000
10000
5000
0
alpha (n=25)
clade20C (n=1)
delta (n=13)
omicron (n=6)
neg (n=72)
NA
WHOvariant
oteobacteria.c__Gammaproteobacteria.o__Cardiobacteriales.f__Car

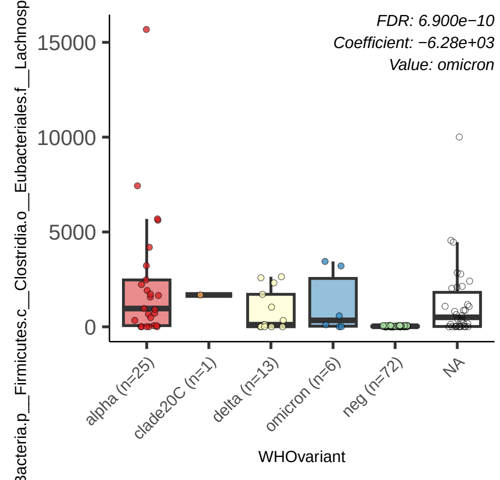

FDR: 6.900e−10
Coefficient: −6.28e+03
Value: omicron
15000
10000
5000
0
Bacteria.p__Firmicutes.c__Clostridia.o__Eubacteriales.f__Lachnosp
alpha (n=25)
clade20C (n=1)
delta (n=13)
omicron (n=6)
neg (n=72)
NA
WHOvariant

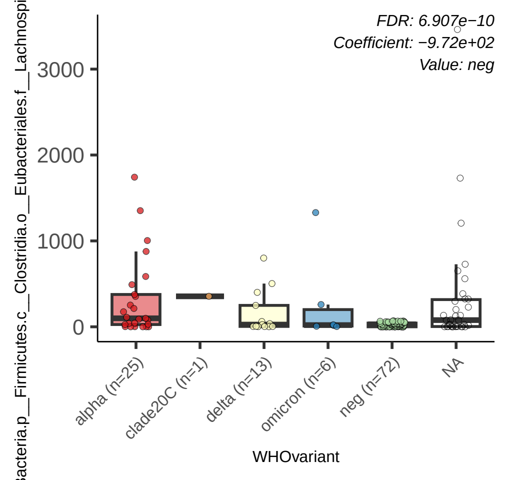

FDR: 6.907e−10
Coefficient: −9.72e+02
Value: neg
3000
2000
1000
0
Bacteria.p__Firmicutes.c__Clostridia.o__Eubacteriales.f__Lachnospi
alpha (n=25)
clade20C (n=1)
delta (n=13)
omicron (n=6)
neg (n=72)
NA
WHOvariant

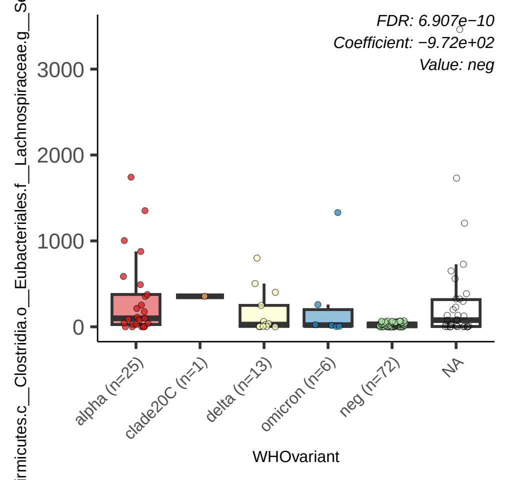

FDR: 6.907e−10
Coefficient: −9.72e+02
Value: neg
3000
2000
1000
0
alpha (n=25)
clade20C (n=1)
delta (n=13)
omicron (n=6)
neg (n=72)
NA
WHOvariant
irmicutes.c__Clostridia.o__Eubacteriales.f__Lachnospiraceae.g__Sc

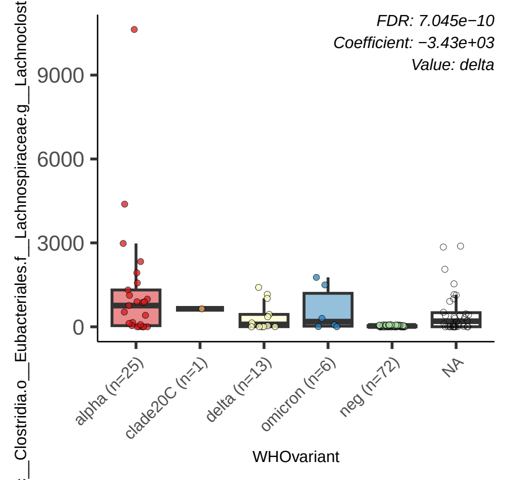

FDR: 7.045e−10
Coefficient: −3.43e+03
Value: delta
9000
6000
3000
0
Clostridia.o__Eubacteriales.f__Lachnospiraceae.g__Lachnoclost
alpha (n=25)
clade20C (n=1)
delta (n=13)
omicron (n=6)
neg (n=72)
NA
WHOvariant

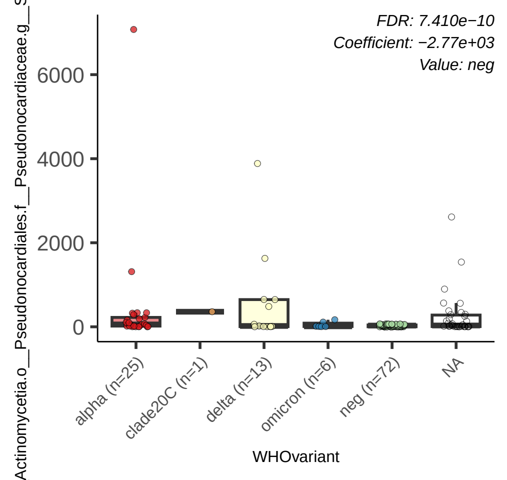
FDR: 7.410e−10
Coefficient: −2.77e+03
Value: neg
Actinomycetia.o__Pseudonocardiales.f__Pseudonocardiaceae.g__
0
2000
4000
6000
alpha (n=25)
clade20C (n=1)
delta (n=13)
omicron (n=6)
neg (n=72)
NA
WHOvariant

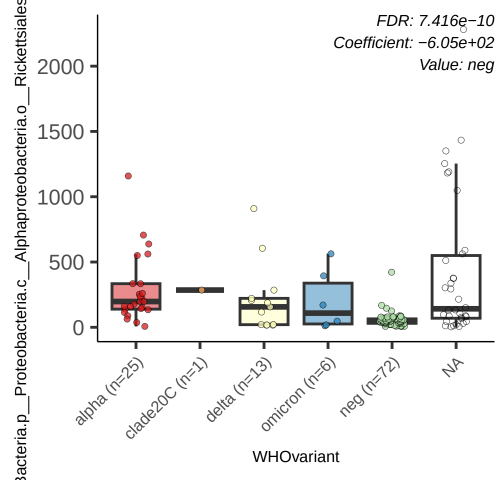
FDR: 7.416e−10
Coefficient: −6.05e+02
Value: neg
Bacteria.p__Proteobacteria.c__Alphaproteobacteria.o__Rickettsiales
2000
1500
1000
500
0
alpha (n=25)
clade20C (n=1)
delta (n=13)
omicron (n=6)
neg (n=72)
NA
WHOvariant

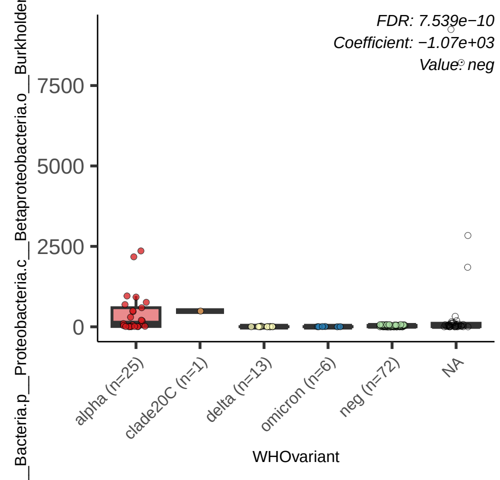

FDR: 7.539e−10
Coefficient: −1.07e+03
Value: neg
7500
5000
2500
0
_Bacteria.p__Proteobacteria.c__Betaproteobacteria.o__Burkholder
alpha (n=25)
clade20C (n=1)
delta (n=13)
omicron (n=6)
neg (n=72)
NA
WHOvariant

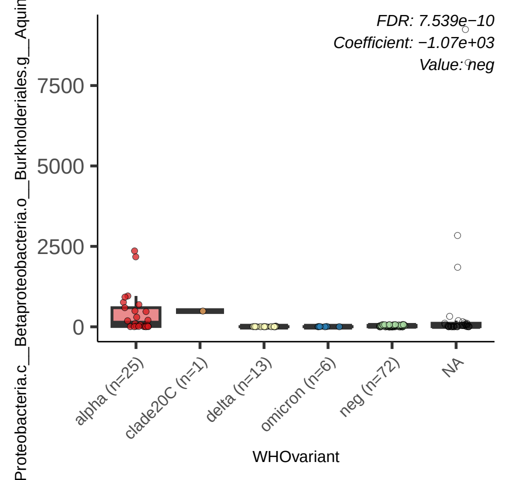

FDR: 7.539e−10
Coefficient: −1.07e+03
Value: neg
7500
5000
2500
0
alpha (n=25)
clade20C (n=1)
delta (n=13)
omicron (n=6)
neg (n=72)
NA
WHOvariant
Proteobacteria.c__Betaproteobacteria.o__Burkholderiales.g__Aqui

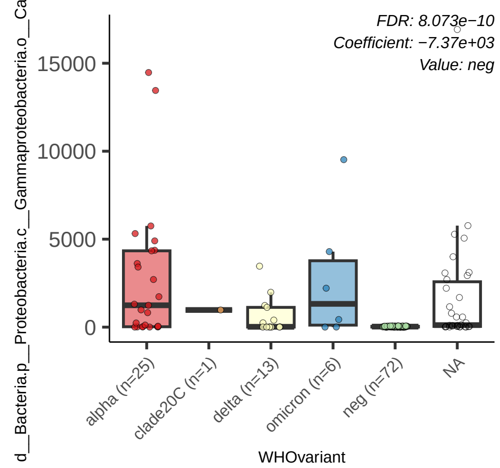
FDR: 8.073e−10
Coefficient: −7.37e+03
Value: neg
d__Bacteria.p__Proteobacteria.c__Gammaproteobacteria.o__Ca
15000
10000
5000
0
alpha (n=25)
clade20C (n=1)
delta (n=13)
omicron (n=6)
neg (n=72)
NA
WHOvariant

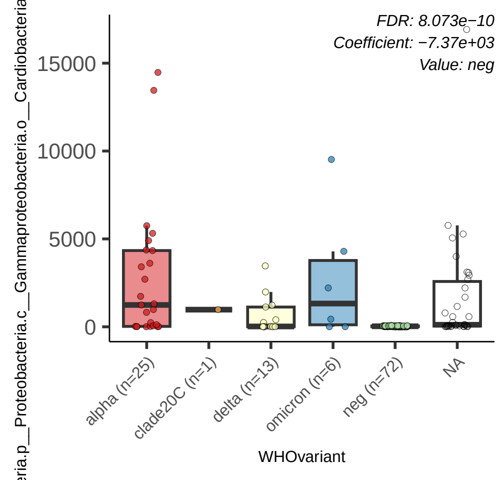

FDR: 8.073e−10
Coefficient: −7.37e+03
Value: neg
15000
10000
5000
0
eria.p__Proteobacteria.c__Gammaproteobacteria.o__Cardiobacteria
alpha (n=25)
clade20C (n=1)
delta (n=13)
omicron (n=6)
neg (n=72)
NA
WHOvariant

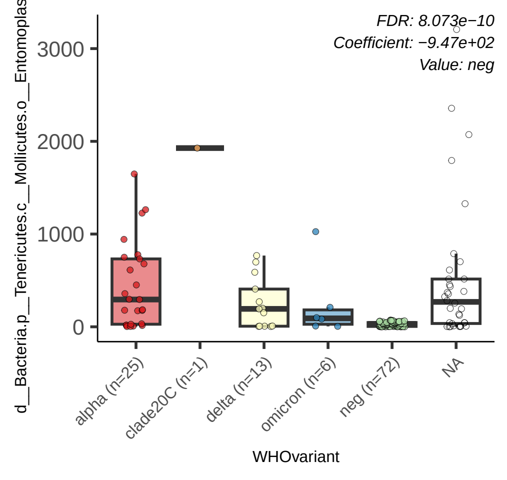
FDR: 8.073e−10
Coefficient: −9.47e+02
Value: neg
d__Bacteria.p__Tenericutes.c__Mollicutes.o__Entomoplas
3000
2000
1000
0
alpha (n=25)
clade20C (n=1)
delta (n=13)
omicron (n=6)
neg (n=72)
NA
WHOvariant

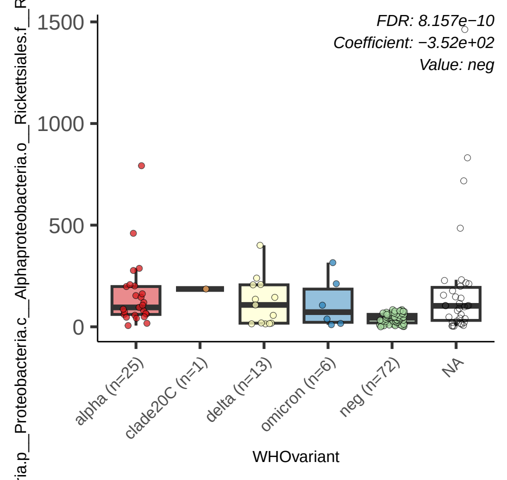
FDR: 8.157e−10
Coefficient: −3.52e+02
Value: neg
1500
1000
500
0
ria.p__Proteobacteria.c__Alphaproteobacteria.o__Rickettsiales.f__R
alpha (n=25)
clade20C (n=1)
delta (n=13)
omicron (n=6)
neg (n=72)
NA
WHOvariant

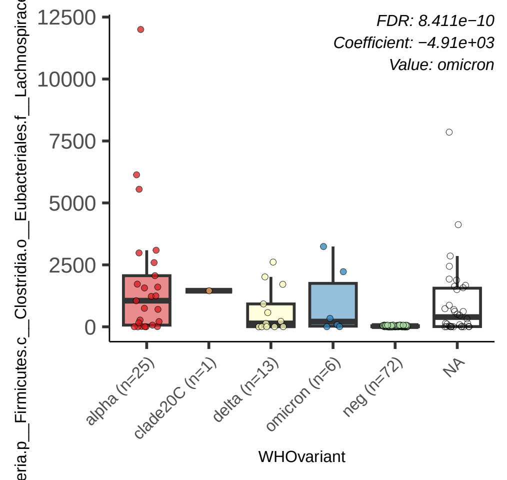
FDR: 8.411e−10
Coefficient: −4.91e+03
Value: omicron
12500
10000
7500
5000
2500
0
alpha (n=25)
clade20C (n=1)
delta (n=13)
omicron (n=6)
neg (n=72)
NA
WHOvariant

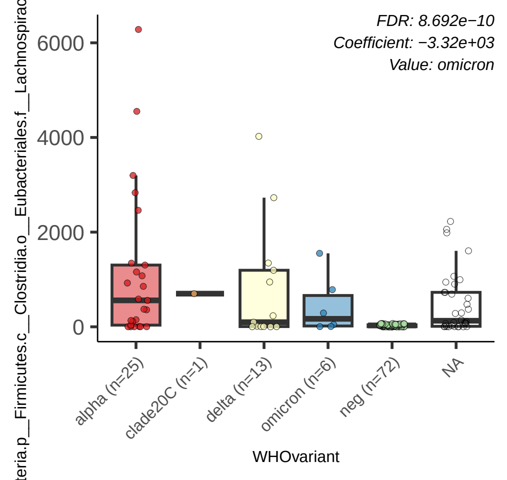

FDR: 8.692e−10
Coefficient: −3.32e+03
Value: omicron
teria.p__Firmicutes.c__Clostridia.o__Eubacteriales.f__Lachnospirac
6000
4000
2000
0
alpha (n=25)
clade20C (n=1)
delta (n=13)
omicron (n=6)
neg (n=72)
NA
WHOvariant

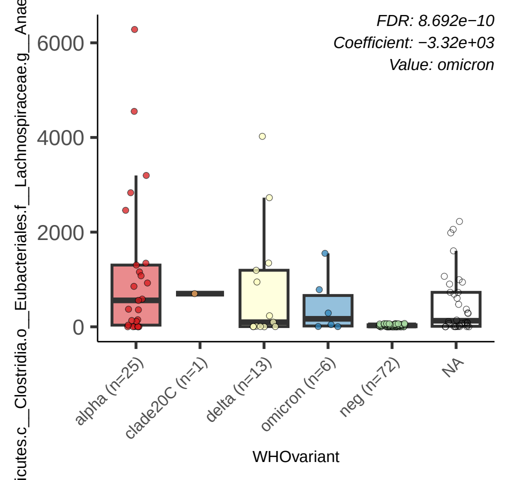

FDR: 8.692e−10
Coefficient: −3.32e+03
Value: omicron
6000
4000
2000
0
alpha (n=25)
clade20C (n=1)
delta (n=13)
omicron (n=6)
neg (n=72)
NA
WHOvariant
icutes.c__Clostridia.o__Eubacteriales.f__Lachnospiraceae.g__Anae

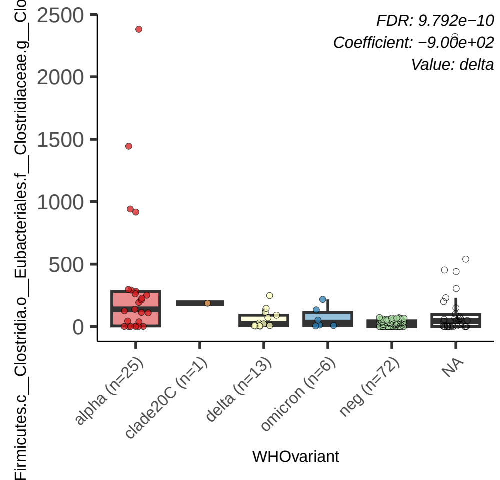

FDR: 9.792e−10
Coefficient: −9.00e+02
Value: delta
Firmicutes.c__Clostridia.o__Eubacteriales.f__Clostridiaceae.g__Clo
2500
2000
1500
1000
500
0
alpha (n=25)
clade20C (n=1)
delta (n=13)
omicron (n=6)
neg (n=72)
NA
WHOvariant

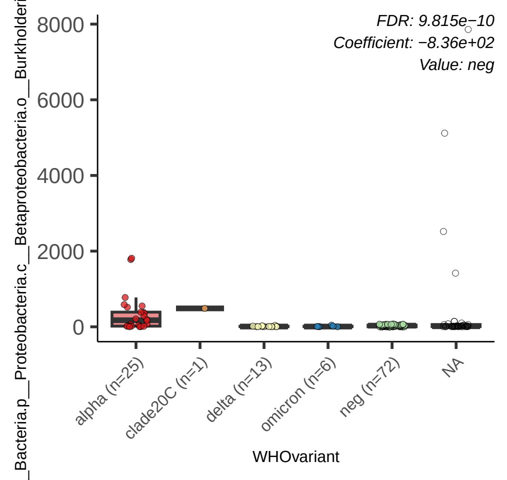
FDR: 9.815e−10
Coefficient: −8.36e+02
Value: neg
_Bacteria.p__Proteobacteria.c__Betaproteobacteria.o__Burkholderi
8000
6000
4000
2000
0
alpha (n=25)
clade20C (n=1)
delta (n=13)
omicron (n=6)
neg (n=72)
NA
WHOvariant

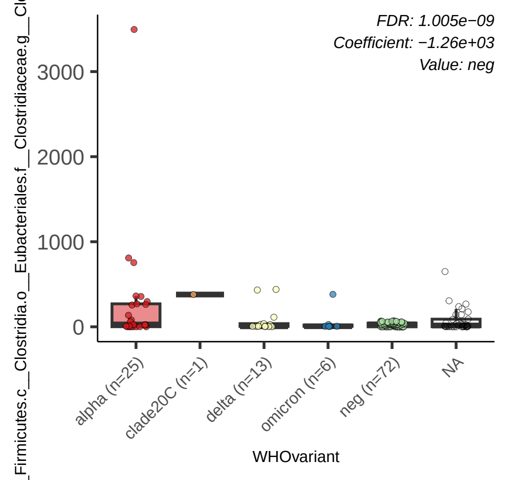
FDR: 1.005e−09
Coefficient: −1.26e+03
Value: neg
3000
2000
1000
0
Firmicutes.c__Clostridia.o__Eubacteriales.f__Clostridiaceae.g__Cl
alpha (n=25)
clade20C (n=1)
delta (n=13)
omicron (n=6)
neg (n=72)
NA
WHOvariant

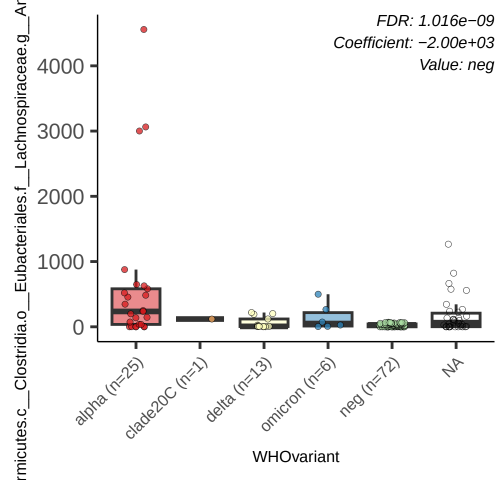
FDR: 1.016e−09
Coefficient: −2.00e+03
Value: neg
rmicutes.c__Clostridia.o__Eubacteriales.f__Lachnospiraceae.g__Ar
4000
3000
2000
1000
0
alpha (n=25)
clade20C (n=1)
delta (n=13)
omicron (n=6)
neg (n=72)
NA
WHOvariant

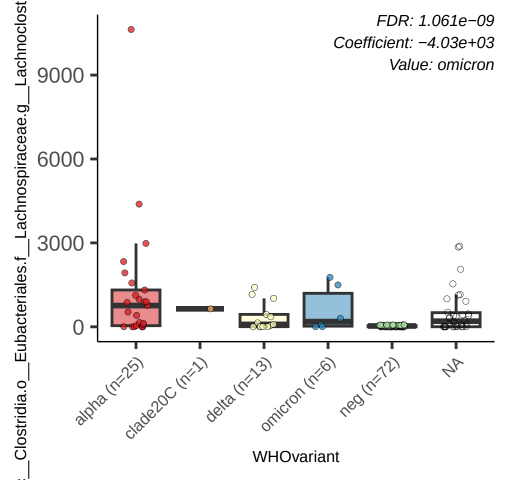

FDR: 1.061e−09
Coefficient: −4.03e+03
Value: omicron
9000
6000
3000
0
Clostridia.o__Eubacteriales.f__Lachnospiraceae.g__Lachnoclost
alpha (n=25)
clade20C (n=1)
delta (n=13)
omicron (n=6)
neg (n=72)
NA
WHOvariant

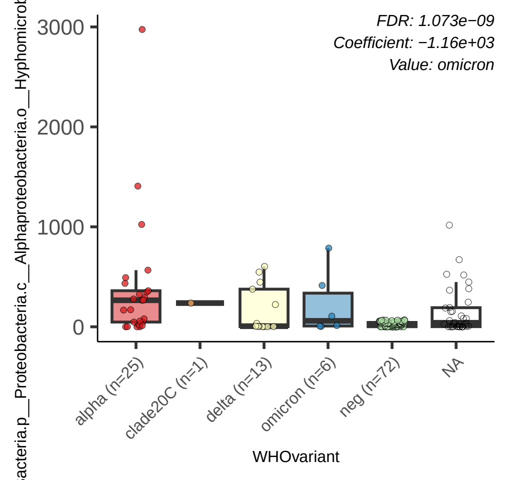

FDR: 1.073e−09
Coefficient: −1.16e+03
Value: omicron
3000
2000
1000
0
Bacteria.p__Proteobacteria.c__Alphaproteobacteria.o__Hyphomicrob
alpha (n=25)
clade20C (n=1)
delta (n=13)
omicron (n=6)
neg (n=72)
NA
WHOvariant

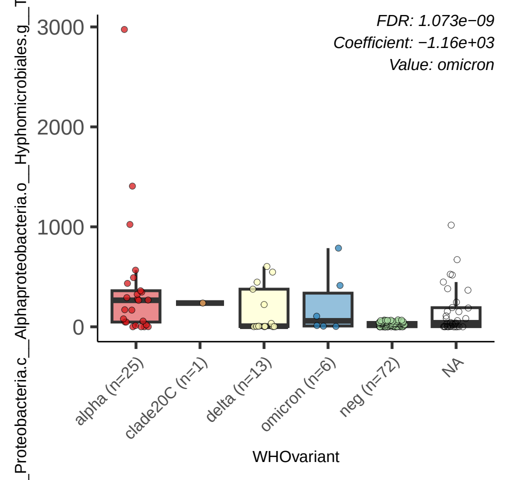

FDR: 1.073e−09
Coefficient: −1.16e+03
Value: omicron
_Proteobacteria.c__Alphaproteobacteria.o__Hyphomicrobiales.g__T
3000
2000
1000
0
alpha (n=25)
clade20C (n=1)
delta (n=13)
omicron (n=6)
neg (n=72)
NA
WHOvariant

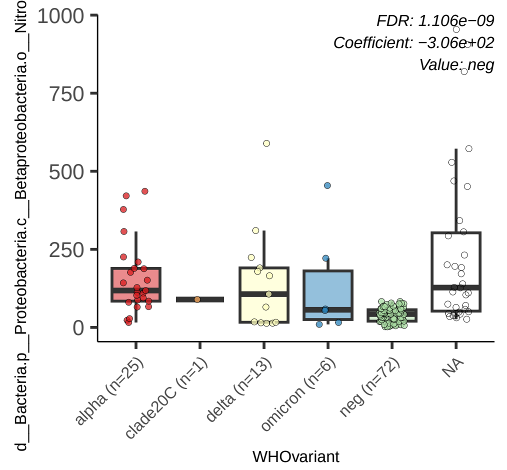

FDR: 1.106e−09
Coefficient: −3.06e+02
Value: neg
d__Bacteria.p__Proteobacteria.c__Betaproteobacteria.o__Nitro
1000
750
500
250
0
alpha (n=25)
clade20C (n=1)
delta (n=13)
omicron (n=6)
neg (n=72)
NA
WHOvariant

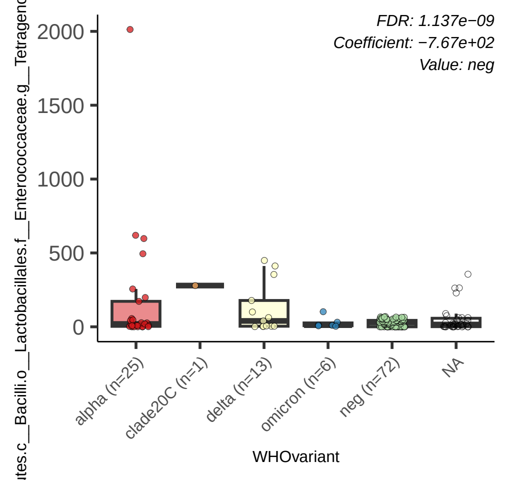

FDR: 1.137e−09
Coefficient: −7.67e+02
Value: neg
utes.c__Bacilli.o__Lactobacillales.f__Enterococcaceae.g__Tetragenc
2000
1500
1000
500
0
alpha (n=25)
clade20C (n=1)
delta (n=13)
omicron (n=6)
neg (n=72)
NA
WHOvariant

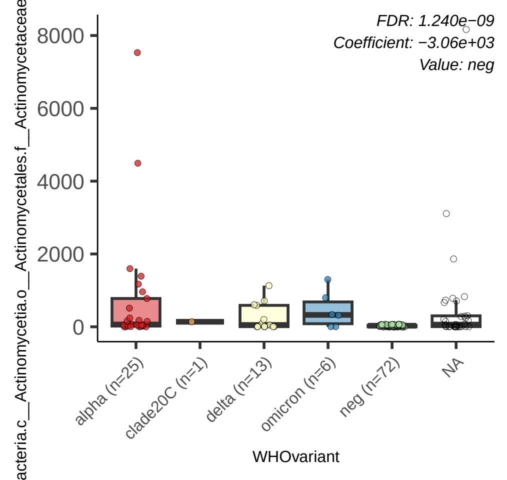

FDR: 1.240e−09
Coefficient: −3.06e+03
Value: neg
acteria.c__Actinomycetia.o__Actinomycetales.f__Actinomycetaceae
8000
6000
4000
2000
0
alpha (n=25)
clade20C (n=1)
delta (n=13)
omicron (n=6)
neg (n=72)
NA
WHOvariant

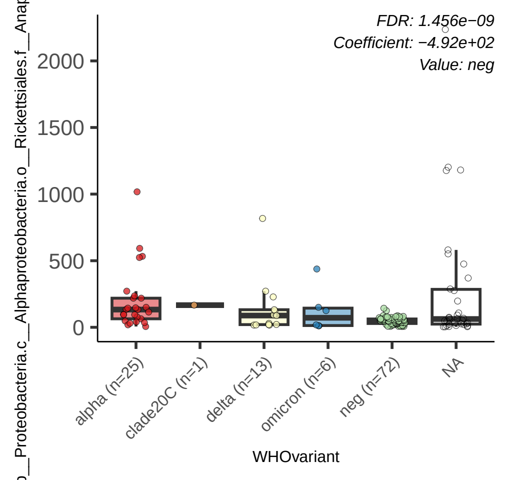

FDR: 1.456e−09
Coefficient: −4.92e+02
Value: neg
0
500
1000
1500
2000
o__Proteobacteria.c__Alphaproteobacteria.o__Rickettsiales.f__Anap
alpha (n=25)
clade20C (n=1)
delta (n=13)
omicron (n=6)
neg (n=72)
NA
WHOvariant

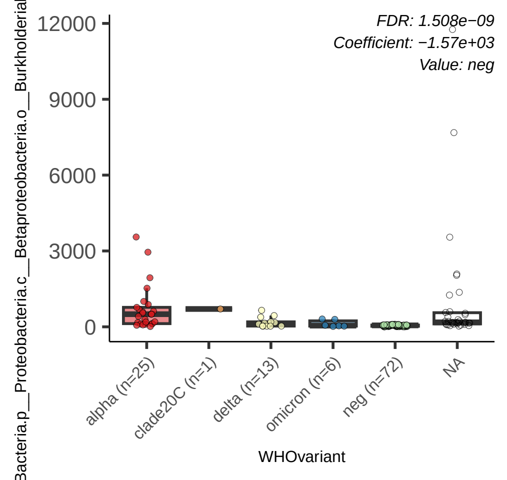

FDR: 1.508e−09
Coefficient: −1.57e+03
Value: neg
Bacteria.p__Proteobacteria.c__Betaproteobacteria.o__Burkholderial
12000
9000
6000
3000
0
alpha (n=25)
clade20C (n=1)
delta (n=13)
omicron (n=6)
neg (n=72)
NA
WHOvariant

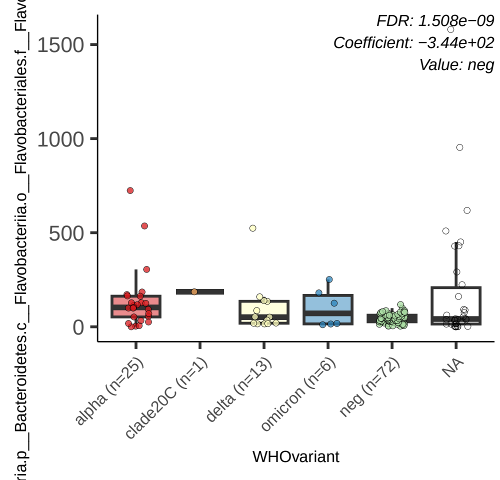

FDR: 1.508e−09
Coefficient: −3.44e+02
Value: neg
1500
1000
500
0
ria.p__Bacteroidetes.c__Flavobacteriia.o__Flavobacteriales.f__Flavo
alpha (n=25)
clade20C (n=1)
delta (n=13)
omicron (n=6)
neg (n=72)
NA
WHOvariant

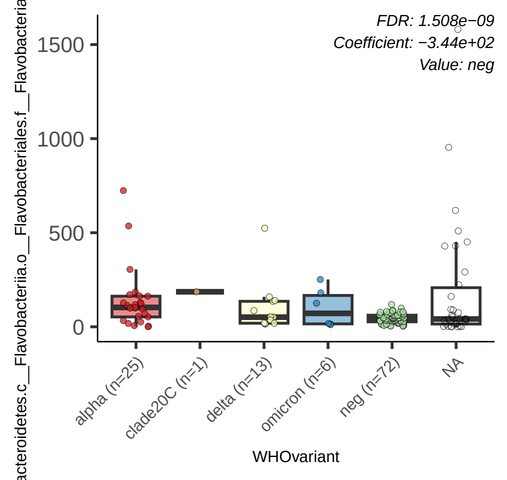
FDR: 1.508e−09
Coefficient: −3.44e+02
Value: neg
1500
1000
500
0
acteroidetes.c__Flavobacteriia.o__Flavobacteriales.f__Flavobacteria
alpha (n=25)
clade20C (n=1)
delta (n=13)
omicron (n=6)
neg (n=72)
NA
WHOvariant

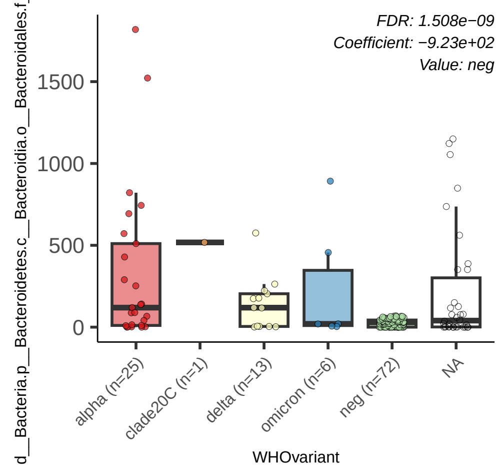

FDR: 1.508e−09
Coefficient: −9.23e+02
Value: neg
d__Bacteria.p__Bacteroidetes.c__Bacteroidia.o__Bacteroidales.f__
0
500
1000
1500
alpha (n=25)
clade20C (n=1)
delta (n=13)
omicron (n=6)
neg (n=72)
NA
WHOvariant

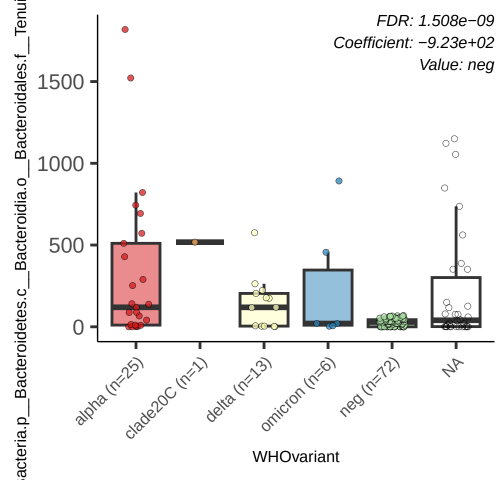
FDR: 1.508e−09
Coefficient: −9.23e+02
Value: neg
1500
1000
500
0
alpha (n=25)
clade20C (n=1)
delta (n=13)
omicron (n=6)
neg (n=72)
NA
WHOvariant

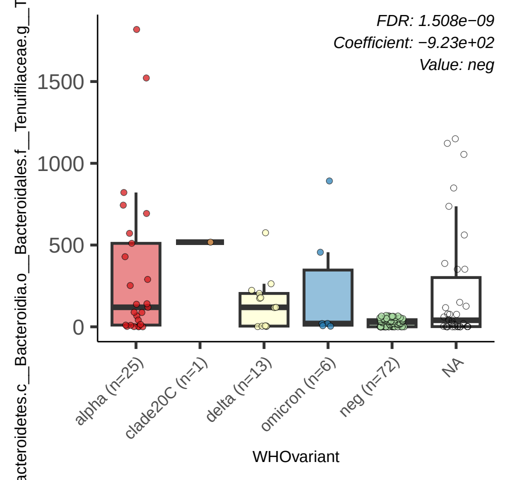

FDR: 1.508e−09
Coefficient: −9.23e+02
Value: neg
acteroidetes.c__Bacteroidia.o__Bacteroidales.f__Tenuifilaceae.g__T
1500
1000
500
0
alpha (n=25)
clade20C (n=1)
delta (n=13)
omicron (n=6)
neg (n=72)
NA
WHOvariant

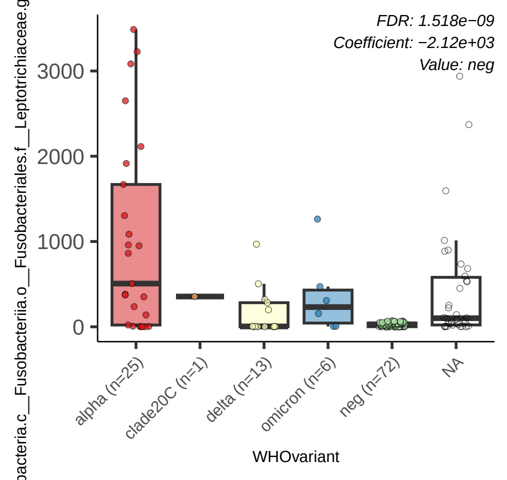

FDR: 1.518e−09
Coefficient: −2.12e+03
Value: neg
bacteria.c__Fusobacteriia.o__Fusobacteriales.f__Leptotrichiaceae.g
3000
2000
1000
0
alpha (n=25)
clade20C (n=1)
delta (n=13)
omicron (n=6)
neg (n=72)
NA
WHOvariant

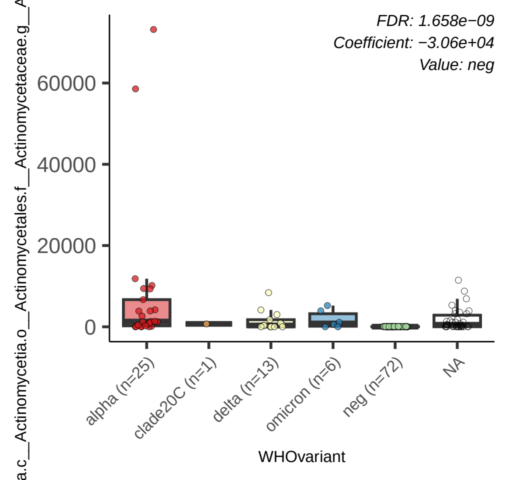
FDR: 1.658e−09
Coefficient: −3.06e+04
Value: neg
60000
40000
20000
0
alpha (n=25)
clade20C (n=1)
delta (n=13)
omicron (n=6)
neg (n=72)
NA
WHOvariant
a.c__Actinomycetia.o__Actinomycetales.f__Actinomycetaceae.g__A

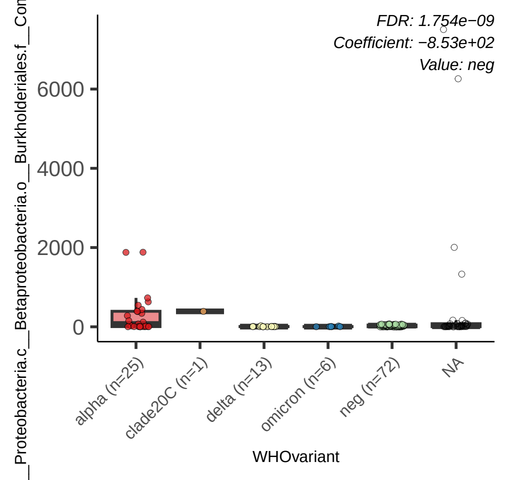

FDR: 1.754e−09
Coefficient: −8.53e+02
Value: neg
Proteobacteria.c__Betaproteobacteria.o__Burkholderiales.f__Com
6000
4000
2000
0
alpha (n=25)
clade20C (n=1)
delta (n=13)
omicron (n=6)
neg (n=72)
NA
WHOvariant

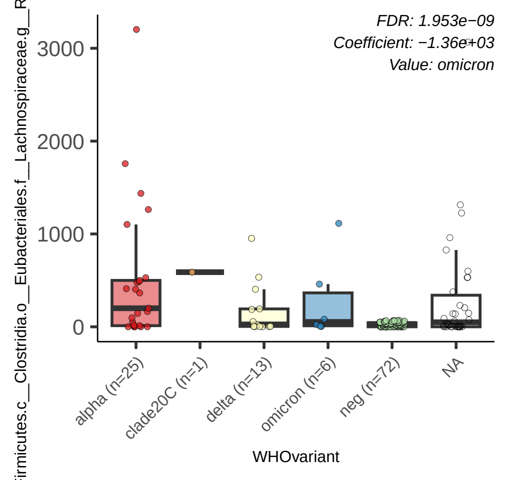

FDR: 1.953e−09
Coefficient: −1.36e+03
Value: omicron
3000
2000
1000
0
alpha (n=25)
clade20C (n=1)
delta (n=13)
omicron (n=6)
neg (n=72)
NA
WHOvariant
irmicutes.c__Clostridia.o__Eubacteriales.f__Lachnospiraceae.g__R

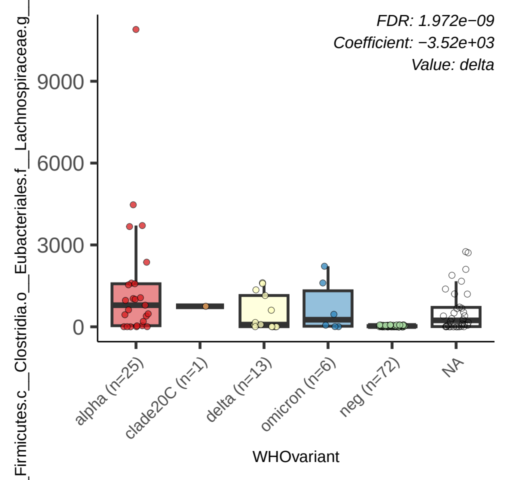

FDR: 1.972e−09
Coefficient: −3.52e+03
Value: delta
Firmicutes.c__Clostridia.o__Eubacteriales.f__Lachnospiraceae.g__
0
3000
6000
9000
alpha (n=25)
clade20C (n=1)
delta (n=13)
omicron (n=6)
neg (n=72)
NA
WHOvariant

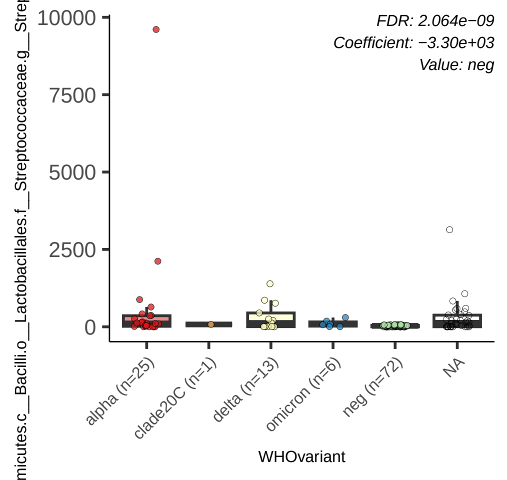

FDR: 2.064e−09
Coefficient: −3.30e+03
Value: neg
10000
7500
5000
2500
0
micutes.c__Bacilli.o__Lactobacillales.f__Streptococcaceae.g__Strep
alpha (n=25)
clade20C (n=1)
delta (n=13)
omicron (n=6)
neg (n=72)
NA
WHOvariant

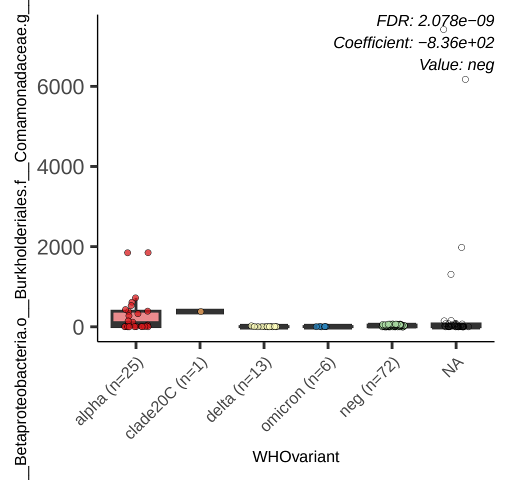

FDR: 2.078e−09
Coefficient: −8.36e+02
Value: neg
Betaproteobacteria.o__Burkholderiales.f__Comamonadaceae.g__
0
2000
4000
6000
alpha (n=25)
clade20C (n=1)
delta (n=13)
omicron (n=6)
neg (n=72)
NA
WHOvariant

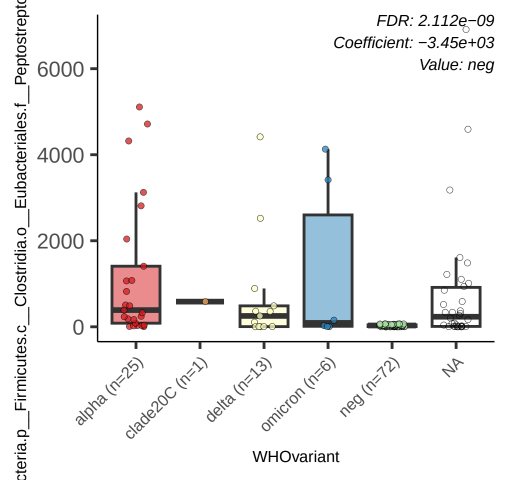

FDR: 2.112e−09
Coefficient: −3.45e+03
Value: neg
cteria.p__Firmicutes.c__Clostridia.o__Eubacteriales.f__Peptostrepto
0
2000
4000
6000
alpha (n=25)
clade20C (n=1)
delta (n=13)
omicron (n=6)
neg (n=72)
NA
WHOvariant

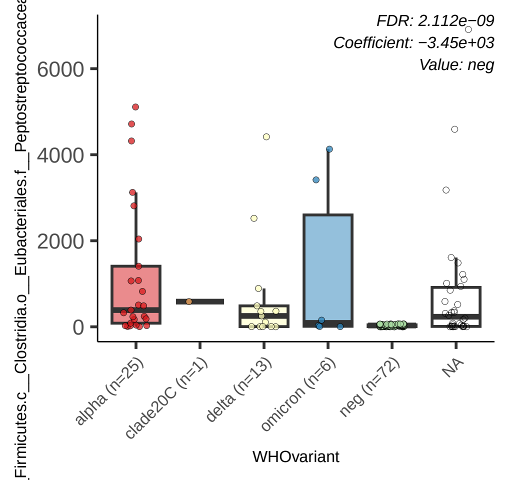
FDR: 2.112e−09
Coefficient: −3.45e+03
Value: neg
Firmicutes.c__Clostridia.o__Eubacteriales.f__Peptostreptococcaceae
0
2000
4000
6000
alpha (n=25)
clade20C (n=1)
delta (n=13)
omicron (n=6)
neg (n=72)
NA
WHOvariant

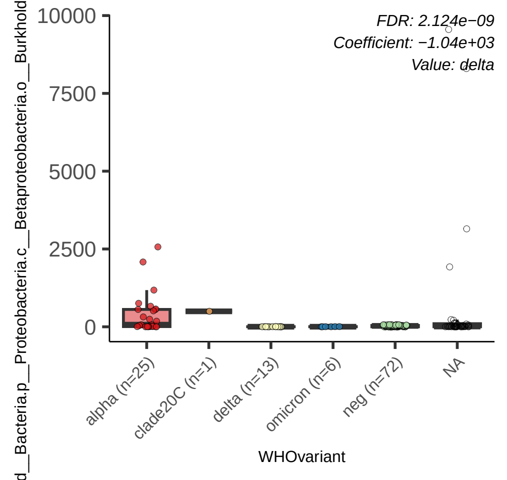
FDR: 2.124e−09
Coefficient: −1.04e+03
Value: delta
10000
7500
5000
2500
0
d__Bacteria.p__Proteobacteria.c__Betaproteobacteria.o__Burkhold
alpha (n=25)
clade20C (n=1)
delta (n=13)
omicron (n=6)
neg (n=72)
NA
WHOvariant

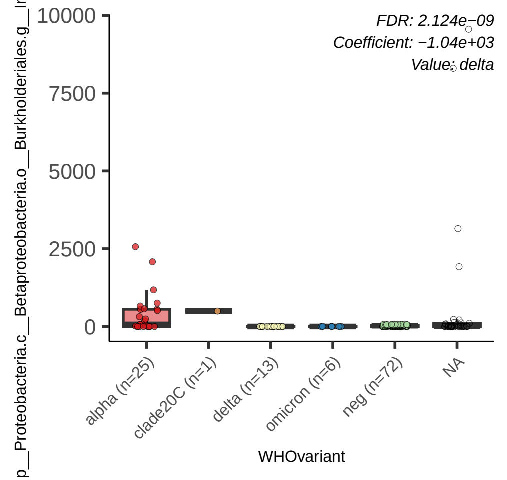

FDR: 2.124e−09
Coefficient: −1.04e+03
Value: delta
10000
7500
5000
2500
0
p__Proteobacteria.c__Betaproteobacteria.o__Burkholderiales.g__In
alpha (n=25)
clade20C (n=1)
delta (n=13)
omicron (n=6)
neg (n=72)
NA
WHOvariant

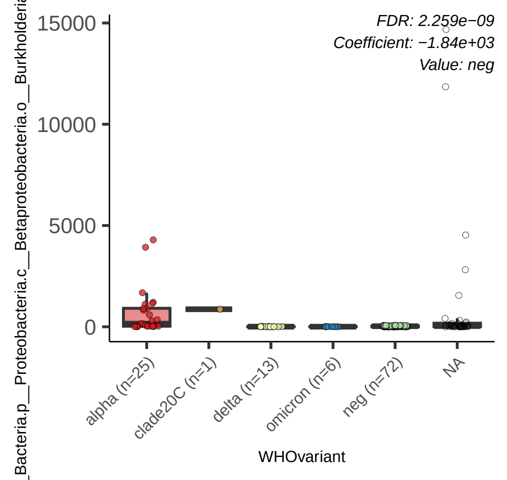

FDR: 2.259e−09
Coefficient: −1.84e+03
Value: neg
15000
10000
5000
0
Bacteria.p__Proteobacteria.c__Betaproteobacteria.o__Burkholderia
alpha (n=25)
clade20C (n=1)
delta (n=13)
omicron (n=6)
neg (n=72)
NA
WHOvariant

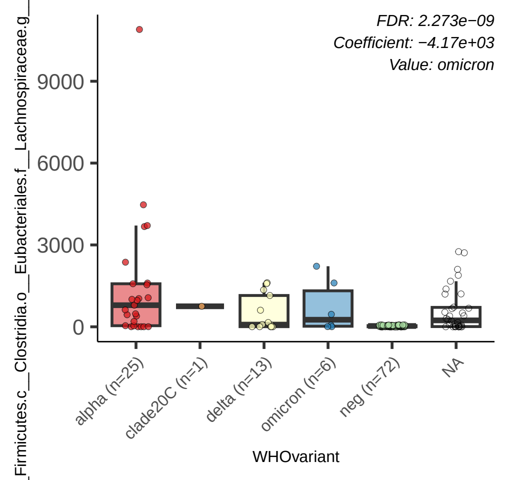

FDR: 2.273e−09
Coefficient: −4.17e+03
Value: omicron
Firmicutes.c__Clostridia.o__Eubacteriales.f__Lachnospiraceae.g__
9000
6000
3000
0
alpha (n=25)
clade20C (n=1)
delta (n=13)
omicron (n=6)
neg (n=72)
NA
WHOvariant

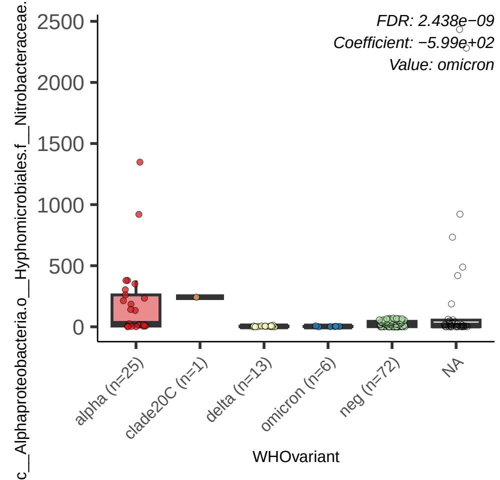

FDR: 2.438e−09
Coefficient: −5.99e+02
Value: omicron
c__Alphaproteobacteria.o__Hyphomicrobiales.f__Nitrobacteraceae.
2500
2000
1500
1000
500
0
alpha (n=25)
clade20C (n=1)
delta (n=13)
omicron (n=6)
neg (n=72)
NA
WHOvariant

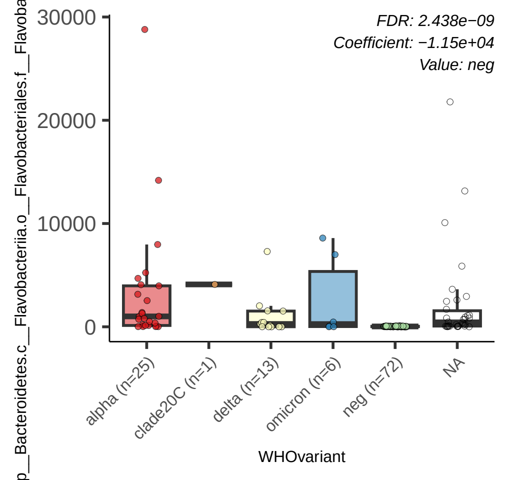

FDR: 2.438e−09
Coefficient: −1.15e+04
Value: neg
30000
20000
10000
0
p__Bacteroidetes.c__Flavobacteriia.o__Flavobacteriales.f__Flavoba
alpha (n=25)
clade20C (n=1)
delta (n=13)
omicron (n=6)
neg (n=72)
NA
WHOvariant

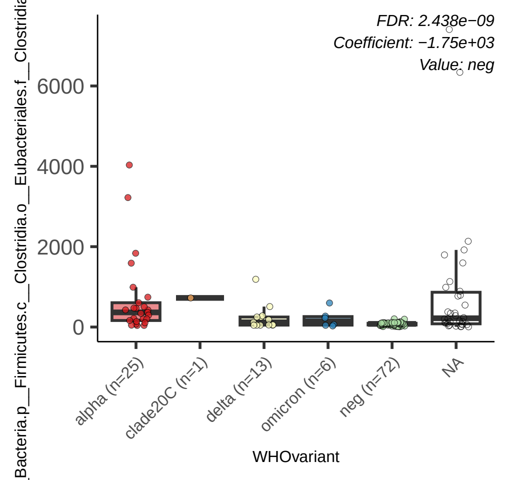
FDR: 2.438e−09
Coefficient: −1.75e+03
Value: neg
Bacteria.p__Firmicutes.c__Clostridia.o__Eubacteriales.f__Clostridia
6000
4000
2000
0
alpha (n=25)
clade20C (n=1)
delta (n=13)
omicron (n=6)
neg (n=72)
NA
WHOvariant

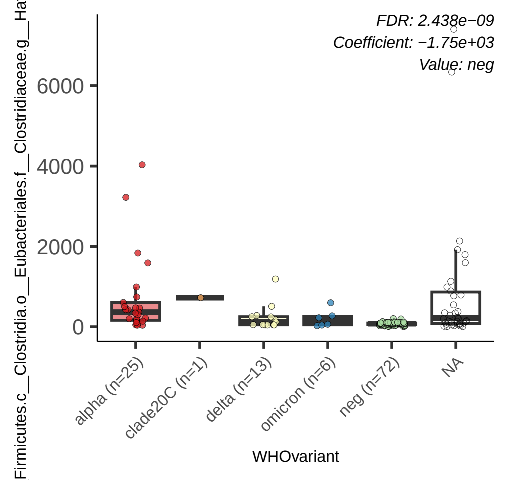
FDR: 2.438e−09
Coefficient: −1.75e+03
Value: neg
6000
4000
2000
0
alpha (n=25)
clade20C (n=1)
delta (n=13)
omicron (n=6)
neg (n=72)
NA
WHOvariant
Firmicutes.c__Clostridia.o__Eubacteriales.f__Clostridiaceae.g__Ha

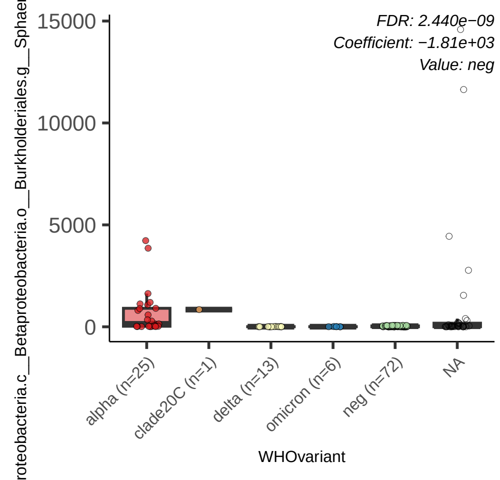

FDR: 2.440e−09
Coefficient: −1.81e+03
Value: neg
15000
10000
5000
0
alpha (n=25)
clade20C (n=1)
delta (n=13)
omicron (n=6)
neg (n=72)
NA
WHOvariant

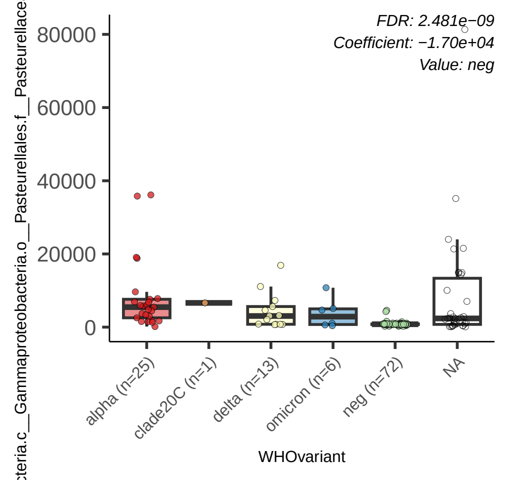
FDR: 2.481e−09
Coefficient: −1.70e+04
Value: neg
80000
60000
40000
20000
0
cteria.c__Gammaproteobacteria.o__Pasteurellales.f__Pasteurellace
alpha (n=25)
clade20C (n=1)
delta (n=13)
omicron (n=6)
neg (n=72)
NA
WHOvariant

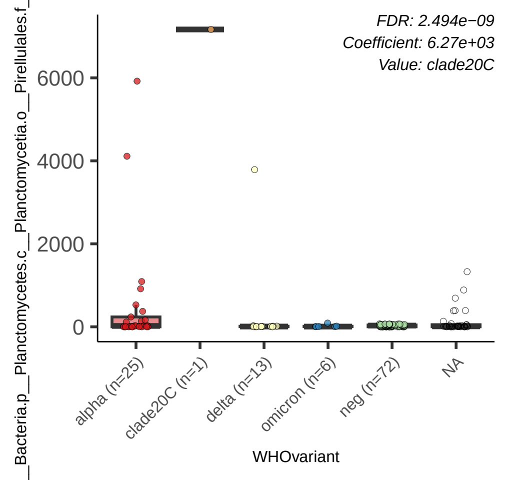

FDR: 2.494e−09
Coefficient: 6.27e+03
Value: clade20C
Bacteria.p__Planctomycetes.c__Planctomycetia.o__Pirellulales.f__
0
2000
4000
6000
alpha (n=25)
clade20C (n=1)
delta (n=13)
omicron (n=6)
neg (n=72)
NA
WHOvariant

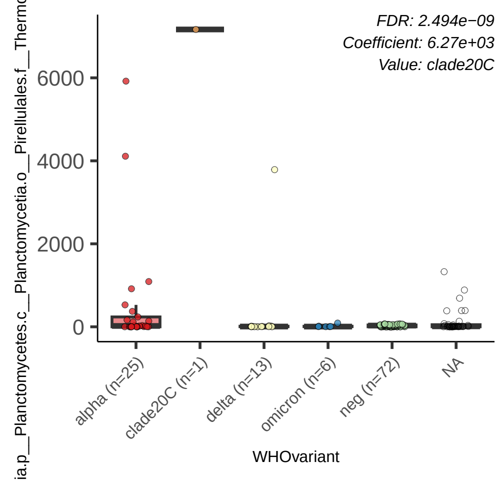
FDR: 2.494e−09
Coefficient: 6.27e+03
Value: clade20C
6000
4000
2000
0
alpha (n=25)
clade20C (n=1)
delta (n=13)
omicron (n=6)
neg (n=72)
NA
WHOvariant
ia.p__Planctomycetes.c__Planctomycetia.o__Pirellulales.f__Thermo

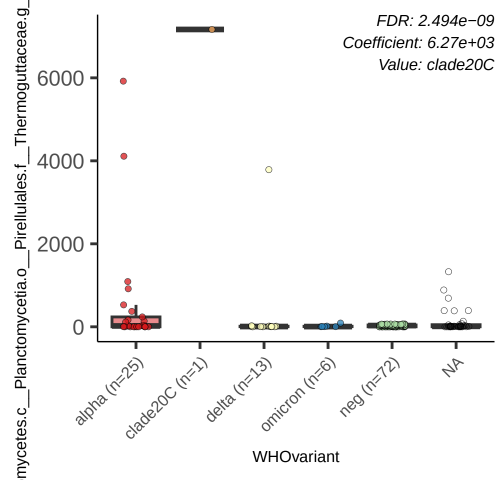
FDR: 2.494e−09
Coefficient: 6.27e+03
Value: clade20C
mycetes.c__Planctomycetia.o__Pirellulales.f__Thermoguttaceae.g_
6000
4000
2000
0
alpha (n=25)
clade20C (n=1)
delta (n=13)
omicron (n=6)
neg (n=72)
NA
WHOvariant

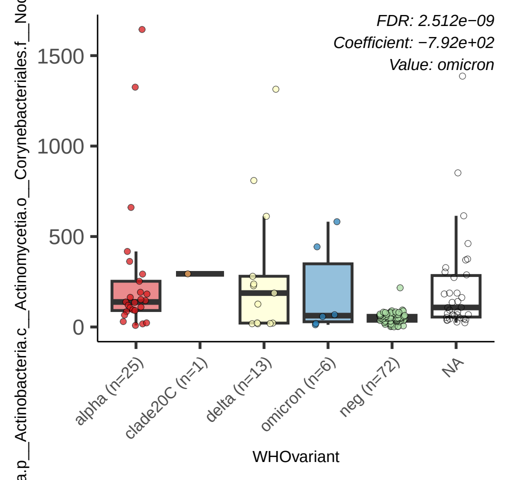
FDR: 2.512e−09
Coefficient: −7.92e+02
Value: omicron
0
500
1000
1500
a.p__Actinobacteria.c__Actinomycetia.o__Corynebacteriales.f__Noc
alpha (n=25)
clade20C (n=1)
delta (n=13)
omicron (n=6)
neg (n=72)
NA
WHOvariant

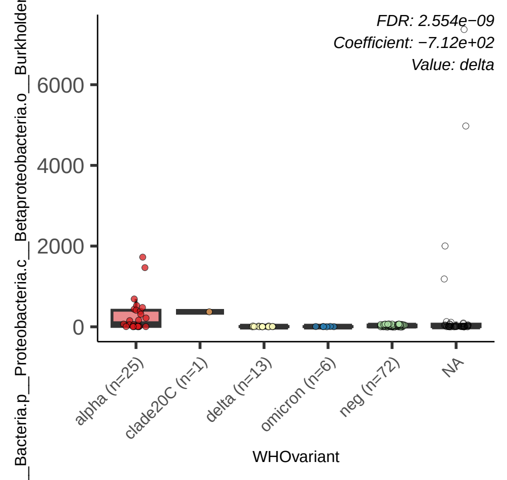
FDR: 2.554e−09
Coefficient: −7.12e+02
Value: delta
Bacteria.p__Proteobacteria.c__Betaproteobacteria.o__Burkholder
6000
4000
2000
0
alpha (n=25)
clade20C (n=1)
delta (n=13)
omicron (n=6)
neg (n=72)
NA
WHOvariant

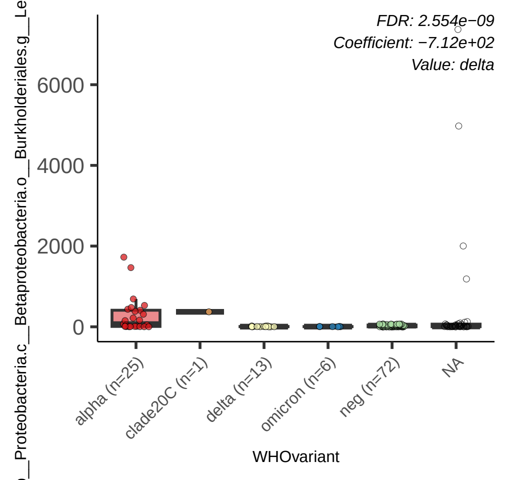

FDR: 2.554e−09
Coefficient: −7.12e+02
Value: delta
6000
4000
2000
0
o__Proteobacteria.c__Betaproteobacteria.o__Burkholderiales.g__Le
alpha (n=25)
clade20C (n=1)
delta (n=13)
omicron (n=6)
neg (n=72)
NA
WHOvariant

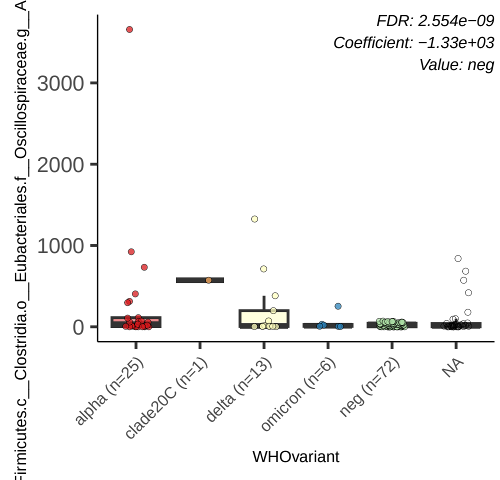

FDR: 2.554e−09
Coefficient: −1.33e+03
Value: neg
Firmicutes.c__Clostridia.o__Eubacteriales.f__Oscillospiraceae.g__A
3000
2000
1000
0
alpha (n=25)
clade20C (n=1)
delta (n=13)
omicron (n=6)
neg (n=72)
NA
WHOvariant

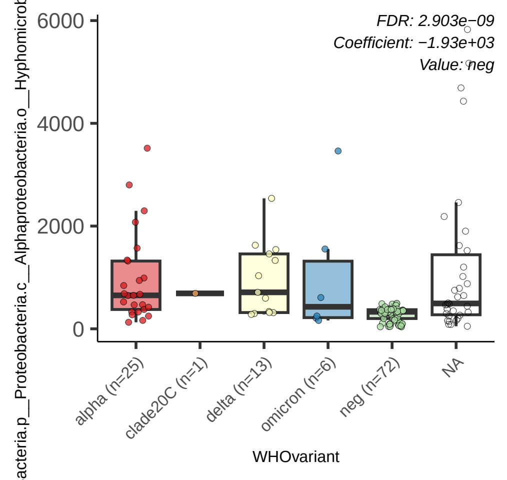

FDR: 2.903e−09
Coefficient: −1.93e+03
Value: neg
6000
4000
2000
0
acteria.p__Proteobacteria.c__Alphaproteobacteria.o__Hyphomicrob
alpha (n=25)
clade20C (n=1)
delta (n=13)
omicron (n=6)
neg (n=72)
NA
WHOvariant

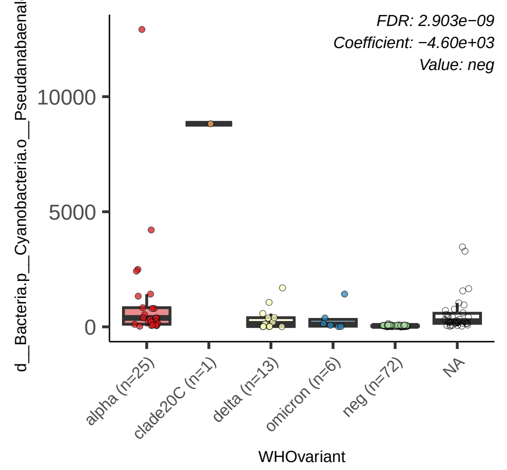

FDR: 2.903e−09
Coefficient: −4.60e+03
Value: neg
d__Bacteria.p__Cyanobacteria.o__Pseudanabaenal
10000
5000
0
alpha (n=25)
clade20C (n=1)
delta (n=13)
omicron (n=6)
neg (n=72)
NA
WHOvariant

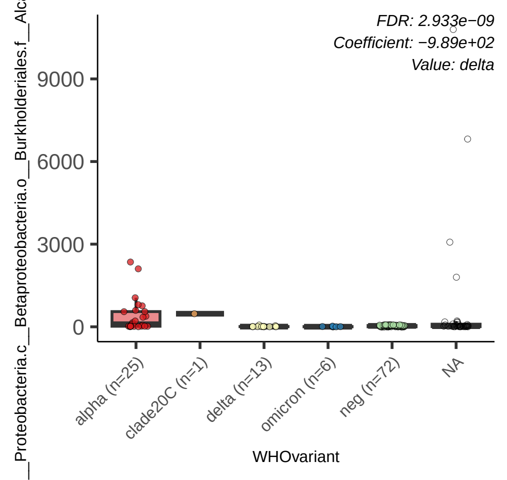

FDR: 2.933e−09
Coefficient: −9.89e+02
Value: delta
9000
6000
3000
0
_Proteobacteria.c__Betaproteobacteria.o__Burkholderiales.f__Alca
alpha (n=25)
clade20C (n=1)
delta (n=13)
omicron (n=6)
neg (n=72)
NA
WHOvariant

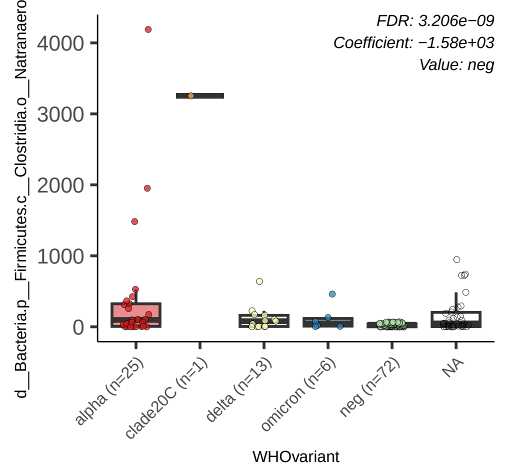

FDR: 3.206e−09
Coefficient: −1.58e+03
Value: neg
d__Bacteria.p__Firmicutes.c__Clostridia.o__Natranaero
0
1000
2000
3000
4000
alpha (n=25)
clade20C (n=1)
delta (n=13)
omicron (n=6)
neg (n=72)
NA
WHOvariant

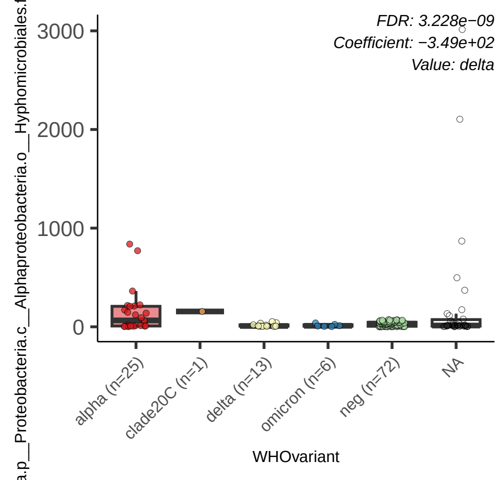

FDR: 3.228e−09
Coefficient: −3.49e+02
Value: delta
3000
2000
1000
0
a.p__Proteobacteria.c__Alphaproteobacteria.o__Hyphomicrobiales.f
alpha (n=25)
clade20C (n=1)
delta (n=13)
omicron (n=6)
neg (n=72)
NA
WHOvariant

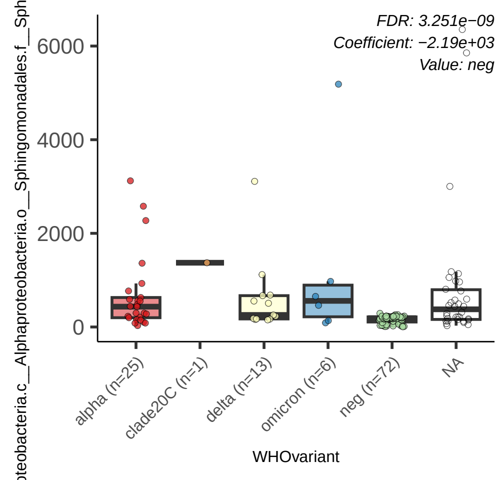
FDR: 3.251e−09
Coefficient: −2.19e+03
Value: neg
6000
4000
2000
0
alpha (n=25)
clade20C (n=1)
delta (n=13)
omicron (n=6)
neg (n=72)
NA
WHOvariant

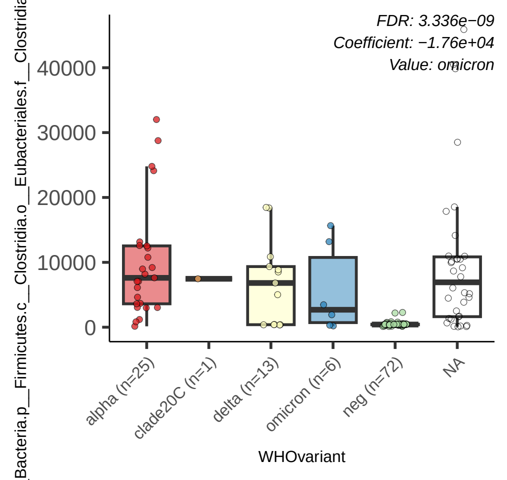

FDR: 3.336e−09
Coefficient: −1.76e+04
Value: omicron
Bacteria.p__Firmicutes.c__Clostridia.o__Eubacteriales.f__Clostridia
40000
30000
20000
10000
0
alpha (n=25)
clade20C (n=1)
delta (n=13)
omicron (n=6)
neg (n=72)
NA
WHOvariant

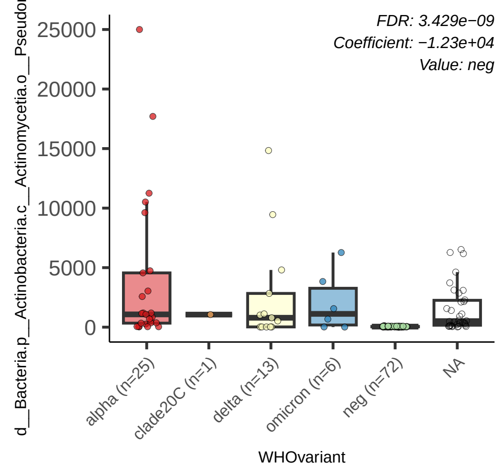

FDR: 3.429e−09
Coefficient: −1.23e+04
Value: neg
d__Bacteria.p__Actinobacteria.c__Actinomycetia.o__Pseudo
25000
20000
15000
10000
5000
0
alpha (n=25)
clade20C (n=1)
delta (n=13)
omicron (n=6)
neg (n=72)
NA
WHOvariant

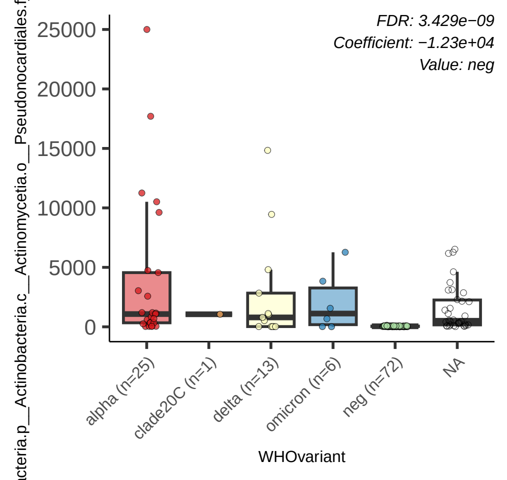

FDR: 3.429e−09
Coefficient: −1.23e+04
Value: neg
25000
20000
15000
10000
5000
0
acteria.p__Actinobacteria.c__Actinomycetia.o__Pseudonocardiales.f
alpha (n=25)
clade20C (n=1)
delta (n=13)
omicron (n=6)
neg (n=72)
NA
WHOvariant

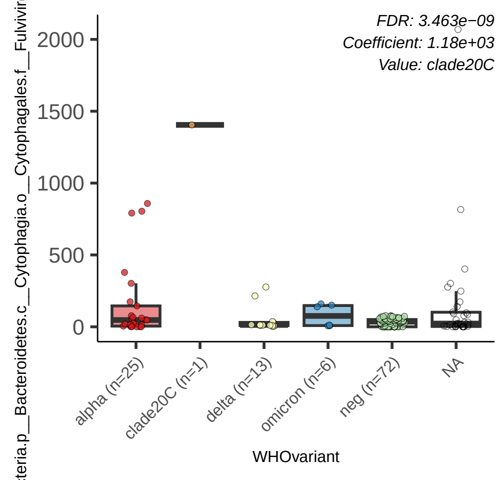

FDR: 3.463e−09
Coefficient: 1.18e+03
Value: clade20C
2000
1500
1000
500
0
alpha (n=25)
clade20C (n=1)
delta (n=13)
omicron (n=6)
neg (n=72)
NA
WHOvariant

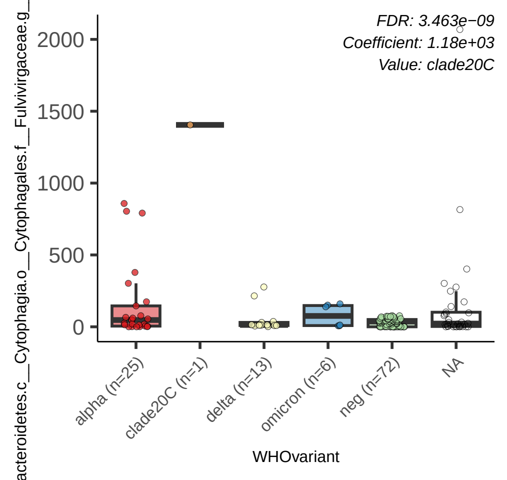
FDR: 3.463e−09
Coefficient: 1.18e+03
Value: clade20C
Bacteroidetes.c__Cytophagia.o__Cytophagales.f__Fulvivirgaceae.g__
2000
1500
1000
500
0
alpha (n=25)
clade20C (n=1)
delta (n=13)
omicron (n=6)
neg (n=72)
NA
WHOvariant

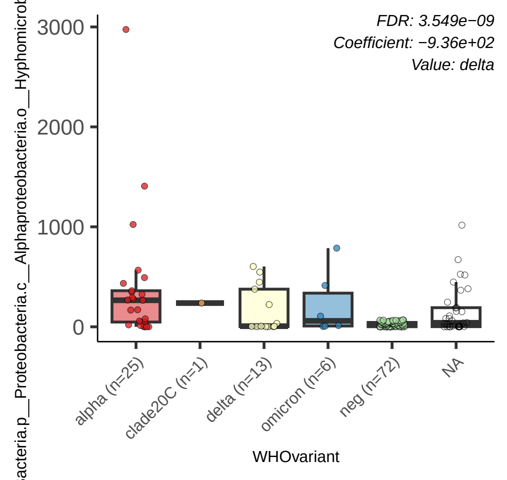
FDR: 3.549e−09
Coefficient: −9.36e+02
Value: delta
3000
2000
1000
0
Bacteria.p__Proteobacteria.c__Alphaproteobacteria.o__Hyphomicrob
alpha (n=25)
clade20C (n=1)
delta (n=13)
omicron (n=6)
neg (n=72)
NA
WHOvariant

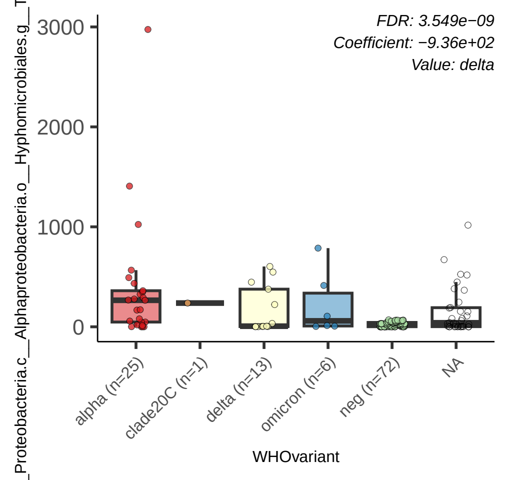

FDR: 3.549e−09
Coefficient: −9.36e+02
Value: delta
3000
2000
1000
0
_Proteobacteria.c__Alphaproteobacteria.o__Hyphomicrobiales.g__T
alpha (n=25)
clade20C (n=1)
delta (n=13)
omicron (n=6)
neg (n=72)
NA
WHOvariant

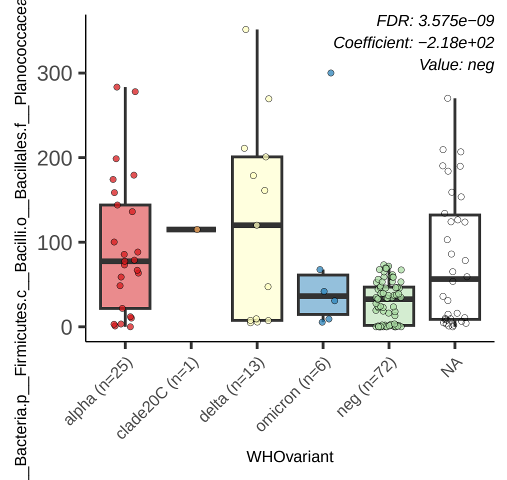

FDR: 3.575e−09
Coefficient: −2.18e+02
Value: neg
Bacteria.p__Firmicutes.c__Bacilli.o__Bacillales.f__Planococcaceae
300
200
100
0
alpha (n=25)
clade20C (n=1)
delta (n=13)
omicron (n=6)
neg (n=72)
NA
WHOvariant

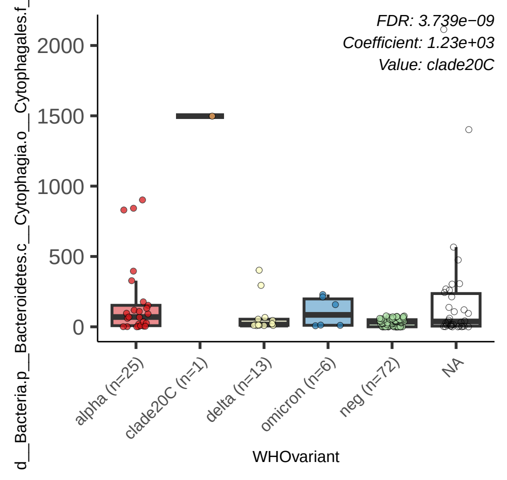

FDR: 3.739e−09
Coefficient: 1.23e+03
Value: clade20C
d__Bacteria.p__Bacteroidetes.c__Cytophagia.o__Cytophagales.f__
2000
1500
1000
500
0
alpha (n=25)
clade20C (n=1)
delta (n=13)
omicron (n=6)
neg (n=72)
NA
WHOvariant

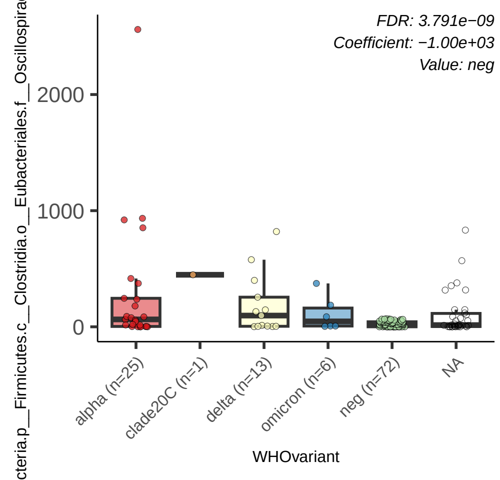
FDR: 3.791e−09
Coefficient: −1.00e+03
Value: neg
cteria.p__Firmicutes.c__Clostridia.o__Eubacteriales.f__Oscillospira
2000
1000
0
alpha (n=25)
clade20C (n=1)
delta (n=13)
omicron (n=6)
neg (n=72)
NA
WHOvariant

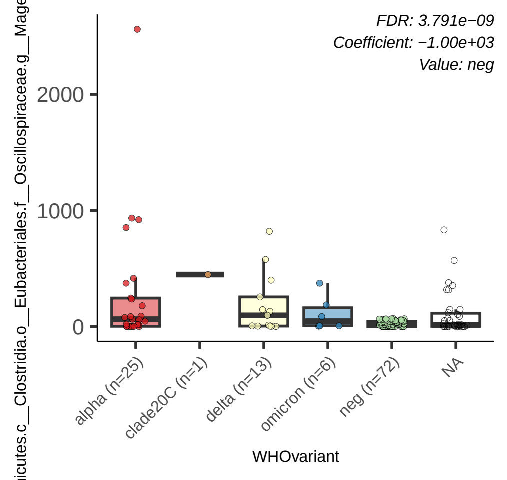

FDR: 3.791e−09
Coefficient: −1.00e+03
Value: neg
2000
1000
0
alpha (n=25)
clade20C (n=1)
delta (n=13)
omicron (n=6)
neg (n=72)
NA
WHOvariant
icutes.c__Clostridia.o__Eubacteriales.f__Oscillospiraceae.g__Mage

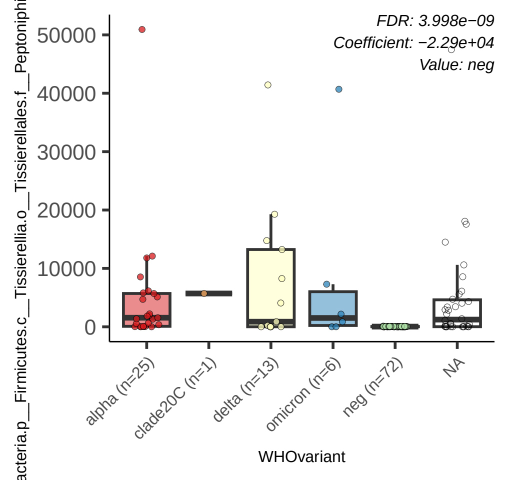
FDR: 3.998e−09
Coefficient: −2.29e+04
Value: neg
acteria.p__Firmicutes.c__Tissierellia.o__Tissierellales.f__Peptoniphi
50000
40000
30000
20000
10000
0
alpha (n=25)
clade20C (n=1)
delta (n=13)
omicron (n=6)
neg (n=72)
NA
WHOvariant

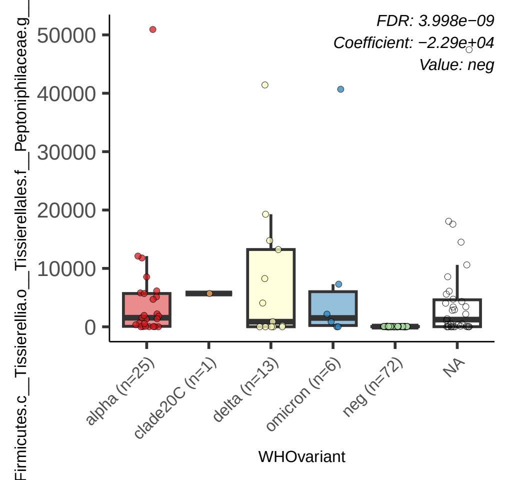

FDR: 3.998e−09
Coefficient: −2.29e+04
Value: neg
Firmicutes.c__Tissierellia.o__Tissierellales.f__Peptoniphilaceae.g__
50000
40000
30000
20000
10000
0
alpha (n=25)
clade20C (n=1)
delta (n=13)
omicron (n=6)
neg (n=72)
NA
WHOvariant

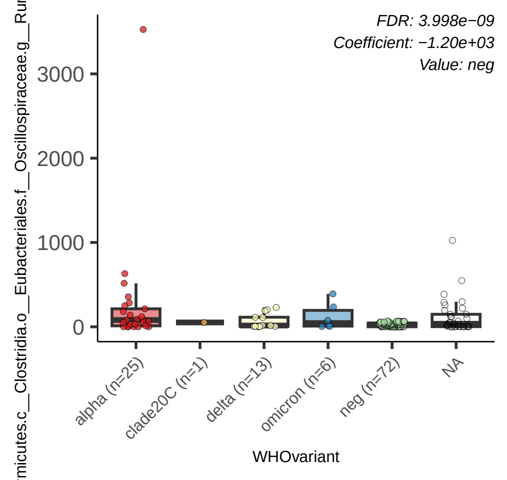
FDR: 3.998e−09
Coefficient: −1.20e+03
Value: neg
rmicutes.c__Clostridia.o__Eubacteriales.f__Oscillospiraceae.g__Ru
3000
2000
1000
0
alpha (n=25)
clade20C (n=1)
delta (n=13)
omicron (n=6)
neg (n=72)
NA
WHOvariant

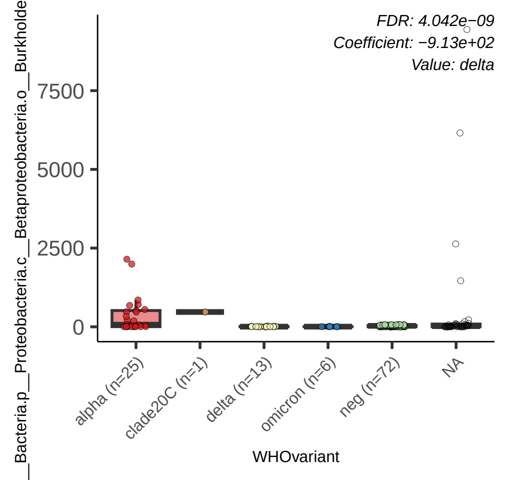
FDR: 4.042e−09
Coefficient: −9.13e+02
Value: delta
Bacteria.p__Proteobacteria.c__Betaproteobacteria.o__Burkholde
7500
5000
2500
0
alpha (n=25)
clade20C (n=1)
delta (n=13)
omicron (n=6)
neg (n=72)
NA
WHOvariant

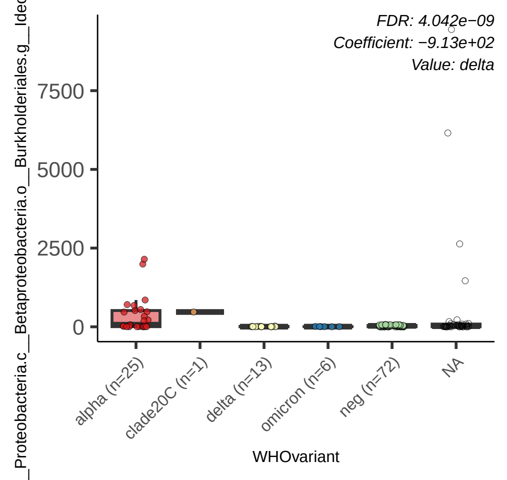

FDR: 4.042e−09
Coefficient: −9.13e+02
Value: delta
_Proteobacteria.c__Betaproteobacteria.o__Burkholderiales.g__Ideo
7500
5000
2500
0
alpha (n=25)
clade20C (n=1)
delta (n=13)
omicron (n=6)
neg (n=72)
NA
WHOvariant

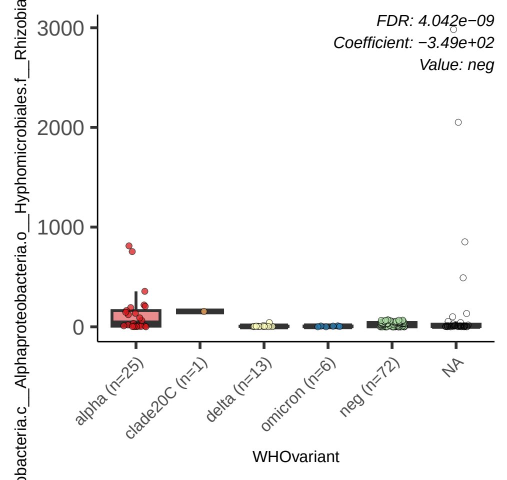

FDR: 4.042e−09
Coefficient: −3.49e+02
Value: neg
3000
2000
1000
0
obacteria.c__Alphaproteobacteria.o__Hyphomicrobiales.f__Rhizobia
alpha (n=25)
clade20C (n=1)
delta (n=13)
omicron (n=6)
neg (n=72)
NA
WHOvariant

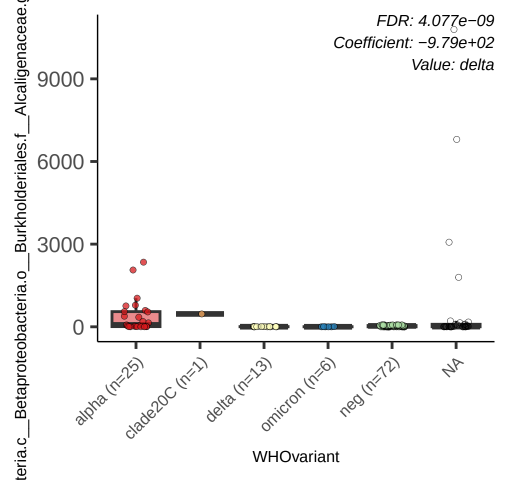

FDR: 4.077e−09
Coefficient: −9.79e+02
Value: delta
teria.c__Betaproteobacteria.o__Burkholderiales.f__Alcaligenaceae.g
9000
6000
3000
0
alpha (n=25)
clade20C (n=1)
delta (n=13)
omicron (n=6)
neg (n=72)
NA
WHOvariant

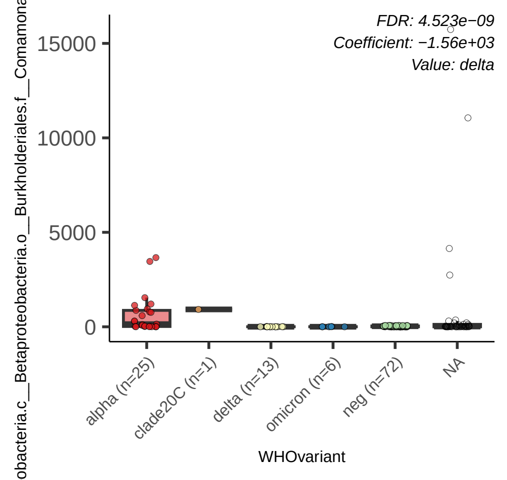

FDR: 4.523e−09
Coefficient: −1.56e+03
Value: delta
15000
10000
5000
0
obacteria.c__Betaproteobacteria.o__Burkholderiales.f__Comamona
alpha (n=25)
clade20C (n=1)
delta (n=13)
omicron (n=6)
neg (n=72)
NA
WHOvariant

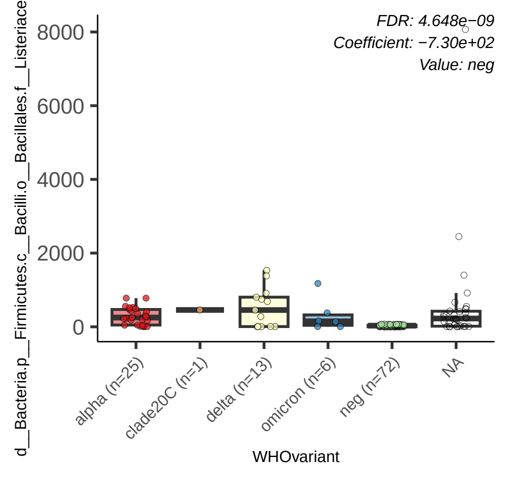
FDR: 4.648e−09
Coefficient: −7.30e+02
Value: neg
d__Bacteria.p__Firmicutes.c__Bacilli.o__Bacillales.f__Listeriace
8000
6000
4000
2000
0
alpha (n=25)
clade20C (n=1)
delta (n=13)
omicron (n=6)
neg (n=72)
NA
WHOvariant

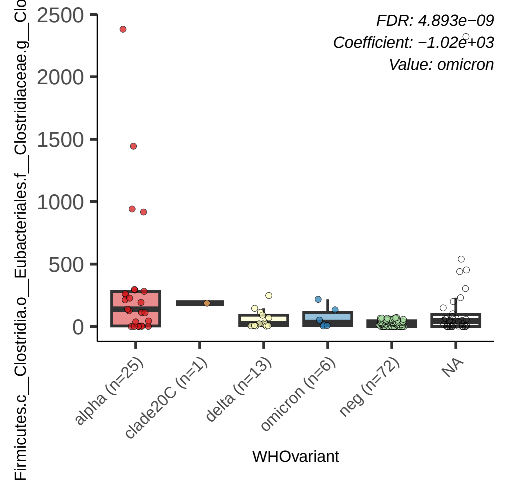

FDR: 4.893e−09
Coefficient: −1.02e+03
Value: omicron
Firmicutes.c__Clostridia.o__Eubacteriales.f__Clostridiaceae.g__Clo
2500
2000
1500
1000
500
0
alpha (n=25)
clade20C (n=1)
delta (n=13)
omicron (n=6)
neg (n=72)
NA
WHOvariant

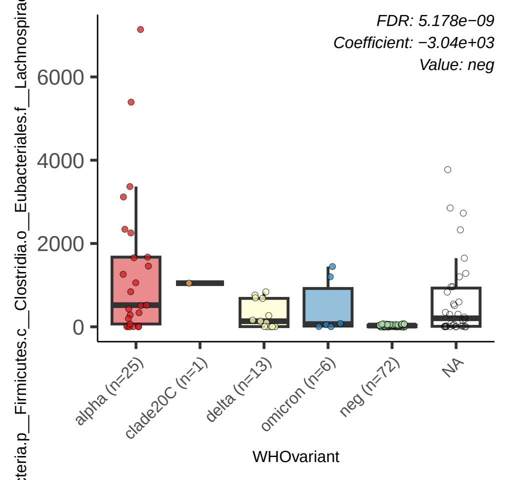

FDR: 5.178e−09
Coefficient: −3.04e+03
Value: neg
0
2000
4000
6000
cteria.p__Firmicutes.c__Clostridia.o__Eubacteriales.f__Lachnospirac
alpha (n=25)
clade20C (n=1)
delta (n=13)
omicron (n=6)
neg (n=72)
NA
WHOvariant

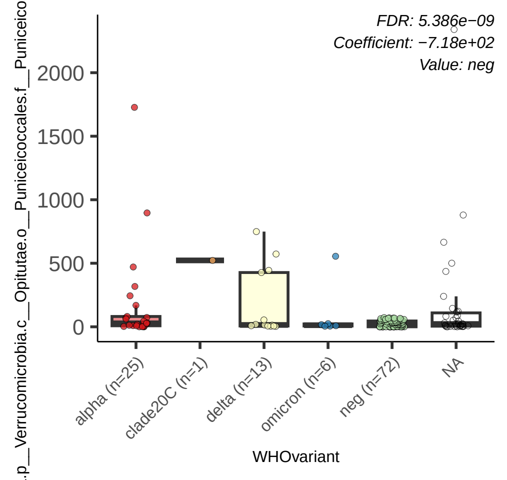

FDR: 5.386e−09
Coefficient: −7.18e+02
Value: neg
p__Verrucomicrobia.c__Opitutae.o__Puniceicoccales.f__Puniceico
2000
1500
1000
500
0
alpha (n=25)
clade20C (n=1)
delta (n=13)
omicron (n=6)
neg (n=72)
NA
WHOvariant

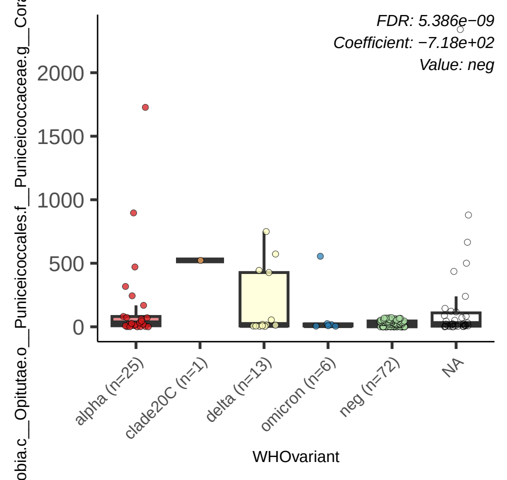

FDR: 5.386e−09
Coefficient: −7.18e+02
Value: neg
obia.c__Opitutae.o__Puniceicoccales.f__Puniceicoccaceae.g__Cora
2000
1500
1000
500
0
alpha (n=25)
clade20C (n=1)
delta (n=13)
omicron (n=6)
neg (n=72)
NA
WHOvariant

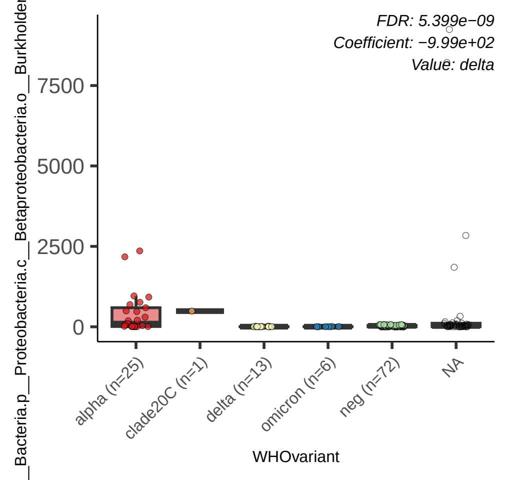
FDR: 5.399e−09
Coefficient: −9.99e+02
Value: delta
_Bacteria.p__Proteobacteria.c__Betaproteobacteria.o__Burkholder
7500
5000
2500
0
alpha (n=25)
clade20C (n=1)
delta (n=13)
omicron (n=6)
neg (n=72)
NA
WHOvariant

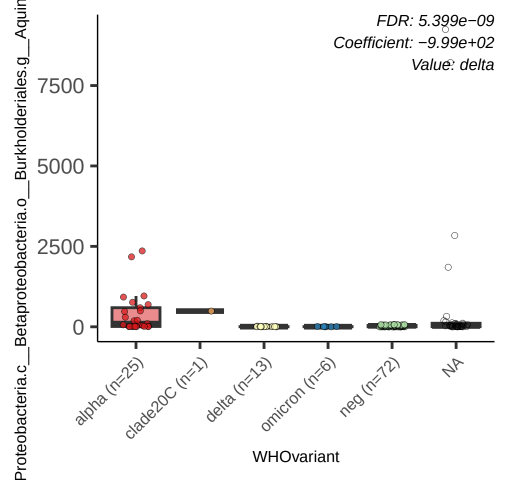

FDR: 5.399e−09
Coefficient: −9.99e+02
Value: delta
Proteobacteria.c__Betaproteobacteria.o__Burkholderiales.g__Aquin
7500
5000
2500
0
alpha (n=25)
clade20C (n=1)
delta (n=13)
omicron (n=6)
neg (n=72)
NA
WHOvariant

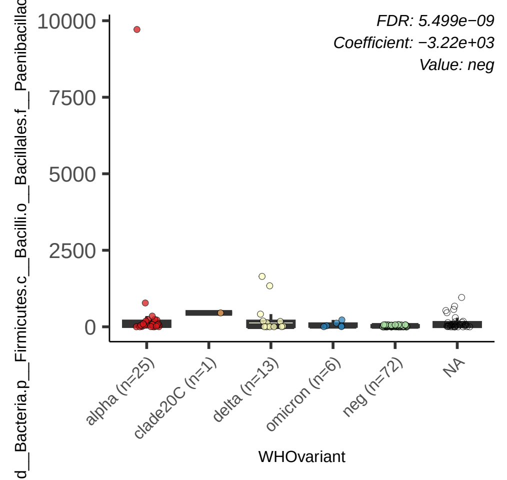

FDR: 5.499e−09
Coefficient: −3.22e+03
Value: neg
10000
7500
5000
2500
0
d__Bacteria.p__Firmicutes.c__Bacilli.o__Bacillales.f__Paenibacillac
alpha (n=25)
clade20C (n=1)
delta (n=13)
omicron (n=6)
neg (n=72)
NA
WHOvariant

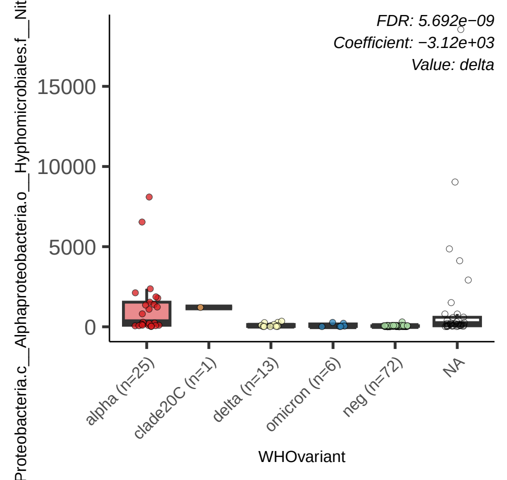

FDR: 5.692e−09
Coefficient: −3.12e+03
Value: delta
15000
10000
5000
0
Proteobacteria.c__Alphaproteobacteria.o__Hyphomicrobiales.f__Nit
alpha (n=25)
clade20C (n=1)
delta (n=13)
omicron (n=6)
neg (n=72)
NA
WHOvariant

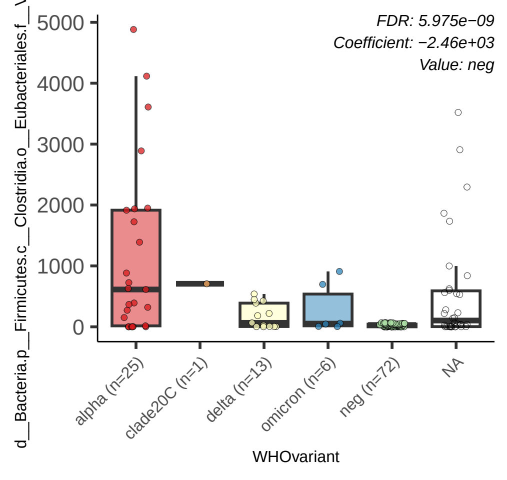
FDR: 5.975e−09
Coefficient: −2.46e+03
Value: neg
d__Bacteria.p__Firmicutes.c__Clostridia.o__Eubacteriales.f__
5000
4000
3000
2000
1000
0
alpha (n=25)
clade20C (n=1)
delta (n=13)
omicron (n=6)
neg (n=72)
NA
WHOvariant

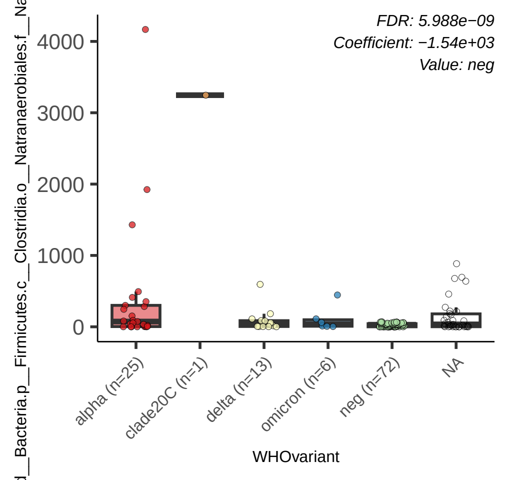

FDR: 5.988e−09
Coefficient: −1.54e+03
Value: neg
4000
3000
2000
1000
0
alpha (n=25)
clade20C (n=1)
delta (n=13)
omicron (n=6)
neg (n=72)
NA
WHOvariant
d__Bacteria.p__Firmicutes.c__Clostridia.o__Natranaerobiales.f__Na

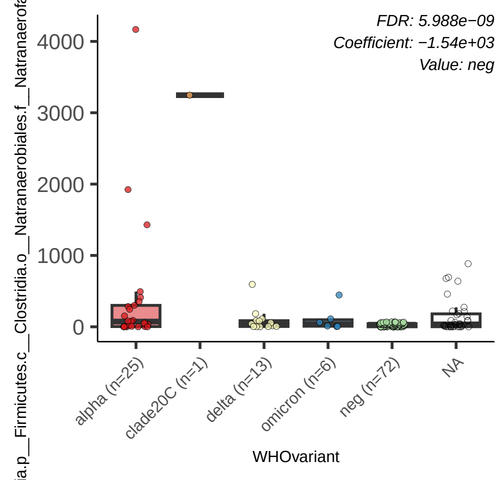

FDR: 5.988e−09
Coefficient: −1.54e+03
Value: neg
ia.p__Firmicutes.c__Clostridia.o__Natranaerobiales.f__Natranaerofa
0
1000
2000
3000
4000
alpha (n=25)
clade20C (n=1)
delta (n=13)
omicron (n=6)
neg (n=72)
NA
WHOvariant

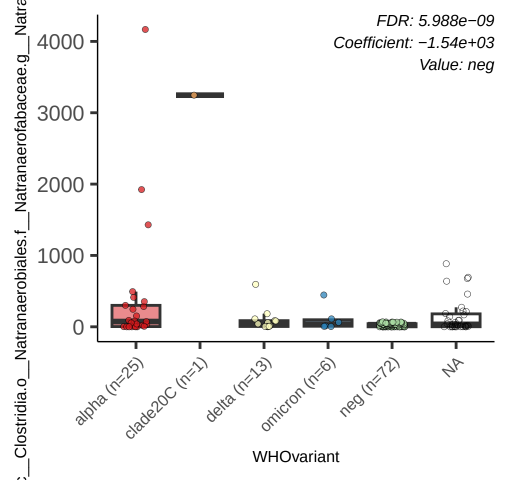

FDR: 5.988e−09
Coefficient: −1.54e+03
Value: neg
c__Clostridia.o__Natranaerobiales.f__Natranaerofabaceae.g__Natra
4000
3000
2000
1000
0
alpha (n=25)
clade20C (n=1)
delta (n=13)
omicron (n=6)
neg (n=72)
NA
WHOvariant

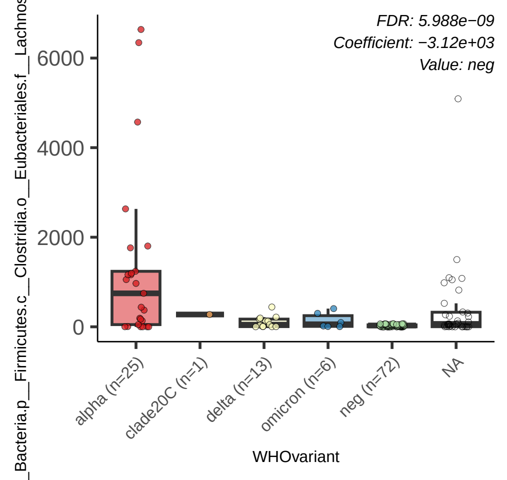

FDR: 5.988e−09
Coefficient: −3.12e+03
Value: neg
0
2000
4000
6000
alpha (n=25)
clade20C (n=1)
delta (n=13)
omicron (n=6)
neg (n=72)
NA
WHOvariant
Bacteria.p__Firmicutes.c__Clostridia.o__Eubacteriales.f__Lachnos

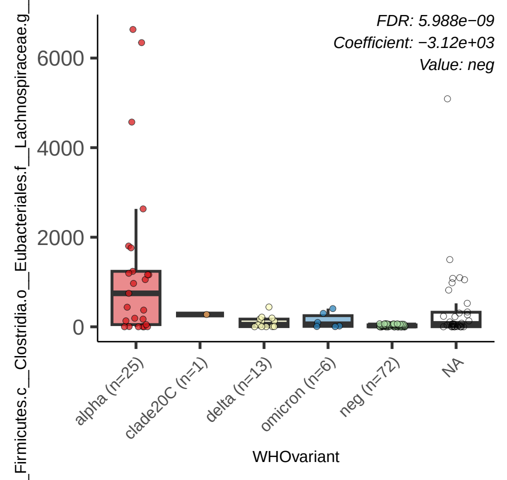

FDR: 5.988e−09
Coefficient: −3.12e+03
Value: neg
Firmicutes.c__Clostridia.o__Eubacteriales.f__Lachnospiraceae.g__
6000
4000
2000
0
alpha (n=25)
clade20C (n=1)
delta (n=13)
omicron (n=6)
neg (n=72)
NA
WHOvariant

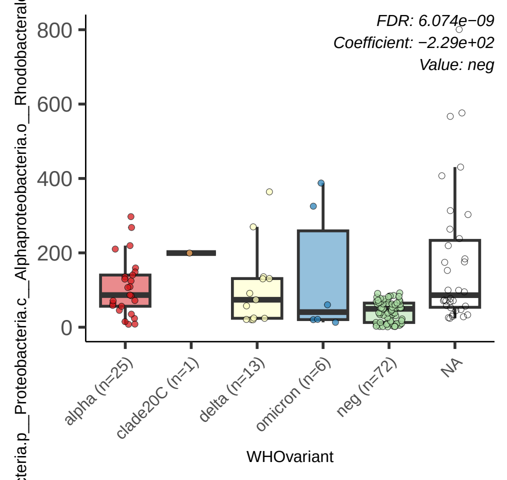

FDR: 6.074e−09
Coefficient: −2.29e+02
Value: neg
800
600
400
200
0
cteria.p__Proteobacteria.c__Alphaproteobacteria.o__Rhodobacteral
alpha (n=25)
clade20C (n=1)
delta (n=13)
omicron (n=6)
neg (n=72)
NA
WHOvariant

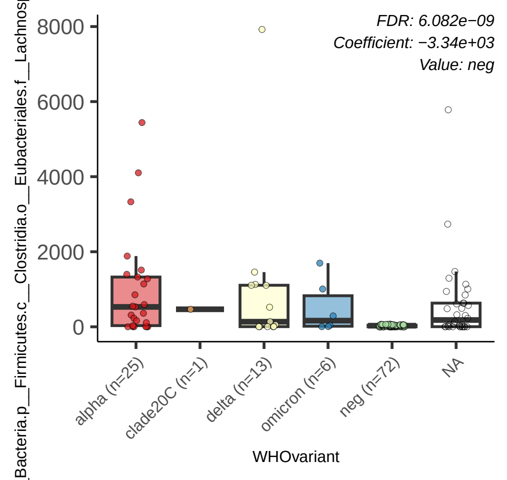

FDR: 6.082e−09
Coefficient: −3.34e+03
Value: neg
8000
6000
4000
2000
0
Bacteria.p__Firmicutes.c__Clostridia.o__Eubacteriales.f__Lachnos
alpha (n=25)
clade20C (n=1)
delta (n=13)
omicron (n=6)
neg (n=72)
NA
WHOvariant

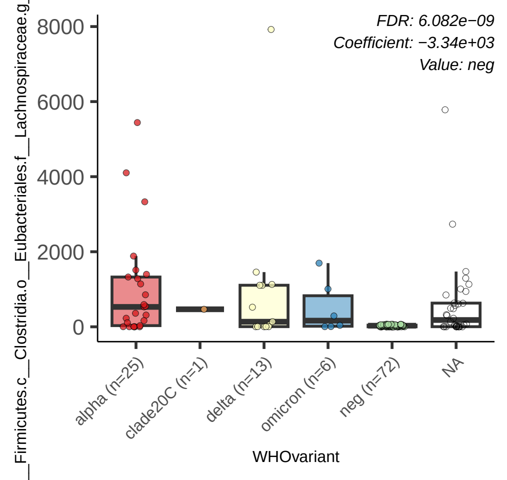

FDR: 6.082e−09
Coefficient: −3.34e+03
Value: neg
Firmicutes.c__Clostridia.o__Eubacteriales.f__Lachnospiraceae.g_
8000
6000
4000
2000
0
alpha (n=25)
clade20C (n=1)
delta (n=13)
omicron (n=6)
neg (n=72)
NA
WHOvariant

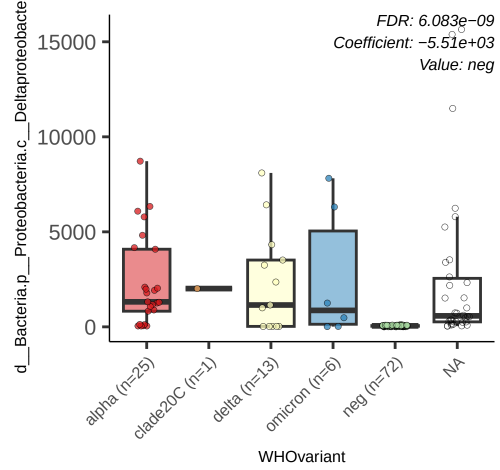

FDR: 6.083e−09
Coefficient: −5.51e+03
Value: neg
d__Bacteria.p__Proteobacteria.c__Deltaproteobacte
15000
10000
5000
0
alpha (n=25)
clade20C (n=1)
delta (n=13)
omicron (n=6)
neg (n=72)
NA
WHOvariant

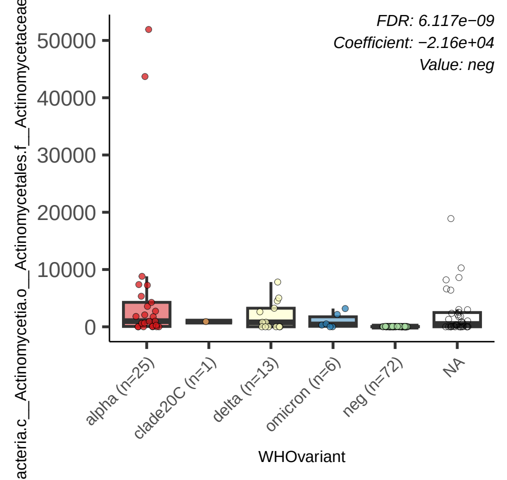
FDR: 6.117e−09
Coefficient: −2.16e+04
Value: neg
acteria.c__Actinomycetia.o__Actinomycetales.f__Actinomycetaceae
50000
40000
30000
20000
10000
0
alpha (n=25)
clade20C (n=1)
delta (n=13)
omicron (n=6)
neg (n=72)
NA
WHOvariant

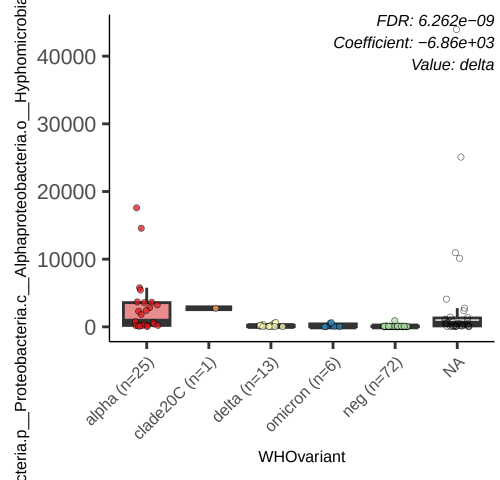
FDR: 6.262e−09
Coefficient: −6.86e+03
Value: delta
40000
30000
20000
10000
0
cteria.p__Proteobacteria.c__Alphaproteobacteria.o__Hyphomicrobia
alpha (n=25)
clade20C (n=1)
delta (n=13)
omicron (n=6)
neg (n=72)
NA
WHOvariant

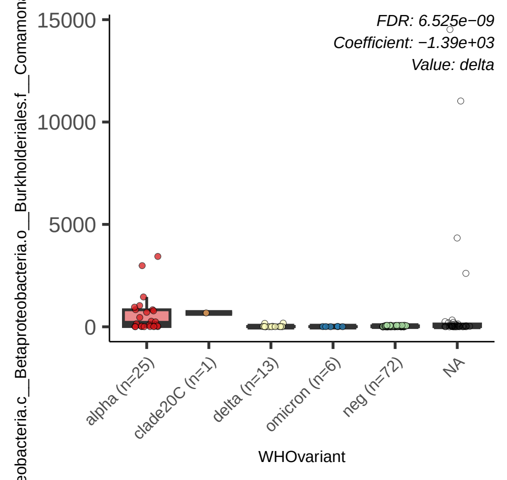

FDR: 6.525e−09
Coefficient: −1.39e+03
Value: delta
15000
10000
5000
0
eobacteria.c__Betaproteobacteria.o__Burkholderiales.f__Comamon
alpha (n=25)
clade20C (n=1)
delta (n=13)
omicron (n=6)
neg (n=72)
NA
WHOvariant

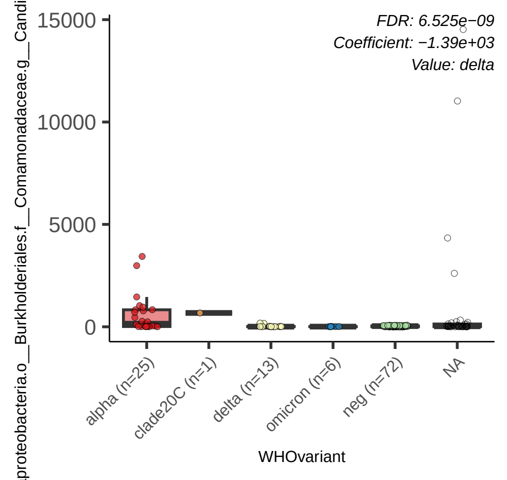

FDR: 6.525e−09
Coefficient: −1.39e+03
Value: delta
15000
10000
5000
0
proteobacteria.o__Burkholderiales.f__Comamonadaceae.g__Candi
alpha (n=25)
clade20C (n=1)
delta (n=13)
omicron (n=6)
neg (n=72)
NA
WHOvariant

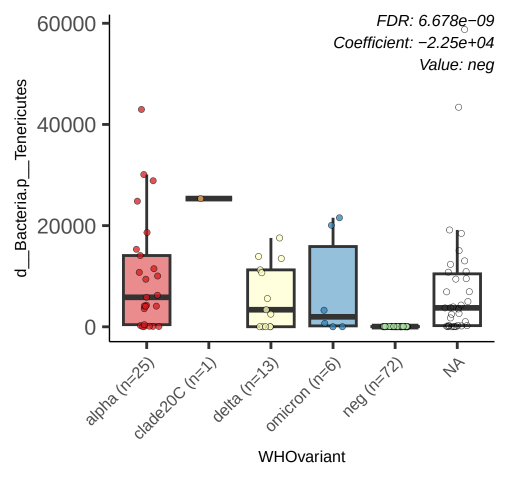
FDR: 6.678e−09
Coefficient: −2.25e+04
Value: neg
60000
40000
20000
0
d__Bacteria.p__Tenericutes
alpha (n=25)
clade20C (n=1)
delta (n=13)
omicron (n=6)
neg (n=72)
NA
WHOvariant

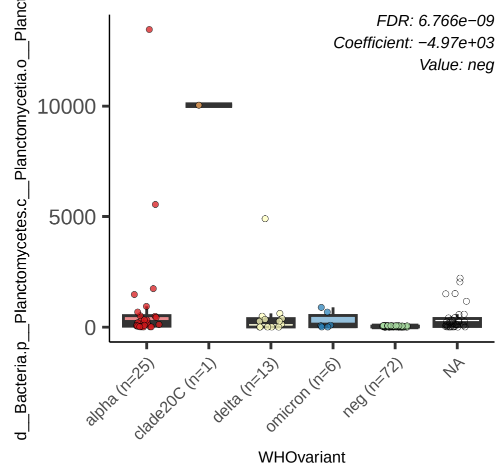

FDR: 6.766e−09
Coefficient: −4.97e+03
Value: neg
d__Bacteria.p__Planctomycetes.c__Planctomycetia.o__Planc
10000
5000
0
alpha (n=25)
clade20C (n=1)
delta (n=13)
omicron (n=6)
neg (n=72)
NA
WHOvariant

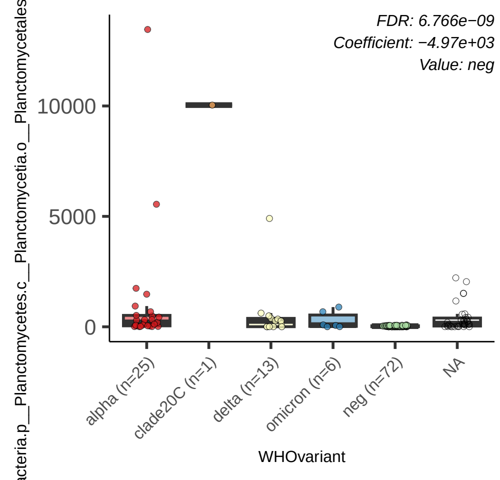

FDR: 6.766e−09
Coefficient: −4.97e+03
Value: neg
10000
5000
0
acteria.p__Planctomycetes.c__Planctomycetia.o__Planctomycetales
alpha (n=25)
clade20C (n=1)
delta (n=13)
omicron (n=6)
neg (n=72)
NA
WHOvariant

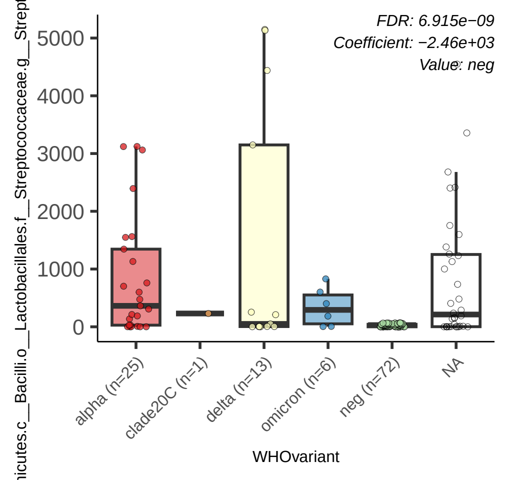
FDR: 6.915e−09
Coefficient: −2.46e+03
Value: neg
5000
4000
3000
2000
1000
0
alpha (n=25)
clade20C (n=1)
delta (n=13)
omicron (n=6)
neg (n=72)
NA
WHOvariant
nicutes.c__Bacilli.o__Lactobacillales.f__Streptococcaceae.g__Strep

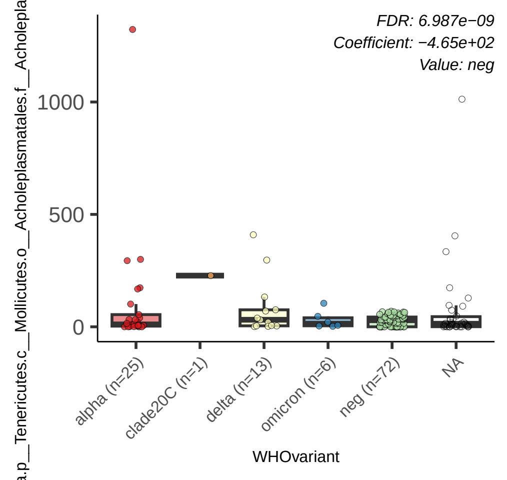

FDR: 6.987e−09
Coefficient: −4.65e+02
Value: neg
1000
500
0
alpha (n=25)
clade20C (n=1)
delta (n=13)
omicron (n=6)
neg (n=72)
NA
WHOvariant
a.p__Tenericutes.c__Mollicutes.o__Acholeplasmatales.f__Acholepla

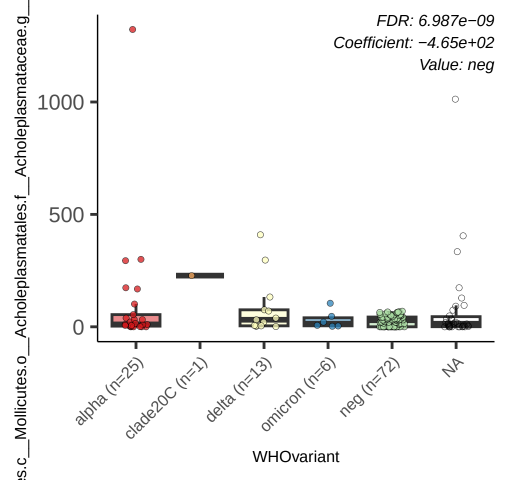

FDR: 6.987e−09
Coefficient: −4.65e+02
Value: neg
1000
500
0
es.c__Mollicutes.o__Acholeplasmatales.f__Acholeplasmataceae.g__
alpha (n=25)
clade20C (n=1)
delta (n=13)
omicron (n=6)
neg (n=72)
NA
WHOvariant

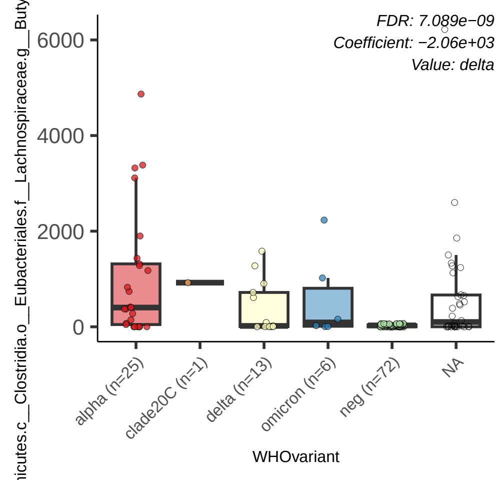

FDR: 7.089e−09
Coefficient: −2.06e+03
Value: delta
6000
4000
2000
0
nicutes.c__Clostridia.o__Eubacteriales.f__Lachnospiraceae.g__Buty
alpha (n=25)
clade20C (n=1)
delta (n=13)
omicron (n=6)
neg (n=72)
NA
WHOvariant

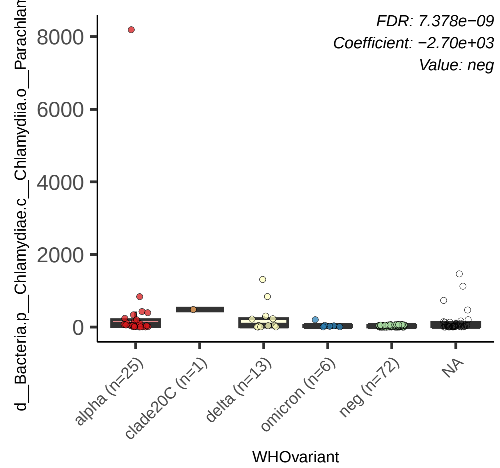
FDR: 7.378e−09
Coefficient: −2.70e+03
Value: neg
d__Bacteria.p__Chlamydiae.c__Chlamydiia.o__Parachlam
8000
6000
4000
2000
0
alpha (n=25)
clade20C (n=1)
delta (n=13)
omicron (n=6)
neg (n=72)
NA
WHOvariant

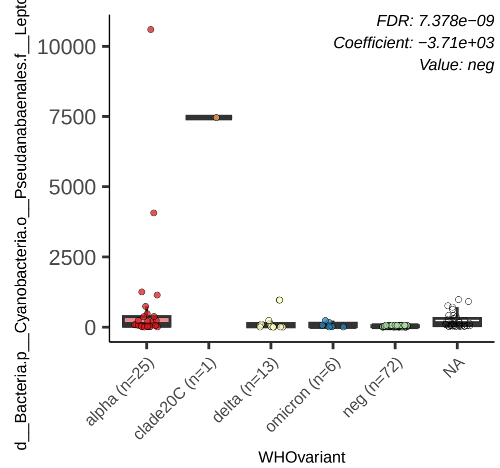

FDR: 7.378e−09
Coefficient: −3.71e+03
Value: neg
d__Bacteria.p__Cyanobacteria.o__Pseudanabaenales.f__Lepto
10000
7500
5000
2500
0
alpha (n=25)
clade20C (n=1)
delta (n=13)
omicron (n=6)
neg (n=72)
NA
WHOvariant

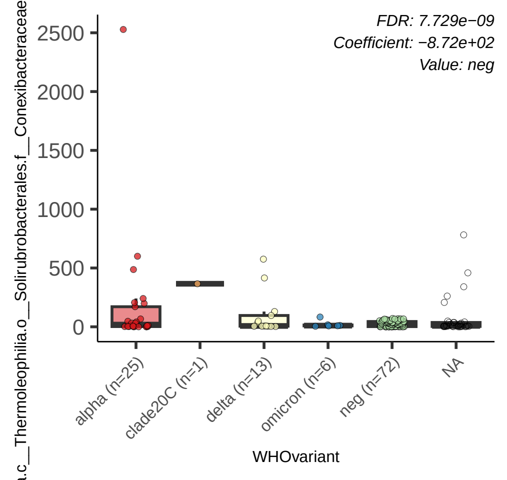
FDR: 7.729e−09
Coefficient: −8.72e+02
Value: neg
Thermoleophilia.c__Thermoleophilia.o__Solirubrobacterales.f__Conexibacteraceae
2500
2000
1500
1000
500
0
alpha (n=25)
clade20C (n=1)
delta (n=13)
omicron (n=6)
neg (n=72)
NA
WHOvariant

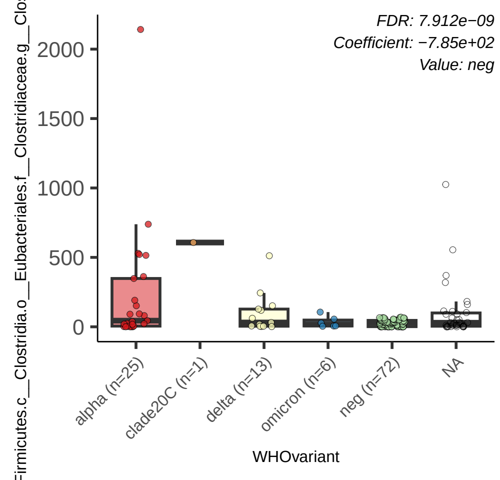
FDR: 7.912e−09
Coefficient: −7.85e+02
Value: neg
Firmicutes.c__Clostridia.o__Eubacteriales.f__Clostridiaceae.g__Clos
2000
1500
1000
500
0
alpha (n=25)
clade20C (n=1)
delta (n=13)
omicron (n=6)
neg (n=72)
NA
WHOvariant

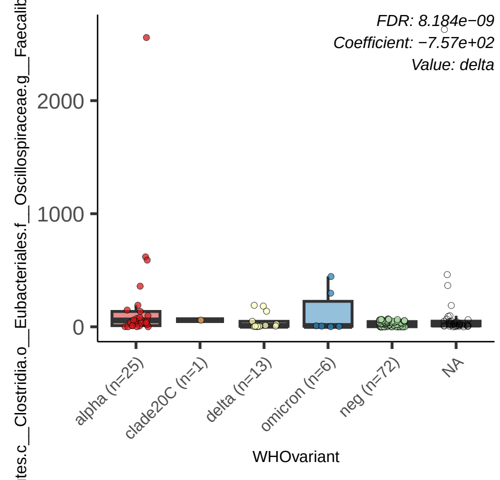
FDR: 8.184e−09
Coefficient: −7.57e+02
Value: delta
2000
1000
0
alpha (n=25)
clade20C (n=1)
delta (n=13)
omicron (n=6)
neg (n=72)
NA
WHOvariant
utes.c__Clostridia.o__Eubacteriales.f__Oscillospiraceae.g__Faecalib

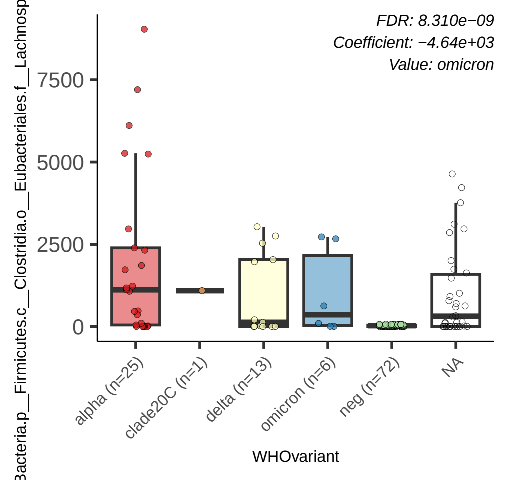

FDR: 8.310e−09
Coefficient: −4.64e+03
Value: omicron
Bacteria.p__Firmicutes.c__Clostridia.o__Eubacteriales.f__Lachnosp
7500
5000
2500
0
alpha (n=25)
clade20C (n=1)
delta (n=13)
omicron (n=6)
neg (n=72)
NA
WHOvariant

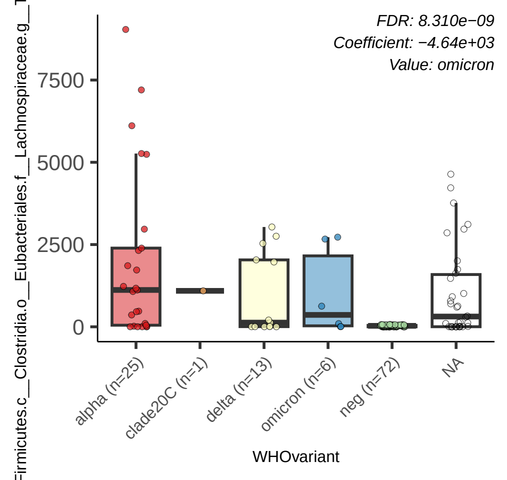

FDR: 8.310e−09
Coefficient: −4.64e+03
Value: omicron
Firmicutes.c__Clostridia.o__Eubacteriales.f__Lachnospiraceae.g__
7500
5000
2500
0
alpha (n=25)
clade20C (n=1)
delta (n=13)
omicron (n=6)
neg (n=72)
NA
WHOvariant

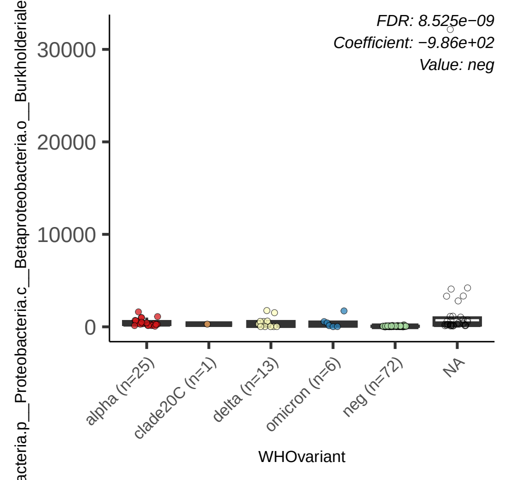

FDR: 8.525e−09
Coefficient: −9.86e+02
Value: neg
30000
20000
10000
0
acteria.p__Proteobacteria.c__Betaproteobacteria.o__Burkholderiale
alpha (n=25)
clade20C (n=1)
delta (n=13)
omicron (n=6)
neg (n=72)
NA
WHOvariant

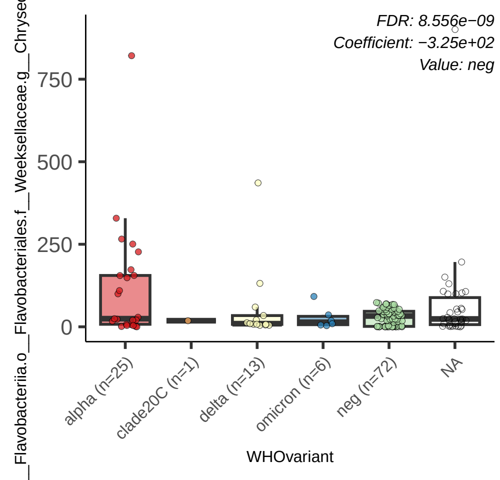
FDR: 8.556e−09
Coefficient: −3.25e+02
Value: neg
0
250
500
750
Flavobacteriia.o__Flavobacteriales.f__Weeksellaceae.g__Chryse
alpha (n=25)
clade20C (n=1)
delta (n=13)
omicron (n=6)
neg (n=72)
NA
WHOvariant

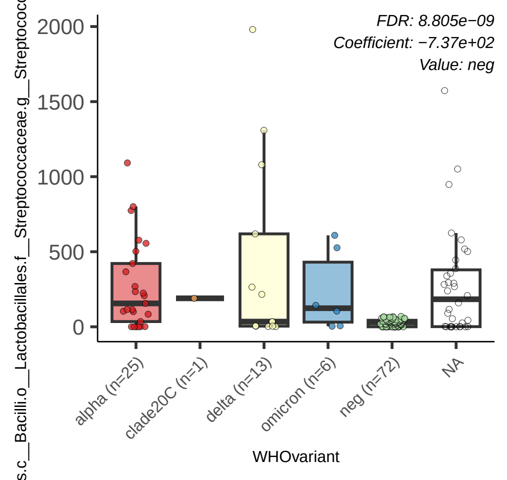

FDR: 8.805e−09
Coefficient: −7.37e+02
Value: neg
s.c__Bacilli.o__Lactobacillales.f__Streptococcaceae.g__Streptococc
2000
1500
1000
500
0
alpha (n=25)
clade20C (n=1)
delta (n=13)
omicron (n=6)
neg (n=72)
NA
WHOvariant

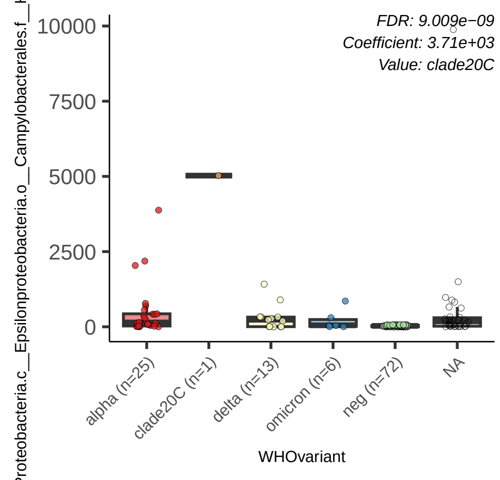
FDR: 9.009e−09
Coefficient: 3.71e+03
Value: clade20C
10000
7500
5000
2500
0
Proteobacteria.c__Epsilonproteobacteria.o__Campylobacterales.f__H
alpha (n=25)
clade20C (n=1)
delta (n=13)
omicron (n=6)
neg (n=72)
NA
WHOvariant

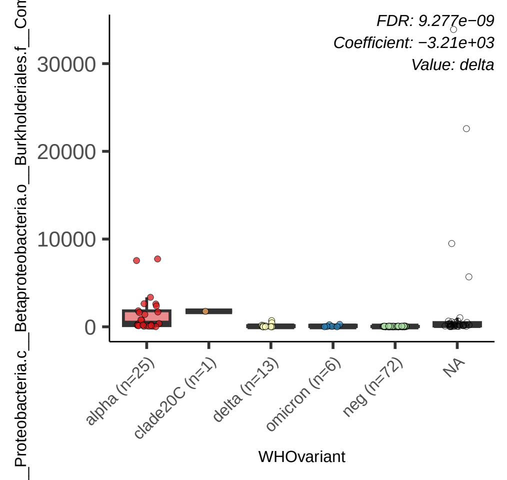
FDR: 9.277e−09
Coefficient: −3.21e+03
Value: delta
Proteobacteria.c__Betaproteobacteria.o__Burkholderiales.f__Com
30000
20000
10000
0
alpha (n=25)
clade20C (n=1)
delta (n=13)
omicron (n=6)
neg (n=72)
NA
WHOvariant

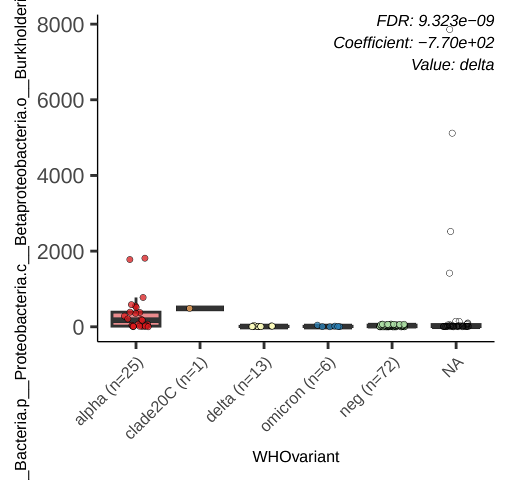

FDR: 9.323e−09
Coefficient: −7.70e+02
Value: delta
Bacteria.p__Proteobacteria.c__Betaproteobacteria.o__Burkholderi
8000
6000
4000
2000
0
alpha (n=25)
clade20C (n=1)
delta (n=13)
omicron (n=6)
neg (n=72)
NA
WHOvariant

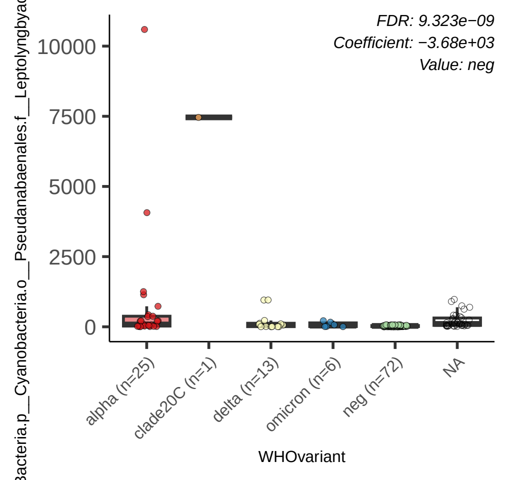

FDR: 9.323e−09
Coefficient: −3.68e+03
Value: neg
10000
7500
5000
2500
0
alpha (n=25)
clade20C (n=1)
delta (n=13)
omicron (n=6)
neg (n=72)
NA
WHOvariant
Bacteria.p__Cyanobacteria.o__Pseudanabaenales.f__Leptolyngbyac

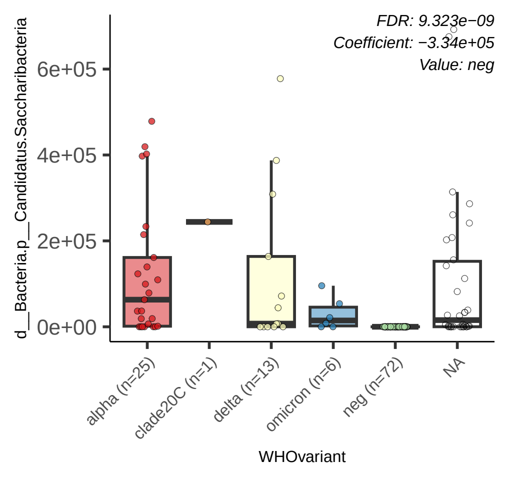

FDR: 9.323e−09
Coefficient: −3.34e+05
Value: neg
d__Bacteria.p__Candidatus.Saccharibacteria
6e+05
4e+05
2e+05
0e+00
alpha (n=25)
clade20C (n=1)
delta (n=13)
omicron (n=6)
neg (n=72)
NA
WHOvariant

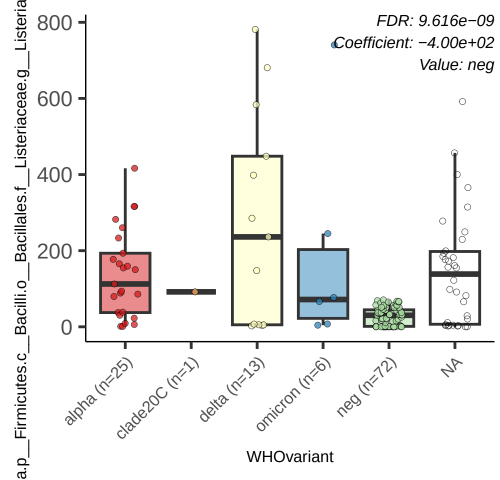

FDR: 9.616e−09
Coefficient: −4.00e+02
Value: neg
800
600
400
200
0
a.p__Firmicutes.c__Bacilli.o__Bacillales.f__Listeriaceae.g__Listeria
alpha (n=25)
clade20C (n=1)
delta (n=13)
omicron (n=6)
neg (n=72)
NA
WHOvariant

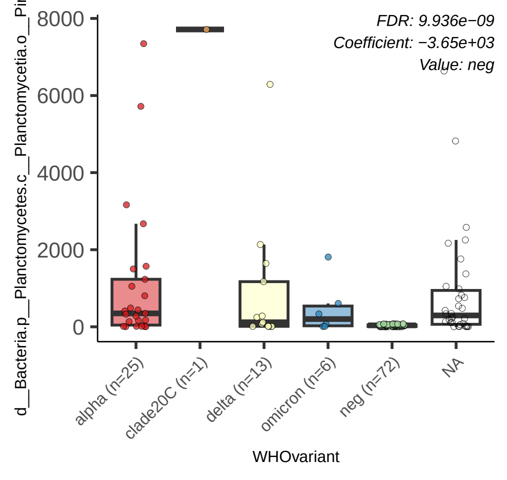

FDR: 9.936e−09
Coefficient: −3.65e+03
Value: neg
d__Bacteria.p__Planctomycetes.c__Planctomycetia.o__Pi
8000
6000
4000
2000
0
alpha (n=25)
clade20C (n=1)
delta (n=13)
omicron (n=6)
neg (n=72)
NA
WHOvariant

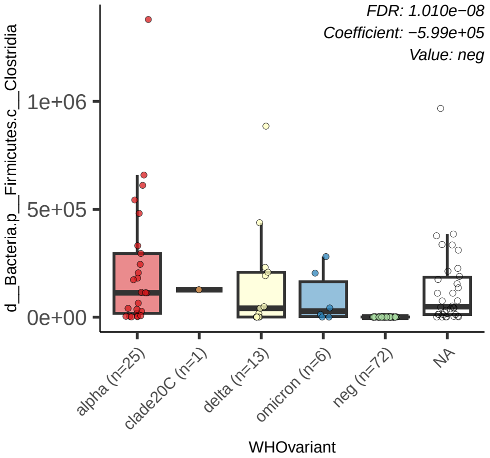

FDR: 1.010e−08
Coefficient: −5.99e+05
Value: neg
d__Bacteria.p__Firmicutes.c__Clostridia
1e+06
5e+05
0e+00
alpha (n=25)
clade20C (n=1)
delta (n=13)
omicron (n=6)
neg (n=72)
NA
WHOvariant

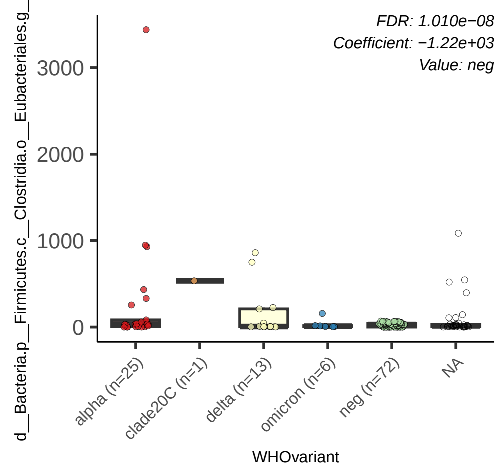
FDR: 1.010e−08
Coefficient: −1.22e+03
Value: neg
d__Bacteria.p__Firmicutes.c__Clostridia.o__Eubacteriales.g__
3000
2000
1000
0
alpha (n=25)
clade20C (n=1)
delta (n=13)
omicron (n=6)
neg (n=72)
NA
WHOvariant

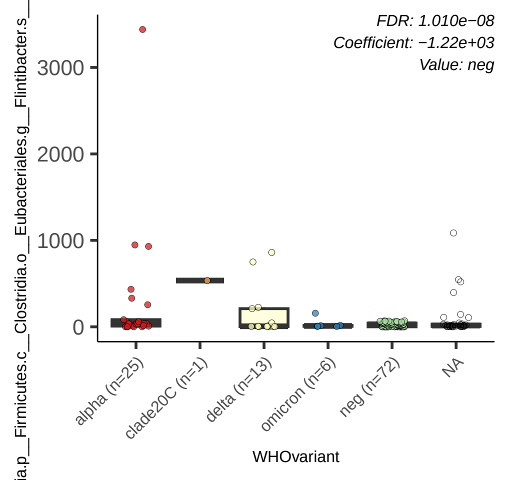

FDR: 1.010e−08
Coefficient: −1.22e+03
Value: neg
ia.p__Firmicutes.c__Clostridia.o__Eubacteriales.g__Flintibacter.s__
3000
2000
1000
0
alpha (n=25)
clade20C (n=1)
delta (n=13)
omicron (n=6)
neg (n=72)
NA
WHOvariant

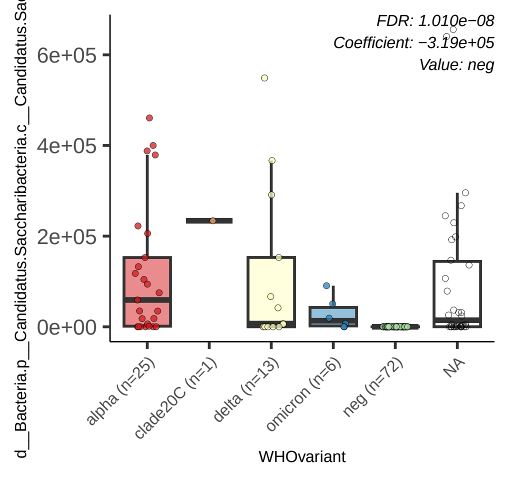
FDR: 1.010e−08
Coefficient: −3.19e+05
Value: neg
6e+05
4e+05
2e+05
0e+00
d__Bacteria.p__Candidatus.Saccharibacteria.c__Candidatus.Sac
alpha (n=25)
clade20C (n=1)
delta (n=13)
omicron (n=6)
neg (n=72)
NA
WHOvariant

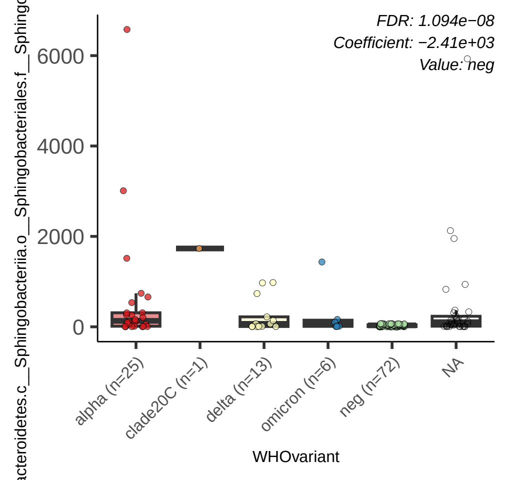
FDR: 1.094e−08
Coefficient: −2.41e+03
Value: neg
6000
4000
2000
0
acteroidetes.c__Sphingobacteriia.o__Sphingobacteriales.f__Sphingo
alpha (n=25)
clade20C (n=1)
delta (n=13)
omicron (n=6)
neg (n=72)
NA
WHOvariant

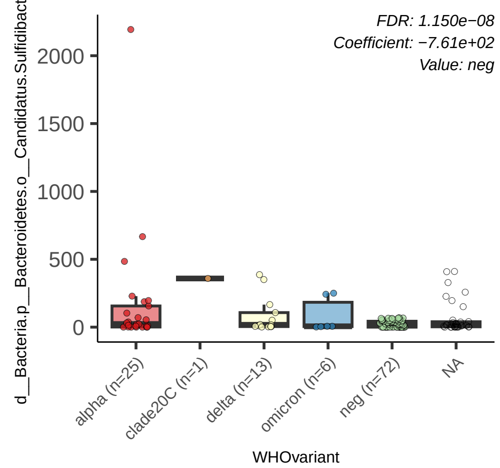
FDR: 1.150e−08
Coefficient: −7.61e+02
Value: neg
d__Bacteria.p__Bacteroidetes.o__Candidatus.Sulfidibact
2000
1500
1000
500
0
alpha (n=25)
clade20C (n=1)
delta (n=13)
omicron (n=6)
neg (n=72)
NA
WHOvariant

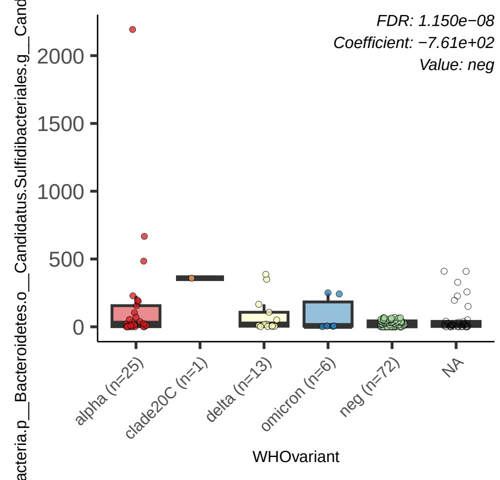

FDR: 1.150e−08
Coefficient: −7.61e+02
Value: neg
acteria.p__Bacteroidetes.o__Candidatus.Sulfidibacteriales.g__Cand
0
500
1000
1500
2000
alpha (n=25)
clade20C (n=1)
delta (n=13)
omicron (n=6)
neg (n=72)
NA
WHOvariant

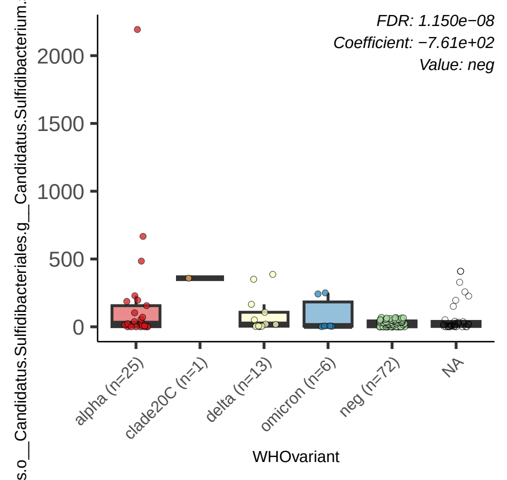
FDR: 1.150e−08
Coefficient: −7.61e+02
Value: neg
s.o__Candidatus.Sulfidibacteriales.g__Candidatus.Sulfidibacterium.s
0
500
1000
1500
2000
alpha (n=25)
clade20C (n=1)
delta (n=13)
omicron (n=6)
neg (n=72)
NA
WHOvariant

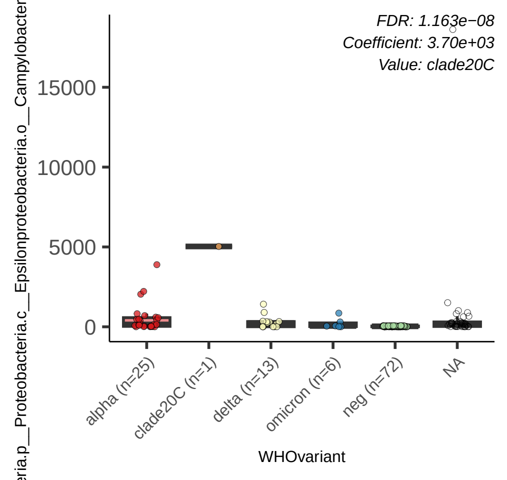
FDR: 1.163e−08
Coefficient: 3.70e+03
Value: clade20C
15000
10000
5000
0
alpha (n=25)
clade20C (n=1)
delta (n=13)
omicron (n=6)
neg (n=72)
NA
WHOvariant
eria.p__Proteobacteria.c__Epsilonproteobacteria.o__Campylobacter

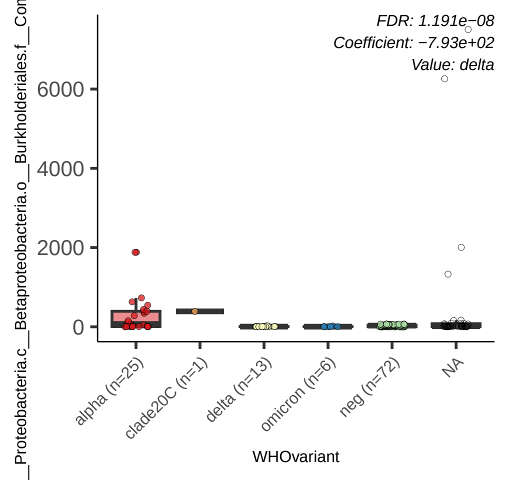

FDR: 1.191e−08
Coefficient: −7.93e+02
Value: delta
0
2000
4000
6000
alpha (n=25)
clade20C (n=1)
delta (n=13)
omicron (n=6)
neg (n=72)
NA
WHOvariant
__Proteobacteria.c__Betaproteobacteria.o__Burkholderiales.f__Con

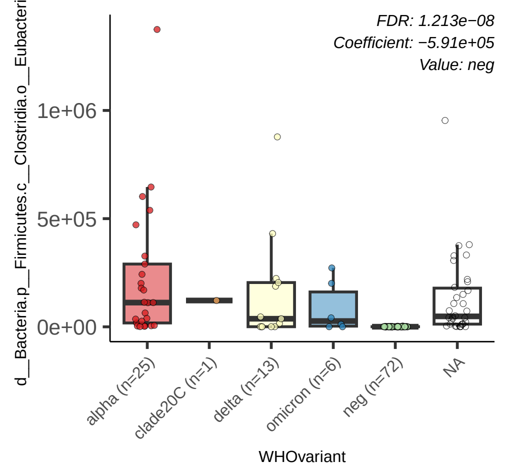
FDR: 1.213e−08
Coefficient: −5.91e+05
Value: neg
d__Bacteria.p__Firmicutes.c__Clostridia.o__Eubacteri
1e+06
5e+05
0e+00
alpha (n=25)
clade20C (n=1)
delta (n=13)
omicron (n=6)
neg (n=72)
NA
WHOvariant

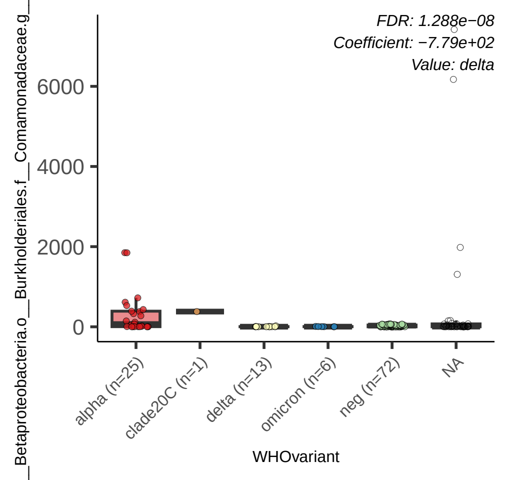
FDR: 1.288e−08
Coefficient: −7.79e+02
Value: delta
Betaproteobacteria.o__Burkholderiales.f__Comamonadaceae.g__
6000
4000
2000
0
alpha (n=25)
clade20C (n=1)
delta (n=13)
omicron (n=6)
neg (n=72)
NA
WHOvariant

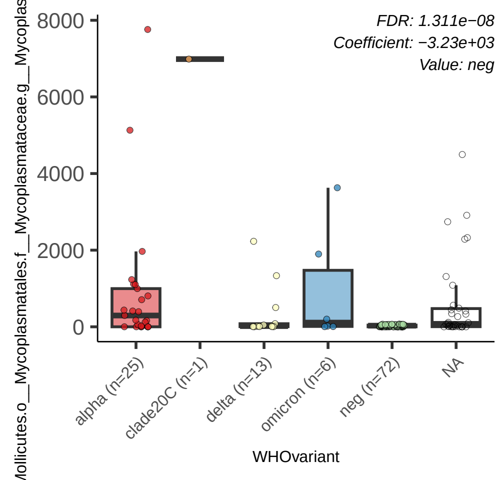

FDR: 1.311e−08
Coefficient: −3.23e+03
Value: neg
8000
6000
4000
2000
0
ollicutes.o__Mycoplasmatales.f__Mycoplasmataceae.g__Mycoplas
alpha (n=25)
clade20C (n=1)
delta (n=13)
omicron (n=6)
neg (n=72)
NA
WHOvariant

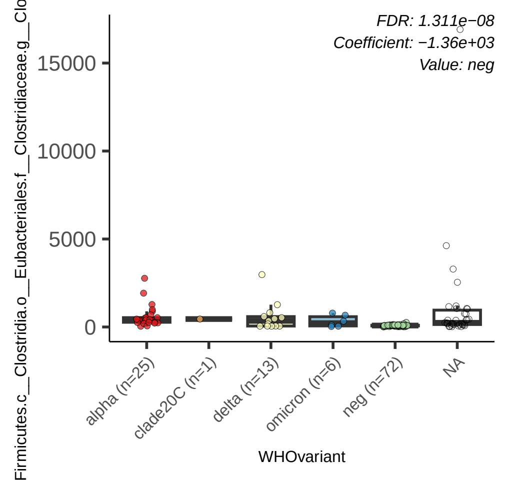
FDR: 1.311e−08
Coefficient: −1.36e+03
Value: neg
Firmicutes.c__Clostridia.o__Eubacteriales.f__Clostridiaceae.g__Clo
15000
10000
5000
0
alpha (n=25)
clade20C (n=1)
delta (n=13)
omicron (n=6)
neg (n=72)
NA
WHOvariant

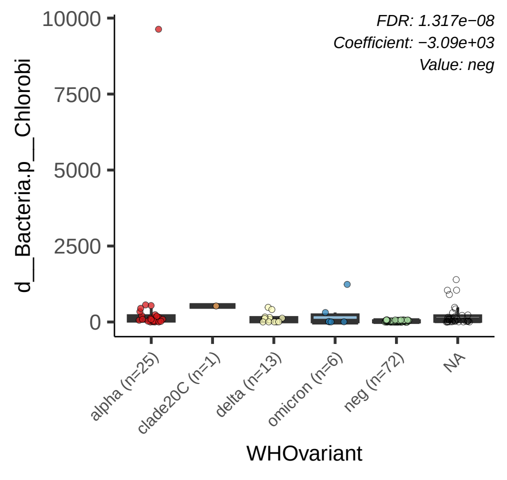
FDR: 1.317e−08
Coefficient: −3.09e+03
Value: neg
d__Bacteria.p__Chlorobi
10000
7500
5000
2500
0
alpha (n=25)
clade20C (n=1)
delta (n=13)
omicron (n=6)
neg (n=72)
NA
WHOvariant

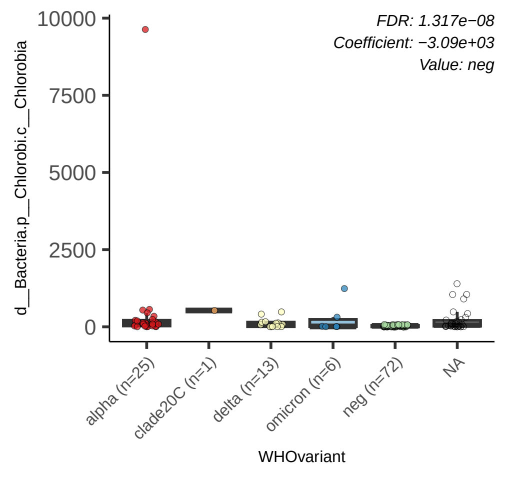

FDR: 1.317e−08
Coefficient: −3.09e+03
Value: neg
d__Bacteria.p__Chlorobi.c__Chlorobia
10000
7500
5000
2500
0
alpha (n=25)
clade20C (n=1)
delta (n=13)
omicron (n=6)
neg (n=72)
NA
WHOvariant

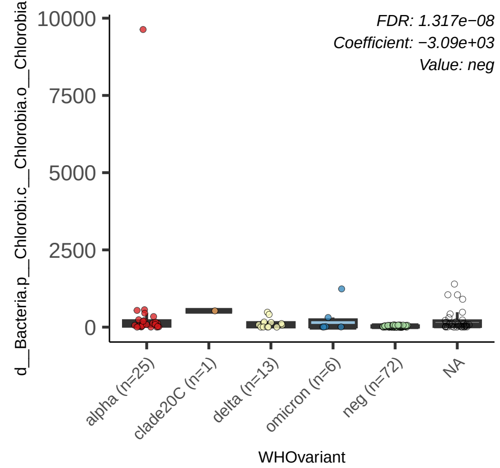

FDR: 1.317e−08
Coefficient: −3.09e+03
Value: neg
d__Bacteria.p__Chlorobi.c__Chlorobia.o__Chlorobia
10000
7500
5000
2500
0
alpha (n=25)
clade20C (n=1)
delta (n=13)
omicron (n=6)
neg (n=72)
NA
WHOvariant

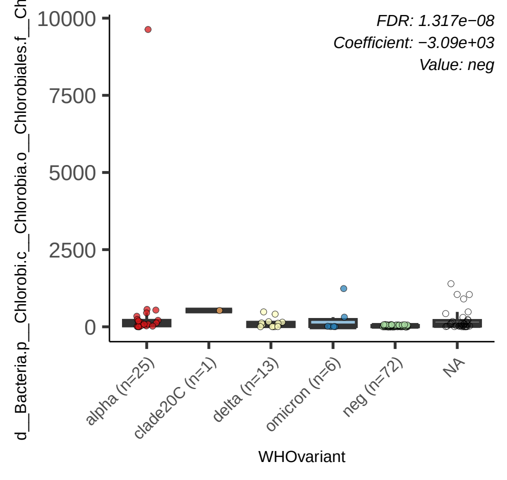

FDR: 1.317e−08
Coefficient: −3.09e+03
Value: neg
d__Bacteria.p__Chlorobi.c__Chlorobia.o__Chlorobiales.f__Ch
10000
7500
5000
2500
0
alpha (n=25)
clade20C (n=1)
delta (n=13)
omicron (n=6)
neg (n=72)
NA
WHOvariant

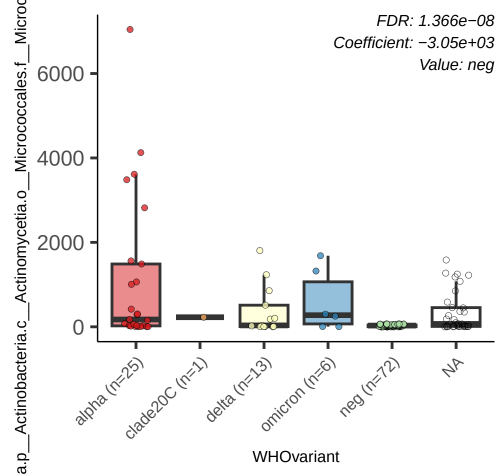

FDR: 1.366e−08
Coefficient: −3.05e+03
Value: neg
6000
4000
2000
0
ria.p__Actinobacteria.c__Actinomycetia.o__Micrococcales.f__Microc
alpha (n=25)
clade20C (n=1)
delta (n=13)
omicron (n=6)
neg (n=72)
NA
WHOvariant

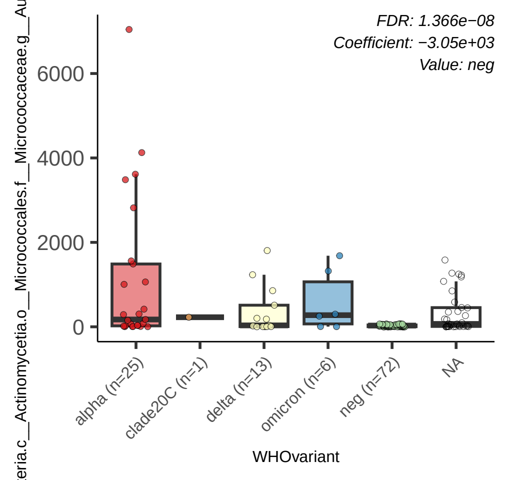

FDR: 1.366e−08
Coefficient: −3.05e+03
Value: neg
6000
4000
2000
0
alpha (n=25)
clade20C (n=1)
delta (n=13)
omicron (n=6)
neg (n=72)
NA
WHOvariant
teria.c__Actinomycetia.o__Micrococcales.f__Micrococcaceae.g__Au

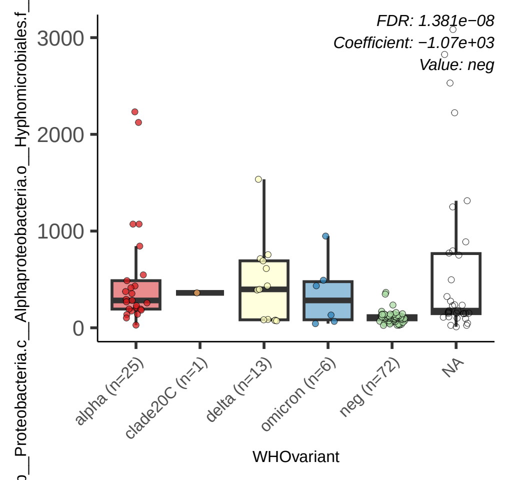

FDR: 1.381e−08
Coefficient: −1.07e+03
Value: neg
o__Proteobacteria.c__Alphaproteobacteria.o__Hyphomicrobiales.f
3000
2000
1000
0
alpha (n=25)
clade20C (n=1)
delta (n=13)
omicron (n=6)
neg (n=72)
NA
WHOvariant

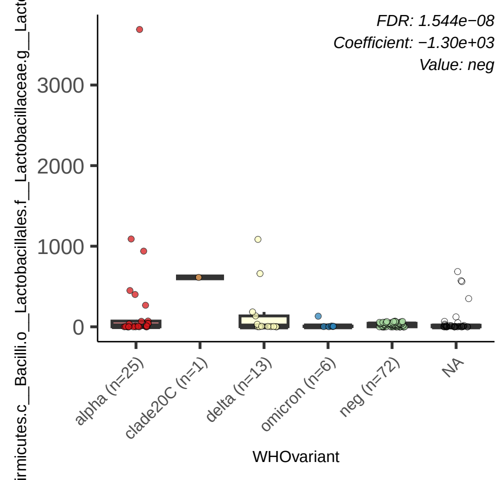

FDR: 1.544e−08
Coefficient: −1.30e+03
Value: neg
irmicutes.c__Bacilli.o__Lactobacillales.f__Lactobacillaceae.g__Lacto
3000
2000
1000
0
alpha (n=25)
clade20C (n=1)
delta (n=13)
omicron (n=6)
neg (n=72)
NA
WHOvariant

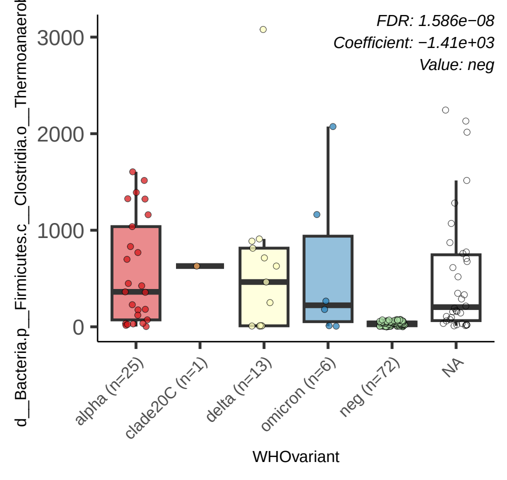

FDR: 1.586e−08
Coefficient: −1.41e+03
Value: neg
d__Bacteria.p__Firmicutes.c__Clostridia.o__Thermoanaerob
3000
2000
1000
0
alpha (n=25)
clade20C (n=1)
delta (n=13)
omicron (n=6)
neg (n=72)
NA
WHOvariant

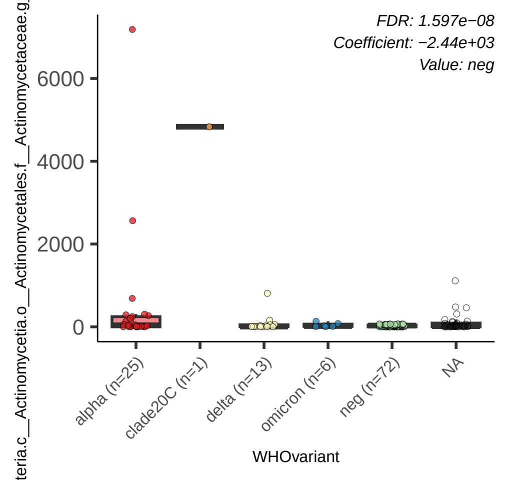
FDR: 1.597e−08
Coefficient: −2.44e+03
Value: neg
teria.c__Actinomycetia.o__Actinomycetales.f__Actinomycetaceae.g_
6000
4000
2000
0
alpha (n=25)
clade20C (n=1)
delta (n=13)
omicron (n=6)
neg (n=72)
NA
WHOvariant

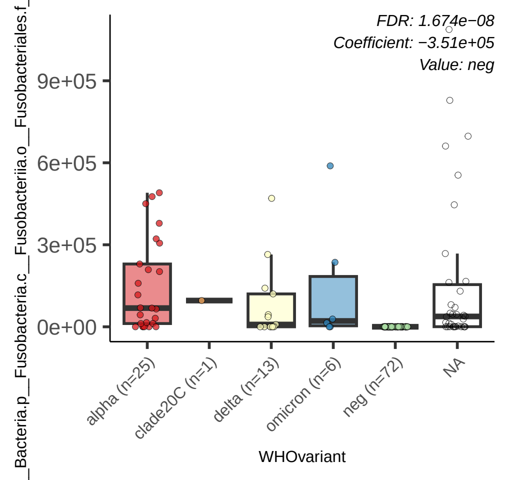

FDR: 1.674e−08
Coefficient: −3.51e+05
Value: neg
9e+05
6e+05
3e+05
0e+00
_Bacteria.p__Fusobacteria.c__Fusobacteriia.o__Fusobacteriales.f_
alpha (n=25)
clade20C (n=1)
delta (n=13)
omicron (n=6)
neg (n=72)
NA
WHOvariant

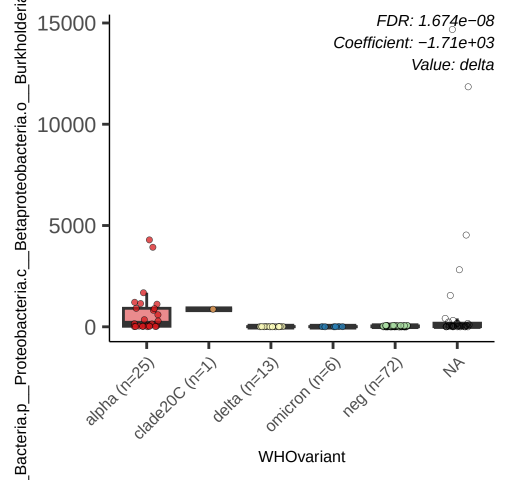

FDR: 1.674e−08
Coefficient: −1.71e+03
Value: delta
15000
10000
5000
0
alpha (n=25)
clade20C (n=1)
delta (n=13)
omicron (n=6)
neg (n=72)
NA
WHOvariant
Bacteria.p__Proteobacteria.c__Betaproteobacteria.o__Burkholderia

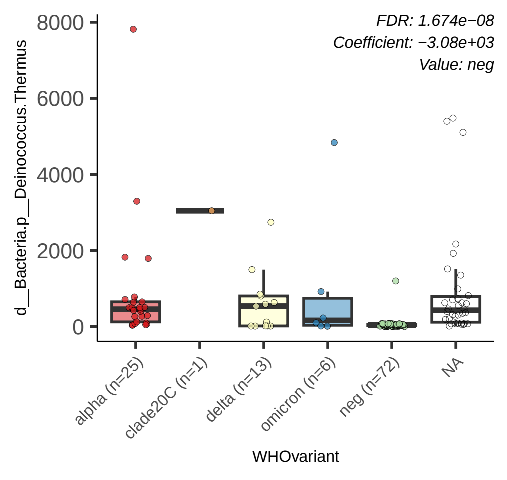
FDR: 1.674e−08
Coefficient: −3.08e+03
Value: neg
d__Bacteria.p__Deinococcus.Thermus
8000
6000
4000
2000
0
alpha (n=25)
clade20C (n=1)
delta (n=13)
omicron (n=6)
neg (n=72)
NA
WHOvariant

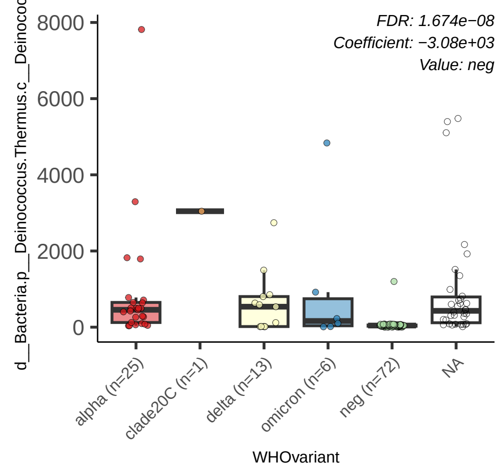

FDR: 1.674e−08
Coefficient: −3.08e+03
Value: neg
d__Bacteria.p__Deinococcus.Thermus.c__Deinococ
8000
6000
4000
2000
0
alpha (n=25)
clade20C (n=1)
delta (n=13)
omicron (n=6)
neg (n=72)
NA
WHOvariant

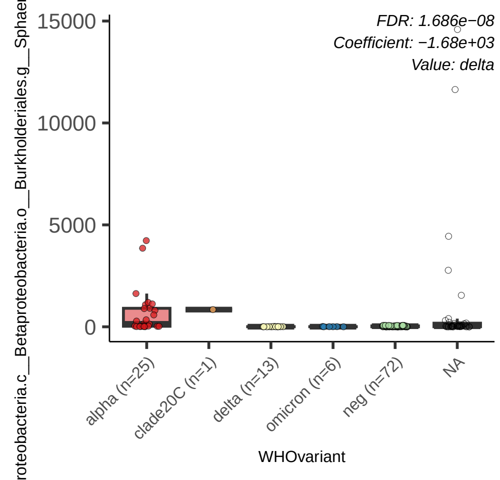

FDR: 1.686e−08
Coefficient: −1.68e+03
Value: delta
15000
10000
5000
0
roteobacteria.c__Betaproteobacteria.o__Burkholderiales.g__Sphae
alpha (n=25)
clade20C (n=1)
delta (n=13)
omicron (n=6)
neg (n=72)
NA
WHOvariant

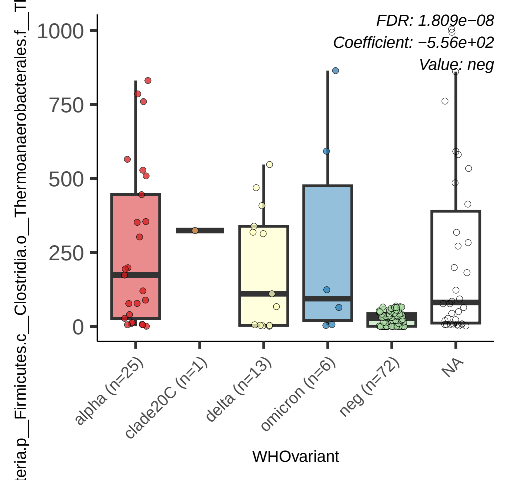

FDR: 1.809e−08
Coefficient: −5.56e+02
Value: neg
teria.p__Firmicutes.c__Clostridia.o__Thermoanaerobacterales.f__Th
1000
750
500
250
0
alpha (n=25)
clade20C (n=1)
delta (n=13)
omicron (n=6)
neg (n=72)
NA
WHOvariant

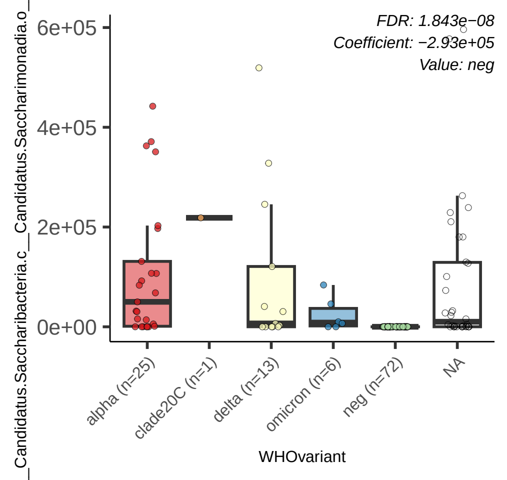

FDR: 1.843e−08
Coefficient: −2.93e+05
Value: neg
Candidatus.Saccharibacteria.c__Candidatus.Saccharimonadia.o
6e+05
4e+05
2e+05
0e+00
alpha (n=25)
clade20C (n=1)
delta (n=13)
omicron (n=6)
neg (n=72)
NA
WHOvariant

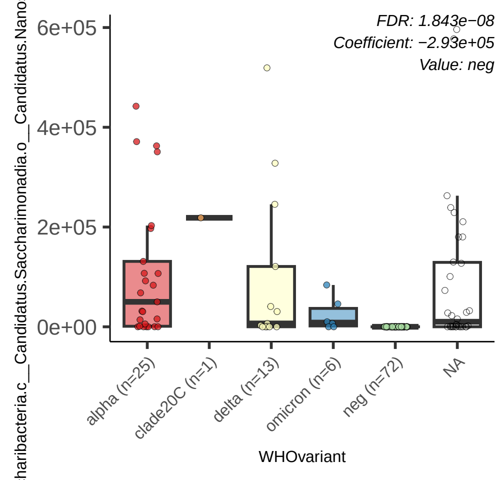
FDR: 1.843e−08
Coefficient: −2.93e+05
Value: neg
6e+05
4e+05
2e+05
0e+00
charibacteria.c__Candidatus.Saccharimonadia.o__Candidatus.Nano
alpha (n=25)
clade20C (n=1)
delta (n=13)
omicron (n=6)
neg (n=72)
NA
WHOvariant

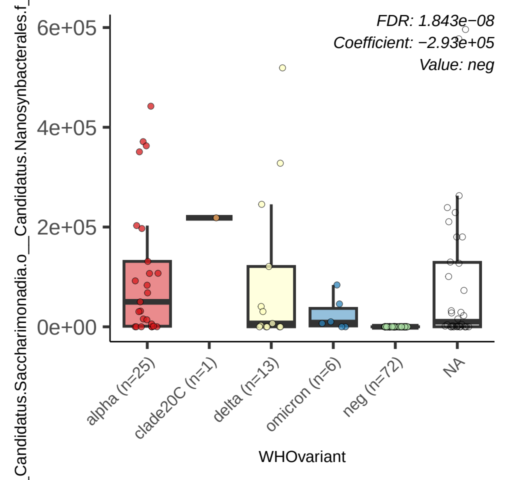

FDR: 1.843e−08
Coefficient: −2.93e+05
Value: neg
Candidatus.Saccharimonadia.o__Candidatus.Nanosynbacterales.f_
6e+05
4e+05
2e+05
0e+00
alpha (n=25)
clade20C (n=1)
delta (n=13)
omicron (n=6)
neg (n=72)
NA
WHOvariant

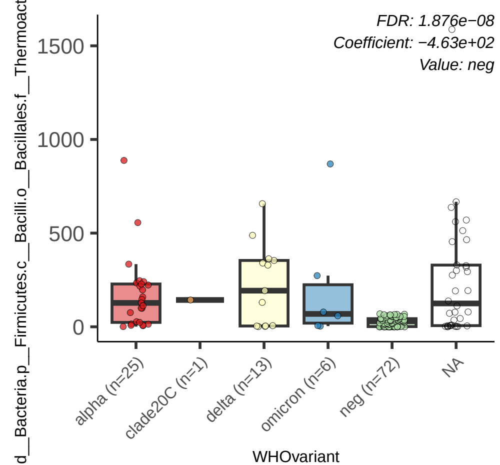
FDR: 1.876e−08
Coefficient: −4.63e+02
Value: neg
d__Bacteria.p__Firmicutes.c__Bacilli.o__Bacillales.f__Thermoact
1500
1000
500
0
alpha (n=25)
clade20C (n=1)
delta (n=13)
omicron (n=6)
neg (n=72)
NA
WHOvariant

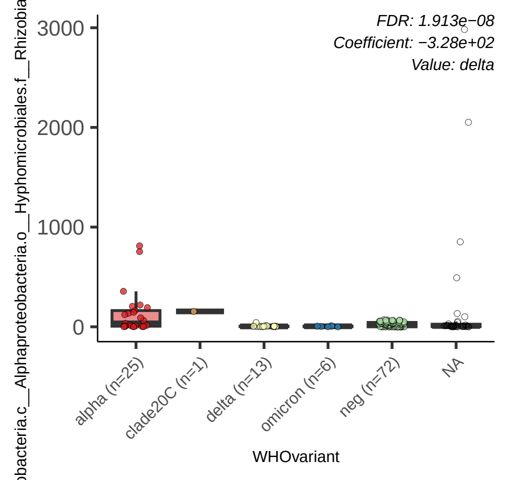

FDR: 1.913e−08
Coefficient: −3.28e+02
Value: delta
3000
2000
1000
0
obacteria.c__Alphaproteobacteria.o__Hyphomicrobiales.f__Rhizobia
alpha (n=25)
clade20C (n=1)
delta (n=13)
omicron (n=6)
neg (n=72)
NA
WHOvariant

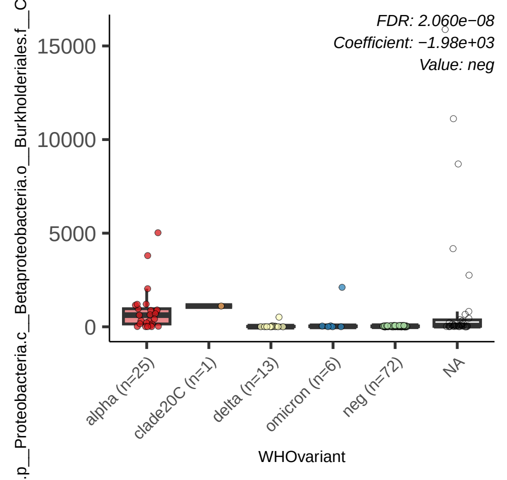

FDR: 2.060e−08
Coefficient: −1.98e+03
Value: neg
15000
10000
5000
0
.p__Proteobacteria.c__Betaproteobacteria.o__Burkholderiales.f__C
alpha (n=25)
clade20C (n=1)
delta (n=13)
omicron (n=6)
neg (n=72)
NA
WHOvariant

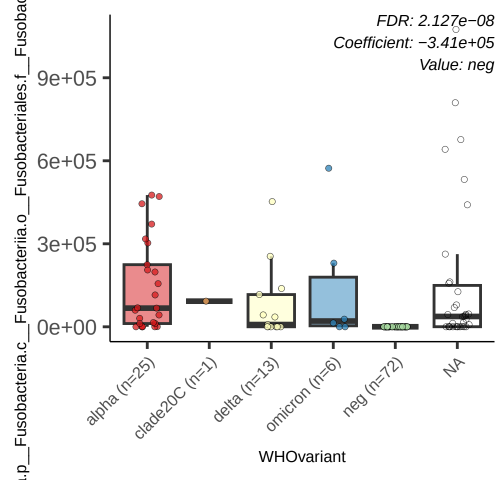
FDR: 2.127e−08
Coefficient: −3.41e+05
Value: neg
9e+05
6e+05
3e+05
0e+00
alpha (n=25)
clade20C (n=1)
delta (n=13)
omicron (n=6)
neg (n=72)
NA
WHOvariant
a.p__Fusobacteria.c__Fusobacteriia.o__Fusobacteriales.f__Fusobac

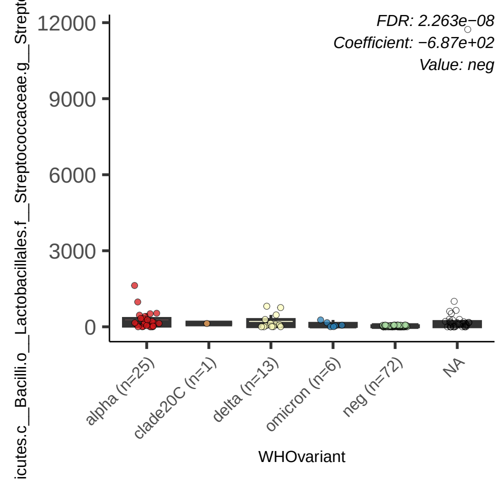

FDR: 2.263e−08
Coefficient: −6.87e+02
Value: neg
12000
9000
6000
3000
0
icutes.c__Bacilli.o__Lactobacillales.f__Streptococcaceae.g__Strept
alpha (n=25)
clade20C (n=1)
delta (n=13)
omicron (n=6)
neg (n=72)
NA
WHOvariant

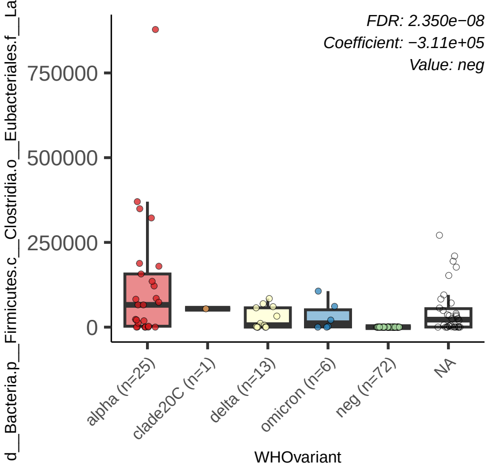

d__Bacteria.p__Firmicutes.c__Clostridia.o__Eubacteriales.f__La
FDR: 2.350e−08
Coefficient: −3.11e+05
Value: neg
750000
500000
250000
0
alpha (n=25)
clade20C (n=1)
delta (n=13)
omicron (n=6)
neg (n=72)
NA
WHOvariant

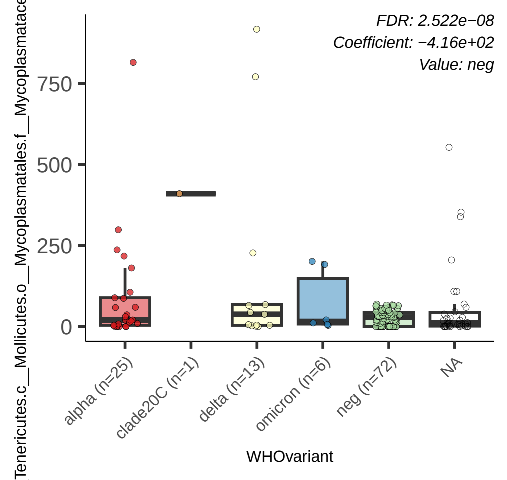
FDR: 2.522e−08
Coefficient: −4.16e+02
Value: neg
Tenericutes.c__Mollicutes.o__Mycoplasmatales.f__Mycoplasmatace
0
250
500
750
alpha (n=25)
clade20C (n=1)
delta (n=13)
omicron (n=6)
neg (n=72)
NA
WHOvariant

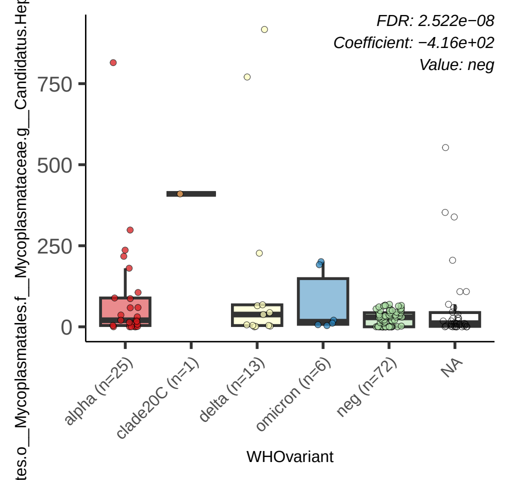

FDR: 2.522e−08
Coefficient: −4.16e+02
Value: neg
0
250
500
750
tes.o__Mycoplasmatales.f__Mycoplasmataceae.g__Candidatus.Hep
alpha (n=25)
clade20C (n=1)
delta (n=13)
omicron (n=6)
neg (n=72)
NA
WHOvariant

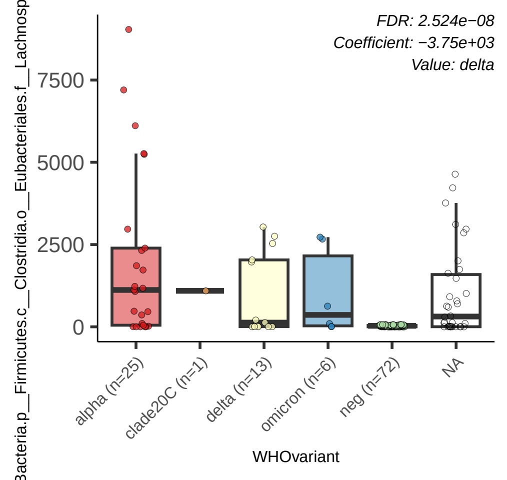

FDR: 2.524e−08
Coefficient: −3.75e+03
Value: delta
Bacteria.p__Firmicutes.c__Clostridia.o__Eubacteriales.f__Lachnosp
0
2500
5000
7500
alpha (n=25)
clade20C (n=1)
delta (n=13)
omicron (n=6)
neg (n=72)
NA
WHOvariant

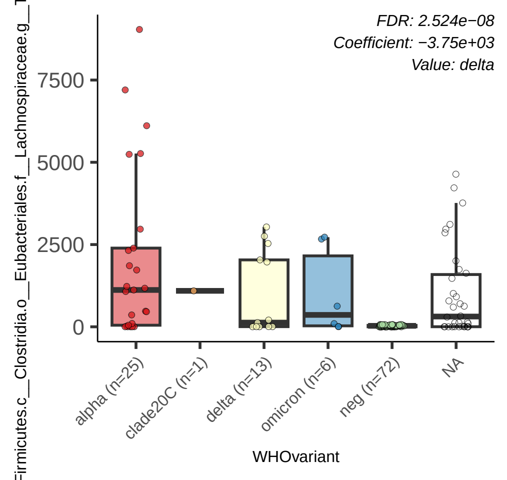

FDR: 2.524e−08
Coefficient: −3.75e+03
Value: delta
Firmicutes.c__Clostridia.o__Eubacteriales.f__Lachnospiraceae.g__
7500
5000
2500
0
alpha (n=25)
clade20C (n=1)
delta (n=13)
omicron (n=6)
neg (n=72)
NA
WHOvariant

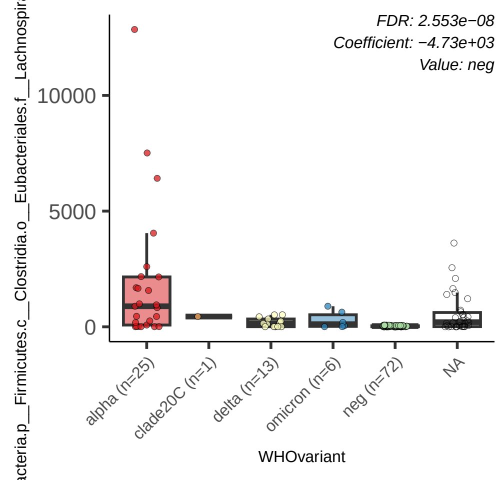

FDR: 2.553e−08
Coefficient: −4.73e+03
Value: neg
acteria.p__Firmicutes.c__Clostridia.o__Eubacteriales.f__Lachnospira
10000
5000
0
alpha (n=25)
clade20C (n=1)
delta (n=13)
omicron (n=6)
neg (n=72)
NA
WHOvariant

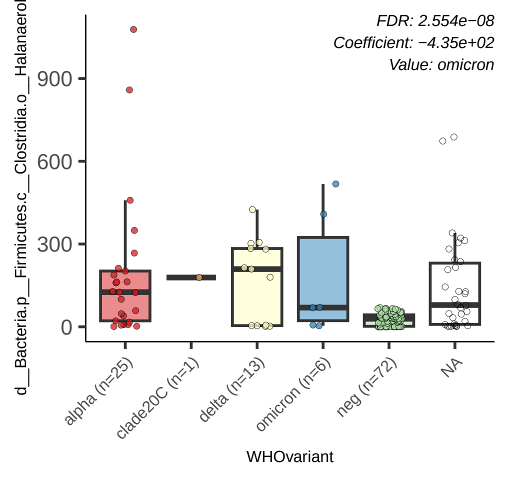

FDR: 2.554e−08
Coefficient: −4.35e+02
Value: omicron
d__Bacteria.p__Firmicutes.c__Clostridia.o__Halanaerob
0
300
600
900
alpha (n=25)
clade20C (n=1)
delta (n=13)
omicron (n=6)
neg (n=72)
NA
WHOvariant

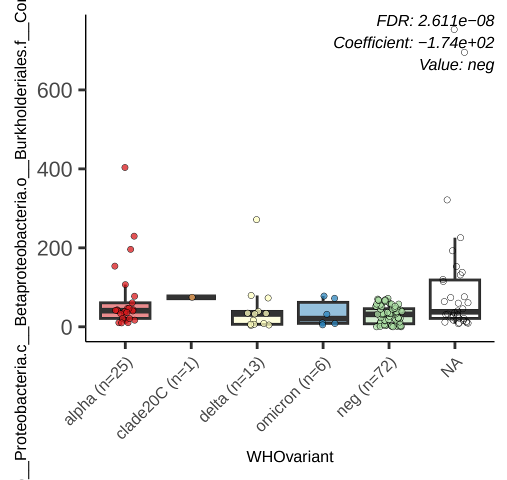
FDR: 2.611e−08
Coefficient: −1.74e+02
Value: neg
Proteobacteria.c__Betaproteobacteria.o__Burkholderiales.f__Co
600
400
200
0
alpha (n=25)
clade20C (n=1)
delta (n=13)
omicron (n=6)
neg (n=72)
NA
WHOvariant

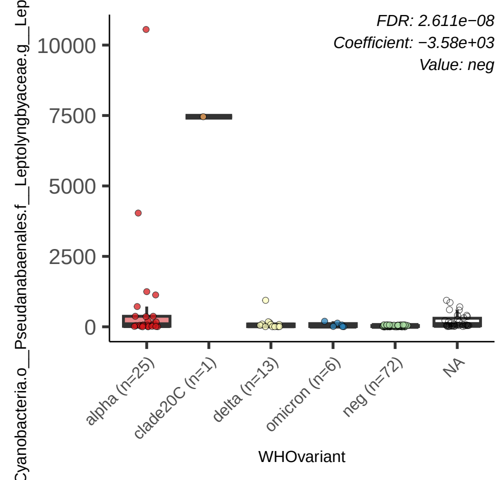
FDR: 2.611e−08
Coefficient: −3.58e+03
Value: neg
Cyanobacteria.o__Pseudanabaenales.f__Leptolyngbyaceae.g__Lep
10000
7500
5000
2500
0
alpha (n=25)
clade20C (n=1)
delta (n=13)
omicron (n=6)
neg (n=72)
NA
WHOvariant

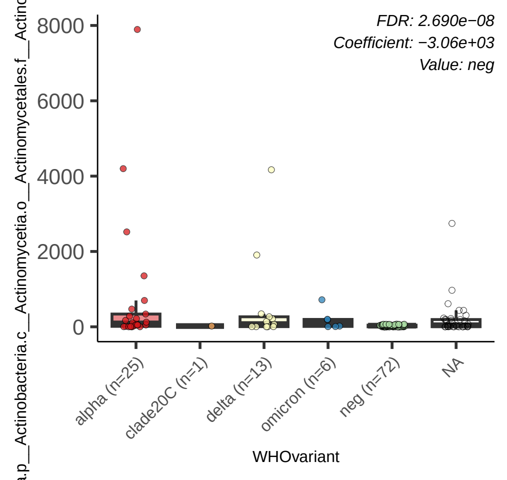
FDR: 2.690e−08
Coefficient: −3.06e+03
Value: neg
8000
6000
4000
2000
0
alpha (n=25)
clade20C (n=1)
delta (n=13)
omicron (n=6)
neg (n=72)
NA
WHOvariant

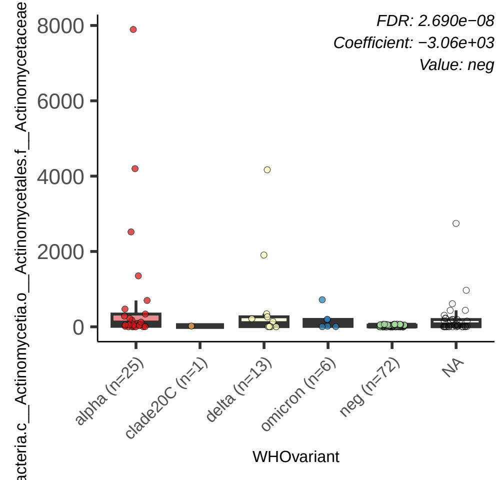

FDR: 2.690e−08
Coefficient: −3.06e+03
Value: neg
8000
6000
4000
2000
0
acteria.c__Actinomycetia.o__Actinomycetales.f__Actinomycetaceae
alpha (n=25)
clade20C (n=1)
delta (n=13)
omicron (n=6)
neg (n=72)
NA
WHOvariant

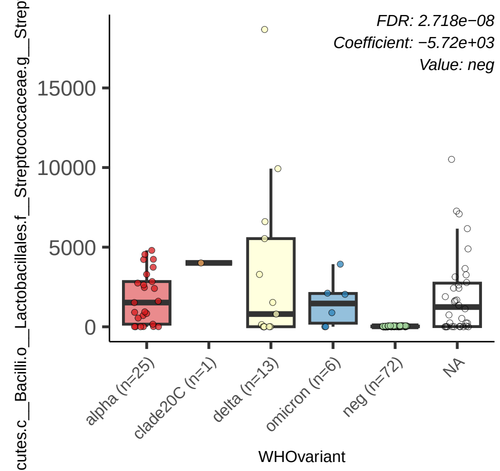
FDR: 2.718e−08
Coefficient: −5.72e+03
Value: neg
15000
10000
5000
0
alpha (n=25)
clade20C (n=1)
delta (n=13)
omicron (n=6)
neg (n=72)
NA
WHOvariant

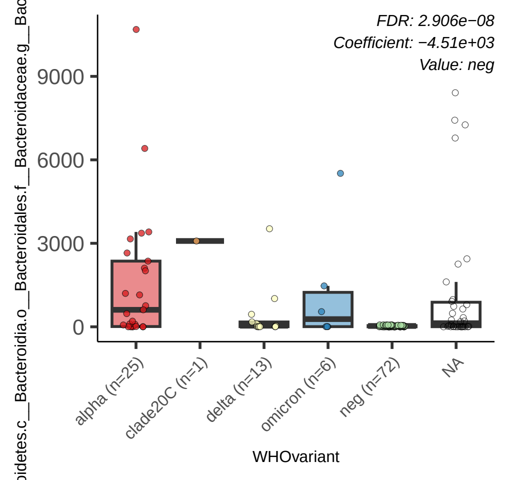
FDR: 2.906e−08
Coefficient: −4.51e+03
Value: neg
oidetes.c__Bacteroidia.o__Bacteroidales.f__Bacteroidaceae.g__Bac
9000
6000
3000
0
alpha (n=25)
clade20C (n=1)
delta (n=13)
omicron (n=6)
neg (n=72)
NA
WHOvariant

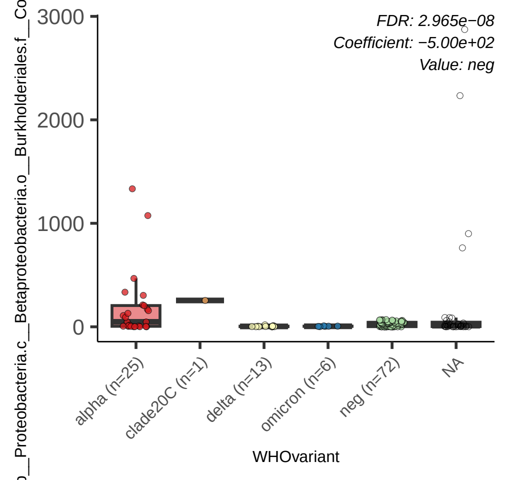

FDR: 2.965e−08
Coefficient: −5.00e+02
Value: neg
3000
2000
1000
0
o__Proteobacteria.c__Betaproteobacteria.o__Burkholderiales.f__Co
alpha (n=25)
clade20C (n=1)
delta (n=13)
omicron (n=6)
neg (n=72)
NA
WHOvariant

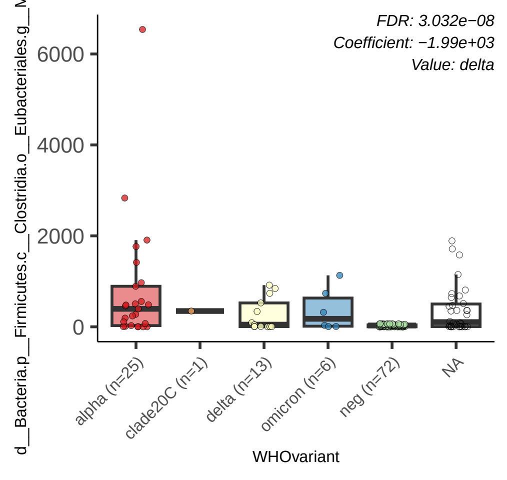

FDR: 3.032e−08
Coefficient: −1.99e+03
Value: delta
d__Bacteria.p__Firmicutes.c__Clostridia.o__Eubacteriales.g__M
6000
4000
2000
0
alpha (n=25)
clade20C (n=1)
delta (n=13)
omicron (n=6)
neg (n=72)
NA
WHOvariant

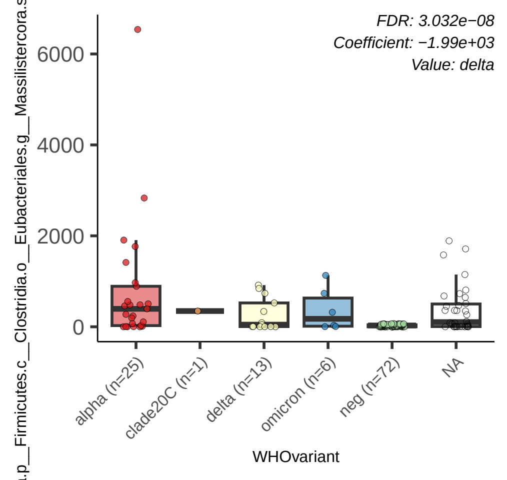

FDR: 3.032e−08
Coefficient: −1.99e+03
Value: delta
Firmicutes.c__Clostridia.o__Eubacteriales.g__Massilistercora.s
6000
4000
2000
0
alpha (n=25)
clade20C (n=1)
delta (n=13)
omicron (n=6)
neg (n=72)
NA
WHOvariant

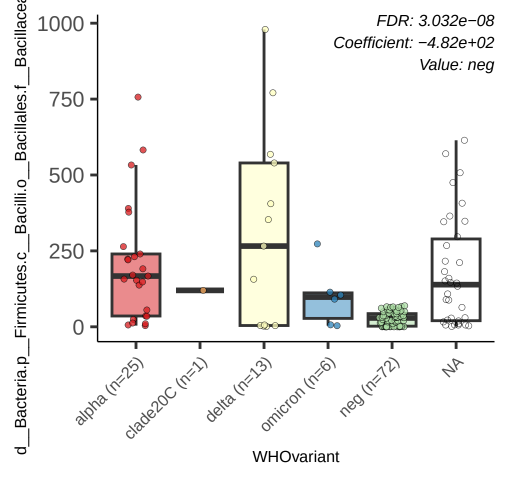

FDR: 3.032e−08
Coefficient: −4.82e+02
Value: neg
d__Bacteria.p__Firmicutes.c__Bacilli.o__Bacillales.f__Bacillacea
1000
750
500
250
0
alpha (n=25)
clade20C (n=1)
delta (n=13)
omicron (n=6)
neg (n=72)
NA
WHOvariant

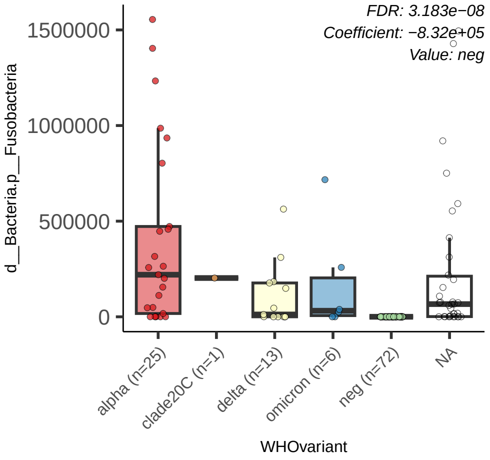

FDR: 3.183e−08
Coefficient: −8.32e+05
Value: neg
d__Bacteria.p__Fusobacteria
1500000
1000000
500000
0
alpha (n=25)
clade20C (n=1)
delta (n=13)
omicron (n=6)
neg (n=72)
NA
WHOvariant

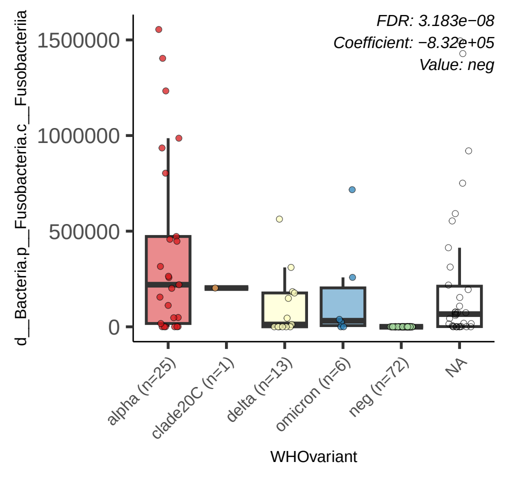
FDR: 3.183e−08
Coefficient: −8.32e+05
Value: neg
d__Bacteria.p__Fusobacteria.c__Fusobacteriia
1500000
1000000
500000
0
alpha (n=25)
clade20C (n=1)
delta (n=13)
omicron (n=6)
neg (n=72)
NA
WHOvariant

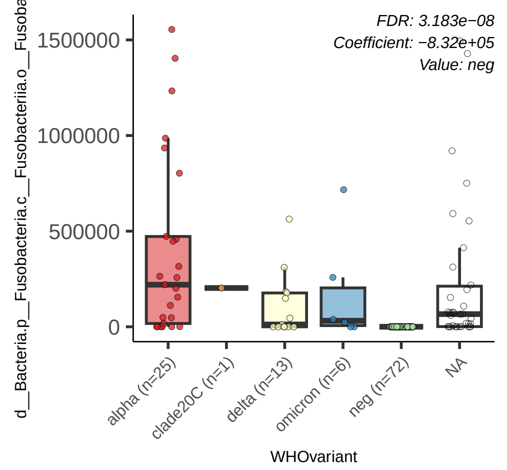

FDR: 3.183e−08
Coefficient: −8.32e+05
Value: neg
d__Bacteria.p__Fusobacteria.c__Fusobacteriia.o__Fusoba
1500000
1000000
500000
0
alpha (n=25)
clade20C (n=1)
delta (n=13)
omicron (n=6)
neg (n=72)
NA
WHOvariant

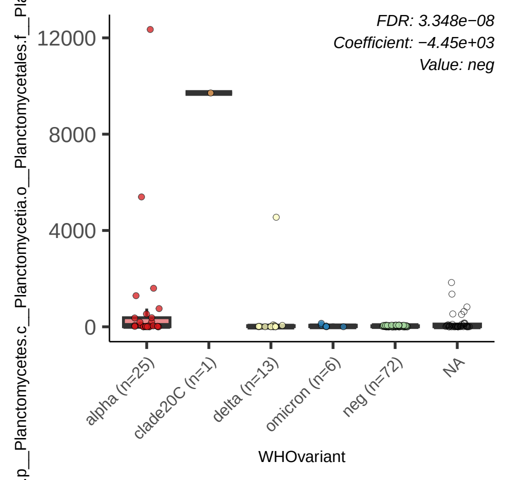

FDR: 3.348e−08
Coefficient: −4.45e+03
Value: neg
12000
8000
4000
0
alpha (n=25)
clade20C (n=1)
delta (n=13)
omicron (n=6)
neg (n=72)
NA
WHOvariant
p__Planctomycetes.c__Planctomycetia.o__Planctomycetales.f__Pla

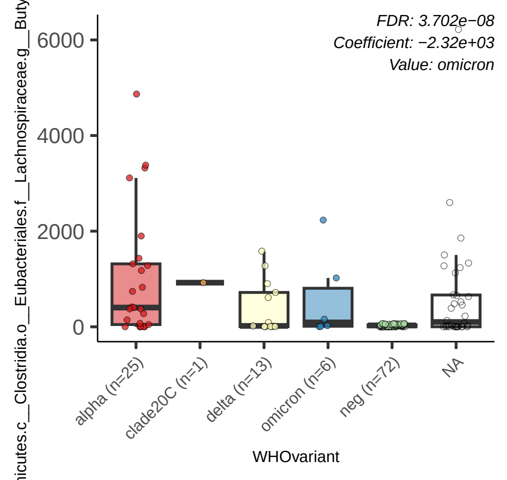

FDR: 3.702e−08
Coefficient: −2.32e+03
Value: omicron
6000
4000
2000
0
alpha (n=25)
clade20C (n=1)
delta (n=13)
omicron (n=6)
neg (n=72)
NA
WHOvariant
nicutes.c__Clostridia.o__Eubacteriales.f__Lachnospiraceae.g__Buty

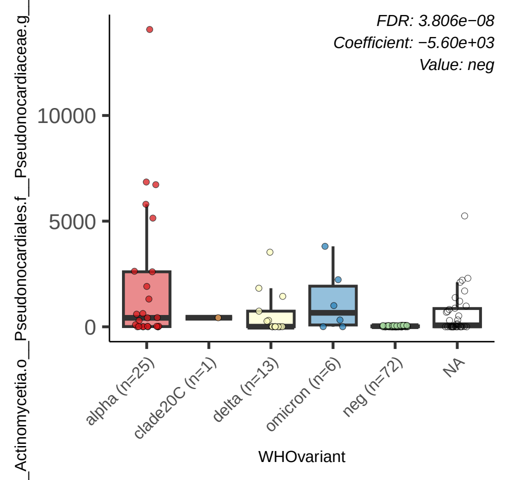
FDR: 3.806e−08
Coefficient: −5.60e+03
Value: neg
Actinomycetia.o__Pseudonocardiales.f__Pseudonocardiaceae.g__
0
5000
10000
alpha (n=25)
clade20C (n=1)
delta (n=13)
omicron (n=6)
neg (n=72)
NA
WHOvariant

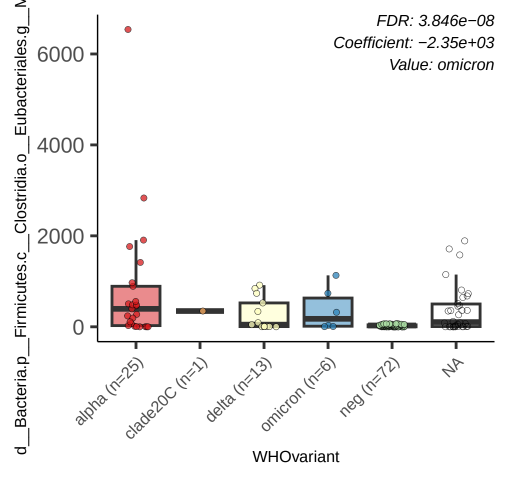

FDR: 3.846e−08
Coefficient: −2.35e+03
Value: omicron
d__Bacteria.p__Firmicutes.c__Clostridia.o__Eubacteriales.g__M
6000
4000
2000
0
alpha (n=25)
clade20C (n=1)
delta (n=13)
omicron (n=6)
neg (n=72)
NA
WHOvariant

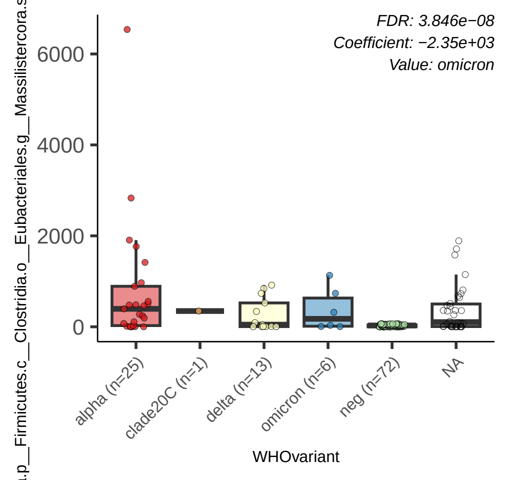

FDR: 3.846e−08
Coefficient: −2.35e+03
Value: omicron
6000
4000
2000
0
a.p__Firmicutes.c__Clostridia.o__Eubacteriales.g__Massilistercora.s
alpha (n=25)
clade20C (n=1)
delta (n=13)
omicron (n=6)
neg (n=72)
NA
WHOvariant

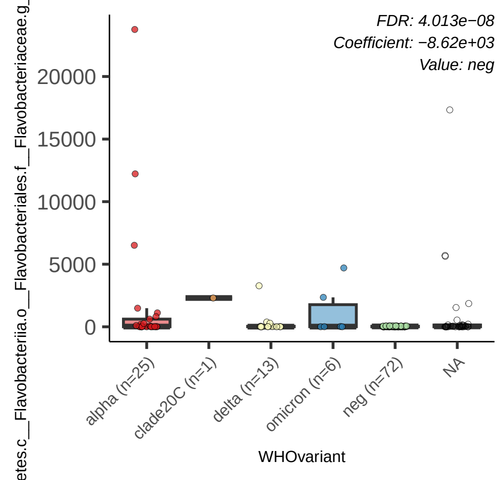

FDR: 4.013e−08
Coefficient: −8.62e+03
Value: neg
20000
15000
10000
5000
0
alpha (n=25)
clade20C (n=1)
delta (n=13)
omicron (n=6)
neg (n=72)
NA
WHOvariant
etes.c__Flavobacteriia.o__Flavobacteriales.f__Flavobacteriaceae.g_

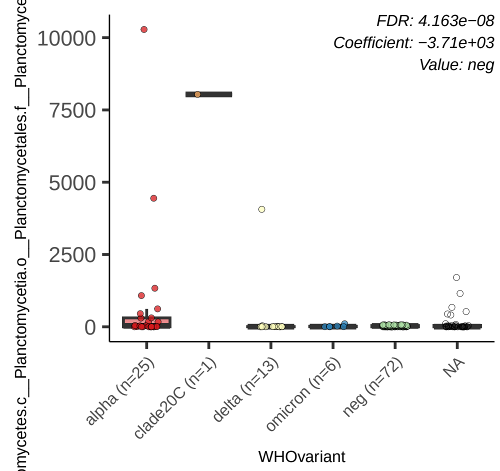

FDR: 4.163e−08
Coefficient: −3.71e+03
Value: neg
10000
7500
5000
2500
0
alpha (n=25)
clade20C (n=1)
delta (n=13)
omicron (n=6)
neg (n=72)
NA
WHOvariant
tomycetes.c__Planctomycetia.o__Planctomycetales.f__Planctomyce

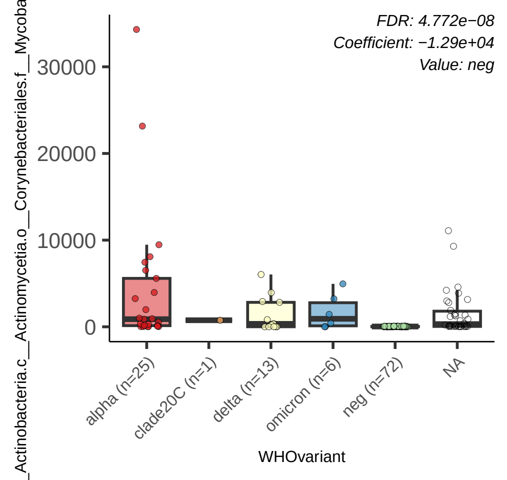

FDR: 4.772e−08
Coefficient: −1.29e+04
Value: neg
_Actinobacteria.c__Actinomycetia.o__Corynebacteriales.f__Mycoba
30000
20000
10000
0
alpha (n=25)
clade20C (n=1)
delta (n=13)
omicron (n=6)
neg (n=72)
NA
WHOvariant

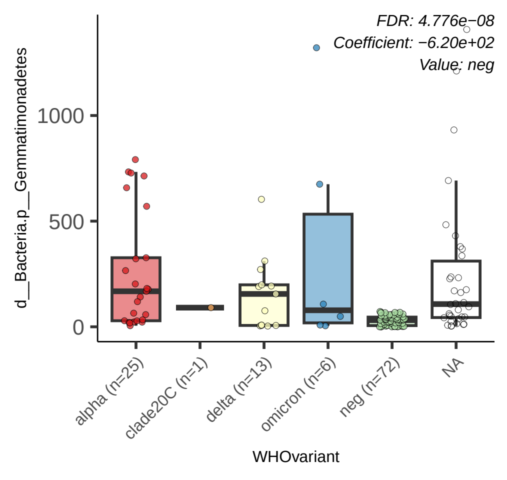
FDR: 4.776e−08
Coefficient: −6.20e+02
Value: neg
d__Bacteria.p__Gemmatimonadetes
1000
500
0
alpha (n=25)
clade20C (n=1)
delta (n=13)
omicron (n=6)
neg (n=72)
NA
WHOvariant

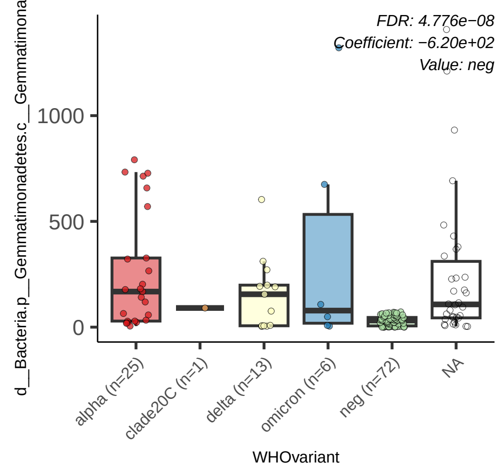

FDR: 4.776e−08
Coefficient: −6.20e+02
Value: neg
d__Bacteria.p__Gemmatimonadetes.c__Gemmatimona
1000
500
0
alpha (n=25)
clade20C (n=1)
delta (n=13)
omicron (n=6)
neg (n=72)
NA
WHOvariant

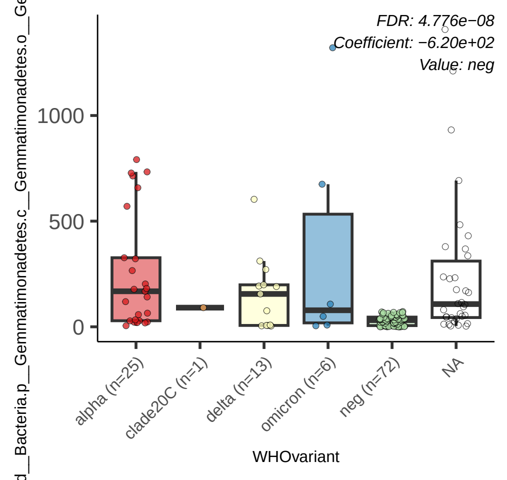

FDR: 4.776e−08
Coefficient: −6.20e+02
Value: neg
d__Bacteria.p__Gemmatimonadetes.c__Gemmatimonadetes.o__Ge
1000
500
0
alpha (n=25)
clade20C (n=1)
delta (n=13)
omicron (n=6)
neg (n=72)
NA
WHOvariant

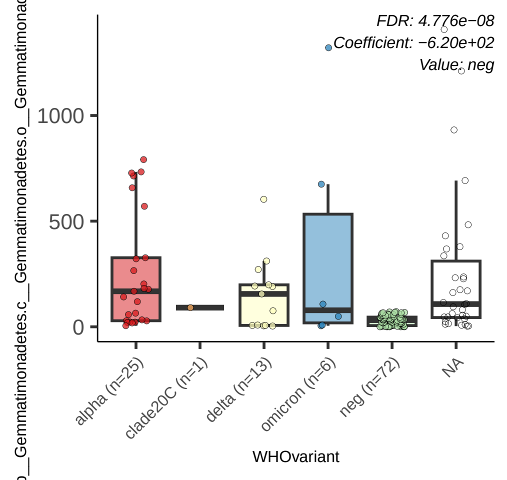

FDR: 4.776e−08
Coefficient: −6.20e+02
Value: neg
0
500
1000
alpha (n=25)
clade20C (n=1)
delta (n=13)
omicron (n=6)
neg (n=72)
NA
WHOvariant
o__Gemmatimonadetes.c__Gemmatimonadetes.o__Gemmatimonad

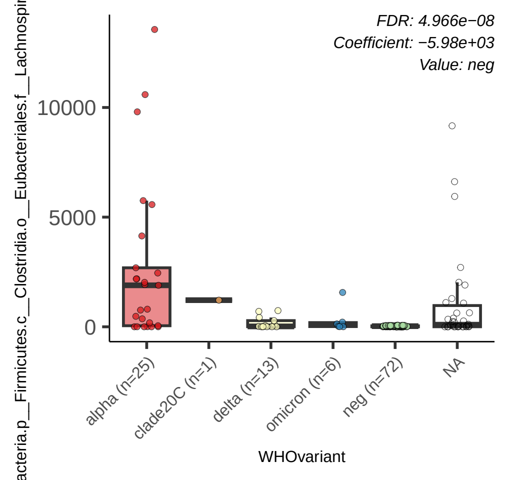

FDR: 4.966e−08
Coefficient: −5.98e+03
Value: neg
acteria.p__Firmicutes.c__Clostridia.o__Eubacteriales.f__Lachnospi
10000
5000
0
alpha (n=25)
clade20C (n=1)
delta (n=13)
omicron (n=6)
neg (n=72)
NA
WHOvariant

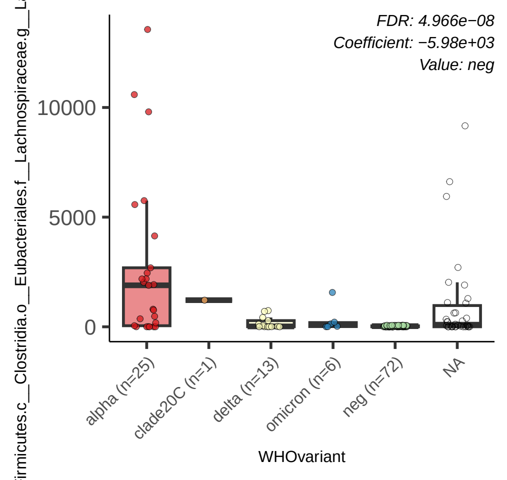

FDR: 4.966e−08
Coefficient: −5.98e+03
Value: neg
irmicutes.c__Clostridia.o__Eubacteriales.f__Lachnospiraceae.g__La
10000
5000
0
alpha (n=25)
clade20C (n=1)
delta (n=13)
omicron (n=6)
neg (n=72)
NA
WHOvariant

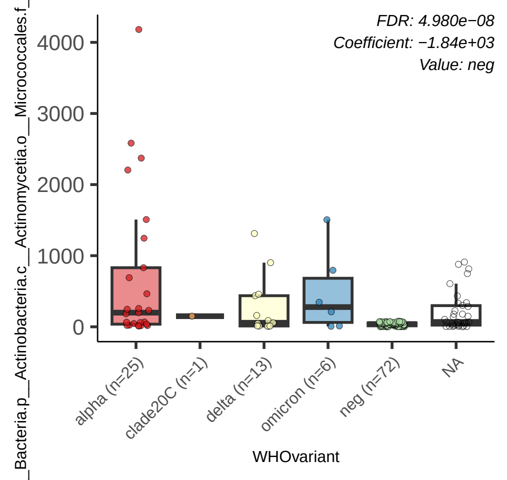

FDR: 4.980e−08
Coefficient: −1.84e+03
Value: neg
_Bacteria.p__Actinobacteria.c__Actinomycetia.o__Micrococcales.f_
4000
3000
2000
1000
0
alpha (n=25)
clade20C (n=1)
delta (n=13)
omicron (n=6)
neg (n=72)
NA
WHOvariant

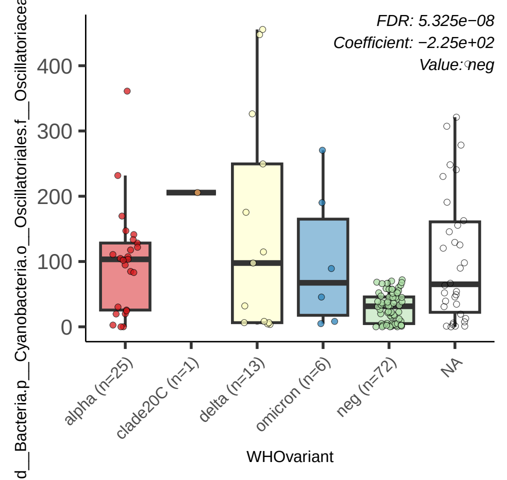

FDR: 5.325e−08
Coefficient: −2.25e+02
Value: neg
d__Bacteria.p__Cyanobacteria.o__Oscillatoriales.f__Oscillatoriacea
0
100
200
300
400
alpha (n=25)
clade20C (n=1)
delta (n=13)
omicron (n=6)
neg (n=72)
NA
WHOvariant

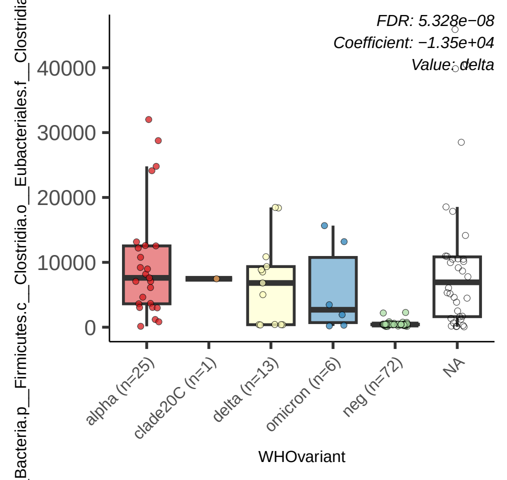
FDR: 5.328e−08
Coefficient: −1.35e+04
Value: delta
Bacteria.p__Firmicutes.c__Clostridia.o__Eubacteriales.f__Clostridia
40000
30000
20000
10000
0
alpha (n=25)
clade20C (n=1)
delta (n=13)
omicron (n=6)
neg (n=72)
NA
WHOvariant

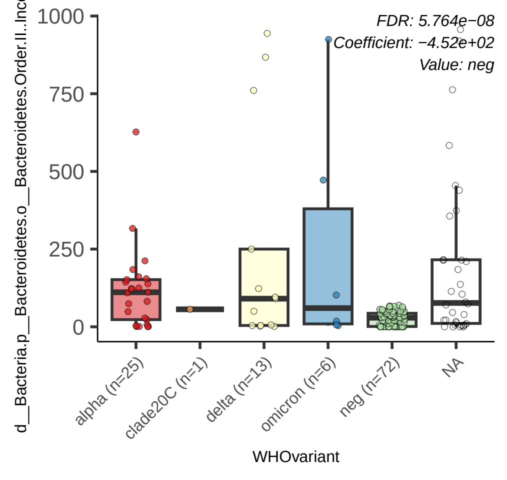

FDR: 5.764e−08
Coefficient: −4.52e+02
Value: neg
d__Bacteria.p__Bacteroidetes.o__Bacteroidetes.Order.II..Inc
1000
750
500
250
0
alpha (n=25)
clade20C (n=1)
delta (n=13)
omicron (n=6)
neg (n=72)
NA
WHOvariant

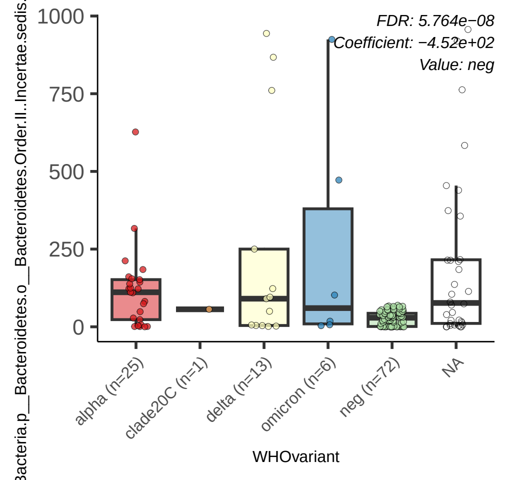

FDR: 5.764e−08
Coefficient: −4.52e+02
Value: neg
1000
750
500
250
0
Bacteria.p__Bacteroidetes.o__Bacteroidetes.Order.II..Incertae.sedis.
alpha (n=25)
clade20C (n=1)
delta (n=13)
omicron (n=6)
neg (n=72)
NA
WHOvariant

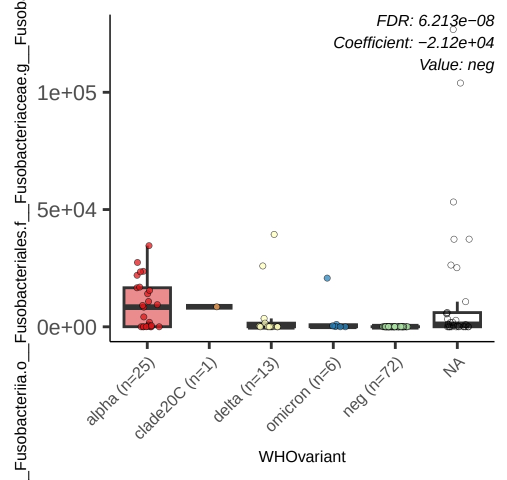

FDR: 6.213e−08
Coefficient: −2.12e+04
Value: neg
Fusobacteriia.o__Fusobacteriales.f__Fusobacteriaceae.g__Fusob
1e+05
5e+04
0e+00
alpha (n=25)
clade20C (n=1)
delta (n=13)
omicron (n=6)
neg (n=72)
NA
WHOvariant

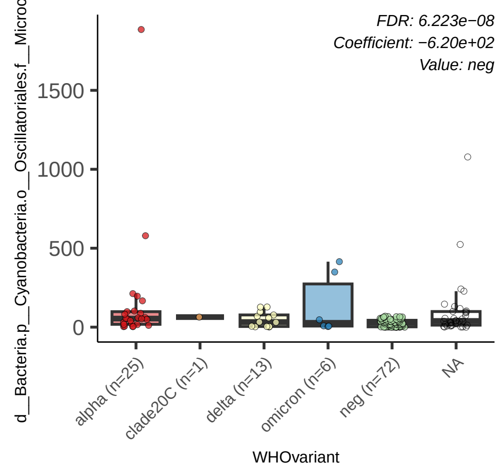

FDR: 6.223e−08
Coefficient: −6.20e+02
Value: neg
1500
1000
500
0
d__Bacteria.p__Cyanobacteria.o__Oscillatoriales.f__Microc
alpha (n=25)
clade20C (n=1)
delta (n=13)
omicron (n=6)
neg (n=72)
NA
WHOvariant

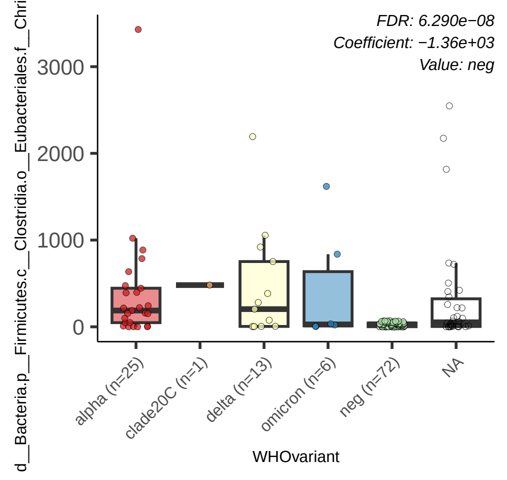

FDR: 6.290e−08
Coefficient: −1.36e+03
Value: neg
d__Bacteria.p__Firmicutes.c__Clostridia.o__Eubacteriales.f__Chri
3000
2000
1000
0
alpha (n=25)
clade20C (n=1)
delta (n=13)
omicron (n=6)
neg (n=72)
NA
WHOvariant

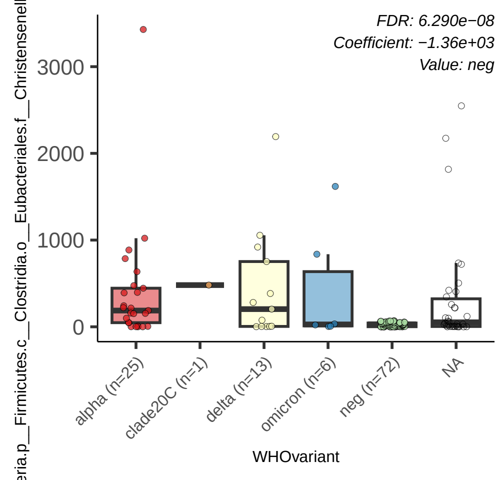
FDR: 6.290e−08
Coefficient: −1.36e+03
Value: neg
3000
2000
1000
0
alpha (n=25)
clade20C (n=1)
delta (n=13)
omicron (n=6)
neg (n=72)
NA
WHOvariant
eria.p__Firmicutes.c__Clostridia.o__Eubacteriales.f__Christensenell

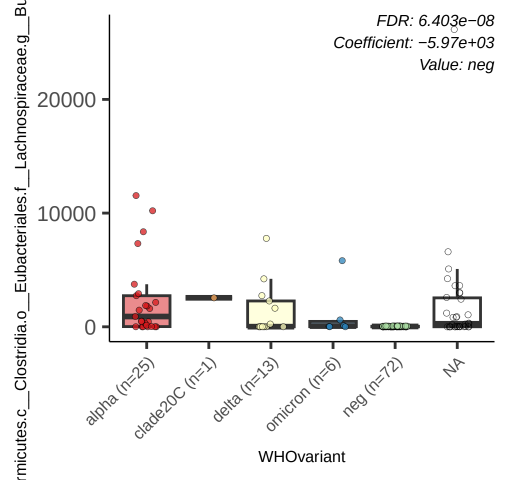

FDR: 6.403e−08
Coefficient: −5.97e+03
Value: neg
rmicutes.c__Clostridia.o__Eubacteriales.f__Lachnospiraceae.g__Bu
20000
10000
0
alpha (n=25)
clade20C (n=1)
delta (n=13)
omicron (n=6)
neg (n=72)
NA
WHOvariant

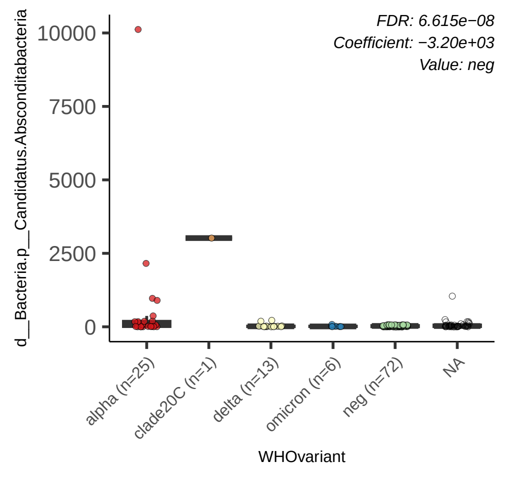

FDR: 6.615e−08
Coefficient: −3.20e+03
Value: neg
d__Bacteria.p__Candidatus.Abscondita bacteria
10000
7500
5000
2500
0
alpha (n=25)
clade20C (n=1)
delta (n=13)
omicron (n=6)
neg (n=72)
NA
WHOvariant

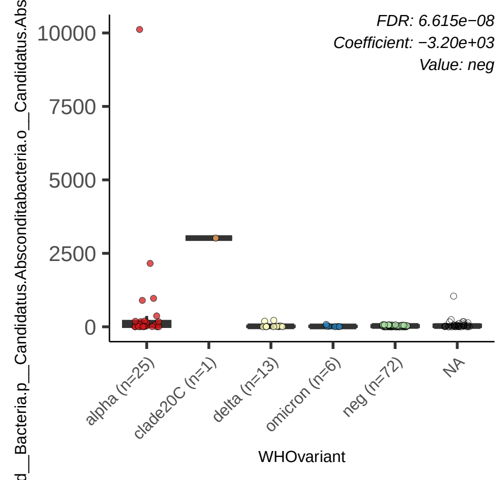

FDR: 6.615e−08
Coefficient: −3.20e+03
Value: neg
10000
7500
5000
2500
0
d__Bacteria.p__Candidatus.Absconditabacteria.o__Candidatus.Abs
alpha (n=25)
clade20C (n=1)
delta (n=13)
omicron (n=6)
neg (n=72)
NA
WHOvariant

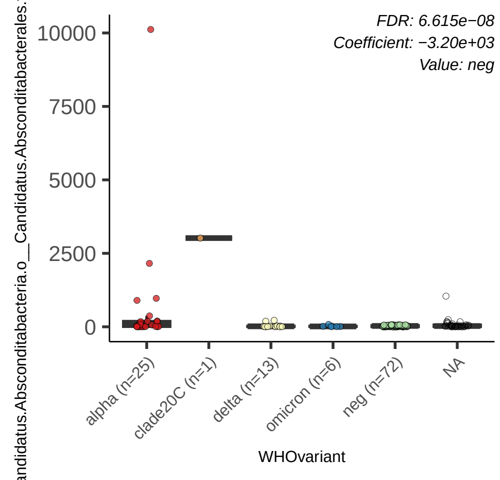
FDR: 6.615e−08
Coefficient: −3.20e+03
Value: neg
10000
7500
5000
2500
0
alpha (n=25)
clade20C (n=1)
delta (n=13)
omicron (n=6)
neg (n=72)
NA
WHOvariant

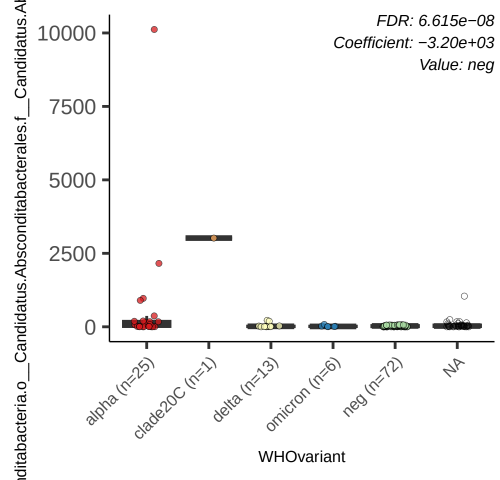

FDR: 6.615e−08
Coefficient: −3.20e+03
Value: neg
10000
7500
5000
2500
0
alpha (n=25)
clade20C (n=1)
delta (n=13)
omicron (n=6)
neg (n=72)
NA
WHOvariant
nditabacteria.o__Candidatus.Absconditabacterales.f__Candidatus.Al

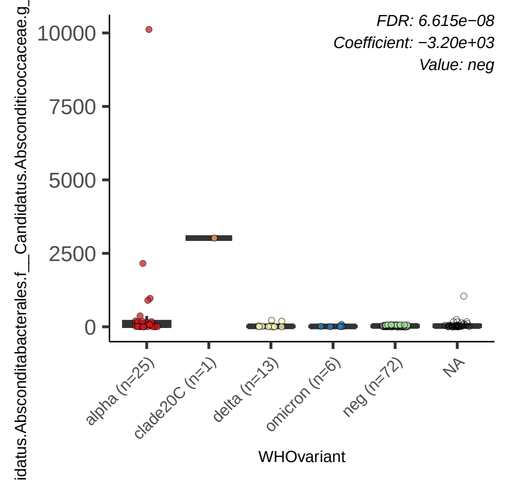

FDR: 6.615e−08
Coefficient: −3.20e+03
Value: neg
10000
7500
5000
2500
0
idatus.Absconditabacterales.f__Candidatus.Absconditicoccaceae.g_
alpha (n=25)
clade20C (n=1)
delta (n=13)
omicron (n=6)
neg (n=72)
NA
WHOvariant

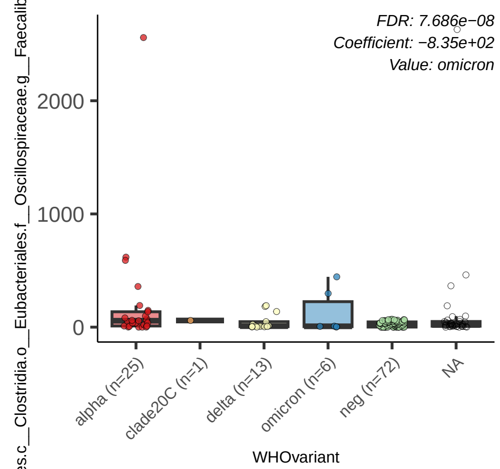

FDR: 7.686e−08
Coefficient: −8.35e+02
Value: omicron
tes.c__Clostridia.o__Eubacteriales.f__Oscillospiraceae.g__Faecalib
2000
1000
0
alpha (n=25)
clade20C (n=1)
delta (n=13)
omicron (n=6)
neg (n=72)
NA
WHOvariant

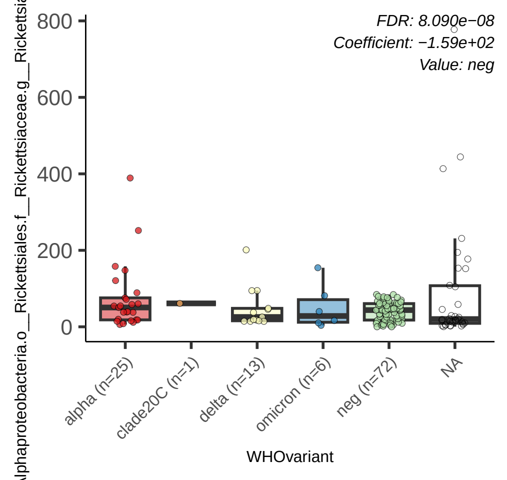

FDR: 8.090e−08
Coefficient: −1.59e+02
Value: neg
800
600
400
200
0
Alphaproteobacteria.o__Rickettsiales.f__Rickettsiaceae.g__Rickettsia
alpha (n=25)
clade20C (n=1)
delta (n=13)
omicron (n=6)
neg (n=72)
NA
WHOvariant

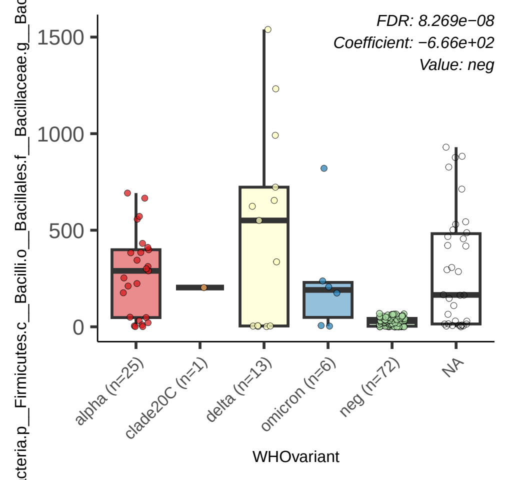

FDR: 8.269e−08
Coefficient: −6.66e+02
Value: neg
0
500
1000
1500
cteria.p__Firmicutes.c__Bacilli.o__Bacillales.f__Bacillaceae.g__Bac
alpha (n=25)
clade20C (n=1)
delta (n=13)
omicron (n=6)
neg (n=72)
NA
WHOvariant

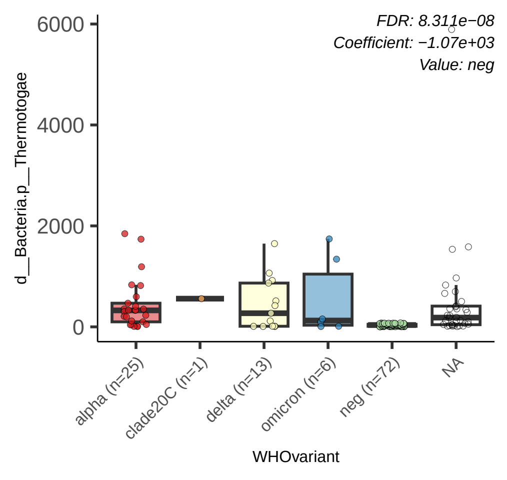

FDR: 8.311e−08
Coefficient: −1.07e+03
Value: neg
6000
4000
2000
0
d__Bacteria.p__Thermotogae
alpha (n=25)
clade20C (n=1)
delta (n=13)
omicron (n=6)
neg (n=72)
NA
WHOvariant

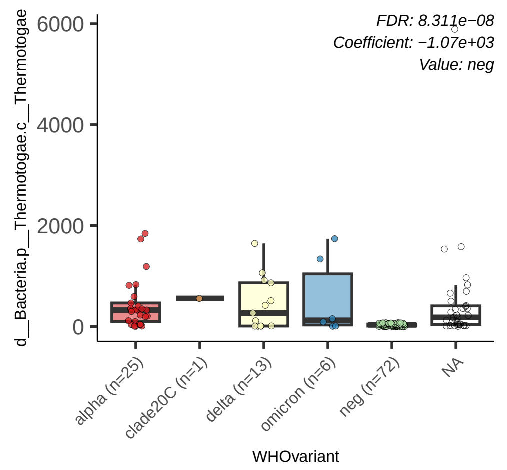

FDR: 8.311e−08
Coefficient: −1.07e+03
Value: neg
d__Bacteria.p__Thermotogae.c__Thermotogae
6000
4000
2000
0
alpha (n=25)
clade20C (n=1)
delta (n=13)
omicron (n=6)
neg (n=72)
NA
WHOvariant

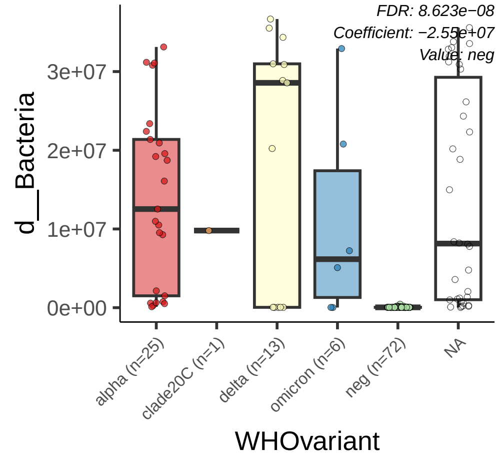
FDR: 8.623e−08
Coefficient: −2.55e+07
Value: neg
d__Bacteria
3e+07
2e+07
1e+07
0e+00
alpha (n=25)
clade20C (n=1)
delta (n=13)
omicron (n=6)
neg (n=72)
NA
WHOvariant

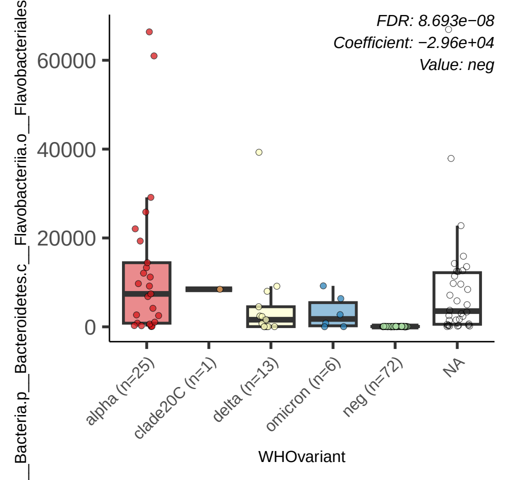

FDR: 8.693e−08
Coefficient: −2.96e+04
Value: neg
60000
40000
20000
0
__Bacteria.p__Bacteroidetes.c__Flavobacteriia.o__Flavobacteriales
alpha (n=25)
clade20C (n=1)
delta (n=13)
omicron (n=6)
neg (n=72)
NA
WHOvariant

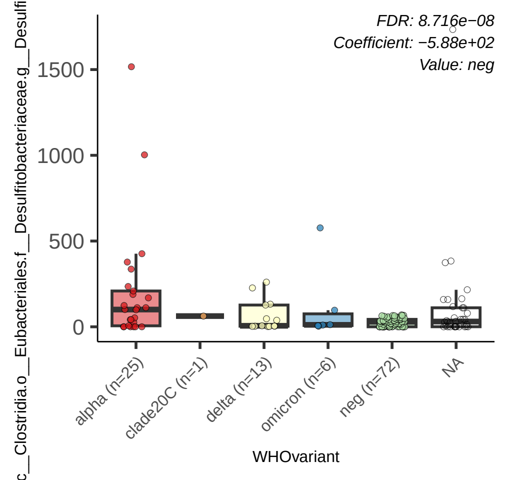

FDR: 8.716e−08
Coefficient: −5.88e+02
Value: neg
c__Clostridia.o__Eubacteriales.f__Desulfitobacteriaceae.g__Desulfi
0
500
1000
1500
alpha (n=25)
clade20C (n=1)
delta (n=13)
omicron (n=6)
neg (n=72)
NA
WHOvariant

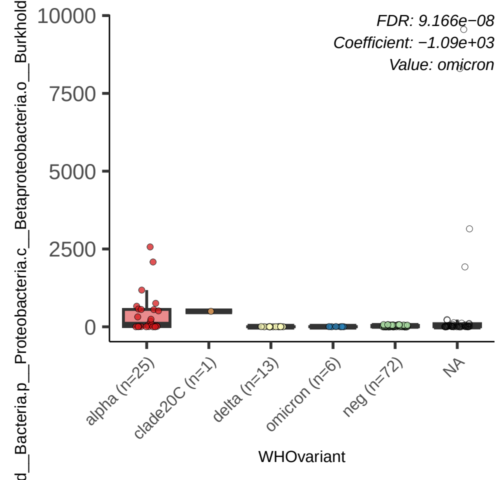

FDR: 9.166e−08
Coefficient: −1.09e+03
Value: omicron
10000
7500
5000
2500
0
d__Bacteria.p__Proteobacteria.c__Betaproteobacteria.o__Burkhold
alpha (n=25)
clade20C (n=1)
delta (n=13)
omicron (n=6)
neg (n=72)
NA
WHOvariant

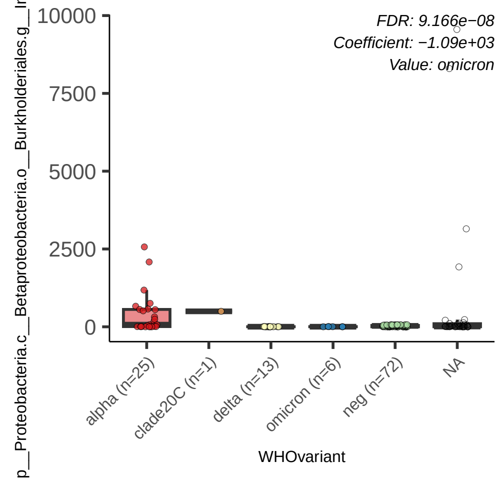

p__Proteobacteria.c__Betaproteobacteria.o__Burkholderiales.g__In
10000
7500
5000
2500
0
FDR: 9.166e−08
Coefficient: −1.09e+03
Value: omicron
alpha (n=25)
clade20C (n=1)
delta (n=13)
omicron (n=6)
neg (n=72)
NA
WHOvariant

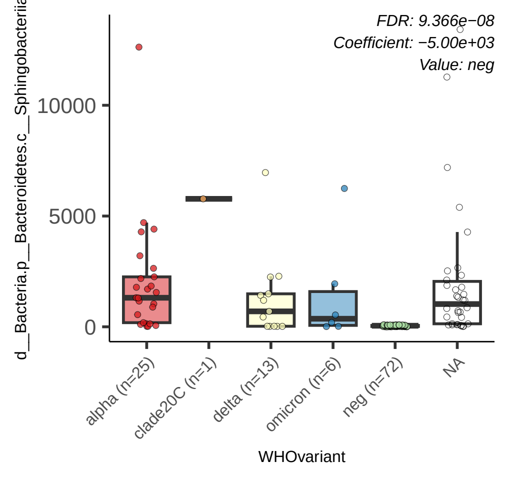
FDR: 9.366e−08
Coefficient: −5.00e+03
Value: neg
d__Bacteria.p__Bacteroidetes.c__Sphingobacteriia
10000
5000
0
alpha (n=25)
clade20C (n=1)
delta (n=13)
omicron (n=6)
neg (n=72)
NA
WHOvariant

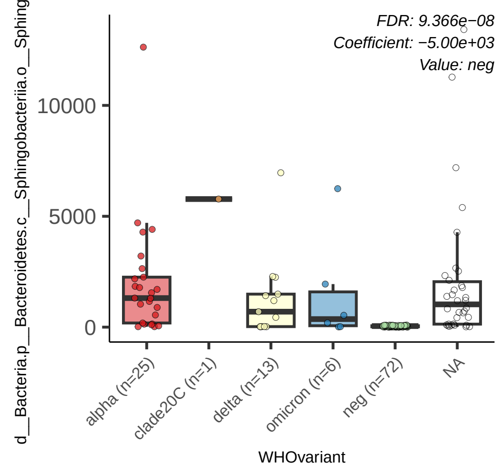

FDR: 9.366e−08
Coefficient: −5.00e+03
Value: neg
d__Bacteria.p__Bacteroidetes.c__Sphingobacteriia.o__Sphing
10000
5000
0
alpha (n=25)
clade20C (n=1)
delta (n=13)
omicron (n=6)
neg (n=72)
NA
WHOvariant

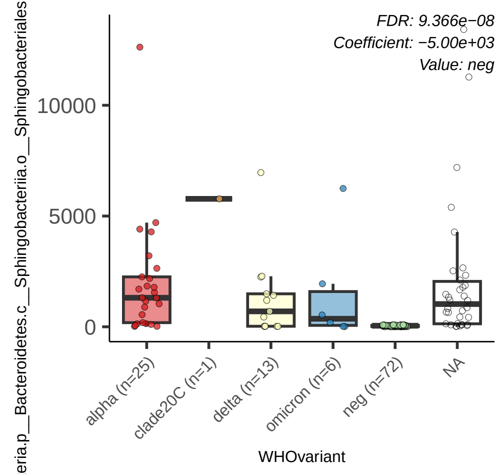
FDR: 9.366e−08
Coefficient: −5.00e+03
Value: neg
10000
5000
0
cteria.p__Bacteroidetes.c__Sphingobacteriia.o__Sphingobacteriales
alpha (n=25)
clade20C (n=1)
delta (n=13)
omicron (n=6)
neg (n=72)
NA
WHOvariant

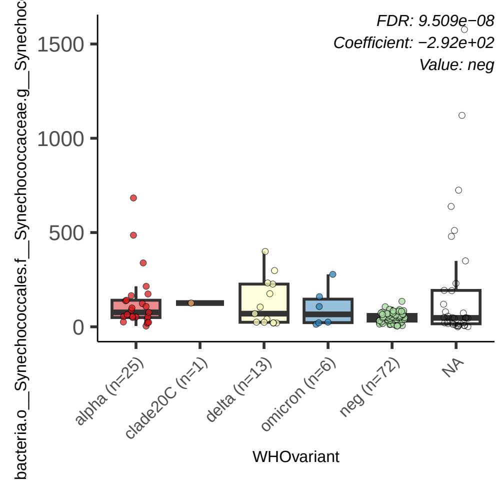

FDR: 9.509e−08
Coefficient: −2.92e+02
Value: neg
1500
1000
500
0
bacteria.o__Synechococcales.f__Synechococcaceae.g__Synechoc
alpha (n=25)
clade20C (n=1)
delta (n=13)
omicron (n=6)
neg (n=72)
NA
WHOvariant

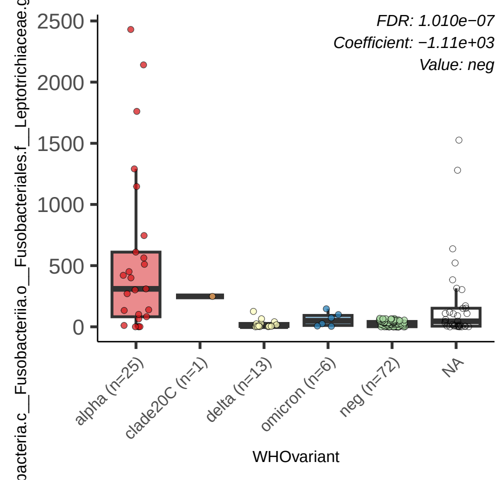

FDR: 1.010e−07
Coefficient: −1.11e+03
Value: neg
2500
2000
1500
1000
500
0
bacteria.c__Fusobacteriia.o__Fusobacteriales.f__Leptotrichiaceae.g
alpha (n=25)
clade20C (n=1)
delta (n=13)
omicron (n=6)
neg (n=72)
NA
WHOvariant

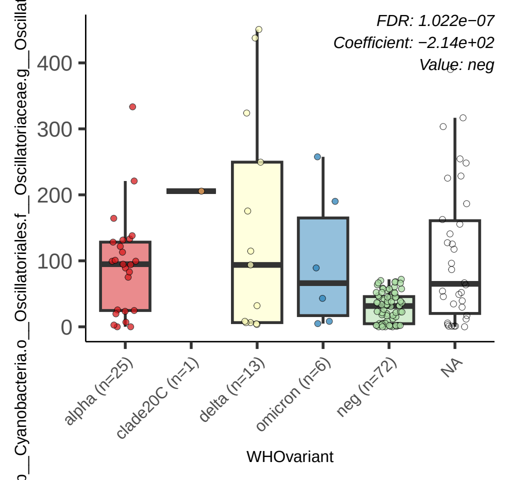

FDR: 1.022e−07
Coefficient: −2.14e+02
Value: neg
o__Cyanobacteria.o__Oscillatoriales.f__Oscillatoriaceae.g__Oscillat
0
100
200
300
400
alpha (n=25)
clade20C (n=1)
delta (n=13)
omicron (n=6)
neg (n=72)
NA
WHOvariant

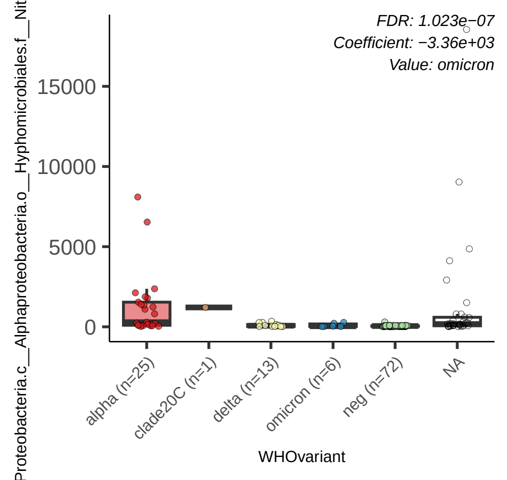

FDR: 1.023e−07
Coefficient: −3.36e+03
Value: omicron
Proteobacteria.c__Alphaproteobacteria.o__Hyphomicrobiales.f__Nit
15000
10000
5000
0
alpha (n=25)
clade20C (n=1)
delta (n=13)
omicron (n=6)
neg (n=72)
NA
WHOvariant

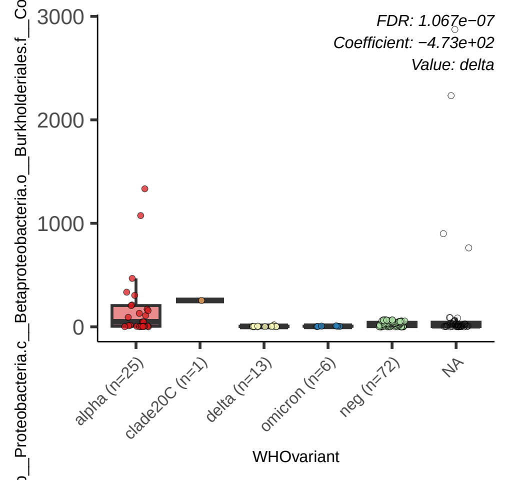

FDR: 1.067e−07
Coefficient: −4.73e+02
Value: delta
3000
2000
1000
0
o__Proteobacteria.c__Betaproteobacteria.o__Burkholderiales.f__Co
alpha (n=25)
clade20C (n=1)
delta (n=13)
omicron (n=6)
neg (n=72)
NA
WHOvariant

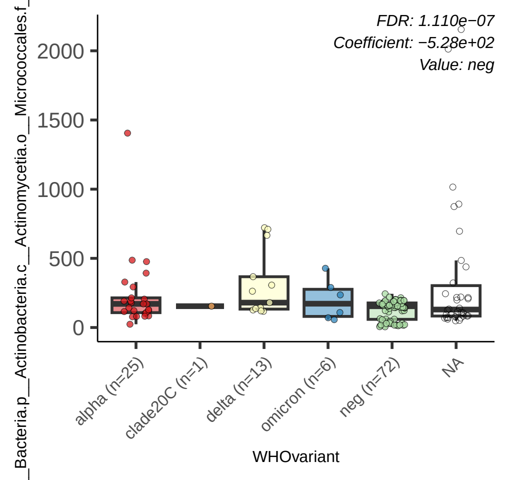

FDR: 1.110e−07
Coefficient: −5.28e+02
Value: neg
Bacteria.p__Actinobacteria.c__Actinomycetia.o__Micrococcales.f__
2000
1500
1000
500
0
alpha (n=25)
clade20C (n=1)
delta (n=13)
omicron (n=6)
neg (n=72)
NA
WHOvariant

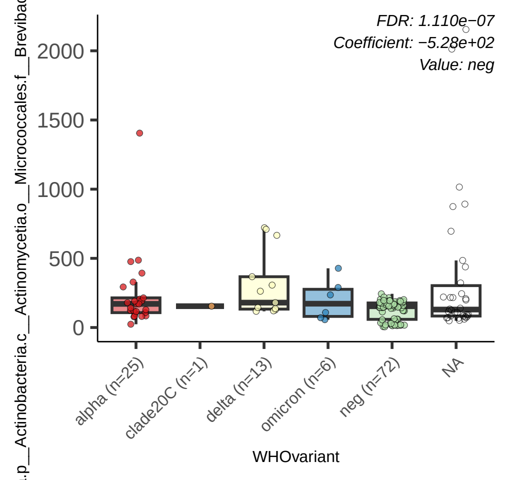

FDR: 1.110e−07
Coefficient: −5.28e+02
Value: neg
p__Actinobacteria.c__Actinomycetia.o__Micrococcales.f__Brevibac
2000
1500
1000
500
0
alpha (n=25)
clade20C (n=1)
delta (n=13)
omicron (n=6)
neg (n=72)
NA
WHOvariant

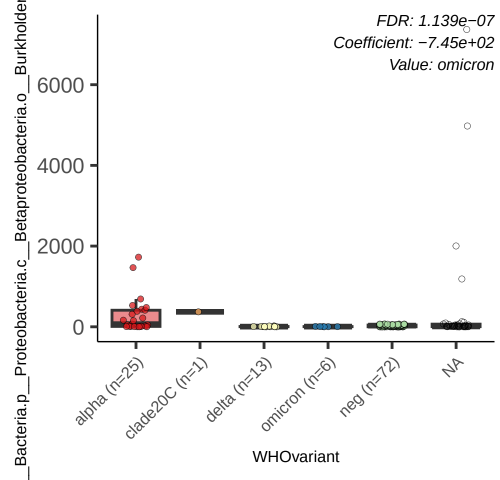
FDR: 1.139e−07
Coefficient: −7.45e+02
Value: omicron
0
2000
4000
6000
_Bacteria.p__Proteobacteria.c__Betaproteobacteria.o__Burkholder
alpha (n=25)
clade20C (n=1)
delta (n=13)
omicron (n=6)
neg (n=72)
NA
WHOvariant

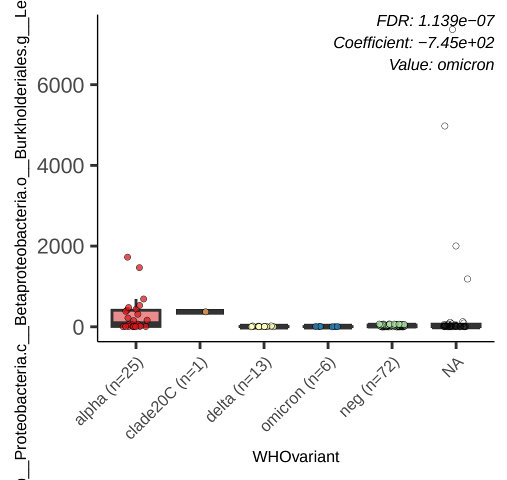

FDR: 1.139e−07
Coefficient: −7.45e+02
Value: omicron
o__Proteobacteria.c__Betaproteobacteria.o__Burkholderiales.g__Le
6000
4000
2000
0
alpha (n=25)
clade20C (n=1)
delta (n=13)
omicron (n=6)
neg (n=72)
NA
WHOvariant

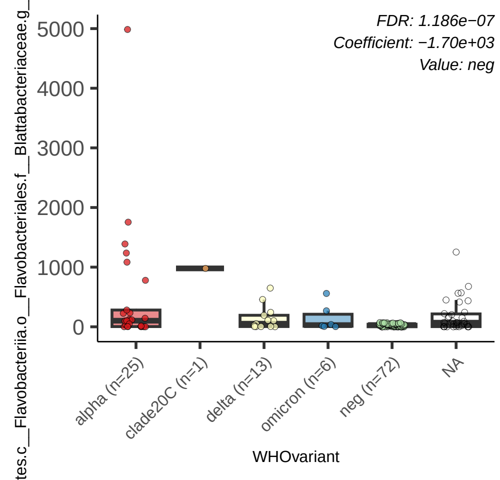

FDR: 1.186e−07
Coefficient: −1.70e+03
Value: neg
tes.c__Flavobacteriia.o__Flavobacteriales.f__Blattabacteriaceae.g__
5000
4000
3000
2000
1000
0
alpha (n=25)
clade20C (n=1)
delta (n=13)
omicron (n=6)
neg (n=72)
NA
WHOvariant

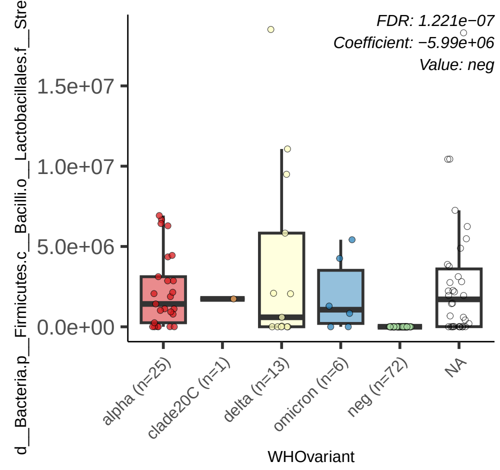
FDR: 1.221e−07
Coefficient: −5.99e+06
Value: neg
d__Bacteria.p__Firmicutes.c__Bacilli.o__Lactobacillales.f__Stre
1.5e+07
1.0e+07
5.0e+06
0.0e+00
alpha (n=25)
clade20C (n=1)
delta (n=13)
omicron (n=6)
neg (n=72)
NA
WHOvariant

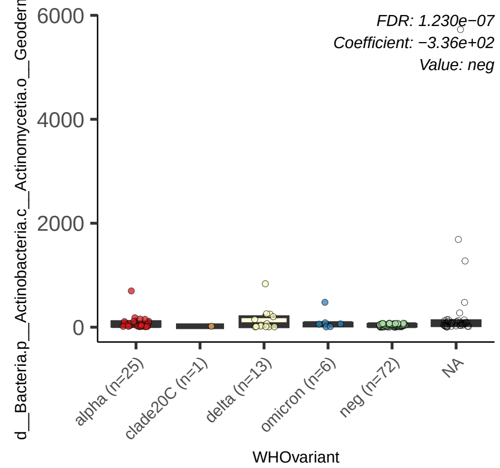

FDR: 1.230e−07
Coefficient: −3.36e+02
Value: neg
d__Bacteria.p__Actinobacteria.c__Actinomycetia.o__Geoderm
6000
4000
2000
0
alpha (n=25)
clade20C (n=1)
delta (n=13)
omicron (n=6)
neg (n=72)
NA
WHOvariant

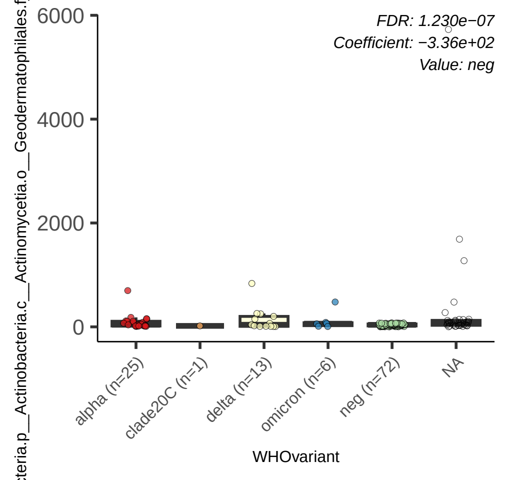

FDR: 1.230e−07
Coefficient: −3.36e+02
Value: neg
6000
4000
2000
0
cteria.p__Actinobacteria.c__Actinomycetia.o__Geodermatophilales.f
alpha (n=25)
clade20C (n=1)
delta (n=13)
omicron (n=6)
neg (n=72)
NA
WHOvariant

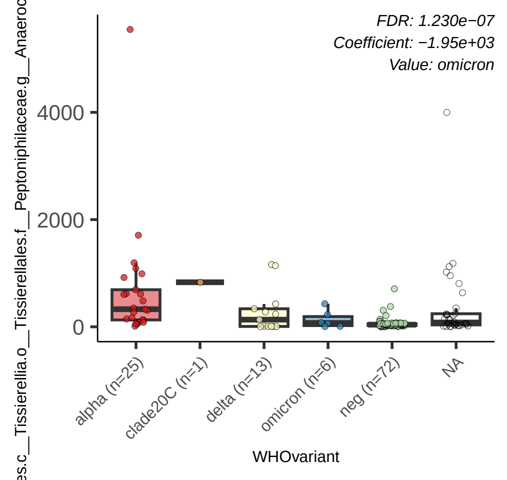
FDR: 1.230e−07
Coefficient: −1.95e+03
Value: omicron
es.c__Tissierellia.o__Tissierellales.f__Peptoniphilaceae.g__Anaeroc
0
2000
4000
alpha (n=25)
clade20C (n=1)
delta (n=13)
omicron (n=6)
neg (n=72)
NA
WHOvariant

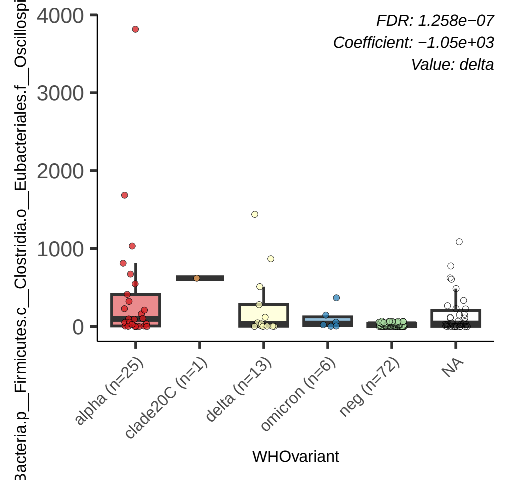

FDR: 1.258e−07
Coefficient: −1.05e+03
Value: delta
4000
3000
2000
1000
0
Bacteria.p__Firmicutes.c__Clostridia.o__Eubacteriales.f__Oscillospi
alpha (n=25)
clade20C (n=1)
delta (n=13)
omicron (n=6)
neg (n=72)
NA
WHOvariant

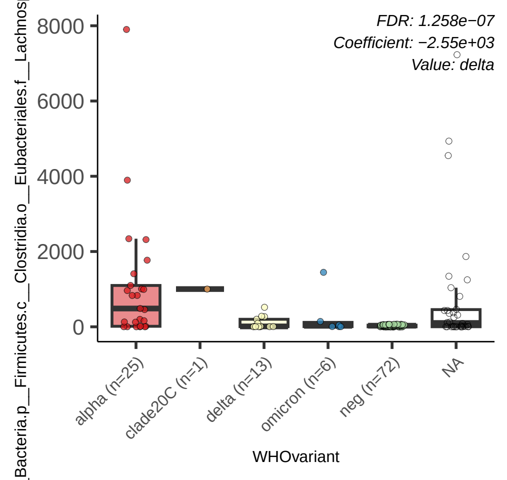

FDR: 1.258e−07
Coefficient: −2.55e+03
Value: delta
Bacteria.p__Firmicutes.c__Clostridia.o__Eubacteriales.f__Lachnos
8000
6000
4000
2000
0
alpha (n=25)
clade20C (n=1)
delta (n=13)
omicron (n=6)
neg (n=72)
NA
WHOvariant

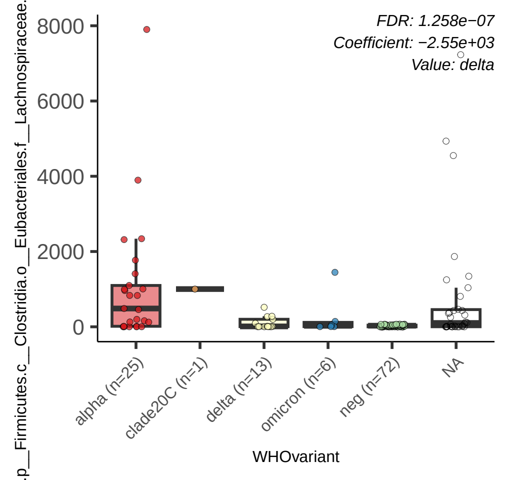

FDR: 1.258e−07
Coefficient: −2.55e+03
Value: delta
p__Firmicutes.c__Clostridia.o__Eubacteriales.f__Lachnospiraceae.
8000
6000
4000
2000
0
alpha (n=25)
clade20C (n=1)
delta (n=13)
omicron (n=6)
neg (n=72)
NA
WHOvariant

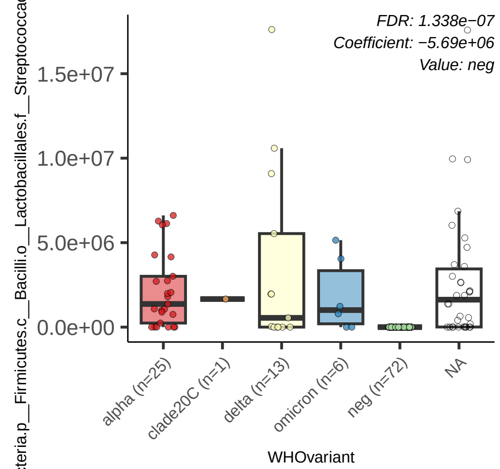

FDR: 1.338e−07
Coefficient: −5.69e+06
Value: neg
acteria.p__Firmicutes.c__Bacilli.o__Lactobacillales.f__Streptococcac
1.5e+07
1.0e+07
5.0e+06
0.0e+00
alpha (n=25)
clade20C (n=1)
delta (n=13)
omicron (n=6)
neg (n=72)
NA
WHOvariant

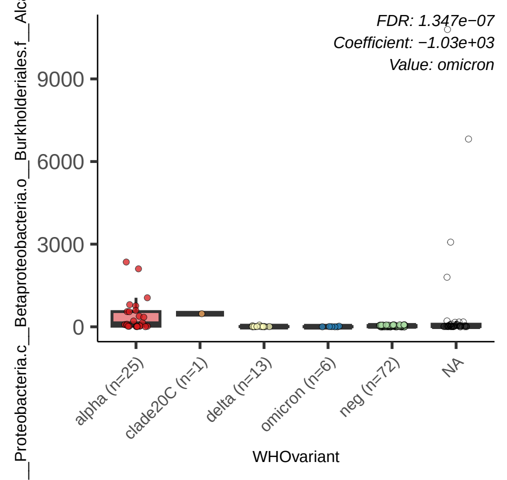
FDR: 1.347e−07
Coefficient: −1.03e+03
Value: omicron
9000
6000
3000
0
alpha (n=25)
clade20C (n=1)
delta (n=13)
omicron (n=6)
neg (n=72)
NA
WHOvariant
Proteobacteria.c__Betaproteobacteria.o__Burkholderiales.f__Alc

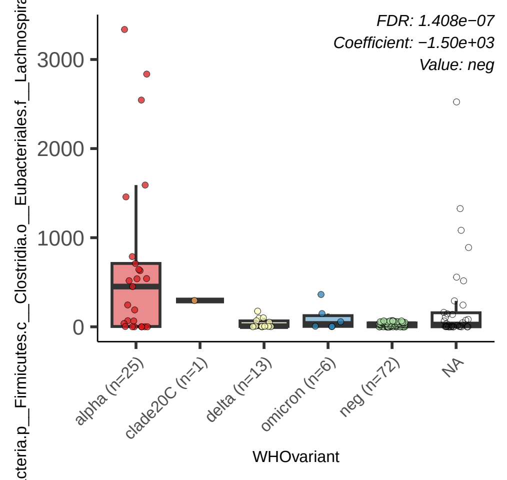

FDR: 1.408e−07
Coefficient: −1.50e+03
Value: neg
3000
2000
1000
0
alpha (n=25)
clade20C (n=1)
delta (n=13)
omicron (n=6)
neg (n=72)
NA
WHOvariant
cteria.p__Firmicutes.c__Clostridia.o__Eubacteriales.f__Lachnospira

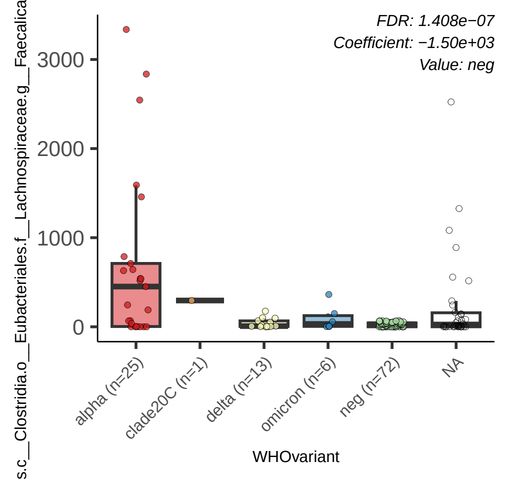
FDR: 1.408e−07
Coefficient: −1.50e+03
Value: neg
s.c__Clostridia.o__Eubacteriales.f__Lachnospiraceae.g__Faecalica
3000
2000
1000
0
alpha (n=25)
clade20C (n=1)
delta (n=13)
omicron (n=6)
neg (n=72)
NA
WHOvariant

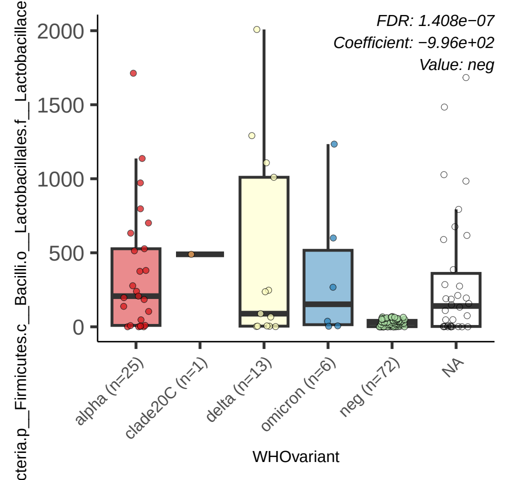
FDR: 1.408e−07
Coefficient: −9.96e+02
Value: neg
2000
1500
1000
500
0
cteria.p__Firmicutes.c__Bacilli.o__Lactobacillales.f__Lactobacillace
alpha (n=25)
clade20C (n=1)
delta (n=13)
omicron (n=6)
neg (n=72)
NA
WHOvariant

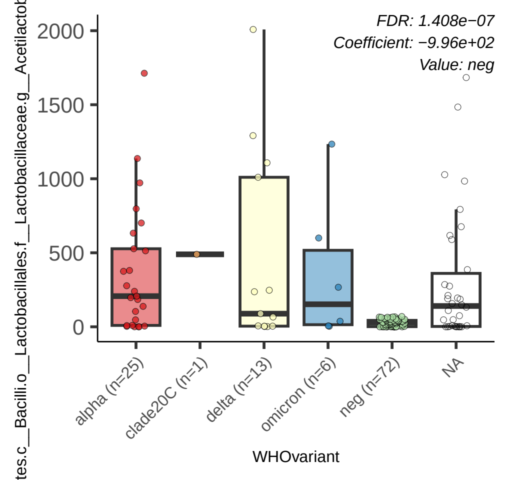
FDR: 1.408e−07
Coefficient: −9.96e+02
Value: neg
tes.c__Bacilli.o__Lactobacillales.f__Lactobacillaceae.g__Acetilactob
2000
1500
1000
500
0
alpha (n=25)
clade20C (n=1)
delta (n=13)
omicron (n=6)
neg (n=72)
NA
WHOvariant

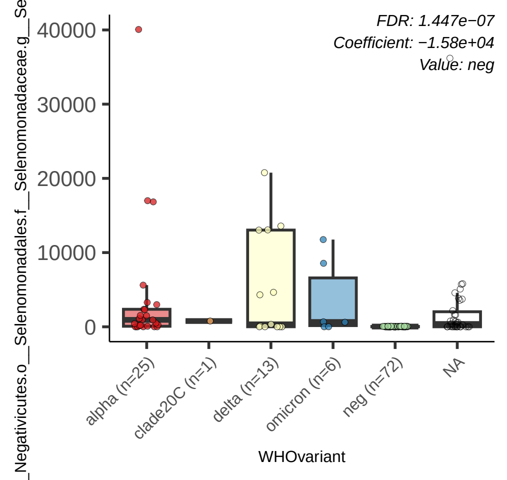

FDR: 1.447e−07
Coefficient: −1.58e+04
Value: neg
Negativicutes.o__Selenomonadales.f__Selenomonadaceae.g__Se
40000
30000
20000
10000
0
alpha (n=25)
clade20C (n=1)
delta (n=13)
omicron (n=6)
neg (n=72)
NA
WHOvariant

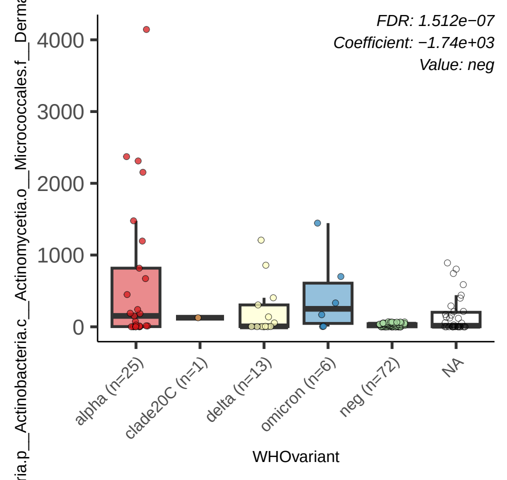
FDR: 1.512e−07
Coefficient: −1.74e+03
Value: neg
ria.p__Actinobacteria.c__Actinomycetia.o__Micrococcales.f__Derma
4000
3000
2000
1000
0
alpha (n=25)
clade20C (n=1)
delta (n=13)
omicron (n=6)
neg (n=72)
NA
WHOvariant

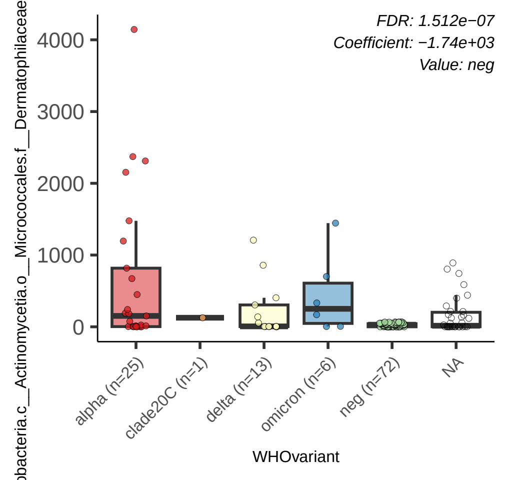

FDR: 1.512e−07
Coefficient: −1.74e+03
Value: neg
obacteria.c__Actinomycetia.o__Micrococcales.f__Dermatophilaceae
4000
3000
2000
1000
0
alpha (n=25)
clade20C (n=1)
delta (n=13)
omicron (n=6)
neg (n=72)
NA
WHOvariant

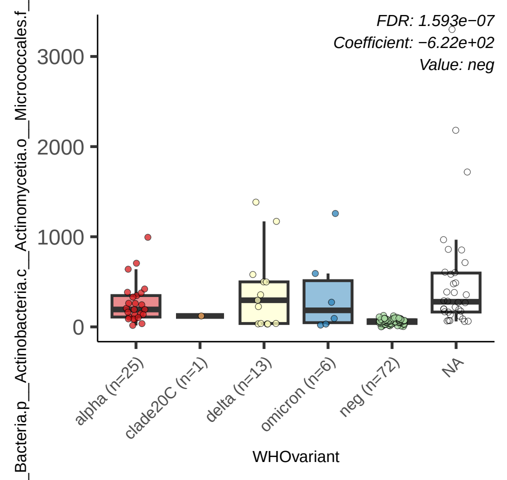
FDR: 1.593e−07
Coefficient: −6.22e+02
Value: neg
Bacteria.p__Actinobacteria.c__Actinomycetia.o__Micrococcales.f
3000
2000
1000
0
alpha (n=25)
clade20C (n=1)
delta (n=13)
omicron (n=6)
neg (n=72)
NA
WHOvariant

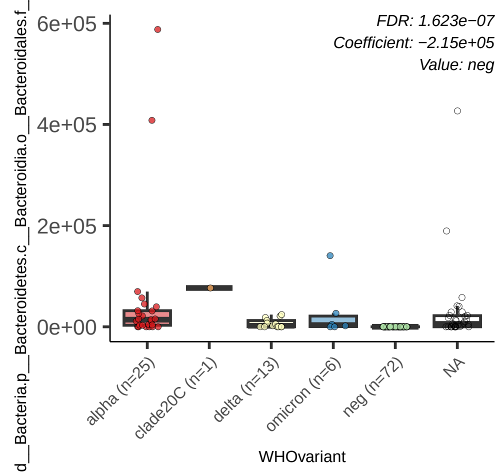

d__Bacteria.p__Bacteroidetes.c__Bacteroidia.o__Bacteroidales.f
6e+05
4e+05
2e+05
0e+00
FDR: 1.623e−07
Coefficient: −2.15e+05
Value: neg
alpha (n=25)
clade20C (n=1)
delta (n=13)
omicron (n=6)
neg (n=72)
NA
WHOvariant

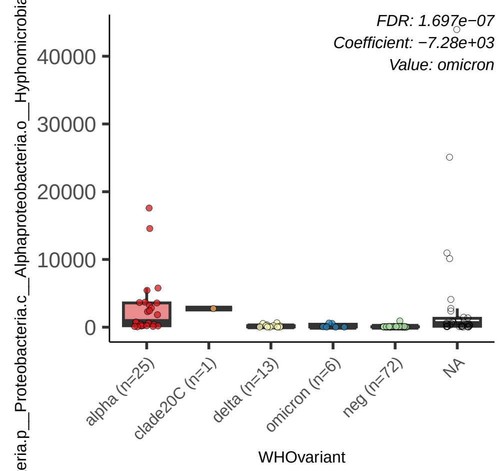

FDR: 1.697e−07
Coefficient: −7.28e+03
Value: omicron
0
10000
20000
30000
40000
cteria.p__Proteobacteria.c__Alphaproteobacteria.o__Hyphomicrobia
alpha (n=25)
clade20C (n=1)
delta (n=13)
omicron (n=6)
neg (n=72)
NA
WHOvariant

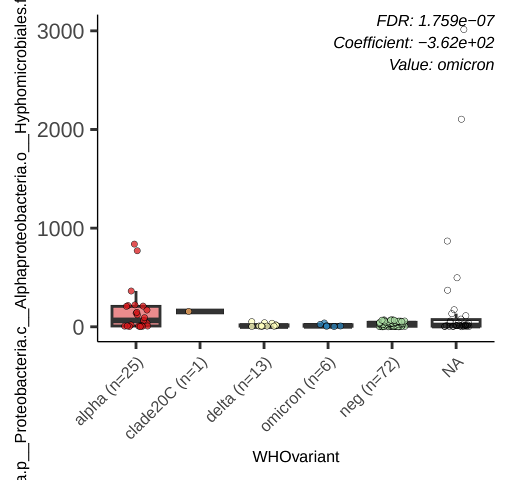

FDR: 1.759e−07
Coefficient: −3.62e+02
Value: omicron
3000
2000
1000
0
a.p__Proteobacteria.c__Alphaproteobacteria.o__Hyphomicrobiales.f
alpha (n=25)
clade20C (n=1)
delta (n=13)
omicron (n=6)
neg (n=72)
NA
WHOvariant

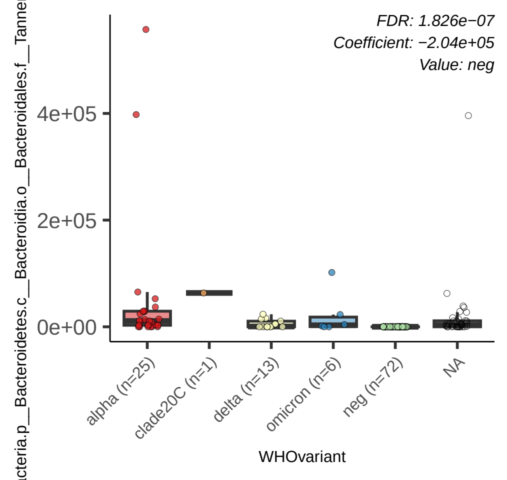

FDR: 1.826e−07
Coefficient: −2.04e+05
Value: neg
4e+05
2e+05
0e+00
alpha (n=25)
clade20C (n=1)
delta (n=13)
omicron (n=6)
neg (n=72)
NA
WHOvariant
acteria.p__Bacteroidetes.c__Bacteroidia.o__Bacteroidales.f__Tanner

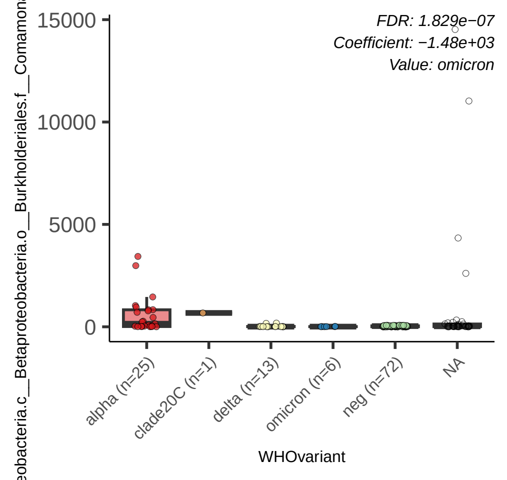

FDR: 1.829e−07
Coefficient: −1.48e+03
Value: omicron
15000
10000
5000
0
eobacteria.c__Betaproteobacteria.o__Burkholderiales.f__Comamona
alpha (n=25)
clade20C (n=1)
delta (n=13)
omicron (n=6)
neg (n=72)
NA
WHOvariant

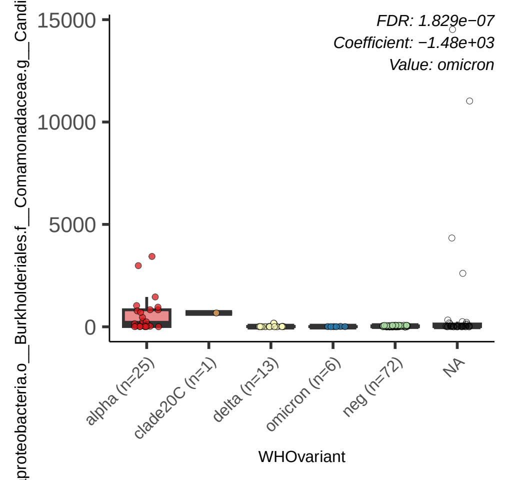

FDR: 1.829e−07
Coefficient: −1.48e+03
Value: omicron
15000
10000
5000
0
proteobacteria.o__Burkholderiales.f__Comamonadaceae.g__Candi
alpha (n=25)
clade20C (n=1)
delta (n=13)
omicron (n=6)
neg (n=72)
NA
WHOvariant

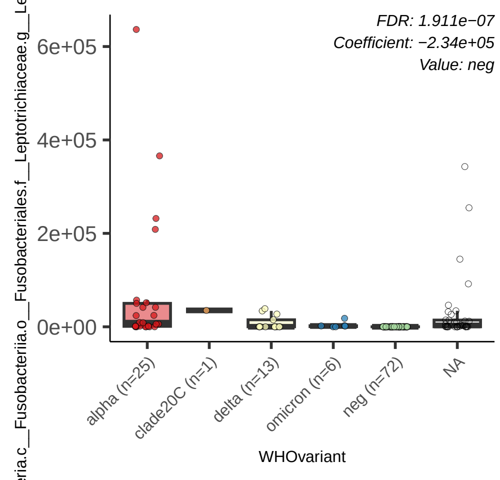

FDR: 1.911e−07
Coefficient: −2.34e+05
Value: neg
6e+05
4e+05
2e+05
0e+00
alpha (n=25)
clade20C (n=1)
delta (n=13)
omicron (n=6)
neg (n=72)
NA
WHOvariant
Fusobacteriales.f__Leptotrichiaceae.g__Le
Fusobacteriia.o__Fusobacteriales.f__Leptotrichiaceae.g__Le

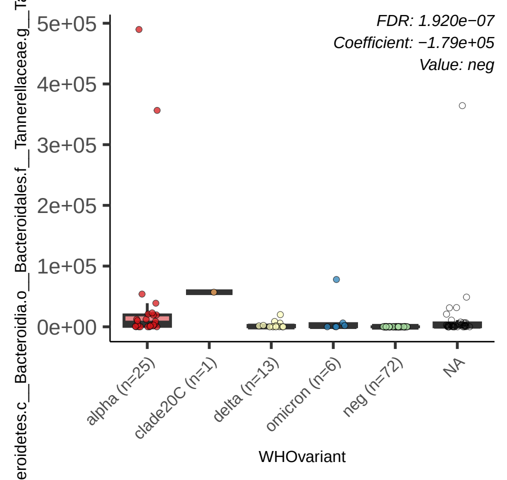

FDR: 1.920e−07
Coefficient: −1.79e+05
Value: neg
5e+05
4e+05
3e+05
2e+05
1e+05
0e+00
alpha (n=25)
clade20C (n=1)
delta (n=13)
omicron (n=6)
neg (n=72)
NA
WHOvariant
eroidetes.c__Bacteroidia.o__Bacteroidales.f__Tannerellaceae.g__Ta

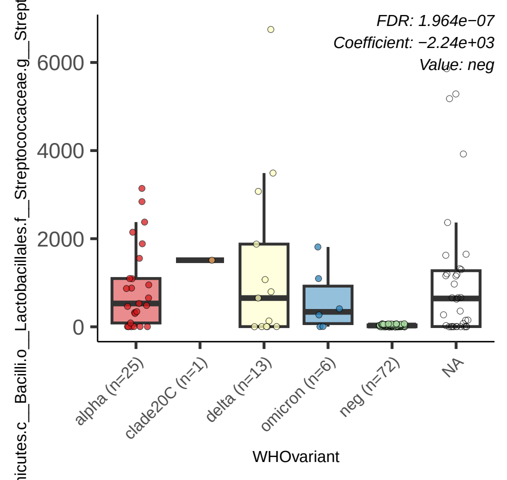
FDR: 1.964e−07
Coefficient: −2.24e+03
Value: neg
6000
4000
2000
0
micutes.c__Bacilli.o__Lactobacillales.f__Streptococcaceae.g__Strept
alpha (n=25)
clade20C (n=1)
delta (n=13)
omicron (n=6)
neg (n=72)
NA
WHOvariant

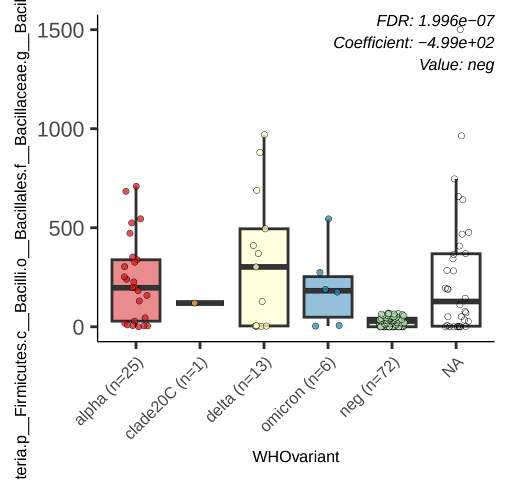
FDR: 1.996e−07
Coefficient: −4.99e+02
Value: neg
1500
1000
500
0
teria.p__Firmicutes.c__Bacilli.o__Bacillales.f__Bacillaceae.g__Bacil
alpha (n=25)
clade20C (n=1)
delta (n=13)
omicron (n=6)
neg (n=72)
NA
WHOvariant

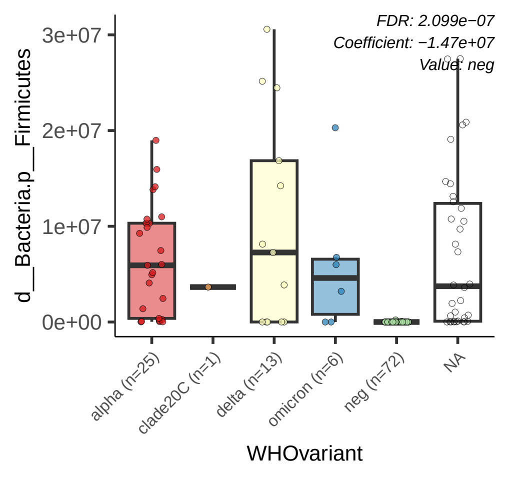

FDR: 2.099e−07
Coefficient: −1.47e+07
Value: neg
d__Bacteria.p__Firmicutes
3e+07
2e+07
1e+07
0e+00
alpha (n=25)
clade20C (n=1)
delta (n=13)
omicron (n=6)
neg (n=72)
NA
WHOvariant

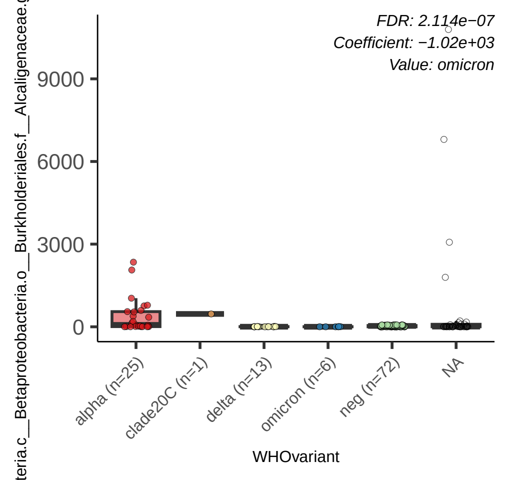

FDR: 2.114e−07
Coefficient: −1.02e+03
Value: omicron
9000
6000
3000
0
teria.c__Betaproteobacteria.o__Burkholderiales.f__Alcaligenaceae.g
alpha (n=25)
clade20C (n=1)
delta (n=13)
omicron (n=6)
neg (n=72)
NA
WHOvariant

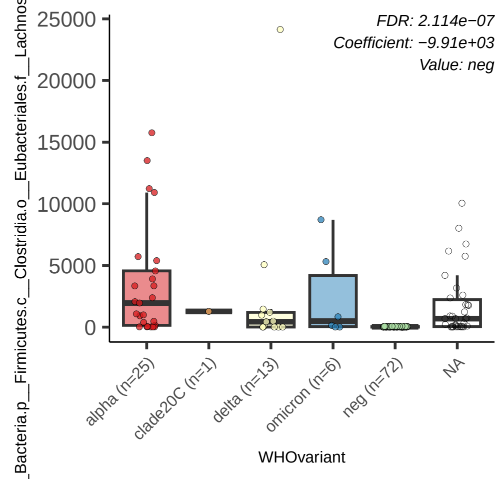

FDR: 2.114e−07
Coefficient: −9.91e+03
Value: neg
Bacteria.p__Firmicutes.c__Clostridia.o__Eubacteriales.f__Lachnos
25000
20000
15000
10000
5000
0
alpha (n=25)
clade20C (n=1)
delta (n=13)
omicron (n=6)
neg (n=72)
NA
WHOvariant

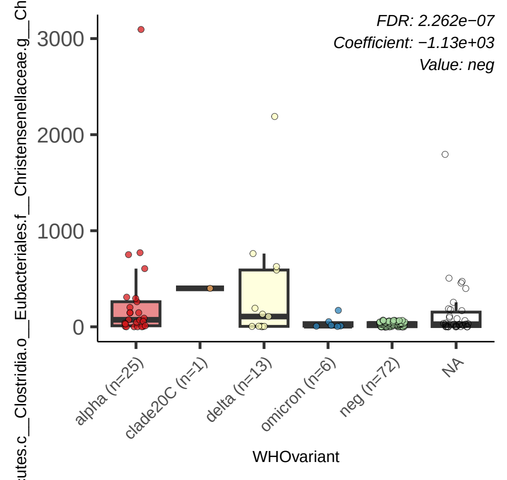

FDR: 2.262e−07
Coefficient: −1.13e+03
Value: neg
3000
2000
1000
0
cutes.c__Clostridia.o__Eubacteriales.f__Christensenellaceae.g__Ch
alpha (n=25)
clade20C (n=1)
delta (n=13)
omicron (n=6)
neg (n=72)
NA
WHOvariant

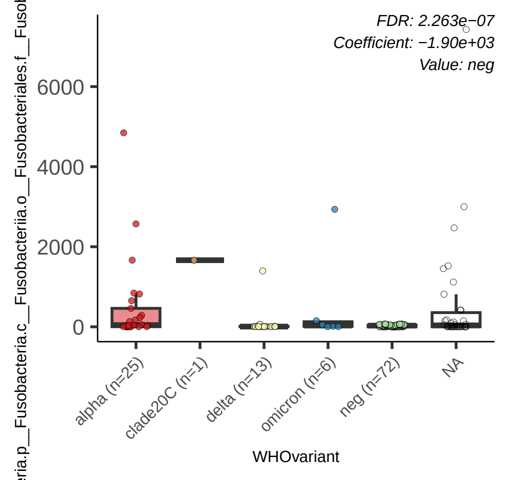
FDR: 2.263e−07
Coefficient: −1.90e+03
Value: neg
6000
4000
2000
0
alpha (n=25)
clade20C (n=1)
delta (n=13)
omicron (n=6)
neg (n=72)
NA
WHOvariant
eria.p__Fusobacteria.c__Fusobacteriia.o__Fusobacteriales.f__Fusob

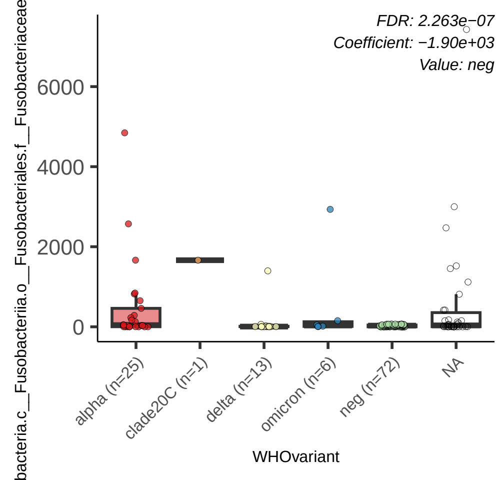

FDR: 2.263e−07
Coefficient: −1.90e+03
Value: neg
6000
4000
2000
0
bacteria.c__Fusobacteriia.o__Fusobacteriales.f__Fusobacteriaceae
alpha (n=25)
clade20C (n=1)
delta (n=13)
omicron (n=6)
neg (n=72)
NA
WHOvariant

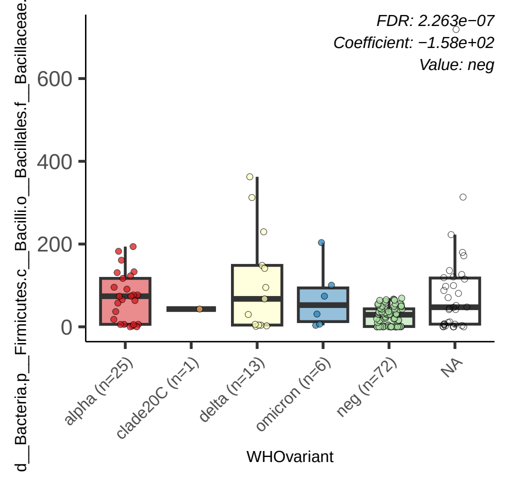

FDR: 2.263e−07
Coefficient: −1.58e+02
Value: neg
d__Bacteria.p__Firmicutes.c__Bacilli.o__Bacillales.f__Bacillaceae.
0
200
400
600
alpha (n=25)
clade20C (n=1)
delta (n=13)
omicron (n=6)
neg (n=72)
NA
WHOvariant

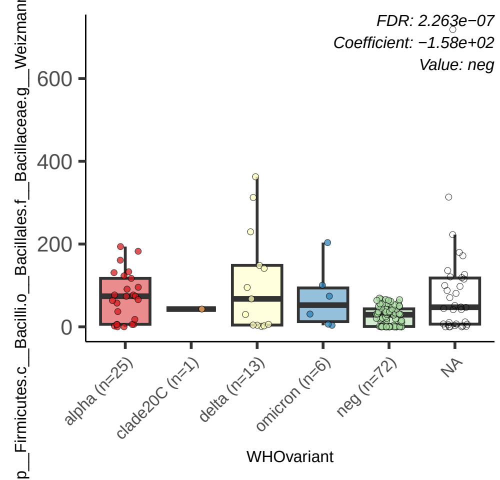

FDR: 2.263e−07
Coefficient: −1.58e+02
Value: neg
p__Firmicutes.c__Bacilli.o__Bacillales.f__Bacillaceae.g__Weizmann
600
400
200
0
alpha (n=25)
clade20C (n=1)
delta (n=13)
omicron (n=6)
neg (n=72)
NA
WHOvariant

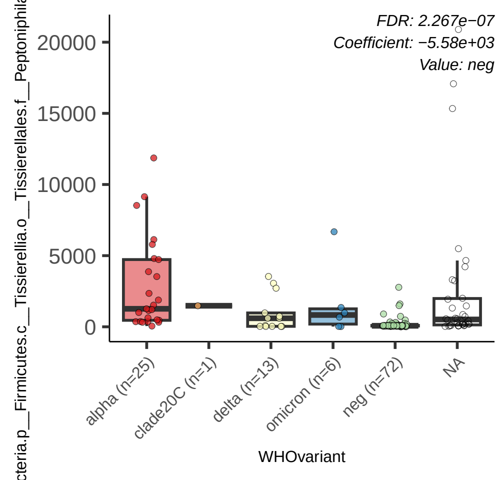

FDR: 2.267e−07
Coefficient: −5.58e+03
Value: neg
20000
15000
10000
5000
0
alpha (n=25)
clade20C (n=1)
delta (n=13)
omicron (n=6)
neg (n=72)
NA
WHOvariant
cteria.p__Firmicutes.c__Tissierellia.o__Tissierellales.f__Peptoniphila

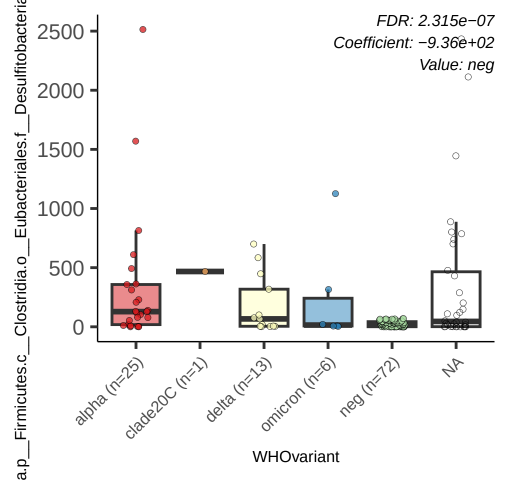
FDR: 2.315e−07
Coefficient: −9.36e+02
Value: neg
Firmicutes.c__Clostridia.o__Eubacteriales.f__Desulfitobacteria
2500
2000
1500
1000
500
0
alpha (n=25)
clade20C (n=1)
delta (n=13)
omicron (n=6)
neg (n=72)
NA
WHOvariant

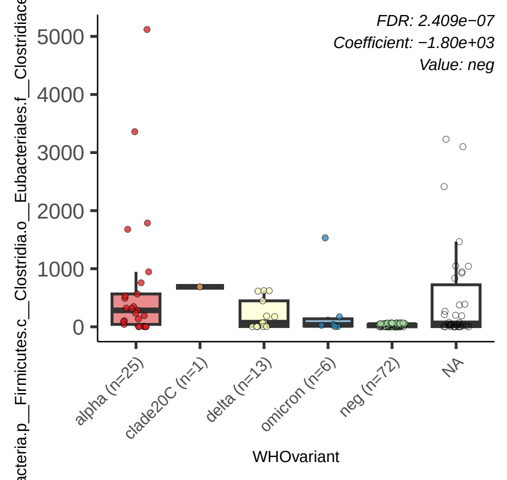
FDR: 2.409e−07
Coefficient: −1.80e+03
Value: neg
5000
4000
3000
2000
1000
0
acteria.p__Firmicutes.c__Clostridia.o__Eubacteriales.f__Clostridiace
alpha (n=25)
clade20C (n=1)
delta (n=13)
omicron (n=6)
neg (n=72)
NA
WHOvariant

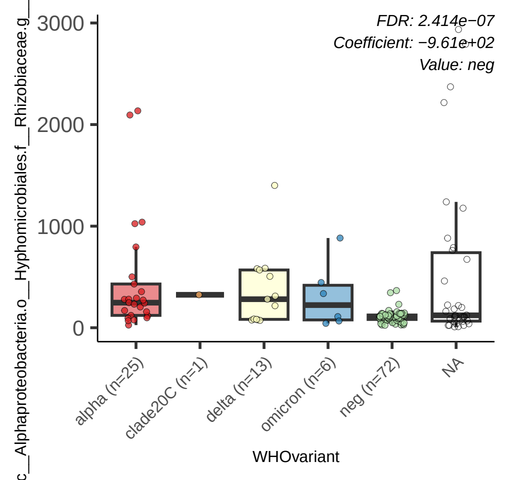
FDR: 2.414e−07
Coefficient: −9.61e+02
Value: neg
3000
2000
1000
0
c__Alphaproteobacteria.o__Hyphomicrobiales.f__Rhizobiaceae.g__
alpha (n=25)
clade20C (n=1)
delta (n=13)
omicron (n=6)
neg (n=72)
NA
WHOvariant

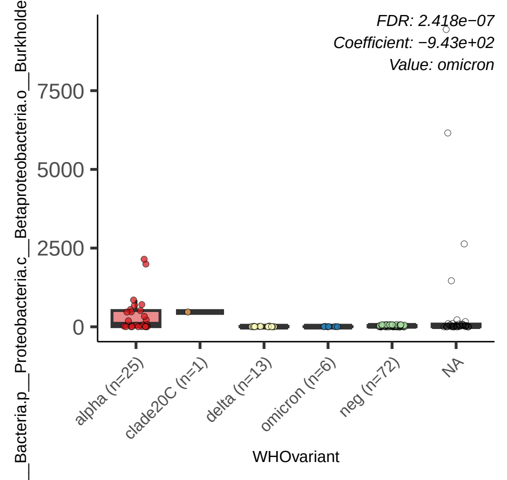

FDR: 2.418e−07
Coefficient: −9.43e+02
Value: omicron
7500
5000
2500
0
alpha (n=25)
clade20C (n=1)
delta (n=13)
omicron (n=6)
neg (n=72)
NA
WHOvariant
Bacteria.p__Proteobacteria.c__Betaproteobacteria.o__Burkholde

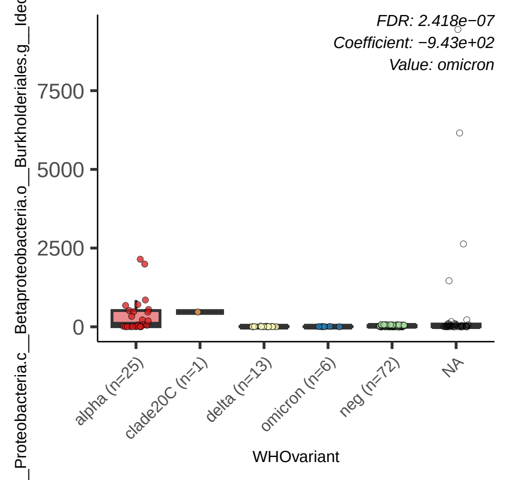

FDR: 2.418e−07
Coefficient: −9.43e+02
Value: omicron
7500
5000
2500
0
_Proteobacteria.c__Betaproteobacteria.o__Burkholderiales.g__Ideo
alpha (n=25)
clade20C (n=1)
delta (n=13)
omicron (n=6)
neg (n=72)
NA
WHOvariant

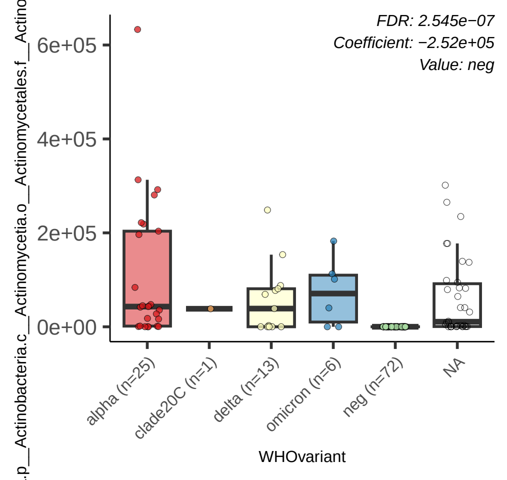
FDR: 2.545e−07
Coefficient: −2.52e+05
Value: neg
p__Actinobacteria.c__Actinomycetia.o__Actinomycetales.f__Actino
6e+05
4e+05
2e+05
0e+00
alpha (n=25)
clade20C (n=1)
delta (n=13)
omicron (n=6)
neg (n=72)
NA
WHOvariant

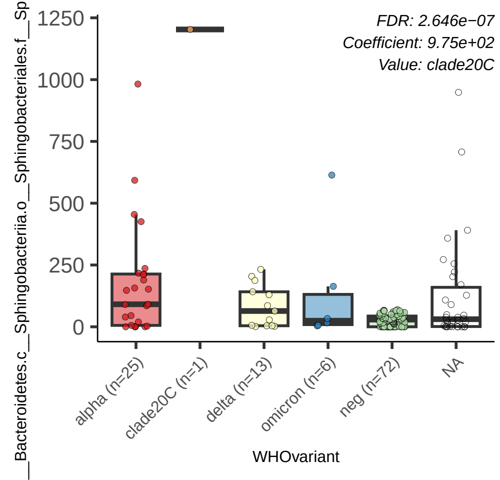
FDR: 2.646e−07
Coefficient: 9.75e+02
Value: clade20C
1250
1000
750
500
250
0
Bacteroidetes.c__Sphingobacteriia.o__Sphingobacteriales.f__Sp
alpha (n=25)
clade20C (n=1)
delta (n=13)
omicron (n=6)
neg (n=72)
NA
WHOvariant

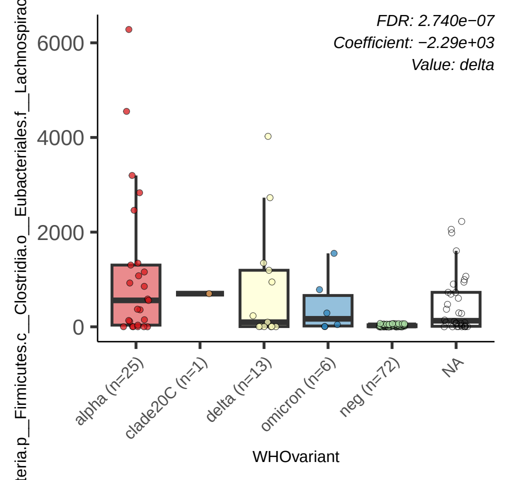
FDR: 2.740e−07
Coefficient: −2.29e+03
Value: delta
teria.p__Firmicutes.c__Clostridia.o__Eubacteriales.f__Lachnospirac
6000
4000
2000
0
alpha (n=25)
clade20C (n=1)
delta (n=13)
omicron (n=6)
neg (n=72)
NA
WHOvariant

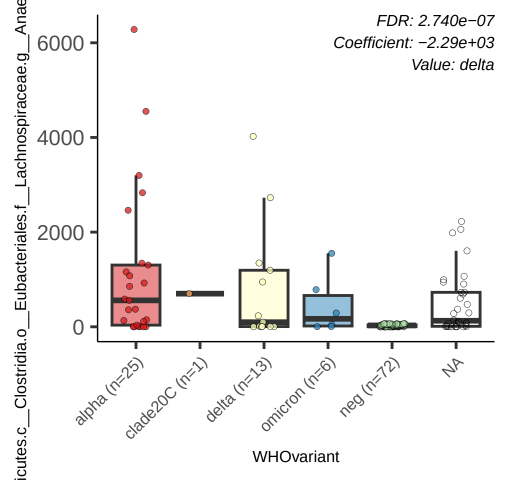

FDR: 2.740e−07
Coefficient: −2.29e+03
Value: delta
icutes.c__Clostridia.o__Eubacteriales.f__Lachnospiraceae.g__Anae
6000
4000
2000
0
alpha (n=25)
clade20C (n=1)
delta (n=13)
omicron (n=6)
neg (n=72)
NA
WHOvariant

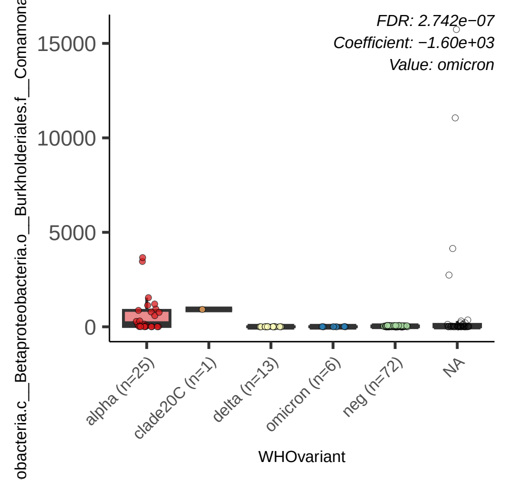

FDR: 2.742e−07
Coefficient: −1.60e+03
Value: omicron
15000
10000
5000
0
obacteria.c__Betaproteobacteria.o__Burkholderiales.f__Comamona
alpha (n=25)
clade20C (n=1)
delta (n=13)
omicron (n=6)
neg (n=72)
NA
WHOvariant

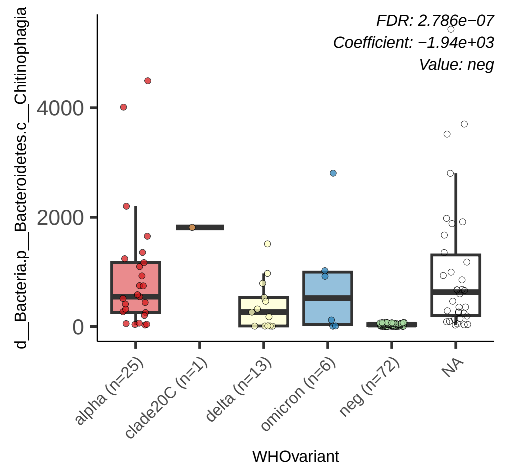

FDR: 2.786e−07
Coefficient: −1.94e+03
Value: neg
d__Bacteria.p__Bacteroidetes.c__Chitinophagia
4000
2000
0
alpha (n=25)
clade20C (n=1)
delta (n=13)
omicron (n=6)
neg (n=72)
NA
WHOvariant

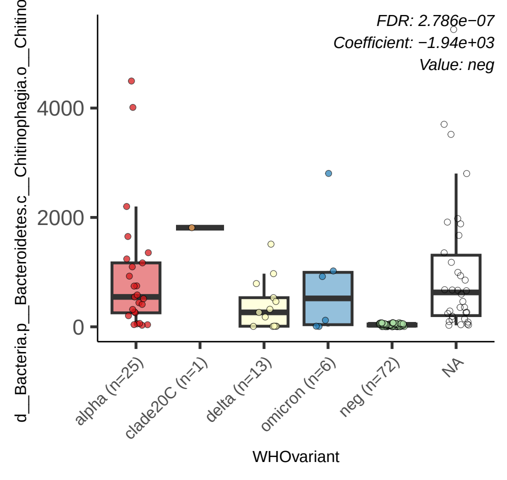
FDR: 2.786e−07
Coefficient: −1.94e+03
Value: neg
d__Bacteria.p__Bacteroidetes.c__Chitinophagia.o__Chitino
4000
2000
0
alpha (n=25)
clade20C (n=1)
delta (n=13)
omicron (n=6)
neg (n=72)
NA
WHOvariant

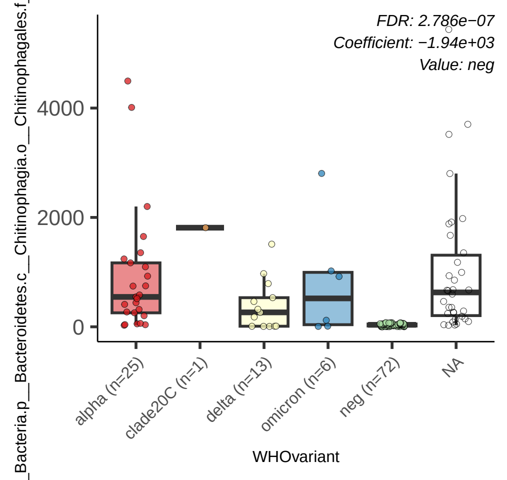
FDR: 2.786e−07
Coefficient: −1.94e+03
Value: neg
_Bacteria.p__Bacteroidetes.c__Chitinophagia.o__Chitinophagales.f_
4000
2000
0
alpha (n=25)
clade20C (n=1)
delta (n=13)
omicron (n=6)
neg (n=72)
NA
WHOvariant

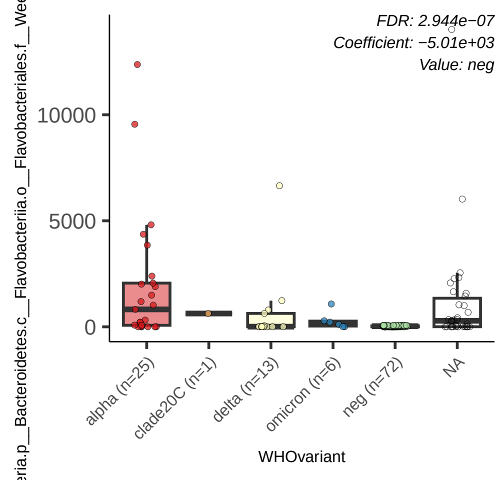
FDR: 2.944e−07
Coefficient: −5.01e+03
Value: neg
10000
5000
0
alpha (n=25)
clade20C (n=1)
delta (n=13)
omicron (n=6)
neg (n=72)
NA
WHOvariant

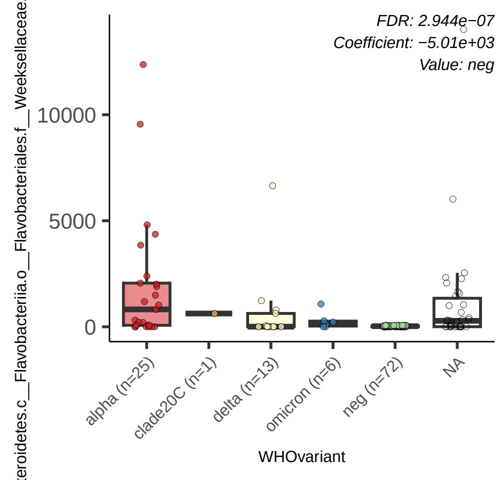
FDR: 2.944e−07
Coefficient: −5.01e+03
Value: neg
teroidetes.c__Flavobacteriia.o__Flavobacteriales.f__Weeksellaceae.
10000
5000
0
alpha (n=25)
clade20C (n=1)
delta (n=13)
omicron (n=6)
neg (n=72)
NA
WHOvariant

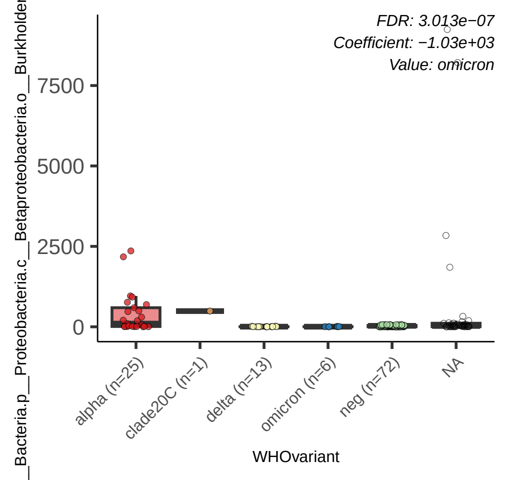
FDR: 3.013e−07
Coefficient: −1.03e+03
Value: omicron
_Bacteria.p__Proteobacteria.c__Betaproteobacteria.o__Burkholder
7500
5000
2500
0
alpha (n=25)
clade20C (n=1)
delta (n=13)
omicron (n=6)
neg (n=72)
NA
WHOvariant

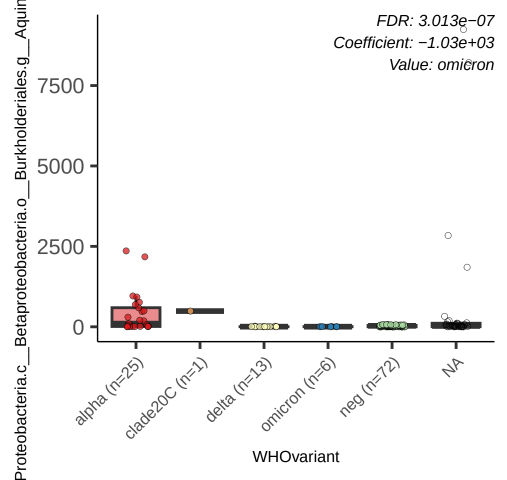
FDR: 3.013e−07
Coefficient: −1.03e+03
Value: omicron
Proteobacteria.c__Betaproteobacteria.o__Burkholderiales.g__Aquin
7500
5000
2500
0
alpha (n=25)
clade20C (n=1)
delta (n=13)
omicron (n=6)
neg (n=72)
NA
WHOvariant

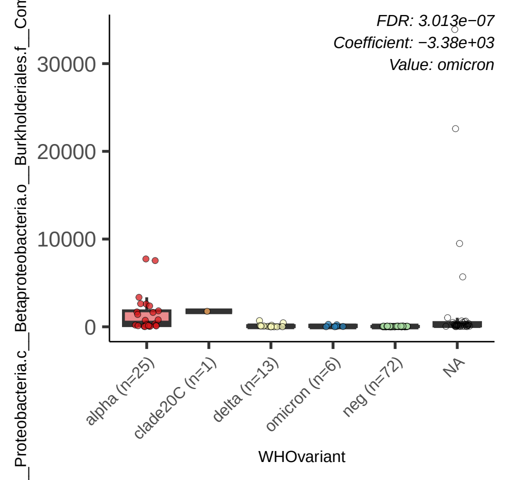

FDR: 3.013e−07
Coefficient: −3.38e+03
Value: omicron
30000
20000
10000
0
_Proteobacteria.c__Betaproteobacteria.o__Burkholderiales.f__Com
alpha (n=25)
clade20C (n=1)
delta (n=13)
omicron (n=6)
neg (n=72)
NA
WHOvariant

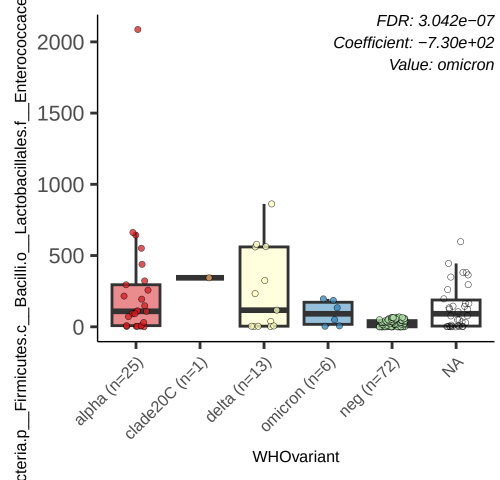
FDR: 3.042e−07
Coefficient: −7.30e+02
Value: omicron
2000
1500
1000
500
0
Firmicutes.c__Bacilli.o__Lactobacillales.f__Enterococcace
alpha (n=25)
clade20C (n=1)
delta (n=13)
omicron (n=6)
neg (n=72)
NA
WHOvariant

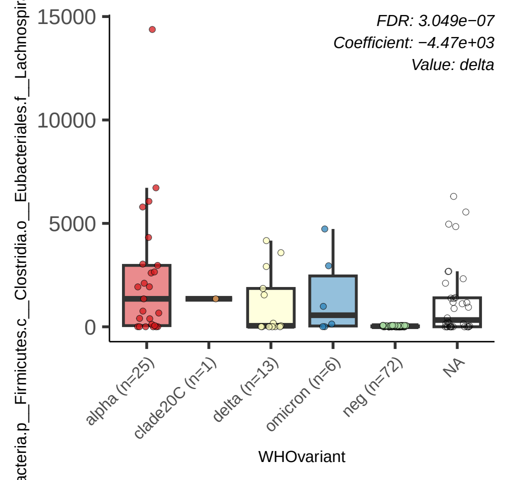

FDR: 3.049e−07
Coefficient: −4.47e+03
Value: delta
15000
10000
5000
0
acteria.p__Firmicutes.c__Clostridia.o__Eubacteriales.f__Lachnospi
alpha (n=25)
clade20C (n=1)
delta (n=13)
omicron (n=6)
neg (n=72)
NA
WHOvariant

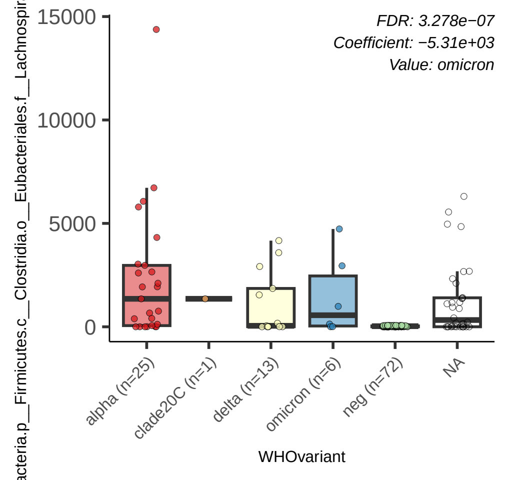

Firmicutes.c__Clostridia.o__Eubacteriales.f__Lachnospir
FDR: 3.278e−07
Coefficient: −5.31e+03
Value: omicron
15000
10000
5000
0
alpha (n=25)
clade20C (n=1)
delta (n=13)
omicron (n=6)
neg (n=72)
NA
WHOvariant

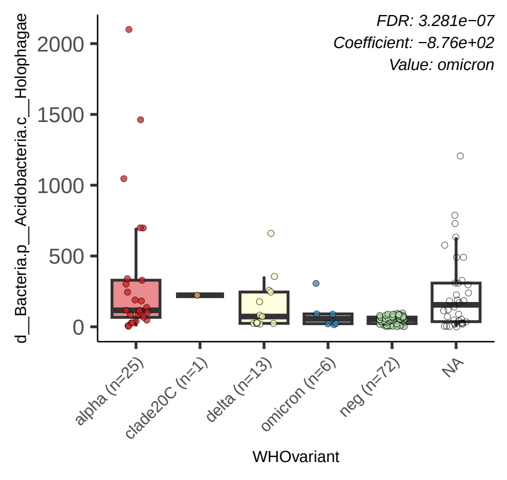

FDR: 3.281e−07
Coefficient: −8.76e+02
Value: omicron
d__Bacteria.p__Acidobacteria.c__Holophagae
0
500
1000
1500
2000
alpha (n=25)
clade20C (n=1)
delta (n=13)
omicron (n=6)
neg (n=72)
NA
WHOvariant

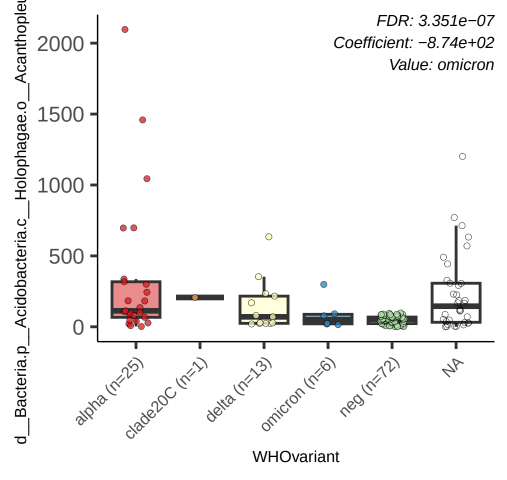

FDR: 3.351e−07
Coefficient: −8.74e+02
Value: omicron
d__Bacteria.p__Acidobacteria.c__Holophagae.o__Acanthopleu
2000
1500
1000
500
0
alpha (n=25)
clade20C (n=1)
delta (n=13)
omicron (n=6)
neg (n=72)
NA
WHOvariant

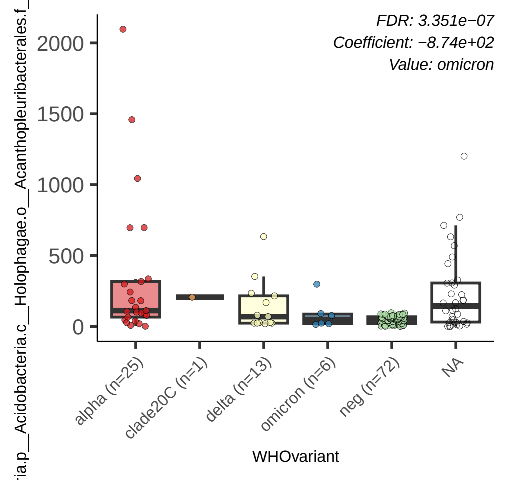

FDR: 3.351e−07
Coefficient: −8.74e+02
Value: omicron
ria.p__Acidobacteria.c__Holophagae.o__Acanthopleuribacterales.f__
2000
1500
1000
500
0
alpha (n=25)
clade20C (n=1)
delta (n=13)
omicron (n=6)
neg (n=72)
NA
WHOvariant

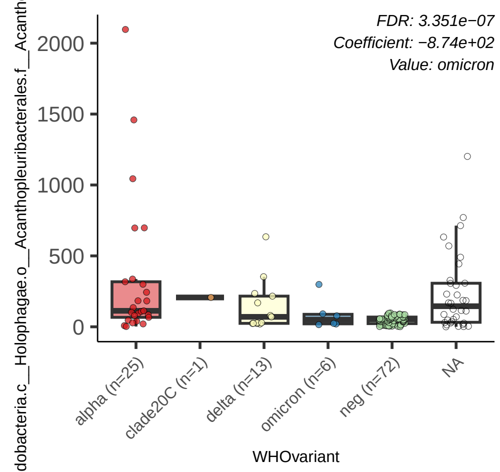

FDR: 3.351e−07
Coefficient: −8.74e+02
Value: omicron
cidobacteria.c__Holophagae.o__Acanthopleuribacterales.f__Acanth
2000
1500
1000
500
0
alpha (n=25)
clade20C (n=1)
delta (n=13)
omicron (n=6)
neg (n=72)
NA
WHOvariant

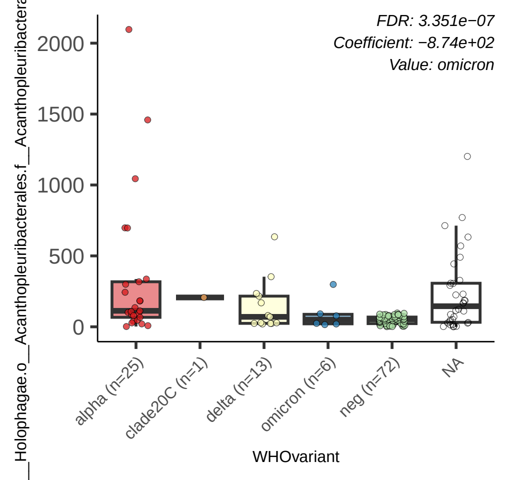

FDR: 3.351e−07
Coefficient: −8.74e+02
Value: omicron
2000
1500
1000
500
0
Holophagae.o__Acanthopleuribacterales.f__Acanthopleuribactera
alpha (n=25)
clade20C (n=1)
delta (n=13)
omicron (n=6)
neg (n=72)
NA
WHOvariant

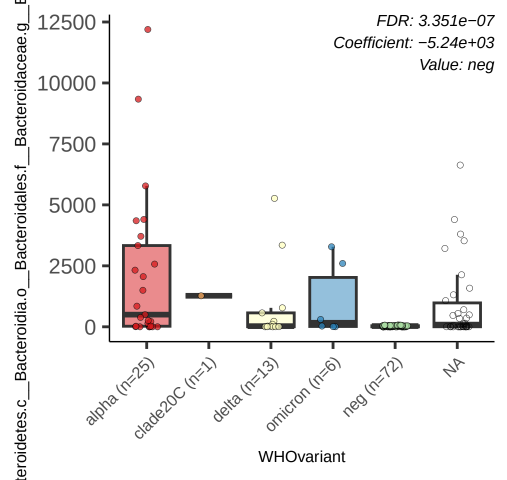
FDR: 3.351e−07
Coefficient: −5.24e+03
Value: neg
12500
10000
7500
5000
2500
0
alpha (n=25)
clade20C (n=1)
delta (n=13)
omicron (n=6)
neg (n=72)
NA
WHOvariant
teroidetes.c__Bacteroidia.o__Bacteroidales.f__Bacteroidaceae.g__B

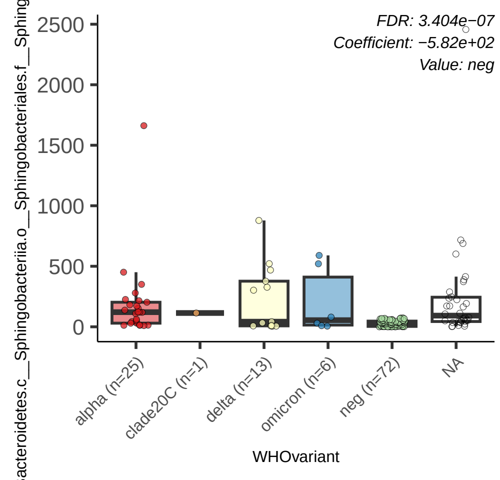
FDR: 3.404e−07
Coefficient: −5.82e+02
Value: neg
2500
2000
1500
1000
500
0
alpha (n=25)
clade20C (n=1)
delta (n=13)
omicron (n=6)
neg (n=72)
NA
WHOvariant

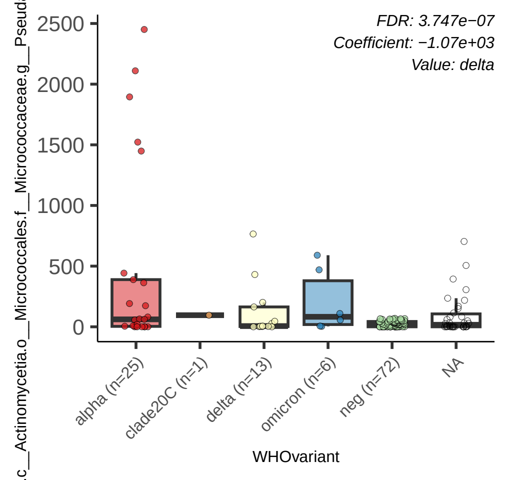

FDR: 3.747e−07
Coefficient: −1.07e+03
Value: delta
c__Actinomycetia.o__Micrococcales.f__Micrococcaceae.g__Pseud
2500
2000
1500
1000
500
0
alpha (n=25)
clade20C (n=1)
delta (n=13)
omicron (n=6)
neg (n=72)
NA
WHOvariant

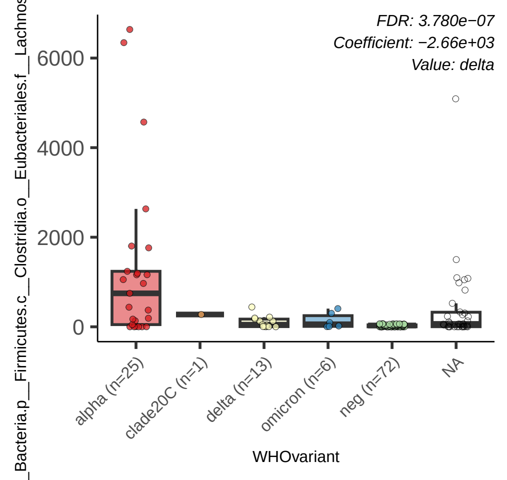

FDR: 3.780e−07
Coefficient: −2.66e+03
Value: delta
6000
4000
2000
0
_Bacteria.p__Firmicutes.c__Clostridia.o__Eubacteriales.f__Lachnos
alpha (n=25)
clade20C (n=1)
delta (n=13)
omicron (n=6)
neg (n=72)
NA
WHOvariant

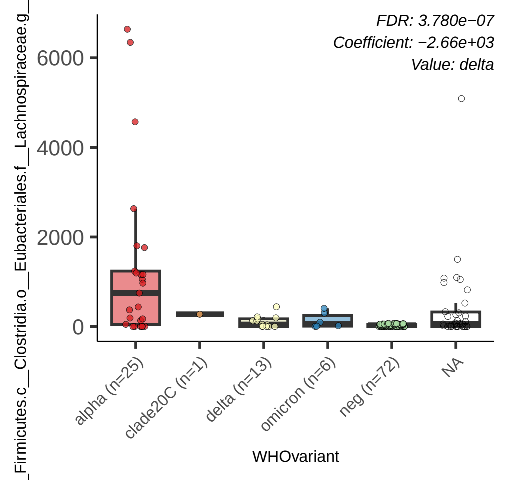
FDR: 3.780e−07
Coefficient: −2.66e+03
Value: delta
Firmicutes.c__Clostridia.o__Eubacteriales.f__Lachnospiraceae.g__
6000
4000
2000
0
alpha (n=25)
clade20C (n=1)
delta (n=13)
omicron (n=6)
neg (n=72)
NA
WHOvariant

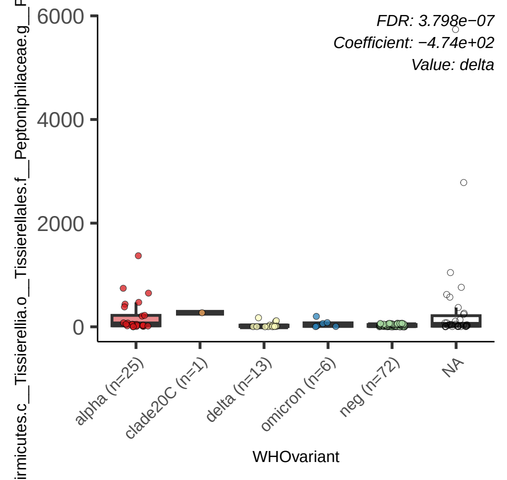

irmicutes.c__Tissierellia.o__Tissierellales.f__Peptoniphilaceae.g__F
FDR: 3.798e−07
Coefficient: −4.74e+02
Value: delta
6000
4000
2000
0
alpha (n=25)
clade20C (n=1)
delta (n=13)
omicron (n=6)
neg (n=72)
NA
WHOvariant

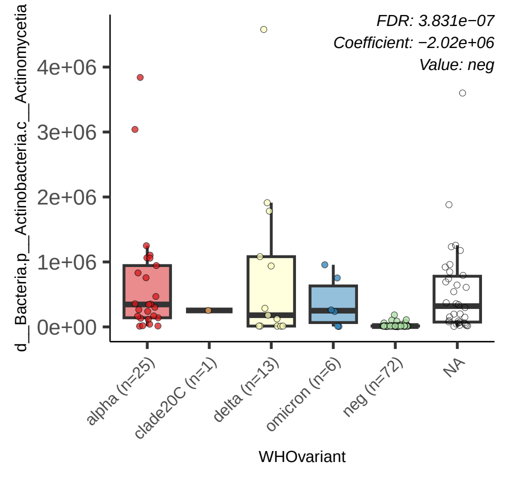
FDR: 3.831e−07
Coefficient: −2.02e+06
Value: neg
d__Bacteria.p__Actinobacteria.c__Actinomycetia
0e+00
1e+06
2e+06
3e+06
4e+06
alpha (n=25)
clade20C (n=1)
delta (n=13)
omicron (n=6)
neg (n=72)
NA
WHOvariant

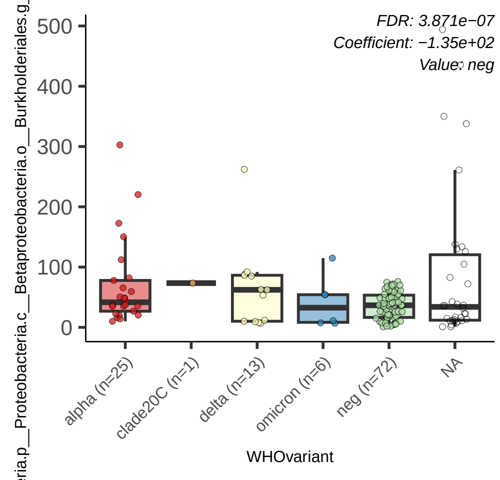

FDR: 3.871e−07
Coefficient: −1.35e+02
Value: neg
eria.p__Proteobacteria.c__Betaproteobacteria.o__Burkholderiales.g_
0
100
200
300
400
500
alpha (n=25)
clade20C (n=1)
delta (n=13)
omicron (n=6)
neg (n=72)
NA
WHOvariant

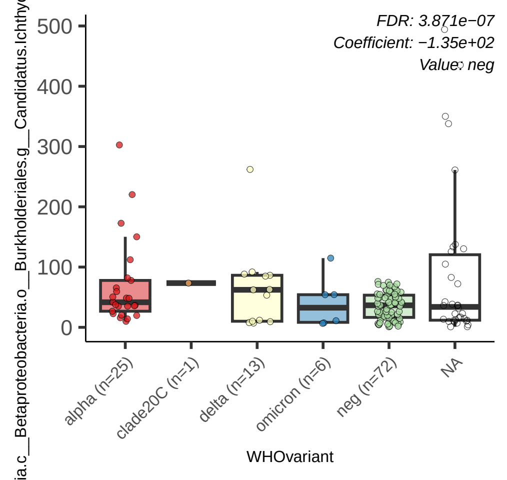

FDR: 3.871e−07
Coefficient: −1.35e+02
Value: neg
ia.c__Betaproteobacteria.o__Burkholderiales.g__Candidatus.Ichthyo
500
400
300
200
100
0
alpha (n=25)
clade20C (n=1)
delta (n=13)
omicron (n=6)
neg (n=72)
NA
WHOvariant

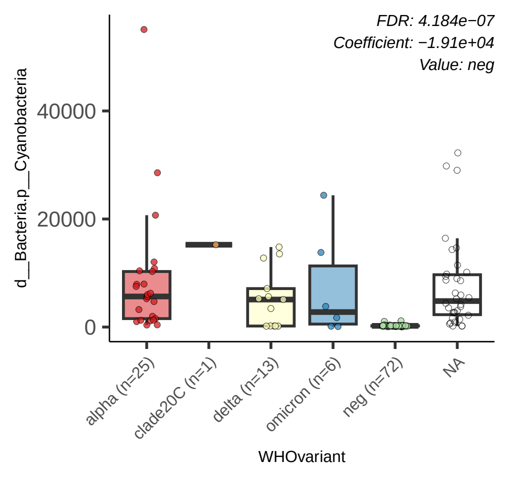

FDR: 4.184e−07
Coefficient: −1.91e+04
Value: neg
d__Bacteria.p__Cyanobacteria
40000
20000
0
alpha (n=25)
clade20C (n=1)
delta (n=13)
omicron (n=6)
neg (n=72)
NA
WHOvariant

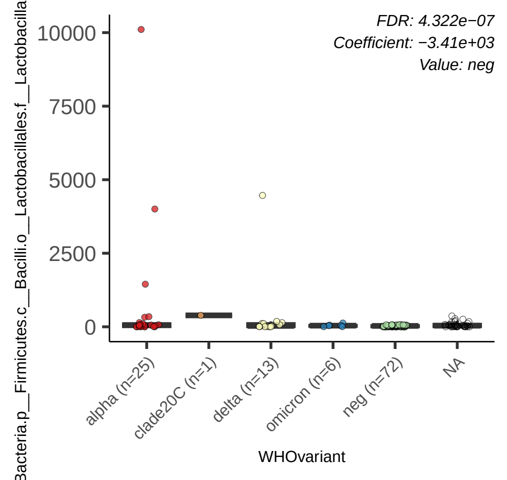
FDR: 4.322e−07
Coefficient: −3.41e+03
Value: neg
Bacteria.p__Firmicutes.c__Bacilli.o__Lactobacillales.f__Lactobacilla
10000
7500
5000
2500
0
alpha (n=25)
clade20C (n=1)
delta (n=13)
omicron (n=6)
neg (n=72)
NA
WHOvariant

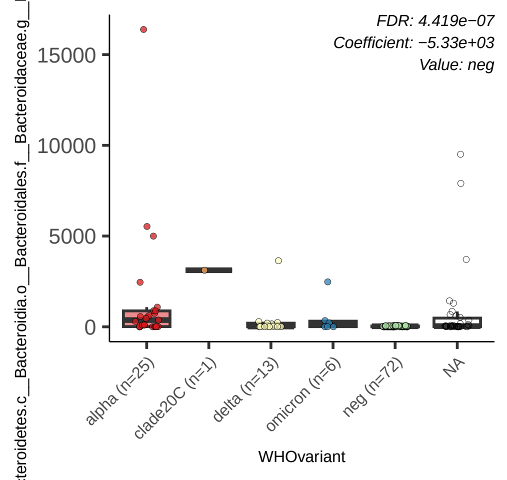

FDR: 4.419e−07
Coefficient: −5.33e+03
Value: neg
15000
10000
5000
0
alpha (n=25)
clade20C (n=1)
delta (n=13)
omicron (n=6)
neg (n=72)
NA
WHOvariant

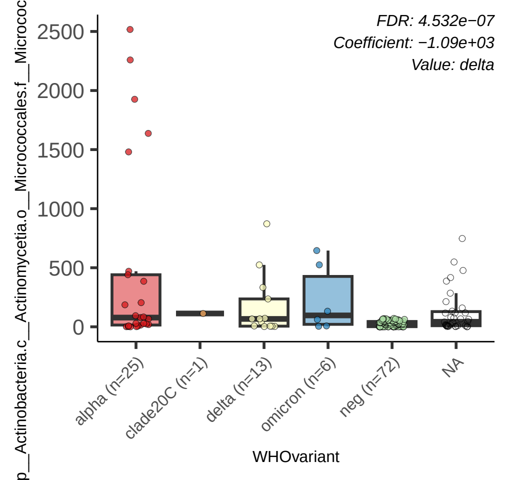

FDR: 4.532e−07
Coefficient: −1.09e+03
Value: delta
p__Actinobacteria.c__Actinomycetia.o__Micrococcales.f__Micrococ
2500
2000
1500
1000
500
0
alpha (n=25)
clade20C (n=1)
delta (n=13)
omicron (n=6)
neg (n=72)
NA
WHOvariant

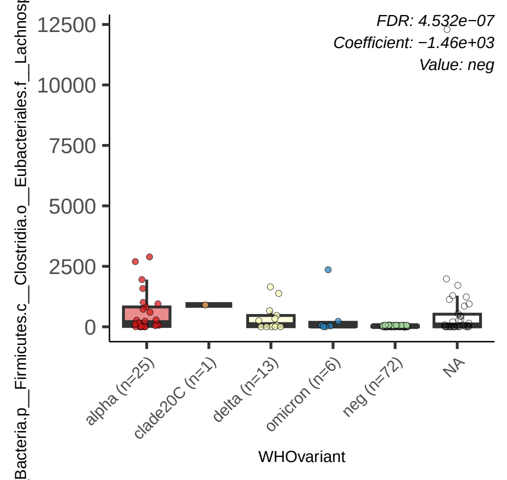
FDR: 4.532e−07
Coefficient: −1.46e+03
Value: neg
Bacteria.p__Firmicutes.c__Clostridia.o__Eubacteriales.f__Lachnosp
12500
10000
7500
5000
2500
0
alpha (n=25)
clade20C (n=1)
delta (n=13)
omicron (n=6)
neg (n=72)
NA
WHOvariant

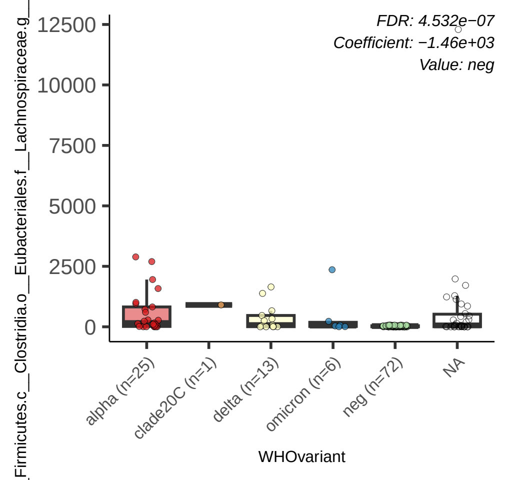
FDR: 4.532e−07
Coefficient: −1.46e+03
Value: neg
Firmicutes.c__Clostridia.o__Eubacteriales.f__Lachnospiraceae.g__
12500
10000
7500
5000
2500
0
alpha (n=25)
clade20C (n=1)
delta (n=13)
omicron (n=6)
neg (n=72)
NA
WHOvariant

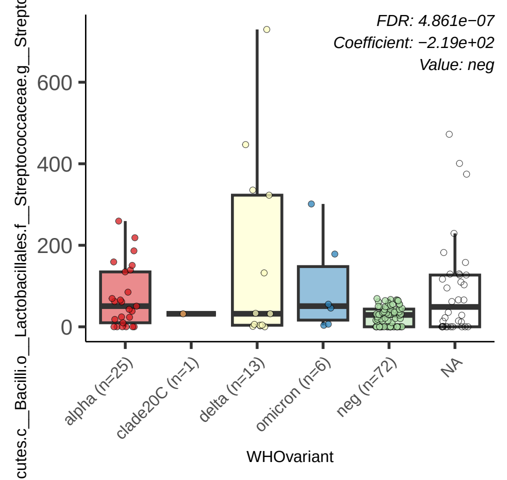
FDR: 4.861e−07
Coefficient: −2.19e+02
Value: neg
cutes.c__Bacilli.o__Lactobacillales.f__Streptococcaceae.g__Strepto
0
200
400
600
alpha (n=25)
clade20C (n=1)
delta (n=13)
omicron (n=6)
neg (n=72)
NA
WHOvariant

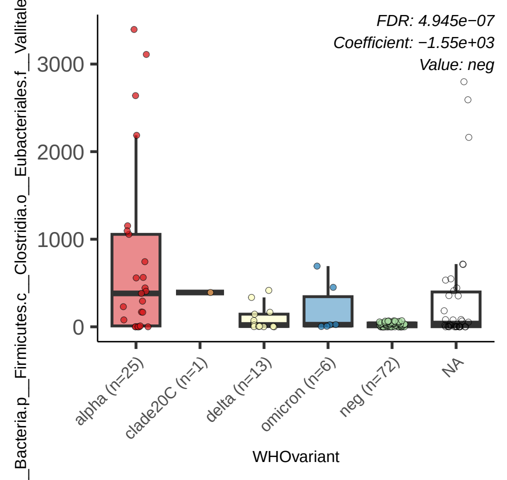

FDR: 4.945e−07
Coefficient: −1.55e+03
Value: neg
Bacteria.p__Firmicutes.c__Clostridia.o__Eubacteriales.f__Vallitale
3000
2000
1000
0
alpha (n=25)
clade20C (n=1)
delta (n=13)
omicron (n=6)
neg (n=72)
NA
WHOvariant

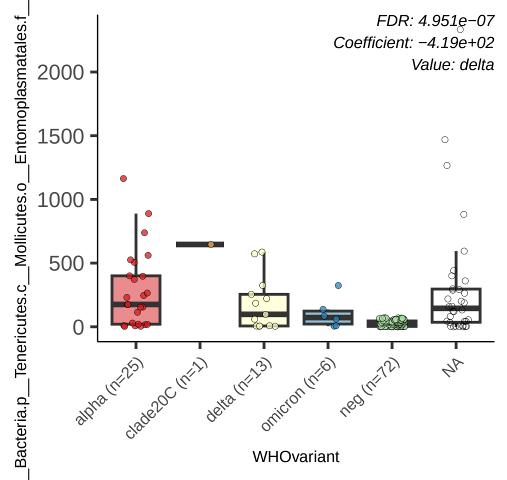

FDR: 4.951e−07
Coefficient: −4.19e+02
Value: delta
Bacteria.p__Tenericutes.c__Mollicutes.o__Entomoplasmatales.f
2000
1500
1000
500
0
alpha (n=25)
clade20C (n=1)
delta (n=13)
omicron (n=6)
neg (n=72)
NA
WHOvariant

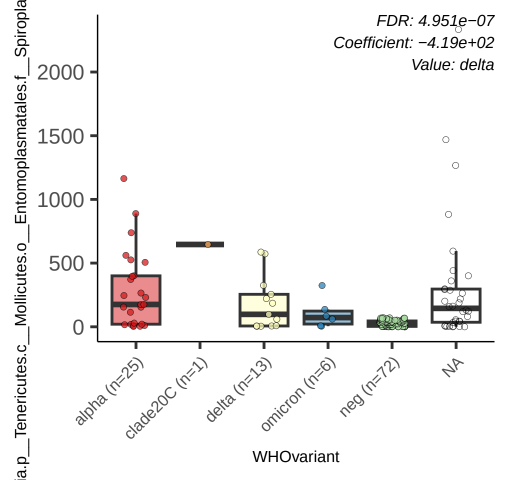

FDR: 4.951e−07
Coefficient: −4.19e+02
Value: delta
ia.p__Tenericutes.c__Mollicutes.o__Entomoplasmatales.f__Spiropla
2000
1500
1000
500
0
alpha (n=25)
clade20C (n=1)
delta (n=13)
omicron (n=6)
neg (n=72)
NA
WHOvariant

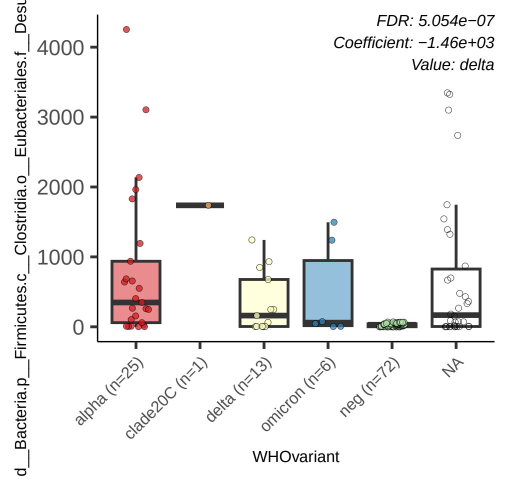

FDR: 5.054e−07
Coefficient: −1.46e+03
Value: delta
d__Bacteria.p__Firmicutes.c__Clostridia.o__Eubacteriales.f__Desu
4000
3000
2000
1000
0
alpha (n=25)
clade20C (n=1)
delta (n=13)
omicron (n=6)
neg (n=72)
NA
WHOvariant

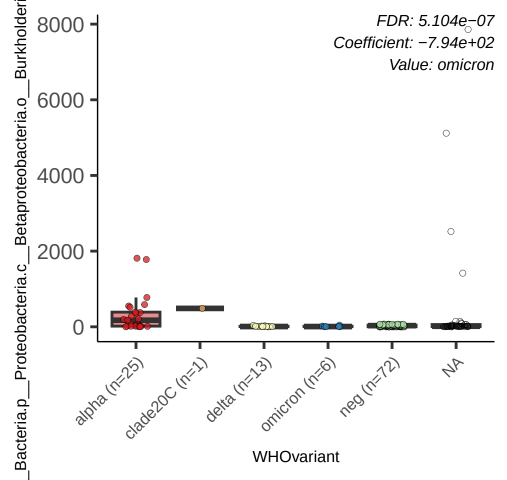
FDR: 5.104e−07
Coefficient: −7.94e+02
Value: omicron
Bacteria.p__Proteobacteria.c__Betaproteobacteria.o__Burkholderi
8000
6000
4000
2000
0
alpha (n=25)
clade20C (n=1)
delta (n=13)
omicron (n=6)
neg (n=72)
NA
WHOvariant

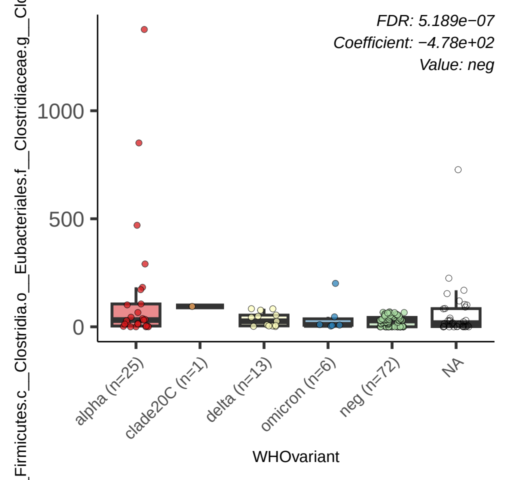

FDR: 5.189e−07
Coefficient: −4.78e+02
Value: neg
1000
500
0
Firmicutes.c__Clostridia.o__Eubacteriales.f__Clostridiaceae.g__Clo
alpha (n=25)
clade20C (n=1)
delta (n=13)
omicron (n=6)
neg (n=72)
NA
WHOvariant

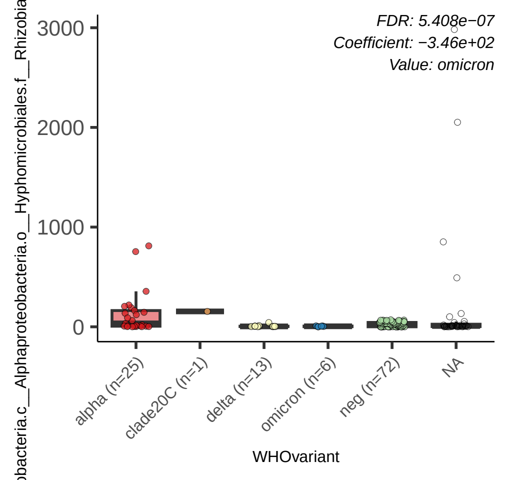
FDR: 5.408e−07
Coefficient: −3.46e+02
Value: omicron
3000
2000
1000
0
obacteria.c__Alphaproteobacteria.o__Hyphomicrobiales.f__Rhizobia
alpha (n=25)
clade20C (n=1)
delta (n=13)
omicron (n=6)
neg (n=72)
NA
WHOvariant

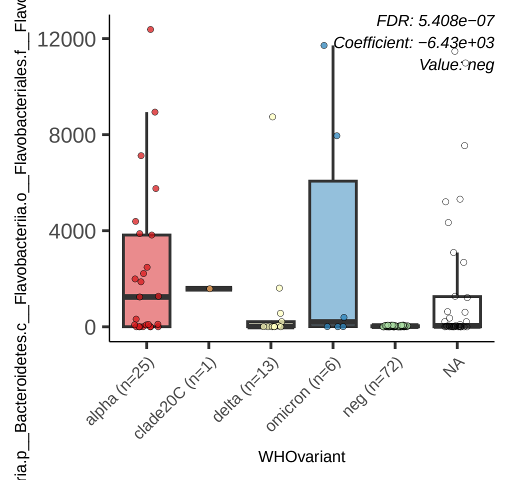
FDR: 5.408e−07
Coefficient: −6.43e+03
Value: neg
12000
8000
4000
0
ria.p__Bacteroidetes.c__Flavobacteriia.o__Flavobacteriales.f__Flavo
alpha (n=25)
clade20C (n=1)
delta (n=13)
omicron (n=6)
neg (n=72)
NA
WHOvariant

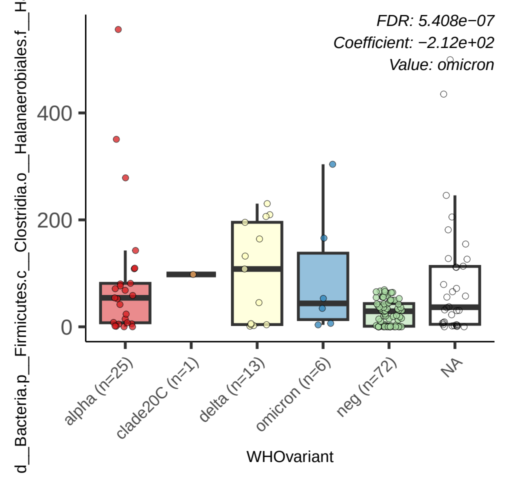

FDR: 5.408e−07
Coefficient: −2.12e+02
Value: omicron
d__Bacteria.p__Firmicutes.c__Clostridia.o__Halanaerobiales.f__H
0
200
400
alpha (n=25)
clade20C (n=1)
delta (n=13)
omicron (n=6)
neg (n=72)
NA
WHOvariant

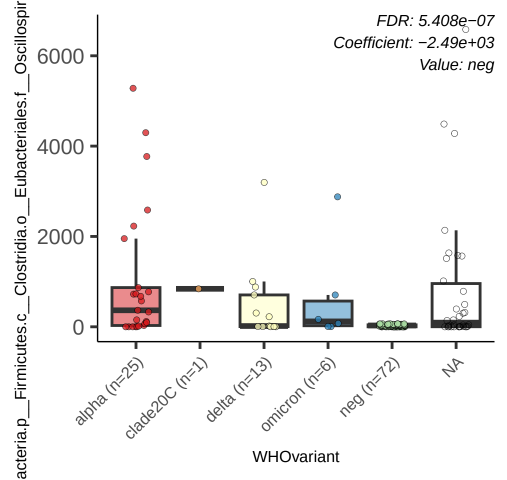

FDR: 5.408e−07
Coefficient: −2.49e+03
Value: neg
6000
4000
2000
0
acteria.p__Firmicutes.c__Clostridia.o__Eubacteriales.f__Oscillospir
alpha (n=25)
clade20C (n=1)
delta (n=13)
omicron (n=6)
neg (n=72)
NA
WHOvariant

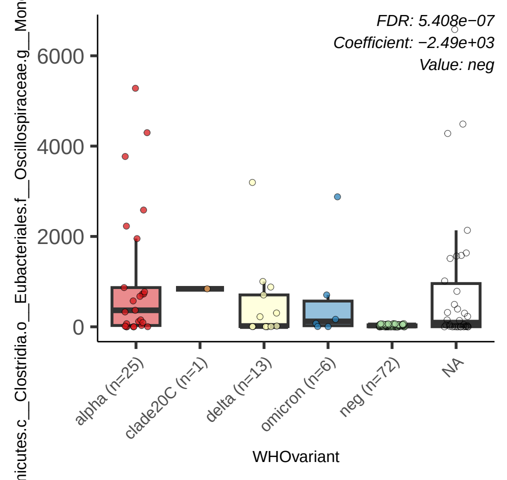

FDR: 5.408e−07
Coefficient: −2.49e+03
Value: neg
6000
4000
2000
0
alpha (n=25)
clade20C (n=1)
delta (n=13)
omicron (n=6)
neg (n=72)
NA
WHOvariant

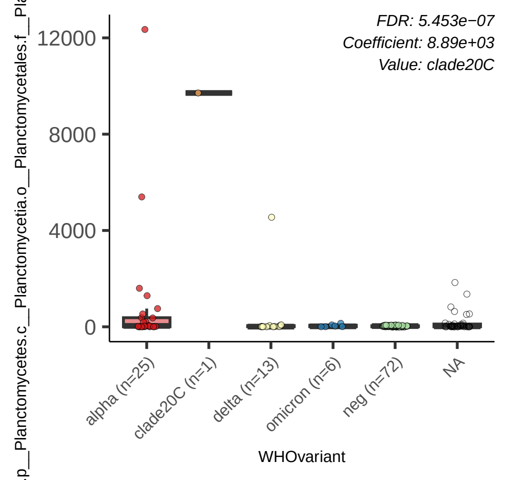

FDR: 5.453e−07
Coefficient: 8.89e+03
Value: clade20C
12000
8000
4000
0
alpha (n=25)
clade20C (n=1)
delta (n=13)
omicron (n=6)
neg (n=72)
NA
WHOvariant
Planctomycetes.c__Planctomycetia.o__Planctomycetales.f__

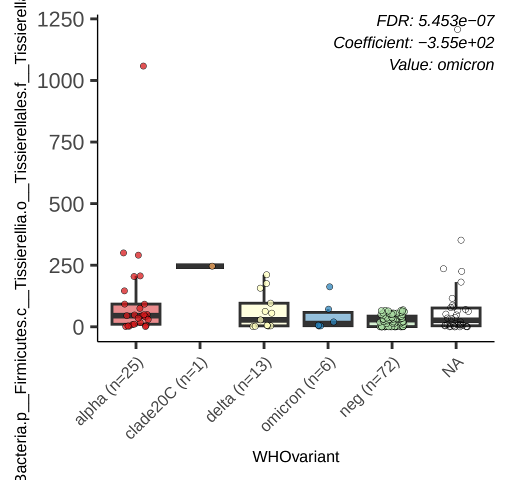

Bacteria.p__Firmicutes.c__Tissierellia.o__Tissierellales.f__Tissierella
FDR: 5.453e−07
Coefficient: −3.55e+02
Value: omicron
1250
1000
750
500
250
0
alpha (n=25)
clade20C (n=1)
delta (n=13)
omicron (n=6)
neg (n=72)
NA
WHOvariant

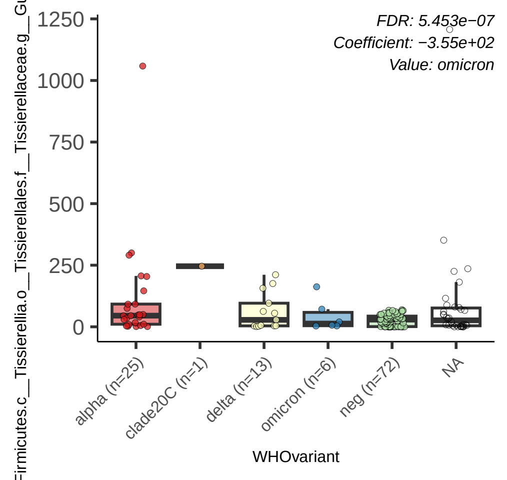

FDR: 5.453e−07
Coefficient: −3.55e+02
Value: omicron
Firmicutes.c__Tissierellia.o__Tissierellales.f__Tissierellaceae.g__Gu
1250
1000
750
500
250
0
alpha (n=25)
clade20C (n=1)
delta (n=13)
omicron (n=6)
neg (n=72)
NA
WHOvariant

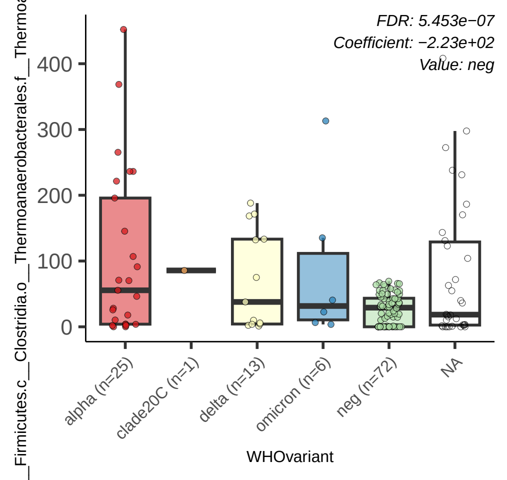

FDR: 5.453e−07
Coefficient: −2.23e+02
Value: neg
Firmicutes.c__Clostridia.o__Thermoanaerobacterales.f__Thermoanaerobacteraceae
0
100
200
300
400
alpha (n=25)
clade20C (n=1)
delta (n=13)
omicron (n=6)
neg (n=72)
NA
WHOvariant

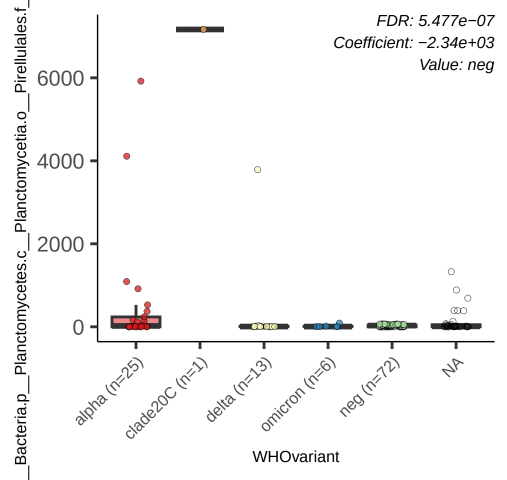

FDR: 5.477e−07
Coefficient: −2.34e+03
Value: neg
Bacteria.p__Planctomycetes.c__Planctomycetia.o__Pirellulales.f
0
2000
4000
6000
alpha (n=25)
clade20C (n=1)
delta (n=13)
omicron (n=6)
neg (n=72)
NA
WHOvariant

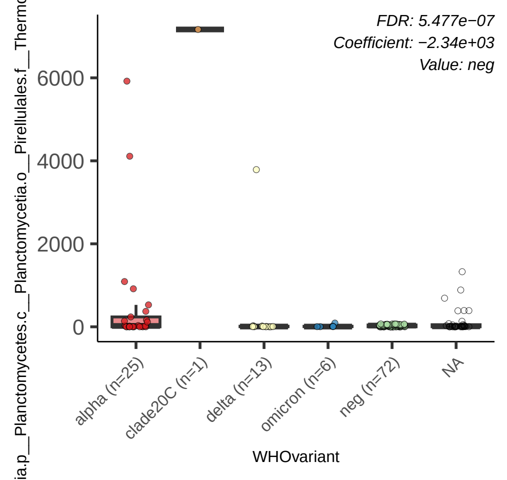
FDR: 5.477e−07
Coefficient: −2.34e+03
Value: neg
ia.p__Planctomycetes.c__Planctomycetia.o__Pirellulales.f__Thermo
6000
4000
2000
0
alpha (n=25)
clade20C (n=1)
delta (n=13)
omicron (n=6)
neg (n=72)
NA
WHOvariant

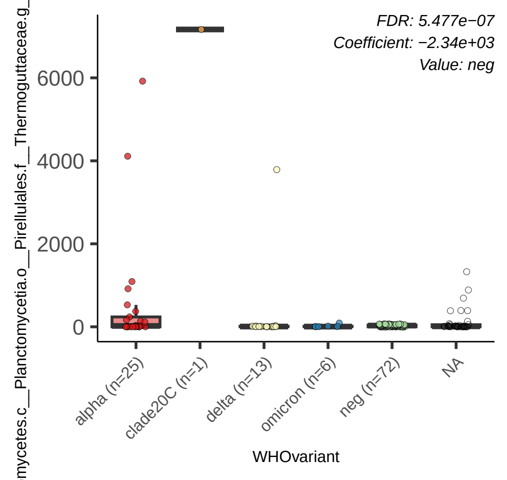

FDR: 5.477e−07
Coefficient: −2.34e+03
Value: neg
mycetes.c__Planctomycetia.o__Pirellulales.f__Thermoguttaceae.g__
6000
4000
2000
0
alpha (n=25)
clade20C (n=1)
delta (n=13)
omicron (n=6)
neg (n=72)
NA
WHOvariant

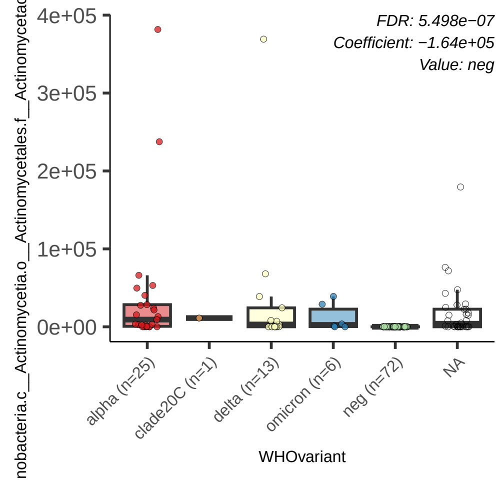

FDR: 5.498e−07
Coefficient: −1.64e+05
Value: neg
4e+05
3e+05
2e+05
1e+05
0e+00
nobacteria.c__Actinomycetia.o__Actinomycetales.f__Actinomycetac
alpha (n=25)
clade20C (n=1)
delta (n=13)
omicron (n=6)
neg (n=72)
NA
WHOvariant

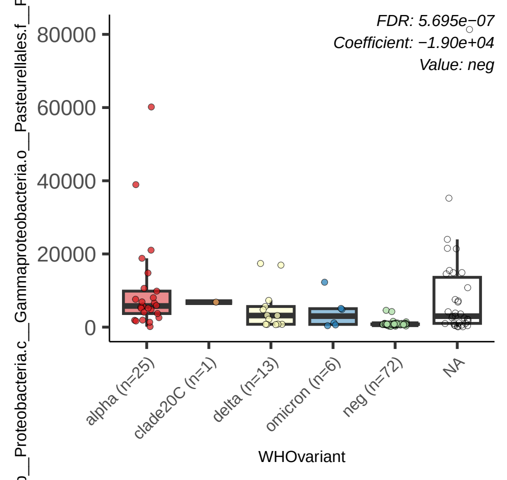

FDR: 5.695e−07
Coefficient: −1.90e+04
Value: neg
80000
60000
40000
20000
0
Proteobacteria.c__Gammaproteobacteria.o__Pasteurellales.f__
alpha (n=25)
clade20C (n=1)
delta (n=13)
omicron (n=6)
neg (n=72)
NA
WHOvariant

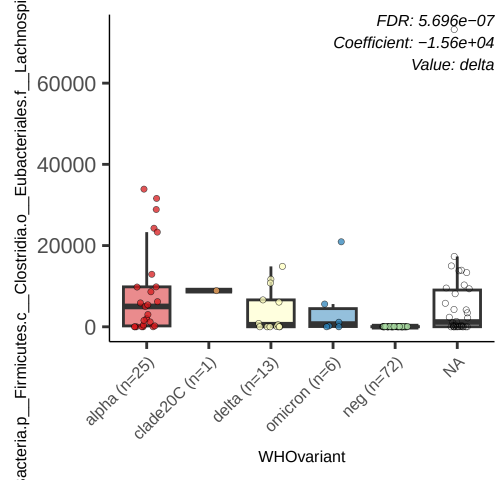

FDR: 5.696e−07
Coefficient: −1.56e+04
Value: delta
60000
40000
20000
0
acteria.p__Firmicutes.c__Clostridia.o__Eubacteriales.f__Lachnospi
alpha (n=25)
clade20C (n=1)
delta (n=13)
omicron (n=6)
neg (n=72)
NA
WHOvariant

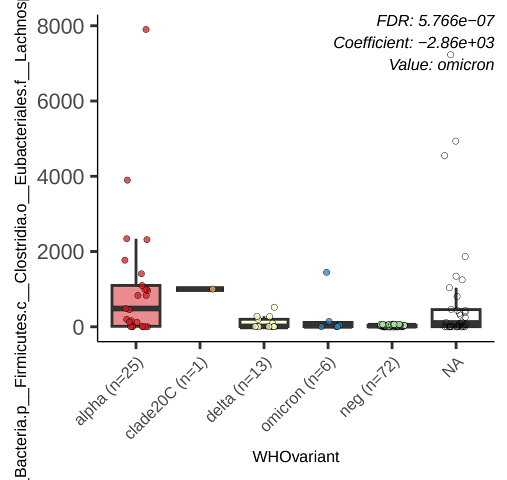
FDR: 5.766e−07
Coefficient: −2.86e+03
Value: omicron
Bacteria.p__Firmicutes.c__Clostridia.o__Eubacteriales.f__Lachnosp
8000
6000
4000
2000
0
alpha (n=25)
clade20C (n=1)
delta (n=13)
omicron (n=6)
neg (n=72)
NA
WHOvariant

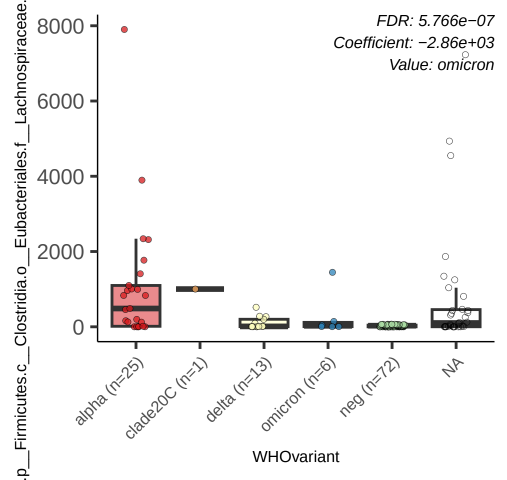
FDR: 5.766e−07
Coefficient: −2.86e+03
Value: omicron
8000
6000
4000
2000
0
alpha (n=25)
clade20C (n=1)
delta (n=13)
omicron (n=6)
neg (n=72)
NA
WHOvariant

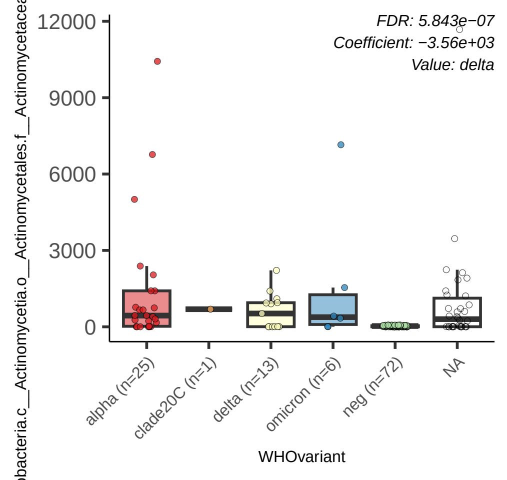

FDR: 5.843e−07
Coefficient: −3.56e+03
Value: delta
12000
9000
6000
3000
0
bacteria.c__Actinomycetia.o__Actinomycetales.f__Actinomycetacea
alpha (n=25)
clade20C (n=1)
delta (n=13)
omicron (n=6)
neg (n=72)
NA
WHOvariant

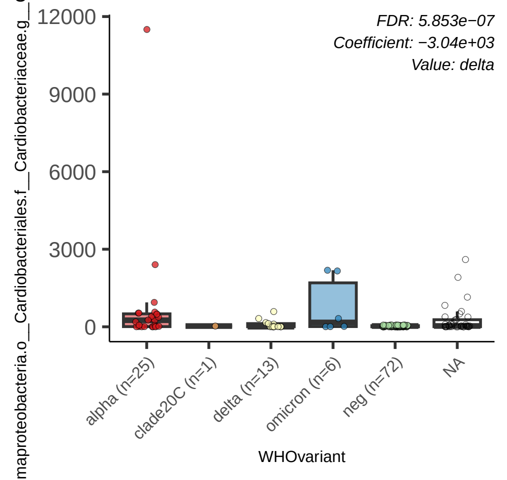

FDR: 5.853e−07
Coefficient: −3.04e+03
Value: delta
maproteobacteria.o__Cardiobacteriales.f__Cardiobacteriaceae.g__
12000
9000
6000
3000
0
alpha (n=25)
clade20C (n=1)
delta (n=13)
omicron (n=6)
neg (n=72)
NA
WHOvariant

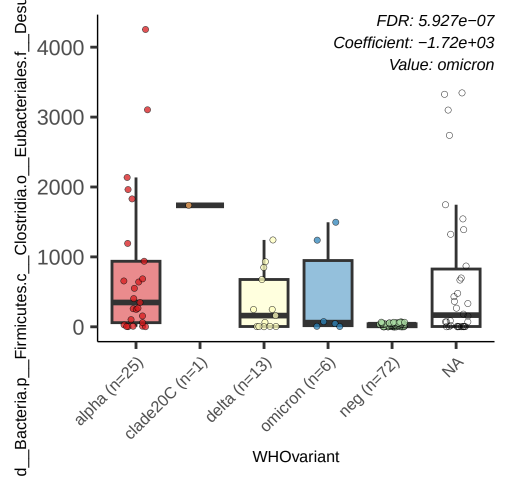

FDR: 5.927e−07
Coefficient: −1.72e+03
Value: omicron
d__Bacteria.p__Firmicutes.c__Clostridia.o__Eubacteriales.f__Desu
4000
3000
2000
1000
0
alpha (n=25)
clade20C (n=1)
delta (n=13)
omicron (n=6)
neg (n=72)
NA
WHOvariant

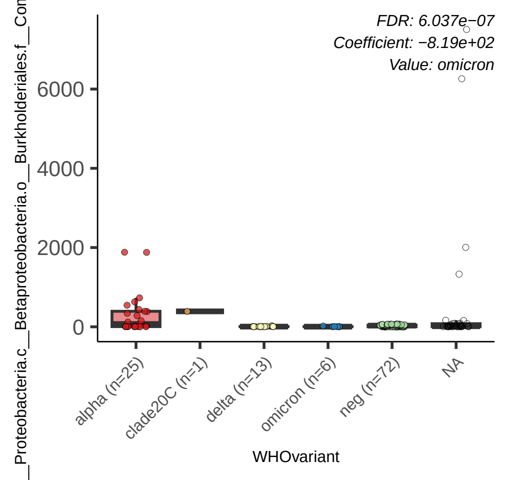

FDR: 6.037e−07
Coefficient: −8.19e+02
Value: omicron
6000
4000
2000
0
_Proteobacteria.c__Betaproteobacteria.o__Burkholderiales.f__Con
alpha (n=25)
clade20C (n=1)
delta (n=13)
omicron (n=6)
neg (n=72)
NA
WHOvariant

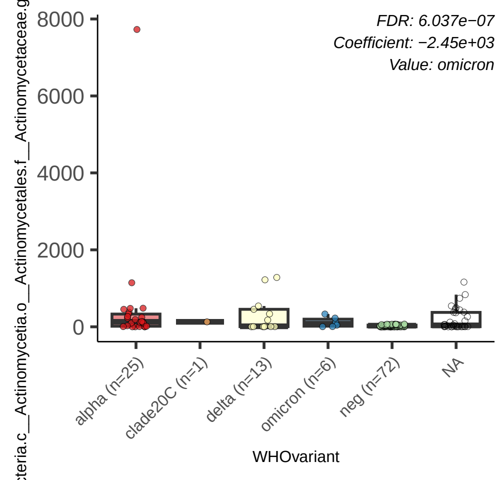

FDR: 6.037e−07
Coefficient: −2.45e+03
Value: omicron
8000
6000
4000
2000
0
cteria.c__Actinomycetia.o__Actinomycetales.f__Actinomycetaceae.g
alpha (n=25)
clade20C (n=1)
delta (n=13)
omicron (n=6)
neg (n=72)
NA
WHOvariant

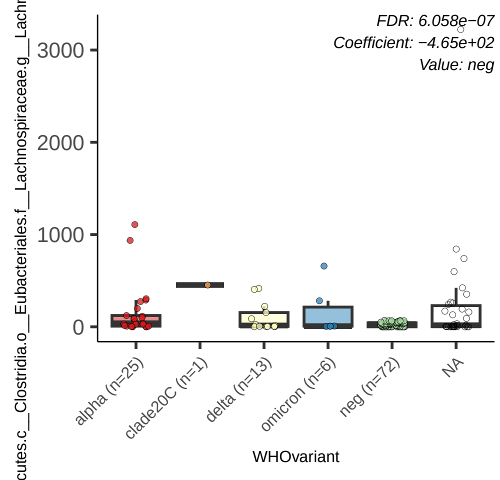

FDR: 6.058e−07
Coefficient: −4.65e+02
Value: neg
3000
2000
1000
0
cutes.c__Clostridia.o__Eubacteriales.f__Lachnospiraceae.g__Lachn
alpha (n=25)
clade20C (n=1)
delta (n=13)
omicron (n=6)
neg (n=72)
NA
WHOvariant

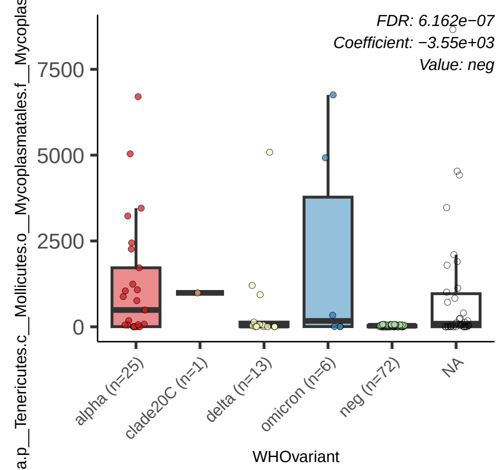

FDR: 6.162e−07
Coefficient: −3.55e+03
Value: neg
7500
5000
2500
0
ria.p__Tenericutes.c__Mollicutes.o__Mycoplasmatales.f__Mycoplas
alpha (n=25)
clade20C (n=1)
delta (n=13)
omicron (n=6)
neg (n=72)
NA
WHOvariant

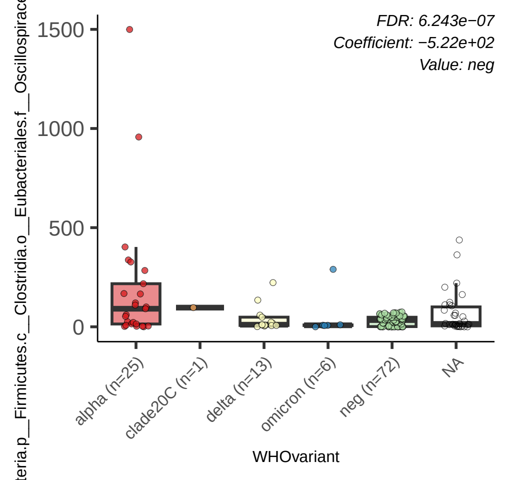

FDR: 6.243e−07
Coefficient: −5.22e+02
Value: neg
1500
1000
500
0
alpha (n=25)
clade20C (n=1)
delta (n=13)
omicron (n=6)
neg (n=72)
NA
WHOvariant
teria.p__Firmicutes.c__Clostridia.o__Eubacteriales.f__Oscillospirace

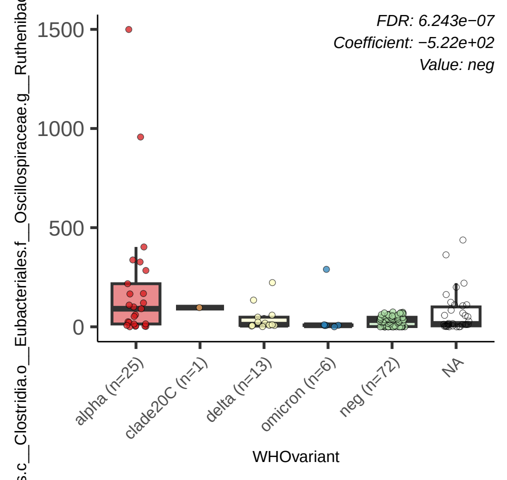

FDR: 6.243e−07
Coefficient: −5.22e+02
Value: neg
1500
1000
500
0
s.c__Clostridia.o__Eubacteriales.f__Oscillospiraceae.g__Ruthenibac
alpha (n=25)
clade20C (n=1)
delta (n=13)
omicron (n=6)
neg (n=72)
NA
WHOvariant

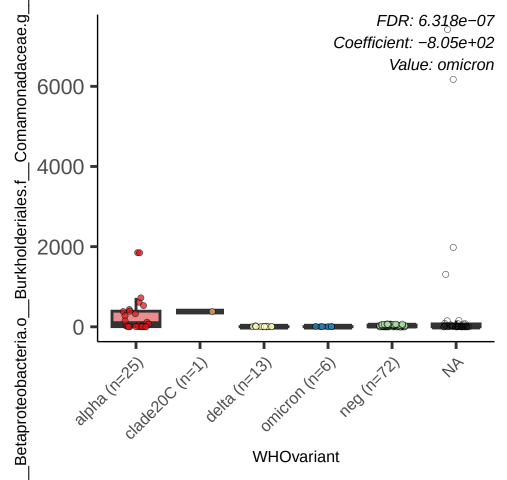

FDR: 6.318e−07
Coefficient: −8.05e+02
Value: omicron
Betaproteobacteria.o__Burkholderiales.f__Comamonadaceae.g__
6000
4000
2000
0
alpha (n=25)
clade20C (n=1)
delta (n=13)
omicron (n=6)
neg (n=72)
NA
WHOvariant

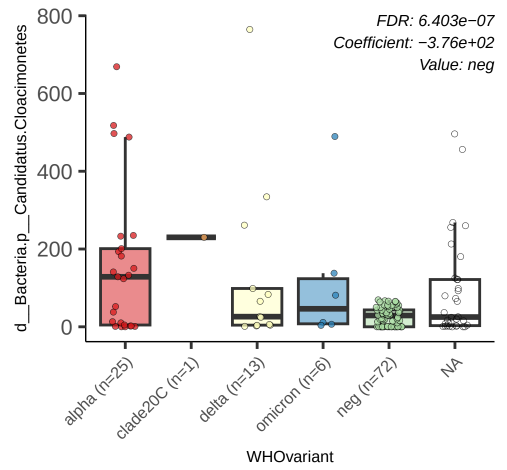
FDR: 6.403e−07
Coefficient: −3.76e+02
Value: neg
d__Bacteria.p__Candidatus.Cloacimonetes
800
600
400
200
0
alpha (n=25)
clade20C (n=1)
delta (n=13)
omicron (n=6)
neg (n=72)
NA
WHOvariant

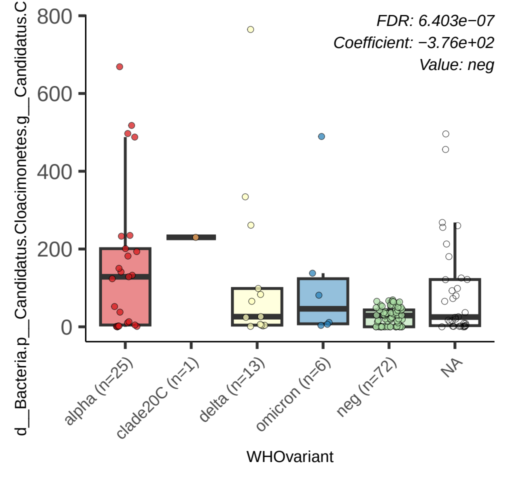

FDR: 6.403e−07
Coefficient: −3.76e+02
Value: neg
d__Bacteria.p__Candidatus.Cloacimonetes.g__Candidatus.C
800
600
400
200
0
alpha (n=25)
clade20C (n=1)
delta (n=13)
omicron (n=6)
neg (n=72)
NA
WHOvariant

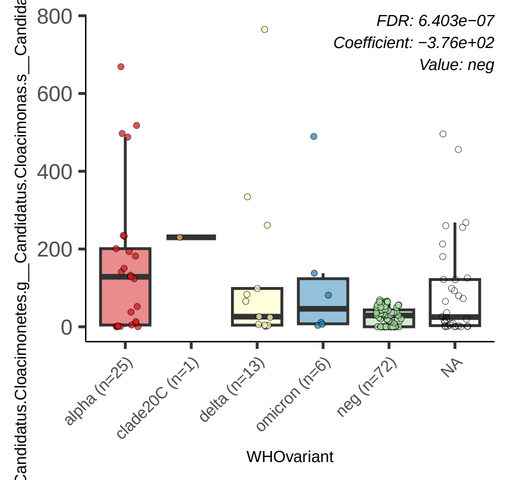

Candidatus.Cloacimonetes.g__Candidatus.Cloacimonas.s__Candida
FDR: 6.403e−07
Coefficient: −3.76e+02
Value: neg
800
600
400
200
0
alpha (n=25)
clade20C (n=1)
delta (n=13)
omicron (n=6)
neg (n=72)
NA
WHOvariant

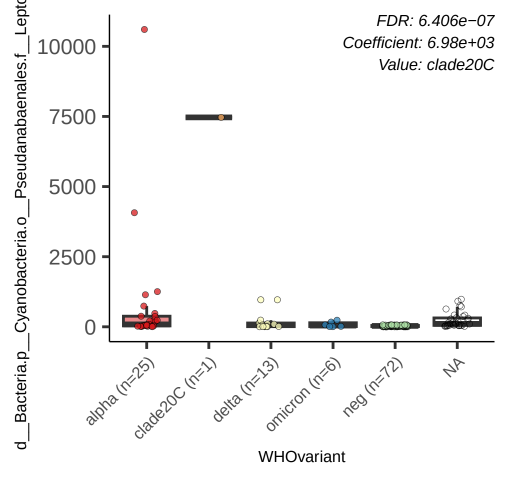
FDR: 6.406e−07
Coefficient: 6.98e+03
Value: clade20C
d__Bacteria.p__Cyanobacteria.o__Pseudanabaenales.f__Lepto
10000
7500
5000
2500
0
alpha (n=25)
clade20C (n=1)
delta (n=13)
omicron (n=6)
neg (n=72)
NA
WHOvariant

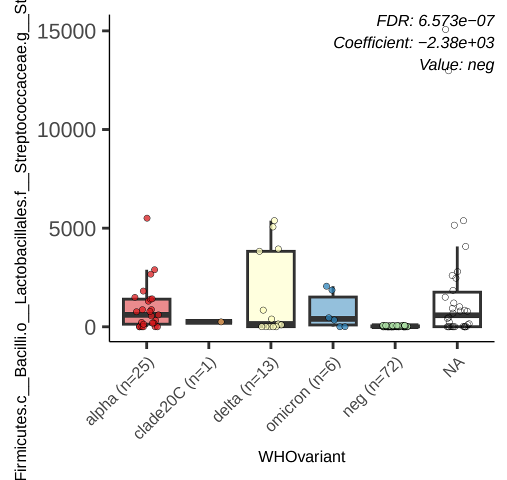
FDR: 6.573e−07
Coefficient: −2.38e+03
Value: neg
Firmicutes.c__Bacilli.o__Lactobacillales.f__Streptococcaceae.g__St
15000
10000
5000
0
alpha (n=25)
clade20C (n=1)
delta (n=13)
omicron (n=6)
neg (n=72)
NA
WHOvariant

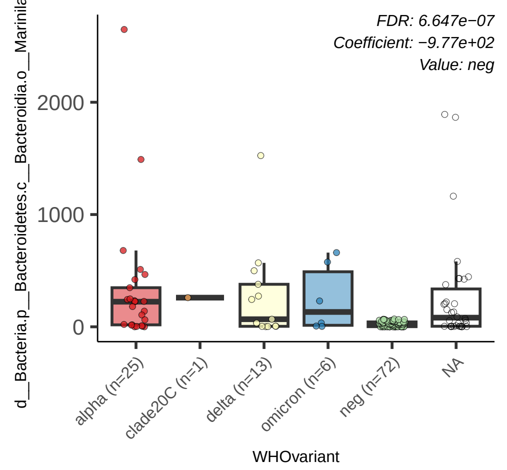

FDR: 6.647e−07
Coefficient: −9.77e+02
Value: neg
2000
1000
0
d__Bacteria.p__Bacteroidetes.c__Bacteroidia.o__Marinila
alpha (n=25)
clade20C (n=1)
delta (n=13)
omicron (n=6)
neg (n=72)
NA
WHOvariant

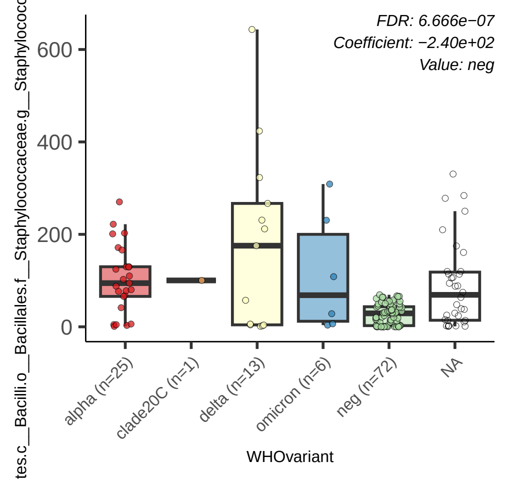
FDR: 6.666e−07
Coefficient: −2.40e+02
Value: neg
0
200
400
600
tes.c__Bacilli.o__Bacillales.f__Staphylococcaceae.g__Staphylococc
alpha (n=25)
clade20C (n=1)
delta (n=13)
omicron (n=6)
neg (n=72)
NA
WHOvariant

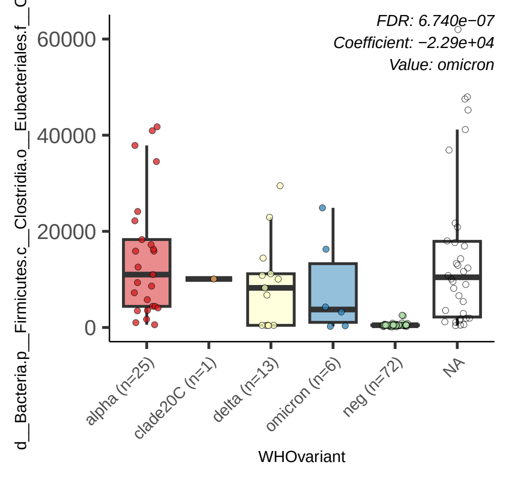
FDR: 6.740e−07
Coefficient: −2.29e+04
Value: omicron
d__Bacteria.p__Firmicutes.c__Clostridia.o__Eubacteriales.f__C
60000
40000
20000
0
alpha (n=25)
clade20C (n=1)
delta (n=13)
omicron (n=6)
neg (n=72)
NA
WHOvariant

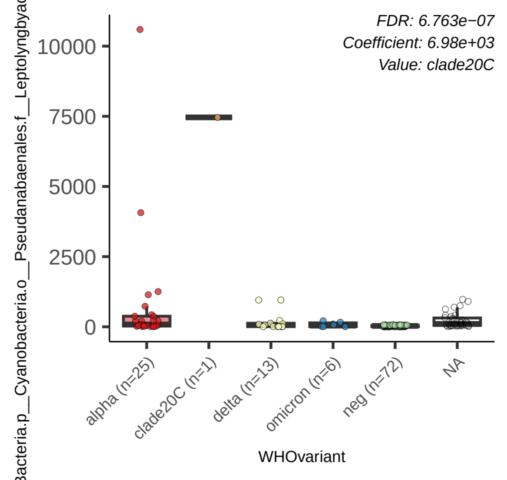

FDR: 6.763e−07
Coefficient: 6.98e+03
Value: clade20C
Bacteria.p__Cyanobacteria.o__Pseudanabaenales.f__Leptolyngbyac
10000
7500
5000
2500
0
alpha (n=25)
clade20C (n=1)
delta (n=13)
omicron (n=6)
neg (n=72)
NA
WHOvariant

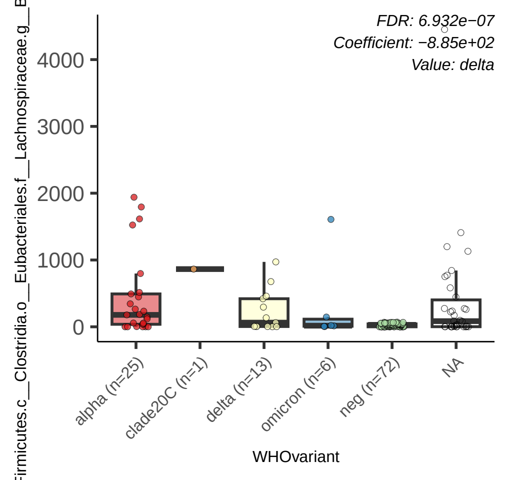

FDR: 6.932e−07
Coefficient: −8.85e+02
Value: delta
Firmicutes.c__Clostridia.o__Eubacteriales.f__Lachnospiraceae.g__B
4000
3000
2000
1000
0
alpha (n=25)
clade20C (n=1)
delta (n=13)
omicron (n=6)
neg (n=72)
NA
WHOvariant

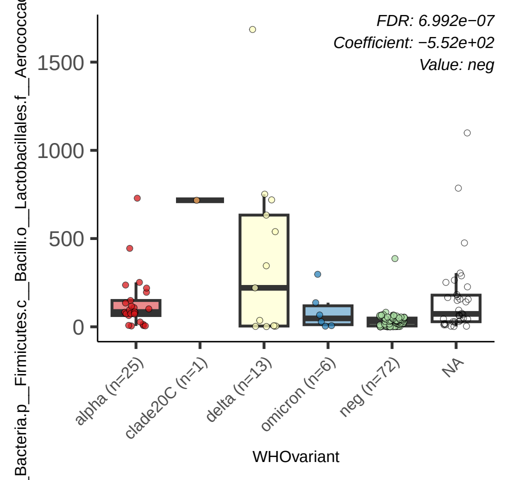
FDR: 6.992e−07
Coefficient: −5.52e+02
Value: neg
_Bacteria.p__Firmicutes.c__Bacilli.o__Lactobacillales.f__Aerococcac
0
500
1000
1500
alpha (n=25)
clade20C (n=1)
delta (n=13)
omicron (n=6)
neg (n=72)
NA
WHOvariant

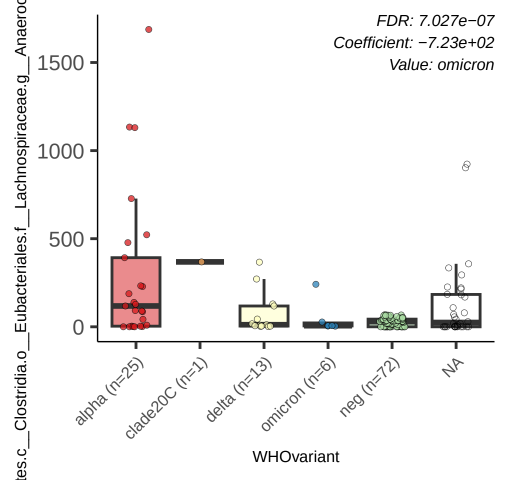

FDR: 7.027e−07
Coefficient: −7.23e+02
Value: omicron
tes.c__Clostridia.o__Eubacteriales.f__Lachnospiraceae.g__Anaeroc
0
500
1000
1500
alpha (n=25)
clade20C (n=1)
delta (n=13)
omicron (n=6)
neg (n=72)
NA
WHOvariant

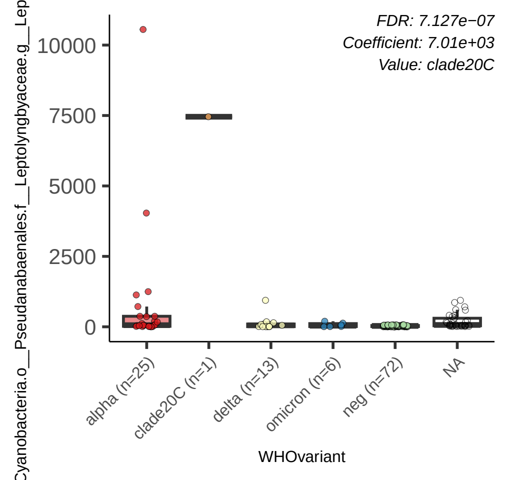

FDR: 7.127e−07
Coefficient: 7.01e+03
Value: clade20C
Cyanobacteria.o__Pseudanabaenales.f__Leptolyngbyaceae.g__Lep
10000
7500
5000
2500
0
alpha (n=25)
clade20C (n=1)
delta (n=13)
omicron (n=6)
neg (n=72)
NA
WHOvariant

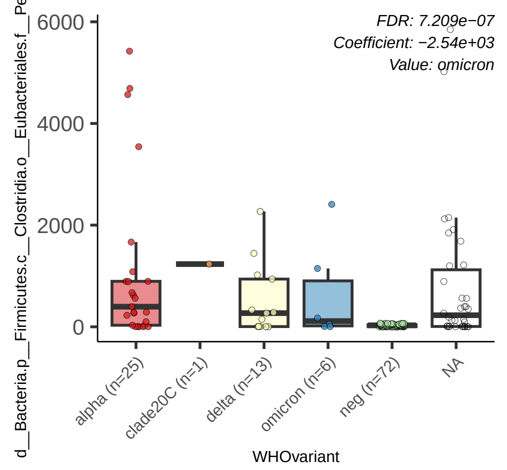
FDR: 7.209e−07
Coefficient: −2.54e+03
Value: omicron
d__Bacteria.p__Firmicutes.c__Clostridia.o__Eubacteriales.f__Pe
6000
4000
2000
0
alpha (n=25)
clade20C (n=1)
delta (n=13)
omicron (n=6)
neg (n=72)
NA
WHOvariant

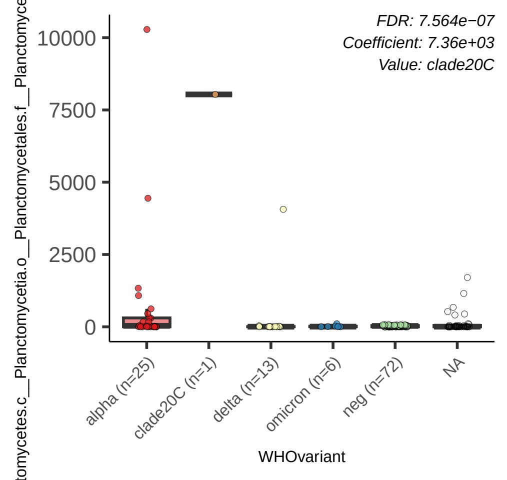
FDR: 7.564e−07
Coefficient: 7.36e+03
Value: clade20C
10000
7500
5000
2500
0
alpha (n=25)
clade20C (n=1)
delta (n=13)
omicron (n=6)
neg (n=72)
NA
WHOvariant
tomycetes.c__Planctomycetia.o__Planctomycetales.f__Planctomyce

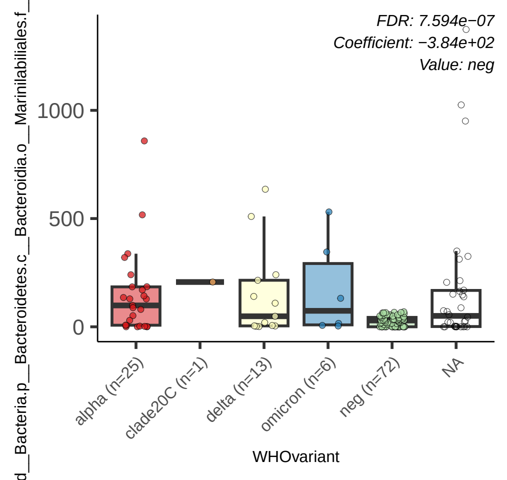

FDR: 7.594e−07
Coefficient: −3.84e+02
Value: neg
d__Bacteria.p__Bacteroidetes.c__Bacteroidia.o__Marinilabiliales.f
1000
500
0
alpha (n=25)
clade20C (n=1)
delta (n=13)
omicron (n=6)
neg (n=72)
NA
WHOvariant

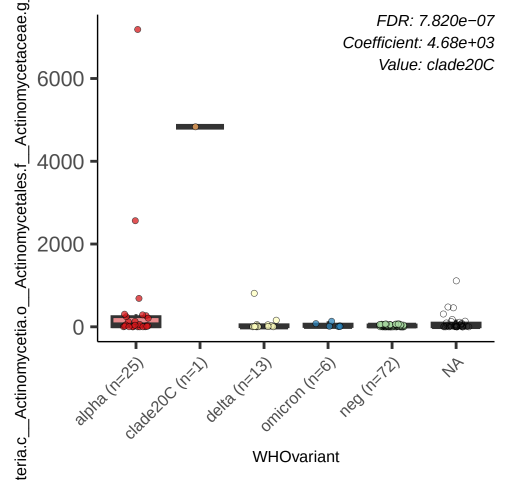

FDR: 7.820e−07
Coefficient: 4.68e+03
Value: clade20C
teria.c__Actinomycetia.o__Actinomycetales.f__Actinomycetaceae.g
6000
4000
2000
0
alpha (n=25)
clade20C (n=1)
delta (n=13)
omicron (n=6)
neg (n=72)
NA
WHOvariant

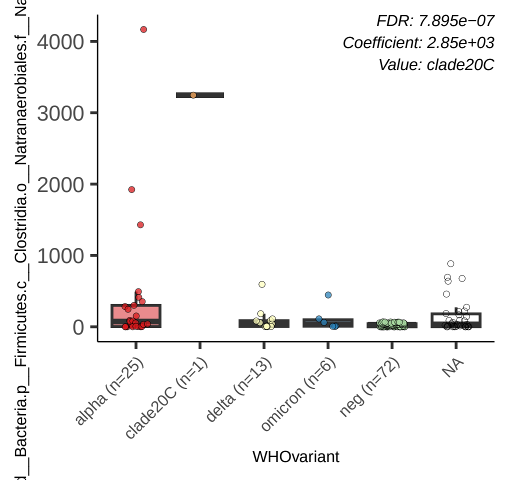

FDR: 7.895e−07
Coefficient: 2.85e+03
Value: clade20C
d__Bacteria.p__Firmicutes.c__Clostridia.o__Natranaerobiales.f__Na
4000
3000
2000
1000
0
alpha (n=25)
clade20C (n=1)
delta (n=13)
omicron (n=6)
neg (n=72)
NA
WHOvariant

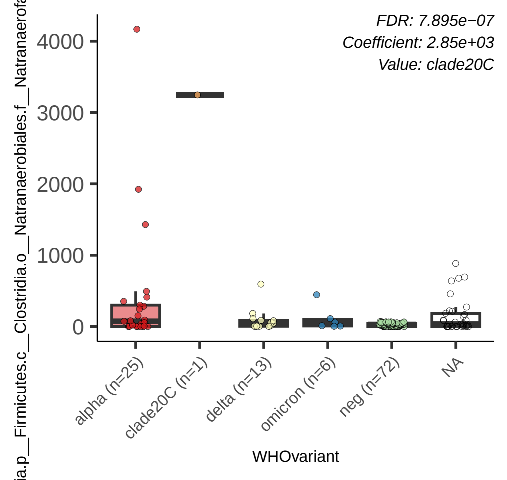
FDR: 7.895e−07
Coefficient: 2.85e+03
Value: clade20C
4000
3000
2000
1000
0
alpha (n=25)
clade20C (n=1)
delta (n=13)
omicron (n=6)
neg (n=72)
NA
WHOvariant

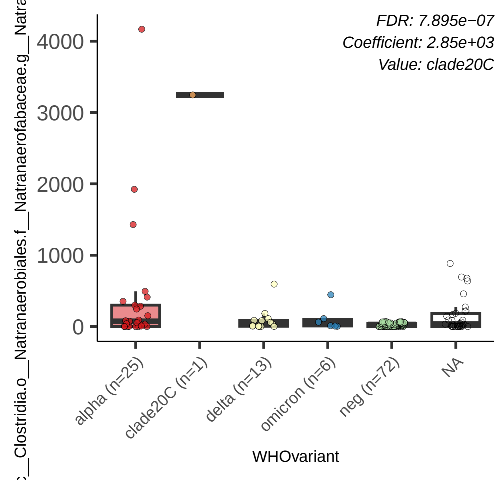

FDR: 7.895e−07
Coefficient: 2.85e+03
Value: clade20C
c__Clostridia.o__Natranaerobiales.f__Natranaerofabaceae.g__Natra
4000
3000
2000
1000
0
alpha (n=25)
clade20C (n=1)
delta (n=13)
omicron (n=6)
neg (n=72)
NA
WHOvariant

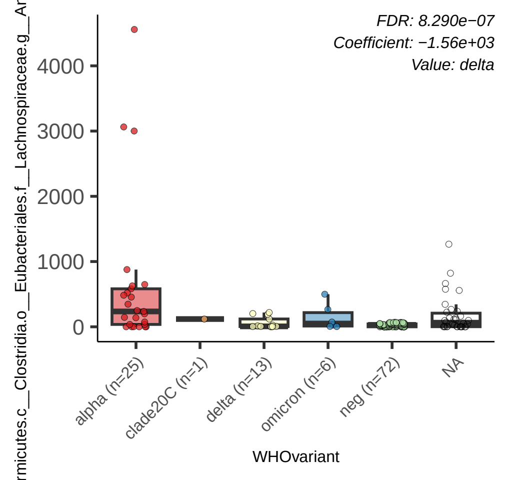

FDR: 8.290e−07
Coefficient: −1.56e+03
Value: delta
4000
3000
2000
1000
0
alpha (n=25)
clade20C (n=1)
delta (n=13)
omicron (n=6)
neg (n=72)
NA
WHOvariant
rmicutes.c__Clostridia.o__Eubacteriales.f__Lachnospiraceae.g__Ar

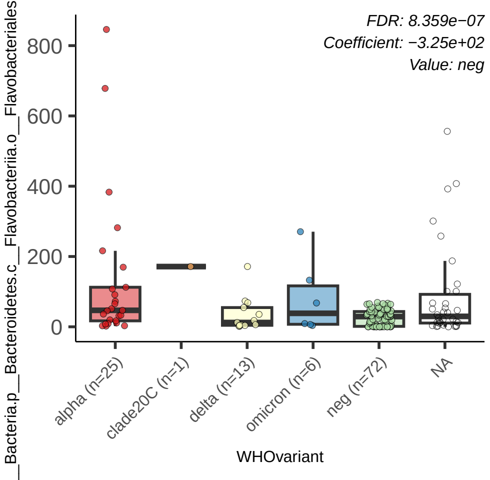

FDR: 8.359e−07
Coefficient: −3.25e+02
Value: neg
Bacteria.p__Bacteroidetes.c__Flavobacteriia.o__Flavobacteriales
0
200
400
600
800
alpha (n=25)
clade20C (n=1)
delta (n=13)
omicron (n=6)
neg (n=72)
NA
WHOvariant

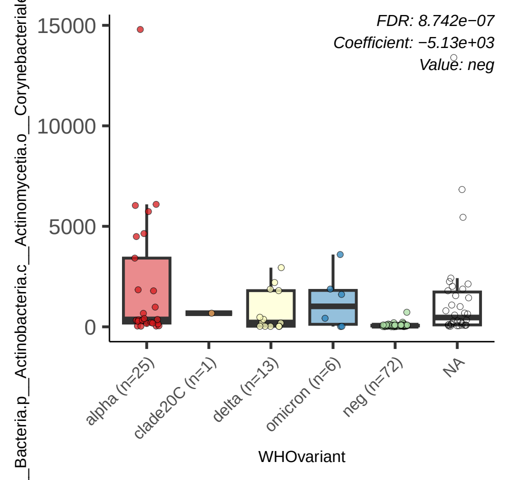

FDR: 8.742e−07
Coefficient: −5.13e+03
Value: neg
15000
10000
5000
0
alpha (n=25)
clade20C (n=1)
delta (n=13)
omicron (n=6)
neg (n=72)
NA
WHOvariant
Bacteria.p__Actinobacteria.c__Actinomycetia.o__Corynebacteriale

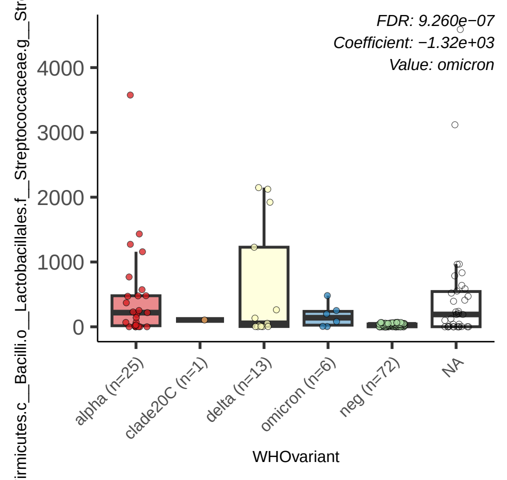

FDR: 9.260e−07
Coefficient: −1.32e+03
Value: omicron
irmicutes.c__Bacilli.o__Lactobacillales.f__Streptococcaceae.g__Str
0
1000
2000
3000
4000
alpha (n=25)
clade20C (n=1)
delta (n=13)
omicron (n=6)
neg (n=72)
NA
WHOvariant

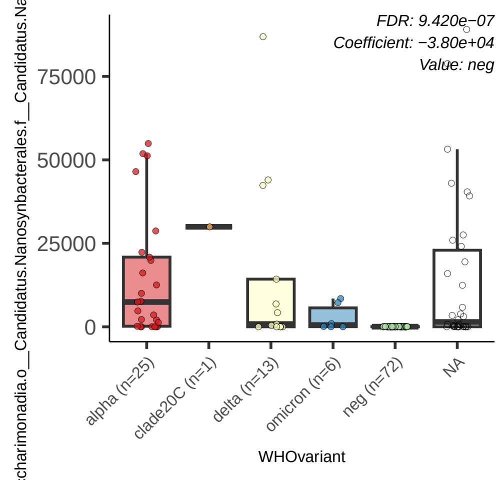
FDR: 9.420e−07
Coefficient: −3.80e+04
Value: neg
75000
50000
25000
0
alpha (n=25)
clade20C (n=1)
delta (n=13)
omicron (n=6)
neg (n=72)
NA
WHOvariant
echarimonadia.o__Candidatus.Nanosynbacterales.f__Candidatus.Na

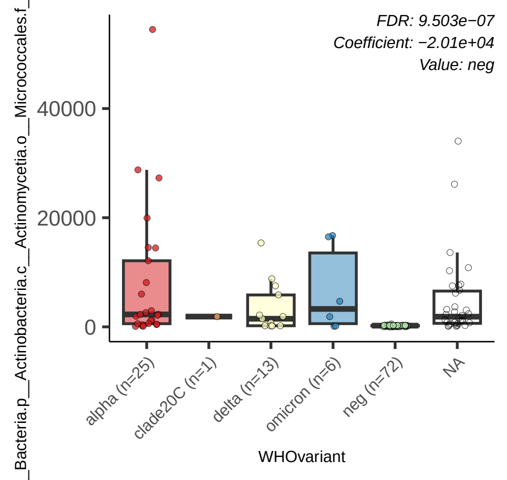

FDR: 9.503e−07
Coefficient: −2.01e+04
Value: neg
Bacteria.p__Actinobacteria.c__Actinomycetia.o__Micrococcales.f__
40000
20000
0
alpha (n=25)
clade20C (n=1)
delta (n=13)
omicron (n=6)
neg (n=72)
NA
WHOvariant

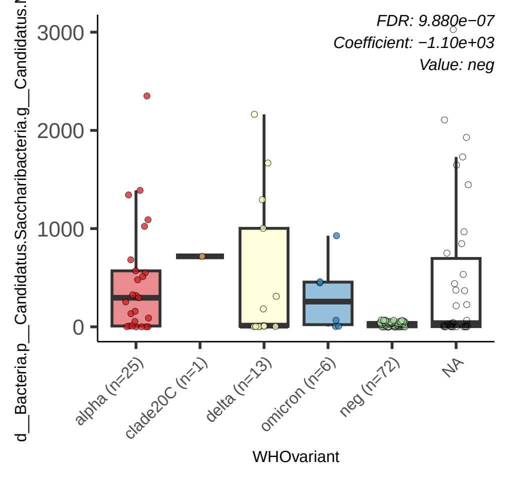
FDR: 9.880e−07
Coefficient: −1.10e+03
Value: neg
d__Bacteria.p__Candidatus.Saccharibacteria.g__Candidatus.N
3000
2000
1000
0
alpha (n=25)
clade20C (n=1)
delta (n=13)
omicron (n=6)
neg (n=72)
NA
WHOvariant

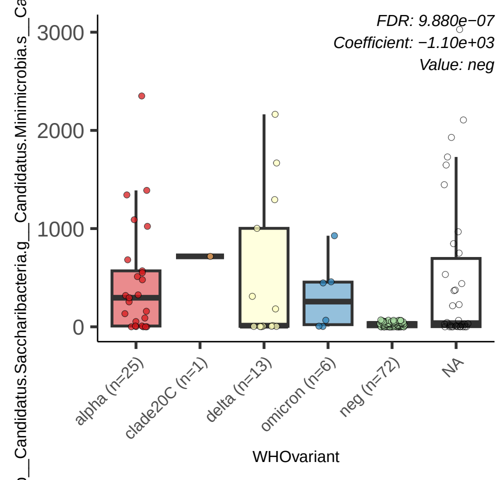

FDR: 9.880e−07
Coefficient: −1.10e+03
Value: neg
3000
2000
1000
0
alpha (n=25)
clade20C (n=1)
delta (n=13)
omicron (n=6)
neg (n=72)
NA
WHOvariant

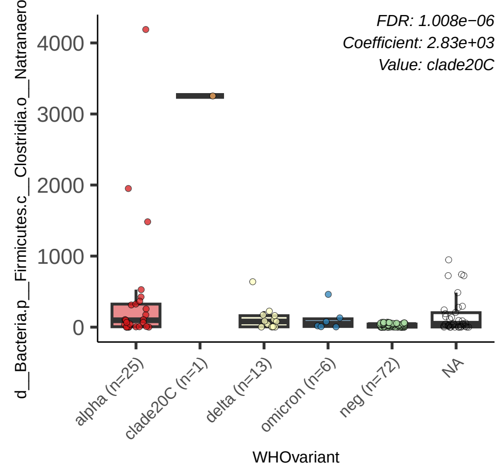
FDR: 1.008e−06
Coefficient: 2.83e+03
Value: clade20C
d__Bacteria.p__Firmicutes.c__Clostridia.o__Natranaero
4000
3000
2000
1000
0
alpha (n=25)
clade20C (n=1)
delta (n=13)
omicron (n=6)
neg (n=72)
NA
WHOvariant

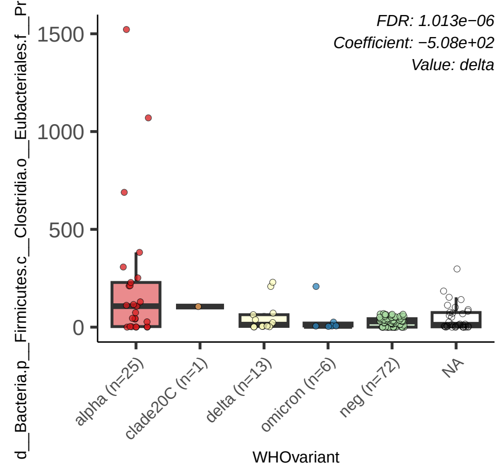

FDR: 1.013e−06
Coefficient: −5.08e+02
Value: delta
d__Bacteria.p__Firmicutes.c__Clostridia.o__Eubacteriales.f__Pr
1500
1000
500
0
alpha (n=25)
clade20C (n=1)
delta (n=13)
omicron (n=6)
neg (n=72)
NA
WHOvariant

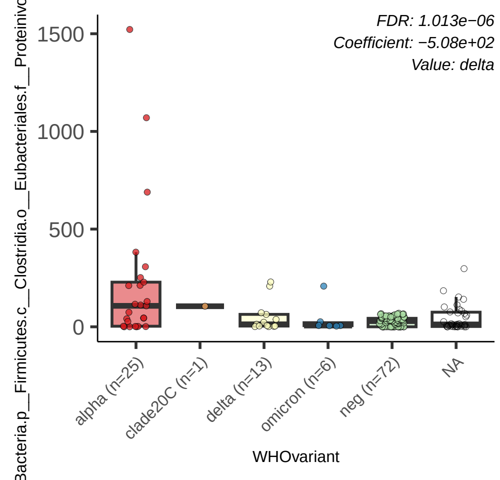

FDR: 1.013e−06
Coefficient: −5.08e+02
Value: delta
Bacteria.p__Firmicutes.c__Clostridia.o__Eubacteriales.f__Proteinivo
1500
1000
500
0
alpha (n=25)
clade20C (n=1)
delta (n=13)
omicron (n=6)
neg (n=72)
NA
WHOvariant

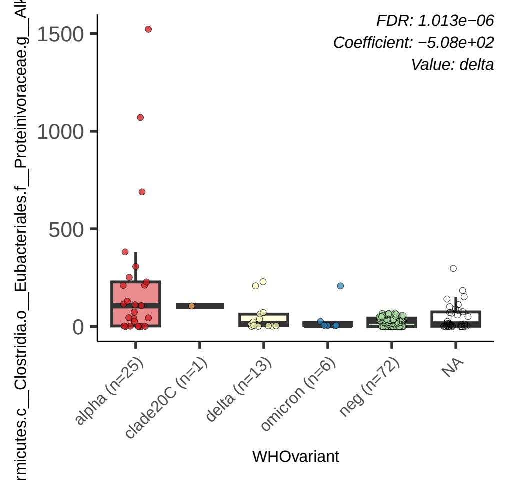

FDR: 1.013e−06
Coefficient: −5.08e+02
Value: delta
rmicutes.c__Clostridia.o__Eubacteriales.f__Proteinivoraceae.g__Alk
1500
1000
500
0
alpha (n=25)
clade20C (n=1)
delta (n=13)
omicron (n=6)
neg (n=72)
NA
WHOvariant

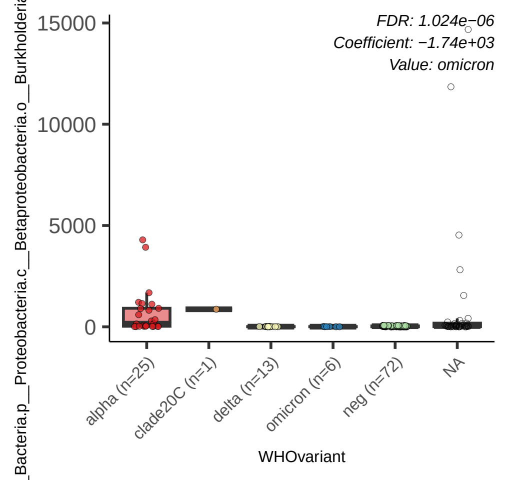
FDR: 1.024e−06
Coefficient: −1.74e+03
Value: omicron
15000
10000
5000
0
Bacteria.p__Proteobacteria.c__Betaproteobacteria.o__Burkholderia
alpha (n=25)
clade20C (n=1)
delta (n=13)
omicron (n=6)
neg (n=72)
NA
WHOvariant

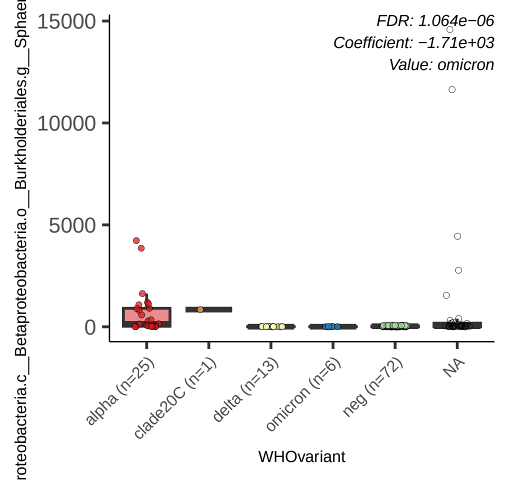
FDR: 1.064e−06
Coefficient: −1.71e+03
Value: omicron
15000
10000
5000
0
roteobacteria.c__Betaproteobacteria.o__Burkholderiales.g__Sphae
alpha (n=25)
clade20C (n=1)
delta (n=13)
omicron (n=6)
neg (n=72)
NA
WHOvariant

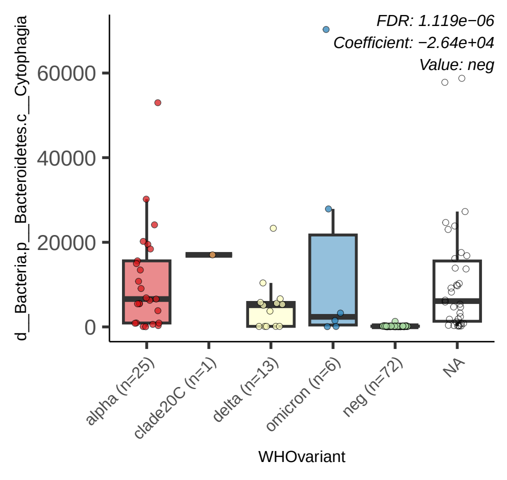

FDR: 1.119e−06
Coefficient: −2.64e+04
Value: neg
d__Bacteria.p__Bacteroidetes.c__Cytophagia
60000
40000
20000
0
alpha (n=25)
clade20C (n=1)
delta (n=13)
omicron (n=6)
neg (n=72)
NA
WHOvariant

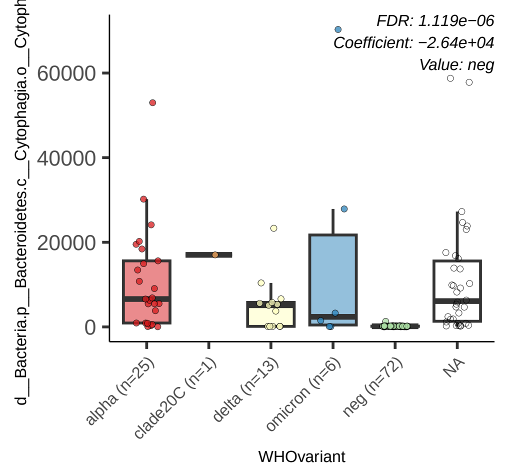

FDR: 1.119e−06
Coefficient: −2.64e+04
Value: neg
d__Bacteria.p__Bacteroidetes.c__Cytophagia.o__Cytoph
60000
40000
20000
0
alpha (n=25)
clade20C (n=1)
delta (n=13)
omicron (n=6)
neg (n=72)
NA
WHOvariant

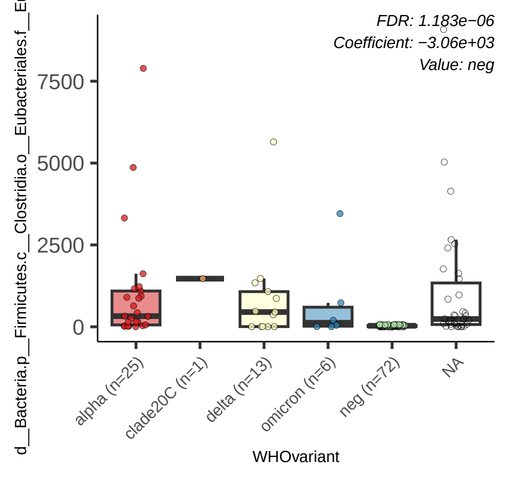

FDR: 1.183e−06
Coefficient: −3.06e+03
Value: neg
d__Bacteria.p__Firmicutes.c__Clostridia.o__Eubacteriales.f__Eu
0
2500
5000
7500
alpha (n=25)
clade20C (n=1)
delta (n=13)
omicron (n=6)
neg (n=72)
NA
WHOvariant

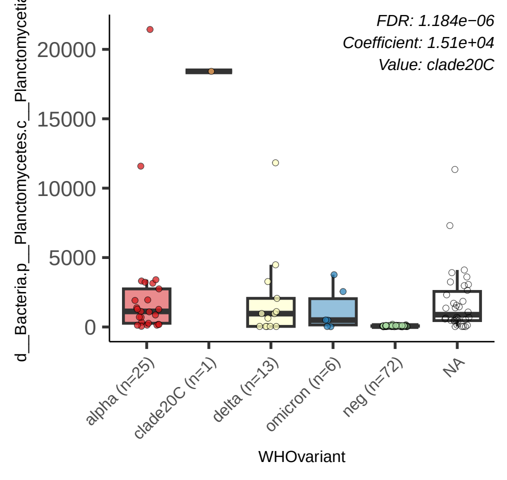

FDR: 1.184e−06
Coefficient: 1.51e+04
Value: clade20C
d__Bacteria.p__Planctomycetes.c__Planctomycetia
20000
15000
10000
5000
0
alpha (n=25)
clade20C (n=1)
delta (n=13)
omicron (n=6)
neg (n=72)
NA
WHOvariant

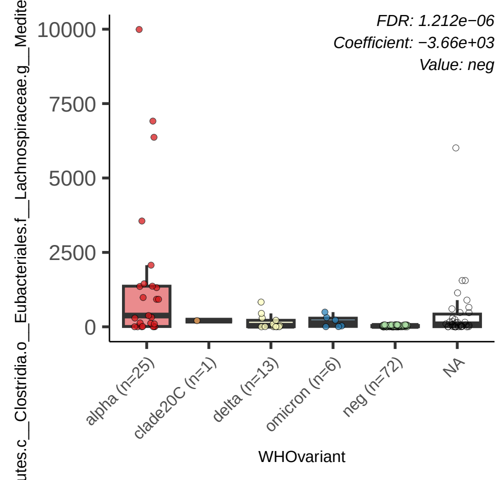

FDR: 1.212e−06
Coefficient: −3.66e+03
Value: neg
utes.c__Clostridia.o__Eubacteriales.f__Lachnospiraceae.g__Medite
10000
7500
5000
2500
0
alpha (n=25)
clade20C (n=1)
delta (n=13)
omicron (n=6)
neg (n=72)
NA
WHOvariant

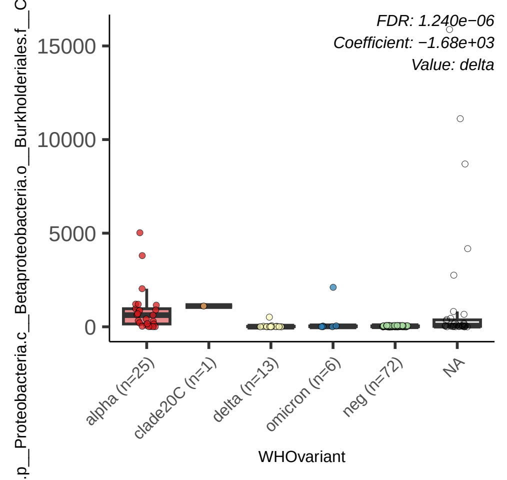
FDR: 1.240e−06
Coefficient: −1.68e+03
Value: delta
p__Proteobacteria.c__Betaproteobacteria.o__Burkholderiales.f__C
15000
10000
5000
0
alpha (n=25)
clade20C (n=1)
delta (n=13)
omicron (n=6)
neg (n=72)
NA
WHOvariant

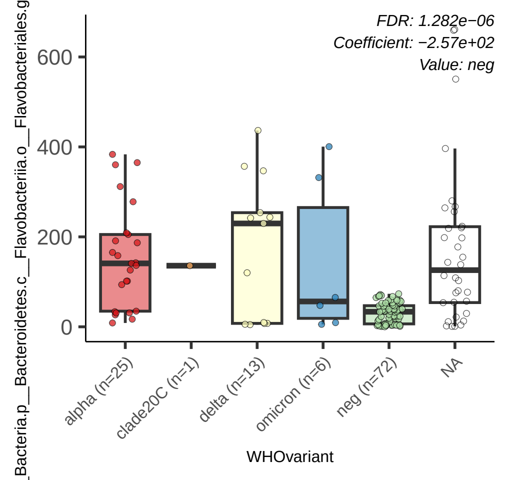

FDR: 1.282e−06
Coefficient: −2.57e+02
Value: neg
0
200
400
600
alpha (n=25)
clade20C (n=1)
delta (n=13)
omicron (n=6)
neg (n=72)
NA
WHOvariant
Bacteria.p__Bacteroidetes.c__Flavobacteriia.o__Flavobacteriales.g

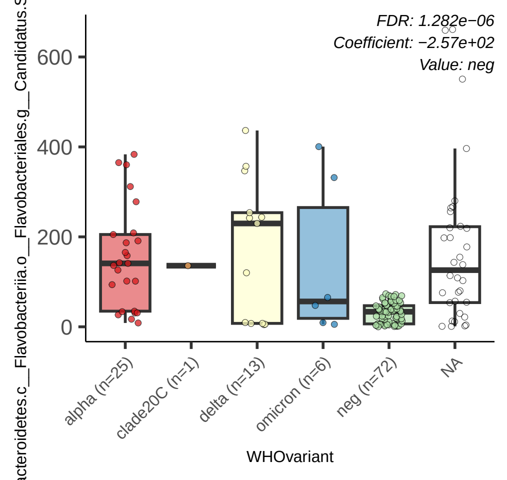

FDR: 1.282e−06
Coefficient: −2.57e+02
Value: neg
acteroidetes.c__Flavobacteriia.o__Flavobacteriales.g__Candidatus.S
0
200
400
600
alpha (n=25)
clade20C (n=1)
delta (n=13)
omicron (n=6)
neg (n=72)
NA
WHOvariant

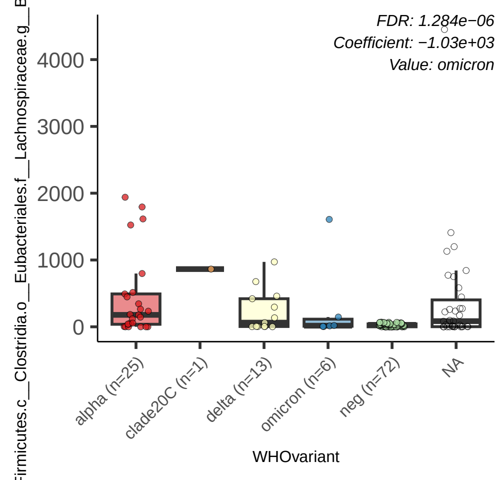

FDR: 1.284e−06
Coefficient: −1.03e+03
Value: omicron
Firmicutes.c__Clostridia.o__Eubacteriales.f__Lachnospiraceae.g__B
4000
3000
2000
1000
0
alpha (n=25)
clade20C (n=1)
delta (n=13)
omicron (n=6)
neg (n=72)
NA
WHOvariant

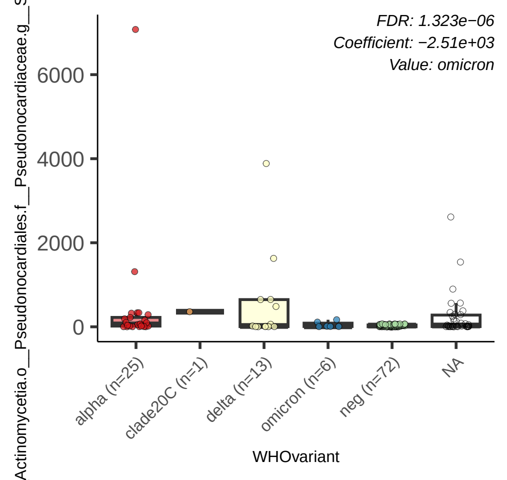
FDR: 1.323e−06
Coefficient: −2.51e+03
Value: omicron
Actinomycetia.o__Pseudonocardiales.f__Pseudonocardiaceae.g__
6000
4000
2000
0
alpha (n=25)
clade20C (n=1)
delta (n=13)
omicron (n=6)
neg (n=72)
NA
WHOvariant

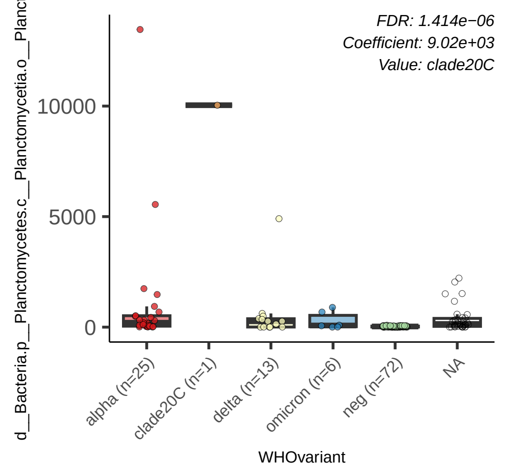
FDR: 1.414e−06
Coefficient: 9.02e+03
Value: clade20C
d__Bacteria.p__Planctomycetes.c__Planctomycetia.o__Planc
10000
5000
0
alpha (n=25)
clade20C (n=1)
delta (n=13)
omicron (n=6)
neg (n=72)
NA
WHOvariant

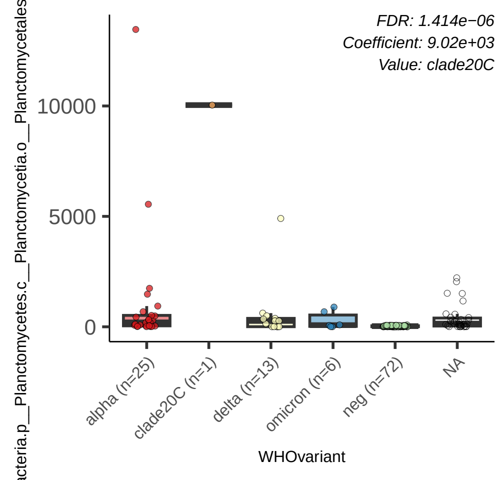

FDR: 1.414e−06
Coefficient: 9.02e+03
Value: clade20C
10000
5000
0
alpha (n=25)
clade20C (n=1)
delta (n=13)
omicron (n=6)
neg (n=72)
NA
WHOvariant
acteria.p__Planctomycetes.c__Planctomycetia.o__Planctomycetales

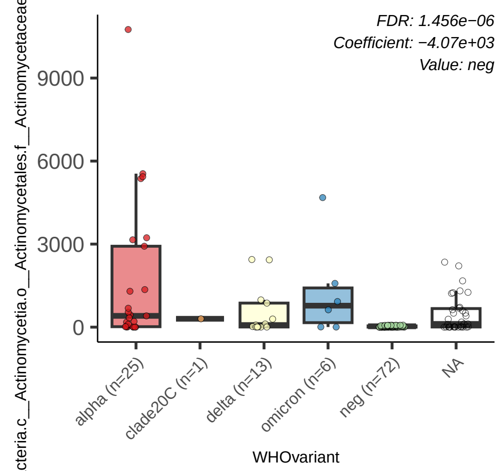
FDR: 1.456e−06
Coefficient: −4.07e+03
Value: neg
9000
6000
3000
0
acteria.c__Actinomycetia.o__Actinomycetales.f__Actinomycetaceae
alpha (n=25)
clade20C (n=1)
delta (n=13)
omicron (n=6)
neg (n=72)
NA
WHOvariant

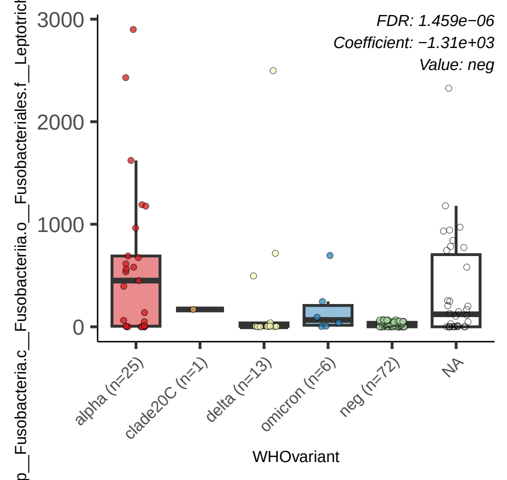

FDR: 1.459e−06
Coefficient: −1.31e+03
Value: neg
3000
2000
1000
0
p__Fusobacteria.c__Fusobacteriia.o__Fusobacteriales.f__Leptotrich
alpha (n=25)
clade20C (n=1)
delta (n=13)
omicron (n=6)
neg (n=72)
NA
WHOvariant

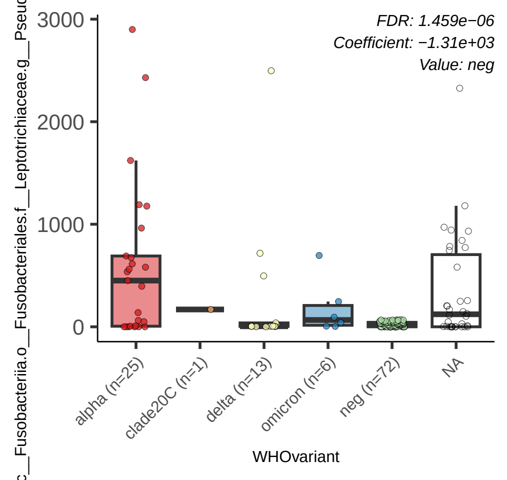

FDR: 1.459e−06
Coefficient: −1.31e+03
Value: neg
3000
2000
1000
0
c__Fusobacteriia.o__Fusobacteriales.f__Leptotrichiaceae.g__Pseud
alpha (n=25)
clade20C (n=1)
delta (n=13)
omicron (n=6)
neg (n=72)
NA
WHOvariant

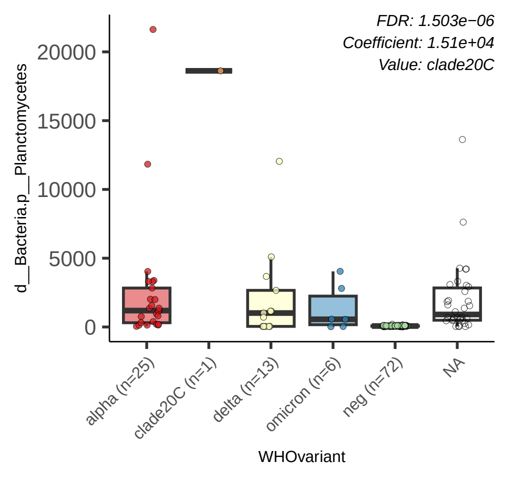

FDR: 1.503e−06
Coefficient: 1.51e+04
Value: clade20C
d__Bacteria.p__Planctomycetes
20000
15000
10000
5000
0
alpha (n=25)
clade20C (n=1)
delta (n=13)
omicron (n=6)
neg (n=72)
NA
WHOvariant

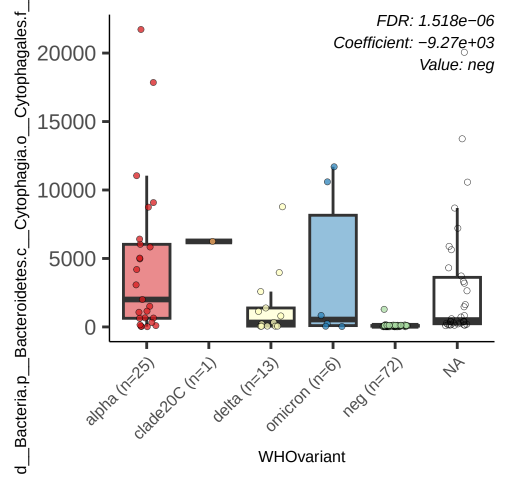

FDR: 1.518e−06
Coefficient: −9.27e+03
Value: neg
d__Bacteria.p__Bacteroidetes.c__Cytophagia.o__Cytophagales.f__
0
5000
10000
15000
20000
alpha (n=25)
clade20C (n=1)
delta (n=13)
omicron (n=6)
neg (n=72)
NA
WHOvariant

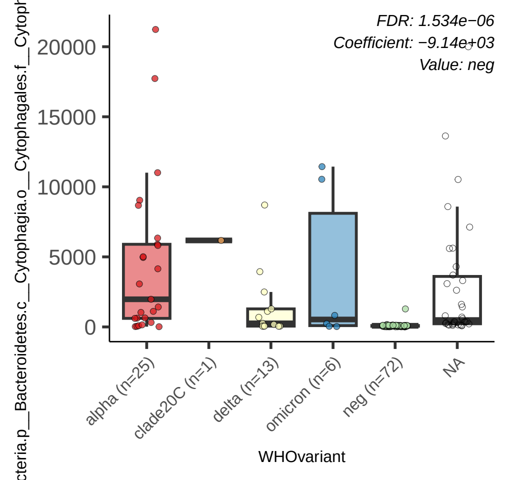

FDR: 1.534e−06
Coefficient: −9.14e+03
Value: neg
cteria.p__Bacteroidetes.c__Cytophagia.o__Cytophagales.f__Cytoph
20000
15000
10000
5000
0
alpha (n=25)
clade20C (n=1)
delta (n=13)
omicron (n=6)
neg (n=72)
NA
WHOvariant

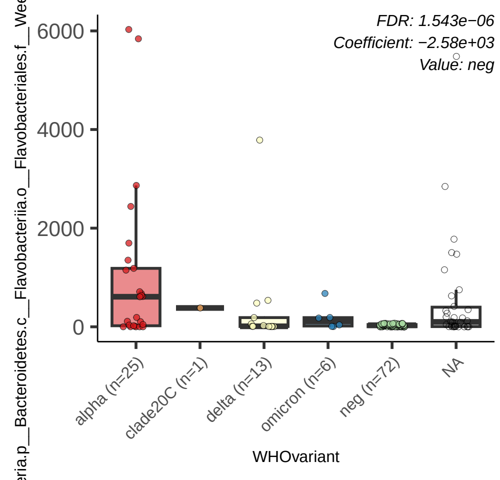

FDR: 1.543e−06
Coefficient: −2.58e+03
Value: neg
6000
4000
2000
0
alpha (n=25)
clade20C (n=1)
delta (n=13)
omicron (n=6)
neg (n=72)
NA
WHOvariant

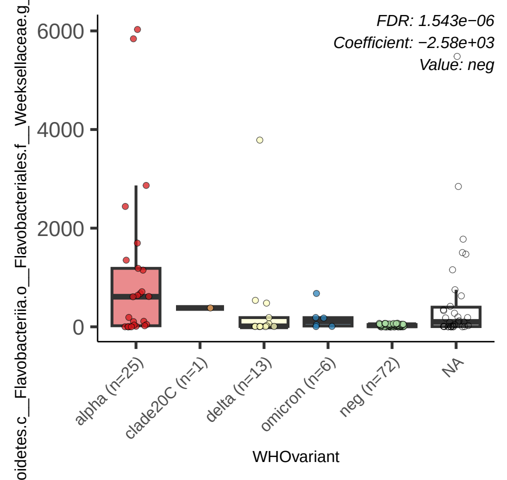

FDR: 1.543e−06
Coefficient: −2.58e+03
Value: neg
oidetes.c__Flavobacteriia.o__Flavobacteriales.f__Weeksellaceae.g_
6000
4000
2000
0
alpha (n=25)
clade20C (n=1)
delta (n=13)
omicron (n=6)
neg (n=72)
NA
WHOvariant

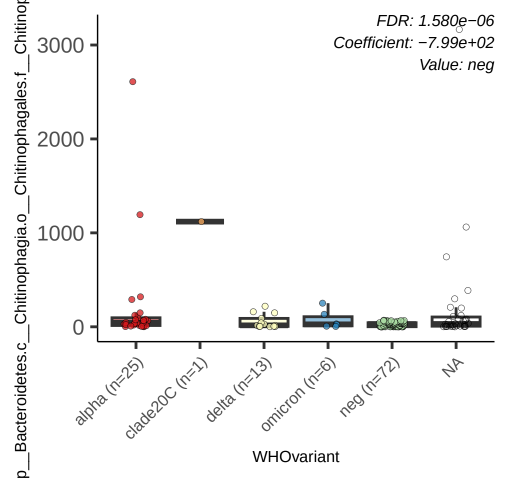
FDR: 1.580e−06
Coefficient: −7.99e+02
Value: neg
p__Bacteroidetes.c__Chitinophagia.o__Chitinophagales.f__Chitinop
3000
2000
1000
0
alpha (n=25)
clade20C (n=1)
delta (n=13)
omicron (n=6)
neg (n=72)
NA
WHOvariant

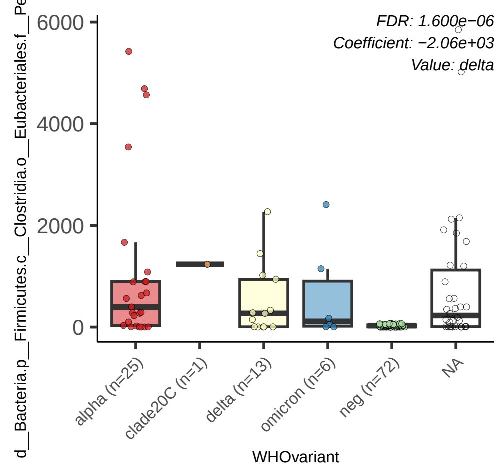

FDR: 1.600e−06
Coefficient: −2.06e+03
Value: delta
d__Bacteria.p__Firmicutes.c__Clostridia.o__Eubacteriales.f__Pe
6000
4000
2000
0
alpha (n=25)
clade20C (n=1)
delta (n=13)
omicron (n=6)
neg (n=72)
NA
WHOvariant

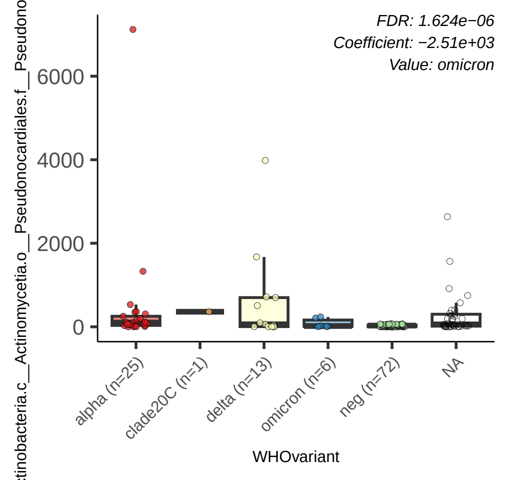

FDR: 1.624e−06
Coefficient: −2.51e+03
Value: omicron
ctinobacteria.c__Actinomycetia.o__Pseudonocardiales.f__Pseudono
6000
4000
2000
0
alpha (n=25)
clade20C (n=1)
delta (n=13)
omicron (n=6)
neg (n=72)
NA
WHOvariant

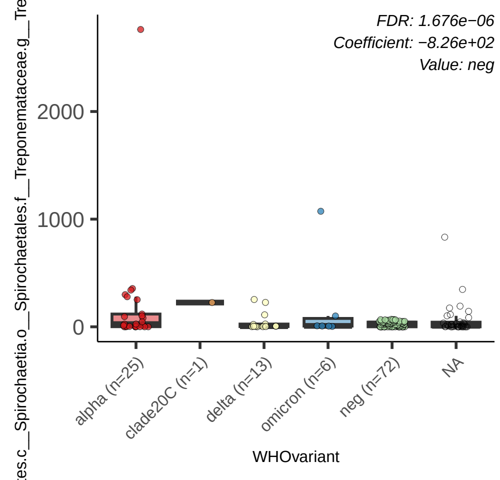

FDR: 1.676e−06
Coefficient: −8.26e+02
Value: neg
2000
1000
0
alpha (n=25)
clade20C (n=1)
delta (n=13)
omicron (n=6)
neg (n=72)
NA
WHOvariant

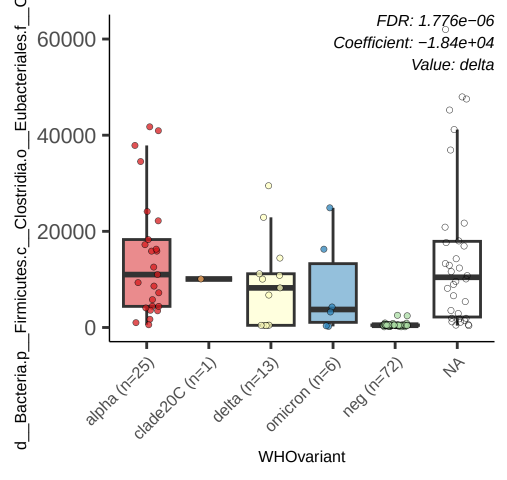

FDR: 1.776e−06
Coefficient: −1.84e+04
Value: delta
d__Bacteria.p__Firmicutes.c__Clostridia.o__Eubacteriales.f__C
60000
40000
20000
0
alpha (n=25)
clade20C (n=1)
delta (n=13)
omicron (n=6)
neg (n=72)
NA
WHOvariant

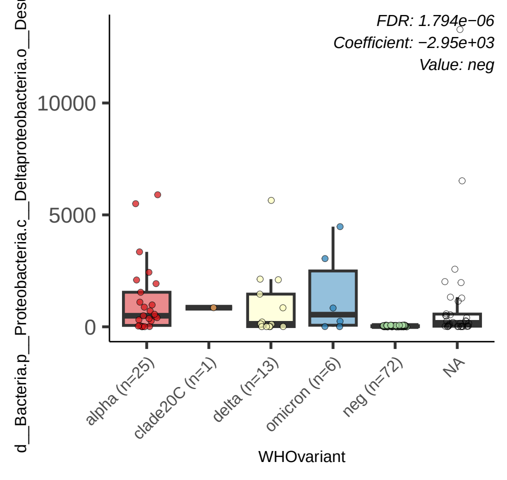
FDR: 1.794e−06
Coefficient: −2.95e+03
Value: neg
d__Bacteria.p__Proteobacteria.c__Deltaproteobacteria.o__Desu
10000
5000
0
alpha (n=25)
clade20C (n=1)
delta (n=13)
omicron (n=6)
neg (n=72)
NA
WHOvariant

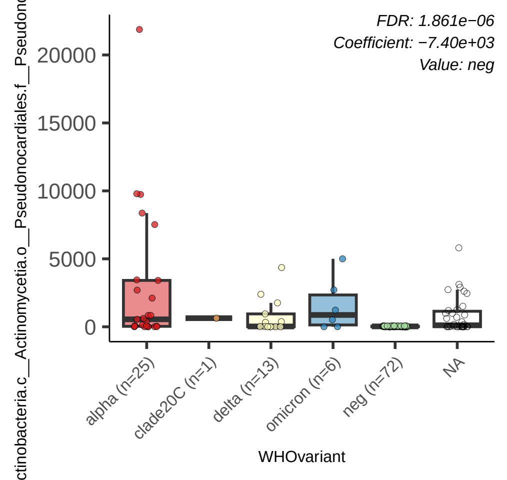
FDR: 1.861e−06
Coefficient: −7.40e+03
Value: neg
ctinobacteria.c__Actinomycetia.o__Pseudonocardiales.f__Pseudonoc
20000
15000
10000
5000
0
alpha (n=25)
clade20C (n=1)
delta (n=13)
omicron (n=6)
neg (n=72)
NA
WHOvariant

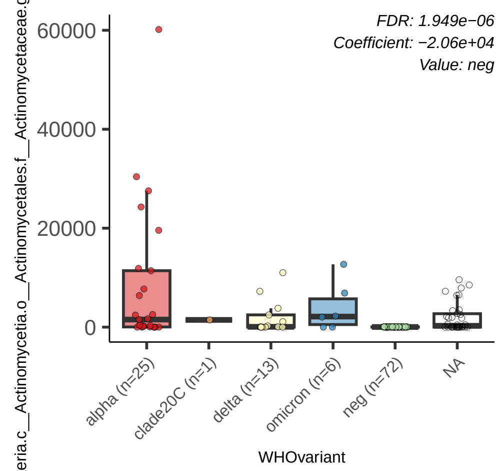

FDR: 1.949e−06
Coefficient: −2.06e+04
Value: neg
60000
40000
20000
0
alpha (n=25)
clade20C (n=1)
delta (n=13)
omicron (n=6)
neg (n=72)
NA
WHOvariant
cteria.c__Actinomycetia.o__Actinomycetales.f__Actinomycetaceae.g

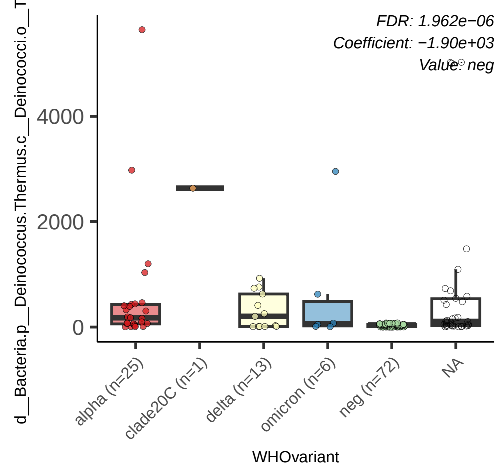

FDR: 1.962e−06
Coefficient: −1.90e+03
Value: neg
d__Bacteria.p__Deinococcus.Thermus.c__Deinococci.o__T
0
2000
4000
alpha (n=25)
clade20C (n=1)
delta (n=13)
omicron (n=6)
neg (n=72)
NA
WHOvariant

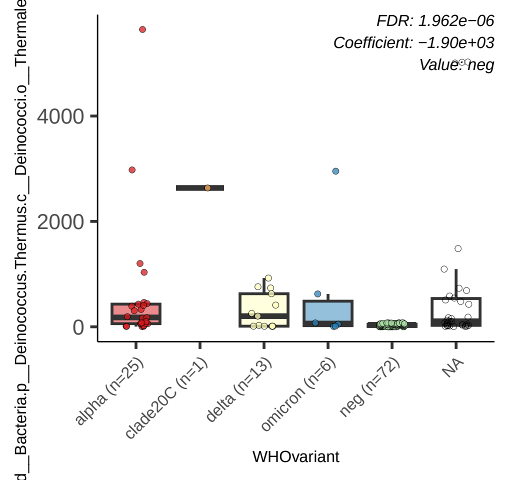

FDR: 1.962e−06
Coefficient: −1.90e+03
Value: neg
d__Bacteria.p__Deinococcus.Thermus.c__Deinococci.o__Thermale
0
2000
4000
alpha (n=25)
clade20C (n=1)
delta (n=13)
omicron (n=6)
neg (n=72)
NA
WHOvariant

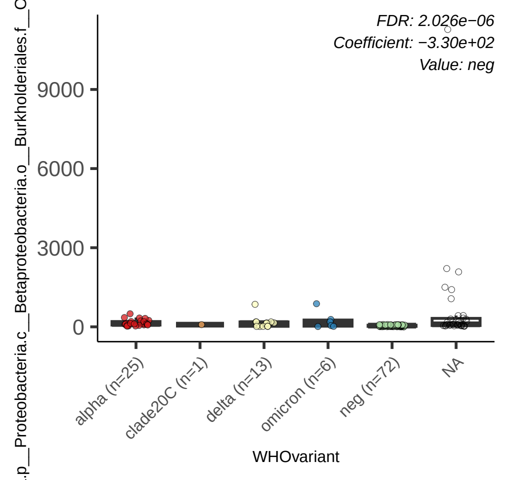

FDR: 2.026e−06
Coefficient: −3.30e+02
Value: neg
9000
6000
3000
0
p__Proteobacteria.c__Betaproteobacteria.o__Burkholderiales.f__C
alpha (n=25)
clade20C (n=1)
delta (n=13)
omicron (n=6)
neg (n=72)
NA
WHOvariant

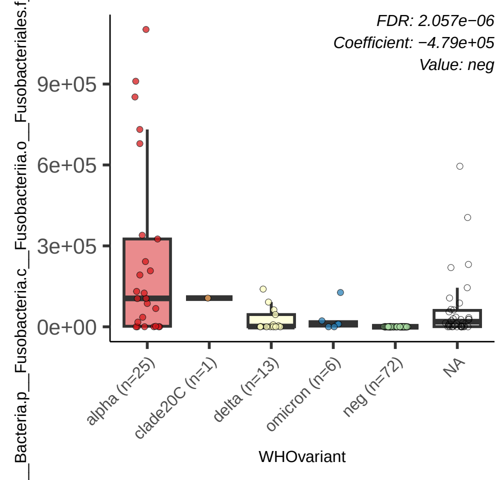

FDR: 2.057e−06
Coefficient: −4.79e+05
Value: neg
__Bacteria.p__Fusobacteria.c__Fusobacteriia.o__Fusobacteriales.f__
9e+05
6e+05
3e+05
0e+00
alpha (n=25)
clade20C (n=1)
delta (n=13)
omicron (n=6)
neg (n=72)
NA
WHOvariant

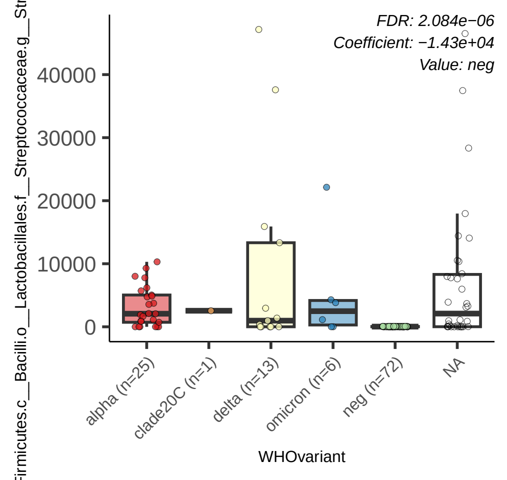

FDR: 2.084e−06
Coefficient: −1.43e+04
Value: neg
40000
30000
20000
10000
0
Firmicutes.c__Bacilli.o__Lactobacillales.f__Streptococcaceae.g__Str
alpha (n=25)
clade20C (n=1)
delta (n=13)
omicron (n=6)
neg (n=72)
NA
WHOvariant

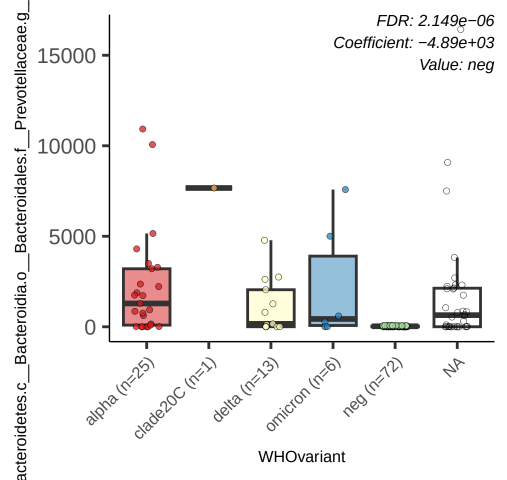
FDR: 2.149e−06
Coefficient: −4.89e+03
Value: neg
15000
10000
5000
0
acteroidetes.c__Bacteroidia.o__Bacteroidales.f__Prevotellaceae.g__
alpha (n=25)
clade20C (n=1)
delta (n=13)
omicron (n=6)
neg (n=72)
NA
WHOvariant

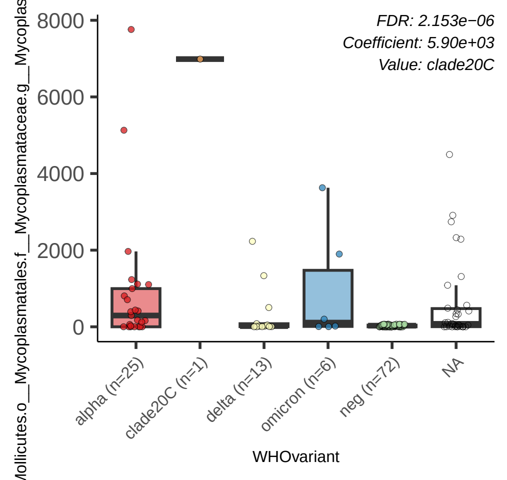

FDR: 2.153e−06
Coefficient: 5.90e+03
Value: clade20C
8000
6000
4000
2000
0
Mollicutes.o__Mycoplasmatales.f__Mycoplasmataceae.g__Mycoplas
alpha (n=25)
clade20C (n=1)
delta (n=13)
omicron (n=6)
neg (n=72)
NA
WHOvariant

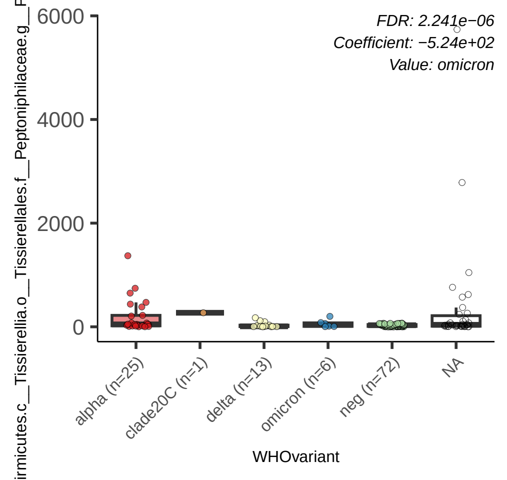

FDR: 2.241e−06
Coefficient: −5.24e+02
Value: omicron
6000
4000
2000
0
irmicutes.c__Tissierellia.o__Tissierellales.f__Peptoniphilaceae.g__F
alpha (n=25)
clade20C (n=1)
delta (n=13)
omicron (n=6)
neg (n=72)
NA
WHOvariant

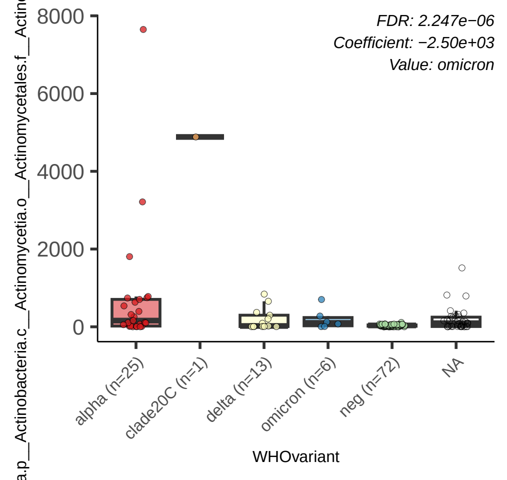

FDR: 2.247e−06
Coefficient: −2.50e+03
Value: omicron
8000
6000
4000
2000
0
a.p__Actinobacteria.c__Actinomycetia.o__Actinomycetales.f__Actino
alpha (n=25)
clade20C (n=1)
delta (n=13)
omicron (n=6)
neg (n=72)
NA
WHOvariant

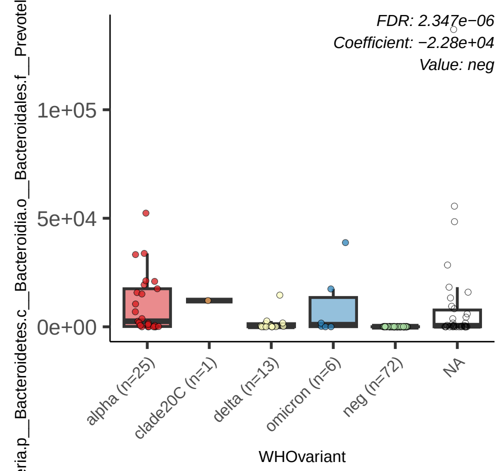

FDR: 2.347e−06
Coefficient: −2.28e+04
Value: neg
1e+05
5e+04
0e+00
teria.p__Bacteroidetes.c__Bacteroidia.o__Bacteroidales.f__Prevotel
alpha (n=25)
clade20C (n=1)
delta (n=13)
omicron (n=6)
neg (n=72)
NA
WHOvariant

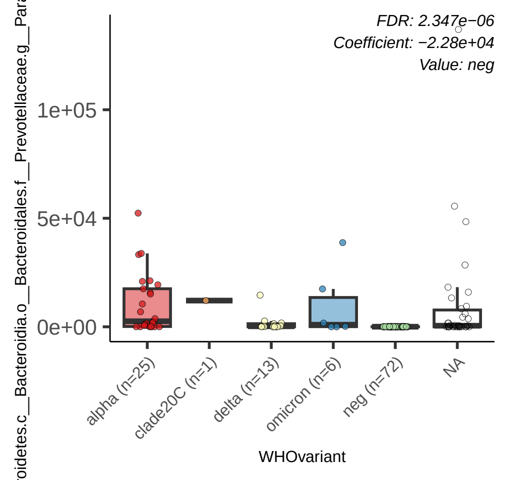

FDR: 2.347e−06
Coefficient: −2.28e+04
Value: neg
1e+05
5e+04
0e+00
oidetes.c__Bacteroidia.o__Bacteroidales.f__Prevotellaceae.g__Para
alpha (n=25)
clade20C (n=1)
delta (n=13)
omicron (n=6)
neg (n=72)
NA
WHOvariant

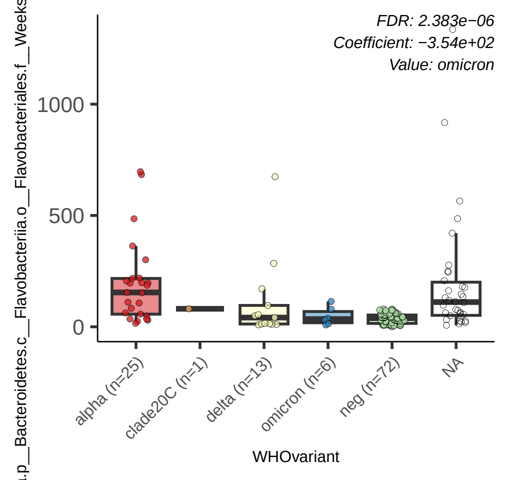

FDR: 2.383e−06
Coefficient: −3.54e+02
Value: omicron
1000
500
0
alpha (n=25)
clade20C (n=1)
delta (n=13)
omicron (n=6)
neg (n=72)
NA
WHOvariant
a.p__Bacteroidetes.c__Flavobacteriia.o__Flavobacteriales.f__Weeks

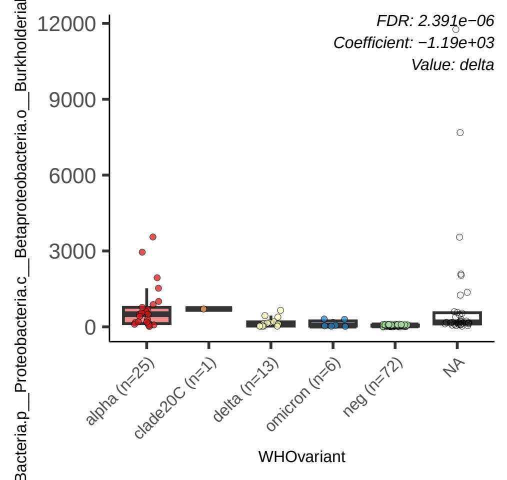

FDR: 2.391e−06
Coefficient: −1.19e+03
Value: delta
12000
9000
6000
3000
0
Bacteria.p__Proteobacteria.c__Betaproteobacteria.o__Burkholderial
alpha (n=25)
clade20C (n=1)
delta (n=13)
omicron (n=6)
neg (n=72)
NA
WHOvariant

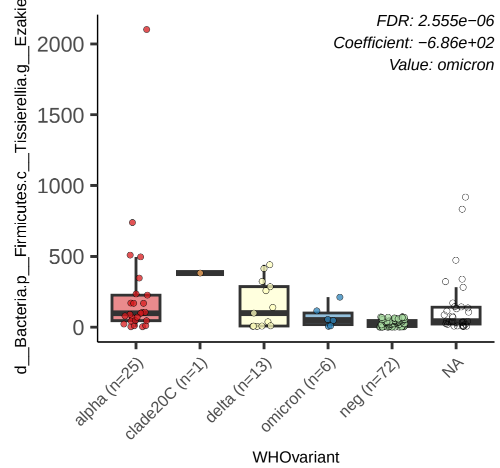

FDR: 2.555e−06
Coefficient: −6.86e+02
Value: omicron
d__Bacteria.p__Firmicutes.c__Tissierellia.g__Ezakie
2000
1500
1000
500
0
alpha (n=25)
clade20C (n=1)
delta (n=13)
omicron (n=6)
neg (n=72)
NA
WHOvariant

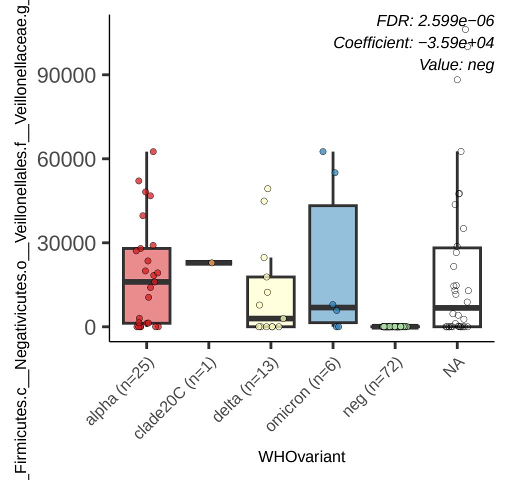
FDR: 2.599e−06
Coefficient: −3.59e+04
Value: neg
Firmicutes.c__Negativicutes.o__Veillonellales.f__Veillonellaceae.g__
0
30000
60000
90000
alpha (n=25)
clade20C (n=1)
delta (n=13)
omicron (n=6)
neg (n=72)
NA
WHOvariant

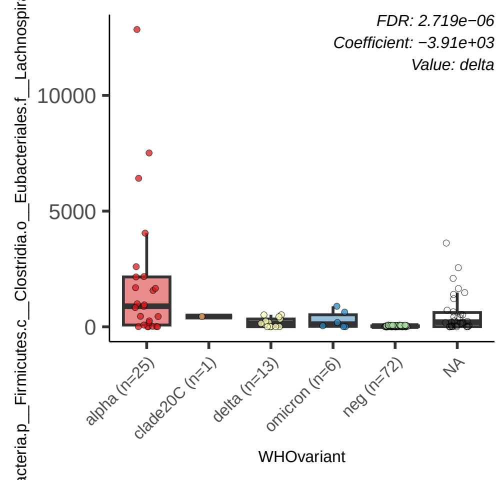

acteria.p__Firmicutes.c__Clostridia.o__Eubacteriales.f__Lachnospira
FDR: 2.719e−06
Coefficient: −3.91e+03
Value: delta
10000
5000
0
alpha (n=25)
clade20C (n=1)
delta (n=13)
omicron (n=6)
neg (n=72)
NA
WHOvariant

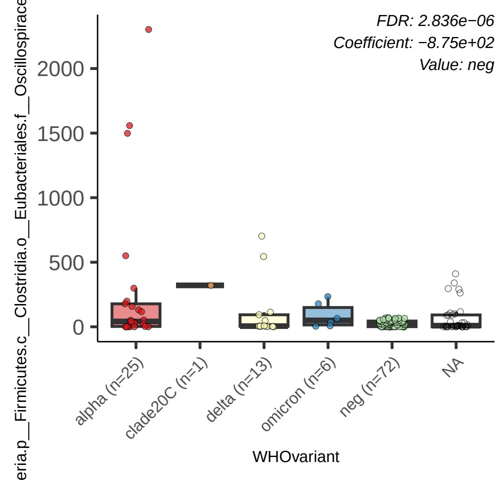

FDR: 2.836e−06
Coefficient: −8.75e+02
Value: neg
eria.p__Firmicutes.c__Clostridia.o__Eubacteriales.f__Oscillospirace
2000
1500
1000
500
0
alpha (n=25)
clade20C (n=1)
delta (n=13)
omicron (n=6)
neg (n=72)
NA
WHOvariant

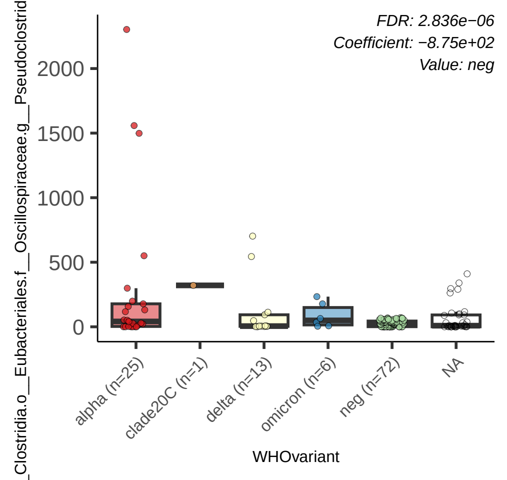

FDR: 2.836e−06
Coefficient: −8.75e+02
Value: neg
2000
1500
1000
500
0
Clostridia.o__Eubacteriales.f__Oscillospiraceae.g__Pseudoclostrid
alpha (n=25)
clade20C (n=1)
delta (n=13)
omicron (n=6)
neg (n=72)
NA
WHOvariant

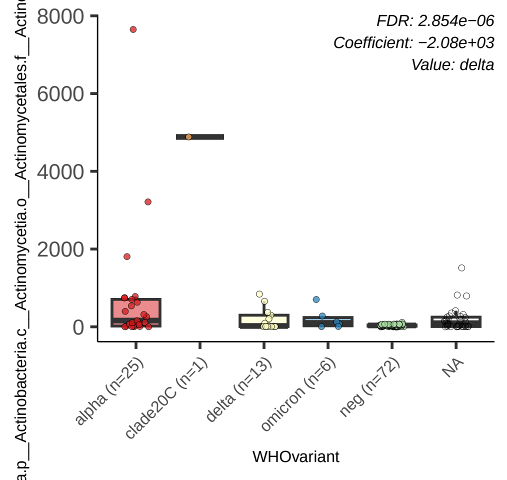

FDR: 2.854e−06
Coefficient: −2.08e+03
Value: delta
a.p__Actinobacteria.c__Actinomycetia.o__Actinomycetales.f__Actino
8000
6000
4000
2000
0
alpha (n=25)
clade20C (n=1)
delta (n=13)
omicron (n=6)
neg (n=72)
NA
WHOvariant

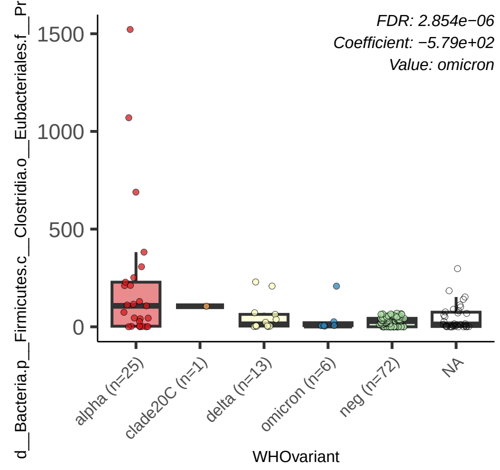
FDR: 2.854e−06
Coefficient: −5.79e+02
Value: omicron
d__Bacteria.p__Firmicutes.c__Clostridia.o__Eubacteriales.f__Pr
0
500
1000
1500
alpha (n=25)
clade20C (n=1)
delta (n=13)
omicron (n=6)
neg (n=72)
NA
WHOvariant

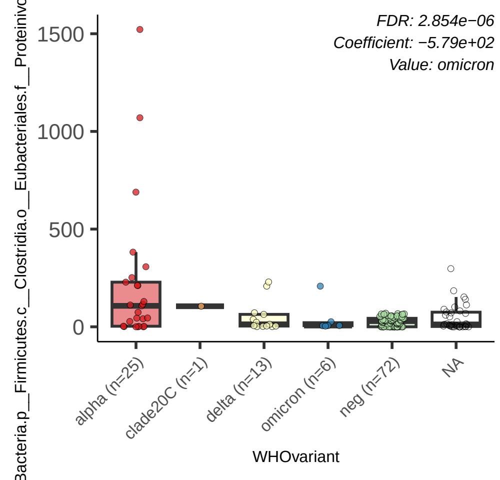

FDR: 2.854e−06
Coefficient: −5.79e+02
Value: omicron
Bacteria.p__Firmicutes.c__Clostridia.o__Eubacteriales.f__Proteinivo
1500
1000
500
0
alpha (n=25)
clade20C (n=1)
delta (n=13)
omicron (n=6)
neg (n=72)
NA
WHOvariant

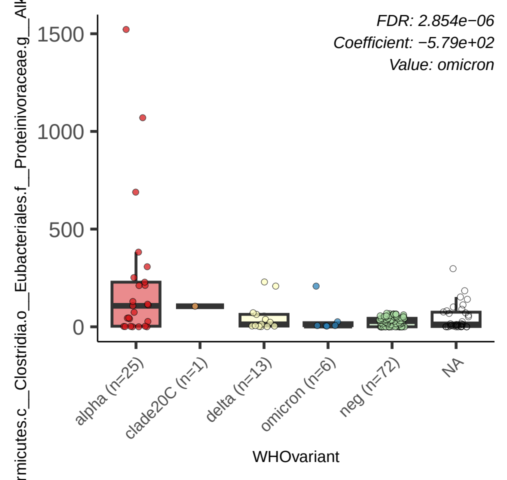

FDR: 2.854e−06
Coefficient: −5.79e+02
Value: omicron
rmicutes.c__Clostridia.o__Eubacteriales.f__Proteinivoraceae.g__Alk
1500
1000
500
0
alpha (n=25)
clade20C (n=1)
delta (n=13)
omicron (n=6)
neg (n=72)
NA
WHOvariant

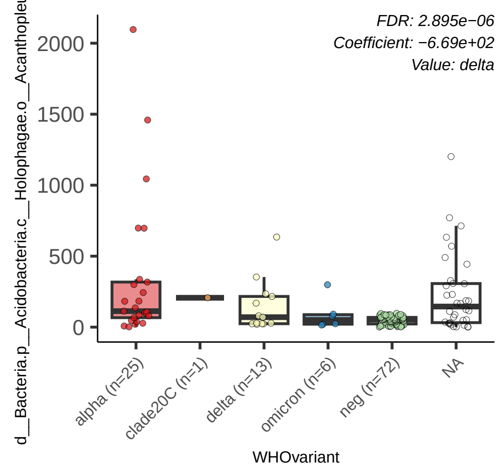

FDR: 2.895e−06
Coefficient: −6.69e+02
Value: delta
d__Bacteria.p__Acidobacteria.c__Holophagae.o__Acanthopleu
2000
1500
1000
500
0
alpha (n=25)
clade20C (n=1)
delta (n=13)
omicron (n=6)
neg (n=72)
NA
WHOvariant

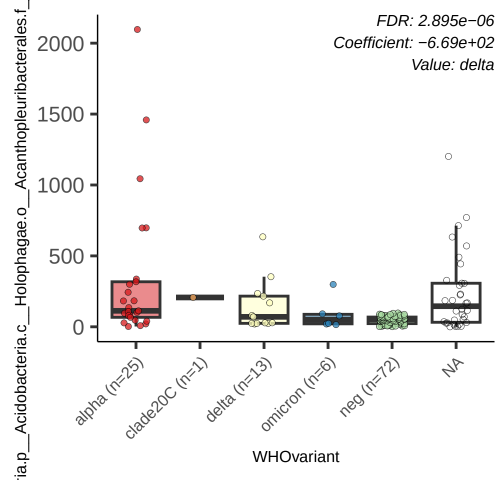

FDR: 2.895e−06
Coefficient: −6.69e+02
Value: delta
ria.p__Acidobacteria.c__Holophagae.o__Acanthopleuribacterales.f__
2000
1500
1000
500
0
alpha (n=25)
clade20C (n=1)
delta (n=13)
omicron (n=6)
neg (n=72)
NA
WHOvariant

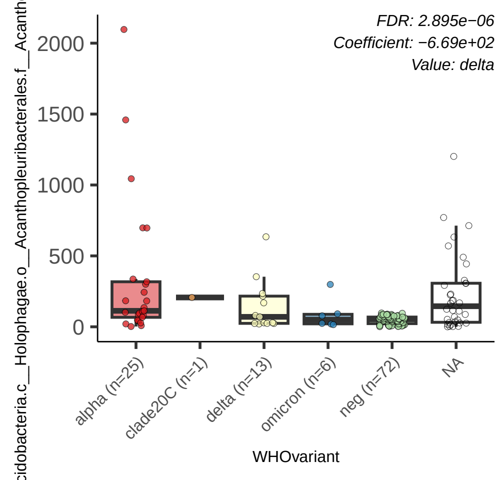

FDR: 2.895e−06
Coefficient: −6.69e+02
Value: delta
cidobacteria.c__Holophagae.o__Acanthopleuribacterales.f__Acanth
2000
1500
1000
500
0
alpha (n=25)
clade20C (n=1)
delta (n=13)
omicron (n=6)
neg (n=72)
NA
WHOvariant

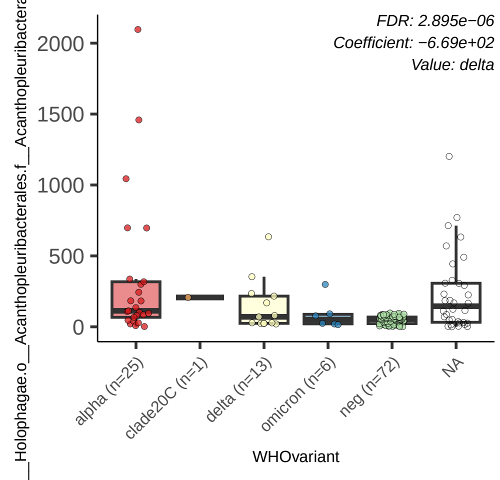

FDR: 2.895e−06
Coefficient: −6.69e+02
Value: delta
Holophagae.o__Acanthopleuribacterales.f__Acanthopleuribactera
2000
1500
1000
500
0
alpha (n=25)
clade20C (n=1)
delta (n=13)
omicron (n=6)
neg (n=72)
NA
WHOvariant

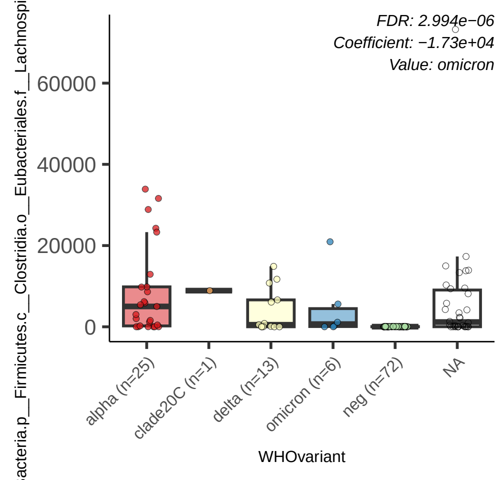

FDR: 2.994e−06
Coefficient: −1.73e+04
Value: omicron
acteria.p__Firmicutes.c__Clostridia.o__Eubacteriales.f__Lachnospi
60000
40000
20000
0
alpha (n=25)
clade20C (n=1)
delta (n=13)
omicron (n=6)
neg (n=72)
NA
WHOvariant

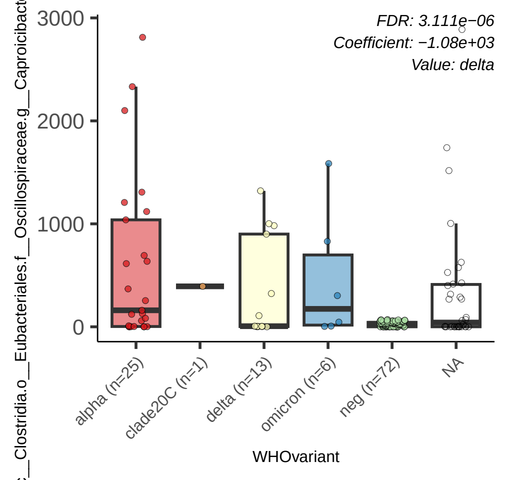

FDR: 3.111e−06
Coefficient: −1.08e+03
Value: delta
3000
2000
1000
0
Clostridia.o__Eubacteriales.f__Oscillospiraceae.g__Caproicibacte
alpha (n=25)
clade20C (n=1)
delta (n=13)
omicron (n=6)
neg (n=72)
NA
WHOvariant

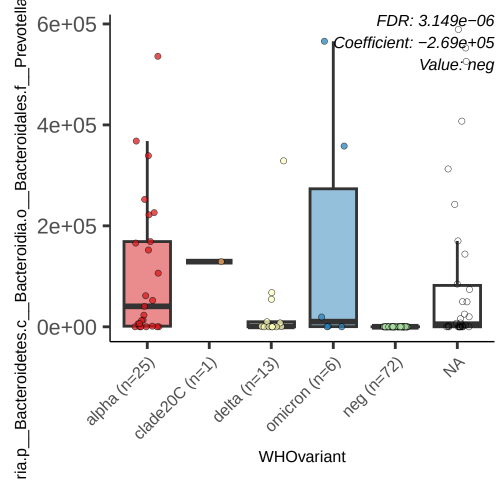

FDR: 3.149e−06
Coefficient: −2.69e+05
Value: neg
6e+05
4e+05
2e+05
0e+00
ria.p__Bacteroidetes.c__Bacteroidia.o__Bacteroidales.f__Prevotella
alpha (n=25)
clade20C (n=1)
delta (n=13)
omicron (n=6)
neg (n=72)
NA
WHOvariant

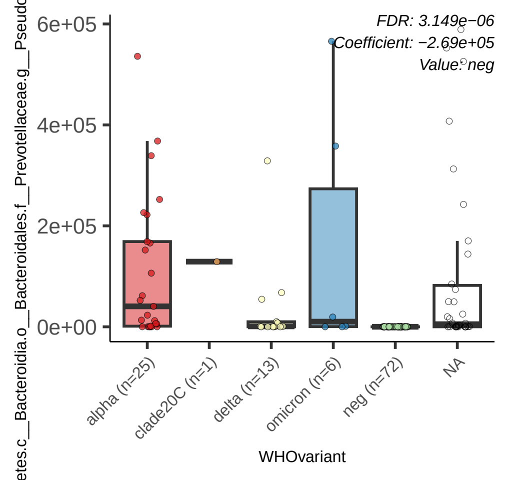

FDR: 3.149e−06
Coefficient: −2.69e+05
Value: neg
6e+05
4e+05
2e+05
0e+00
etes.c__Bacteroidia.o__Bacteroidales.f__Prevotellaceae.g__Pseudo
alpha (n=25)
clade20C (n=1)
delta (n=13)
omicron (n=6)
neg (n=72)
NA
WHOvariant

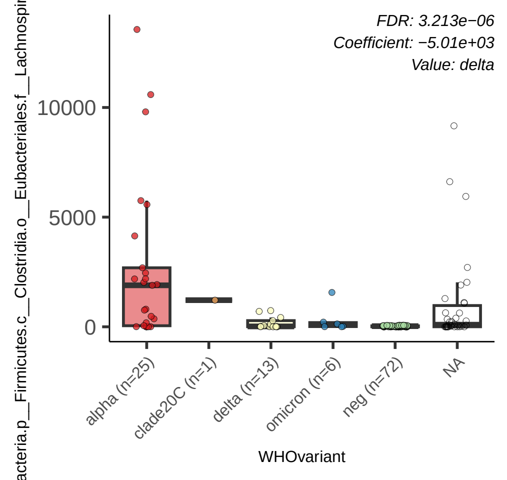

FDR: 3.213e−06
Coefficient: −5.01e+03
Value: delta
acteria.p__Firmicutes.c__Clostridia.o__Eubacteriales.f__Lachnospi
10000
5000
0
alpha (n=25)
clade20C (n=1)
delta (n=13)
omicron (n=6)
neg (n=72)
NA
WHOvariant

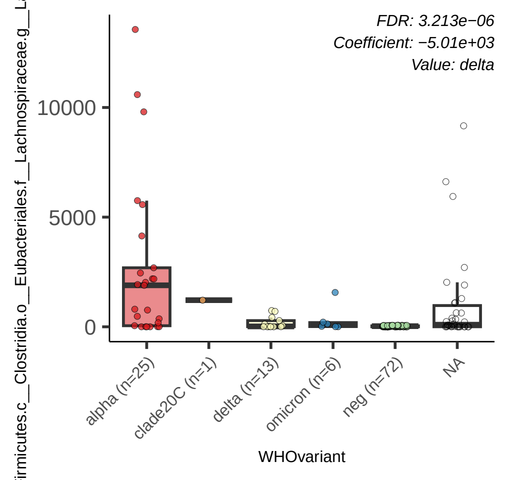

FDR: 3.213e−06
Coefficient: −5.01e+03
Value: delta
10000
5000
0
alpha (n=25)
clade20C (n=1)
delta (n=13)
omicron (n=6)
neg (n=72)
NA
WHOvariant
irmicutes.c__Clostridia.o__Eubacteriales.f__Lachnospiraceae.g__L

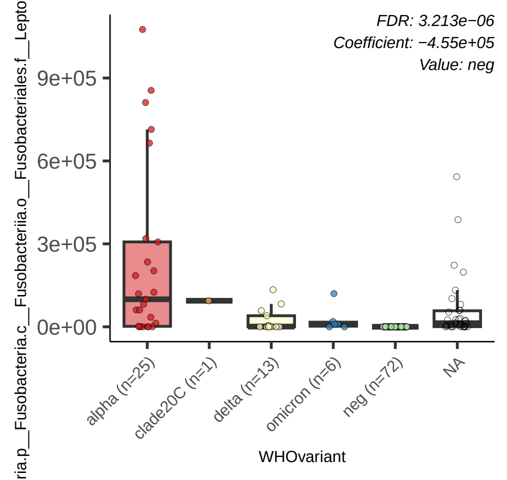

FDR: 3.213e−06
Coefficient: −4.55e+05
Value: neg
0e+00
3e+05
6e+05
9e+05
ria.p__Fusobacteria.c__Fusobacteriia.o__Fusobacteriales.f__Lepto
alpha (n=25)
clade20C (n=1)
delta (n=13)
omicron (n=6)
neg (n=72)
NA
WHOvariant

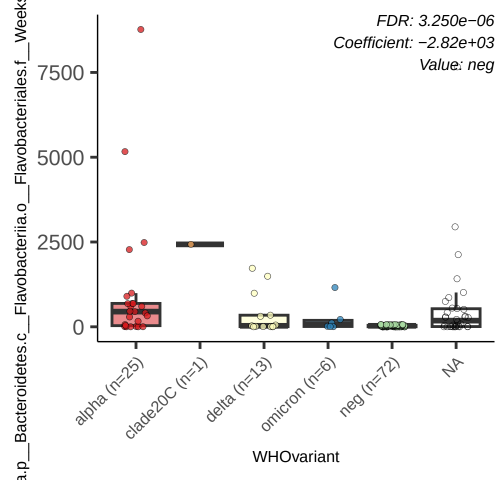

FDR: 3.250e−06
Coefficient: −2.82e+03
Value: neg
7500
5000
2500
0
a.p__Bacteroidetes.c__Flavobacteriia.o__Flavobacteriales.f__Weeks
alpha (n=25)
clade20C (n=1)
delta (n=13)
omicron (n=6)
neg (n=72)
NA
WHOvariant

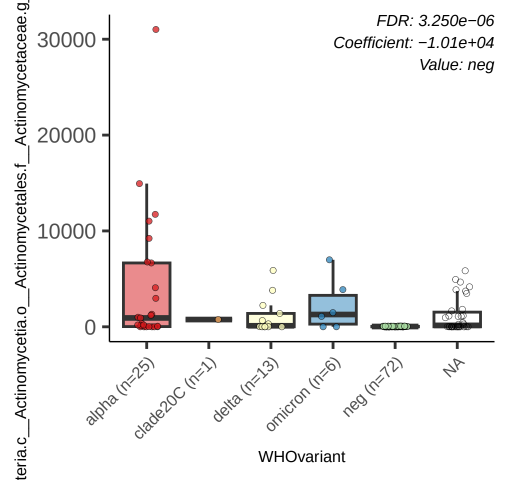

FDR: 3.250e−06
Coefficient: −1.01e+04
Value: neg
teria.c__Actinomycetia.o__Actinomycetales.f__Actinomycetaceae.g
30000
20000
10000
0
alpha (n=25)
clade20C (n=1)
delta (n=13)
omicron (n=6)
neg (n=72)
NA
WHOvariant

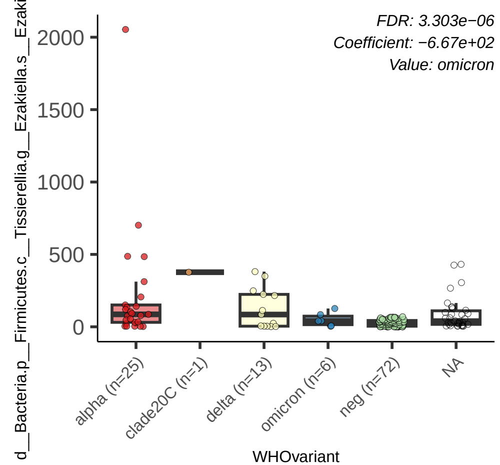
FDR: 3.303e−06
Coefficient: −6.67e+02
Value: omicron
d__Bacteria.p__Firmicutes.c__Tissierellia.g__Ezakiella.s__Ezaki
2000
1500
1000
500
0
alpha (n=25)
clade20C (n=1)
delta (n=13)
omicron (n=6)
neg (n=72)
NA
WHOvariant

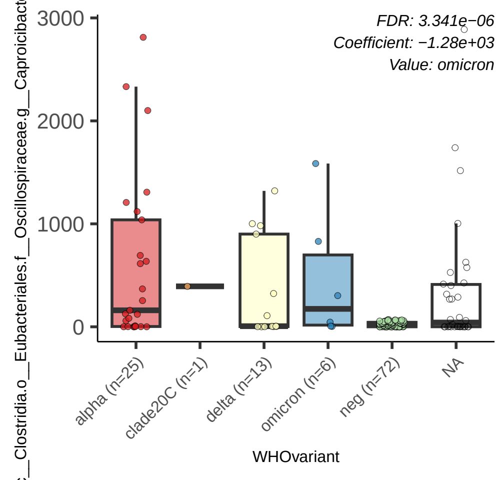
FDR: 3.341e−06
Coefficient: −1.28e+03
Value: omicron
3000
2000
1000
0
Clostridia.o__Eubacteriales.f__Oscillospiraceae.g__Caproicibacte
alpha (n=25)
clade20C (n=1)
delta (n=13)
omicron (n=6)
neg (n=72)
NA
WHOvariant

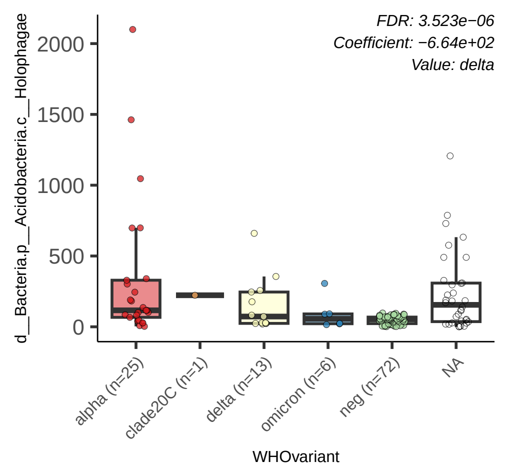

FDR: 3.523e−06
Coefficient: −6.64e+02
Value: delta
d__Bacteria.p__Acidobacteria.c__Holophagae
2000
1500
1000
500
0
alpha (n=25)
clade20C (n=1)
delta (n=13)
omicron (n=6)
neg (n=72)
NA
WHOvariant

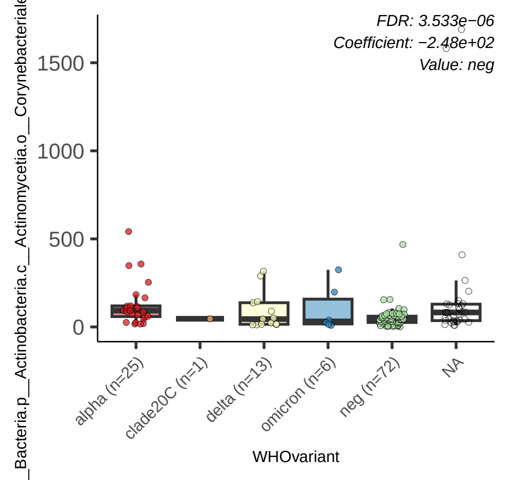

FDR: 3.533e−06
Coefficient: −2.48e+02
Value: neg
Bacteria.p__Actinobacteria.c__Actinomycetia.o__Corynebacteriale
0
500
1000
1500
alpha (n=25)
clade20C (n=1)
delta (n=13)
omicron (n=6)
neg (n=72)
NA
WHOvariant

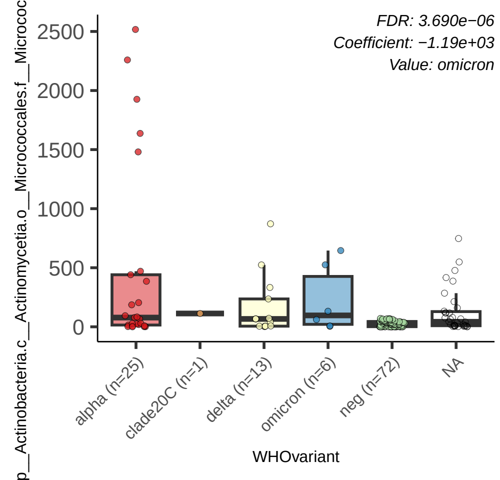

FDR: 3.690e−06
Coefficient: −1.19e+03
Value: omicron
p__Actinobacteria.c__Actinomycetia.o__Micrococcales.f__Micrococ
2500
2000
1500
1000
500
0
alpha (n=25)
clade20C (n=1)
delta (n=13)
omicron (n=6)
neg (n=72)
NA
WHOvariant

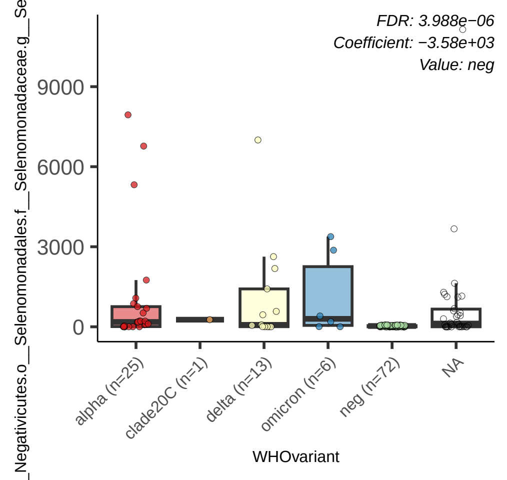

FDR: 3.988e−06
Coefficient: −3.58e+03
Value: neg
9000
6000
3000
0
Negativicutes.o__Selenomonadales.f__Selenomonadaceae.g__Se
alpha (n=25)
clade20C (n=1)
delta (n=13)
omicron (n=6)
neg (n=72)
NA
WHOvariant

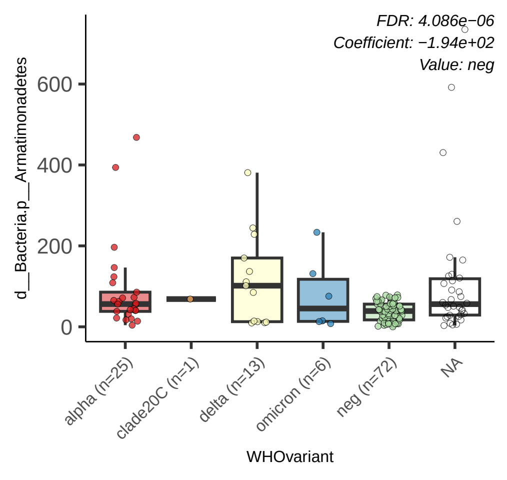

FDR: 4.086e−06
Coefficient: −1.94e+02
Value: neg
d__Bacteria.p__Armatimonadetes
600
400
200
0
alpha (n=25)
clade20C (n=1)
delta (n=13)
omicron (n=6)
neg (n=72)
NA
WHOvariant

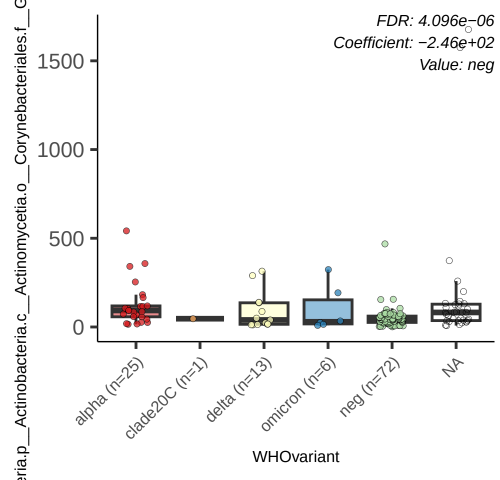

FDR: 4.096e−06
Coefficient: −2.46e+02
Value: neg
Actinobacteria.p__Actinobacteria.c__Actinomycetia.o__Corynebacteriales.f__G
1500
1000
500
0
alpha (n=25)
clade20C (n=1)
delta (n=13)
omicron (n=6)
neg (n=72)
NA
WHOvariant

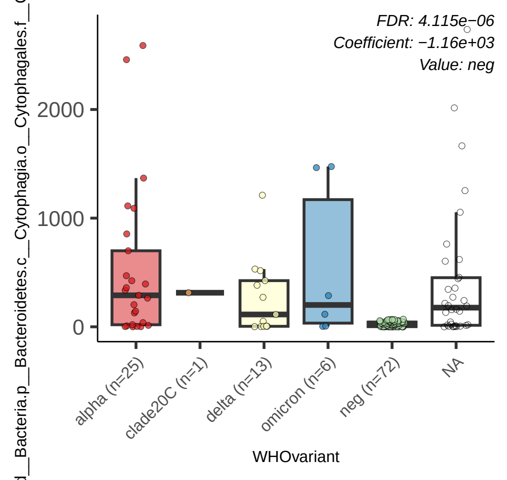
FDR: 4.115e−06
Coefficient: −1.16e+03
Value: neg
d__Bacteria.p__Bacteroidetes.c__Cytophagia.o__Cytophagales.f__
2000
1000
0
alpha (n=25)
clade20C (n=1)
delta (n=13)
omicron (n=6)
neg (n=72)
NA
WHOvariant

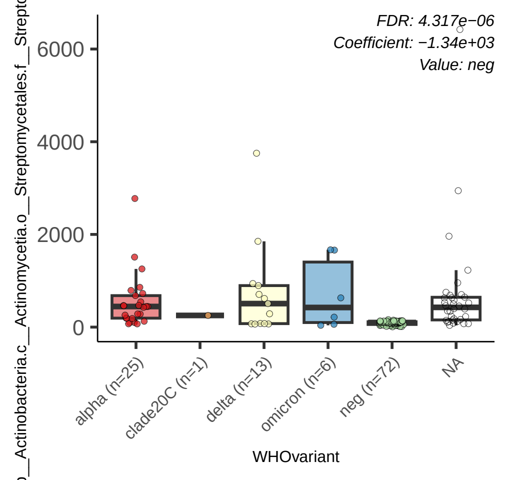

FDR: 4.317e−06
Coefficient: −1.34e+03
Value: neg
6000
4000
2000
0
o__Actinobacteria.c__Actinomycetia.o__Streptomycetales.f__Streptomycetaceae.g__Strepto
alpha (n=25)
clade20C (n=1)
delta (n=13)
omicron (n=6)
neg (n=72)
NA
WHOvariant

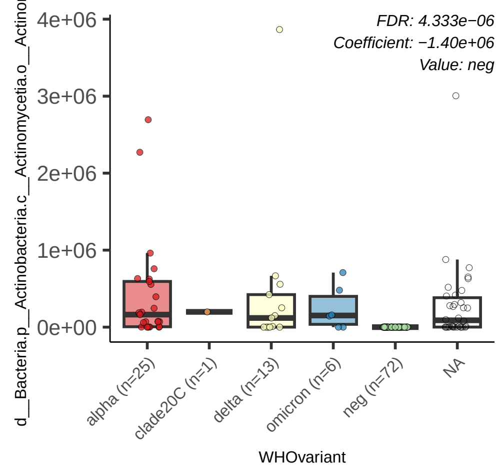

FDR: 4.333e−06
Coefficient: −1.40e+06
Value: neg
d__Bacteria.p__Actinobacteria.c__Actinomycetia.o__Actino
4e+06
3e+06
2e+06
1e+06
0e+00
alpha (n=25)
clade20C (n=1)
delta (n=13)
omicron (n=6)
neg (n=72)
NA
WHOvariant

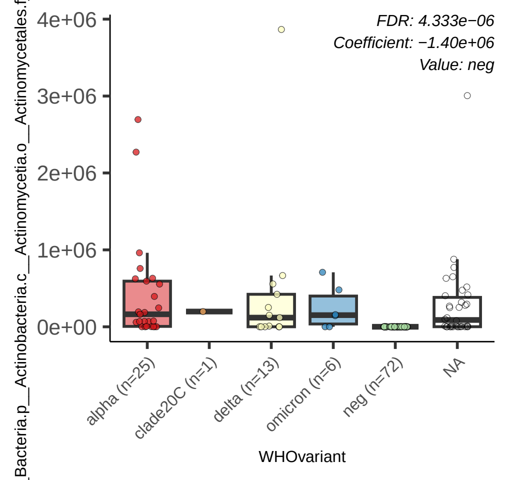
FDR: 4.333e−06
Coefficient: −1.40e+06
Value: neg
_Bacteria.p__Actinobacteria.c__Actinomycetia.o__Actinomycetales.f
4e+06
3e+06
2e+06
1e+06
0e+00
alpha (n=25)
clade20C (n=1)
delta (n=13)
omicron (n=6)
neg (n=72)
NA
WHOvariant

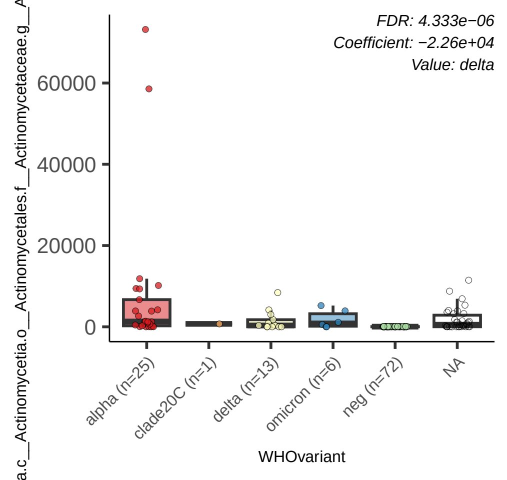
FDR: 4.333e−06
Coefficient: −2.26e+04
Value: delta
a.c__Actinomycetia.o__Actinomycetales.f__Actinomycetaceae.g__A
60000
40000
20000
0
alpha (n=25)
clade20C (n=1)
delta (n=13)
omicron (n=6)
neg (n=72)
NA
WHOvariant

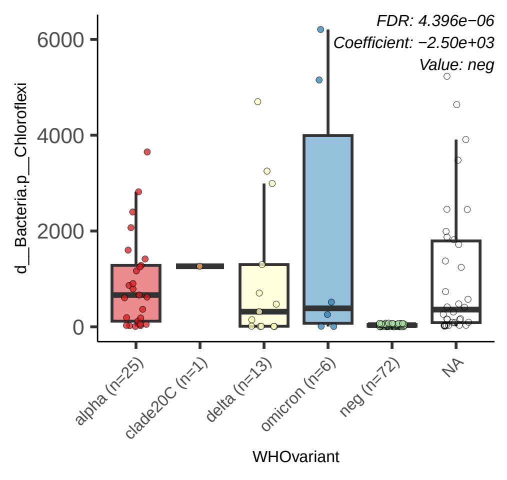
FDR: 4.396e−06
Coefficient: −2.50e+03
Value: neg
6000
4000
2000
0
d__Bacteria.p__Chloroflexi
alpha (n=25)
clade20C (n=1)
delta (n=13)
omicron (n=6)
neg (n=72)
NA
WHOvariant

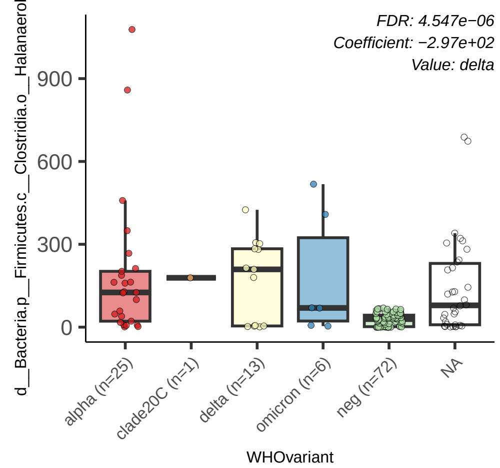

FDR: 4.547e−06
Coefficient: −2.97e+02
Value: delta
d__Bacteria.p__Firmicutes.c__Clostridia.o__Halanaerob
0
300
600
900
alpha (n=25)
clade20C (n=1)
delta (n=13)
omicron (n=6)
neg (n=72)
NA
WHOvariant

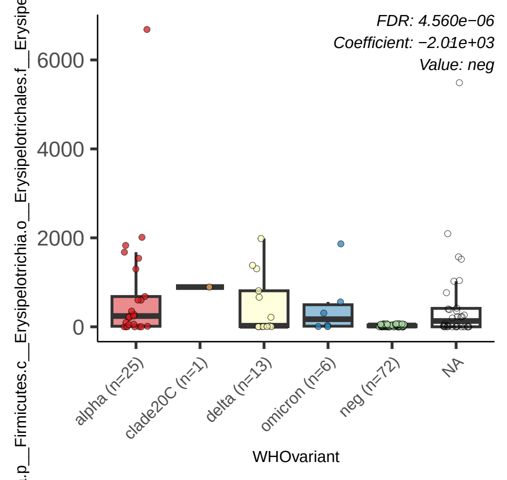

FDR: 4.560e−06
Coefficient: −2.01e+03
Value: neg
p__Firmicutes.c__Erysipelotrichia.o__Erysipelotrichales.f__Erysipe
6000
4000
2000
0
alpha (n=25)
clade20C (n=1)
delta (n=13)
omicron (n=6)
neg (n=72)
NA
WHOvariant

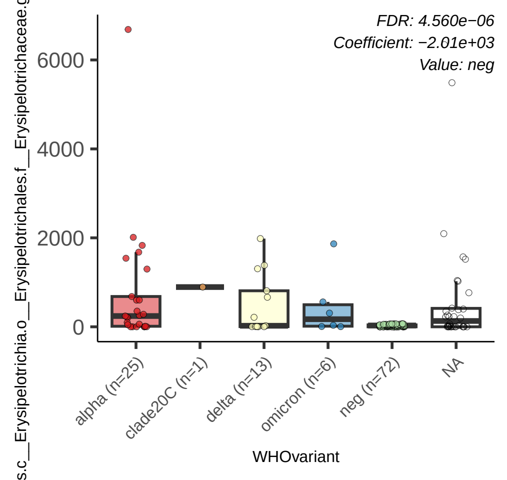

FDR: 4.560e−06
Coefficient: −2.01e+03
Value: neg
s.c__Erysipelotrichia.o__Erysipelotrichales.f__Erysipelotrichaceae.g
6000
4000
2000
0
alpha (n=25)
clade20C (n=1)
delta (n=13)
omicron (n=6)
neg (n=72)
NA
WHOvariant

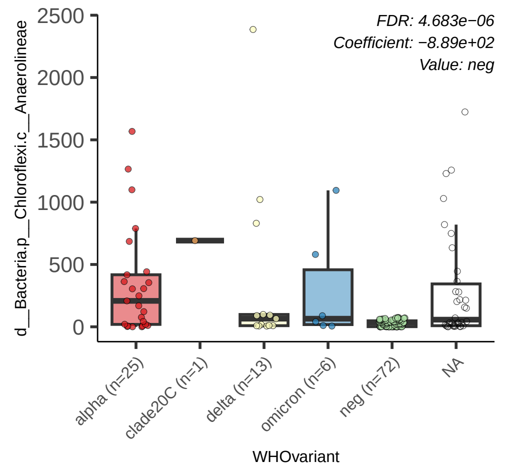
FDR: 4.683e−06
Coefficient: −8.89e+02
Value: neg
d__Bacteria.p__Chloroflexi.c__Anaerolineae
2500
2000
1500
1000
500
0
alpha (n=25)
clade20C (n=1)
delta (n=13)
omicron (n=6)
neg (n=72)
NA
WHOvariant

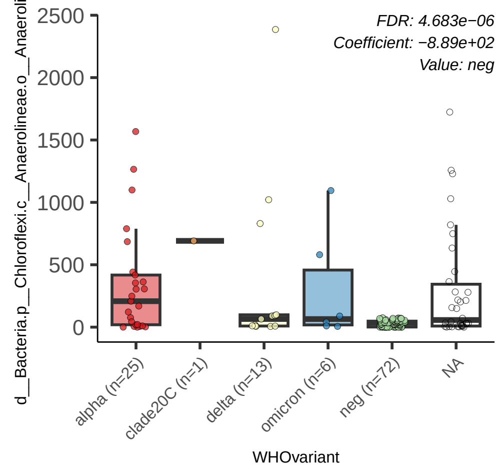

FDR: 4.683e−06
Coefficient: −8.89e+02
Value: neg
d__Bacteria.p__Chloroflexi.c__Anaerolineae.o__Anaeroli
2500
2000
1500
1000
500
0
alpha (n=25)
clade20C (n=1)
delta (n=13)
omicron (n=6)
neg (n=72)
NA
WHOvariant

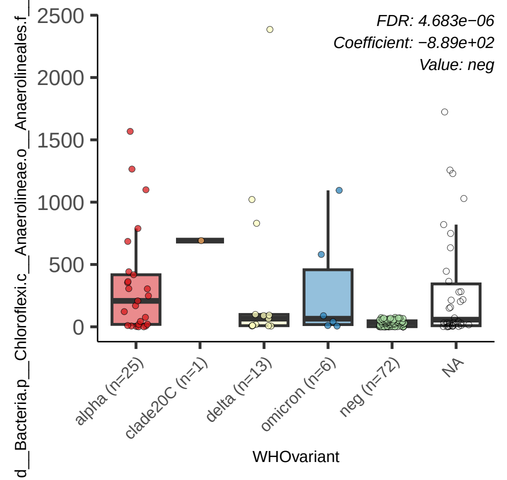

FDR: 4.683e−06
Coefficient: −8.89e+02
Value: neg
d__Bacteria.p__Chloroflexi.c__Anaerolineae.o__Anaerolineales.f
2500
2000
1500
1000
500
0
alpha (n=25)
clade20C (n=1)
delta (n=13)
omicron (n=6)
neg (n=72)
NA
WHOvariant

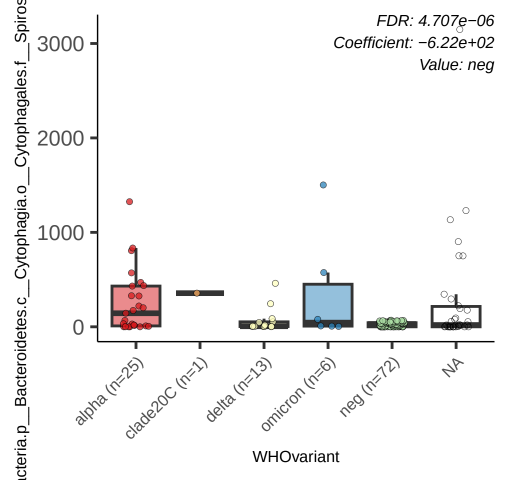

FDR: 4.707e−06
Coefficient: −6.22e+02
Value: neg
3000
2000
1000
0
acteria.p__Bacteroidetes.c__Cytophagia.o__Cytophagales.f__Spiros
alpha (n=25)
clade20C (n=1)
delta (n=13)
omicron (n=6)
neg (n=72)
NA
WHOvariant

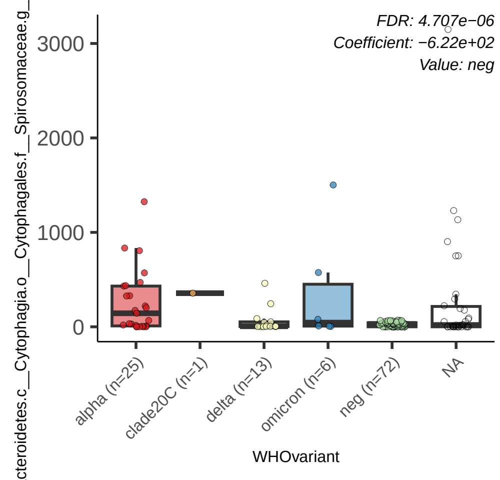

FDR: 4.707e−06
Coefficient: −6.22e+02
Value: neg
3000
2000
1000
0
cteroidetes.c__Cytophagia.o__Cytophagales.f__Spirosomaceae.g__
alpha (n=25)
clade20C (n=1)
delta (n=13)
omicron (n=6)
neg (n=72)
NA
WHOvariant

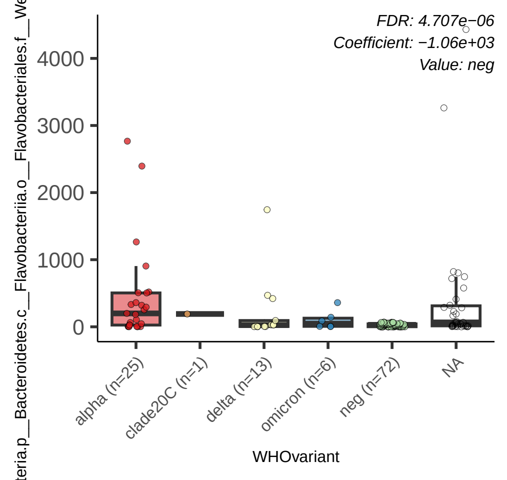

FDR: 4.707e−06
Coefficient: −1.06e+03
Value: neg
4000
3000
2000
1000
0
alpha (n=25)
clade20C (n=1)
delta (n=13)
omicron (n=6)
neg (n=72)
NA
WHOvariant
teria.p__Bacteroidetes.c__Flavobacteriia.o__Flavobacteriales.f__We

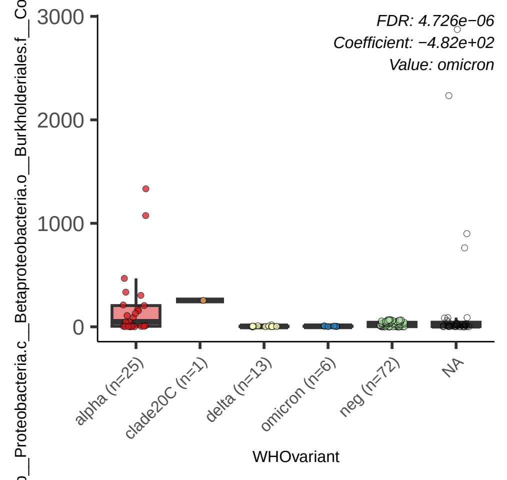

Proteobacteria.c__Betaproteobacteria.o__Burkholderiales.f__Co
FDR: 4.726e−06
Coefficient: −4.82e+02
Value: omicron
3000
2000
1000
0
alpha (n=25)
clade20C (n=1)
delta (n=13)
omicron (n=6)
neg (n=72)
NA
WHOvariant

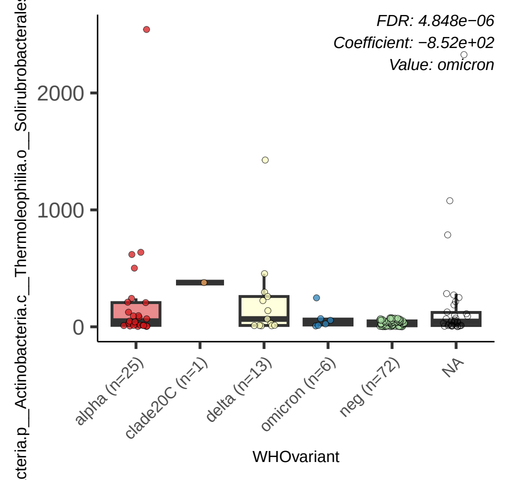

FDR: 4.848e−06
Coefficient: −8.52e+02
Value: omicron
2000
1000
0
cteria.p__Actinobacteria.c__Thermoleophilia.o__Solirubrobacterales
alpha (n=25)
clade20C (n=1)
delta (n=13)
omicron (n=6)
neg (n=72)
NA
WHOvariant

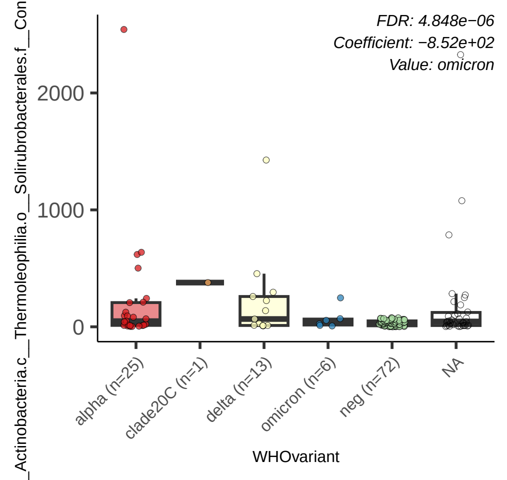
FDR: 4.848e−06
Coefficient: −8.52e+02
Value: omicron
Actinobacteria.c__Thermoleophilia.o__Solirubrobacterales.f__Con
2000
1000
0
alpha (n=25)
clade20C (n=1)
delta (n=13)
omicron (n=6)
neg (n=72)
NA
WHOvariant

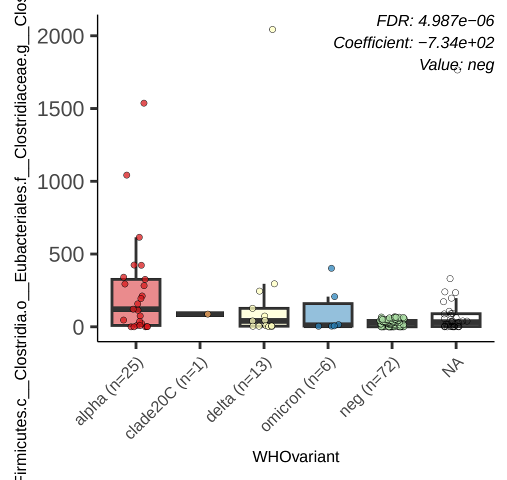
FDR: 4.987e−06
Coefficient: −7.34e+02
Value: neg
Firmicutes.c__Clostridia.o__Eubacteriales.f__Clostridiaceae.g__Clos
2000
1500
1000
500
0
alpha (n=25)
clade20C (n=1)
delta (n=13)
omicron (n=6)
neg (n=72)
NA
WHOvariant

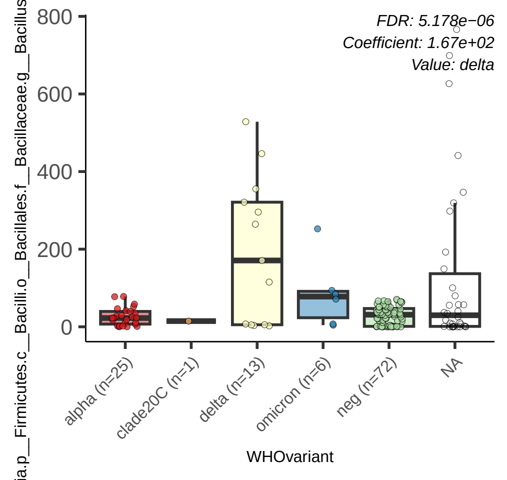

FDR: 5.178e−06
Coefficient: 1.67e+02
Value: delta
ia.p__Firmicutes.c__Bacilli.o__Bacillales.f__Bacillaceae.g__Bacillus
800
600
400
200
0
alpha (n=25)
clade20C (n=1)
delta (n=13)
omicron (n=6)
neg (n=72)
NA
WHOvariant

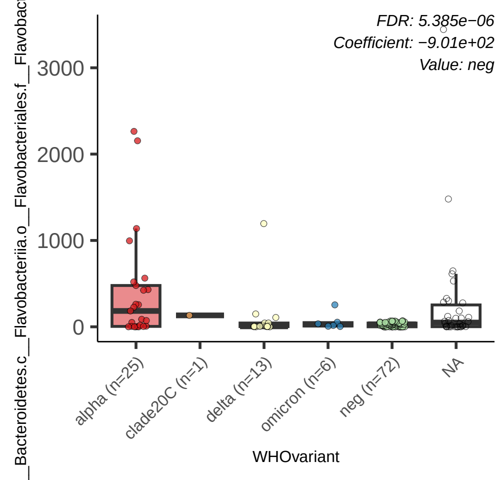

FDR: 5.385e−06
Coefficient: −9.01e+02
Value: neg
3000
2000
1000
0
alpha (n=25)
clade20C (n=1)
delta (n=13)
omicron (n=6)
neg (n=72)
NA
WHOvariant
_Bacteroidetes.c__Flavobacteriia.o__Flavobacteriales.f__Flavobact

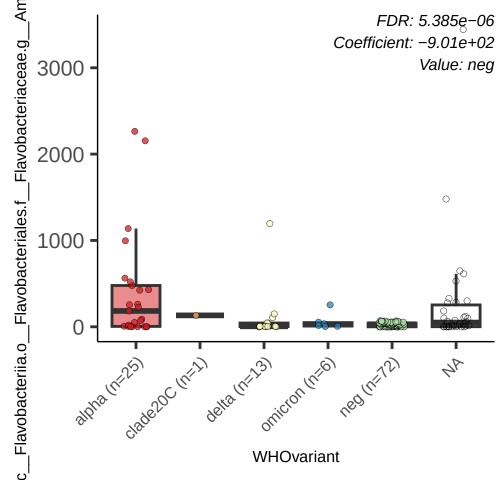

FDR: 5.385e−06
Coefficient: −9.01e+02
Value: neg
c__Flavobacteriia.o__Flavobacteriales.f__Flavobacteriaceae.g__Am
3000
2000
1000
0
alpha (n=25)
clade20C (n=1)
delta (n=13)
omicron (n=6)
neg (n=72)
NA
WHOvariant

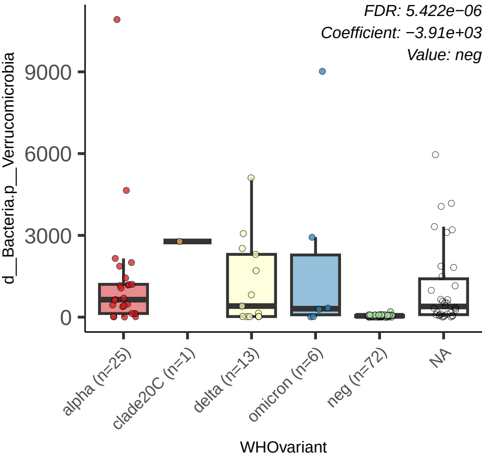

FDR: 5.422e−06
Coefficient: −3.91e+03
Value: neg
d__Bacteria.p__Verrucomicrobia
9000
6000
3000
0
alpha (n=25)
clade20C (n=1)
delta (n=13)
omicron (n=6)
neg (n=72)
NA
WHOvariant

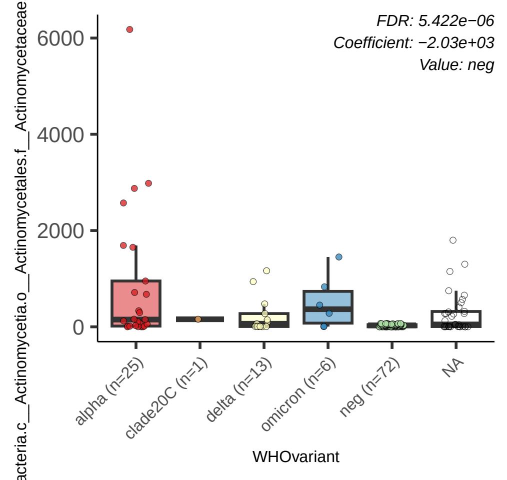

FDR: 5.422e−06
Coefficient: −2.03e+03
Value: neg
6000
4000
2000
0
acteria.c__Actinomycetia.o__Actinomycetales.f__Actinomycetaceae
alpha (n=25)
clade20C (n=1)
delta (n=13)
omicron (n=6)
neg (n=72)
NA
WHOvariant

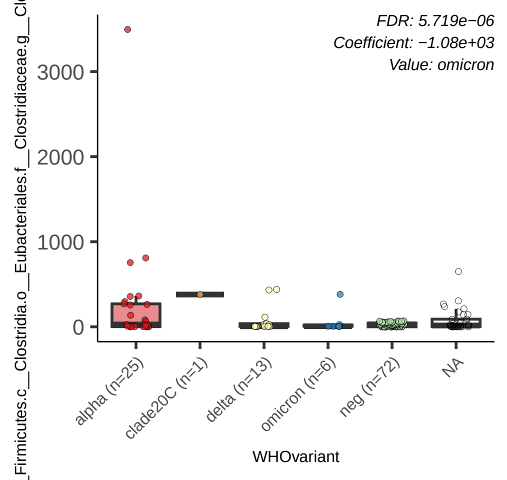
FDR: 5.719e−06
Coefficient: −1.08e+03
Value: omicron
Firmicutes.c__Clostridia.o__Eubacteriales.f__Clostridiaceae.g__Clo
3000
2000
1000
0
alpha (n=25)
clade20C (n=1)
delta (n=13)
omicron (n=6)
neg (n=72)
NA
WHOvariant

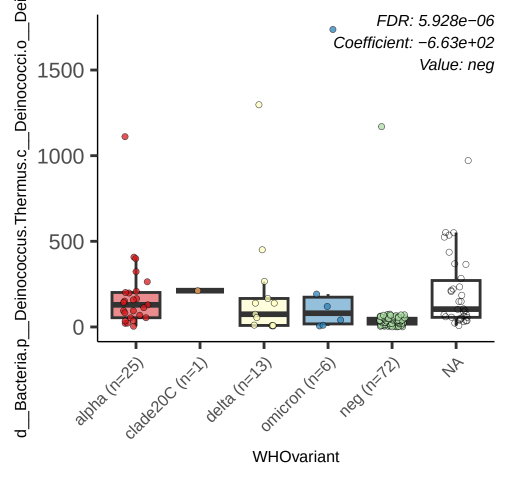

FDR: 5.928e−06
Coefficient: −6.63e+02
Value: neg
d__Bacteria.p__Deinococcus.Thermus.c__Deinococci.o__Dei
0
500
1000
1500
alpha (n=25)
clade20C (n=1)
delta (n=13)
omicron (n=6)
neg (n=72)
NA
WHOvariant

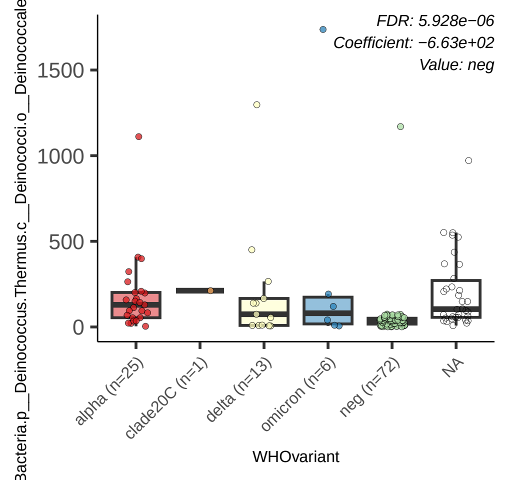

FDR: 5.928e−06
Coefficient: −6.63e+02
Value: neg
Bacteria.p__Deinococcus.Thermus.c__Deinococci.o__Deinococcale
0
500
1000
1500
alpha (n=25)
clade20C (n=1)
delta (n=13)
omicron (n=6)
neg (n=72)
NA
WHOvariant

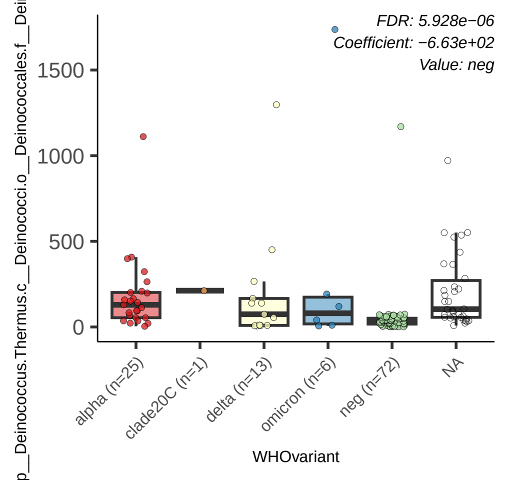
FDR: 5.928e−06
Coefficient: −6.63e+02
Value: neg
0
500
1000
1500
p__Deinococcus.Thermus.c__Deinococci.o__Deinococcales.f__Dei
alpha (n=25)
clade20C (n=1)
delta (n=13)
omicron (n=6)
neg (n=72)
NA
WHOvariant

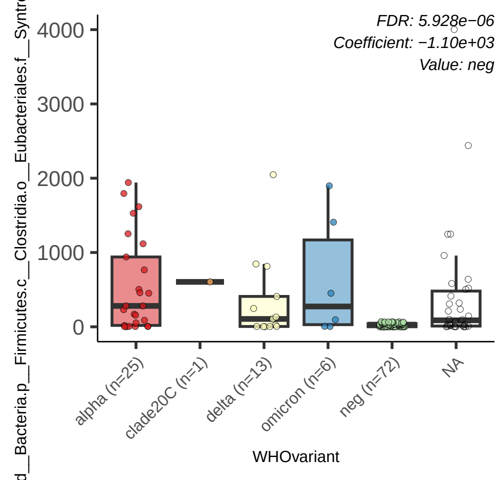
d__Bacteria.p__Firmicutes.c__Clostridia.o__Eubacteriales.f__Syntro
FDR: 5.928e−06
Coefficient: −1.10e+03
Value: neg
4000
3000
2000
1000
0
alpha (n=25)
clade20C (n=1)
delta (n=13)
omicron (n=6)
neg (n=72)
NA
WHOvariant

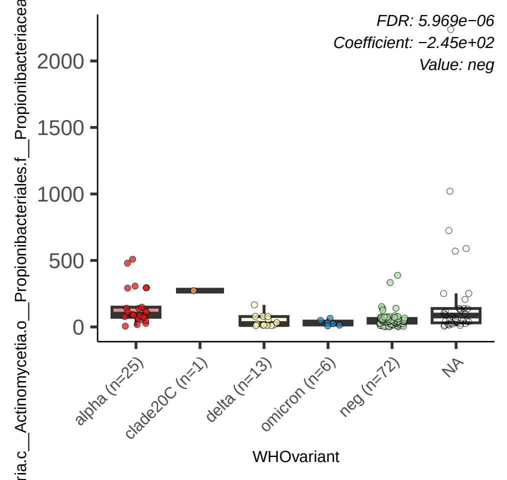

FDR: 5.969e−06
Coefficient: −2.45e+02
Value: neg
ria.c__Actinomycetia.o__Propionibacteriales.f__Propionibacteriacea
2000
1500
1000
500
0
alpha (n=25)
clade20C (n=1)
delta (n=13)
omicron (n=6)
neg (n=72)
NA
WHOvariant

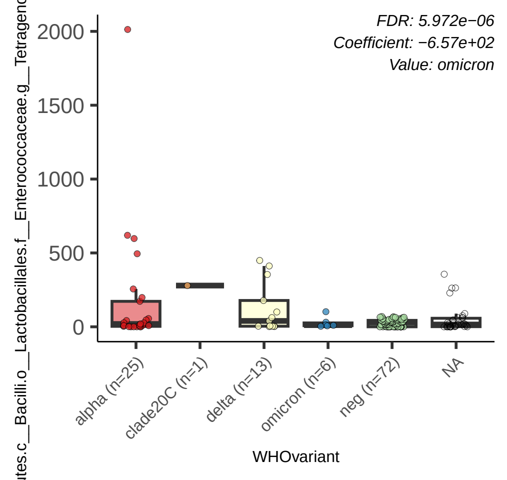
FDR: 5.972e−06
Coefficient: −6.57e+02
Value: omicron
2000
1500
1000
500
0
utes.c__Bacilli.o__Lactobacillales.f__Enterococcaceae.g__Tetragenc
alpha (n=25)
clade20C (n=1)
delta (n=13)
omicron (n=6)
neg (n=72)
NA
WHOvariant

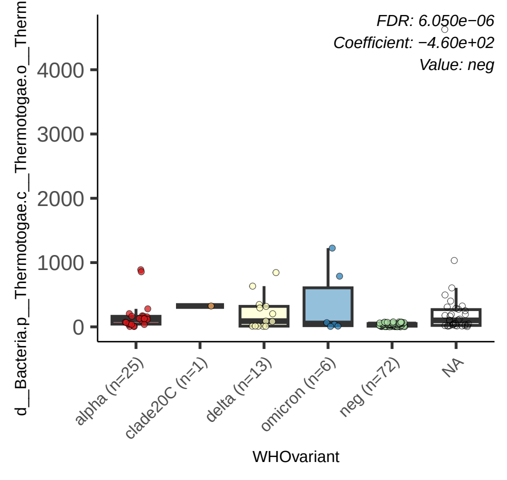

FDR: 6.050e−06
Coefficient: −4.60e+02
Value: neg
d__Bacteria.p__Thermotogae.c__Thermotogae.o__Therm
4000
3000
2000
1000
0
alpha (n=25)
clade20C (n=1)
delta (n=13)
omicron (n=6)
neg (n=72)
NA
WHOvariant

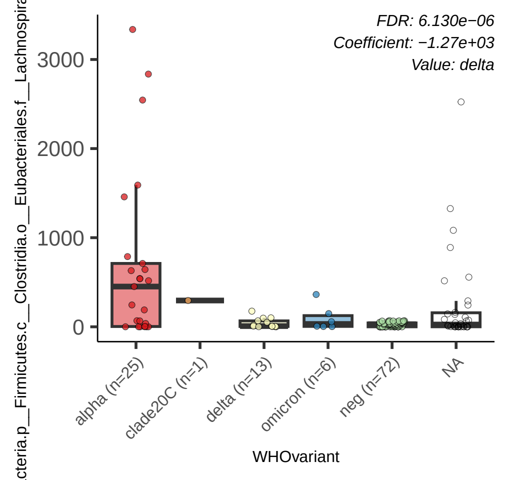

FDR: 6.130e−06
Coefficient: −1.27e+03
Value: delta
3000
2000
1000
0
cteria.p__Firmicutes.c__Clostridia.o__Eubacteriales.f__Lachnospira
alpha (n=25)
clade20C (n=1)
delta (n=13)
omicron (n=6)
neg (n=72)
NA
WHOvariant

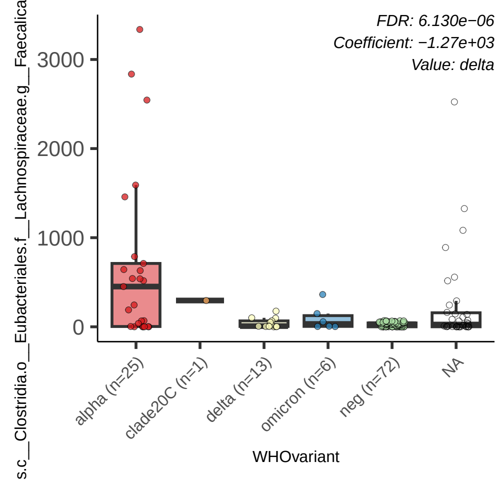

FDR: 6.130e−06
Coefficient: −1.27e+03
Value: delta
s.c__Clostridia.o__Eubacteriales.f__Lachnospiraceae.g__Faecalica
3000
2000
1000
0
alpha (n=25)
clade20C (n=1)
delta (n=13)
omicron (n=6)
neg (n=72)
NA
WHOvariant

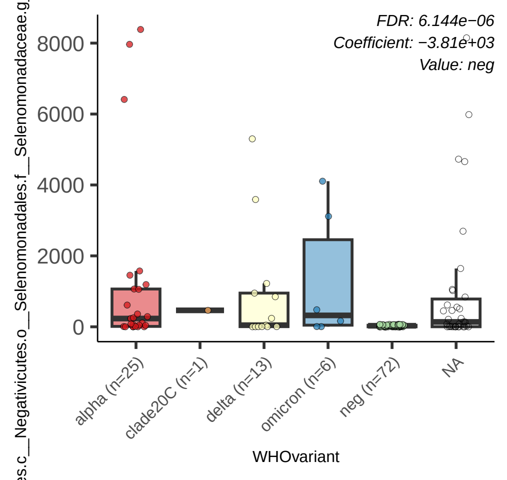

FDR: 6.144e−06
Coefficient: −3.81e+03
Value: neg
8000
6000
4000
2000
0
alpha (n=25)
clade20C (n=1)
delta (n=13)
omicron (n=6)
neg (n=72)
NA
WHOvariant
es.c__Negativicutes.o__Selenomonadales.f__Selenomonadaceae.g_

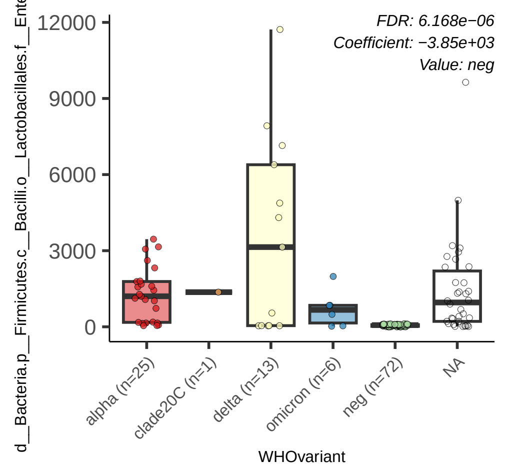

FDR: 6.168e−06
Coefficient: −3.85e+03
Value: neg
12000
9000
6000
3000
0
d__Bacteria.p__Firmicutes.c__Bacilli.o__Lactobacillales.f__Ente
alpha (n=25)
clade20C (n=1)
delta (n=13)
omicron (n=6)
neg (n=72)
NA
WHOvariant

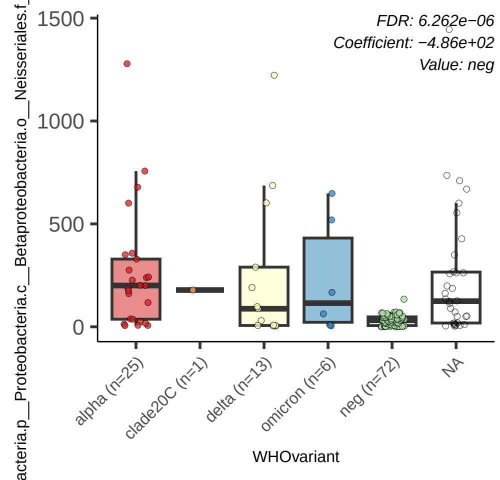

FDR: 6.262e−06
Coefficient: −4.86e+02
Value: neg
1500
1000
500
0
acteria.p__Proteobacteria.c__Betaproteobacteria.o__Neisseriales.f_
alpha (n=25)
clade20C (n=1)
delta (n=13)
omicron (n=6)
neg (n=72)
NA
WHOvariant

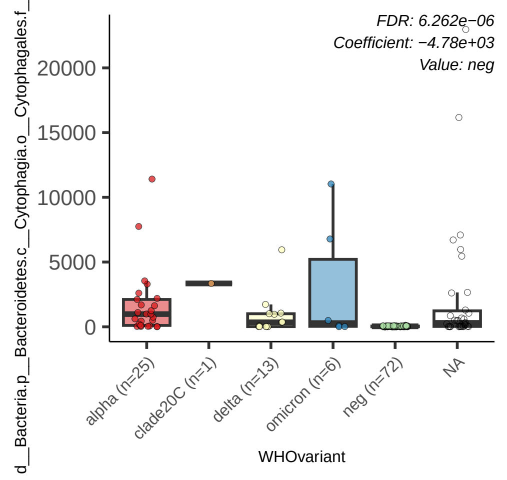

FDR: 6.262e−06
Coefficient: −4.78e+03
Value: neg
d__Bacteria.p__Bacteroidetes.c__Cytophagia.o__Cytophagales.f__
20000
15000
10000
5000
0
alpha (n=25)
clade20C (n=1)
delta (n=13)
omicron (n=6)
neg (n=72)
NA
WHOvariant

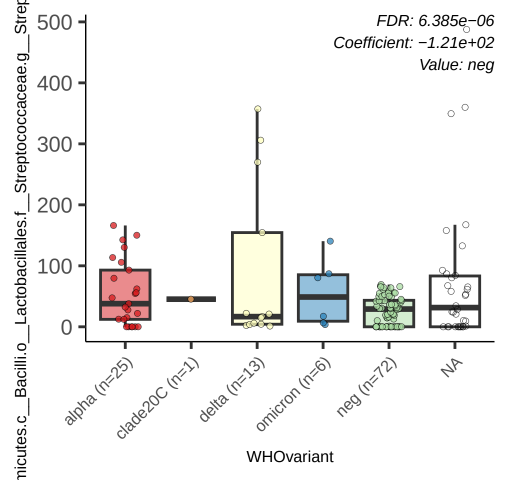

FDR: 6.385e−06
Coefficient: −1.21e+02
Value: neg
micutes.c__Bacilli.o__Lactobacillales.f__Streptococcaceae.g__Strep
500
400
300
200
100
0
alpha (n=25)
clade20C (n=1)
delta (n=13)
omicron (n=6)
neg (n=72)
NA
WHOvariant

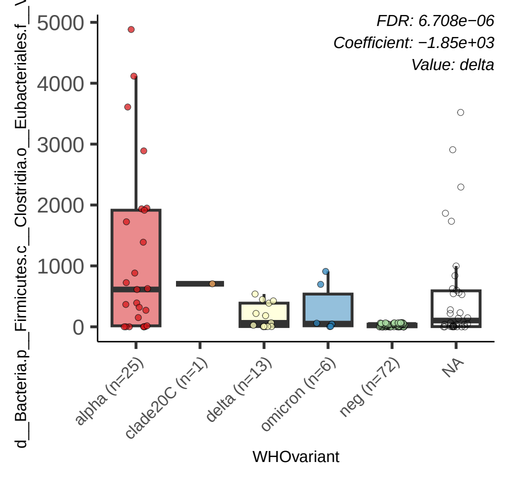

FDR: 6.708e−06
Coefficient: −1.85e+03
Value: delta
d__Bacteria.p__Firmicutes.c__Clostridia.o__Eubacteriales.f__
5000
4000
3000
2000
1000
0
alpha (n=25)
clade20C (n=1)
delta (n=13)
omicron (n=6)
neg (n=72)
NA
WHOvariant

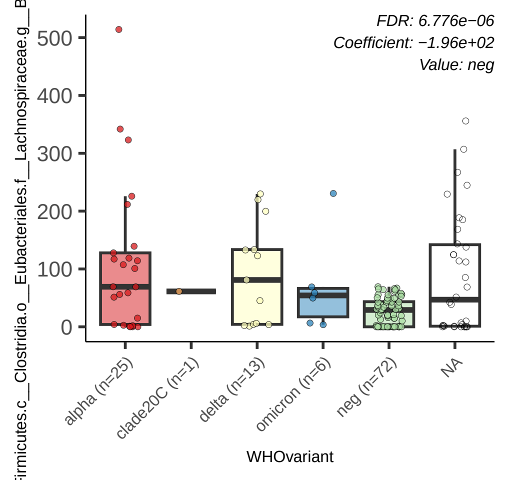
FDR: 6.776e−06
Coefficient: −1.96e+02
Value: neg
Firmicutes.c__Clostridia.o__Eubacteriales.f__Lachnospiraceae.g__B
500
400
300
200
100
0
alpha (n=25)
clade20C (n=1)
delta (n=13)
omicron (n=6)
neg (n=72)
NA
WHOvariant

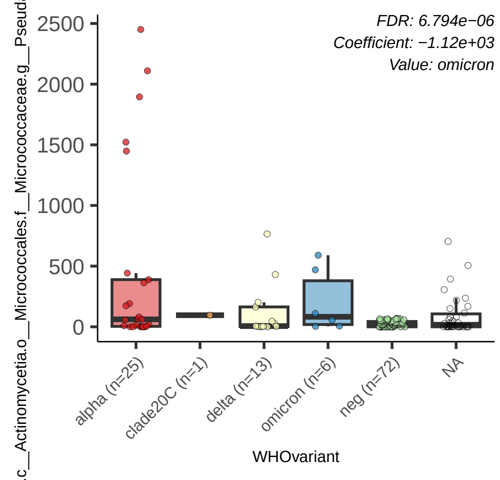
FDR: 6.794e−06
Coefficient: −1.12e+03
Value: omicron
2500
2000
1500
1000
500
0
alpha (n=25)
clade20C (n=1)
delta (n=13)
omicron (n=6)
neg (n=72)
NA
WHOvariant

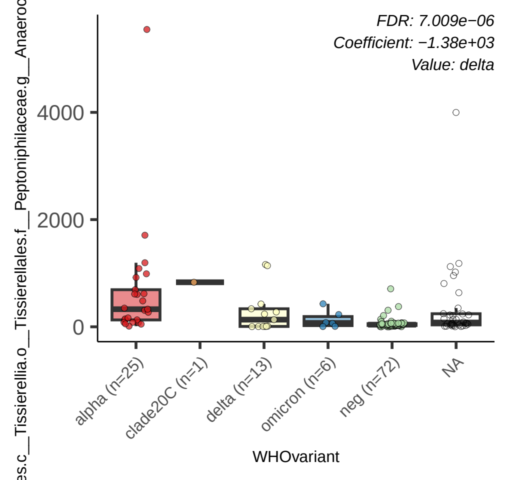

FDR: 7.009e−06
Coefficient: −1.38e+03
Value: delta
es.c__Tissierellia.o__Tissierellales.f__Peptoniphilaceae.g__Anaeroc
4000
2000
0
alpha (n=25)
clade20C (n=1)
delta (n=13)
omicron (n=6)
neg (n=72)
NA
WHOvariant

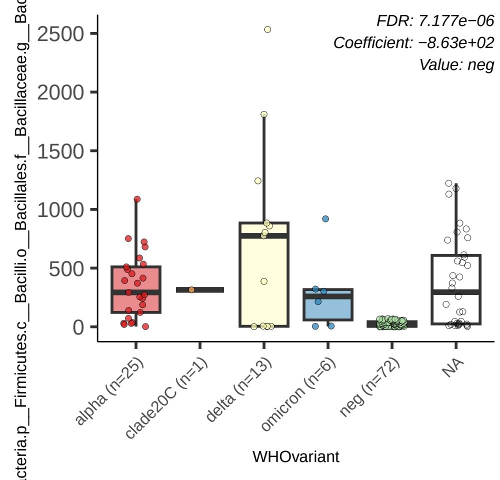

FDR: 7.177e−06
Coefficient: −8.63e+02
Value: neg
2500
2000
1500
1000
500
0
alpha (n=25)
clade20C (n=1)
delta (n=13)
omicron (n=6)
neg (n=72)
NA
WHOvariant
cteria.p__Firmicutes.c__Bacilli.o__Bacillales.f__Bacillaceae.g__Bac

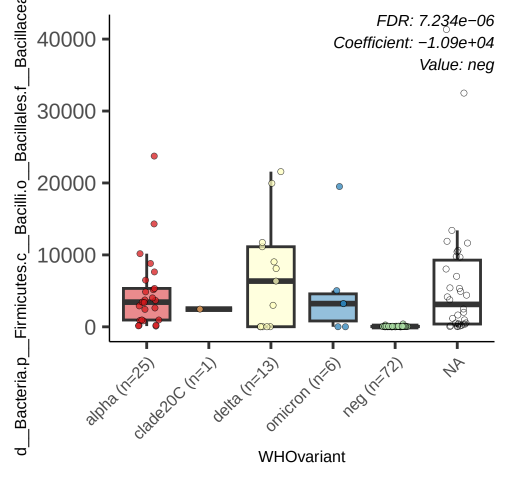
FDR: 7.234e−06
Coefficient: −1.09e+04
Value: neg
d__Bacteria.p__Firmicutes.c__Bacilli.o__Bacillales.f__Bacillacea
40000
30000
20000
10000
0
alpha (n=25)
clade20C (n=1)
delta (n=13)
omicron (n=6)
neg (n=72)
NA
WHOvariant

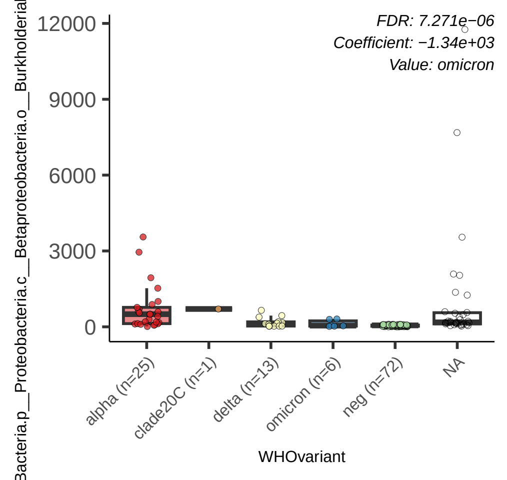
FDR: 7.271e−06
Coefficient: −1.34e+03
Value: omicron
12000
9000
6000
3000
0
alpha (n=25)
clade20C (n=1)
delta (n=13)
omicron (n=6)
neg (n=72)
NA
WHOvariant
Bacteria.p__Proteobacteria.c__Betaproteobacteria.o__Burkholderial

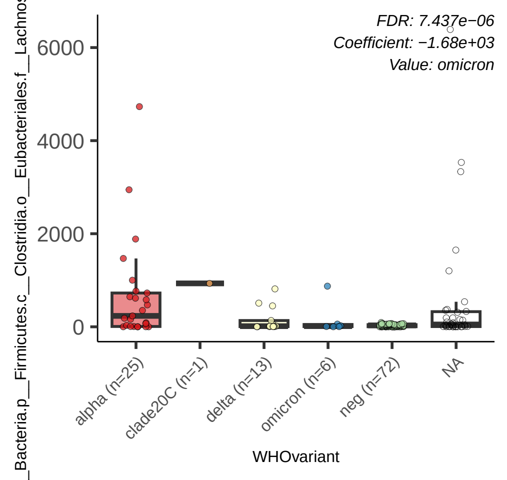

FDR: 7.437e−06
Coefficient: −1.68e+03
Value: omicron
6000
4000
2000
0
Bacteria.p__Firmicutes.c__Clostridia.o__Eubacteriales.f__Lachnos
alpha (n=25)
clade20C (n=1)
delta (n=13)
omicron (n=6)
neg (n=72)
NA
WHOvariant

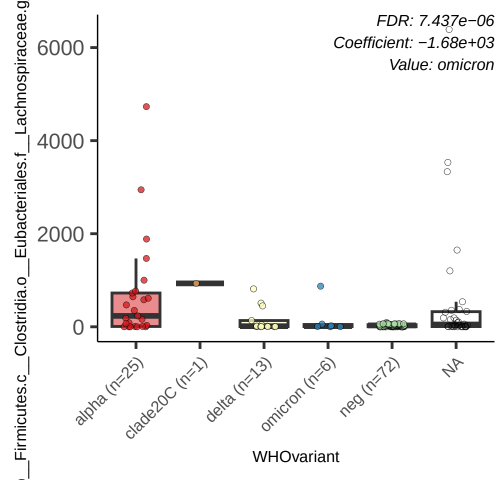

FDR: 7.437e−06
Coefficient: −1.68e+03
Value: omicron
Firmicutes.c__Clostridia.o__Eubacteriales.f__Lachnospiraceae.g
6000
4000
2000
0
alpha (n=25)
clade20C (n=1)
delta (n=13)
omicron (n=6)
neg (n=72)
NA
WHOvariant

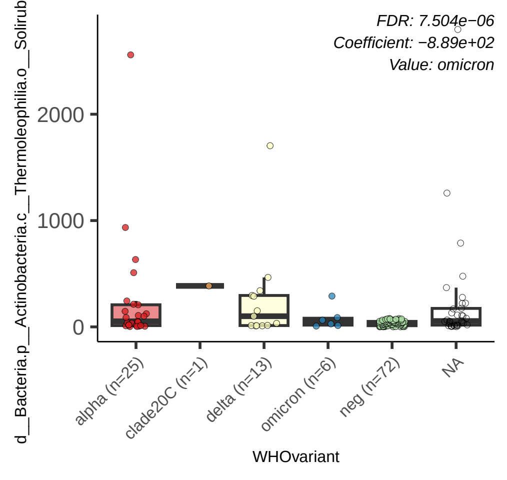
FDR: 7.504e−06
Coefficient: −8.89e+02
Value: omicron
d__Bacteria.p__Actinobacteria.c__Thermoleophilia.o__Solirub
0
1000
2000
alpha (n=25)
clade20C (n=1)
delta (n=13)
omicron (n=6)
neg (n=72)
NA
WHOvariant

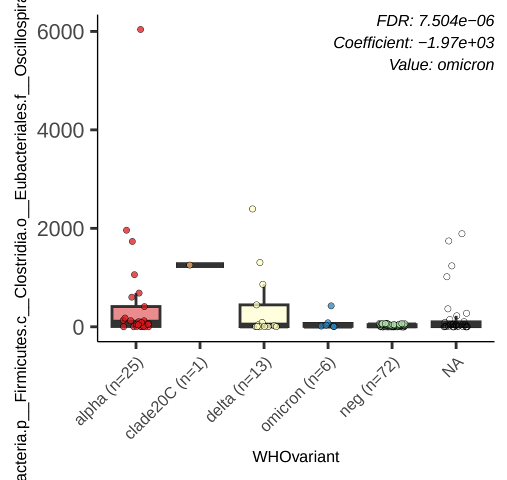
FDR: 7.504e−06
Coefficient: −1.97e+03
Value: omicron
6000
4000
2000
0
alpha (n=25)
clade20C (n=1)
delta (n=13)
omicron (n=6)
neg (n=72)
NA
WHOvariant
acteria.p__Firmicutes.c__Clostridia.o__Eubacteriales.f__Oscillospira

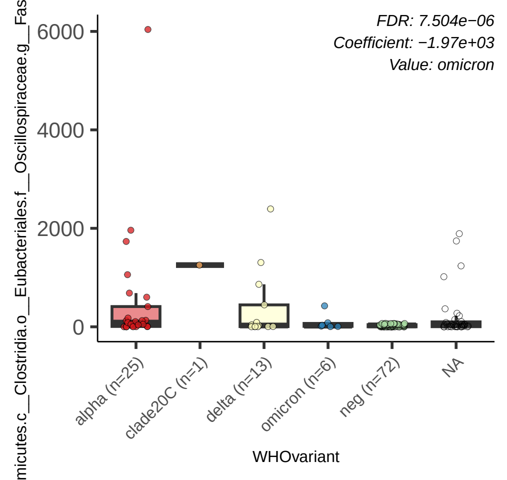

FDR: 7.504e−06
Coefficient: −1.97e+03
Value: omicron
micutes.c__Clostridia.o__Eubacteriales.f__Oscillospiraceae.g__Fas
6000
4000
2000
0
alpha (n=25)
clade20C (n=1)
delta (n=13)
omicron (n=6)
neg (n=72)
NA
WHOvariant

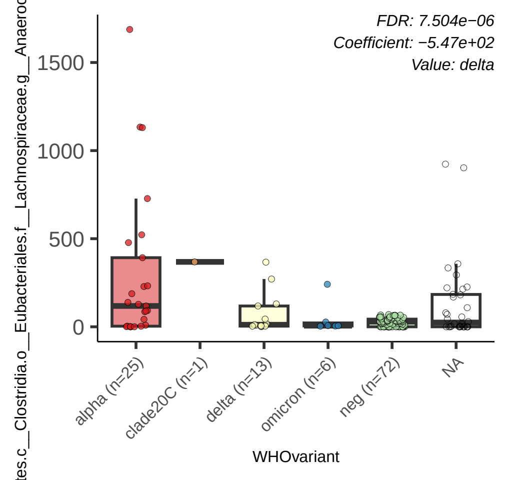

FDR: 7.504e−06
Coefficient: −5.47e+02
Value: delta
tes.c__Clostridia.o__Eubacteriales.f__Lachnospiraceae.g__Anaeroc
0
500
1000
1500
alpha (n=25)
clade20C (n=1)
delta (n=13)
omicron (n=6)
neg (n=72)
NA
WHOvariant

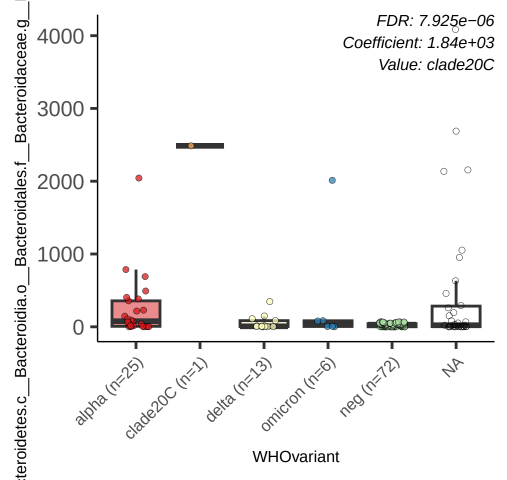

FDR: 7.925e−06
Coefficient: 1.84e+03
Value: clade20C
4000
3000
2000
1000
0
acteroidetes.c__Bacteroidia.o__Bacteroidales.f__Bacteroidaceae.g__
alpha (n=25)
clade20C (n=1)
delta (n=13)
omicron (n=6)
neg (n=72)
NA
WHOvariant

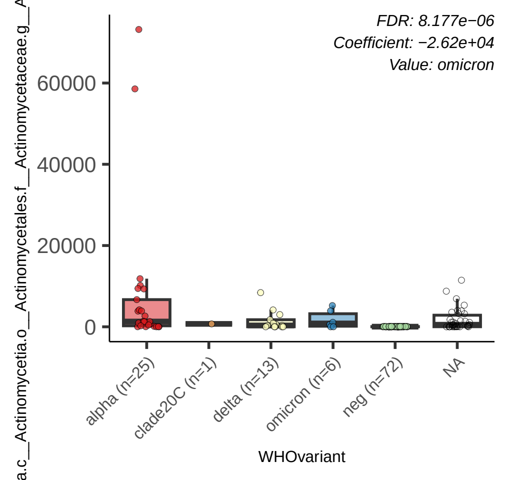

FDR: 8.177e−06
Coefficient: −2.62e+04
Value: omicron
60000
40000
20000
0
a.c__Actinomycetia.o__Actinomycetales.f__Actinomycetaceae.g__A
alpha (n=25)
clade20C (n=1)
delta (n=13)
omicron (n=6)
neg (n=72)
NA
WHOvariant

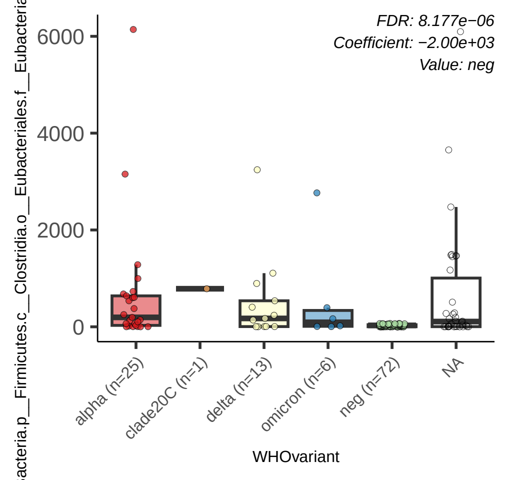

FDR: 8.177e−06
Coefficient: −2.00e+03
Value: neg
6000
4000
2000
0
acteria.p__Firmicutes.c__Clostridia.o__Eubacteriales.f__Eubacteria
alpha (n=25)
clade20C (n=1)
delta (n=13)
omicron (n=6)
neg (n=72)
NA
WHOvariant

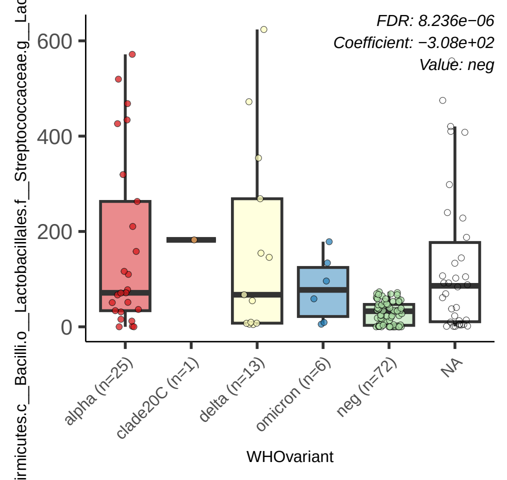
FDR: 8.236e−06
Coefficient: −3.08e+02
Value: neg
600
400
200
0
alpha (n=25)
clade20C (n=1)
delta (n=13)
omicron (n=6)
neg (n=72)
NA
WHOvariant
irmicutes.c__Bacilli.o__Lactobacillales.f__Streptococcaceae.g__Lac

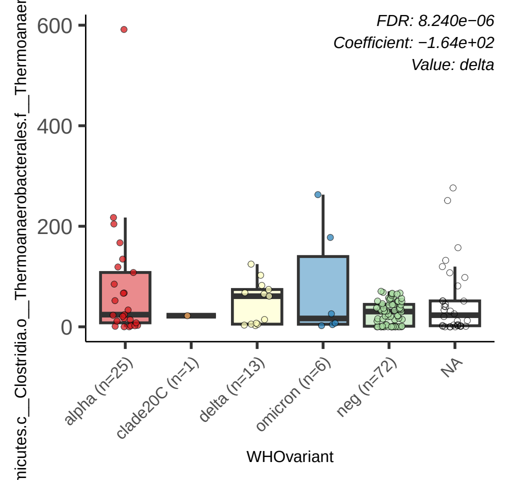

FDR: 8.240e−06
Coefficient: −1.64e+02
Value: delta
micutes.c__Clostridia.o__Thermoanaerobacterales.f__Thermoanaer
600
400
200
0
alpha (n=25)
clade20C (n=1)
delta (n=13)
omicron (n=6)
neg (n=72)
NA
WHOvariant

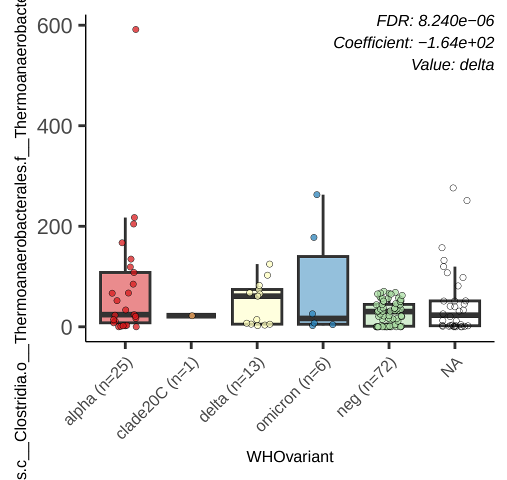

FDR: 8.240e−06
Coefficient: −1.64e+02
Value: delta
600
400
200
0
s.c__Clostridia.o__Thermoanaerobacterales.f__Thermoanaerobact
alpha (n=25)
clade20C (n=1)
delta (n=13)
omicron (n=6)
neg (n=72)
NA
WHOvariant

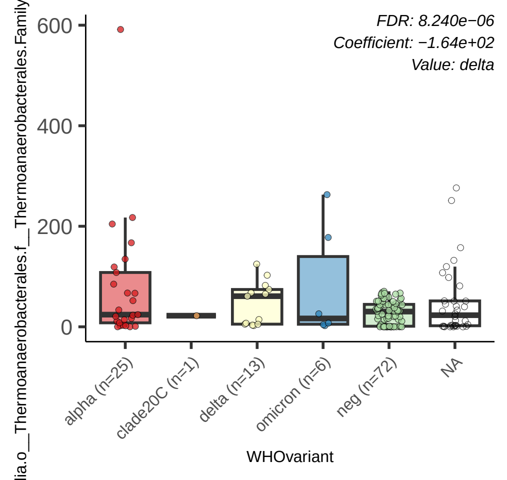
FDR: 8.240e−06
Coefficient: −1.64e+02
Value: delta
600
400
200
0
lia.o__Thermoanaerobacterales.f__Thermoanaerobacterales.Family
alpha (n=25)
clade20C (n=1)
delta (n=13)
omicron (n=6)
neg (n=72)
NA
WHOvariant

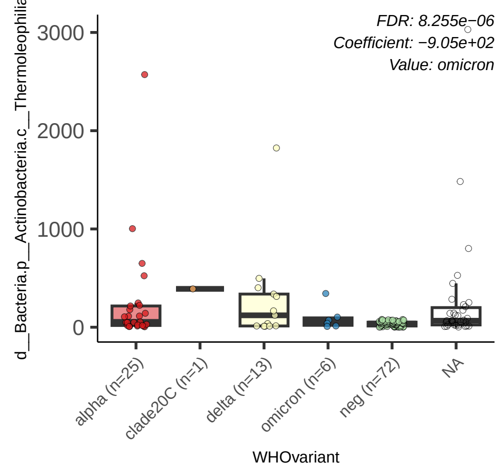

FDR: 8.255e−06
Coefficient: −9.05e+02
Value: omicron
d__Bacteria.p__Actinobacteria.c__Thermoleophilia
3000
2000
1000
0
alpha (n=25)
clade20C (n=1)
delta (n=13)
omicron (n=6)
neg (n=72)
NA
WHOvariant

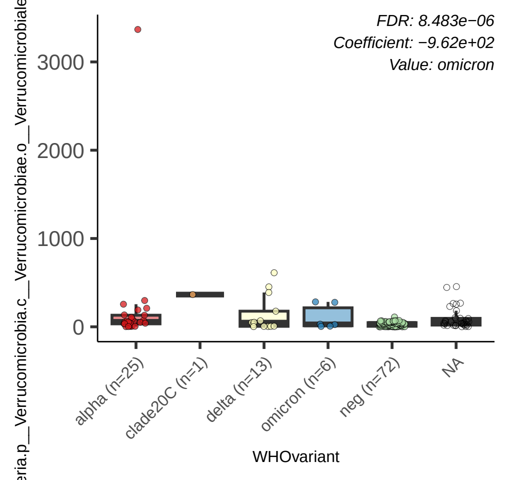

FDR: 8.483e−06
Coefficient: −9.62e+02
Value: omicron
eria.p__Verrucomicrobia.c__Verrucomicrobiae.o__Verrucomicrobiale
3000
2000
1000
0
alpha (n=25)
clade20C (n=1)
delta (n=13)
omicron (n=6)
neg (n=72)
NA
WHOvariant

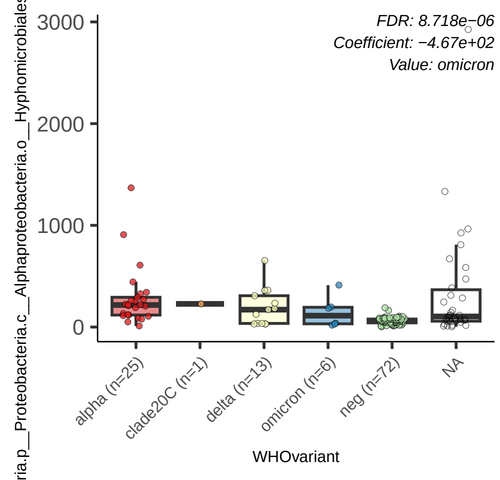

FDR: 8.718e−06
Coefficient: −4.67e+02
Value: omicron
3000
2000
1000
0
ria.p__Proteobacteria.c__Alphaproteobacteria.o__Hyphomicrobiales
alpha (n=25)
clade20C (n=1)
delta (n=13)
omicron (n=6)
neg (n=72)
NA
WHOvariant

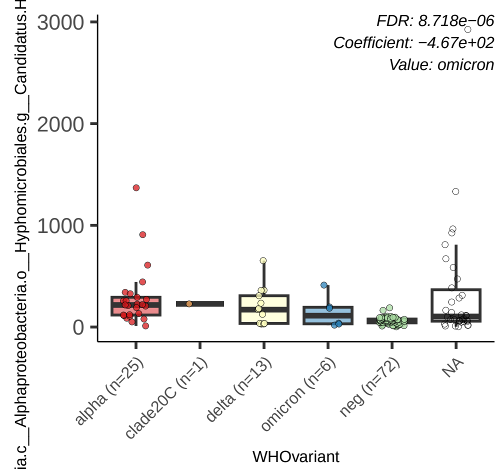

FDR: 8.718e−06
Coefficient: −4.67e+02
Value: omicron
3000
2000
1000
0
eria.c__Alphaproteobacteria.o__Hyphomicrobiales.g__Candidatus.H
alpha (n=25)
clade20C (n=1)
delta (n=13)
omicron (n=6)
neg (n=72)
NA
WHOvariant

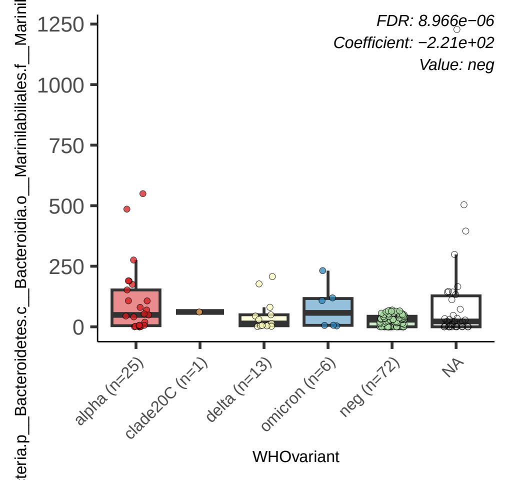
FDR: 8.966e−06
Coefficient: −2.21e+02
Value: neg
1250
1000
750
500
250
0
alpha (n=25)
clade20C (n=1)
delta (n=13)
omicron (n=6)
neg (n=72)
NA
WHOvariant
cteria.p__Bacteroidetes.c__Bacteroidia.o__Marinilabiliales.f__Marinil

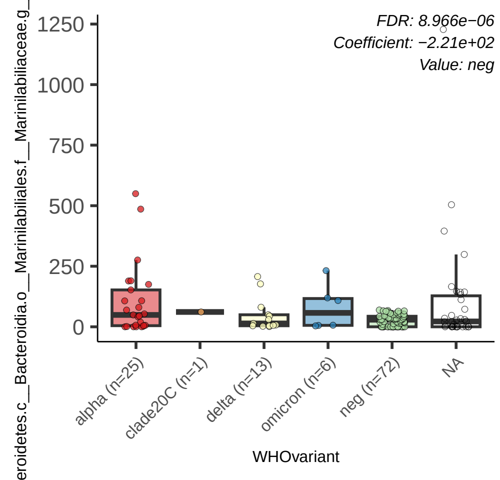

FDR: 8.966e−06
Coefficient: −2.21e+02
Value: neg
1250
1000
750
500
250
0
eroidetes.c__Bacteroidia.o__Marinilabiliales.f__Marinilabiliaceae.g__
alpha (n=25)
clade20C (n=1)
delta (n=13)
omicron (n=6)
neg (n=72)
NA
WHOvariant

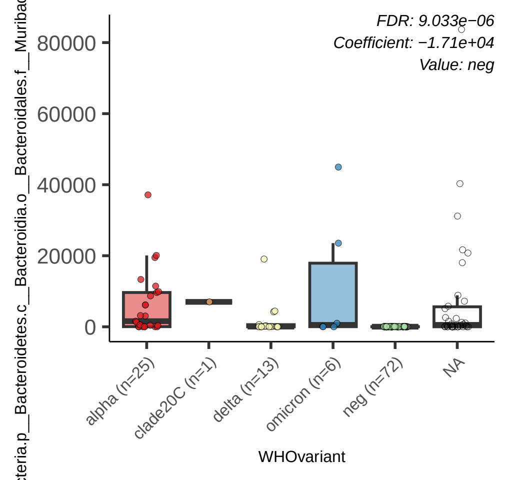
FDR: 9.033e−06
Coefficient: −1.71e+04
Value: neg
80000
60000
40000
20000
0
alpha (n=25)
clade20C (n=1)
delta (n=13)
omicron (n=6)
neg (n=72)
NA
WHOvariant

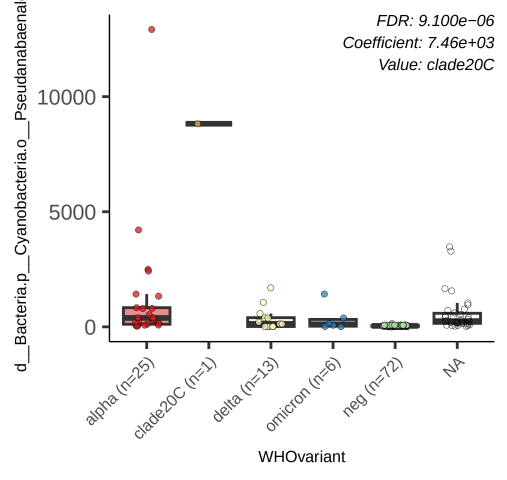

FDR: 9.100e−06
Coefficient: 7.46e+03
Value: clade20C
d__Bacteria.p__Cyanobacteria.o__Pseudanabaenal
10000
5000
0
alpha (n=25)
clade20C (n=1)
delta (n=13)
omicron (n=6)
neg (n=72)
NA
WHOvariant

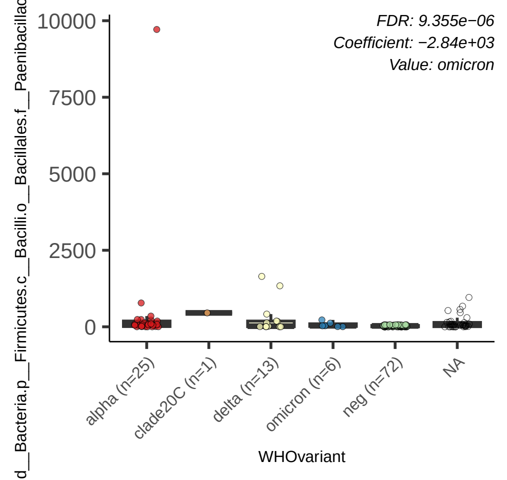

FDR: 9.355e−06
Coefficient: −2.84e+03
Value: omicron
10000
7500
5000
2500
0
alpha (n=25)
clade20C (n=1)
delta (n=13)
omicron (n=6)
neg (n=72)
NA
WHOvariant
d__Bacteria.p__Firmicutes.c__Bacilli.o__Bacillales.f__Paenibacillac

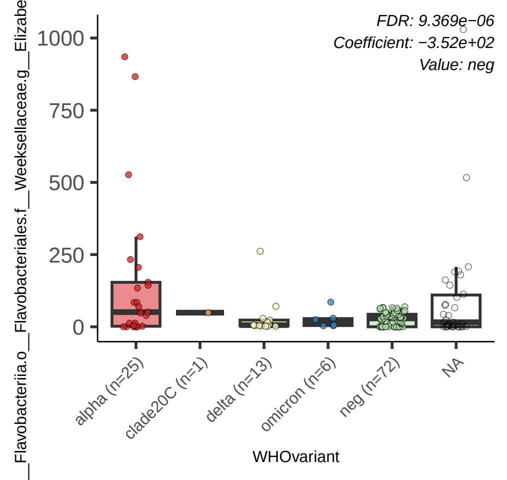

FDR: 9.369e−06
Coefficient: −3.52e+02
Value: neg
Flavobacteriia.o__Flavobacteriales.f__Weeksellaceae.g__Elizabe
1000
750
500
250
0
alpha (n=25)
clade20C (n=1)
delta (n=13)
omicron (n=6)
neg (n=72)
NA
WHOvariant

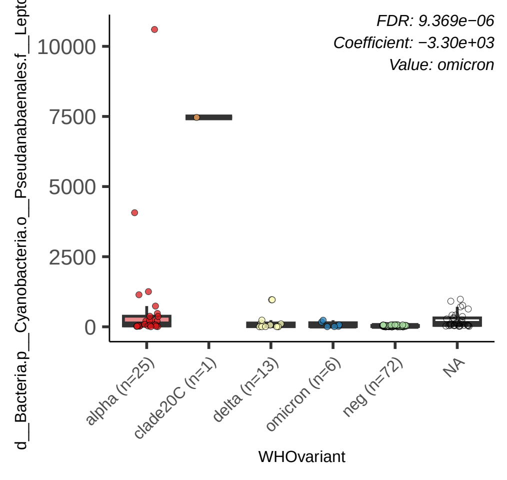
FDR: 9.369e−06
Coefficient: −3.30e+03
Value: omicron
d__Bacteria.p__Cyanobacteria.o__Pseudanabaenales.f__Lepto
10000
7500
5000
2500
0
alpha (n=25)
clade20C (n=1)
delta (n=13)
omicron (n=6)
neg (n=72)
NA
WHOvariant

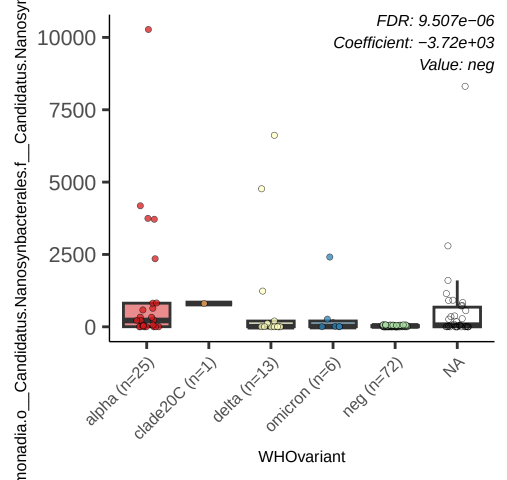

FDR: 9.507e−06
Coefficient: −3.72e+03
Value: neg
10000
7500
5000
2500
0
alpha (n=25)
clade20C (n=1)
delta (n=13)
omicron (n=6)
neg (n=72)
NA
WHOvariant
monadia.o__Candidatus.Nanosynbacterales.f__Candidatus.Nanosyn

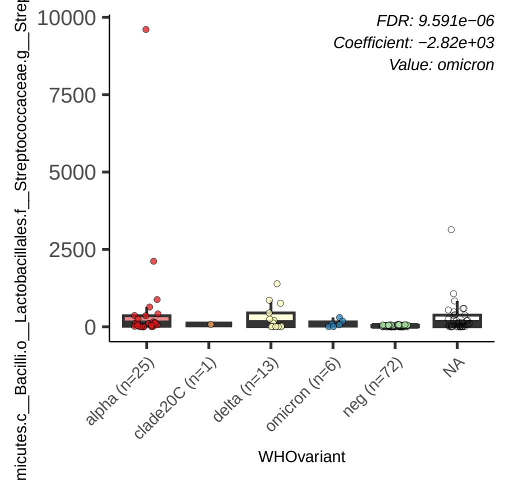

FDR: 9.591e−06
Coefficient: −2.82e+03
Value: omicron
micutes.c__Bacilli.o__Lactobacillales.f__Streptococcaceae.g__Strep
10000
7500
5000
2500
0
alpha (n=25)
clade20C (n=1)
delta (n=13)
omicron (n=6)
neg (n=72)
NA
WHOvariant

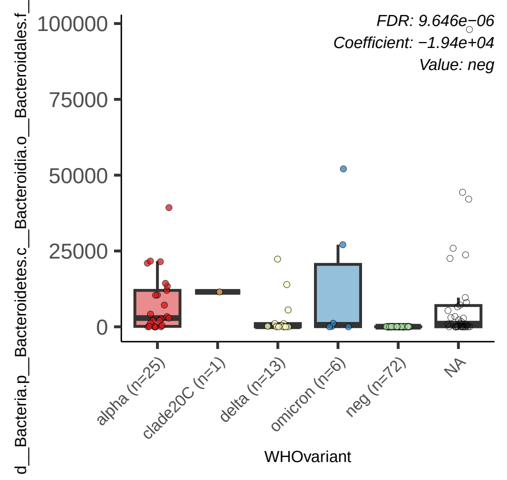

FDR: 9.646e−06
Coefficient: −1.94e+04
Value: neg
d__Bacteria.p__Bacteroidetes.c__Bacteroidia.o__Bacteroidales.f__
100000
75000
50000
25000
0
alpha (n=25)
clade20C (n=1)
delta (n=13)
omicron (n=6)
neg (n=72)
NA
WHOvariant

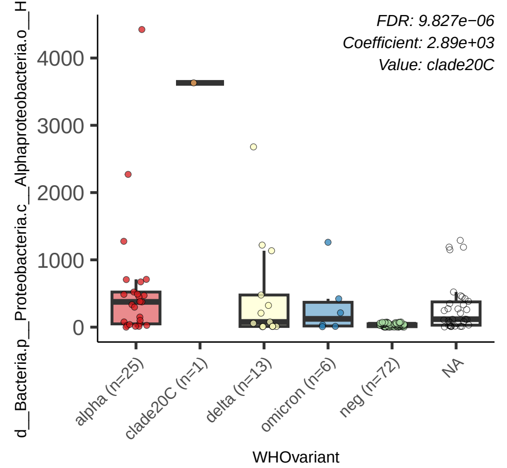

FDR: 9.827e−06
Coefficient: 2.89e+03
Value: clade20C
d__Bacteria.p__Proteobacteria.c__Alphaproteobacteria.o__H
4000
3000
2000
1000
0
alpha (n=25)
clade20C (n=1)
delta (n=13)
omicron (n=6)
neg (n=72)
NA
WHOvariant

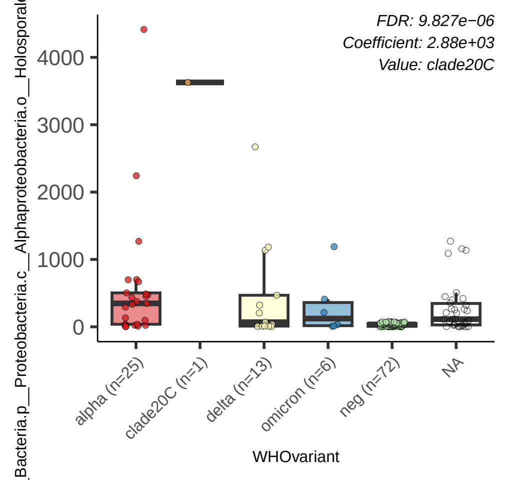

FDR: 9.827e−06
Coefficient: 2.88e+03
Value: clade20C
Bacteria.p__Proteobacteria.c__Alphaproteobacteria.o__Holosporale
4000
3000
2000
1000
0
alpha (n=25)
clade20C (n=1)
delta (n=13)
omicron (n=6)
neg (n=72)
NA
WHOvariant

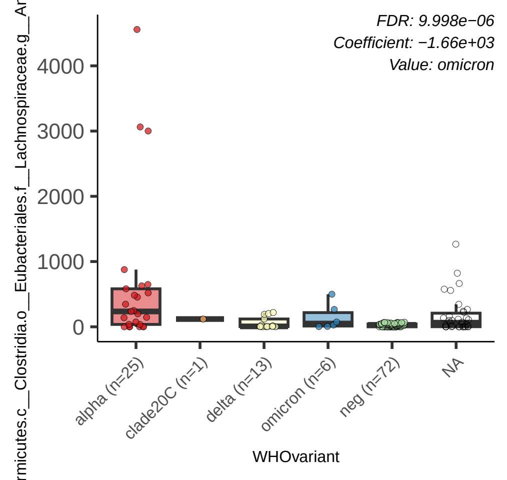

FDR: 9.998e−06
Coefficient: −1.66e+03
Value: omicron
4000
3000
2000
1000
0
alpha (n=25)
clade20C (n=1)
delta (n=13)
omicron (n=6)
neg (n=72)
NA
WHOvariant
rmicutes.c__Clostridia.o__Eubacteriales.f__Lachnospiraceae.g__Ar

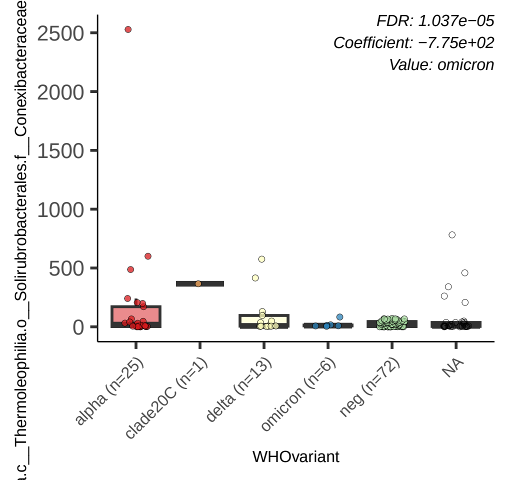

FDR: 1.037e−05
Coefficient: −7.75e+02
Value: omicron
2500
2000
1500
1000
500
0
a.c__Thermoleophilia.o__Solirubrobacterales.f__Conexibacteraceae
alpha (n=25)
clade20C (n=1)
delta (n=13)
omicron (n=6)
neg (n=72)
NA
WHOvariant

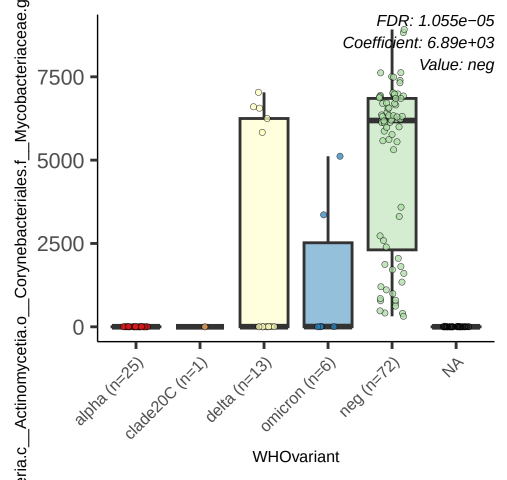
FDR: 1.055e−05
Coefficient: 6.89e+03
Value: neg
7500
5000
2500
0
alpha (n=25)
clade20C (n=1)
delta (n=13)
omicron (n=6)
neg (n=72)
NA
WHOvariant
eria.c__Actinomycetia.o__Corynebacteriales.f__Mycobacteriaceae.g

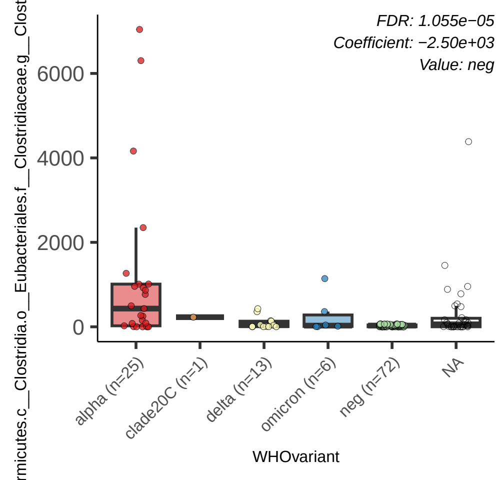

FDR: 1.055e−05
Coefficient: −2.50e+03
Value: neg
0
2000
4000
6000
rmicutes.c__Clostridia.o__Eubacteriales.f__Clostridiaceae.g__Clost
alpha (n=25)
clade20C (n=1)
delta (n=13)
omicron (n=6)
neg (n=72)
NA
WHOvariant

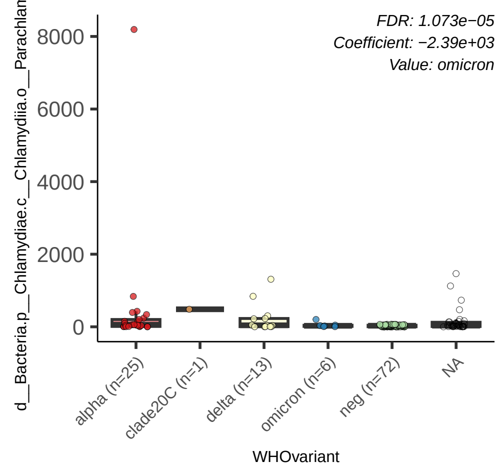

FDR: 1.073e−05
Coefficient: −2.39e+03
Value: omicron
d__Bacteria.p__Chlamydiae.c__Chlamydiia.o__Parachlam
8000
6000
4000
2000
0
alpha (n=25)
clade20C (n=1)
delta (n=13)
omicron (n=6)
neg (n=72)
NA
WHOvariant

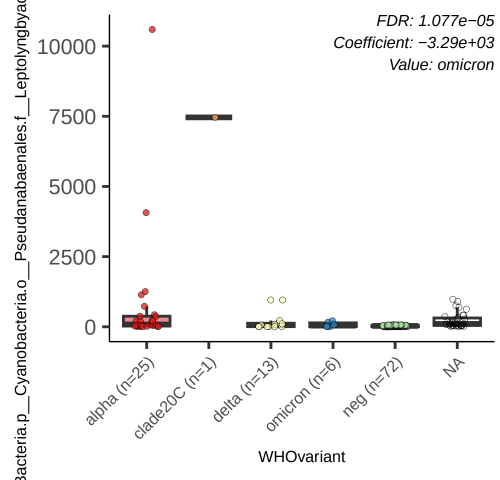

FDR: 1.077e−05
Coefficient: −3.29e+03
Value: omicron
10000
7500
5000
2500
0
alpha (n=25)
clade20C (n=1)
delta (n=13)
omicron (n=6)
neg (n=72)
NA
WHOvariant
Bacteria.p__Cyanobacteria.o__Pseudanabaenales.f__Leptolyngbyac

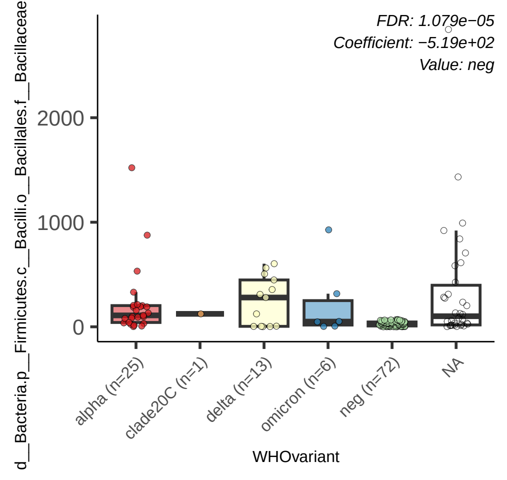
FDR: 1.079e−05
Coefficient: −5.19e+02
Value: neg
d__Bacteria.p__Firmicutes.c__Bacilli.o__Bacillales.f__Bacillaceae
0
1000
2000
alpha (n=25)
clade20C (n=1)
delta (n=13)
omicron (n=6)
neg (n=72)
NA
WHOvariant

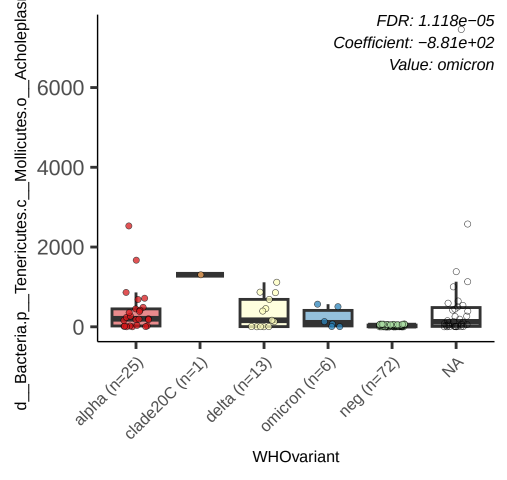
FDR: 1.118e−05
Coefficient: −8.81e+02
Value: omicron
d__Bacteria.p__Tenericutes.c__Mollicutes.o__Acholeplas
6000
4000
2000
0
alpha (n=25)
clade20C (n=1)
delta (n=13)
omicron (n=6)
neg (n=72)
NA
WHOvariant

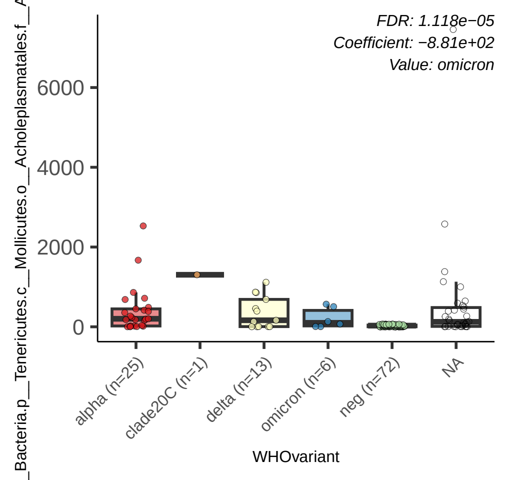

FDR: 1.118e−05
Coefficient: −8.81e+02
Value: omicron
6000
4000
2000
0
Bacteria.p__Tenericutes.c__Mollicutes.o__Acholeplasmatales.f__A
alpha (n=25)
clade20C (n=1)
delta (n=13)
omicron (n=6)
neg (n=72)
NA
WHOvariant

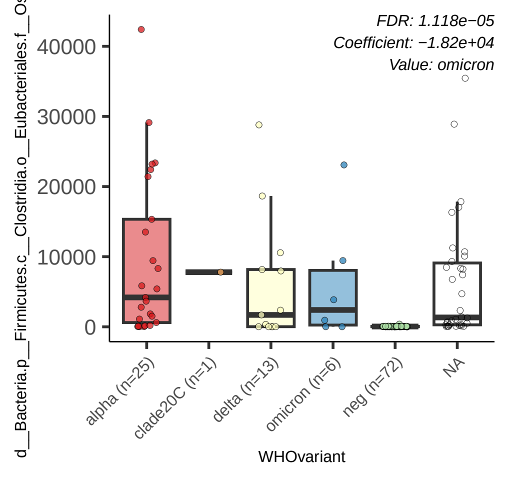

d__Bacteria.p__Firmicutes.c__Clostridia.o__Eubacteriales.f__Os
FDR: 1.118e−05
Coefficient: −1.82e+04
Value: omicron
40000
30000
20000
10000
0
alpha (n=25)
clade20C (n=1)
delta (n=13)
omicron (n=6)
neg (n=72)
NA
WHOvariant

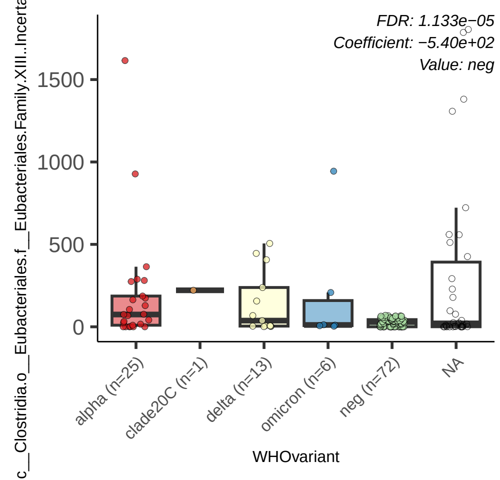

FDR: 1.133e−05
Coefficient: −5.40e+02
Value: neg
c__Clostridia.o__Eubacteriales.f__Eubacteriales.Family.XIII..Incerta
1500
1000
500
0
alpha (n=25)
clade20C (n=1)
delta (n=13)
omicron (n=6)
neg (n=72)
NA
WHOvariant

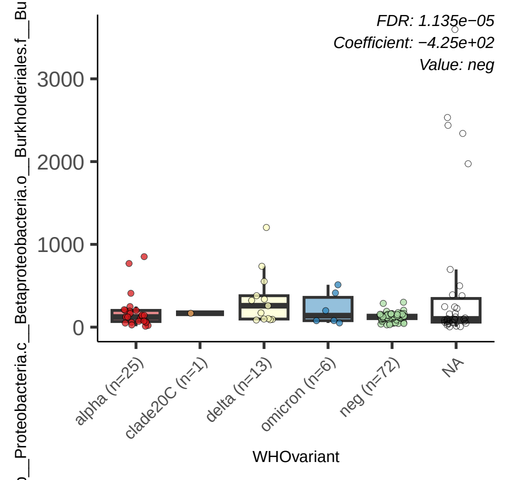

FDR: 1.135e−05
Coefficient: −4.25e+02
Value: neg
3000
2000
1000
0
o__Proteobacteria.c__Betaproteobacteria.o__Burkholderiales.f__Bu
alpha (n=25)
clade20C (n=1)
delta (n=13)
omicron (n=6)
neg (n=72)
NA
WHOvariant

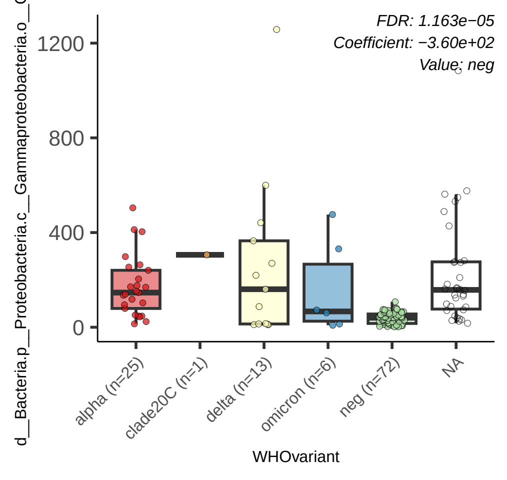

FDR: 1.163e−05
Coefficient: −3.60e+02
Value: neg
1200
800
400
0
d__Bacteria.p__Proteobacteria.c__Gammaproteobacteria.o__C
alpha (n=25)
clade20C (n=1)
delta (n=13)
omicron (n=6)
neg (n=72)
NA
WHOvariant

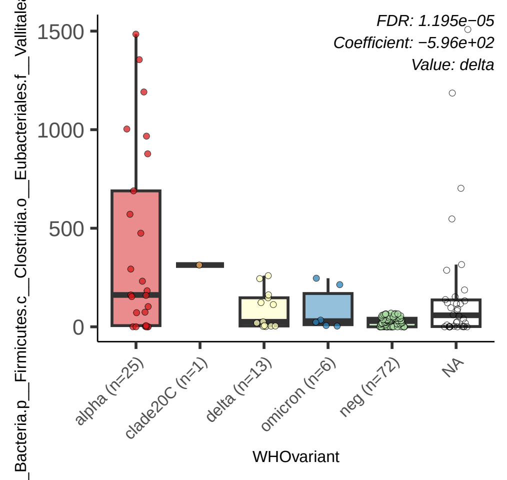

FDR: 1.195e−05
Coefficient: −5.96e+02
Value: delta
Bacteria.p__Firmicutes.c__Clostridia.o__Eubacteriales.f__Vallitale
1500
1000
500
0
alpha (n=25)
clade20C (n=1)
delta (n=13)
omicron (n=6)
neg (n=72)
NA
WHOvariant

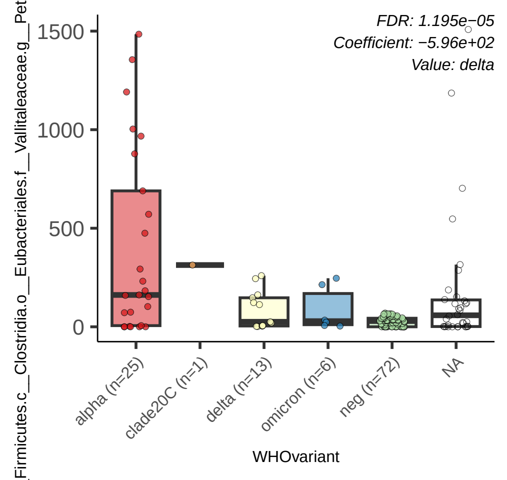

FDR: 1.195e−05
Coefficient: −5.96e+02
Value: delta
Firmicutes.c__Clostridia.o__Eubacteriales.f__Vallitaleaceae.g__Pet
0
500
1000
1500
alpha (n=25)
clade20C (n=1)
delta (n=13)
omicron (n=6)
neg (n=72)
NA
WHOvariant

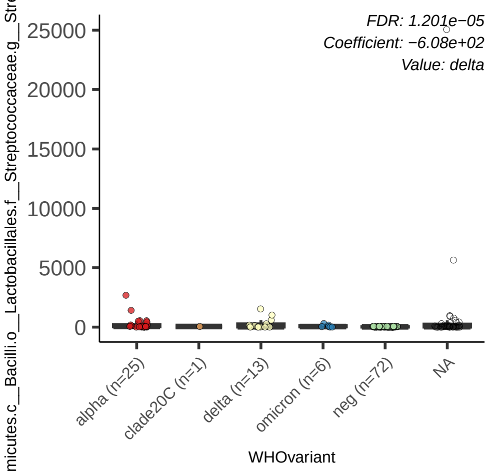

FDR: 1.201e−05
Coefficient: −6.08e+02
Value: delta
25000
20000
15000
10000
5000
0
alpha (n=25)
clade20C (n=1)
delta (n=13)
omicron (n=6)
neg (n=72)
NA
WHOvariant

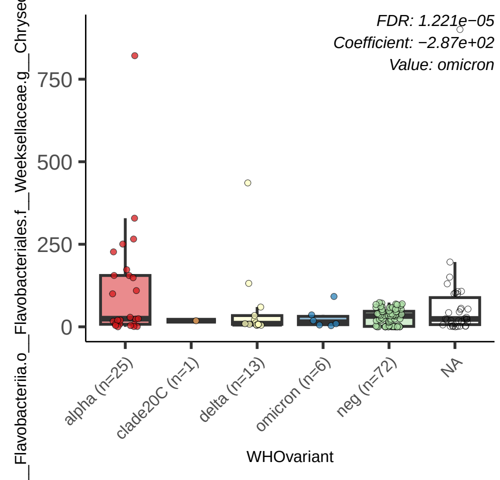

FDR: 1.221e−05
Coefficient: −2.87e+02
Value: omicron
_Flavobacteriia.o__Flavobacteriales.f__Weeksellaceae.g__Chryse
0
250
500
750
alpha (n=25)
clade20C (n=1)
delta (n=13)
omicron (n=6)
neg (n=72)
NA
WHOvariant

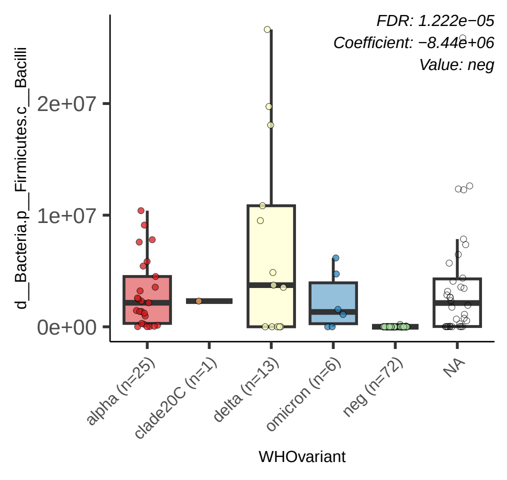

FDR: 1.222e−05
Coefficient: −8.44e+06
Value: neg
d__Bacteria.p__Firmicutes.c__Bacilli
2e+07
1e+07
0e+00
alpha (n=25)
clade20C (n=1)
delta (n=13)
omicron (n=6)
neg (n=72)
NA
WHOvariant

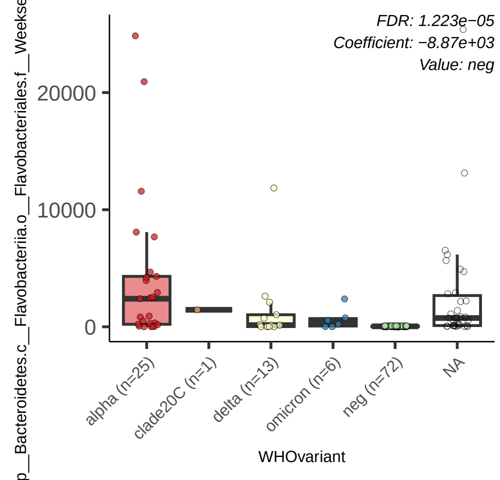

FDR: 1.223e−05
Coefficient: −8.87e+03
Value: neg
p__Bacteroidetes.c__Flavobacteriia.o__Flavobacteriales.f__Weekse
20000
10000
0
alpha (n=25)
clade20C (n=1)
delta (n=13)
omicron (n=6)
neg (n=72)
NA
WHOvariant

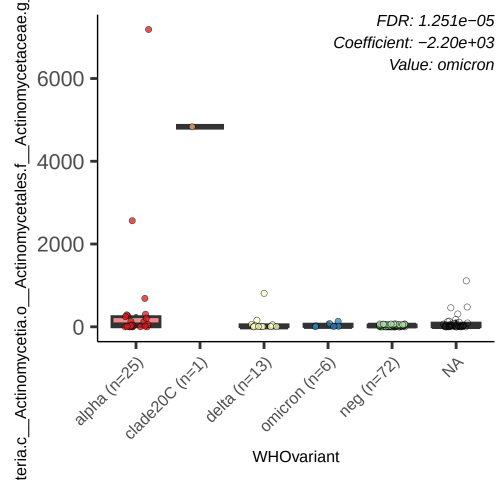

FDR: 1.251e−05
Coefficient: −2.20e+03
Value: omicron
teria.c__Actinomycetia.o__Actinomycetales.f__Actinomycetaceae.g_
6000
4000
2000
0
alpha (n=25)
clade20C (n=1)
delta (n=13)
omicron (n=6)
neg (n=72)
NA
WHOvariant

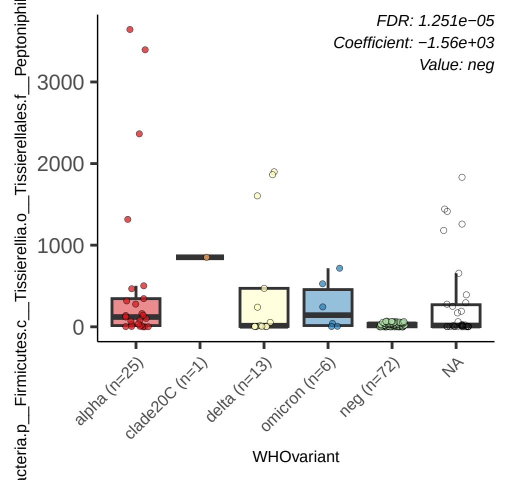
FDR: 1.251e−05
Coefficient: −1.56e+03
Value: neg
acteria.p__Firmicutes.c__Tissierellia.o__Tissierellales.f__Peptoniphil
3000
2000
1000
0
alpha (n=25)
clade20C (n=1)
delta (n=13)
omicron (n=6)
neg (n=72)
NA
WHOvariant

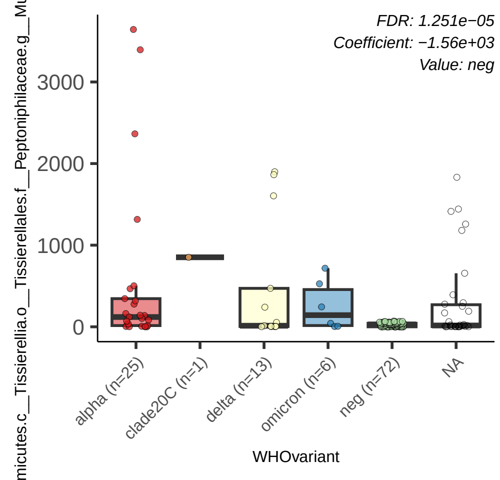

FDR: 1.251e−05
Coefficient: −1.56e+03
Value: neg
micutes.c__Tissierellia.o__Tissierellales.f__Peptoniphilaceae.g__Mu
3000
2000
1000
0
alpha (n=25)
clade20C (n=1)
delta (n=13)
omicron (n=6)
neg (n=72)
NA
WHOvariant

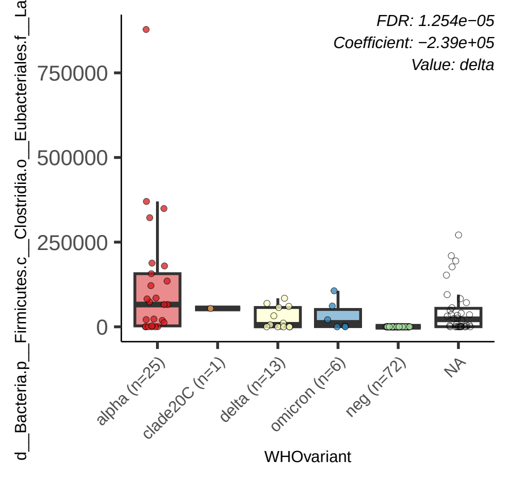
FDR: 1.254e−05
Coefficient: −2.39e+05
Value: delta
d__Bacteria.p__Firmicutes.c__Clostridia.o__Eubacteriales.f__La
750000
500000
250000
0
alpha (n=25)
clade20C (n=1)
delta (n=13)
omicron (n=6)
neg (n=72)
NA
WHOvariant

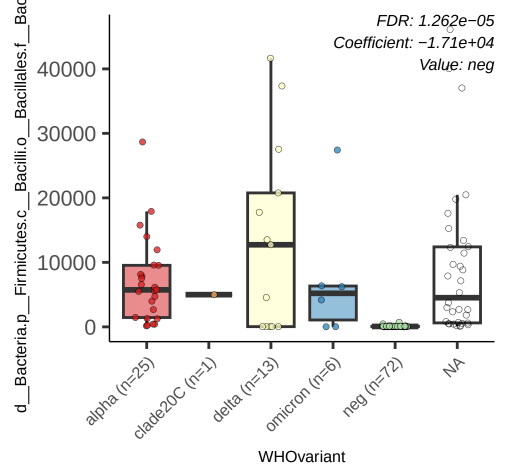

FDR: 1.262e−05
Coefficient: −1.71e+04
Value: neg
d__Bacteria.p__Firmicutes.c__Bacilli.o__Bacillales.f__Bac
40000
30000
20000
10000
0
alpha (n=25)
clade20C (n=1)
delta (n=13)
omicron (n=6)
neg (n=72)
NA
WHOvariant

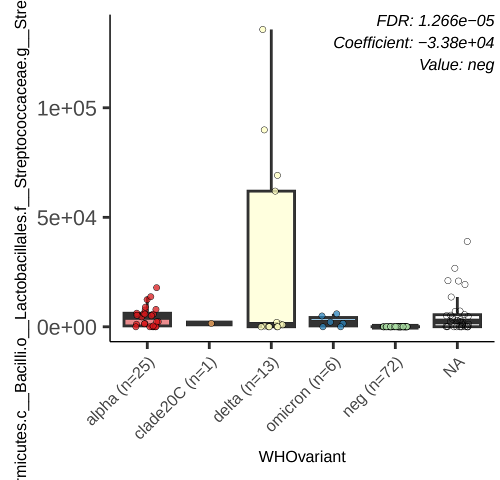

FDR: 1.266e−05
Coefficient: −3.38e+04
Value: neg
1e+05
5e+04
0e+00
alpha (n=25)
clade20C (n=1)
delta (n=13)
omicron (n=6)
neg (n=72)
NA
WHOvariant
rmicutes.c__Bacilli.o__Lactobacillales.f__Streptococcaceae.g__Stre

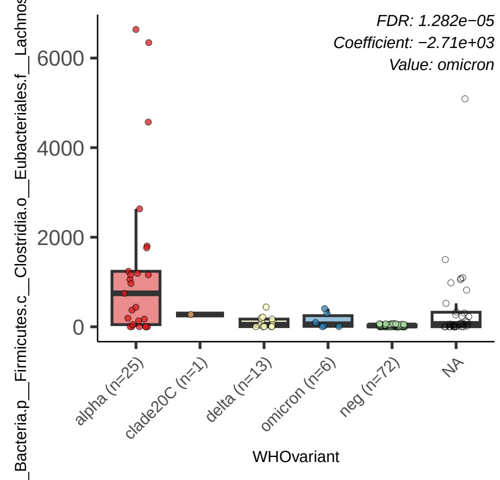

FDR: 1.282e−05
Coefficient: −2.71e+03
Value: omicron
6000
4000
2000
0
_Bacteria.p__Firmicutes.c__Clostridia.o__Eubacteriales.f__Lachnos
alpha (n=25)
clade20C (n=1)
delta (n=13)
omicron (n=6)
neg (n=72)
NA
WHOvariant

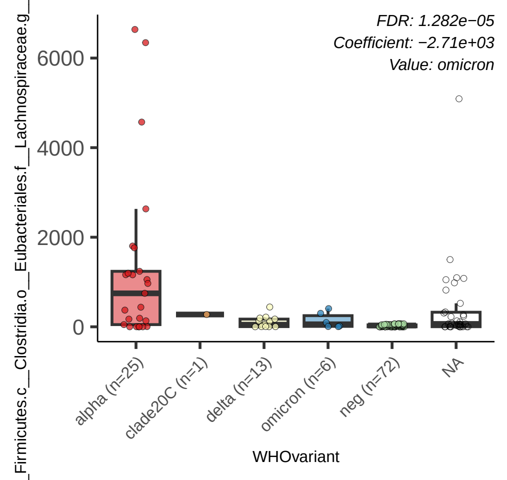

FDR: 1.282e−05
Coefficient: −2.71e+03
Value: omicron
Firmicutes.c__Clostridia.o__Eubacteriales.f__Lachnospiraceae.g__
6000
4000
2000
0
alpha (n=25)
clade20C (n=1)
delta (n=13)
omicron (n=6)
neg (n=72)
NA
WHOvariant

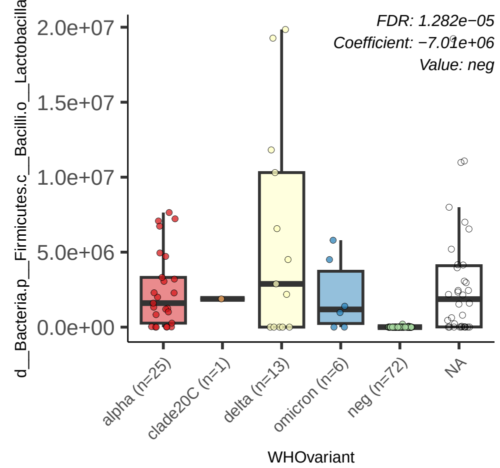
FDR: 1.282e−05
Coefficient: −7.01e+06
Value: neg
d__Bacteria.p__Firmicutes.c__Bacilli.o__Lactobacilla
2.0e+07
1.5e+07
1.0e+07
5.0e+06
0.0e+00
alpha (n=25)
clade20C (n=1)
delta (n=13)
omicron (n=6)
neg (n=72)
NA
WHOvariant

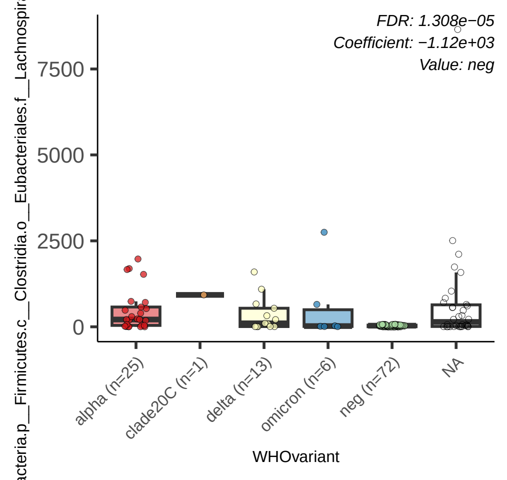

FDR: 1.308e−05
Coefficient: −1.12e+03
Value: neg
acteria.p__Firmicutes.c__Clostridia.o__Eubacteriales.f__Lachnospira
7500
5000
2500
0
alpha (n=25)
clade20C (n=1)
delta (n=13)
omicron (n=6)
neg (n=72)
NA
WHOvariant

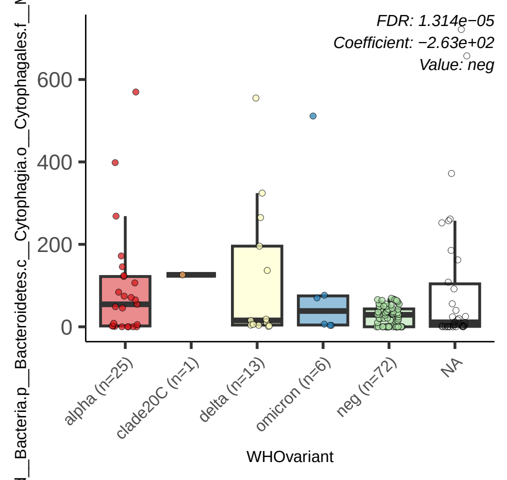

FDR: 1.314e−05
Coefficient: −2.63e+02
Value: neg
d__Bacteria.p__Bacteroidetes.c__Cytophagia.o__Cytophagales.f__M
0
200
400
600
alpha (n=25)
clade20C (n=1)
delta (n=13)
omicron (n=6)
neg (n=72)
NA
WHOvariant

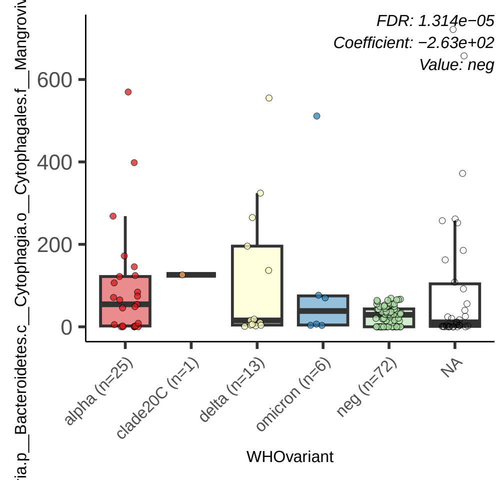

FDR: 1.314e−05
Coefficient: −2.63e+02
Value: neg
ria.p__Bacteroidetes.c__Cytophagia.o__Cytophagales.f__Mangrovi
600
400
200
0
alpha (n=25)
clade20C (n=1)
delta (n=13)
omicron (n=6)
neg (n=72)
NA
WHOvariant

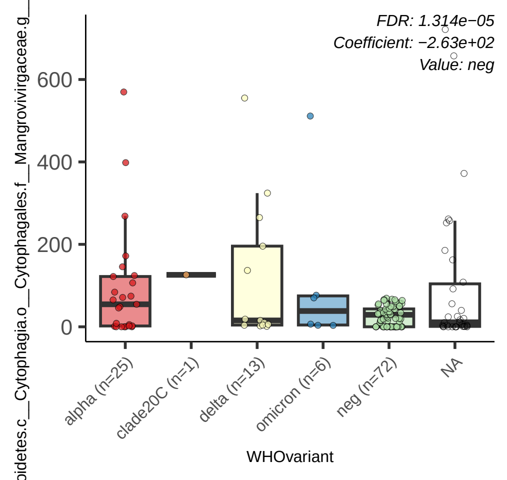

FDR: 1.314e−05
Coefficient: −2.63e+02
Value: neg
oidetes.c__Cytophagia.o__Cytophagales.f__Mangrovivirgaceae.g__
0
200
400
600
alpha (n=25)
clade20C (n=1)
delta (n=13)
omicron (n=6)
neg (n=72)
NA
WHOvariant

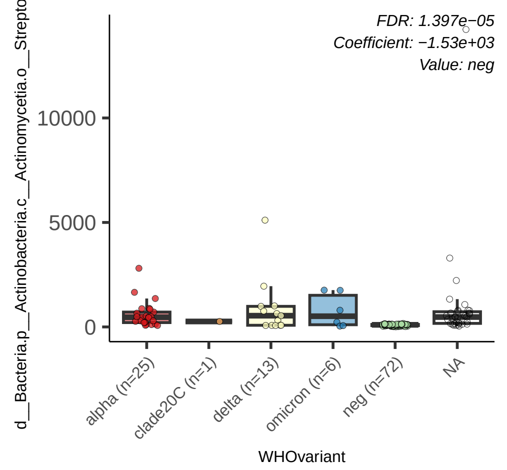

d__Bacteria.p__Actinobacteria.c__Actinomycetia.o__Strepto
FDR: 1.397e−05
Coefficient: −1.53e+03
Value: neg
10000
5000
0
alpha (n=25)
clade20C (n=1)
delta (n=13)
omicron (n=6)
neg (n=72)
NA
WHOvariant

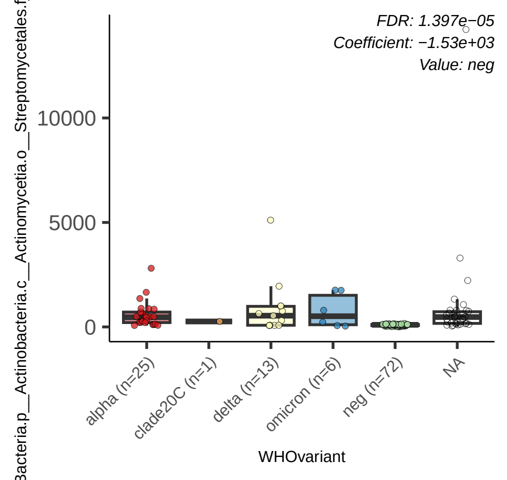

FDR: 1.397e−05
Coefficient: −1.53e+03
Value: neg
10000
5000
0
Bacteria.p__Actinobacteria.c__Actinomycetia.o__Streptomycetales.f
alpha (n=25)
clade20C (n=1)
delta (n=13)
omicron (n=6)
neg (n=72)
NA
WHOvariant

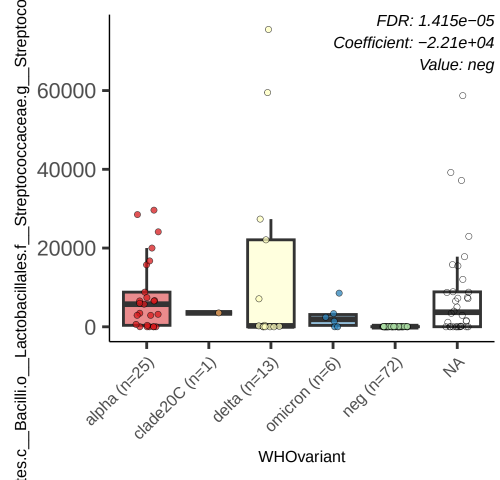
FDR: 1.415e−05
Coefficient: −2.21e+04
Value: neg
60000
40000
20000
0
alpha (n=25)
clade20C (n=1)
delta (n=13)
omicron (n=6)
neg (n=72)
NA
WHOvariant
tes.c__Bacilli.o__Lactobacillales.f__Streptococcaceae.g__Streptoco

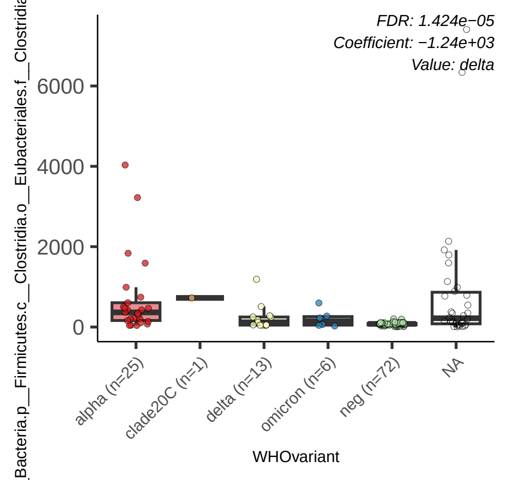

FDR: 1.424e−05
Coefficient: −1.24e+03
Value: delta
Bacteria.p__Firmicutes.c__Clostridia.o__Eubacteriales.f__Clostridia
6000
4000
2000
0
alpha (n=25)
clade20C (n=1)
delta (n=13)
omicron (n=6)
neg (n=72)
NA
WHOvariant

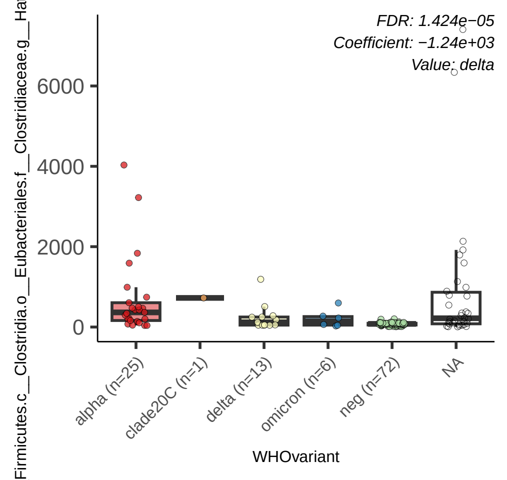

FDR: 1.424e−05
Coefficient: −1.24e+03
Value: delta
6000
4000
2000
0
Firmicutes.c__Clostridia.o__Eubacteriales.f__Clostridiaceae.g__Ha
alpha (n=25)
clade20C (n=1)
delta (n=13)
omicron (n=6)
neg (n=72)
NA
WHOvariant

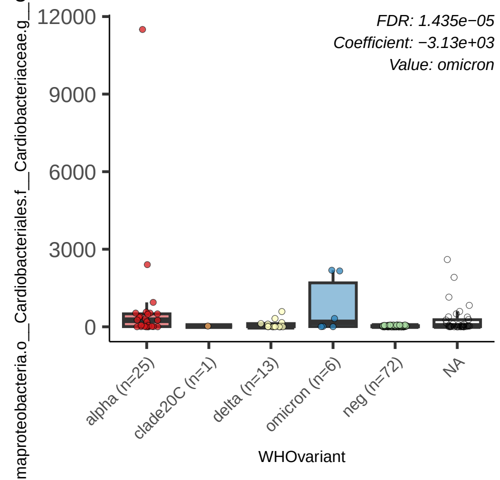
FDR: 1.435e−05
Coefficient: −3.13e+03
Value: omicron
12000
9000
6000
3000
0
maproteobacteria.o__Cardiobacteriales.f__Cardiobacteriaceae.g__
alpha (n=25)
clade20C (n=1)
delta (n=13)
omicron (n=6)
neg (n=72)
NA
WHOvariant

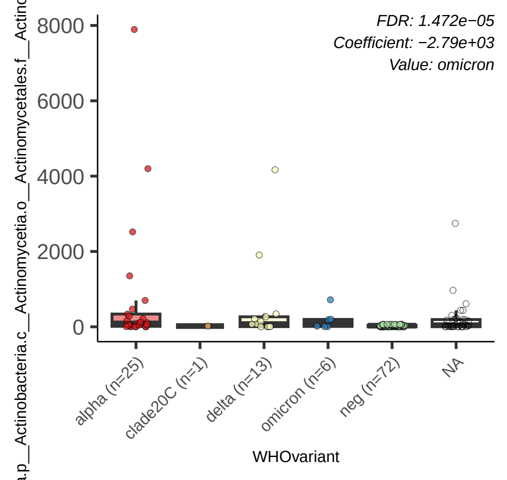
FDR: 1.472e−05
Coefficient: −2.79e+03
Value: omicron
8000
6000
4000
2000
0
alpha (n=25)
clade20C (n=1)
delta (n=13)
omicron (n=6)
neg (n=72)
NA
WHOvariant
a.p__Actinobacteria.c__Actinomycetia.o__Actinomycetales.f__Actino

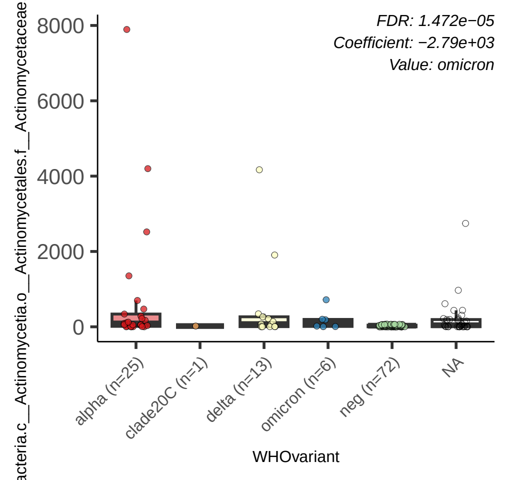

FDR: 1.472e−05
Coefficient: −2.79e+03
Value: omicron
acteria.c__Actinomycetia.o__Actinomycetales.f__Actinomycetaceae
8000
6000
4000
2000
0
alpha (n=25)
clade20C (n=1)
delta (n=13)
omicron (n=6)
neg (n=72)
NA
WHOvariant

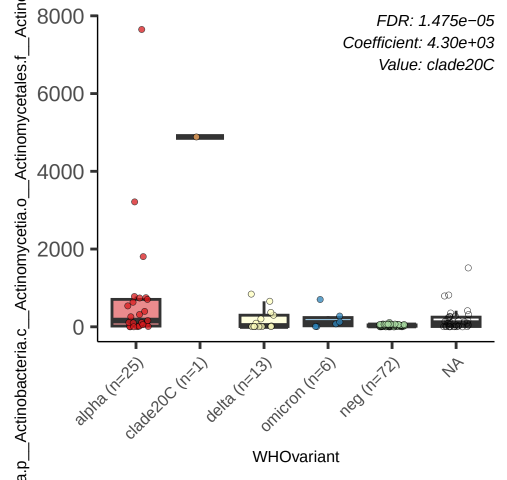

FDR: 1.475e−05
Coefficient: 4.30e+03
Value: clade20C
a.p__Actinobacteria.c__Actinomycetia.o__Actinomycetales.f__Actino
8000
6000
4000
2000
0
alpha (n=25)
clade20C (n=1)
delta (n=13)
omicron (n=6)
neg (n=72)
NA
WHOvariant

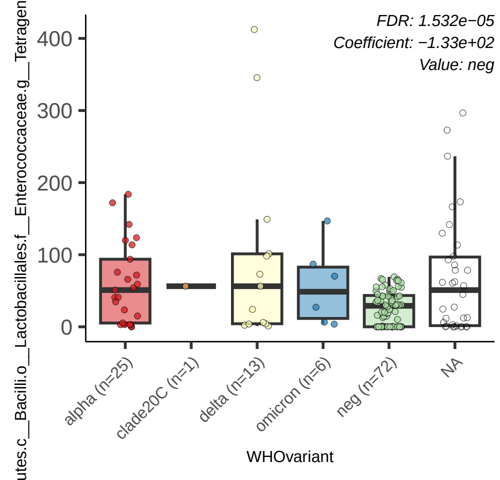

FDR: 1.532e−05
Coefficient: −1.33e+02
Value: neg
utes.c__Bacilli.o__Lactobacillales.f__Enterococcaceae.g__Tetragen
400
300
200
100
0
alpha (n=25)
clade20C (n=1)
delta (n=13)
omicron (n=6)
neg (n=72)
NA
WHOvariant

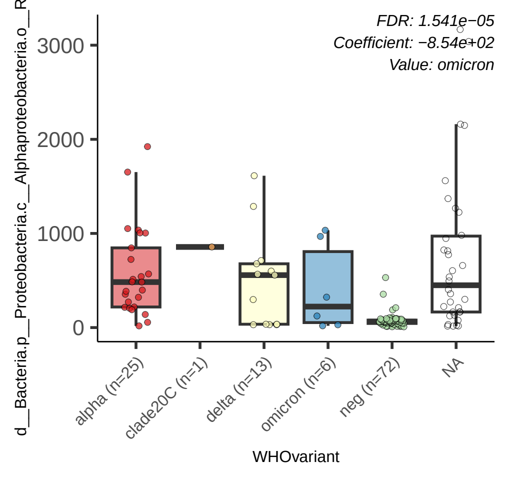

FDR: 1.541e−05
Coefficient: −8.54e+02
Value: omicron
d__Bacteria.p__Proteobacteria.c__Alphaproteobacteria.o__R
3000
2000
1000
0
alpha (n=25)
clade20C (n=1)
delta (n=13)
omicron (n=6)
neg (n=72)
NA
WHOvariant

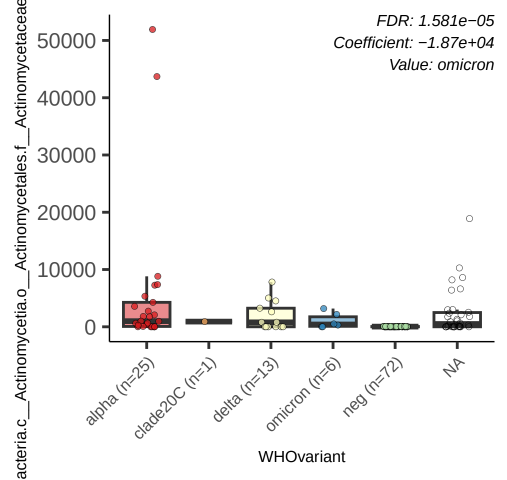

FDR: 1.581e−05
Coefficient: −1.87e+04
Value: omicron
acteria.c__Actinomycetia.o__Actinomycetales.f__Actinomycetaceae
50000
40000
30000
20000
10000
0
alpha (n=25)
clade20C (n=1)
delta (n=13)
omicron (n=6)
neg (n=72)
NA
WHOvariant

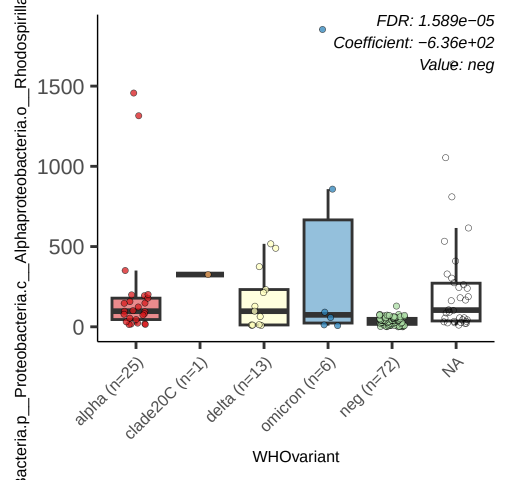
FDR: 1.589e−05
Coefficient: −6.36e+02
Value: neg
Bacteria.p__Proteobacteria.c__Alphaproteobacteria.o__Rhodospirilla
1500
1000
500
0
alpha (n=25)
clade20C (n=1)
delta (n=13)
omicron (n=6)
neg (n=72)
NA
WHOvariant

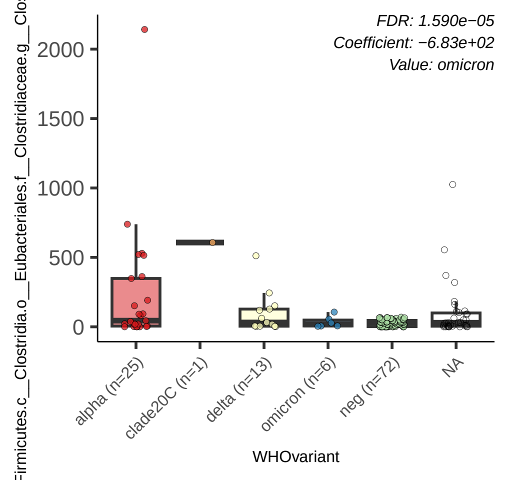

FDR: 1.590e−05
Coefficient: −6.83e+02
Value: omicron
Firmicutes.c__Clostridia.o__Eubacteriales.f__Clostridiaceae.g__Clo
0
500
1000
1500
2000
alpha (n=25)
clade20C (n=1)
delta (n=13)
omicron (n=6)
neg (n=72)
NA
WHOvariant

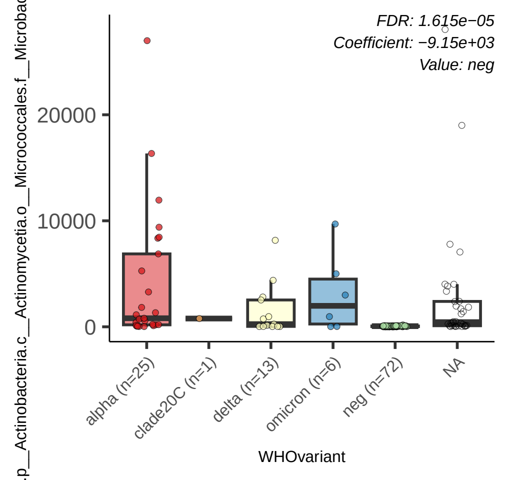
FDR: 1.615e−05
Coefficient: −9.15e+03
Value: neg
p__Actinobacteria.c__Actinomycetia.o__Micrococcales.f__Microbac
20000
10000
0
alpha (n=25)
clade20C (n=1)
delta (n=13)
omicron (n=6)
neg (n=72)
NA
WHOvariant

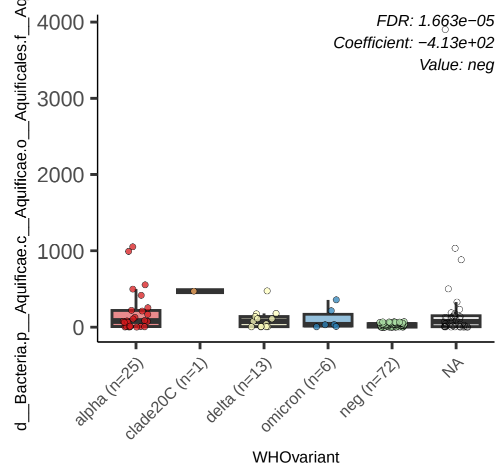

FDR: 1.663e−05
Coefficient: −4.13e+02
Value: neg
d__Bacteria.p__Aquificae.c__Aquificae.o__Aquificales.f__Aq
4000
3000
2000
1000
0
alpha (n=25)
clade20C (n=1)
delta (n=13)
omicron (n=6)
neg (n=72)
NA
WHOvariant

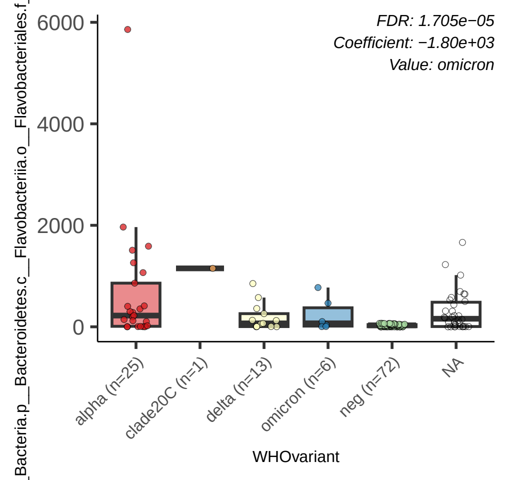

FDR: 1.705e−05
Coefficient: −1.80e+03
Value: omicron
Bacteria.p__Bacteroidetes.c__Flavobacteriia.o__Flavobacteriales.f_
6000
4000
2000
0
alpha (n=25)
clade20C (n=1)
delta (n=13)
omicron (n=6)
neg (n=72)
NA
WHOvariant

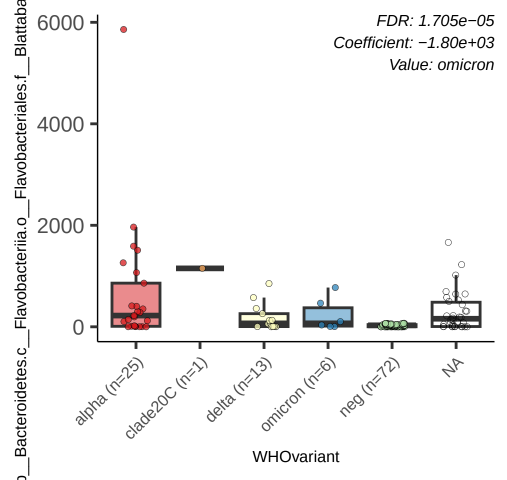
FDR: 1.705e−05
Coefficient: −1.80e+03
Value: omicron
6000
4000
2000
0
o__Bacteroidetes.c__Flavobacteriia.o__Flavobacteriales.f__Blattaba
alpha (n=25)
clade20C (n=1)
delta (n=13)
omicron (n=6)
neg (n=72)
NA
WHOvariant

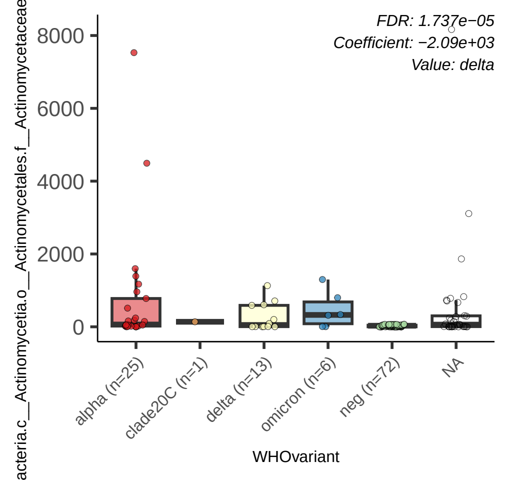
FDR: 1.737e−05
Coefficient: −2.09e+03
Value: delta
acteria.c__Actinomycetia.o__Actinomycetales.f__Actinomycetaceae
8000
6000
4000
2000
0
alpha (n=25)
clade20C (n=1)
delta (n=13)
omicron (n=6)
neg (n=72)
NA
WHOvariant

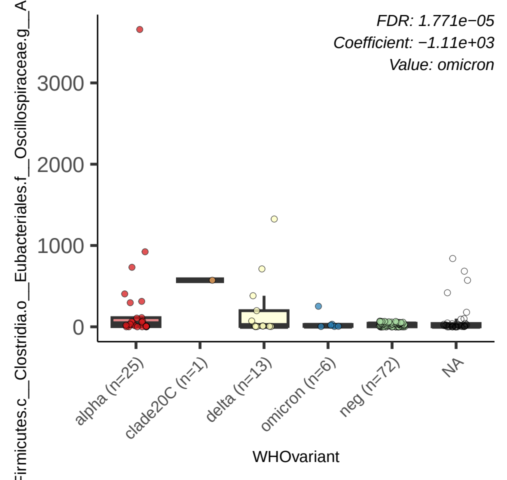

FDR: 1.771e−05
Coefficient: −1.11e+03
Value: omicron
Firmicutes.c__Clostridia.o__Eubacteriales.f__Oscillospiraceae.g__A
3000
2000
1000
0
alpha (n=25)
clade20C (n=1)
delta (n=13)
omicron (n=6)
neg (n=72)
NA
WHOvariant

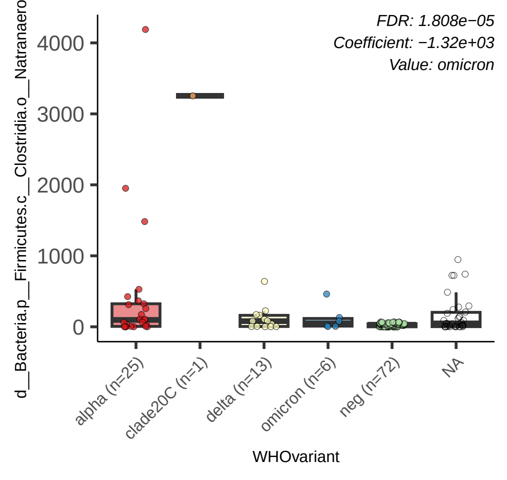

FDR: 1.808e−05
Coefficient: −1.32e+03
Value: omicron
d__Bacteria.p__Firmicutes.c__Clostridia.o__Natranaero
4000
3000
2000
1000
0
alpha (n=25)
clade20C (n=1)
delta (n=13)
omicron (n=6)
neg (n=72)
NA
WHOvariant

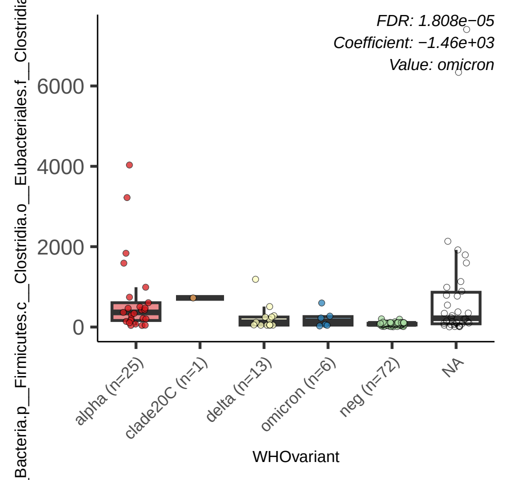
FDR: 1.808e−05
Coefficient: −1.46e+03
Value: omicron
Bacteria.p__Firmicutes.c__Clostridia.o__Eubacteriales.f__Clostridia
6000
4000
2000
0
alpha (n=25)
clade20C (n=1)
delta (n=13)
omicron (n=6)
neg (n=72)
NA
WHOvariant

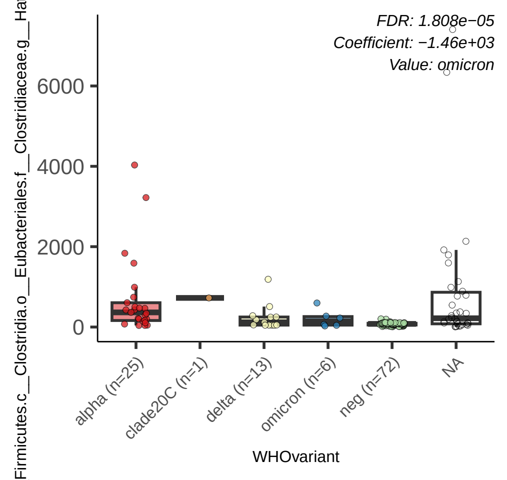

FDR: 1.808e−05
Coefficient: −1.46e+03
Value: omicron
Firmicutes.c__Clostridia.o__Eubacteriales.f__Clostridiaceae.g__Ha
6000
4000
2000
0
alpha (n=25)
clade20C (n=1)
delta (n=13)
omicron (n=6)
neg (n=72)
NA
WHOvariant

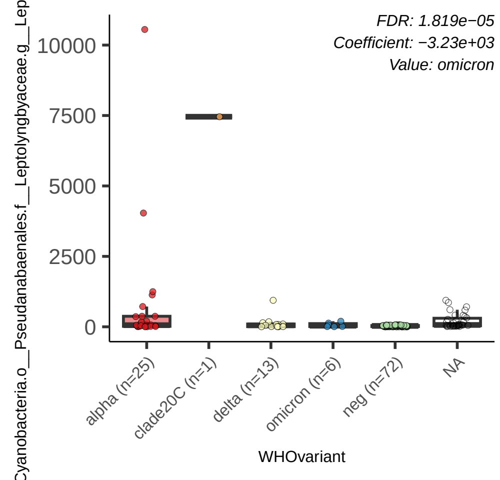

FDR: 1.819e−05
Coefficient: −3.23e+03
Value: omicron
10000
7500
5000
2500
0
Cyanobacteria.o__Pseudanabaenales.f__Leptolyngbyaceae.g__Lep
alpha (n=25)
clade20C (n=1)
delta (n=13)
omicron (n=6)
neg (n=72)
NA
WHOvariant

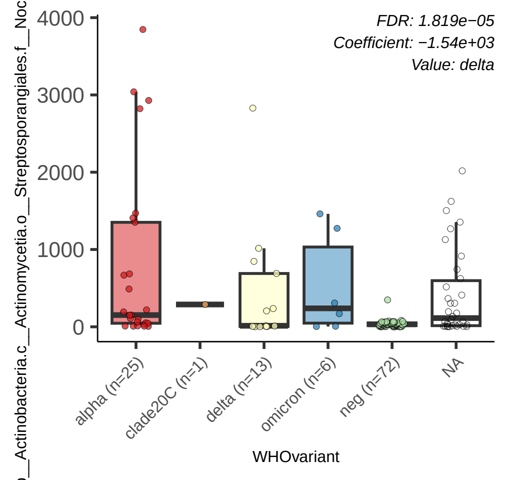

FDR: 1.819e−05
Coefficient: −1.54e+03
Value: delta
Actinobacteria.c__Actinomycetia.o__Streptosporangiales.f__Noc
4000
3000
2000
1000
0
alpha (n=25)
clade20C (n=1)
delta (n=13)
omicron (n=6)
neg (n=72)
NA
WHOvariant

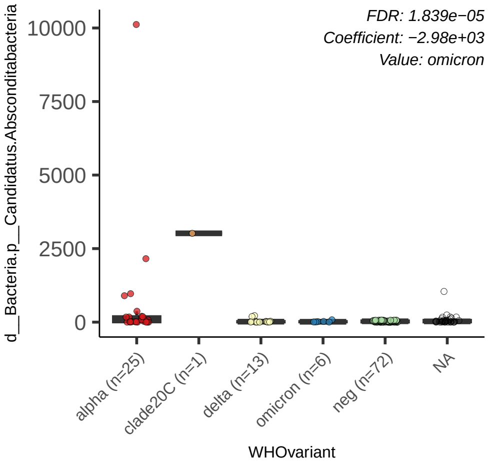

FDR: 1.839e−05
Coefficient: −2.98e+03
Value: omicron
d__Bacteria.p__Candidatus.Abscondita bacteria
10000
7500
5000
2500
0
alpha (n=25)
clade20C (n=1)
delta (n=13)
omicron (n=6)
neg (n=72)
NA
WHOvariant

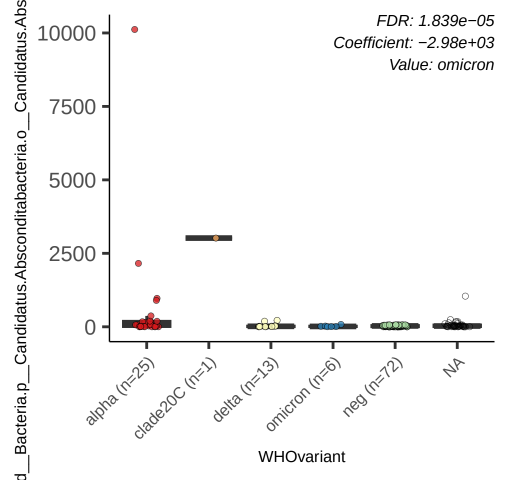
FDR: 1.839e−05
Coefficient: −2.98e+03
Value: omicron
d__Bacteria.p__Candidatus.Abscondita bacteria.o__Candidatus.Abs
10000
7500
5000
2500
0
alpha (n=25)
clade20C (n=1)
delta (n=13)
omicron (n=6)
neg (n=72)
NA
WHOvariant

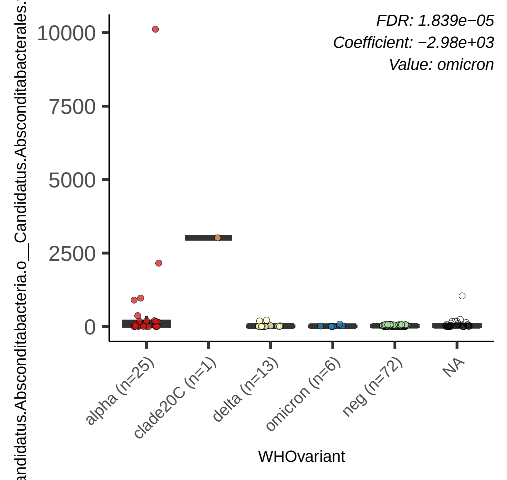

FDR: 1.839e−05
Coefficient: −2.98e+03
Value: omicron
andidatus.Absconditabacteria.o__Candidatus.Absconditabacterales.
10000
7500
5000
2500
0
alpha (n=25)
clade20C (n=1)
delta (n=13)
omicron (n=6)
neg (n=72)
NA
WHOvariant

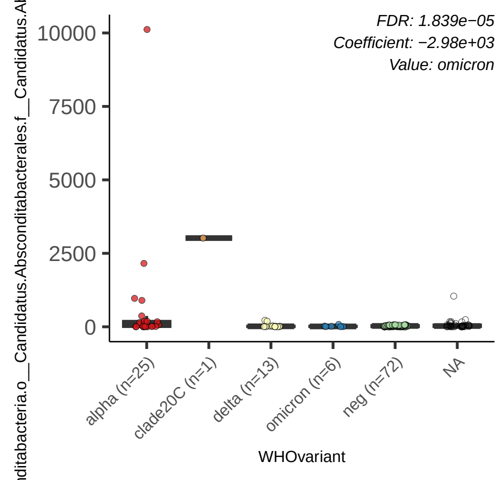
FDR: 1.839e−05
Coefficient: −2.98e+03
Value: omicron
10000
7500
5000
2500
0
alpha (n=25)
clade20C (n=1)
delta (n=13)
omicron (n=6)
neg (n=72)
NA
WHOvariant
nditabacteria.o__Candidatus.Absconditabacterales.f__Candidatus.Ab

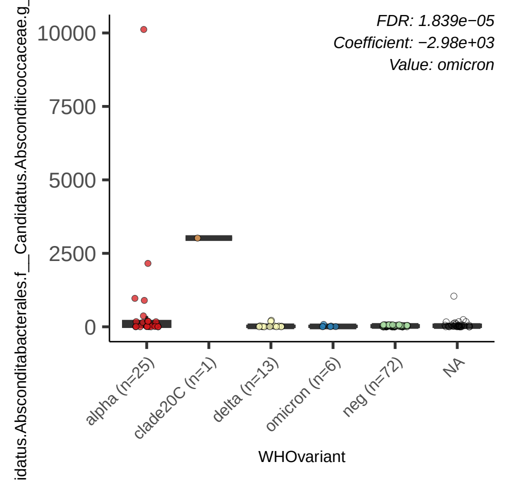

FDR: 1.839e−05
Coefficient: −2.98e+03
Value: omicron
idatus.Abscönditabacterales.f__Candidatus.Absconditicoccaceae.g_
10000
7500
5000
2500
0
alpha (n=25)
clade20C (n=1)
delta (n=13)
omicron (n=6)
neg (n=72)
NA
WHOvariant

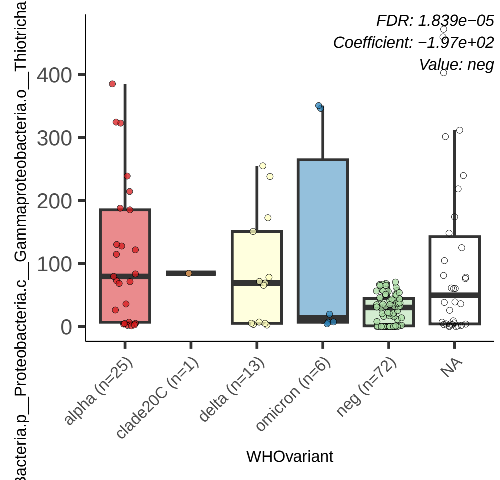

FDR: 1.839e−05
Coefficient: −1.97e+02
Value: neg
Bacteria.p__Proteobacteria.c__Gammaproteobacteria.o__Thiotrichal
0
100
200
300
400
alpha (n=25)
clade20C (n=1)
delta (n=13)
omicron (n=6)
neg (n=72)
NA
WHOvariant

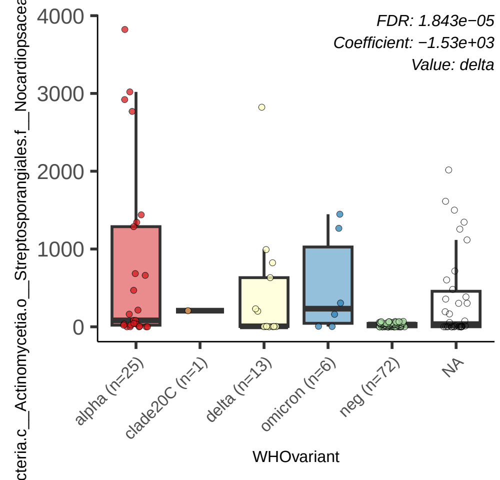

FDR: 1.843e−05
Coefficient: −1.53e+03
Value: delta
cteria.c__Actinomycetia.o__Streptosporangiales.f__Nocardiopsacea
4000
3000
2000
1000
0
alpha (n=25)
clade20C (n=1)
delta (n=13)
omicron (n=6)
neg (n=72)
NA
WHOvariant

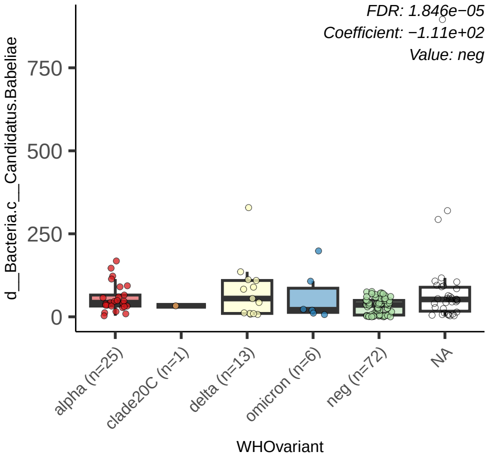
FDR: 1.846e−05
Coefficient: −1.11e+02
Value: neg
d__Bacteria.c__Candidatus.Babeliae
750
500
250
0
alpha (n=25)
clade20C (n=1)
delta (n=13)
omicron (n=6)
neg (n=72)
NA
WHOvariant

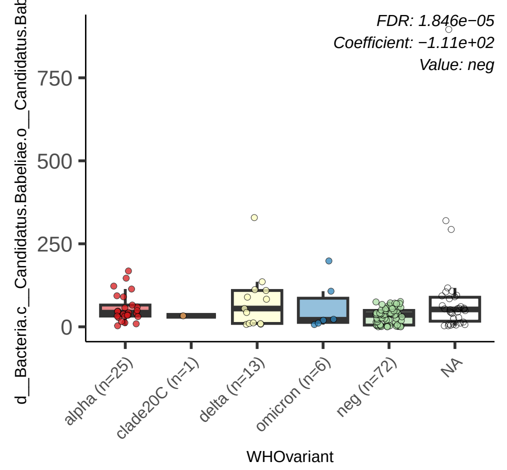

FDR: 1.846e−05
Coefficient: −1.11e+02
Value: neg
d__Bacteria.c__Candidatus.Babeliae.o__Candidatus.Bab
0
250
500
750
alpha (n=25)
clade20C (n=1)
delta (n=13)
omicron (n=6)
neg (n=72)
NA
WHOvariant

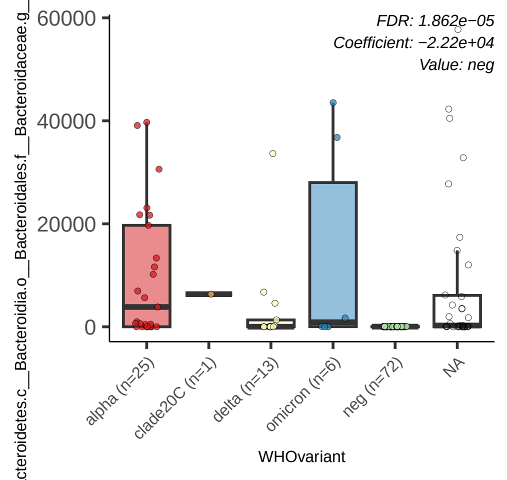

FDR: 1.862e−05
Coefficient: −2.22e+04
Value: neg
60000
40000
20000
0
cteroidetes.c__Bacteroidia.o__Bacteroidales.f__Bacteroidaceae.g_
alpha (n=25)
clade20C (n=1)
delta (n=13)
omicron (n=6)
neg (n=72)
NA
WHOvariant

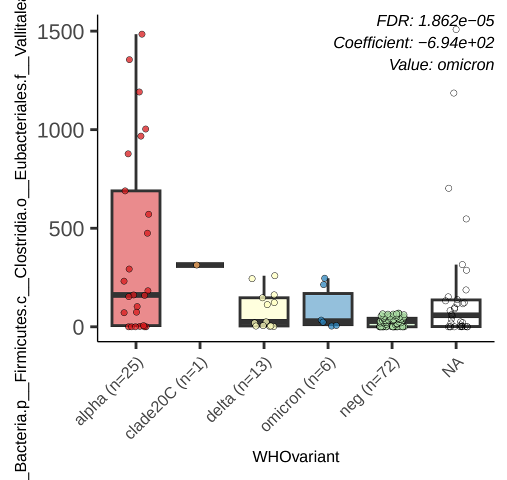
FDR: 1.862e−05
Coefficient: −6.94e+02
Value: omicron
1500
1000
500
0
alpha (n=25)
clade20C (n=1)
delta (n=13)
omicron (n=6)
neg (n=72)
NA
WHOvariant
Bacteria.p__Firmicutes.c__Clostridia.o__Eubacteriales.f__Vallitale

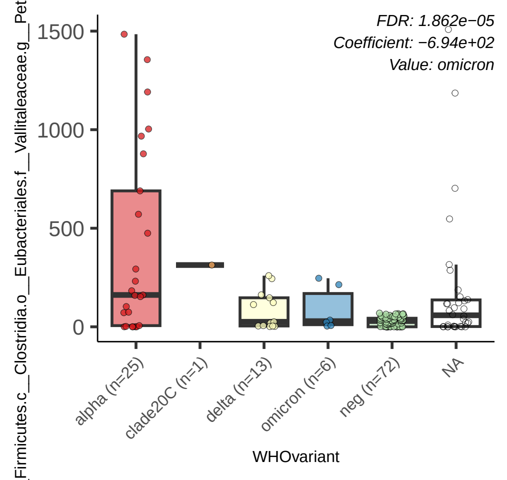
FDR: 1.862e−05
Coefficient: −6.94e+02
Value: omicron
Firmicutes.c__Clostridia.o__Eubacteriales.f__Vallitaleaceae.g__Pet
1500
1000
500
0
alpha (n=25)
clade20C (n=1)
delta (n=13)
omicron (n=6)
neg (n=72)
NA
WHOvariant

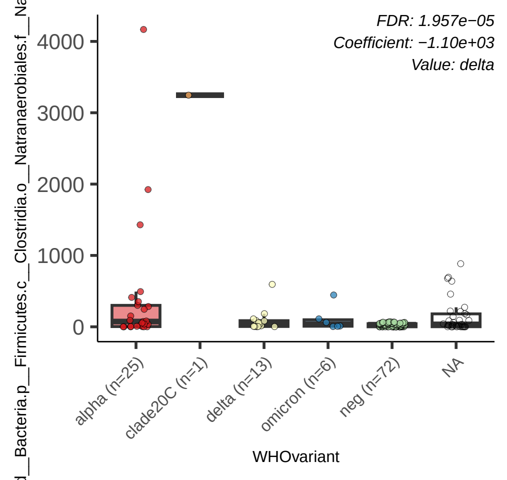

FDR: 1.957e−05
Coefficient: −1.10e+03
Value: delta
4000
3000
2000
1000
0
d__Bacteria.p__Firmicutes.c__Clostridia.o__Natranaerobiales.f__Na
alpha (n=25)
clade20C (n=1)
delta (n=13)
omicron (n=6)
neg (n=72)
NA
WHOvariant

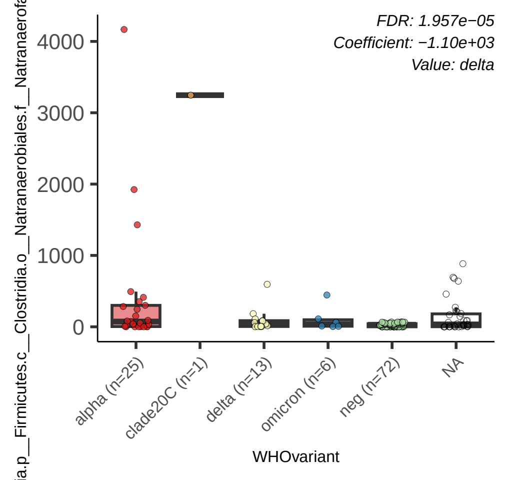

FDR: 1.957e−05
Coefficient: −1.10e+03
Value: delta
4000
3000
2000
1000
0
ia.p__Firmicutes.c__Clostridia.o__Natranaerobiales.f__Natranaerofa
alpha (n=25)
clade20C (n=1)
delta (n=13)
omicron (n=6)
neg (n=72)
NA
WHOvariant

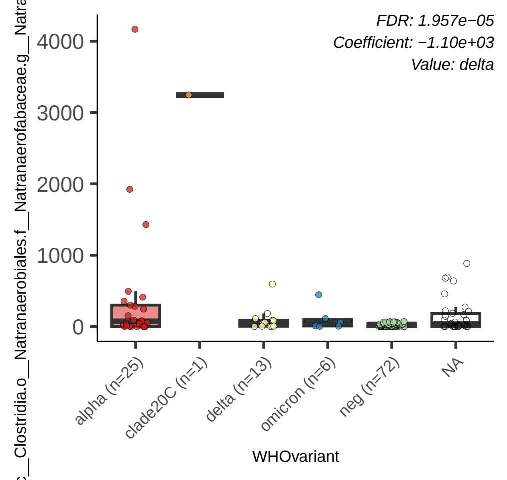

FDR: 1.957e−05
Coefficient: −1.10e+03
Value: delta
4000
3000
2000
1000
0
c__Clostridia.o__Natranaerobiales.f__Natranaerofabaceae.g__Natra
alpha (n=25)
clade20C (n=1)
delta (n=13)
omicron (n=6)
neg (n=72)
NA
WHOvariant

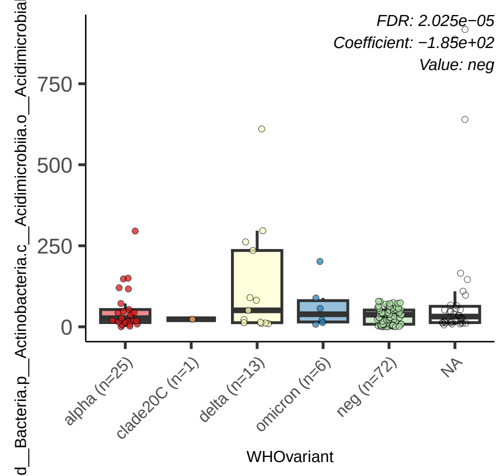
FDR: 2.025e−05
Coefficient: −1.85e+02
Value: neg
d__Bacteria.p__Actinobacteria.c__Acidimicrobiia.o__Acidimicrobial
750
500
250
0
alpha (n=25)
clade20C (n=1)
delta (n=13)
omicron (n=6)
neg (n=72)
NA
WHOvariant

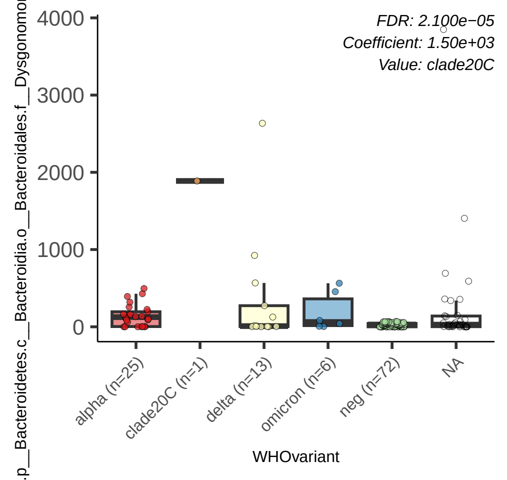

.p__Bacteroidetes.c__Bacteroidia.o__Bacteroidales.f__Dysgonomo
4000
3000
2000
1000
0
FDR: 2.100e−05
Coefficient: 1.50e+03
Value: clade20C
alpha (n=25)
clade20C (n=1)
delta (n=13)
omicron (n=6)
neg (n=72)
NA
WHOvariant

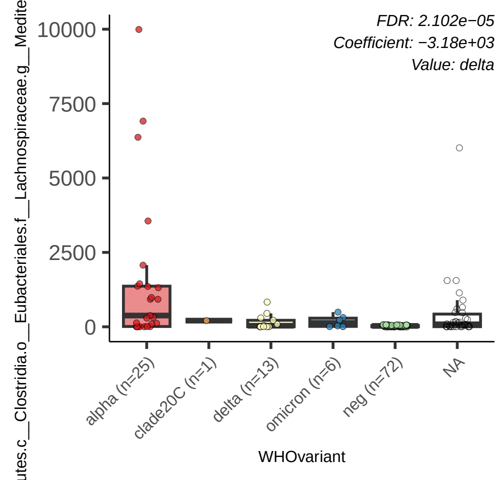

FDR: 2.102e−05
Coefficient: −3.18e+03
Value: delta
10000
7500
5000
2500
0
utes.c__Clostridia.o__Eubacteriales.f__Lachnospiraceae.g__Medite
alpha (n=25)
clade20C (n=1)
delta (n=13)
omicron (n=6)
neg (n=72)
NA
WHOvariant

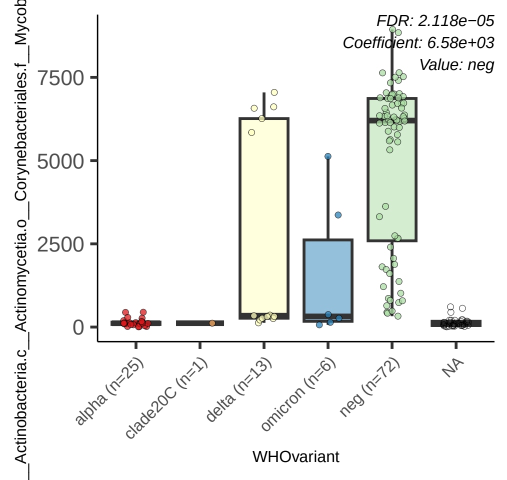

FDR: 2.118e−05
Coefficient: 6.58e+03
Value: neg
Actinobacteria.c__Actinomycetia.o__Corynebacteriales.f__Mycob
7500
5000
2500
0
alpha (n=25)
clade20C (n=1)
delta (n=13)
omicron (n=6)
neg (n=72)
NA
WHOvariant

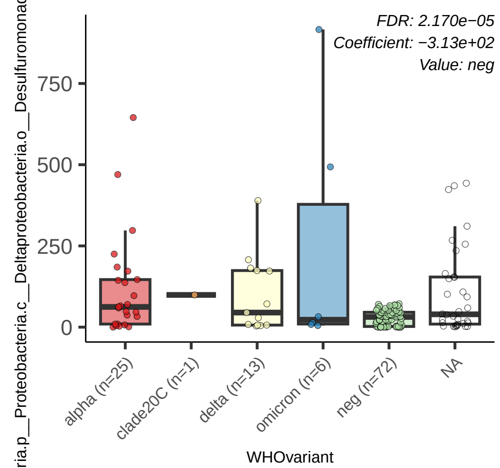

FDR: 2.170e−05
Coefficient: −3.13e+02
Value: neg
teria.p__Proteobacteria.c__Deltaproteobacteria.o__Desulfuromonad
750
500
250
0
alpha (n=25)
clade20C (n=1)
delta (n=13)
omicron (n=6)
neg (n=72)
NA
WHOvariant

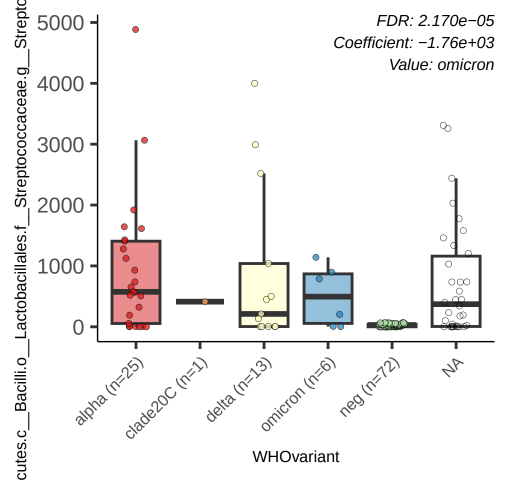

FDR: 2.170e−05
Coefficient: −1.76e+03
Value: omicron
5000
4000
3000
2000
1000
0
cutes.c__Bacilli.o__Lactobacillales.f__Streptococcaceae.g__Strepto
alpha (n=25)
clade20C (n=1)
delta (n=13)
omicron (n=6)
neg (n=72)
NA
WHOvariant

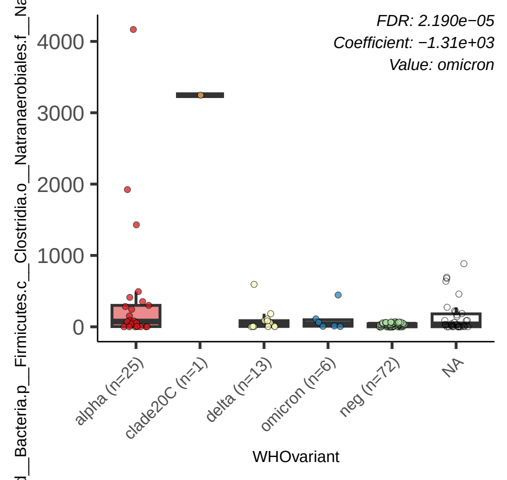

FDR: 2.190e−05
Coefficient: −1.31e+03
Value: omicron
d__Bacteria.p__Firmicutes.c__Clostridia.o__Natranaerobiales.f__Na
0
1000
2000
3000
4000
alpha (n=25)
clade20C (n=1)
delta (n=13)
omicron (n=6)
neg (n=72)
NA
WHOvariant

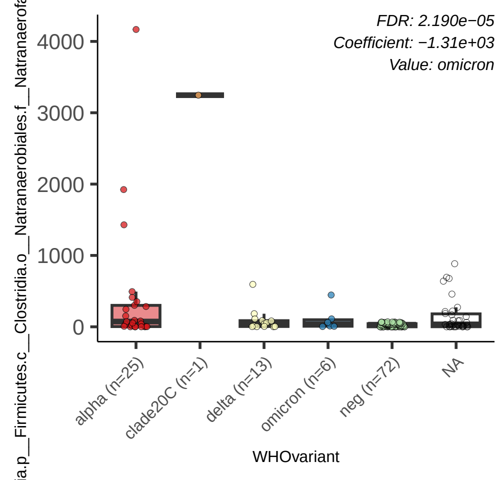

FDR: 2.190e−05
Coefficient: −1.31e+03
Value: omicron
4000
3000
2000
1000
0
alpha (n=25)
clade20C (n=1)
delta (n=13)
omicron (n=6)
neg (n=72)
NA
WHOvariant

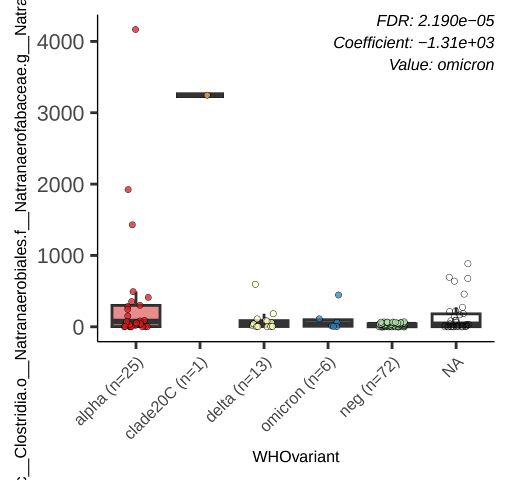
FDR: 2.190e−05
Coefficient: −1.31e+03
Value: omicron
4000
3000
2000
1000
0
c__Clostridia.o__Natranaerobiales.f__Natranaerofabaceae.g__Natra
alpha (n=25)
clade20C (n=1)
delta (n=13)
omicron (n=6)
neg (n=72)
NA
WHOvariant

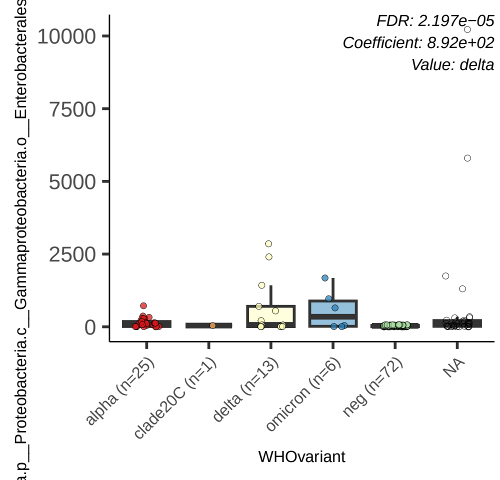

FDR: 2.197e−05
Coefficient: 8.92e+02
Value: delta
a.p__Proteobacteria.c__Gammaproteobacteria.o__Enterobacterales
10000
7500
5000
2500
0
alpha (n=25)
clade20C (n=1)
delta (n=13)
omicron (n=6)
neg (n=72)
NA
WHOvariant

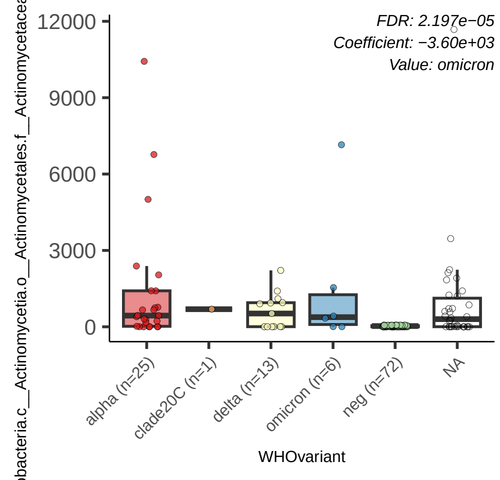
FDR: 2.197e−05
Coefficient: −3.60e+03
Value: omicron
12000
9000
6000
3000
0
obacteria.c__Actinomycetia.o__Actinomycetales.f__Actinomycetacea
alpha (n=25)
clade20C (n=1)
delta (n=13)
omicron (n=6)
neg (n=72)
NA
WHOvariant

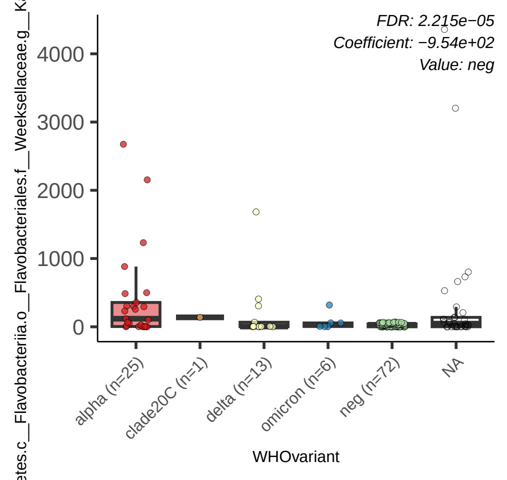

FDR: 2.215e−05
Coefficient: −9.54e+02
Value: neg
4000
3000
2000
1000
0
alpha (n=25)
clade20C (n=1)
delta (n=13)
omicron (n=6)
neg (n=72)
NA
WHOvariant
etes.c__Flavobacteriia.o__Flavobacteriales.f__Weeksellaceae.g__K

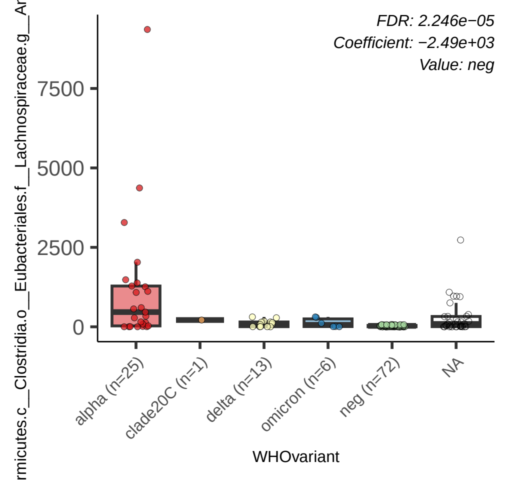

FDR: 2.246e−05
Coefficient: −2.49e+03
Value: neg
7500
5000
2500
0
rmicutes.c__Clostridia.o__Eubacteriales.f__Lachnospiraceae.g__Ar
alpha (n=25)
clade20C (n=1)
delta (n=13)
omicron (n=6)
neg (n=72)
NA
WHOvariant

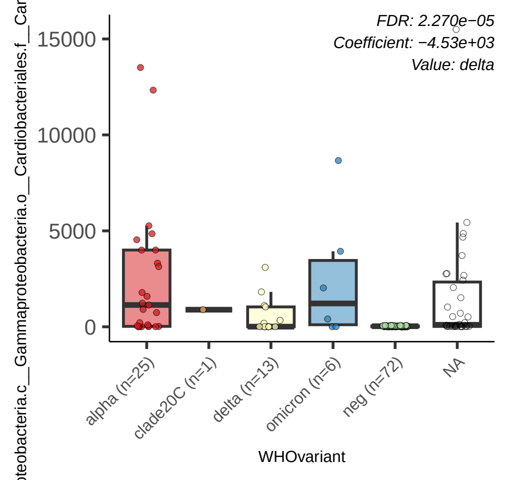

FDR: 2.270e−05
Coefficient: −4.53e+03
Value: delta
15000
10000
5000
0
alpha (n=25)
clade20C (n=1)
delta (n=13)
omicron (n=6)
neg (n=72)
NA
WHOvariant
oteobacteria.c__Gammaproteobacteria.o__Cardiobacteriales.f__Car

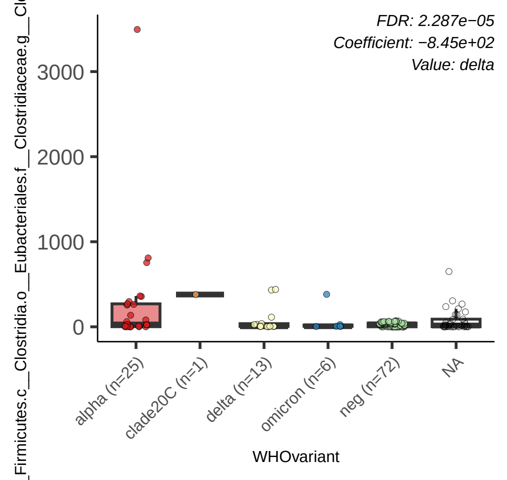

FDR: 2.287e−05
Coefficient: −8.45e+02
Value: delta
3000
2000
1000
0
Firmicutes.c__Clostridia.o__Eubacteriales.f__Clostridiaceae.g__Clo
alpha (n=25)
clade20C (n=1)
delta (n=13)
omicron (n=6)
neg (n=72)
NA
WHOvariant

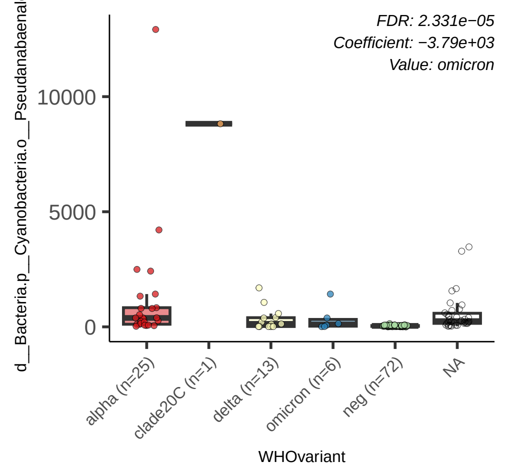
FDR: 2.331e−05
Coefficient: −3.79e+03
Value: omicron
d__Bacteria.p__Cyanobacteria.o__Pseudanabaenal
10000
5000
0
alpha (n=25)
clade20C (n=1)
delta (n=13)
omicron (n=6)
neg (n=72)
NA
WHOvariant

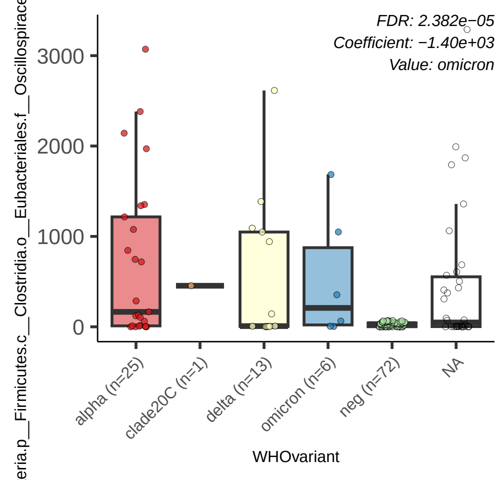

FDR: 2.382e−05
Coefficient: −1.40e+03
Value: omicron
3000
2000
1000
0
teria.p__Firmicutes.c__Clostridia.o__Eubacteriales.f__Oscillospirace
alpha (n=25)
clade20C (n=1)
delta (n=13)
omicron (n=6)
neg (n=72)
NA
WHOvariant

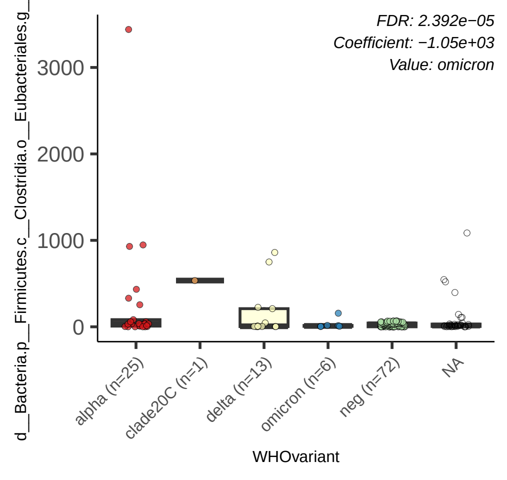
FDR: 2.392e−05
Coefficient: −1.05e+03
Value: omicron
d__Bacteria.p__Firmicutes.c__Clostridia.o__Eubacteriales.g__
3000
2000
1000
0
alpha (n=25)
clade20C (n=1)
delta (n=13)
omicron (n=6)
neg (n=72)
NA
WHOvariant

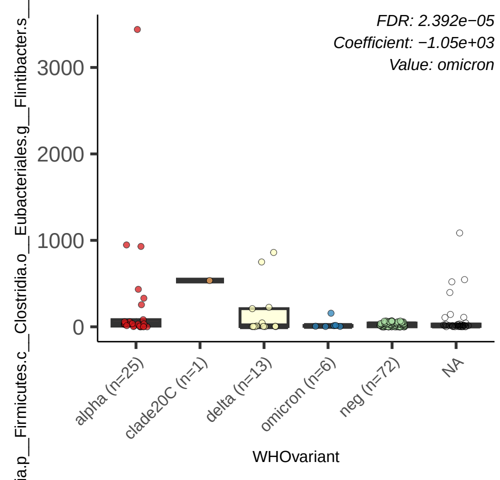
FDR: 2.392e−05
Coefficient: −1.05e+03
Value: omicron
ia.p__Firmicutes.c__Clostridia.o__Eubacteriales.g__Flintibacter.s__
3000
2000
1000
0
alpha (n=25)
clade20C (n=1)
delta (n=13)
omicron (n=6)
neg (n=72)
NA
WHOvariant

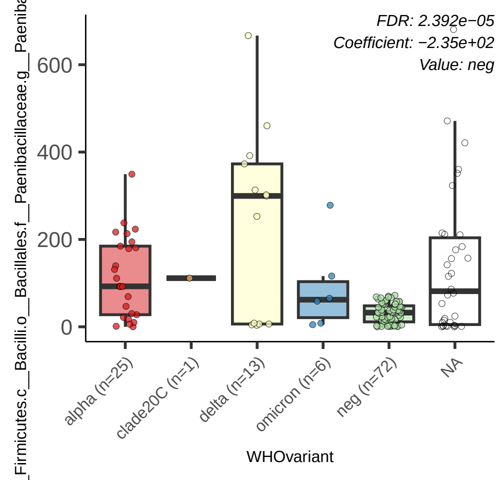

FDR: 2.392e−05
Coefficient: −2.35e+02
Value: neg
Firmicutes.c__Bacilli.o__Bacillales.f__Paenibacillaceae.g__Paeniba
0
200
400
600
alpha (n=25)
clade20C (n=1)
delta (n=13)
omicron (n=6)
neg (n=72)
NA
WHOvariant

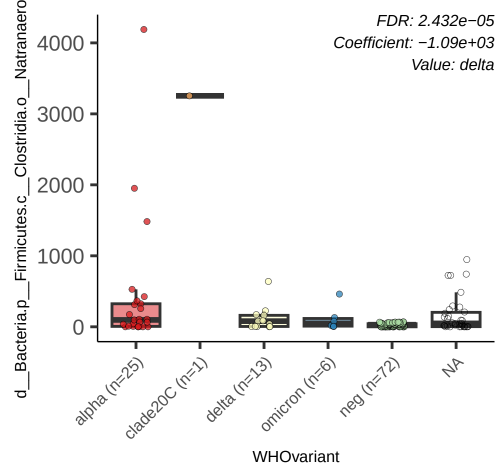

FDR: 2.432e−05
Coefficient: −1.09e+03
Value: delta
d__Bacteria.p__Firmicutes.c__Clostridia.o__Natranaero
4000
3000
2000
1000
0
alpha (n=25)
clade20C (n=1)
delta (n=13)
omicron (n=6)
neg (n=72)
NA
WHOvariant

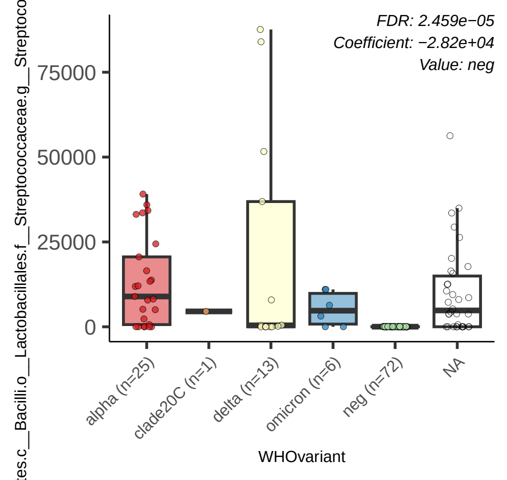
FDR: 2.459e−05
Coefficient: −2.82e+04
Value: neg
75000
50000
25000
0
tes.c__Bacilli.o__Lactobacillales.f__Streptococcaceae.g__Streptoco
alpha (n=25)
clade20C (n=1)
delta (n=13)
omicron (n=6)
neg (n=72)
NA
WHOvariant

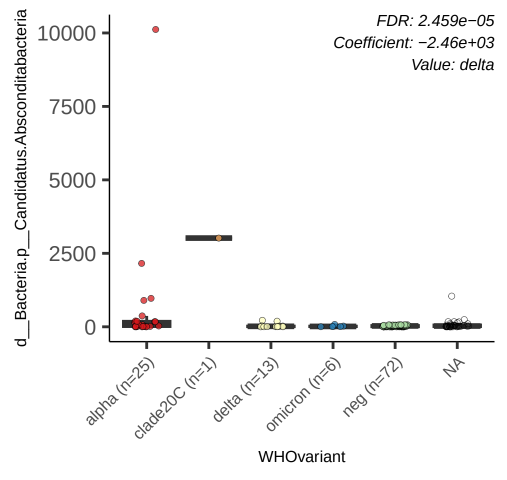

FDR: 2.459e−05
Coefficient: −2.46e+03
Value: delta
d__Bacteria.p__Candidatus.Absconditabacteria
10000
7500
5000
2500
0
alpha (n=25)
clade20C (n=1)
delta (n=13)
omicron (n=6)
neg (n=72)
NA
WHOvariant

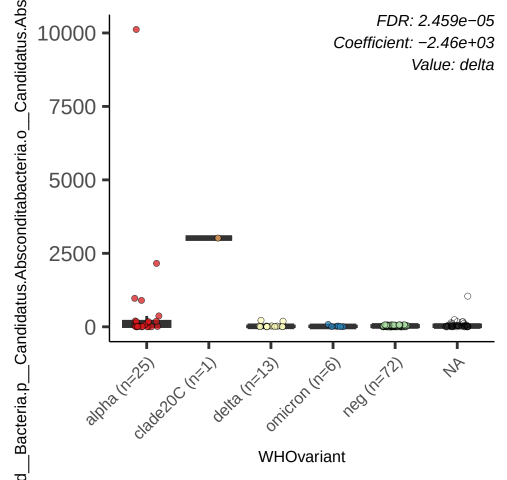

FDR: 2.459e−05
Coefficient: −2.46e+03
Value: delta
10000
7500
5000
2500
0
d__Bacteria.p__Candidatus.Absconditabacteria.o__Candidatus.Abs
alpha (n=25)
clade20C (n=1)
delta (n=13)
omicron (n=6)
neg (n=72)
NA
WHOvariant

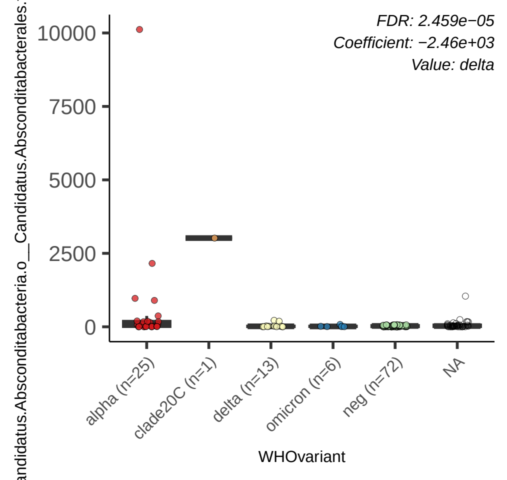

FDR: 2.459e−05
Coefficient: −2.46e+03
Value: delta
10000
7500
5000
2500
0
Candidatus.Absconditabacteria.o__Candidatus.Absconditabacterales.
alpha (n=25)
clade20C (n=1)
delta (n=13)
omicron (n=6)
neg (n=72)
NA
WHOvariant

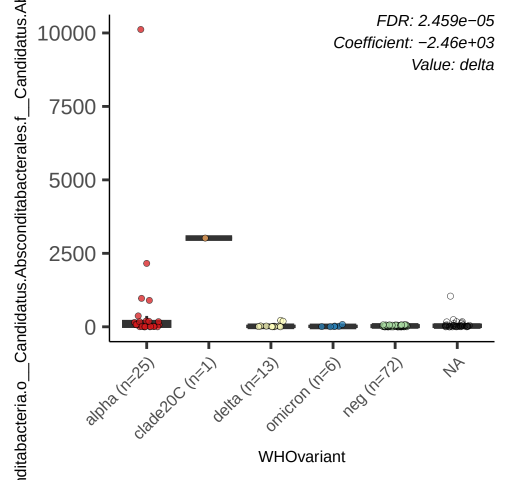
FDR: 2.459e−05
Coefficient: −2.46e+03
Value: delta
10000
7500
5000
2500
0
alpha (n=25)
clade20C (n=1)
delta (n=13)
omicron (n=6)
neg (n=72)
NA
WHOvariant

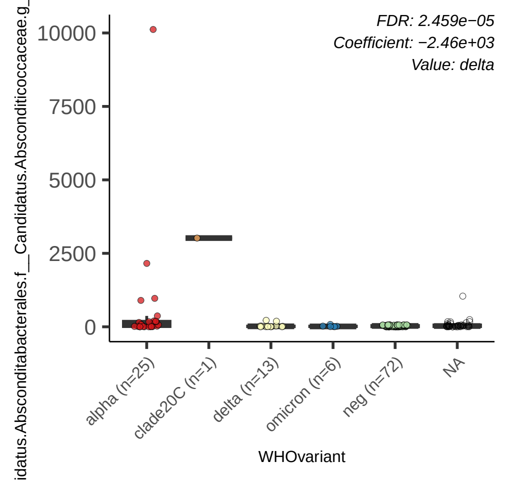

FDR: 2.459e−05
Coefficient: −2.46e+03
Value: delta
10000
7500
5000
2500
0
idatus.Absconditabacterales.f__Candidatus.Absconditicoccaceae.g_
alpha (n=25)
clade20C (n=1)
delta (n=13)
omicron (n=6)
neg (n=72)
NA
WHOvariant

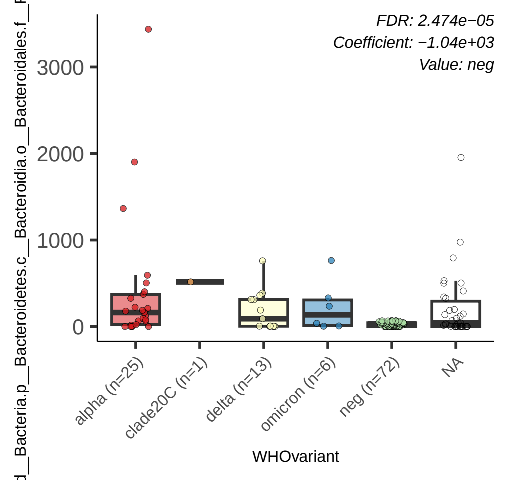

FDR: 2.474e−05
Coefficient: −1.04e+03
Value: neg
3000
2000
1000
0
alpha (n=25)
clade20C (n=1)
delta (n=13)
omicron (n=6)
neg (n=72)
NA
WHOvariant
Bacteria.p__Bacteroidetes.c__Bacteroidia.o__Bacteroidales.f__

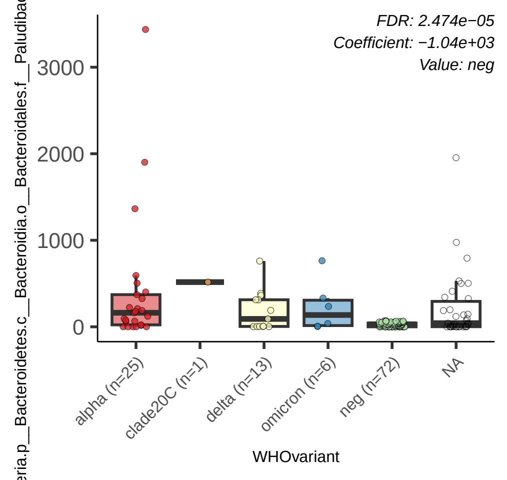

FDR: 2.474e−05
Coefficient: −1.04e+03
Value: neg
3000
2000
1000
0
alpha (n=25)
clade20C (n=1)
delta (n=13)
omicron (n=6)
neg (n=72)
NA
WHOvariant
eria.p__Bacteroidetes.c__Bacteroidia.o__Bacteroidales.f__Paludibac

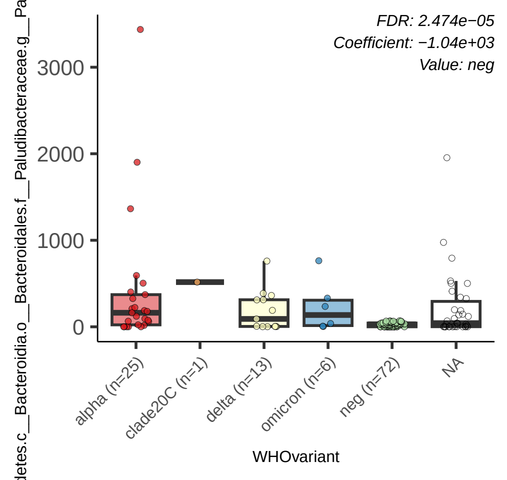

FDR: 2.474e−05
Coefficient: −1.04e+03
Value: neg
detes.c__Bacteroidia.o__Bacteroidales.f__Paludibacteraceae.g__Pa
3000
2000
1000
0
alpha (n=25)
clade20C (n=1)
delta (n=13)
omicron (n=6)
neg (n=72)
NA
WHOvariant

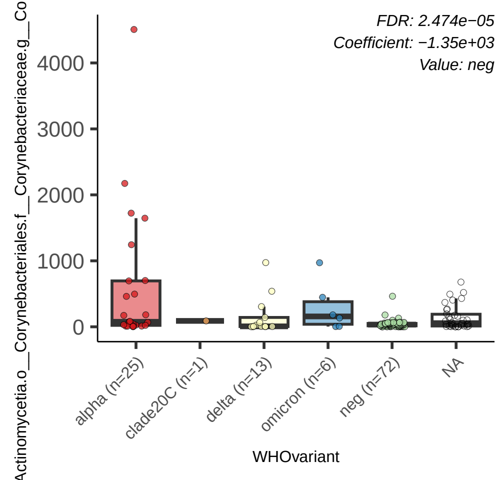
FDR: 2.474e−05
Coefficient: −1.35e+03
Value: neg
Actinomycetia.o__Corynebacteriales.f__Corynebacteriaceae.g__Co
4000
3000
2000
1000
0
alpha (n=25)
clade20C (n=1)
delta (n=13)
omicron (n=6)
neg (n=72)
NA
WHOvariant

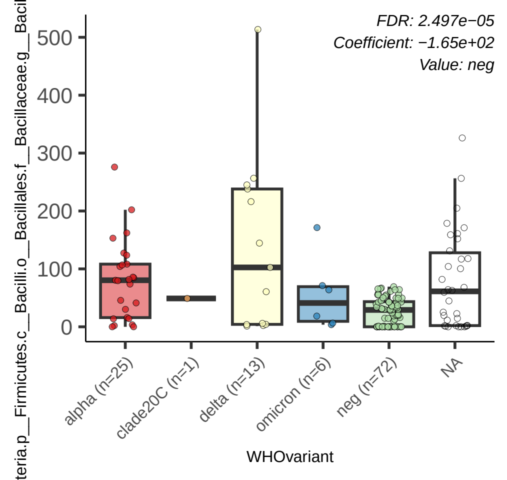

FDR: 2.497e−05
Coefficient: −1.65e+02
Value: neg
teria.p__Firmicutes.c__Bacilli.o__Bacillales.f__Bacillaceae.g__Bacil
0
100
200
300
400
500
alpha (n=25)
clade20C (n=1)
delta (n=13)
omicron (n=6)
neg (n=72)
NA
WHOvariant

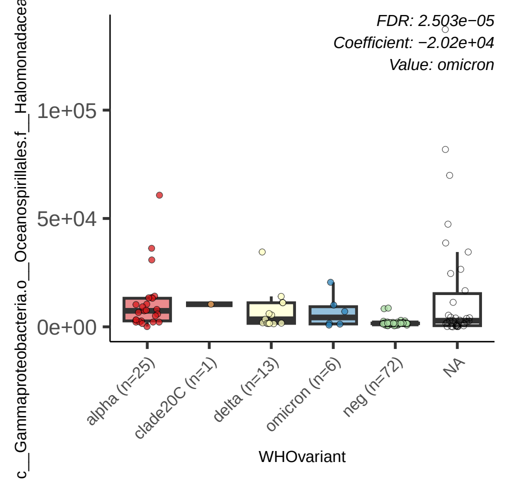
FDR: 2.503e−05
Coefficient: −2.02e+04
Value: omicron
c__Gammaproteobacteria.o__Oceanospirillales.f__Halomonadaceae
1e+05
5e+04
0e+00
alpha (n=25)
clade20C (n=1)
delta (n=13)
omicron (n=6)
neg (n=72)
NA
WHOvariant

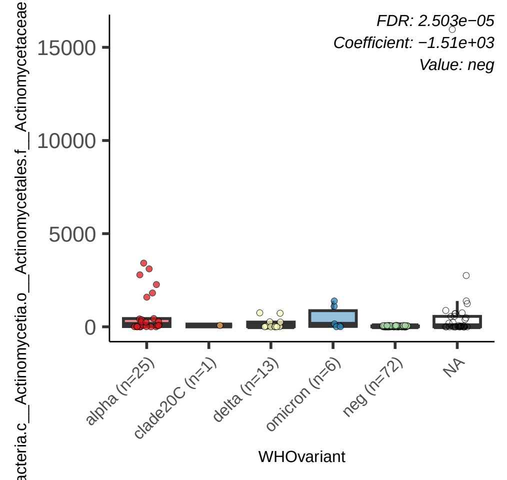

FDR: 2.503e−05
Coefficient: −1.51e+03
Value: neg
15000
10000
5000
0
acteria.c__Actinomycetia.o__Actinomycetales.f__Actinomycetaceae
alpha (n=25)
clade20C (n=1)
delta (n=13)
omicron (n=6)
neg (n=72)
NA
WHOvariant

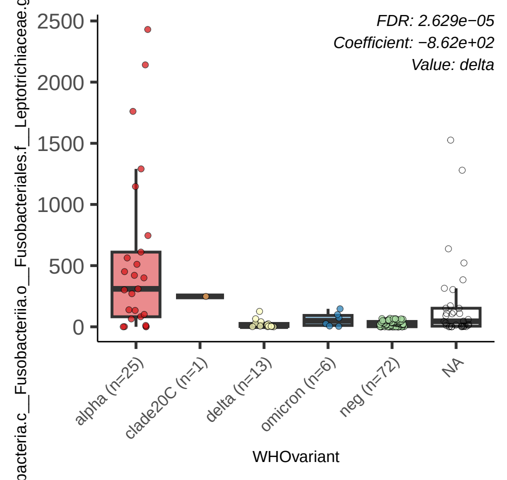
FDR: 2.629e−05
Coefficient: −8.62e+02
Value: delta
bacteria.c__Fusobacteriia.o__Fusobacteriales.f__Leptotrichiaceae.g
2500
2000
1500
1000
500
0
alpha (n=25)
clade20C (n=1)
delta (n=13)
omicron (n=6)
neg (n=72)
NA
WHOvariant

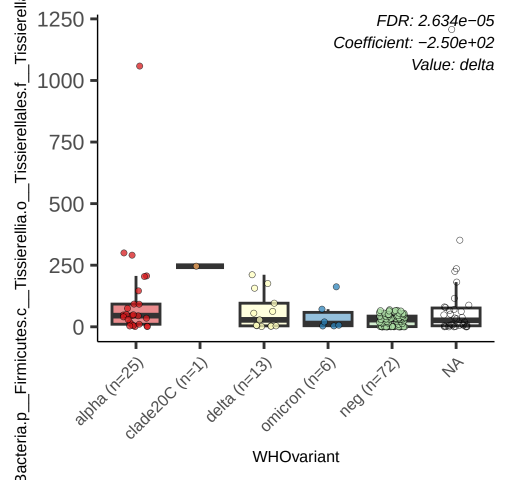

FDR: 2.634e−05
Coefficient: −2.50e+02
Value: delta
Bacteria.p__Firmicutes.c__Tissierellia.o__Tissierellales.f__Tissierella
0
250
500
750
1000
1250
alpha (n=25)
clade20C (n=1)
delta (n=13)
omicron (n=6)
neg (n=72)
NA
WHOvariant

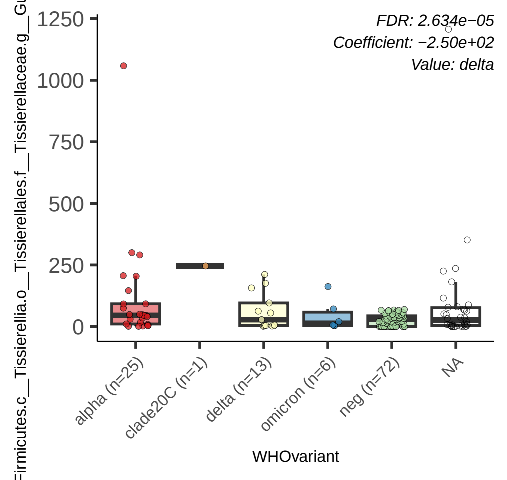
FDR: 2.634e−05
Coefficient: −2.50e+02
Value: delta
Firmicutes.c__Tissierellia.o__Tissierellales.f__Tissierellaceae.g__Gu
1250
1000
750
500
250
0
alpha (n=25)
clade20C (n=1)
delta (n=13)
omicron (n=6)
neg (n=72)
NA
WHOvariant

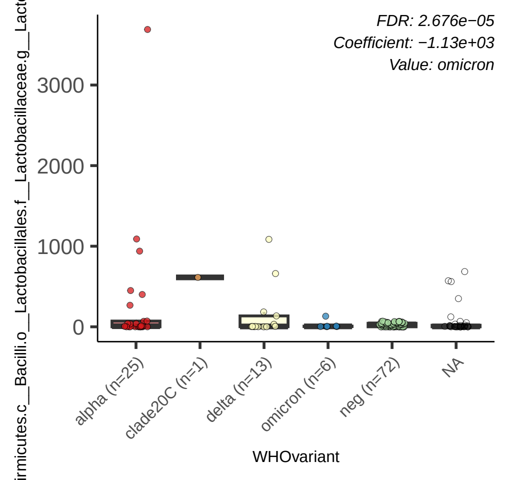

FDR: 2.676e−05
Coefficient: −1.13e+03
Value: omicron
3000
2000
1000
0
irmicutes.c__Bacilli.o__Lactobacillales.f__Lactobacillaceae.g__Lacto
alpha (n=25)
clade20C (n=1)
delta (n=13)
omicron (n=6)
neg (n=72)
NA
WHOvariant

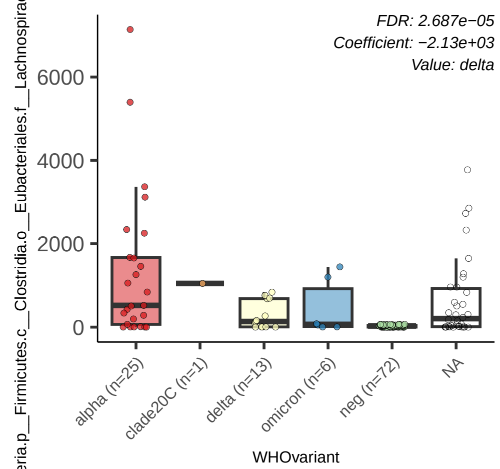

FDR: 2.687e−05
Coefficient: −2.13e+03
Value: delta
6000
4000
2000
0
cteria.p__Firmicutes.c__Clostridia.o__Eubacteriales.f__Lachnospirac
alpha (n=25)
clade20C (n=1)
delta (n=13)
omicron (n=6)
neg (n=72)
NA
WHOvariant

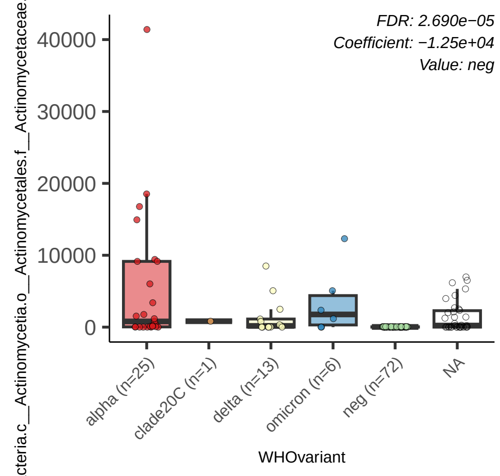

FDR: 2.690e−05
Coefficient: −1.25e+04
Value: neg
acteria.c__Actinomycetia.o__Actinomycetales.f__Actinomycetaceae.
40000
30000
20000
10000
0
alpha (n=25)
clade20C (n=1)
delta (n=13)
omicron (n=6)
neg (n=72)
NA
WHOvariant

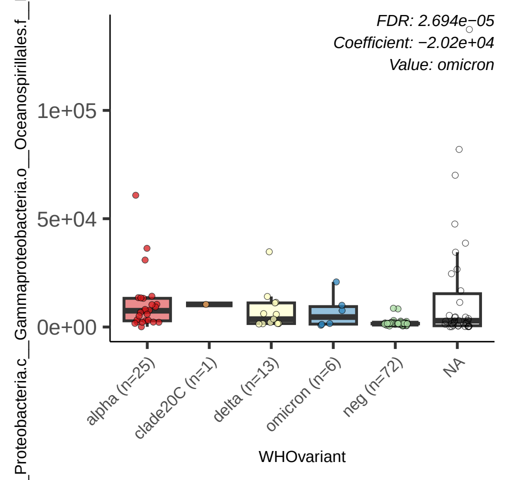
FDR: 2.694e−05
Coefficient: −2.02e+04
Value: omicron
Proteobacteria.c__Gammaproteobacteria.o__Oceanospirillales.f__
1e+05
5e+04
0e+00
alpha (n=25)
clade20C (n=1)
delta (n=13)
omicron (n=6)
neg (n=72)
NA
WHOvariant

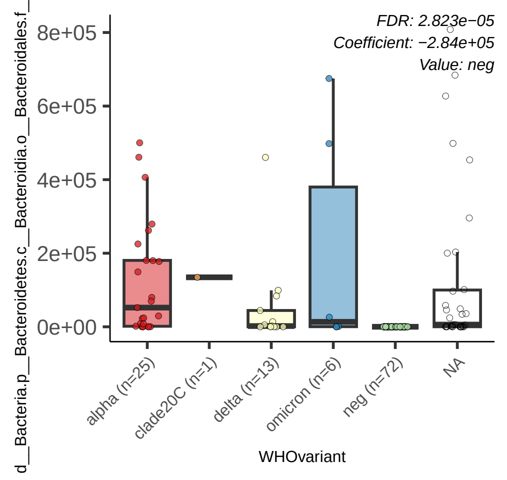

FDR: 2.823e−05
Coefficient: −2.84e+05
Value: neg
d__Bacteria.p__Bacteroidetes.c__Bacteroidia.o__Bacteroidales.f__
8e+05
6e+05
4e+05
2e+05
0e+00
alpha (n=25)
clade20C (n=1)
delta (n=13)
omicron (n=6)
neg (n=72)
NA
WHOvariant

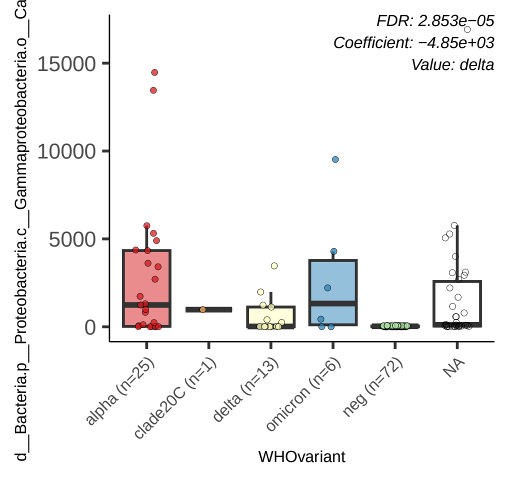

FDR: 2.853e−05
Coefficient: −4.85e+03
Value: delta
15000
10000
5000
0
d__Bacteria.p__Proteobacteria.c__Gammaproteobacteria.o__Ca
alpha (n=25)
clade20C (n=1)
delta (n=13)
omicron (n=6)
neg (n=72)
NA
WHOvariant

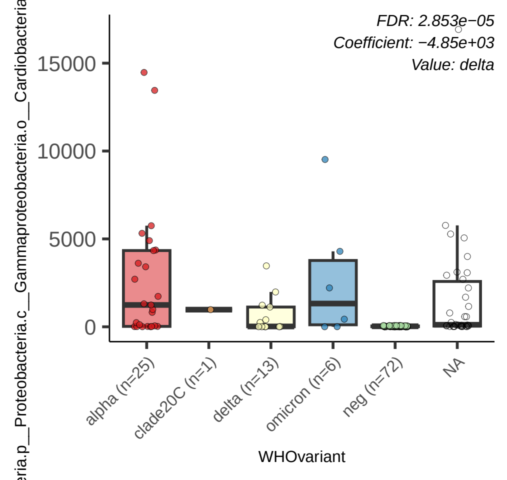
FDR: 2.853e−05
Coefficient: −4.85e+03
Value: delta
15000
10000
5000
0
eria.p__Proteobacteria.c__Gammaproteobacteria.o__Cardiobacteria
alpha (n=25)
clade20C (n=1)
delta (n=13)
omicron (n=6)
neg (n=72)
NA
WHOvariant

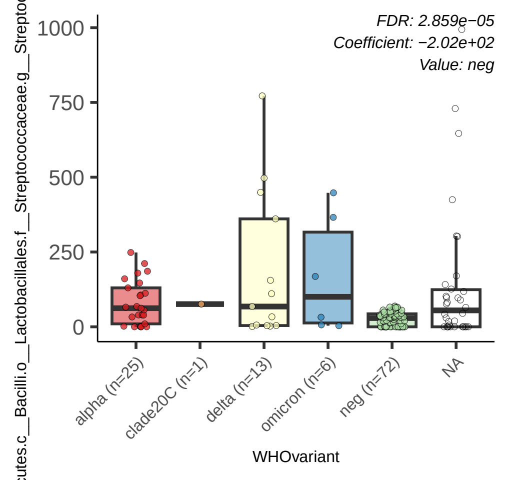
FDR: 2.859e−05
Coefficient: −2.02e+02
Value: neg
1000
750
500
250
0
alpha (n=25)
clade20C (n=1)
delta (n=13)
omicron (n=6)
neg (n=72)
NA
WHOvariant
cutes.c__Bacilli.o__Lactobacillales.f__Streptococcaceae.g__Streptoc

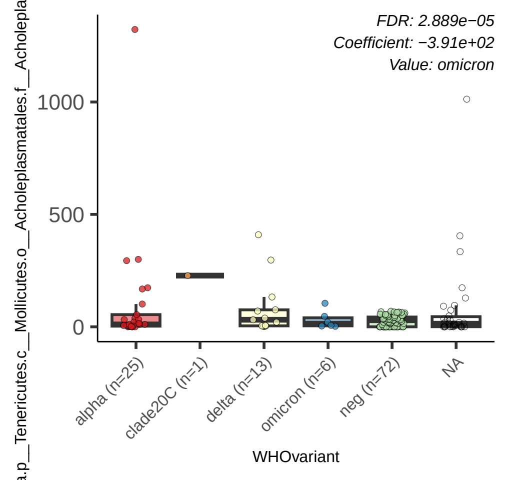
FDR: 2.889e−05
Coefficient: −3.91e+02
Value: omicron
a.p__Tenericutes.c__Mollicutes.o__Acholeplasmatales.f__Acholepla
0
500
1000
alpha (n=25)
clade20C (n=1)
delta (n=13)
omicron (n=6)
neg (n=72)
NA
WHOvariant

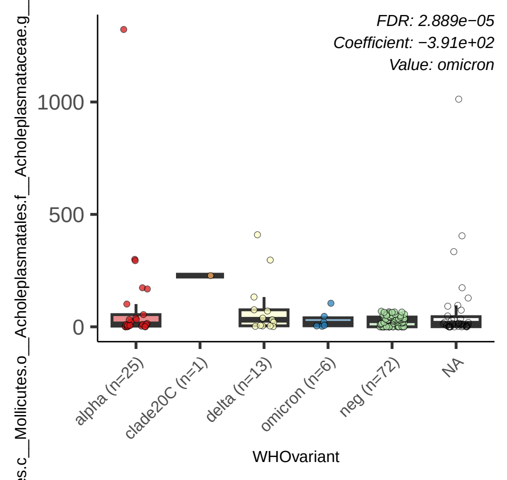
FDR: 2.889e−05
Coefficient: −3.91e+02
Value: omicron
es.c__Mollicutes.o__Acholeplasmatales.f__Acholeplasmataceae.g__
1000
500
0
alpha (n=25)
clade20C (n=1)
delta (n=13)
omicron (n=6)
neg (n=72)
NA
WHOvariant

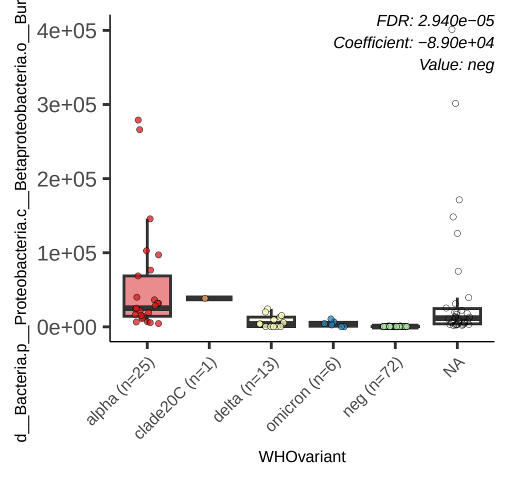

FDR: 2.940e−05
Coefficient: −8.90e+04
Value: neg
d__Bacteria.p__Proteobacteria.c__Betaproteobacteria.o__Bur
4e+05
3e+05
2e+05
1e+05
0e+00
alpha (n=25)
clade20C (n=1)
delta (n=13)
omicron (n=6)
neg (n=72)
NA
WHOvariant

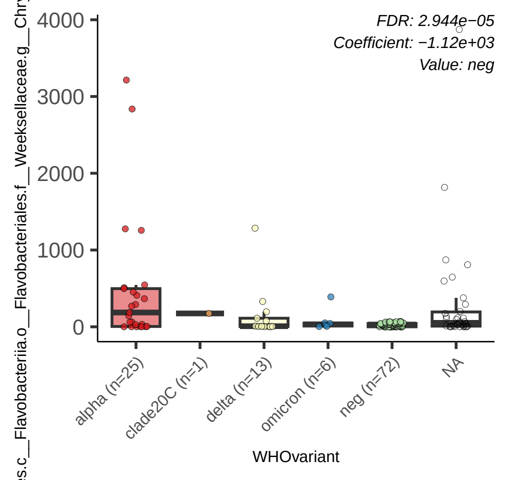

FDR: 2.944e−05
Coefficient: −1.12e+03
Value: neg
4000
3000
2000
1000
0
es.c__Flavobacteriia.o__Flavobacteriales.f__Weeksellaceae.g__Chry
alpha (n=25)
clade20C (n=1)
delta (n=13)
omicron (n=6)
neg (n=72)
NA
WHOvariant

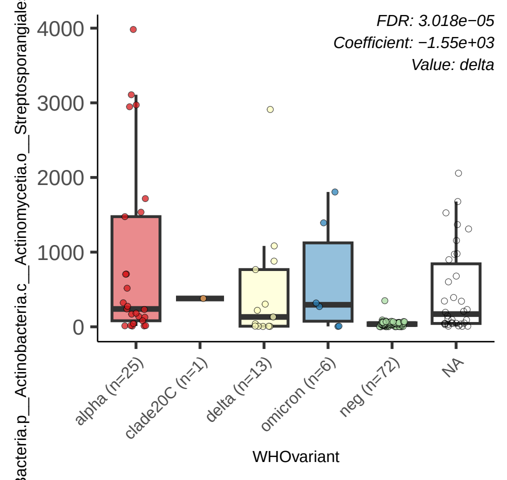

FDR: 3.018e−05
Coefficient: −1.55e+03
Value: delta
Bacteria.p__Actinobacteria.c__Actinomycetia.o__Streptosporangiales
4000
3000
2000
1000
0
alpha (n=25)
clade20C (n=1)
delta (n=13)
omicron (n=6)
neg (n=72)
NA
WHOvariant

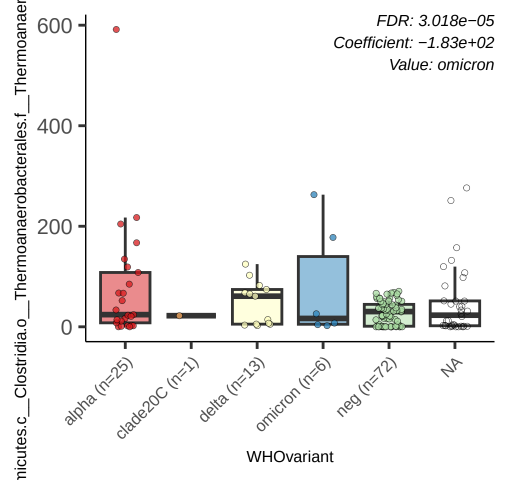
FDR: 3.018e−05
Coefficient: −1.83e+02
Value: omicron
600
400
200
0
micutes.c__Clostridia.o__Thermoanaerobacterales.f__Thermoanaer
alpha (n=25)
clade20C (n=1)
delta (n=13)
omicron (n=6)
neg (n=72)
NA
WHOvariant

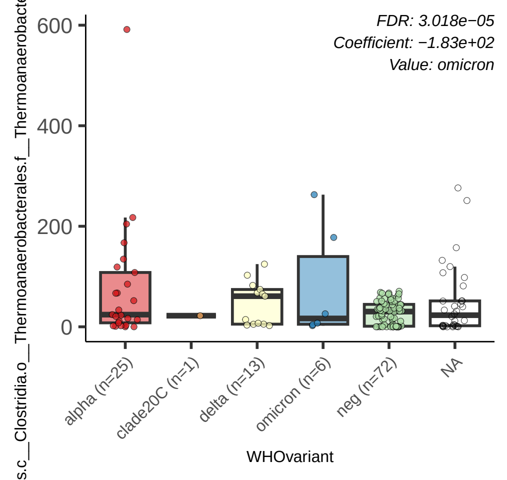

FDR: 3.018e−05
Coefficient: −1.83e+02
Value: omicron
s.c__Clostridia.o__Thermoanaerobacterales.f__Thermoanaerobacte
600
400
200
0
alpha (n=25)
clade20C (n=1)
delta (n=13)
omicron (n=6)
neg (n=72)
NA
WHOvariant

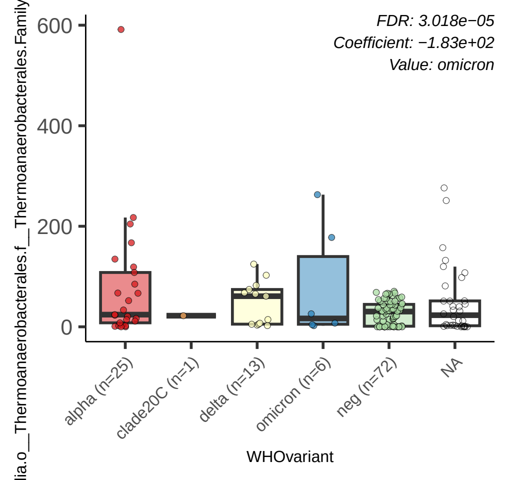
FDR: 3.018e−05
Coefficient: −1.83e+02
Value: omicron
lia.o__Thermoanaerobacterales.f__Thermoanaerobacterales.Family
600
400
200
0
alpha (n=25)
clade20C (n=1)
delta (n=13)
omicron (n=6)
neg (n=72)
NA
WHOvariant

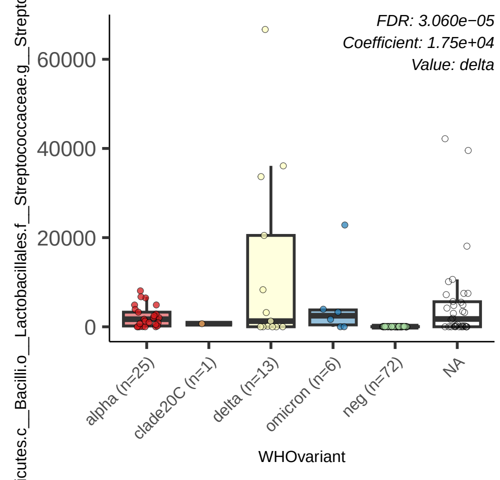

FDR: 3.060e−05
Coefficient: 1.75e+04
Value: delta
60000
40000
20000
0
alpha (n=25)
clade20C (n=1)
delta (n=13)
omicron (n=6)
neg (n=72)
NA
WHOvariant

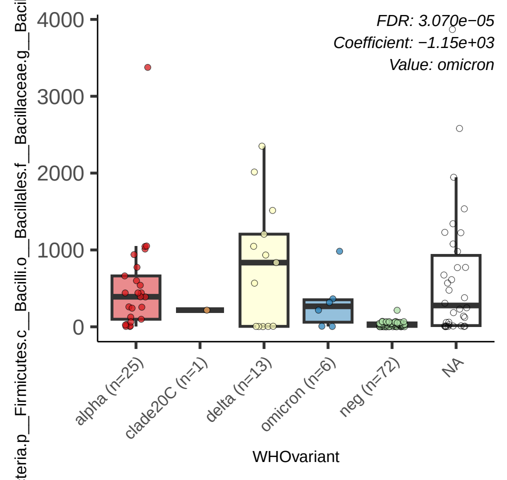

FDR: 3.070e−05
Coefficient: −1.15e+03
Value: omicron
4000
3000
2000
1000
0
teria.p__Firmicutes.c__Bacilli.o__Bacillales.f__Bacillaceae.g__Bacil
alpha (n=25)
clade20C (n=1)
delta (n=13)
omicron (n=6)
neg (n=72)
NA
WHOvariant

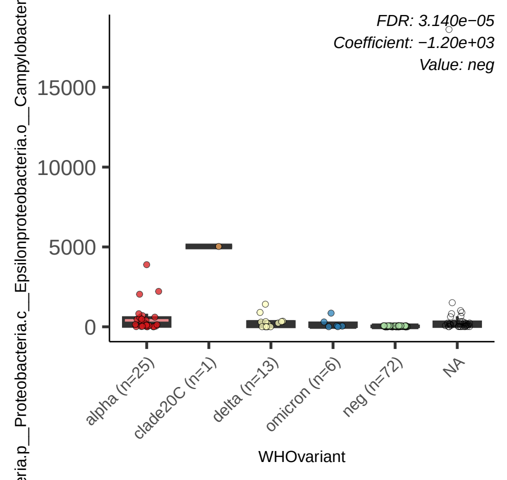

FDR: 3.140e−05
Coefficient: −1.20e+03
Value: neg
15000
10000
5000
0
alpha (n=25)
clade20C (n=1)
delta (n=13)
omicron (n=6)
neg (n=72)
NA
WHOvariant
eria.p__Proteobacteria.c__Epsilonproteobacteria.o__Campylobacter

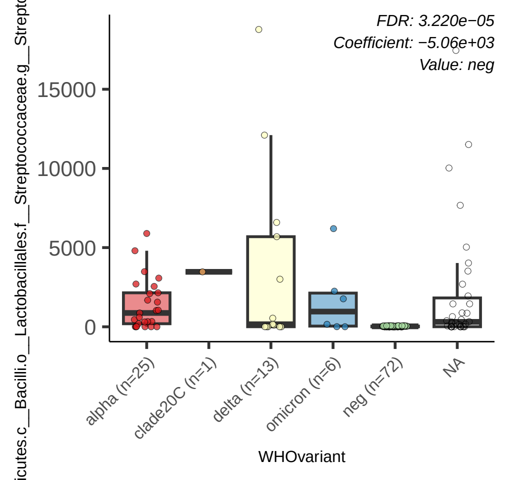

FDR: 3.220e−05
Coefficient: −5.06e+03
Value: neg
15000
10000
5000
0
icutes.c__Bacilli.o__Lactobacillales.f__Streptococcaceae.g__Strept
alpha (n=25)
clade20C (n=1)
delta (n=13)
omicron (n=6)
neg (n=72)
NA
WHOvariant

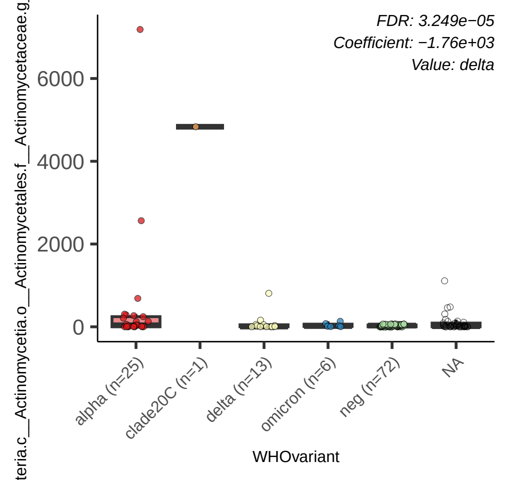
FDR: 3.249e−05
Coefficient: −1.76e+03
Value: delta
6000
4000
2000
0
teria.c__Actinomycetia.o__Actinomycetales.f__Actinomycetaceae.g
alpha (n=25)
clade20C (n=1)
delta (n=13)
omicron (n=6)
neg (n=72)
NA
WHOvariant

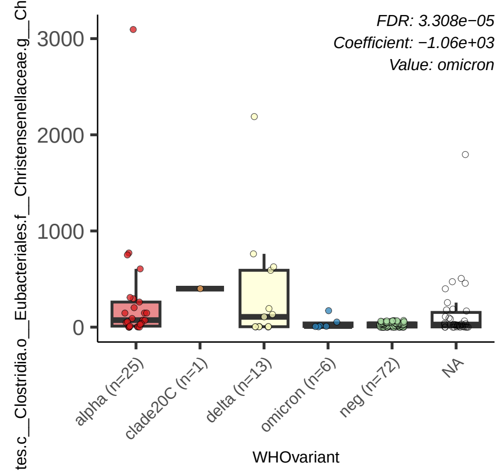

FDR: 3.308e−05
Coefficient: −1.06e+03
Value: omicron
3000
2000
1000
0
cutes.c__Clostridia.o__Eubacteriales.f__Christensenellaceae.g__Ch
alpha (n=25)
clade20C (n=1)
delta (n=13)
omicron (n=6)
neg (n=72)
NA
WHOvariant

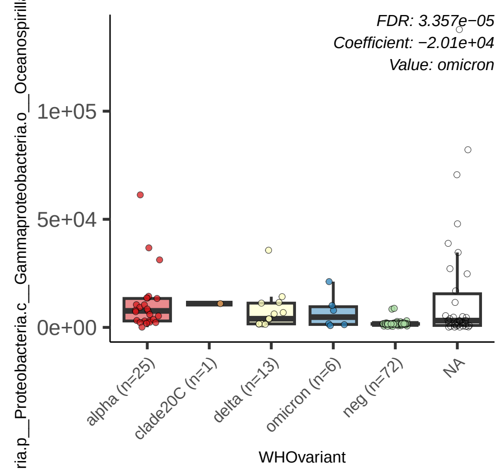
FDR: 3.357e−05
Coefficient: −2.01e+04
Value: omicron
1e+05
5e+04
0e+00
teria.p__Proteobacteria.c__Gammaproteobacteria.o__Oceanospirill
alpha (n=25)
clade20C (n=1)
delta (n=13)
omicron (n=6)
neg (n=72)
NA
WHOvariant

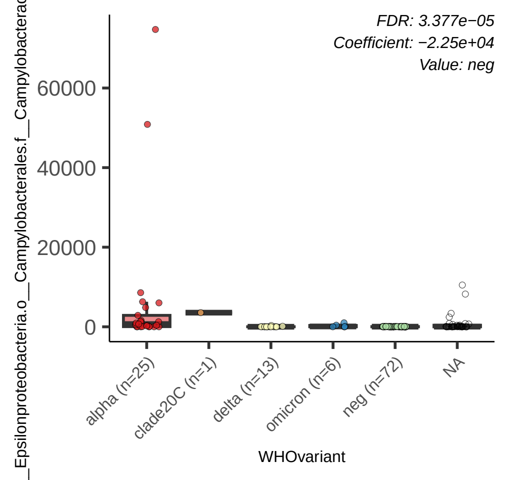

FDR: 3.377e−05
Coefficient: −2.25e+04
Value: neg
60000
40000
20000
0
_Epsilonproteobacteria.o__Campylobacterales.f__Campylobacterac
alpha (n=25)
clade20C (n=1)
delta (n=13)
omicron (n=6)
neg (n=72)
NA
WHOvariant

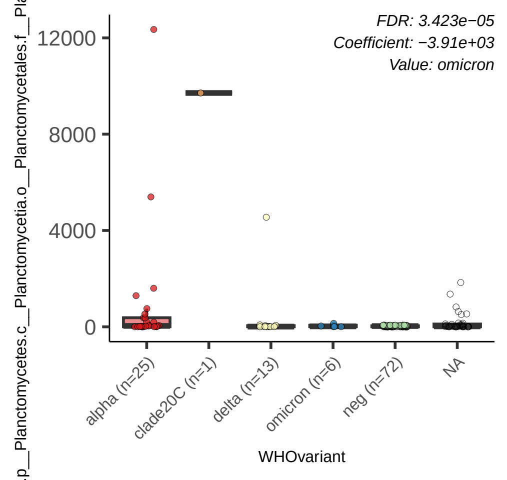

FDR: 3.423e−05
Coefficient: −3.91e+03
Value: omicron
12000
8000
4000
0
p__Planctomycetes.c__Planctomycetia.o__Planctomycetales.f__Pla
alpha (n=25)
clade20C (n=1)
delta (n=13)
omicron (n=6)
neg (n=72)
NA
WHOvariant

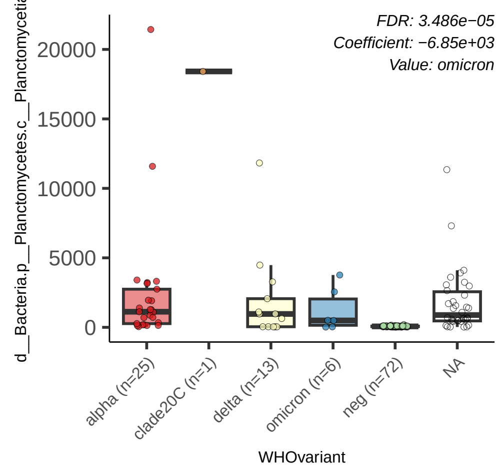

FDR: 3.486e−05
Coefficient: −6.85e+03
Value: omicron
d__Bacteria.p__Planctomycetes.c__Planctomycetia
20000
15000
10000
5000
0
alpha (n=25)
clade20C (n=1)
delta (n=13)
omicron (n=6)
neg (n=72)
NA
WHOvariant

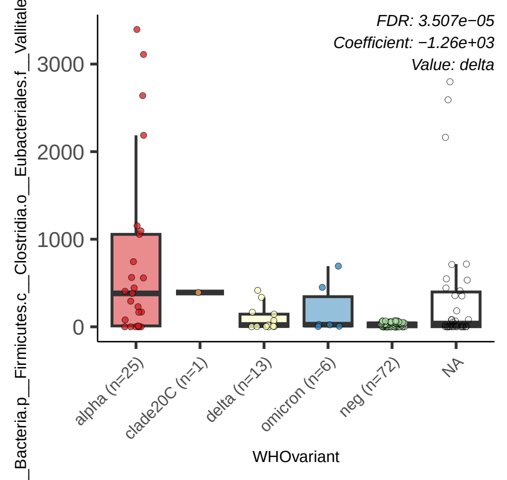
FDR: 3.507e−05
Coefficient: −1.26e+03
Value: delta
_Bacteria.p__Firmicutes.c__Clostridia.o__Eubacteriales.f__Vallitale
3000
2000
1000
0
alpha (n=25)
clade20C (n=1)
delta (n=13)
omicron (n=6)
neg (n=72)
NA
WHOvariant

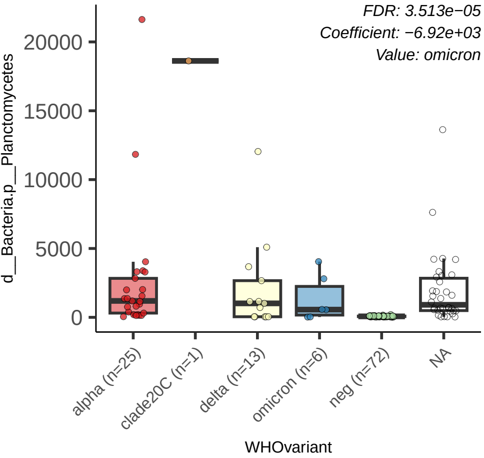

FDR: 3.513e−05
Coefficient: −6.92e+03
Value: omicron
d__Bacteria.p__Planctomycetes
20000
15000
10000
5000
0
alpha (n=25)
clade20C (n=1)
delta (n=13)
omicron (n=6)
neg (n=72)
NA
WHOvariant

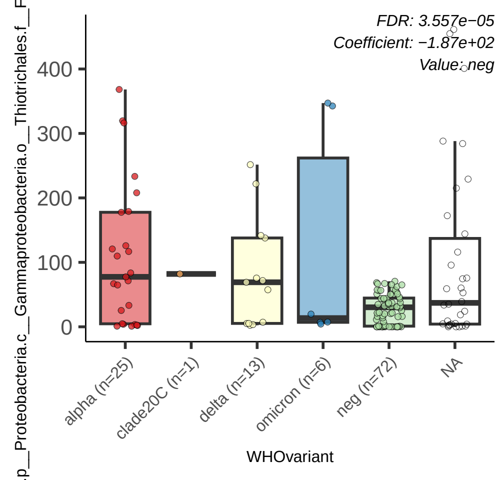

FDR: 3.557e−05
Coefficient: −1.87e+02
Value: neg
p__Proteobacteria.c__Gammaproteobacteria.o__Thiotrichales.f__F
0
100
200
300
400
alpha (n=25)
clade20C (n=1)
delta (n=13)
omicron (n=6)
neg (n=72)
NA
WHOvariant

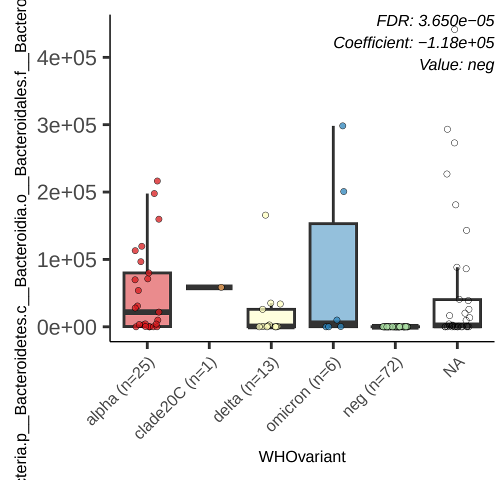
FDR: 3.650e−05
Coefficient: −1.18e+05
Value: neg
cteria.p__Bacteroidetes.c__Bacteroidia.o__Bacteroidales.f__Bactero
4e+05
3e+05
2e+05
1e+05
0e+00
alpha (n=25)
clade20C (n=1)
delta (n=13)
omicron (n=6)
neg (n=72)
NA
WHOvariant

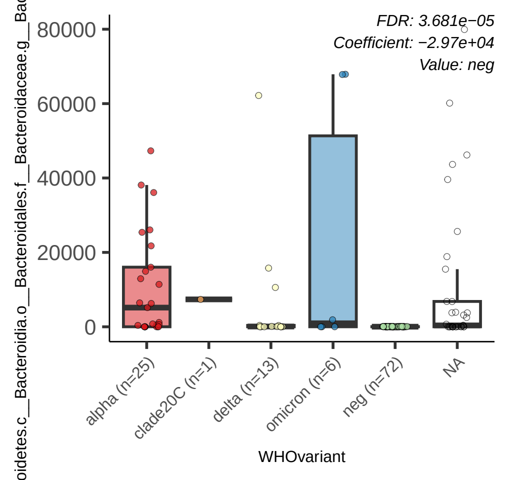

FDR: 3.681e−05
Coefficient: −2.97e+04
Value: neg
80000
60000
40000
20000
0
oidetes.c__Bacteroidia.o__Bacteroidales.f__Bacteroidaceae.g__Bac
alpha (n=25)
clade20C (n=1)
delta (n=13)
omicron (n=6)
neg (n=72)
NA
WHOvariant

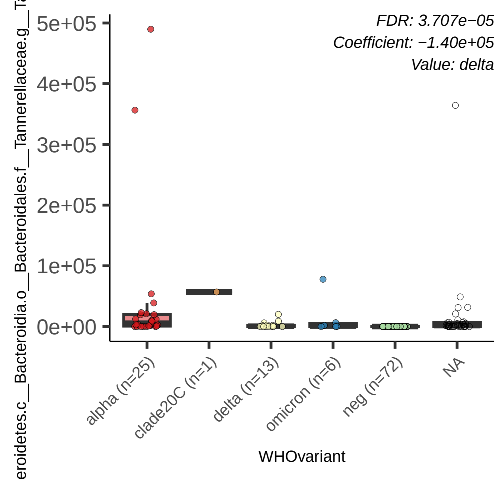

FDR: 3.707e−05
Coefficient: −1.40e+05
Value: delta
eroidetes.c__Bacteroidia.o__Bacteroidales.f__Tannerellaceae.g__Ta
5e+05
4e+05
3e+05
2e+05
1e+05
0e+00
alpha (n=25)
clade20C (n=1)
delta (n=13)
omicron (n=6)
neg (n=72)
NA
WHOvariant

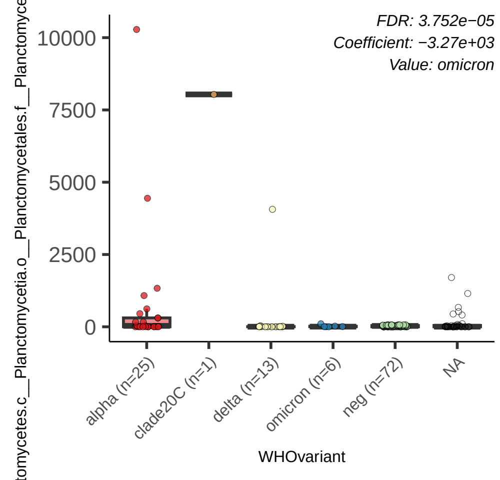

FDR: 3.752e−05
Coefficient: −3.27e+03
Value: omicron
10000
7500
5000
2500
0
alpha (n=25)
clade20C (n=1)
delta (n=13)
omicron (n=6)
neg (n=72)
NA
WHOvariant
tomycetes.c__Planctomycetia.o__Planctomycetales.f__Planctomyce

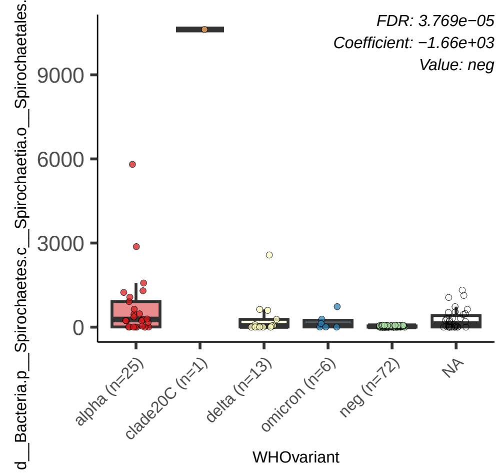
FDR: 3.769e−05
Coefficient: −1.66e+03
Value: neg
d__Bacteria.p__Spirochaetes.c__Spirochaetia.o__Spirochaetales.
0
3000
6000
9000
alpha (n=25)
clade20C (n=1)
delta (n=13)
omicron (n=6)
neg (n=72)
NA
WHOvariant

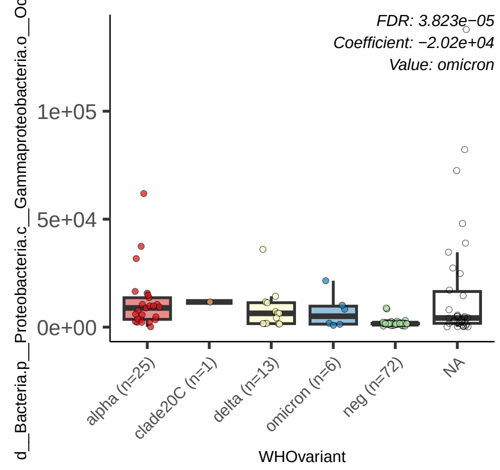

FDR: 3.823e−05
Coefficient: −2.02e+04
Value: omicron
d__Bacteria.p__Proteobacteria.c__Gammaproteobacteria.o__Oc
1e+05
5e+04
0e+00
alpha (n=25)
clade20C (n=1)
delta (n=13)
omicron (n=6)
neg (n=72)
NA
WHOvariant

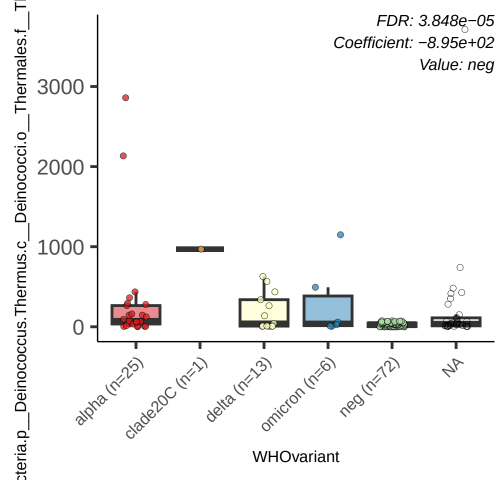
FDR: 3.848e−05
Coefficient: −8.95e+02
Value: neg
3000
2000
1000
0
cteria.p__Deinococcus.Thermus.c__Deinococci.o__Thermales.f__Th
alpha (n=25)
clade20C (n=1)
delta (n=13)
omicron (n=6)
neg (n=72)
NA
WHOvariant

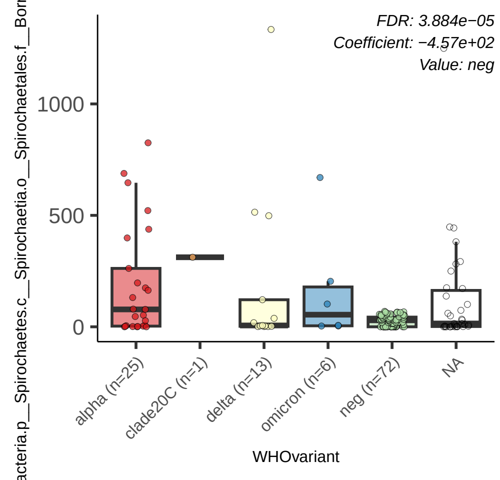

FDR: 3.884e−05
Coefficient: −4.57e+02
Value: neg
acteria.p__Spirochaetes.c__Spirochaetia.o__Spirochaetales.f__Borr
1000
500
0
alpha (n=25)
clade20C (n=1)
delta (n=13)
omicron (n=6)
neg (n=72)
NA
WHOvariant

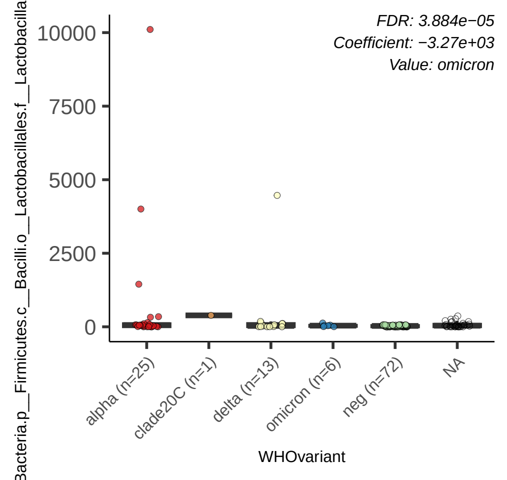
FDR: 3.884e−05
Coefficient: −3.27e+03
Value: omicron
10000
7500
5000
2500
0
alpha (n=25)
clade20C (n=1)
delta (n=13)
omicron (n=6)
neg (n=72)
NA
WHOvariant
Bacteria.p__Firmicutes.c__Bacilli.o__Lactobacillales.f__Lactobacilla

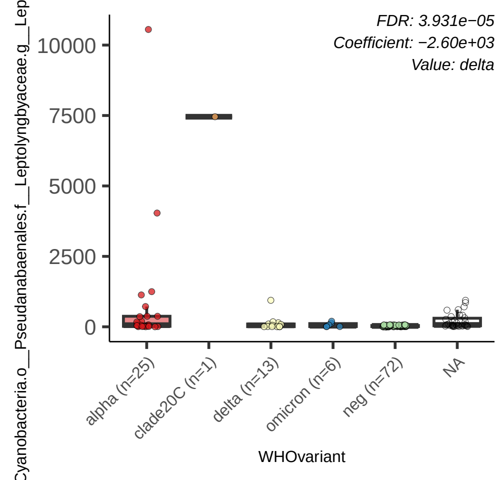

FDR: 3.931e−05
Coefficient: −2.60e+03
Value: delta
Cyanobacteria.o__Pseudanabaenales.f__Leptolyngbyaceae.g__Lep
0
2500
5000
7500
10000
alpha (n=25)
clade20C (n=1)
delta (n=13)
omicron (n=6)
neg (n=72)
NA
WHOvariant

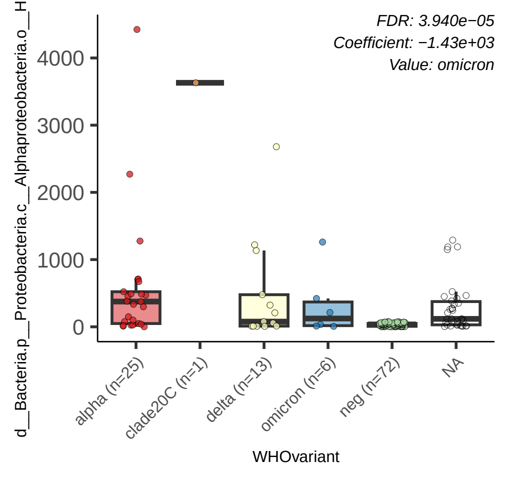
FDR: 3.940e−05
Coefficient: −1.43e+03
Value: omicron
d__Bacteria.p__Proteobacteria.c__Alphaproteobacteria.o__H
0
1000
2000
3000
4000
alpha (n=25)
clade20C (n=1)
delta (n=13)
omicron (n=6)
neg (n=72)
NA
WHOvariant

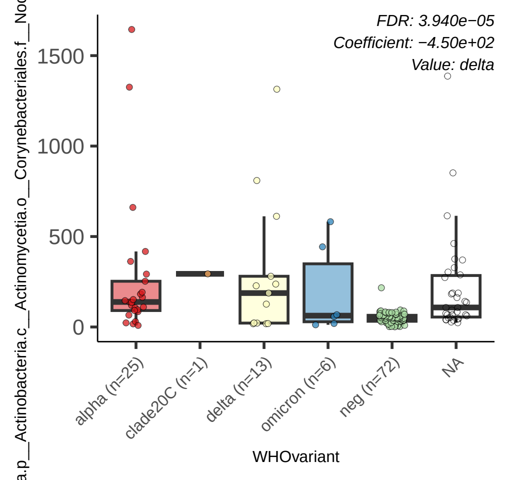

FDR: 3.940e−05
Coefficient: −4.50e+02
Value: delta
0
500
1000
1500
Actinobacteria.p__Actinobacteria.c__Actinomycetia.o__Corynebacteriales.f__Noc
alpha (n=25)
clade20C (n=1)
delta (n=13)
omicron (n=6)
neg (n=72)
NA
WHOvariant

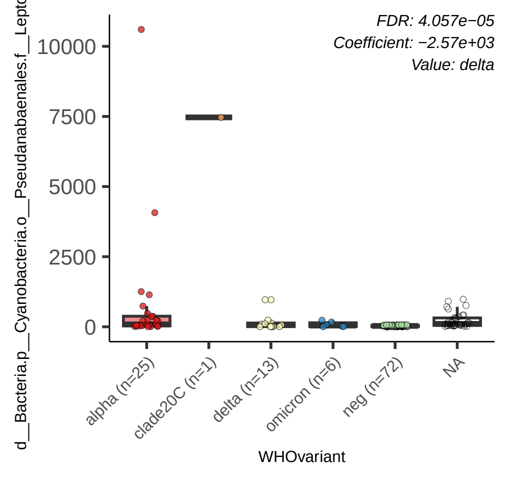
FDR: 4.057e−05
Coefficient: −2.57e+03
Value: delta
d__Bacteria.p__Cyanobacteria.o__Pseudanabaenales.f__Lepto
10000
7500
5000
2500
0
alpha (n=25)
clade20C (n=1)
delta (n=13)
omicron (n=6)
neg (n=72)
NA
WHOvariant

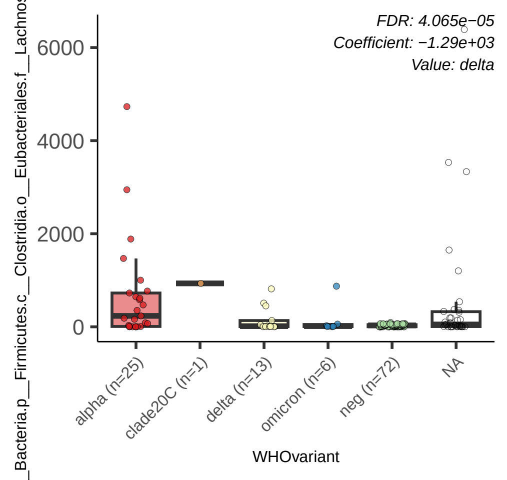
FDR: 4.065e−05
Coefficient: −1.29e+03
Value: delta
6000
4000
2000
0
alpha (n=25)
clade20C (n=1)
delta (n=13)
omicron (n=6)
neg (n=72)
NA
WHOvariant
Bacteria.p__Firmicutes.c__Clostridia.o__Eubacteriales.f__Lachnos

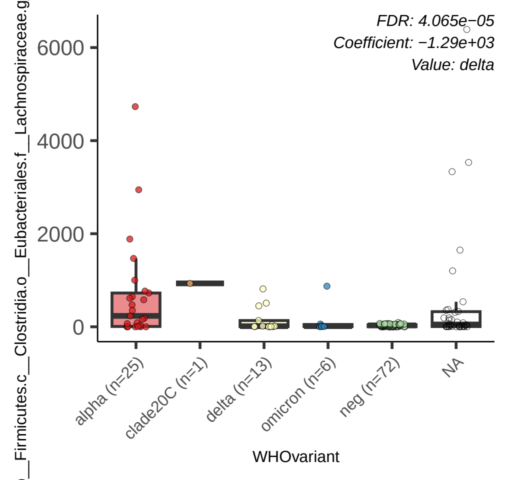
FDR: 4.065e−05
Coefficient: −1.29e+03
Value: delta
6000
4000
2000
0
Firmicutes.c__Clostridia.o__Eubacteriales.f__Lachnospiraceae.g
alpha (n=25)
clade20C (n=1)
delta (n=13)
omicron (n=6)
neg (n=72)
NA
WHOvariant

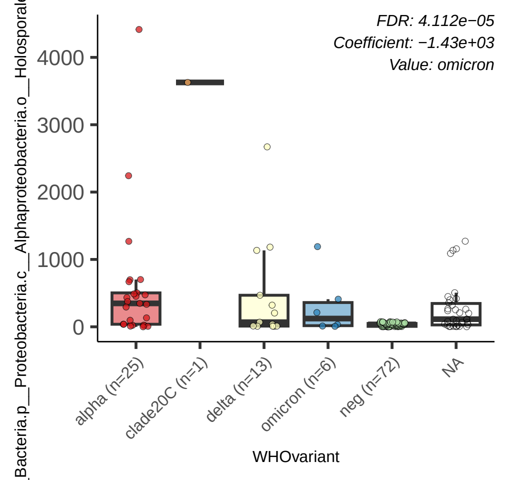

FDR: 4.112e−05
Coefficient: −1.43e+03
Value: omicron
Bacteria.p__Proteobacteria.c__Alphaproteobacteria.o__Holosporale
4000
3000
2000
1000
0
alpha (n=25)
clade20C (n=1)
delta (n=13)
omicron (n=6)
neg (n=72)
NA
WHOvariant

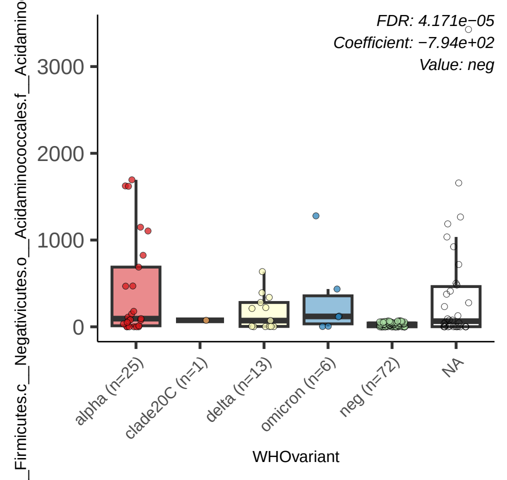

FDR: 4.171e−05
Coefficient: −7.94e+02
Value: neg
Firmicutes.c__Negativicutes.o__Acidaminococcales.f__Acidaminoc
3000
2000
1000
0
alpha (n=25)
clade20C (n=1)
delta (n=13)
omicron (n=6)
neg (n=72)
NA
WHOvariant

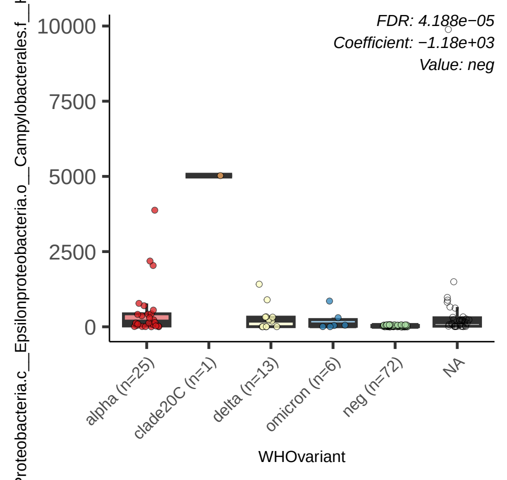

FDR: 4.188e−05
Coefficient: −1.18e+03
Value: neg
10000
7500
5000
2500
0
Proteobacteria.c__Epsilonproteobacteria.o__Campylobacterales.f__H
alpha (n=25)
clade20C (n=1)
delta (n=13)
omicron (n=6)
neg (n=72)
NA
WHOvariant

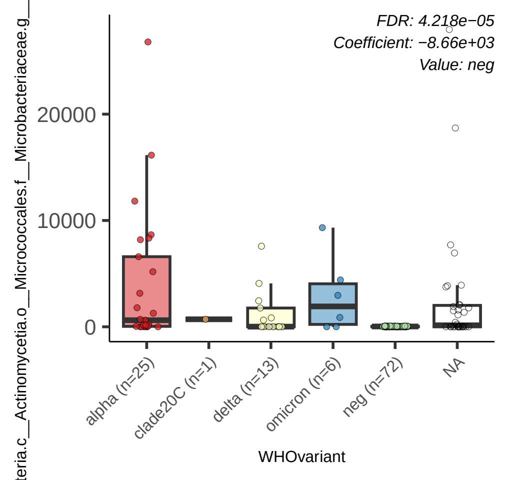

FDR: 4.218e−05
Coefficient: −8.66e+03
Value: neg
teria.c__Actinomycetia.o__Micrococcales.f__Microbacteriaceae.g__
20000
10000
0
alpha (n=25)
clade20C (n=1)
delta (n=13)
omicron (n=6)
neg (n=72)
NA
WHOvariant

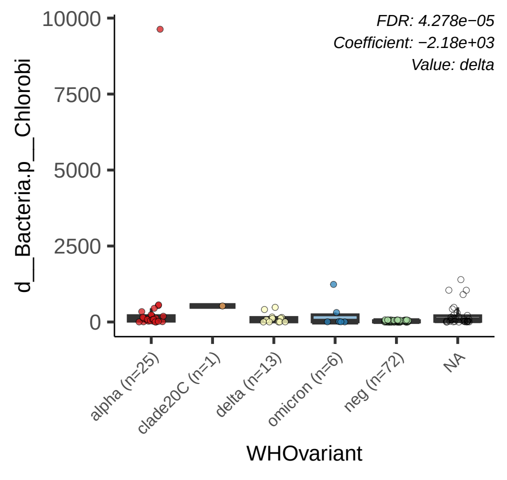

FDR: 4.278e−05
Coefficient: −2.18e+03
Value: delta
10000
7500
5000
2500
0
d__Bacteria.p__Chlorobi
alpha (n=25)
clade20C (n=1)
delta (n=13)
omicron (n=6)
neg (n=72)
NA
WHOvariant

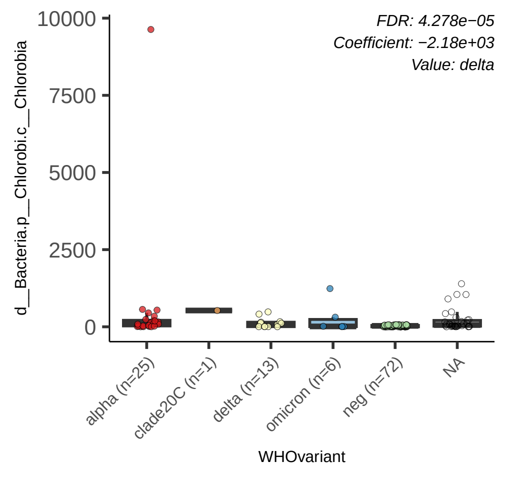

FDR: 4.278e−05
Coefficient: −2.18e+03
Value: delta
10000
7500
5000
2500
0
d__Bacteria.p__Chlorobi.c__Chlorobia
alpha (n=25)
clade20C (n=1)
delta (n=13)
omicron (n=6)
neg (n=72)
NA
WHOvariant

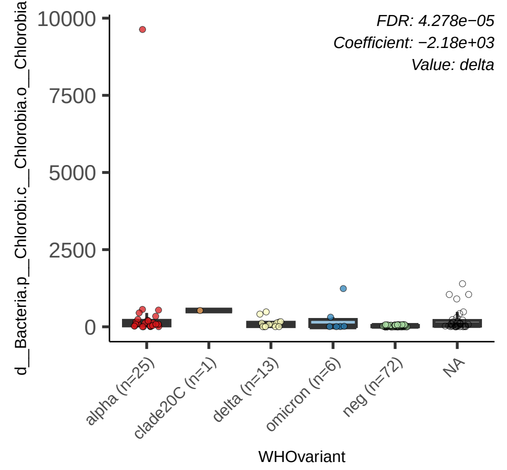

FDR: 4.278e−05
Coefficient: −2.18e+03
Value: delta
10000
7500
5000
2500
0
d__Bacteria.p__Chlorobi.c__Chlorobia.o__Chlorobia
alpha (n=25)
clade20C (n=1)
delta (n=13)
omicron (n=6)
neg (n=72)
NA
WHOvariant

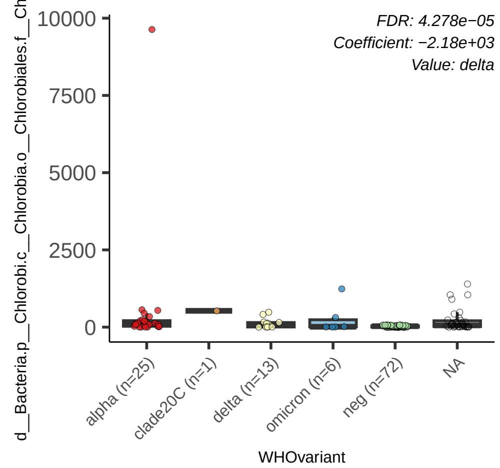

d__Bacteria.p__Chlorobi.c__Chlorobia.o__Chlorobiales.f__Ch
FDR: 4.278e−05
Coefficient: −2.18e+03
Value: delta
10000
7500
5000
2500
0
alpha (n=25)
clade20C (n=1)
delta (n=13)
omicron (n=6)
neg (n=72)
NA
WHOvariant

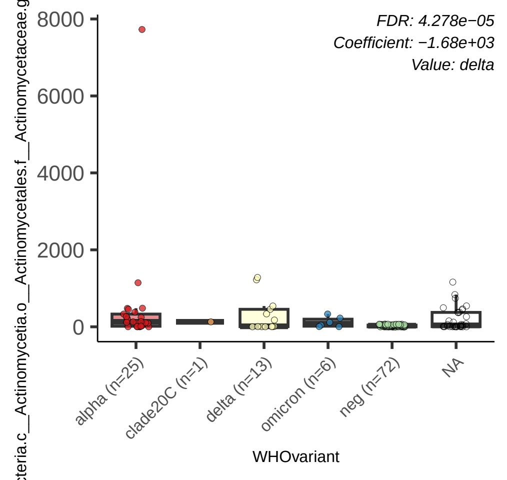

FDR: 4.278e−05
Coefficient: −1.68e+03
Value: delta
8000
6000
4000
2000
0
cteria.c__Actinomycetia.o__Actinomycetales.f__Actinomycetaceae.g
alpha (n=25)
clade20C (n=1)
delta (n=13)
omicron (n=6)
neg (n=72)
NA
WHOvariant

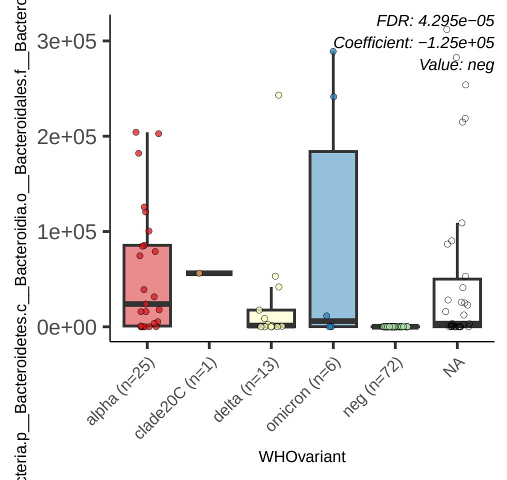
FDR: 4.295e−05
Coefficient: −1.25e+05
Value: neg
3e+05
2e+05
1e+05
0e+00
alpha (n=25)
clade20C (n=1)
delta (n=13)
omicron (n=6)
neg (n=72)
NA
WHOvariant

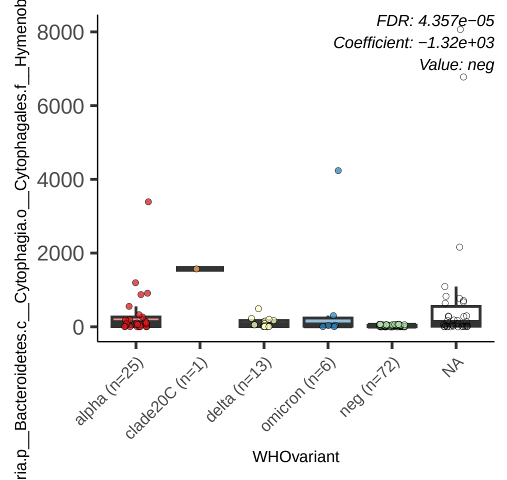

FDR: 4.357e−05
Coefficient: −1.32e+03
Value: neg
8000
6000
4000
2000
0
ria.p__Bacteroidetes.c__Cytophagia.o__Cytophagales.f__Hymenob
alpha (n=25)
clade20C (n=1)
delta (n=13)
omicron (n=6)
neg (n=72)
NA
WHOvariant

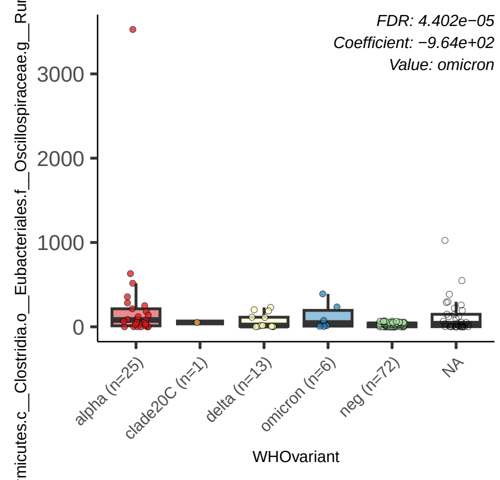
FDR: 4.402e−05
Coefficient: −9.64e+02
Value: omicron
3000
2000
1000
0
alpha (n=25)
clade20C (n=1)
delta (n=13)
omicron (n=6)
neg (n=72)
NA
WHOvariant

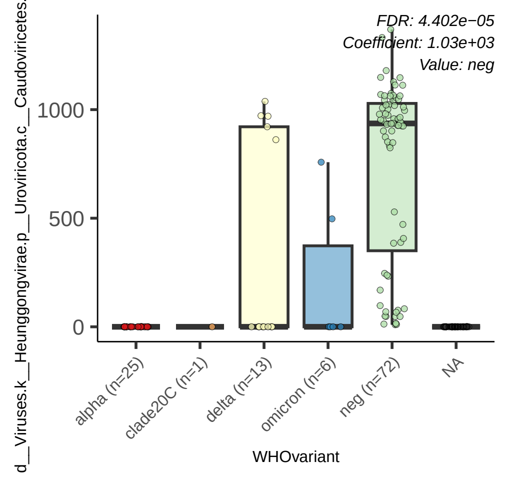
FDR: 4.402e−05
Coefficient: 1.03e+03
Value: neg
d__Viruses.k__Heunggongvirae.p__Uroviricota.c__Caudoviricetes.
1000
500
0
alpha (n=25)
clade20C (n=1)
delta (n=13)
omicron (n=6)
neg (n=72)
NA
WHOvariant

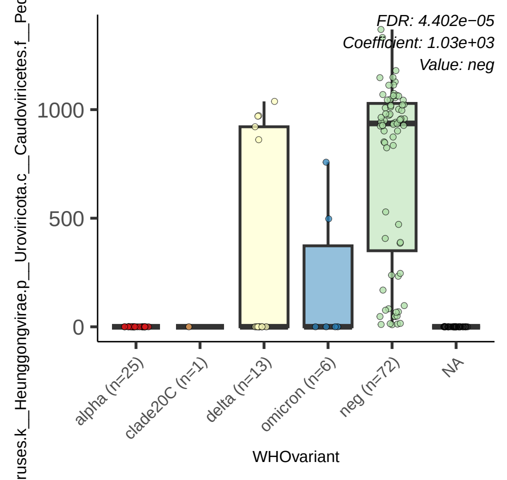

FDR: 4.402e−05
Coefficient: 1.03e+03
Value: neg
1000
500
0
alpha (n=25)
clade20C (n=1)
delta (n=13)
omicron (n=6)
neg (n=72)
NA
WHOvariant
ruses.k__Heunggongvirae.p__Uroviricota.c__Caudoviricetes.f__Pe

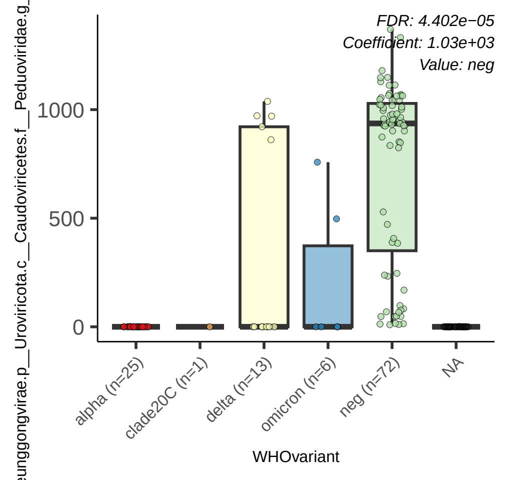

FDR: 4.402e−05
Coefficient: 1.03e+03
Value: neg
eunggongvirae.p__Uroviricota.c__Caudoviricetes.f__Peduoviridae.g_
1000
500
0
alpha (n=25)
clade20C (n=1)
delta (n=13)
omicron (n=6)
neg (n=72)
NA
WHOvariant

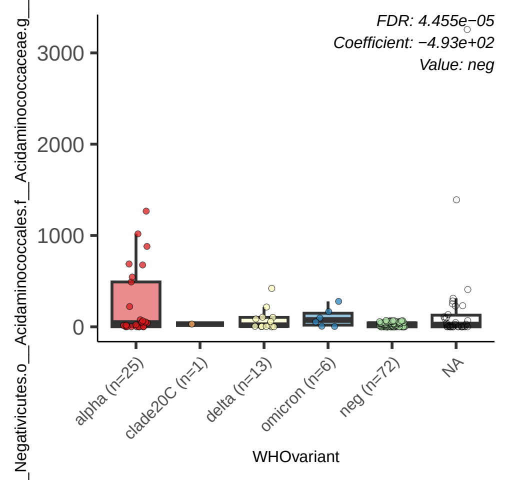

FDR: 4.455e−05
Coefficient: −4.93e+02
Value: neg
Negativicutes.o__Acidaminococcales.f__Acidaminococcaceae.g__
3000
2000
1000
0
alpha (n=25)
clade20C (n=1)
delta (n=13)
omicron (n=6)
neg (n=72)
NA
WHOvariant

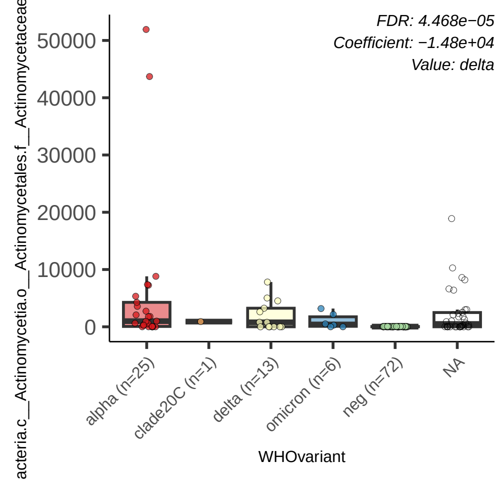
FDR: 4.468e−05
Coefficient: −1.48e+04
Value: delta
acteria.c__Actinomycetia.o__Actinomycetales.f__Actinomycetaceae
50000
40000
30000
20000
10000
0
alpha (n=25)
clade20C (n=1)
delta (n=13)
omicron (n=6)
neg (n=72)
NA
WHOvariant

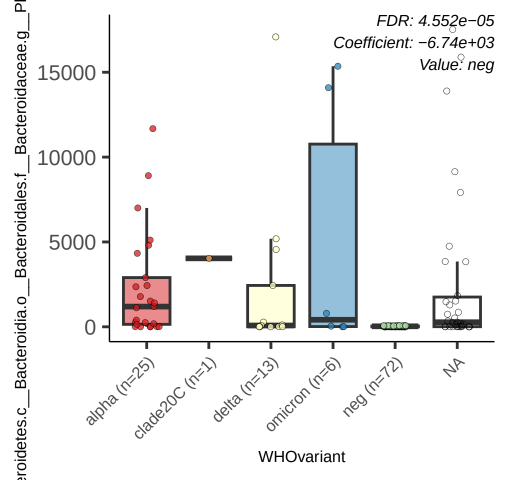

FDR: 4.552e−05
Coefficient: −6.74e+03
Value: neg
15000
10000
5000
0
alpha (n=25)
clade20C (n=1)
delta (n=13)
omicron (n=6)
neg (n=72)
NA
WHOvariant
eroidetes.c__Bacteroidia.o__Bacteroidales.f__Bacteroidaceae.g__P

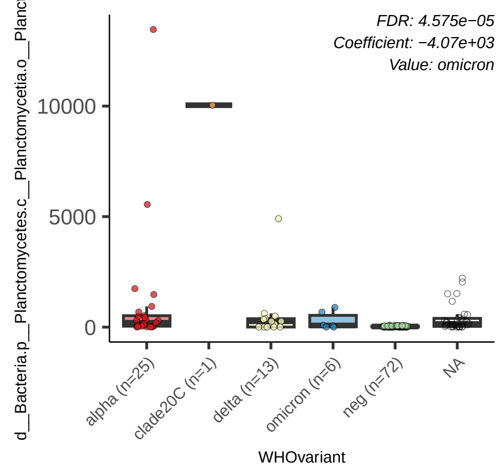

FDR: 4.575e−05
Coefficient: −4.07e+03
Value: omicron
d__Bacteria.p__Planctomycetes.c__Planctomycetia.o__Planc
10000
5000
0
alpha (n=25)
clade20C (n=1)
delta (n=13)
omicron (n=6)
neg (n=72)
NA
WHOvariant

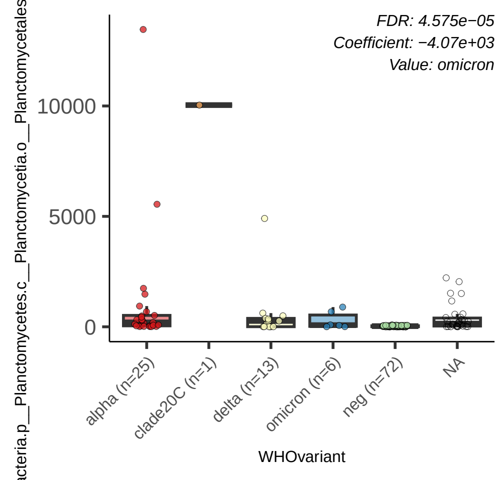

FDR: 4.575e−05
Coefficient: −4.07e+03
Value: omicron
10000
5000
0
acteria.p__Planctomycetes.c__Planctomycetia.o__Planctomycetales
alpha (n=25)
clade20C (n=1)
delta (n=13)
omicron (n=6)
neg (n=72)
NA
WHOvariant

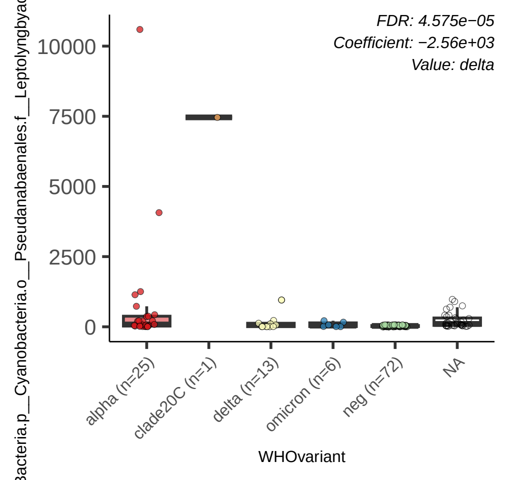

FDR: 4.575e−05
Coefficient: −2.56e+03
Value: delta
10000
7500
5000
2500
0
alpha (n=25)
clade20C (n=1)
delta (n=13)
omicron (n=6)
neg (n=72)
NA
WHOvariant
Bacteria.p__Cyanobacteria.o__Pseudanabaenales.f__Leptolyngbyac

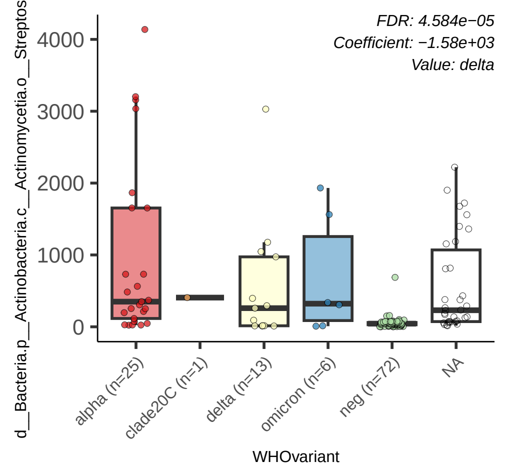

FDR: 4.584e−05
Coefficient: −1.58e+03
Value: delta
d__Bacteria.p__Actinobacteria.c__Actinomycetia.o__Streptos
4000
3000
2000
1000
0
alpha (n=25)
clade20C (n=1)
delta (n=13)
omicron (n=6)
neg (n=72)
NA
WHOvariant

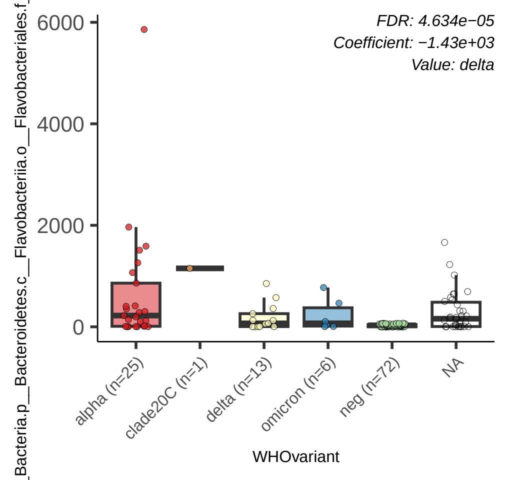

FDR: 4.634e−05
Coefficient: −1.43e+03
Value: delta
6000
4000
2000
0
Bacteria.p__Bacteroidetes.c__Flavobacteriia.o__Flavobacteriales.f_
alpha (n=25)
clade20C (n=1)
delta (n=13)
omicron (n=6)
neg (n=72)
NA
WHOvariant

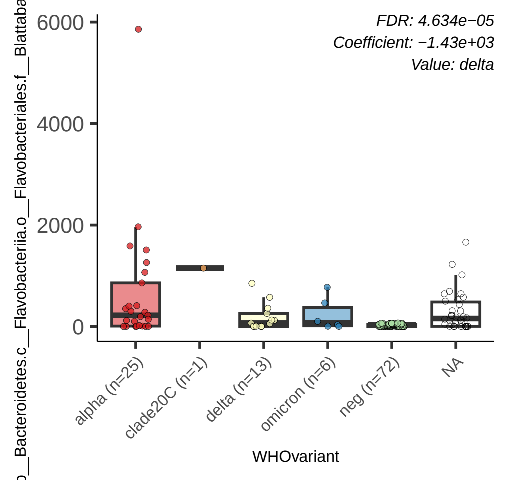
FDR: 4.634e−05
Coefficient: −1.43e+03
Value: delta
o__Bacteroidetes.c__Flavobacteriia.o__Flavobacteriales.f__Blattaba
6000
4000
2000
0
alpha (n=25)
clade20C (n=1)
delta (n=13)
omicron (n=6)
neg (n=72)
NA
WHOvariant

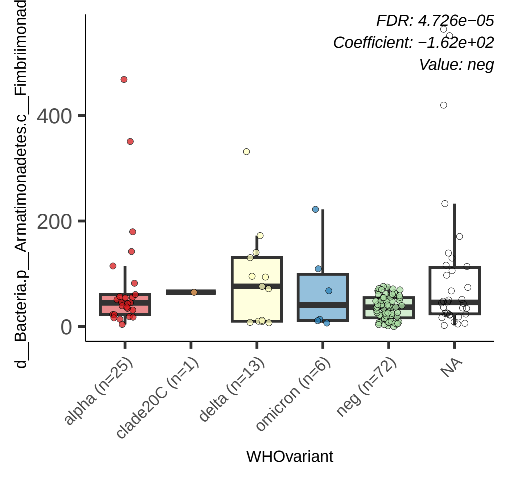

FDR: 4.726e−05
Coefficient: −1.62e+02
Value: neg
d__Bacteria.p__Armatimonadetes.c__Fimbriimonad
0
200
400
alpha (n=25)
clade20C (n=1)
delta (n=13)
omicron (n=6)
neg (n=72)
NA
WHOvariant

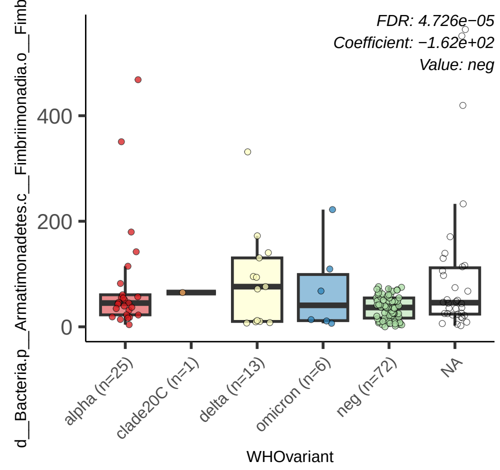

FDR: 4.726e−05
Coefficient: −1.62e+02
Value: neg
d__Bacteria.p__Armatimonadetes.c__Fimbriimonadia.o__Fimb
0
200
400
alpha (n=25)
clade20C (n=1)
delta (n=13)
omicron (n=6)
neg (n=72)
NA
WHOvariant

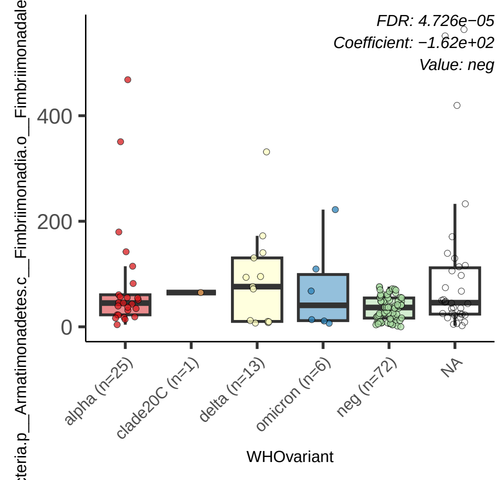
FDR: 4.726e−05
Coefficient: −1.62e+02
Value: neg
cteria.p__Armatimonadetes.c__Fimbriimonadia.o__Fimbriimonadale
0
200
400
alpha (n=25)
clade20C (n=1)
delta (n=13)
omicron (n=6)
neg (n=72)
NA
WHOvariant

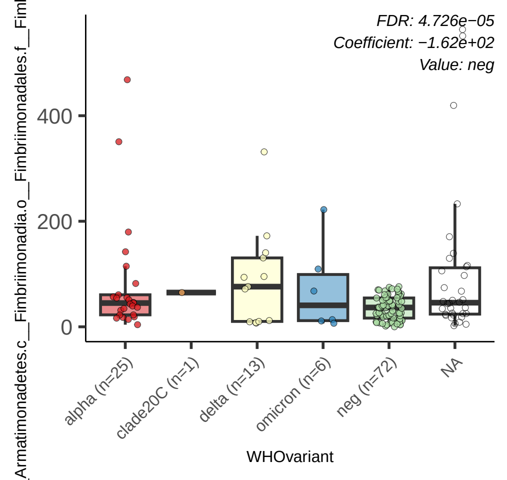

FDR: 4.726e−05
Coefficient: −1.62e+02
Value: neg
Armatimonadetes.c__Fimbriimonadia.o__Fimbriimonadales.f__Fiml
0
200
400
alpha (n=25)
clade20C (n=1)
delta (n=13)
omicron (n=6)
neg (n=72)
NA
WHOvariant

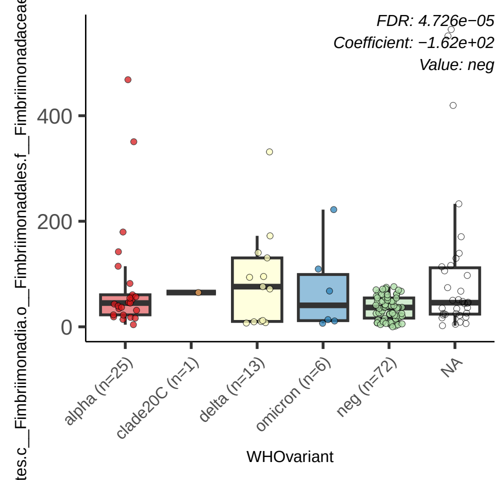

FDR: 4.726e−05
Coefficient: −1.62e+02
Value: neg
tes.c__Fimbriimonadia.o__Fimbriimonadales.f__Fimbriimonadaceae
0
200
400
alpha (n=25)
clade20C (n=1)
delta (n=13)
omicron (n=6)
neg (n=72)
NA
WHOvariant

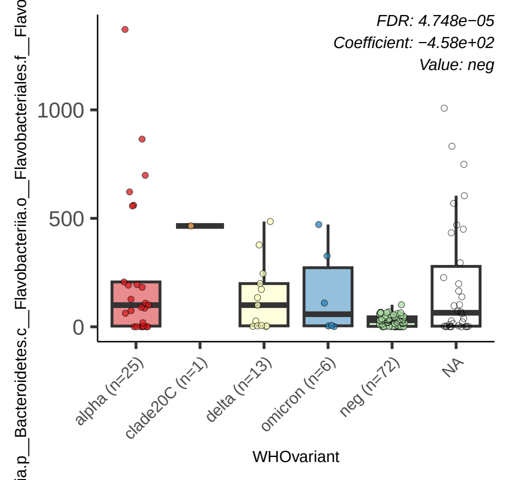
FDR: 4.748e−05
Coefficient: −4.58e+02
Value: neg
1000
500
0
alpha (n=25)
clade20C (n=1)
delta (n=13)
omicron (n=6)
neg (n=72)
NA
WHOvariant
ia.p__Bacteroidetes.c__Flavobacteriia.o__Flavobacteriales.f__Flavo

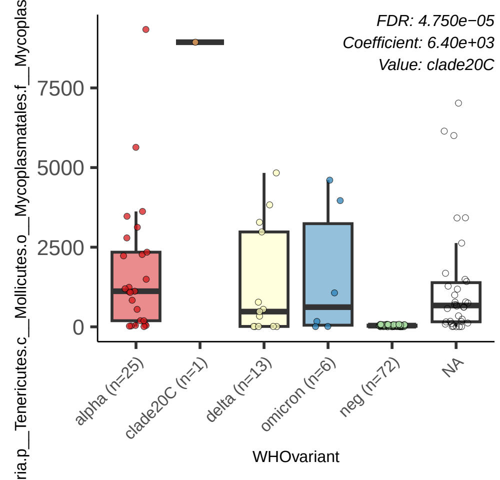

FDR: 4.750e−05
Coefficient: 6.40e+03
Value: clade20C
ria.p__Tenericutes.c__Mollicutes.o__Mycoplasmatales.f__Mycoplas
7500
5000
2500
0
alpha (n=25)
clade20C (n=1)
delta (n=13)
omicron (n=6)
neg (n=72)
NA
WHOvariant

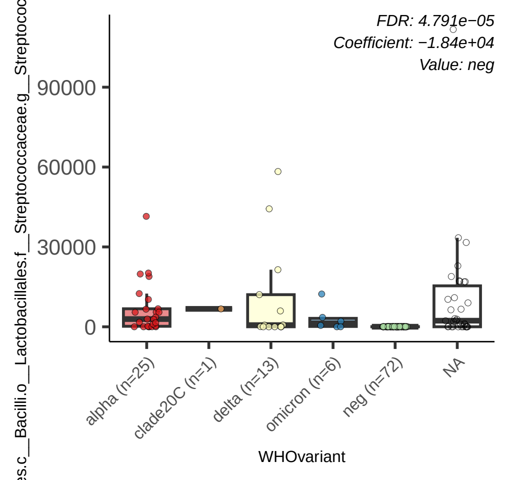

FDR: 4.791e−05
Coefficient: −1.84e+04
Value: neg
90000
60000
30000
0
alpha (n=25)
clade20C (n=1)
delta (n=13)
omicron (n=6)
neg (n=72)
NA
WHOvariant
es.c__Bacilli.o__Lactobacillales.f__Streptococcaceae.g__Streptococ

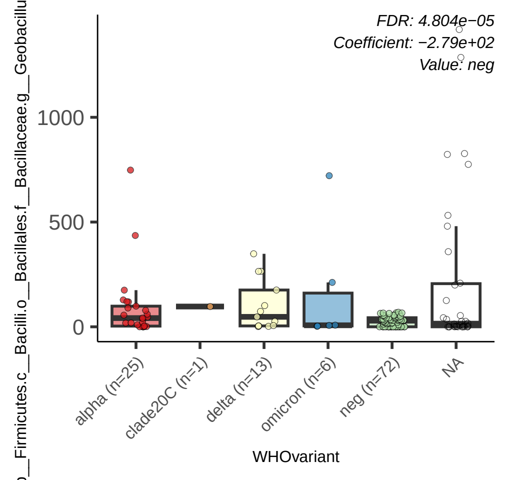
FDR: 4.804e−05
Coefficient: −2.79e+02
Value: neg
1000
500
0
alpha (n=25)
clade20C (n=1)
delta (n=13)
omicron (n=6)
neg (n=72)
NA
WHOvariant
o__Firmicutes.c__Bacilli.o__Bacillales.f__Bacillaceae.g__Geobacillu

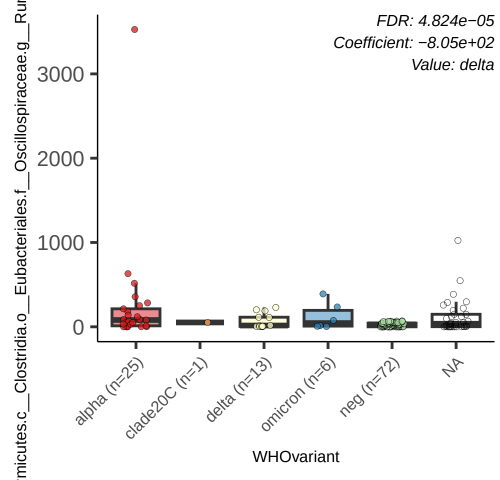

FDR: 4.824e−05
Coefficient: −8.05e+02
Value: delta
3000
2000
1000
0
micutes.c__Clostridia.o__Eubacteriales.f__Oscillospiraceae.g__Ru
alpha (n=25)
clade20C (n=1)
delta (n=13)
omicron (n=6)
neg (n=72)
NA
WHOvariant

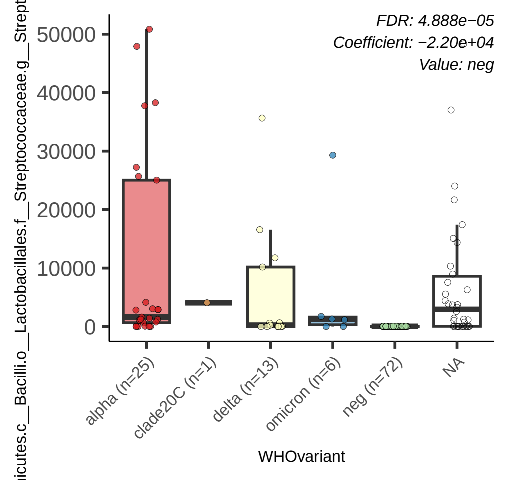

FDR: 4.888e−05
Coefficient: −2.20e+04
Value: neg
50000
40000
30000
20000
10000
0
alpha (n=25)
clade20C (n=1)
delta (n=13)
omicron (n=6)
neg (n=72)
NA
WHOvariant

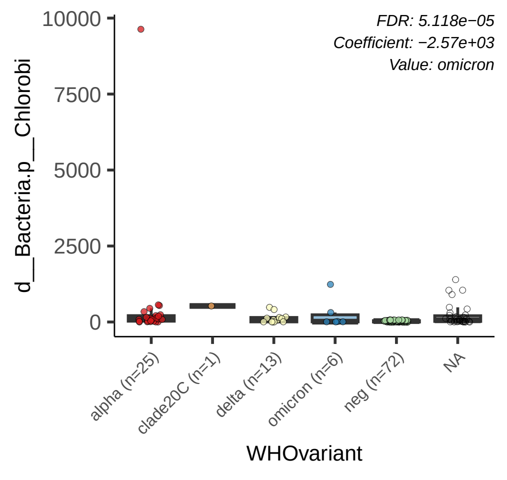
FDR: 5.118e−05
Coefficient: −2.57e+03
Value: omicron
d__Bacteria.p__Chlorobi
10000
7500
5000
2500
0
alpha (n=25)
clade20C (n=1)
delta (n=13)
omicron (n=6)
neg (n=72)
NA
WHOvariant

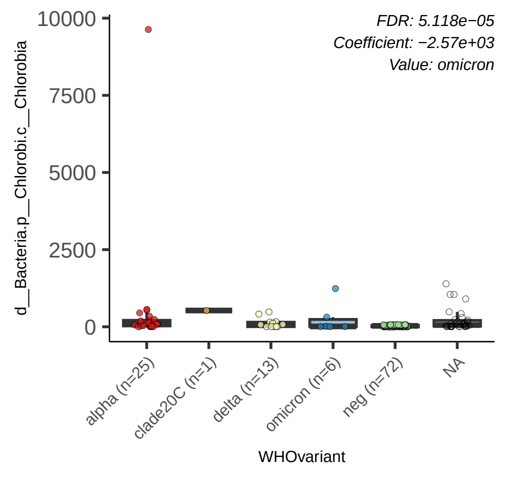

FDR: 5.118e−05
Coefficient: −2.57e+03
Value: omicron
d__Bacteria.p__Chlorobi.c__Chlorobia
10000
7500
5000
2500
0
alpha (n=25)
clade20C (n=1)
delta (n=13)
omicron (n=6)
neg (n=72)
NA
WHOvariant

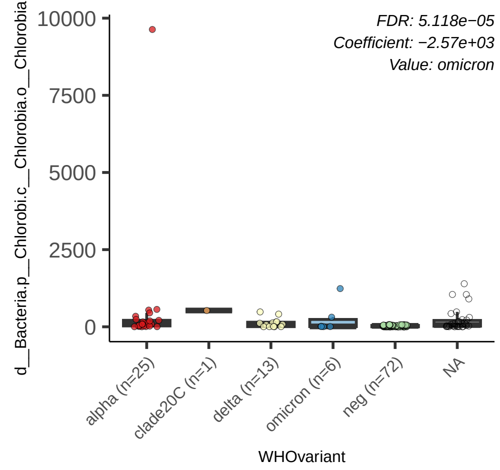

FDR: 5.118e−05
Coefficient: −2.57e+03
Value: omicron
d__Bacteria.p__Chlorobi.c__Chlorobia.o__Chlorobia
10000
7500
5000
2500
0
alpha (n=25)
clade20C (n=1)
delta (n=13)
omicron (n=6)
neg (n=72)
NA
WHOvariant

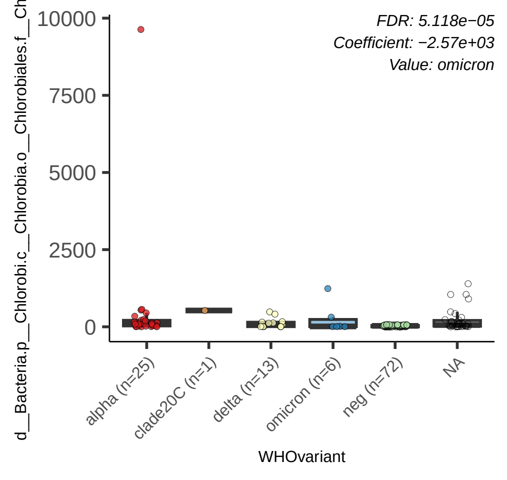

FDR: 5.118e−05
Coefficient: −2.57e+03
Value: omicron
d__Bacteria.p__Chlorobi.c__Chlorobia.o__Chlorobiales.f__Ch
10000
7500
5000
2500
0
alpha (n=25)
clade20C (n=1)
delta (n=13)
omicron (n=6)
neg (n=72)
NA
WHOvariant

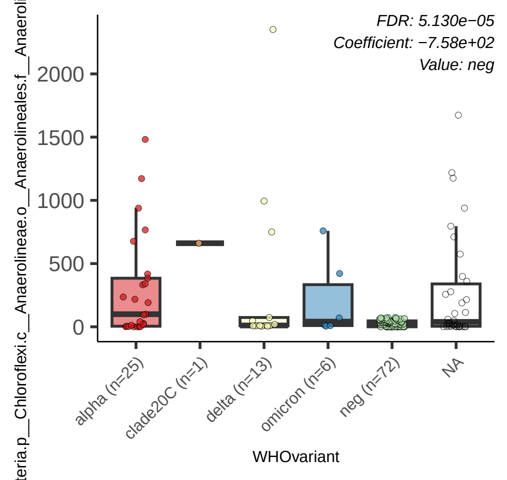
FDR: 5.130e−05
Coefficient: −7.58e+02
Value: neg
teria.p__Chloroflexi.c__Anaerolineae.o__Anaerolineales.f__Anaeroli
2000
1500
1000
500
0
alpha (n=25)
clade20C (n=1)
delta (n=13)
omicron (n=6)
neg (n=72)
NA
WHOvariant

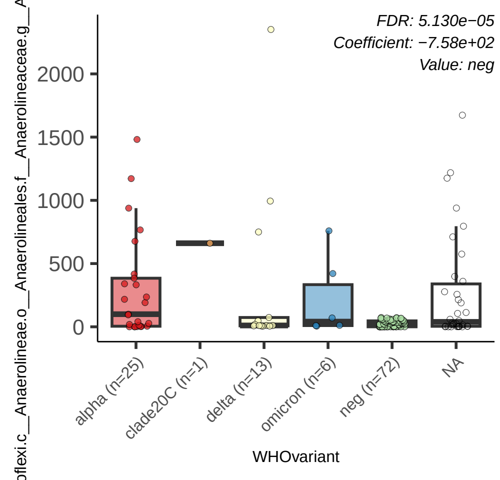

FDR: 5.130e−05
Coefficient: −7.58e+02
Value: neg
2000
1500
1000
500
0
alpha (n=25)
clade20C (n=1)
delta (n=13)
omicron (n=6)
neg (n=72)
NA
WHOvariant
oflexi.c__Anaerolineae.o__Anaerolineales.f__Anaerolineaceae.g__A

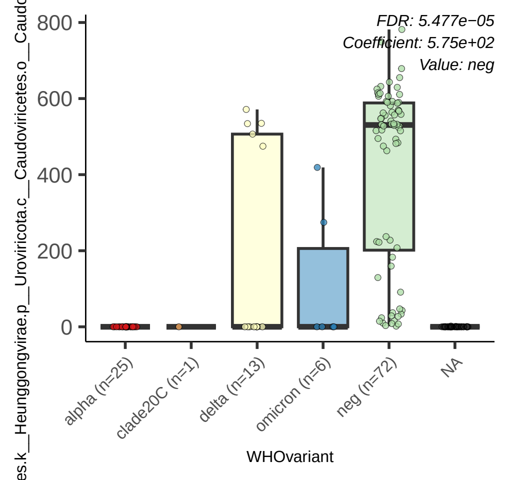

es.k__Heunggongvirae.p__Uroviricota.c__Caudoviricetes.o__Caudo
FDR: 5.477e−05
Coefficient: 5.75e+02
Value: neg
800
600
400
200
0
alpha (n=25)
clade20C (n=1)
delta (n=13)
omicron (n=6)
neg (n=72)
NA
WHOvariant

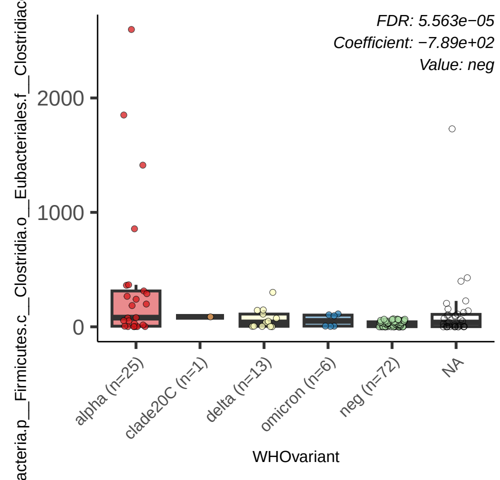

FDR: 5.563e−05
Coefficient: −7.89e+02
Value: neg
acteria.p__Firmicutes.c__Clostridia.o__Eubacteriales.f__Clostridiac
0
1000
2000
alpha (n=25)
clade20C (n=1)
delta (n=13)
omicron (n=6)
neg (n=72)
NA
WHOvariant

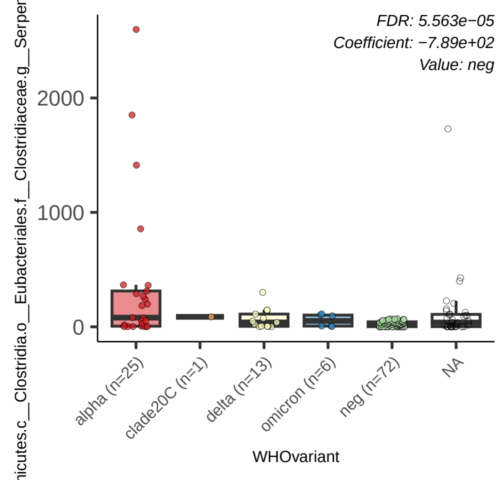

FDR: 5.563e−05
Coefficient: −7.89e+02
Value: neg
2000
1000
0
alpha (n=25)
clade20C (n=1)
delta (n=13)
omicron (n=6)
neg (n=72)
NA
WHOvariant
nicutes.c__Clostridia.o__Eubacteriales.f__Clostridiaceae.g__Serpen

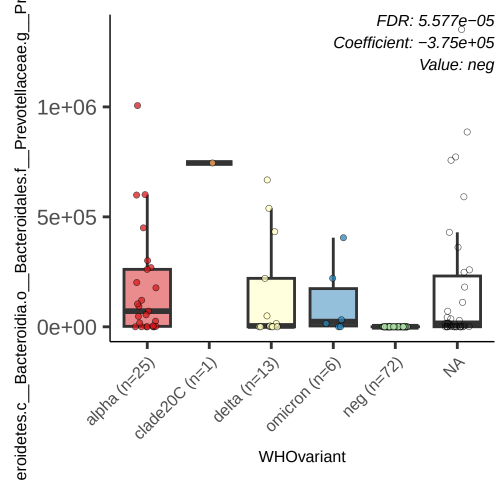

FDR: 5.577e−05
Coefficient: −3.75e+05
Value: neg
eroidetes.c__Bacteroidia.o__Bacteroidales.f__Prevotellaceae.g__Pr
1e+06
5e+05
0e+00
alpha (n=25)
clade20C (n=1)
delta (n=13)
omicron (n=6)
neg (n=72)
NA
WHOvariant

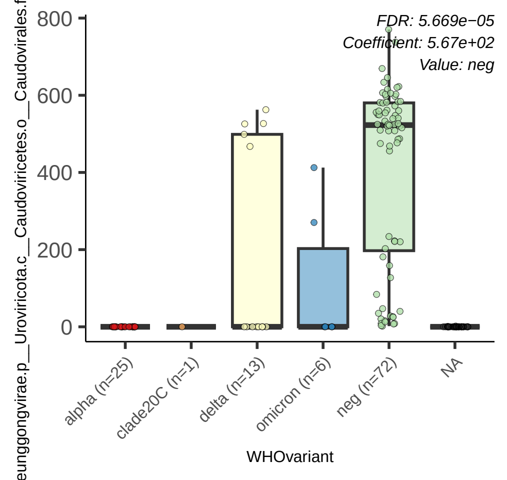

FDR: 5.669e−05
Coefficient: 5.67e+02
Value: neg
800
600
400
200
0
alpha (n=25)
clade20C (n=1)
delta (n=13)
omicron (n=6)
neg (n=72)
NA
WHOvariant

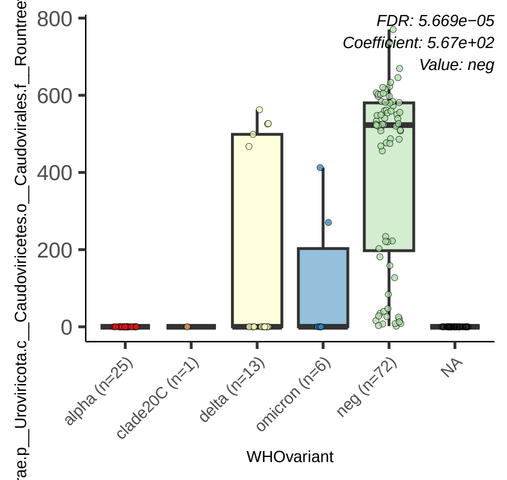

FDR: 5.669e−05
Coefficient: 5.67e+02
Value: neg
0
200
400
600
800
rae.p__Uroviricota.c__Caudoviricetes.o__Caudovirales.f__Rountree
alpha (n=25)
clade20C (n=1)
delta (n=13)
omicron (n=6)
neg (n=72)
NA
WHOvariant

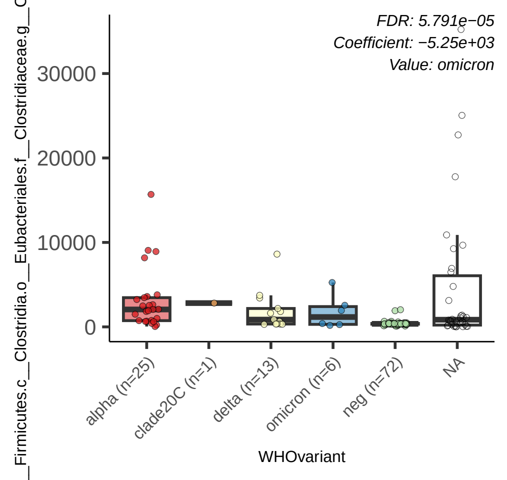
FDR: 5.791e−05
Coefficient: −5.25e+03
Value: omicron
Firmicutes.c__Clostridia.o__Eubacteriales.f__Clostridiaceae.g__C
0
10000
20000
30000
alpha (n=25)
clade20C (n=1)
delta (n=13)
omicron (n=6)
neg (n=72)
NA
WHOvariant

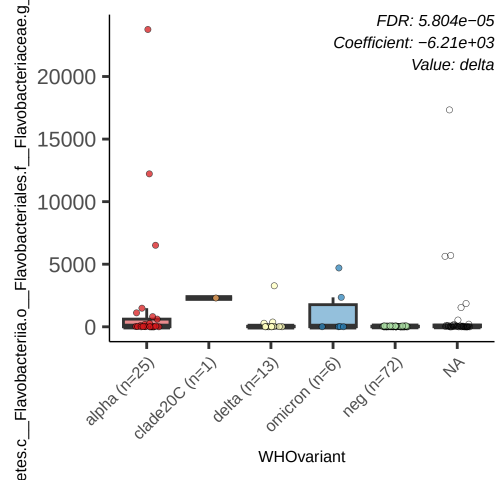
FDR: 5.804e−05
Coefficient: −6.21e+03
Value: delta
etes.c__Flavobacteriia.o__Flavobacteriales.f__Flavobacteriaceae.g_
20000
15000
10000
5000
0
alpha (n=25)
clade20C (n=1)
delta (n=13)
omicron (n=6)
neg (n=72)
NA
WHOvariant

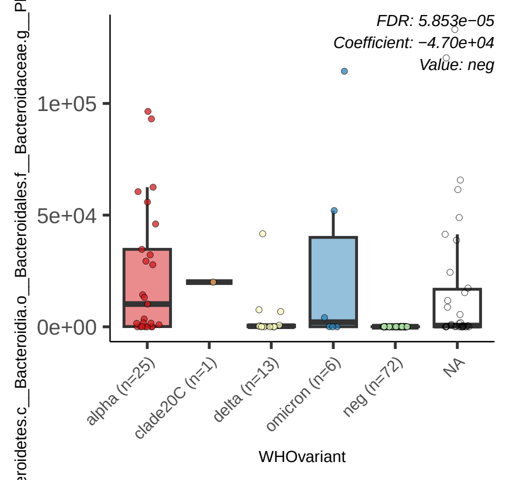

FDR: 5.853e−05
Coefficient: −4.70e+04
Value: neg
1e+05
5e+04
0e+00
eroidetes.c__Bacteroidia.o__Bacteroidales.f__Bacteroidaceae.g__P
alpha (n=25)
clade20C (n=1)
delta (n=13)
omicron (n=6)
neg (n=72)
NA
WHOvariant

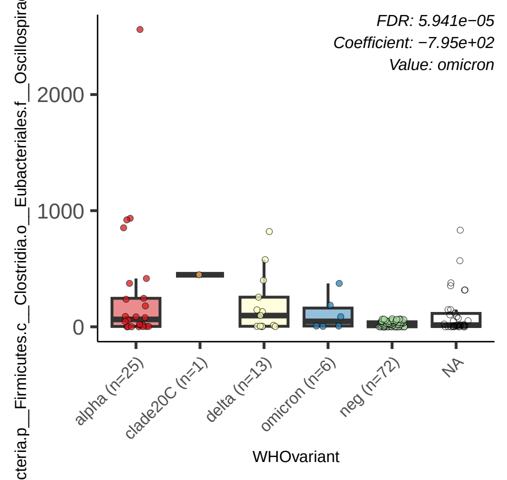

FDR: 5.941e−05
Coefficient: −7.95e+02
Value: omicron
cteria.p__Firmicutes.c__Clostridia.o__Eubacteriales.f__Oscillospirac
2000
1000
0
alpha (n=25)
clade20C (n=1)
delta (n=13)
omicron (n=6)
neg (n=72)
NA
WHOvariant

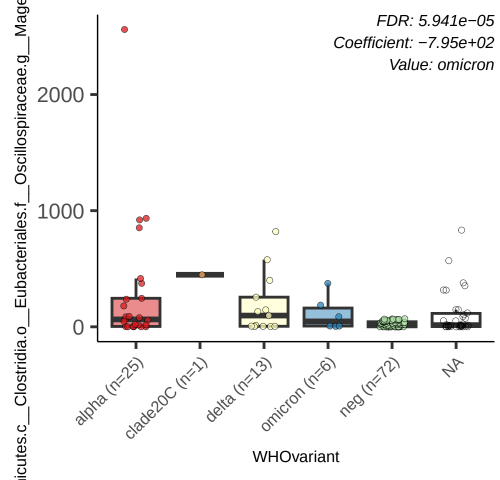

FDR: 5.941e−05
Coefficient: −7.95e+02
Value: omicron
2000
1000
0
alpha (n=25)
clade20C (n=1)
delta (n=13)
omicron (n=6)
neg (n=72)
NA
WHOvariant
icutes.c__Clostridia.o__Eubacteriales.f__Oscillospiraceae.g__Mage

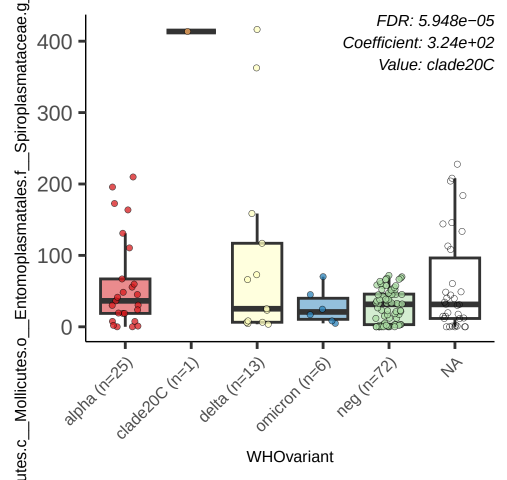

FDR: 5.948e−05
Coefficient: 3.24e+02
Value: clade20C
utes.c__Mollicutes.o__Entomoplasmatales.f__Spiroplasmataceae.g_
400
300
200
100
0
alpha (n=25)
clade20C (n=1)
delta (n=13)
omicron (n=6)
neg (n=72)
NA
WHOvariant

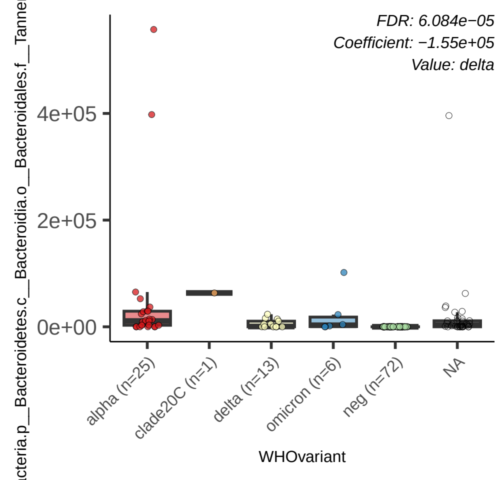

FDR: 6.084e−05
Coefficient: −1.55e+05
Value: delta
4e+05
2e+05
0e+00
acteria.p__Bacteroidetes.c__Bacteroidia.o__Bacteroidales.f__Tanne
alpha (n=25)
clade20C (n=1)
delta (n=13)
omicron (n=6)
neg (n=72)
NA
WHOvariant

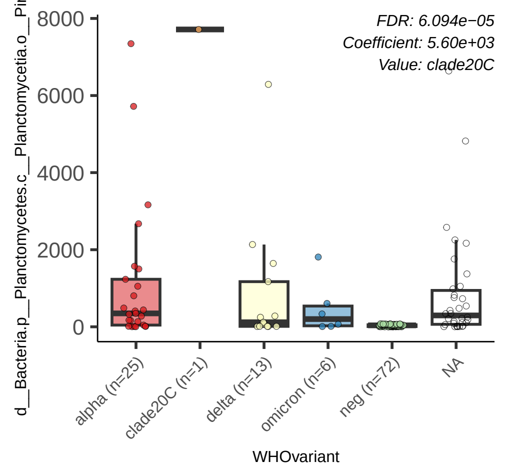
FDR: 6.094e−05
Coefficient: 5.60e+03
Value: clade20C
d__Bacteria.p__Planctomycetes.c__Planctomycetia.o__Pi
0
2000
4000
6000
8000
alpha (n=25)
clade20C (n=1)
delta (n=13)
omicron (n=6)
neg (n=72)
NA
WHOvariant

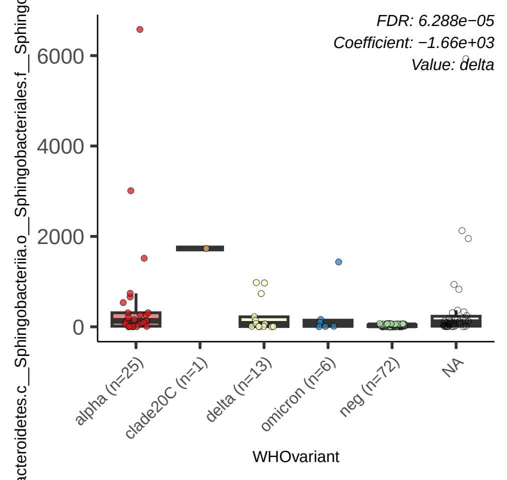

FDR: 6.288e−05
Coefficient: −1.66e+03
Value: delta
6000
4000
2000
0
acteroidetes.c__Sphingobacteriia.o__Sphingobacteriales.f__Sphingo
alpha (n=25)
clade20C (n=1)
delta (n=13)
omicron (n=6)
neg (n=72)
NA
WHOvariant

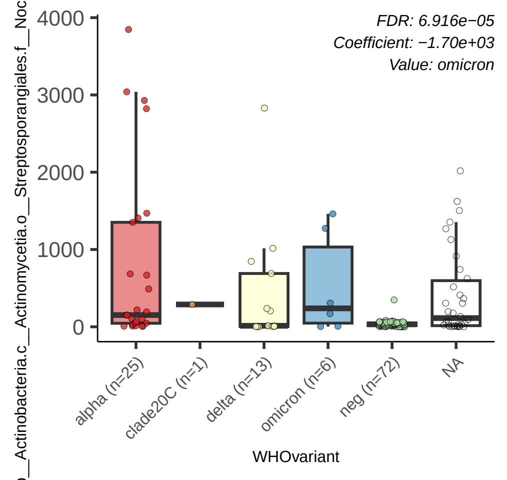

FDR: 6.916e−05
Coefficient: −1.70e+03
Value: omicron
4000
3000
2000
1000
0
o__Actinobacteria.c__Actinomycetia.o__Streptosporangiales.f__Noc
alpha (n=25)
clade20C (n=1)
delta (n=13)
omicron (n=6)
neg (n=72)
NA
WHOvariant

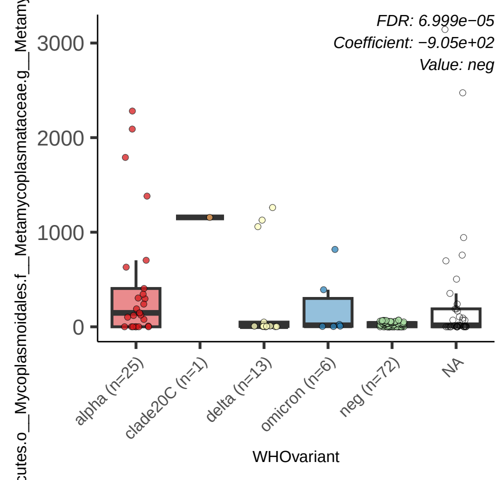

FDR: 6.999e−05
Coefficient: −9.05e+02
Value: neg
3000
2000
1000
0
cutes.o__Mycoplasmoidales.f__Metamycoplasmataceae.g__Metamy
alpha (n=25)
clade20C (n=1)
delta (n=13)
omicron (n=6)
neg (n=72)
NA
WHOvariant

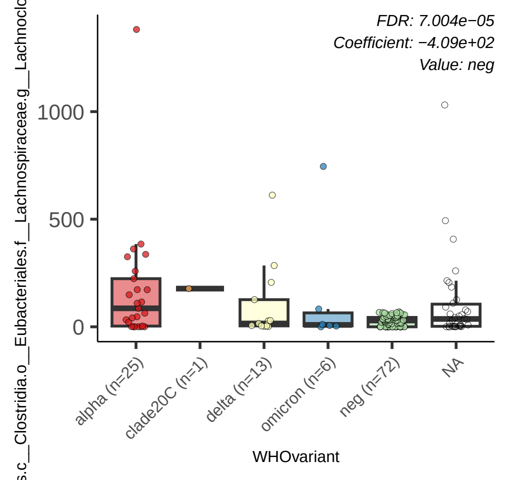
FDR: 7.004e−05
Coefficient: −4.09e+02
Value: neg
1000
500
0
alpha (n=25)
clade20C (n=1)
delta (n=13)
omicron (n=6)
neg (n=72)
NA
WHOvariant
s.c__Clostridia.o__Eubacteriales.f__Lachnospiraceae.g__Lachnocl

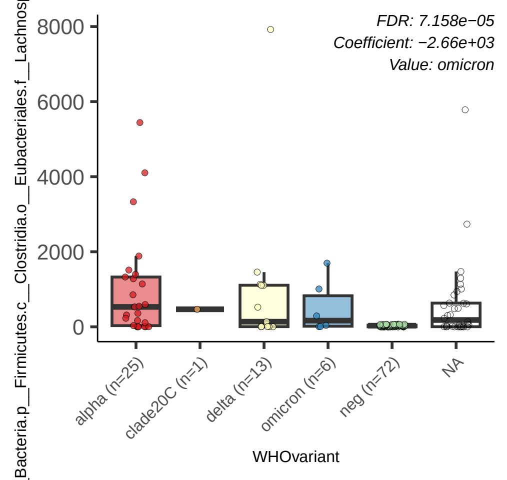

FDR: 7.158e−05
Coefficient: −2.66e+03
Value: omicron
Bacteria.p__Firmicutes.c__Clostridia.o__Eubacteriales.f__Lachnosp
8000
6000
4000
2000
0
alpha (n=25)
clade20C (n=1)
delta (n=13)
omicron (n=6)
neg (n=72)
NA
WHOvariant

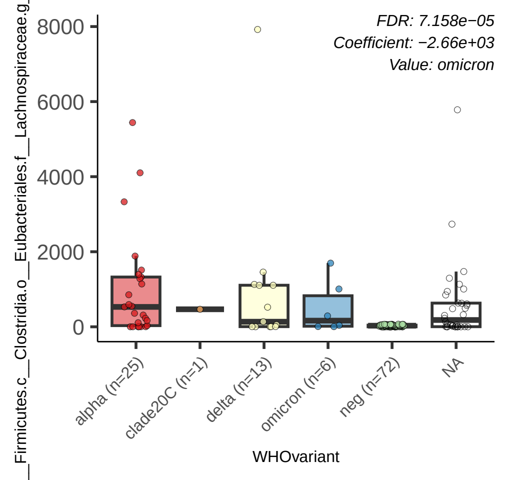

FDR: 7.158e−05
Coefficient: −2.66e+03
Value: omicron
8000
6000
4000
2000
0
Firmicutes.c__Clostridia.o__Eubacteriales.f__Lachnospiraceae.g_
alpha (n=25)
clade20C (n=1)
delta (n=13)
omicron (n=6)
neg (n=72)
NA
WHOvariant

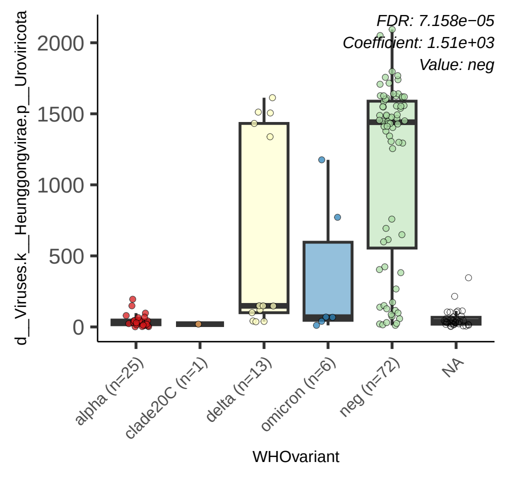

FDR: 7.158e−05
Coefficient: 1.51e+03
Value: neg
d__Viruses.k__Heunggongvirae.p__Uroviricota
0
500
1000
1500
2000
alpha (n=25)
clade20C (n=1)
delta (n=13)
omicron (n=6)
neg (n=72)
NA
WHOvariant

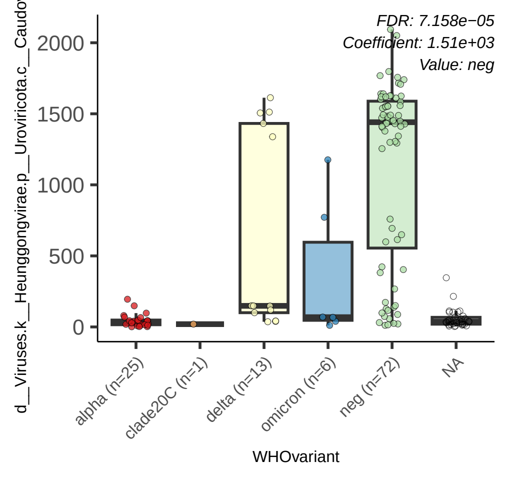

FDR: 7.158e−05
Coefficient: 1.51e+03
Value: neg
d__Viruses.k__Heunggongvirae.p__Uroviricota.c__Caudo
0
500
1000
1500
2000
alpha (n=25)
clade20C (n=1)
delta (n=13)
omicron (n=6)
neg (n=72)
NA
WHOvariant

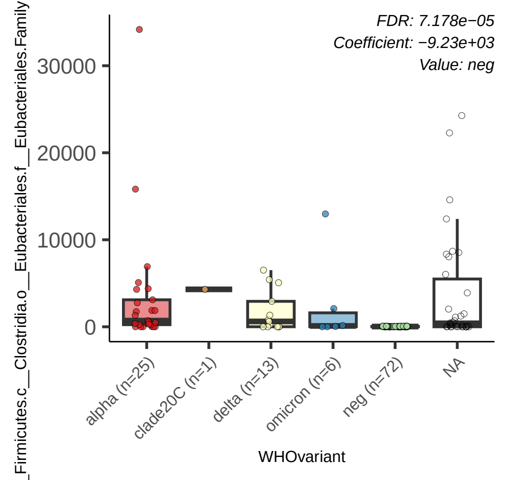
FDR: 7.178e−05
Coefficient: −9.23e+03
Value: neg
30000
20000
10000
0
Firmicutes.c__Clostridia.o__Eubacteriales.f__Eubacteriales.Family
alpha (n=25)
clade20C (n=1)
delta (n=13)
omicron (n=6)
neg (n=72)
NA
WHOvariant

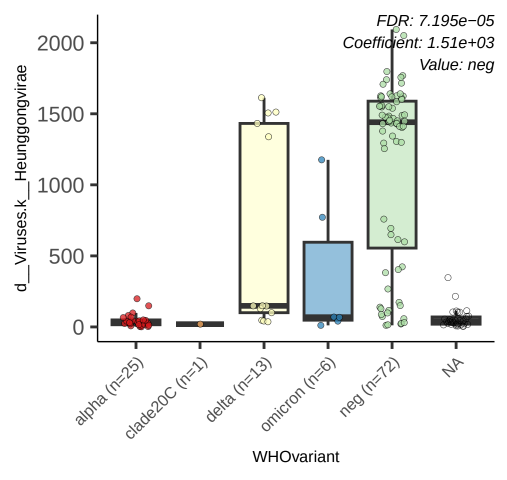
FDR: 7.195e−05
Coefficient: 1.51e+03
Value: neg
d__Viruses.k__Heunggongvirae
0
500
1000
1500
2000
alpha (n=25)
clade20C (n=1)
delta (n=13)
omicron (n=6)
neg (n=72)
NA
WHOvariant

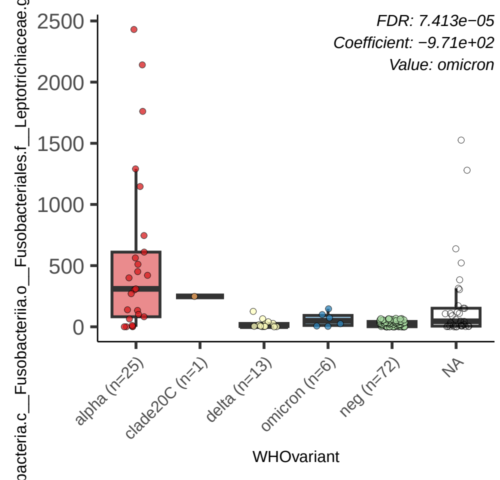
FDR: 7.413e−05
Coefficient: −9.71e+02
Value: omicron
2500
2000
1500
1000
500
0
bacteria.c__Fusobacteriia.o__Fusobacteriales.f__Leptotrichiaceae.g
alpha (n=25)
clade20C (n=1)
delta (n=13)
omicron (n=6)
neg (n=72)
NA
WHOvariant

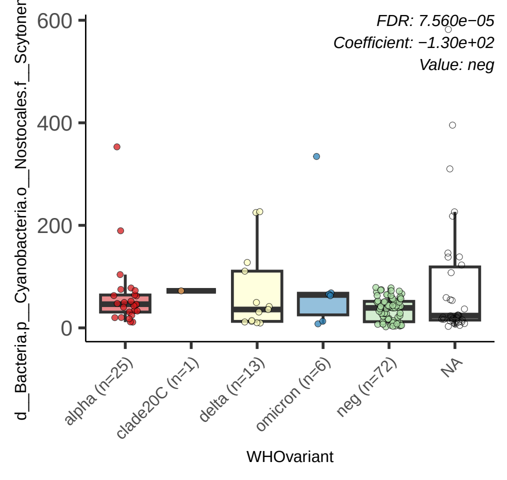

FDR: 7.560e−05
Coefficient: −1.30e+02
Value: neg
d__Bacteria.p__Cyanobacteria.o__Nostocales.f__Scytoner
600
400
200
0
alpha (n=25)
clade20C (n=1)
delta (n=13)
omicron (n=6)
neg (n=72)
NA
WHOvariant

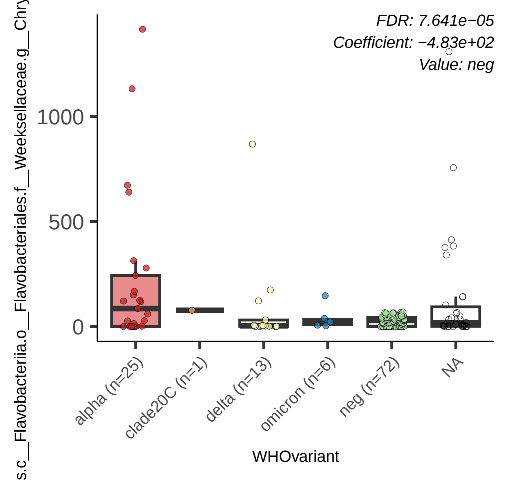

FDR: 7.641e−05
Coefficient: −4.83e+02
Value: neg
1000
500
0
alpha (n=25)
clade20C (n=1)
delta (n=13)
omicron (n=6)
neg (n=72)
NA
WHOvariant
s.c__Flavobacteriia.o__Flavobacteriales.f__Weeksellaceae.g__Chry

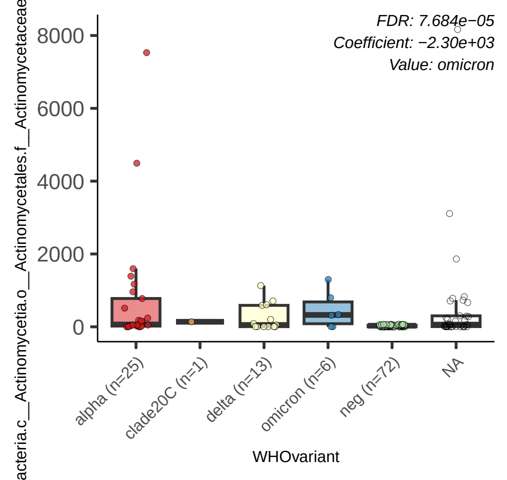
FDR: 7.684e−05
Coefficient: −2.30e+03
Value: omicron
acteria.c__Actinomycetia.o__Actinomycetales.f__Actinomycetaceae
8000
6000
4000
2000
0
alpha (n=25)
clade20C (n=1)
delta (n=13)
omicron (n=6)
neg (n=72)
NA
WHOvariant

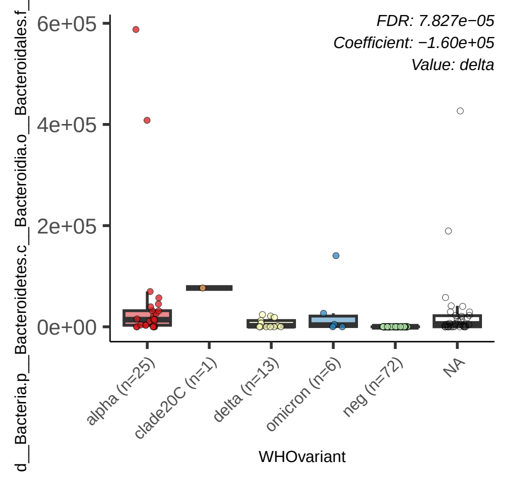

FDR: 7.827e−05
Coefficient: −1.60e+05
Value: delta
d__Bacteria.p__Bacteroidetes.c__Bacteroidia.o__Bacteroidales.f
6e+05
4e+05
2e+05
0e+00
alpha (n=25)
clade20C (n=1)
delta (n=13)
omicron (n=6)
neg (n=72)
NA
WHOvariant

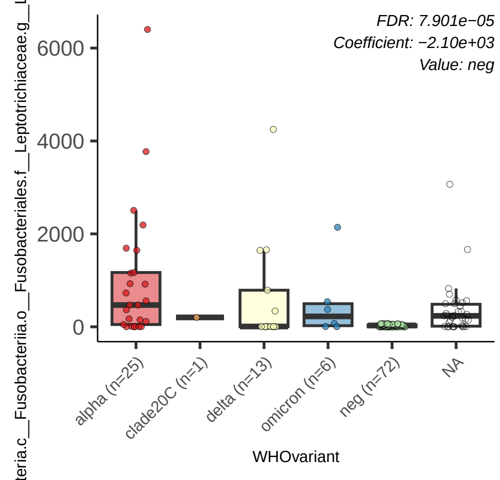
FDR: 7.901e−05
Coefficient: −2.10e+03
Value: neg
teria.c__Fusobacteriia.o__Fusobacteriales.f__Leptotrichiaceae.g__L
6000
4000
2000
0
alpha (n=25)
clade20C (n=1)
delta (n=13)
omicron (n=6)
neg (n=72)
NA
WHOvariant

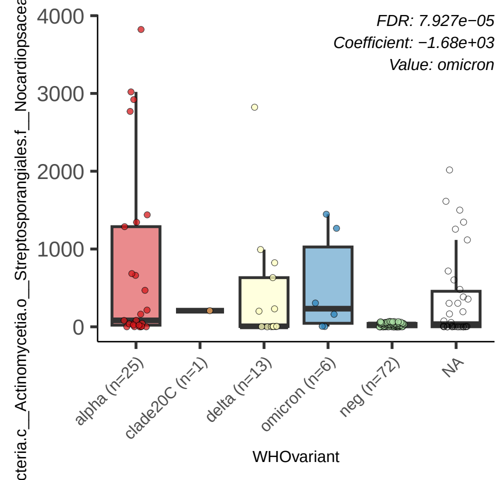

FDR: 7.927e−05
Coefficient: −1.68e+03
Value: omicron
4000
3000
2000
1000
0
cteria.c__Actinomycetia.o__Streptosporangiales.f__Nocardiopsacea
alpha (n=25)
clade20C (n=1)
delta (n=13)
omicron (n=6)
neg (n=72)
NA
WHOvariant

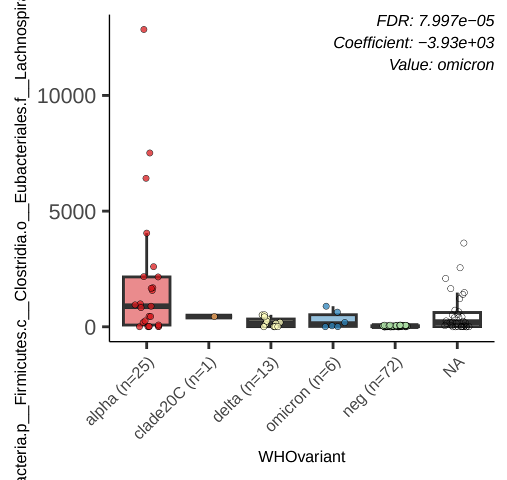

FDR: 7.997e−05
Coefficient: −3.93e+03
Value: omicron
acteria.p__Firmicutes.c__Clostridia.o__Eubacteriales.f__Lachnospira
10000
5000
0
alpha (n=25)
clade20C (n=1)
delta (n=13)
omicron (n=6)
neg (n=72)
NA
WHOvariant

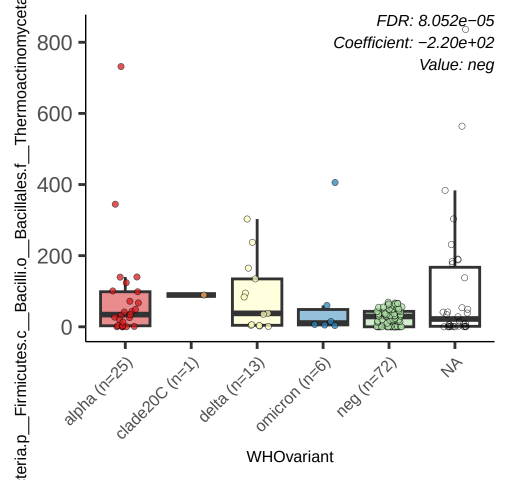

FDR: 8.052e−05
Coefficient: −2.20e+02
Value: neg
0
200
400
600
800
teria.p__Firmicutes.c__Bacilli.o__Bacillales.f__Thermoactinomyceta
alpha (n=25)
clade20C (n=1)
delta (n=13)
omicron (n=6)
neg (n=72)
NA
WHOvariant

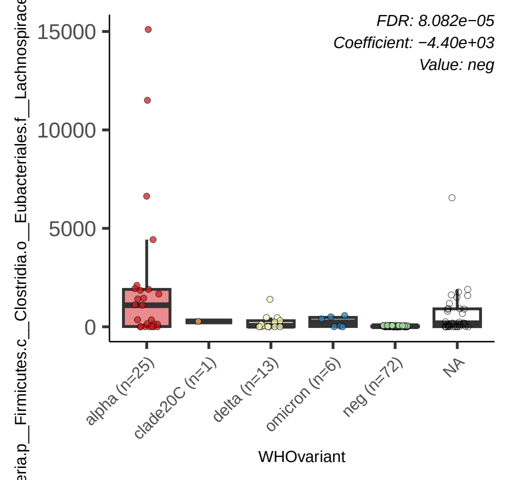
FDR: 8.082e−05
Coefficient: −4.40e+03
Value: neg
15000
10000
5000
0
eria.p__Firmicutes.c__Clostridia.o__Eubacteriales.f__Lachnospirace
alpha (n=25)
clade20C (n=1)
delta (n=13)
omicron (n=6)
neg (n=72)
NA
WHOvariant

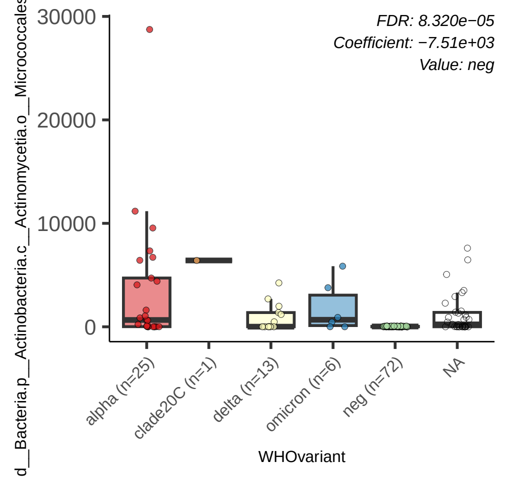
FDR: 8.320e−05
Coefficient: −7.51e+03
Value: neg
d__Bacteria.p__Actinobacteria.c__Actinomycetia.o__Micrococcales
30000
20000
10000
0
alpha (n=25)
clade20C (n=1)
delta (n=13)
omicron (n=6)
neg (n=72)
NA
WHOvariant

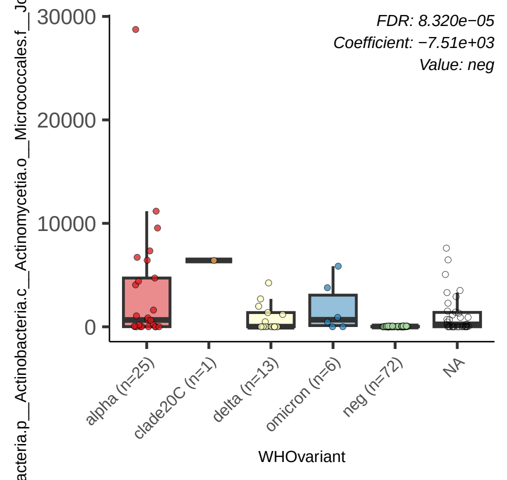

FDR: 8.320e−05
Coefficient: −7.51e+03
Value: neg
30000
20000
10000
0
alpha (n=25)
clade20C (n=1)
delta (n=13)
omicron (n=6)
neg (n=72)
NA
WHOvariant
acteria.p__Actinobacteria.c__Actinomycetia.o__Micrococcales.f__Jo

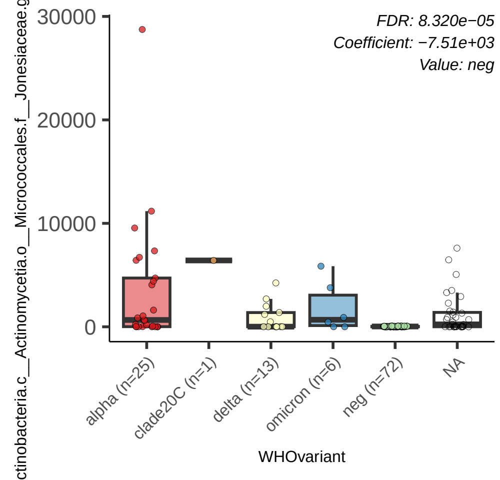

FDR: 8.320e−05
Coefficient: −7.51e+03
Value: neg
ctinobacteria.c__Actinomycetia.o__Micrococcales.f__Jonesiaceae.g
30000
20000
10000
0
alpha (n=25)
clade20C (n=1)
delta (n=13)
omicron (n=6)
neg (n=72)
NA
WHOvariant

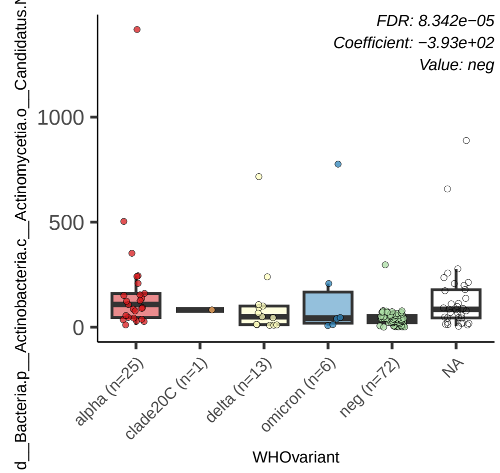

FDR: 8.342e−05
Coefficient: −3.93e+02
Value: neg
d__Bacteria.p__Actinobacteria.c__Actinomycetia.o__Candidatus.N
0
500
1000
alpha (n=25)
clade20C (n=1)
delta (n=13)
omicron (n=6)
neg (n=72)
NA
WHOvariant

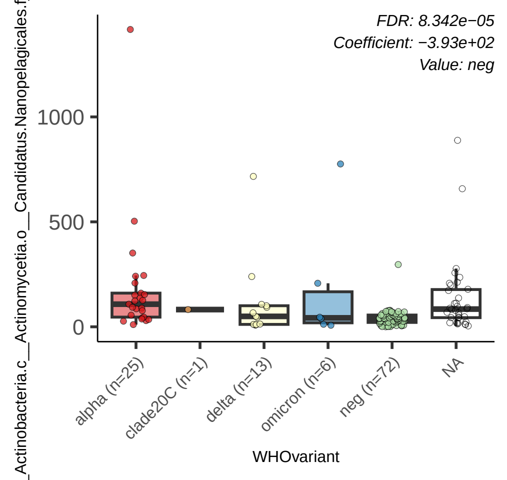

FDR: 8.342e−05
Coefficient: −3.93e+02
Value: neg
Actinobacteria.c__Actinomycetia.o__Candidatus.Nanopelagicales.f
1000
500
0
alpha (n=25)
clade20C (n=1)
delta (n=13)
omicron (n=6)
neg (n=72)
NA
WHOvariant

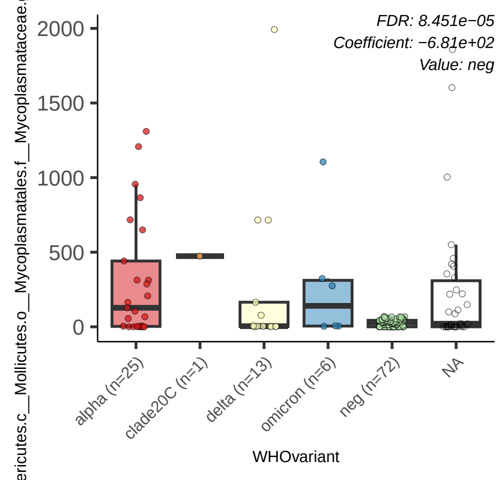
FDR: 8.451e−05
Coefficient: −6.81e+02
Value: neg
2000
1500
1000
500
0
ericutes.c__Mollicutes.o__Mycoplasmatales.f__Mycoplasmataceae.
alpha (n=25)
clade20C (n=1)
delta (n=13)
omicron (n=6)
neg (n=72)
NA
WHOvariant

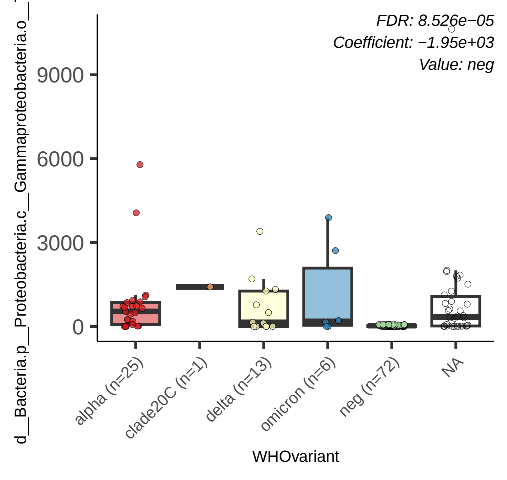

FDR: 8.526e−05
Coefficient: −1.95e+03
Value: neg
d__Bacteria.p__Proteobacteria.c__Gammaproteobacteria.o__
9000
6000
3000
0
alpha (n=25)
clade20C (n=1)
delta (n=13)
omicron (n=6)
neg (n=72)
NA
WHOvariant

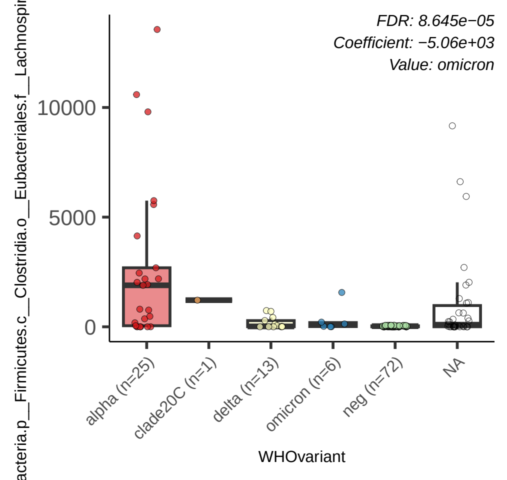
FDR: 8.645e−05
Coefficient: −5.06e+03
Value: omicron
acteria.p__Firmicutes.c__Clostridia.o__Eubacteriales.f__Lachnospi
10000
5000
0
alpha (n=25)
clade20C (n=1)
delta (n=13)
omicron (n=6)
neg (n=72)
NA
WHOvariant

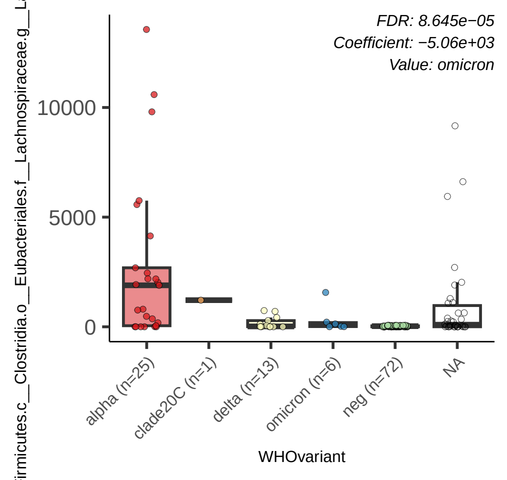

FDR: 8.645e−05
Coefficient: −5.06e+03
Value: omicron
10000
5000
0
alpha (n=25)
clade20C (n=1)
delta (n=13)
omicron (n=6)
neg (n=72)
NA
WHOvariant
irmicutes.c__Clostridia.o__Eubacteriales.f__Lachnospiraceae.g__L

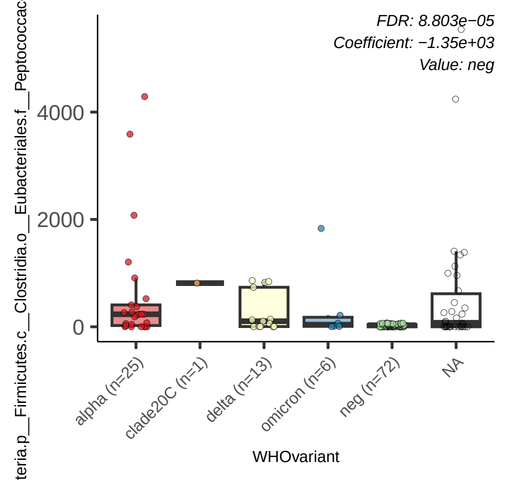

FDR: 8.803e−05
Coefficient: −1.35e+03
Value: neg
teria.p__Firmicutes.c__Clostridia.o__Eubacteriales.f__Peptococcac
4000
2000
0
alpha (n=25)
clade20C (n=1)
delta (n=13)
omicron (n=6)
neg (n=72)
NA
WHOvariant

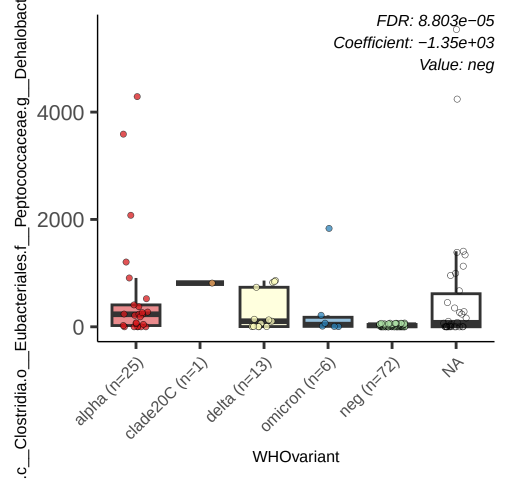
FDR: 8.803e−05
Coefficient: −1.35e+03
Value: neg
c__Clostridia.o__Eubacteriales.f__Peptococcaceae.g__Dehalobact
4000
2000
0
alpha (n=25)
clade20C (n=1)
delta (n=13)
omicron (n=6)
neg (n=72)
NA
WHOvariant

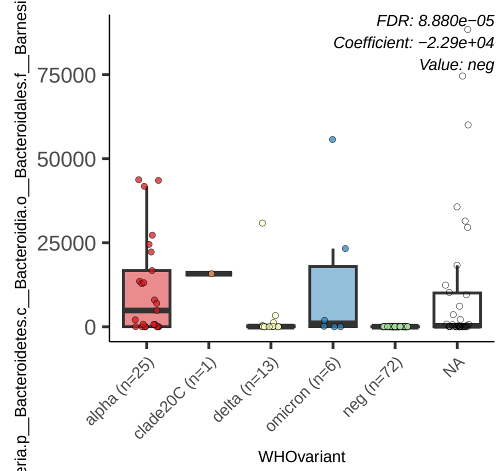
FDR: 8.880e−05
Coefficient: −2.29e+04
Value: neg
teria.p__Bacteroidetes.c__Bacteroidia.o__Bacteroidales.f__Barnesi
75000
50000
25000
0
alpha (n=25)
clade20C (n=1)
delta (n=13)
omicron (n=6)
neg (n=72)
NA
WHOvariant

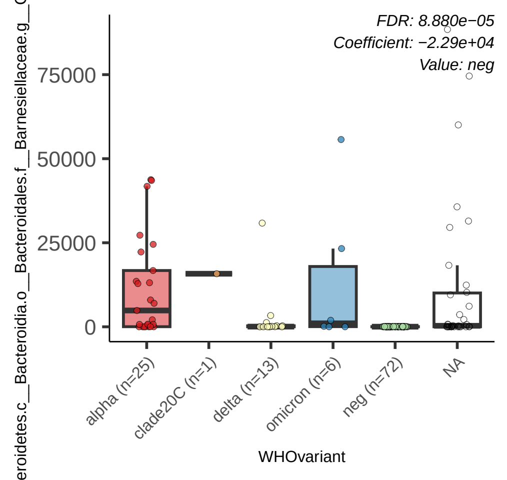

FDR: 8.880e−05
Coefficient: −2.29e+04
Value: neg
eroidetes.c__Bacteroidia.o__Bacteroidales.f__Barnesiellaceae.g__C
75000
50000
25000
0
alpha (n=25)
clade20C (n=1)
delta (n=13)
omicron (n=6)
neg (n=72)
NA
WHOvariant

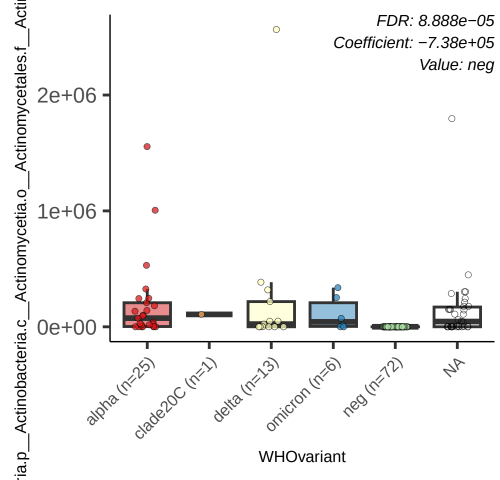

FDR: 8.888e−05
Coefficient: −7.38e+05
Value: neg
2e+06
1e+06
0e+00
ria.p__Actinobacteria.c__Actinomycetia.o__Actinomycetales.f__Acti
alpha (n=25)
clade20C (n=1)
delta (n=13)
omicron (n=6)
neg (n=72)
NA
WHOvariant

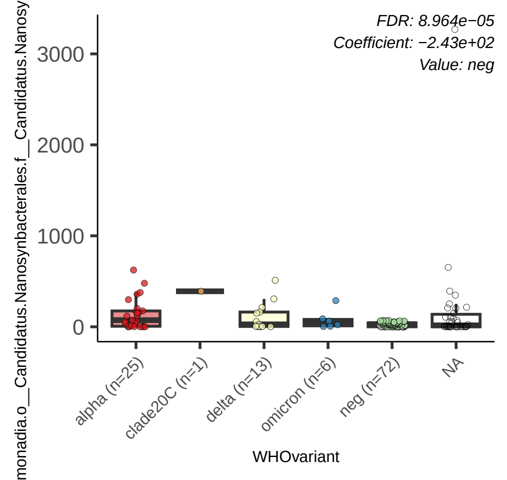

FDR: 8.964e−05
Coefficient: −2.43e+02
Value: neg
3000
2000
1000
0
monadia.o__Candidatus.Nanosynbacterales.f__Candidatus.Nanosy
alpha (n=25)
clade20C (n=1)
delta (n=13)
omicron (n=6)
neg (n=72)
NA
WHOvariant

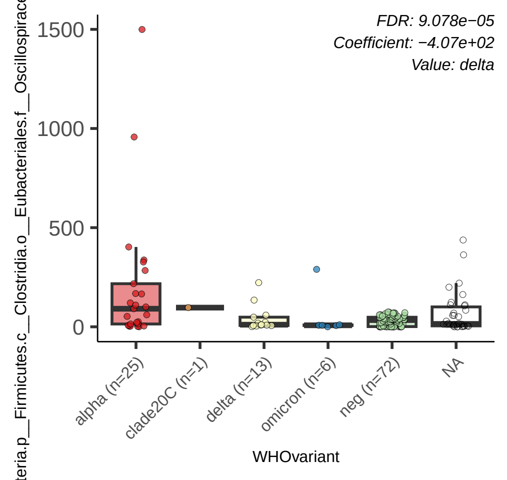
FDR: 9.078e−05
Coefficient: −4.07e+02
Value: delta
1500
1000
500
0
alpha (n=25)
clade20C (n=1)
delta (n=13)
omicron (n=6)
neg (n=72)
NA
WHOvariant
teria.p__Firmicutes.c__Clostridia.o__Eubacteriales.f__Oscillospirace

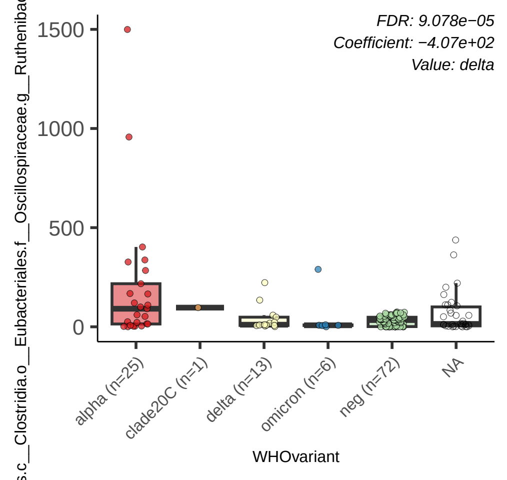
FDR: 9.078e−05
Coefficient: −4.07e+02
Value: delta
1500
1000
500
0
s.c__Clostridia.o__Eubacteriales.f__Oscillospiraceae.g__Ruthenibac
alpha (n=25)
clade20C (n=1)
delta (n=13)
omicron (n=6)
neg (n=72)
NA
WHOvariant

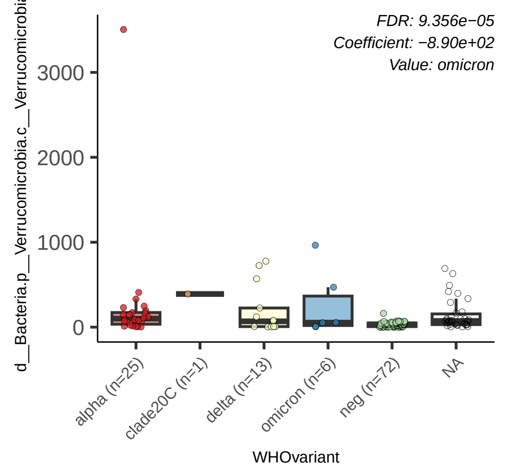

FDR: 9.356e−05
Coefficient: −8.90e+02
Value: omicron
d__Bacteria.p__Verrucomicrobia.c__Verrucomicrobia
3000
2000
1000
0
alpha (n=25)
clade20C (n=1)
delta (n=13)
omicron (n=6)
neg (n=72)
NA
WHOvariant

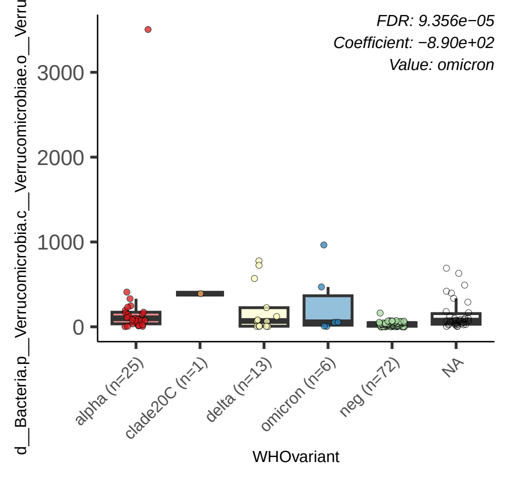
FDR: 9.356e−05
Coefficient: −8.90e+02
Value: omicron
d__Bacteria.p__Verrucomicrobia.c__Verrucomicrobiae.o__Verru
3000
2000
1000
0
alpha (n=25)
clade20C (n=1)
delta (n=13)
omicron (n=6)
neg (n=72)
NA
WHOvariant

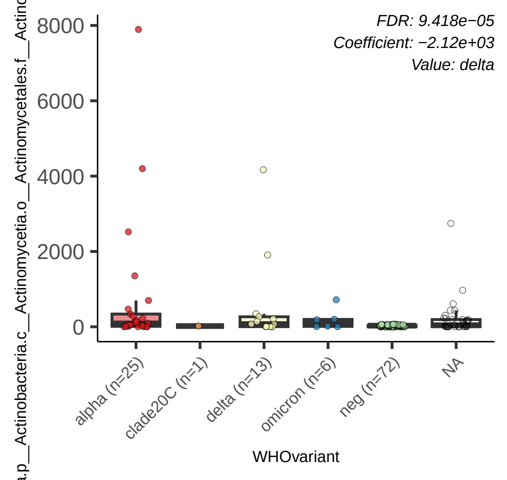

FDR: 9.418e−05
Coefficient: −2.12e+03
Value: delta
8000
6000
4000
2000
0
alpha (n=25)
clade20C (n=1)
delta (n=13)
omicron (n=6)
neg (n=72)
NA
WHOvariant

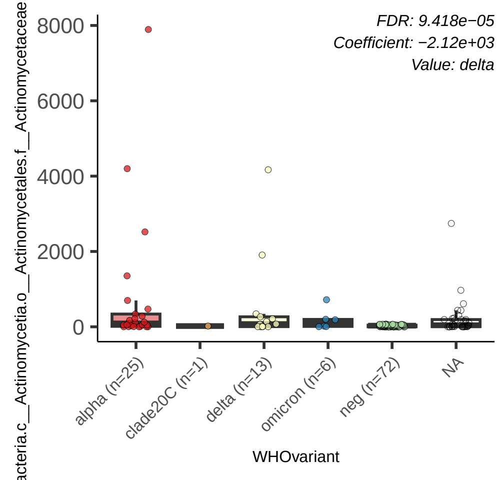

FDR: 9.418e−05
Coefficient: −2.12e+03
Value: delta
8000
6000
4000
2000
0
acteria.c__Actinomycetia.o__Actinomycetales.f__Actinomycetaceae
alpha (n=25)
clade20C (n=1)
delta (n=13)
omicron (n=6)
neg (n=72)
NA
WHOvariant

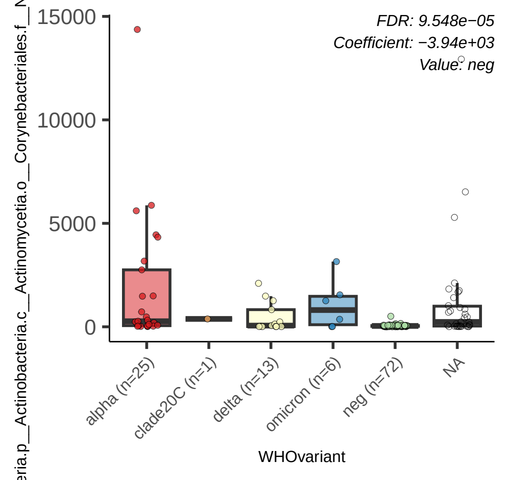

FDR: 9.548e−05
Coefficient: −3.94e+03
Value: neg
15000
10000
5000
0
alpha (n=25)
clade20C (n=1)
delta (n=13)
omicron (n=6)
neg (n=72)
NA
WHOvariant

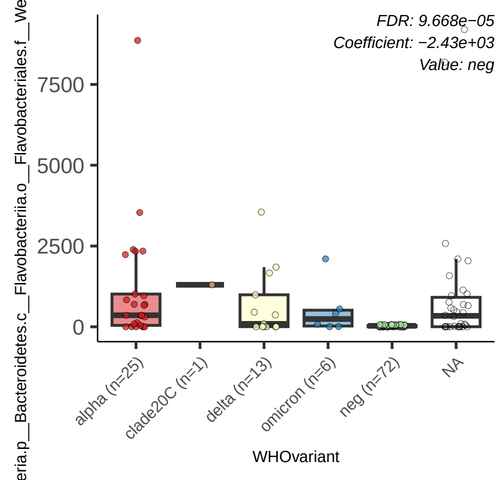

FDR: 9.668e−05
Coefficient: −2.43e+03
Value: neg
7500
5000
2500
0
alpha (n=25)
clade20C (n=1)
delta (n=13)
omicron (n=6)
neg (n=72)
NA
WHOvariant
eria.p__Bacteroidetes.c__Flavobacteriia.o__Flavobacteriales.f__We

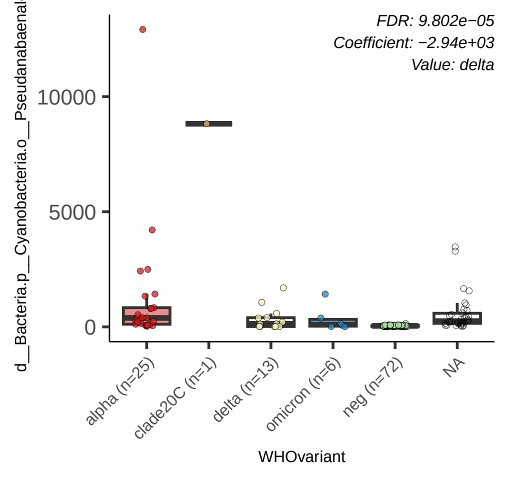

FDR: 9.802e−05
Coefficient: −2.94e+03
Value: delta
d__Bacteria.p__Cyanobacteria.o__Pseudanabaenales
0
5000
10000
alpha (n=25)
clade20C (n=1)
delta (n=13)
omicron (n=6)
neg (n=72)
NA
WHOvariant

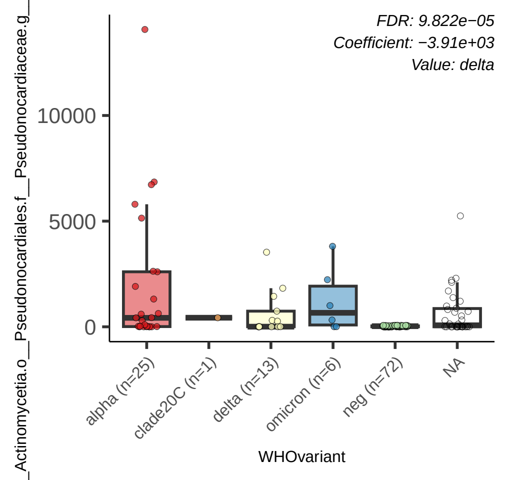
FDR: 9.822e−05
Coefficient: −3.91e+03
Value: delta
Actinomycetia.o__Pseudonocardiales.f__Pseudonocardiaceae.g__
10000
5000
0
alpha (n=25)
clade20C (n=1)
delta (n=13)
omicron (n=6)
neg (n=72)
NA
WHOvariant

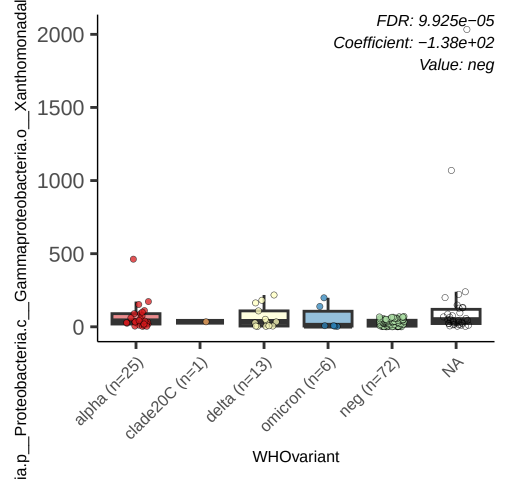

FDR: 9.925e−05
Coefficient: −1.38e+02
Value: neg
ia.p__Proteobacteria.c__Gammaproteobacteria.o__Xanthomonadal
2000
1500
1000
500
0
alpha (n=25)
clade20C (n=1)
delta (n=13)
omicron (n=6)
neg (n=72)
NA
WHOvariant

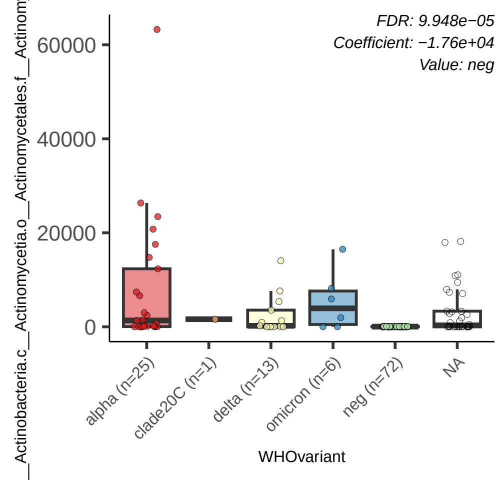

FDR: 9.948e−05
Coefficient: −1.76e+04
Value: neg
Actinobacteria.c__Actinomycetia.o__Actinomycetales.f__Actinomy
60000
40000
20000
0
alpha (n=25)
clade20C (n=1)
delta (n=13)
omicron (n=6)
neg (n=72)
NA
WHOvariant

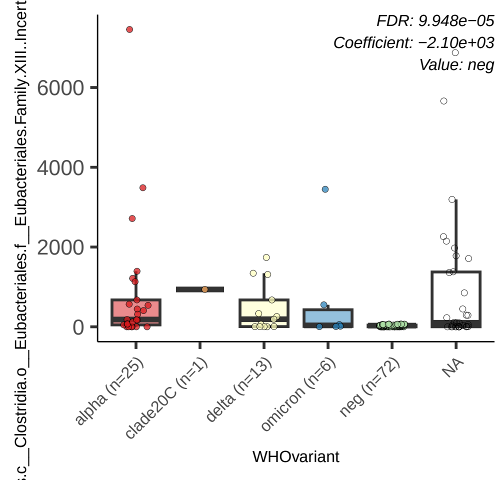

FDR: 9.948e−05
Coefficient: −2.10e+03
Value: neg
6000
4000
2000
0
s.c__Clostridia.o__Eubacteriales.f__Eubacteriales.Family.XIII..Incert
alpha (n=25)
clade20C (n=1)
delta (n=13)
omicron (n=6)
neg (n=72)
NA
WHOvariant

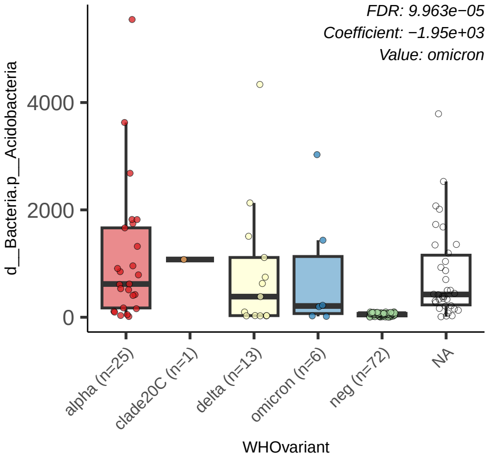
FDR: 9.963e−05
Coefficient: −1.95e+03
Value: omicron
d__Bacteria.p__Acidobacteria
4000
2000
0
alpha (n=25)
clade20C (n=1)
delta (n=13)
omicron (n=6)
neg (n=72)
NA
WHOvariant

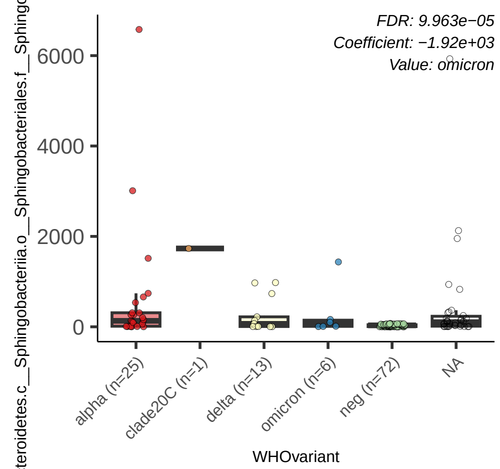

FDR: 9.963e−05
Coefficient: −1.92e+03
Value: omicron
6000
4000
2000
0
alpha (n=25)
clade20C (n=1)
delta (n=13)
omicron (n=6)
neg (n=72)
NA
WHOvariant
acteroidetes.c__Sphingobacteriia.o__Sphingobacteriales.f__Sphingo

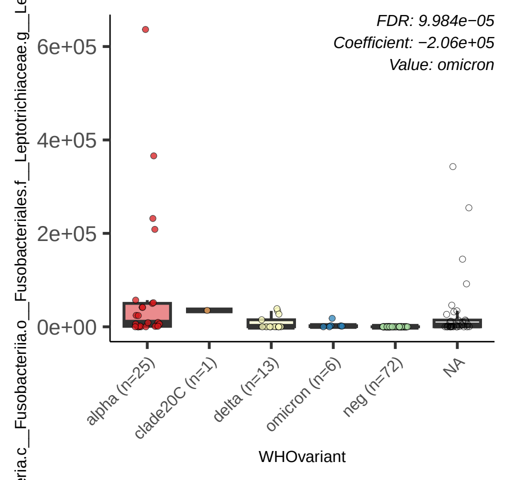

FDR: 9.984e−05
Coefficient: −2.06e+05
Value: omicron
6e+05
4e+05
2e+05
0e+00
alpha (n=25)
clade20C (n=1)
delta (n=13)
omicron (n=6)
neg (n=72)
NA
WHOvariant
eria.c__Fusobacteriia.o__Fusobacteriales.f__Leptotrichiaceae.g__Le

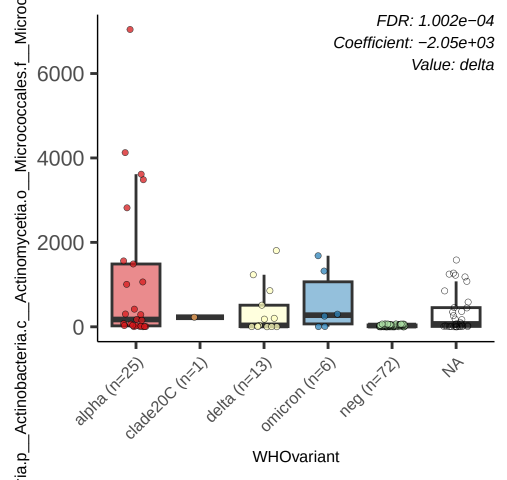

FDR: 1.002e−04
Coefficient: −2.05e+03
Value: delta
ria.p__Actinobacteria.c__Actinomycetia.o__Micrococcales.f__Microc
6000
4000
2000
0
alpha (n=25)
clade20C (n=1)
delta (n=13)
omicron (n=6)
neg (n=72)
NA
WHOvariant

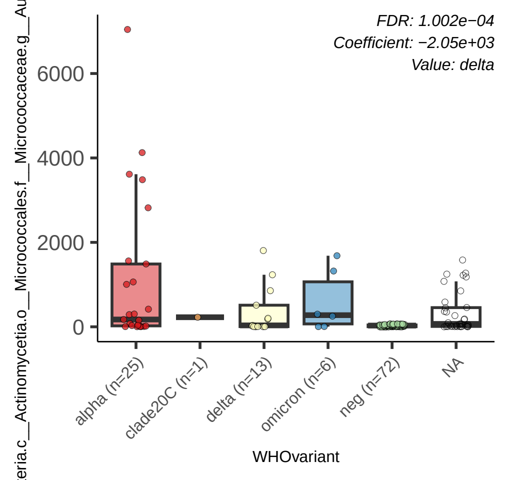
FDR: 1.002e−04
Coefficient: −2.05e+03
Value: delta
6000
4000
2000
0
teria.c__Actinomycetia.o__Micrococcales.f__Micrococcaceae.g__Au
alpha (n=25)
clade20C (n=1)
delta (n=13)
omicron (n=6)
neg (n=72)
NA
WHOvariant

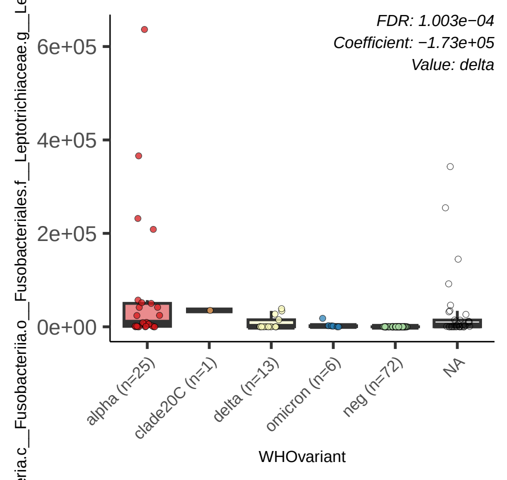
FDR: 1.003e−04
Coefficient: −1.73e+05
Value: delta
0e+00
2e+05
4e+05
6e+05
Fusobacteriia.c__Fusobacteriia.o__Fusobacteriales.f__Leptotrichiaceae.g__Le
alpha (n=25)
clade20C (n=1)
delta (n=13)
omicron (n=6)
neg (n=72)
NA
WHOvariant

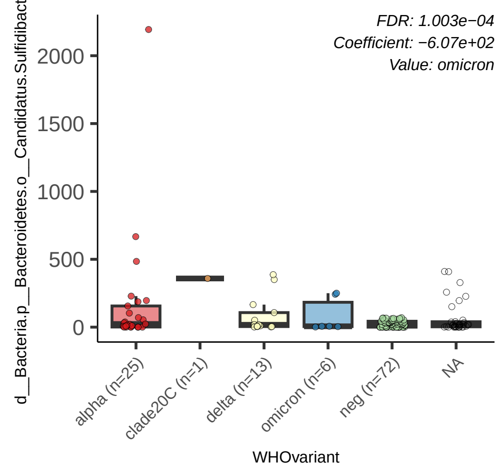
FDR: 1.003e−04
Coefficient: −6.07e+02
Value: omicron
d__Bacteria.p__Bacteroidetes.o__Candidatus.Sulfidibact
2000
1500
1000
500
0
alpha (n=25)
clade20C (n=1)
delta (n=13)
omicron (n=6)
neg (n=72)
NA
WHOvariant

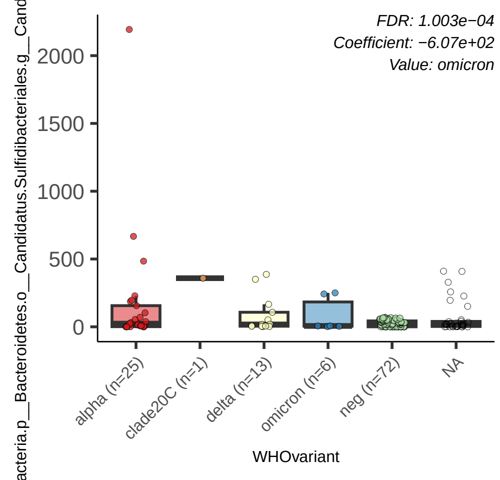

FDR: 1.003e−04
Coefficient: −6.07e+02
Value: omicron
acteria.p__Bacteroidetes.o__Candidatus.Sulfidibacteriales.g__Cand
2000
1500
1000
500
0
alpha (n=25)
clade20C (n=1)
delta (n=13)
omicron (n=6)
neg (n=72)
NA
WHOvariant

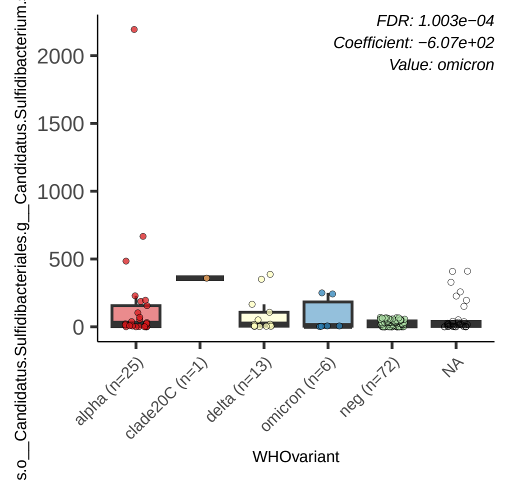

FDR: 1.003e−04
Coefficient: −6.07e+02
Value: omicron
s.o__Candidatus.Sulfidibacteriales.g__Candidatus.Sulfidibacterium.s
0
500
1000
1500
2000
alpha (n=25)
clade20C (n=1)
delta (n=13)
omicron (n=6)
neg (n=72)
NA
WHOvariant

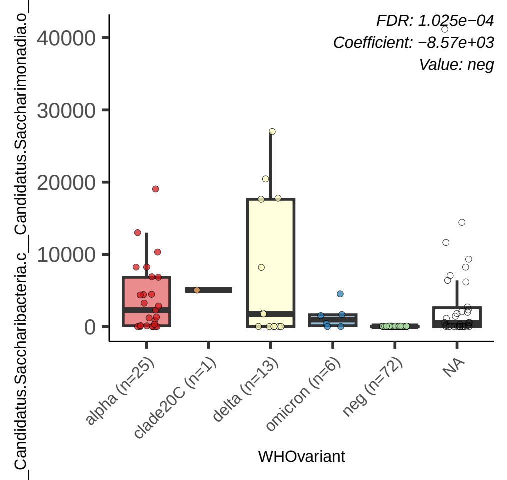

FDR: 1.025e−04
Coefficient: −8.57e+03
Value: neg
Candidatus.Saccharibacteria.c__Candidatus.Saccharimonadia.o__
40000
30000
20000
10000
0
alpha (n=25)
clade20C (n=1)
delta (n=13)
omicron (n=6)
neg (n=72)
NA
WHOvariant

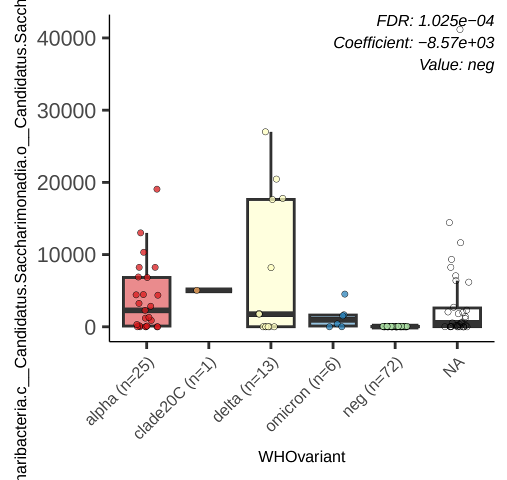
FDR: 1.025e−04
Coefficient: −8.57e+03
Value: neg
40000
30000
20000
10000
0
naribacteria.c__Candidatus.Saccharimonadia.o__Candidatus.Sacch
alpha (n=25)
clade20C (n=1)
delta (n=13)
omicron (n=6)
neg (n=72)
NA
WHOvariant

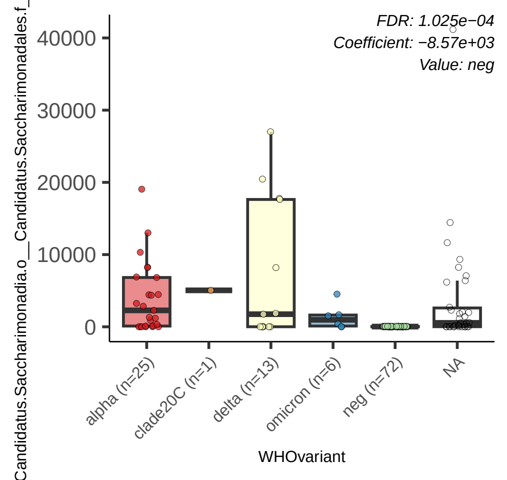

FDR: 1.025e−04
Coefficient: −8.57e+03
Value: neg
Candidatus.Saccharimonadia.o__Candidatus.Saccharimonadales.f__
40000
30000
20000
10000
0
alpha (n=25)
clade20C (n=1)
delta (n=13)
omicron (n=6)
neg (n=72)
NA
WHOvariant

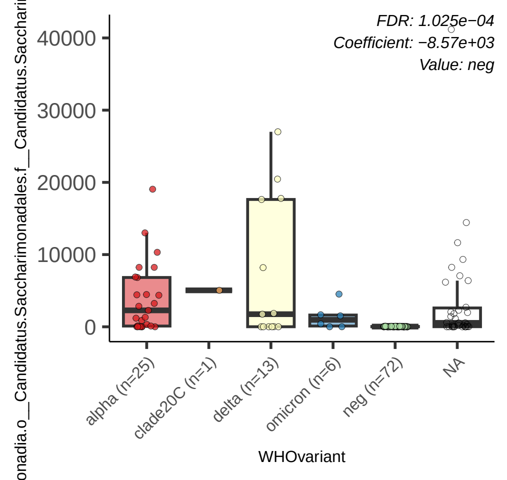

FDR: 1.025e−04
Coefficient: −8.57e+03
Value: neg
onadia.o__Candidatus.Saccharimonadales.f__Candidatus.Sacchari
40000
30000
20000
10000
0
alpha (n=25)
clade20C (n=1)
delta (n=13)
omicron (n=6)
neg (n=72)
NA
WHOvariant

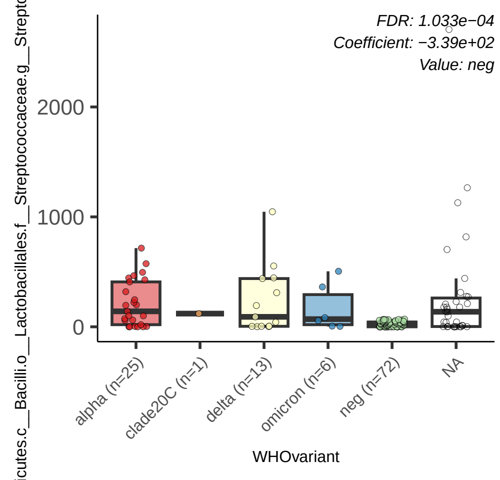
FDR: 1.033e−04
Coefficient: −3.39e+02
Value: neg
2000
1000
0
alpha (n=25)
clade20C (n=1)
delta (n=13)
omicron (n=6)
neg (n=72)
NA
WHOvariant

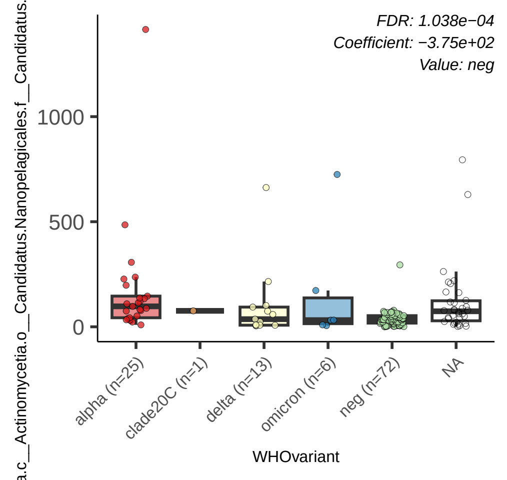

FDR: 1.038e−04
Coefficient: −3.75e+02
Value: neg
a.c__Actinomycetia.o__Candidatus.Nanopelagicales.f__Candidatus.
1000
500
0
alpha (n=25)
clade20C (n=1)
delta (n=13)
omicron (n=6)
neg (n=72)
NA
WHOvariant

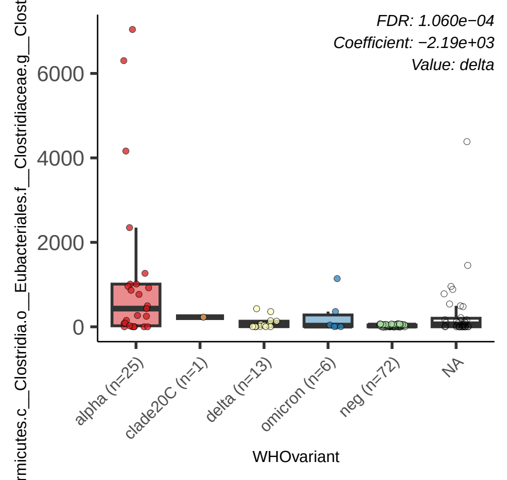

FDR: 1.060e−04
Coefficient: −2.19e+03
Value: delta
0
2000
4000
6000
rmicutes.c__Clostridia.o__Eubacteriales.f__Clostridiaceae.g__Clost
alpha (n=25)
clade20C (n=1)
delta (n=13)
omicron (n=6)
neg (n=72)
NA
WHOvariant

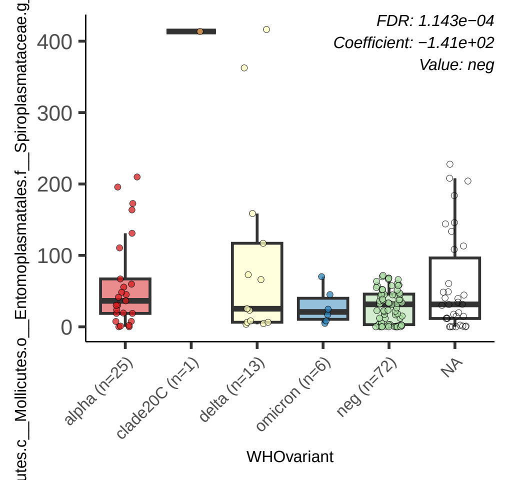

FDR: 1.143e−04
Coefficient: −1.41e+02
Value: neg
utes.c__Mollicutes.o__Entomoplasmatales.f__Spiroplasmataceae.g_
0
100
200
300
400
alpha (n=25)
clade20C (n=1)
delta (n=13)
omicron (n=6)
neg (n=72)
NA
WHOvariant

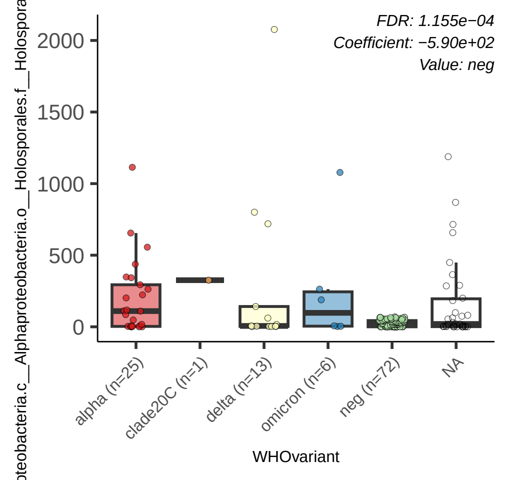
FDR: 1.155e−04
Coefficient: −5.90e+02
Value: neg
oteobacteria.c__Alphaproteobacteria.o__Holosporales.f__Holospora
2000
1500
1000
500
0
alpha (n=25)
clade20C (n=1)
delta (n=13)
omicron (n=6)
neg (n=72)
NA
WHOvariant

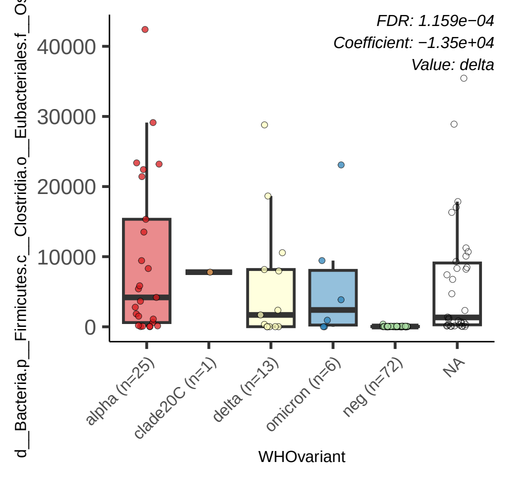

FDR: 1.159e−04
Coefficient: −1.35e+04
Value: delta
d__Bacteria.p__Firmicutes.c__Clostridia.o__Eubacteriales.f__Os
40000
30000
20000
10000
0
alpha (n=25)
clade20C (n=1)
delta (n=13)
omicron (n=6)
neg (n=72)
NA
WHOvariant

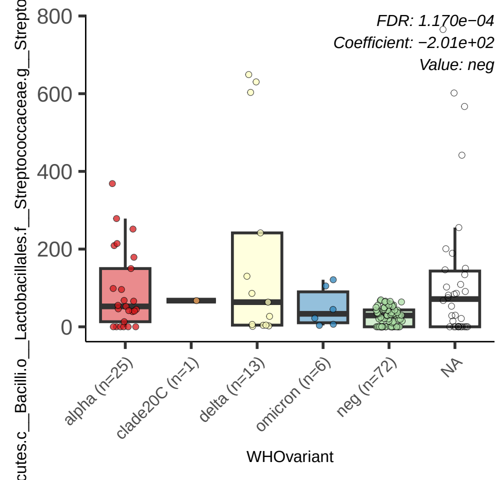
FDR: 1.170e−04
Coefficient: −2.01e+02
Value: neg
800
600
400
200
0
cutes.c__Bacilli.o__Lactobacillales.f__Streptococcaceae.g__Strepto
alpha (n=25)
clade20C (n=1)
delta (n=13)
omicron (n=6)
neg (n=72)
NA
WHOvariant

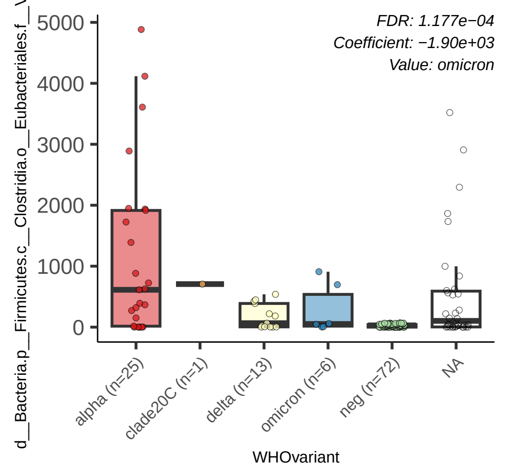

FDR: 1.177e−04
Coefficient: −1.90e+03
Value: omicron
d__Bacteria.p__Firmicutes.c__Clostridia.o__Eubacteriales.f__
5000
4000
3000
2000
1000
0
alpha (n=25)
clade20C (n=1)
delta (n=13)
omicron (n=6)
neg (n=72)
NA
WHOvariant

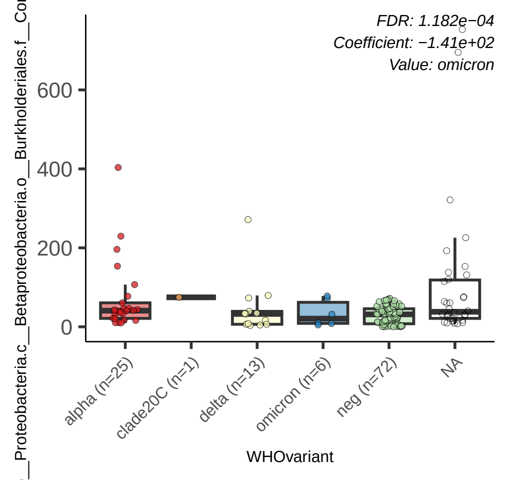
FDR: 1.182e−04
Coefficient: −1.41e+02
Value: omicron
Proteobacteria.c__Betaproteobacteria.o__Burkholderiales.f__Co
600
400
200
0
alpha (n=25)
clade20C (n=1)
delta (n=13)
omicron (n=6)
neg (n=72)
NA
WHOvariant

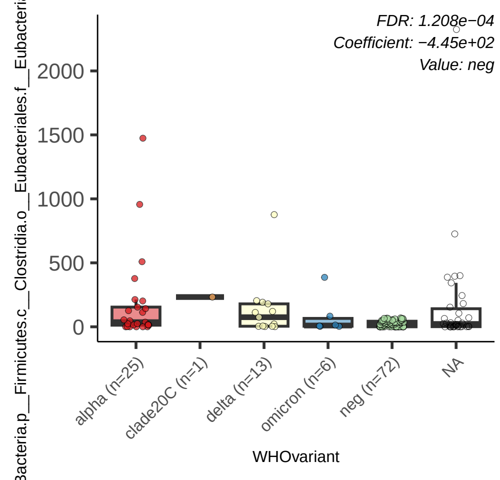

FDR: 1.208e−04
Coefficient: −4.45e+02
Value: neg
Bacteria.p__Firmicutes.c__Clostridia.o__Eubacteriales.f__Eubacteri
2000
1500
1000
500
0
alpha (n=25)
clade20C (n=1)
delta (n=13)
omicron (n=6)
neg (n=72)
NA
WHOvariant

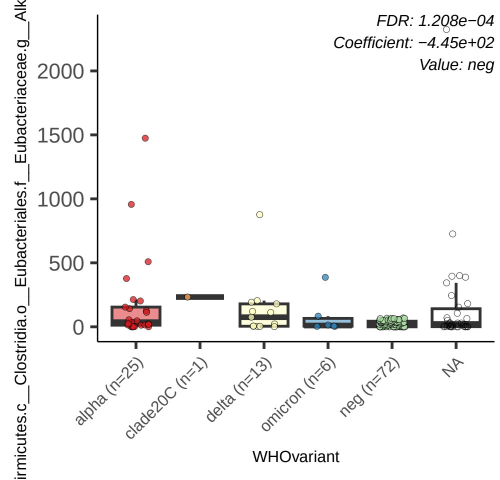
FDR: 1.208e−04
Coefficient: −4.45e+02
Value: neg
irmicutes.c__Clostridia.o__Eubacteriales.f__Eubacteriaceae.g__Alk
2000
1500
1000
500
0
alpha (n=25)
clade20C (n=1)
delta (n=13)
omicron (n=6)
neg (n=72)
NA
WHOvariant

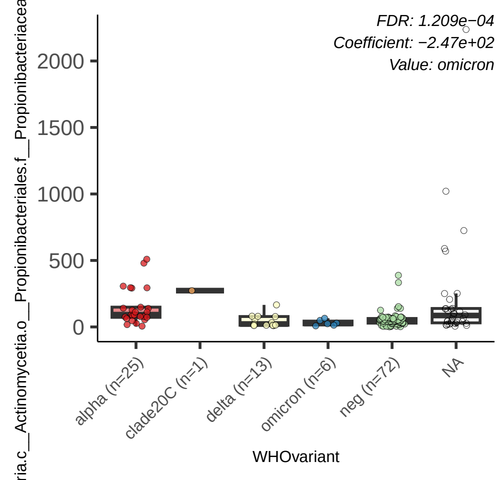

FDR: 1.209e−04
Coefficient: −2.47e+02
Value: omicron
ria.c__Actinomycetia.o__Propionibacteriales.f__Propionibacteriacea
0
500
1000
1500
2000
alpha (n=25)
clade20C (n=1)
delta (n=13)
omicron (n=6)
neg (n=72)
NA
WHOvariant

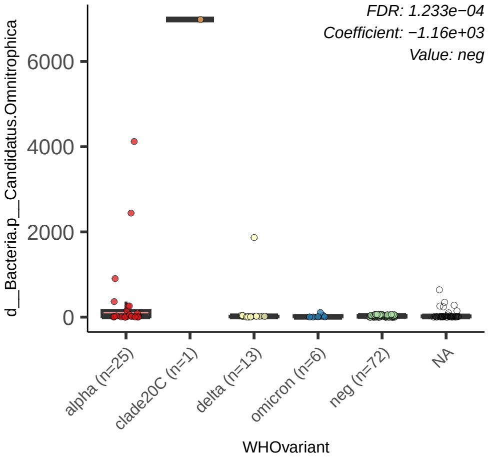
FDR: 1.233e−04
Coefficient: −1.16e+03
Value: neg
d__Bacteria.p__Candidatus.Omnitrophica
6000
4000
2000
0
alpha (n=25)
clade20C (n=1)
delta (n=13)
omicron (n=6)
neg (n=72)
NA
WHOvariant

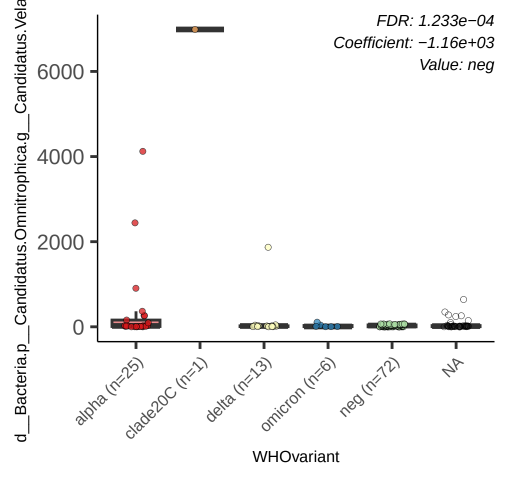

FDR: 1.233e−04
Coefficient: −1.16e+03
Value: neg
d__Bacteria.p__Candidatus.Omnitrophica.g__Candidatus.Vela
6000
4000
2000
0
alpha (n=25)
clade20C (n=1)
delta (n=13)
omicron (n=6)
neg (n=72)
NA
WHOvariant

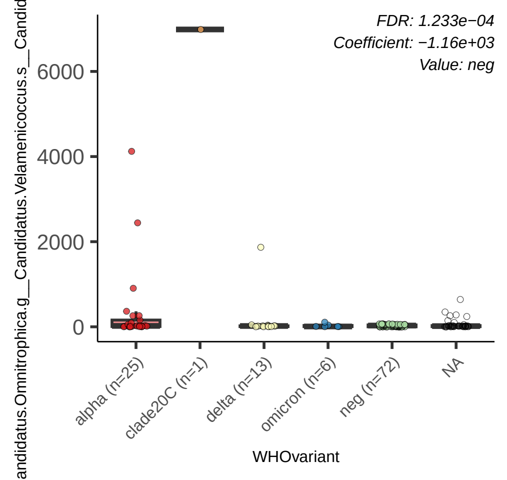

FDR: 1.233e−04
Coefficient: −1.16e+03
Value: neg
6000
4000
2000
0
andidatus.Omnitrophica.g__Candidatus.Velamenicoccus.s__Candid
alpha (n=25)
clade20C (n=1)
delta (n=13)
omicron (n=6)
neg (n=72)
NA
WHOvariant

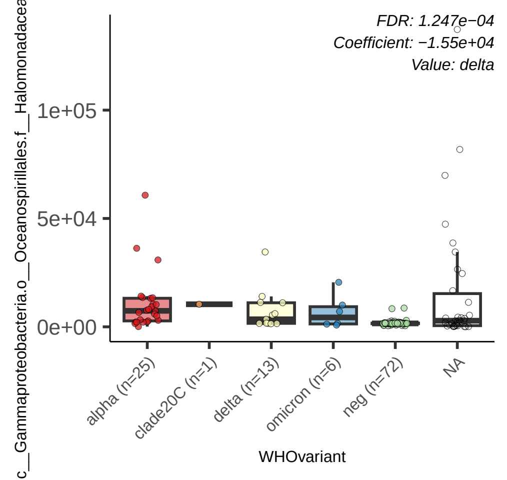
FDR: 1.247e−04
Coefficient: −1.55e+04
Value: delta
c__Gammaproteobacteria.o__Oceanospirillales.f__Halomonadaceae
1e+05
5e+04
0e+00
alpha (n=25)
clade20C (n=1)
delta (n=13)
omicron (n=6)
neg (n=72)
NA
WHOvariant

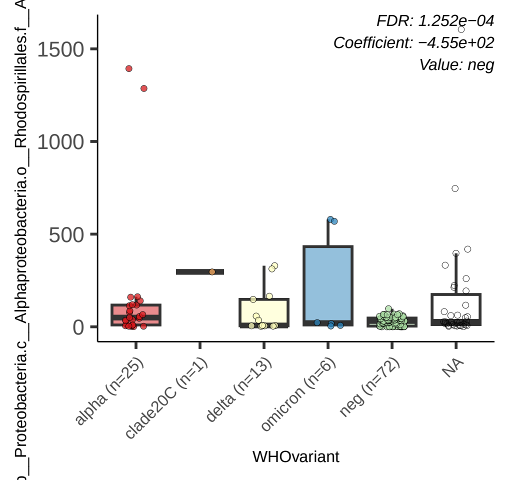

FDR: 1.252e−04
Coefficient: −4.55e+02
Value: neg
1500
1000
500
0
alpha (n=25)
clade20C (n=1)
delta (n=13)
omicron (n=6)
neg (n=72)
NA
WHOvariant

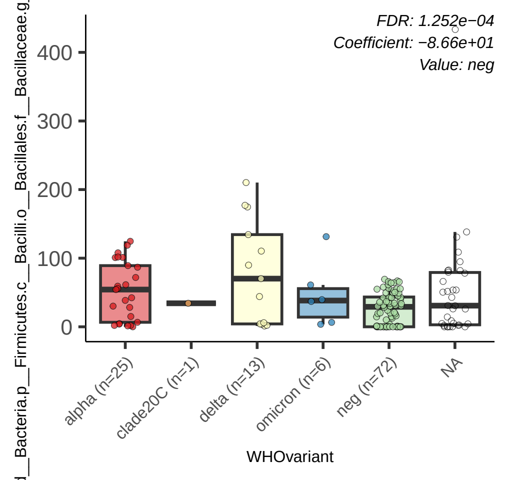
FDR: 1.252e−04
Coefficient: −8.66e+01
Value: neg
d__Bacteria.p__Firmicutes.c__Bacilli.o__Bacillales.f__Bacillaceae.g__
400
300
200
100
0
alpha (n=25)
clade20C (n=1)
delta (n=13)
omicron (n=6)
neg (n=72)
NA
WHOvariant

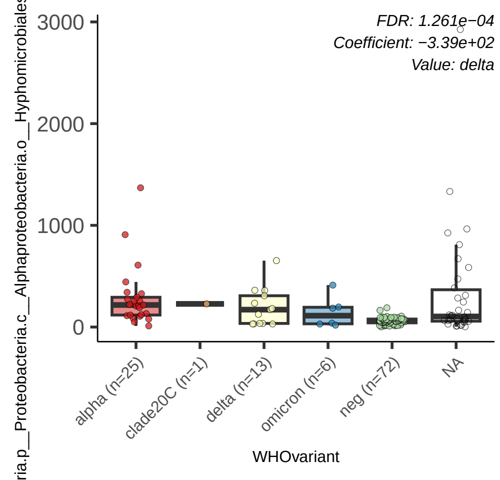

FDR: 1.261e−04
Coefficient: −3.39e+02
Value: delta
ria.p__Proteobacteria.c__Alphaproteobacteria.o__Hyphomicrobiales
3000
2000
1000
0
alpha (n=25)
clade20C (n=1)
delta (n=13)
omicron (n=6)
neg (n=72)
NA
WHOvariant

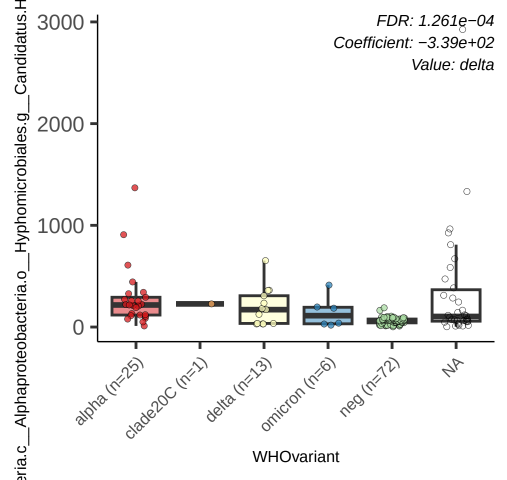

FDR: 1.261e−04
Coefficient: −3.39e+02
Value: delta
3000
2000
1000
0
alpha (n=25)
clade20C (n=1)
delta (n=13)
omicron (n=6)
neg (n=72)
NA
WHOvariant
eria.c__Alphaproteobacteria.o__Hyphomicrobiales.g__Candidatus.H

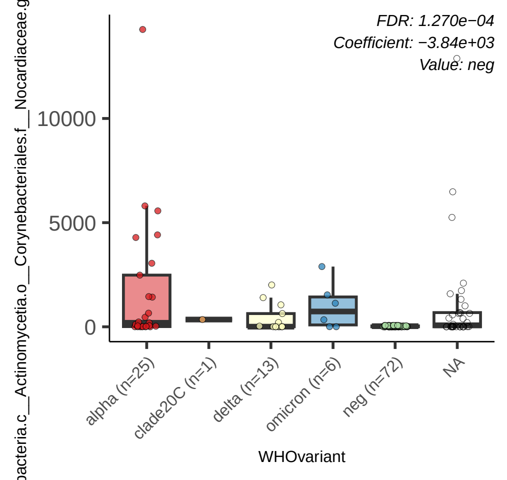
FDR: 1.270e−04
Coefficient: −3.84e+03
Value: neg
10000
5000
0
bacteria.c__Actinomycetia.o__Corynebacteriales.f__Nocardiaceae.g
alpha (n=25)
clade20C (n=1)
delta (n=13)
omicron (n=6)
neg (n=72)
NA
WHOvariant

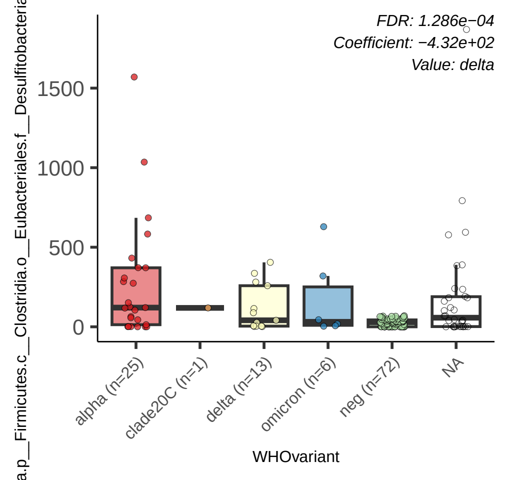

FDR: 1.286e−04
Coefficient: −4.32e+02
Value: delta
1500
1000
500
0
Firmicutes.c__Clostridia.o__Eubacteriales.f__Desulfitobacteria
alpha (n=25)
clade20C (n=1)
delta (n=13)
omicron (n=6)
neg (n=72)
NA
WHOvariant

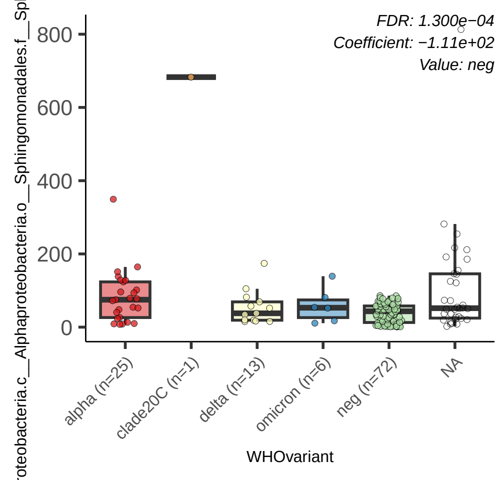

FDR: 1.300e−04
Coefficient: −1.11e+02
Value: neg
0
200
400
600
800
roteobacteria.c__Alphaproteobacteria.o__Sphingomonadales.f__Sph
alpha (n=25)
clade20C (n=1)
delta (n=13)
omicron (n=6)
neg (n=72)
NA
WHOvariant

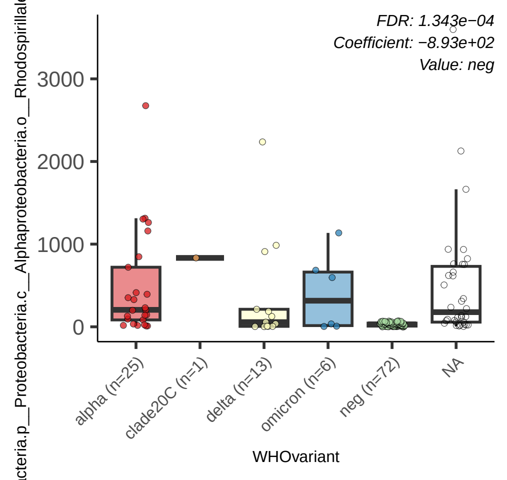
FDR: 1.343e−04
Coefficient: −8.93e+02
Value: neg
3000
2000
1000
0
alpha (n=25)
clade20C (n=1)
delta (n=13)
omicron (n=6)
neg (n=72)
NA
WHOvariant
acteria.p__Proteobacteria.c__Alphaproteobacteria.o__Rhodospirillale

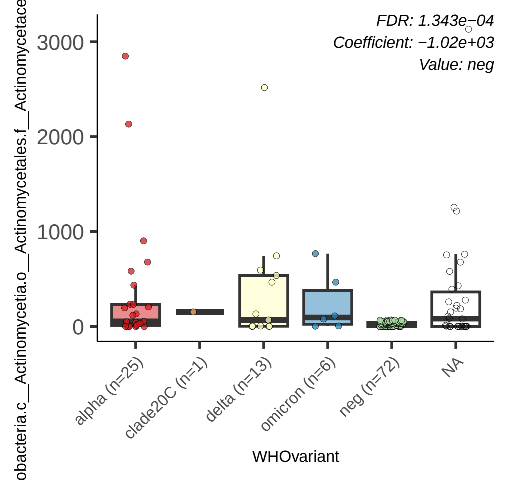
FDR: 1.343e−04
Coefficient: −1.02e+03
Value: neg
3000
2000
1000
0
alpha (n=25)
clade20C (n=1)
delta (n=13)
omicron (n=6)
neg (n=72)
NA
WHOvariant
obacteria.c__Actinomycetia.o__Actinomycetales.f__Actinomycetace

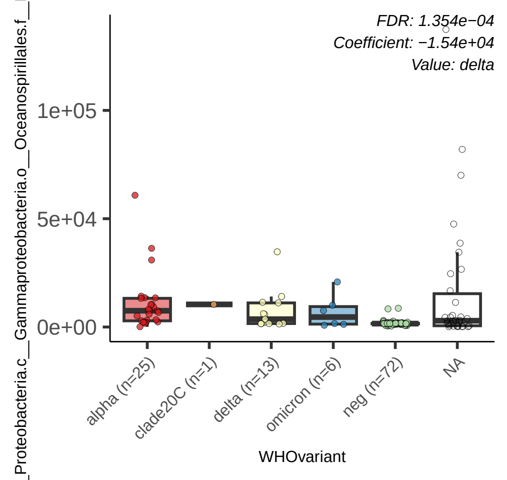
FDR: 1.354e−04
Coefficient: −1.54e+04
Value: delta
Proteobacteria.c__Gammaproteobacteria.o__Oceanospirillales.f__
1e+05
5e+04
0e+00
alpha (n=25)
clade20C (n=1)
delta (n=13)
omicron (n=6)
neg (n=72)
NA
WHOvariant

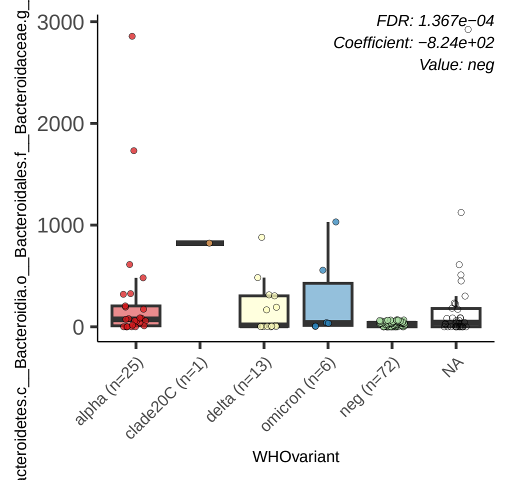

FDR: 1.367e−04
Coefficient: −8.24e+02
Value: neg
3000
2000
1000
0
acteroidetes.c__Bacteroidia.o__Bacteroidales.f__Bacteroidaceae.g__
alpha (n=25)
clade20C (n=1)
delta (n=13)
omicron (n=6)
neg (n=72)
NA
WHOvariant

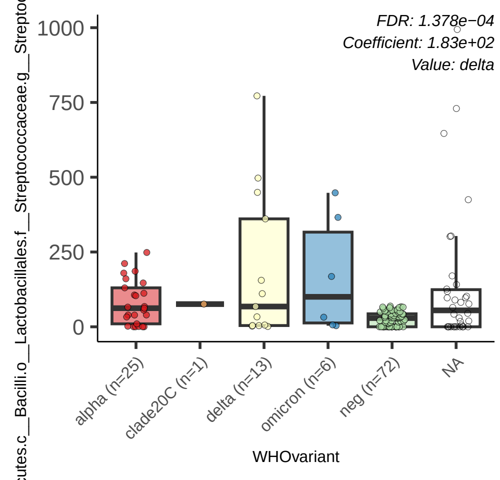

cutes.c__Bacilli.o__Lactobacillales.f__Streptococcaceae.g__Streptoc
FDR: 1.378e−04
Coefficient: 1.83e+02
Value: delta
1000
750
500
250
0
alpha (n=25)
clade20C (n=1)
delta (n=13)
omicron (n=6)
neg (n=72)
NA
WHOvariant

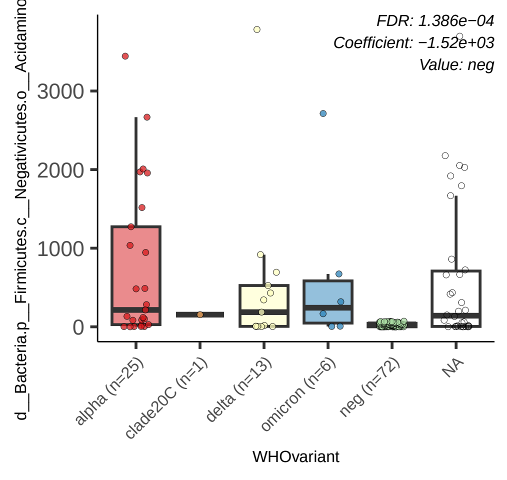

FDR: 1.386e−04
Coefficient: −1.52e+03
Value: neg
d__Bacteria.p__Firmicutes.c__Negativicutes.o__Acidamino
3000
2000
1000
0
alpha (n=25)
clade20C (n=1)
delta (n=13)
omicron (n=6)
neg (n=72)
NA
WHOvariant

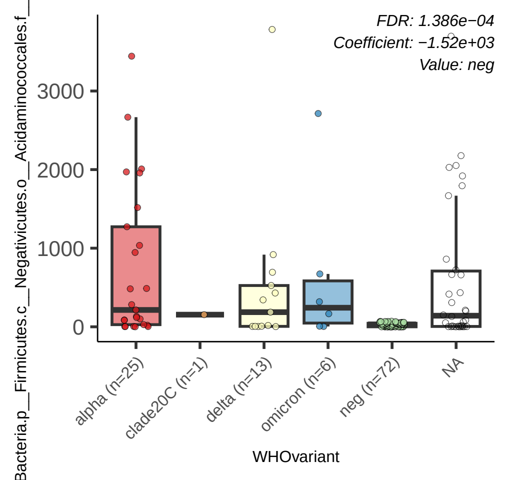

FDR: 1.386e−04
Coefficient: −1.52e+03
Value: neg
Bacteria.p__Firmicutes.c__Negativicutes.o__Acidaminococcales.f__
3000
2000
1000
0
alpha (n=25)
clade20C (n=1)
delta (n=13)
omicron (n=6)
neg (n=72)
NA
WHOvariant

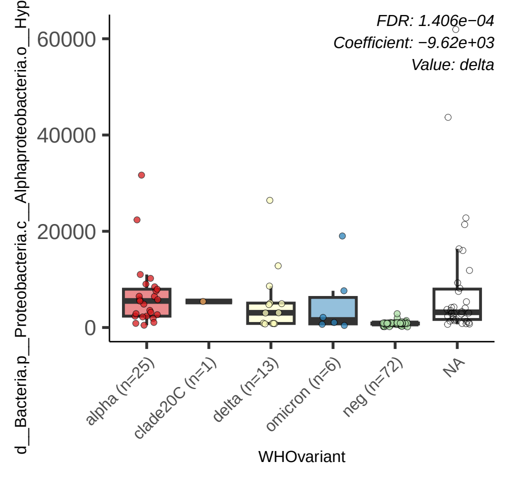

FDR: 1.406e−04
Coefficient: −9.62e+03
Value: delta
d__Bacteria.p__Proteobacteria.c__Alphaproteobacteria.o__Hyp
60000
40000
20000
0
alpha (n=25)
clade20C (n=1)
delta (n=13)
omicron (n=6)
neg (n=72)
NA
WHOvariant

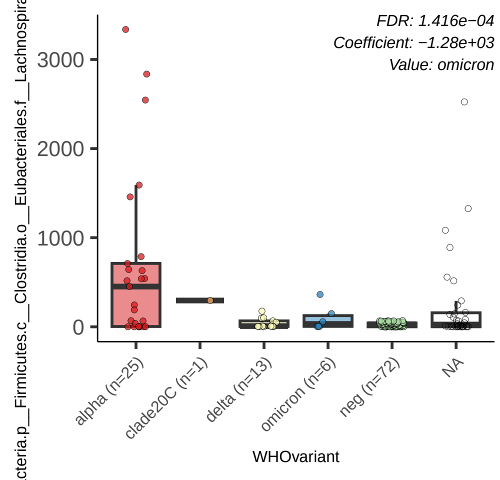
FDR: 1.416e−04
Coefficient: −1.28e+03
Value: omicron
3000
2000
1000
0
cteria.p__Firmicutes.c__Clostridia.o__Eubacteriales.f__Lachnospira
alpha (n=25)
clade20C (n=1)
delta (n=13)
omicron (n=6)
neg (n=72)
NA
WHOvariant

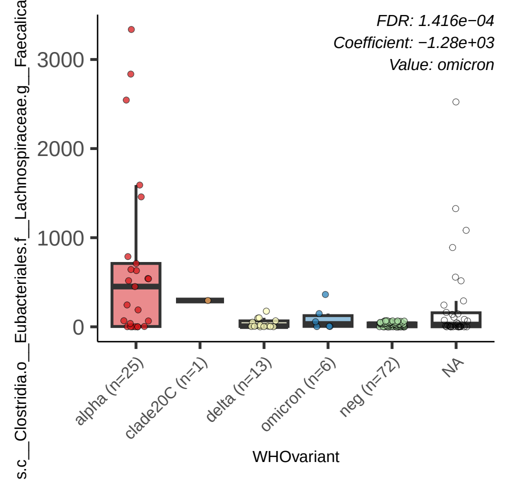
FDR: 1.416e−04
Coefficient: −1.28e+03
Value: omicron
s.c__Clostridia.o__Eubacteriales.f__Lachnospiraceae.g__Faecalica
0
1000
2000
3000
alpha (n=25)
clade20C (n=1)
delta (n=13)
omicron (n=6)
neg (n=72)
NA
WHOvariant

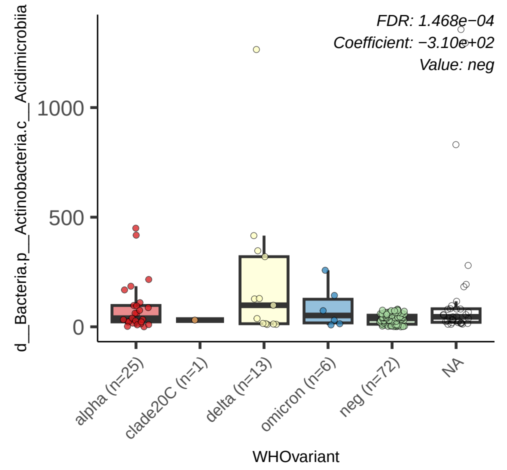

FDR: 1.468e−04
Coefficient: −3.10e+02
Value: neg
d__Bacteria.p__Actinobacteria.c__Acidimicrobiia
1000
500
0
alpha (n=25)
clade20C (n=1)
delta (n=13)
omicron (n=6)
neg (n=72)
NA
WHOvariant

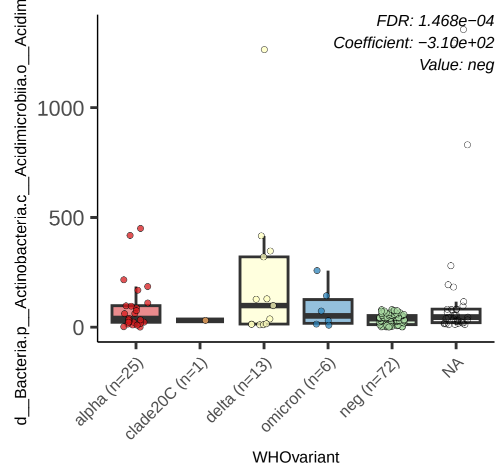

FDR: 1.468e−04
Coefficient: −3.10e+02
Value: neg
d__Bacteria.p__Actinobacteria.c__Acidimicrobiia.o__Acidim
0
500
1000
alpha (n=25)
clade20C (n=1)
delta (n=13)
omicron (n=6)
neg (n=72)
NA
WHOvariant

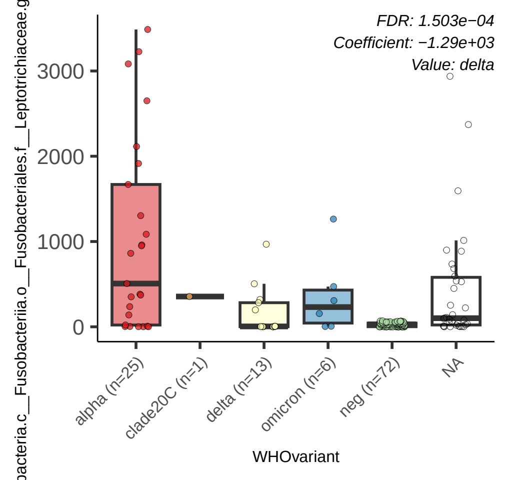

FDR: 1.503e−04
Coefficient: −1.29e+03
Value: delta
3000
2000
1000
0
alpha (n=25)
clade20C (n=1)
delta (n=13)
omicron (n=6)
neg (n=72)
NA
WHOvariant

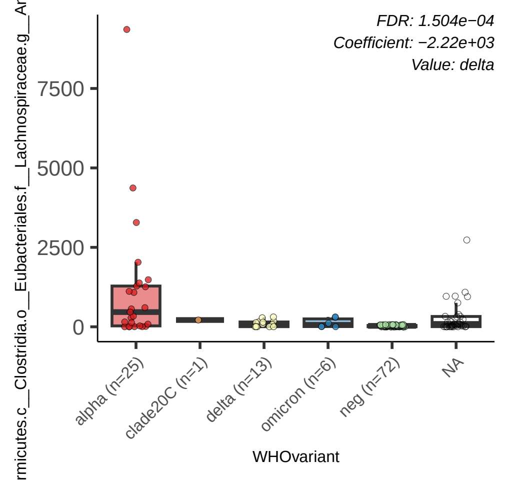

FDR: 1.504e−04
Coefficient: −2.22e+03
Value: delta
rmicutes.c__Clostridia.o__Eubacteriales.f__Lachnospiraceae.g__Ar
7500
5000
2500
0
alpha (n=25)
clade20C (n=1)
delta (n=13)
omicron (n=6)
neg (n=72)
NA
WHOvariant

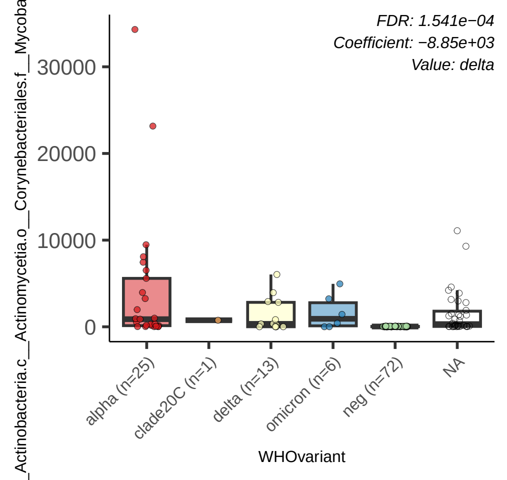

FDR: 1.541e−04
Coefficient: −8.85e+03
Value: delta
_Actinobacteria.c__Actinomycetia.o__Corynebacteriales.f__Mycoba
30000
20000
10000
0
alpha (n=25)
clade20C (n=1)
delta (n=13)
omicron (n=6)
neg (n=72)
NA
WHOvariant

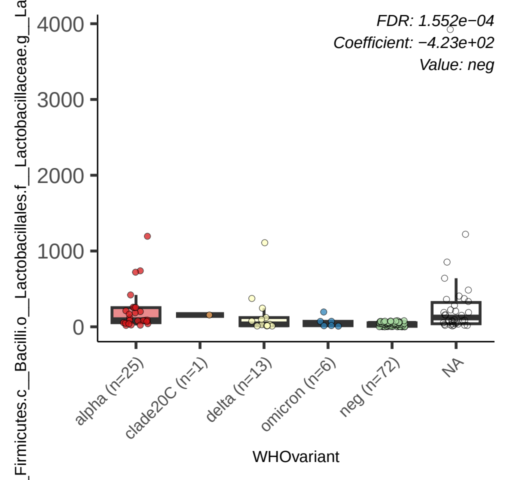
FDR: 1.552e−04
Coefficient: −4.23e+02
Value: neg
4000
3000
2000
1000
0
Firmicutes.c__Bacilli.o__Lactobacillales.f__Lactobacillaceae.g__La
alpha (n=25)
clade20C (n=1)
delta (n=13)
omicron (n=6)
neg (n=72)
NA
WHOvariant

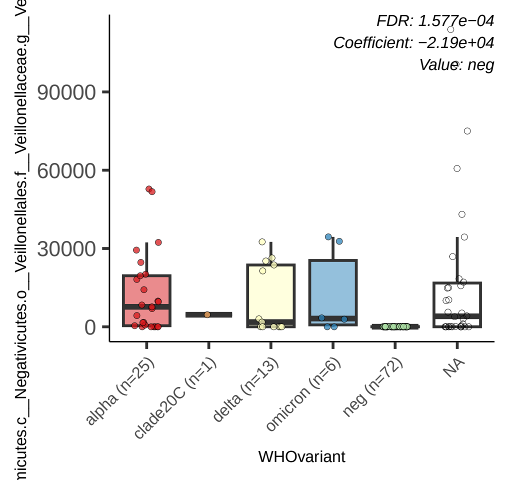

FDR: 1.577e−04
Coefficient: −2.19e+04
Value: neg
micutes.c__Negativicutes.o__Veillonellales.f__Veillonellaceae.g__Ve
90000
60000
30000
0
alpha (n=25)
clade20C (n=1)
delta (n=13)
omicron (n=6)
neg (n=72)
NA
WHOvariant

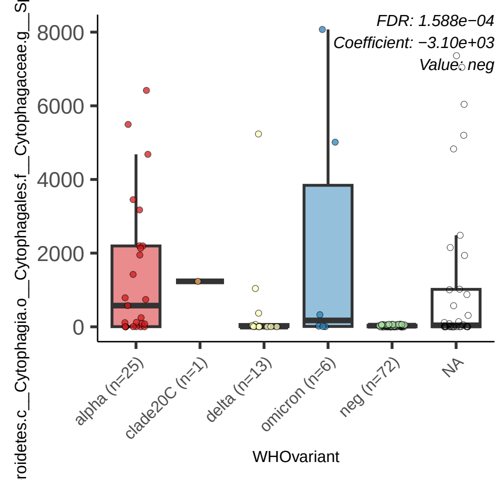
FDR: 1.588e−04
Coefficient: −3.10e+03
Value: neg
8000
6000
4000
2000
0
alpha (n=25)
clade20C (n=1)
delta (n=13)
omicron (n=6)
neg (n=72)
NA
WHOvariant
roidetes.c__Cytophagia.o__Cytophagales.f__Cytophagaceae.g__Sp

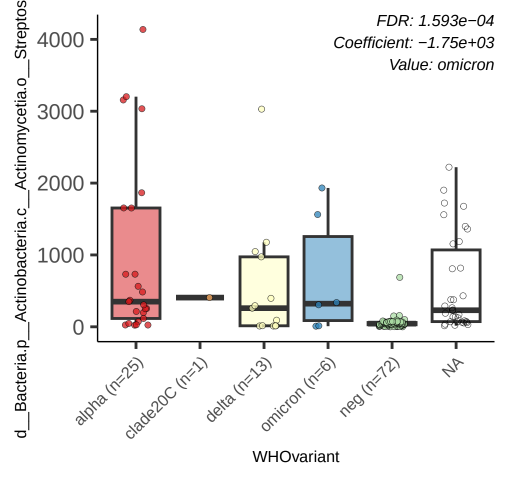

FDR: 1.593e−04
Coefficient: −1.75e+03
Value: omicron
d__Bacteria.p__Actinobacteria.c__Actinomycetia.o__Streptos
4000
3000
2000
1000
0
alpha (n=25)
clade20C (n=1)
delta (n=13)
omicron (n=6)
neg (n=72)
NA
WHOvariant

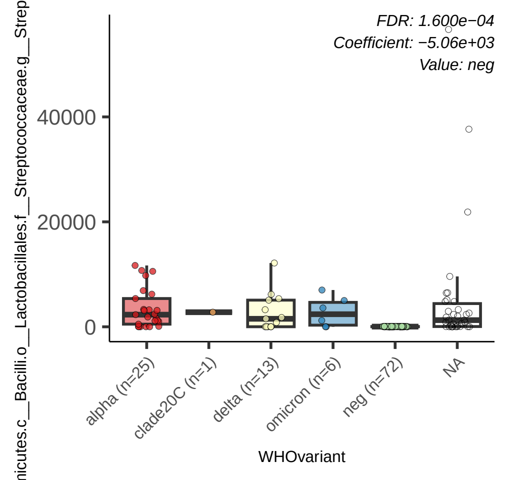

FDR: 1.600e−04
Coefficient: −5.06e+03
Value: neg
40000
20000
0
alpha (n=25)
clade20C (n=1)
delta (n=13)
omicron (n=6)
neg (n=72)
NA
WHOvariant
nicutes.c__Bacilli.o__Lactobacillales.f__Streptococcaceae.g__Strep

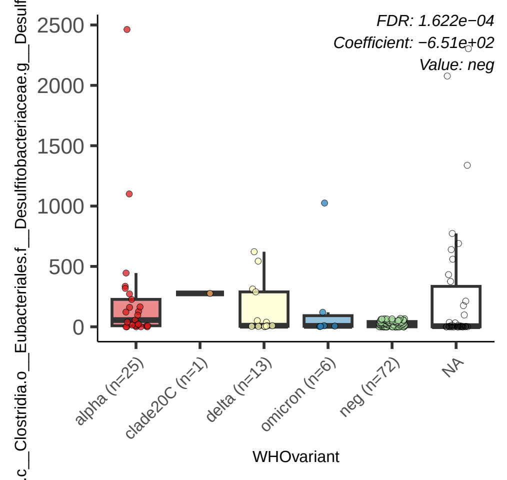

FDR: 1.622e−04
Coefficient: −6.51e+02
Value: neg
c__Clostridia.o__Eubacteriales.f__Desulfitobacteriaceae.g__Desulf
2500
2000
1500
1000
500
0
alpha (n=25)
clade20C (n=1)
delta (n=13)
omicron (n=6)
neg (n=72)
NA
WHOvariant

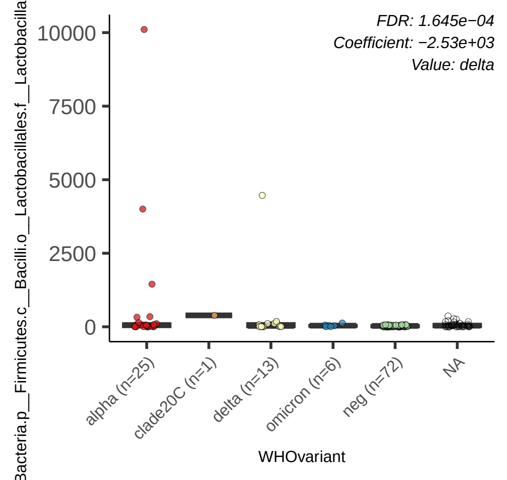

FDR: 1.645e−04
Coefficient: −2.53e+03
Value: delta
Bacteria.p__Firmicutes.c__Bacilli.o__Lactobacillales.f__Lactobacilla
10000
7500
5000
2500
0
alpha (n=25)
clade20C (n=1)
delta (n=13)
omicron (n=6)
neg (n=72)
NA
WHOvariant

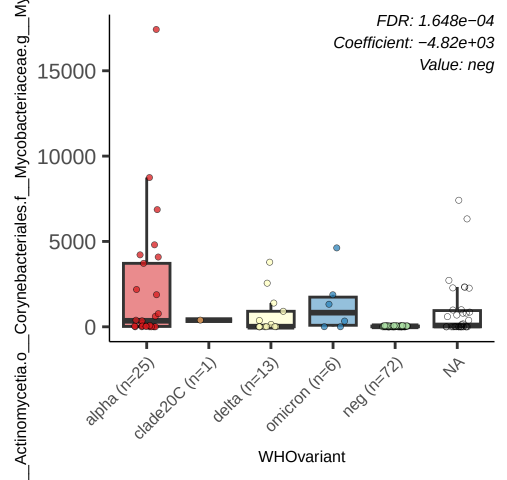
FDR: 1.648e−04
Coefficient: −4.82e+03
Value: neg
__Actinomycetia.o__Corynebacteriales.f__Mycobacteriaceae.g__My
15000
10000
5000
0
alpha (n=25)
clade20C (n=1)
delta (n=13)
omicron (n=6)
neg (n=72)
NA
WHOvariant

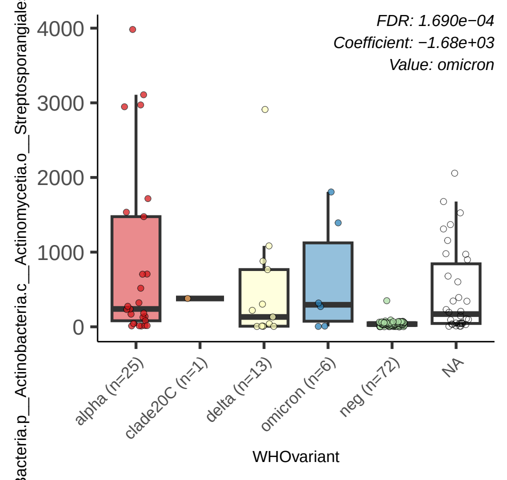

FDR: 1.690e−04
Coefficient: −1.68e+03
Value: omicron
Bacteria.p__Actinobacteria.c__Actinomycetia.o__Streptosporangiales
4000
3000
2000
1000
0
alpha (n=25)
clade20C (n=1)
delta (n=13)
omicron (n=6)
neg (n=72)
NA
WHOvariant

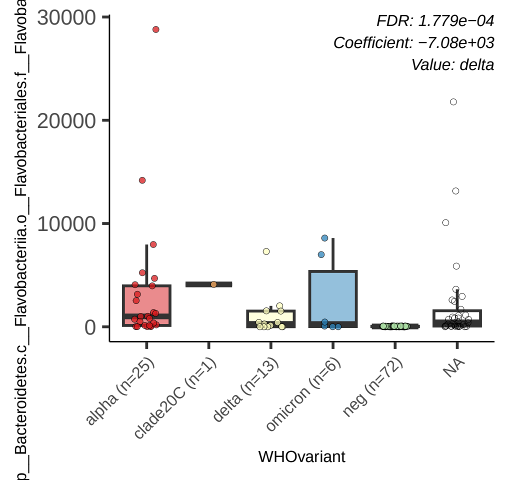

FDR: 1.779e−04
Coefficient: −7.08e+03
Value: delta
p__Bacteroidetes.c__Flavobacteriia.o__Flavobacteriales.f__Flavoba
30000
20000
10000
0
alpha (n=25)
clade20C (n=1)
delta (n=13)
omicron (n=6)
neg (n=72)
NA
WHOvariant

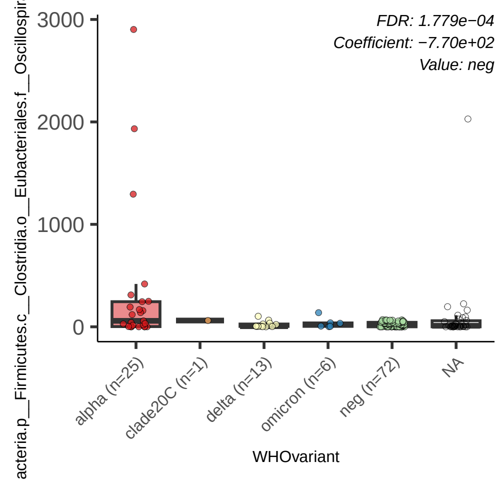

FDR: 1.779e−04
Coefficient: −7.70e+02
Value: neg
3000
2000
1000
0
acteria.p__Firmicutes.c__Clostridia.o__Eubacteriales.f__Oscillospir
alpha (n=25)
clade20C (n=1)
delta (n=13)
omicron (n=6)
neg (n=72)
NA
WHOvariant

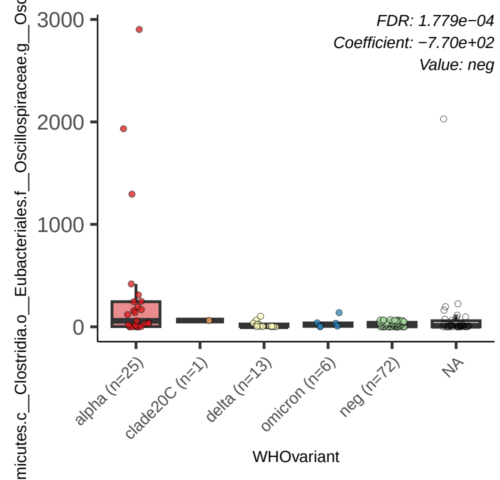

micutes.c__Clostridia.o__Eubacteriales.f__Oscillospiraceae.g__Osc
FDR: 1.779e−04
Coefficient: −7.70e+02
Value: neg
3000
2000
1000
0
alpha (n=25)
clade20C (n=1)
delta (n=13)
omicron (n=6)
neg (n=72)
NA
WHOvariant

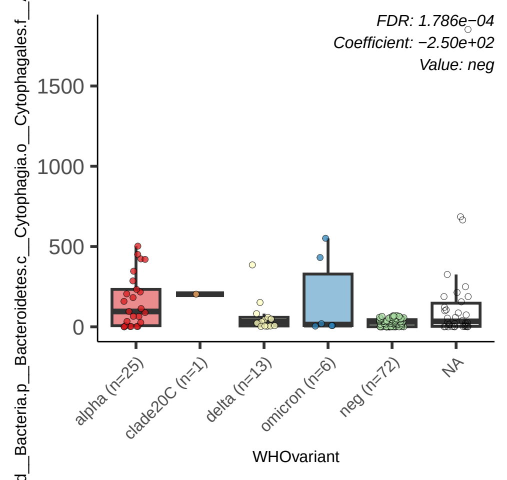
FDR: 1.786e−04
Coefficient: −2.50e+02
Value: neg
d__Bacteria.p__Bacteroidetes.c__Cytophagia.o__Cytophagales.f__
1500
1000
500
0
alpha (n=25)
clade20C (n=1)
delta (n=13)
omicron (n=6)
neg (n=72)
NA
WHOvariant

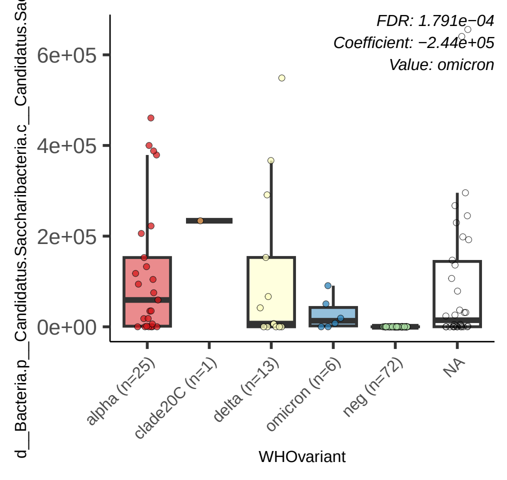

FDR: 1.791e−04
Coefficient: −2.44e+05
Value: omicron
6e+05
4e+05
2e+05
0e+00
d__Bacteria.p__Candidatus.Saccharibacteria.c__Candidatus.Sac
alpha (n=25)
clade20C (n=1)
delta (n=13)
omicron (n=6)
neg (n=72)
NA
WHOvariant

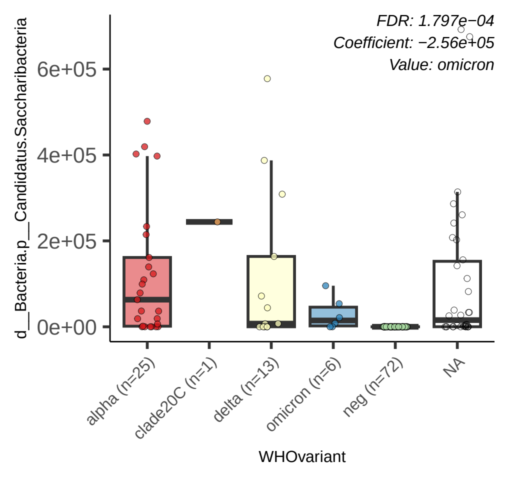
FDR: 1.797e−04
Coefficient: −2.56e+05
Value: omicron
d__Bacteria.p__Candidatus.Saccharibacteria
6e+05
4e+05
2e+05
0e+00
alpha (n=25)
clade20C (n=1)
delta (n=13)
omicron (n=6)
neg (n=72)
NA
WHOvariant

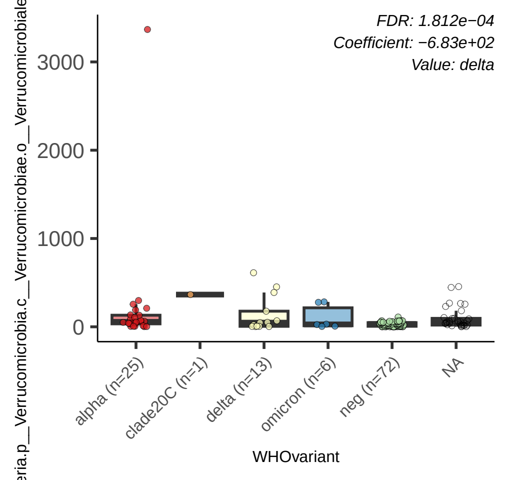
FDR: 1.812e−04
Coefficient: −6.83e+02
Value: delta
eria.p__Verrucomicrobia.c__Verrucomicrobiae.o__Verrucomicrobiale
3000
2000
1000
0
alpha (n=25)
clade20C (n=1)
delta (n=13)
omicron (n=6)
neg (n=72)
NA
WHOvariant

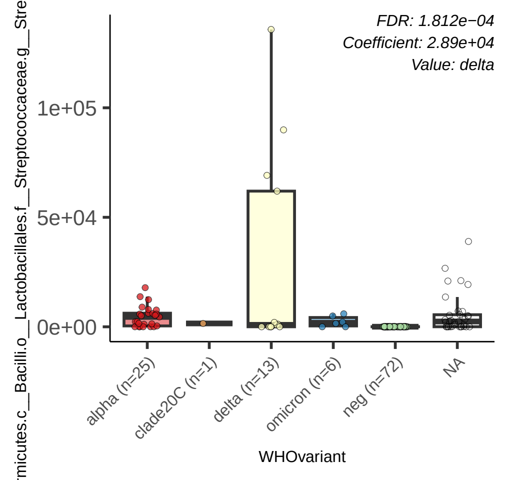

FDR: 1.812e−04
Coefficient: 2.89e+04
Value: delta
1e+05
5e+04
0e+00
micutes.c__Bacilli.o__Lactobacillales.f__Streptococcaceae.g__Stre
alpha (n=25)
clade20C (n=1)
delta (n=13)
omicron (n=6)
neg (n=72)
NA
WHOvariant

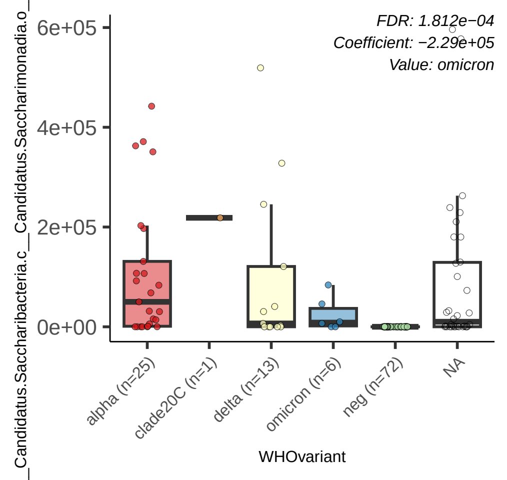
FDR: 1.812e−04
Coefficient: −2.29e+05
Value: omicron
Candidatus.Saccharibacteria.c__Candidatus.Saccharimonadia.o__
6e+05
4e+05
2e+05
0e+00
alpha (n=25)
clade20C (n=1)
delta (n=13)
omicron (n=6)
neg (n=72)
NA
WHOvariant

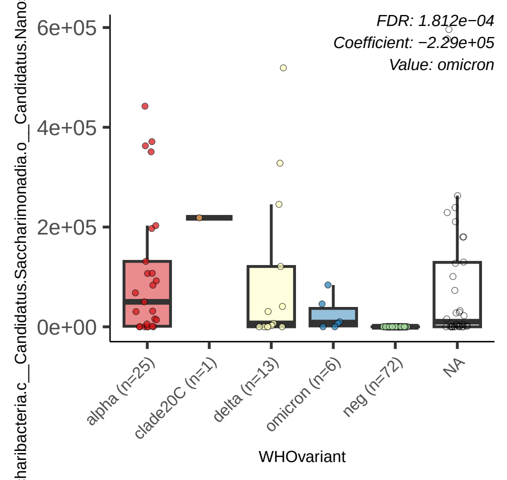
FDR: 1.812e−04
Coefficient: −2.29e+05
Value: omicron
6e+05
4e+05
2e+05
0e+00
charibacteria.c__Candidatus.Saccharimonadia.o__Candidatus.Nano
alpha (n=25)
clade20C (n=1)
delta (n=13)
omicron (n=6)
neg (n=72)
NA
WHOvariant

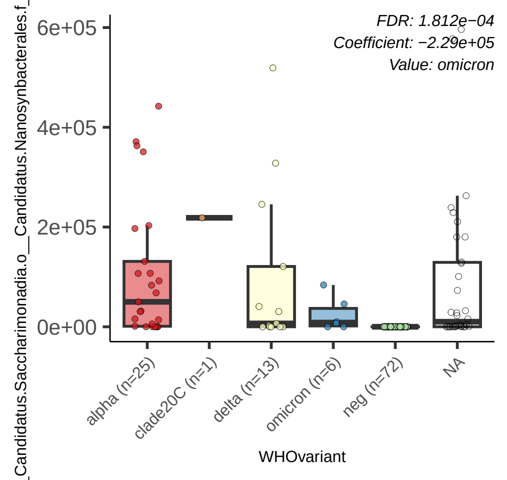
FDR: 1.812e−04
Coefficient: −2.29e+05
Value: omicron
_Candidatus.Saccharimonadia.o__Candidatus.Nanosynbacterales.f_
6e+05
4e+05
2e+05
0e+00
alpha (n=25)
clade20C (n=1)
delta (n=13)
omicron (n=6)
neg (n=72)
NA
WHOvariant

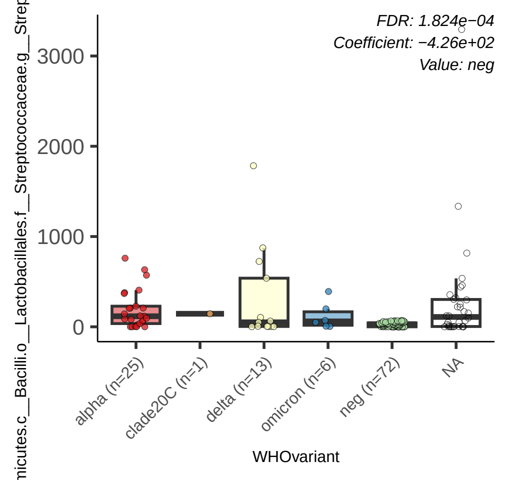

FDR: 1.824e−04
Coefficient: −4.26e+02
Value: neg
0
1000
2000
3000
micutes.c__Bacilli.o__Lactobacillales.f__Streptococcaceae.g__Strep
alpha (n=25)
clade20C (n=1)
delta (n=13)
omicron (n=6)
neg (n=72)
NA
WHOvariant

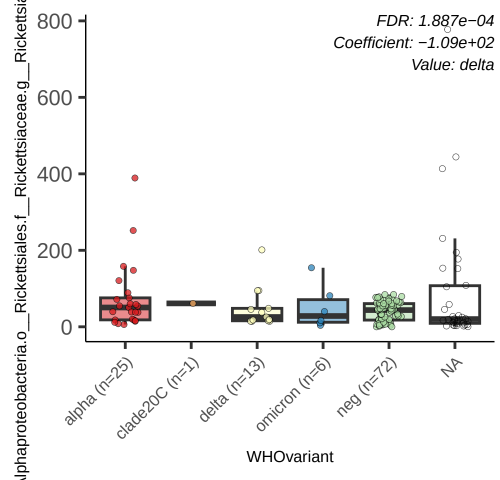

FDR: 1.887e−04
Coefficient: −1.09e+02
Value: delta
Alphaproteobacteria.o__Rickettsiales.f__Rickettsiaceae.g__Rickettsia
800
600
400
200
0
alpha (n=25)
clade20C (n=1)
delta (n=13)
omicron (n=6)
neg (n=72)
NA
WHOvariant

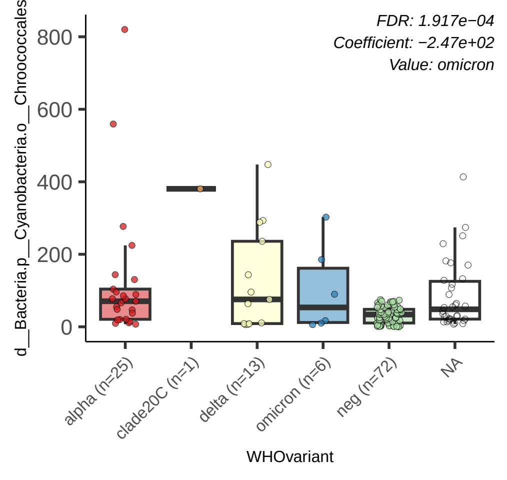
FDR: 1.917e−04
Coefficient: −2.47e+02
Value: omicron
d__Bacteria.p__Cyanobacteria.o__Chroococcales
800
600
400
200
0
alpha (n=25)
clade20C (n=1)
delta (n=13)
omicron (n=6)
neg (n=72)
NA
WHOvariant

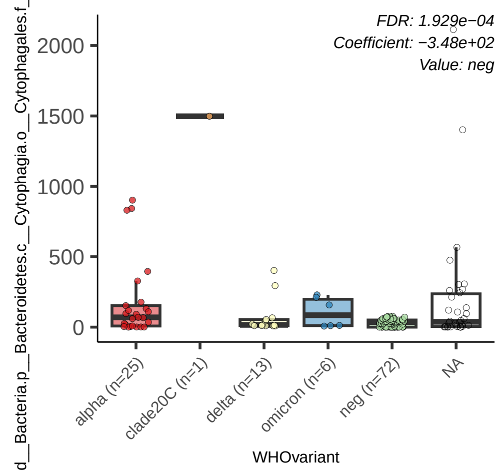
FDR: 1.929e−04
Coefficient: −3.48e+02
Value: neg
d__Bacteria.p__Bacteroidetes.c__Cytophagia.o__Cytophagales.f__
0
500
1000
1500
2000
alpha (n=25)
clade20C (n=1)
delta (n=13)
omicron (n=6)
neg (n=72)
NA
WHOvariant

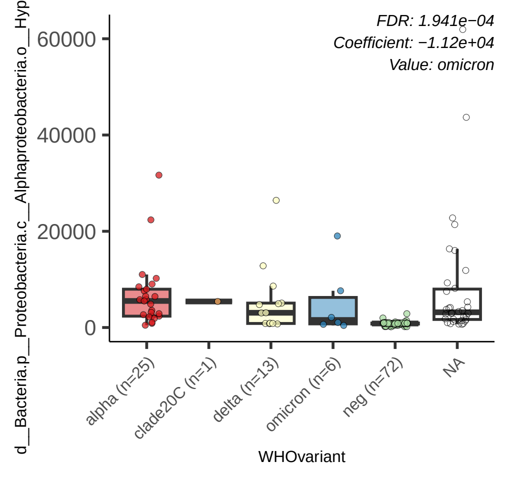

FDR: 1.941e−04
Coefficient: −1.12e+04
Value: omicron
d__Bacteria.p__Proteobacteria.c__Alphaproteobacteria.o__Hyp
60000
40000
20000
0
alpha (n=25)
clade20C (n=1)
delta (n=13)
omicron (n=6)
neg (n=72)
NA
WHOvariant

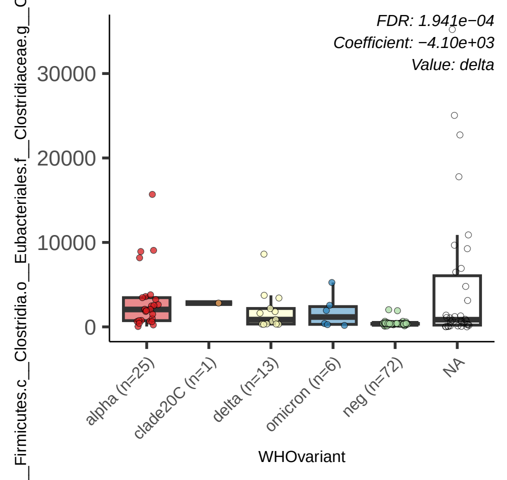

FDR: 1.941e−04
Coefficient: −4.10e+03
Value: delta
30000
20000
10000
0
Firmicutes.c__Clostridia.o__Eubacteriales.f__Clostridiaceae.g__C
alpha (n=25)
clade20C (n=1)
delta (n=13)
omicron (n=6)
neg (n=72)
NA
WHOvariant

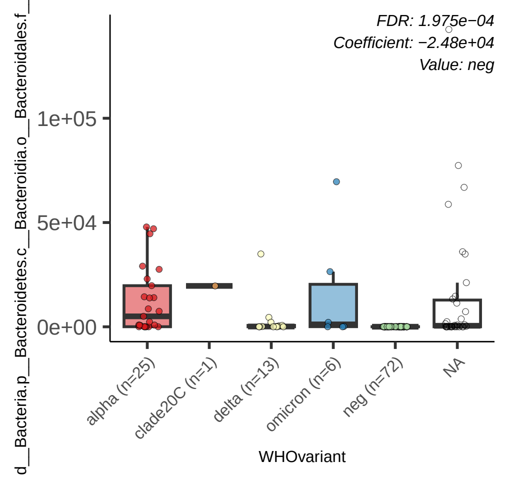

FDR: 1.975e−04
Coefficient: −2.48e+04
Value: neg
d__Bacteria.p__Bacteroidetes.c__Bacteroidia.o__Bacteroidales.f__
1e+05
5e+04
0e+00
alpha (n=25)
clade20C (n=1)
delta (n=13)
omicron (n=6)
neg (n=72)
NA
WHOvariant

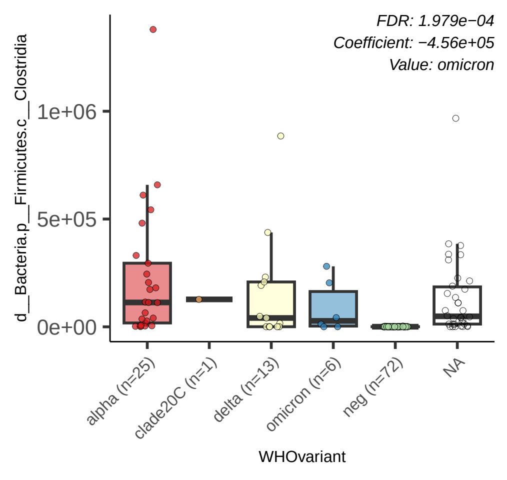

FDR: 1.979e−04
Coefficient: −4.56e+05
Value: omicron
d__Bacteria.p__Firmicutes.c__Clostridia
1e+06
5e+05
0e+00
alpha (n=25)
clade20C (n=1)
delta (n=13)
omicron (n=6)
neg (n=72)
NA
WHOvariant

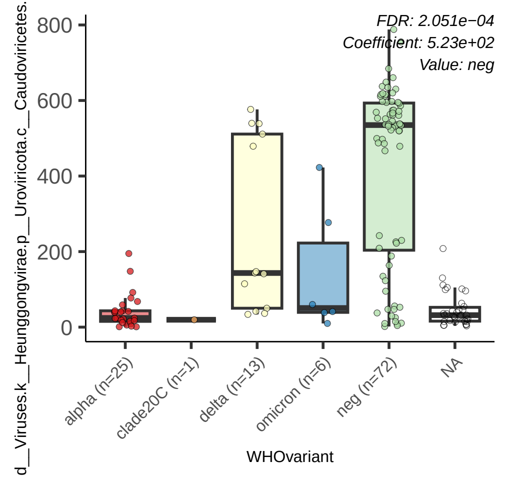

FDR: 2.051e−04
Coefficient: 5.23e+02
Value: neg
d__Viruses.k__Heunggongvirae.p__Uroviricota.c__Caudoviricetes.
0
200
400
600
800
alpha (n=25)
clade20C (n=1)
delta (n=13)
omicron (n=6)
neg (n=72)
NA
WHOvariant

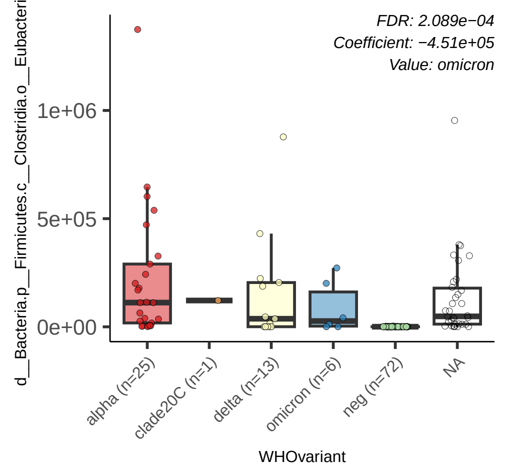

FDR: 2.089e−04
Coefficient: −4.51e+05
Value: omicron
d__Bacteria.p__Firmicutes.c__Clostridia.o__Eubacteri
1e+06
5e+05
0e+00
alpha (n=25)
clade20C (n=1)
delta (n=13)
omicron (n=6)
neg (n=72)
NA
WHOvariant

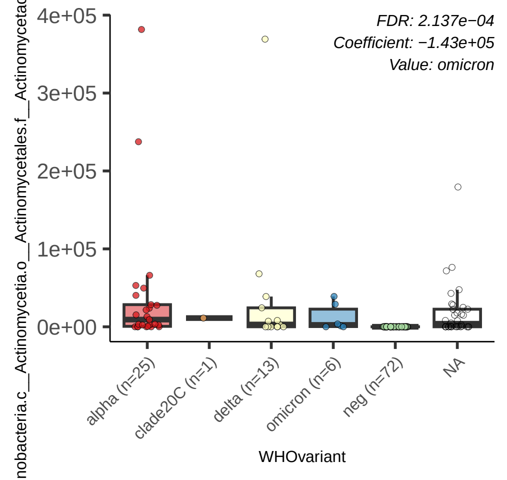

FDR: 2.137e−04
Coefficient: −1.43e+05
Value: omicron
nobacteria.c__Actinomycetia.o__Actinomycetales.f__Actinomycetac
4e+05
3e+05
2e+05
1e+05
0e+00
alpha (n=25)
clade20C (n=1)
delta (n=13)
omicron (n=6)
neg (n=72)
NA
WHOvariant

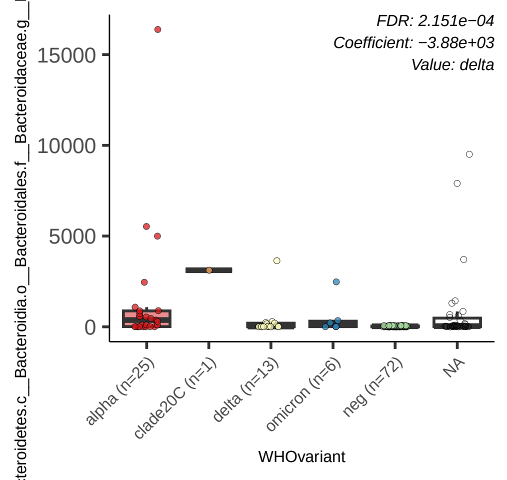

FDR: 2.151e−04
Coefficient: −3.88e+03
Value: delta
15000
10000
5000
0
alpha (n=25)
clade20C (n=1)
delta (n=13)
omicron (n=6)
neg (n=72)
NA
WHOvariant

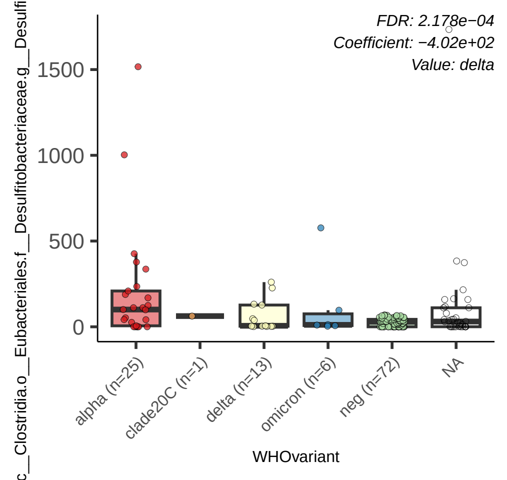

FDR: 2.178e−04
Coefficient: −4.02e+02
Value: delta
c__Clostridia.o__Eubacteriales.f__Desulfitobacteriaceae.g__Desulfi
1500
1000
500
0
alpha (n=25)
clade20C (n=1)
delta (n=13)
omicron (n=6)
neg (n=72)
NA
WHOvariant

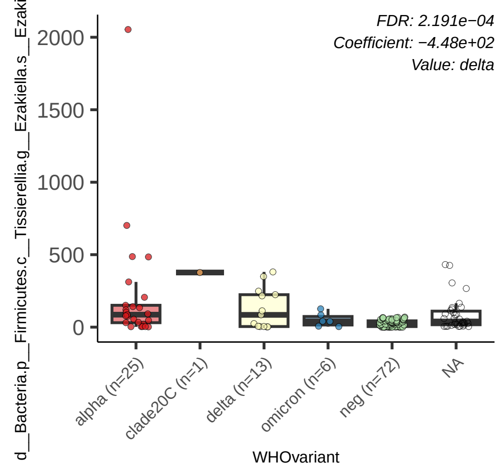

FDR: 2.191e−04
Coefficient: −4.48e+02
Value: delta
d__Bacteria.p__Firmicutes.c__Tissierellia.g__Ezakiella.s__Ezaki
2000
1500
1000
500
0
alpha (n=25)
clade20C (n=1)
delta (n=13)
omicron (n=6)
neg (n=72)
NA
WHOvariant

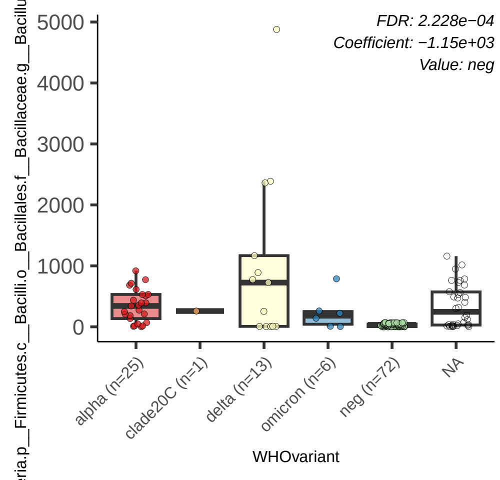

eria.p__Firmicutes.c__Bacilli.o__Bacillales.f__Bacillaceae.g__Bacillu
FDR: 2.228e−04
Coefficient: −1.15e+03
Value: neg
5000
4000
3000
2000
1000
0
alpha (n=25)
clade20C (n=1)
delta (n=13)
omicron (n=6)
neg (n=72)
NA
WHOvariant

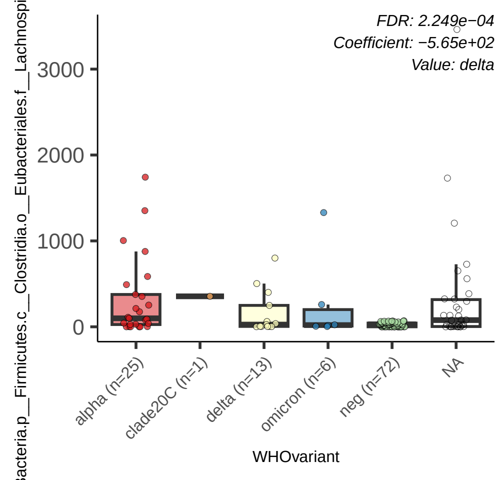
FDR: 2.249e−04
Coefficient: −5.65e+02
Value: delta
Bacteria.p__Firmicutes.c__Clostridia.o__Eubacteriales.f__Lachnospi
3000
2000
1000
0
alpha (n=25)
clade20C (n=1)
delta (n=13)
omicron (n=6)
neg (n=72)
NA
WHOvariant

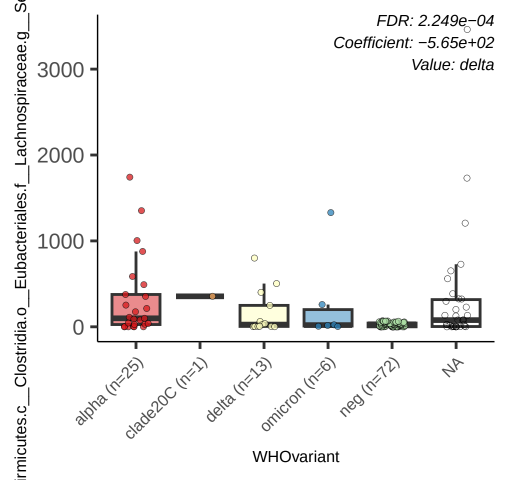
FDR: 2.249e−04
Coefficient: −5.65e+02
Value: delta
irmicutes.c__Clostridia.o__Eubacteriales.f__Lachnospiraceae.g__Sc
3000
2000
1000
0
alpha (n=25)
clade20C (n=1)
delta (n=13)
omicron (n=6)
neg (n=72)
NA
WHOvariant

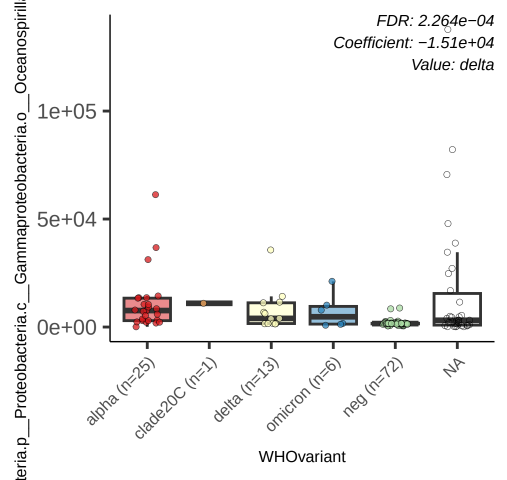

FDR: 2.264e−04
Coefficient: −1.51e+04
Value: delta
1e+05
5e+04
0e+00
teria.p__Proteobacteria.c__Gammaproteobacteria.o__Oceanospirilla
alpha (n=25)
clade20C (n=1)
delta (n=13)
omicron (n=6)
neg (n=72)
NA
WHOvariant

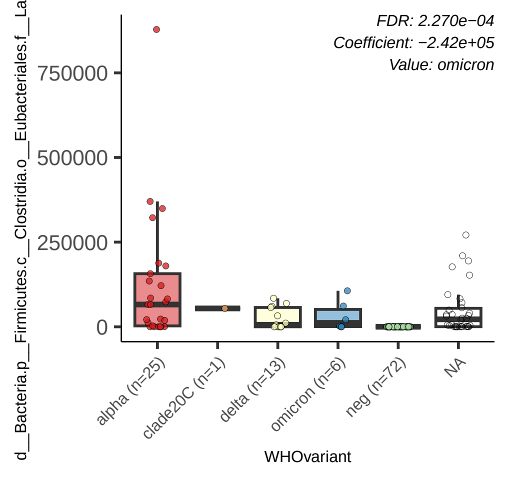

FDR: 2.270e−04
Coefficient: −2.42e+05
Value: omicron
d__Bacteria.p__Firmicutes.c__Clostridia.o__Eubacteriales.f__La
750000
500000
250000
0
alpha (n=25)
clade20C (n=1)
delta (n=13)
omicron (n=6)
neg (n=72)
NA
WHOvariant

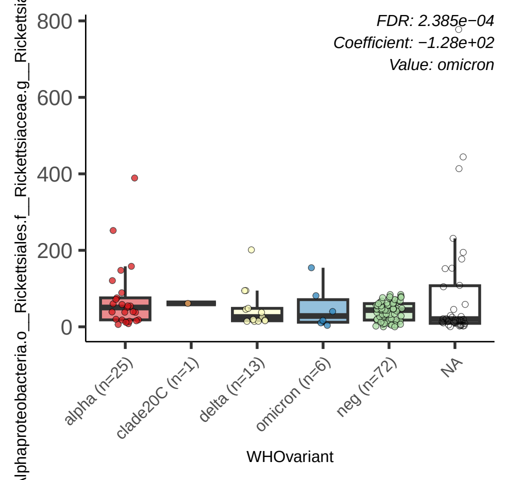
FDR: 2.385e−04
Coefficient: −1.28e+02
Value: omicron
Alphaproteobacteria.o__Rickettsiales.f__Rickettsiaceae.g__Rickettsia
800
600
400
200
0
alpha (n=25)
clade20C (n=1)
delta (n=13)
omicron (n=6)
neg (n=72)
NA
WHOvariant

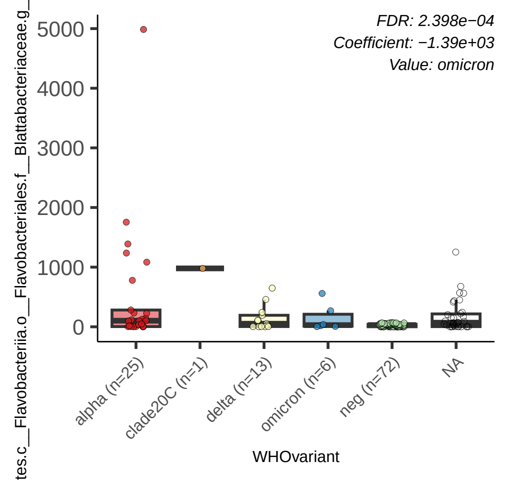
FDR: 2.398e−04
Coefficient: −1.39e+03
Value: omicron
tes.c__Flavobacteriia.o__Flavobacteriales.f__Blattabacteriaceae.g__
5000
4000
3000
2000
1000
0
alpha (n=25)
clade20C (n=1)
delta (n=13)
omicron (n=6)
neg (n=72)
NA
WHOvariant

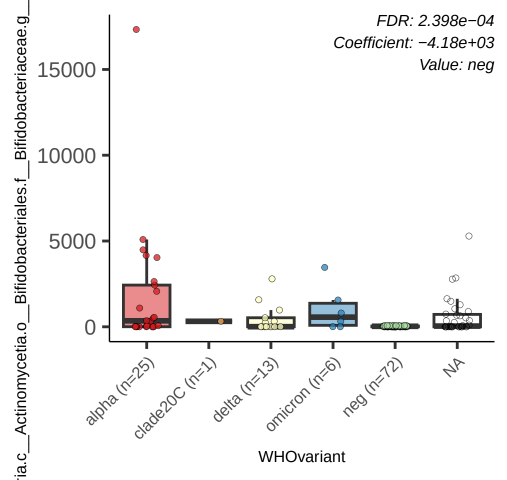

FDR: 2.398e−04
Coefficient: −4.18e+03
Value: neg
ria.c__Actinomycetia.o__Bifidobacteriales.f__Bifidobacteriaceae.g__
15000
10000
5000
0
alpha (n=25)
clade20C (n=1)
delta (n=13)
omicron (n=6)
neg (n=72)
NA
WHOvariant

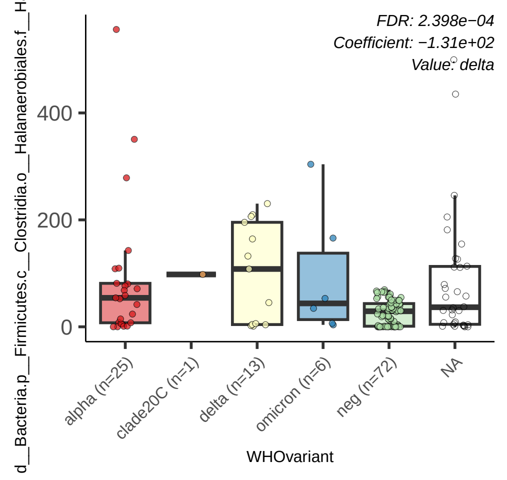

FDR: 2.398e−04
Coefficient: −1.31e+02
Value: delta
d__Bacteria.p__Firmicutes.c__Clostridia.o__Halanaerobiales.f__H
0
200
400
alpha (n=25)
clade20C (n=1)
delta (n=13)
omicron (n=6)
neg (n=72)
NA
WHOvariant

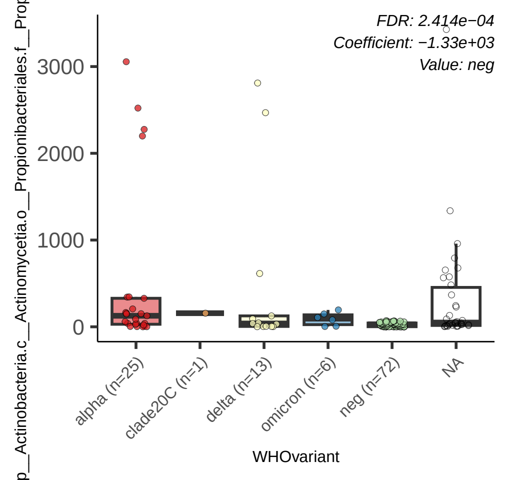

FDR: 2.414e−04
Coefficient: −1.33e+03
Value: neg
p__Actinobacteria.c__Actinomycetia.o__Propionibacteriales.f__Prop
3000
2000
1000
0
alpha (n=25)
clade20C (n=1)
delta (n=13)
omicron (n=6)
neg (n=72)
NA
WHOvariant

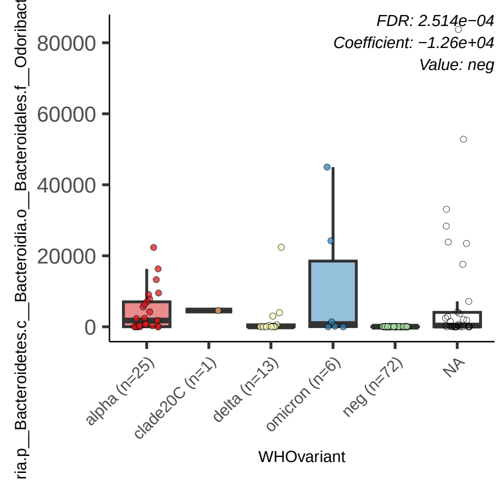

FDR: 2.514e−04
Coefficient: −1.26e+04
Value: neg
ria.p__Bacteroidetes.c__Bacteroidia.o__Bacteroidales.f__Odoribact
80000
60000
40000
20000
0
alpha (n=25)
clade20C (n=1)
delta (n=13)
omicron (n=6)
neg (n=72)
NA
WHOvariant

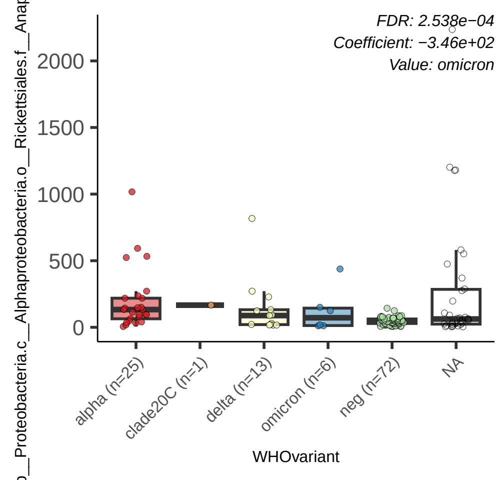

FDR: 2.538e−04
Coefficient: −3.46e+02
Value: omicron
p__Proteobacteria.c__Alphaproteobacteria.o__Rickettsiales.f__Anap
2000
1500
1000
500
0
alpha (n=25)
clade20C (n=1)
delta (n=13)
omicron (n=6)
neg (n=72)
NA
WHOvariant

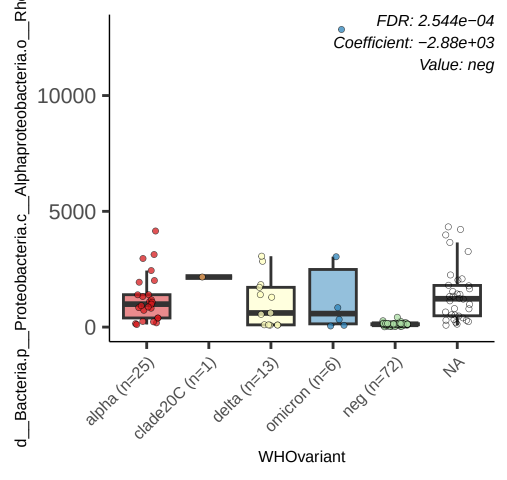
FDR: 2.544e−04
Coefficient: −2.88e+03
Value: neg
10000
5000
0
d__Bacteria.p__Proteobacteria.c__Alphaproteobacteria.o__Rh
alpha (n=25)
clade20C (n=1)
delta (n=13)
omicron (n=6)
neg (n=72)
NA
WHOvariant

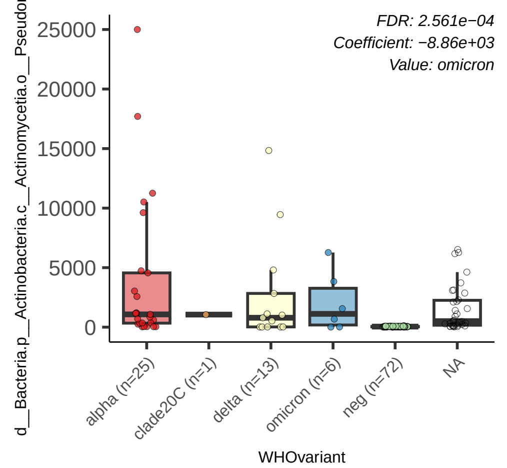

FDR: 2.561e−04
Coefficient: −8.86e+03
Value: omicron
d__Bacteria.p__Actinobacteria.c__Actinomycetia.o__Pseudo
25000
20000
15000
10000
5000
0
alpha (n=25)
clade20C (n=1)
delta (n=13)
omicron (n=6)
neg (n=72)
NA
WHOvariant

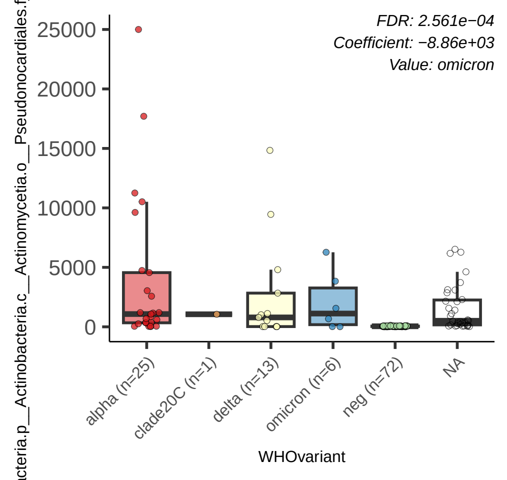

FDR: 2.561e−04
Coefficient: −8.86e+03
Value: omicron
25000
20000
15000
10000
5000
0
acteria.p__Actinobacteria.c__Actinomycetia.o__Pseudonocardiales.f
alpha (n=25)
clade20C (n=1)
delta (n=13)
omicron (n=6)
neg (n=72)
NA
WHOvariant

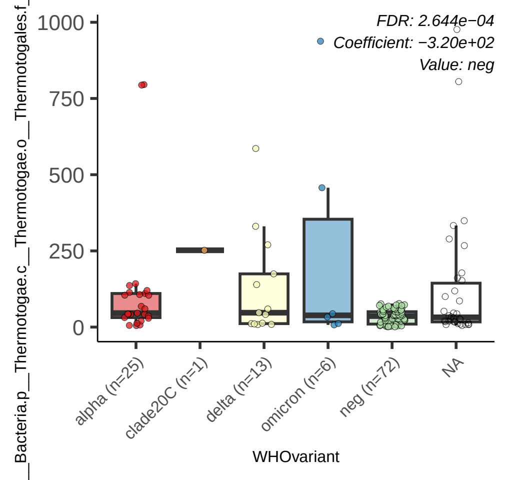

FDR: 2.644e−04
Coefficient: −3.20e+02
Value: neg
Bacteria.p__Thermotogae.c__Thermotogae.o__Thermotogales.f__
1000
750
500
250
0
alpha (n=25)
clade20C (n=1)
delta (n=13)
omicron (n=6)
neg (n=72)
NA
WHOvariant

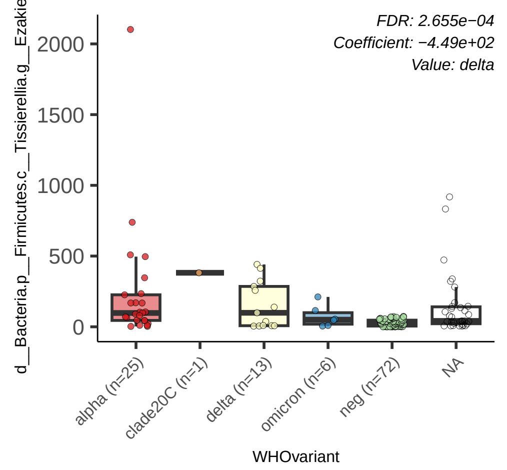

FDR: 2.655e−04
Coefficient: −4.49e+02
Value: delta
d__Bacteria.p__Firmicutes.c__Tissierellia.g__Ezakie
2000
1500
1000
500
0
alpha (n=25)
clade20C (n=1)
delta (n=13)
omicron (n=6)
neg (n=72)
NA
WHOvariant

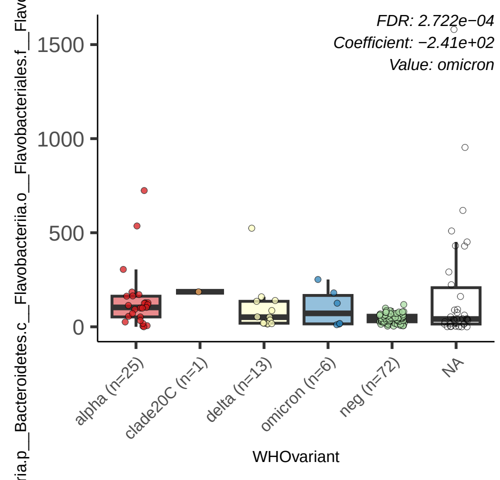

FDR: 2.722e−04
Coefficient: −2.41e+02
Value: omicron
Bacteroidetes.c__Flavobacteriia.o__Flavobacteriales.f__Flavo
1500
1000
500
0
alpha (n=25)
clade20C (n=1)
delta (n=13)
omicron (n=6)
neg (n=72)
NA
WHOvariant

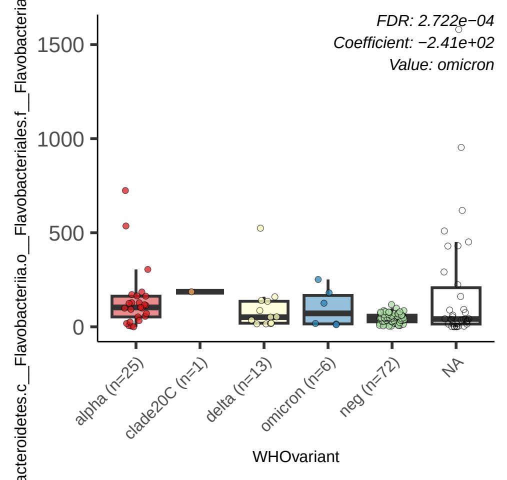
FDR: 2.722e−04
Coefficient: −2.41e+02
Value: omicron
acteroidetes.c__Flavobacteriia.o__Flavobacteriales.f__Flavobacteria
0
500
1000
1500
alpha (n=25)
clade20C (n=1)
delta (n=13)
omicron (n=6)
neg (n=72)
NA
WHOvariant

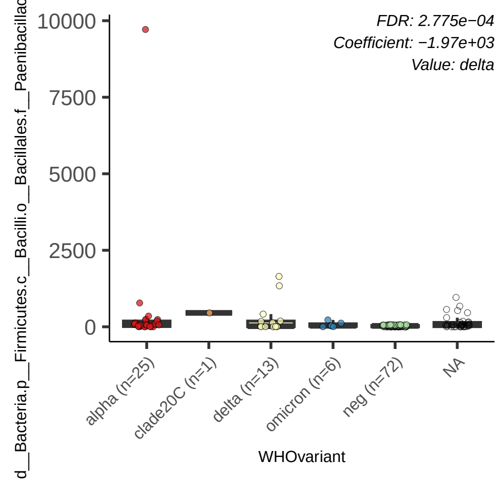
FDR: 2.775e−04
Coefficient: −1.97e+03
Value: delta
d__Bacteria.p__Firmicutes.c__Bacilli.o__Bacillales.f__Paenibacillac
10000
7500
5000
2500
0
alpha (n=25)
clade20C (n=1)
delta (n=13)
omicron (n=6)
neg (n=72)
NA
WHOvariant

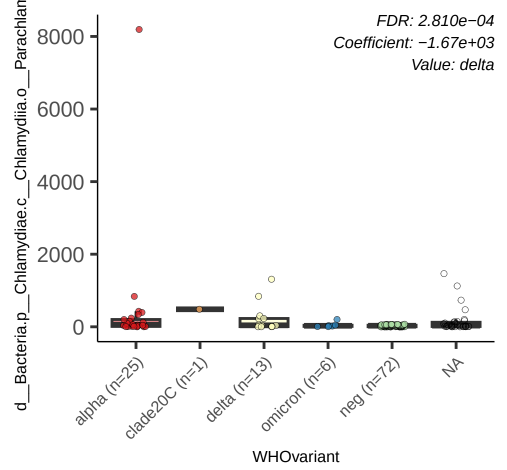
FDR: 2.810e−04
Coefficient: −1.67e+03
Value: delta
d__Bacteria.p__Chlamydiae.c__Chlamydiia.o__Parachlam
8000
6000
4000
2000
0
alpha (n=25)
clade20C (n=1)
delta (n=13)
omicron (n=6)
neg (n=72)
NA
WHOvariant

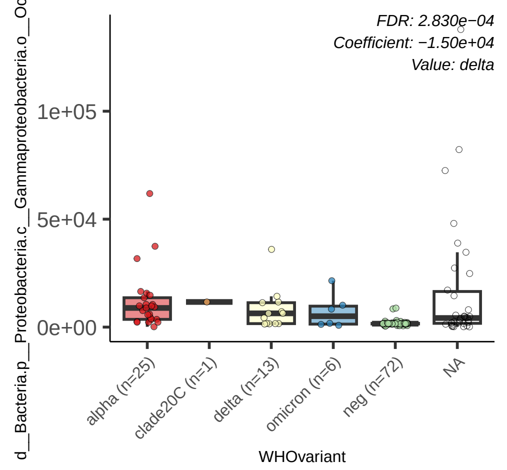

FDR: 2.830e−04
Coefficient: −1.50e+04
Value: delta
d__Bacteria.p__Proteobacteria.c__Gammaproteobacteria.o__Oc
1e+05
5e+04
0e+00
alpha (n=25)
clade20C (n=1)
delta (n=13)
omicron (n=6)
neg (n=72)
NA
WHOvariant

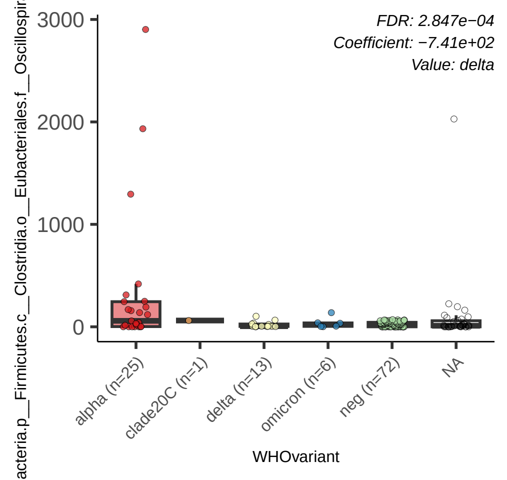

FDR: 2.847e−04
Coefficient: −7.41e+02
Value: delta
3000
2000
1000
0
acteria.p__Firmicutes.c__Clostridia.o__Eubacteriales.f__Oscillospir
alpha (n=25)
clade20C (n=1)
delta (n=13)
omicron (n=6)
neg (n=72)
NA
WHOvariant

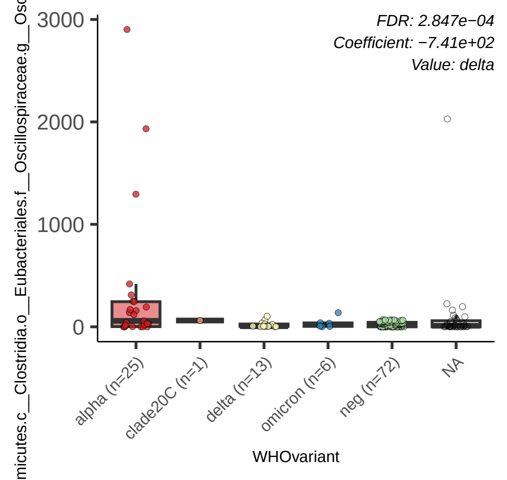

FDR: 2.847e−04
Coefficient: −7.41e+02
Value: delta
3000
2000
1000
0
micutes.c__Clostridia.o__Eubacteriales.f__Oscillospiraceae.g__Osc
alpha (n=25)
clade20C (n=1)
delta (n=13)
omicron (n=6)
neg (n=72)
NA
WHOvariant

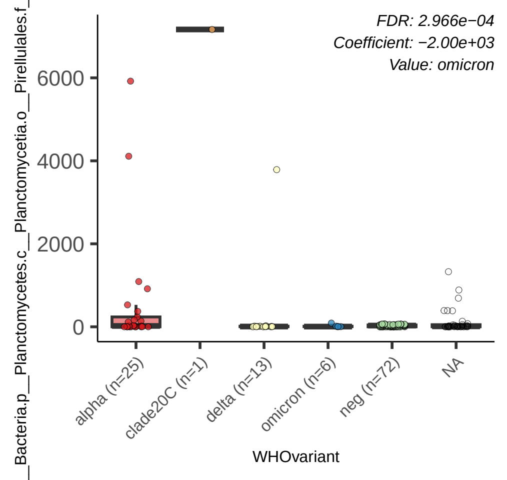

FDR: 2.966e−04
Coefficient: −2.00e+03
Value: omicron
Bacteria.p__Planctomycetes.c__Planctomycetia.o__Pirellulales.f
6000
4000
2000
0
alpha (n=25)
clade20C (n=1)
delta (n=13)
omicron (n=6)
neg (n=72)
NA
WHOvariant

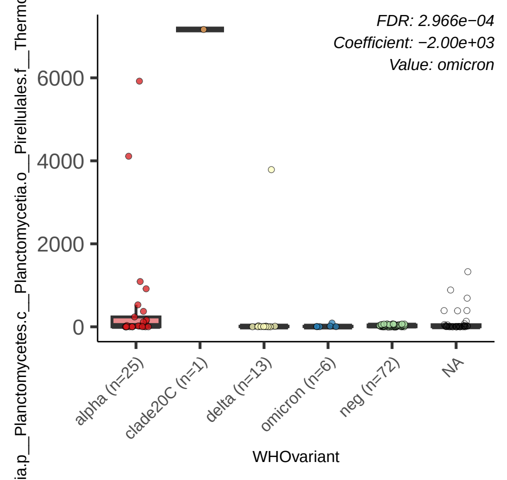

FDR: 2.966e−04
Coefficient: −2.00e+03
Value: omicron
ia.p__Planctomycetes.c__Planctomycetia.o__Pirellulales.f__Thermo
6000
4000
2000
0
alpha (n=25)
clade20C (n=1)
delta (n=13)
omicron (n=6)
neg (n=72)
NA
WHOvariant

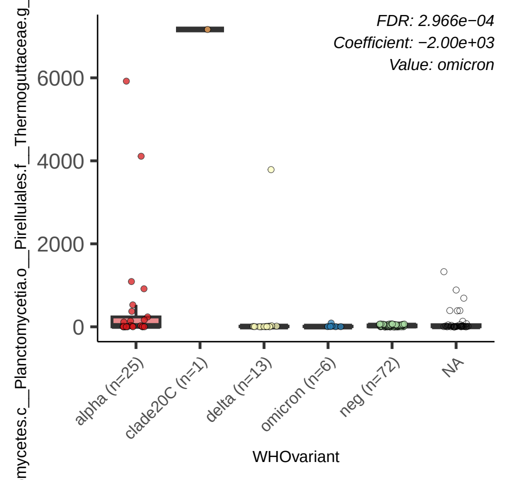
FDR: 2.966e−04
Coefficient: −2.00e+03
Value: omicron
mycetes.c__Planctomycetia.o__Pirellulales.f__Thermoguttaceae.g__
0
2000
4000
6000
alpha (n=25)
clade20C (n=1)
delta (n=13)
omicron (n=6)
neg (n=72)
NA
WHOvariant

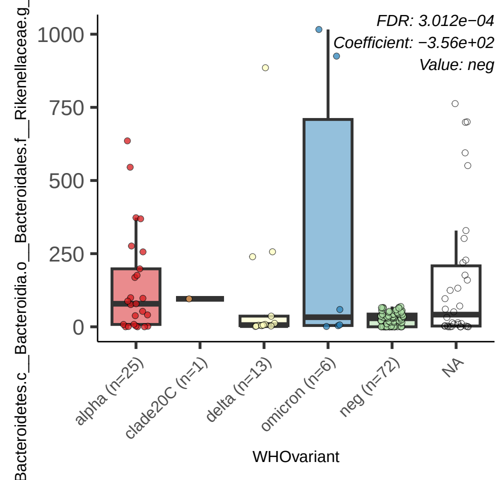
FDR: 3.012e−04
Coefficient: −3.56e+02
Value: neg
Bacteroidetes.c__Bacteroidia.o__Bacteroidales.f__Rikenellaceae.g__
1000
750
500
250
0
alpha (n=25)
clade20C (n=1)
delta (n=13)
omicron (n=6)
neg (n=72)
NA
WHOvariant

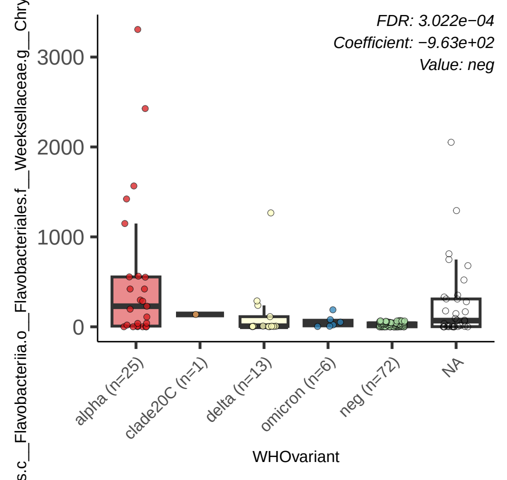

FDR: 3.022e−04
Coefficient: −9.63e+02
Value: neg
s.c__Flavobacteriia.o__Flavobacteriales.f__Weeksellaceae.g__Chry
3000
2000
1000
0
alpha (n=25)
clade20C (n=1)
delta (n=13)
omicron (n=6)
neg (n=72)
NA
WHOvariant

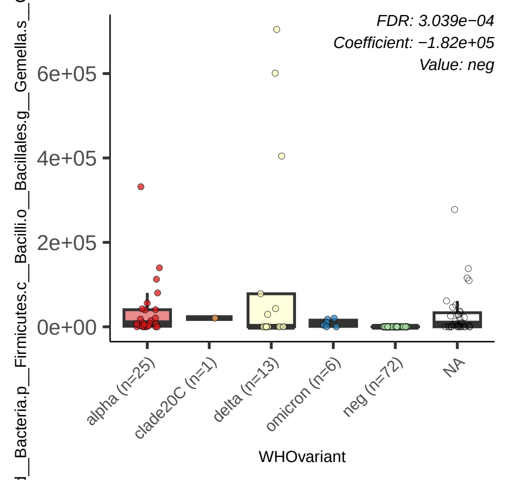

FDR: 3.039e−04
Coefficient: −1.82e+05
Value: neg
d__Bacteria.p__Firmicutes.c__Bacilli.o__Bacillales.g__Gemella.s__
6e+05
4e+05
2e+05
0e+00
alpha (n=25)
clade20C (n=1)
delta (n=13)
omicron (n=6)
neg (n=72)
NA
WHOvariant

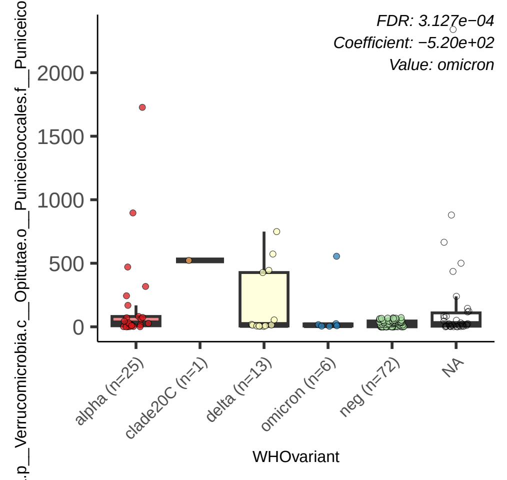

FDR: 3.127e−04
Coefficient: −5.20e+02
Value: omicron
p__Verrucomicrobia.c__Opitutae.o__Puniceicoccales.f__Puniceicoccaceae
2000
1500
1000
500
0
alpha (n=25)
clade20C (n=1)
delta (n=13)
omicron (n=6)
neg (n=72)
NA
WHOvariant

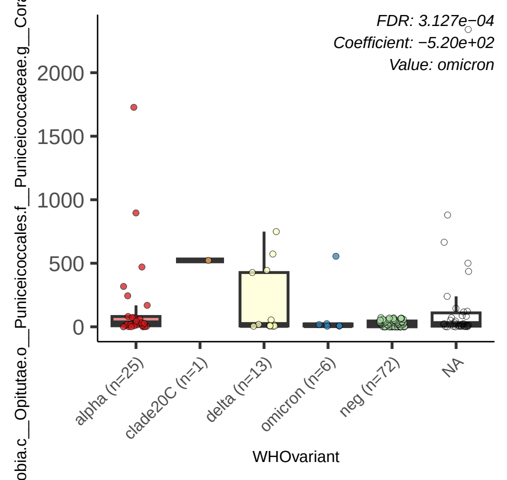

FDR: 3.127e−04
Coefficient: −5.20e+02
Value: omicron
obia.c__Opitutae.o__Puniceicoccales.f__Puniceicoccaceae.g__Cora
2000
1500
1000
500
0
alpha (n=25)
clade20C (n=1)
delta (n=13)
omicron (n=6)
neg (n=72)
NA
WHOvariant

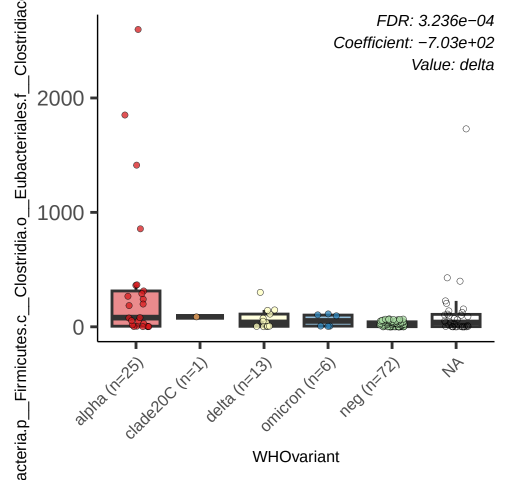
FDR: 3.236e−04
Coefficient: −7.03e+02
Value: delta
acteria.p__Firmicutes.c__Clostridia.o__Eubacteriales.f__Clostridiac
2000
1000
0
alpha (n=25)
clade20C (n=1)
delta (n=13)
omicron (n=6)
neg (n=72)
NA
WHOvariant

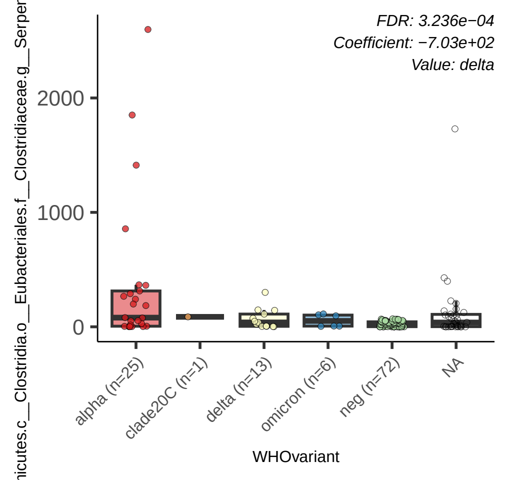

FDR: 3.236e−04
Coefficient: −7.03e+02
Value: delta
2000
1000
0
alpha (n=25)
clade20C (n=1)
delta (n=13)
omicron (n=6)
neg (n=72)
NA
WHOvariant
nicutes.c__Clostridia.o__Eubacteriales.f__Clostridiaceae.g__Serpen

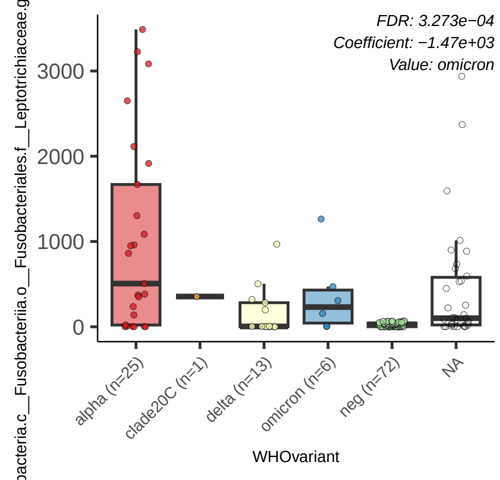

FDR: 3.273e−04
Coefficient: −1.47e+03
Value: omicron
bacteria.c__Fusobacteriia.o__Fusobacteriales.f__Leptotrichiaceae.g
3000
2000
1000
0
alpha (n=25)
clade20C (n=1)
delta (n=13)
omicron (n=6)
neg (n=72)
NA
WHOvariant

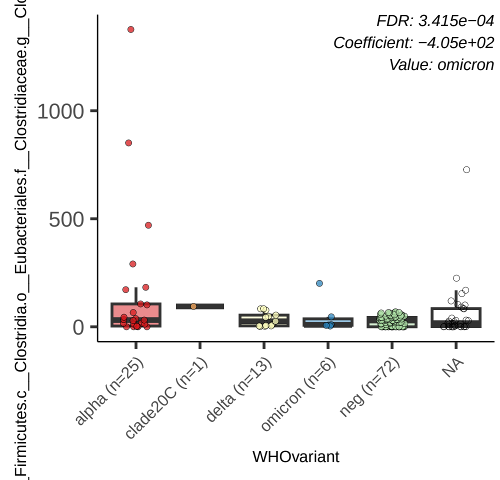

FDR: 3.415e−04
Coefficient: −4.05e+02
Value: omicron
1000
500
0
Firmicutes.c__Clostridia.o__Eubacteriales.f__Clostridiaceae.g__Clo
alpha (n=25)
clade20C (n=1)
delta (n=13)
omicron (n=6)
neg (n=72)
NA
WHOvariant

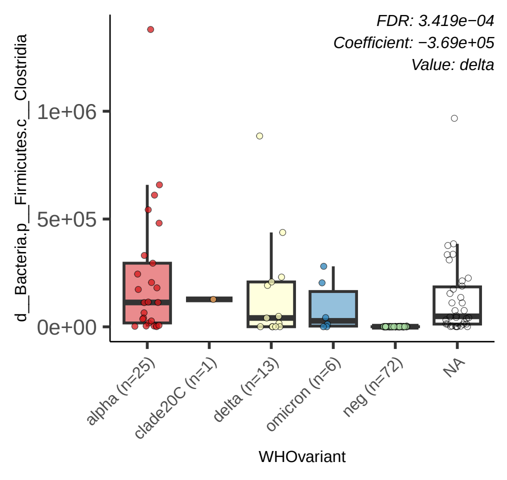

FDR: 3.419e−04
Coefficient: −3.69e+05
Value: delta
d__Bacteria.p__Firmicutes.c__Clostridia
1e+06
5e+05
0e+00
alpha (n=25)
clade20C (n=1)
delta (n=13)
omicron (n=6)
neg (n=72)
NA
WHOvariant

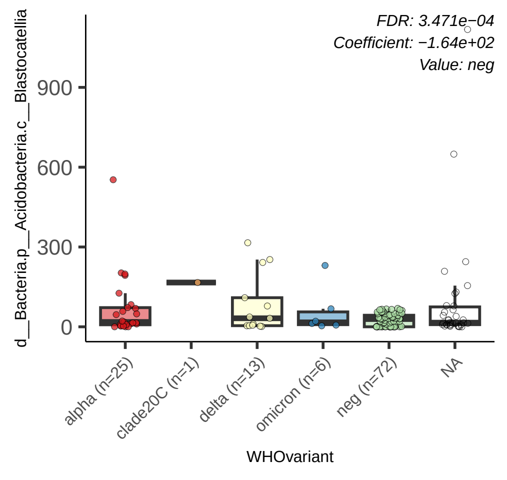
FDR: 3.471e−04
Coefficient: −1.64e+02
Value: neg
d__Bacteria.p__Acidobacteria.c__Blastocatellia
900
600
300
0
alpha (n=25)
clade20C (n=1)
delta (n=13)
omicron (n=6)
neg (n=72)
NA
WHOvariant

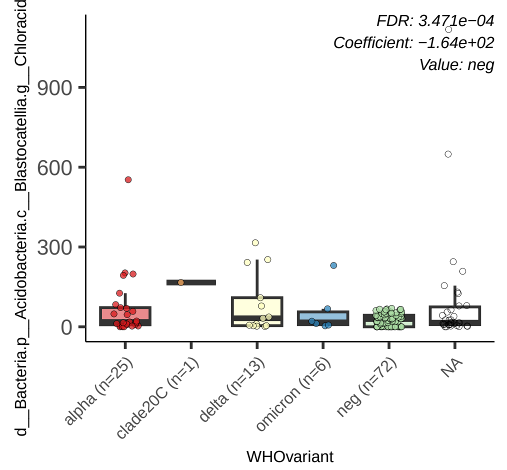
FDR: 3.471e−04
Coefficient: −1.64e+02
Value: neg
d__Bacteria.p__Acidobacteria.c__Blastocatellia.g__Chloracid
0
300
600
900
alpha (n=25)
clade20C (n=1)
delta (n=13)
omicron (n=6)
neg (n=72)
NA
WHOvariant

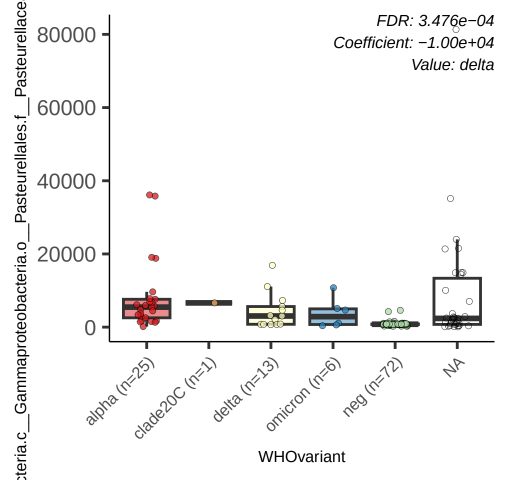

FDR: 3.476e−04
Coefficient: −1.00e+04
Value: delta
80000
60000
40000
20000
0
cteria.c__Gammaproteobacteria.o__Pasteurellales.f__Pasteurellace
alpha (n=25)
clade20C (n=1)
delta (n=13)
omicron (n=6)
neg (n=72)
NA
WHOvariant

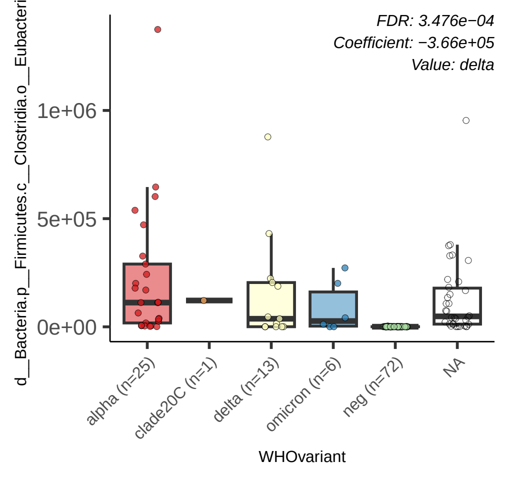

FDR: 3.476e−04
Coefficient: −3.66e+05
Value: delta
d__Bacteria.p__Firmicutes.c__Clostridia.o__Eubacteri
1e+06
5e+05
0e+00
alpha (n=25)
clade20C (n=1)
delta (n=13)
omicron (n=6)
neg (n=72)
NA
WHOvariant

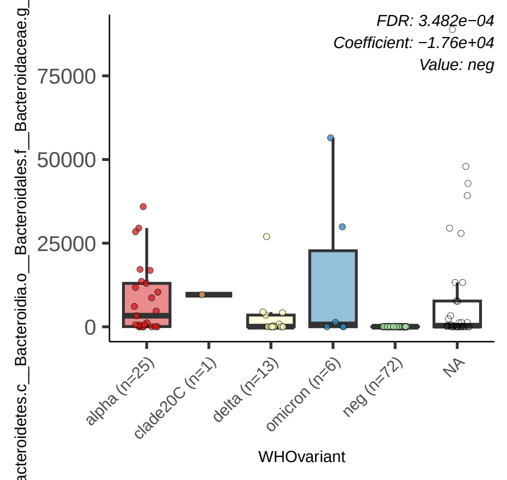
FDR: 3.482e−04
Coefficient: −1.76e+04
Value: neg
acteroidetes.c__Bacteroidia.o__Bacteroidales.f__Bacteroidaceae.g_
75000
50000
25000
0
alpha (n=25)
clade20C (n=1)
delta (n=13)
omicron (n=6)
neg (n=72)
NA
WHOvariant

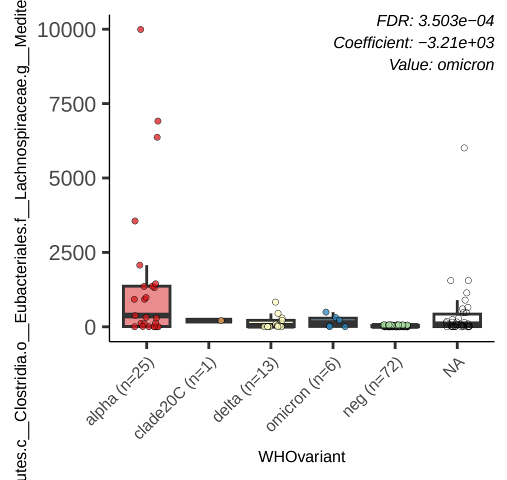

FDR: 3.503e−04
Coefficient: −3.21e+03
Value: omicron
utes.c__Clostridia.o__Eubacteriales.f__Lachnospiraceae.g__Medite
10000
7500
5000
2500
0
alpha (n=25)
clade20C (n=1)
delta (n=13)
omicron (n=6)
neg (n=72)
NA
WHOvariant

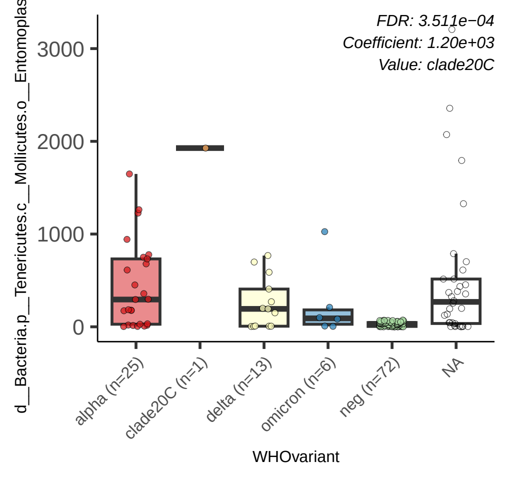
FDR: 3.511e−04
Coefficient: 1.20e+03
Value: clade20C
d__Bacteria.p__Tenericutes.c__Mollicutes.o__Entomoplas
3000
2000
1000
0
alpha (n=25)
clade20C (n=1)
delta (n=13)
omicron (n=6)
neg (n=72)
NA
WHOvariant

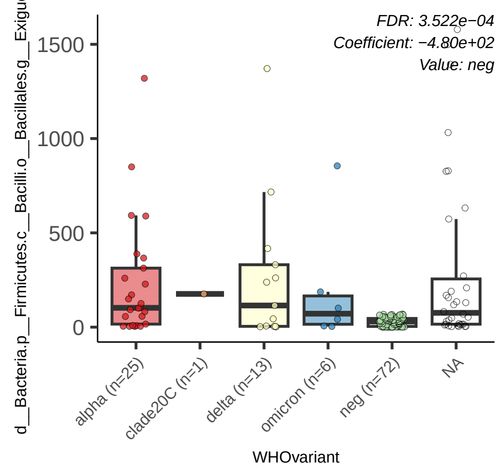

d__Bacteria.p__Firmicutes.c__Bacilli.o__Bacillales.g__Exigu
FDR: 3.522e−04
Coefficient: −4.80e+02
Value: neg
1500
1000
500
0
alpha (n=25)
clade20C (n=1)
delta (n=13)
omicron (n=6)
neg (n=72)
NA
WHOvariant

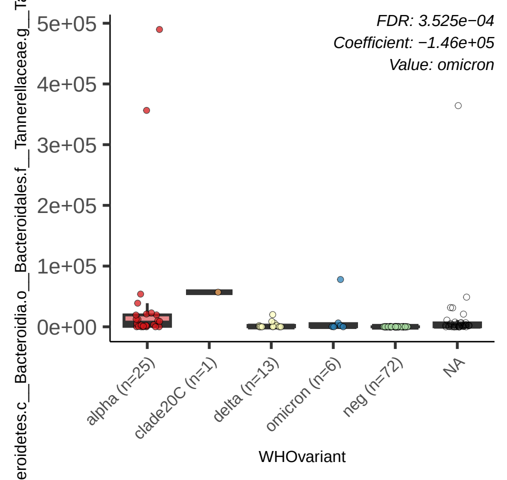

FDR: 3.525e−04
Coefficient: −1.46e+05
Value: omicron
eroidetes.c__Bacteroidia.o__Bacteroidales.f__Tannerellaceae.g__Ta
5e+05
4e+05
3e+05
2e+05
1e+05
0e+00
alpha (n=25)
clade20C (n=1)
delta (n=13)
omicron (n=6)
neg (n=72)
NA
WHOvariant

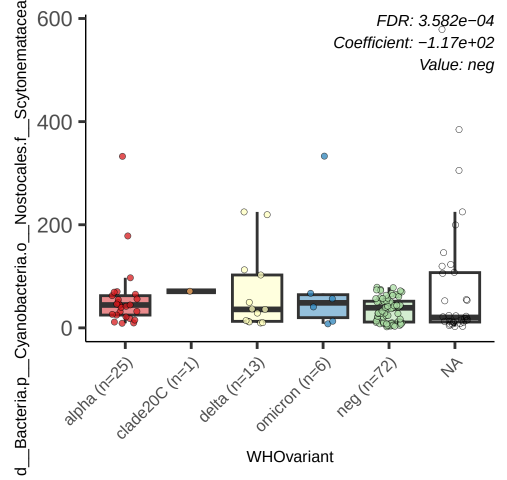
FDR: 3.582e−04
Coefficient: −1.17e+02
Value: neg
d__Bacteria.p__Cyanobacteria.o__Nostocales.f__Scytonematacea
600
400
200
0
alpha (n=25)
clade20C (n=1)
delta (n=13)
omicron (n=6)
neg (n=72)
NA
WHOvariant

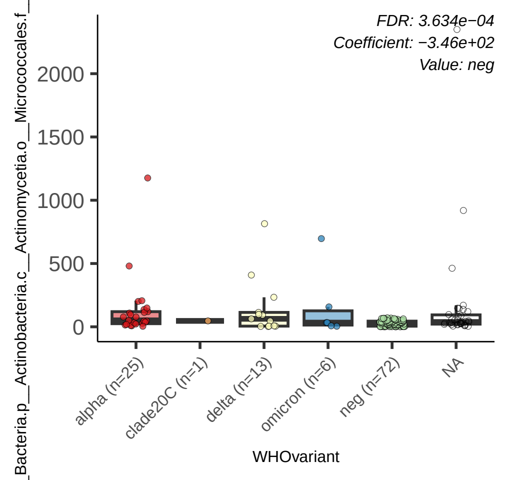
FDR: 3.634e−04
Coefficient: −3.46e+02
Value: neg
Bacteria.p__Actinobacteria.c__Actinomycetia.o__Micrococcales.f__
2000
1500
1000
500
0
alpha (n=25)
clade20C (n=1)
delta (n=13)
omicron (n=6)
neg (n=72)
NA
WHOvariant

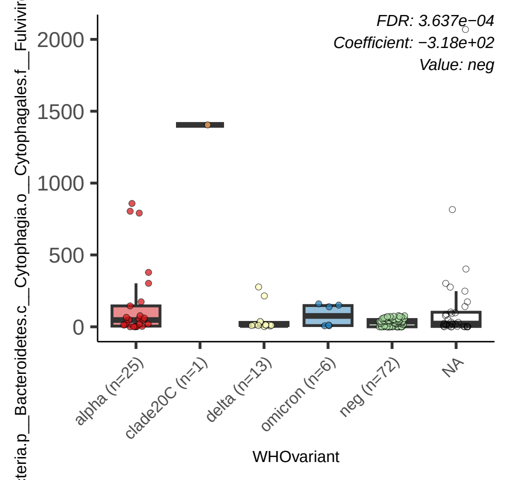
FDR: 3.637e−04
Coefficient: −3.18e+02
Value: neg
2000
1500
1000
500
0
cteria.p__Bacteroidetes.c__Cytophagia.o__Cytophagales.f__Fulvivir
alpha (n=25)
clade20C (n=1)
delta (n=13)
omicron (n=6)
neg (n=72)
NA
WHOvariant

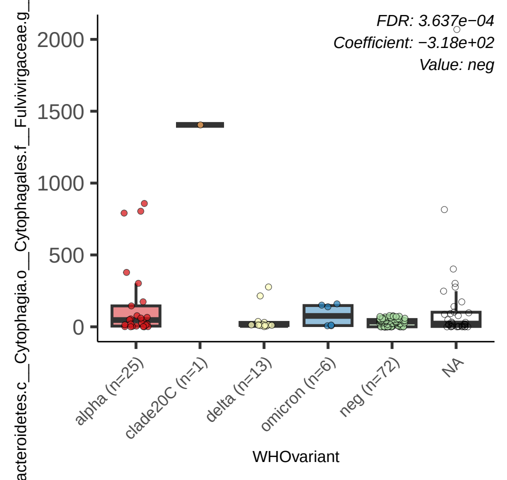

FDR: 3.637e−04
Coefficient: −3.18e+02
Value: neg
acteroidetes.c__Cytophagia.o__Cytophagales.f__Fulvivirgaceae.g__
2000
1500
1000
500
0
alpha (n=25)
clade20C (n=1)
delta (n=13)
omicron (n=6)
neg (n=72)
NA
WHOvariant

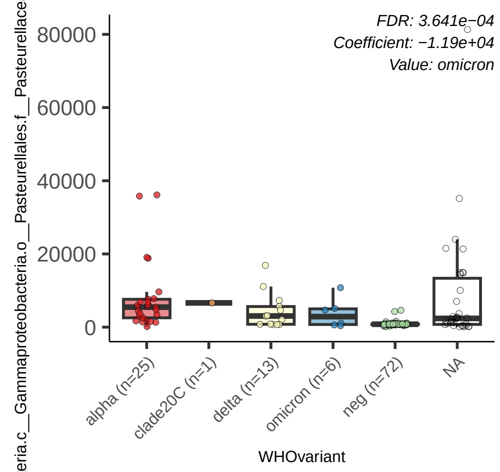

FDR: 3.641e−04
Coefficient: −1.19e+04
Value: omicron
80000
60000
40000
20000
0
cteria.c__Gammaproteobacteria.o__Pasteurellales.f__Pasteurellace
alpha (n=25)
clade20C (n=1)
delta (n=13)
omicron (n=6)
neg (n=72)
NA
WHOvariant

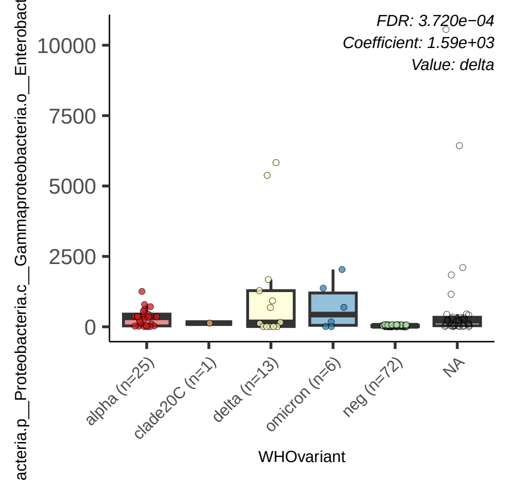

FDR: 3.720e−04
Coefficient: 1.59e+03
Value: delta
10000
7500
5000
2500
0
alpha (n=25)
clade20C (n=1)
delta (n=13)
omicron (n=6)
neg (n=72)
NA
WHOvariant
acteria.p__Proteobacteria.c__Gammaproteobacteria.o__Enterobact

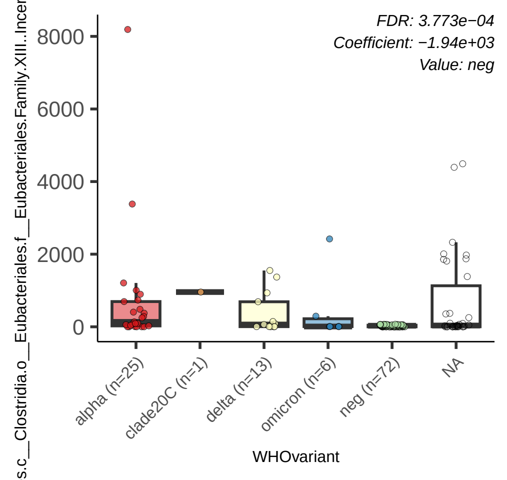
FDR: 3.773e−04
Coefficient: −1.94e+03
Value: neg
s.c__Clostridia.o__Eubacteriales.f__Eubacteriales.Family.XIII..Incer
8000
6000
4000
2000
0
alpha (n=25)
clade20C (n=1)
delta (n=13)
omicron (n=6)
neg (n=72)
NA
WHOvariant

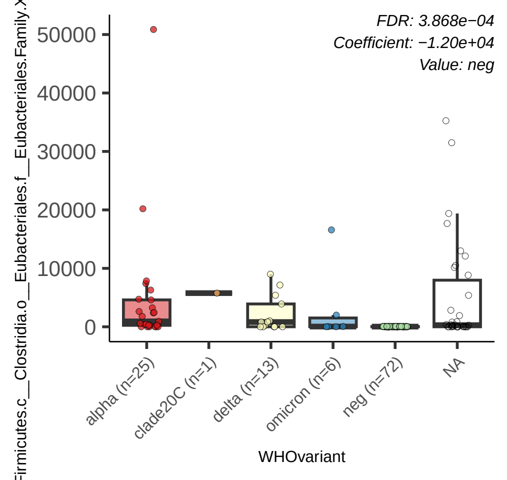

FDR: 3.868e−04
Coefficient: −1.20e+04
Value: neg
Firmicutes.c__Clostridia.o__Eubacteriales.f__Eubacteriales.Family.X
0
10000
20000
30000
40000
50000
alpha (n=25)
clade20C (n=1)
delta (n=13)
omicron (n=6)
neg (n=72)
NA
WHOvariant

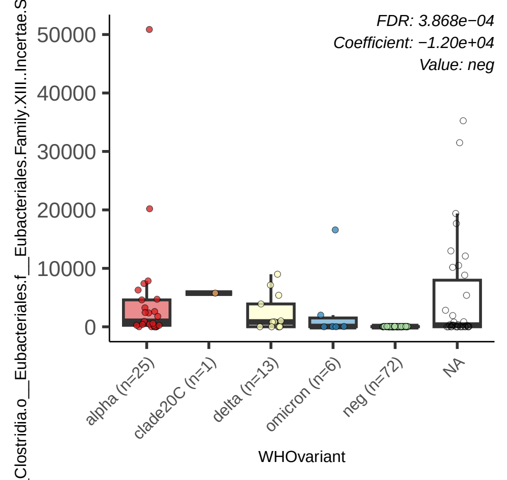
FDR: 3.868e−04
Coefficient: −1.20e+04
Value: neg
Clostridia.o__Eubacteriales.f__Eubacteriales.Family.XIII..Incertae.S
50000
40000
30000
20000
10000
0
alpha (n=25)
clade20C (n=1)
delta (n=13)
omicron (n=6)
neg (n=72)
NA
WHOvariant

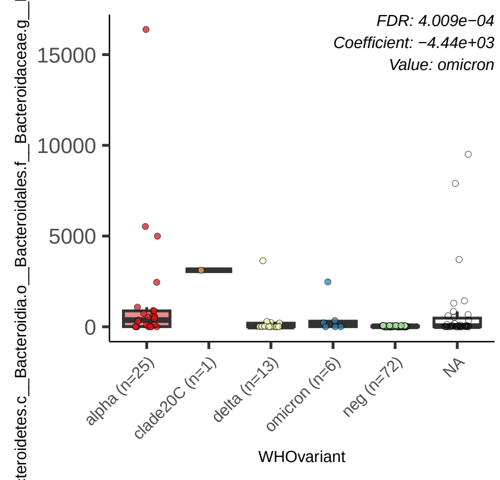
FDR: 4.009e−04
Coefficient: −4.44e+03
Value: omicron
Bacteroidetes.c__Bacteroidia.o__Bacteroidales.f__Bacteroidaceae.g__
15000
10000
5000
0
alpha (n=25)
clade20C (n=1)
delta (n=13)
omicron (n=6)
neg (n=72)
NA
WHOvariant

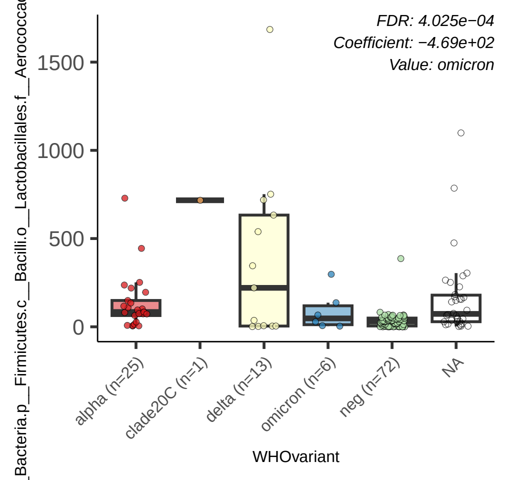

FDR: 4.025e−04
Coefficient: −4.69e+02
Value: omicron
1500
1000
500
0
Bacteria.p__Firmicutes.c__Bacilli.o__Lactobacillales.f__Aerococcac
alpha (n=25)
clade20C (n=1)
delta (n=13)
omicron (n=6)
neg (n=72)
NA
WHOvariant

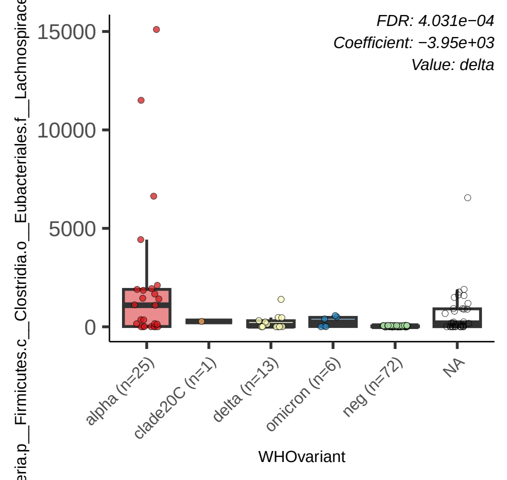
FDR: 4.031e−04
Coefficient: −3.95e+03
Value: delta
15000
10000
5000
0
eria.p__Firmicutes.c__Clostridia.o__Eubacteriales.f__Lachnospirace
alpha (n=25)
clade20C (n=1)
delta (n=13)
omicron (n=6)
neg (n=72)
NA
WHOvariant

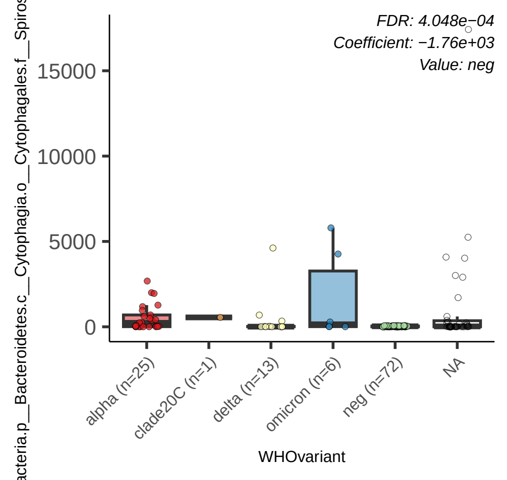
FDR: 4.048e−04
Coefficient: −1.76e+03
Value: neg
15000
10000
5000
0
acteria.p__Bacteroidetes.c__Cytophagia.o__Cytophagales.f__Spiros
alpha (n=25)
clade20C (n=1)
delta (n=13)
omicron (n=6)
neg (n=72)
NA
WHOvariant

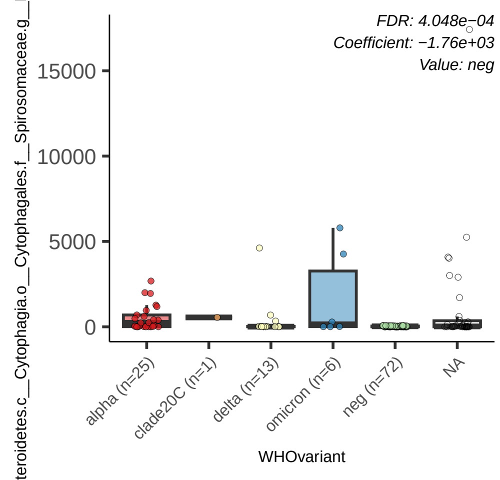

FDR: 4.048e−04
Coefficient: −1.76e+03
Value: neg
teroidetes.c__Cytophagia.o__Cytophagales.f__Spirosomaceae.g__
15000
10000
5000
0
alpha (n=25)
clade20C (n=1)
delta (n=13)
omicron (n=6)
neg (n=72)
NA
WHOvariant

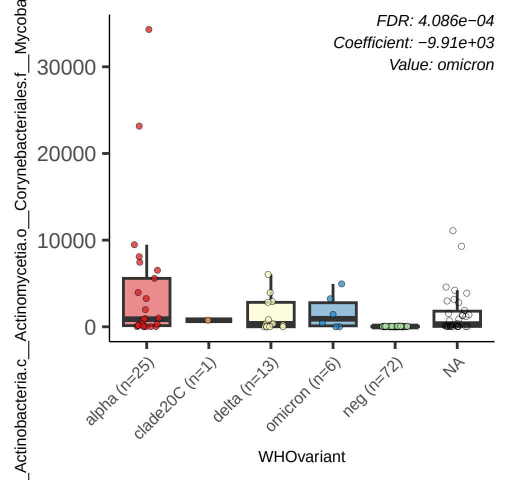
FDR: 4.086e−04
Coefficient: −9.91e+03
Value: omicron
Actinobacteria.c__Actinomycetia.o__Corynebacteriales.f__Mycoba
30000
20000
10000
0
alpha (n=25)
clade20C (n=1)
delta (n=13)
omicron (n=6)
neg (n=72)
NA
WHOvariant

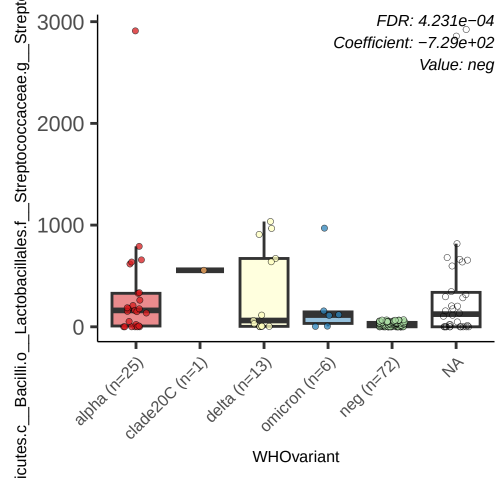

FDR: 4.231e−04
Coefficient: −7.29e+02
Value: neg
3000
2000
1000
0
icutes.c__Bacilli.o__Lactobacillales.f__Streptococcaceae.g__Strept
alpha (n=25)
clade20C (n=1)
delta (n=13)
omicron (n=6)
neg (n=72)
NA
WHOvariant

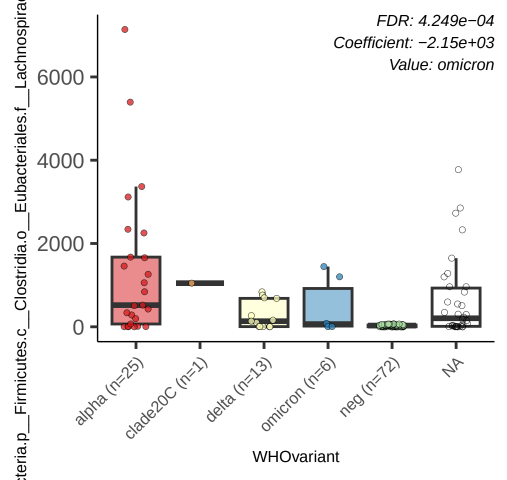

FDR: 4.249e−04
Coefficient: −2.15e+03
Value: omicron
0
2000
4000
6000
cteria.p__Firmicutes.c__Clostridia.o__Eubacteriales.f__Lachnospirac
alpha (n=25)
clade20C (n=1)
delta (n=13)
omicron (n=6)
neg (n=72)
NA
WHOvariant

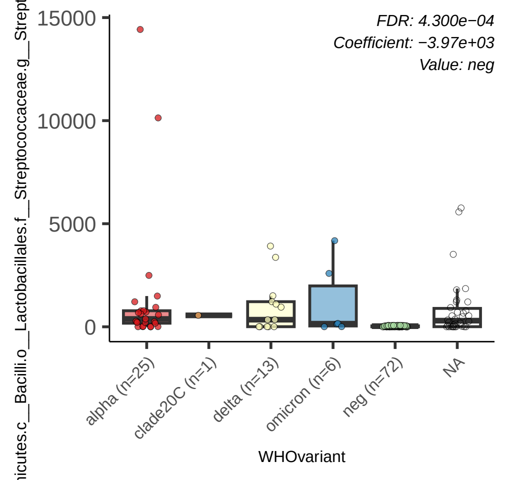
FDR: 4.300e−04
Coefficient: −3.97e+03
Value: neg
15000
10000
5000
0
nicutes.c__Bacilli.o__Lactobacillales.f__Streptococcaceae.g__Strept
alpha (n=25)
clade20C (n=1)
delta (n=13)
omicron (n=6)
neg (n=72)
NA
WHOvariant

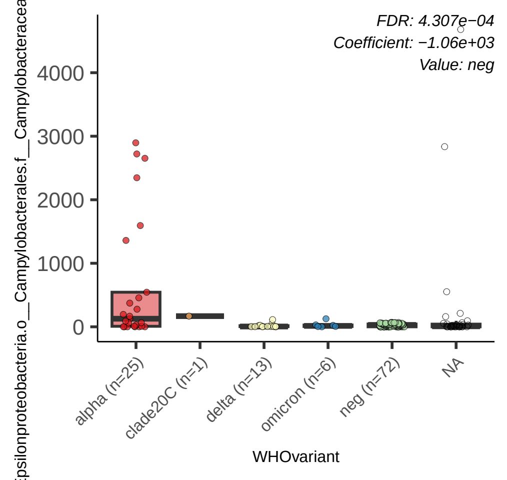
FDR: 4.307e−04
Coefficient: −1.06e+03
Value: neg
0
1000
2000
3000
4000
alpha (n=25)
clade20C (n=1)
delta (n=13)
omicron (n=6)
neg (n=72)
NA
WHOvariant
psilonproteobacteria.o__Campylobacterales.f__Campylobacteracea

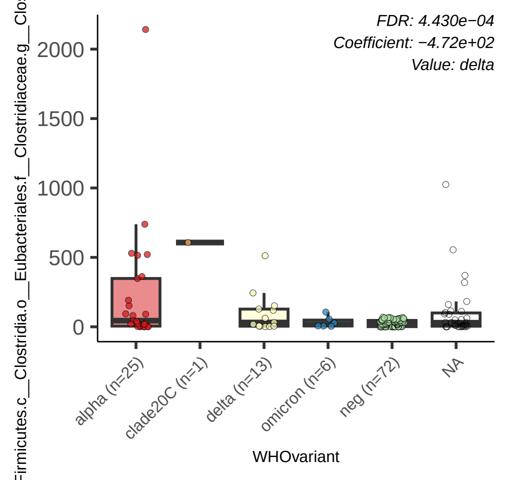

FDR: 4.430e−04
Coefficient: −4.72e+02
Value: delta
Firmicutes.c__Clostridia.o__Eubacteriales.f__Clostridiaceae.g__Clos
2000
1500
1000
500
0
alpha (n=25)
clade20C (n=1)
delta (n=13)
omicron (n=6)
neg (n=72)
NA
WHOvariant

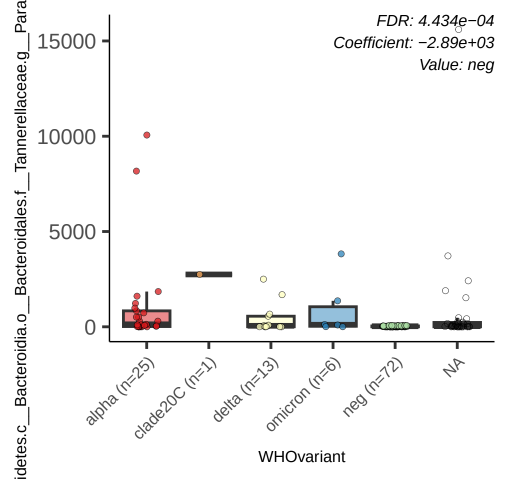

FDR: 4.434e−04
Coefficient: −2.89e+03
Value: neg
15000
10000
5000
0
idetes.c__Bacteroidia.o__Bacteroidales.f__Tannerellaceae.g__Para
alpha (n=25)
clade20C (n=1)
delta (n=13)
omicron (n=6)
neg (n=72)
NA
WHOvariant

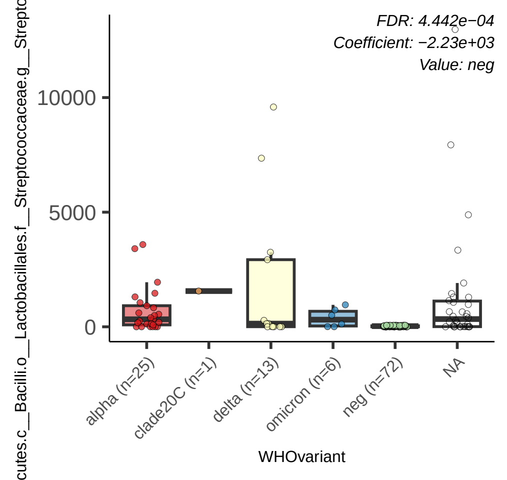
FDR: 4.442e−04
Coefficient: −2.23e+03
Value: neg
10000
5000
0
cutes.c__Bacilli.o__Lactobacillales.f__Streptococcaceae.g__Strepto
alpha (n=25)
clade20C (n=1)
delta (n=13)
omicron (n=6)
neg (n=72)
NA
WHOvariant

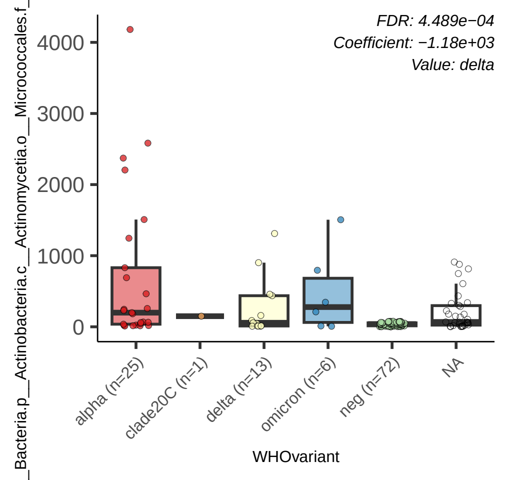

FDR: 4.489e−04
Coefficient: −1.18e+03
Value: delta
Bacteria.p__Actinobacteria.c__Actinomycetia.o__Micrococcales.f__
4000
3000
2000
1000
0
alpha (n=25)
clade20C (n=1)
delta (n=13)
omicron (n=6)
neg (n=72)
NA
WHOvariant

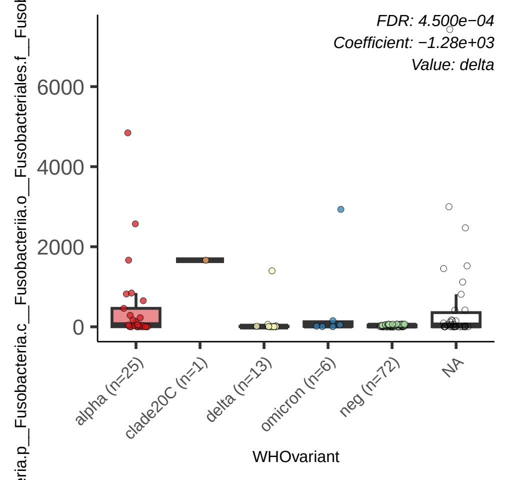

FDR: 4.500e−04
Coefficient: −1.28e+03
Value: delta
6000
4000
2000
0
alpha (n=25)
clade20C (n=1)
delta (n=13)
omicron (n=6)
neg (n=72)
NA
WHOvariant
eria.p__Fusobacteria.c__Fusobacteriia.o__Fusobacteriales.f__Fusob

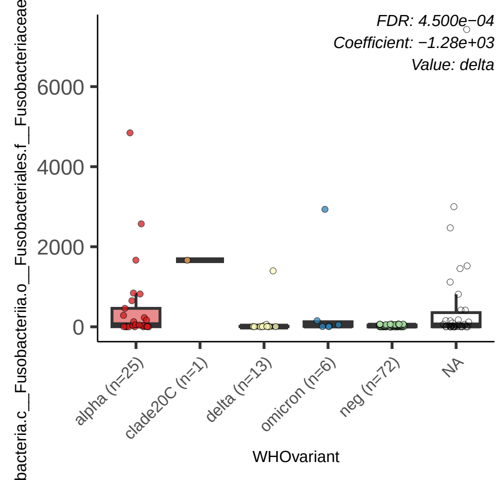
FDR: 4.500e−04
Coefficient: −1.28e+03
Value: delta
bacteria.c__Fusobacteriia.o__Fusobacteriales.f__Fusobacteriaceae
6000
4000
2000
0
alpha (n=25)
clade20C (n=1)
delta (n=13)
omicron (n=6)
neg (n=72)
NA
WHOvariant

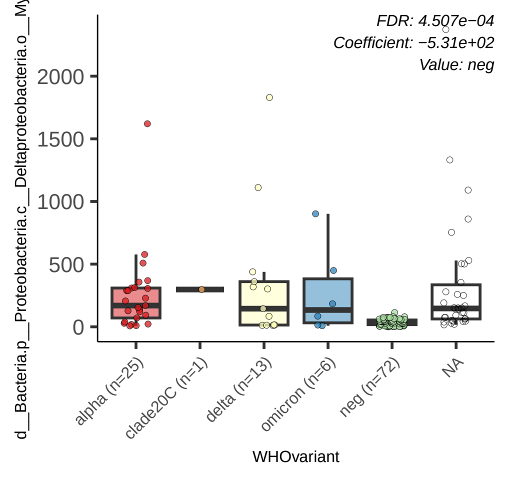

FDR: 4.507e−04
Coefficient: −5.31e+02
Value: neg
d__Bacteria.p__Proteobacteria.c__Deltaproteobacteria.o__My
2000
1500
1000
500
0
alpha (n=25)
clade20C (n=1)
delta (n=13)
omicron (n=6)
neg (n=72)
NA
WHOvariant

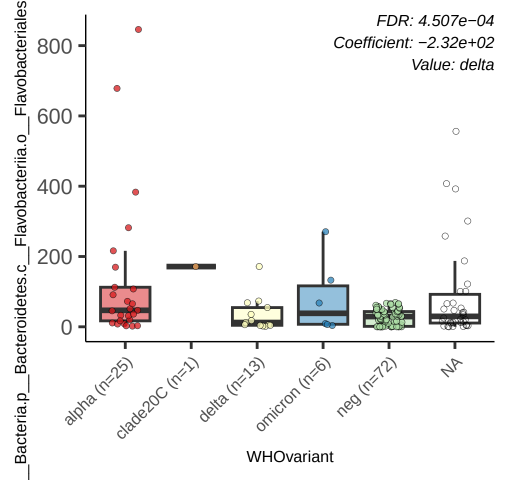
FDR: 4.507e−04
Coefficient: −2.32e+02
Value: delta
0
200
400
600
800
Bacteria.p__Bacteroidetes.c__Flavobacteriia.o__Flavobacteriales
alpha (n=25)
clade20C (n=1)
delta (n=13)
omicron (n=6)
neg (n=72)
NA
WHOvariant

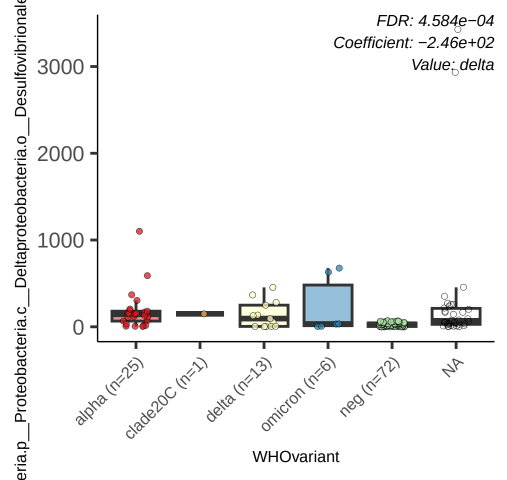

FDR: 4.584e−04
Coefficient: −2.46e+02
Value: delta
3000
2000
1000
0
alpha (n=25)
clade20C (n=1)
delta (n=13)
omicron (n=6)
neg (n=72)
NA
WHOvariant
teria.p__Proteobacteria.c__Deltaproteobacteria.o__Desulfovibrionale

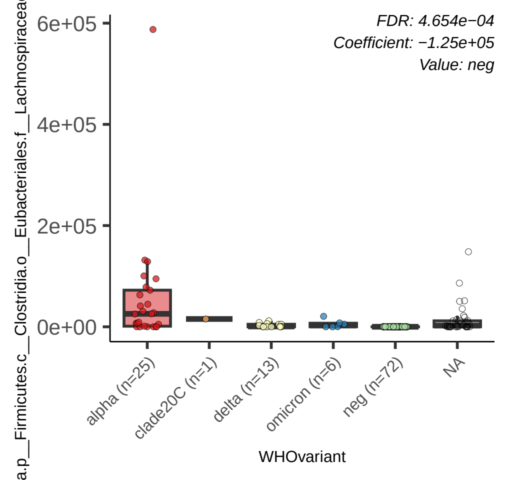

FDR: 4.654e−04
Coefficient: −1.25e+05
Value: neg
6e+05
4e+05
2e+05
0e+00
a.p__Firmicutes.c__Clostridia.o__Eubacteriales.f__Lachnospiracea
alpha (n=25)
clade20C (n=1)
delta (n=13)
omicron (n=6)
neg (n=72)
NA
WHOvariant

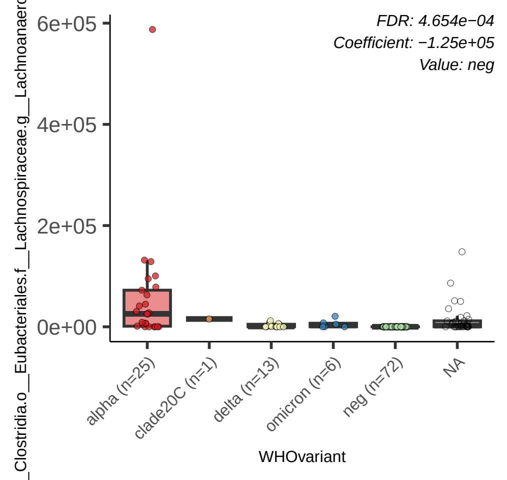
FDR: 4.654e−04
Coefficient: −1.25e+05
Value: neg
Clostridia.o__Eubacteriales.f__Lachnospiraceae.g__Lachnoanaero
6e+05
4e+05
2e+05
0e+00
alpha (n=25)
clade20C (n=1)
delta (n=13)
omicron (n=6)
neg (n=72)
NA
WHOvariant

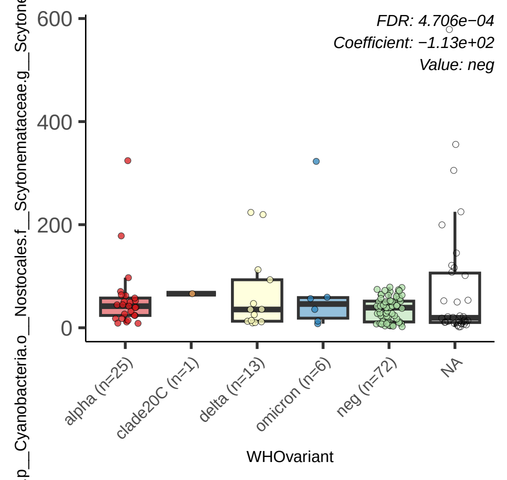

FDR: 4.706e−04
Coefficient: −1.13e+02
Value: neg
600
400
200
0
p__Cyanobacteria.o__Nostocales.f__Scytonemataceae.g__Scytone
alpha (n=25)
clade20C (n=1)
delta (n=13)
omicron (n=6)
neg (n=72)
NA
WHOvariant

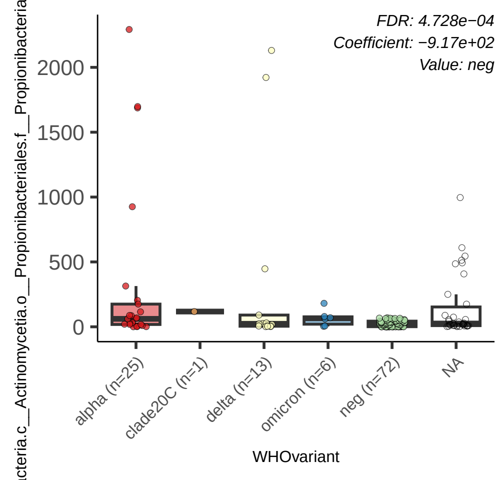
FDR: 4.728e−04
Coefficient: −9.17e+02
Value: neg
2000
1500
1000
500
0
acteria.c__Actinomycetia.o__Propionibacteriales.f__Propionibacteria
alpha (n=25)
clade20C (n=1)
delta (n=13)
omicron (n=6)
neg (n=72)
NA
WHOvariant

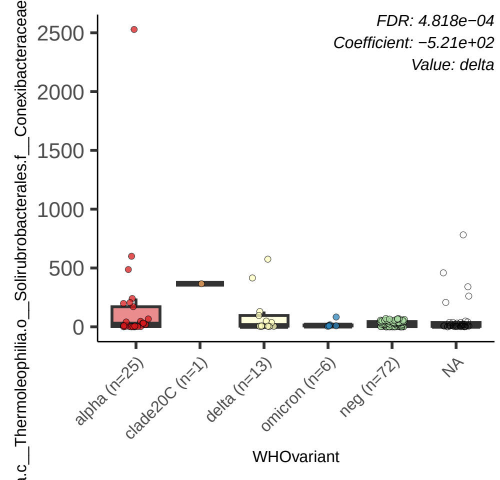
FDR: 4.818e−04
Coefficient: −5.21e+02
Value: delta
2500
2000
1500
1000
500
0
a.c__Thermoleophilia.o__Solirubrobacterales.f__Conexibacteraceae
alpha (n=25)
clade20C (n=1)
delta (n=13)
omicron (n=6)
neg (n=72)
NA
WHOvariant

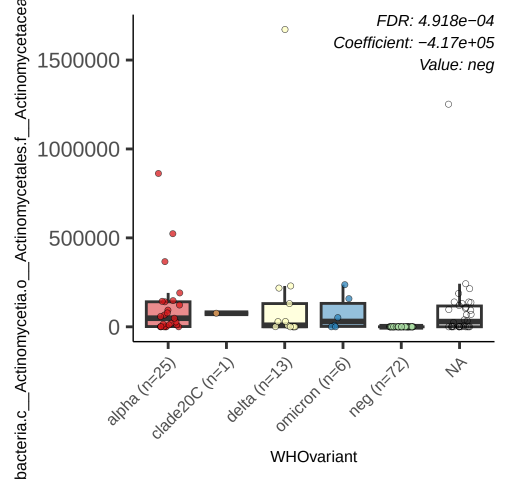

FDR: 4.918e−04
Coefficient: −4.17e+05
Value: neg
bacteria.c__Actinomycetia.o__Actinomycetales.f__Actinomycetacea
1500000
1000000
500000
0
alpha (n=25)
clade20C (n=1)
delta (n=13)
omicron (n=6)
neg (n=72)
NA
WHOvariant

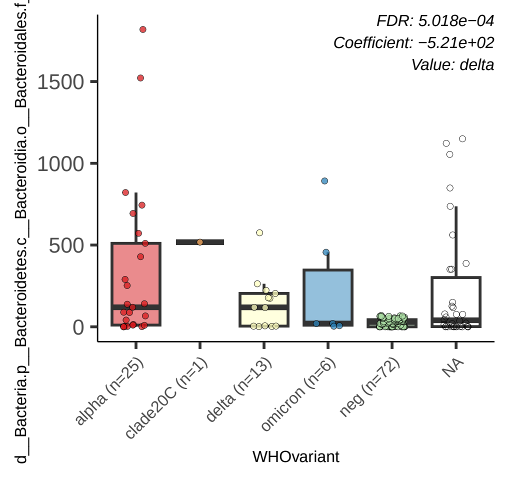

FDR: 5.018e−04
Coefficient: −5.21e+02
Value: delta
d__Bacteria.p__Bacteroidetes.c__Bacteroidia.o__Bacteroidales.f_
0
500
1000
1500
alpha (n=25)
clade20C (n=1)
delta (n=13)
omicron (n=6)
neg (n=72)
NA
WHOvariant

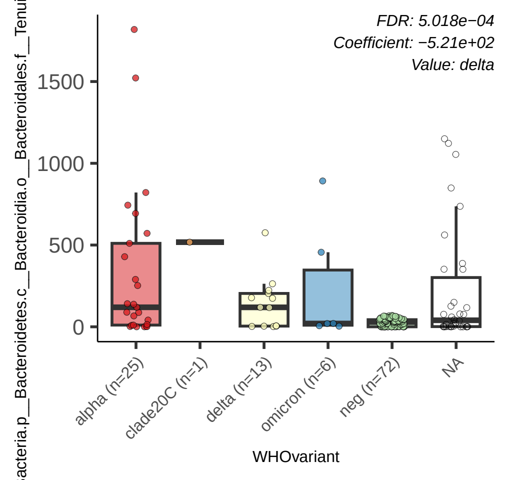
FDR: 5.018e−04
Coefficient: −5.21e+02
Value: delta
Bacteria.p__Bacteroidetes.c__Bacteroidia.o__Bacteroidales.f__Tenui
1500
1000
500
0
alpha (n=25)
clade20C (n=1)
delta (n=13)
omicron (n=6)
neg (n=72)
NA
WHOvariant

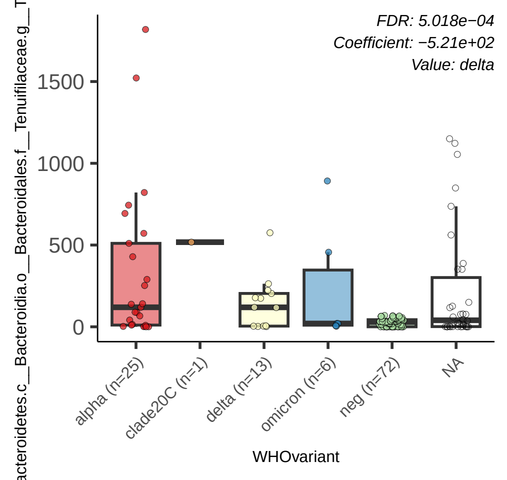
FDR: 5.018e−04
Coefficient: −5.21e+02
Value: delta
acteroidetes.c__Bacteroidia.o__Bacteroidales.f__Tenuifilaceae.g__T
1500
1000
500
0
alpha (n=25)
clade20C (n=1)
delta (n=13)
omicron (n=6)
neg (n=72)
NA
WHOvariant

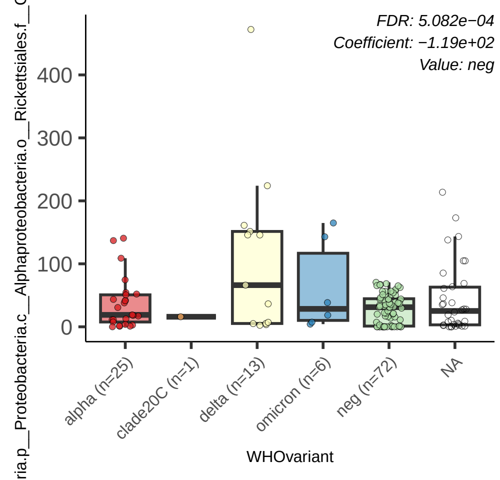

FDR: 5.082e−04
Coefficient: −1.19e+02
Value: neg
0
100
200
300
400
ria.p__Proteobacteria.c__Alphaproteobacteria.o__Rickettsiales.f__C
alpha (n=25)
clade20C (n=1)
delta (n=13)
omicron (n=6)
neg (n=72)
NA
WHOvariant

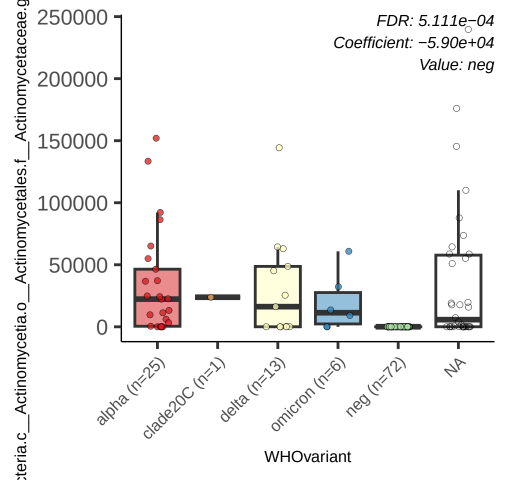

FDR: 5.111e−04
Coefficient: −5.90e+04
Value: neg
250000
200000
150000
100000
50000
0
alpha (n=25)
clade20C (n=1)
delta (n=13)
omicron (n=6)
neg (n=72)
NA
WHOvariant
cteria.c__Actinomycetia.o__Actinomycetales.f__Actinomycetaceae.g

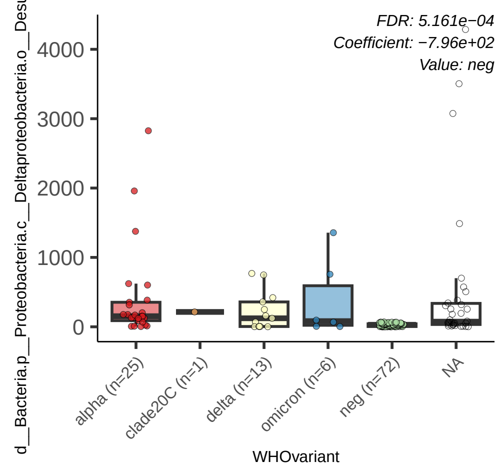

FDR: 5.161e−04
Coefficient: −7.96e+02
Value: neg
d__Bacteria.p__Proteobacteria.c__Deltaproteobacteria.o__Desu
4000
3000
2000
1000
0
alpha (n=25)
clade20C (n=1)
delta (n=13)
omicron (n=6)
neg (n=72)
NA
WHOvariant

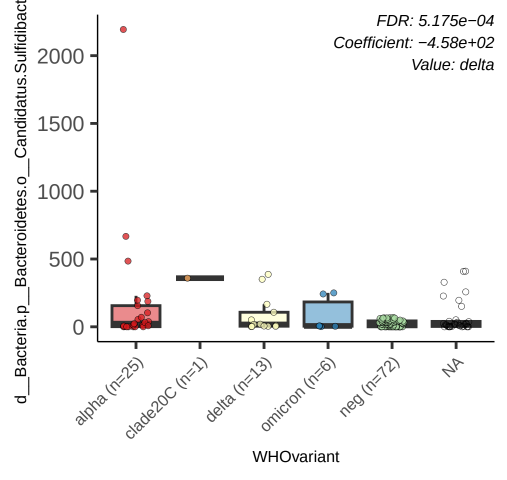
FDR: 5.175e−04
Coefficient: −4.58e+02
Value: delta
d__Bacteria.p__Bacteroidetes.o__Candidatus.Sulfidibact
2000
1500
1000
500
0
alpha (n=25)
clade20C (n=1)
delta (n=13)
omicron (n=6)
neg (n=72)
NA
WHOvariant

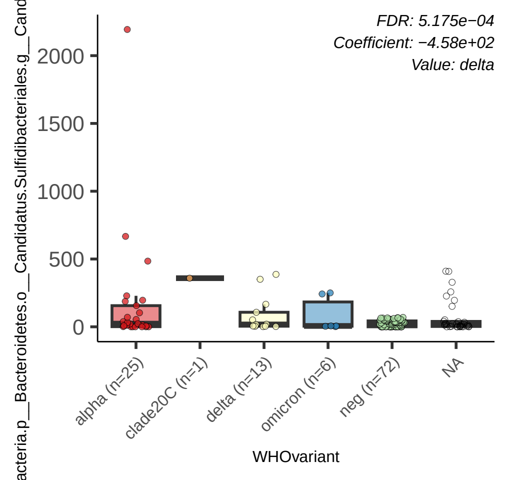
FDR: 5.175e−04
Coefficient: −4.58e+02
Value: delta
acteria.p__Bacteroidetes.o__Candidatus.Sulfidibacteriales.g__Cand
2000
1500
1000
500
0
alpha (n=25)
clade20C (n=1)
delta (n=13)
omicron (n=6)
neg (n=72)
NA
WHOvariant

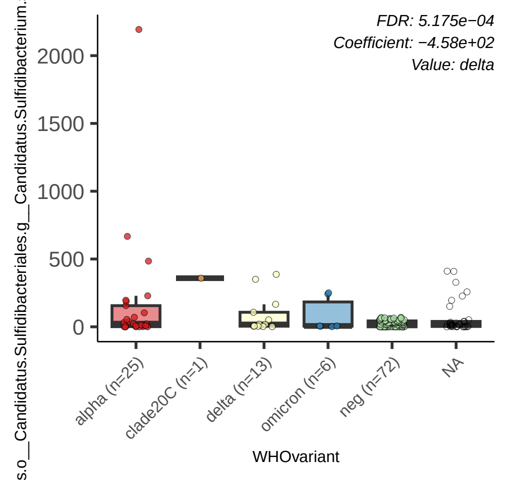
FDR: 5.175e−04
Coefficient: −4.58e+02
Value: delta
s.o__Candidatus.Sulfidibacteriales.g__Candidatus.Sulfidibacterium.s
2000
1500
1000
500
0
alpha (n=25)
clade20C (n=1)
delta (n=13)
omicron (n=6)
neg (n=72)
NA
WHOvariant

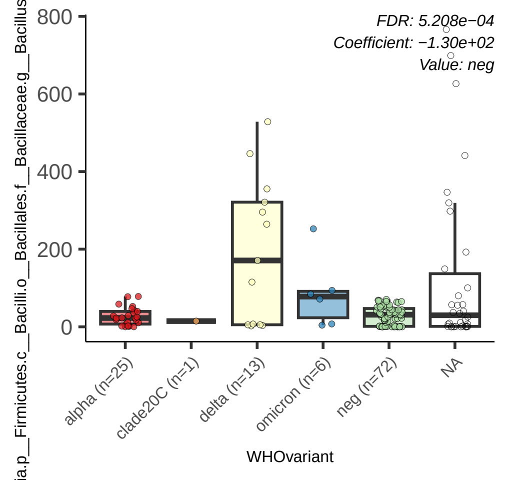

FDR: 5.208e−04
Coefficient: −1.30e+02
Value: neg
800
600
400
200
0
ia.p__Firmicutes.c__Bacilli.o__Bacillales.f__Bacillaceae.g__Bacillus
alpha (n=25)
clade20C (n=1)
delta (n=13)
omicron (n=6)
neg (n=72)
NA
WHOvariant

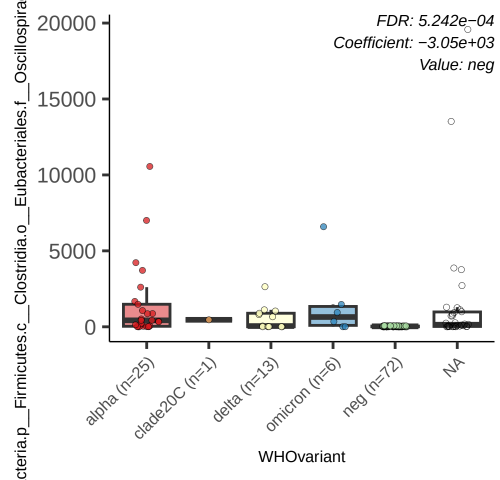

FDR: 5.242e−04
Coefficient: −3.05e+03
Value: neg
20000
15000
10000
5000
0
cteria.p__Firmicutes.c__Clostridia.o__Eubacteriales.f__Oscillospira
alpha (n=25)
clade20C (n=1)
delta (n=13)
omicron (n=6)
neg (n=72)
NA
WHOvariant

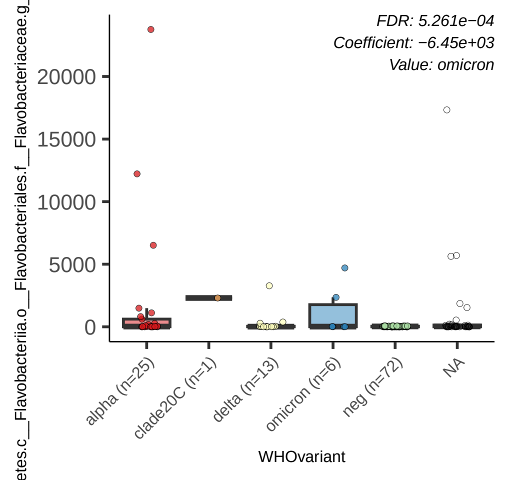

FDR: 5.261e−04
Coefficient: −6.45e+03
Value: omicron
etes.c__Flavobacteriia.o__Flavobacteriales.f__Flavobacteriaceae.g_
0
5000
10000
15000
20000
alpha (n=25)
clade20C (n=1)
delta (n=13)
omicron (n=6)
neg (n=72)
NA
WHOvariant

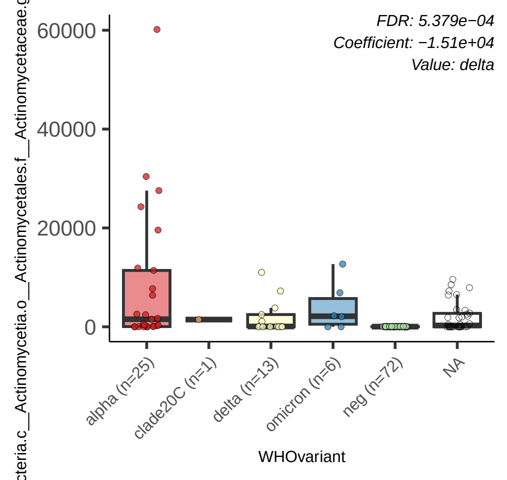

FDR: 5.379e−04
Coefficient: −1.51e+04
Value: delta
60000
40000
20000
0
cteria.c__Actinomycetia.o__Actinomycetales.f__Actinomycetaceae.g
alpha (n=25)
clade20C (n=1)
delta (n=13)
omicron (n=6)
neg (n=72)
NA
WHOvariant

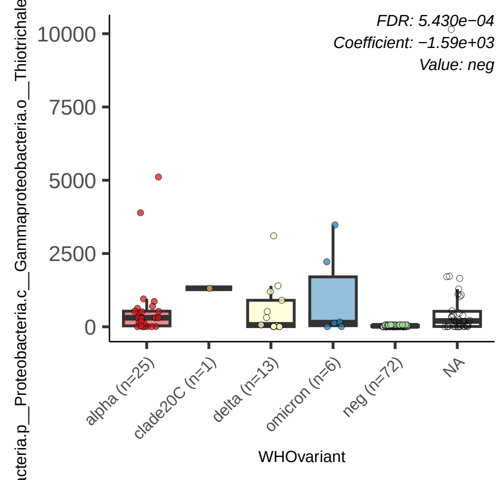
FDR: 5.430e−04
Coefficient: −1.59e+03
Value: neg
10000
7500
5000
2500
0
acteria.p__Proteobacteria.c__Gammaproteobacteria.o__Thiotrichale
alpha (n=25)
clade20C (n=1)
delta (n=13)
omicron (n=6)
neg (n=72)
NA
WHOvariant

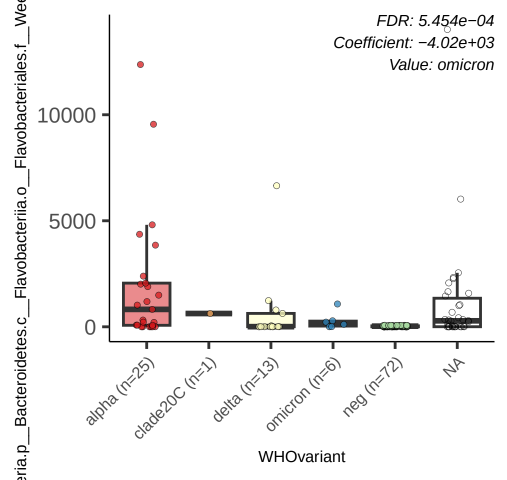

FDR: 5.454e−04
Coefficient: −4.02e+03
Value: omicron
10000
5000
0
alpha (n=25)
clade20C (n=1)
delta (n=13)
omicron (n=6)
neg (n=72)
NA
WHOvariant

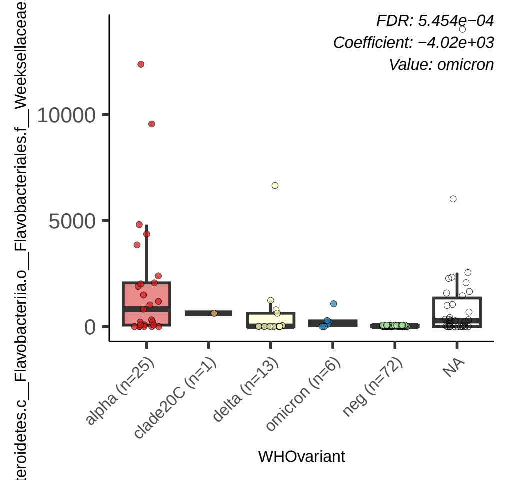

FDR: 5.454e−04
Coefficient: −4.02e+03
Value: omicron
10000
5000
0
alpha (n=25)
clade20C (n=1)
delta (n=13)
omicron (n=6)
neg (n=72)
NA
WHOvariant
teroidetes.c__Flavobacteriia.o__Flavobacteriales.f__Weeksellaceae.

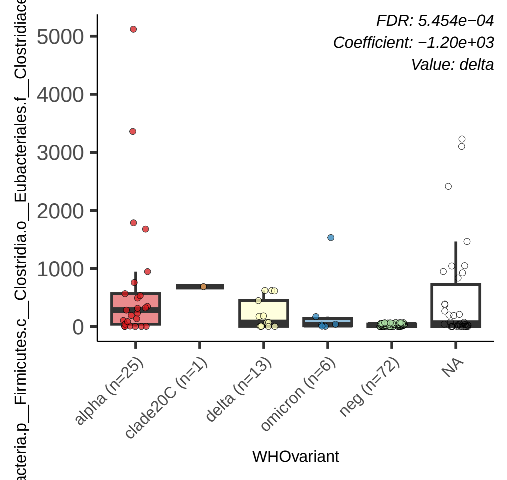

FDR: 5.454e−04
Coefficient: −1.20e+03
Value: delta
acteria.p__Firmicutes.c__Clostridia.o__Eubacteriales.f__Clostridiace
5000
4000
3000
2000
1000
0
alpha (n=25)
clade20C (n=1)
delta (n=13)
omicron (n=6)
neg (n=72)
NA
WHOvariant

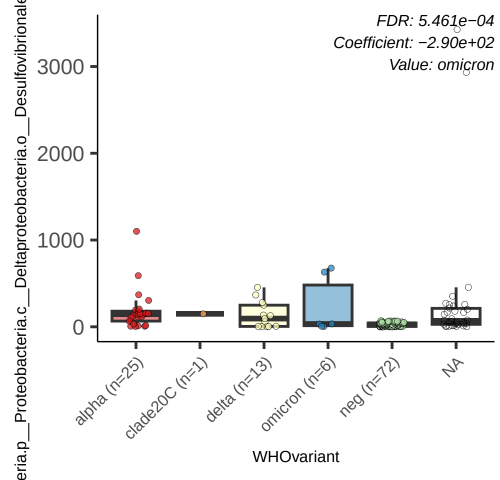

FDR: 5.461e−04
Coefficient: −2.90e+02
Value: omicron
3000
2000
1000
0
eria.p__Proteobacteria.c__Deltaproteobacteria.o__Desulfovibrionale
alpha (n=25)
clade20C (n=1)
delta (n=13)
omicron (n=6)
neg (n=72)
NA
WHOvariant

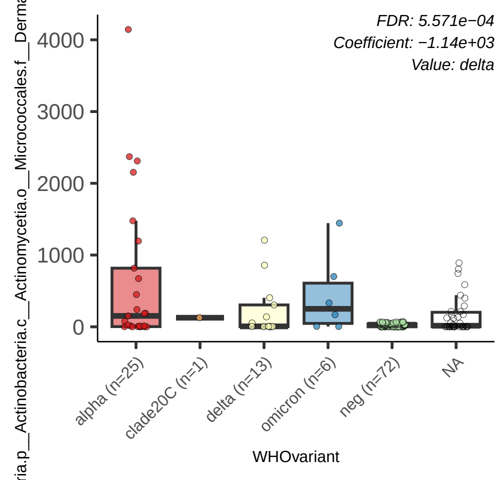

FDR: 5.571e−04
Coefficient: −1.14e+03
Value: delta
4000
3000
2000
1000
0
ria.p__Actinobacteria.c__Actinomycetia.o__Micrococcales.f__Derma
alpha (n=25)
clade20C (n=1)
delta (n=13)
omicron (n=6)
neg (n=72)
NA
WHOvariant

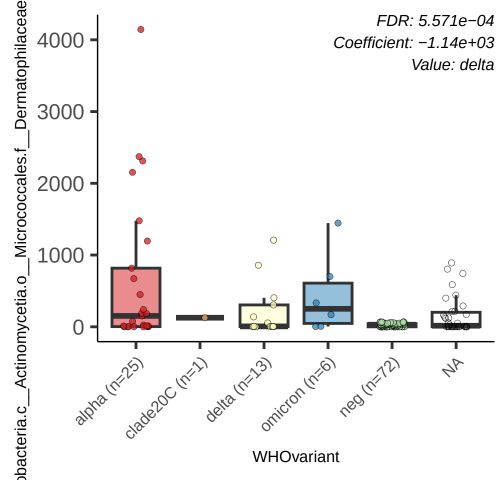

FDR: 5.571e−04
Coefficient: −1.14e+03
Value: delta
4000
3000
2000
1000
0
bacteria.c__Actinomycetia.o__Micrococcales.f__Dermatophilaceae
alpha (n=25)
clade20C (n=1)
delta (n=13)
omicron (n=6)
neg (n=72)
NA
WHOvariant

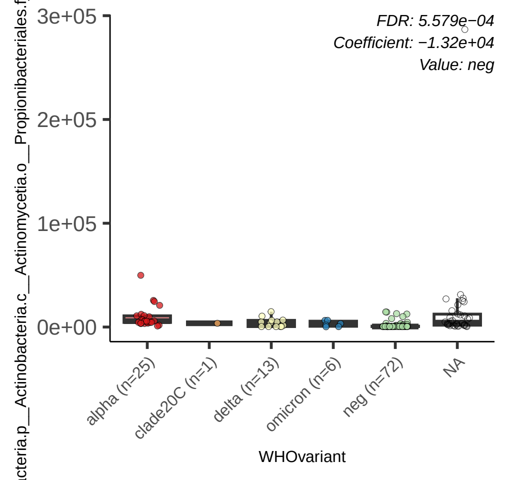

FDR: 5.579e−04
Coefficient: −1.32e+04
Value: neg
3e+05
2e+05
1e+05
0e+00
cteria.p__Actinobacteria.c__Actinomycetia.o__Propionibacteriales.f
alpha (n=25)
clade20C (n=1)
delta (n=13)
omicron (n=6)
neg (n=72)
NA
WHOvariant

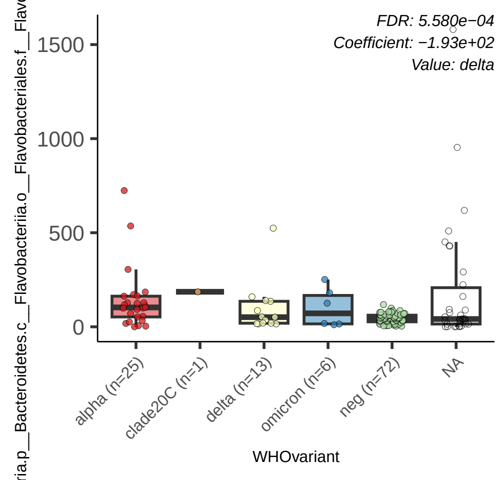
FDR: 5.580e−04
Coefficient: −1.93e+02
Value: delta
0
500
1000
1500
ria.p__Bacteroidetes.c__Flavobacteriia.o__Flavobacteriales.f__Flavo
alpha (n=25)
clade20C (n=1)
delta (n=13)
omicron (n=6)
neg (n=72)
NA
WHOvariant

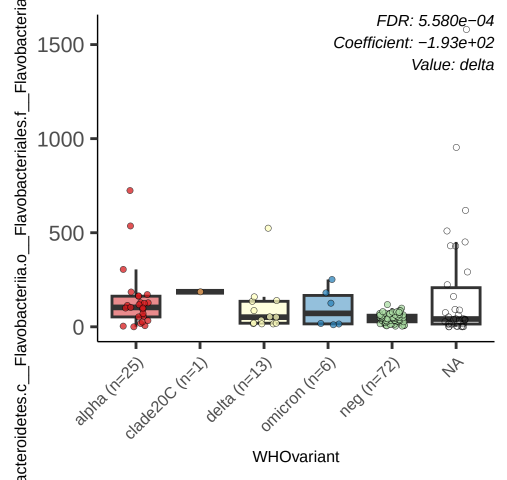

FDR: 5.580e−04
Coefficient: −1.93e+02
Value: delta
1500
1000
500
0
acteroidetes.c__Flavobacteriia.o__Flavobacteriales.f__Flavobacteria
alpha (n=25)
clade20C (n=1)
delta (n=13)
omicron (n=6)
neg (n=72)
NA
WHOvariant

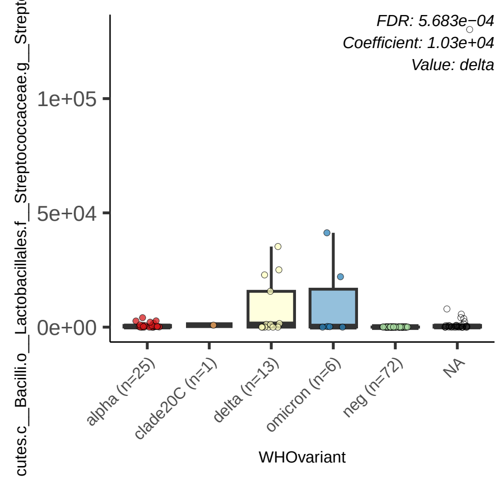

icutes.c__Bacilli.o__Lactobacillales.f__Streptococcaceae.g__Strept
FDR: 5.683e−04
Coefficient: 1.03e+04
Value: delta
1e+05
5e+04
0e+00
alpha (n=25)
clade20C (n=1)
delta (n=13)
omicron (n=6)
neg (n=72)
NA
WHOvariant

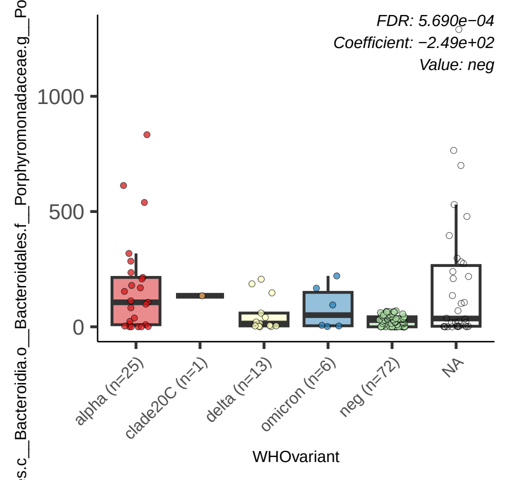

FDR: 5.690e−04
Coefficient: −2.49e+02
Value: neg
1000
500
0
alpha (n=25)
clade20C (n=1)
delta (n=13)
omicron (n=6)
neg (n=72)
NA
WHOvariant
s.c__Bacteroidia.o__Bacteroidales.f__Porphyromonadaceae.g__Po

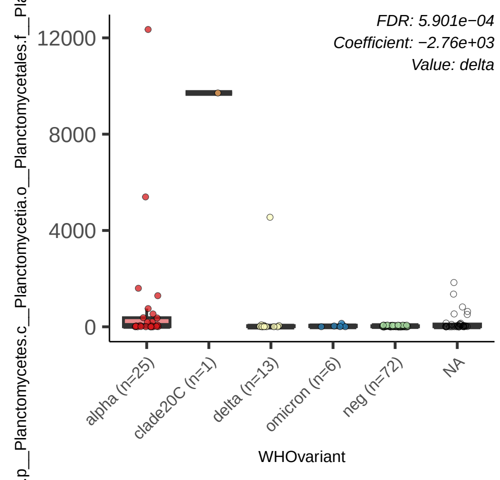

FDR: 5.901e−04
Coefficient: −2.76e+03
Value: delta
12000
8000
4000
0
alpha (n=25)
clade20C (n=1)
delta (n=13)
omicron (n=6)
neg (n=72)
NA
WHOvariant

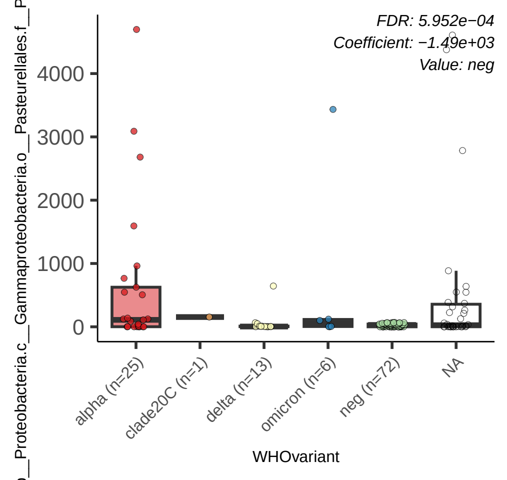

FDR: 5.952e−04
Coefficient: −1.49e+03
Value: neg
Proteobacteria.c__Gammaproteobacteria.o__Pasteurellales.f__P
4000
3000
2000
1000
0
alpha (n=25)
clade20C (n=1)
delta (n=13)
omicron (n=6)
neg (n=72)
NA
WHOvariant

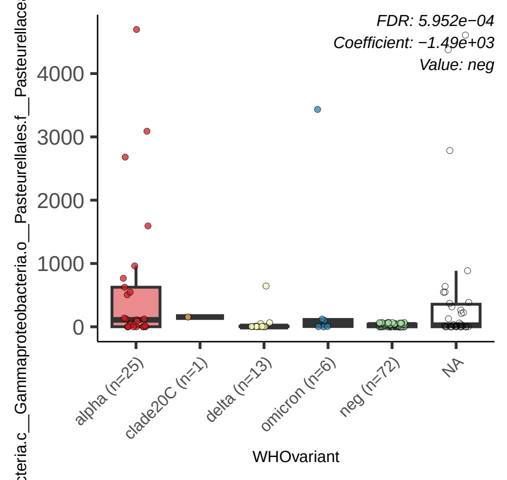

FDR: 5.952e−04
Coefficient: −1.49e+03
Value: neg
4000
3000
2000
1000
0
alpha (n=25)
clade20C (n=1)
delta (n=13)
omicron (n=6)
neg (n=72)
NA
WHOvariant
cteria.c__Gammaproteobacteria.o__Pasteurellales.f__Pasteurellace

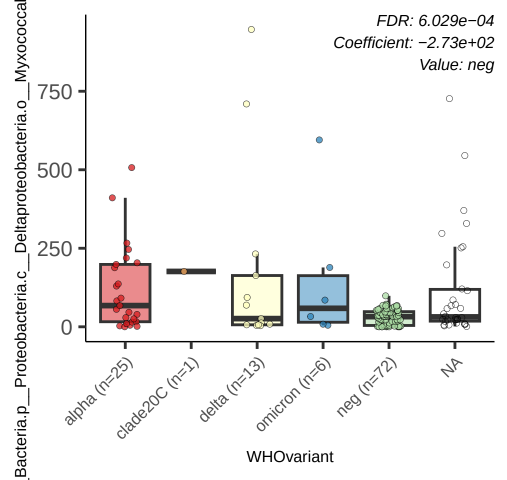
FDR: 6.029e−04
Coefficient: −2.73e+02
Value: neg
Bacteria.p__Proteobacteria.c__Deltaproteobacteria.o__Myxococcal
0
250
500
750
alpha (n=25)
clade20C (n=1)
delta (n=13)
omicron (n=6)
neg (n=72)
NA
WHOvariant

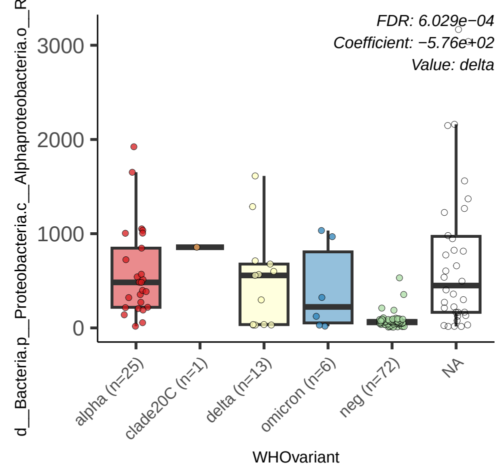

FDR: 6.029e−04
Coefficient: −5.76e+02
Value: delta
d__Bacteria.p__Proteobacteria.c__Alphaproteobacteria.o__R
3000
2000
1000
0
alpha (n=25)
clade20C (n=1)
delta (n=13)
omicron (n=6)
neg (n=72)
NA
WHOvariant

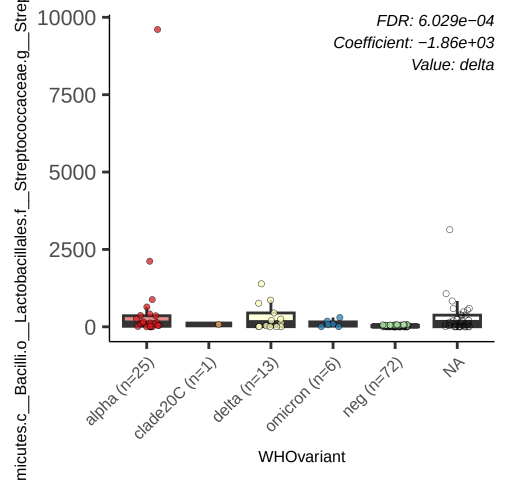

FDR: 6.029e−04
Coefficient: −1.86e+03
Value: delta
10000
7500
5000
2500
0
micutes.c__Bacilli.o__Lactobacillales.f__Streptococcaceae.g__Strep
alpha (n=25)
clade20C (n=1)
delta (n=13)
omicron (n=6)
neg (n=72)
NA
WHOvariant

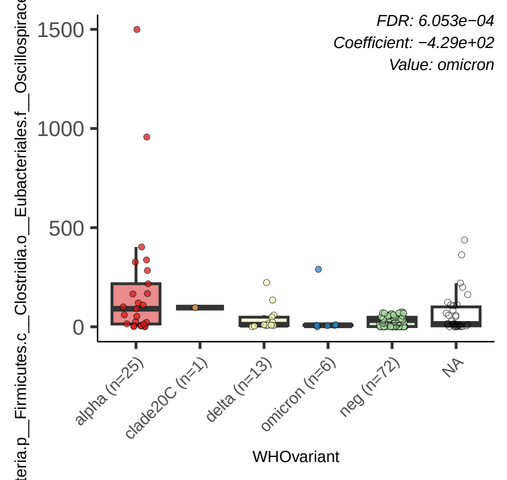

FDR: 6.053e−04
Coefficient: −4.29e+02
Value: omicron
1500
1000
500
0
alpha (n=25)
clade20C (n=1)
delta (n=13)
omicron (n=6)
neg (n=72)
NA
WHOvariant
teria.p__Firmicutes.c__Clostridia.o__Eubacteriales.f__Oscillospirac

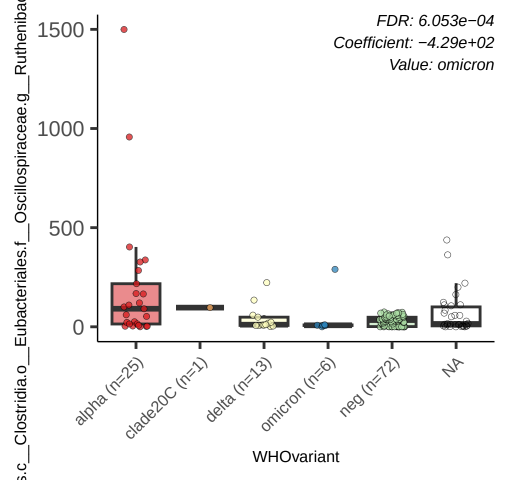

FDR: 6.053e−04
Coefficient: −4.29e+02
Value: omicron
1500
1000
500
0
s.c__Clostridia.o__Eubacteriales.f__Oscillospiraceae.g__Ruthenibac
alpha (n=25)
clade20C (n=1)
delta (n=13)
omicron (n=6)
neg (n=72)
NA
WHOvariant

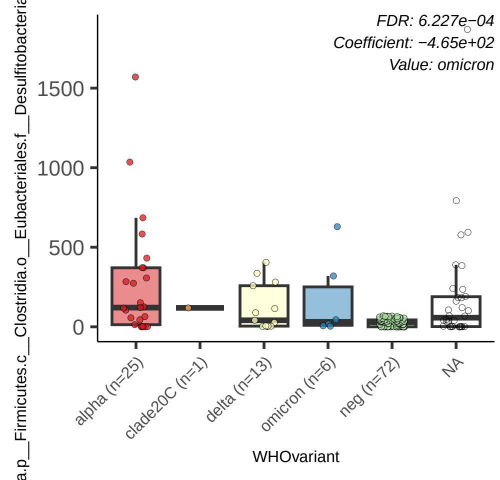

FDR: 6.227e−04
Coefficient: −4.65e+02
Value: omicron
a.p__Firmicutes.c__Clostridia.o__Eubacteriales.f__Desulfitobacteria
1500
1000
500
0
alpha (n=25)
clade20C (n=1)
delta (n=13)
omicron (n=6)
neg (n=72)
NA
WHOvariant

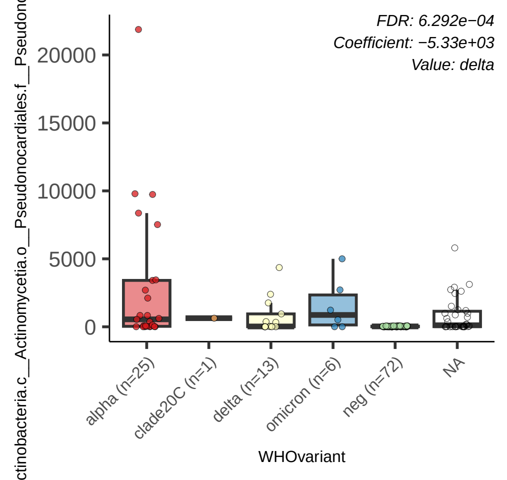

FDR: 6.292e−04
Coefficient: −5.33e+03
Value: delta
20000
15000
10000
5000
0
ctinobacteria.c__Actinomycetia.o__Pseudonocardiales.f__Pseudonoc
alpha (n=25)
clade20C (n=1)
delta (n=13)
omicron (n=6)
neg (n=72)
NA
WHOvariant

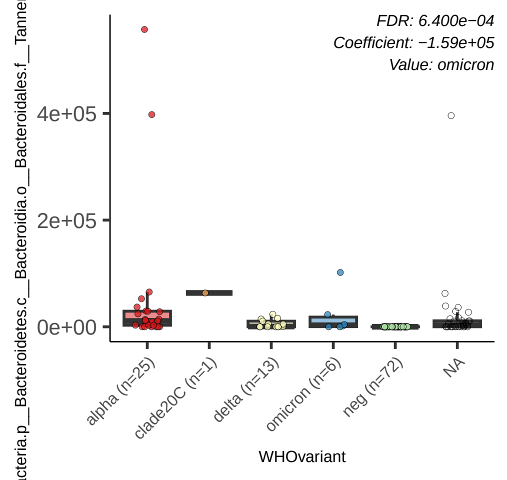

FDR: 6.400e−04
Coefficient: −1.59e+05
Value: omicron
4e+05
2e+05
0e+00
alpha (n=25)
clade20C (n=1)
delta (n=13)
omicron (n=6)
neg (n=72)
NA
WHOvariant
acteria.p__Bacteroidetes.c__Bacteroidia.o__Bacteroidales.f__Tanne

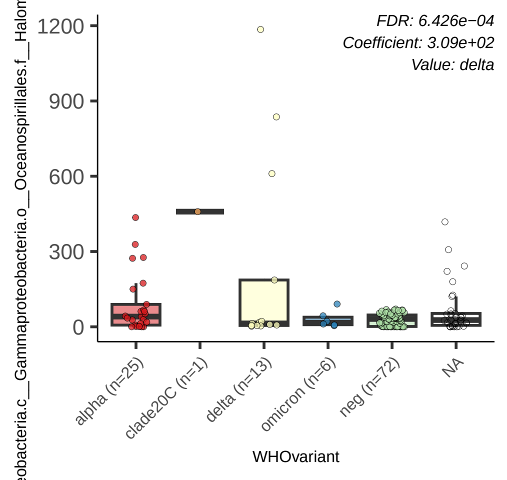

FDR: 6.426e−04
Coefficient: 3.09e+02
Value: delta
1200
900
600
300
0
eobacteria.c__Gammaproteobacteria.o__Oceanospirillales.f__Halom
alpha (n=25)
clade20C (n=1)
delta (n=13)
omicron (n=6)
neg (n=72)
NA
WHOvariant

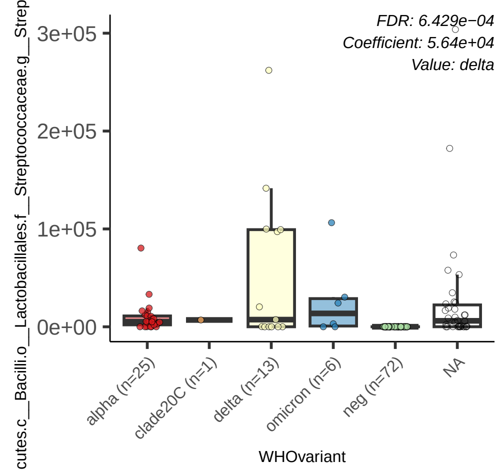

FDR: 6.429e−04
Coefficient: 5.64e+04
Value: delta
3e+05
2e+05
1e+05
0e+00
alpha (n=25)
clade20C (n=1)
delta (n=13)
omicron (n=6)
neg (n=72)
NA
WHOvariant

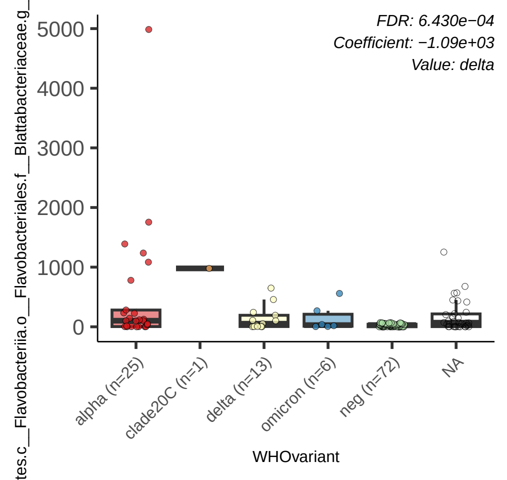

FDR: 6.430e−04
Coefficient: −1.09e+03
Value: delta
tes.c__Flavobacteriia.o__Flavobacteriales.f__Blattabacteriaceae.g__
5000
4000
3000
2000
1000
0
alpha (n=25)
clade20C (n=1)
delta (n=13)
omicron (n=6)
neg (n=72)
NA
WHOvariant

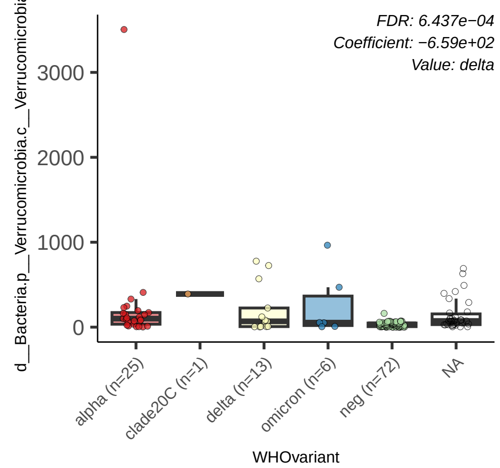

FDR: 6.437e−04
Coefficient: −6.59e+02
Value: delta
3000
2000
1000
0
d__Bacteria.p__Verrucomicrobia.c__Verrucomicrobia
alpha (n=25)
clade20C (n=1)
delta (n=13)
omicron (n=6)
neg (n=72)
NA
WHOvariant

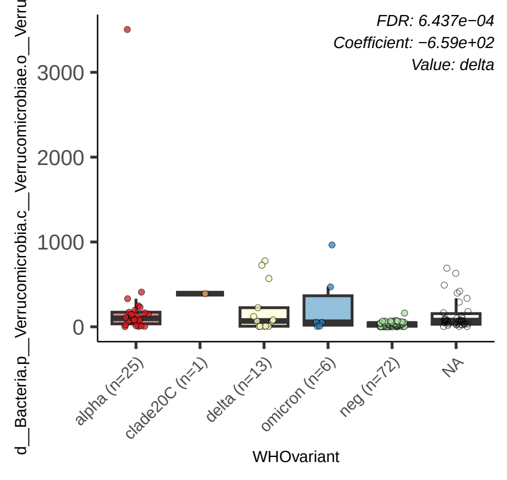
FDR: 6.437e−04
Coefficient: −6.59e+02
Value: delta
d__Bacteria.p__Verrucomicrobia.c__Verrucomicrobiae.o__Verru
3000
2000
1000
0
alpha (n=25)
clade20C (n=1)
delta (n=13)
omicron (n=6)
neg (n=72)
NA
WHOvariant

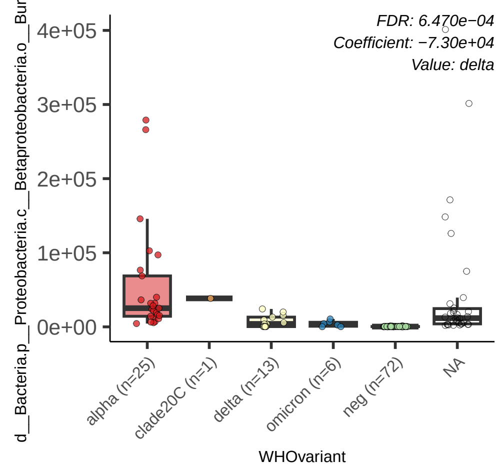

FDR: 6.470e−04
Coefficient: −7.30e+04
Value: delta
d__Bacteria.p__Proteobacteria.c__Betaproteobacteria.o__Bur
4e+05
3e+05
2e+05
1e+05
0e+00
alpha (n=25)
clade20C (n=1)
delta (n=13)
omicron (n=6)
neg (n=72)
NA
WHOvariant

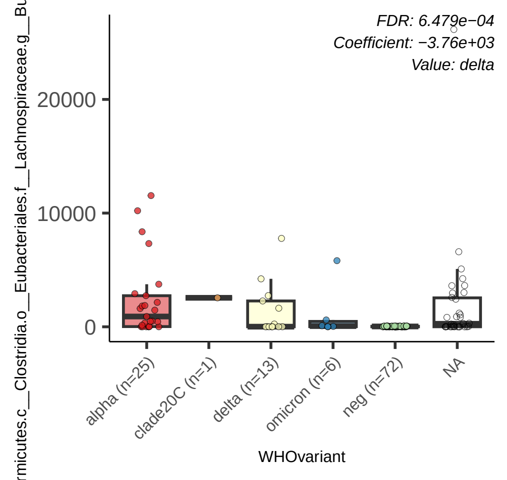
FDR: 6.479e−04
Coefficient: −3.76e+03
Value: delta
20000
10000
0
rmicutes.c__Clostridia.o__Eubacteriales.f__Lachnospiraceae.g__Bu
alpha (n=25)
clade20C (n=1)
delta (n=13)
omicron (n=6)
neg (n=72)
NA
WHOvariant

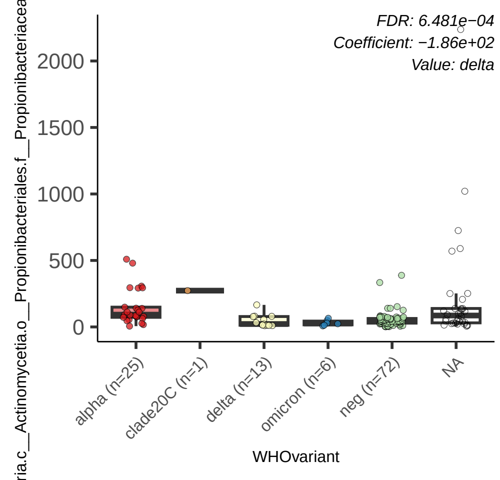

FDR: 6.481e−04
Coefficient: −1.86e+02
Value: delta
ria.c__Actinomycetia.o__Propionibacteriales.f__Propionibacteriacea
0
500
1000
1500
2000
alpha (n=25)
clade20C (n=1)
delta (n=13)
omicron (n=6)
neg (n=72)
NA
WHOvariant

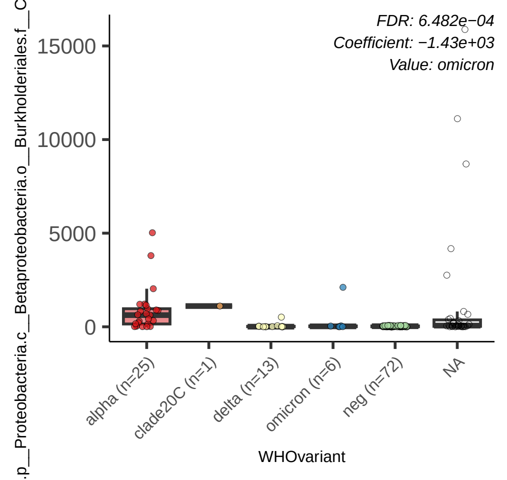
FDR: 6.482e−04
Coefficient: −1.43e+03
Value: omicron
15000
10000
5000
0
p__Proteobacteria.c__Betaproteobacteria.o__Burkholderiales.f__C
alpha (n=25)
clade20C (n=1)
delta (n=13)
omicron (n=6)
neg (n=72)
NA
WHOvariant

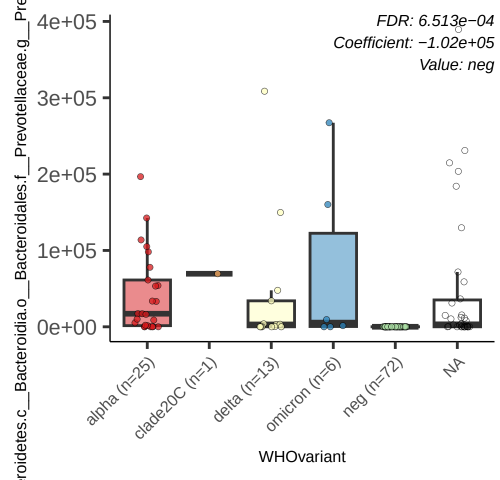

FDR: 6.513e−04
Coefficient: −1.02e+05
Value: neg
4e+05
3e+05
2e+05
1e+05
0e+00
alpha (n=25)
clade20C (n=1)
delta (n=13)
omicron (n=6)
neg (n=72)
NA
WHOvariant

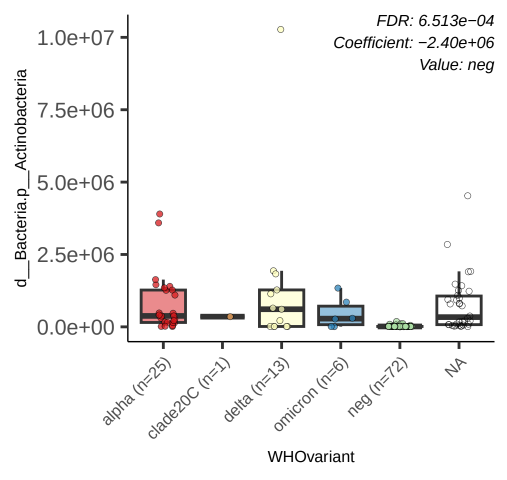
FDR: 6.513e−04
Coefficient: −2.40e+06
Value: neg
d__Bacteria.p__Actinobacteria
1.0e+07
7.5e+06
5.0e+06
2.5e+06
0.0e+00
alpha (n=25)
clade20C (n=1)
delta (n=13)
omicron (n=6)
neg (n=72)
NA
WHOvariant

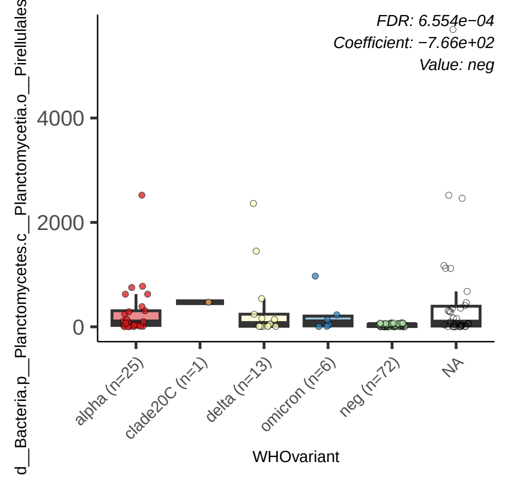

FDR: 6.554e−04
Coefficient: −7.66e+02
Value: neg
d__Bacteria.p__Planctomycetes.c__Planctomycetia.o__Pirellulales
4000
2000
0
alpha (n=25)
clade20C (n=1)
delta (n=13)
omicron (n=6)
neg (n=72)
NA
WHOvariant

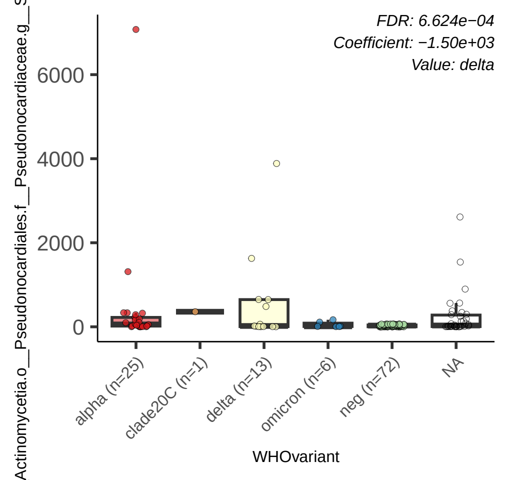
FDR: 6.624e−04
Coefficient: −1.50e+03
Value: delta
Actinomycetia.o__Pseudonocardiales.f__Pseudonocardiaceae.g__
6000
4000
2000
0
alpha (n=25)
clade20C (n=1)
delta (n=13)
omicron (n=6)
neg (n=72)
NA
WHOvariant

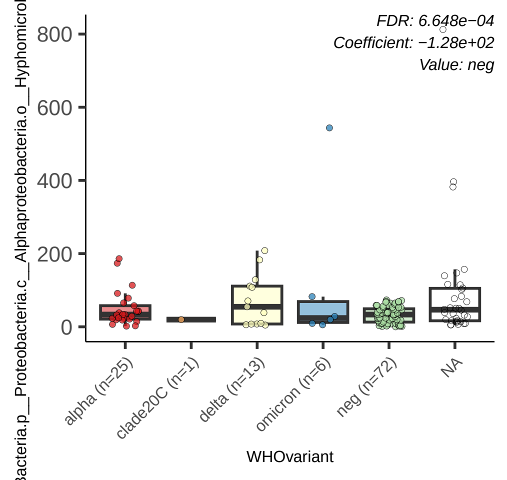

FDR: 6.648e−04
Coefficient: −1.28e+02
Value: neg
800
600
400
200
0
Bacteria.p__Proteobacteria.c__Alphaproteobacteria.o__Hyphomicrob
alpha (n=25)
clade20C (n=1)
delta (n=13)
omicron (n=6)
neg (n=72)
NA
WHOvariant

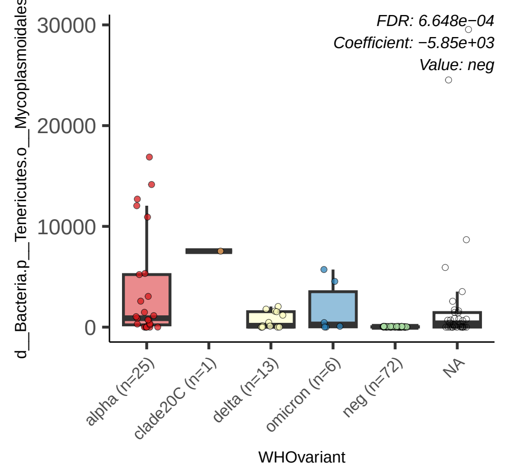

FDR: 6.648e−04
Coefficient: −5.85e+03
Value: neg
d__Bacteria.p__Tenericutes.o__Mycoplasmoidales
30000
20000
10000
0
alpha (n=25)
clade20C (n=1)
delta (n=13)
omicron (n=6)
neg (n=72)
NA
WHOvariant

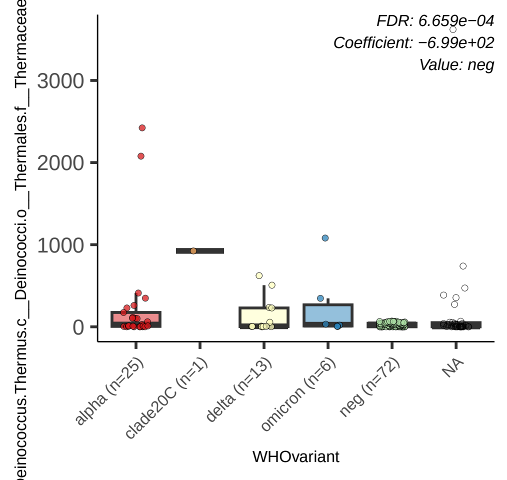

FDR: 6.659e−04
Coefficient: −6.99e+02
Value: neg
0
1000
2000
3000
alpha (n=25)
clade20C (n=1)
delta (n=13)
omicron (n=6)
neg (n=72)
NA
WHOvariant
Deinococcus.Thermus.c__Deinococci.o__Thermales.f__Thermaceae

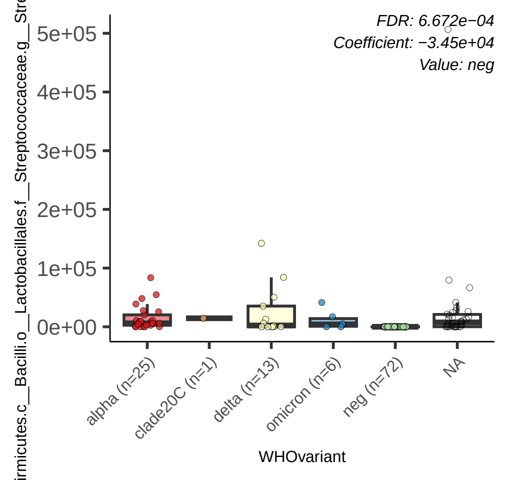
FDR: 6.672e−04
Coefficient: −3.45e+04
Value: neg
5e+05
4e+05
3e+05
2e+05
1e+05
0e+00
alpha (n=25)
clade20C (n=1)
delta (n=13)
omicron (n=6)
neg (n=72)
NA
WHOvariant
irmicutes.c__Bacilli.o__Lactobacillales.f__Streptococcaceae.g__Stre

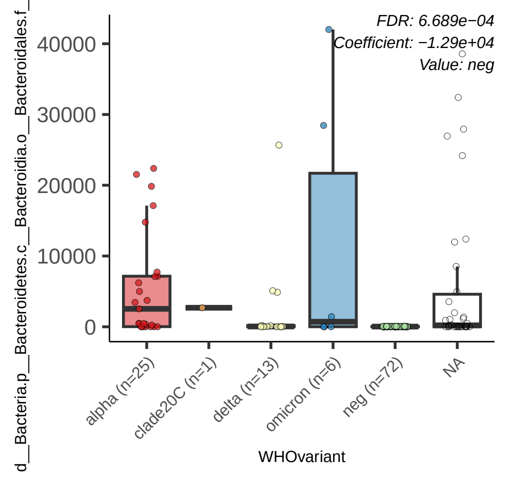

FDR: 6.689e−04
Coefficient: −1.29e+04
Value: neg
d__Bacteria.p__Bacteroidetes.c__Bacteroidia.o__Bacteroidales.f__
40000
30000
20000
10000
0
alpha (n=25)
clade20C (n=1)
delta (n=13)
omicron (n=6)
neg (n=72)
NA
WHOvariant

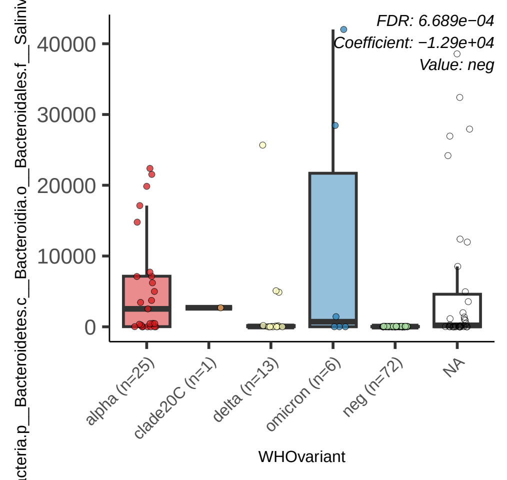

FDR: 6.689e−04
Coefficient: −1.29e+04
Value: neg
40000
30000
20000
10000
0
alpha (n=25)
clade20C (n=1)
delta (n=13)
omicron (n=6)
neg (n=72)
NA
WHOvariant
acteria.p__Bacteroidetes.c__Bacteroidia.o__Bacteroidales.f__Saliniv

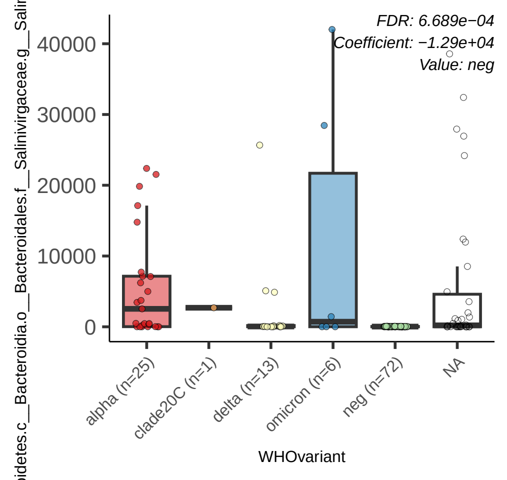

FDR: 6.689e−04
Coefficient: −1.29e+04
Value: neg
40000
30000
20000
10000
0
oidetes.c__Bacteroidia.o__Bacteroidales.f__Salinivirgaceae.g__Salin
alpha (n=25)
clade20C (n=1)
delta (n=13)
omicron (n=6)
neg (n=72)
NA
WHOvariant

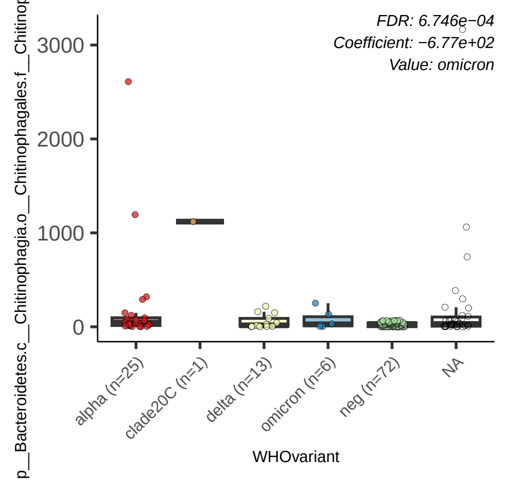

FDR: 6.746e−04
Coefficient: −6.77e+02
Value: omicron
p__Bacteroidetes.c__Chitinophagia.o__Chitinophagales.f__Chitinop
3000
2000
1000
0
alpha (n=25)
clade20C (n=1)
delta (n=13)
omicron (n=6)
neg (n=72)
NA
WHOvariant

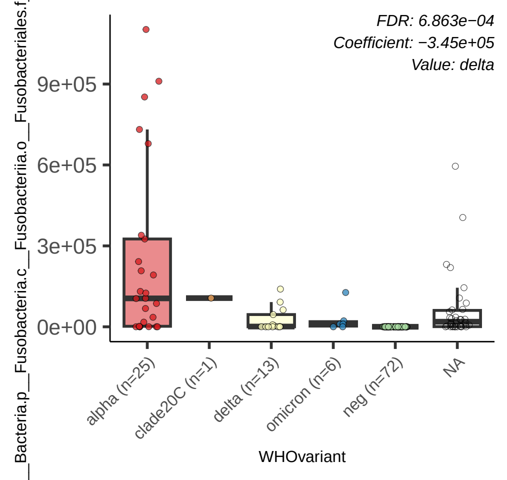
FDR: 6.863e−04
Coefficient: −3.45e+05
Value: delta
9e+05
6e+05
3e+05
0e+00
alpha (n=25)
clade20C (n=1)
delta (n=13)
omicron (n=6)
neg (n=72)
NA
WHOvariant

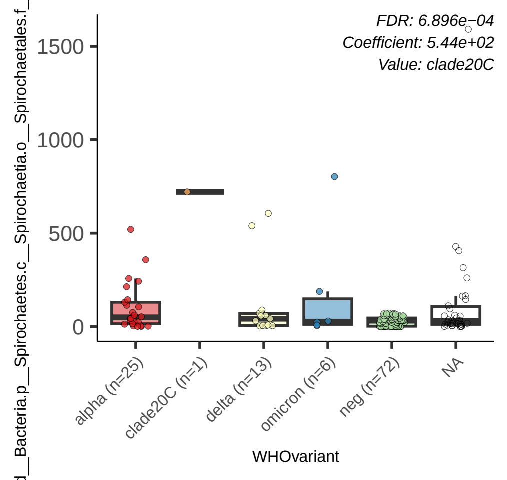

FDR: 6.896e−04
Coefficient: 5.44e+02
Value: clade20C
d__Bacteria.p__Spirochaetes.c__Spirochaetia.o__Spirochaetales.f__
1500
1000
500
0
alpha (n=25)
clade20C (n=1)
delta (n=13)
omicron (n=6)
neg (n=72)
NA
WHOvariant

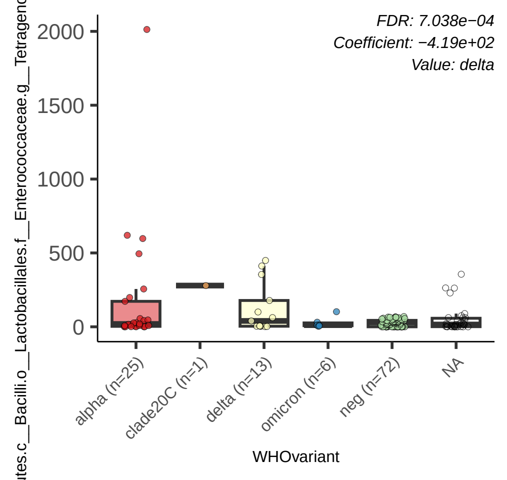

FDR: 7.038e−04
Coefficient: −4.19e+02
Value: delta
2000
1500
1000
500
0
alpha (n=25)
clade20C (n=1)
delta (n=13)
omicron (n=6)
neg (n=72)
NA
WHOvariant
utes.c__Bacilli.o__Lactobacillales.f__Enterococcaceae.g__Tetragenc

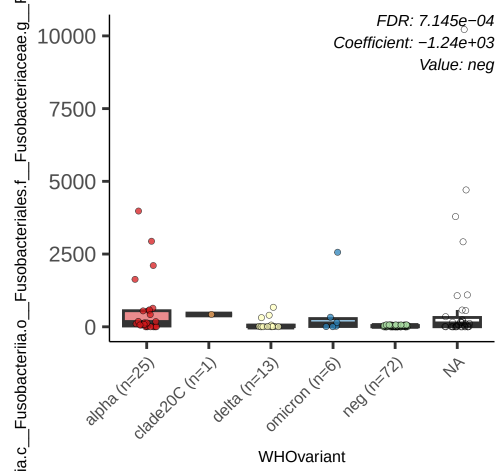

FDR: 7.145e−04
Coefficient: −1.24e+03
Value: neg
10000
7500
5000
2500
0
alpha (n=25)
clade20C (n=1)
delta (n=13)
omicron (n=6)
neg (n=72)
NA
WHOvariant

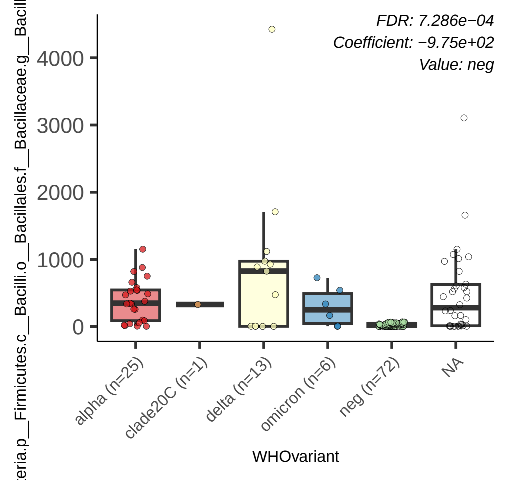

FDR: 7.286e−04
Coefficient: −9.75e+02
Value: neg
4000
3000
2000
1000
0
alpha (n=25)
clade20C (n=1)
delta (n=13)
omicron (n=6)
neg (n=72)
NA
WHOvariant

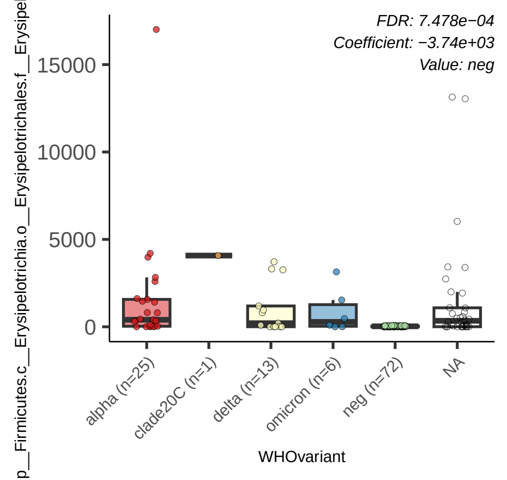

FDR: 7.478e−04
Coefficient: −3.74e+03
Value: neg
p__Firmicutes.c__Erysipelotrichia.o__Erysipelotrichales.f__Erysipel
15000
10000
5000
0
alpha (n=25)
clade20C (n=1)
delta (n=13)
omicron (n=6)
neg (n=72)
NA
WHOvariant

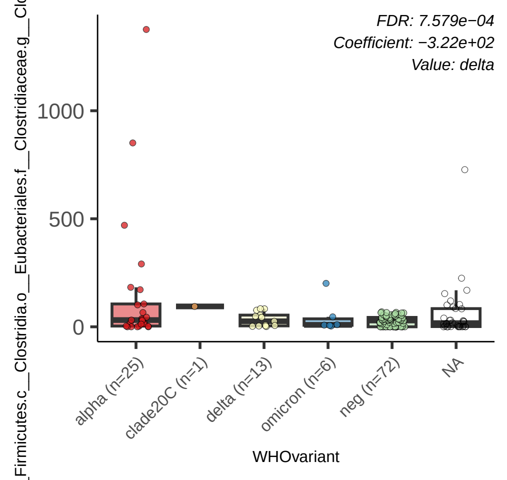
FDR: 7.579e−04
Coefficient: −3.22e+02
Value: delta
Firmicutes.c__Clostridia.o__Eubacteriales.f__Clostridiaceae.g__Clo
1000
500
0
alpha (n=25)
clade20C (n=1)
delta (n=13)
omicron (n=6)
neg (n=72)
NA
WHOvariant

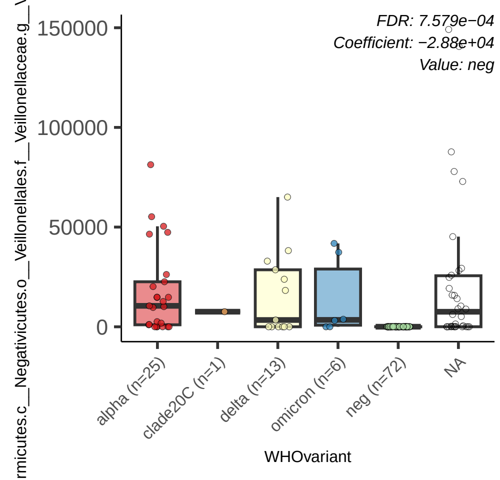
FDR: 7.579e−04
Coefficient: −2.88e+04
Value: neg
150000
100000
50000
0
rmicutes.c__Negativicutes.o__Veillonellales.f__Veillonellaceae.g__
alpha (n=25)
clade20C (n=1)
delta (n=13)
omicron (n=6)
neg (n=72)
NA
WHOvariant

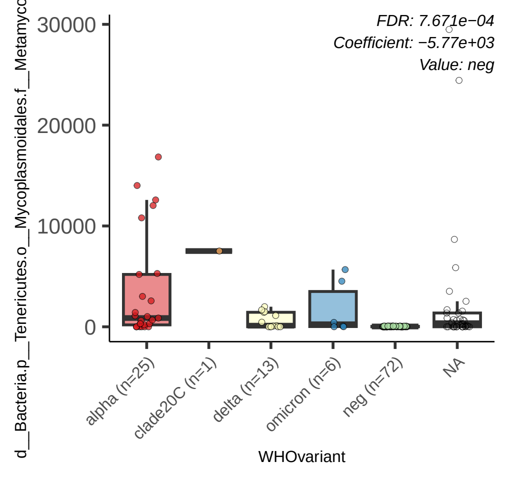
FDR: 7.671e−04
Coefficient: −5.77e+03
Value: neg
d__Bacteria.p__Tenericutes.o__Mycoplasmoidales.f__Metamyco
30000
20000
10000
0
alpha (n=25)
clade20C (n=1)
delta (n=13)
omicron (n=6)
neg (n=72)
NA
WHOvariant

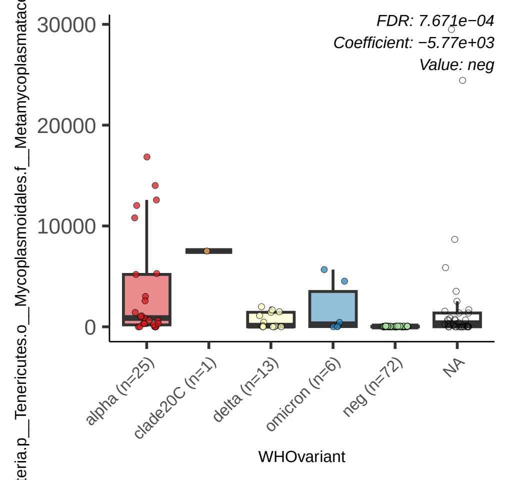

FDR: 7.671e−04
Coefficient: −5.77e+03
Value: neg
30000
20000
10000
0
teria.p__Tenericutes.o__Mycoplasmoidales.f__Metamycoplasmatac
alpha (n=25)
clade20C (n=1)
delta (n=13)
omicron (n=6)
neg (n=72)
NA
WHOvariant

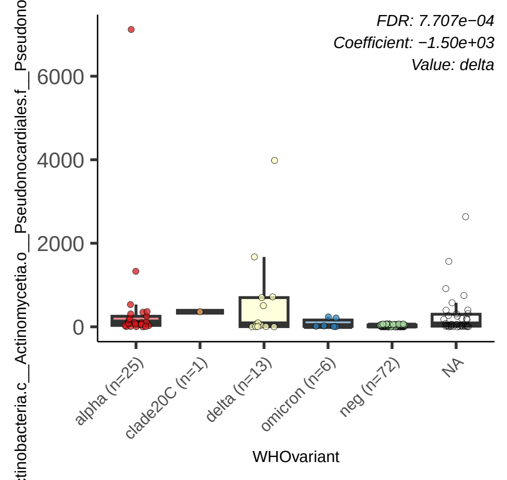

FDR: 7.707e−04
Coefficient: −1.50e+03
Value: delta
Actinobacteria.c__Actinomycetia.o__Pseudonocardiales.f__Pseudono
6000
4000
2000
0
alpha (n=25)
clade20C (n=1)
delta (n=13)
omicron (n=6)
neg (n=72)
NA
WHOvariant

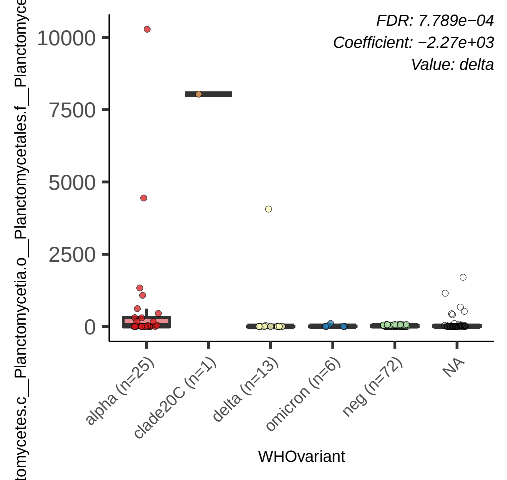
FDR: 7.789e−04
Coefficient: −2.27e+03
Value: delta
10000
7500
5000
2500
0
tomycetes.c__Planctomycetia.o__Planctomycetales.f__Planctomyce
alpha (n=25)
clade20C (n=1)
delta (n=13)
omicron (n=6)
neg (n=72)
NA
WHOvariant

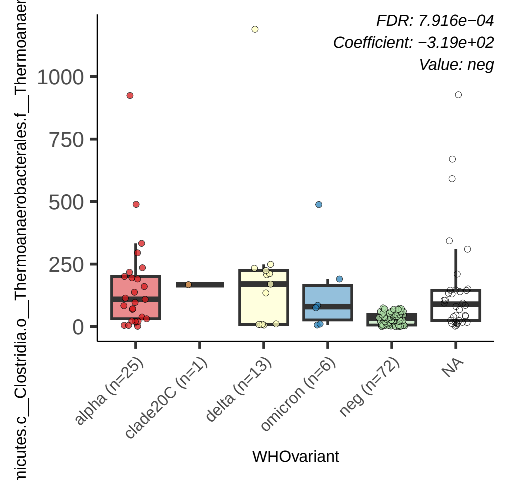

FDR: 7.916e−04
Coefficient: −3.19e+02
Value: neg
1000
750
500
250
0
micutes.c__Clostridia.o__Thermoanaerobacterales.f__Thermoanaer
alpha (n=25)
clade20C (n=1)
delta (n=13)
omicron (n=6)
neg (n=72)
NA
WHOvariant

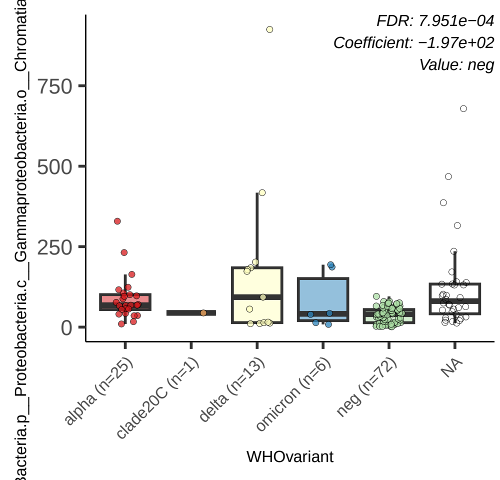

FDR: 7.951e−04
Coefficient: −1.97e+02
Value: neg
750
500
250
0
Bacteria.p__Proteobacteria.c__Gammaproteobacteria.o__Chromatia
alpha (n=25)
clade20C (n=1)
delta (n=13)
omicron (n=6)
neg (n=72)
NA
WHOvariant

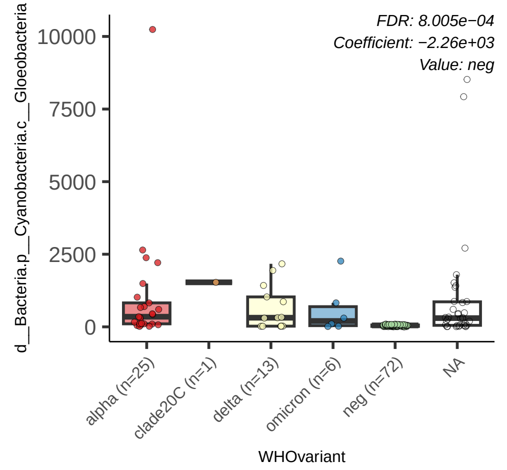

FDR: 8.005e−04
Coefficient: −2.26e+03
Value: neg
d__Bacteria.p__Cyanobacteria.c__Gloeobacteria
10000
7500
5000
2500
0
alpha (n=25)
clade20C (n=1)
delta (n=13)
omicron (n=6)
neg (n=72)
NA
WHOvariant

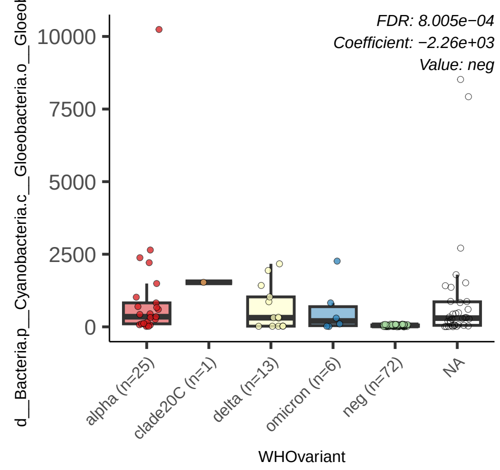

FDR: 8.005e−04
Coefficient: −2.26e+03
Value: neg
d__Bacteria.p__Cyanobacteria.c__Gloeobacteria.o__Gloeob
10000
7500
5000
2500
0
alpha (n=25)
clade20C (n=1)
delta (n=13)
omicron (n=6)
neg (n=72)
NA
WHOvariant

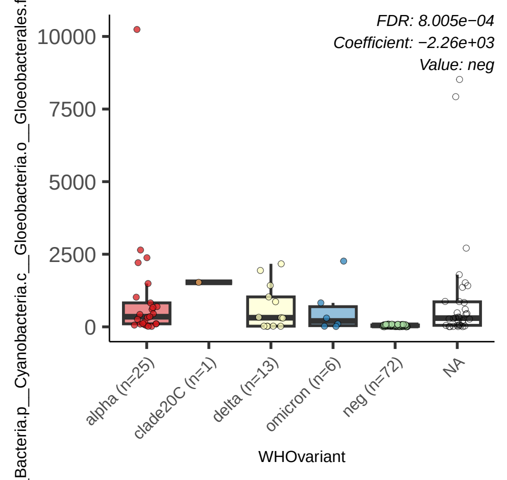
FDR: 8.005e−04
Coefficient: −2.26e+03
Value: neg
10000
7500
5000
2500
0
alpha (n=25)
clade20C (n=1)
delta (n=13)
omicron (n=6)
neg (n=72)
NA
WHOvariant
Bacteria.p__Cyanobacteria.c__Gloeobacteria.o__Gloeobacterales.f

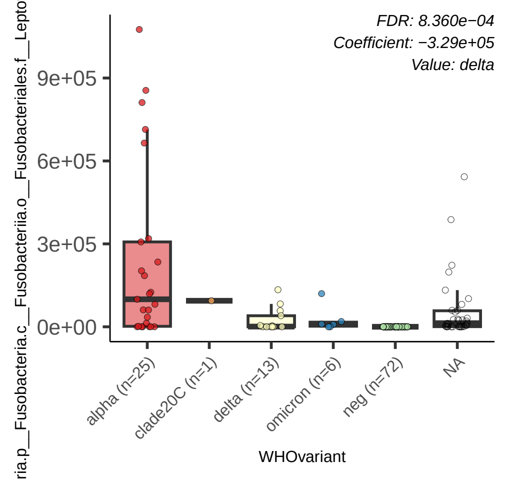
FDR: 8.360e−04
Coefficient: −3.29e+05
Value: delta
9e+05
6e+05
3e+05
0e+00
ria.p__Fusobacteria.c__Fusobacteriia.o__Fusobacteriales.f__Lepto
alpha (n=25)
clade20C (n=1)
delta (n=13)
omicron (n=6)
neg (n=72)
NA
WHOvariant

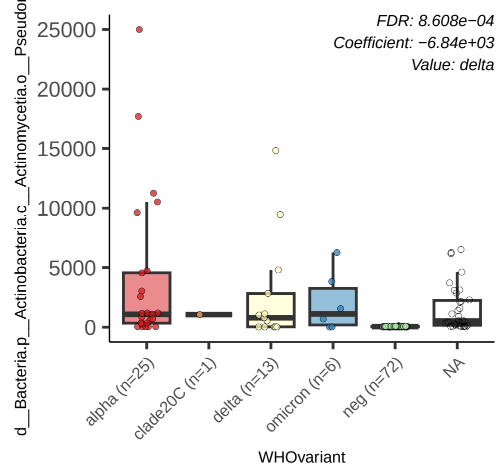

FDR: 8.608e−04
Coefficient: −6.84e+03
Value: delta
d__Bacteria.p__Actinobacteria.c__Actinomycetia.o__Pseudo
25000
20000
15000
10000
5000
0
alpha (n=25)
clade20C (n=1)
delta (n=13)
omicron (n=6)
neg (n=72)
NA
WHOvariant

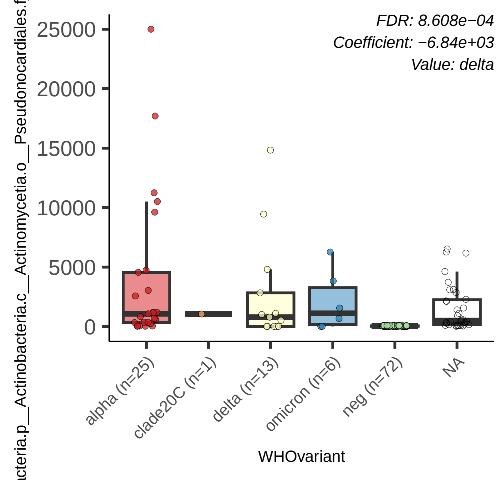
FDR: 8.608e−04
Coefficient: −6.84e+03
Value: delta
25000
20000
15000
10000
5000
0
alpha (n=25)
clade20C (n=1)
delta (n=13)
omicron (n=6)
neg (n=72)
NA
WHOvariant

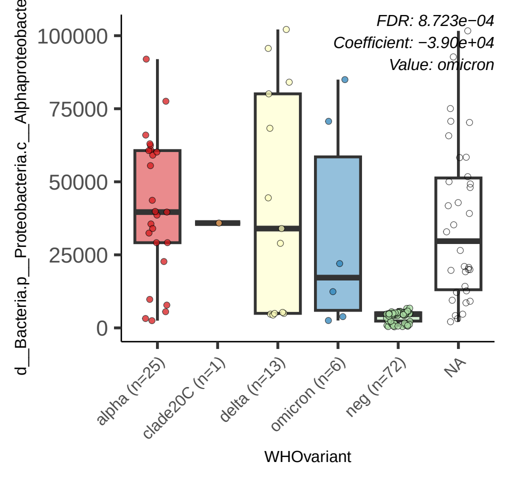

FDR: 8.723e−04
Coefficient: −3.90e+04
Value: omicron
d__Bacteria.p__Proteobacteria.c__Alphaproteobacte
100000
75000
50000
25000
0
alpha (n=25)
clade20C (n=1)
delta (n=13)
omicron (n=6)
neg (n=72)
NA
WHOvariant

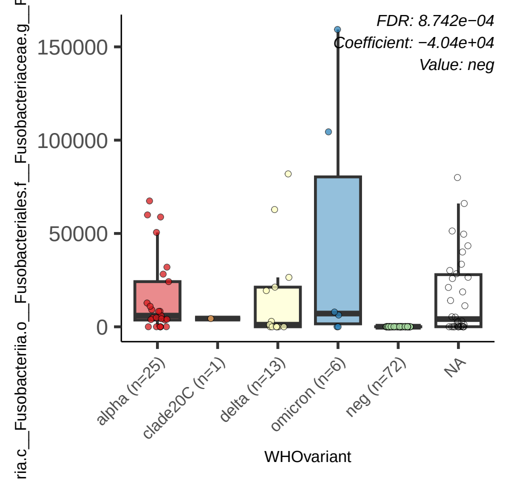

FDR: 8.742e−04
Coefficient: −4.04e+04
Value: neg
150000
100000
50000
0
ria.c__Fusobacteriia.o__Fusobacteriales.f__Fusobacteriaceae.g__F
alpha (n=25)
clade20C (n=1)
delta (n=13)
omicron (n=6)
neg (n=72)
NA
WHOvariant

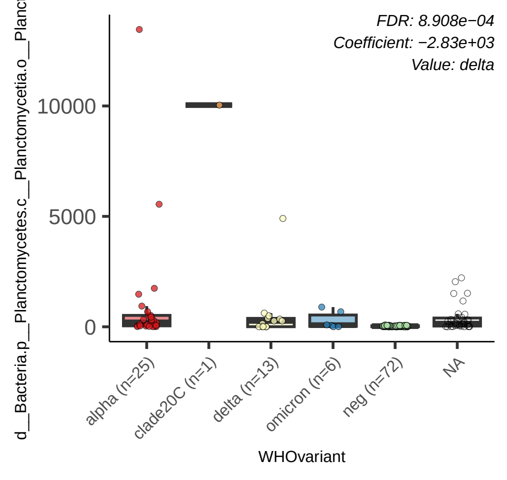

FDR: 8.908e−04
Coefficient: −2.83e+03
Value: delta
d__Bacteria.p__Planctomycetes.c__Planctomycetia.o__Planc
10000
5000
0
alpha (n=25)
clade20C (n=1)
delta (n=13)
omicron (n=6)
neg (n=72)
NA
WHOvariant

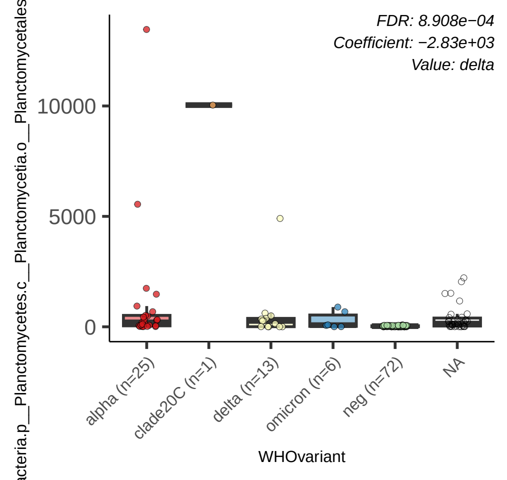

FDR: 8.908e−04
Coefficient: −2.83e+03
Value: delta
10000
5000
0
alpha (n=25)
clade20C (n=1)
delta (n=13)
omicron (n=6)
neg (n=72)
NA
WHOvariant
acteria.p__Planctomycetes.c__Planctomycetia.o__Planctomycetales

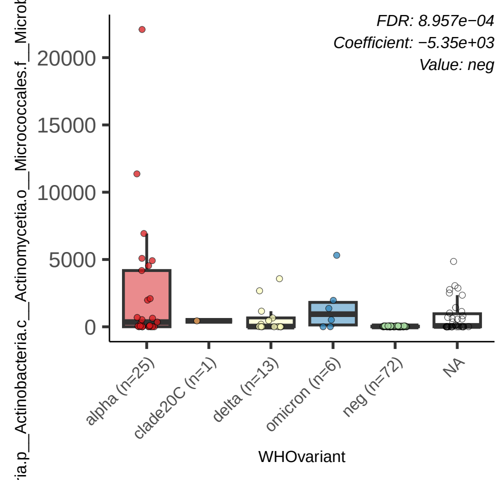
FDR: 8.957e−04
Coefficient: −5.35e+03
Value: neg
20000
15000
10000
5000
0
ria.p__Actinobacteria.c__Actinomycetia.o__Micrococcales.f__Microb
alpha (n=25)
clade20C (n=1)
delta (n=13)
omicron (n=6)
neg (n=72)
NA
WHOvariant

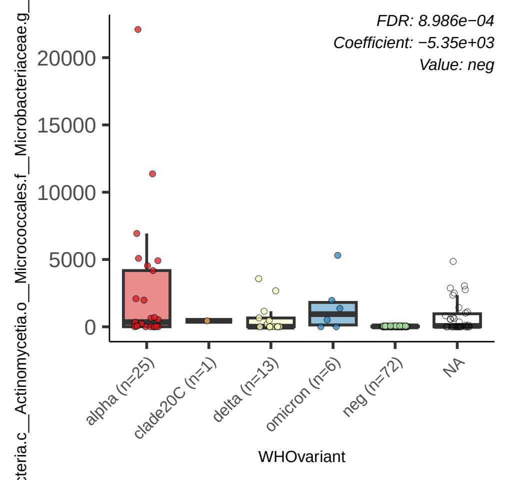

FDR: 8.986e−04
Coefficient: −5.35e+03
Value: neg
20000
15000
10000
5000
0
cteria.c__Actinomycetia.o__Micrococcales.f__Microbacteriaceae.g__
alpha (n=25)
clade20C (n=1)
delta (n=13)
omicron (n=6)
neg (n=72)
NA
WHOvariant

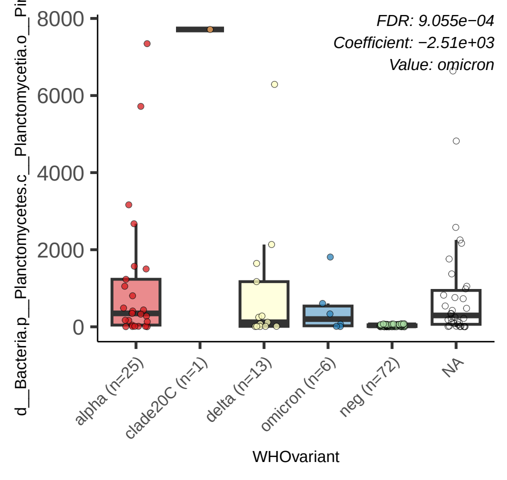

d__Bacteria.p__Planctomycetes.c__Planctomycetia.o__Pi
8000
6000
4000
2000
0
FDR: 9.055e−04
Coefficient: −2.51e+03
Value: omicron
alpha (n=25)
clade20C (n=1)
delta (n=13)
omicron (n=6)
neg (n=72)
NA
WHOvariant

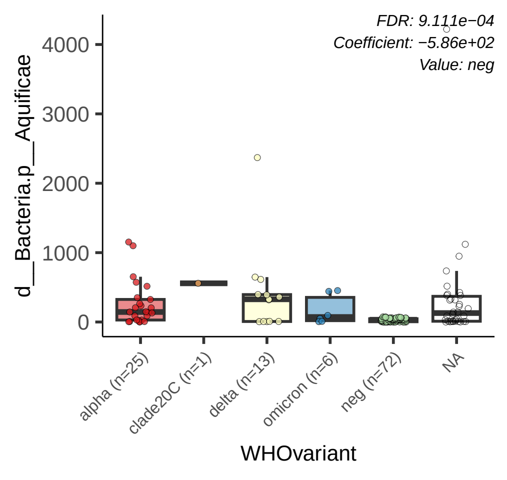
FDR: 9.111e−04
Coefficient: −5.86e+02
Value: neg
d__Bacteria.p__Aquificae
4000
3000
2000
1000
0
alpha (n=25)
clade20C (n=1)
delta (n=13)
omicron (n=6)
neg (n=72)
NA
WHOvariant

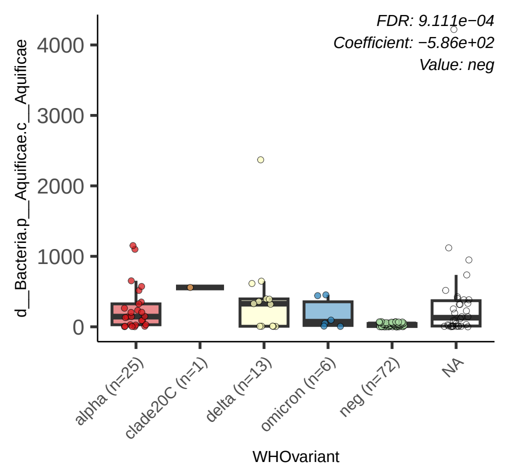

FDR: 9.111e−04
Coefficient: −5.86e+02
Value: neg
d__Bacteria.p__Aquificae.c__Aquificae
4000
3000
2000
1000
0
alpha (n=25)
clade20C (n=1)
delta (n=13)
omicron (n=6)
neg (n=72)
NA
WHOvariant

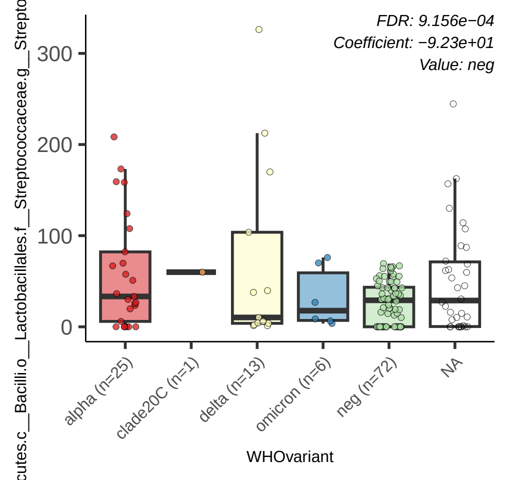

FDR: 9.156e−04
Coefficient: −9.23e+01
Value: neg
0
100
200
300
cutes.c__Bacilli.o__Lactobacillales.f__Streptococcaceae.g__Strepto
alpha (n=25)
clade20C (n=1)
delta (n=13)
omicron (n=6)
neg (n=72)
NA
WHOvariant

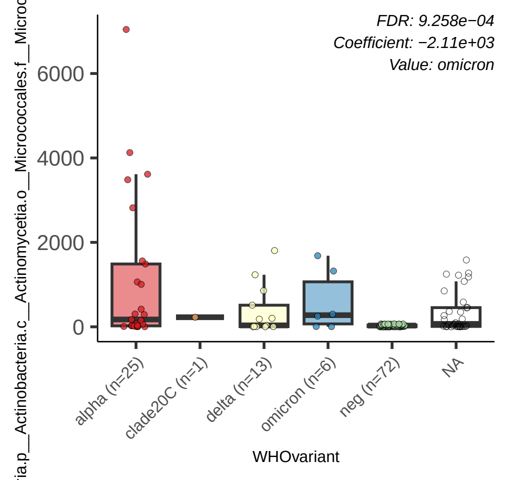

FDR: 9.258e−04
Coefficient: −2.11e+03
Value: omicron
0
2000
4000
6000
alpha (n=25)
clade20C (n=1)
delta (n=13)
omicron (n=6)
neg (n=72)
NA
WHOvariant

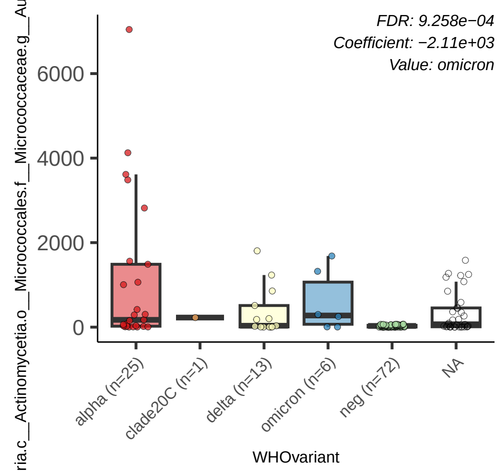

FDR: 9.258e−04
Coefficient: −2.11e+03
Value: omicron
6000
4000
2000
0
alpha (n=25)
clade20C (n=1)
delta (n=13)
omicron (n=6)
neg (n=72)
NA
WHOvariant
teria.c__Actinomycetia.o__Micrococcales.f__Micrococcaceae.g__Au

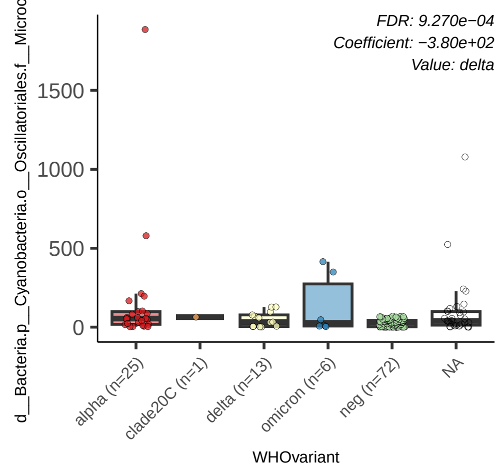

FDR: 9.270e−04
Coefficient: −3.80e+02
Value: delta
d__Bacteria.p__Cyanobacteria.o__Oscillatoriales.f__Microc
1500
1000
500
0
alpha (n=25)
clade20C (n=1)
delta (n=13)
omicron (n=6)
neg (n=72)
NA
WHOvariant

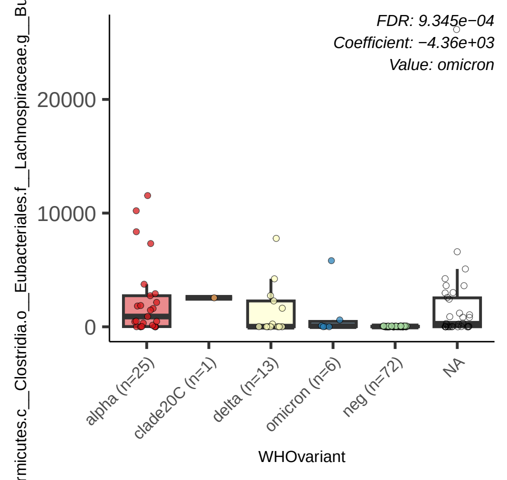

FDR: 9.345e−04
Coefficient: −4.36e+03
Value: omicron
rmicutes.c__Clostridia.o__Eubacteriales.f__Lachnospiraceae.g__Bu
20000
10000
0
alpha (n=25)
clade20C (n=1)
delta (n=13)
omicron (n=6)
neg (n=72)
NA
WHOvariant

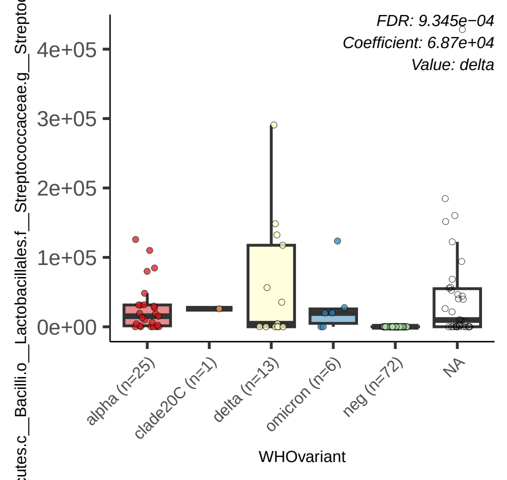

FDR: 9.345e−04
Coefficient: 6.87e+04
Value: delta
4e+05
3e+05
2e+05
1e+05
0e+00
alpha (n=25)
clade20C (n=1)
delta (n=13)
omicron (n=6)
neg (n=72)
NA
WHOvariant
cutes.c__Bacilli.o__Lactobacillales.f__Streptococcaceae.g__Streptoc

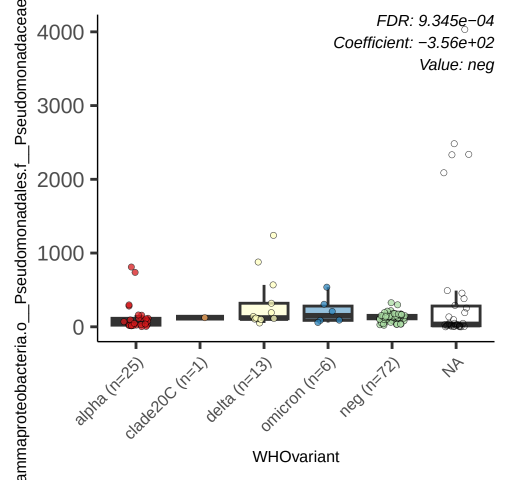
FDR: 9.345e−04
Coefficient: −3.56e+02
Value: neg
ammaproteobacteria.o__Pseudomonadales.f__Pseudomonadaceae
4000
3000
2000
1000
0
alpha (n=25)
clade20C (n=1)
delta (n=13)
omicron (n=6)
neg (n=72)
NA
WHOvariant

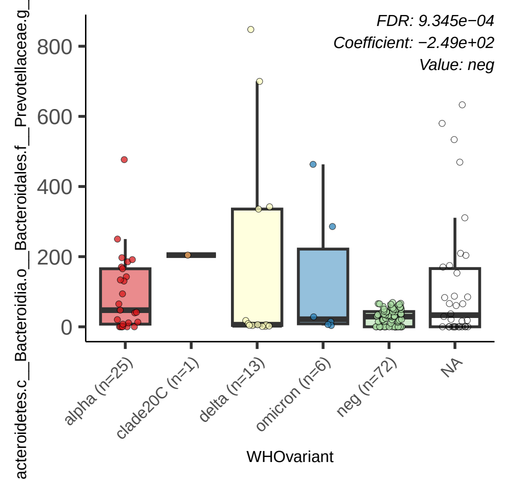

acteroidetes.c__Bacteroidia.o__Bacteroidales.f__Prevotellaceae.g__
FDR: 9.345e−04
Coefficient: −2.49e+02
Value: neg
0
200
400
600
800
alpha (n=25)
clade20C (n=1)
delta (n=13)
omicron (n=6)
neg (n=72)
NA
WHOvariant

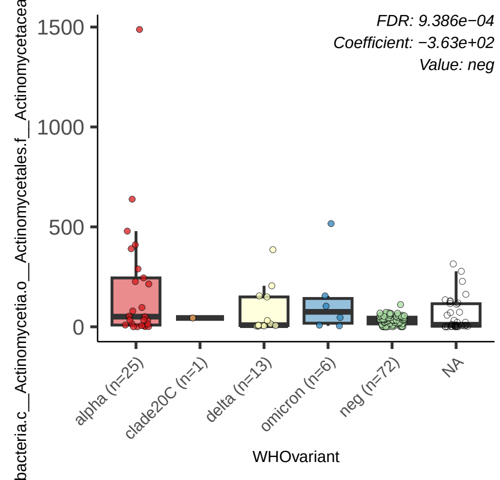
FDR: 9.386e−04
Coefficient: −3.63e+02
Value: neg
1500
1000
500
0
bacteria.c__Actinomycetia.o__Actinomycetales.f__Actinomycetacea
alpha (n=25)
clade20C (n=1)
delta (n=13)
omicron (n=6)
neg (n=72)
NA
WHOvariant

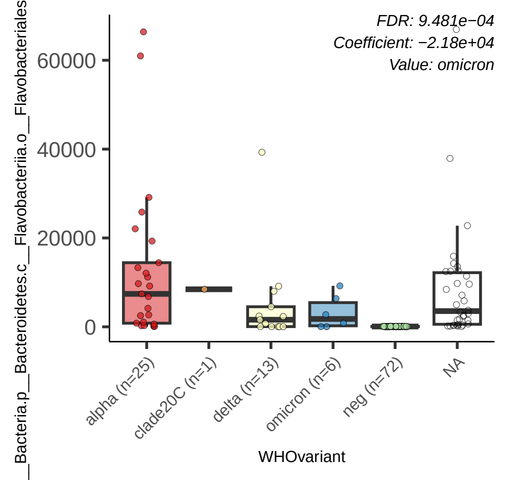

FDR: 9.481e−04
Coefficient: −2.18e+04
Value: omicron
Bacteria.p__Bacteroidetes.c__Flavobacteriia.o__Flavobacteriales
60000
40000
20000
0
alpha (n=25)
clade20C (n=1)
delta (n=13)
omicron (n=6)
neg (n=72)
NA
WHOvariant

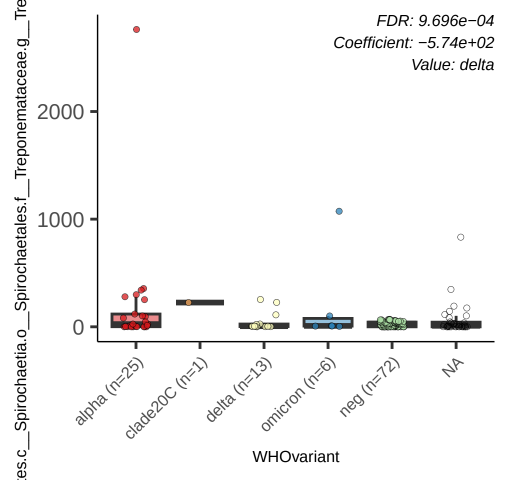

FDR: 9.696e−04
Coefficient: −5.74e+02
Value: delta
2000
1000
0
alpha (n=25)
clade20C (n=1)
delta (n=13)
omicron (n=6)
neg (n=72)
NA
WHOvariant

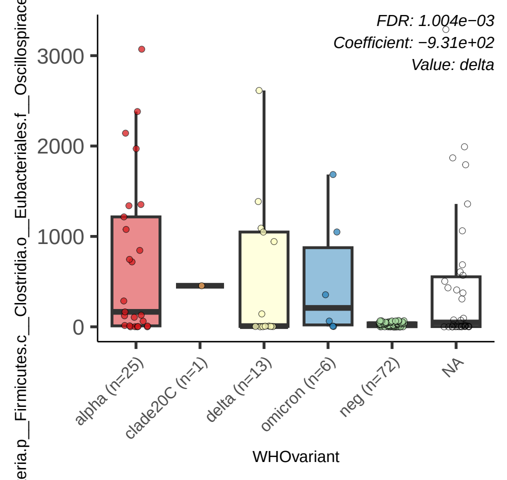
FDR: 1.004e−03
Coefficient: −9.31e+02
Value: delta
0
1000
2000
3000
alpha (n=25)
clade20C (n=1)
delta (n=13)
omicron (n=6)
neg (n=72)
NA
WHOvariant
teria.p__Firmicutes.c__Clostridia.o__Eubacteriales.f__Oscillospirace

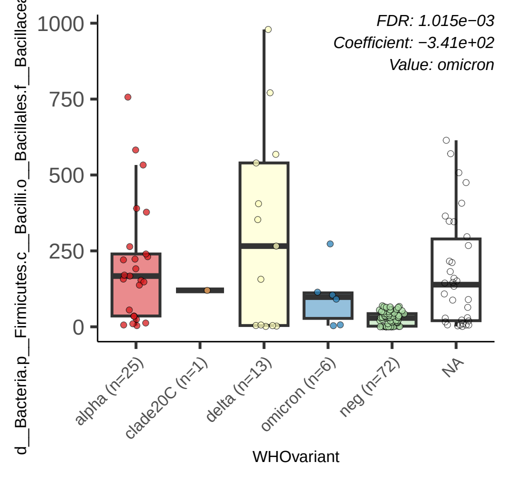

FDR: 1.015e−03
Coefficient: −3.41e+02
Value: omicron
d__Bacteria.p__Firmicutes.c__Bacilli.o__Bacillales.f__Bacillacea
1000
750
500
250
0
alpha (n=25)
clade20C (n=1)
delta (n=13)
omicron (n=6)
neg (n=72)
NA
WHOvariant

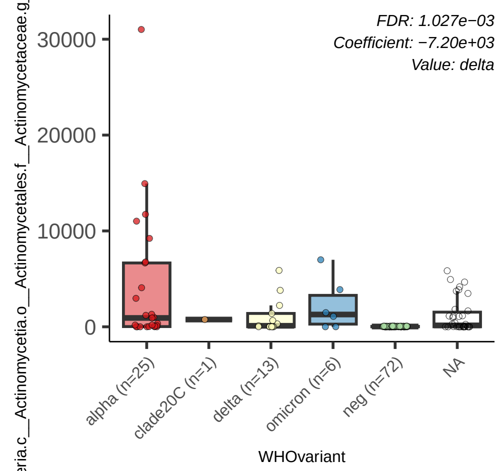

FDR: 1.027e−03
Coefficient: −7.20e+03
Value: delta
teria.c__Actinomycetia.o__Actinomycetales.f__Actinomycetaceae.g
30000
20000
10000
0
alpha (n=25)
clade20C (n=1)
delta (n=13)
omicron (n=6)
neg (n=72)
NA
WHOvariant

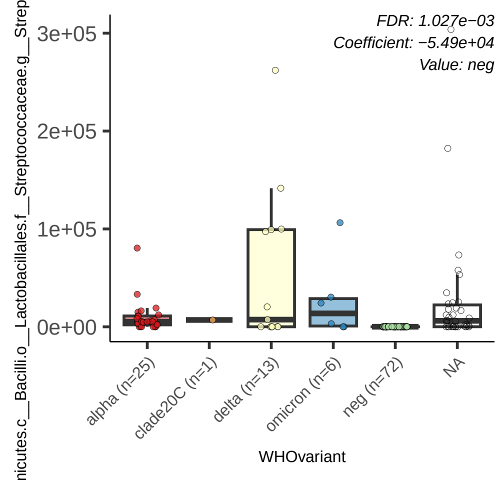

FDR: 1.027e−03
Coefficient: −5.49e+04
Value: neg
nicutes.c__Bacilli.o__Lactobacillales.f__Streptococcaceae.g__Strep
3e+05
2e+05
1e+05
0e+00
alpha (n=25)
clade20C (n=1)
delta (n=13)
omicron (n=6)
neg (n=72)
NA
WHOvariant

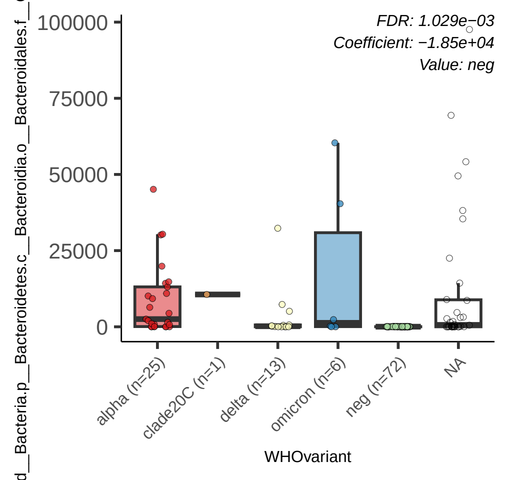

d__Bacteria.p__Bacteroidetes.c__Bacteroidia.o__Bacteroidales.f__
FDR: 1.029e−03
Coefficient: −1.85e+04
Value: neg
100000
75000
50000
25000
0
alpha (n=25)
clade20C (n=1)
delta (n=13)
omicron (n=6)
neg (n=72)
NA
WHOvariant

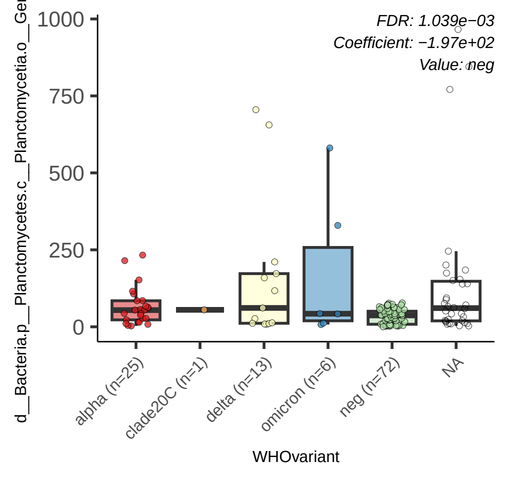

FDR: 1.039e−03
Coefficient: −1.97e+02
Value: neg
d__Bacteria.p__Planctomycetes.c__Planctomycetia.o__Ge
1000
750
500
250
0
alpha (n=25)
clade20C (n=1)
delta (n=13)
omicron (n=6)
neg (n=72)
NA
WHOvariant

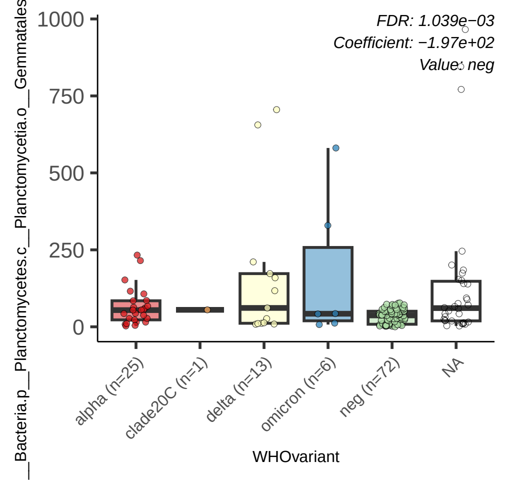

FDR: 1.039e−03
Coefficient: −1.97e+02
Value: neg
1000
750
500
250
0
_Bacteria.p__Planctomycetes.c__Planctomycetia.o__Gemmatales
alpha (n=25)
clade20C (n=1)
delta (n=13)
omicron (n=6)
neg (n=72)
NA
WHOvariant

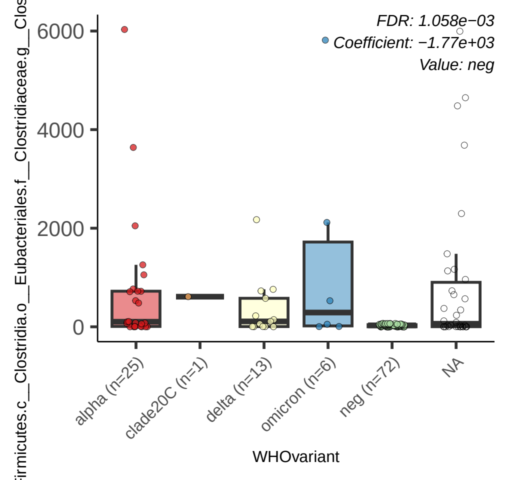

FDR: 1.058e−03
Coefficient: −1.77e+03
Value: neg
6000
4000
2000
0
irmicutes.c__Clostridia.o__Eubacteriales.f__Clostridiaceae.g__Clos
alpha (n=25)
clade20C (n=1)
delta (n=13)
omicron (n=6)
neg (n=72)
NA
WHOvariant

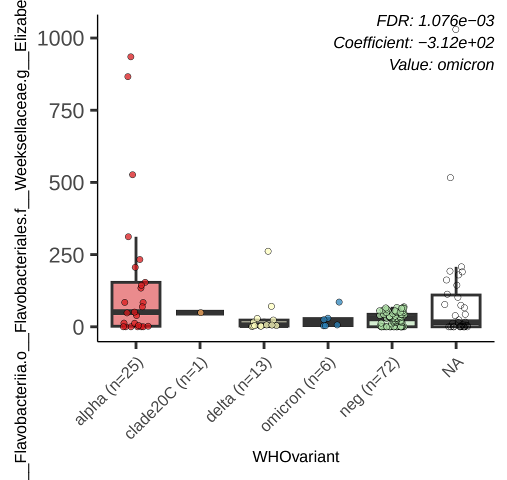

FDR: 1.076e−03
Coefficient: −3.12e+02
Value: omicron
Flavobacteriia.o__Flavobacteriales.f__Weeksellaceae.g__Elizabe
1000
750
500
250
0
alpha (n=25)
clade20C (n=1)
delta (n=13)
omicron (n=6)
neg (n=72)
NA
WHOvariant

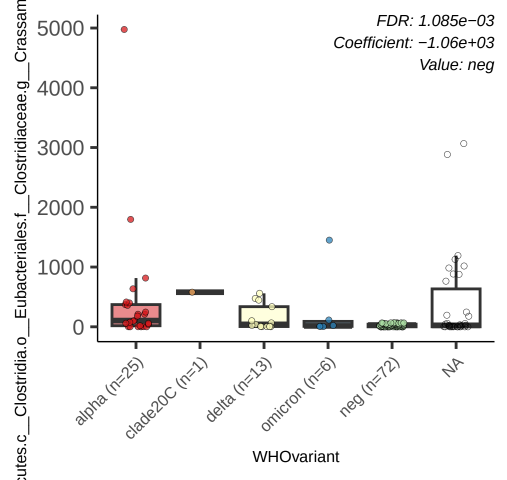

FDR: 1.085e−03
Coefficient: −1.06e+03
Value: neg
5000
4000
3000
2000
1000
0
cutes.c__Clostridia.o__Eubacteriales.f__Clostridiaceae.g__Crassam
alpha (n=25)
clade20C (n=1)
delta (n=13)
omicron (n=6)
neg (n=72)
NA
WHOvariant

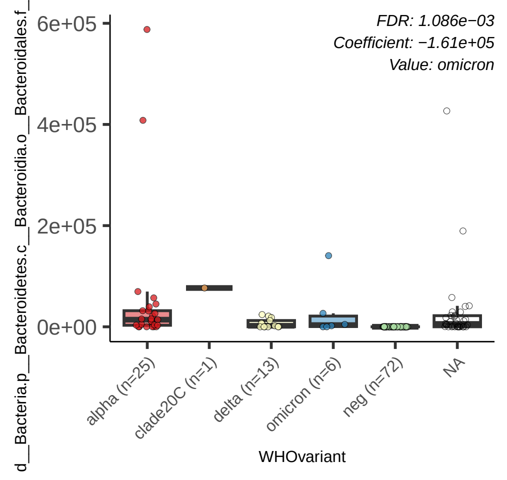

FDR: 1.086e−03
Coefficient: −1.61e+05
Value: omicron
d__Bacteria.p__Bacteroidetes.c__Bacteroidia.o__Bacteroidales.f
6e+05
4e+05
2e+05
0e+00
alpha (n=25)
clade20C (n=1)
delta (n=13)
omicron (n=6)
neg (n=72)
NA
WHOvariant

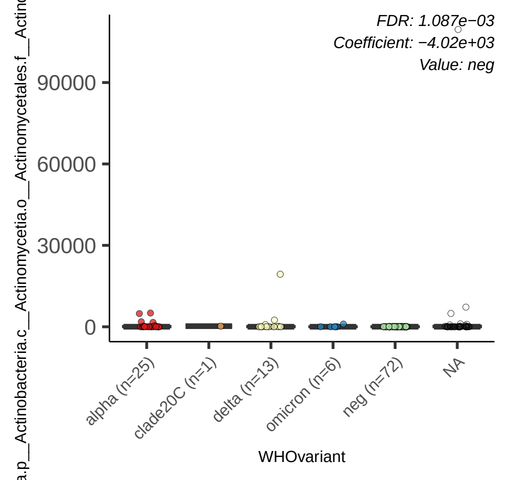

FDR: 1.087e−03
Coefficient: −4.02e+03
Value: neg
90000
60000
30000
0
alpha (n=25)
clade20C (n=1)
delta (n=13)
omicron (n=6)
neg (n=72)
NA
WHOvariant
a.p__Actinobacteria.c__Actinomycetia.o__Actinomycetales.f__Actino

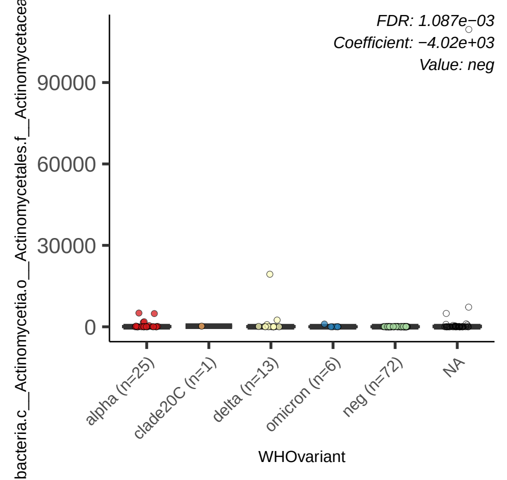

FDR: 1.087e−03
Coefficient: −4.02e+03
Value: neg
90000
60000
30000
0
bacteria.c__Actinomycetia.o__Actinomycetales.f__Actinomycetacea
alpha (n=25)
clade20C (n=1)
delta (n=13)
omicron (n=6)
neg (n=72)
NA
WHOvariant

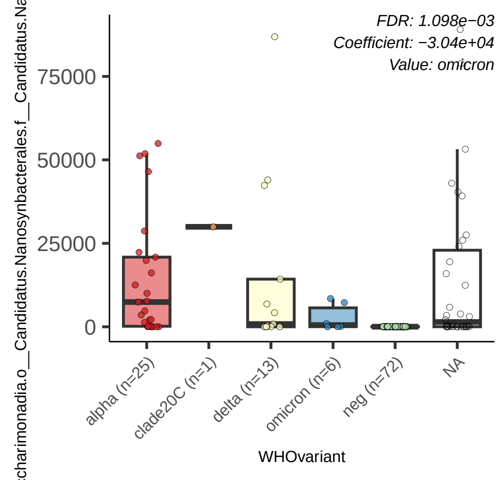

FDR: 1.098e−03
Coefficient: −3.04e+04
Value: omicron
75000
50000
25000
0
alpha (n=25)
clade20C (n=1)
delta (n=13)
omicron (n=6)
neg (n=72)
NA
WHOvariant

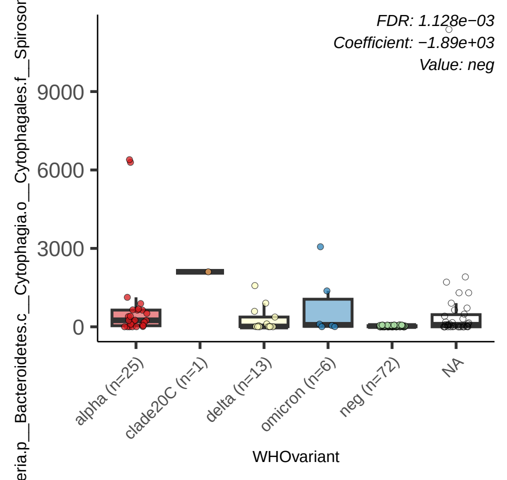

FDR: 1.128e−03
Coefficient: −1.89e+03
Value: neg
9000
6000
3000
0
eria.p__Bacteroidetes.c__Cytophagia.o__Cytophagales.f__Spirosor
alpha (n=25)
clade20C (n=1)
delta (n=13)
omicron (n=6)
neg (n=72)
NA
WHOvariant

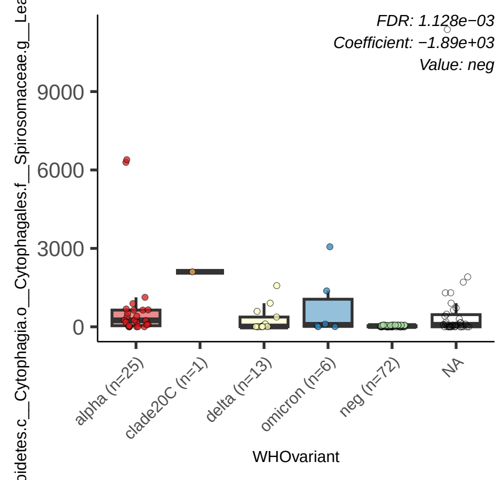

FDR: 1.128e−03
Coefficient: −1.89e+03
Value: neg
9000
6000
3000
0
oidetes.c__Cytophagia.o__Cytophagales.f__Spirosomaceae.g__Lea
alpha (n=25)
clade20C (n=1)
delta (n=13)
omicron (n=6)
neg (n=72)
NA
WHOvariant

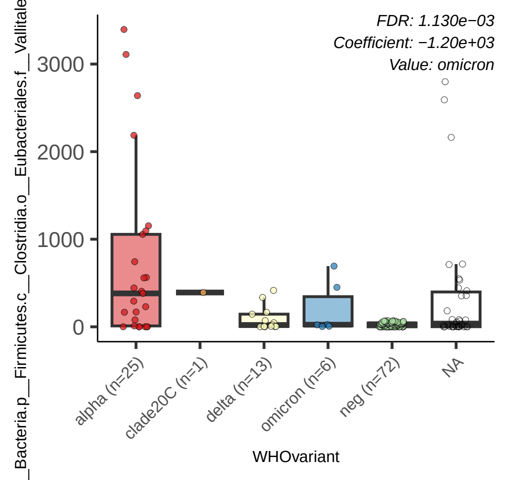

FDR: 1.130e−03
Coefficient: −1.20e+03
Value: omicron
Bacteria.p__Firmicutes.c__Clostridia.o__Eubacteriales.f__Vallitale
3000
2000
1000
0
alpha (n=25)
clade20C (n=1)
delta (n=13)
omicron (n=6)
neg (n=72)
NA
WHOvariant

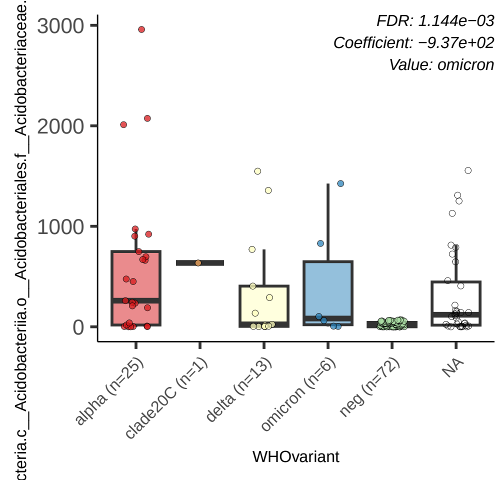

FDR: 1.144e−03
Coefficient: −9.37e+02
Value: omicron
cteria.c__Acidobacteriia.o__Acidobacteriales.f__Acidobacteriaceae.
3000
2000
1000
0
alpha (n=25)
clade20C (n=1)
delta (n=13)
omicron (n=6)
neg (n=72)
NA
WHOvariant

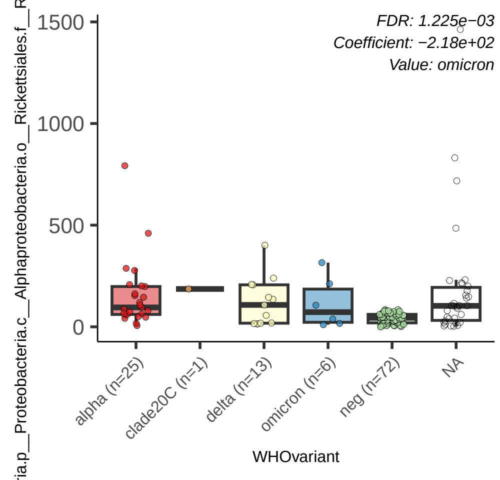

FDR: 1.225e−03
Coefficient: −2.18e+02
Value: omicron
1500
1000
500
0
ria.p__Proteobacteria.c__Alphaproteobacteria.o__Rickettsiales.f__R
alpha (n=25)
clade20C (n=1)
delta (n=13)
omicron (n=6)
neg (n=72)
NA
WHOvariant

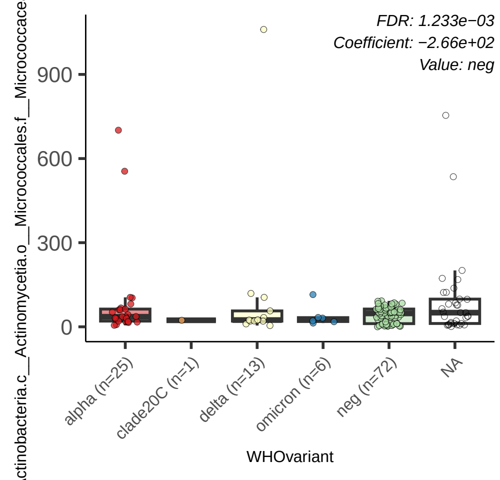

FDR: 1.233e−03
Coefficient: −2.66e+02
Value: neg
Actinobacteria.c__Actinomycetia.o__Micrococcales.f__Micrococcace
900
600
300
0
alpha (n=25)
clade20C (n=1)
delta (n=13)
omicron (n=6)
neg (n=72)
NA
WHOvariant

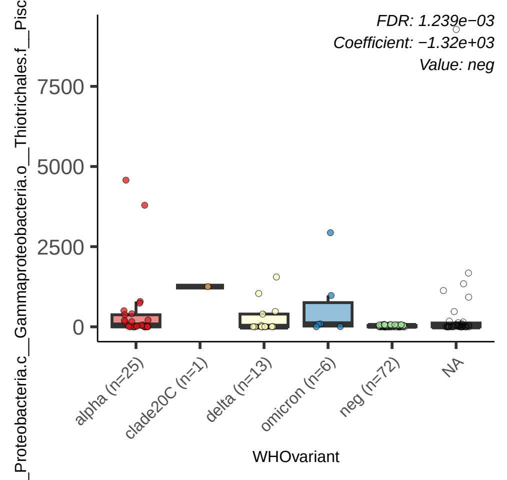

Proteobacteria.c__Gammaproteobacteria.o__Thiotrichales.f__Pisc
FDR: 1.239e−03
Coefficient: −1.32e+03
Value: neg
7500
5000
2500
0
alpha (n=25)
clade20C (n=1)
delta (n=13)
omicron (n=6)
neg (n=72)
NA
WHOvariant

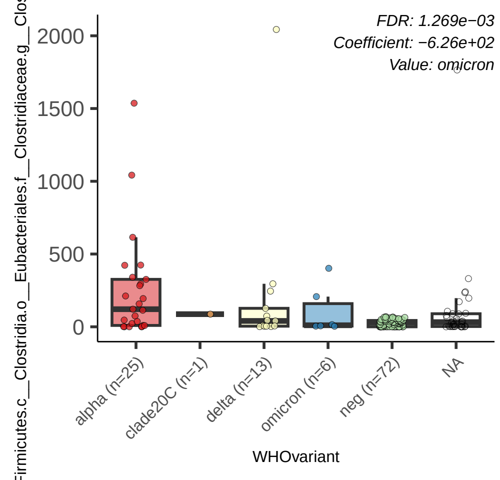
FDR: 1.269e−03
Coefficient: −6.26e+02
Value: omicron
Firmicutes.c__Clostridia.o__Eubacteriales.f__Clostridiaceae.g__Clos
2000
1500
1000
500
0
alpha (n=25)
clade20C (n=1)
delta (n=13)
omicron (n=6)
neg (n=72)
NA
WHOvariant

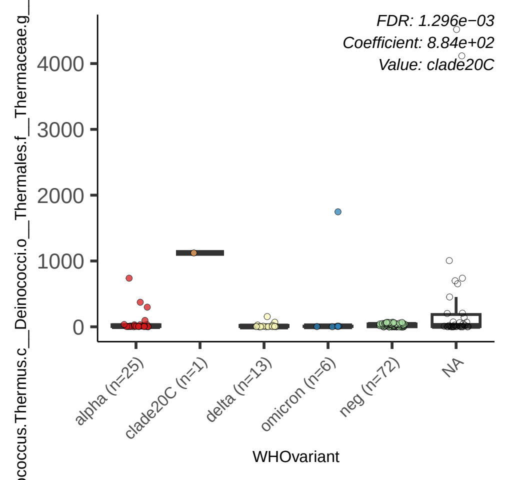

FDR: 1.296e−03
Coefficient: 8.84e+02
Value: clade20C
0
1000
2000
3000
4000
occus.Thermus.c__Deinococci.o__Thermales.f__Thermaceae.g__
alpha (n=25)
clade20C (n=1)
delta (n=13)
omicron (n=6)
neg (n=72)
NA
WHOvariant

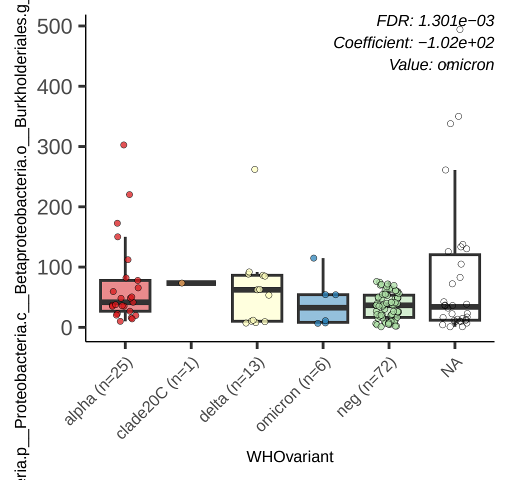
FDR: 1.301e−03
Coefficient: −1.02e+02
Value: omicron
Proteobacteria.c__Betaproteobacteria.o__Burkholderiales.g_
0
100
200
300
400
500
alpha (n=25)
clade20C (n=1)
delta (n=13)
omicron (n=6)
neg (n=72)
NA
WHOvariant

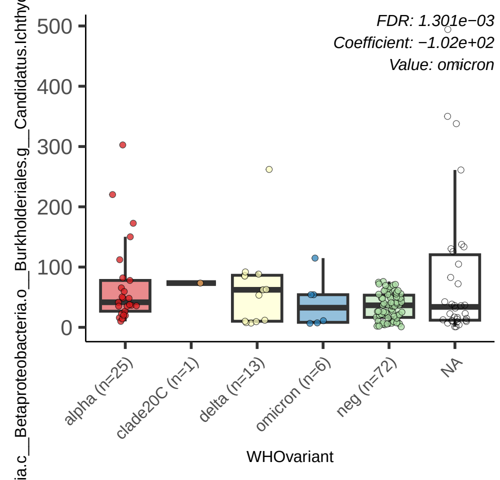

FDR: 1.301e−03
Coefficient: −1.02e+02
Value: omicron
ia.c__Betaproteobacteria.o__Burkholderiales.g__Candidatus.Ichthyo
500
400
300
200
100
0
alpha (n=25)
clade20C (n=1)
delta (n=13)
omicron (n=6)
neg (n=72)
NA
WHOvariant

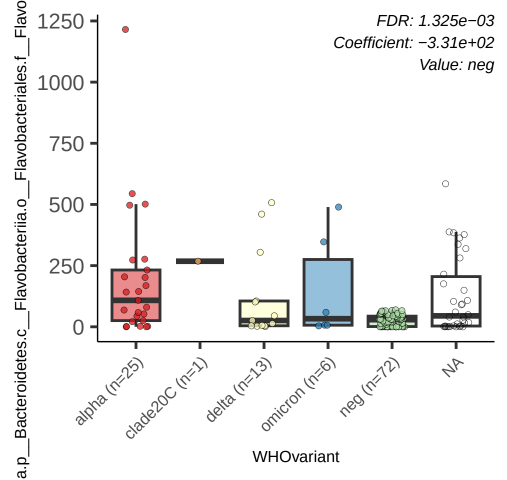

FDR: 1.325e−03
Coefficient: −3.31e+02
Value: neg
a.p__Bacteroidetes.c__Flavobacteriia.o__Flavobacteriales.f__Flavo
1250
1000
750
500
250
0
alpha (n=25)
clade20C (n=1)
delta (n=13)
omicron (n=6)
neg (n=72)
NA
WHOvariant

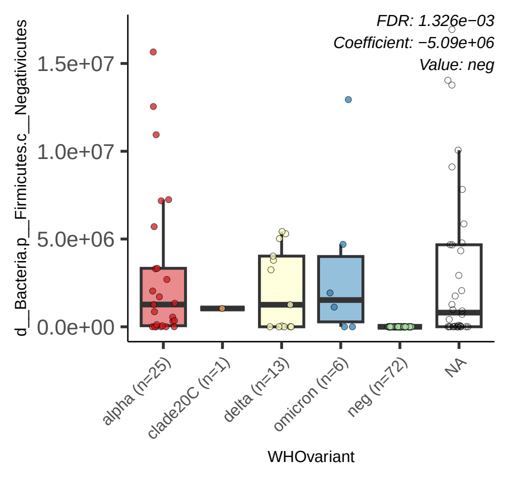

FDR: 1.326e−03
Coefficient: −5.09e+06
Value: neg
d__Bacteria.p__Firmicutes.c__Negativicutes
1.5e+07
1.0e+07
5.0e+06
0.0e+00
alpha (n=25)
clade20C (n=1)
delta (n=13)
omicron (n=6)
neg (n=72)
NA
WHOvariant

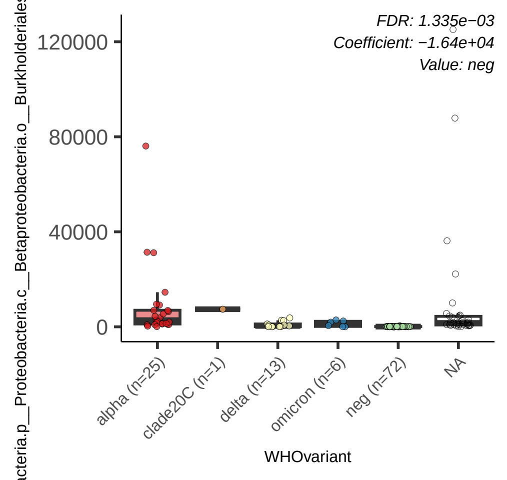

FDR: 1.335e−03
Coefficient: −1.64e+04
Value: neg
120000
80000
40000
0
acteria.p__Proteobacteria.c__Betaproteobacteria.o__Burkholderiales
alpha (n=25)
clade20C (n=1)
delta (n=13)
omicron (n=6)
neg (n=72)
NA
WHOvariant

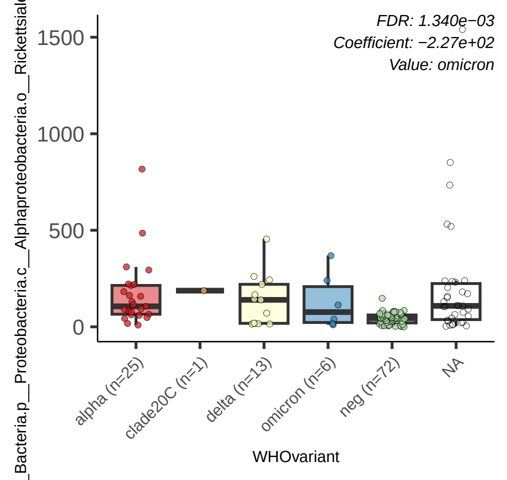

FDR: 1.340e−03
Coefficient: −2.27e+02
Value: omicron
Bacteria.p__Proteobacteria.c__Alphaproteobacteria.o__Rickettsiale
1500
1000
500
0
alpha (n=25)
clade20C (n=1)
delta (n=13)
omicron (n=6)
neg (n=72)
NA
WHOvariant

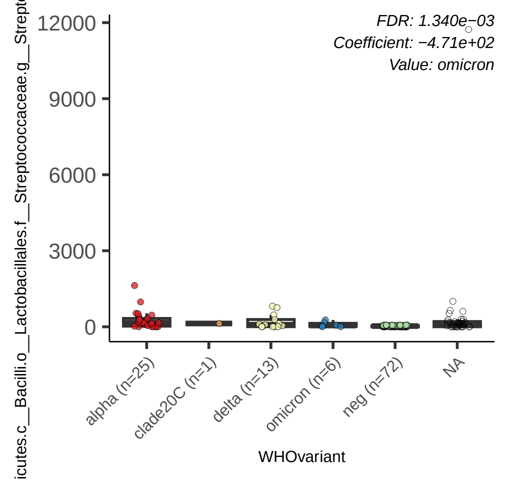
FDR: 1.340e−03
Coefficient: −4.71e+02
Value: omicron
12000
9000
6000
3000
0
icutes.c__Bacilli.o__Lactobacillales.f__Streptococcaceae.g__Strept
alpha (n=25)
clade20C (n=1)
delta (n=13)
omicron (n=6)
neg (n=72)
NA
WHOvariant

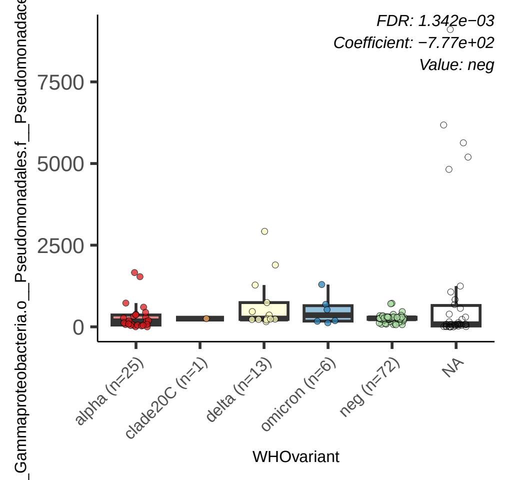

FDR: 1.342e−03
Coefficient: −7.77e+02
Value: neg
_Gammaproteobacteria.o__Pseudomonadales.f__Pseudomonadace
7500
5000
2500
0
alpha (n=25)
clade20C (n=1)
delta (n=13)
omicron (n=6)
neg (n=72)
NA
WHOvariant

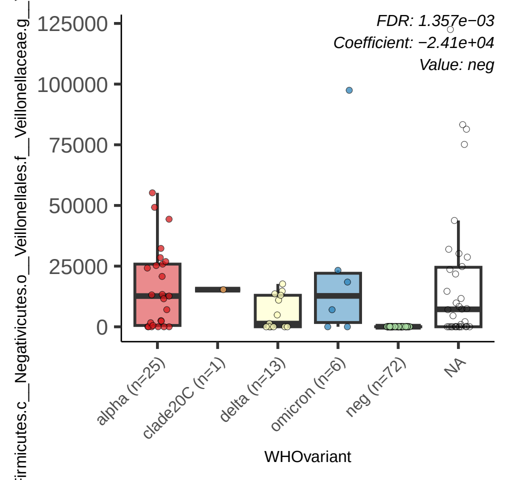

FDR: 1.357e−03
Coefficient: −2.41e+04
Value: neg
125000
100000
75000
50000
25000
0
irmicutes.c__Negativicutes.o__Veillonellales.f__Veillonellaceae.g__
alpha (n=25)
clade20C (n=1)
delta (n=13)
omicron (n=6)
neg (n=72)
NA
WHOvariant

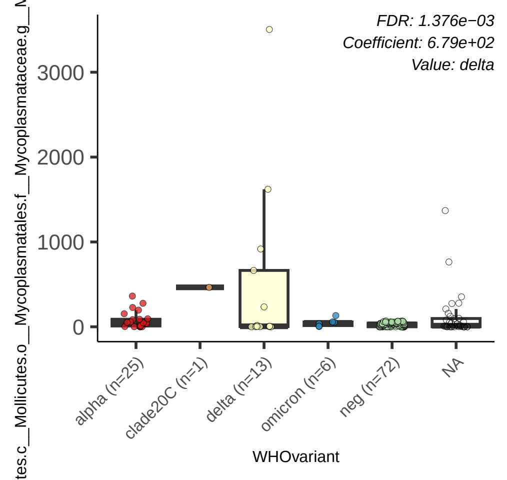

FDR: 1.376e−03
Coefficient: 6.79e+02
Value: delta
3000
2000
1000
0
tes.c__Mollicutes.o__Mycoplasmatales.f__Mycoplasmataceae.g__M
alpha (n=25)
clade20C (n=1)
delta (n=13)
omicron (n=6)
neg (n=72)
NA
WHOvariant

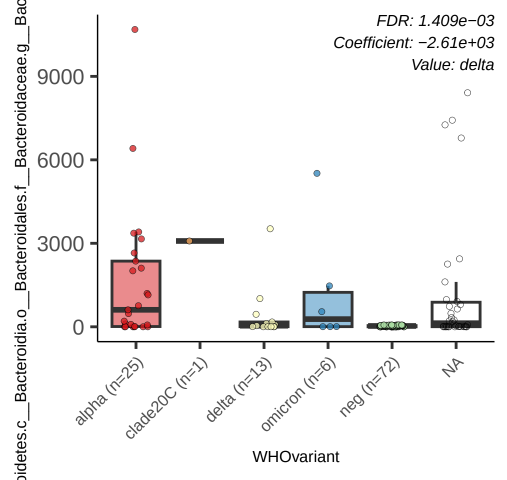

FDR: 1.409e−03
Coefficient: −2.61e+03
Value: delta
9000
6000
3000
0
alpha (n=25)
clade20C (n=1)
delta (n=13)
omicron (n=6)
neg (n=72)
NA
WHOvariant

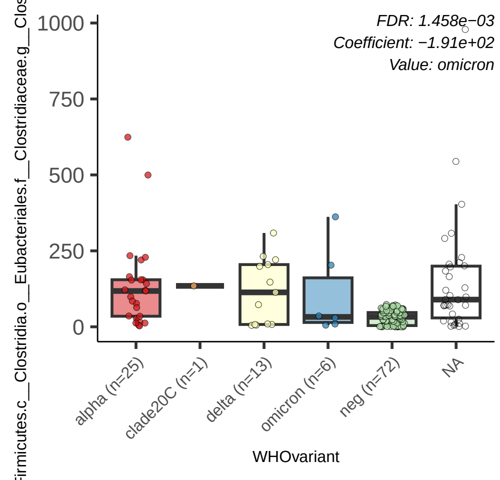

FDR: 1.458e−03
Coefficient: −1.91e+02
Value: omicron
Firmicutes.c__Clostridia.o__Eubacteriales.f__Clostridiaceae.g__Clos
1000
750
500
250
0
alpha (n=25)
clade20C (n=1)
delta (n=13)
omicron (n=6)
neg (n=72)
NA
WHOvariant

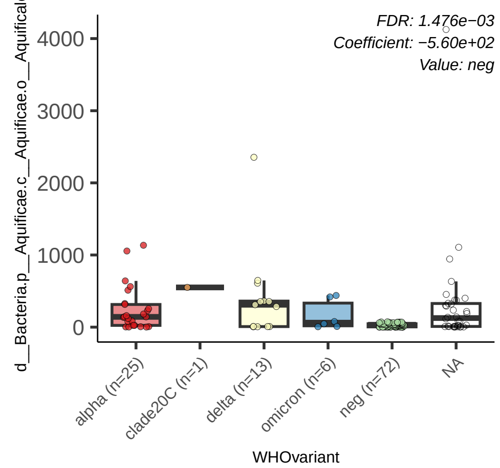

FDR: 1.476e−03
Coefficient: −5.60e+02
Value: neg
d__Bacteria.p__Aquificae.c__Aquificae.o__Aquificale
4000
3000
2000
1000
0
alpha (n=25)
clade20C (n=1)
delta (n=13)
omicron (n=6)
neg (n=72)
NA
WHOvariant

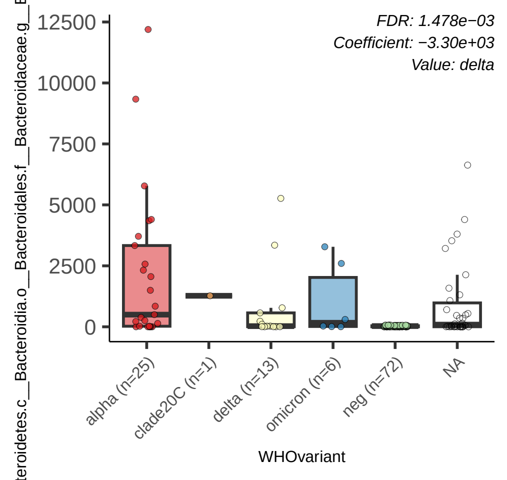
FDR: 1.478e−03
Coefficient: −3.30e+03
Value: delta
12500
10000
7500
5000
2500
0
alpha (n=25)
clade20C (n=1)
delta (n=13)
omicron (n=6)
neg (n=72)
NA
WHOvariant

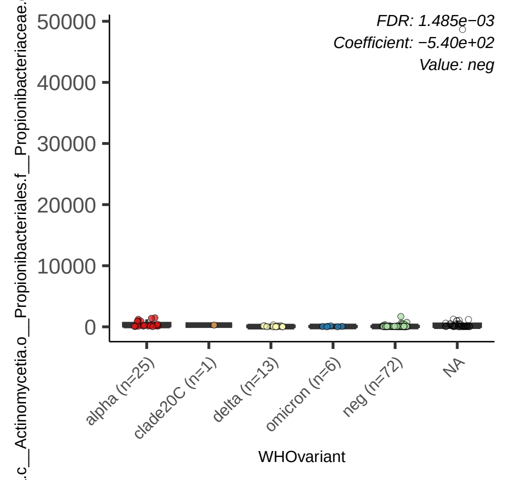

FDR: 1.485e−03
Coefficient: −5.40e+02
Value: neg
50000
40000
30000
20000
10000
0
.c__Actinomycetia.o__Propionibacteriales.f__Propionibacteriaceae.
alpha (n=25)
clade20C (n=1)
delta (n=13)
omicron (n=6)
neg (n=72)
NA
WHOvariant

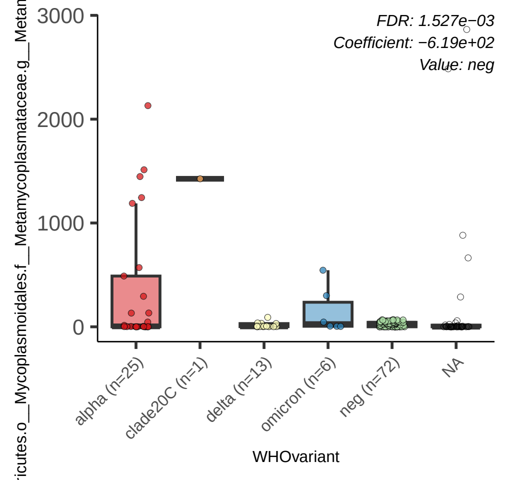

FDR: 1.527e−03
Coefficient: −6.19e+02
Value: neg
3000
2000
1000
0
ricutes.o__Mycoplasmoidales.f__Metamycoplasmataceae.g__Metan
alpha (n=25)
clade20C (n=1)
delta (n=13)
omicron (n=6)
neg (n=72)
NA
WHOvariant

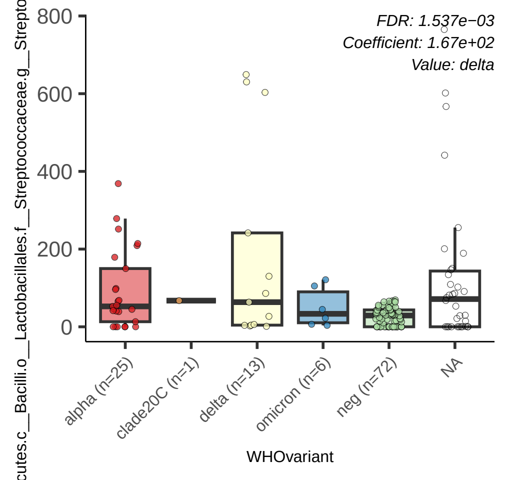

FDR: 1.537e−03
Coefficient: 1.67e+02
Value: delta
800
600
400
200
0
cutes.c__Bacilli.o__Lactobacillales.f__Streptococcaceae.g__Strepto
alpha (n=25)
clade20C (n=1)
delta (n=13)
omicron (n=6)
neg (n=72)
NA
WHOvariant

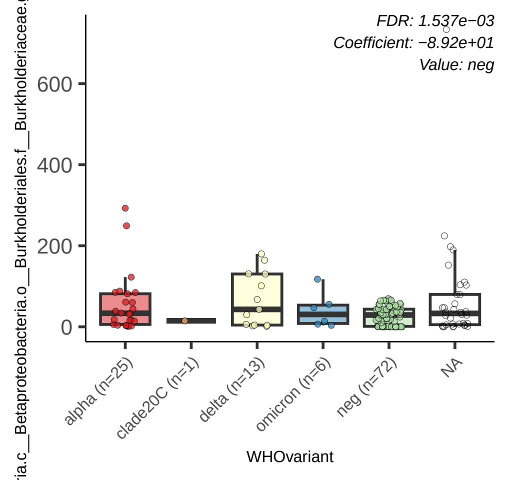

FDR: 1.537e−03
Coefficient: −8.92e+01
Value: neg
0
200
400
600
ria.c__Betaproteobacteria.o__Burkholderiales.f__Burkholderiaceae.g
alpha (n=25)
clade20C (n=1)
delta (n=13)
omicron (n=6)
neg (n=72)
NA
WHOvariant

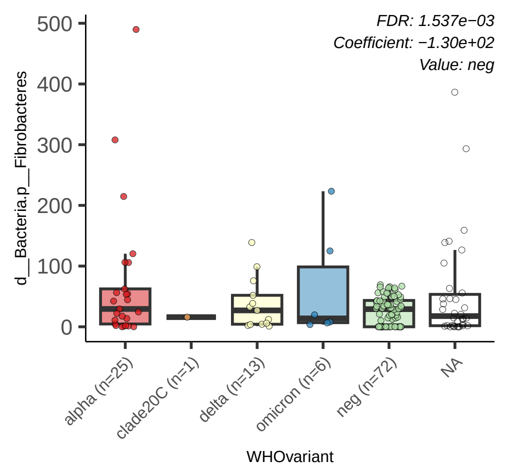

FDR: 1.537e−03
Coefficient: −1.30e+02
Value: neg
d__Bacteria.p__Fibrobacteres
500
400
300
200
100
0
alpha (n=25)
clade20C (n=1)
delta (n=13)
omicron (n=6)
neg (n=72)
NA
WHOvariant

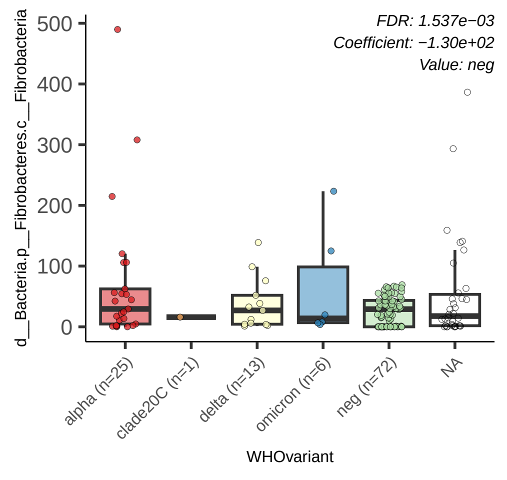
FDR: 1.537e−03
Coefficient: −1.30e+02
Value: neg
d__Bacteria.p__Fibrobacteres.c__Fibrobacteria
500
400
300
200
100
0
alpha (n=25)
clade20C (n=1)
delta (n=13)
omicron (n=6)
neg (n=72)
NA
WHOvariant

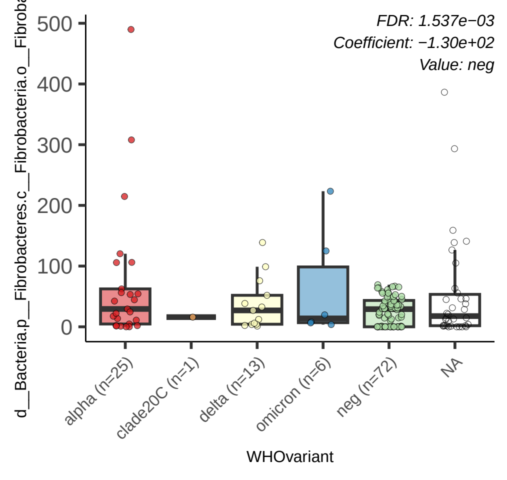

FDR: 1.537e−03
Coefficient: −1.30e+02
Value: neg
d__Bacteria.p__Fibrobacteres.c__Fibrobacteria.o__Fibroba
500
400
300
200
100
0
alpha (n=25)
clade20C (n=1)
delta (n=13)
omicron (n=6)
neg (n=72)
NA
WHOvariant

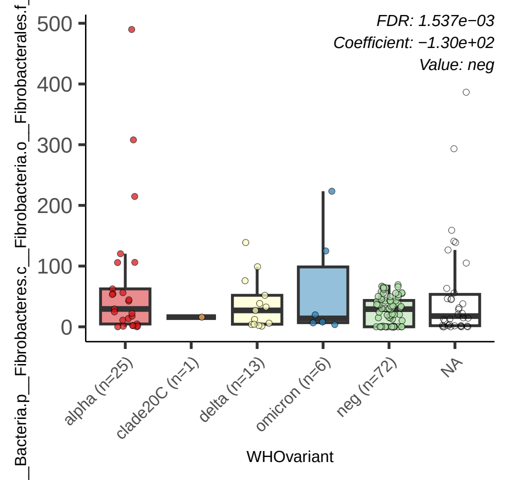

FDR: 1.537e−03
Coefficient: −1.30e+02
Value: neg
Bacteria.p__Fibrobacteres.c__Fibrobacteria.o__Fibrobacterales.f_
500
400
300
200
100
0
alpha (n=25)
clade20C (n=1)
delta (n=13)
omicron (n=6)
neg (n=72)
NA
WHOvariant

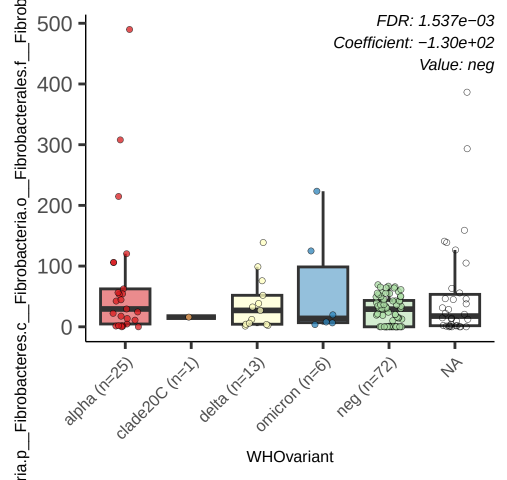

FDR: 1.537e−03
Coefficient: −1.30e+02
Value: neg
0
100
200
300
400
500
Fibrobacteria.p__Fibrobacteres.c__Fibrobacteria.o__Fibrobacterales.f__Fibrob
alpha (n=25)
clade20C (n=1)
delta (n=13)
omicron (n=6)
neg (n=72)
NA
WHOvariant

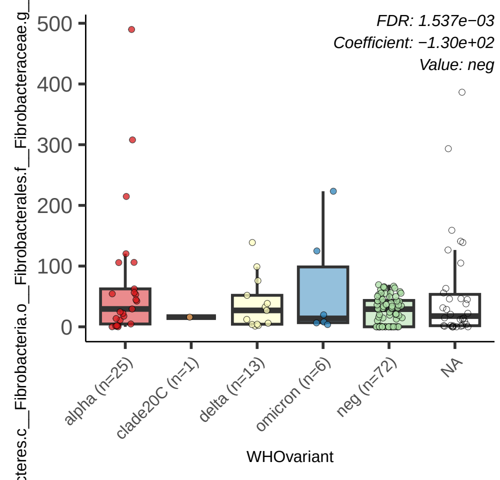

FDR: 1.537e−03
Coefficient: −1.30e+02
Value: neg
0
100
200
300
400
500
cteres.c__Fibrobacteria.o__Fibrobacterales.f__Fibrobacteraceae.g__
alpha (n=25)
clade20C (n=1)
delta (n=13)
omicron (n=6)
neg (n=72)
NA
WHOvariant

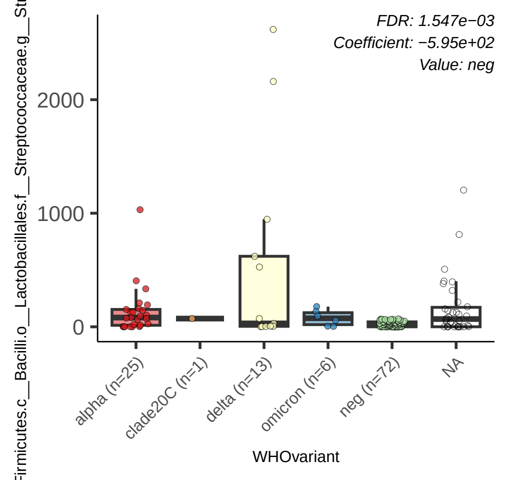

FDR: 1.547e−03
Coefficient: −5.95e+02
Value: neg
Firmicutes.c__Bacilli.o__Lactobacillales.f__Streptococcaceae.g__St
2000
1000
0
alpha (n=25)
clade20C (n=1)
delta (n=13)
omicron (n=6)
neg (n=72)
NA
WHOvariant

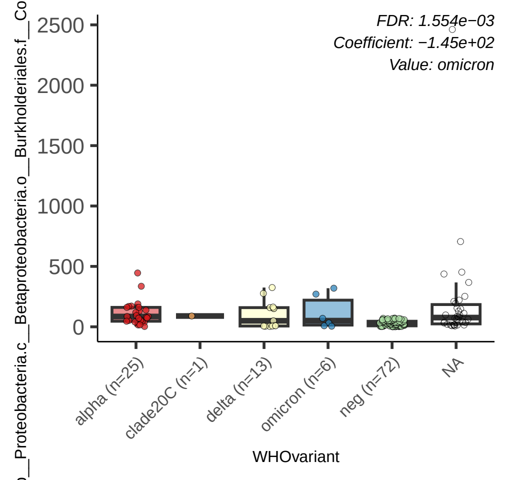

FDR: 1.554e−03
Coefficient: −1.45e+02
Value: omicron
Proteobacteria.c__Betaproteobacteria.o__Burkholderiales.f__Co
2500
2000
1500
1000
500
0
alpha (n=25)
clade20C (n=1)
delta (n=13)
omicron (n=6)
neg (n=72)
NA
WHOvariant

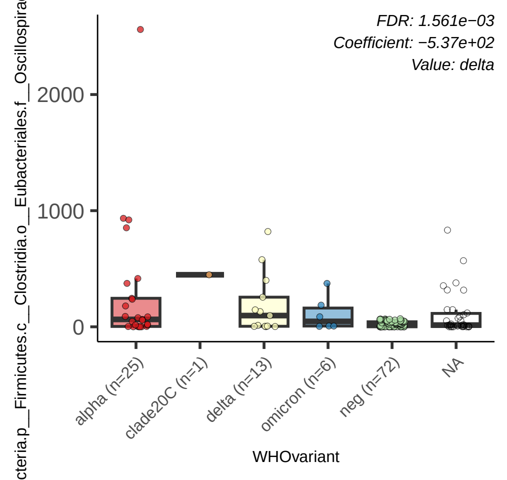

FDR: 1.561e−03
Coefficient: −5.37e+02
Value: delta
2000
1000
0
alpha (n=25)
clade20C (n=1)
delta (n=13)
omicron (n=6)
neg (n=72)
NA
WHOvariant
cteria.p__Firmicutes.c__Clostridia.o__Eubacteriales.f__Oscillospira

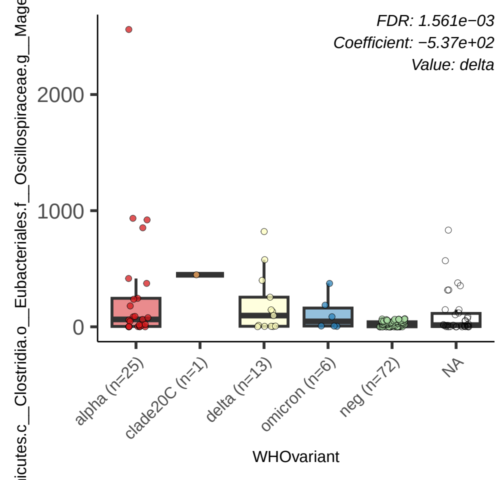

FDR: 1.561e−03
Coefficient: −5.37e+02
Value: delta
2000
1000
0
alpha (n=25)
clade20C (n=1)
delta (n=13)
omicron (n=6)
neg (n=72)
NA
WHOvariant
icutes.c__Clostridia.o__Eubacteriales.f__Oscillospiraceae.g__Mage

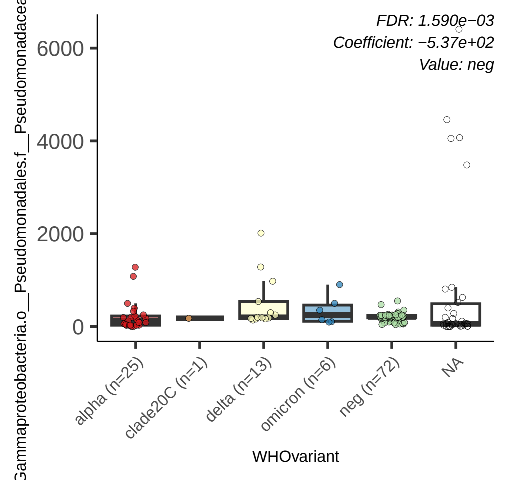
FDR: 1.590e−03
Coefficient: −5.37e+02
Value: neg
6000
4000
2000
0
Gammaproteobacteria.o__Pseudomonadales.f__Pseudomonadacea
alpha (n=25)
clade20C (n=1)
delta (n=13)
omicron (n=6)
neg (n=72)
NA
WHOvariant

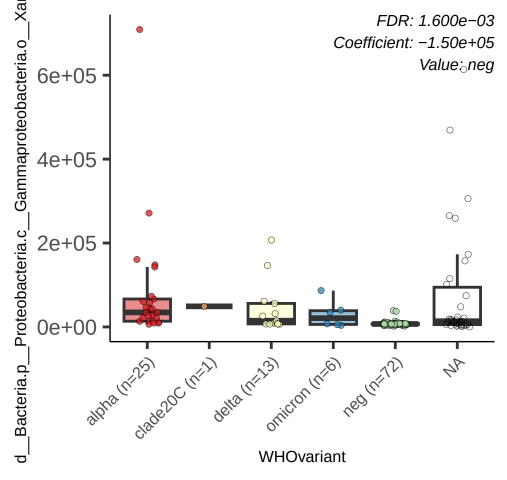

FDR: 1.600e−03
Coefficient: −1.50e+05
Value: neg
d__Bacteria.p__Proteobacteria.c__Gammaproteobacteria.o__Xa
6e+05
4e+05
2e+05
0e+00
alpha (n=25)
clade20C (n=1)
delta (n=13)
omicron (n=6)
neg (n=72)
NA
WHOvariant

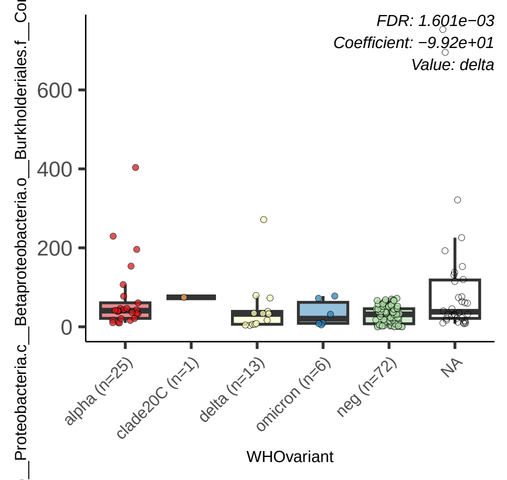

FDR: 1.601e−03
Coefficient: −9.92e+01
Value: delta
Proteobacteria.c__Betaproteobacteria.o__Burkholderiales.f__Co
0
200
400
600
alpha (n=25)
clade20C (n=1)
delta (n=13)
omicron (n=6)
neg (n=72)
NA
WHOvariant

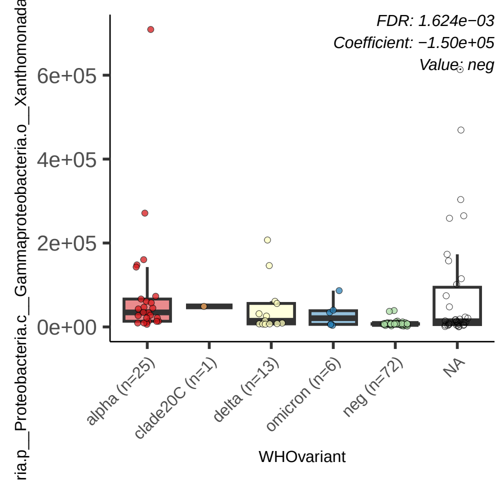

FDR: 1.624e−03
Coefficient: −1.50e+05
Value: neg
ria.p__Proteobacteria.c__Gammaproteobacteria.o__Xanthomonada
6e+05
4e+05
2e+05
0e+00
alpha (n=25)
clade20C (n=1)
delta (n=13)
omicron (n=6)
neg (n=72)
NA
WHOvariant

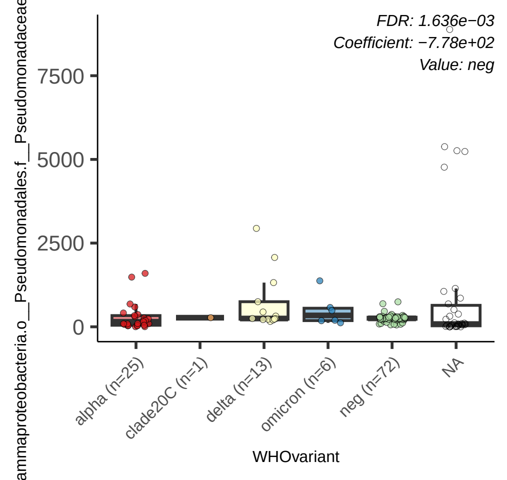

FDR: 1.636e−03
Coefficient: −7.78e+02
Value: neg
7500
5000
2500
0
ammaproteobacteria.o__Pseudomonadales.f__Pseudomonadaceae
alpha (n=25)
clade20C (n=1)
delta (n=13)
omicron (n=6)
neg (n=72)
NA
WHOvariant

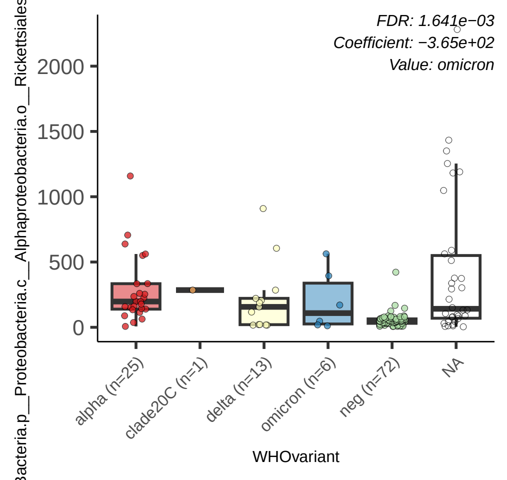

FDR: 1.641e−03
Coefficient: −3.65e+02
Value: omicron
Bacteria.p__Proteobacteria.c__Alphaproteobacteria.o__Rickettsiales
2000
1500
1000
500
0
alpha (n=25)
clade20C (n=1)
delta (n=13)
omicron (n=6)
neg (n=72)
NA
WHOvariant

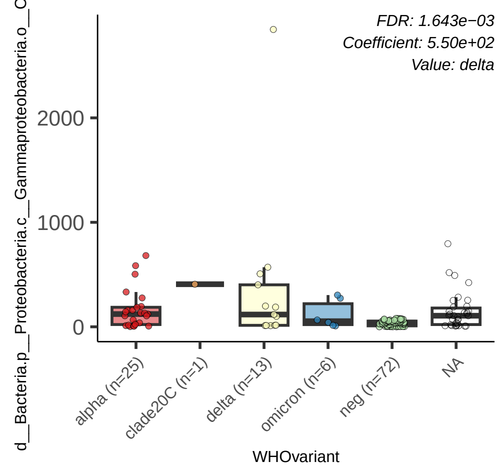

FDR: 1.643e−03
Coefficient: 5.50e+02
Value: delta
2000
1000
0
d__Bacteria.p__Proteobacteria.c__Gammaproteobacteria.o__C
alpha (n=25)
clade20C (n=1)
delta (n=13)
omicron (n=6)
neg (n=72)
NA
WHOvariant

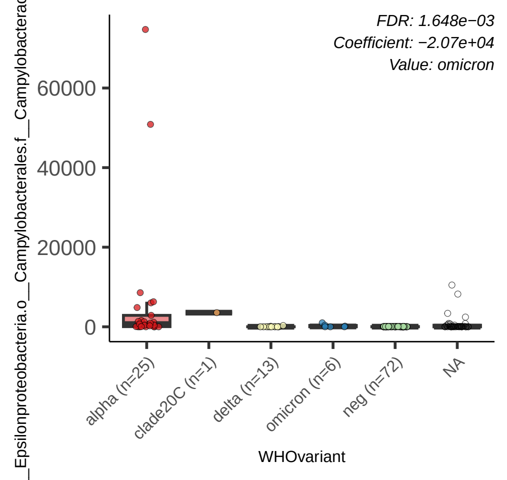
FDR: 1.648e−03
Coefficient: −2.07e+04
Value: omicron
_Epsilonproteobacteria.o__Campylobacterales.f__Campylobacterac
60000
40000
20000
0
alpha (n=25)
clade20C (n=1)
delta (n=13)
omicron (n=6)
neg (n=72)
NA
WHOvariant

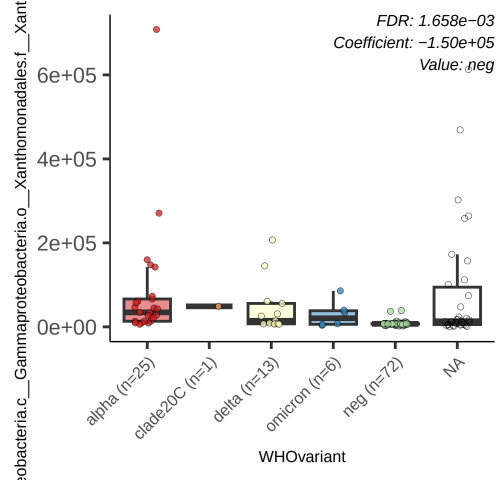
FDR: 1.658e−03
Coefficient: −1.50e+05
Value: neg
eobacteria.c__Gammaproteobacteria.o__Xanthomonadales.f__Xant
6e+05
4e+05
2e+05
0e+00
alpha (n=25)
clade20C (n=1)
delta (n=13)
omicron (n=6)
neg (n=72)
NA
WHOvariant

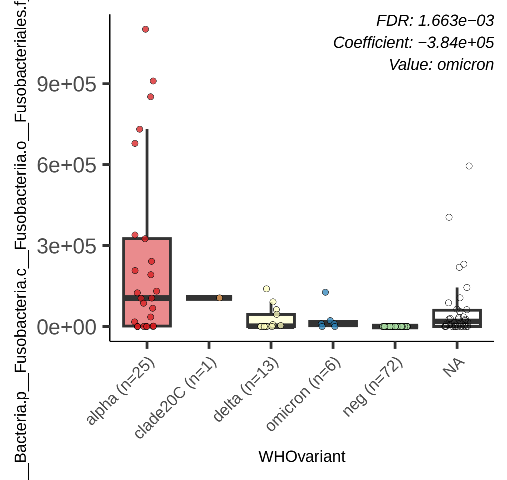

FDR: 1.663e−03
Coefficient: −3.84e+05
Value: omicron
__Bacteria.p__Fusobacteria.c__Fusobacteriia.o__Fusobacteriales.f_
9e+05
6e+05
3e+05
0e+00
alpha (n=25)
clade20C (n=1)
delta (n=13)
omicron (n=6)
neg (n=72)
NA
WHOvariant

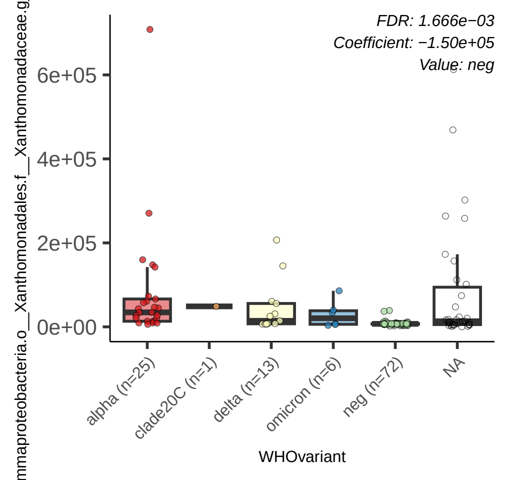
FDR: 1.666e−03
Coefficient: −1.50e+05
Value: neg
6e+05
4e+05
2e+05
0e+00
alpha (n=25)
clade20C (n=1)
delta (n=13)
omicron (n=6)
neg (n=72)
NA
WHOvariant
mmaproteobacteria.o__Xanthomonadales.f__Xanthomonadaceae.g

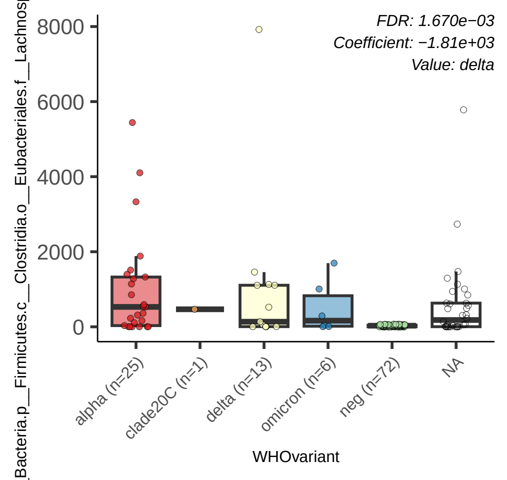
FDR: 1.670e−03
Coefficient: −1.81e+03
Value: delta
8000
6000
4000
2000
0
Bacteria.p__Firmicutes.c__Clostridia.o__Eubacteriales.f__Lachnos
alpha (n=25)
clade20C (n=1)
delta (n=13)
omicron (n=6)
neg (n=72)
NA
WHOvariant

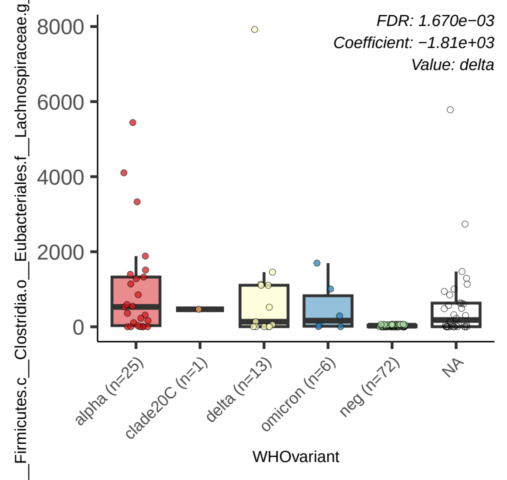
FDR: 1.670e−03
Coefficient: −1.81e+03
Value: delta
Firmicutes.c__Clostridia.o__Eubacteriales.f__Lachnospiraceae.g_
8000
6000
4000
2000
0
alpha (n=25)
clade20C (n=1)
delta (n=13)
omicron (n=6)
neg (n=72)
NA
WHOvariant

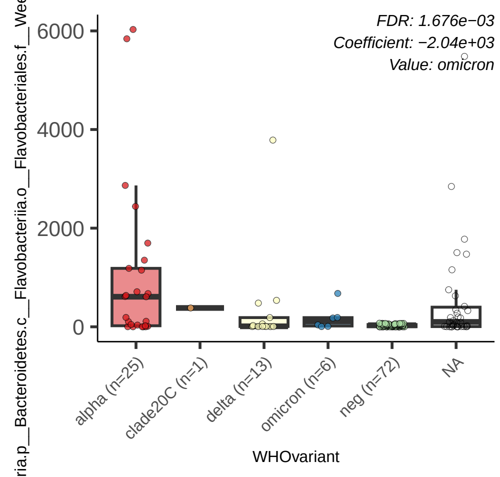

FDR: 1.676e−03
Coefficient: −2.04e+03
Value: omicron
6000
4000
2000
0
eria.p__Bacteroidetes.c__Flavobacteriia.o__Flavobacteriales.f__Wee
alpha (n=25)
clade20C (n=1)
delta (n=13)
omicron (n=6)
neg (n=72)
NA
WHOvariant

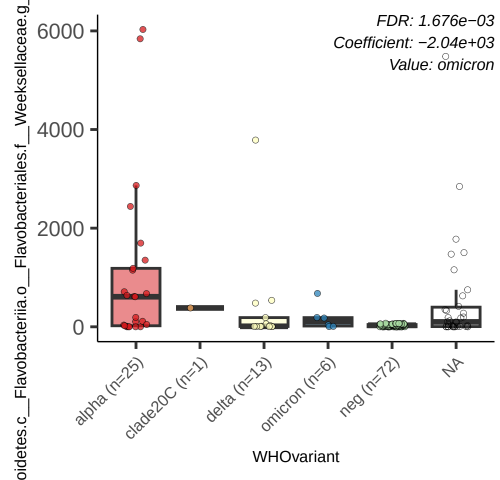

FDR: 1.676e−03
Coefficient: −2.04e+03
Value: omicron
oidetes.c__Flavobacteriia.o__Flavobacteriales.f__Weeksellaceae.g_
6000
4000
2000
0
alpha (n=25)
clade20C (n=1)
delta (n=13)
omicron (n=6)
neg (n=72)
NA
WHOvariant

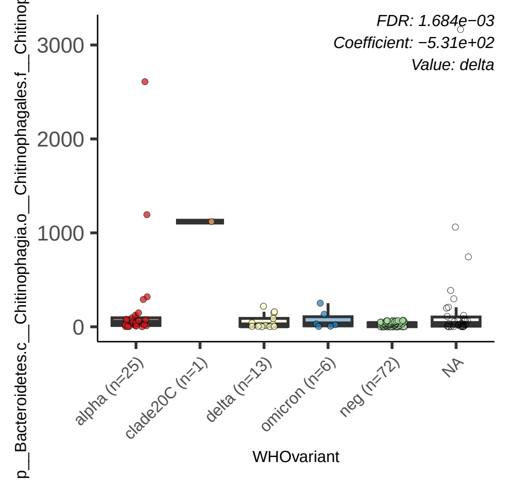

FDR: 1.684e−03
Coefficient: −5.31e+02
Value: delta
p__Bacteroidetes.c__Chitinophagia.o__Chitinophagales.f__Chitinop
3000
2000
1000
0
alpha (n=25)
clade20C (n=1)
delta (n=13)
omicron (n=6)
neg (n=72)
NA
WHOvariant

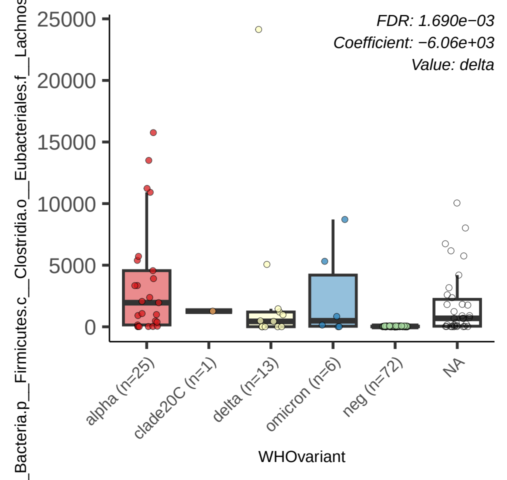

FDR: 1.690e−03
Coefficient: −6.06e+03
Value: delta
_Bacteria.p__Firmicutes.c__Clostridia.o__Eubacteriales.f__Lachnos
25000
20000
15000
10000
5000
0
alpha (n=25)
clade20C (n=1)
delta (n=13)
omicron (n=6)
neg (n=72)
NA
WHOvariant

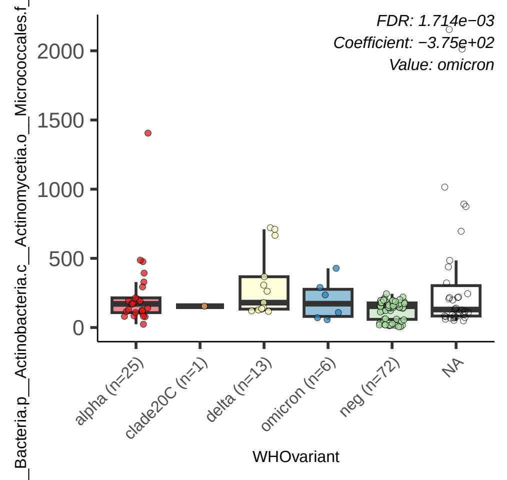

FDR: 1.714e−03
Coefficient: −3.75e+02
Value: omicron
Bacteria.p__Actinobacteria.c__Actinomycetia.o__Micrococcales.f__
0
500
1000
1500
2000
alpha (n=25)
clade20C (n=1)
delta (n=13)
omicron (n=6)
neg (n=72)
NA
WHOvariant

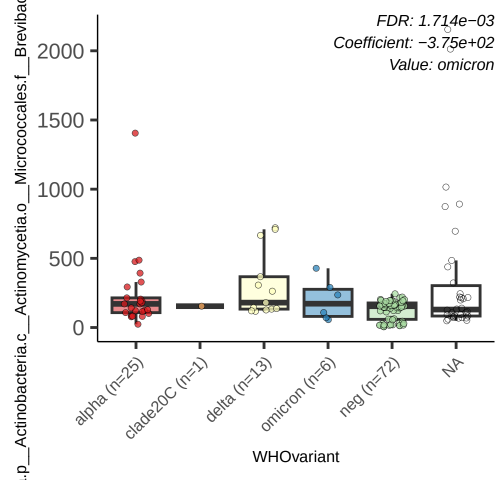
FDR: 1.714e−03
Coefficient: −3.75e+02
Value: omicron
p__Actinobacteria.c__Actinomycetia.o__Micrococcales.f__Brevibac
0
500
1000
1500
2000
alpha (n=25)
clade20C (n=1)
delta (n=13)
omicron (n=6)
neg (n=72)
NA
WHOvariant

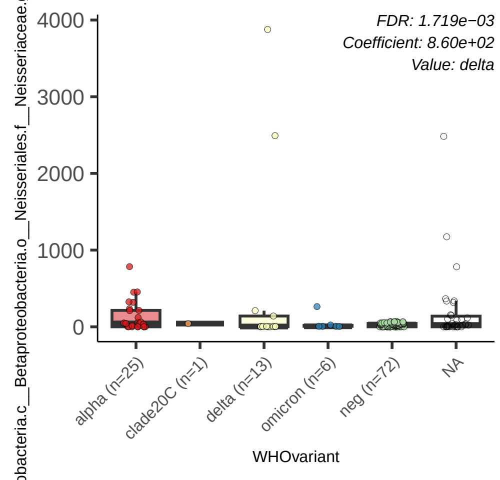

FDR: 1.719e−03
Coefficient: 8.60e+02
Value: delta
4000
3000
2000
1000
0
obacteria.c__Betaproteobacteria.o__Neisseriales.f__Neisseriaceae.
alpha (n=25)
clade20C (n=1)
delta (n=13)
omicron (n=6)
neg (n=72)
NA
WHOvariant

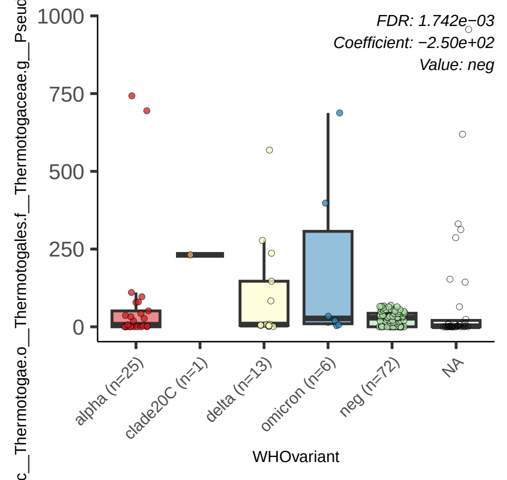

c__Thermotogae.o__Thermotogales.f__Thermotogaceae.g__Pseud
1000
750
500
250
0
FDR: 1.742e−03
Coefficient: −2.50e+02
Value: neg
alpha (n=25)
clade20C (n=1)
delta (n=13)
omicron (n=6)
neg (n=72)
NA
WHOvariant

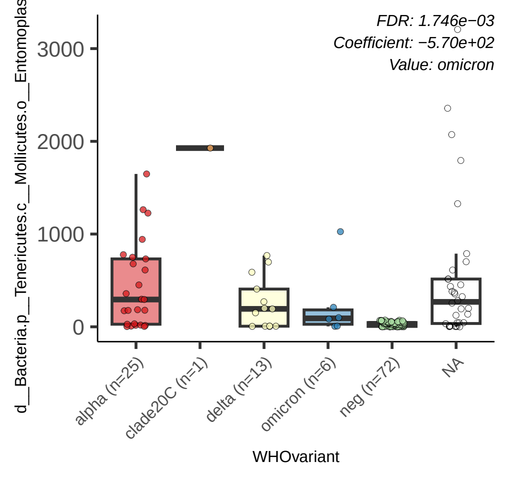

FDR: 1.746e−03
Coefficient: −5.70e+02
Value: omicron
d__Bacteria.p__Tenericutes.c__Mollicutes.o__Entomoplas
3000
2000
1000
0
alpha (n=25)
clade20C (n=1)
delta (n=13)
omicron (n=6)
neg (n=72)
NA
WHOvariant

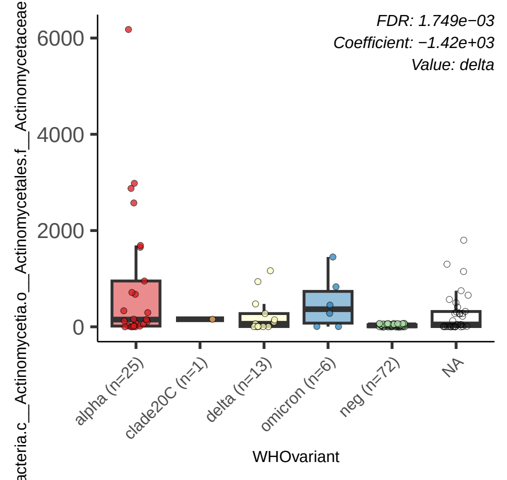

FDR: 1.749e−03
Coefficient: −1.42e+03
Value: delta
6000
4000
2000
0
acteria.c__Actinomycetia.o__Actinomycetales.f__Actinomycetaceae
alpha (n=25)
clade20C (n=1)
delta (n=13)
omicron (n=6)
neg (n=72)
NA
WHOvariant

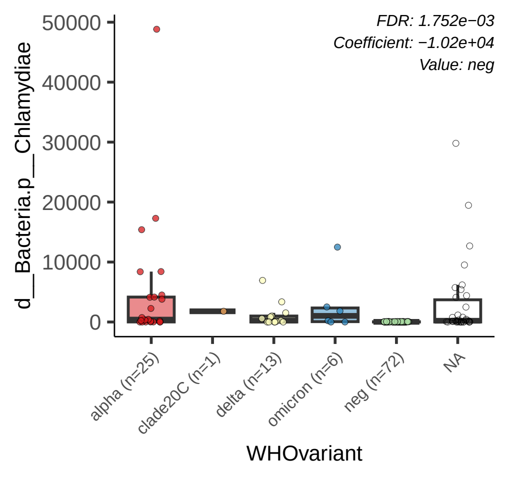
FDR: 1.752e−03
Coefficient: −1.02e+04
Value: neg
d__Bacteria.p__Chlamydiae
50000
40000
30000
20000
10000
0
alpha (n=25)
clade20C (n=1)
delta (n=13)
omicron (n=6)
neg (n=72)
NA
WHOvariant

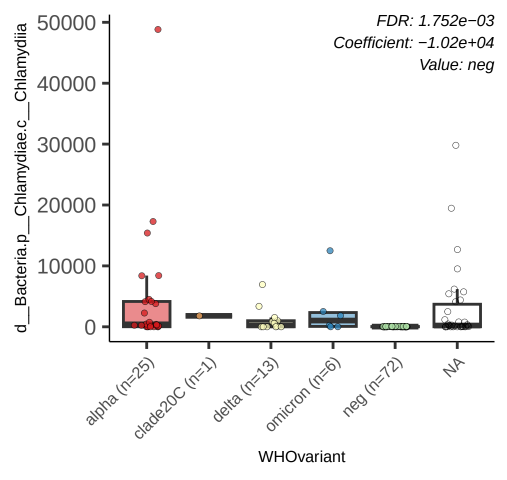

FDR: 1.752e−03
Coefficient: −1.02e+04
Value: neg
d__Bacteria.p__Chlamydiae.c__Chlamydiia
50000
40000
30000
20000
10000
0
alpha (n=25)
clade20C (n=1)
delta (n=13)
omicron (n=6)
neg (n=72)
NA
WHOvariant

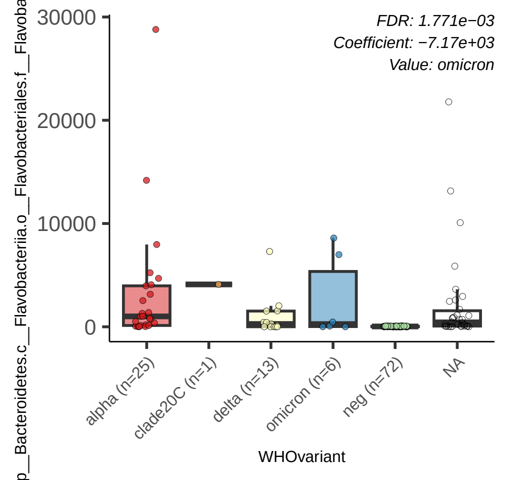
FDR: 1.771e−03
Coefficient: −7.17e+03
Value: omicron
30000
20000
10000
0
p__Bacteroidetes.c__Flavobacteriia.o__Flavobacteriales.f__Flavoba
alpha (n=25)
clade20C (n=1)
delta (n=13)
omicron (n=6)
neg (n=72)
NA
WHOvariant

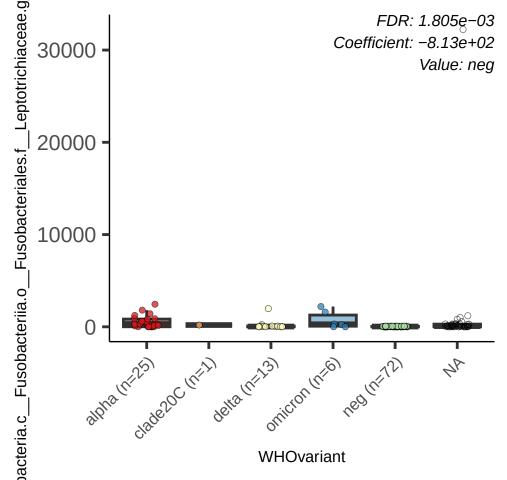

FDR: 1.805e−03
Coefficient: −8.13e+02
Value: neg
30000
20000
10000
0
bacteria.c__Fusobacteriia.o__Fusobacteriales.f__Leptotrichiaceae.g
alpha (n=25)
clade20C (n=1)
delta (n=13)
omicron (n=6)
neg (n=72)
NA
WHOvariant

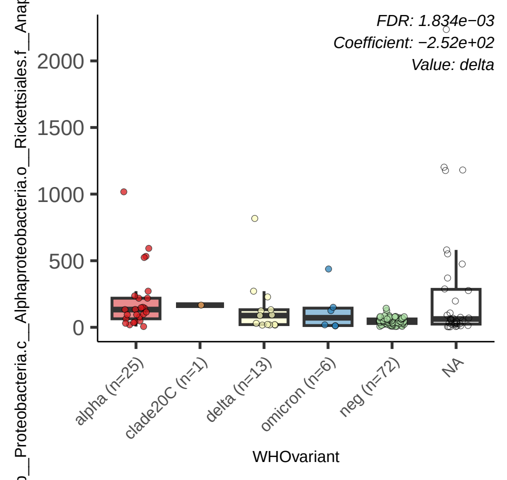

FDR: 1.834e−03
Coefficient: −2.52e+02
Value: delta
o__Proteobacteria.c__Alphaproteobacteria.o__Rickettsiales.f__Anap
2000
1500
1000
500
0
alpha (n=25)
clade20C (n=1)
delta (n=13)
omicron (n=6)
neg (n=72)
NA
WHOvariant

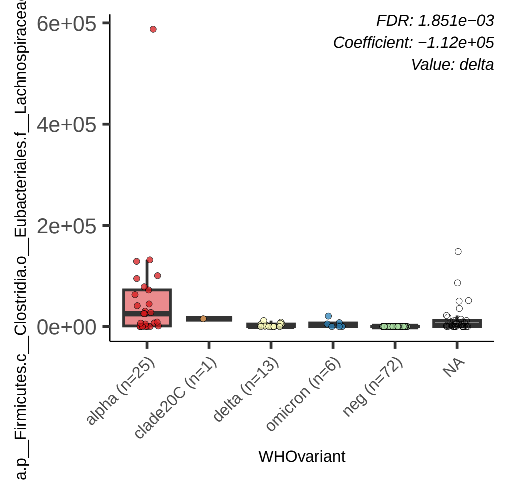

FDR: 1.851e−03
Coefficient: −1.12e+05
Value: delta
6e+05
4e+05
2e+05
0e+00
a.p__Firmicutes.c__Clostridia.o__Eubacteriales.f__Lachnospiracea
alpha (n=25)
clade20C (n=1)
delta (n=13)
omicron (n=6)
neg (n=72)
NA
WHOvariant

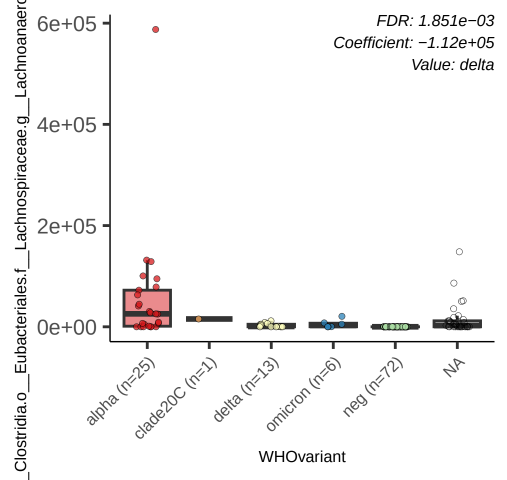

FDR: 1.851e−03
Coefficient: −1.12e+05
Value: delta
6e+05
4e+05
2e+05
0e+00
_Clostridia.o__Eubacteriales.f__Lachnospiraceae.g__Lachnoanaero
alpha (n=25)
clade20C (n=1)
delta (n=13)
omicron (n=6)
neg (n=72)
NA
WHOvariant

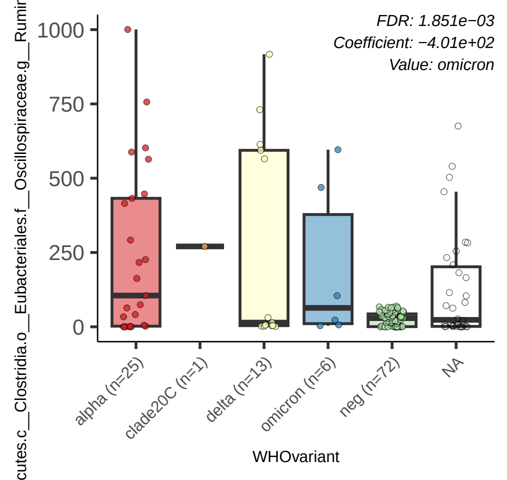

FDR: 1.851e−03
Coefficient: −4.01e+02
Value: omicron
cutes.c__Clostridia.o__Eubacteriales.f__Oscillospiraceae.g__Rumi
1000
750
500
250
0
alpha (n=25)
clade20C (n=1)
delta (n=13)
omicron (n=6)
neg (n=72)
NA
WHOvariant

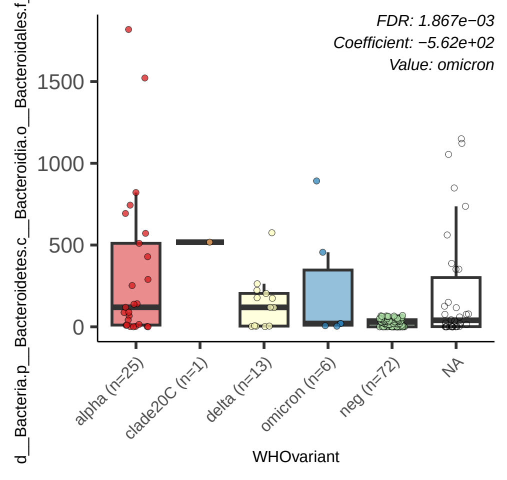

FDR: 1.867e−03
Coefficient: −5.62e+02
Value: omicron
d__Bacteria.p__Bacteroidetes.c__Bacteroidia.o__Bacteroidales.f_
0
500
1000
1500
alpha (n=25)
clade20C (n=1)
delta (n=13)
omicron (n=6)
neg (n=72)
NA
WHOvariant

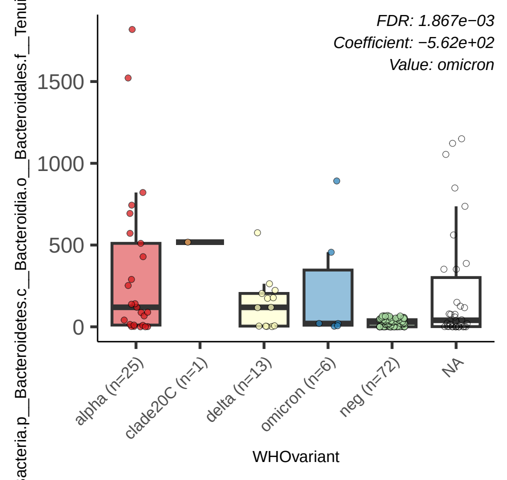

FDR: 1.867e−03
Coefficient: −5.62e+02
Value: omicron
Bacteria.p__Bacteroidetes.c__Bacteroidia.o__Bacteroidales.f__Tenu
1500
1000
500
0
alpha (n=25)
clade20C (n=1)
delta (n=13)
omicron (n=6)
neg (n=72)
NA
WHOvariant

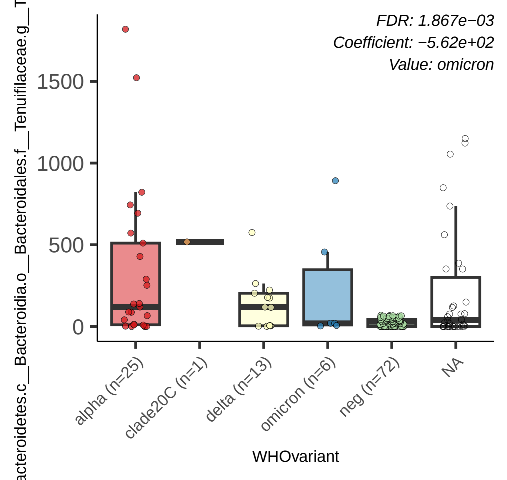

FDR: 1.867e−03
Coefficient: −5.62e+02
Value: omicron
acteroidetes.c__Bacteroidia.o__Bacteroidales.f__Tenuifilaceae.g__T
0
500
1000
1500
alpha (n=25)
clade20C (n=1)
delta (n=13)
omicron (n=6)
neg (n=72)
NA
WHOvariant

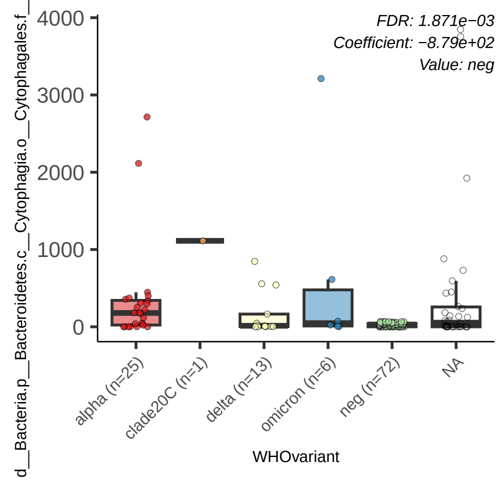

FDR: 1.871e−03
Coefficient: −8.79e+02
Value: neg
d__Bacteria.p__Bacteroidetes.c__Cytophagia.o__Cytophagales.f
4000
3000
2000
1000
0
alpha (n=25)
clade20C (n=1)
delta (n=13)
omicron (n=6)
neg (n=72)
NA
WHOvariant

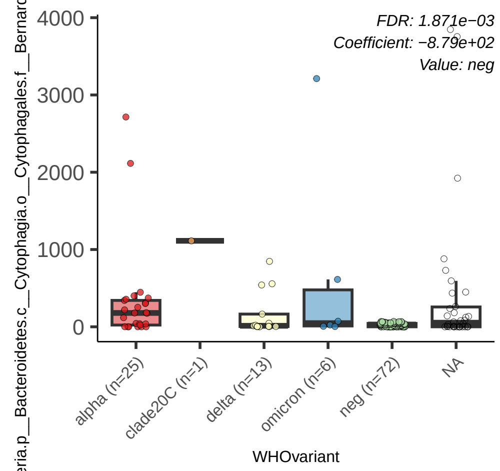
FDR: 1.871e−03
Coefficient: −8.79e+02
Value: neg
teria.p__Bacteroidetes.c__Cytophagia.o__Cytophagales.f__Bernard
4000
3000
2000
1000
0
alpha (n=25)
clade20C (n=1)
delta (n=13)
omicron (n=6)
neg (n=72)
NA
WHOvariant

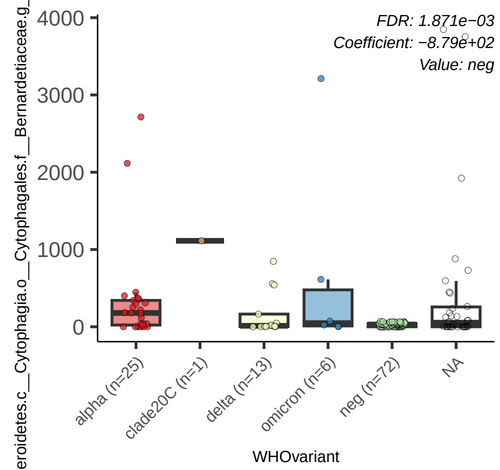

FDR: 1.871e−03
Coefficient: −8.79e+02
Value: neg
4000
3000
2000
1000
0
cteroidetes.c__Cytophagia.o__Cytophagales.f__Bernardetiaceae.g_
alpha (n=25)
clade20C (n=1)
delta (n=13)
omicron (n=6)
neg (n=72)
NA
WHOvariant

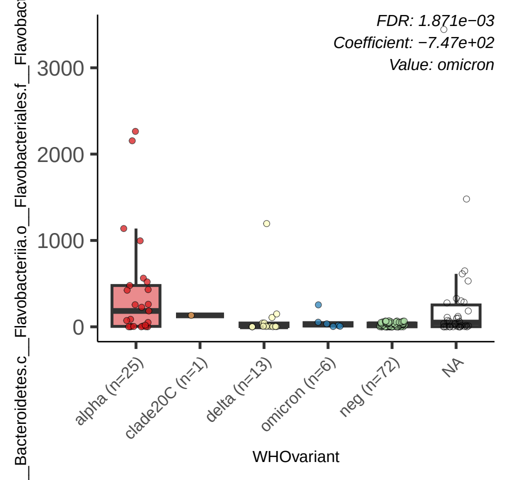
FDR: 1.871e−03
Coefficient: −7.47e+02
Value: omicron
3000
2000
1000
0
alpha (n=25)
clade20C (n=1)
delta (n=13)
omicron (n=6)
neg (n=72)
NA
WHOvariant
Bacteroidetes.c__Flavobacteriia.o__Flavobacteriales.f__Flavobact

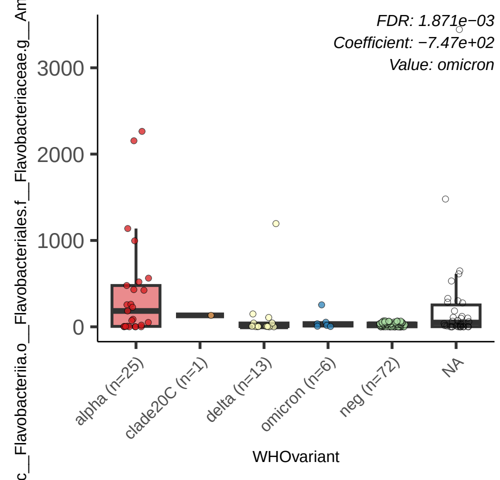

FDR: 1.871e−03
Coefficient: −7.47e+02
Value: omicron
c__Flavobacteriia.o__Flavobacteriales.f__Flavobacteriaceae.g__Am
3000
2000
1000
0
alpha (n=25)
clade20C (n=1)
delta (n=13)
omicron (n=6)
neg (n=72)
NA
WHOvariant

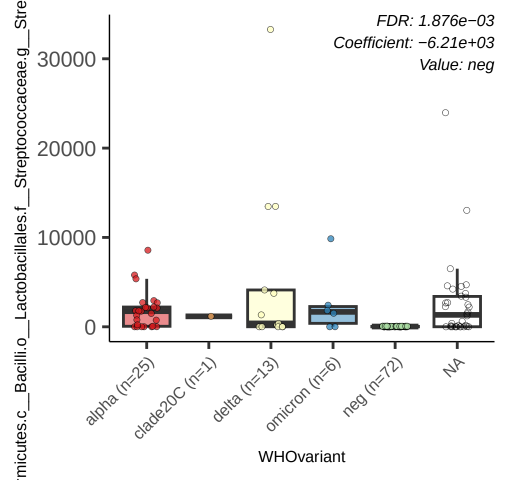

FDR: 1.876e−03
Coefficient: −6.21e+03
Value: neg
30000
20000
10000
0
rmicutes.c__Bacilli.o__Lactobacillales.f__Streptococcaceae.g__Stre
alpha (n=25)
clade20C (n=1)
delta (n=13)
omicron (n=6)
neg (n=72)
NA
WHOvariant

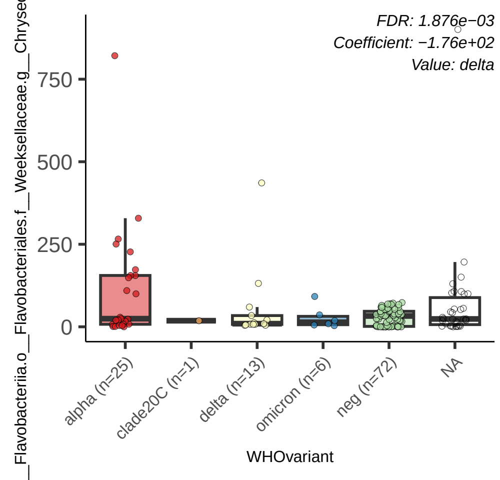

FDR: 1.876e−03
Coefficient: −1.76e+02
Value: delta
Flavobacteriia.o__Flavobacteriales.f__Weeksellaceae.g__Chryse
750
500
250
0
alpha (n=25)
clade20C (n=1)
delta (n=13)
omicron (n=6)
neg (n=72)
NA
WHOvariant

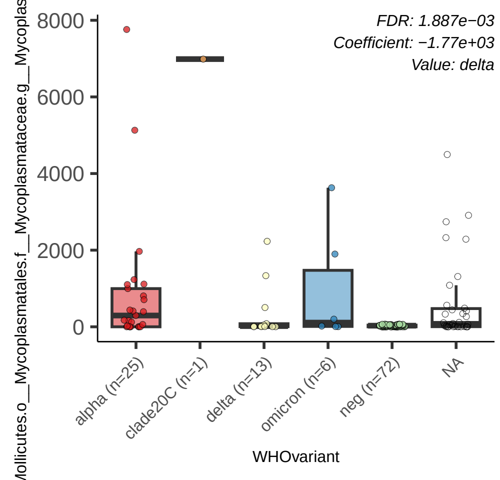

FDR: 1.887e−03
Coefficient: −1.77e+03
Value: delta
8000
6000
4000
2000
0
Mollicutes.o__Mycoplasmatales.f__Mycoplasmataceae.g__Mycoplas
alpha (n=25)
clade20C (n=1)
delta (n=13)
omicron (n=6)
neg (n=72)
NA
WHOvariant

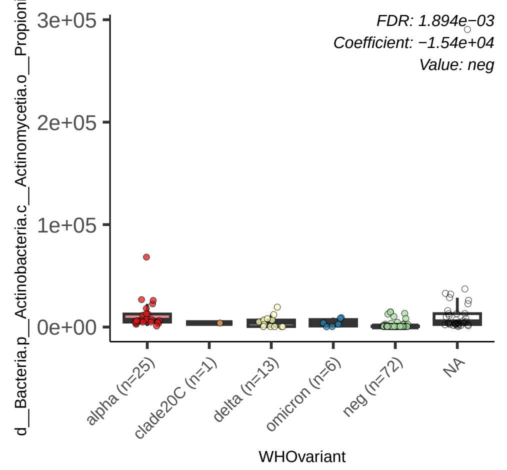

FDR: 1.894e−03
Coefficient: −1.54e+04
Value: neg
d__Bacteria.p__Actinobacteria.c__Actinomycetia.o__Propioni
3e+05
2e+05
1e+05
0e+00
alpha (n=25)
clade20C (n=1)
delta (n=13)
omicron (n=6)
neg (n=72)
NA
WHOvariant

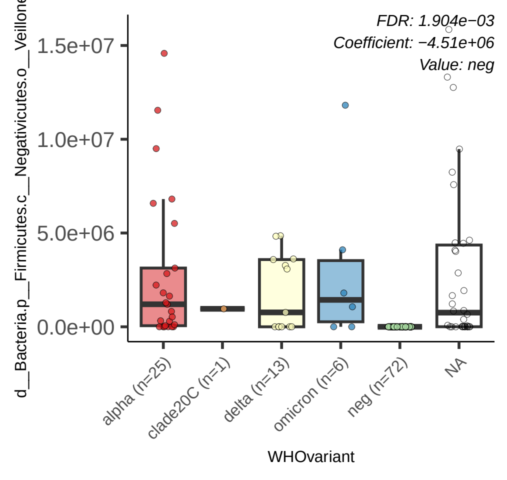

FDR: 1.904e−03
Coefficient: −4.51e+06
Value: neg
d__Bacteria.p__Firmicutes.c__Negativicutes.o__Veillone
1.5e+07
1.0e+07
5.0e+06
0.0e+00
alpha (n=25)
clade20C (n=1)
delta (n=13)
omicron (n=6)
neg (n=72)
NA
WHOvariant

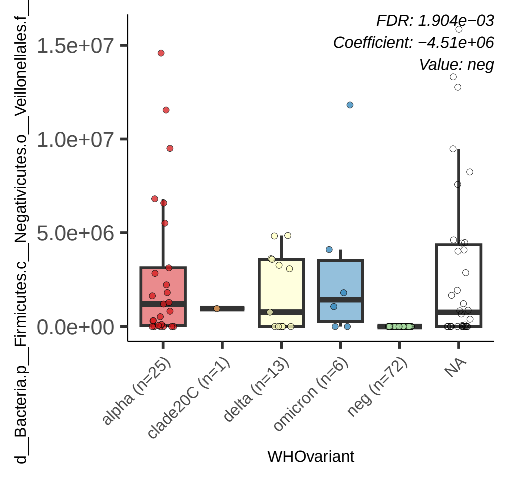

FDR: 1.904e−03
Coefficient: −4.51e+06
Value: neg
d__Bacteria.p__Firmicutes.c__Negativicutes.o__Veillonellales.f__
0.0e+00
5.0e+06
1.0e+07
1.5e+07
alpha (n=25)
clade20C (n=1)
delta (n=13)
omicron (n=6)
neg (n=72)
NA
WHOvariant

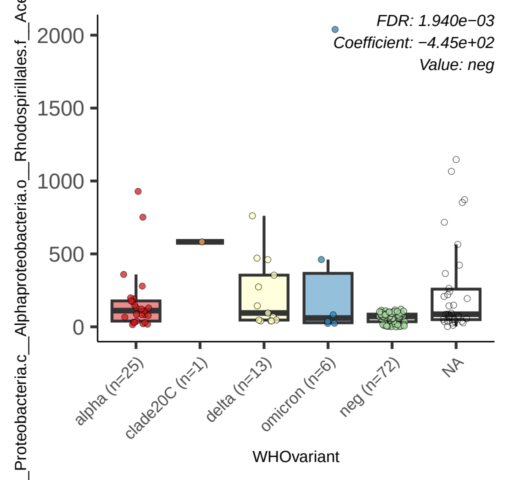
FDR: 1.940e−03
Coefficient: −4.45e+02
Value: neg
_Proteobacteria.c__Alphaproteobacteria.o__Rhodospirillales.f__Ace
2000
1500
1000
500
0
alpha (n=25)
clade20C (n=1)
delta (n=13)
omicron (n=6)
neg (n=72)
NA
WHOvariant

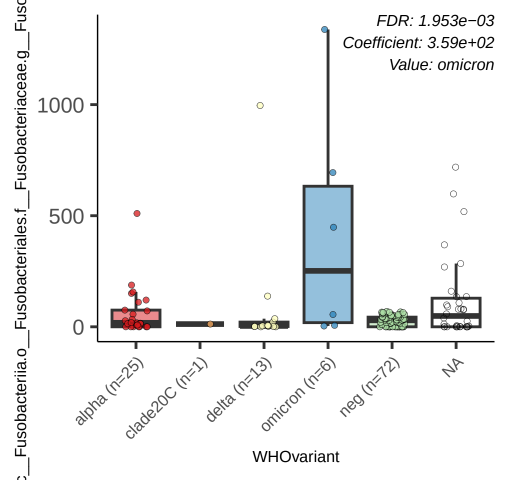

FDR: 1.953e−03
Coefficient: 3.59e+02
Value: omicron
1000
500
0
alpha (n=25)
clade20C (n=1)
delta (n=13)
omicron (n=6)
neg (n=72)
NA
WHOvariant
c__Fusobacteriia.o__Fusobacteriales.f__Fusobacteriaceae.g__Fuso

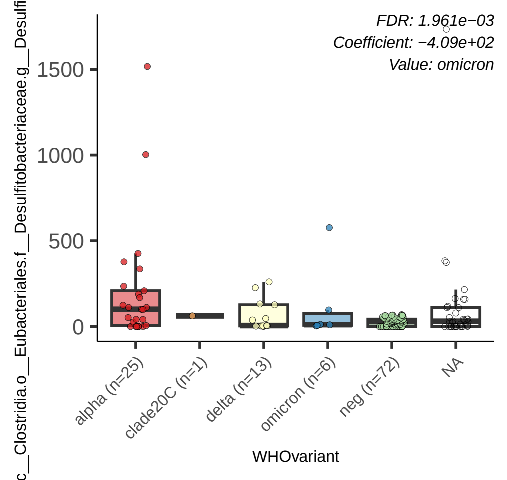
FDR: 1.961e−03
Coefficient: −4.09e+02
Value: omicron
c__Clostridia.o__Eubacteriales.f__Desulfitobacteriaceae.g__Desulfi
1500
1000
500
0
alpha (n=25)
clade20C (n=1)
delta (n=13)
omicron (n=6)
neg (n=72)
NA
WHOvariant

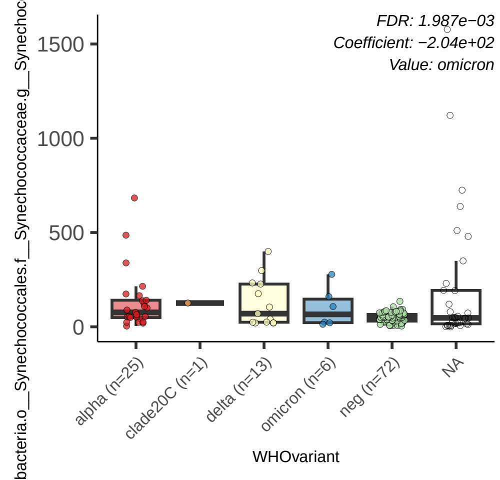

FDR: 1.987e−03
Coefficient: −2.04e+02
Value: omicron
1500
1000
500
0
bacteria.o__Synechococcales.f__Synechococcaceae.g__Synechoc
alpha (n=25)
clade20C (n=1)
delta (n=13)
omicron (n=6)
neg (n=72)
NA
WHOvariant

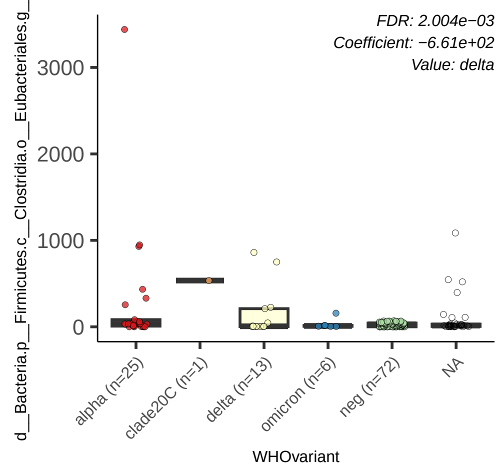

FDR: 2.004e−03
Coefficient: −6.61e+02
Value: delta
d__Bacteria.p__Firmicutes.c__Clostridia.o__Eubacteriales.g__
3000
2000
1000
0
alpha (n=25)
clade20C (n=1)
delta (n=13)
omicron (n=6)
neg (n=72)
NA
WHOvariant

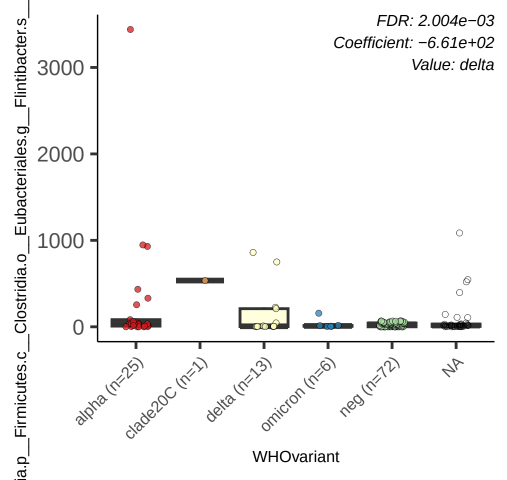

FDR: 2.004e−03
Coefficient: −6.61e+02
Value: delta
3000
2000
1000
0
ia.p__Firmicutes.c__Clostridia.o__Eubacteriales.g__Flintibacter.s__
alpha (n=25)
clade20C (n=1)
delta (n=13)
omicron (n=6)
neg (n=72)
NA
WHOvariant

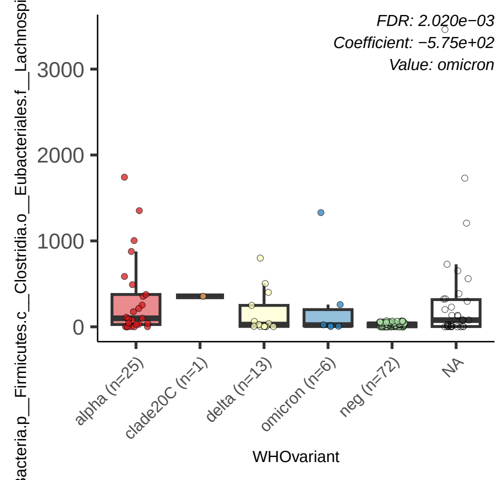
FDR: 2.020e−03
Coefficient: −5.75e+02
Value: omicron
Bacteria.p__Firmicutes.c__Clostridia.o__Eubacteriales.f__Lachnospi
3000
2000
1000
0
alpha (n=25)
clade20C (n=1)
delta (n=13)
omicron (n=6)
neg (n=72)
NA
WHOvariant

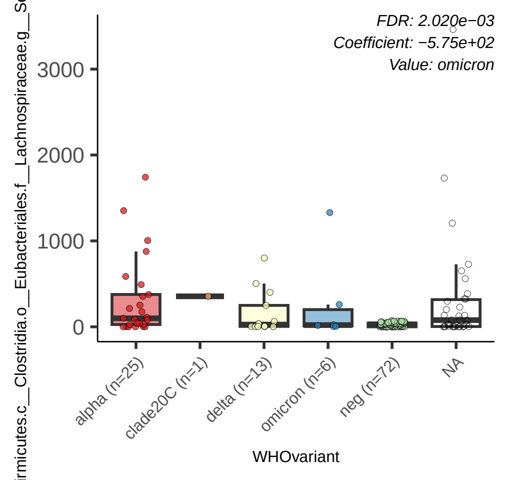

FDR: 2.020e−03
Coefficient: −5.75e+02
Value: omicron
3000
2000
1000
0
alpha (n=25)
clade20C (n=1)
delta (n=13)
omicron (n=6)
neg (n=72)
NA
WHOvariant

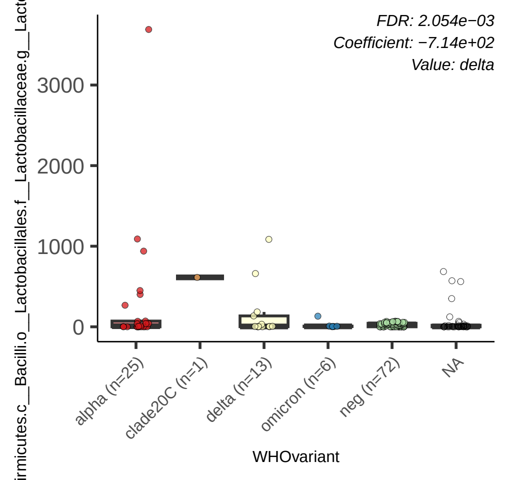

FDR: 2.054e−03
Coefficient: −7.14e+02
Value: delta
3000
2000
1000
0
irmicutes.c__Bacilli.o__Lactobacillales.f__Lactobacillaceae.g__Lacto
alpha (n=25)
clade20C (n=1)
delta (n=13)
omicron (n=6)
neg (n=72)
NA
WHOvariant

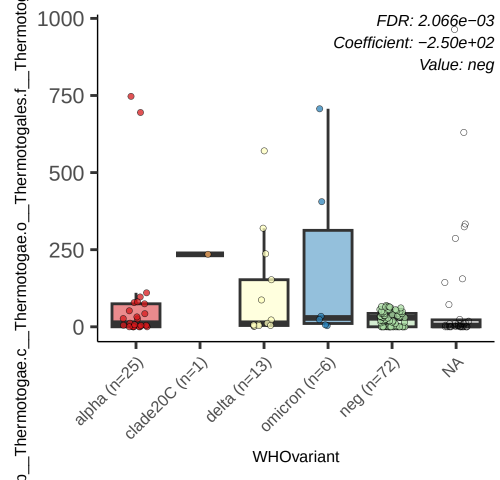

FDR: 2.066e−03
Coefficient: −2.50e+02
Value: neg
1000
750
500
250
0
alpha (n=25)
clade20C (n=1)
delta (n=13)
omicron (n=6)
neg (n=72)
NA
WHOvariant

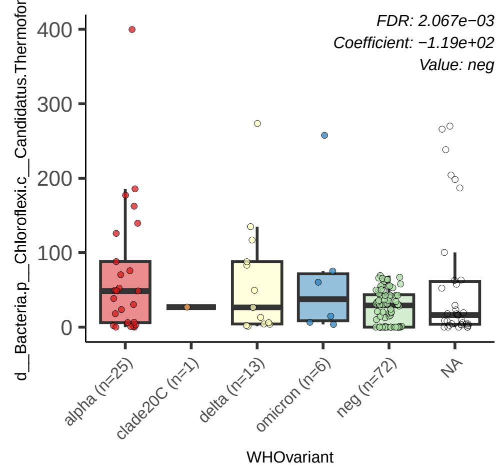
FDR: 2.067e−03
Coefficient: −1.19e+02
Value: neg
d__Bacteria.p__Chloroflexi.c__Candidatus.Thermofor
400
300
200
100
0
alpha (n=25)
clade20C (n=1)
delta (n=13)
omicron (n=6)
neg (n=72)
NA
WHOvariant

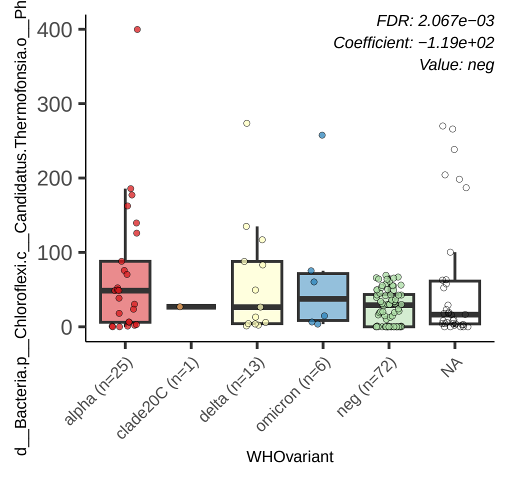

FDR: 2.067e−03
Coefficient: −1.19e+02
Value: neg
d__Bacteria.p__Chloroflexi.c__Candidatus.Thermofonsia.o__Ph
400
300
200
100
0
alpha (n=25)
clade20C (n=1)
delta (n=13)
omicron (n=6)
neg (n=72)
NA
WHOvariant

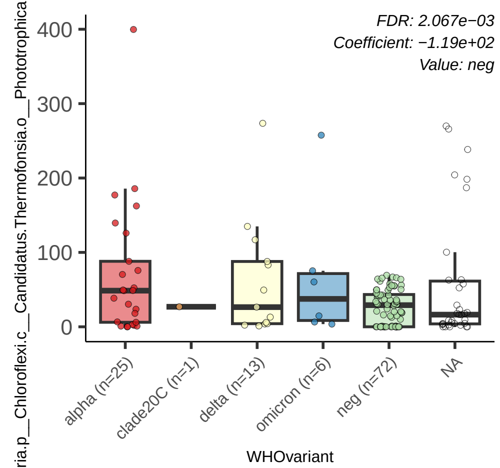

FDR: 2.067e−03
Coefficient: −1.19e+02
Value: neg
teria.p__Chloroflexi.c__Candidatus.Thermofonsia.o__Phototrophica
400
300
200
100
0
alpha (n=25)
clade20C (n=1)
delta (n=13)
omicron (n=6)
neg (n=72)
NA
WHOvariant

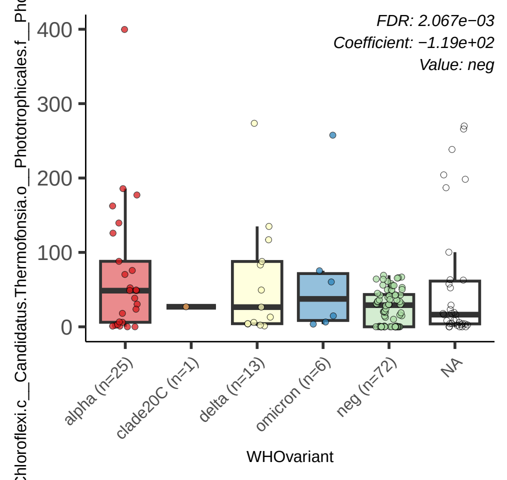

FDR: 2.067e−03
Coefficient: −1.19e+02
Value: neg
Chloroflexi.c__Candidatus.Thermofonsia.o__Phototrophicales.f__Pho
400
300
200
100
0
alpha (n=25)
clade20C (n=1)
delta (n=13)
omicron (n=6)
neg (n=72)
NA
WHOvariant

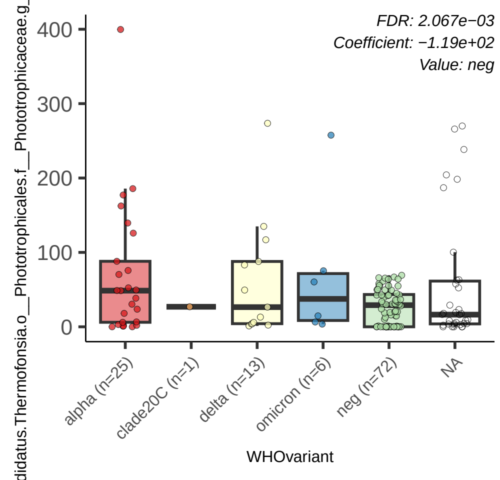

FDR: 2.067e−03
Coefficient: −1.19e+02
Value: neg
didatus.Thermofonsia.o__Phototrophicales.f__Phototrophicaceae.g_
400
300
200
100
0
alpha (n=25)
clade20C (n=1)
delta (n=13)
omicron (n=6)
neg (n=72)
NA
WHOvariant

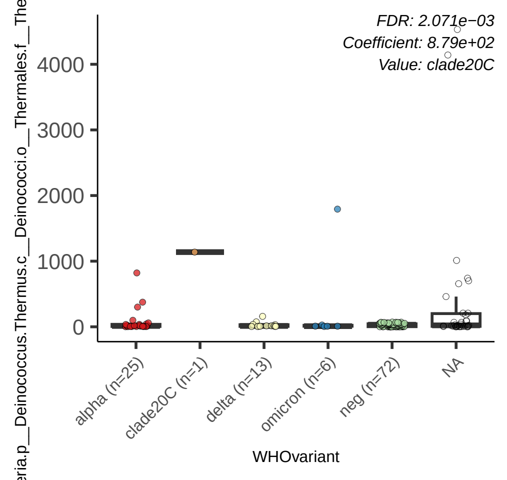

FDR: 2.071e−03
Coefficient: 8.79e+02
Value: clade20C
4000
3000
2000
1000
0
alpha (n=25)
clade20C (n=1)
delta (n=13)
omicron (n=6)
neg (n=72)
NA
WHOvariant
eria.p__Deinococcus.Thermus.c__Deinococci.o__Thermales.f__The

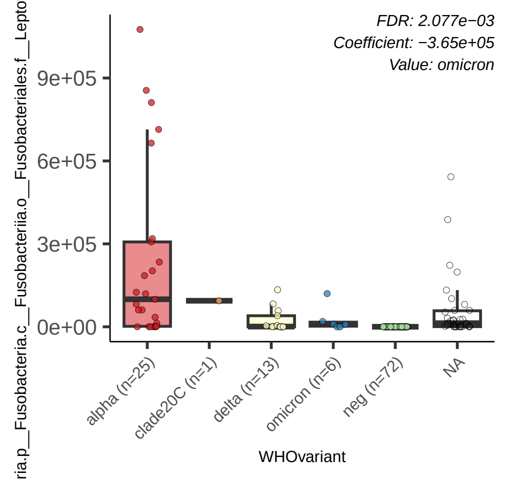
FDR: 2.077e−03
Coefficient: −3.65e+05
Value: omicron
ria.p__Fusobacteria.c__Fusobacteriia.o__Fusobacteriales.f__Lepto
9e+05
6e+05
3e+05
0e+00
alpha (n=25)
clade20C (n=1)
delta (n=13)
omicron (n=6)
neg (n=72)
NA
WHOvariant

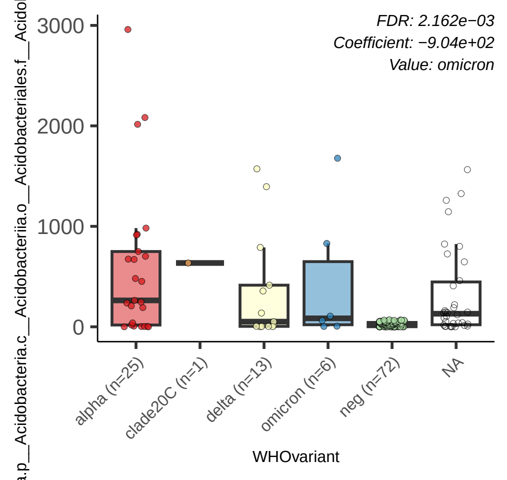

FDR: 2.162e−03
Coefficient: −9.04e+02
Value: omicron
a.p__Acidobacteria.c__Acidobacteriia.o__Acidobacteriales.f__Acidob
3000
2000
1000
0
alpha (n=25)
clade20C (n=1)
delta (n=13)
omicron (n=6)
neg (n=72)
NA
WHOvariant

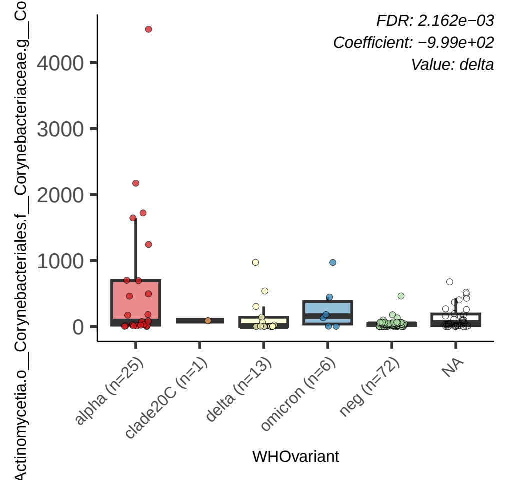
FDR: 2.162e−03
Coefficient: −9.99e+02
Value: delta
Actinomycetia.o__Corynebacteriales.f__Corynebacteriaceae.g__Co
0
1000
2000
3000
4000
alpha (n=25)
clade20C (n=1)
delta (n=13)
omicron (n=6)
neg (n=72)
NA
WHOvariant

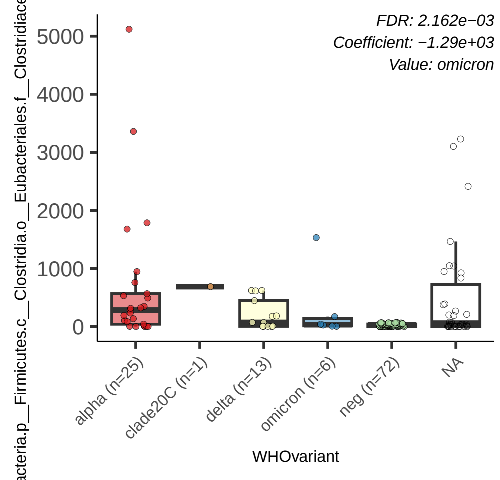

FDR: 2.162e−03
Coefficient: −1.29e+03
Value: omicron
5000
4000
3000
2000
1000
0
acteria.p__Firmicutes.c__Clostridia.o__Eubacteriales.f__Clostridiace
alpha (n=25)
clade20C (n=1)
delta (n=13)
omicron (n=6)
neg (n=72)
NA
WHOvariant

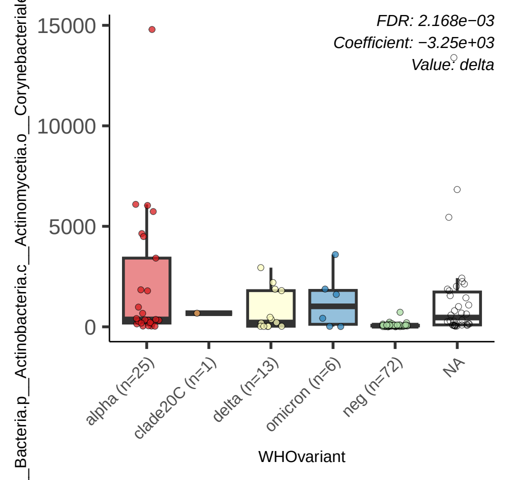
FDR: 2.168e−03
Coefficient: −3.25e+03
Value: delta
15000
10000
5000
0
alpha (n=25)
clade20C (n=1)
delta (n=13)
omicron (n=6)
neg (n=72)
NA
WHOvariant
Bacteria.p__Actinobacteria.c__Actinomycetia.o__Corynebacteriale

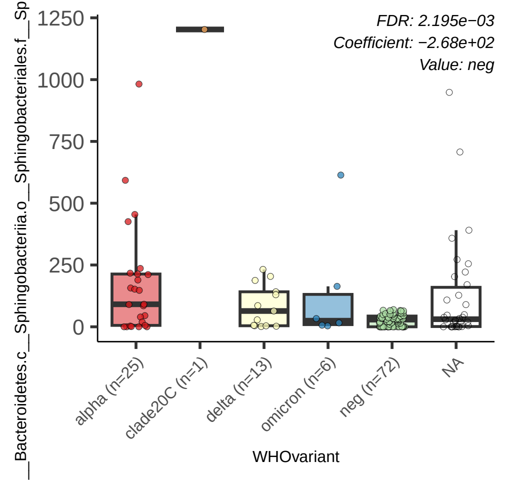
FDR: 2.195e−03
Coefficient: −2.68e+02
Value: neg
1250
1000
750
500
250
0
Bacteroidetes.c__Sphingobacteriia.o__Sphingobacteriales.f__Sp
alpha (n=25)
clade20C (n=1)
delta (n=13)
omicron (n=6)
neg (n=72)
NA
WHOvariant

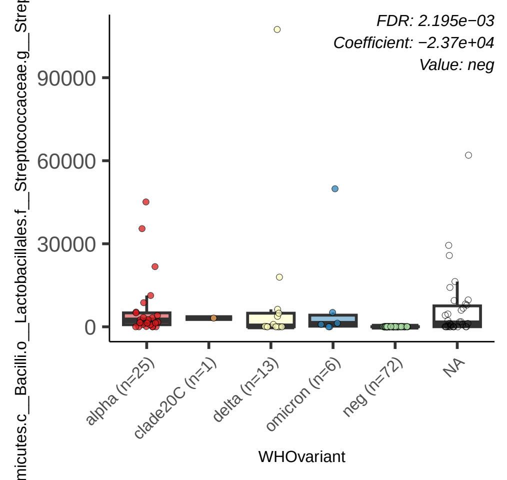

FDR: 2.195e−03
Coefficient: −2.37e+04
Value: neg
micutes.c__Bacilli.o__Lactobacillales.f__Streptococcaceae.g__Strep
90000
60000
30000
0
alpha (n=25)
clade20C (n=1)
delta (n=13)
omicron (n=6)
neg (n=72)
NA
WHOvariant

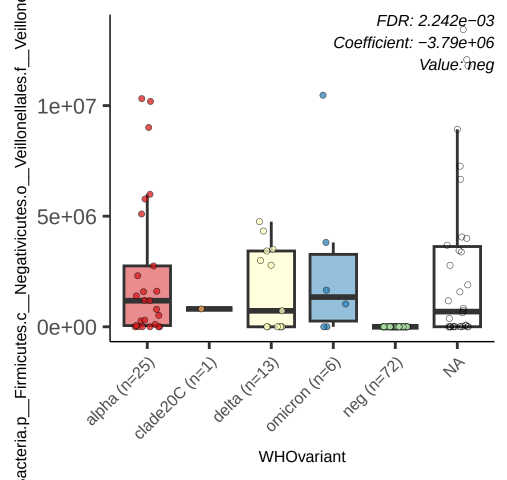

FDR: 2.242e−03
Coefficient: −3.79e+06
Value: neg
1e+07
5e+06
0e+00
Firmicutes.c__Negativicutes.o__Veillonellales.f__Veillone
acteria.p__
alpha (n=25)
clade20C (n=1)
delta (n=13)
omicron (n=6)
neg (n=72)
NA
WHOvariant

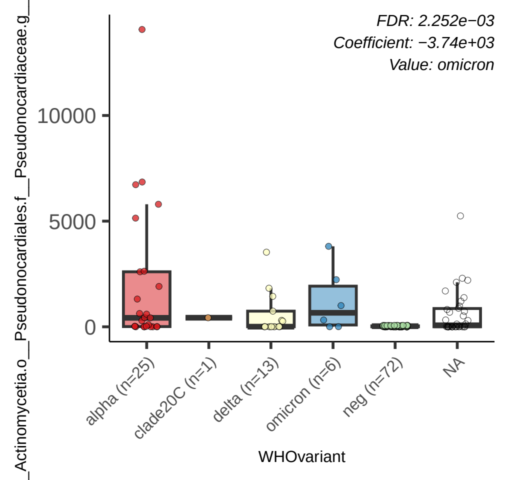

FDR: 2.252e−03
Coefficient: −3.74e+03
Value: omicron
Actinomycetia.o__Pseudonocardiales.f__Pseudonocardiaceae.g__
10000
5000
0
alpha (n=25)
clade20C (n=1)
delta (n=13)
omicron (n=6)
neg (n=72)
NA
WHOvariant

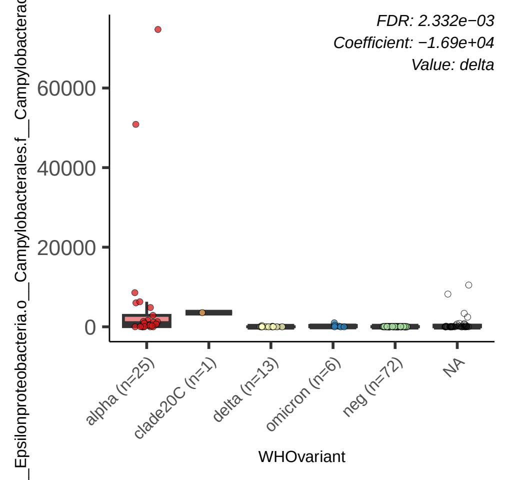

FDR: 2.332e−03
Coefficient: −1.69e+04
Value: delta
60000
40000
20000
0
_Epsilonproteobacteria.o__Campylobacterales.f__Campylobacterac
alpha (n=25)
clade20C (n=1)
delta (n=13)
omicron (n=6)
neg (n=72)
NA
WHOvariant

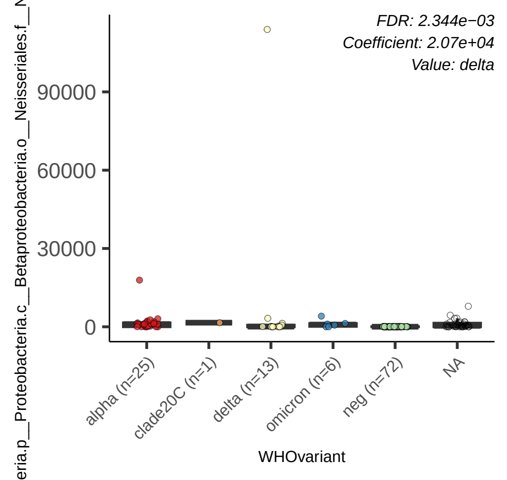

FDR: 2.344e−03
Coefficient: 2.07e+04
Value: delta
eria.p__Proteobacteria.c__Betaproteobacteria.o__Neisseriales.f__N
90000
60000
30000
0
alpha (n=25)
clade20C (n=1)
delta (n=13)
omicron (n=6)
neg (n=72)
NA
WHOvariant

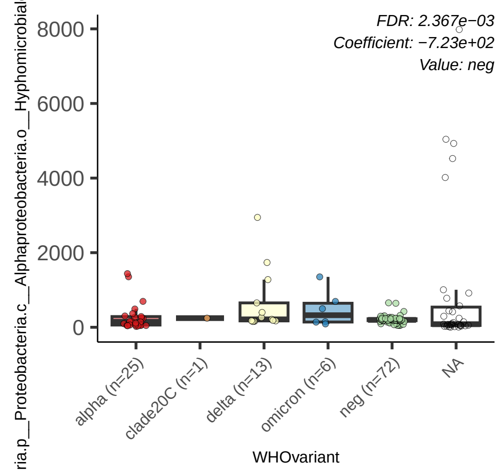
FDR: 2.367e−03
Coefficient: −7.23e+02
Value: neg
8000
6000
4000
2000
0
teria.p__Proteobacteria.c__Alphaproteobacteria.o__Hyphomicrobial
alpha (n=25)
clade20C (n=1)
delta (n=13)
omicron (n=6)
neg (n=72)
NA
WHOvariant

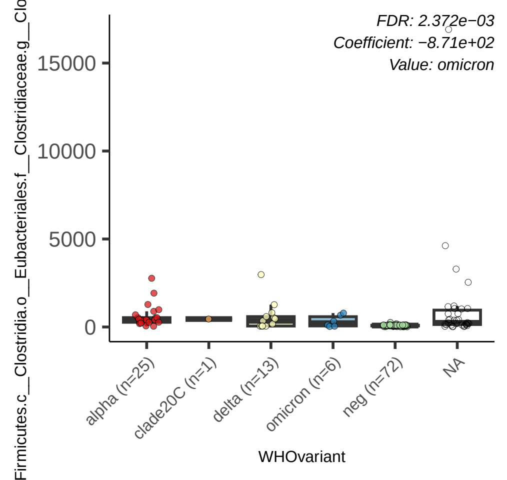

FDR: 2.372e−03
Coefficient: −8.71e+02
Value: omicron
Firmicutes.c__Clostridia.o__Eubacteriales.f__Clostridiaceae.g__Clo
15000
10000
5000
0
alpha (n=25)
clade20C (n=1)
delta (n=13)
omicron (n=6)
neg (n=72)
NA
WHOvariant

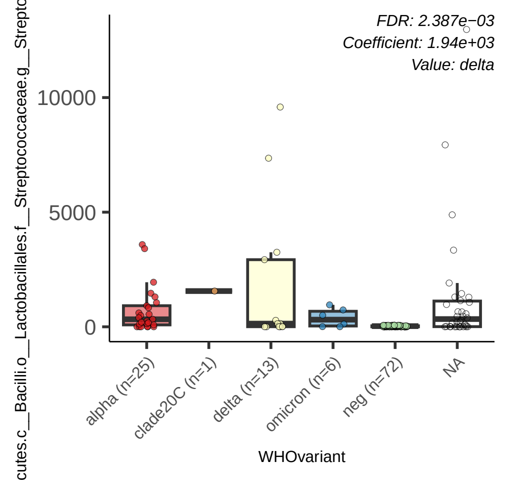
FDR: 2.387e−03
Coefficient: 1.94e+03
Value: delta
cutes.c__Bacilli.o__Lactobacillales.f__Streptococcaceae.g__Strepto
10000
5000
0
alpha (n=25)
clade20C (n=1)
delta (n=13)
omicron (n=6)
neg (n=72)
NA
WHOvariant

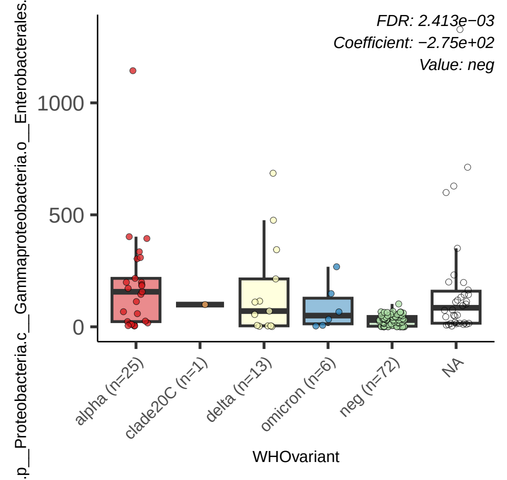
FDR: 2.413e−03
Coefficient: −2.75e+02
Value: neg
p__Proteobacteria.c__Gammaproteobacteria.o__Enterobacterales.
1000
500
0
alpha (n=25)
clade20C (n=1)
delta (n=13)
omicron (n=6)
neg (n=72)
NA
WHOvariant

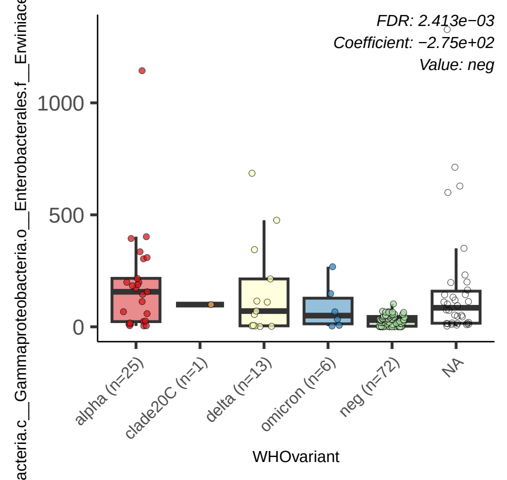

FDR: 2.413e−03
Coefficient: −2.75e+02
Value: neg
acteria.c__Gammaproteobacteria.o__Enterobacterales.f__Erwiniace
1000
500
0
alpha (n=25)
clade20C (n=1)
delta (n=13)
omicron (n=6)
neg (n=72)
NA
WHOvariant

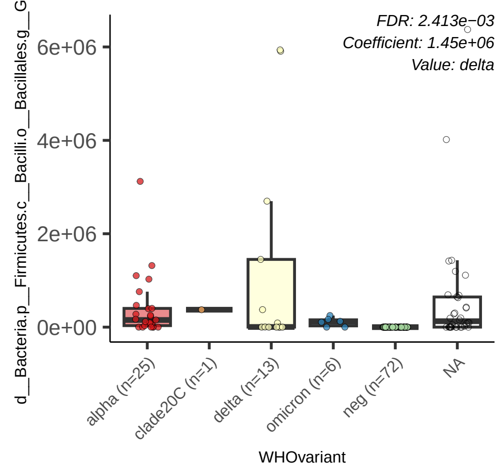

FDR: 2.413e−03
Coefficient: 1.45e+06
Value: delta
6e+06
4e+06
2e+06
0e+00
d__Bacteria.p__Firmicutes.c__Bacilli.o__Bacillales.g__G
alpha (n=25)
clade20C (n=1)
delta (n=13)
omicron (n=6)
neg (n=72)
NA
WHOvariant

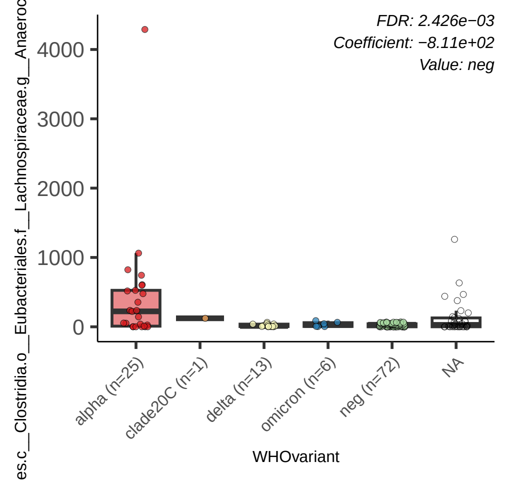

FDR: 2.426e−03
Coefficient: −8.11e+02
Value: neg
es.c__Clostridia.o__Eubacteriales.f__Lachnospiraceae.g__Anaeroc
4000
3000
2000
1000
0
alpha (n=25)
clade20C (n=1)
delta (n=13)
omicron (n=6)
neg (n=72)
NA
WHOvariant

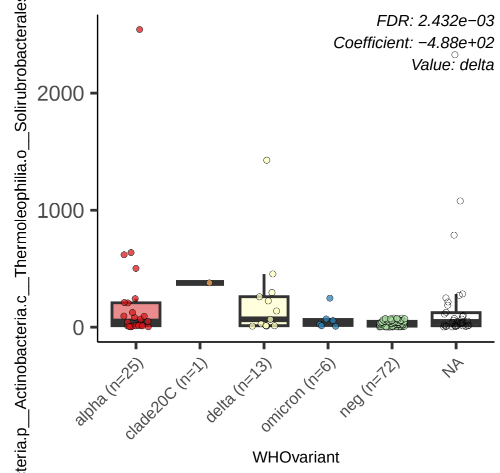

FDR: 2.432e−03
Coefficient: −4.88e+02
Value: delta
cteria.p__Actinobacteria.c__Thermoleophilia.o__Solirubrobacterales
0
1000
2000
alpha (n=25)
clade20C (n=1)
delta (n=13)
omicron (n=6)
neg (n=72)
NA
WHOvariant

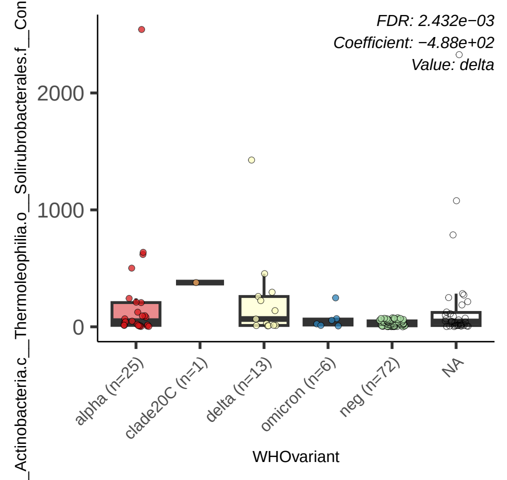

FDR: 2.432e−03
Coefficient: −4.88e+02
Value: delta
Actinobacteria.c__Thermoleophilia.o__Solirubrobacterales.f__Con
2000
1000
0
alpha (n=25)
clade20C (n=1)
delta (n=13)
omicron (n=6)
neg (n=72)
NA
WHOvariant

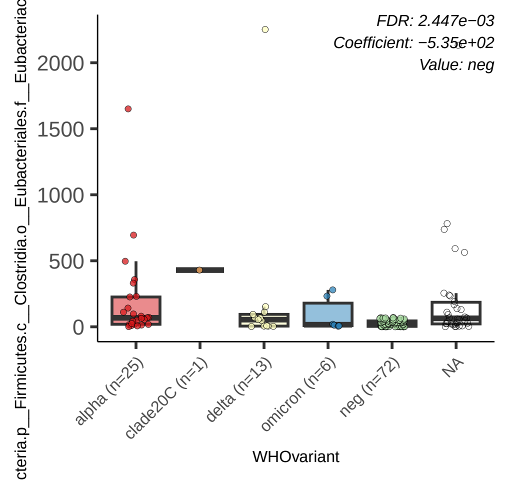

FDR: 2.447e−03
Coefficient: −5.35e+02
Value: neg
cteria.p__Firmicutes.c__Clostridia.o__Eubacteriales.f__Eubacteriac
2000
1500
1000
500
0
alpha (n=25)
clade20C (n=1)
delta (n=13)
omicron (n=6)
neg (n=72)
NA
WHOvariant

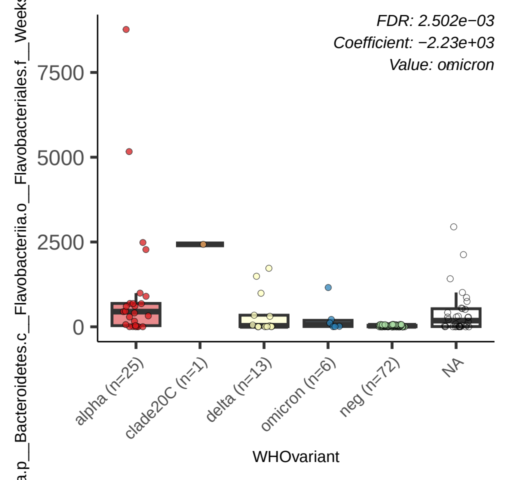

FDR: 2.502e−03
Coefficient: −2.23e+03
Value: omicron
a.p__Bacteroidetes.c__Flavobacteriia.o__Flavobacteriales.f__Weeks
7500
5000
2500
0
alpha (n=25)
clade20C (n=1)
delta (n=13)
omicron (n=6)
neg (n=72)
NA
WHOvariant

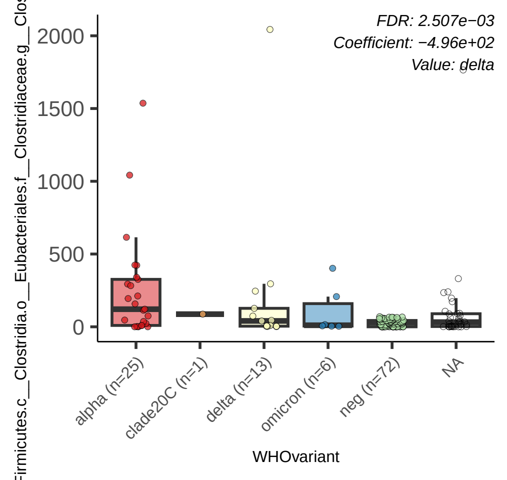
FDR: 2.507e−03
Coefficient: −4.96e+02
Value: delta
2000
1500
1000
500
0
Firmicutes.c__Clostridia.o__Eubacteriales.f__Clostridiaceae.g__Clos
alpha (n=25)
clade20C (n=1)
delta (n=13)
omicron (n=6)
neg (n=72)
NA
WHOvariant

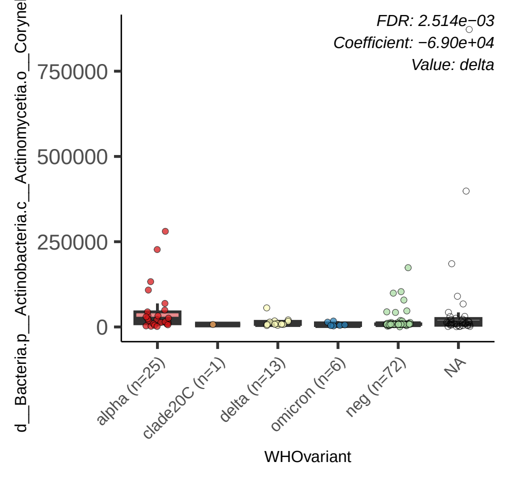
FDR: 2.514e−03
Coefficient: −6.90e+04
Value: delta
d__Bacteria.p__Actinobacteria.c__Actinomycetia.o__Coryne
750000
500000
250000
0
alpha (n=25)
clade20C (n=1)
delta (n=13)
omicron (n=6)
neg (n=72)
NA
WHOvariant

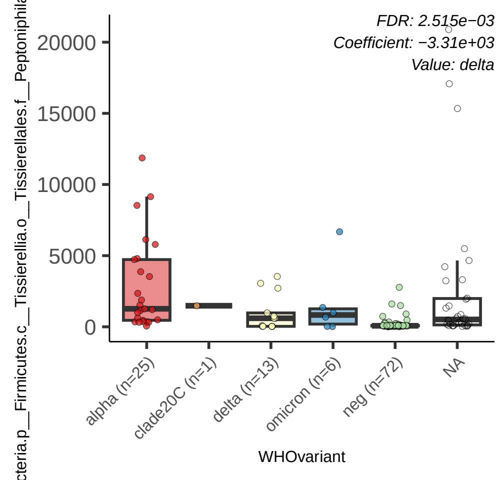
FDR: 2.515e−03
Coefficient: −3.31e+03
Value: delta
20000
15000
10000
5000
0
cteria.p__Firmicutes.c__Tissierellia.o__Tissierellales.f__Peptoniphila
alpha (n=25)
clade20C (n=1)
delta (n=13)
omicron (n=6)
neg (n=72)
NA
WHOvariant

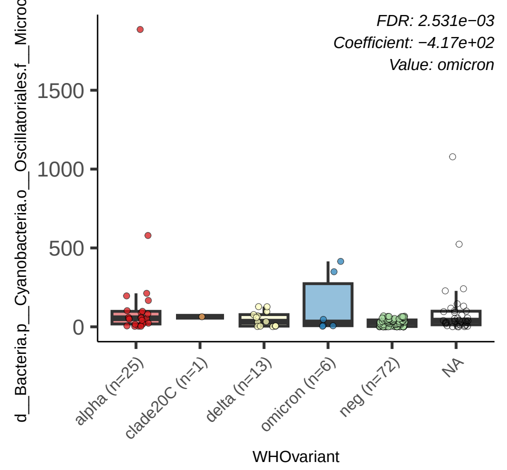

FDR: 2.531e−03
Coefficient: −4.17e+02
Value: omicron
d__Bacteria.p__Cyanobacteria.o__Oscillatoriales.f__Microc
1500
1000
500
0
alpha (n=25)
clade20C (n=1)
delta (n=13)
omicron (n=6)
neg (n=72)
NA
WHOvariant

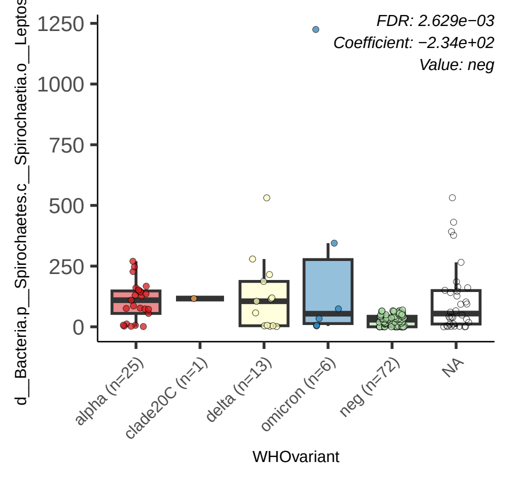
FDR: 2.629e−03
Coefficient: −2.34e+02
Value: neg
d__Bacteria.p__Spirochaetes.c__Spirochaetia.o__Leptos
1250
1000
750
500
250
0
alpha (n=25)
clade20C (n=1)
delta (n=13)
omicron (n=6)
neg (n=72)
NA
WHOvariant

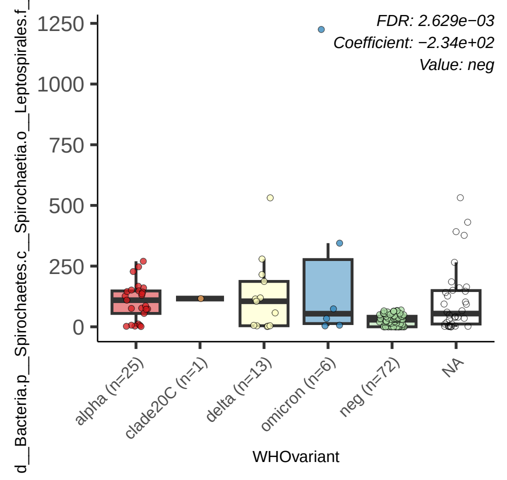
FDR: 2.629e−03
Coefficient: −2.34e+02
Value: neg
d__Bacteria.p__Spirochaetes.c__Spirochaetia.o__Leptospirales.f__
1250
1000
750
500
250
0
alpha (n=25)
clade20C (n=1)
delta (n=13)
omicron (n=6)
neg (n=72)
NA
WHOvariant

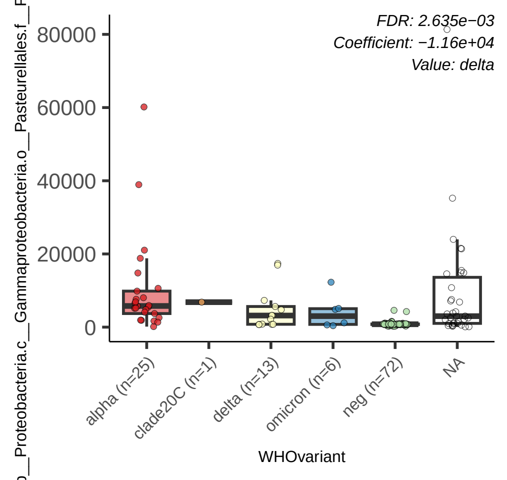
FDR: 2.635e−03
Coefficient: −1.16e+04
Value: delta
80000
60000
40000
20000
0
alpha (n=25)
clade20C (n=1)
delta (n=13)
omicron (n=6)
neg (n=72)
NA
WHOvariant
p__Proteobacteria.c__Gammaproteobacteria.o__Pasteurellales.f__P

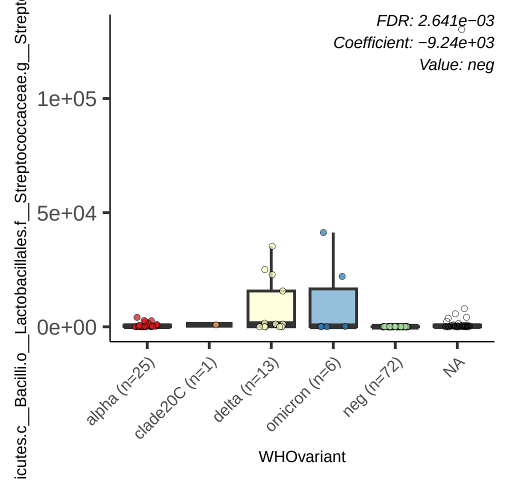

FDR: 2.641e−03
Coefficient: −9.24e+03
Value: neg
1e+05
5e+04
0e+00
icutes.c__Bacilli.o__Lactobacillales.f__Streptococcaceae.g__Strept
alpha (n=25)
clade20C (n=1)
delta (n=13)
omicron (n=6)
neg (n=72)
NA
WHOvariant

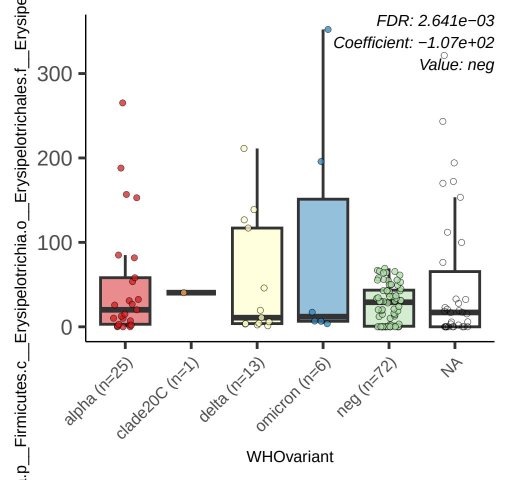

FDR: 2.641e−03
Coefficient: −1.07e+02
Value: neg
300
200
100
0
Firmicutes.c__Erysipelotrichia.o__Erysipelotrichales.f__Erysipe
alpha (n=25)
clade20C (n=1)
delta (n=13)
omicron (n=6)
neg (n=72)
NA
WHOvariant

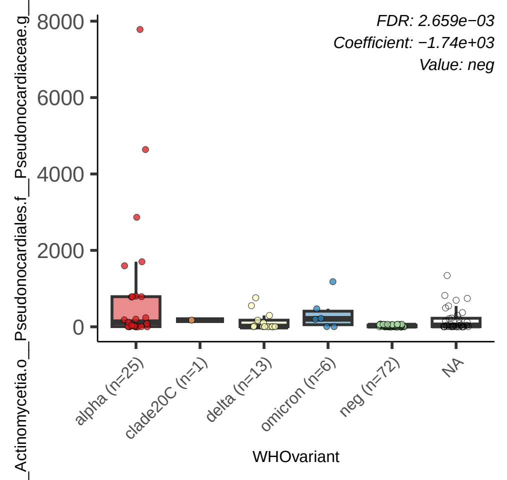

FDR: 2.659e−03
Coefficient: −1.74e+03
Value: neg
Actinomycetia.o__Pseudonocardiales.f__Pseudonocardiaceae.g__
8000
6000
4000
2000
0
alpha (n=25)
clade20C (n=1)
delta (n=13)
omicron (n=6)
neg (n=72)
NA
WHOvariant

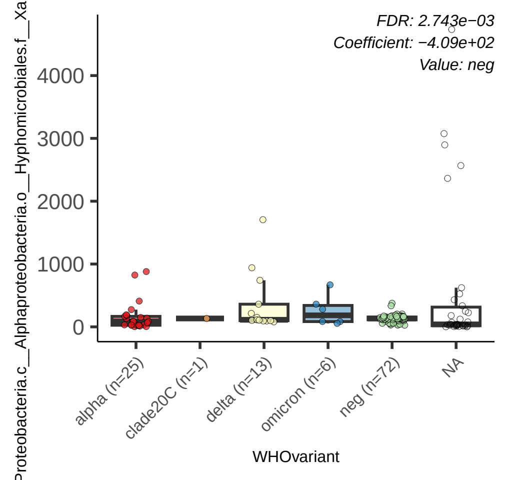

FDR: 2.743e−03
Coefficient: −4.09e+02
Value: neg
Proteobacteria.c__Alphaproteobacteria.o__Hyphomicrobiales.f__Xa
0
1000
2000
3000
4000
alpha (n=25)
clade20C (n=1)
delta (n=13)
omicron (n=6)
neg (n=72)
NA
WHOvariant

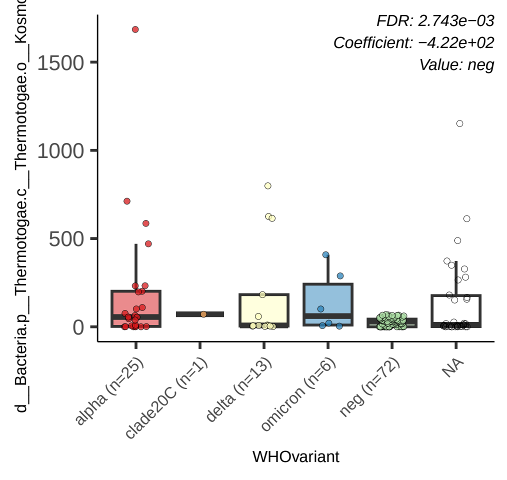
FDR: 2.743e−03
Coefficient: −4.22e+02
Value: neg
d__Bacteria.p__Thermotogae.c__Thermotogae.o__Kosmo
1500
1000
500
0
alpha (n=25)
clade20C (n=1)
delta (n=13)
omicron (n=6)
neg (n=72)
NA
WHOvariant

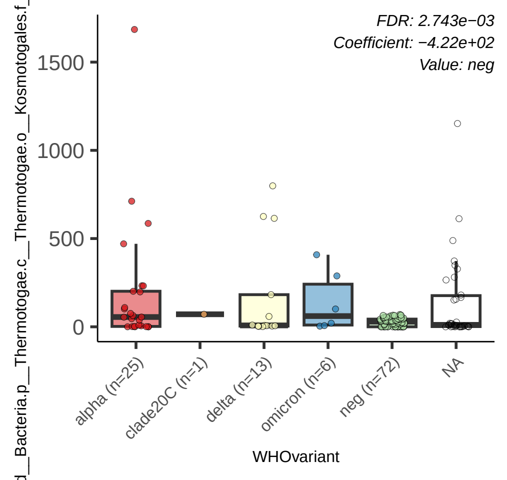

FDR: 2.743e−03
Coefficient: −4.22e+02
Value: neg
d__Bacteria.p__Thermotogae.c__Thermotogae.o__Kosmotogales.f__
0
500
1000
1500
alpha (n=25)
clade20C (n=1)
delta (n=13)
omicron (n=6)
neg (n=72)
NA
WHOvariant

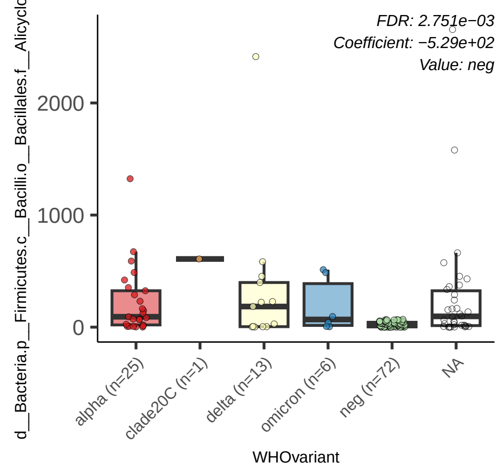

FDR: 2.751e−03
Coefficient: −5.29e+02
Value: neg
d__Bacteria.p__Firmicutes.c__Bacilli.o__Bacillales.f__Alicyclo
2000
1000
0
alpha (n=25)
clade20C (n=1)
delta (n=13)
omicron (n=6)
neg (n=72)
NA
WHOvariant

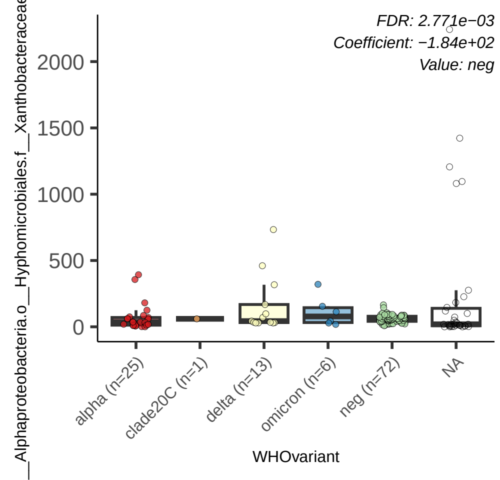

FDR: 2.771e−03
Coefficient: −1.84e+02
Value: neg
__Alphaproteobacteria.o__Hyphomicrobiales.f__Xanthobacteraceae
2000
1500
1000
500
0
alpha (n=25)
clade20C (n=1)
delta (n=13)
omicron (n=6)
neg (n=72)
NA
WHOvariant

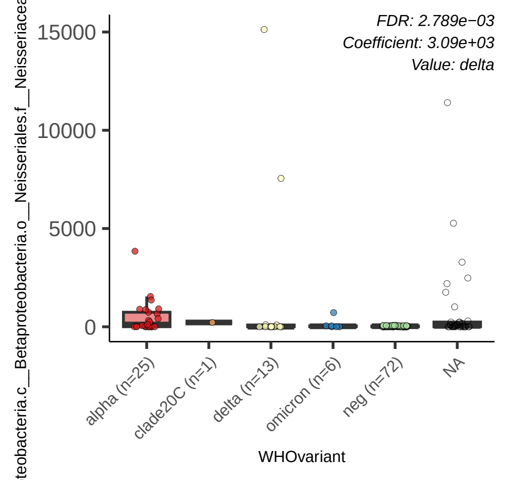

FDR: 2.789e−03
Coefficient: 3.09e+03
Value: delta
15000
10000
5000
0
teobacteria.c__Betaproteobacteria.o__Neisseriales.f__Neisseriacea
alpha (n=25)
clade20C (n=1)
delta (n=13)
omicron (n=6)
neg (n=72)
NA
WHOvariant

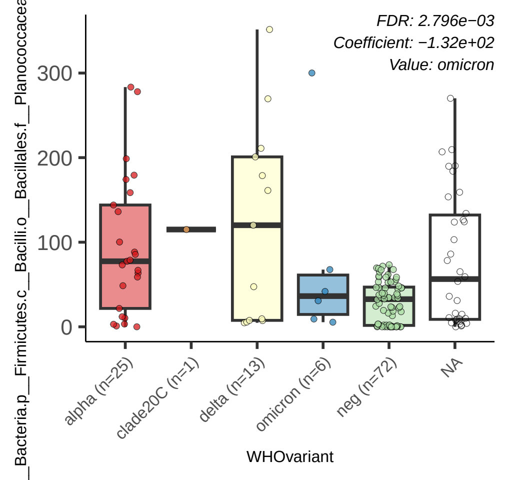

FDR: 2.796e−03
Coefficient: −1.32e+02
Value: omicron
Bacteria.p__Firmicutes.c__Bacilli.o__Bacillales.f__Planococcacea
300
200
100
0
alpha (n=25)
clade20C (n=1)
delta (n=13)
omicron (n=6)
neg (n=72)
NA
WHOvariant

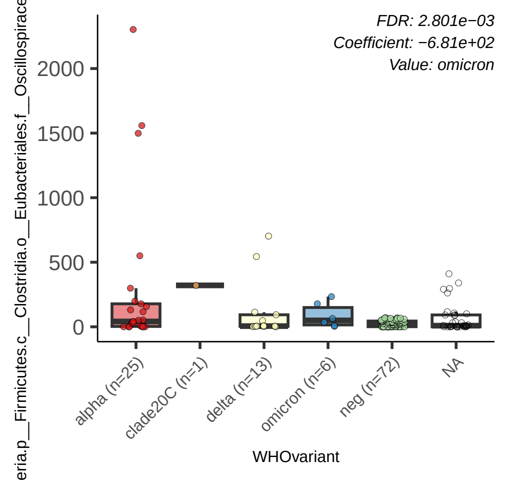

FDR: 2.801e−03
Coefficient: −6.81e+02
Value: omicron
eria.p__Firmicutes.c__Clostridia.o__Eubacteriales.f__Oscillospirace
2000
1500
1000
500
0
alpha (n=25)
clade20C (n=1)
delta (n=13)
omicron (n=6)
neg (n=72)
NA
WHOvariant

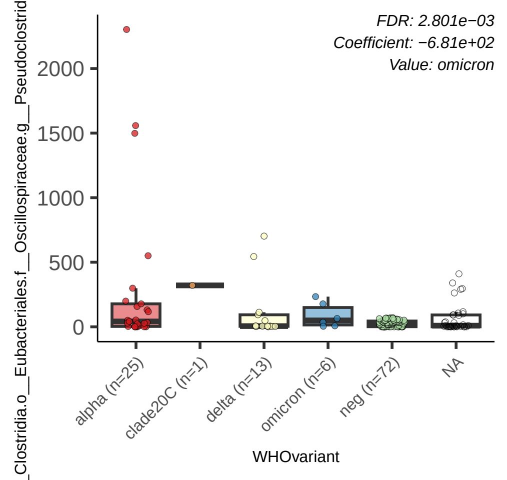

FDR: 2.801e−03
Coefficient: −6.81e+02
Value: omicron
Clostridia.o__Eubacteriales.f__Oscillospiraceae.g__Pseudoclostrid
2000
1500
1000
500
0
alpha (n=25)
clade20C (n=1)
delta (n=13)
omicron (n=6)
neg (n=72)
NA
WHOvariant

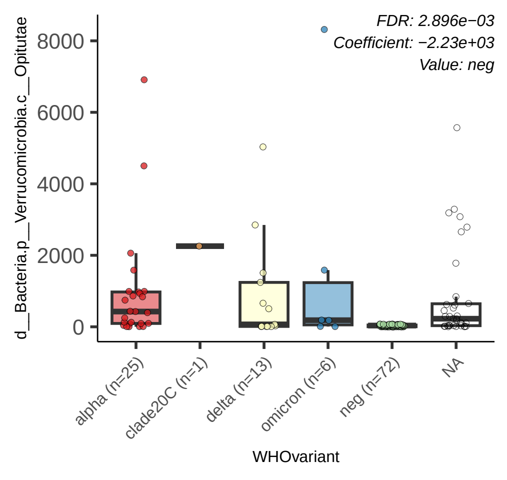

FDR: 2.896e−03
Coefficient: −2.23e+03
Value: neg
d__Bacteria.p__Verrucomicrobia.c__Opitutae
8000
6000
4000
2000
0
alpha (n=25)
clade20C (n=1)
delta (n=13)
omicron (n=6)
neg (n=72)
NA
WHOvariant

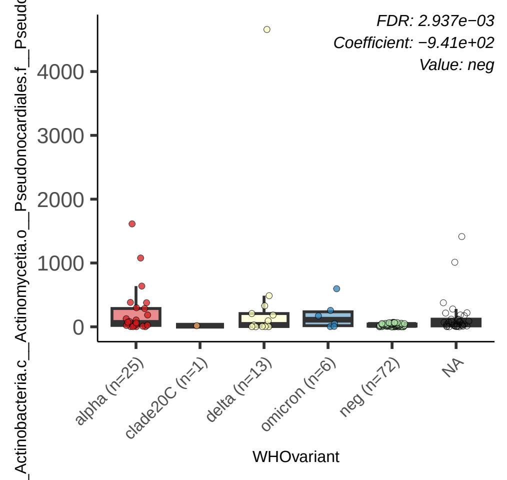
FDR: 2.937e−03
Coefficient: −9.41e+02
Value: neg
Actinobacteria.c__Actinomycetia.o__Pseudonocardiales.f__Pseudo
4000
3000
2000
1000
0
alpha (n=25)
clade20C (n=1)
delta (n=13)
omicron (n=6)
neg (n=72)
NA
WHOvariant

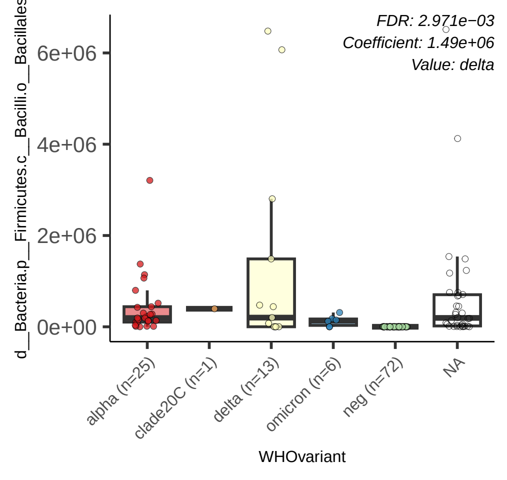

FDR: 2.971e−03
Coefficient: 1.49e+06
Value: delta
d__Bacteria.p__Firmicutes.c__Bacilli.o__Bacillales
6e+06
4e+06
2e+06
0e+00
alpha (n=25)
clade20C (n=1)
delta (n=13)
omicron (n=6)
neg (n=72)
NA
WHOvariant

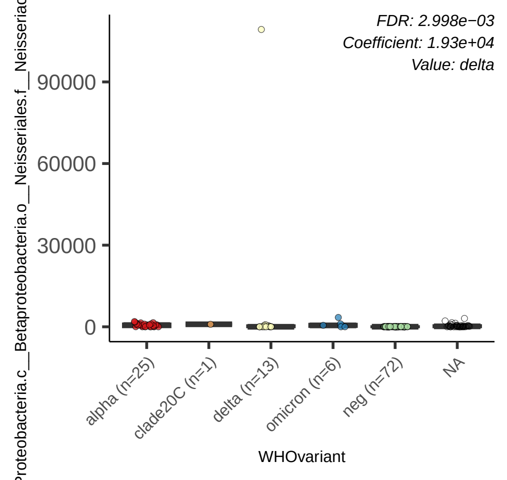
FDR: 2.998e−03
Coefficient: 1.93e+04
Value: delta
Proteobacteria.c__Betaproteobacteria.o__Neisseriales.f__Neisseriac
90000
60000
30000
0
alpha (n=25)
clade20C (n=1)
delta (n=13)
omicron (n=6)
neg (n=72)
NA
WHOvariant

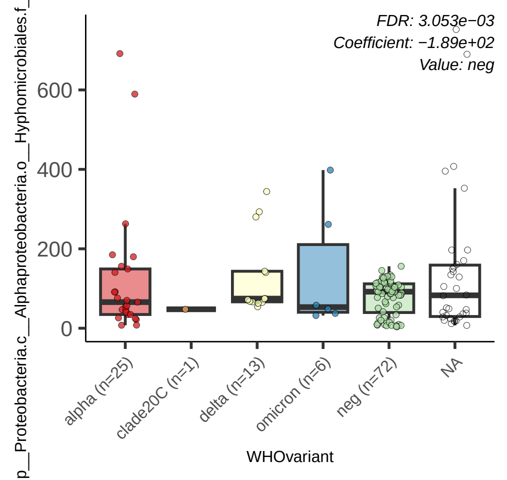
FDR: 3.053e−03
Coefficient: −1.89e+02
Value: neg
p__Proteobacteria.c__Alphaproteobacteria.o__Hyphomicrobiales.f__
0
200
400
600
alpha (n=25)
clade20C (n=1)
delta (n=13)
omicron (n=6)
neg (n=72)
NA
WHOvariant

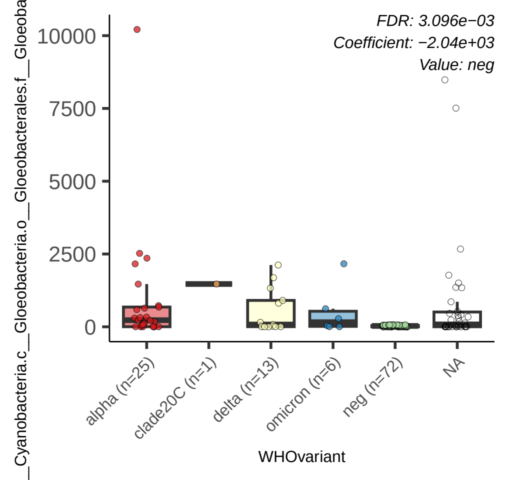

FDR: 3.096e−03
Coefficient: −2.04e+03
Value: neg
10000
7500
5000
2500
0
Cyanobacteria.c__Gloeobacteria.o__Gloeobacterales.f__Gloeoba
alpha (n=25)
clade20C (n=1)
delta (n=13)
omicron (n=6)
neg (n=72)
NA
WHOvariant

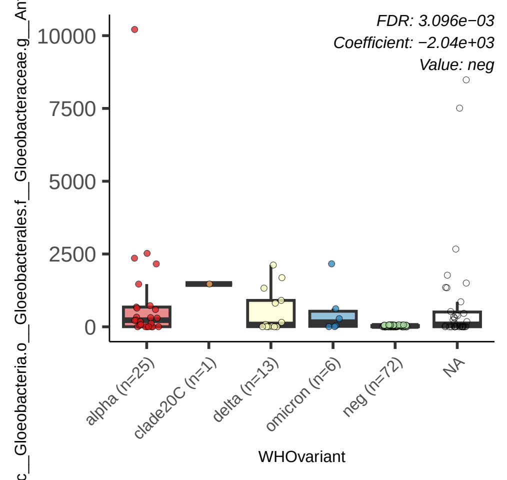

FDR: 3.096e−03
Coefficient: −2.04e+03
Value: neg
c__Gloeobacteria.o__Gloeobacterales.f__Gloeobacteraceae.g__An
10000
7500
5000
2500
0
alpha (n=25)
clade20C (n=1)
delta (n=13)
omicron (n=6)
neg (n=72)
NA
WHOvariant

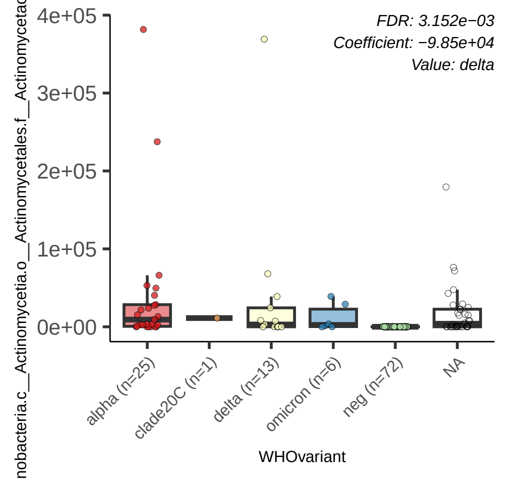

FDR: 3.152e−03
Coefficient: −9.85e+04
Value: delta
nobacteria.c__Actinomycetia.o__Actinomycetales.f__Actinomycetac
4e+05
3e+05
2e+05
1e+05
0e+00
alpha (n=25)
clade20C (n=1)
delta (n=13)
omicron (n=6)
neg (n=72)
NA
WHOvariant

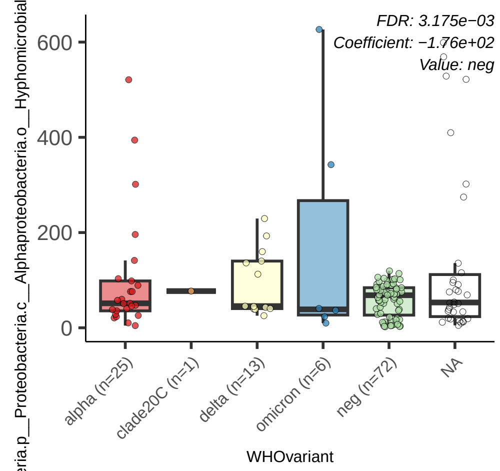
FDR: 3.175e−03
Coefficient: −1.76e+02
Value: neg
teria.p__Proteobacteria.c__Alphaproteobacteria.o__Hyphomicrobial
600
400
200
0
alpha (n=25)
clade20C (n=1)
delta (n=13)
omicron (n=6)
neg (n=72)
NA
WHOvariant

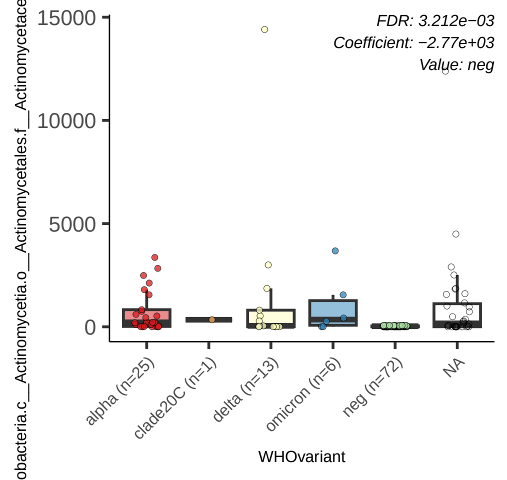

FDR: 3.212e−03
Coefficient: −2.77e+03
Value: neg
15000
10000
5000
0
obacteria.c__Actinomycetia.o__Actinomycetales.f__Actinomycetace
alpha (n=25)
clade20C (n=1)
delta (n=13)
omicron (n=6)
neg (n=72)
NA
WHOvariant

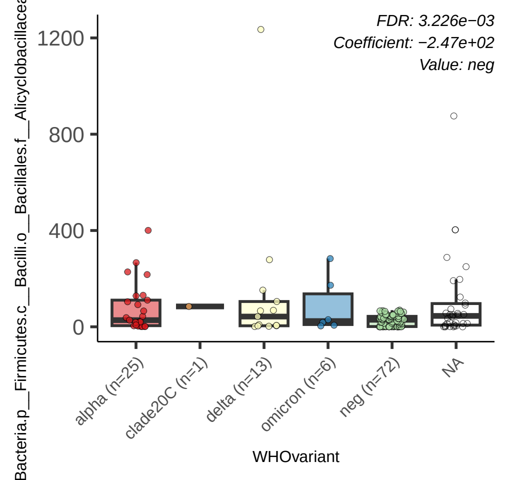

FDR: 3.226e−03
Coefficient: −2.47e+02
Value: neg
Bacteria.p__Firmicutes.c__Bacilli.o__Bacillales.f__Alicyclobacillacea
1200
800
400
0
alpha (n=25)
clade20C (n=1)
delta (n=13)
omicron (n=6)
neg (n=72)
NA
WHOvariant

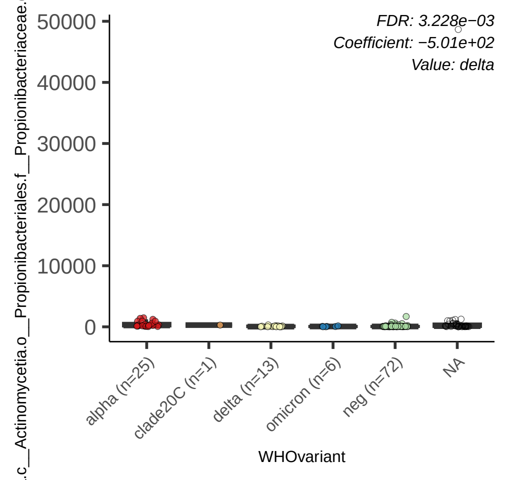

FDR: 3.228e−03
Coefficient: −5.01e+02
Value: delta
50000
40000
30000
20000
10000
0
.c__Actinomycetia.o__Propionibacteriales.f__Propionibacteriaceae.
alpha (n=25)
clade20C (n=1)
delta (n=13)
omicron (n=6)
neg (n=72)
NA
WHOvariant

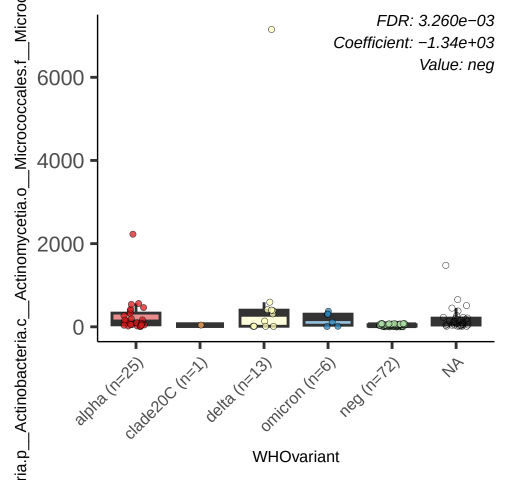
FDR: 3.260e−03
Coefficient: −1.34e+03
Value: neg
6000
4000
2000
0
ria.p__Actinobacteria.c__Actinomycetia.o__Micrococcales.f__Microc
alpha (n=25)
clade20C (n=1)
delta (n=13)
omicron (n=6)
neg (n=72)
NA
WHOvariant

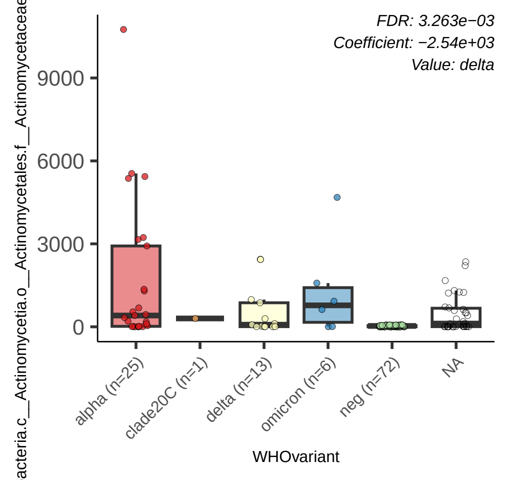

FDR: 3.263e−03
Coefficient: −2.54e+03
Value: delta
9000
6000
3000
0
alpha (n=25)
clade20C (n=1)
delta (n=13)
omicron (n=6)
neg (n=72)
NA
WHOvariant
acteria.c__Actinomycetia.o__Actinomycetales.f__Actinomycetaceae

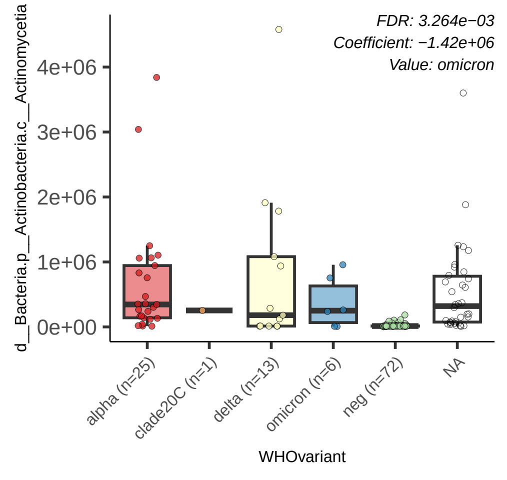

FDR: 3.264e−03
Coefficient: −1.42e+06
Value: omicron
d__Bacteria.p__Actinobacteria.c__Actinomycetia
0e+00
1e+06
2e+06
3e+06
4e+06
alpha (n=25)
clade20C (n=1)
delta (n=13)
omicron (n=6)
neg (n=72)
NA
WHOvariant

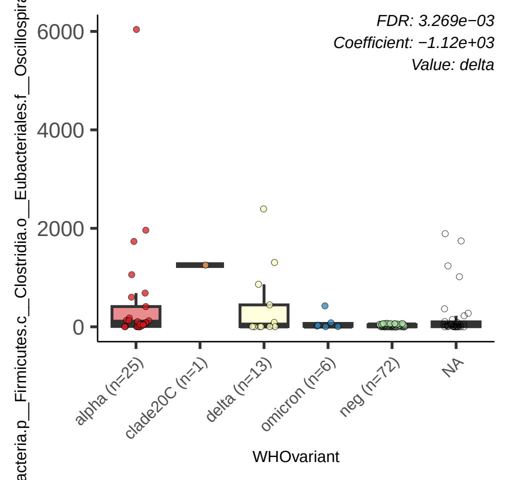

FDR: 3.269e−03
Coefficient: −1.12e+03
Value: delta
6000
4000
2000
0
alpha (n=25)
clade20C (n=1)
delta (n=13)
omicron (n=6)
neg (n=72)
NA
WHOvariant

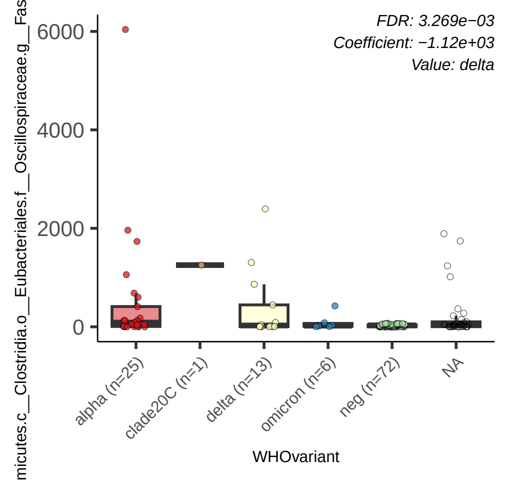

micutes.c__Clostridia.o__Eubacteriales.f__Oscillospiraceae.g__Fas
FDR: 3.269e−03
Coefficient: −1.12e+03
Value: delta
6000
4000
2000
0
alpha (n=25)
clade20C (n=1)
delta (n=13)
omicron (n=6)
neg (n=72)
NA
WHOvariant

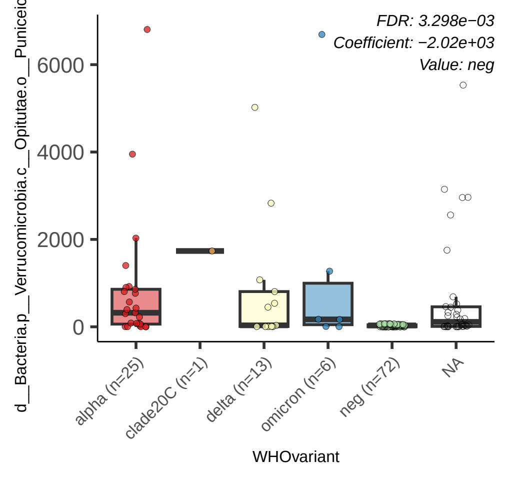

FDR: 3.298e−03
Coefficient: −2.02e+03
Value: neg
d__Bacteria.p__Verrucomicrobia.c__Opitutae.o__Puniceic
0
2000
4000
6000
alpha (n=25)
clade20C (n=1)
delta (n=13)
omicron (n=6)
neg (n=72)
NA
WHOvariant

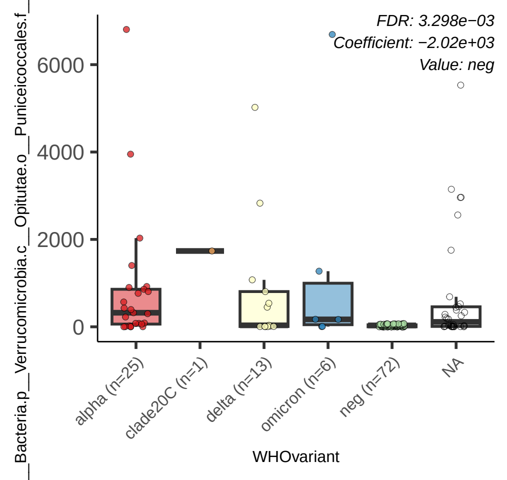
FDR: 3.298e−03
Coefficient: −2.02e+03
Value: neg
Bacteria.p__Verrucomicrobia.c__Opitutae.o__Puniceicoccales.f
0
2000
4000
6000
alpha (n=25)
clade20C (n=1)
delta (n=13)
omicron (n=6)
neg (n=72)
NA
WHOvariant

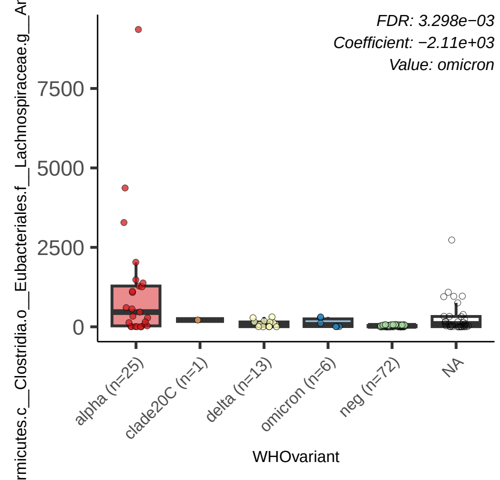

FDR: 3.298e−03
Coefficient: −2.11e+03
Value: omicron
rmicutes.c__Clostridia.o__Eubacteriales.f__Lachnospiraceae.g__Ar
7500
5000
2500
0
alpha (n=25)
clade20C (n=1)
delta (n=13)
omicron (n=6)
neg (n=72)
NA
WHOvariant

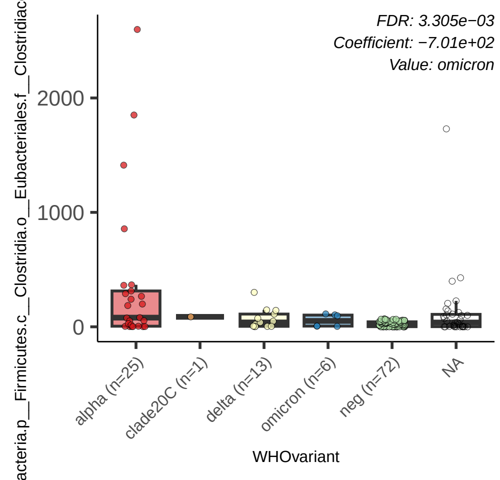

FDR: 3.305e−03
Coefficient: −7.01e+02
Value: omicron
acteria.p__Firmicutes.c__Clostridia.o__Eubacteriales.f__Clostridiac
2000
1000
0
alpha (n=25)
clade20C (n=1)
delta (n=13)
omicron (n=6)
neg (n=72)
NA
WHOvariant

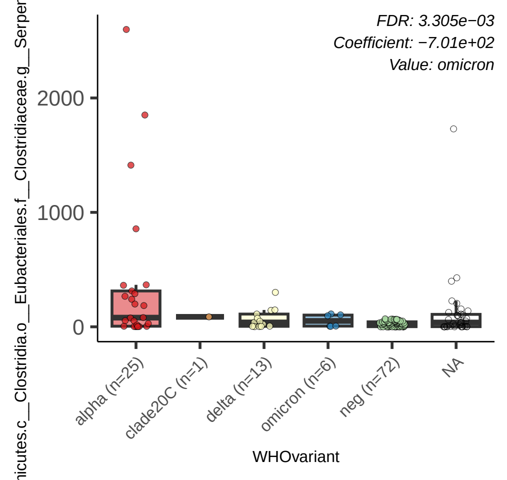

FDR: 3.305e−03
Coefficient: −7.01e+02
Value: omicron
2000
1000
0
alpha (n=25)
clade20C (n=1)
delta (n=13)
omicron (n=6)
neg (n=72)
NA
WHOvariant
nicutes.c__Clostridia.o__Eubacteriales.f__Clostridiaceae.g__Serpen

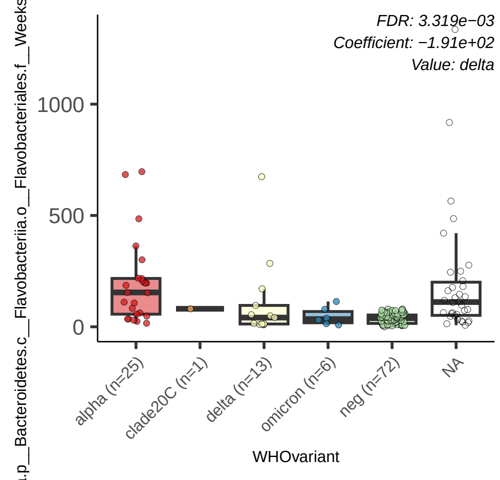

FDR: 3.319e−03
Coefficient: −1.91e+02
Value: delta
1000
500
0
alpha (n=25)
clade20C (n=1)
delta (n=13)
omicron (n=6)
neg (n=72)
NA
WHOvariant
Bacteroidetes.c__Flavobacteriia.o__Flavobacteriales.f__Weeks

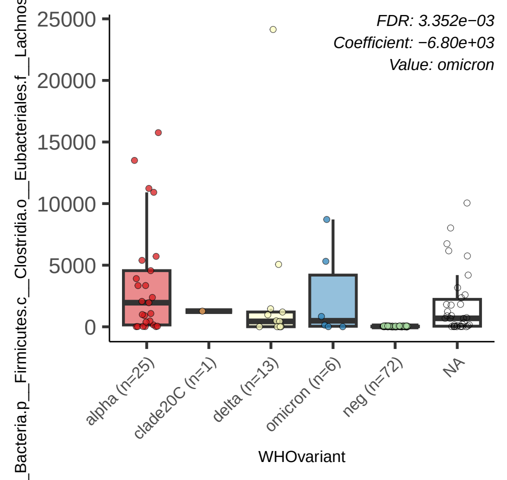

FDR: 3.352e−03
Coefficient: −6.80e+03
Value: omicron
Bacteria.p__Firmicutes.c__Clostridia.o__Eubacteriales.f__Lachnos
25000
20000
15000
10000
5000
0
alpha (n=25)
clade20C (n=1)
delta (n=13)
omicron (n=6)
neg (n=72)
NA
WHOvariant

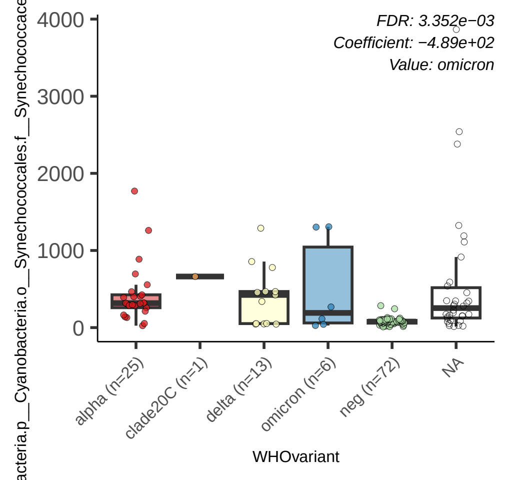

FDR: 3.352e−03
Coefficient: −4.89e+02
Value: omicron
4000
3000
2000
1000
0
bacteria.p__Cyanobacteria.o__Synechococcales.f__Synechococcace
alpha (n=25)
clade20C (n=1)
delta (n=13)
omicron (n=6)
neg (n=72)
NA
WHOvariant

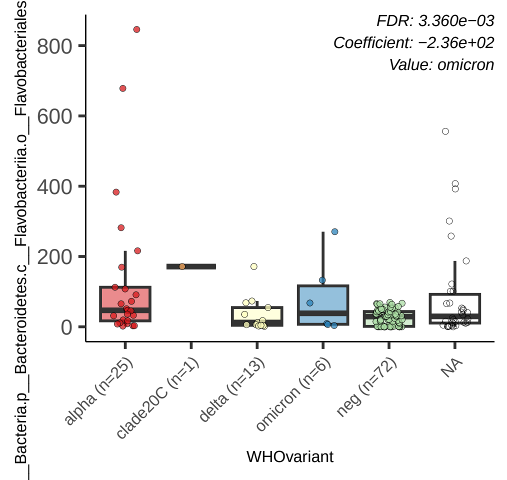

FDR: 3.360e−03
Coefficient: −2.36e+02
Value: omicron
Bacteria.p__Bacteroidetes.c__Flavobacteriia.o__Flavobacteriales
0
200
400
600
800
alpha (n=25)
clade20C (n=1)
delta (n=13)
omicron (n=6)
neg (n=72)
NA
WHOvariant

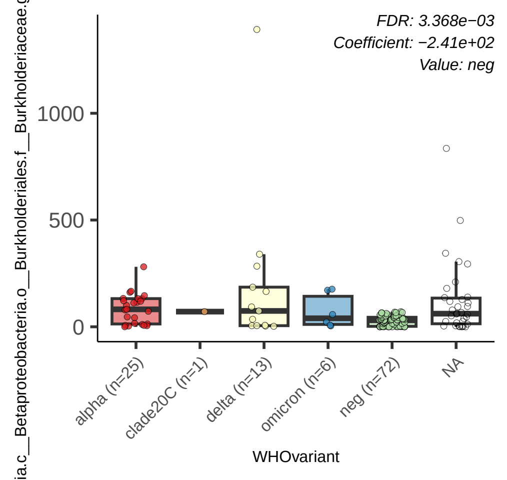
FDR: 3.368e−03
Coefficient: −2.41e+02
Value: neg
1000
500
0
ia.c__Betaproteobacteria.o__Burkholderiales.f__Burkholderiaceae.g
alpha (n=25)
clade20C (n=1)
delta (n=13)
omicron (n=6)
neg (n=72)
NA
WHOvariant

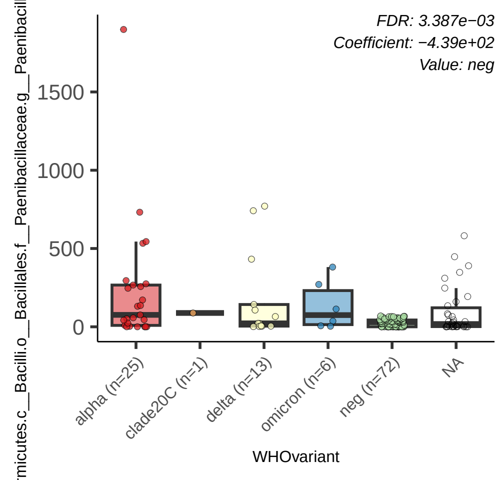

FDR: 3.387e−03
Coefficient: −4.39e+02
Value: neg
1500
1000
500
0
alpha (n=25)
clade20C (n=1)
delta (n=13)
omicron (n=6)
neg (n=72)
NA
WHOvariant
rmicutes.c__Bacilli.o__Bacillales.f__Paenibacillaceae.g__Paenibacil

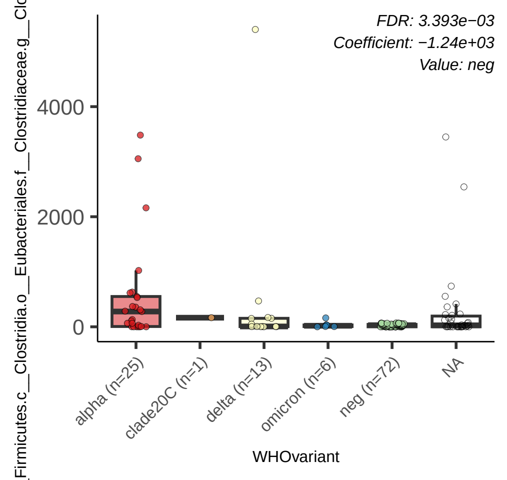

FDR: 3.393e−03
Coefficient: −1.24e+03
Value: neg
Firmicutes.c__Clostridia.o__Eubacteriales.f__Clostridiaceae.g__Clo
4000
2000
0
alpha (n=25)
clade20C (n=1)
delta (n=13)
omicron (n=6)
neg (n=72)
NA
WHOvariant

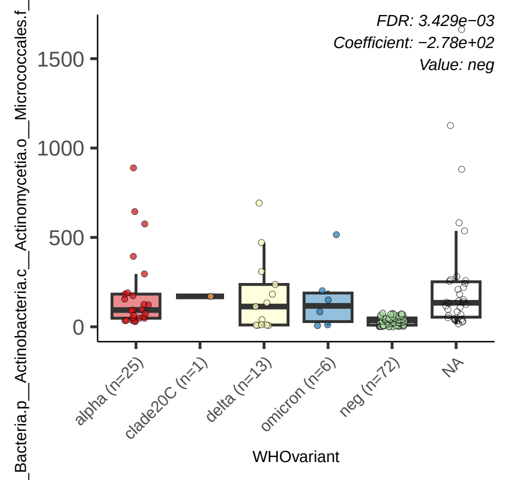

Bacteria.p__Actinobacteria.c__Actinomycetia.o__Micrococcales.f__
FDR: 3.429e−03
Coefficient: −2.78e+02
Value: neg
1500
1000
500
0
alpha (n=25)
clade20C (n=1)
delta (n=13)
omicron (n=6)
neg (n=72)
NA
WHOvariant

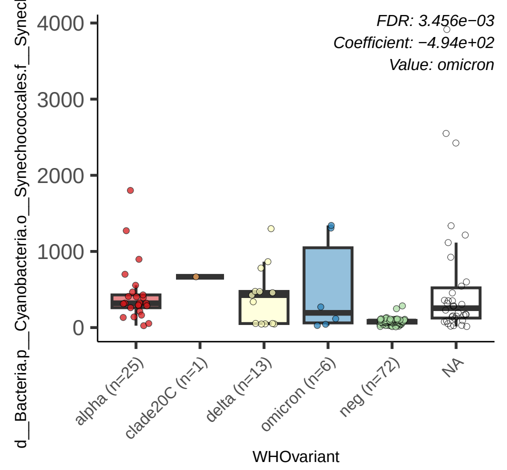

FDR: 3.456e−03
Coefficient: −4.94e+02
Value: omicron
d__Bacteria.p__Cyanobacteria.o__Synechococcales.f__Synec
4000
3000
2000
1000
0
alpha (n=25)
clade20C (n=1)
delta (n=13)
omicron (n=6)
neg (n=72)
NA
WHOvariant

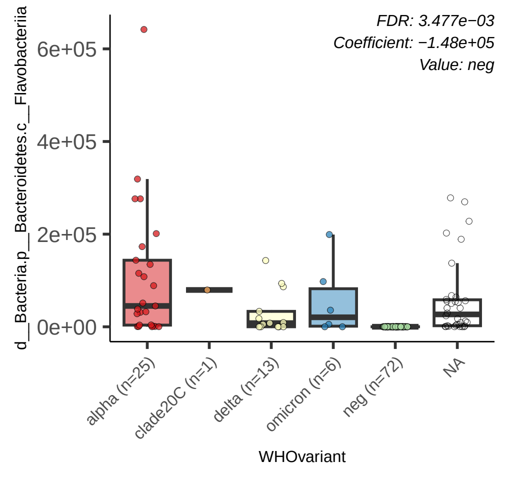

FDR: 3.477e−03
Coefficient: −1.48e+05
Value: neg
d__Bacteria.p__Bacteroidetes.c__Flavobacteriia
6e+05
4e+05
2e+05
0e+00
alpha (n=25)
clade20C (n=1)
delta (n=13)
omicron (n=6)
neg (n=72)
NA
WHOvariant

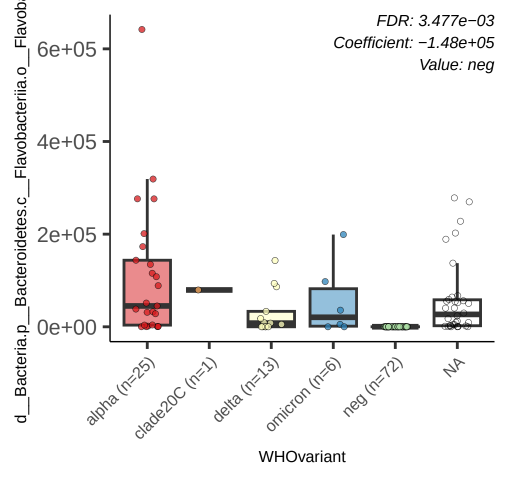

FDR: 3.477e−03
Coefficient: −1.48e+05
Value: neg
d__Bacteria.p__Bacteroidetes.c__Flavobacteriia.o__Flavoba
6e+05
4e+05
2e+05
0e+00
alpha (n=25)
clade20C (n=1)
delta (n=13)
omicron (n=6)
neg (n=72)
NA
WHOvariant

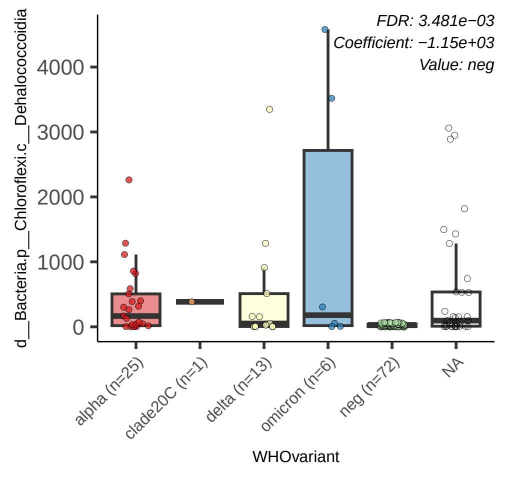

FDR: 3.481e−03
Coefficient: −1.15e+03
Value: neg
d__Bacteria.p__Chloroflexi.c__Dehalococcoidia
4000
3000
2000
1000
0
alpha (n=25)
clade20C (n=1)
delta (n=13)
omicron (n=6)
neg (n=72)
NA
WHOvariant

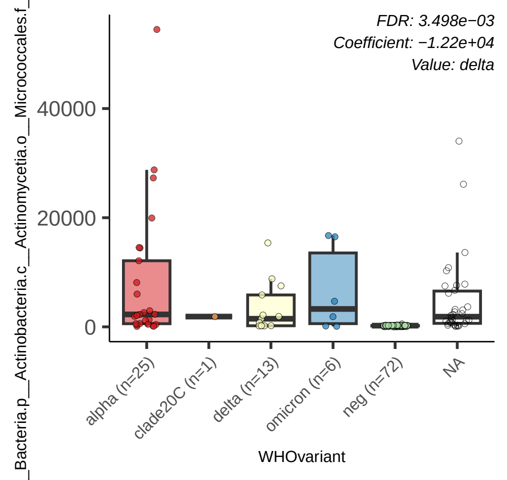

FDR: 3.498e−03
Coefficient: −1.22e+04
Value: delta
Bacteria.p__Actinobacteria.c__Actinomycetia.o__Micrococcales.f__
0
20000
40000
alpha (n=25)
clade20C (n=1)
delta (n=13)
omicron (n=6)
neg (n=72)
NA
WHOvariant

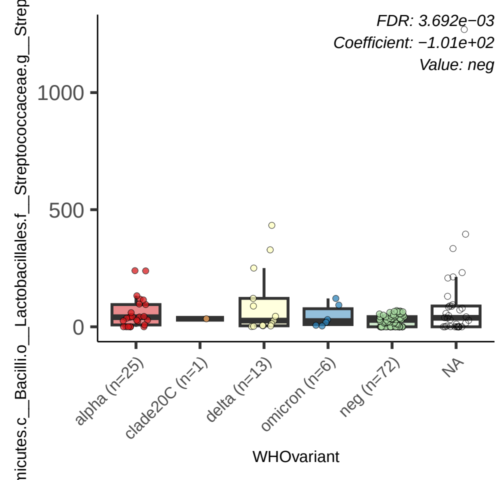
FDR: 3.692e−03
Coefficient: −1.01e+02
Value: neg
1000
500
0
alpha (n=25)
clade20C (n=1)
delta (n=13)
omicron (n=6)
neg (n=72)
NA
WHOvariant
micutes.c__Bacilli.o__Lactobacillales.f__Streptococcaceae.g__Strep

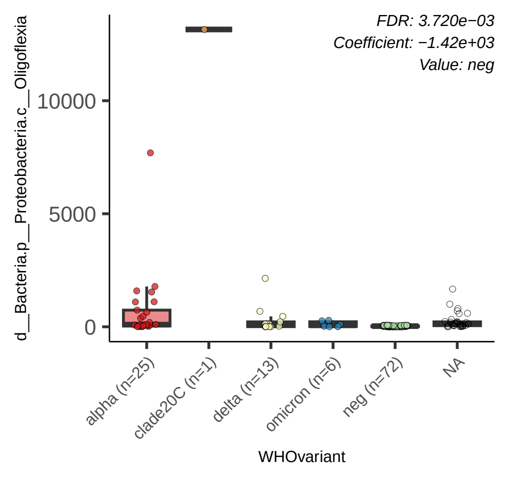

FDR: 3.720e−03
Coefficient: −1.42e+03
Value: neg
d__Bacteria.p__Proteobacteria.c__Oligoflexia
10000
5000
0
alpha (n=25)
clade20C (n=1)
delta (n=13)
omicron (n=6)
neg (n=72)
NA
WHOvariant

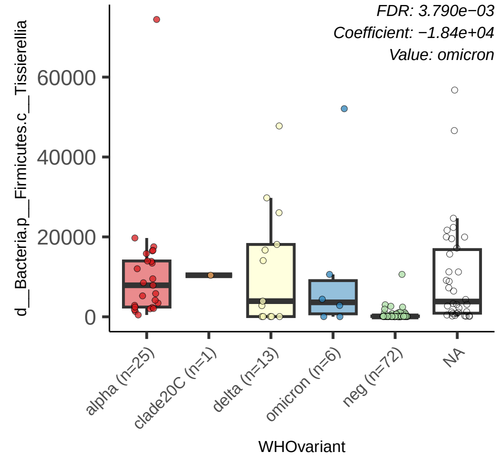

FDR: 3.790e−03
Coefficient: −1.84e+04
Value: omicron
d__Bacteria.p__Firmicutes.c__Tissierellia
0
20000
40000
60000
alpha (n=25)
clade20C (n=1)
delta (n=13)
omicron (n=6)
neg (n=72)
NA
WHOvariant

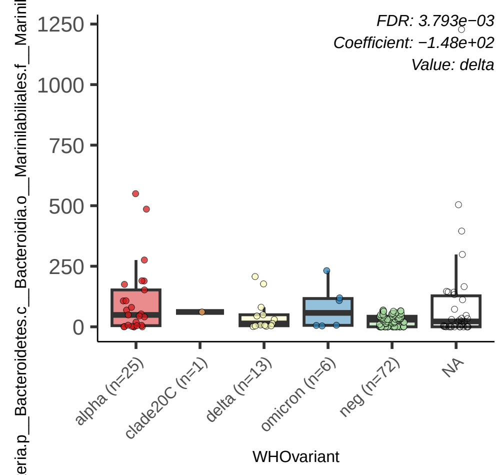

FDR: 3.793e−03
Coefficient: −1.48e+02
Value: delta
1250
1000
750
500
250
0
alpha (n=25)
clade20C (n=1)
delta (n=13)
omicron (n=6)
neg (n=72)
NA
WHOvariant
cteria.p__Bacteroidetes.c__Bacteroidia.o__Marinilabiliales.f__Marinil

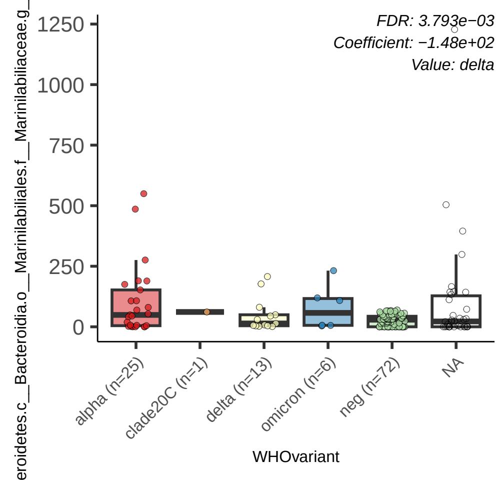

FDR: 3.793e−03
Coefficient: −1.48e+02
Value: delta
1250
1000
750
500
250
0
eroidetes.c__Bacteroidia.o__Marinilabiliales.f__Marinilabiliaceae.g__
alpha (n=25)
clade20C (n=1)
delta (n=13)
omicron (n=6)
neg (n=72)
NA
WHOvariant

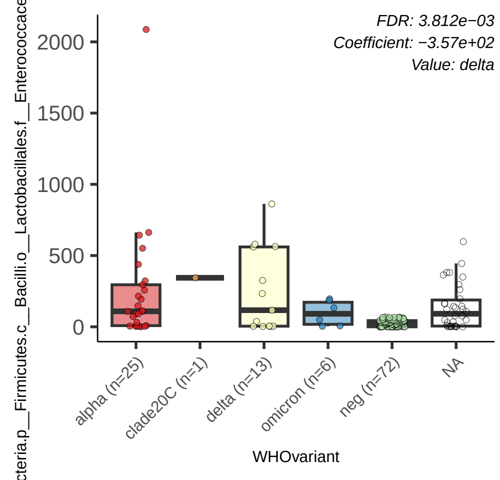

FDR: 3.812e−03
Coefficient: −3.57e+02
Value: delta
2000
1500
1000
500
0
cteria.p__Firmicutes.c__Bacilli.o__Lactobacillales.f__Enterococcace
alpha (n=25)
clade20C (n=1)
delta (n=13)
omicron (n=6)
neg (n=72)
NA
WHOvariant

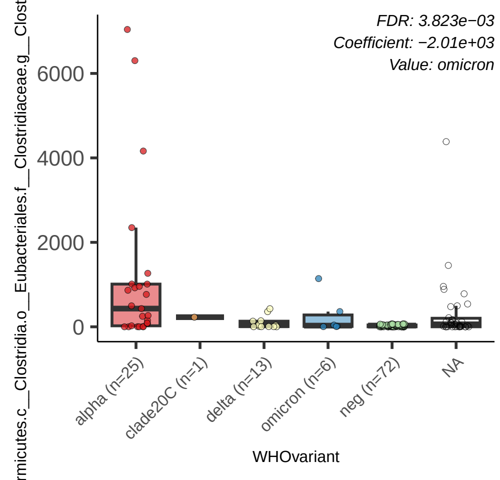

FDR: 3.823e−03
Coefficient: −2.01e+03
Value: omicron
rmicutes.c__Clostridia.o__Eubacteriales.f__Clostridiaceae.g__Clost
6000
4000
2000
0
alpha (n=25)
clade20C (n=1)
delta (n=13)
omicron (n=6)
neg (n=72)
NA
WHOvariant

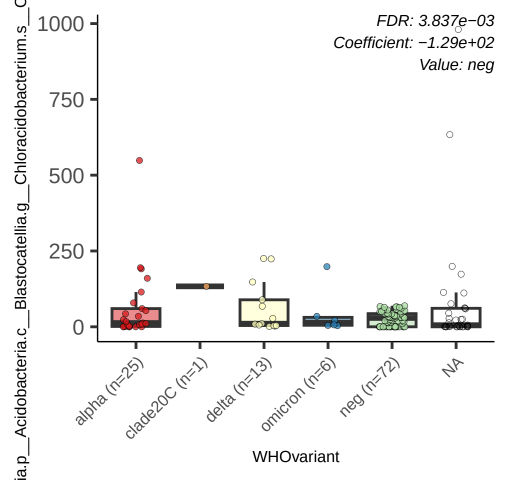

FDR: 3.837e−03
Coefficient: −1.29e+02
Value: neg
ia.p__Acidobacteria.c__Blastocatellia.g__Chloracidobacterium.s__C
1000
750
500
250
0
alpha (n=25)
clade20C (n=1)
delta (n=13)
omicron (n=6)
neg (n=72)
NA
WHOvariant

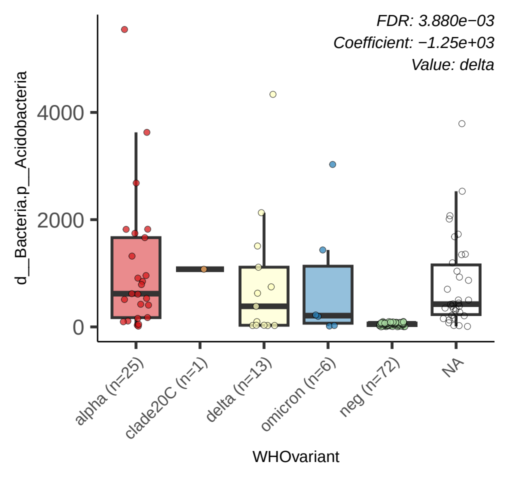
FDR: 3.880e−03
Coefficient: −1.25e+03
Value: delta
d__Bacteria.p__Acidobacteria
4000
2000
0
alpha (n=25)
clade20C (n=1)
delta (n=13)
omicron (n=6)
neg (n=72)
NA
WHOvariant

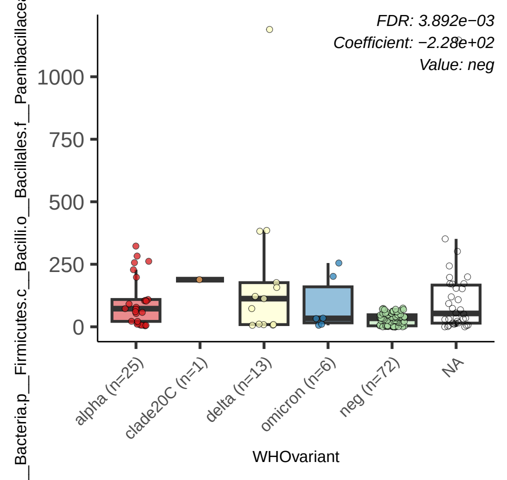
FDR: 3.892e−03
Coefficient: −2.28e+02
Value: neg
Bacteria.p__Firmicutes.c__Bacilli.o__Bacillales.f__Paenibacillace
1000
750
500
250
0
alpha (n=25)
clade20C (n=1)
delta (n=13)
omicron (n=6)
neg (n=72)
NA
WHOvariant

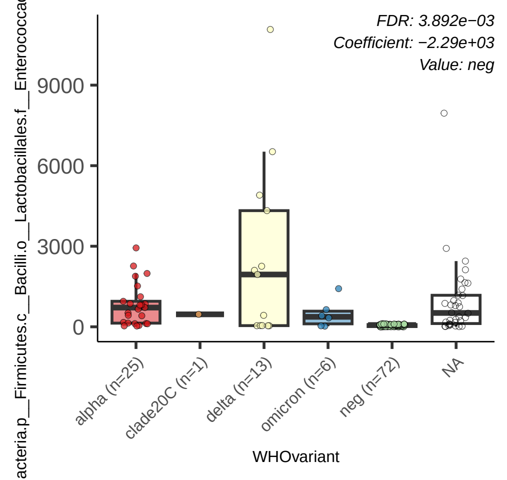

FDR: 3.892e−03
Coefficient: −2.29e+03
Value: neg
acteria.p__Firmicutes.c__Bacilli.o__Lactobacillales.f__Enterococcac
9000
6000
3000
0
alpha (n=25)
clade20C (n=1)
delta (n=13)
omicron (n=6)
neg (n=72)
NA
WHOvariant

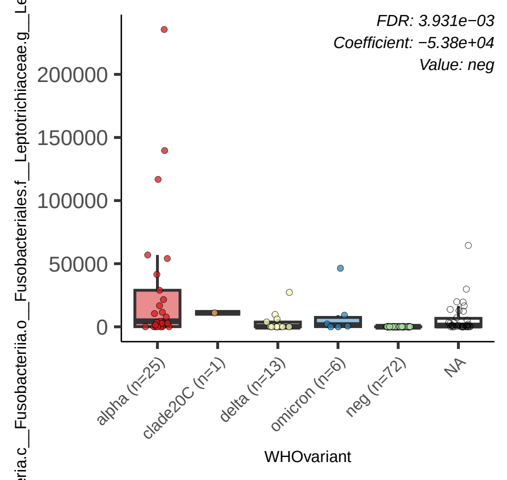
FDR: 3.931e−03
Coefficient: −5.38e+04
Value: neg
eria.c__Fusobacteriia.o__Fusobacteriales.f__Leptotrichiaceae.g__Le
200000
150000
100000
50000
0
alpha (n=25)
clade20C (n=1)
delta (n=13)
omicron (n=6)
neg (n=72)
NA
WHOvariant

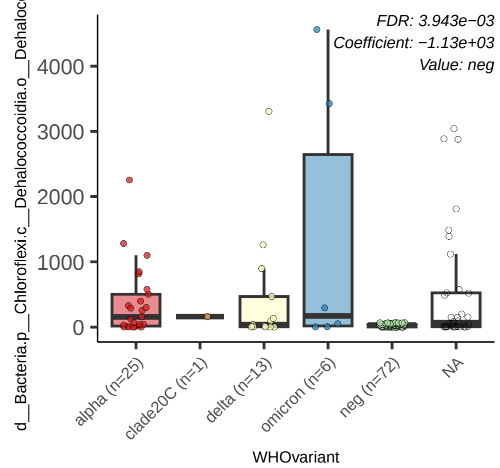

FDR: 3.943e−03
Coefficient: −1.13e+03
Value: neg
d__Bacteria.p__Chloroflexi.c__Dehalococcoidia.o__Dehaloc
4000
3000
2000
1000
0
alpha (n=25)
clade20C (n=1)
delta (n=13)
omicron (n=6)
neg (n=72)
NA
WHOvariant

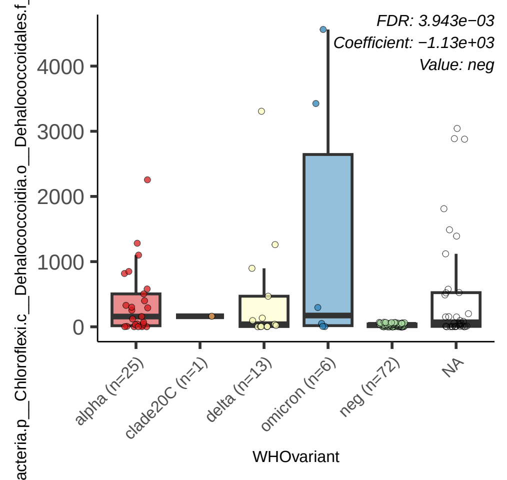

FDR: 3.943e−03
Coefficient: −1.13e+03
Value: neg
4000
3000
2000
1000
0
acteria.p__Chloroflexi.c__Dehalococcoidia.o__Dehalococcoidales.f_
alpha (n=25)
clade20C (n=1)
delta (n=13)
omicron (n=6)
neg (n=72)
NA
WHOvariant

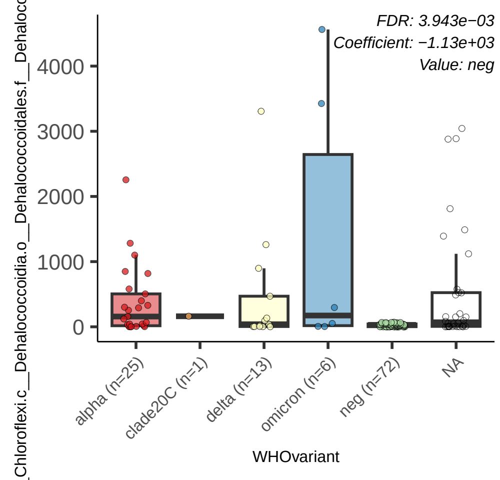
FDR: 3.943e−03
Coefficient: −1.13e+03
Value: neg
4000
3000
2000
1000
0
Chloroflexi.c__Dehalococcoidia.o__Dehalococcoidales.f__Dehaloco
alpha (n=25)
clade20C (n=1)
delta (n=13)
omicron (n=6)
neg (n=72)
NA
WHOvariant

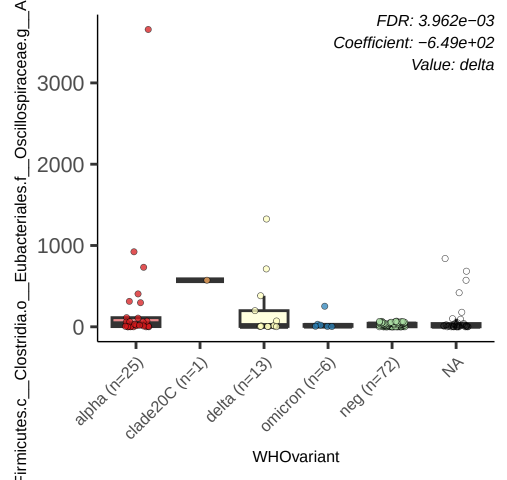

FDR: 3.962e−03
Coefficient: −6.49e+02
Value: delta
Firmicutes.c__Clostridia.o__Eubacteriales.f__Oscillospiraceae.g__A
3000
2000
1000
0
alpha (n=25)
clade20C (n=1)
delta (n=13)
omicron (n=6)
neg (n=72)
NA
WHOvariant

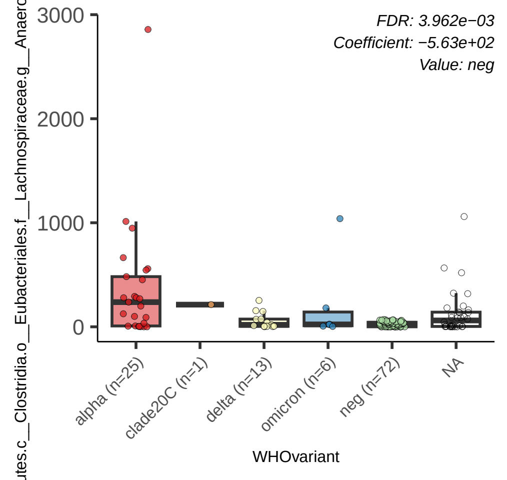

utes.c__Clostridia.o__Eubacteriales.f__Lachnospiraceae.g__Anaero
FDR: 3.962e−03
Coefficient: −5.63e+02
Value: neg
3000
2000
1000
0
alpha (n=25)
clade20C (n=1)
delta (n=13)
omicron (n=6)
neg (n=72)
NA
WHOvariant

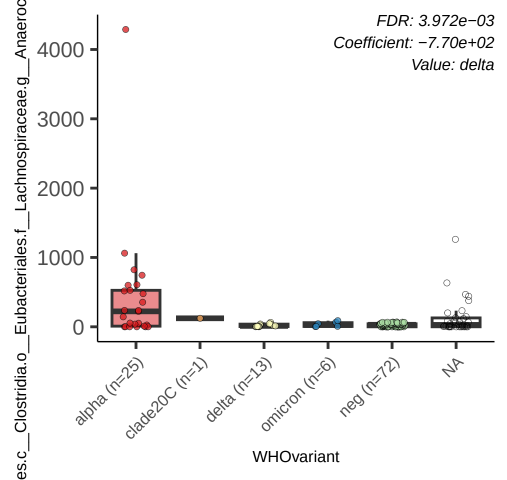

FDR: 3.972e−03
Coefficient: −7.70e+02
Value: delta
es.c__Clostridia.o__Eubacteriales.f__Lachnospiraceae.g__Anaeroc
4000
3000
2000
1000
0
alpha (n=25)
clade20C (n=1)
delta (n=13)
omicron (n=6)
neg (n=72)
NA
WHOvariant

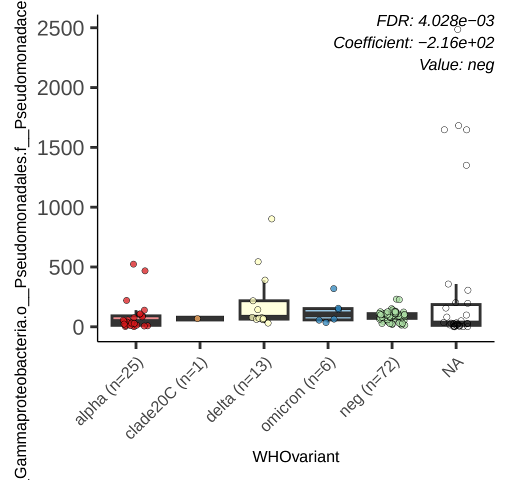

FDR: 4.028e−03
Coefficient: −2.16e+02
Value: neg
Gammaproteobacteria.o__Pseudomonadales.f__Pseudomonadace
2500
2000
1500
1000
500
0
alpha (n=25)
clade20C (n=1)
delta (n=13)
omicron (n=6)
neg (n=72)
NA
WHOvariant

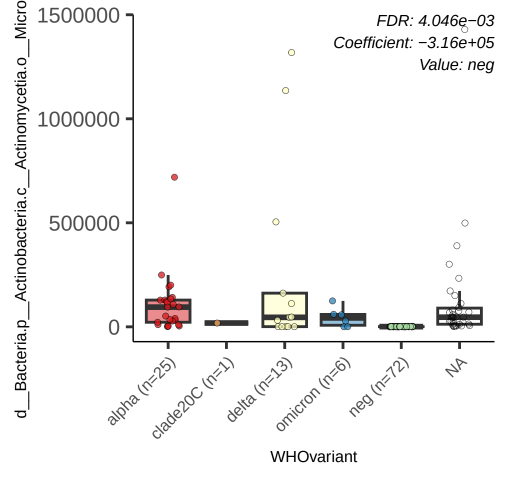

FDR: 4.046e−03
Coefficient: −3.16e+05
Value: neg
d__Bacteria.p__Actinobacteria.c__Actinomycetia.o__Micro
1500000
1000000
500000
0
alpha (n=25)
clade20C (n=1)
delta (n=13)
omicron (n=6)
neg (n=72)
NA
WHOvariant

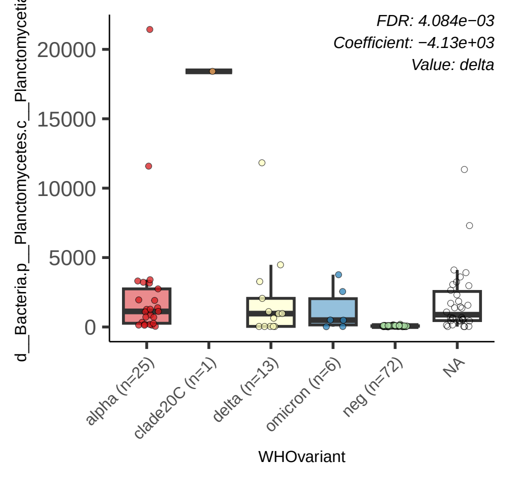

FDR: 4.084e−03
Coefficient: −4.13e+03
Value: delta
d__Bacteria.p__Planctomycetes.c__Planctomycetia
20000
15000
10000
5000
0
alpha (n=25)
clade20C (n=1)
delta (n=13)
omicron (n=6)
neg (n=72)
NA
WHOvariant

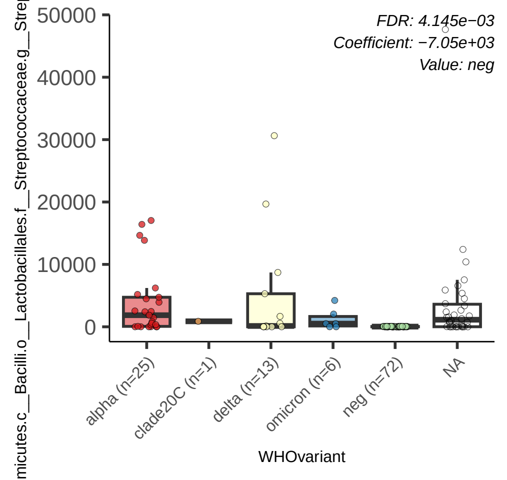
FDR: 4.145e−03
Coefficient: −7.05e+03
Value: neg
micutes.c__Bacilli.o__Lactobacillales.f__Streptococcaceae.g__Strep
50000
40000
30000
20000
10000
0
alpha (n=25)
clade20C (n=1)
delta (n=13)
omicron (n=6)
neg (n=72)
NA
WHOvariant

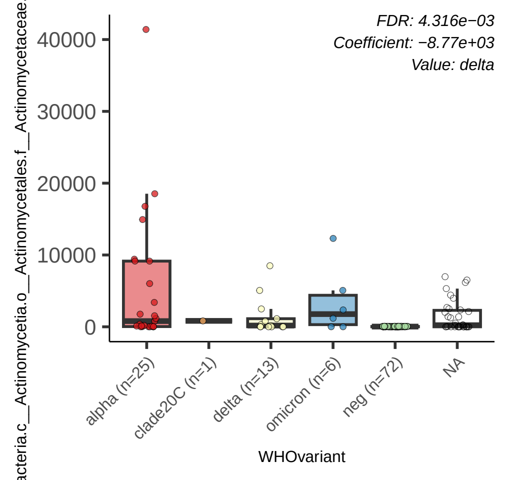

FDR: 4.316e−03
Coefficient: −8.77e+03
Value: delta
40000
30000
20000
10000
0
acteria.c__Actinomycetia.o__Actinomycetales.f__Actinomycetaceae.
alpha (n=25)
clade20C (n=1)
delta (n=13)
omicron (n=6)
neg (n=72)
NA
WHOvariant

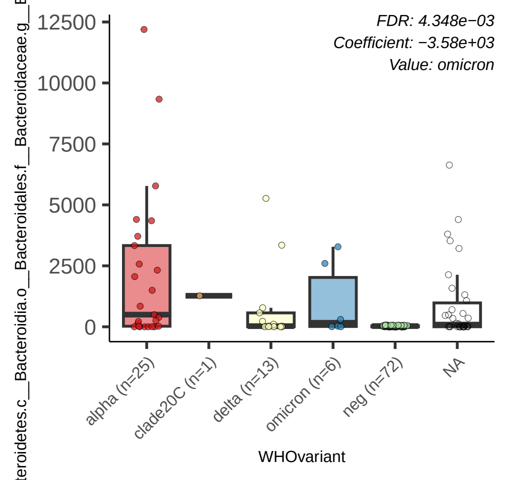

FDR: 4.348e−03
Coefficient: −3.58e+03
Value: omicron
12500
10000
7500
5000
2500
0
alpha (n=25)
clade20C (n=1)
delta (n=13)
omicron (n=6)
neg (n=72)
NA
WHOvariant

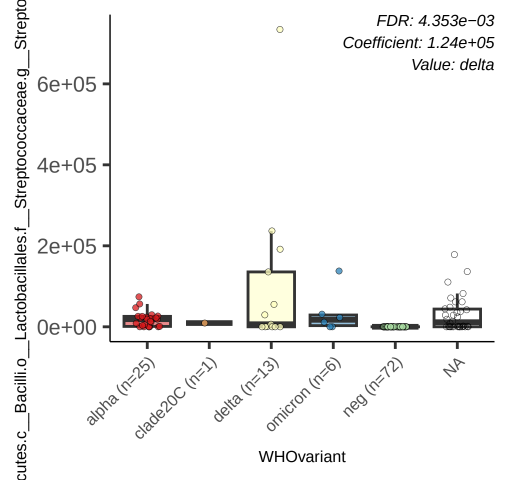

FDR: 4.353e−03
Coefficient: 1.24e+05
Value: delta
6e+05
4e+05
2e+05
0e+00
cutes.c__Bacilli.o__Lactobacillales.f__Streptococcaceae.g__Strepto
alpha (n=25)
clade20C (n=1)
delta (n=13)
omicron (n=6)
neg (n=72)
NA
WHOvariant

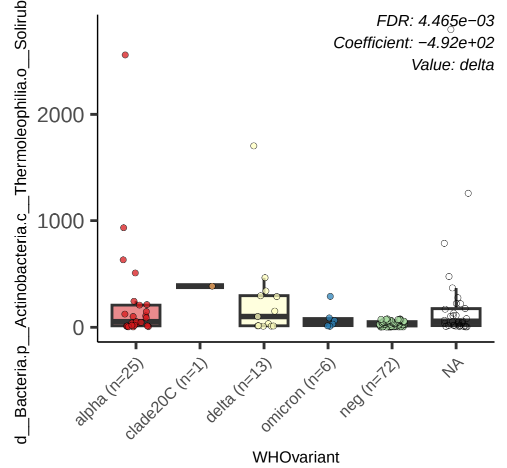

FDR: 4.465e−03
Coefficient: −4.92e+02
Value: delta
d__Bacteria.p__Actinobacteria.c__Thermoleophilia.o__Solirub
2000
1000
0
alpha (n=25)
clade20C (n=1)
delta (n=13)
omicron (n=6)
neg (n=72)
NA
WHOvariant

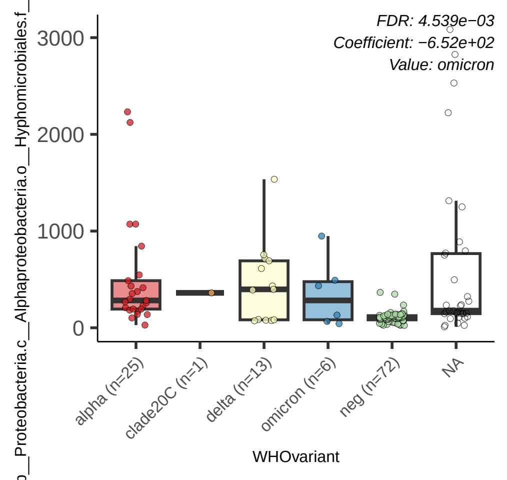
FDR: 4.539e−03
Coefficient: −6.52e+02
Value: omicron
o__Proteobacteria.c__Alphaproteobacteria.o__Hyphomicrobiales.f
0
1000
2000
3000
alpha (n=25)
clade20C (n=1)
delta (n=13)
omicron (n=6)
neg (n=72)
NA
WHOvariant

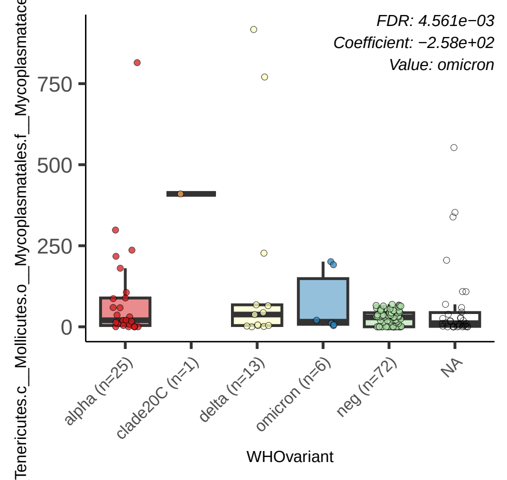

FDR: 4.561e−03
Coefficient: −2.58e+02
Value: omicron
Tenericutes.c__Mollicutes.o__Mycoplasmatales.f__Mycoplasmatace
0
250
500
750
alpha (n=25)
clade20C (n=1)
delta (n=13)
omicron (n=6)
neg (n=72)
NA
WHOvariant

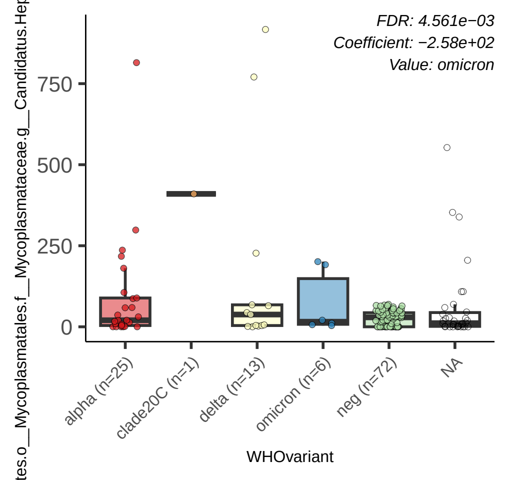

FDR: 4.561e−03
Coefficient: −2.58e+02
Value: omicron
750
500
250
0
tes.o__Mycoplasmatales.f__Mycoplasmataceae.g__Candidatus.Hep
alpha (n=25)
clade20C (n=1)
delta (n=13)
omicron (n=6)
neg (n=72)
NA
WHOvariant

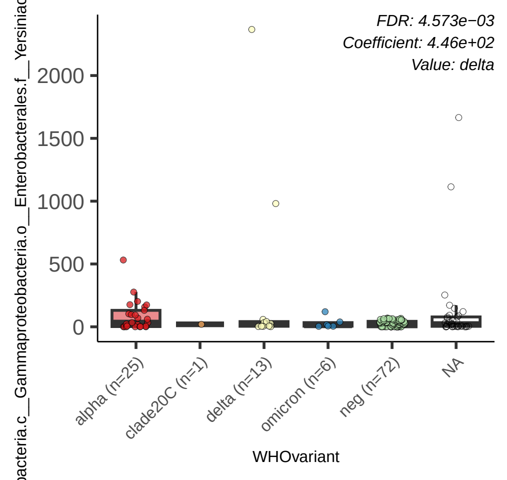

FDR: 4.573e−03
Coefficient: 4.46e+02
Value: delta
bacteria.c__Gammaproteobacteria.o__Enterobacterales.f__Yersiniac
2000
1500
1000
500
0
alpha (n=25)
clade20C (n=1)
delta (n=13)
omicron (n=6)
neg (n=72)
NA
WHOvariant

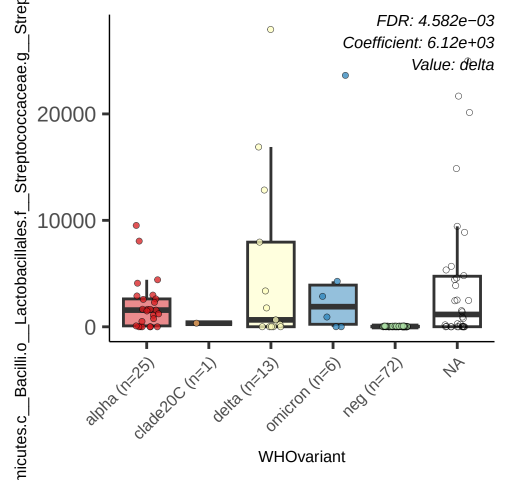

FDR: 4.582e−03
Coefficient: 6.12e+03
Value: delta
20000
10000
0
alpha (n=25)
clade20C (n=1)
delta (n=13)
omicron (n=6)
neg (n=72)
NA
WHOvariant
micutes.c__Bacilli.o__Lactobacillales.f__Streptococcaceae.g__Strep

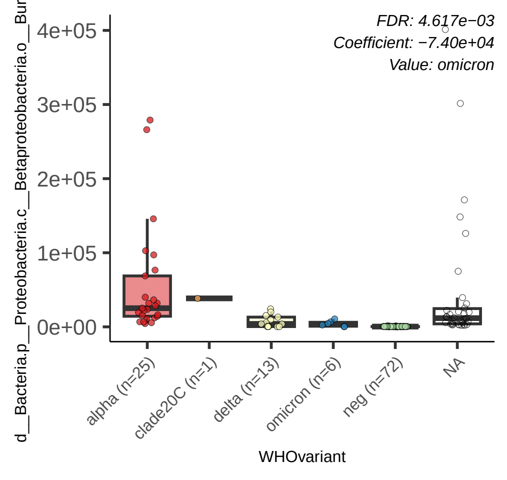

FDR: 4.617e−03
Coefficient: −7.40e+04
Value: omicron
d__Bacteria.p__Proteobacteria.c__Betaproteobacteria.o__Bur
4e+05
3e+05
2e+05
1e+05
0e+00
alpha (n=25)
clade20C (n=1)
delta (n=13)
omicron (n=6)
neg (n=72)
NA
WHOvariant

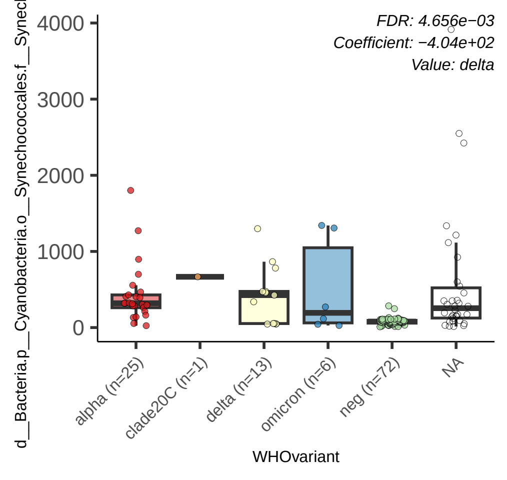
FDR: 4.656e−03
Coefficient: −4.04e+02
Value: delta
d__Bacteria.p__Cyanobacteria.o__Synechococcales.f__Synec
4000
3000
2000
1000
0
alpha (n=25)
clade20C (n=1)
delta (n=13)
omicron (n=6)
neg (n=72)
NA
WHOvariant

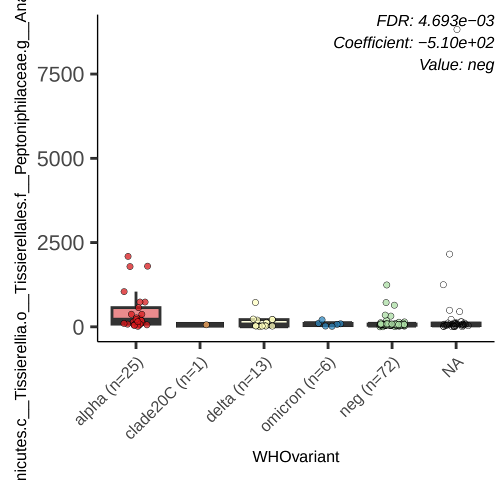

FDR: 4.693e−03
Coefficient: −5.10e+02
Value: neg
7500
5000
2500
0
nicutes.c__Tissierellia.o__Tissierellales.f__Peptoniphilaceae.g__Ana
alpha (n=25)
clade20C (n=1)
delta (n=13)
omicron (n=6)
neg (n=72)
NA
WHOvariant

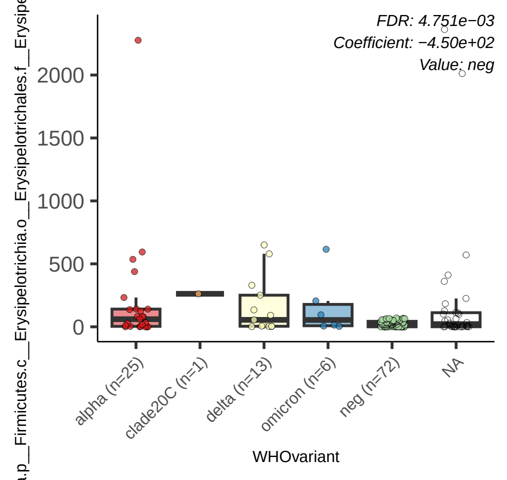
FDR: 4.751e−03
Coefficient: −4.50e+02
Value: neg
Firmicutes.c__Erysipelotrichia.o__Erysipelotrichales.f__Erysipe
0
500
1000
1500
2000
alpha (n=25)
clade20C (n=1)
delta (n=13)
omicron (n=6)
neg (n=72)
NA
WHOvariant

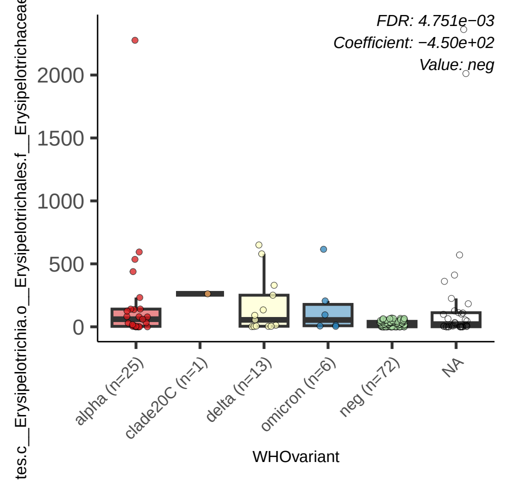

FDR: 4.751e−03
Coefficient: −4.50e+02
Value: neg
tes.c__Erysipelotrichia.o__Erysipelotrichales.f__Erysipelotrichaceae
2000
1500
1000
500
0
alpha (n=25)
clade20C (n=1)
delta (n=13)
omicron (n=6)
neg (n=72)
NA
WHOvariant

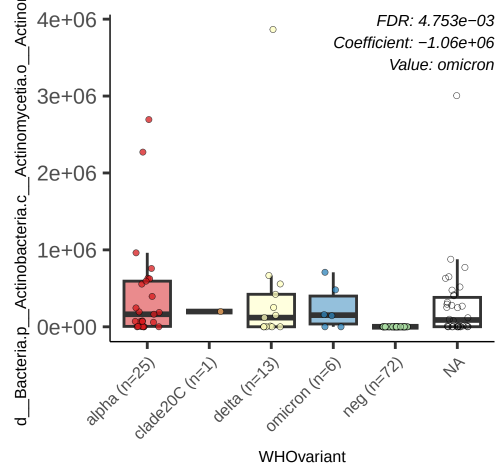

FDR: 4.753e−03
Coefficient: −1.06e+06
Value: omicron
d__Bacteria.p__Actinobacteria.c__Actinomycetia.o__Actino
4e+06
3e+06
2e+06
1e+06
0e+00
alpha (n=25)
clade20C (n=1)
delta (n=13)
omicron (n=6)
neg (n=72)
NA
WHOvariant

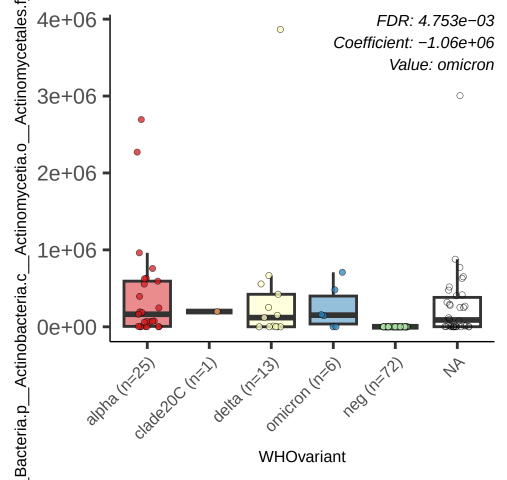

FDR: 4.753e−03
Coefficient: −1.06e+06
Value: omicron
4e+06
3e+06
2e+06
1e+06
0e+00
Bacteria.p__Actinobacteria.c__Actinomycetia.o__Actinomycetales.f
alpha (n=25)
clade20C (n=1)
delta (n=13)
omicron (n=6)
neg (n=72)
NA
WHOvariant

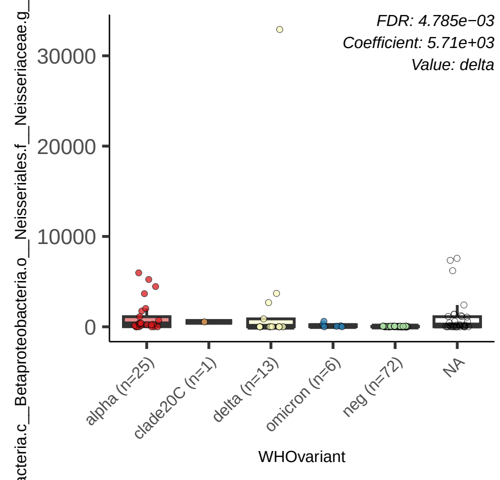

FDR: 4.785e−03
Coefficient: 5.71e+03
Value: delta
30000
20000
10000
0
alpha (n=25)
clade20C (n=1)
delta (n=13)
omicron (n=6)
neg (n=72)
NA
WHOvariant
acteria.c__Betaproteobacteria.o__Neisseriales.f__Neisseriaceae.g__

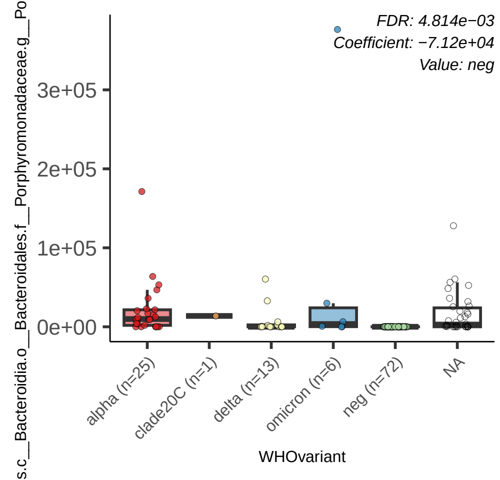
FDR: 4.814e−03
Coefficient: −7.12e+04
Value: neg
s.c__Bacteroidia.o__Bacteroidales.f__Porphyromonadaceae.g__Po
3e+05
2e+05
1e+05
0e+00
alpha (n=25)
clade20C (n=1)
delta (n=13)
omicron (n=6)
neg (n=72)
NA
WHOvariant

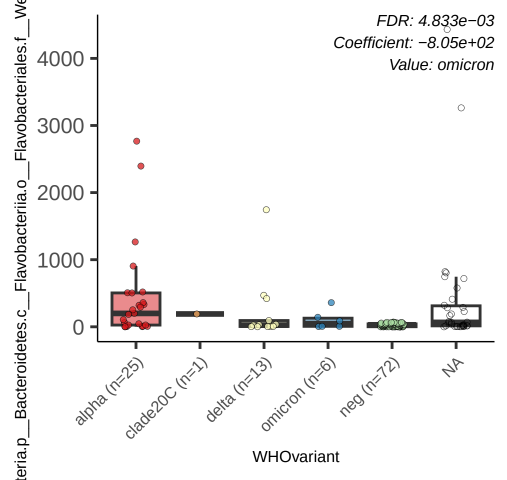

FDR: 4.833e−03
Coefficient: −8.05e+02
Value: omicron
4000
3000
2000
1000
0
alpha (n=25)
clade20C (n=1)
delta (n=13)
omicron (n=6)
neg (n=72)
NA
WHOvariant

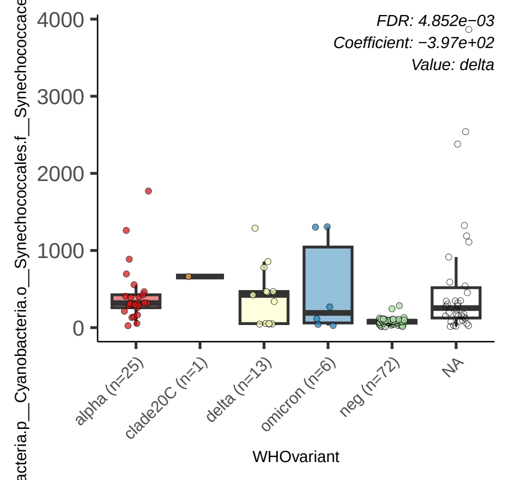

FDR: 4.852e−03
Coefficient: −3.97e+02
Value: delta
4000
3000
2000
1000
0
acteria.p__Cyanobacteria.o__Synechococcales.f__Synechococcace
alpha (n=25)
clade20C (n=1)
delta (n=13)
omicron (n=6)
neg (n=72)
NA
WHOvariant

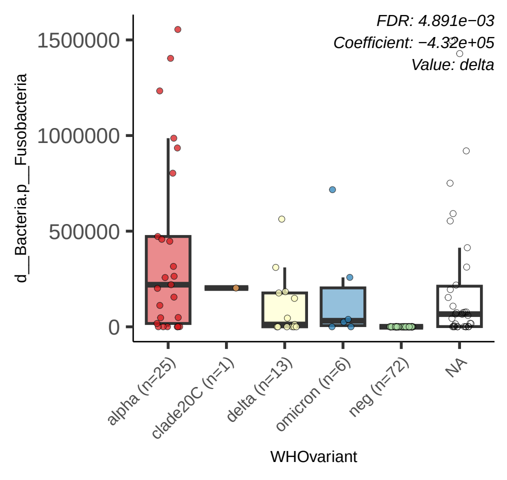
FDR: 4.891e−03
Coefficient: −4.32e+05
Value: delta
d__Bacteria.p__Fusobacteria
1500000
1000000
500000
0
alpha (n=25)
clade20C (n=1)
delta (n=13)
omicron (n=6)
neg (n=72)
NA
WHOvariant

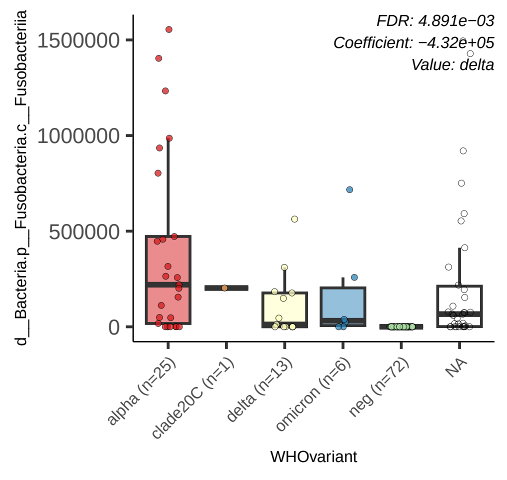
FDR: 4.891e−03
Coefficient: −4.32e+05
Value: delta
d__Bacteria.p__Fusobacteria.c__Fusobacteriia
1500000
1000000
500000
0
alpha (n=25)
clade20C (n=1)
delta (n=13)
omicron (n=6)
neg (n=72)
NA
WHOvariant

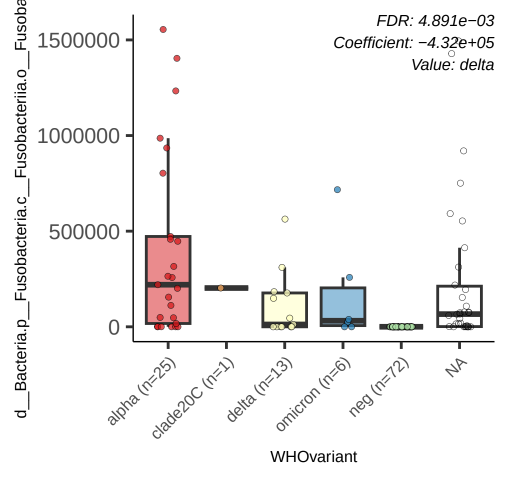
FDR: 4.891e−03
Coefficient: −4.32e+05
Value: delta
d__Bacteria.p__Fusobacteria.c__Fusobacteriia.o__Fusoba
1500000
1000000
500000
0
alpha (n=25)
clade20C (n=1)
delta (n=13)
omicron (n=6)
neg (n=72)
NA
WHOvariant

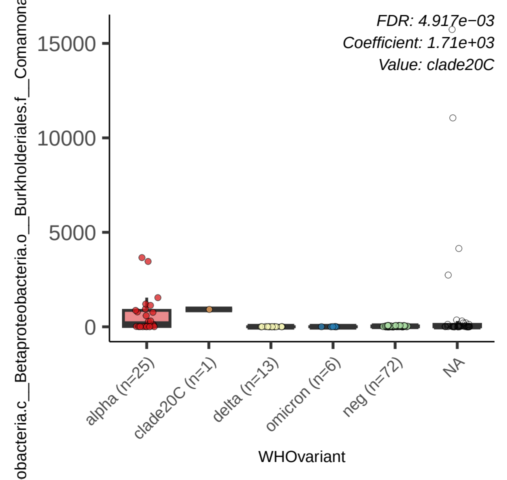

FDR: 4.917e−03
Coefficient: 1.71e+03
Value: clade20C
15000
10000
5000
0
obacteria.c__Betaproteobacteria.o__Burkholderiales.f__Comamona
alpha (n=25)
clade20C (n=1)
delta (n=13)
omicron (n=6)
neg (n=72)
NA
WHOvariant

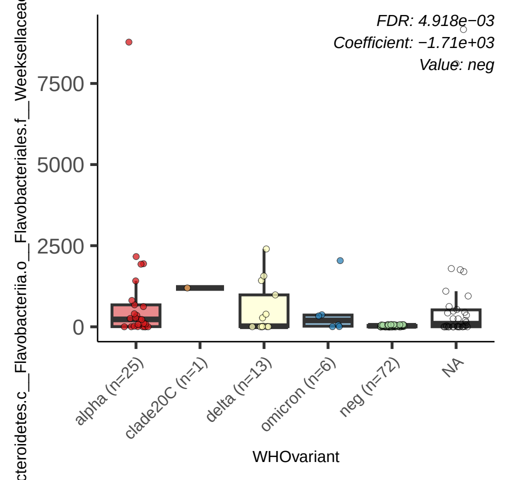

FDR: 4.918e−03
Coefficient: −1.71e+03
Value: neg
0
2500
5000
7500
cteroidetes.c__Flavobacteriia.o__Flavobacteriales.f__Weeksellacea
alpha (n=25)
clade20C (n=1)
delta (n=13)
omicron (n=6)
neg (n=72)
NA
WHOvariant

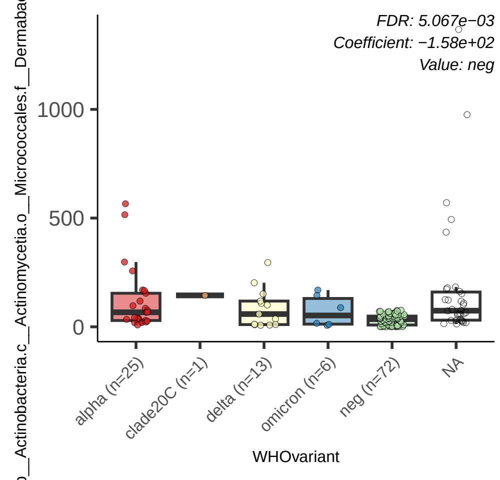

FDR: 5.067e−03
Coefficient: −1.58e+02
Value: neg
1000
500
0
o__Actinobacteria.c__Actinomycetia.o__Micrococcales.f__Dermabac
alpha (n=25)
clade20C (n=1)
delta (n=13)
omicron (n=6)
neg (n=72)
NA
WHOvariant

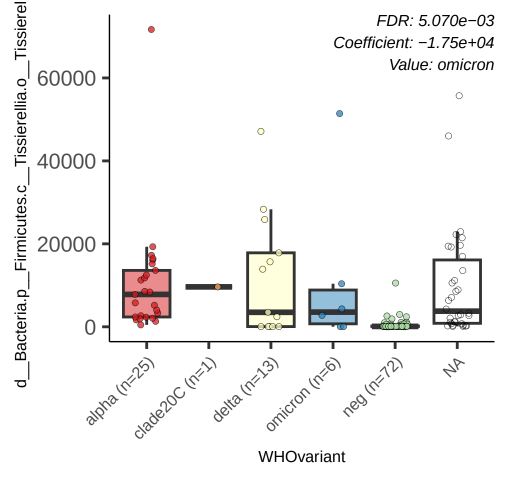

FDR: 5.070e−03
Coefficient: −1.75e+04
Value: omicron
d__Bacteria.p__Firmicutes.c__Tissierellia.o__Tissierel
60000
40000
20000
0
alpha (n=25)
clade20C (n=1)
delta (n=13)
omicron (n=6)
neg (n=72)
NA
WHOvariant

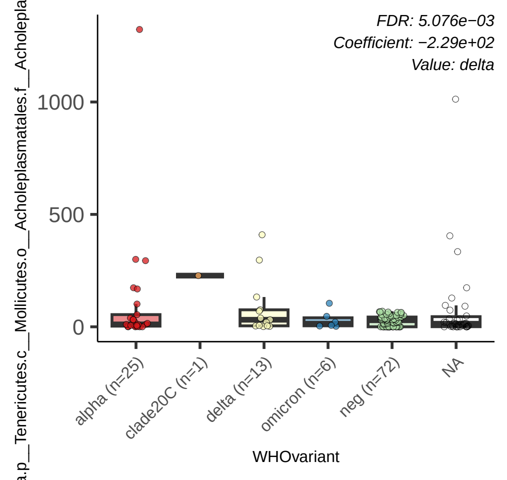

FDR: 5.076e−03
Coefficient: −2.29e+02
Value: delta
1000
500
0
a.p__Tenericutes.c__Mollicutes.o__Acholeplasmatales.f__Acholepla
alpha (n=25)
clade20C (n=1)
delta (n=13)
omicron (n=6)
neg (n=72)
NA
WHOvariant

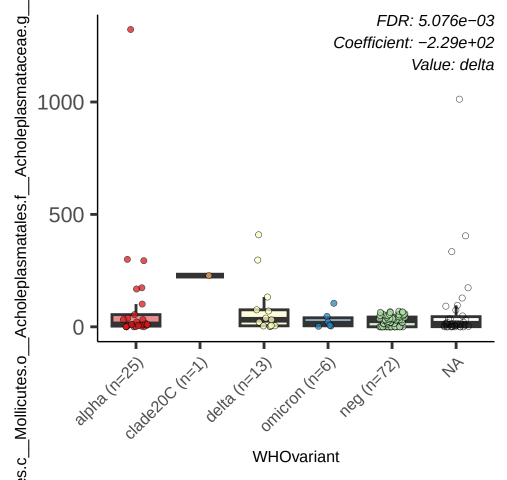

FDR: 5.076e−03
Coefficient: −2.29e+02
Value: delta
es.c__Mollicutes.o__Acholeplasmatales.f__Acholeplasmataceae.g__
1000
500
0
alpha (n=25)
clade20C (n=1)
delta (n=13)
omicron (n=6)
neg (n=72)
NA
WHOvariant

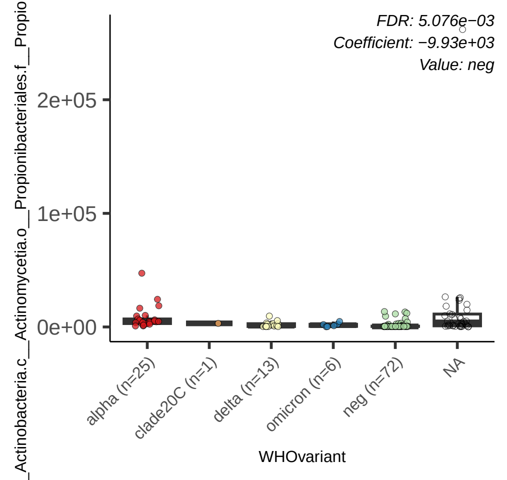

FDR: 5.076e−03
Coefficient: −9.93e+03
Value: neg
Actinobacteria.c__Actinomycetia.o__Propionibacteriales.f__Propio
2e+05
1e+05
0e+00
alpha (n=25)
clade20C (n=1)
delta (n=13)
omicron (n=6)
neg (n=72)
NA
WHOvariant

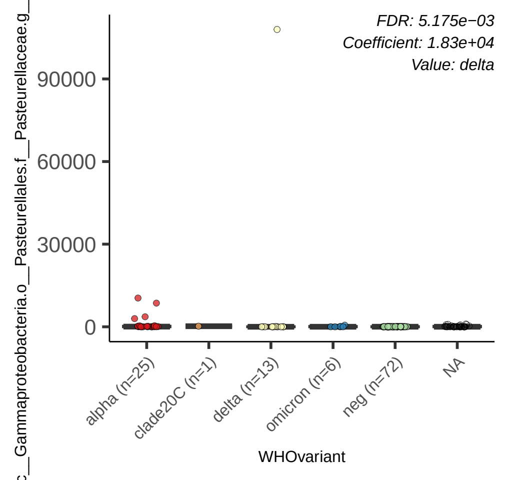

FDR: 5.175e−03
Coefficient: 1.83e+04
Value: delta
90000
60000
30000
0
c__Gammaproteobacteria.o__Pasteurellales.f__Pasteurellaceae.g__
alpha (n=25)
clade20C (n=1)
delta (n=13)
omicron (n=6)
neg (n=72)
NA
WHOvariant

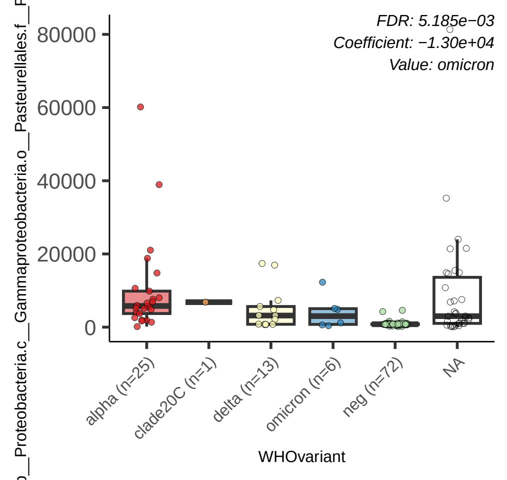

FDR: 5.185e−03
Coefficient: −1.30e+04
Value: omicron
80000
60000
40000
20000
0
p__Proteobacteria.c__Gammaproteobacteria.o__Pasteurellales.f__P
alpha (n=25)
clade20C (n=1)
delta (n=13)
omicron (n=6)
neg (n=72)
NA
WHOvariant

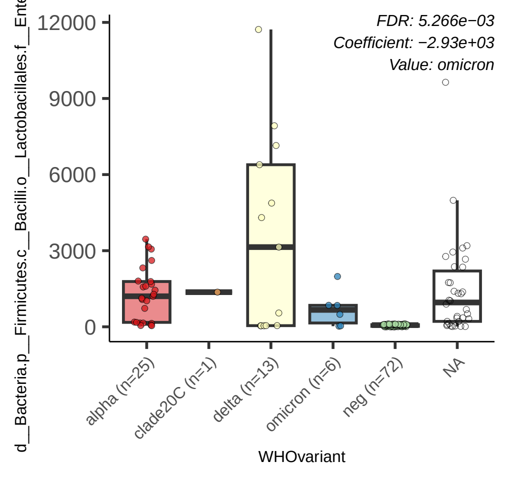
FDR: 5.266e−03
Coefficient: −2.93e+03
Value: omicron
d__Bacteria.p__Firmicutes.c__Bacilli.o__Lactobacillales.f__Ente
12000
9000
6000
3000
0
alpha (n=25)
clade20C (n=1)
delta (n=13)
omicron (n=6)
neg (n=72)
NA
WHOvariant

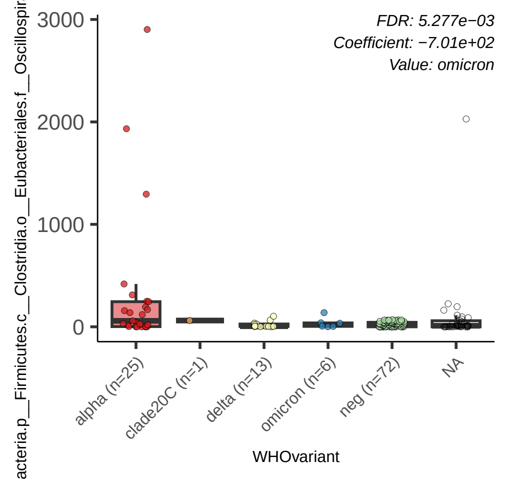

FDR: 5.277e−03
Coefficient: −7.01e+02
Value: omicron
3000
2000
1000
0
acteria.p__Firmicutes.c__Clostridia.o__Eubacteriales.f__Oscillospir
alpha (n=25)
clade20C (n=1)
delta (n=13)
omicron (n=6)
neg (n=72)
NA
WHOvariant

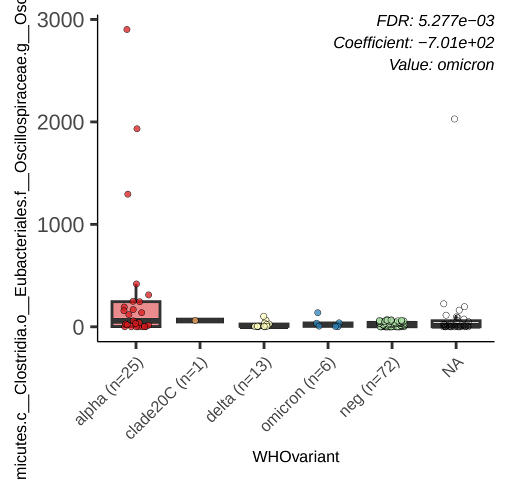

FDR: 5.277e−03
Coefficient: −7.01e+02
Value: omicron
3000
2000
1000
0
micutes.c__Clostridia.o__Eubacteriales.f__Oscillospiraceae.g__Osc
alpha (n=25)
clade20C (n=1)
delta (n=13)
omicron (n=6)
neg (n=72)
NA
WHOvariant

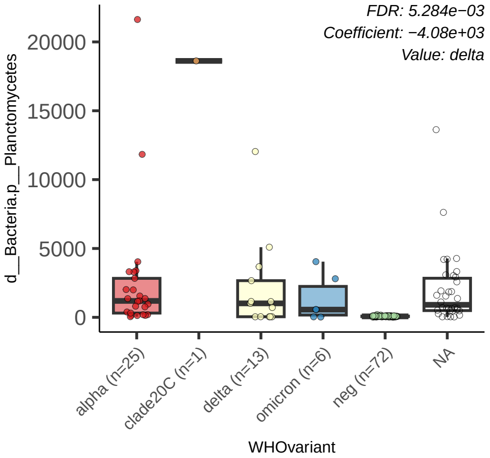
FDR: 5.284e−03
Coefficient: −4.08e+03
Value: delta
d__Bacteria.p__Planctomycetes
20000
15000
10000
5000
0
alpha (n=25)
clade20C (n=1)
delta (n=13)
omicron (n=6)
neg (n=72)
NA
WHOvariant

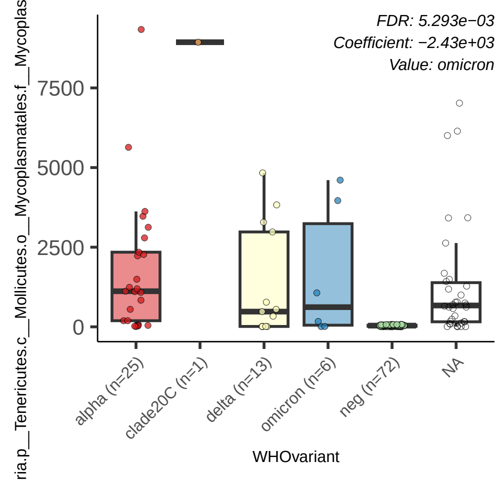
FDR: 5.293e−03
Coefficient: −2.43e+03
Value: omicron
ria.p__Tenericutes.c__Mollicutes.o__Mycoplasmatales.f__Mycoplas
7500
5000
2500
0
alpha (n=25)
clade20C (n=1)
delta (n=13)
omicron (n=6)
neg (n=72)
NA
WHOvariant

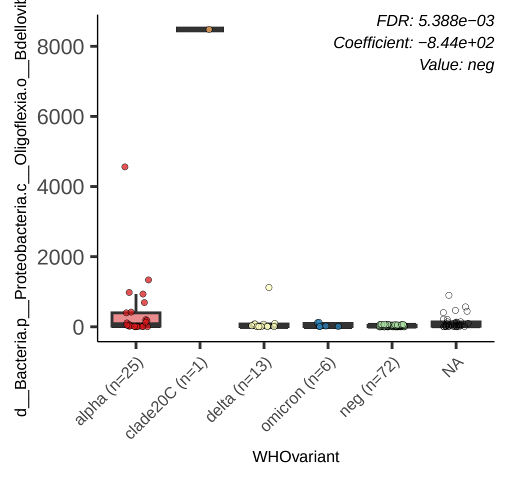
FDR: 5.388e−03
Coefficient: −8.44e+02
Value: neg
d__Bacteria.p__Proteobacteria.c__Oligoflexia.o__Bdellovib
8000
6000
4000
2000
0
alpha (n=25)
clade20C (n=1)
delta (n=13)
omicron (n=6)
neg (n=72)
NA
WHOvariant

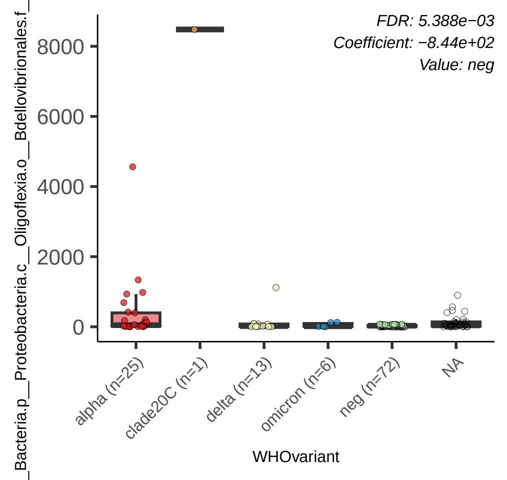

FDR: 5.388e−03
Coefficient: −8.44e+02
Value: neg
Bacteria.p__Proteobacteria.c__Oligoflexia.o__Bdellovibrionales.f
8000
6000
4000
2000
0
alpha (n=25)
clade20C (n=1)
delta (n=13)
omicron (n=6)
neg (n=72)
NA
WHOvariant

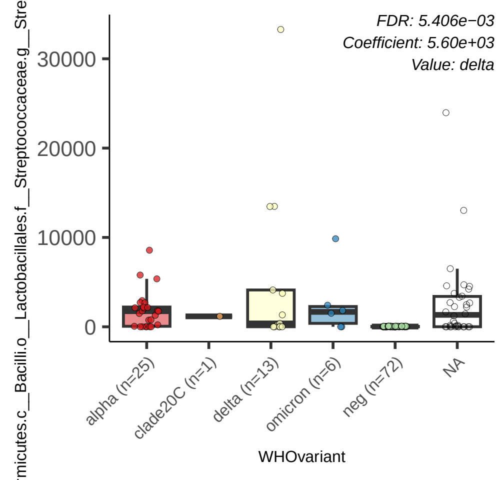

FDR: 5.406e−03
Coefficient: 5.60e+03
Value: delta
30000
20000
10000
0
alpha (n=25)
clade20C (n=1)
delta (n=13)
omicron (n=6)
neg (n=72)
NA
WHOvariant

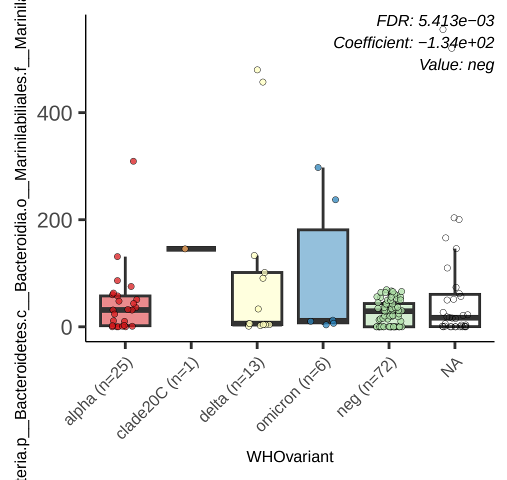
FDR: 5.413e−03
Coefficient: −1.34e+02
Value: neg
teria.p__Bacteroidetes.c__Bacteroidia.o__Marinilabiliales.f__Marinil
0
200
400
alpha (n=25)
clade20C (n=1)
delta (n=13)
omicron (n=6)
neg (n=72)
NA
WHOvariant

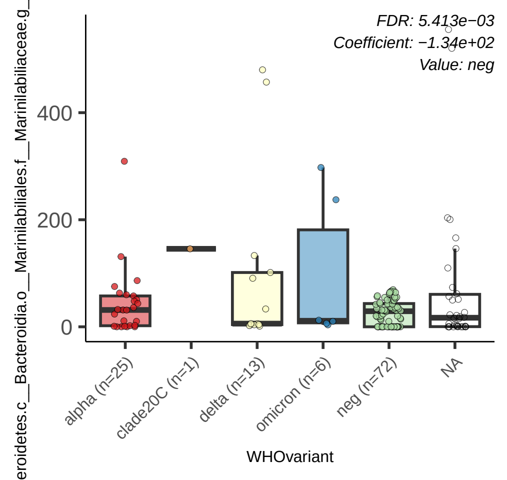
FDR: 5.413e−03
Coefficient: −1.34e+02
Value: neg
eroidetes.c__Bacteroidia.o__Marinilabiliales.f__Marinilabiliaceae.g__
0
200
400
alpha (n=25)
clade20C (n=1)
delta (n=13)
omicron (n=6)
neg (n=72)
NA
WHOvariant

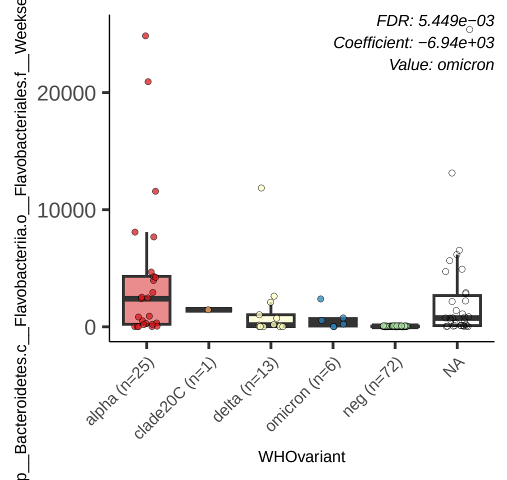
FDR: 5.449e−03
Coefficient: −6.94e+03
Value: omicron
20000
10000
0
p__Bacteroidetes.c__Flavobacteriia.o__Flavobacteriales.f__Weekse
alpha (n=25)
clade20C (n=1)
delta (n=13)
omicron (n=6)
neg (n=72)
NA
WHOvariant

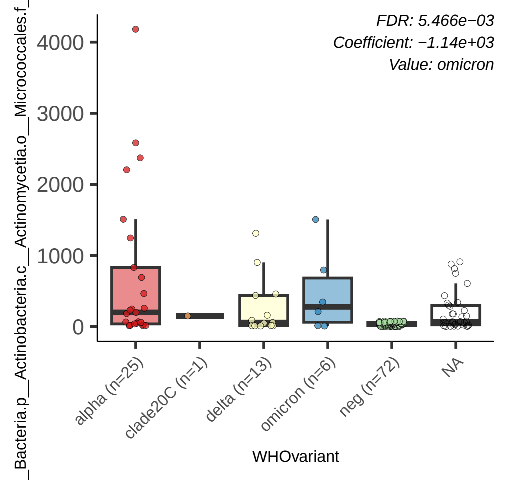

FDR: 5.466e−03
Coefficient: −1.14e+03
Value: omicron
Bacteria.p__Actinobacteria.c__Actinomycetia.o__Micrococcales.f__
4000
3000
2000
1000
0
alpha (n=25)
clade20C (n=1)
delta (n=13)
omicron (n=6)
neg (n=72)
NA
WHOvariant

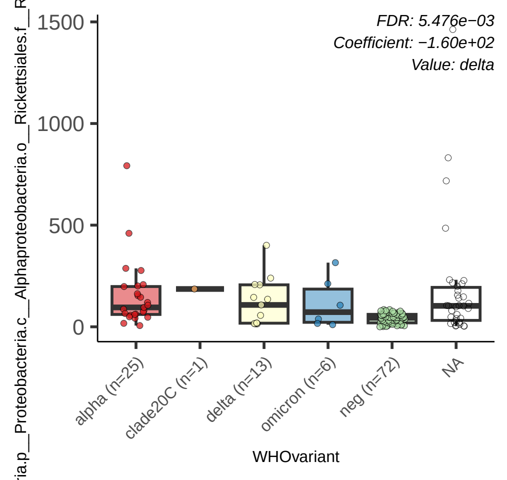
FDR: 5.476e−03
Coefficient: −1.60e+02
Value: delta
ria.p__Proteobacteria.c__Alphaproteobacteria.o__Rickettsiales.f__R
1500
1000
500
0
alpha (n=25)
clade20C (n=1)
delta (n=13)
omicron (n=6)
neg (n=72)
NA
WHOvariant

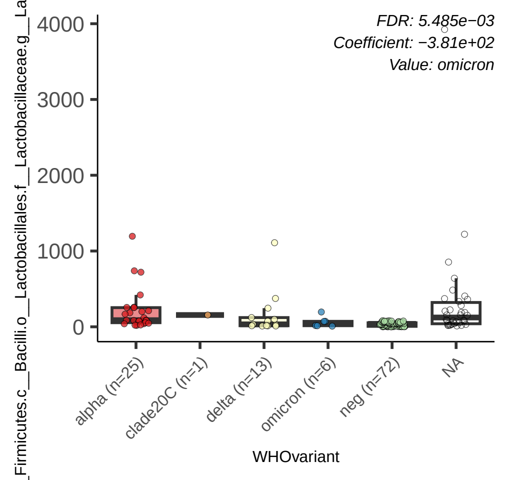
FDR: 5.485e−03
Coefficient: −3.81e+02
Value: omicron
Firmicutes.c__Bacilli.o__Lactobacillales.f__Lactobacillaceae.g__La
4000
3000
2000
1000
0
alpha (n=25)
clade20C (n=1)
delta (n=13)
omicron (n=6)
neg (n=72)
NA
WHOvariant

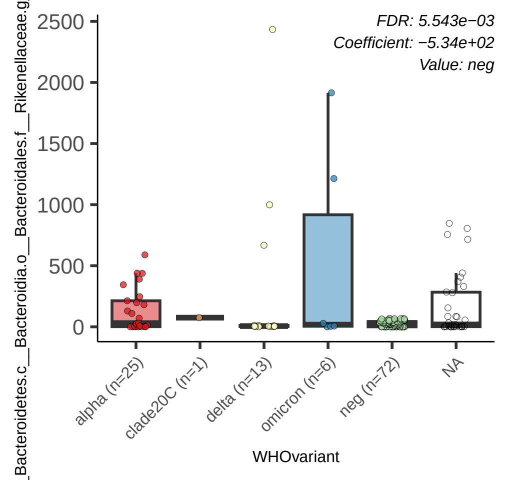
FDR: 5.543e−03
Coefficient: −5.34e+02
Value: neg
Bacteroidetes.c__Bacteroidia.o__Bacteroidales.f__Rikenellaceae.g
2500
2000
1500
1000
500
0
alpha (n=25)
clade20C (n=1)
delta (n=13)
omicron (n=6)
neg (n=72)
NA
WHOvariant

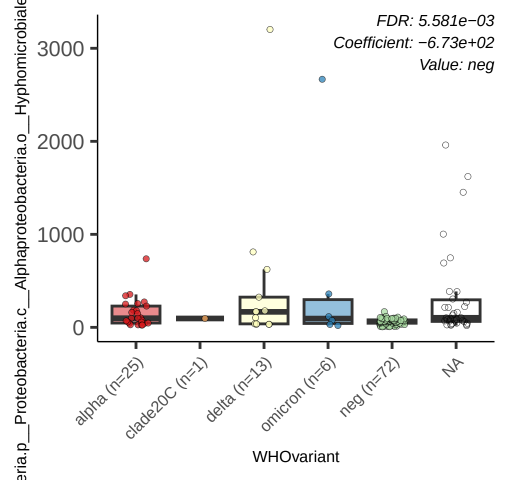

FDR: 5.581e−03
Coefficient: −6.73e+02
Value: neg
3000
2000
1000
0
eria.p__Proteobacteria.c__Alphaproteobacteria.o__Hyphomicrobiale
alpha (n=25)
clade20C (n=1)
delta (n=13)
omicron (n=6)
neg (n=72)
NA
WHOvariant

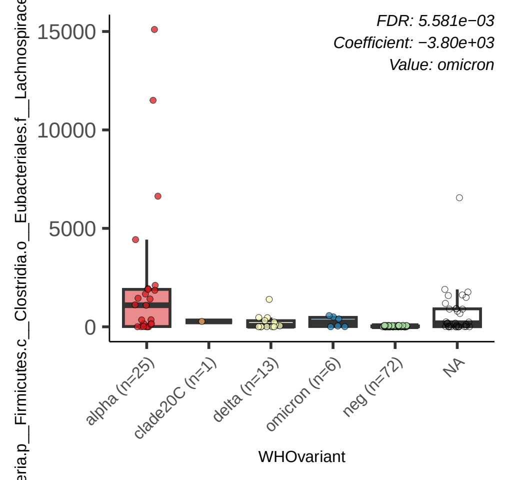

FDR: 5.581e−03
Coefficient: −3.80e+03
Value: omicron
0
5000
10000
15000
eria.p__Firmicutes.c__Clostridia.o__Eubacteriales.f__Lachnospirace
alpha (n=25)
clade20C (n=1)
delta (n=13)
omicron (n=6)
neg (n=72)
NA
WHOvariant

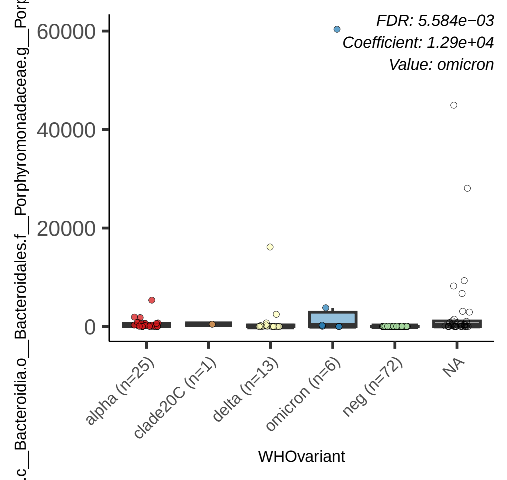

FDR: 5.584e−03
Coefficient: 1.29e+04
Value: omicron
60000
40000
20000
0
c__Bacteroidia.o__Bacteroidales.f__Porphyromonadaceae.g__Porp
alpha (n=25)
clade20C (n=1)
delta (n=13)
omicron (n=6)
neg (n=72)
NA
WHOvariant

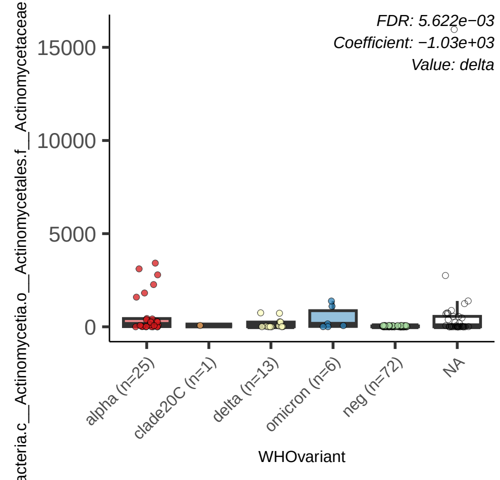

FDR: 5.622e−03
Coefficient: −1.03e+03
Value: delta
15000
10000
5000
0
acteria.c__Actinomycetia.o__Actinomycetales.f__Actinomycetaceae
alpha (n=25)
clade20C (n=1)
delta (n=13)
omicron (n=6)
neg (n=72)
NA
WHOvariant

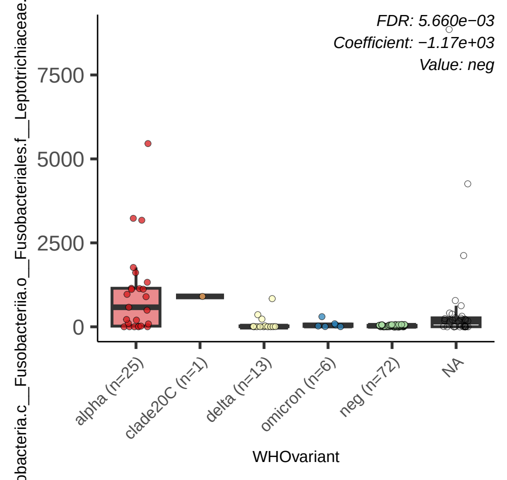

FDR: 5.660e−03
Coefficient: −1.17e+03
Value: neg
obacteria.c__Fusobacteriia.o__Fusobacteriales.f__Leptotrichiaceae.
7500
5000
2500
0
alpha (n=25)
clade20C (n=1)
delta (n=13)
omicron (n=6)
neg (n=72)
NA
WHOvariant

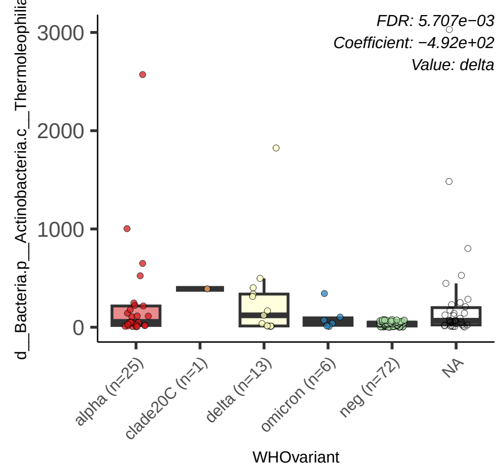

FDR: 5.707e−03
Coefficient: −4.92e+02
Value: delta
d__Bacteria.p__Actinobacteria.c__Thermoleophilia
3000
2000
1000
0
alpha (n=25)
clade20C (n=1)
delta (n=13)
omicron (n=6)
neg (n=72)
NA
WHOvariant

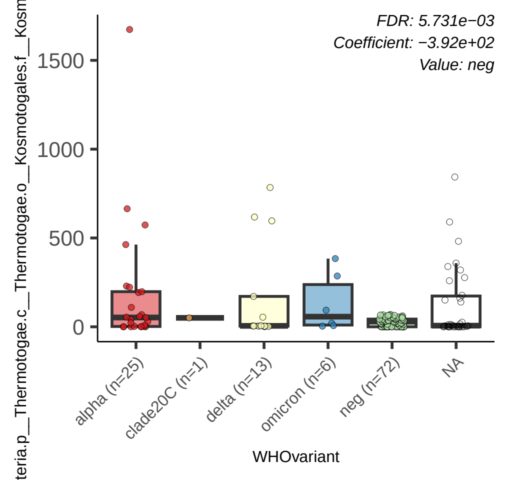

FDR: 5.731e−03
Coefficient: −3.92e+02
Value: neg
teria.p__Thermotogae.c__Thermotogae.o__Kosmotogales.f__Kosm
1500
1000
500
0
alpha (n=25)
clade20C (n=1)
delta (n=13)
omicron (n=6)
neg (n=72)
NA
WHOvariant

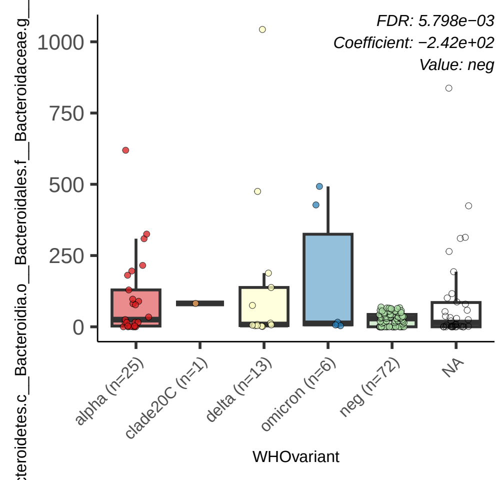

FDR: 5.798e−03
Coefficient: −2.42e+02
Value: neg
cteroidetes.c__Bacteroidia.o__Bacteroidales.f__Bacteroidaceae.g__
1000
750
500
250
0
alpha (n=25)
clade20C (n=1)
delta (n=13)
omicron (n=6)
neg (n=72)
NA
WHOvariant

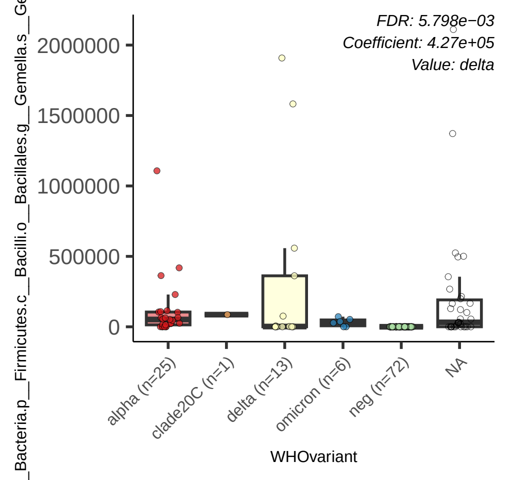

FDR: 5.798e−03
Coefficient: 4.27e+05
Value: delta
2000000
1500000
1000000
500000
0
alpha (n=25)
clade20C (n=1)
delta (n=13)
omicron (n=6)
neg (n=72)
NA
WHOvariant
Bacteria.p__Firmicutes.c__Bacilli.o__Bacillales.g__Gemella.s__Ge

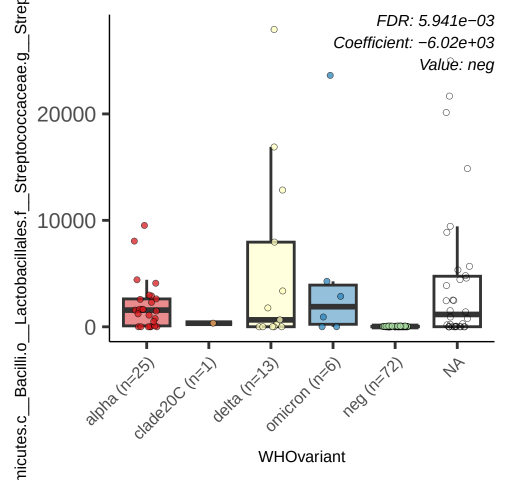

FDR: 5.941e−03
Coefficient: −6.02e+03
Value: neg
20000
10000
0
micutes.c__Bacilli.o__Lactobacillales.f__Streptococcaceae.g__Strep
alpha (n=25)
clade20C (n=1)
delta (n=13)
omicron (n=6)
neg (n=72)
NA
WHOvariant

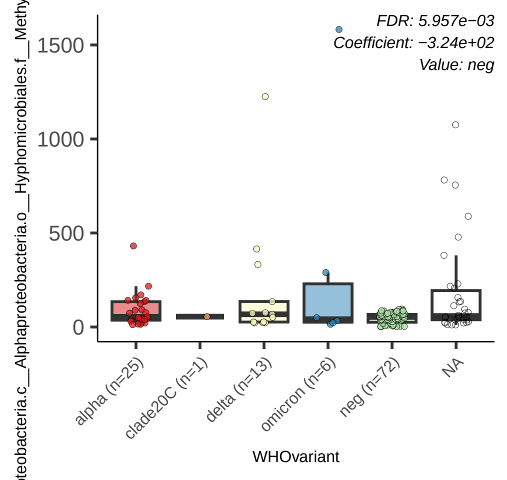

FDR: 5.957e−03
Coefficient: −3.24e+02
Value: neg
1500
1000
500
0
alpha (n=25)
clade20C (n=1)
delta (n=13)
omicron (n=6)
neg (n=72)
NA
WHOvariant
oteobacteria.c__Alphaproteobacteria.o__Hyphomicrobiales.f__Methy

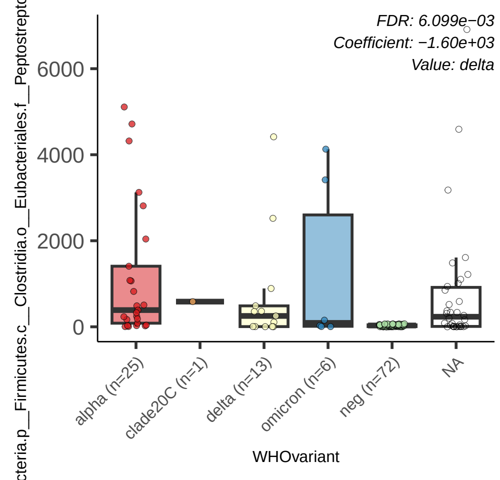

FDR: 6.099e−03
Coefficient: −1.60e+03
Value: delta
6000
4000
2000
0
cteria.p__Firmicutes.c__Clostridia.o__Eubacteriales.f__Peptostrepto
alpha (n=25)
clade20C (n=1)
delta (n=13)
omicron (n=6)
neg (n=72)
NA
WHOvariant

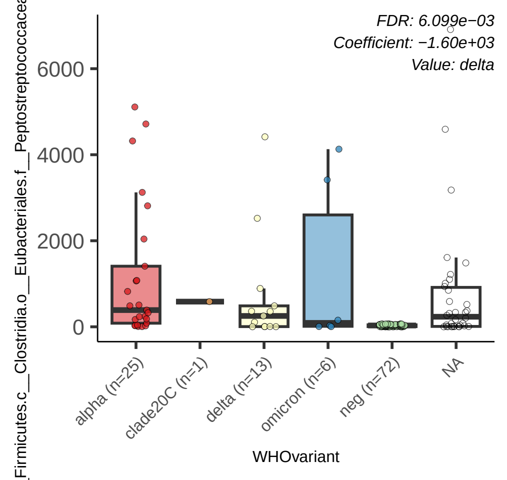

FDR: 6.099e−03
Coefficient: −1.60e+03
Value: delta
6000
4000
2000
0
Firmicutes.c__Clostridia.o__Eubacteriales.f__Peptostreptococcacea
alpha (n=25)
clade20C (n=1)
delta (n=13)
omicron (n=6)
neg (n=72)
NA
WHOvariant

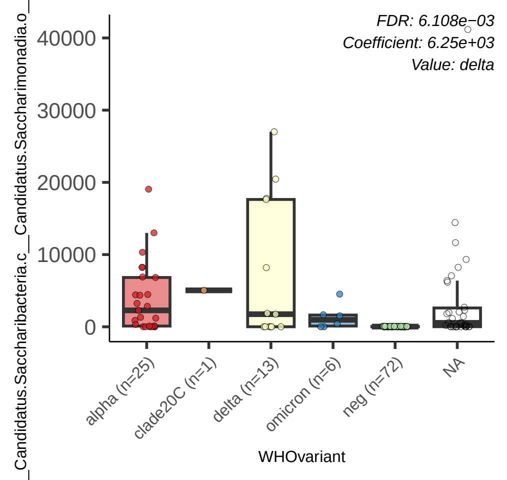
FDR: 6.108e−03
Coefficient: 6.25e+03
Value: delta
Candidatus.Saccharibacteria.c__Candidatus.Saccharimonadia.o
40000
30000
20000
10000
0
alpha (n=25)
clade20C (n=1)
delta (n=13)
omicron (n=6)
neg (n=72)
NA
WHOvariant

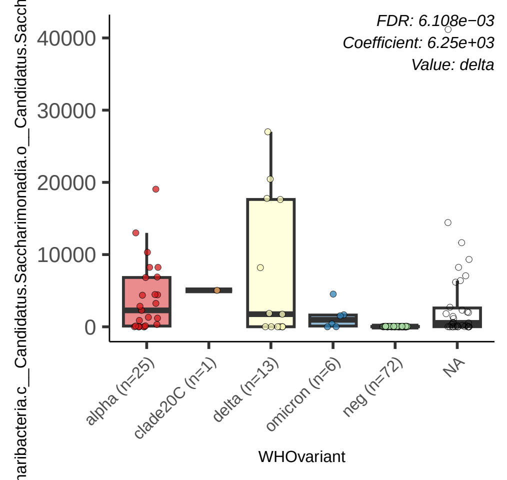
FDR: 6.108e−03
Coefficient: 6.25e+03
Value: delta
40000
30000
20000
10000
0
alpha (n=25)
clade20C (n=1)
delta (n=13)
omicron (n=6)
neg (n=72)
NA
WHOvariant
haribacteria.c__Candidatus.Saccharimonadia.o__Candidatus.Sacch

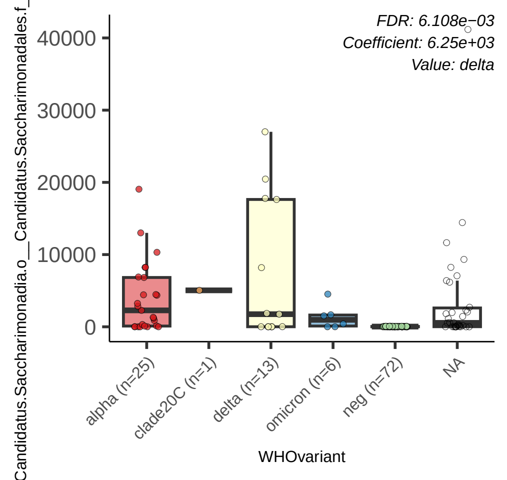

FDR: 6.108e−03
Coefficient: 6.25e+03
Value: delta
Candidatus.Saccharimonadia.o__Candidatus.Saccharimonadales.f_
40000
30000
20000
10000
0
alpha (n=25)
clade20C (n=1)
delta (n=13)
omicron (n=6)
neg (n=72)
NA
WHOvariant

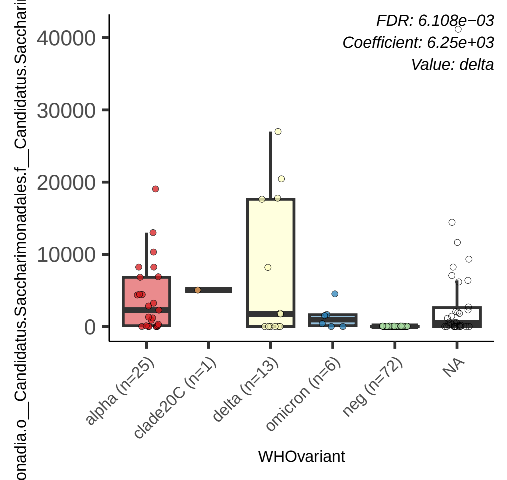

FDR: 6.108e−03
Coefficient: 6.25e+03
Value: delta
40000
30000
20000
10000
0
onadia.o__Candidatus.Saccharimonadales.f__Candidatus.Sacchari
alpha (n=25)
clade20C (n=1)
delta (n=13)
omicron (n=6)
neg (n=72)
NA
WHOvariant

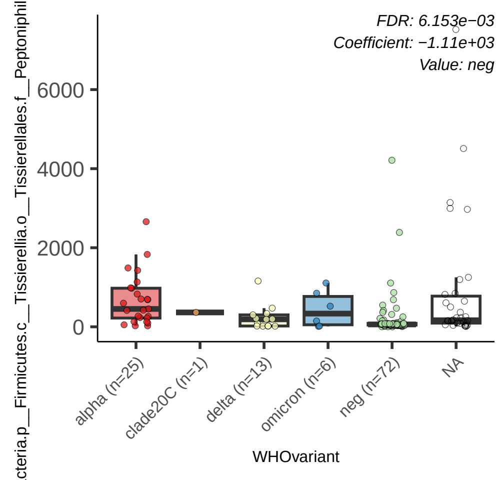

FDR: 6.153e−03
Coefficient: −1.11e+03
Value: neg
6000
4000
2000
0
cteria.p__Firmicutes.c__Tissierellia.o__Tissierellales.f__Peptoniphil
alpha (n=25)
clade20C (n=1)
delta (n=13)
omicron (n=6)
neg (n=72)
NA
WHOvariant

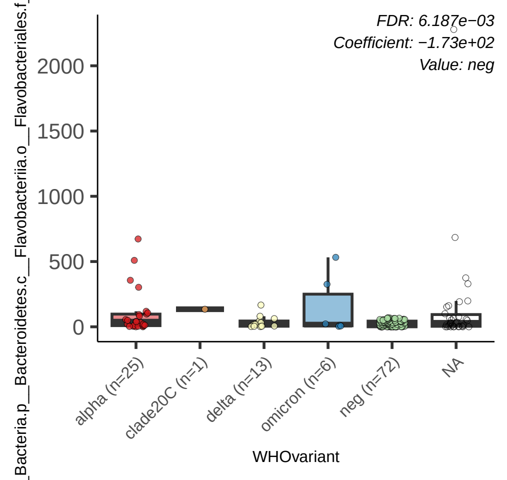

FDR: 6.187e−03
Coefficient: −1.73e+02
Value: neg
Bacteria.p__Bacteroidetes.c__Flavobacteriia.o__Flavobacteriales.f
2000
1500
1000
500
0
alpha (n=25)
clade20C (n=1)
delta (n=13)
omicron (n=6)
neg (n=72)
NA
WHOvariant

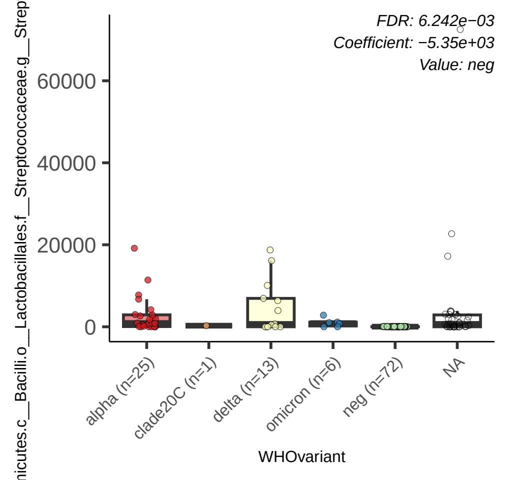

FDR: 6.242e−03
Coefficient: −5.35e+03
Value: neg
60000
40000
20000
0
alpha (n=25)
clade20C (n=1)
delta (n=13)
omicron (n=6)
neg (n=72)
NA
WHOvariant

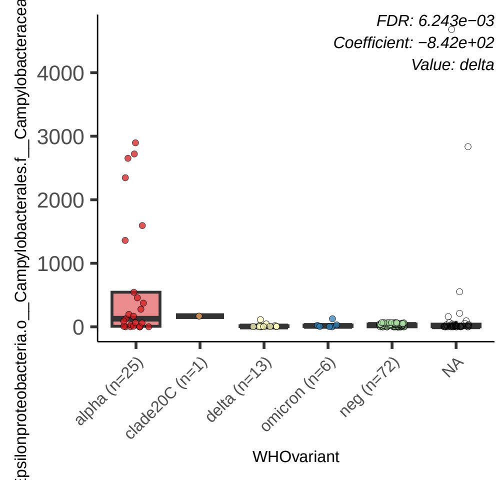

FDR: 6.243e−03
Coefficient: −8.42e+02
Value: delta
4000
3000
2000
1000
0
psilonproteobacteria.o__Campylobacterales.f__Campylobacteracea
alpha (n=25)
clade20C (n=1)
delta (n=13)
omicron (n=6)
neg (n=72)
NA
WHOvariant

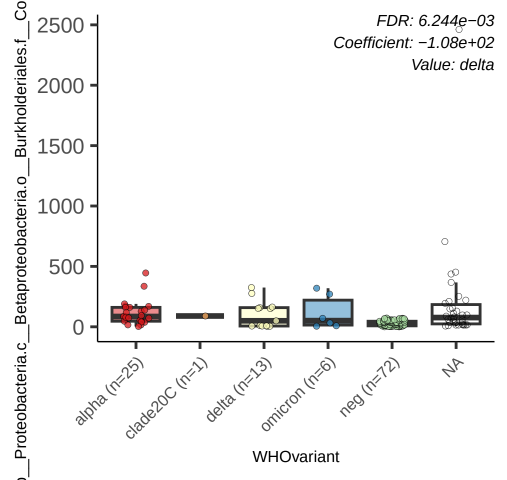

FDR: 6.244e−03
Coefficient: −1.08e+02
Value: delta
o__Proteobacteria.c__Betaproteobacteria.o__Burkholderiales.f__Co
2500
2000
1500
1000
500
0
alpha (n=25)
clade20C (n=1)
delta (n=13)
omicron (n=6)
neg (n=72)
NA
WHOvariant

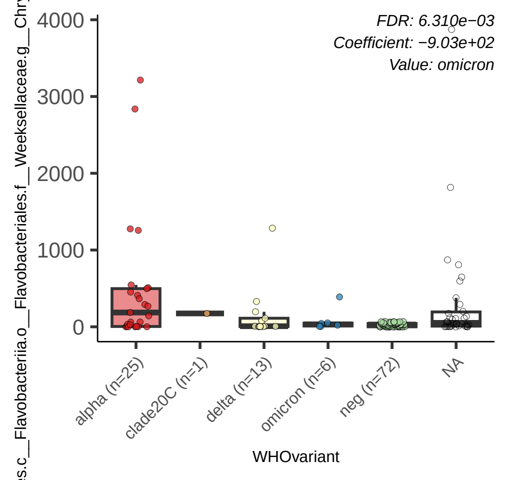

FDR: 6.310e−03
Coefficient: −9.03e+02
Value: omicron
4000
3000
2000
1000
0
es.c__Flavobacteriia.o__Flavobacteriales.f__Weeksellaceae.g__Chry
alpha (n=25)
clade20C (n=1)
delta (n=13)
omicron (n=6)
neg (n=72)
NA
WHOvariant

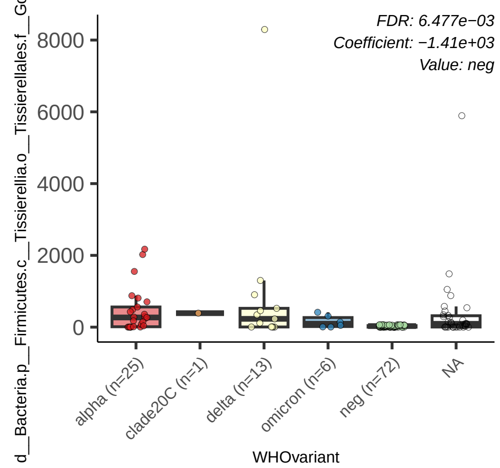

FDR: 6.477e−03
Coefficient: −1.41e+03
Value: neg
d__Bacteria.p__Firmicutes.c__Tissierellia.o__Tissierellales.f__Go
8000
6000
4000
2000
0
alpha (n=25)
clade20C (n=1)
delta (n=13)
omicron (n=6)
neg (n=72)
NA
WHOvariant

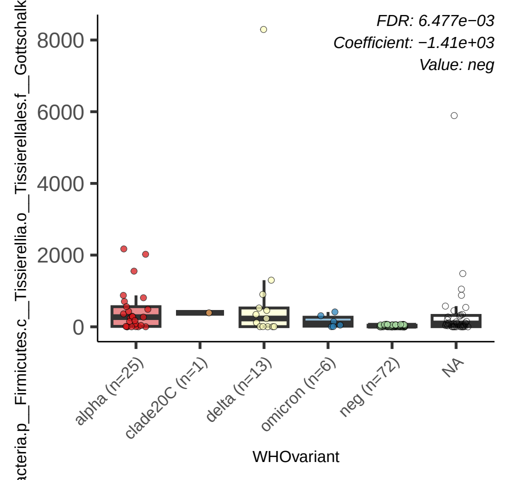

FDR: 6.477e−03
Coefficient: −1.41e+03
Value: neg
8000
6000
4000
2000
0
alpha (n=25)
clade20C (n=1)
delta (n=13)
omicron (n=6)
neg (n=72)
NA
WHOvariant
acteria.p__Firmicutes.c__Tissierellia.o__Tissierellales.f__Gottschalk

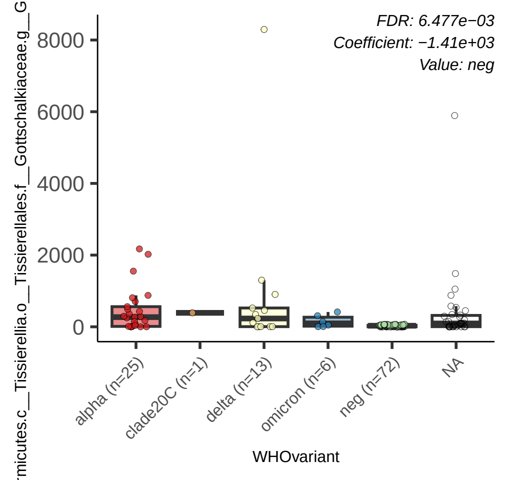

FDR: 6.477e−03
Coefficient: −1.41e+03
Value: neg
8000
6000
4000
2000
0
alpha (n=25)
clade20C (n=1)
delta (n=13)
omicron (n=6)
neg (n=72)
NA
WHOvariant
rmicutes.c__Tissierellia.o__Tissierellales.f__Gottschalkiaceae.g__G

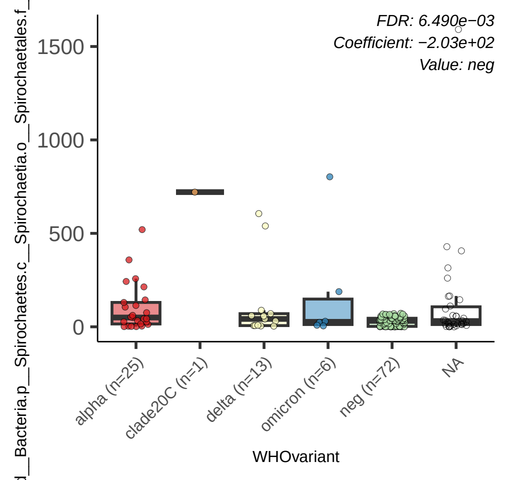

FDR: 6.490e−03
Coefficient: −2.03e+02
Value: neg
d__Bacteria.p__Spirochaetes.c__Spirochaetia.o__Spirochaetales.f__
1500
1000
500
0
alpha (n=25)
clade20C (n=1)
delta (n=13)
omicron (n=6)
neg (n=72)
NA
WHOvariant

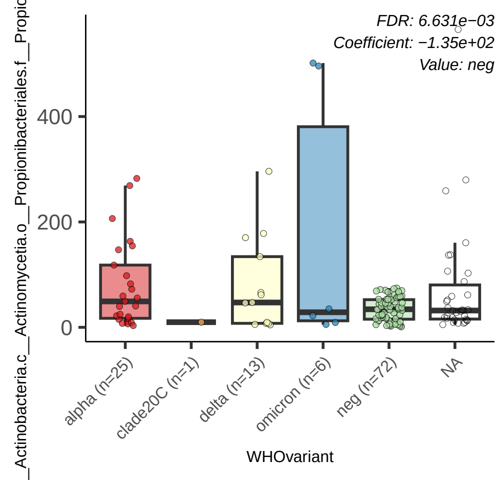

FDR: 6.631e−03
Coefficient: −1.35e+02
Value: neg
0
200
400
_Actinobacteria.c__Actinomycetia.o__Propionibacteriales.f__Propic
alpha (n=25)
clade20C (n=1)
delta (n=13)
omicron (n=6)
neg (n=72)
NA
WHOvariant

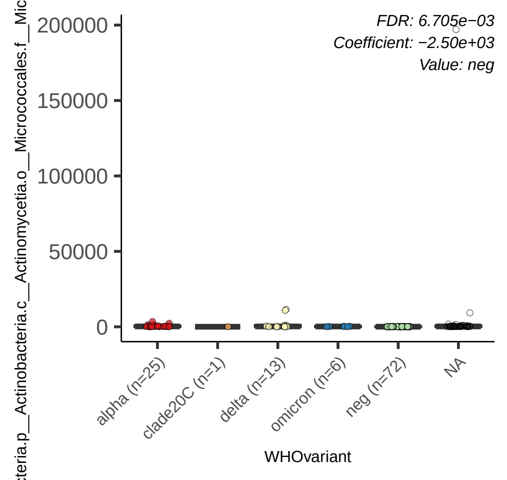

FDR: 6.705e−03
Coefficient: −2.50e+03
Value: neg
200000
150000
100000
50000
0
alpha (n=25)
clade20C (n=1)
delta (n=13)
omicron (n=6)
neg (n=72)
NA
WHOvariant
cteria.p__Actinobacteria.c__Actinomycetia.o__Micrococcales.f__Mic

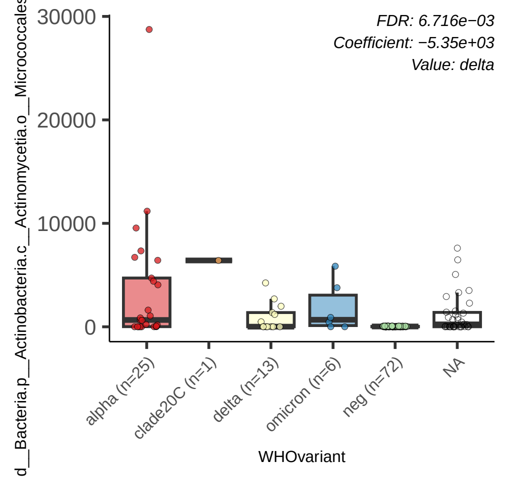

FDR: 6.716e−03
Coefficient: −5.35e+03
Value: delta
d__Bacteria.p__Actinobacteria.c__Actinomycetia.o__Micrococcales
30000
20000
10000
0
alpha (n=25)
clade20C (n=1)
delta (n=13)
omicron (n=6)
neg (n=72)
NA
WHOvariant

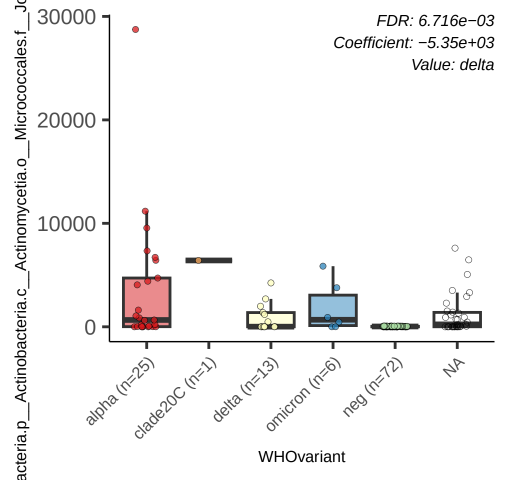

FDR: 6.716e−03
Coefficient: −5.35e+03
Value: delta
30000
20000
10000
0
acteria.p__Actinobacteria.c__Actinomycetia.o__Micrococcales.f__Jo
alpha (n=25)
clade20C (n=1)
delta (n=13)
omicron (n=6)
neg (n=72)
NA
WHOvariant

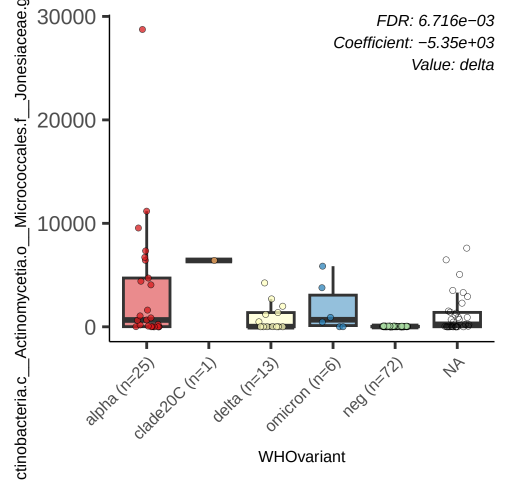

ctinobacteria.c__Actinomycetia.o__Micrococcales.f__Jonesiaceae.g
FDR: 6.716e−03
Coefficient: −5.35e+03
Value: delta
30000
20000
10000
0
alpha (n=25)
clade20C (n=1)
delta (n=13)
omicron (n=6)
neg (n=72)
NA
WHOvariant

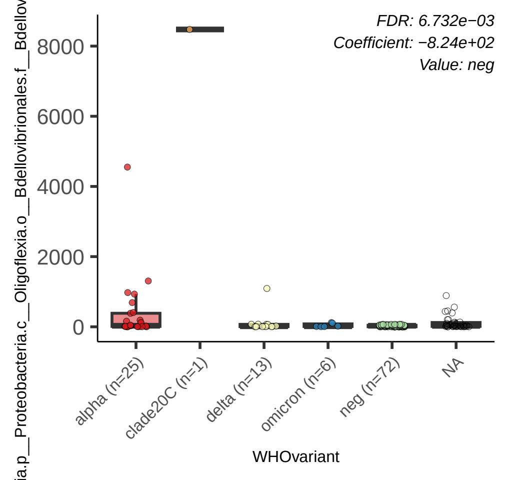
FDR: 6.732e−03
Coefficient: −8.24e+02
Value: neg
ia.p__Proteobacteria.c__Oligoflexia.o__Bdellovibrionales.f__Bdellov
8000
6000
4000
2000
0
alpha (n=25)
clade20C (n=1)
delta (n=13)
omicron (n=6)
neg (n=72)
NA
WHOvariant

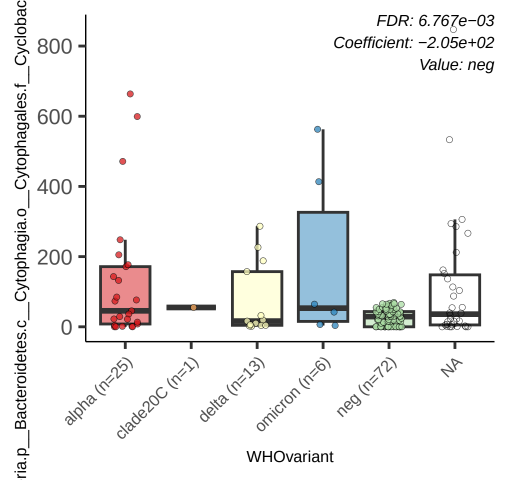

FDR: 6.767e−03
Coefficient: −2.05e+02
Value: neg
800
600
400
200
0
ria.p__Bacteroidetes.c__Cytophagia.o__Cytophagales.f__Cyclobac
alpha (n=25)
clade20C (n=1)
delta (n=13)
omicron (n=6)
neg (n=72)
NA
WHOvariant

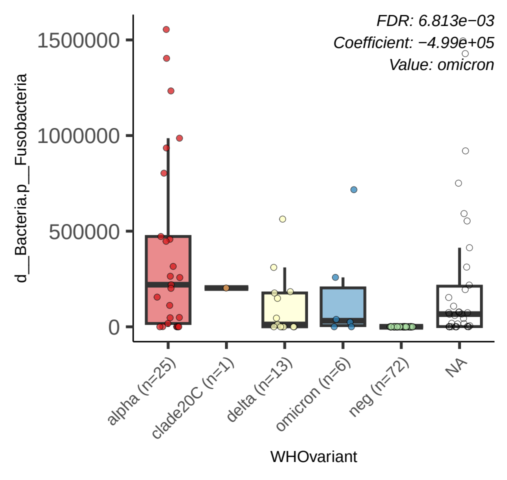

FDR: 6.813e−03
Coefficient: −4.99e+05
Value: omicron
d__Bacteria.p__Fusobacteria
1500000
1000000
500000
0
alpha (n=25)
clade20C (n=1)
delta (n=13)
omicron (n=6)
neg (n=72)
NA
WHOvariant

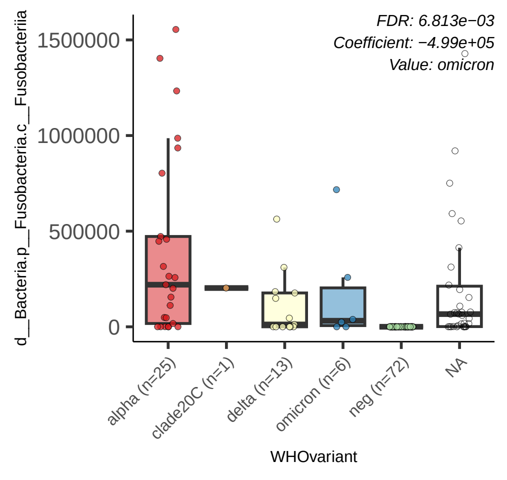

FDR: 6.813e−03
Coefficient: −4.99e+05
Value: omicron
d__Bacteria.p__Fusobacteria.c__Fusobacteriia
1500000
1000000
500000
0
alpha (n=25)
clade20C (n=1)
delta (n=13)
omicron (n=6)
neg (n=72)
NA
WHOvariant

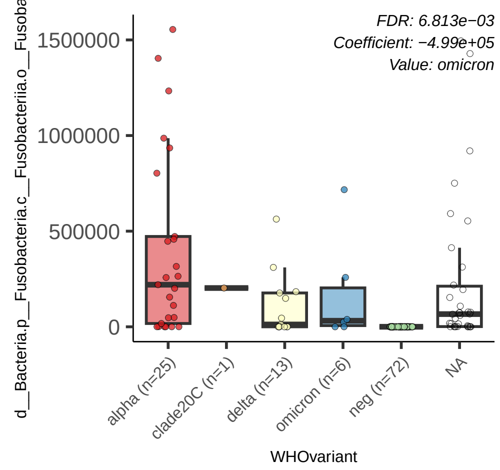

FDR: 6.813e−03
Coefficient: −4.99e+05
Value: omicron
d__Bacteria.p__Fusobacteria.c__Fusobacteriia.o__Fusoba
1500000
1000000
500000
0
alpha (n=25)
clade20C (n=1)
delta (n=13)
omicron (n=6)
neg (n=72)
NA
WHOvariant

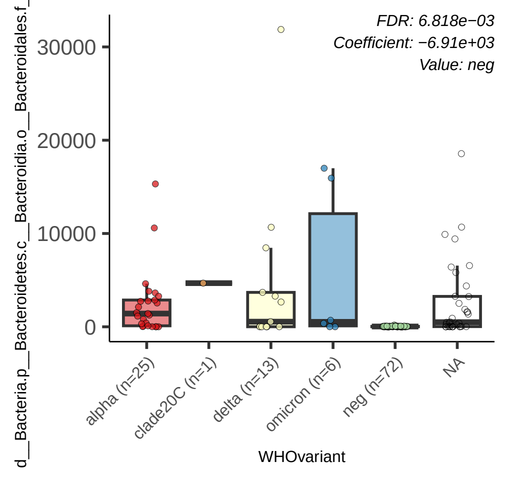
FDR: 6.818e−03
Coefficient: −6.91e+03
Value: neg
d__Bacteria.p__Bacteroidetes.c__Bacteroidia.o__Bacteroidales.f__
30000
20000
10000
0
alpha (n=25)
clade20C (n=1)
delta (n=13)
omicron (n=6)
neg (n=72)
NA
WHOvariant

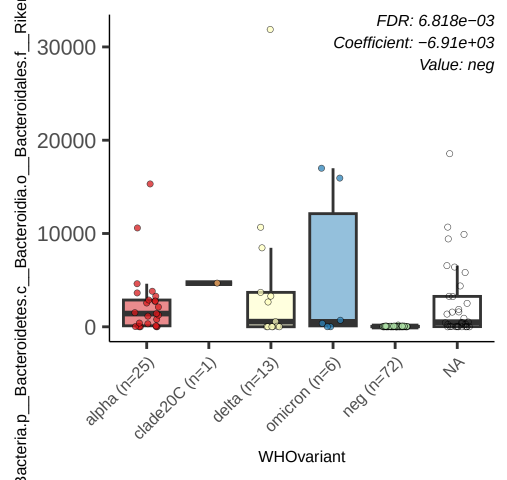

FDR: 6.818e−03
Coefficient: −6.91e+03
Value: neg
Bacteria.p__Bacteroidetes.c__Bacteroidia.o__Bacteroidales.f__Riker
30000
20000
10000
0
alpha (n=25)
clade20C (n=1)
delta (n=13)
omicron (n=6)
neg (n=72)
NA
WHOvariant

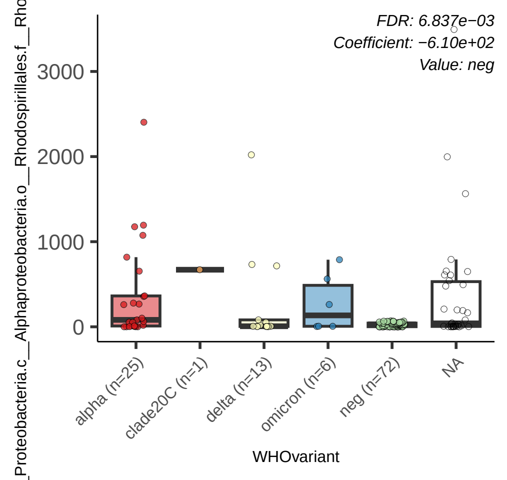
FDR: 6.837e−03
Coefficient: −6.10e+02
Value: neg
Proteobacteria.c__Alphaproteobacteria.o__Rhodospirillales.f__Rho
3000
2000
1000
0
alpha (n=25)
clade20C (n=1)
delta (n=13)
omicron (n=6)
neg (n=72)
NA
WHOvariant

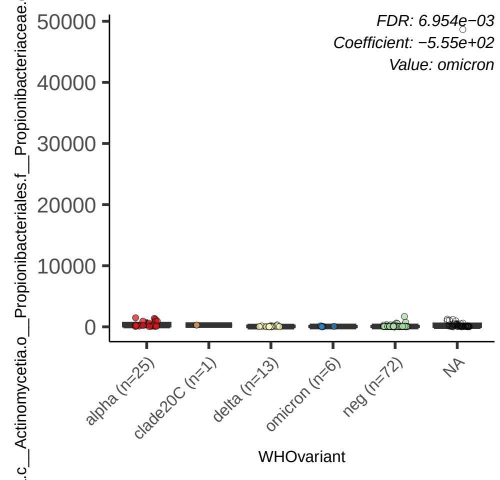

FDR: 6.954e−03
Coefficient: −5.55e+02
Value: omicron
50000
40000
30000
20000
10000
0
alpha (n=25)
clade20C (n=1)
delta (n=13)
omicron (n=6)
neg (n=72)
NA
WHOvariant
.c__Actinomycetia.o__Propionibacteriales.f__Propionibacteriaceae.

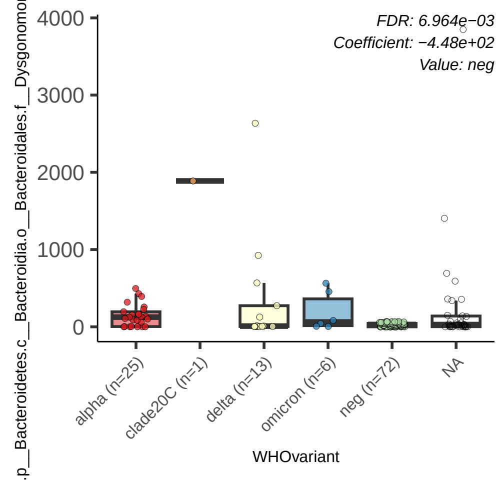
FDR: 6.964e−03
Coefficient: −4.48e+02
Value: neg
p__Bacteroidetes.c__Bacteroidia.o__Bacteroidales.f__Dysgonomo
4000
3000
2000
1000
0
alpha (n=25)
clade20C (n=1)
delta (n=13)
omicron (n=6)
neg (n=72)
NA
WHOvariant

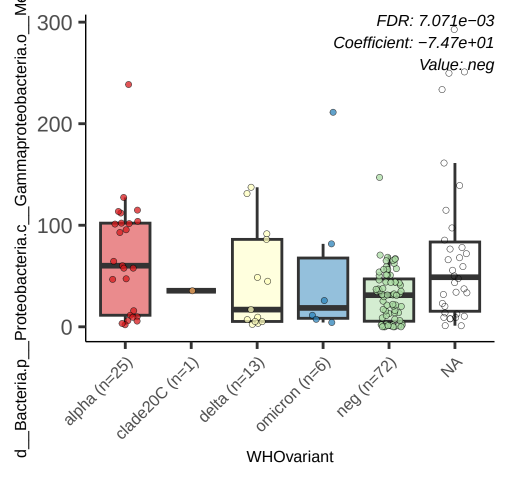
FDR: 7.071e−03
Coefficient: −7.47e+01
Value: neg
d__Bacteria.p__Proteobacteria.c__Gammaproteobacteria.o__Me
300
200
100
0
alpha (n=25)
clade20C (n=1)
delta (n=13)
omicron (n=6)
neg (n=72)
NA
WHOvariant

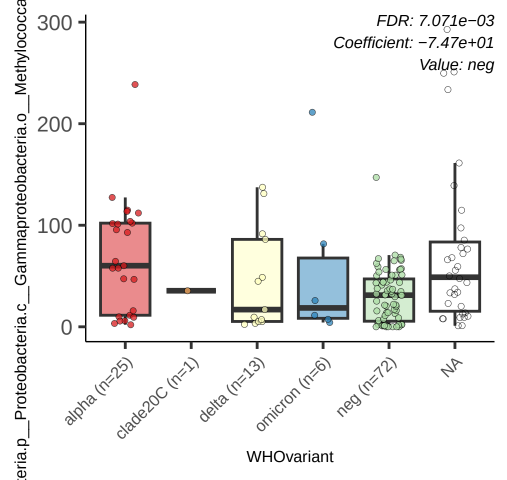

FDR: 7.071e−03
Coefficient: −7.47e+01
Value: neg
300
200
100
0
teria.p__Proteobacteria.c__Gammaproteobacteria.o__Methylococca
alpha (n=25)
clade20C (n=1)
delta (n=13)
omicron (n=6)
neg (n=72)
NA
WHOvariant

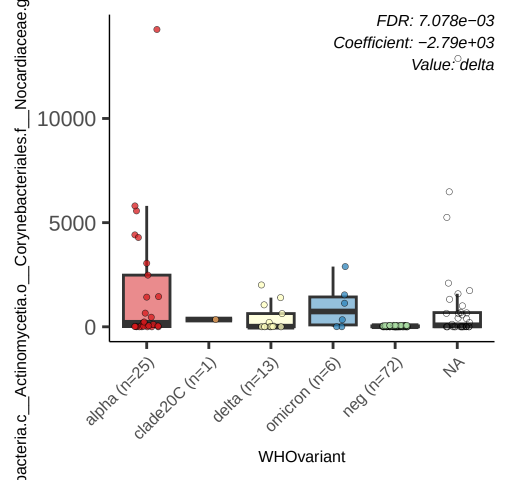
FDR: 7.078e−03
Coefficient: −2.79e+03
Value: delta
bacteria.c__Actinomycetia.o__Corynebacteriales.f__Nocardiaceae.g
10000
5000
0
alpha (n=25)
clade20C (n=1)
delta (n=13)
omicron (n=6)
neg (n=72)
NA
WHOvariant

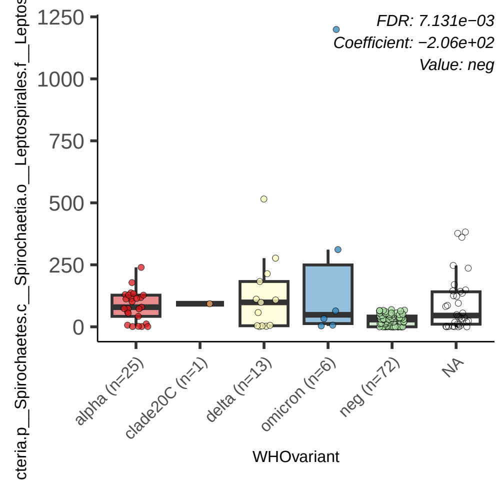

FDR: 7.131e−03
Coefficient: −2.06e+02
Value: neg
1250
1000
750
500
250
0
cteria.p__Spirochaetes.c__Spirochaetia.o__Leptospirales.f__Leptos
alpha (n=25)
clade20C (n=1)
delta (n=13)
omicron (n=6)
neg (n=72)
NA
WHOvariant

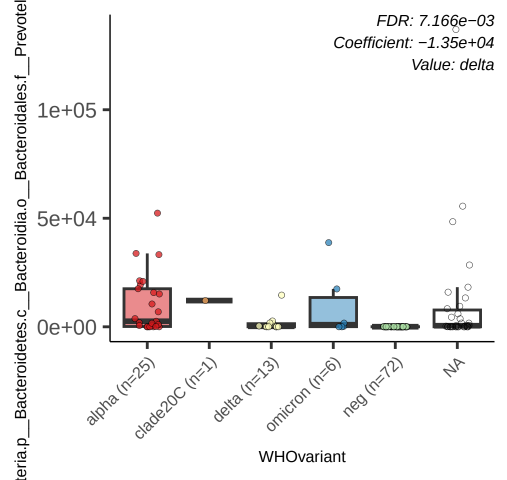
FDR: 7.166e−03
Coefficient: −1.35e+04
Value: delta
teria.p__Bacteroidetes.c__Bacteroidia.o__Bacteroidales.f__Prevotel
1e+05
5e+04
0e+00
alpha (n=25)
clade20C (n=1)
delta (n=13)
omicron (n=6)
neg (n=72)
NA
WHOvariant

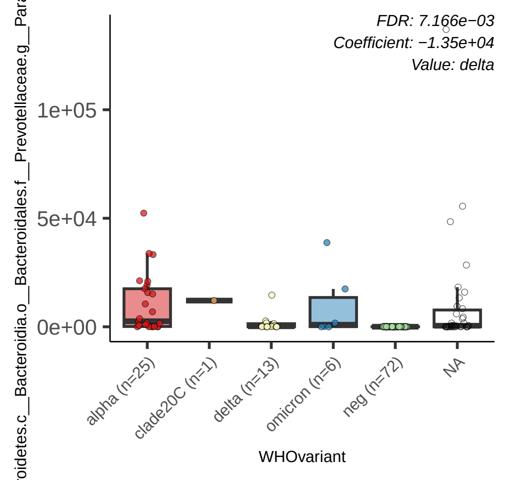

FDR: 7.166e−03
Coefficient: −1.35e+04
Value: delta
roidetes.c__Bacteroidia.o__Bacteroidales.f__Prevotellaceae.g__Para
1e+05
5e+04
0e+00
alpha (n=25)
clade20C (n=1)
delta (n=13)
omicron (n=6)
neg (n=72)
NA
WHOvariant

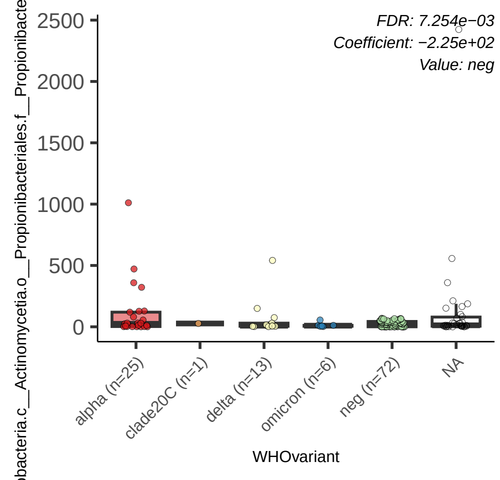

FDR: 7.254e−03
Coefficient: −2.25e+02
Value: neg
2500
2000
1500
1000
500
0
obacteria.c__Actinomycetia.o__Propionibacteriales.f__Propionibacte
alpha (n=25)
clade20C (n=1)
delta (n=13)
omicron (n=6)
neg (n=72)
NA
WHOvariant

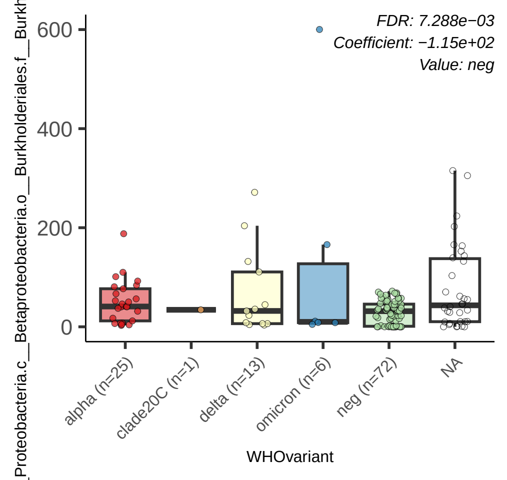
FDR: 7.288e−03
Coefficient: −1.15e+02
Value: neg
600
400
200
0
Proteobacteria.c__Betaproteobacteria.o__Burkholderiales.f__Burkh
alpha (n=25)
clade20C (n=1)
delta (n=13)
omicron (n=6)
neg (n=72)
NA
WHOvariant

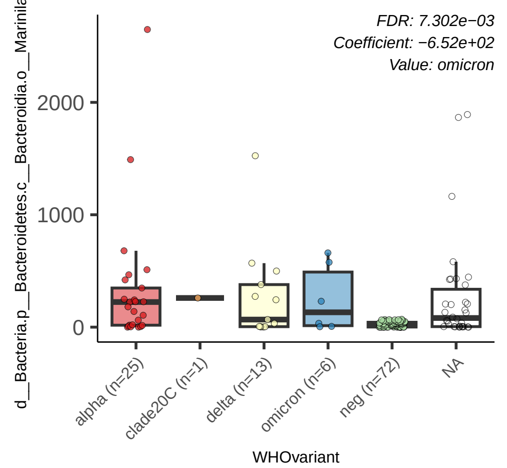

FDR: 7.302e−03
Coefficient: −6.52e+02
Value: omicron
d__Bacteria.p__Bacteroidetes.c__Bacteroidia.o__Marinila
2000
1000
0
alpha (n=25)
clade20C (n=1)
delta (n=13)
omicron (n=6)
neg (n=72)
NA
WHOvariant

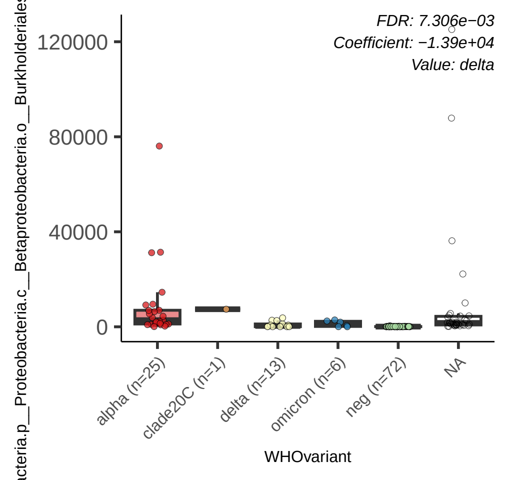

FDR: 7.306e−03
Coefficient: −1.39e+04
Value: delta
120000
80000
40000
0
acteria.p__Proteobacteria.c__Betaproteobacteria.o__Burkholderiales
alpha (n=25)
clade20C (n=1)
delta (n=13)
omicron (n=6)
neg (n=72)
NA
WHOvariant

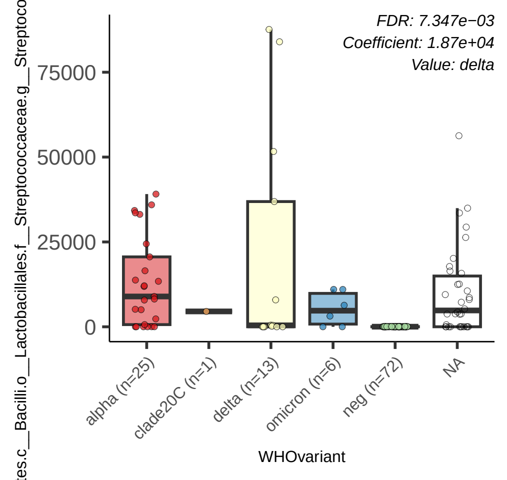
FDR: 7.347e−03
Coefficient: 1.87e+04
Value: delta
75000
50000
25000
0
tes.c__Bacilli.o__Lactobacillales.f__Streptococcaceae.g__Streptoco
alpha (n=25)
clade20C (n=1)
delta (n=13)
omicron (n=6)
neg (n=72)
NA
WHOvariant

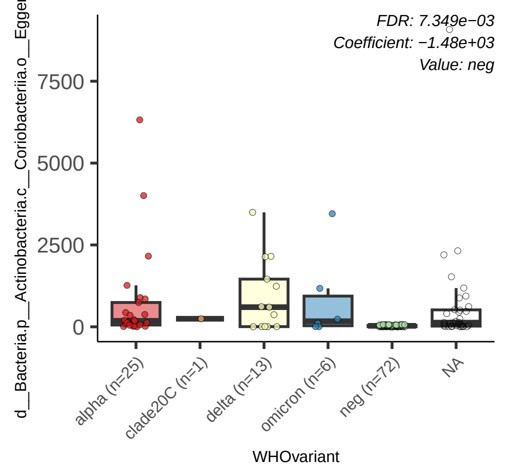
FDR: 7.349e−03
Coefficient: −1.48e+03
Value: neg
d__Bacteria.p__Actinobacteria.c__Coriobacteriia.o__Egger
7500
5000
2500
0
alpha (n=25)
clade20C (n=1)
delta (n=13)
omicron (n=6)
neg (n=72)
NA
WHOvariant

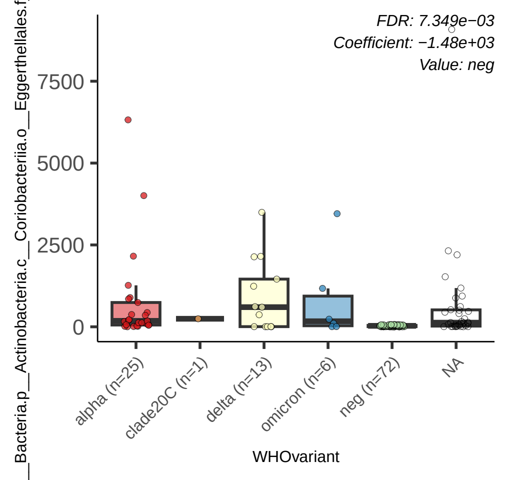

FDR: 7.349e−03
Coefficient: −1.48e+03
Value: neg
_Bacteria.p__Actinobacteria.c__Coriobacteriia.o__Eggerthellales.f_
7500
5000
2500
0
alpha (n=25)
clade20C (n=1)
delta (n=13)
omicron (n=6)
neg (n=72)
NA
WHOvariant

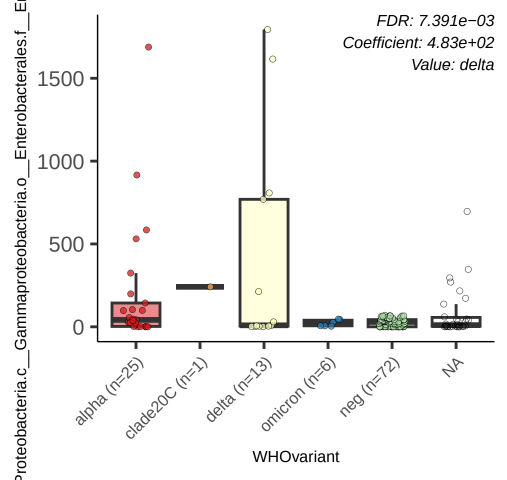

FDR: 7.391e−03
Coefficient: 4.83e+02
Value: delta
Proteobacteria.c__Gammaproteobacteria.o__Enterobacterales.f__E
0
500
1000
1500
alpha (n=25)
clade20C (n=1)
delta (n=13)
omicron (n=6)
neg (n=72)
NA
WHOvariant

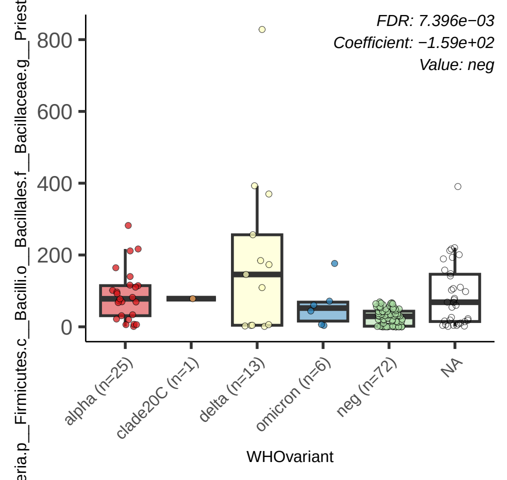
FDR: 7.396e−03
Coefficient: −1.59e+02
Value: neg
800
600
400
200
0
eria.p__Firmicutes.c__Bacilli.o__Bacillales.f__Bacillaceae.g__Priest
alpha (n=25)
clade20C (n=1)
delta (n=13)
omicron (n=6)
neg (n=72)
NA
WHOvariant

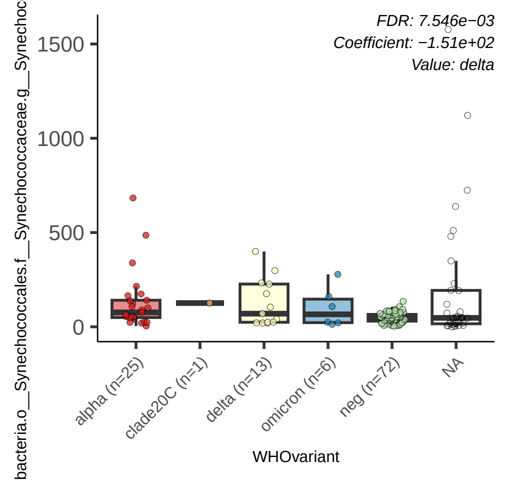

FDR: 7.546e−03
Coefficient: −1.51e+02
Value: delta
1500
1000
500
0
bacteria.o__Synechococcales.f__Synechococcaceae.g__Synechoc
alpha (n=25)
clade20C (n=1)
delta (n=13)
omicron (n=6)
neg (n=72)
NA
WHOvariant

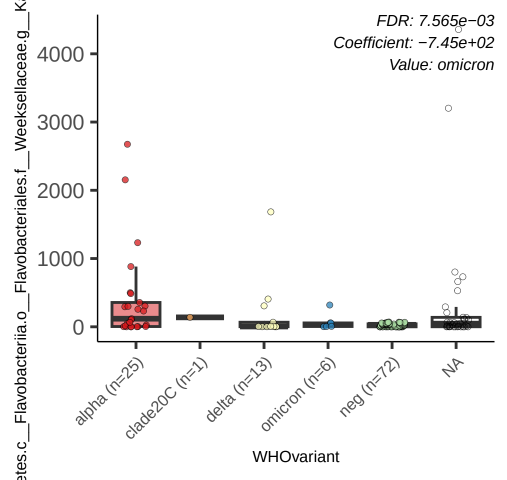

FDR: 7.565e−03
Coefficient: −7.45e+02
Value: omicron
4000
3000
2000
1000
0
etes.c__Flavobacteriia.o__Flavobacteriales.f__Weeksellaceae.g__K
alpha (n=25)
clade20C (n=1)
delta (n=13)
omicron (n=6)
neg (n=72)
NA
WHOvariant

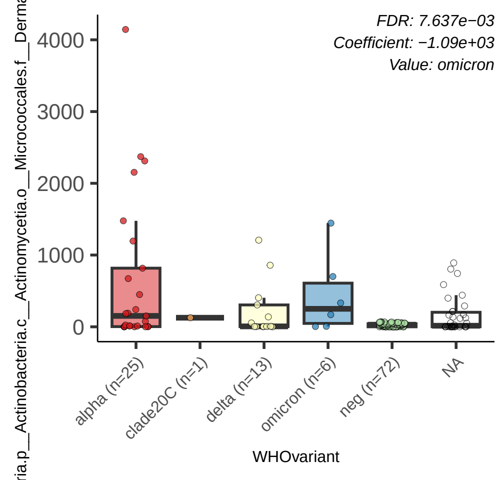

FDR: 7.637e−03
Coefficient: −1.09e+03
Value: omicron
4000
3000
2000
1000
0
ria.p__Actinobacteria.c__Actinomycetia.o__Micrococcales.f__Derma
alpha (n=25)
clade20C (n=1)
delta (n=13)
omicron (n=6)
neg (n=72)
NA
WHOvariant

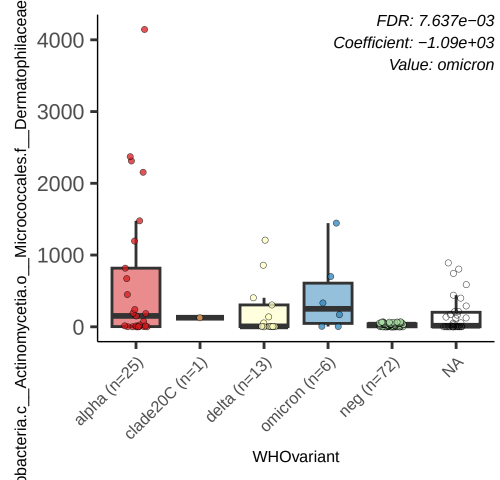

FDR: 7.637e−03
Coefficient: −1.09e+03
Value: omicron
bacteria.c__Actinomycetia.o__Micrococcales.f__Dermatophilaceae
4000
3000
2000
1000
0
alpha (n=25)
clade20C (n=1)
delta (n=13)
omicron (n=6)
neg (n=72)
NA
WHOvariant

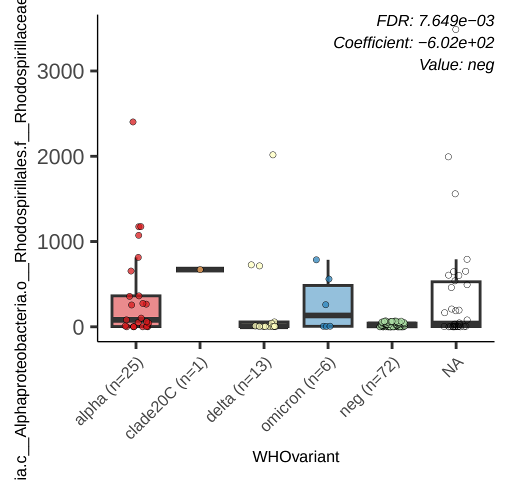

FDR: 7.649e−03
Coefficient: −6.02e+02
Value: neg
ia.c__Alphaproteobacteria.o__Rhodospirillales.f__Rhodospirillaceae
3000
2000
1000
0
alpha (n=25)
clade20C (n=1)
delta (n=13)
omicron (n=6)
neg (n=72)
NA
WHOvariant

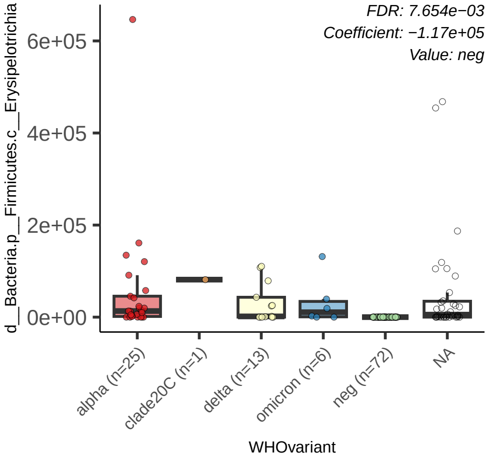

FDR: 7.654e−03
Coefficient: −1.17e+05
Value: neg
d__Bacteria.p__Firmicutes.c__Erysipelotrichia
6e+05
4e+05
2e+05
0e+00
alpha (n=25)
clade20C (n=1)
delta (n=13)
omicron (n=6)
neg (n=72)
NA
WHOvariant

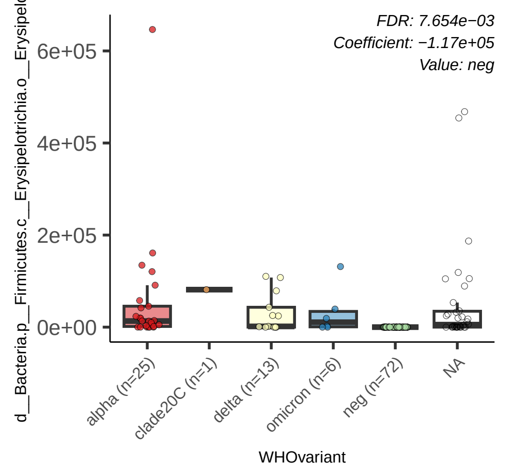

FDR: 7.654e−03
Coefficient: −1.17e+05
Value: neg
d__Bacteria.p__Firmicutes.c__Erysipelotrichia.o__Erysipelo
6e+05
4e+05
2e+05
0e+00
alpha (n=25)
clade20C (n=1)
delta (n=13)
omicron (n=6)
neg (n=72)
NA
WHOvariant

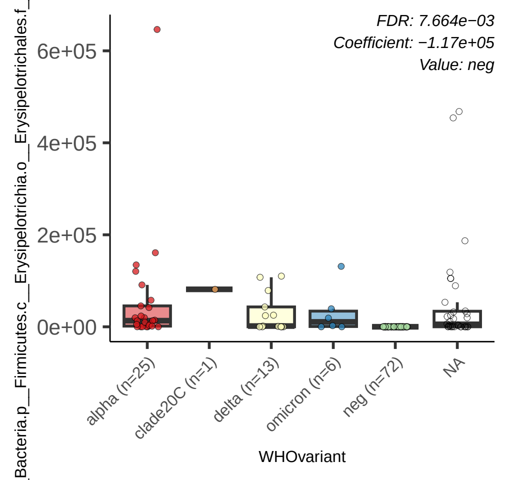
FDR: 7.664e−03
Coefficient: −1.17e+05
Value: neg
Bacteria.p__Firmicutes.c__Erysipelotrichia.o__Erysipelotrichales.f__
6e+05
4e+05
2e+05
0e+00
alpha (n=25)
clade20C (n=1)
delta (n=13)
omicron (n=6)
neg (n=72)
NA
WHOvariant

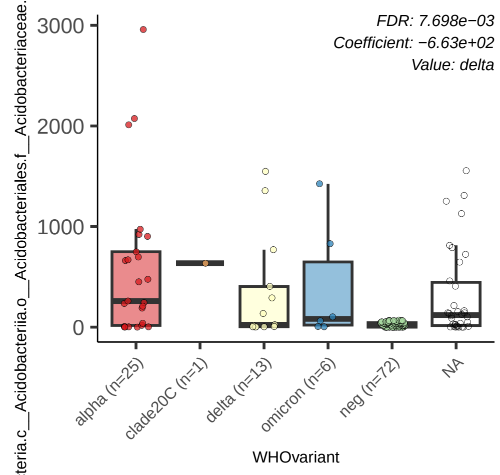

FDR: 7.698e−03
Coefficient: −6.63e+02
Value: delta
cteria.c__Acidobacteriia.o__Acidobacteriales.f__Acidobacteriaceae.
3000
2000
1000
0
alpha (n=25)
clade20C (n=1)
delta (n=13)
omicron (n=6)
neg (n=72)
NA
WHOvariant

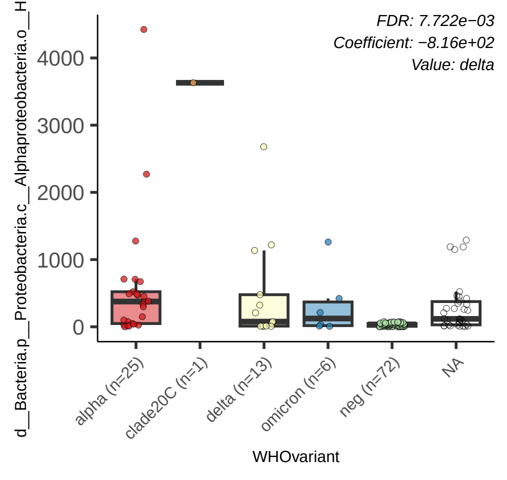

FDR: 7.722e−03
Coefficient: −8.16e+02
Value: delta
d__Bacteria.p__Proteobacteria.c__Alphaproteobacteria.o__H
4000
3000
2000
1000
0
alpha (n=25)
clade20C (n=1)
delta (n=13)
omicron (n=6)
neg (n=72)
NA
WHOvariant

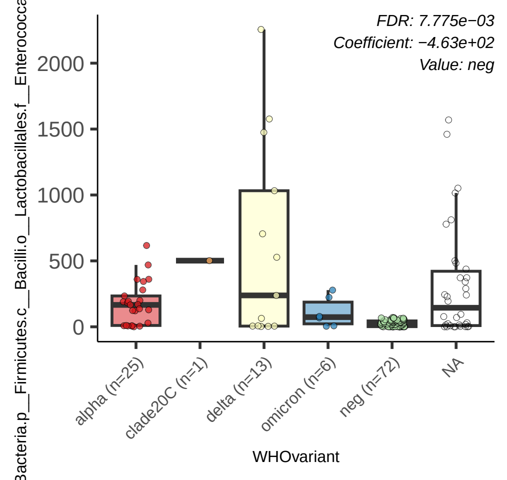
FDR: 7.775e−03
Coefficient: −4.63e+02
Value: neg
Bacteria.p__Firmicutes.c__Bacilli.o__Lactobacillales.f__Enterococca
2000
1500
1000
500
0
alpha (n=25)
clade20C (n=1)
delta (n=13)
omicron (n=6)
neg (n=72)
NA
WHOvariant

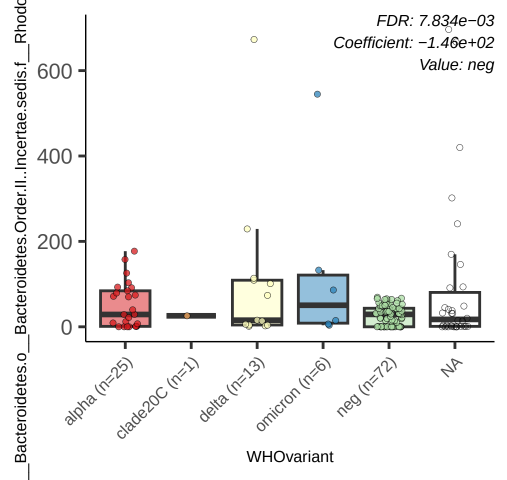

FDR: 7.834e−03
Coefficient: −1.46e+02
Value: neg
600
400
200
0
Bacteroidetes.o__Bacteroidetes.Order.II..Incertae.sedis.f__Rhodo
alpha (n=25)
clade20C (n=1)
delta (n=13)
omicron (n=6)
neg (n=72)
NA
WHOvariant

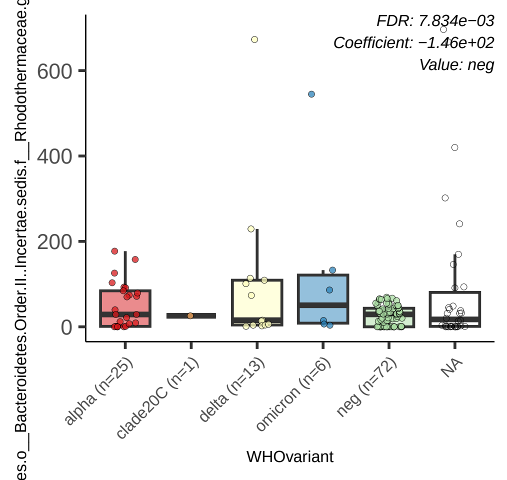

FDR: 7.834e−03
Coefficient: −1.46e+02
Value: neg
0
200
400
600
es.o__Bacteroidetes.Order.II..Incertae.sedis.f__Rhodothermaceae.g
alpha (n=25)
clade20C (n=1)
delta (n=13)
omicron (n=6)
neg (n=72)
NA
WHOvariant

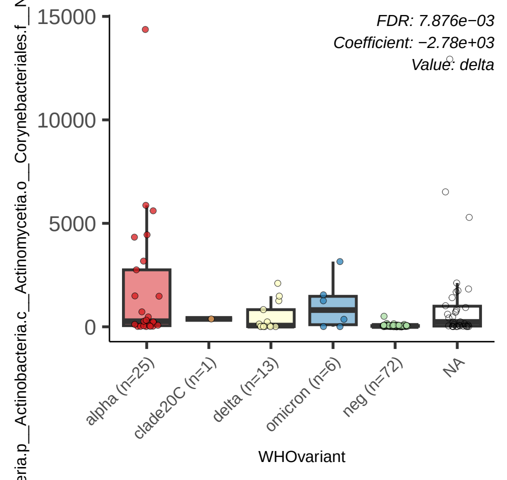

FDR: 7.876e−03
Coefficient: −2.78e+03
Value: delta
15000
10000
5000
0
Actinobacteria.p__Actinobacteria.c__Actinomycetia.o__Corynebacteriales.f__N
alpha (n=25)
clade20C (n=1)
delta (n=13)
omicron (n=6)
neg (n=72)
NA
WHOvariant

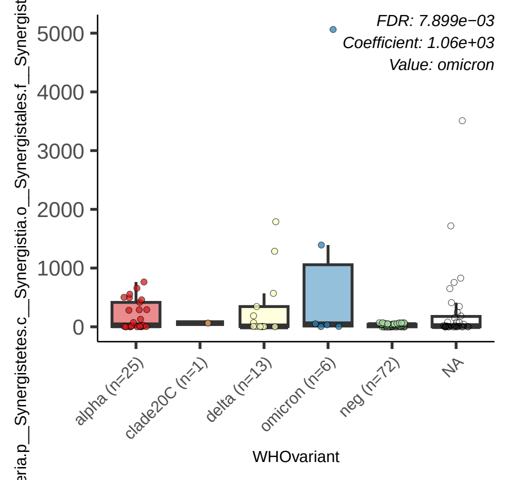
FDR: 7.899e−03
Coefficient: 1.06e+03
Value: omicron
eria.p__Synergistetes.c__Synergistia.o__Synergistales.f__Synergist
5000
4000
3000
2000
1000
0
alpha (n=25)
clade20C (n=1)
delta (n=13)
omicron (n=6)
neg (n=72)
NA
WHOvariant

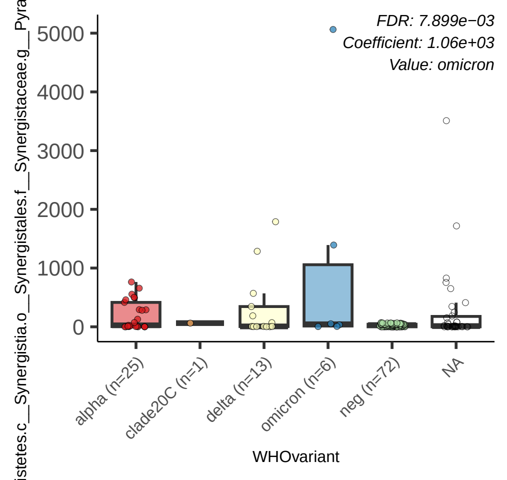
FDR: 7.899e−03
Coefficient: 1.06e+03
Value: omicron
5000
4000
3000
2000
1000
0
alpha (n=25)
clade20C (n=1)
delta (n=13)
omicron (n=6)
neg (n=72)
NA
WHOvariant
istetes.c__Synergistia.o__Synergistales.f__Synergistaceae.g__Pyra

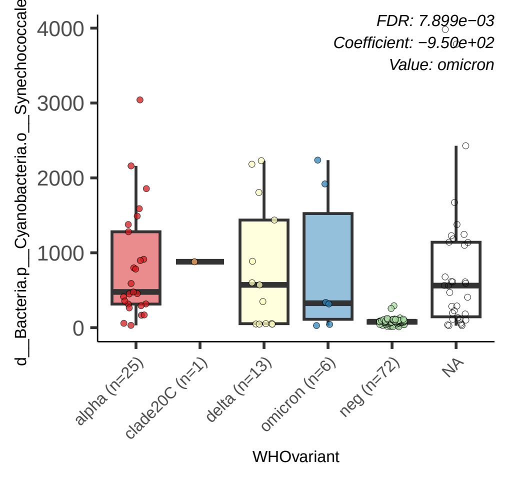

FDR: 7.899e−03
Coefficient: −9.50e+02
Value: omicron
d__Bacteria.p__Cyanobacteria.o__Synechococcales
4000
3000
2000
1000
0
alpha (n=25)
clade20C (n=1)
delta (n=13)
omicron (n=6)
neg (n=72)
NA
WHOvariant

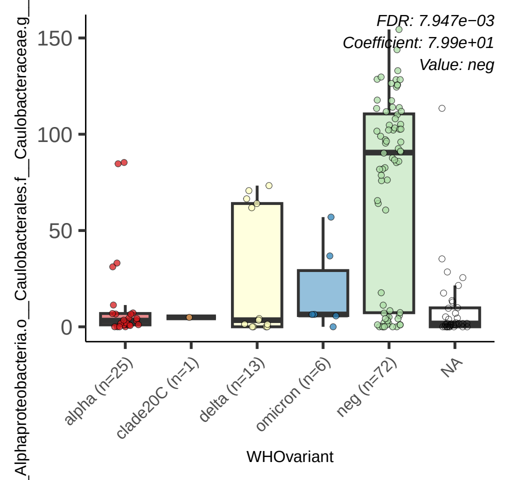

FDR: 7.947e−03
Coefficient: 7.99e+01
Value: neg
Alphaproteobacteria.o__Caulobacterales.f__Caulobacteraceae.g__
0
50
100
150
alpha (n=25)
clade20C (n=1)
delta (n=13)
omicron (n=6)
neg (n=72)
NA
WHOvariant

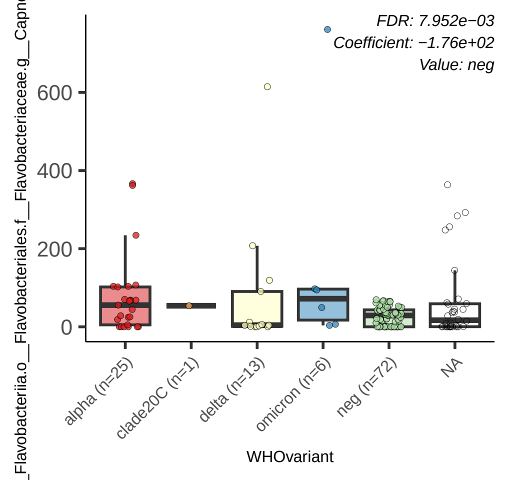

FDR: 7.952e−03
Coefficient: −1.76e+02
Value: neg
Flavobacteriia.o__Flavobacteriales.f__Flavobacteriaceae.g__Capno
0
200
400
600
alpha (n=25)
clade20C (n=1)
delta (n=13)
omicron (n=6)
neg (n=72)
NA
WHOvariant

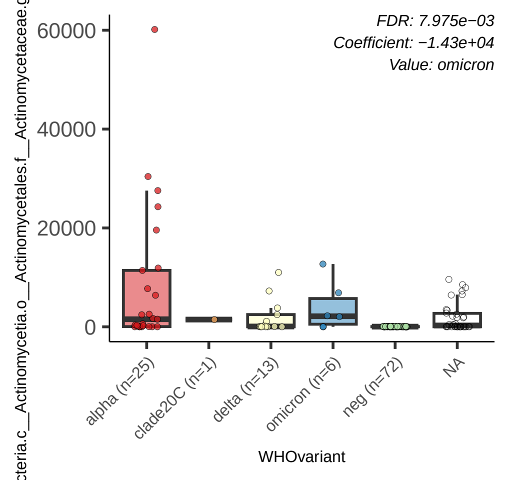
FDR: 7.975e−03
Coefficient: −1.43e+04
Value: omicron
cteria.c__Actinomycetia.o__Actinomycetales.f__Actinomycetaceae.g
60000
40000
20000
0
alpha (n=25)
clade20C (n=1)
delta (n=13)
omicron (n=6)
neg (n=72)
NA
WHOvariant

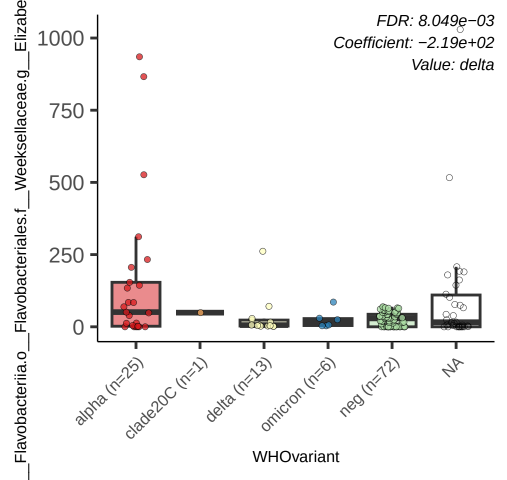

FDR: 8.049e−03
Coefficient: −2.19e+02
Value: delta
Flavobacteriia.o__Flavobacteriales.f__Weeksellaceae.g__Elizabe
1000
750
500
250
0
alpha (n=25)
clade20C (n=1)
delta (n=13)
omicron (n=6)
neg (n=72)
NA
WHOvariant

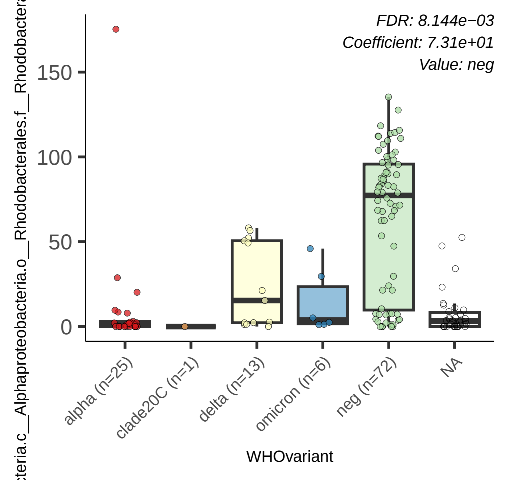

FDR: 8.144e−03
Coefficient: 7.31e+01
Value: neg
150
100
50
0
cteria.c__Alphaproteobacteria.o__Rhodobacterales.f__Rhodobacter
alpha (n=25)
clade20C (n=1)
delta (n=13)
omicron (n=6)
neg (n=72)
NA
WHOvariant

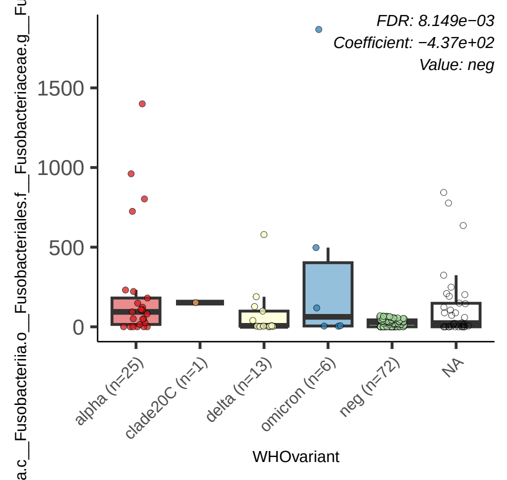
FDR: 8.149e−03
Coefficient: −4.37e+02
Value: neg
a.c__Fusobacteriia.o__Fusobacteriales.f__Fusobacteriaceae.g__Fu
0
500
1000
1500
alpha (n=25)
clade20C (n=1)
delta (n=13)
omicron (n=6)
neg (n=72)
NA
WHOvariant

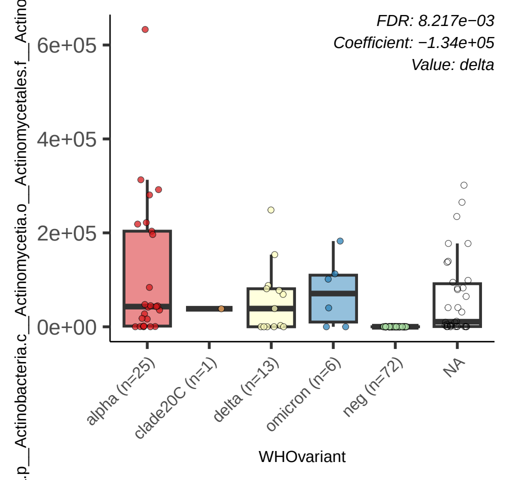
FDR: 8.217e−03
Coefficient: −1.34e+05
Value: delta
0e+00
2e+05
4e+05
6e+05
.p__Actinobacteria.c__Actinomycetia.o__Actinomycetales.f__Actino
alpha (n=25)
clade20C (n=1)
delta (n=13)
omicron (n=6)
neg (n=72)
NA
WHOvariant

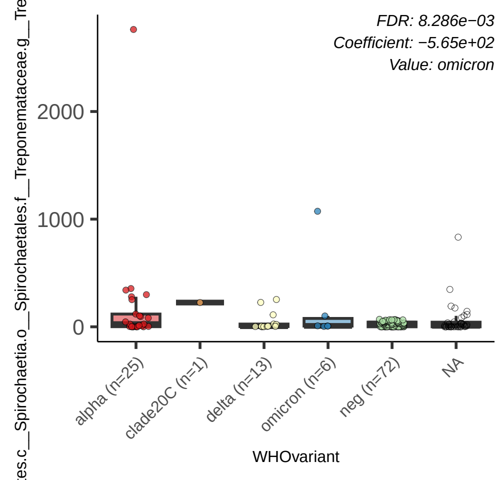

FDR: 8.286e−03
Coefficient: −5.65e+02
Value: omicron
2000
1000
0
alpha (n=25)
clade20C (n=1)
delta (n=13)
omicron (n=6)
neg (n=72)
NA
WHOvariant

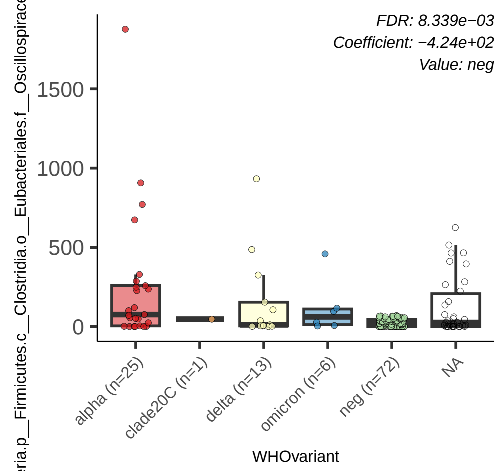

FDR: 8.339e−03
Coefficient: −4.24e+02
Value: neg
teria.p__Firmicutes.c__Clostridia.o__Eubacteriales.f__Oscillospirace
0
500
1000
1500
alpha (n=25)
clade20C (n=1)
delta (n=13)
omicron (n=6)
neg (n=72)
NA
WHOvariant

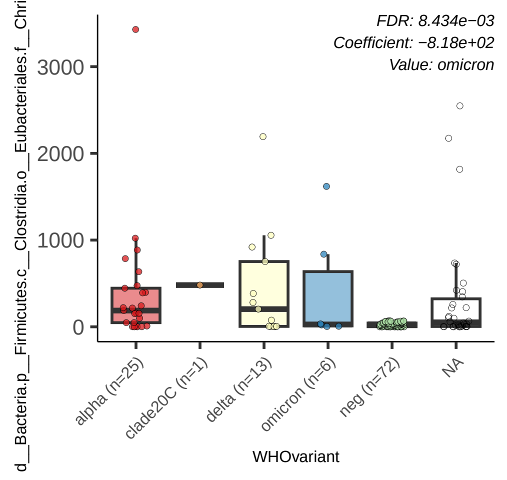
FDR: 8.434e−03
Coefficient: −8.18e+02
Value: omicron
d__Bacteria.p__Firmicutes.c__Clostridia.o__Eubacteriales.f__Chri
3000
2000
1000
0
alpha (n=25)
clade20C (n=1)
delta (n=13)
omicron (n=6)
neg (n=72)
NA
WHOvariant

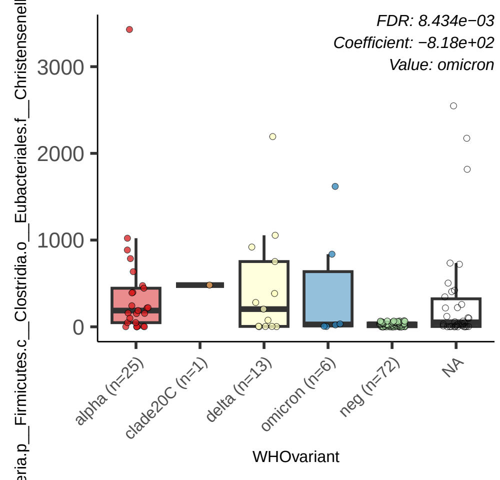
FDR: 8.434e−03
Coefficient: −8.18e+02
Value: omicron
3000
2000
1000
0
alpha (n=25)
clade20C (n=1)
delta (n=13)
omicron (n=6)
neg (n=72)
NA
WHOvariant
eria.p__Firmicutes.c__Clostridia.o__Eubacteriales.f__Christensenell

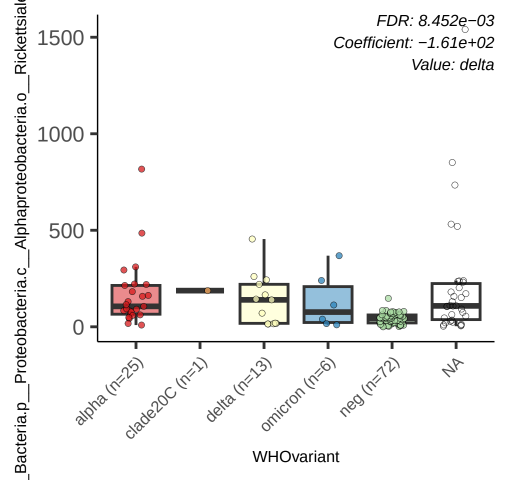
FDR: 8.452e−03
Coefficient: −1.61e+02
Value: delta
1500
1000
500
0
alpha (n=25)
clade20C (n=1)
delta (n=13)
omicron (n=6)
neg (n=72)
NA
WHOvariant
Bacteria.p__Proteobacteria.c__Alphaproteobacteria.o__Rickettsiale

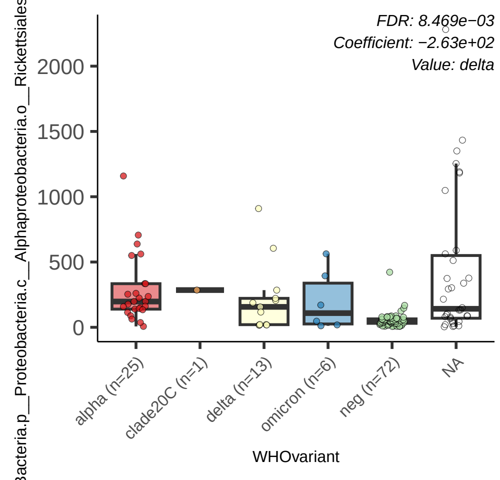
FDR: 8.469e−03
Coefficient: −2.63e+02
Value: delta
Bacteria.p__Proteobacteria.c__Alphaproteobacteria.o__Rickettsiales
2000
1500
1000
500
0
alpha (n=25)
clade20C (n=1)
delta (n=13)
omicron (n=6)
neg (n=72)
NA
WHOvariant

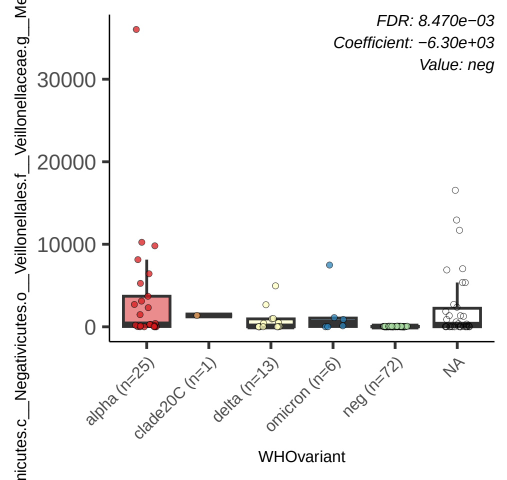

FDR: 8.470e−03
Coefficient: −6.30e+03
Value: neg
30000
20000
10000
0
nicutes.c__Negativicutes.o__Veillonellales.f__Veillonellaceae.g__Me
alpha (n=25)
clade20C (n=1)
delta (n=13)
omicron (n=6)
neg (n=72)
NA
WHOvariant

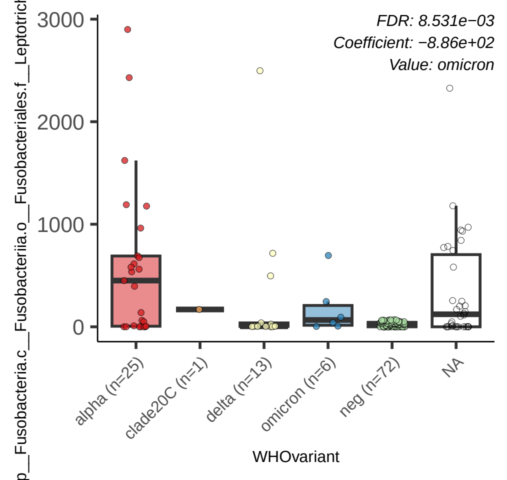

FDR: 8.531e−03
Coefficient: −8.86e+02
Value: omicron
3000
2000
1000
0
p__Fusobacteria.c__Fusobacteriia.o__Fusobacteriales.f__Leptotrich
alpha (n=25)
clade20C (n=1)
delta (n=13)
omicron (n=6)
neg (n=72)
NA
WHOvariant

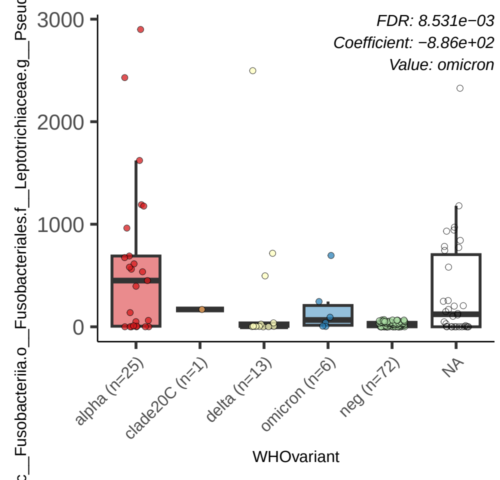
FDR: 8.531e−03
Coefficient: −8.86e+02
Value: omicron
c__Fusobacteriia.o__Fusobacteriales.f__Leptotrichiaceae.g__Pseud
3000
2000
1000
0
alpha (n=25)
clade20C (n=1)
delta (n=13)
omicron (n=6)
neg (n=72)
NA
WHOvariant

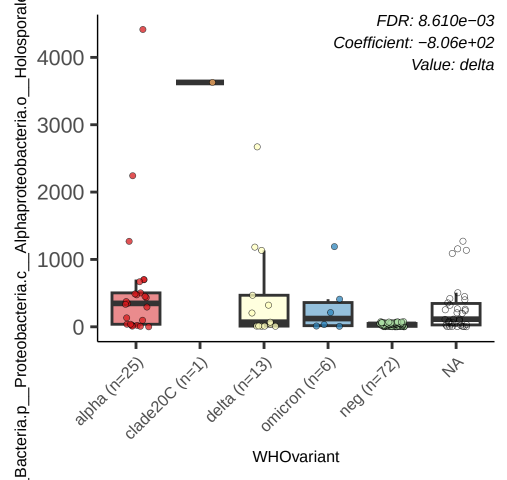

FDR: 8.610e−03
Coefficient: −8.06e+02
Value: delta
Bacteria.p__Proteobacteria.c__Alphaproteobacteria.o__Holosporale
4000
3000
2000
1000
0
alpha (n=25)
clade20C (n=1)
delta (n=13)
omicron (n=6)
neg (n=72)
NA
WHOvariant

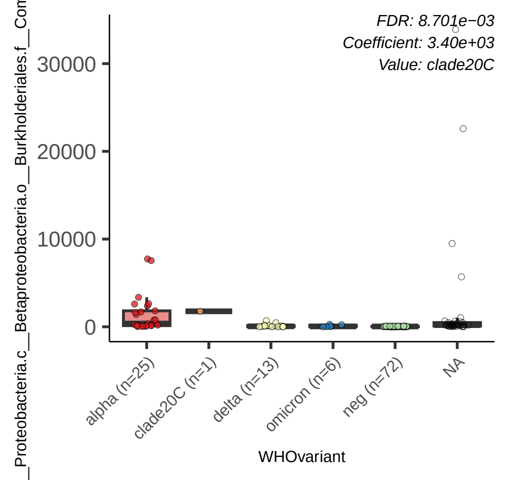
FDR: 8.701e−03
Coefficient: 3.40e+03
Value: clade20C
30000
20000
10000
0
alpha (n=25)
clade20C (n=1)
delta (n=13)
omicron (n=6)
neg (n=72)
NA
WHOvariant
_Proteobacteria.c__Betaproteobacteria.o__Burkholderiales.f__Com

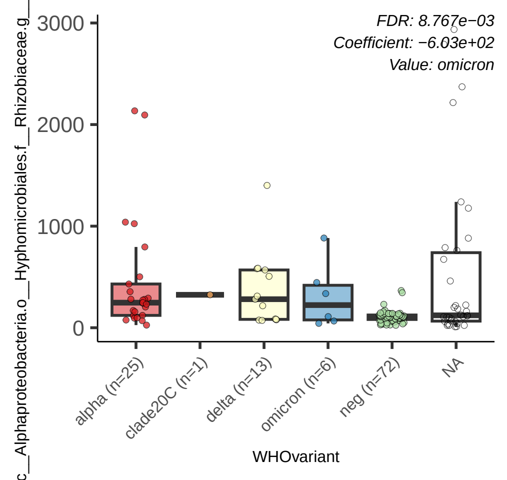
FDR: 8.767e−03
Coefficient: −6.03e+02
Value: omicron
c__Alphaproteobacteria.o__Hyphomicrobiales.f__Rhizobiaceae.g__
3000
2000
1000
0
alpha (n=25)
clade20C (n=1)
delta (n=13)
omicron (n=6)
neg (n=72)
NA
WHOvariant

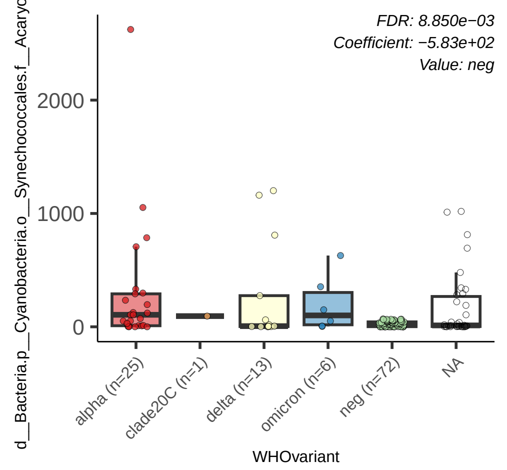
FDR: 8.850e−03
Coefficient: −5.83e+02
Value: neg
d__Bacteria.p__Cyanobacteria.o__Synechococcales.f__Acaryo
2000
1000
0
alpha (n=25)
clade20C (n=1)
delta (n=13)
omicron (n=6)
neg (n=72)
NA
WHOvariant

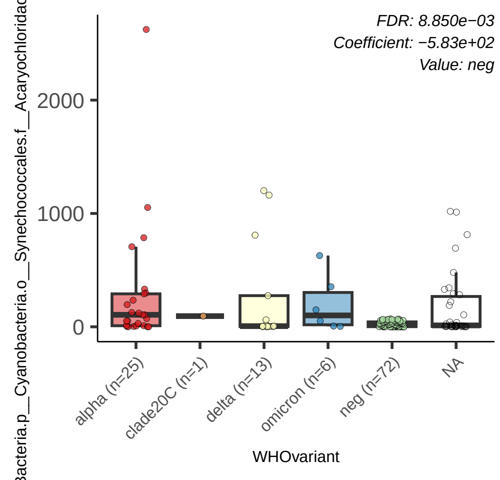

FDR: 8.850e−03
Coefficient: −5.83e+02
Value: neg
2000
1000
0
Bacteria.p__Cyanobacteria.o__Synechococcales.f__Acaryochloridac
alpha (n=25)
clade20C (n=1)
delta (n=13)
omicron (n=6)
neg (n=72)
NA
WHOvariant

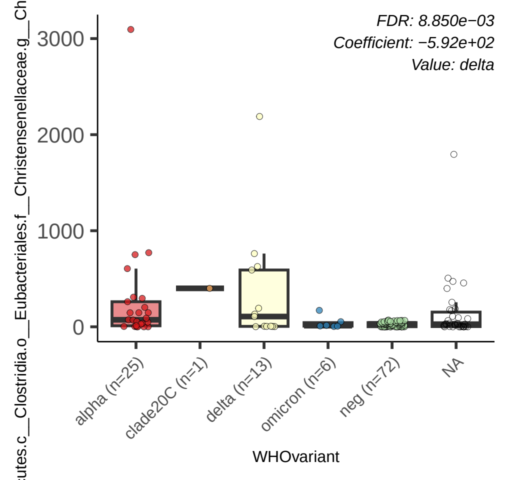
FDR: 8.850e−03
Coefficient: −5.92e+02
Value: delta
3000
2000
1000
0
alpha (n=25)
clade20C (n=1)
delta (n=13)
omicron (n=6)
neg (n=72)
NA
WHOvariant
cutes.c__Clostridia.o__Eubacteriales.f__Christensenellaceae.g__Ch

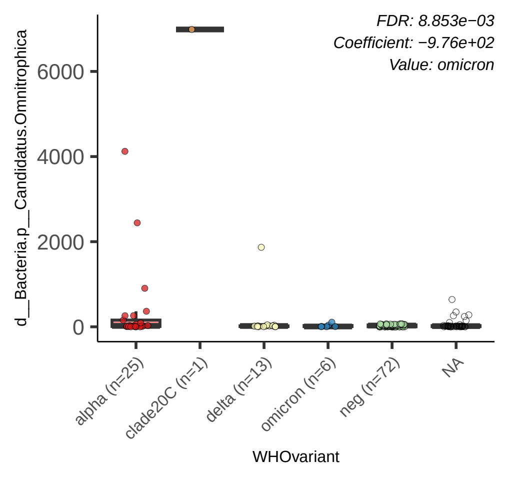

FDR: 8.853e−03
Coefficient: −9.76e+02
Value: omicron
d__Bacteria.p__Candidatus.Omnitrophica
6000
4000
2000
0
alpha (n=25)
clade20C (n=1)
delta (n=13)
omicron (n=6)
neg (n=72)
NA
WHOvariant

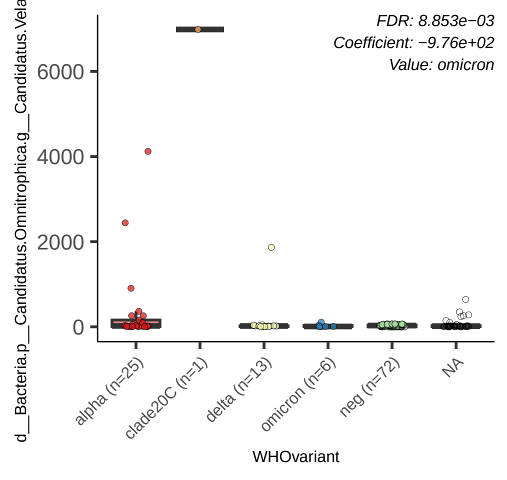
FDR: 8.853e−03
Coefficient: −9.76e+02
Value: omicron
d__Bacteria.p__Candidatus.Omnitrophica.g__Candidatus.Vela
6000
4000
2000
0
alpha (n=25)
clade20C (n=1)
delta (n=13)
omicron (n=6)
neg (n=72)
NA
WHOvariant

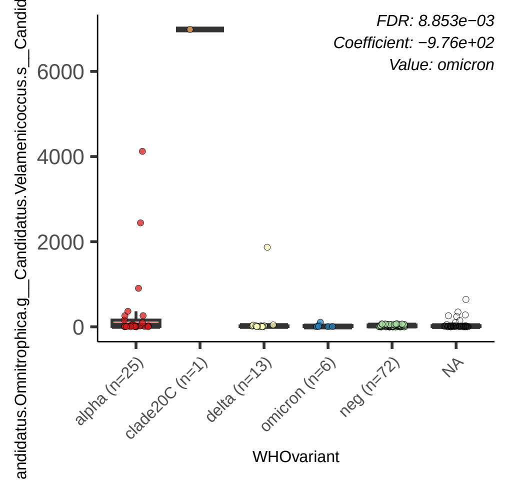

FDR: 8.853e−03
Coefficient: −9.76e+02
Value: omicron
6000
4000
2000
0
andidatus.Omnitrophica.g__Candidatus.Velamenicoccus.s__Candid
alpha (n=25)
clade20C (n=1)
delta (n=13)
omicron (n=6)
neg (n=72)
NA
WHOvariant

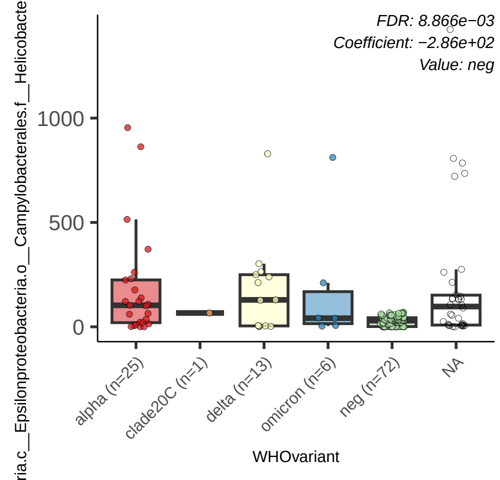

FDR: 8.866e−03
Coefficient: −2.86e+02
Value: neg
ria.c__Epsilonproteobacteria.o__Campylobacterales.f__Helicobacte
1000
500
0
alpha (n=25)
clade20C (n=1)
delta (n=13)
omicron (n=6)
neg (n=72)
NA
WHOvariant

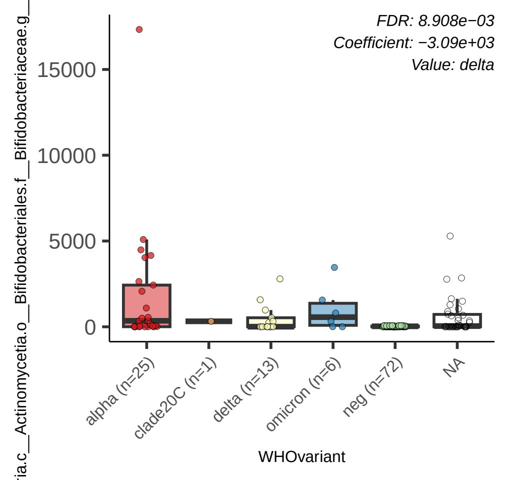

FDR: 8.908e−03
Coefficient: −3.09e+03
Value: delta
ria.c__Actinomycetia.o__Bifidobacteriales.f__Bifidobacteriaceae.g__
15000
10000
5000
0
alpha (n=25)
clade20C (n=1)
delta (n=13)
omicron (n=6)
neg (n=72)
NA
WHOvariant

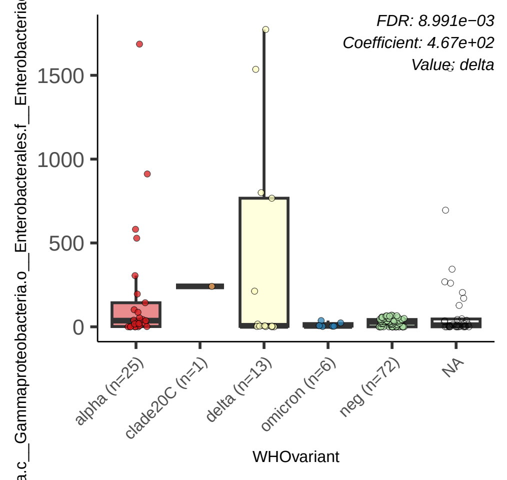
FDR: 8.991e−03
Coefficient: 4.67e+02
Value: delta
1500
1000
500
0
a.c__Gammaproteobacteria.o__Enterobacterales.f__Enterobacteria
alpha (n=25)
clade20C (n=1)
delta (n=13)
omicron (n=6)
neg (n=72)
NA
WHOvariant

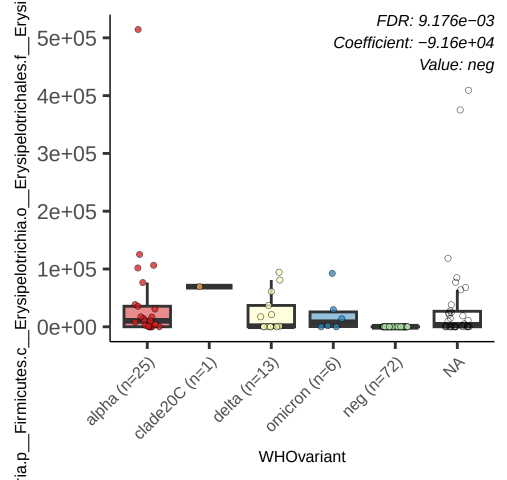
FDR: 9.176e−03
Coefficient: −9.16e+04
Value: neg
5e+05
4e+05
3e+05
2e+05
1e+05
0e+00
ria.p__Firmicutes.c__Erysipelotrichia.o__Erysipelotrichales.f__Erysi
alpha (n=25)
clade20C (n=1)
delta (n=13)
omicron (n=6)
neg (n=72)
NA
WHOvariant

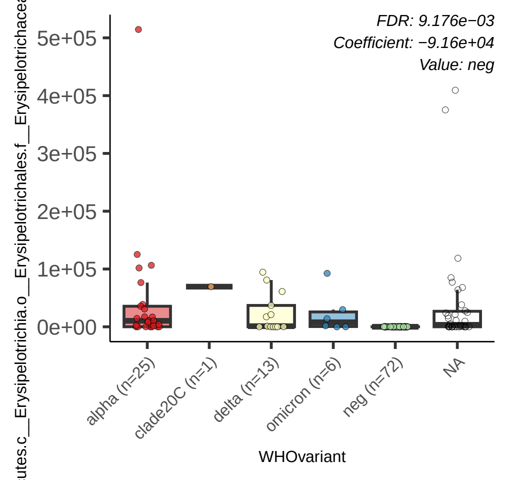

FDR: 9.176e−03
Coefficient: −9.16e+04
Value: neg
5e+05
4e+05
3e+05
2e+05
1e+05
0e+00
utes.c__Erysipelotrichia.o__Erysipelotrichales.f__Erysipelotrichacea
alpha (n=25)
clade20C (n=1)
delta (n=13)
omicron (n=6)
neg (n=72)
NA
WHOvariant

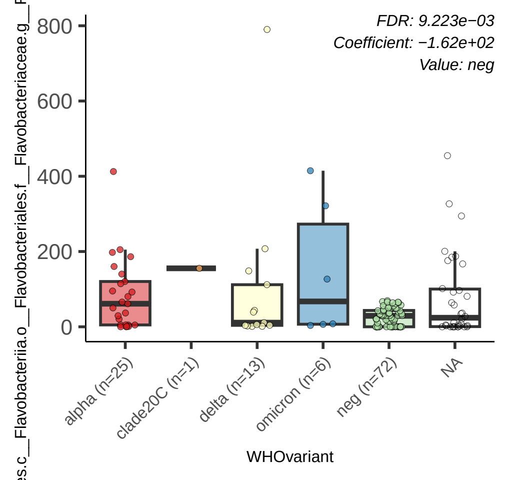
FDR: 9.223e−03
Coefficient: −1.62e+02
Value: neg
800
600
400
200
0
es.c__Flavobacteriia.o__Flavobacteriales.f__Flavobacteriaceae.g__F
alpha (n=25)
clade20C (n=1)
delta (n=13)
omicron (n=6)
neg (n=72)
NA
WHOvariant

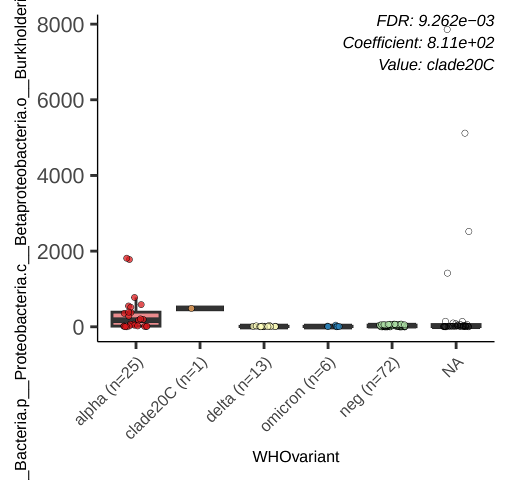

FDR: 9.262e−03
Coefficient: 8.11e+02
Value: clade20C
8000
6000
4000
2000
0
_Bacteria.p__Proteobacteria.c__Betaproteobacteria.o__Burkholderi
alpha (n=25)
clade20C (n=1)
delta (n=13)
omicron (n=6)
neg (n=72)
NA
WHOvariant

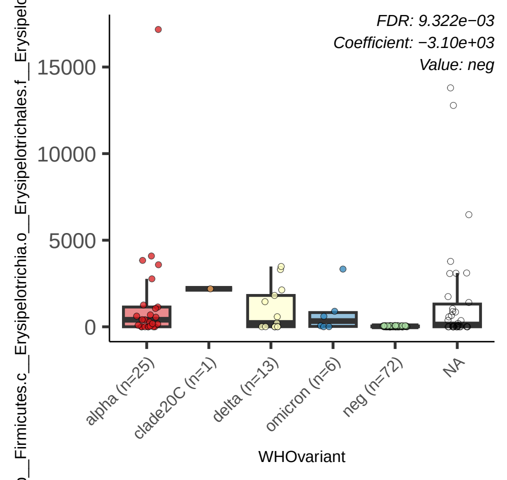

FDR: 9.322e−03
Coefficient: −3.10e+03
Value: neg
o__Firmicutes.c__Erysipelotrichia.o__Erysipelotrichales.f__Erysipelo
15000
10000
5000
0
alpha (n=25)
clade20C (n=1)
delta (n=13)
omicron (n=6)
neg (n=72)
NA
WHOvariant

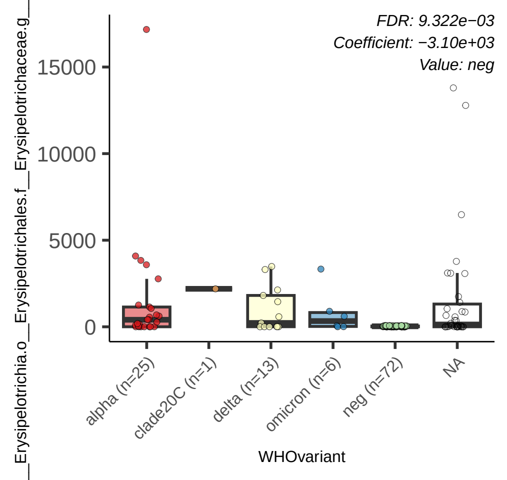

FDR: 9.322e−03
Coefficient: −3.10e+03
Value: neg
Erysipelotrichia.o__Erysipelotrichales.f__Erysipelotrichaceae.g__
15000
10000
5000
0
alpha (n=25)
clade20C (n=1)
delta (n=13)
omicron (n=6)
neg (n=72)
NA
WHOvariant

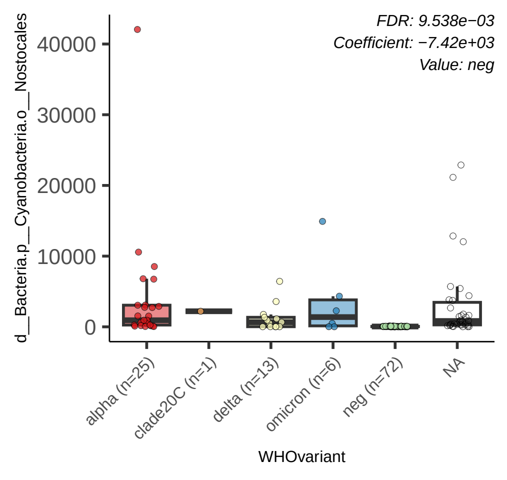
FDR: 9.538e−03
Coefficient: −7.42e+03
Value: neg
d__Bacteria.p__Cyanobacteria.o__Nostocales
40000
30000
20000
10000
0
alpha (n=25)
clade20C (n=1)
delta (n=13)
omicron (n=6)
neg (n=72)
NA
WHOvariant

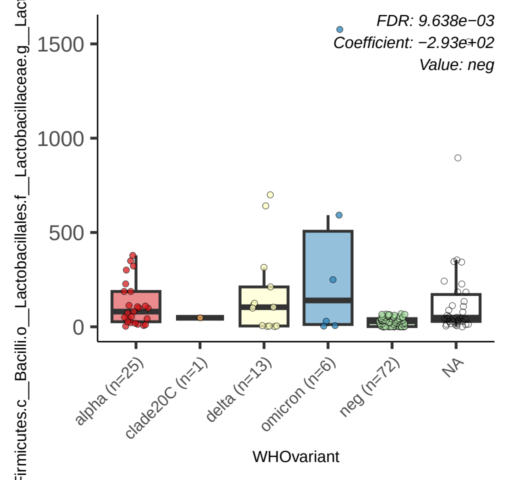
FDR: 9.638e−03
Coefficient: −2.93e+02
Value: neg
Firmicutes.c__Bacilli.o__Lactobacillales.f__Lactobacillaceae.g__Lact
1500
1000
500
0
alpha (n=25)
clade20C (n=1)
delta (n=13)
omicron (n=6)
neg (n=72)
NA
WHOvariant

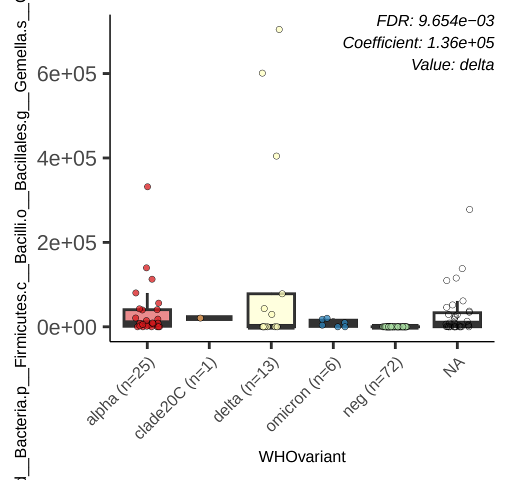

d__Bacteria.p__Firmicutes.c__Bacilli.o__Bacillales.g__Gemella.s__G
FDR: 9.654e−03
Coefficient: 1.36e+05
Value: delta
6e+05
4e+05
2e+05
0e+00
alpha (n=25)
clade20C (n=1)
delta (n=13)
omicron (n=6)
neg (n=72)
NA
WHOvariant

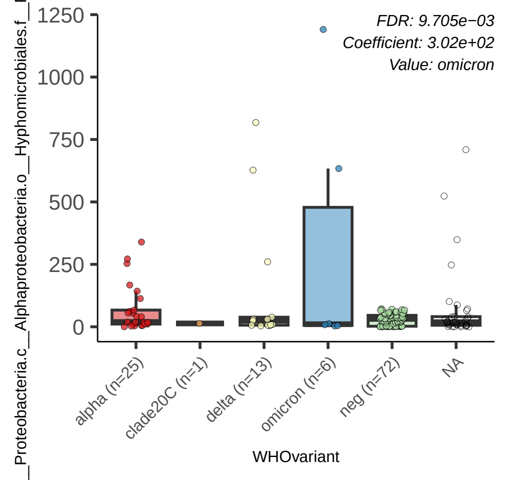

FDR: 9.705e−03
Coefficient: 3.02e+02
Value: omicron
Proteobacteria.c__Alphaproteobacteria.o__Hyphomicrobiales.f__
1250
1000
750
500
250
0
alpha (n=25)
clade20C (n=1)
delta (n=13)
omicron (n=6)
neg (n=72)
NA
WHOvariant

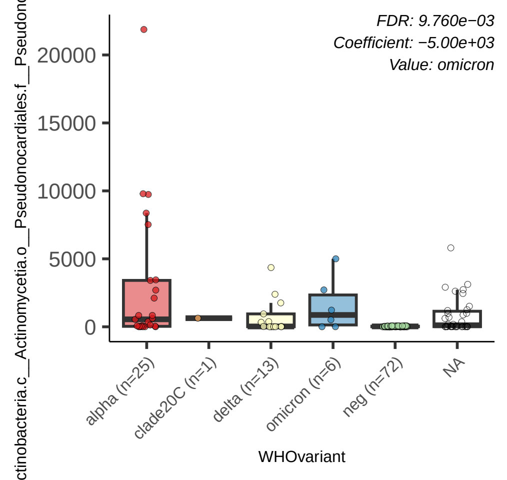

FDR: 9.760e−03
Coefficient: −5.00e+03
Value: omicron
0
5000
10000
15000
20000
ctinobacteria.c__Actinomycetia.o__Pseudonocardiales.f__Pseudonoc
alpha (n=25)
clade20C (n=1)
delta (n=13)
omicron (n=6)
neg (n=72)
NA
WHOvariant

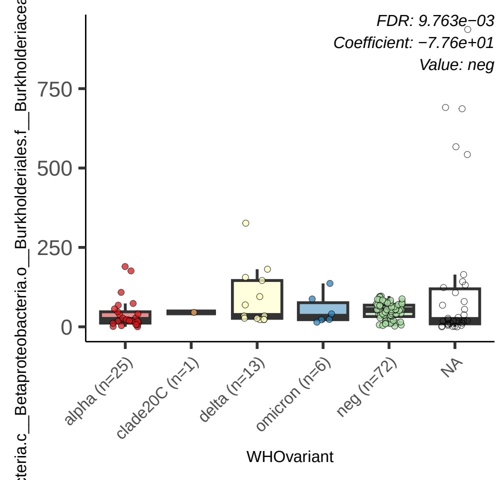

FDR: 9.763e−03
Coefficient: −7.76e+01
Value: neg
cteria.c__Betaproteobacteria.o__Burkholderiales.f__Burkholderiacea
750
500
250
0
alpha (n=25)
clade20C (n=1)
delta (n=13)
omicron (n=6)
neg (n=72)
NA
WHOvariant

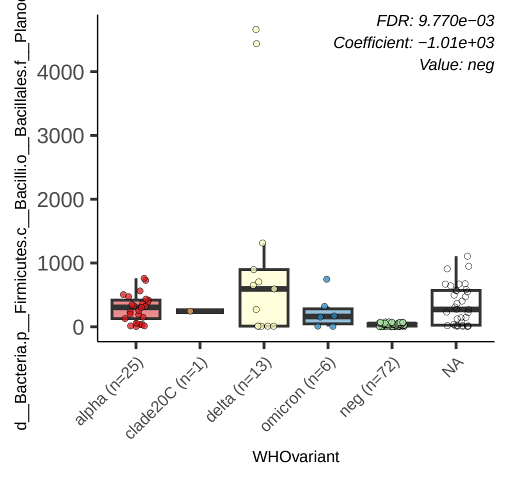

FDR: 9.770e−03
Coefficient: −1.01e+03
Value: neg
d__Bacteria.p__Firmicutes.c__Bacilli.o__Bacillales.f__Planoc
4000
3000
2000
1000
0
alpha (n=25)
clade20C (n=1)
delta (n=13)
omicron (n=6)
neg (n=72)
NA
WHOvariant

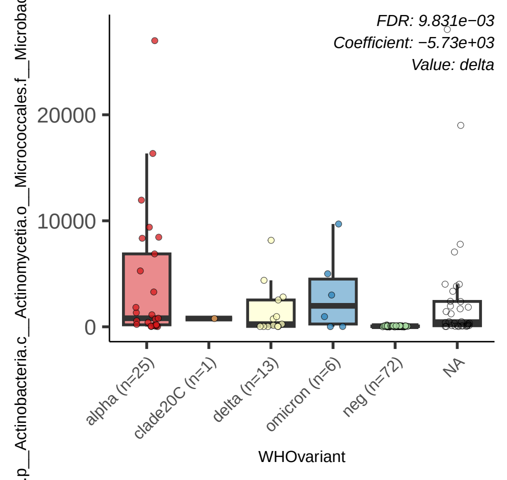

FDR: 9.831e−03
Coefficient: −5.73e+03
Value: delta
20000
10000
0
p__Actinobacteria.c__Actinomycetia.o__Micrococcales.f__Microbac
alpha (n=25)
clade20C (n=1)
delta (n=13)
omicron (n=6)
neg (n=72)
NA
WHOvariant

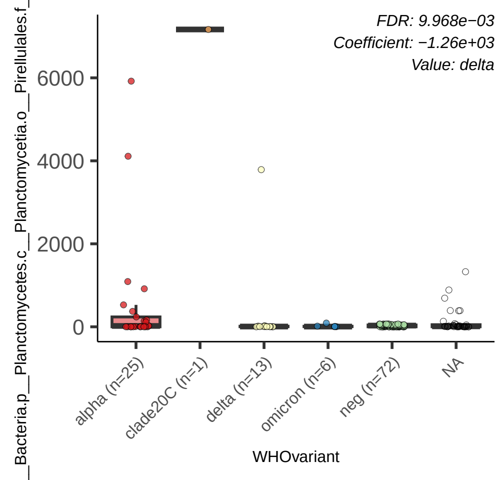

FDR: 9.968e−03
Coefficient: −1.26e+03
Value: delta
_Bacteria.p__Planctomycetes.c__Planctomycetia.o__Pirellulales.f_
6000
4000
2000
0
alpha (n=25)
clade20C (n=1)
delta (n=13)
omicron (n=6)
neg (n=72)
NA
WHOvariant

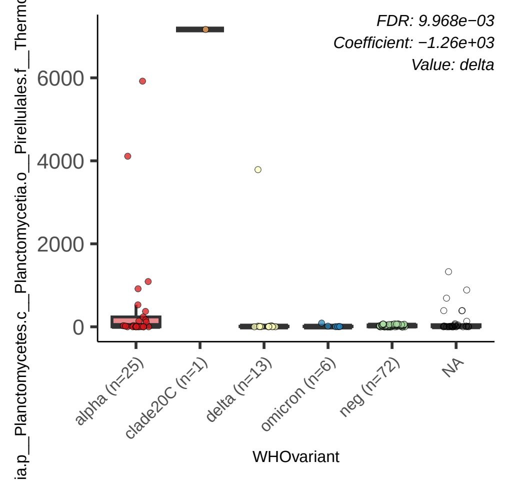
FDR: 9.968e−03
Coefficient: −1.26e+03
Value: delta
6000
4000
2000
0
ia.p__Planctomycetes.c__Planctomycetia.o__Pirellulales.f__Thermo
alpha (n=25)
clade20C (n=1)
delta (n=13)
omicron (n=6)
neg (n=72)
NA
WHOvariant

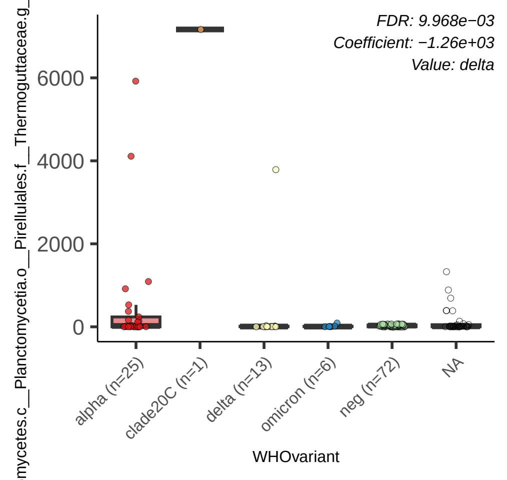

FDR: 9.968e−03
Coefficient: −1.26e+03
Value: delta
mycetes.c__Planctomycetia.o__Pirellulales.f__Thermoguttaceae.g__
6000
4000
2000
0
alpha (n=25)
clade20C (n=1)
delta (n=13)
omicron (n=6)
neg (n=72)
NA
WHOvariant

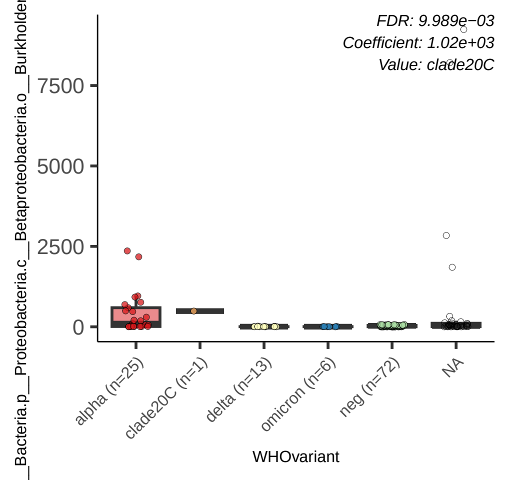

FDR: 9.989e−03
Coefficient: 1.02e+03
Value: clade20C
7500
5000
2500
0
_Bacteria.p__Proteobacteria.c__Betaproteobacteria.o__Burkholder
alpha (n=25)
clade20C (n=1)
delta (n=13)
omicron (n=6)
neg (n=72)
NA
WHOvariant

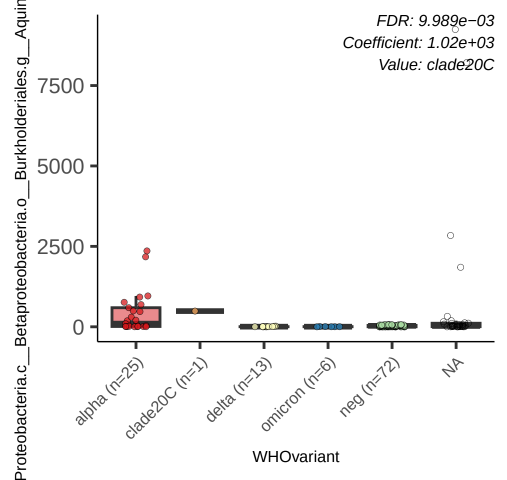

FDR: 9.989e−03
Coefficient: 1.02e+03
Value: clade20C
Proteobacteria.c__Betaproteobacteria.o__Burkholderiales.g__Aquin
7500
5000
2500
0
alpha (n=25)
clade20C (n=1)
delta (n=13)
omicron (n=6)
neg (n=72)
NA
WHOvariant

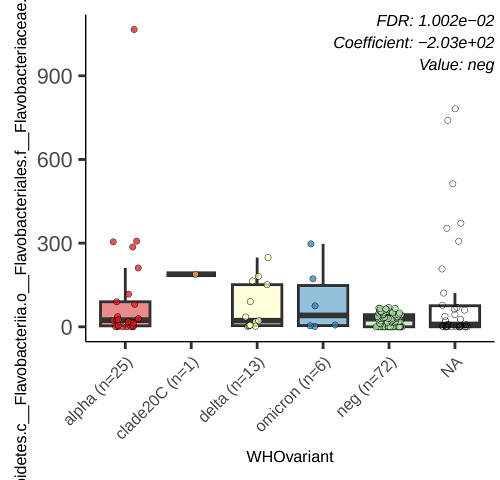

FDR: 1.002e−02
Coefficient: −2.03e+02
Value: neg
0
300
600
900
alpha (n=25)
clade20C (n=1)
delta (n=13)
omicron (n=6)
neg (n=72)
NA
WHOvariant
oidetes.c__Flavobacteriia.o__Flavobacteriales.f__Flavobacteriaceae.

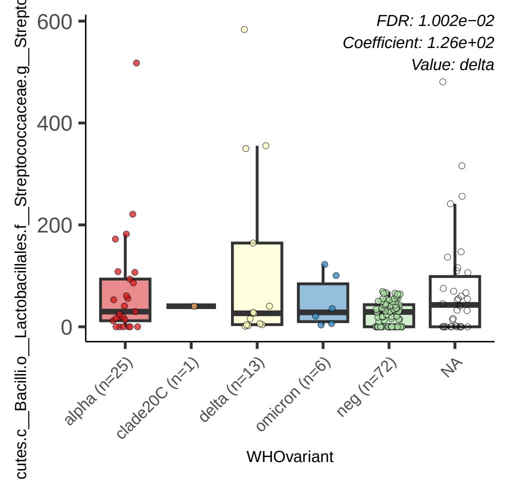

FDR: 1.002e−02
Coefficient: 1.26e+02
Value: delta
600
400
200
0
cutes.c__Bacilli.o__Lactobacillales.f__Streptococcaceae.g__Strepto
alpha (n=25)
clade20C (n=1)
delta (n=13)
omicron (n=6)
neg (n=72)
NA
WHOvariant

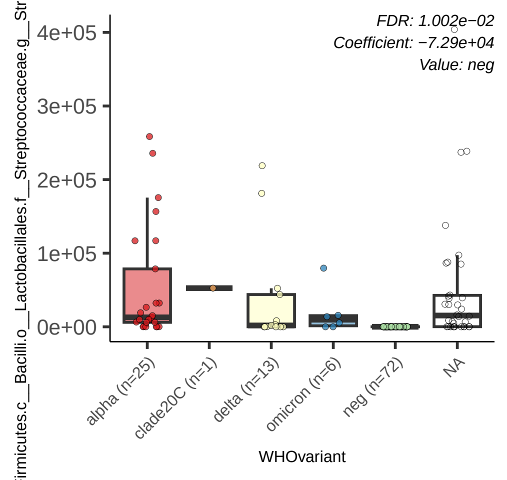

FDR: 1.002e−02
Coefficient: −7.29e+04
Value: neg
4e+05
3e+05
2e+05
1e+05
0e+00
irmicutes.c__Bacilli.o__Lactobacillales.f__Streptococcaceae.g__Str
alpha (n=25)
clade20C (n=1)
delta (n=13)
omicron (n=6)
neg (n=72)
NA
WHOvariant

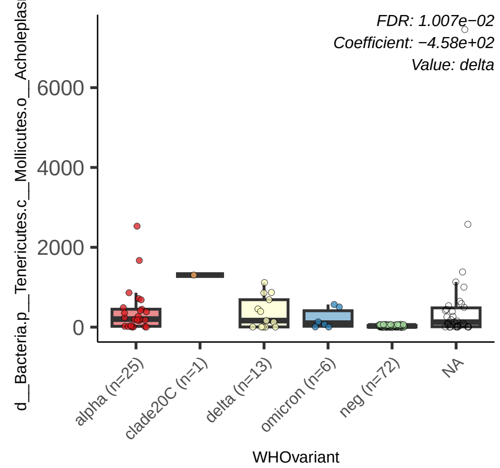

FDR: 1.007e−02
Coefficient: −4.58e+02
Value: delta
d__Bacteria.p__Tenericutes.c__Mollicutes.o__Acholeplas
0
2000
4000
6000
alpha (n=25)
clade20C (n=1)
delta (n=13)
omicron (n=6)
neg (n=72)
NA
WHOvariant

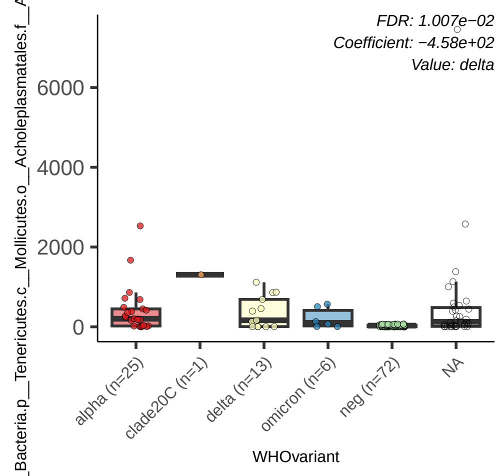

FDR: 1.007e−02
Coefficient: −4.58e+02
Value: delta
6000
4000
2000
0
Bacteria.p__Tenericutes.c__Mollicutes.o__Acholeplasmatales.f__A
alpha (n=25)
clade20C (n=1)
delta (n=13)
omicron (n=6)
neg (n=72)
NA
WHOvariant

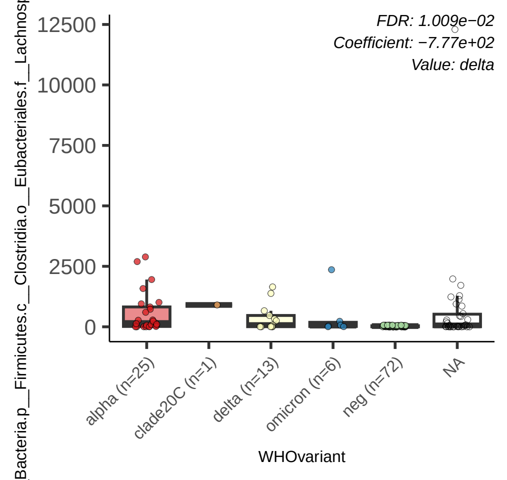

FDR: 1.009e−02
Coefficient: −7.77e+02
Value: delta
Bacteria.p__Firmicutes.c__Clostridia.o__Eubacteriales.f__Lachnosp
12500
10000
7500
5000
2500
0
alpha (n=25)
clade20C (n=1)
delta (n=13)
omicron (n=6)
neg (n=72)
NA
WHOvariant

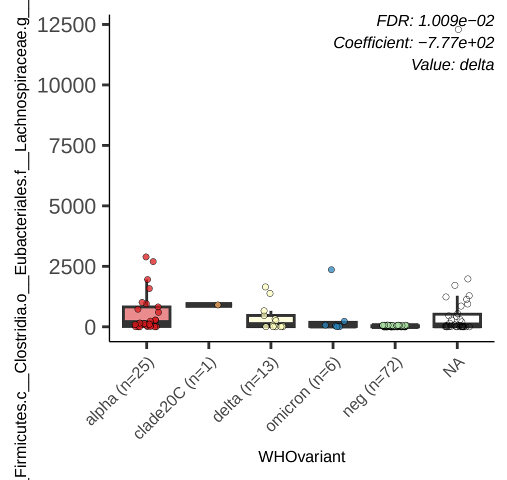

FDR: 1.009e−02
Coefficient: −7.77e+02
Value: delta
Firmicutes.c__Clostridia.o__Eubacteriales.f__Lachnospiraceae.g__
12500
10000
7500
5000
2500
0
alpha (n=25)
clade20C (n=1)
delta (n=13)
omicron (n=6)
neg (n=72)
NA
WHOvariant

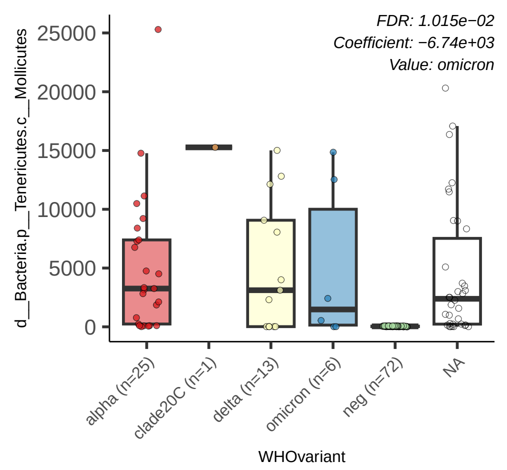

FDR: 1.015e−02
Coefficient: −6.74e+03
Value: omicron
d__Bacteria.p__Tenericutes.c__Mollicutes
25000
20000
15000
10000
5000
0
alpha (n=25)
clade20C (n=1)
delta (n=13)
omicron (n=6)
neg (n=72)
NA
WHOvariant

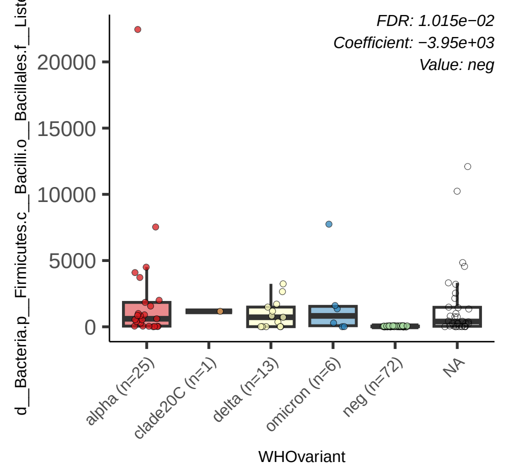

FDR: 1.015e−02
Coefficient: −3.95e+03
Value: neg
d__Bacteria.p__Firmicutes.c__Bacilli.o__Bacillales.f__Listo
20000
15000
10000
5000
0
alpha (n=25)
clade20C (n=1)
delta (n=13)
omicron (n=6)
neg (n=72)
NA
WHOvariant

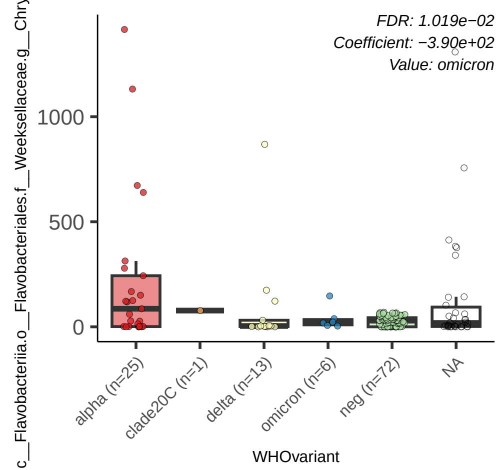

FDR: 1.019e−02
Coefficient: −3.90e+02
Value: omicron
1000
500
0
alpha (n=25)
clade20C (n=1)
delta (n=13)
omicron (n=6)
neg (n=72)
NA
WHOvariant
s.c__Flavobacteriia.o__Flavobacteriales.f__Weeksellaceae.g__Chry

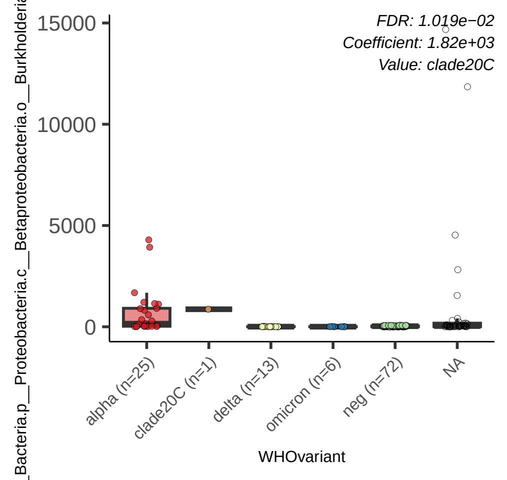

FDR: 1.019e−02
Coefficient: 1.82e+03
Value: clade20C
15000
10000
5000
0
Bacteria.p__Proteobacteria.c__Betaproteobacteria.o__Burkholderia
alpha (n=25)
clade20C (n=1)
delta (n=13)
omicron (n=6)
neg (n=72)
NA
WHOvariant

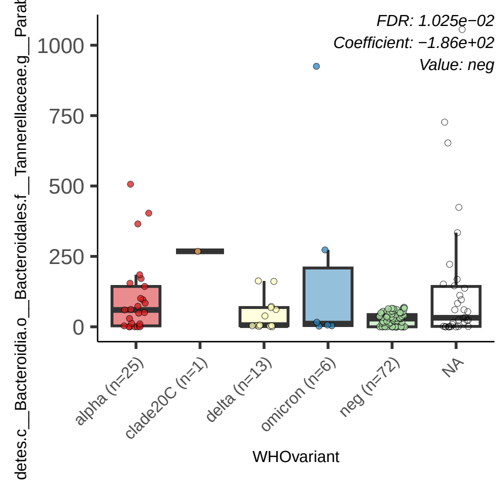

FDR: 1.025e−02
Coefficient: −1.86e+02
Value: neg
detes.c__Bacteroidia.o__Bacteroidales.f__Tannerellaceae.g__Parab
1000
750
500
250
0
alpha (n=25)
clade20C (n=1)
delta (n=13)
omicron (n=6)
neg (n=72)
NA
WHOvariant

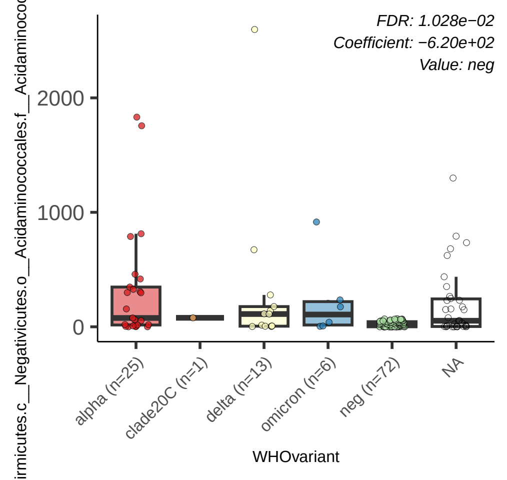

FDR: 1.028e−02
Coefficient: −6.20e+02
Value: neg
2000
1000
0
irmicutes.c__Negativicutes.o__Acidaminococcales.f__Acidaminococ
alpha (n=25)
clade20C (n=1)
delta (n=13)
omicron (n=6)
neg (n=72)
NA
WHOvariant

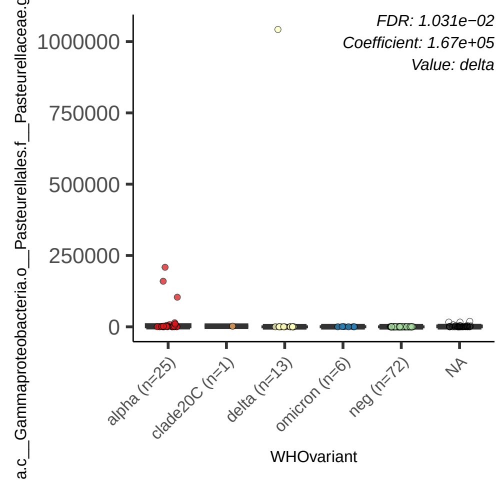

FDR: 1.031e−02
Coefficient: 1.67e+05
Value: delta
1000000
750000
500000
250000
0
a.c__Gammaproteobacteria.o__Pasteurellales.f__Pasteurellaceae.g
alpha (n=25)
clade20C (n=1)
delta (n=13)
omicron (n=6)
neg (n=72)
NA
WHOvariant

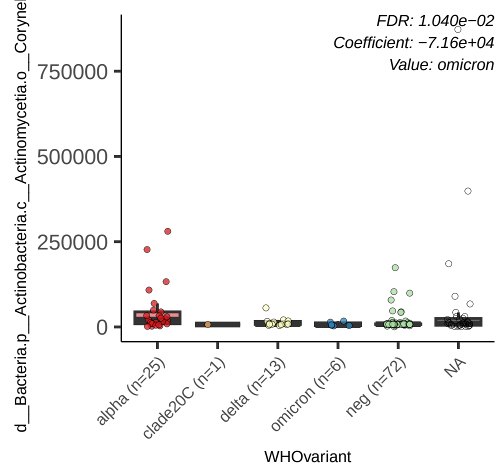

FDR: 1.040e−02
Coefficient: −7.16e+04
Value: omicron
d__Bacteria.p__Actinobacteria.c__Actinomycetia.o__Coryne
750000
500000
250000
0
alpha (n=25)
clade20C (n=1)
delta (n=13)
omicron (n=6)
neg (n=72)
NA
WHOvariant

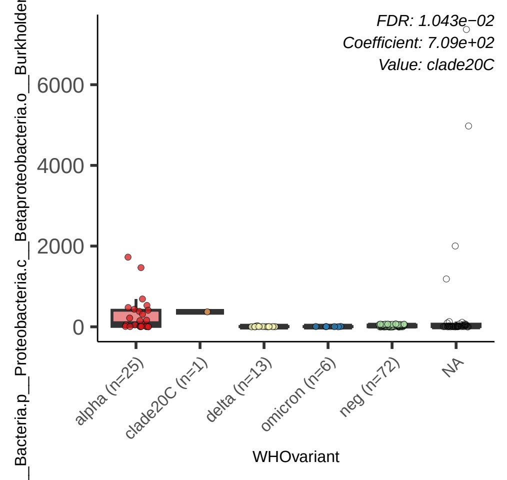

FDR: 1.043e−02
Coefficient: 7.09e+02
Value: clade20C
6000
4000
2000
0
alpha (n=25)
clade20C (n=1)
delta (n=13)
omicron (n=6)
neg (n=72)
NA
WHOvariant
Bacteria.p__Proteobacteria.c__Betaproteobacteria.o__Burkholder

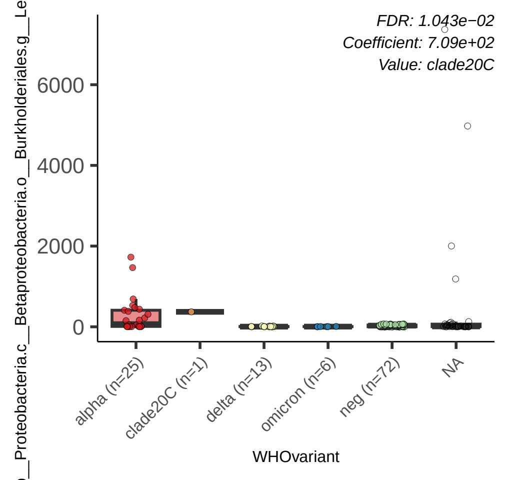

FDR: 1.043e−02
Coefficient: 7.09e+02
Value: clade20C
o__Proteobacteria.c__Betaproteobacteria.o__Burkholderiales.g__Le
6000
4000
2000
0
alpha (n=25)
clade20C (n=1)
delta (n=13)
omicron (n=6)
neg (n=72)
NA
WHOvariant

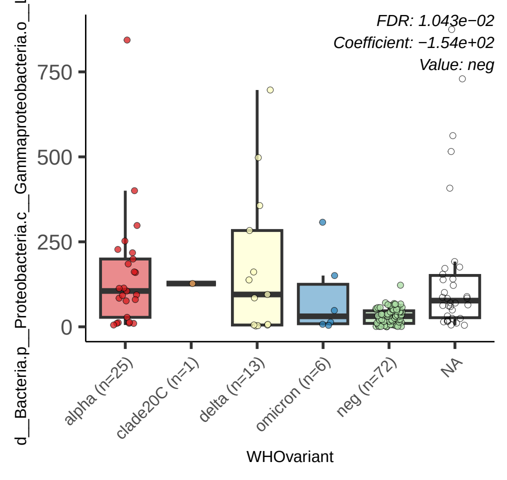

FDR: 1.043e−02
Coefficient: −1.54e+02
Value: neg
d__Bacteria.p__Proteobacteria.c__Gammaproteobacteria.o__
0
250
500
750
alpha (n=25)
clade20C (n=1)
delta (n=13)
omicron (n=6)
neg (n=72)
NA
WHOvariant

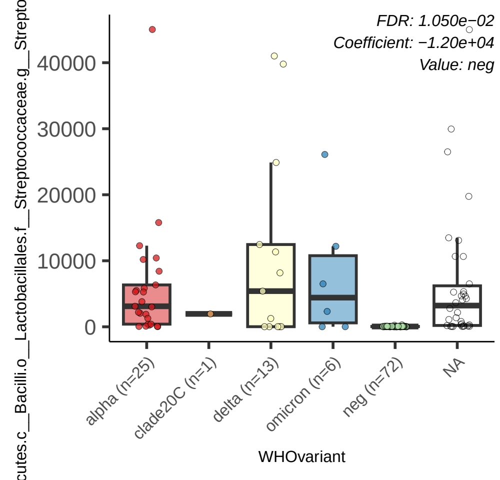

FDR: 1.050e−02
Coefficient: −1.20e+04
Value: neg
cutes.c__Bacilli.o__Lactobacillales.f__Streptococcaceae.g__Strepto
40000
30000
20000
10000
0
alpha (n=25)
clade20C (n=1)
delta (n=13)
omicron (n=6)
neg (n=72)
NA
WHOvariant

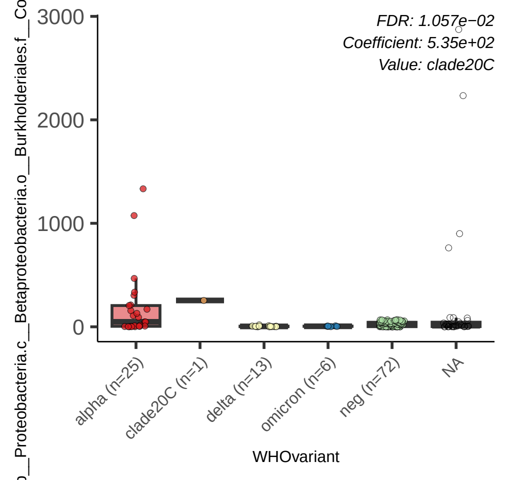

o__Proteobacteria.c__Betaproteobacteria.o__Burkholderiales.f__Co
3000
2000
1000
0
FDR: 1.057e−02
Coefficient: 5.35e+02
Value: clade20C
alpha (n=25)
clade20C (n=1)
delta (n=13)
omicron (n=6)
neg (n=72)
NA
WHOvariant

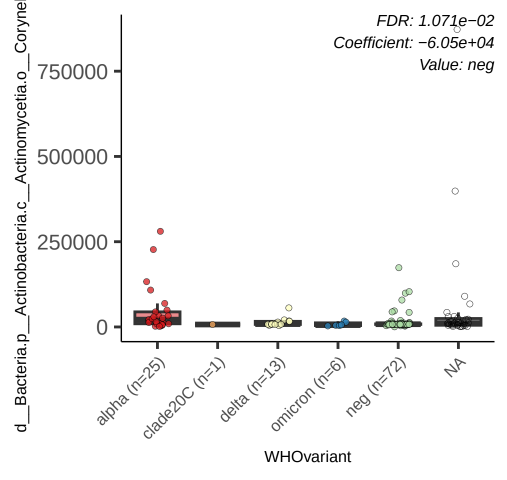

FDR: 1.071e−02
Coefficient: −6.05e+04
Value: neg
d__Bacteria.p__Actinobacteria.c__Actinomycetia.o__Coryne
750000
500000
250000
0
alpha (n=25)
clade20C (n=1)
delta (n=13)
omicron (n=6)
neg (n=72)
NA
WHOvariant

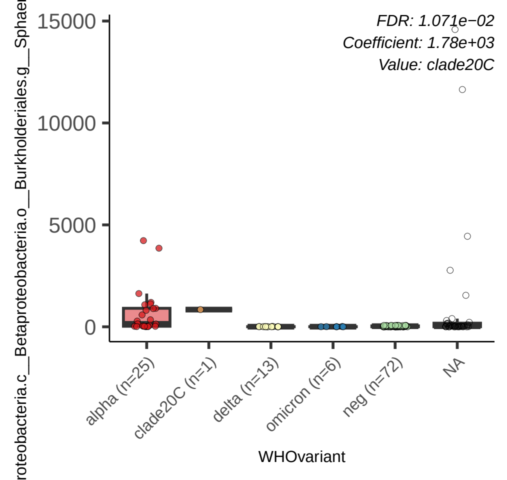

FDR: 1.071e−02
Coefficient: 1.78e+03
Value: clade20C
15000
10000
5000
0
roteobacteria.c__Betaproteobacteria.o__Burkholderiales.g__Sphaer
alpha (n=25)
clade20C (n=1)
delta (n=13)
omicron (n=6)
neg (n=72)
NA
WHOvariant

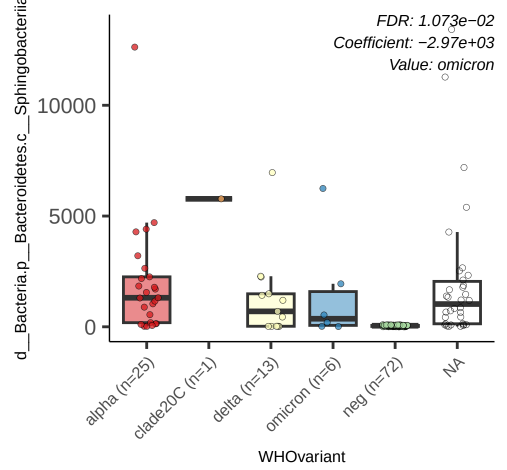

FDR: 1.073e−02
Coefficient: −2.97e+03
Value: omicron
d__Bacteria.p__Bacteroidetes.c__Sphingobacteriia
10000
5000
0
alpha (n=25)
clade20C (n=1)
delta (n=13)
omicron (n=6)
neg (n=72)
NA
WHOvariant

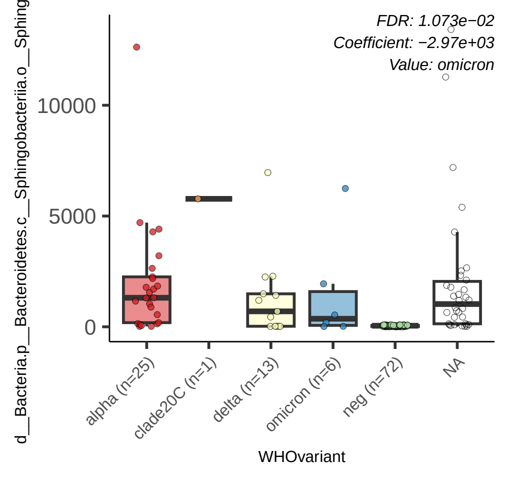
FDR: 1.073e−02
Coefficient: −2.97e+03
Value: omicron
d__Bacteria.p__Bacteroidetes.c__Sphingobacteriia.o__Sphing
10000
5000
0
alpha (n=25)
clade20C (n=1)
delta (n=13)
omicron (n=6)
neg (n=72)
NA
WHOvariant

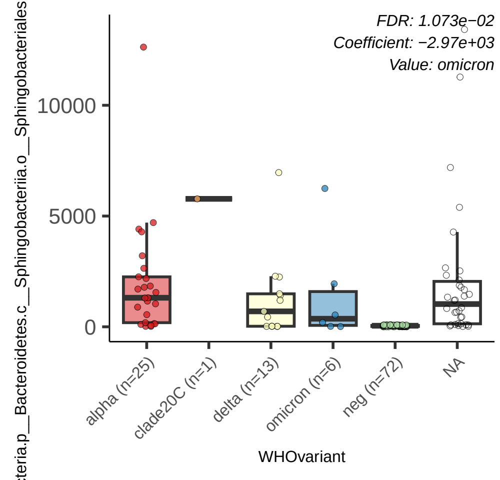
FDR: 1.073e−02
Coefficient: −2.97e+03
Value: omicron
cteria.p__Bacteroidetes.c__Sphingobacteriia.o__Sphingobacteriales
10000
5000
0
alpha (n=25)
clade20C (n=1)
delta (n=13)
omicron (n=6)
neg (n=72)
NA
WHOvariant

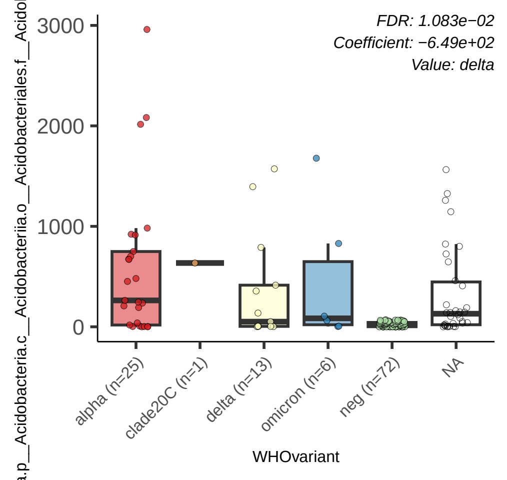

FDR: 1.083e−02
Coefficient: −6.49e+02
Value: delta
a.p__Acidobacteria.c__Acidobacteriia.o__Acidobacteriales.f__Acidob
3000
2000
1000
0
alpha (n=25)
clade20C (n=1)
delta (n=13)
omicron (n=6)
neg (n=72)
NA
WHOvariant

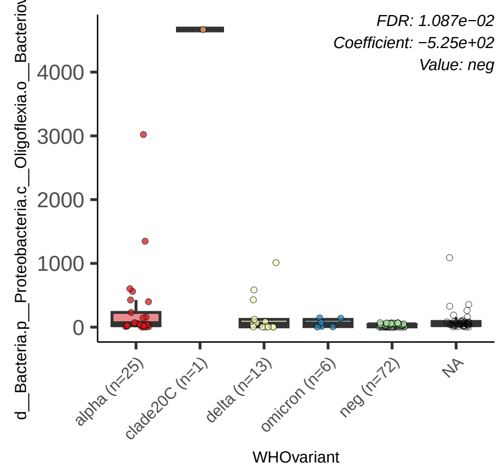
FDR: 1.087e−02
Coefficient: −5.25e+02
Value: neg
d__Bacteria.p__Proteobacteria.c__Oligoflexia.o__Bacteriov
4000
3000
2000
1000
0
alpha (n=25)
clade20C (n=1)
delta (n=13)
omicron (n=6)
neg (n=72)
NA
WHOvariant

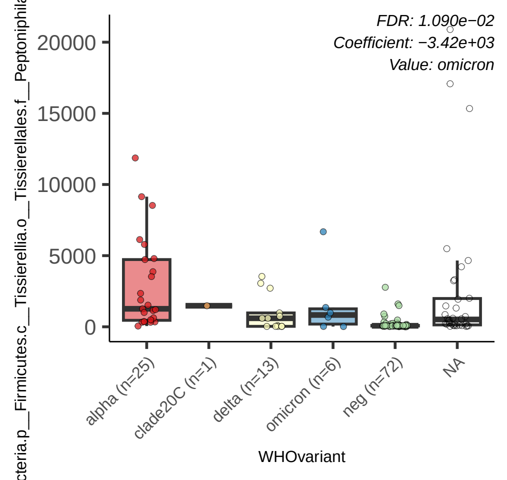

FDR: 1.090e−02
Coefficient: −3.42e+03
Value: omicron
20000
15000
10000
5000
0
alpha (n=25)
clade20C (n=1)
delta (n=13)
omicron (n=6)
neg (n=72)
NA
WHOvariant
cteria.p__Firmicutes.c__Tissierellia.o__Tissierellales.f__Peptoniphila

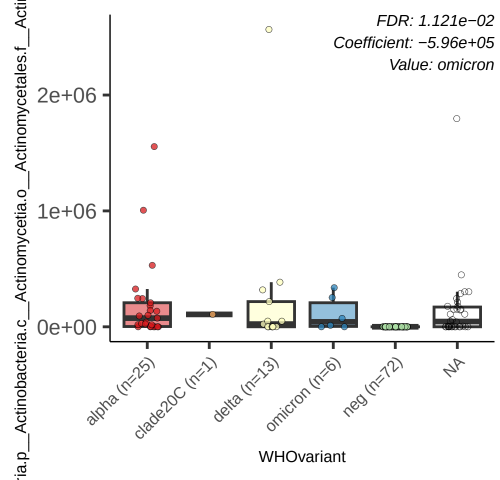

FDR: 1.121e−02
Coefficient: −5.96e+05
Value: omicron
ria.p__Actinobacteria.c__Actinomycetia.o__Actinomycetales.f__Acti
2e+06
1e+06
0e+00
alpha (n=25)
clade20C (n=1)
delta (n=13)
omicron (n=6)
neg (n=72)
NA
WHOvariant

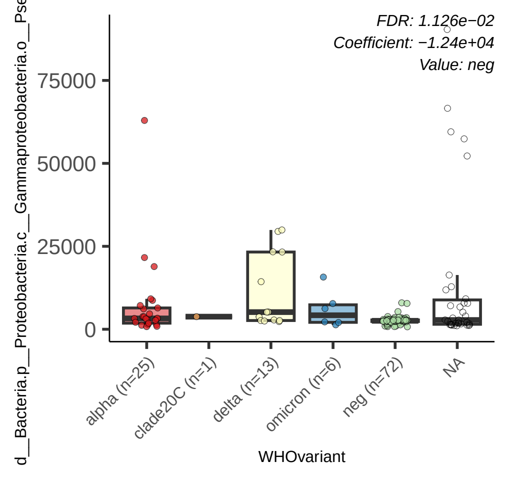

FDR: 1.126e−02
Coefficient: −1.24e+04
Value: neg
75000
50000
25000
0
d__Bacteria.p__Proteobacteria.c__Gammaproteobacteria.o__Pse
alpha (n=25)
clade20C (n=1)
delta (n=13)
omicron (n=6)
neg (n=72)
NA
WHOvariant

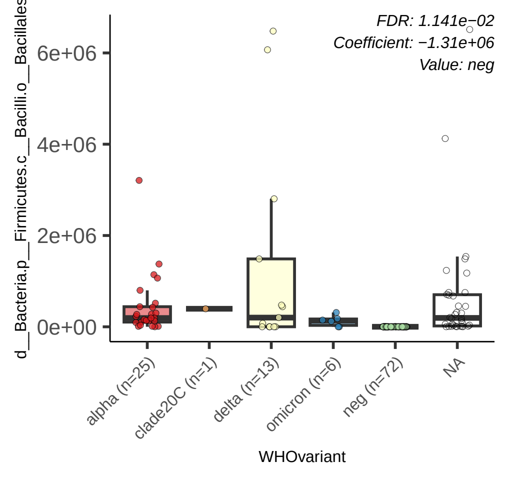
FDR: 1.141e−02
Coefficient: −1.31e+06
Value: neg
d__Bacteria.p__Firmicutes.c__Bacilli.o__Bacillales
6e+06
4e+06
2e+06
0e+00
alpha (n=25)
clade20C (n=1)
delta (n=13)
omicron (n=6)
neg (n=72)
NA
WHOvariant

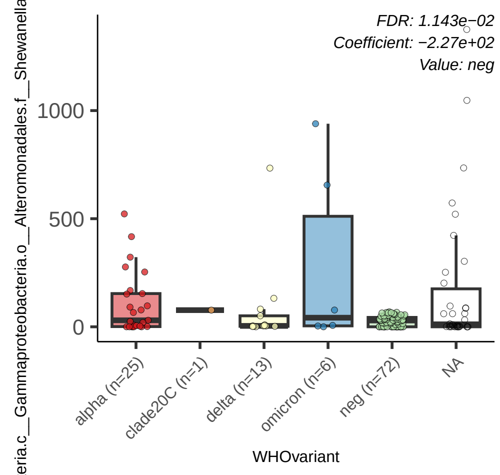

Gammaproteobacteria.c__Gammaproteobacteria.o__Alteromonadales.f__Shewanella
FDR: 1.143e−02
Coefficient: −2.27e+02
Value: neg
1000
500
0
alpha (n=25)
clade20C (n=1)
delta (n=13)
omicron (n=6)
neg (n=72)
NA
WHOvariant

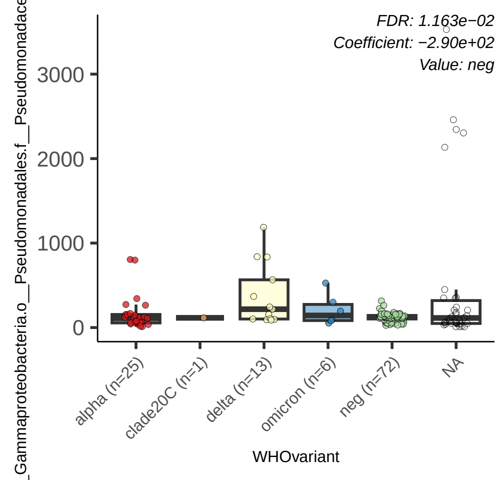

FDR: 1.163e−02
Coefficient: −2.90e+02
Value: neg
_Gammaproteobacteria.o__Pseudomonadales.f__Pseudomonadace
3000
2000
1000
0
alpha (n=25)
clade20C (n=1)
delta (n=13)
omicron (n=6)
neg (n=72)
NA
WHOvariant

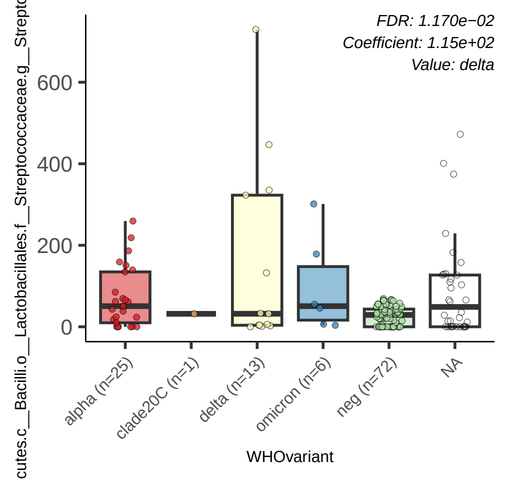
FDR: 1.170e−02
Coefficient: 1.15e+02
Value: delta
600
400
200
0
cutes.c__Bacilli.o__Lactobacillales.f__Streptococcaceae.g__Strepto
alpha (n=25)
clade20C (n=1)
delta (n=13)
omicron (n=6)
neg (n=72)
NA
WHOvariant

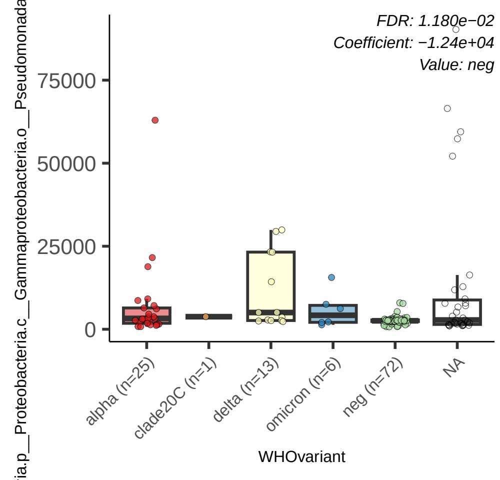

FDR: 1.180e−02
Coefficient: −1.24e+04
Value: neg
75000
50000
25000
0
ia.p__Proteobacteria.c__Gammaproteobacteria.o__Pseudomonada
alpha (n=25)
clade20C (n=1)
delta (n=13)
omicron (n=6)
neg (n=72)
NA
WHOvariant

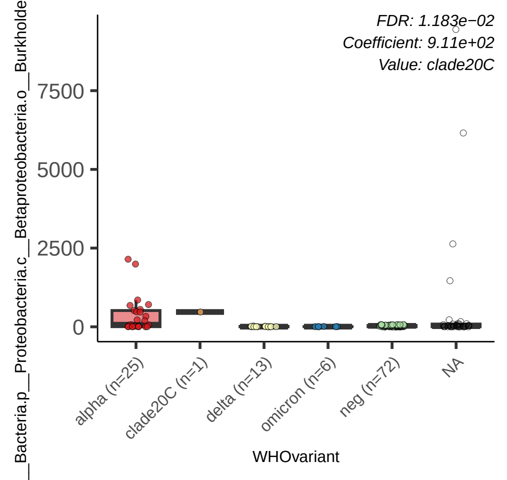

FDR: 1.183e−02
Coefficient: 9.11e+02
Value: clade20C
Bacteria.p__Proteobacteria.c__Betaproteobacteria.o__Burkholde
7500
5000
2500
0
alpha (n=25)
clade20C (n=1)
delta (n=13)
omicron (n=6)
neg (n=72)
NA
WHOvariant

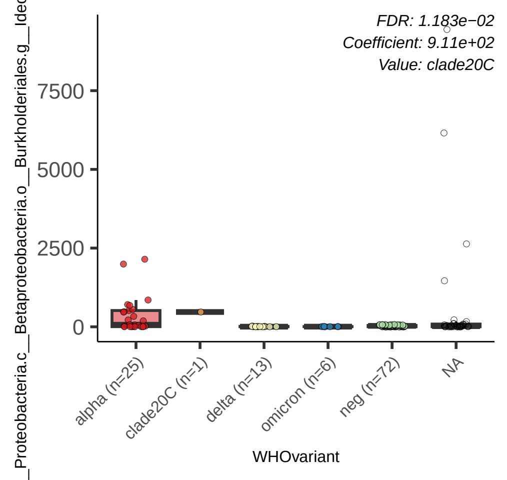

FDR: 1.183e−02
Coefficient: 9.11e+02
Value: clade20C
7500
5000
2500
0
alpha (n=25)
clade20C (n=1)
delta (n=13)
omicron (n=6)
neg (n=72)
NA
WHOvariant
Proteobacteria.c__Betaproteobacteria.o__Burkholderiales.g__Ideo

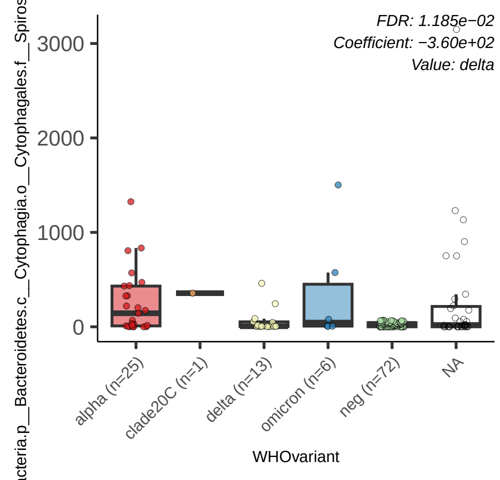

FDR: 1.185e−02
Coefficient: −3.60e+02
Value: delta
3000
2000
1000
0
acteria.p__Bacteroidetes.c__Cytophagia.o__Cytophagales.f__Spiros
alpha (n=25)
clade20C (n=1)
delta (n=13)
omicron (n=6)
neg (n=72)
NA
WHOvariant

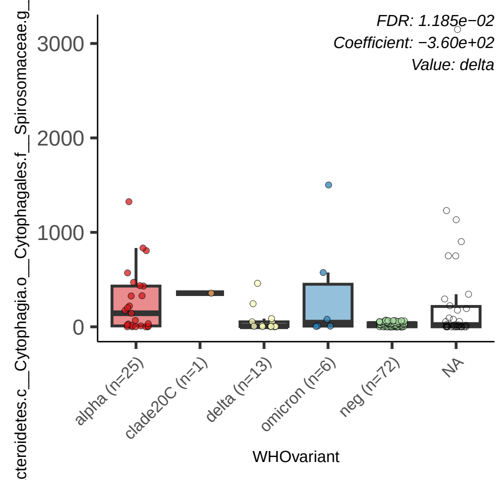

FDR: 1.185e−02
Coefficient: −3.60e+02
Value: delta
cteroidetes.c__Cytophagia.o__Cytophagales.f__Spirosomaceae.g__
3000
2000
1000
0
alpha (n=25)
clade20C (n=1)
delta (n=13)
omicron (n=6)
neg (n=72)
NA
WHOvariant

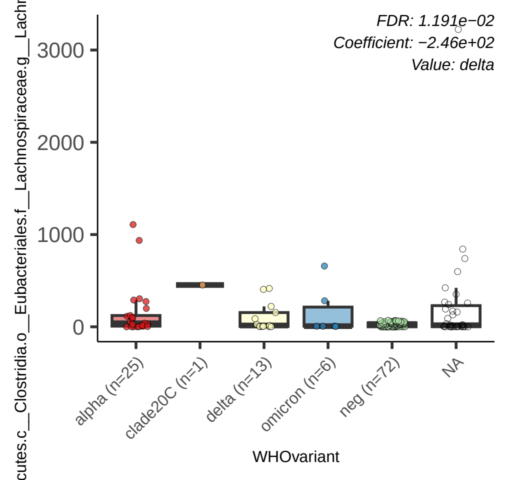

FDR: 1.191e−02
Coefficient: −2.46e+02
Value: delta
3000
2000
1000
0
cutes.c__Clostridia.o__Eubacteriales.f__Lachnospiraceae.g__Lachn
alpha (n=25)
clade20C (n=1)
delta (n=13)
omicron (n=6)
neg (n=72)
NA
WHOvariant

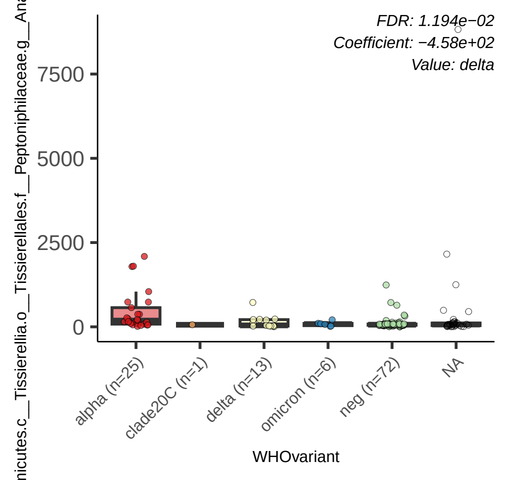
FDR: 1.194e−02
Coefficient: −4.58e+02
Value: delta
7500
5000
2500
0
nicutes.c__Tissierellia.o__Tissierellales.f__Peptoniphilaceae.g__Ana
alpha (n=25)
clade20C (n=1)
delta (n=13)
omicron (n=6)
neg (n=72)
NA
WHOvariant

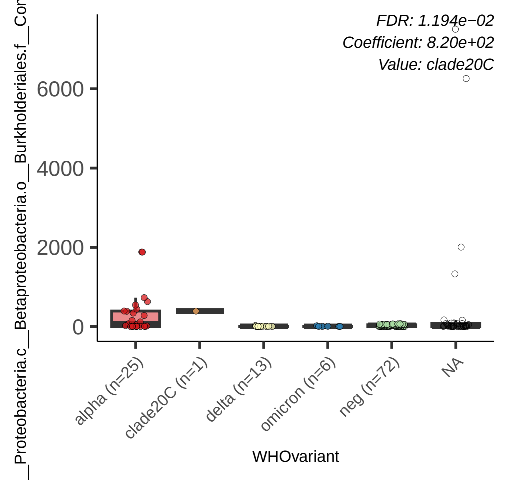
FDR: 1.194e−02
Coefficient: 8.20e+02
Value: clade20C
6000
4000
2000
0
Proteobacteria.c__Betaproteobacteria.o__Burkholderiales.f__Con
alpha (n=25)
clade20C (n=1)
delta (n=13)
omicron (n=6)
neg (n=72)
NA
WHOvariant

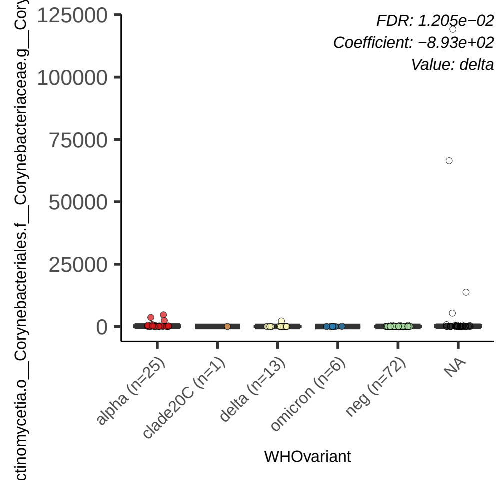

FDR: 1.205e−02
Coefficient: −8.93e+02
Value: delta
125000
100000
75000
50000
25000
0
alpha (n=25)
clade20C (n=1)
delta (n=13)
omicron (n=6)
neg (n=72)
NA
WHOvariant
ctinomycetia.o__Corynebacteriales.f__Corynebacteriaceae.g__Cory

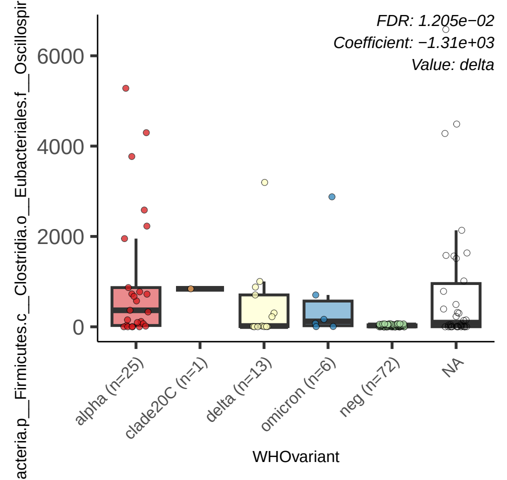

FDR: 1.205e−02
Coefficient: −1.31e+03
Value: delta
6000
4000
2000
0
acteria.p__Firmicutes.c__Clostridia.o__Eubacteriales.f__Oscillospir
alpha (n=25)
clade20C (n=1)
delta (n=13)
omicron (n=6)
neg (n=72)
NA
WHOvariant

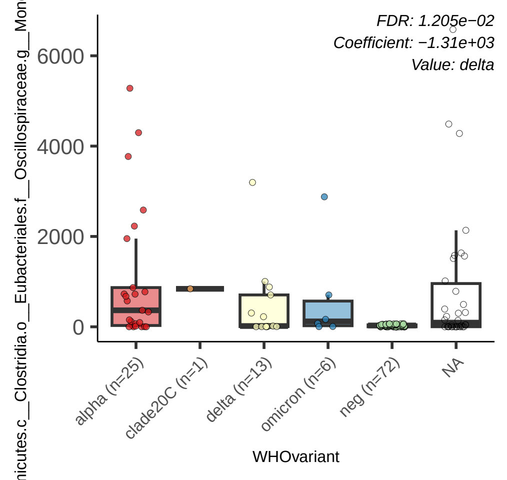

FDR: 1.205e−02
Coefficient: −1.31e+03
Value: delta
6000
4000
2000
0
nicutes.c__Clostridia.o__Eubacteriales.f__Oscillospiraceae.g__Mon
alpha (n=25)
clade20C (n=1)
delta (n=13)
omicron (n=6)
neg (n=72)
NA
WHOvariant

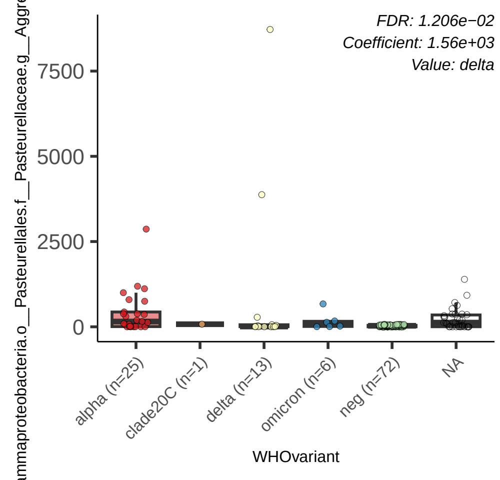

FDR: 1.206e−02
Coefficient: 1.56e+03
Value: delta
7500
5000
2500
0
alpha (n=25)
clade20C (n=1)
delta (n=13)
omicron (n=6)
neg (n=72)
NA
WHOvariant
ammaproteobacteria.o__Pasteurellales.f__Pasteurellaceae.g__Aggre

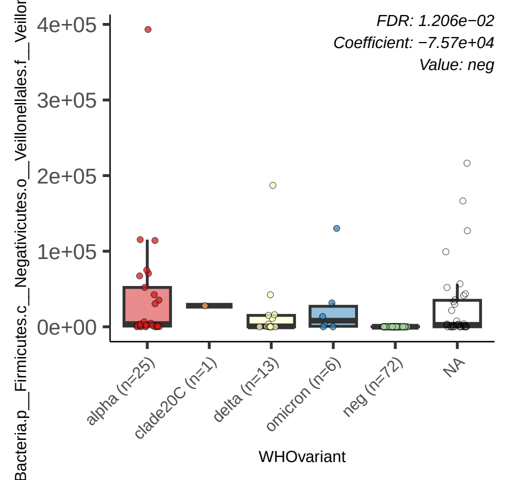

FDR: 1.206e−02
Coefficient: −7.57e+04
Value: neg
Bacteria.p__Firmicutes.c__Negativicutes.o__Veillonellales.f__Veillon
4e+05
3e+05
2e+05
1e+05
0e+00
alpha (n=25)
clade20C (n=1)
delta (n=13)
omicron (n=6)
neg (n=72)
NA
WHOvariant

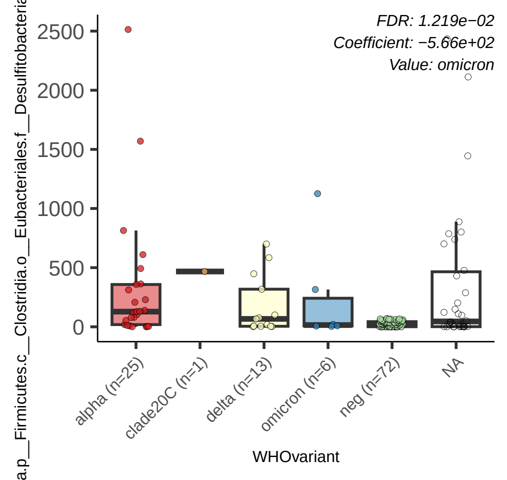

FDR: 1.219e−02
Coefficient: −5.66e+02
Value: omicron
a.p__Firmicutes.c__Clostridia.o__Eubacteriales.f__Desulfitobacteria
2500
2000
1500
1000
500
0
alpha (n=25)
clade20C (n=1)
delta (n=13)
omicron (n=6)
neg (n=72)
NA
WHOvariant

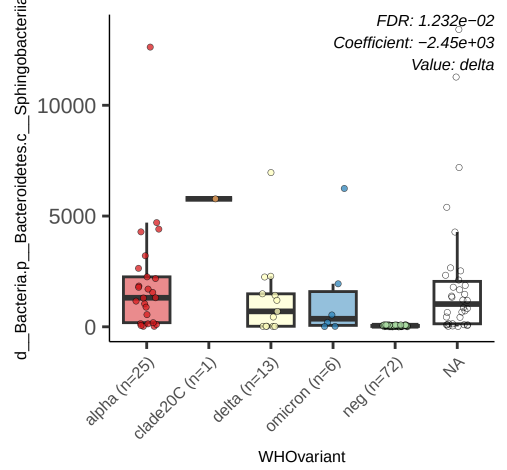

FDR: 1.232e−02
Coefficient: −2.45e+03
Value: delta
d__Bacteria.p__Bacteroidetes.c__Sphingobacteriia
0
5000
10000
alpha (n=25)
clade20C (n=1)
delta (n=13)
omicron (n=6)
neg (n=72)
NA
WHOvariant

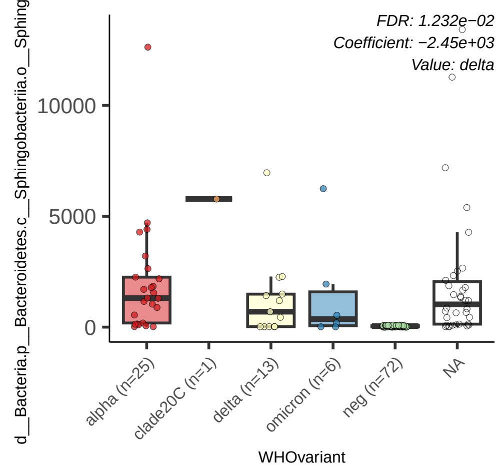

FDR: 1.232e−02
Coefficient: −2.45e+03
Value: delta
d__Bacteria.p__Bacteroidetes.c__Sphingobacteriia.o__Sphing
10000
5000
0
alpha (n=25)
clade20C (n=1)
delta (n=13)
omicron (n=6)
neg (n=72)
NA
WHOvariant

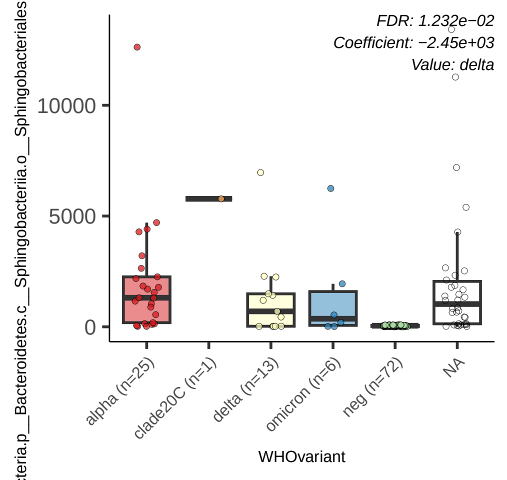

FDR: 1.232e−02
Coefficient: −2.45e+03
Value: delta
10000
5000
0
[illegible]cteria.p__Bacteroidetes.c__Sphingobacteriia.o__Sphingobacteriales
alpha (n=25)
clade20C (n=1)
delta (n=13)
omicron (n=6)
neg (n=72)
NA
WHOvariant

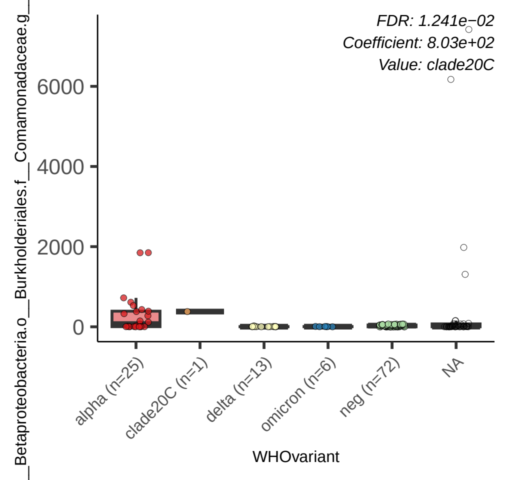

FDR: 1.241e−02
Coefficient: 8.03e+02
Value: clade20C
Betaproteobacteria.o__Burkholderiales.f__Comamonadaceae.g__
6000
4000
2000
0
alpha (n=25)
clade20C (n=1)
delta (n=13)
omicron (n=6)
neg (n=72)
NA
WHOvariant

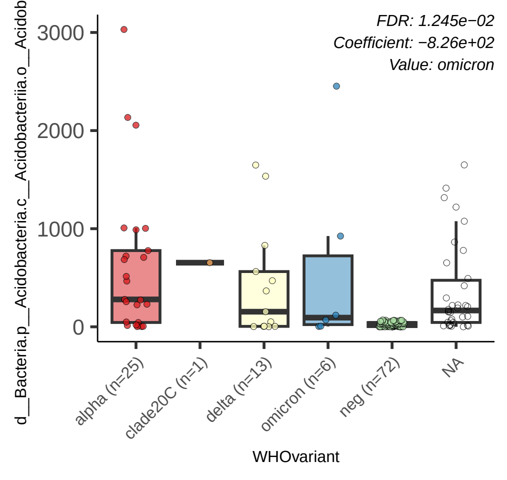

FDR: 1.245e−02
Coefficient: −8.26e+02
Value: omicron
d__Bacteria.p__Acidobacteria.c__Acidobacteriia.o__Acidob
3000
2000
1000
0
alpha (n=25)
clade20C (n=1)
delta (n=13)
omicron (n=6)
neg (n=72)
NA
WHOvariant

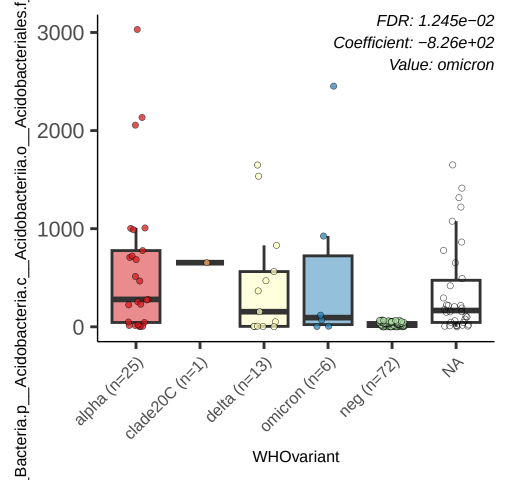

FDR: 1.245e−02
Coefficient: −8.26e+02
Value: omicron
Bacteria.p__Acidobacteria.c__Acidobacteriia.o__Acidobacteriales.f_
0
1000
2000
3000
alpha (n=25)
clade20C (n=1)
delta (n=13)
omicron (n=6)
neg (n=72)
NA
WHOvariant

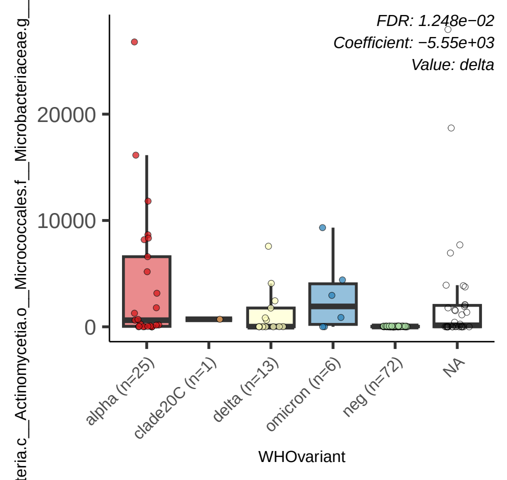

FDR: 1.248e−02
Coefficient: −5.55e+03
Value: delta
20000
10000
0
alpha (n=25)
clade20C (n=1)
delta (n=13)
omicron (n=6)
neg (n=72)
NA
WHOvariant
teria.c__Actinomycetia.o__Micrococcales.f__Microbacteriaceae.g__

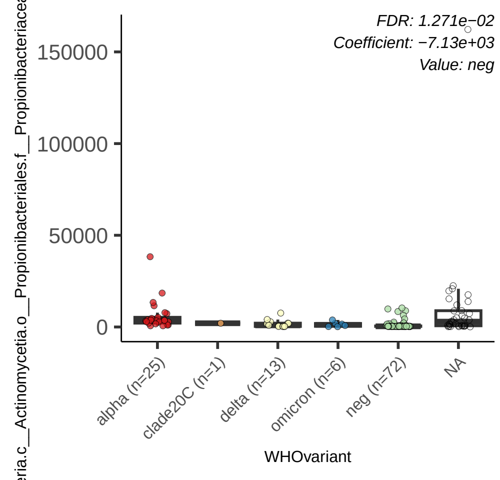

FDR: 1.271e−02
Coefficient: −7.13e+03
Value: neg
150000
100000
50000
0
eria.c__Actinomycetia.o__Propionibacteriales.f__Propionibacteriacea
alpha (n=25)
clade20C (n=1)
delta (n=13)
omicron (n=6)
neg (n=72)
NA
WHOvariant

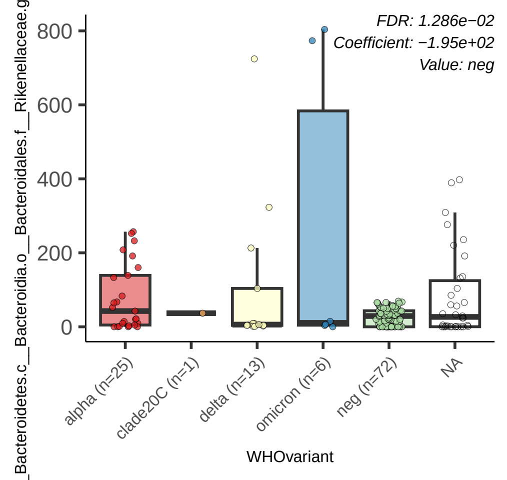

FDR: 1.286e−02
Coefficient: −1.95e+02
Value: neg
Bacteroidetes.c__Bacteroidia.o__Bacteroidales.f__Rikenellaceae.g
800
600
400
200
0
alpha (n=25)
clade20C (n=1)
delta (n=13)
omicron (n=6)
neg (n=72)
NA
WHOvariant

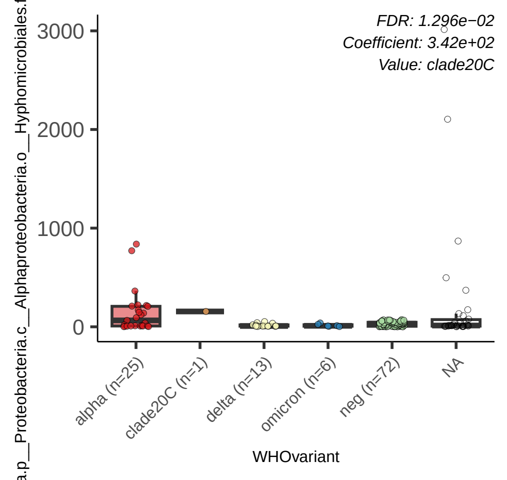

FDR: 1.296e−02
Coefficient: 3.42e+02
Value: clade20C
3000
2000
1000
0
a.p__Proteobacteria.c__Alphaproteobacteria.o__Hyphomicrobiales.f
alpha (n=25)
clade20C (n=1)
delta (n=13)
omicron (n=6)
neg (n=72)
NA
WHOvariant

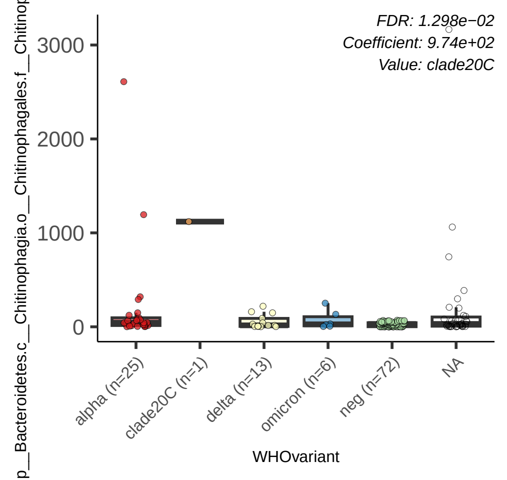

FDR: 1.298e−02
Coefficient: 9.74e+02
Value: clade20C
3000
2000
1000
0
p__Bacteroidetes.c__Chitinophagia.o__Chitinophagales.f__Chitinop
alpha (n=25)
clade20C (n=1)
delta (n=13)
omicron (n=6)
neg (n=72)
NA
WHOvariant

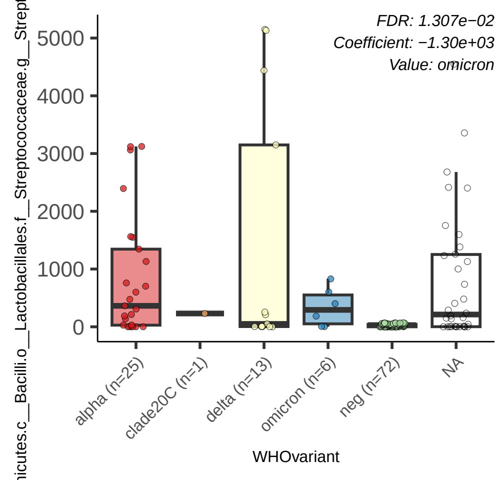

FDR: 1.307e−02
Coefficient: −1.30e+03
Value: omicron
5000
4000
3000
2000
1000
0
alpha (n=25)
clade20C (n=1)
delta (n=13)
omicron (n=6)
neg (n=72)
NA
WHOvariant

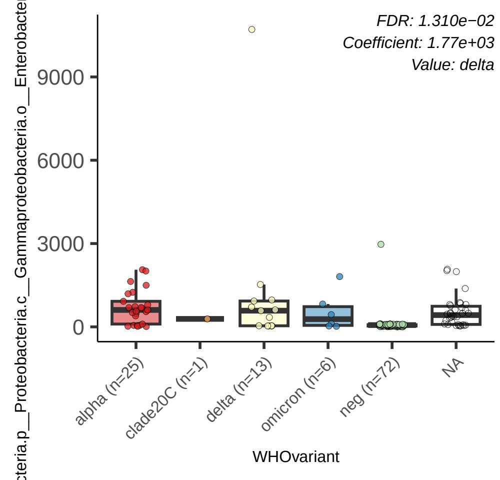

FDR: 1.310e−02
Coefficient: 1.77e+03
Value: delta
9000
6000
3000
0
alpha (n=25)
clade20C (n=1)
delta (n=13)
omicron (n=6)
neg (n=72)
NA
WHOvariant
cteria.p__Proteobacteria.c__Gammaproteobacteria.o__Enterobacte

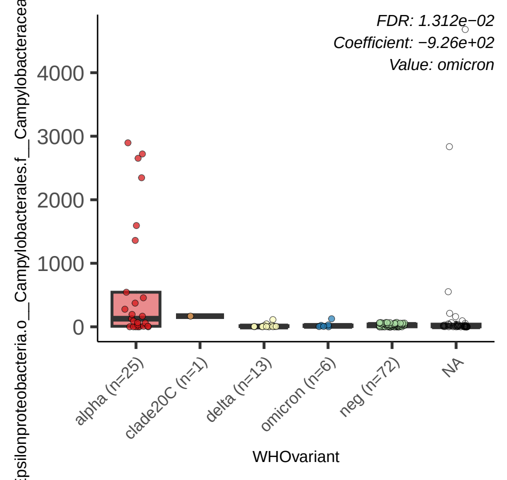
FDR: 1.312e−02
Coefficient: −9.26e+02
Value: omicron
4000
3000
2000
1000
0
tpsilonproteobacteria.o__Campylobacterales.f__Campylobacterace
alpha (n=25)
clade20C (n=1)
delta (n=13)
omicron (n=6)
neg (n=72)
NA
WHOvariant

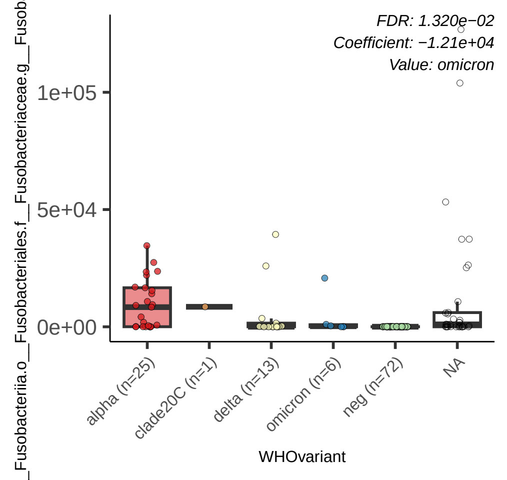

FDR: 1.320e−02
Coefficient: −1.21e+04
Value: omicron
Fusobacteriia.o__Fusobacteriales.f__Fusobacteriaceae.g__Fusob
1e+05
5e+04
0e+00
alpha (n=25)
clade20C (n=1)
delta (n=13)
omicron (n=6)
neg (n=72)
NA
WHOvariant

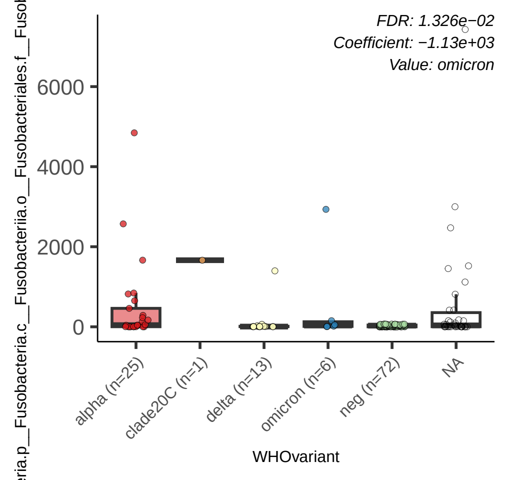

FDR: 1.326e−02
Coefficient: −1.13e+03
Value: omicron
6000
4000
2000
0
alpha (n=25)
clade20C (n=1)
delta (n=13)
omicron (n=6)
neg (n=72)
NA
WHOvariant
eria.p__Fusobacteria.c__Fusobacteriia.o__Fusobacteriales.f__Fusob

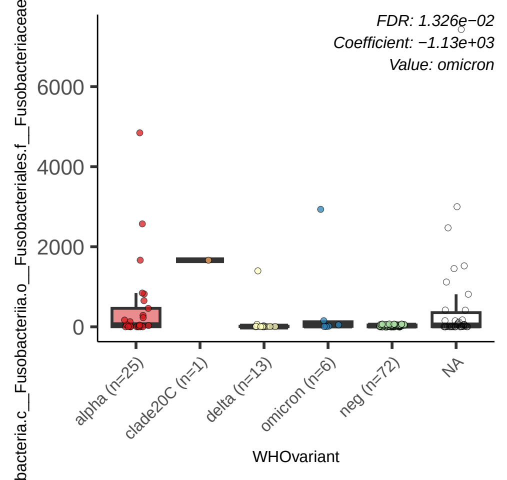
FDR: 1.326e−02
Coefficient: −1.13e+03
Value: omicron
6000
4000
2000
0
bacteria.c__Fusobacteriia.o__Fusobacteriales.f__Fusobacteriaceae
alpha (n=25)
clade20C (n=1)
delta (n=13)
omicron (n=6)
neg (n=72)
NA
WHOvariant

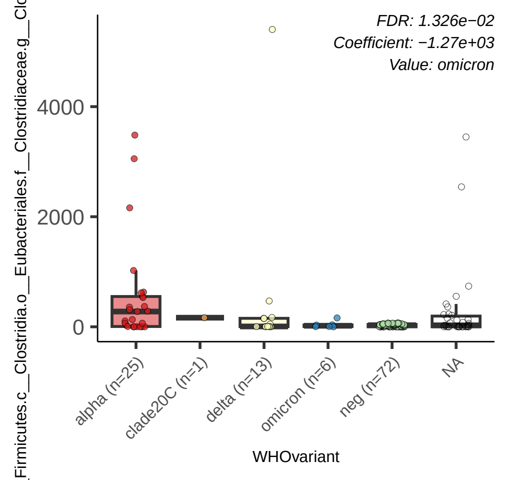
FDR: 1.326e−02
Coefficient: −1.27e+03
Value: omicron
4000
2000
0
alpha (n=25)
clade20C (n=1)
delta (n=13)
omicron (n=6)
neg (n=72)
NA
WHOvariant
Firmicutes.c__Clostridia.o__Eubacteriales.f__Clostridiaceae.g__Clo

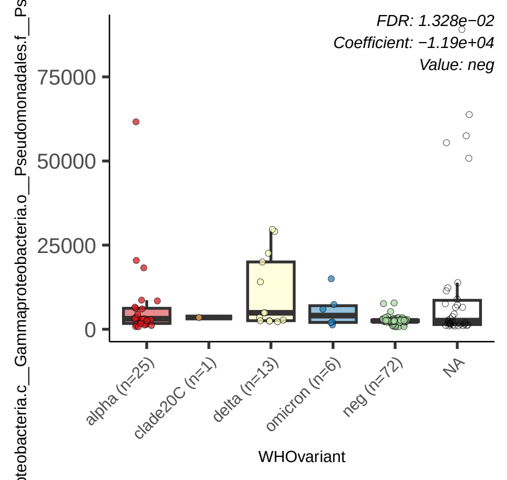
FDR: 1.328e−02
Coefficient: −1.19e+04
Value: neg
oteobacteria.c__Gammaproteobacteria.o__Pseudomonadales.f__Ps
75000
50000
25000
0
alpha (n=25)
clade20C (n=1)
delta (n=13)
omicron (n=6)
neg (n=72)
NA
WHOvariant

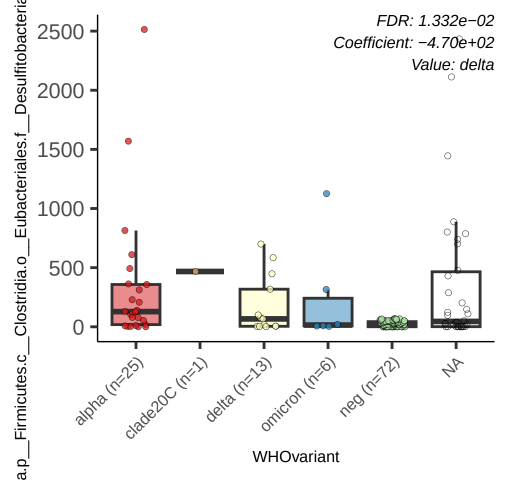

FDR: 1.332e−02
Coefficient: −4.70e+02
Value: delta
2500
2000
1500
1000
500
0
a.p__Firmicutes.c__Clostridia.o__Eubacteriales.f__Desulfitobacteria
alpha (n=25)
clade20C (n=1)
delta (n=13)
omicron (n=6)
neg (n=72)
NA
WHOvariant

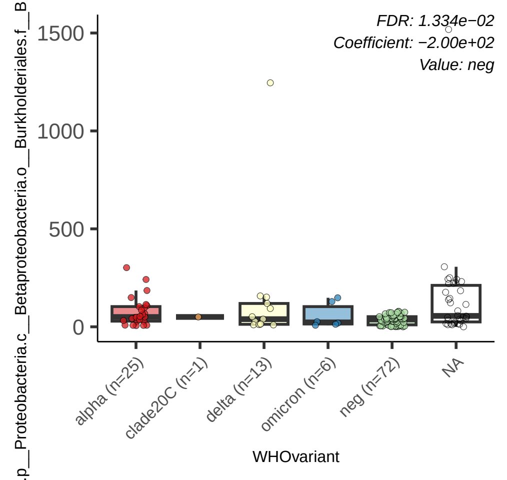

FDR: 1.334e−02
Coefficient: −2.00e+02
Value: neg
p__Proteobacteria.c__Betaproteobacteria.o__Burkholderiales.f__B
1500
1000
500
0
alpha (n=25)
clade20C (n=1)
delta (n=13)
omicron (n=6)
neg (n=72)
NA
WHOvariant

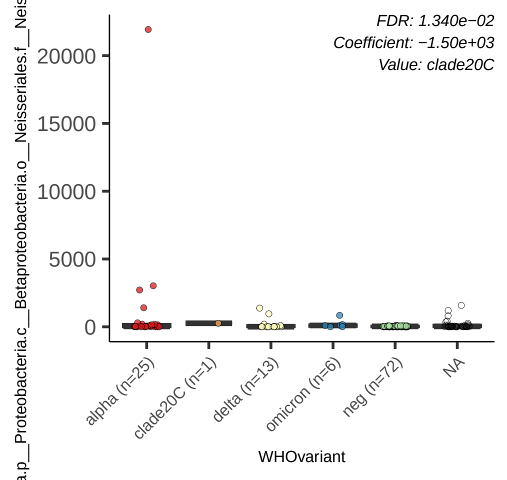

FDR: 1.340e−02
Coefficient: −1.50e+03
Value: clade20C
20000
15000
10000
5000
0
a.p__Proteobacteria.c__Betaproteobacteria.o__Neisseriales.f__Neis
alpha (n=25)
clade20C (n=1)
delta (n=13)
omicron (n=6)
neg (n=72)
NA
WHOvariant

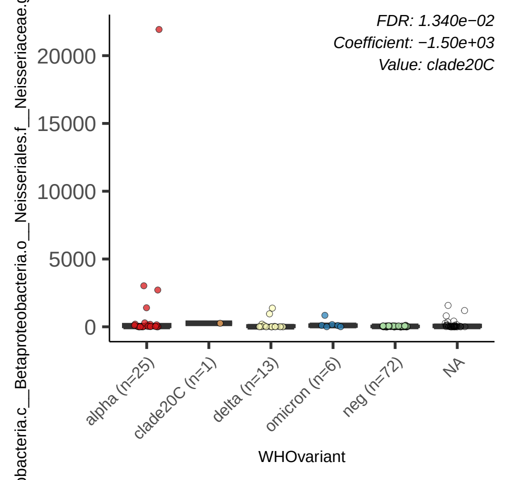
FDR: 1.340e−02
Coefficient: −1.50e+03
Value: clade20C
obacteria.c__Betaproteobacteria.o__Neisseriales.f__Neisseriaceae.g
20000
15000
10000
5000
0
alpha (n=25)
clade20C (n=1)
delta (n=13)
omicron (n=6)
neg (n=72)
NA
WHOvariant

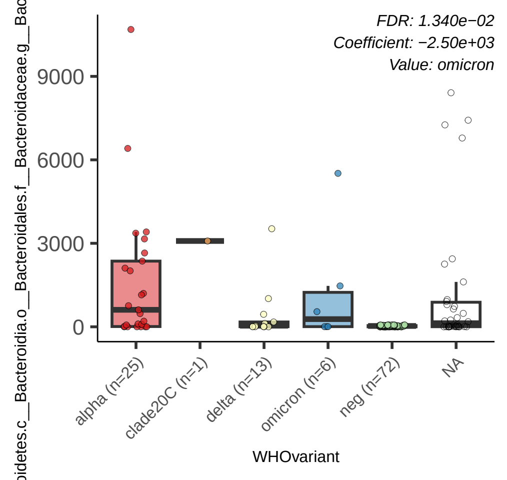

FDR: 1.340e−02
Coefficient: −2.50e+03
Value: omicron
0
3000
6000
9000
oidetes.c__Bacteroidia.o__Bacteroidales.f__Bacteroidaceae.g__Bac
alpha (n=25)
clade20C (n=1)
delta (n=13)
omicron (n=6)
neg (n=72)
NA
WHOvariant

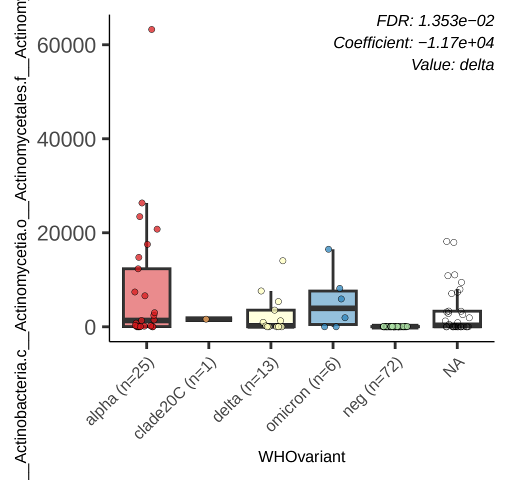
FDR: 1.353e−02
Coefficient: −1.17e+04
Value: delta
60000
40000
20000
0
__Actinobacteria.c__Actinomycetia.o__Actinomycetales.f__Actinomy
alpha (n=25)
clade20C (n=1)
delta (n=13)
omicron (n=6)
neg (n=72)
NA
WHOvariant

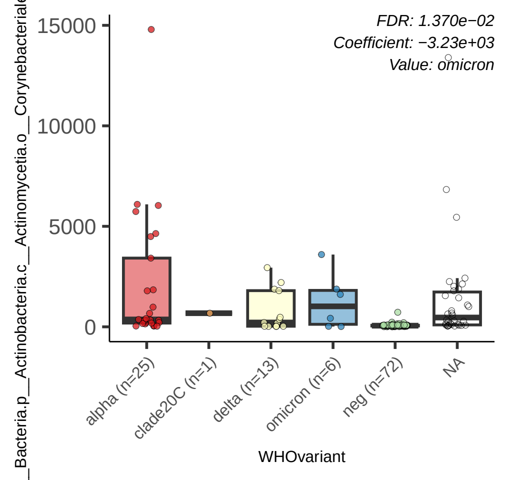

FDR: 1.370e−02
Coefficient: −3.23e+03
Value: omicron
15000
10000
5000
0
alpha (n=25)
clade20C (n=1)
delta (n=13)
omicron (n=6)
neg (n=72)
NA
WHOvariant
Bacteria.p__Actinobacteria.c__Actinomycetia.o__Corynebacteriale

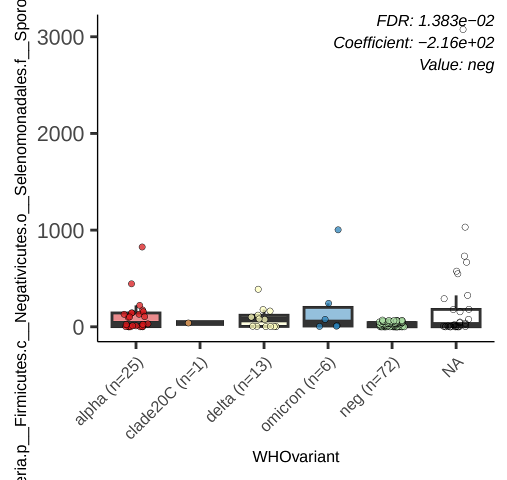
FDR: 1.383e−02
Coefficient: −2.16e+02
Value: neg
3000
2000
1000
0
eria.p__Firmicutes.c__Negativicutes.o__Selenomonadales.f__Sporo
alpha (n=25)
clade20C (n=1)
delta (n=13)
omicron (n=6)
neg (n=72)
NA
WHOvariant

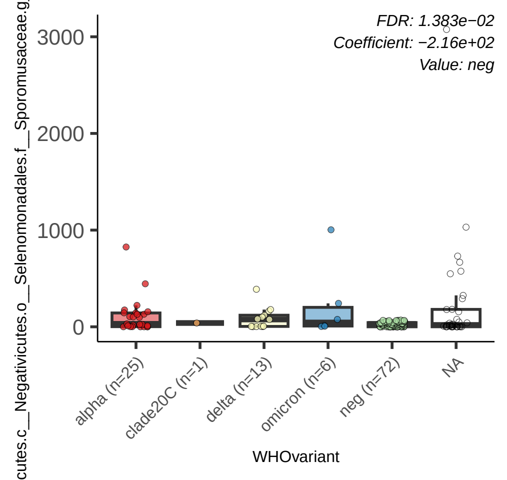

FDR: 1.383e−02
Coefficient: −2.16e+02
Value: neg
3000
2000
1000
0
cutes.c__Negativicutes.o__Selenomonadales.f__Sporomusaceae.g
alpha (n=25)
clade20C (n=1)
delta (n=13)
omicron (n=6)
neg (n=72)
NA
WHOvariant

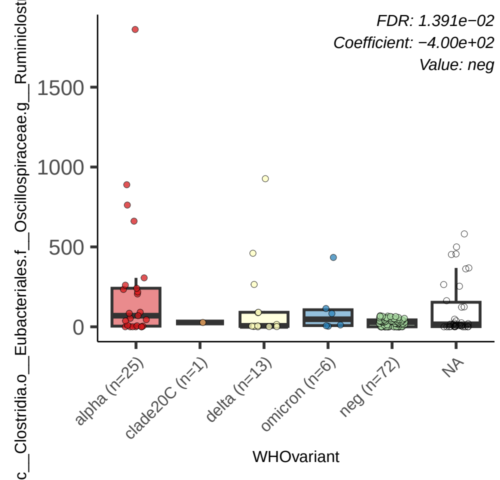

FDR: 1.391e−02
Coefficient: −4.00e+02
Value: neg
c__Clostridia.o__Eubacteriales.f__Oscillospiraceae.g__Ruminiclost
1500
1000
500
0
alpha (n=25)
clade20C (n=1)
delta (n=13)
omicron (n=6)
neg (n=72)
NA
WHOvariant

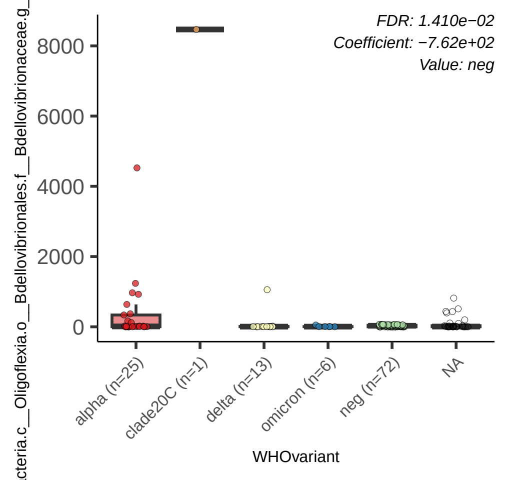

FDR: 1.410e−02
Coefficient: −7.62e+02
Value: neg
cteria.c__Oligoflexia.o__Bdellovibrionales.f__Bdellovibrionaceae.g_
8000
6000
4000
2000
0
alpha (n=25)
clade20C (n=1)
delta (n=13)
omicron (n=6)
neg (n=72)
NA
WHOvariant

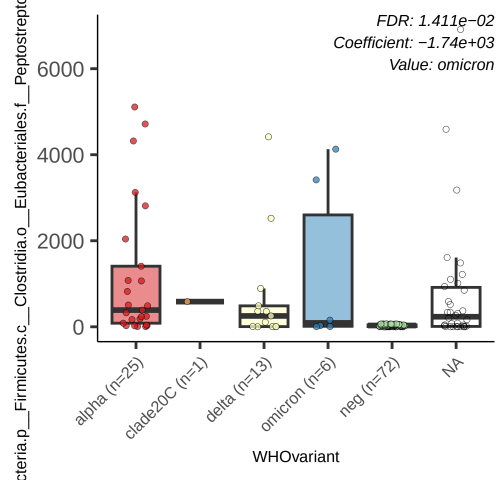
FDR: 1.411e−02
Coefficient: −1.74e+03
Value: omicron
cteria.p__Firmicutes.c__Clostridia.o__Eubacteriales.f__Peptostrepto
6000
4000
2000
0
alpha (n=25)
clade20C (n=1)
delta (n=13)
omicron (n=6)
neg (n=72)
NA
WHOvariant

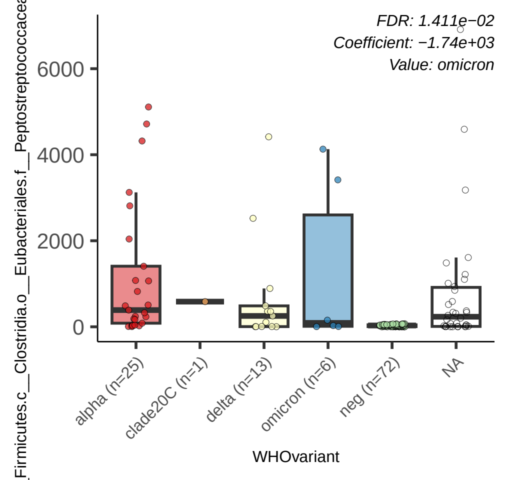
FDR: 1.411e−02
Coefficient: −1.74e+03
Value: omicron
Firmicutes.c__Clostridia.o__Eubacteriales.f__Peptostreptococcacea
6000
4000
2000
0
alpha (n=25)
clade20C (n=1)
delta (n=13)
omicron (n=6)
neg (n=72)
NA
WHOvariant

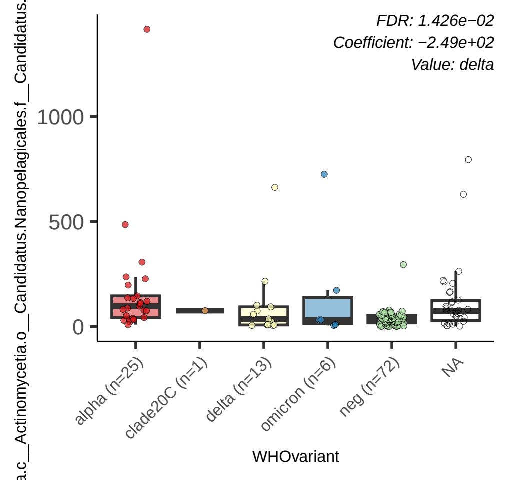

FDR: 1.426e−02
Coefficient: −2.49e+02
Value: delta
a.c__Actinomycetia.o__Candidatus.Nanopelagicales.f__Candidatus.
1000
500
0
alpha (n=25)
clade20C (n=1)
delta (n=13)
omicron (n=6)
neg (n=72)
NA
WHOvariant

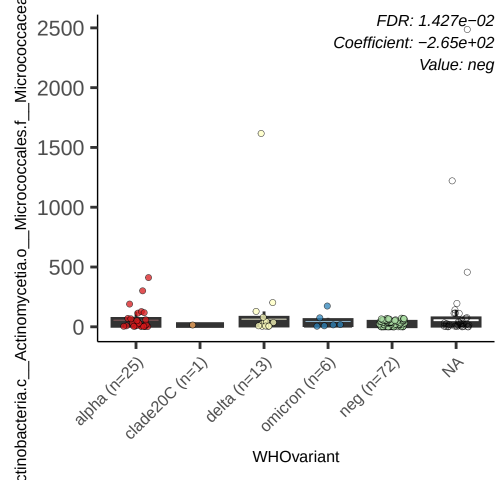

FDR: 1.427e−02
Coefficient: −2.65e+02
Value: neg
2500
2000
1500
1000
500
0
alpha (n=25)
clade20C (n=1)
delta (n=13)
omicron (n=6)
neg (n=72)
NA
WHOvariant

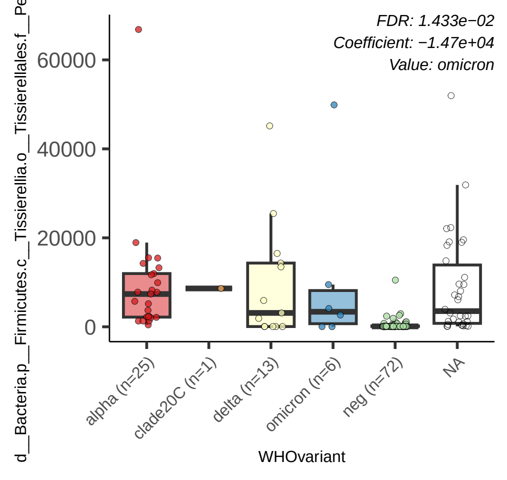
FDR: 1.433e−02
Coefficient: −1.47e+04
Value: omicron
d__Bacteria.p__Firmicutes.c__Tissierellia.o__Tissierellales.f__Pe
0
20000
40000
60000
alpha (n=25)
clade20C (n=1)
delta (n=13)
omicron (n=6)
neg (n=72)
NA
WHOvariant

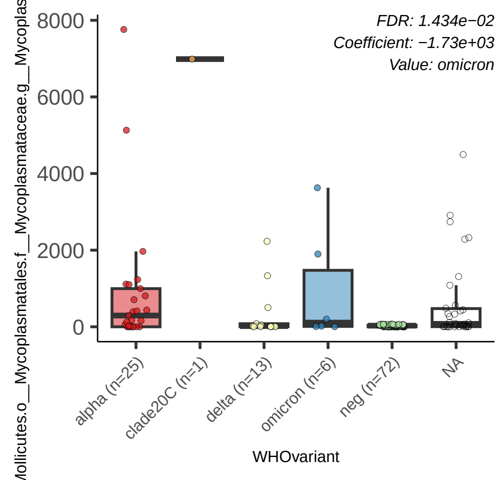

FDR: 1.434e−02
Coefficient: −1.73e+03
Value: omicron
8000
6000
4000
2000
0
Mollicutes.o__Mycoplasmatales.f__Mycoplasmataceae.g__Mycoplas
alpha (n=25)
clade20C (n=1)
delta (n=13)
omicron (n=6)
neg (n=72)
NA
WHOvariant

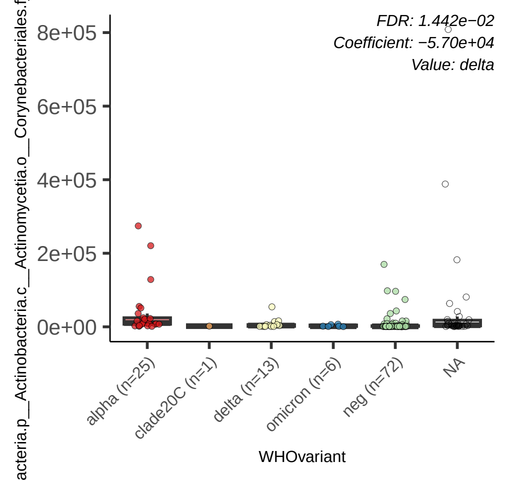

FDR: 1.442e−02
Coefficient: −5.70e+04
Value: delta
0e+00
2e+05
4e+05
6e+05
8e+05
alpha (n=25)
clade20C (n=1)
delta (n=13)
omicron (n=6)
neg (n=72)
NA
WHOvariant
acteria.p__Actinobacteria.c__Actinomycetia.o__Corynebacteriales.f

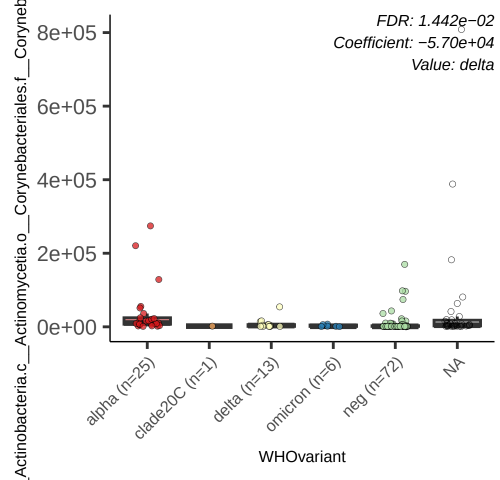

FDR: 1.442e−02
Coefficient: −5.70e+04
Value: delta
8e+05
6e+05
4e+05
2e+05
0e+00
Actinobacteria.c__Actinomycetia.o__Corynebacteriales.f__Coryneb
alpha (n=25)
clade20C (n=1)
delta (n=13)
omicron (n=6)
neg (n=72)
NA
WHOvariant

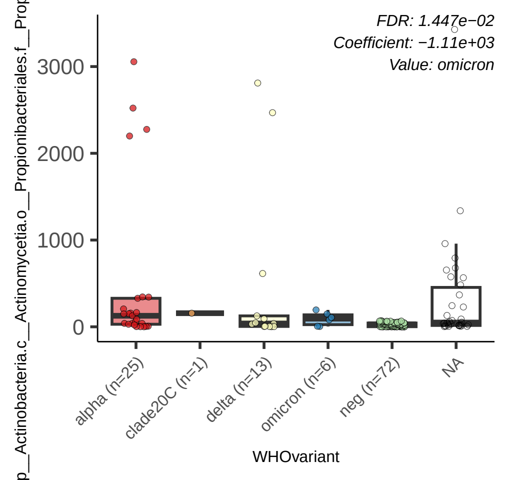
FDR: 1.447e−02
Coefficient: −1.11e+03
Value: omicron
p__Actinobacteria.c__Actinomycetia.o__Propionibacteriales.f__Prop
3000
2000
1000
0
alpha (n=25)
clade20C (n=1)
delta (n=13)
omicron (n=6)
neg (n=72)
NA
WHOvariant

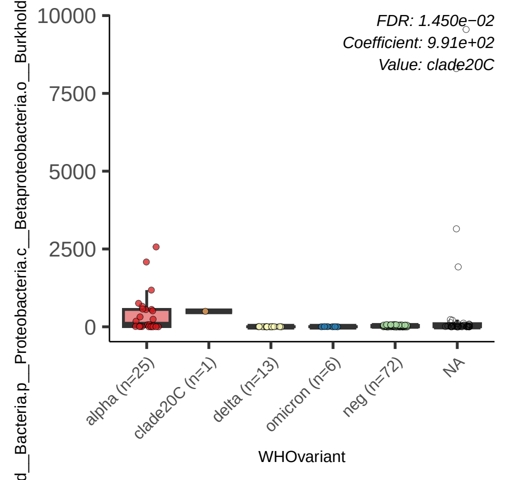
FDR: 1.450e−02
Coefficient: 9.91e+02
Value: clade20C
10000
7500
5000
2500
0
d__Bacteria.p__Proteobacteria.c__Betaproteobacteria.o__Burkhold
alpha (n=25)
clade20C (n=1)
delta (n=13)
omicron (n=6)
neg (n=72)
NA
WHOvariant

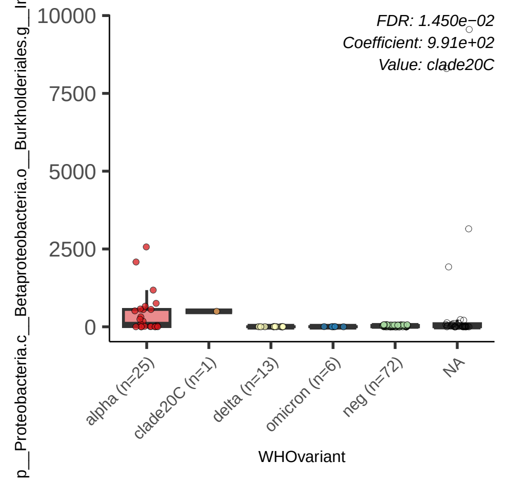

FDR: 1.450e−02
Coefficient: 9.91e+02
Value: clade20C
p__Proteobacteria.c__Betaproteobacteria.o__Burkholderiales.g__In
10000
7500
5000
2500
0
alpha (n=25)
clade20C (n=1)
delta (n=13)
omicron (n=6)
neg (n=72)
NA
WHOvariant

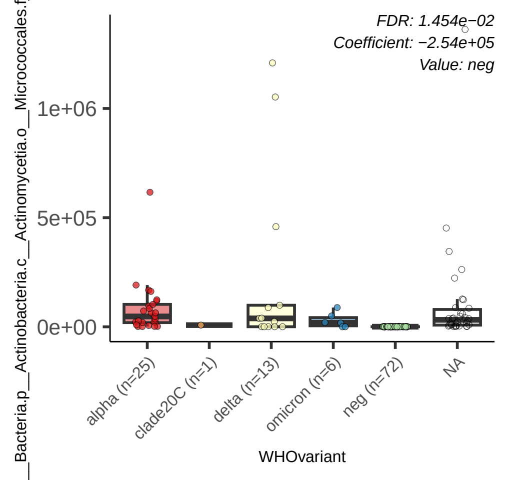

FDR: 1.454e−02
Coefficient: −2.54e+05
Value: neg
Bacteria.p__Actinobacteria.c__Actinomycetia.o__Micrococcales.f
1e+06
5e+05
0e+00
alpha (n=25)
clade20C (n=1)
delta (n=13)
omicron (n=6)
neg (n=72)
NA
WHOvariant

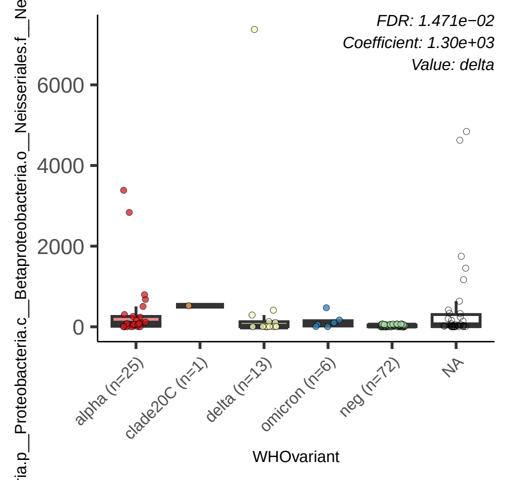

FDR: 1.471e−02
Coefficient: 1.30e+03
Value: delta
ria.p__Proteobacteria.c__Betaproteobacteria.o__Neisseriales.f__Ne
6000
4000
2000
0
alpha (n=25)
clade20C (n=1)
delta (n=13)
omicron (n=6)
neg (n=72)
NA
WHOvariant

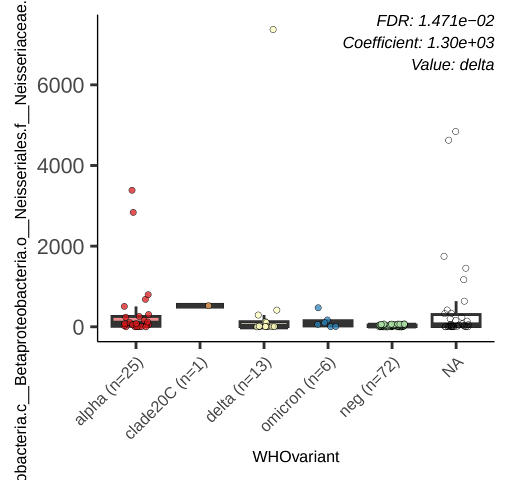

FDR: 1.471e−02
Coefficient: 1.30e+03
Value: delta
obacteria.c__Betaproteobacteria.o__Neisseriales.f__Neisseriaceae.
0
2000
4000
6000
alpha (n=25)
clade20C (n=1)
delta (n=13)
omicron (n=6)
neg (n=72)
NA
WHOvariant

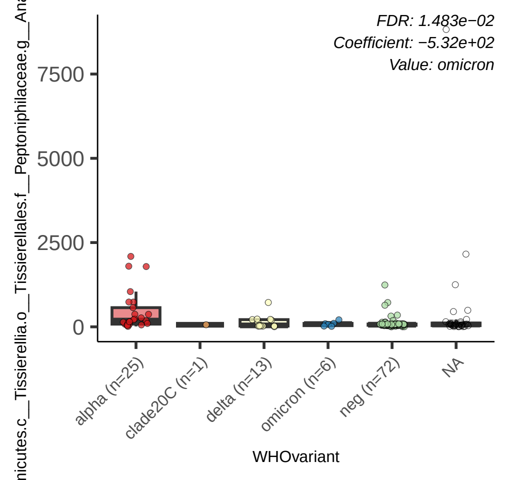

FDR: 1.483e−02
Coefficient: −5.32e+02
Value: omicron
nicutes.c__Tissierellia.o__Tissierellales.f__Peptoniphilaceae.g__Ana
7500
5000
2500
0
alpha (n=25)
clade20C (n=1)
delta (n=13)
omicron (n=6)
neg (n=72)
NA
WHOvariant

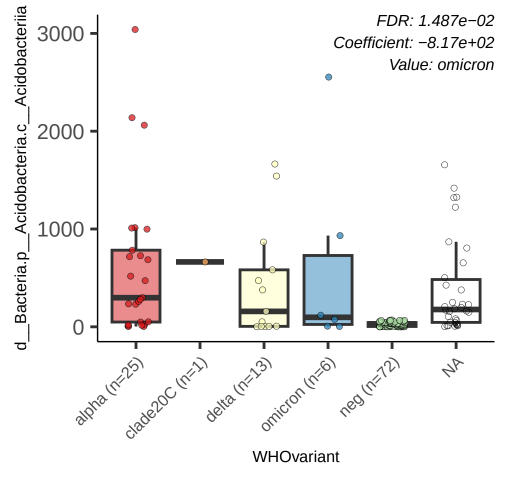

FDR: 1.487e−02
Coefficient: −8.17e+02
Value: omicron
d__Bacteria.p__Acidobacteria.c__Acidobacteriia
3000
2000
1000
0
alpha (n=25)
clade20C (n=1)
delta (n=13)
omicron (n=6)
neg (n=72)
NA
WHOvariant

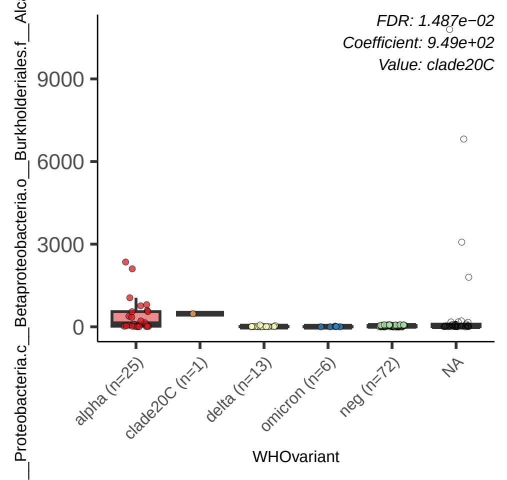

FDR: 1.487e−02
Coefficient: 9.49e+02
Value: clade20C
9000
6000
3000
0
_Proteobacteria.c__Betaproteobacteria.o__Burkholderiales.f__Alc
alpha (n=25)
clade20C (n=1)
delta (n=13)
omicron (n=6)
neg (n=72)
NA
WHOvariant

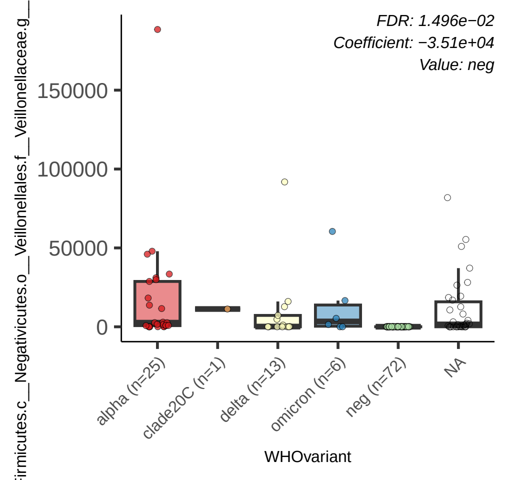
FDR: 1.496e−02
Coefficient: −3.51e+04
Value: neg
150000
100000
50000
0
Firmicutes.c__Negativicutes.o__Veillonellales.f__Veillonellaceae.g__
alpha (n=25)
clade20C (n=1)
delta (n=13)
omicron (n=6)
neg (n=72)
NA
WHOvariant

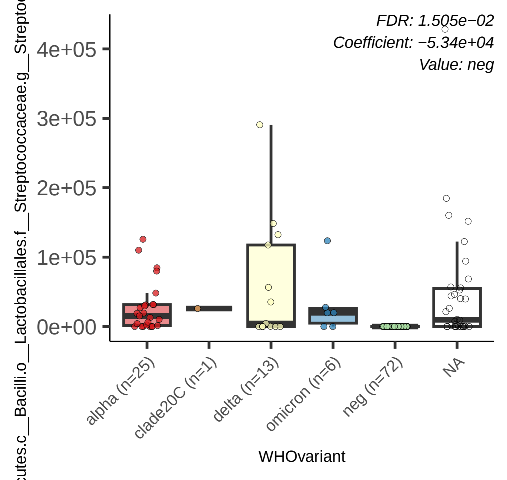
FDR: 1.505e−02
Coefficient: −5.34e+04
Value: neg
4e+05
3e+05
2e+05
1e+05
0e+00
alpha (n=25)
clade20C (n=1)
delta (n=13)
omicron (n=6)
neg (n=72)
NA
WHOvariant
cutes.c__Bacilli.o__Lactobacillales.f__Streptococcaceae.g__Streptoc

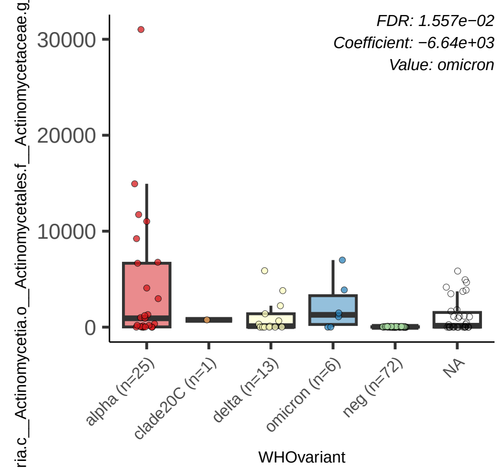

FDR: 1.557e−02
Coefficient: −6.64e+03
Value: omicron
30000
20000
10000
0
teria.c__Actinomycetia.o__Actinomycetales.f__Actinomycetaceae.g
alpha (n=25)
clade20C (n=1)
delta (n=13)
omicron (n=6)
neg (n=72)
NA
WHOvariant

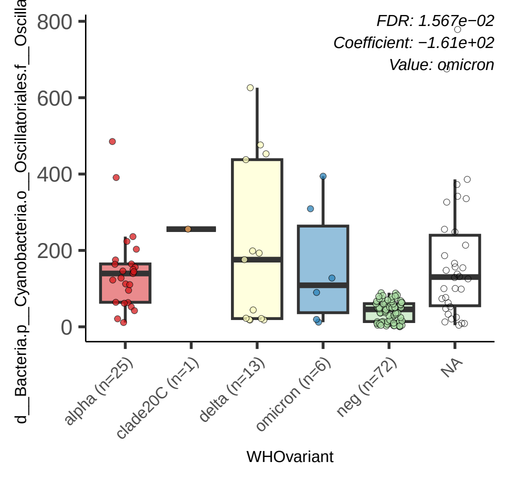

FDR: 1.567e−02
Coefficient: −1.61e+02
Value: omicron
d__Bacteria.p__Cyanobacteria.o__Oscillatoriales.f__Oscilla
800
600
400
200
0
alpha (n=25)
clade20C (n=1)
delta (n=13)
omicron (n=6)
neg (n=72)
NA
WHOvariant

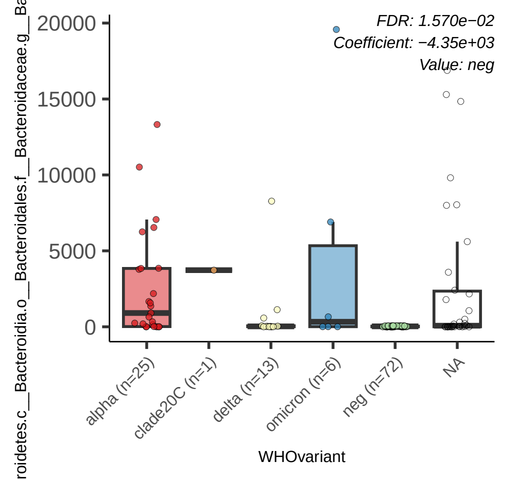

FDR: 1.570e−02
Coefficient: −4.35e+03
Value: neg
roidetes.c__Bacteroidia.o__Bacteroidales.f__Bacteroidaceae.g__Ba
20000
15000
10000
5000
0
alpha (n=25)
clade20C (n=1)
delta (n=13)
omicron (n=6)
neg (n=72)
NA
WHOvariant

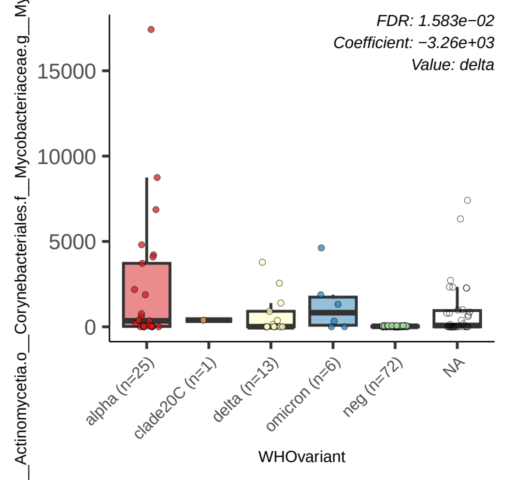

FDR: 1.583e−02
Coefficient: −3.26e+03
Value: delta
15000
10000
5000
0
Actinomycetia.o__Corynebacteriales.f__Mycobacteriaceae.g__My
alpha (n=25)
clade20C (n=1)
delta (n=13)
omicron (n=6)
neg (n=72)
NA
WHOvariant

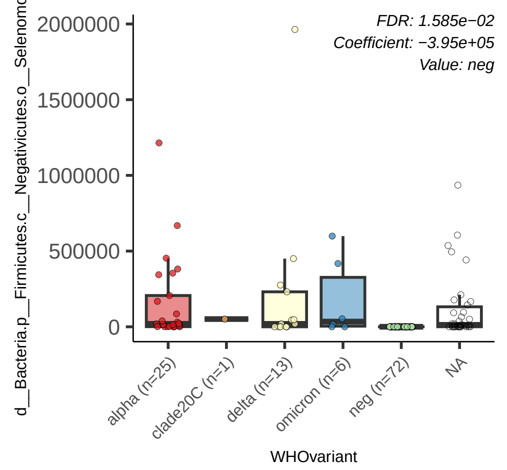

FDR: 1.585e−02
Coefficient: −3.95e+05
Value: neg
2000000
1500000
1000000
500000
0
d__Bacteria.p__Firmicutes.c__Negativicutes.o__Selenomo
alpha (n=25)
clade20C (n=1)
delta (n=13)
omicron (n=6)
neg (n=72)
NA
WHOvariant

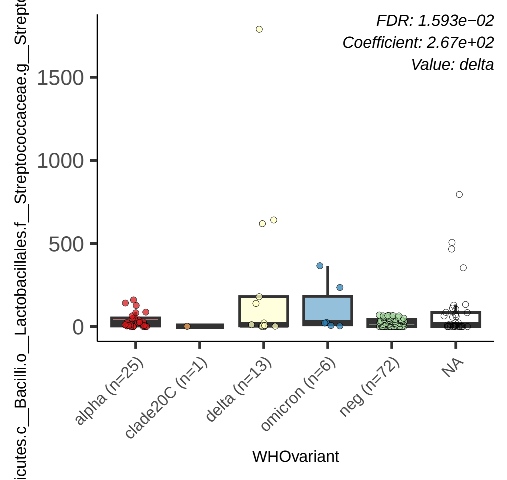

FDR: 1.593e−02
Coefficient: 2.67e+02
Value: delta
1500
1000
500
0
alpha (n=25)
clade20C (n=1)
delta (n=13)
omicron (n=6)
neg (n=72)
NA
WHOvariant

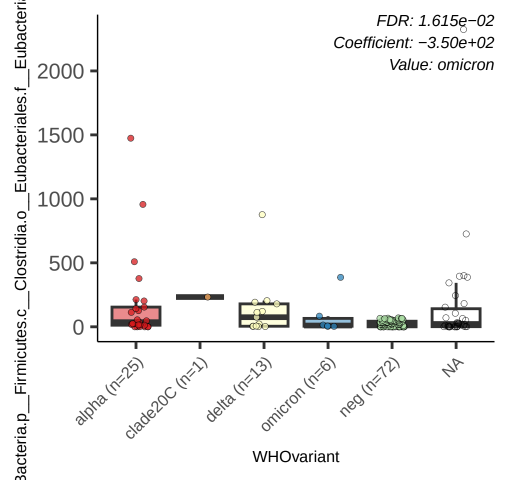

FDR: 1.615e−02
Coefficient: −3.50e+02
Value: omicron
Bacteria.p__Firmicutes.c__Clostridia.o__Eubacteriales.f__Eubacteri
2000
1500
1000
500
0
alpha (n=25)
clade20C (n=1)
delta (n=13)
omicron (n=6)
neg (n=72)
NA
WHOvariant

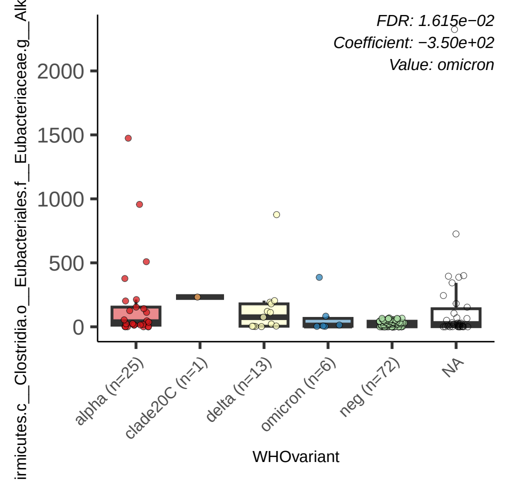

FDR: 1.615e−02
Coefficient: −3.50e+02
Value: omicron
2000
1500
1000
500
0
alpha (n=25)
clade20C (n=1)
delta (n=13)
omicron (n=6)
neg (n=72)
NA
WHOvariant
irmicutes.c__Clostridia.o__Eubacteriales.f__Eubacteriaceae.g__Alk

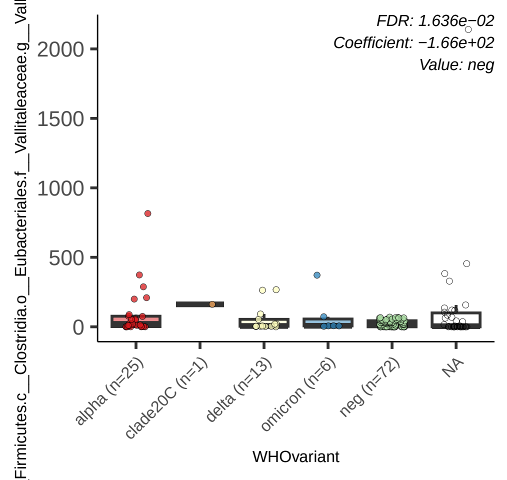
FDR: 1.636e−02
Coefficient: −1.66e+02
Value: neg
Firmicutes.c__Clostridia.o__Eubacteriales.f__Vallitaleaceae.g__Vall
2000
1500
1000
500
0
alpha (n=25)
clade20C (n=1)
delta (n=13)
omicron (n=6)
neg (n=72)
NA
WHOvariant

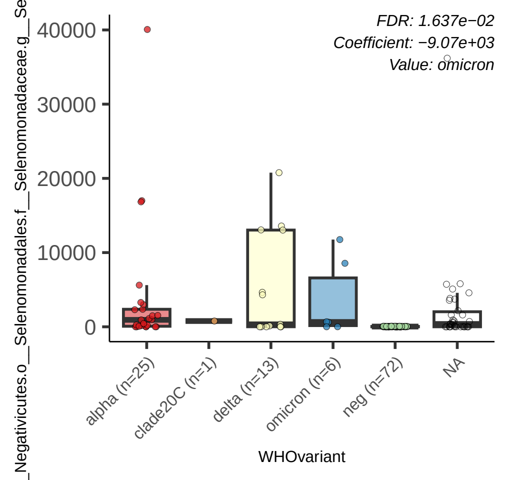

FDR: 1.637e−02
Coefficient: −9.07e+03
Value: omicron
Negativicutes.o__Selenomonadales.f__Selenomonadaceae.g__Se
40000
30000
20000
10000
0
alpha (n=25)
clade20C (n=1)
delta (n=13)
omicron (n=6)
neg (n=72)
NA
WHOvariant

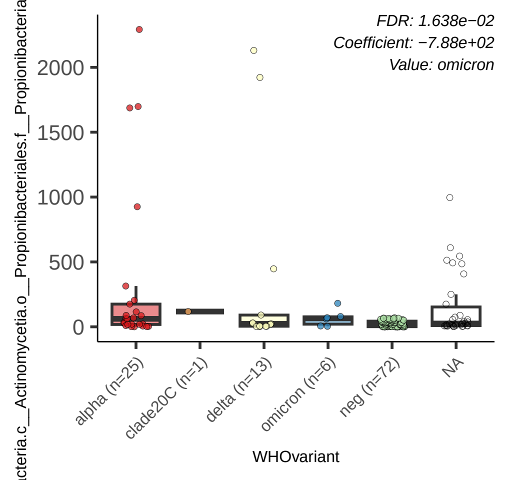
FDR: 1.638e−02
Coefficient: −7.88e+02
Value: omicron
2000
1500
1000
500
0
alpha (n=25)
clade20C (n=1)
delta (n=13)
omicron (n=6)
neg (n=72)
NA
WHOvariant
acteria.c__Actinomycetia.o__Propionibacteriales.f__Propionibacteria

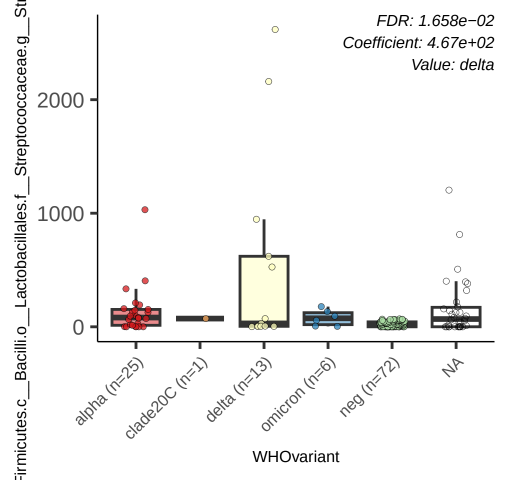
FDR: 1.658e−02
Coefficient: 4.67e+02
Value: delta
2000
1000
0
Firmicutes.c__Bacilli.o__Lactobacillales.f__Streptococcaceae.g__St
alpha (n=25)
clade20C (n=1)
delta (n=13)
omicron (n=6)
neg (n=72)
NA
WHOvariant

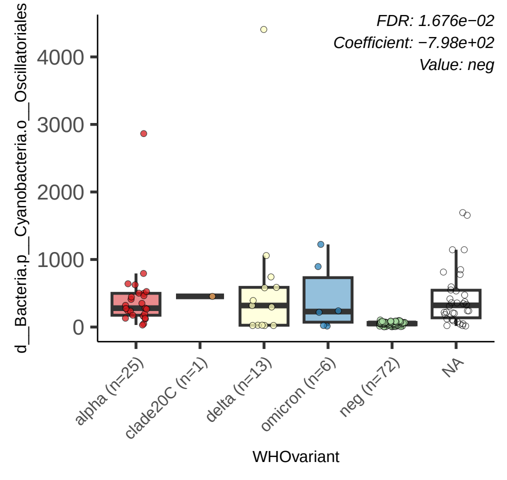

FDR: 1.676e−02
Coefficient: −7.98e+02
Value: neg
d__Bacteria.p__Cyanobacteria.o__Oscillatoriales
4000
3000
2000
1000
0
alpha (n=25)
clade20C (n=1)
delta (n=13)
omicron (n=6)
neg (n=72)
NA
WHOvariant

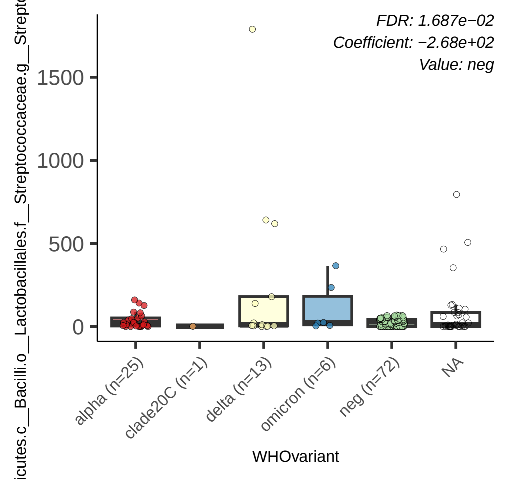

FDR: 1.687e−02
Coefficient: −2.68e+02
Value: neg
1500
1000
500
0
icutes.c__Bacilli.o__Lactobacillales.f__Streptococcaceae.g__Strepto
alpha (n=25)
clade20C (n=1)
delta (n=13)
omicron (n=6)
neg (n=72)
NA
WHOvariant

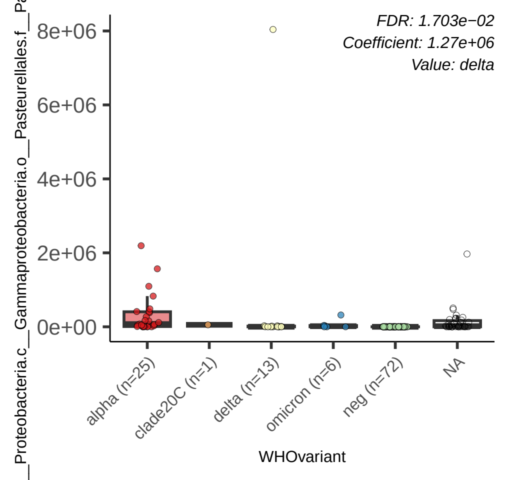

FDR: 1.703e−02
Coefficient: 1.27e+06
Value: delta
8e+06
6e+06
4e+06
2e+06
0e+00
Proteobacteria.c__Gammaproteobacteria.o__Pasteurellales.f__Pa
alpha (n=25)
clade20C (n=1)
delta (n=13)
omicron (n=6)
neg (n=72)
NA
WHOvariant

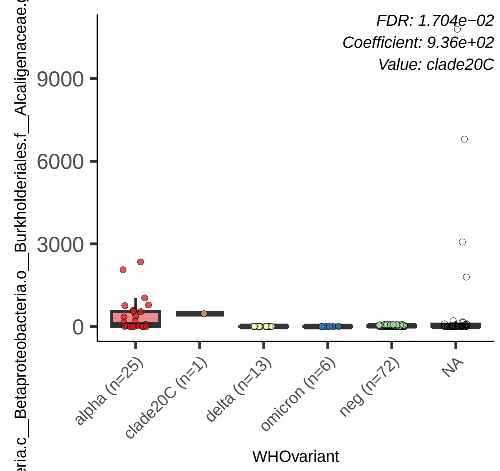

FDR: 1.704e−02
Coefficient: 9.36e+02
Value: clade20C
teria.c__Betaproteobacteria.o__Burkholderiales.f__Alcaligenaceae.g
9000
6000
3000
0
alpha (n=25)
clade20C (n=1)
delta (n=13)
omicron (n=6)
neg (n=72)
NA
WHOvariant

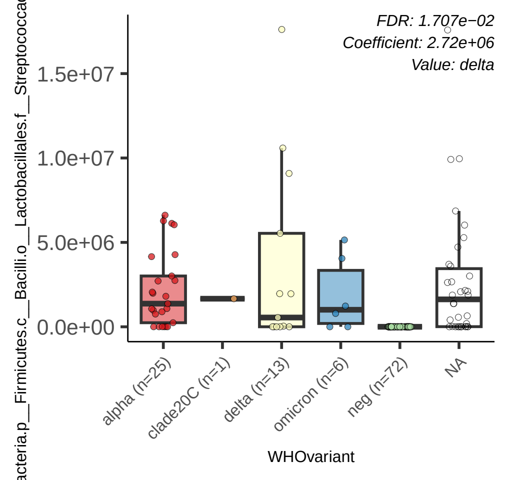

FDR: 1.707e−02
Coefficient: 2.72e+06
Value: delta
1.5e+07
1.0e+07
5.0e+06
0.0e+00
alpha (n=25)
clade20C (n=1)
delta (n=13)
omicron (n=6)
neg (n=72)
NA
WHOvariant

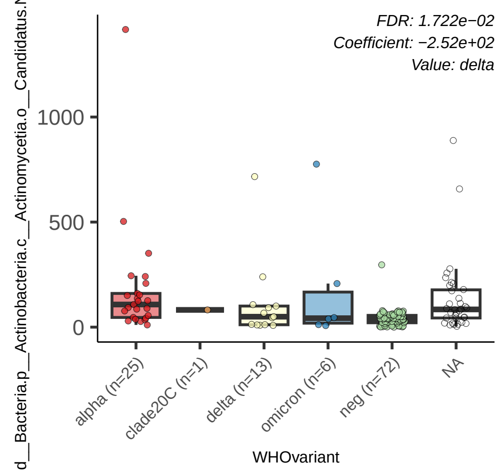

FDR: 1.722e−02
Coefficient: −2.52e+02
Value: delta
d__Bacteria.p__Actinobacteria.c__Actinomycetia.o__Candidatus.N
0
500
1000
alpha (n=25)
clade20C (n=1)
delta (n=13)
omicron (n=6)
neg (n=72)
NA
WHOvariant

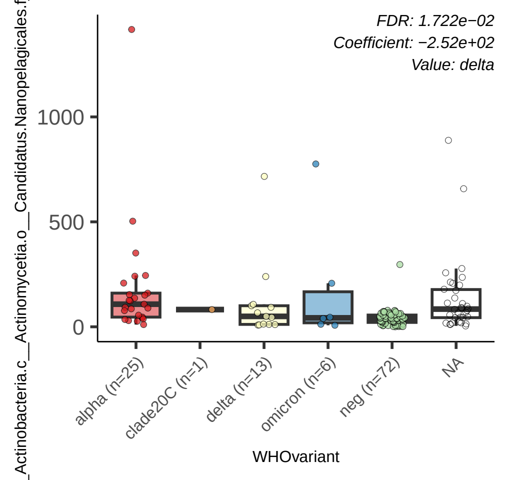

FDR: 1.722e−02
Coefficient: −2.52e+02
Value: delta
_Actinobacteria.c__Actinomycetia.o__Candidatus.Nanopelagicales.f_
1000
500
0
alpha (n=25)
clade20C (n=1)
delta (n=13)
omicron (n=6)
neg (n=72)
NA
WHOvariant

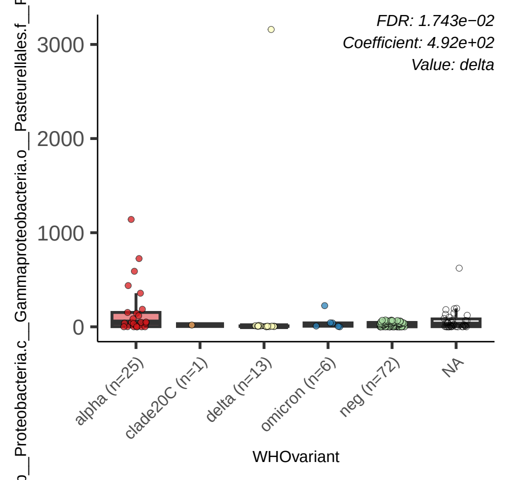

FDR: 1.743e−02
Coefficient: 4.92e+02
Value: delta
3000
2000
1000
0
o__Proteobacteria.c__Gammaproteobacteria.o__Pasteurellales.f__P
alpha (n=25)
clade20C (n=1)
delta (n=13)
omicron (n=6)
neg (n=72)
NA
WHOvariant

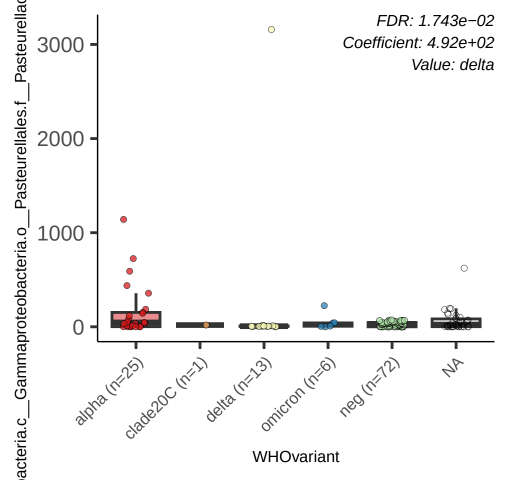

FDR: 1.743e−02
Coefficient: 4.92e+02
Value: delta
3000
2000
1000
0
bacteria.c__Gammaproteobacteria.o__Pasteurellales.f__Pasteurellac
alpha (n=25)
clade20C (n=1)
delta (n=13)
omicron (n=6)
neg (n=72)
NA
WHOvariant

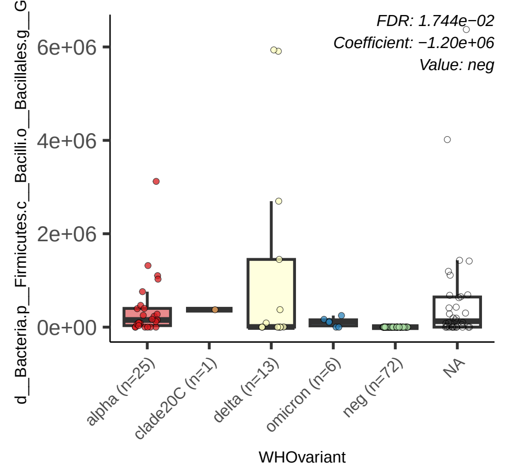

FDR: 1.744e−02
Coefficient: −1.20e+06
Value: neg
d__Bacteria.p__Firmicutes.c__Bacilli.o__Bacillales.g__G
6e+06
4e+06
2e+06
0e+00
alpha (n=25)
clade20C (n=1)
delta (n=13)
omicron (n=6)
neg (n=72)
NA
WHOvariant

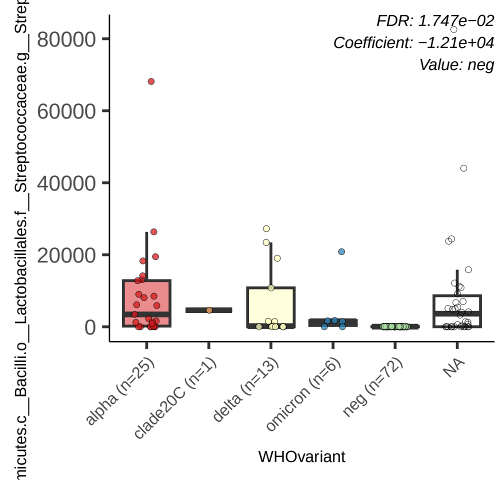

FDR: 1.747e−02
Coefficient: −1.21e+04
Value: neg
micutes.c__Bacilli.o__Lactobacillales.f__Streptococcaceae.g__Strep
80000
60000
40000
20000
0
alpha (n=25)
clade20C (n=1)
delta (n=13)
omicron (n=6)
neg (n=72)
NA
WHOvariant

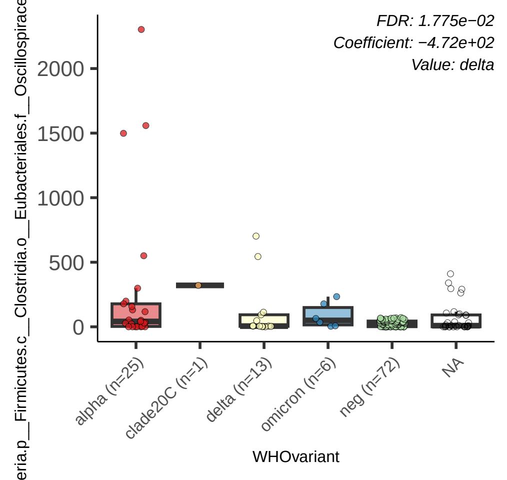

FDR: 1.775e−02
Coefficient: −4.72e+02
Value: delta
eria.p__Firmicutes.c__Clostridia.o__Eubacteriales.f__Oscillospirace
2000
1500
1000
500
0
alpha (n=25)
clade20C (n=1)
delta (n=13)
omicron (n=6)
neg (n=72)
NA
WHOvariant

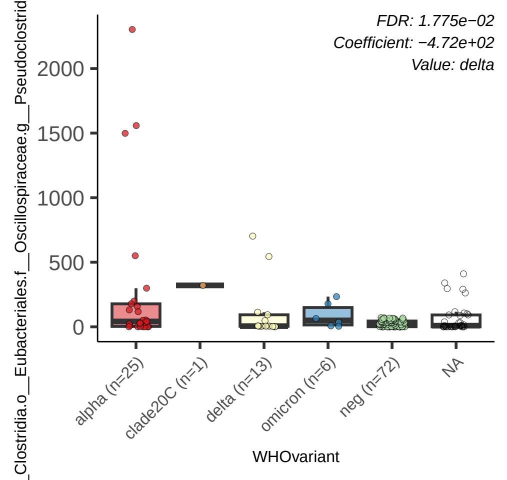

FDR: 1.775e−02
Coefficient: −4.72e+02
Value: delta
Clostridia.o__Eubacteriales.f__Oscillospiraceae.g__Pseudoclostrid
2000
1500
1000
500
0
alpha (n=25)
clade20C (n=1)
delta (n=13)
omicron (n=6)
neg (n=72)
NA
WHOvariant

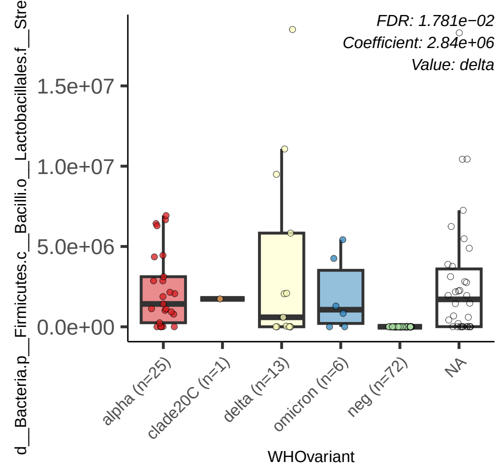

FDR: 1.781e−02
Coefficient: 2.84e+06
Value: delta
d__Bacteria.p__Firmicutes.c__Bacilli.o__Lactobacillales.f__Stre
1.5e+07
1.0e+07
5.0e+06
0.0e+00
alpha (n=25)
clade20C (n=1)
delta (n=13)
omicron (n=6)
neg (n=72)
NA
WHOvariant

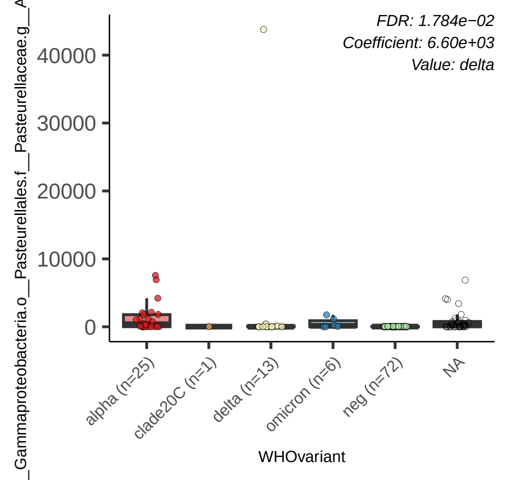

FDR: 1.784e−02
Coefficient: 6.60e+03
Value: delta
40000
30000
20000
10000
0
_Gammaproteobacteria.o__Pasteurellales.f__Pasteurellaceae.g__A
alpha (n=25)
clade20C (n=1)
delta (n=13)
omicron (n=6)
neg (n=72)
NA
WHOvariant

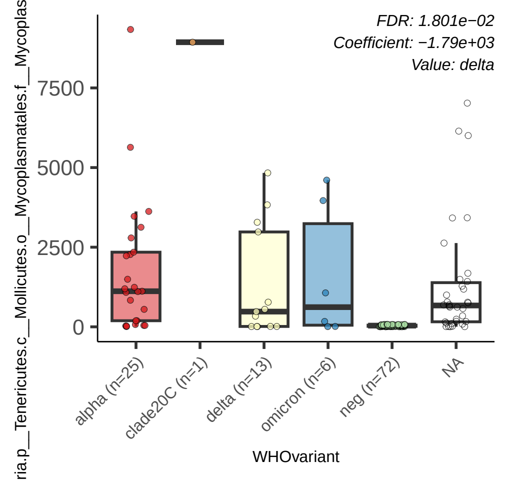
FDR: 1.801e−02
Coefficient: −1.79e+03
Value: delta
7500
5000
2500
0
ria.p__Tenericutes.c__Mollicutes.o__Mycoplasmatales.f__Mycoplas
alpha (n=25)
clade20C (n=1)
delta (n=13)
omicron (n=6)
neg (n=72)
NA
WHOvariant

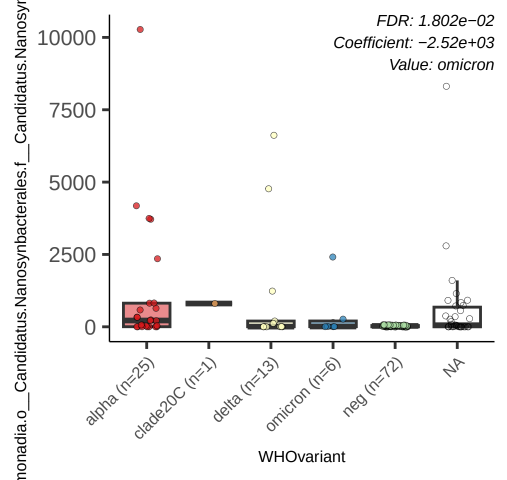

FDR: 1.802e−02
Coefficient: −2.52e+03
Value: omicron
10000
7500
5000
2500
0
monadia.o__Candidatus.Nanosynbacterales.f__Candidatus.Nanosyn
alpha (n=25)
clade20C (n=1)
delta (n=13)
omicron (n=6)
neg (n=72)
NA
WHOvariant

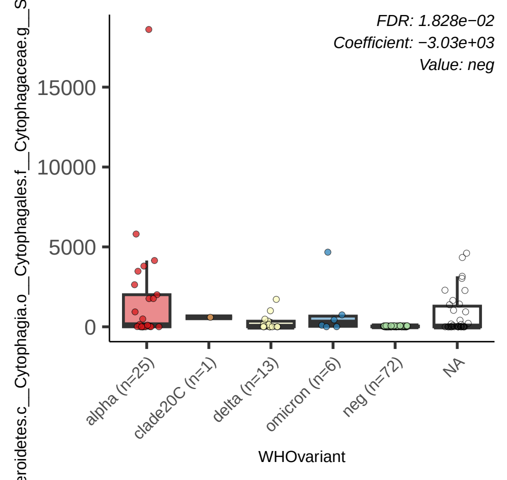

FDR: 1.828e−02
Coefficient: −3.03e+03
Value: neg
15000
10000
5000
0
eroidetes.c__Cytophagia.o__Cytophagales.f__Cytophagaceae.g__S
alpha (n=25)
clade20C (n=1)
delta (n=13)
omicron (n=6)
neg (n=72)
NA
WHOvariant

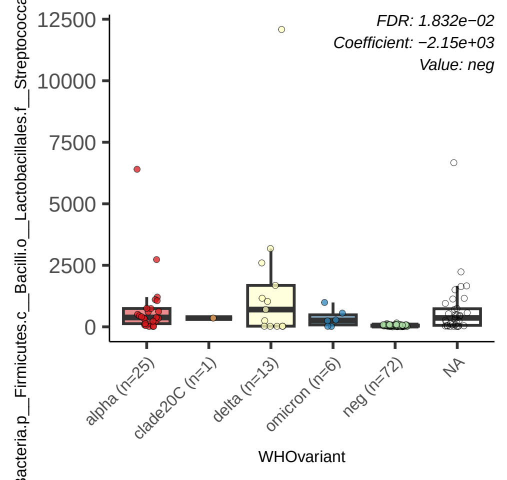
FDR: 1.832e−02
Coefficient: −2.15e+03
Value: neg
Bacteria.p__Firmicutes.c__Bacilli.o__Lactobacillales.f__Streptococca
12500
10000
7500
5000
2500
0
alpha (n=25)
clade20C (n=1)
delta (n=13)
omicron (n=6)
neg (n=72)
NA
WHOvariant

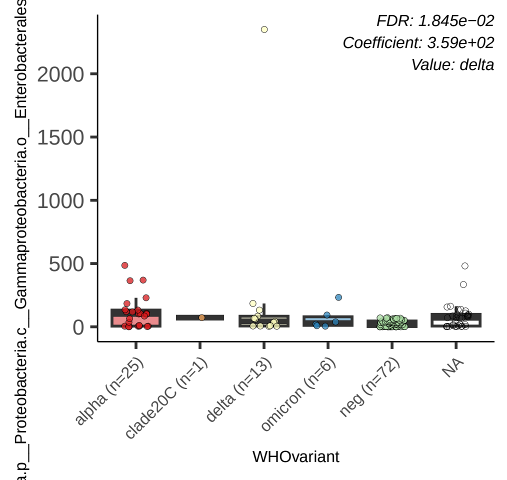

FDR: 1.845e−02
Coefficient: 3.59e+02
Value: delta
2000
1500
1000
500
0
alpha (n=25)
clade20C (n=1)
delta (n=13)
omicron (n=6)
neg (n=72)
NA
WHOvariant
a.p__Proteobacteria.c__Gammaproteobacteria.o__Enterobacterales

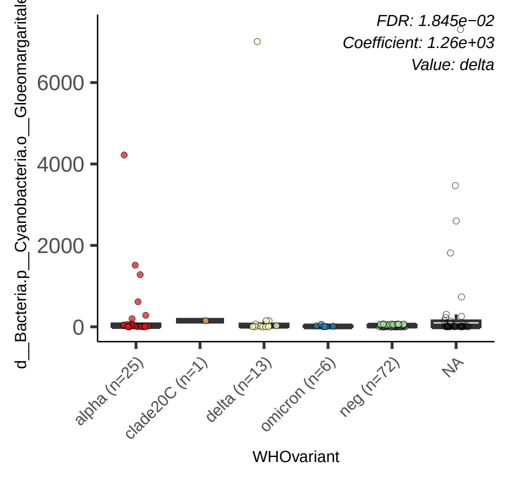

FDR: 1.845e−02
Coefficient: 1.26e+03
Value: delta
d__Bacteria.p__Cyanobacteria.o__Gloeomargaritale
6000
4000
2000
0
alpha (n=25)
clade20C (n=1)
delta (n=13)
omicron (n=6)
neg (n=72)
NA
WHOvariant

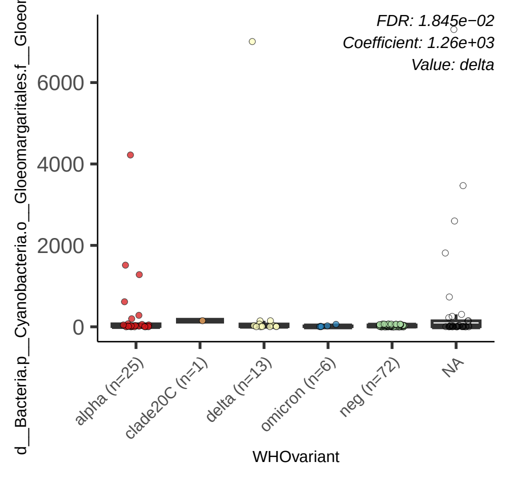
FDR: 1.845e−02
Coefficient: 1.26e+03
Value: delta
d__Bacteria.p__Cyanobacteria.o__Gloeomargaritales.f__Gloeor
6000
4000
2000
0
alpha (n=25)
clade20C (n=1)
delta (n=13)
omicron (n=6)
neg (n=72)
NA
WHOvariant

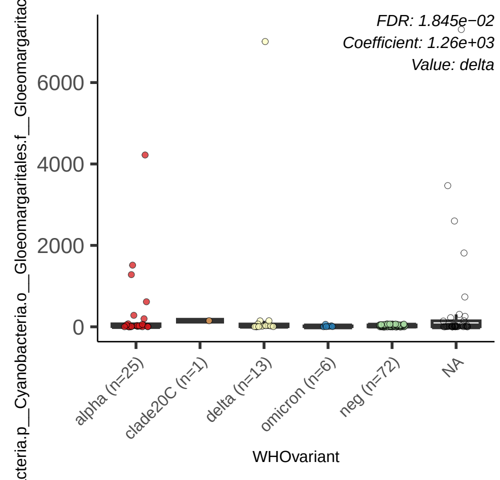

FDR: 1.845e−02
Coefficient: 1.26e+03
Value: delta
6000
4000
2000
0
alpha (n=25)
clade20C (n=1)
delta (n=13)
omicron (n=6)
neg (n=72)
NA
WHOvariant

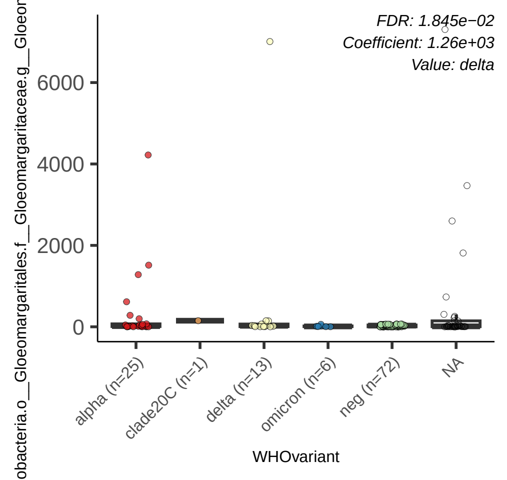
FDR: 1.845e−02
Coefficient: 1.26e+03
Value: delta
obacteria.o__Gloeomargaritales.f__Gloeomargaritaceae.g__Gloeon
6000
4000
2000
0
alpha (n=25)
clade20C (n=1)
delta (n=13)
omicron (n=6)
neg (n=72)
NA
WHOvariant

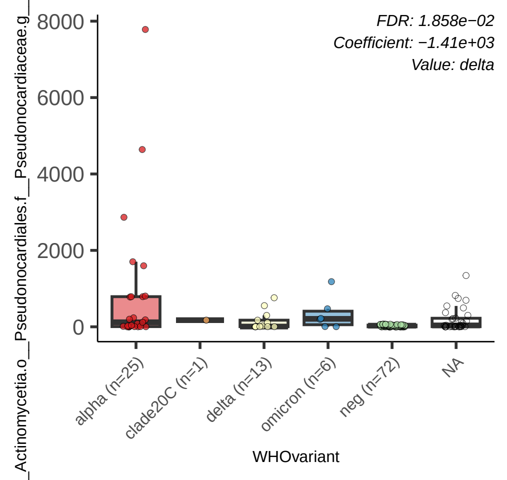

FDR: 1.858e−02
Coefficient: −1.41e+03
Value: delta
Actinomycetia.o__Pseudonocardiales.f__Pseudonocardiaceae.g__
8000
6000
4000
2000
0
alpha (n=25)
clade20C (n=1)
delta (n=13)
omicron (n=6)
neg (n=72)
NA
WHOvariant

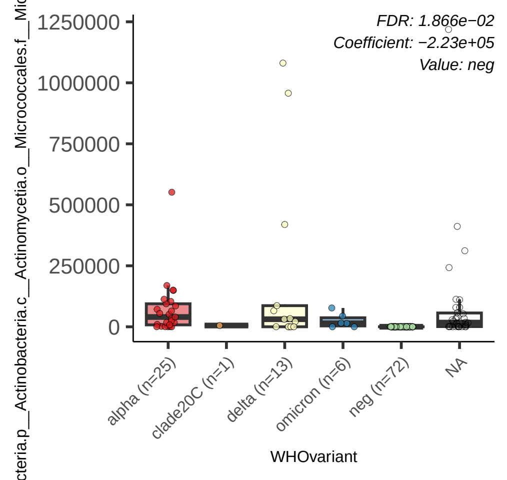
FDR: 1.866e−02
Coefficient: −2.23e+05
Value: neg
1250000
1000000
750000
500000
250000
0
alpha (n=25)
clade20C (n=1)
delta (n=13)
omicron (n=6)
neg (n=72)
NA
WHOvariant
cteria.p__Actinobacteria.c__Actinomycetia.o__Micrococcales.f__Mic

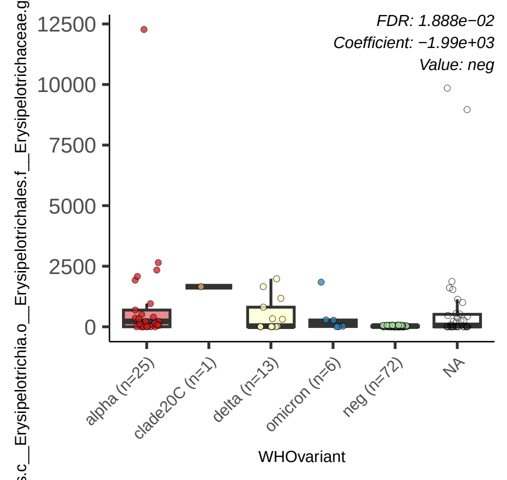

FDR: 1.888e−02
Coefficient: −1.99e+03
Value: neg
12500
10000
7500
5000
2500
0
s.c__Erysipelotrichia.o__Erysipelotrichales.f__Erysipelotrichaceae.g
alpha (n=25)
clade20C (n=1)
delta (n=13)
omicron (n=6)
neg (n=72)
NA
WHOvariant

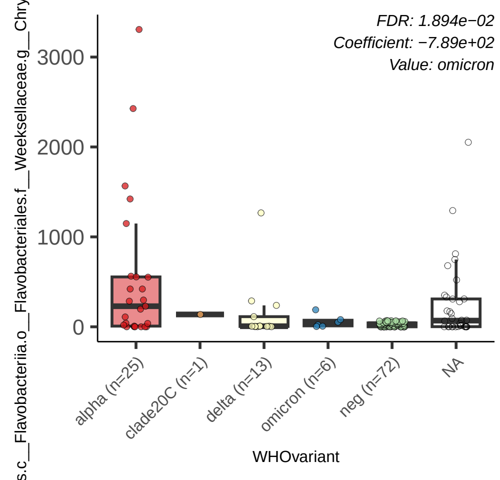

FDR: 1.894e−02
Coefficient: −7.89e+02
Value: omicron
3000
2000
1000
0
s.c__Flavobacteriia.o__Flavobacteriales.f__Weeksellaceae.g__Chry
alpha (n=25)
clade20C (n=1)
delta (n=13)
omicron (n=6)
neg (n=72)
NA
WHOvariant

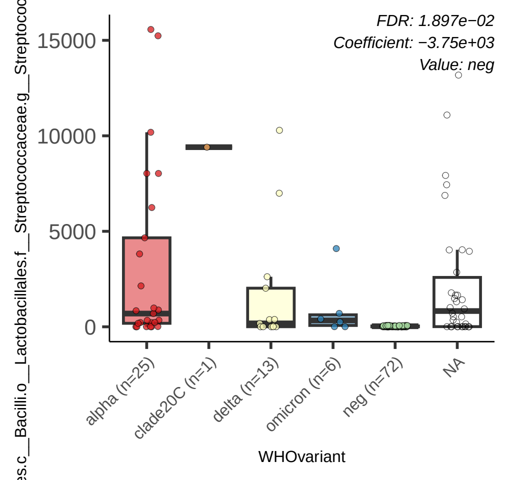

FDR: 1.897e−02
Coefficient: −3.75e+03
Value: neg
es.c__Bacilli.o__Lactobacillales.f__Streptococcaceae.g__Streptococ
15000
10000
5000
0
alpha (n=25)
clade20C (n=1)
delta (n=13)
omicron (n=6)
neg (n=72)
NA
WHOvariant

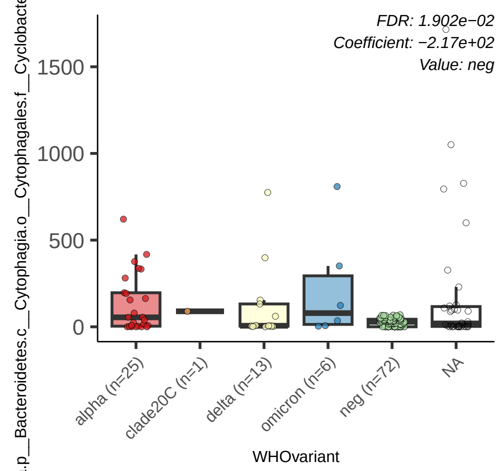
FDR: 1.902e−02
Coefficient: −2.17e+02
Value: neg
1500
1000
500
0
ia.p__Bacteroidetes.c__Cytophagia.o__Cytophagales.f__Cyclobacte
alpha (n=25)
clade20C (n=1)
delta (n=13)
omicron (n=6)
neg (n=72)
NA
WHOvariant

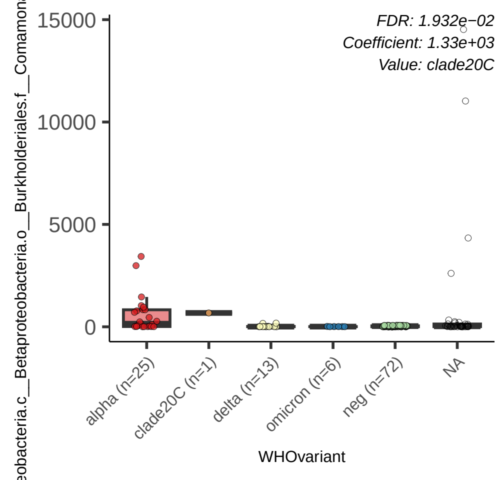
FDR: 1.932e−02
Coefficient: 1.33e+03
Value: clade20C
15000
10000
5000
0
eobacteria.c__Betaproteobacteria.o__Burkholderiales.f__Comamona
alpha (n=25)
clade20C (n=1)
delta (n=13)
omicron (n=6)
neg (n=72)
NA
WHOvariant

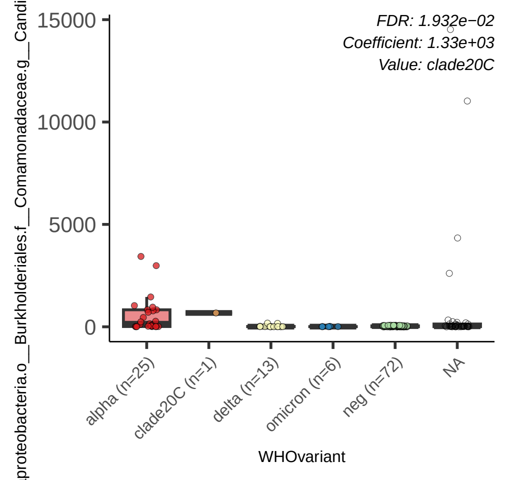

FDR: 1.932e−02
Coefficient: 1.33e+03
Value: clade20C
15000
10000
5000
0
proteobacteria.o__Burkholderiales.f__Comamonadaceae.g__Candi
alpha (n=25)
clade20C (n=1)
delta (n=13)
omicron (n=6)
neg (n=72)
NA
WHOvariant

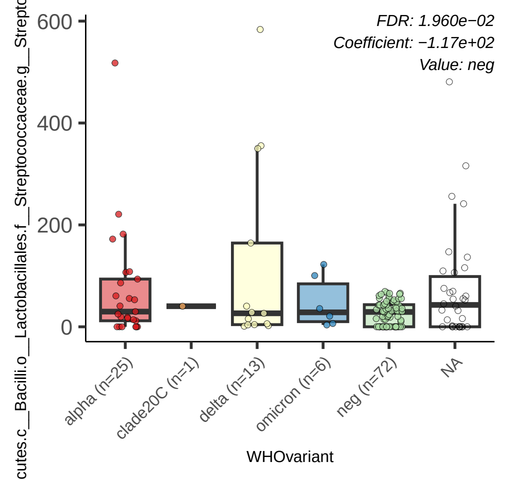

FDR: 1.960e−02
Coefficient: −1.17e+02
Value: neg
600
400
200
0
cutes.c__Bacilli.o__Lactobacillales.f__Streptococcaceae.g__Strepto
alpha (n=25)
clade20C (n=1)
delta (n=13)
omicron (n=6)
neg (n=72)
NA
WHOvariant

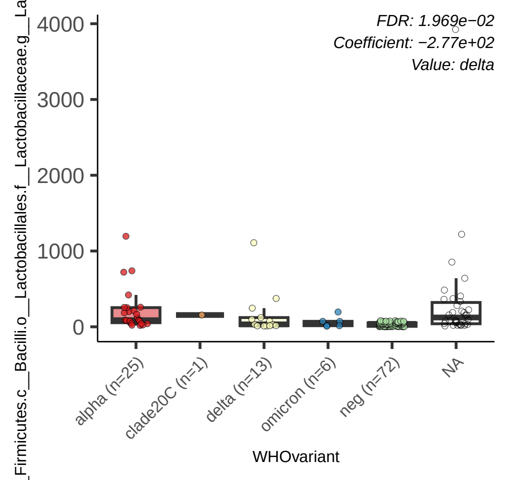
FDR: 1.969e−02
Coefficient: −2.77e+02
Value: delta
4000
3000
2000
1000
0
alpha (n=25)
clade20C (n=1)
delta (n=13)
omicron (n=6)
neg (n=72)
NA
WHOvariant
Firmicutes.c__Bacilli.o__Lactobacillales.f__Lactobacillaceae.g__La

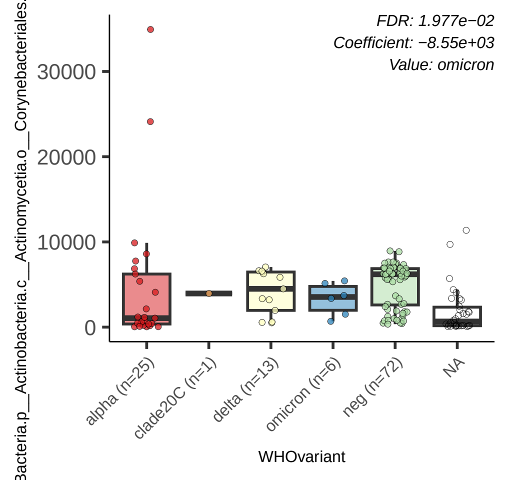
FDR: 1.977e−02
Coefficient: −8.55e+03
Value: omicron
Bacteria.p__Actinobacteria.c__Actinomycetia.o__Corynebacteriales.
30000
20000
10000
0
alpha (n=25)
clade20C (n=1)
delta (n=13)
omicron (n=6)
neg (n=72)
NA
WHOvariant

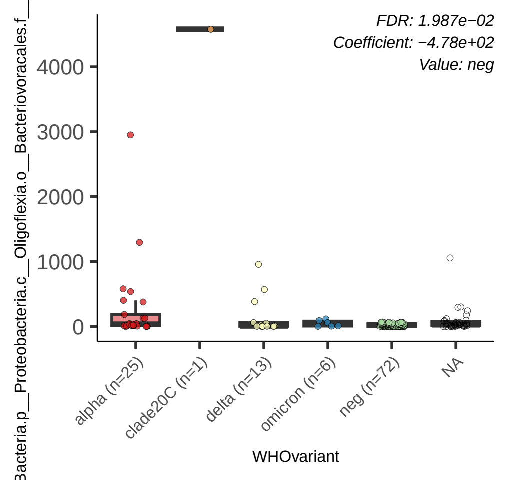
FDR: 1.987e−02
Coefficient: −4.78e+02
Value: neg
Bacteria.p__Proteobacteria.c__Oligoflexia.o__Bacteriovoracales.f__
4000
3000
2000
1000
0
alpha (n=25)
clade20C (n=1)
delta (n=13)
omicron (n=6)
neg (n=72)
NA
WHOvariant

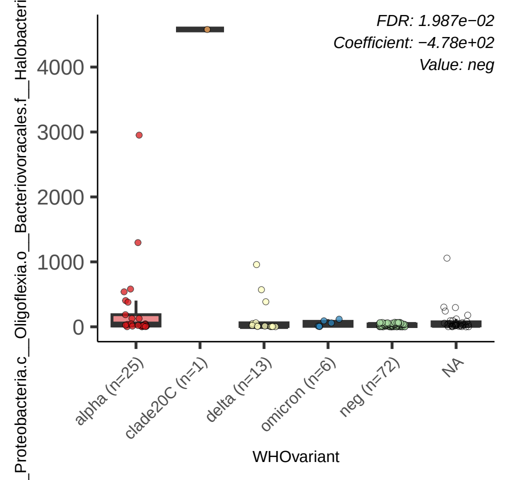

FDR: 1.987e−02
Coefficient: −4.78e+02
Value: neg
Proteobacteria.c__Oligoflexia.o__Bacteriovoracales.f__Halobacteri
4000
3000
2000
1000
0
alpha (n=25)
clade20C (n=1)
delta (n=13)
omicron (n=6)
neg (n=72)
NA
WHOvariant

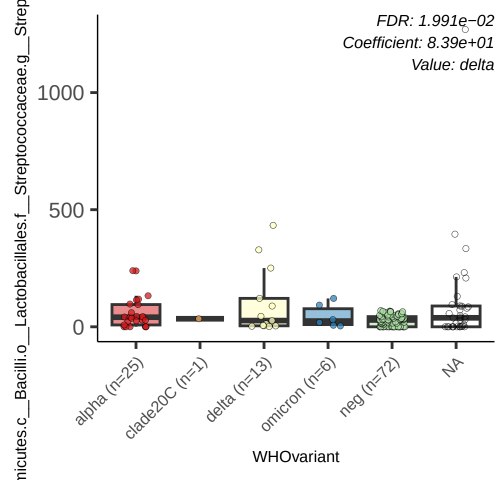

FDR: 1.991e−02
Coefficient: 8.39e+01
Value: delta
1000
500
0
micutes.c__Bacilli.o__Lactobacillales.f__Streptococcaceae.g__Strep
alpha (n=25)
clade20C (n=1)
delta (n=13)
omicron (n=6)
neg (n=72)
NA
WHOvariant

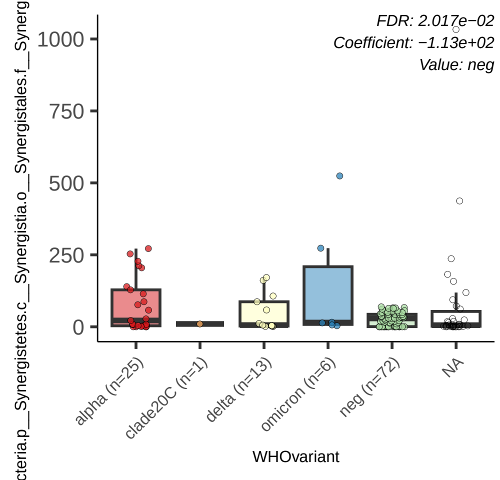
FDR: 2.017e−02
Coefficient: −1.13e+02
Value: neg
1000
750
500
250
0
alpha (n=25)
clade20C (n=1)
delta (n=13)
omicron (n=6)
neg (n=72)
NA
WHOvariant
cteria.p__Synergistetes.c__Synergistia.o__Synergistales.f__Synerg

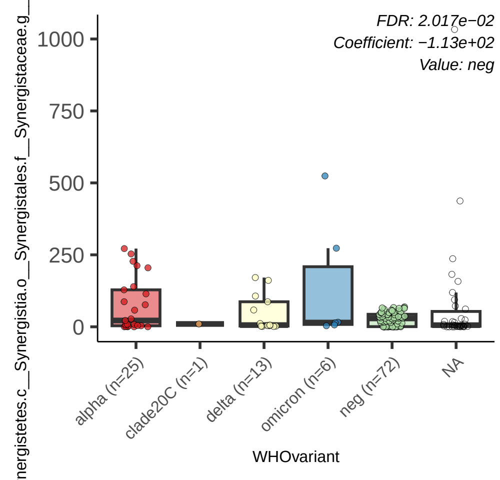

FDR: 2.017e−02
Coefficient: −1.13e+02
Value: neg
ynergistetes.c__Synergistia.o__Synergistales.f__Synergistaceae.g__
1000
750
500
250
0
alpha (n=25)
clade20C (n=1)
delta (n=13)
omicron (n=6)
neg (n=72)
NA
WHOvariant

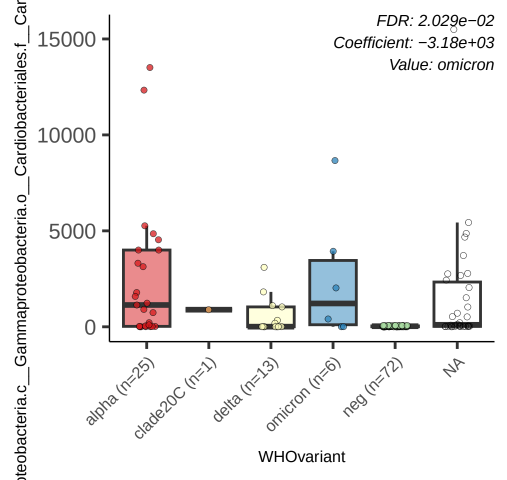
FDR: 2.029e−02
Coefficient: −3.18e+03
Value: omicron
15000
10000
5000
0
alpha (n=25)
clade20C (n=1)
delta (n=13)
omicron (n=6)
neg (n=72)
NA
WHOvariant
oteobacteria.c__Gammaproteobacteria.o__Cardiobacteriales.f__Car

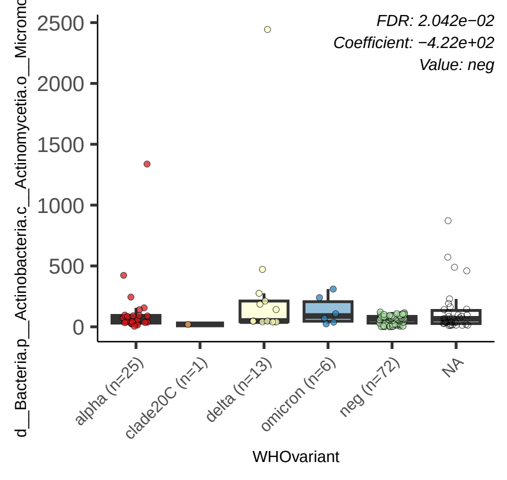

FDR: 2.042e−02
Coefficient: −4.22e+02
Value: neg
d__Bacteria.p__Actinobacteria.c__Actinomycetia.o__Micromo
2500
2000
1500
1000
500
0
alpha (n=25)
clade20C (n=1)
delta (n=13)
omicron (n=6)
neg (n=72)
NA
WHOvariant

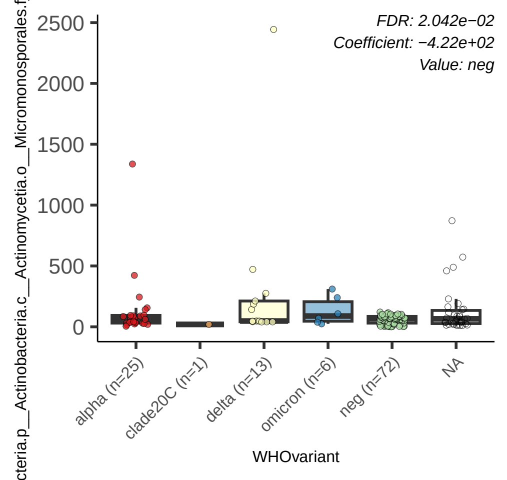
FDR: 2.042e−02
Coefficient: −4.22e+02
Value: neg
2500
2000
1500
1000
500
0
cteria.p__Actinobacteria.c__Actinomycetia.o__Micromonosporales.f
alpha (n=25)
clade20C (n=1)
delta (n=13)
omicron (n=6)
neg (n=72)
NA
WHOvariant

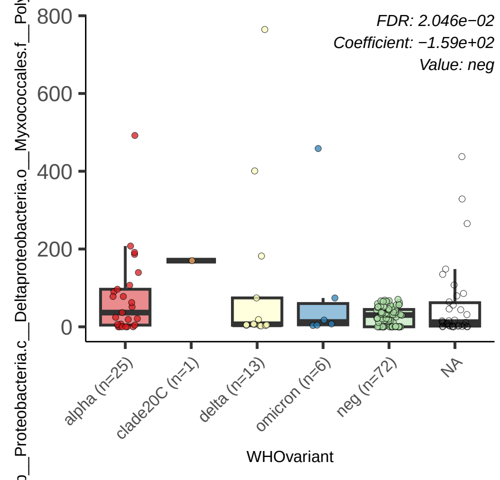

FDR: 2.046e−02
Coefficient: −1.59e+02
Value: neg
0
200
400
600
800
alpha (n=25)
clade20C (n=1)
delta (n=13)
omicron (n=6)
neg (n=72)
NA
WHOvariant

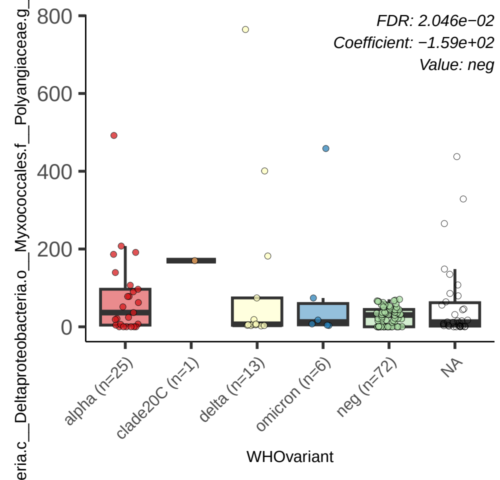

FDR: 2.046e−02
Coefficient: −1.59e+02
Value: neg
eria.c__Deltaproteobacteria.o__Myxococcales.f__Polyangiaceae.g__
800
600
400
200
0
alpha (n=25)
clade20C (n=1)
delta (n=13)
omicron (n=6)
neg (n=72)
NA
WHOvariant

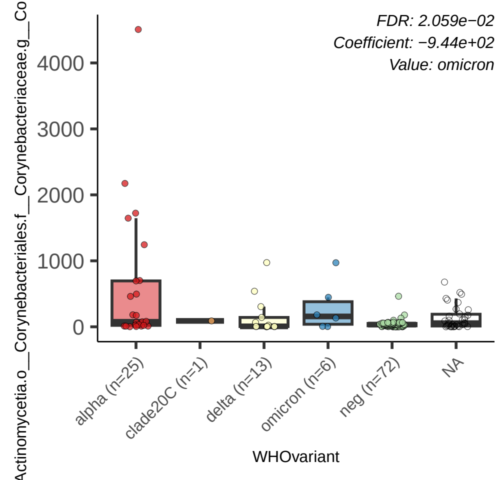

FDR: 2.059e−02
Coefficient: −9.44e+02
Value: omicron
Actinomycetia.o__Corynebacteriales.f__Corynebacteriaceae.g__Co
4000
3000
2000
1000
0
alpha (n=25)
clade20C (n=1)
delta (n=13)
omicron (n=6)
neg (n=72)
NA
WHOvariant

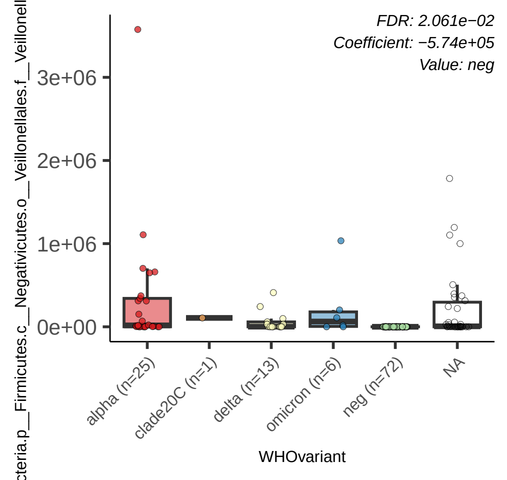
FDR: 2.061e−02
Coefficient: −5.74e+05
Value: neg
cteria.p__Firmicutes.c__Negativicutes.o__Veillonellales.f__Veillonell
3e+06
2e+06
1e+06
0e+00
alpha (n=25)
clade20C (n=1)
delta (n=13)
omicron (n=6)
neg (n=72)
NA
WHOvariant

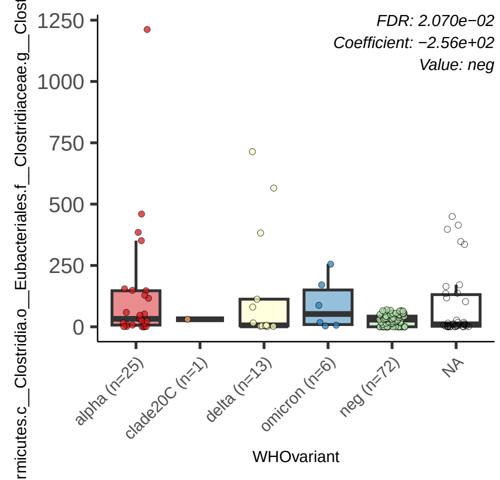

FDR: 2.070e−02
Coefficient: −2.56e+02
Value: neg
rmicutes.c__Clostridia.o__Eubacteriales.f__Clostridiaceae.g__Clost
1250
1000
750
500
250
0
alpha (n=25)
clade20C (n=1)
delta (n=13)
omicron (n=6)
neg (n=72)
NA
WHOvariant

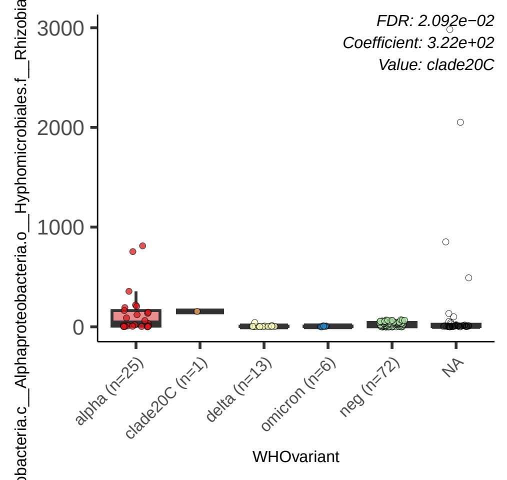
FDR: 2.092e−02
Coefficient: 3.22e+02
Value: clade20C
3000
2000
1000
0
obacteria.c__Alphaproteobacteria.o__Hyphomicrobiales.f__Rhizobia
alpha (n=25)
clade20C (n=1)
delta (n=13)
omicron (n=6)
neg (n=72)
NA
WHOvariant

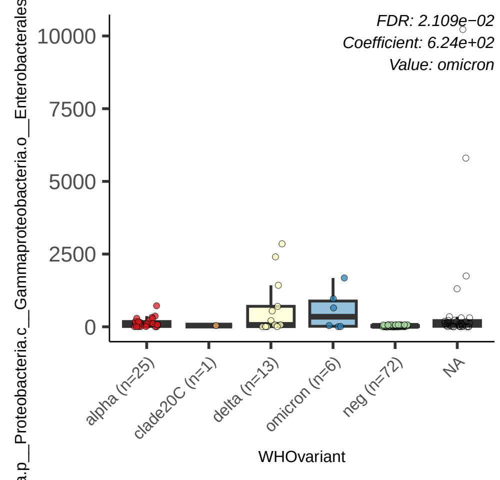
FDR: 2.109e−02
Coefficient: 6.24e+02
Value: omicron
10000
7500
5000
2500
0
a.p__Proteobacteria.c__Gammaproteobacteria.o__Enterobacterales
alpha (n=25)
clade20C (n=1)
delta (n=13)
omicron (n=6)
neg (n=72)
NA
WHOvariant

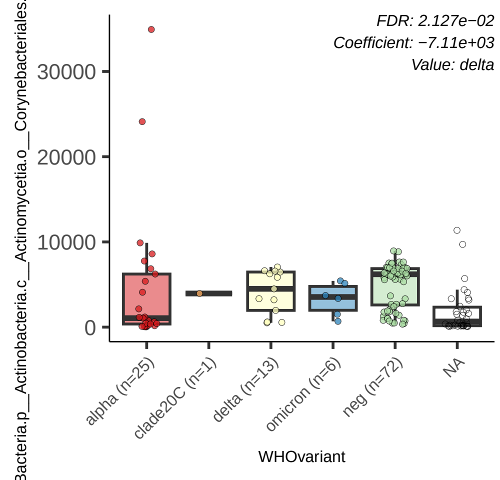

FDR: 2.127e−02
Coefficient: −7.11e+03
Value: delta
Bacteria.p__Actinobacteria.c__Actinomycetia.o__Corynebacteriales.
30000
20000
10000
0
alpha (n=25)
clade20C (n=1)
delta (n=13)
omicron (n=6)
neg (n=72)
NA
WHOvariant

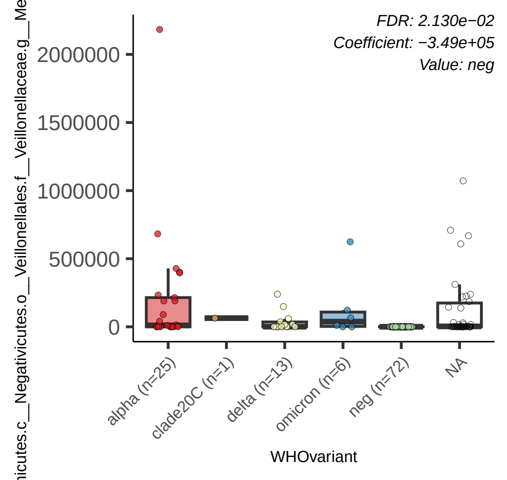

FDR: 2.130e−02
Coefficient: −3.49e+05
Value: neg
icutes.c__Negativicutes.o__Veillonellales.f__Veillonellaceae.g__Me
2000000
1500000
1000000
500000
0
alpha (n=25)
clade20C (n=1)
delta (n=13)
omicron (n=6)
neg (n=72)
NA
WHOvariant

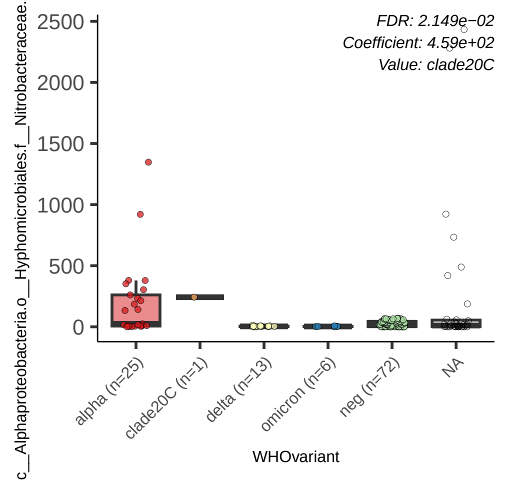

FDR: 2.149e−02
Coefficient: 4.59e+02
Value: clade20C
c__Alphaproteobacteria.o__Hyphomicrobiales.f__Nitrobacteraceae.
2500
2000
1500
1000
500
0
alpha (n=25)
clade20C (n=1)
delta (n=13)
omicron (n=6)
neg (n=72)
NA
WHOvariant

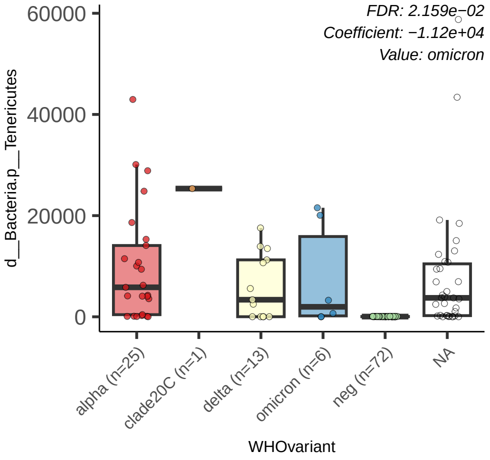
FDR: 2.159e−02
Coefficient: −1.12e+04
Value: omicron
d__Bacteria.p__Tenericutes
60000
40000
20000
0
alpha (n=25)
clade20C (n=1)
delta (n=13)
omicron (n=6)
neg (n=72)
NA
WHOvariant

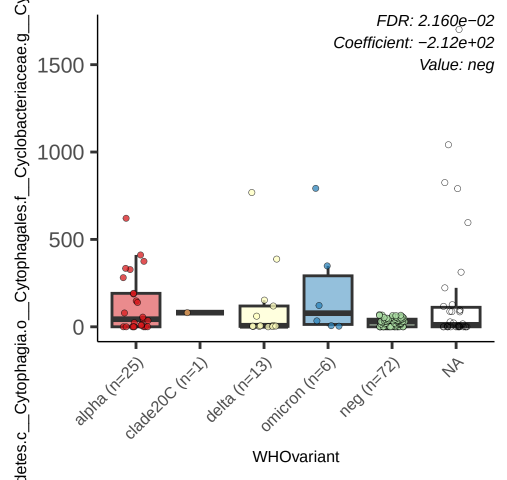

FDR: 2.160e−02
Coefficient: −2.12e+02
Value: neg
1500
1000
500
0
detes.c__Cytophagia.o__Cytophagales.f__Cyclobacteriaceae.g__Cy
alpha (n=25)
clade20C (n=1)
delta (n=13)
omicron (n=6)
neg (n=72)
NA
WHOvariant

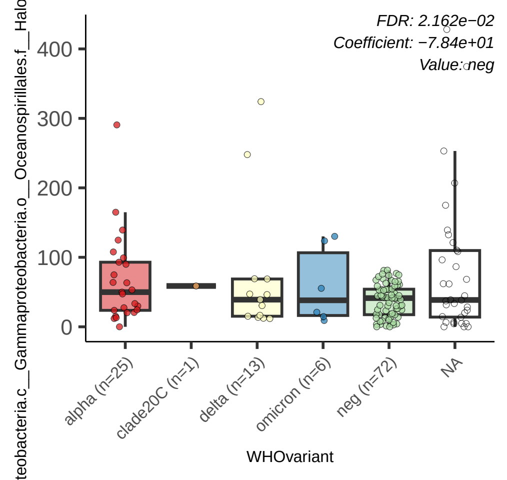

FDR: 2.162e−02
Coefficient: −7.84e+01
Value: neg
0
100
200
300
400
teobacteria.c__Gammaproteobacteria.o__Oceanospirillales.f__Halo
alpha (n=25)
clade20C (n=1)
delta (n=13)
omicron (n=6)
neg (n=72)
NA
WHOvariant

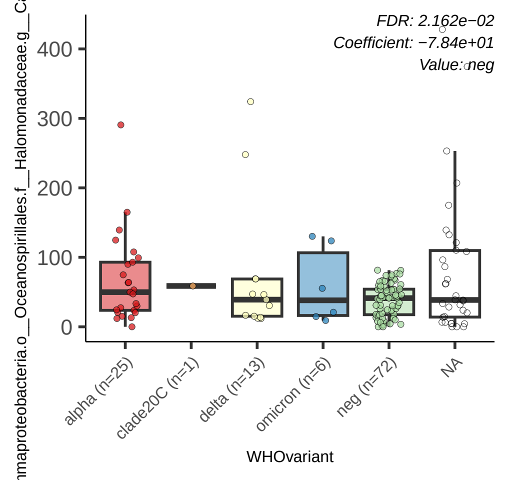

FDR: 2.162e−02
Coefficient: −7.84e+01
Value: neg
0
100
200
300
400
alpha (n=25)
clade20C (n=1)
delta (n=13)
omicron (n=6)
neg (n=72)
NA
WHOvariant

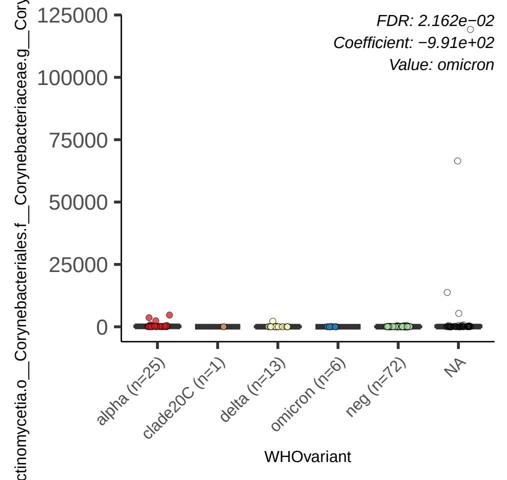

FDR: 2.162e−02
Coefficient: −9.91e+02
Value: omicron
125000
100000
75000
50000
25000
0
ctinomycetia.o__Corynebacteriales.f__Corynebacteriaceae.g__Cory
alpha (n=25)
clade20C (n=1)
delta (n=13)
omicron (n=6)
neg (n=72)
NA
WHOvariant

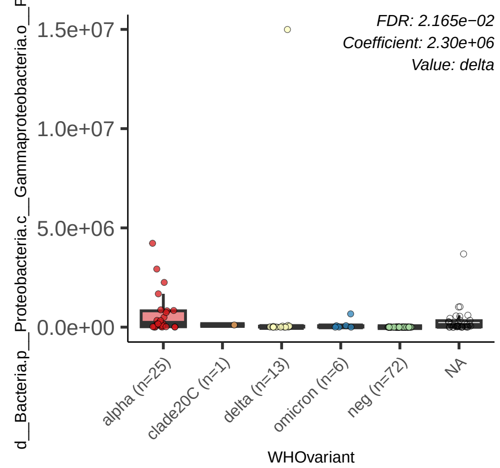

FDR: 2.165e−02
Coefficient: 2.30e+06
Value: delta
d__Bacteria.p__Proteobacteria.c__Gammaproteobacteria.o__F
1.5e+07
1.0e+07
5.0e+06
0.0e+00
alpha (n=25)
clade20C (n=1)
delta (n=13)
omicron (n=6)
neg (n=72)
NA
WHOvariant

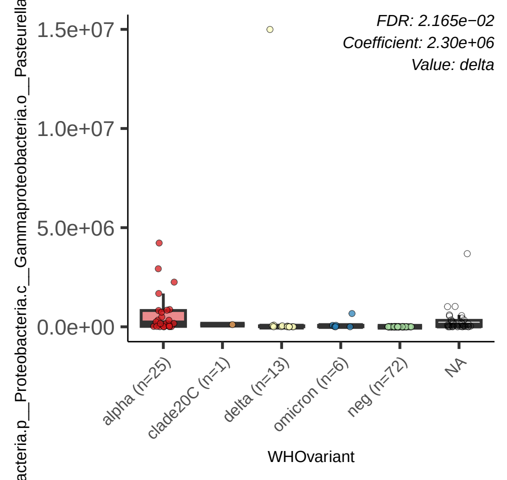

FDR: 2.165e−02
Coefficient: 2.30e+06
Value: delta
1.5e+07
1.0e+07
5.0e+06
0.0e+00
acteria.p__Proteobacteria.c__Gammaproteobacteria.o__Pasteurella
alpha (n=25)
clade20C (n=1)
delta (n=13)
omicron (n=6)
neg (n=72)
NA
WHOvariant

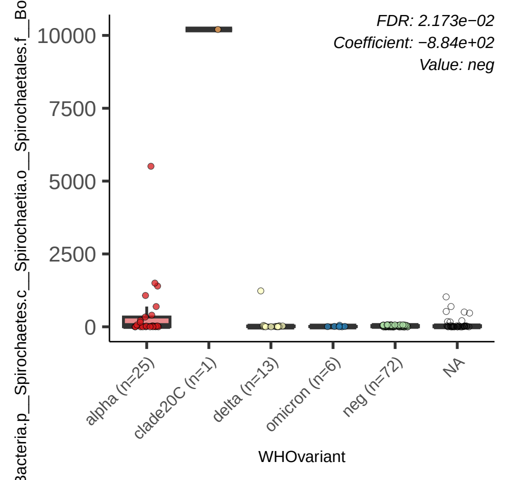

FDR: 2.173e−02
Coefficient: −8.84e+02
Value: neg
Bacteria.p__Spirochaetes.c__Spirochaetia.o__Spirochaetales.f__Bo
10000
7500
5000
2500
0
alpha (n=25)
clade20C (n=1)
delta (n=13)
omicron (n=6)
neg (n=72)
NA
WHOvariant

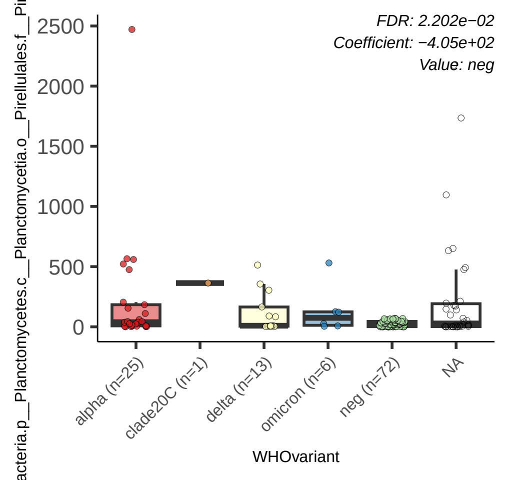

FDR: 2.202e−02
Coefficient: −4.05e+02
Value: neg
acteria.p__Planctomycetes.c__Planctomycetia.o__Pirellulales.f__Pi
2500
2000
1500
1000
500
0
alpha (n=25)
clade20C (n=1)
delta (n=13)
omicron (n=6)
neg (n=72)
NA
WHOvariant

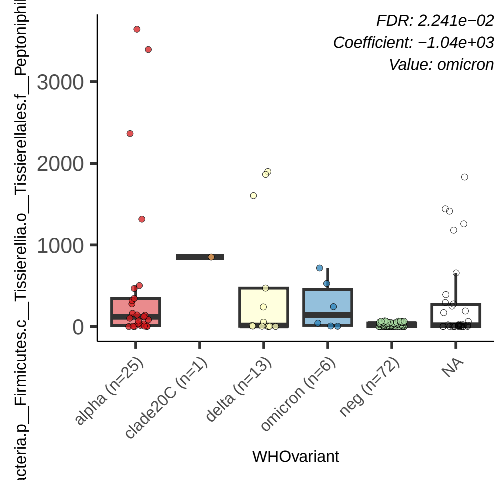
FDR: 2.241e−02
Coefficient: −1.04e+03
Value: omicron
acteria.p__Firmicutes.c__Tissierellia.o__Tissierellales.f__Peptoniphil
0
1000
2000
3000
alpha (n=25)
clade20C (n=1)
delta (n=13)
omicron (n=6)
neg (n=72)
NA
WHOvariant

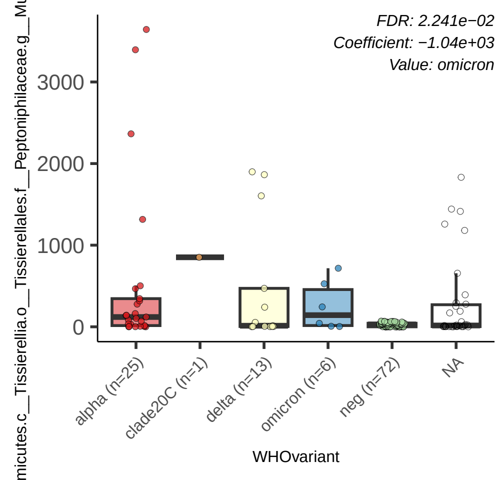

FDR: 2.241e−02
Coefficient: −1.04e+03
Value: omicron
micutes.c__Tissierellia.o__Tissierellales.f__Peptoniphilaceae.g__Mu
0
1000
2000
3000
alpha (n=25)
clade20C (n=1)
delta (n=13)
omicron (n=6)
neg (n=72)
NA
WHOvariant

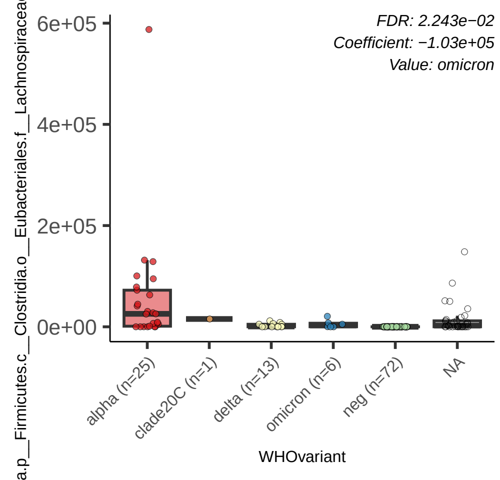

a.p__Firmicutes.c__Clostridia.o__Eubacteriales.f__Lachnospiraceae
FDR: 2.243e−02
Coefficient: −1.03e+05
Value: omicron
6e+05
4e+05
2e+05
0e+00
alpha (n=25)
clade20C (n=1)
delta (n=13)
omicron (n=6)
neg (n=72)
NA
WHOvariant

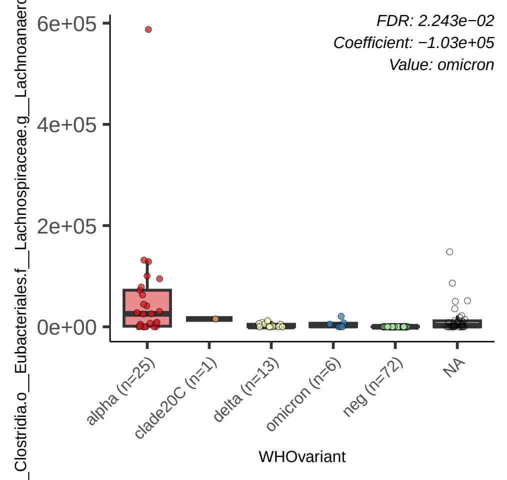

FDR: 2.243e−02
Coefficient: −1.03e+05
Value: omicron
6e+05
4e+05
2e+05
0e+00
_Clostridia.o__Eubacteriales.f__Lachnospiraceae.g__Lachnoanaero
alpha (n=25)
clade20C (n=1)
delta (n=13)
omicron (n=6)
neg (n=72)
NA
WHOvariant

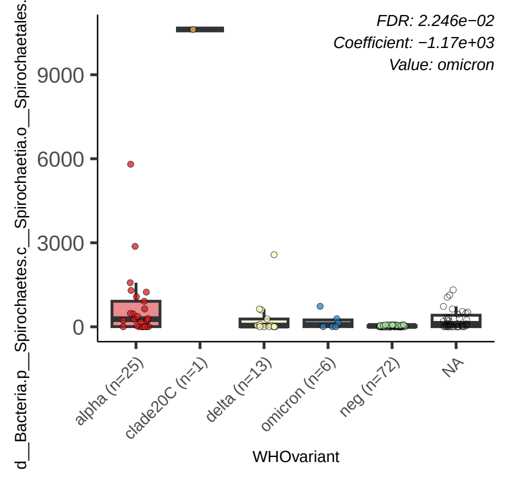
FDR: 2.246e−02
Coefficient: −1.17e+03
Value: omicron
9000
6000
3000
0
d__Bacteria.p__Spirochaetes.c__Spirochaetia.o__Spirochaetales.
alpha (n=25)
clade20C (n=1)
delta (n=13)
omicron (n=6)
neg (n=72)
NA
WHOvariant

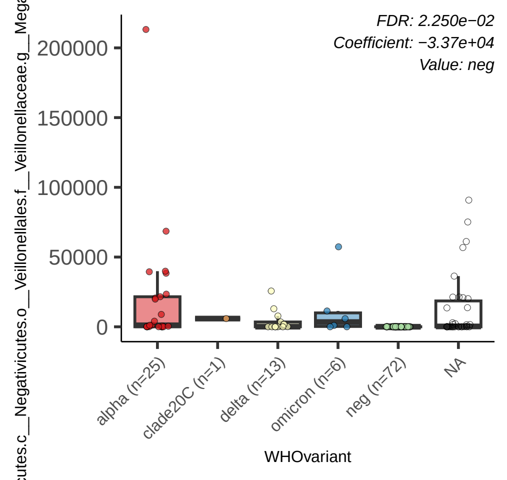
FDR: 2.250e−02
Coefficient: −3.37e+04
Value: neg
200000
150000
100000
50000
0
cutes.c__Negativicutes.o__Veillonellales.f__Veillonellaceae.g__Mega
alpha (n=25)
clade20C (n=1)
delta (n=13)
omicron (n=6)
neg (n=72)
NA
WHOvariant

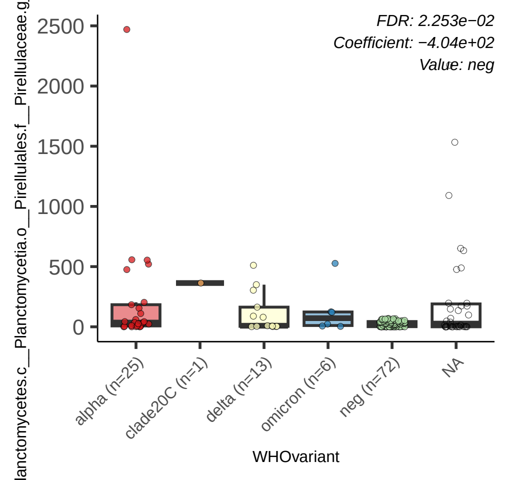

FDR: 2.253e−02
Coefficient: −4.04e+02
Value: neg
2500
2000
1500
1000
500
0
lanctomycetes.c__Planctomycetia.o__Pirellulales.f__Pirellulaceae.g.
alpha (n=25)
clade20C (n=1)
delta (n=13)
omicron (n=6)
neg (n=72)
NA
WHOvariant

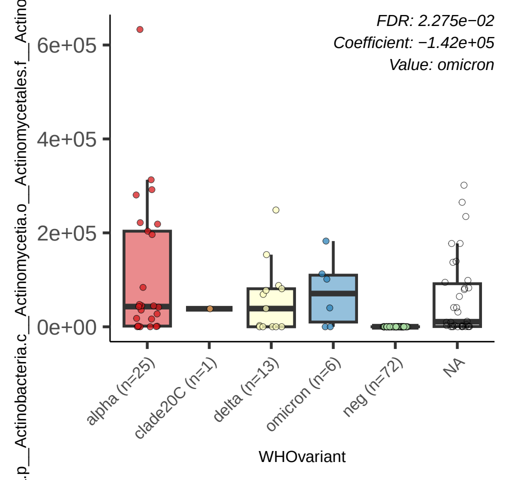
FDR: 2.275e−02
Coefficient: −1.42e+05
Value: omicron
p__Actinobacteria.c__Actinomycetia.o__Actinomycetales.f__Actino
6e+05
4e+05
2e+05
0e+00
alpha (n=25)
clade20C (n=1)
delta (n=13)
omicron (n=6)
neg (n=72)
NA
WHOvariant

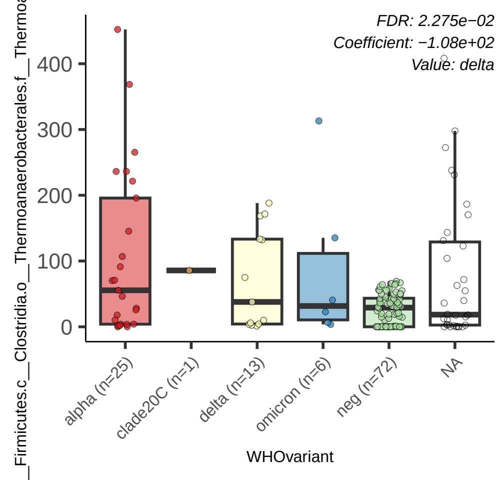

FDR: 2.275e−02
Coefficient: −1.08e+02
Value: delta
_Firmicutes.c__Clostridia.o__Thermoanaerobacterales.f__Thermoa
0
100
200
300
400
alpha (n=25)
clade20C (n=1)
delta (n=13)
omicron (n=6)
neg (n=72)
NA
WHOvariant

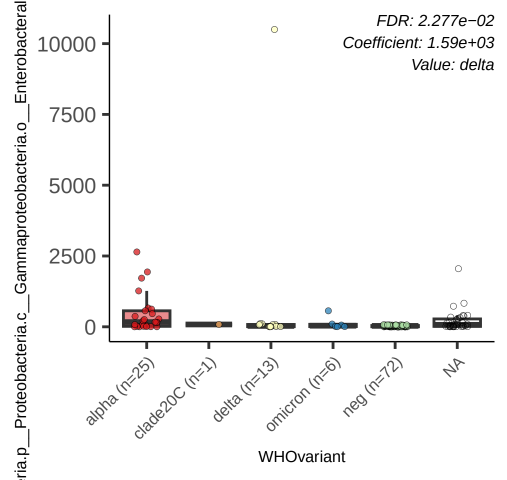
FDR: 2.277e−02
Coefficient: 1.59e+03
Value: delta
10000
7500
5000
2500
0
ria.p__Proteobacteria.c__Gammaproteobacteria.o__Enterobacteral
alpha (n=25)
clade20C (n=1)
delta (n=13)
omicron (n=6)
neg (n=72)
NA
WHOvariant

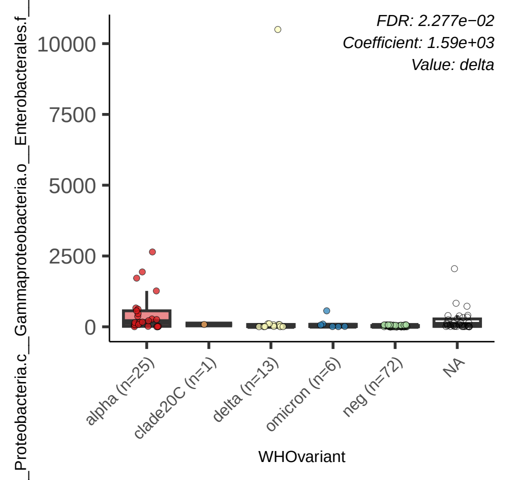

FDR: 2.277e−02
Coefficient: 1.59e+03
Value: delta
Proteobacteria.c__Gammaproteobacteria.o__Enterobacterales.f
10000
7500
5000
2500
0
alpha (n=25)
clade20C (n=1)
delta (n=13)
omicron (n=6)
neg (n=72)
NA
WHOvariant

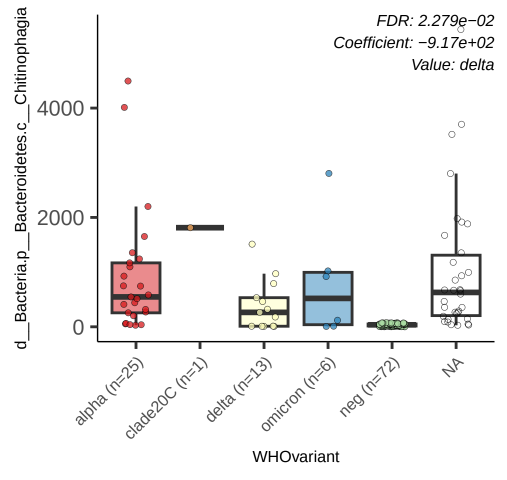

FDR: 2.279e−02
Coefficient: −9.17e+02
Value: delta
d__Bacteria.p__Bacteroidetes.c__Chitinophagia
4000
2000
0
alpha (n=25)
clade20C (n=1)
delta (n=13)
omicron (n=6)
neg (n=72)
NA
WHOvariant

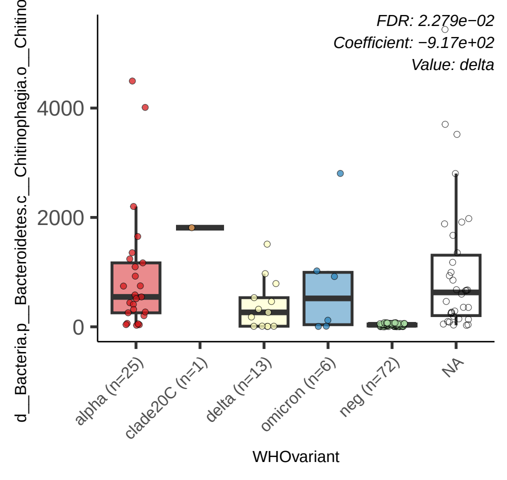

FDR: 2.279e−02
Coefficient: −9.17e+02
Value: delta
d__Bacteria.p__Bacteroidetes.c__Chitinophagia.o__Chitino
4000
2000
0
alpha (n=25)
clade20C (n=1)
delta (n=13)
omicron (n=6)
neg (n=72)
NA
WHOvariant

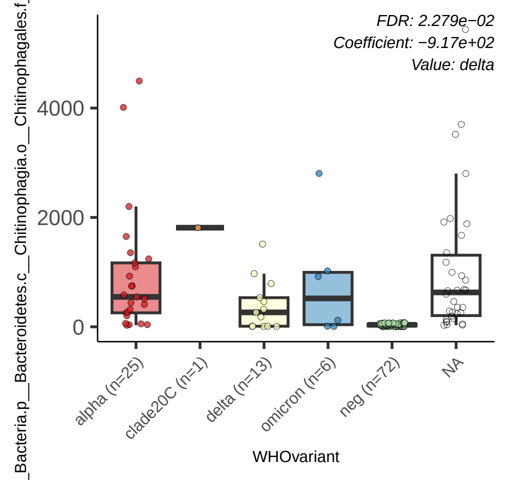

FDR: 2.279e−02
Coefficient: −9.17e+02
Value: delta
Bacteria.p__Bacteroidetes.c__Chitinophagia.o__Chitinophagales.f_
0
2000
4000
alpha (n=25)
clade20C (n=1)
delta (n=13)
omicron (n=6)
neg (n=72)
NA
WHOvariant

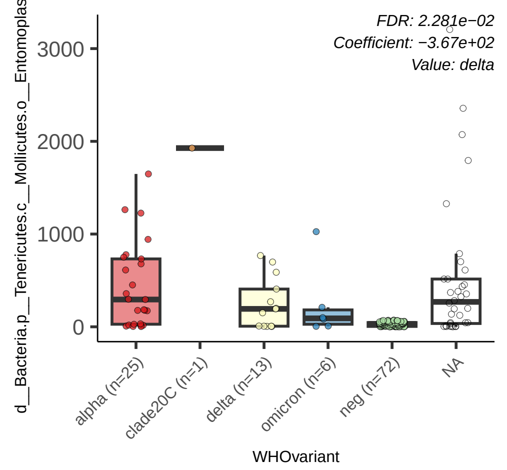

FDR: 2.281e−02
Coefficient: −3.67e+02
Value: delta
3000
2000
1000
0
d__Bacteria.p__Tenericutes.c__Mollicutes.o__Entomoplas
alpha (n=25)
clade20C (n=1)
delta (n=13)
omicron (n=6)
neg (n=72)
NA
WHOvariant

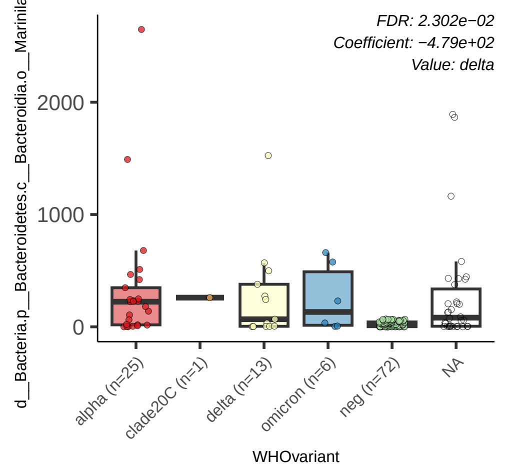

FDR: 2.302e−02
Coefficient: −4.79e+02
Value: delta
d__Bacteria.p__Bacteroidetes.c__Bacteroidia.o__Marinila
2000
1000
0
alpha (n=25)
clade20C (n=1)
delta (n=13)
omicron (n=6)
neg (n=72)
NA
WHOvariant

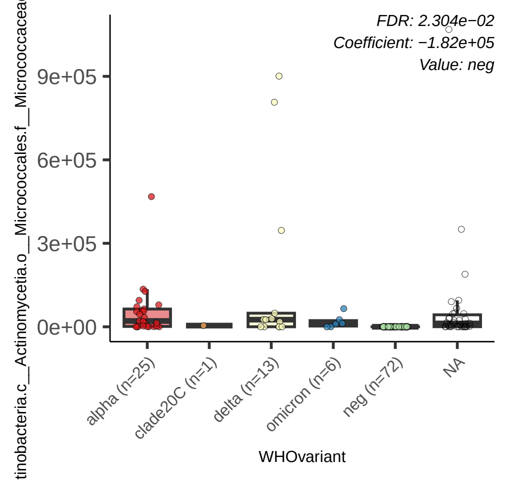

FDR: 2.304e−02
Coefficient: −1.82e+05
Value: neg
9e+05
6e+05
3e+05
0e+00
tinobacteria.c__Actinomycetia.o__Micrococcales.f__Micrococcacea
alpha (n=25)
clade20C (n=1)
delta (n=13)
omicron (n=6)
neg (n=72)
NA
WHOvariant

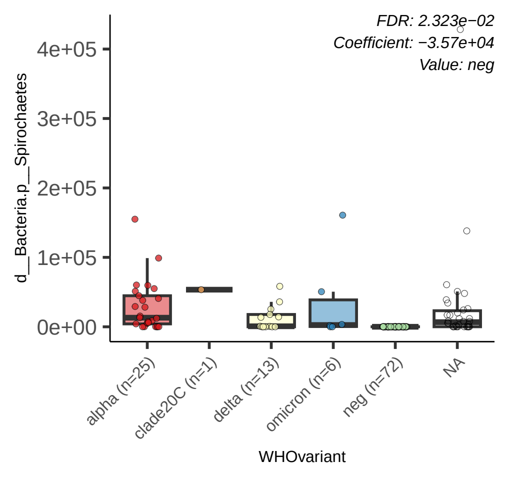
FDR: 2.323e−02
Coefficient: −3.57e+04
Value: neg
d__Bacteria.p__Spirochaetes
4e+05
3e+05
2e+05
1e+05
0e+00
alpha (n=25)
clade20C (n=1)
delta (n=13)
omicron (n=6)
neg (n=72)
NA
WHOvariant

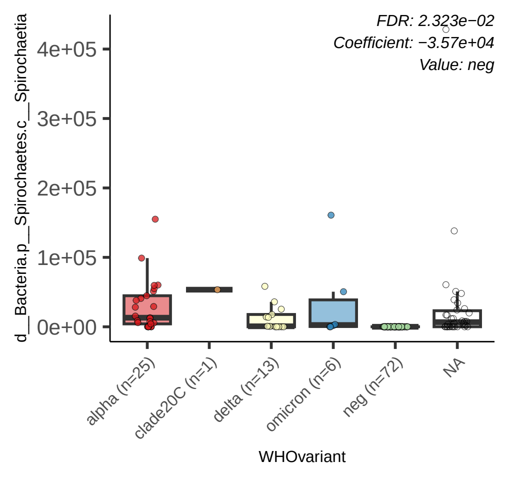

FDR: 2.323e−02
Coefficient: −3.57e+04
Value: neg
d__Bacteria.p__Spirochaetes.c__Spirochaetia
4e+05
3e+05
2e+05
1e+05
0e+00
alpha (n=25)
clade20C (n=1)
delta (n=13)
omicron (n=6)
neg (n=72)
NA
WHOvariant

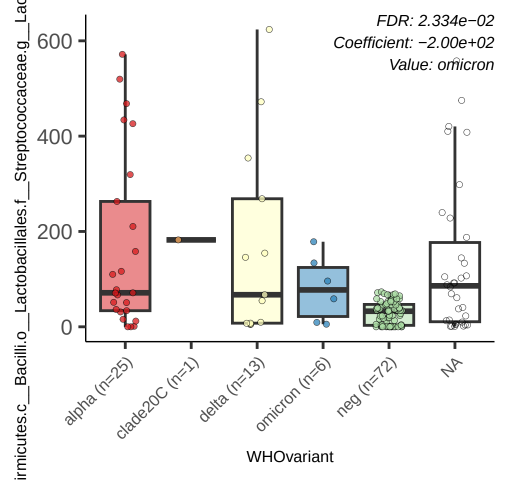

FDR: 2.334e−02
Coefficient: −2.00e+02
Value: omicron
irmicutes.c__Bacilli.o__Lactobacillales.f__Streptococcaceae.g__Lac
600
400
200
0
alpha (n=25)
clade20C (n=1)
delta (n=13)
omicron (n=6)
neg (n=72)
NA
WHOvariant

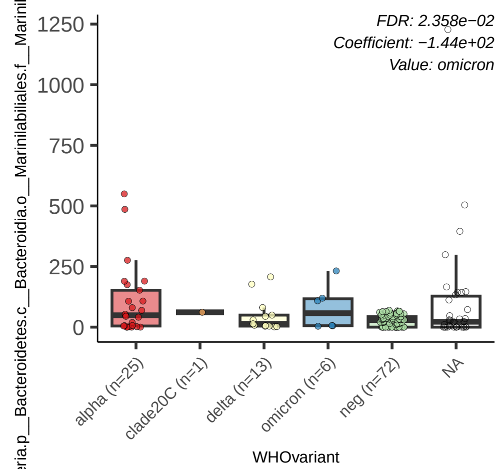
FDR: 2.358e−02
Coefficient: −1.44e+02
Value: omicron
1250
1000
750
500
250
0
alpha (n=25)
clade20C (n=1)
delta (n=13)
omicron (n=6)
neg (n=72)
NA
WHOvariant
cteria.p__Bacteroidetes.c__Bacteroidia.o__Marinilabiliales.f__Marinil

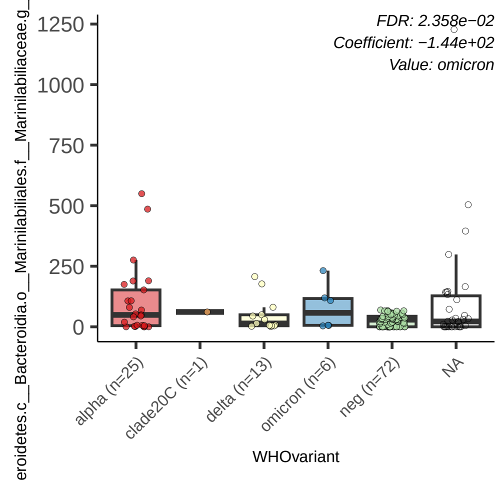
FDR: 2.358e−02
Coefficient: −1.44e+02
Value: omicron
eroidetes.c__Bacteroidia.o__Marinilabiliales.f__Marinilabiliaceae.g__
1250
1000
750
500
250
0
alpha (n=25)
clade20C (n=1)
delta (n=13)
omicron (n=6)
neg (n=72)
NA
WHOvariant

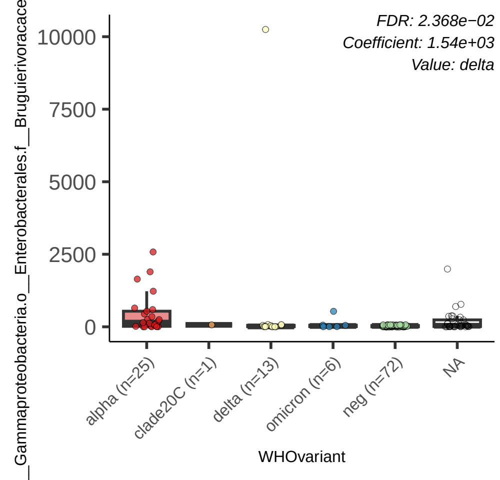
FDR: 2.368e−02
Coefficient: 1.54e+03
Value: delta
10000
7500
5000
2500
0
alpha (n=25)
clade20C (n=1)
delta (n=13)
omicron (n=6)
neg (n=72)
NA
WHOvariant

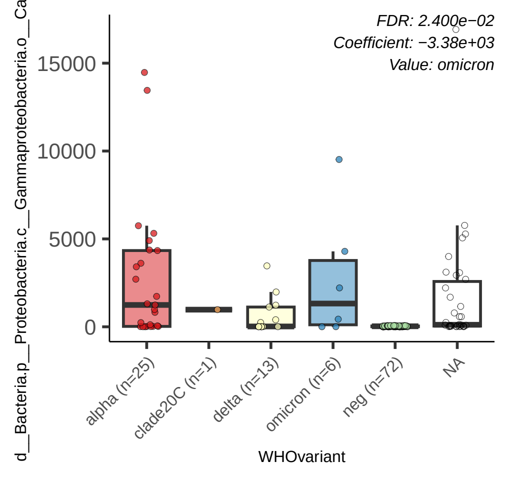
FDR: 2.400e−02
Coefficient: −3.38e+03
Value: omicron
15000
10000
5000
0
d__Bacteria.p__Proteobacteria.c__Gammaproteobacteria.o__Ca
alpha (n=25)
clade20C (n=1)
delta (n=13)
omicron (n=6)
neg (n=72)
NA
WHOvariant

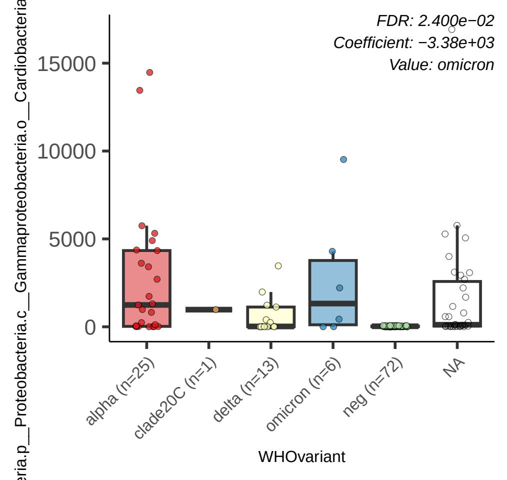

FDR: 2.400e−02
Coefficient: −3.38e+03
Value: omicron
15000
10000
5000
0
alpha (n=25)
clade20C (n=1)
delta (n=13)
omicron (n=6)
neg (n=72)
NA
WHOvariant
eria.p__Proteobacteria.c__Gammaproteobacteria.o__Cardiobacteria

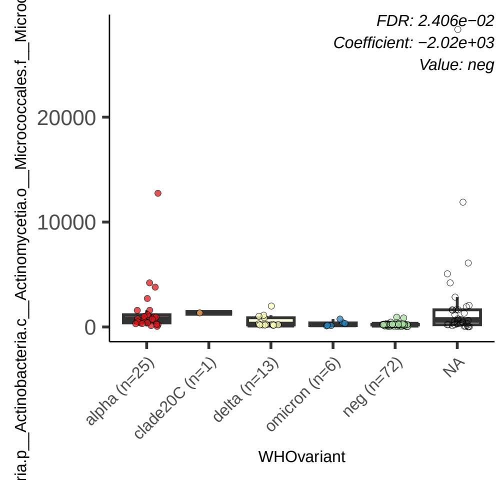
FDR: 2.406e−02
Coefficient: −2.02e+03
Value: neg
ria.p__Actinobacteria.c__Actinomycetia.o__Micrococcales.f__Microc
20000
10000
0
alpha (n=25)
clade20C (n=1)
delta (n=13)
omicron (n=6)
neg (n=72)
NA
WHOvariant

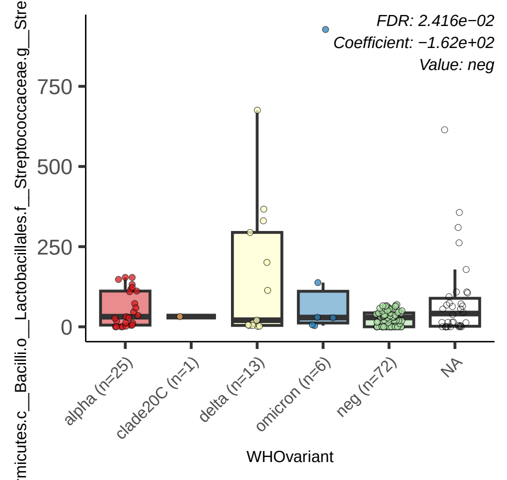
FDR: 2.416e−02
Coefficient: −1.62e+02
Value: neg
0
250
500
750
alpha (n=25)
clade20C (n=1)
delta (n=13)
omicron (n=6)
neg (n=72)
NA
WHOvariant
micutes.c__Bacilli.o__Lactobacillales.f__Streptococcaceae.g__Stre

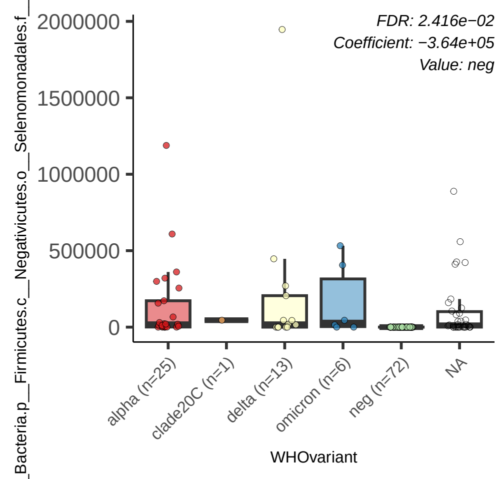

FDR: 2.416e−02
Coefficient: −3.64e+05
Value: neg
Bacteria.p__Firmicutes.c__Negativicutes.o__Selenomonadales.f
2000000
1500000
1000000
500000
0
alpha (n=25)
clade20C (n=1)
delta (n=13)
omicron (n=6)
neg (n=72)
NA
WHOvariant

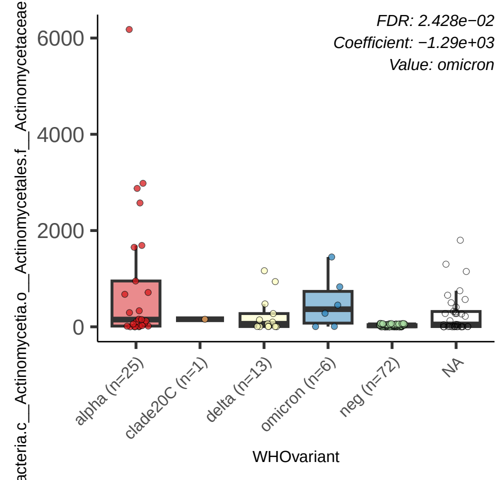

FDR: 2.428e−02
Coefficient: −1.29e+03
Value: omicron
acteria.c__Actinomycetia.o__Actinomycetales.f__Actinomycetaceae
6000
4000
2000
0
alpha (n=25)
clade20C (n=1)
delta (n=13)
omicron (n=6)
neg (n=72)
NA
WHOvariant

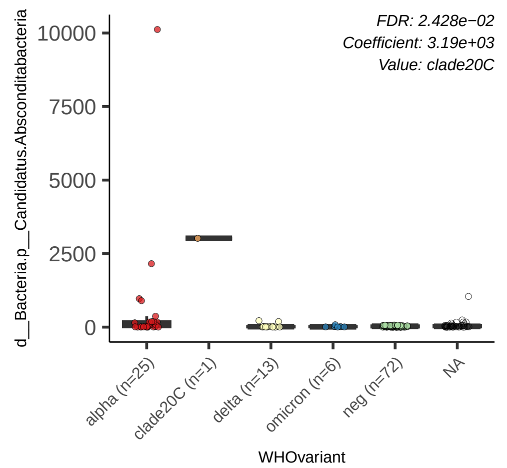

FDR: 2.428e−02
Coefficient: 3.19e+03
Value: clade20C
d__Bacteria.p__Candidatus.Abscondita bacteria
10000
7500
5000
2500
0
alpha (n=25)
clade20C (n=1)
delta (n=13)
omicron (n=6)
neg (n=72)
NA
WHOvariant

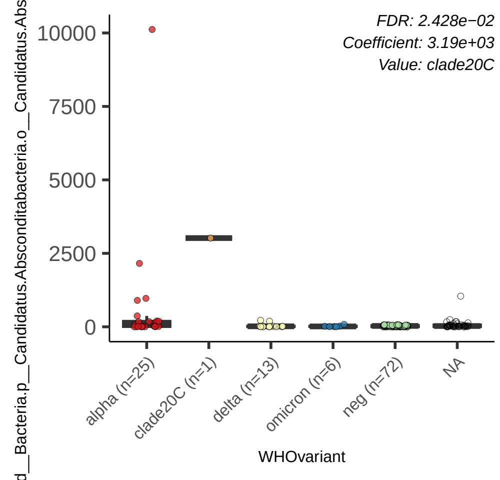

FDR: 2.428e−02
Coefficient: 3.19e+03
Value: clade20C
10000
7500
5000
2500
0
d__Bacteria.p__Candidatus.Absconditabacteria.o__Candidatus.Abs
alpha (n=25)
clade20C (n=1)
delta (n=13)
omicron (n=6)
neg (n=72)
NA
WHOvariant

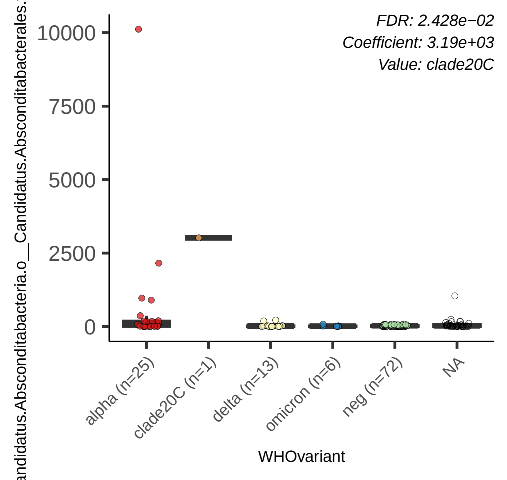

FDR: 2.428e−02
Coefficient: 3.19e+03
Value: clade20C
10000
7500
5000
2500
0
andidatus.Abscondit abacteria.o__Candidatus.Abscondit abacterales.
alpha (n=25)
clade20C (n=1)
delta (n=13)
omicron (n=6)
neg (n=72)
NA
WHOvariant

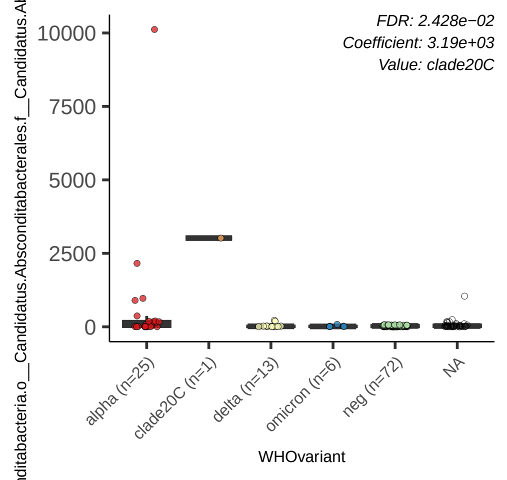

FDR: 2.428e−02
Coefficient: 3.19e+03
Value: clade20C
10000
7500
5000
2500
0
alpha (n=25)
clade20C (n=1)
delta (n=13)
omicron (n=6)
neg (n=72)
NA
WHOvariant
nditabacteria.o__Candidatus.Absconditabacterales.f__Candidatus.Ab

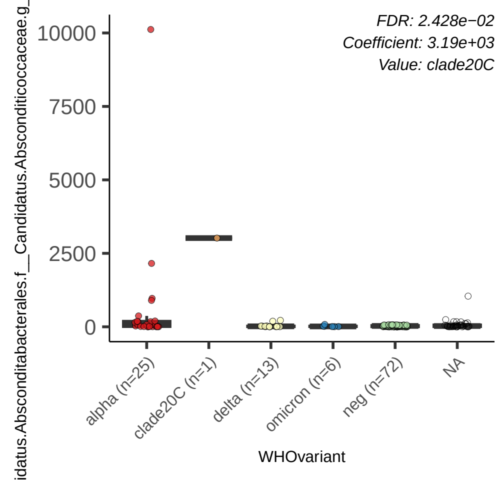

FDR: 2.428e−02
Coefficient: 3.19e+03
Value: clade20C
10000
7500
5000
2500
0
idatus.Absconditabacterales.f__Candidatus.Absconditicoccaceae.g_
alpha (n=25)
clade20C (n=1)
delta (n=13)
omicron (n=6)
neg (n=72)
NA
WHOvariant

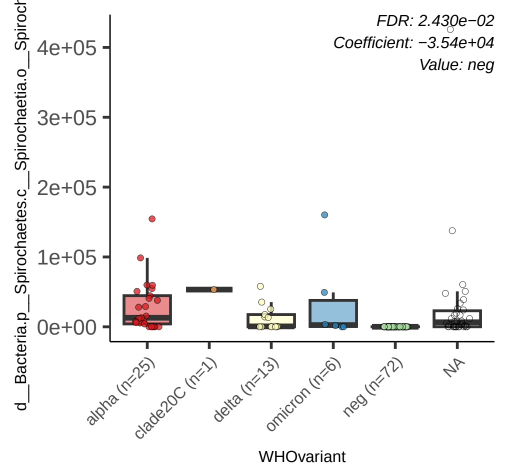
FDR: 2.430e−02
Coefficient: −3.54e+04
Value: neg
d__Bacteria.p__Spirochaetes.c__Spirochaetia.o__Spiroch
4e+05
3e+05
2e+05
1e+05
0e+00
alpha (n=25)
clade20C (n=1)
delta (n=13)
omicron (n=6)
neg (n=72)
NA
WHOvariant

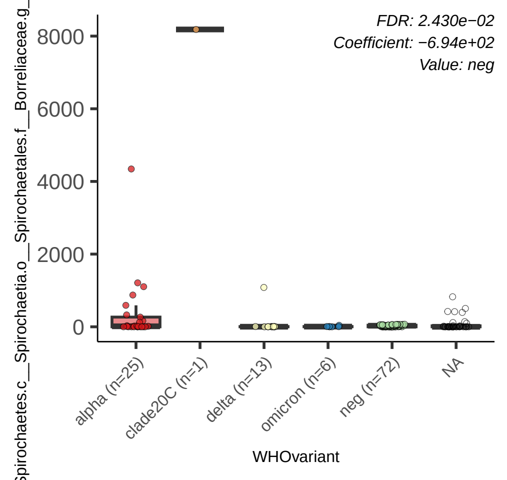

FDR: 2.430e−02
Coefficient: −6.94e+02
Value: neg
Spirochaetes.c__Spirochaetia.o__Spirochaetales.f__Borreliaceae.g_
8000
6000
4000
2000
0
alpha (n=25)
clade20C (n=1)
delta (n=13)
omicron (n=6)
neg (n=72)
NA
WHOvariant

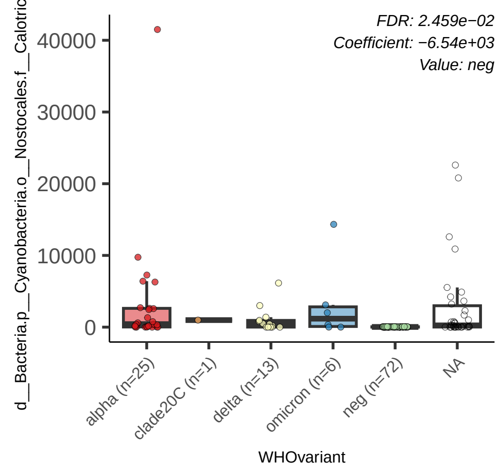

FDR: 2.459e−02
Coefficient: −6.54e+03
Value: neg
d__Bacteria.p__Cyanobacteria.o__Nostocales.f__Calotric
40000
30000
20000
10000
0
alpha (n=25)
clade20C (n=1)
delta (n=13)
omicron (n=6)
neg (n=72)
NA
WHOvariant

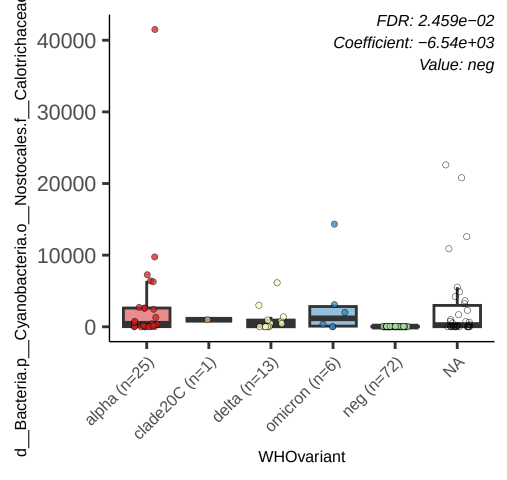
FDR: 2.459e−02
Coefficient: −6.54e+03
Value: neg
d__Bacteria.p__Cyanobacteria.o__Nostocales.f__Calotrichaceae
40000
30000
20000
10000
0
alpha (n=25)
clade20C (n=1)
delta (n=13)
omicron (n=6)
neg (n=72)
NA
WHOvariant

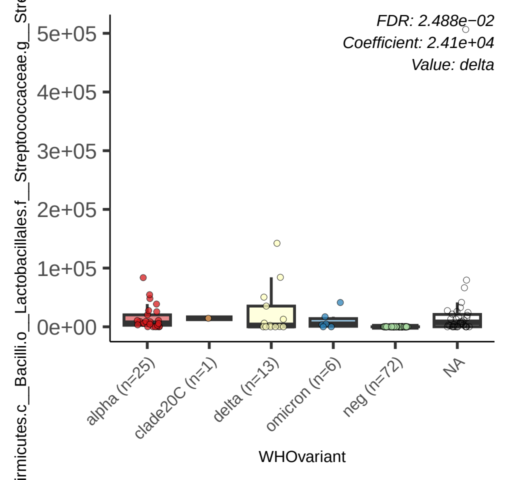

FDR: 2.488e−02
Coefficient: 2.41e+04
Value: delta
5e+05
4e+05
3e+05
2e+05
1e+05
0e+00
alpha (n=25)
clade20C (n=1)
delta (n=13)
omicron (n=6)
neg (n=72)
NA
WHOvariant

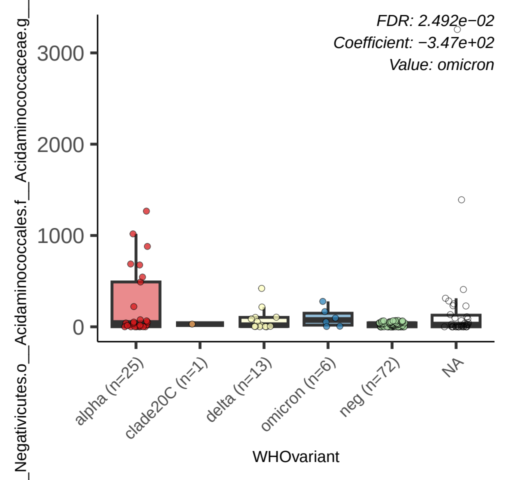

FDR: 2.492e−02
Coefficient: −3.47e+02
Value: omicron
Negativicutes.o__Acidaminococcales.f__Acidaminococcaceae.g__
0
1000
2000
3000
alpha (n=25)
clade20C (n=1)
delta (n=13)
omicron (n=6)
neg (n=72)
NA
WHOvariant

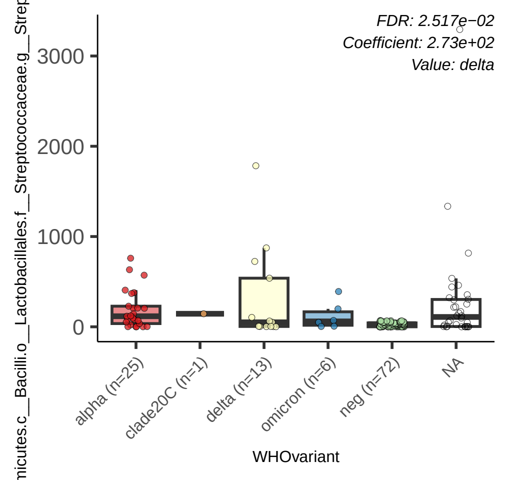

FDR: 2.517e−02
Coefficient: 2.73e+02
Value: delta
micutes.c__Bacilli.o__Lactobacillales.f__Streptococcaceae.g__Strep
3000
2000
1000
0
alpha (n=25)
clade20C (n=1)
delta (n=13)
omicron (n=6)
neg (n=72)
NA
WHOvariant

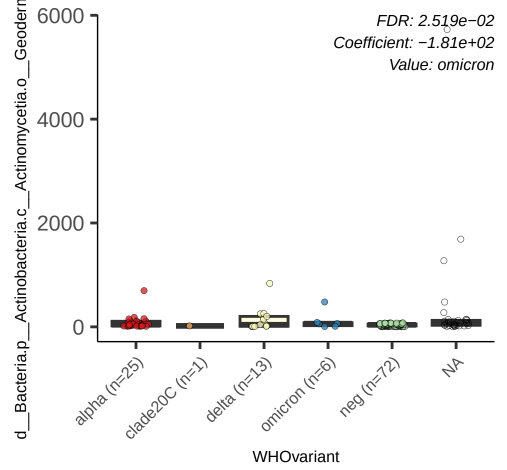

FDR: 2.519e−02
Coefficient: −1.81e+02
Value: omicron
6000
4000
2000
0
d__Bacteria.p__Actinobacteria.c__Actinomycetia.o__Geoderm
alpha (n=25)
clade20C (n=1)
delta (n=13)
omicron (n=6)
neg (n=72)
NA
WHOvariant

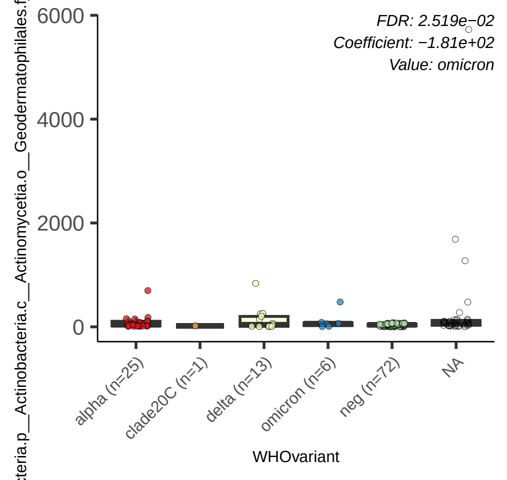

FDR: 2.519e−02
Coefficient: −1.81e+02
Value: omicron
6000
4000
2000
0
cteria.p__Actinobacteria.c__Actinomycetia.o__Geodermatophilales.f
alpha (n=25)
clade20C (n=1)
delta (n=13)
omicron (n=6)
neg (n=72)
NA
WHOvariant

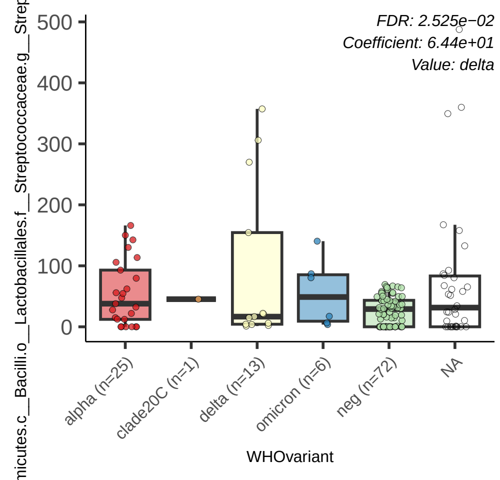

FDR: 2.525e−02
Coefficient: 6.44e+01
Value: delta
micutes.c__Bacilli.o__Lactobacillales.f__Streptococcaceae.g__Strep
500
400
300
200
100
0
alpha (n=25)
clade20C (n=1)
delta (n=13)
omicron (n=6)
neg (n=72)
NA
WHOvariant

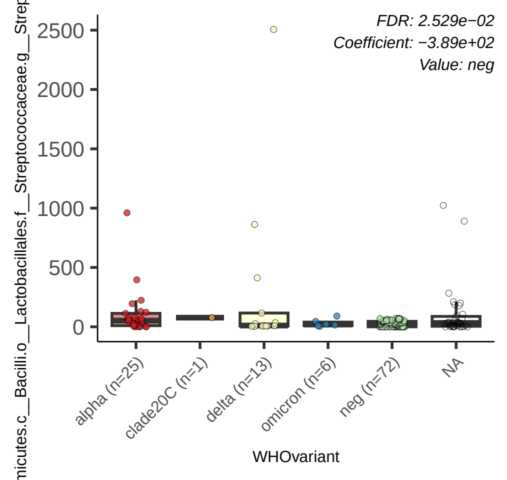

FDR: 2.529e−02
Coefficient: −3.89e+02
Value: neg
2500
2000
1500
1000
500
0
micutes.c__Bacilli.o__Lactobacillales.f__Streptococcaceae.g__Strep
alpha (n=25)
clade20C (n=1)
delta (n=13)
omicron (n=6)
neg (n=72)
NA
WHOvariant

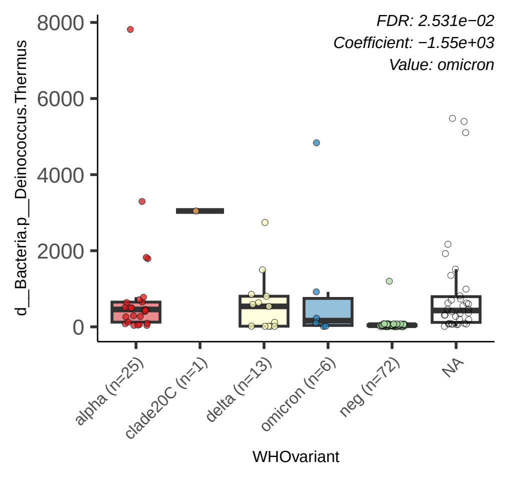
FDR: 2.531e−02
Coefficient: −1.55e+03
Value: omicron
8000
6000
4000
2000
0
d__Bacteria.p__Deinococcus.Thermus
alpha (n=25)
clade20C (n=1)
delta (n=13)
omicron (n=6)
neg (n=72)
NA
WHOvariant

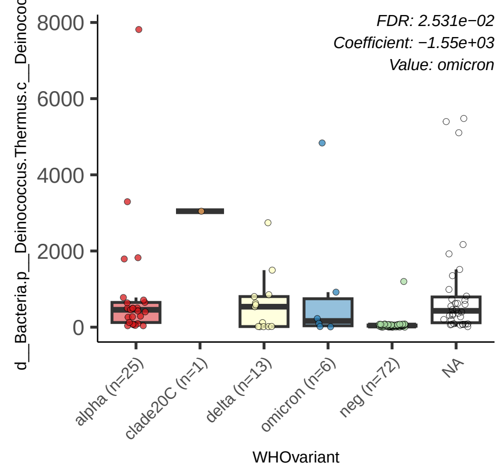

FDR: 2.531e−02
Coefficient: −1.55e+03
Value: omicron
d__Bacteria.p__Deinococcus.Thermus.c__Deinococ
8000
6000
4000
2000
0
alpha (n=25)
clade20C (n=1)
delta (n=13)
omicron (n=6)
neg (n=72)
NA
WHOvariant

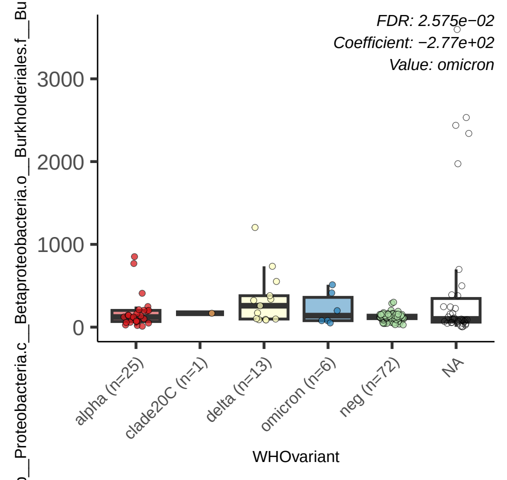

FDR: 2.575e−02
Coefficient: −2.77e+02
Value: omicron
3000
2000
1000
0
o__Proteobacteria.c__Betaproteobacteria.o__Burkholderiales.f__Bu
alpha (n=25)
clade20C (n=1)
delta (n=13)
omicron (n=6)
neg (n=72)
NA
WHOvariant

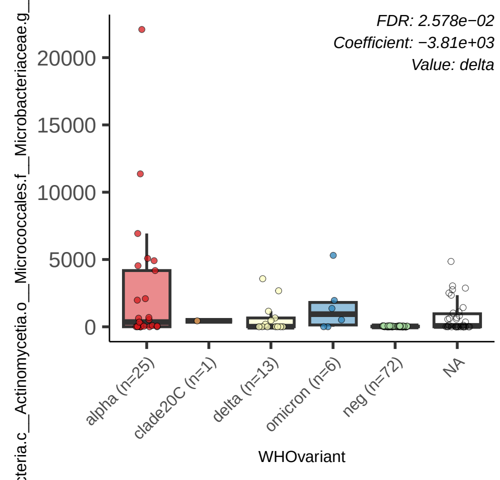

FDR: 2.578e−02
Coefficient: −3.81e+03
Value: delta
20000
15000
10000
5000
0
cteria.c__Actinomycetia.o__Micrococcales.f__Microbacteriaceae.g__
alpha (n=25)
clade20C (n=1)
delta (n=13)
omicron (n=6)
neg (n=72)
NA
WHOvariant

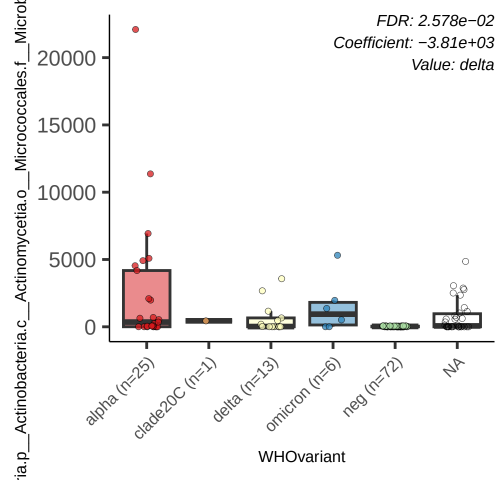
FDR: 2.578e−02
Coefficient: −3.81e+03
Value: delta
20000
15000
10000
5000
0
ria.p__Actinobacteria.c__Actinomycetia.o__Micrococcales.f__Microb
alpha (n=25)
clade20C (n=1)
delta (n=13)
omicron (n=6)
neg (n=72)
NA
WHOvariant

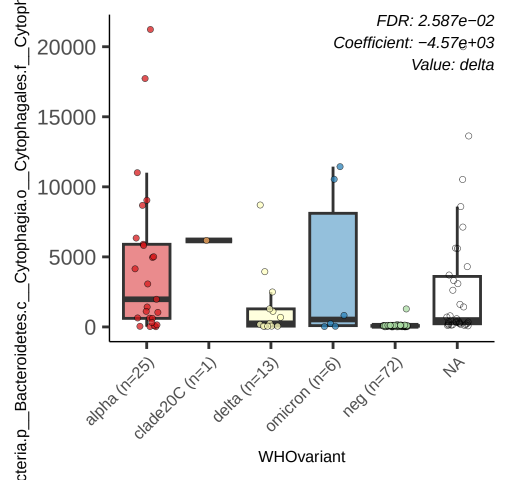

FDR: 2.587e−02
Coefficient: −4.57e+03
Value: delta
20000
15000
10000
5000
0
cteria.p__Bacteroidetes.c__Cytophagia.o__Cytophagales.f__Cytoph
alpha (n=25)
clade20C (n=1)
delta (n=13)
omicron (n=6)
neg (n=72)
NA
WHOvariant

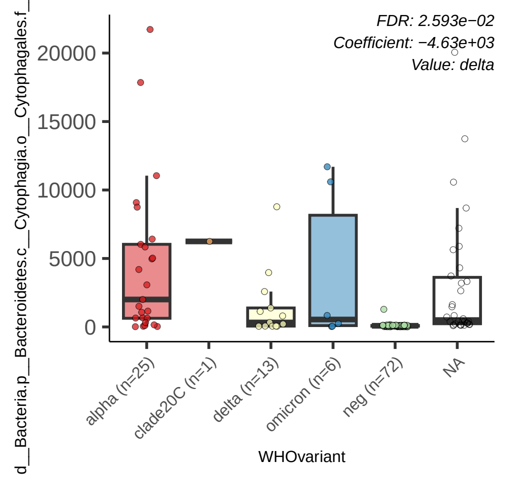

FDR: 2.593e−02
Coefficient: −4.63e+03
Value: delta
d__Bacteria.p__Bacteroidetes.c__Cytophagia.o__Cytophagales.f__
20000
15000
10000
5000
0
alpha (n=25)
clade20C (n=1)
delta (n=13)
omicron (n=6)
neg (n=72)
NA
WHOvariant

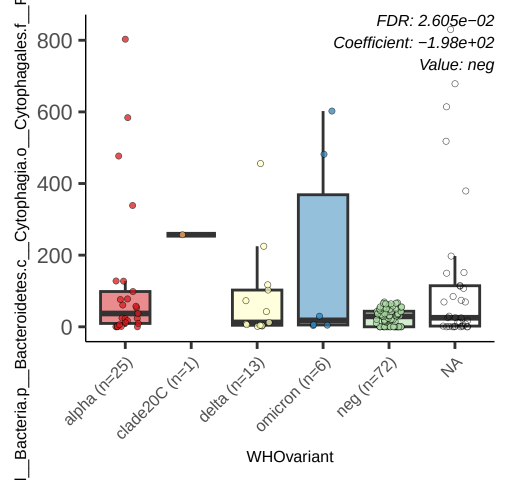

FDR: 2.605e−02
Coefficient: −1.98e+02
Value: neg
800
600
400
200
0
Bacteria.p__Bacteroidetes.c__Cytophagia.o__Cytophagales.f__
alpha (n=25)
clade20C (n=1)
delta (n=13)
omicron (n=6)
neg (n=72)
NA
WHOvariant

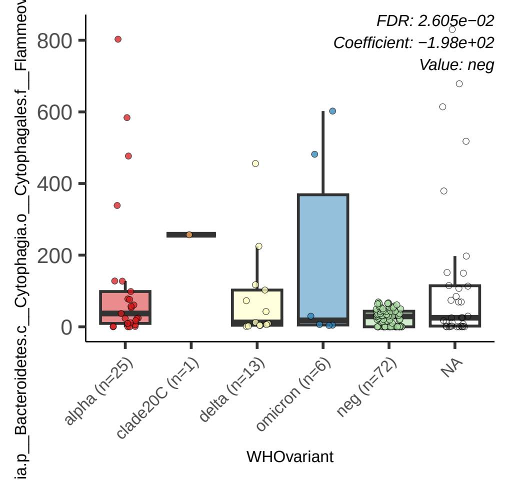

FDR: 2.605e−02
Coefficient: −1.98e+02
Value: neg
800
600
400
200
0
ia.p__Bacteroidetes.c__Cytophagia.o__Cytophagales.f__Flammeov
alpha (n=25)
clade20C (n=1)
delta (n=13)
omicron (n=6)
neg (n=72)
NA
WHOvariant

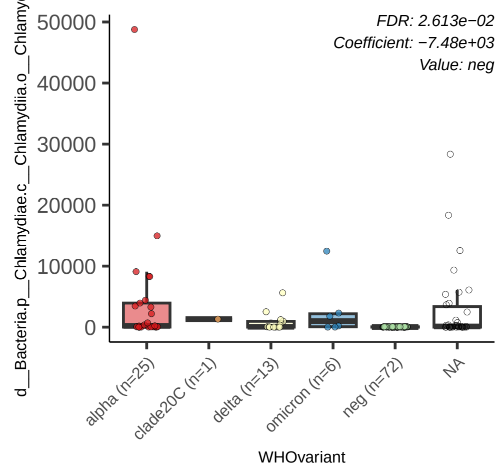

FDR: 2.613e−02
Coefficient: −7.48e+03
Value: neg
d__Bacteria.p__Chlamydiae.c__Chlamydiia.o__Chlamy
50000
40000
30000
20000
10000
0
alpha (n=25)
clade20C (n=1)
delta (n=13)
omicron (n=6)
neg (n=72)
NA
WHOvariant

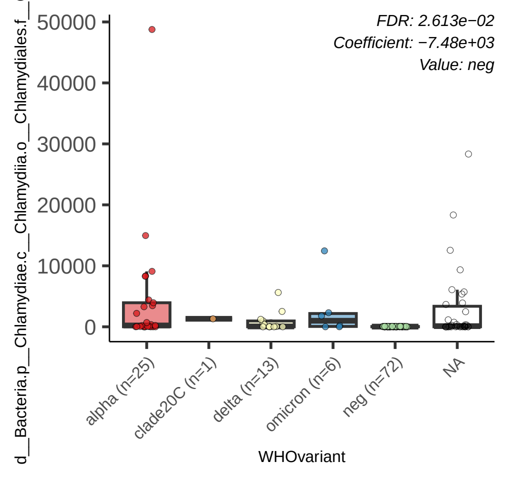
FDR: 2.613e−02
Coefficient: −7.48e+03
Value: neg
d__Bacteria.p__Chlamydiae.c__Chlamydiia.o__Chlamydiales.f__
50000
40000
30000
20000
10000
0
alpha (n=25)
clade20C (n=1)
delta (n=13)
omicron (n=6)
neg (n=72)
NA
WHOvariant

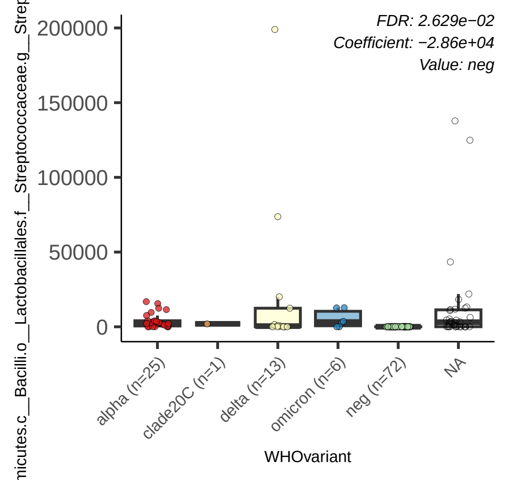

FDR: 2.629e−02
Coefficient: −2.86e+04
Value: neg
200000
150000
100000
50000
0
micutes.c__Bacilli.o__Lactobacillales.f__Streptococcaceae.g__Strep
alpha (n=25)
clade20C (n=1)
delta (n=13)
omicron (n=6)
neg (n=72)
NA
WHOvariant

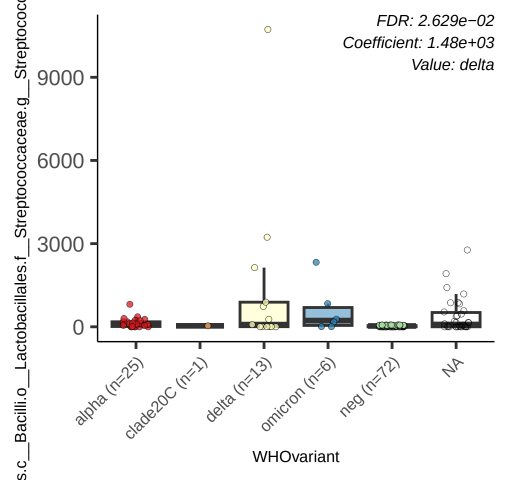

FDR: 2.629e−02
Coefficient: 1.48e+03
Value: delta
s.c__Bacilli.o__Lactobacillales.f__Streptococcaceae.g__Streptococc
9000
6000
3000
0
alpha (n=25)
clade20C (n=1)
delta (n=13)
omicron (n=6)
neg (n=72)
NA
WHOvariant

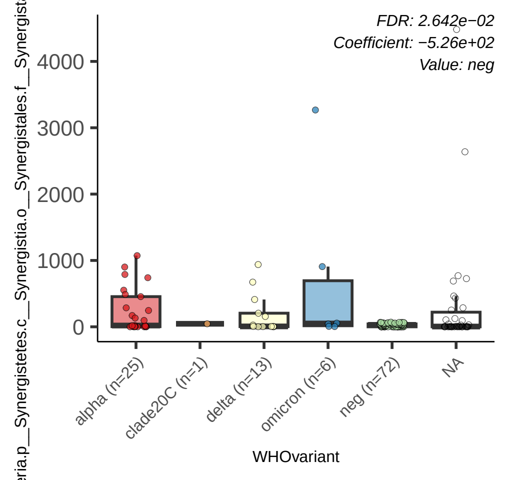

FDR: 2.642e−02
Coefficient: −5.26e+02
Value: neg
4000
3000
2000
1000
0
eria.p__Synergistetes.c__Synergistia.o__Synergistales.f__Synergist
alpha (n=25)
clade20C (n=1)
delta (n=13)
omicron (n=6)
neg (n=72)
NA
WHOvariant

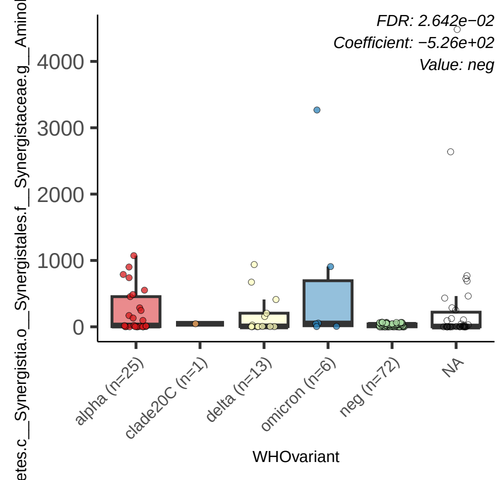

FDR: 2.642e−02
Coefficient: −5.26e+02
Value: neg
etes.c__Synergistia.o__Synergistales.f__Synergistaceae.g__Aminob
0
1000
2000
3000
4000
alpha (n=25)
clade20C (n=1)
delta (n=13)
omicron (n=6)
neg (n=72)
NA
WHOvariant

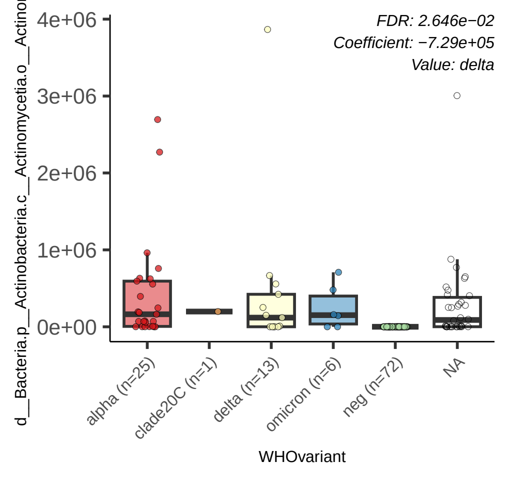
FDR: 2.646e−02
Coefficient: −7.29e+05
Value: delta
d__Bacteria.p__Actinobacteria.c__Actinomycetia.o__Actino
4e+06
3e+06
2e+06
1e+06
0e+00
alpha (n=25)
clade20C (n=1)
delta (n=13)
omicron (n=6)
neg (n=72)
NA
WHOvariant

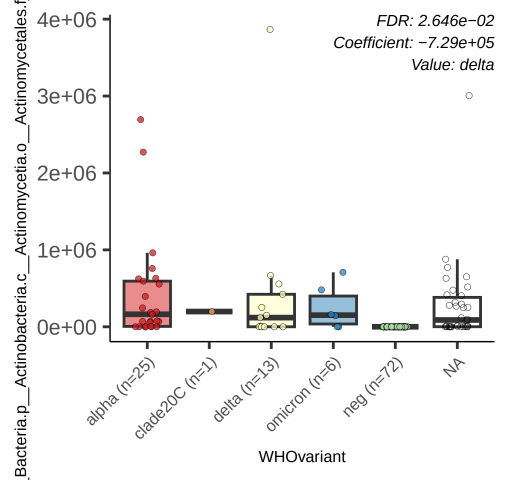
FDR: 2.646e−02
Coefficient: −7.29e+05
Value: delta
4e+06
3e+06
2e+06
1e+06
0e+00
_Bacteria.p__Actinobacteria.c__Actinomycetia.o__Actinomycetales.f
alpha (n=25)
clade20C (n=1)
delta (n=13)
omicron (n=6)
neg (n=72)
NA
WHOvariant

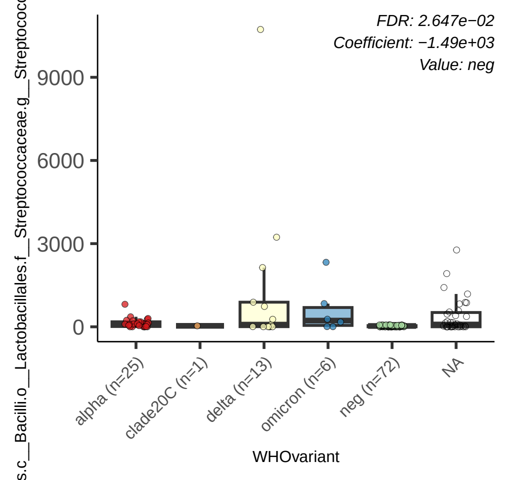

FDR: 2.647e−02
Coefficient: −1.49e+03
Value: neg
s.c__Bacilli.o__Lactobacillales.f__Streptococcaceae.g__Streptococc
9000
6000
3000
0
alpha (n=25)
clade20C (n=1)
delta (n=13)
omicron (n=6)
neg (n=72)
NA
WHOvariant

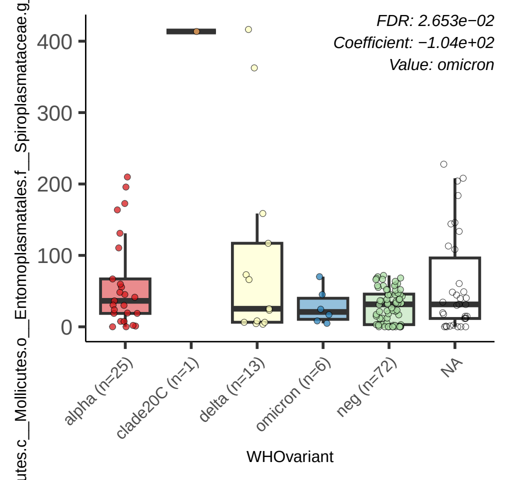
FDR: 2.653e−02
Coefficient: −1.04e+02
Value: omicron
utes.c__Mollicutes.o__Entomoplasmatales.f__Spiroplasmataceae.g_
0
100
200
300
400
alpha (n=25)
clade20C (n=1)
delta (n=13)
omicron (n=6)
neg (n=72)
NA
WHOvariant

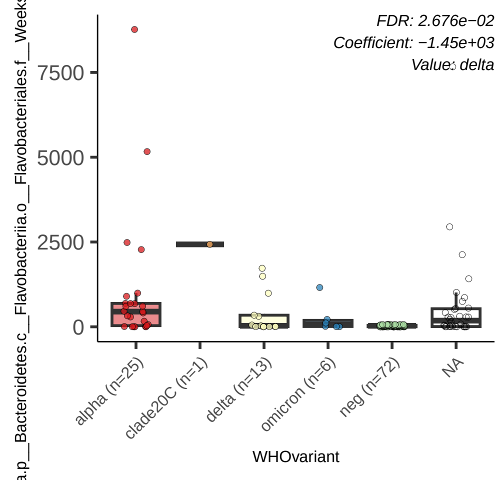

FDR: 2.676e−02
Coefficient: −1.45e+03
Value: delta
a.p__Bacteroidetes.c__Flavobacteriia.o__Flavobacteriales.f__Weeks
7500
5000
2500
0
alpha (n=25)
clade20C (n=1)
delta (n=13)
omicron (n=6)
neg (n=72)
NA
WHOvariant

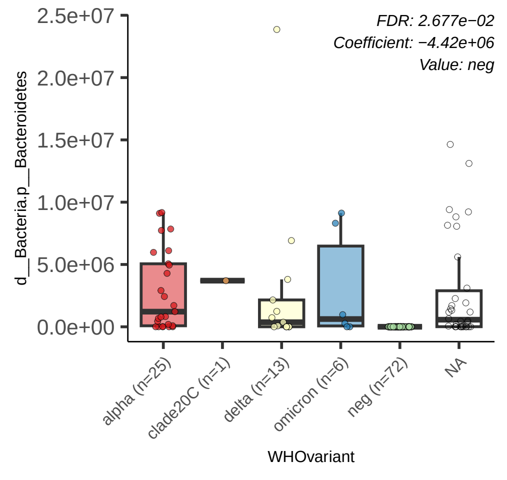
FDR: 2.677e−02
Coefficient: −4.42e+06
Value: neg
d__Bacteria.p__Bacteroidetes
2.5e+07
2.0e+07
1.5e+07
1.0e+07
5.0e+06
0.0e+00
alpha (n=25)
clade20C (n=1)
delta (n=13)
omicron (n=6)
neg (n=72)
NA
WHOvariant

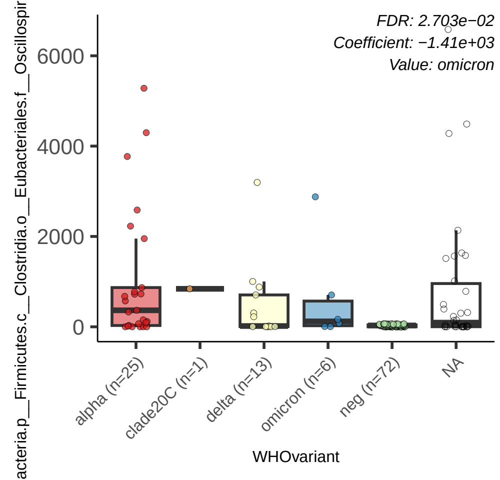

FDR: 2.703e−02
Coefficient: −1.41e+03
Value: omicron
acteria.p__Firmicutes.c__Clostridia.o__Eubacteriales.f__Oscillospir
0
2000
4000
6000
alpha (n=25)
clade20C (n=1)
delta (n=13)
omicron (n=6)
neg (n=72)
NA
WHOvariant

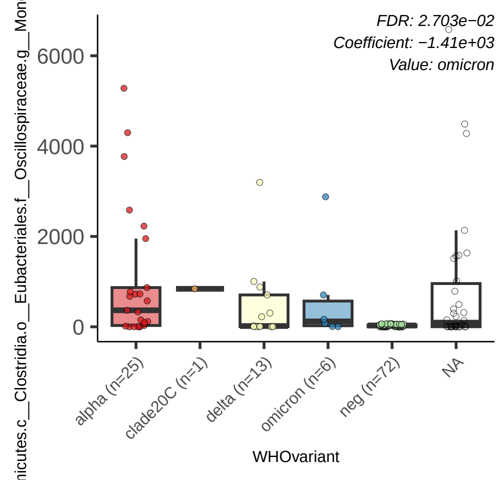
FDR: 2.703e−02
Coefficient: −1.41e+03
Value: omicron
6000
4000
2000
0
nicutes.c__Clostridia.o__Eubacteriales.f__Oscillospiraceae.g__Mon
alpha (n=25)
clade20C (n=1)
delta (n=13)
omicron (n=6)
neg (n=72)
NA
WHOvariant

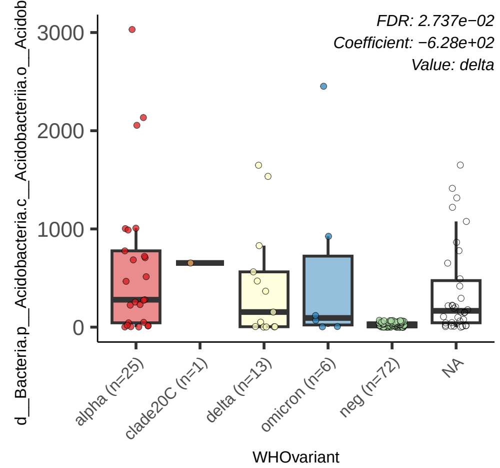

FDR: 2.737e−02
Coefficient: −6.28e+02
Value: delta
d__Bacteria.p__Acidobacteria.c__Acidobacteria.o__Acidob
3000
2000
1000
0
alpha (n=25)
clade20C (n=1)
delta (n=13)
omicron (n=6)
neg (n=72)
NA
WHOvariant

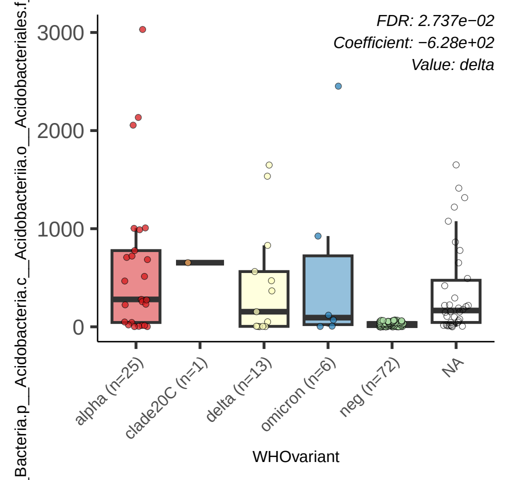

FDR: 2.737e−02
Coefficient: −6.28e+02
Value: delta
3000
2000
1000
0
Bacteria.p__Acidobacteria.c__Acidobacteriia.o__Acidobacteriales.f_
alpha (n=25)
clade20C (n=1)
delta (n=13)
omicron (n=6)
neg (n=72)
NA
WHOvariant

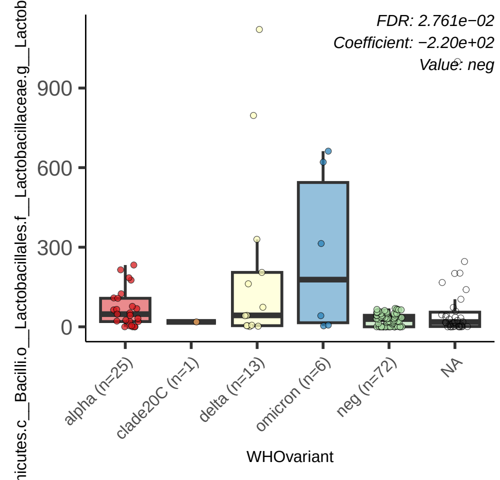

FDR: 2.761e−02
Coefficient: −2.20e+02
Value: neg
900
600
300
0
alpha (n=25)
clade20C (n=1)
delta (n=13)
omicron (n=6)
neg (n=72)
NA
WHOvariant

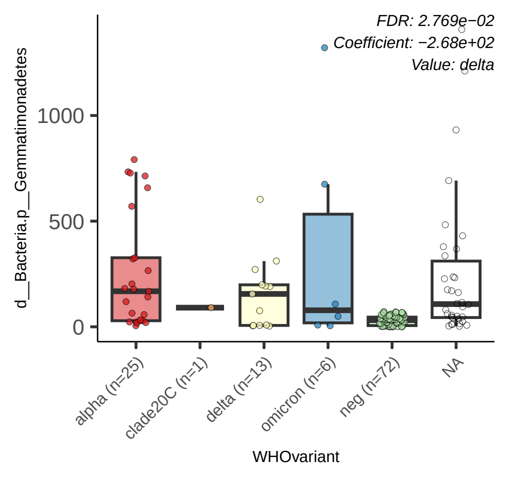

FDR: 2.769e−02
Coefficient: −2.68e+02
Value: delta
d__Bacteria.p__Gemmatimonadetes
1000
500
0
alpha (n=25)
clade20C (n=1)
delta (n=13)
omicron (n=6)
neg (n=72)
NA
WHOvariant

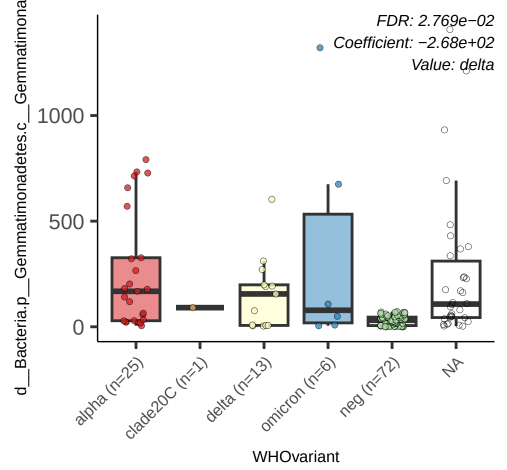

FDR: 2.769e−02
Coefficient: −2.68e+02
Value: delta
d__Bacteria.p__Gemmatimonadetes.c__Gemmatimona
1000
500
0
alpha (n=25)
clade20C (n=1)
delta (n=13)
omicron (n=6)
neg (n=72)
NA
WHOvariant

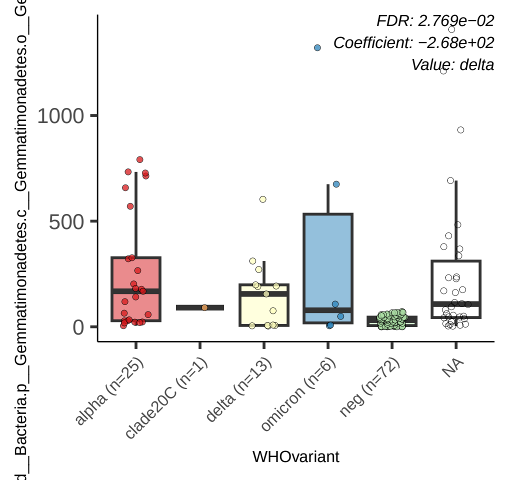

FDR: 2.769e−02
Coefficient: −2.68e+02
Value: delta
d__Bacteria.p__Gemmatimonadetes.c__Gemmatimonadetes.o__Ge
1000
500
0
alpha (n=25)
clade20C (n=1)
delta (n=13)
omicron (n=6)
neg (n=72)
NA
WHOvariant

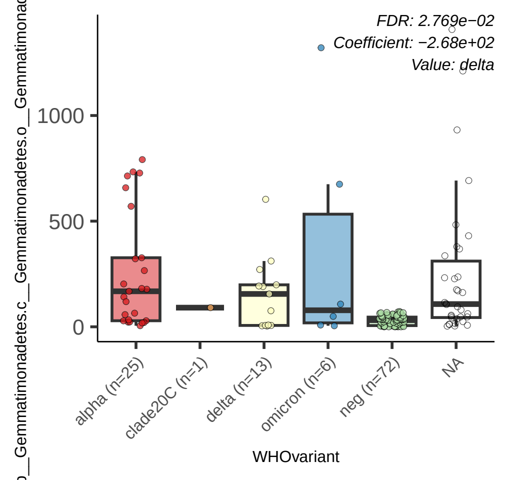

FDR: 2.769e−02
Coefficient: −2.68e+02
Value: delta
0
500
1000
alpha (n=25)
clade20C (n=1)
delta (n=13)
omicron (n=6)
neg (n=72)
NA
WHOvariant
o__Gemmatimonadetes.c__Gemmatimonadetes.o__Gemmatimonad

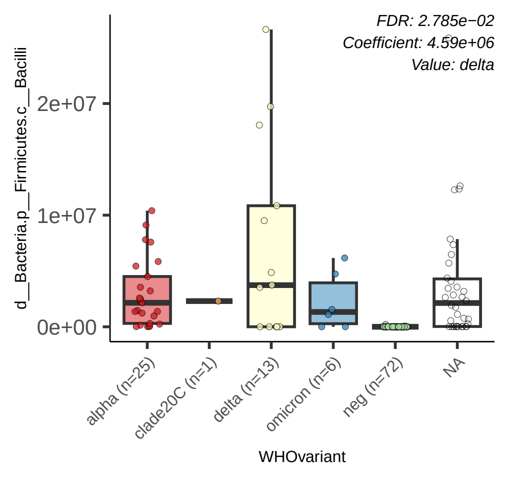
FDR: 2.785e−02
Coefficient: 4.59e+06
Value: delta
d__Bacteria.p__Firmicutes.c__Bacilli
2e+07
1e+07
0e+00
alpha (n=25)
clade20C (n=1)
delta (n=13)
omicron (n=6)
neg (n=72)
NA
WHOvariant

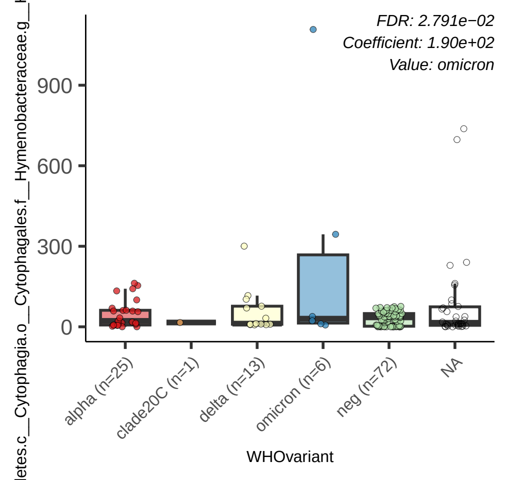

FDR: 2.791e−02
Coefficient: 1.90e+02
Value: omicron
letes.c__Cytophagia.o__Cytophagales.f__Hymenobacteraceae.g__H
900
600
300
0
alpha (n=25)
clade20C (n=1)
delta (n=13)
omicron (n=6)
neg (n=72)
NA
WHOvariant

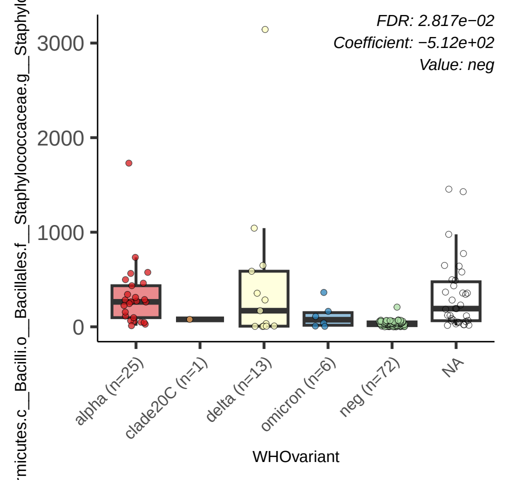
FDR: 2.817e−02
Coefficient: −5.12e+02
Value: neg
3000
2000
1000
0
alpha (n=25)
clade20C (n=1)
delta (n=13)
omicron (n=6)
neg (n=72)
NA
WHOvariant
rmicutes.c__Bacilli.o__Bacillales.f__Staphylococcaceae.g__Staphylo

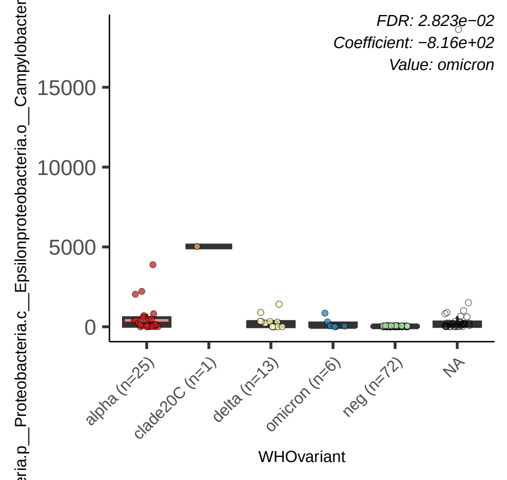

FDR: 2.823e−02
Coefficient: −8.16e+02
Value: omicron
15000
10000
5000
0
alpha (n=25)
clade20C (n=1)
delta (n=13)
omicron (n=6)
neg (n=72)
NA
WHOvariant
eria.p__Proteobacteria.c__Epsilonproteobacteria.o__Campylobacter

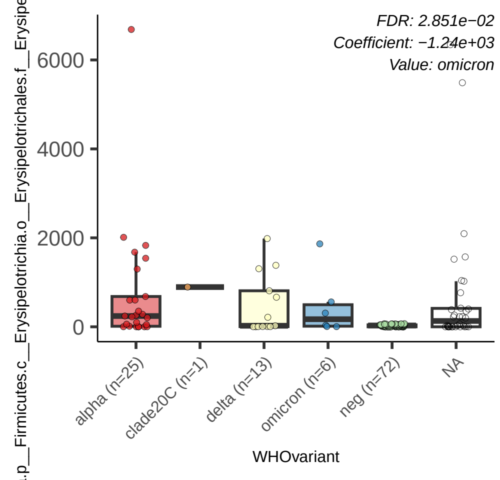

FDR: 2.851e−02
Coefficient: −1.24e+03
Value: omicron
Firmicutes.c__Erysipelotrichia.o__Erysipelotrichales.f__Erysipe
0
2000
4000
6000
alpha (n=25)
clade20C (n=1)
delta (n=13)
omicron (n=6)
neg (n=72)
NA
WHOvariant

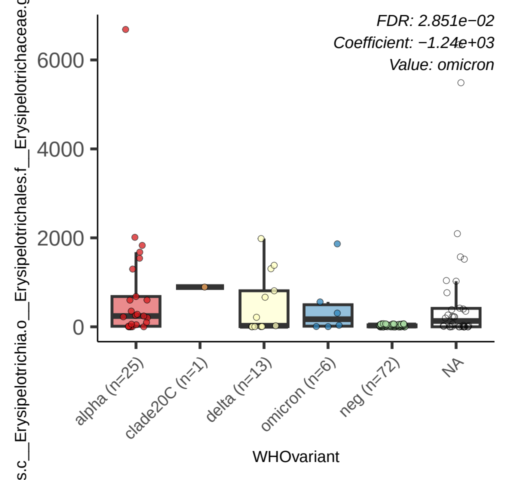

FDR: 2.851e−02
Coefficient: −1.24e+03
Value: omicron
6000
4000
2000
0
s.c__Erysipelotrichia.o__Erysipelotrichales.f__Erysipelotrichaceae.g
alpha (n=25)
clade20C (n=1)
delta (n=13)
omicron (n=6)
neg (n=72)
NA
WHOvariant

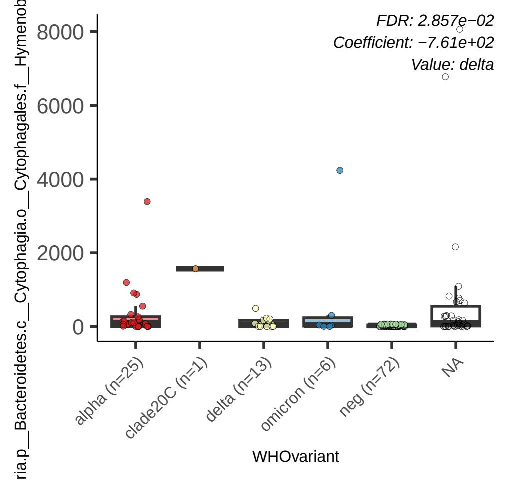
FDR: 2.857e−02
Coefficient: −7.61e+02
Value: delta
8000
6000
4000
2000
0
ria.p__Bacteroidetes.c__Cytophagia.o__Cytophagales.f__Hymenob
alpha (n=25)
clade20C (n=1)
delta (n=13)
omicron (n=6)
neg (n=72)
NA
WHOvariant

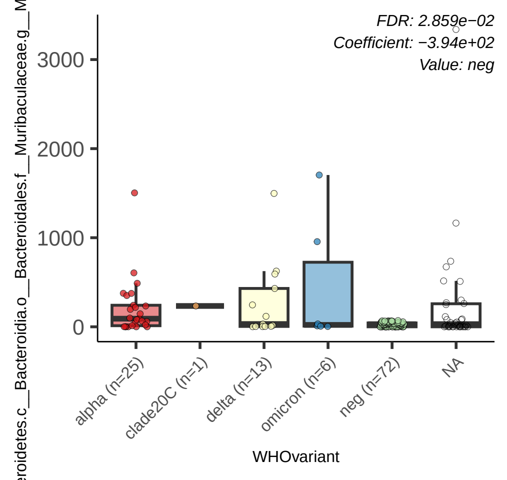
FDR: 2.859e−02
Coefficient: −3.94e+02
Value: neg
3000
2000
1000
0
alpha (n=25)
clade20C (n=1)
delta (n=13)
omicron (n=6)
neg (n=72)
NA
WHOvariant
eroidetes.c__Bacteroidia.o__Bacteroidales.f__Muribaculaceae.g__M

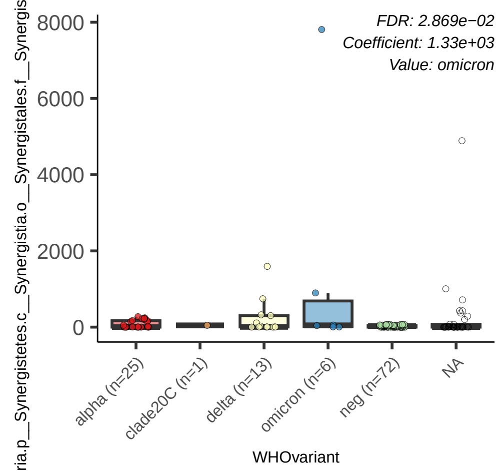

FDR: 2.869e−02
Coefficient: 1.33e+03
Value: omicron
8000
6000
4000
2000
0
alpha (n=25)
clade20C (n=1)
delta (n=13)
omicron (n=6)
neg (n=72)
NA
WHOvariant

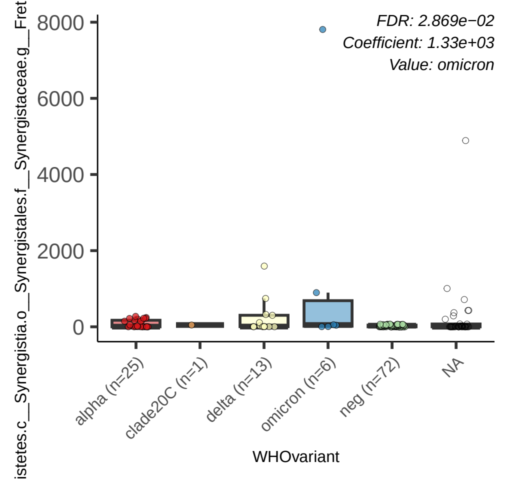

istetes.c__Synergistia.o__Synergistales.f__Synergistaceae.g__Fret
8000
6000
4000
2000
0
FDR: 2.869e−02
Coefficient: 1.33e+03
Value: omicron
alpha (n=25)
clade20C (n=1)
delta (n=13)
omicron (n=6)
neg (n=72)
NA
WHOvariant

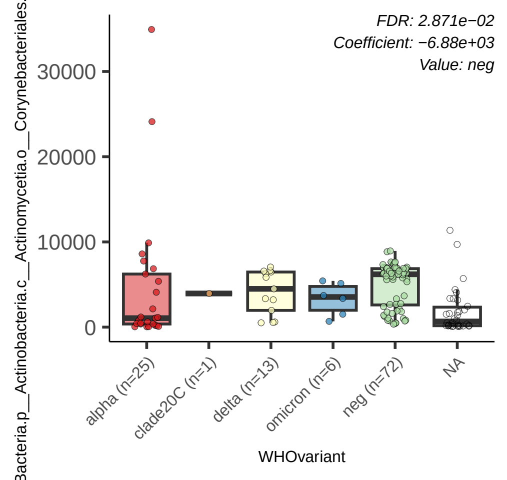
FDR: 2.871e−02
Coefficient: −6.88e+03
Value: neg
Bacteria.p__Actinobacteria.c__Actinomycetia.o__Corynebacteriales.
30000
20000
10000
0
alpha (n=25)
clade20C (n=1)
delta (n=13)
omicron (n=6)
neg (n=72)
NA
WHOvariant

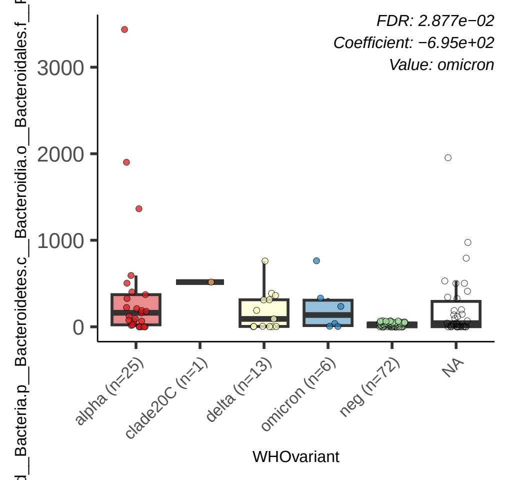
FDR: 2.877e−02
Coefficient: −6.95e+02
Value: omicron
3000
2000
1000
0
d__Bacteria.p__Bacteroidetes.c__Bacteroidia.o__Bacteroidales.f__
alpha (n=25)
clade20C (n=1)
delta (n=13)
omicron (n=6)
neg (n=72)
NA
WHOvariant

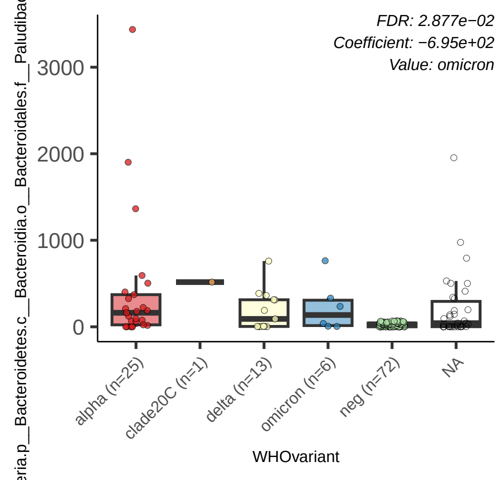

FDR: 2.877e−02
Coefficient: −6.95e+02
Value: omicron
3000
2000
1000
0
eria.p__Bacteroidetes.c__Bacteroidia.o__Bacteroidales.f__Paludibac
alpha (n=25)
clade20C (n=1)
delta (n=13)
omicron (n=6)
neg (n=72)
NA
WHOvariant

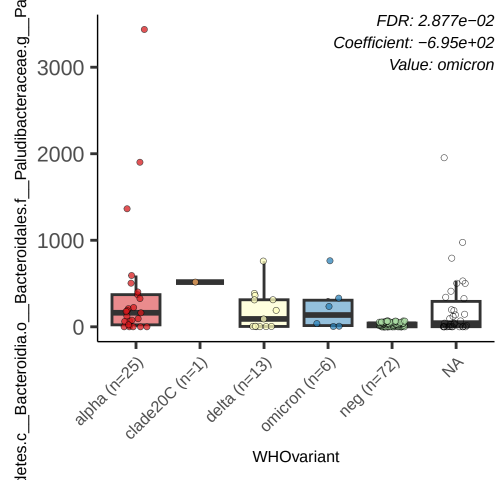

FDR: 2.877e−02
Coefficient: −6.95e+02
Value: omicron
3000
2000
1000
0
detes.c__Bacteroidia.o__Bacteroidales.f__Paludibacteraceae.g__Pa
alpha (n=25)
clade20C (n=1)
delta (n=13)
omicron (n=6)
neg (n=72)
NA
WHOvariant

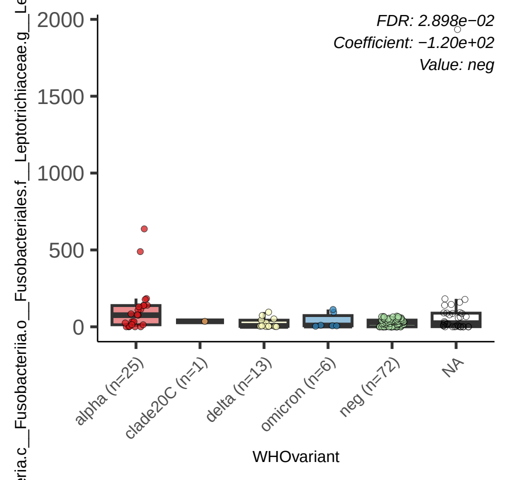

FDR: 2.898e−02
Coefficient: −1.20e+02
Value: neg
2000
1500
1000
500
0
Le
Leptotrichiaceae.g__
Fusobacteriales.f__
Fusobacteriia.o__
Fusobacteriia.c__
eria.c__
alpha (n=25)
clade20C (n=1)
delta (n=13)
omicron (n=6)
neg (n=72)
NA
WHOvariant

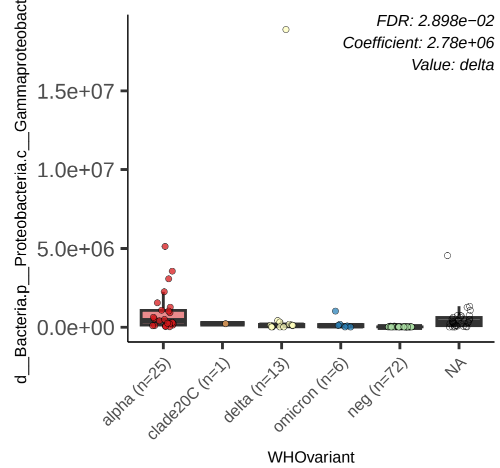

FDR: 2.898e−02
Coefficient: 2.78e+06
Value: delta
d__Bacteria.p__Proteobacteria.c__Gammaproteobact
1.5e+07
1.0e+07
5.0e+06
0.0e+00
alpha (n=25)
clade20C (n=1)
delta (n=13)
omicron (n=6)
neg (n=72)
NA
WHOvariant

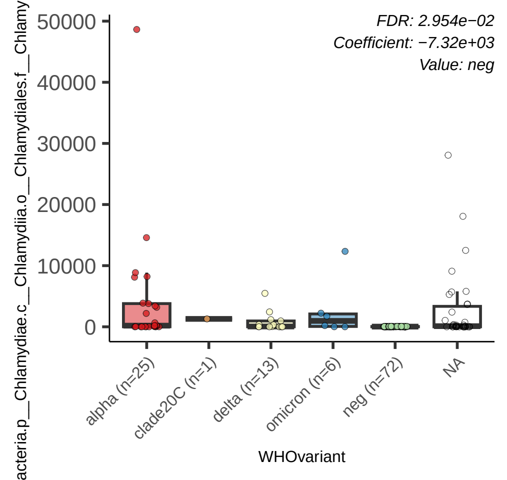

FDR: 2.954e−02
Coefficient: −7.32e+03
Value: neg
50000
40000
30000
20000
10000
0
acteria.p__Chlamydiae.c__Chlamydiia.o__Chlamydiales.f__Chlamy
alpha (n=25)
clade20C (n=1)
delta (n=13)
omicron (n=6)
neg (n=72)
NA
WHOvariant

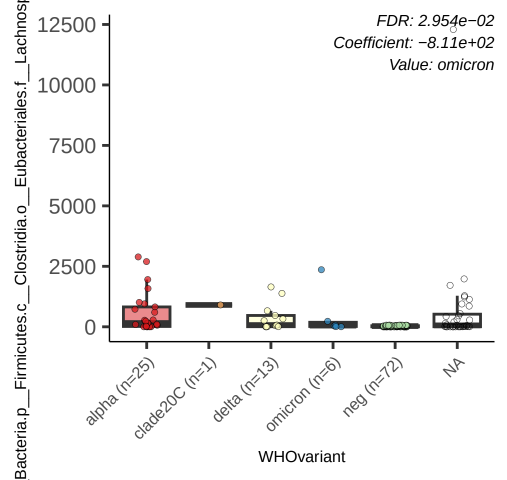

FDR: 2.954e−02
Coefficient: −8.11e+02
Value: omicron
12500
10000
7500
5000
2500
0
Bacteria.p__Firmicutes.c__Clostridia.o__Eubacteriales.f__Lachnosp
alpha (n=25)
clade20C (n=1)
delta (n=13)
omicron (n=6)
neg (n=72)
NA
WHOvariant

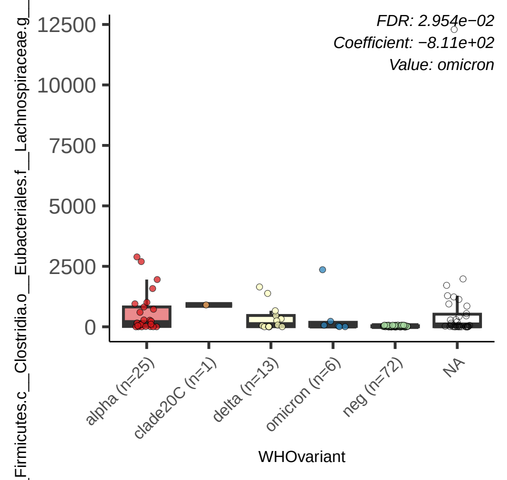

FDR: 2.954e−02
Coefficient: −8.11e+02
Value: omicron
Firmicutes.c__Clostridia.o__Eubacteriales.f__Lachnospiraceae.g__
12500
10000
7500
5000
2500
0
alpha (n=25)
clade20C (n=1)
delta (n=13)
omicron (n=6)
neg (n=72)
NA
WHOvariant

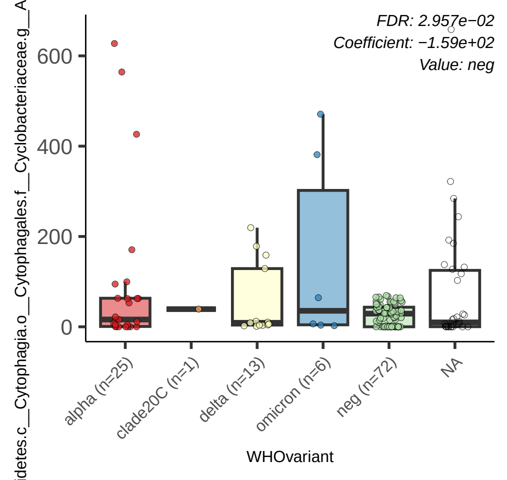

FDR: 2.957e−02
Coefficient: −1.59e+02
Value: neg
0
200
400
600
idetes.c__Cytophagia.o__Cytophagales.f__Cyclobacteriaceae.g__A
alpha (n=25)
clade20C (n=1)
delta (n=13)
omicron (n=6)
neg (n=72)
NA
WHOvariant

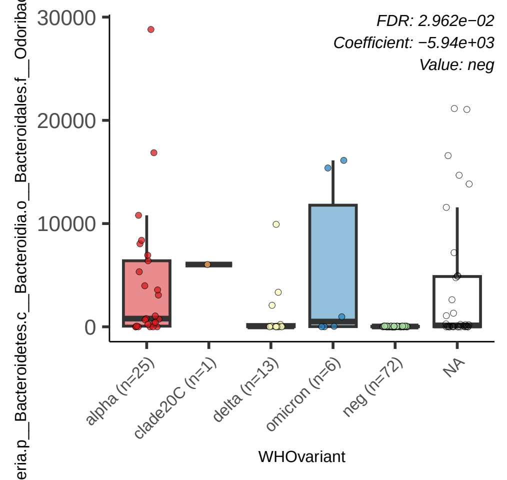

FDR: 2.962e−02
Coefficient: −5.94e+03
Value: neg
30000
20000
10000
0
eria.p__Bacteroidetes.c__Bacteroidia.o__Bacteroidales.f__Odoribac
alpha (n=25)
clade20C (n=1)
delta (n=13)
omicron (n=6)
neg (n=72)
NA
WHOvariant

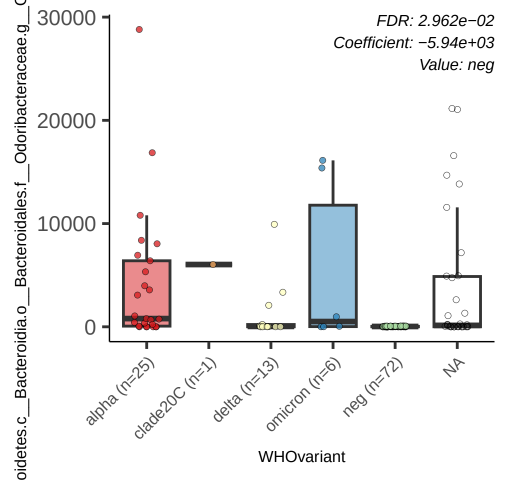

FDR: 2.962e−02
Coefficient: −5.94e+03
Value: neg
30000
20000
10000
0
oidetes.c__Bacteroidia.o__Bacteroidales.f__Odoribacteraceae.g__O
alpha (n=25)
clade20C (n=1)
delta (n=13)
omicron (n=6)
neg (n=72)
NA
WHOvariant

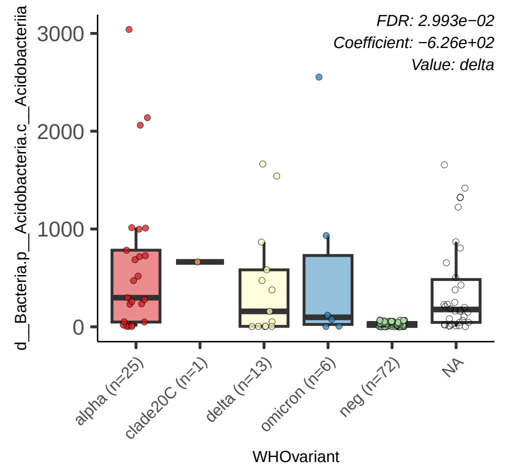

FDR: 2.993e−02
Coefficient: −6.26e+02
Value: delta
d__Bacteria.p__Acidobacteria.c__Acidobacteriia
3000
2000
1000
0
alpha (n=25)
clade20C (n=1)
delta (n=13)
omicron (n=6)
neg (n=72)
NA
WHOvariant

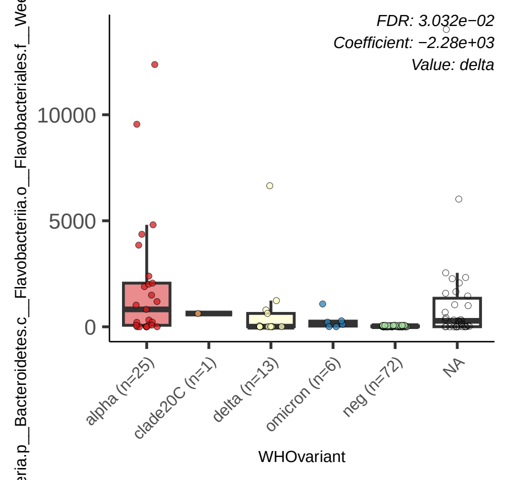

FDR: 3.032e−02
Coefficient: −2.28e+03
Value: delta
10000
5000
0
alpha (n=25)
clade20C (n=1)
delta (n=13)
omicron (n=6)
neg (n=72)
NA
WHOvariant
eria.p__Bacteroidetes.c__Flavobacteriia.o__Flavobacteriales.f__Wee

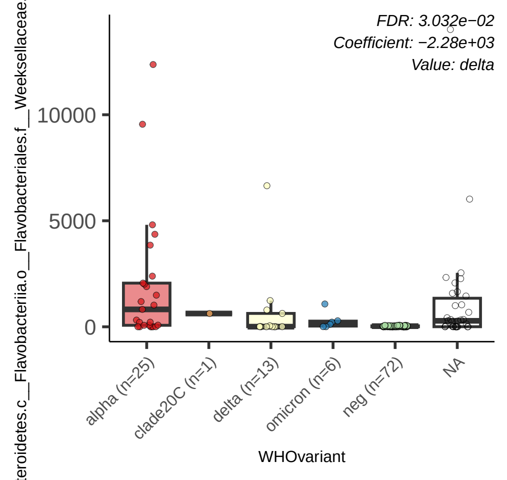

FDR: 3.032e−02
Coefficient: −2.28e+03
Value: delta
10000
5000
0
alpha (n=25)
clade20C (n=1)
delta (n=13)
omicron (n=6)
neg (n=72)
NA
WHOvariant

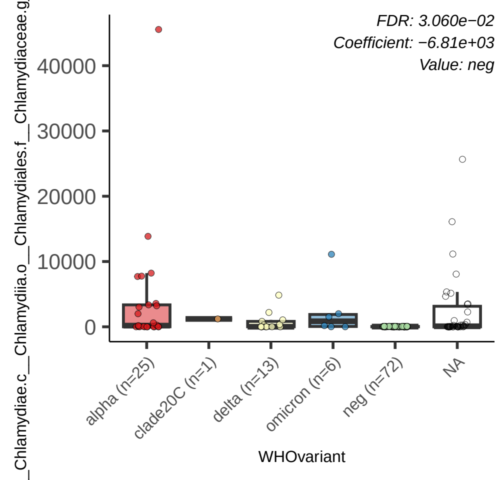
FDR: 3.060e−02
Coefficient: −6.81e+03
Value: neg
_Chlamydiae.c__Chlamydiia.o__Chlamydiales.f__Chlamydiaceae.g
40000
30000
20000
10000
0
alpha (n=25)
clade20C (n=1)
delta (n=13)
omicron (n=6)
neg (n=72)
NA
WHOvariant

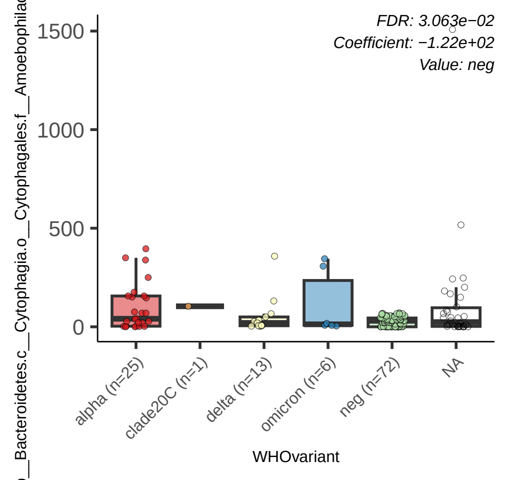
FDR: 3.063e−02
Coefficient: −1.22e+02
Value: neg
1500
1000
500
0
Bacteroidetes.c__Cytophagia.o__Cytophagales.f__Amoebophila
alpha (n=25)
clade20C (n=1)
delta (n=13)
omicron (n=6)
neg (n=72)
NA
WHOvariant

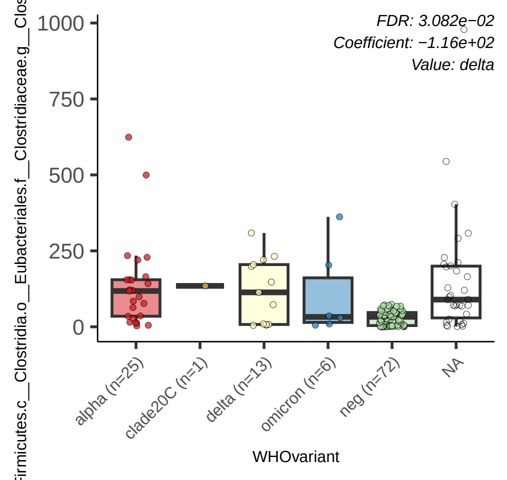
FDR: 3.082e−02
Coefficient: −1.16e+02
Value: delta
1000
750
500
250
0
alpha (n=25)
clade20C (n=1)
delta (n=13)
omicron (n=6)
neg (n=72)
NA
WHOvariant

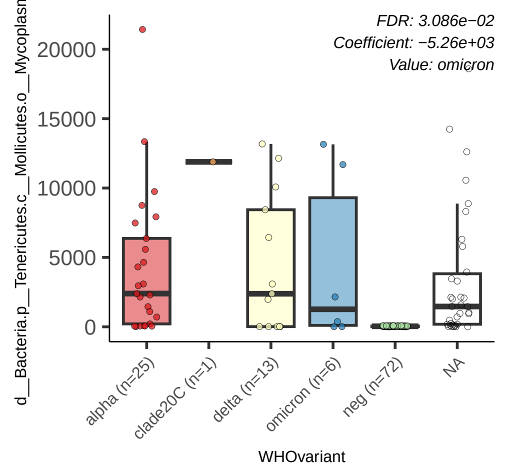
FDR: 3.086e−02
Coefficient: −5.26e+03
Value: omicron
d__Bacteria.p__Tenericutes.c__Mollicutes.o__Mycoplasm
20000
15000
10000
5000
0
alpha (n=25)
clade20C (n=1)
delta (n=13)
omicron (n=6)
neg (n=72)
NA
WHOvariant

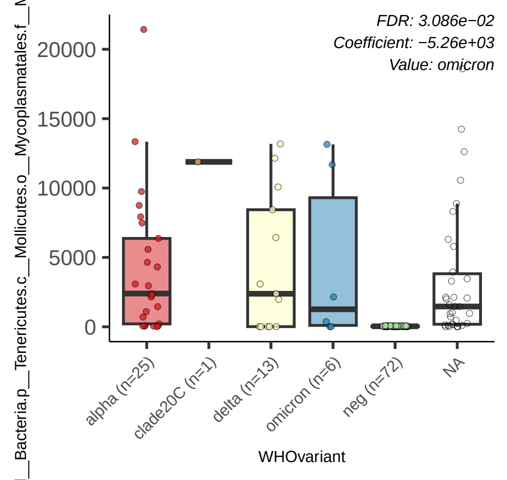

FDR: 3.086e−02
Coefficient: −5.26e+03
Value: omicron
__Bacteria.p__Tenericutes.c__Mollicutes.o__Mycoplasmatales.f__M
20000
15000
10000
5000
0
alpha (n=25)
clade20C (n=1)
delta (n=13)
omicron (n=6)
neg (n=72)
NA
WHOvariant

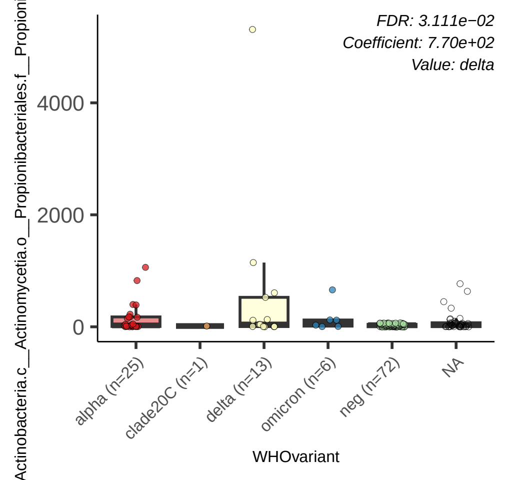
FDR: 3.111e−02
Coefficient: 7.70e+02
Value: delta
Actinobacteria.c__Actinomycetia.o__Propionibacteriales.f__Propioni
4000
2000
0
alpha (n=25)
clade20C (n=1)
delta (n=13)
omicron (n=6)
neg (n=72)
NA
WHOvariant

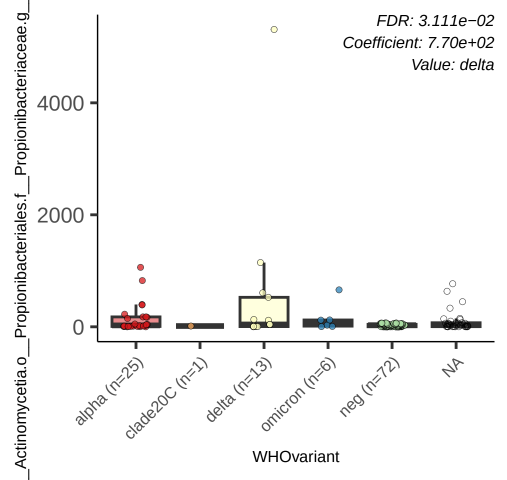

FDR: 3.111e−02
Coefficient: 7.70e+02
Value: delta
Actinomycetia.o__Propionibacteriales.f__Propionibacteriaceae.g__
4000
2000
0
alpha (n=25)
clade20C (n=1)
delta (n=13)
omicron (n=6)
neg (n=72)
NA
WHOvariant

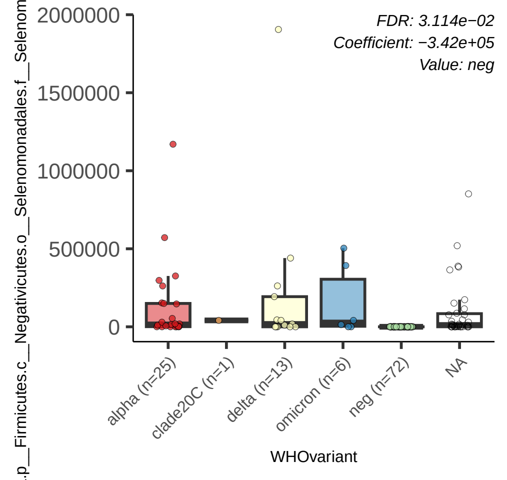
FDR: 3.114e−02
Coefficient: −3.42e+05
Value: neg
2000000
1500000
1000000
500000
0
.p__Firmicutes.c__Negativicutes.o__Selenomonadales.f__Selenom
alpha (n=25)
clade20C (n=1)
delta (n=13)
omicron (n=6)
neg (n=72)
NA
WHOvariant

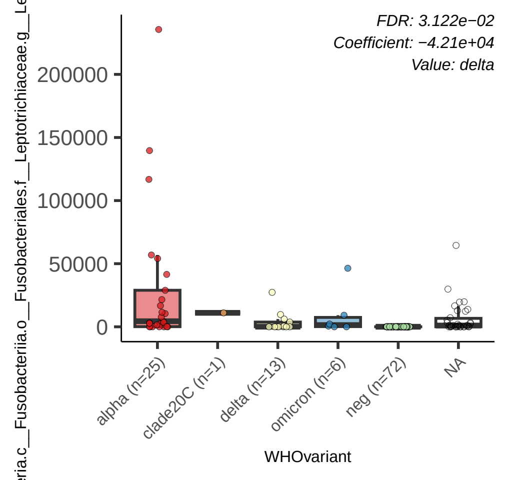

FDR: 3.122e−02
Coefficient: −4.21e+04
Value: delta
200000
150000
100000
50000
0
Fusobacteriia.o__Fusobacteriales.f__Leptotrichiaceae.g__Le
eria.c__
alpha (n=25)
clade20C (n=1)
delta (n=13)
omicron (n=6)
neg (n=72)
NA
WHOvariant

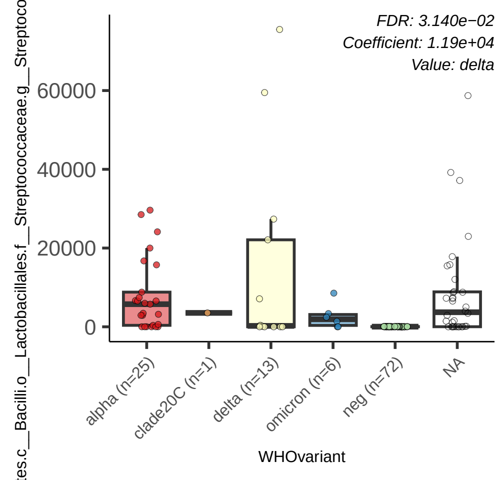

tes.c__Bacilli.o__Lactobacillales.f__Streptococcaceae.g__Streptoco
FDR: 3.140e−02
Coefficient: 1.19e+04
Value: delta
60000
40000
20000
0
alpha (n=25)
clade20C (n=1)
delta (n=13)
omicron (n=6)
neg (n=72)
NA
WHOvariant

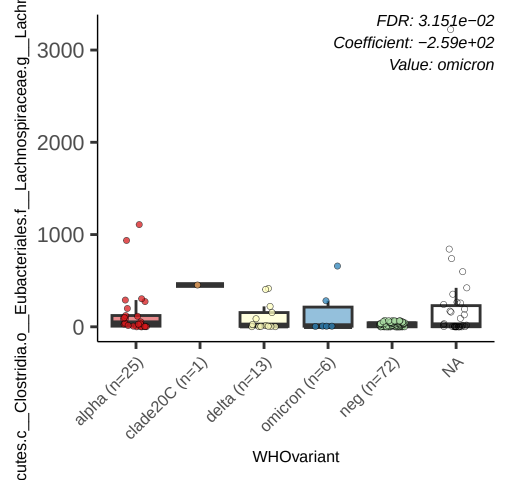

FDR: 3.151e−02
Coefficient: −2.59e+02
Value: omicron
cutes.c__Clostridia.o__Eubacteriales.f__Lachnospiraceae.g__Lachn
3000
2000
1000
0
alpha (n=25)
clade20C (n=1)
delta (n=13)
omicron (n=6)
neg (n=72)
NA
WHOvariant

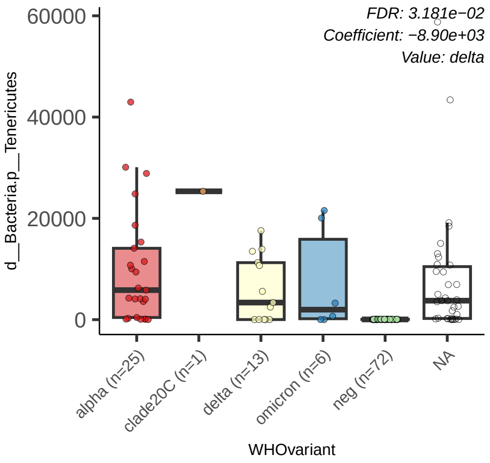
FDR: 3.181e−02
Coefficient: −8.90e+03
Value: delta
60000
40000
20000
0
d__Bacteria.p__Tenericutes
alpha (n=25)
clade20C (n=1)
delta (n=13)
omicron (n=6)
neg (n=72)
NA
WHOvariant

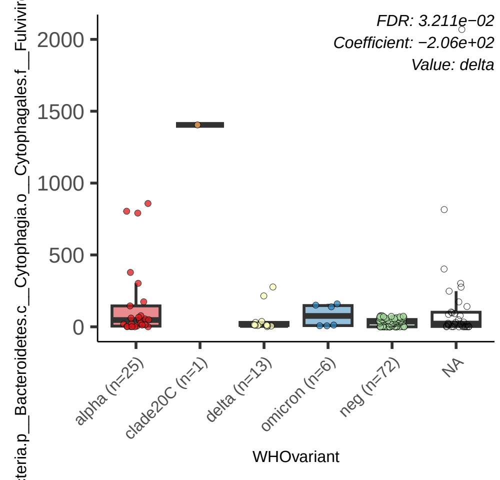

FDR: 3.211e−02
Coefficient: −2.06e+02
Value: delta
2000
1500
1000
500
0
alpha (n=25)
clade20C (n=1)
delta (n=13)
omicron (n=6)
neg (n=72)
NA
WHOvariant

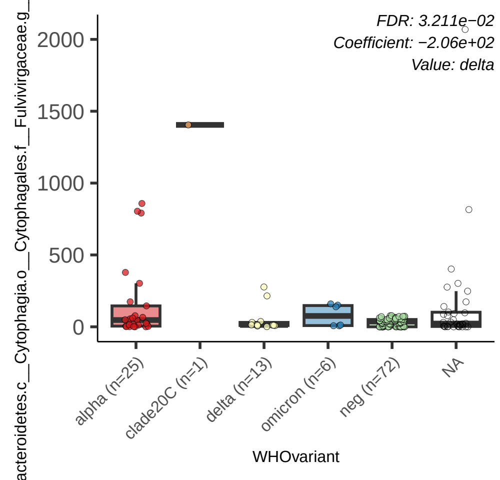

FDR: 3.211e−02
Coefficient: −2.06e+02
Value: delta
acteroidetes.c__Cytophagia.o__Cytophagales.f__Fulvivirgaceae.g__
2000
1500
1000
500
0
alpha (n=25)
clade20C (n=1)
delta (n=13)
omicron (n=6)
neg (n=72)
NA
WHOvariant

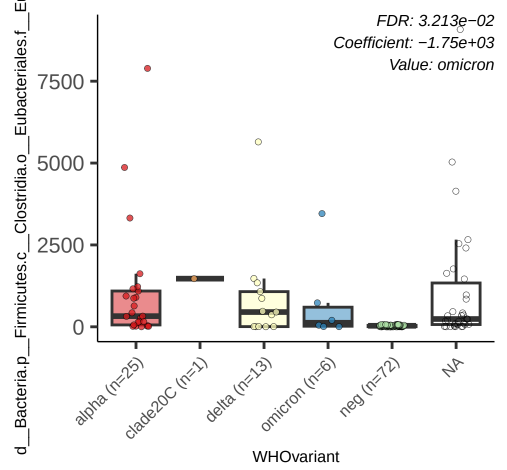

FDR: 3.213e−02
Coefficient: −1.75e+03
Value: omicron
d__Bacteria.p__Firmicutes.c__Clostridia.o__Eubacteriales.f__Eu
7500
5000
2500
0
alpha (n=25)
clade20C (n=1)
delta (n=13)
omicron (n=6)
neg (n=72)
NA
WHOvariant

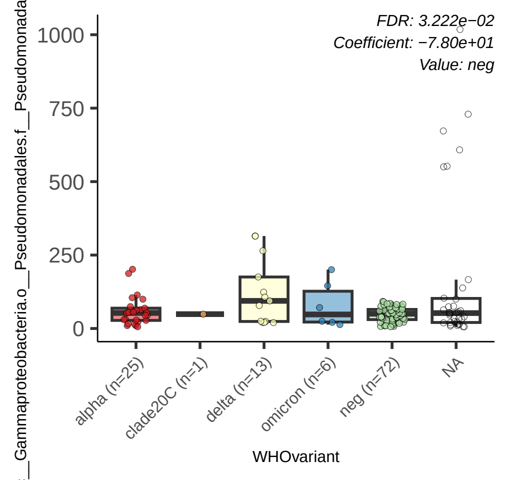

FDR: 3.222e−02
Coefficient: −7.80e+01
Value: neg
1000
750
500
250
0
Gammaproteobacteria.o__Pseudomonadales.f__Pseudomonada
alpha (n=25)
clade20C (n=1)
delta (n=13)
omicron (n=6)
neg (n=72)
NA
WHOvariant

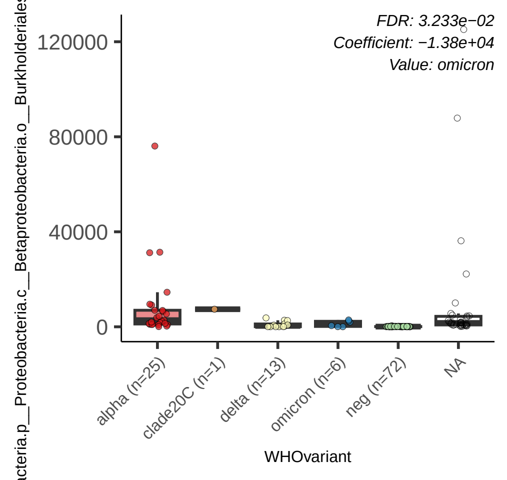

FDR: 3.233e−02
Coefficient: −1.38e+04
Value: omicron
120000
80000
40000
0
acteria.p__Proteobacteria.c__Betaproteobacteria.o__Burkholderiales
alpha (n=25)
clade20C (n=1)
delta (n=13)
omicron (n=6)
neg (n=72)
NA
WHOvariant

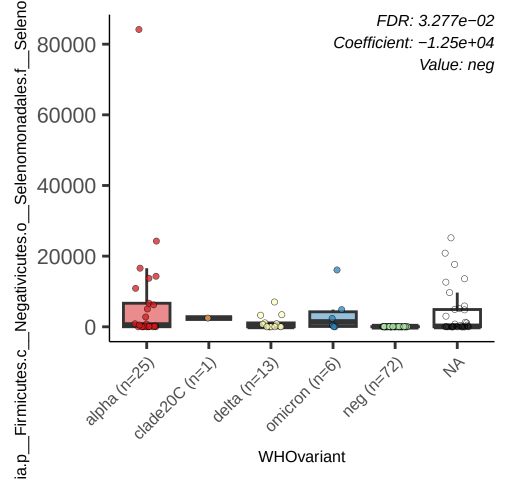

FDR: 3.277e−02
Coefficient: −1.25e+04
Value: neg
ia.p__Firmicutes.c__Negativicutes.o__Selenomonadales.f__Seleno
80000
60000
40000
20000
0
alpha (n=25)
clade20C (n=1)
delta (n=13)
omicron (n=6)
neg (n=72)
NA
WHOvariant

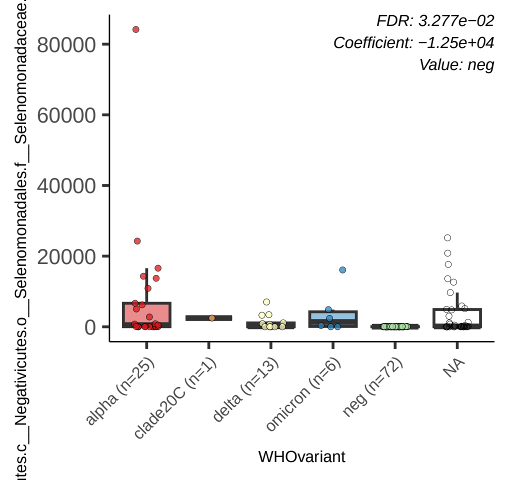

FDR: 3.277e−02
Coefficient: −1.25e+04
Value: neg
80000
60000
40000
20000
0
utes.c__Negativicutes.o__Selenomonadales.f__Selenomonadaceae.
alpha (n=25)
clade20C (n=1)
delta (n=13)
omicron (n=6)
neg (n=72)
NA
WHOvariant

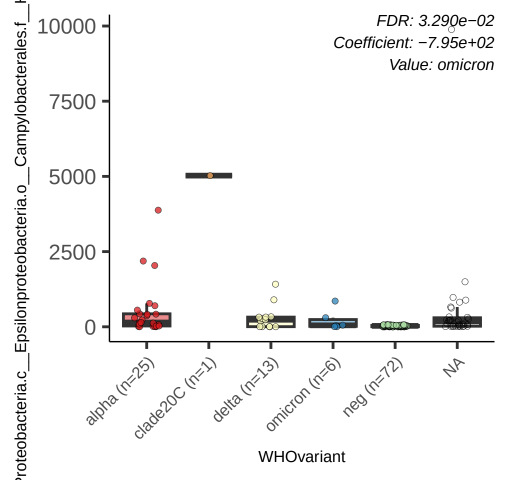
FDR: 3.290e−02
Coefficient: −7.95e+02
Value: omicron
10000
7500
5000
2500
0
Proteobacteria.c__Epsilonproteobacteria.o__Campylobacterales.f__H
alpha (n=25)
clade20C (n=1)
delta (n=13)
omicron (n=6)
neg (n=72)
NA
WHOvariant

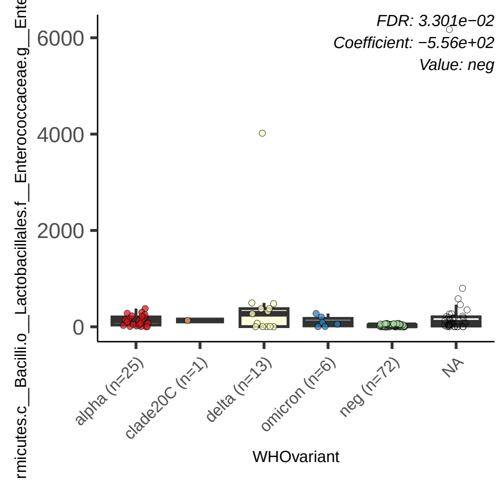
FDR: 3.301e−02
Coefficient: −5.56e+02
Value: neg
rmicutes.c__Bacilli.o__Lactobacillales.f__Enterococcaceae.g__Ente
6000
4000
2000
0
alpha (n=25)
clade20C (n=1)
delta (n=13)
omicron (n=6)
neg (n=72)
NA
WHOvariant

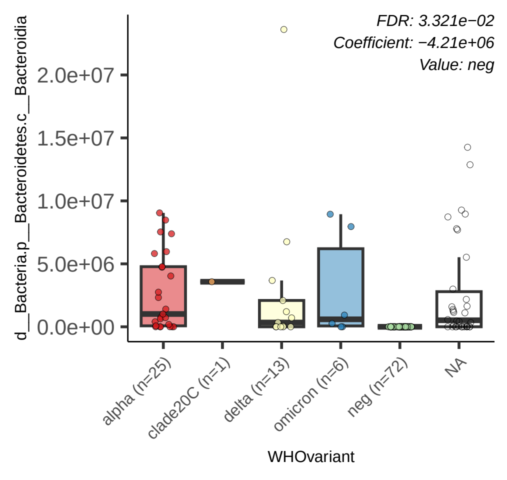

FDR: 3.321e−02
Coefficient: −4.21e+06
Value: neg
d__Bacteria.p__Bacteroidetes.c__Bacteroidia
2.0e+07
1.5e+07
1.0e+07
5.0e+06
0.0e+00
alpha (n=25)
clade20C (n=1)
delta (n=13)
omicron (n=6)
neg (n=72)
NA
WHOvariant

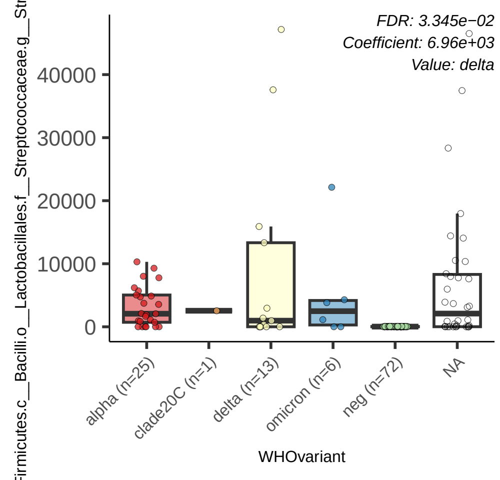

FDR: 3.345e−02
Coefficient: 6.96e+03
Value: delta
Firmicutes.c__Bacilli.o__Lactobacillales.f__Streptococcaceae.g__Str
40000
30000
20000
10000
0
alpha (n=25)
clade20C (n=1)
delta (n=13)
omicron (n=6)
neg (n=72)
NA
WHOvariant

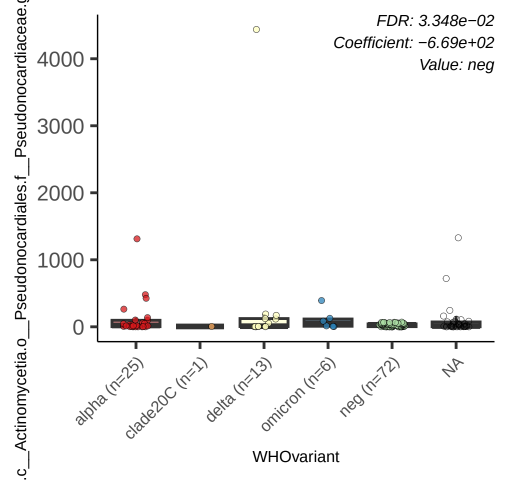

FDR: 3.348e−02
Coefficient: −6.69e+02
Value: neg
4000
3000
2000
1000
0
alpha (n=25)
clade20C (n=1)
delta (n=13)
omicron (n=6)
neg (n=72)
NA
WHOvariant
c__Actinomycetia.o__Pseudonocardiales.f__Pseudonocardiaceae.g

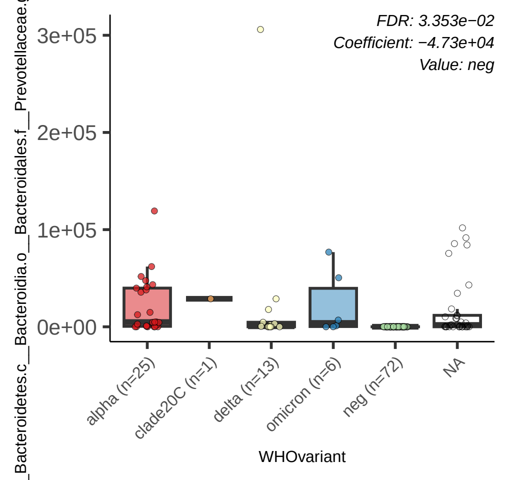
FDR: 3.353e−02
Coefficient: −4.73e+04
Value: neg
_Bacteroidetes.c__Bacteroidia.o__Bacteroidales.f__Prevotellaceae.g
3e+05
2e+05
1e+05
0e+00
alpha (n=25)
clade20C (n=1)
delta (n=13)
omicron (n=6)
neg (n=72)
NA
WHOvariant

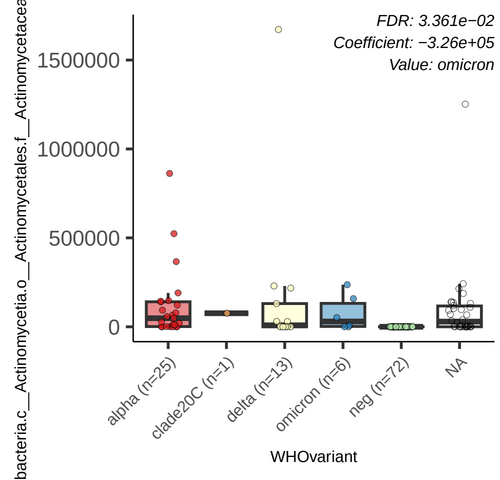

FDR: 3.361e−02
Coefficient: −3.26e+05
Value: omicron
bacteria.c__Actinomycetia.o__Actinomycetales.f__Actinomycetacea
1500000
1000000
500000
0
alpha (n=25)
clade20C (n=1)
delta (n=13)
omicron (n=6)
neg (n=72)
NA
WHOvariant

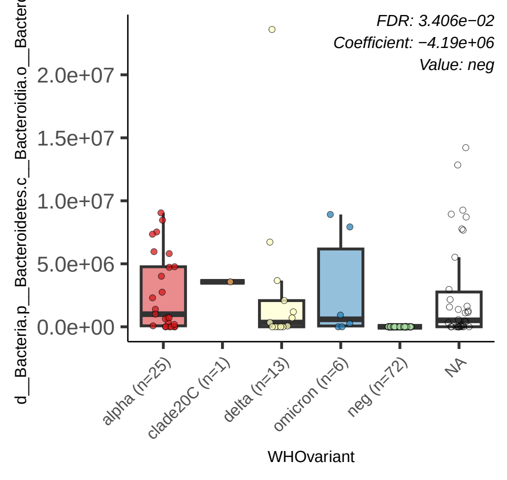

FDR: 3.406e−02
Coefficient: −4.19e+06
Value: neg
d__Bacteria.p__Bacteroidetes.c__Bacteroidia.o__Bacter
2.0e+07
1.5e+07
1.0e+07
5.0e+06
0.0e+00
alpha (n=25)
clade20C (n=1)
delta (n=13)
omicron (n=6)
neg (n=72)
NA
WHOvariant

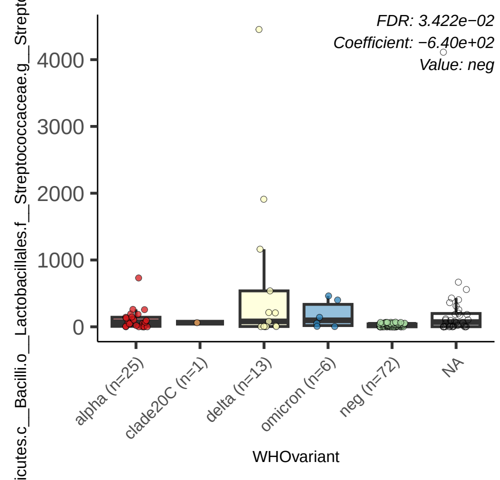
FDR: 3.422e−02
Coefficient: −6.40e+02
Value: neg
icutes.c__Bacilli.o__Lactobacillales.f__Streptococcaceae.g__Strept
4000
3000
2000
1000
0
alpha (n=25)
clade20C (n=1)
delta (n=13)
omicron (n=6)
neg (n=72)
NA
WHOvariant

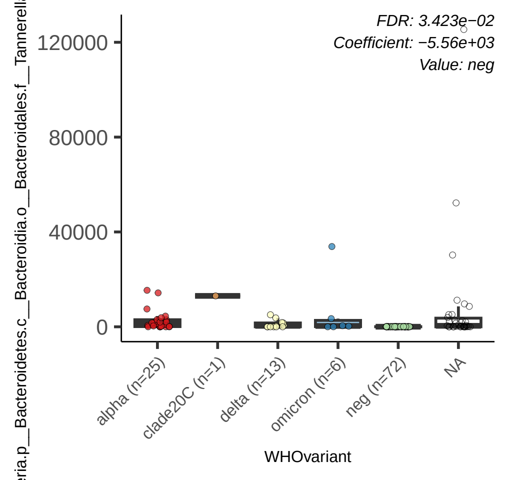

FDR: 3.423e−02
Coefficient: −5.56e+03
Value: neg
120000
80000
40000
0
eria.p__Bacteroidetes.c__Bacteroidia.o__Bacteroidales.f__Tannerella
alpha (n=25)
clade20C (n=1)
delta (n=13)
omicron (n=6)
neg (n=72)
NA
WHOvariant

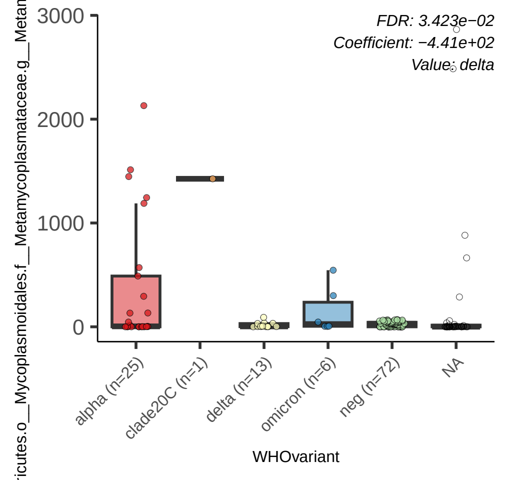

FDR: 3.423e−02
Coefficient: −4.41e+02
Value: delta
3000
2000
1000
0
ricutes.o__Mycoplasmoidales.f__Metamycoplasmataceae.g__Metan
alpha (n=25)
clade20C (n=1)
delta (n=13)
omicron (n=6)
neg (n=72)
NA
WHOvariant

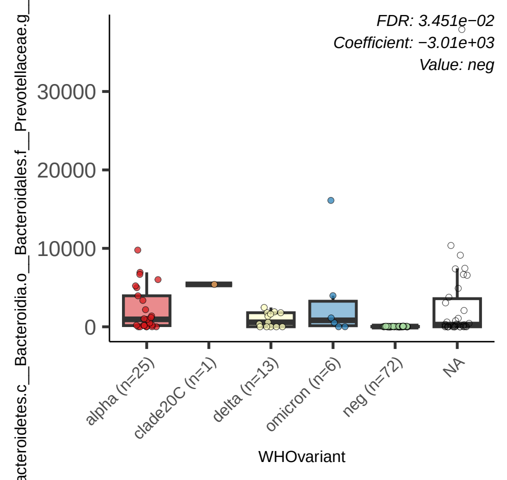

FDR: 3.451e−02
Coefficient: −3.01e+03
Value: neg
acteroidetes.c__Bacteroidia.o__Bacteroidales.f__Prevotellaceae.g__
30000
20000
10000
0
alpha (n=25)
clade20C (n=1)
delta (n=13)
omicron (n=6)
neg (n=72)
NA
WHOvariant

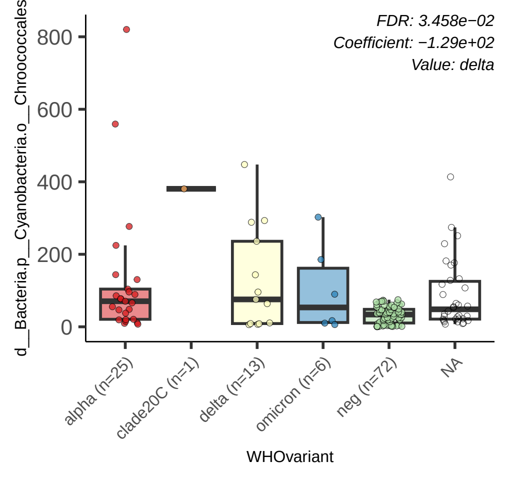
FDR: 3.458e−02
Coefficient: −1.29e+02
Value: delta
d__Bacteria.p__Cyanobacteria.o__Chroococcales
800
600
400
200
0
alpha (n=25)
clade20C (n=1)
delta (n=13)
omicron (n=6)
neg (n=72)
NA
WHOvariant

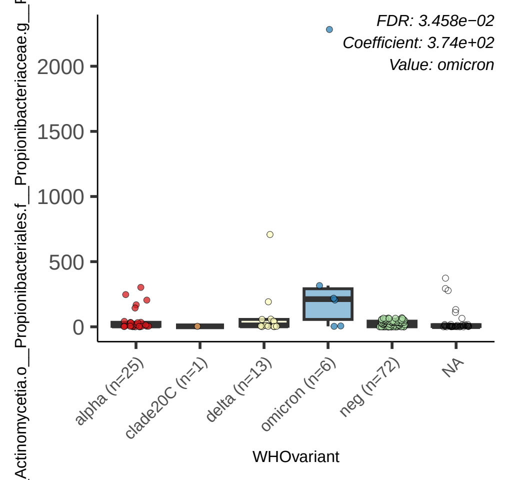

FDR: 3.458e−02
Coefficient: 3.74e+02
Value: omicron
Actinomycetia.o__Propionibacteriales.f__Propionibacteriaceae.g__P
2000
1500
1000
500
0
alpha (n=25)
clade20C (n=1)
delta (n=13)
omicron (n=6)
neg (n=72)
NA
WHOvariant

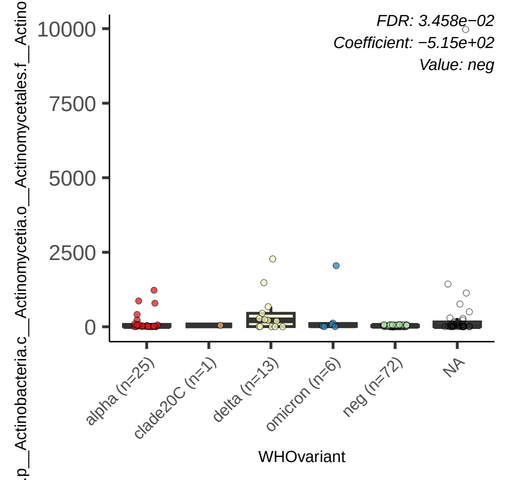
FDR: 3.458e−02
Coefficient: −5.15e+02
Value: neg
10000
7500
5000
2500
0
.p__Actinobacteria.c__Actinomycetia.o__Actinomycetales.f__Actino
alpha (n=25)
clade20C (n=1)
delta (n=13)
omicron (n=6)
neg (n=72)
NA
WHOvariant

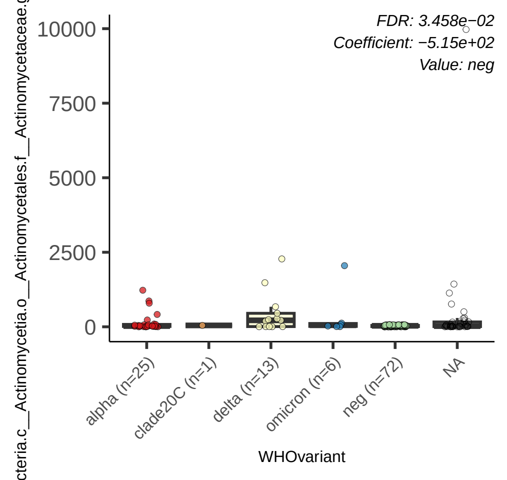

FDR: 3.458e−02
Coefficient: −5.15e+02
Value: neg
10000
7500
5000
2500
0
cteria.c__Actinomycetia.o__Actinomycetales.f__Actinomycetaceae.g
alpha (n=25)
clade20C (n=1)
delta (n=13)
omicron (n=6)
neg (n=72)
NA
WHOvariant

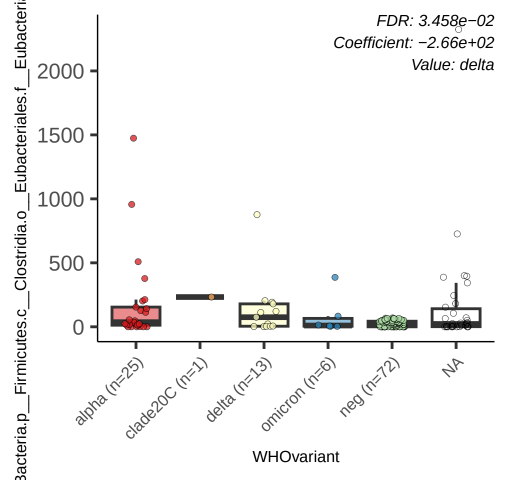

FDR: 3.458e−02
Coefficient: −2.66e+02
Value: delta
Bacteria.p__Firmicutes.c__Clostridia.o__Eubacteriales.f__Eubacteri
2000
1500
1000
500
0
alpha (n=25)
clade20C (n=1)
delta (n=13)
omicron (n=6)
neg (n=72)
NA
WHOvariant

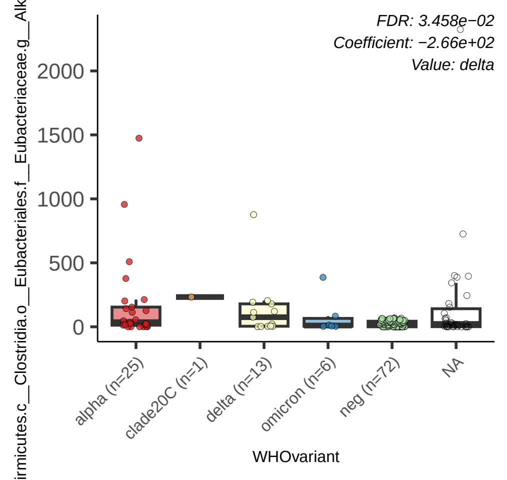

FDR: 3.458e−02
Coefficient: −2.66e+02
Value: delta
2000
1500
1000
500
0
irmicutes.c__Clostridia.o__Eubacteriales.f__Eubacteriaceae.g__Alk
alpha (n=25)
clade20C (n=1)
delta (n=13)
omicron (n=6)
neg (n=72)
NA
WHOvariant

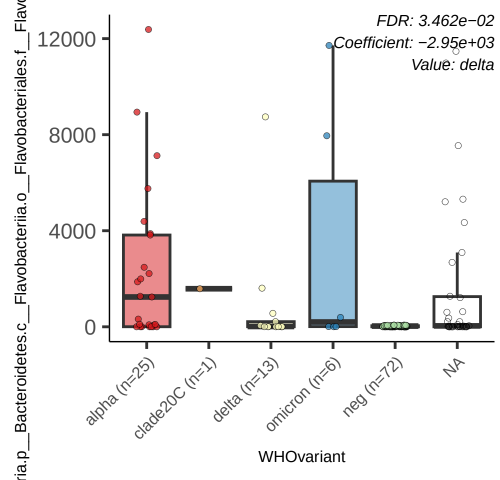

FDR: 3.462e−02
Coefficient: −2.95e+03
Value: delta
12000
8000
4000
0
ria.p__Bacteroidetes.c__Flavobacteriia.o__Flavobacteriales.f__Flavo
alpha (n=25)
clade20C (n=1)
delta (n=13)
omicron (n=6)
neg (n=72)
NA
WHOvariant

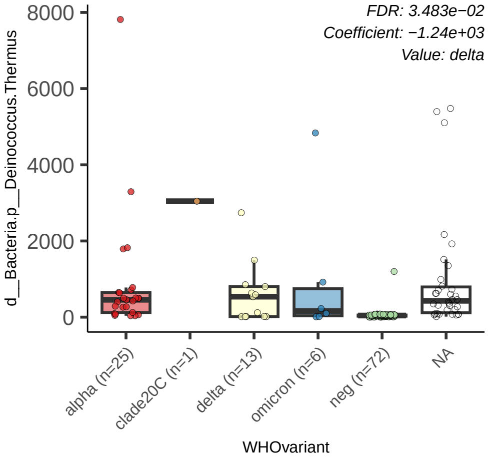
FDR: 3.483e−02
Coefficient: −1.24e+03
Value: delta
8000
6000
4000
2000
0
d__Bacteria.p__Deinococcus.Thermus
alpha (n=25)
clade20C (n=1)
delta (n=13)
omicron (n=6)
neg (n=72)
NA
WHOvariant

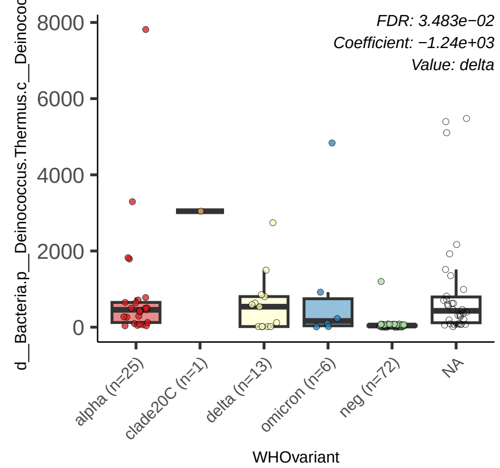

FDR: 3.483e−02
Coefficient: −1.24e+03
Value: delta
d__Bacteria.p__Deinococcus.Thermus.c__Deinococ
8000
6000
4000
2000
0
alpha (n=25)
clade20C (n=1)
delta (n=13)
omicron (n=6)
neg (n=72)
NA
WHOvariant

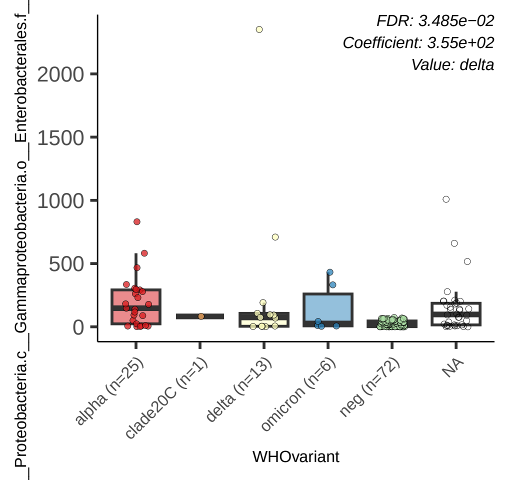

FDR: 3.485e−02
Coefficient: 3.55e+02
Value: delta
Proteobacteria.c__Gammaproteobacteria.o__Enterobacterales.f
0
500
1000
1500
2000
alpha (n=25)
clade20C (n=1)
delta (n=13)
omicron (n=6)
neg (n=72)
NA
WHOvariant

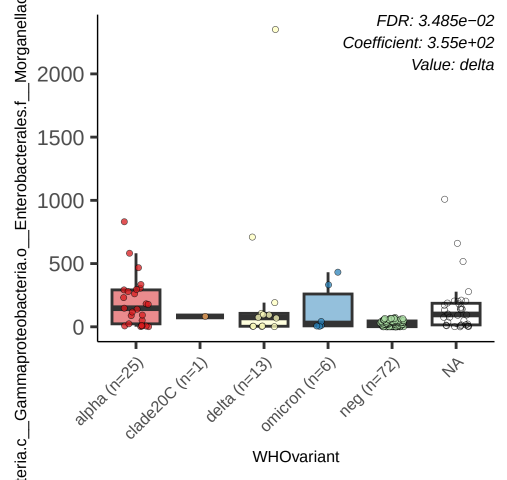

FDR: 3.485e−02
Coefficient: 3.55e+02
Value: delta
2000
1500
1000
500
0
teria.c__Gammaproteobacteria.o__Enterobacterales.f__Morganellac
alpha (n=25)
clade20C (n=1)
delta (n=13)
omicron (n=6)
neg (n=72)
NA
WHOvariant

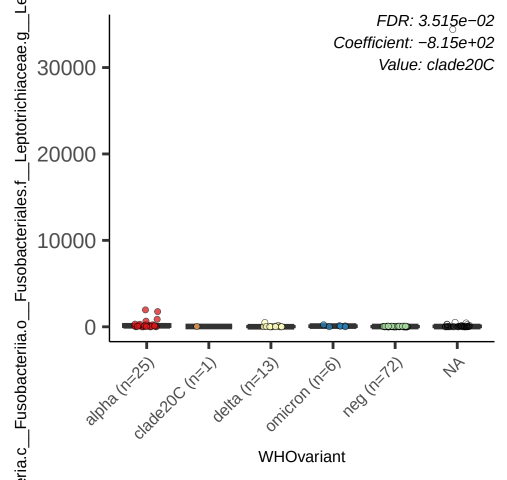

FDR: 3.515e−02
Coefficient: −8.15e+02
Value: clade20C
30000
20000
10000
0
alpha (n=25)
clade20C (n=1)
delta (n=13)
omicron (n=6)
neg (n=72)
NA
WHOvariant

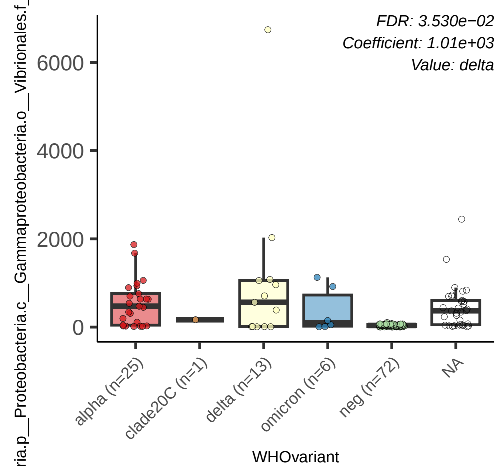
FDR: 3.530e−02
Coefficient: 1.01e+03
Value: delta
6000
4000
2000
0
teria.p__Proteobacteria.c__Gammaproteobacteria.o__Vibrionales.f_
alpha (n=25)
clade20C (n=1)
delta (n=13)
omicron (n=6)
neg (n=72)
NA
WHOvariant

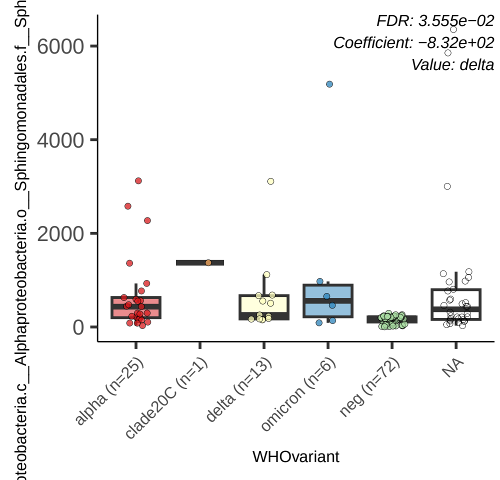
FDR: 3.555e−02
Coefficient: −8.32e+02
Value: delta
oteobacteria.c__Alphaproteobacteria.o__Sphingomonadales.f__Sph
6000
4000
2000
0
alpha (n=25)
clade20C (n=1)
delta (n=13)
omicron (n=6)
neg (n=72)
NA
WHOvariant

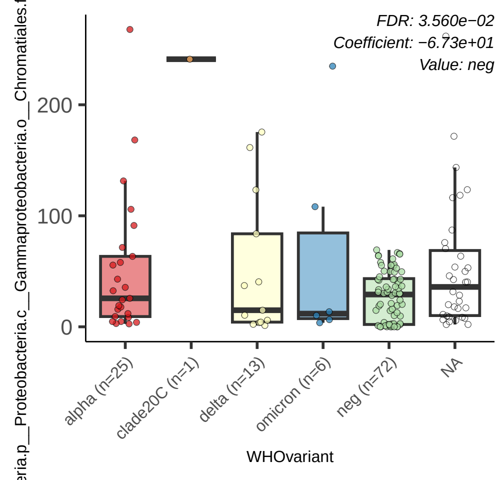

FDR: 3.560e−02
Coefficient: −6.73e+01
Value: neg
eria.p__Proteobacteria.c__Gammaproteobacteria.o__Chromatiales.f
0
100
200
alpha (n=25)
clade20C (n=1)
delta (n=13)
omicron (n=6)
neg (n=72)
NA
WHOvariant

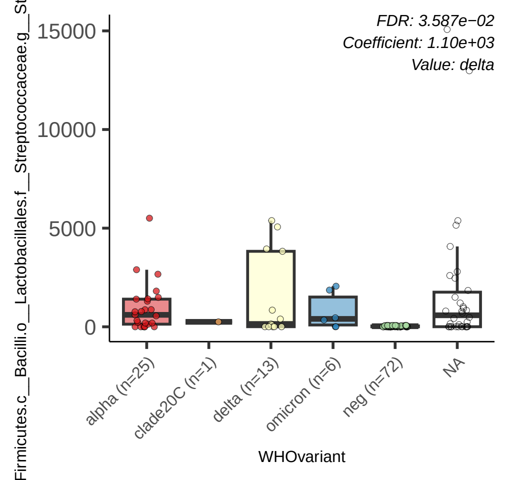

FDR: 3.587e−02
Coefficient: 1.10e+03
Value: delta
Firmicutes.c__Bacilli.o__Lactobacillales.f__Streptococcaceae.g__St
15000
10000
5000
0
alpha (n=25)
clade20C (n=1)
delta (n=13)
omicron (n=6)
neg (n=72)
NA
WHOvariant

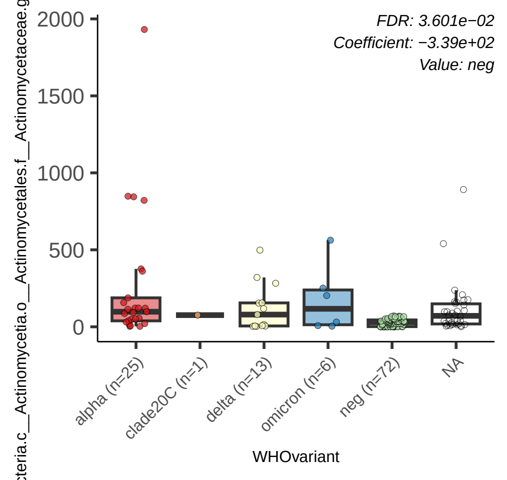

FDR: 3.601e−02
Coefficient: −3.39e+02
Value: neg
cteria.c__Actinomycetia.o__Actinomycetales.f__Actinomycetaceae.g
2000
1500
1000
500
0
alpha (n=25)
clade20C (n=1)
delta (n=13)
omicron (n=6)
neg (n=72)
NA
WHOvariant

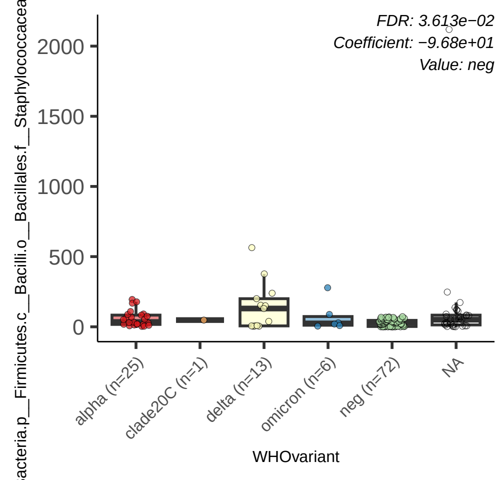

FDR: 3.613e−02
Coefficient: −9.68e+01
Value: neg
2000
1500
1000
500
0
alpha (n=25)
clade20C (n=1)
delta (n=13)
omicron (n=6)
neg (n=72)
NA
WHOvariant

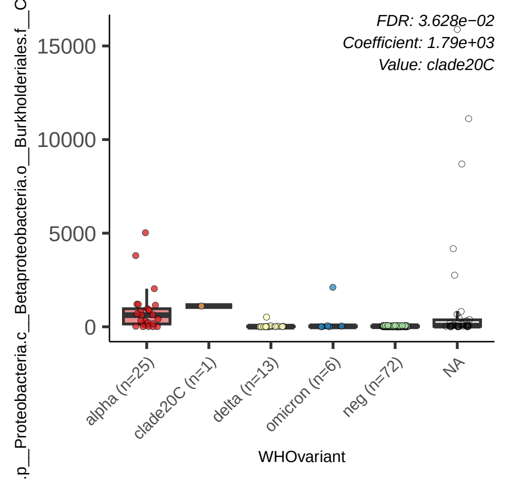

FDR: 3.628e−02
Coefficient: 1.79e+03
Value: clade20C
15000
10000
5000
0
p__Proteobacteria.c__Betaproteobacteria.o__Burkholderiales.f__C
alpha (n=25)
clade20C (n=1)
delta (n=13)
omicron (n=6)
neg (n=72)
NA
WHOvariant

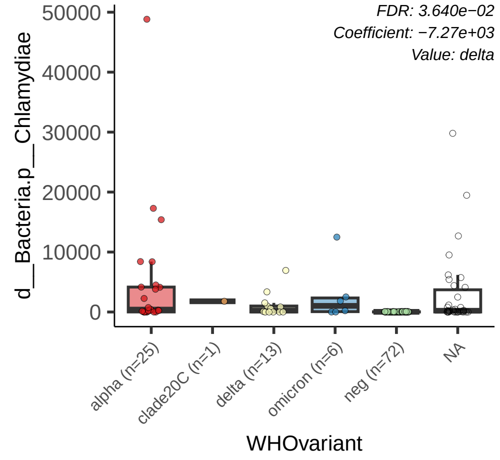

FDR: 3.640e−02
Coefficient: −7.27e+03
Value: delta
d__Bacteria.p__Chlamydiae
50000
40000
30000
20000
10000
0
alpha (n=25)
clade20C (n=1)
delta (n=13)
omicron (n=6)
neg (n=72)
NA
WHOvariant

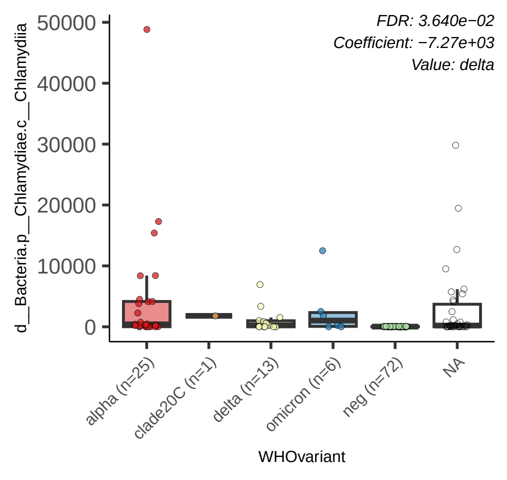

FDR: 3.640e−02
Coefficient: −7.27e+03
Value: delta
d__Bacteria.p__Chlamydiae.c__Chlamydiia
50000
40000
30000
20000
10000
0
alpha (n=25)
clade20C (n=1)
delta (n=13)
omicron (n=6)
neg (n=72)
NA
WHOvariant

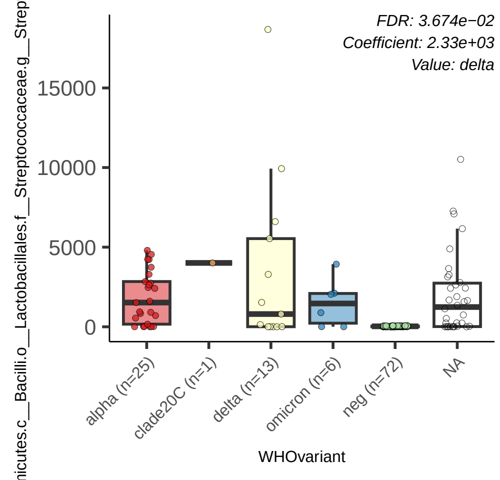

FDR: 3.674e−02
Coefficient: 2.33e+03
Value: delta
nicutes.c__Bacilli.o__Lactobacillales.f__Streptococcaceae.g__Strep
15000
10000
5000
0
alpha (n=25)
clade20C (n=1)
delta (n=13)
omicron (n=6)
neg (n=72)
NA
WHOvariant

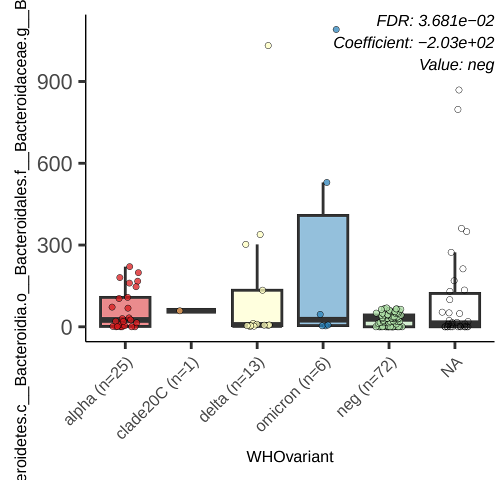

FDR: 3.681e−02
Coefficient: −2.03e+02
Value: neg
900
600
300
0
eroidetes.c__Bacteroidia.o__Bacteroidales.f__Bacteroidaceae.g__B
alpha (n=25)
clade20C (n=1)
delta (n=13)
omicron (n=6)
neg (n=72)
NA
WHOvariant

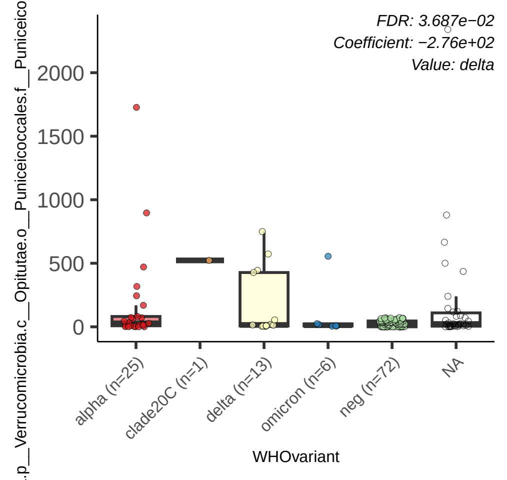

FDR: 3.687e−02
Coefficient: −2.76e+02
Value: delta
p__Verrucomicrobia.c__Opitutae.o__Puniceicoccales.f__Puniceicoccaceae
0
500
1000
1500
2000
alpha (n=25)
clade20C (n=1)
delta (n=13)
omicron (n=6)
neg (n=72)
NA
WHOvariant

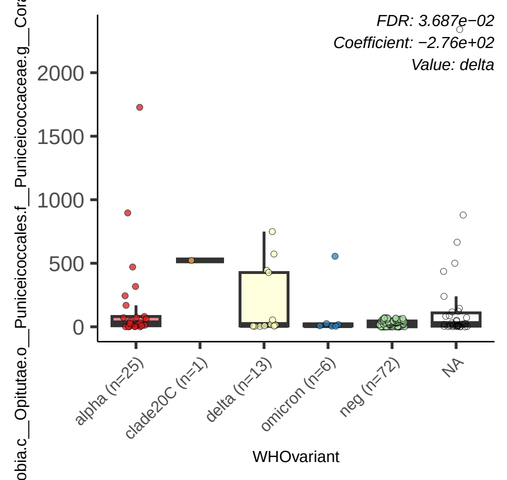

FDR: 3.687e−02
Coefficient: −2.76e+02
Value: delta
0
500
1000
1500
2000
obia.c__Opitutae.o__Puniceicoccales.f__Puniceicoccaceae.g__Cora
alpha (n=25)
clade20C (n=1)
delta (n=13)
omicron (n=6)
neg (n=72)
NA
WHOvariant

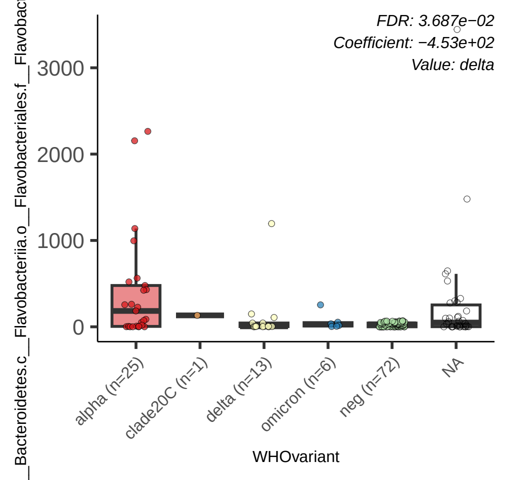

FDR: 3.687e−02
Coefficient: −4.53e+02
Value: delta
3000
2000
1000
0
Bacteroidetes.c__Flavobacteriia.o__Flavobacteriales.f__Flavobact
alpha (n=25)
clade20C (n=1)
delta (n=13)
omicron (n=6)
neg (n=72)
NA
WHOvariant

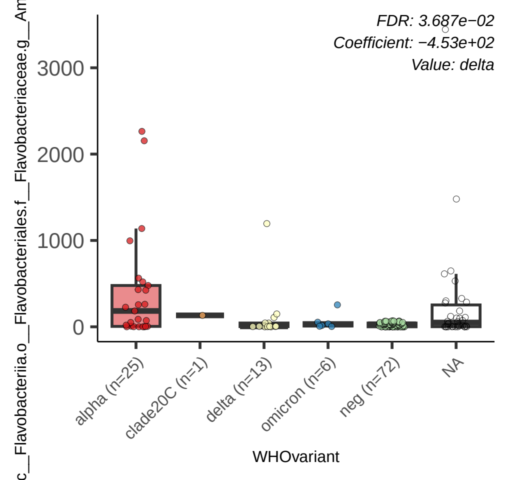

FDR: 3.687e−02
Coefficient: −4.53e+02
Value: delta
3000
2000
1000
0
c__Flavobacteriia.o__Flavobacteriales.f__Flavobacteriaceae.g__Am
alpha (n=25)
clade20C (n=1)
delta (n=13)
omicron (n=6)
neg (n=72)
NA
WHOvariant

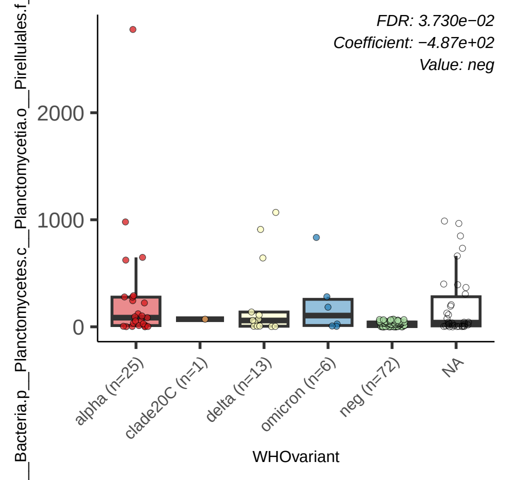

FDR: 3.730e−02
Coefficient: −4.87e+02
Value: neg
Bacteria.p__Planctomycetes.c__Planctomycetia.o__Pirellulales.f__
2000
1000
0
alpha (n=25)
clade20C (n=1)
delta (n=13)
omicron (n=6)
neg (n=72)
NA
WHOvariant

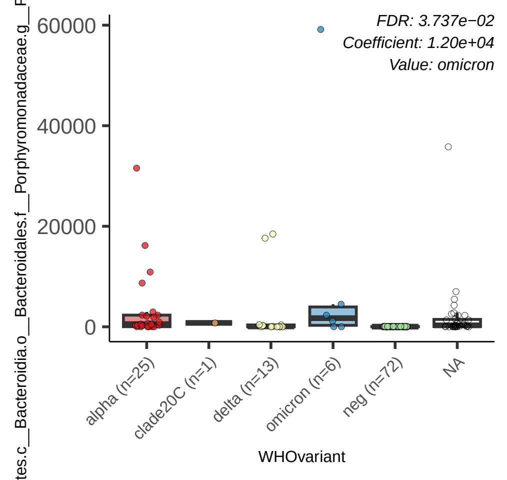
FDR: 3.737e−02
Coefficient: 1.20e+04
Value: omicron
60000
40000
20000
0
alpha (n=25)
clade20C (n=1)
delta (n=13)
omicron (n=6)
neg (n=72)
NA
WHOvariant
tes.c__Bacteroidia.o__Bacteroidales.f__Porphyromonadaceae.g__P

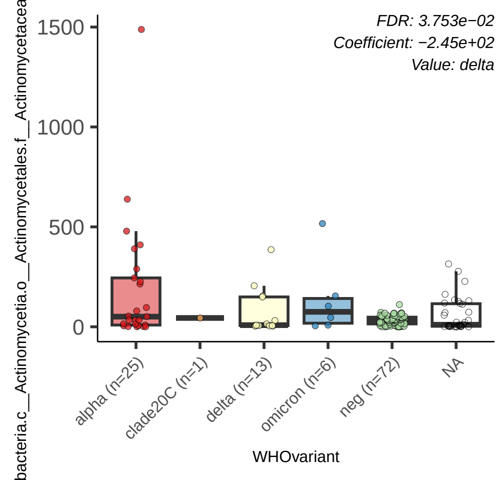
FDR: 3.753e−02
Coefficient: −2.45e+02
Value: delta
1500
1000
500
0
bacteria.c__Actinomycetia.o__Actinomycetales.f__Actinomycetacea
alpha (n=25)
clade20C (n=1)
delta (n=13)
omicron (n=6)
neg (n=72)
NA
WHOvariant

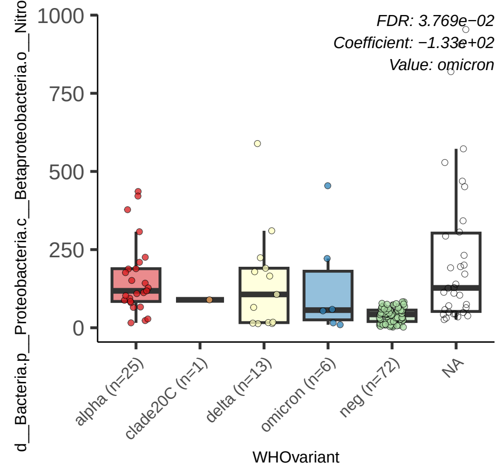

FDR: 3.769e−02
Coefficient: −1.33e+02
Value: omicron
d__Bacteria.p__Proteobacteria.c__Betaproteobacteria.o__Nitro
1000
750
500
250
0
alpha (n=25)
clade20C (n=1)
delta (n=13)
omicron (n=6)
neg (n=72)
NA
WHOvariant

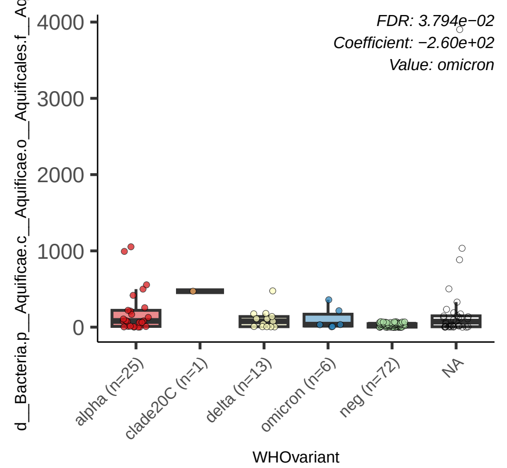

FDR: 3.794e−02
Coefficient: −2.60e+02
Value: omicron
d__Bacteria.p__Aquificae.c__Aquificae.o__Aquificales.f__Aq
4000
3000
2000
1000
0
alpha (n=25)
clade20C (n=1)
delta (n=13)
omicron (n=6)
neg (n=72)
NA
WHOvariant

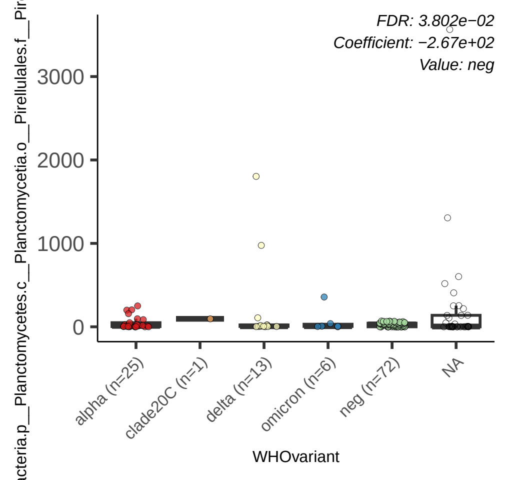

FDR: 3.802e−02
Coefficient: −2.67e+02
Value: neg
3000
2000
1000
0
alpha (n=25)
clade20C (n=1)
delta (n=13)
omicron (n=6)
neg (n=72)
NA
WHOvariant
acteria.p__Planctomycetes.c__Planctomycetia.o__Pirellulales.f__Pir

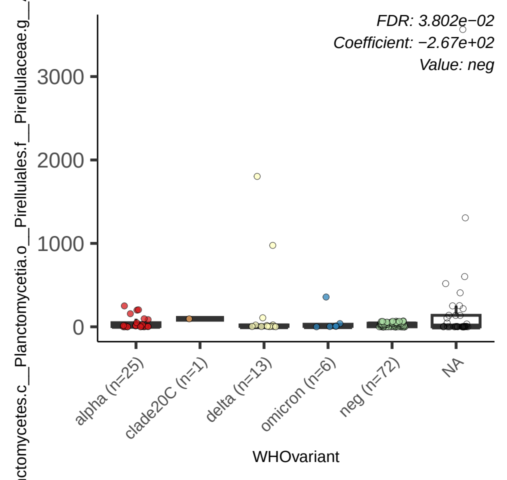
FDR: 3.802e−02
Coefficient: −2.67e+02
Value: neg
3000
2000
1000
0
alpha (n=25)
clade20C (n=1)
delta (n=13)
omicron (n=6)
neg (n=72)
NA
WHOvariant
ctomycetes.c__Planctomycetia.o__Pirellulales.f__Pirellulaceae.g__

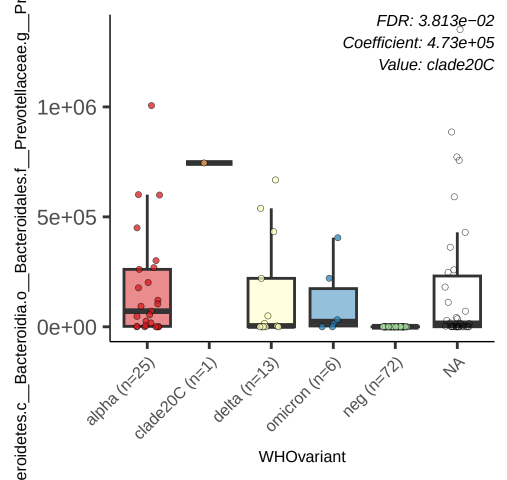

FDR: 3.813e−02
Coefficient: 4.73e+05
Value: clade20C
eroidetes.c__Bacteroidia.o__Bacteroidales.f__Prevotellaceae.g__Pr
1e+06
5e+05
0e+00
alpha (n=25)
clade20C (n=1)
delta (n=13)
omicron (n=6)
neg (n=72)
NA
WHOvariant

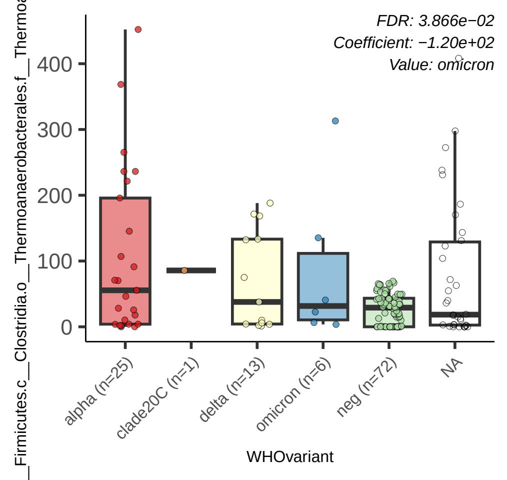
FDR: 3.866e−02
Coefficient: −1.20e+02
Value: omicron
Firmicutes.c__Clostridia.o__Thermoanaerobacterales.f__Thermoa
0
100
200
300
400
alpha (n=25)
clade20C (n=1)
delta (n=13)
omicron (n=6)
neg (n=72)
NA
WHOvariant

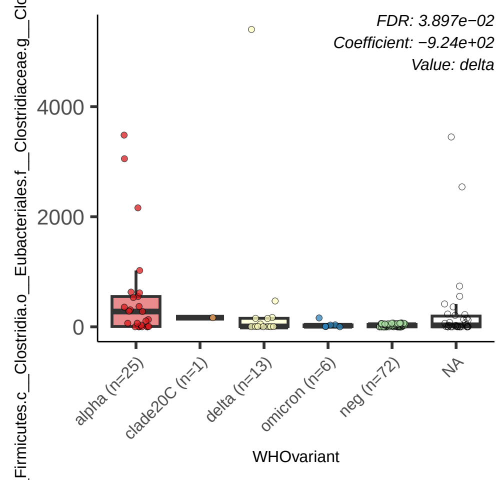

Firmicutes.c__Clostridia.o__Eubacteriales.f__Clostridiaceae.g__Clo
FDR: 3.897e−02
Coefficient: −9.24e+02
Value: delta
4000
2000
0
alpha (n=25)
clade20C (n=1)
delta (n=13)
omicron (n=6)
neg (n=72)
NA
WHOvariant

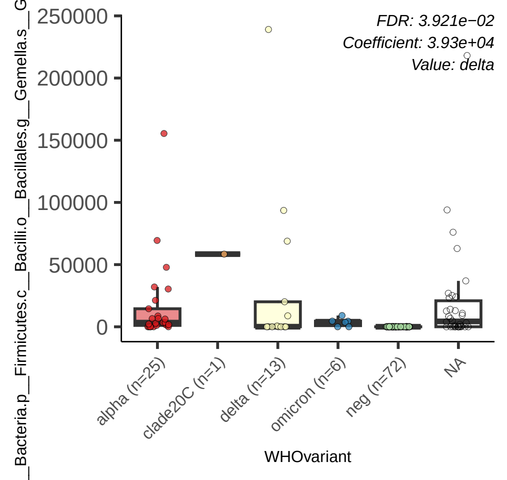
FDR: 3.921e−02
Coefficient: 3.93e+04
Value: delta
Bacteria.p__Firmicutes.c__Bacilli.o__Bacillales.g__Gemella.s__G
250000
200000
150000
100000
50000
0
alpha (n=25)
clade20C (n=1)
delta (n=13)
omicron (n=6)
neg (n=72)
NA
WHOvariant

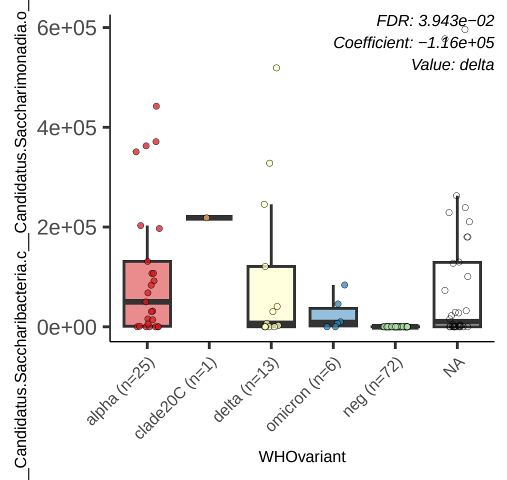

FDR: 3.943e−02
Coefficient: −1.16e+05
Value: delta
Candidatus.Saccharibacteria.c__Candidatus.Saccharimonadia.o__
6e+05
4e+05
2e+05
0e+00
alpha (n=25)
clade20C (n=1)
delta (n=13)
omicron (n=6)
neg (n=72)
NA
WHOvariant

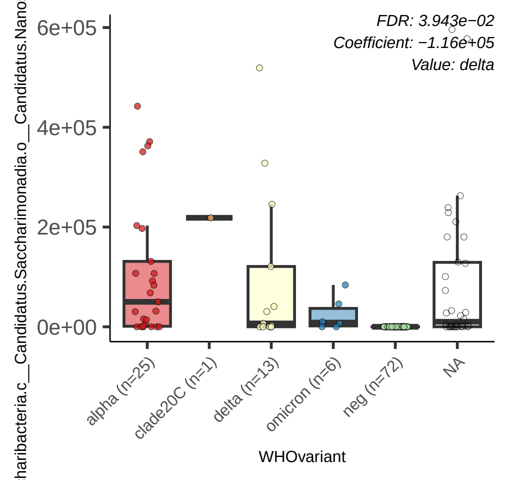

FDR: 3.943e−02
Coefficient: −1.16e+05
Value: delta
charibacteria.c__Candidatus.Saccharimonadia.o__Candidatus.Nano
6e+05
4e+05
2e+05
0e+00
alpha (n=25)
clade20C (n=1)
delta (n=13)
omicron (n=6)
neg (n=72)
NA
WHOvariant

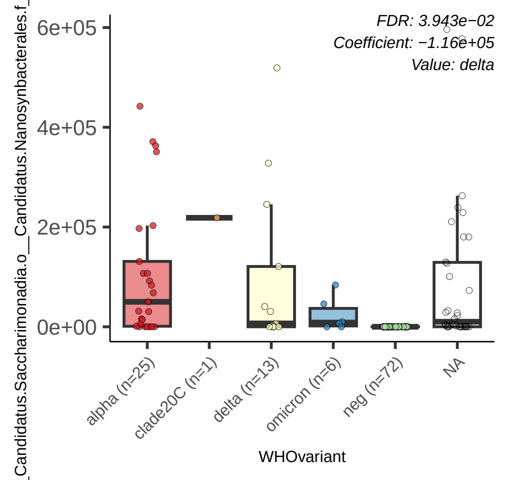

FDR: 3.943e−02
Coefficient: −1.16e+05
Value: delta
Candidatus.Saccharimonadia.o__Candidatus.Nanosynbacterales.f_
6e+05
4e+05
2e+05
0e+00
alpha (n=25)
clade20C (n=1)
delta (n=13)
omicron (n=6)
neg (n=72)
NA
WHOvariant

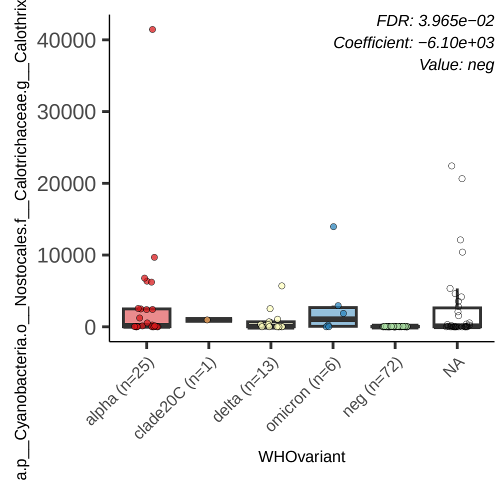

FDR: 3.965e−02
Coefficient: −6.10e+03
Value: neg
a.p__Cyanobacteria.o__Nostocales.f__Calotrichaceae.g__Calothrix
40000
30000
20000
10000
0
alpha (n=25)
clade20C (n=1)
delta (n=13)
omicron (n=6)
neg (n=72)
NA
WHOvariant

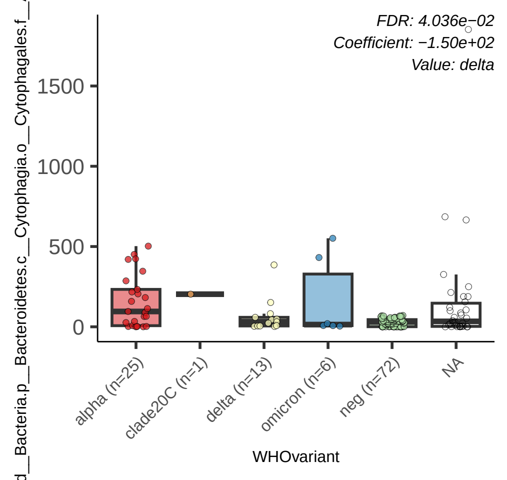

FDR: 4.036e−02
Coefficient: −1.50e+02
Value: delta
d__Bacteria.p__Bacteroidetes.c__Cytophagia.o__Cytophagales.f__
1500
1000
500
0
alpha (n=25)
clade20C (n=1)
delta (n=13)
omicron (n=6)
neg (n=72)
NA
WHOvariant

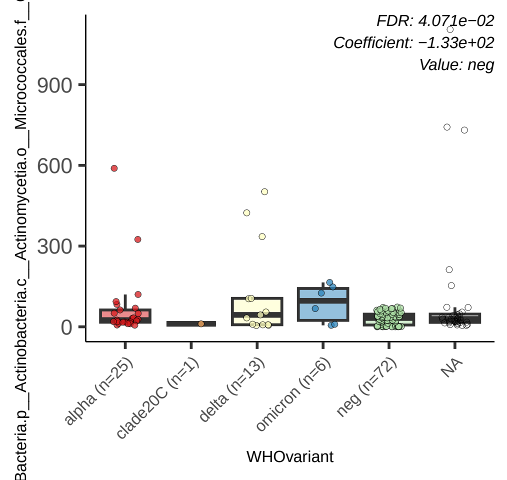

FDR: 4.071e−02
Coefficient: −1.33e+02
Value: neg
Bacteria.p__Actinobacteria.c__Actinomycetia.o__Micrococcales.f__
0
300
600
900
alpha (n=25)
clade20C (n=1)
delta (n=13)
omicron (n=6)
neg (n=72)
NA
WHOvariant

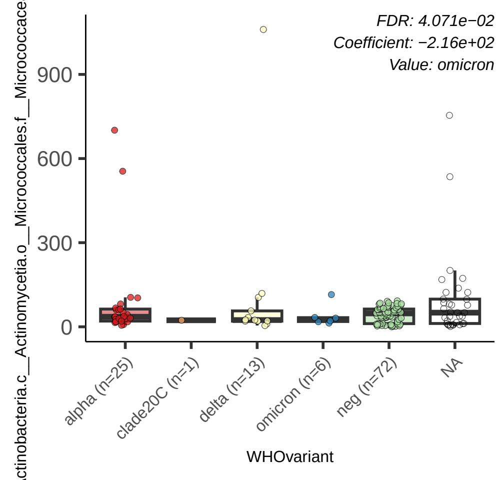

FDR: 4.071e−02
Coefficient: −2.16e+02
Value: omicron
0
300
600
900
Actinobacteria.c__Actinomycetia.o__Micrococcales.f__Micrococcace
alpha (n=25)
clade20C (n=1)
delta (n=13)
omicron (n=6)
neg (n=72)
NA
WHOvariant

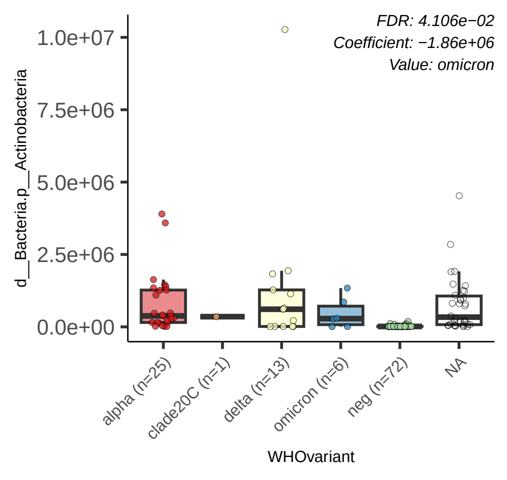
FDR: 4.106e−02
Coefficient: −1.86e+06
Value: omicron
1.0e+07
7.5e+06
5.0e+06
2.5e+06
0.0e+00
d__Bacteria.p__Actinobacteria
alpha (n=25)
clade20C (n=1)
delta (n=13)
omicron (n=6)
neg (n=72)
NA
WHOvariant

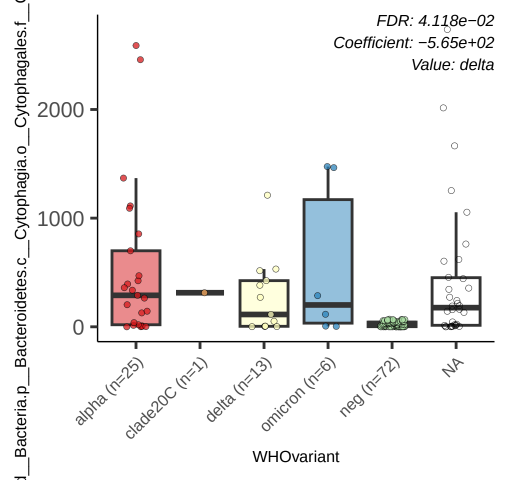

FDR: 4.118e−02
Coefficient: −5.65e+02
Value: delta
d__Bacteria.p__Bacteroidetes.c__Cytophagia.o__Cytophagales.f__C
0
1000
2000
alpha (n=25)
clade20C (n=1)
delta (n=13)
omicron (n=6)
neg (n=72)
NA
WHOvariant

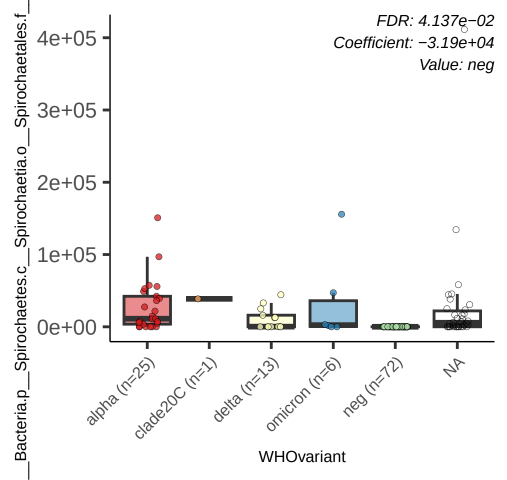

FDR: 4.137e−02
Coefficient: −3.19e+04
Value: neg
Bacteria.p__Spirochaetes.c__Spirochaetia.o__Spirochaetales.f__
4e+05
3e+05
2e+05
1e+05
0e+00
alpha (n=25)
clade20C (n=1)
delta (n=13)
omicron (n=6)
neg (n=72)
NA
WHOvariant

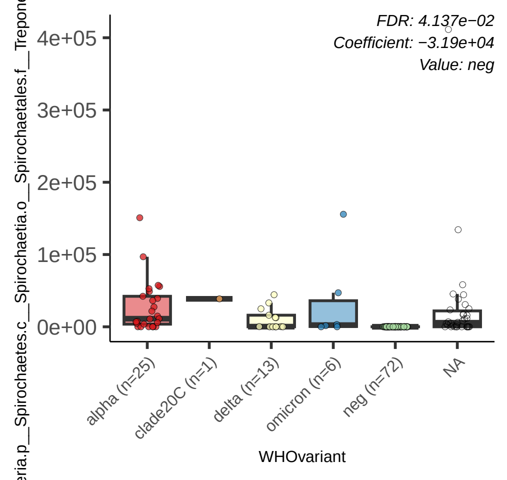

FDR: 4.137e−02
Coefficient: −3.19e+04
Value: neg
4e+05
3e+05
2e+05
1e+05
0e+00
alpha (n=25)
clade20C (n=1)
delta (n=13)
omicron (n=6)
neg (n=72)
NA
WHOvariant

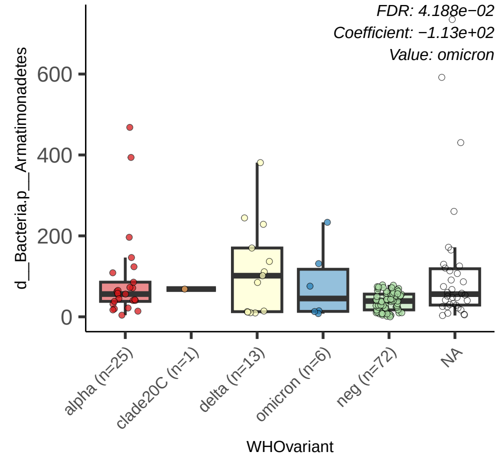

FDR: 4.188e−02
Coefficient: −1.13e+02
Value: omicron
d__Bacteria.p__Armatimonadetes
600
400
200
0
alpha (n=25)
clade20C (n=1)
delta (n=13)
omicron (n=6)
neg (n=72)
NA
WHOvariant

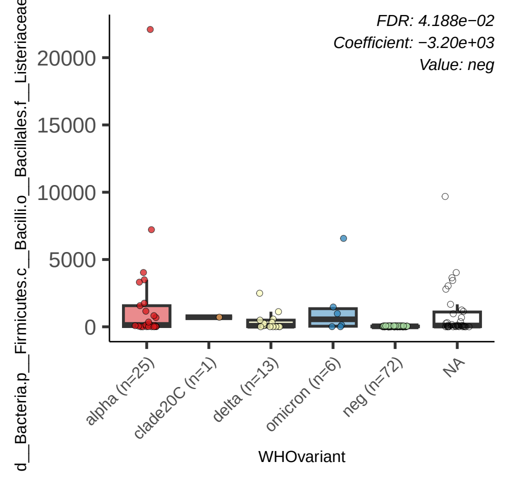

FDR: 4.188e−02
Coefficient: −3.20e+03
Value: neg
d__Bacteria.p__Firmicutes.c__Bacilli.o__Bacillales.f__Listeriaceae
20000
15000
10000
5000
0
alpha (n=25)
clade20C (n=1)
delta (n=13)
omicron (n=6)
neg (n=72)
NA
WHOvariant

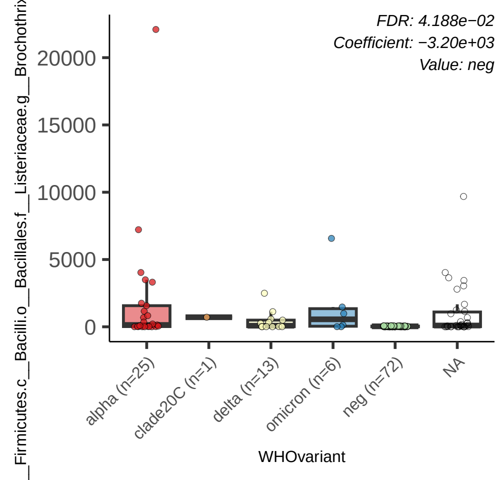
FDR: 4.188e−02
Coefficient: −3.20e+03
Value: neg
Firmicutes.c__Bacilli.o__Bacillales.f__Listeriaceae.g__Brochothrix
20000
15000
10000
5000
0
alpha (n=25)
clade20C (n=1)
delta (n=13)
omicron (n=6)
neg (n=72)
NA
WHOvariant

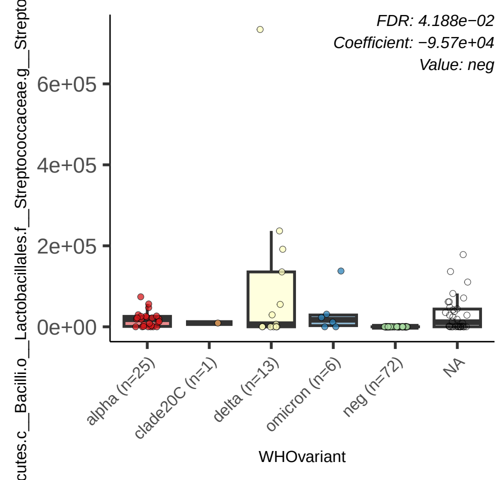
FDR: 4.188e−02
Coefficient: −9.57e+04
Value: neg
cutes.c__Bacilli.o__Lactobacillales.f__Streptococcaceae.g__Strepto
6e+05
4e+05
2e+05
0e+00
alpha (n=25)
clade20C (n=1)
delta (n=13)
omicron (n=6)
neg (n=72)
NA
WHOvariant

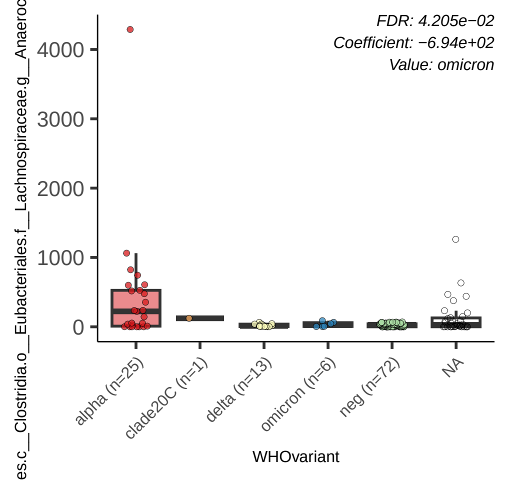

FDR: 4.205e−02
Coefficient: −6.94e+02
Value: omicron
es.c__Clostridia.o__Eubacteriales.f__Lachnospiraceae.g__Anaeroc
4000
3000
2000
1000
0
alpha (n=25)
clade20C (n=1)
delta (n=13)
omicron (n=6)
neg (n=72)
NA
WHOvariant

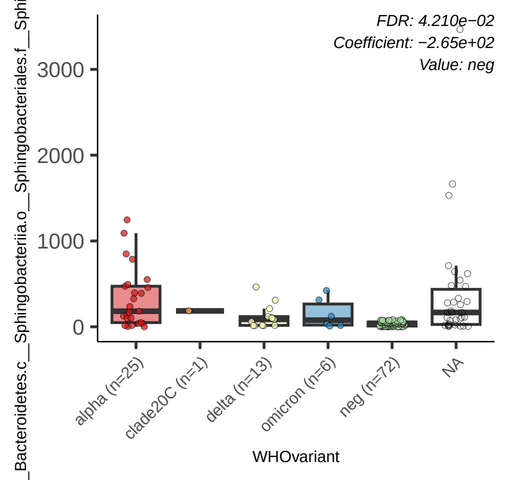

FDR: 4.210e−02
Coefficient: −2.65e+02
Value: neg
3000
2000
1000
0
Bacteroidetes.c__Sphingobacteriia.o__Sphingobacteriales.f__Sphi
alpha (n=25)
clade20C (n=1)
delta (n=13)
omicron (n=6)
neg (n=72)
NA
WHOvariant

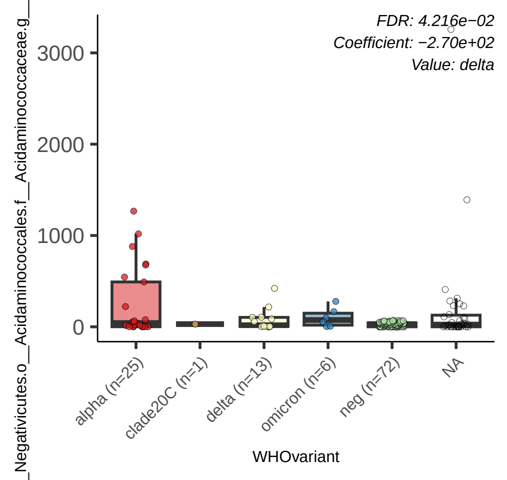

FDR: 4.216e−02
Coefficient: −2.70e+02
Value: delta
Negativicutes.o__Acidaminococcales.f__Acidaminococcaceae.g__
3000
2000
1000
0
alpha (n=25)
clade20C (n=1)
delta (n=13)
omicron (n=6)
neg (n=72)
NA
WHOvariant

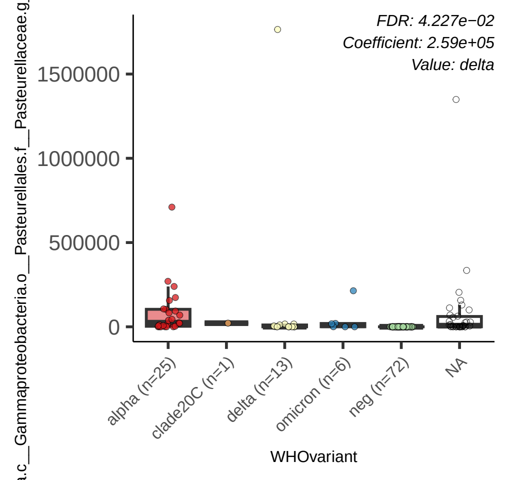

FDR: 4.227e−02
Coefficient: 2.59e+05
Value: delta
1500000
1000000
500000
0
a.c__Gammaproteobacteria.o__Pasteurellales.f__Pasteurellaceae.g__
alpha (n=25)
clade20C (n=1)
delta (n=13)
omicron (n=6)
neg (n=72)
NA
WHOvariant

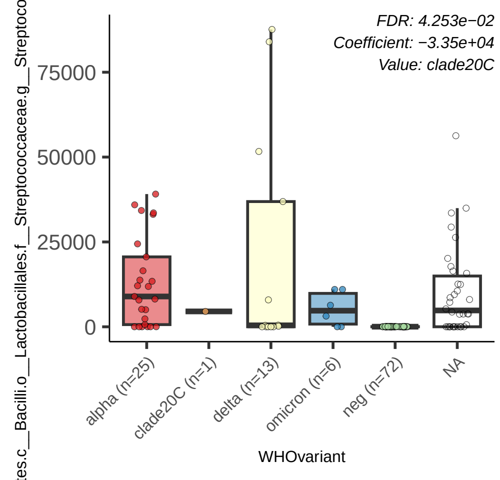
FDR: 4.253e−02
Coefficient: −3.35e+04
Value: clade20C
75000
50000
25000
0
tes.c__Bacilli.o__Lactobacillales.f__Streptococcaceae.g__Streptoco
alpha (n=25)
clade20C (n=1)
delta (n=13)
omicron (n=6)
neg (n=72)
NA
WHOvariant

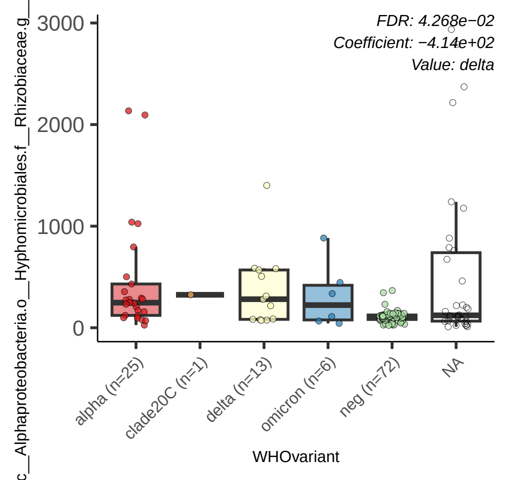

FDR: 4.268e−02
Coefficient: −4.14e+02
Value: delta
c__Alphaproteobacteria.o__Hyphomicrobiales.f__Rhizobiaceae.g__
3000
2000
1000
0
alpha (n=25)
clade20C (n=1)
delta (n=13)
omicron (n=6)
neg (n=72)
NA
WHOvariant

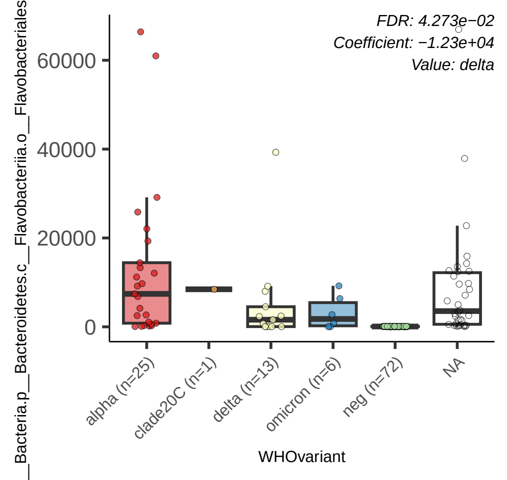

FDR: 4.273e−02
Coefficient: −1.23e+04
Value: delta
_Bacteria.p__Bacteroidetes.c__Flavobacteriia.o__Flavobacteriales
60000
40000
20000
0
alpha (n=25)
clade20C (n=1)
delta (n=13)
omicron (n=6)
neg (n=72)
NA
WHOvariant

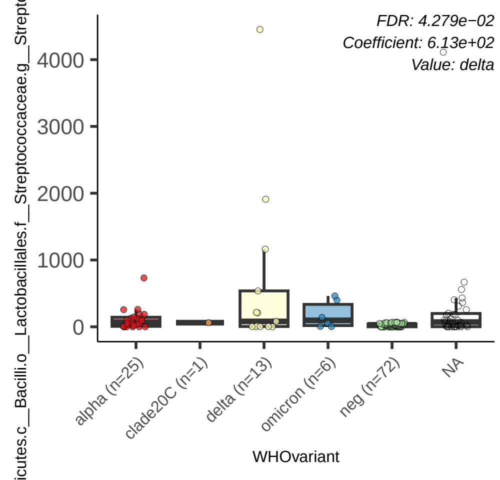

FDR: 4.279e−02
Coefficient: 6.13e+02
Value: delta
icutes.c__Bacilli.o__Lactobacillales.f__Streptococcaceae.g__Strept
0
1000
2000
3000
4000
alpha (n=25)
clade20C (n=1)
delta (n=13)
omicron (n=6)
neg (n=72)
NA
WHOvariant

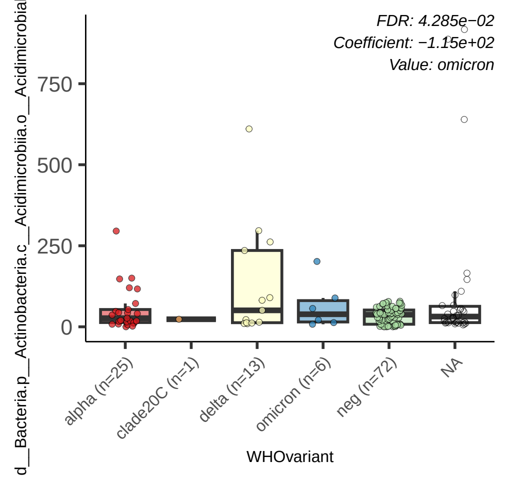

FDR: 4.285e−02
Coefficient: −1.15e+02
Value: omicron
d__Bacteria.p__Actinobacteria.c__Acidimicrobiia.o__Acidimicrobial
0
250
500
750
alpha (n=25)
clade20C (n=1)
delta (n=13)
omicron (n=6)
neg (n=72)
NA
WHOvariant

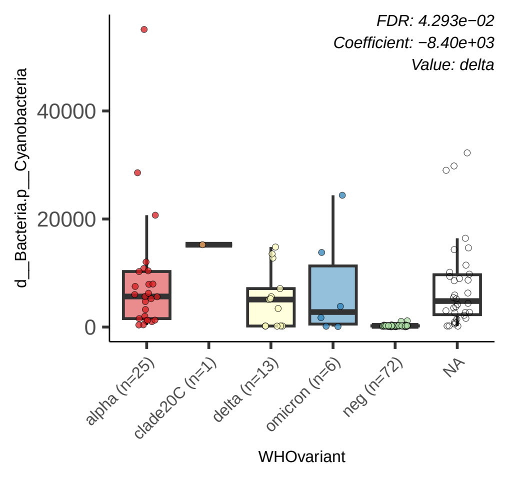

FDR: 4.293e−02
Coefficient: −8.40e+03
Value: delta
d__Bacteria.p__Cyanobacteria
40000
20000
0
alpha (n=25)
clade20C (n=1)
delta (n=13)
omicron (n=6)
neg (n=72)
NA
WHOvariant

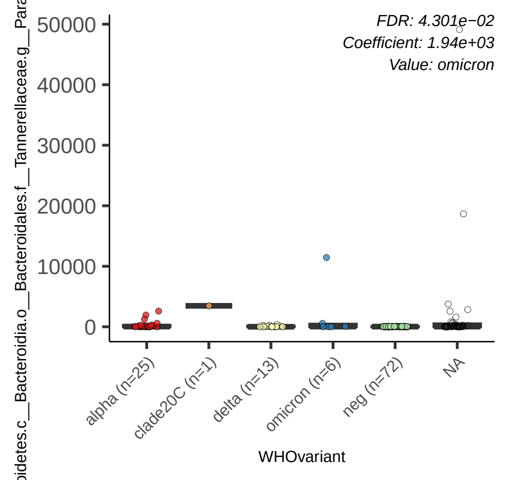

FDR: 4.301e−02
Coefficient: 1.94e+03
Value: omicron
oidetes.c__Bacteroidia.o__Bacteroidales.f__Tannerellaceae.g__Para
50000
40000
30000
20000
10000
0
alpha (n=25)
clade20C (n=1)
delta (n=13)
omicron (n=6)
neg (n=72)
NA
WHOvariant

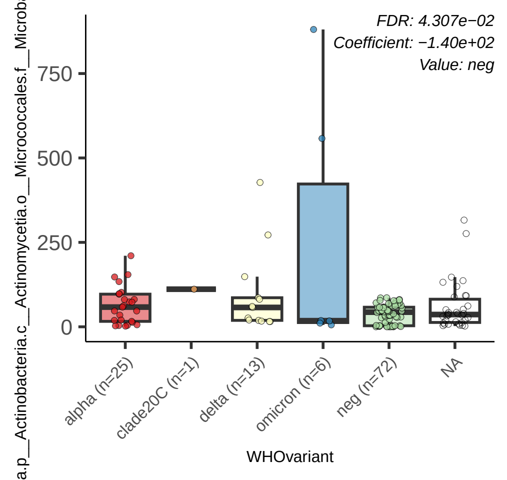
FDR: 4.307e−02
Coefficient: −1.40e+02
Value: neg
0
250
500
750
a.p__Actinobacteria.c__Actinomycetia.o__Micrococcales.f__Microb
alpha (n=25)
clade20C (n=1)
delta (n=13)
omicron (n=6)
neg (n=72)
NA
WHOvariant

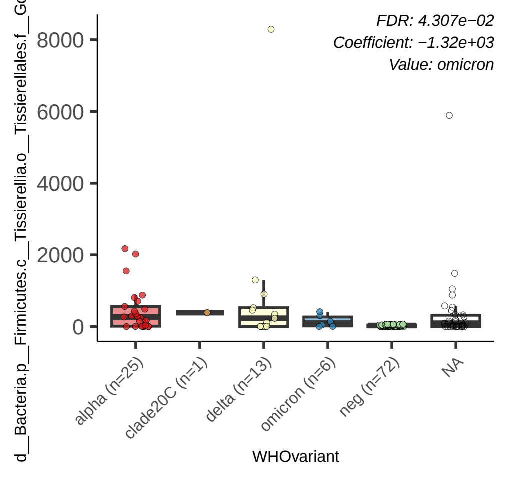

FDR: 4.307e−02
Coefficient: −1.32e+03
Value: omicron
d__Bacteria.p__Firmicutes.c__Tissierellia.o__Tissierellales.f__Go
8000
6000
4000
2000
0
alpha (n=25)
clade20C (n=1)
delta (n=13)
omicron (n=6)
neg (n=72)
NA
WHOvariant

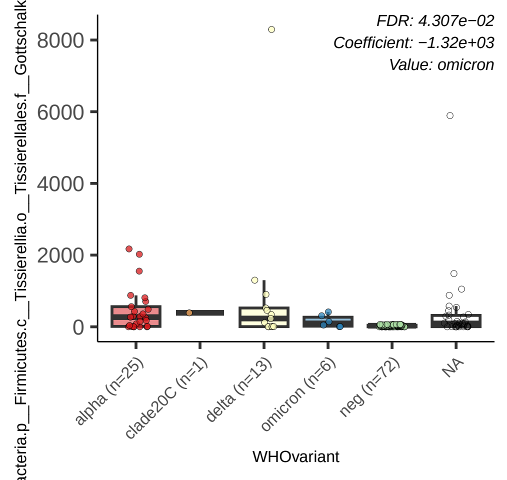
FDR: 4.307e−02
Coefficient: −1.32e+03
Value: omicron
8000
6000
4000
2000
0
acteria.p__Firmicutes.c__Tissierellia.o__Tissierellales.f__Gottschalk
alpha (n=25)
clade20C (n=1)
delta (n=13)
omicron (n=6)
neg (n=72)
NA
WHOvariant

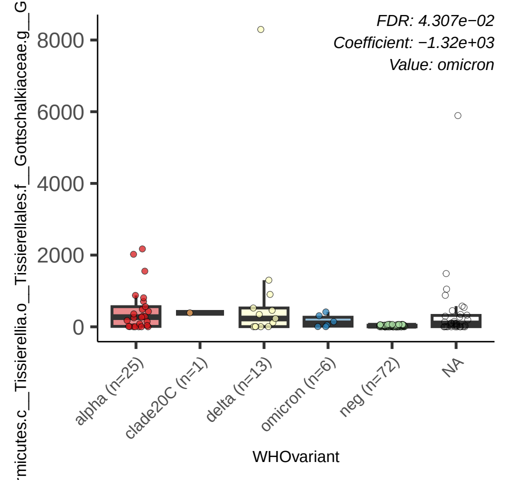

FDR: 4.307e−02
Coefficient: −1.32e+03
Value: omicron
8000
6000
4000
2000
0
Firmicutes.c__Tissierellia.o__Tissierellales.f__Gottschalkiaceae.g__G
alpha (n=25)
clade20C (n=1)
delta (n=13)
omicron (n=6)
neg (n=72)
NA
WHOvariant

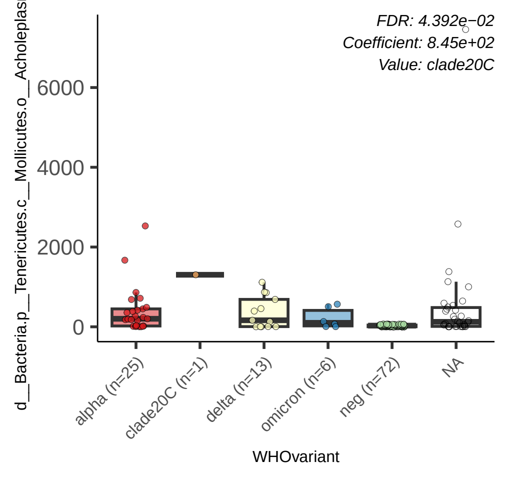

FDR: 4.392e−02
Coefficient: 8.45e+02
Value: clade20C
d__Bacteria.p__Tenericutes.c__Mollicutes.o__Acholeplas
6000
4000
2000
0
alpha (n=25)
clade20C (n=1)
delta (n=13)
omicron (n=6)
neg (n=72)
NA
WHOvariant

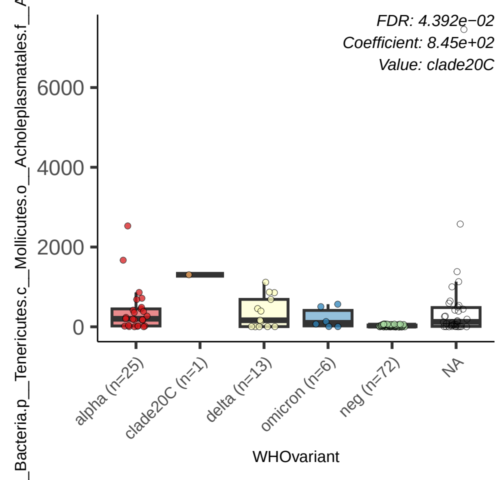
FDR: 4.392e−02
Coefficient: 8.45e+02
Value: clade20C
_Bacteria.p__Tenericutes.c__Mollicutes.o__Acholeplasmatales.f__A
6000
4000
2000
0
alpha (n=25)
clade20C (n=1)
delta (n=13)
omicron (n=6)
neg (n=72)
NA
WHOvariant

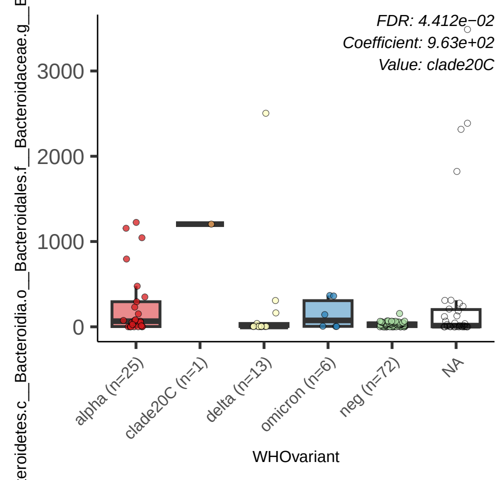

FDR: 4.412e−02
Coefficient: 9.63e+02
Value: clade20C
0
1000
2000
3000
alpha (n=25)
clade20C (n=1)
delta (n=13)
omicron (n=6)
neg (n=72)
NA
WHOvariant

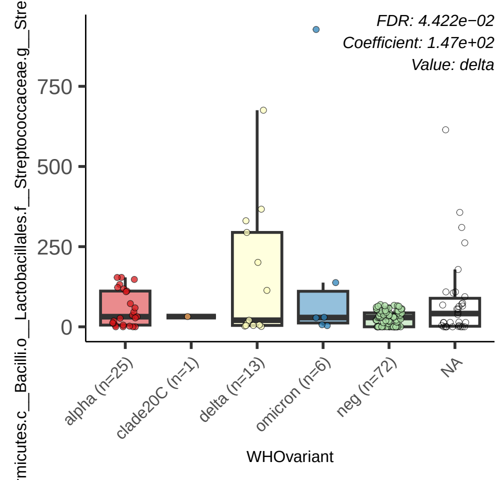

FDR: 4.422e−02
Coefficient: 1.47e+02
Value: delta
0
250
500
750
micutes.c__Bacilli.o__Lactobacillales.f__Streptococcaceae.g__Stre
alpha (n=25)
clade20C (n=1)
delta (n=13)
omicron (n=6)
neg (n=72)
NA
WHOvariant

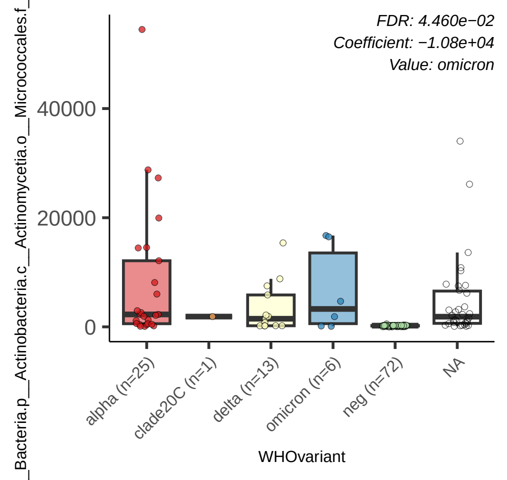
FDR: 4.460e−02
Coefficient: −1.08e+04
Value: omicron
Bacteria.p__Actinobacteria.c__Actinomycetia.o__Micrococcales.f__
0
20000
40000
alpha (n=25)
clade20C (n=1)
delta (n=13)
omicron (n=6)
neg (n=72)
NA
WHOvariant

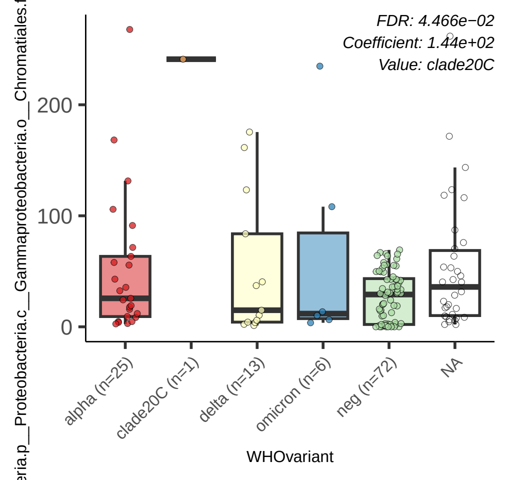

FDR: 4.466e−02
Coefficient: 1.44e+02
Value: clade20C
eria.p__Proteobacteria.c__Gammaproteobacteria.o__Chromatiales.f
0
100
200
alpha (n=25)
clade20C (n=1)
delta (n=13)
omicron (n=6)
neg (n=72)
NA
WHOvariant

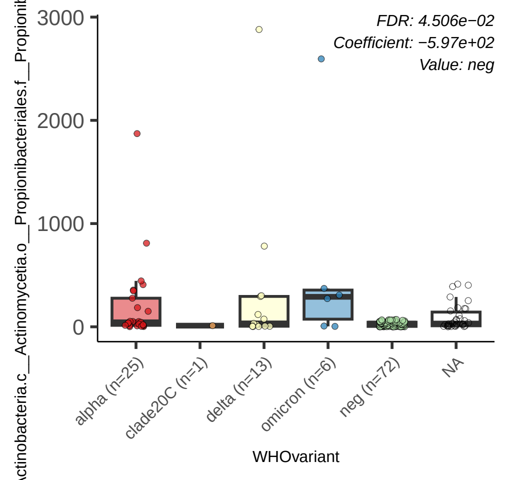

FDR: 4.506e−02
Coefficient: −5.97e+02
Value: neg
Actinobacteria.c__Actinomycetia.o__Propionibacteriales.f__Propionib
3000
2000
1000
0
alpha (n=25)
clade20C (n=1)
delta (n=13)
omicron (n=6)
neg (n=72)
NA
WHOvariant

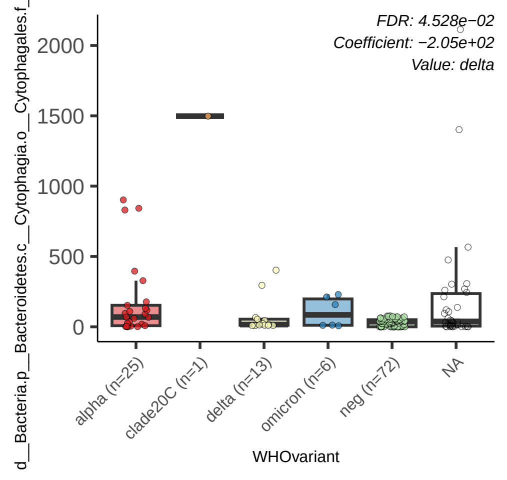
FDR: 4.528e−02
Coefficient: −2.05e+02
Value: delta
d__Bacteria.p__Bacteroidetes.c__Cytophagia.o__Cytophagales.f__
2000
1500
1000
500
0
alpha (n=25)
clade20C (n=1)
delta (n=13)
omicron (n=6)
neg (n=72)
NA
WHOvariant

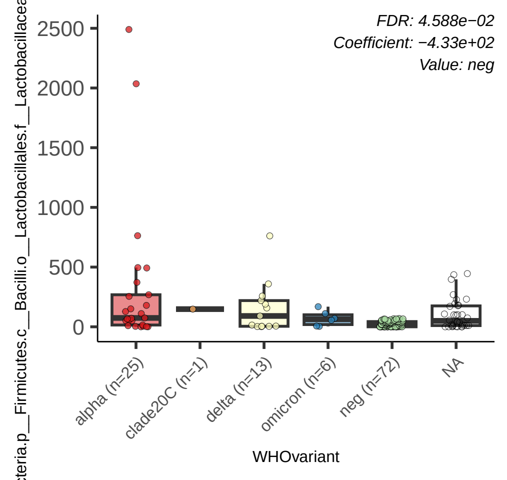
FDR: 4.588e−02
Coefficient: −4.33e+02
Value: neg
2500
2000
1500
1000
500
0
cteria.p__Firmicutes.c__Bacilli.o__Lactobacillales.f__Lactobacillacea
alpha (n=25)
clade20C (n=1)
delta (n=13)
omicron (n=6)
neg (n=72)
NA
WHOvariant

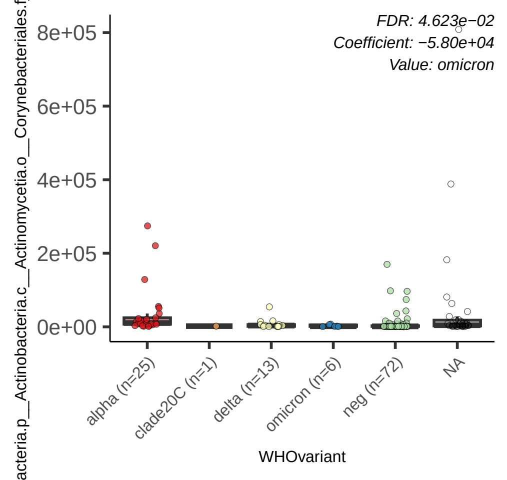

FDR: 4.623e−02
Coefficient: −5.80e+04
Value: omicron
8e+05
6e+05
4e+05
2e+05
0e+00
acteria.p__Actinobacteria.c__Actinomycetia.o__Corynebacteriales.f
alpha (n=25)
clade20C (n=1)
delta (n=13)
omicron (n=6)
neg (n=72)
NA
WHOvariant

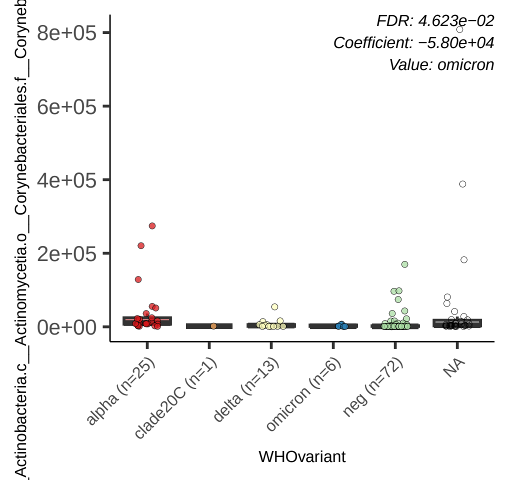
FDR: 4.623e−02
Coefficient: −5.80e+04
Value: omicron
Actinobacteria.c__Actinomycetia.o__Corynebacteriales.f__Coryneb
8e+05
6e+05
4e+05
2e+05
0e+00
alpha (n=25)
clade20C (n=1)
delta (n=13)
omicron (n=6)
neg (n=72)
NA
WHOvariant

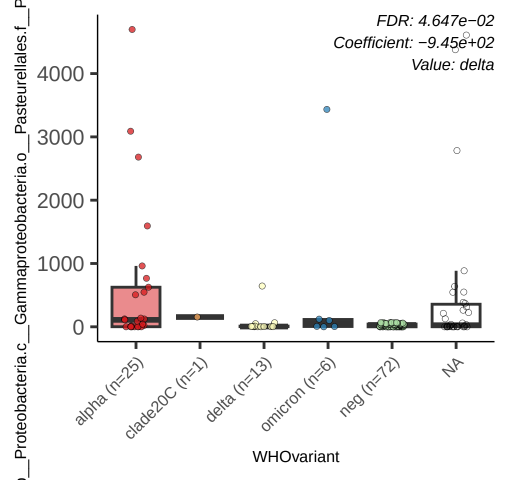

FDR: 4.647e−02
Coefficient: −9.45e+02
Value: delta
0
1000
2000
3000
4000
o__Proteobacteria.c__Gammaproteobacteria.o__Pasteurellales.f__P
alpha (n=25)
clade20C (n=1)
delta (n=13)
omicron (n=6)
neg (n=72)
NA
WHOvariant

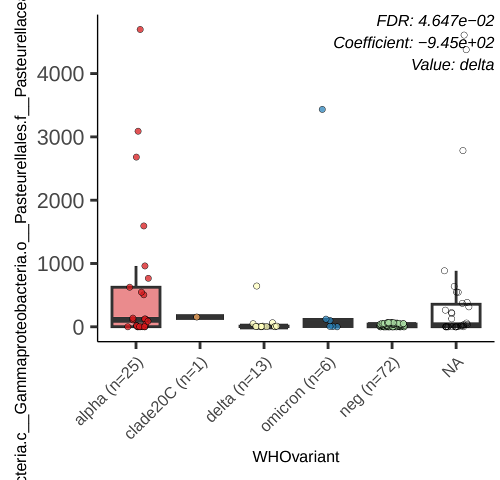

FDR: 4.647e−02
Coefficient: −9.45e+02
Value: delta
4000
3000
2000
1000
0
alpha (n=25)
clade20C (n=1)
delta (n=13)
omicron (n=6)
neg (n=72)
NA
WHOvariant
cteria.c__Gammaproteobacteria.o__Pasteurellales.f__Pasteurellace

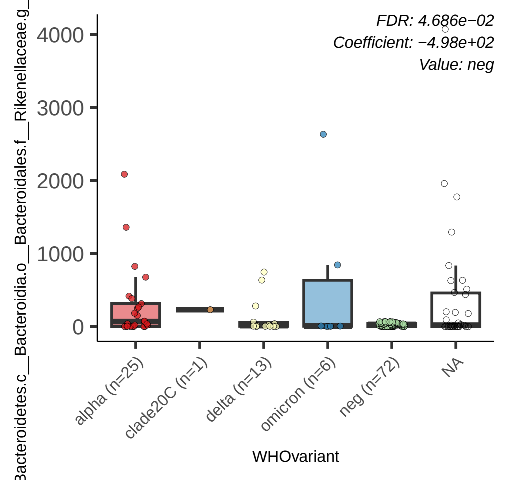

FDR: 4.686e−02
Coefficient: −4.98e+02
Value: neg
Bacteroidetes.c__Bacteroidia.o__Bacteroidales.f__Rikenellaceae.g__
4000
3000
2000
1000
0
alpha (n=25)
clade20C (n=1)
delta (n=13)
omicron (n=6)
neg (n=72)
NA
WHOvariant

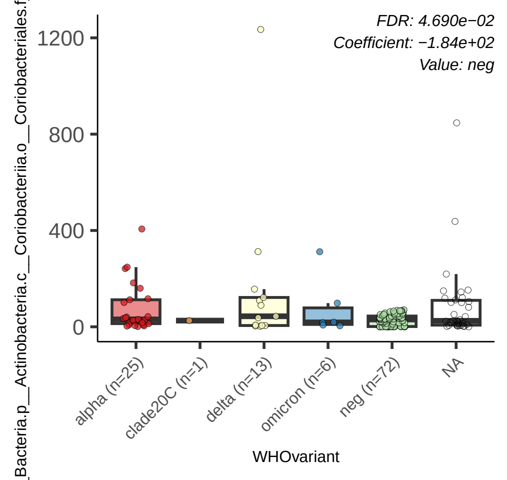

FDR: 4.690e−02
Coefficient: −1.84e+02
Value: neg
1200
800
400
0
Bacteria.p__Actinobacteria.c__Coriobacteriia.o__Coriobacteriales.f
alpha (n=25)
clade20C (n=1)
delta (n=13)
omicron (n=6)
neg (n=72)
NA
WHOvariant

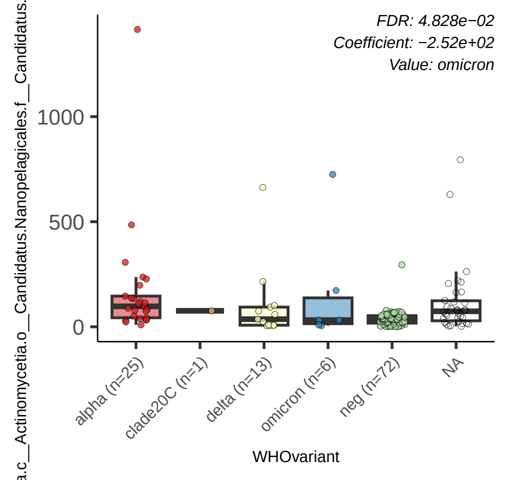
FDR: 4.828e−02
Coefficient: −2.52e+02
Value: omicron
a.c__Actinomycetia.o__Candidatus.Nanopelagicales.f__Candidatus.
0
500
1000
alpha (n=25)
clade20C (n=1)
delta (n=13)
omicron (n=6)
neg (n=72)
NA
WHOvariant

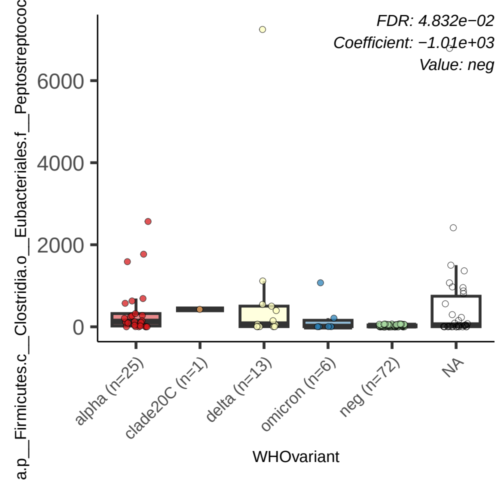

FDR: 4.832e−02
Coefficient: −1.01e+03
Value: neg
0
2000
4000
6000
a.p__Firmicutes.c__Clostridia.o__Eubacteriales.f__Peptostreptococ
alpha (n=25)
clade20C (n=1)
delta (n=13)
omicron (n=6)
neg (n=72)
NA
WHOvariant

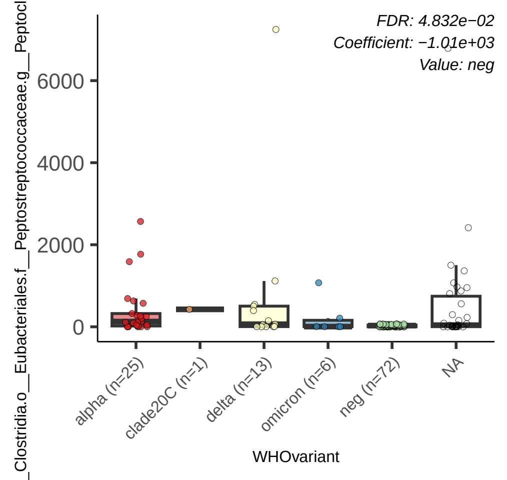

FDR: 4.832e−02
Coefficient: −1.01e+03
Value: neg
6000
4000
2000
0
alpha (n=25)
clade20C (n=1)
delta (n=13)
omicron (n=6)
neg (n=72)
NA
WHOvariant
_Clostridia.o__Eubacteriales.f__Peptostreptococcaceae.g__Peptocl

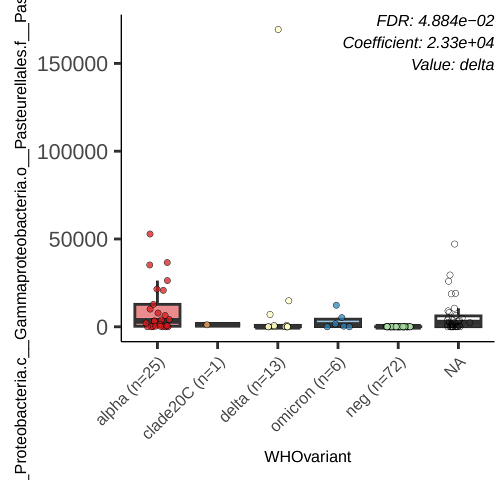

FDR: 4.884e−02
Coefficient: 2.33e+04
Value: delta
Proteobacteria.c__Gammaproteobacteria.o__Pasteurellales.f__Pas
150000
100000
50000
0
alpha (n=25)
clade20C (n=1)
delta (n=13)
omicron (n=6)
neg (n=72)
NA
WHOvariant

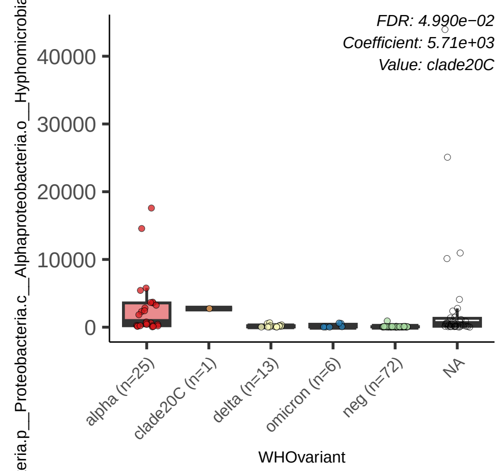

FDR: 4.990e−02
Coefficient: 5.71e+03
Value: clade20C
40000
30000
20000
10000
0
cteria.p__Proteobacteria.c__Alphaproteobacteria.o__Hyphomicrobia
alpha (n=25)
clade20C (n=1)
delta (n=13)
omicron (n=6)
neg (n=72)
NA
WHOvariant

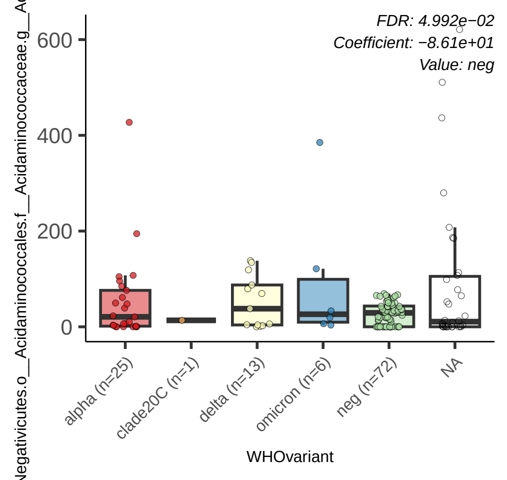

FDR: 4.992e−02
Coefficient: −8.61e+01
Value: neg
Negativicutes.o__Acidaminococcales.f__Acidaminococcaceae.g__A
0
200
400
600
alpha (n=25)
clade20C (n=1)
delta (n=13)
omicron (n=6)
neg (n=72)
NA
WHOvariant

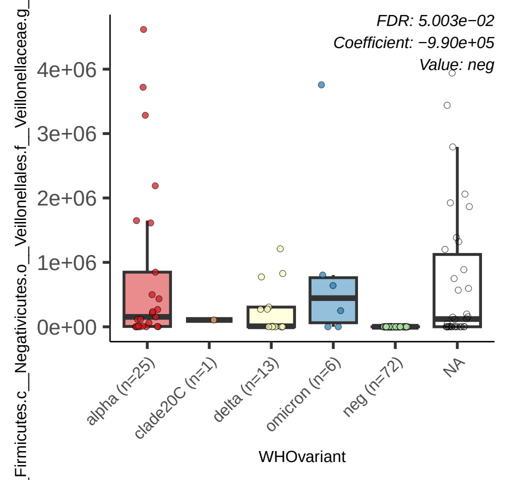

FDR: 5.003e−02
Coefficient: −9.90e+05
Value: neg
Firmicutes.c__Negativicutes.o__Veillonellales.f__Veillonellaceae.g__
4e+06
3e+06
2e+06
1e+06
0e+00
alpha (n=25)
clade20C (n=1)
delta (n=13)
omicron (n=6)
neg (n=72)
NA
WHOvariant

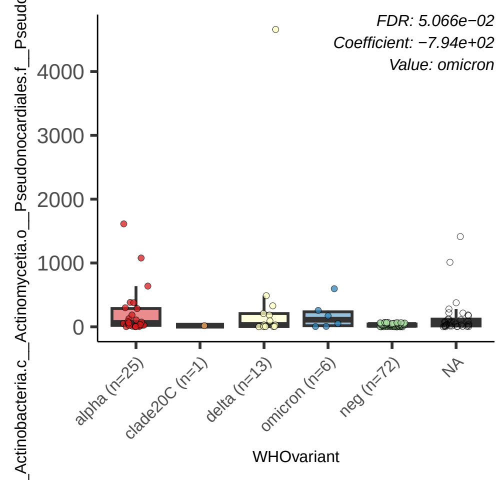

FDR: 5.066e−02
Coefficient: −7.94e+02
Value: omicron
_Actinobacteria.c__Actinomycetia.o__Pseudonocardiales.f__Pseudo
4000
3000
2000
1000
0
alpha (n=25)
clade20C (n=1)
delta (n=13)
omicron (n=6)
neg (n=72)
NA
WHOvariant

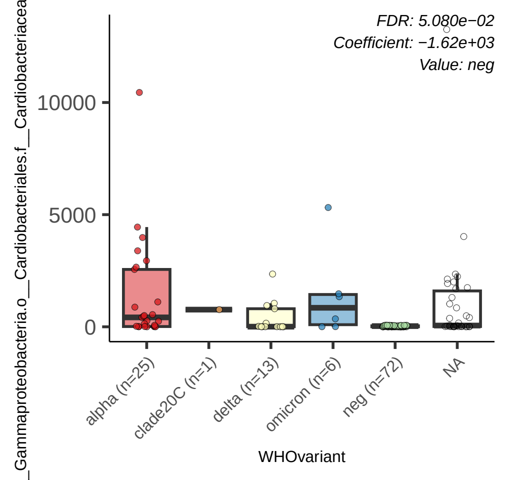
FDR: 5.080e−02
Coefficient: −1.62e+03
Value: neg
10000
5000
0
_Gammaproteobacteria.o__Cardiobacteriales.f__Cardiobacteriacea
alpha (n=25)
clade20C (n=1)
delta (n=13)
omicron (n=6)
neg (n=72)
NA
WHOvariant

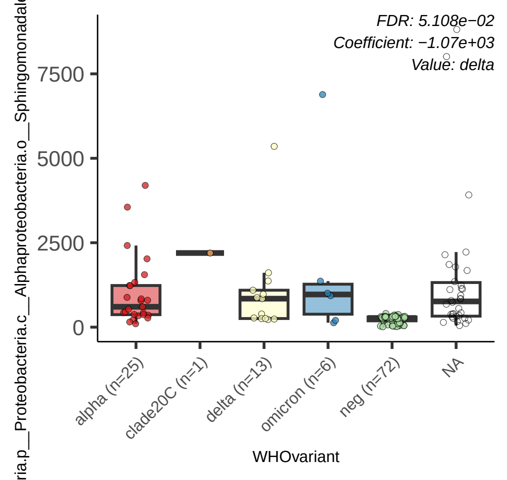
FDR: 5.108e−02
Coefficient: −1.07e+03
Value: delta
7500
5000
2500
0
ria.p__Proteobacteria.c__Alphaproteobacteria.o__Sphingomonadale
alpha (n=25)
clade20C (n=1)
delta (n=13)
omicron (n=6)
neg (n=72)
NA
WHOvariant

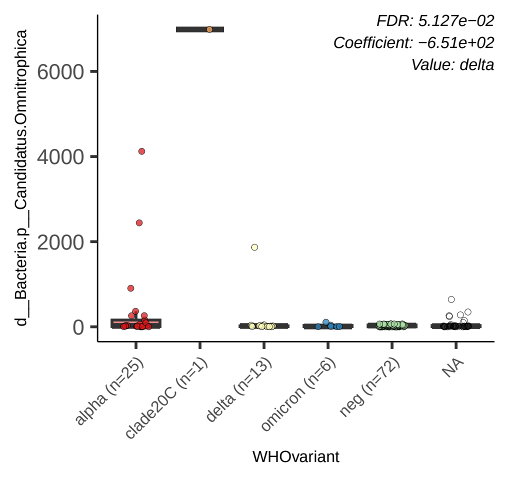

FDR: 5.127e−02
Coefficient: −6.51e+02
Value: delta
d__Bacteria.p__Candidatus.Omnitrophica
6000
4000
2000
0
alpha (n=25)
clade20C (n=1)
delta (n=13)
omicron (n=6)
neg (n=72)
NA
WHOvariant

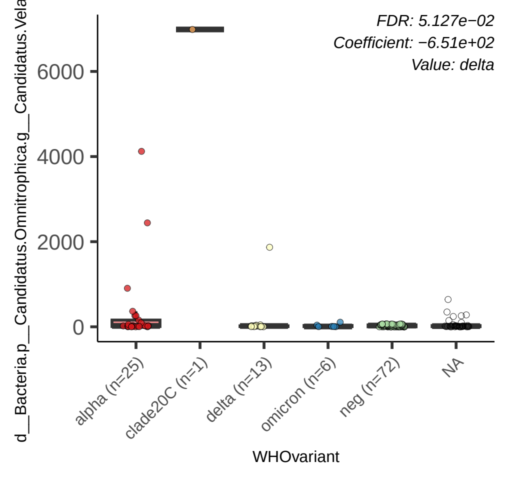
FDR: 5.127e−02
Coefficient: −6.51e+02
Value: delta
d__Bacteria.p__Candidatus.Omnitrophica.g__Candidatus.Vela
6000
4000
2000
0
alpha (n=25)
clade20C (n=1)
delta (n=13)
omicron (n=6)
neg (n=72)
NA
WHOvariant

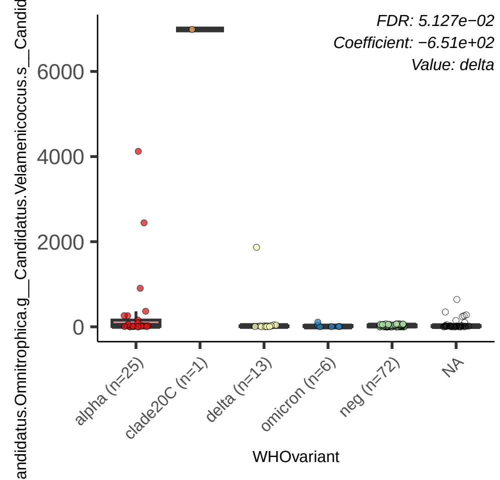
FDR: 5.127e−02
Coefficient: −6.51e+02
Value: delta
andidatus.Omnitrophica.g__Candidatus.Velamenicoccus.s__Candid
6000
4000
2000
0
alpha (n=25)
clade20C (n=1)
delta (n=13)
omicron (n=6)
neg (n=72)
NA
WHOvariant

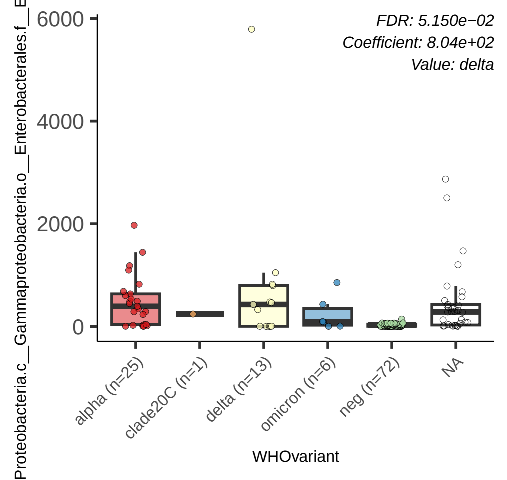

FDR: 5.150e−02
Coefficient: 8.04e+02
Value: delta
6000
4000
2000
0
Proteobacteria.c__Gammaproteobacteria.o__Enterobacterales.f__E
alpha (n=25)
clade20C (n=1)
delta (n=13)
omicron (n=6)
neg (n=72)
NA
WHOvariant

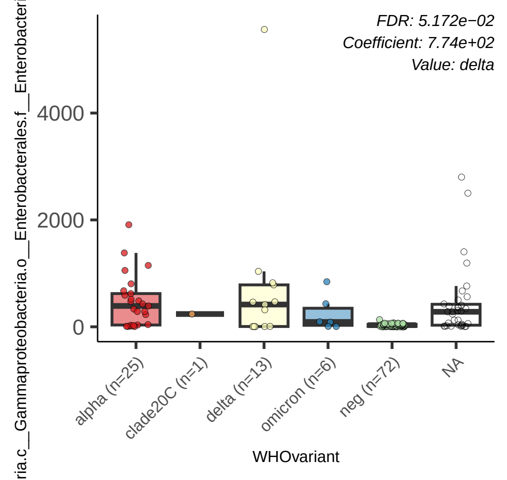

FDR: 5.172e−02
Coefficient: 7.74e+02
Value: delta
ria.c__Gammaproteobacteria.o__Enterobacterales.f__Enterobacteri
4000
2000
0
alpha (n=25)
clade20C (n=1)
delta (n=13)
omicron (n=6)
neg (n=72)
NA
WHOvariant

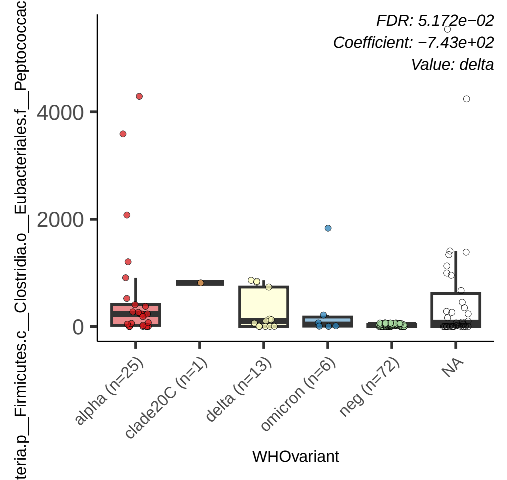
FDR: 5.172e−02
Coefficient: −7.43e+02
Value: delta
4000
2000
0
teria.p__Firmicutes.c__Clostridia.o__Eubacteriales.f__Peptococcac
alpha (n=25)
clade20C (n=1)
delta (n=13)
omicron (n=6)
neg (n=72)
NA
WHOvariant

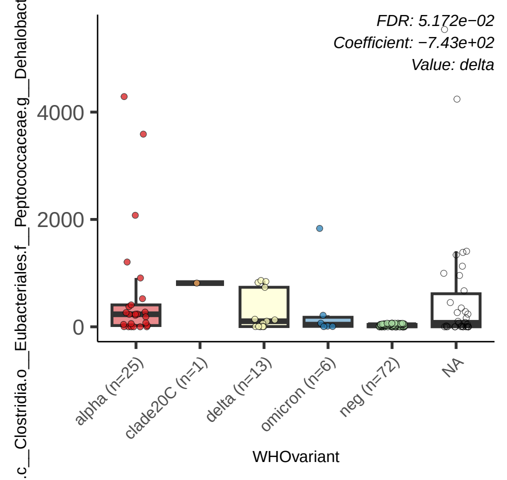

FDR: 5.172e−02
Coefficient: −7.43e+02
Value: delta
.c__Clostridia.o__Eubacteriales.f__Peptococcaceae.g__Dehalobact
4000
2000
0
alpha (n=25)
clade20C (n=1)
delta (n=13)
omicron (n=6)
neg (n=72)
NA
WHOvariant

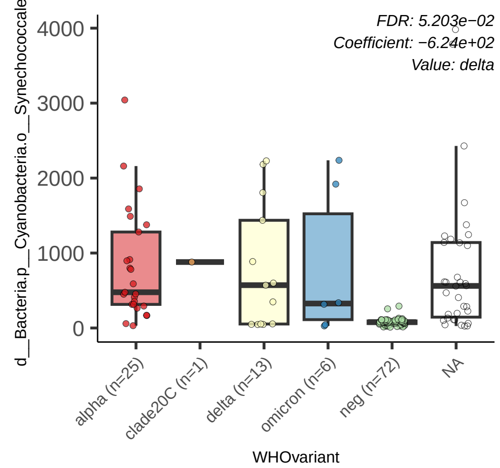

FDR: 5.203e−02
Coefficient: −6.24e+02
Value: delta
d__Bacteria.p__Cyanobacteria.o__Synechococcale
4000
3000
2000
1000
0
alpha (n=25)
clade20C (n=1)
delta (n=13)
omicron (n=6)
neg (n=72)
NA
WHOvariant

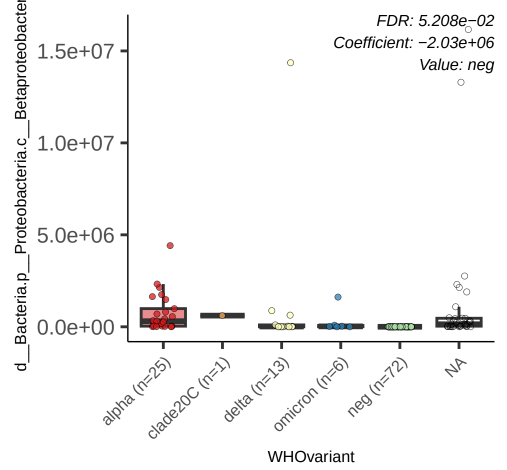
FDR: 5.208e−02
Coefficient: −2.03e+06
Value: neg
d__Bacteria.p__Proteobacteria.c__Betaproteobacter
1.5e+07
1.0e+07
5.0e+06
0.0e+00
alpha (n=25)
clade20C (n=1)
delta (n=13)
omicron (n=6)
neg (n=72)
NA
WHOvariant

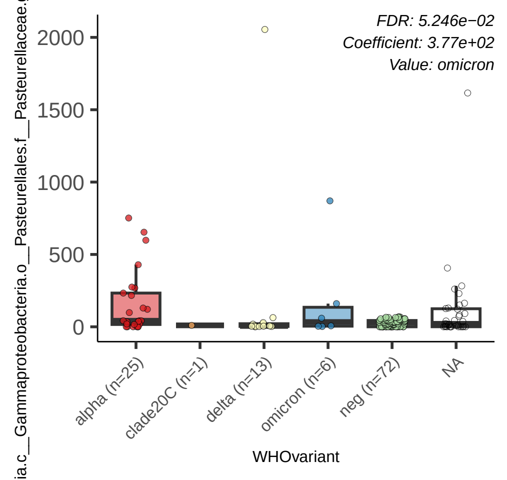

FDR: 5.246e−02
Coefficient: 3.77e+02
Value: omicron
ia.c__Gammaproteobacteria.o__Pasteurellales.f__Pasteurellaceae.g
2000
1500
1000
500
0
alpha (n=25)
clade20C (n=1)
delta (n=13)
omicron (n=6)
neg (n=72)
NA
WHOvariant

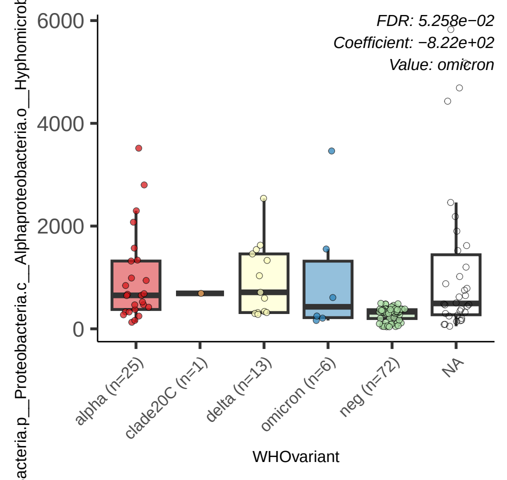
FDR: 5.258e−02
Coefficient: −8.22e+02
Value: omicron
6000
4000
2000
0
acteria.p__Proteobacteria.c__Alphaproteobacteria.o__Hyphomicrob
alpha (n=25)
clade20C (n=1)
delta (n=13)
omicron (n=6)
neg (n=72)
NA
WHOvariant

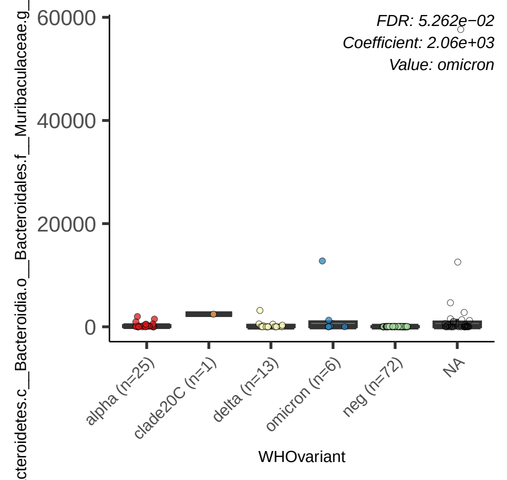
FDR: 5.262e−02
Coefficient: 2.06e+03
Value: omicron
acteroidetes.c__Bacteroidia.o__Bacteroidales.f__Muribaculaceae.g__
60000
40000
20000
0
alpha (n=25)
clade20C (n=1)
delta (n=13)
omicron (n=6)
neg (n=72)
NA
WHOvariant

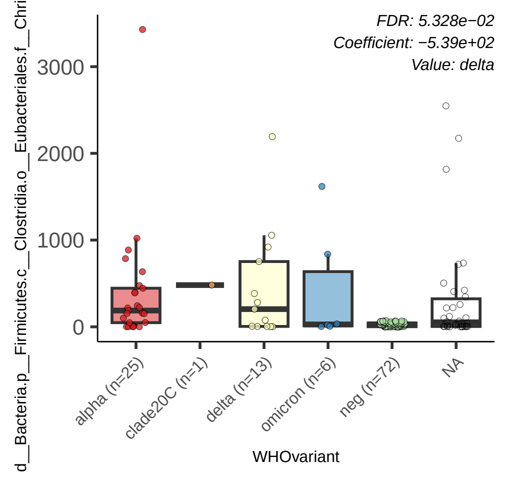
FDR: 5.328e−02
Coefficient: −5.39e+02
Value: delta
d__Bacteria.p__Firmicutes.c__Clostridia.o__Eubacteriales.f__Chri
3000
2000
1000
0
alpha (n=25)
clade20C (n=1)
delta (n=13)
omicron (n=6)
neg (n=72)
NA
WHOvariant

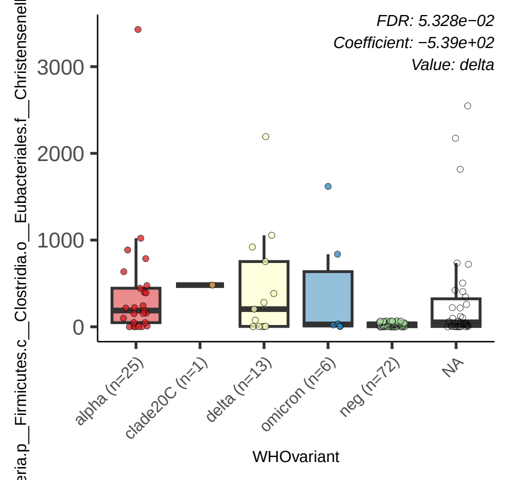

FDR: 5.328e−02
Coefficient: −5.39e+02
Value: delta
3000
2000
1000
0
eria.p__Firmicutes.c__Clostridia.o__Eubacteriales.f__Christensenell
alpha (n=25)
clade20C (n=1)
delta (n=13)
omicron (n=6)
neg (n=72)
NA
WHOvariant

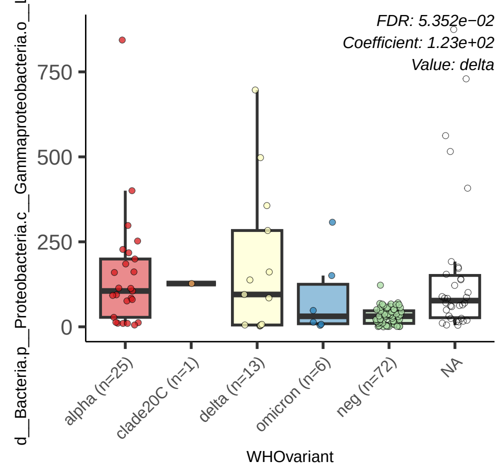

FDR: 5.352e−02
Coefficient: 1.23e+02
Value: delta
d__Bacteria.p__Proteobacteria.c__Gammaproteobacteria.o__L
0
250
500
750
alpha (n=25)
clade20C (n=1)
delta (n=13)
omicron (n=6)
neg (n=72)
NA
WHOvariant

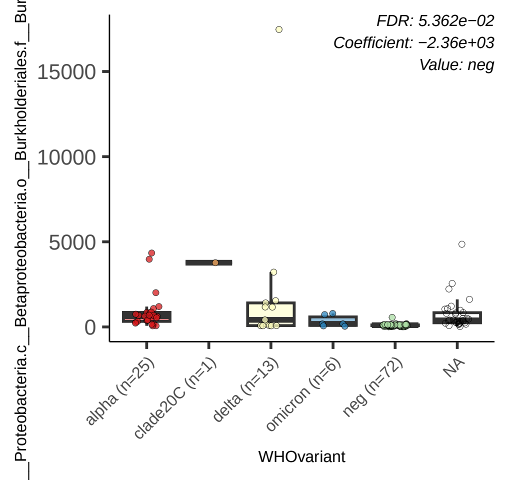
FDR: 5.362e−02
Coefficient: −2.36e+03
Value: neg
_Proteobacteria.c__Betaproteobacteria.o__Burkholderiales.f__Bur
0
5000
10000
15000
alpha (n=25)
clade20C (n=1)
delta (n=13)
omicron (n=6)
neg (n=72)
NA
WHOvariant

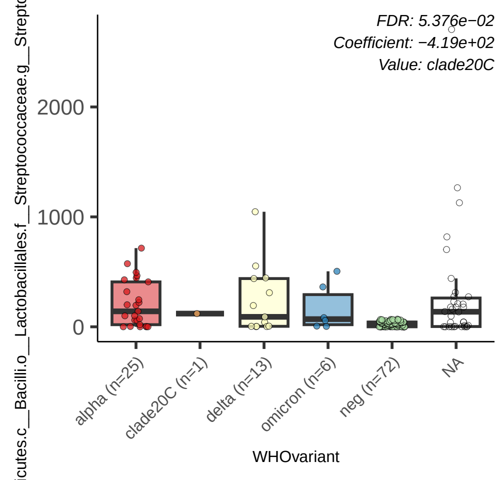

FDR: 5.376e−02
Coefficient: −4.19e+02
Value: clade20C
2000
1000
0
alpha (n=25)
clade20C (n=1)
delta (n=13)
omicron (n=6)
neg (n=72)
NA
WHOvariant
icutes.c__Bacilli.o__Lactobacillales.f__Streptococcaceae.g__Strepto

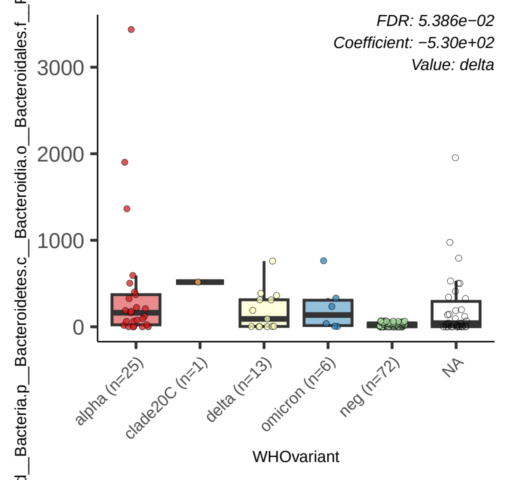
FDR: 5.386e−02
Coefficient: −5.30e+02
Value: delta
3000
2000
1000
0
alpha (n=25)
clade20C (n=1)
delta (n=13)
omicron (n=6)
neg (n=72)
NA
WHOvariant
d__Bacteria.p__Bacteroidetes.c__Bacteroidia.o__Bacteroidales.f__

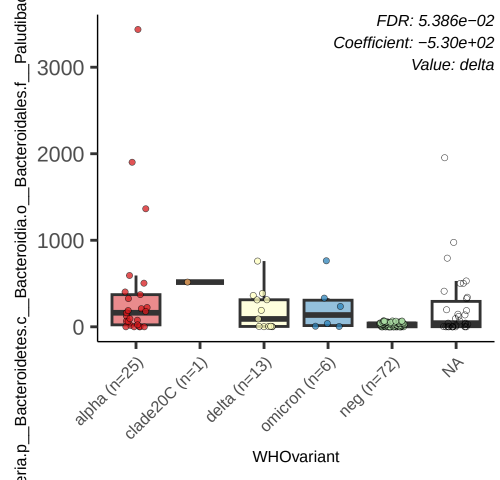

FDR: 5.386e−02
Coefficient: −5.30e+02
Value: delta
3000
2000
1000
0
alpha (n=25)
clade20C (n=1)
delta (n=13)
omicron (n=6)
neg (n=72)
NA
WHOvariant
eria.p__Bacteroidetes.c__Bacteroidia.o__Bacteroidales.f__Paludibac

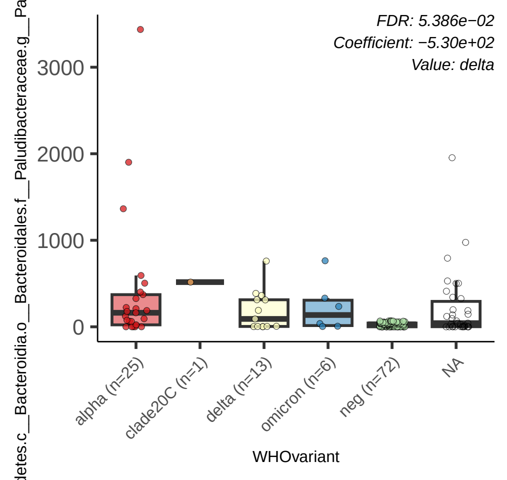

FDR: 5.386e−02
Coefficient: −5.30e+02
Value: delta
3000
2000
1000
0
detes.c__Bacteroidia.o__Bacteroidales.f__Paludibacteraceae.g__Pa
alpha (n=25)
clade20C (n=1)
delta (n=13)
omicron (n=6)
neg (n=72)
NA
WHOvariant

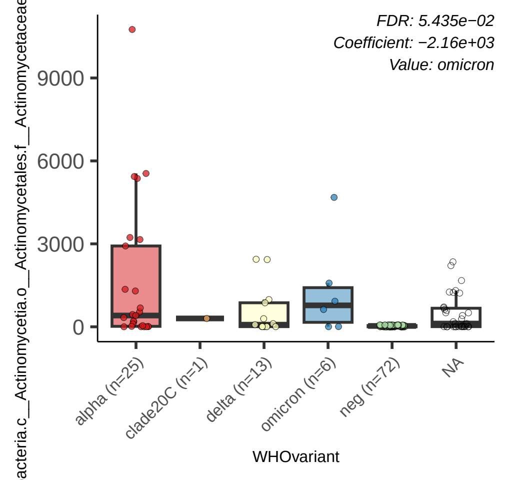
FDR: 5.435e−02
Coefficient: −2.16e+03
Value: omicron
acteria.c__Actinomycetia.o__Actinomycetales.f__Actinomycetaceae
9000
6000
3000
0
alpha (n=25)
clade20C (n=1)
delta (n=13)
omicron (n=6)
neg (n=72)
NA
WHOvariant

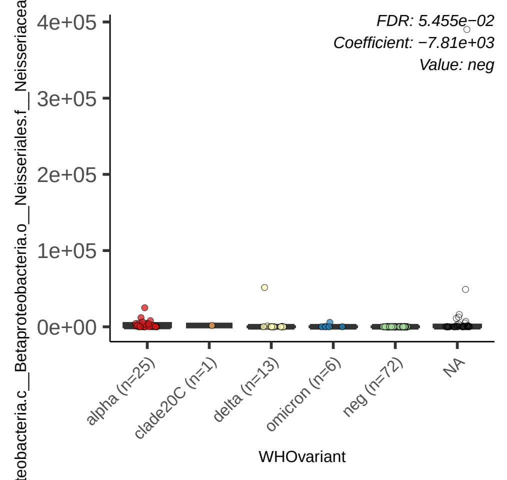

FDR: 5.455e−02
Coefficient: −7.81e+03
Value: neg
4e+05
3e+05
2e+05
1e+05
0e+00
teobacteria.c__Betaproteobacteria.o__Neisseriales.f__Neisseriacea
alpha (n=25)
clade20C (n=1)
delta (n=13)
omicron (n=6)
neg (n=72)
NA
WHOvariant

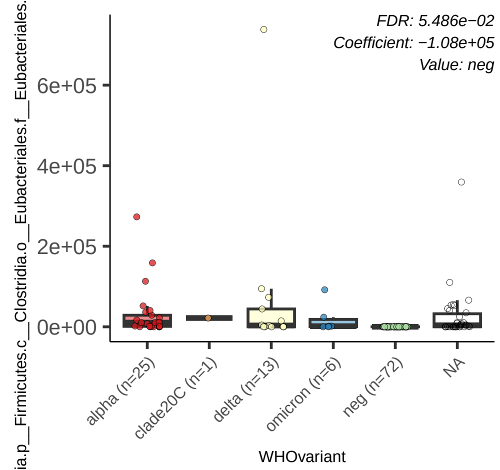

FDR: 5.486e−02
Coefficient: −1.08e+05
Value: neg
6e+05
4e+05
2e+05
0e+00
alpha (n=25)
clade20C (n=1)
delta (n=13)
omicron (n=6)
neg (n=72)
NA
WHOvariant

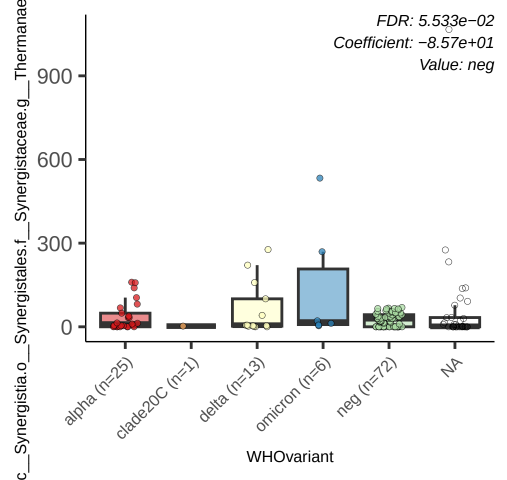
FDR: 5.533e−02
Coefficient: −8.57e+01
Value: neg
c__Synergistia.o__Synergistales.f__Synergistaceae.g__Thermanae
0
300
600
900
alpha (n=25)
clade20C (n=1)
delta (n=13)
omicron (n=6)
neg (n=72)
NA
WHOvariant

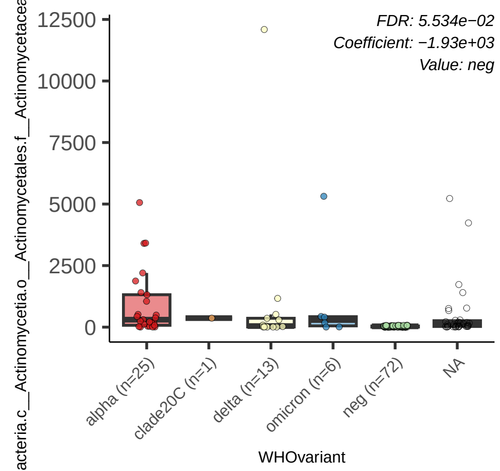

FDR: 5.534e−02
Coefficient: −1.93e+03
Value: neg
12500
10000
7500
5000
2500
0
bacteria.c__Actinomycetia.o__Actinomycetales.f__Actinomycetacea
alpha (n=25)
clade20C (n=1)
delta (n=13)
omicron (n=6)
neg (n=72)
NA
WHOvariant

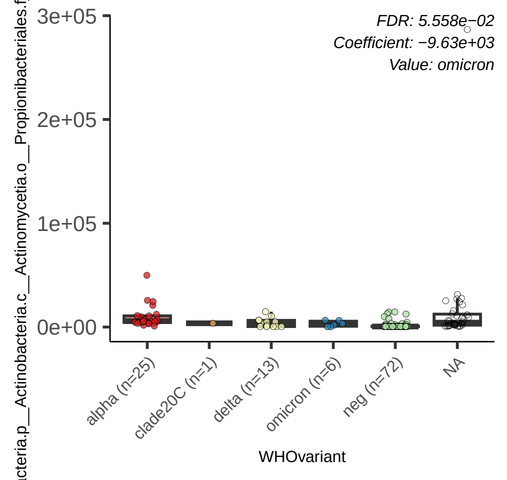

FDR: 5.558e−02
Coefficient: −9.63e+03
Value: omicron
3e+05
2e+05
1e+05
0e+00
cteria.p__Actinobacteria.c__Actinomycetia.o__Propionibacteriales.f
alpha (n=25)
clade20C (n=1)
delta (n=13)
omicron (n=6)
neg (n=72)
NA
WHOvariant

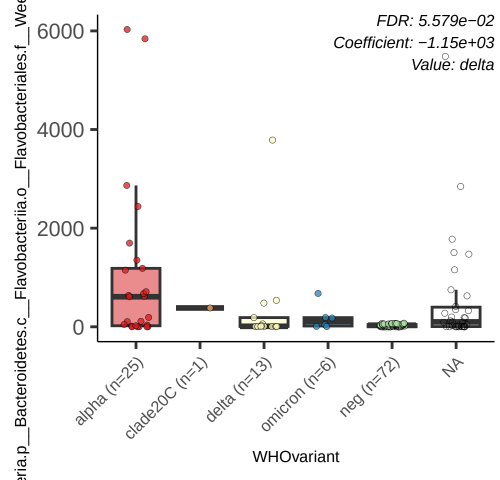

FDR: 5.579e−02
Coefficient: −1.15e+03
Value: delta
6000
4000
2000
0
Bacteroidetes.c__Flavobacteriia.o__Flavobacteriales.f__Wee
alpha (n=25)
clade20C (n=1)
delta (n=13)
omicron (n=6)
neg (n=72)
NA
WHOvariant

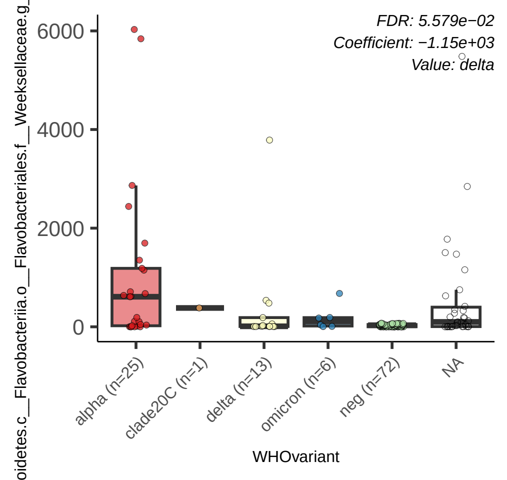

FDR: 5.579e−02
Coefficient: −1.15e+03
Value: delta
oidetes.c__Flavobacteriia.o__Flavobacteriales.f__Weeksellaceae.g_
6000
4000
2000
0
alpha (n=25)
clade20C (n=1)
delta (n=13)
omicron (n=6)
neg (n=72)
NA
WHOvariant

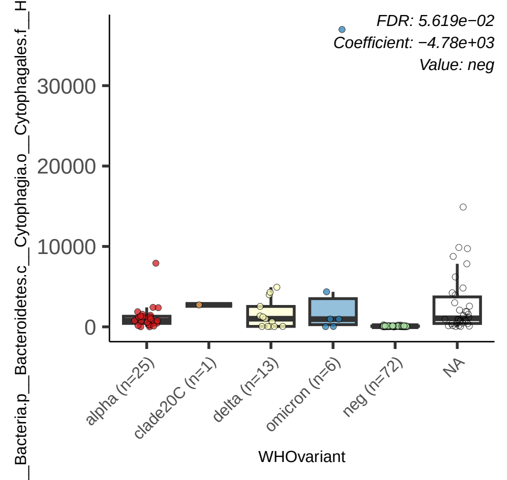

Bacteria.p__Bacteroidetes.c__Cytophagia.o__Cytophagales.f__H
FDR: 5.619e−02
Coefficient: −4.78e+03
Value: neg
30000
20000
10000
0
alpha (n=25)
clade20C (n=1)
delta (n=13)
omicron (n=6)
neg (n=72)
NA
WHOvariant

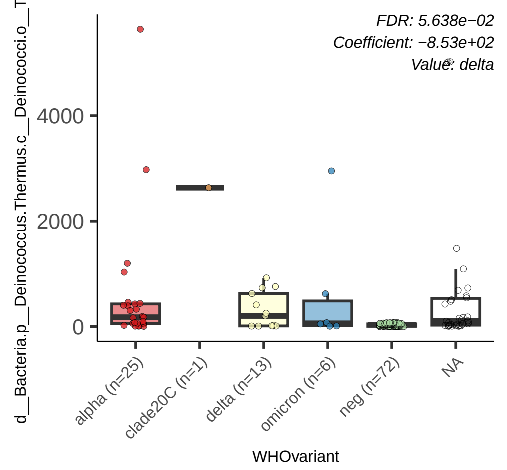

FDR: 5.638e−02
Coefficient: −8.53e+02
Value: delta
d__Bacteria.p__Deinococcus.Thermus.c__Deinococci.o__T
4000
2000
0
alpha (n=25)
clade20C (n=1)
delta (n=13)
omicron (n=6)
neg (n=72)
NA
WHOvariant

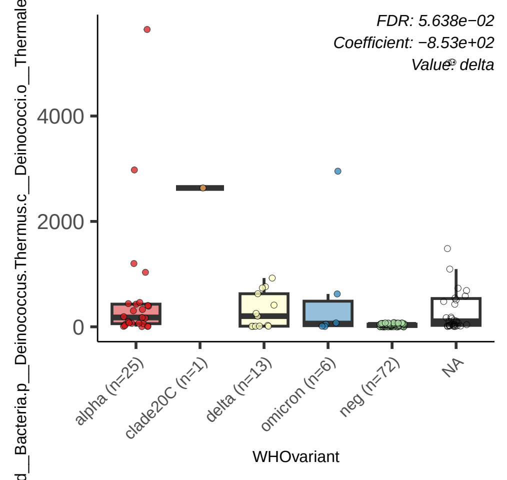

FDR: 5.638e−02
Coefficient: −8.53e+02
Value: delta
d__Bacteria.p__Deinococcus.Thermus.c__Deinococci.o__Thermale
4000
2000
0
alpha (n=25)
clade20C (n=1)
delta (n=13)
omicron (n=6)
neg (n=72)
NA
WHOvariant

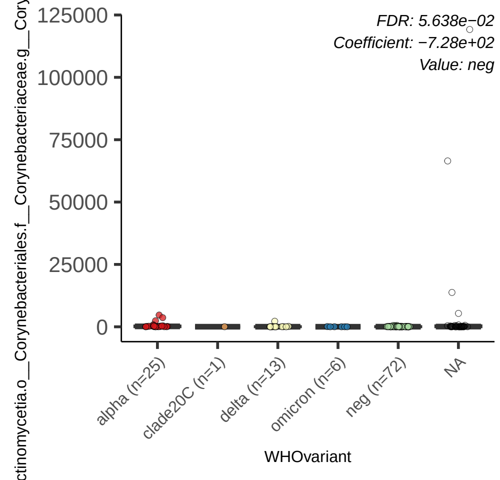

FDR: 5.638e−02
Coefficient: −7.28e+02
Value: neg
125000
100000
75000
50000
25000
0
ctinomycetia.o__Corynebacteriales.f__Corynebacteriaceae.g__Cory
alpha (n=25)
clade20C (n=1)
delta (n=13)
omicron (n=6)
neg (n=72)
NA
WHOvariant

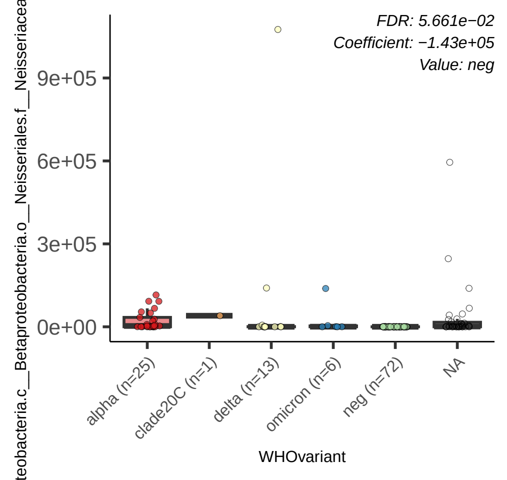

FDR: 5.661e−02
Coefficient: −1.43e+05
Value: neg
9e+05
6e+05
3e+05
0e+00
teobacteria.c__Betaproteobacteria.o__Neisseriales.f__Neisseriacea
alpha (n=25)
clade20C (n=1)
delta (n=13)
omicron (n=6)
neg (n=72)
NA
WHOvariant

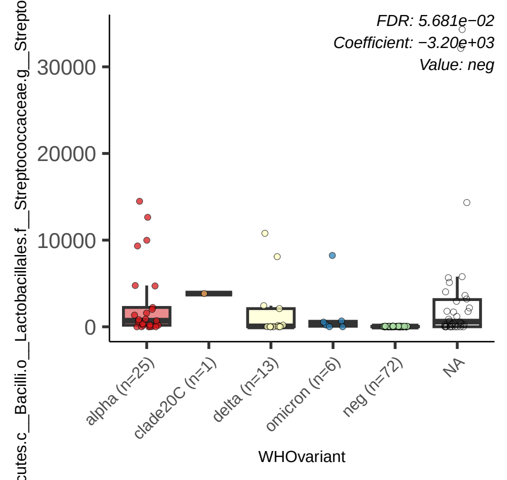

FDR: 5.681e−02
Coefficient: −3.20e+03
Value: neg
30000
20000
10000
0
cutes.c__Bacilli.o__Lactobacillales.f__Streptococcaceae.g__Strepto
alpha (n=25)
clade20C (n=1)
delta (n=13)
omicron (n=6)
neg (n=72)
NA
WHOvariant

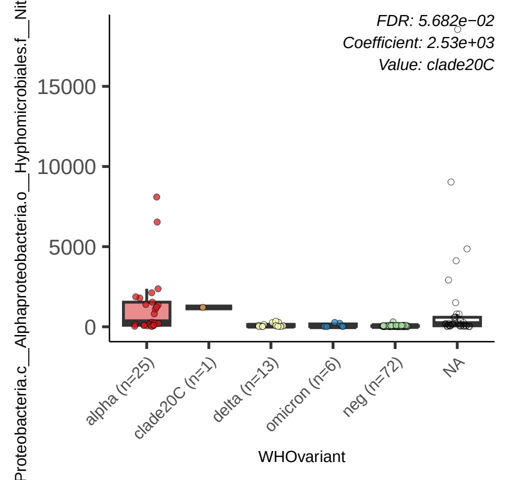

FDR: 5.682e−02
Coefficient: 2.53e+03
Value: clade20C
15000
10000
5000
0
Proteobacteria.c__Alphaproteobacteria.o__Hyphomicrobiales.f__Nit
alpha (n=25)
clade20C (n=1)
delta (n=13)
omicron (n=6)
neg (n=72)
NA
WHOvariant

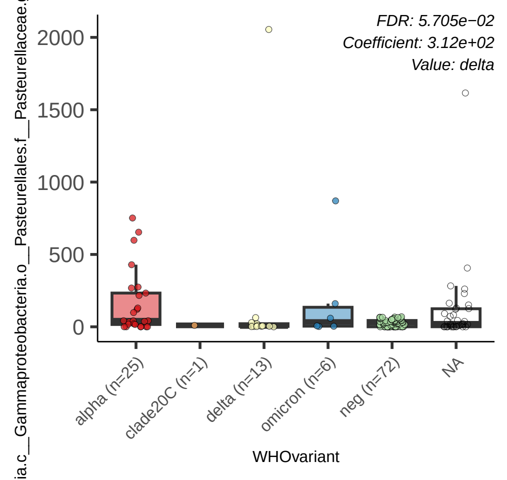

FDR: 5.705e−02
Coefficient: 3.12e+02
Value: delta
ia.c__Gammaproteobacteria.o__Pasteurellales.f__Pasteurellaceae.g
2000
1500
1000
500
0
alpha (n=25)
clade20C (n=1)
delta (n=13)
omicron (n=6)
neg (n=72)
NA
WHOvariant

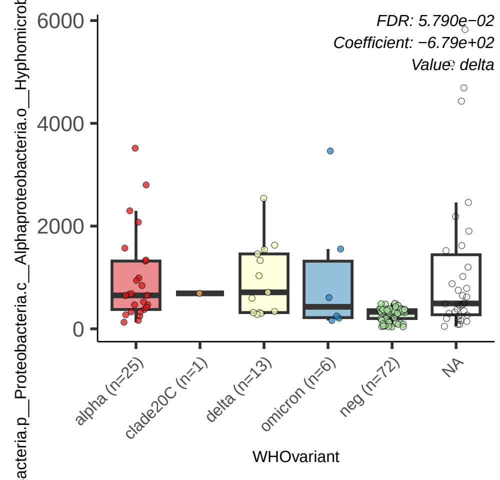
FDR: 5.790e−02
Coefficient: −6.79e+02
Value: delta
acteria.p__Proteobacteria.c__Alphaproteobacteria.o__Hyphomicrob
6000
4000
2000
0
alpha (n=25)
clade20C (n=1)
delta (n=13)
omicron (n=6)
neg (n=72)
NA
WHOvariant

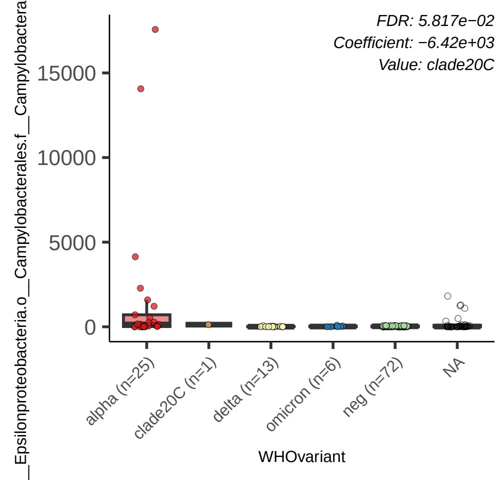
FDR: 5.817e−02
Coefficient: −6.42e+03
Value: clade20C
__Epsilonproteobacteria.o__Campylobacterales.f__Campylobactera
15000
10000
5000
0
alpha (n=25)
clade20C (n=1)
delta (n=13)
omicron (n=6)
neg (n=72)
NA
WHOvariant

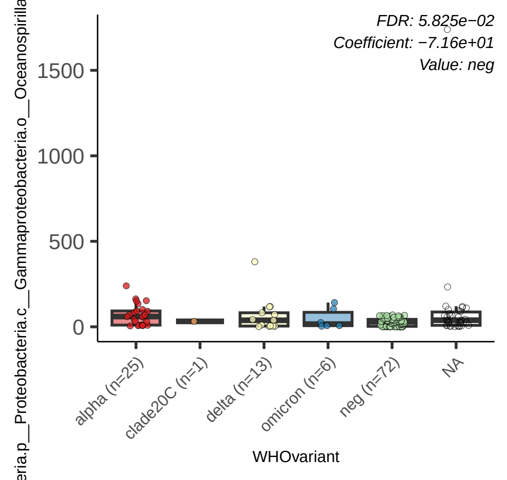
FDR: 5.825e−02
Coefficient: −7.16e+01
Value: neg
1500
1000
500
0
eria.p__Proteobacteria.c__Gammaproteobacteria.o__Oceanospirilla
alpha (n=25)
clade20C (n=1)
delta (n=13)
omicron (n=6)
neg (n=72)
NA
WHOvariant

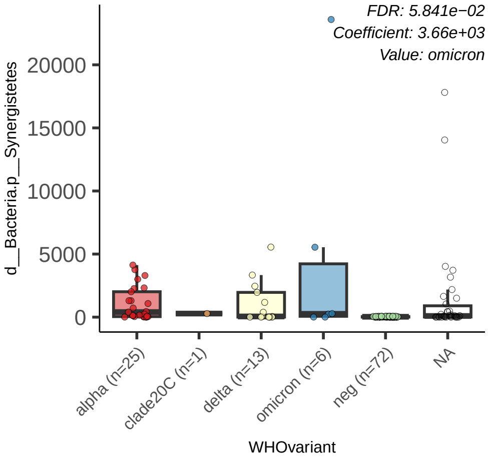

FDR: 5.841e−02
Coefficient: 3.66e+03
Value: omicron
d__Bacteria.p__Synergistetes
20000
15000
10000
5000
0
alpha (n=25)
clade20C (n=1)
delta (n=13)
omicron (n=6)
neg (n=72)
NA
WHOvariant

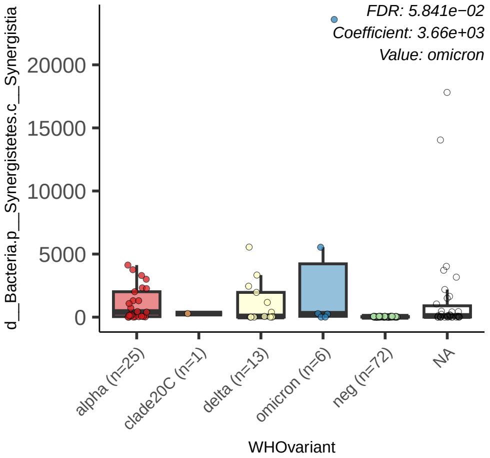

FDR: 5.841e−02
Coefficient: 3.66e+03
Value: omicron
d__Bacteria.p__Synergistetes.c__Synergistia
20000
15000
10000
5000
0
alpha (n=25)
clade20C (n=1)
delta (n=13)
omicron (n=6)
neg (n=72)
NA
WHOvariant

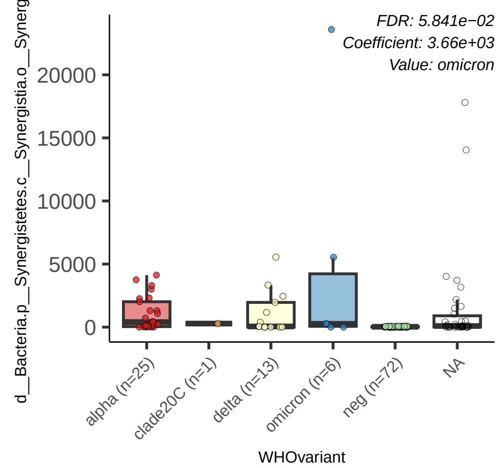

FDR: 5.841e−02
Coefficient: 3.66e+03
Value: omicron
d__Bacteria.p__Synergistetes.c__Synergistia.o__Synerg
20000
15000
10000
5000
0
alpha (n=25)
clade20C (n=1)
delta (n=13)
omicron (n=6)
neg (n=72)
NA
WHOvariant

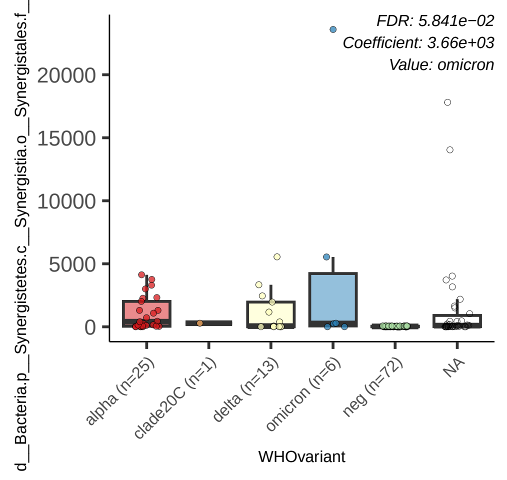
FDR: 5.841e−02
Coefficient: 3.66e+03
Value: omicron
d__Bacteria.p__Synergistetes.c__Synergistia.o__Synergistales.f__
0
5000
10000
15000
20000
alpha (n=25)
clade20C (n=1)
delta (n=13)
omicron (n=6)
neg (n=72)
NA
WHOvariant

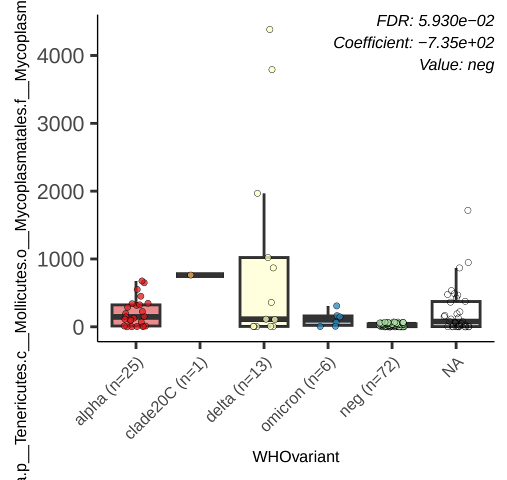
FDR: 5.930e−02
Coefficient: −7.35e+02
Value: neg
0
1000
2000
3000
4000
a.p__Tenericutes.c__Mollicutes.o__Mycoplasmatales.f__Mycoplasm
alpha (n=25)
clade20C (n=1)
delta (n=13)
omicron (n=6)
neg (n=72)
NA
WHOvariant

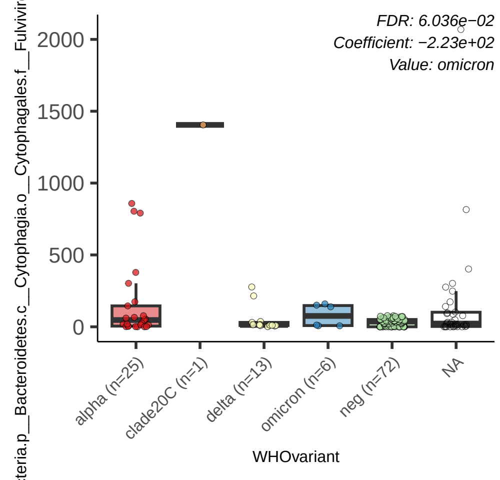

FDR: 6.036e−02
Coefficient: −2.23e+02
Value: omicron
2000
1500
1000
500
0
cteria.p__Bacteroidetes.c__Cytophagia.o__Cytophagales.f__Fulvivir
alpha (n=25)
clade20C (n=1)
delta (n=13)
omicron (n=6)
neg (n=72)
NA
WHOvariant

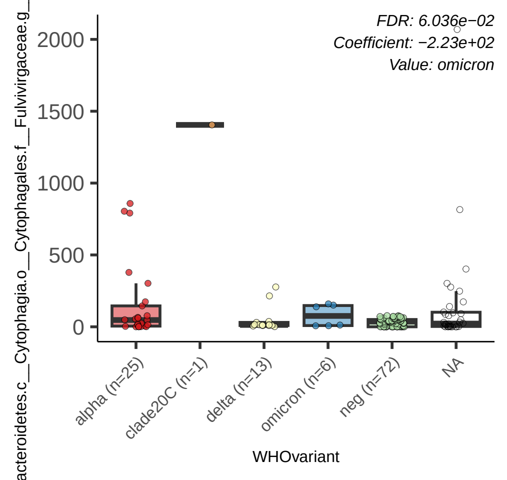

FDR: 6.036e−02
Coefficient: −2.23e+02
Value: omicron
acteroidetes.c__Cytophagia.o__Cytophagales.f__Fulvivirgaceae.g__
2000
1500
1000
500
0
alpha (n=25)
clade20C (n=1)
delta (n=13)
omicron (n=6)
neg (n=72)
NA
WHOvariant

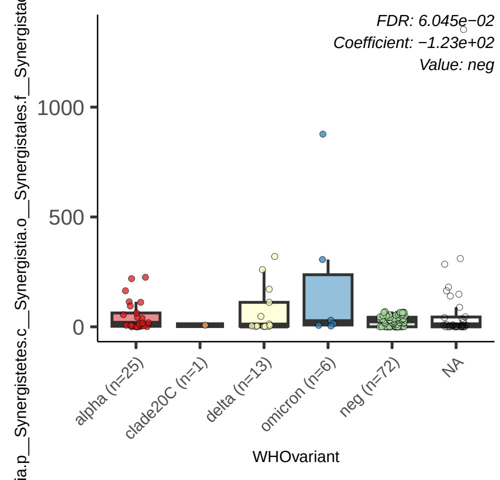
FDR: 6.045e−02
Coefficient: −1.23e+02
Value: neg
1000
500
0
alpha (n=25)
clade20C (n=1)
delta (n=13)
omicron (n=6)
neg (n=72)
NA
WHOvariant
ia.p__Synergistetes.c__Synergistia.o__Synergistales.f__Synergistac

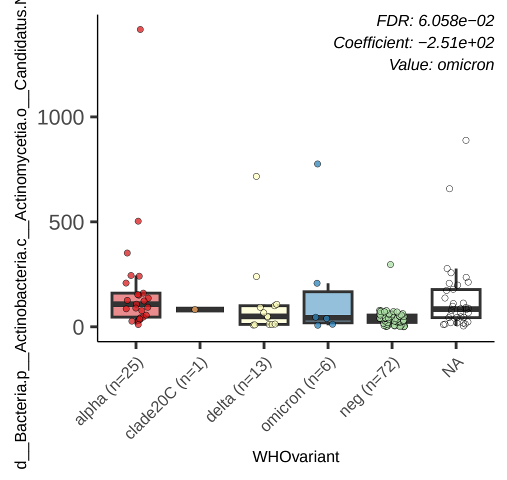

FDR: 6.058e−02
Coefficient: −2.51e+02
Value: omicron
1000
500
0
alpha (n=25)
clade20C (n=1)
delta (n=13)
omicron (n=6)
neg (n=72)
NA
WHOvariant
d__Bacteria.p__Actinobacteria.c__Actinomycetia.o__Candidatus.N

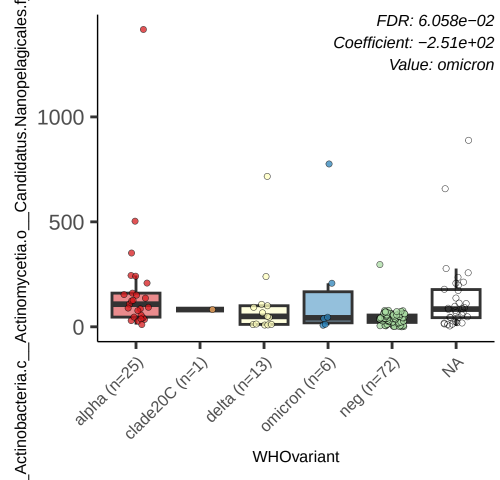

FDR: 6.058e−02
Coefficient: −2.51e+02
Value: omicron
1000
500
0
alpha (n=25)
clade20C (n=1)
delta (n=13)
omicron (n=6)
neg (n=72)
NA
WHOvariant
Actinobacteria.c__Actinomycetia.o__Candidatus.Nanopelagicales.f

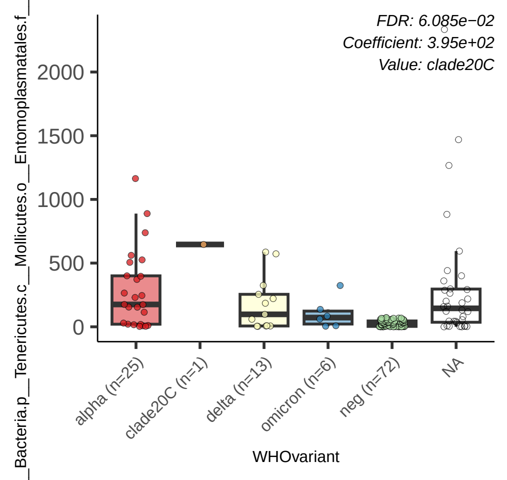
FDR: 6.085e−02
Coefficient: 3.95e+02
Value: clade20C
Bacteria.p__Tenericutes.c__Mollicutes.o__Entomoplasmatales.f
2000
1500
1000
500
0
alpha (n=25)
clade20C (n=1)
delta (n=13)
omicron (n=6)
neg (n=72)
NA
WHOvariant

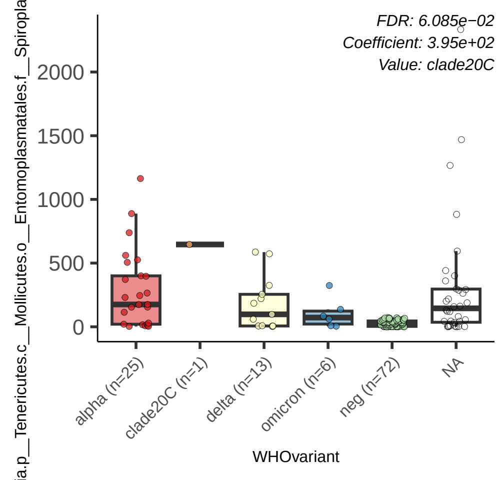

FDR: 6.085e−02
Coefficient: 3.95e+02
Value: clade20C
ia.p__Tenericutes.c__Mollicutes.o__Entomoplasmatales.f__Spiropla
0
500
1000
1500
2000
alpha (n=25)
clade20C (n=1)
delta (n=13)
omicron (n=6)
neg (n=72)
NA
WHOvariant

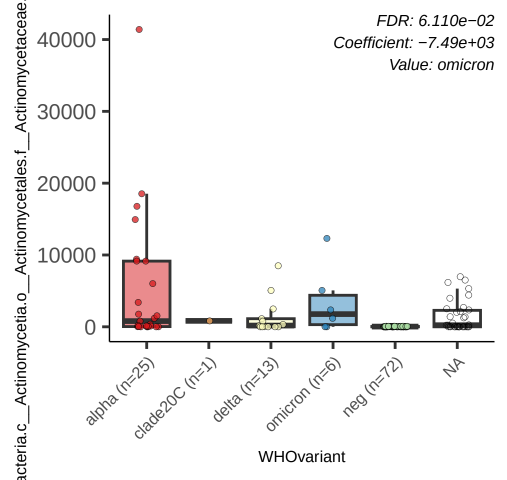
FDR: 6.110e−02
Coefficient: −7.49e+03
Value: omicron
acteria.c__Actinomycetia.o__Actinomycetales.f__Actinomycetaceae.
40000
30000
20000
10000
0
alpha (n=25)
clade20C (n=1)
delta (n=13)
omicron (n=6)
neg (n=72)
NA
WHOvariant

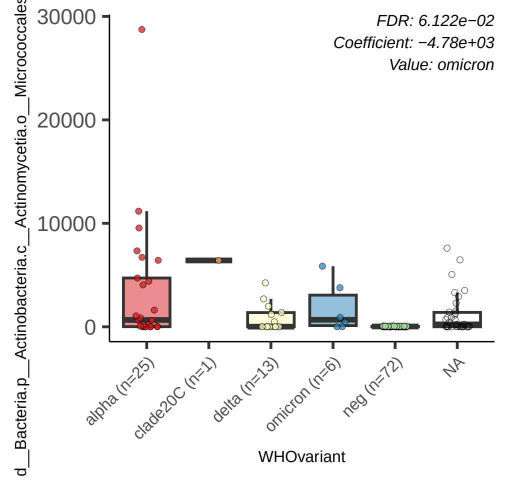
FDR: 6.122e−02
Coefficient: −4.78e+03
Value: omicron
d__Bacteria.p__Actinobacteria.c__Actinomycetia.o__Micrococcales
30000
20000
10000
0
alpha (n=25)
clade20C (n=1)
delta (n=13)
omicron (n=6)
neg (n=72)
NA
WHOvariant

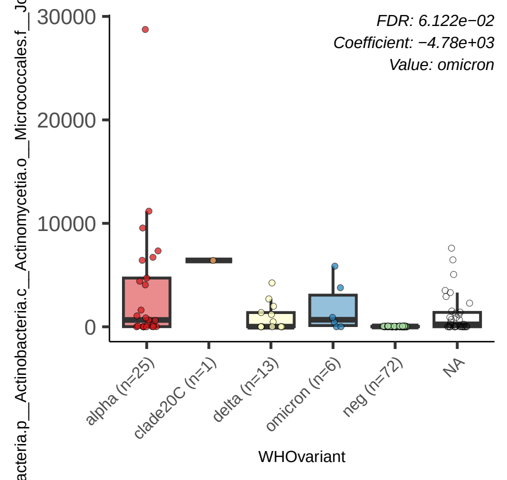
FDR: 6.122e−02
Coefficient: −4.78e+03
Value: omicron
30000
20000
10000
0
alpha (n=25)
clade20C (n=1)
delta (n=13)
omicron (n=6)
neg (n=72)
NA
WHOvariant
acteria.p__Actinobacteria.c__Actinomycetia.o__Micrococcales.f__Jo

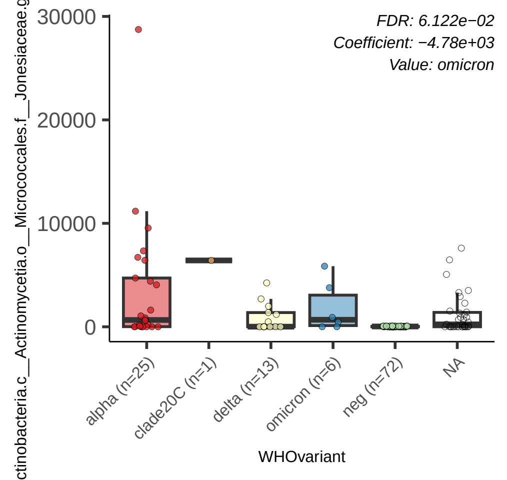

FDR: 6.122e−02
Coefficient: −4.78e+03
Value: omicron
30000
20000
10000
0
ctinobacteria.c__Actinomycetia.o__Micrococcales.f__Jonesiaceae.g
alpha (n=25)
clade20C (n=1)
delta (n=13)
omicron (n=6)
neg (n=72)
NA
WHOvariant

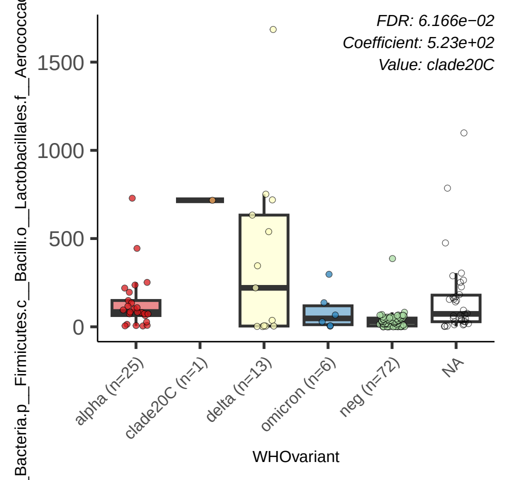

FDR: 6.166e−02
Coefficient: 5.23e+02
Value: clade20C
Bacteria.p__Firmicutes.c__Bacilli.o__Lactobacillales.f__Aerococcac
1500
1000
500
0
alpha (n=25)
clade20C (n=1)
delta (n=13)
omicron (n=6)
neg (n=72)
NA
WHOvariant

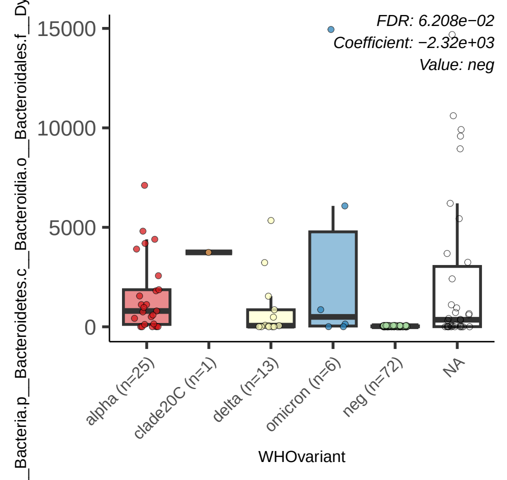

FDR: 6.208e−02
Coefficient: −2.32e+03
Value: neg
15000
10000
5000
0
Bacteria.p__Bacteroidetes.c__Bacteroidia.o__Bacteroidales.f__Dy
alpha (n=25)
clade20C (n=1)
delta (n=13)
omicron (n=6)
neg (n=72)
NA
WHOvariant

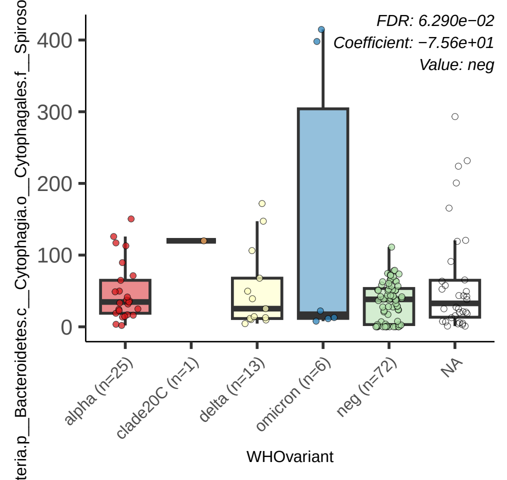
FDR: 6.290e−02
Coefficient: −7.56e+01
Value: neg
teria.p__Bacteroidetes.c__Cytophagia.o__Cytophagales.f__Spiroso
0
100
200
300
400
alpha (n=25)
clade20C (n=1)
delta (n=13)
omicron (n=6)
neg (n=72)
NA
WHOvariant

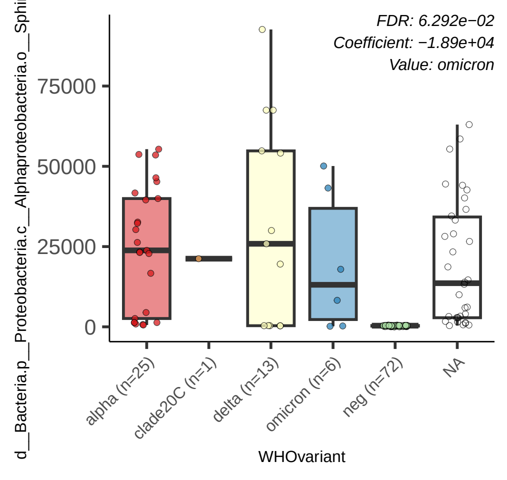

FDR: 6.292e−02
Coefficient: −1.89e+04
Value: omicron
d__Bacteria.p__Proteobacteria.c__Alphaproteobacteria.o__Sphi
0
25000
50000
75000
alpha (n=25)
clade20C (n=1)
delta (n=13)
omicron (n=6)
neg (n=72)
NA
WHOvariant

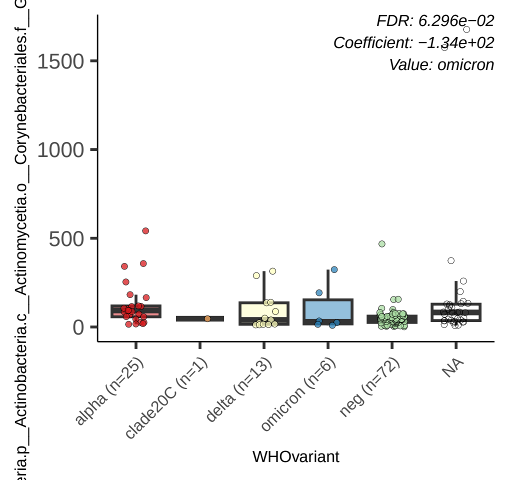

FDR: 6.296e−02
Coefficient: −1.34e+02
Value: omicron
1500
1000
500
0
Actinobacteria.p__Actinobacteria.c__Actinomycetia.o__Corynebacteriales.f__G
alpha (n=25)
clade20C (n=1)
delta (n=13)
omicron (n=6)
neg (n=72)
NA
WHOvariant

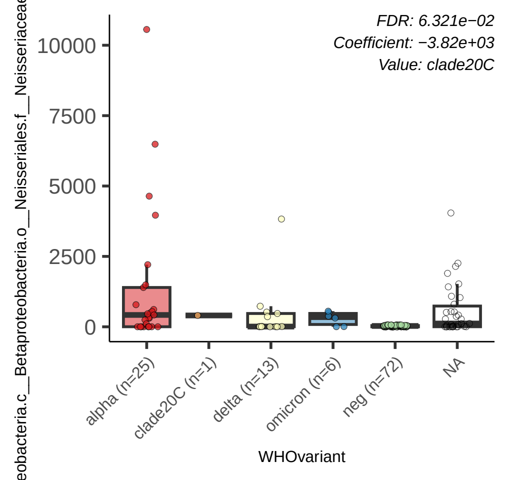

FDR: 6.321e−02
Coefficient: −3.82e+03
Value: clade20C
10000
7500
5000
2500
0
eobacteria.c__Betaproteobacteria.o__Neisseriales.f__Neisseriaceae
alpha (n=25)
clade20C (n=1)
delta (n=13)
omicron (n=6)
neg (n=72)
NA
WHOvariant

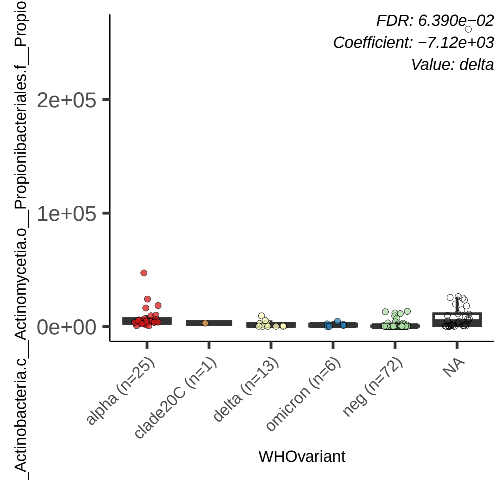

FDR: 6.390e−02
Coefficient: −7.12e+03
Value: delta
2e+05
1e+05
0e+00
alpha (n=25)
clade20C (n=1)
delta (n=13)
omicron (n=6)
neg (n=72)
NA
WHOvariant
Actinobacteria.c__Actinomycetia.o__Propionibacteriales.f__Propio

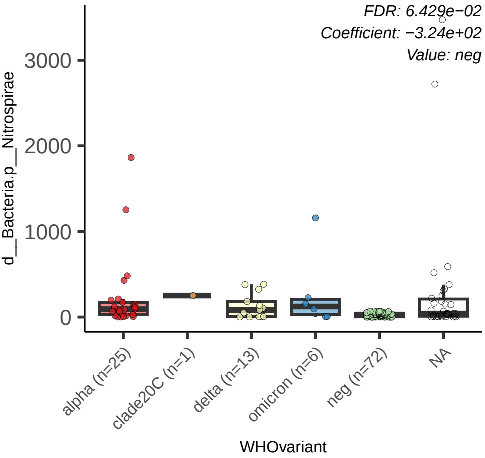

FDR: 6.429e−02
Coefficient: −3.24e+02
Value: neg
d__Bacteria.p__Nitrospirae
3000
2000
1000
0
alpha (n=25)
clade20C (n=1)
delta (n=13)
omicron (n=6)
neg (n=72)
NA
WHOvariant

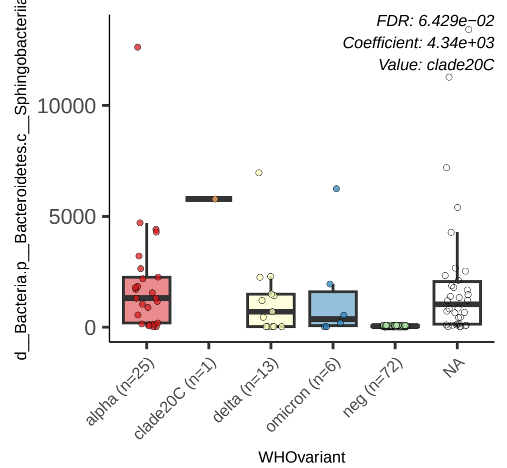
FDR: 6.429e−02
Coefficient: 4.34e+03
Value: clade20C
d__Bacteria.p__Bacteroidetes.c__Sphingobacteriia
10000
5000
0
alpha (n=25)
clade20C (n=1)
delta (n=13)
omicron (n=6)
neg (n=72)
NA
WHOvariant

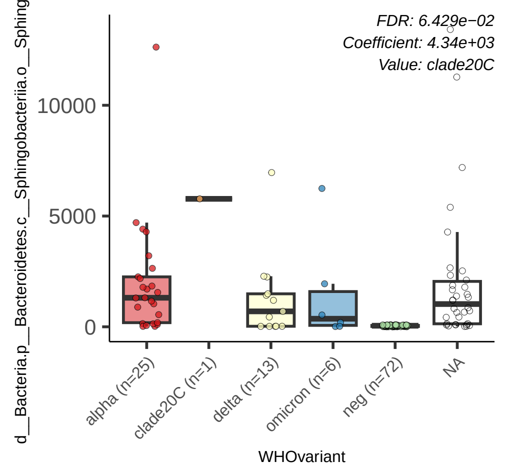

FDR: 6.429e−02
Coefficient: 4.34e+03
Value: clade20C
d__Bacteria.p__Bacteroidetes.c__Sphingobacteriia.o__Sphing
10000
5000
0
alpha (n=25)
clade20C (n=1)
delta (n=13)
omicron (n=6)
neg (n=72)
NA
WHOvariant

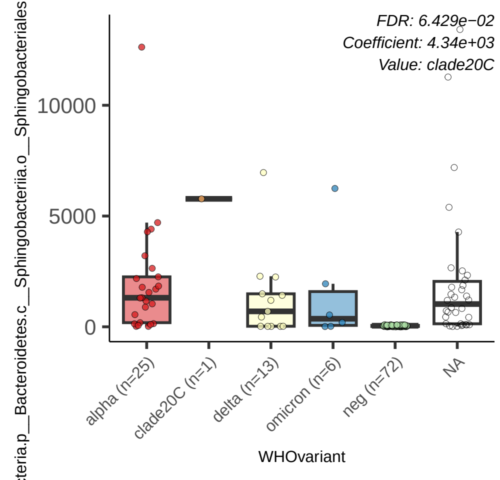

FDR: 6.429e−02
Coefficient: 4.34e+03
Value: clade20C
10000
5000
0
acteria.p__Bacteroidetes.c__Sphingobacteriia.o__Sphingobacteriales
alpha (n=25)
clade20C (n=1)
delta (n=13)
omicron (n=6)
neg (n=72)
NA
WHOvariant

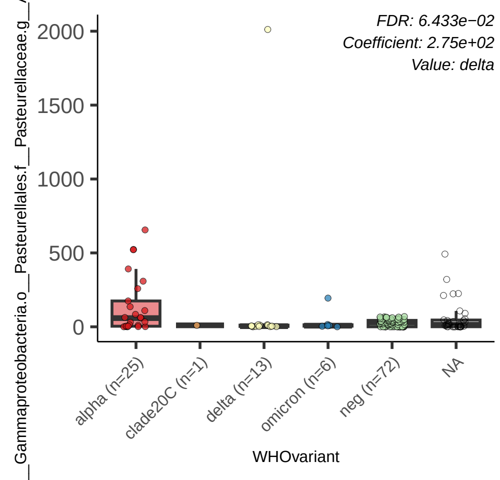

FDR: 6.433e−02
Coefficient: 2.75e+02
Value: delta
__Gammaproteobacteria.o__Pasteurellales.f__Pasteurellaceae.g__
2000
1500
1000
500
0
alpha (n=25)
clade20C (n=1)
delta (n=13)
omicron (n=6)
neg (n=72)
NA
WHOvariant

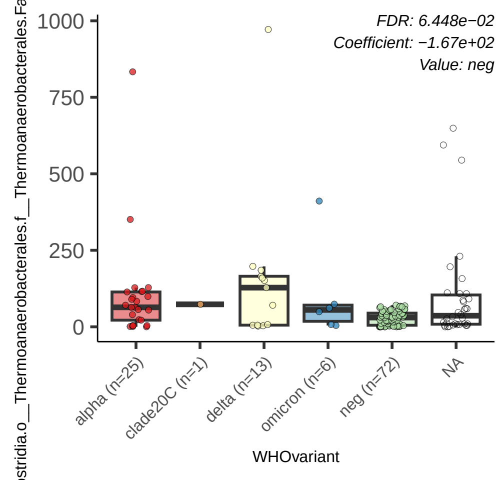

FDR: 6.448e−02
Coefficient: −1.67e+02
Value: neg
1000
750
500
250
0
ostridia.o__Thermoanaerobacterales.f__Thermoanaerobacterales.Fa
alpha (n=25)
clade20C (n=1)
delta (n=13)
omicron (n=6)
neg (n=72)
NA
WHOvariant

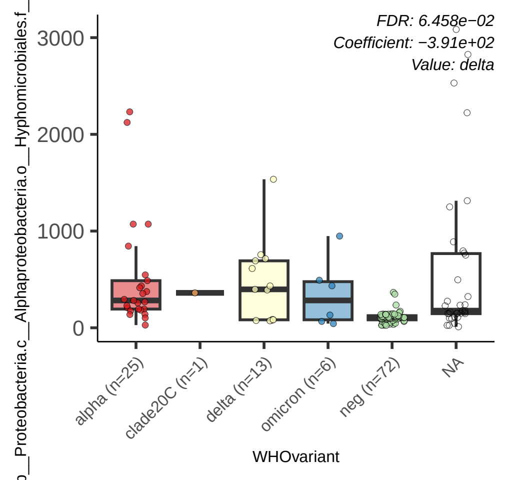

FDR: 6.458e−02
Coefficient: −3.91e+02
Value: delta
3000
2000
1000
0
o__Proteobacteria.c__Alphaproteobacteria.o__Hyphomicrobiales.f__
alpha (n=25)
clade20C (n=1)
delta (n=13)
omicron (n=6)
neg (n=72)
NA
WHOvariant

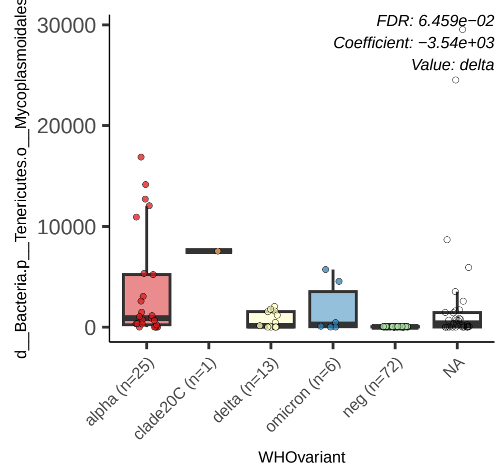

FDR: 6.459e−02
Coefficient: −3.54e+03
Value: delta
30000
20000
10000
0
d__Bacteria.p__Tenericutes.o__Mycoplasmoidales
alpha (n=25)
clade20C (n=1)
delta (n=13)
omicron (n=6)
neg (n=72)
NA
WHOvariant

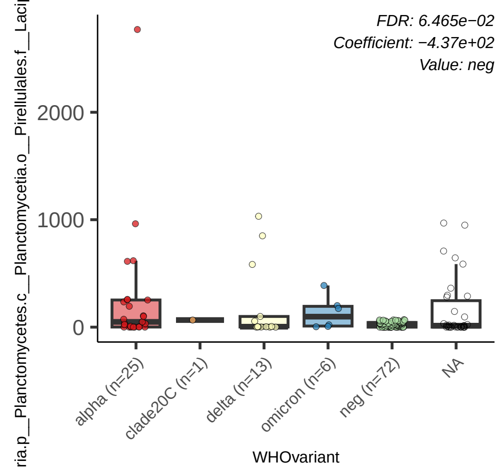

FDR: 6.465e−02
Coefficient: −4.37e+02
Value: neg
2000
1000
0
teria.p__Planctomycetes.c__Planctomycetia.o__Pirellulales.f__Lacip
alpha (n=25)
clade20C (n=1)
delta (n=13)
omicron (n=6)
neg (n=72)
NA
WHOvariant

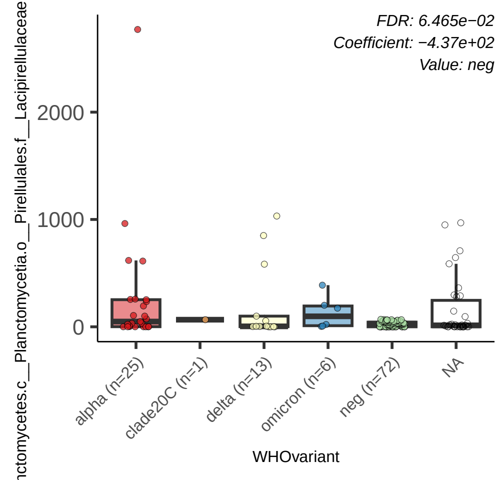

FDR: 6.465e−02
Coefficient: −4.37e+02
Value: neg
nctomycetes.c__Planctomycetia.o__Pirellulales.f__Lacipirellulaceae
2000
1000
0
alpha (n=25)
clade20C (n=1)
delta (n=13)
omicron (n=6)
neg (n=72)
NA
WHOvariant

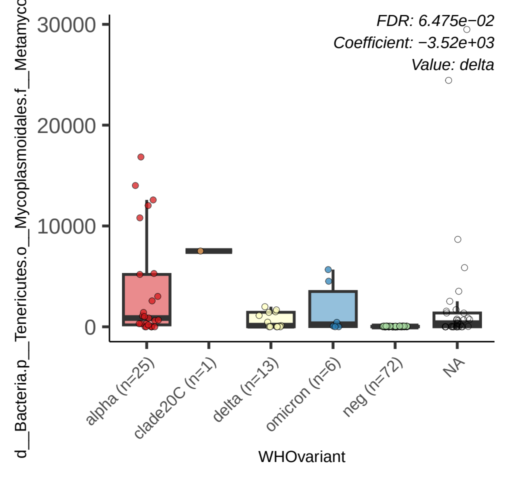

FDR: 6.475e−02
Coefficient: −3.52e+03
Value: delta
30000
20000
10000
0
d__Bacteria.p__Tenericutes.o__Mycoplasmoidales.f__Metamyco
alpha (n=25)
clade20C (n=1)
delta (n=13)
omicron (n=6)
neg (n=72)
NA
WHOvariant

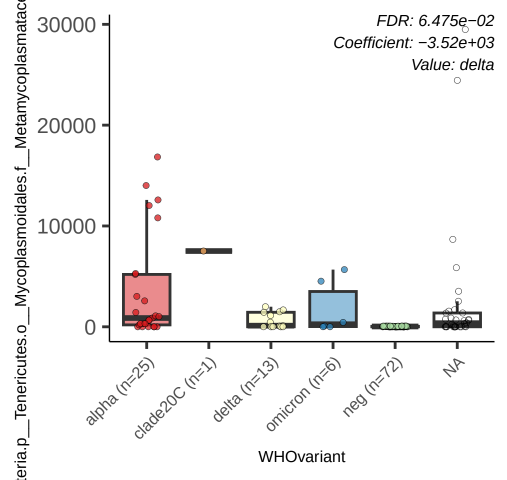

FDR: 6.475e−02
Coefficient: −3.52e+03
Value: delta
30000
20000
10000
0
alpha (n=25)
clade20C (n=1)
delta (n=13)
omicron (n=6)
neg (n=72)
NA
WHOvariant
teria.p__Tenericutes.o__Mycoplasmoidales.f__Metamycoplasmatac

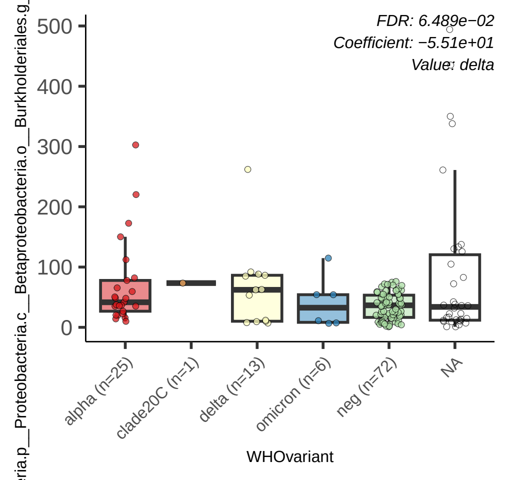
FDR: 6.489e−02
Coefficient: −5.51e+01
Value: delta
0
100
200
300
400
500
alpha (n=25)
clade20C (n=1)
delta (n=13)
omicron (n=6)
neg (n=72)
NA
WHOvariant
eria.p__Proteobacteria.c__Betaproteobacteria.o__Burkholderiales.g_

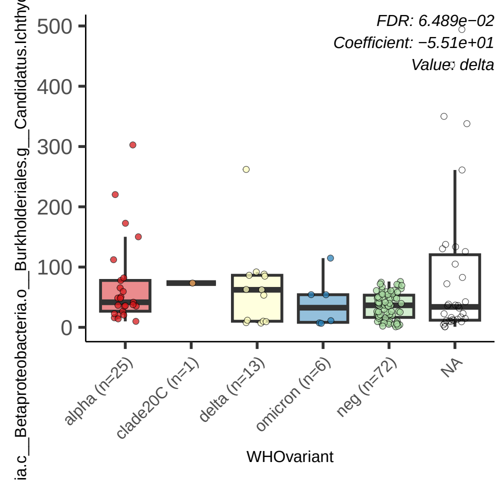

FDR: 6.489e−02
Coefficient: −5.51e+01
Value: delta
500
400
300
200
100
0
ia.c__Betaproteobacteria.o__Burkholderiales.g__Candidatus.Ichthyo
alpha (n=25)
clade20C (n=1)
delta (n=13)
omicron (n=6)
neg (n=72)
NA
WHOvariant

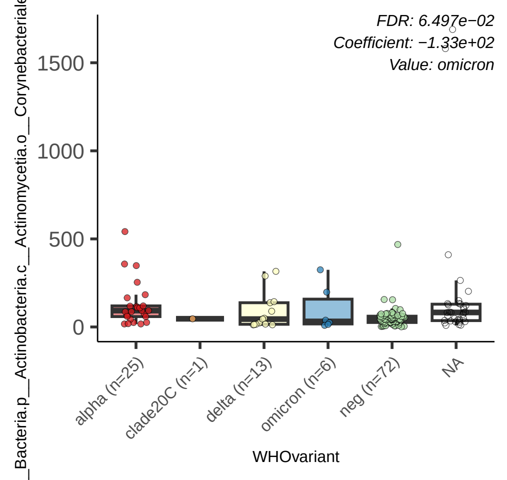
FDR: 6.497e−02
Coefficient: −1.33e+02
Value: omicron
_Bacteria.p__Actinobacteria.c__Actinomycetia.o__Corynebacteriale
0
500
1000
1500
alpha (n=25)
clade20C (n=1)
delta (n=13)
omicron (n=6)
neg (n=72)
NA
WHOvariant

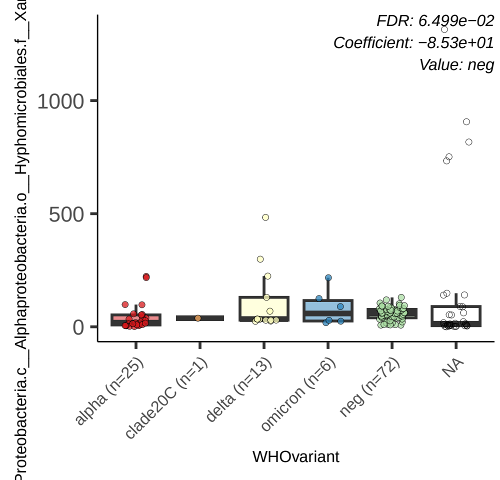
FDR: 6.499e−02
Coefficient: −8.53e+01
Value: neg
1000
500
0
Proteobacteria.c__Alphaproteobacteria.o__Hyphomicrobiales.f__Xa
alpha (n=25)
clade20C (n=1)
delta (n=13)
omicron (n=6)
neg (n=72)
NA
WHOvariant

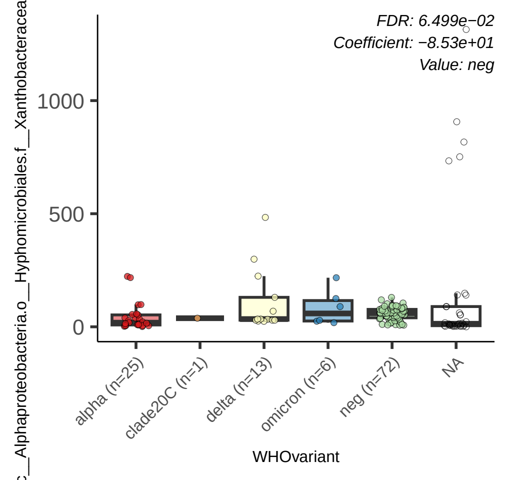

FDR: 6.499e−02
Coefficient: −8.53e+01
Value: neg
1000
500
0
c__Alphaproteobacteria.o__Hyphomicrobiales.f__Xanthobacteracea
alpha (n=25)
clade20C (n=1)
delta (n=13)
omicron (n=6)
neg (n=72)
NA
WHOvariant

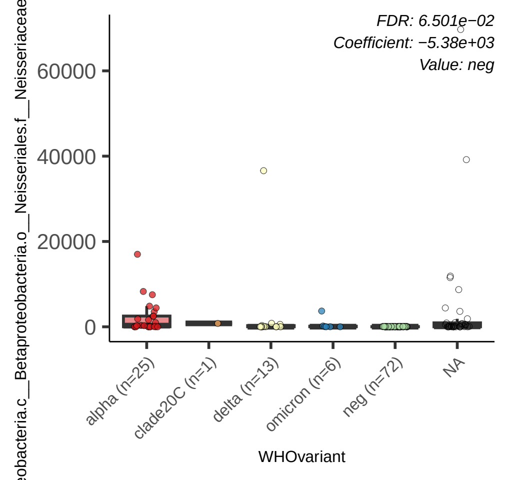

FDR: 6.501e−02
Coefficient: −5.38e+03
Value: neg
60000
40000
20000
0
eobacteria.c__Betaproteobacteria.o__Neisseriales.f__Neisseriaceae
alpha (n=25)
clade20C (n=1)
delta (n=13)
omicron (n=6)
neg (n=72)
NA
WHOvariant

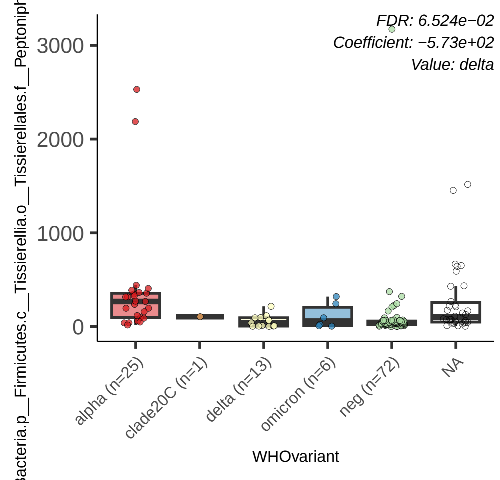

FDR: 6.524e−02
Coefficient: −5.73e+02
Value: delta
3000
2000
1000
0
alpha (n=25)
clade20C (n=1)
delta (n=13)
omicron (n=6)
neg (n=72)
NA
WHOvariant
Bacteria.p__Firmicutes.c__Tissierellia.o__Tissierellales.f__Peptoniph

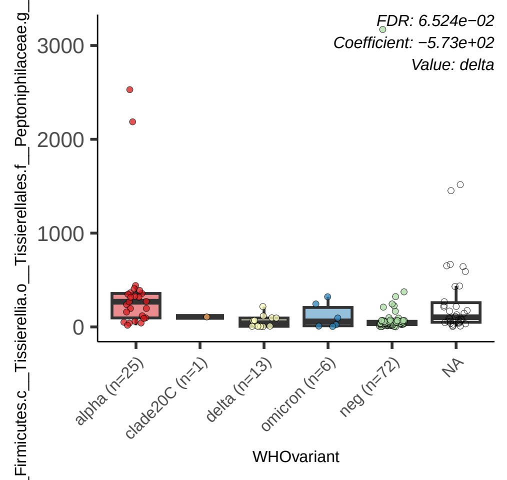
FDR: 6.524e−02
Coefficient: −5.73e+02
Value: delta
Firmicutes.c__Tissierellia.o__Tissierellales.f__Peptoniphilaceae.g__
3000
2000
1000
0
alpha (n=25)
clade20C (n=1)
delta (n=13)
omicron (n=6)
neg (n=72)
NA
WHOvariant

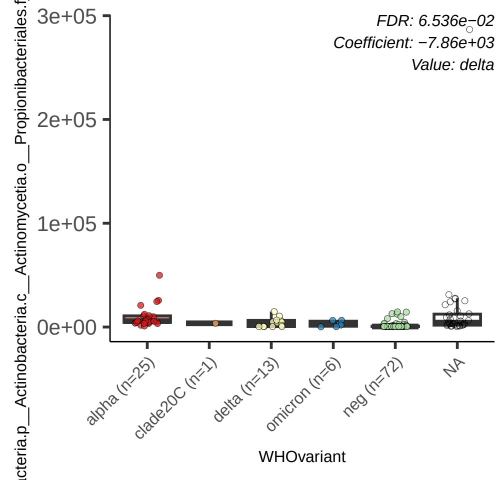

FDR: 6.536e−02
Coefficient: −7.86e+03
Value: delta
3e+05
2e+05
1e+05
0e+00
acteria.p__Actinobacteria.c__Actinomycetia.o__Propionibacteriales.f
alpha (n=25)
clade20C (n=1)
delta (n=13)
omicron (n=6)
neg (n=72)
NA
WHOvariant

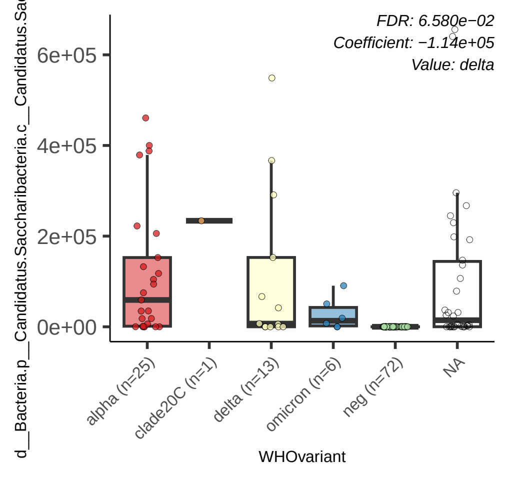
FDR: 6.580e−02
Coefficient: −1.14e+05
Value: delta
d__Bacteria.p__Candidatus.Saccharibacteria.c__Candidatus.Sac
6e+05
4e+05
2e+05
0e+00
alpha (n=25)
clade20C (n=1)
delta (n=13)
omicron (n=6)
neg (n=72)
NA
WHOvariant

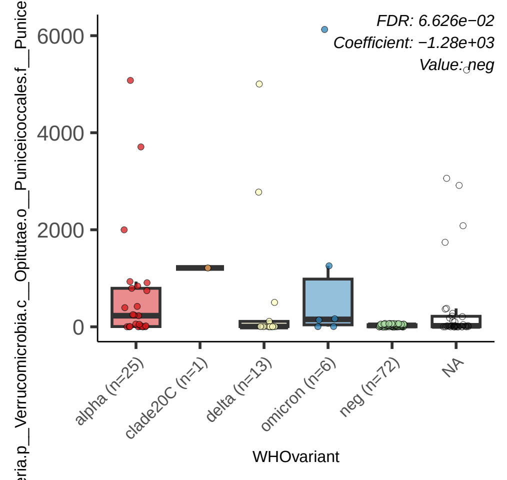

FDR: 6.626e−02
Coefficient: −1.28e+03
Value: neg
6000
4000
2000
0
alpha (n=25)
clade20C (n=1)
delta (n=13)
omicron (n=6)
neg (n=72)
NA
WHOvariant
eria.p__Verrucomicrobia.c__Opitutae.o__Puniceicoccales.f__Punice

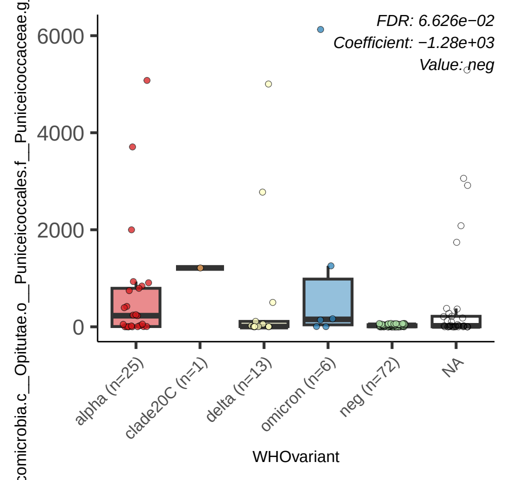
FDR: 6.626e−02
Coefficient: −1.28e+03
Value: neg
6000
4000
2000
0
alpha (n=25)
clade20C (n=1)
delta (n=13)
omicron (n=6)
neg (n=72)
NA
WHOvariant
comicrobia.c__Opitutae.o__Puniceicoccales.f__Puniceicoccaceae.g

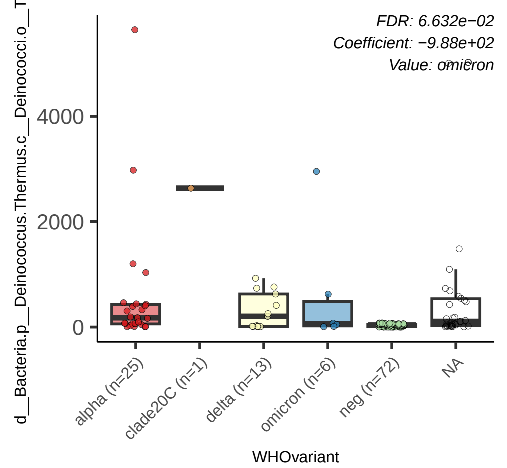

FDR: 6.632e−02
Coefficient: −9.88e+02
Value: omicron
d__Bacteria.p__Deinococcus.Thermus.c__Deinococci.o__T
4000
2000
0
alpha (n=25)
clade20C (n=1)
delta (n=13)
omicron (n=6)
neg (n=72)
NA
WHOvariant

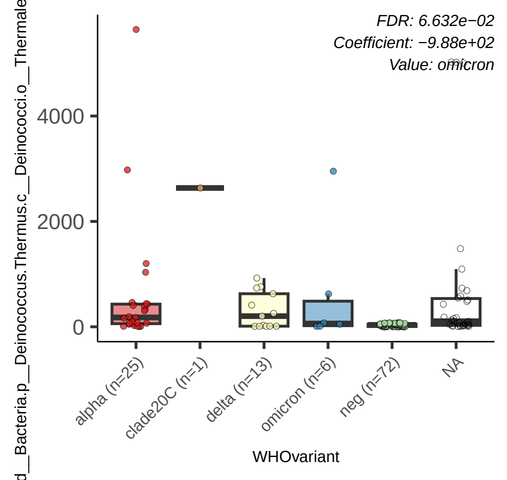
FDR: 6.632e−02
Coefficient: −9.88e+02
Value: omicron
4000
2000
0
d__Bacteria.p__Deinococcus.Thermus.c__Deinococci.o__Thermale
alpha (n=25)
clade20C (n=1)
delta (n=13)
omicron (n=6)
neg (n=72)
NA
WHOvariant

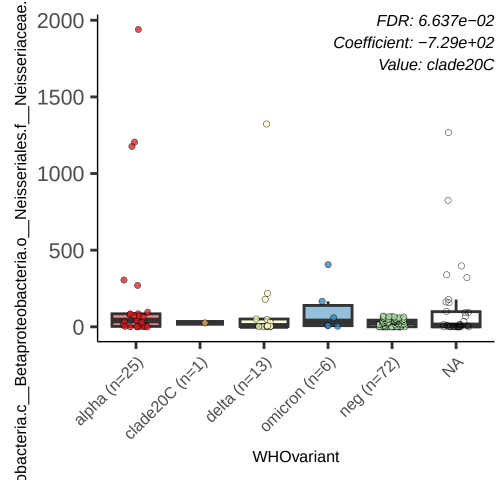
FDR: 6.637e−02
Coefficient: −7.29e+02
Value: clade20C
obacteria.c__Betaproteobacteria.o__Neisseriales.f__Neisseriaceae.
2000
1500
1000
500
0
alpha (n=25)
clade20C (n=1)
delta (n=13)
omicron (n=6)
neg (n=72)
NA
WHOvariant

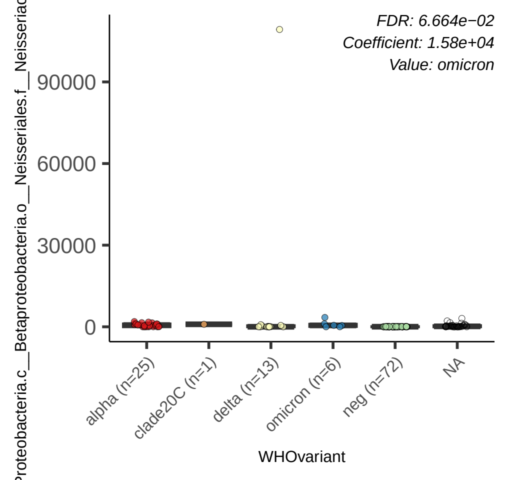

FDR: 6.664e−02
Coefficient: 1.58e+04
Value: omicron
90000
60000
30000
0
Proteobacteria.c__Betaproteobacteria.o__Neisseriales.f__Neisseriac
alpha (n=25)
clade20C (n=1)
delta (n=13)
omicron (n=6)
neg (n=72)
NA
WHOvariant

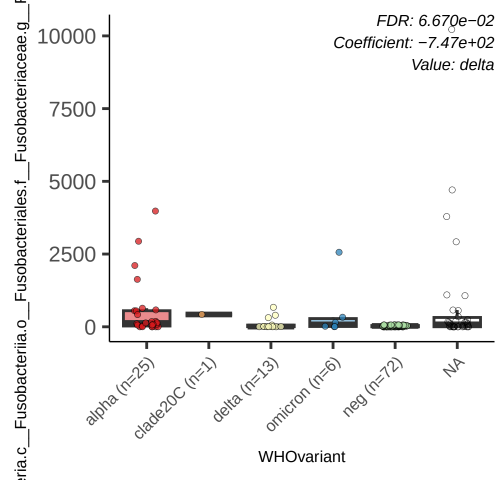

FDR: 6.670e−02
Coefficient: −7.47e+02
Value: delta
10000
7500
5000
2500
0
alpha (n=25)
clade20C (n=1)
delta (n=13)
omicron (n=6)
neg (n=72)
NA
WHOvariant
eria.c__Fusobacteriia.o__Fusobacteriales.f__Fusobacteriaceae.g__F

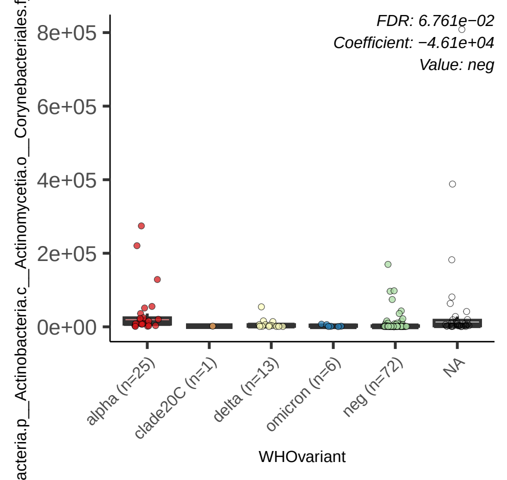

FDR: 6.761e−02
Coefficient: −4.61e+04
Value: neg
8e+05
6e+05
4e+05
2e+05
0e+00
acteria.p__Actinobacteria.c__Actinomycetia.o__Corynebacteriales.f
alpha (n=25)
clade20C (n=1)
delta (n=13)
omicron (n=6)
neg (n=72)
NA
WHOvariant

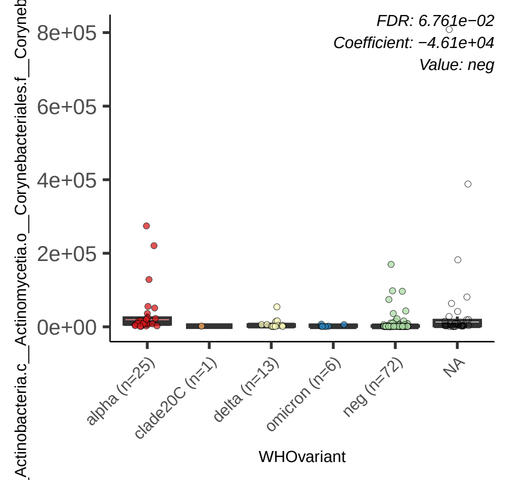

FDR: 6.761e−02
Coefficient: −4.61e+04
Value: neg
8e+05
6e+05
4e+05
2e+05
0e+00
Actinobacteria.c__Actinomycetia.o__Corynebacteriales.f__Coryneb
alpha (n=25)
clade20C (n=1)
delta (n=13)
omicron (n=6)
neg (n=72)
NA
WHOvariant

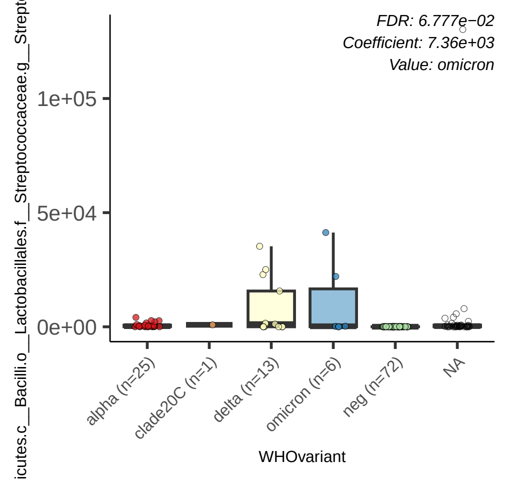
FDR: 6.777e−02
Coefficient: 7.36e+03
Value: omicron
1e+05
5e+04
0e+00
icutes.c__Bacilli.o__Lactobacillales.f__Streptococcaceae.g__Strept
alpha (n=25)
clade20C (n=1)
delta (n=13)
omicron (n=6)
neg (n=72)
NA
WHOvariant

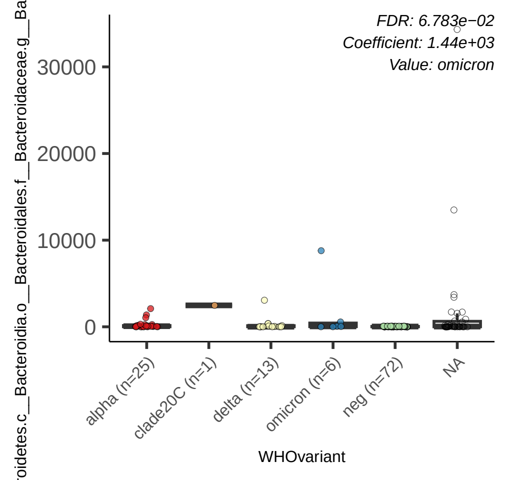

FDR: 6.783e−02
Coefficient: 1.44e+03
Value: omicron
30000
20000
10000
0
alpha (n=25)
clade20C (n=1)
delta (n=13)
omicron (n=6)
neg (n=72)
NA
WHOvariant

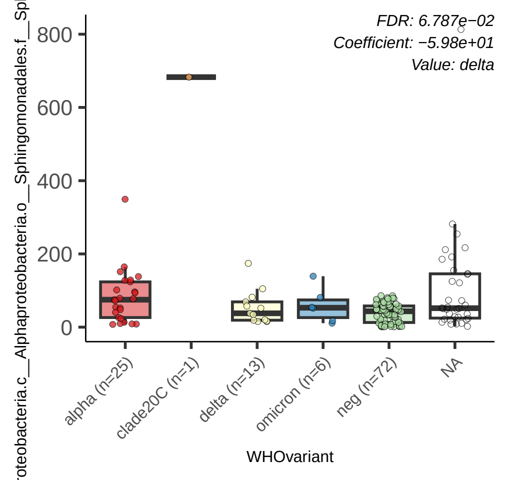

FDR: 6.787e−02
Coefficient: −5.98e+01
Value: delta
800
600
400
200
0
roteobacteria.c__Alphaproteobacteria.o__Sphingomonadales.f__Sph
alpha (n=25)
clade20C (n=1)
delta (n=13)
omicron (n=6)
neg (n=72)
NA
WHOvariant

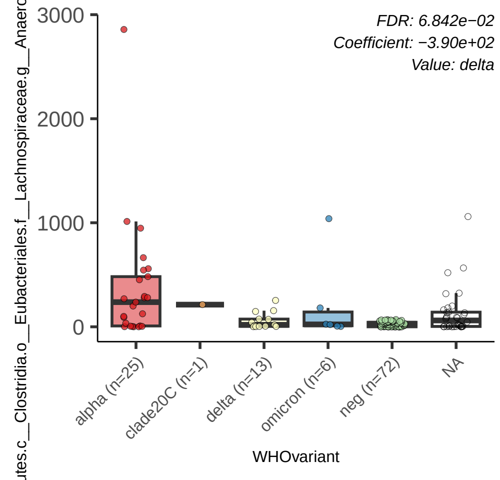
FDR: 6.842e−02
Coefficient: −3.90e+02
Value: delta
3000
2000
1000
0
utes.c__Clostridia.o__Eubacteriales.f__Lachnospiraceae.g__Anaero
alpha (n=25)
clade20C (n=1)
delta (n=13)
omicron (n=6)
neg (n=72)
NA
WHOvariant

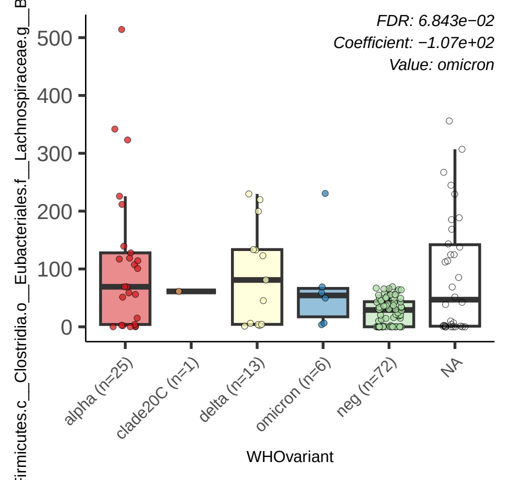

FDR: 6.843e−02
Coefficient: −1.07e+02
Value: omicron
Firmicutes.c__Clostridia.o__Eubacteriales.f__Lachnospiraceae.g__B
0
100
200
300
400
500
alpha (n=25)
clade20C (n=1)
delta (n=13)
omicron (n=6)
neg (n=72)
NA
WHOvariant

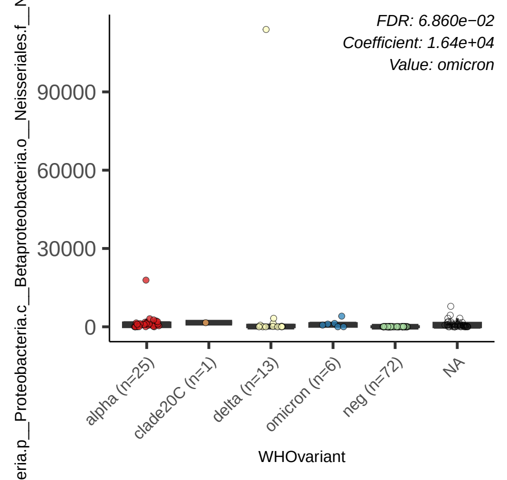
FDR: 6.860e−02
Coefficient: 1.64e+04
Value: omicron
eria.p__Proteobacteria.c__Betaproteobacteria.o__Neisseriales.f__N
90000
60000
30000
0
alpha (n=25)
clade20C (n=1)
delta (n=13)
omicron (n=6)
neg (n=72)
NA
WHOvariant

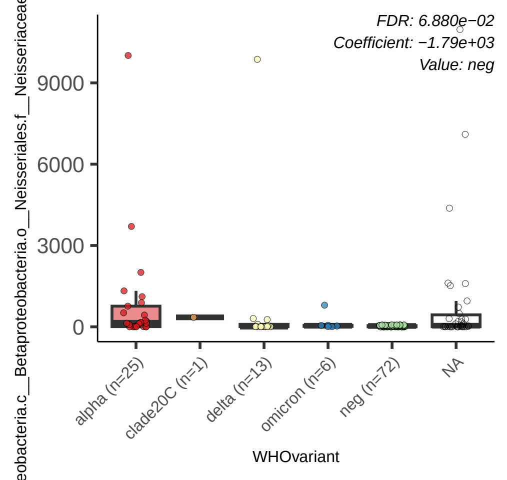

FDR: 6.880e−02
Coefficient: −1.79e+03
Value: neg
eobacteria.c__Betaproteobacteria.o__Neisseriales.f__Neisseriaceae
9000
6000
3000
0
alpha (n=25)
clade20C (n=1)
delta (n=13)
omicron (n=6)
neg (n=72)
NA
WHOvariant

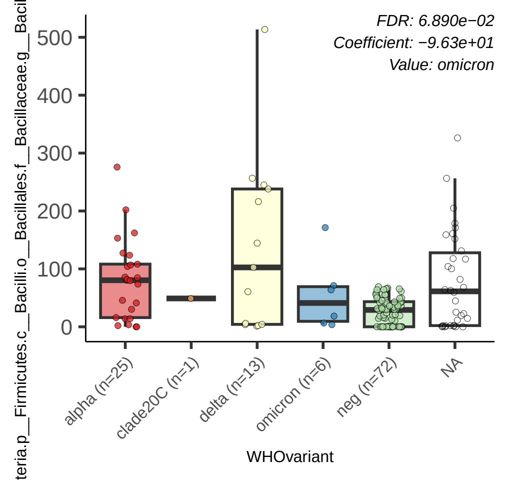

FDR: 6.890e−02
Coefficient: −9.63e+01
Value: omicron
teria.p__Firmicutes.c__Bacilli.o__Bacillales.f__Bacillaceae.g__Bacil
0
100
200
300
400
500
alpha (n=25)
clade20C (n=1)
delta (n=13)
omicron (n=6)
neg (n=72)
NA
WHOvariant

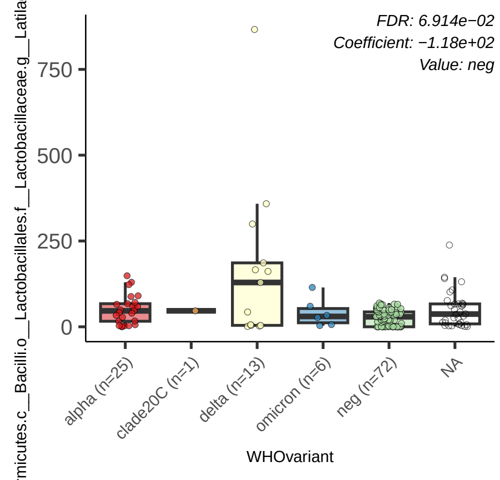

FDR: 6.914e−02
Coefficient: −1.18e+02
Value: neg
micutes.c__Bacilli.o__Lactobacillales.f__Lactobacillaceae.g__Latila
750
500
250
0
alpha (n=25)
clade20C (n=1)
delta (n=13)
omicron (n=6)
neg (n=72)
NA
WHOvariant

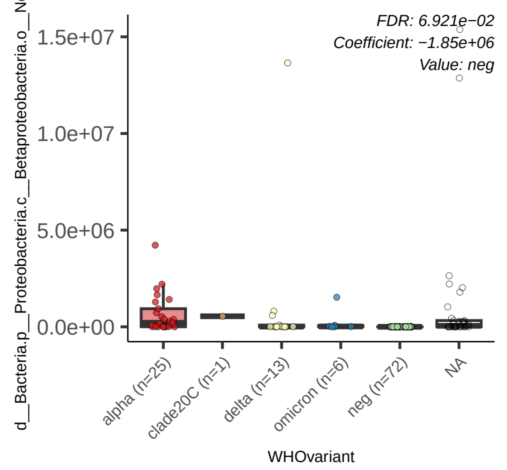

FDR: 6.921e−02
Coefficient: −1.85e+06
Value: neg
d__Bacteria.p__Proteobacteria.c__Betaproteobacteria.o__N
1.5e+07
1.0e+07
5.0e+06
0.0e+00
alpha (n=25)
clade20C (n=1)
delta (n=13)
omicron (n=6)
neg (n=72)
NA
WHOvariant

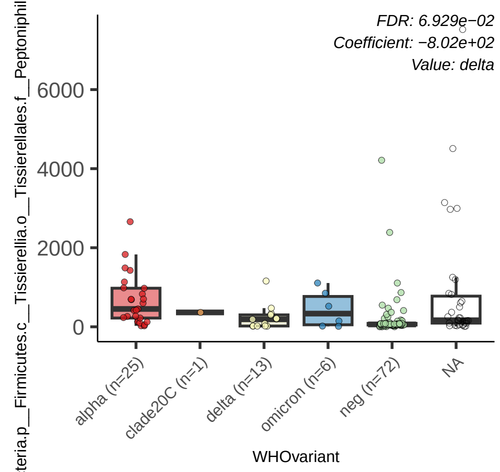

FDR: 6.929e−02
Coefficient: −8.02e+02
Value: delta
6000
4000
2000
0
cteria.p__Firmicutes.c__Tissierellia.o__Tissierellales.f__Peptoniphil
alpha (n=25)
clade20C (n=1)
delta (n=13)
omicron (n=6)
neg (n=72)
NA
WHOvariant

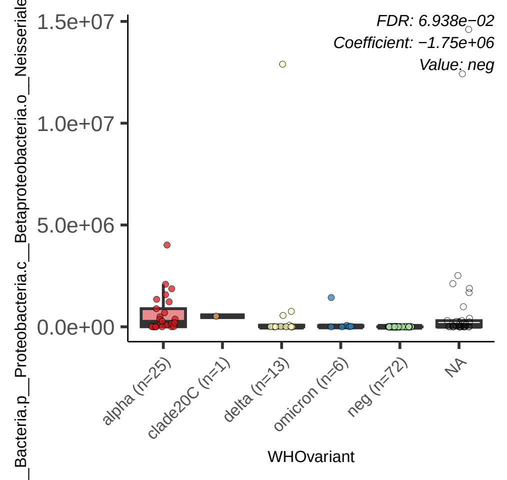

FDR: 6.938e−02
Coefficient: −1.75e+06
Value: neg
1.5e+07
1.0e+07
5.0e+06
0.0e+00
__Bacteria.p__Proteobacteria.c__Betaproteobacteria.o__Neisseriale
alpha (n=25)
clade20C (n=1)
delta (n=13)
omicron (n=6)
neg (n=72)
NA
WHOvariant

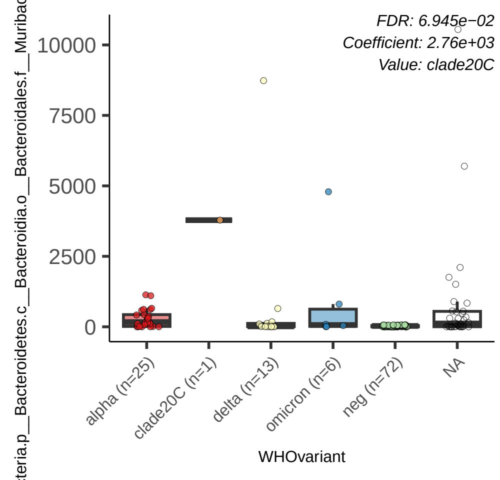

FDR: 6.945e−02
Coefficient: 2.76e+03
Value: clade20C
10000
7500
5000
2500
0
alpha (n=25)
clade20C (n=1)
delta (n=13)
omicron (n=6)
neg (n=72)
NA
WHOvariant
cteria.p__Bacteroidetes.c__Bacteroidia.o__Bacteroidales.f__Muribac

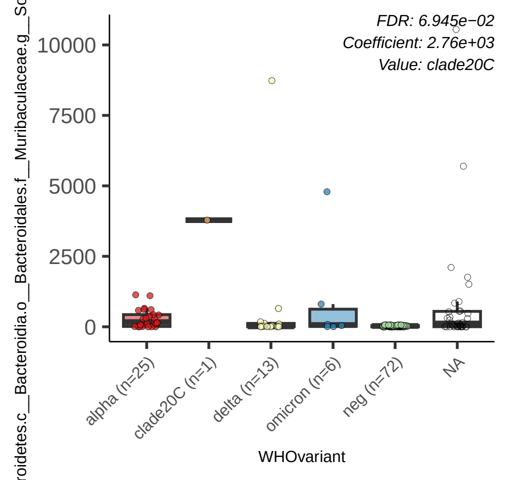

FDR: 6.945e−02
Coefficient: 2.76e+03
Value: clade20C
10000
7500
5000
2500
0
roidetes.c__Bacteroidia.o__Bacteroidales.f__Muribaculaceae.g__Sc
alpha (n=25)
clade20C (n=1)
delta (n=13)
omicron (n=6)
neg (n=72)
NA
WHOvariant

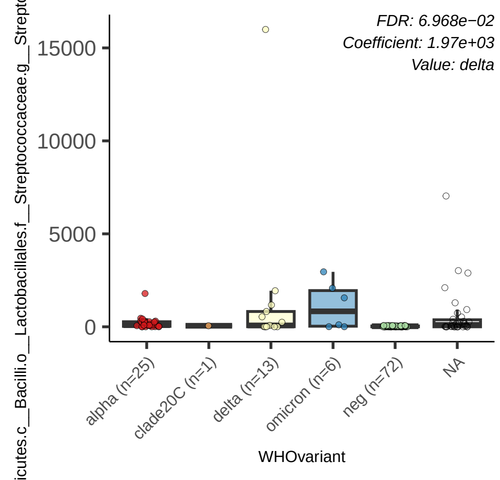

FDR: 6.968e−02
Coefficient: 1.97e+03
Value: delta
15000
10000
5000
0
icutes.c__Bacilli.o__Lactobacillales.f__Streptococcaceae.g__Strept
alpha (n=25)
clade20C (n=1)
delta (n=13)
omicron (n=6)
neg (n=72)
NA
WHOvariant

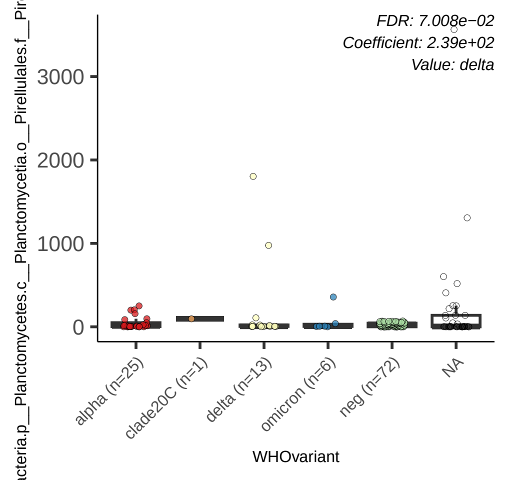

FDR: 7.008e−02
Coefficient: 2.39e+02
Value: delta
3000
2000
1000
0
alpha (n=25)
clade20C (n=1)
delta (n=13)
omicron (n=6)
neg (n=72)
NA
WHOvariant
acteria.p__Planctomycetes.c__Planctomycetia.o__Pirellulales.f__Pir

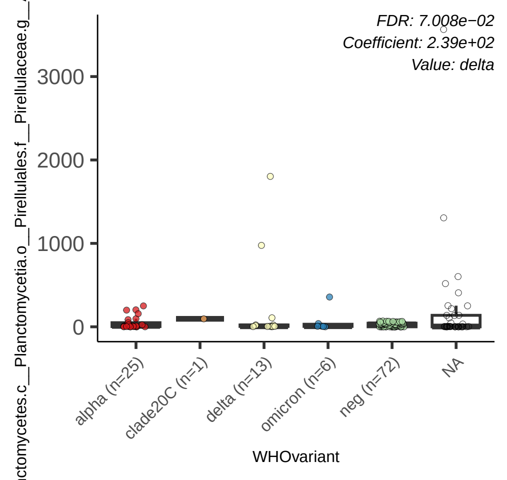

FDR: 7.008e−02
Coefficient: 2.39e+02
Value: delta
ctomycetes.c__Planctomycetia.o__Pirellulales.f__Pirellulaceae.g__
3000
2000
1000
0
alpha (n=25)
clade20C (n=1)
delta (n=13)
omicron (n=6)
neg (n=72)
NA
WHOvariant

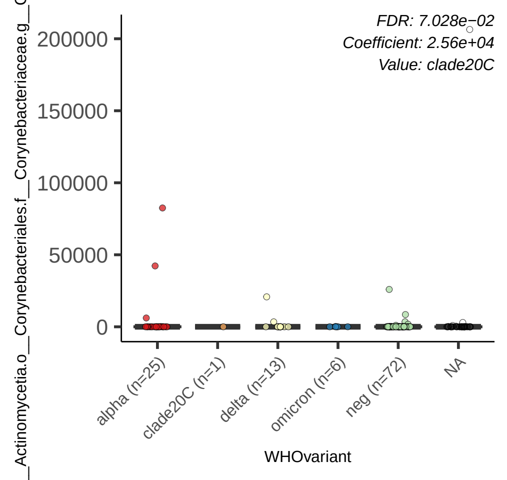

FDR: 7.028e−02
Coefficient: 2.56e+04
Value: clade20C
_Actinomycetia.o__Corynebacteriales.f__Corynebacteriaceae.g__C
200000
150000
100000
50000
0
alpha (n=25)
clade20C (n=1)
delta (n=13)
omicron (n=6)
neg (n=72)
NA
WHOvariant

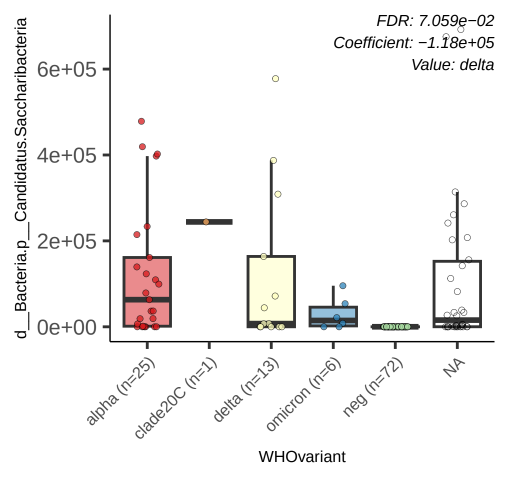
FDR: 7.059e−02
Coefficient: −1.18e+05
Value: delta
d__Bacteria.p__Candidatus.Saccharibacteria
6e+05
4e+05
2e+05
0e+00
alpha (n=25)
clade20C (n=1)
delta (n=13)
omicron (n=6)
neg (n=72)
NA
WHOvariant

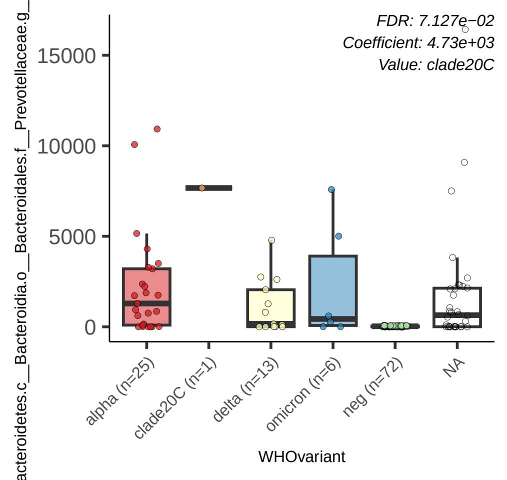

FDR: 7.127e−02
Coefficient: 4.73e+03
Value: clade20C
acteroidetes.c__Bacteroidia.o__Bacteroidales.f__Prevotellaceae.g__
15000
10000
5000
0
alpha (n=25)
clade20C (n=1)
delta (n=13)
omicron (n=6)
neg (n=72)
NA
WHOvariant

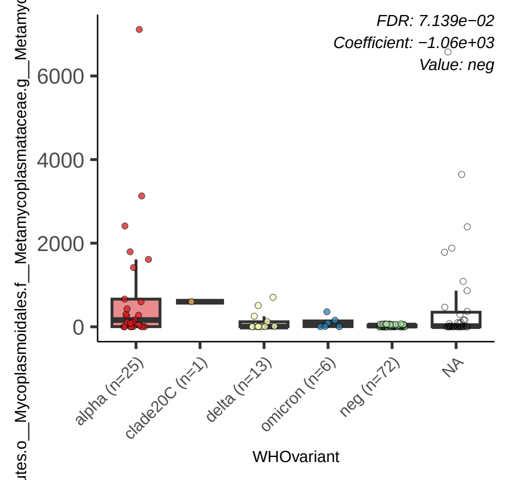

FDR: 7.139e−02
Coefficient: −1.06e+03
Value: neg
0
2000
4000
6000
utes.o__Mycoplasmoidales.f__Metamycoplasmataceae.g__Metamyc
alpha (n=25)
clade20C (n=1)
delta (n=13)
omicron (n=6)
neg (n=72)
NA
WHOvariant

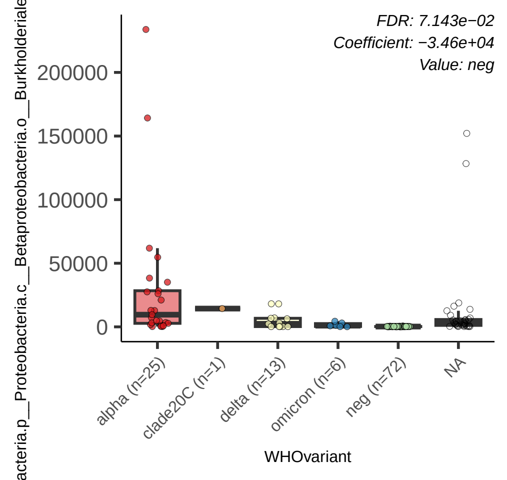

FDR: 7.143e−02
Coefficient: −3.46e+04
Value: neg
acteria.p__Proteobacteria.c__Betaproteobacteria.o__Burkholderiale
200000
150000
100000
50000
0
alpha (n=25)
clade20C (n=1)
delta (n=13)
omicron (n=6)
neg (n=72)
NA
WHOvariant

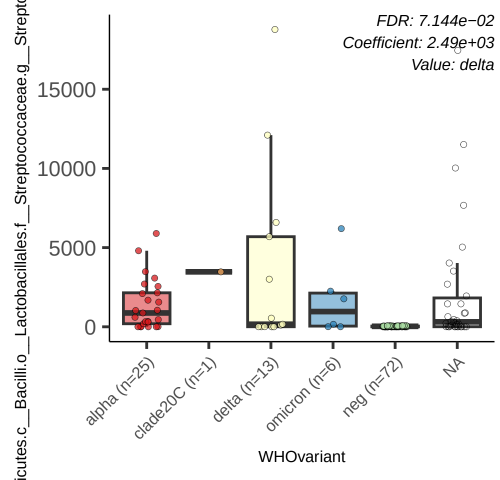

FDR: 7.144e−02
Coefficient: 2.49e+03
Value: delta
15000
10000
5000
0
icutes.c__Bacilli.o__Lactobacillales.f__Streptococcaceae.g__Strepto
alpha (n=25)
clade20C (n=1)
delta (n=13)
omicron (n=6)
neg (n=72)
NA
WHOvariant

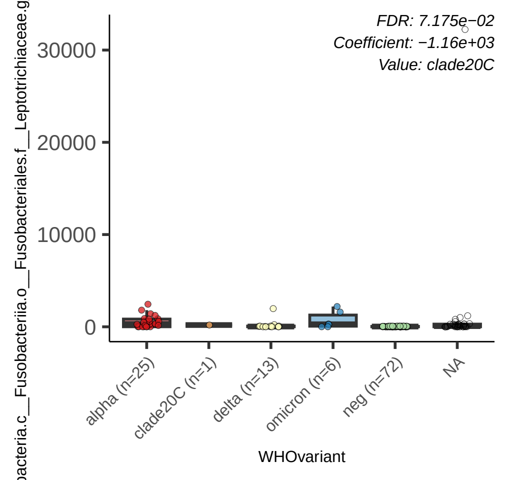

FDR: 7.175e−02
Coefficient: −1.16e+03
Value: clade20C
30000
20000
10000
0
bacteria.c__Fusobacteriia.o__Fusobacteriales.f__Leptotrichiaceae.g
alpha (n=25)
clade20C (n=1)
delta (n=13)
omicron (n=6)
neg (n=72)
NA
WHOvariant

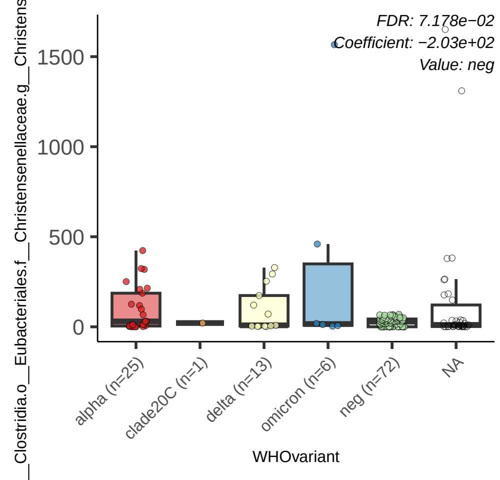
FDR: 7.178e−02
Coefficient: −2.03e+02
Value: neg
1500
1000
500
0
__Clostridia.o__Eubacteriales.f__Christensenellaceae.g__Christens
alpha (n=25)
clade20C (n=1)
delta (n=13)
omicron (n=6)
neg (n=72)
NA
WHOvariant

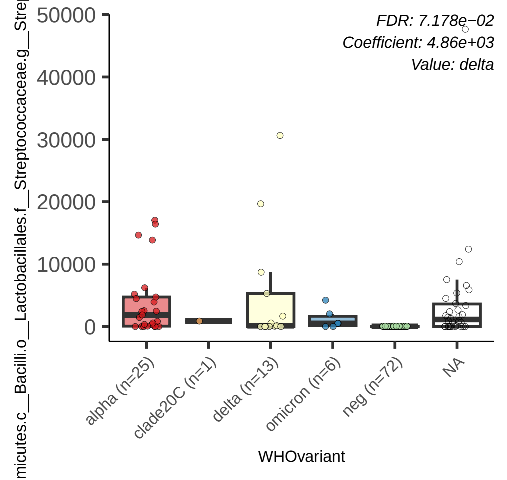

FDR: 7.178e−02
Coefficient: 4.86e+03
Value: delta
50000
40000
30000
20000
10000
0
micutes.c__Bacilli.o__Lactobacillales.f__Streptococcaceae.g__Strep
alpha (n=25)
clade20C (n=1)
delta (n=13)
omicron (n=6)
neg (n=72)
NA
WHOvariant

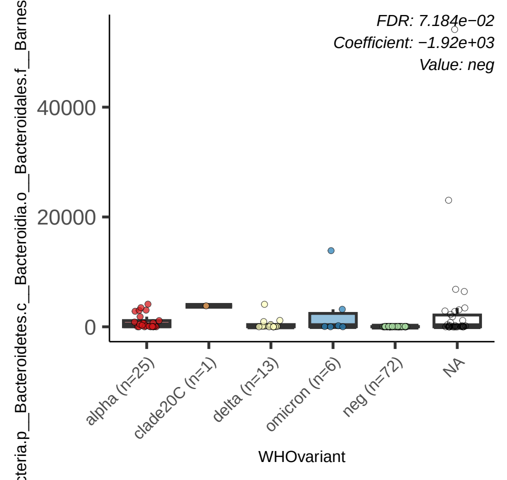

FDR: 7.184e−02
Coefficient: −1.92e+03
Value: neg
0
20000
40000
cteria.p__Bacteroidetes.c__Bacteroidia.o__Bacteroidales.f__Barnes
alpha (n=25)
clade20C (n=1)
delta (n=13)
omicron (n=6)
neg (n=72)
NA
WHOvariant

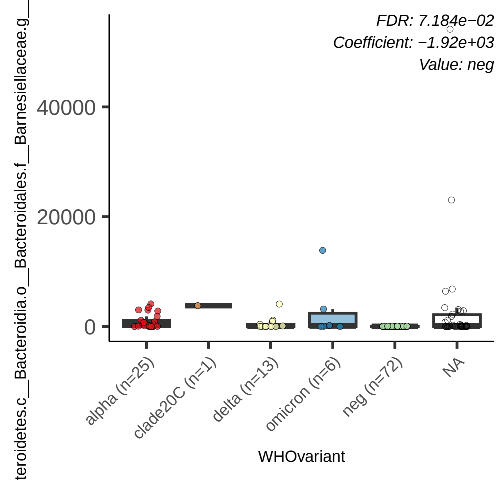
FDR: 7.184e−02
Coefficient: −1.92e+03
Value: neg
40000
20000
0
teroidetes.c__Bacteroidia.o__Bacteroidales.f__Barnesiellaceae.g__
alpha (n=25)
clade20C (n=1)
delta (n=13)
omicron (n=6)
neg (n=72)
NA
WHOvariant

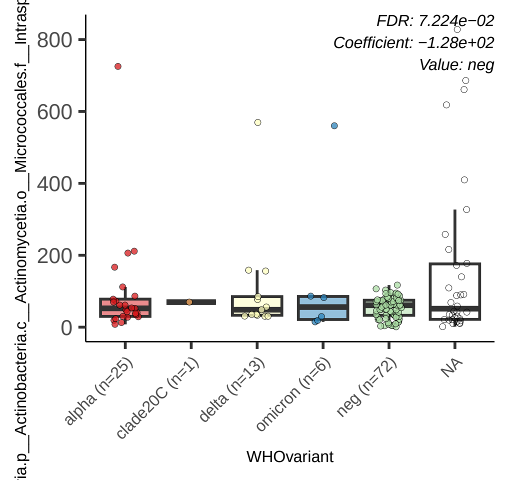

FDR: 7.224e−02
Coefficient: −1.28e+02
Value: neg
ia.p__Actinobacteria.c__Actinomycetia.o__Micrococcales.f__Intrasp
800
600
400
200
0
alpha (n=25)
clade20C (n=1)
delta (n=13)
omicron (n=6)
neg (n=72)
NA
WHOvariant

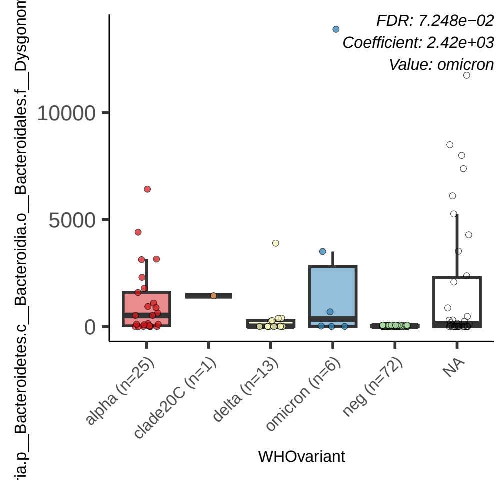

FDR: 7.248e−02
Coefficient: 2.42e+03
Value: omicron
10000
5000
0
alpha (n=25)
clade20C (n=1)
delta (n=13)
omicron (n=6)
neg (n=72)
NA
WHOvariant
ria.p__Bacteroidetes.c__Bacteroidia.o__Bacteroidales.f__Dysgonom

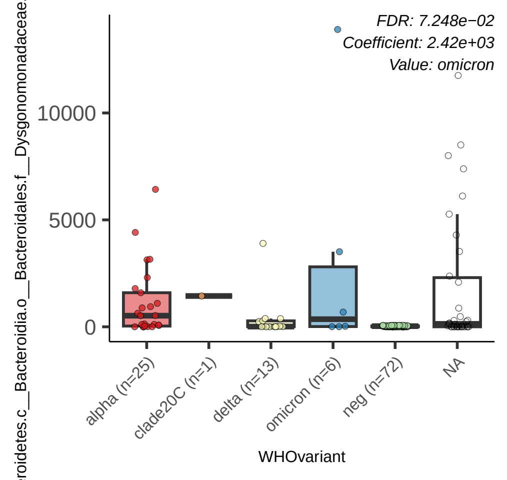

FDR: 7.248e−02
Coefficient: 2.42e+03
Value: omicron
10000
5000
0
roidetes.c__Bacteroidia.o__Bacteroidales.f__Dysgonomonadaceae.
alpha (n=25)
clade20C (n=1)
delta (n=13)
omicron (n=6)
neg (n=72)
NA
WHOvariant

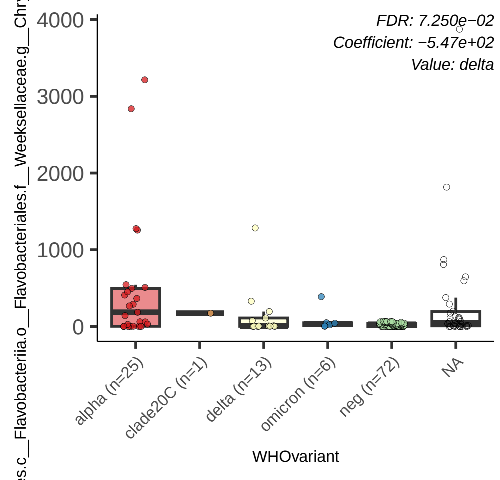

FDR: 7.250e−02
Coefficient: −5.47e+02
Value: delta
4000
3000
2000
1000
0
es.c__Flavobacteriia.o__Flavobacteriales.f__Weeksellaceae.g__Chry
alpha (n=25)
clade20C (n=1)
delta (n=13)
omicron (n=6)
neg (n=72)
NA
WHOvariant

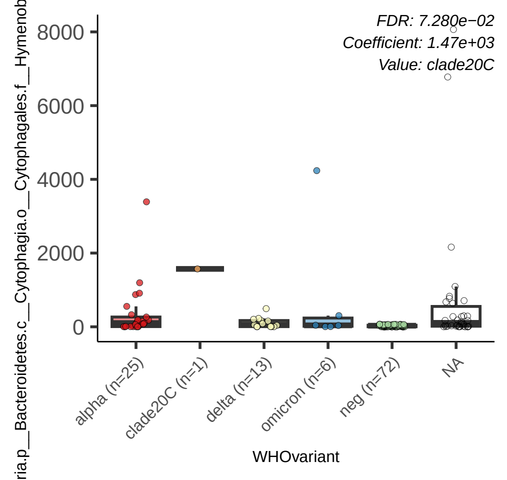
FDR: 7.280e−02
Coefficient: 1.47e+03
Value: clade20C
8000
6000
4000
2000
0
ria.p__Bacteroidetes.c__Cytophagia.o__Cytophagales.f__Hymenob
alpha (n=25)
clade20C (n=1)
delta (n=13)
omicron (n=6)
neg (n=72)
NA
WHOvariant

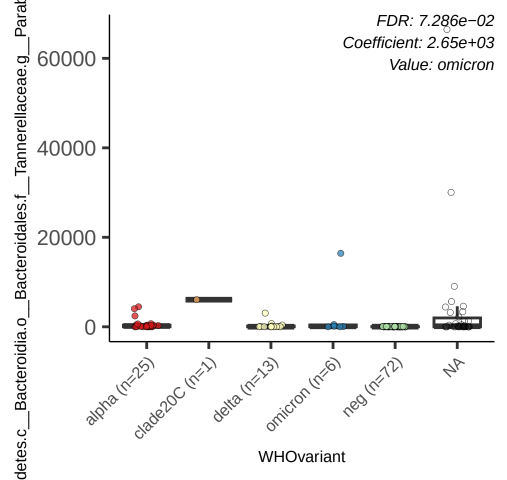

FDR: 7.286e−02
Coefficient: 2.65e+03
Value: omicron
60000
40000
20000
0
alpha (n=25)
clade20C (n=1)
delta (n=13)
omicron (n=6)
neg (n=72)
NA
WHOvariant
detes.c__Bacteroidia.o__Bacteroidales.f__Tannerellaceae.g__Parab

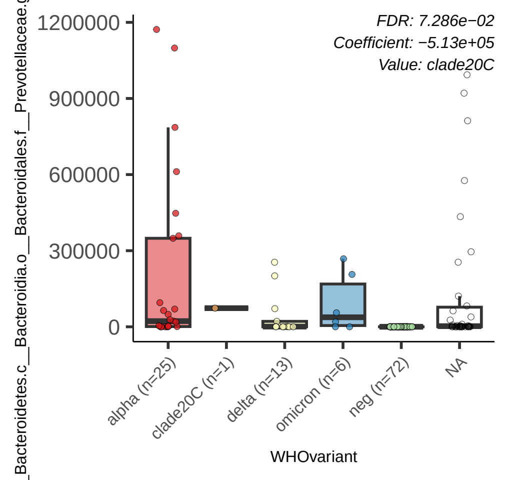

FDR: 7.286e−02
Coefficient: −5.13e+05
Value: clade20C
1200000
900000
600000
300000
0
Bacteroidetes.c__Bacteroidia.o__Bacteroidales.f__Prevotellaceae.g
alpha (n=25)
clade20C (n=1)
delta (n=13)
omicron (n=6)
neg (n=72)
NA
WHOvariant

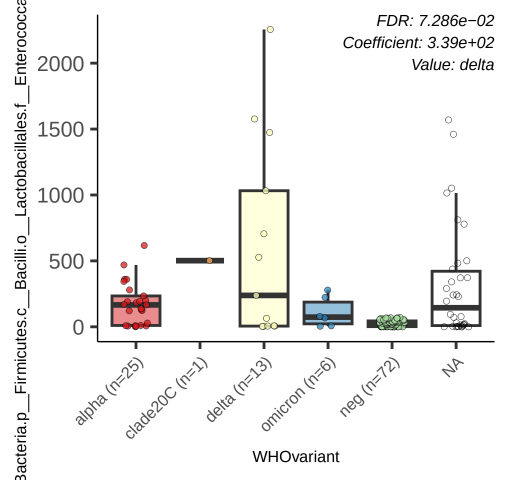
FDR: 7.286e−02
Coefficient: 3.39e+02
Value: delta
Bacteria.p__Firmicutes.c__Bacilli.o__Lactobacillales.f__Enterococca
0
500
1000
1500
2000
alpha (n=25)
clade20C (n=1)
delta (n=13)
omicron (n=6)
neg (n=72)
NA
WHOvariant

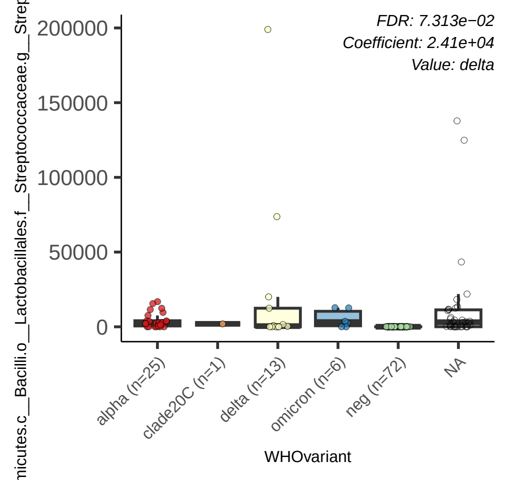
FDR: 7.313e−02
Coefficient: 2.41e+04
Value: delta
200000
150000
100000
50000
0
micutes.c__Bacilli.o__Lactobacillales.f__Streptococcaceae.g__Strep
alpha (n=25)
clade20C (n=1)
delta (n=13)
omicron (n=6)
neg (n=72)
NA
WHOvariant

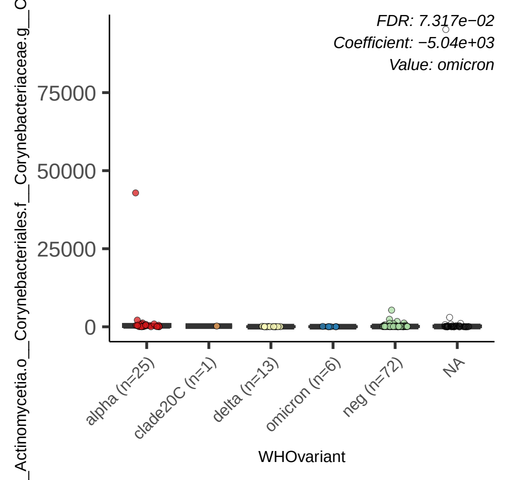

FDR: 7.317e−02
Coefficient: −5.04e+03
Value: omicron
75000
50000
25000
0
Actinomycetia.o__Corynebacteriales.f__Corynebacteriaceae.g__C
alpha (n=25)
clade20C (n=1)
delta (n=13)
omicron (n=6)
neg (n=72)
NA
WHOvariant

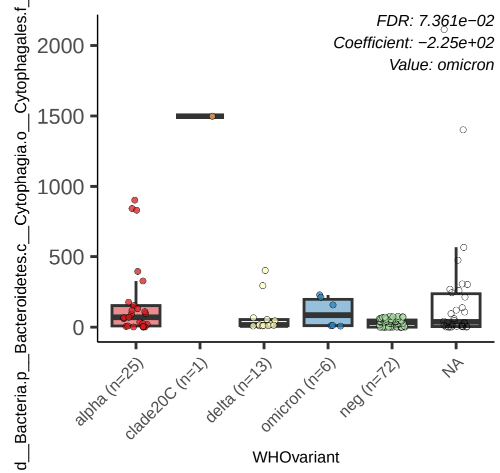

FDR: 7.361e−02
Coefficient: −2.25e+02
Value: omicron
d__Bacteria.p__Bacteroidetes.c__Cytophagia.o__Cytophagales.f__
0
500
1000
1500
2000
alpha (n=25)
clade20C (n=1)
delta (n=13)
omicron (n=6)
neg (n=72)
NA
WHOvariant

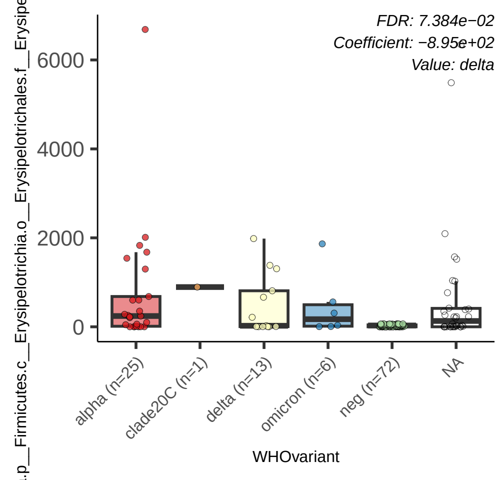

FDR: 7.384e−02
Coefficient: −8.95e+02
Value: delta
Firmicutes.c__Erysipelotrichia.o__Erysipelotrichales.f__Erysipe
6000
4000
2000
0
alpha (n=25)
clade20C (n=1)
delta (n=13)
omicron (n=6)
neg (n=72)
NA
WHOvariant

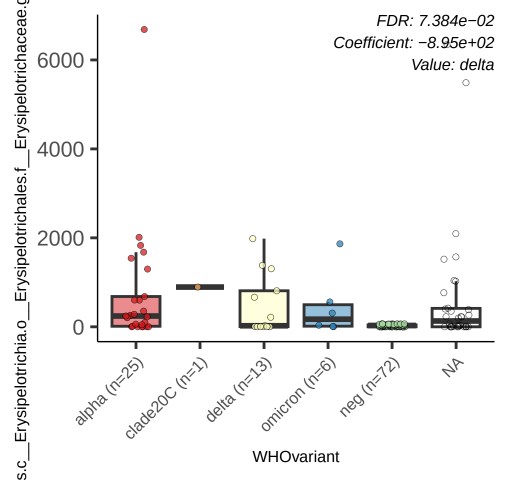

FDR: 7.384e−02
Coefficient: −8.95e+02
Value: delta
s.c__Erysipelotrichia.o__Erysipelotrichales.f__Erysipelotrichaceae.g
0
2000
4000
6000
alpha (n=25)
clade20C (n=1)
delta (n=13)
omicron (n=6)
neg (n=72)
NA
WHOvariant

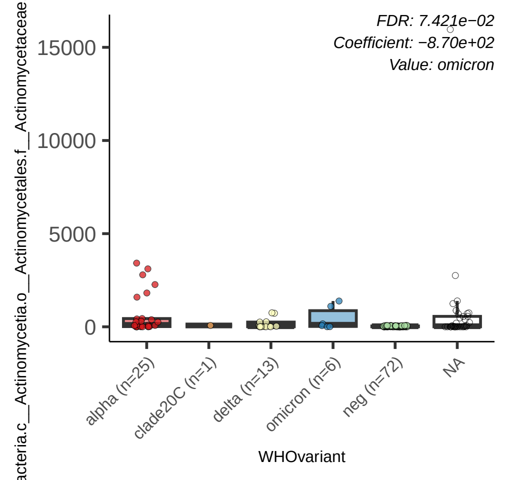

FDR: 7.421e−02
Coefficient: −8.70e+02
Value: omicron
15000
10000
5000
0
alpha (n=25)
clade20C (n=1)
delta (n=13)
omicron (n=6)
neg (n=72)
NA
WHOvariant
acteria.c__Actinomycetia.o__Actinomycetales.f__Actinomycetaceae

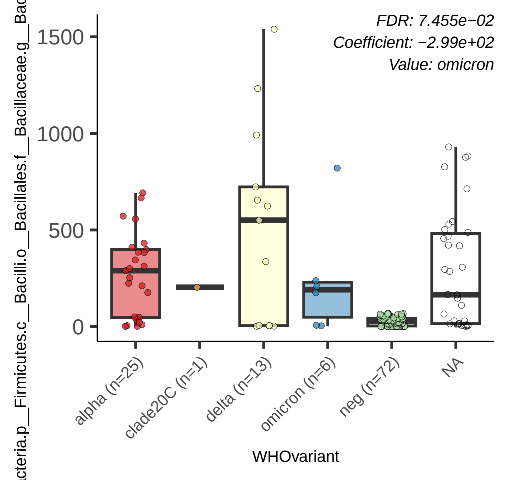

FDR: 7.455e−02
Coefficient: −2.99e+02
Value: omicron
1500
1000
500
0
alpha (n=25)
clade20C (n=1)
delta (n=13)
omicron (n=6)
neg (n=72)
NA
WHOvariant
cteria.p__Firmicutes.c__Bacilli.o__Bacillales.f__Bacillaceae.g__Bac

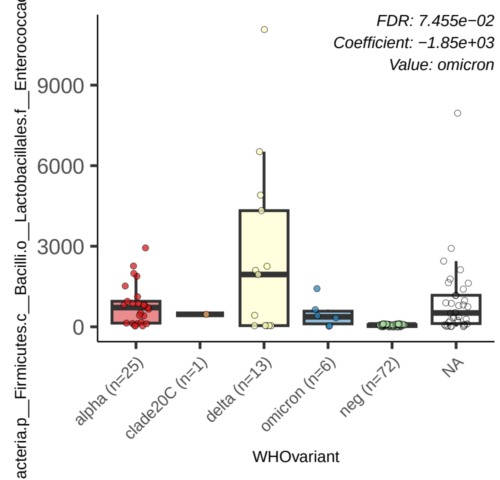

FDR: 7.455e−02
Coefficient: −1.85e+03
Value: omicron
acteria.p__Firmicutes.c__Bacilli.o__Lactobacillales.f__Enterococcac
9000
6000
3000
0
alpha (n=25)
clade20C (n=1)
delta (n=13)
omicron (n=6)
neg (n=72)
NA
WHOvariant

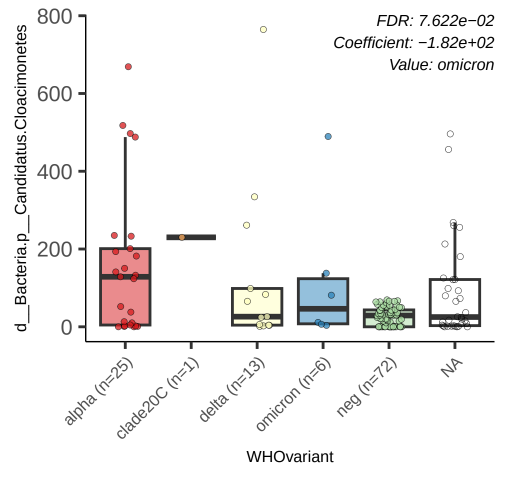

FDR: 7.622e−02
Coefficient: −1.82e+02
Value: omicron
800
600
400
200
0
d__Bacteria.p__Candidatus.Cloacimonetes
alpha (n=25)
clade20C (n=1)
delta (n=13)
omicron (n=6)
neg (n=72)
NA
WHOvariant

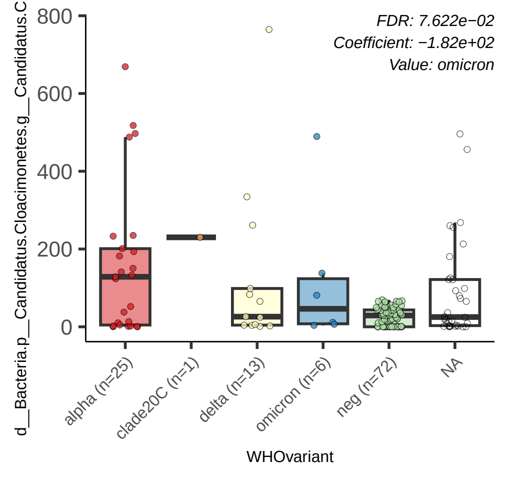

FDR: 7.622e−02
Coefficient: −1.82e+02
Value: omicron
d__Bacteria.p__Candidatus.Cloacimonetes.g__Candidatus.C
800
600
400
200
0
alpha (n=25)
clade20C (n=1)
delta (n=13)
omicron (n=6)
neg (n=72)
NA
WHOvariant

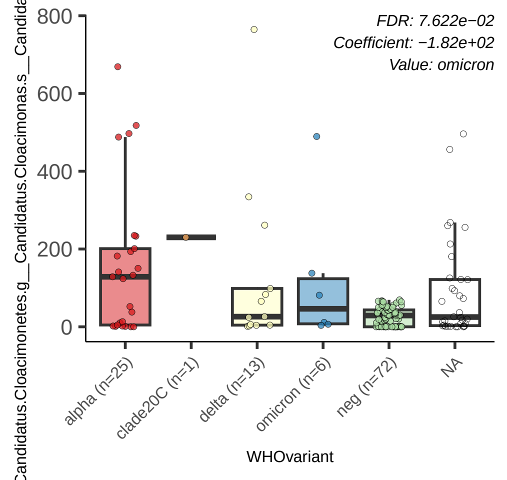

FDR: 7.622e−02
Coefficient: −1.82e+02
Value: omicron
800
600
400
200
0
Candidatus.Cloacimonetes.g__Candidatus.Cloacimonas.s__Candida
alpha (n=25)
clade20C (n=1)
delta (n=13)
omicron (n=6)
neg (n=72)
NA
WHOvariant

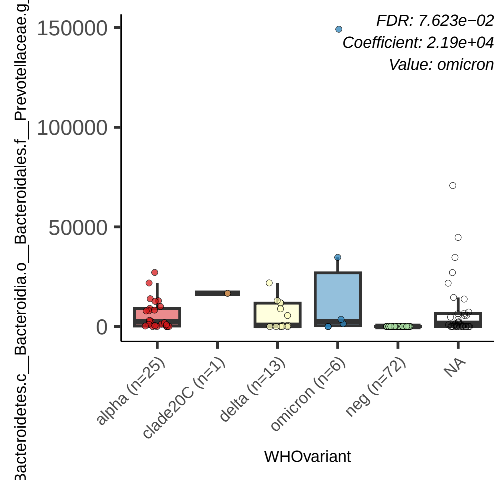
FDR: 7.623e−02
Coefficient: 2.19e+04
Value: omicron
Bacteroidetes.c__Bacteroidia.o__Bacteroidales.f__Prevotellaceae.g_
150000
100000
50000
0
alpha (n=25)
clade20C (n=1)
delta (n=13)
omicron (n=6)
neg (n=72)
NA
WHOvariant

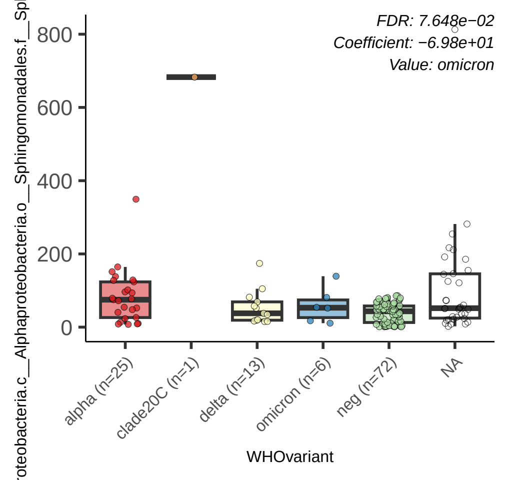

FDR: 7.648e−02
Coefficient: −6.98e+01
Value: omicron
800
600
400
200
0
roteobacteria.c__Alphaproteobacteria.o__Sphingomonadales.f__Sph
alpha (n=25)
clade20C (n=1)
delta (n=13)
omicron (n=6)
neg (n=72)
NA
WHOvariant

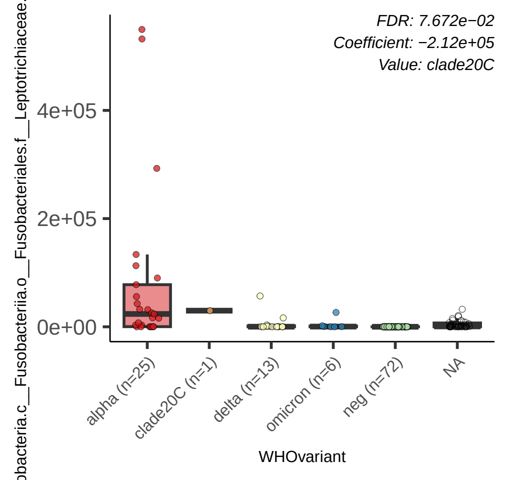
FDR: 7.672e−02
Coefficient: −2.12e+05
Value: clade20C
obacteria.c__Fusobacteriia.o__Fusobacteriales.f__Leptotrichiaceae.
4e+05
2e+05
0e+00
alpha (n=25)
clade20C (n=1)
delta (n=13)
omicron (n=6)
neg (n=72)
NA
WHOvariant

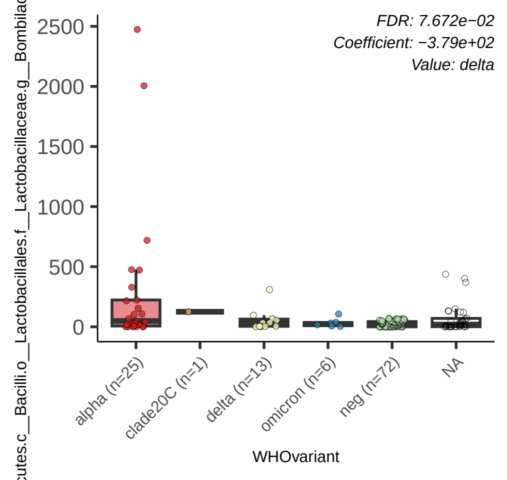

FDR: 7.672e−02
Coefficient: −3.79e+02
Value: delta
cutes.c__Bacilli.o__Lactobacillales.f__Lactobacillaceae.g__Bombilac
2500
2000
1500
1000
500
0
alpha (n=25)
clade20C (n=1)
delta (n=13)
omicron (n=6)
neg (n=72)
NA
WHOvariant

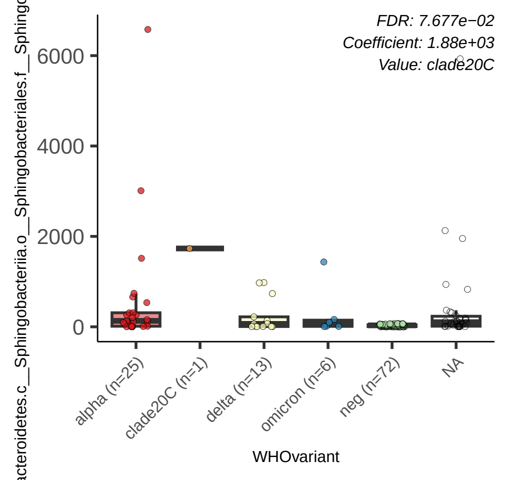
FDR: 7.677e−02
Coefficient: 1.88e+03
Value: clade20C
acteroidetes.c__Sphingobacteriia.o__Sphingobacteriales.f__Sphingo
6000
4000
2000
0
alpha (n=25)
clade20C (n=1)
delta (n=13)
omicron (n=6)
neg (n=72)
NA
WHOvariant

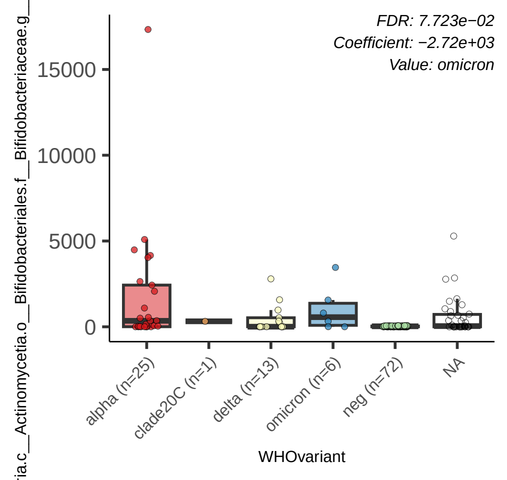

FDR: 7.723e−02
Coefficient: −2.72e+03
Value: omicron
15000
10000
5000
0
ria.c__Actinomycetia.o__Bifidobacteriales.f__Bifidobacteriaceae.g__
alpha (n=25)
clade20C (n=1)
delta (n=13)
omicron (n=6)
neg (n=72)
NA
WHOvariant

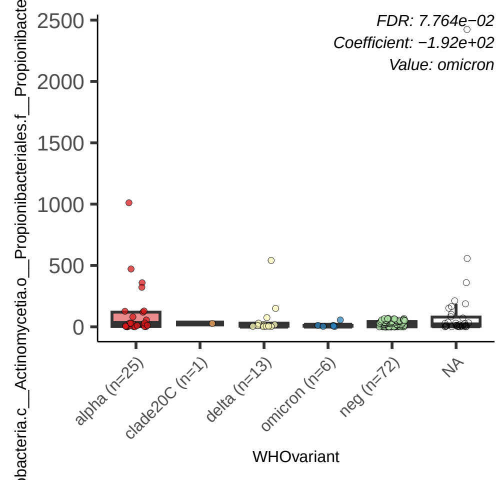

FDR: 7.764e−02
Coefficient: −1.92e+02
Value: omicron
2500
2000
1500
1000
500
0
bacteria.c__Actinomycetia.o__Propionibacteriales.f__Propionibacte
alpha (n=25)
clade20C (n=1)
delta (n=13)
omicron (n=6)
neg (n=72)
NA
WHOvariant

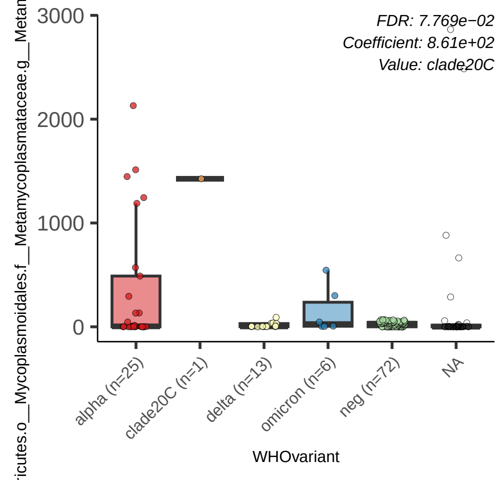

FDR: 7.769e−02
Coefficient: 8.61e+02
Value: clade20C
3000
2000
1000
0
ricutes.o__Mycoplasmoidales.f__Metamycoplasmataceae.g__Metan
alpha (n=25)
clade20C (n=1)
delta (n=13)
omicron (n=6)
neg (n=72)
NA
WHOvariant

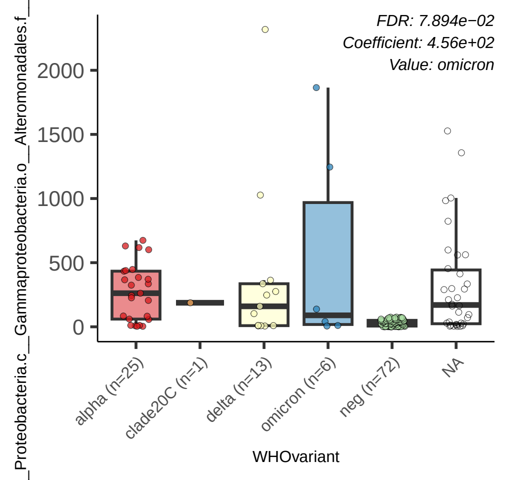
FDR: 7.894e−02
Coefficient: 4.56e+02
Value: omicron
_Proteobacteria.c__Gammaproteobacteria.o__Alteromonadales.f
0
500
1000
1500
2000
alpha (n=25)
clade20C (n=1)
delta (n=13)
omicron (n=6)
neg (n=72)
NA
WHOvariant

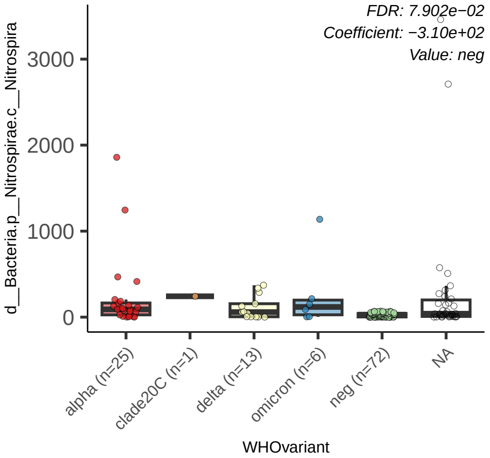
FDR: 7.902e−02
Coefficient: −3.10e+02
Value: neg
d__Bacteria.p__Nitrospirae.c__Nitrospira
3000
2000
1000
0
alpha (n=25)
clade20C (n=1)
delta (n=13)
omicron (n=6)
neg (n=72)
NA
WHOvariant

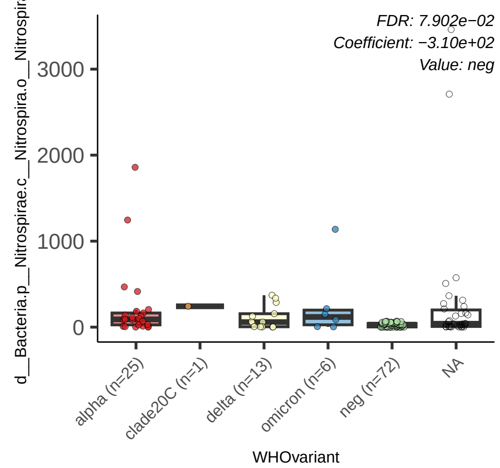

FDR: 7.902e−02
Coefficient: −3.10e+02
Value: neg
d__Bacteria.p__Nitrospirae.c__Nitrospira.o__Nitrospira
0
1000
2000
3000
alpha (n=25)
clade20C (n=1)
delta (n=13)
omicron (n=6)
neg (n=72)
NA
WHOvariant

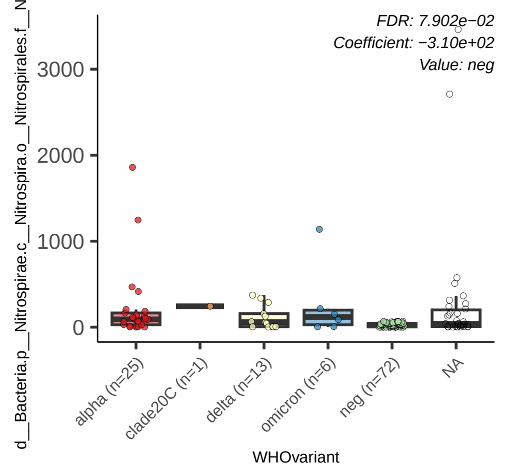

FDR: 7.902e−02
Coefficient: −3.10e+02
Value: neg
d__Bacteria.p__Nitrospirae.c__Nitrospira.o__Nitrospirales.f__N
3000
2000
1000
0
alpha (n=25)
clade20C (n=1)
delta (n=13)
omicron (n=6)
neg (n=72)
NA
WHOvariant

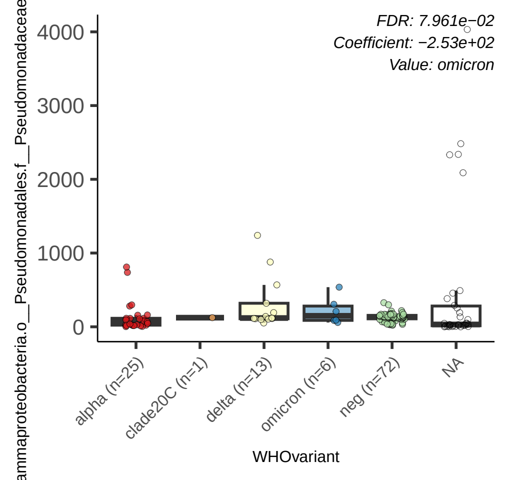

FDR: 7.961e−02
Coefficient: −2.53e+02
Value: omicron
ammaproteobacteria.o__Pseudomonadales.f__Pseudomonadaceae
4000
3000
2000
1000
0
alpha (n=25)
clade20C (n=1)
delta (n=13)
omicron (n=6)
neg (n=72)
NA
WHOvariant

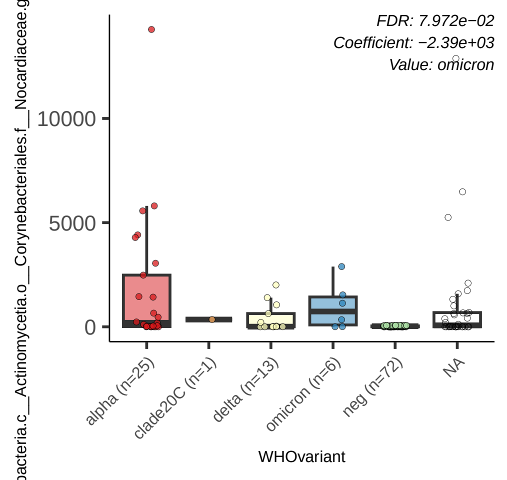
FDR: 7.972e−02
Coefficient: −2.39e+03
Value: omicron
10000
5000
0
bacteria.c__Actinomycetia.o__Corynebacteriales.f__Nocardiaceae.g
alpha (n=25)
clade20C (n=1)
delta (n=13)
omicron (n=6)
neg (n=72)
NA
WHOvariant

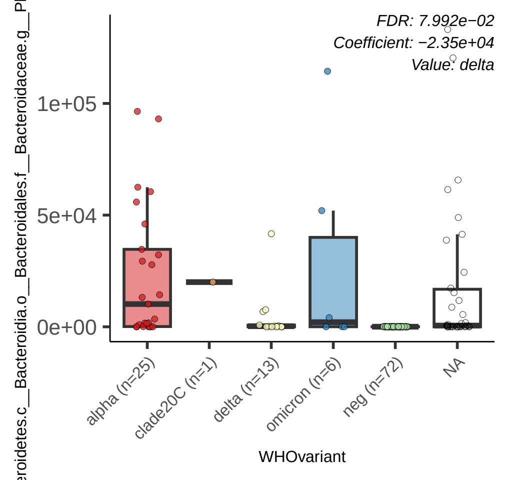

FDR: 7.992e−02
Coefficient: −2.35e+04
Value: delta
1e+05
5e+04
0e+00
eroidetes.c__Bacteroidia.o__Bacteroidales.f__Bacteroidaceae.g__P
alpha (n=25)
clade20C (n=1)
delta (n=13)
omicron (n=6)
neg (n=72)
NA
WHOvariant

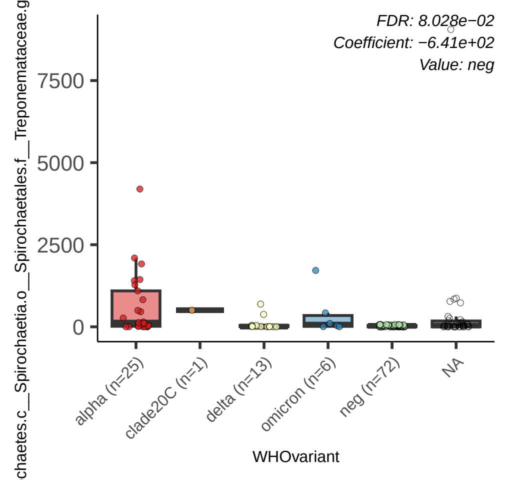

FDR: 8.028e−02
Coefficient: −6.41e+02
Value: neg
chaetes.c__Spirochaetia.o__Spirochaetales.f__Treponemataceae.g
7500
5000
2500
0
alpha (n=25)
clade20C (n=1)
delta (n=13)
omicron (n=6)
neg (n=72)
NA
WHOvariant

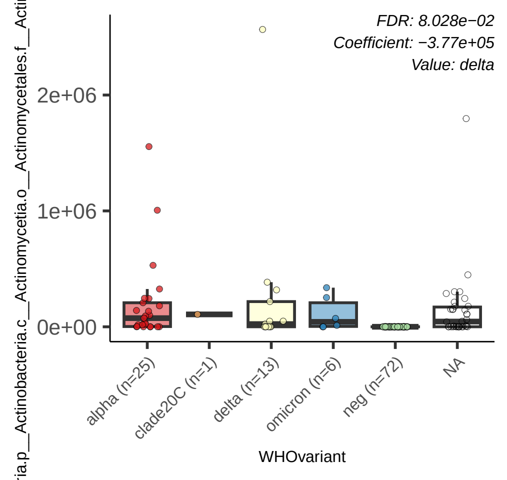
FDR: 8.028e−02
Coefficient: −3.77e+05
Value: delta
2e+06
1e+06
0e+00
alpha (n=25)
clade20C (n=1)
delta (n=13)
omicron (n=6)
neg (n=72)
NA
WHOvariant

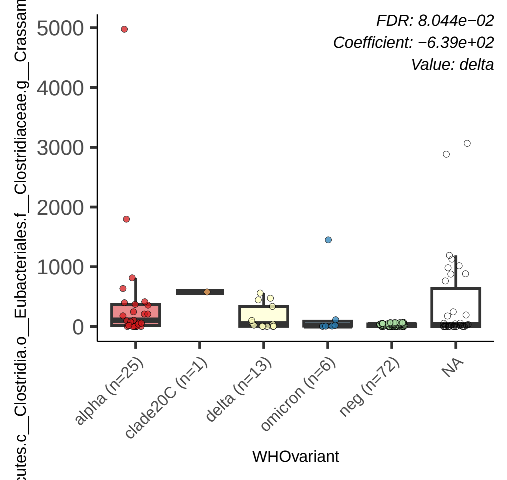
FDR: 8.044e−02
Coefficient: −6.39e+02
Value: delta
5000
4000
3000
2000
1000
0
cutes.c__Clostridia.o__Eubacteriales.f__Clostridiaceae.g__Crassam
alpha (n=25)
clade20C (n=1)
delta (n=13)
omicron (n=6)
neg (n=72)
NA
WHOvariant

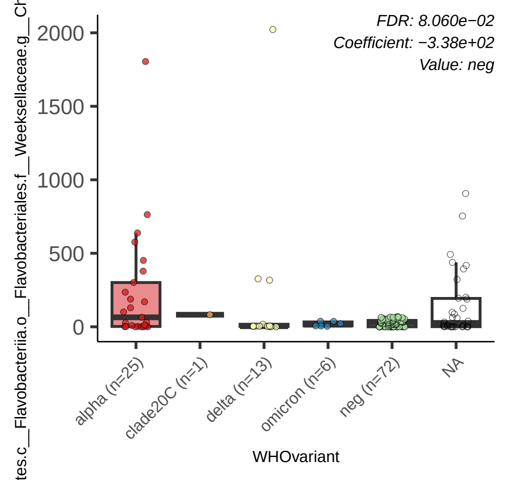

FDR: 8.060e−02
Coefficient: −3.38e+02
Value: neg
2000
1500
1000
500
0
tes.c__Flavobacteriia.o__Flavobacteriales.f__Weeksellaceae.g__Ch
alpha (n=25)
clade20C (n=1)
delta (n=13)
omicron (n=6)
neg (n=72)
NA
WHOvariant

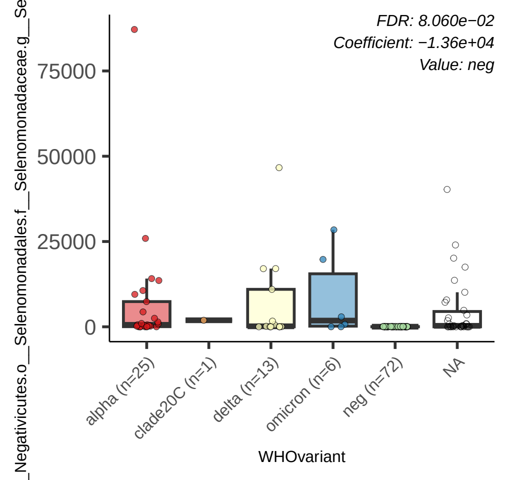
FDR: 8.060e−02
Coefficient: −1.36e+04
Value: neg
75000
50000
25000
0
_Negativicutes.o__Selenomonadales.f__Selenomonadaceae.g__Se
alpha (n=25)
clade20C (n=1)
delta (n=13)
omicron (n=6)
neg (n=72)
NA
WHOvariant

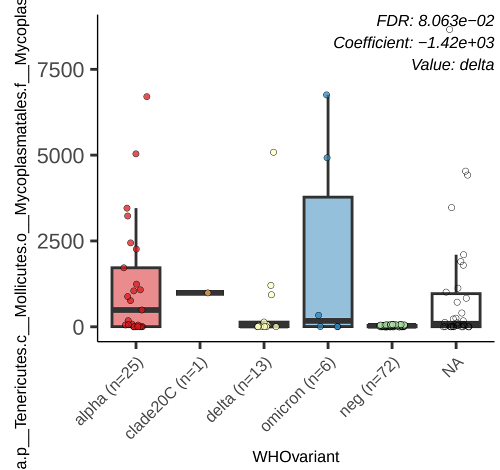

FDR: 8.063e−02
Coefficient: −1.42e+03
Value: delta
ria.p__Tenericutes.c__Mollicutes.o__Mycoplasmatales.f__Mycoplas
7500
5000
2500
0
alpha (n=25)
clade20C (n=1)
delta (n=13)
omicron (n=6)
neg (n=72)
NA
WHOvariant

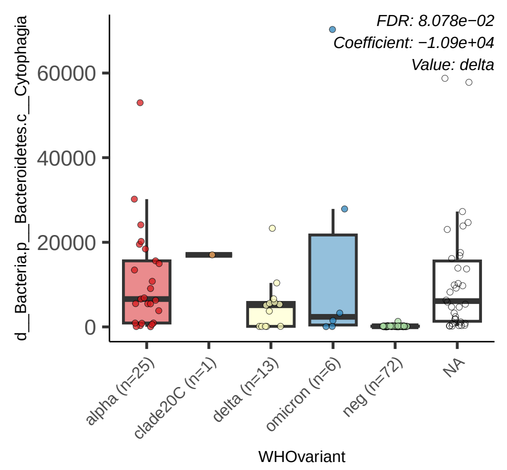
FDR: 8.078e−02
Coefficient: −1.09e+04
Value: delta
d__Bacteria.p__Bacteroidetes.c__Cytophagia
60000
40000
20000
0
alpha (n=25)
clade20C (n=1)
delta (n=13)
omicron (n=6)
neg (n=72)
NA
WHOvariant

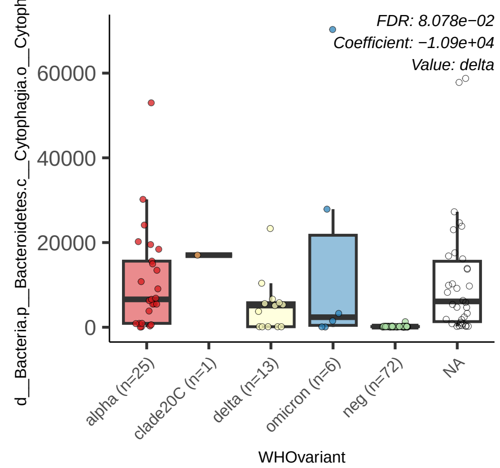

FDR: 8.078e−02
Coefficient: −1.09e+04
Value: delta
d__Bacteria.p__Bacteroidetes.c__Cytophagia.o__Cytoph
60000
40000
20000
0
alpha (n=25)
clade20C (n=1)
delta (n=13)
omicron (n=6)
neg (n=72)
NA
WHOvariant

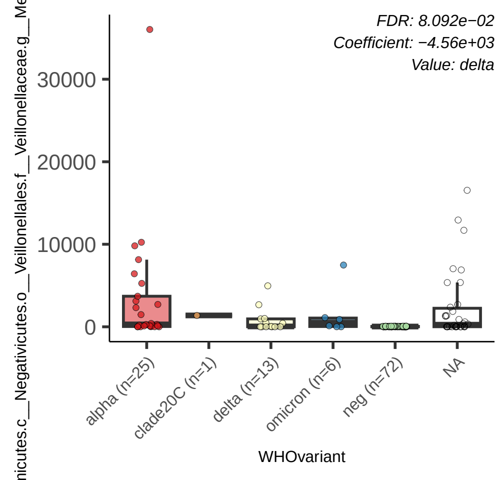

FDR: 8.092e−02
Coefficient: −4.56e+03
Value: delta
30000
20000
10000
0
alpha (n=25)
clade20C (n=1)
delta (n=13)
omicron (n=6)
neg (n=72)
NA
WHOvariant
nicutes.c__Negativicutes.o__Veillonellales.f__Veillonellaceae.g__Me

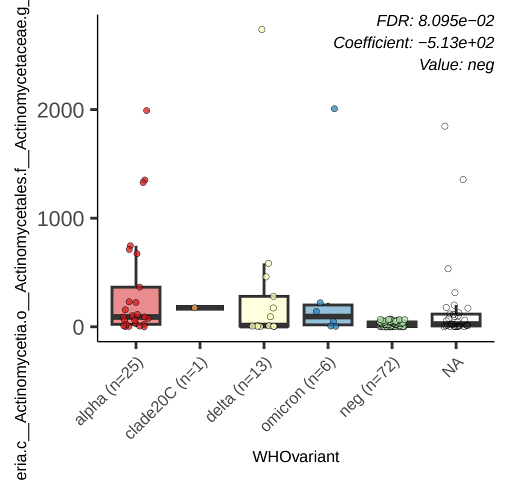
FDR: 8.095e−02
Coefficient: −5.13e+02
Value: neg
eria.c__Actinomycetia.o__Actinomycetales.f__Actinomycetaceae.g__
2000
1000
0
alpha (n=25)
clade20C (n=1)
delta (n=13)
omicron (n=6)
neg (n=72)
NA
WHOvariant

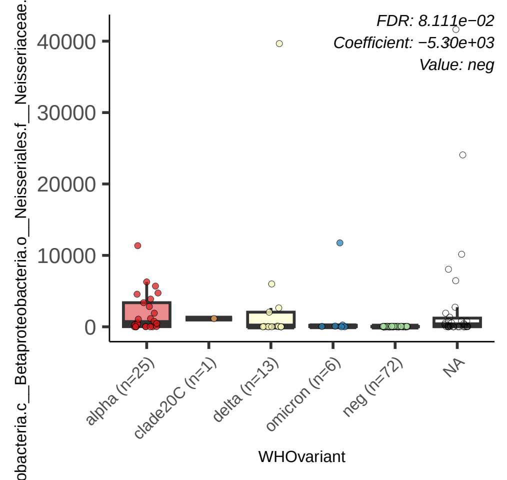

FDR: 8.111e−02
Coefficient: −5.30e+03
Value: neg
obacteria.c__Betaproteobacteria.o__Neisseriales.f__Neisseriaceae.
40000
30000
20000
10000
0
alpha (n=25)
clade20C (n=1)
delta (n=13)
omicron (n=6)
neg (n=72)
NA
WHOvariant

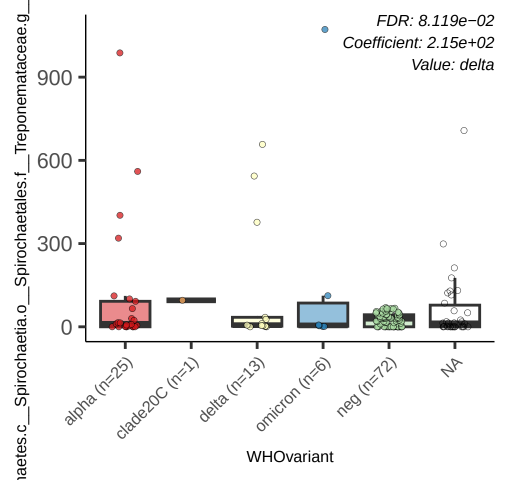
FDR: 8.119e−02
Coefficient: 2.15e+02
Value: delta
900
600
300
0
alpha (n=25)
clade20C (n=1)
delta (n=13)
omicron (n=6)
neg (n=72)
NA
WHOvariant

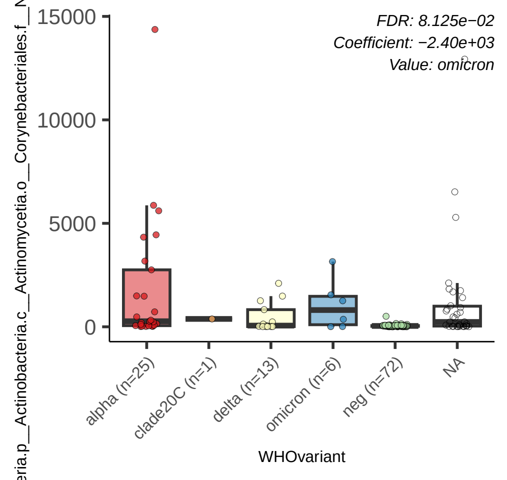

FDR: 8.125e−02
Coefficient: −2.40e+03
Value: omicron
15000
10000
5000
0
alpha (n=25)
clade20C (n=1)
delta (n=13)
omicron (n=6)
neg (n=72)
NA
WHOvariant
Actinobacteria.c__Actinomycetia.o__Corynebacteriales.f__N

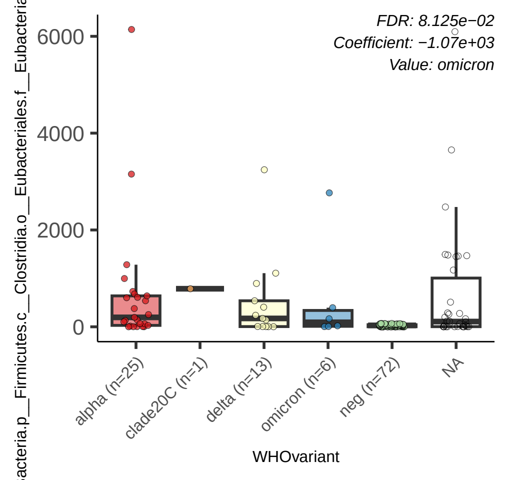
FDR: 8.125e−02
Coefficient: −1.07e+03
Value: omicron
6000
4000
2000
0
acteria.p__Firmicutes.c__Clostridia.o__Eubacteriales.f__Eubacteria
alpha (n=25)
clade20C (n=1)
delta (n=13)
omicron (n=6)
neg (n=72)
NA
WHOvariant

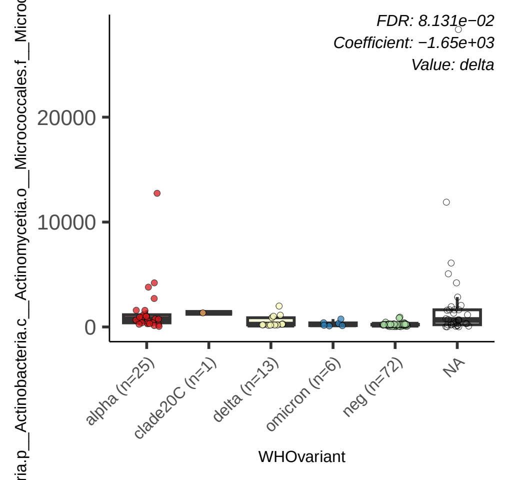
FDR: 8.131e−02
Coefficient: −1.65e+03
Value: delta
ria.p__Actinobacteria.c__Actinomycetia.o__Micrococcales.f__Microc
20000
10000
0
alpha (n=25)
clade20C (n=1)
delta (n=13)
omicron (n=6)
neg (n=72)
NA
WHOvariant

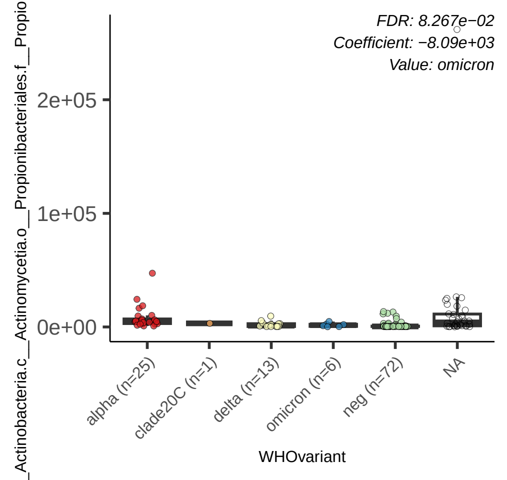
FDR: 8.267e−02
Coefficient: −8.09e+03
Value: omicron
_Actinobacteria.c__Actinomycetia.o__Propionibacteriales.f__Propio
2e+05
1e+05
0e+00
alpha (n=25)
clade20C (n=1)
delta (n=13)
omicron (n=6)
neg (n=72)
NA
WHOvariant

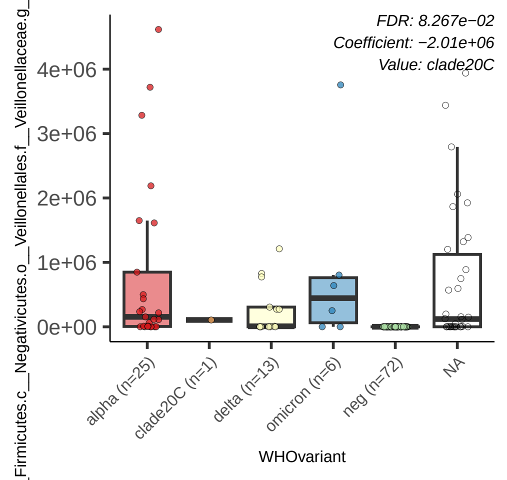
FDR: 8.267e−02
Coefficient: −2.01e+06
Value: clade20C
Firmicutes.c__Negativicutes.o__Veillonellales.f__Veillonellaceae.g__
4e+06
3e+06
2e+06
1e+06
0e+00
alpha (n=25)
clade20C (n=1)
delta (n=13)
omicron (n=6)
neg (n=72)
NA
WHOvariant

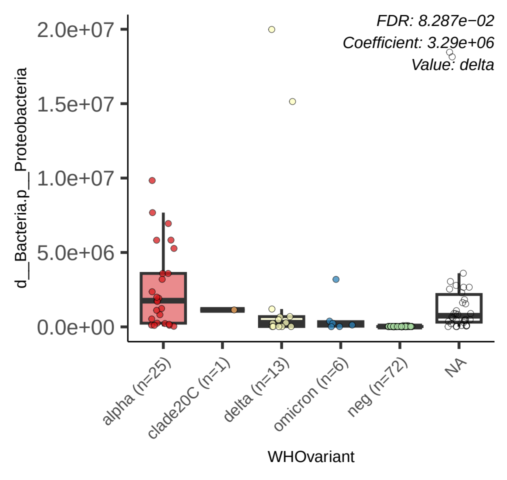
FDR: 8.287e−02
Coefficient: 3.29e+06
Value: delta
d__Bacteria.p__Proteobacteria
2.0e+07
1.5e+07
1.0e+07
5.0e+06
0.0e+00
alpha (n=25)
clade20C (n=1)
delta (n=13)
omicron (n=6)
neg (n=72)
NA
WHOvariant

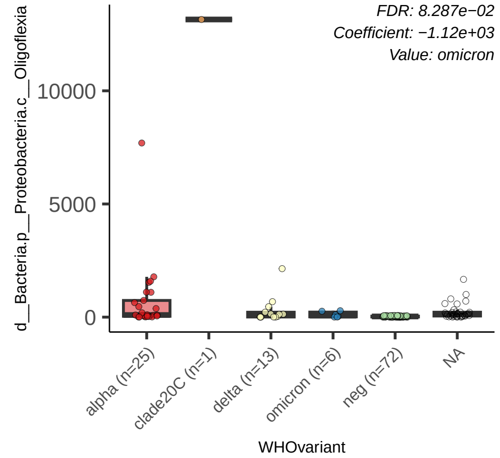
FDR: 8.287e−02
Coefficient: −1.12e+03
Value: omicron
d__Bacteria.p__Proteobacteria.c__Oligoflexia
10000
5000
0
alpha (n=25)
clade20C (n=1)
delta (n=13)
omicron (n=6)
neg (n=72)
NA
WHOvariant

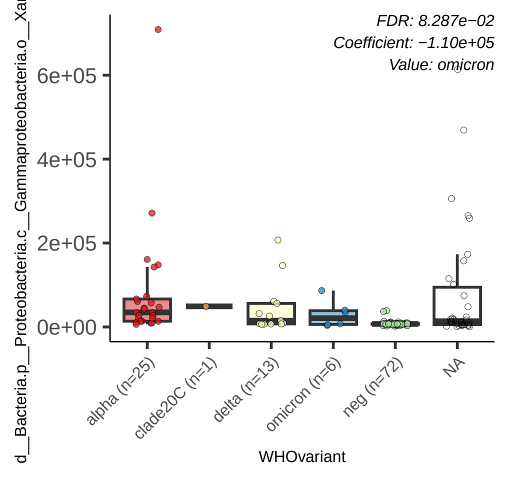

FDR: 8.287e−02
Coefficient: −1.10e+05
Value: omicron
6e+05
4e+05
2e+05
0e+00
d__Bacteria.p__Proteobacteria.c__Gammaproteobacteria.o__Xa
alpha (n=25)
clade20C (n=1)
delta (n=13)
omicron (n=6)
neg (n=72)
NA
WHOvariant

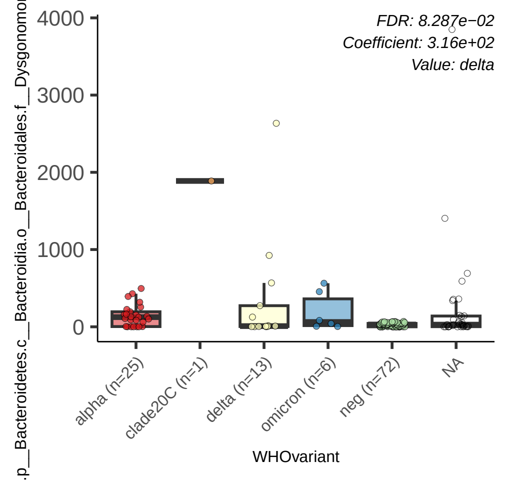

FDR: 8.287e−02
Coefficient: 3.16e+02
Value: delta
4000
3000
2000
1000
0
p__Bacteroidetes.c__Bacteroidia.o__Bacteroidales.f__Dysgonomo
alpha (n=25)
clade20C (n=1)
delta (n=13)
omicron (n=6)
neg (n=72)
NA
WHOvariant

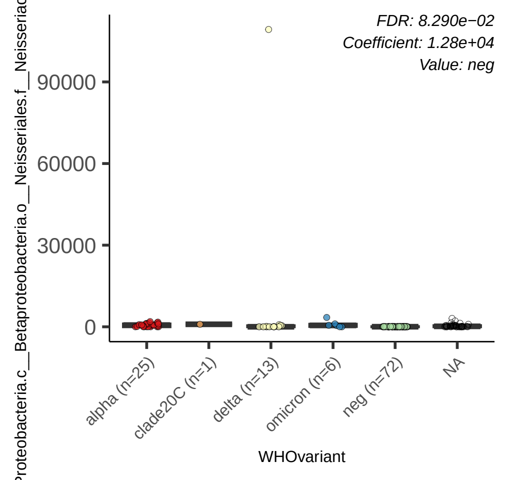
FDR: 8.290e−02
Coefficient: 1.28e+04
Value: neg
Proteobacteria.c__Betaproteobacteria.o__Neisseriales.f__Neisseriac
90000
60000
30000
0
alpha (n=25)
clade20C (n=1)
delta (n=13)
omicron (n=6)
neg (n=72)
NA
WHOvariant

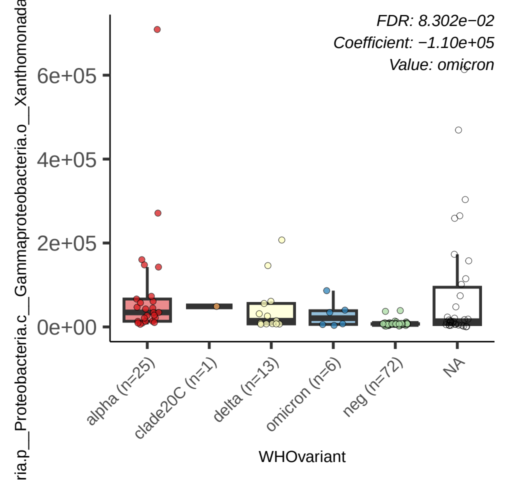

FDR: 8.302e−02
Coefficient: −1.10e+05
Value: omicron
ria.p__Proteobacteria.c__Gammaproteobacteria.o__Xanthomonada
6e+05
4e+05
2e+05
0e+00
alpha (n=25)
clade20C (n=1)
delta (n=13)
omicron (n=6)
neg (n=72)
NA
WHOvariant

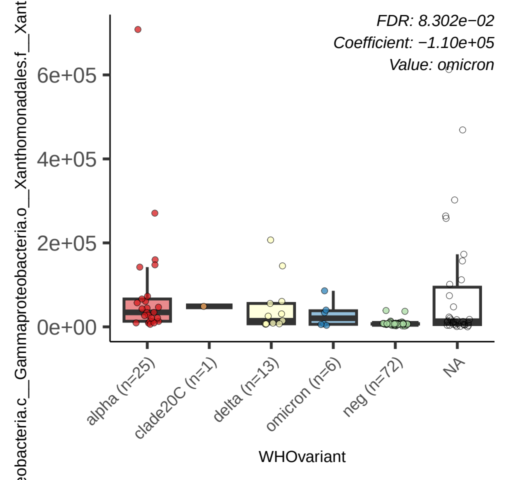

FDR: 8.302e−02
Coefficient: −1.10e+05
Value: omicron
eobacteria.c__Gammaproteobacteria.o__Xanthomonadales.f__Xant
6e+05
4e+05
2e+05
0e+00
alpha (n=25)
clade20C (n=1)
delta (n=13)
omicron (n=6)
neg (n=72)
NA
WHOvariant

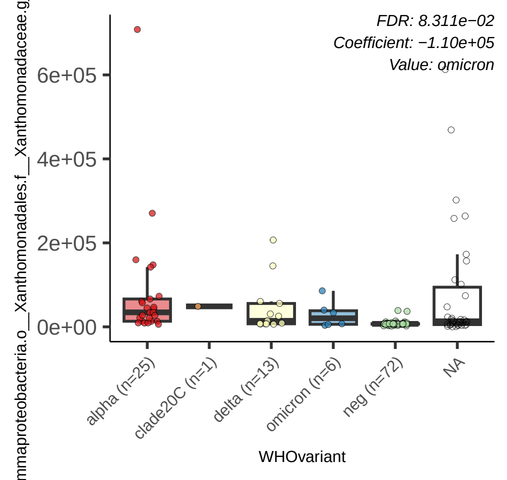

FDR: 8.311e−02
Coefficient: −1.10e+05
Value: omicron
mmaproteobacteria.o__Xanthomonadales.f__Xanthomonadaceae.g
6e+05
4e+05
2e+05
0e+00
alpha (n=25)
clade20C (n=1)
delta (n=13)
omicron (n=6)
neg (n=72)
NA
WHOvariant

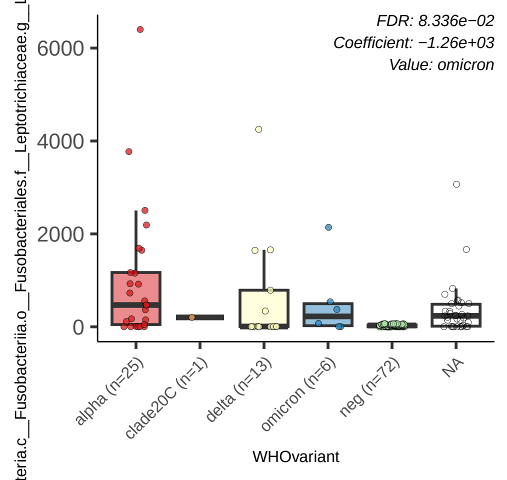

FDR: 8.336e−02
Coefficient: −1.26e+03
Value: omicron
6000
4000
2000
0
alpha (n=25)
clade20C (n=1)
delta (n=13)
omicron (n=6)
neg (n=72)
NA
WHOvariant
teria.c__Fusobacteriia.o__Fusobacteriales.f__Leptotrichiaceae.g__L

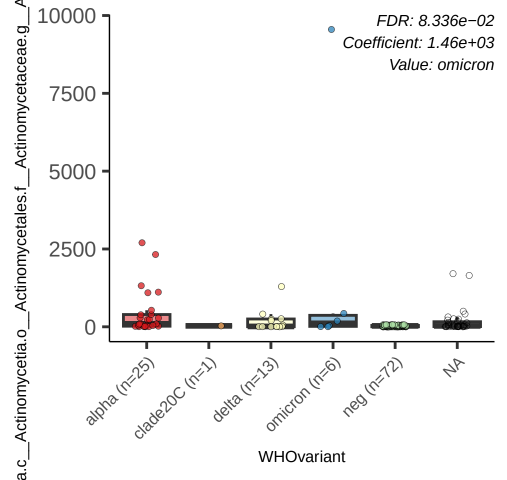
FDR: 8.336e−02
Coefficient: 1.46e+03
Value: omicron
10000
7500
5000
2500
0
alpha (n=25)
clade20C (n=1)
delta (n=13)
omicron (n=6)
neg (n=72)
NA
WHOvariant
a.c__Actinomycetia.o__Actinomycetales.f__Actinomycetaceae.g__A

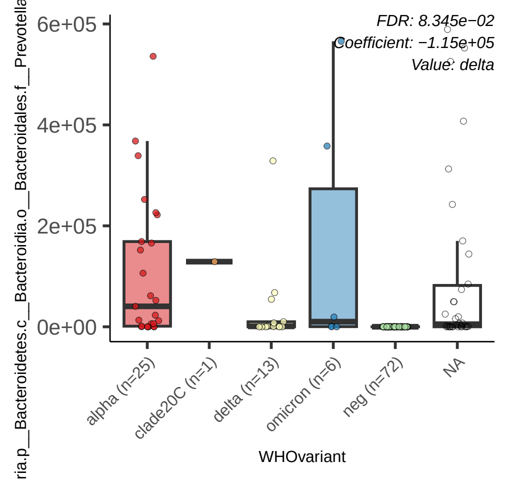
FDR: 8.345e−02
Coefficient: −1.15e+05
Value: delta
6e+05
4e+05
2e+05
0e+00
ria.p__Bacteroidetes.c__Bacteroidia.o__Bacteroidales.f__Prevotella
alpha (n=25)
clade20C (n=1)
delta (n=13)
omicron (n=6)
neg (n=72)
NA
WHOvariant

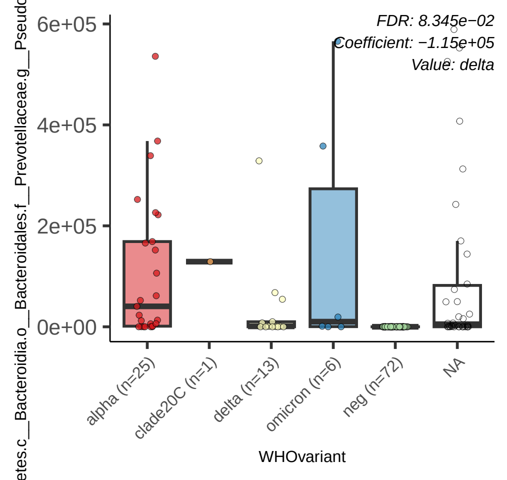

FDR: 8.345e−02
Coefficient: −1.15e+05
Value: delta
6e+05
4e+05
2e+05
0e+00
etes.c__Bacteroidia.o__Bacteroidales.f__Prevotellaceae.g__Pseudo
alpha (n=25)
clade20C (n=1)
delta (n=13)
omicron (n=6)
neg (n=72)
NA
WHOvariant

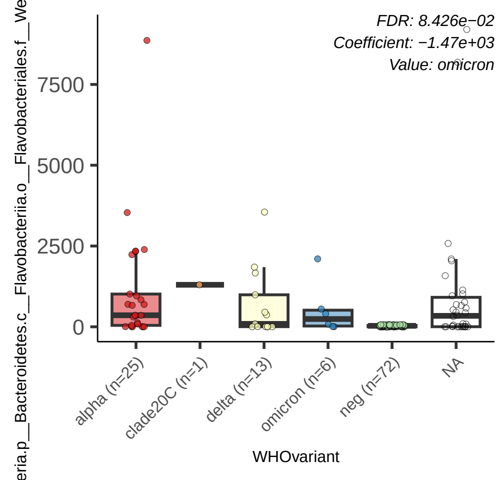
FDR: 8.426e−02
Coefficient: −1.47e+03
Value: omicron
7500
5000
2500
0
alpha (n=25)
clade20C (n=1)
delta (n=13)
omicron (n=6)
neg (n=72)
NA
WHOvariant
eria.p__Bacteroidetes.c__Flavobacteriia.o__Flavobacteriales.f__We

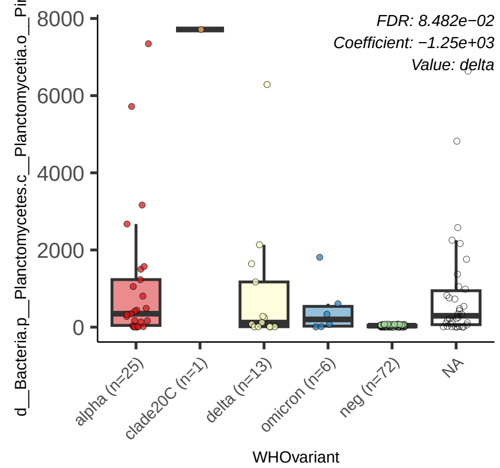

FDR: 8.482e−02
Coefficient: −1.25e+03
Value: delta
d__Bacteria.p__Planctomycetes.c__Planctomycetia.o__Pi
8000
6000
4000
2000
0
alpha (n=25)
clade20C (n=1)
delta (n=13)
omicron (n=6)
neg (n=72)
NA
WHOvariant

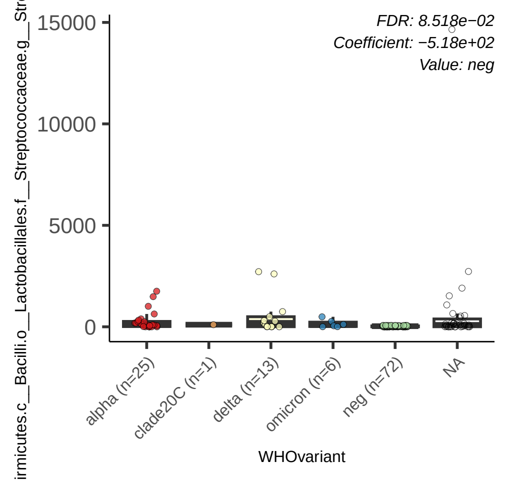

FDR: 8.518e−02
Coefficient: −5.18e+02
Value: neg
15000
10000
5000
0
irmicutes.c__Bacilli.o__Lactobacillales.f__Streptococcaceae.g__Str
alpha (n=25)
clade20C (n=1)
delta (n=13)
omicron (n=6)
neg (n=72)
NA
WHOvariant

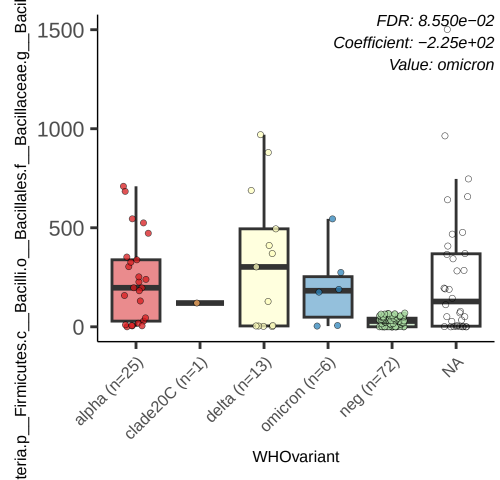

FDR: 8.550e−02
Coefficient: −2.25e+02
Value: omicron
teria.p__Firmicutes.c__Bacilli.o__Bacillales.f__Bacillaceae.g__Bacil
1500
1000
500
0
alpha (n=25)
clade20C (n=1)
delta (n=13)
omicron (n=6)
neg (n=72)
NA
WHOvariant

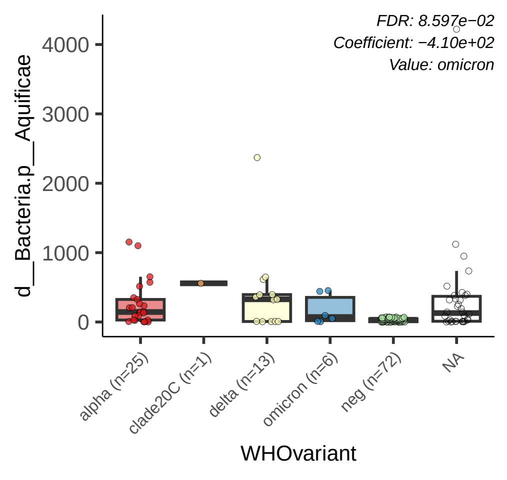

FDR: 8.597e−02
Coefficient: −4.10e+02
Value: omicron
d__Bacteria.p__Aquificae
4000
3000
2000
1000
0
alpha (n=25)
clade20C (n=1)
delta (n=13)
omicron (n=6)
neg (n=72)
NA
WHOvariant

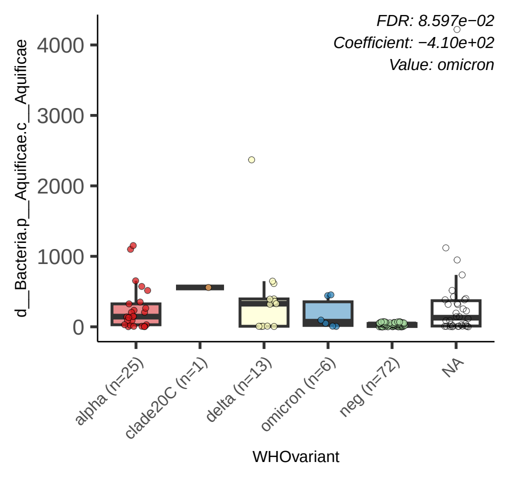

FDR: 8.597e−02
Coefficient: −4.10e+02
Value: omicron
d__Bacteria.p__Aquificae.c__Aquificae
4000
3000
2000
1000
0
alpha (n=25)
clade20C (n=1)
delta (n=13)
omicron (n=6)
neg (n=72)
NA
WHOvariant

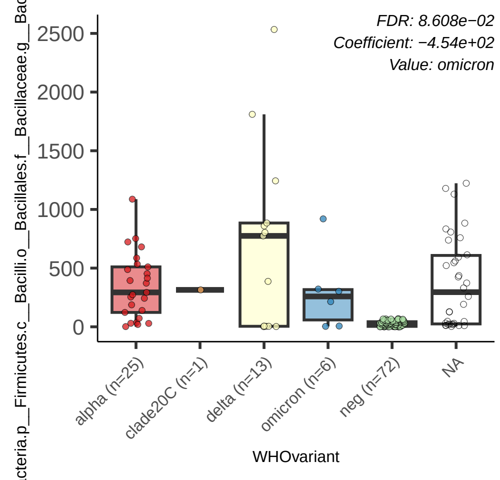
FDR: 8.608e−02
Coefficient: −4.54e+02
Value: omicron
2500
2000
1500
1000
500
0
alpha (n=25)
clade20C (n=1)
delta (n=13)
omicron (n=6)
neg (n=72)
NA
WHOvariant

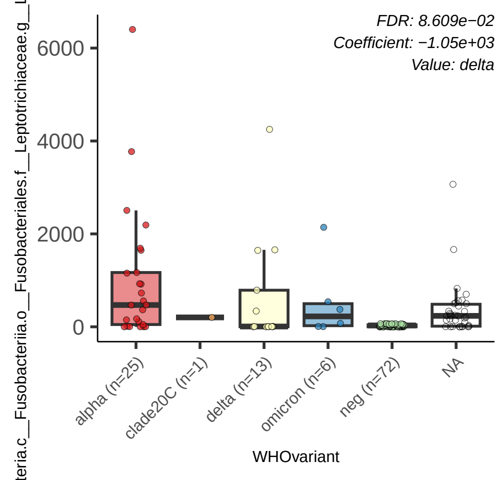

FDR: 8.609e−02
Coefficient: −1.05e+03
Value: delta
teria.c__Fusobacteriia.o__Fusobacteriales.f__Leptotrichiaceae.g__L
6000
4000
2000
0
alpha (n=25)
clade20C (n=1)
delta (n=13)
omicron (n=6)
neg (n=72)
NA
WHOvariant

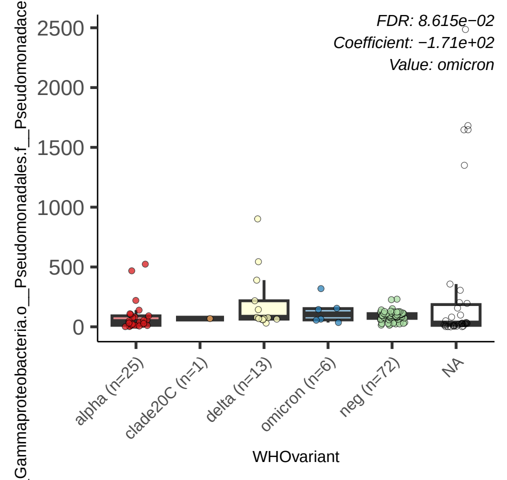
FDR: 8.615e−02
Coefficient: −1.71e+02
Value: omicron
Gammaproteobacteria.o__Pseudomonadales.f__Pseudomonadace
2500
2000
1500
1000
500
0
alpha (n=25)
clade20C (n=1)
delta (n=13)
omicron (n=6)
neg (n=72)
NA
WHOvariant

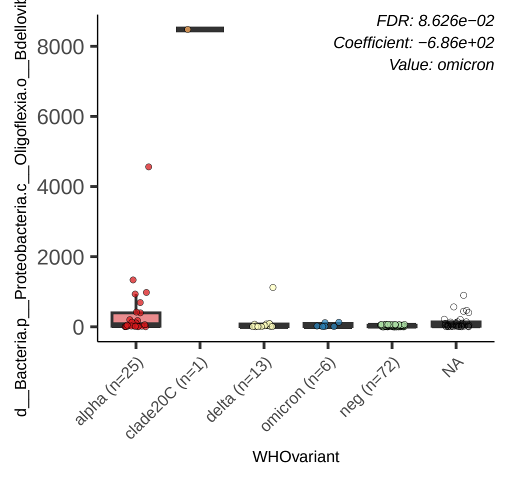

FDR: 8.626e−02
Coefficient: −6.86e+02
Value: omicron
d__Bacteria.p__Proteobacteria.c__Oligoflexia.o__Bdellovib
0
2000
4000
6000
8000
alpha (n=25)
clade20C (n=1)
delta (n=13)
omicron (n=6)
neg (n=72)
NA
WHOvariant

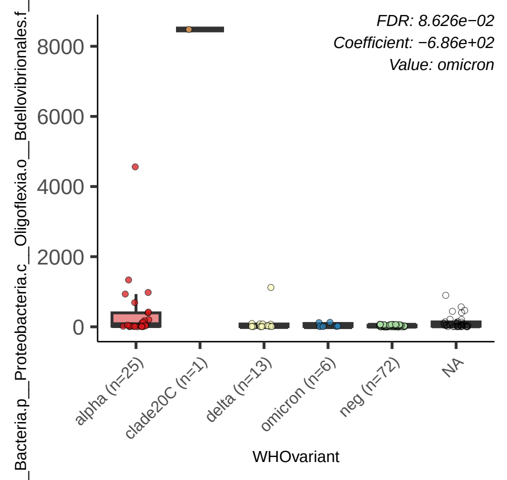

FDR: 8.626e−02
Coefficient: −6.86e+02
Value: omicron
Bacteria.p__Proteobacteria.c__Oligoflexia.o__Bdellovibrionales.f
8000
6000
4000
2000
0
alpha (n=25)
clade20C (n=1)
delta (n=13)
omicron (n=6)
neg (n=72)
NA
WHOvariant

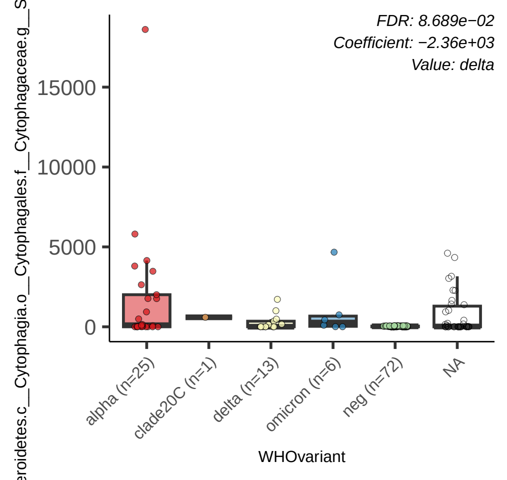

FDR: 8.689e−02
Coefficient: −2.36e+03
Value: delta
15000
10000
5000
0
eroidetes.c__Cytophagia.o__Cytophagales.f__Cytophagaceae.g__S
alpha (n=25)
clade20C (n=1)
delta (n=13)
omicron (n=6)
neg (n=72)
NA
WHOvariant

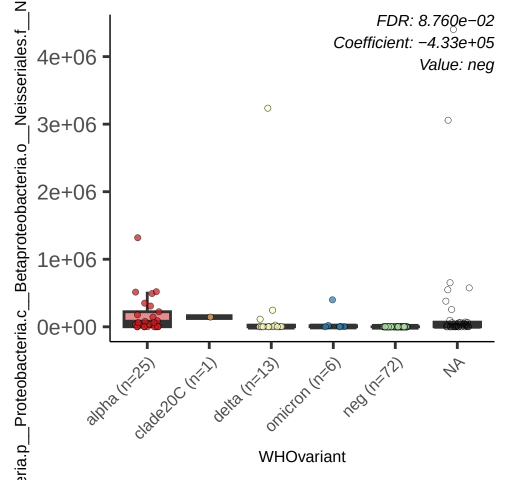

FDR: 8.760e−02
Coefficient: −4.33e+05
Value: neg
4e+06
3e+06
2e+06
1e+06
0e+00
alpha (n=25)
clade20C (n=1)
delta (n=13)
omicron (n=6)
neg (n=72)
NA
WHOvariant

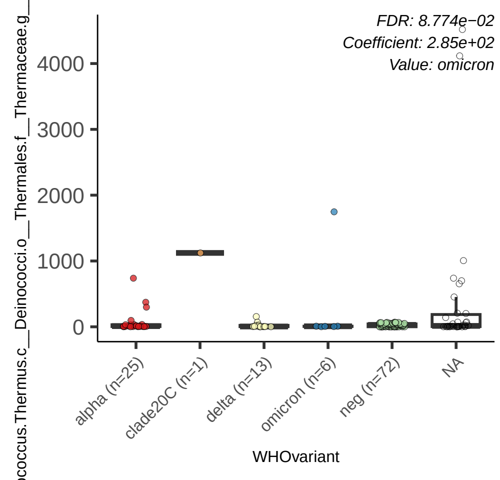

FDR: 8.774e−02
Coefficient: 2.85e+02
Value: omicron
4000
3000
2000
1000
0
alpha (n=25)
clade20C (n=1)
delta (n=13)
omicron (n=6)
neg (n=72)
NA
WHOvariant
occus.Thermus.c__Deinococci.o__Thermales.f__Thermaceae.g__

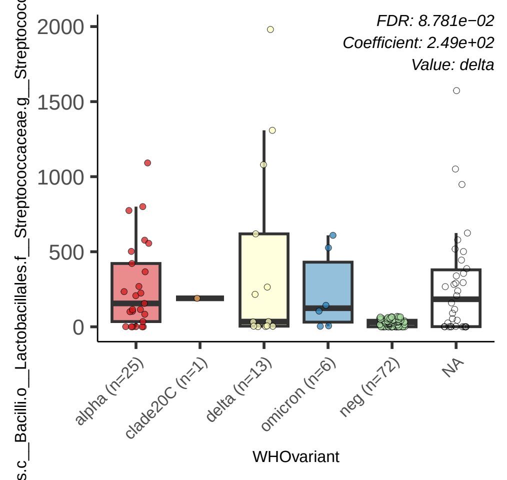

FDR: 8.781e−02
Coefficient: 2.49e+02
Value: delta
2000
1500
1000
500
0
s.c__Bacilli.o__Lactobacillales.f__Streptococcaceae.g__Streptococc
alpha (n=25)
clade20C (n=1)
delta (n=13)
omicron (n=6)
neg (n=72)
NA
WHOvariant

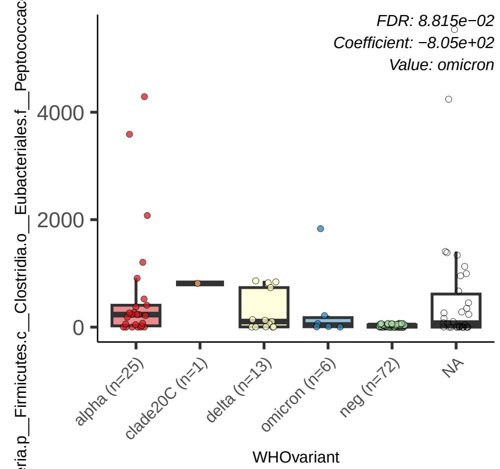
FDR: 8.815e−02
Coefficient: −8.05e+02
Value: omicron
teria.p__Firmicutes.c__Clostridia.o__Eubacteriales.f__Peptococcac
4000
2000
0
alpha (n=25)
clade20C (n=1)
delta (n=13)
omicron (n=6)
neg (n=72)
NA
WHOvariant

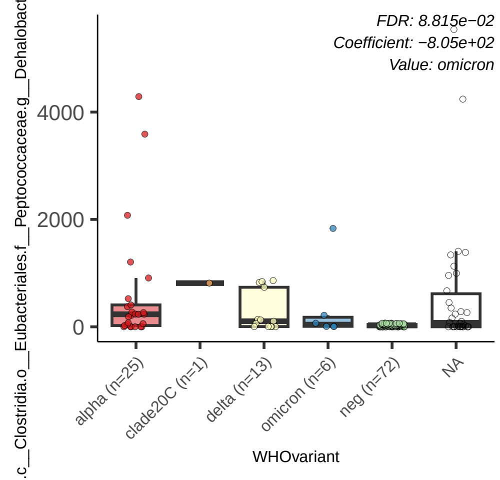

FDR: 8.815e−02
Coefficient: −8.05e+02
Value: omicron
c__Clostridia.o__Eubacteriales.f__Peptococcaceae.g__Dehalobact
0
2000
4000
alpha (n=25)
clade20C (n=1)
delta (n=13)
omicron (n=6)
neg (n=72)
NA
WHOvariant

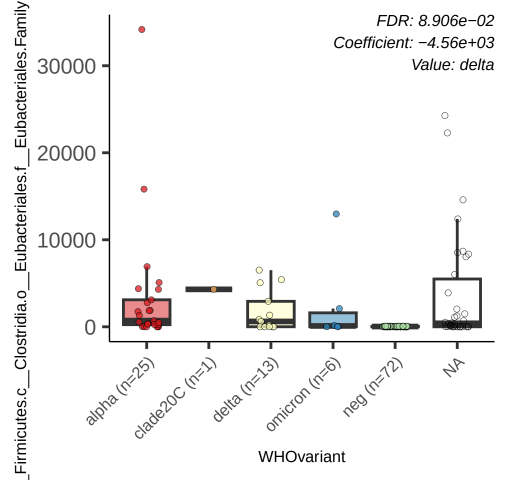

FDR: 8.906e−02
Coefficient: −4.56e+03
Value: delta
Firmicutes.c__Clostridia.o__Eubacteriales.f__Eubacteriales.Family
30000
20000
10000
0
alpha (n=25)
clade20C (n=1)
delta (n=13)
omicron (n=6)
neg (n=72)
NA
WHOvariant

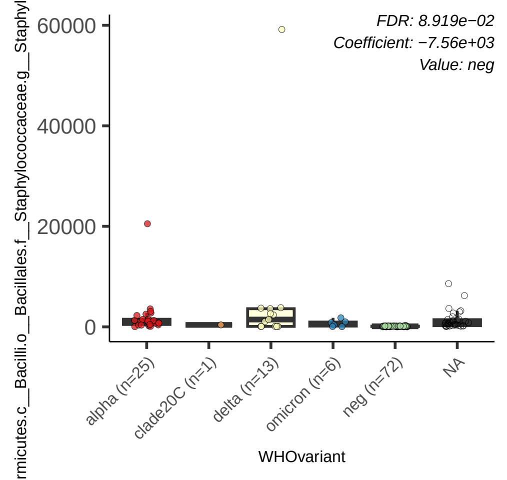
FDR: 8.919e−02
Coefficient: −7.56e+03
Value: neg
rmicutes.c__Bacilli.o__Bacillales.f__Staphylococcaceae.g__Staphyl
60000
40000
20000
0
alpha (n=25)
clade20C (n=1)
delta (n=13)
omicron (n=6)
neg (n=72)
NA
WHOvariant

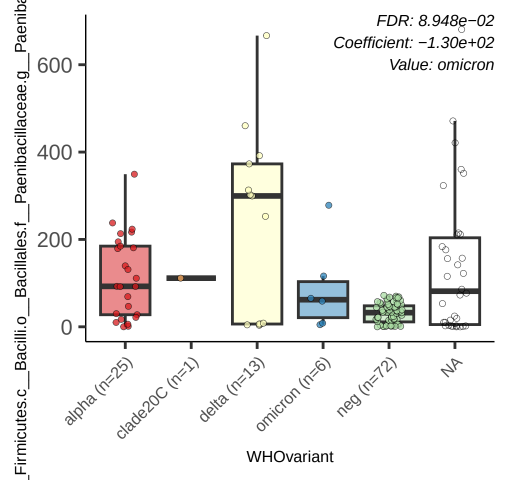
FDR: 8.948e−02
Coefficient: −1.30e+02
Value: omicron
Firmicutes.c__Bacilli.o__Bacillales.f__Paenibacillaceae.g__Paeniba
0
200
400
600
alpha (n=25)
clade20C (n=1)
delta (n=13)
omicron (n=6)
neg (n=72)
NA
WHOvariant

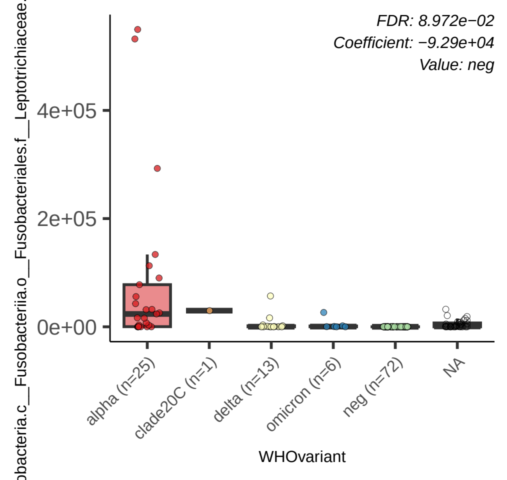

FDR: 8.972e−02
Coefficient: −9.29e+04
Value: neg
obacteria.c__Fusobacteriia.o__Fusobacteriales.f__Leptotrichiaceae.
4e+05
2e+05
0e+00
alpha (n=25)
clade20C (n=1)
delta (n=13)
omicron (n=6)
neg (n=72)
NA
WHOvariant

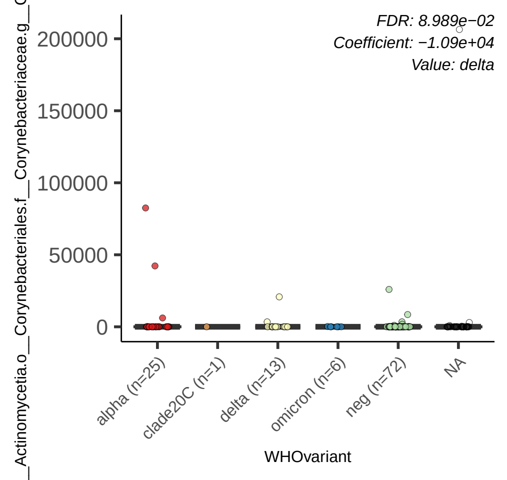
FDR: 8.989e−02
Coefficient: −1.09e+04
Value: delta
200000
150000
100000
50000
0
_Actinomycetia.o__Corynebacteriales.f__Corynebacteriaceae.g__C
alpha (n=25)
clade20C (n=1)
delta (n=13)
omicron (n=6)
neg (n=72)
NA
WHOvariant

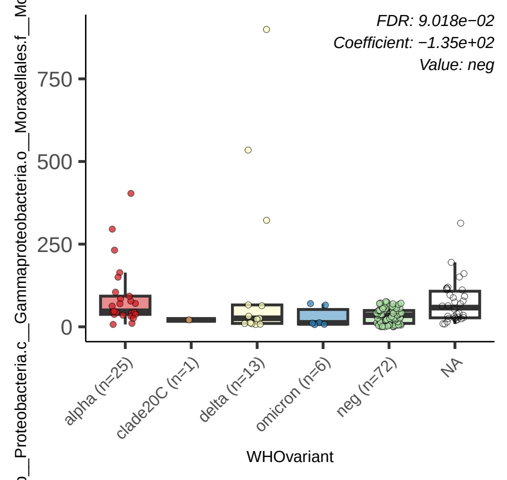

FDR: 9.018e−02
Coefficient: −1.35e+02
Value: neg
0
250
500
750
alpha (n=25)
clade20C (n=1)
delta (n=13)
omicron (n=6)
neg (n=72)
NA
WHOvariant

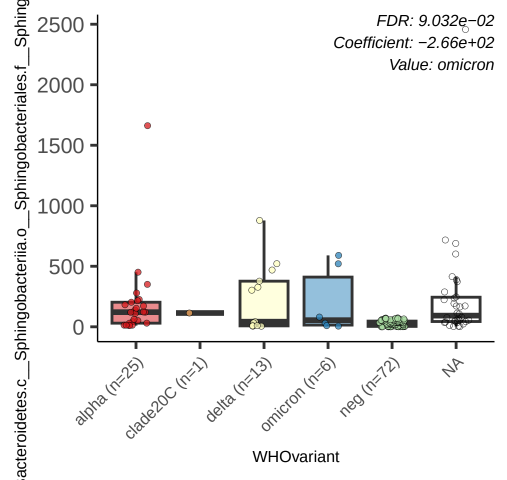

FDR: 9.032e−02
Coefficient: −2.66e+02
Value: omicron
Sphingo
Bacteroidetes.c__Sphingobacteriia.o__Sphingobacteriales.f__Sphing
2500
2000
1500
1000
500
0
alpha (n=25)
clade20C (n=1)
delta (n=13)
omicron (n=6)
neg (n=72)
NA
WHOvariant

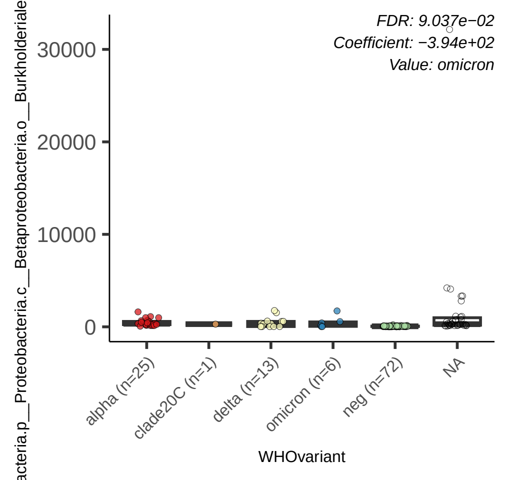

FDR: 9.037e−02
Coefficient: −3.94e+02
Value: omicron
30000
20000
10000
0
acteria.p__Proteobacteria.c__Betaproteobacteria.o__Burkholderiale
alpha (n=25)
clade20C (n=1)
delta (n=13)
omicron (n=6)
neg (n=72)
NA
WHOvariant

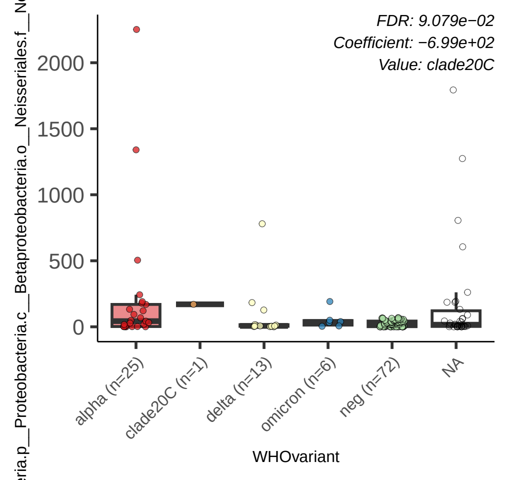

FDR: 9.079e−02
Coefficient: −6.99e+02
Value: clade20C
0
500
1000
1500
2000
alpha (n=25)
clade20C (n=1)
delta (n=13)
omicron (n=6)
neg (n=72)
NA
WHOvariant
eria.p__Proteobacteria.c__Betaproteobacteria.o__Neisseriales.f__Ne

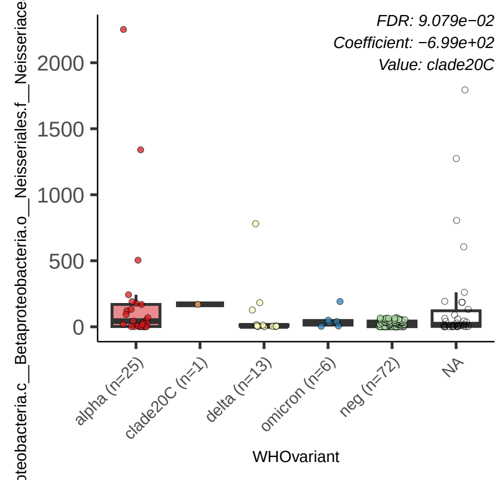
FDR: 9.079e−02
Coefficient: −6.99e+02
Value: clade20C
2000
1500
1000
500
0
alpha (n=25)
clade20C (n=1)
delta (n=13)
omicron (n=6)
neg (n=72)
NA
WHOvariant

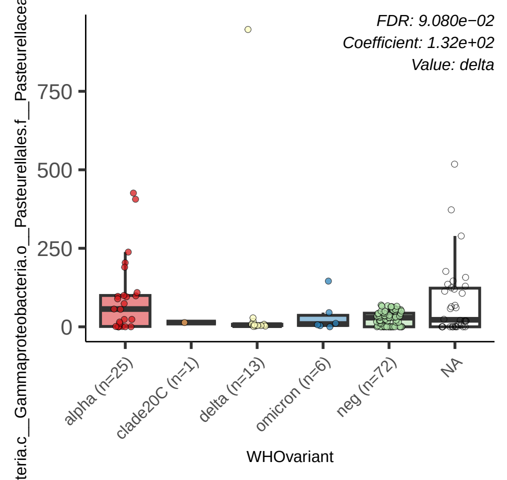

FDR: 9.080e−02
Coefficient: 1.32e+02
Value: delta
teria.c__Gammaproteobacteria.o__Pasteurellales.f__Pasteurellacea
750
500
250
0
alpha (n=25)
clade20C (n=1)
delta (n=13)
omicron (n=6)
neg (n=72)
NA
WHOvariant

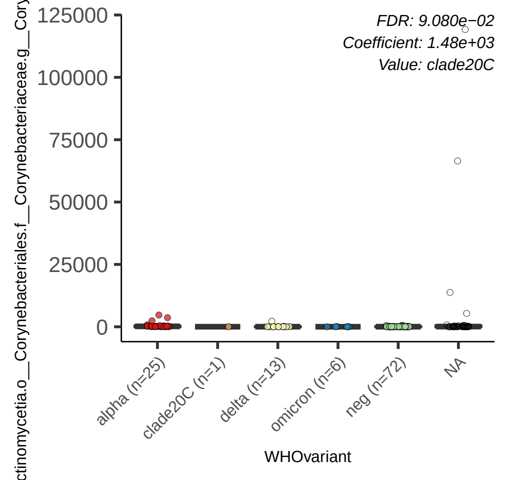

FDR: 9.080e−02
Coefficient: 1.48e+03
Value: clade20C
125000
100000
75000
50000
25000
0
ctinomycetia.o__Corynebacteriales.f__Corynebacteriaceae.g__Cory
alpha (n=25)
clade20C (n=1)
delta (n=13)
omicron (n=6)
neg (n=72)
NA
WHOvariant

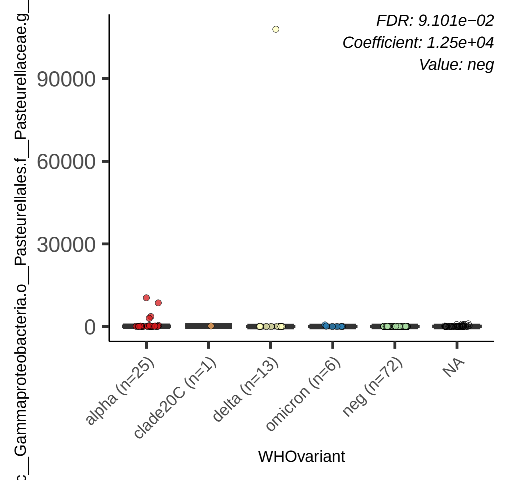

FDR: 9.101e−02
Coefficient: 1.25e+04
Value: neg
c__Gammaproteobacteria.o__Pasteurellales.f__Pasteurellaceae.g__
90000
60000
30000
0
alpha (n=25)
clade20C (n=1)
delta (n=13)
omicron (n=6)
neg (n=72)
NA
WHOvariant

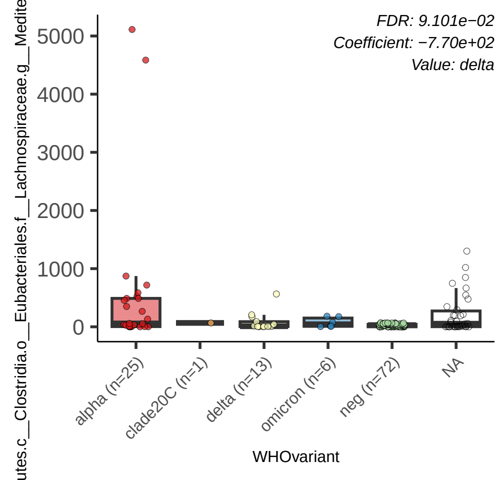
FDR: 9.101e−02
Coefficient: −7.70e+02
Value: delta
5000
4000
3000
2000
1000
0
utes.c__Clostridia.o__Eubacteriales.f__Lachnospiraceae.g__Medite
alpha (n=25)
clade20C (n=1)
delta (n=13)
omicron (n=6)
neg (n=72)
NA
WHOvariant

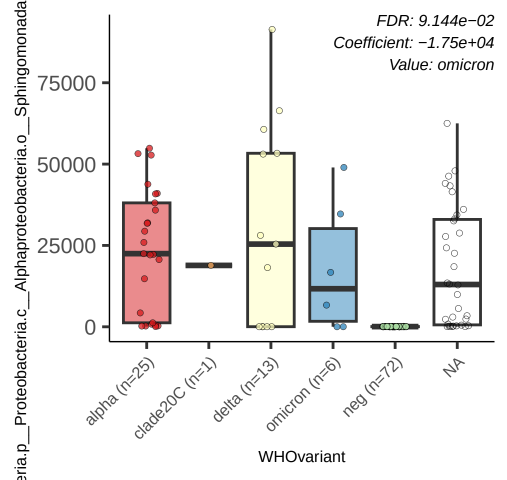
FDR: 9.144e−02
Coefficient: −1.75e+04
Value: omicron
75000
50000
25000
0
eria.p__Proteobacteria.c__Alphaproteobacteria.o__Sphingomonada
alpha (n=25)
clade20C (n=1)
delta (n=13)
omicron (n=6)
neg (n=72)
NA
WHOvariant

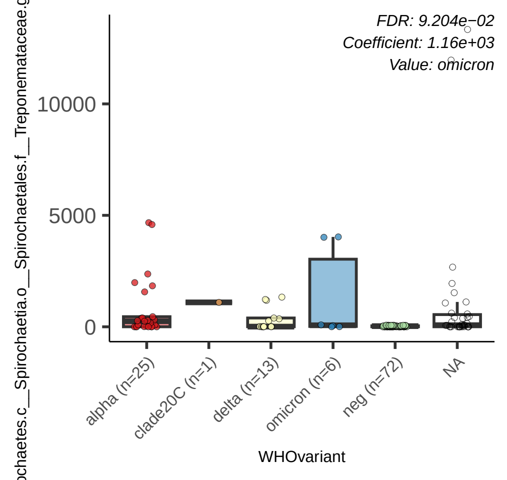

FDR: 9.204e−02
Coefficient: 1.16e+03
Value: omicron
10000
5000
0
ochaetes.c__Spirochaetia.o__Spirochaetales.f__Treponemataceae.g
alpha (n=25)
clade20C (n=1)
delta (n=13)
omicron (n=6)
neg (n=72)
NA
WHOvariant

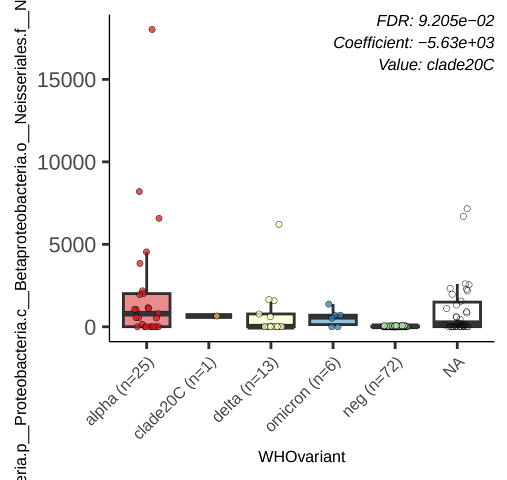

FDR: 9.205e−02
Coefficient: −5.63e+03
Value: clade20C
15000
10000
5000
0
eria.p__Proteobacteria.c__Betaproteobacteria.o__Neisseriales.f__N
alpha (n=25)
clade20C (n=1)
delta (n=13)
omicron (n=6)
neg (n=72)
NA
WHOvariant

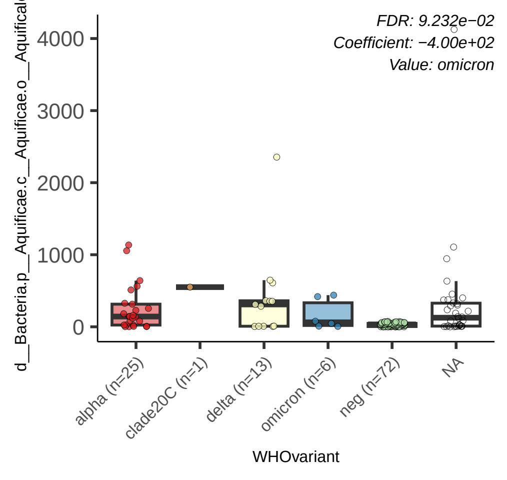
FDR: 9.232e−02
Coefficient: −4.00e+02
Value: omicron
d__Bacteria.p__Aquificae.c__Aquificae.o__Aquificales
4000
3000
2000
1000
0
alpha (n=25)
clade20C (n=1)
delta (n=13)
omicron (n=6)
neg (n=72)
NA
WHOvariant

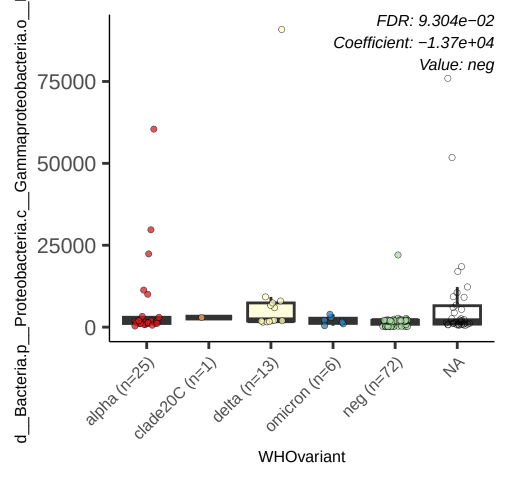

FDR: 9.304e−02
Coefficient: −1.37e+04
Value: neg
d__Bacteria.p__Proteobacteria.c__Gammaproteobacteria.o__
75000
50000
25000
0
alpha (n=25)
clade20C (n=1)
delta (n=13)
omicron (n=6)
neg (n=72)
NA
WHOvariant

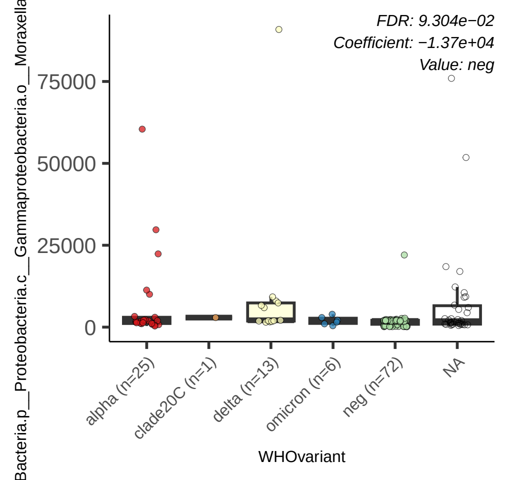

FDR: 9.304e−02
Coefficient: −1.37e+04
Value: neg
Bacteria.p__Proteobacteria.c__Gammaproteobacteria.o__Moraxella
0
25000
50000
75000
alpha (n=25)
clade20C (n=1)
delta (n=13)
omicron (n=6)
neg (n=72)
NA
WHOvariant

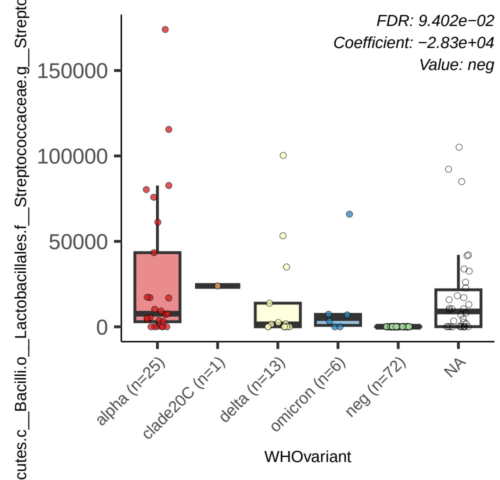

FDR: 9.402e−02
Coefficient: −2.83e+04
Value: neg
cutes.c__Bacilli.o__Lactobacillales.f__Streptococcaceae.g__Strepto
150000
100000
50000
0
alpha (n=25)
clade20C (n=1)
delta (n=13)
omicron (n=6)
neg (n=72)
NA
WHOvariant

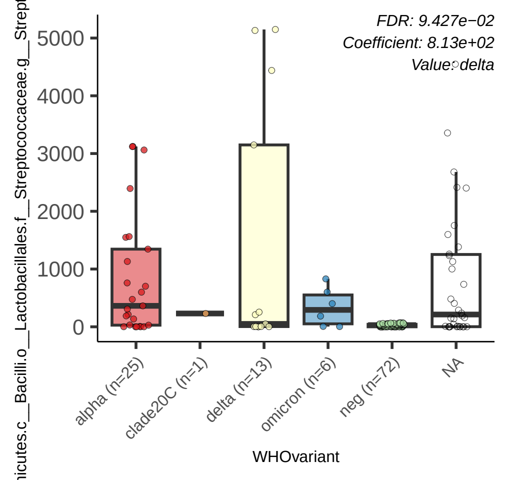

FDR: 9.427e−02
Coefficient: 8.13e+02
Value: delta
5000
4000
3000
2000
1000
0
micutes.c__Bacilli.o__Lactobacillales.f__Streptococcaceae.g__Strep
alpha (n=25)
clade20C (n=1)
delta (n=13)
omicron (n=6)
neg (n=72)
NA
WHOvariant

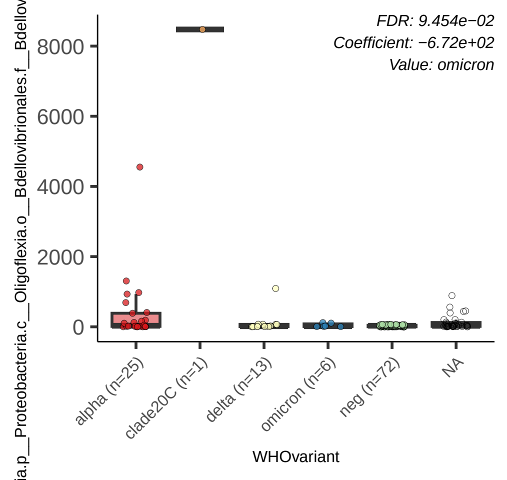

FDR: 9.454e−02
Coefficient: −6.72e+02
Value: omicron
ia.p__Proteobacteria.c__Oligoflexia.o__Bdellovibrionales.f__Bdellov
8000
6000
4000
2000
0
alpha (n=25)
clade20C (n=1)
delta (n=13)
omicron (n=6)
neg (n=72)
NA
WHOvariant

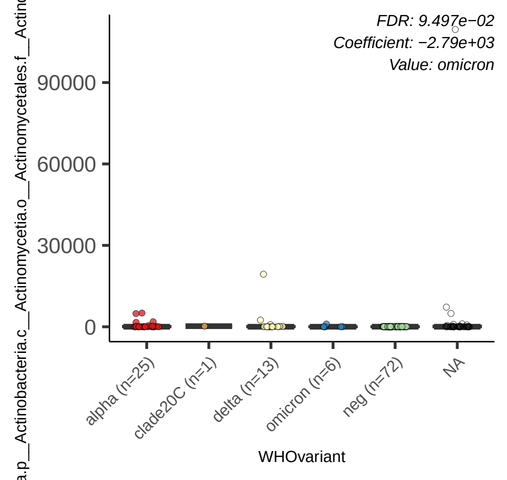

FDR: 9.497e−02
Coefficient: −2.79e+03
Value: omicron
a.p__Actinobacteria.c__Actinomycetia.o__Actinomycetales.f__Actino
90000
60000
30000
0
alpha (n=25)
clade20C (n=1)
delta (n=13)
omicron (n=6)
neg (n=72)
NA
WHOvariant

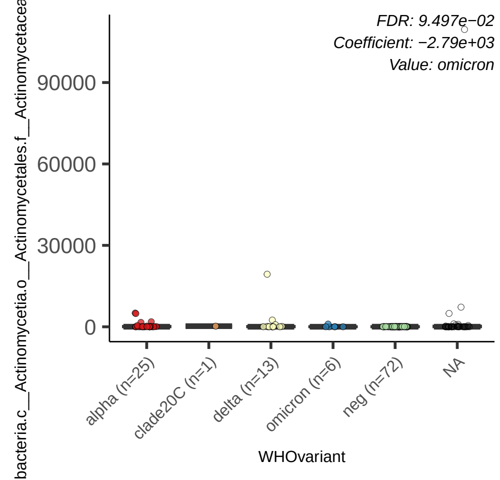

FDR: 9.497e−02
Coefficient: −2.79e+03
Value: omicron
90000
60000
30000
0
bacteria.c__Actinomycetia.o__Actinomycetales.f__Actinomycetacea
alpha (n=25)
clade20C (n=1)
delta (n=13)
omicron (n=6)
neg (n=72)
NA
WHOvariant

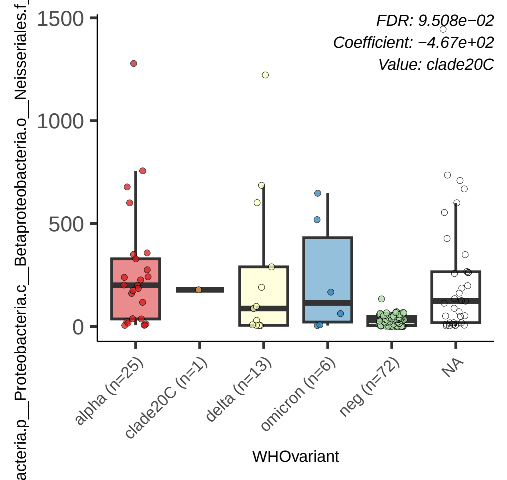

FDR: 9.508e−02
Coefficient: −4.67e+02
Value: clade20C
1500
1000
500
0
acteria.p__Proteobacteria.c__Betaproteobacteria.o__Neisseriales.f_
alpha (n=25)
clade20C (n=1)
delta (n=13)
omicron (n=6)
neg (n=72)
NA
WHOvariant

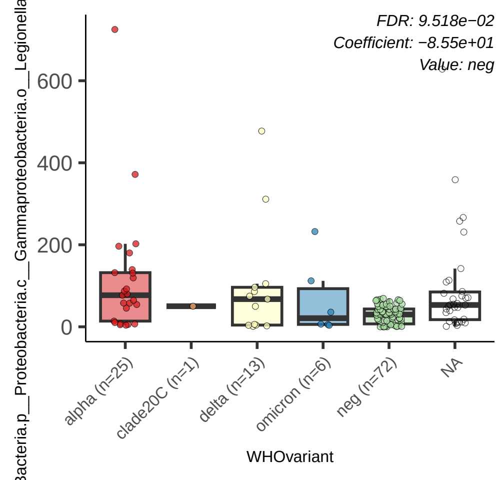
FDR: 9.518e−02
Coefficient: −8.55e+01
Value: neg
Bacteria.p__Proteobacteria.c__Gammaproteobacteria.o__Legionella
0
200
400
600
alpha (n=25)
clade20C (n=1)
delta (n=13)
omicron (n=6)
neg (n=72)
NA
WHOvariant

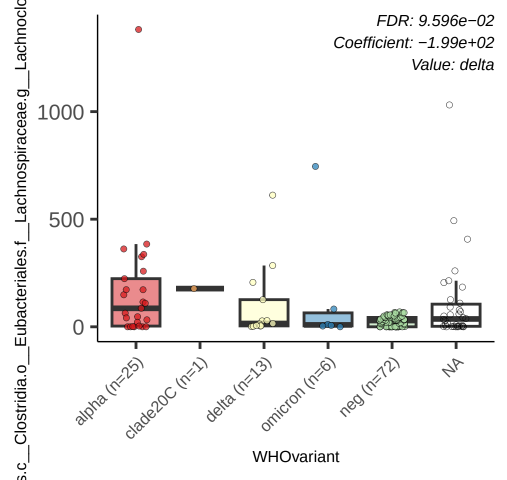

FDR: 9.596e−02
Coefficient: −1.99e+02
Value: delta
s.c__Clostridia.o__Eubacteriales.f__Lachnospiraceae.g__Lachnoclo
1000
500
0
alpha (n=25)
clade20C (n=1)
delta (n=13)
omicron (n=6)
neg (n=72)
NA
WHOvariant

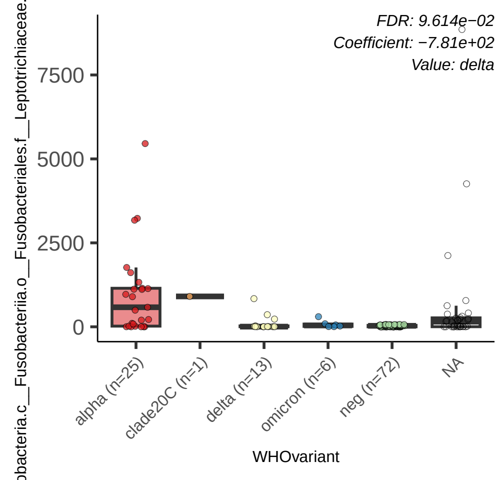

FDR: 9.614e−02
Coefficient: −7.81e+02
Value: delta
7500
5000
2500
0
obacteria.c__Fusobacteriia.o__Fusobacteriales.f__Leptotrichiaceae.
alpha (n=25)
clade20C (n=1)
delta (n=13)
omicron (n=6)
neg (n=72)
NA
WHOvariant

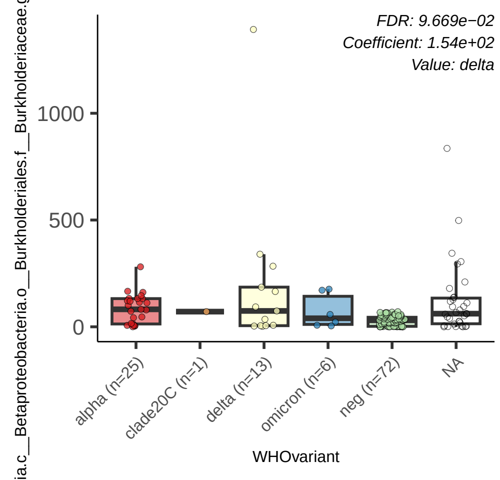

FDR: 9.669e−02
Coefficient: 1.54e+02
Value: delta
1000
500
0
ia.c__Betaproteobacteria.o__Burkholderiales.f__Burkholderiaceae.g
alpha (n=25)
clade20C (n=1)
delta (n=13)
omicron (n=6)
neg (n=72)
NA
WHOvariant

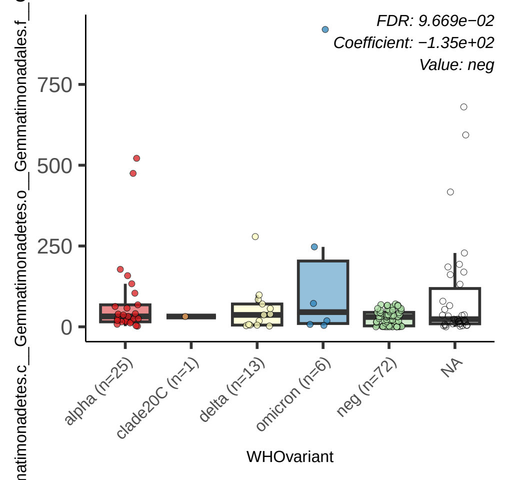

FDR: 9.669e−02
Coefficient: −1.35e+02
Value: neg
750
500
250
0
matimonadetes.c__Gemmatimonadetes.o__Gemmatimonadales.f__
alpha (n=25)
clade20C (n=1)
delta (n=13)
omicron (n=6)
neg (n=72)
NA
WHOvariant

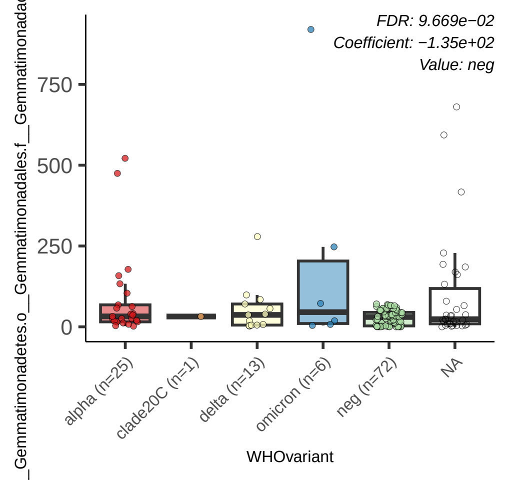
FDR: 9.669e−02
Coefficient: −1.35e+02
Value: neg
750
500
250
0
_Gemmatimonadetes.o__Gemmatimonadales.f__Gemmatimonada
alpha (n=25)
clade20C (n=1)
delta (n=13)
omicron (n=6)
neg (n=72)
NA
WHOvariant

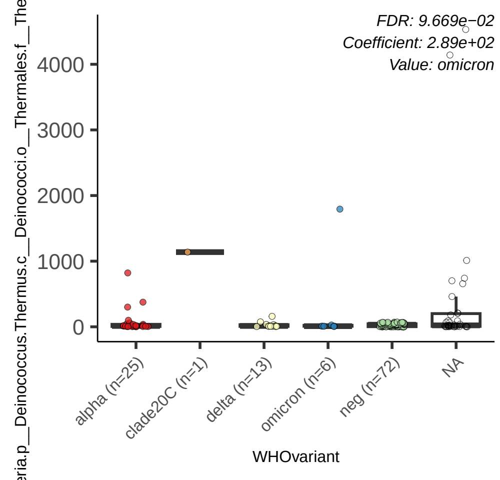

FDR: 9.669e−02
Coefficient: 2.89e+02
Value: omicron
4000
3000
2000
1000
0
alpha (n=25)
clade20C (n=1)
delta (n=13)
omicron (n=6)
neg (n=72)
NA
WHOvariant
eria.p__Deinococcus.Thermus.c__Deinococci.o__Thermales.f__The

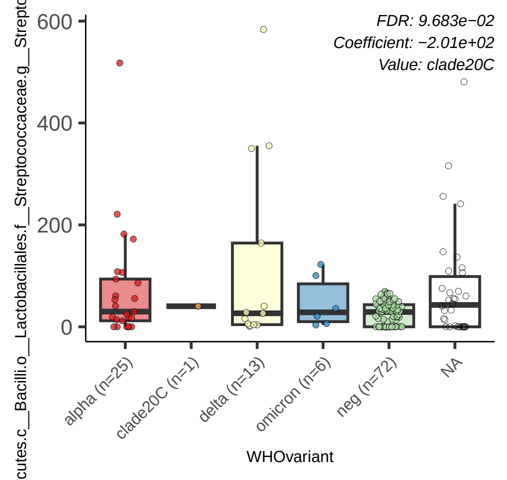

FDR: 9.683e−02
Coefficient: −2.01e+02
Value: clade20C
0
200
400
600
cutes.c__Bacilli.o__Lactobacillales.f__Streptococcaceae.g__Strepto
alpha (n=25)
clade20C (n=1)
delta (n=13)
omicron (n=6)
neg (n=72)
NA
WHOvariant

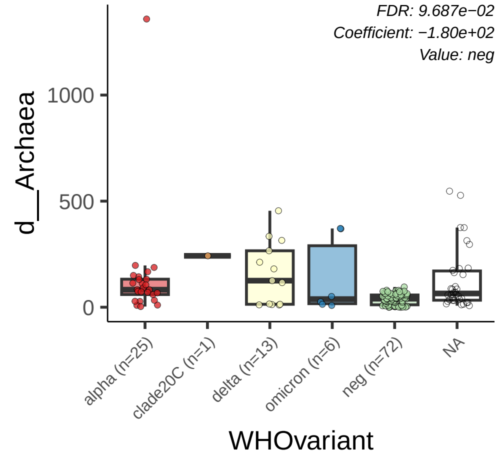

FDR: 9.687e−02
Coefficient: −1.80e+02
Value: neg
d__Archaea
1000
500
0
alpha (n=25)
clade20C (n=1)
delta (n=13)
omicron (n=6)
neg (n=72)
NA
WHOvariant

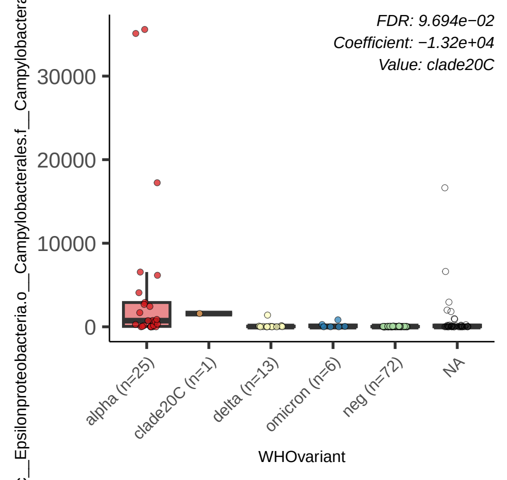

FDR: 9.694e−02
Coefficient: −1.32e+04
Value: clade20C
30000
20000
10000
0
c__Epsilonproteobacteria.o__Campylobacterales.f__Campylobactera
alpha (n=25)
clade20C (n=1)
delta (n=13)
omicron (n=6)
neg (n=72)
NA
WHOvariant

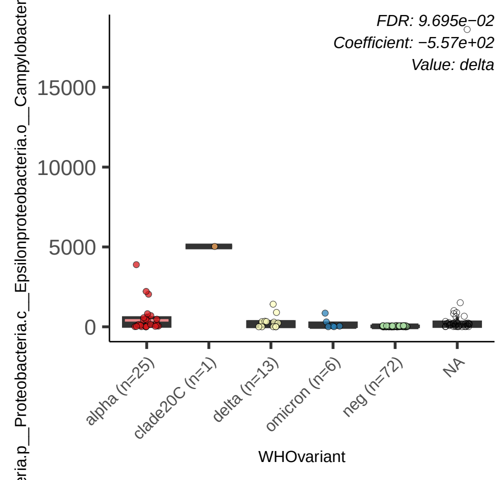

FDR: 9.695e−02
Coefficient: −5.57e+02
Value: delta
eria.p__Proteobacteria.c__Epsilonproteobacteria.o__Campylobacter
15000
10000
5000
0
alpha (n=25)
clade20C (n=1)
delta (n=13)
omicron (n=6)
neg (n=72)
NA
WHOvariant

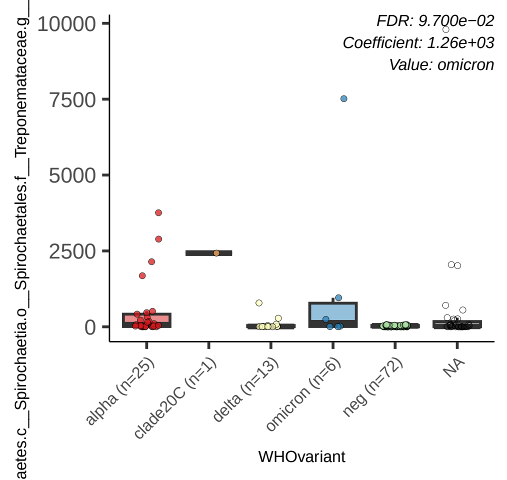
FDR: 9.700e−02
Coefficient: 1.26e+03
Value: omicron
10000
7500
5000
2500
0
aetes.c__Spirochaetia.o__Spirochaetales.f__Treponemataceae.g__
alpha (n=25)
clade20C (n=1)
delta (n=13)
omicron (n=6)
neg (n=72)
NA
WHOvariant

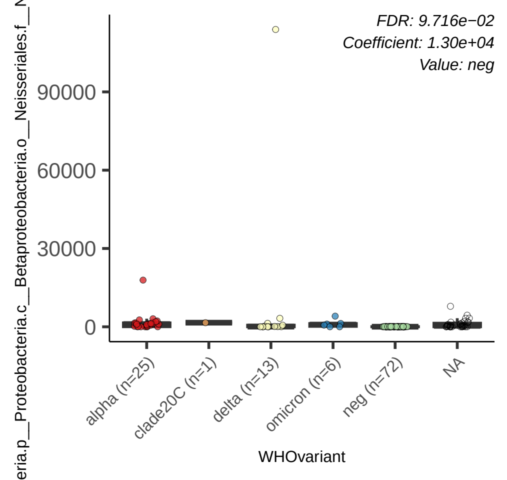
FDR: 9.716e−02
Coefficient: 1.30e+04
Value: neg
90000
60000
30000
0
eria.p__Proteobacteria.c__Betaproteobacteria.o__Neisseriales.f__N
alpha (n=25)
clade20C (n=1)
delta (n=13)
omicron (n=6)
neg (n=72)
NA
WHOvariant

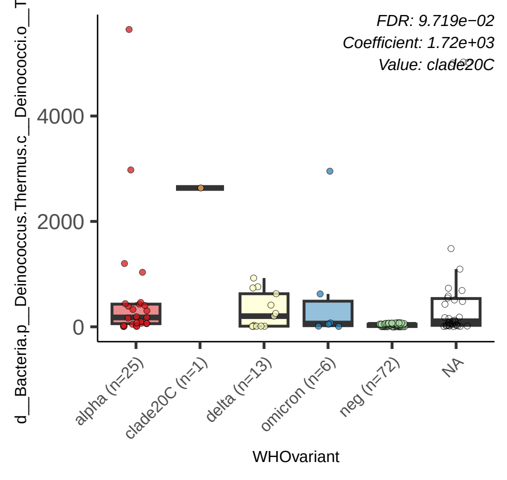

FDR: 9.719e−02
Coefficient: 1.72e+03
Value: clade20C
d__Bacteria.p__Deinococcus.Thermus.c__Deinococci.o__T
4000
2000
0
alpha (n=25)
clade20C (n=1)
delta (n=13)
omicron (n=6)
neg (n=72)
NA
WHOvariant

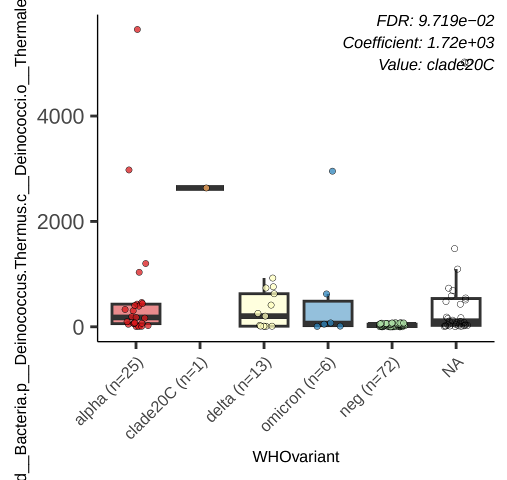

FDR: 9.719e−02
Coefficient: 1.72e+03
Value: clade20C
d__Bacteria.p__Deinococcus.Thermus.c__Deinococci.o__Thermale
4000
2000
0
alpha (n=25)
clade20C (n=1)
delta (n=13)
omicron (n=6)
neg (n=72)
NA
WHOvariant

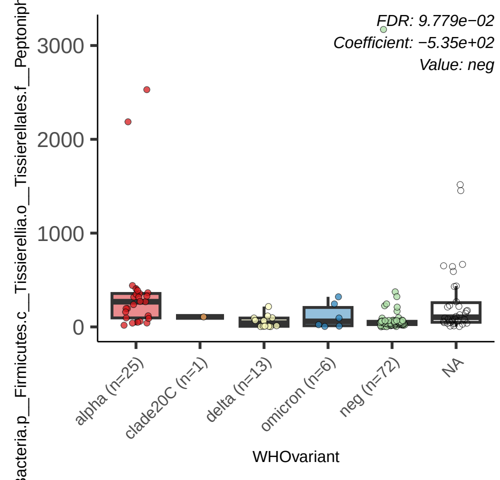
FDR: 9.779e−02
Coefficient: −5.35e+02
Value: neg
3000
2000
1000
0
Bacteria.p__Firmicutes.c__Tissierellia.o__Tissierellales.f__Peptoniph
alpha (n=25)
clade20C (n=1)
delta (n=13)
omicron (n=6)
neg (n=72)
NA
WHOvariant

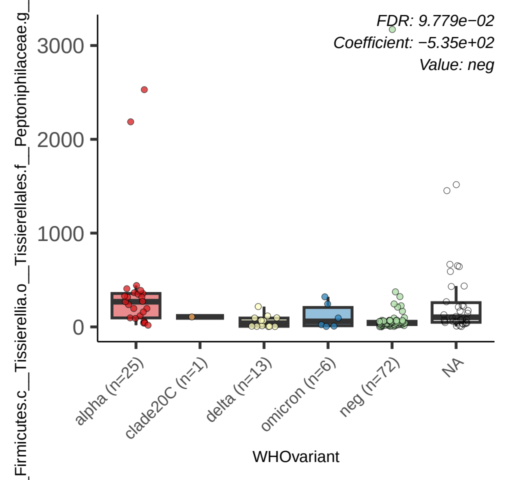

FDR: 9.779e−02
Coefficient: −5.35e+02
Value: neg
Firmicutes.c__Tissierellia.o__Tissierellales.f__Peptoniphilaceae.g__
3000
2000
1000
0
alpha (n=25)
clade20C (n=1)
delta (n=13)
omicron (n=6)
neg (n=72)
NA
WHOvariant

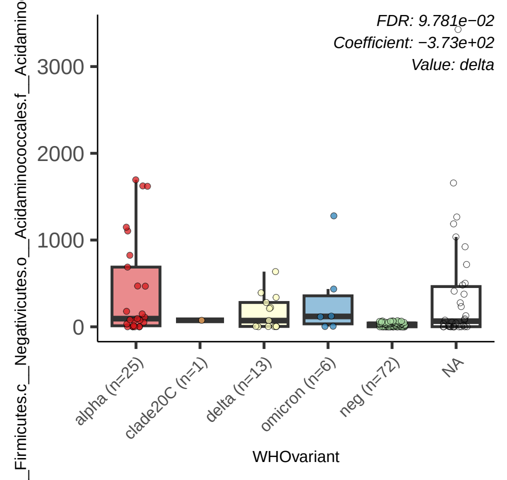

FDR: 9.781e−02
Coefficient: −3.73e+02
Value: delta
Firmicutes.c__Negativicutes.o__Acidaminococcales.f__Acidaminoc
3000
2000
1000
0
alpha (n=25)
clade20C (n=1)
delta (n=13)
omicron (n=6)
neg (n=72)
NA
WHOvariant

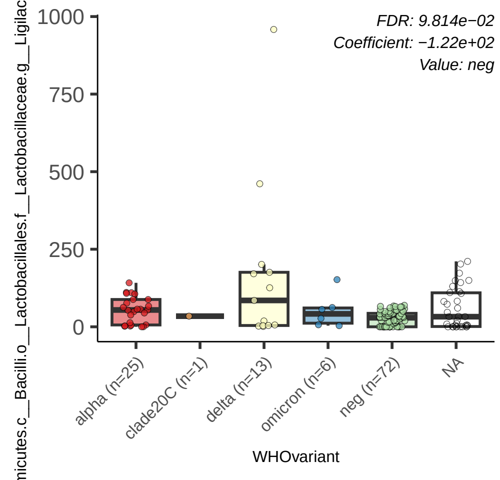

FDR: 9.814e−02
Coefficient: −1.22e+02
Value: neg
micutes.c__Bacilli.o__Lactobacillales.f__Lactobacillaceae.g__Ligilac
1000
750
500
250
0
alpha (n=25)
clade20C (n=1)
delta (n=13)
omicron (n=6)
neg (n=72)
NA
WHOvariant

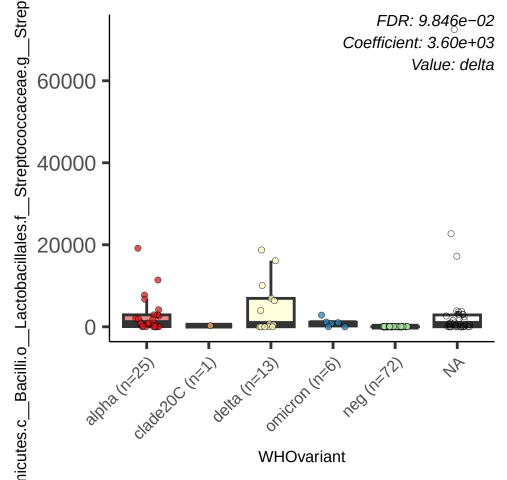
FDR: 9.846e−02
Coefficient: 3.60e+03
Value: delta
60000
40000
20000
0
alpha (n=25)
clade20C (n=1)
delta (n=13)
omicron (n=6)
neg (n=72)
NA
WHOvariant
nicutes.c__Bacilli.o__Lactobacillales.f__Streptococcaceae.g__Strep

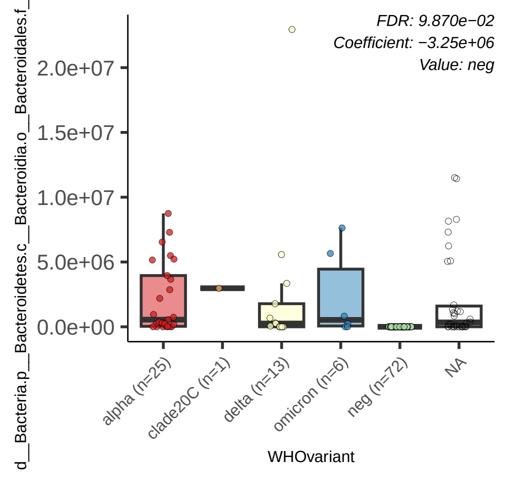

FDR: 9.870e−02
Coefficient: −3.25e+06
Value: neg
d__Bacteria.p__Bacteroidetes.c__Bacteroidia.o__Bacteroidales.f__
2.0e+07
1.5e+07
1.0e+07
5.0e+06
0.0e+00
alpha (n=25)
clade20C (n=1)
delta (n=13)
omicron (n=6)
neg (n=72)
NA
WHOvariant

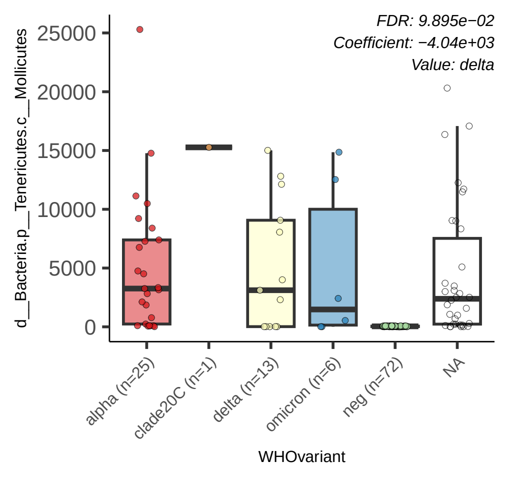

FDR: 9.895e−02
Coefficient: −4.04e+03
Value: delta
d__Bacteria.p__Tenericutes.c__Mollicutes
25000
20000
15000
10000
5000
0
alpha (n=25)
clade20C (n=1)
delta (n=13)
omicron (n=6)
neg (n=72)
NA
WHOvariant

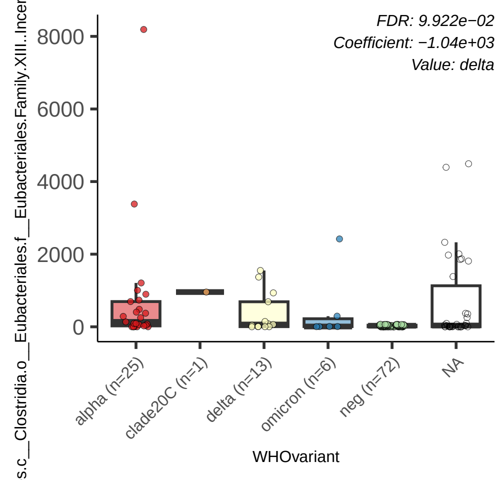

FDR: 9.922e−02
Coefficient: −1.04e+03
Value: delta
s.c__Clostridia.o__Eubacteriales.f__Eubacteriales.Family.XIII..Incer
8000
6000
4000
2000
0
alpha (n=25)
clade20C (n=1)
delta (n=13)
omicron (n=6)
neg (n=72)
NA
WHOvariant

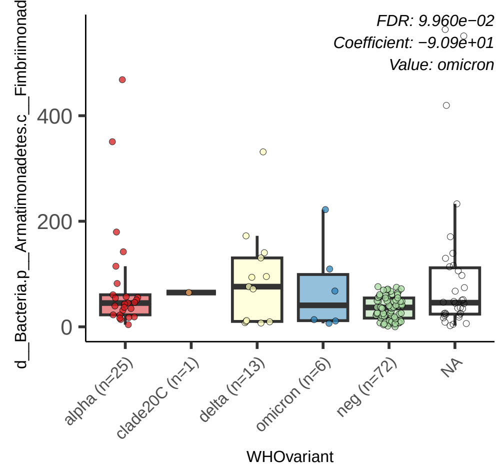

FDR: 9.960e−02
Coefficient: −9.09e+01
Value: omicron
d__Bacteria.p__Armatimonadetes.c__Fimbriimonad
0
200
400
alpha (n=25)
clade20C (n=1)
delta (n=13)
omicron (n=6)
neg (n=72)
NA
WHOvariant

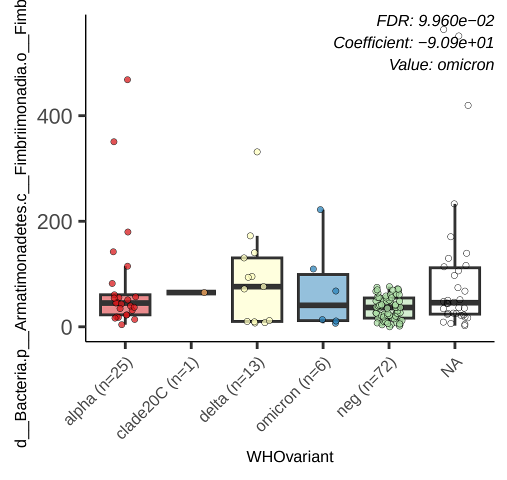
FDR: 9.960e−02
Coefficient: −9.09e+01
Value: omicron
d__Bacteria.p__Armatimonadetes.c__Fimbriimonadia.o__Fimb
400
200
0
alpha (n=25)
clade20C (n=1)
delta (n=13)
omicron (n=6)
neg (n=72)
NA
WHOvariant

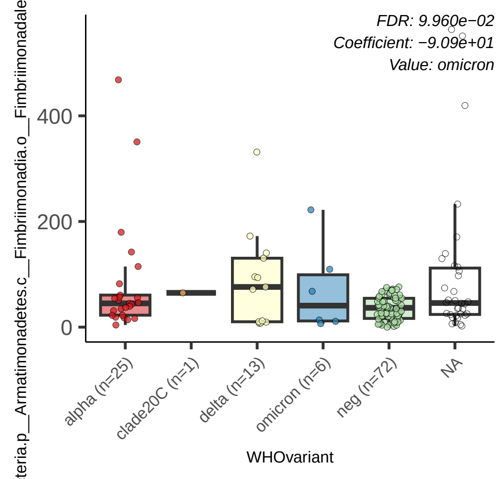

FDR: 9.960e−02
Coefficient: −9.09e+01
Value: omicron
cteria.p__Armatimonadetes.c__Fimbriimonadia.o__Fimbriimonadale
400
200
0
alpha (n=25)
clade20C (n=1)
delta (n=13)
omicron (n=6)
neg (n=72)
NA
WHOvariant

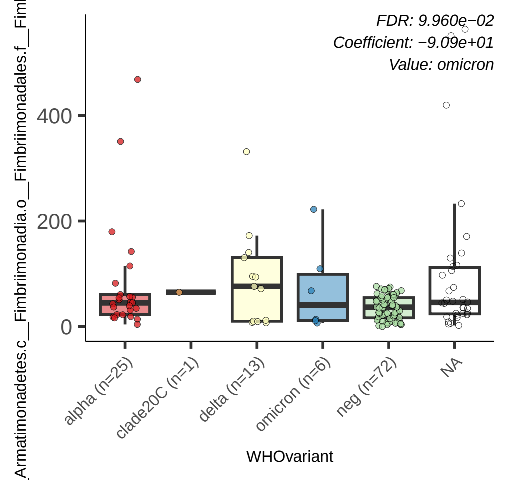

FDR: 9.960e−02
Coefficient: −9.09e+01
Value: omicron
Armatimonadetes.c__Fimbriimonadia.o__Fimbriimonadales.f__Fimb
0
200
400
alpha (n=25)
clade20C (n=1)
delta (n=13)
omicron (n=6)
neg (n=72)
NA
WHOvariant

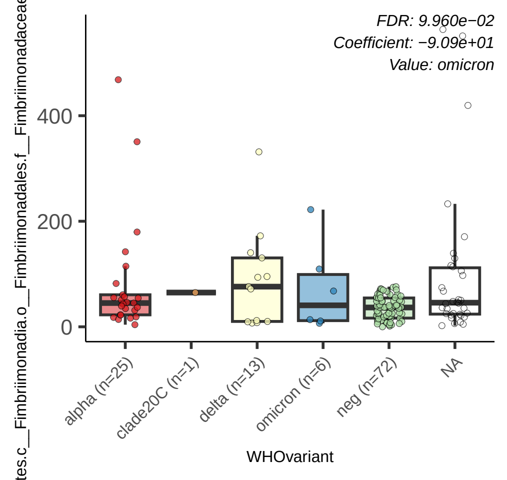
FDR: 9.960e−02
Coefficient: −9.09e+01
Value: omicron
tes.c__Fimbriimonadia.o__Fimbriimonadales.f__Fimbriimonadaceae
400
200
0
alpha (n=25)
clade20C (n=1)
delta (n=13)
omicron (n=6)
neg (n=72)
NA
WHOvariant

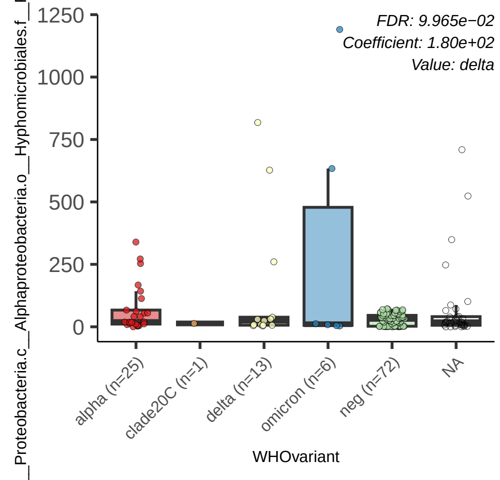

FDR: 9.965e−02
Coefficient: 1.80e+02
Value: delta
Proteobacteria.c__Alphaproteobacteria.o__Hyphomicrobiales.f__
1250
1000
750
500
250
0
alpha (n=25)
clade20C (n=1)
delta (n=13)
omicron (n=6)
neg (n=72)
NA
WHOvariant

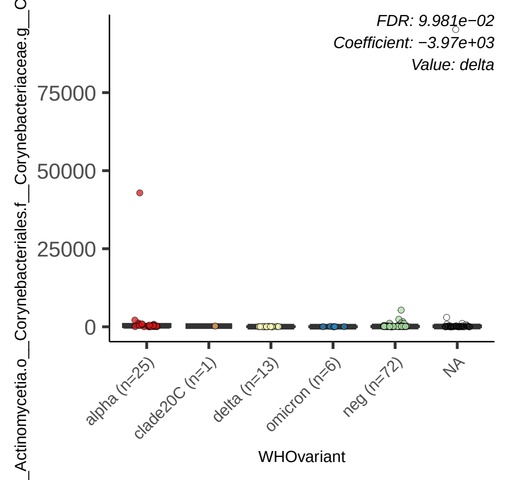
FDR: 9.981e−02
Coefficient: −3.97e+03
Value: delta
Actinomycetia.o__Corynebacteriales.f__Corynebacteriaceae.g__C
75000
50000
25000
0
alpha (n=25)
clade20C (n=1)
delta (n=13)
omicron (n=6)
neg (n=72)
NA
WHOvariant

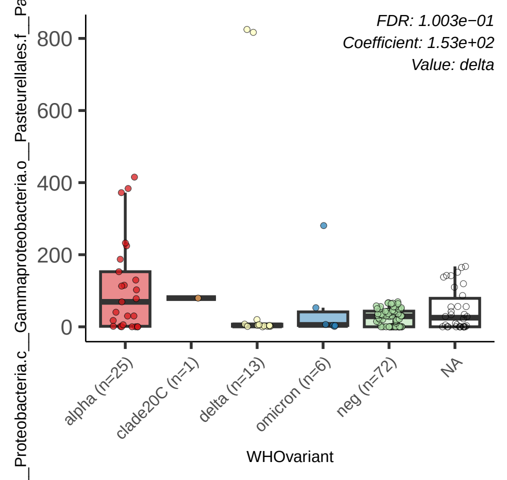

FDR: 1.003e−01
Coefficient: 1.53e+02
Value: delta
Proteobacteria.c__Gammaproteobacteria.o__Pasteurellales.f__Pa
800
600
400
200
0
alpha (n=25)
clade20C (n=1)
delta (n=13)
omicron (n=6)
neg (n=72)
NA
WHOvariant

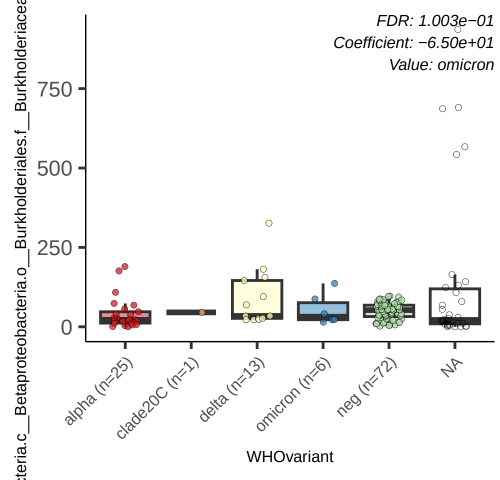

FDR: 1.003e−01
Coefficient: −6.50e+01
Value: omicron
750
500
250
0
cteria.c__Betaproteobacteria.o__Burkholderiales.f__Burkholderiacea
alpha (n=25)
clade20C (n=1)
delta (n=13)
omicron (n=6)
neg (n=72)
NA
WHOvariant

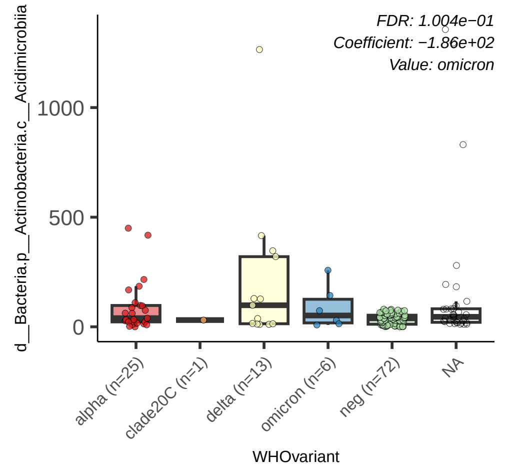

FDR: 1.004e−01
Coefficient: −1.86e+02
Value: omicron
d__Bacteria.p__Actinobacteria.c__Acidimicrobia
1000
500
0
alpha (n=25)
clade20C (n=1)
delta (n=13)
omicron (n=6)
neg (n=72)
NA
WHOvariant

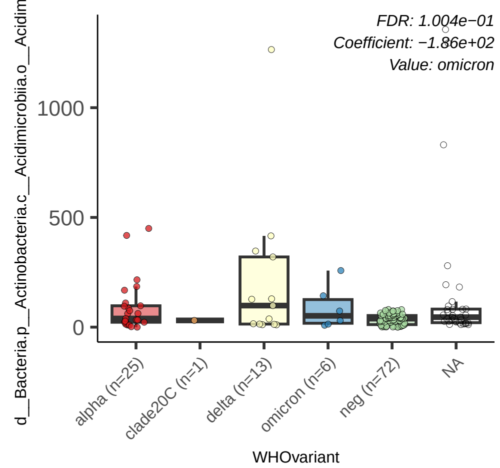
FDR: 1.004e−01
Coefficient: −1.86e+02
Value: omicron
d__Bacteria.p__Actinobacteria.c__Acidimicrobiia.o__Acidim
1000
500
0
alpha (n=25)
clade20C (n=1)
delta (n=13)
omicron (n=6)
neg (n=72)
NA
WHOvariant

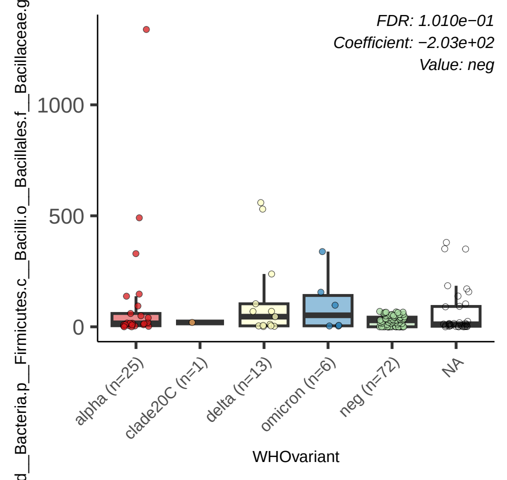
FDR: 1.010e−01
Coefficient: −2.03e+02
Value: neg
d__Bacteria.p__Firmicutes.c__Bacilli.o__Bacillales.f__Bacillaceae.g
1000
500
0
alpha (n=25)
clade20C (n=1)
delta (n=13)
omicron (n=6)
neg (n=72)
NA
WHOvariant

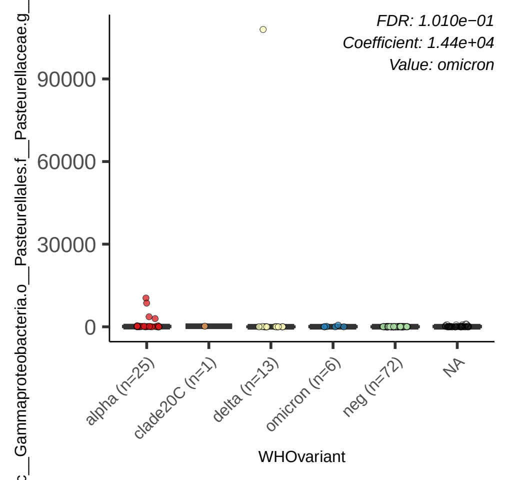
FDR: 1.010e−01
Coefficient: 1.44e+04
Value: omicron
90000
60000
30000
0
c__Gammaproteobacteria.o__Pasteurellales.f__Pasteurellaceae.g__
alpha (n=25)
clade20C (n=1)
delta (n=13)
omicron (n=6)
neg (n=72)
NA
WHOvariant

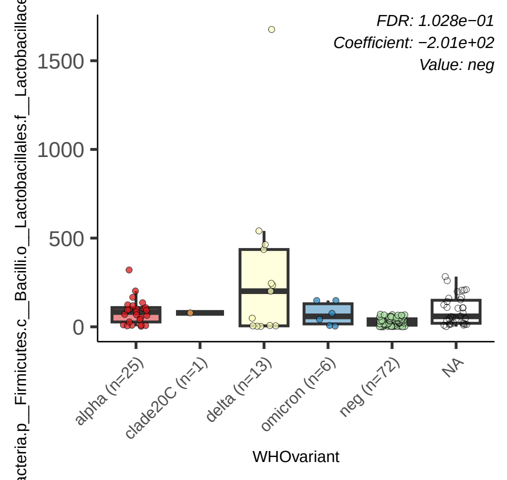
FDR: 1.028e−01
Coefficient: −2.01e+02
Value: neg
1500
1000
500
0
acteria.p__Firmicutes.c__Bacilli.o__Lactobacillales.f__Lactobacillace
alpha (n=25)
clade20C (n=1)
delta (n=13)
omicron (n=6)
neg (n=72)
NA
WHOvariant

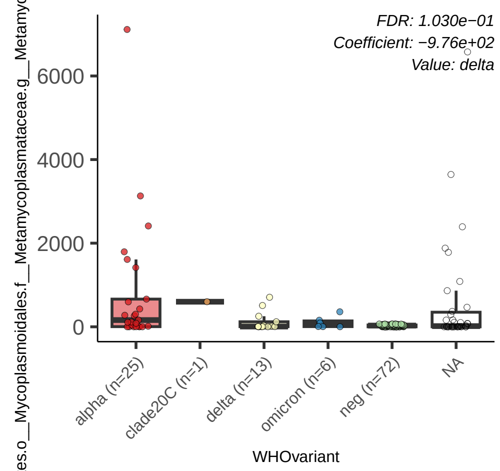
FDR: 1.030e−01
Coefficient: −9.76e+02
Value: delta
6000
4000
2000
0
utes.o__Mycoplasmoidales.f__Metamycoplasmataceae.g__Metamyc
alpha (n=25)
clade20C (n=1)
delta (n=13)
omicron (n=6)
neg (n=72)
NA
WHOvariant

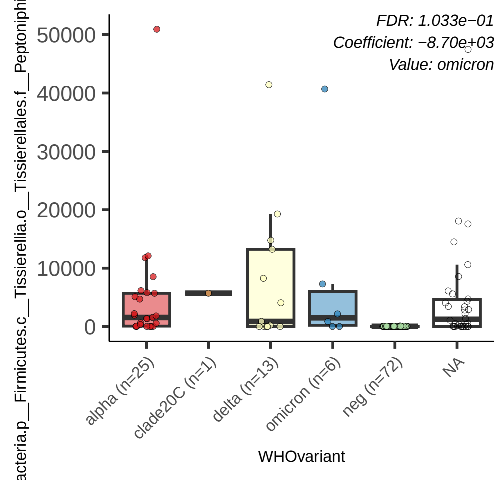

FDR: 1.033e−01
Coefficient: −8.70e+03
Value: omicron
acteria.p__Firmicutes.c__Tissierellia.o__Tissierellales.f__Peptoniphi
50000
40000
30000
20000
10000
0
alpha (n=25)
clade20C (n=1)
delta (n=13)
omicron (n=6)
neg (n=72)
NA
WHOvariant

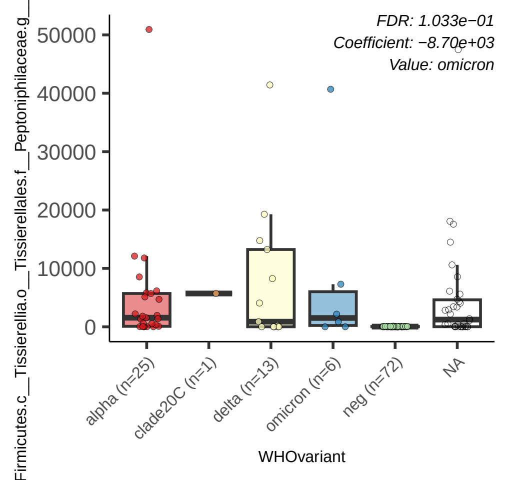

FDR: 1.033e−01
Coefficient: −8.70e+03
Value: omicron
Firmicutes.c__Tissierellia.o__Tissierellales.f__Peptoniphilaceae.g__
50000
40000
30000
20000
10000
0
alpha (n=25)
clade20C (n=1)
delta (n=13)
omicron (n=6)
neg (n=72)
NA
WHOvariant

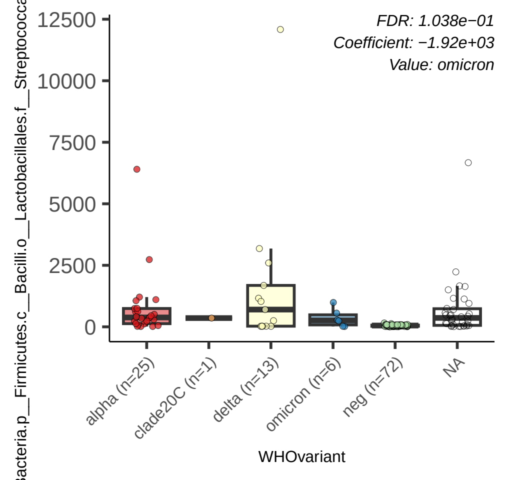

FDR: 1.038e−01
Coefficient: −1.92e+03
Value: omicron
Bacteria.p__Firmicutes.c__Bacilli.o__Lactobacillales.f__Streptococca
12500
10000
7500
5000
2500
0
alpha (n=25)
clade20C (n=1)
delta (n=13)
omicron (n=6)
neg (n=72)
NA
WHOvariant

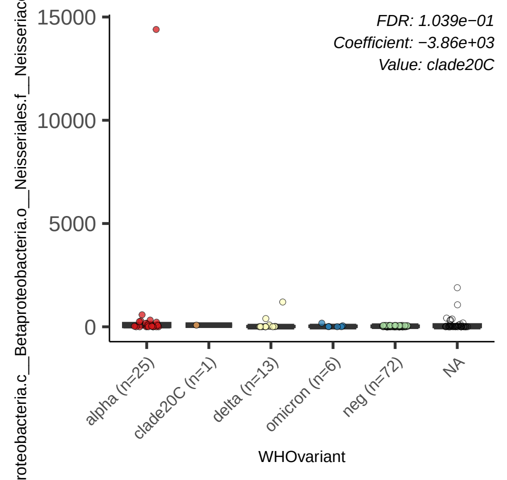

FDR: 1.039e−01
Coefficient: −3.86e+03
Value: clade20C
15000
10000
5000
0
roteobacteria.c__Betaproteobacteria.o__Neisseriales.f__Neisseriac
alpha (n=25)
clade20C (n=1)
delta (n=13)
omicron (n=6)
neg (n=72)
NA
WHOvariant

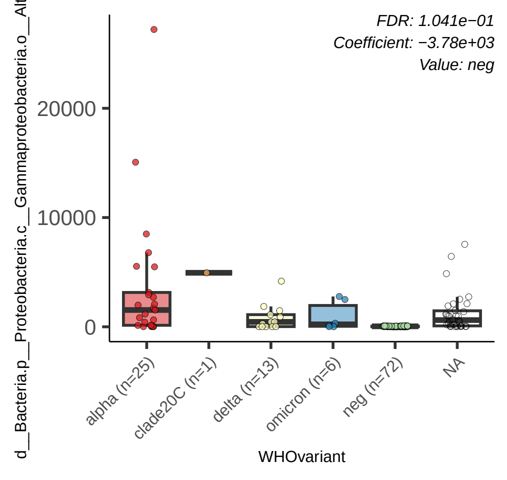

FDR: 1.041e−01
Coefficient: −3.78e+03
Value: neg
20000
10000
0
d__Bacteria.p__Proteobacteria.c__Gammaproteobacteria.o__Alt
alpha (n=25)
clade20C (n=1)
delta (n=13)
omicron (n=6)
neg (n=72)
NA
WHOvariant

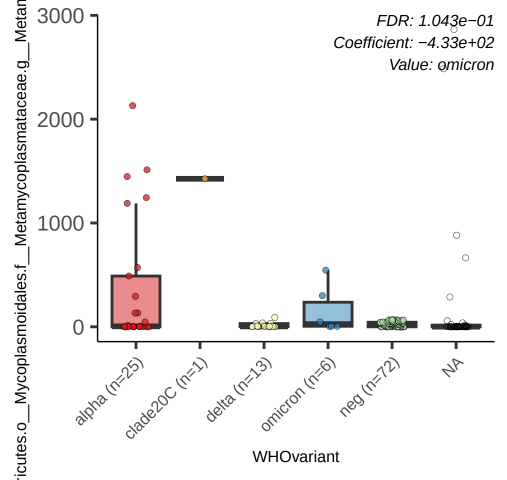

FDR: 1.043e−01
Coefficient: −4.33e+02
Value: omicron
ricutes.o__Mycoplasmoidales.f__Metamycoplasmataceae.g__Metan
3000
2000
1000
0
alpha (n=25)
clade20C (n=1)
delta (n=13)
omicron (n=6)
neg (n=72)
NA
WHOvariant

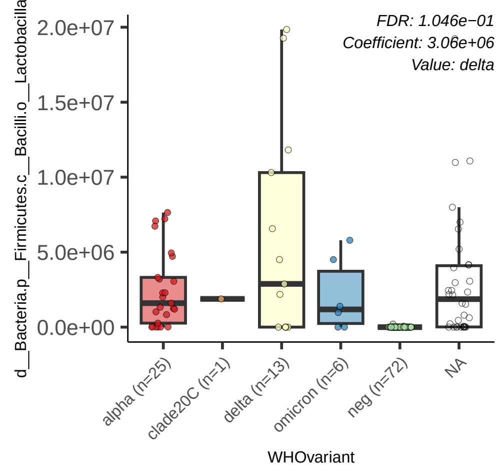

FDR: 1.046e−01
Coefficient: 3.06e+06
Value: delta
d__Bacteria.p__Firmicutes.c__Bacilli.o__Lactobacilla
2.0e+07
1.5e+07
1.0e+07
5.0e+06
0.0e+00
alpha (n=25)
clade20C (n=1)
delta (n=13)
omicron (n=6)
neg (n=72)
NA
WHOvariant

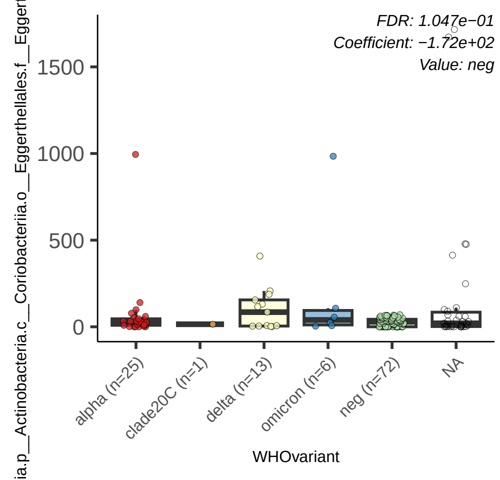

FDR: 1.047e−01
Coefficient: −1.72e+02
Value: neg
ia.p__Actinobacteria.c__Coriobacteriia.o__Eggerthellales.f__Eggert
0
500
1000
1500
alpha (n=25)
clade20C (n=1)
delta (n=13)
omicron (n=6)
neg (n=72)
NA
WHOvariant

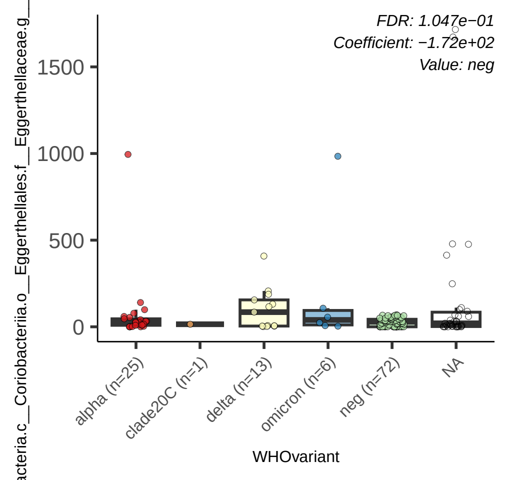
FDR: 1.047e−01
Coefficient: −1.72e+02
Value: neg
acteria.c__Coriobacteriia.o__Eggerthellales.f__Eggerthellaceae.g__
1500
1000
500
0
alpha (n=25)
clade20C (n=1)
delta (n=13)
omicron (n=6)
neg (n=72)
NA
WHOvariant

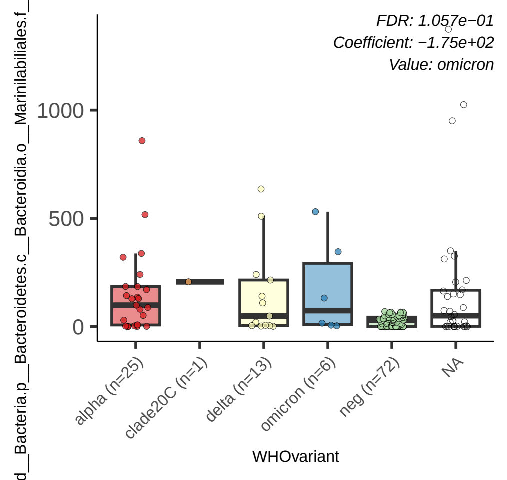

FDR: 1.057e−01
Coefficient: −1.75e+02
Value: omicron
d__Bacteria.p__Bacteroidetes.c__Bacteroidia.o__Marinilabiliales.f
1000
500
0
alpha (n=25)
clade20C (n=1)
delta (n=13)
omicron (n=6)
neg (n=72)
NA
WHOvariant

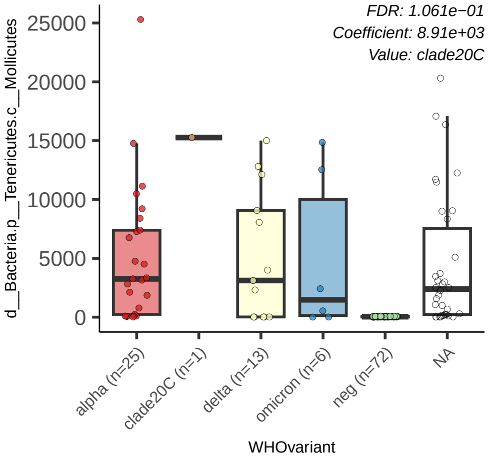
FDR: 1.061e−01
Coefficient: 8.91e+03
Value: clade20C
d__Bacteria.p__Tenericutes.c__Mollicutes
25000
20000
15000
10000
5000
0
alpha (n=25)
clade20C (n=1)
delta (n=13)
omicron (n=6)
neg (n=72)
NA
WHOvariant

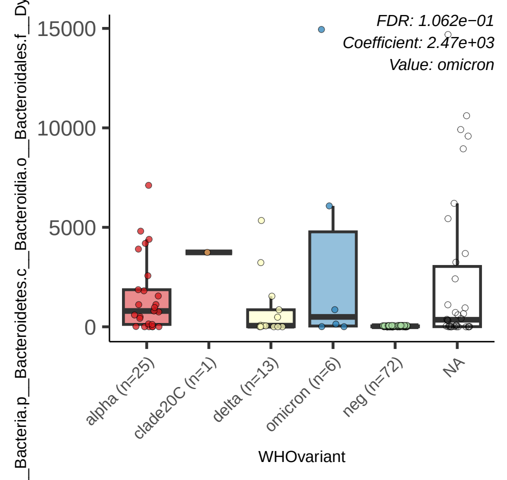

FDR: 1.062e−01
Coefficient: 2.47e+03
Value: omicron
15000
10000
5000
0
Bacteria.p__Bacteroidetes.c__Bacteroidia.o__Bacteroidales.f__Dy
alpha (n=25)
clade20C (n=1)
delta (n=13)
omicron (n=6)
neg (n=72)
NA
WHOvariant

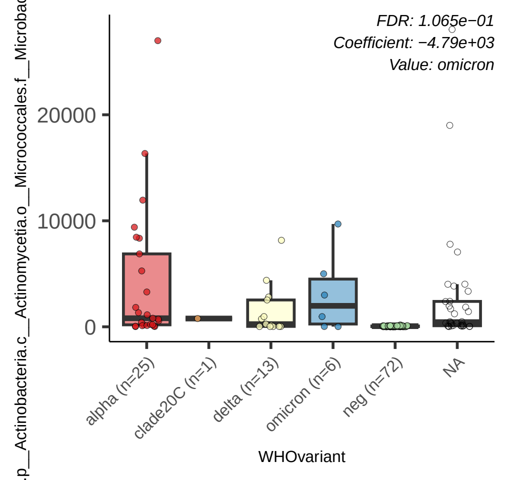

FDR: 1.065e−01
Coefficient: −4.79e+03
Value: omicron
p__Actinobacteria.c__Actinomycetia.o__Micrococcales.f__Microbac
20000
10000
0
alpha (n=25)
clade20C (n=1)
delta (n=13)
omicron (n=6)
neg (n=72)
NA
WHOvariant

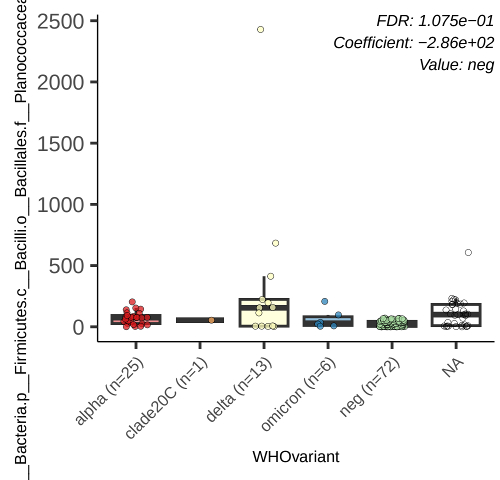
FDR: 1.075e−01
Coefficient: −2.86e+02
Value: neg
Bacteria.p__Firmicutes.c__Bacilli.o__Bacillales.f__Planococcacea
2500
2000
1500
1000
500
0
alpha (n=25)
clade20C (n=1)
delta (n=13)
omicron (n=6)
neg (n=72)
NA
WHOvariant

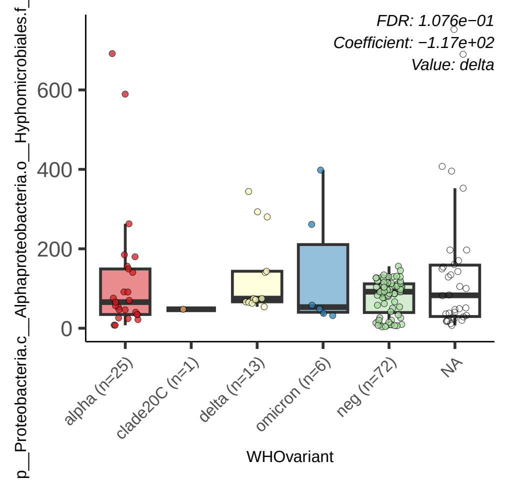

FDR: 1.076e−01
Coefficient: −1.17e+02
Value: delta
p__Proteobacteria.c__Alphaproteobacteria.o__Hyphomicrobiales.f__
0
200
400
600
alpha (n=25)
clade20C (n=1)
delta (n=13)
omicron (n=6)
neg (n=72)
NA
WHOvariant

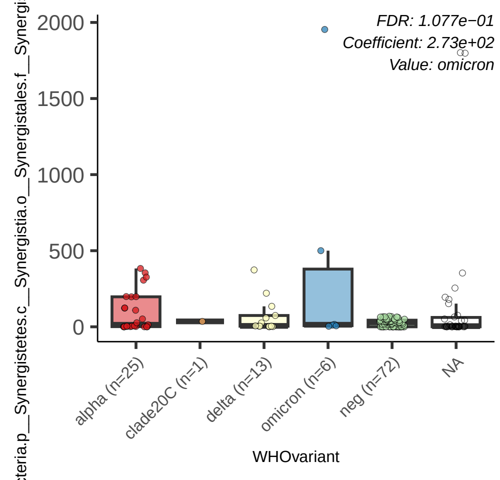

FDR: 1.077e−01
Coefficient: 2.73e+02
Value: omicron
2000
1500
1000
500
0
cteria.p__Synergistetes.c__Synergistia.o__Synergistales.f__Synergi
alpha (n=25)
clade20C (n=1)
delta (n=13)
omicron (n=6)
neg (n=72)
NA
WHOvariant

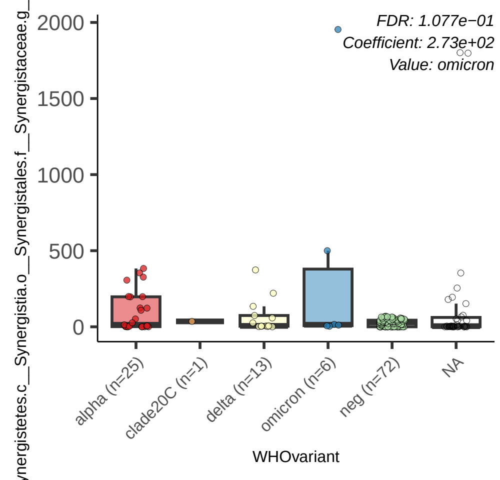

FDR: 1.077e−01
Coefficient: 2.73e+02
Value: omicron
2000
1500
1000
500
0
Synergistetes.c__Synergistia.o__Synergistales.f__Synergistaceae.g__
alpha (n=25)
clade20C (n=1)
delta (n=13)
omicron (n=6)
neg (n=72)
NA
WHOvariant

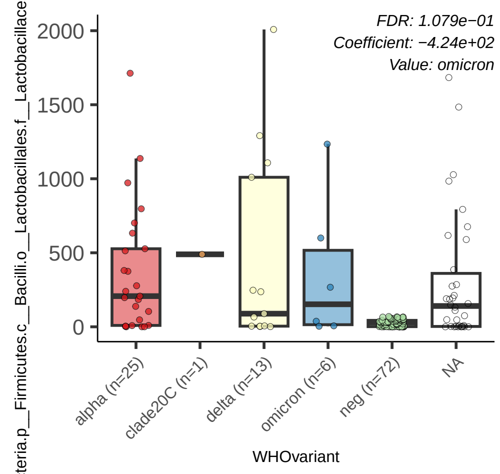

FDR: 1.079e−01
Coefficient: −4.24e+02
Value: omicron
cteria.p__Firmicutes.c__Bacilli.o__Lactobacillales.f__Lactobacillace
2000
1500
1000
500
0
alpha (n=25)
clade20C (n=1)
delta (n=13)
omicron (n=6)
neg (n=72)
NA
WHOvariant

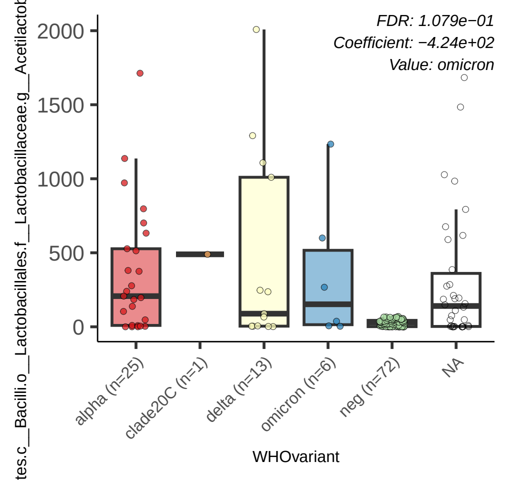

FDR: 1.079e−01
Coefficient: −4.24e+02
Value: omicron
tes.c__Bacilli.o__Lactobacillales.f__Lactobacillaceae.g__Acetilactob
2000
1500
1000
500
0
alpha (n=25)
clade20C (n=1)
delta (n=13)
omicron (n=6)
neg (n=72)
NA
WHOvariant

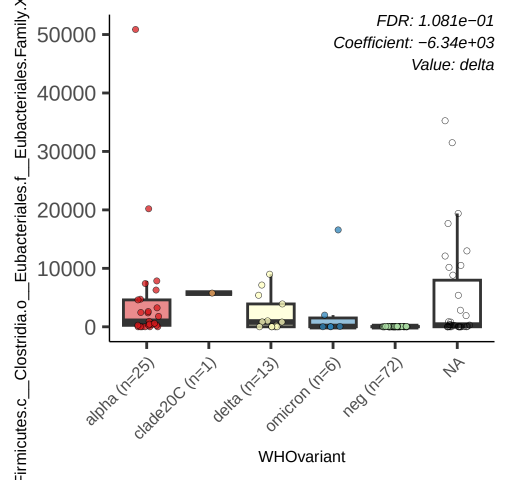
FDR: 1.081e−01
Coefficient: −6.34e+03
Value: delta
Firmicutes.c__Clostridia.o__Eubacteriales.f__Eubacteriales.Family.X
50000
40000
30000
20000
10000
0
alpha (n=25)
clade20C (n=1)
delta (n=13)
omicron (n=6)
neg (n=72)
NA
WHOvariant

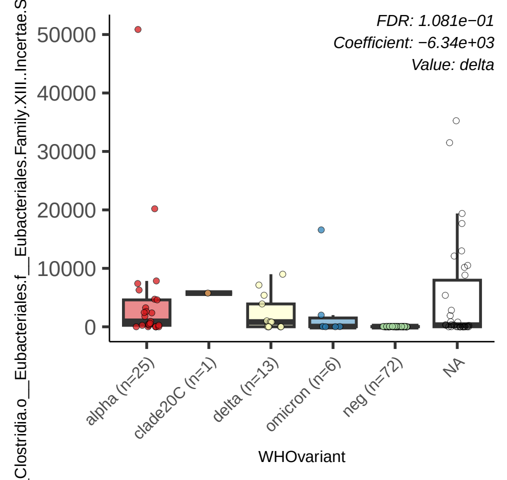

FDR: 1.081e−01
Coefficient: −6.34e+03
Value: delta
50000
40000
30000
20000
10000
0
Clostridia.o__Eubacteriales.f__Eubacteriales.Family.XIII..Incertae.S
alpha (n=25)
clade20C (n=1)
delta (n=13)
omicron (n=6)
neg (n=72)
NA
WHOvariant

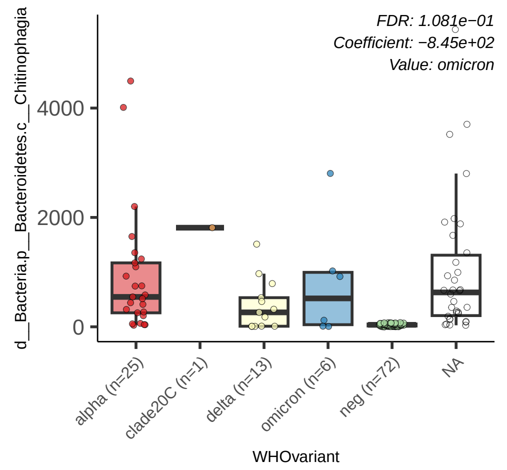

FDR: 1.081e−01
Coefficient: −8.45e+02
Value: omicron
d__Bacteria.p__Bacteroidetes.c__Chitinophagia
4000
2000
0
alpha (n=25)
clade20C (n=1)
delta (n=13)
omicron (n=6)
neg (n=72)
NA
WHOvariant

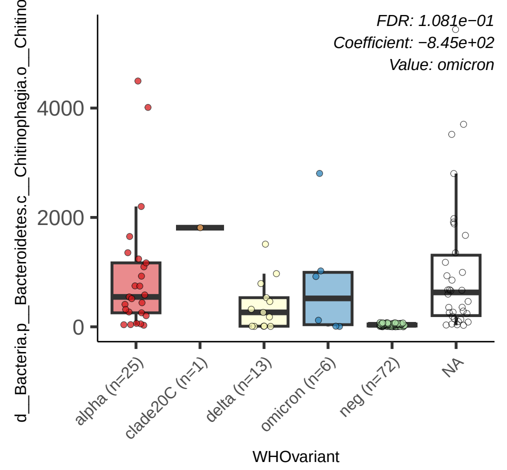

FDR: 1.081e−01
Coefficient: −8.45e+02
Value: omicron
d__Bacteria.p__Bacteroidetes.c__Chitinophagia.o__Chitino
4000
2000
0
alpha (n=25)
clade20C (n=1)
delta (n=13)
omicron (n=6)
neg (n=72)
NA
WHOvariant

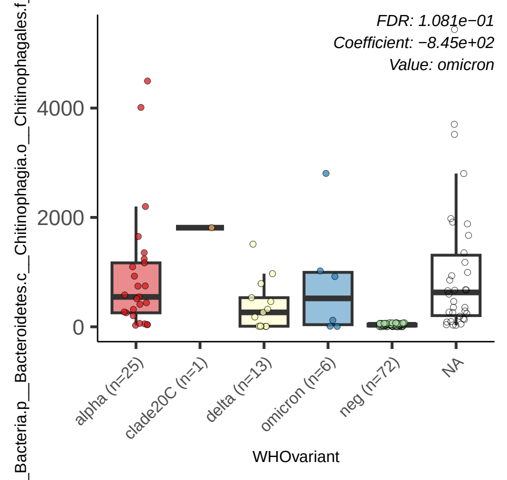

FDR: 1.081e−01
Coefficient: −8.45e+02
Value: omicron
Bacteria.p__Bacteroidetes.c__Chitinophagia.o__Chitinophagales.f__
4000
2000
0
alpha (n=25)
clade20C (n=1)
delta (n=13)
omicron (n=6)
neg (n=72)
NA
WHOvariant

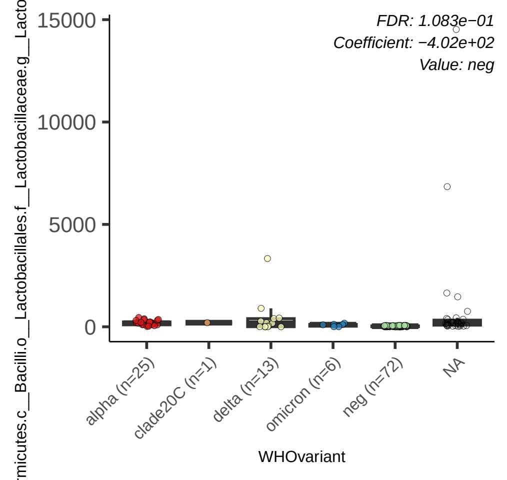

FDR: 1.083e−01
Coefficient: −4.02e+02
Value: neg
15000
10000
5000
0
rmicutes.c__Bacilli.o__Lactobacillales.f__Lactobacillaceae.g__Lacto
alpha (n=25)
clade20C (n=1)
delta (n=13)
omicron (n=6)
neg (n=72)
NA
WHOvariant

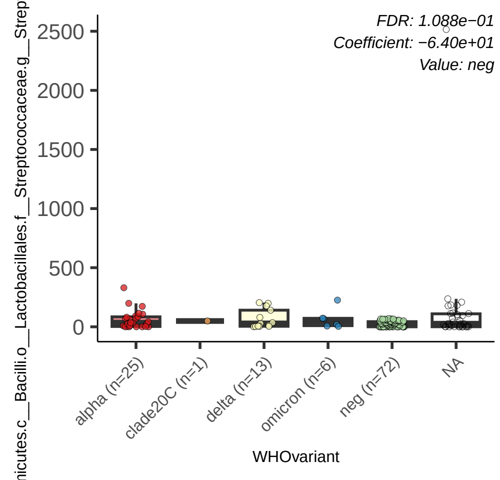

FDR: 1.088e−01
Coefficient: −6.40e+01
Value: neg
2500
2000
1500
1000
500
0
nicutes.c__Bacilli.o__Lactobacillales.f__Streptococcaceae.g__Strep
alpha (n=25)
clade20C (n=1)
delta (n=13)
omicron (n=6)
neg (n=72)
NA
WHOvariant

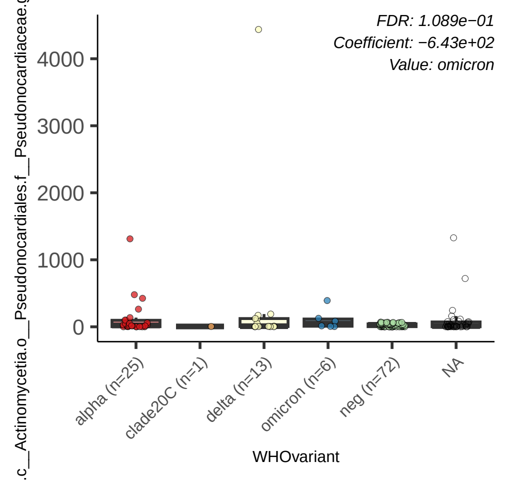

FDR: 1.089e−01
Coefficient: −6.43e+02
Value: omicron
c__Actinomycetia.o__Pseudonocardiales.f__Pseudonocardiaceae.g
0
1000
2000
3000
4000
alpha (n=25)
clade20C (n=1)
delta (n=13)
omicron (n=6)
neg (n=72)
NA
WHOvariant

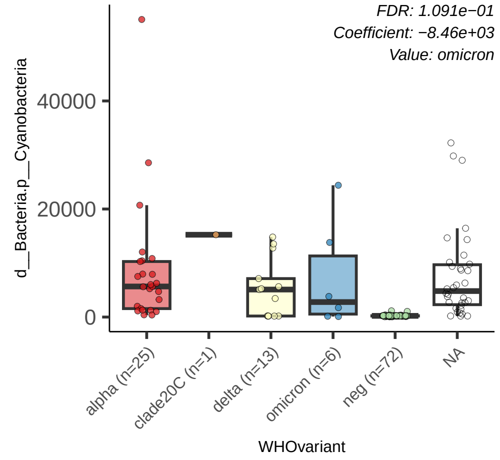

FDR: 1.091e−01
Coefficient: −8.46e+03
Value: omicron
d__Bacteria.p__Cyanobacteria
0
20000
40000
alpha (n=25)
clade20C (n=1)
delta (n=13)
omicron (n=6)
neg (n=72)
NA
WHOvariant

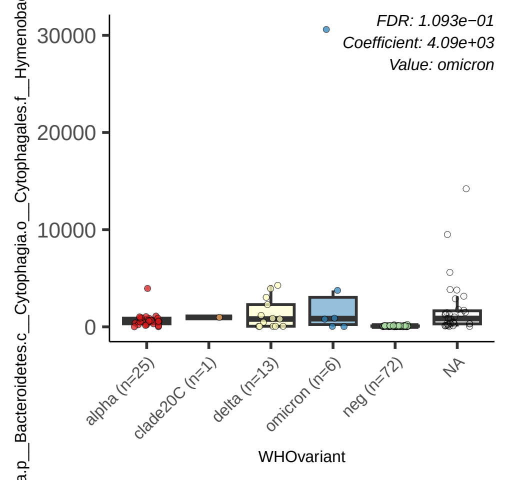

FDR: 1.093e−01
Coefficient: 4.09e+03
Value: omicron
a.p__Bacteroidetes.c__Cytophagia.o__Cytophagales.f__Hymenoba
30000
20000
10000
0
alpha (n=25)
clade20C (n=1)
delta (n=13)
omicron (n=6)
neg (n=72)
NA
WHOvariant

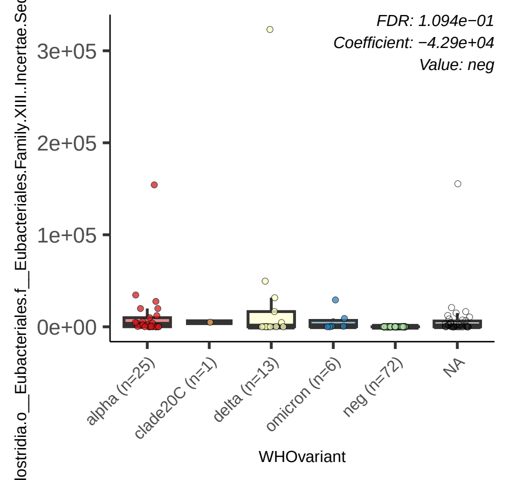
FDR: 1.094e−01
Coefficient: −4.29e+04
Value: neg
3e+05
2e+05
1e+05
0e+00
lostridia.o__Eubacteriales.f__Eubacteriales.Family.XIII..Incertae.Sed
alpha (n=25)
clade20C (n=1)
delta (n=13)
omicron (n=6)
neg (n=72)
NA
WHOvariant

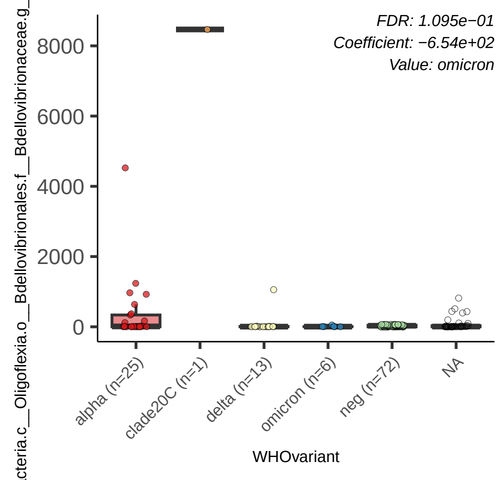
FDR: 1.095e−01
Coefficient: −6.54e+02
Value: omicron
8000
6000
4000
2000
0
cteria.c__Oligoflexia.o__Bdellovibrionales.f__Bdellovibrionaceae.g_
alpha (n=25)
clade20C (n=1)
delta (n=13)
omicron (n=6)
neg (n=72)
NA
WHOvariant

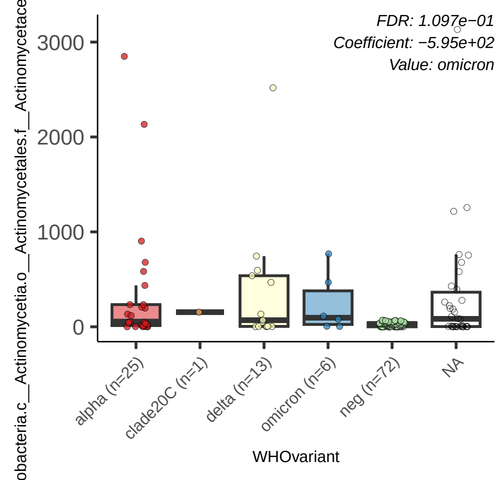

FDR: 1.097e−01
Coefficient: −5.95e+02
Value: omicron
3000
2000
1000
0
obacteria.c__Actinomycetia.o__Actinomycetales.f__Actinomycetace
alpha (n=25)
clade20C (n=1)
delta (n=13)
omicron (n=6)
neg (n=72)
NA
WHOvariant

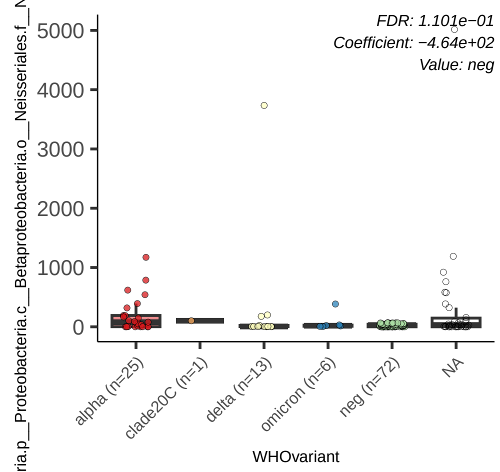

FDR: 1.101e−01
Coefficient: −4.64e+02
Value: neg
5000
4000
3000
2000
1000
0
eria.p__Proteobacteria.c__Betaproteobacteria.o__Neisseriales.f__N
alpha (n=25)
clade20C (n=1)
delta (n=13)
omicron (n=6)
neg (n=72)
NA
WHOvariant

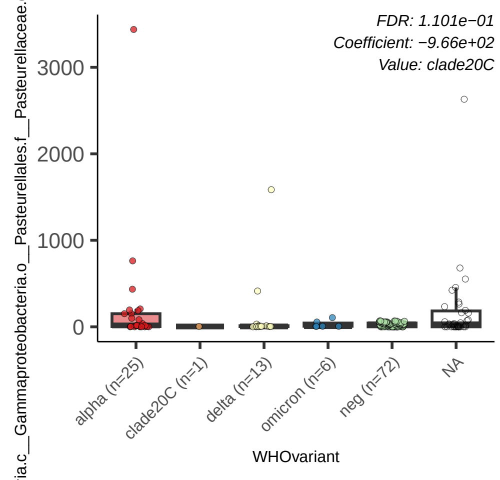

FDR: 1.101e−01
Coefficient: −9.66e+02
Value: clade20C
ria.c__Gammaproteobacteria.o__Pasteurellales.f__Pasteurellaceae.
0
1000
2000
3000
alpha (n=25)
clade20C (n=1)
delta (n=13)
omicron (n=6)
neg (n=72)
NA
WHOvariant

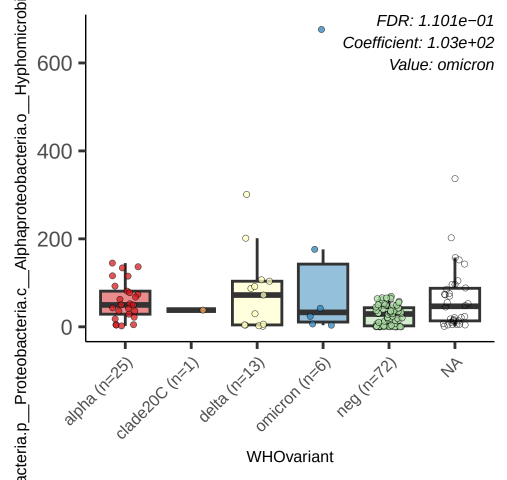

FDR: 1.101e−01
Coefficient: 1.03e+02
Value: omicron
0
200
400
600
acteria.p__Proteobacteria.c__Alphaproteobacteria.o__Hyphomicrobi
alpha (n=25)
clade20C (n=1)
delta (n=13)
omicron (n=6)
neg (n=72)
NA
WHOvariant

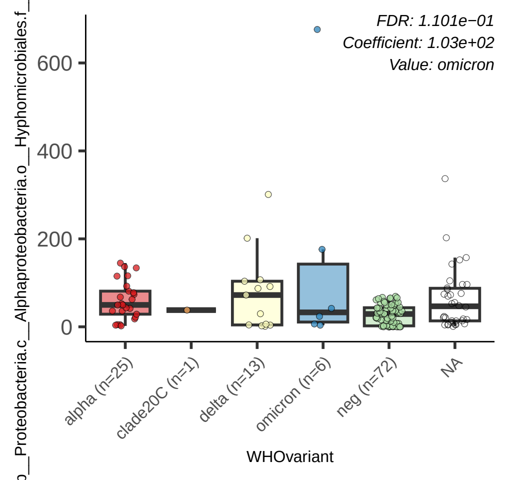

FDR: 1.101e−01
Coefficient: 1.03e+02
Value: omicron
o__Proteobacteria.c__Alphaproteobacteria.o__Hyphomicrobiales.f__
0
200
400
600
alpha (n=25)
clade20C (n=1)
delta (n=13)
omicron (n=6)
neg (n=72)
NA
WHOvariant

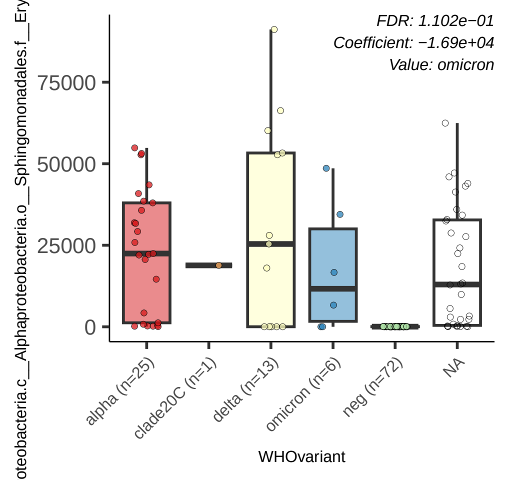

FDR: 1.102e−01
Coefficient: −1.69e+04
Value: omicron
oteobacteria.c__Alphaproteobacteria.o__Sphingomonadales.f__Ery
75000
50000
25000
0
alpha (n=25)
clade20C (n=1)
delta (n=13)
omicron (n=6)
neg (n=72)
NA
WHOvariant

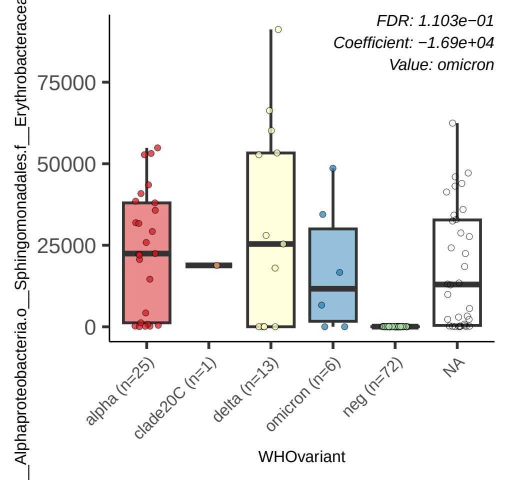

FDR: 1.103e−01
Coefficient: −1.69e+04
Value: omicron
__Alphaproteobacteria.o__Sphingomonadales.f__Erythrobacteraceae
0
25000
50000
75000
alpha (n=25)
clade20C (n=1)
delta (n=13)
omicron (n=6)
neg (n=72)
NA
WHOvariant

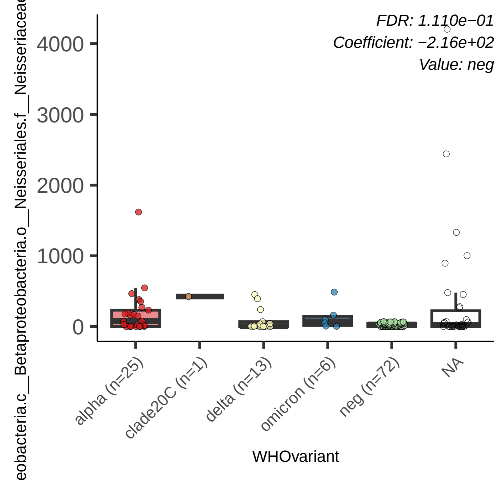

FDR: 1.110e−01
Coefficient: −2.16e+02
Value: neg
4000
3000
2000
1000
0
eobacteria.c__Betaproteobacteria.o__Neisseriales.f__Neisseriaceae
alpha (n=25)
clade20C (n=1)
delta (n=13)
omicron (n=6)
neg (n=72)
NA
WHOvariant

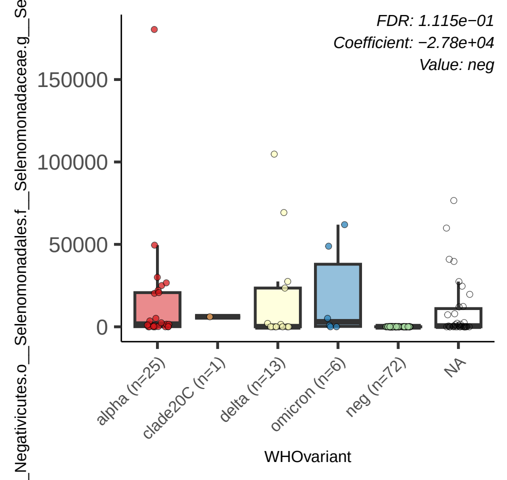

FDR: 1.115e−01
Coefficient: −2.78e+04
Value: neg
Negativicutes.o__Selenomonadales.f__Selenomonadaceae.g__Se
150000
100000
50000
0
alpha (n=25)
clade20C (n=1)
delta (n=13)
omicron (n=6)
neg (n=72)
NA
WHOvariant

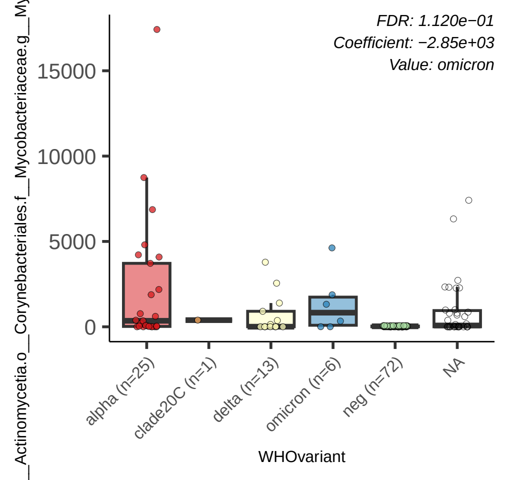

FDR: 1.120e−01
Coefficient: −2.85e+03
Value: omicron
Actinomycetia.o__Corynebacteriales.f__Mycobacteriaceae.g__My
15000
10000
5000
0
alpha (n=25)
clade20C (n=1)
delta (n=13)
omicron (n=6)
neg (n=72)
NA
WHOvariant

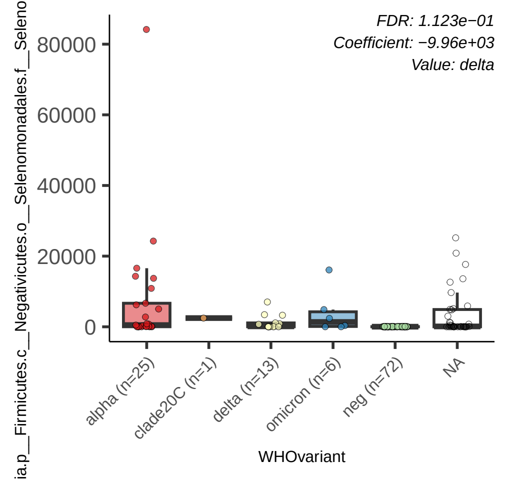

FDR: 1.123e−01
Coefficient: −9.96e+03
Value: delta
ia.p__Firmicutes.c__Negativicutes.o__Selenomonadales.f__Seleno
80000
60000
40000
20000
0
alpha (n=25)
clade20C (n=1)
delta (n=13)
omicron (n=6)
neg (n=72)
NA
WHOvariant

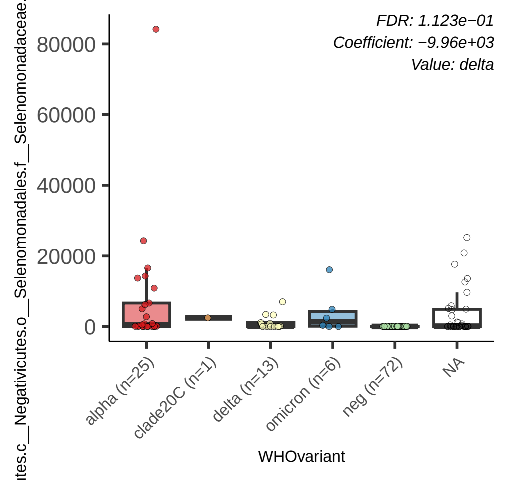

FDR: 1.123e−01
Coefficient: −9.96e+03
Value: delta
80000
60000
40000
20000
0
alpha (n=25)
clade20C (n=1)
delta (n=13)
omicron (n=6)
neg (n=72)
NA
WHOvariant
utes.c__Negativicutes.o__Selenomonadales.f__Selenomonadaceae.

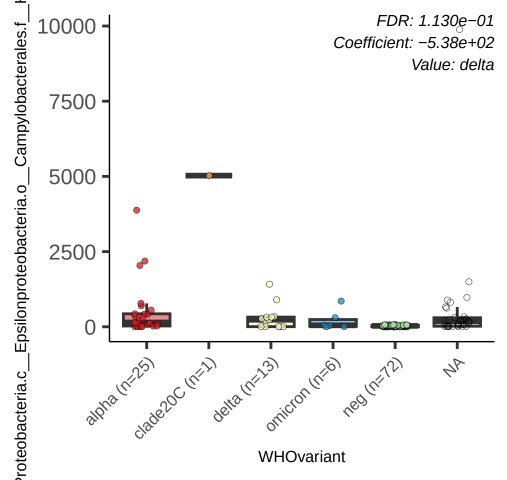

FDR: 1.130e−01
Coefficient: −5.38e+02
Value: delta
Proteobacteria.c__Epsilonproteobacteria.o__Campylobacterales.f__H
10000
7500
5000
2500
0
alpha (n=25)
clade20C (n=1)
delta (n=13)
omicron (n=6)
neg (n=72)
NA
WHOvariant

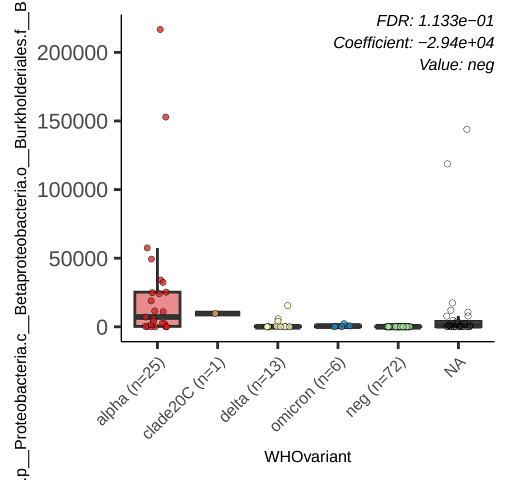

FDR: 1.133e−01
Coefficient: −2.94e+04
Value: neg
p__Proteobacteria.c__Betaproteobacteria.o__Burkholderiales.f__B
200000
150000
100000
50000
0
alpha (n=25)
clade20C (n=1)
delta (n=13)
omicron (n=6)
neg (n=72)
NA
WHOvariant

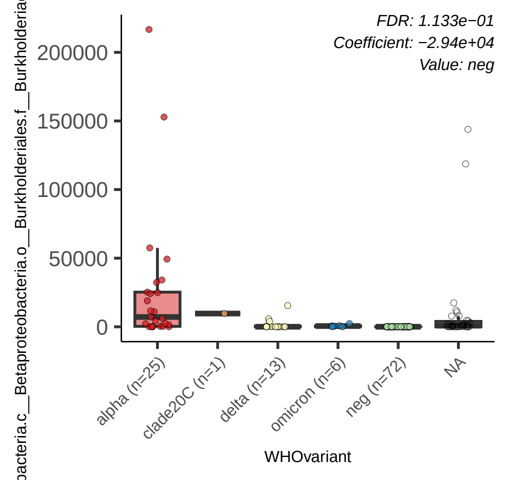

FDR: 1.133e−01
Coefficient: −2.94e+04
Value: neg
200000
150000
100000
50000
0
bacteria.c__Betaproteobacteria.o__Burkholderiales.f__Burkholderia
alpha (n=25)
clade20C (n=1)
delta (n=13)
omicron (n=6)
neg (n=72)
NA
WHOvariant

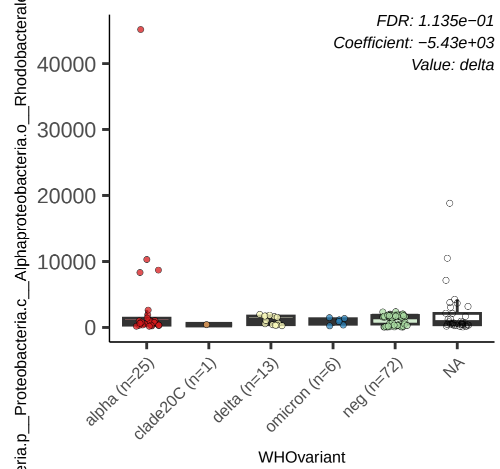
FDR: 1.135e−01
Coefficient: −5.43e+03
Value: delta
cteria.p__Proteobacteria.c__Alphaproteobacteria.o__Rhodobacterale
40000
30000
20000
10000
0
alpha (n=25)
clade20C (n=1)
delta (n=13)
omicron (n=6)
neg (n=72)
NA
WHOvariant

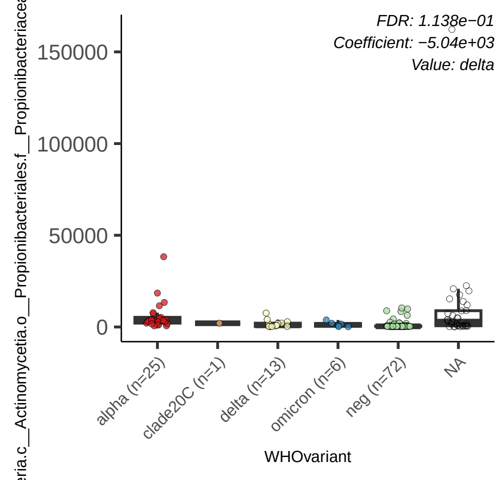

FDR: 1.138e−01
Coefficient: −5.04e+03
Value: delta
150000
100000
50000
0
eria.c__Actinomycetia.o__Propionibacteriales.f__Propionibacteriacea
alpha (n=25)
clade20C (n=1)
delta (n=13)
omicron (n=6)
neg (n=72)
NA
WHOvariant

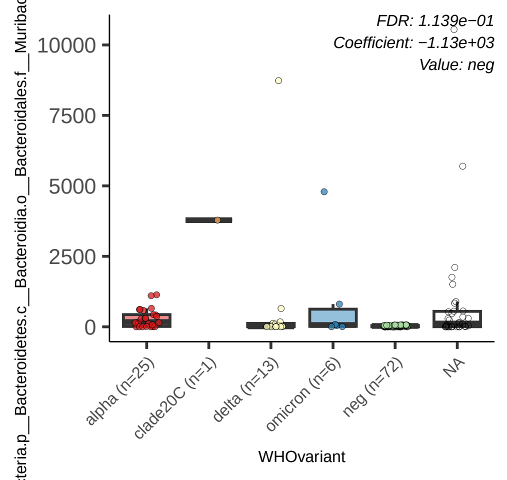

FDR: 1.139e−01
Coefficient: −1.13e+03
Value: neg
10000
7500
5000
2500
0
cteria.p__Bacteroidetes.c__Bacteroidia.o__Bacteroidales.f__Muribac
alpha (n=25)
clade20C (n=1)
delta (n=13)
omicron (n=6)
neg (n=72)
NA
WHOvariant

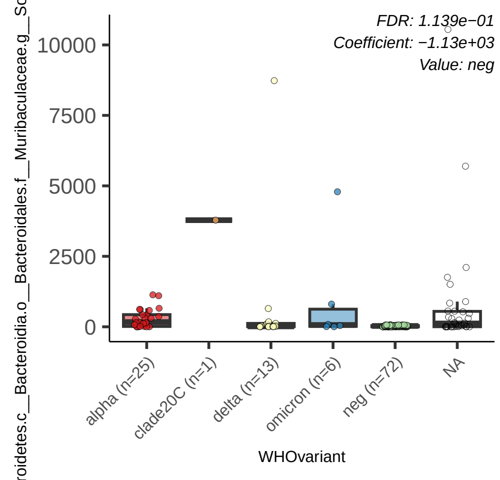

FDR: 1.139e−01
Coefficient: −1.13e+03
Value: neg
roidetes.c__Bacteroidia.o__Bacteroidales.f__Muribaculaceae.g__Sc
10000
7500
5000
2500
0
alpha (n=25)
clade20C (n=1)
delta (n=13)
omicron (n=6)
neg (n=72)
NA
WHOvariant

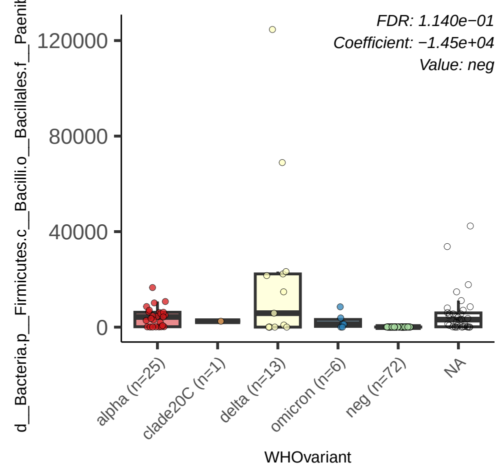

FDR: 1.140e−01
Coefficient: −1.45e+04
Value: neg
120000
80000
40000
0
d__Bacteria.p__Firmicutes.c__Bacilli.o__Bacillales.f__Paenib
alpha (n=25)
clade20C (n=1)
delta (n=13)
omicron (n=6)
neg (n=72)
NA
WHOvariant

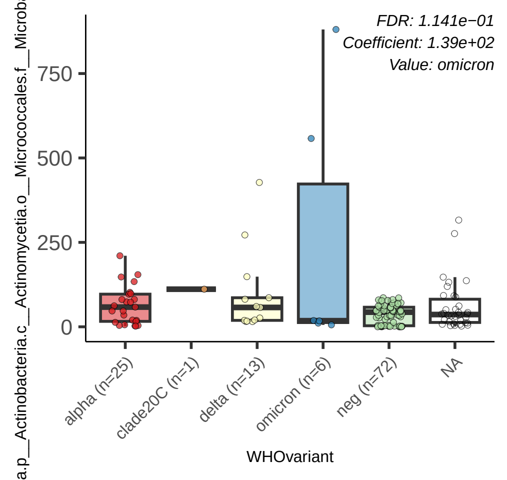

FDR: 1.141e−01
Coefficient: 1.39e+02
Value: omicron
a.p__Actinobacteria.c__Actinomycetia.o__Micrococcales.f__Microb
0
250
500
750
alpha (n=25)
clade20C (n=1)
delta (n=13)
omicron (n=6)
neg (n=72)
NA
WHOvariant

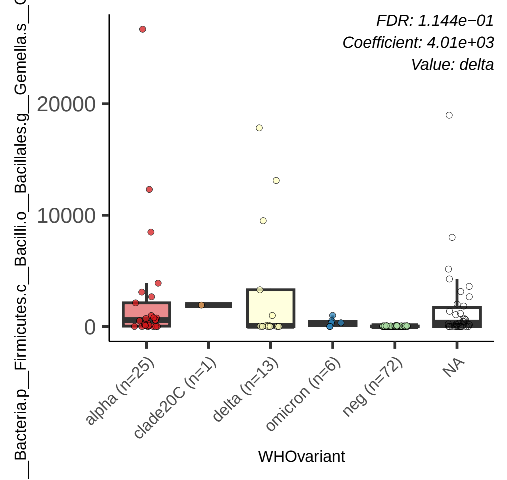

FDR: 1.144e−01
Coefficient: 4.01e+03
Value: delta
20000
10000
0
alpha (n=25)
clade20C (n=1)
delta (n=13)
omicron (n=6)
neg (n=72)
NA
WHOvariant
Bacteria.p__Firmicutes.c__Bacilli.o__Bacillales.g__Gemella.s__

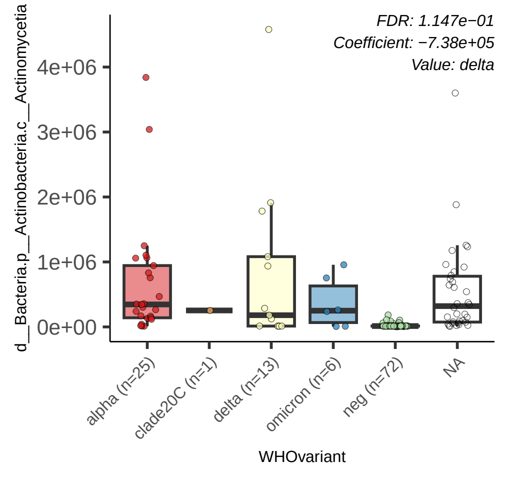

FDR: 1.147e−01
Coefficient: −7.38e+05
Value: delta
d__Bacteria.p__Actinobacteria.c__Actinomycetia
4e+06
3e+06
2e+06
1e+06
0e+00
alpha (n=25)
clade20C (n=1)
delta (n=13)
omicron (n=6)
neg (n=72)
NA
WHOvariant

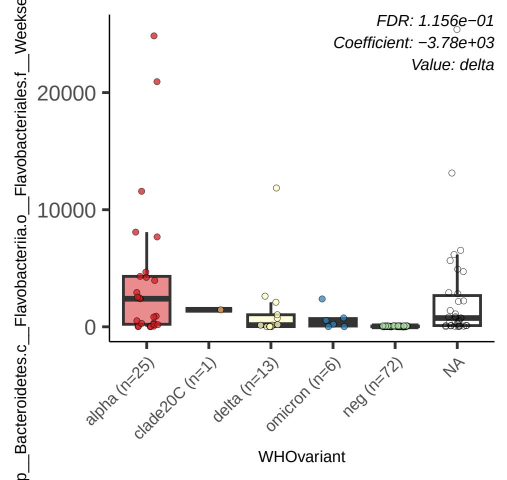

FDR: 1.156e−01
Coefficient: −3.78e+03
Value: delta
20000
10000
0
p__Bacteroidetes.c__Flavobacteriia.o__Flavobacteriales.f__Weekse
alpha (n=25)
clade20C (n=1)
delta (n=13)
omicron (n=6)
neg (n=72)
NA
WHOvariant

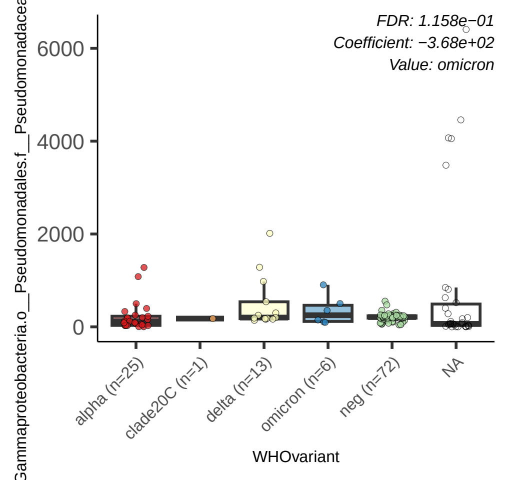

FDR: 1.158e−01
Coefficient: −3.68e+02
Value: omicron
Gammaproteobacteria.o__Pseudomonadales.f__Pseudomonadacea
6000
4000
2000
0
alpha (n=25)
clade20C (n=1)
delta (n=13)
omicron (n=6)
neg (n=72)
NA
WHOvariant

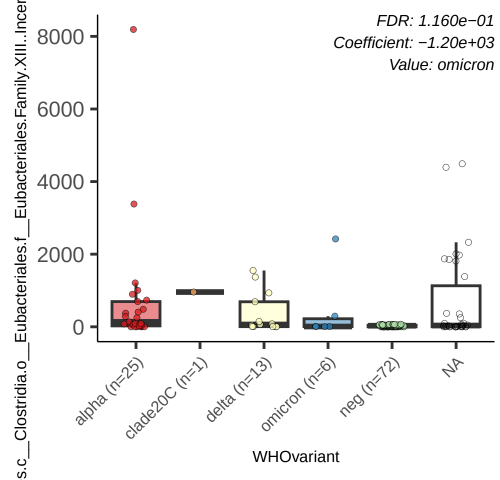

FDR: 1.160e−01
Coefficient: −1.20e+03
Value: omicron
8000
6000
4000
2000
0
s.c__Clostridia.o__Eubacteriales.f__Eubacteriales.Family.XIII..Incer
alpha (n=25)
clade20C (n=1)
delta (n=13)
omicron (n=6)
neg (n=72)
NA
WHOvariant

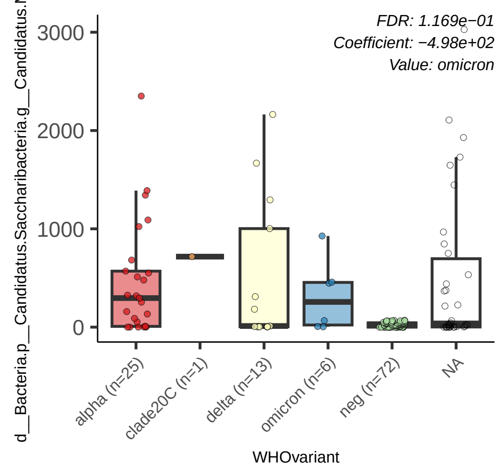

FDR: 1.169e−01
Coefficient: −4.98e+02
Value: omicron
d__Bacteria.p__Candidatus.Saccharibacteria.g__Candidatus.N
3000
2000
1000
0
alpha (n=25)
clade20C (n=1)
delta (n=13)
omicron (n=6)
neg (n=72)
NA
WHOvariant

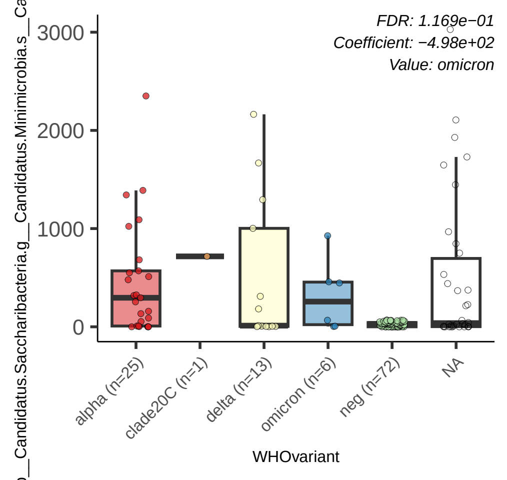

FDR: 1.169e−01
Coefficient: −4.98e+02
Value: omicron
Candidatus.Saccharibacteria.g__Candidatus.Minimicrobia.s__Ca
0
1000
2000
3000
alpha (n=25)
clade20C (n=1)
delta (n=13)
omicron (n=6)
neg (n=72)
NA
WHOvariant

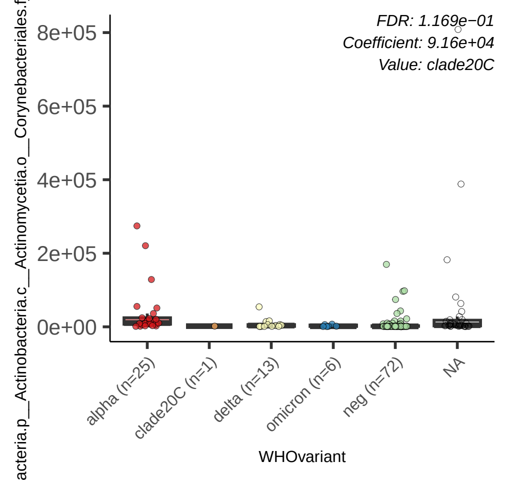

FDR: 1.169e−01
Coefficient: 9.16e+04
Value: clade20C
acteria.p__Actinobacteria.c__Actinomycetia.o__Corynebacteriales.f
8e+05
6e+05
4e+05
2e+05
0e+00
alpha (n=25)
clade20C (n=1)
delta (n=13)
omicron (n=6)
neg (n=72)
NA
WHOvariant

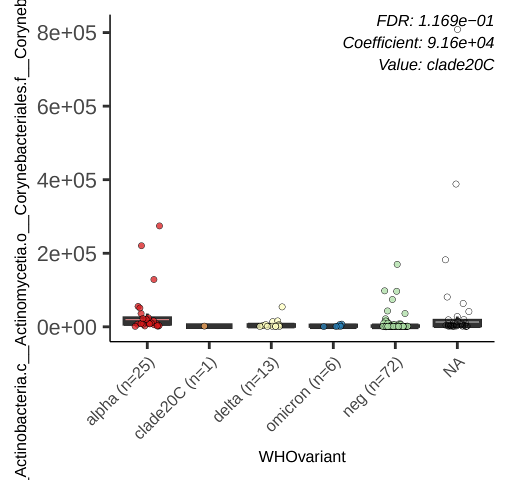

FDR: 1.169e−01
Coefficient: 9.16e+04
Value: clade20C
8e+05
6e+05
4e+05
2e+05
0e+00
Actinobacteria.c__Actinomycetia.o__Corynebacteriales.f__Coryneb
alpha (n=25)
clade20C (n=1)
delta (n=13)
omicron (n=6)
neg (n=72)
NA
WHOvariant

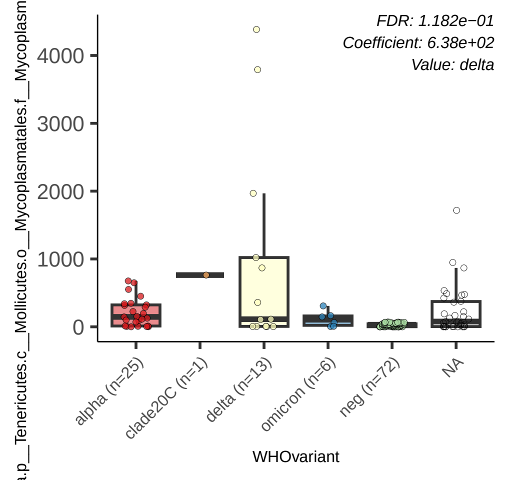

FDR: 1.182e−01
Coefficient: 6.38e+02
Value: delta
4000
3000
2000
1000
0
Tenericutes.c__Mollicutes.o__Mycoplasmatales.f__Mycoplasm
alpha (n=25)
clade20C (n=1)
delta (n=13)
omicron (n=6)
neg (n=72)
NA
WHOvariant

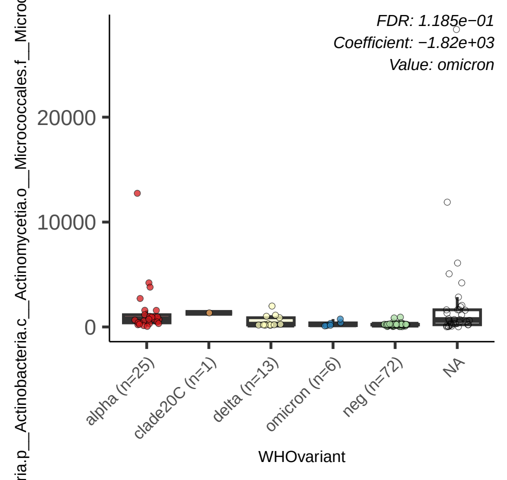

FDR: 1.185e−01
Coefficient: −1.82e+03
Value: omicron
20000
10000
0
alpha (n=25)
clade20C (n=1)
delta (n=13)
omicron (n=6)
neg (n=72)
NA
WHOvariant
ria.p__Actinobacteria.c__Actinomycetia.o__Micrococcales.f__Microc

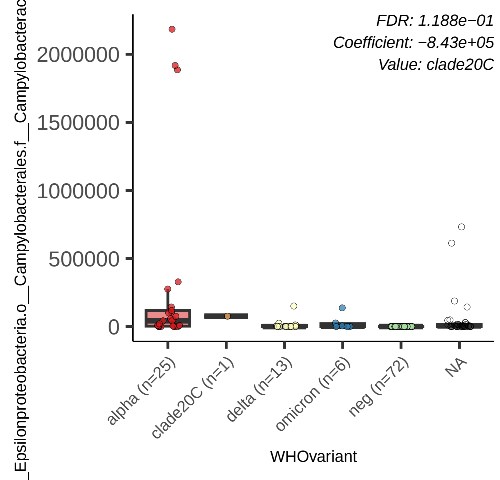
FDR: 1.188e−01
Coefficient: −8.43e+05
Value: clade20C
_Epsilonproteobacteria.o__Campylobacterales.f__Campylobacterac
2000000
1500000
1000000
500000
0
alpha (n=25)
clade20C (n=1)
delta (n=13)
omicron (n=6)
neg (n=72)
NA
WHOvariant

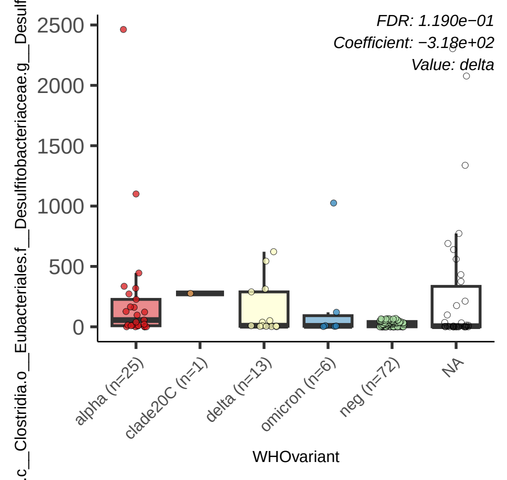

FDR: 1.190e−01
Coefficient: −3.18e+02
Value: delta
c__Clostridia.o__Eubacteriales.f__Desulfitobacteriaceae.g__Desulf
2500
2000
1500
1000
500
0
alpha (n=25)
clade20C (n=1)
delta (n=13)
omicron (n=6)
neg (n=72)
NA
WHOvariant

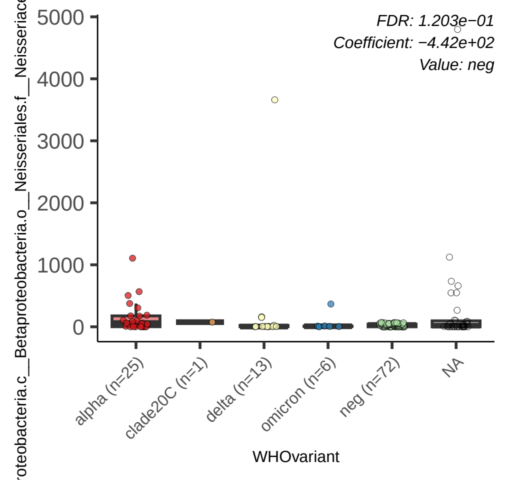
FDR: 1.203e−01
Coefficient: −4.42e+02
Value: neg
5000
4000
3000
2000
1000
0
roteobacteria.c__Betaproteobacteria.o__Neisseriales.f__Neisseriace
alpha (n=25)
clade20C (n=1)
delta (n=13)
omicron (n=6)
neg (n=72)
NA
WHOvariant

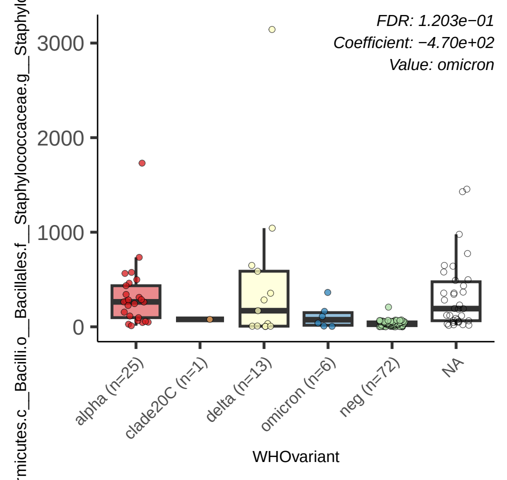

FDR: 1.203e−01
Coefficient: −4.70e+02
Value: omicron
3000
2000
1000
0
rmicutes.c__Bacilli.o__Bacillales.f__Staphylococcaceae.g__Staphylo
alpha (n=25)
clade20C (n=1)
delta (n=13)
omicron (n=6)
neg (n=72)
NA
WHOvariant

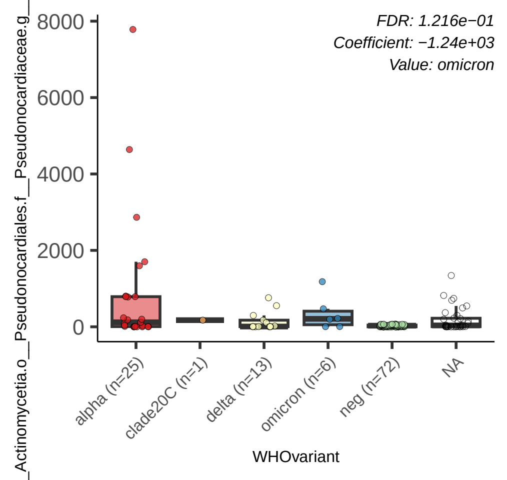

FDR: 1.216e−01
Coefficient: −1.24e+03
Value: omicron
Actinomycetia.o__Pseudonocardiales.f__Pseudonocardiaceae.g__
8000
6000
4000
2000
0
alpha (n=25)
clade20C (n=1)
delta (n=13)
omicron (n=6)
neg (n=72)
NA
WHOvariant

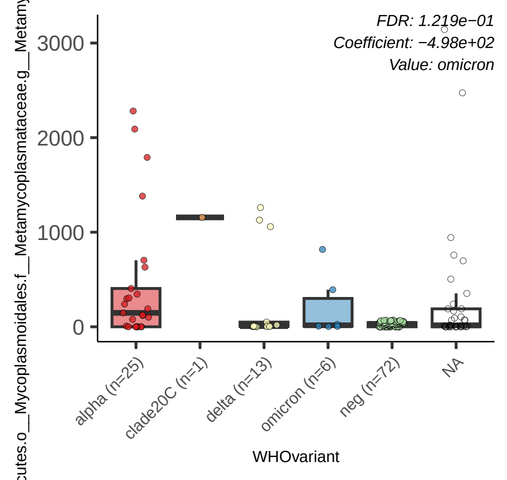

FDR: 1.219e−01
Coefficient: −4.98e+02
Value: omicron
cutes.o__Mycoplasmoidales.f__Metamycoplasmataceae.g__Metamy
3000
2000
1000
0
alpha (n=25)
clade20C (n=1)
delta (n=13)
omicron (n=6)
neg (n=72)
NA
WHOvariant

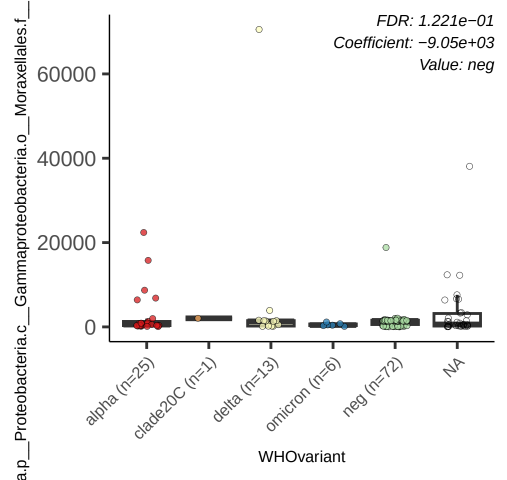

FDR: 1.221e−01
Coefficient: −9.05e+03
Value: neg
a.p__Proteobacteria.c__Gammaproteobacteria.o__Moraxellales.f__
60000
40000
20000
0
alpha (n=25)
clade20C (n=1)
delta (n=13)
omicron (n=6)
neg (n=72)
NA
WHOvariant

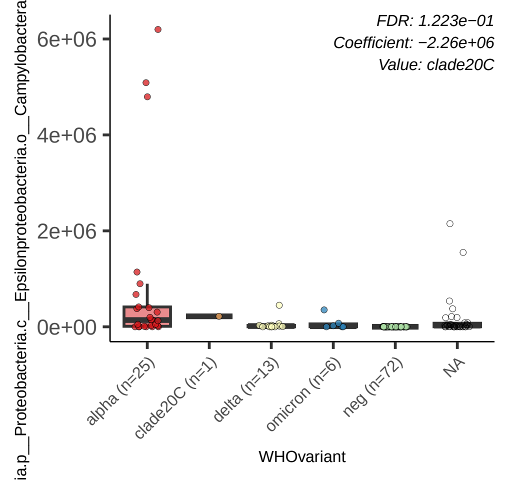
FDR: 1.223e−01
Coefficient: −2.26e+06
Value: clade20C
6e+06
4e+06
2e+06
0e+00
ia.p__Proteobacteria.c__Epsilonproteobacteria.o__Campylobacteria
alpha (n=25)
clade20C (n=1)
delta (n=13)
omicron (n=6)
neg (n=72)
NA
WHOvariant

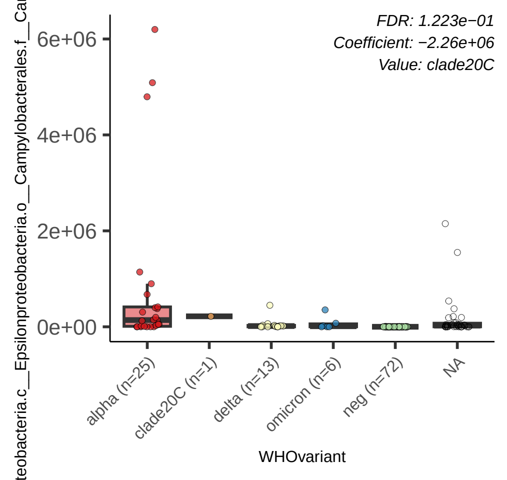
FDR: 1.223e−01
Coefficient: −2.26e+06
Value: clade20C
teobacteria.c__Epsilonproteobacteria.o__Campylobacterales.f__Ca
6e+06
4e+06
2e+06
0e+00
alpha (n=25)
clade20C (n=1)
delta (n=13)
omicron (n=6)
neg (n=72)
NA
WHOvariant

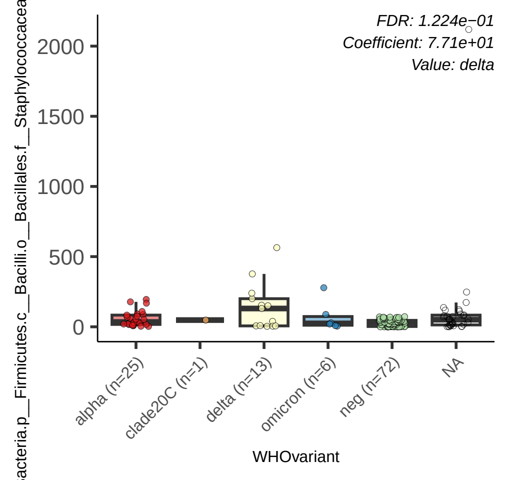

FDR: 1.224e−01
Coefficient: 7.71e+01
Value: delta
Bacteria.p__Firmicutes.c__Bacilli.o__Bacillales.f__Staphylococcacea
2000
1500
1000
500
0
alpha (n=25)
clade20C (n=1)
delta (n=13)
omicron (n=6)
neg (n=72)
NA
WHOvariant

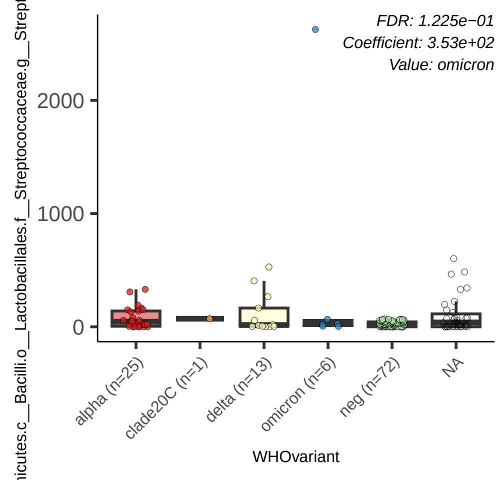

FDR: 1.225e−01
Coefficient: 3.53e+02
Value: omicron
2000
1000
0
micutes.c__Bacilli.o__Lactobacillales.f__Streptococcaceae.g__Strept
alpha (n=25)
clade20C (n=1)
delta (n=13)
omicron (n=6)
neg (n=72)
NA
WHOvariant

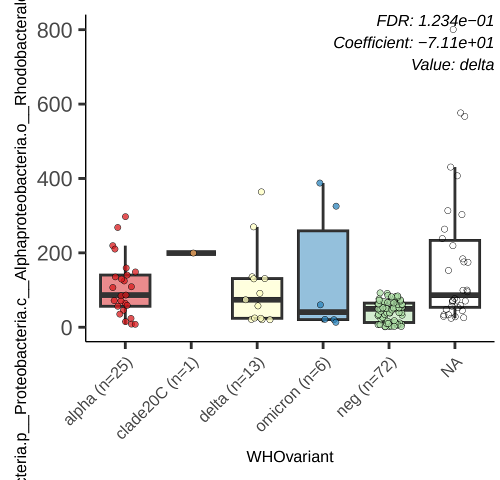

FDR: 1.234e−01
Coefficient: −7.11e+01
Value: delta
800
600
400
200
0
cteria.p__Proteobacteria.c__Alphaproteobacteria.o__Rhodobacterale
alpha (n=25)
clade20C (n=1)
delta (n=13)
omicron (n=6)
neg (n=72)
NA
WHOvariant

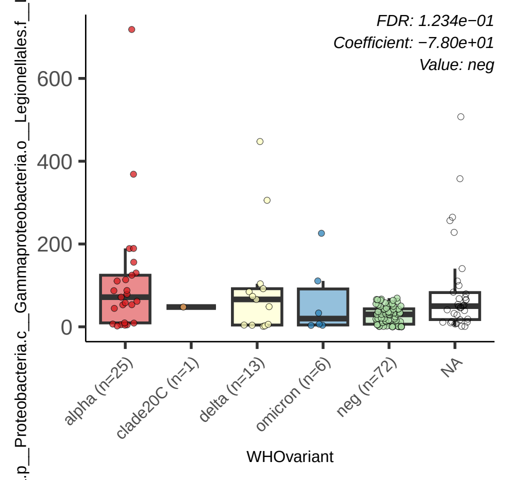

FDR: 1.234e−01
Coefficient: −7.80e+01
Value: neg
p__Proteobacteria.c__Gammaproteobacteria.o__Legionellales.f__
0
200
400
600
alpha (n=25)
clade20C (n=1)
delta (n=13)
omicron (n=6)
neg (n=72)
NA
WHOvariant

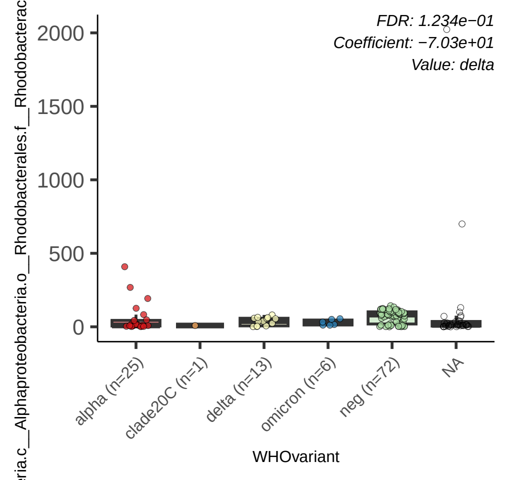

FDR: 1.234e−01
Coefficient: −7.03e+01
Value: delta
2000
1500
1000
500
0
ria.c__Alphaproteobacteria.o__Rhodobacterales.f__Rhodobacterac
alpha (n=25)
clade20C (n=1)
delta (n=13)
omicron (n=6)
neg (n=72)
NA
WHOvariant

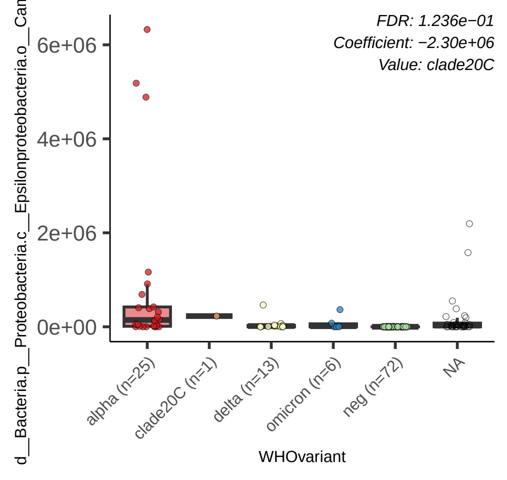

FDR: 1.236e−01
Coefficient: −2.30e+06
Value: clade20C
d__Bacteria.p__Proteobacteria.c__Epsilonproteobacteria.o__Can
6e+06
4e+06
2e+06
0e+00
alpha (n=25)
clade20C (n=1)
delta (n=13)
omicron (n=6)
neg (n=72)
NA
WHOvariant

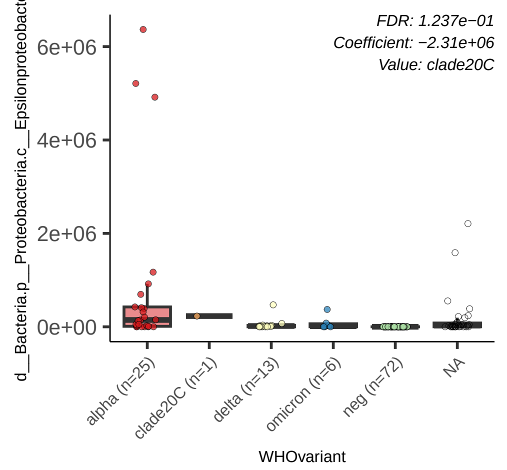

FDR: 1.237e−01
Coefficient: −2.31e+06
Value: clade20C
d__Bacteria.p__Proteobacteria.c__Epsilonproteobacte
6e+06
4e+06
2e+06
0e+00
alpha (n=25)
clade20C (n=1)
delta (n=13)
omicron (n=6)
neg (n=72)
NA
WHOvariant

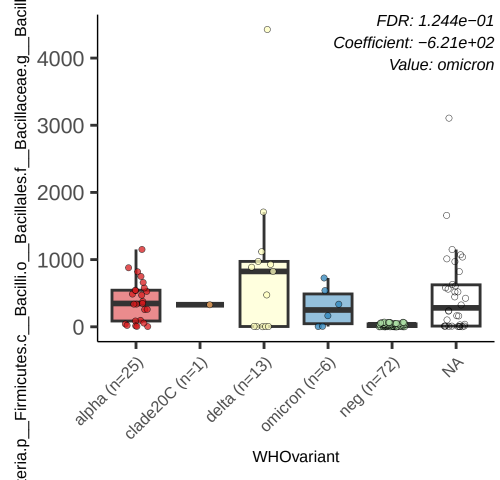
FDR: 1.244e−01
Coefficient: −6.21e+02
Value: omicron
4000
3000
2000
1000
0
alpha (n=25)
clade20C (n=1)
delta (n=13)
omicron (n=6)
neg (n=72)
NA
WHOvariant

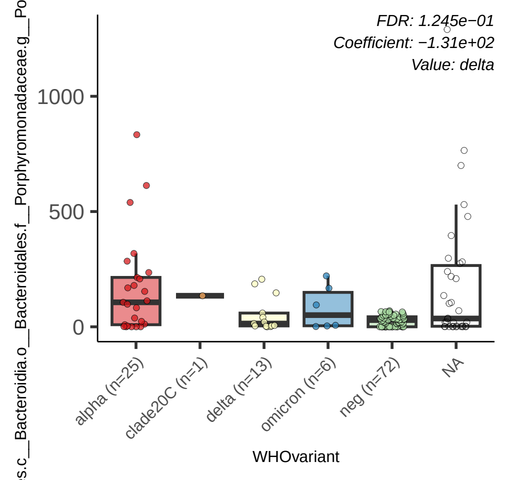

FDR: 1.245e−01
Coefficient: −1.31e+02
Value: delta
s.c__Bacteroidia.o__Bacteroidales.f__Porphyromonadaceae.g__Po
1000
500
0
alpha (n=25)
clade20C (n=1)
delta (n=13)
omicron (n=6)
neg (n=72)
NA
WHOvariant

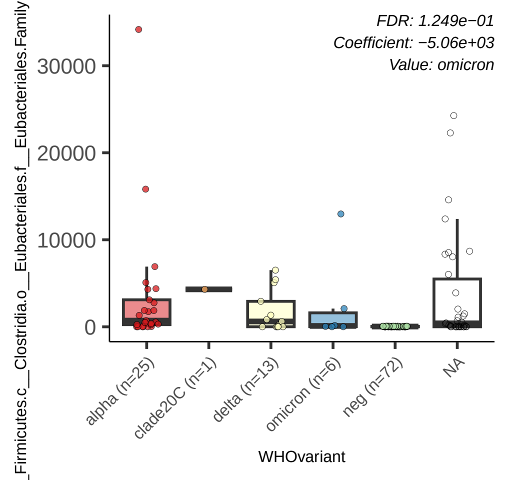
FDR: 1.249e−01
Coefficient: −5.06e+03
Value: omicron
Firmicutes.c__Clostridia.o__Eubacteriales.f__Eubacteriales.Family
30000
20000
10000
0
alpha (n=25)
clade20C (n=1)
delta (n=13)
omicron (n=6)
neg (n=72)
NA
WHOvariant

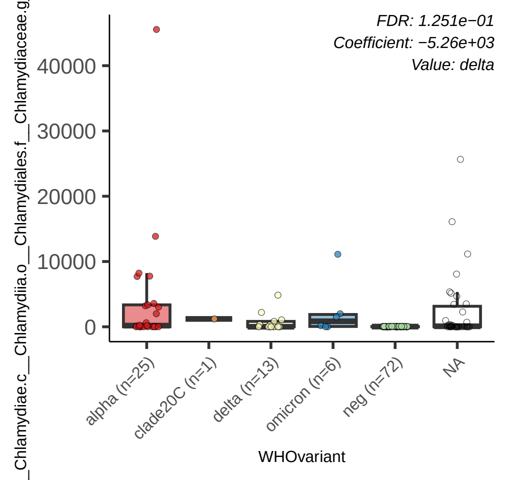

FDR: 1.251e−01
Coefficient: −5.26e+03
Value: delta
40000
30000
20000
10000
0
_Chlamydiae.c__Chlamydiia.o__Chlamydiales.f__Chlamydiaceae.g
alpha (n=25)
clade20C (n=1)
delta (n=13)
omicron (n=6)
neg (n=72)
NA
WHOvariant

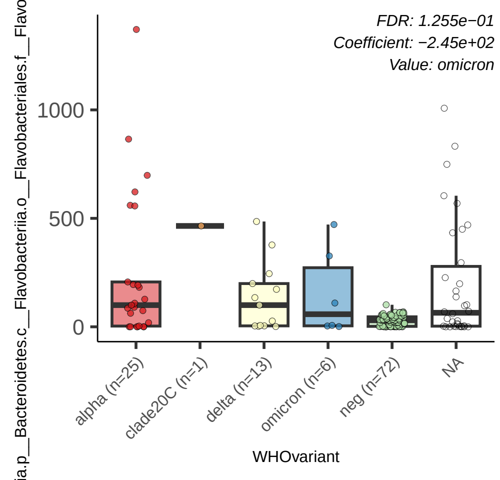

FDR: 1.255e−01
Coefficient: −2.45e+02
Value: omicron
ia.p__Bacteroidetes.c__Flavobacteriia.o__Flavobacteriales.f__Flavo
1000
500
0
alpha (n=25)
clade20C (n=1)
delta (n=13)
omicron (n=6)
neg (n=72)
NA
WHOvariant

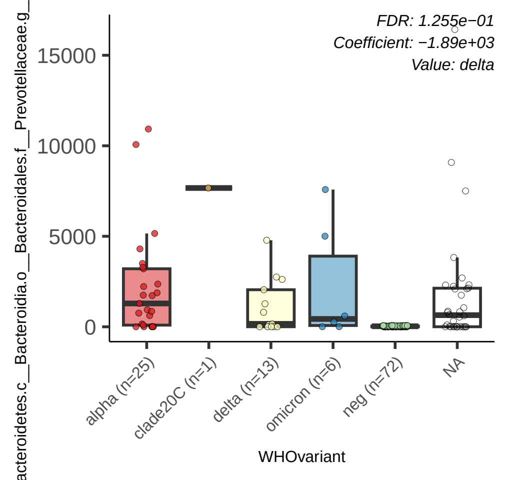

FDR: 1.255e−01
Coefficient: −1.89e+03
Value: delta
15000
10000
5000
0
acteroidetes.c__Bacteroidia.o__Bacteroidales.f__Prevotellaceae.g__
alpha (n=25)
clade20C (n=1)
delta (n=13)
omicron (n=6)
neg (n=72)
NA
WHOvariant

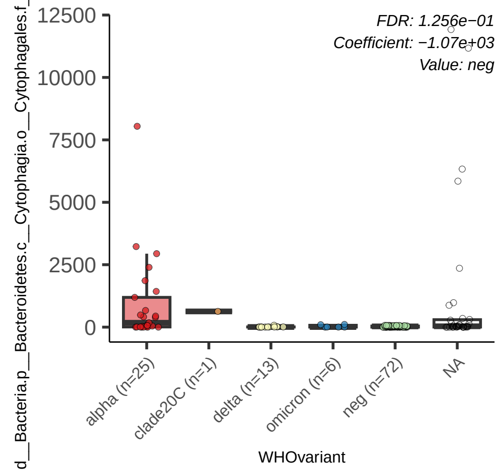

FDR: 1.256e−01
Coefficient: −1.07e+03
Value: neg
d__Bacteria.p__Bacteroidetes.c__Cytophagia.o__Cytophagales.f_
12500
10000
7500
5000
2500
0
alpha (n=25)
clade20C (n=1)
delta (n=13)
omicron (n=6)
neg (n=72)
NA
WHOvariant

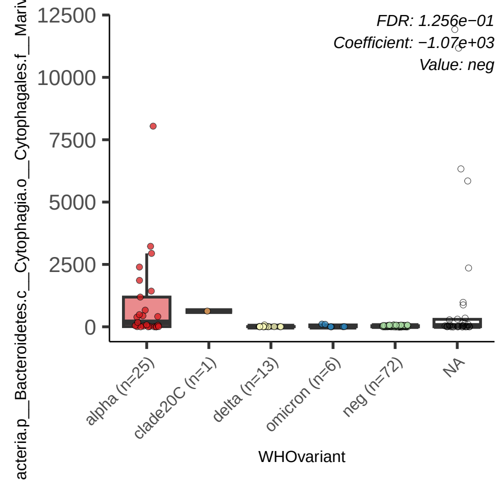

acteria.p__Bacteroidetes.c__Cytophagia.o__Cytophagales.f__Mariv
FDR: 1.256e−01
Coefficient: −1.07e+03
Value: neg
12500
10000
7500
5000
2500
0
alpha (n=25)
clade20C (n=1)
delta (n=13)
omicron (n=6)
neg (n=72)
NA
WHOvariant

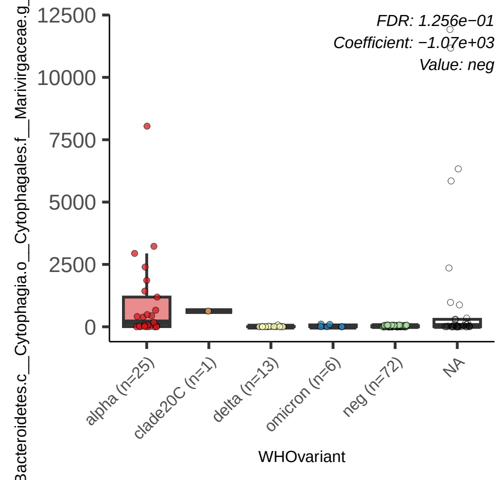
FDR: 1.256e−01
Coefficient: −1.07e+03
Value: neg
Bacteroidetes.c__Cytophagia.o__Cytophagales.f__Marivirgaceae.g_
12500
10000
7500
5000
2500
0
alpha (n=25)
clade20C (n=1)
delta (n=13)
omicron (n=6)
neg (n=72)
NA
WHOvariant

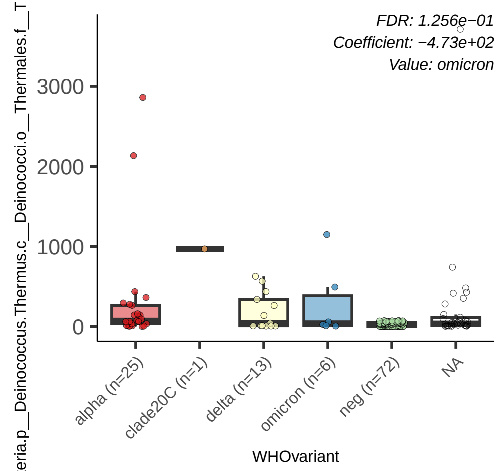

FDR: 1.256e−01
Coefficient: −4.73e+02
Value: omicron
p__Deinococcus.Thermus.c__Deinococci.o__Thermales.f__T
3000
2000
1000
0
alpha (n=25)
clade20C (n=1)
delta (n=13)
omicron (n=6)
neg (n=72)
NA
WHOvariant

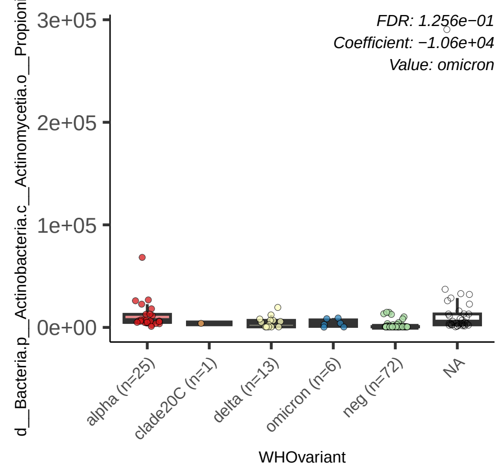

FDR: 1.256e−01
Coefficient: −1.06e+04
Value: omicron
3e+05
2e+05
1e+05
0e+00
d__Bacteria.p__Actinobacteria.c__Actinomycetia.o__Propioni
alpha (n=25)
clade20C (n=1)
delta (n=13)
omicron (n=6)
neg (n=72)
NA
WHOvariant

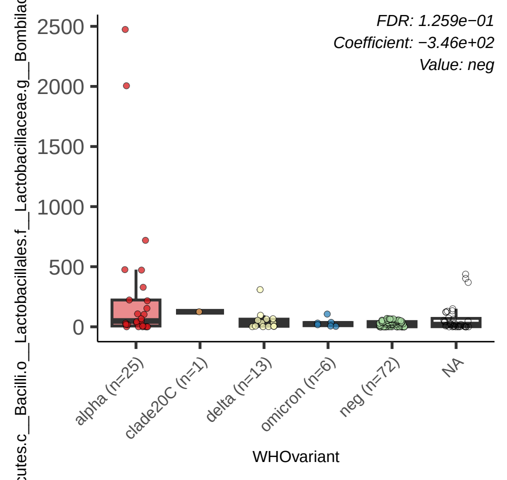

FDR: 1.259e−01
Coefficient: −3.46e+02
Value: neg
cutes.c__Bacilli.o__Lactobacillales.f__Lactobacillaceae.g__Bombilac
2500
2000
1500
1000
500
0
alpha (n=25)
clade20C (n=1)
delta (n=13)
omicron (n=6)
neg (n=72)
NA
WHOvariant

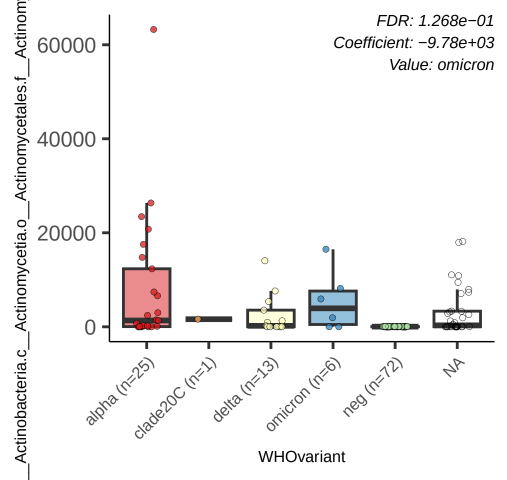

FDR: 1.268e−01
Coefficient: −9.78e+03
Value: omicron
__Actinobacteria.c__Actinomycetia.o__Actinomycetales.f__Actinomy
60000
40000
20000
0
alpha (n=25)
clade20C (n=1)
delta (n=13)
omicron (n=6)
neg (n=72)
NA
WHOvariant

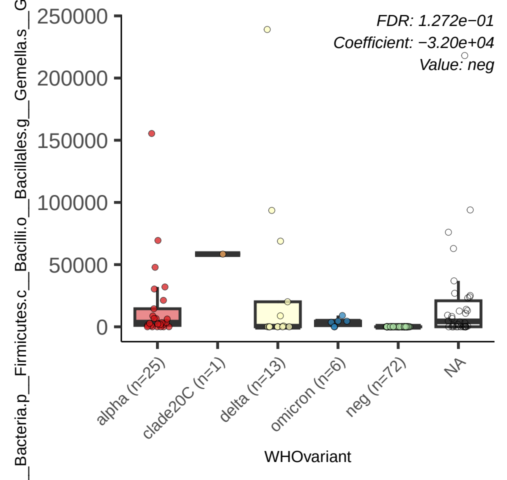

FDR: 1.272e−01
Coefficient: −3.20e+04
Value: neg
_Bacteria.p__Firmicutes.c__Bacilli.o__Bacillales.g__Gemella.s__G
250000
200000
150000
100000
50000
0
alpha (n=25)
clade20C (n=1)
delta (n=13)
omicron (n=6)
neg (n=72)
NA
WHOvariant

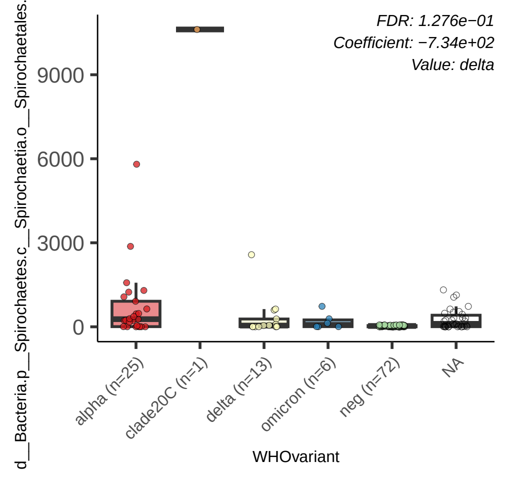
FDR: 1.276e−01
Coefficient: −7.34e+02
Value: delta
d__Bacteria.p__Spirochaetes.c__Spirochaetia.o__Spirochaetales.
9000
6000
3000
0
alpha (n=25)
clade20C (n=1)
delta (n=13)
omicron (n=6)
neg (n=72)
NA
WHOvariant

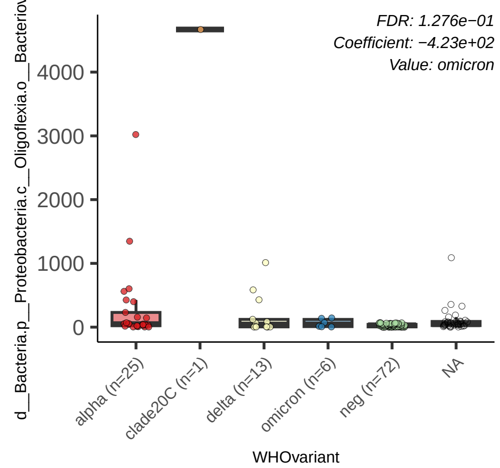

FDR: 1.276e−01
Coefficient: −4.23e+02
Value: omicron
d__Bacteria.p__Proteobacteria.c__Oligoflexia.o__Bacterio
4000
3000
2000
1000
0
alpha (n=25)
clade20C (n=1)
delta (n=13)
omicron (n=6)
neg (n=72)
NA
WHOvariant

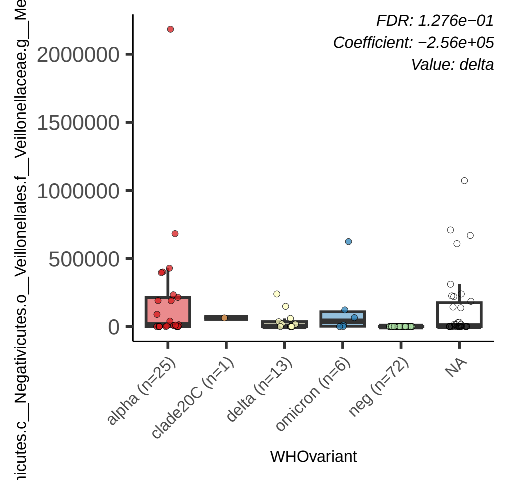

FDR: 1.276e−01
Coefficient: −2.56e+05
Value: delta
2000000
1500000
1000000
500000
0
alpha (n=25)
clade20C (n=1)
delta (n=13)
omicron (n=6)
neg (n=72)
NA
WHOvariant
icutes.c__Negativicutes.o__Veillonellales.f__Veillonellaceae.g__Me

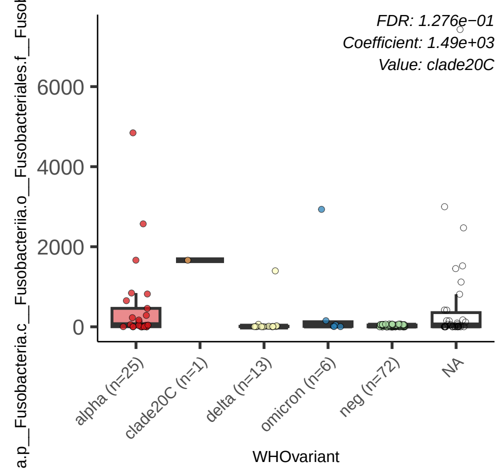
FDR: 1.276e−01
Coefficient: 1.49e+03
Value: clade20C
6000
4000
2000
0
alpha (n=25)
clade20C (n=1)
delta (n=13)
omicron (n=6)
neg (n=72)
NA
WHOvariant

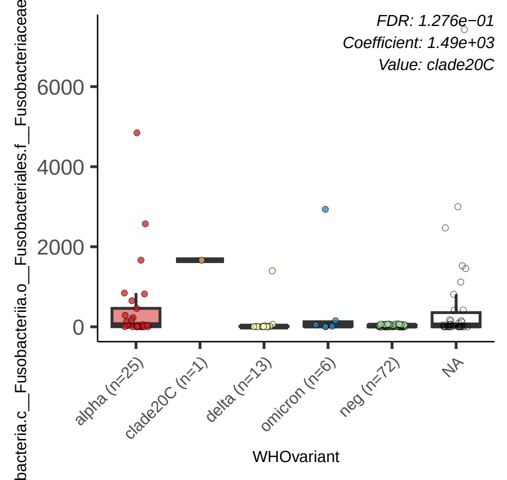

FDR: 1.276e−01
Coefficient: 1.49e+03
Value: clade20C
bacteria.c__Fusobacteriia.o__Fusobacteriales.f__Fusobacteriaceae
0
2000
4000
6000
alpha (n=25)
clade20C (n=1)
delta (n=13)
omicron (n=6)
neg (n=72)
NA
WHOvariant

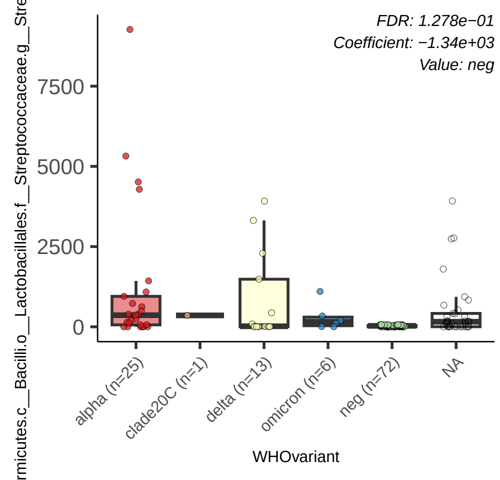

FDR: 1.278e−01
Coefficient: −1.34e+03
Value: neg
7500
5000
2500
0
alpha (n=25)
clade20C (n=1)
delta (n=13)
omicron (n=6)
neg (n=72)
NA
WHOvariant

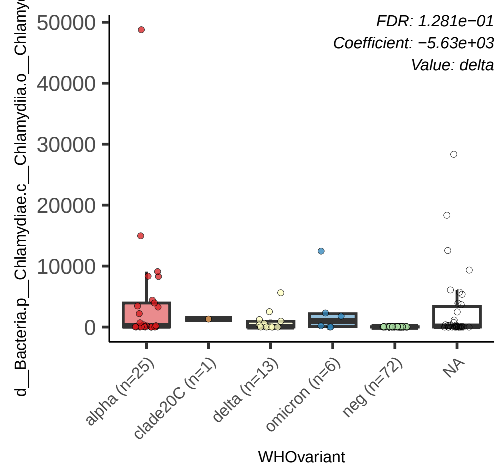
FDR: 1.281e−01
Coefficient: −5.63e+03
Value: delta
d__Bacteria.p__Chlamydiae.c__Chlamydiia.o__Chlamy
50000
40000
30000
20000
10000
0
alpha (n=25)
clade20C (n=1)
delta (n=13)
omicron (n=6)
neg (n=72)
NA
WHOvariant

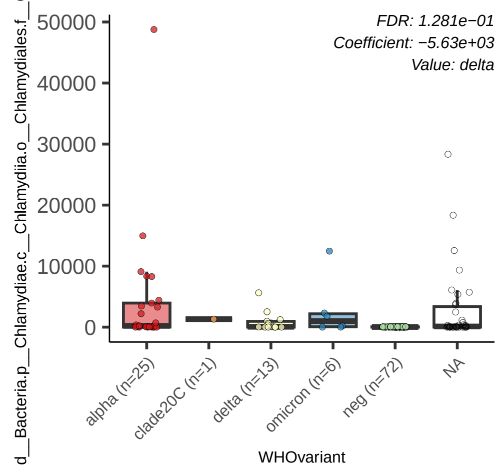

FDR: 1.281e−01
Coefficient: −5.63e+03
Value: delta
d__Bacteria.p__Chlamydiae.c__Chlamydiia.o__Chlamydiales.f__
50000
40000
30000
20000
10000
0
alpha (n=25)
clade20C (n=1)
delta (n=13)
omicron (n=6)
neg (n=72)
NA
WHOvariant

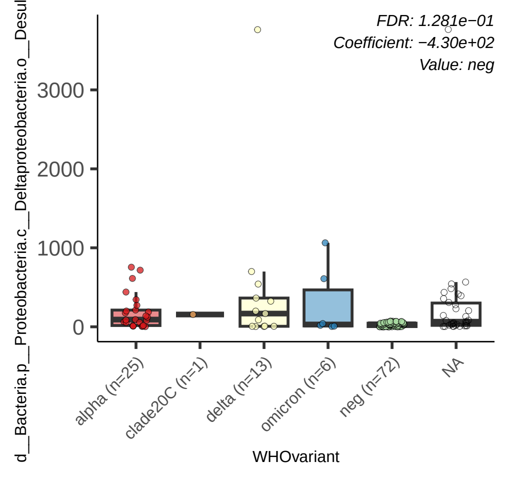

FDR: 1.281e−01
Coefficient: −4.30e+02
Value: neg
3000
2000
1000
0
d__Bacteria.p__Proteobacteria.c__Deltaproteobacteria.o__Desul
alpha (n=25)
clade20C (n=1)
delta (n=13)
omicron (n=6)
neg (n=72)
NA
WHOvariant

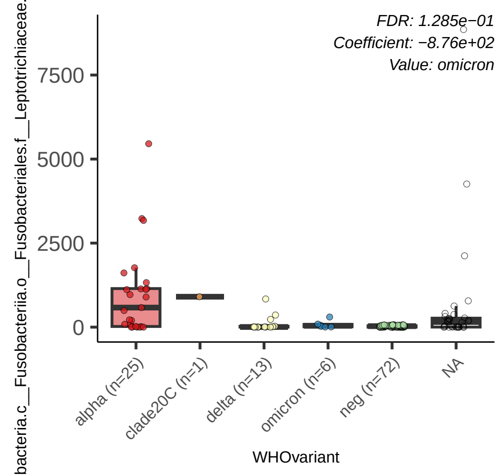

FDR: 1.285e−01
Coefficient: −8.76e+02
Value: omicron
obacteria.c__Fusobacteriia.o__Fusobacteriales.f__Leptotrichiaceae.
7500
5000
2500
0
alpha (n=25)
clade20C (n=1)
delta (n=13)
omicron (n=6)
neg (n=72)
NA
WHOvariant

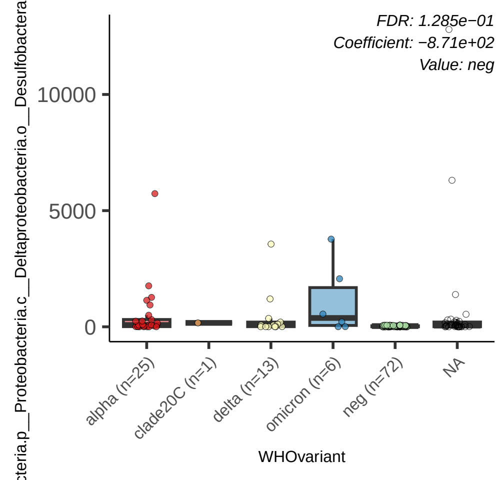
FDR: 1.285e−01
Coefficient: −8.71e+02
Value: neg
10000
5000
0
cteria.p__Proteobacteria.c__Deltaproteobacteria.o__Desulfobactera
alpha (n=25)
clade20C (n=1)
delta (n=13)
omicron (n=6)
neg (n=72)
NA
WHOvariant

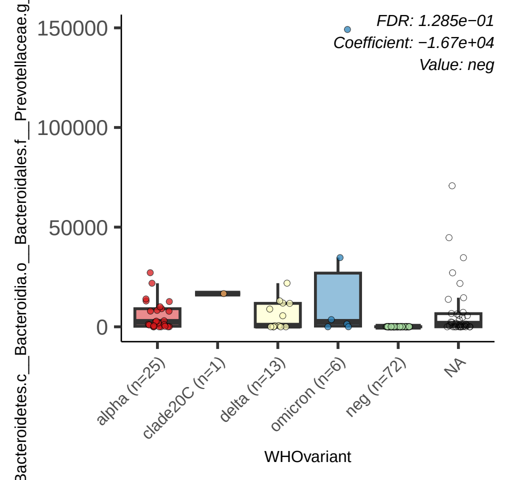

FDR: 1.285e−01
Coefficient: −1.67e+04
Value: neg
Bacteroidetes.c__Bacteroidia.o__Bacteroidales.f__Prevotellaceae.g_
150000
100000
50000
0
alpha (n=25)
clade20C (n=1)
delta (n=13)
omicron (n=6)
neg (n=72)
NA
WHOvariant

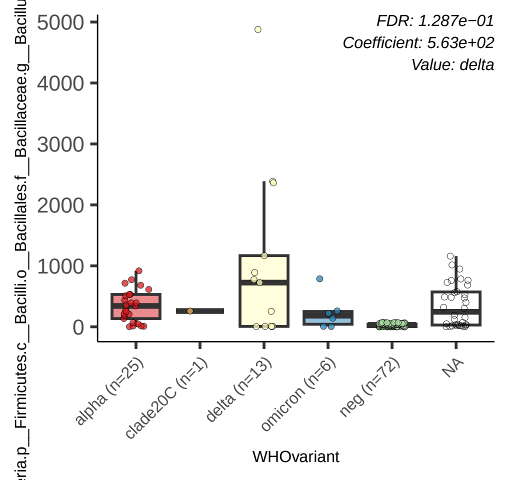

FDR: 1.287e−01
Coefficient: 5.63e+02
Value: delta
5000
4000
3000
2000
1000
0
alpha (n=25)
clade20C (n=1)
delta (n=13)
omicron (n=6)
neg (n=72)
NA
WHOvariant
eria.p__Firmicutes.c__Bacilli.o__Bacillales.f__Bacillaceae.g__Bacillu

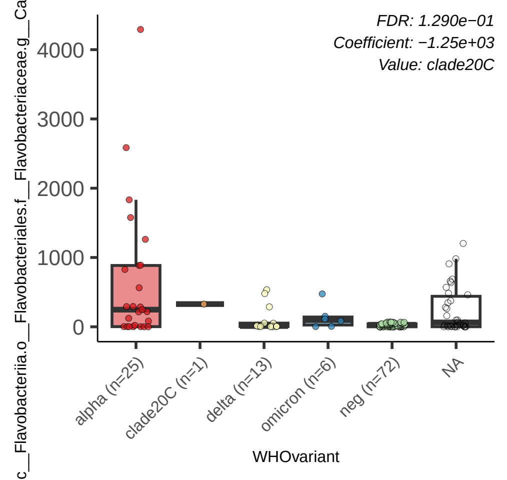

FDR: 1.290e−01
Coefficient: −1.25e+03
Value: clade20C
c__Flavobacteriia.o__Flavobacteriales.f__Flavobacteriaceae.g__Ca
4000
3000
2000
1000
0
alpha (n=25)
clade20C (n=1)
delta (n=13)
omicron (n=6)
neg (n=72)
NA
WHOvariant

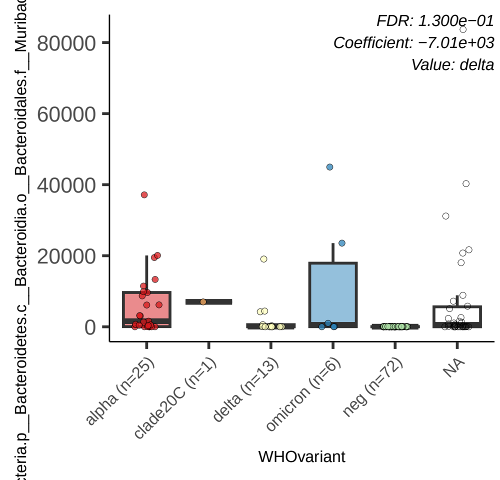

FDR: 1.300e−01
Coefficient: −7.01e+03
Value: delta
80000
60000
40000
20000
0
acteria.p__Bacteroidetes.c__Bacteroidia.o__Bacteroidales.f__Muribac
alpha (n=25)
clade20C (n=1)
delta (n=13)
omicron (n=6)
neg (n=72)
NA
WHOvariant

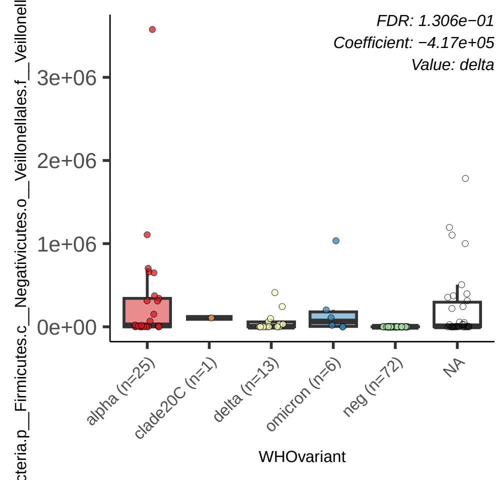

FDR: 1.306e−01
Coefficient: −4.17e+05
Value: delta
0e+00
1e+06
2e+06
3e+06
cteria.p__Firmicutes.c__Negativicutes.o__Veillonellales.f__Veillonell
alpha (n=25)
clade20C (n=1)
delta (n=13)
omicron (n=6)
neg (n=72)
NA
WHOvariant

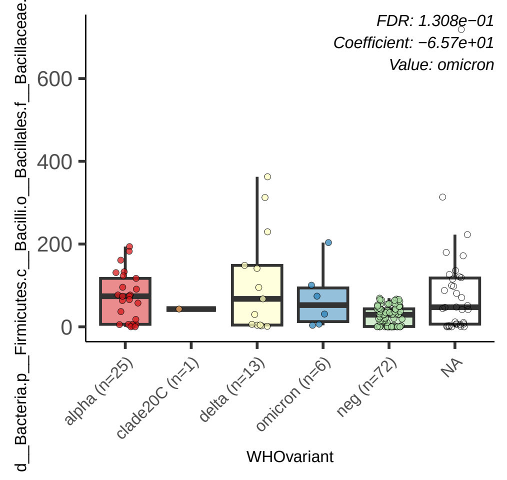

FDR: 1.308e−01
Coefficient: −6.57e+01
Value: omicron
d__Bacteria.p__Firmicutes.c__Bacilli.o__Bacillales.f__Bacillaceae.
0
200
400
600
alpha (n=25)
clade20C (n=1)
delta (n=13)
omicron (n=6)
neg (n=72)
NA
WHOvariant

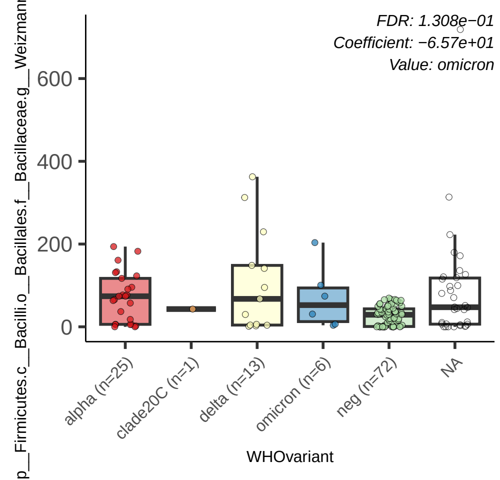

FDR: 1.308e−01
Coefficient: −6.57e+01
Value: omicron
p__Firmicutes.c__Bacilli.o__Bacillales.f__Bacillaceae.g__Weizmann
0
200
400
600
alpha (n=25)
clade20C (n=1)
delta (n=13)
omicron (n=6)
neg (n=72)
NA
WHOvariant

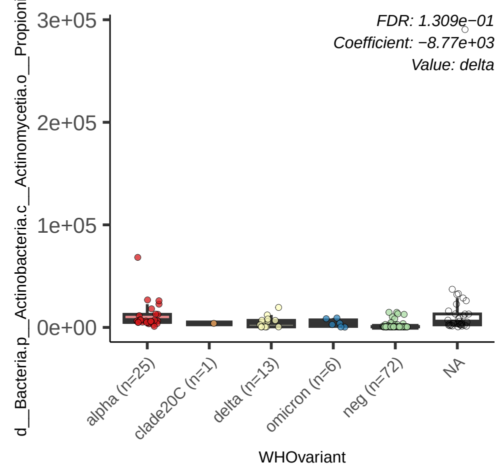

FDR: 1.309e−01
Coefficient: −8.77e+03
Value: delta
3e+05
2e+05
1e+05
0e+00
d__Bacteria.p__Actinobacteria.c__Actinomycetia.o__Propioni
alpha (n=25)
clade20C (n=1)
delta (n=13)
omicron (n=6)
neg (n=72)
NA
WHOvariant

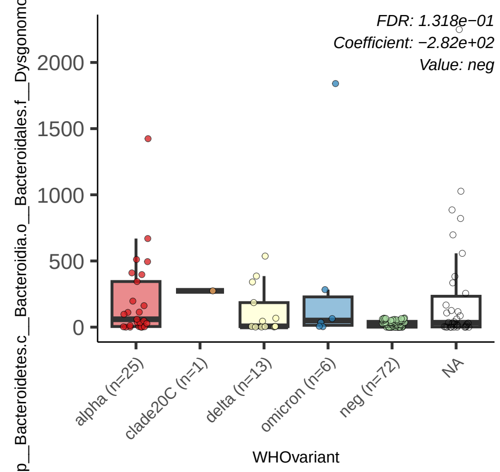

FDR: 1.318e−01
Coefficient: −2.82e+02
Value: neg
2000
1500
1000
500
0
alpha (n=25)
clade20C (n=1)
delta (n=13)
omicron (n=6)
neg (n=72)
NA
WHOvariant

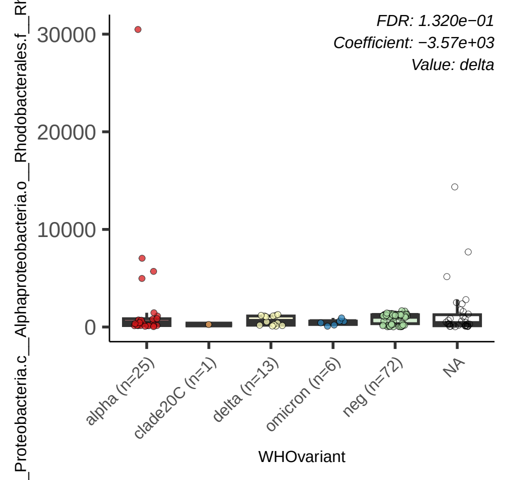

FDR: 1.320e−01
Coefficient: −3.57e+03
Value: delta
_Proteobacteria.c__Alphaproteobacteria.o__Rhodobacterales.f__Rh
30000
20000
10000
0
alpha (n=25)
clade20C (n=1)
delta (n=13)
omicron (n=6)
neg (n=72)
NA
WHOvariant

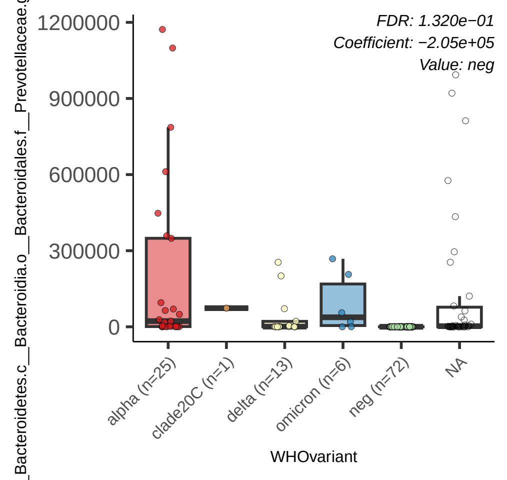

FDR: 1.320e−01
Coefficient: −2.05e+05
Value: neg
1200000
900000
600000
300000
0
Bacteroidetes.c__Bacteroidia.o__Bacteroidales.f__Prevotellaceae.g
alpha (n=25)
clade20C (n=1)
delta (n=13)
omicron (n=6)
neg (n=72)
NA
WHOvariant

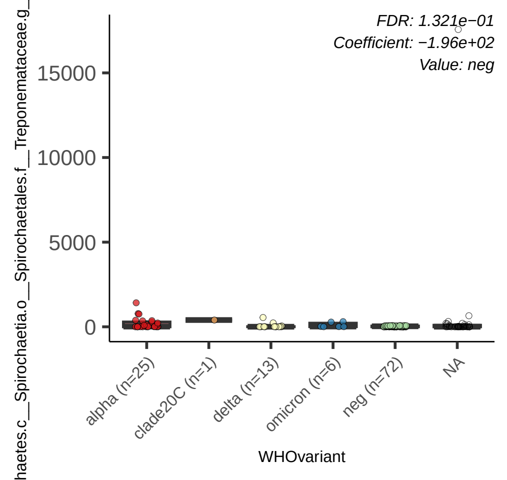
FDR: 1.321e−01
Coefficient: −1.96e+02
Value: neg
15000
10000
5000
0
alpha (n=25)
clade20C (n=1)
delta (n=13)
omicron (n=6)
neg (n=72)
NA
WHOvariant
haetes.c__Spirochaetia.o__Spirochaetales.f__Treponemataceae.g_

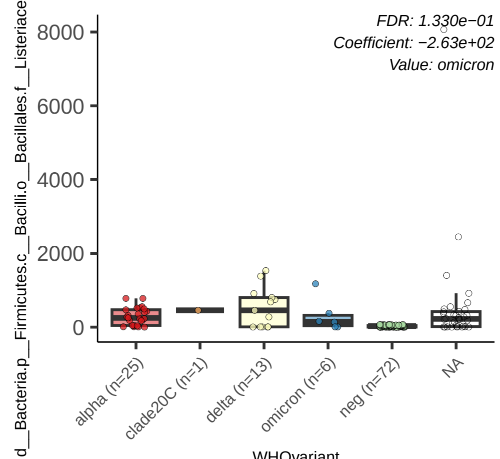

FDR: 1.330e−01
Coefficient: −2.63e+02
Value: omicron
d__Bacteria.p__Firmicutes.c__Bacilli.o__Bacillales.f__Listeriace
8000
6000
4000
2000
0
alpha (n=25)
clade20C (n=1)
delta (n=13)
omicron (n=6)
neg (n=72)
NA
WHOvariant

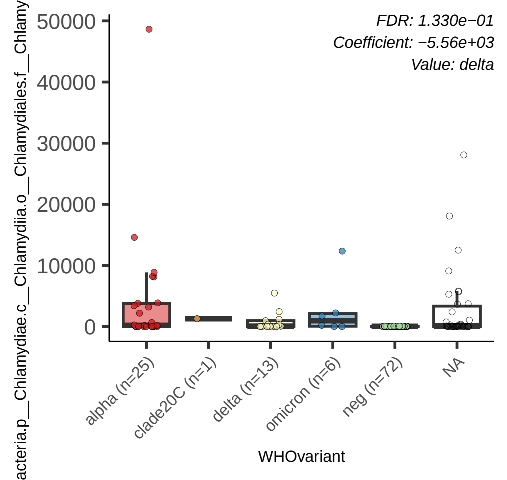

FDR: 1.330e−01
Coefficient: −5.56e+03
Value: delta
50000
40000
30000
20000
10000
0
acteria.p__Chlamydiae.c__Chlamydiia.o__Chlamydiales.f__Chlamy
alpha (n=25)
clade20C (n=1)
delta (n=13)
omicron (n=6)
neg (n=72)
NA
WHOvariant

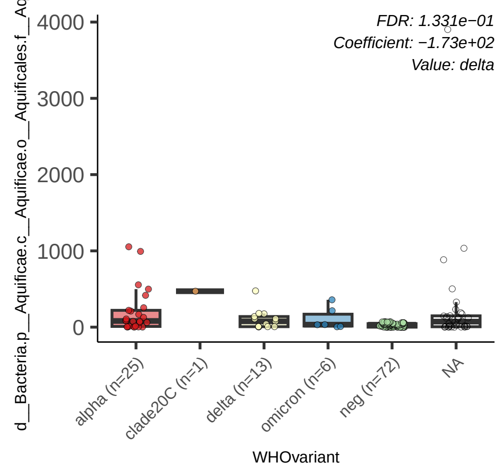

FDR: 1.331e−01
Coefficient: −1.73e+02
Value: delta
d__Bacteria.p__Aquificae.c__Aquificae.o__Aquificales.f__Aq
4000
3000
2000
1000
0
alpha (n=25)
clade20C (n=1)
delta (n=13)
omicron (n=6)
neg (n=72)
NA
WHOvariant

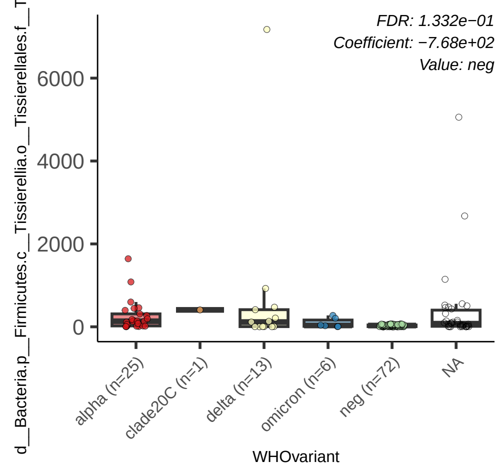

FDR: 1.332e−01
Coefficient: −7.68e+02
Value: neg
d__Bacteria.p__Firmicutes.c__Tissierellia.o__Tissierellales.f__T
6000
4000
2000
0
alpha (n=25)
clade20C (n=1)
delta (n=13)
omicron (n=6)
neg (n=72)
NA
WHOvariant

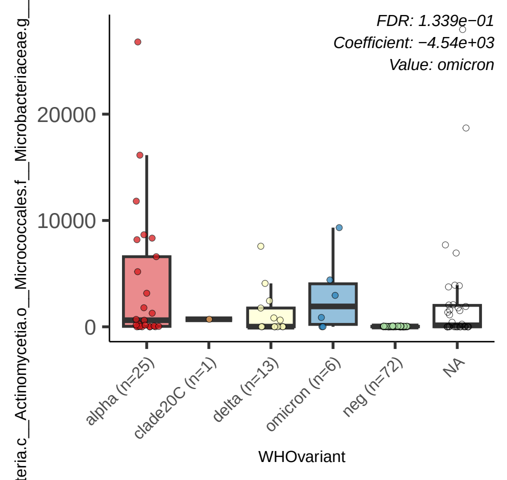

FDR: 1.339e−01
Coefficient: −4.54e+03
Value: omicron
teria.c__Actinomycetia.o__Micrococcales.f__Microbacteriaceae.g__
20000
10000
0
alpha (n=25)
clade20C (n=1)
delta (n=13)
omicron (n=6)
neg (n=72)
NA
WHOvariant

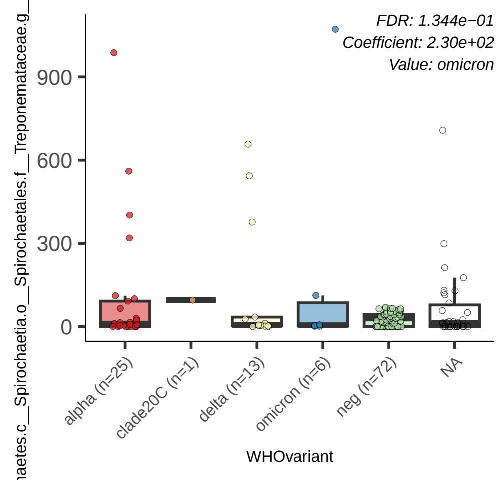
FDR: 1.344e−01
Coefficient: 2.30e+02
Value: omicron
haetes.c__Spirochaetia.o__Spirochaetales.f__Treponemataceae.g__
900
600
300
0
alpha (n=25)
clade20C (n=1)
delta (n=13)
omicron (n=6)
neg (n=72)
NA
WHOvariant

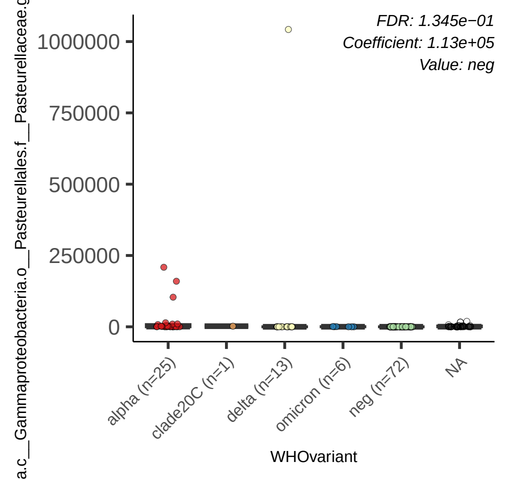
FDR: 1.345e−01
Coefficient: 1.13e+05
Value: neg
a.c__Gammaproteobacteria.o__Pasteurellales.f__Pasteurellaceae.g
1000000
750000
500000
250000
0
alpha (n=25)
clade20C (n=1)
delta (n=13)
omicron (n=6)
neg (n=72)
NA
WHOvariant

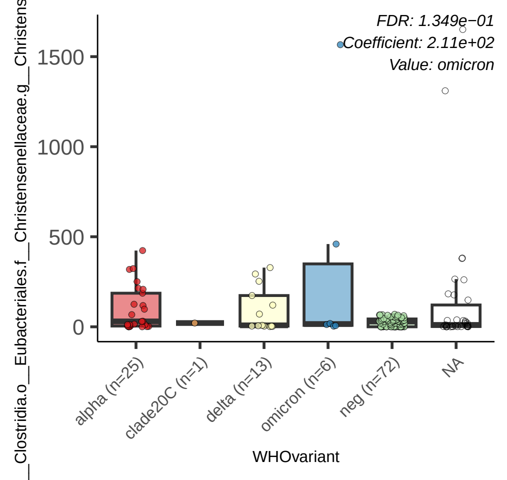

FDR: 1.349e−01
Coefficient: 2.11e+02
Value: omicron
__Clostridia.o__Eubacteriales.f__Christensenellaceae.g__Christens
1500
1000
500
0
alpha (n=25)
clade20C (n=1)
delta (n=13)
omicron (n=6)
neg (n=72)
NA
WHOvariant

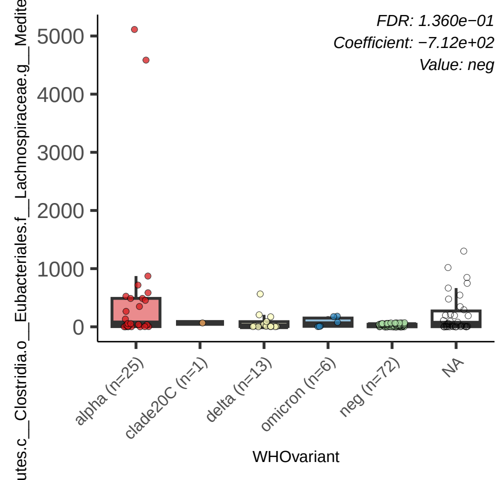

FDR: 1.360e−01
Coefficient: −7.12e+02
Value: neg
utes.c__Clostridia.o__Eubacteriales.f__Lachnospiraceae.g__Medite
5000
4000
3000
2000
1000
0
alpha (n=25)
clade20C (n=1)
delta (n=13)
omicron (n=6)
neg (n=72)
NA
WHOvariant

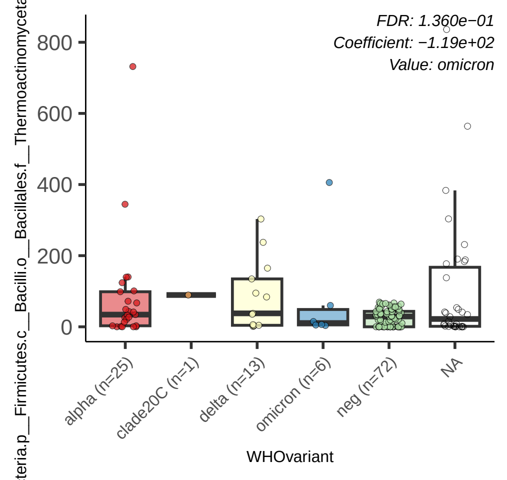
FDR: 1.360e−01
Coefficient: −1.19e+02
Value: omicron
0
200
400
600
800
alpha (n=25)
clade20C (n=1)
delta (n=13)
omicron (n=6)
neg (n=72)
NA
WHOvariant

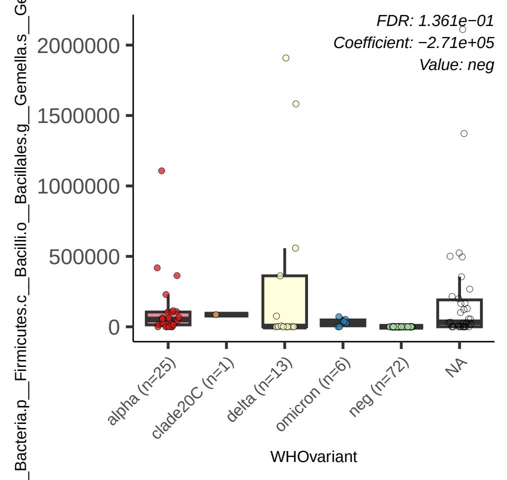

FDR: 1.361e−01
Coefficient: −2.71e+05
Value: neg
2000000
1500000
1000000
500000
0
alpha (n=25)
clade20C (n=1)
delta (n=13)
omicron (n=6)
neg (n=72)
NA
WHOvariant
Bacteria.p__Firmicutes.c__Bacilli.o__Bacillales.g__Gemella.s__Ge

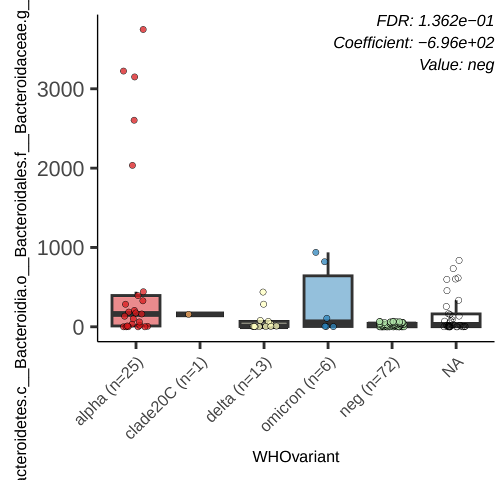

FDR: 1.362e−01
Coefficient: −6.96e+02
Value: neg
3000
2000
1000
0
acteroidetes.c__Bacteroidia.o__Bacteroidales.f__Bacteroidaceae.g_
alpha (n=25)
clade20C (n=1)
delta (n=13)
omicron (n=6)
neg (n=72)
NA
WHOvariant

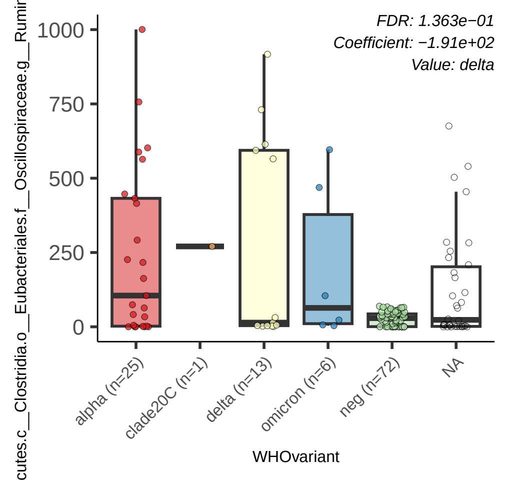

FDR: 1.363e−01
Coefficient: −1.91e+02
Value: delta
cutes.c__Clostridia.o__Eubacteriales.f__Oscillospiraceae.g__Rumi
1000
750
500
250
0
alpha (n=25)
clade20C (n=1)
delta (n=13)
omicron (n=6)
neg (n=72)
NA
WHOvariant

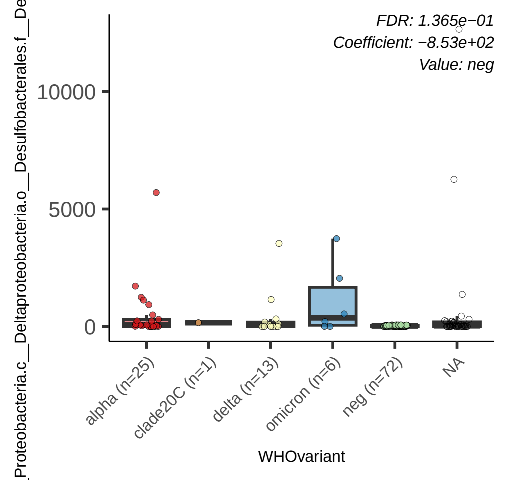

FDR: 1.365e−01
Coefficient: −8.53e+02
Value: neg
10000
5000
0
Proteobacteria.c__Deltaproteobacteria.o__Desulfobacterales.f__De
alpha (n=25)
clade20C (n=1)
delta (n=13)
omicron (n=6)
neg (n=72)
NA
WHOvariant

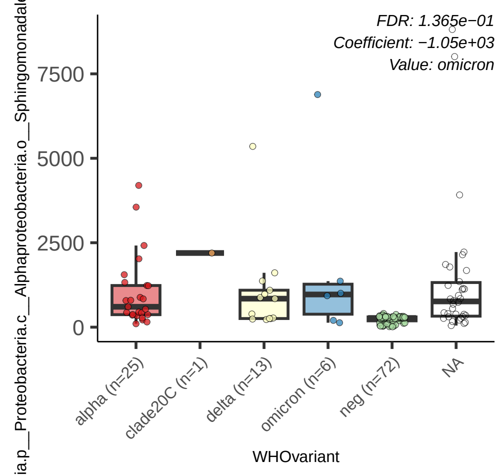

FDR: 1.365e−01
Coefficient: −1.05e+03
Value: omicron
ria.p__Proteobacteria.c__Alphaproteobacteria.o__Sphingomonadale
7500
5000
2500
0
alpha (n=25)
clade20C (n=1)
delta (n=13)
omicron (n=6)
neg (n=72)
NA
WHOvariant

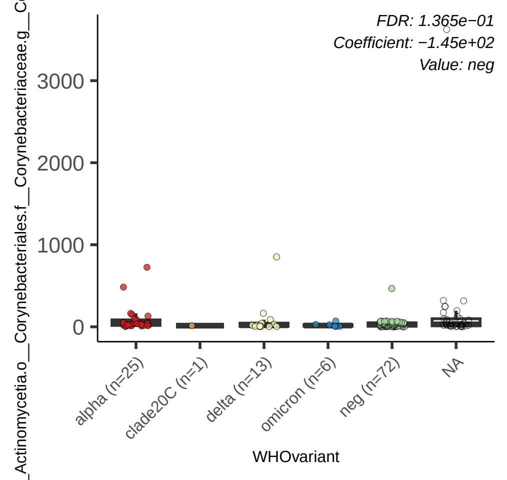
FDR: 1.365e−01
Coefficient: −1.45e+02
Value: neg
Actinomycetia.o__Corynebacteriales.f__Corynebacteriaceae.g__Co
3000
2000
1000
0
alpha (n=25)
clade20C (n=1)
delta (n=13)
omicron (n=6)
neg (n=72)
NA
WHOvariant

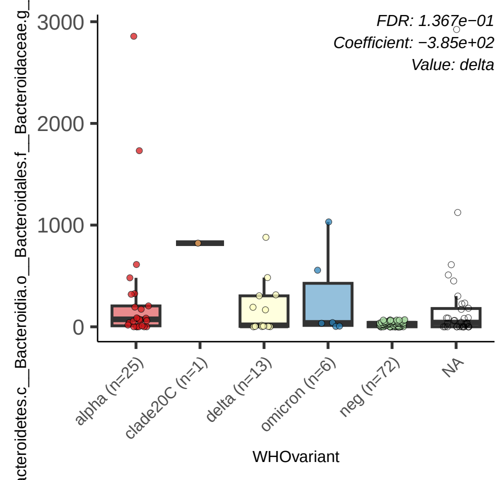

FDR: 1.367e−01
Coefficient: −3.85e+02
Value: delta
acteroidetes.c__Bacteroidia.o__Bacteroidales.f__Bacteroidaceae.g__
3000
2000
1000
0
alpha (n=25)
clade20C (n=1)
delta (n=13)
omicron (n=6)
neg (n=72)
NA
WHOvariant

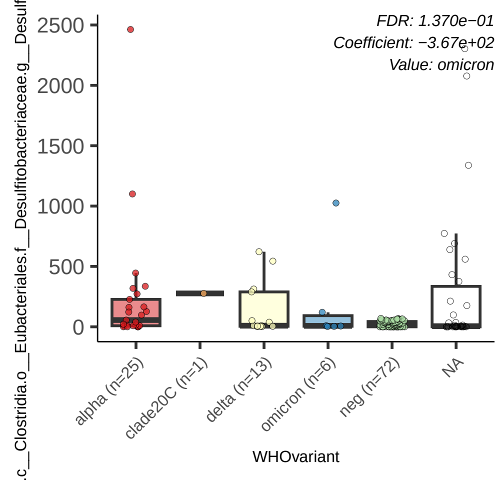

FDR: 1.370e−01
Coefficient: −3.67e+02
Value: omicron
c__Clostridia.o__Eubacteriales.f__Desulfitobacteriaceae.g__Desulf
2500
2000
1500
1000
500
0
alpha (n=25)
clade20C (n=1)
delta (n=13)
omicron (n=6)
neg (n=72)
NA
WHOvariant

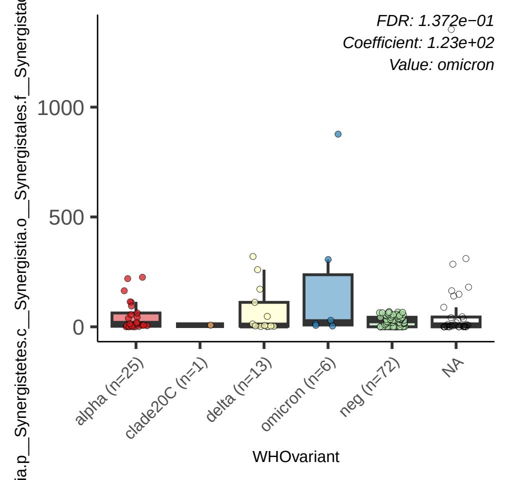

FDR: 1.372e−01
Coefficient: 1.23e+02
Value: omicron
1000
500
0
alpha (n=25)
clade20C (n=1)
delta (n=13)
omicron (n=6)
neg (n=72)
NA
WHOvariant
ia.p__Synergistetes.c__Synergistia.o__Synergistales.f__Synergistac

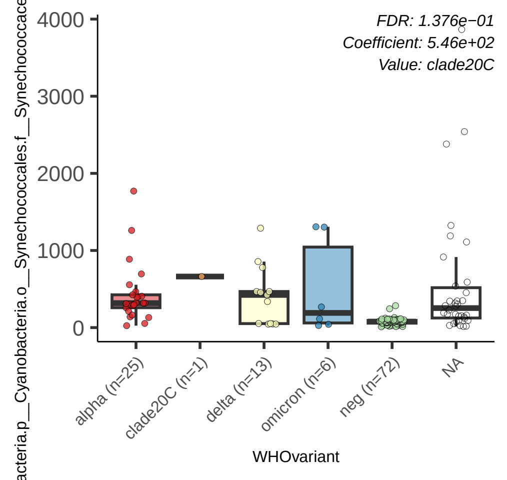
FDR: 1.376e−01
Coefficient: 5.46e+02
Value: clade20C
4000
3000
2000
1000
0
bacteria.p__Cyanobacteria.o__Synechococcales.f__Synechococcace
alpha (n=25)
clade20C (n=1)
delta (n=13)
omicron (n=6)
neg (n=72)
NA
WHOvariant

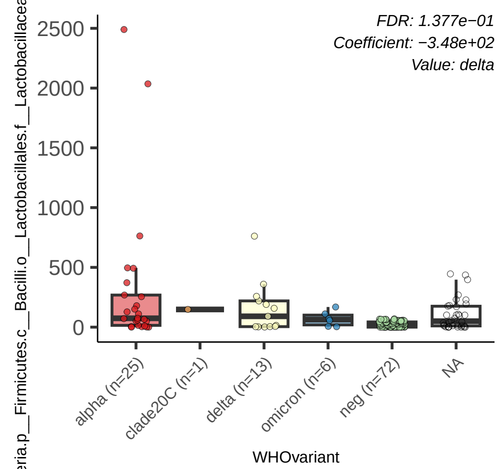
FDR: 1.377e−01
Coefficient: −3.48e+02
Value: delta
2500
2000
1500
1000
500
0
cteria.p__Firmicutes.c__Bacilli.o__Lactobacillales.f__Lactobacillacea
alpha (n=25)
clade20C (n=1)
delta (n=13)
omicron (n=6)
neg (n=72)
NA
WHOvariant

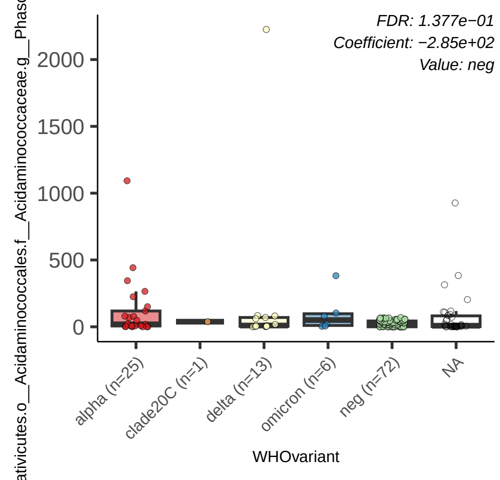

FDR: 1.377e−01
Coefficient: −2.85e+02
Value: neg
0
500
1000
1500
2000
atìvicutes.o__Acidaminococcales.f__Acidaminococcaceae.g__Phasc
alpha (n=25)
clade20C (n=1)
delta (n=13)
omicron (n=6)
neg (n=72)
NA
WHOvariant

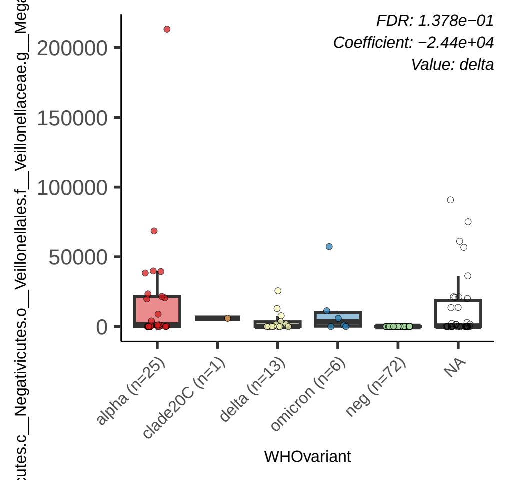

FDR: 1.378e−01
Coefficient: −2.44e+04
Value: delta
cutes.c__Negativicutes.o__Veillonellales.f__Veillonellaceae.g__Mega
200000
150000
100000
50000
0
alpha (n=25)
clade20C (n=1)
delta (n=13)
omicron (n=6)
neg (n=72)
NA
WHOvariant

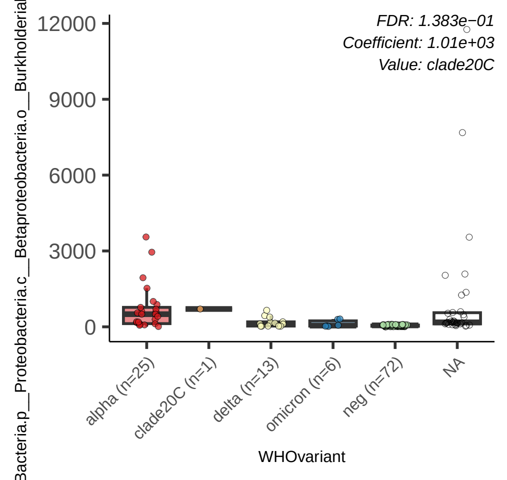

FDR: 1.383e−01
Coefficient: 1.01e+03
Value: clade20C
12000
9000
6000
3000
0
Bacteria.p__Proteobacteria.c__Betaproteobacteria.o__Burkholderial
alpha (n=25)
clade20C (n=1)
delta (n=13)
omicron (n=6)
neg (n=72)
NA
WHOvariant

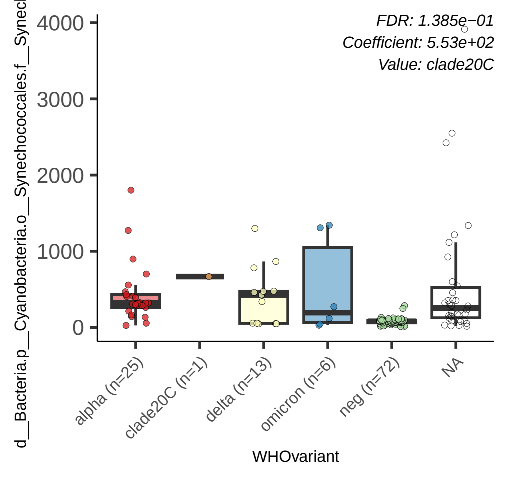

FDR: 1.385e−01
Coefficient: 5.53e+02
Value: clade20C
d__Bacteria.p__Cyanobacteria.o__Synechococcales.f__Synec
4000
3000
2000
1000
0
alpha (n=25)
clade20C (n=1)
delta (n=13)
omicron (n=6)
neg (n=72)
NA
WHOvariant

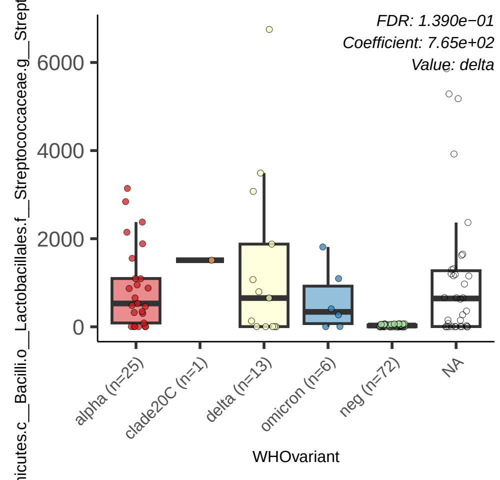
FDR: 1.390e−01
Coefficient: 7.65e+02
Value: delta
6000
4000
2000
0
alpha (n=25)
clade20C (n=1)
delta (n=13)
omicron (n=6)
neg (n=72)
NA
WHOvariant

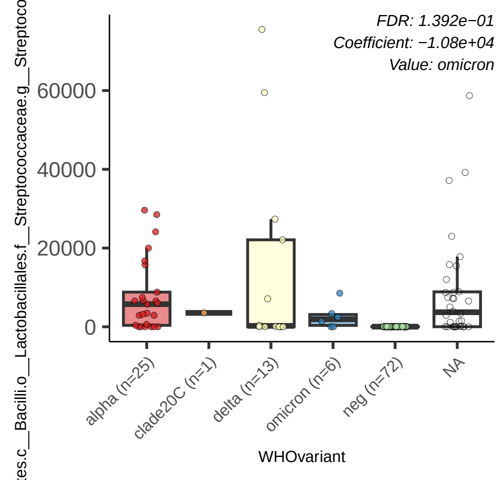
FDR: 1.392e−01
Coefficient: −1.08e+04
Value: omicron
tes.c__Bacilli.o__Lactobacillales.f__Streptococcaceae.g__Streptoco
60000
40000
20000
0
alpha (n=25)
clade20C (n=1)
delta (n=13)
omicron (n=6)
neg (n=72)
NA
WHOvariant

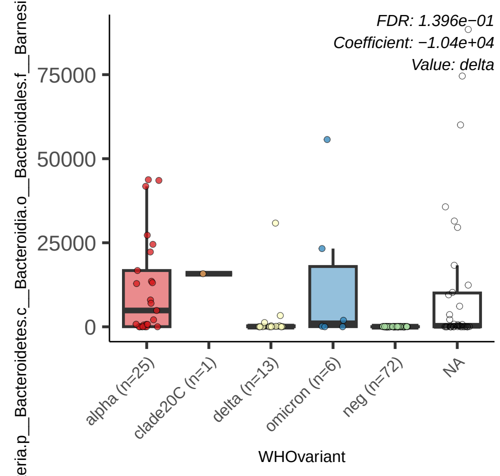
FDR: 1.396e−01
Coefficient: −1.04e+04
Value: delta
75000
50000
25000
0
teria.p__Bacteroidetes.c__Bacteroidia.o__Bacteroidales.f__Barnesi
alpha (n=25)
clade20C (n=1)
delta (n=13)
omicron (n=6)
neg (n=72)
NA
WHOvariant

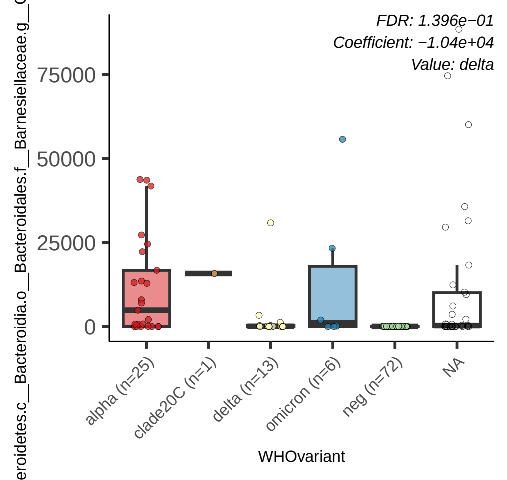

FDR: 1.396e−01
Coefficient: −1.04e+04
Value: delta
eroidetes.c__Bacteroidia.o__Bacteroidales.f__Barnesiellaceae.g__C
75000
50000
25000
0
alpha (n=25)
clade20C (n=1)
delta (n=13)
omicron (n=6)
neg (n=72)
NA
WHOvariant

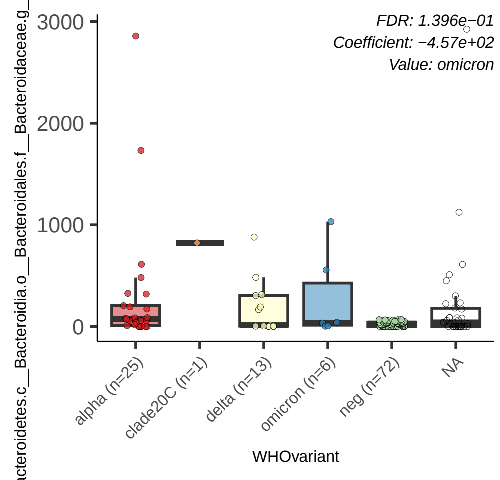
FDR: 1.396e−01
Coefficient: −4.57e+02
Value: omicron
acteroidetes.c__Bacteroidia.o__Bacteroidales.f__Bacteroidaceae.g__
3000
2000
1000
0
alpha (n=25)
clade20C (n=1)
delta (n=13)
omicron (n=6)
neg (n=72)
NA
WHOvariant

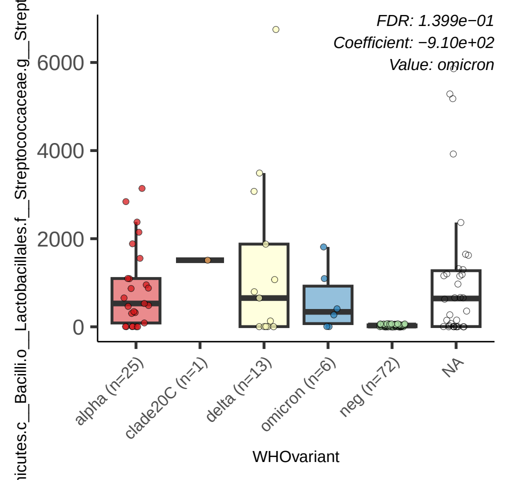

FDR: 1.399e−01
Coefficient: −9.10e+02
Value: omicron
0
2000
4000
6000
nicutes.c__Bacilli.o__Lactobacillales.f__Streptococcaceae.g__Strept
alpha (n=25)
clade20C (n=1)
delta (n=13)
omicron (n=6)
neg (n=72)
NA
WHOvariant

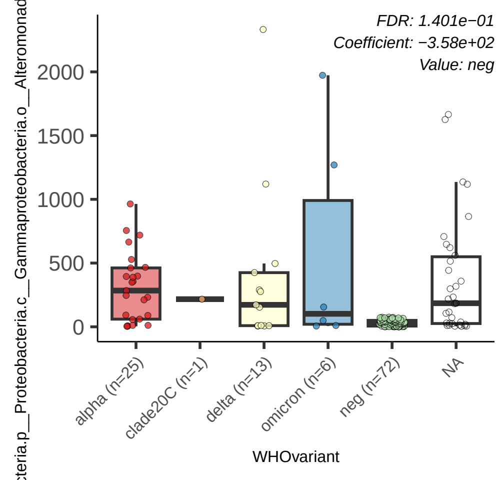
FDR: 1.401e−01
Coefficient: −3.58e+02
Value: neg
cteria.p__Proteobacteria.c__Gammaproteobacteria.o__Alteromonad
0
500
1000
1500
2000
alpha (n=25)
clade20C (n=1)
delta (n=13)
omicron (n=6)
neg (n=72)
NA
WHOvariant

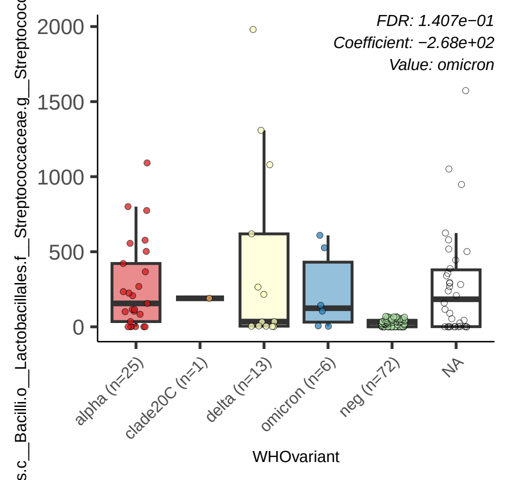

FDR: 1.407e−01
Coefficient: −2.68e+02
Value: omicron
s.c__Bacilli.o__Lactobacillales.f__Streptococcaceae.g__Streptococc
2000
1500
1000
500
0
alpha (n=25)
clade20C (n=1)
delta (n=13)
omicron (n=6)
neg (n=72)
NA
WHOvariant

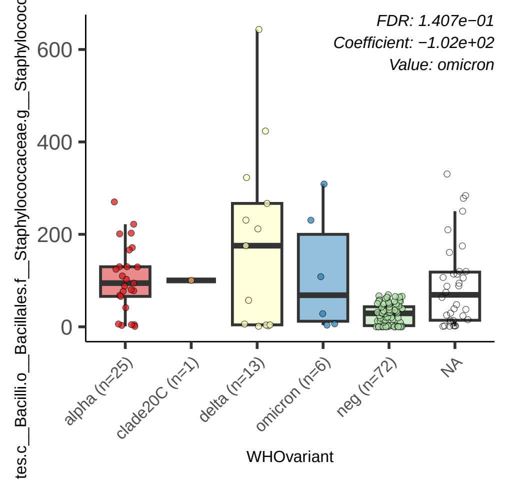
FDR: 1.407e−01
Coefficient: −1.02e+02
Value: omicron
600
400
200
0
alpha (n=25)
clade20C (n=1)
delta (n=13)
omicron (n=6)
neg (n=72)
NA
WHOvariant
tes.c__Bacilli.o__Bacillales.f__Staphylococcaceae.g__Staphylococc

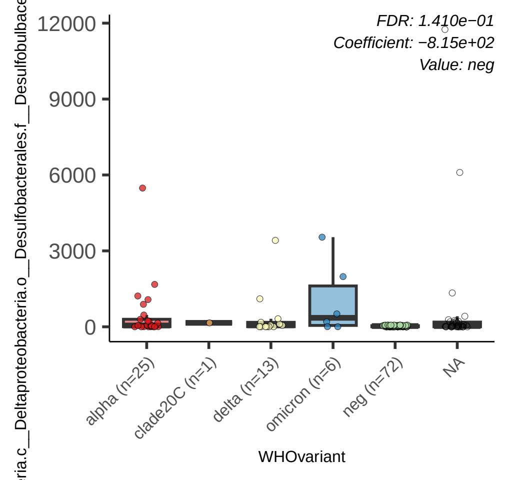

FDR: 1.410e−01
Coefficient: −8.15e+02
Value: neg
12000
9000
6000
3000
0
alpha (n=25)
clade20C (n=1)
delta (n=13)
omicron (n=6)
neg (n=72)
NA
WHOvariant
ria.c__Deltaproteobacteria.o__Desulfobacterales.f__Desulfobulbace

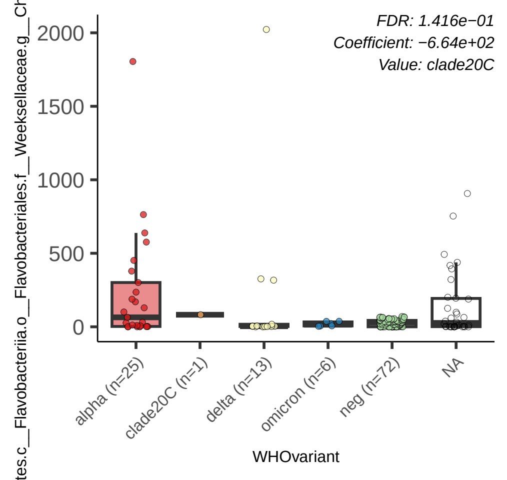
FDR: 1.416e−01
Coefficient: −6.64e+02
Value: clade20C
tes.c__Flavobacteriia.o__Flavobacteriales.f__Weeksellaceae.g__Ch
2000
1500
1000
500
0
alpha (n=25)
clade20C (n=1)
delta (n=13)
omicron (n=6)
neg (n=72)
NA
WHOvariant

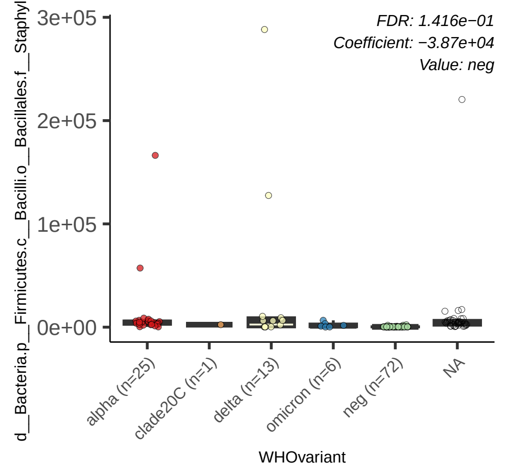

d__Bacteria.p__Firmicutes.c__Bacilli.o__Bacillales.f__Staphyl
3e+05
2e+05
1e+05
0e+00
FDR: 1.416e−01
Coefficient: −3.87e+04
Value: neg
alpha (n=25)
clade20C (n=1)
delta (n=13)
omicron (n=6)
neg (n=72)
NA
WHOvariant

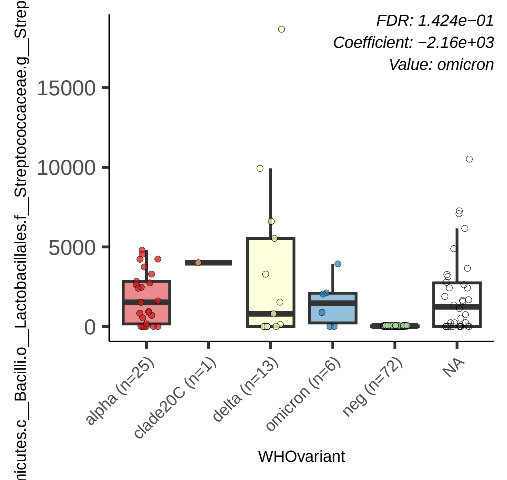

FDR: 1.424e−01
Coefficient: −2.16e+03
Value: omicron
15000
10000
5000
0
alpha (n=25)
clade20C (n=1)
delta (n=13)
omicron (n=6)
neg (n=72)
NA
WHOvariant
nicutes.c__Bacilli.o__Lactobacillales.f__Streptococcaceae.g__Strep

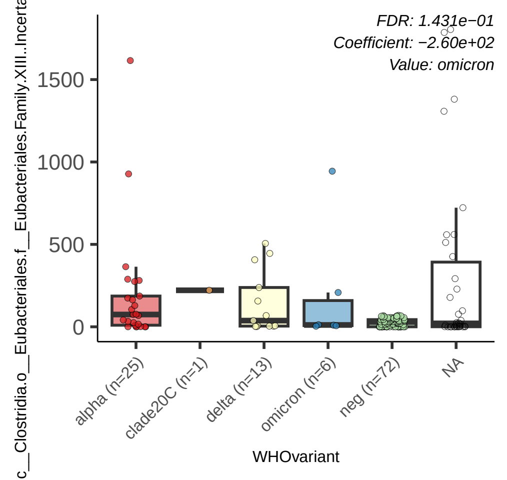

FDR: 1.431e−01
Coefficient: −2.60e+02
Value: omicron
c__Clostridia.o__Eubacteriales.f__Eubacteriales.Family.XIII..Incerta
1500
1000
500
0
alpha (n=25)
clade20C (n=1)
delta (n=13)
omicron (n=6)
neg (n=72)
NA
WHOvariant

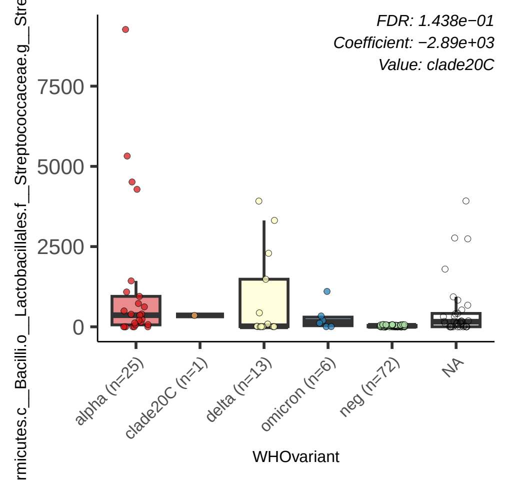

FDR: 1.438e−01
Coefficient: −2.89e+03
Value: clade20C
7500
5000
2500
0
rmicutes.c__Bacilli.o__Lactobacillales.f__Streptococcaceae.g__Stre
alpha (n=25)
clade20C (n=1)
delta (n=13)
omicron (n=6)
neg (n=72)
NA
WHOvariant

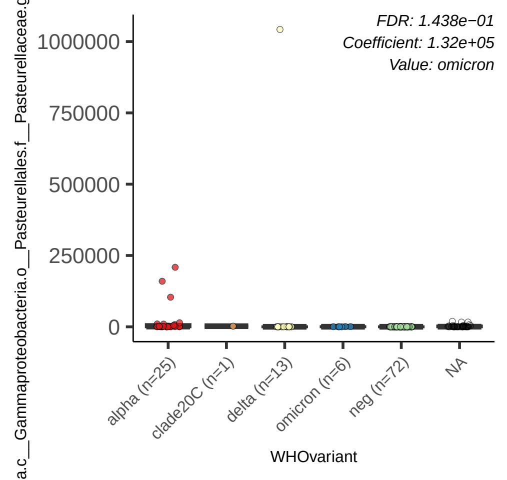

FDR: 1.438e−01
Coefficient: 1.32e+05
Value: omicron
a.c__Gammaproteobacteria.o__Pasteurellales.f__Pasteurellaceae.g
1000000
750000
500000
250000
0
alpha (n=25)
clade20C (n=1)
delta (n=13)
omicron (n=6)
neg (n=72)
NA
WHOvariant

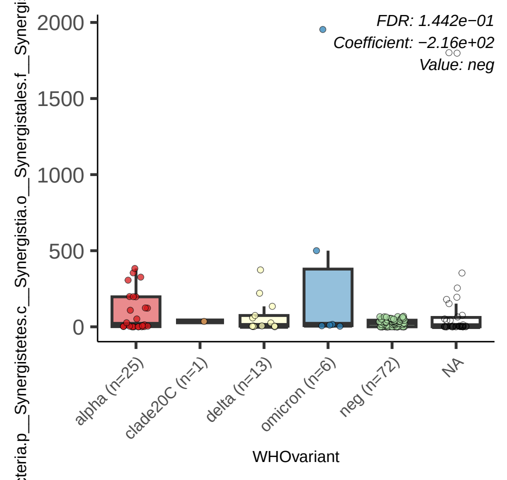
FDR: 1.442e−01
Coefficient: −2.16e+02
Value: neg
2000
1500
1000
500
0
alpha (n=25)
clade20C (n=1)
delta (n=13)
omicron (n=6)
neg (n=72)
NA
WHOvariant
cteria.p__Synergistetes.c__Synergistia.o__Synergistales.f__Synergi

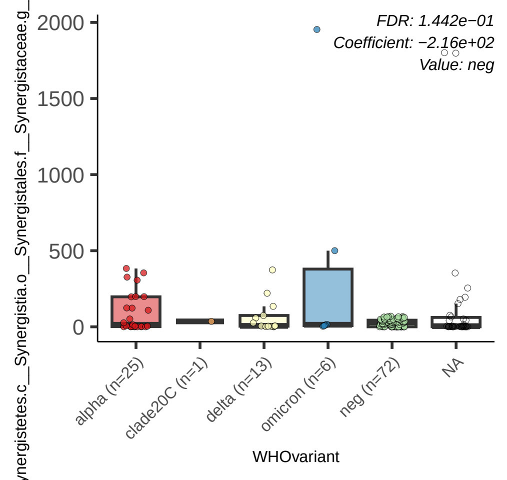

FDR: 1.442e−01
Coefficient: −2.16e+02
Value: neg
2000
1500
1000
500
0
Synergistetes.c__Synergistia.o__Synergistales.f__Synergistaceae.g__
alpha (n=25)
clade20C (n=1)
delta (n=13)
omicron (n=6)
neg (n=72)
NA
WHOvariant

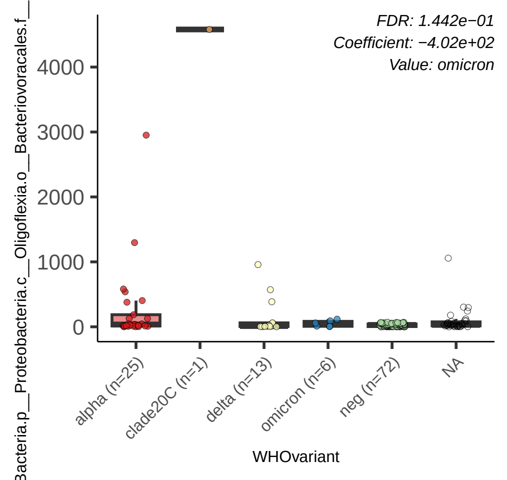
FDR: 1.442e−01
Coefficient: −4.02e+02
Value: omicron
Bacteria.p__Proteobacteria.c__Oligoflexia.o__Bacteriovoracales.f__
0
1000
2000
3000
4000
alpha (n=25)
clade20C (n=1)
delta (n=13)
omicron (n=6)
neg (n=72)
NA
WHOvariant

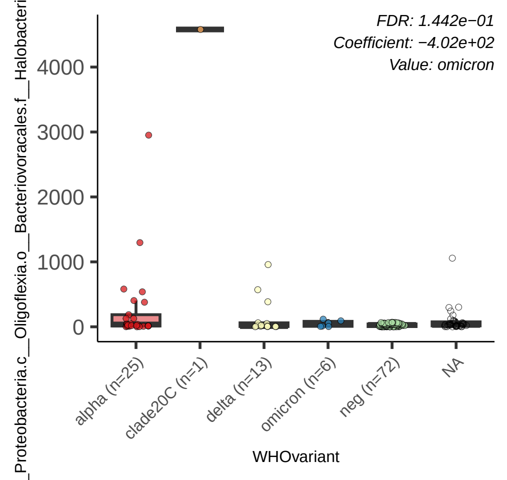
FDR: 1.442e−01
Coefficient: −4.02e+02
Value: omicron
Proteobacteria.c__Oligoflexia.o__Bacteriovoracales.f__Halobacteri
4000
3000
2000
1000
0
alpha (n=25)
clade20C (n=1)
delta (n=13)
omicron (n=6)
neg (n=72)
NA
WHOvariant

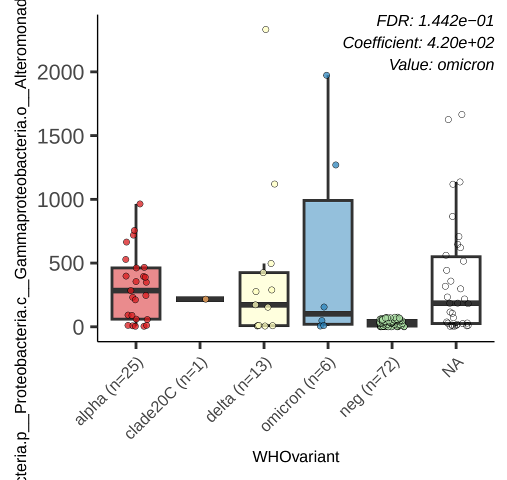
FDR: 1.442e−01
Coefficient: 4.20e+02
Value: omicron
cteria.p__Proteobacteria.c__Gammaproteobacteria.o__Alteromonad
2000
1500
1000
500
0
alpha (n=25)
clade20C (n=1)
delta (n=13)
omicron (n=6)
neg (n=72)
NA
WHOvariant

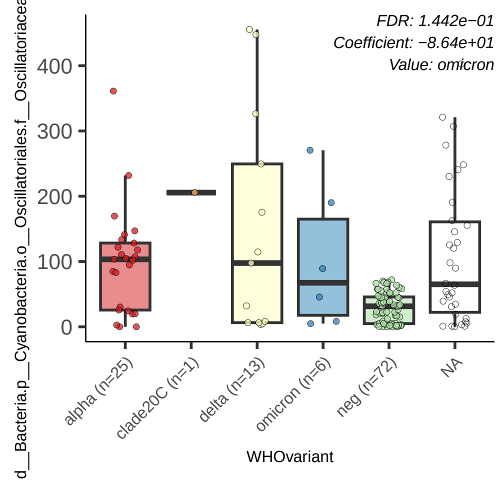

FDR: 1.442e−01
Coefficient: −8.64e+01
Value: omicron
d__Bacteria.p__Cyanobacteria.o__Oscillatoriales.f__Oscillatoriacea
0
100
200
300
400
alpha (n=25)
clade20C (n=1)
delta (n=13)
omicron (n=6)
neg (n=72)
NA
WHOvariant

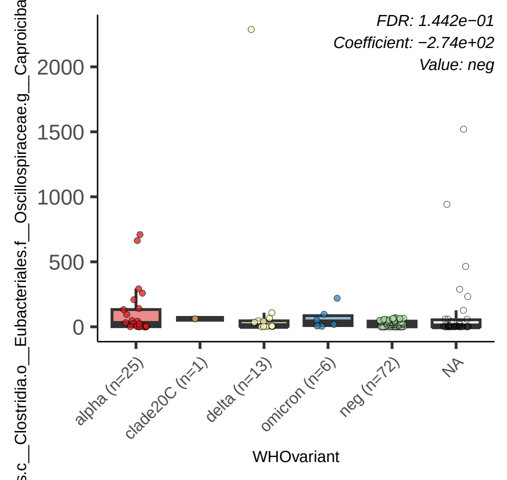

FDR: 1.442e−01
Coefficient: −2.74e+02
Value: neg
s.c__Clostridia.o__Eubacteriales.f__Oscillospiraceae.g__Caproiciba
2000
1500
1000
500
0
alpha (n=25)
clade20C (n=1)
delta (n=13)
omicron (n=6)
neg (n=72)
NA
WHOvariant

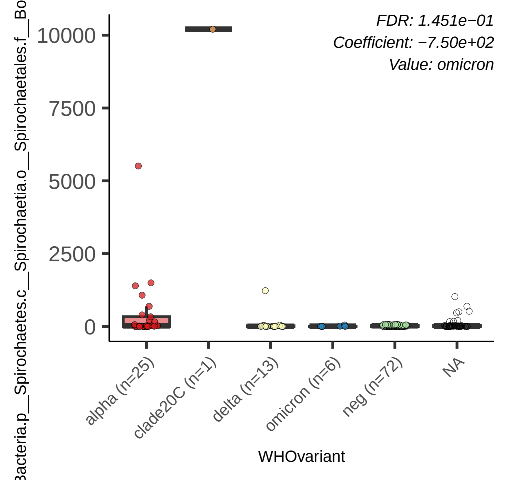
FDR: 1.451e−01
Coefficient: −7.50e+02
Value: omicron
10000
7500
5000
2500
0
alpha (n=25)
clade20C (n=1)
delta (n=13)
omicron (n=6)
neg (n=72)
NA
WHOvariant
Bacteria.p__Spirochaetes.c__Spirochaetia.o__Spirochaetales.f__Bo

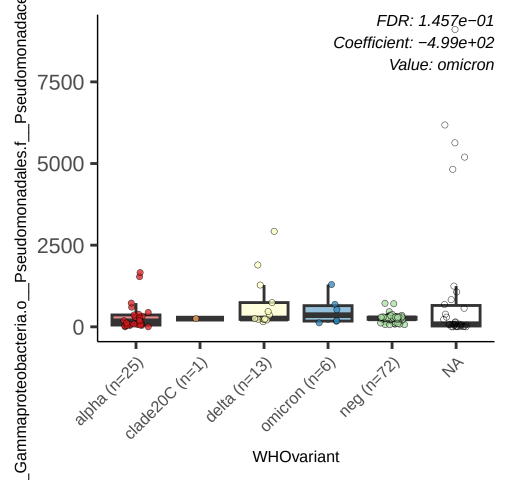

FDR: 1.457e−01
Coefficient: −4.99e+02
Value: omicron
7500
5000
2500
0
Gammaproteobacteria.o__Pseudomonadales.f__Pseudomonadace
alpha (n=25)
clade20C (n=1)
delta (n=13)
omicron (n=6)
neg (n=72)
NA
WHOvariant

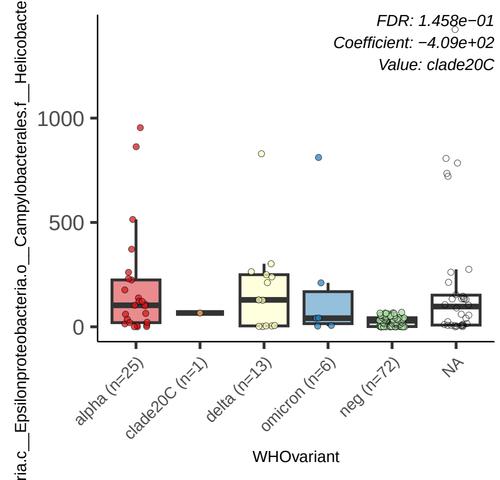

FDR: 1.458e−01
Coefficient: −4.09e+02
Value: clade20C
ria.c__Epsilonproteobacteria.o__Campylobacterales.f__Helicobacte
1000
500
0
alpha (n=25)
clade20C (n=1)
delta (n=13)
omicron (n=6)
neg (n=72)
NA
WHOvariant

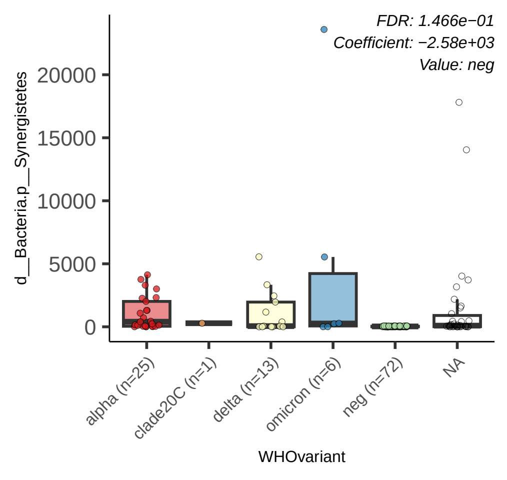
FDR: 1.466e−01
Coefficient: −2.58e+03
Value: neg
d__Bacteria.p__Synergistetes
20000
15000
10000
5000
0
alpha (n=25)
clade20C (n=1)
delta (n=13)
omicron (n=6)
neg (n=72)
NA
WHOvariant

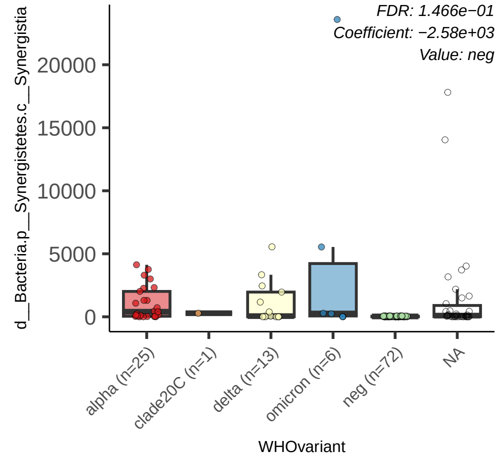
FDR: 1.466e−01
Coefficient: −2.58e+03
Value: neg
d__Bacteria.p__Synergistetes.c__Synergistia
20000
15000
10000
5000
0
alpha (n=25)
clade20C (n=1)
delta (n=13)
omicron (n=6)
neg (n=72)
NA
WHOvariant

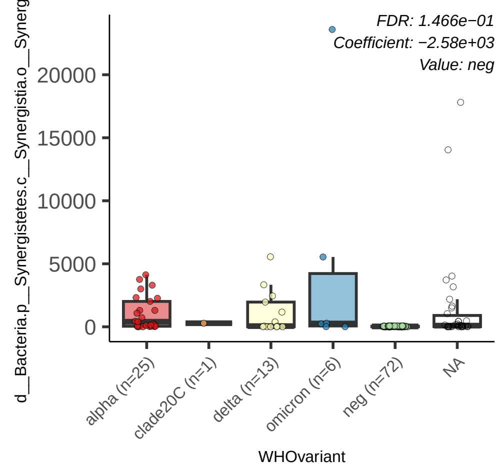

FDR: 1.466e−01
Coefficient: −2.58e+03
Value: neg
d__Bacteria.p__Synergistetes.c__Synergistia.o__Synerg
20000
15000
10000
5000
0
alpha (n=25)
clade20C (n=1)
delta (n=13)
omicron (n=6)
neg (n=72)
NA
WHOvariant

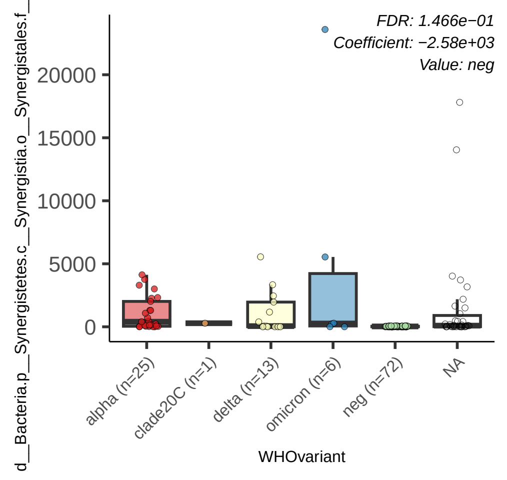

FDR: 1.466e−01
Coefficient: −2.58e+03
Value: neg
d__Bacteria.p__Synergistetes.c__Synergistia.o__Synergistales.f__
20000
15000
10000
5000
0
alpha (n=25)
clade20C (n=1)
delta (n=13)
omicron (n=6)
neg (n=72)
NA
WHOvariant

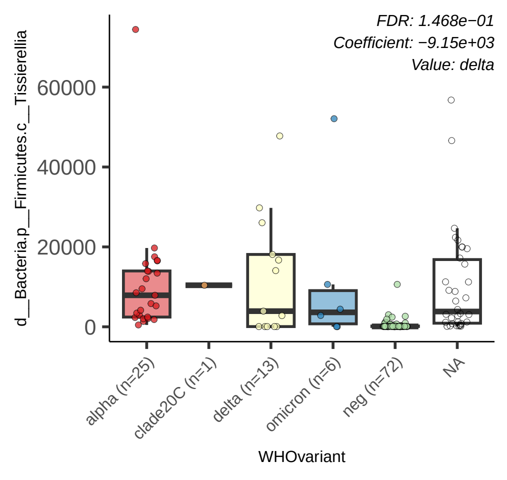

FDR: 1.468e−01
Coefficient: −9.15e+03
Value: delta
d__Bacteria.p__Firmicutes.c__Tissierellia
60000
40000
20000
0
alpha (n=25)
clade20C (n=1)
delta (n=13)
omicron (n=6)
neg (n=72)
NA
WHOvariant

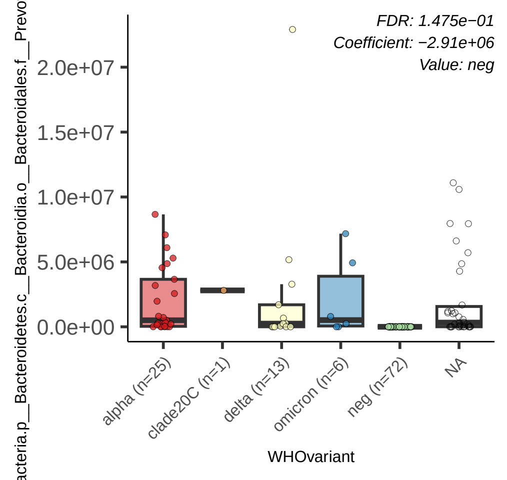

FDR: 1.475e−01
Coefficient: −2.91e+06
Value: neg
2.0e+07
1.5e+07
1.0e+07
5.0e+06
0.0e+00
acteria.p__Bacteroidetes.c__Bacteroidia.o__Bacteroidales.f__Prevo
alpha (n=25)
clade20C (n=1)
delta (n=13)
omicron (n=6)
neg (n=72)
NA
WHOvariant

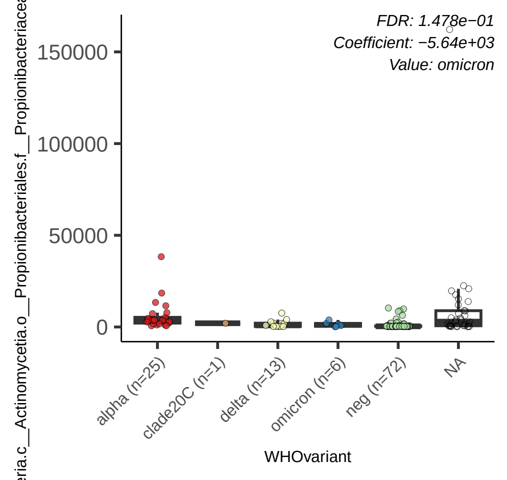
FDR: 1.478e−01
Coefficient: −5.64e+03
Value: omicron
150000
100000
50000
0
alpha (n=25)
clade20C (n=1)
delta (n=13)
omicron (n=6)
neg (n=72)
NA
WHOvariant
eria.c__Actinomycetia.o__Propionibacteriales.f__Propionibacteriacea

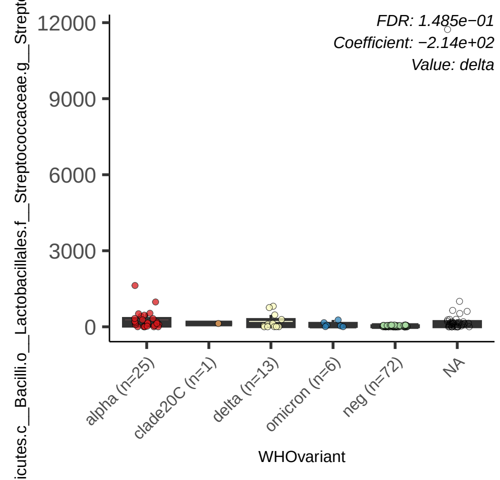

FDR: 1.485e−01
Coefficient: −2.14e+02
Value: delta
12000
9000
6000
3000
0
icutes.c__Bacilli.o__Lactobacillales.f__Streptococcaceae.g__Strept
alpha (n=25)
clade20C (n=1)
delta (n=13)
omicron (n=6)
neg (n=72)
NA
WHOvariant

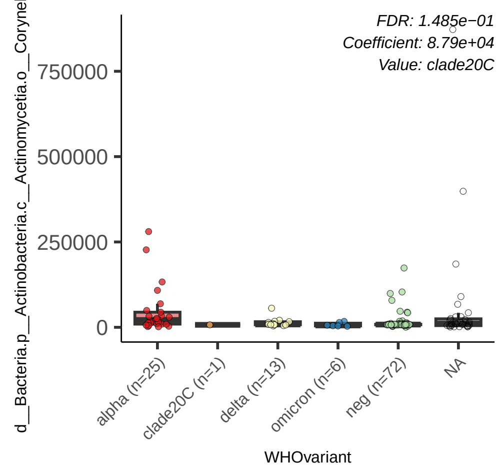

FDR: 1.485e−01
Coefficient: 8.79e+04
Value: clade20C
d__Bacteria.p__Actinobacteria.c__Actinomycetia.o__Coryne
750000
500000
250000
0
alpha (n=25)
clade20C (n=1)
delta (n=13)
omicron (n=6)
neg (n=72)
NA
WHOvariant

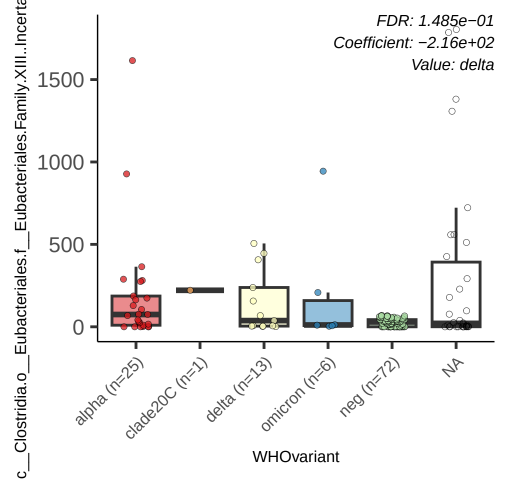
FDR: 1.485e−01
Coefficient: −2.16e+02
Value: delta
c__Clostridia.o__Eubacteriales.f__Eubacteriales.Family.XIII..Incerta
1500
1000
500
0
alpha (n=25)
clade20C (n=1)
delta (n=13)
omicron (n=6)
neg (n=72)
NA
WHOvariant

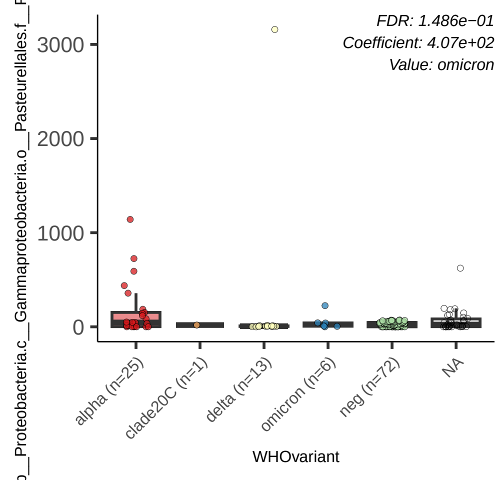

FDR: 1.486e−01
Coefficient: 4.07e+02
Value: omicron
3000
2000
1000
0
alpha (n=25)
clade20C (n=1)
delta (n=13)
omicron (n=6)
neg (n=72)
NA
WHOvariant
Proteobacteria.c__Gammaproteobacteria.o__Pasteurellales.f__P

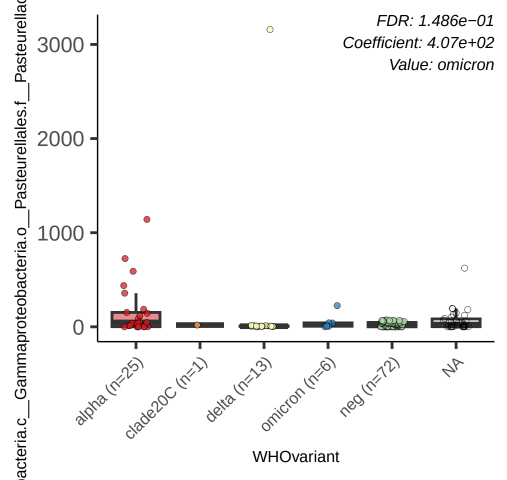
FDR: 1.486e−01
Coefficient: 4.07e+02
Value: omicron
3000
2000
1000
0
alpha (n=25)
clade20C (n=1)
delta (n=13)
omicron (n=6)
neg (n=72)
NA
WHOvariant
bacteria.c__Gammaproteobacteria.o__Pasteurellales.f__Pasteurellac

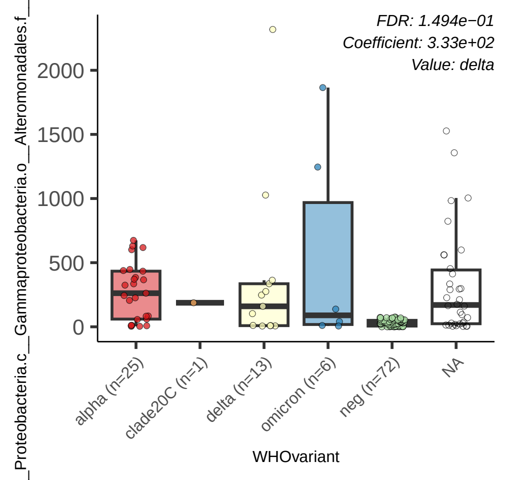

FDR: 1.494e−01
Coefficient: 3.33e+02
Value: delta
Proteobacteria.c__Gammaproteobacteria.o__Alteromonadales.f
2000
1500
1000
500
0
alpha (n=25)
clade20C (n=1)
delta (n=13)
omicron (n=6)
neg (n=72)
NA
WHOvariant

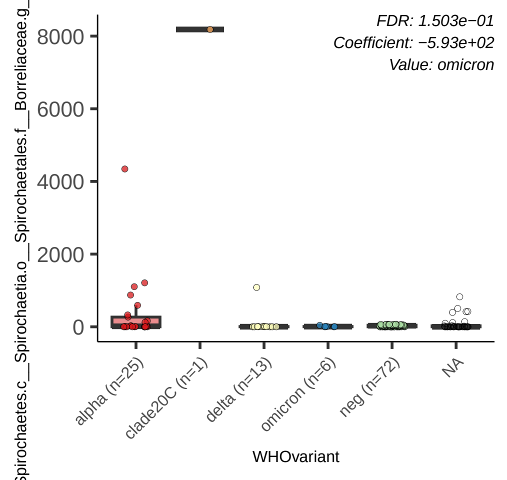
FDR: 1.503e−01
Coefficient: −5.93e+02
Value: omicron
Spirochaetes.c__Spirochaetia.o__Spirochaetales.f__Borreliaceae.g__
8000
6000
4000
2000
0
alpha (n=25)
clade20C (n=1)
delta (n=13)
omicron (n=6)
neg (n=72)
NA
WHOvariant

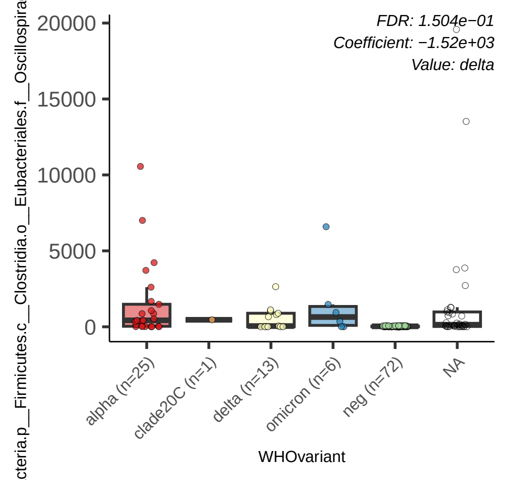

FDR: 1.504e−01
Coefficient: −1.52e+03
Value: delta
20000
15000
10000
5000
0
cteria.p__Firmicutes.c__Clostridia.o__Eubacteriales.f__Oscillospira
alpha (n=25)
clade20C (n=1)
delta (n=13)
omicron (n=6)
neg (n=72)
NA
WHOvariant

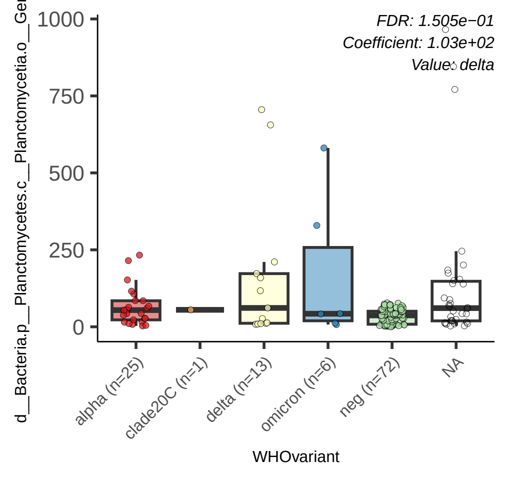

FDR: 1.505e−01
Coefficient: 1.03e+02
Value: delta
d__Bacteria.p__Planctomycetes.c__Planctomycetia.o__Gen
1000
750
500
250
0
alpha (n=25)
clade20C (n=1)
delta (n=13)
omicron (n=6)
neg (n=72)
NA
WHOvariant

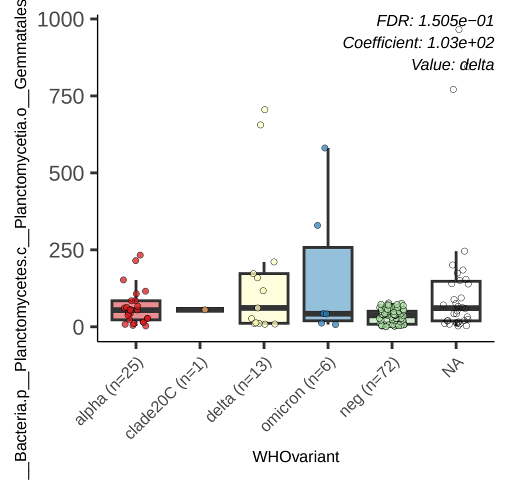

FDR: 1.505e−01
Coefficient: 1.03e+02
Value: delta
1000
750
500
250
0
_Bacteria.p__Planctomycetes.c__Planctomycetia.o__Gemmatales
alpha (n=25)
clade20C (n=1)
delta (n=13)
omicron (n=6)
neg (n=72)
NA
WHOvariant

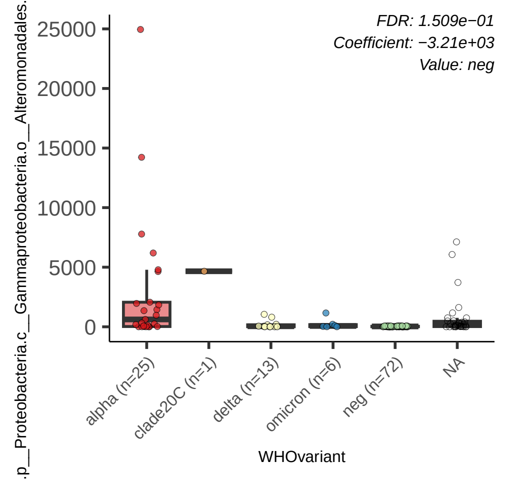

FDR: 1.509e−01
Coefficient: −3.21e+03
Value: neg
p__Proteobacteria.c__Gammaproteobacteria.o__Alteromonadales.
25000
20000
15000
10000
5000
0
alpha (n=25)
clade20C (n=1)
delta (n=13)
omicron (n=6)
neg (n=72)
NA
WHOvariant

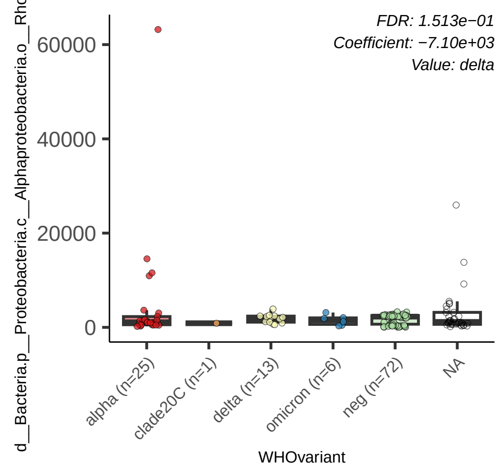
FDR: 1.513e−01
Coefficient: −7.10e+03
Value: delta
d__Bacteria.p__Proteobacteria.c__Alphaproteobacteria.o__Rho
60000
40000
20000
0
alpha (n=25)
clade20C (n=1)
delta (n=13)
omicron (n=6)
neg (n=72)
NA
WHOvariant

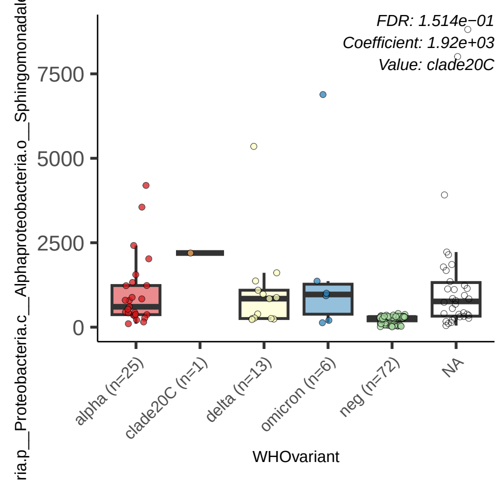

FDR: 1.514e−01
Coefficient: 1.92e+03
Value: clade20C
ria.p__Proteobacteria.c__Alphaproteobacteria.o__Sphingomonadal
7500
5000
2500
0
alpha (n=25)
clade20C (n=1)
delta (n=13)
omicron (n=6)
neg (n=72)
NA
WHOvariant

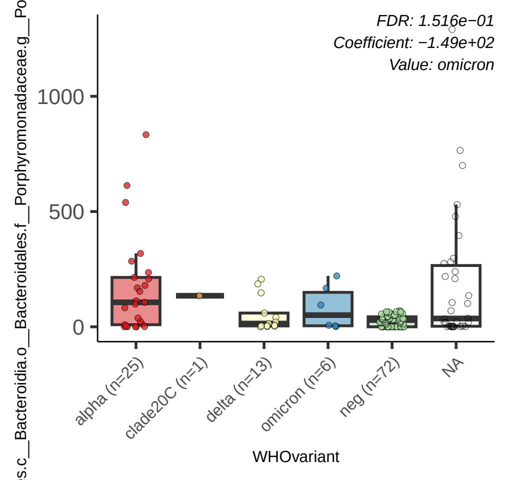

FDR: 1.516e−01
Coefficient: −1.49e+02
Value: omicron
s.c__Bacteroidia.o__Bacteroidales.f__Porphyromonadaceae.g__Po
1000
500
0
alpha (n=25)
clade20C (n=1)
delta (n=13)
omicron (n=6)
neg (n=72)
NA
WHOvariant

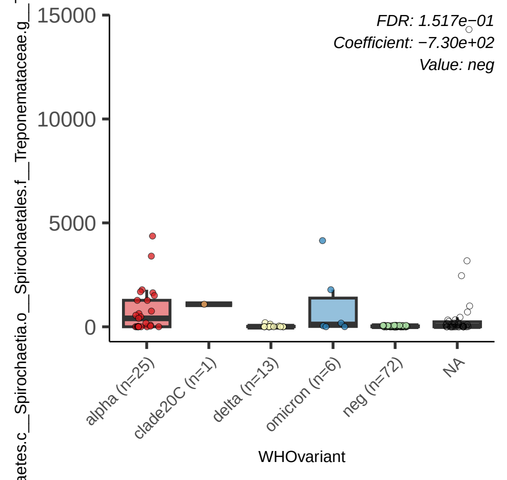

FDR: 1.517e−01
Coefficient: −7.30e+02
Value: neg
15000
10000
5000
0
aetes.c__Spirochaetia.o__Spirochaetales.f__Treponemataceae.g__
alpha (n=25)
clade20C (n=1)
delta (n=13)
omicron (n=6)
neg (n=72)
NA
WHOvariant

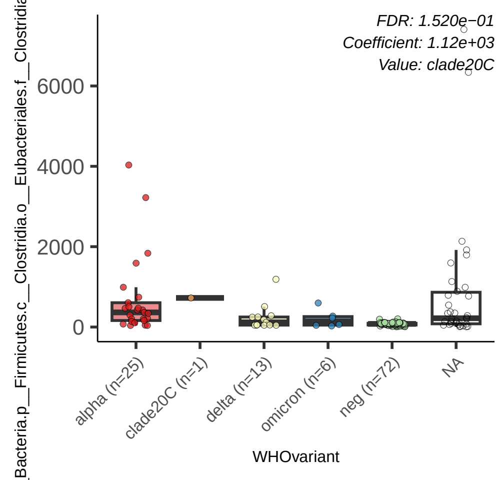

FDR: 1.520e−01
Coefficient: 1.12e+03
Value: clade20C
6000
4000
2000
0
Bacteria.p__Firmicutes.c__Clostridia.o__Eubacteriales.f__Clostridia
alpha (n=25)
clade20C (n=1)
delta (n=13)
omicron (n=6)
neg (n=72)
NA
WHOvariant

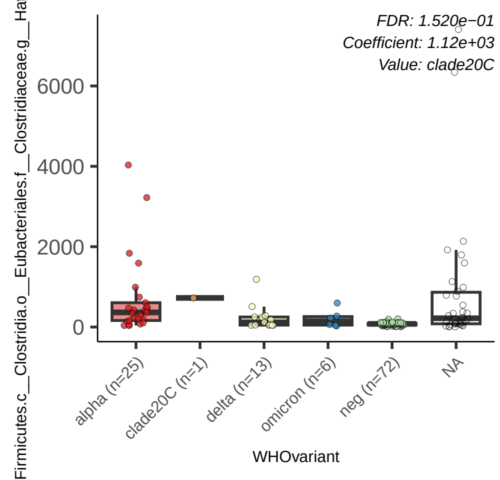

FDR: 1.520e−01
Coefficient: 1.12e+03
Value: clade20C
Firmicutes.c__Clostridia.o__Eubacteriales.f__Clostridiaceae.g__Ha
6000
4000
2000
0
alpha (n=25)
clade20C (n=1)
delta (n=13)
omicron (n=6)
neg (n=72)
NA
WHOvariant

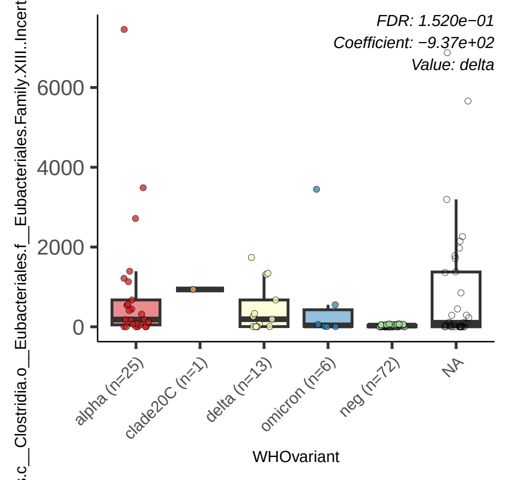

FDR: 1.520e−01
Coefficient: −9.37e+02
Value: delta
s.c__Clostridia.o__Eubacteriales.f__Eubacteriales.Family.XIII..Incert
0
2000
4000
6000
alpha (n=25)
clade20C (n=1)
delta (n=13)
omicron (n=6)
neg (n=72)
NA
WHOvariant

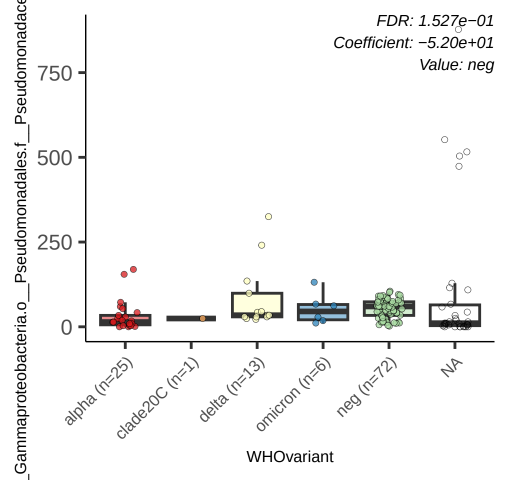

FDR: 1.527e−01
Coefficient: −5.20e+01
Value: neg
_Gammaproteobacteria.o__Pseudomonadales.f__Pseudomonadace
750
500
250
0
alpha (n=25)
clade20C (n=1)
delta (n=13)
omicron (n=6)
neg (n=72)
NA
WHOvariant

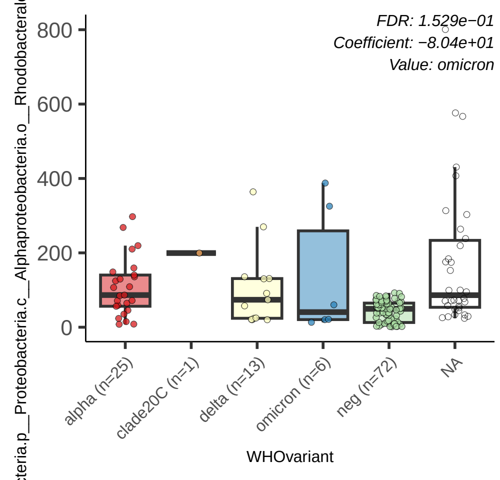
FDR: 1.529e−01
Coefficient: −8.04e+01
Value: omicron
800
600
400
200
0
alpha (n=25)
clade20C (n=1)
delta (n=13)
omicron (n=6)
neg (n=72)
NA
WHOvariant
cteria.p__Proteobacteria.c__Alphaproteobacteria.o__Rhodobacterale

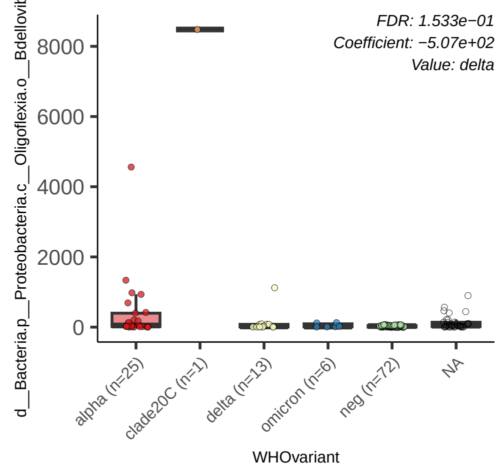
FDR: 1.533e−01
Coefficient: −5.07e+02
Value: delta
d__Bacteria.p__Proteobacteria.c__Oligoflexia.o__Bdellovib
8000
6000
4000
2000
0
alpha (n=25)
clade20C (n=1)
delta (n=13)
omicron (n=6)
neg (n=72)
NA
WHOvariant

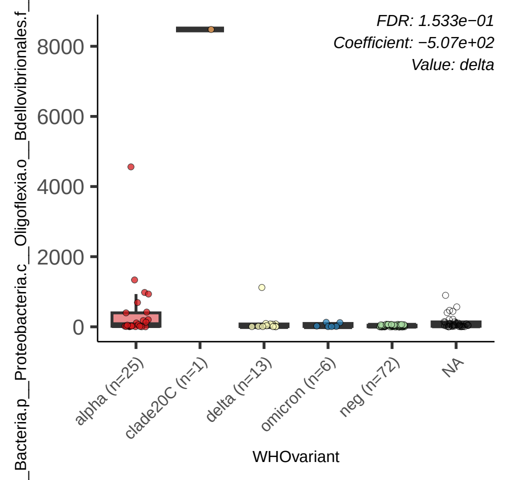

FDR: 1.533e−01
Coefficient: −5.07e+02
Value: delta
Bacteria.p__Proteobacteria.c__Oligoflexia.o__Bdellovibrionales.f
8000
6000
4000
2000
0
alpha (n=25)
clade20C (n=1)
delta (n=13)
omicron (n=6)
neg (n=72)
NA
WHOvariant

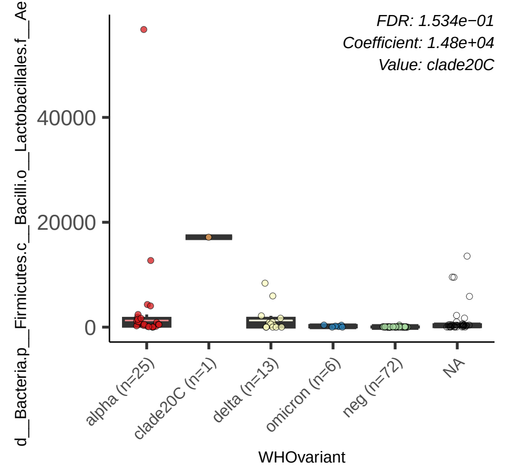

FDR: 1.534e−01
Coefficient: 1.48e+04
Value: clade20C
d__Bacteria.p__Firmicutes.c__Bacilli.o__Lactobacillales.f__Ae
40000
20000
0
alpha (n=25)
clade20C (n=1)
delta (n=13)
omicron (n=6)
neg (n=72)
NA
WHOvariant

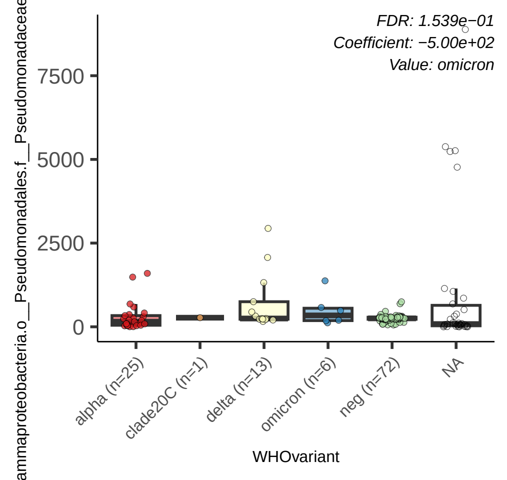
FDR: 1.539e−01
Coefficient: −5.00e+02
Value: omicron
ammaproteobacteria.o__Pseudomonadales.f__Pseudomonadaceae
7500
5000
2500
0
alpha (n=25)
clade20C (n=1)
delta (n=13)
omicron (n=6)
neg (n=72)
NA
WHOvariant

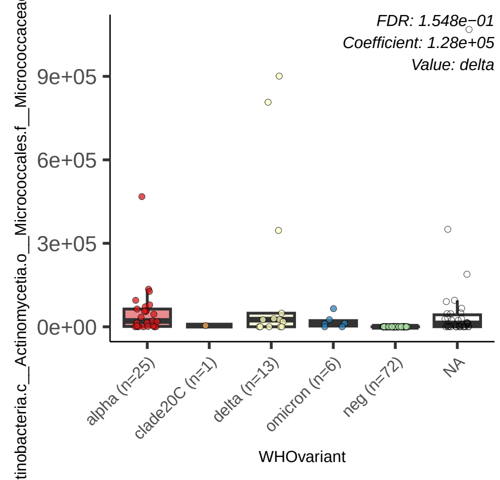
FDR: 1.548e−01
Coefficient: 1.28e+05
Value: delta
9e+05
6e+05
3e+05
0e+00
tinobacteria.c__Actinomycetia.o__Micrococcales.f__Micrococcacea
alpha (n=25)
clade20C (n=1)
delta (n=13)
omicron (n=6)
neg (n=72)
NA
WHOvariant

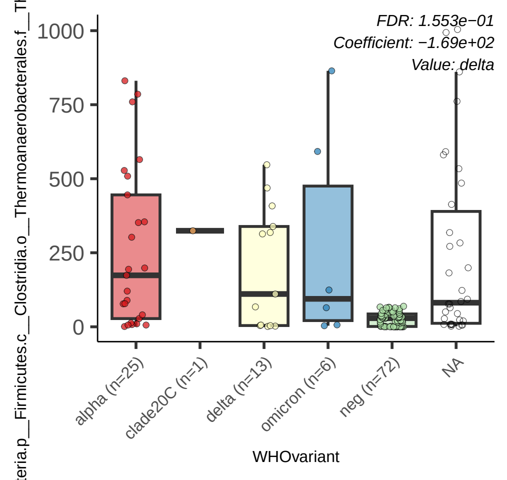
FDR: 1.553e−01
Coefficient: −1.69e+02
Value: delta
1000
750
500
250
0
alpha (n=25)
clade20C (n=1)
delta (n=13)
omicron (n=6)
neg (n=72)
NA
WHOvariant

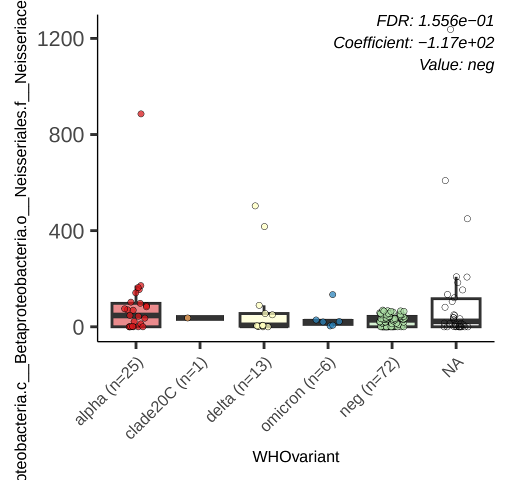

FDR: 1.556e−01
Coefficient: −1.17e+02
Value: neg
1200
800
400
0
oteobacteria.c__Betaproteobacteria.o__Neisseriales.f__Neisseriace
alpha (n=25)
clade20C (n=1)
delta (n=13)
omicron (n=6)
neg (n=72)
NA
WHOvariant

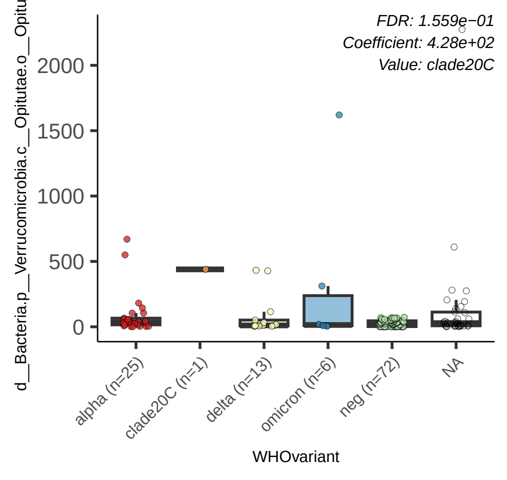
FDR: 1.559e−01
Coefficient: 4.28e+02
Value: clade20C
d__Bacteria.p__Verrucomicrobia.c__Opitutae.o__Opitu
2000
1500
1000
500
0
alpha (n=25)
clade20C (n=1)
delta (n=13)
omicron (n=6)
neg (n=72)
NA
WHOvariant

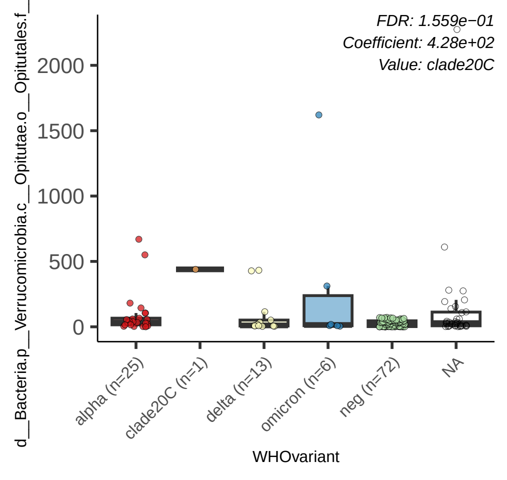

FDR: 1.559e−01
Coefficient: 4.28e+02
Value: clade20C
d__Bacteria.p__Verrucomicrobia.c__Opitutae.o__Opitutales.f__
2000
1500
1000
500
0
alpha (n=25)
clade20C (n=1)
delta (n=13)
omicron (n=6)
neg (n=72)
NA
WHOvariant

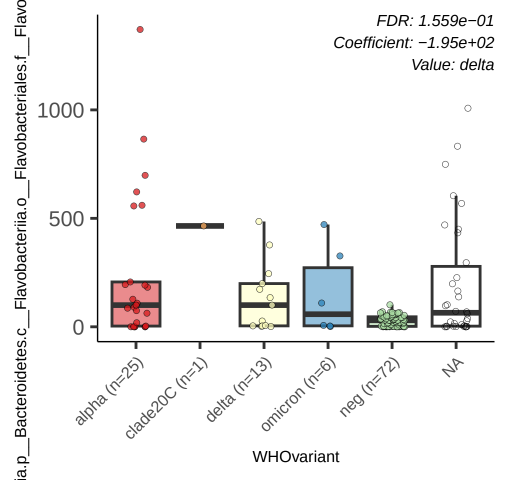

FDR: 1.559e−01
Coefficient: −1.95e+02
Value: delta
1000
500
0
ia.p__Bacteroidetes.c__Flavobacteriia.o__Flavobacteriales.f__Flavo
alpha (n=25)
clade20C (n=1)
delta (n=13)
omicron (n=6)
neg (n=72)
NA
WHOvariant

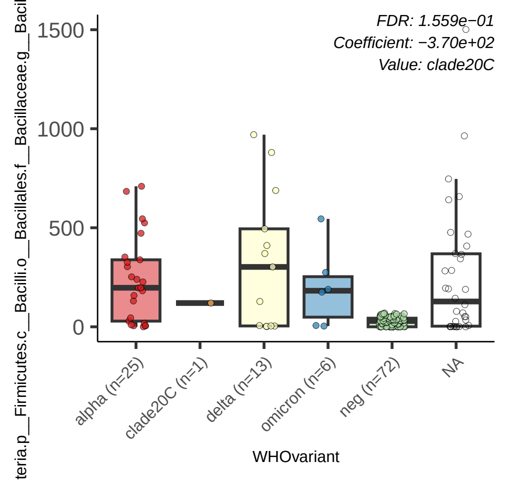

teria.p__Firmicutes.c__Bacilli.o__Bacillales.f__Bacillaceae.g__Bacil
FDR: 1.559e−01
Coefficient: −3.70e+02
Value: clade20C
1500
1000
500
0
alpha (n=25)
clade20C (n=1)
delta (n=13)
omicron (n=6)
neg (n=72)
NA
WHOvariant

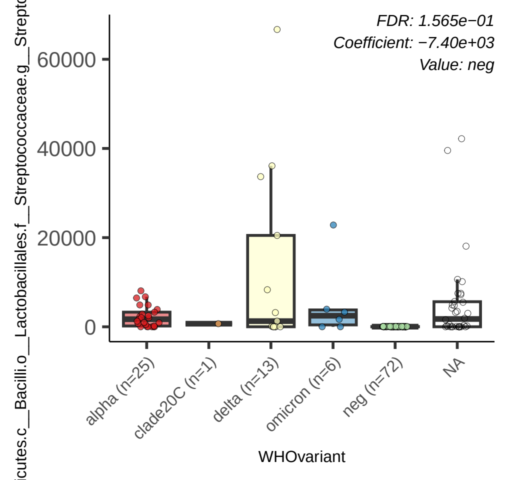
FDR: 1.565e−01
Coefficient: −7.40e+03
Value: neg
icutes.c__Bacilli.o__Lactobacillales.f__Streptococcaceae.g__Strept
60000
40000
20000
0
alpha (n=25)
clade20C (n=1)
delta (n=13)
omicron (n=6)
neg (n=72)
NA
WHOvariant

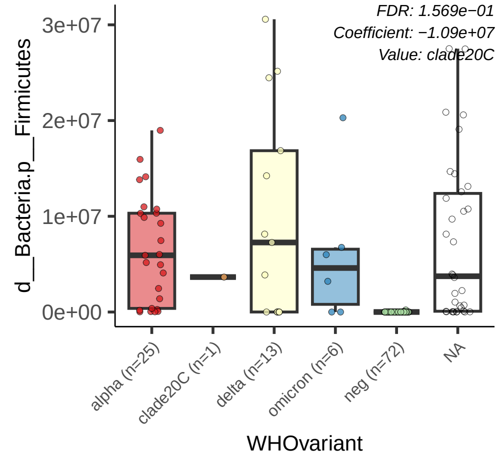
FDR: 1.569e−01
Coefficient: −1.09e+07
Value: clade20C
d__Bacteria.p__Firmicutes
3e+07
2e+07
1e+07
0e+00
alpha (n=25)
clade20C (n=1)
delta (n=13)
omicron (n=6)
neg (n=72)
NA
WHOvariant

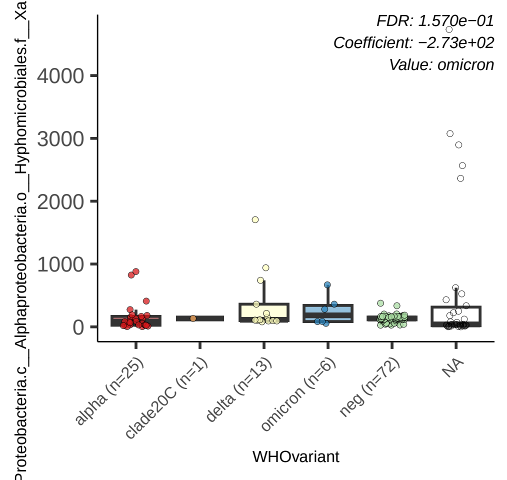

FDR: 1.570e−01
Coefficient: −2.73e+02
Value: omicron
Proteobacteria.c__Alphaproteobacteria.o__Hyphomicrobiales.f__Xa
0
1000
2000
3000
4000
alpha (n=25)
clade20C (n=1)
delta (n=13)
omicron (n=6)
neg (n=72)
NA
WHOvariant

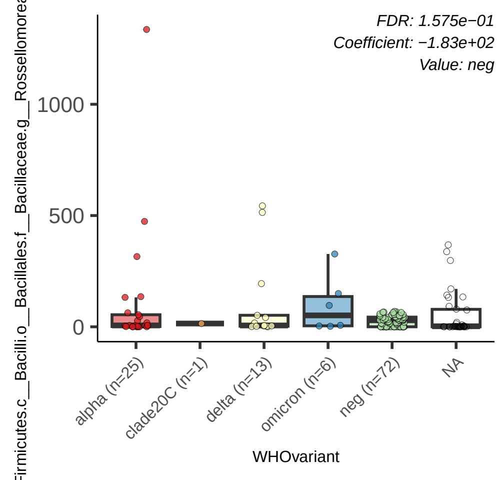

FDR: 1.575e−01
Coefficient: −1.83e+02
Value: neg
Firmicutes.c__Bacilli.o__Bacillales.f__Bacillaceae.g__Rossellomorea
1000
500
0
alpha (n=25)
clade20C (n=1)
delta (n=13)
omicron (n=6)
neg (n=72)
NA
WHOvariant

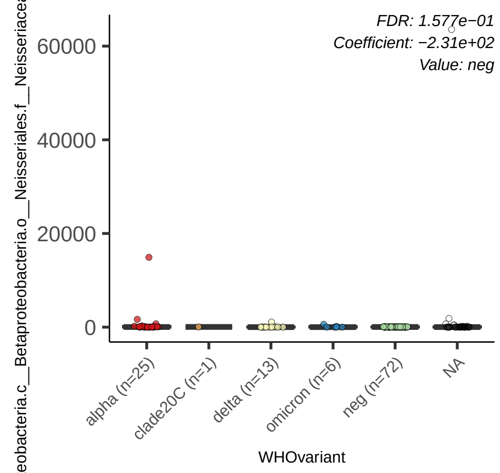

FDR: 1.577e−01
Coefficient: −2.31e+02
Value: neg
60000
40000
20000
0
oteobacteria.c__Betaproteobacteria.o__Neisseriales.f__Neisseriacea
alpha (n=25)
clade20C (n=1)
delta (n=13)
omicron (n=6)
neg (n=72)
NA
WHOvariant

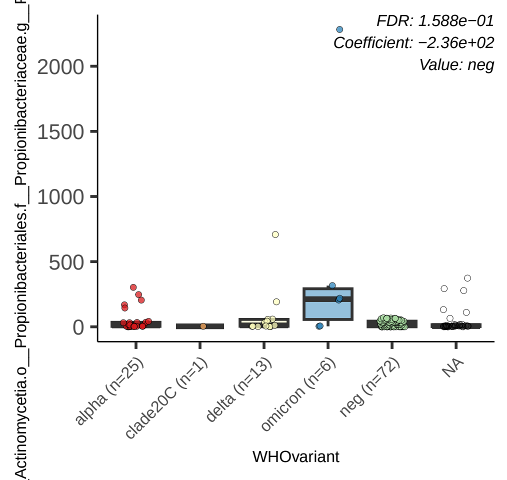

FDR: 1.588e−01
Coefficient: −2.36e+02
Value: neg
Actinomycetia.o__Propionibacteriales.f__Propionibacteriaceae.g__P
0
500
1000
1500
2000
alpha (n=25)
clade20C (n=1)
delta (n=13)
omicron (n=6)
neg (n=72)
NA
WHOvariant

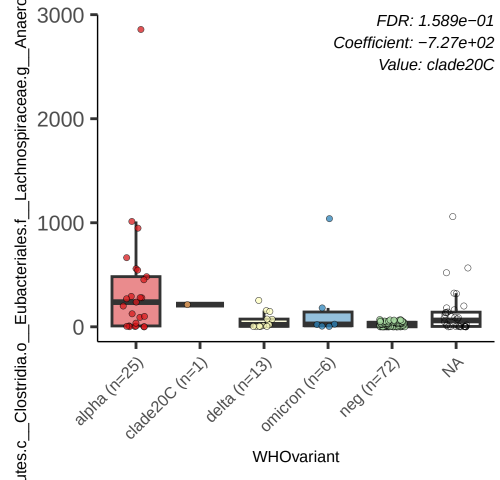

FDR: 1.589e−01
Coefficient: −7.27e+02
Value: clade20C
3000
2000
1000
0
utes.c__Clostridia.o__Eubacteriales.f__Lachnospiraceae.g__Anaero
alpha (n=25)
clade20C (n=1)
delta (n=13)
omicron (n=6)
neg (n=72)
NA
WHOvariant

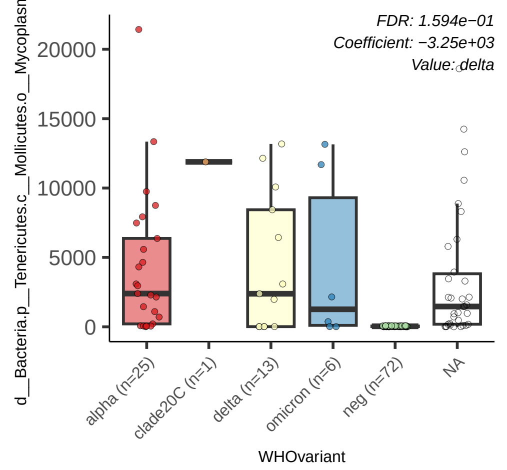
FDR: 1.594e−01
Coefficient: −3.25e+03
Value: delta
d__Bacteria.p__Tenericutes.c__Mollicutes.o__Mycoplasm
20000
15000
10000
5000
0
alpha (n=25)
clade20C (n=1)
delta (n=13)
omicron (n=6)
neg (n=72)
NA
WHOvariant

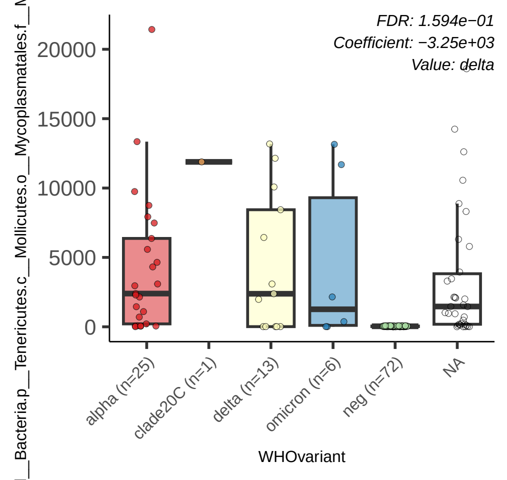
FDR: 1.594e−01
Coefficient: −3.25e+03
Value: delta
__Bacteria.p__Tenericutes.c__Mollicutes.o__Mycoplasmatales.f__M
20000
15000
10000
5000
0
alpha (n=25)
clade20C (n=1)
delta (n=13)
omicron (n=6)
neg (n=72)
NA
WHOvariant

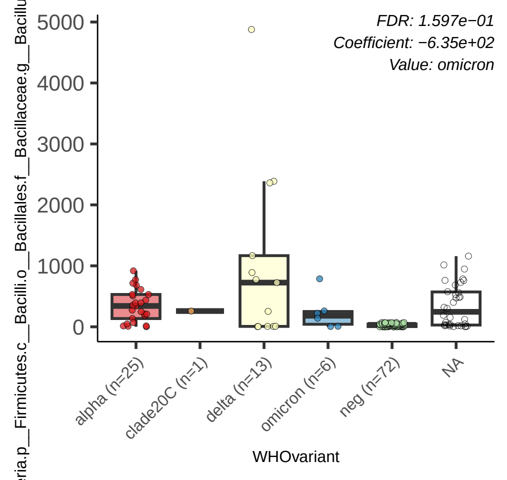

FDR: 1.597e−01
Coefficient: −6.35e+02
Value: omicron
5000
4000
3000
2000
1000
0
eria.p__Firmicutes.c__Bacilli.o__Bacillales.f__Bacillaceae.g__Bacillu
alpha (n=25)
clade20C (n=1)
delta (n=13)
omicron (n=6)
neg (n=72)
NA
WHOvariant

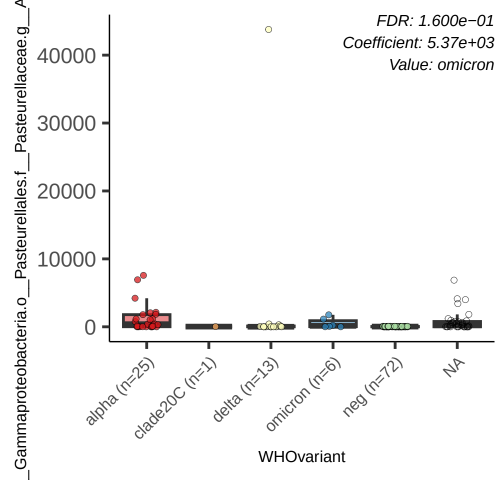

FDR: 1.600e−01
Coefficient: 5.37e+03
Value: omicron
40000
30000
20000
10000
0
alpha (n=25)
clade20C (n=1)
delta (n=13)
omicron (n=6)
neg (n=72)
NA
WHOvariant
_Gammaproteobacteria.o__Pasteurellales.f__Pasteurellaceae.g__A

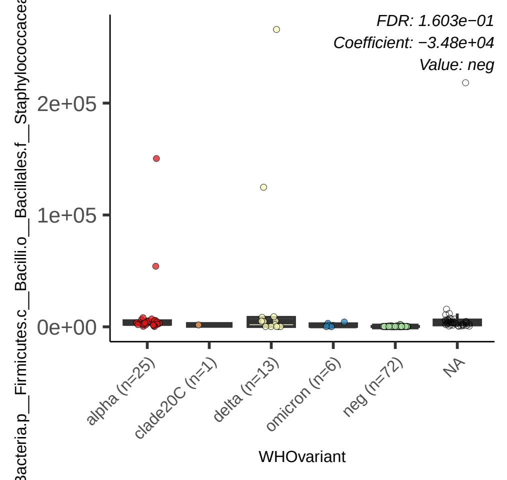
FDR: 1.603e−01
Coefficient: −3.48e+04
Value: neg
Bacteria.p__Firmicutes.c__Bacilli.o__Bacillales.f__Staphylococcacea
2e+05
1e+05
0e+00
alpha (n=25)
clade20C (n=1)
delta (n=13)
omicron (n=6)
neg (n=72)
NA
WHOvariant

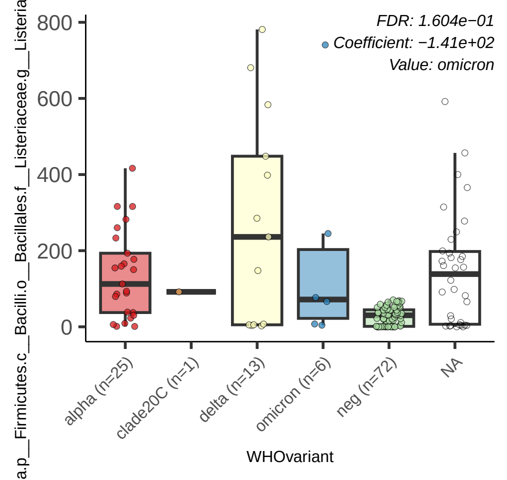

FDR: 1.604e−01
Coefficient: −1.41e+02
Value: omicron
800
600
400
200
0
a.p__Firmicutes.c__Bacilli.o__Bacillales.f__Listeriaceae.g__Listeria
alpha (n=25)
clade20C (n=1)
delta (n=13)
omicron (n=6)
neg (n=72)
NA
WHOvariant

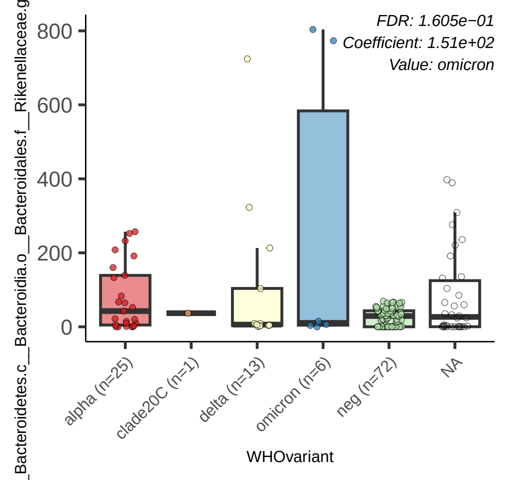

FDR: 1.605e−01
Coefficient: 1.51e+02
Value: omicron
800
600
400
200
0
Bacteroidetes.c__Bacteroidia.o__Bacteroidales.f__Rikenellaceae.g
alpha (n=25)
clade20C (n=1)
delta (n=13)
omicron (n=6)
neg (n=72)
NA
WHOvariant

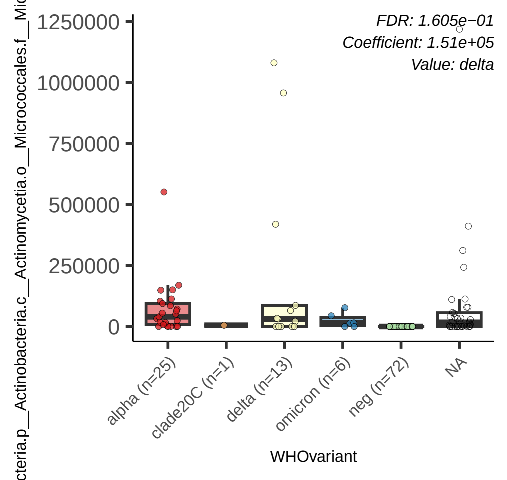

FDR: 1.605e−01
Coefficient: 1.51e+05
Value: delta
cteria.p__Actinobacteria.c__Actinomycetia.o__Micrococcales.f__Mic
1250000
1000000
750000
500000
250000
0
alpha (n=25)
clade20C (n=1)
delta (n=13)
omicron (n=6)
neg (n=72)
NA
WHOvariant

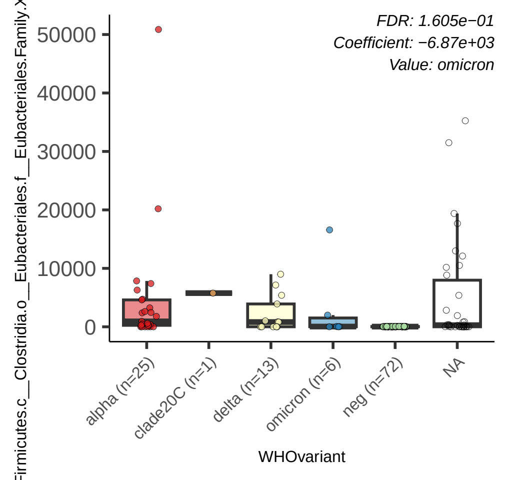
FDR: 1.605e−01
Coefficient: −6.87e+03
Value: omicron
Firmicutes.c__Clostridia.o__Eubacteriales.f__Eubacteriales.Family.X
50000
40000
30000
20000
10000
0
alpha (n=25)
clade20C (n=1)
delta (n=13)
omicron (n=6)
neg (n=72)
NA
WHOvariant

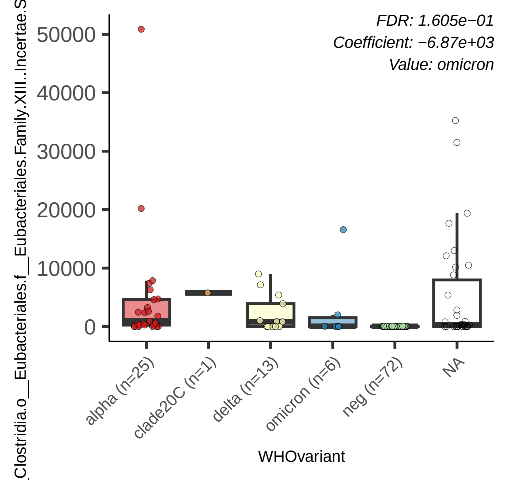
FDR: 1.605e−01
Coefficient: −6.87e+03
Value: omicron
Clostridia.o__Eubacteriales.f__Eubacteriales.Family.XIII..Incertae.S
50000
40000
30000
20000
10000
0
alpha (n=25)
clade20C (n=1)
delta (n=13)
omicron (n=6)
neg (n=72)
NA
WHOvariant

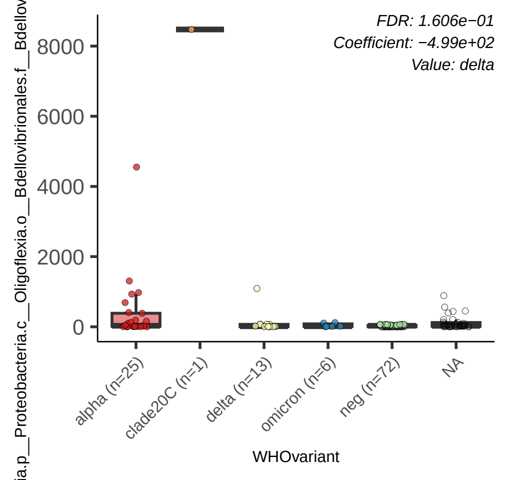
FDR: 1.606e−01
Coefficient: −4.99e+02
Value: delta
8000
6000
4000
2000
0
ia.p__Proteobacteria.c__Oligoflexia.o__Bdellovibrionales.f__Bdellov
alpha (n=25)
clade20C (n=1)
delta (n=13)
omicron (n=6)
neg (n=72)
NA
WHOvariant

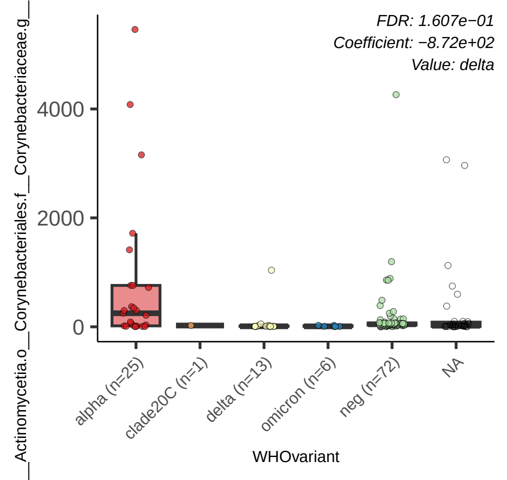
FDR: 1.607e−01
Coefficient: −8.72e+02
Value: delta
Actinomycetia.o__Corynebacteriales.f__Corynebacteriaceae.g__
4000
2000
0
alpha (n=25)
clade20C (n=1)
delta (n=13)
omicron (n=6)
neg (n=72)
NA
WHOvariant

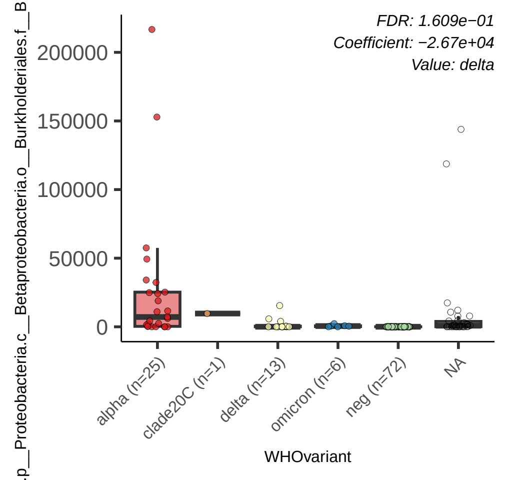

FDR: 1.609e−01
Coefficient: −2.67e+04
Value: delta
p__Proteobacteria.c__Betaproteobacteria.o__Burkholderiales.f__B
200000
150000
100000
50000
0
alpha (n=25)
clade20C (n=1)
delta (n=13)
omicron (n=6)
neg (n=72)
NA
WHOvariant

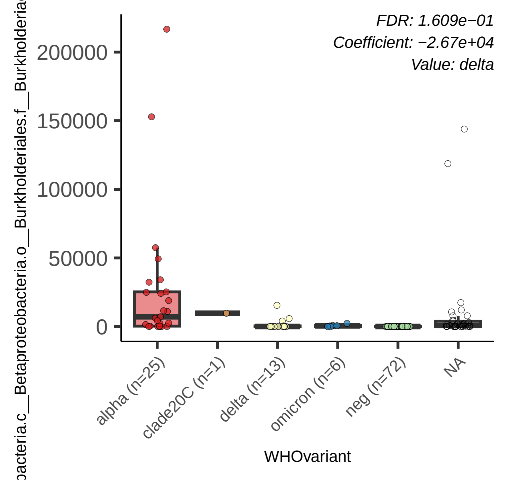
FDR: 1.609e−01
Coefficient: −2.67e+04
Value: delta
200000
150000
100000
50000
0
alpha (n=25)
clade20C (n=1)
delta (n=13)
omicron (n=6)
neg (n=72)
NA
WHOvariant
bacteria.c__Betaproteobacteria.o__Burkholderiales.f__Burkholderia

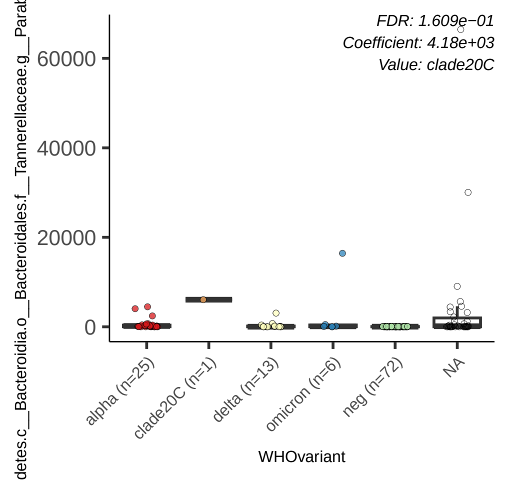

FDR: 1.609e−01
Coefficient: 4.18e+03
Value: clade20C
60000
40000
20000
0
detes.c__Bacteroidia.o__Bacteroidales.f__Tannerellaceae.g__Parab
alpha (n=25)
clade20C (n=1)
delta (n=13)
omicron (n=6)
neg (n=72)
NA
WHOvariant

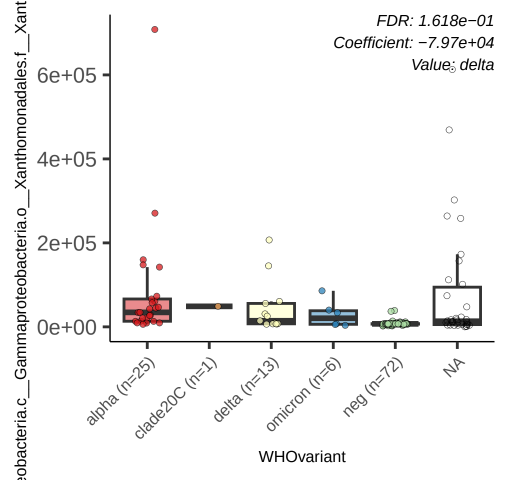

FDR: 1.618e−01
Coefficient: −7.97e+04
Value: delta
6e+05
4e+05
2e+05
0e+00
eobacteria.c__Gammaproteobacteria.o__Xanthomonadales.f__Xant
alpha (n=25)
clade20C (n=1)
delta (n=13)
omicron (n=6)
neg (n=72)
NA
WHOvariant

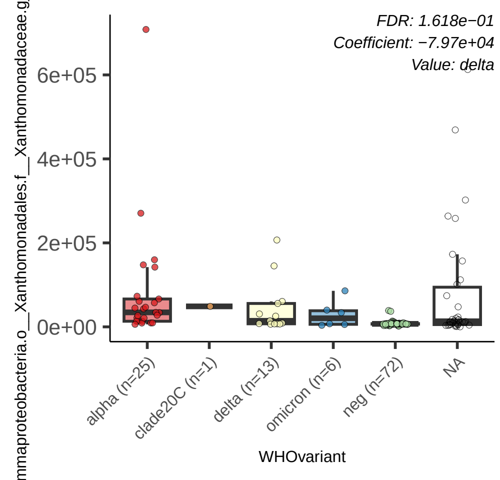
FDR: 1.618e−01
Coefficient: −7.97e+04
Value: delta
mmaproteobacteria.o__Xanthomonadales.f__Xanthomonadaceae.g
6e+05
4e+05
2e+05
0e+00
alpha (n=25)
clade20C (n=1)
delta (n=13)
omicron (n=6)
neg (n=72)
NA
WHOvariant

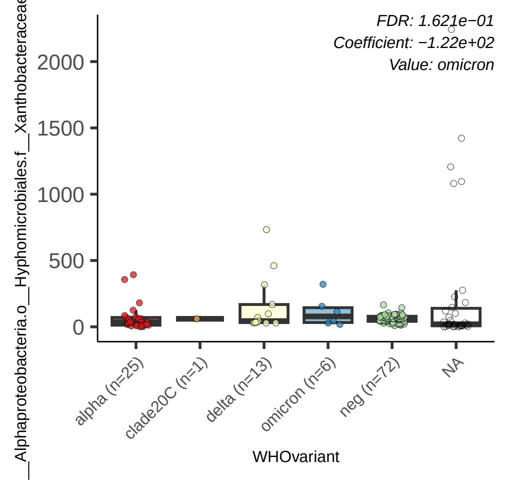

FDR: 1.621e−01
Coefficient: −1.22e+02
Value: omicron
__Alphaproteobacteria.o__Hyphomicrobiales.f__Xanthobacteraceae
0
500
1000
1500
2000
alpha (n=25)
clade20C (n=1)
delta (n=13)
omicron (n=6)
neg (n=72)
NA
WHOvariant

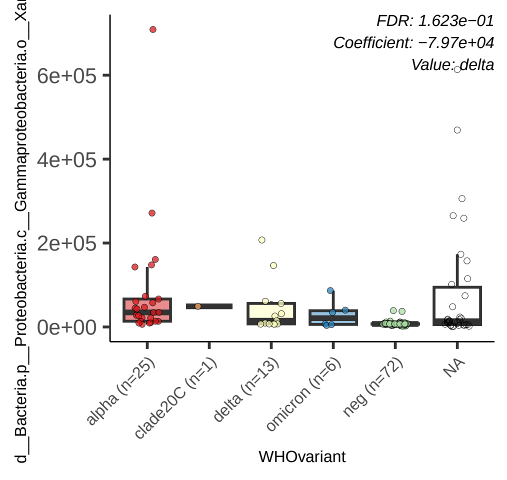

FDR: 1.623e−01
Coefficient: −7.97e+04
Value: delta
d__Bacteria.p__Proteobacteria.c__Gammaproteobacteria.o__Xa
6e+05
4e+05
2e+05
0e+00
alpha (n=25)
clade20C (n=1)
delta (n=13)
omicron (n=6)
neg (n=72)
NA
WHOvariant

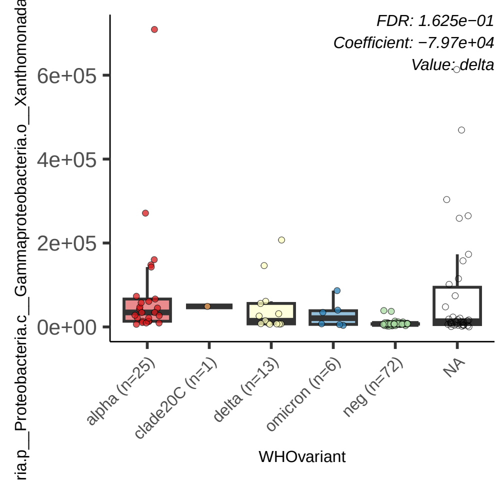

FDR: 1.625e−01
Coefficient: −7.97e+04
Value: delta
ria.p__Proteobacteria.c__Gammaproteobacteria.o__Xanthomonada
6e+05
4e+05
2e+05
0e+00
alpha (n=25)
clade20C (n=1)
delta (n=13)
omicron (n=6)
neg (n=72)
NA
WHOvariant

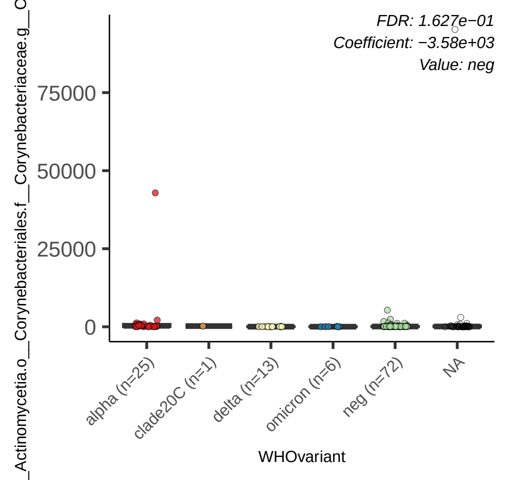
FDR: 1.627e−01
Coefficient: −3.58e+03
Value: neg
75000
50000
25000
0
_Actinomycetia.o__Corynebacteriales.f__Corynebacteriaceae.g__C
alpha (n=25)
clade20C (n=1)
delta (n=13)
omicron (n=6)
neg (n=72)
NA
WHOvariant

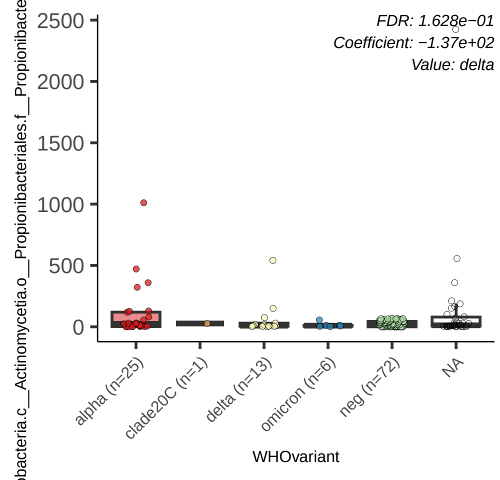

FDR: 1.628e−01
Coefficient: −1.37e+02
Value: delta
2500
2000
1500
1000
500
0
obacteria.c__Actinomycetia.o__Propionibacteriales.f__Propionibacte
alpha (n=25)
clade20C (n=1)
delta (n=13)
omicron (n=6)
neg (n=72)
NA
WHOvariant

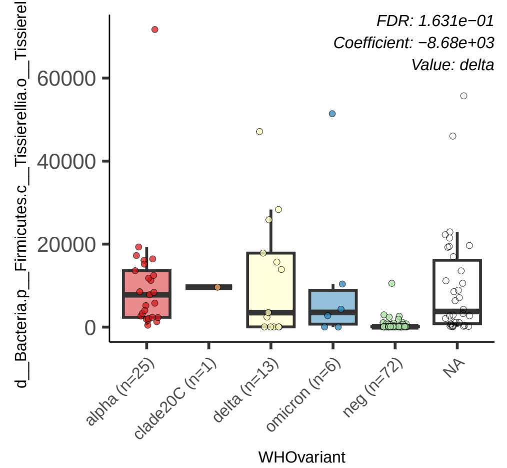

FDR: 1.631e−01
Coefficient: −8.68e+03
Value: delta
d__Bacteria.p__Firmicutes.c__Tissierellia.o__Tissierel
60000
40000
20000
0
alpha (n=25)
clade20C (n=1)
delta (n=13)
omicron (n=6)
neg (n=72)
NA
WHOvariant

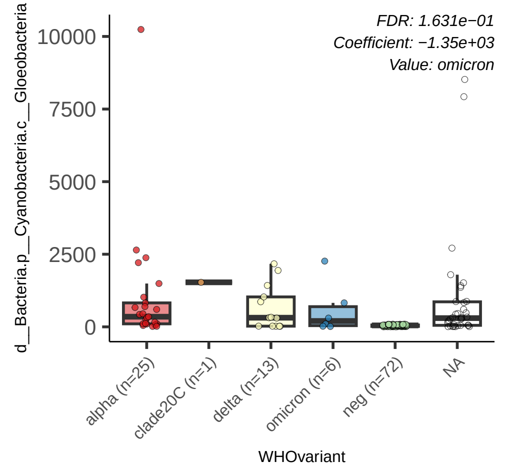

FDR: 1.631e−01
Coefficient: −1.35e+03
Value: omicron
d__Bacteria.p__Cyanobacteria.c__Gloeobacteria
10000
7500
5000
2500
0
alpha (n=25)
clade20C (n=1)
delta (n=13)
omicron (n=6)
neg (n=72)
NA
WHOvariant

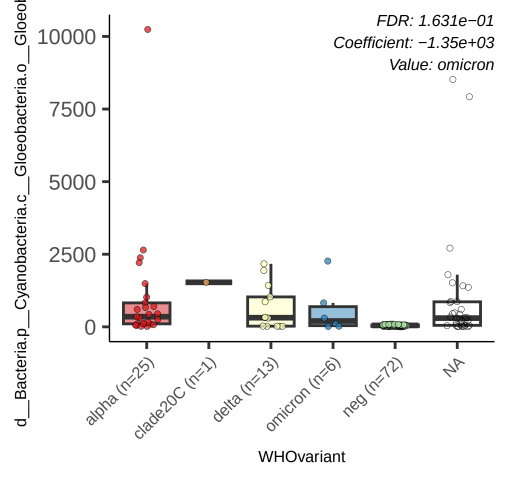

FDR: 1.631e−01
Coefficient: −1.35e+03
Value: omicron
d__Bacteria.p__Cyanobacteria.c__Gloeobacteria.o__Gloeob
10000
7500
5000
2500
0
alpha (n=25)
clade20C (n=1)
delta (n=13)
omicron (n=6)
neg (n=72)
NA
WHOvariant

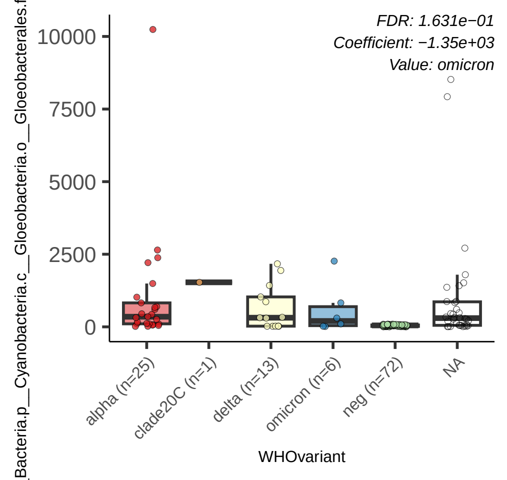

FDR: 1.631e−01
Coefficient: −1.35e+03
Value: omicron
10000
7500
5000
2500
0
Bacteria.p__Cyanobacteria.c__Gloeobacteria.o__Gloeobacterales.f
alpha (n=25)
clade20C (n=1)
delta (n=13)
omicron (n=6)
neg (n=72)
NA
WHOvariant

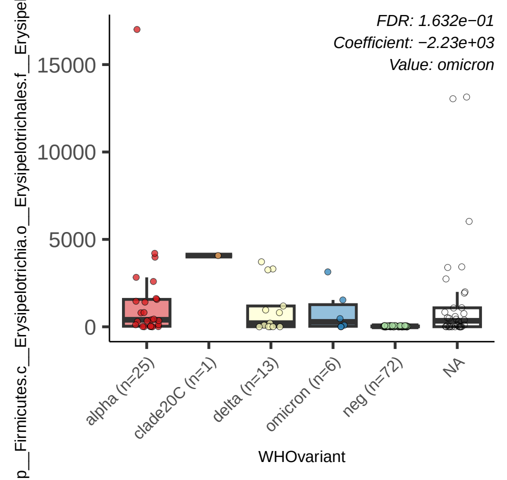
FDR: 1.632e−01
Coefficient: −2.23e+03
Value: omicron
p__Firmicutes.c__Erysipelotrichia.o__Erysipelotrichales.f__Erysipel
15000
10000
5000
0
alpha (n=25)
clade20C (n=1)
delta (n=13)
omicron (n=6)
neg (n=72)
NA
WHOvariant

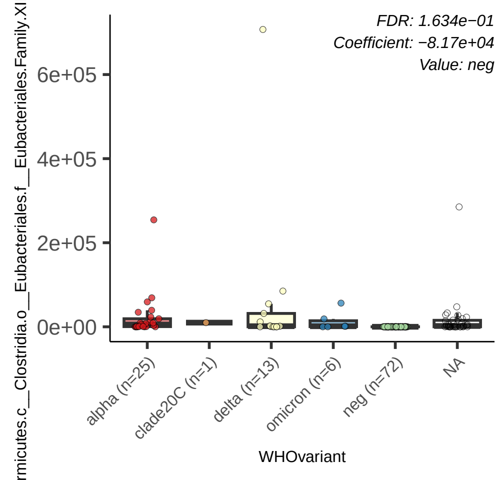

FDR: 1.634e−01
Coefficient: −8.17e+04
Value: neg
rmicutes.c__Clostridia.o__Eubacteriales.f__Eubacteriales.Family.XI
6e+05
4e+05
2e+05
0e+00
alpha (n=25)
clade20C (n=1)
delta (n=13)
omicron (n=6)
neg (n=72)
NA
WHOvariant

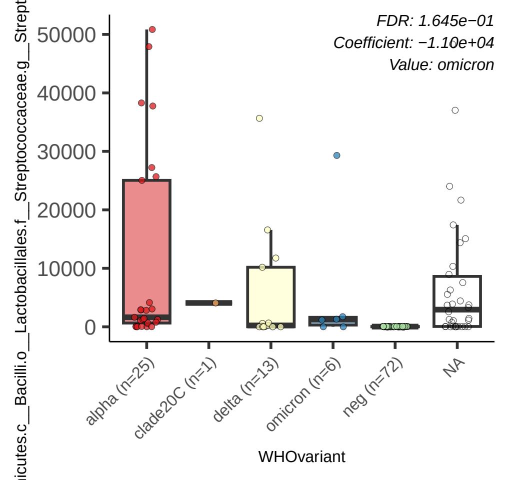

FDR: 1.645e−01
Coefficient: −1.10e+04
Value: omicron
50000
40000
30000
20000
10000
0
icutes.c__Bacilli.o__Lactobacillales.f__Streptococcaceae.g__Strept
alpha (n=25)
clade20C (n=1)
delta (n=13)
omicron (n=6)
neg (n=72)
NA
WHOvariant

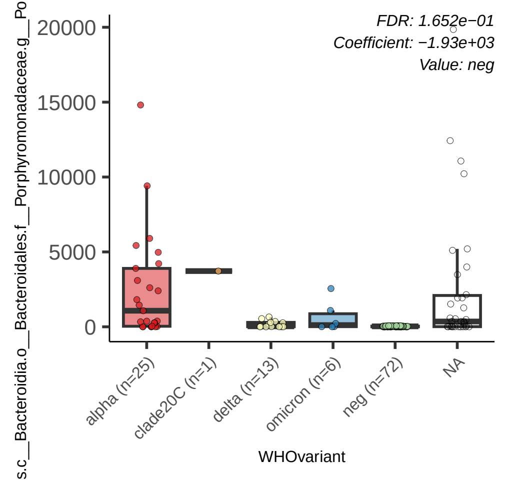

FDR: 1.652e−01
Coefficient: −1.93e+03
Value: neg
20000
15000
10000
5000
0
s.c__Bacteroidia.o__Bacteroidales.f__Porphyromonadaceae.g__Po
alpha (n=25)
clade20C (n=1)
delta (n=13)
omicron (n=6)
neg (n=72)
NA
WHOvariant

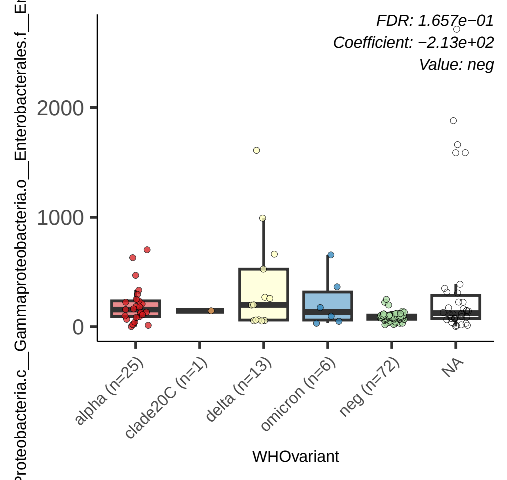
FDR: 1.657e−01
Coefficient: −2.13e+02
Value: neg
Proteobacteria.c__Gammaproteobacteria.o__Enterobacterales.f__En
2000
1000
0
alpha (n=25)
clade20C (n=1)
delta (n=13)
omicron (n=6)
neg (n=72)
NA
WHOvariant

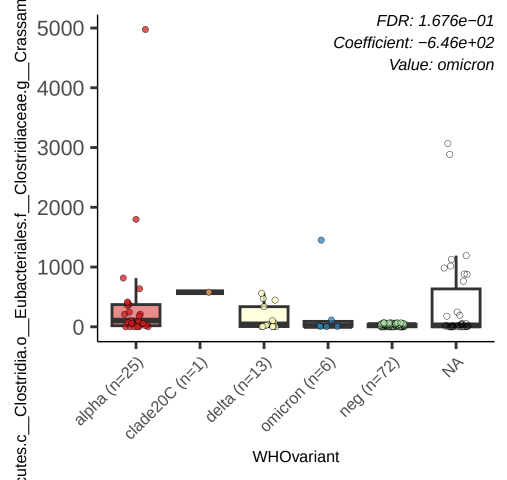

FDR: 1.676e−01
Coefficient: −6.46e+02
Value: omicron
cutes.c__Clostridia.o__Eubacteriales.f__Clostridiaceae.g__Crassam
5000
4000
3000
2000
1000
0
alpha (n=25)
clade20C (n=1)
delta (n=13)
omicron (n=6)
neg (n=72)
NA
WHOvariant

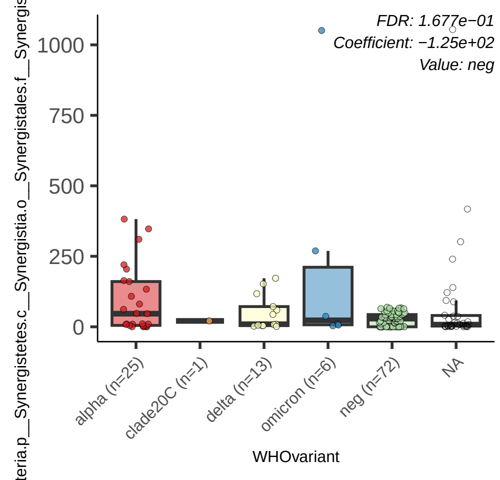

FDR: 1.677e−01
Coefficient: −1.25e+02
Value: neg
1000
750
500
250
0
teria.p__Synergistetes.c__Synergistia.o__Synergistales.f__Synergis
alpha (n=25)
clade20C (n=1)
delta (n=13)
omicron (n=6)
neg (n=72)
NA
WHOvariant

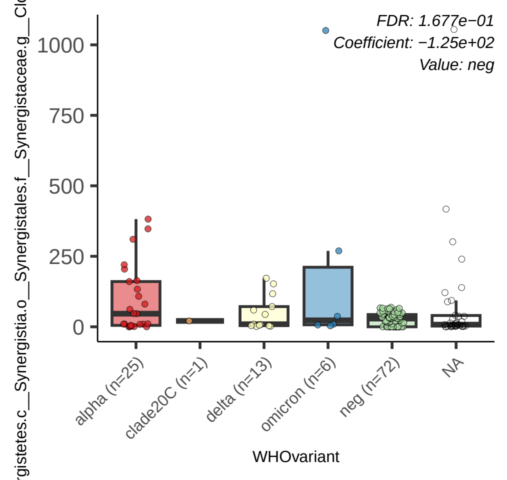

FDR: 1.677e−01
Coefficient: −1.25e+02
Value: neg
1000
750
500
250
0
rgistetes.c__Synergistia.o__Synergistales.f__Synergistaceae.g__Clo
alpha (n=25)
clade20C (n=1)
delta (n=13)
omicron (n=6)
neg (n=72)
NA
WHOvariant

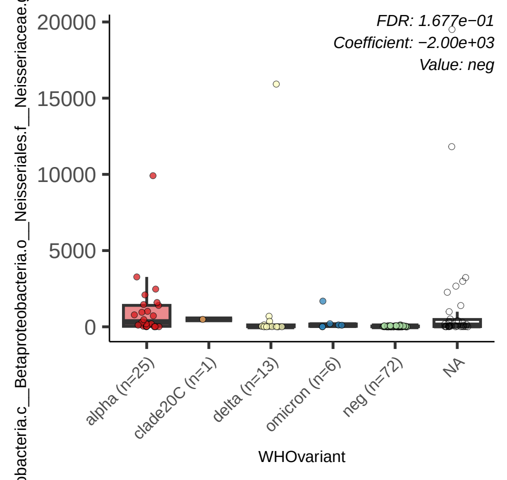

FDR: 1.677e−01
Coefficient: −2.00e+03
Value: neg
20000
15000
10000
5000
0
obacteria.c__Betaproteobacteria.o__Neisseriales.f__Neisseriaceae.g
alpha (n=25)
clade20C (n=1)
delta (n=13)
omicron (n=6)
neg (n=72)
NA
WHOvariant

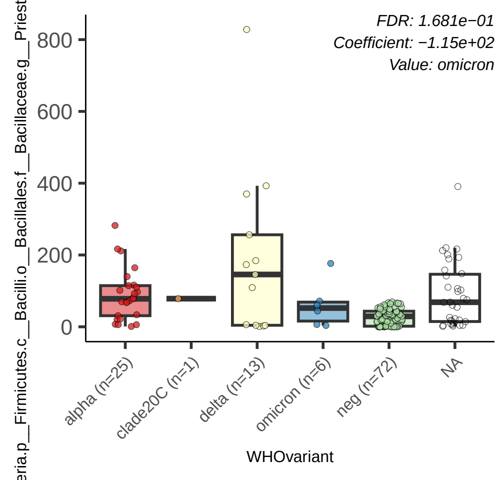

FDR: 1.681e−01
Coefficient: −1.15e+02
Value: omicron
eria.p__Firmicutes.c__Bacilli.o__Bacillales.f__Bacillaceae.g__Priest
0
200
400
600
800
alpha (n=25)
clade20C (n=1)
delta (n=13)
omicron (n=6)
neg (n=72)
NA
WHOvariant

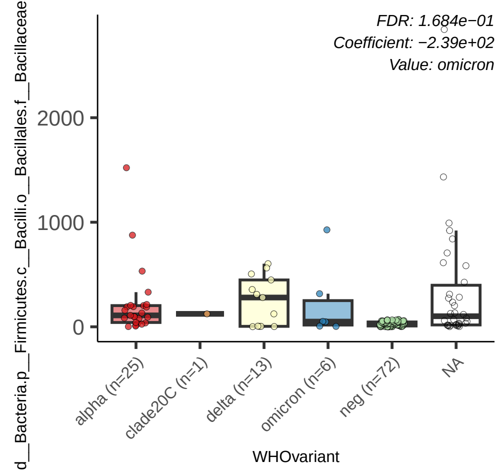
FDR: 1.684e−01
Coefficient: −2.39e+02
Value: omicron
d__Bacteria.p__Firmicutes.c__Bacilli.o__Bacillales.f__Bacillaceae
2000
1000
0
alpha (n=25)
clade20C (n=1)
delta (n=13)
omicron (n=6)
neg (n=72)
NA
WHOvariant

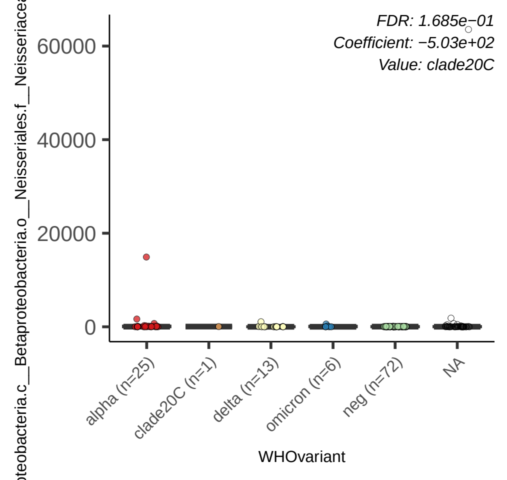
FDR: 1.685e−01
Coefficient: −5.03e+02
Value: clade20C
60000
40000
20000
0
alpha (n=25)
clade20C (n=1)
delta (n=13)
omicron (n=6)
neg (n=72)
NA
WHOvariant
oteobacteria.c__Betaproteobacteria.o__Neisseriales.f__Neisseriace

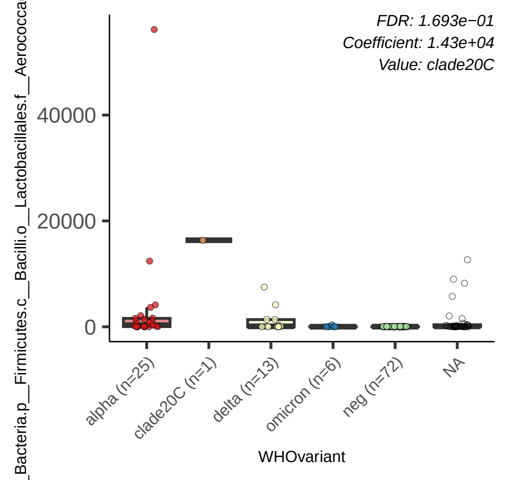

FDR: 1.693e−01
Coefficient: 1.43e+04
Value: clade20C
Bacteria.p__Firmicutes.c__Bacilli.o__Lactobacillales.f__Aerococca
40000
20000
0
alpha (n=25)
clade20C (n=1)
delta (n=13)
omicron (n=6)
neg (n=72)
NA
WHOvariant

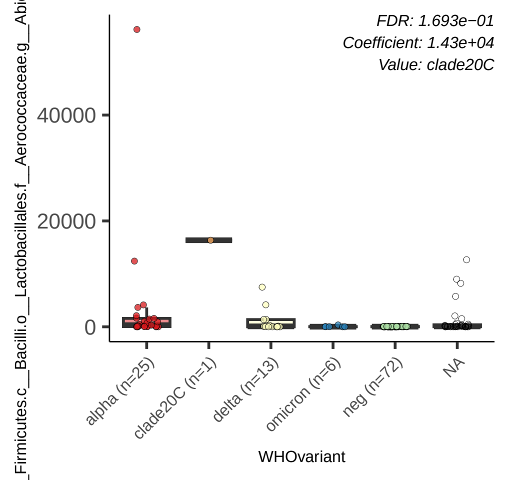

FDR: 1.693e−01
Coefficient: 1.43e+04
Value: clade20C
_Firmicutes.c__Bacilli.o__Lactobacillales.f__Aerococcaceae.g__Abi
40000
20000
0
alpha (n=25)
clade20C (n=1)
delta (n=13)
omicron (n=6)
neg (n=72)
NA
WHOvariant

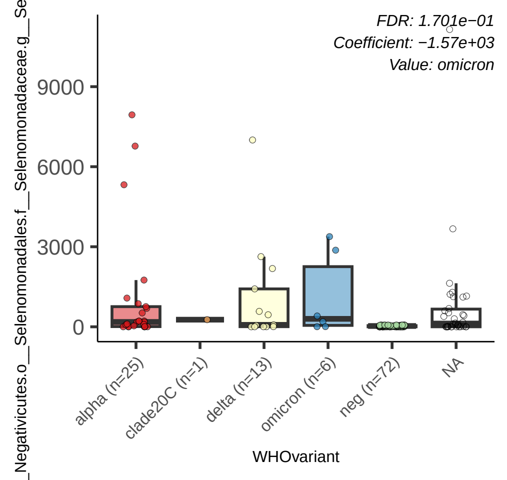
FDR: 1.701e−01
Coefficient: −1.57e+03
Value: omicron
_Negativicutes.o__Selenomonadales.f__Selenomonadaceae.g__Se
9000
6000
3000
0
alpha (n=25)
clade20C (n=1)
delta (n=13)
omicron (n=6)
neg (n=72)
NA
WHOvariant

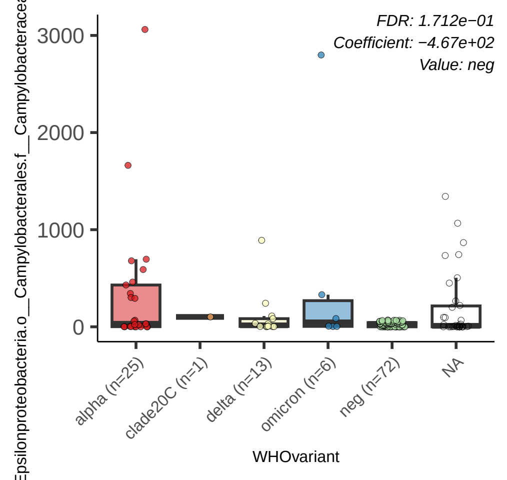

FDR: 1.712e−01
Coefficient: −4.67e+02
Value: neg
3000
2000
1000
0
Epsilonproteobacteria.o__Campylobacterales.f__Campylobacterace
alpha (n=25)
clade20C (n=1)
delta (n=13)
omicron (n=6)
neg (n=72)
NA
WHOvariant

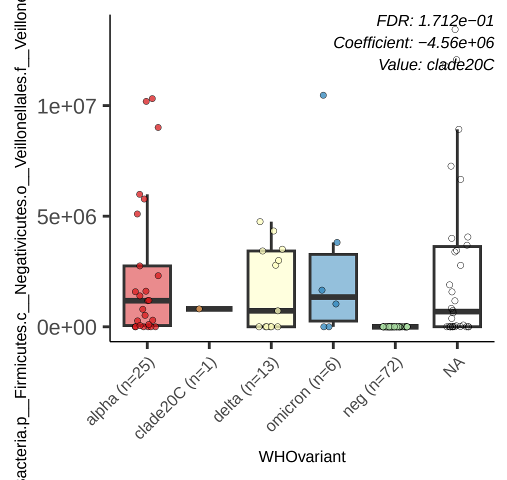
FDR: 1.712e−01
Coefficient: −4.56e+06
Value: clade20C
1e+07
5e+06
0e+00
Bacteria.p__Firmicutes.c__Negativicutes.o__Veillonellales.f__Veillone
alpha (n=25)
clade20C (n=1)
delta (n=13)
omicron (n=6)
neg (n=72)
NA
WHOvariant

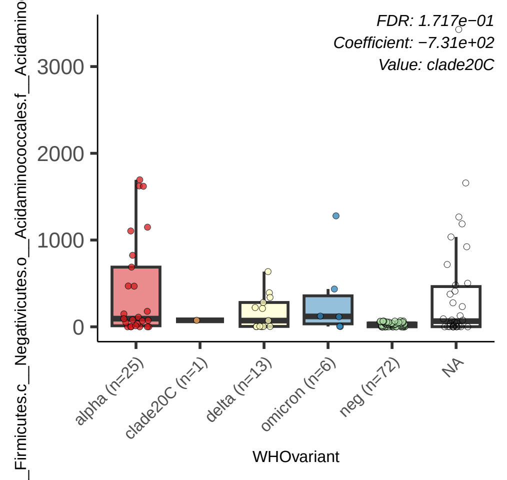

FDR: 1.717e−01
Coefficient: −7.31e+02
Value: clade20C
Firmicutes.c__Negativicutes.o__Acidaminococcales.f__Acidaminoc
3000
2000
1000
0
alpha (n=25)
clade20C (n=1)
delta (n=13)
omicron (n=6)
neg (n=72)
NA
WHOvariant

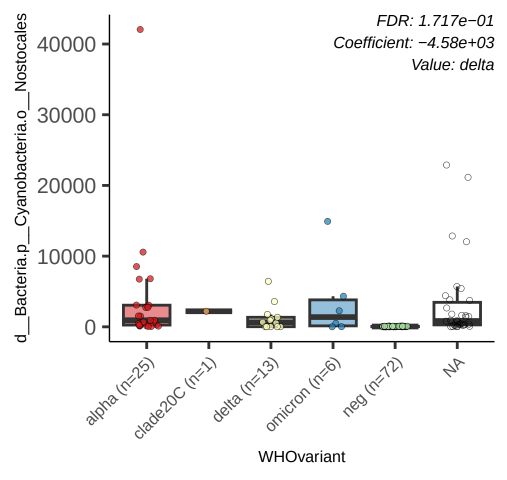

FDR: 1.717e−01
Coefficient: −4.58e+03
Value: delta
d__Bacteria.p__Cyanobacteria.o__Nostocales
40000
30000
20000
10000
0
alpha (n=25)
clade20C (n=1)
delta (n=13)
omicron (n=6)
neg (n=72)
NA
WHOvariant

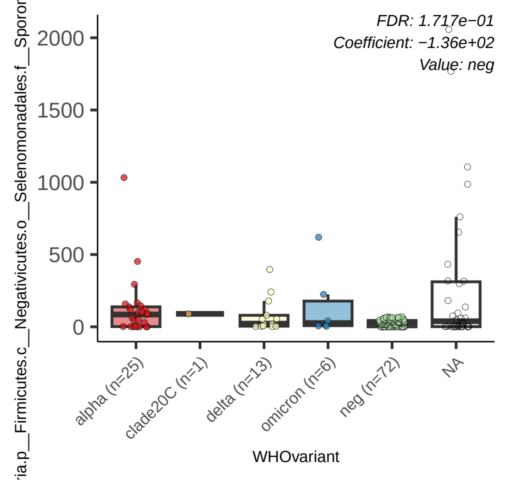
FDR: 1.717e−01
Coefficient: −1.36e+02
Value: neg
ria.p__Firmicutes.c__Negativicutes.o__Selenomonadales.f__Sporor
2000
1500
1000
500
0
alpha (n=25)
clade20C (n=1)
delta (n=13)
omicron (n=6)
neg (n=72)
NA
WHOvariant

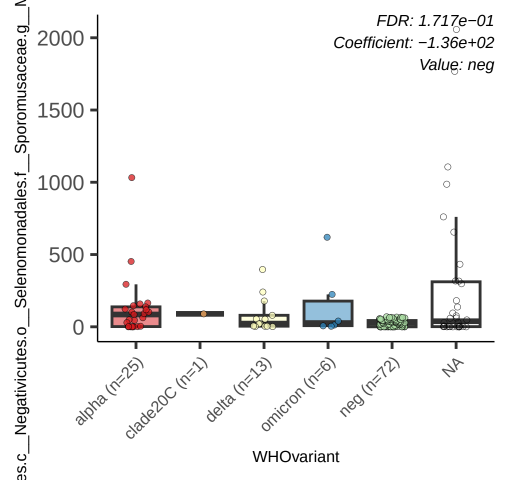
FDR: 1.717e−01
Coefficient: −1.36e+02
Value: neg
2000
1500
1000
500
0
es.c__Negativicutes.o__Selenomonadales.f__Sporomusaceae.g__M
alpha (n=25)
clade20C (n=1)
delta (n=13)
omicron (n=6)
neg (n=72)
NA
WHOvariant

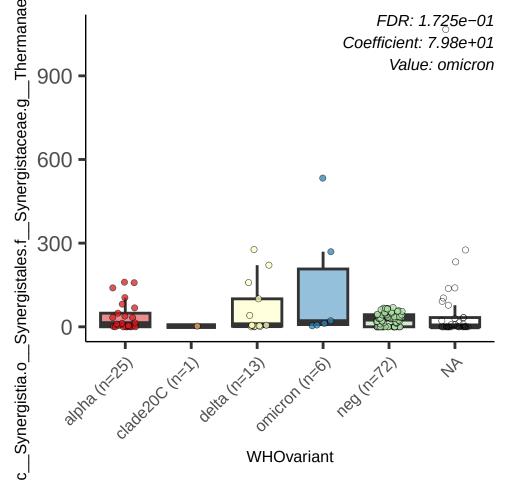

FDR: 1.725e−01
Coefficient: 7.98e+01
Value: omicron
c__Synergistia.o__Synergistales.f__Synergistaceae.g__Thermanae
900
600
300
0
alpha (n=25)
clade20C (n=1)
delta (n=13)
omicron (n=6)
neg (n=72)
NA
WHOvariant

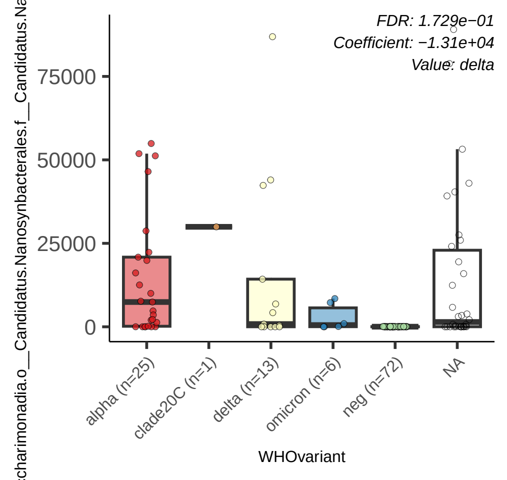

FDR: 1.729e−01
Coefficient: −1.31e+04
Value: delta
75000
50000
25000
0
alpha (n=25)
clade20C (n=1)
delta (n=13)
omicron (n=6)
neg (n=72)
NA
WHOvariant

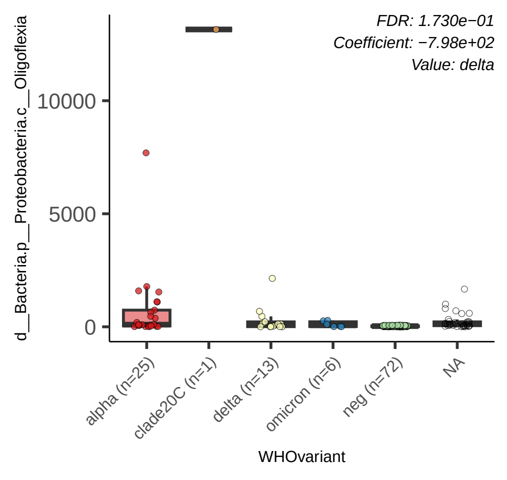

FDR: 1.730e−01
Coefficient: −7.98e+02
Value: delta
d__Bacteria.p__Proteobacteria.c__Oligoflexia
10000
5000
0
alpha (n=25)
clade20C (n=1)
delta (n=13)
omicron (n=6)
neg (n=72)
NA
WHOvariant

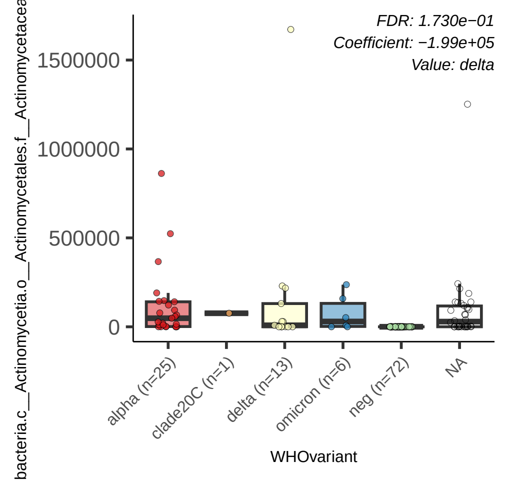
FDR: 1.730e−01
Coefficient: −1.99e+05
Value: delta
1500000
1000000
500000
0
bacteria.c__Actinomycetia.o__Actinomycetales.f__Actinomycetacea
alpha (n=25)
clade20C (n=1)
delta (n=13)
omicron (n=6)
neg (n=72)
NA
WHOvariant

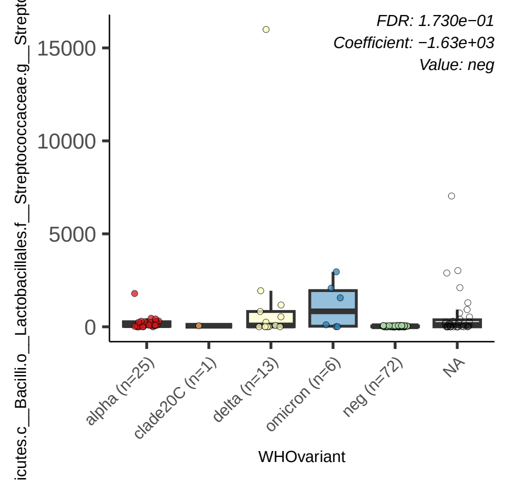
FDR: 1.730e−01
Coefficient: −1.63e+03
Value: neg
icutes.c__Bacilli.o__Lactobacillales.f__Streptococcaceae.g__Strept
15000
10000
5000
0
alpha (n=25)
clade20C (n=1)
delta (n=13)
omicron (n=6)
neg (n=72)
NA
WHOvariant

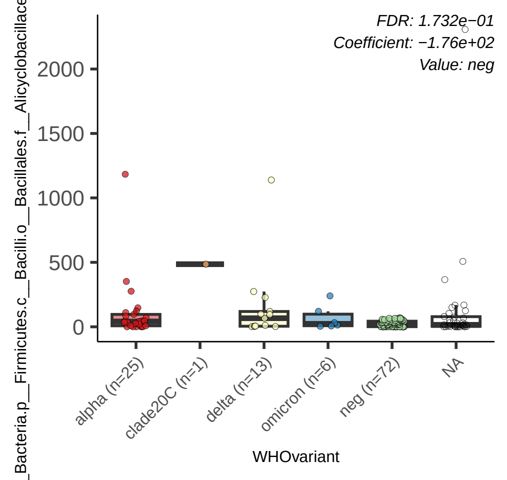

FDR: 1.732e−01
Coefficient: −1.76e+02
Value: neg
Bacteria.p__Firmicutes.c__Bacilli.o__Bacillales.f__Alicyclobacillace
2000
1500
1000
500
0
alpha (n=25)
clade20C (n=1)
delta (n=13)
omicron (n=6)
neg (n=72)
NA
WHOvariant

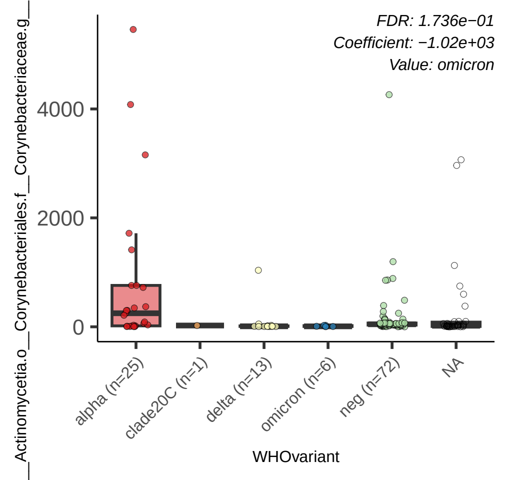

FDR: 1.736e−01
Coefficient: −1.02e+03
Value: omicron
__Actinomycetia.o__Corynebacteriales.f__Corynebacteriaceae.g__
0
2000
4000
alpha (n=25)
clade20C (n=1)
delta (n=13)
omicron (n=6)
neg (n=72)
NA
WHOvariant

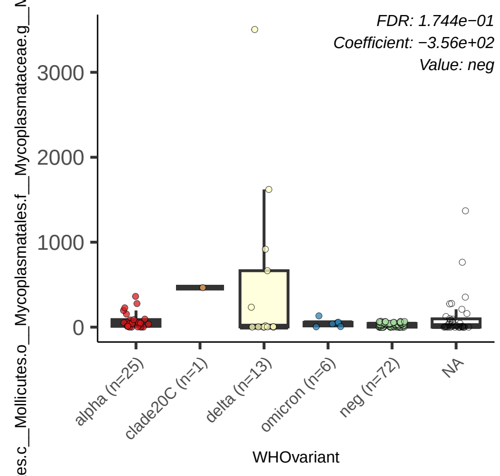

FDR: 1.744e−01
Coefficient: −3.56e+02
Value: neg
3000
2000
1000
0
tes.c__Mollicutes.o__Mycoplasmatales.f__Mycoplasmataceae.g__M
alpha (n=25)
clade20C (n=1)
delta (n=13)
omicron (n=6)
neg (n=72)
NA
WHOvariant

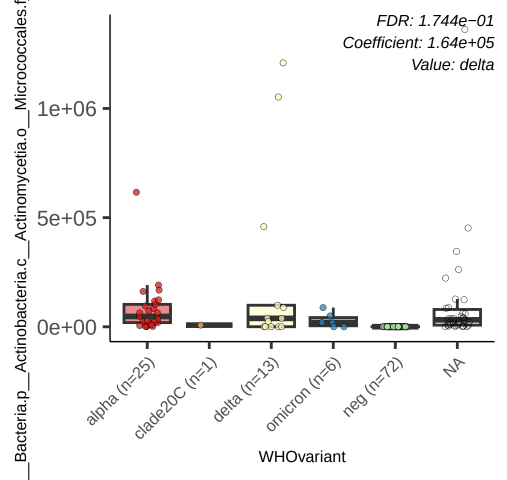

FDR: 1.744e−01
Coefficient: 1.64e+05
Value: delta
Bacteria.p__Actinobacteria.c__Actinomycetia.o__Micrococcales.f
1e+06
5e+05
0e+00
alpha (n=25)
clade20C (n=1)
delta (n=13)
omicron (n=6)
neg (n=72)
NA
WHOvariant

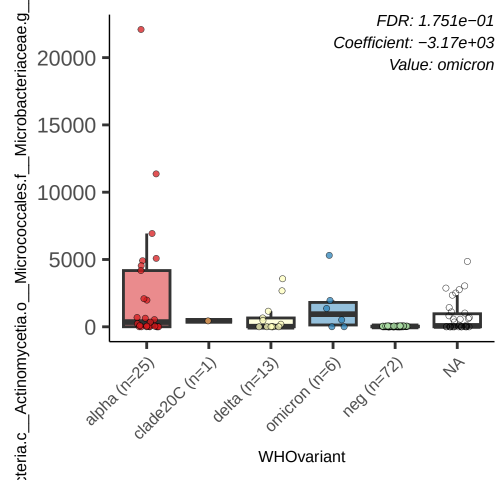

FDR: 1.751e−01
Coefficient: −3.17e+03
Value: omicron
cteria.c__Actinomycetia.o__Micrococcales.f__Microbacteriaceae.g__
20000
15000
10000
5000
0
alpha (n=25)
clade20C (n=1)
delta (n=13)
omicron (n=6)
neg (n=72)
NA
WHOvariant

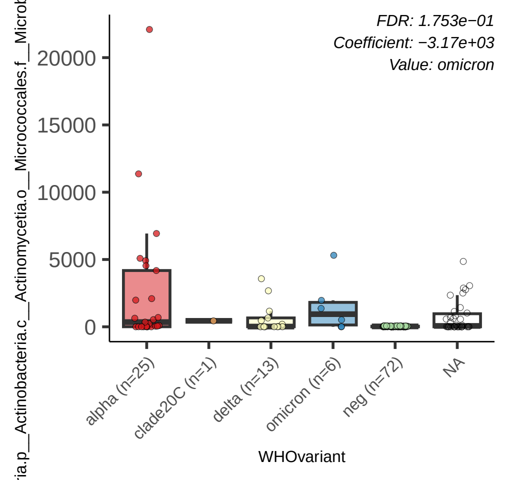
FDR: 1.753e−01
Coefficient: −3.17e+03
Value: omicron
0
5000
10000
15000
20000
ria.p__Actinobacteria.c__Actinomycetia.o__Micrococcales.f__Microb
alpha (n=25)
clade20C (n=1)
delta (n=13)
omicron (n=6)
neg (n=72)
NA
WHOvariant

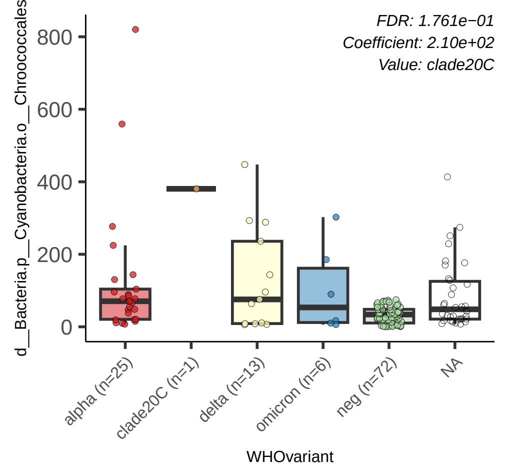
FDR: 1.761e−01
Coefficient: 2.10e+02
Value: clade20C
d__Bacteria.p__Cyanobacteria.o__Chroococcales
800
600
400
200
0
alpha (n=25)
clade20C (n=1)
delta (n=13)
omicron (n=6)
neg (n=72)
NA
WHOvariant

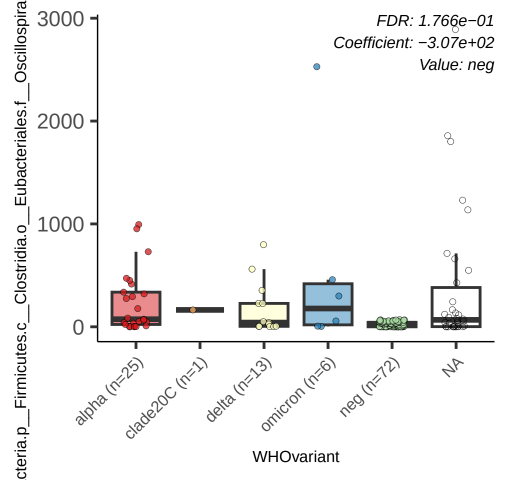
FDR: 1.766e−01
Coefficient: −3.07e+02
Value: neg
cteria.p__Firmicutes.c__Clostridia.o__Eubacteriales.f__Oscillospira
3000
2000
1000
0
alpha (n=25)
clade20C (n=1)
delta (n=13)
omicron (n=6)
neg (n=72)
NA
WHOvariant

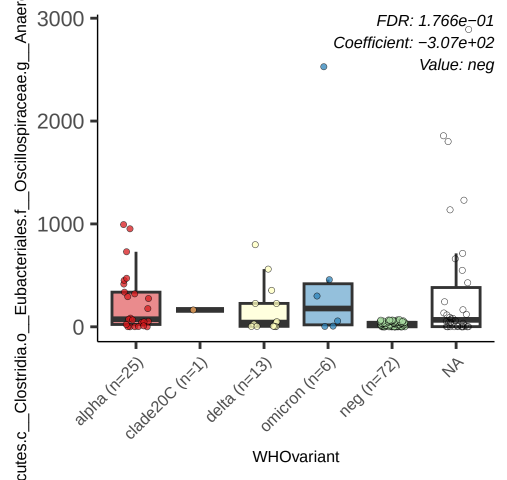
FDR: 1.766e−01
Coefficient: −3.07e+02
Value: neg
3000
2000
1000
0
cutes.c__Clostridia.o__Eubacteriales.f__Oscillospiraceae.g__Anaer
alpha (n=25)
clade20C (n=1)
delta (n=13)
omicron (n=6)
neg (n=72)
NA
WHOvariant

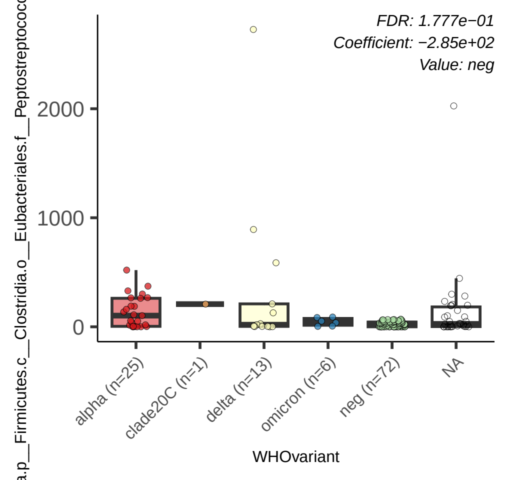
FDR: 1.777e−01
Coefficient: −2.85e+02
Value: neg
a.p__Firmicutes.c__Clostridia.o__Eubacteriales.f__Peptostreptococ
0
1000
2000
alpha (n=25)
clade20C (n=1)
delta (n=13)
omicron (n=6)
neg (n=72)
NA
WHOvariant

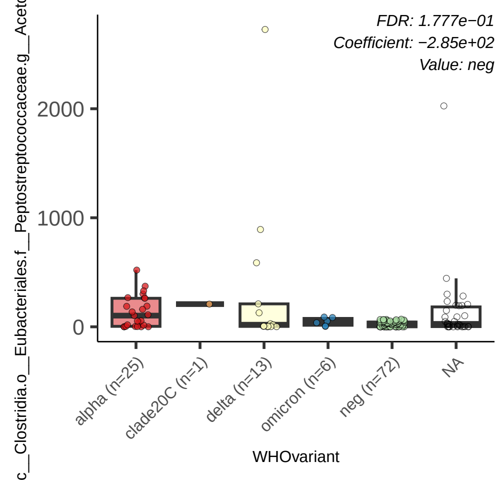

FDR: 1.777e−01
Coefficient: −2.85e+02
Value: neg
c__Clostridia.o__Eubacteriales.f__Peptostreptococcaceae.g__Aceto
2000
1000
0
alpha (n=25)
clade20C (n=1)
delta (n=13)
omicron (n=6)
neg (n=72)
NA
WHOvariant

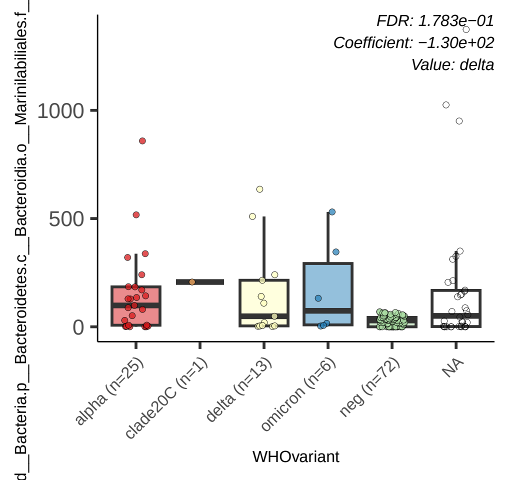

d__Bacteria.p__Bacteroidetes.c__Bacteroidia.o__Marinilabiliales.f
FDR: 1.783e−01
Coefficient: −1.30e+02
Value: delta
1000
500
0
alpha (n=25)
clade20C (n=1)
delta (n=13)
omicron (n=6)
neg (n=72)
NA
WHOvariant

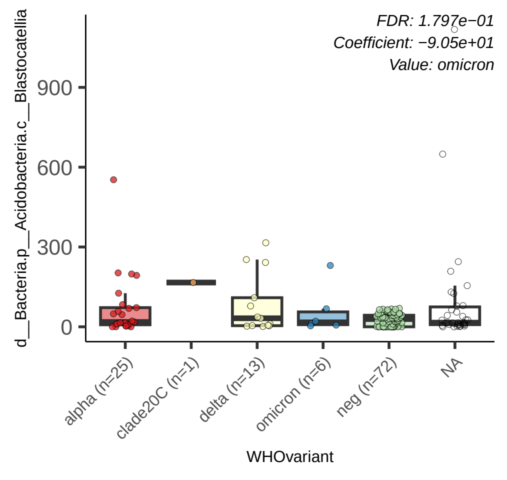
FDR: 1.797e−01
Coefficient: −9.05e+01
Value: omicron
d__Bacteria.p__Acidobacteria.c__Blastocatellia
900
600
300
0
alpha (n=25)
clade20C (n=1)
delta (n=13)
omicron (n=6)
neg (n=72)
NA
WHOvariant

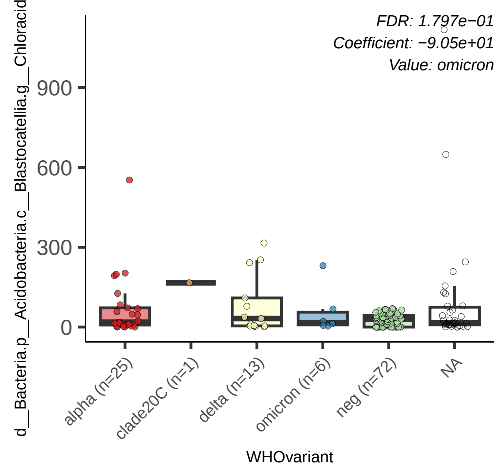
FDR: 1.797e−01
Coefficient: −9.05e+01
Value: omicron
d__Bacteria.p__Acidobacteria.c__Blastocatellia.g__Chloracid
900
600
300
0
alpha (n=25)
clade20C (n=1)
delta (n=13)
omicron (n=6)
neg (n=72)
NA
WHOvariant

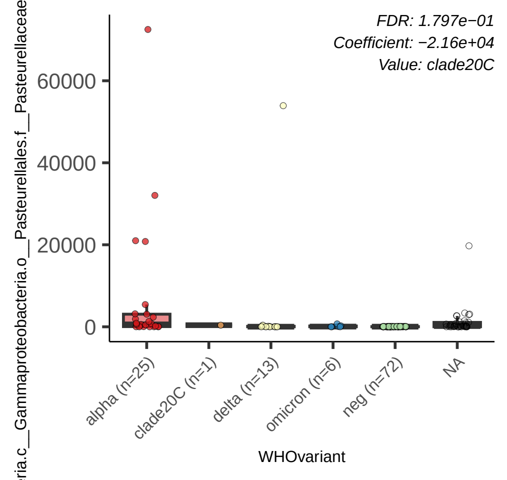

FDR: 1.797e−01
Coefficient: −2.16e+04
Value: clade20C
60000
40000
20000
0
ria.c__Gammaproteobacteria.o__Pasteurellales.f__Pasteurellaceae
alpha (n=25)
clade20C (n=1)
delta (n=13)
omicron (n=6)
neg (n=72)
NA
WHOvariant

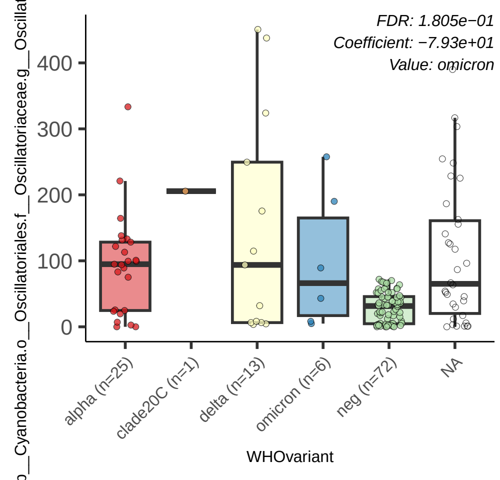

FDR: 1.805e−01
Coefficient: −7.93e+01
Value: omicron
o__Cyanobacteria.o__Oscillatoriales.f__Oscillatoriaceae.g__Oscillat
0
100
200
300
400
alpha (n=25)
clade20C (n=1)
delta (n=13)
omicron (n=6)
neg (n=72)
NA
WHOvariant

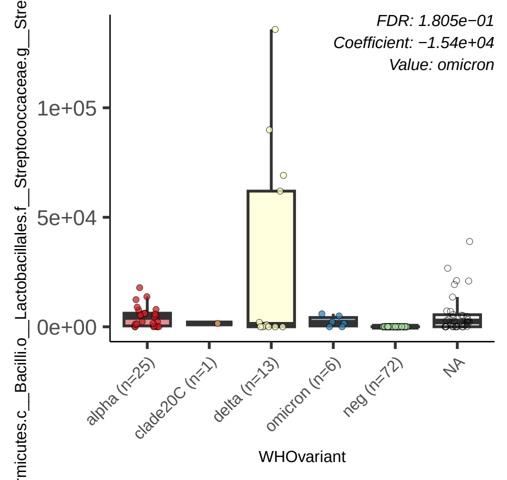

FDR: 1.805e−01
Coefficient: −1.54e+04
Value: omicron
1e+05
5e+04
0e+00
alpha (n=25)
clade20C (n=1)
delta (n=13)
omicron (n=6)
neg (n=72)
NA
WHOvariant
rmicutes.c__Bacilli.o__Lactobacillales.f__Streptococcaceae.g__Stre

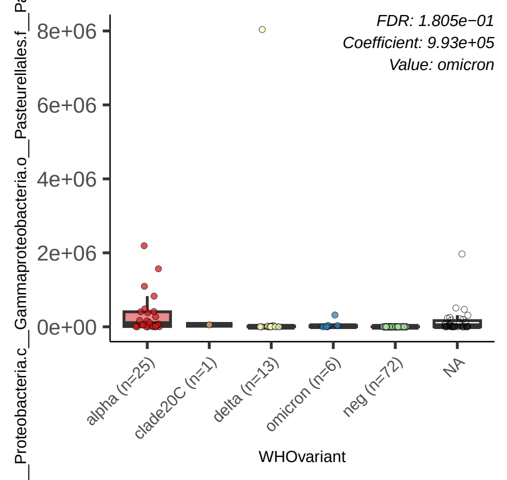

FDR: 1.805e−01
Coefficient: 9.93e+05
Value: omicron
8e+06
6e+06
4e+06
2e+06
0e+00
Proteobacteria.c__Gammaproteobacteria.o__Pasteurellales.f__Pa
alpha (n=25)
clade20C (n=1)
delta (n=13)
omicron (n=6)
neg (n=72)
NA
WHOvariant

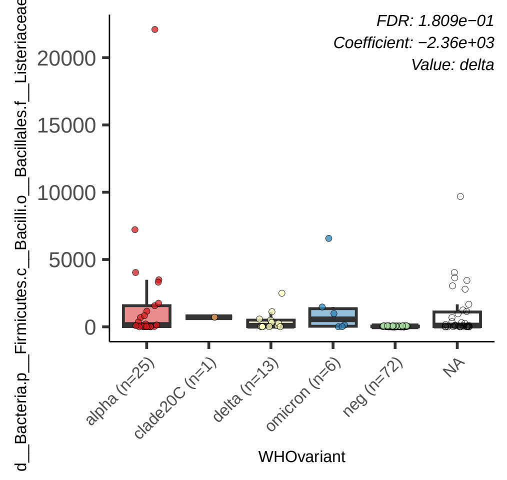
FDR: 1.809e−01
Coefficient: −2.36e+03
Value: delta
d__Bacteria.p__Firmicutes.c__Bacilli.o__Bacillales.f__Listeriaceae
20000
15000
10000
5000
0
alpha (n=25)
clade20C (n=1)
delta (n=13)
omicron (n=6)
neg (n=72)
NA
WHOvariant

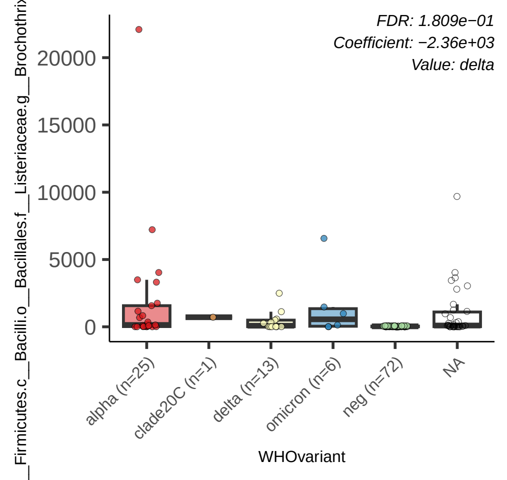

FDR: 1.809e−01
Coefficient: −2.36e+03
Value: delta
Firmicutes.c__Bacilli.o__Bacillales.f__Listeriaceae.g__Brochothrix
20000
15000
10000
5000
0
alpha (n=25)
clade20C (n=1)
delta (n=13)
omicron (n=6)
neg (n=72)
NA
WHOvariant

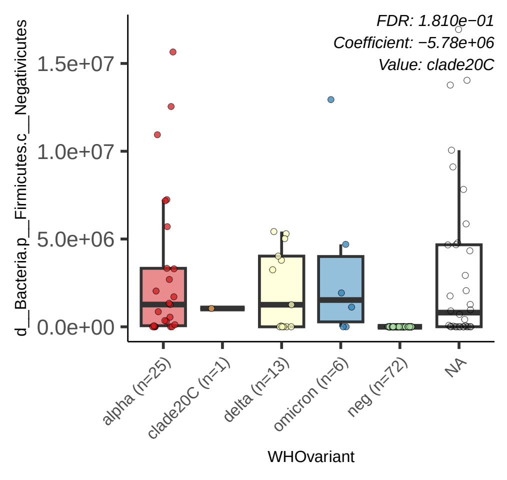
FDR: 1.810e−01
Coefficient: −5.78e+06
Value: clade20C
d__Bacteria.p__Firmicutes.c__Negativicutes
1.5e+07
1.0e+07
5.0e+06
0.0e+00
alpha (n=25)
clade20C (n=1)
delta (n=13)
omicron (n=6)
neg (n=72)
NA
WHOvariant

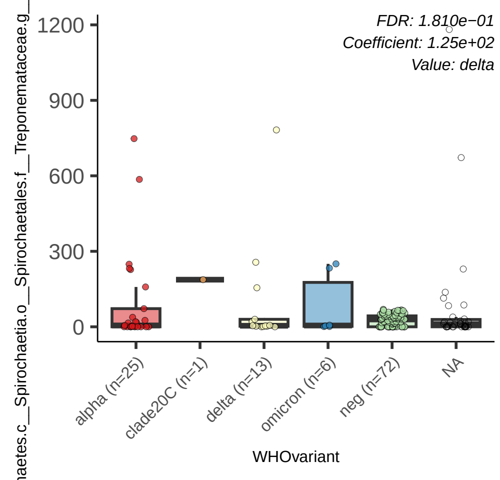
FDR: 1.810e−01
Coefficient: 1.25e+02
Value: delta
1200
900
600
300
0
haetes.c__Spirochaetia.o__Spirochaetales.f__Treponemataceae.g__
alpha (n=25)
clade20C (n=1)
delta (n=13)
omicron (n=6)
neg (n=72)
NA
WHOvariant

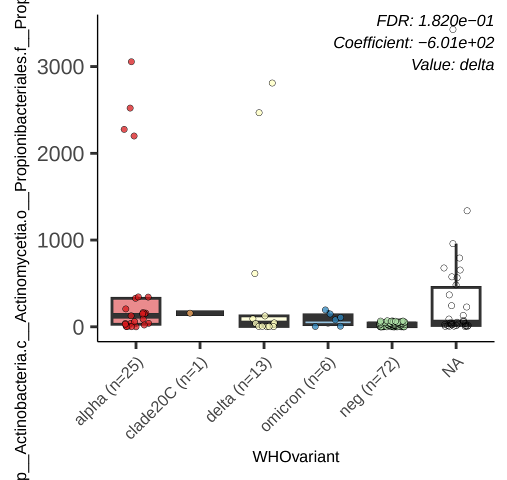
FDR: 1.820e−01
Coefficient: −6.01e+02
Value: delta
p__Actinobacteria.c__Actinomycetia.o__Propionibacteriales.f__Prop
3000
2000
1000
0
alpha (n=25)
clade20C (n=1)
delta (n=13)
omicron (n=6)
neg (n=72)
NA
WHOvariant

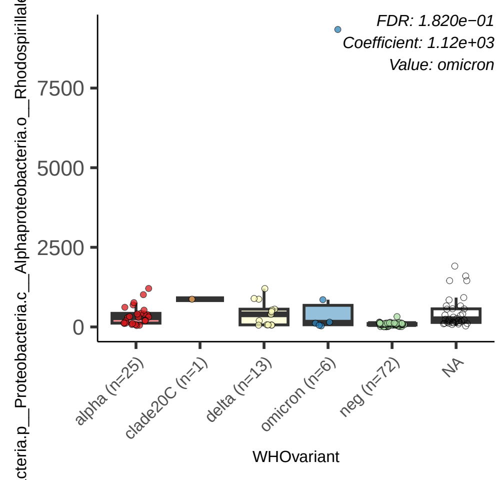
FDR: 1.820e−01
Coefficient: 1.12e+03
Value: omicron
7500
5000
2500
0
alpha (n=25)
clade20C (n=1)
delta (n=13)
omicron (n=6)
neg (n=72)
NA
WHOvariant
cteria.p__Proteobacteria.c__Alphaproteobacteria.o__Rhodospirillale

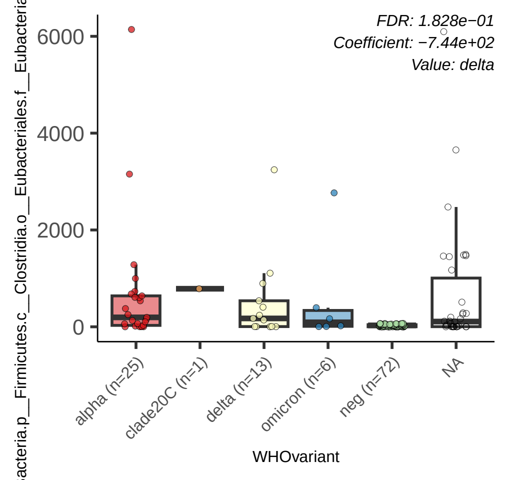

FDR: 1.828e−01
Coefficient: −7.44e+02
Value: delta
6000
4000
2000
0
acteria.p__Firmicutes.c__Clostridia.o__Eubacteriales.f__Eubacteria
alpha (n=25)
clade20C (n=1)
delta (n=13)
omicron (n=6)
neg (n=72)
NA
WHOvariant

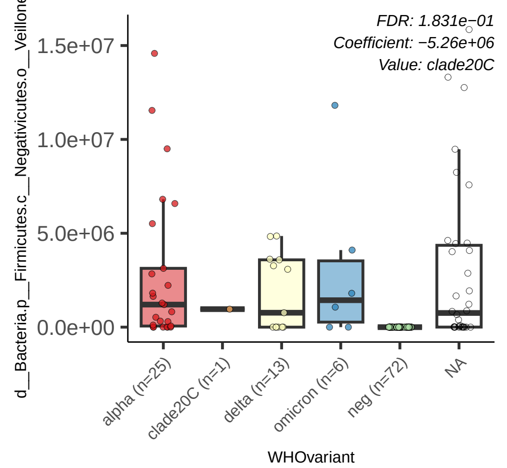

FDR: 1.831e−01
Coefficient: −5.26e+06
Value: clade20C
d__Bacteria.p__Firmicutes.c__Negativicutes.o__Veillone
1.5e+07
1.0e+07
5.0e+06
0.0e+00
alpha (n=25)
clade20C (n=1)
delta (n=13)
omicron (n=6)
neg (n=72)
NA
WHOvariant

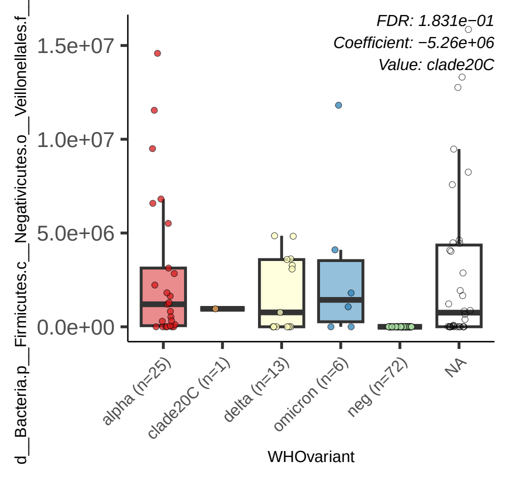

FDR: 1.831e−01
Coefficient: −5.26e+06
Value: clade20C
d__Bacteria.p__Firmicutes.c__Negativicutes.o__Veillonellales.f
1.5e+07
1.0e+07
5.0e+06
0.0e+00
alpha (n=25)
clade20C (n=1)
delta (n=13)
omicron (n=6)
neg (n=72)
NA
WHOvariant

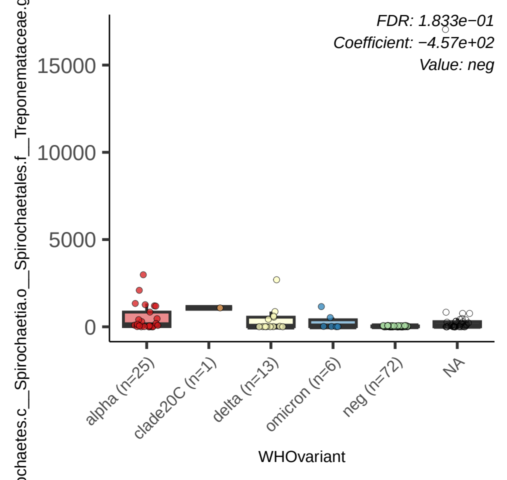

FDR: 1.833e−01
Coefficient: −4.57e+02
Value: neg
15000
10000
5000
0
ochaetes.c__Spirochaetia.o__Spirochaetales.f__Treponemataceae.g
alpha (n=25)
clade20C (n=1)
delta (n=13)
omicron (n=6)
neg (n=72)
NA
WHOvariant

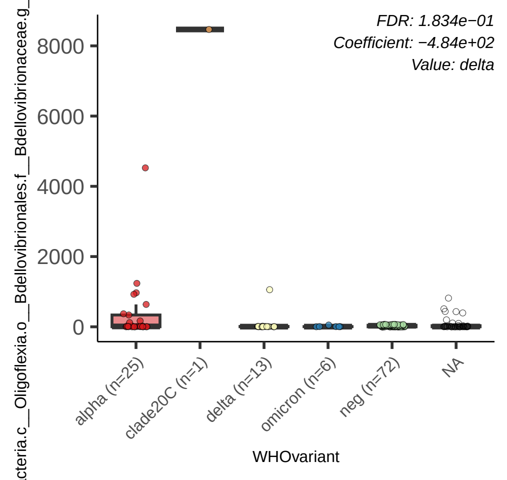

FDR: 1.834e−01
Coefficient: −4.84e+02
Value: delta
8000
6000
4000
2000
0
cteria.c__Oligoflexia.o__Bdellovibrionales.f__Bdellovibrionaceae.g_
alpha (n=25)
clade20C (n=1)
delta (n=13)
omicron (n=6)
neg (n=72)
NA
WHOvariant

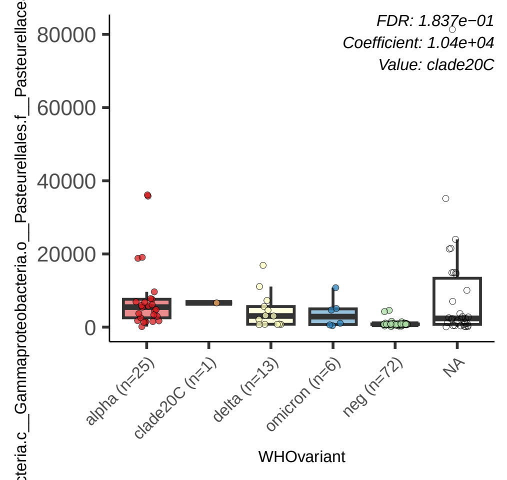
FDR: 1.837e−01
Coefficient: 1.04e+04
Value: clade20C
80000
60000
40000
20000
0
cteria.c__Gammaproteobacteria.o__Pasteurellales.f__Pasteurellace
alpha (n=25)
clade20C (n=1)
delta (n=13)
omicron (n=6)
neg (n=72)
NA
WHOvariant

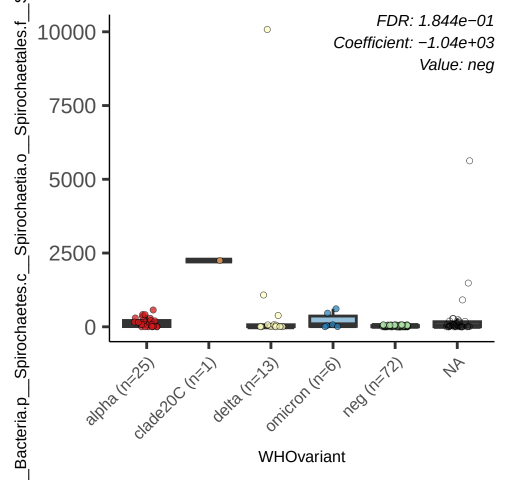
FDR: 1.844e−01
Coefficient: −1.04e+03
Value: neg
10000
7500
5000
2500
0
alpha (n=25)
clade20C (n=1)
delta (n=13)
omicron (n=6)
neg (n=72)
NA
WHOvariant
Bacteria.p__Spirochaetes.c__Spirochaetia.o__Spirochaetales.f__

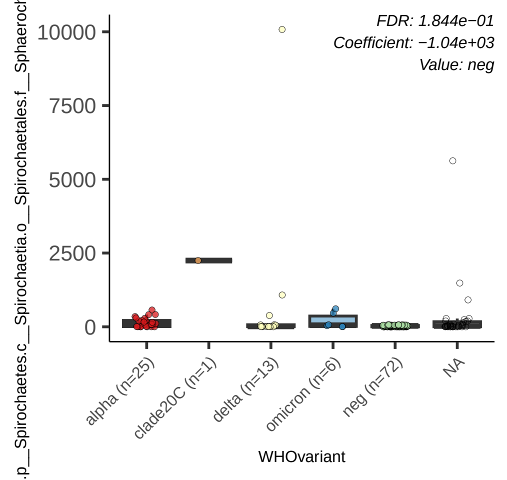

FDR: 1.844e−01
Coefficient: −1.04e+03
Value: neg
10000
7500
5000
2500
0
alpha (n=25)
clade20C (n=1)
delta (n=13)
omicron (n=6)
neg (n=72)
NA
WHOvariant
.p__Spirochaetes.c__Spirochaetia.o__Spirochaetales.f__Sphaeroch

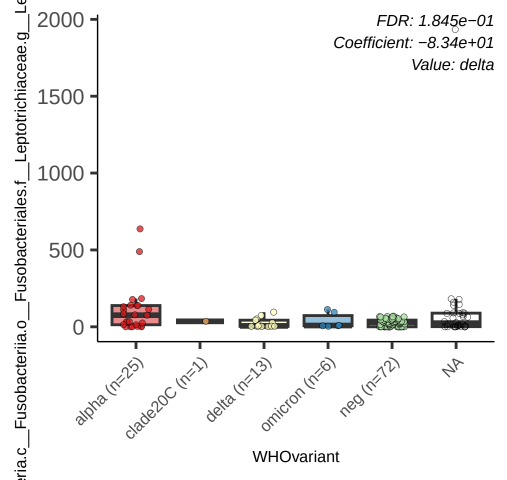

FDR: 1.845e−01
Coefficient: −8.34e+01
Value: delta
2000
1500
1000
500
0
Fusobacteriia.c__Fusobacteriia.o__Fusobacteriales.f__Leptotrichiaceae.g__Le
alpha (n=25)
clade20C (n=1)
delta (n=13)
omicron (n=6)
neg (n=72)
NA
WHOvariant

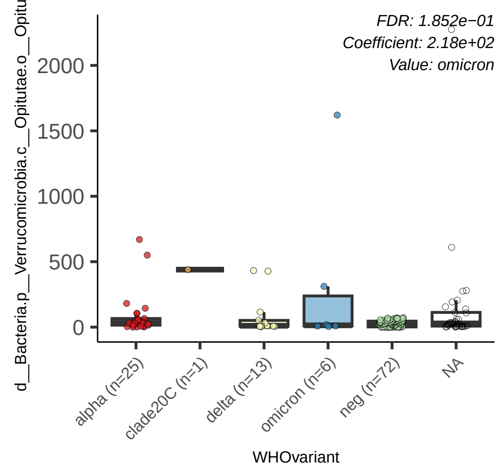
FDR: 1.852e−01
Coefficient: 2.18e+02
Value: omicron
d__Bacteria.p__Verrucomicrobia.c__Opitutae.o__Opitu
0
500
1000
1500
2000
alpha (n=25)
clade20C (n=1)
delta (n=13)
omicron (n=6)
neg (n=72)
NA
WHOvariant

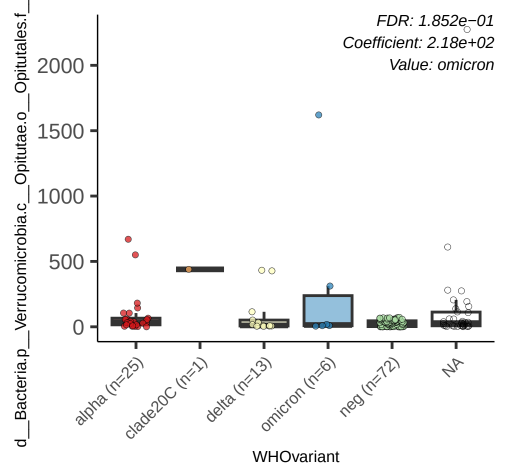

FDR: 1.852e−01
Coefficient: 2.18e+02
Value: omicron
d__Bacteria.p__Verrucomicrobia.c__Opitutae.o__Opitutales.f__
0
500
1000
1500
2000
alpha (n=25)
clade20C (n=1)
delta (n=13)
omicron (n=6)
neg (n=72)
NA
WHOvariant

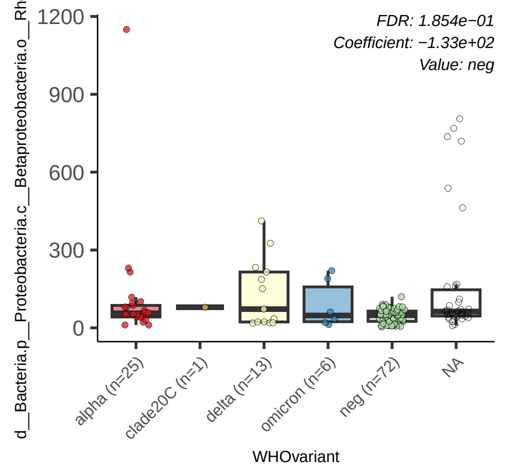
FDR: 1.854e−01
Coefficient: −1.33e+02
Value: neg
d__Bacteria.p__Proteobacteria.c__Betaproteobacteria.o__Rh
1200
900
600
300
0
alpha (n=25)
clade20C (n=1)
delta (n=13)
omicron (n=6)
neg (n=72)
NA
WHOvariant

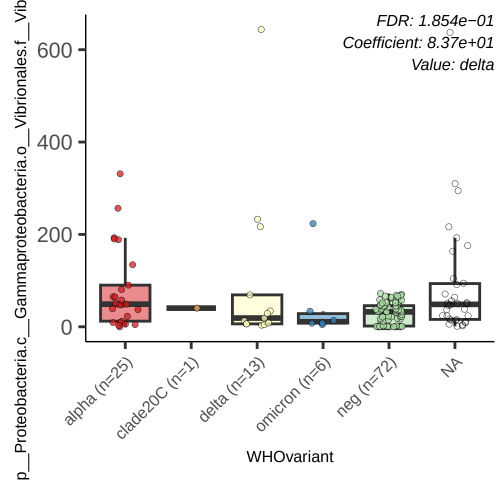

FDR: 1.854e−01
Coefficient: 8.37e+01
Value: delta
p__Proteobacteria.c__Gammaproteobacteria.o__Vibrionales.f__Vib
0
200
400
600
alpha (n=25)
clade20C (n=1)
delta (n=13)
omicron (n=6)
neg (n=72)
NA
WHOvariant

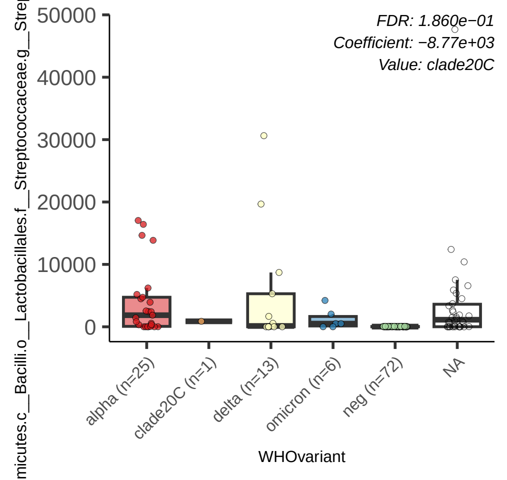

FDR: 1.860e−01
Coefficient: −8.77e+03
Value: clade20C
micutes.c__Bacilli.o__Lactobacillales.f__Streptococcaceae.g__Strep
50000
40000
30000
20000
10000
0
alpha (n=25)
clade20C (n=1)
delta (n=13)
omicron (n=6)
neg (n=72)
NA
WHOvariant

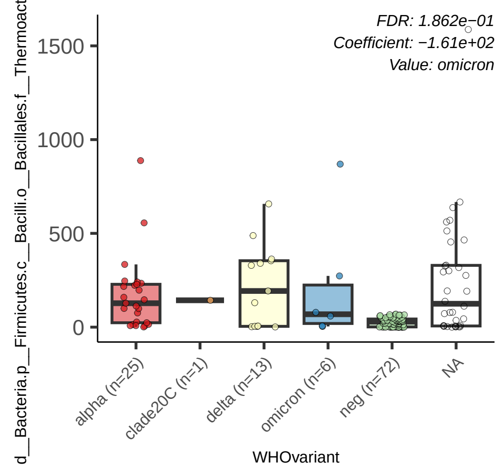

FDR: 1.862e−01
Coefficient: −1.61e+02
Value: omicron
d__Bacteria.p__Firmicutes.c__Bacilli.o__Bacillales.f__Thermoact
1500
1000
500
0
alpha (n=25)
clade20C (n=1)
delta (n=13)
omicron (n=6)
neg (n=72)
NA
WHOvariant

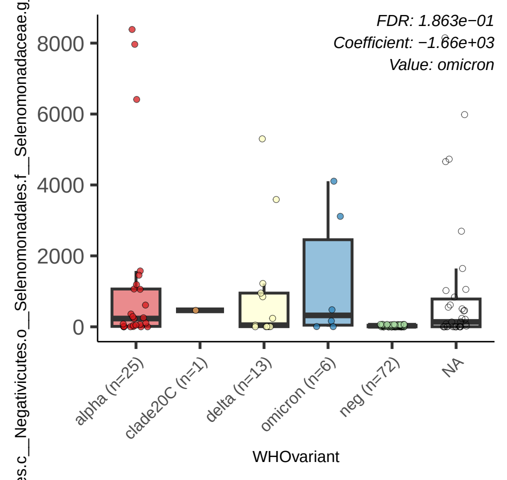
FDR: 1.863e−01
Coefficient: −1.66e+03
Value: omicron
es.c__Negativicutes.o__Selenomonadales.f__Selenomonadaceae.g_
8000
6000
4000
2000
0
alpha (n=25)
clade20C (n=1)
delta (n=13)
omicron (n=6)
neg (n=72)
NA
WHOvariant

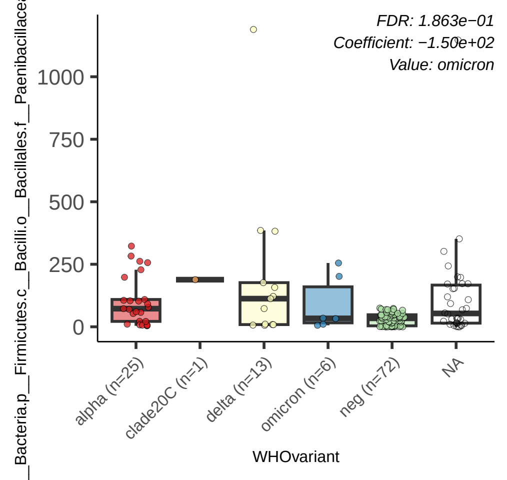

FDR: 1.863e−01
Coefficient: −1.50e+02
Value: omicron
Bacteria.p__Firmicutes.c__Bacilli.o__Bacillales.f__Paenibacillace
1000
750
500
250
0
alpha (n=25)
clade20C (n=1)
delta (n=13)
omicron (n=6)
neg (n=72)
NA
WHOvariant

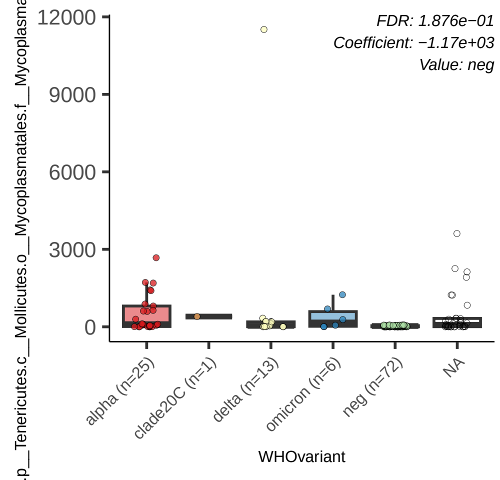

FDR: 1.876e−01
Coefficient: −1.17e+03
Value: neg
12000
9000
6000
3000
0
.p__Tenericutes.c__Mollicutes.o__Mycoplasmatales.f__Mycoplasma
alpha (n=25)
clade20C (n=1)
delta (n=13)
omicron (n=6)
neg (n=72)
NA
WHOvariant

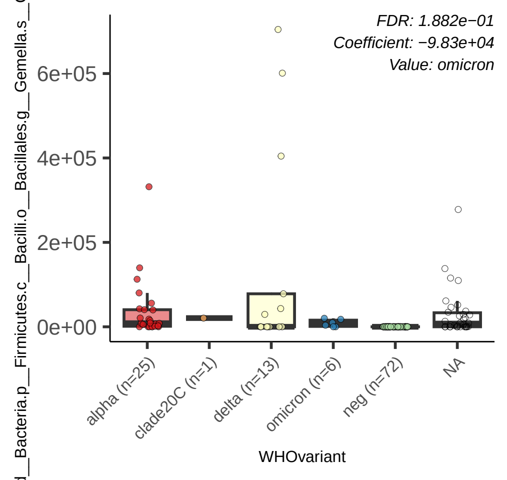

FDR: 1.882e−01
Coefficient: −9.83e+04
Value: omicron
d__Bacteria.p__Firmicutes.c__Bacilli.o__Bacillales.g__Gemella.s__G
6e+05
4e+05
2e+05
0e+00
alpha (n=25)
clade20C (n=1)
delta (n=13)
omicron (n=6)
neg (n=72)
NA
WHOvariant

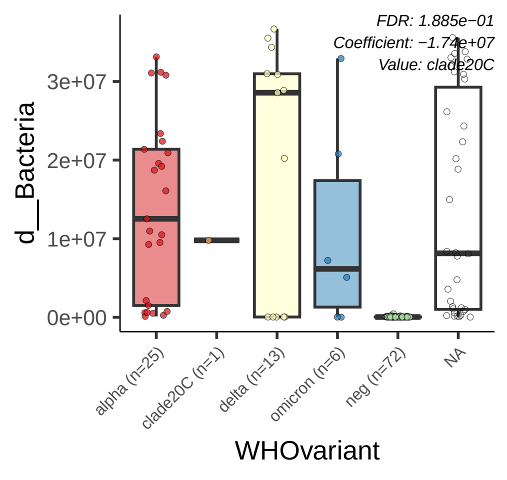
FDR: 1.885e−01
Coefficient: −1.74e+07
Value: clade20C
d__Bacteria
3e+07
2e+07
1e+07
0e+00
alpha (n=25)
clade20C (n=1)
delta (n=13)
omicron (n=6)
neg (n=72)
NA
WHOvariant

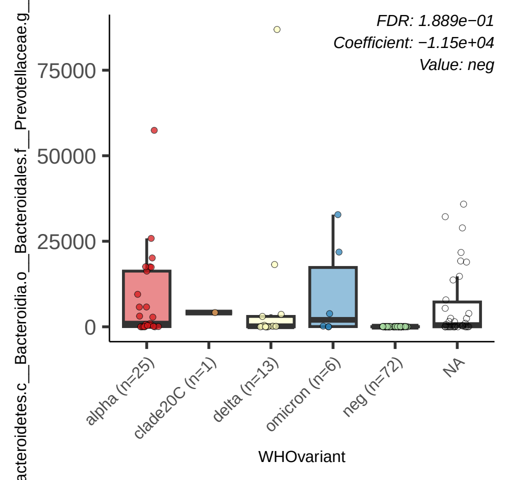
FDR: 1.889e−01
Coefficient: −1.15e+04
Value: neg
acteroidetes.c__Bacteroidia.o__Bacteroidales.f__Prevotellaceae.g__
75000
50000
25000
0
alpha (n=25)
clade20C (n=1)
delta (n=13)
omicron (n=6)
neg (n=72)
NA
WHOvariant

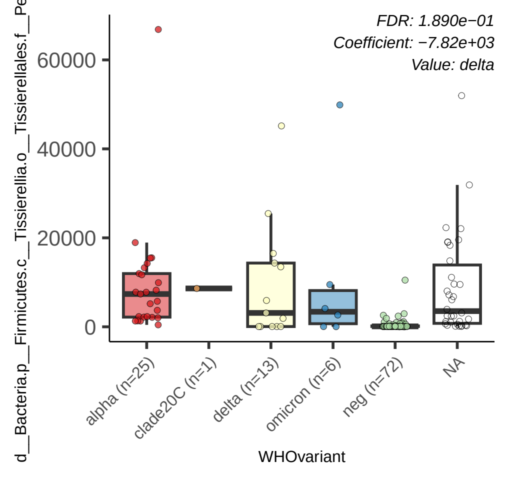

FDR: 1.890e−01
Coefficient: −7.82e+03
Value: delta
d__Bacteria.p__Firmicutes.c__Tissierellia.o__Tissierellales.f__Pe
60000
40000
20000
0
alpha (n=25)
clade20C (n=1)
delta (n=13)
omicron (n=6)
neg (n=72)
NA
WHOvariant

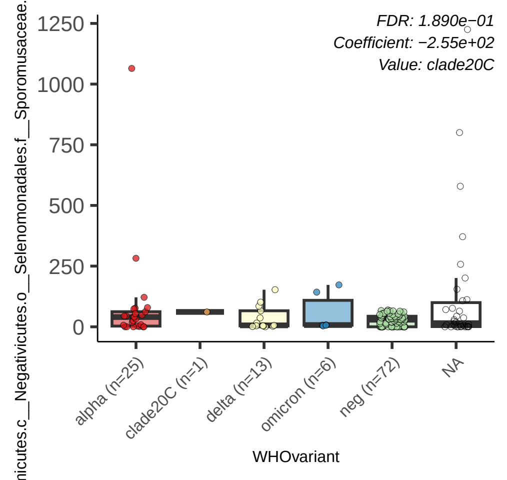

FDR: 1.890e−01
Coefficient: −2.55e+02
Value: clade20C
nicutes.c__Negativicutes.o__Selenomonadales.f__Sporomusaceae.
1250
1000
750
500
250
0
alpha (n=25)
clade20C (n=1)
delta (n=13)
omicron (n=6)
neg (n=72)
NA
WHOvariant

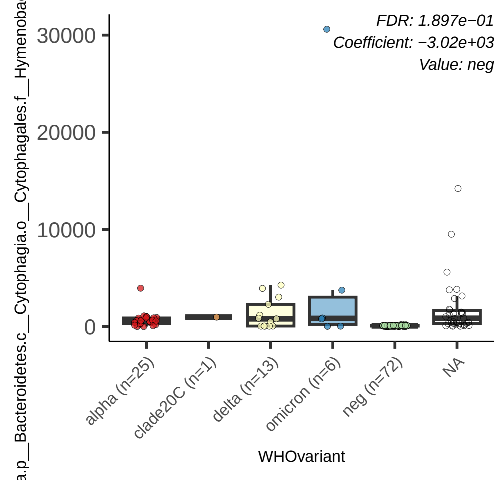

FDR: 1.897e−01
Coefficient: −3.02e+03
Value: neg
30000
20000
10000
0
a.p__Bacteroidetes.c__Cytophagia.o__Cytophagales.f__Hymenoba
alpha (n=25)
clade20C (n=1)
delta (n=13)
omicron (n=6)
neg (n=72)
NA
WHOvariant

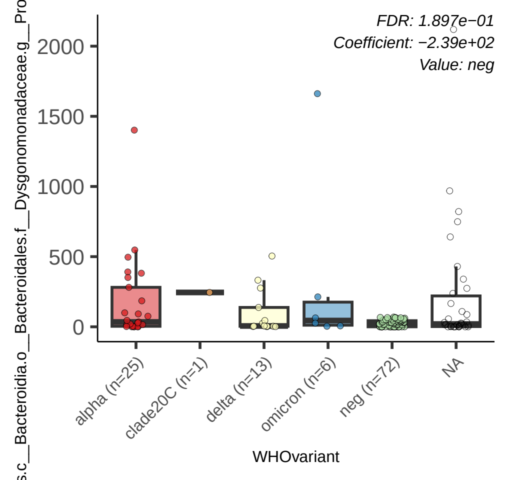

FDR: 1.897e−01
Coefficient: −2.39e+02
Value: neg
s.c__Bacteroidia.o__Bacteroidales.f__Dysgonomonadaceae.g__Pro
0
500
1000
1500
2000
alpha (n=25)
clade20C (n=1)
delta (n=13)
omicron (n=6)
neg (n=72)
NA
WHOvariant

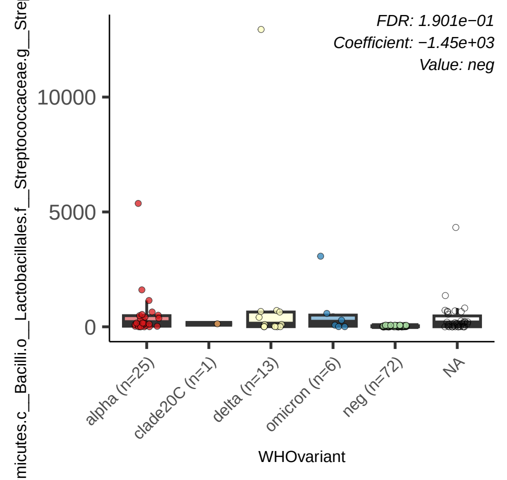
FDR: 1.901e−01
Coefficient: −1.45e+03
Value: neg
10000
5000
0
alpha (n=25)
clade20C (n=1)
delta (n=13)
omicron (n=6)
neg (n=72)
NA
WHOvariant
micutes.c__Bacilli.o__Lactobacillales.f__Streptococcaceae.g__Stre

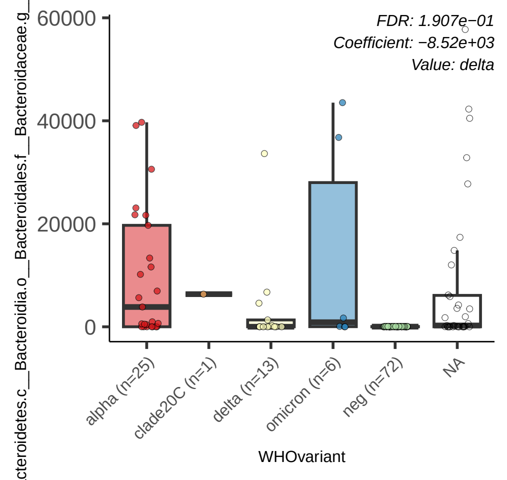
FDR: 1.907e−01
Coefficient: −8.52e+03
Value: delta
60000
40000
20000
0
cteroidetes.c__Bacteroidia.o__Bacteroidales.f__Bacteroidaceae.g__
alpha (n=25)
clade20C (n=1)
delta (n=13)
omicron (n=6)
neg (n=72)
NA
WHOvariant

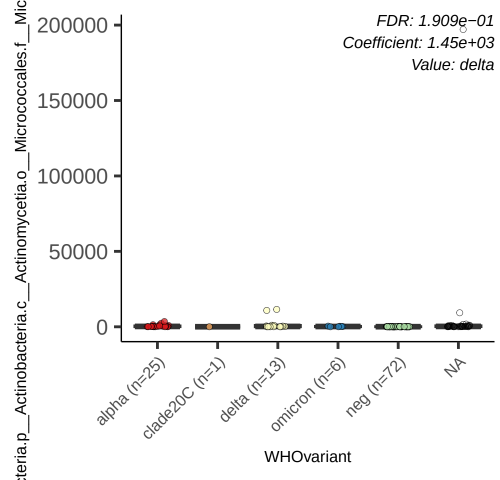

cteria.p__Actinobacteria.c__Actinomycetia.o__Micrococcales.f__Mic
FDR: 1.909e−01
Coefficient: 1.45e+03
Value: delta
200000
150000
100000
50000
0
alpha (n=25)
clade20C (n=1)
delta (n=13)
omicron (n=6)
neg (n=72)
NA
WHOvariant

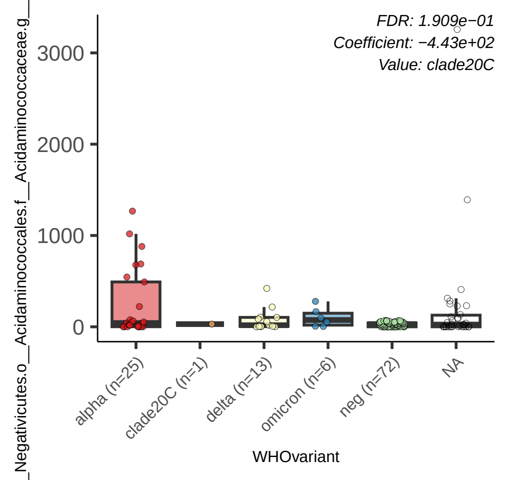

FDR: 1.909e−01
Coefficient: −4.43e+02
Value: clade20C
Negativicutes.o__Acidaminococcales.f__Acidaminococcaceae.g__
3000
2000
1000
0
alpha (n=25)
clade20C (n=1)
delta (n=13)
omicron (n=6)
neg (n=72)
NA
WHOvariant

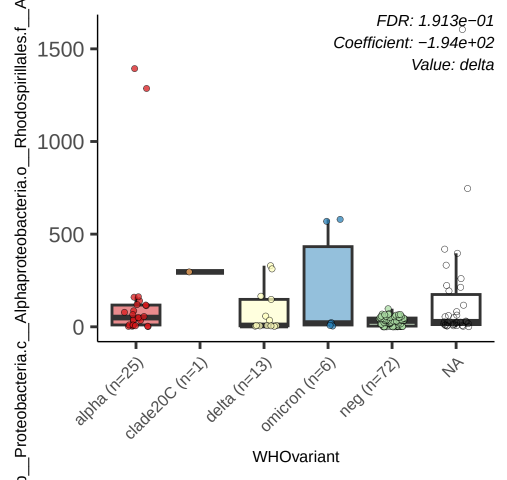
FDR: 1.913e−01
Coefficient: −1.94e+02
Value: delta
1500
1000
500
0
Proteobacteria.c__Alphaproteobacteria.o__Rhodospirillales.f__A
alpha (n=25)
clade20C (n=1)
delta (n=13)
omicron (n=6)
neg (n=72)
NA
WHOvariant

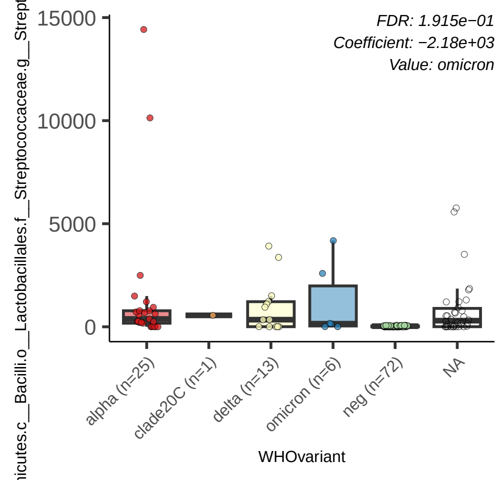
FDR: 1.915e−01
Coefficient: −2.18e+03
Value: omicron
15000
10000
5000
0
alpha (n=25)
clade20C (n=1)
delta (n=13)
omicron (n=6)
neg (n=72)
NA
WHOvariant
nicutes.c__Bacilli.o__Lactobacillales.f__Streptococcaceae.g__Strept

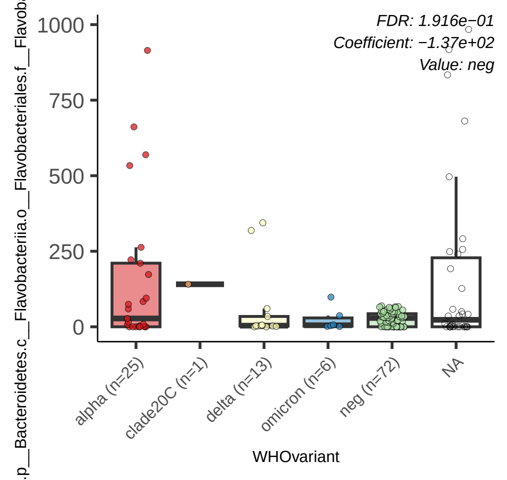

FDR: 1.916e−01
Coefficient: −1.37e+02
Value: neg
1000
750
500
250
0
p__Bacteroidetes.c__Flavobacteriia.o__Flavobacteriales.f__Flavob
alpha (n=25)
clade20C (n=1)
delta (n=13)
omicron (n=6)
neg (n=72)
NA
WHOvariant

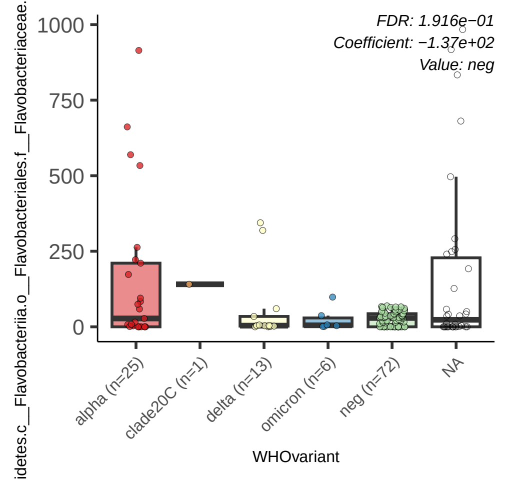

FDR: 1.916e−01
Coefficient: −1.37e+02
Value: neg
1000
750
500
250
0
idetes.c__Flavobacteriia.o__Flavobacteriales.f__Flavobacteriaceae.
alpha (n=25)
clade20C (n=1)
delta (n=13)
omicron (n=6)
neg (n=72)
NA
WHOvariant

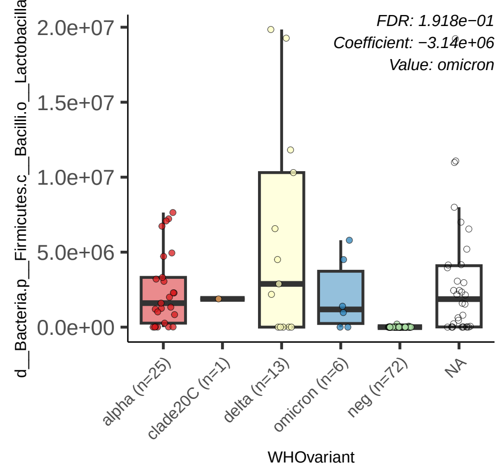

FDR: 1.918e−01
Coefficient: −3.14e+06
Value: omicron
d__Bacteria.p__Firmicutes.c__Bacilli.o__Lactobacilla
2.0e+07
1.5e+07
1.0e+07
5.0e+06
0.0e+00
alpha (n=25)
clade20C (n=1)
delta (n=13)
omicron (n=6)
neg (n=72)
NA
WHOvariant

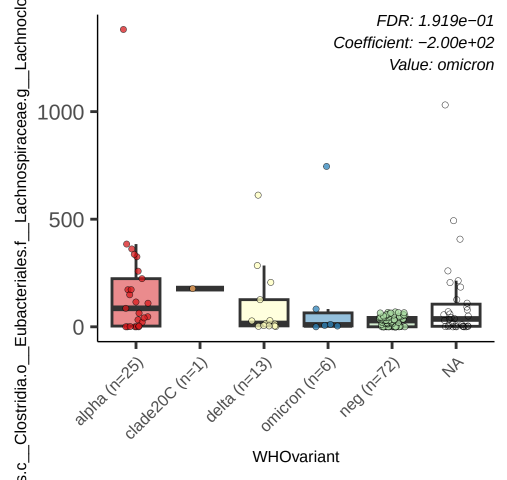

FDR: 1.919e−01
Coefficient: −2.00e+02
Value: omicron
s.c__Clostridia.o__Eubacteriales.f__Lachnospiraceae.g__Lachnoclo
1000
500
0
alpha (n=25)
clade20C (n=1)
delta (n=13)
omicron (n=6)
neg (n=72)
NA
WHOvariant

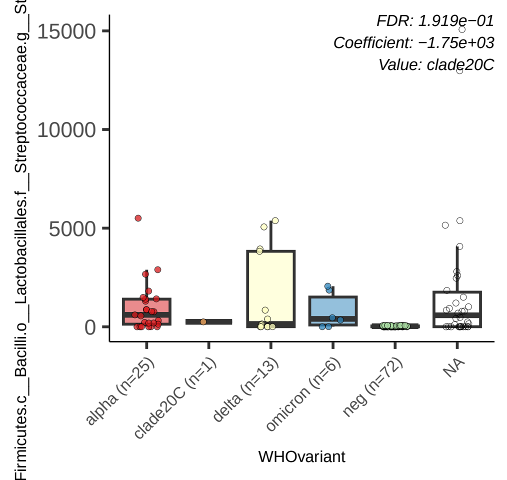
FDR: 1.919e−01
Coefficient: −1.75e+03
Value: clade20C
15000
10000
5000
0
Firmicutes.c__Bacilli.o__Lactobacillales.f__Streptococcaceae.g__St
alpha (n=25)
clade20C (n=1)
delta (n=13)
omicron (n=6)
neg (n=72)
NA
WHOvariant

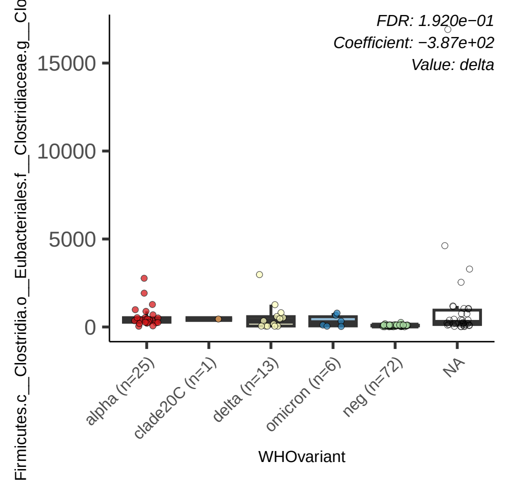

FDR: 1.920e−01
Coefficient: −3.87e+02
Value: delta
15000
10000
5000
0
Firmicutes.c__Clostridia.o__Eubacteriales.f__Clostridiaceae.g__Clo
alpha (n=25)
clade20C (n=1)
delta (n=13)
omicron (n=6)
neg (n=72)
NA
WHOvariant

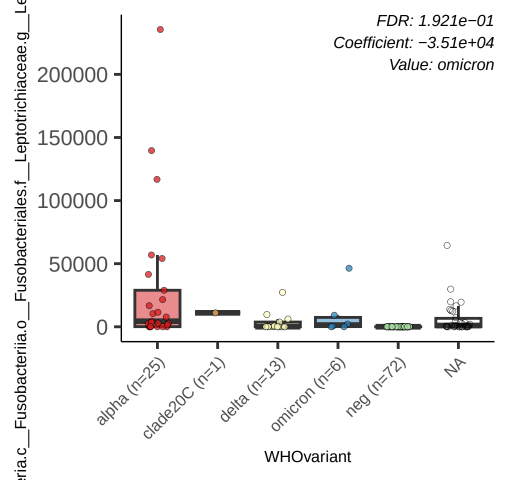

FDR: 1.921e−01
Coefficient: −3.51e+04
Value: omicron
eria.c__Fusobacteriia.o__Fusobacteriales.f__Leptotrichiaceae.g__Le
200000
150000
100000
50000
0
alpha (n=25)
clade20C (n=1)
delta (n=13)
omicron (n=6)
neg (n=72)
NA
WHOvariant

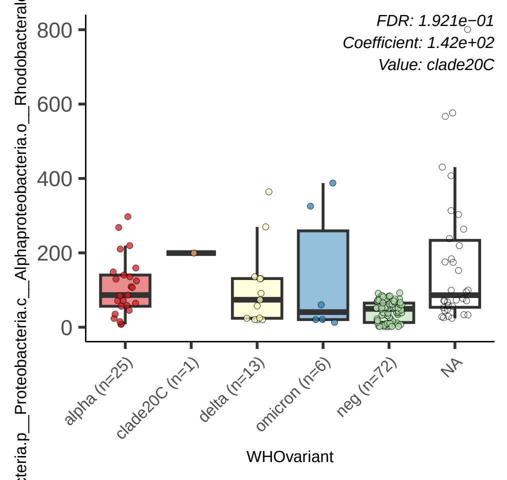

FDR: 1.921e−01
Coefficient: 1.42e+02
Value: clade20C
0
200
400
600
800
cteria.p__Proteobacteria.c__Alphaproteobacteria.o__Rhodobacterale
alpha (n=25)
clade20C (n=1)
delta (n=13)
omicron (n=6)
neg (n=72)
NA
WHOvariant

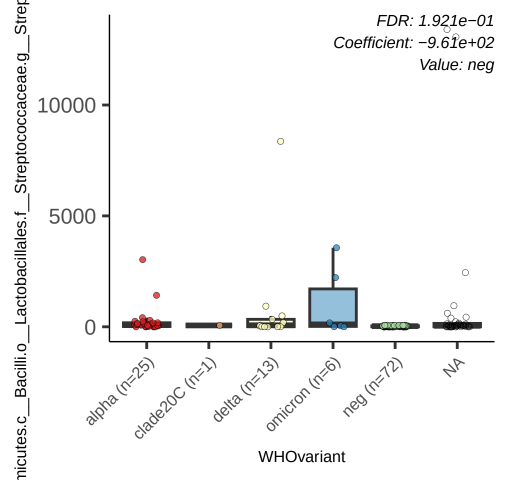

FDR: 1.921e−01
Coefficient: −9.61e+02
Value: neg
10000
5000
0
alpha (n=25)
clade20C (n=1)
delta (n=13)
omicron (n=6)
neg (n=72)
NA
WHOvariant
micutes.c__Bacilli.o__Lactobacillales.f__Streptococcaceae.g__Strep

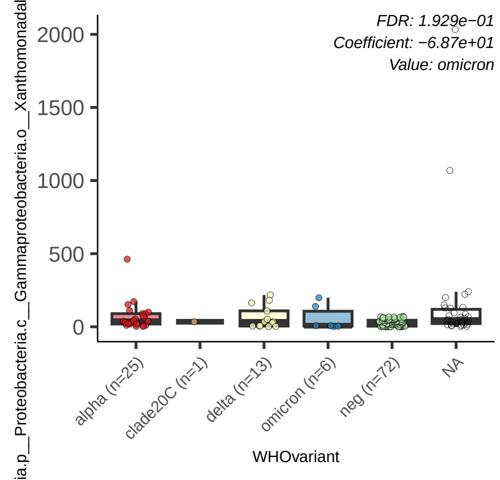
FDR: 1.929e−01
Coefficient: −6.87e+01
Value: omicron
2000
1500
1000
500
0
ia.p__Proteobacteria.c__Gammaproteobacteria.o__Xanthomonadal
alpha (n=25)
clade20C (n=1)
delta (n=13)
omicron (n=6)
neg (n=72)
NA
WHOvariant

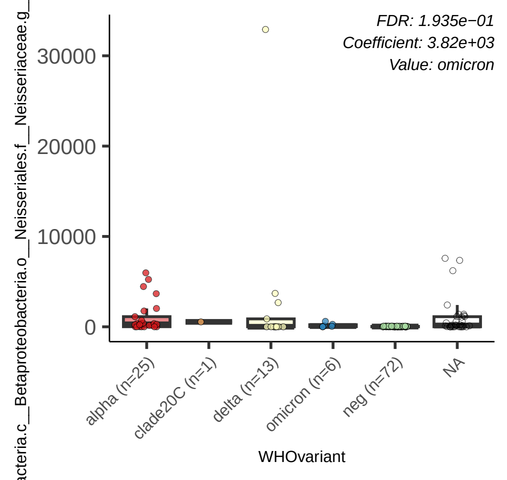

FDR: 1.935e−01
Coefficient: 3.82e+03
Value: omicron
30000
20000
10000
0
alpha (n=25)
clade20C (n=1)
delta (n=13)
omicron (n=6)
neg (n=72)
NA
WHOvariant
acteria.c__Betaproteobacteria.o__Neisseriales.f__Neisseriaceae.g__

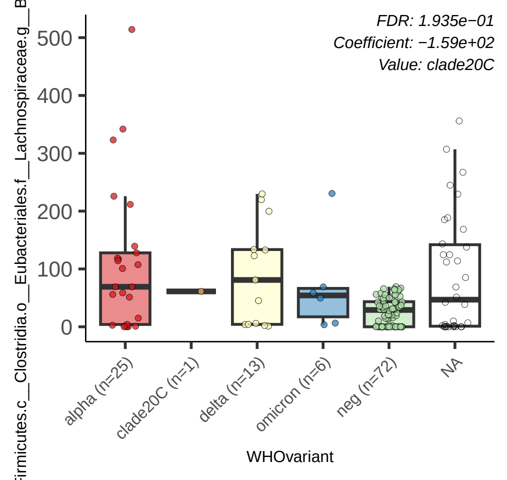

FDR: 1.935e−01
Coefficient: −1.59e+02
Value: clade20C
Firmicutes.c__Clostridia.o__Eubacteriales.f__Lachnospiraceae.g__B
0
100
200
300
400
500
alpha (n=25)
clade20C (n=1)
delta (n=13)
omicron (n=6)
neg (n=72)
NA
WHOvariant

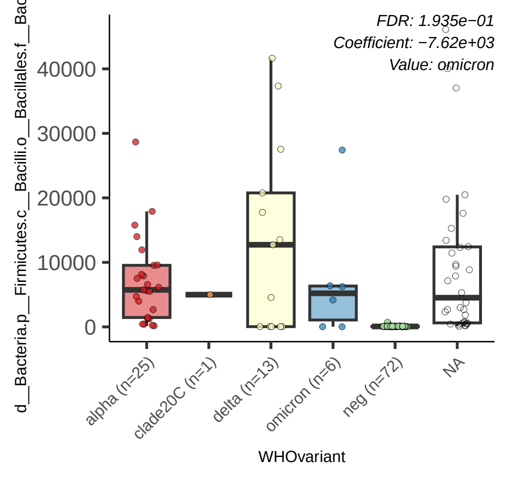

FDR: 1.935e−01
Coefficient: −7.62e+03
Value: omicron
d__Bacteria.p__Firmicutes.c__Bacilli.o__Bacillales.f__Bac
40000
30000
20000
10000
0
alpha (n=25)
clade20C (n=1)
delta (n=13)
omicron (n=6)
neg (n=72)
NA
WHOvariant

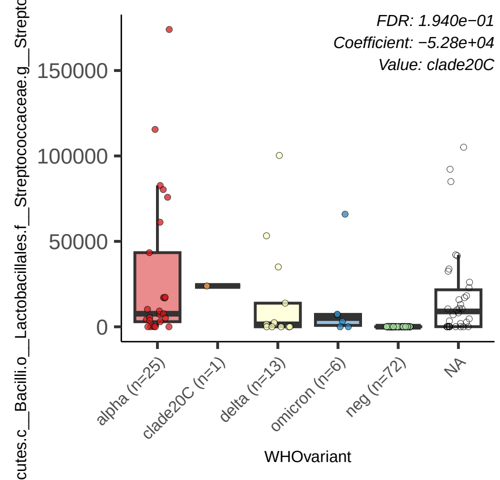

FDR: 1.940e−01
Coefficient: −5.28e+04
Value: clade20C
150000
100000
50000
0
alpha (n=25)
clade20C (n=1)
delta (n=13)
omicron (n=6)
neg (n=72)
NA
WHOvariant
cutes.c__Bacilli.o__Lactobacillales.f__Streptococcaceae.g__Strepto

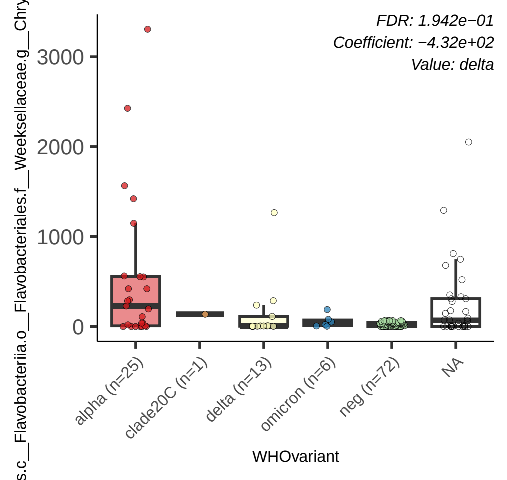

FDR: 1.942e−01
Coefficient: −4.32e+02
Value: delta
3000
2000
1000
0
s.c__Flavobacteriia.o__Flavobacteriales.f__Weeksellaceae.g__Chry
alpha (n=25)
clade20C (n=1)
delta (n=13)
omicron (n=6)
neg (n=72)
NA
WHOvariant

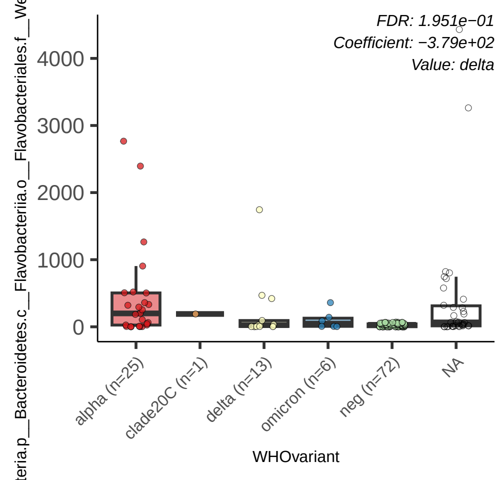

FDR: 1.951e−01
Coefficient: −3.79e+02
Value: delta
4000
3000
2000
1000
0
alpha (n=25)
clade20C (n=1)
delta (n=13)
omicron (n=6)
neg (n=72)
NA
WHOvariant
teria.p__Bacteroidetes.c__Flavobacteriia.o__Flavobacteriales.f__We

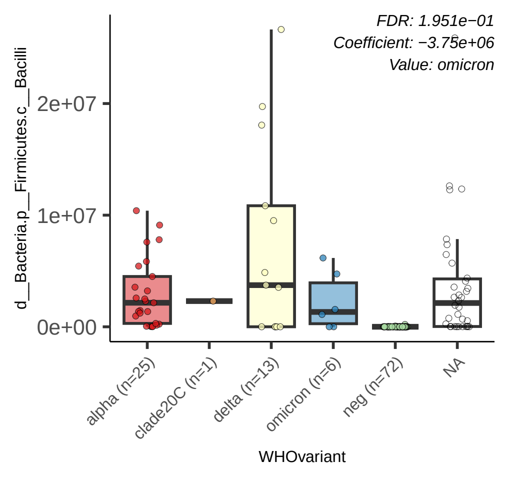
FDR: 1.951e−01
Coefficient: −3.75e+06
Value: omicron
d__Bacteria.p__Firmicutes.c__Bacilli
2e+07
1e+07
0e+00
alpha (n=25)
clade20C (n=1)
delta (n=13)
omicron (n=6)
neg (n=72)
NA
WHOvariant

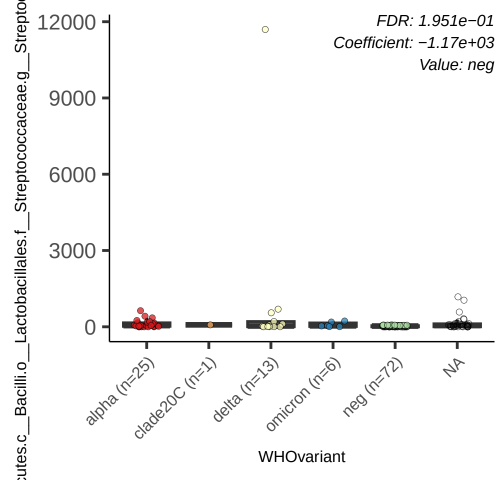
FDR: 1.951e−01
Coefficient: −1.17e+03
Value: neg
12000
9000
6000
3000
0
alpha (n=25)
clade20C (n=1)
delta (n=13)
omicron (n=6)
neg (n=72)
NA
WHOvariant
cutes.c__Bacilli.o__Lactobacillales.f__Streptococcaceae.g__Strepto

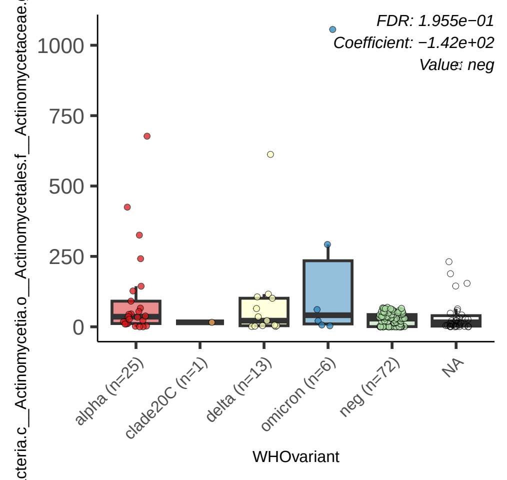

FDR: 1.955e−01
Coefficient: −1.42e+02
Value: neg
1000
750
500
250
0
cteria.c__Actinomycetia.o__Actinomycetales.f__Actinomycetaceae.
alpha (n=25)
clade20C (n=1)
delta (n=13)
omicron (n=6)
neg (n=72)
NA
WHOvariant

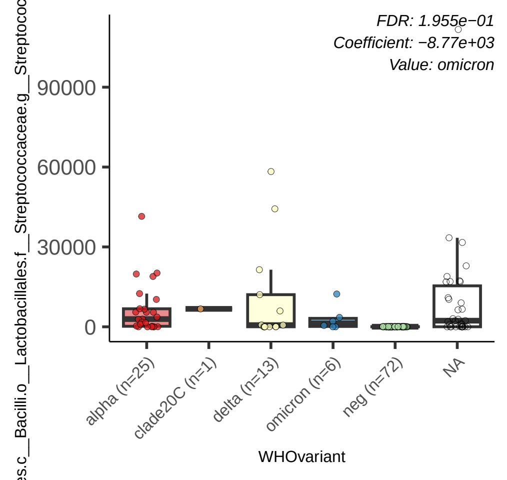

FDR: 1.955e−01
Coefficient: −8.77e+03
Value: omicron
es.c__Bacilli.o__Lactobacillales.f__Streptococcaceae.g__Streptococ
90000
60000
30000
0
alpha (n=25)
clade20C (n=1)
delta (n=13)
omicron (n=6)
neg (n=72)
NA
WHOvariant

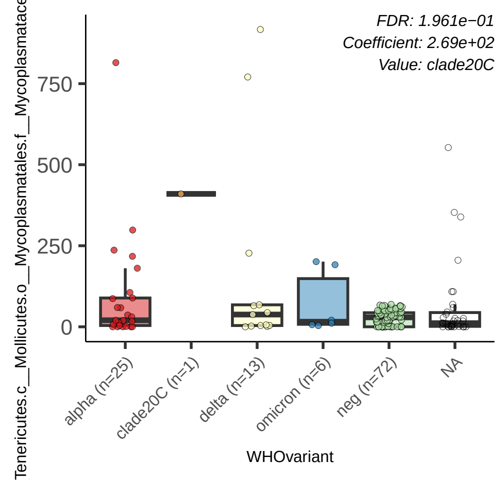
FDR: 1.961e−01
Coefficient: 2.69e+02
Value: clade20C
Tenericutes.c__Mollicutes.o__Mycoplasmatales.f__Mycoplasmatace
750
500
250
0
alpha (n=25)
clade20C (n=1)
delta (n=13)
omicron (n=6)
neg (n=72)
NA
WHOvariant

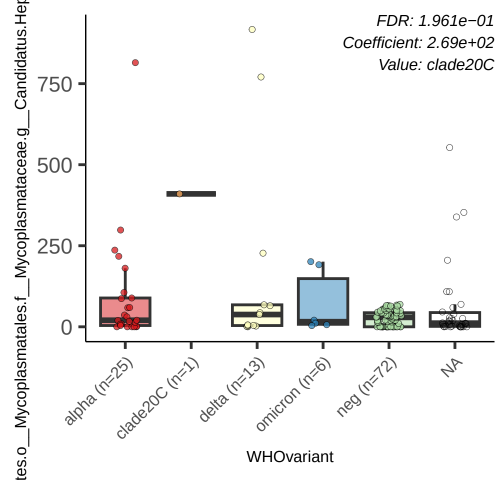
FDR: 1.961e−01
Coefficient: 2.69e+02
Value: clade20C
tes.o__Mycoplasmatales.f__Mycoplasmataceae.g__Candidatus.Hep
0
250
500
750
alpha (n=25)
clade20C (n=1)
delta (n=13)
omicron (n=6)
neg (n=72)
NA
WHOvariant

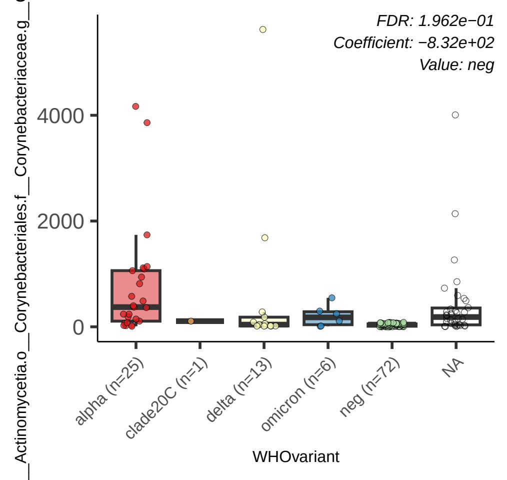

FDR: 1.962e−01
Coefficient: −8.32e+02
Value: neg
Actinomycetia.o__Corynebacteriales.f__Corynebacteriaceae.g__
4000
2000
0
alpha (n=25)
clade20C (n=1)
delta (n=13)
omicron (n=6)
neg (n=72)
NA
WHOvariant

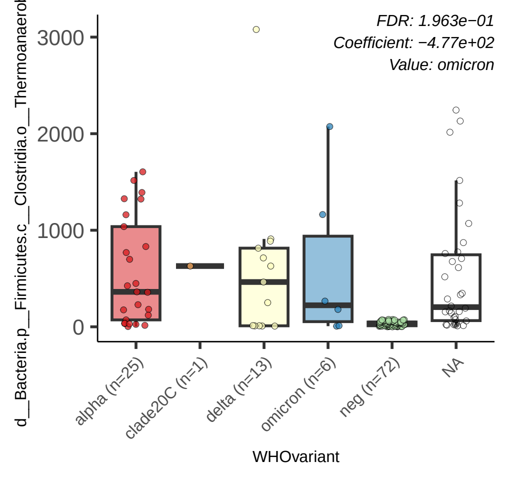

FDR: 1.963e−01
Coefficient: −4.77e+02
Value: omicron
d__Bacteria.p__Firmicutes.c__Clostridia.o__Thermoanaerob
3000
2000
1000
0
alpha (n=25)
clade20C (n=1)
delta (n=13)
omicron (n=6)
neg (n=72)
NA
WHOvariant

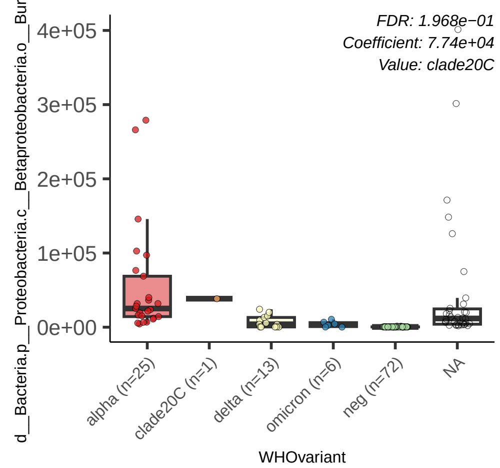

FDR: 1.968e−01
Coefficient: 7.74e+04
Value: clade20C
d__Bacteria.p__Proteobacteria.c__Betaproteobacteria.o__Bur
4e+05
3e+05
2e+05
1e+05
0e+00
alpha (n=25)
clade20C (n=1)
delta (n=13)
omicron (n=6)
neg (n=72)
NA
WHOvariant

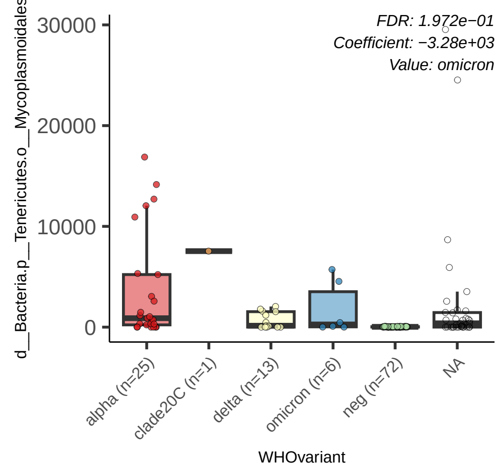

FDR: 1.972e−01
Coefficient: −3.28e+03
Value: omicron
d__Bacteria.p__Tenericutes.o__Mycoplasmoidales
30000
20000
10000
0
alpha (n=25)
clade20C (n=1)
delta (n=13)
omicron (n=6)
neg (n=72)
NA
WHOvariant

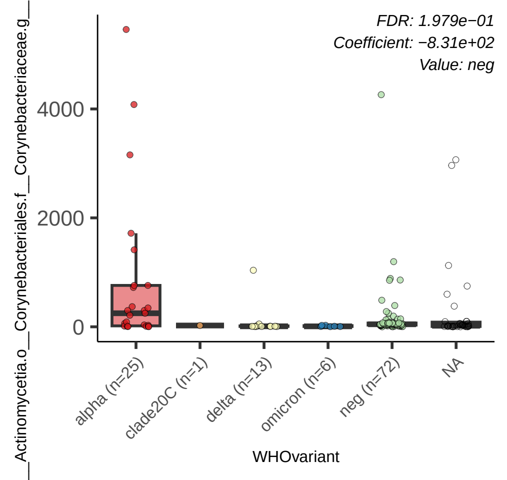

FDR: 1.979e−01
Coefficient: −8.31e+02
Value: neg
Actinomycetia.o__Corynebacteriales.f__Corynebacteriaceae.g__
4000
2000
0
alpha (n=25)
clade20C (n=1)
delta (n=13)
omicron (n=6)
neg (n=72)
NA
WHOvariant

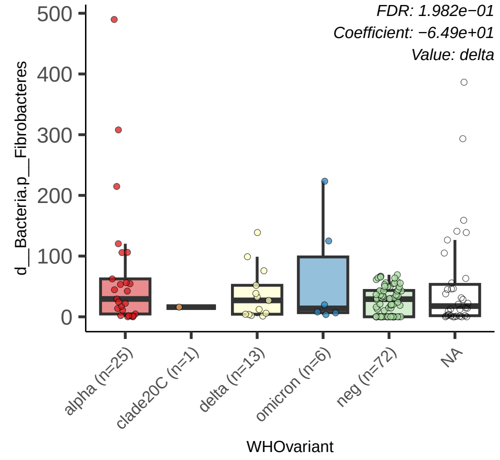

FDR: 1.982e−01
Coefficient: −6.49e+01
Value: delta
d__Bacteria.p__Fibrobacteres
0
100
200
300
400
500
alpha (n=25)
clade20C (n=1)
delta (n=13)
omicron (n=6)
neg (n=72)
NA
WHOvariant

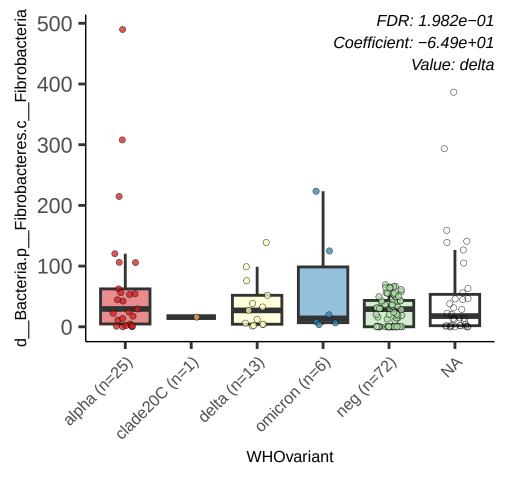

FDR: 1.982e−01
Coefficient: −6.49e+01
Value: delta
d__Bacteria.p__Fibrobacteres.c__Fibrobacteria
500
400
300
200
100
0
alpha (n=25)
clade20C (n=1)
delta (n=13)
omicron (n=6)
neg (n=72)
NA
WHOvariant

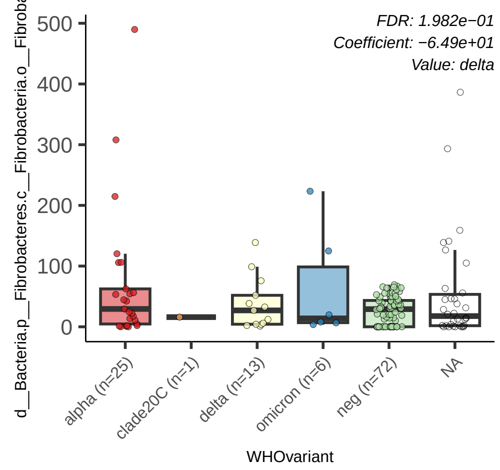

FDR: 1.982e−01
Coefficient: −6.49e+01
Value: delta
d__Bacteria.p__Fibrobacteres.c__Fibrobacteria.o__Fibroba
500
400
300
200
100
0
alpha (n=25)
clade20C (n=1)
delta (n=13)
omicron (n=6)
neg (n=72)
NA
WHOvariant

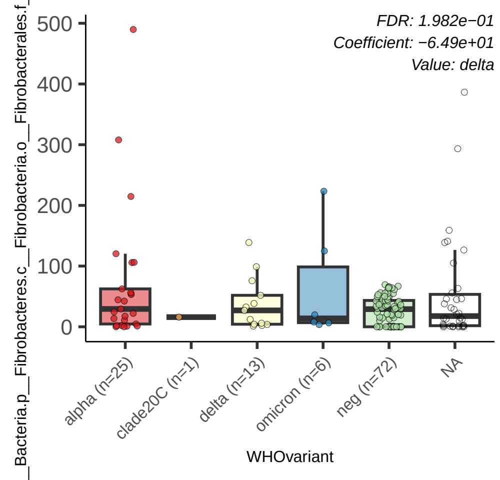

FDR: 1.982e−01
Coefficient: −6.49e+01
Value: delta
_Bacteria.p__Fibrobacteres.c__Fibrobacteria.o__Fibrobacterales.f
500
400
300
200
100
0
alpha (n=25)
clade20C (n=1)
delta (n=13)
omicron (n=6)
neg (n=72)
NA
WHOvariant

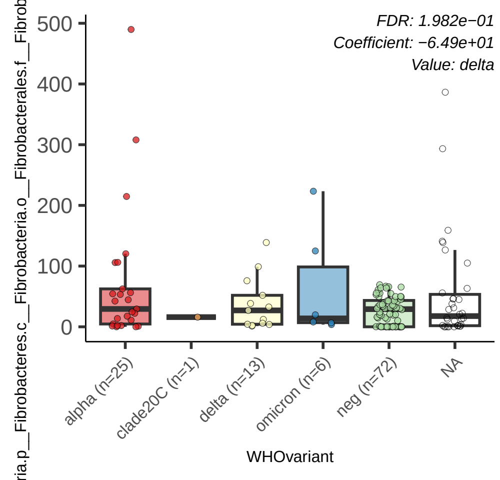

FDR: 1.982e−01
Coefficient: −6.49e+01
Value: delta
Fibrobacteres.c__Fibrobacteria.o__Fibrobacterales.f__Fibrob
ria.p__Fibrobacteres.c__Fibrobacteria.o__Fibrobacterales.f__Fibrob
500
400
300
200
100
0
alpha (n=25)
clade20C (n=1)
delta (n=13)
omicron (n=6)
neg (n=72)
NA
WHOvariant

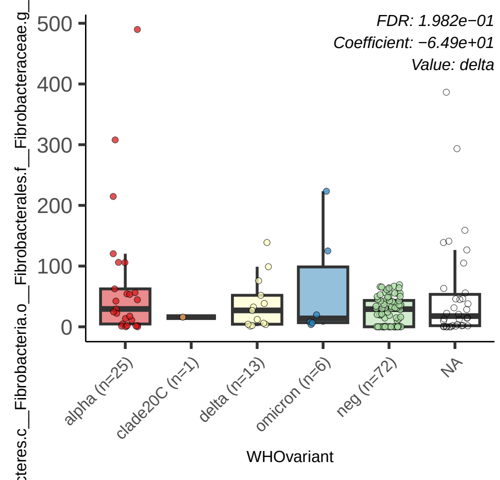

FDR: 1.982e−01
Coefficient: −6.49e+01
Value: delta
cteres.c__Fibrobacteria.o__Fibrobacterales.f__Fibrobacteraceae.g__
0
100
200
300
400
500
alpha (n=25)
clade20C (n=1)
delta (n=13)
omicron (n=6)
neg (n=72)
NA
WHOvariant

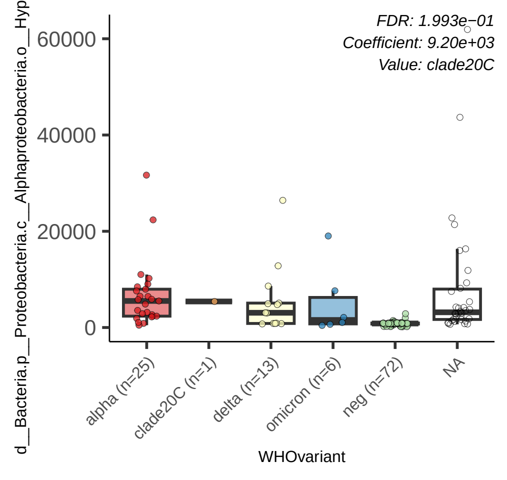

FDR: 1.993e−01
Coefficient: 9.20e+03
Value: clade20C
d__Bacteria.p__Proteobacteria.c__Alphaproteobacteria.o__Hyp
60000
40000
20000
0
alpha (n=25)
clade20C (n=1)
delta (n=13)
omicron (n=6)
neg (n=72)
NA
WHOvariant

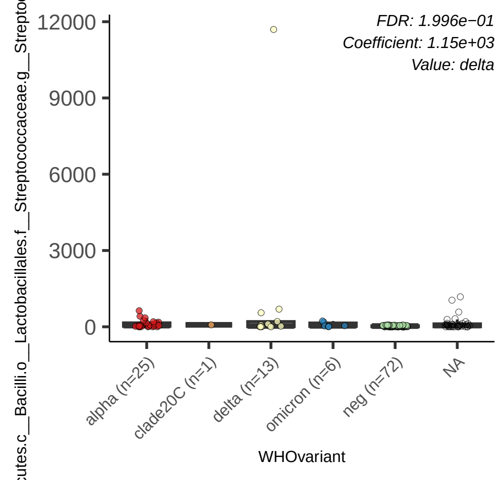
FDR: 1.996e−01
Coefficient: 1.15e+03
Value: delta
12000
9000
6000
3000
0
alpha (n=25)
clade20C (n=1)
delta (n=13)
omicron (n=6)
neg (n=72)
NA
WHOvariant
cutes.c__Bacilli.o__Lactobacillales.f__Streptococcaceae.g__Strepto

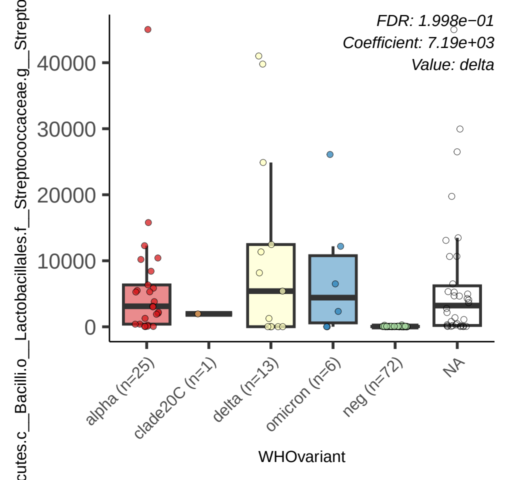

FDR: 1.998e−01
Coefficient: 7.19e+03
Value: delta
cutes.c__Bacilli.o__Lactobacillales.f__Streptococcaceae.g__Strepto
0
10000
20000
30000
40000
alpha (n=25)
clade20C (n=1)
delta (n=13)
omicron (n=6)
neg (n=72)
NA
WHOvariant

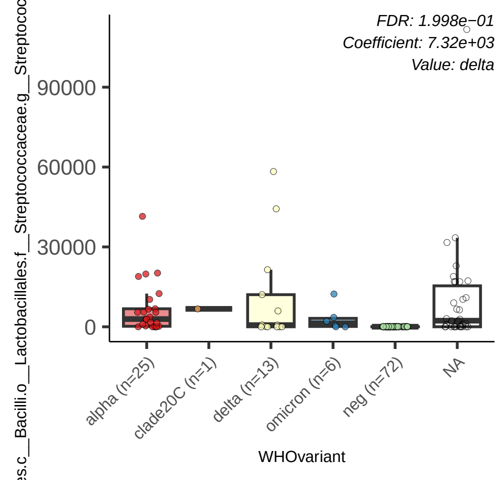
FDR: 1.998e−01
Coefficient: 7.32e+03
Value: delta
90000
60000
30000
0
alpha (n=25)
clade20C (n=1)
delta (n=13)
omicron (n=6)
neg (n=72)
NA
WHOvariant
es.c__Bacilli.o__Lactobacillales.f__Streptococcaceae.g__Streptococ

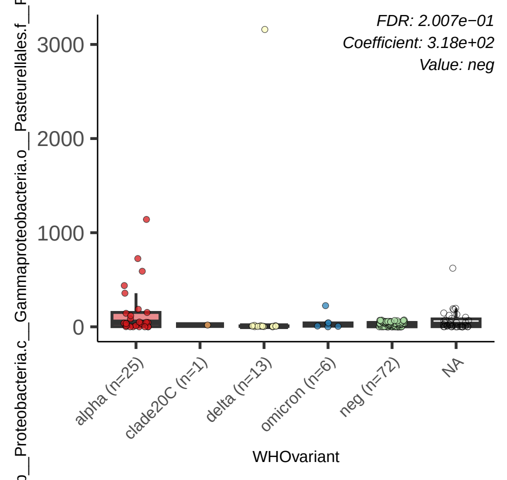

FDR: 2.007e−01
Coefficient: 3.18e+02
Value: neg
3000
2000
1000
0
alpha (n=25)
clade20C (n=1)
delta (n=13)
omicron (n=6)
neg (n=72)
NA
WHOvariant
o__Proteobacteria.c__Gammaproteobacteria.o__Pasteurellales.f__F

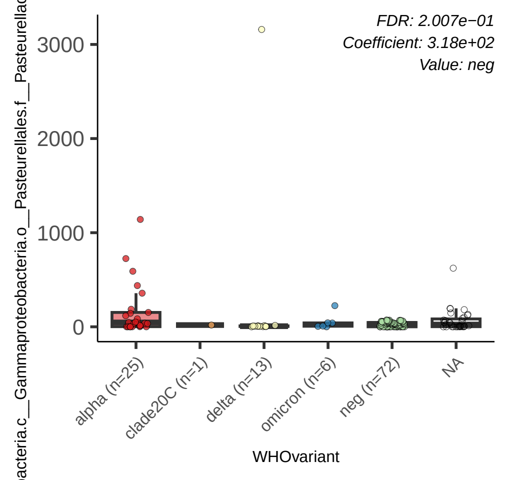
FDR: 2.007e−01
Coefficient: 3.18e+02
Value: neg
3000
2000
1000
0
bacteria.c__Gammaproteobacteria.o__Pasteurellales.f__Pasteurellac
alpha (n=25)
clade20C (n=1)
delta (n=13)
omicron (n=6)
neg (n=72)
NA
WHOvariant

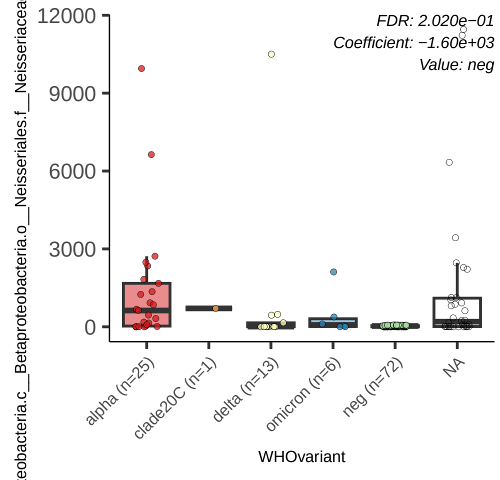

FDR: 2.020e−01
Coefficient: −1.60e+03
Value: neg
12000
9000
6000
3000
0
eobacteria.c__Betaproteobacteria.o__Neisseriales.f__Neisseriacea
alpha (n=25)
clade20C (n=1)
delta (n=13)
omicron (n=6)
neg (n=72)
NA
WHOvariant

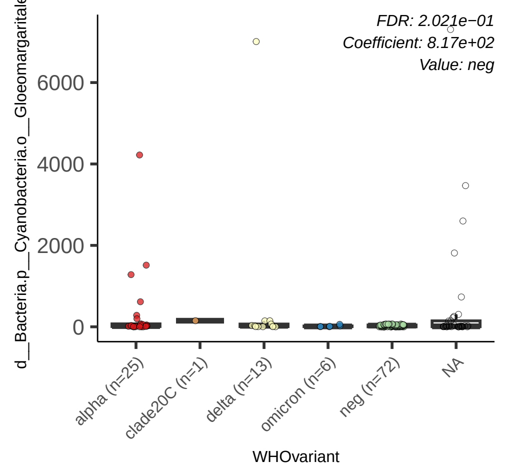

FDR: 2.021e−01
Coefficient: 8.17e+02
Value: neg
d__Bacteria.p__Cyanobacteria.o__Gloeomargaritale
6000
4000
2000
0
alpha (n=25)
clade20C (n=1)
delta (n=13)
omicron (n=6)
neg (n=72)
NA
WHOvariant

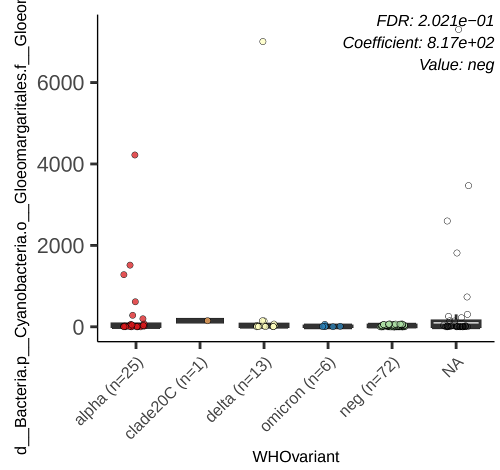

FDR: 2.021e−01
Coefficient: 8.17e+02
Value: neg
d__Bacteria.p__Cyanobacteria.o__Gloeomargaritales.f__Gloeor
6000
4000
2000
0
alpha (n=25)
clade20C (n=1)
delta (n=13)
omicron (n=6)
neg (n=72)
NA
WHOvariant

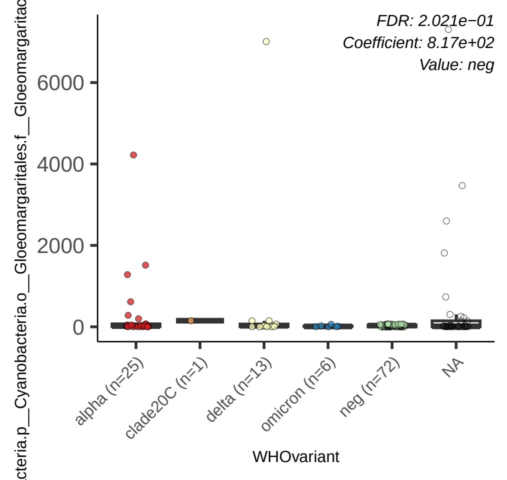

FDR: 2.021e−01
Coefficient: 8.17e+02
Value: neg
6000
4000
2000
0
acteria.p__Cyanobacteria.o__Gloeomargaritales.f__Gloeomargaritac
alpha (n=25)
clade20C (n=1)
delta (n=13)
omicron (n=6)
neg (n=72)
NA
WHOvariant

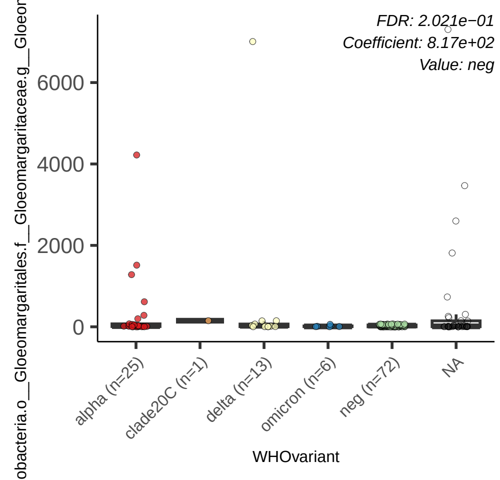

FDR: 2.021e−01
Coefficient: 8.17e+02
Value: neg
6000
4000
2000
0
obacteria.o__Gloeomargaritales.f__Gloeomargaritaceae.g__Gloeon
alpha (n=25)
clade20C (n=1)
delta (n=13)
omicron (n=6)
neg (n=72)
NA
WHOvariant

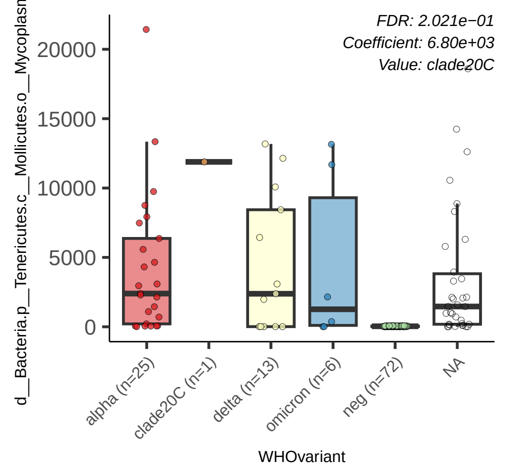
FDR: 2.021e−01
Coefficient: 6.80e+03
Value: clade20C
d__Bacteria.p__Tenericutes.c__Mollicutes.o__Mycoplasm
20000
15000
10000
5000
0
alpha (n=25)
clade20C (n=1)
delta (n=13)
omicron (n=6)
neg (n=72)
NA
WHOvariant

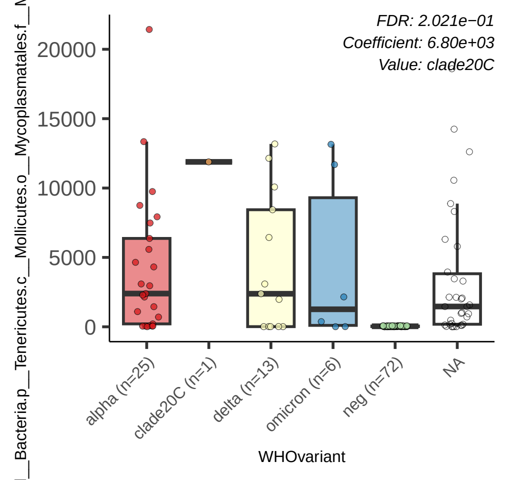

FDR: 2.021e−01
Coefficient: 6.80e+03
Value: clade20C
__Bacteria.p__Tenericutes.c__Mollicutes.o__Mycoplasmatales.f__M
20000
15000
10000
5000
0
alpha (n=25)
clade20C (n=1)
delta (n=13)
omicron (n=6)
neg (n=72)
NA
WHOvariant

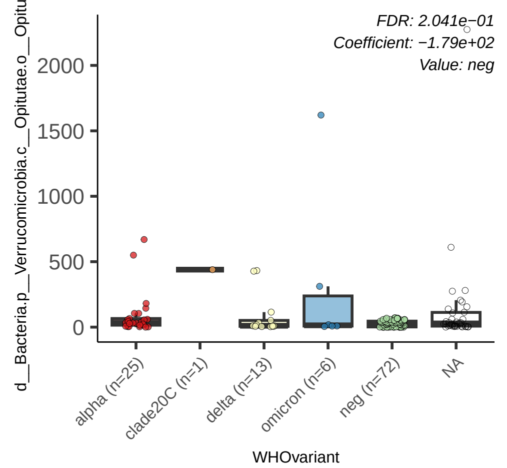
FDR: 2.041e−01
Coefficient: −1.79e+02
Value: neg
d__Bacteria.p__Verrucomicrobia.c__Opitutae.o__Opitu
2000
1500
1000
500
0
alpha (n=25)
clade20C (n=1)
delta (n=13)
omicron (n=6)
neg (n=72)
NA
WHOvariant

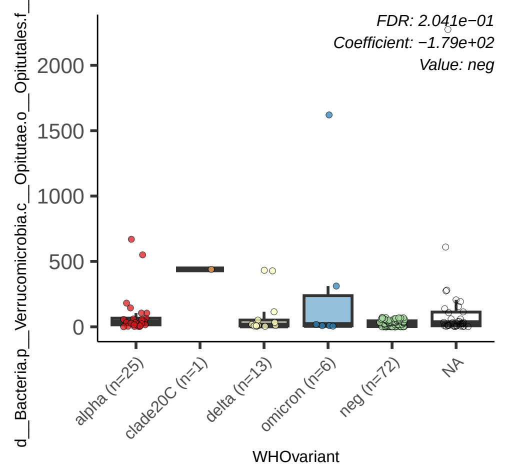

FDR: 2.041e−01
Coefficient: −1.79e+02
Value: neg
d__Bacteria.p__Verrucomicrobia.c__Opitutae.o__Opitutales.f__
0
500
1000
1500
2000
alpha (n=25)
clade20C (n=1)
delta (n=13)
omicron (n=6)
neg (n=72)
NA
WHOvariant

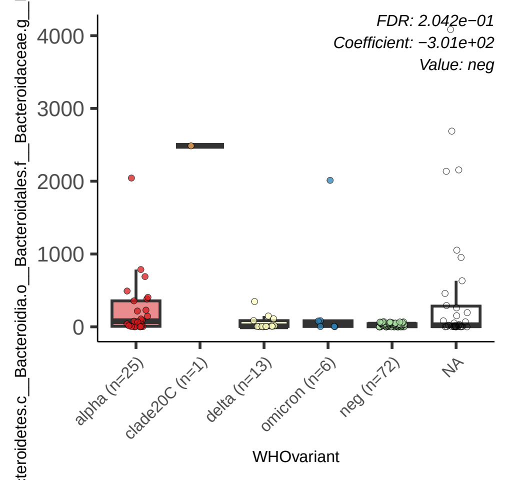

FDR: 2.042e−01
Coefficient: −3.01e+02
Value: neg
4000
3000
2000
1000
0
Bacteroidetes.c__Bacteroidia.o__Bacteroidales.f__Bacteroidaceae.g__
alpha (n=25)
clade20C (n=1)
delta (n=13)
omicron (n=6)
neg (n=72)
NA
WHOvariant

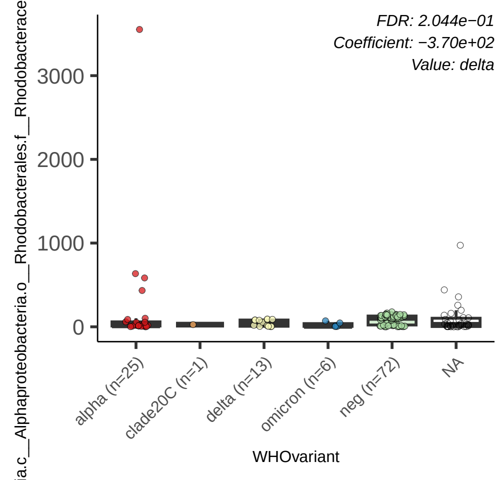

FDR: 2.044e−01
Coefficient: −3.70e+02
Value: delta
ia.c__Alphaproteobacteria.o__Rhodobacterales.f__Rhodobacterace
3000
2000
1000
0
alpha (n=25)
clade20C (n=1)
delta (n=13)
omicron (n=6)
neg (n=72)
NA
WHOvariant

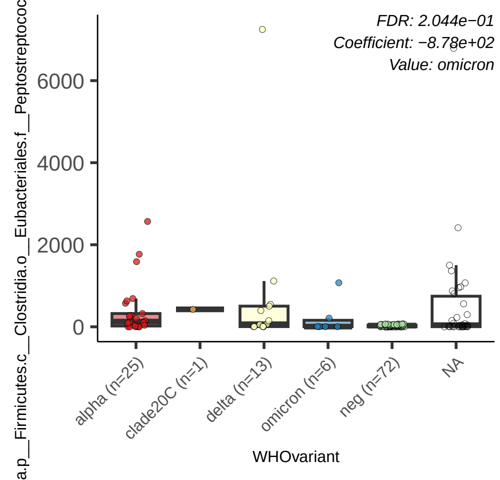

FDR: 2.044e−01
Coefficient: −8.78e+02
Value: omicron
a.p__Firmicutes.c__Clostridia.o__Eubacteriales.f__Peptostreptococ
0
2000
4000
6000
alpha (n=25)
clade20C (n=1)
delta (n=13)
omicron (n=6)
neg (n=72)
NA
WHOvariant

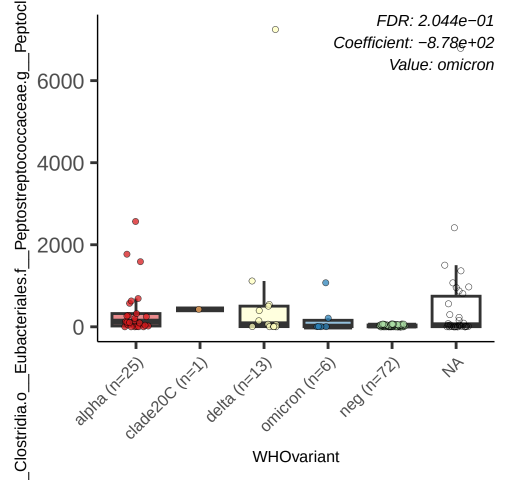
FDR: 2.044e−01
Coefficient: −8.78e+02
Value: omicron
_Clostridia.o__Eubacteriales.f__Peptostreptococcaceae.g__Peptoc
6000
4000
2000
0
alpha (n=25)
clade20C (n=1)
delta (n=13)
omicron (n=6)
neg (n=72)
NA
WHOvariant

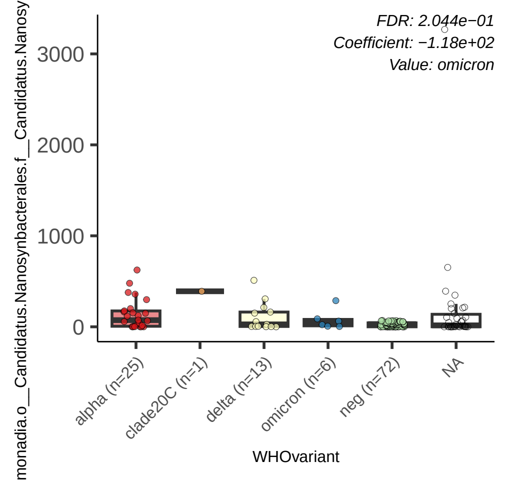
FDR: 2.044e−01
Coefficient: −1.18e+02
Value: omicron
monadia.o__Candidatus.Nanosynbacterales.f__Candidatus.Nanosy
3000
2000
1000
0
alpha (n=25)
clade20C (n=1)
delta (n=13)
omicron (n=6)
neg (n=72)
NA
WHOvariant

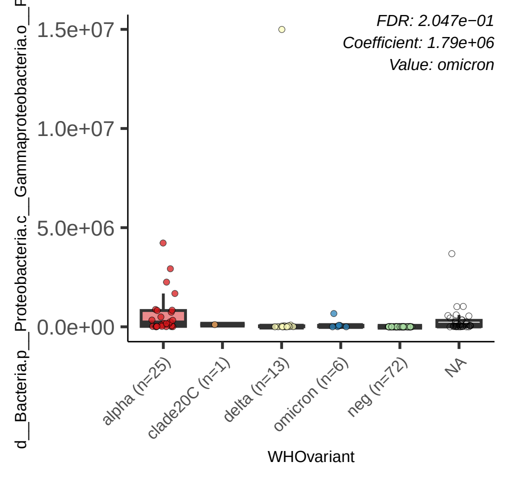
FDR: 2.047e−01
Coefficient: 1.79e+06
Value: omicron
1.5e+07
1.0e+07
5.0e+06
0.0e+00
d__Bacteria.p__Proteobacteria.c__Gammaproteobacteria.o__F
alpha (n=25)
clade20C (n=1)
delta (n=13)
omicron (n=6)
neg (n=72)
NA
WHOvariant

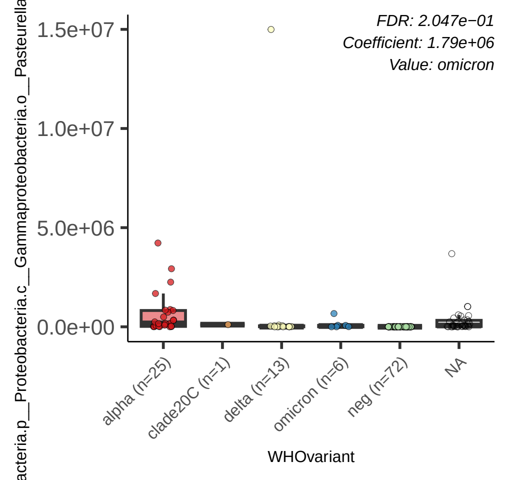
FDR: 2.047e−01
Coefficient: 1.79e+06
Value: omicron
1.5e+07
1.0e+07
5.0e+06
0.0e+00
acteria.p__Proteobacteria.c__Gammaproteobacteria.o__Pasteurella
alpha (n=25)
clade20C (n=1)
delta (n=13)
omicron (n=6)
neg (n=72)
NA
WHOvariant

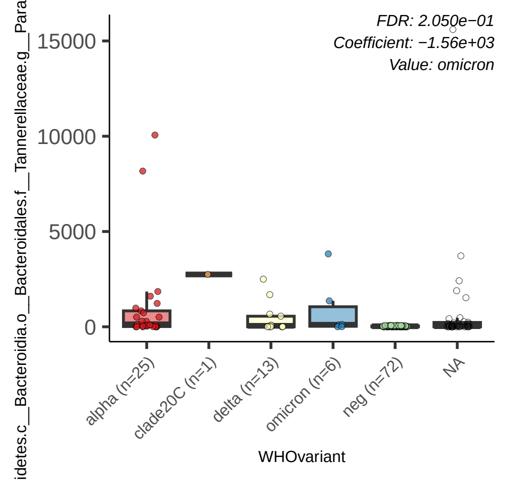

FDR: 2.050e−01
Coefficient: −1.56e+03
Value: omicron
15000
10000
5000
0
idetes.c__Bacteroidia.o__Bacteroidales.f__Tannerellaceae.g__Para
alpha (n=25)
clade20C (n=1)
delta (n=13)
omicron (n=6)
neg (n=72)
NA
WHOvariant

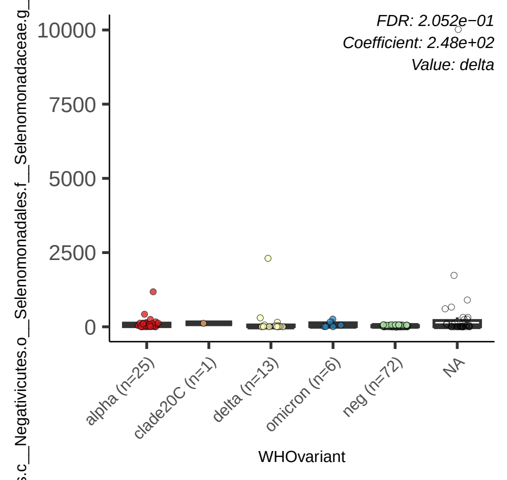

FDR: 2.052e−01
Coefficient: 2.48e+02
Value: delta
10000
7500
5000
2500
0
s.c__Negativicutes.o__Selenomonadales.f__Selenomonadaceae.g__
alpha (n=25)
clade20C (n=1)
delta (n=13)
omicron (n=6)
neg (n=72)
NA
WHOvariant

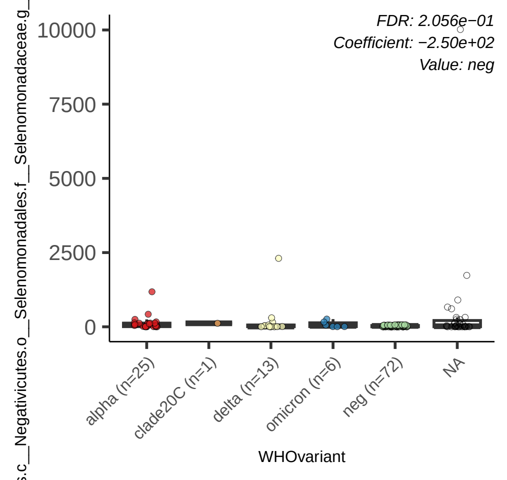

FDR: 2.056e−01
Coefficient: −2.50e+02
Value: neg
10000
7500
5000
2500
0
s.c__Negativicutes.o__Selenomonadales.f__Selenomonadaceae.g__
alpha (n=25)
clade20C (n=1)
delta (n=13)
omicron (n=6)
neg (n=72)
NA
WHOvariant

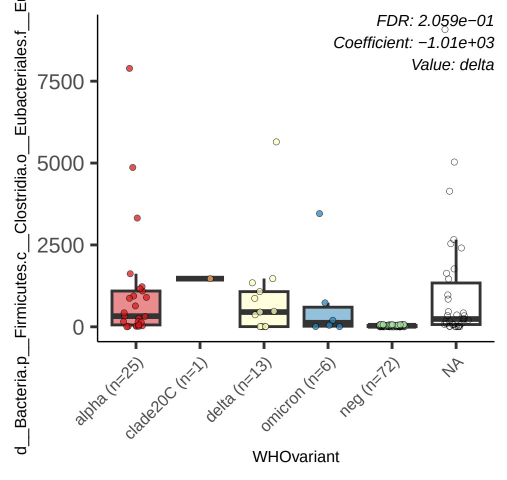

FDR: 2.059e−01
Coefficient: −1.01e+03
Value: delta
d__Bacteria.p__Firmicutes.c__Clostridia.o__Eubacteriales.f__E
7500
5000
2500
0
alpha (n=25)
clade20C (n=1)
delta (n=13)
omicron (n=6)
neg (n=72)
NA
WHOvariant

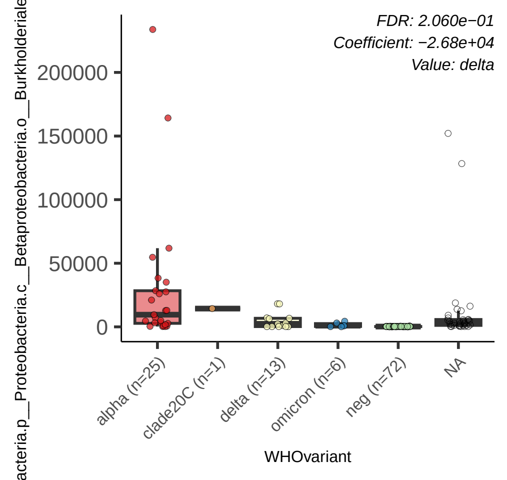

FDR: 2.060e−01
Coefficient: −2.68e+04
Value: delta
200000
150000
100000
50000
0
acteria.p__Proteobacteria.c__Betaproteobacteria.o__Burkholderiale
alpha (n=25)
clade20C (n=1)
delta (n=13)
omicron (n=6)
neg (n=72)
NA
WHOvariant

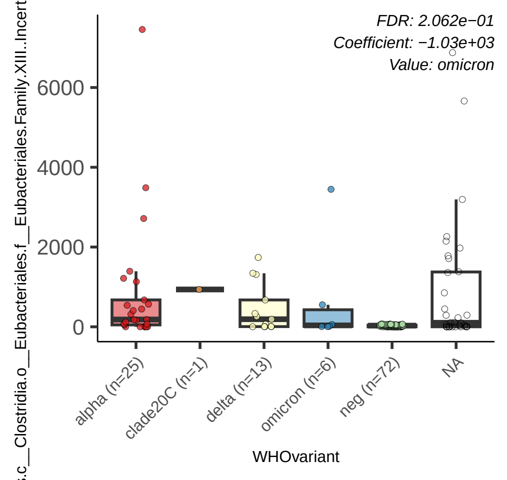
FDR: 2.062e−01
Coefficient: −1.03e+03
Value: omicron
s.c__Clostridia.o__Eubacteriales.f__Eubacteriales.Family.XIII..Incert
6000
4000
2000
0
alpha (n=25)
clade20C (n=1)
delta (n=13)
omicron (n=6)
neg (n=72)
NA
WHOvariant

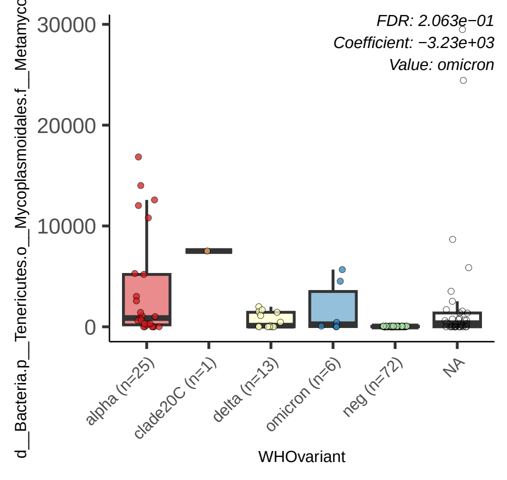

FDR: 2.063e−01
Coefficient: −3.23e+03
Value: omicron
30000
20000
10000
0
alpha (n=25)
clade20C (n=1)
delta (n=13)
omicron (n=6)
neg (n=72)
NA
WHOvariant
d__Bacteria.p__Tenericutes.o__Mycoplasmoidales.f__Metamycc

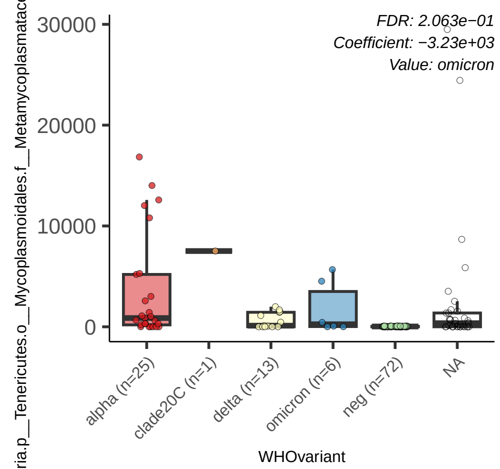

FDR: 2.063e−01
Coefficient: −3.23e+03
Value: omicron
30000
20000
10000
0
teria.p__Tenericutes.o__Mycoplasmoidales.f__Metamycoplasmatac
alpha (n=25)
clade20C (n=1)
delta (n=13)
omicron (n=6)
neg (n=72)
NA
WHOvariant

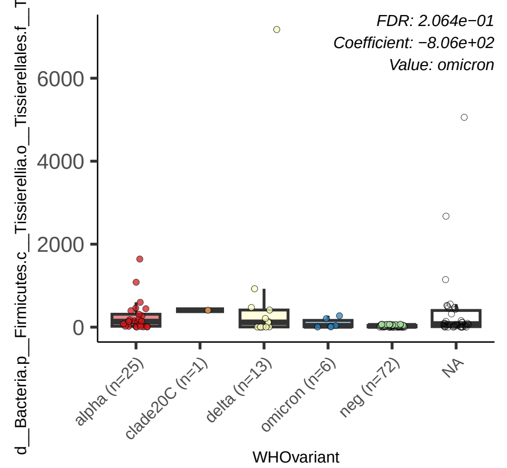

FDR: 2.064e−01
Coefficient: −8.06e+02
Value: omicron
6000
4000
2000
0
d__Bacteria.p__Firmicutes.c__Tissierellia.o__Tissierellales.f__T
alpha (n=25)
clade20C (n=1)
delta (n=13)
omicron (n=6)
neg (n=72)
NA
WHOvariant

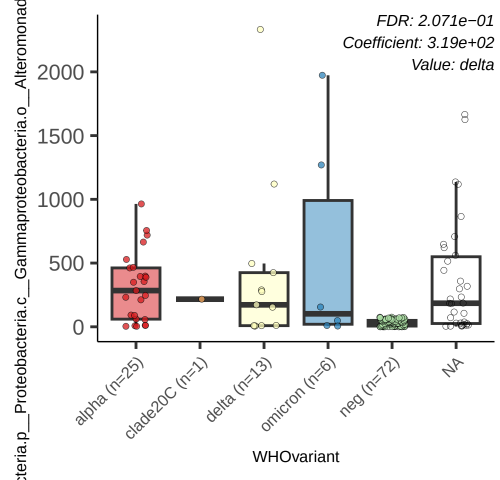

FDR: 2.071e−01
Coefficient: 3.19e+02
Value: delta
cteria.p__Proteobacteria.c__Gammaproteobacteria.o__Alteromonad
2000
1500
1000
500
0
alpha (n=25)
clade20C (n=1)
delta (n=13)
omicron (n=6)
neg (n=72)
NA
WHOvariant

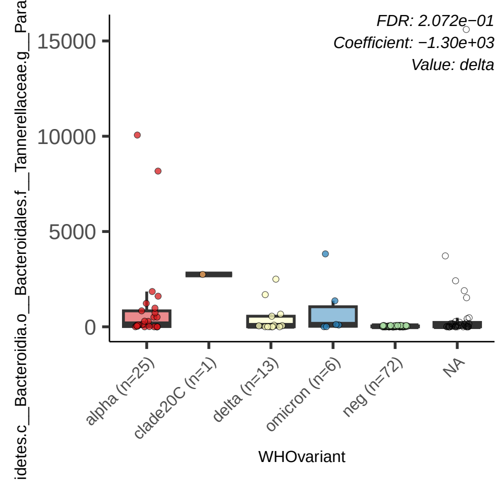

FDR: 2.072e−01
Coefficient: −1.30e+03
Value: delta
15000
10000
5000
0
idetes.c__Bacteroidia.o__Bacteroidales.f__Tannerellaceae.g__Para
alpha (n=25)
clade20C (n=1)
delta (n=13)
omicron (n=6)
neg (n=72)
NA
WHOvariant

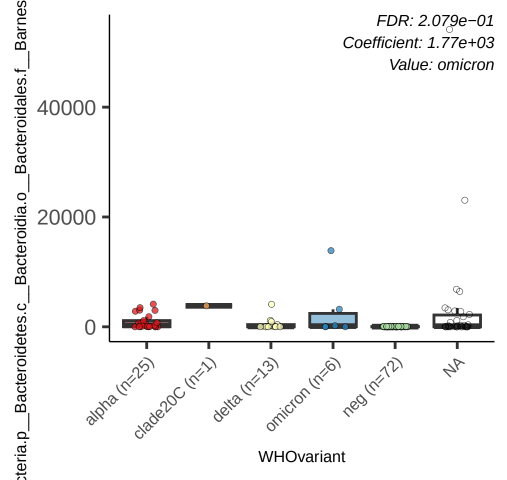
FDR: 2.079e−01
Coefficient: 1.77e+03
Value: omicron
cteria.p__Bacteroidetes.c__Bacteroidia.o__Bacteroidales.f__Barnes
40000
20000
0
alpha (n=25)
clade20C (n=1)
delta (n=13)
omicron (n=6)
neg (n=72)
NA
WHOvariant

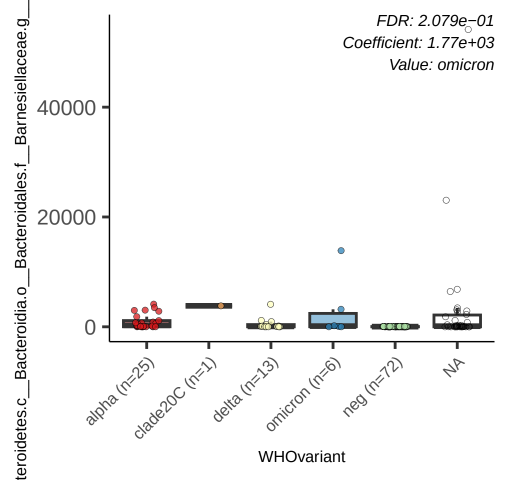
FDR: 2.079e−01
Coefficient: 1.77e+03
Value: omicron
40000
20000
0
teroidetes.c__Bacteroidia.o__Bacteroidales.f__Barnesiellaceae.g__
alpha (n=25)
clade20C (n=1)
delta (n=13)
omicron (n=6)
neg (n=72)
NA
WHOvariant

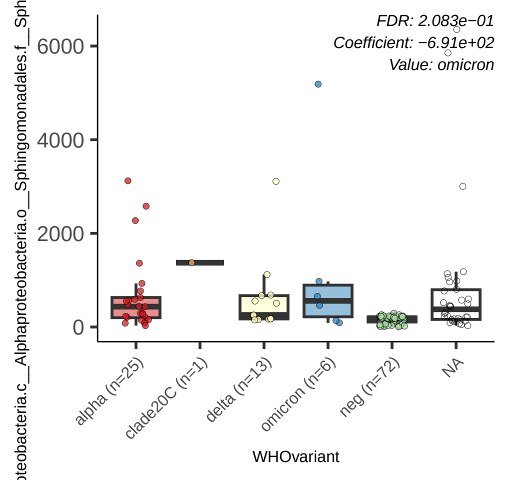

FDR: 2.083e−01
Coefficient: −6.91e+02
Value: omicron
6000
4000
2000
0
oteobacteria.c__Alphaproteobacteria.o__Sphingomonadales.f__Sph
alpha (n=25)
clade20C (n=1)
delta (n=13)
omicron (n=6)
neg (n=72)
NA
WHOvariant

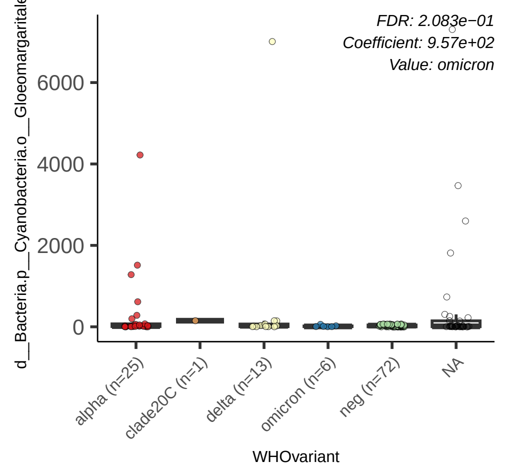

FDR: 2.083e−01
Coefficient: 9.57e+02
Value: omicron
d__Bacteria.p__Cyanobacteria.o__Gloeomargaritale
6000
4000
2000
0
alpha (n=25)
clade20C (n=1)
delta (n=13)
omicron (n=6)
neg (n=72)
NA
WHOvariant

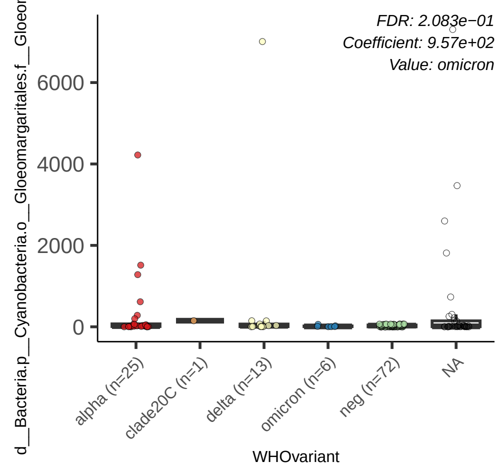

FDR: 2.083e−01
Coefficient: 9.57e+02
Value: omicron
d__Bacteria.p__Cyanobacteria.o__Gloeomargaritales.f__Gloeor
6000
4000
2000
0
alpha (n=25)
clade20C (n=1)
delta (n=13)
omicron (n=6)
neg (n=72)
NA
WHOvariant

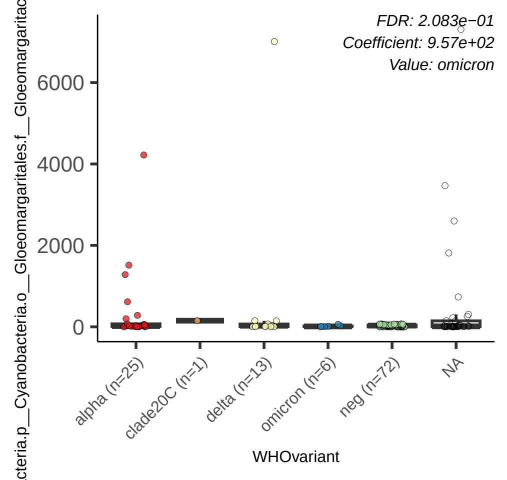

FDR: 2.083e−01
Coefficient: 9.57e+02
Value: omicron
6000
4000
2000
0
cteria.p__Cyanobacteria.o__Gloeomargaritales.f__Gloeomargaritac
alpha (n=25)
clade20C (n=1)
delta (n=13)
omicron (n=6)
neg (n=72)
NA
WHOvariant

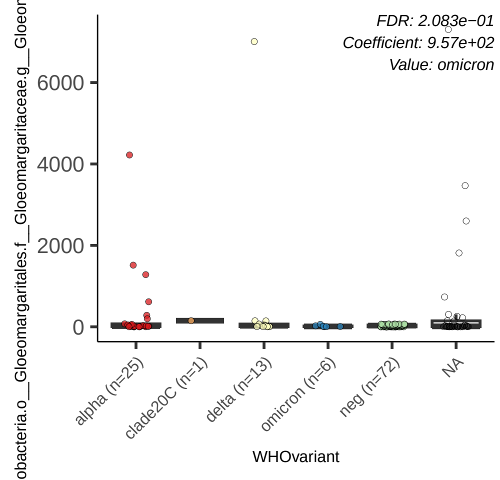

FDR: 2.083e−01
Coefficient: 9.57e+02
Value: omicron
obacteria.o__Gloeomargaritales.f__Gloeomargaritaceae.g__Gloeo
6000
4000
2000
0
alpha (n=25)
clade20C (n=1)
delta (n=13)
omicron (n=6)
neg (n=72)
NA
WHOvariant

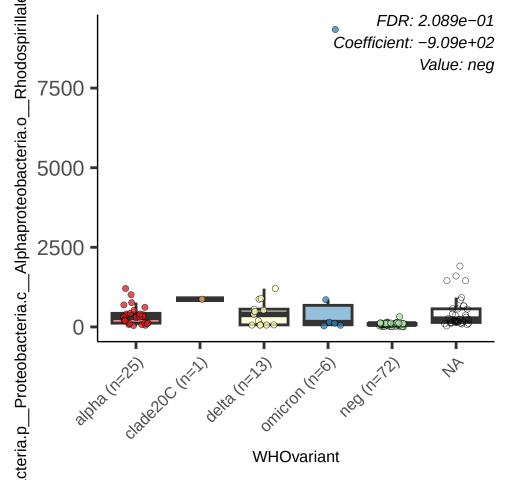

FDR: 2.089e−01
Coefficient: −9.09e+02
Value: neg
7500
5000
2500
0
cteria.p__Proteobacteria.c__Alphaproteobacteria.o__Rhodospirillale
alpha (n=25)
clade20C (n=1)
delta (n=13)
omicron (n=6)
neg (n=72)
NA
WHOvariant

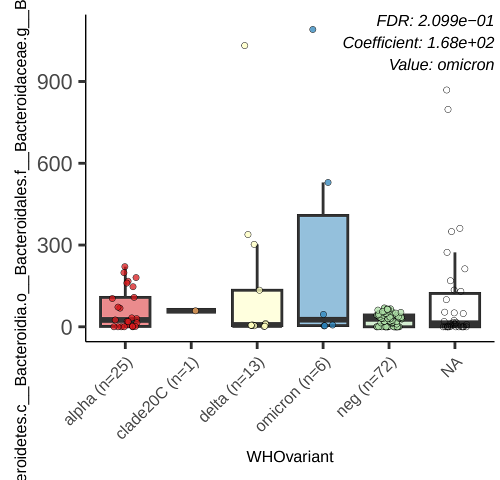

FDR: 2.099e−01
Coefficient: 1.68e+02
Value: omicron
eroidetes.c__Bacteroidia.o__Bacteroidales.f__Bacteroidaceae.g__B
900
600
300
0
alpha (n=25)
clade20C (n=1)
delta (n=13)
omicron (n=6)
neg (n=72)
NA
WHOvariant

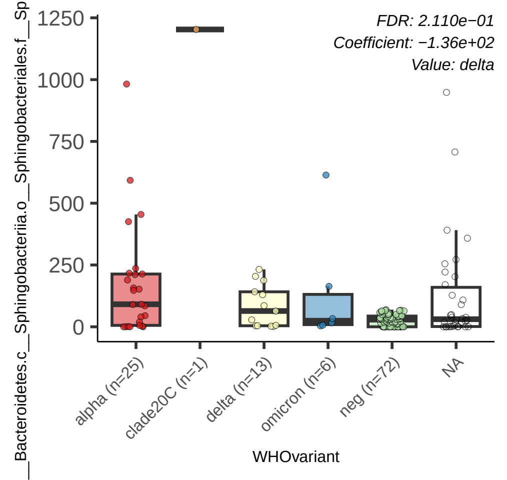
FDR: 2.110e−01
Coefficient: −1.36e+02
Value: delta
1250
1000
750
500
250
0
_Bacteroidetes.c__Sphingobacteriia.o__Sphingobacteriales.f__Sp
alpha (n=25)
clade20C (n=1)
delta (n=13)
omicron (n=6)
neg (n=72)
NA
WHOvariant

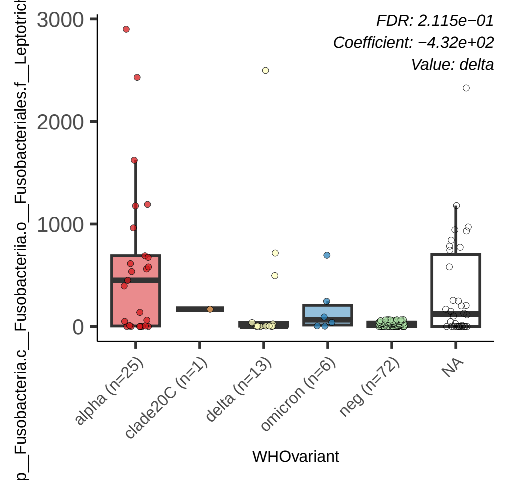

p__Fusobacteria.c__Fusobacteriia.o__Fusobacteriales.f__Leptotrich
FDR: 2.115e−01
Coefficient: −4.32e+02
Value: delta
3000
2000
1000
0
alpha (n=25)
clade20C (n=1)
delta (n=13)
omicron (n=6)
neg (n=72)
NA
WHOvariant

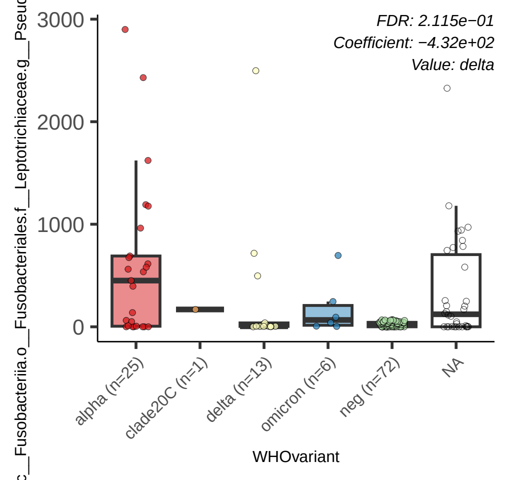

c__Fusobacteriia.o__Fusobacteriales.f__Leptotrichiaceae.g__Pseud
FDR: 2.115e−01
Coefficient: −4.32e+02
Value: delta
3000
2000
1000
0
alpha (n=25)
clade20C (n=1)
delta (n=13)
omicron (n=6)
neg (n=72)
NA
WHOvariant

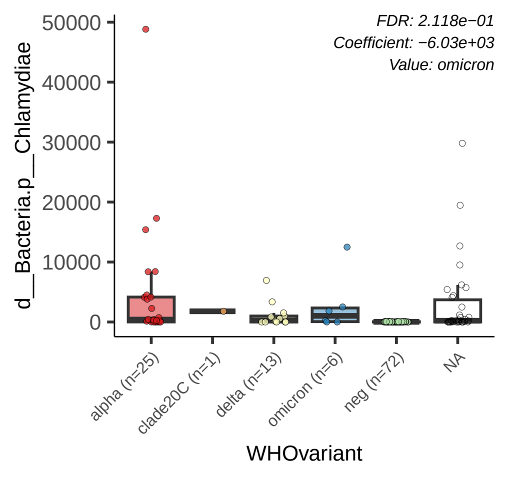
FDR: 2.118e−01
Coefficient: −6.03e+03
Value: omicron
d__Bacteria.p__Chlamydiae
50000
40000
30000
20000
10000
0
alpha (n=25)
clade20C (n=1)
delta (n=13)
omicron (n=6)
neg (n=72)
NA
WHOvariant

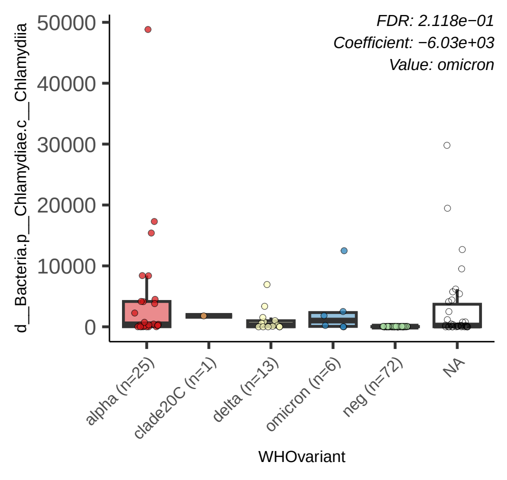
FDR: 2.118e−01
Coefficient: −6.03e+03
Value: omicron
d__Bacteria.p__Chlamydiae.c__Chlamydiia
50000
40000
30000
20000
10000
0
alpha (n=25)
clade20C (n=1)
delta (n=13)
omicron (n=6)
neg (n=72)
NA
WHOvariant

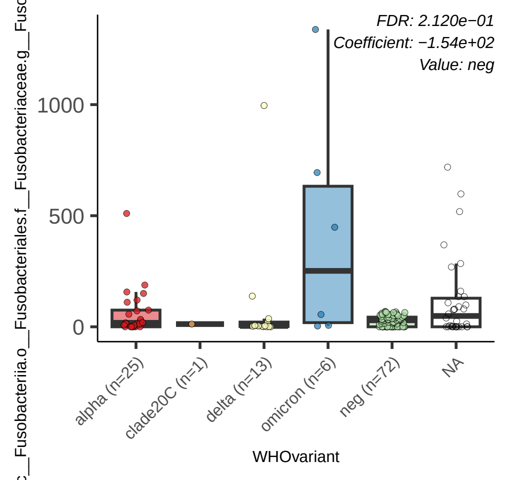

FDR: 2.120e−01
Coefficient: −1.54e+02
Value: neg
c__Fusobacteriia.o__Fusobacteriales.f__Fusobacteriaceae.g__Fuso
1000
500
0
alpha (n=25)
clade20C (n=1)
delta (n=13)
omicron (n=6)
neg (n=72)
NA
WHOvariant

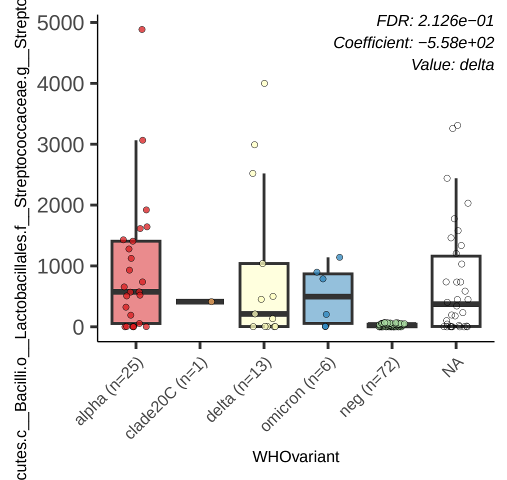

FDR: 2.126e−01
Coefficient: −5.58e+02
Value: delta
cutes.c__Bacilli.o__Lactobacillales.f__Streptococcaceae.g__Strepto
5000
4000
3000
2000
1000
0
alpha (n=25)
clade20C (n=1)
delta (n=13)
omicron (n=6)
neg (n=72)
NA
WHOvariant

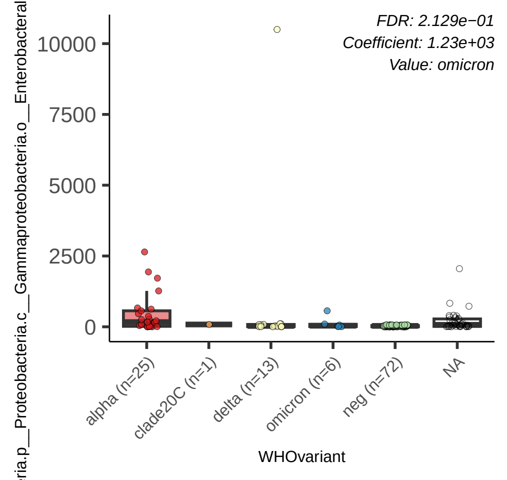

FDR: 2.129e−01
Coefficient: 1.23e+03
Value: omicron
10000
7500
5000
2500
0
ria.p__Proteobacteria.c__Gammaproteobacteria.o__Enterobacteral
alpha (n=25)
clade20C (n=1)
delta (n=13)
omicron (n=6)
neg (n=72)
NA
WHOvariant

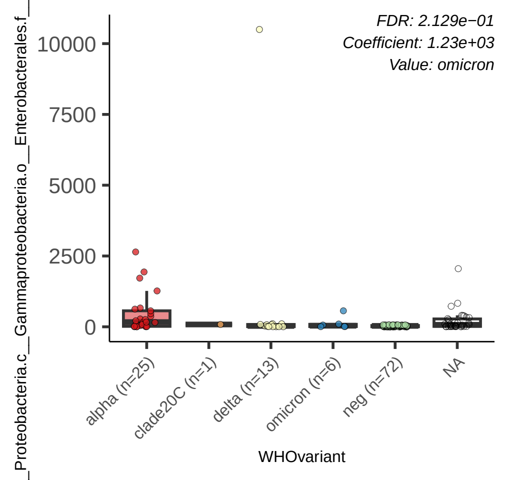
FDR: 2.129e−01
Coefficient: 1.23e+03
Value: omicron
Proteobacteria.c__Gammaproteobacteria.o__Enterobacterales.f
10000
7500
5000
2500
0
alpha (n=25)
clade20C (n=1)
delta (n=13)
omicron (n=6)
neg (n=72)
NA
WHOvariant

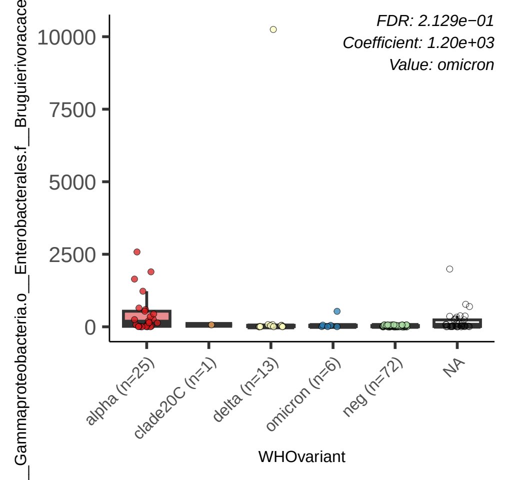
FDR: 2.129e−01
Coefficient: 1.20e+03
Value: omicron
Gammaproteobacteria.o__Enterobacterales.f__Bruguierivoracace
10000
7500
5000
2500
0
alpha (n=25)
clade20C (n=1)
delta (n=13)
omicron (n=6)
neg (n=72)
NA
WHOvariant

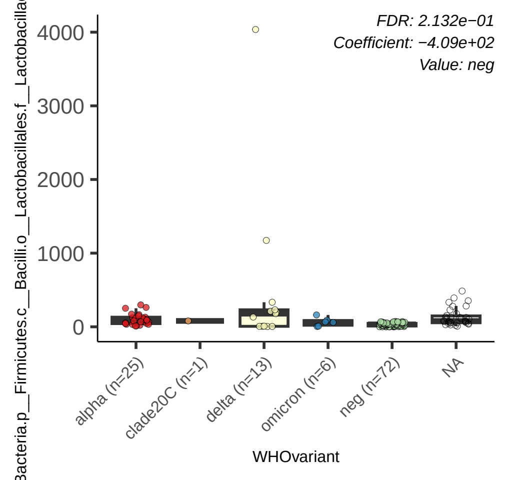
FDR: 2.132e−01
Coefficient: −4.09e+02
Value: neg
4000
3000
2000
1000
0
alpha (n=25)
clade20C (n=1)
delta (n=13)
omicron (n=6)
neg (n=72)
NA
WHOvariant
Bacteria.p__Firmicutes.c__Bacilli.o__Lactobacillales.f__Lactobacillac

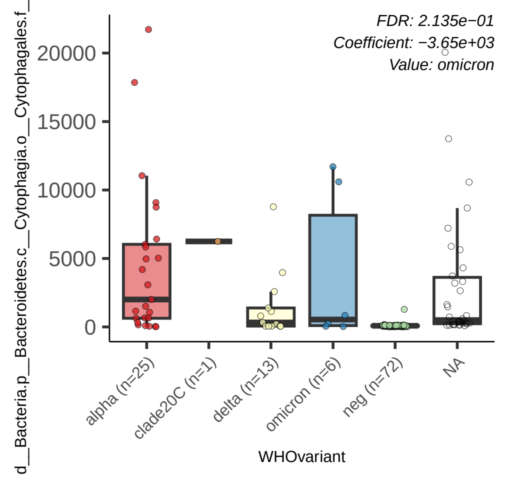
FDR: 2.135e−01
Coefficient: −3.65e+03
Value: omicron
d__Bacteria.p__Bacteroidetes.c__Cytophagia.o__Cytophagales.f__
20000
15000
10000
5000
0
alpha (n=25)
clade20C (n=1)
delta (n=13)
omicron (n=6)
neg (n=72)
NA
WHOvariant

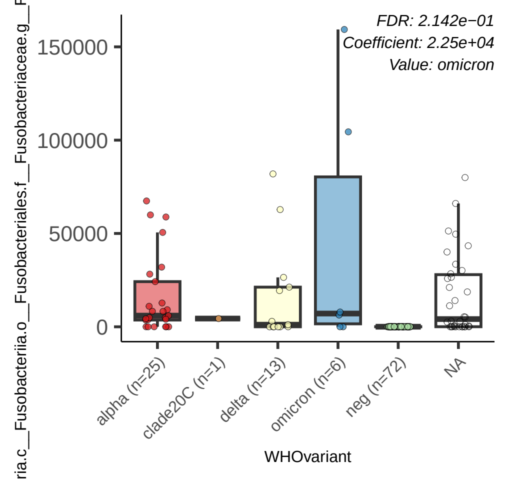

FDR: 2.142e−01
Coefficient: 2.25e+04
Value: omicron
150000
100000
50000
0
ria.c__Fusobacteriia.o__Fusobacteriales.f__Fusobacteriaceae.g__F
alpha (n=25)
clade20C (n=1)
delta (n=13)
omicron (n=6)
neg (n=72)
NA
WHOvariant

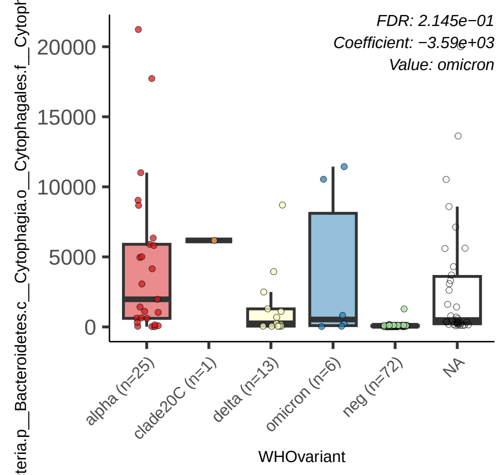

FDR: 2.145e−01
Coefficient: −3.59e+03
Value: omicron
cteria.p__Bacteroidetes.c__Cytophagia.o__Cytophagales.f__Cytoph
20000
15000
10000
5000
0
alpha (n=25)
clade20C (n=1)
delta (n=13)
omicron (n=6)
neg (n=72)
NA
WHOvariant

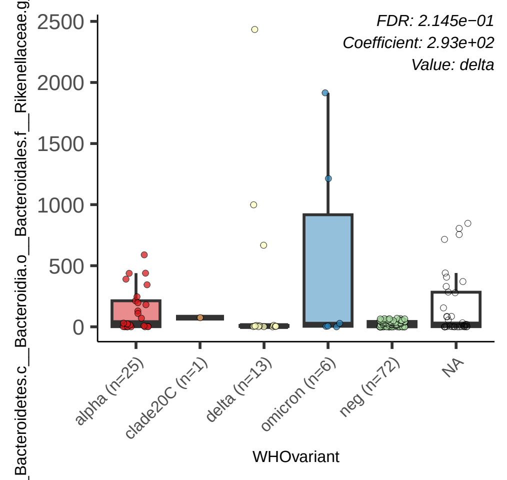
FDR: 2.145e−01
Coefficient: 2.93e+02
Value: delta
Bacteroidetes.c__Bacteroidia.o__Bacteroidales.f__Rikenellaceae.g
2500
2000
1500
1000
500
0
alpha (n=25)
clade20C (n=1)
delta (n=13)
omicron (n=6)
neg (n=72)
NA
WHOvariant

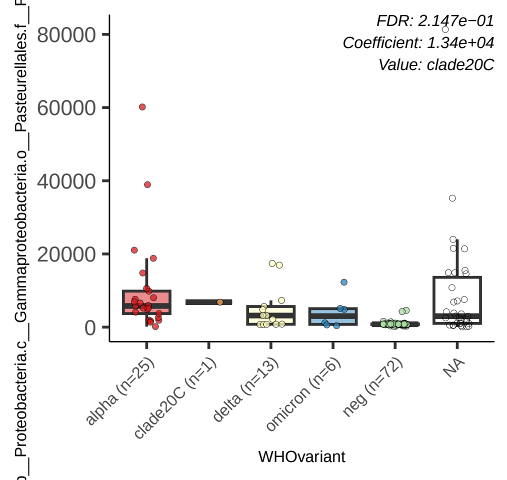

FDR: 2.147e−01
Coefficient: 1.34e+04
Value: clade20C
o__Proteobacteria.c__Gammaproteobacteria.o__Pasteurellales.f__P
80000
60000
40000
20000
0
alpha (n=25)
clade20C (n=1)
delta (n=13)
omicron (n=6)
neg (n=72)
NA
WHOvariant

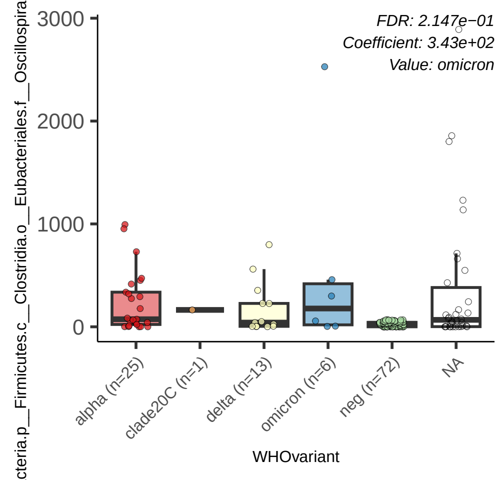

FDR: 2.147e−01
Coefficient: 3.43e+02
Value: omicron
3000
2000
1000
0
cteria.p__Firmicutes.c__Clostridia.o__Eubacteriales.f__Oscillospira
alpha (n=25)
clade20C (n=1)
delta (n=13)
omicron (n=6)
neg (n=72)
NA
WHOvariant

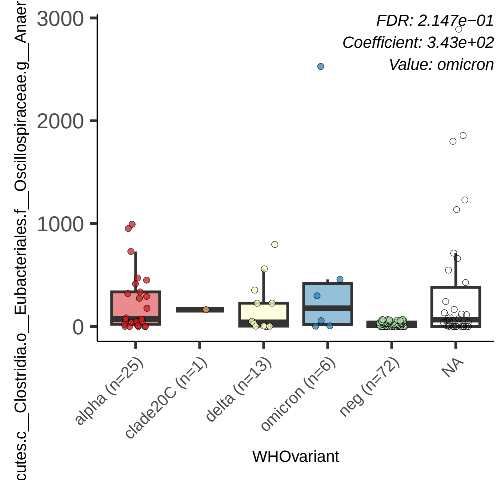

cutes.c__Clostridia.o__Eubacteriales.f__Oscillospiraceae.g__Anaer
3000
2000
1000
0
FDR: 2.147e−01
Coefficient: 3.43e+02
Value: omicron
alpha (n=25)
clade20C (n=1)
delta (n=13)
omicron (n=6)
neg (n=72)
NA
WHOvariant

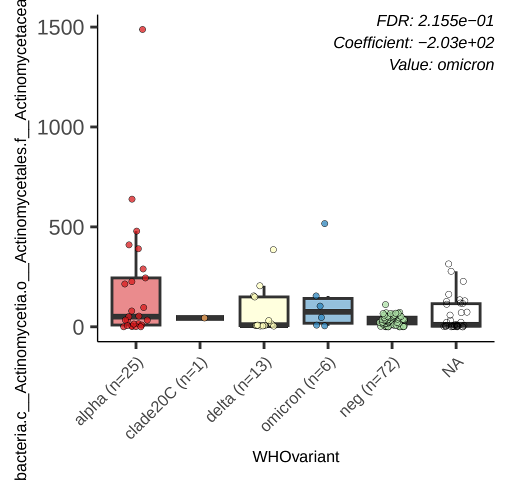

FDR: 2.155e−01
Coefficient: −2.03e+02
Value: omicron
1500
1000
500
0
bacteria.c__Actinomycetia.o__Actinomycetales.f__Actinomycetacea
alpha (n=25)
clade20C (n=1)
delta (n=13)
omicron (n=6)
neg (n=72)
NA
WHOvariant

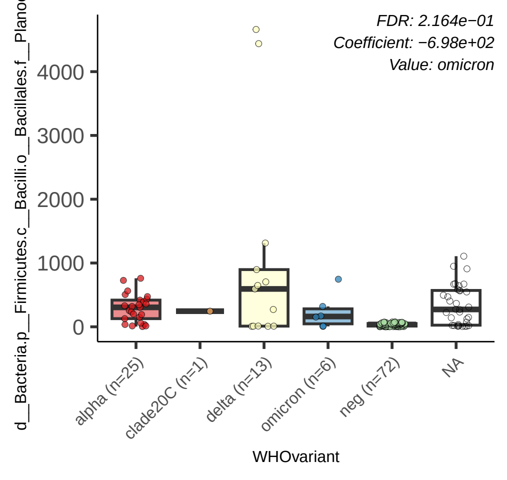

FDR: 2.164e−01
Coefficient: −6.98e+02
Value: omicron
d__Bacteria.p__Firmicutes.c__Bacilli.o__Bacillales.f__Planoc
0
1000
2000
3000
4000
alpha (n=25)
clade20C (n=1)
delta (n=13)
omicron (n=6)
neg (n=72)
NA
WHOvariant

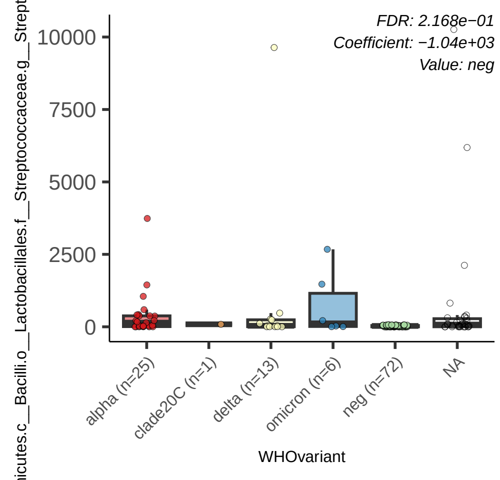

FDR: 2.168e−01
Coefficient: −1.04e+03
Value: neg
10000
7500
5000
2500
0
alpha (n=25)
clade20C (n=1)
delta (n=13)
omicron (n=6)
neg (n=72)
NA
WHOvariant

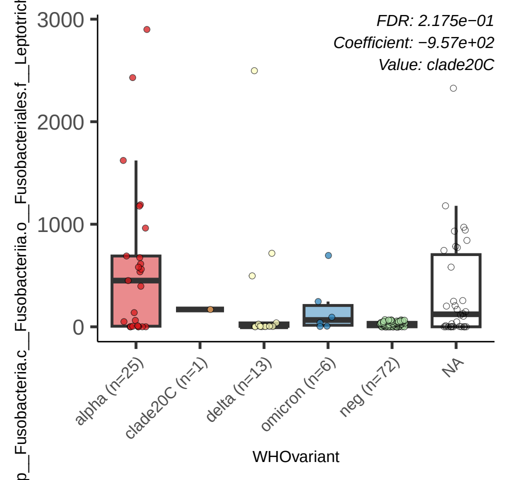

FDR: 2.175e−01
Coefficient: −9.57e+02
Value: clade20C
p__Fusobacteria.c__Fusobacteriia.o__Fusobacteriales.f__Leptotrich
3000
2000
1000
0
alpha (n=25)
clade20C (n=1)
delta (n=13)
omicron (n=6)
neg (n=72)
NA
WHOvariant

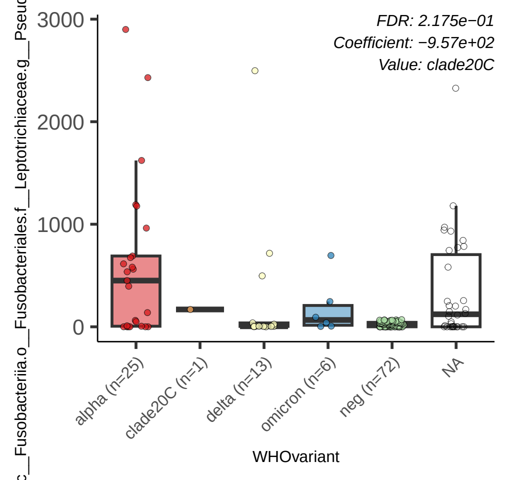

FDR: 2.175e−01
Coefficient: −9.57e+02
Value: clade20C
3000
2000
1000
0
c__Fusobacteriia.o__Fusobacteriales.f__Leptotrichiaceae.g__Pseud
alpha (n=25)
clade20C (n=1)
delta (n=13)
omicron (n=6)
neg (n=72)
NA
WHOvariant

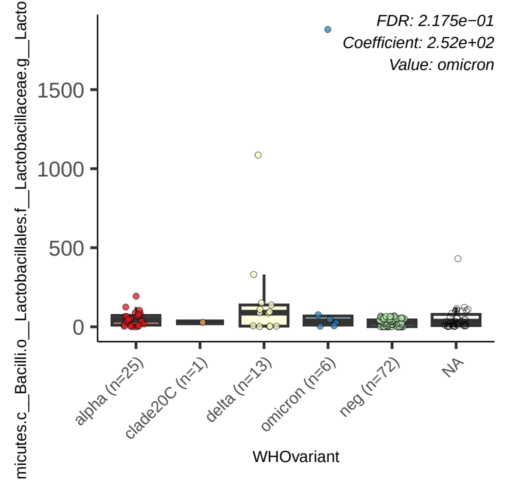
FDR: 2.175e−01
Coefficient: 2.52e+02
Value: omicron
micutes.c__Bacilli.o__Lactobacillales.f__Lactobacillaceae.g__Lacto
1500
1000
500
0
alpha (n=25)
clade20C (n=1)
delta (n=13)
omicron (n=6)
neg (n=72)
NA
WHOvariant

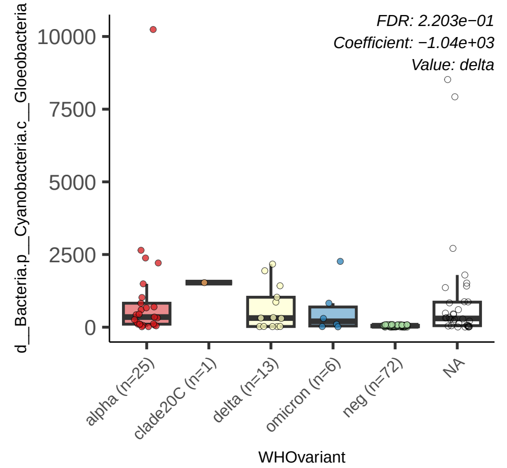

FDR: 2.203e−01
Coefficient: −1.04e+03
Value: delta
d__Bacteria.p__Cyanobacteria.c__Gloeobacteria
10000
7500
5000
2500
0
alpha (n=25)
clade20C (n=1)
delta (n=13)
omicron (n=6)
neg (n=72)
NA
WHOvariant

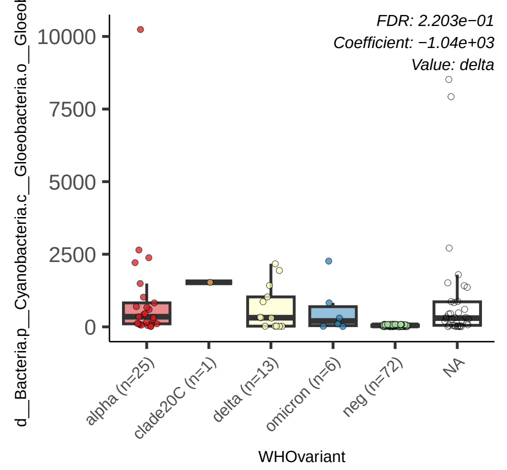

FDR: 2.203e−01
Coefficient: −1.04e+03
Value: delta
d__Bacteria.p__Cyanobacteria.c__Gloeobacteria.o__Gloeob
10000
7500
5000
2500
0
alpha (n=25)
clade20C (n=1)
delta (n=13)
omicron (n=6)
neg (n=72)
NA
WHOvariant

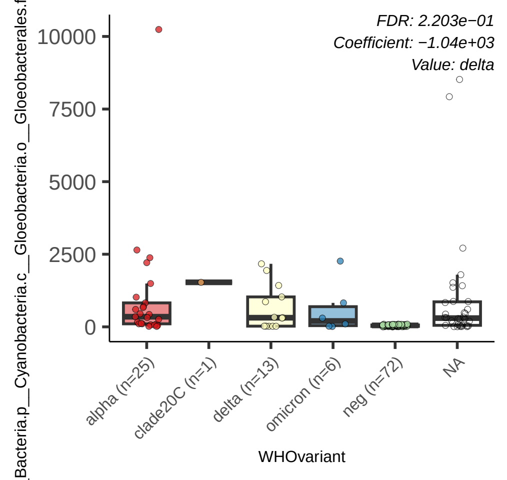
FDR: 2.203e−01
Coefficient: −1.04e+03
Value: delta
10000
7500
5000
2500
0
Bacteria.p__Cyanobacteria.c__Gloeobacteria.o__Gloeobacterales.f
alpha (n=25)
clade20C (n=1)
delta (n=13)
omicron (n=6)
neg (n=72)
NA
WHOvariant

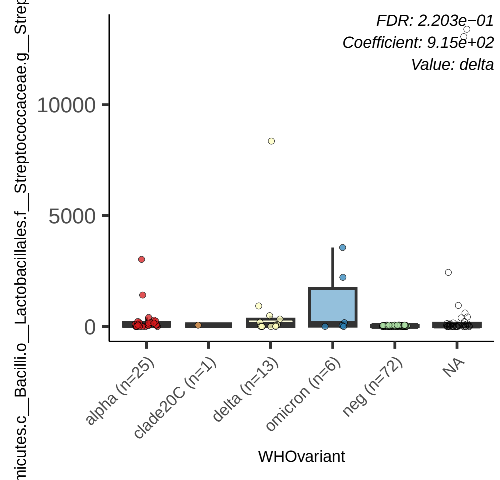
FDR: 2.203e−01
Coefficient: 9.15e+02
Value: delta
10000
5000
0
alpha (n=25)
clade20C (n=1)
delta (n=13)
omicron (n=6)
neg (n=72)
NA
WHOvariant
micutes.c__Bacilli.o__Lactobacillales.f__Streptococcaceae.g__Strep

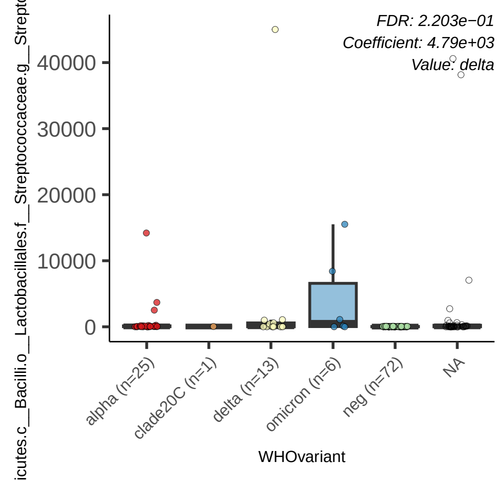

FDR: 2.203e−01
Coefficient: 4.79e+03
Value: delta
icutes.c__Bacilli.o__Lactobacillales.f__Streptococcaceae.g__Strept
40000
30000
20000
10000
0
alpha (n=25)
clade20C (n=1)
delta (n=13)
omicron (n=6)
neg (n=72)
NA
WHOvariant

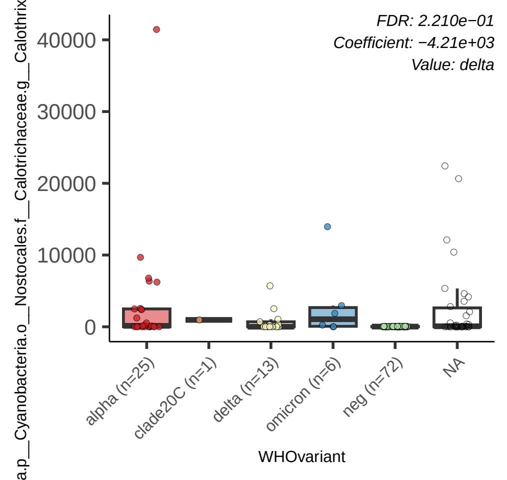

FDR: 2.210e−01
Coefficient: −4.21e+03
Value: delta
40000
30000
20000
10000
0
a.p__Cyanobacteria.o__Nostocales.f__Calotrichaceae.g__Calothrix
alpha (n=25)
clade20C (n=1)
delta (n=13)
omicron (n=6)
neg (n=72)
NA
WHOvariant

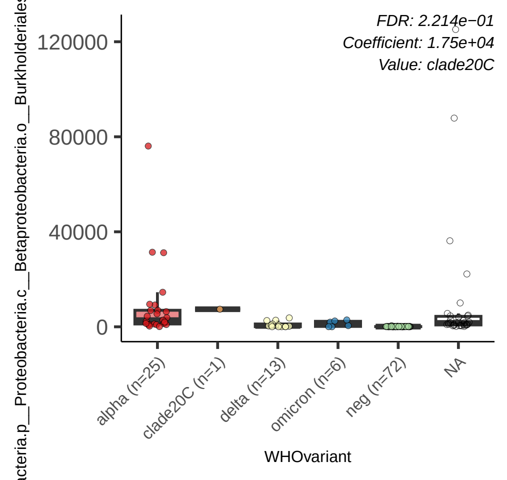
FDR: 2.214e−01
Coefficient: 1.75e+04
Value: clade20C
120000
80000
40000
0
acteria.p__Proteobacteria.c__Betaproteobacteria.o__Burkholderiales
alpha (n=25)
clade20C (n=1)
delta (n=13)
omicron (n=6)
neg (n=72)
NA
WHOvariant

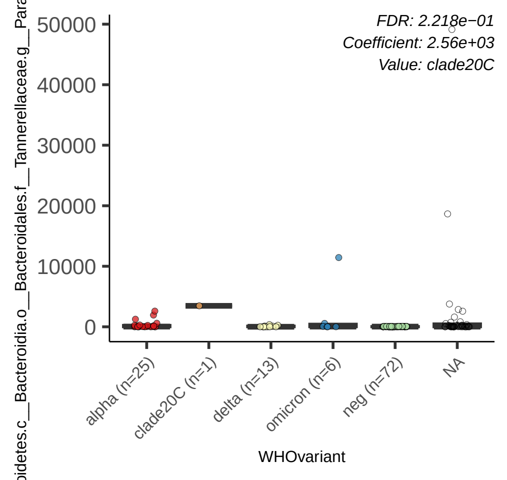
FDR: 2.218e−01
Coefficient: 2.56e+03
Value: clade20C
oidetes.c__Bacteroidia.o__Bacteroidales.f__Tannerellaceae.g__Para
50000
40000
30000
20000
10000
0
alpha (n=25)
clade20C (n=1)
delta (n=13)
omicron (n=6)
neg (n=72)
NA
WHOvariant

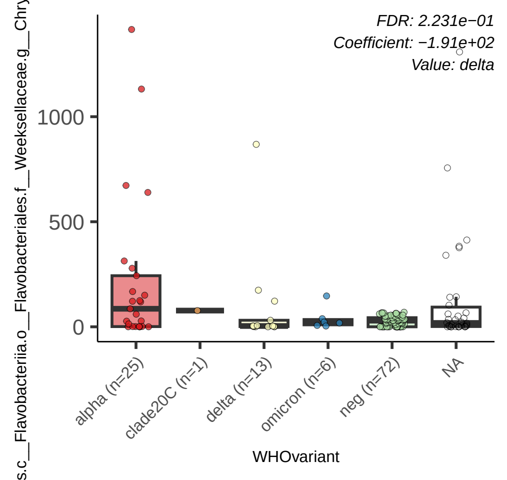
FDR: 2.231e−01
Coefficient: −1.91e+02
Value: delta
s.c__Flavobacteriia.o__Flavobacteriales.f__Weeksellaceae.g__Chry
1000
500
0
alpha (n=25)
clade20C (n=1)
delta (n=13)
omicron (n=6)
neg (n=72)
NA
WHOvariant

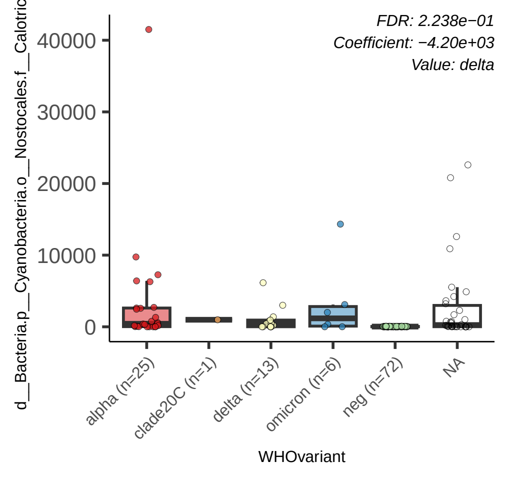

FDR: 2.238e−01
Coefficient: −4.20e+03
Value: delta
d__Bacteria.p__Cyanobacteria.o__Nostocales.f__Calotric
40000
30000
20000
10000
0
alpha (n=25)
clade20C (n=1)
delta (n=13)
omicron (n=6)
neg (n=72)
NA
WHOvariant

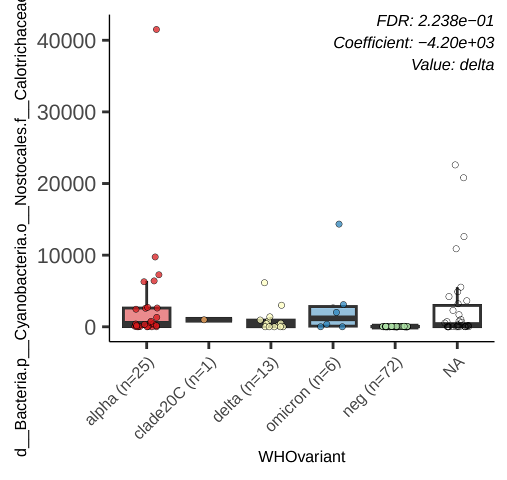

FDR: 2.238e−01
Coefficient: −4.20e+03
Value: delta
d__Bacteria.p__Cyanobacteria.o__Nostocales.f__Calotrichaceae
40000
30000
20000
10000
0
alpha (n=25)
clade20C (n=1)
delta (n=13)
omicron (n=6)
neg (n=72)
NA
WHOvariant

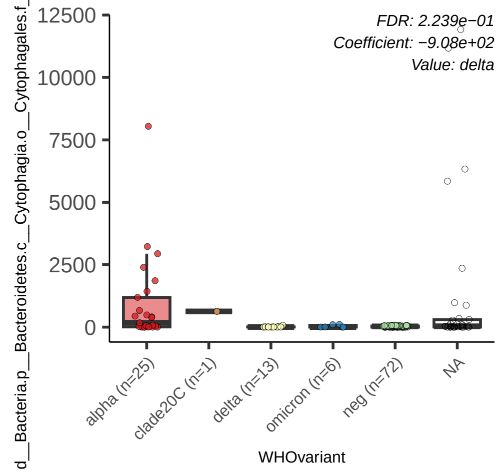

FDR: 2.239e−01
Coefficient: −9.08e+02
Value: delta
d__Bacteria.p__Bacteroidetes.c__Cytophagia.o__Cytophagales.f_
12500
10000
7500
5000
2500
0
alpha (n=25)
clade20C (n=1)
delta (n=13)
omicron (n=6)
neg (n=72)
NA
WHOvariant

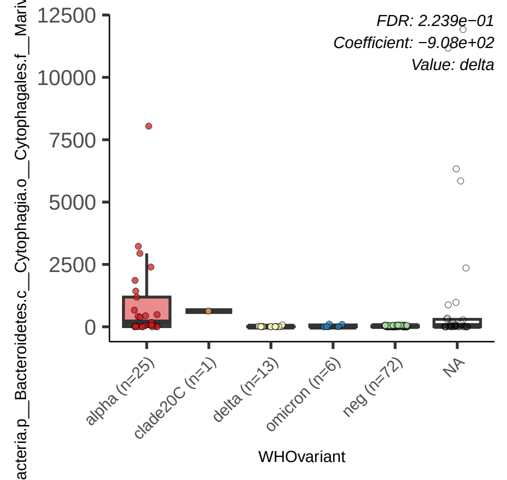

FDR: 2.239e−01
Coefficient: −9.08e+02
Value: delta
12500
10000
7500
5000
2500
0
acteria.p__Bacteroidetes.c__Cytophagia.o__Cytophagales.f__Mariv
alpha (n=25)
clade20C (n=1)
delta (n=13)
omicron (n=6)
neg (n=72)
NA
WHOvariant

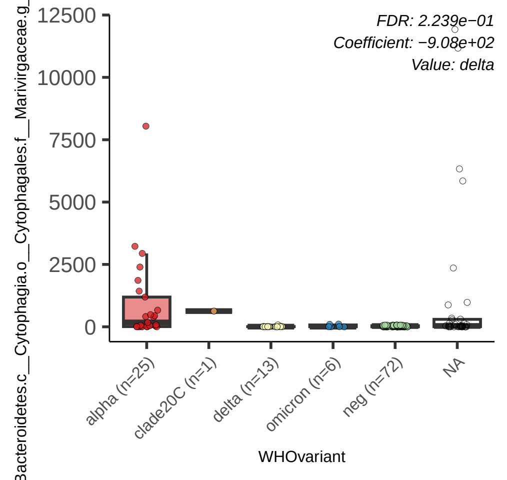

FDR: 2.239e−01
Coefficient: −9.08e+02
Value: delta
Bacteroidetes.c__Cytophagia.o__Cytophagales.f__Marivirgaceae.g_
12500
10000
7500
5000
2500
0
alpha (n=25)
clade20C (n=1)
delta (n=13)
omicron (n=6)
neg (n=72)
NA
WHOvariant

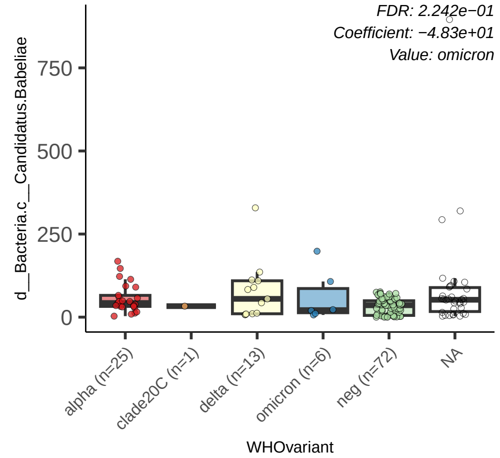

FDR: 2.242e−01
Coefficient: −4.83e+01
Value: omicron
d__Bacteria.c__Candidatus.Babeliae
750
500
250
0
alpha (n=25)
clade20C (n=1)
delta (n=13)
omicron (n=6)
neg (n=72)
NA
WHOvariant

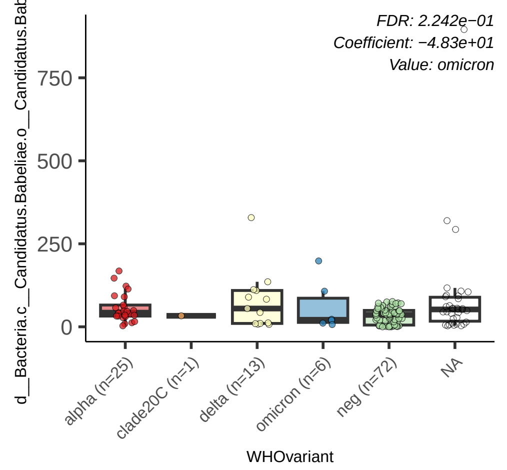

FDR: 2.242e−01
Coefficient: −4.83e+01
Value: omicron
d__Bacteria.c__Candidatus.Babeliae.o__Candidatus.Bab
0
250
500
750
alpha (n=25)
clade20C (n=1)
delta (n=13)
omicron (n=6)
neg (n=72)
NA
WHOvariant

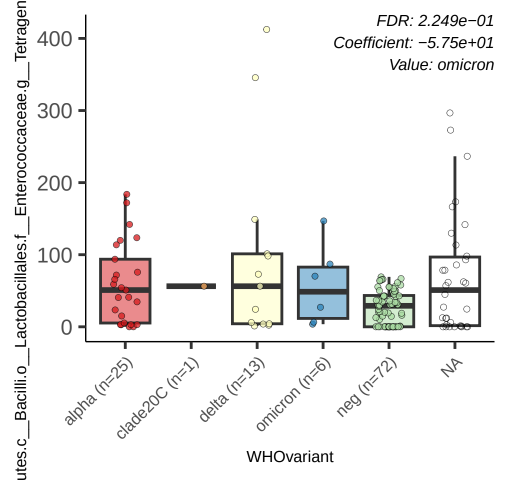
FDR: 2.249e−01
Coefficient: −5.75e+01
Value: omicron
utes.c__Bacilli.o__Lactobacillales.f__Enterococcaceae.g__Tetragen
400
300
200
100
0
alpha (n=25)
clade20C (n=1)
delta (n=13)
omicron (n=6)
neg (n=72)
NA
WHOvariant

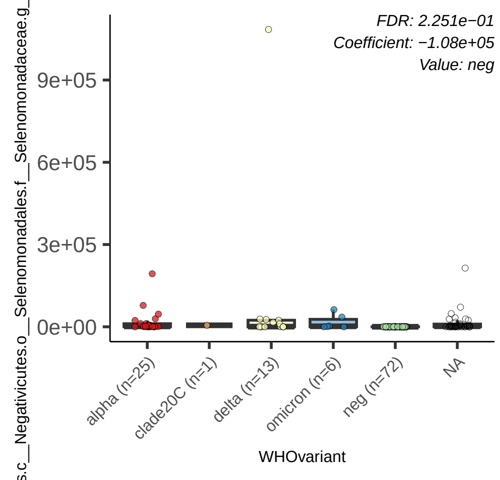

FDR: 2.251e−01
Coefficient: −1.08e+05
Value: neg
9e+05
6e+05
3e+05
0e+00
s.c__Negativicutes.o__Selenomonadales.f__Selenomonadaceae.g__
alpha (n=25)
clade20C (n=1)
delta (n=13)
omicron (n=6)
neg (n=72)
NA
WHOvariant

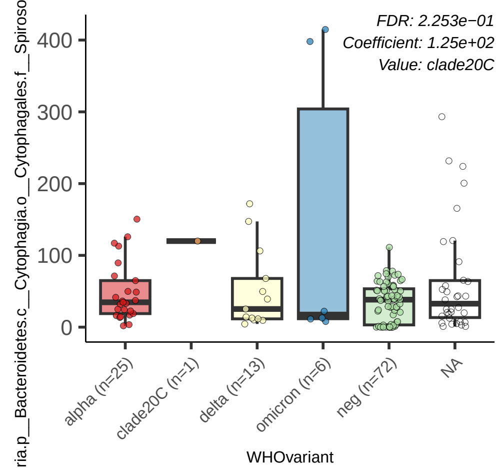

FDR: 2.253e−01
Coefficient: 1.25e+02
Value: clade20C
400
300
200
100
0
teria.p__Bacteroidetes.c__Cytophagia.o__Cytophagales.f__Spiroso
alpha (n=25)
clade20C (n=1)
delta (n=13)
omicron (n=6)
neg (n=72)
NA
WHOvariant

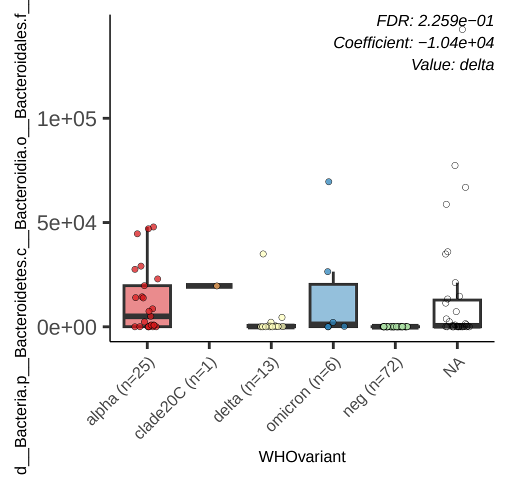
FDR: 2.259e−01
Coefficient: −1.04e+04
Value: delta
d__Bacteria.p__Bacteroidetes.c__Bacteroidia.o__Bacteroidales.f__
1e+05
5e+04
0e+00
alpha (n=25)
clade20C (n=1)
delta (n=13)
omicron (n=6)
neg (n=72)
NA
WHOvariant

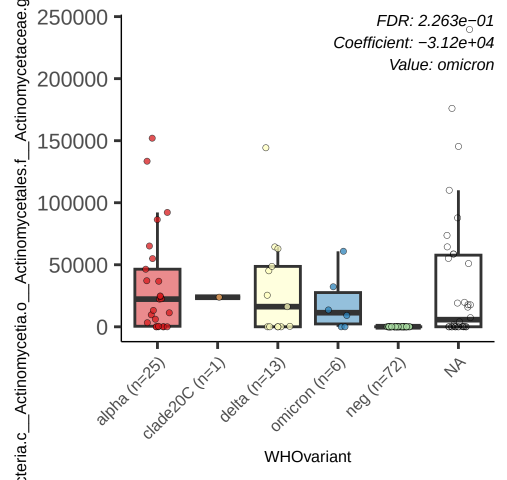

FDR: 2.263e−01
Coefficient: −3.12e+04
Value: omicron
250000
200000
150000
100000
50000
0
alpha (n=25)
clade20C (n=1)
delta (n=13)
omicron (n=6)
neg (n=72)
NA
WHOvariant

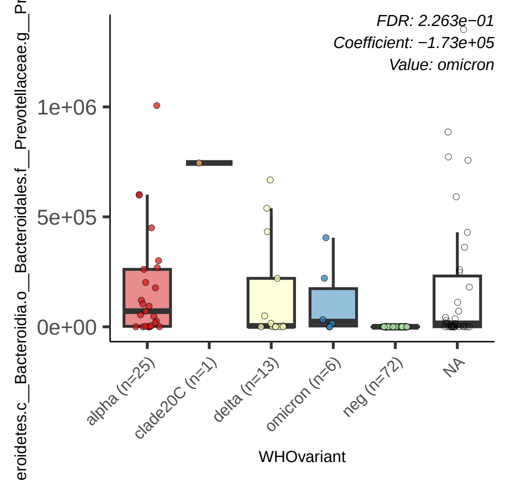

eroidetes.c__Bacteroidia.o__Bacteroidales.f__Prevotellaceae.g__Pre
FDR: 2.263e−01
Coefficient: −1.73e+05
Value: omicron
1e+06
5e+05
0e+00
alpha (n=25)
clade20C (n=1)
delta (n=13)
omicron (n=6)
neg (n=72)
NA
WHOvariant

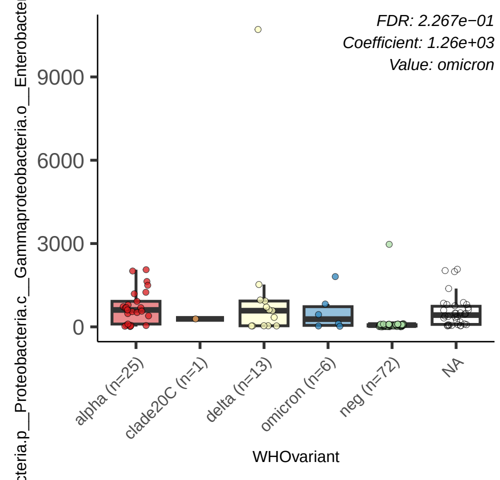

FDR: 2.267e−01
Coefficient: 1.26e+03
Value: omicron
9000
6000
3000
0
alpha (n=25)
clade20C (n=1)
delta (n=13)
omicron (n=6)
neg (n=72)
NA
WHOvariant
cteria.p__Proteobacteria.c__Gammaproteobacteria.o__Enterobacte

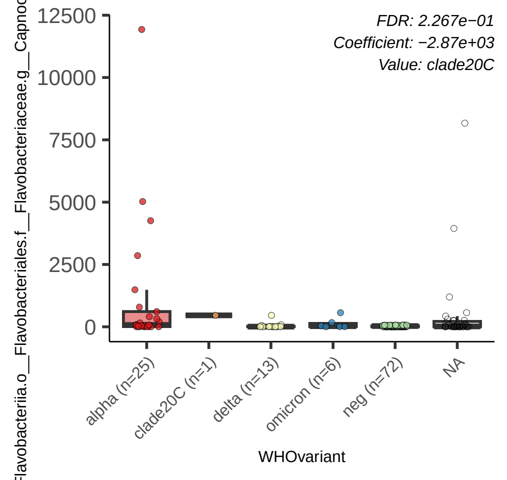
FDR: 2.267e−01
Coefficient: −2.87e+03
Value: clade20C
12500
10000
7500
5000
2500
0
Flavobacteriia.o__Flavobacteriales.f__Flavobacteriaceae.g__Capnoc
alpha (n=25)
clade20C (n=1)
delta (n=13)
omicron (n=6)
neg (n=72)
NA
WHOvariant

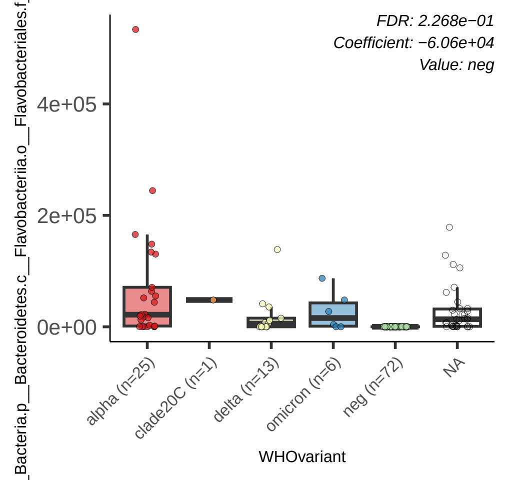

FDR: 2.268e−01
Coefficient: −6.06e+04
Value: neg
Bacteria.p__Bacteroidetes.c__Flavobacteriia.o__Flavobacteriales.f
4e+05
2e+05
0e+00
alpha (n=25)
clade20C (n=1)
delta (n=13)
omicron (n=6)
neg (n=72)
NA
WHOvariant

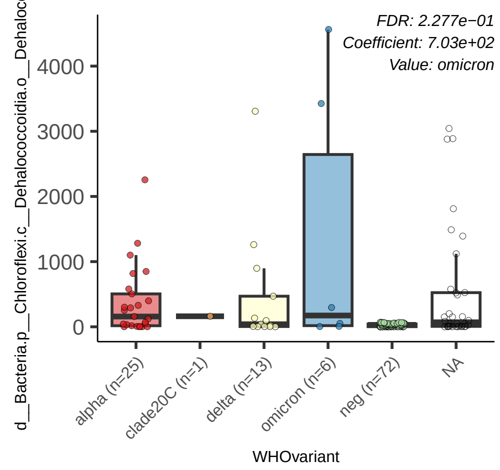

FDR: 2.277e−01
Coefficient: 7.03e+02
Value: omicron
d__Bacteria.p__Chloroflexi.c__Dehalococcoidia.o__Dehaloc
4000
3000
2000
1000
0
alpha (n=25)
clade20C (n=1)
delta (n=13)
omicron (n=6)
neg (n=72)
NA
WHOvariant

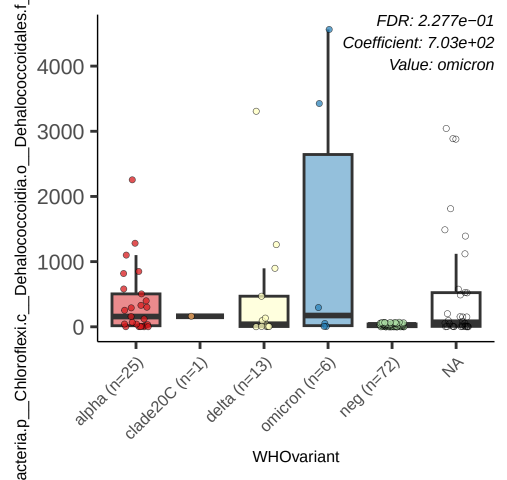

FDR: 2.277e−01
Coefficient: 7.03e+02
Value: omicron
acteria.p__Chloroflexi.c__Dehalococcoidia.o__Dehalococcoidales.f_
0
1000
2000
3000
4000
alpha (n=25)
clade20C (n=1)
delta (n=13)
omicron (n=6)
neg (n=72)
NA
WHOvariant

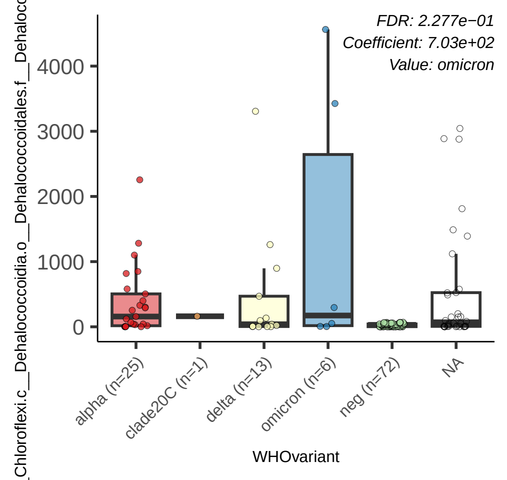
FDR: 2.277e−01
Coefficient: 7.03e+02
Value: omicron
Chloroflexi.c__Dehalococcoidia.o__Dehalococcoidales.f__Dehalococ
4000
3000
2000
1000
0
alpha (n=25)
clade20C (n=1)
delta (n=13)
omicron (n=6)
neg (n=72)
NA
WHOvariant

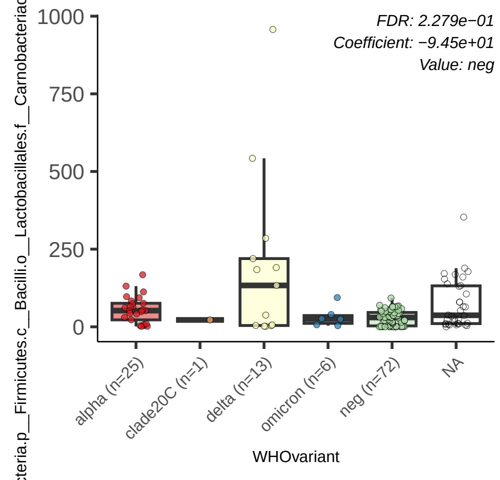

FDR: 2.279e−01
Coefficient: −9.45e+01
Value: neg
Firmicutes.c__Bacilli.o__Lactobacillales.f__Carnobacteriac
1000
750
500
250
0
alpha (n=25)
clade20C (n=1)
delta (n=13)
omicron (n=6)
neg (n=72)
NA
WHOvariant

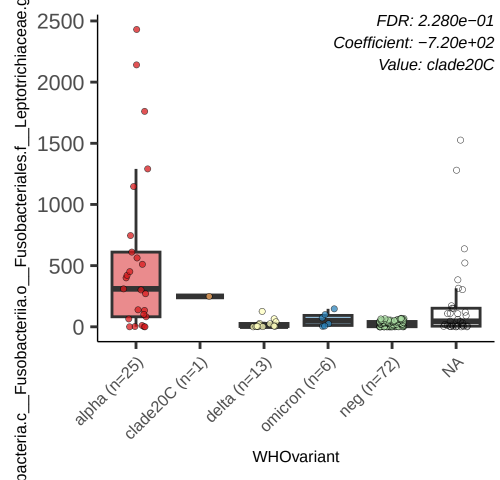

FDR: 2.280e−01
Coefficient: −7.20e+02
Value: clade20C
bacteria.c__Fusobacteriia.o__Fusobacteriales.f__Leptotrichiaceae.g
2500
2000
1500
1000
500
0
alpha (n=25)
clade20C (n=1)
delta (n=13)
omicron (n=6)
neg (n=72)
NA
WHOvariant

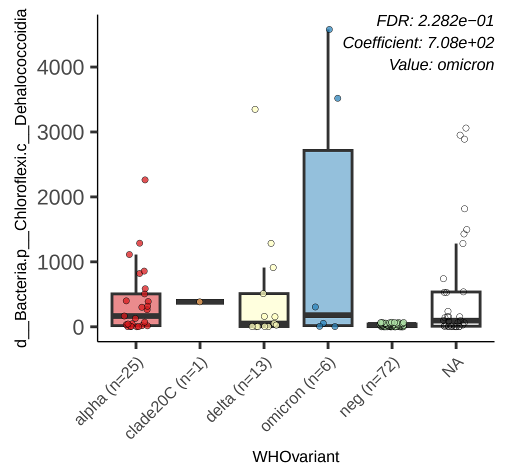
FDR: 2.282e−01
Coefficient: 7.08e+02
Value: omicron
d__Bacteria.p__Chloroflexi.c__Dehalococcoidia
4000
3000
2000
1000
0
alpha (n=25)
clade20C (n=1)
delta (n=13)
omicron (n=6)
neg (n=72)
NA
WHOvariant

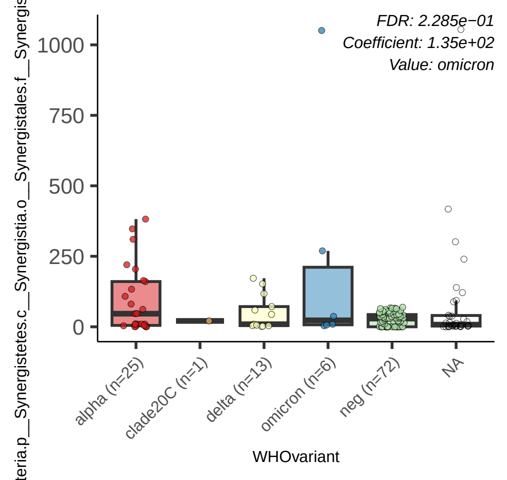
FDR: 2.285e−01
Coefficient: 1.35e+02
Value: omicron
teria.p__Synergistetes.c__Synergistia.o__Synergistales.f__Synergis
1000
750
500
250
0
alpha (n=25)
clade20C (n=1)
delta (n=13)
omicron (n=6)
neg (n=72)
NA
WHOvariant

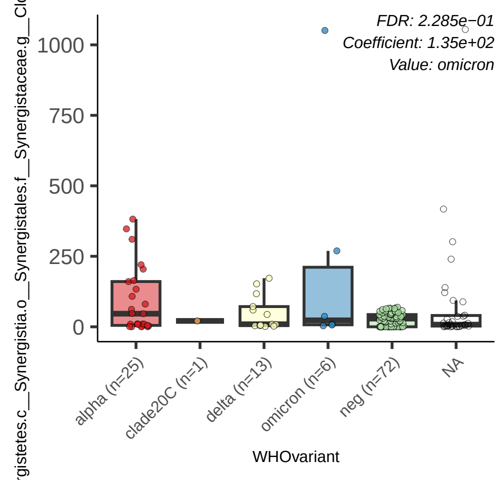

FDR: 2.285e−01
Coefficient: 1.35e+02
Value: omicron
rgistetes.c__Synergistia.o__Synergistales.f__Synergistaceae.g__Cl
1000
750
500
250
0
alpha (n=25)
clade20C (n=1)
delta (n=13)
omicron (n=6)
neg (n=72)
NA
WHOvariant

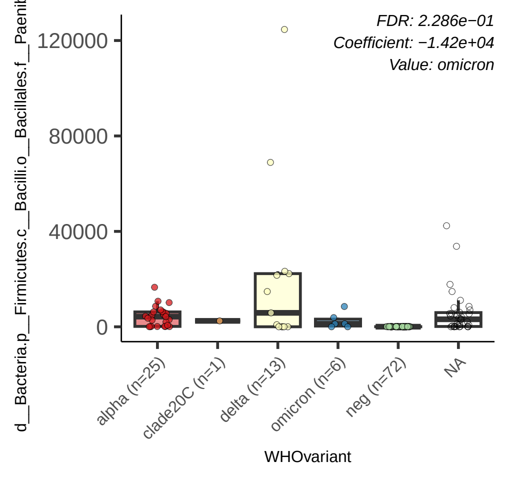
FDR: 2.286e−01
Coefficient: −1.42e+04
Value: omicron
120000
80000
40000
0
d__Bacteria.p__Firmicutes.c__Bacilli.o__Bacillales.f__Paenik
alpha (n=25)
clade20C (n=1)
delta (n=13)
omicron (n=6)
neg (n=72)
NA
WHOvariant

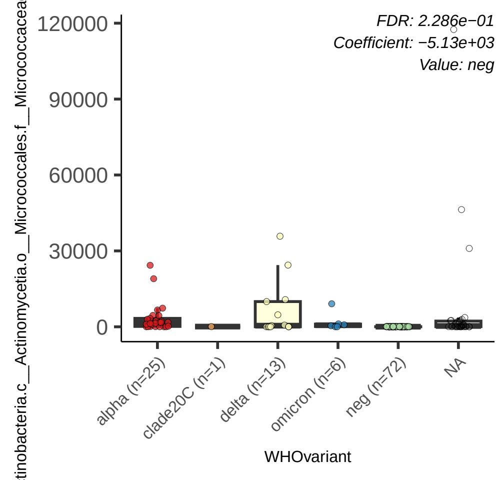
FDR: 2.286e−01
Coefficient: −5.13e+03
Value: neg
120000
90000
60000
30000
0
tinobacteria.c__Actinomycetia.o__Micrococcales.f__Micrococcacea
alpha (n=25)
clade20C (n=1)
delta (n=13)
omicron (n=6)
neg (n=72)
NA
WHOvariant

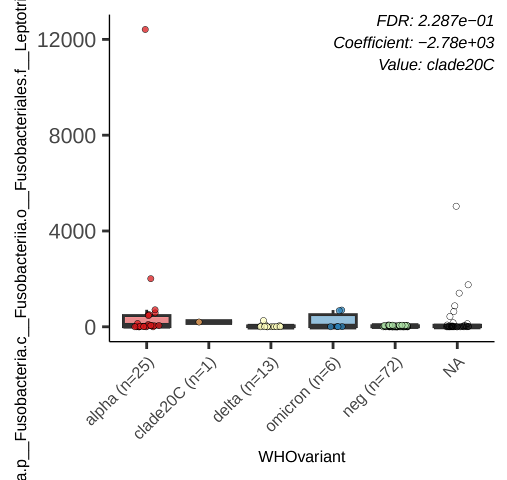

FDR: 2.287e−01
Coefficient: −2.78e+03
Value: clade20C
a.p__Fusobacteria.c__Fusobacteriia.o__Fusobacteriales.f__Leptotri
12000
8000
4000
0
alpha (n=25)
clade20C (n=1)
delta (n=13)
omicron (n=6)
neg (n=72)
NA
WHOvariant

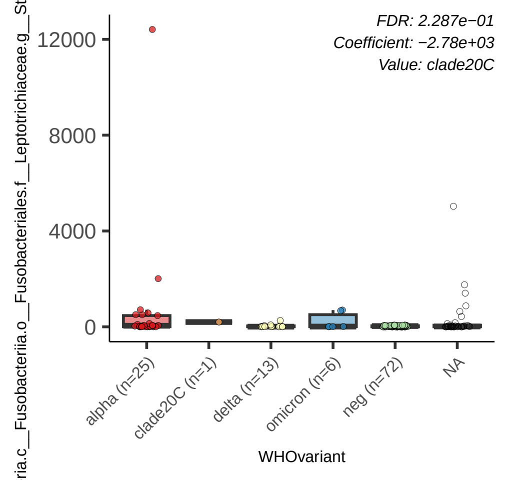

FDR: 2.287e−01
Coefficient: −2.78e+03
Value: clade20C
12000
8000
4000
0
alpha (n=25)
clade20C (n=1)
delta (n=13)
omicron (n=6)
neg (n=72)
NA
WHOvariant
ria.c__Fusobacteriia.o__Fusobacteriales.f__Leptotrichiaceae.g__St

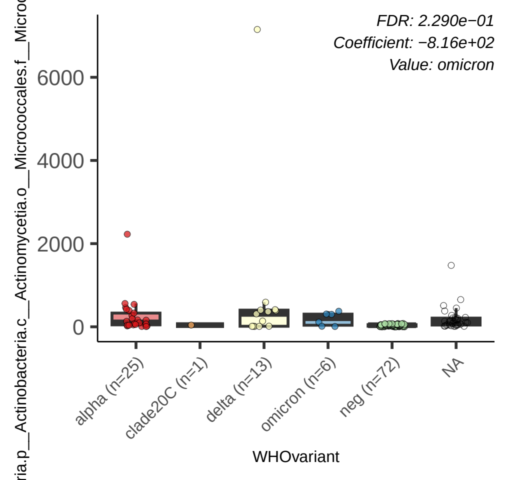

FDR: 2.290e−01
Coefficient: −8.16e+02
Value: omicron
ria.p__Actinobacteria.c__Actinomycetia.o__Micrococcales.f__Microc
6000
4000
2000
0
alpha (n=25)
clade20C (n=1)
delta (n=13)
omicron (n=6)
neg (n=72)
NA
WHOvariant

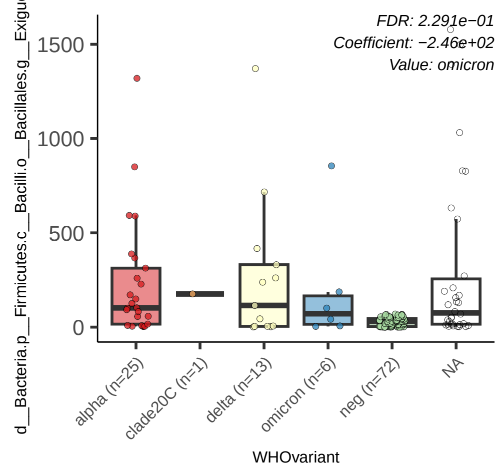

FDR: 2.291e−01
Coefficient: −2.46e+02
Value: omicron
d__Bacteria.p__Firmicutes.c__Bacilli.o__Bacillales.g__Exigu
1500
1000
500
0
alpha (n=25)
clade20C (n=1)
delta (n=13)
omicron (n=6)
neg (n=72)
NA
WHOvariant

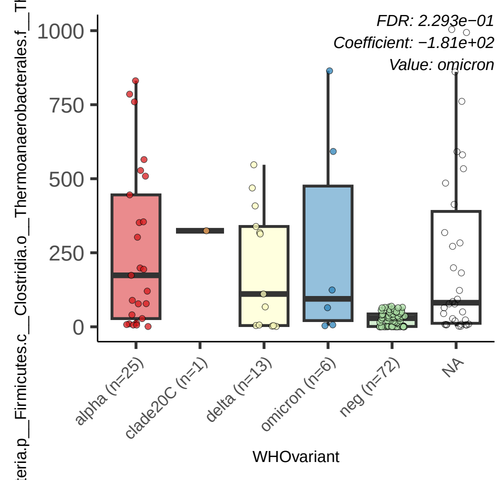

FDR: 2.293e−01
Coefficient: −1.81e+02
Value: omicron
1000
750
500
250
0
alpha (n=25)
clade20C (n=1)
delta (n=13)
omicron (n=6)
neg (n=72)
NA
WHOvariant
Firmicutes.c__Clostridia.o__Thermoanaerobacterales.f__

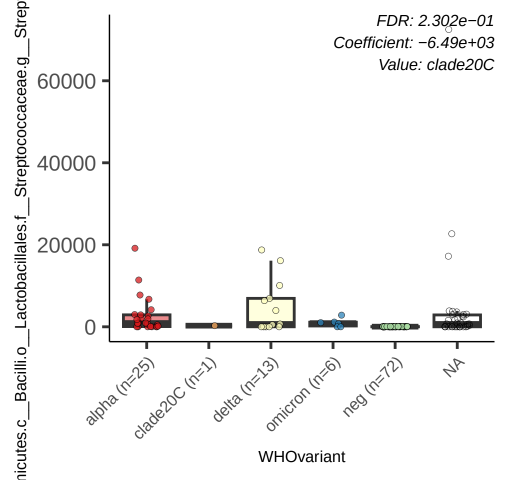

FDR: 2.302e−01
Coefficient: −6.49e+03
Value: clade20C
nicutes.c__Bacilli.o__Lactobacillales.f__Streptococcaceae.g__Strep
60000
40000
20000
0
alpha (n=25)
clade20C (n=1)
delta (n=13)
omicron (n=6)
neg (n=72)
NA
WHOvariant

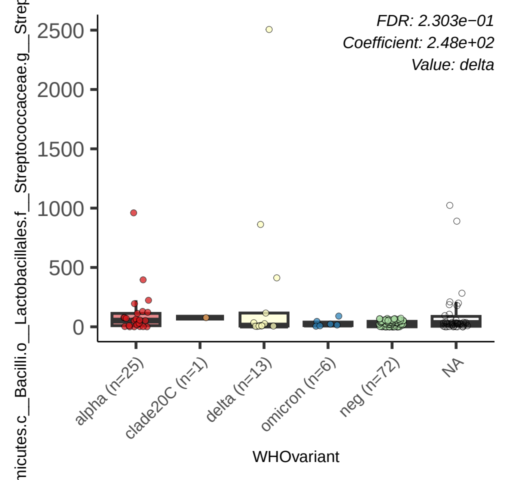

FDR: 2.303e−01
Coefficient: 2.48e+02
Value: delta
micutes.c__Bacilli.o__Lactobacillales.f__Streptococcaceae.g__Strep
2500
2000
1500
1000
500
0
alpha (n=25)
clade20C (n=1)
delta (n=13)
omicron (n=6)
neg (n=72)
NA
WHOvariant

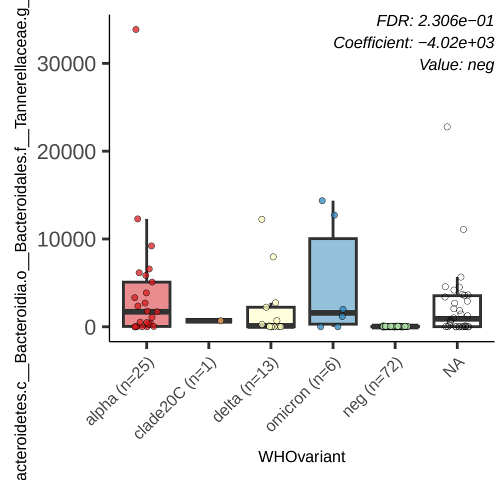

FDR: 2.306e−01
Coefficient: −4.02e+03
Value: neg
acteroidetes.c__Bacteroidia.o__Bacteroidales.f__Tannerellaceae.g__
30000
20000
10000
0
alpha (n=25)
clade20C (n=1)
delta (n=13)
omicron (n=6)
neg (n=72)
NA
WHOvariant

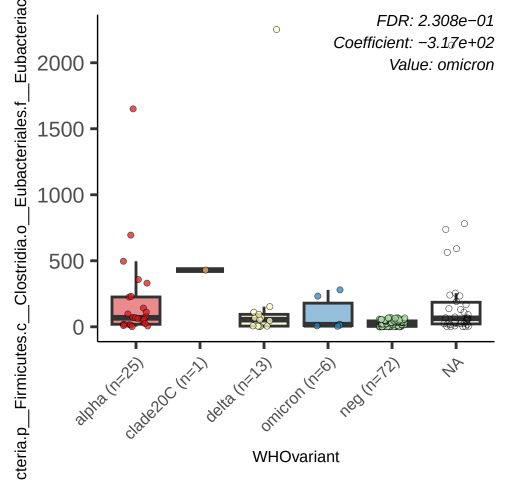

FDR: 2.308e−01
Coefficient: −3.17e+02
Value: omicron
0
500
1000
1500
2000
cteria.p__Firmicutes.c__Clostridia.o__Eubacteriales.f__Eubacteriac
alpha (n=25)
clade20C (n=1)
delta (n=13)
omicron (n=6)
neg (n=72)
NA
WHOvariant

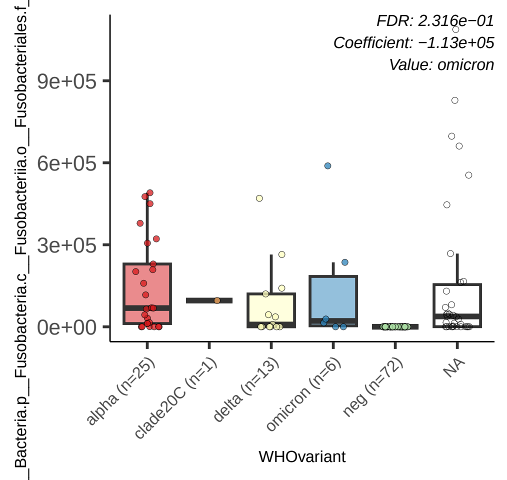

FDR: 2.316e−01
Coefficient: −1.13e+05
Value: omicron
9e+05
6e+05
3e+05
0e+00
_Bacteria.p__Fusobacteria.c__Fusobacteriia.o__Fusobacteriales.f_
alpha (n=25)
clade20C (n=1)
delta (n=13)
omicron (n=6)
neg (n=72)
NA
WHOvariant

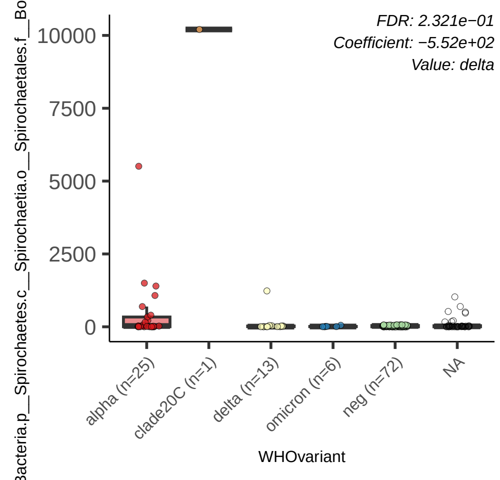

FDR: 2.321e−01
Coefficient: −5.52e+02
Value: delta
10000
7500
5000
2500
0
Bacteria.p__Spirochaetes.c__Spirochaetia.o__Spirochaetales.f__Bo
alpha (n=25)
clade20C (n=1)
delta (n=13)
omicron (n=6)
neg (n=72)
NA
WHOvariant

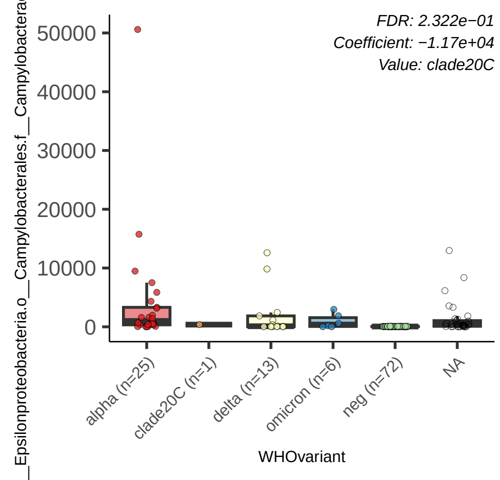

FDR: 2.322e−01
Coefficient: −1.17e+04
Value: clade20C
Epsilonproteobacteria.o__Campylobacterales.f__Campylobactera
50000
40000
30000
20000
10000
0
alpha (n=25)
clade20C (n=1)
delta (n=13)
omicron (n=6)
neg (n=72)
NA
WHOvariant

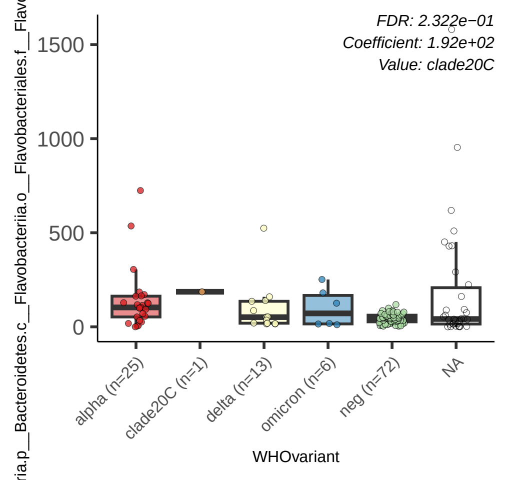

FDR: 2.322e−01
Coefficient: 1.92e+02
Value: clade20C
1500
1000
500
0
ria.p__Bacteroidetes.c__Flavobacteriia.o__Flavobacteriales.f__Flavo
alpha (n=25)
clade20C (n=1)
delta (n=13)
omicron (n=6)
neg (n=72)
NA
WHOvariant

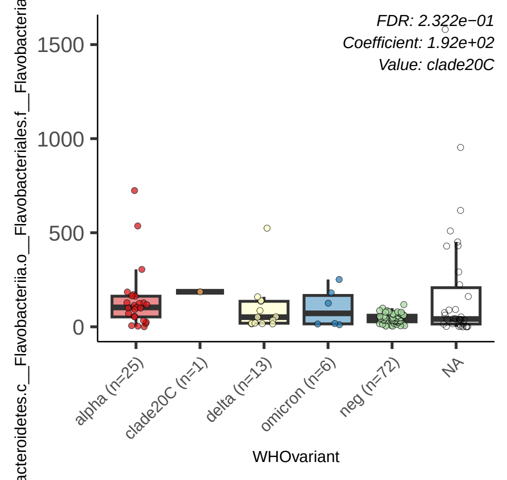

FDR: 2.322e−01
Coefficient: 1.92e+02
Value: clade20C
1500
1000
500
0
acteroidetes.c__Flavobacteriia.o__Flavobacteriales.f__Flavobacteria
alpha (n=25)
clade20C (n=1)
delta (n=13)
omicron (n=6)
neg (n=72)
NA
WHOvariant

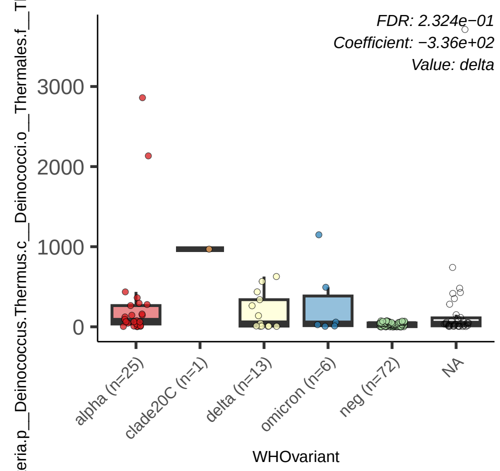

FDR: 2.324e−01
Coefficient: −3.36e+02
Value: delta
0
1000
2000
3000
cteria.p__Deinococcus.Thermus.c__Deinococci.o__Thermales.f__Th
alpha (n=25)
clade20C (n=1)
delta (n=13)
omicron (n=6)
neg (n=72)
NA
WHOvariant

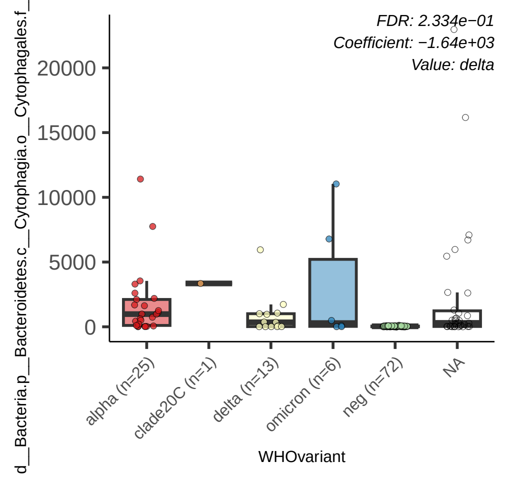

FDR: 2.334e−01
Coefficient: −1.64e+03
Value: delta
d__Bacteria.p__Bacteroidetes.c__Cytophagia.o__Cytophagales.f__
0
5000
10000
15000
20000
alpha (n=25)
clade20C (n=1)
delta (n=13)
omicron (n=6)
neg (n=72)
NA
WHOvariant

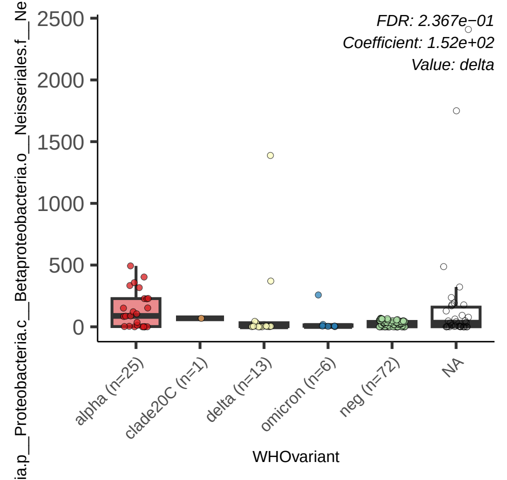

FDR: 2.367e−01
Coefficient: 1.52e+02
Value: delta
ia.p__Proteobacteria.c__Betaproteobacteria.o__Neisseriales.f__Ne
2500
2000
1500
1000
500
0
alpha (n=25)
clade20C (n=1)
delta (n=13)
omicron (n=6)
neg (n=72)
NA
WHOvariant

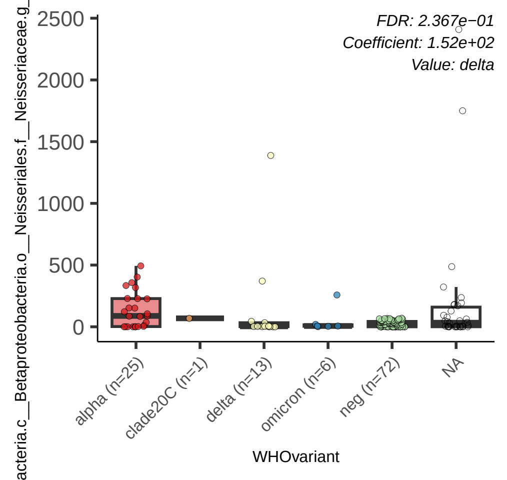
FDR: 2.367e−01
Coefficient: 1.52e+02
Value: delta
2500
2000
1500
1000
500
0
acteria.c__Betaproteobacteria.o__Neisseriales.f__Neisseriaceae.g__
alpha (n=25)
clade20C (n=1)
delta (n=13)
omicron (n=6)
neg (n=72)
NA
WHOvariant

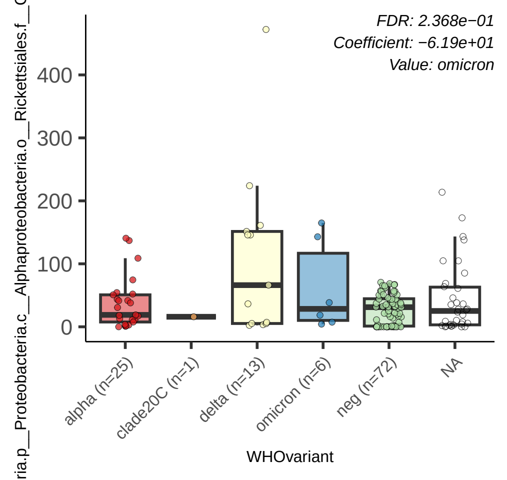

FDR: 2.368e−01
Coefficient: −6.19e+01
Value: omicron
ria.p__Proteobacteria.c__Alphaproteobacteria.o__Rickettsiales.f__C
400
300
200
100
0
alpha (n=25)
clade20C (n=1)
delta (n=13)
omicron (n=6)
neg (n=72)
NA
WHOvariant

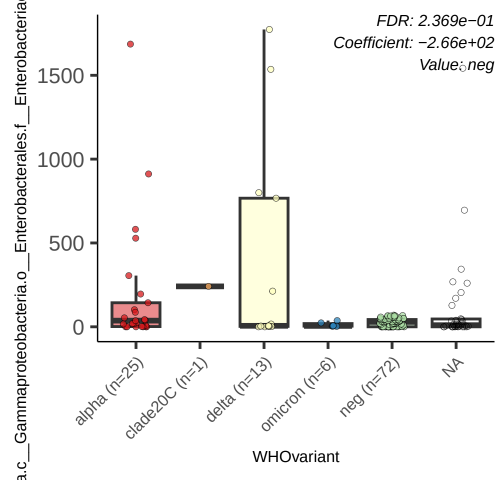

FDR: 2.369e−01
Coefficient: −2.66e+02
Value: neg
1500
1000
500
0
a.c__Gammaproteobacteria.o__Enterobacterales.f__Enterobacteria
alpha (n=25)
clade20C (n=1)
delta (n=13)
omicron (n=6)
neg (n=72)
NA
WHOvariant

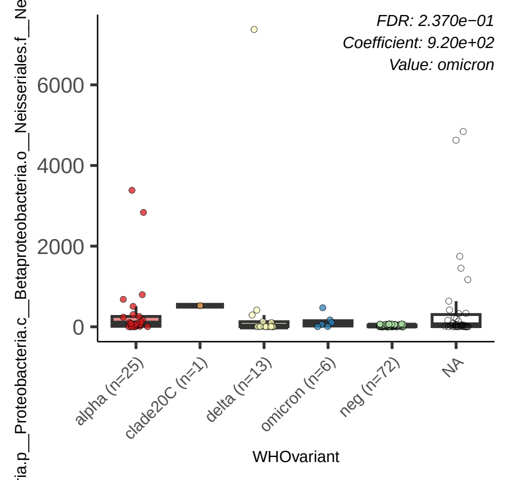

FDR: 2.370e−01
Coefficient: 9.20e+02
Value: omicron
6000
4000
2000
0
ria.p__Proteobacteria.c__Betaproteobacteria.o__Neisseriales.f__Ne
alpha (n=25)
clade20C (n=1)
delta (n=13)
omicron (n=6)
neg (n=72)
NA
WHOvariant

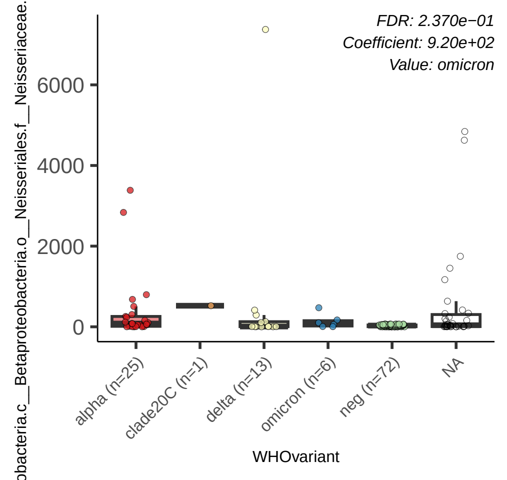
FDR: 2.370e−01
Coefficient: 9.20e+02
Value: omicron
obacteria.c__Betaproteobacteria.o__Neisseriales.f__Neisseriaceae.
6000
4000
2000
0
alpha (n=25)
clade20C (n=1)
delta (n=13)
omicron (n=6)
neg (n=72)
NA
WHOvariant

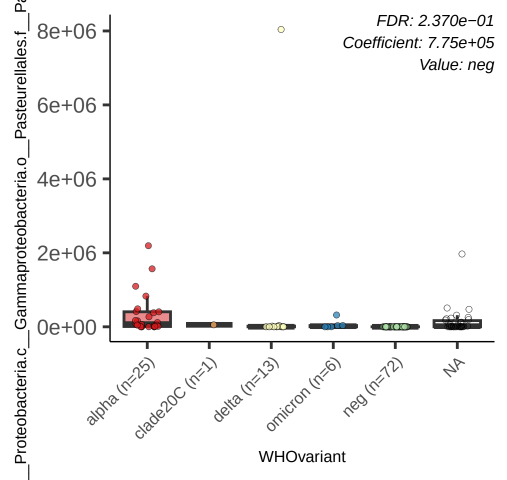

FDR: 2.370e−01
Coefficient: 7.75e+05
Value: neg
8e+06
6e+06
4e+06
2e+06
0e+00
Proteobacteria.c__Gammaproteobacteria.o__Pasteurellales.f__Pa
alpha (n=25)
clade20C (n=1)
delta (n=13)
omicron (n=6)
neg (n=72)
NA
WHOvariant

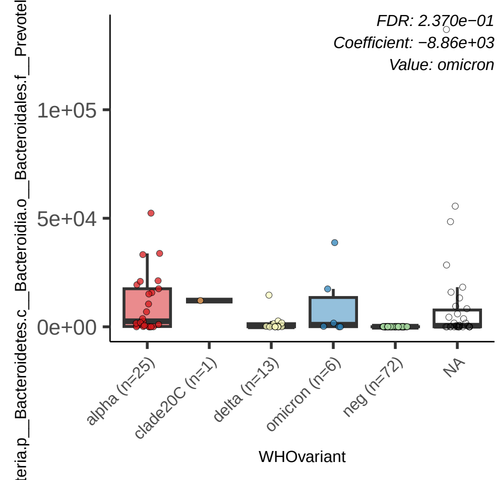
FDR: 2.370e−01
Coefficient: −8.86e+03
Value: omicron
teria.p__Bacteroidetes.c__Bacteroidia.o__Bacteroidales.f__Prevotel
1e+05
5e+04
0e+00
alpha (n=25)
clade20C (n=1)
delta (n=13)
omicron (n=6)
neg (n=72)
NA
WHOvariant

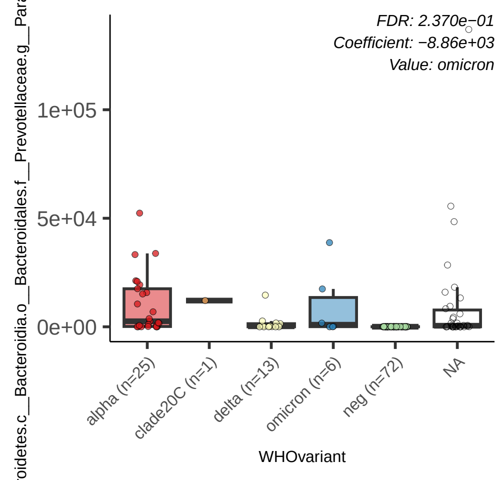

FDR: 2.370e−01
Coefficient: −8.86e+03
Value: omicron
oidetes.c__Bacteroidia.o__Bacteroidales.f__Prevotellaceae.g__Para
1e+05
5e+04
0e+00
alpha (n=25)
clade20C (n=1)
delta (n=13)
omicron (n=6)
neg (n=72)
NA
WHOvariant

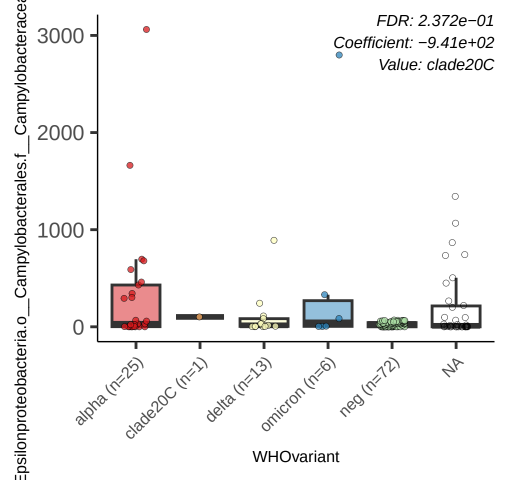

FDR: 2.372e−01
Coefficient: −9.41e+02
Value: clade20C
3000
2000
1000
0
Epsilonproteobacteria.o__Campylobacterales.f__Campylobacteracea
alpha (n=25)
clade20C (n=1)
delta (n=13)
omicron (n=6)
neg (n=72)
NA
WHOvariant

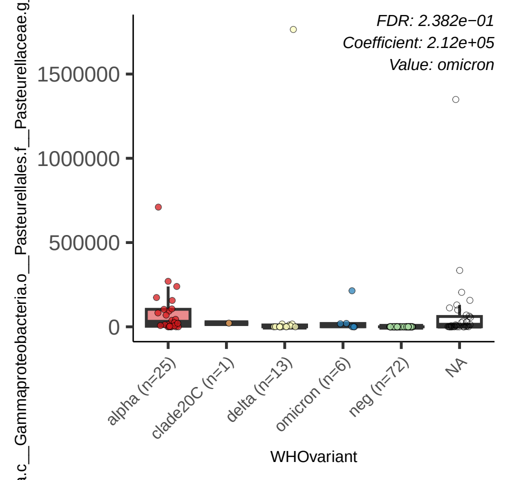

FDR: 2.382e−01
Coefficient: 2.12e+05
Value: omicron
1500000
1000000
500000
0
a.c__Gammaproteobacteria.o__Pasteurellales.f__Pasteurellaceae.g
alpha (n=25)
clade20C (n=1)
delta (n=13)
omicron (n=6)
neg (n=72)
NA
WHOvariant

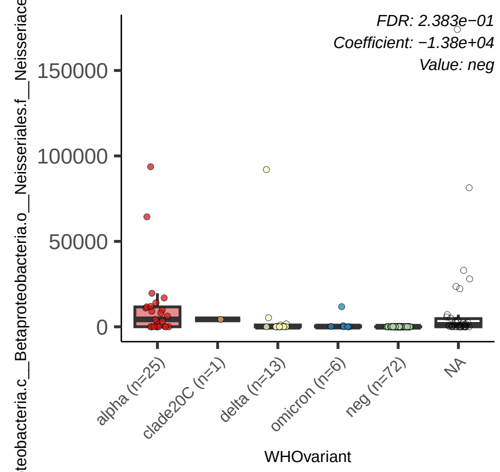
FDR: 2.383e−01
Coefficient: −1.38e+04
Value: neg
oteobacteria.c__Betaproteobacteria.o__Neisseriales.f__Neisseriace
150000
100000
50000
0
alpha (n=25)
clade20C (n=1)
delta (n=13)
omicron (n=6)
neg (n=72)
NA
WHOvariant

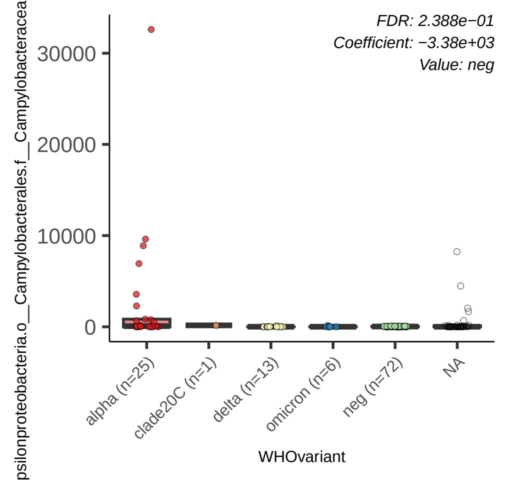

FDR: 2.388e−01
Coefficient: −3.38e+03
Value: neg
psilonproteobacteria.o__Campylobacterales.f__Campylobacteracea
30000
20000
10000
0
alpha (n=25)
clade20C (n=1)
delta (n=13)
omicron (n=6)
neg (n=72)
NA
WHOvariant

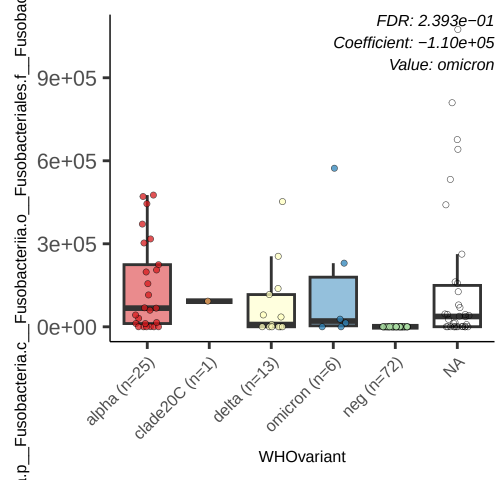

FDR: 2.393e−01
Coefficient: −1.10e+05
Value: omicron
9e+05
6e+05
3e+05
0e+00
alpha (n=25)
clade20C (n=1)
delta (n=13)
omicron (n=6)
neg (n=72)
NA
WHOvariant

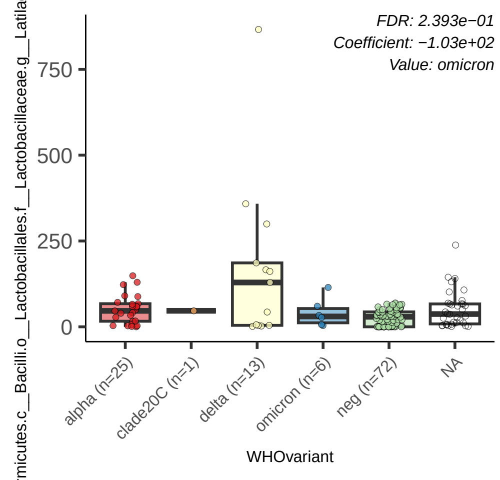
FDR: 2.393e−01
Coefficient: −1.03e+02
Value: omicron
micutes.c__Bacilli.o__Lactobacillales.f__Lactobacillaceae.g__Latila
0
250
500
750
alpha (n=25)
clade20C (n=1)
delta (n=13)
omicron (n=6)
neg (n=72)
NA
WHOvariant

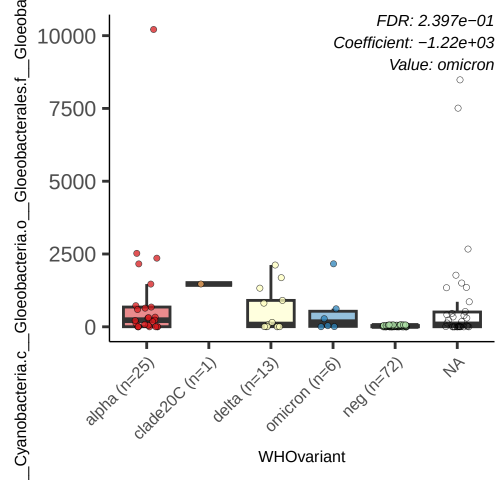
FDR: 2.397e−01
Coefficient: −1.22e+03
Value: omicron
Cyanobacteria.c__Gloeobacteria.o__Gloeobacterales.f__Gloeoba
10000
7500
5000
2500
0
alpha (n=25)
clade20C (n=1)
delta (n=13)
omicron (n=6)
neg (n=72)
NA
WHOvariant

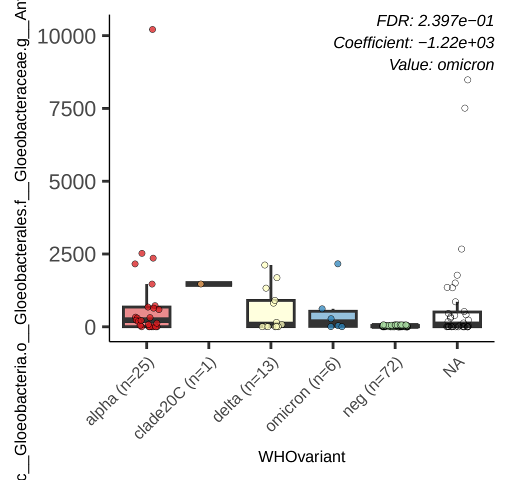

FDR: 2.397e−01
Coefficient: −1.22e+03
Value: omicron
10000
7500
5000
2500
0
alpha (n=25)
clade20C (n=1)
delta (n=13)
omicron (n=6)
neg (n=72)
NA
WHOvariant
c__Gloeobacteria.o__Gloeobacterales.f__Gloeobacteraceae.g__An

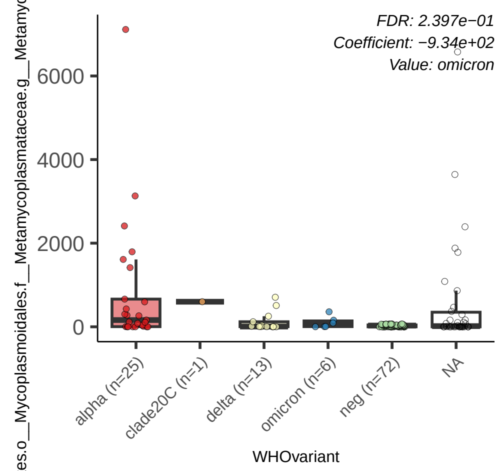

FDR: 2.397e−01
Coefficient: −9.34e+02
Value: omicron
6000
4000
2000
0
alpha (n=25)
clade20C (n=1)
delta (n=13)
omicron (n=6)
neg (n=72)
NA
WHOvariant
utes.o__Mycoplasmoidales.f__Metamycoplasmataceae.g__Metamyc

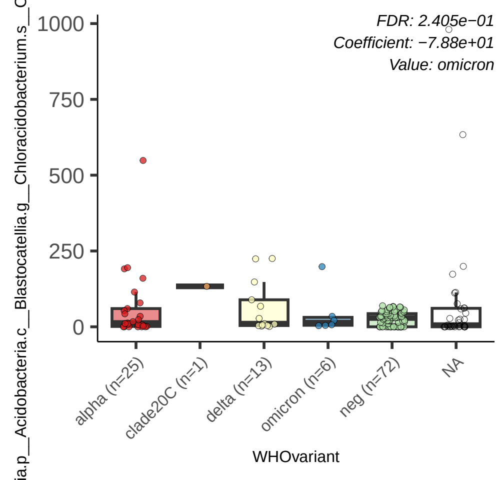

FDR: 2.405e−01
Coefficient: −7.88e+01
Value: omicron
ia.p__Acidobacteria.c__Blastocatellia.g__Chloracidobacterium.s__C
1000
750
500
250
0
alpha (n=25)
clade20C (n=1)
delta (n=13)
omicron (n=6)
neg (n=72)
NA
WHOvariant

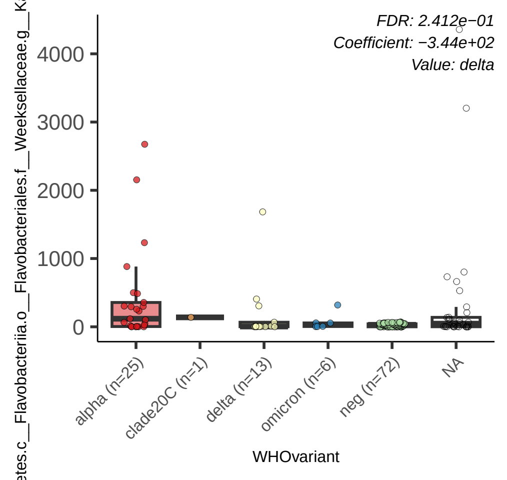

FDR: 2.412e−01
Coefficient: −3.44e+02
Value: delta
4000
3000
2000
1000
0
alpha (n=25)
clade20C (n=1)
delta (n=13)
omicron (n=6)
neg (n=72)
NA
WHOvariant
etes.c__Flavobacteriia.o__Flavobacteriales.f__Weeksellaceae.g__K

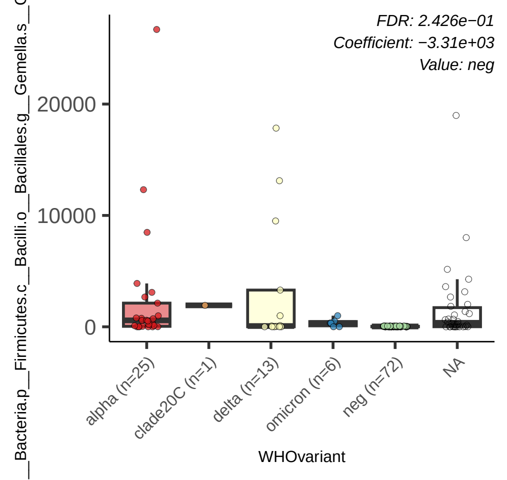
FDR: 2.426e−01
Coefficient: −3.31e+03
Value: neg
20000
10000
0
alpha (n=25)
clade20C (n=1)
delta (n=13)
omicron (n=6)
neg (n=72)
NA
WHOvariant
Bacteria.p__Firmicutes.c__Bacilli.o__Bacillales.g__Gemella.s__

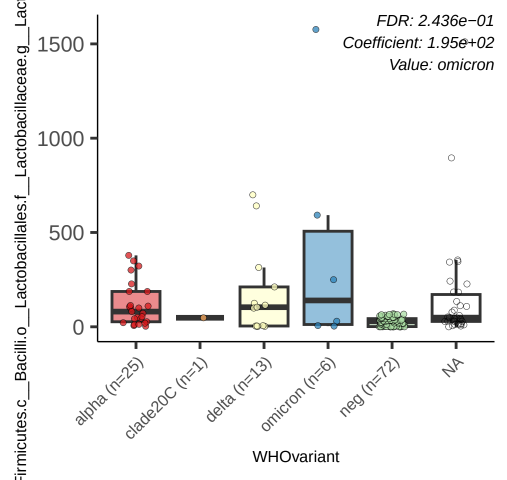
FDR: 2.436e−01
Coefficient: 1.95e+02
Value: omicron
Firmicutes.c__Bacilli.o__Lactobacillales.f__Lactobacillaceae.g__Lact
1500
1000
500
0
alpha (n=25)
clade20C (n=1)
delta (n=13)
omicron (n=6)
neg (n=72)
NA
WHOvariant

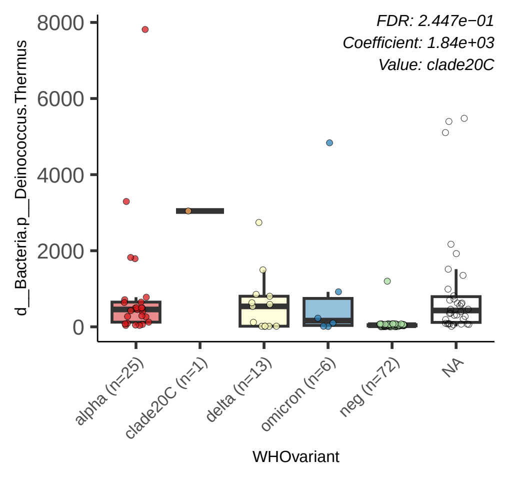

FDR: 2.447e−01
Coefficient: 1.84e+03
Value: clade20C
d__Bacteria.p__Deinococcus.Thermus
8000
6000
4000
2000
0
alpha (n=25)
clade20C (n=1)
delta (n=13)
omicron (n=6)
neg (n=72)
NA
WHOvariant

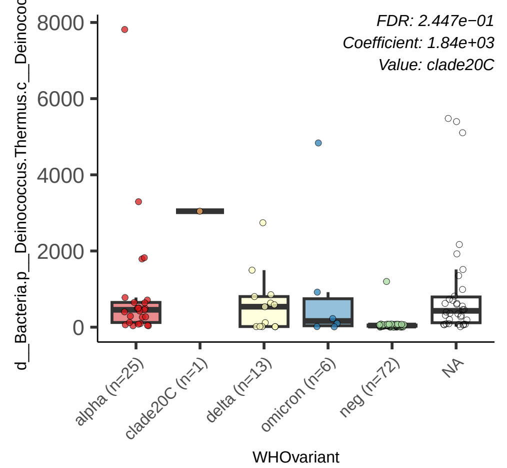

FDR: 2.447e−01
Coefficient: 1.84e+03
Value: clade20C
d__Bacteria.p__Deinococcus.Thermus.c__Deinococ
8000
6000
4000
2000
0
alpha (n=25)
clade20C (n=1)
delta (n=13)
omicron (n=6)
neg (n=72)
NA
WHOvariant

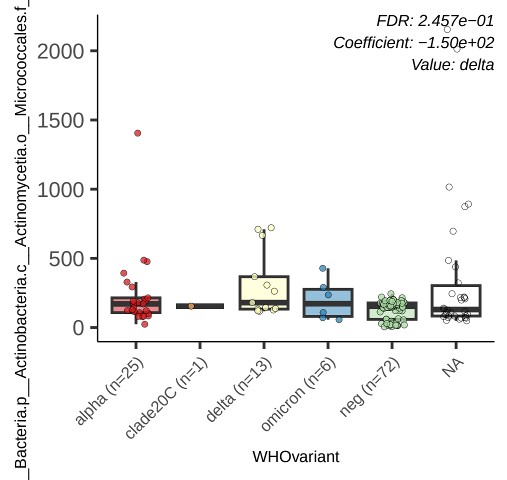
FDR: 2.457e−01
Coefficient: −1.50e+02
Value: delta
Bacteria.p__Actinobacteria.c__Actinomycetia.o__Micrococcales.f__
0
500
1000
1500
2000
alpha (n=25)
clade20C (n=1)
delta (n=13)
omicron (n=6)
neg (n=72)
NA
WHOvariant

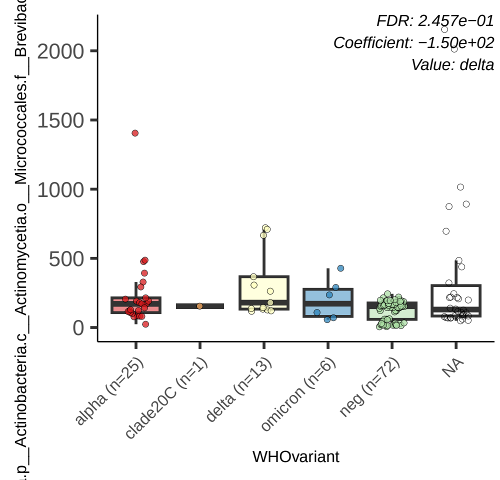

p__Actinobacteria.c__Actinomycetia.o__Micrococcales.f__Brevibac
FDR: 2.457e−01
Coefficient: −1.50e+02
Value: delta
2000
1500
1000
500
0
alpha (n=25)
clade20C (n=1)
delta (n=13)
omicron (n=6)
neg (n=72)
NA
WHOvariant

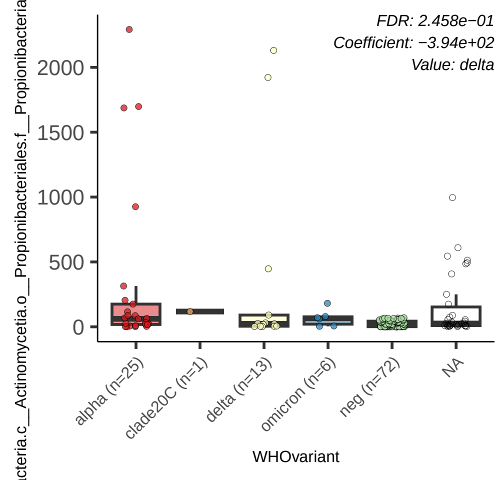

FDR: 2.458e−01
Coefficient: −3.94e+02
Value: delta
acteria.c__Actinomycetia.o__Propionibacteriales.f__Propionibacteriaceae.g__Propionibacteria
2000
1500
1000
500
0
alpha (n=25)
clade20C (n=1)
delta (n=13)
omicron (n=6)
neg (n=72)
NA
WHOvariant

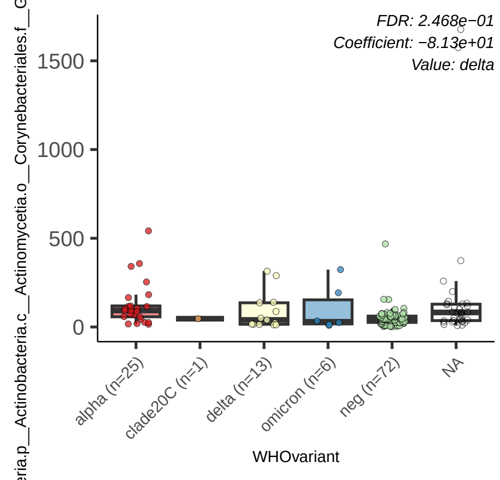

FDR: 2.468e−01
Coefficient: −8.13e+01
Value: delta
1500
1000
500
0
alpha (n=25)
clade20C (n=1)
delta (n=13)
omicron (n=6)
neg (n=72)
NA
WHOvariant
eria.p__Actinobacteria.c__Actinomycetia.o__Corynebacteriales.f__G

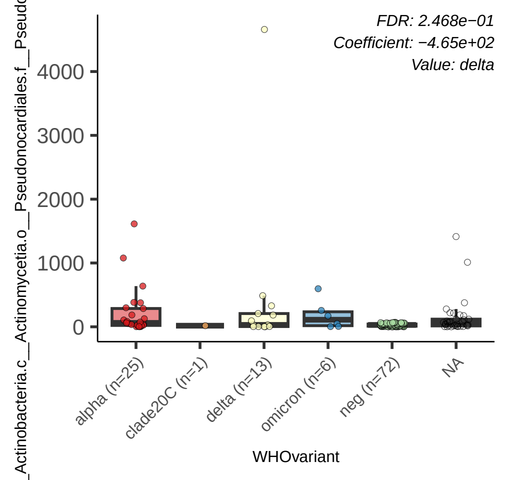

FDR: 2.468e−01
Coefficient: −4.65e+02
Value: delta
Actinobacteria.c__Actinomycetia.o__Pseudonocardiales.f__Pseudo
0
1000
2000
3000
4000
alpha (n=25)
clade20C (n=1)
delta (n=13)
omicron (n=6)
neg (n=72)
NA
WHOvariant

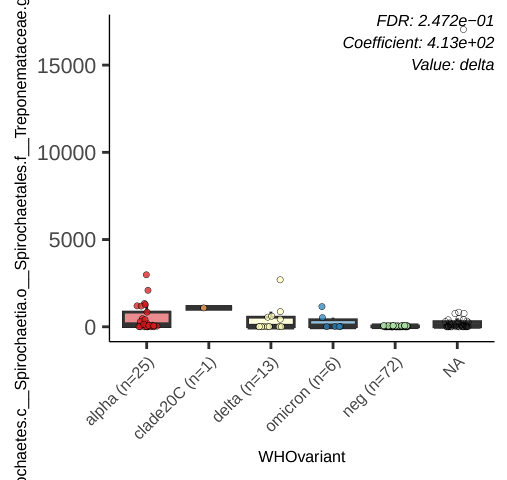
FDR: 2.472e−01
Coefficient: 4.13e+02
Value: delta
15000
10000
5000
0
alpha (n=25)
clade20C (n=1)
delta (n=13)
omicron (n=6)
neg (n=72)
NA
WHOvariant
ochaetes.c__Spirochaetia.o__Spirochaetales.f__Treponemataceae.g

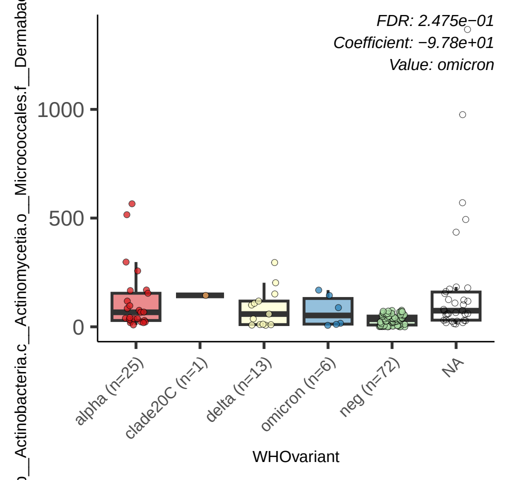

FDR: 2.475e−01
Coefficient: −9.78e+01
Value: omicron
o__Actinobacteria.c__Actinomycetia.o__Micrococcales.f__Dermabac
1000
500
0
alpha (n=25)
clade20C (n=1)
delta (n=13)
omicron (n=6)
neg (n=72)
NA
WHOvariant

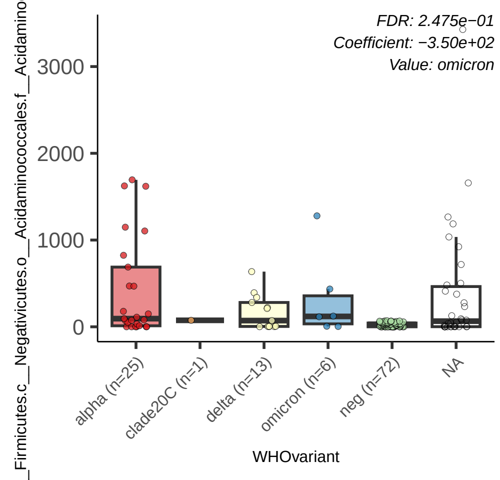

FDR: 2.475e−01
Coefficient: −3.50e+02
Value: omicron
3000
2000
1000
0
Firmicutes.c__Negativicutes.o__Acidaminococcales.f__Acidaminoc
alpha (n=25)
clade20C (n=1)
delta (n=13)
omicron (n=6)
neg (n=72)
NA
WHOvariant

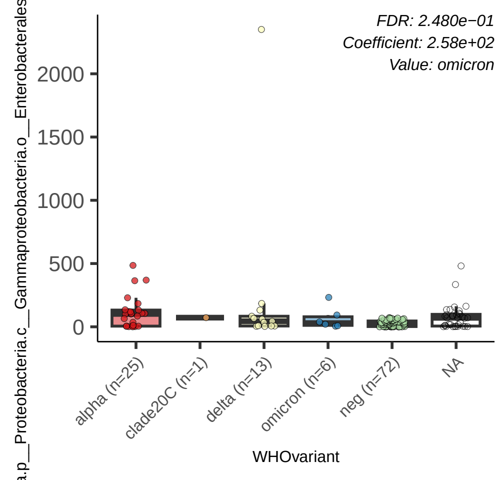

FDR: 2.480e−01
Coefficient: 2.58e+02
Value: omicron
a.p__Proteobacteria.c__Gammaproteobacteria.o__Enterobacterales
0
500
1000
1500
2000
alpha (n=25)
clade20C (n=1)
delta (n=13)
omicron (n=6)
neg (n=72)
NA
WHOvariant

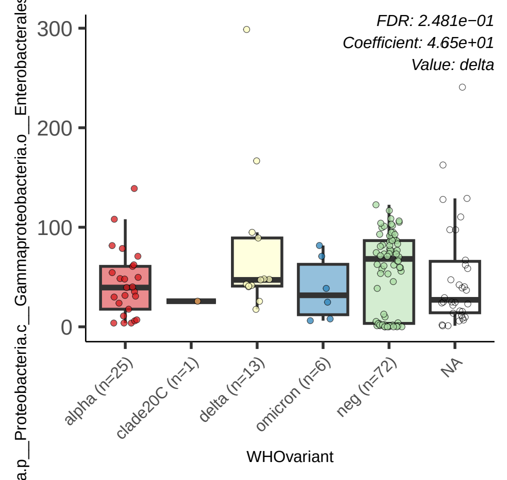
FDR: 2.481e−01
Coefficient: 4.65e+01
Value: delta
a.p__Proteobacteria.c__Gammaproteobacteria.o__Enterobacterales
300
200
100
0
alpha (n=25)
clade20C (n=1)
delta (n=13)
omicron (n=6)
neg (n=72)
NA
WHOvariant

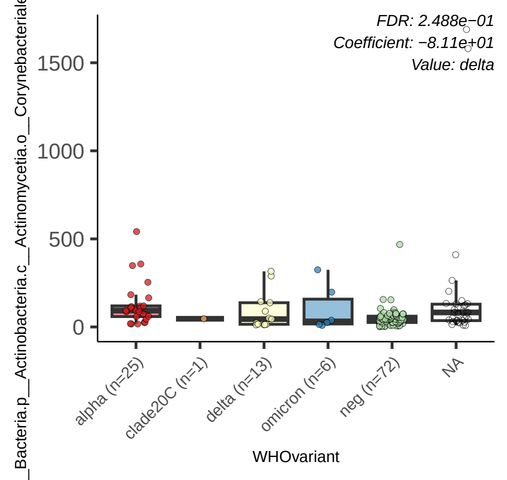

FDR: 2.488e−01
Coefficient: −8.11e+01
Value: delta
0
500
1000
1500
Bacteria.p__Actinobacteria.c__Actinomycetia.o__Corynebacteriale
alpha (n=25)
clade20C (n=1)
delta (n=13)
omicron (n=6)
neg (n=72)
NA
WHOvariant

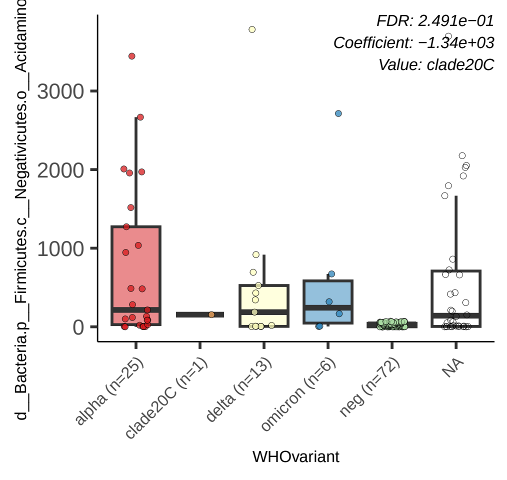

FDR: 2.491e−01
Coefficient: −1.34e+03
Value: clade20C
d__Bacteria.p__Firmicutes.c__Negativicutes.o__Acidamino
3000
2000
1000
0
alpha (n=25)
clade20C (n=1)
delta (n=13)
omicron (n=6)
neg (n=72)
NA
WHOvariant

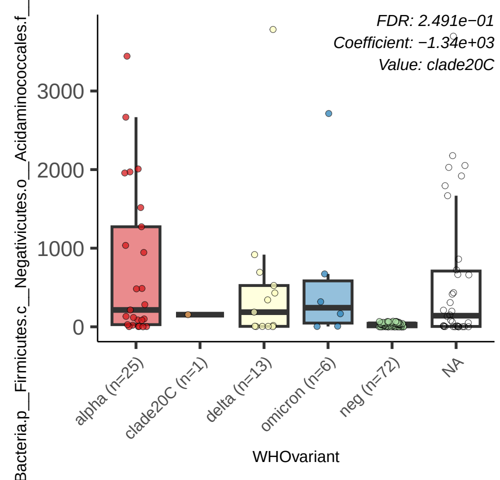
FDR: 2.491e−01
Coefficient: −1.34e+03
Value: clade20C
Bacteria.p__Firmicutes.c__Negativicutes.o__Acidaminococcales.f__
3000
2000
1000
0
alpha (n=25)
clade20C (n=1)
delta (n=13)
omicron (n=6)
neg (n=72)
NA
WHOvariant
