# Supplementary material for: Metatranscriptome Analysis of Nasopharyngeal Swabs across the Varying Severity of COVID-19 Disease Demonstrated Unprecedented Species Diversity
Source: Microorganisms. 2023 Jul 14;11(7):1804. doi: 10.3390/microorganisms11071804 (PMC10384460; doi:10.3390/microorganisms11071804)
Supplement: Supplementary file 1 [file microorganisms-11-01804-s001.zip › Maaslin_results_ONLYhealthy.zip!/Maaslin_results_ONLYhealthy/Age_category.pdf]

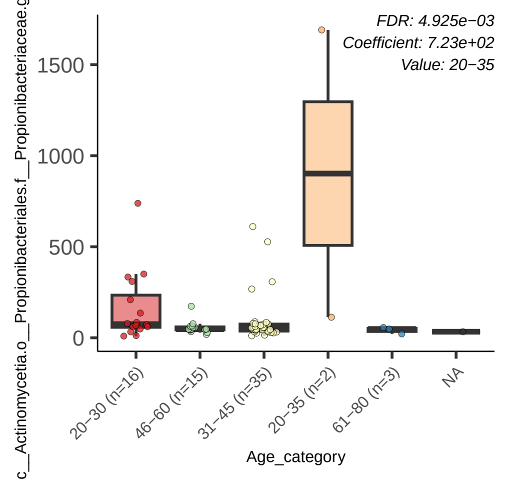
FDR: 4.925e−03
Coefficient: 7.23e+02
Value: 20−35
c__Actinomycetia.o__Propionibacteriales.f__Propionibacteriaceae.g
1500
1000
500
0
20−30 (n=16)
46−60 (n=15)
31−45 (n=35)
20−35 (n=2)
61−80 (n=3)
NA
Age_category

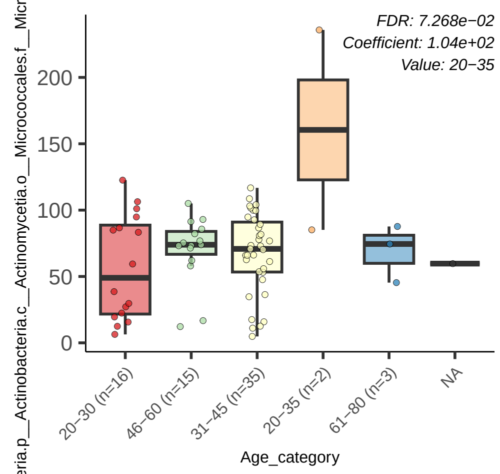
FDR: 7.268e−02
Coefficient: 1.04e+02
Value: 20−35
teria.p__Actinobacteria.c__Actinomycetia.o__Micrococcales.f__Micr
0
50
100
150
200
20−30 (n=16)
46−60 (n=15)
31−45 (n=35)
20−35 (n=2)
61−80 (n=3)
NA
Age_category

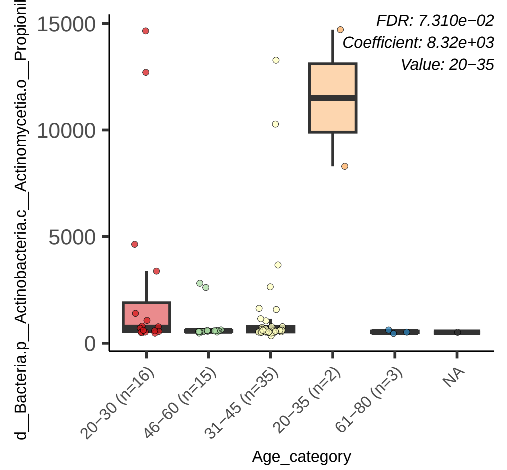
FDR: 7.310e−02
Coefficient: 8.32e+03
Value: 20−35
d__Bacteria.p__Actinobacteria.c__Actinomycetia.o__Propioni
15000
10000
5000
0
20−30 (n=16)
46−60 (n=15)
31−45 (n=35)
20−35 (n=2)
61−80 (n=3)
NA
Age_category

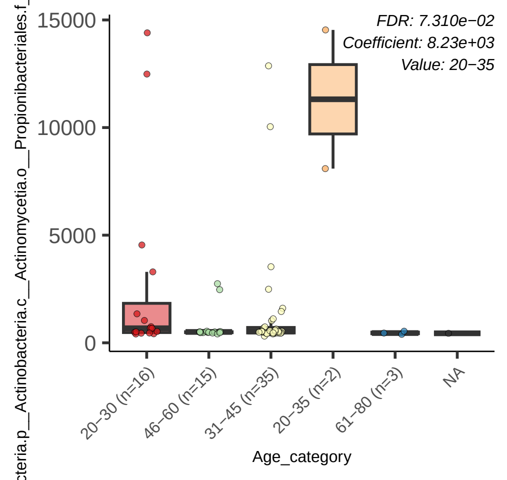
FDR: 7.310e−02
Coefficient: 8.23e+03
Value: 20−35
15000
10000
5000
0
cteria.p__Actinobacteria.c__Actinomycetia.o__Propionibacteriales.f_
20−30 (n=16)
46−60 (n=15)
31−45 (n=35)
20−35 (n=2)
61−80 (n=3)
NA
Age_category

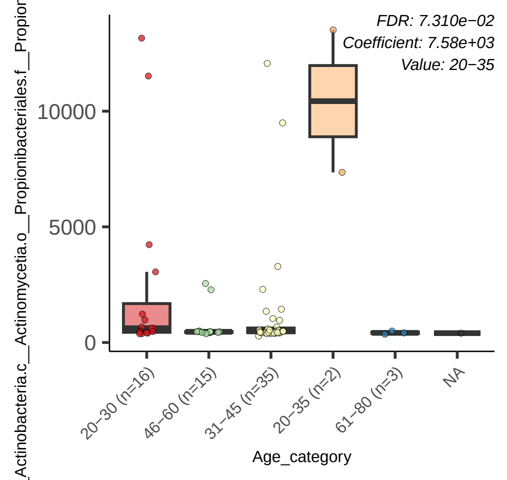

FDR: 7.310e−02
Coefficient: 7.58e+03
Value: 20−35
Actinobacteria.c__Actinomycetia.o__Propionibacteriales.f__Propion
10000
5000
0
20−30 (n=16)
46−60 (n=15)
31−45 (n=35)
20−35 (n=2)
61−80 (n=3)
NA
Age_category

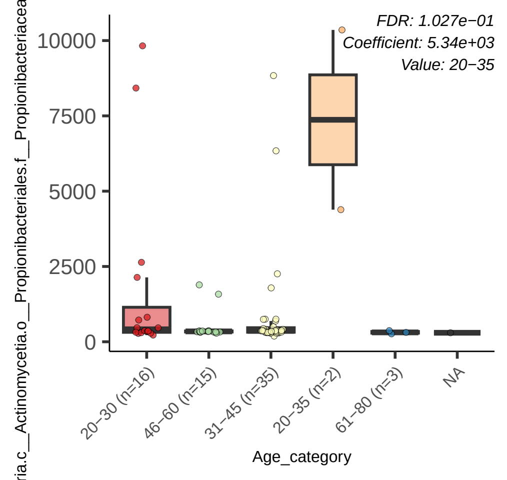
FDR: 1.027e−01
Coefficient: 5.34e+03
Value: 20−35
ria.c__Actinomycetia.o__Propionibacteriales.f__Propionibacteriacea
0
2500
5000
7500
10000
20−30 (n=16)
46−60 (n=15)
31−45 (n=35)
20−35 (n=2)
61−80 (n=3)
NA
Age_category
