# Supplementary material for: Metatranscriptome Analysis of Nasopharyngeal Swabs across the Varying Severity of COVID-19 Disease Demonstrated Unprecedented Species Diversity
Source: Microorganisms. 2023 Jul 14;11(7):1804. doi: 10.3390/microorganisms11071804 (PMC10384460; doi:10.3390/microorganisms11071804)
Supplement: Supplementary file 1 [file microorganisms-11-01804-s001.zip › Maaslin_results_ONLYsevere.zip!/Maaslin_results_ONLYsevere/Age.pdf]

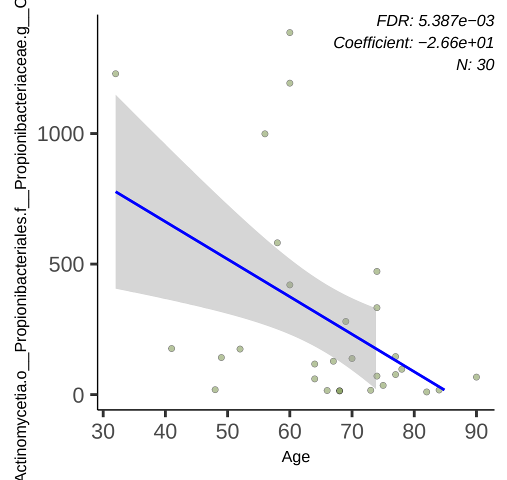

FDR: 5.387e−03
Coefficient: −2.66e+01
N: 30
1000
500
0
30
40
50
60
70
80
90
Age
Actinomycetia.o__Propionibacteriales.f__Propionibacteriaceae.g__C

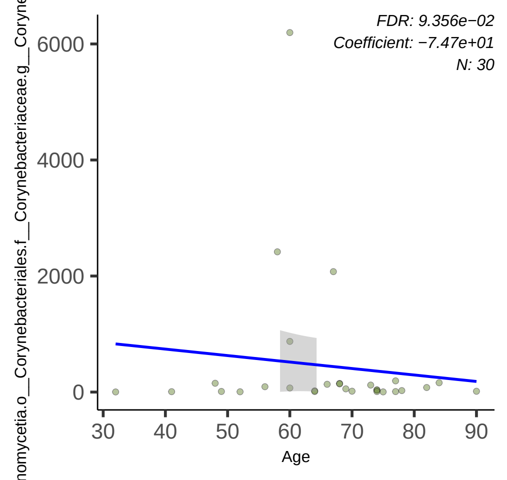
FDR: 9.356e−02
Coefficient: −7.47e+01
N: 30
6000
4000
2000
0
30
40
50
60
70
80
90
Age
nomycetia.o__Corynebacteriales.f__Corynebacteriaceae.g__Coryne

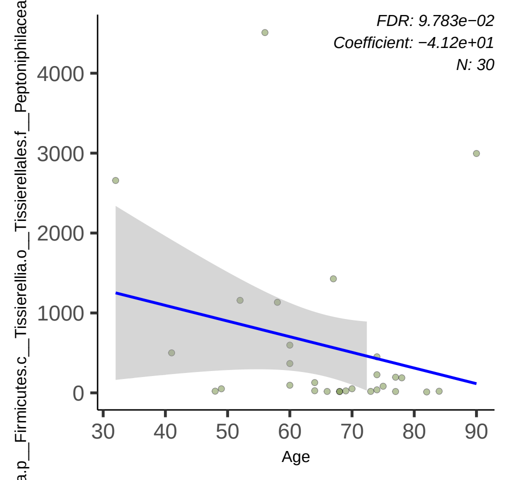

FDR: 9.783e−02
Coefficient: −4.12e+01
N: 30
0
1000
2000
3000
4000
30
40
50
60
70
80
90
Age
a.p__Firmicutes.c__Tissierellia.o__Tissierellales.f__Peptoniphilacea

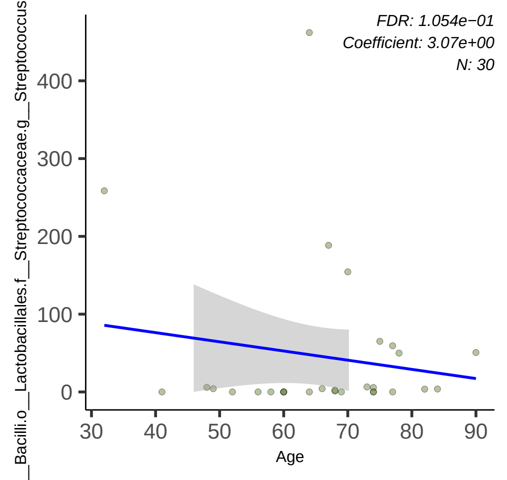
FDR: 1.054e−01
Coefficient: 3.07e+00
N: 30
Bacilli.o__Lactobacillales.f__Streptococcaceae.g__Streptococcus
0
100
200
300
400
30
40
50
60
70
80
90
Age

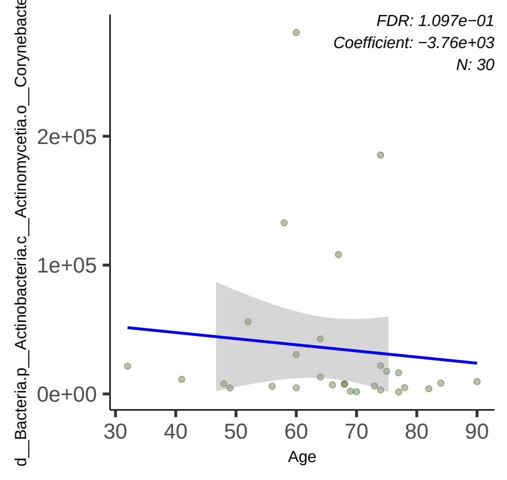

FDR: 1.097e−01
Coefficient: −3.76e+03
N: 30
d__Bacteria.p__Actinobacteria.c__Actinomycetia.o__Corynebacte
2e+05
1e+05
0e+00
30
40
50
60
70
80
90
Age

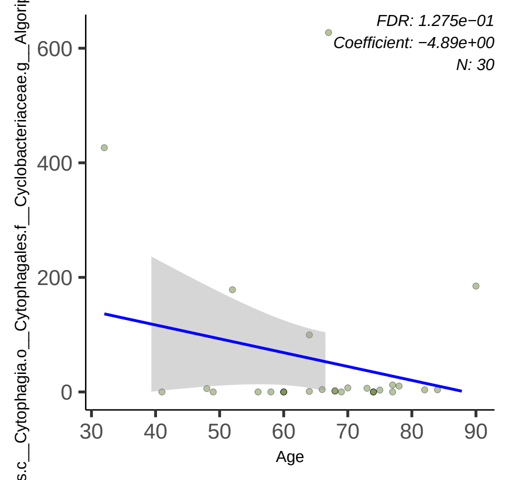
FDR: 1.275e−01
Coefficient: −4.89e+00
N: 30
s.c__Cytophagia.o__Cytophagales.f__Cyclobacteriaceae.g__Algorip
0
200
400
600
30
40
50
60
70
80
90
Age

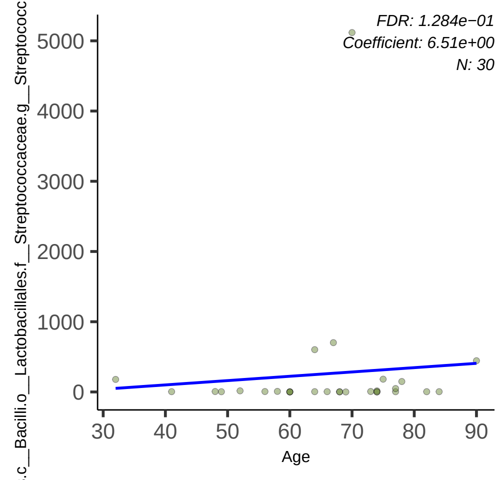
FDR: 1.284e−01
Coefficient: 6.51e+00
N: 30
5000
4000
3000
2000
1000
0
30
40
50
60
70
80
90
Age
Bacilli.o__Lactobacillales.f__Streptococcaceae.g__Streptococc

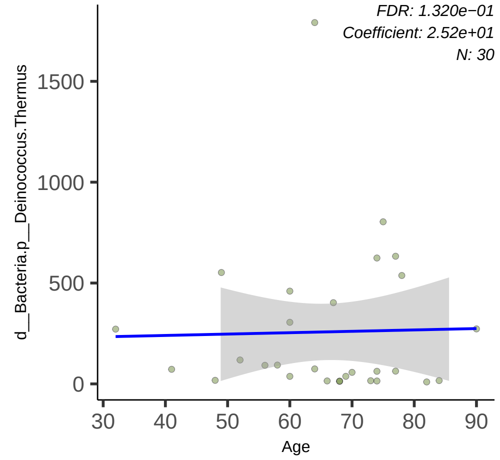

FDR: 1.320e−01
Coefficient: 2.52e+01
N: 30
d__Bacteria.p__Deinococcus.Thermus
1500
1000
500
0
30
40
50
60
70
80
90
Age

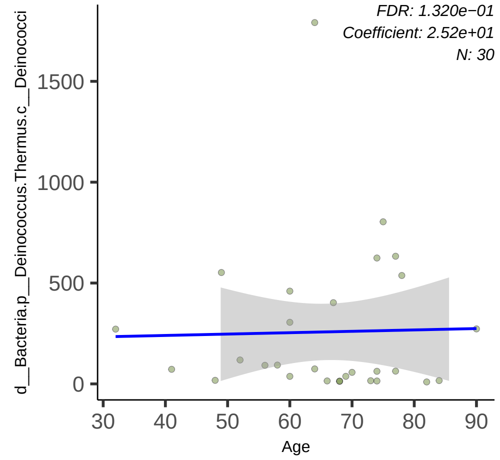
FDR: 1.320e−01
Coefficient: 2.52e+01
N: 30
d__Bacteria.p__Deinococcus.Thermus.c__Deinococci
1500
1000
500
0
30
40
50
60
70
80
90
Age

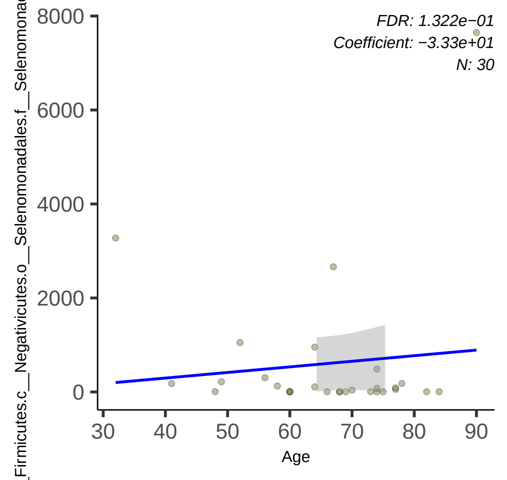
FDR: 1.322e−01
Coefficient: −3.33e+01
N: 30
Firmicutes.c__Negativicutes.o__Selenomonadales.f__Selenomonad
8000
6000
4000
2000
0
30
40
50
60
70
80
90
Age

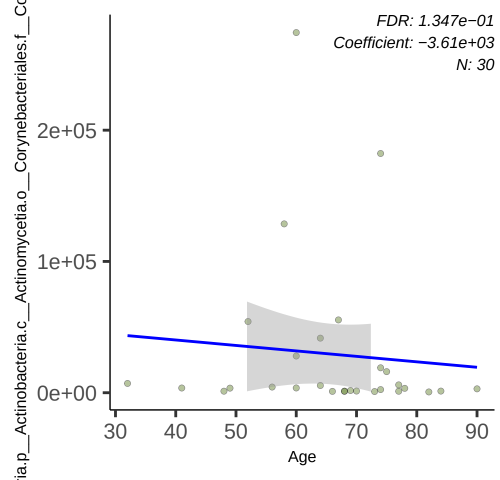
FDR: 1.347e−01
Coefficient: −3.61e+03
N: 30
ia.p__Actinobacteria.c__Actinomycetia.o__Corynebacteriales.f__Co
2e+05
1e+05
0e+00
30
40
50
60
70
80
90
Age

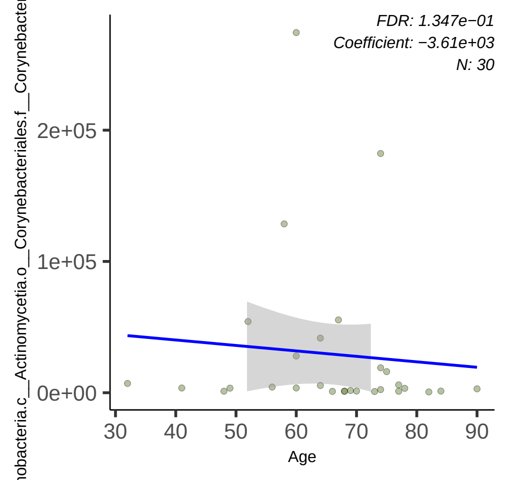
FDR: 1.347e−01
Coefficient: −3.61e+03
N: 30
2e+05
1e+05
0e+00
30
40
50
60
70
80
90
Age
nobacteria.c__Actinomycetia.o__Corynebacteriales.f__Corynebacter

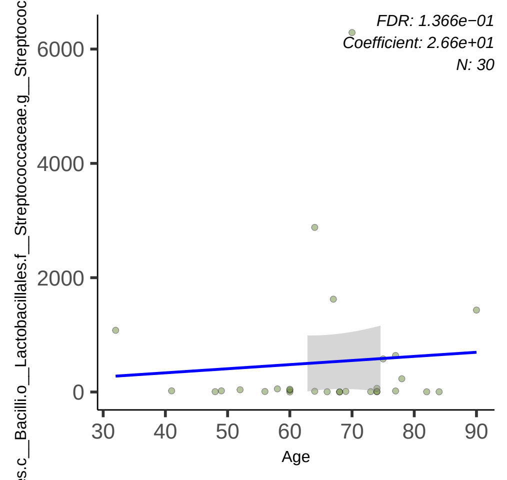

FDR: 1.366e−01
Coefficient: 2.66e+01
N: 30
0
2000
4000
6000
30
40
50
60
70
80
90
Age
es.c__Bacilli.o__Lactobacillales.f__Streptococcaceae.g__Streptococ

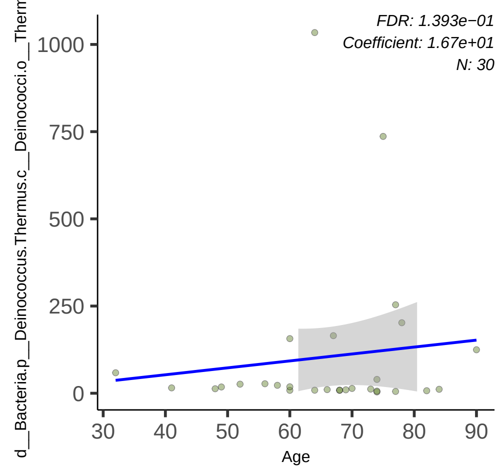
FDR: 1.393e−01
Coefficient: 1.67e+01
N: 30
1000
750
500
250
0
30
40
50
60
70
80
90
Age
d__Bacteria.p__Deinococcus.Thermus.c__Deinococci.o__Therm

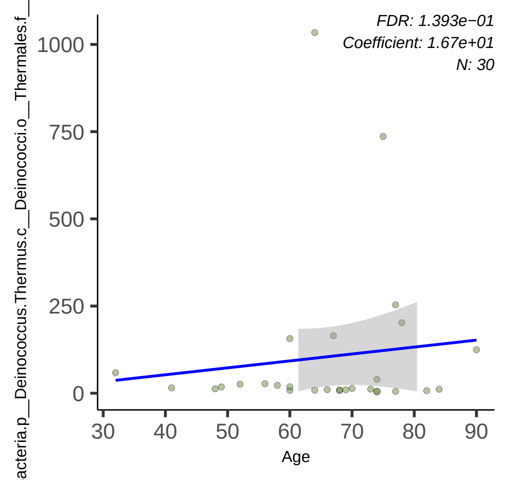

FDR: 1.393e−01
Coefficient: 1.67e+01
N: 30
1000
750
500
250
0
30
40
50
60
70
80
90
Age
acteria.p__Deinococcus.Thermus.c__Deinococci.o__Thermales.f__

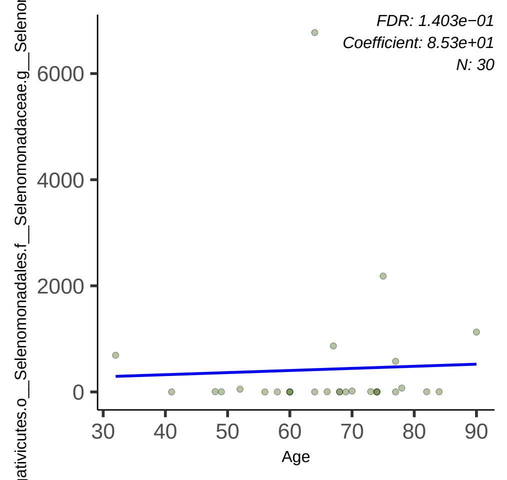
FDR: 1.403e−01
Coefficient: 8.53e+01
N: 30
0
2000
4000
6000
30
40
50
60
70
80
90
Age
gativicutes.o__Selenomonadales.f__Selenomonadaceae.g__Selenor

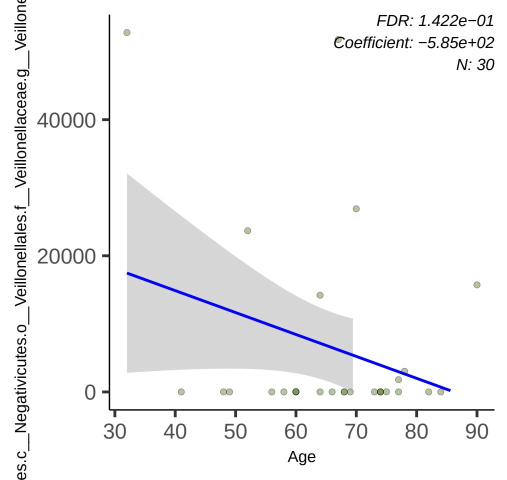

FDR: 1.422e−01
Coefficient: −5.85e+02
N: 30
es.c__Negativicutes.o__Veillonellales.f__Veillonellaceae.g__Veillone
40000
20000
0
30
40
50
60
70
80
90
Age

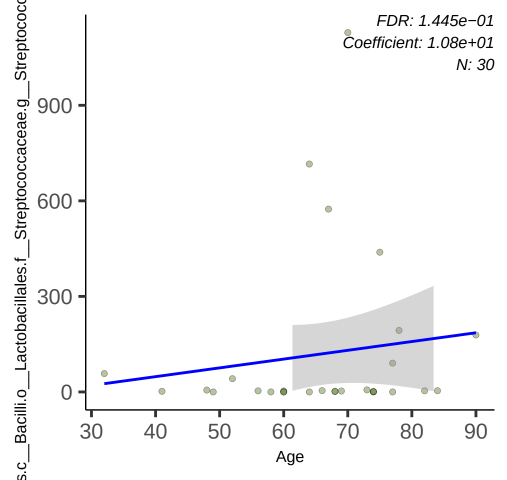

FDR: 1.445e−01
Coefficient: 1.08e+01
N: 30
s.c__Bacilli.o__Lactobacillales.f__Streptococcaceae.g__Streptococ
0
300
600
900
30
40
50
60
70
80
90
Age

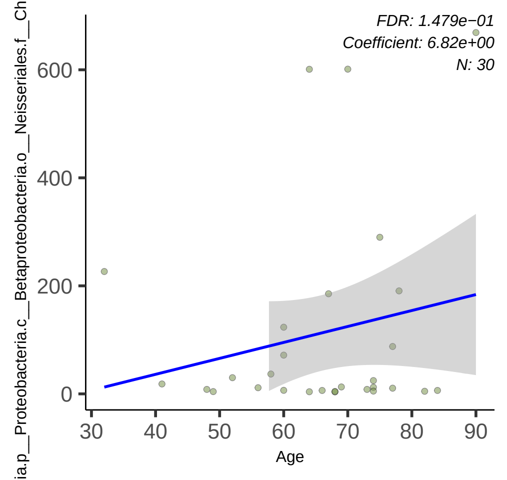
FDR: 1.479e−01
Coefficient: 6.82e+00
N: 30
ia.p__Proteobacteria.c__Betaproteobacteria.o__Neisseriales.f__Ch
0
200
400
600
30
40
50
60
70
80
90
Age

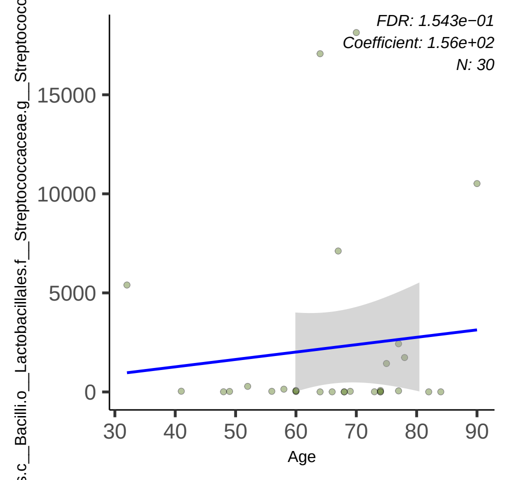

FDR: 1.543e−01
Coefficient: 1.56e+02
N: 30
15000
10000
5000
0
30
40
50
60
70
80
90
Age
s.c__Bacilli.o__Lactobacillales.f__Streptococcaceae.g__Streptococc

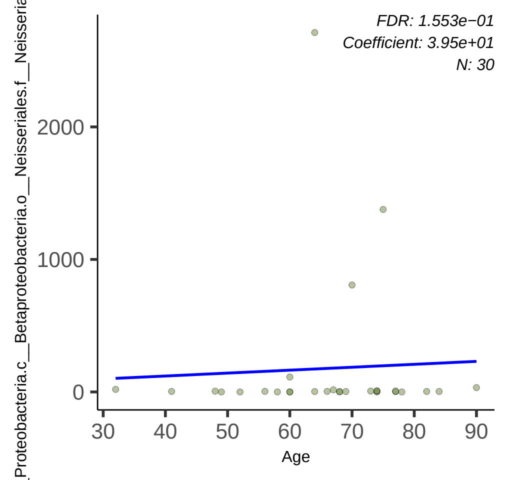

FDR: 1.553e−01
Coefficient: 3.95e+01
N: 30
Proteobacteria.c__Betaproteobacteria.o__Neisseriales.f__Neisseria
2000
1000
0
30
40
50
60
70
80
90
Age

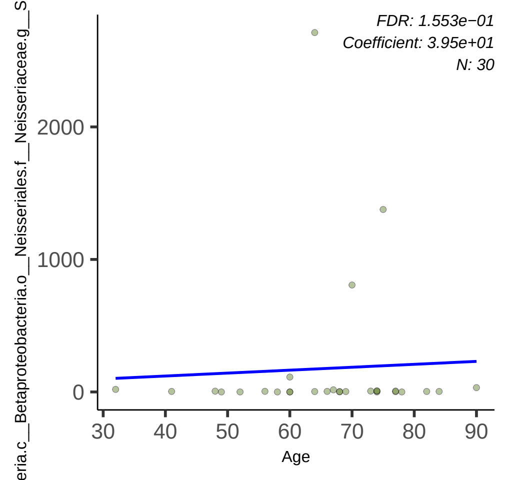

FDR: 1.553e−01
Coefficient: 3.95e+01
N: 30
2000
1000
0
30
40
50
60
70
80
90
Age
eria.c__Betaproteobacteria.o__Neisseriales.f__Neisseriaceae.g__S

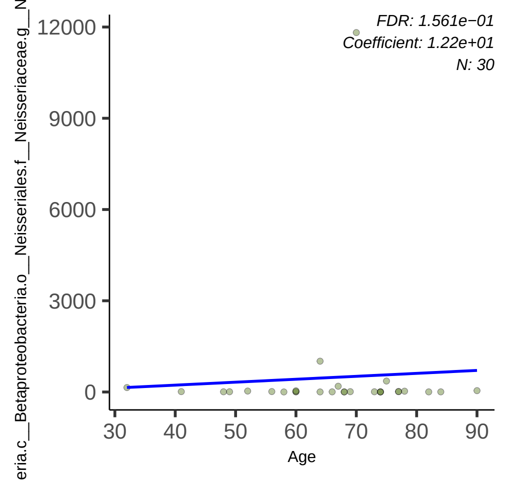

FDR: 1.561e−01
Coefficient: 1.22e+01
N: 30
12000
9000
6000
3000
0
30
40
50
60
70
80
90
Age
eria.c__Betaproteobacteria.o__Neisseriales.f__Neisseriaceae.g__N

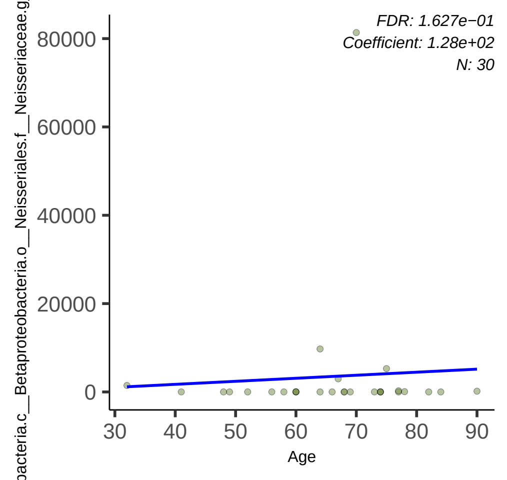

FDR: 1.627e−01
Coefficient: 1.28e+02
N: 30
80000
60000
40000
20000
0
30
40
50
60
70
80
90
Age
obacteria.c__Betaproteobacteria.o__Neisseriales.f__Neisseriaceae.g

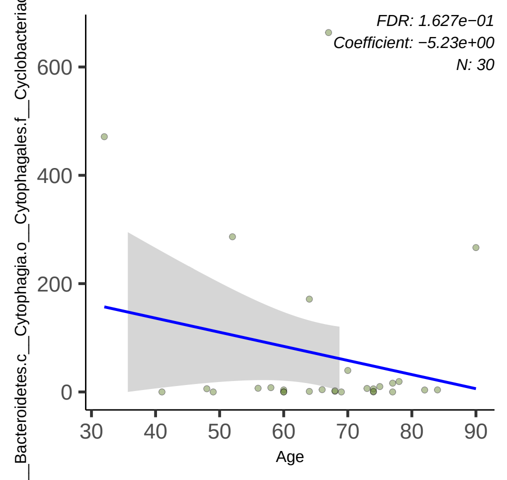
FDR: 1.627e−01
Coefficient: −5.23e+00
N: 30
600
400
200
0
30
40
50
60
70
80
90
Age
Bacteroidetes.c__Cytophagia.o__Cytophagales.f__Cyclobacteriac

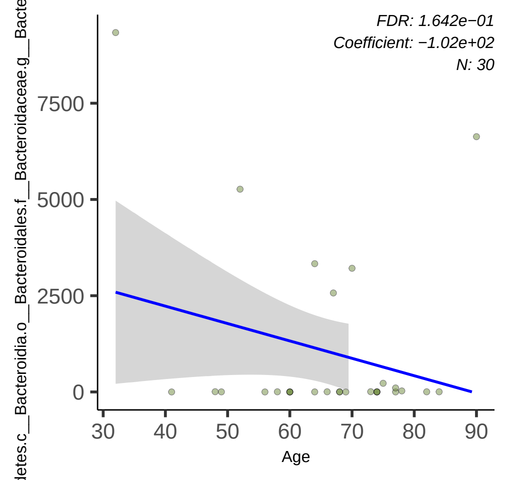

FDR: 1.642e−01
Coefficient: −1.02e+02
N: 30
detes.c__Bacteroidia.o__Bacteroidales.f__Bacteroidaceae.g__Bacte
7500
5000
2500
0
30
40
50
60
70
80
90
Age

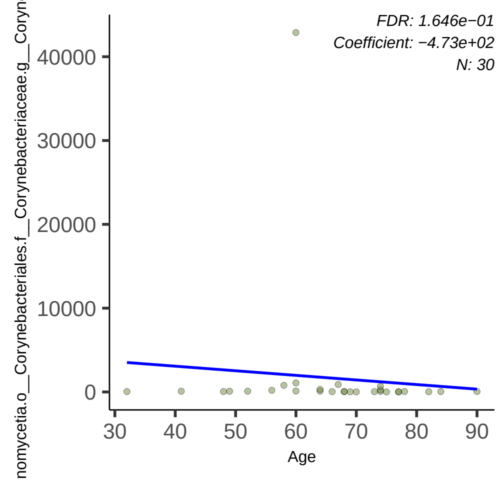

FDR: 1.646e−01
Coefficient: −4.73e+02
N: 30
nomycetia.o__Corynebacteriales.f__Corynebacteriaceae.g__Coryn
40000
30000
20000
10000
0
30
40
50
60
70
80
90
Age

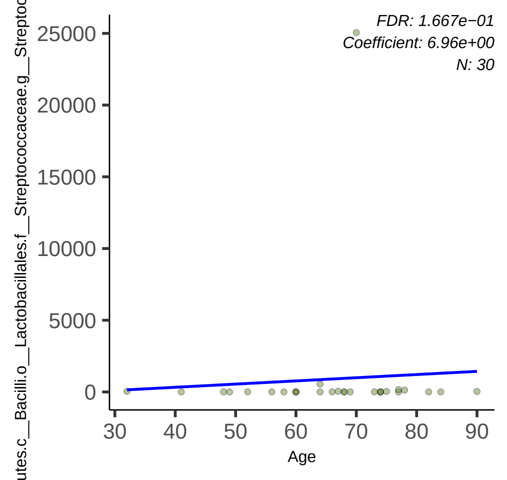

FDR: 1.667e−01
Coefficient: 6.96e+00
N: 30
utes.c__Bacilli.o__Lactobacillales.f__Streptococcaceae.g__Streptoc
25000
20000
15000
10000
5000
0
30
40
50
60
70
80
90
Age

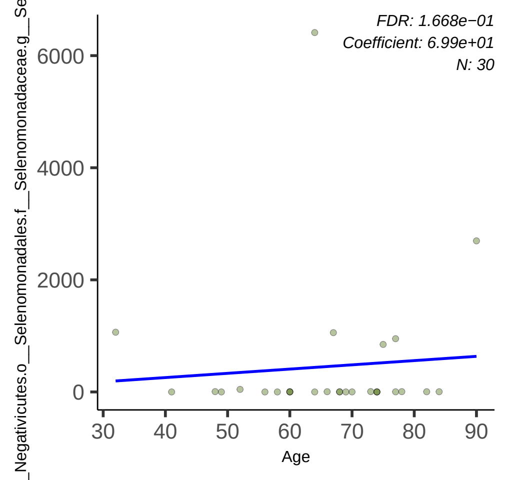

FDR: 1.668e−01
Coefficient: 6.99e+01
N: 30
6000
4000
2000
0
30
40
50
60
70
80
90
Age
Negativicutes.o__Selenomonadales.f__Selenomonadaceae.g__Se

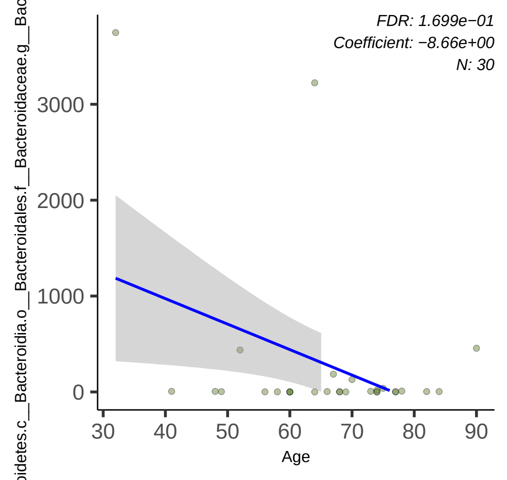

FDR: 1.699e−01
Coefficient: −8.66e+00
N: 30
3000
2000
1000
0
30
40
50
60
70
80
90
Age
oidetes.c__Bacteroidia.o__Bacteroidales.f__Bacteroidaceae.g__Bac

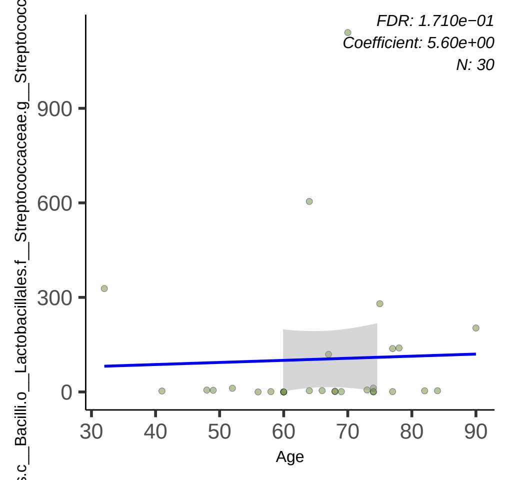

FDR: 1.710e−01
Coefficient: 5.60e+00
N: 30
0
300
600
900
30
40
50
60
70
80
90
Age
s.c__Bacilli.o__Lactobacillales.f__Streptococcaceae.g__Streptococc

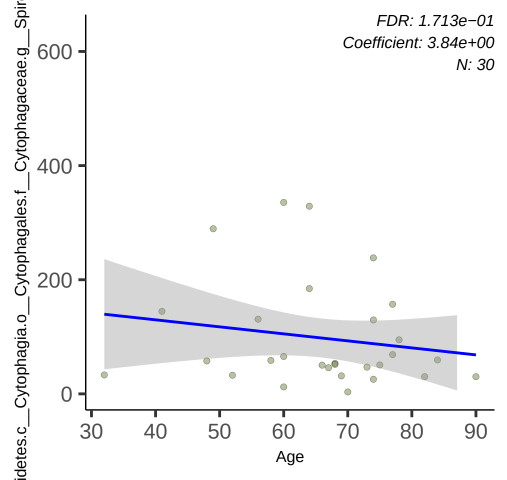

FDR: 1.713e−01
Coefficient: 3.84e+00
N: 30
0
200
400
600
30
40
50
60
70
80
90
Age
idetes.c__Cytophagia.o__Cytophagales.f__Cytophagaceae.g__Spir

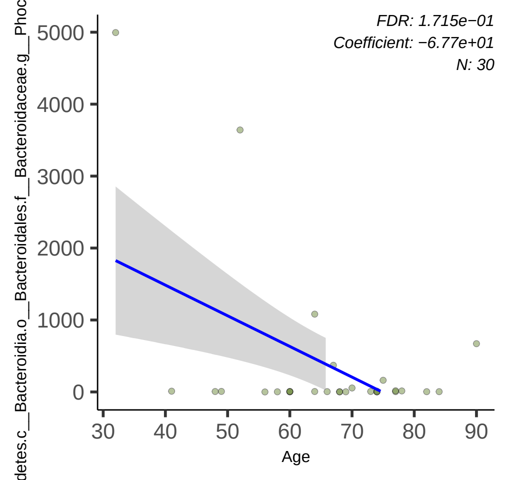
FDR: 1.715e−01
Coefficient: −6.77e+01
N: 30
0
1000
2000
3000
4000
5000
30
40
50
60
70
80
90
Age
detes.c__Bacteroidia.o__Bacteroidales.f__Bacteroidaceae.g__Phoc

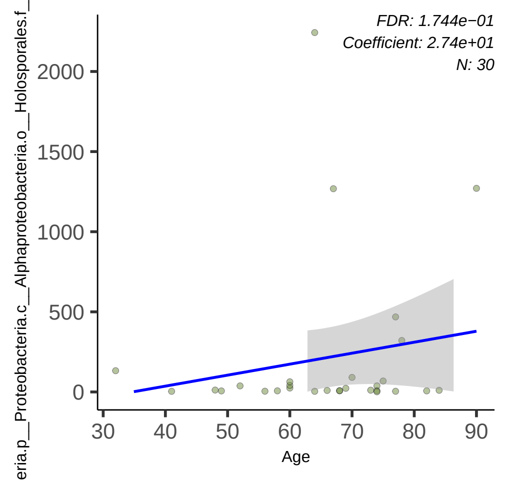
FDR: 1.744e−01
Coefficient: 2.74e+01
N: 30
eria.p__Proteobacteria.c__Alphaproteobacteria.o__Holosporales.f__
2000
1500
1000
500
0
30
40
50
60
70
80
90
Age

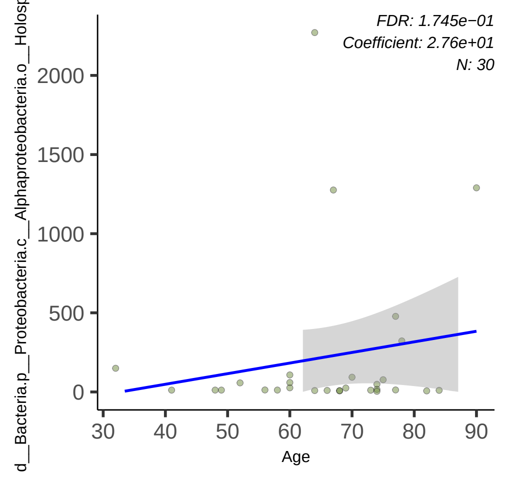

FDR: 1.745e−01
Coefficient: 2.76e+01
N: 30
d__Bacteria.p__Proteobacteria.c__Alphaproteobacteria.o__Holosp
2000
1500
1000
500
0
30
40
50
60
70
80
90
Age

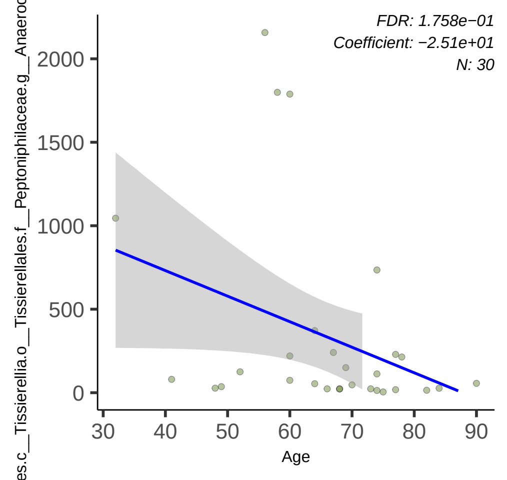
FDR: 1.758e−01
Coefficient: −2.51e+01
N: 30
es.c__Tissierellia.o__Tissierellales.f__Peptoniphilaceae.g__Anaeroc
2000
1500
1000
500
0
30
40
50
60
70
80
90
Age

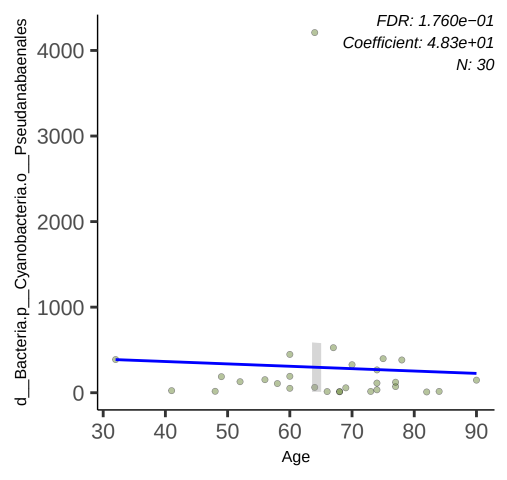
FDR: 1.760e−01
Coefficient: 4.83e+01
N: 30
4000
3000
2000
1000
0
d__Bacteria.p__Cyanobacteria.o__Pseudanabaenales
30
40
50
60
70
80
90
Age

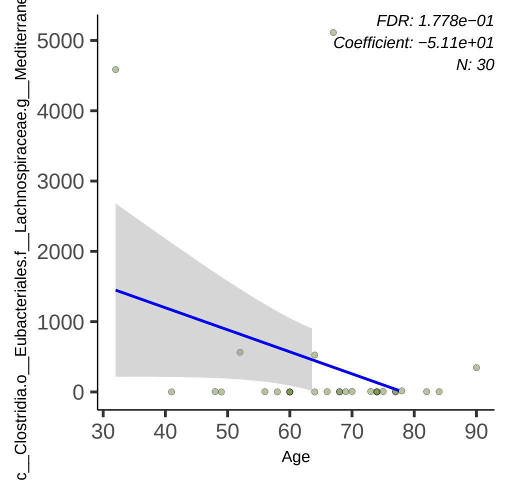

FDR: 1.778e−01
Coefficient: −5.11e+01
N: 30
c__Clostridia.o__Eubacteriales.f__Lachnospiraceae.g__Mediterrane
0
1000
2000
3000
4000
5000
30
40
50
60
70
80
90
Age

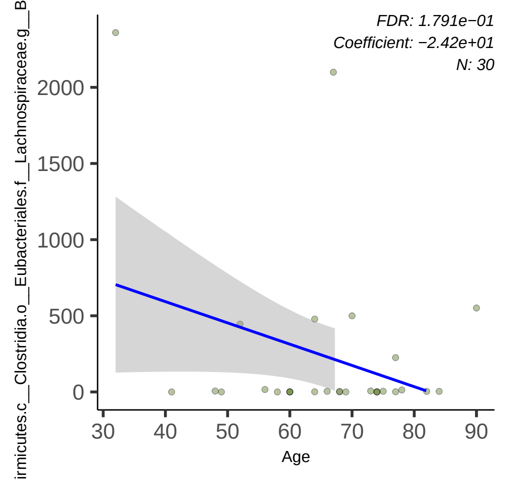

FDR: 1.791e−01
Coefficient: −2.42e+01
N: 30
irmicutes.c__Clostridia.o__Eubacteriales.f__Lachnospiraceae.g__B
0
500
1000
1500
2000
30
40
50
60
70
80
90
Age

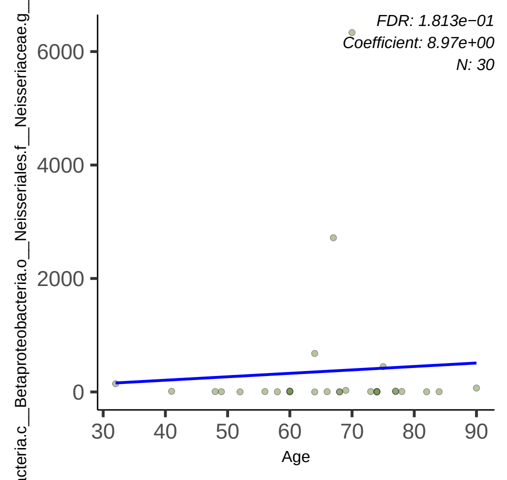
FDR: 1.813e−01
Coefficient: 8.97e+00
N: 30
acteria.c__Betaproteobacteria.o__Neisseriales.f__Neisseriaceae.g__
0
2000
4000
6000
30
40
50
60
70
80
90
Age

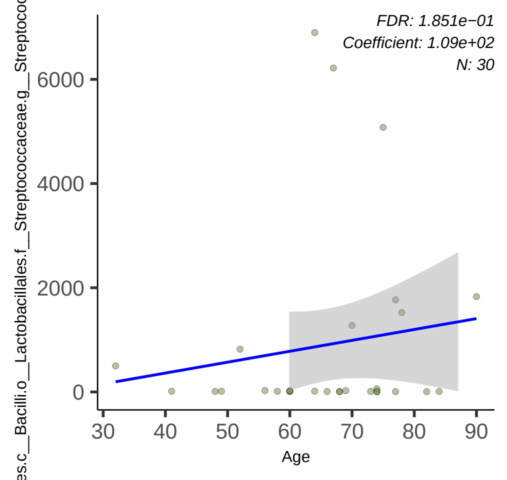

FDR: 1.851e−01
Coefficient: 1.09e+02
N: 30
es.c__Bacilli.o__Lactobacillales.f__Streptococcaceae.g__Streptococ
6000
4000
2000
0
30
40
50
60
70
80
90
Age

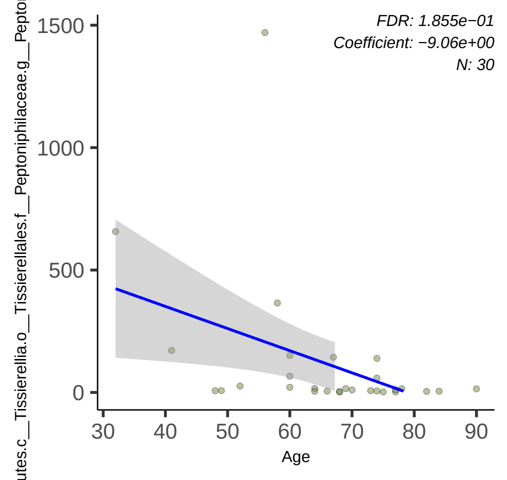

FDR: 1.855e−01
Coefficient: −9.06e+00
N: 30
1500
1000
500
0
30
40
50
60
70
80
90
Age
utes.c__Tissierellia.o__Tissierellales.f__Peptoniphilaceae.g__Pepto

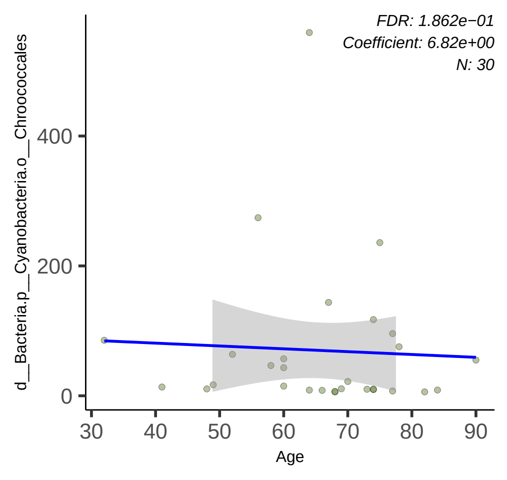

FDR: 1.862e−01
Coefficient: 6.82e+00
N: 30
d__Bacteria.p__Cyanobacteria.o__Chroococcales
0
200
400
30
40
50
60
70
80
90
Age

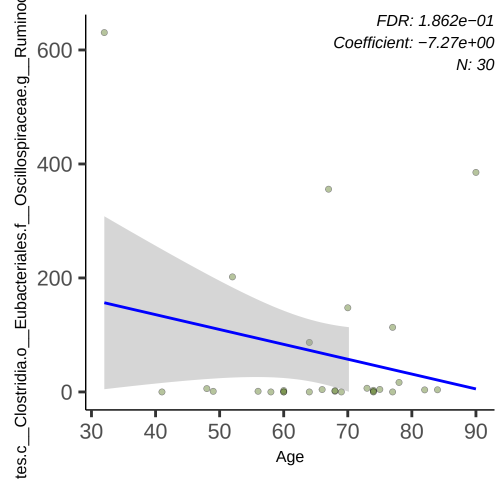

FDR: 1.862e−01
Coefficient: −7.27e+00
N: 30
tes.c__Clostridia.o__Eubacteriales.f__Oscillospiraceae.g__Ruminoc
600
400
200
0
30
40
50
60
70
80
90
Age

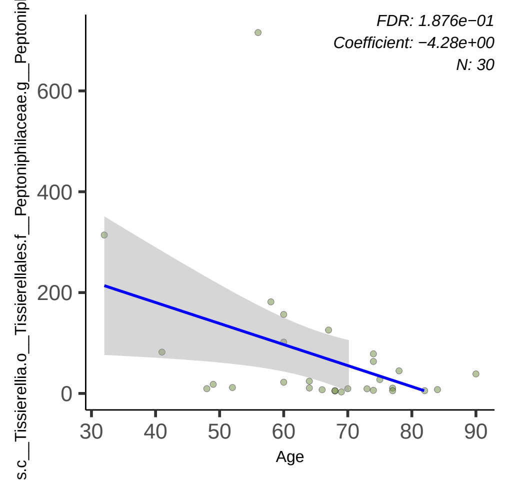

FDR: 1.876e−01
Coefficient: −4.28e+00
N: 30
0
200
400
600
30
40
50
60
70
80
90
Age
s.c__Tissierellia.o__Tissierellales.f__Peptoniphilaceae.g__Peptoniph

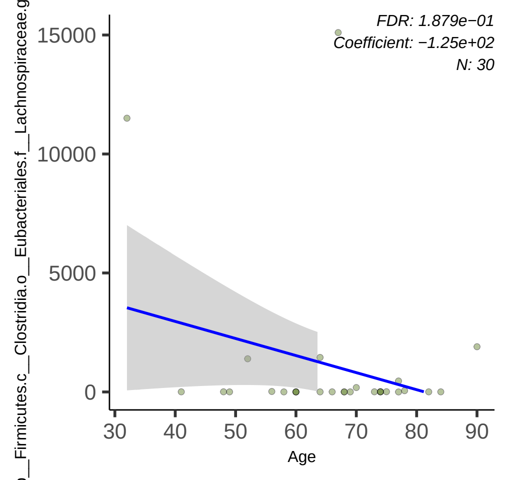

FDR: 1.879e−01
Coefficient: −1.25e+02
N: 30
o__Firmicutes.c__Clostridia.o__Eubacteriales.f__Lachnospiraceae.g
15000
10000
5000
0
30
40
50
60
70
80
90
Age

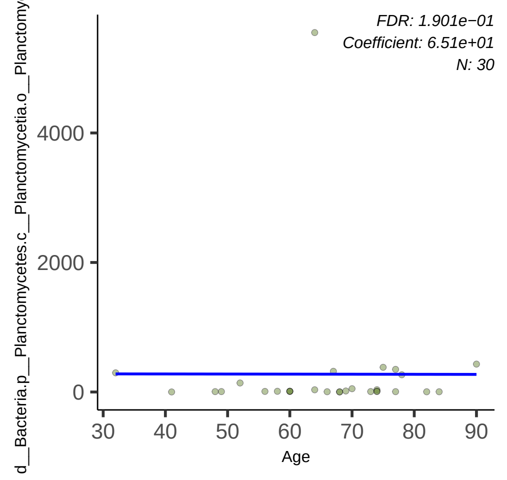

FDR: 1.901e−01
Coefficient: 6.51e+01
N: 30
4000
2000
0
30
40
50
60
70
80
90
Age
d__Bacteria.p__Planctomycetes.c__Planctomycetia.o__Planctomy

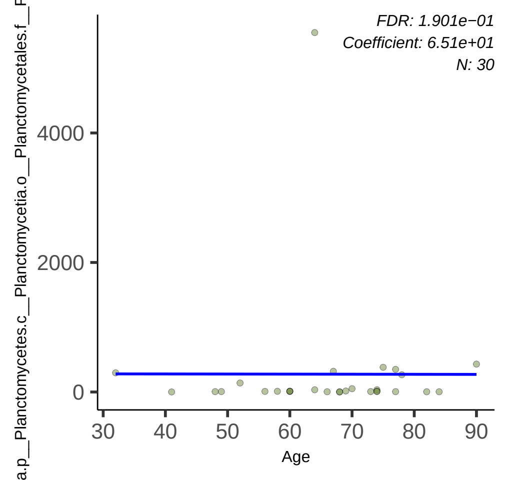

FDR: 1.901e−01
Coefficient: 6.51e+01
N: 30
4000
2000
0
30
40
50
60
70
80
90
Age
a.p__Planctomycetes.c__Planctomycetia.o__Planctomycetales.f__P

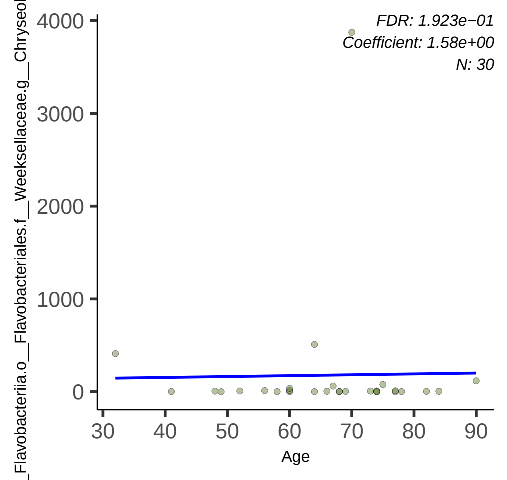

FDR: 1.923e−01
Coefficient: 1.58e+00
N: 30
Flavobacteriia.o__Flavobacteriales.f__Weeksellaceae.g__Chryseob
0
1000
2000
3000
4000
30
40
50
60
70
80
90
Age

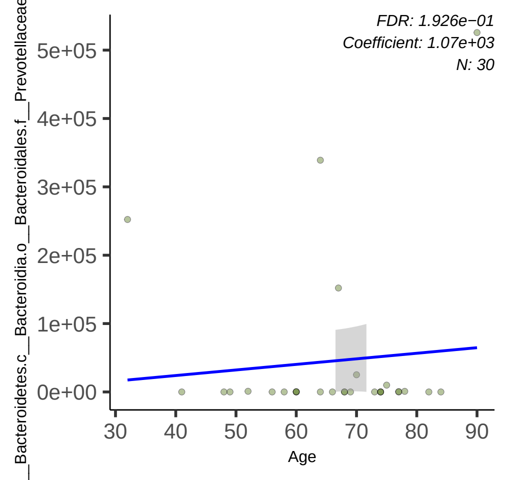
FDR: 1.926e−01
Coefficient: 1.07e+03
N: 30
Bacteroidetes.c__Bacteroidia.o__Bacteroidales.f__Prevotellaceae
5e+05
4e+05
3e+05
2e+05
1e+05
0e+00
30
40
50
60
70
80
90
Age

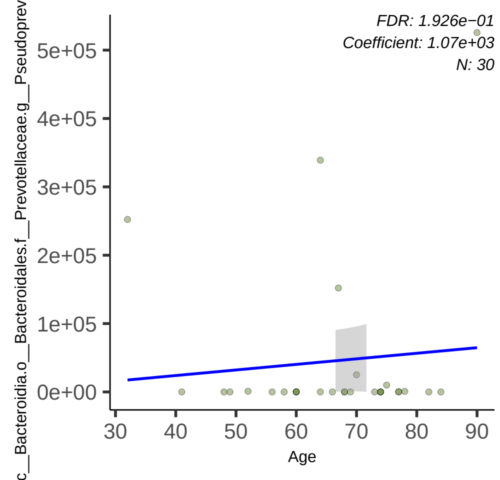

FDR: 1.926e−01
Coefficient: 1.07e+03
N: 30
c__Bacteroidia.o__Bacteroidales.f__Prevotellaceae.g__Pseudoprev
5e+05
4e+05
3e+05
2e+05
1e+05
0e+00
30
40
50
60
70
80
90
Age

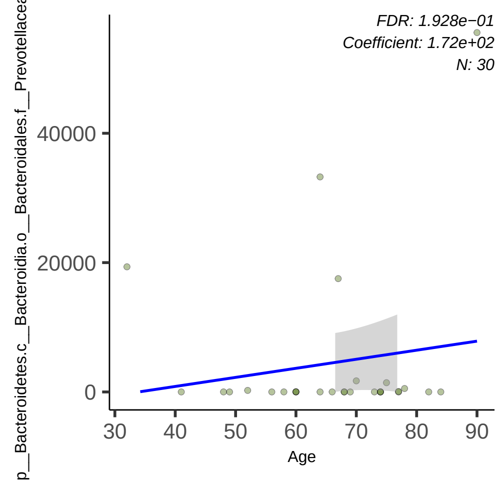
FDR: 1.928e−01
Coefficient: 1.72e+02
N: 30
p__Bacteroidetes.c__Bacteroidia.o__Bacteroidales.f__Prevotellacea
40000
20000
0
30
40
50
60
70
80
90
Age

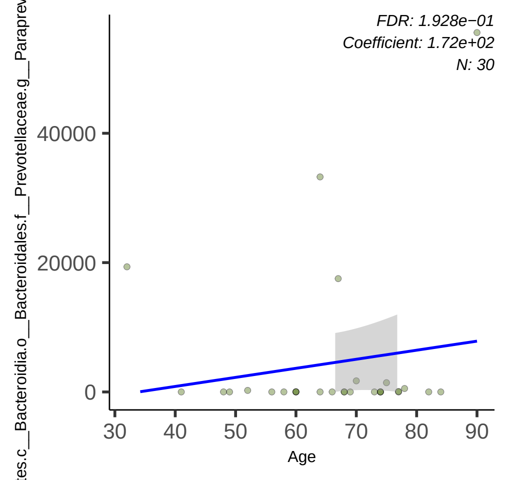
FDR: 1.928e−01
Coefficient: 1.72e+02
N: 30
40000
20000
0
30
40
50
60
70
80
90
Age
tes.c__Bacteroidia.o__Bacteroidales.f__Prevotellaceae.g__Paraprev

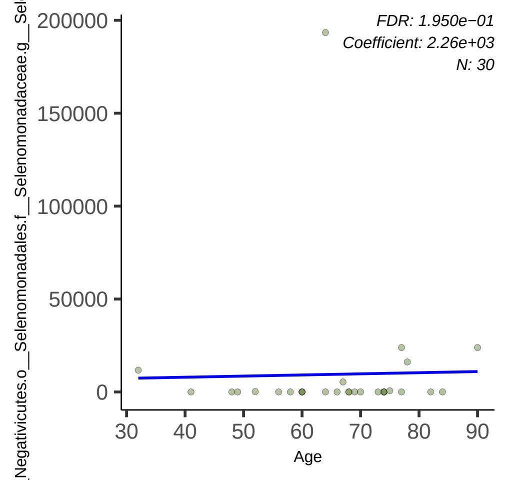

FDR: 1.950e−01
Coefficient: 2.26e+03
N: 30
Negativicutes.o__Selenomonadales.f__Selenomonadaceae.g__Sel
200000
150000
100000
50000
0
30
40
50
60
70
80
90
Age

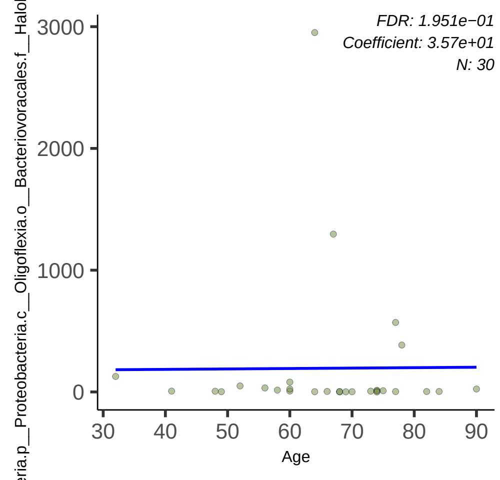

FDR: 1.951e−01
Coefficient: 3.57e+01
N: 30
0
1000
2000
3000
30
40
50
60
70
80
90
Age
eria.p__Proteobacteria.c__Oligoflexia.o__Bacteriovoracales.f__Halob

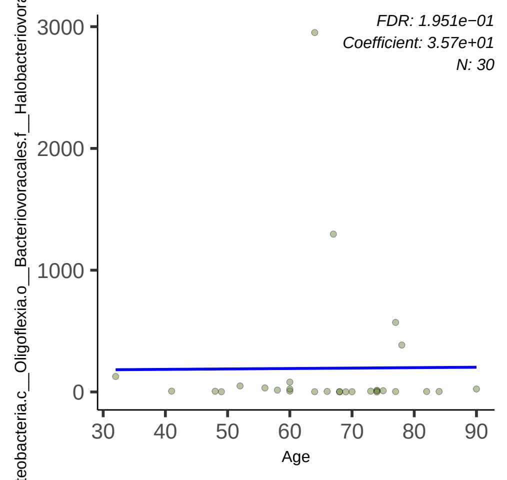
FDR: 1.951e−01
Coefficient: 3.57e+01
N: 30
teobacteria.c__Oligoflexia.o__Bacteriovoracales.f__Halobacteriovora
Age
0
1000
2000
3000
30
40
50
60
70
80
90

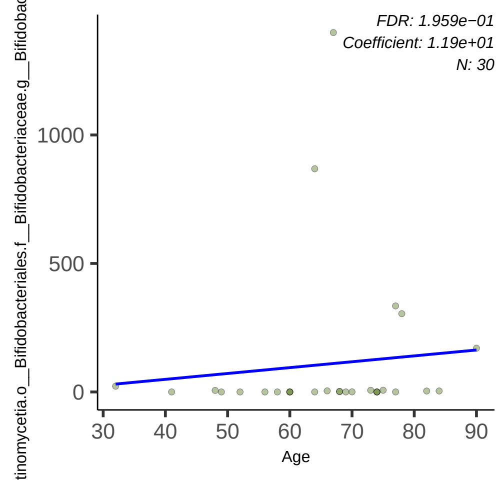

FDR: 1.959e−01
Coefficient: 1.19e+01
N: 30
1000
500
0
30
40
50
60
70
80
90
Age
tinomycetia.o__Bifidobacteriales.f__Bifidobacteriaceae.g__Bifidobac

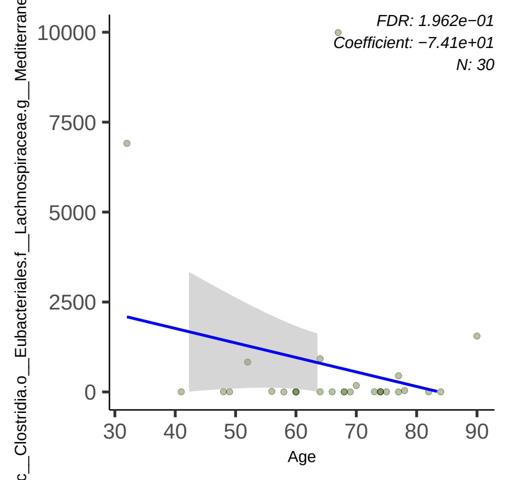
FDR: 1.962e−01
Coefficient: −7.41e+01
N: 30
c__Clostridia.o__Eubacteriales.f__Lachnospiraceae.g__Mediterrane
10000
7500
5000
2500
0
30
40
50
60
70
80
90
Age

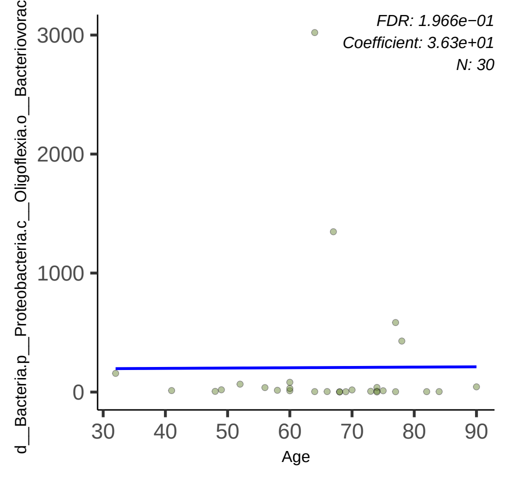
FDR: 1.966e−01
Coefficient: 3.63e+01
N: 30
3000
2000
1000
0
30
40
50
60
70
80
90
Age
d__Bacteria.p__Proteobacteria.c__Oligoflexia.o__Bacteriovorac

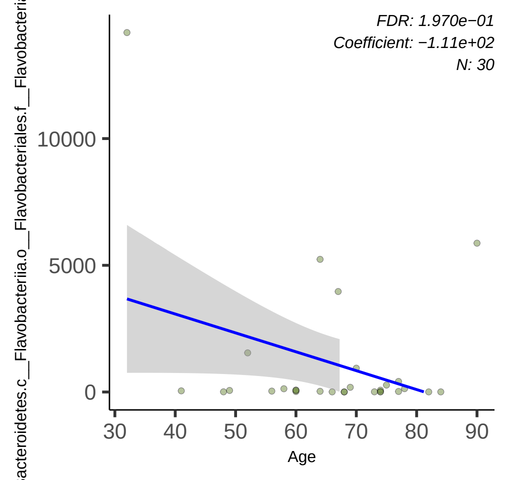

FDR: 1.970e−01
Coefficient: −1.11e+02
N: 30
Bacteroidetes.c__Flavobacteriia.o__Flavobacteriales.f__Flavobacteria
10000
5000
0
30
40
50
60
70
80
90
Age

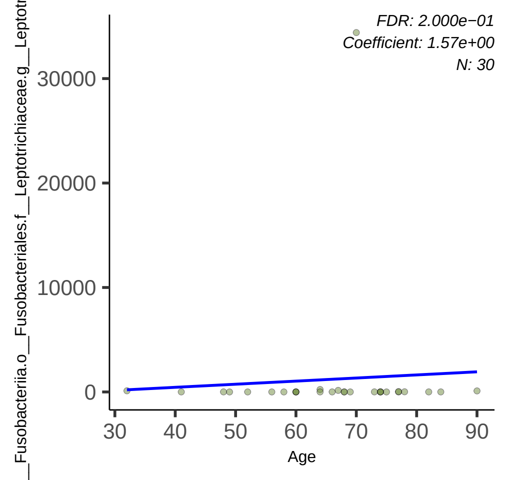
FDR: 2.000e−01
Coefficient: 1.57e+00
N: 30
30000
20000
10000
0
30
40
50
60
70
80
90
Age
Fusobacteriia.o__Fusobacteriales.f__Leptotrichiaceae.g__Leptotr

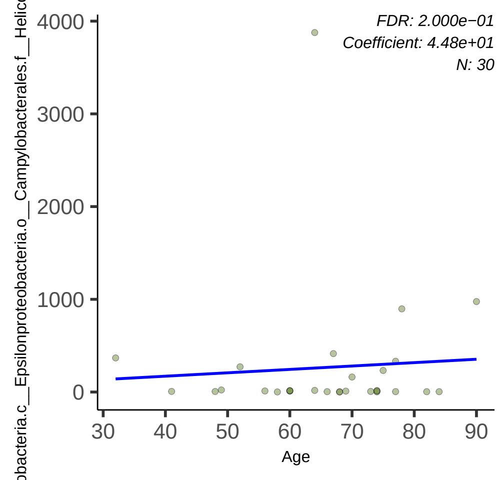

FDR: 2.000e−01
Coefficient: 4.48e+01
N: 30
4000
3000
2000
1000
0
30
40
50
60
70
80
90
Age
obacteria.c__Epsilonproteobacteria.o__Campylobacterales.f__Helico

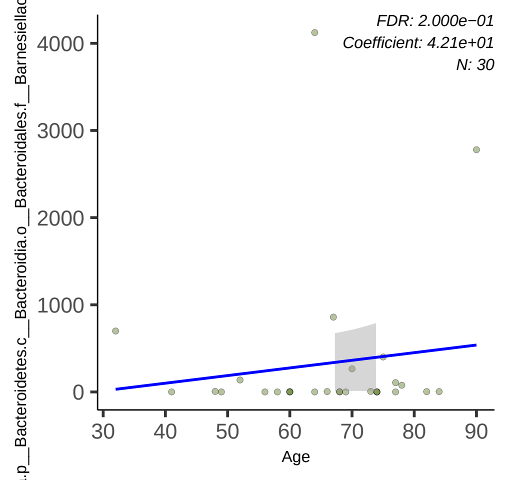
FDR: 2.000e−01
Coefficient: 4.21e+01
N: 30
0
1000
2000
3000
4000
30
40
50
60
70
80
90
Age
p__Bacteroidetes.c__Bacteroidia.o__Bacteroidales.f__Barnesiellac

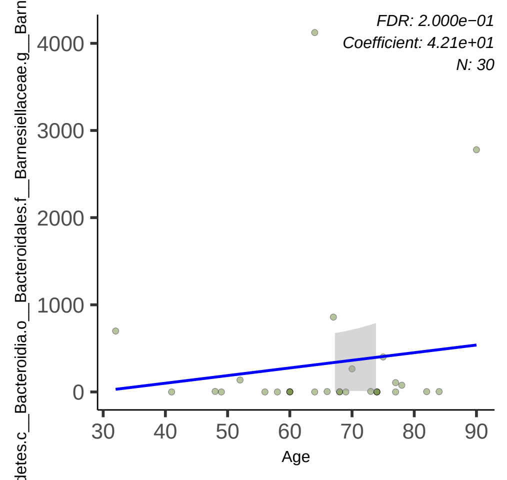

FDR: 2.000e−01
Coefficient: 4.21e+01
N: 30
4000
3000
2000
1000
0
30
40
50
60
70
80
90
Age
detes.c__Bacteroidia.o__Bacteroidales.f__Barnesiellaceae.g__Barn

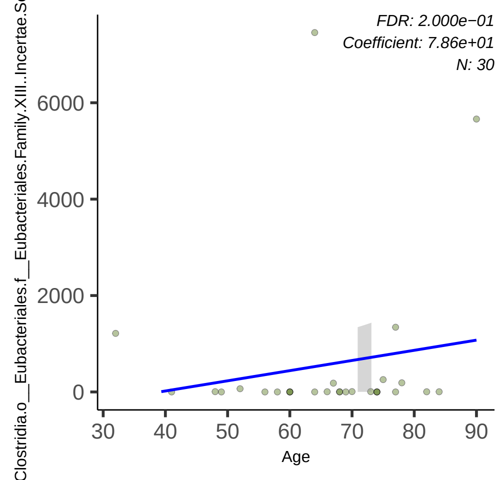
FDR: 2.000e−01
Coefficient: 7.86e+01
N: 30
Clostridia.o__Eubacteriales.f__Eubacteriales.Family.XIII..Incertae.Se
6000
4000
2000
0
30
40
50
60
70
80
90
Age

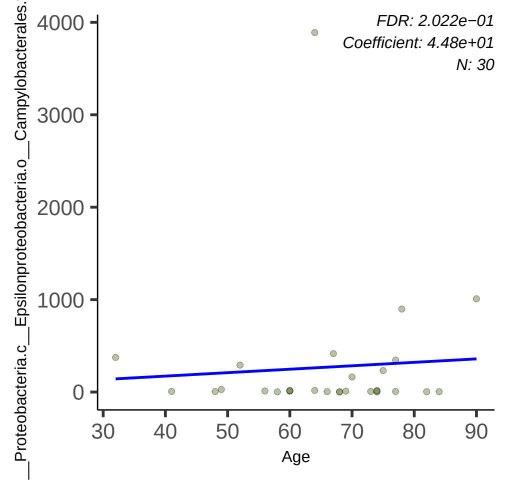

FDR: 2.022e−01
Coefficient: 4.48e+01
N: 30
4000
3000
2000
1000
0
30
40
50
60
70
80
90
Age
__Proteobacteria.c__Epsilonproteobacteria.o__Campylobacterales.

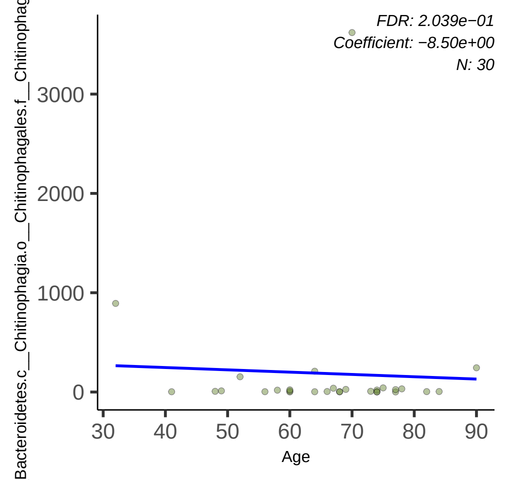

FDR: 2.039e−01
Coefficient: −8.50e+00
N: 30
Bacteroidetes.c__Chitinophagia.o__Chitinophagales.f__Chitinophag
3000
2000
1000
0
30
40
50
60
70
80
90
Age

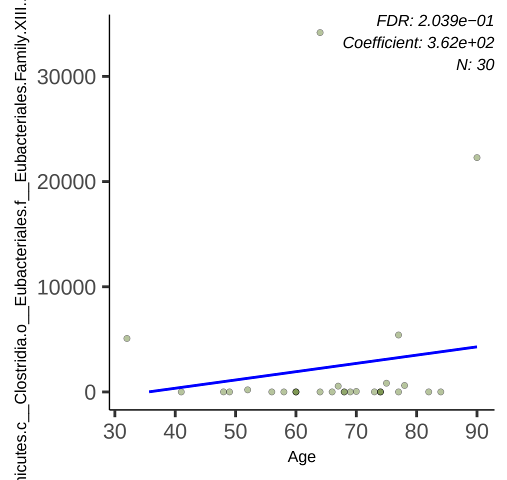

FDR: 2.039e−01
Coefficient: 3.62e+02
N: 30
30000
20000
10000
0
30
40
50
60
70
80
90
Age
nicutes.c__Clostridia.o__Eubacteriales.f__Eubacteriales.Family.XIII.

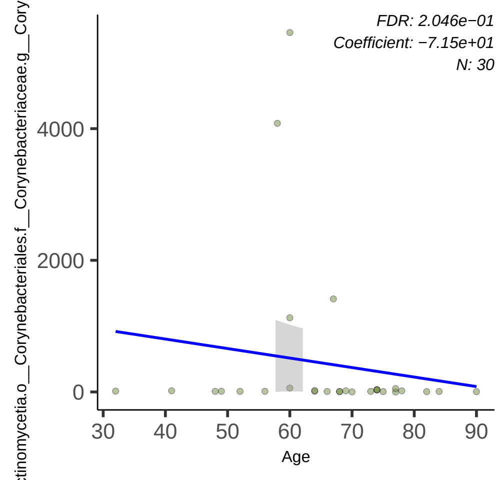

FDR: 2.046e−01
Coefficient: −7.15e+01
N: 30
4000
2000
0
30
40
50
60
70
80
90
Age
ctinomycetia.o__Corynebacteriales.f__Corynebacteriaceae.g__Cory

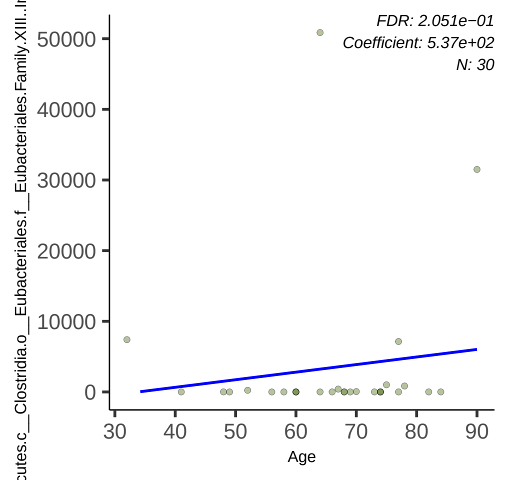

FDR: 2.051e−01
Coefficient: 5.37e+02
N: 30
0
10000
20000
30000
40000
50000
30
40
50
60
70
80
90
Age
cutes.c__Clostridia.o__Eubacteriales.f__Eubacteriales.Family.XIII..In

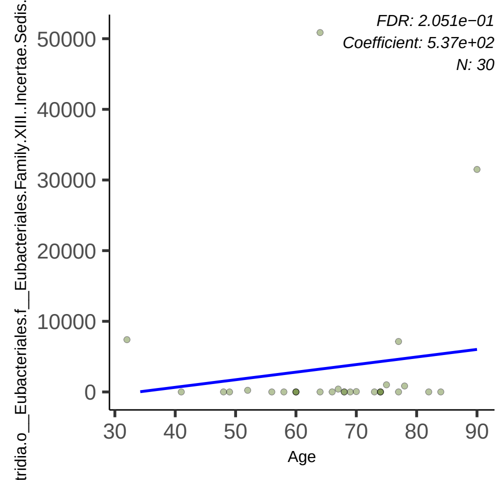
FDR: 2.051e−01
Coefficient: 5.37e+02
N: 30
tridia.o__Eubacteriales.f__Eubacteriales.Family.XIII..Incertae.Sedis.
50000
40000
30000
20000
10000
0
30
40
50
60
70
80
90
Age

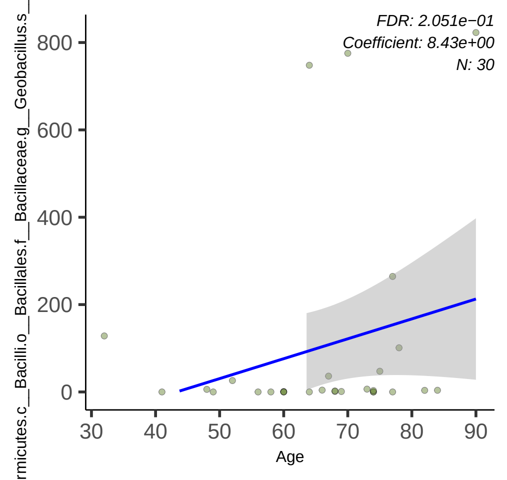
FDR: 2.051e−01
Coefficient: 8.43e+00
N: 30
rmicutes.c__Bacilli.o__Bacillales.f__Bacillaceae.g__Geobacillus.s_
0
200
400
600
800
30
40
50
60
70
80
90
Age

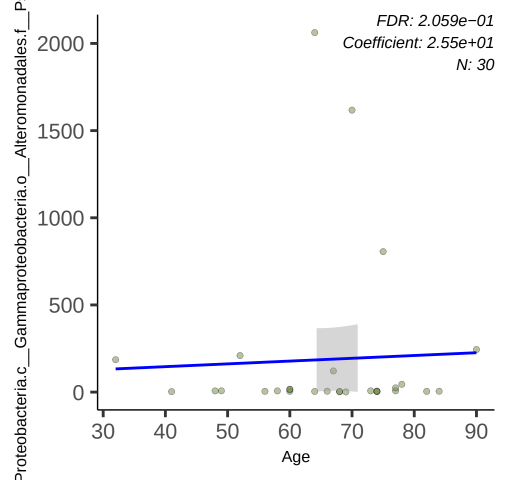
FDR: 2.059e−01
Coefficient: 2.55e+01
N: 30
Proteobacteria.c__Gammaproteobacteria.o__Alteromonadales.f__P
0
500
1000
1500
2000
30
40
50
60
70
80
90
Age

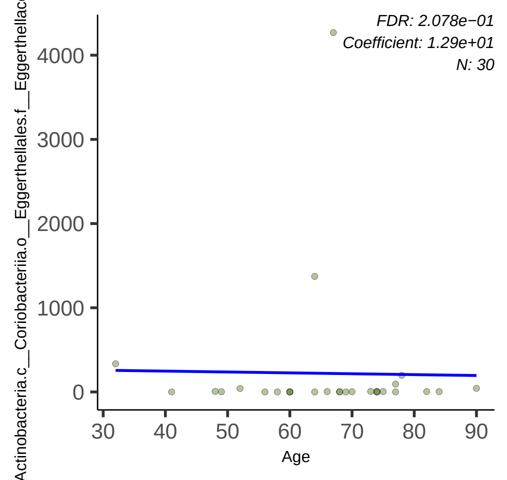

FDR: 2.078e−01
Coefficient: 1.29e+01
N: 30
Actinobacteria.c__Coriobacteriia.o__Eggerthellales.f__Eggerthellac
4000
3000
2000
1000
0
30
40
50
60
70
80
90
Age

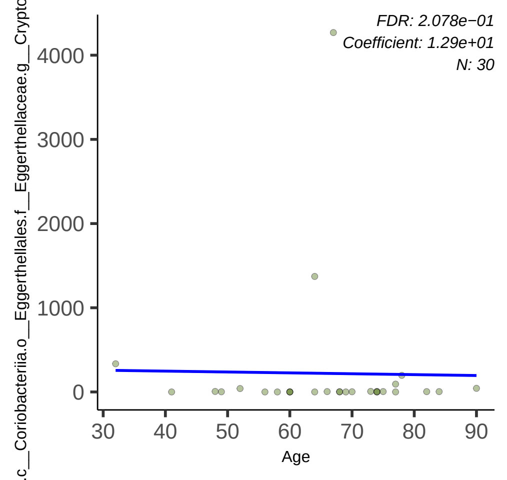

FDR: 2.078e−01
Coefficient: 1.29e+01
N: 30
0
1000
2000
3000
4000
30
40
50
60
70
80
90
Age
c__Coriobacteriia.o__Eggerthellales.f__Eggerthellaceae.g__Crypto

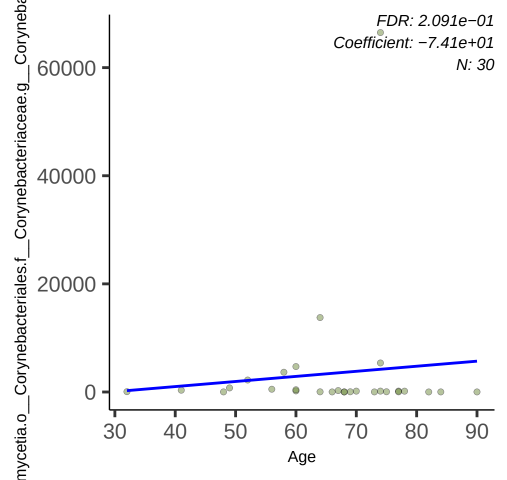

FDR: 2.091e−01
Coefficient: −7.41e+01
N: 30
60000
40000
20000
0
30
40
50
60
70
80
90
Age
mycetia.o__Corynebacteriales.f__Corynebacteriaceae.g__Coryneba

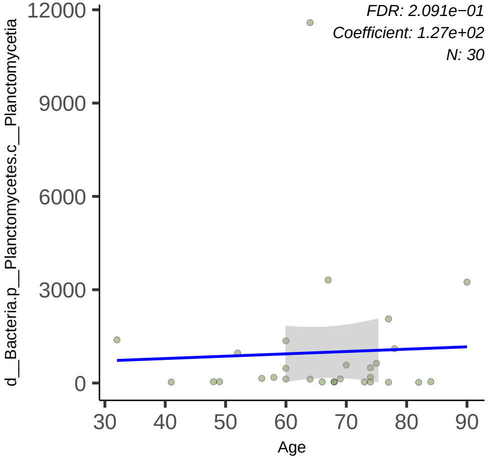
FDR: 2.091e−01
Coefficient: 1.27e+02
N: 30
12000
9000
6000
3000
0
30
40
50
60
70
80
90
Age
d__Bacteria.p__Planctomycetes.c__Planctomycetia

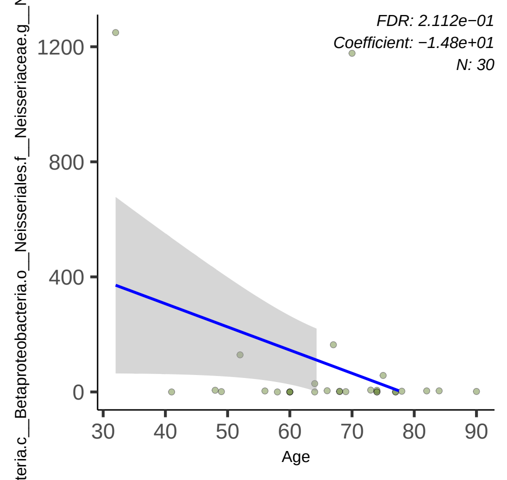

FDR: 2.112e−01
Coefficient: −1.48e+01
N: 30
1200
800
400
0
30
40
50
60
70
80
90
Age
teria.c__Betaproteobacteria.o__Neisseriales.f__Neisseriaceae.g__N

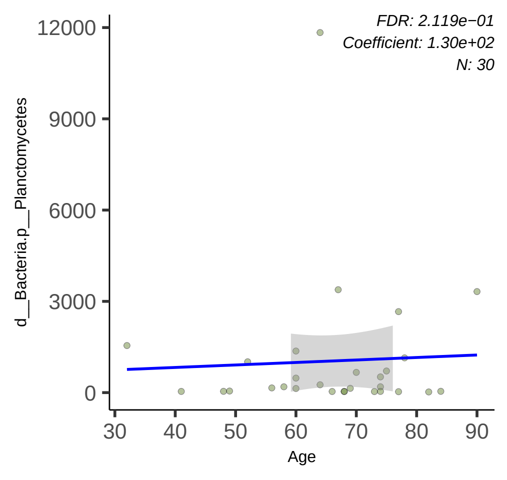

FDR: 2.119e−01
Coefficient: 1.30e+02
N: 30
d__Bacteria.p__Planctomycetes
12000
9000
6000
3000
0
30
40
50
60
70
80
90
Age

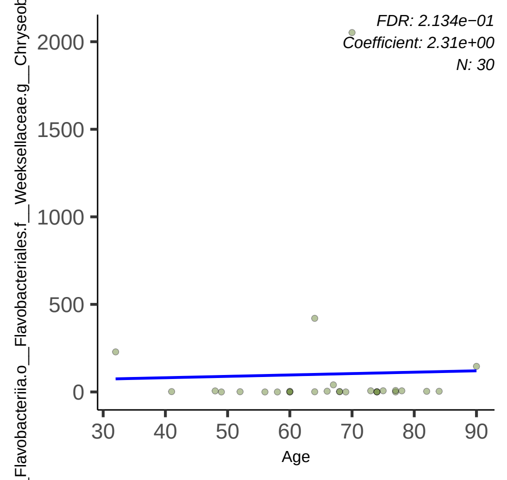

FDR: 2.134e−01
Coefficient: 2.31e+00
N: 30
2000
1500
1000
500
0
30
40
50
60
70
80
90
Age
Flavobacteriia.o__Flavobacteriales.f__Weeksellaceae.g__Chryseob

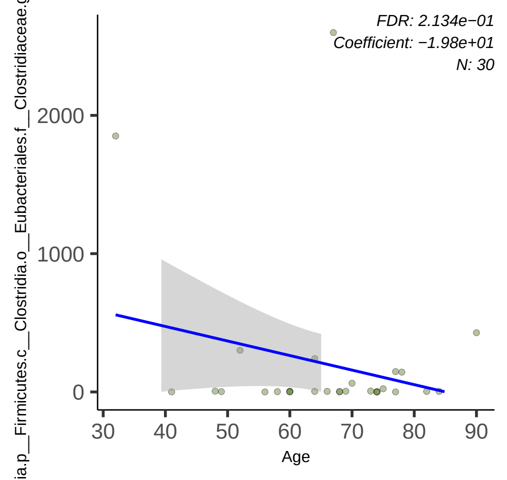
FDR: 2.134e−01
Coefficient: −1.98e+01
N: 30
ia.p__Firmicutes.c__Clostridia.o__Eubacteriales.f__Clostridiaceae.g
2000
1000
0
30
40
50
60
70
80
90
Age

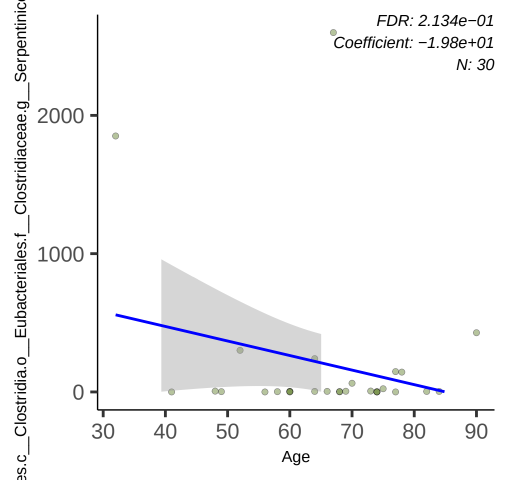

FDR: 2.134e−01
Coefficient: −1.98e+01
N: 30
es.c__Clostridia.o__Eubacteriales.f__Clostridiaceae.g__Serpentinic
2000
1000
0
30
40
50
60
70
80
90
Age

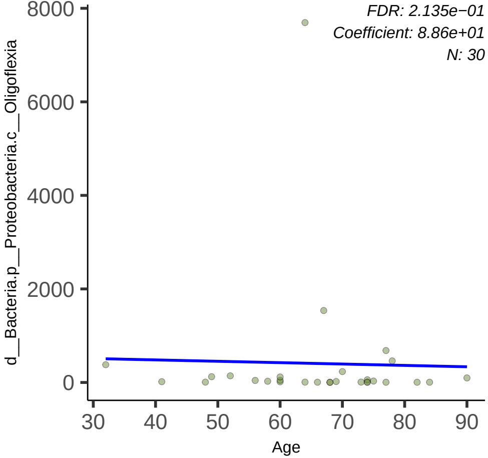

FDR: 2.135e−01
Coefficient: 8.86e+01
N: 30
d__Bacteria.p__Proteobacteria.c__Oligoflexia
8000
6000
4000
2000
0
30
40
50
60
70
80
90
Age

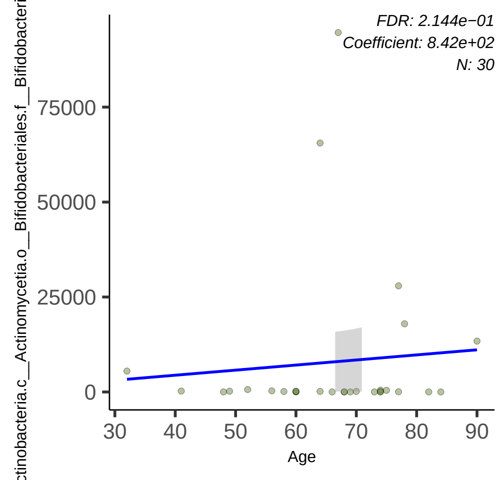

FDR: 2.144e−01
Coefficient: 8.42e+02
N: 30
75000
50000
25000
0
30
40
50
60
70
80
90
Age
ctinobacteria.c__Actinomycetia.o__Bifidobacteriales.f__Bifidobacteri

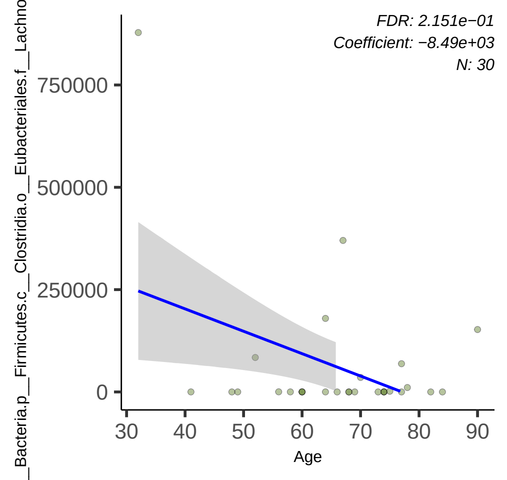

FDR: 2.151e−01
Coefficient: −8.49e+03
N: 30
_Bacteria.p__Firmicutes.c__Clostridia.o__Eubacteriales.f__Lachno
750000
500000
250000
0
30
40
50
60
70
80
90
Age

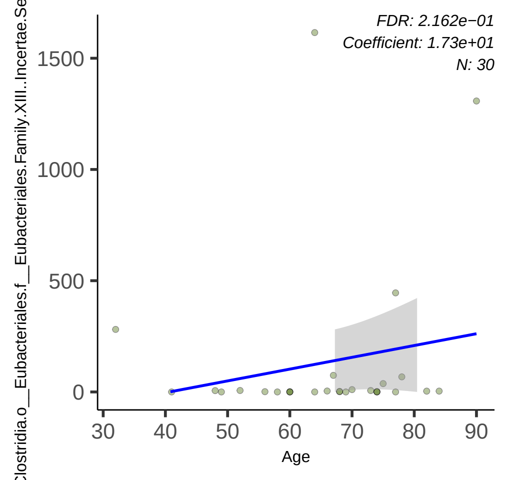

FDR: 2.162e−01
Coefficient: 1.73e+01
N: 30
1500
1000
500
0
30
40
50
60
70
80
90
Age
Clostridia.o__Eubacteriales.f__Eubacteriales.Family.XIII..Incertae.Se

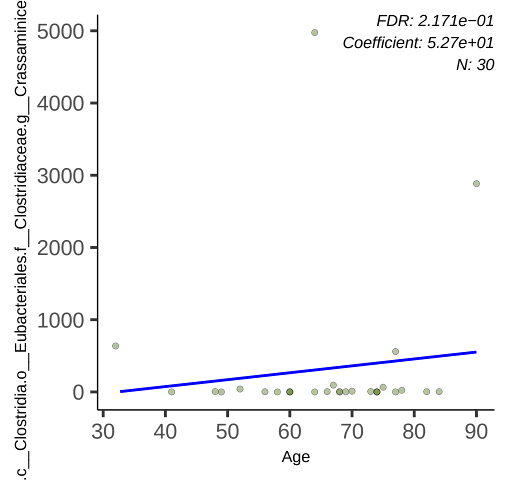
FDR: 2.171e−01
Coefficient: 5.27e+01
N: 30
c__Clostridia.o__Eubacteriales.f__Clostridiaceae.g__Crassaminice
5000
4000
3000
2000
1000
0
30
40
50
60
70
80
90
Age

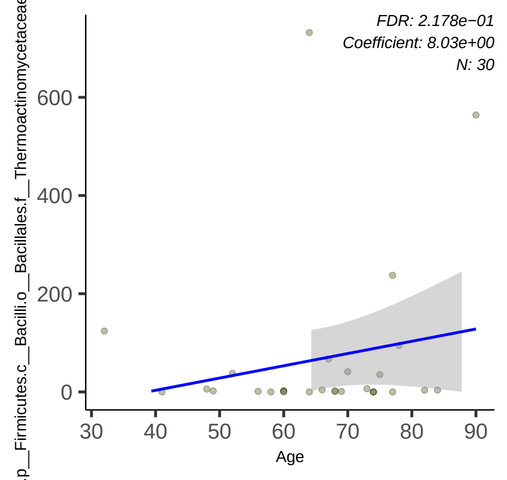
FDR: 2.178e−01
Coefficient: 8.03e+00
N: 30
0
200
400
600
30
40
50
60
70
80
90
Age
p__Firmicutes.c__Bacilli.o__Bacillales.f__Thermoactinomycetaceae

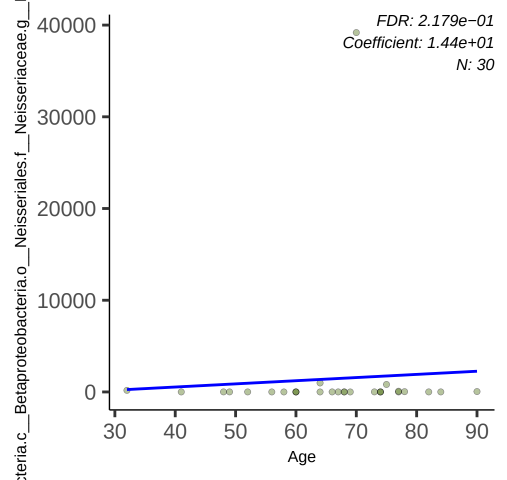
FDR: 2.179e−01
Coefficient: 1.44e+01
N: 30
Betaproteobacteria.o__Neisseriales.f__Neisseriaceae.g
40000
30000
20000
10000
0
30
40
50
60
70
80
90
Age

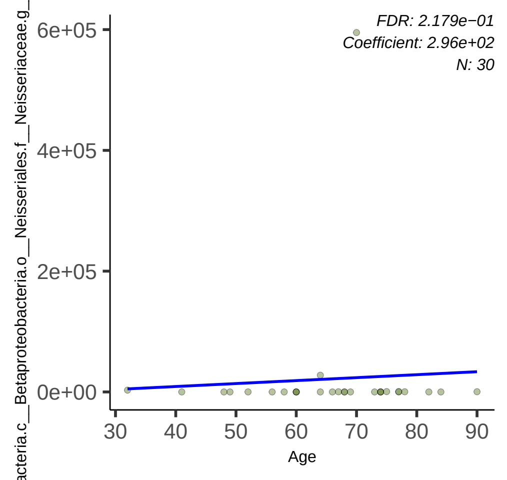

FDR: 2.179e−01
Coefficient: 2.96e+02
N: 30
6e+05
4e+05
2e+05
0e+00
30
40
50
60
70
80
90
Age
acteria.c__Betaproteobacteria.o__Neisseriales.f__Neisseriaceae.g_

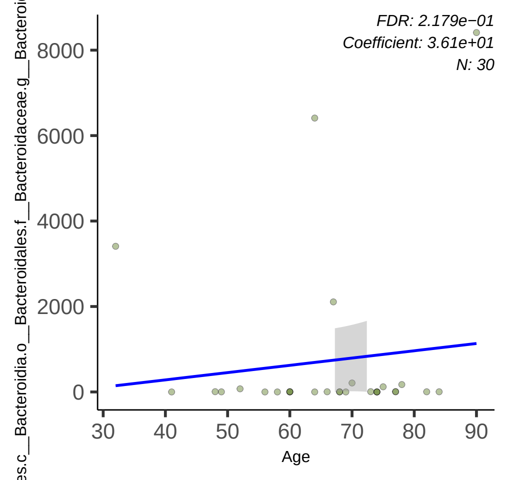
FDR: 2.179e−01
Coefficient: 3.61e+01
N: 30
8000
6000
4000
2000
0
30
40
50
60
70
80
90
Age
es.c__Bacteroidia.o__Bacteroidales.f__Bacteroidaceae.g__Bacteroi

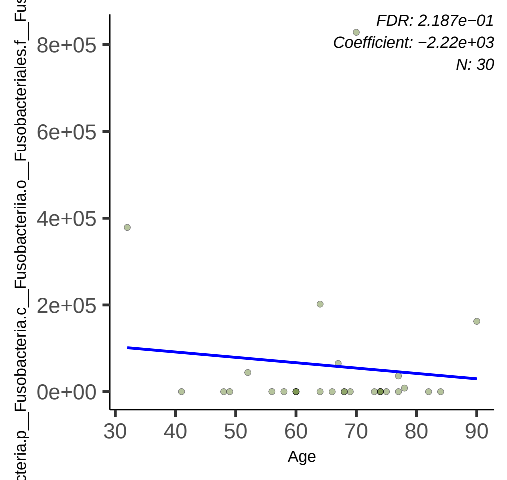

FDR: 2.187e−01
Coefficient: −2.22e+03
N: 30
8e+05
6e+05
4e+05
2e+05
0e+00
30
40
50
60
70
80
90
Age
cteria.p__Fusobacteria.c__Fusobacteriia.o__Fusobacteriales.f__Fus

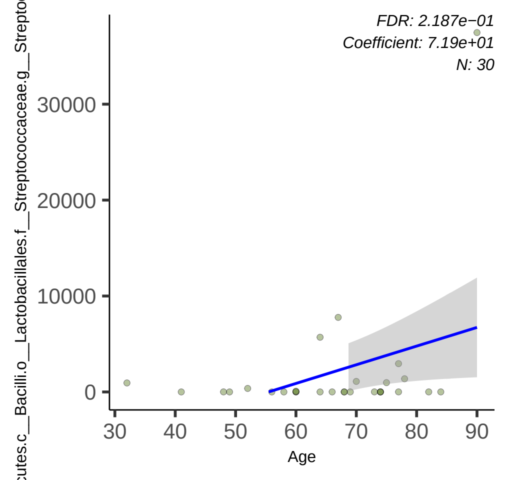

FDR: 2.187e−01
Coefficient: 7.19e+01
N: 30
cutes.c__Bacilli.o__Lactobacillales.f__Streptococcaceae.g__Strepto
30000
20000
10000
0
30
40
50
60
70
80
90
Age

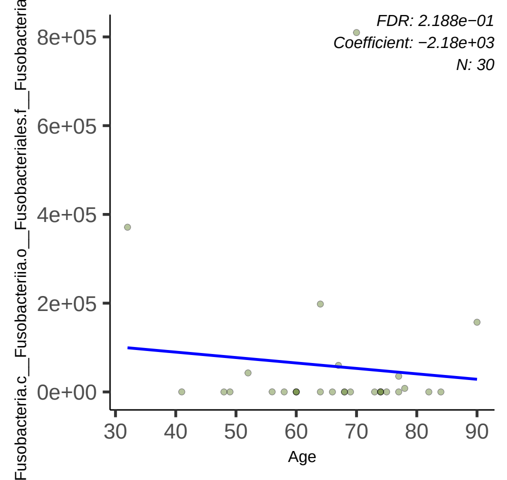

FDR: 2.188e−01
Coefficient: −2.18e+03
N: 30
Fusobacteria.c__Fusobacteriia.o__Fusobacteriales.f__Fusobacteria
8e+05
6e+05
4e+05
2e+05
0e+00
30
40
50
60
70
80
90
Age

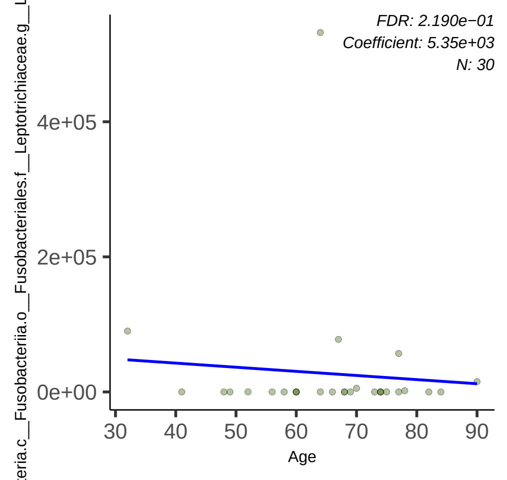
FDR: 2.190e−01
Coefficient: 5.35e+03
N: 30
4e+05
2e+05
0e+00
30
40
50
60
70
80
90
Age
teria.c__Fusobacteriia.o__Fusobacteriales.f__Leptotrichiaceae.g__L

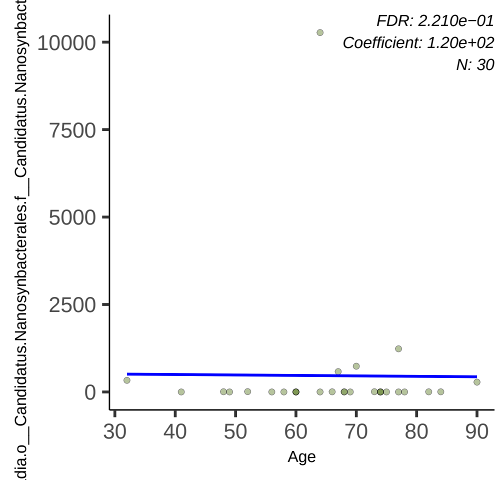
FDR: 2.210e−01
Coefficient: 1.20e+02
N: 30
10000
7500
5000
2500
0
30
40
50
60
70
80
90
Age
dia.o__Candidatus.Nanosynbacterales.f__Candidatus.Nanosynbact

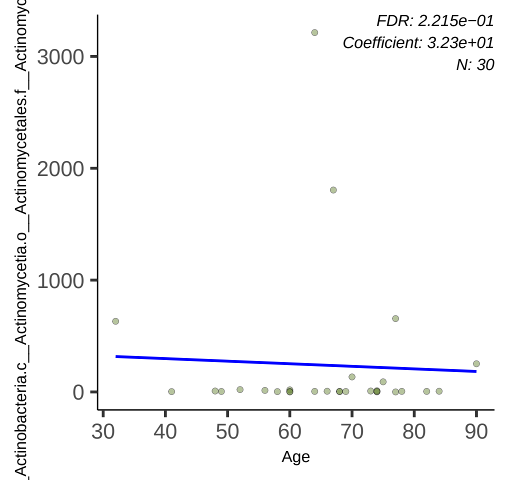
FDR: 2.215e−01
Coefficient: 3.23e+01
N: 30
Actinobacteria.c__Actinomycetia.o__Actinomycetales.f__Actinomyc
3000
2000
1000
0
30
40
50
60
70
80
90
Age

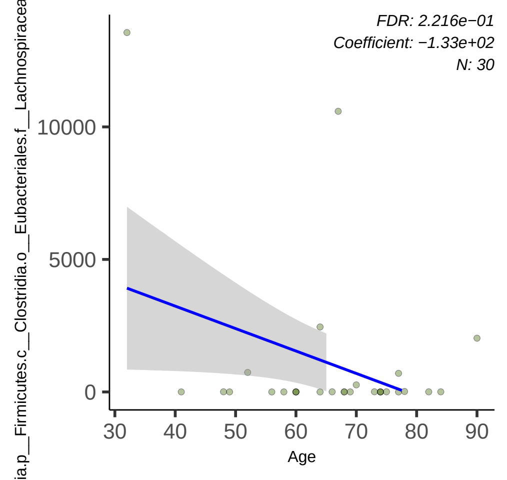

FDR: 2.216e−01
Coefficient: −1.33e+02
N: 30
ia.p__Firmicutes.c__Clostridia.o__Eubacteriales.f__Lachnospiracea
10000
5000
0
30
40
50
60
70
80
90
Age

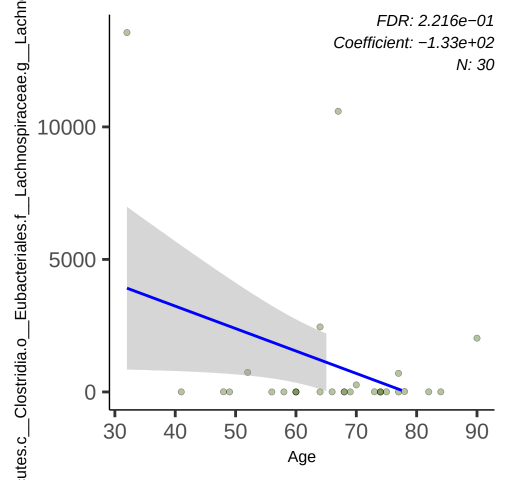
FDR: 2.216e−01
Coefficient: −1.33e+02
N: 30
10000
5000
0
30
40
50
60
70
80
90
Age
utes.c__Clostridia.o__Eubacteriales.f__Lachnospiraceae.g__Lachn

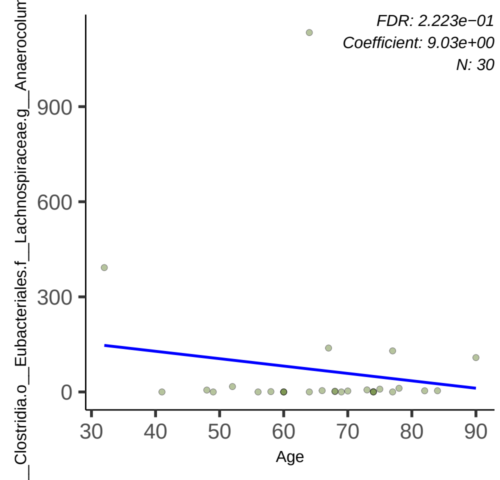

FDR: 2.223e−01
Coefficient: 9.03e+00
N: 30
900
600
300
0
30
40
50
60
70
80
90
Age
Clostridia.o__Eubacteriales.f__Lachnospiraceae.g__Anaerocolum

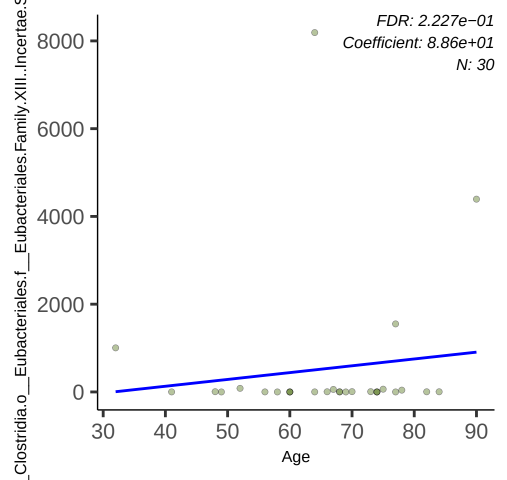
FDR: 2.227e−01
Coefficient: 8.86e+01
N: 30
8000
6000
4000
2000
0
30
40
50
60
70
80
90
Age
_Clostridia.o__Eubacteriales.f__Eubacteriales.Family.XIII..Incertae.S

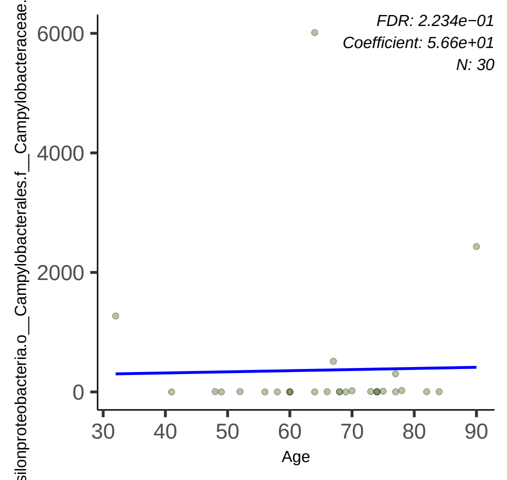

FDR: 2.234e−01
Coefficient: 5.66e+01
N: 30
0
2000
4000
6000
30
40
50
60
70
80
90
Age
silonproteobacteria.o__Campylobacterales.f__Campylobacteraceae.

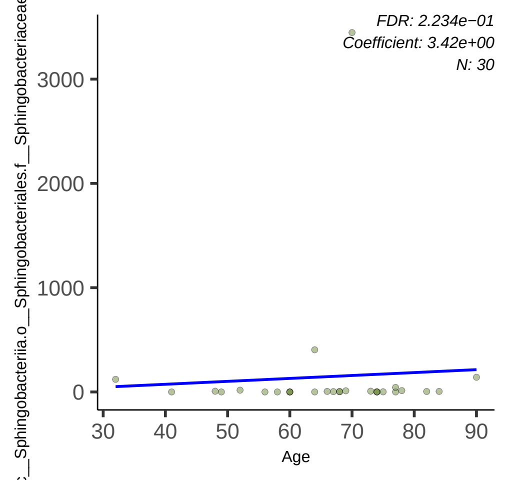
FDR: 2.234e−01
Coefficient: 3.42e+00
N: 30
3000
2000
1000
0
30
40
50
60
70
80
90
Age
c__Sphingobacteriia.o__Sphingobacteriales.f__Sphingobacteriaceae

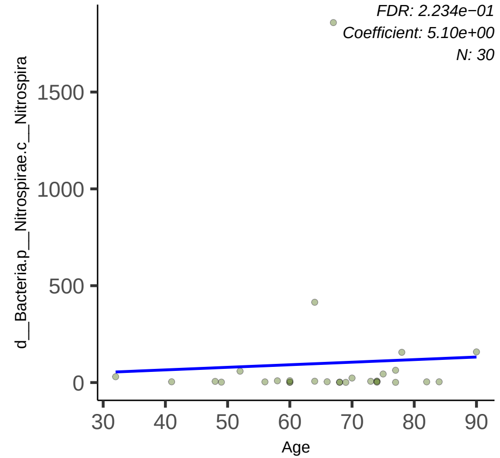
FDR: 2.234e−01
Coefficient: 5.10e+00
N: 30
d__Bacteria.p__Nitrospirae.c__Nitrospira
1500
1000
500
0
30
40
50
60
70
80
90
Age

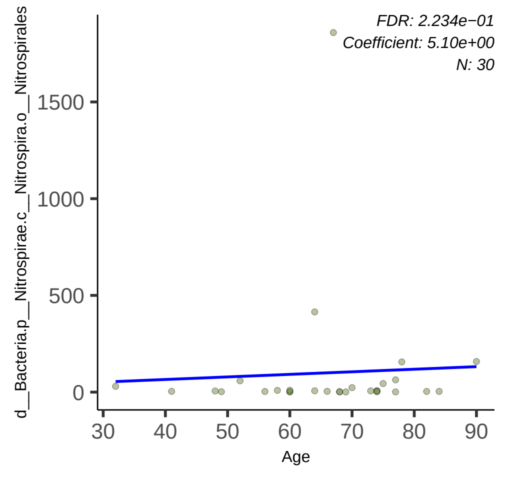
FDR: 2.234e−01
Coefficient: 5.10e+00
N: 30
d__Bacteria.p__Nitrospirae.c__Nitrospira.o__Nitrospirales
1500
1000
500
0
30
40
50
60
70
80
90
Age

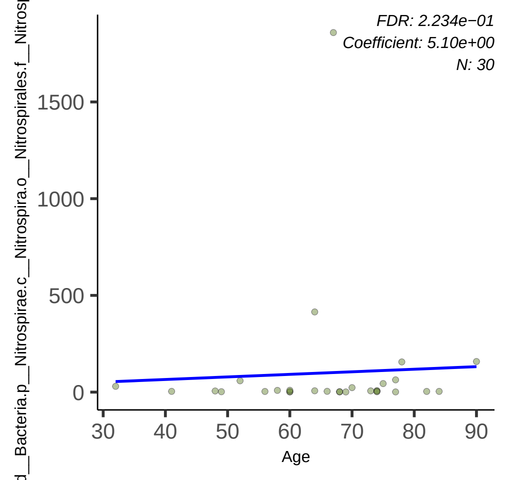
FDR: 2.234e−01
Coefficient: 5.10e+00
N: 30
1500
1000
500
0
30
40
50
60
70
80
90
Age
d__Bacteria.p__Nitrospirae.c__Nitrospira.o__Nitrospirales.f__Nitrosp

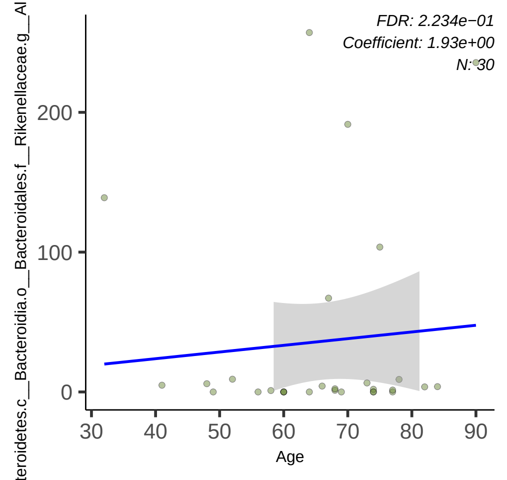

FDR: 2.234e−01
Coefficient: 1.93e+00
N: 30
200
100
0
30
40
50
60
70
80
90
Age
teroidetes.c__Bacteroidia.o__Bacteroidales.f__Rikenellaceae.g__Al

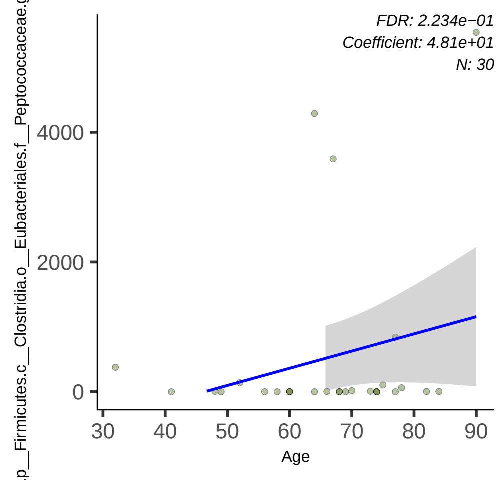

FDR: 2.234e−01
Coefficient: 4.81e+01
N: 30
p__Firmicutes.c__Clostridia.o__Eubacteriales.f__Peptococcaceae.g
4000
2000
0
30
40
50
60
70
80
90
Age

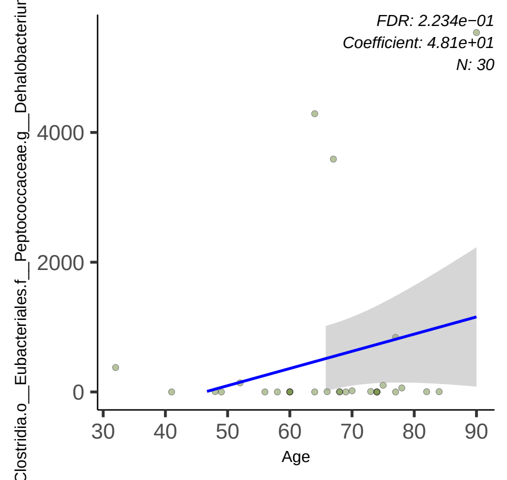
FDR: 2.234e−01
Coefficient: 4.81e+01
N: 30
Clostridia.o__Eubacteriales.f__Peptococcaceae.g__Dehalobacterium
4000
2000
0
30
40
50
60
70
80
90
Age

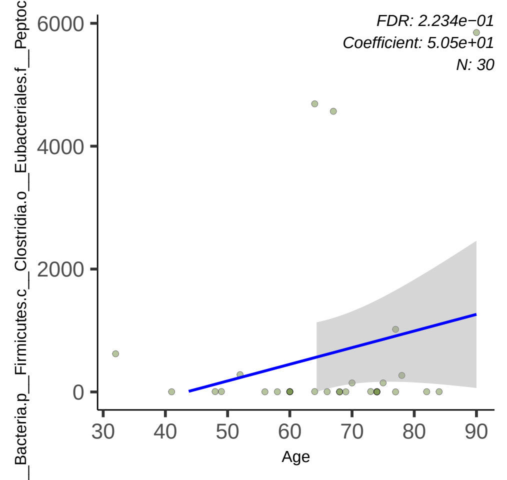

FDR: 2.234e−01
Coefficient: 5.05e+01
N: 30
Bacteria.p__Firmicutes.c__Clostridia.o__Eubacteriales.f__Peptoc
6000
4000
2000
0
30
40
50
60
70
80
90
Age

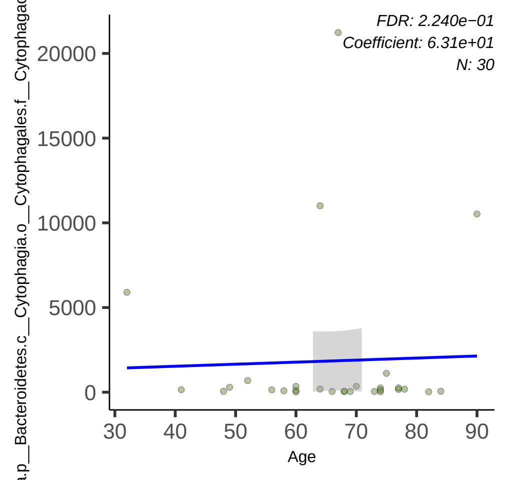
FDR: 2.240e−01
Coefficient: 6.31e+01
N: 30
a.p__Bacteroidetes.c__Cytophagia.o__Cytophagales.f__Cytophaga
20000
15000
10000
5000
0
30
40
50
60
70
80
90
Age

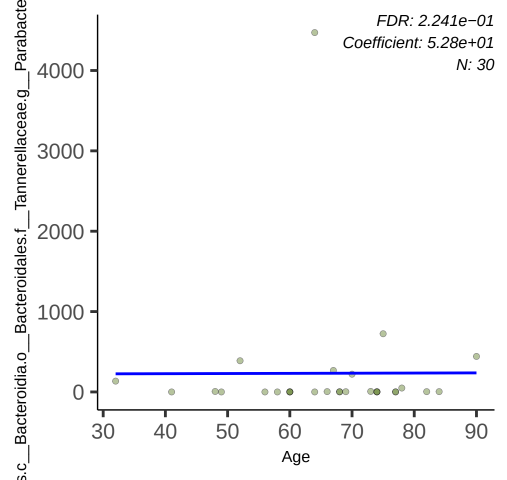
FDR: 2.241e−01
Coefficient: 5.28e+01
N: 30
4000
3000
2000
1000
0
30
40
50
60
70
80
90
Age
s.c__Bacteroidia.o__Bacteroidales.f__Tannerellaceae.g__Parabacte

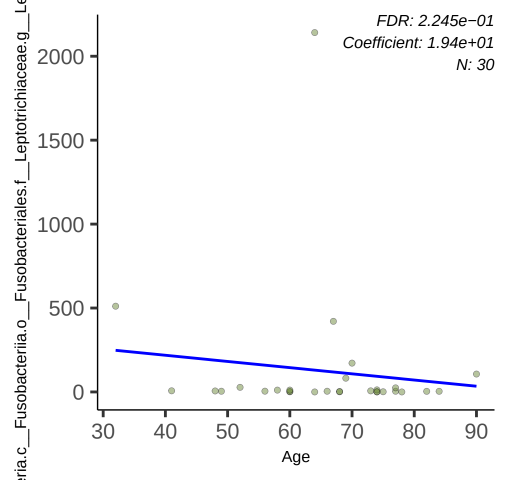

FDR: 2.245e−01
Coefficient: 1.94e+01
N: 30
0
500
1000
1500
2000
30
40
50
60
70
80
90
Age
eria.c__Fusobacteriia.o__Fusobacteriales.f__Leptotrichiaceae.g__Le

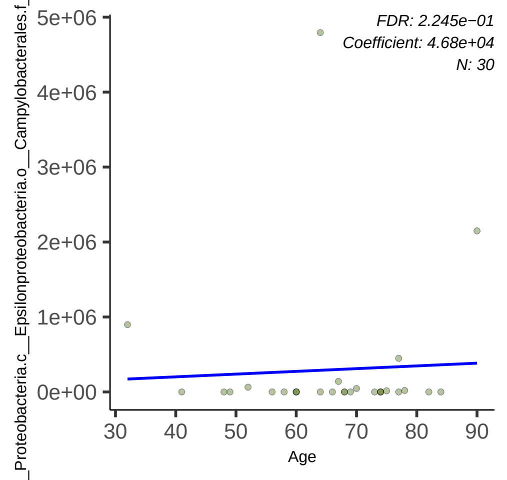
FDR: 2.245e−01
Coefficient: 4.68e+04
N: 30
Proteobacteria.c__Epsilonproteobacteria.o__Campylobacterales.f_
5e+06
4e+06
3e+06
2e+06
1e+06
0e+00
30
40
50
60
70
80
90
Age

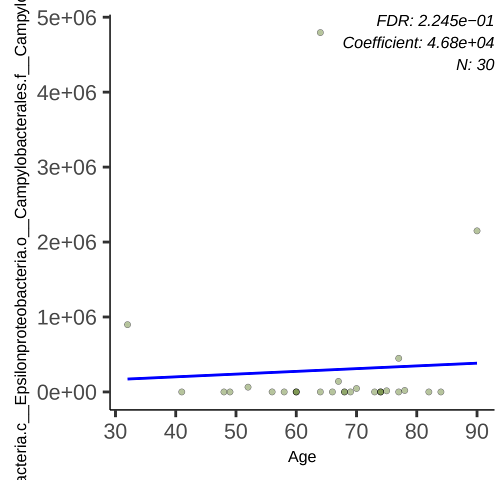
FDR: 2.245e−01
Coefficient: 4.68e+04
N: 30
Epsilonproteobacteria.o__Campylobacterales.f__Campylo
5e+06
4e+06
3e+06
2e+06
1e+06
0e+00
30
40
50
60
70
80
90
Age

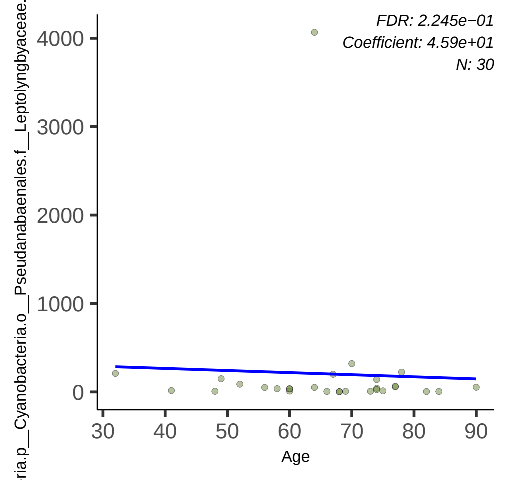
FDR: 2.245e−01
Coefficient: 4.59e+01
N: 30
ria.p__Cyanobacteria.o__Pseudanabaenales.f__Leptolyngbyaceae.
4000
3000
2000
1000
0
30
40
50
60
70
80
90
Age

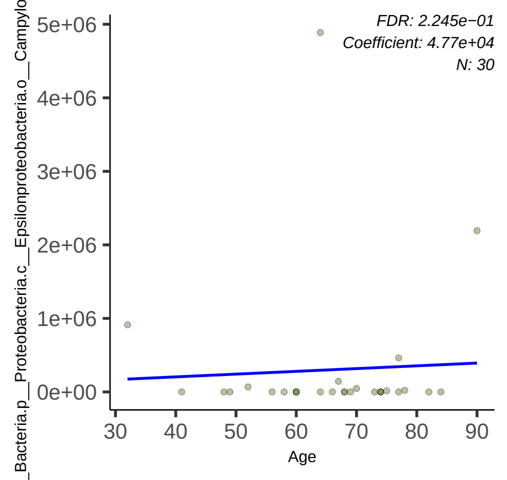
FDR: 2.245e−01
Coefficient: 4.77e+04
N: 30
5e+06
4e+06
3e+06
2e+06
1e+06
0e+00
30
40
50
60
70
80
90
Age
_Bacteria.p__Proteobacteria.c__Epsilonproteobacteria.o__Campylo

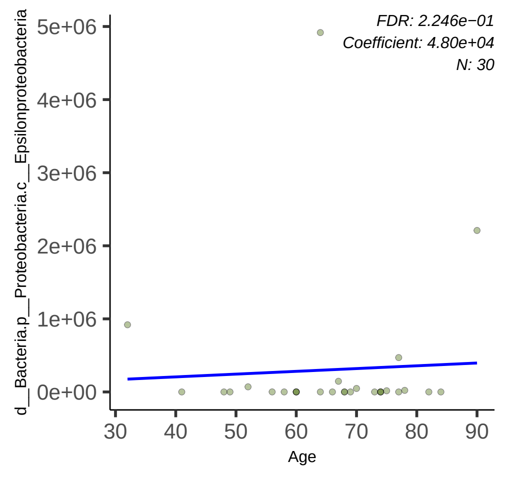

FDR: 2.246e−01
Coefficient: 4.80e+04
N: 30
d__Bacteria.p__Proteobacteria.c__Epsilonproteobacteria
5e+06
4e+06
3e+06
2e+06
1e+06
0e+00
30
40
50
60
70
80
90
Age

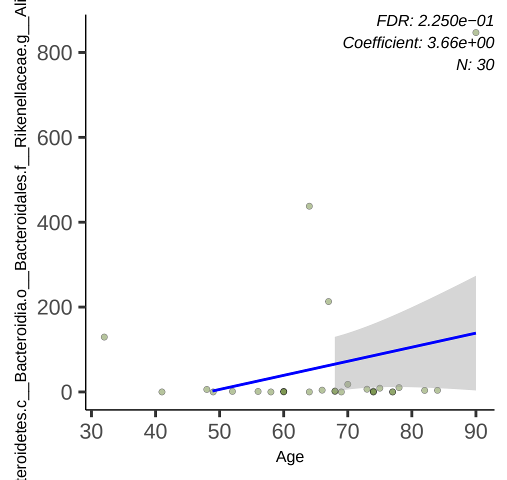
FDR: 2.250e−01
Coefficient: 3.66e+00
N: 30
0
200
400
600
800
30
40
50
60
70
80
90
Age
teroidetes.c__Bacteroidia.o__Bacteroidales.f__Rikenellaceae.g__Ali

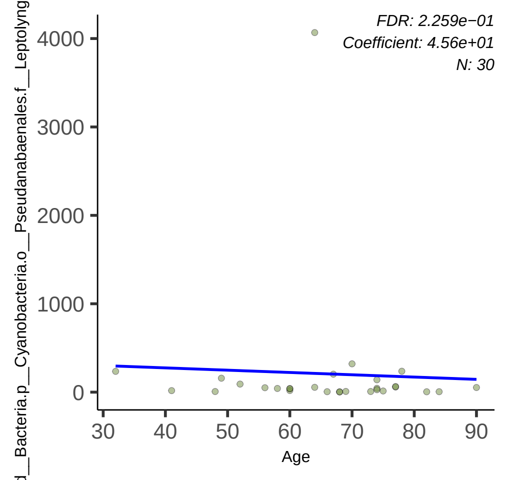

FDR: 2.259e−01
Coefficient: 4.56e+01
N: 30
d__Bacteria.p__Cyanobacteria.o__Pseudanabaenales.f__Leptolyng
4000
3000
2000
1000
0
30
40
50
60
70
80
90
Age

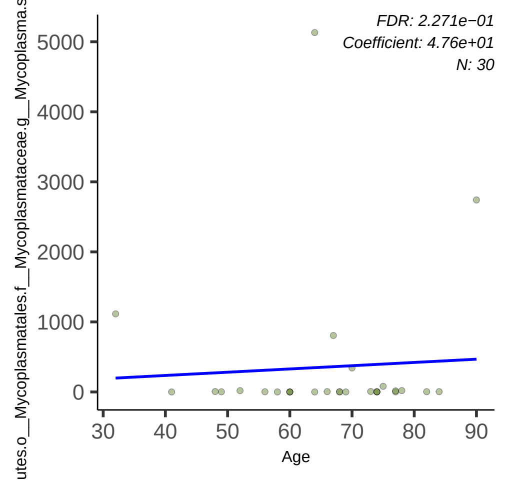
FDR: 2.271e−01
Coefficient: 4.76e+01
N: 30
utes.o__Mycoplasmatales.f__Mycoplasmataceae.g__Mycoplasma.s
0
1000
2000
3000
4000
5000
30
40
50
60
70
80
90
Age

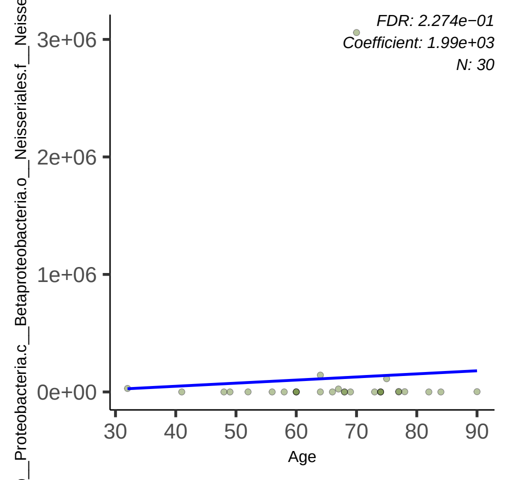
FDR: 2.274e−01
Coefficient: 1.99e+03
N: 30
3e+06
2e+06
1e+06
0e+00
30
40
50
60
70
80
90
Age
Proteobacteria.c__Betaproteobacteria.o__Neisseriales.f__Neisse

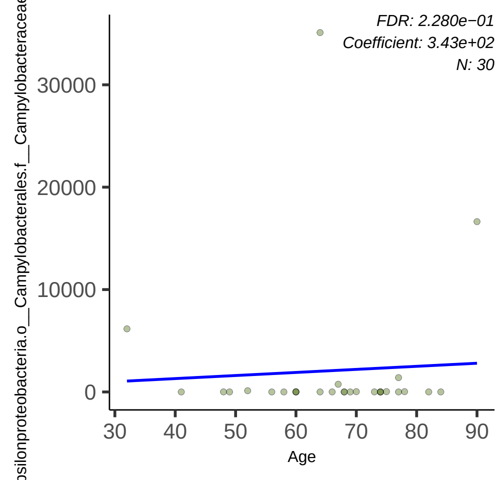

FDR: 2.280e−01
Coefficient: 3.43e+02
N: 30
30000
20000
10000
0
30
40
50
60
70
80
90
Age
psilonproteobacteria.o__Campylobacterales.f__Campylobacteraceae

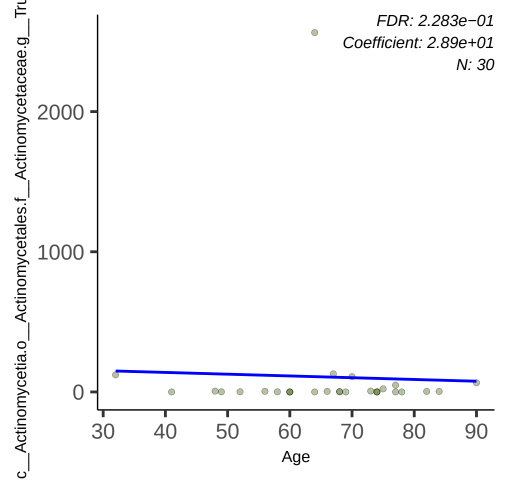

FDR: 2.283e−01
Coefficient: 2.89e+01
N: 30
2000
1000
0
30
40
50
60
70
80
90
Age
c__Actinomycetia.o__Actinomycetales.f__Actinomycetaceae.g__Tru

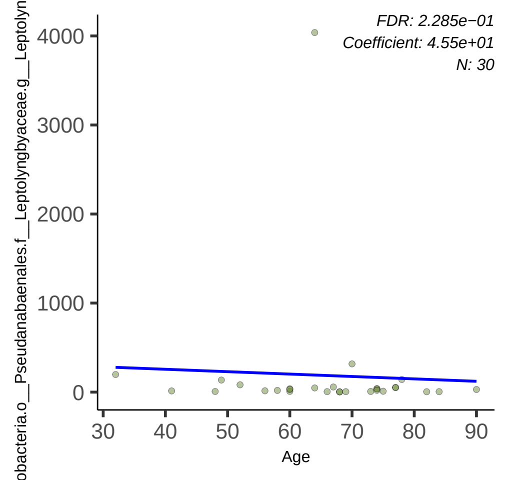
FDR: 2.285e−01
Coefficient: 4.55e+01
N: 30
4000
3000
2000
1000
0
30
40
50
60
70
80
90
Age
obacteria.o__Pseudanabaenales.f__Leptolyngbyaceae.g__Leptolyn

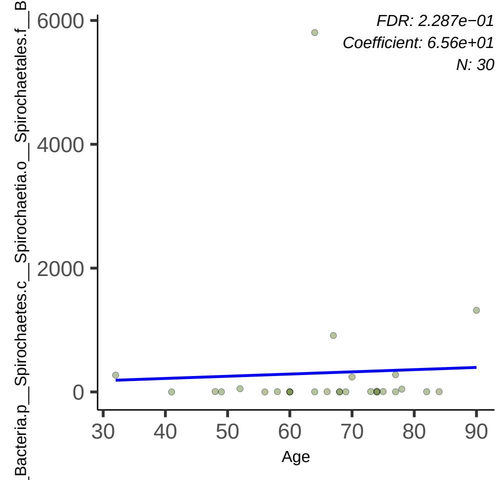

FDR: 2.287e−01
Coefficient: 6.56e+01
N: 30
6000
4000
2000
0
30
40
50
60
70
80
90
Age
_Bacteria.p__Spirochaetes.c__Spirochaetia.o__Spirochaetales.f__B

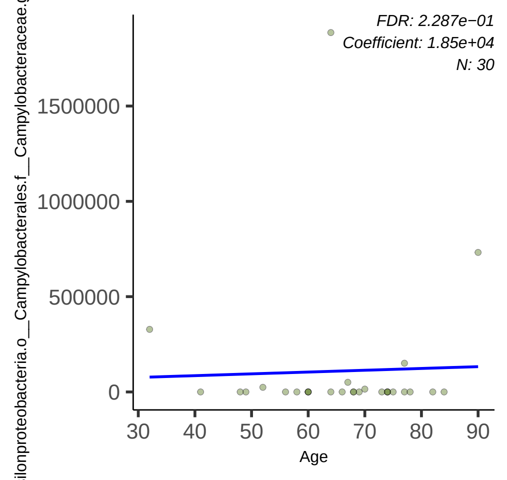

FDR: 2.287e−01
Coefficient: 1.85e+04
N: 30
1500000
1000000
500000
0
30
40
50
60
70
80
90
Age
ilonproteobacteria.o__Campylobacterales.f__Campylobacteraceae.g

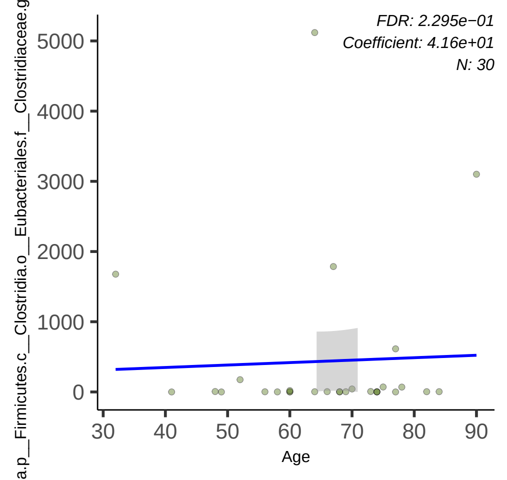
FDR: 2.295e−01
Coefficient: 4.16e+01
N: 30
5000
4000
3000
2000
1000
0
30
40
50
60
70
80
90
Age
a.p__Firmicutes.c__Clostridia.o__Eubacteriales.f__Clostridiaceae.g

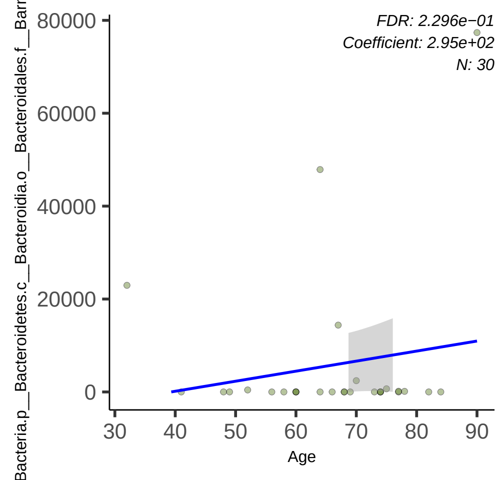
FDR: 2.296e−01
Coefficient: 2.95e+02
N: 30
80000
60000
40000
20000
0
30
40
50
60
70
80
90
Age
Bacteria.p__Bacteroidetes.c__Bacteroidia.o__Bacteroidales.f__Barr

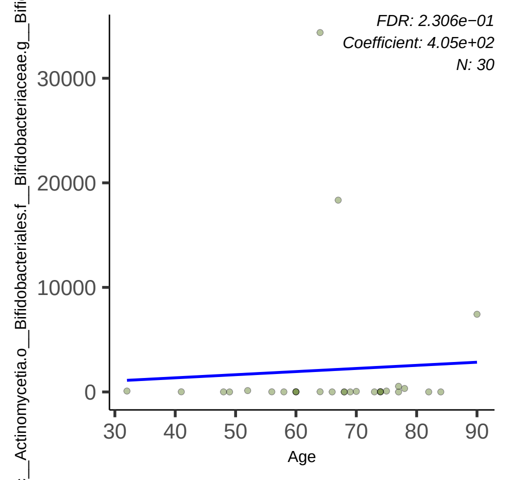

FDR: 2.306e−01
Coefficient: 4.05e+02
N: 30
Actinomycetia.o__Bifidobacteriales.f__Bifidobacteriaceae.g__Bifi
30000
20000
10000
0
30
40
50
60
70
80
90
Age

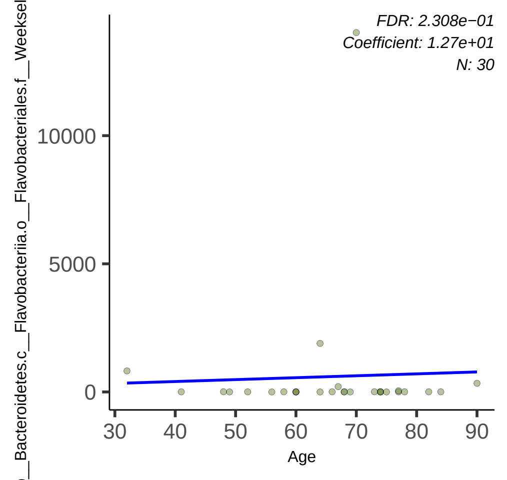
FDR: 2.308e−01
Coefficient: 1.27e+01
N: 30
10000
5000
0
30
40
50
60
70
80
90
Age
Bacteroidetes.c__Flavobacteriia.o__Flavobacteriales.f__Weeksel

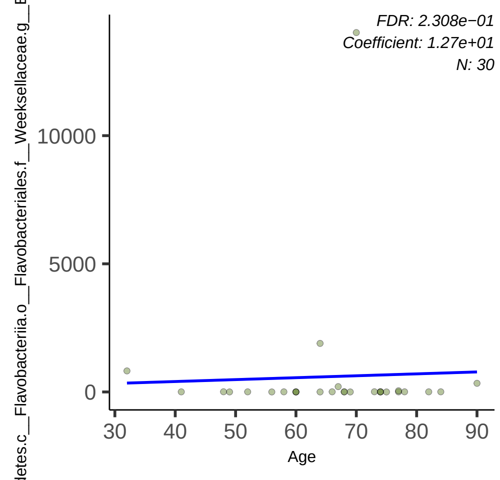

FDR: 2.308e−01
Coefficient: 1.27e+01
N: 30
10000
5000
0
30
40
50
60
70
80
90
Age
detes.c__Flavobacteriia.o__Flavobacteriales.f__Weeksellaceae.g__

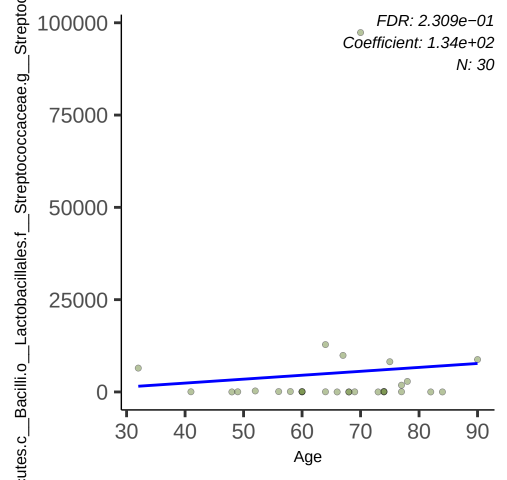
FDR: 2.309e−01
Coefficient: 1.34e+02
N: 30
100000
75000
50000
25000
0
30
40
50
60
70
80
90
Age
cutes.c__Bacilli.o__Lactobacillales.f__Streptococcaceae.g__Streptoc

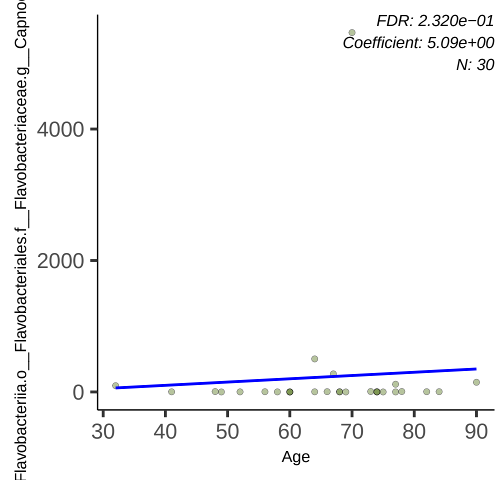
FDR: 2.320e−01
Coefficient: 5.09e+00
N: 30
Flavobacteriia.o__Flavobacteriales.f__Flavobacteriaceae.g__Capno
4000
2000
0
30
40
50
60
70
80
90
Age

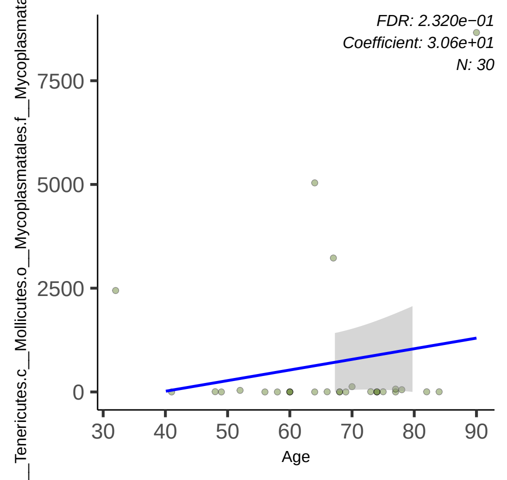
FDR: 2.320e−01
Coefficient: 3.06e+01
N: 30
7500
5000
2500
0
30
40
50
60
70
80
90
Age
__Tenericutes.c__Mollicutes.o__Mycoplasmatales.f__Mycoplasmata

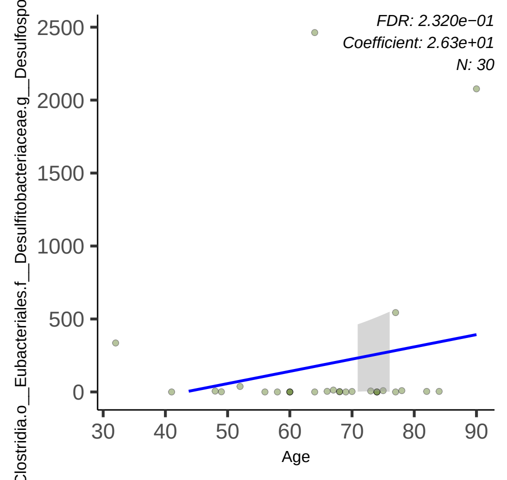
FDR: 2.320e−01
Coefficient: 2.63e+01
N: 30
Clostridia.o__Eubacteriales.f__Desulfitobacteriaceae.g__Desulfospo
Age
2500
2000
1500
1000
500
0
30
40
50
60
70
80
90

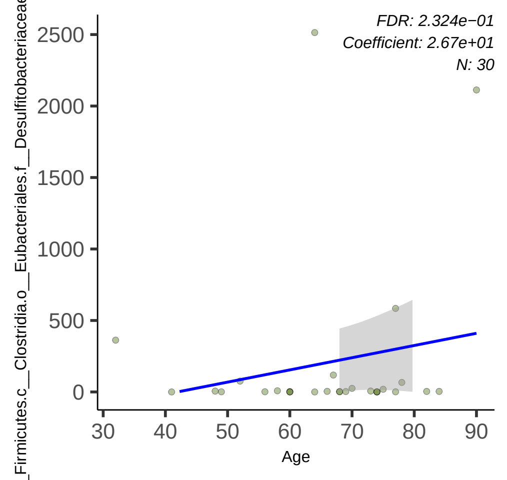
FDR: 2.324e−01
Coefficient: 2.67e+01
N: 30
Firmicutes.c__Clostridia.o__Eubacteriales.f__Desulfitobacteriacea
Age
0
500
1000
1500
2000
2500
30
40
50
60
70
80
90

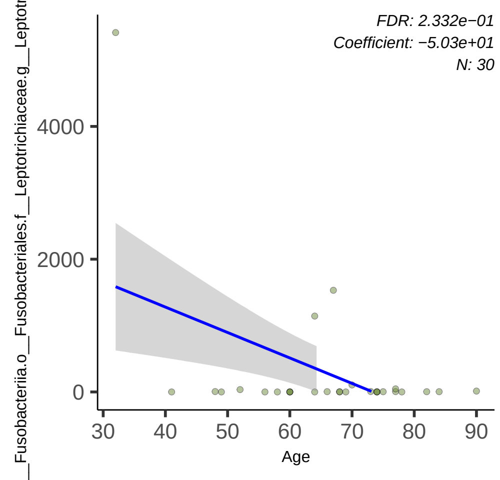

FDR: 2.332e−01
Coefficient: −5.03e+01
N: 30
4000
2000
0
30
40
50
60
70
80
90
Age
__Fusobacteriia.o__Fusobacteriales.f__Leptotrichiaceae.g__Leptotr

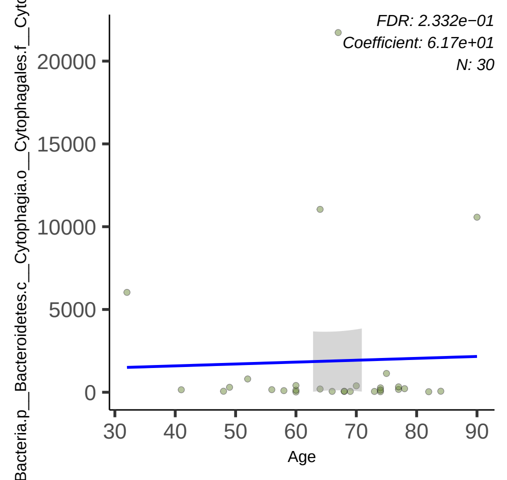

FDR: 2.332e−01
Coefficient: 6.17e+01
N: 30
Bacteria.p__Bacteroidetes.c__Cytophagia.o__Cytophagales.f__Cyto
20000
15000
10000
5000
0
30
40
50
60
70
80
90
Age

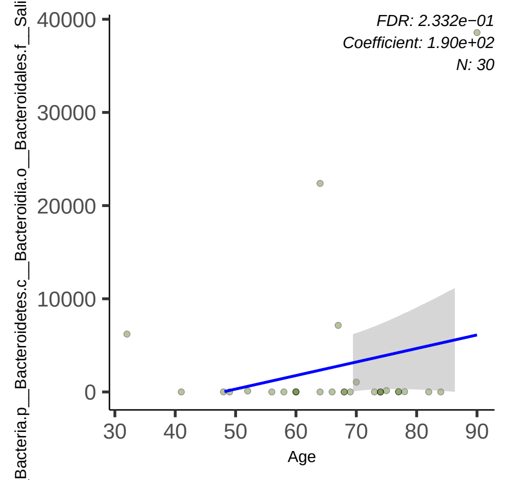
FDR: 2.332e−01
Coefficient: 1.90e+02
N: 30
40000
30000
20000
10000
0
30
40
50
60
70
80
90
Age
Bacteria.p__Bacteroidetes.c__Bacteroidia.o__Bacteroidales.f__Sali

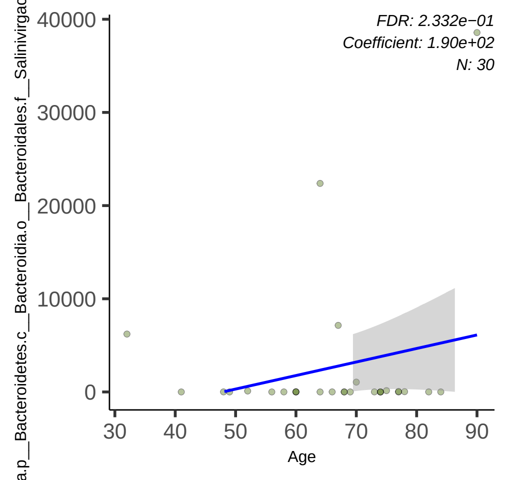
FDR: 2.332e−01
Coefficient: 1.90e+02
N: 30
40000
30000
20000
10000
0
30
40
50
60
70
80
90
Age
a.p__Bacteroidetes.c__Bacteroidia.o__Bacteroidales.f__Salinivirgac

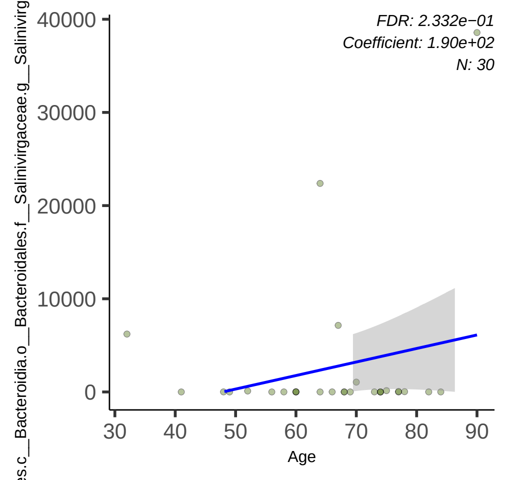

es.c__Bacteroidia.o__Bacteroidales.f__Salinivirgaceae.g__Salinivirg
FDR: 2.332e−01
Coefficient: 1.90e+02
N: 30
40000
30000
20000
10000
0
30
40
50
60
70
80
90
Age

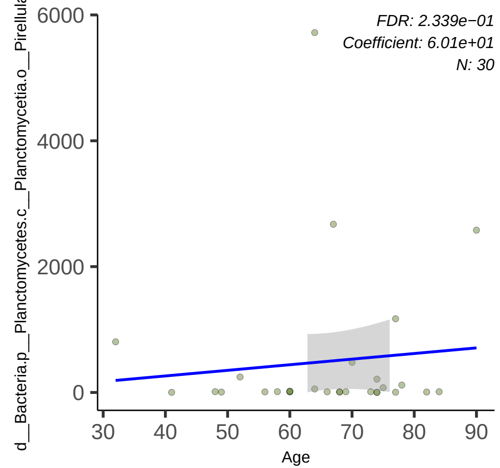
FDR: 2.339e−01
Coefficient: 6.01e+01
N: 30
d__Bacteria.p__Planctomycetes.c__Planctomycetia.o__Pirellula
6000
4000
2000
0
30
40
50
60
70
80
90
Age

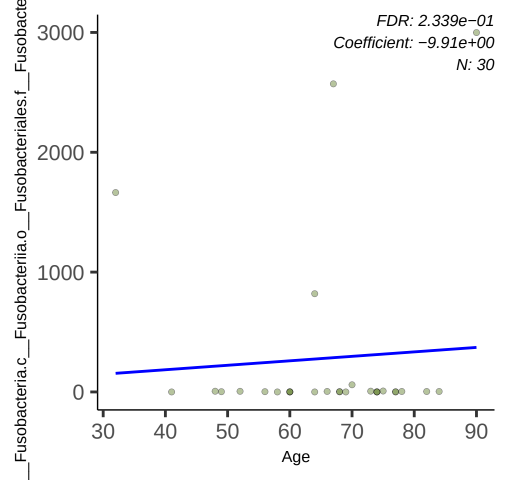
FDR: 2.339e−01
Coefficient: −9.91e+00
N: 30
_Fusobacteria.c__Fusobacteriia.o__Fusobacteriales.f__Fusobacte
3000
2000
1000
0
30
40
50
60
70
80
90
Age

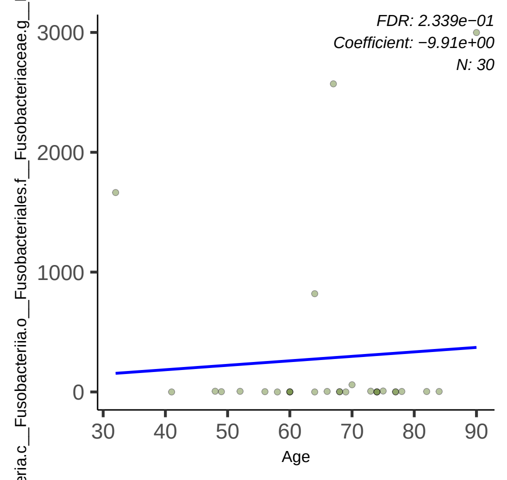

FDR: 2.339e−01
Coefficient: −9.91e+00
N: 30
3000
2000
1000
0
30
40
50
60
70
80
90
Age
eria.c__Fusobacteriia.o__Fusobacteriales.f__Fusobacteriaceae.g__

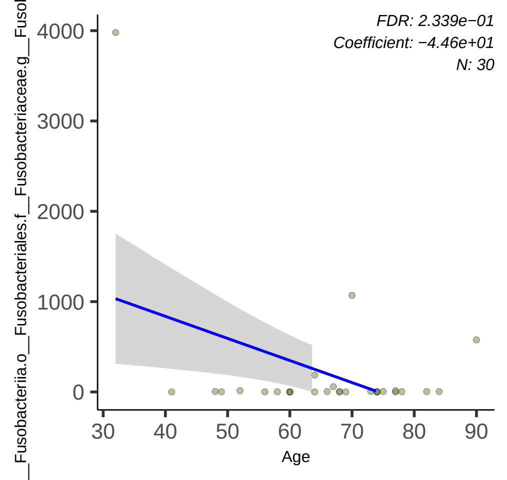

FDR: 2.339e−01
Coefficient: −4.46e+01
N: 30
Fusobacteriia.o__Fusobacteriales.f__Fusobacteriaceae.g__Fusob
4000
3000
2000
1000
0
30
40
50
60
70
80
90
Age

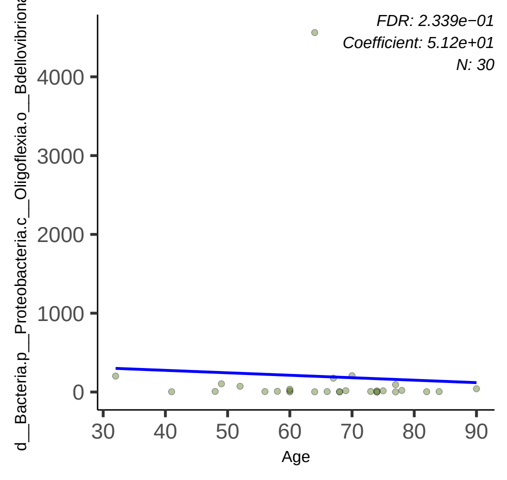

FDR: 2.339e−01
Coefficient: 5.12e+01
N: 30
d__Bacteria.p__Proteobacteria.c__Oligoflexia.o__Bdellovibriona
0
1000
2000
3000
4000
30
40
50
60
70
80
90
Age

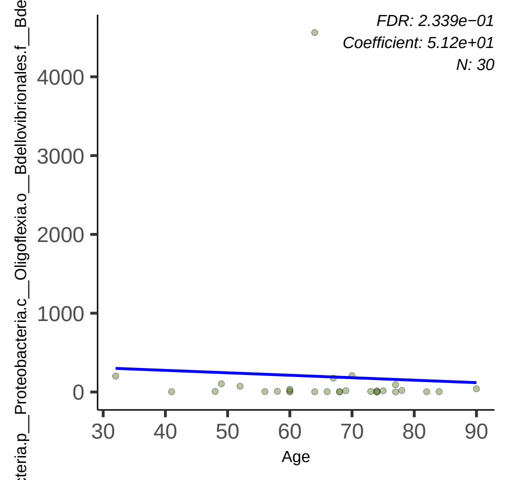
FDR: 2.339e−01
Coefficient: 5.12e+01
N: 30
4000
3000
2000
1000
0
30
40
50
60
70
80
90
Age
cteria.p__Proteobacteria.c__Oligoflexia.o__Bdellovibrionales.f__Bde

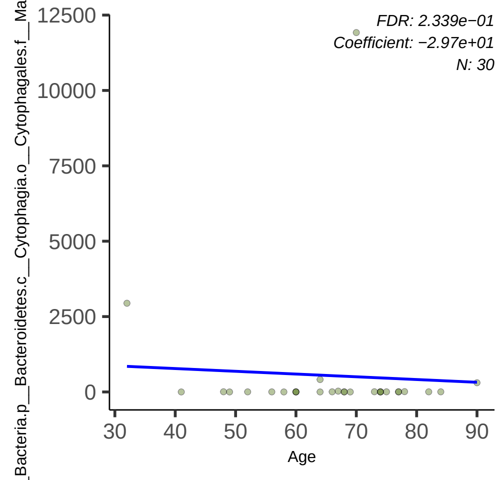
FDR: 2.339e−01
Coefficient: −2.97e+01
N: 30
Bacteria.p__Bacteroidetes.c__Cytophagia.o__Cytophagales.f__Ma
Age
0
2500
5000
7500
10000
12500
30
40
50
60
70
80
90

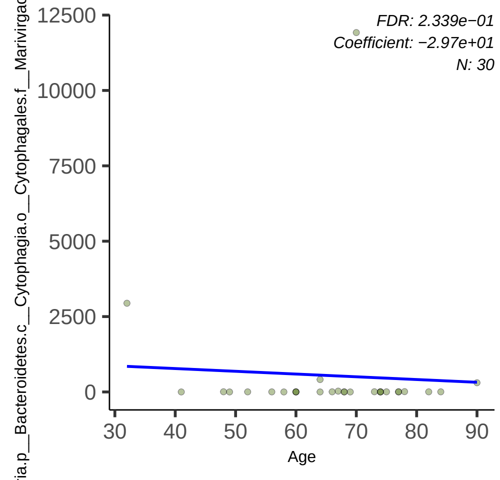

FDR: 2.339e−01
Coefficient: −2.97e+01
N: 30
ia.p__Bacteroidetes.c__Cytophagia.o__Cytophagales.f__Marivirgac
12500
10000
7500
5000
2500
0
30
40
50
60
70
80
90
Age

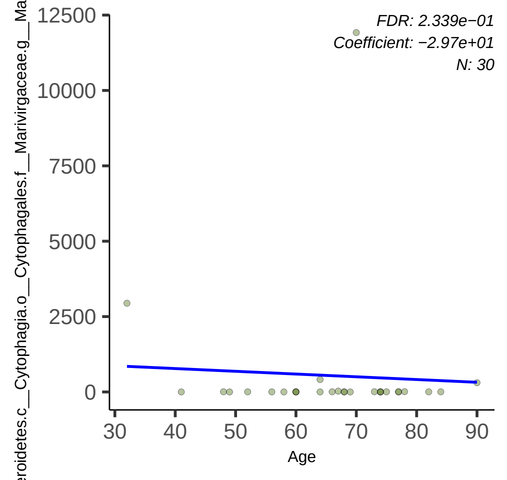

FDR: 2.339e−01
Coefficient: −2.97e+01
N: 30
eroidetes.c__Cytophagia.o__Cytophagales.f__Marivirgaceae.g__Ma
12500
10000
7500
5000
2500
0
30
40
50
60
70
80
90
Age

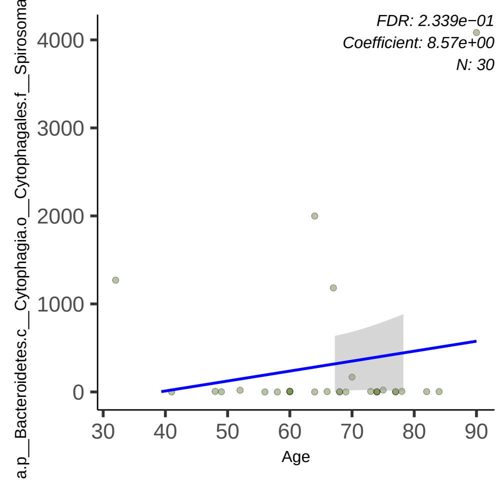

FDR: 2.339e−01
Coefficient: 8.57e+00
N: 30
a.p__Bacteroidetes.c__Cytophagia.o__Cytophagales.f__Spirosoma
0
1000
2000
3000
4000
30
40
50
60
70
80
90
Age

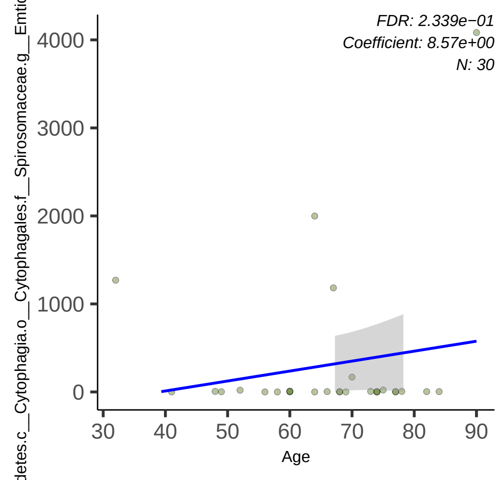
FDR: 2.339e−01
Coefficient: 8.57e+00
N: 30
0
1000
2000
3000
4000
30
40
50
60
70
80
90
Age
detes.c__Cytophagia.o__Cytophagales.f__Spirosomaceae.g__Emtic

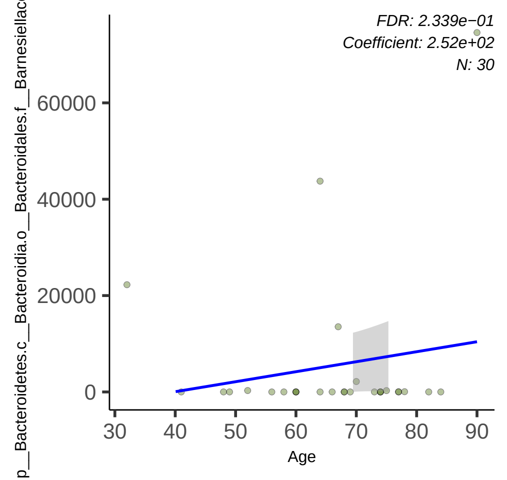

FDR: 2.339e−01
Coefficient: 2.52e+02
N: 30
p__Bacteroidetes.c__Bacteroidia.o__Bacteroidales.f__Barnesiellac
60000
40000
20000
0
30
40
50
60
70
80
90
Age

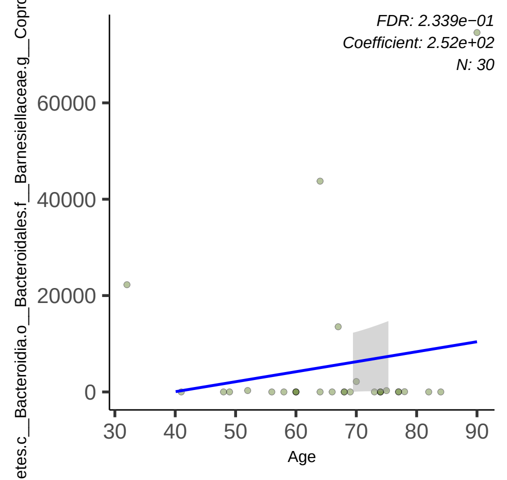
FDR: 2.339e−01
Coefficient: 2.52e+02
N: 30
60000
40000
20000
0
30
40
50
60
70
80
90
Age
etes.c__Bacteroidia.o__Bacteroidales.f__Barnesiellaceae.g__Copro

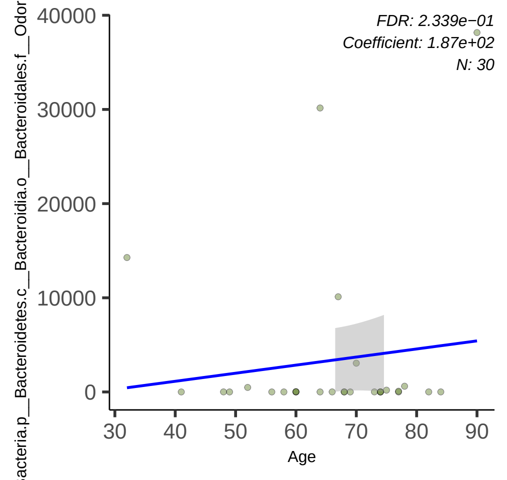
FDR: 2.339e−01
Coefficient: 1.87e+02
N: 30
Age
Bacteria.p__Bacteroidetes.c__Bacteroidia.o__Bacteroidales.f__Odor
0
10000
20000
30000
40000
30
40
50
60
70
80
90

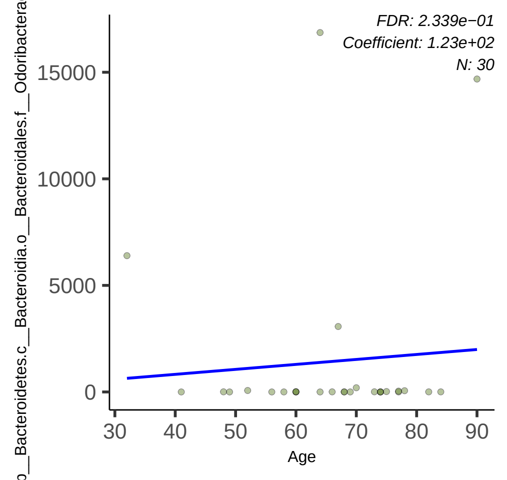
FDR: 2.339e−01
Coefficient: 1.23e+02
N: 30
o__Bacteroidetes.c__Bacteroidia.o__Bacteroidales.f__Odoribactera
15000
10000
5000
0
30
40
50
60
70
80
90
Age

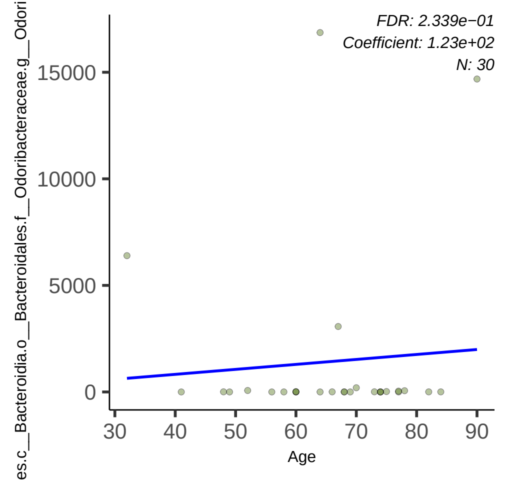

FDR: 2.339e−01
Coefficient: 1.23e+02
N: 30
es.c__Bacteroidia.o__Bacteroidales.f__Odoribacteraceae.g__Odori
15000
10000
5000
0
30
40
50
60
70
80
90
Age

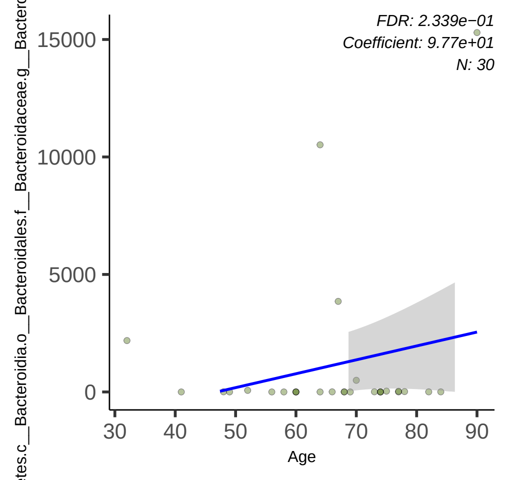
FDR: 2.339e−01
Coefficient: 9.77e+01
N: 30
15000
10000
5000
0
30
40
50
60
70
80
90
Age
etes.c__Bacteroidia.o__Bacteroidales.f__Bacteroidaceae.g__Bacter

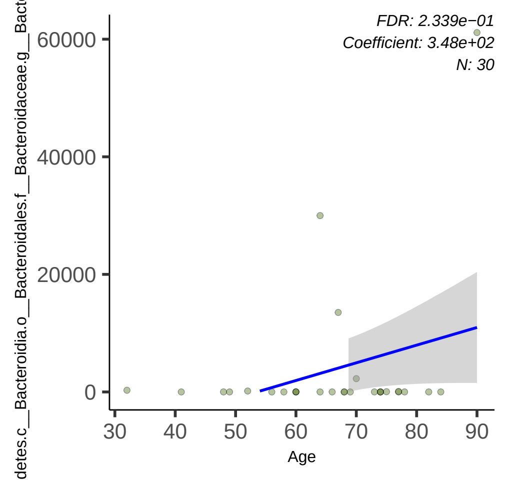

FDR: 2.339e−01
Coefficient: 3.48e+02
N: 30
60000
40000
20000
0
30
40
50
60
70
80
90
Age
detes.c__Bacteroidia.o__Bacteroidales.f__Bacteroidaceae.g__Bact

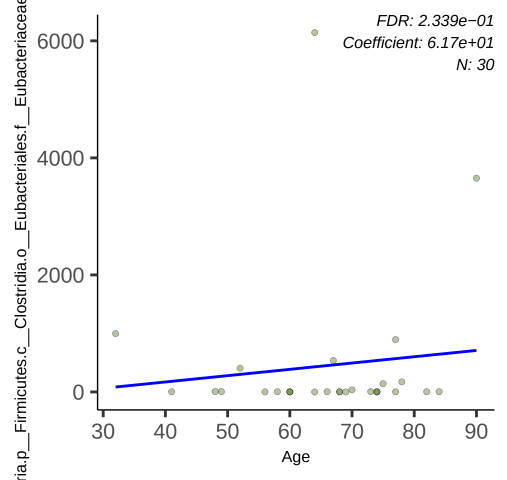

FDR: 2.339e−01
Coefficient: 6.17e+01
N: 30
ria.p__Firmicutes.c__Clostridia.o__Eubacteriales.f__Eubacteriaceae
Age

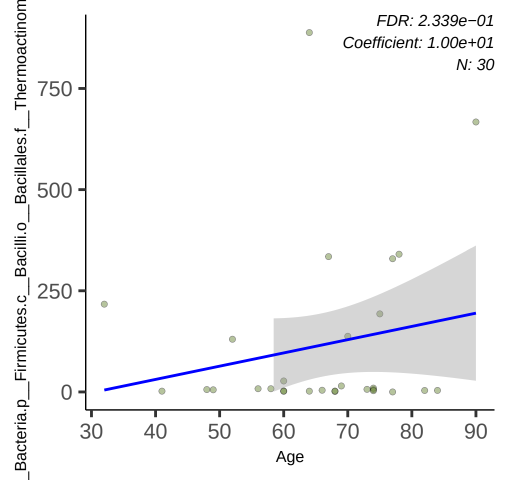

FDR: 2.339e−01
Coefficient: 1.00e+01
N: 30
_Bacteria.p__Firmicutes.c__Bacilli.o__Bacillales.f__Thermoactinom
750
500
250
0
30
40
50
60
70
80
90
Age

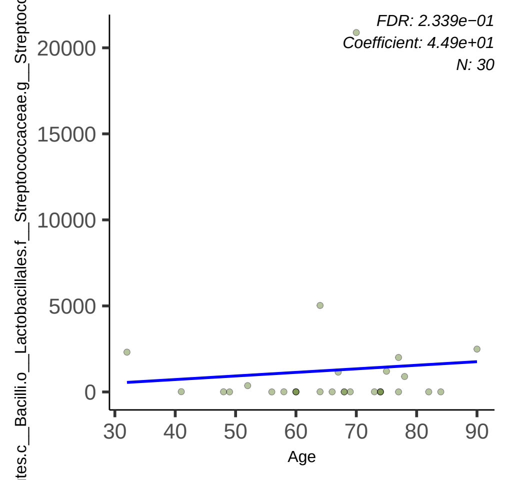

FDR: 2.339e−01
Coefficient: 4.49e+01
N: 30
0
5000
10000
15000
20000
30
40
50
60
70
80
90
Age

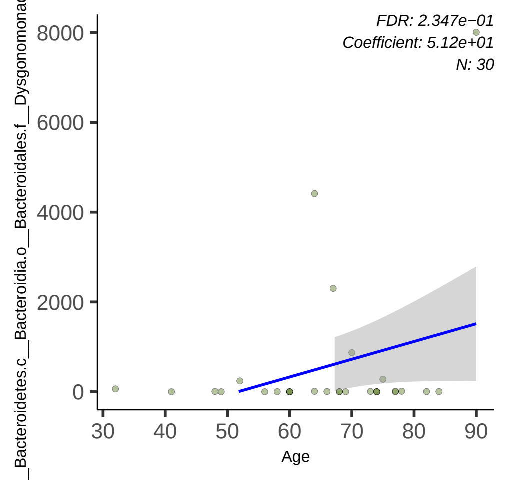

FDR: 2.347e−01
Coefficient: 5.12e+01
N: 30
Bacteroidetes.c__Bacteroidia.o__Bacteroidales.f__Dysgonomonac
8000
6000
4000
2000
0
30
40
50
60
70
80
90
Age

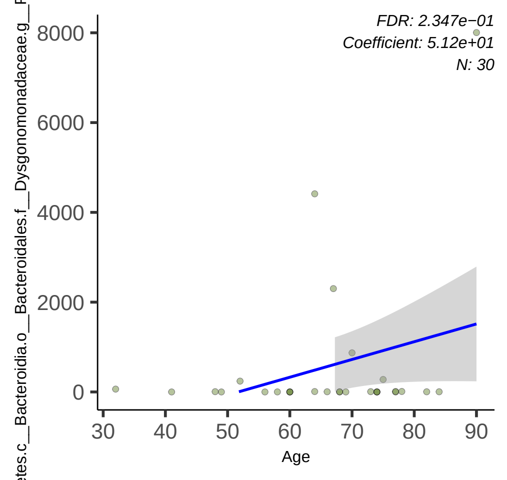

FDR: 2.347e−01
Coefficient: 5.12e+01
N: 30
etes.c__Bacteroidia.o__Bacteroidales.f__Dysgonomonadaceae.g__
8000
6000
4000
2000
0
30
40
50
60
70
80
90
Age

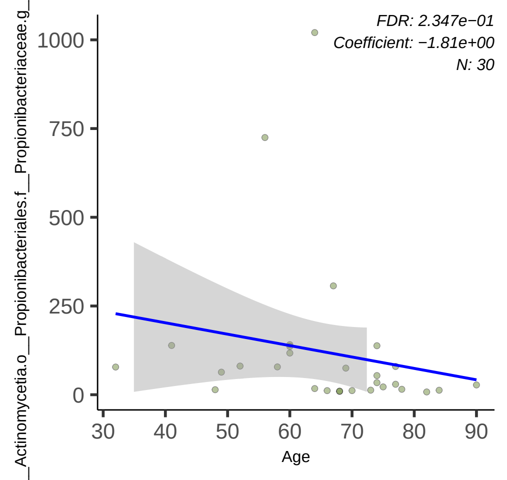

FDR: 2.347e−01
Coefficient: −1.81e+00
N: 30
Actinomycetia.o__Propionibacteriales.f__Propionibacteriaceae.g__
1000
750
500
250
0
30
40
50
60
70
80
90
Age

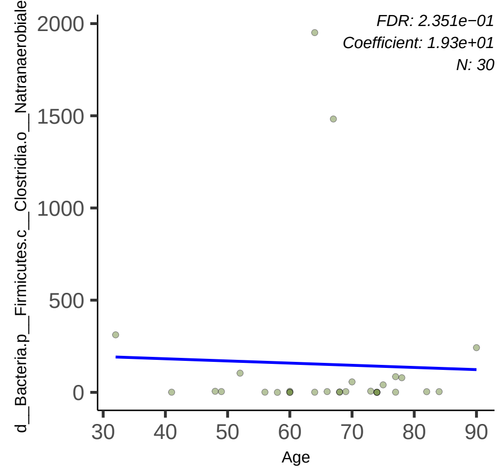

FDR: 2.351e−01
Coefficient: 1.93e+01
N: 30
d__Bacteria.p__Firmicutes.c__Clostridia.o__Natranaerobiale
2000
1500
1000
500
0
30
40
50
60
70
80
90
Age

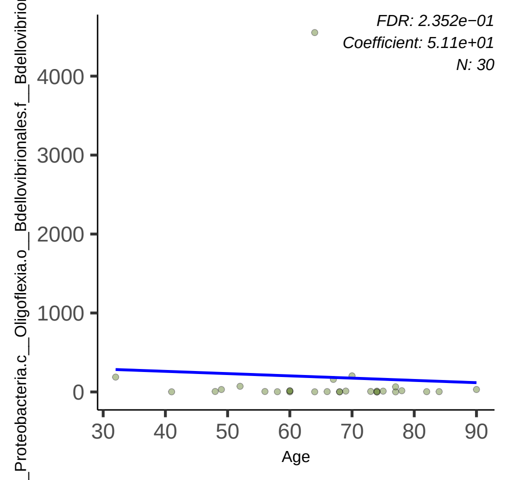
FDR: 2.352e−01
Coefficient: 5.11e+01
N: 30
Proteobacteria.c__Oligoflexia.o__Bdellovibrionales.f__Bdellovibrio
Age

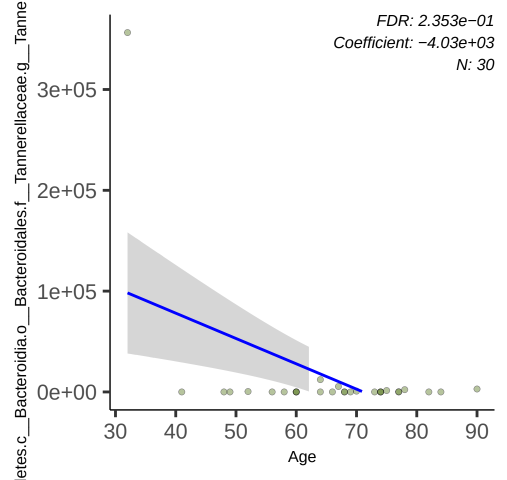

FDR: 2.353e−01
Coefficient: −4.03e+03
N: 30
3e+05
2e+05
1e+05
0e+00
30
40
50
60
70
80
90
Age
letes.c__Bacteroidia.o__Bacteroidales.f__Tannerellaceae.g__Tanne

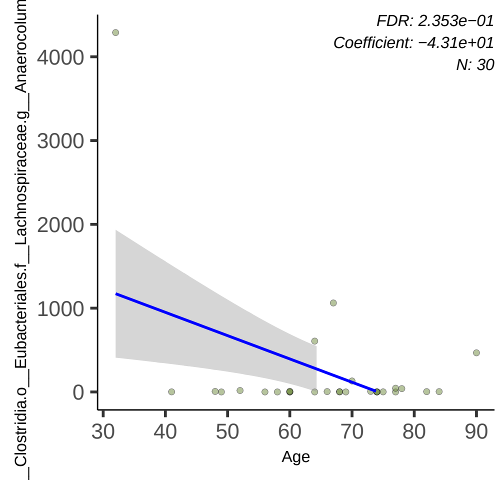
FDR: 2.353e−01
Coefficient: −4.31e+01
N: 30
Clostridia.o__Eubacteriales.f__Lachnospiraceae.g__Anaerocolum
0
1000
2000
3000
4000
30
40
50
60
70
80
90
Age

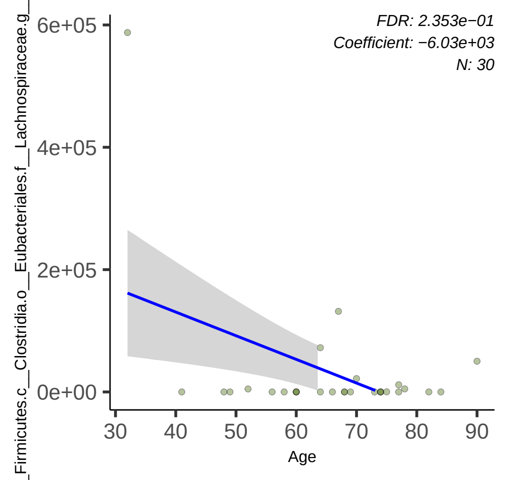

FDR: 2.353e−01
Coefficient: −6.03e+03
N: 30
Firmicutes.c__Clostridia.o__Eubacteriales.f__Lachnospiraceae.g__
6e+05
4e+05
2e+05
0e+00
30
40
50
60
70
80
90
Age

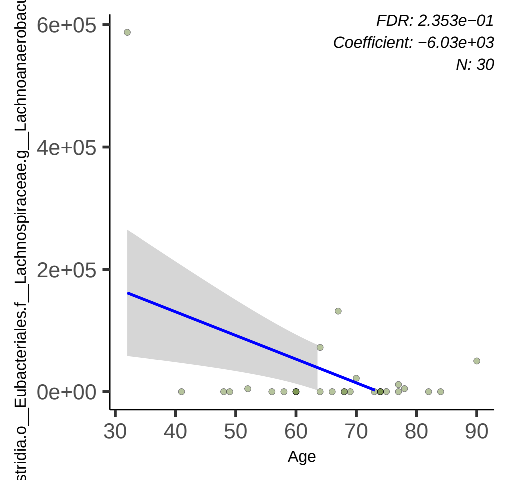
FDR: 2.353e−01
Coefficient: −6.03e+03
N: 30
6e+05
4e+05
2e+05
0e+00
30
40
50
60
70
80
90
Age
stridia.o__Eubacteriales.f__Lachnospiraceae.g__Lachnoanaerobacu

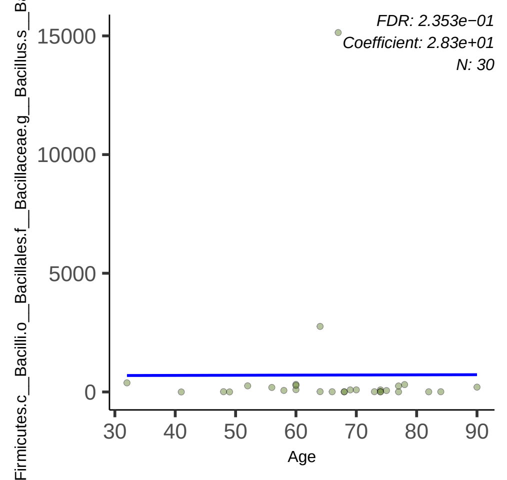

FDR: 2.353e−01
Coefficient: 2.83e+01
N: 30
15000
10000
5000
0
30
40
50
60
70
80
90
Age
Firmicutes.c__Bacilli.o__Bacillales.f__Bacillaceae.g__Bacillus.s__B

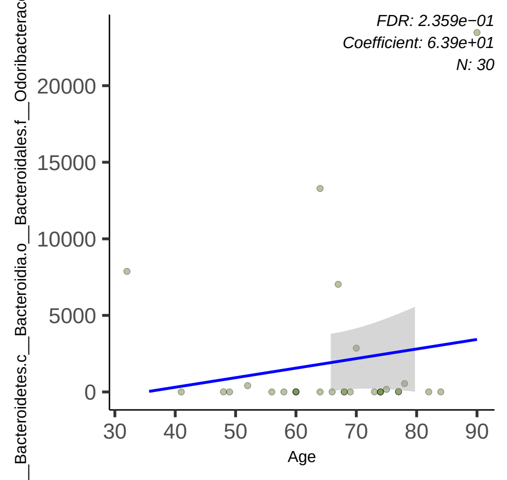

FDR: 2.359e−01
Coefficient: 6.39e+01
N: 30
20000
15000
10000
5000
0
30
40
50
60
70
80
90
Age
Bacteroidetes.c__Bacteroidia.o__Bacteroidales.f__Odoribacterac

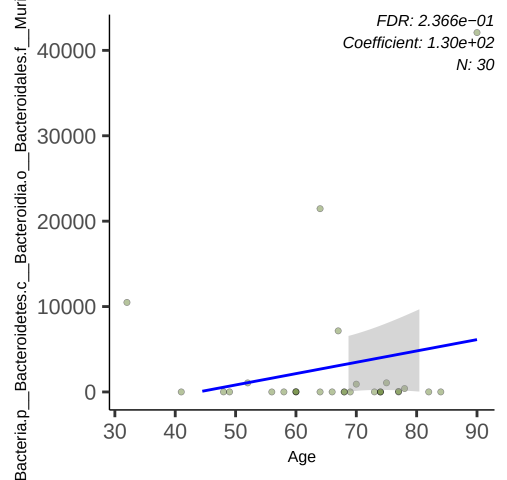

FDR: 2.366e−01
Coefficient: 1.30e+02
N: 30
40000
30000
20000
10000
0
30
40
50
60
70
80
90
Age
Bacteria.p__Bacteroidetes.c__Bacteroidia.o__Bacteroidales.f__Muri

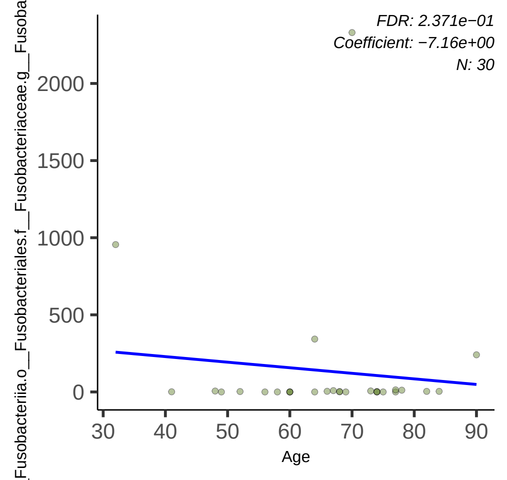

FDR: 2.371e−01
Coefficient: −7.16e+00
N: 30
Fusobacteriia.o__Fusobacteriales.f__Fusobacteriaceae.g__Fusoba
0
500
1000
1500
2000
30
40
50
60
70
80
90
Age

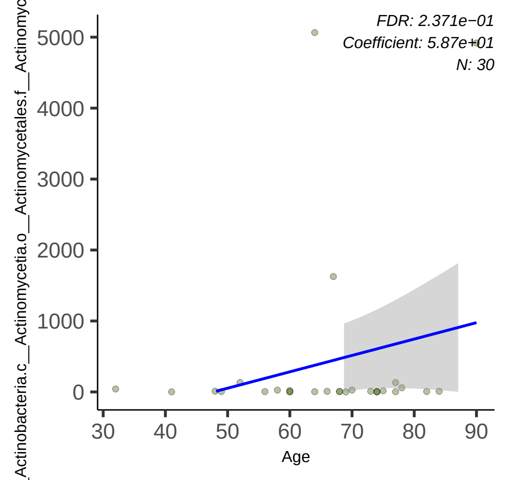

FDR: 2.371e−01
Coefficient: 5.87e+01
N: 30
5000
4000
3000
2000
1000
0
30
40
50
60
70
80
90
Age
Actinobacteria.c__Actinomycetia.o__Actinomycetales.f__Actinomyc

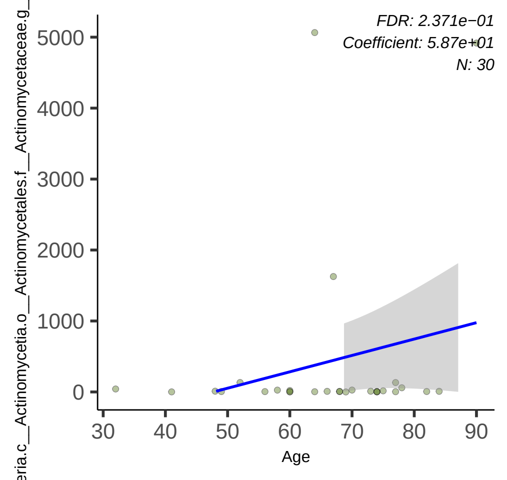

FDR: 2.371e−01
Coefficient: 5.87e+01
N: 30
eria.c__Actinomycetia.o__Actinomycetales.f__Actinomycetaceae.g__
5000
4000
3000
2000
1000
0
30
40
50
60
70
80
90
Age

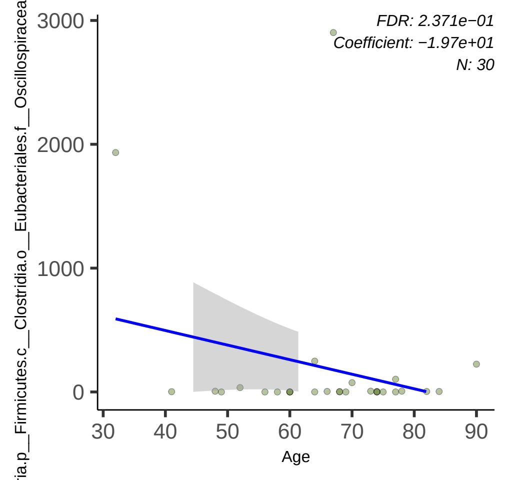

FDR: 2.371e−01
Coefficient: −1.97e+01
N: 30
0
1000
2000
3000
30
40
50
60
70
80
90
Age
ria.p__Firmicutes.c__Clostridia.o__Eubacteriales.f__Oscillospiracea

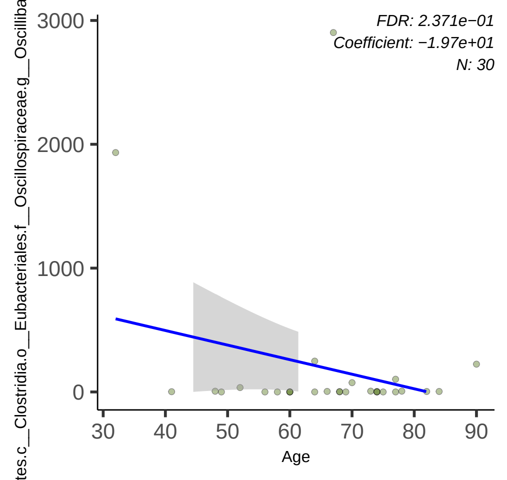

FDR: 2.371e−01
Coefficient: −1.97e+01
N: 30
3000
2000
1000
0
30
40
50
60
70
80
90
Age
tes.c__Clostridia.o__Eubacteriales.f__Oscillospiraceae.g__Oscilliba

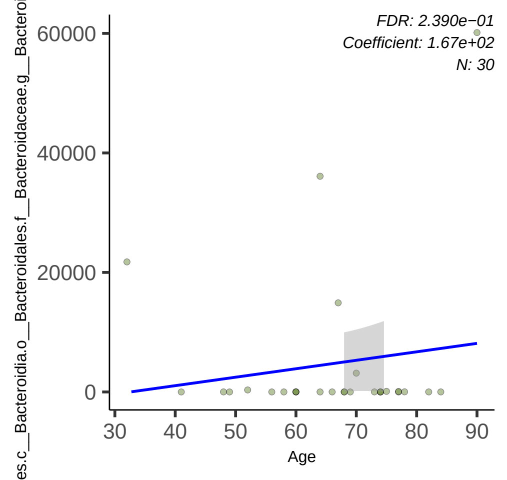
FDR: 2.390e−01
Coefficient: 1.67e+02
N: 30
60000
40000
20000
0
30
40
50
60
70
80
90
Age
es.c__Bacteroidia.o__Bacteroidales.f__Bacteroidaceae.g__Bacteroi

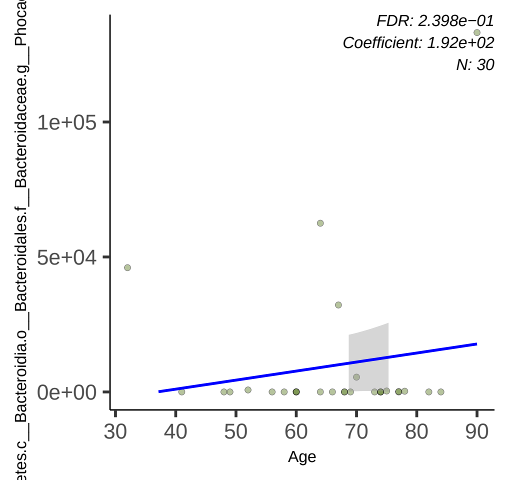
FDR: 2.398e−01
Coefficient: 1.92e+02
N: 30
1e+05
5e+04
0e+00
30
40
50
60
70
80
90
Age
etes.c__Bacteroidia.o__Bacteroidales.f__Bacteroidaceae.g__Phoca

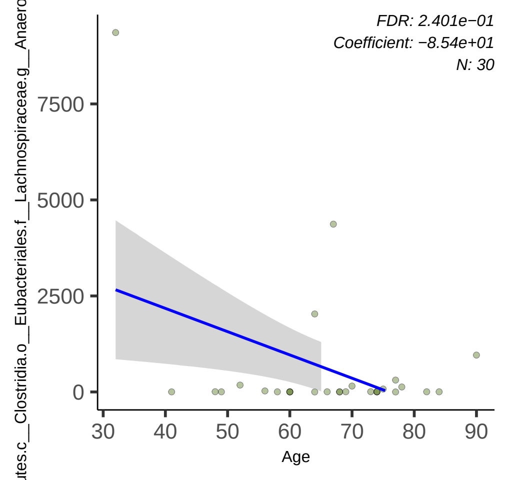
FDR: 2.401e−01
Coefficient: −8.54e+01
N: 30
utes.c__Clostridia.o__Eubacteriales.f__Lachnospiraceae.g__Anaero
7500
5000
2500
0
30
40
50
60
70
80
90
Age

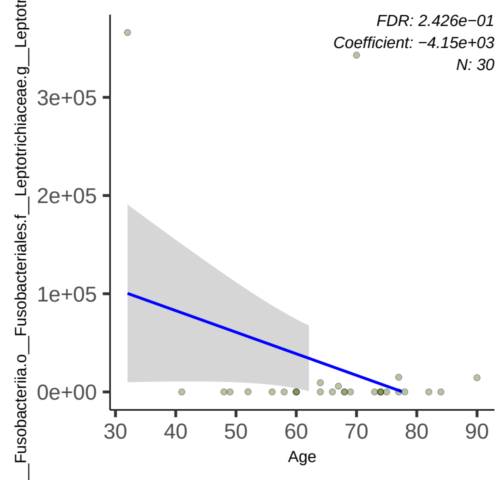
FDR: 2.426e−01
Coefficient: −4.15e+03
N: 30
3e+05
2e+05
1e+05
0e+00
30
40
50
60
70
80
90
Age
Fusobacteriia.o__Fusobacteriales.f__Leptotrichiaceae.g__Leptotr

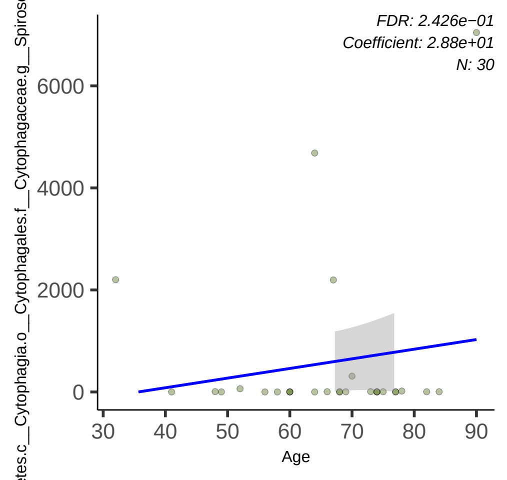

FDR: 2.426e−01
Coefficient: 2.88e+01
N: 30
6000
4000
2000
0
30
40
50
60
70
80
90
Age
etes.c__Cytophagia.o__Cytophagales.f__Cytophagaceae.g__Spiros

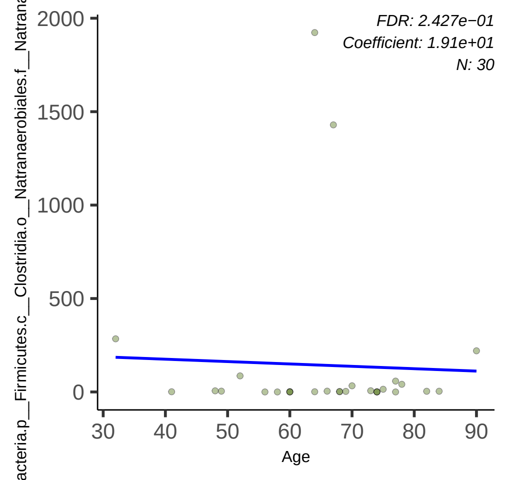
FDR: 2.427e−01
Coefficient: 1.91e+01
N: 30
2000
1500
1000
500
0
30
40
50
60
70
80
90
Age
acteria.p__Firmicutes.c__Clostridia.o__Natranaerobiales.f__Natrana

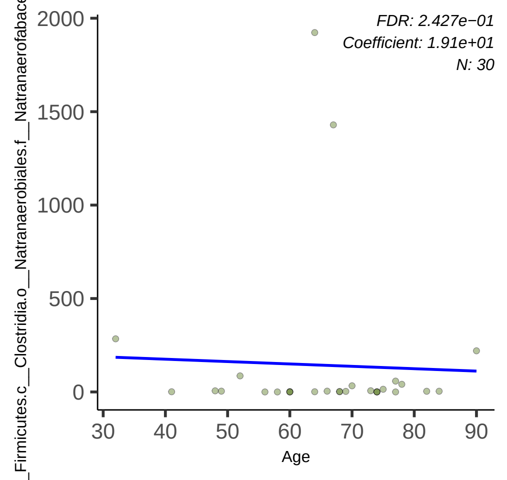

FDR: 2.427e−01
Coefficient: 1.91e+01
N: 30
0
500
1000
1500
2000
30
40
50
60
70
80
90
Age
Firmicutes.c__Clostridia.o__Natranaerobiales.f__Natranaerofabace

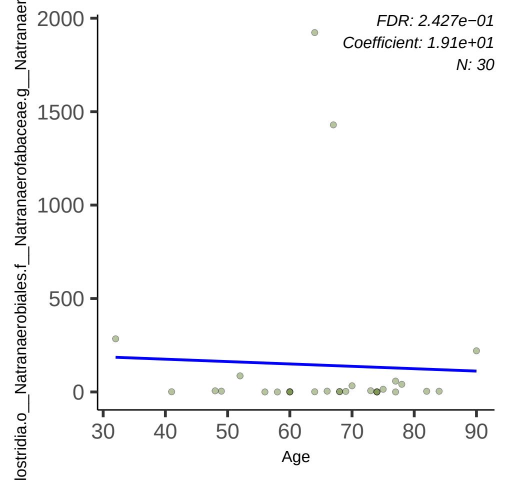

FDR: 2.427e−01
Coefficient: 1.91e+01
N: 30
lostridia.o__Natranaerobiales.f__Natranaerofabaceae.g__Natranaer
2000
1500
1000
500
0
30
40
50
60
70
80
90
Age

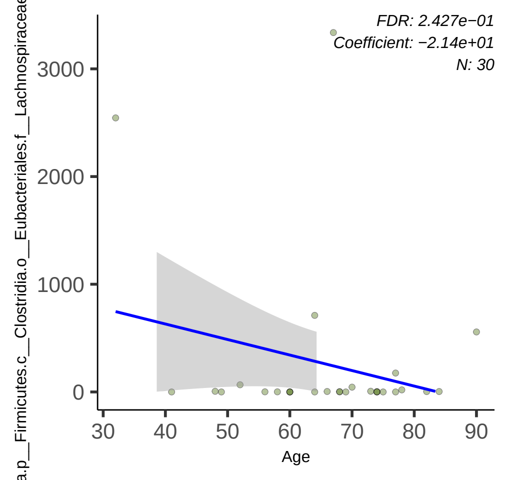

FDR: 2.427e−01
Coefficient: −2.14e+01
N: 30
3000
2000
1000
0
30
40
50
60
70
80
90
Age
a.p__Firmicutes.c__Clostridia.o__Eubacteriales.f__Lachnospiraceae

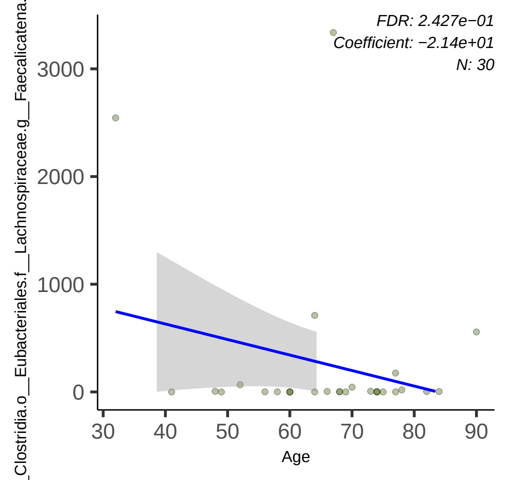

FDR: 2.427e−01
Coefficient: −2.14e+01
N: 30
3000
2000
1000
0
30
40
50
60
70
80
90
Age
_Clostridia.o__Eubacteriales.f__Lachnospiraceae.g__Faecalicatena.

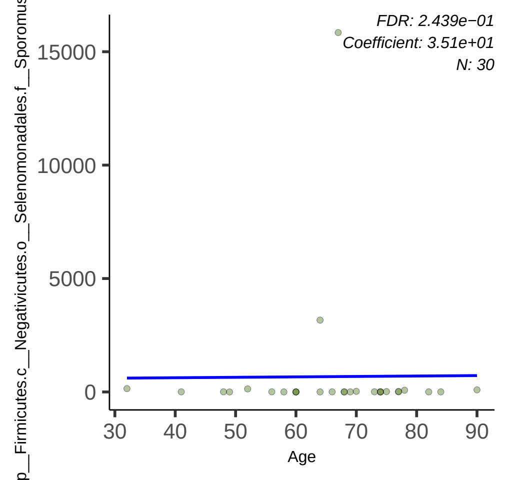
FDR: 2.439e−01
Coefficient: 3.51e+01
N: 30
p__Firmicutes.c__Negativicutes.o__Selenomonadales.f__Sporomus
15000
10000
5000
0
30
40
50
60
70
80
90
Age

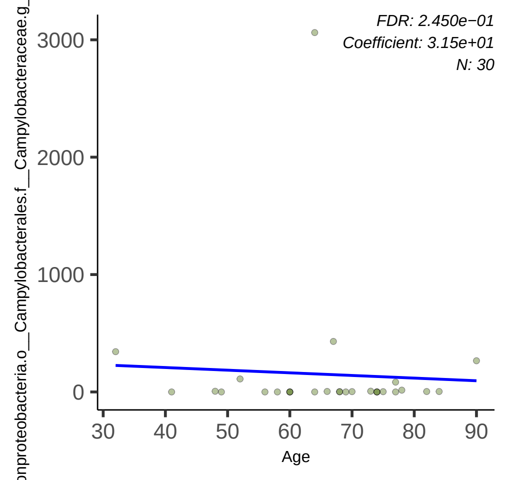
FDR: 2.450e−01
Coefficient: 3.15e+01
N: 30
3000
2000
1000
0
30
40
50
60
70
80
90
Age
onproteobacteria.o__Campylobacterales.f__Campylobacteraceae.g_

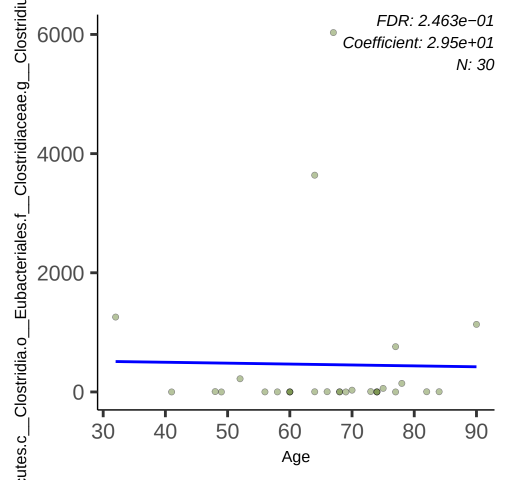

FDR: 2.463e−01
Coefficient: 2.95e+01
N: 30
6000
4000
2000
0
30
40
50
60
70
80
90
Age
cutes.c__Clostridia.o__Eubacteriales.f__Clostridiaceae.g__Clostridiu

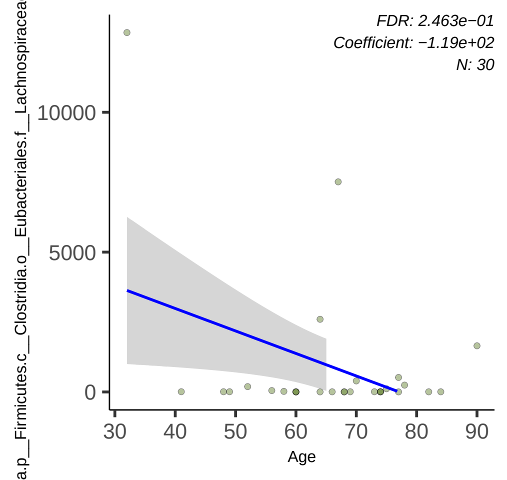

FDR: 2.463e−01
Coefficient: −1.19e+02
N: 30
a.p__Firmicutes.c__Clostridia.o__Eubacteriales.f__Lachnospiraceae
10000
5000
0
30
40
50
60
70
80
90
Age

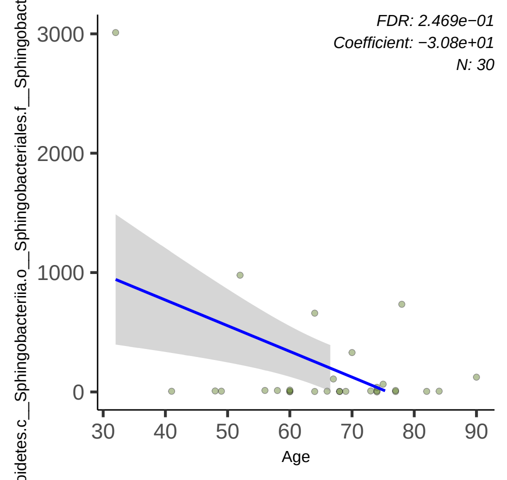
FDR: 2.469e−01
Coefficient: −3.08e+01
N: 30
3000
2000
1000
0
30
40
50
60
70
80
90
Age
oidetes.c__Sphingobacteriia.o__Sphingobacteriales.f__Sphingobact

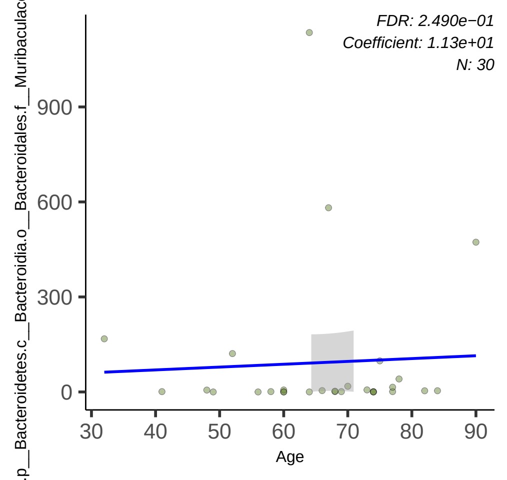

FDR: 2.490e−01
Coefficient: 1.13e+01
N: 30
900
600
300
0
30
40
50
60
70
80
90
Age
.p__Bacteroidetes.c__Bacteroidia.o__Bacteroidales.f__Muribaculac

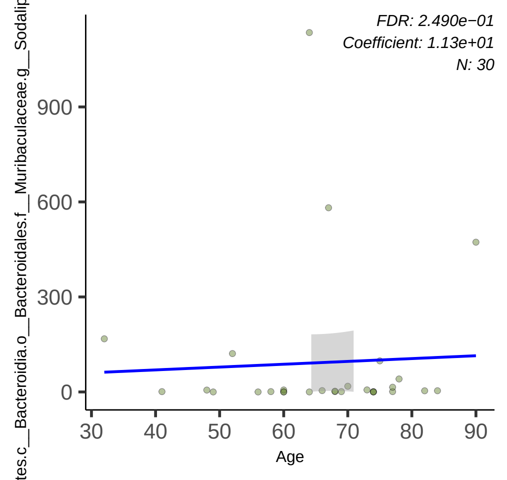

FDR: 2.490e−01
Coefficient: 1.13e+01
N: 30
tes.c__Bacteroidia.o__Bacteroidales.f__Muribaculaceae.g__Sodalip
0
300
600
900
30
40
50
60
70
80
90
Age

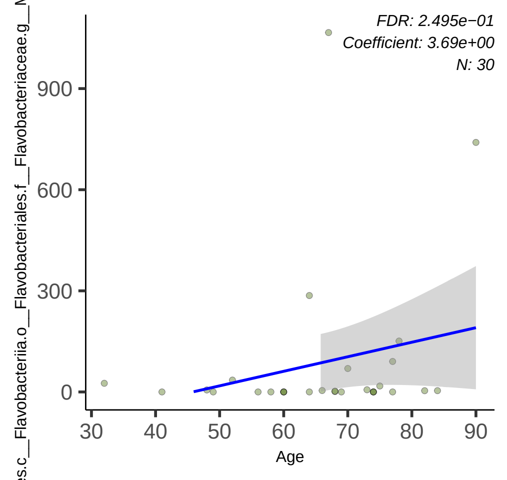

FDR: 2.495e−01
Coefficient: 3.69e+00
N: 30
900
600
300
0
30
40
50
60
70
80
90
Age
Flavobacteriia.o__Flavobacteriales.f__Flavobacteriaceae.g__

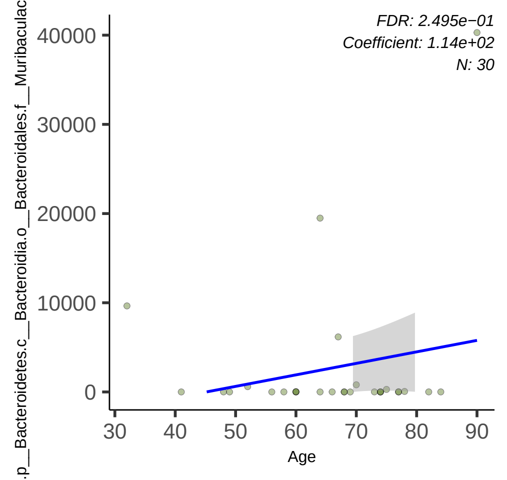
FDR: 2.495e−01
Coefficient: 1.14e+02
N: 30
40000
30000
20000
10000
0
30
40
50
60
70
80
90
Age
