# Supplementary material for: Metatranscriptome Analysis of Nasopharyngeal Swabs across the Varying Severity of COVID-19 Disease Demonstrated Unprecedented Species Diversity
Source: Microorganisms. 2023 Jul 14;11(7):1804. doi: 10.3390/microorganisms11071804 (PMC10384460; doi:10.3390/microorganisms11071804)
Supplement: Supplementary file 1 [file microorganisms-11-01804-s001.zip › Maaslin_results_ONLYsevere.zip!/Maaslin_results_ONLYsevere/Age_category.pdf]

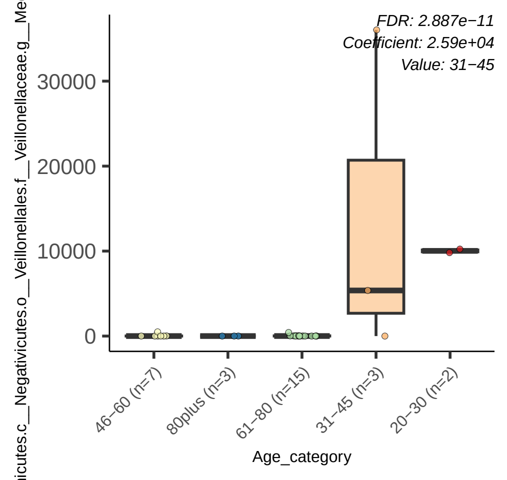
FDR: 2.887e−11
Coefficient: 2.59e+04
Value: 31−45
30000
20000
10000
0
46−60 (n=7)
80plus (n=3)
61−80 (n=15)
31−45 (n=3)
20−30 (n=2)
Age_category
icutes.c__Negativicutes.o__Veillonellales.f__Veillonellaceae.g__Me

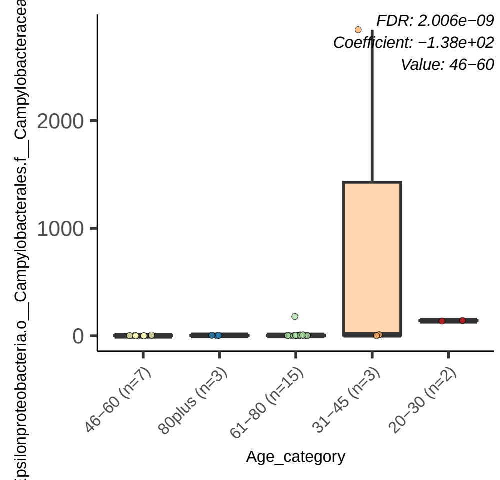

FDR: 2.006e−09
Coefficient: −1.38e+02
Value: 46−60
2000
1000
0
46−60 (n=7)
80plus (n=3)
61−80 (n=15)
31−45 (n=3)
20−30 (n=2)
Age_category
psilonproteobacteria.o__Campylobacterales.f__Campylobacterace

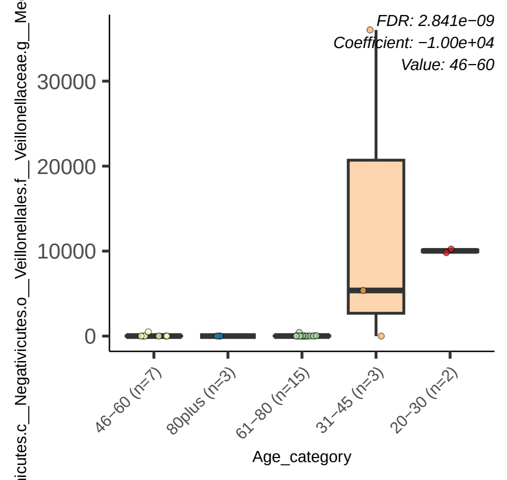

FDR: 2.841e−09
Coefficient: −1.00e+04
Value: 46−60
30000
20000
10000
0
icutes.c__Negativicutes.o__Veillonellales.f__Veillonellaceae.g__Me
46−60 (n=7)
80plus (n=3)
61−80 (n=15)
31−45 (n=3)
20−30 (n=2)
Age_category

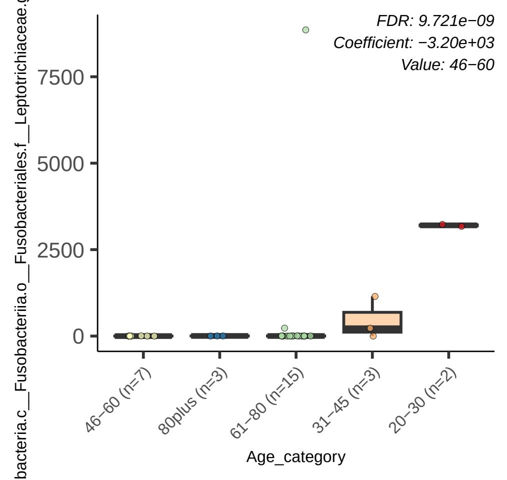

FDR: 9.721e−09
Coefficient: −3.20e+03
Value: 46−60
7500
5000
2500
0
bacteria.c__Fusobacteriia.o__Fusobacteriales.f__Leptotrichiaceae.g
46−60 (n=7)
80plus (n=3)
61−80 (n=15)
31−45 (n=3)
20−30 (n=2)
Age_category

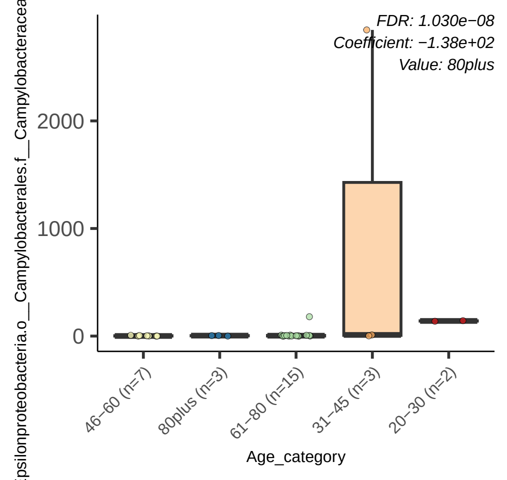

FDR: 1.030e−08
Coefficient: −1.38e+02
Value: 80plus
2000
1000
0
46−60 (n=7)
80plus (n=3)
61−80 (n=15)
31−45 (n=3)
20−30 (n=2)
Age_category
psilonproteobacteria.o__Campylobacterales.f__Campylobacteracea

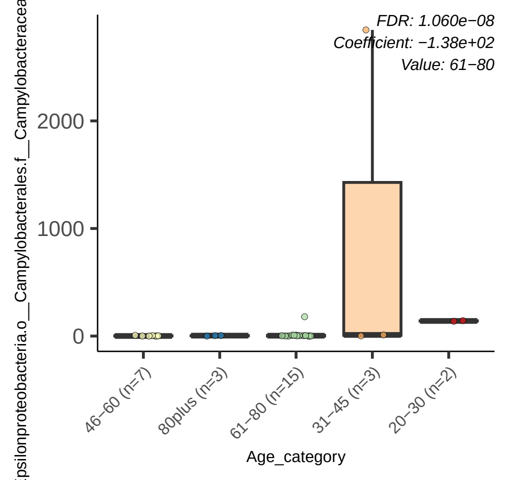
FDR: 1.060e−08
Coefficient: −1.38e+02
Value: 61−80
2000
1000
0
46−60 (n=7)
80plus (n=3)
61−80 (n=15)
31−45 (n=3)
20−30 (n=2)
Age_category
psilonproteobacteria.o__Campylobacterales.f__Campylobacteracea

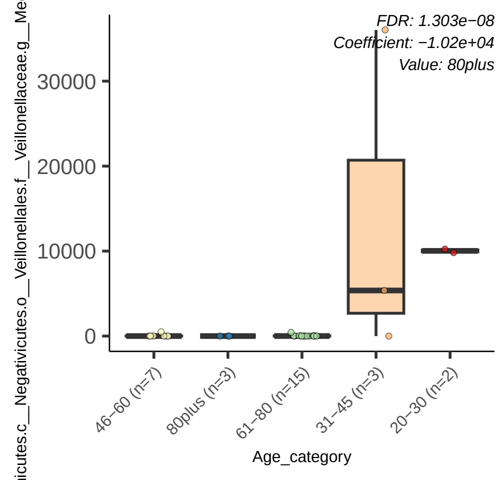

FDR: 1.303e−08
Coefficient: −1.02e+04
Value: 80plus
30000
20000
10000
0
46−60 (n=7)
80plus (n=3)
61−80 (n=15)
31−45 (n=3)
20−30 (n=2)
Age_category
icutes.c__Negativicutes.o__Veillonellales.f__Veillonellaceae.g__Me

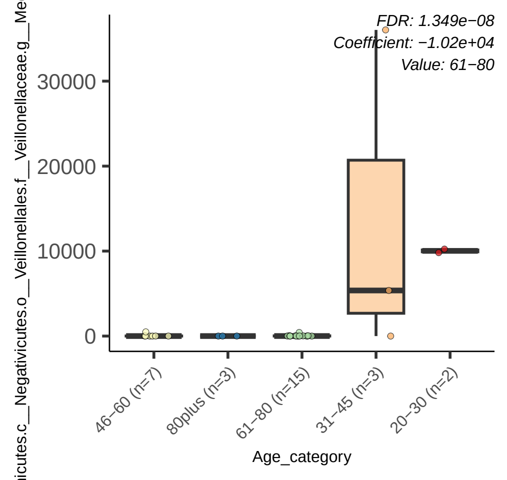

FDR: 1.349e−08
Coefficient: −1.02e+04
Value: 61−80
30000
20000
10000
0
46−60 (n=7)
80plus (n=3)
61−80 (n=15)
31−45 (n=3)
20−30 (n=2)
Age_category
icutes.c__Negativicutes.o__Veillonellales.f__Veillonellaceae.g__Me

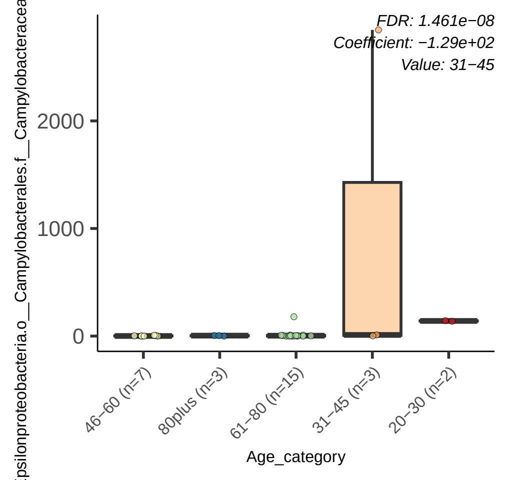

FDR: 1.461e−08
Coefficient: −1.29e+02
Value: 31−45
2000
1000
0
psilonproteobacteria.o__Campylobacterales.f__Campylobacteracea
46−60 (n=7)
80plus (n=3)
61−80 (n=15)
31−45 (n=3)
20−30 (n=2)
Age_category

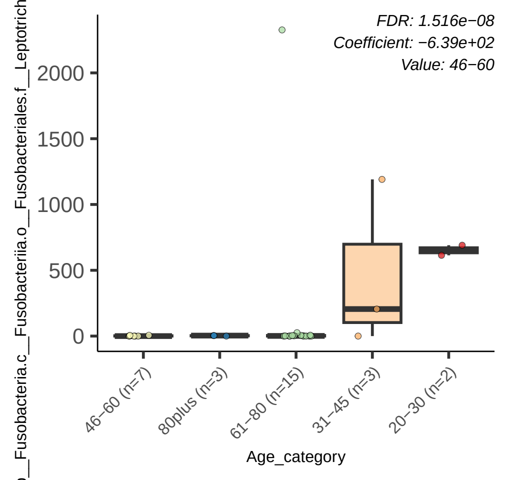

FDR: 1.516e−08
Coefficient: −6.39e+02
Value: 46−60
0
500
1000
1500
2000
46−60 (n=7)
80plus (n=3)
61−80 (n=15)
31−45 (n=3)
20−30 (n=2)
Age_category
Fusobacteria.c__Fusobacteriia.o__Fusobacteriales.f__Leptotrich

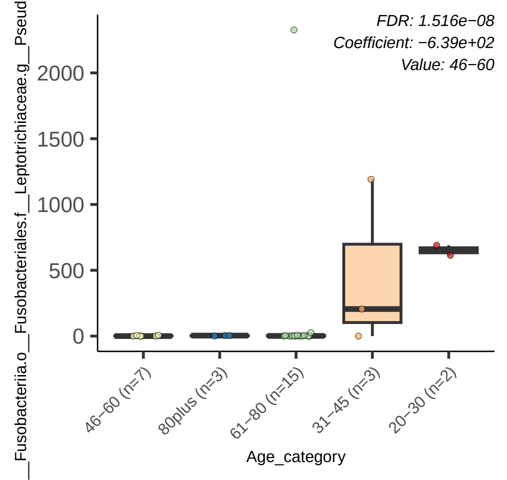

FDR: 1.516e−08
Coefficient: −6.39e+02
Value: 46−60
Fusobacteriia.o__Fusobacteriales.f__Leptotrichiaceae.g__Pseud
2000
1500
1000
500
0
46−60 (n=7)
80plus (n=3)
61−80 (n=15)
31−45 (n=3)
20−30 (n=2)
Age_category

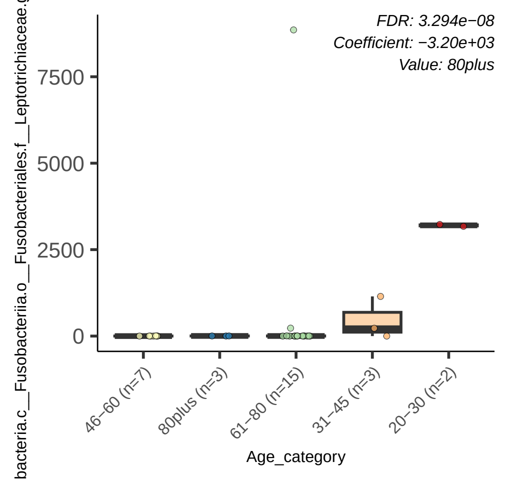

FDR: 3.294e−08
Coefficient: −3.20e+03
Value: 80plus
7500
5000
2500
0
46−60 (n=7)
80plus (n=3)
61−80 (n=15)
31−45 (n=3)
20−30 (n=2)
Age_category
bacteria.c__Fusobacteriia.o__Fusobacteriales.f__Leptotrichiaceae.g

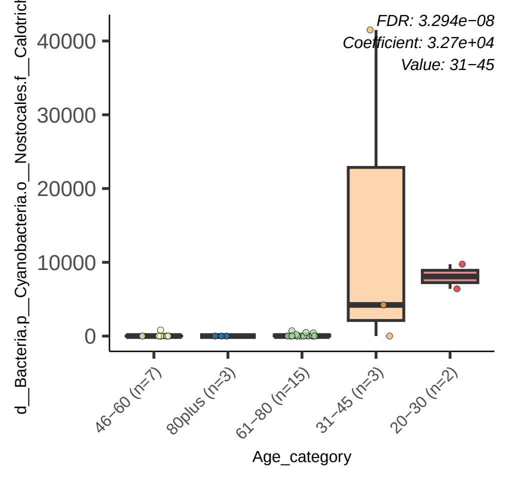

FDR: 3.294e−08
Coefficient: 3.27e+04
Value: 31−45
d__Bacteria.p__Cyanobacteria.o__Nostocales.f__Calotrich
40000
30000
20000
10000
0
46−60 (n=7)
80plus (n=3)
61−80 (n=15)
31−45 (n=3)
20−30 (n=2)
Age_category

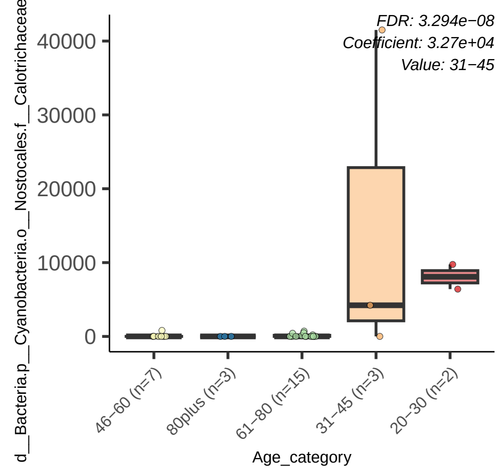

FDR: 3.294e−08
Coefficient: 3.27e+04
Value: 31−45
d__Bacteria.p__Cyanobacteria.o__Nostocales.f__Calotrichaceae
40000
30000
20000
10000
0
46−60 (n=7)
80plus (n=3)
61−80 (n=15)
31−45 (n=3)
20−30 (n=2)
Age_category

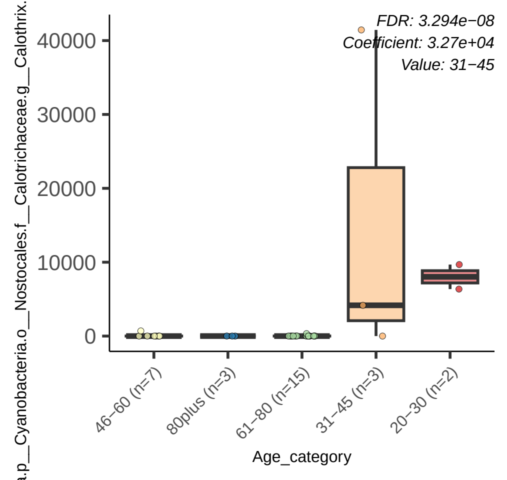

FDR: 3.294e−08
Coefficient: 3.27e+04
Value: 31−45
40000
30000
20000
10000
0
a.p__Cyanobacteria.o__Nostocales.f__Calotrichaceae.g__Calothrix.
46−60 (n=7)
80plus (n=3)
61−80 (n=15)
31−45 (n=3)
20−30 (n=2)
Age_category

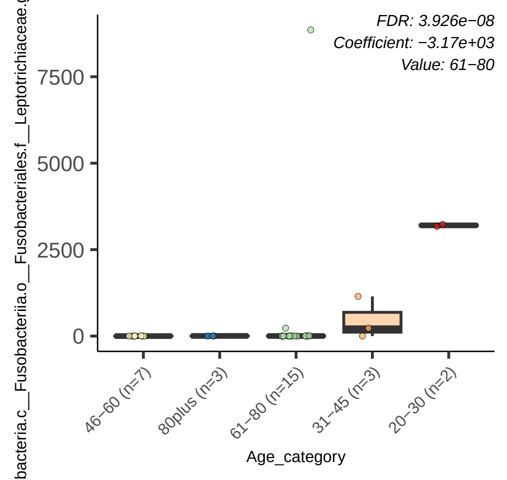

FDR: 3.926e−08
Coefficient: −3.17e+03
Value: 61−80
7500
5000
2500
0
bacteria.c__Fusobacteriia.o__Fusobacteriales.f__Leptotrichiaceae.g
46−60 (n=7)
80plus (n=3)
61−80 (n=15)
31−45 (n=3)
20−30 (n=2)
Age_category

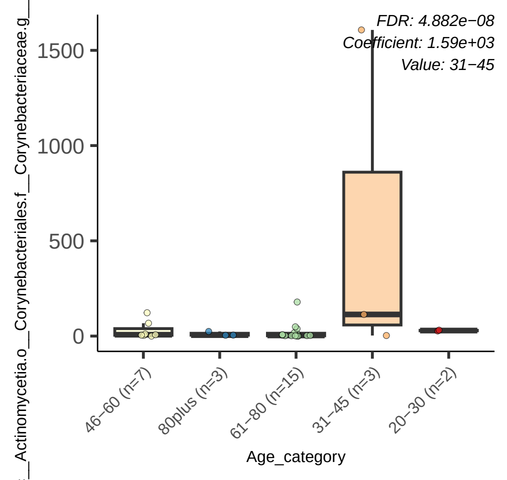

FDR: 4.882e−08
Coefficient: 1.59e+03
Value: 31−45
1500
1000
500
0
__Actinomycetia.o__Corynebacteriales.f__Corynebacteriaceae.g__
46−60 (n=7)
80plus (n=3)
61−80 (n=15)
31−45 (n=3)
20−30 (n=2)
Age_category

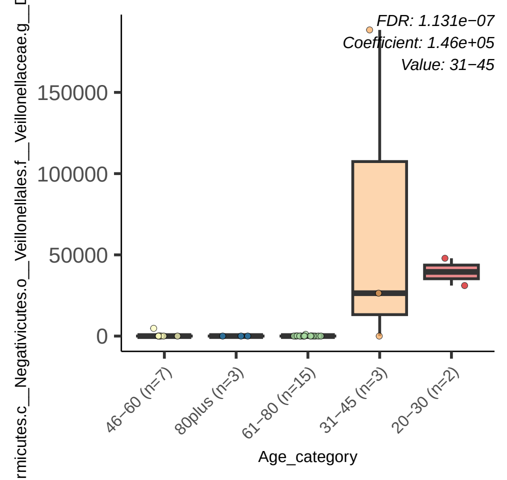

FDR: 1.131e−07
Coefficient: 1.46e+05
Value: 31−45
150000
100000
50000
0
rmicutes.c__Negativicutes.o__Veillonellales.f__Veillonellaceae.g__D
46−60 (n=7)
80plus (n=3)
61−80 (n=15)
31−45 (n=3)
20−30 (n=2)
Age_category

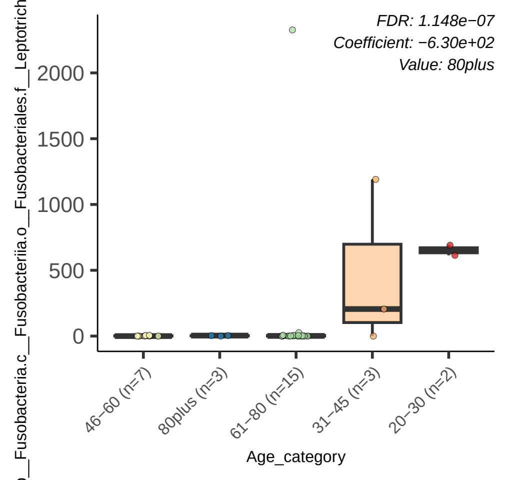

FDR: 1.148e−07
Coefficient: −6.30e+02
Value: 80plus
o__Fusobacteria.c__Fusobacteriia.o__Fusobacteriales.f__Leptotrich
2000
1500
1000
500
0
46−60 (n=7)
80plus (n=3)
61−80 (n=15)
31−45 (n=3)
20−30 (n=2)
Age_category

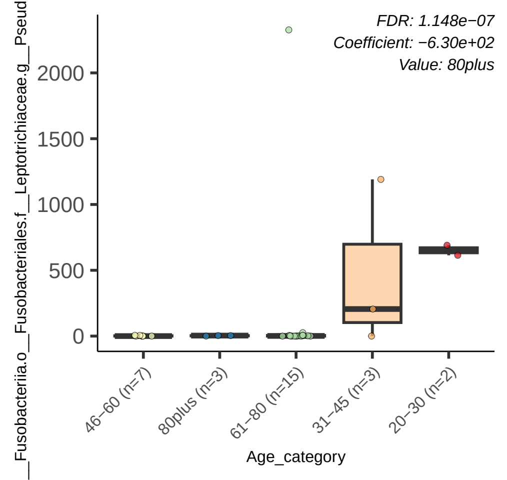

FDR: 1.148e−07
Coefficient: −6.30e+02
Value: 80plus
_Fusobacteriia.o__Fusobacteriales.f__Leptotrichiaceae.g__Pseud
0
500
1000
1500
2000
46−60 (n=7)
80plus (n=3)
61−80 (n=15)
31−45 (n=3)
20−30 (n=2)
Age_category

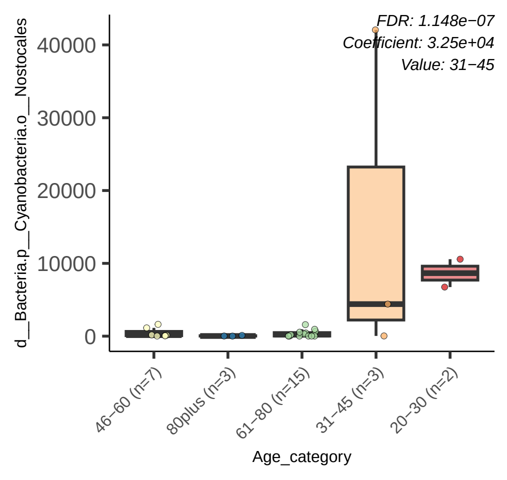

FDR: 1.148e−07
Coefficient: 3.25e+04
Value: 31−45
d__Bacteria.p__Cyanobacteria.o__Nostocales
40000
30000
20000
10000
0
46−60 (n=7)
80plus (n=3)
61−80 (n=15)
31−45 (n=3)
20−30 (n=2)
Age_category

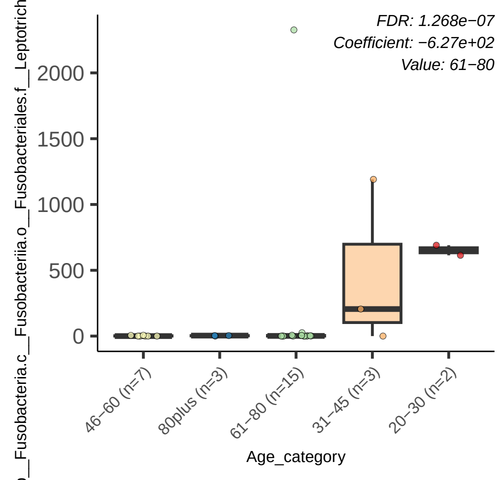
FDR: 1.268e−07
Coefficient: −6.27e+02
Value: 61−80
2000
1500
1000
500
0
46−60 (n=7)
80plus (n=3)
61−80 (n=15)
31−45 (n=3)
20−30 (n=2)
Age_category
o__Fusobacteria.c__Fusobacteriia.o__Fusobacteriales.f__Leptotrich

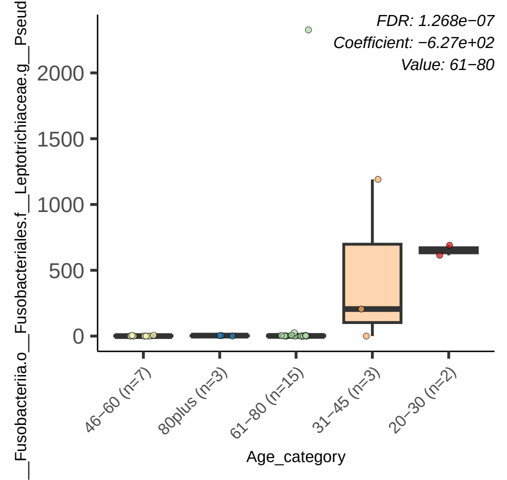

FDR: 1.268e−07
Coefficient: −6.27e+02
Value: 61−80
__Fusobacteriia.o__Fusobacteriales.f__Leptotrichiaceae.g__Pseud
0
500
1000
1500
2000
46−60 (n=7)
80plus (n=3)
61−80 (n=15)
31−45 (n=3)
20−30 (n=2)
Age_category

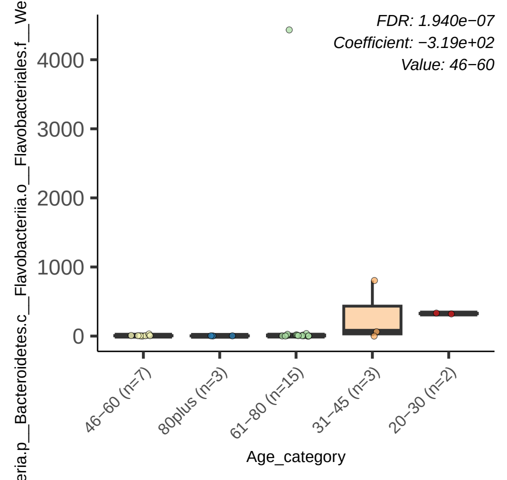

FDR: 1.940e−07
Coefficient: −3.19e+02
Value: 46−60
4000
3000
2000
1000
0
46−60 (n=7)
80plus (n=3)
61−80 (n=15)
31−45 (n=3)
20−30 (n=2)
Age_category
eria.p__Bacteroidetes.c__Flavobacteriia.o__Flavobacteriales.f__We

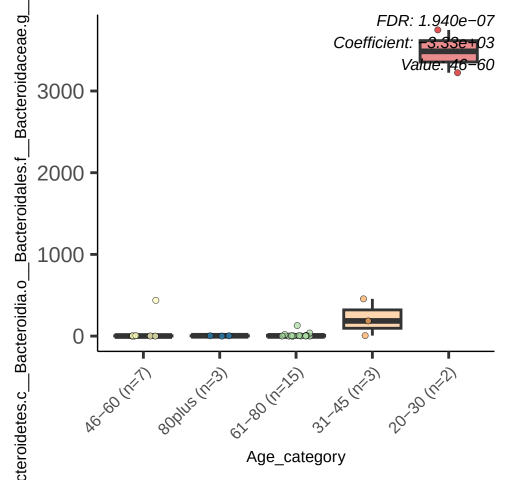

FDR: 1.940e−07
Coefficient: -3.33e+03
Value: 46−60
3000
2000
1000
0
46−60 (n=7)
80plus (n=3)
61−80 (n=15)
31−45 (n=3)
20−30 (n=2)
Age_category
cteroidetes.c__Bacteroidia.o__Bacteroidales.f__Bacteroidaceae.g__

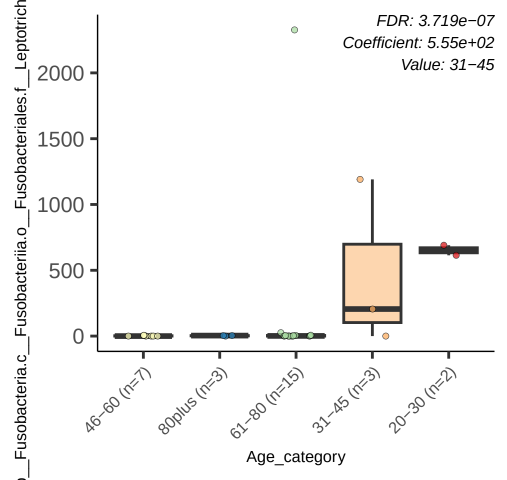

FDR: 3.719e−07
Coefficient: 5.55e+02
Value: 31−45
2000
1500
1000
500
0
46−60 (n=7)
80plus (n=3)
61−80 (n=15)
31−45 (n=3)
20−30 (n=2)
Age_category
o__Fusobacteria.c__Fusobacteriia.o__Fusobacteriales.f__Leptotrich

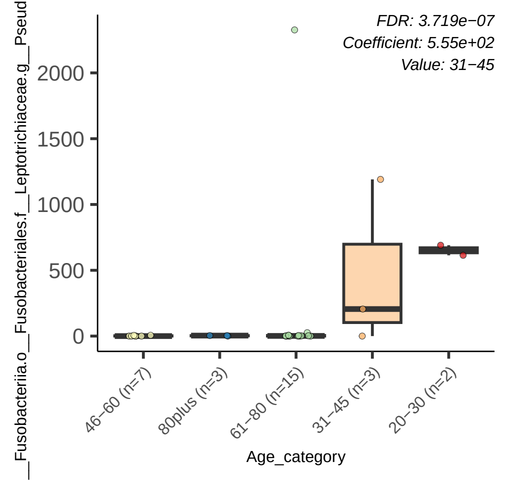
FDR: 3.719e−07
Coefficient: 5.55e+02
Value: 31−45
0
500
1000
1500
2000
46−60 (n=7)
80plus (n=3)
61−80 (n=15)
31−45 (n=3)
20−30 (n=2)
Age_category
_Fusobacteriia.o__Fusobacteriales.f__Leptotrichiaceae.g__Pseud

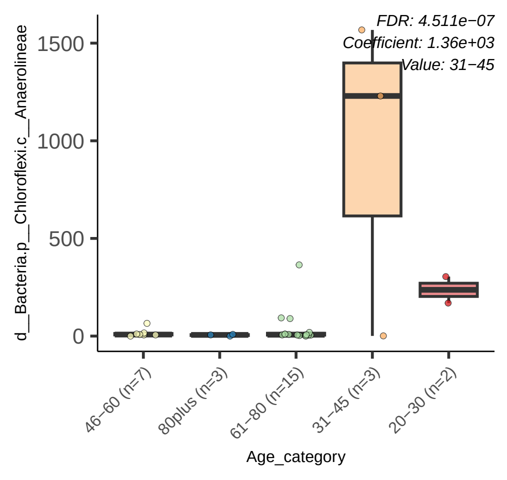

FDR: 4.511e−07
Coefficient: 1.36e+03
Value: 31−45
d__Bacteria.p__Chloroflexi.c__Anaerolineae
1500
1000
500
0
46−60 (n=7)
80plus (n=3)
61−80 (n=15)
31−45 (n=3)
20−30 (n=2)
Age_category

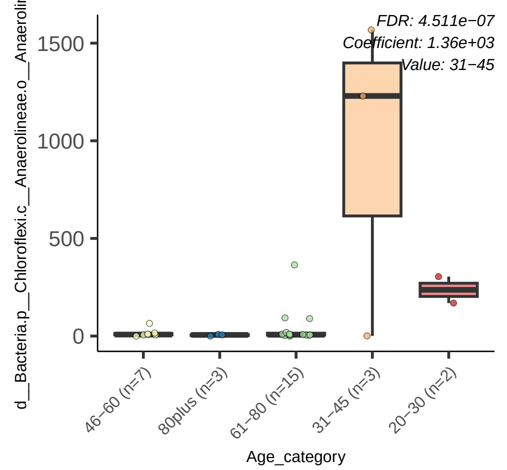

FDR: 4.511e−07
Coefficient: 1.36e+03
Value: 31−45
d__Bacteria.p__Chloroflexi.c__Anaerolineae.o__Anaeroli
1500
1000
500
0
46−60 (n=7)
80plus (n=3)
61−80 (n=15)
31−45 (n=3)
20−30 (n=2)
Age_category

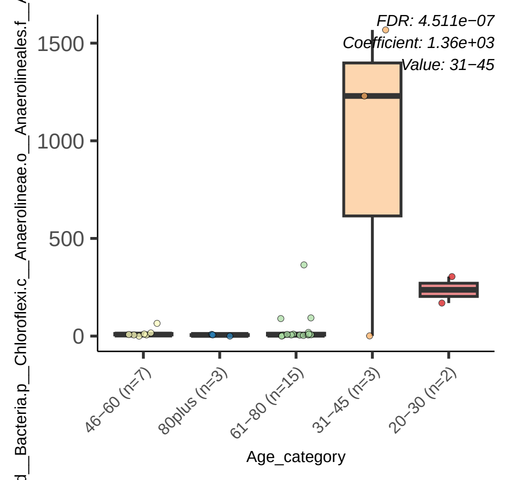

FDR: 4.511e−07
Coefficient: 1.36e+03
Value: 31−45
1500
1000
500
0
46−60 (n=7)
80plus (n=3)
61−80 (n=15)
31−45 (n=3)
20−30 (n=2)
Age_category
d__Bacteria.p__Chloroflexi.c__Anaerolineae.o__Anaerolineales.f__A

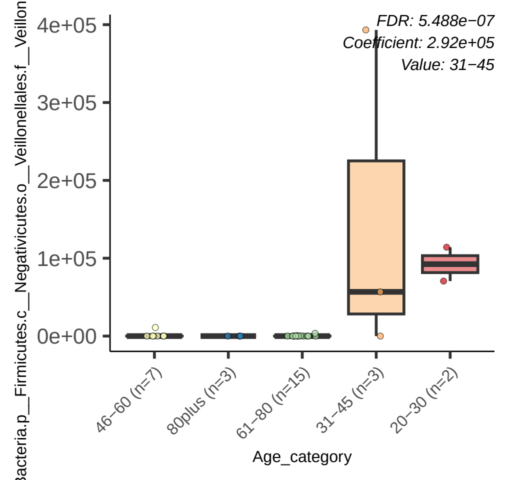

FDR: 5.488e−07
Coefficient: 2.92e+05
Value: 31−45
4e+05
3e+05
2e+05
1e+05
0e+00
Bacteria.p__Firmicutes.c__Negativicutes.o__Veillonellales.f__Veillon
46−60 (n=7)
80plus (n=3)
61−80 (n=15)
31−45 (n=3)
20−30 (n=2)
Age_category

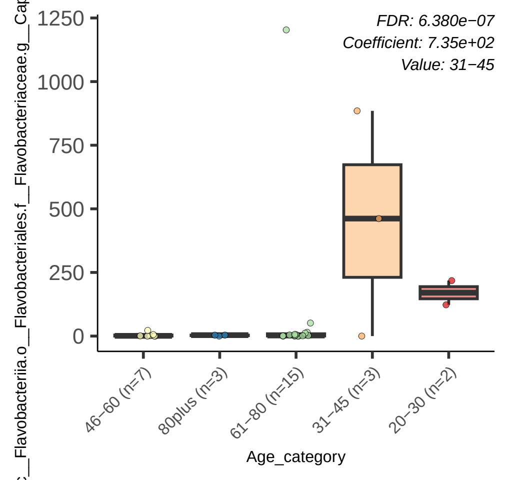

FDR: 6.380e−07
Coefficient: 7.35e+02
Value: 31−45
1250
1000
750
500
250
0
c__Flavobacteriia.o__Flavobacteriales.f__Flavobacteriaceae.g__Cap
46−60 (n=7)
80plus (n=3)
61−80 (n=15)
31−45 (n=3)
20−30 (n=2)
Age_category

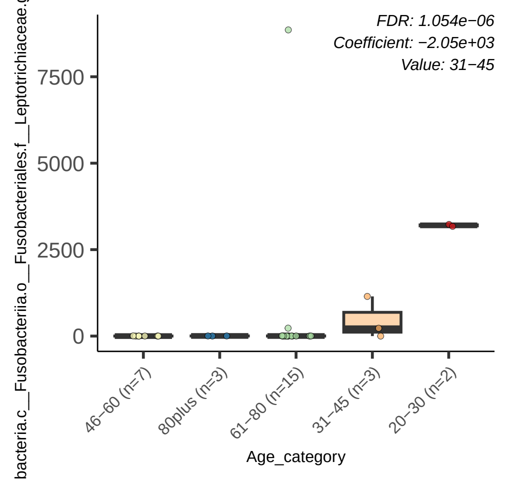

FDR: 1.054e−06
Coefficient: −2.05e+03
Value: 31−45
7500
5000
2500
0
bacteria.c__Fusobacteriia.o__Fusobacteriales.f__Leptotrichiaceae.g
46−60 (n=7)
80plus (n=3)
61−80 (n=15)
31−45 (n=3)
20−30 (n=2)
Age_category

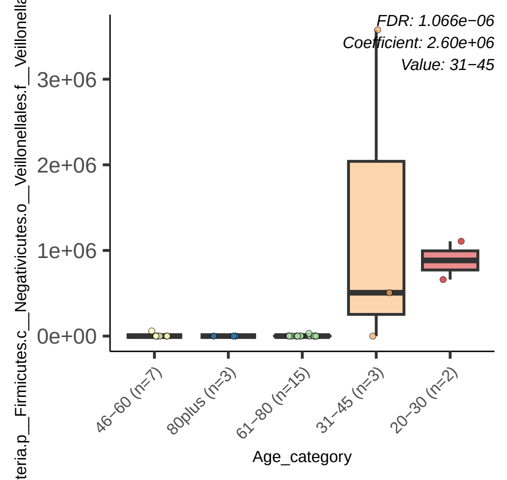

FDR: 1.066e−06
Coefficient: 2.60e+06
Value: 31−45
3e+06
2e+06
1e+06
0e+00
teria.p__Firmicutes.c__Negativicutes.o__Veillonellales.f__Veillonella
46−60 (n=7)
80plus (n=3)
61−80 (n=15)
31−45 (n=3)
20−30 (n=2)
Age_category

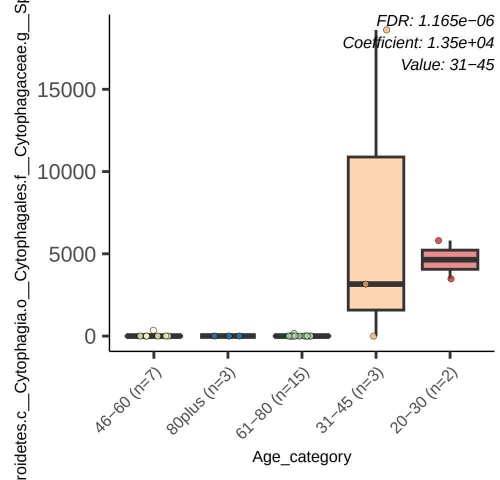

FDR: 1.165e−06
Coefficient: 1.35e+04
Value: 31−45
15000
10000
5000
0
46−60 (n=7)
80plus (n=3)
61−80 (n=15)
31−45 (n=3)
20−30 (n=2)
Age_category
roidetes.c__Cytophagia.o__Cytophagales.f__Cytophagaceae.g__Sp

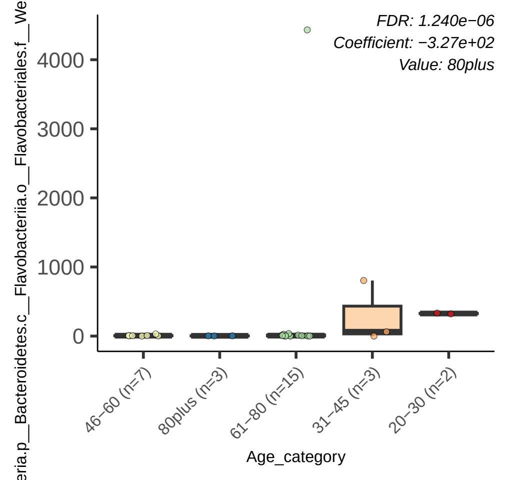

FDR: 1.240e−06
Coefficient: −3.27e+02
Value: 80plus
0
1000
2000
3000
4000
eria.p__Bacteroidetes.c__Flavobacteriia.o__Flavobacteriales.f__We
46−60 (n=7)
80plus (n=3)
61−80 (n=15)
31−45 (n=3)
20−30 (n=2)
Age_category

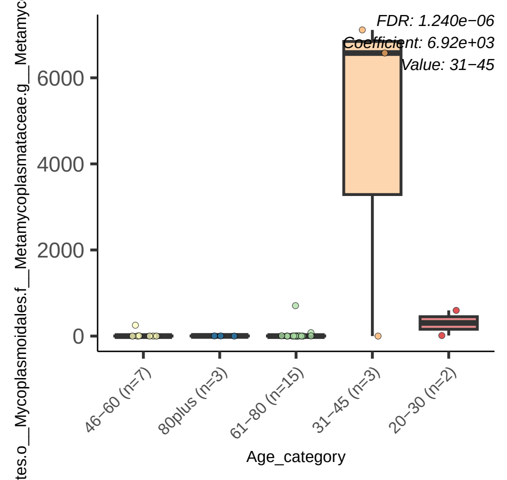

FDR: 1.240e−06
Coefficient: 6.92e+03
Value: 31−45
6000
4000
2000
0
tes.o__Mycoplasmoidales.f__Metamycoplasmataceae.g__Metamyc
46−60 (n=7)
80plus (n=3)
61−80 (n=15)
31−45 (n=3)
20−30 (n=2)
Age_category

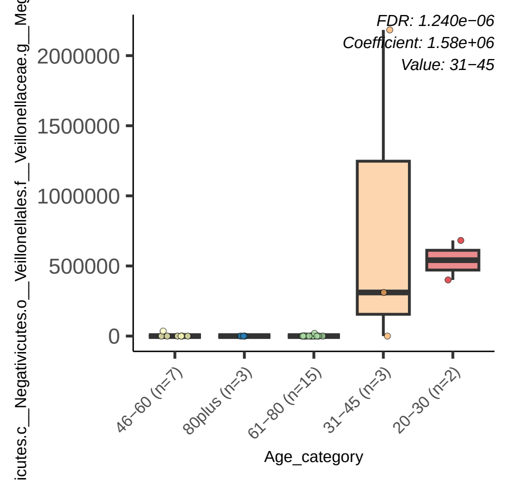

FDR: 1.240e−06
Coefficient: 1.58e+06
Value: 31−45
2000000
1500000
1000000
500000
0
46−60 (n=7)
80plus (n=3)
61−80 (n=15)
31−45 (n=3)
20−30 (n=2)
Age_category
icutes.c__Negativicutes.o__Veillonellales.f__Veillonellaceae.g__Meg

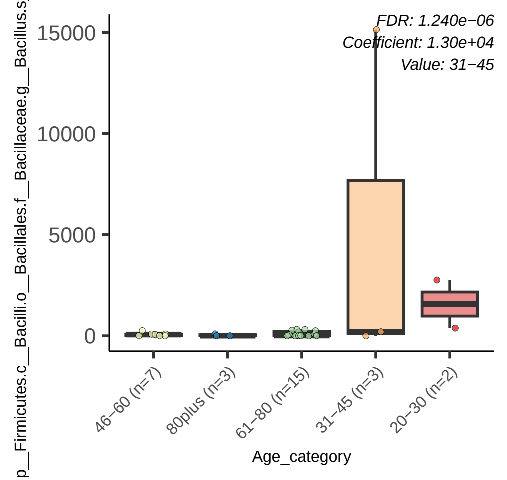

FDR: 1.240e−06
Coefficient: 1.30e+04
Value: 31−45
15000
10000
5000
0
p__Firmicutes.c__Bacilli.o__Bacillales.f__Bacillaceae.g__Bacillus.s_
46−60 (n=7)
80plus (n=3)
61−80 (n=15)
31−45 (n=3)
20−30 (n=2)
Age_category

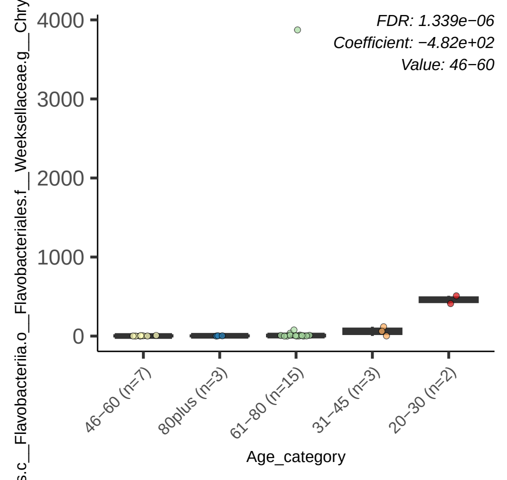

FDR: 1.339e−06
Coefficient: −4.82e+02
Value: 46−60
s.c__Flavobacteriia.o__Flavobacteriales.f__Weeksellaceae.g__Chry
4000
3000
2000
1000
0
46−60 (n=7)
80plus (n=3)
61−80 (n=15)
31−45 (n=3)
20−30 (n=2)
Age_category

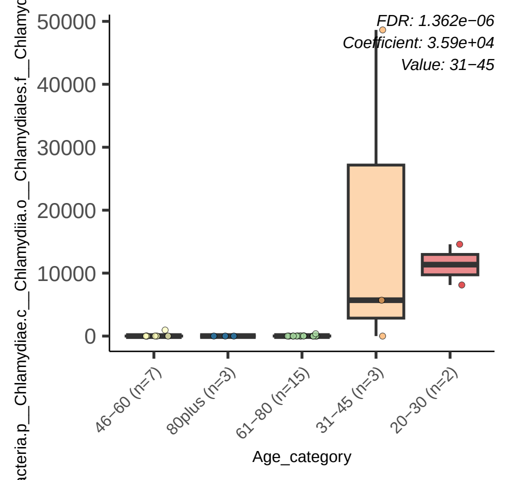

FDR: 1.362e−06
Coefficient: 3.59e+04
Value: 31−45
50000
40000
30000
20000
10000
0
acteria.p__Chlamydiae.c__Chlamydiia.o__Chlamydiales.f__Chlamyd
46−60 (n=7)
80plus (n=3)
61−80 (n=15)
31−45 (n=3)
20−30 (n=2)
Age_category

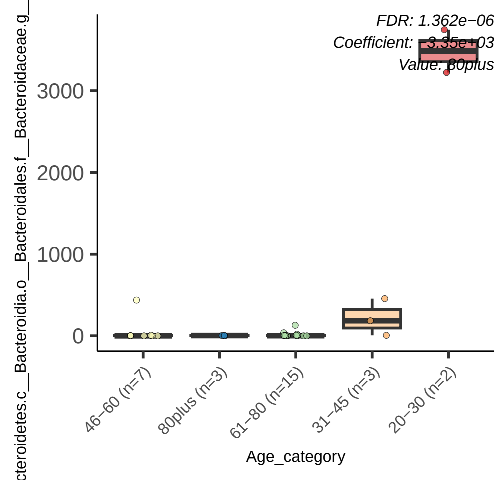
FDR: 1.362e−06
Coefficient: -3.35e+03
Value: 80plus
3000
2000
1000
0
cteroidetes.c__Bacteroidia.o__Bacteroidales.f__Bacteroidaceae.g__
46−60 (n=7)
80plus (n=3)
61−80 (n=15)
31−45 (n=3)
20−30 (n=2)
Age_category

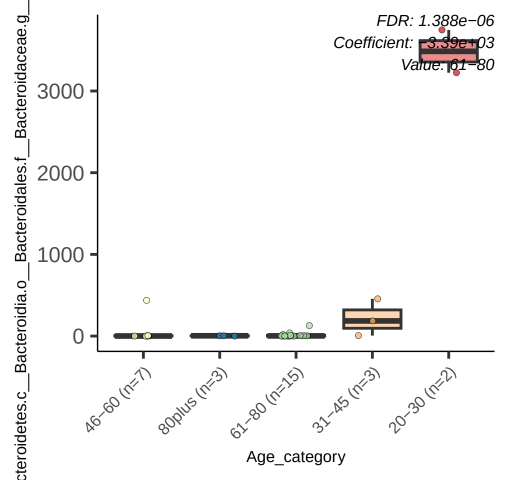
FDR: 1.388e−06
Coefficient: -3.39e+03
Value: 61−80
3000
2000
1000
0
cteroidetes.c__Bacteroidia.o__Bacteroidales.f__Bacteroidaceae.g__
46−60 (n=7)
80plus (n=3)
61−80 (n=15)
31−45 (n=3)
20−30 (n=2)
Age_category

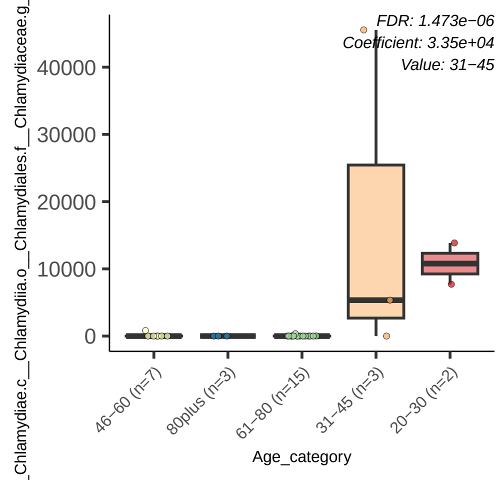
FDR: 1.473e−06
Coefficient: 3.35e+04
Value: 31−45
Chlamydiae.c__Chlamydiia.o__Chlamydiales.f__Chlamydiaceae.g__
40000
30000
20000
10000
0
46−60 (n=7)
80plus (n=3)
61−80 (n=15)
31−45 (n=3)
20−30 (n=2)
Age_category

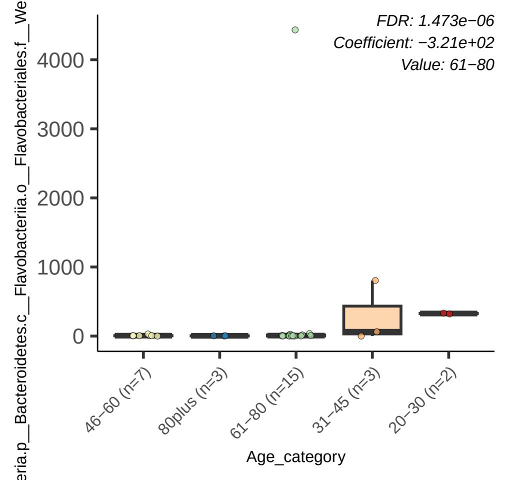

FDR: 1.473e−06
Coefficient: −3.21e+02
Value: 61−80
4000
3000
2000
1000
0
46−60 (n=7)
80plus (n=3)
61−80 (n=15)
31−45 (n=3)
20−30 (n=2)
Age_category
eria.p__Bacteroidetes.c__Flavobacteriia.o__Flavobacteriales.f__We

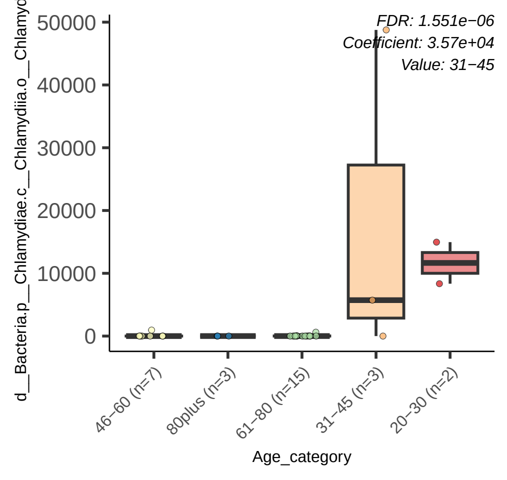

FDR: 1.551e−06
Coefficient: 3.57e+04
Value: 31−45
d__Bacteria.p__Chlamydiae.c__Chlamydiia.o__Chlamyd
50000
40000
30000
20000
10000
0
46−60 (n=7)
80plus (n=3)
61−80 (n=15)
31−45 (n=3)
20−30 (n=2)
Age_category

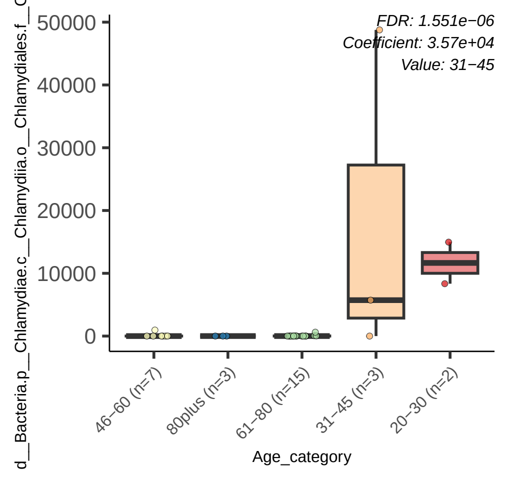
FDR: 1.551e−06
Coefficient: 3.57e+04
Value: 31−45
d__Bacteria.p__Chlamydiae.c__Chlamydiia.o__Chlamydiales.f__C
50000
40000
30000
20000
10000
0
46−60 (n=7)
80plus (n=3)
61−80 (n=15)
31−45 (n=3)
20−30 (n=2)
Age_category

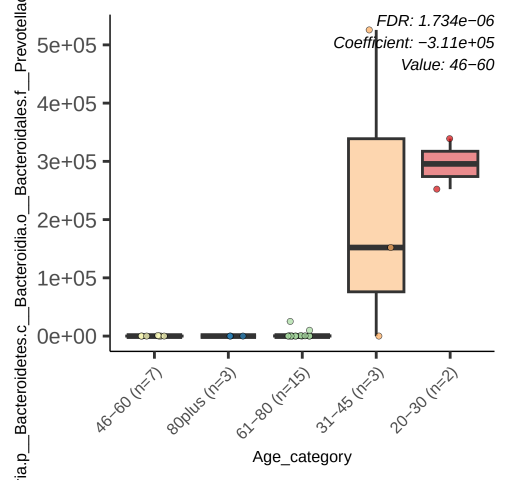
FDR: 1.734e−06
Coefficient: −3.11e+05
Value: 46−60
ria.p__Bacteroidetes.c__Bacteroidia.o__Bacteroidales.f__Prevotellac
5e+05
4e+05
3e+05
2e+05
1e+05
0e+00
46−60 (n=7)
80plus (n=3)
61−80 (n=15)
31−45 (n=3)
20−30 (n=2)
Age_category

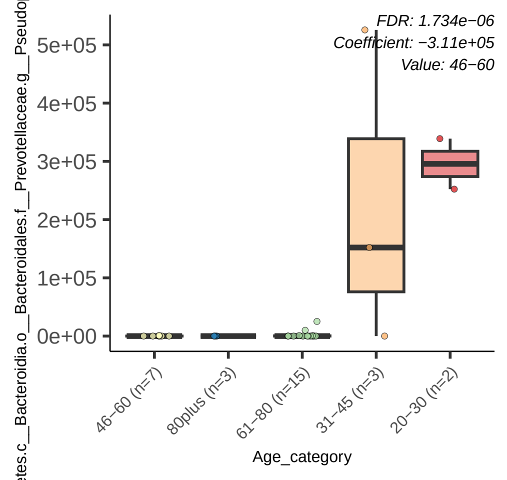

FDR: 1.734e−06
Coefficient: −3.11e+05
Value: 46−60
5e+05
4e+05
3e+05
2e+05
1e+05
0e+00
46−60 (n=7)
80plus (n=3)
61−80 (n=15)
31−45 (n=3)
20−30 (n=2)
Age_category
etes.c__Bacteroidia.o__Bacteroidales.f__Prevotellaceae.g__Pseudo

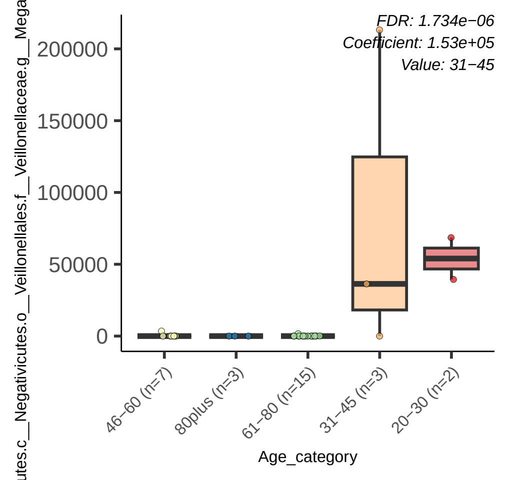

FDR: 1.734e−06
Coefficient: 1.53e+05
Value: 31−45
200000
150000
100000
50000
0
utes.c__Negativicutes.o__Veillonellales.f__Veillonellaceae.g__Mega
46−60 (n=7)
80plus (n=3)
61−80 (n=15)
31−45 (n=3)
20−30 (n=2)
Age_category

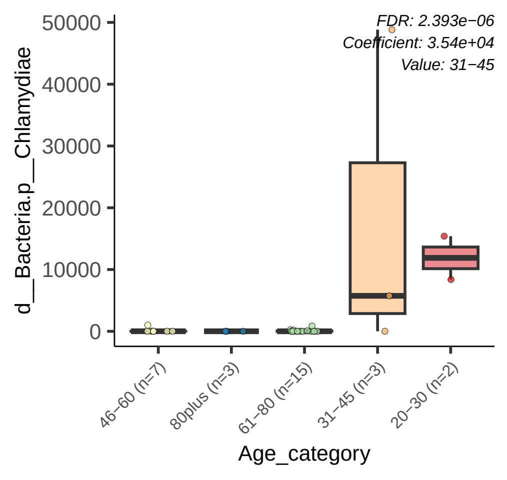
FDR: 2.393e−06
Coefficient: 3.54e+04
Value: 31−45
d__Bacteria.p__Chlamydiae
50000
40000
30000
20000
10000
0
46−60 (n=7)
80plus (n=3)
61−80 (n=15)
31−45 (n=3)
20−30 (n=2)
Age_category

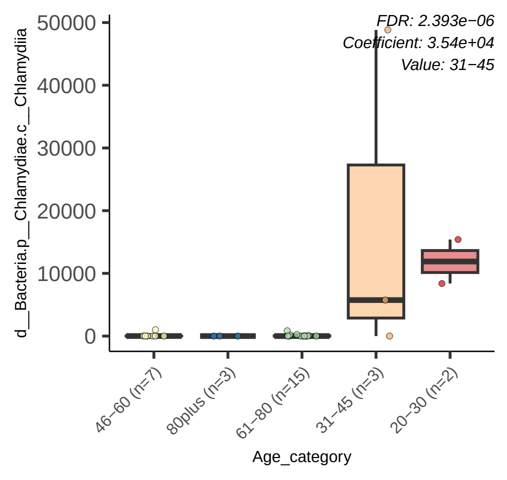
FDR: 2.393e−06
Coefficient: 3.54e+04
Value: 31−45
d__Bacteria.p__Chlamydiae.c__Chlamydiia
50000
40000
30000
20000
10000
0
46−60 (n=7)
80plus (n=3)
61−80 (n=15)
31−45 (n=3)
20−30 (n=2)
Age_category

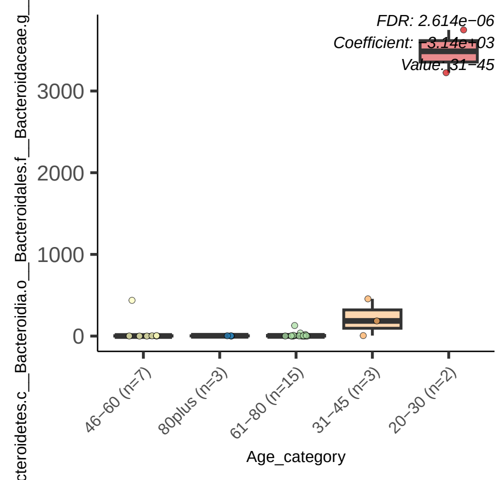

FDR: 2.614e−06
Coefficient: -3.14e+03
Value: 31−45
3000
2000
1000
0
cteroidetes.c__Bacteroidia.o__Bacteroidales.f__Bacteroidaceae.g__
46−60 (n=7)
80plus (n=3)
61−80 (n=15)
31−45 (n=3)
20−30 (n=2)
Age_category

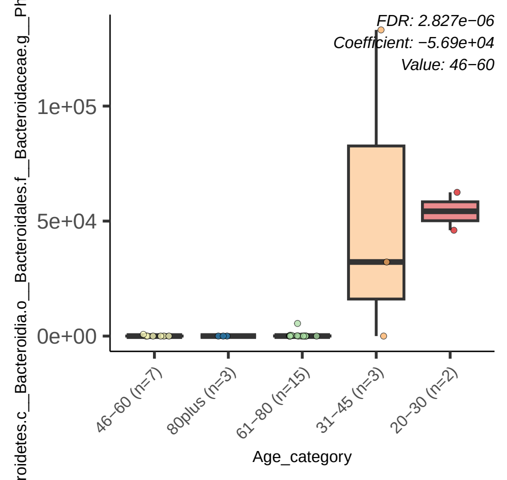

FDR: 2.827e−06
Coefficient: −5.69e+04
Value: 46−60
1e+05
5e+04
0e+00
46−60 (n=7)
80plus (n=3)
61−80 (n=15)
31−45 (n=3)
20−30 (n=2)
Age_category
roidetes.c__Bacteroidia.o__Bacteroidales.f__Bacteroidaceae.g__Ph

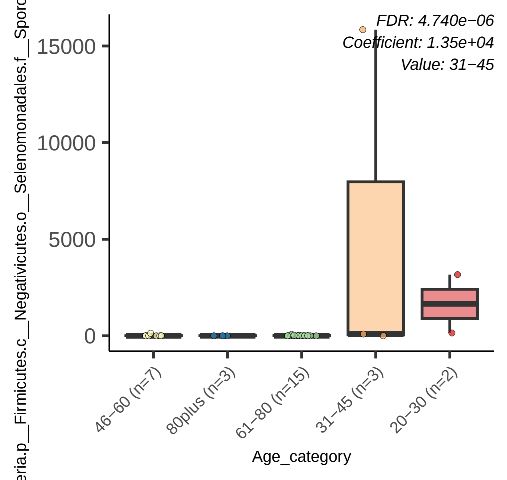

FDR: 4.740e−06
Coefficient: 1.35e+04
Value: 31−45
15000
10000
5000
0
eria.p__Firmicutes.c__Negativicutes.o__Selenomonadales.f__Sporo
46−60 (n=7)
80plus (n=3)
61−80 (n=15)
31−45 (n=3)
20−30 (n=2)
Age_category

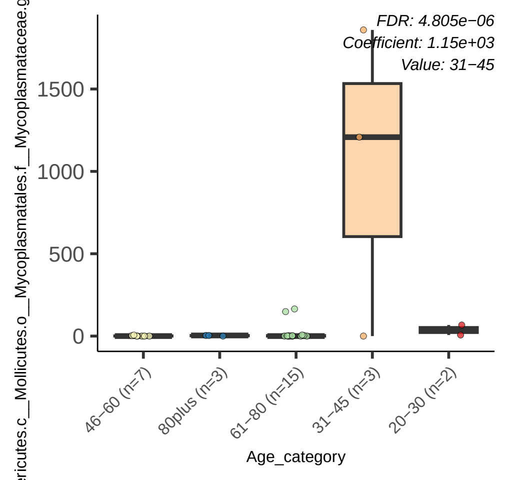

FDR: 4.805e−06
Coefficient: 1.15e+03
Value: 31−45
0
500
1000
1500
ericutes.c__Mollicutes.o__Mycoplasmatales.f__Mycoplasmataceae.g
46−60 (n=7)
80plus (n=3)
61−80 (n=15)
31−45 (n=3)
20−30 (n=2)
Age_category

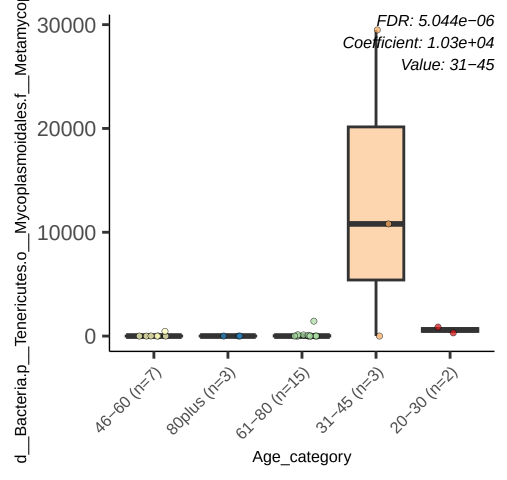

FDR: 5.044e−06
Coefficient: 1.03e+04
Value: 31−45
30000
20000
10000
0
d__Bacteria.p__Tenericutes.o__Mycoplasmoidales.f__Metamycop
46−60 (n=7)
80plus (n=3)
61−80 (n=15)
31−45 (n=3)
20−30 (n=2)
Age_category

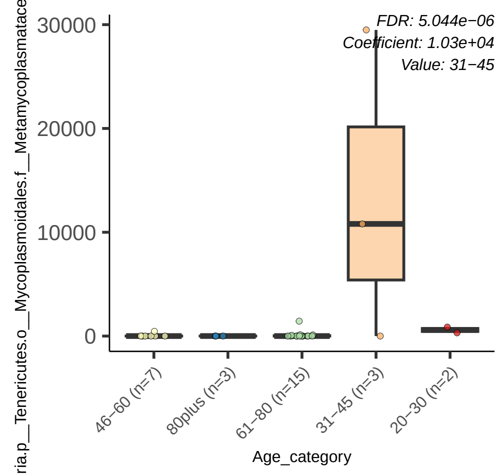
FDR: 5.044e−06
Coefficient: 1.03e+04
Value: 31−45
30000
20000
10000
0
eria.p__Tenericutes.o__Mycoplasmoidales.f__Metamycoplasmatace
46−60 (n=7)
80plus (n=3)
61−80 (n=15)
31−45 (n=3)
20−30 (n=2)
Age_category

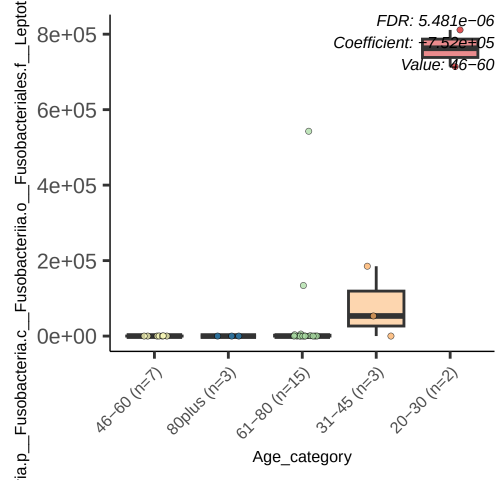

FDR: 5.481e−06
Coefficient: −7.52e+05
Value: 46−60
8e+05
6e+05
4e+05
2e+05
0e+00
ria.p__Fusobacteria.c__Fusobacteriia.o__Fusobacteriales.f__Leptot
46−60 (n=7)
80plus (n=3)
61−80 (n=15)
31−45 (n=3)
20−30 (n=2)
Age_category

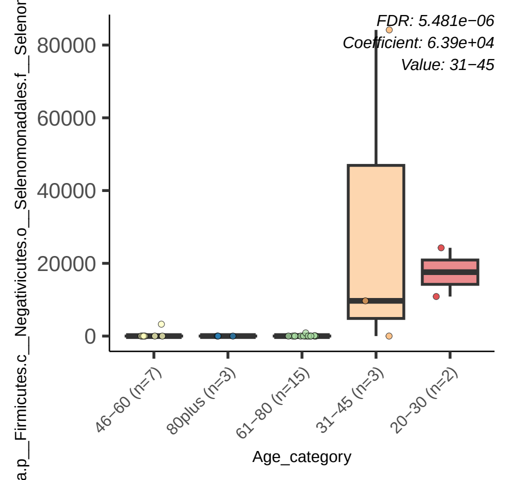
FDR: 5.481e−06
Coefficient: 6.39e+04
Value: 31−45
a.p__Firmicutes.c__Negativicutes.o__Selenomonadales.f__Selenor
80000
60000
40000
20000
0
46−60 (n=7)
80plus (n=3)
61−80 (n=15)
31−45 (n=3)
20−30 (n=2)
Age_category

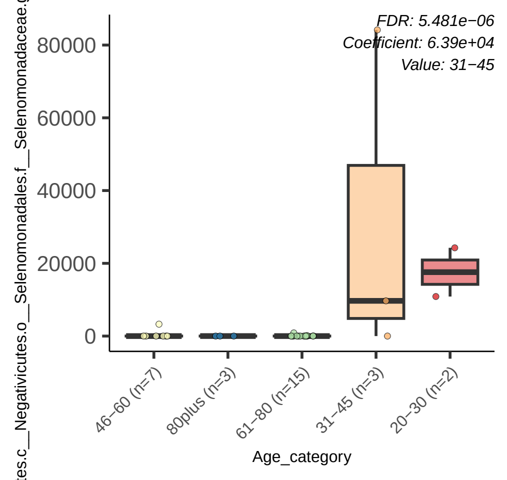

FDR: 5.481e−06
Coefficient: 6.39e+04
Value: 31−45
80000
60000
40000
20000
0
tes.c__Negativicutes.o__Selenomonadales.f__Selenomonadaceae.g
46−60 (n=7)
80plus (n=3)
61−80 (n=15)
31−45 (n=3)
20−30 (n=2)
Age_category

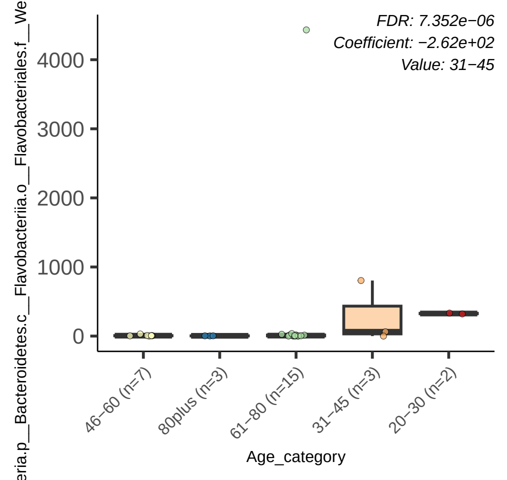

FDR: 7.352e−06
Coefficient: −2.62e+02
Value: 31−45
4000
3000
2000
1000
0
46−60 (n=7)
80plus (n=3)
61−80 (n=15)
31−45 (n=3)
20−30 (n=2)
Age_category
eria.p__Bacteroidetes.c__Flavobacteriia.o__Flavobacteriales.f__We

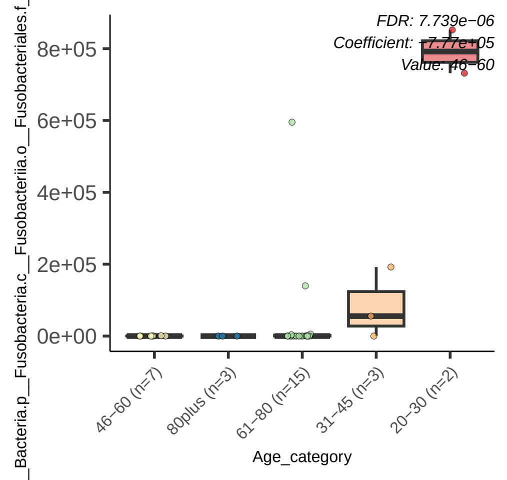

FDR: 7.739e−06
Coefficient: −7.77e+05
Value: 46−60
8e+05
6e+05
4e+05
2e+05
0e+00
_Bacteria.p__Fusobacteria.c__Fusobacteriia.o__Fusobacteriales.f_
46−60 (n=7)
80plus (n=3)
61−80 (n=15)
31−45 (n=3)
20−30 (n=2)
Age_category

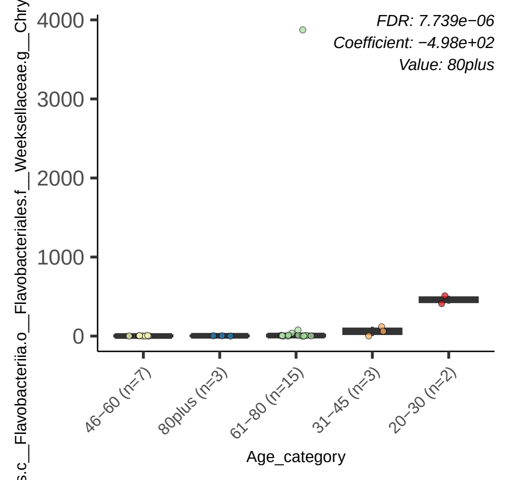

FDR: 7.739e−06
Coefficient: −4.98e+02
Value: 80plus
4000
3000
2000
1000
0
s.c__Flavobacteriia.o__Flavobacteriales.f__Weeksellaceae.g__Chry
46−60 (n=7)
80plus (n=3)
61−80 (n=15)
31−45 (n=3)
20−30 (n=2)
Age_category

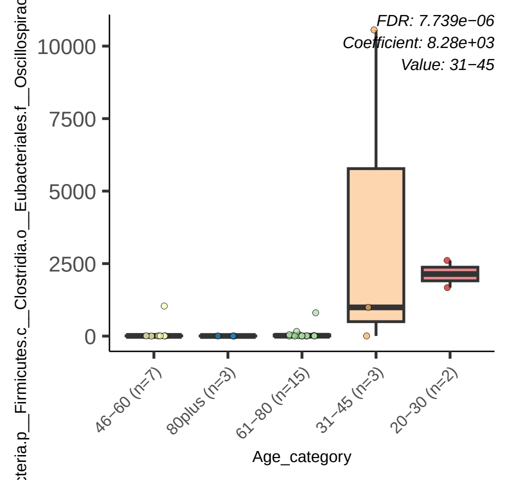

FDR: 7.739e−06
Coefficient: 8.28e+03
Value: 31−45
10000
7500
5000
2500
0
46−60 (n=7)
80plus (n=3)
61−80 (n=15)
31−45 (n=3)
20−30 (n=2)
Age_category

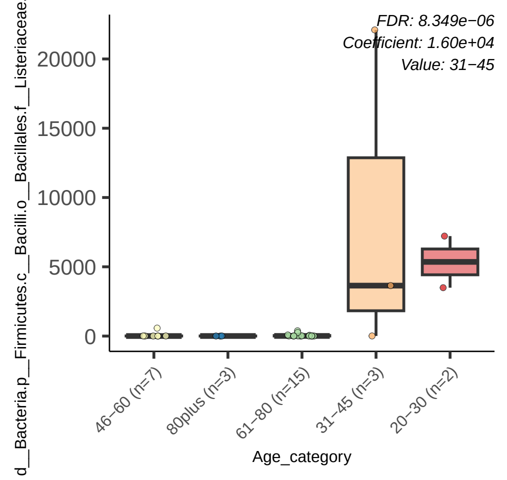
FDR: 8.349e−06
Coefficient: 1.60e+04
Value: 31−45
d__Bacteria.p__Firmicutes.c__Bacilli.o__Bacillales.f__Listeriaceae
20000
15000
10000
5000
0
46−60 (n=7)
80plus (n=3)
61−80 (n=15)
31−45 (n=3)
20−30 (n=2)
Age_category

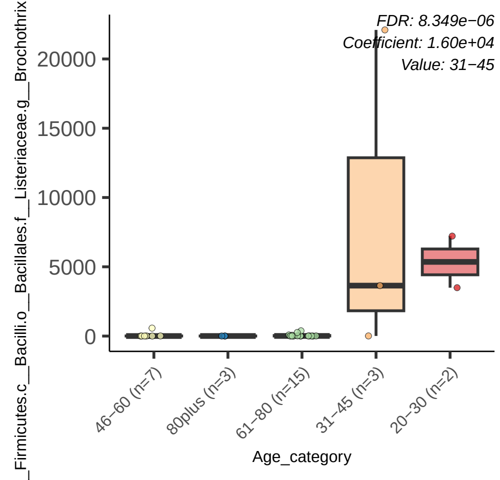
FDR: 8.349e−06
Coefficient: 1.60e+04
Value: 31−45
20000
15000
10000
5000
0
46−60 (n=7)
80plus (n=3)
61−80 (n=15)
31−45 (n=3)
20−30 (n=2)
Age_category
Firmicutes.c__Bacilli.o__Bacillales.f__Listeriaceae.g__Brochothrix

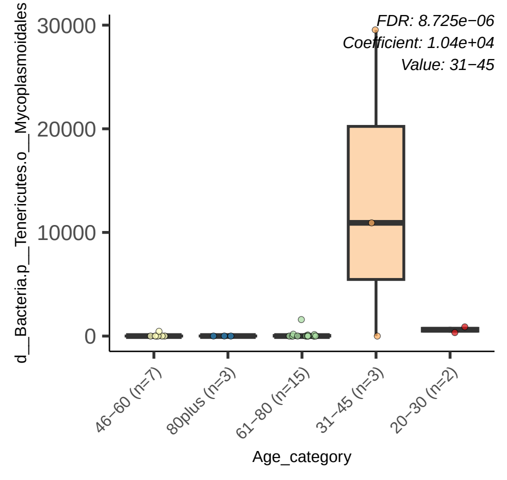

FDR: 8.725e−06
Coefficient: 1.04e+04
Value: 31−45
d__Bacteria.p__Tenericutes.o__Mycoplasmoidales
30000
20000
10000
0
46−60 (n=7)
80plus (n=3)
61−80 (n=15)
31−45 (n=3)
20−30 (n=2)
Age_category

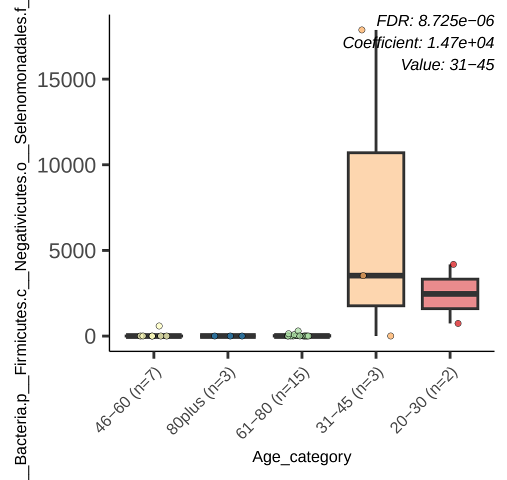
FDR: 8.725e−06
Coefficient: 1.47e+04
Value: 31−45
15000
10000
5000
0
Bacteria.p__Firmicutes.c__Negativicutes.o__Selenomonadales.f
46−60 (n=7)
80plus (n=3)
61−80 (n=15)
31−45 (n=3)
20−30 (n=2)
Age_category

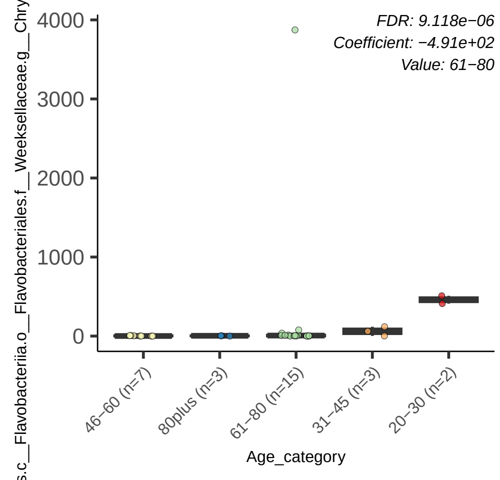

FDR: 9.118e−06
Coefficient: −4.91e+02
Value: 61−80
4000
3000
2000
1000
0
s.c__Flavobacteriia.o__Flavobacteriales.f__Weeksellaceae.g__Chry
46−60 (n=7)
80plus (n=3)
61−80 (n=15)
31−45 (n=3)
20−30 (n=2)
Age_category

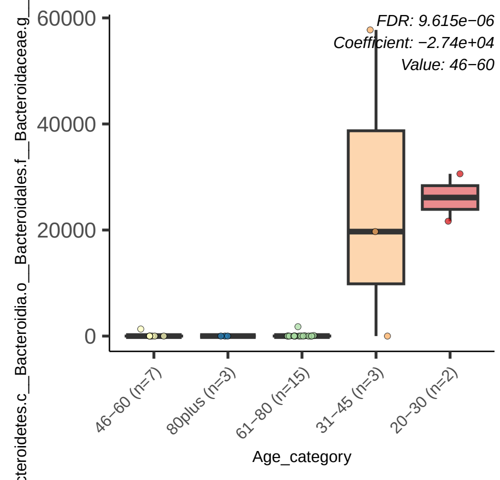

FDR: 9.615e−06
Coefficient: −2.74e+04
Value: 46−60
60000
40000
20000
0
cteroidetes.c__Bacteroidia.o__Bacteroidales.f__Bacteroidaceae.g__
46−60 (n=7)
80plus (n=3)
61−80 (n=15)
31−45 (n=3)
20−30 (n=2)
Age_category

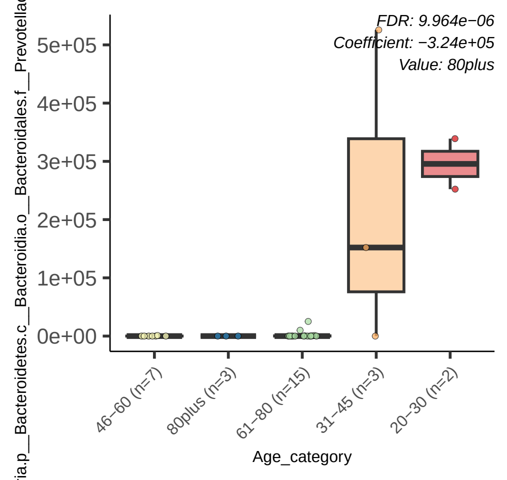

FDR: 9.964e−06
Coefficient: −3.24e+05
Value: 80plus
ria.p__Bacteroidetes.c__Bacteroidia.o__Bacteroidales.f__Prevotellac
5e+05
4e+05
3e+05
2e+05
1e+05
0e+00
46−60 (n=7)
80plus (n=3)
61−80 (n=15)
31−45 (n=3)
20−30 (n=2)
Age_category

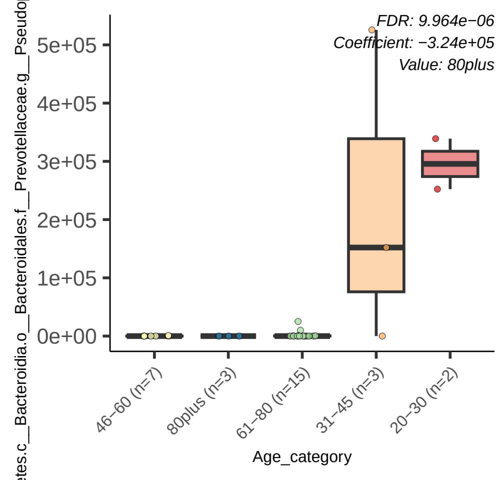
FDR: 9.964e−06
Coefficient: −3.24e+05
Value: 80plus
0e+00
1e+05
2e+05
3e+05
4e+05
5e+05
etes.c__Bacteroidia.o__Bacteroidales.f__Prevotellaceae.g__Pseudo
46−60 (n=7)
80plus (n=3)
61−80 (n=15)
31−45 (n=3)
20−30 (n=2)
Age_category

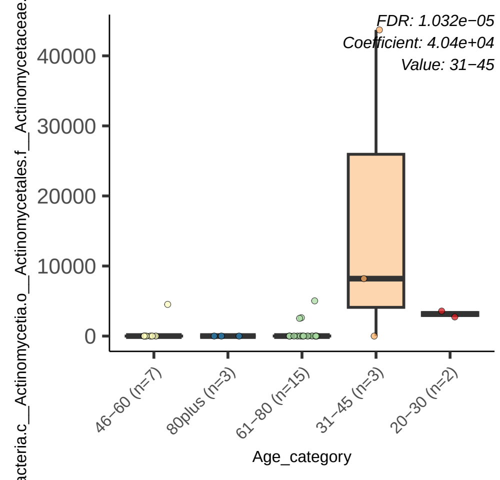

FDR: 1.032e−05
Coefficient: 4.04e+04
Value: 31−45
0
10000
20000
30000
40000
acteria.c__Actinomycetia.o__Actinomycetales.f__Actinomycetaceae.
46−60 (n=7)
80plus (n=3)
61−80 (n=15)
31−45 (n=3)
20−30 (n=2)
Age_category

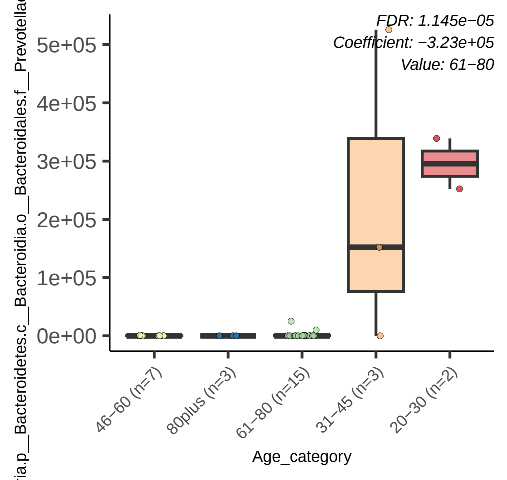

FDR: 1.145e−05
Coefficient: −3.23e+05
Value: 61−80
ia.p__Bacteroidetes.c__Bacteroidia.o__Bacteroidales.f__Prevotellac
5e+05
4e+05
3e+05
2e+05
1e+05
0e+00
46−60 (n=7)
80plus (n=3)
61−80 (n=15)
31−45 (n=3)
20−30 (n=2)
Age_category

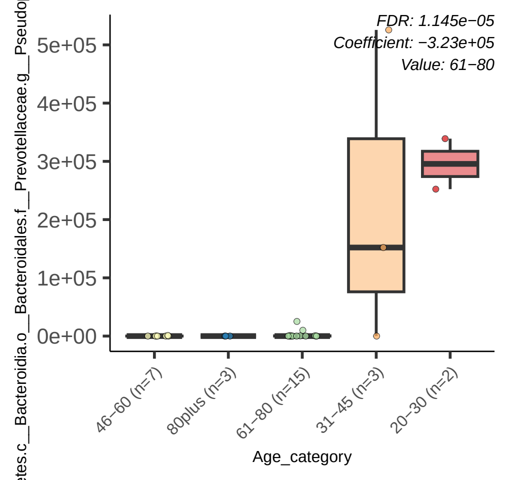
FDR: 1.145e−05
Coefficient: −3.23e+05
Value: 61−80
etes.c__Bacteroidia.o__Bacteroidales.f__Prevotellaceae.g__Pseudo
5e+05
4e+05
3e+05
2e+05
1e+05
0e+00
46−60 (n=7)
80plus (n=3)
61−80 (n=15)
31−45 (n=3)
20−30 (n=2)
Age_category

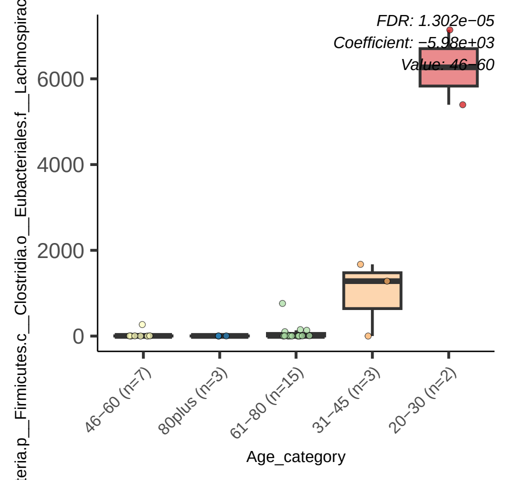

FDR: 1.302e−05
Coefficient: −5.98e+03
Value: 46−60
6000
4000
2000
0
teria.p__Firmicutes.c__Clostridia.o__Eubacteriales.f__Lachnospirac
46−60 (n=7)
80plus (n=3)
61−80 (n=15)
31−45 (n=3)
20−30 (n=2)
Age_category

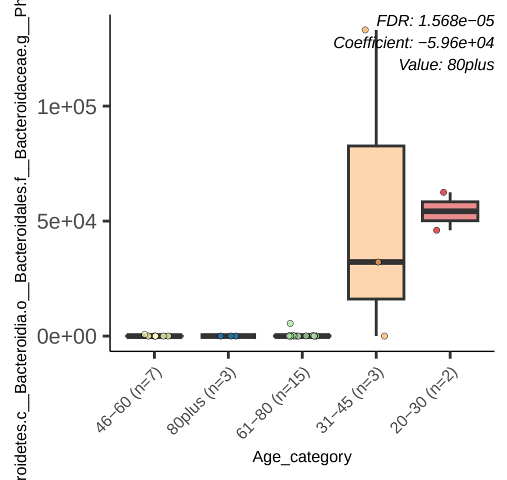
FDR: 1.568e−05
Coefficient: −5.96e+04
Value: 80plus
1e+05
5e+04
0e+00
46−60 (n=7)
80plus (n=3)
61−80 (n=15)
31−45 (n=3)
20−30 (n=2)
Age_category
roidetes.c__Bacteroidia.o__Bacteroidales.f__Bacteroidaceae.g__Ph

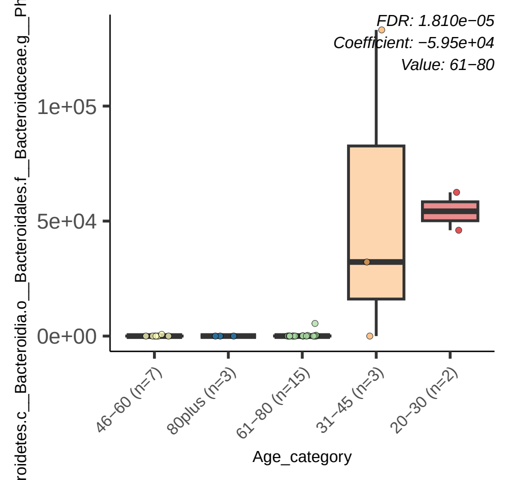

FDR: 1.810e−05
Coefficient: −5.95e+04
Value: 61−80
1e+05
5e+04
0e+00
46−60 (n=7)
80plus (n=3)
61−80 (n=15)
31−45 (n=3)
20−30 (n=2)
Age_category
roidetes.c__Bacteroidia.o__Bacteroidales.f__Bacteroidaceae.g__Ph

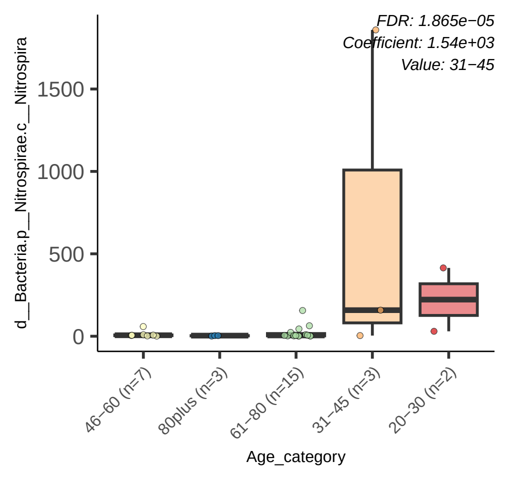
FDR: 1.865e−05
Coefficient: 1.54e+03
Value: 31−45
d__Bacteria.p__Nitrospirae.c__Nitrospira
1500
1000
500
0
46−60 (n=7)
80plus (n=3)
61−80 (n=15)
31−45 (n=3)
20−30 (n=2)
Age_category

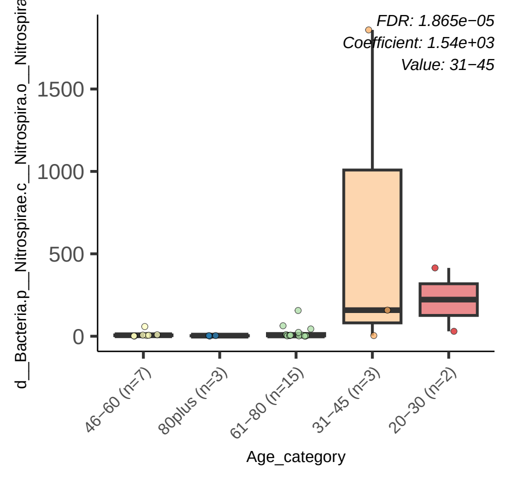

FDR: 1.865e−05
Coefficient: 1.54e+03
Value: 31−45
d__Bacteria.p__Nitrospirae.c__Nitrospira.o__Nitrospira
1500
1000
500
0
46−60 (n=7)
80plus (n=3)
61−80 (n=15)
31−45 (n=3)
20−30 (n=2)
Age_category

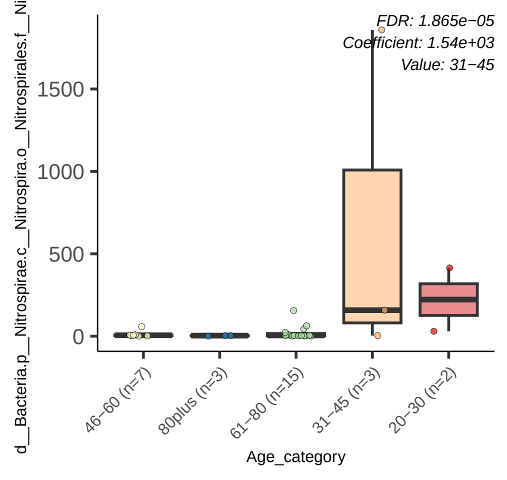

FDR: 1.865e−05
Coefficient: 1.54e+03
Value: 31−45
d__Bacteria.p__Nitrospirae.c__Nitrospira.o__Nitrospirales.f__Ni
1500
1000
500
0
46−60 (n=7)
80plus (n=3)
61−80 (n=15)
31−45 (n=3)
20−30 (n=2)
Age_category

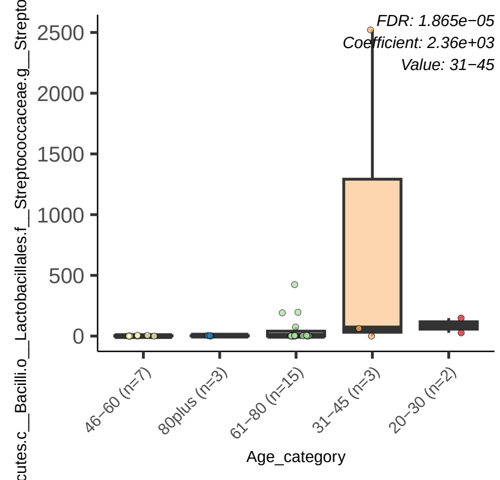
FDR: 1.865e−05
Coefficient: 2.36e+03
Value: 31−45
2500
2000
1500
1000
500
0
cutes.c__Bacilli.o__Lactobacillales.f__Streptococcaceae.g__Strepto
46−60 (n=7)
80plus (n=3)
61−80 (n=15)
31−45 (n=3)
20−30 (n=2)
Age_category

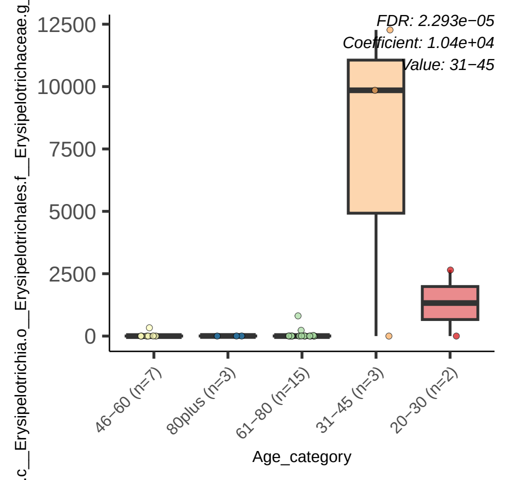

FDR: 2.293e−05
Coefficient: 1.04e+04
Value: 31−45
12500
10000
7500
5000
2500
0
46−60 (n=7)
80plus (n=3)
61−80 (n=15)
31−45 (n=3)
20−30 (n=2)
Age_category
.c__Erysipelotrichia.o__Erysipelotrichales.f__Erysipelotrichaceae.g_

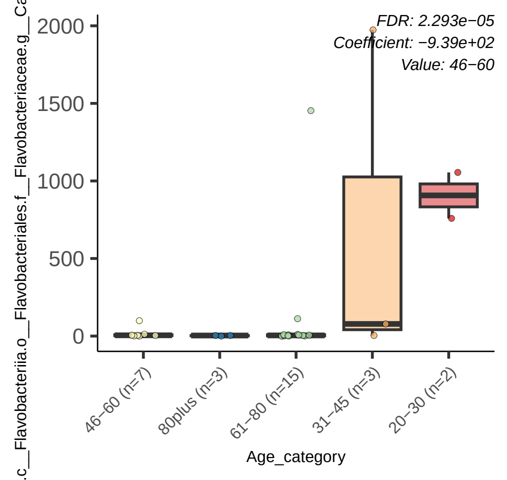

FDR: 2.293e−05
Coefficient: −9.39e+02
Value: 46−60
2000
1500
1000
500
0
46−60 (n=7)
80plus (n=3)
61−80 (n=15)
31−45 (n=3)
20−30 (n=2)
Age_category
c__Flavobacteriia.o__Flavobacteriales.f__Flavobacteriaceae.g__Ca

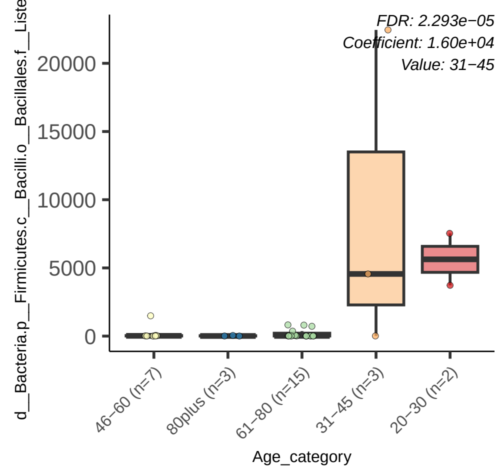

FDR: 2.293e−05
Coefficient: 1.60e+04
Value: 31−45
d__Bacteria.p__Firmicutes.c__Bacilli.o__Bacillales.f__Liste
20000
15000
10000
5000
0
46−60 (n=7)
80plus (n=3)
61−80 (n=15)
31−45 (n=3)
20−30 (n=2)
Age_category

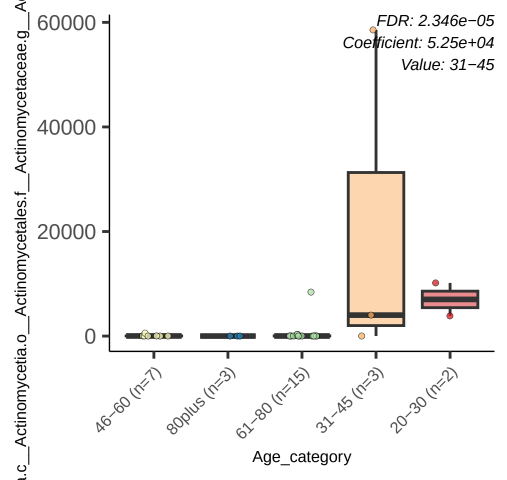
FDR: 2.346e−05
Coefficient: 5.25e+04
Value: 31−45
60000
40000
20000
0
46−60 (n=7)
80plus (n=3)
61−80 (n=15)
31−45 (n=3)
20−30 (n=2)
Age_category
a.c__Actinomycetia.o__Actinomycetales.f__Actinomycetaceae.g__Ac

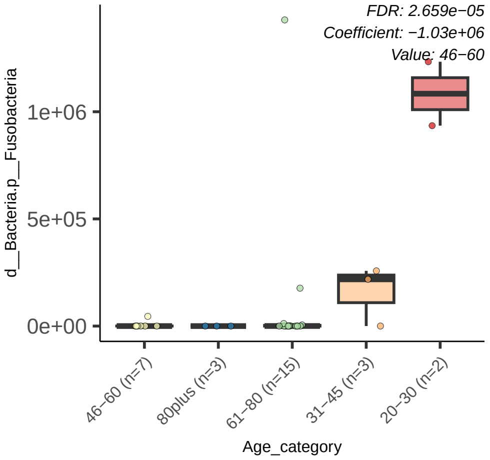

FDR: 2.659e−05
Coefficient: −1.03e+06
Value: 46−60
d__Bacteria.p__Fusobacteria
1e+06
5e+05
0e+00
46−60 (n=7)
80plus (n=3)
61−80 (n=15)
31−45 (n=3)
20−30 (n=2)
Age_category

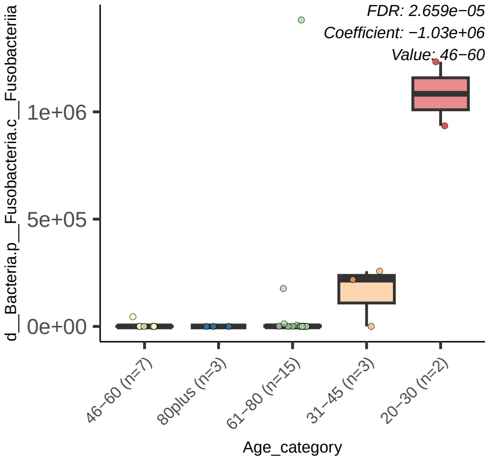

FDR: 2.659e−05
Coefficient: −1.03e+06
Value: 46−60
d__Bacteria.p__Fusobacteria.c__Fusobacteriia
1e+06
5e+05
0e+00
46−60 (n=7)
80plus (n=3)
61−80 (n=15)
31−45 (n=3)
20−30 (n=2)
Age_category

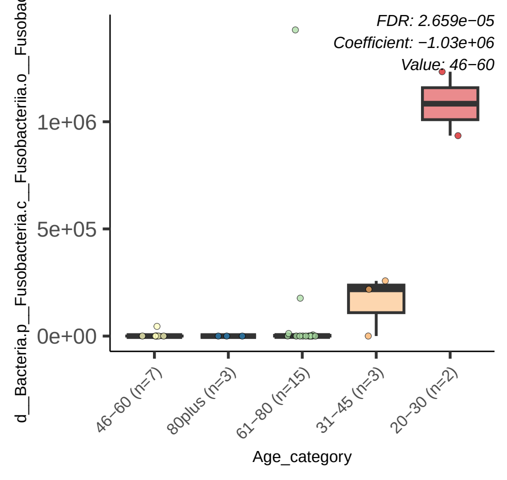

FDR: 2.659e−05
Coefficient: −1.03e+06
Value: 46−60
1e+06
5e+05
0e+00
d__Bacteria.p__Fusobacteria.c__Fusobacteriia.o__Fusobac
46−60 (n=7)
80plus (n=3)
61−80 (n=15)
31−45 (n=3)
20−30 (n=2)
Age_category

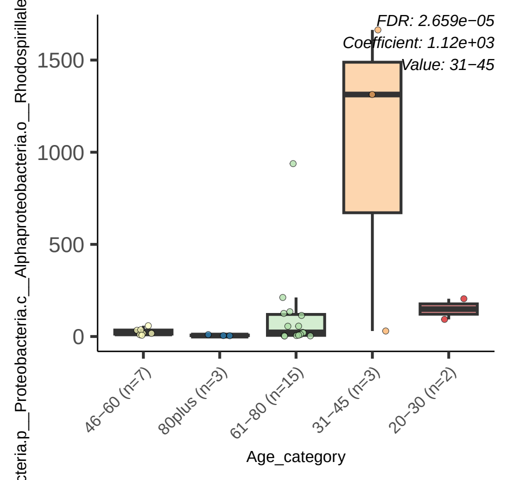
FDR: 2.659e−05
Coefficient: 1.12e+03
Value: 31−45
1500
1000
500
0
cteria.p__Proteobacteria.c__Alphaproteobacteria.o__Rhodospirillale
46−60 (n=7)
80plus (n=3)
61−80 (n=15)
31−45 (n=3)
20−30 (n=2)
Age_category

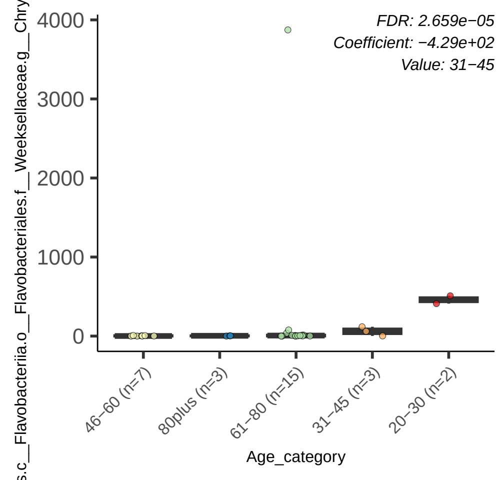

FDR: 2.659e−05
Coefficient: −4.29e+02
Value: 31−45
s.c__Flavobacteriia.o__Flavobacteriales.f__Weeksellaceae.g__Chry
4000
3000
2000
1000
0
46−60 (n=7)
80plus (n=3)
61−80 (n=15)
31−45 (n=3)
20−30 (n=2)
Age_category

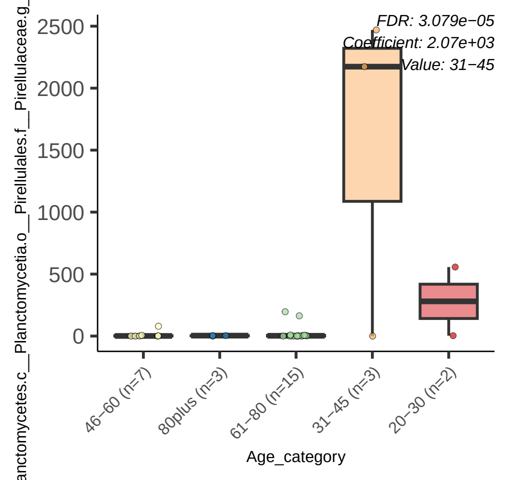

FDR: 3.079e−05
Coefficient: 2.07e+03
Value: 31−45
Planctomycetes.c__Planctomycetia.o__Pirellulales.f__Pirellulaceae.g_
2500
2000
1500
1000
500
0
46−60 (n=7)
80plus (n=3)
61−80 (n=15)
31−45 (n=3)
20−30 (n=2)
Age_category

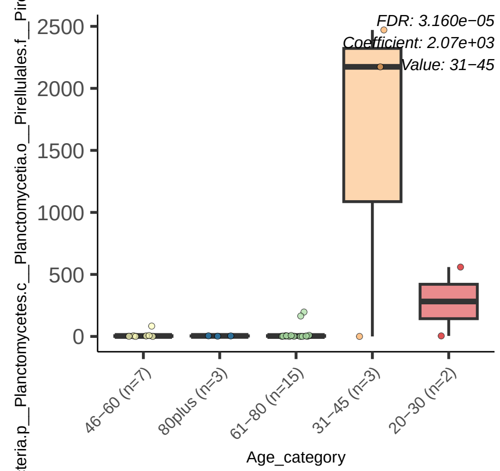

FDR: 3.160e−05
Coefficient: 2.07e+03
Value: 31−45
2500
2000
1500
1000
500
0
46−60 (n=7)
80plus (n=3)
61−80 (n=15)
31−45 (n=3)
20−30 (n=2)
Age_category

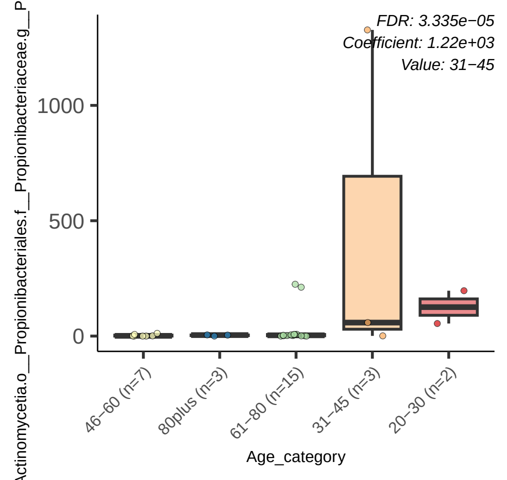
FDR: 3.335e−05
Coefficient: 1.22e+03
Value: 31−45
1000
500
0
Actinomycetia.o__Propionibacteriales.f__Propionibacteriaceae.g__P
46−60 (n=7)
80plus (n=3)
61−80 (n=15)
31−45 (n=3)
20−30 (n=2)
Age_category

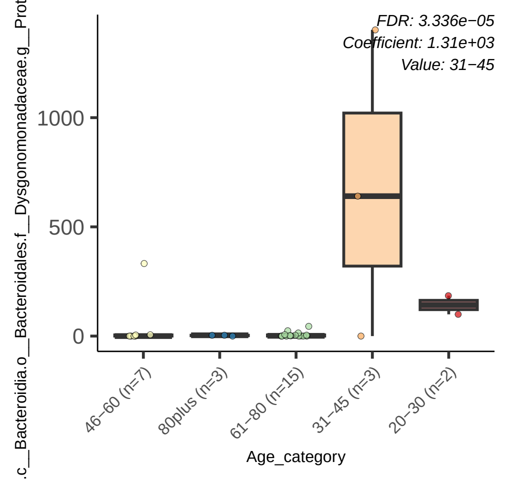

FDR: 3.336e−05
Coefficient: 1.31e+03
Value: 31−45
c__Bacteroidia.o__Bacteroidales.f__Dysgonomonadaceae.g__Prot
1000
500
0
46−60 (n=7)
80plus (n=3)
61−80 (n=15)
31−45 (n=3)
20−30 (n=2)
Age_category

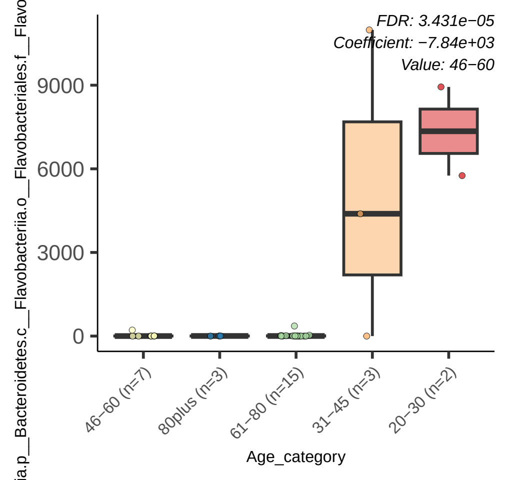
FDR: 3.431e−05
Coefficient: −7.84e+03
Value: 46−60
9000
6000
3000
0
ia.p__Bacteroidetes.c__Flavobacteriia.o__Flavobacteriales.f__Flavo
46−60 (n=7)
80plus (n=3)
61−80 (n=15)
31−45 (n=3)
20−30 (n=2)
Age_category

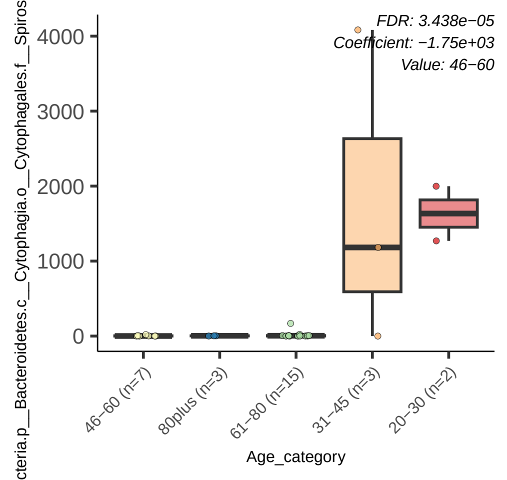

FDR: 3.438e−05
Coefficient: −1.75e+03
Value: 46−60
cteria.p__Bacteroidetes.c__Cytophagia.o__Cytophagales.f__Spiros
4000
3000
2000
1000
0
46−60 (n=7)
80plus (n=3)
61−80 (n=15)
31−45 (n=3)
20−30 (n=2)
Age_category

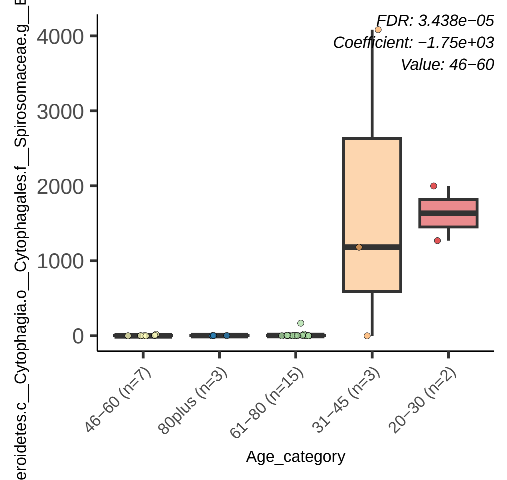

FDR: 3.438e−05
Coefficient: −1.75e+03
Value: 46−60
4000
3000
2000
1000
0
eroidetes.c__Cytophagia.o__Cytophagales.f__Spirosomaceae.g__E
46−60 (n=7)
80plus (n=3)
61−80 (n=15)
31−45 (n=3)
20−30 (n=2)
Age_category

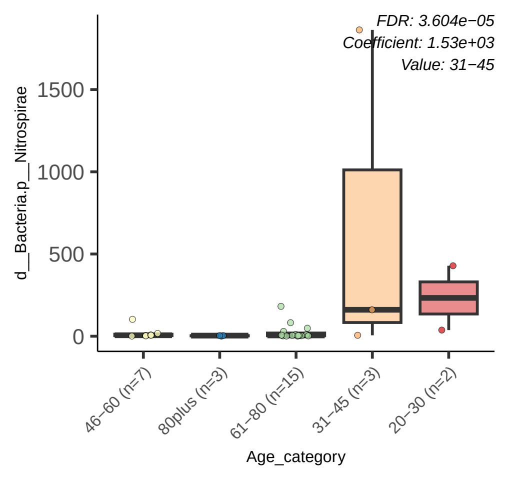
FDR: 3.604e−05
Coefficient: 1.53e+03
Value: 31−45
d__Bacteria.p__Nitrospirae
1500
1000
500
0
46−60 (n=7)
80plus (n=3)
61−80 (n=15)
31−45 (n=3)
20−30 (n=2)
Age_category

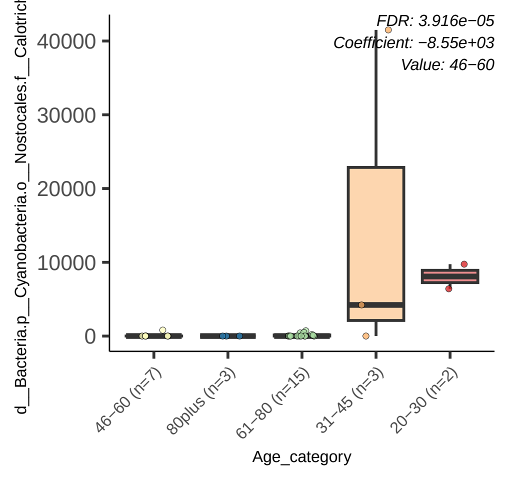

FDR: 3.916e−05
Coefficient: −8.55e+03
Value: 46−60
40000
30000
20000
10000
0
d__Bacteria.p__Cyanobacteria.o__Nostocales.f__Calotrich
46−60 (n=7)
80plus (n=3)
61−80 (n=15)
31−45 (n=3)
20−30 (n=2)
Age_category

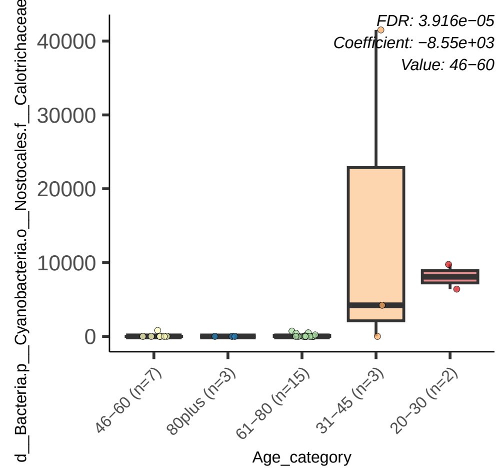
FDR: 3.916e−05
Coefficient: −8.55e+03
Value: 46−60
d__Bacteria.p__Cyanobacteria.o__Nostocales.f__Calotrichaceae
40000
30000
20000
10000
0
46−60 (n=7)
80plus (n=3)
61−80 (n=15)
31−45 (n=3)
20−30 (n=2)
Age_category

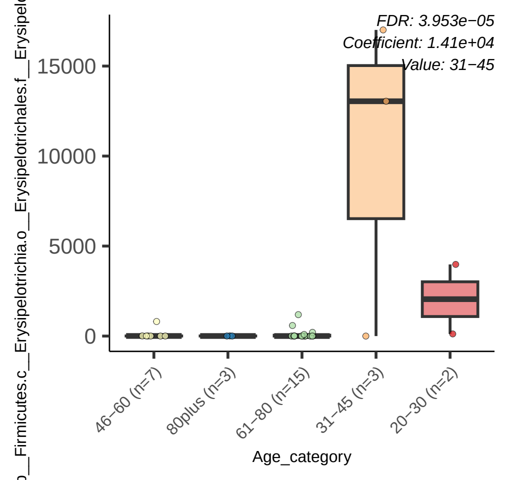

FDR: 3.953e−05
Coefficient: 1.41e+04
Value: 31−45
15000
10000
5000
0
46−60 (n=7)
80plus (n=3)
61−80 (n=15)
31−45 (n=3)
20−30 (n=2)
Age_category
o__Firmicutes.c__Erysipelotrichia.o__Erysipelotrichales.f__Erysipelo

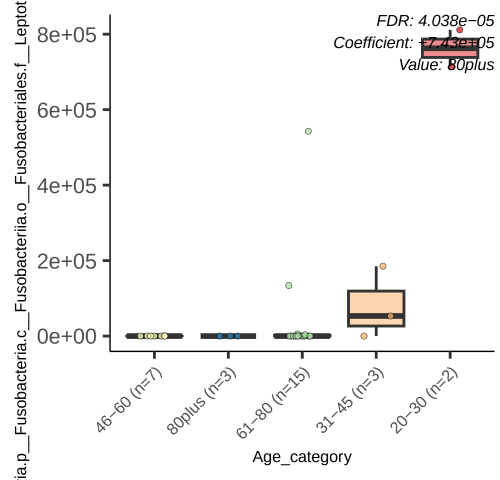

FDR: 4.038e−05
Coefficient: −7.43e+05
Value: 80plus
8e+05
6e+05
4e+05
2e+05
0e+00
ia.p__Fusobacteria.c__Fusobacteriia.o__Fusobacteriales.f__Leptot
46−60 (n=7)
80plus (n=3)
61−80 (n=15)
31−45 (n=3)
20−30 (n=2)
Age_category

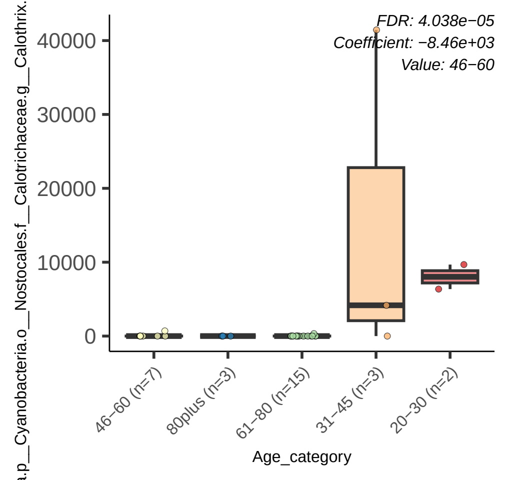
FDR: 4.038e−05
Coefficient: −8.46e+03
Value: 46−60
40000
30000
20000
10000
0
a.p__Cyanobacteria.o__Nostocales.f__Calotrichaceae.g__Calothrix.
46−60 (n=7)
80plus (n=3)
61−80 (n=15)
31−45 (n=3)
20−30 (n=2)
Age_category

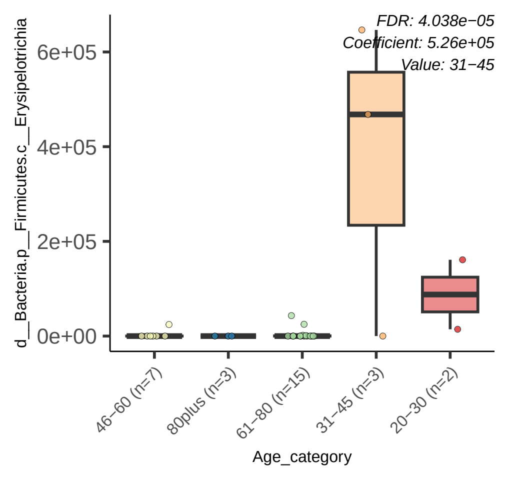

FDR: 4.038e−05
Coefficient: 5.26e+05
Value: 31−45
d__Bacteria.p__Firmicutes.c__Erysipelotrichia
6e+05
4e+05
2e+05
0e+00
46−60 (n=7)
80plus (n=3)
61−80 (n=15)
31−45 (n=3)
20−30 (n=2)
Age_category

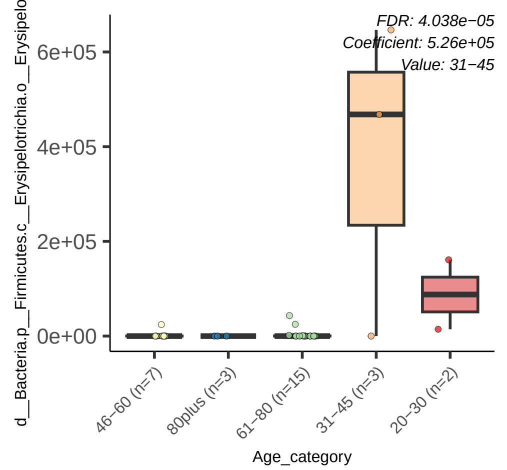

FDR: 4.038e−05
Coefficient: 5.26e+05
Value: 31−45
d__Bacteria.p__Firmicutes.c__Erysipelotrichia.o__Erysipelo
6e+05
4e+05
2e+05
0e+00
46−60 (n=7)
80plus (n=3)
61−80 (n=15)
31−45 (n=3)
20−30 (n=2)
Age_category

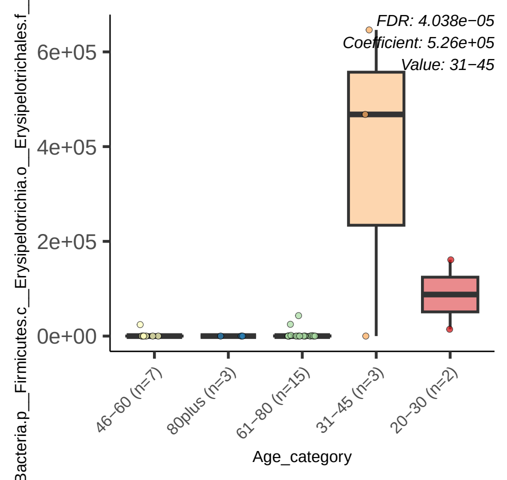
FDR: 4.038e−05
Coefficient: 5.26e+05
Value: 31−45
Bacteria.p__Firmicutes.c__Erysipelotrichia.o__Erysipelotrichales.f__
6e+05
4e+05
2e+05
0e+00
46−60 (n=7)
80plus (n=3)
61−80 (n=15)
31−45 (n=3)
20−30 (n=2)
Age_category

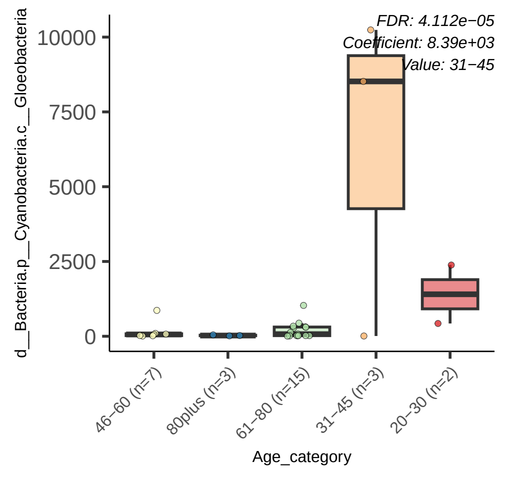

FDR: 4.112e−05
Coefficient: 8.39e+03
Value: 31−45
d__Bacteria.p__Cyanobacteria.c__Gloeobacteria
10000
7500
5000
2500
0
46−60 (n=7)
80plus (n=3)
61−80 (n=15)
31−45 (n=3)
20−30 (n=2)
Age_category

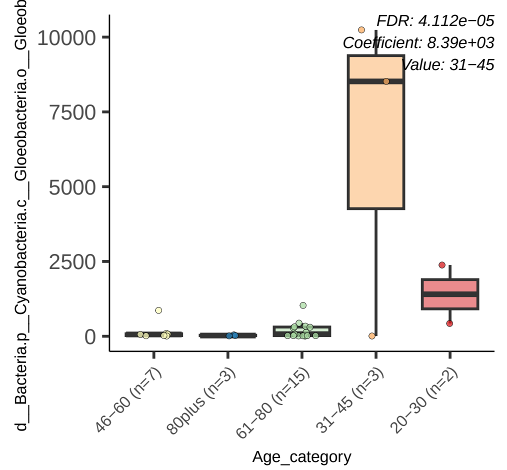

FDR: 4.112e−05
Coefficient: 8.39e+03
Value: 31−45
d__Bacteria.p__Cyanobacteria.c__Gloeobacteria.o__Gloeob
10000
7500
5000
2500
0
46−60 (n=7)
80plus (n=3)
61−80 (n=15)
31−45 (n=3)
20−30 (n=2)
Age_category

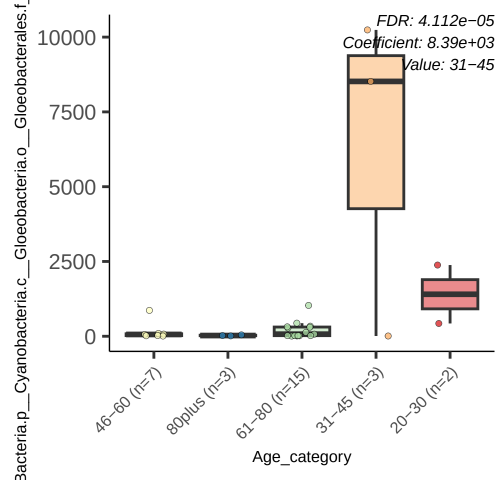

FDR: 4.112e−05
Coefficient: 8.39e+03
Value: 31−45
10000
7500
5000
2500
0
Bacteria.p__Cyanobacteria.c__Gloeobacteria.o__Gloeobacterales.f_
46−60 (n=7)
80plus (n=3)
61−80 (n=15)
31−45 (n=3)
20−30 (n=2)
Age_category

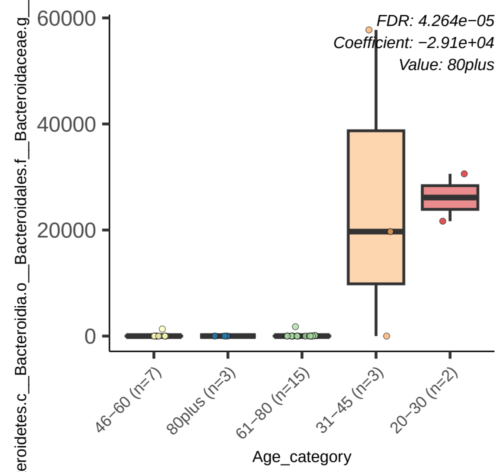
FDR: 4.264e−05
Coefficient: −2.91e+04
Value: 80plus
60000
40000
20000
0
cteroidetes.c__Bacteroidia.o__Bacteroidales.f__Bacteroidaceae.g__
46−60 (n=7)
80plus (n=3)
61−80 (n=15)
31−45 (n=3)
20−30 (n=2)
Age_category

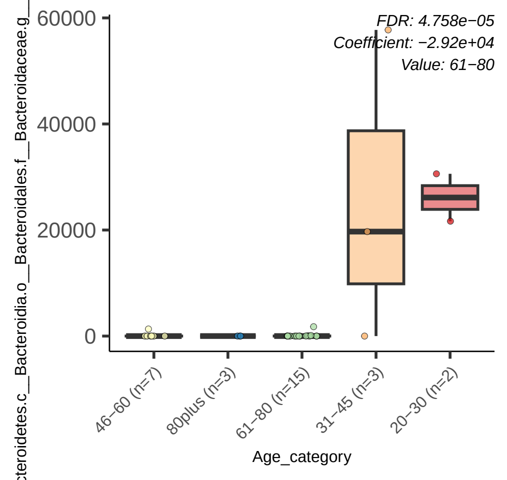

FDR: 4.758e−05
Coefficient: −2.92e+04
Value: 61−80
60000
40000
20000
0
46−60 (n=7)
80plus (n=3)
61−80 (n=15)
31−45 (n=3)
20−30 (n=2)
Age_category
cteroidetes.c__Bacteroidia.o__Bacteroidales.f__Bacteroidaceae.g__

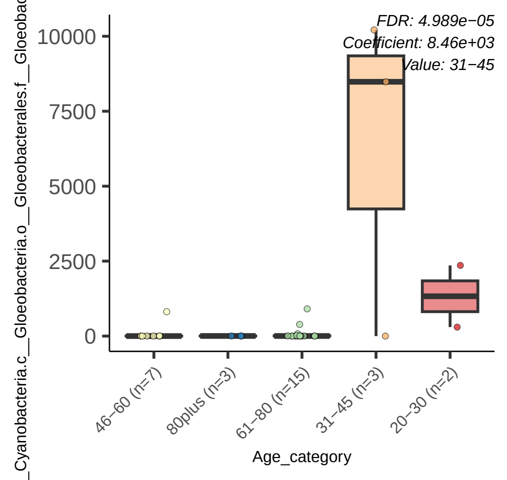

FDR: 4.989e−05
Coefficient: 8.46e+03
Value: 31−45
Cyanobacteria.c__Gloeobacteria.o__Gloeobacterales.f__Gloeobac
10000
7500
5000
2500
0
46−60 (n=7)
80plus (n=3)
61−80 (n=15)
31−45 (n=3)
20−30 (n=2)
Age_category

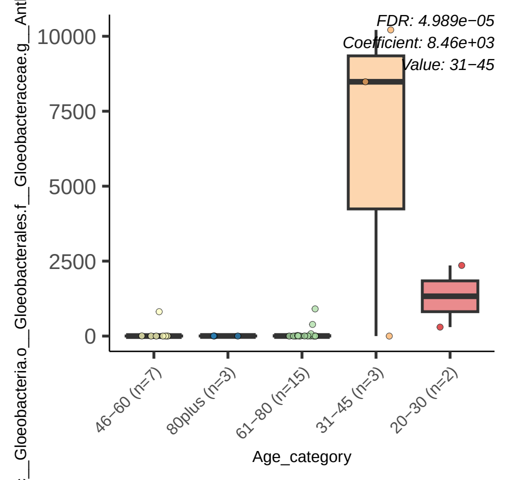

FDR: 4.989e−05
Coefficient: 8.46e+03
Value: 31−45
10000
7500
5000
2500
0
Gloeobacteria.o__Gloeobacterales.f__Gloeobacteraceae.g__Ant
46−60 (n=7)
80plus (n=3)
61−80 (n=15)
31−45 (n=3)
20−30 (n=2)
Age_category

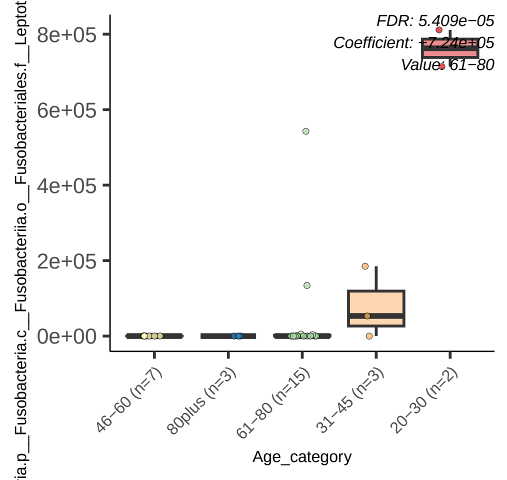
FDR: 5.409e−05
Coefficient: −7.24e+05
Value: 61−80
8e+05
6e+05
4e+05
2e+05
0e+00
ria.p__Fusobacteria.c__Fusobacteriia.o__Fusobacteriales.f__Leptot
46−60 (n=7)
80plus (n=3)
61−80 (n=15)
31−45 (n=3)
20−30 (n=2)
Age_category

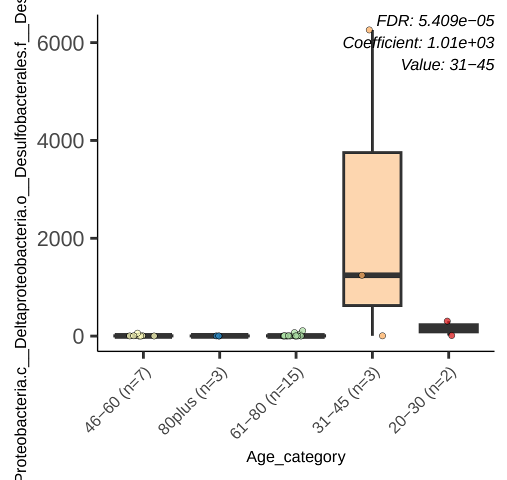

FDR: 5.409e−05
Coefficient: 1.01e+03
Value: 31−45
Proteobacteria.c__Deltaproteobacteria.o__Desulfobacterales.f__Des
6000
4000
2000
0
46−60 (n=7)
80plus (n=3)
61−80 (n=15)
31−45 (n=3)
20−30 (n=2)
Age_category

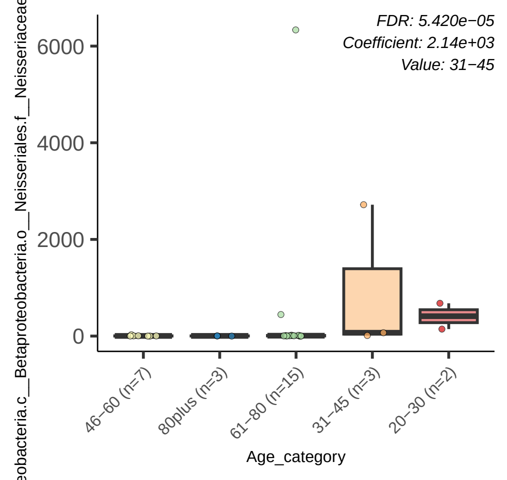

FDR: 5.420e−05
Coefficient: 2.14e+03
Value: 31−45
6000
4000
2000
0
46−60 (n=7)
80plus (n=3)
61−80 (n=15)
31−45 (n=3)
20−30 (n=2)
Age_category
eobacteria.c__Betaproteobacteria.o__Neisseriales.f__Neisseriaceae

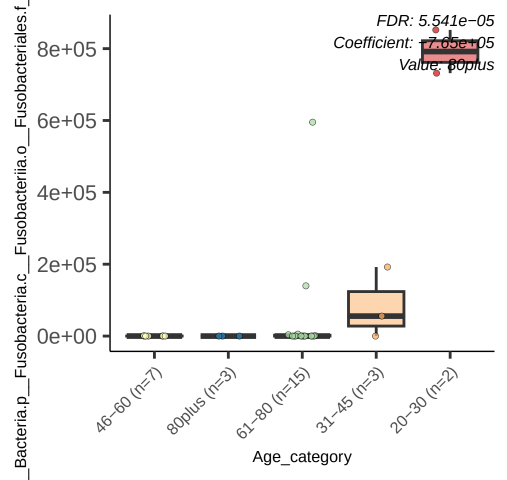

FDR: 5.541e−05
Coefficient: -7.65e+05
Value: 80plus
8e+05
6e+05
4e+05
2e+05
0e+00
Bacteria.p__Fusobacteria.c__Fusobacteriia.o__Fusobacteriales.f__
46−60 (n=7)
80plus (n=3)
61−80 (n=15)
31−45 (n=3)
20−30 (n=2)
Age_category

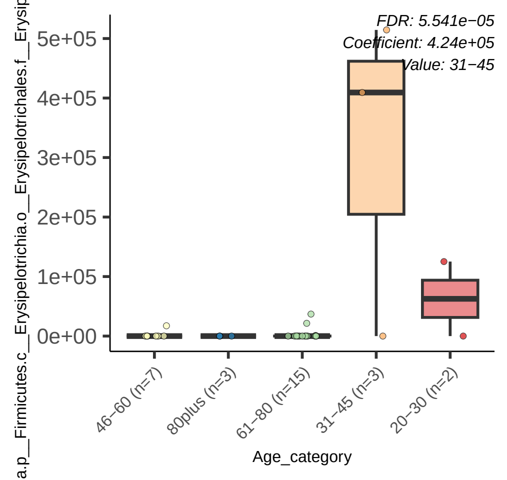

FDR: 5.541e−05
Coefficient: 4.24e+05
Value: 31−45
0e+00
1e+05
2e+05
3e+05
4e+05
5e+05
a.p__Firmicutes.c__Erysipelotrichia.o__Erysipelotrichales.f__Erysip
46−60 (n=7)
80plus (n=3)
61−80 (n=15)
31−45 (n=3)
20−30 (n=2)
Age_category

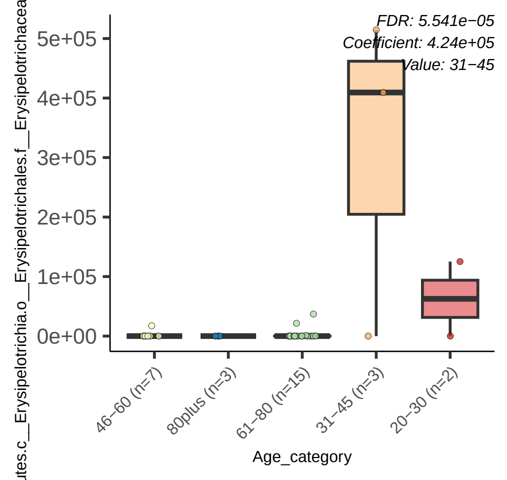

FDR: 5.541e−05
Coefficient: 4.24e+05
Value: 31−45
5e+05
4e+05
3e+05
2e+05
1e+05
0e+00
utes.c__Erysipelotrichia.o__Erysipelotrichales.f__Erysipelotrichacea
46−60 (n=7)
80plus (n=3)
61−80 (n=15)
31−45 (n=3)
20−30 (n=2)
Age_category

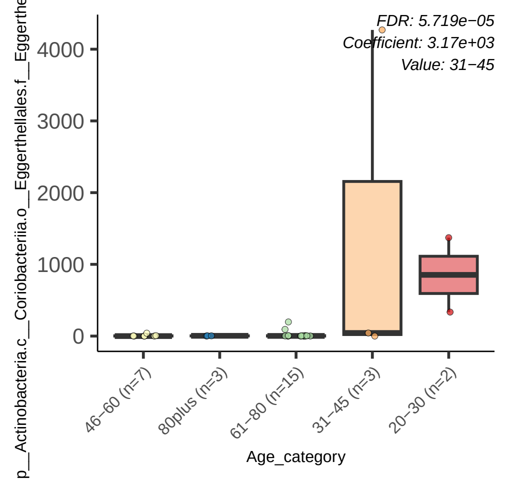
FDR: 5.719e−05
Coefficient: 3.17e+03
Value: 31−45
p__Actinobacteria.c__Coriobacteriia.o__Eggerthellales.f__Eggerthe
4000
3000
2000
1000
0
46−60 (n=7)
80plus (n=3)
61−80 (n=15)
31−45 (n=3)
20−30 (n=2)
Age_category

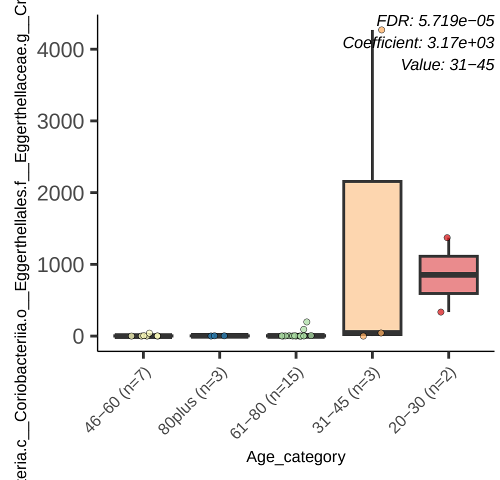

FDR: 5.719e−05
Coefficient: 3.17e+03
Value: 31−45
4000
3000
2000
1000
0
teria.c__Coriobacteriia.o__Eggerthellales.f__Eggerthellaceae.g__Cr
46−60 (n=7)
80plus (n=3)
61−80 (n=15)
31−45 (n=3)
20−30 (n=2)
Age_category

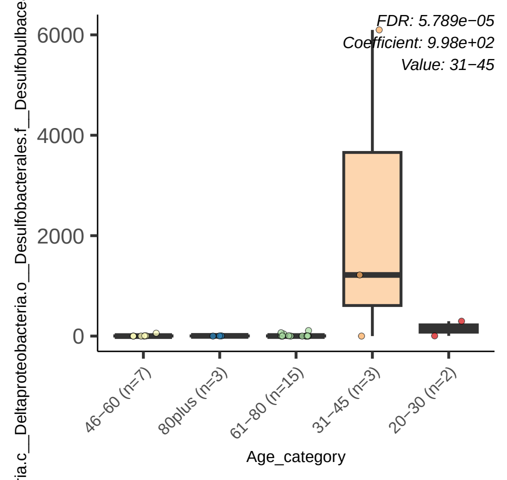

FDR: 5.789e−05
Coefficient: 9.98e+02
Value: 31−45
6000
4000
2000
0
ria.c__Deltaproteobacteria.o__Desulfobacterales.f__Desulfobulbace
46−60 (n=7)
80plus (n=3)
61−80 (n=15)
31−45 (n=3)
20−30 (n=2)
Age_category

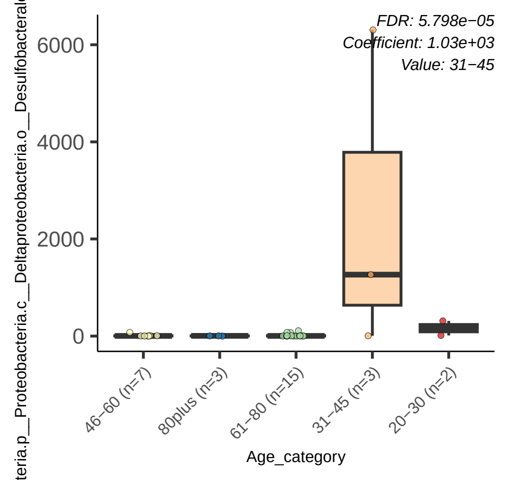

FDR: 5.798e−05
Coefficient: 1.03e+03
Value: 31−45
6000
4000
2000
0
teria.p__Proteobacteria.c__Deltaproteobacteria.o__Desulfobacteral
46−60 (n=7)
80plus (n=3)
61−80 (n=15)
31−45 (n=3)
20−30 (n=2)
Age_category

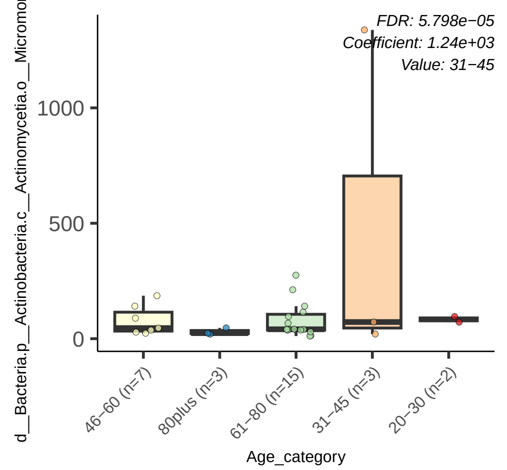

FDR: 5.798e−05
Coefficient: 1.24e+03
Value: 31−45
d__Bacteria.p__Actinobacteria.c__Actinomycetia.o__Micromo
1000
500
0
46−60 (n=7)
80plus (n=3)
61−80 (n=15)
31−45 (n=3)
20−30 (n=2)
Age_category

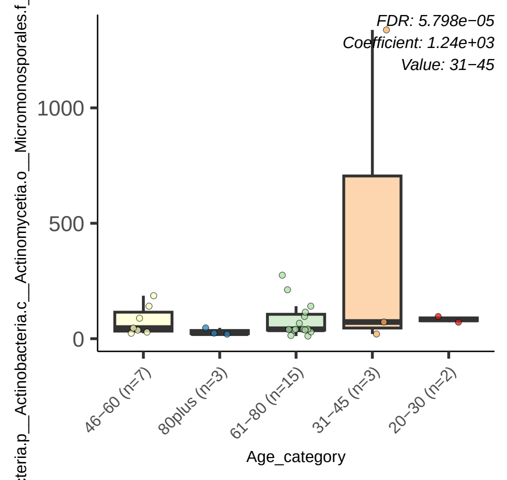
FDR: 5.798e−05
Coefficient: 1.24e+03
Value: 31−45
1000
500
0
46−60 (n=7)
80plus (n=3)
61−80 (n=15)
31−45 (n=3)
20−30 (n=2)
Age_category
cteria.p__Actinobacteria.c__Actinomycetia.o__Micromonosporales.f_

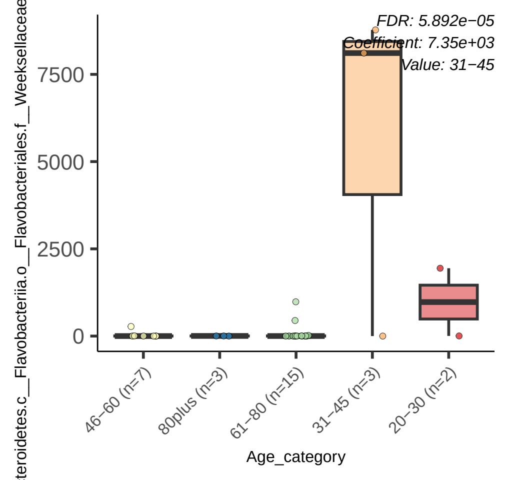
FDR: 5.892e−05
Coefficient: 7.35e+03
Value: 31−45
7500
5000
2500
0
46−60 (n=7)
80plus (n=3)
61−80 (n=15)
31−45 (n=3)
20−30 (n=2)
Age_category
cteroidetes.c__Flavobacteriia.o__Flavobacteriales.f__Weeksellaceae

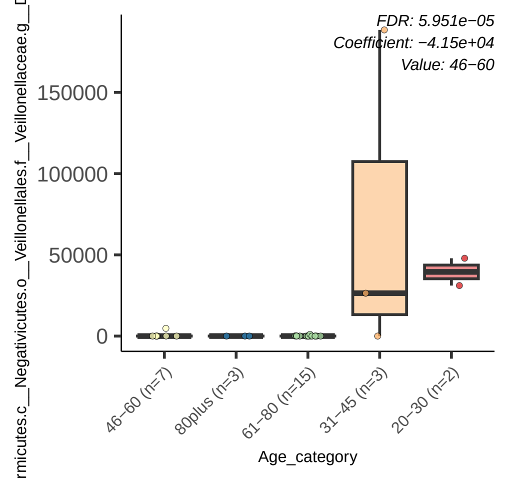

FDR: 5.951e−05
Coefficient: −4.15e+04
Value: 46−60
rmicutes.c__Negativicutes.o__Veillonellales.f__Veillonellaceae.g__D
150000
100000
50000
0
46−60 (n=7)
80plus (n=3)
61−80 (n=15)
31−45 (n=3)
20−30 (n=2)
Age_category

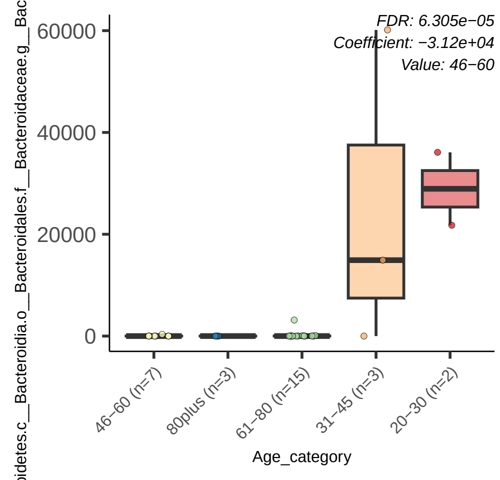

FDR: 6.305e−05
Coefficient: −3.12e+04
Value: 46−60
60000
40000
20000
0
46−60 (n=7)
80plus (n=3)
61−80 (n=15)
31−45 (n=3)
20−30 (n=2)
Age_category
oidetes.c__Bacteroidia.o__Bacteroidales.f__Bacteroidaceae.g__Bac

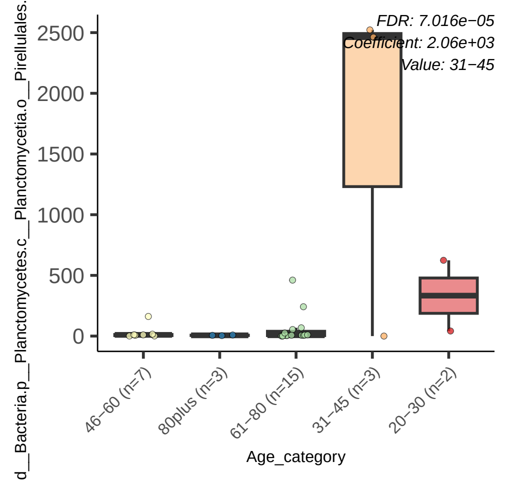

FDR: 7.016e−05
Coefficient: 2.06e+03
Value: 31−45
d__Bacteria.p__Planctomycetes.c__Planctomycetia.o__Pirellulales.
2500
2000
1500
1000
500
0
46−60 (n=7)
80plus (n=3)
61−80 (n=15)
31−45 (n=3)
20−30 (n=2)
Age_category

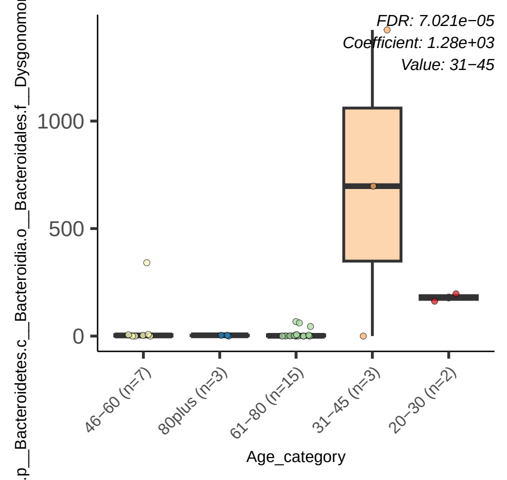

FDR: 7.021e−05
Coefficient: 1.28e+03
Value: 31−45
p__Bacteroidetes.c__Bacteroidia.o__Bacteroidales.f__Dysgonomo
1000
500
0
46−60 (n=7)
80plus (n=3)
61−80 (n=15)
31−45 (n=3)
20−30 (n=2)
Age_category

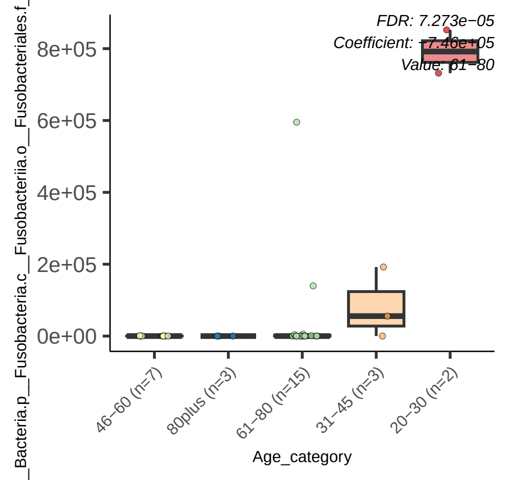
FDR: 7.273e−05
Coefficient: -7.46e+05
Value: 61−80
8e+05
6e+05
4e+05
2e+05
0e+00
_Bacteria.p__Fusobacteria.c__Fusobacteriia.o__Fusobacteriales.f__
46−60 (n=7)
80plus (n=3)
61−80 (n=15)
31−45 (n=3)
20−30 (n=2)
Age_category

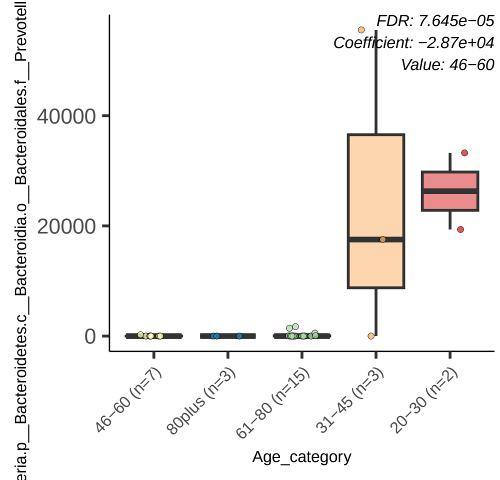
FDR: 7.645e−05
Coefficient: −2.87e+04
Value: 46−60
40000
20000
0
46−60 (n=7)
80plus (n=3)
61−80 (n=15)
31−45 (n=3)
20−30 (n=2)
Age_category
eria.p__Bacteroidetes.c__Bacteroidia.o__Bacteroidales.f__Prevotell

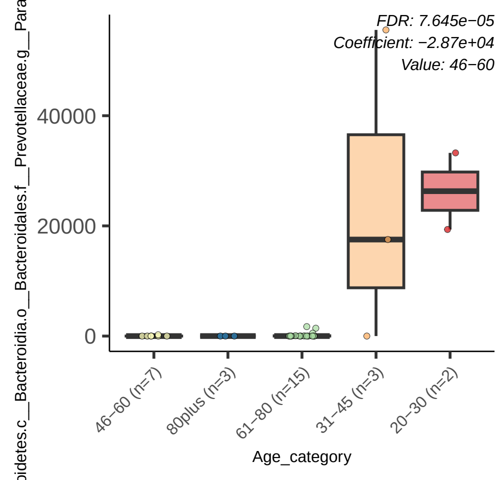

FDR: 7.645e−05
Coefficient: −2.87e+04
Value: 46−60
oidetes.c__Bacteroidia.o__Bacteroidales.f__Prevotellaceae.g__Para
40000
20000
0
46−60 (n=7)
80plus (n=3)
61−80 (n=15)
31−45 (n=3)
20−30 (n=2)
Age_category

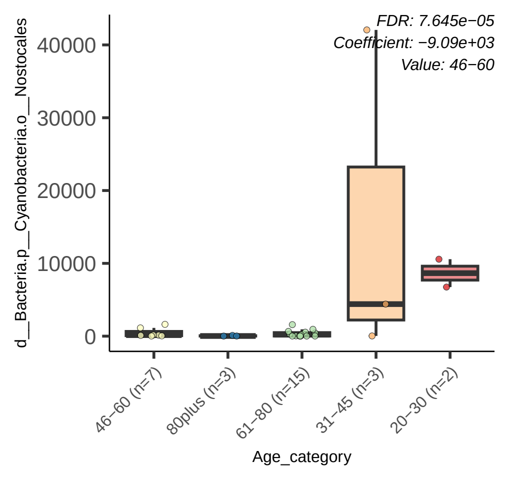
FDR: 7.645e−05
Coefficient: −9.09e+03
Value: 46−60
d__Bacteria.p__Cyanobacteria.o__Nostocales
40000
30000
20000
10000
0
46−60 (n=7)
80plus (n=3)
61−80 (n=15)
31−45 (n=3)
20−30 (n=2)
Age_category

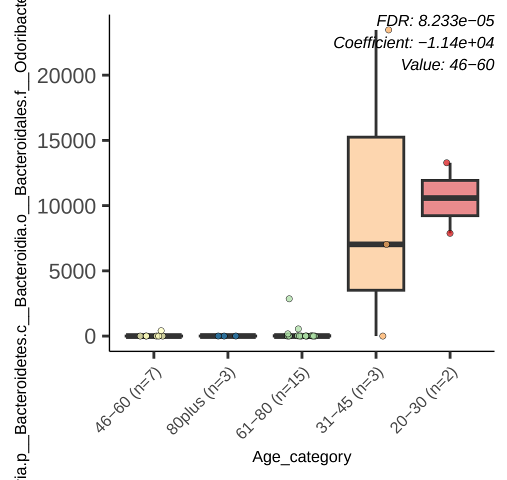

FDR: 8.233e−05
Coefficient: −1.14e+04
Value: 46−60
ia.p__Bacteroidetes.c__Bacteroidia.o__Bacteroidales.f__Odoribacte
20000
15000
10000
5000
0
46−60 (n=7)
80plus (n=3)
61−80 (n=15)
31−45 (n=3)
20−30 (n=2)
Age_category

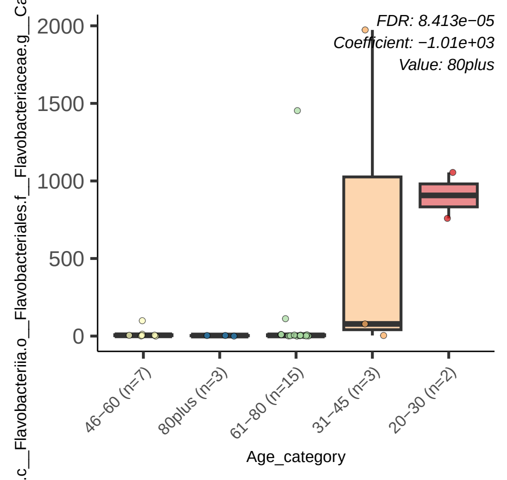

FDR: 8.413e−05
Coefficient: −1.01e+03
Value: 80plus
2000
1500
1000
500
0
c__Flavobacteriia.o__Flavobacteriales.f__Flavobacteriaceae.g__Ca
46−60 (n=7)
80plus (n=3)
61−80 (n=15)
31−45 (n=3)
20−30 (n=2)
Age_category

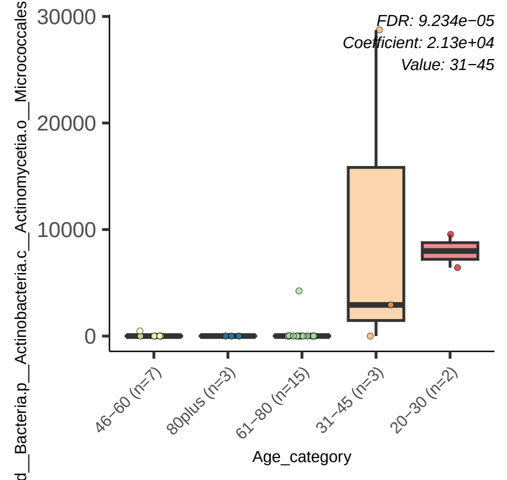

FDR: 9.234e−05
Coefficient: 2.13e+04
Value: 31−45
d__Bacteria.p__Actinobacteria.c__Actinomycetia.o__Micrococcales
30000
20000
10000
0
46−60 (n=7)
80plus (n=3)
61−80 (n=15)
31−45 (n=3)
20−30 (n=2)
Age_category

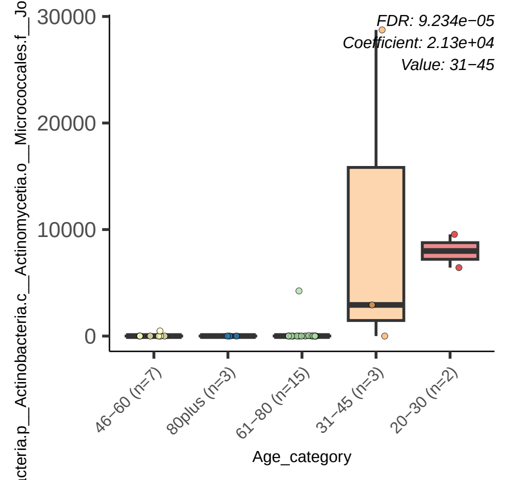

FDR: 9.234e−05
Coefficient: 2.13e+04
Value: 31−45
30000
20000
10000
0
46−60 (n=7)
80plus (n=3)
61−80 (n=15)
31−45 (n=3)
20−30 (n=2)
Age_category
acteria.p__Actinobacteria.c__Actinomycetia.o__Micrococcales.f__Jo

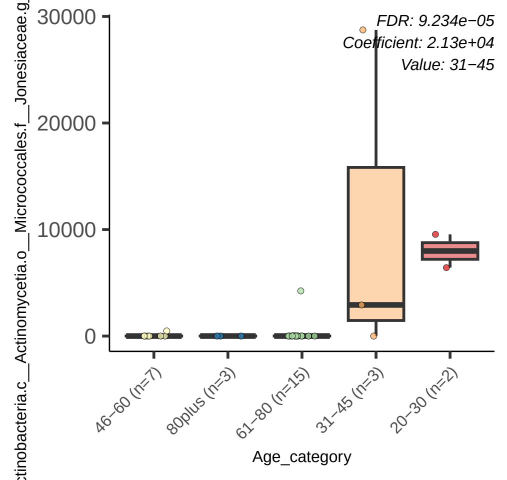
FDR: 9.234e−05
Coefficient: 2.13e+04
Value: 31−45
30000
20000
10000
0
ctinobacteria.c__Actinomycetia.o__Micrococcales.f__Jonesiaceae.g
46−60 (n=7)
80plus (n=3)
61−80 (n=15)
31−45 (n=3)
20−30 (n=2)
Age_category

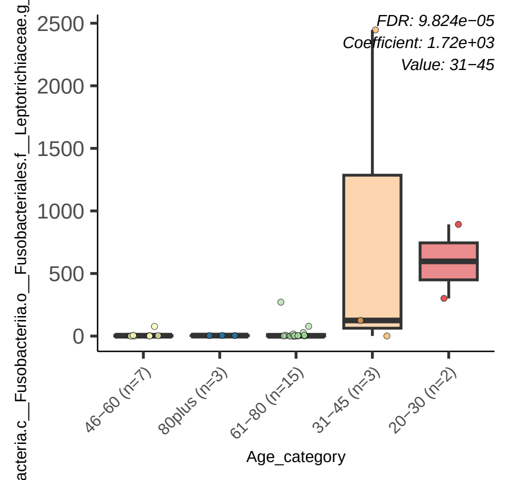

FDR: 9.824e−05
Coefficient: 1.72e+03
Value: 31−45
acteria.c__Fusobacteriia.o__Fusobacteriales.f__Leptotrichiaceae.g_
2500
2000
1500
1000
500
0
46−60 (n=7)
80plus (n=3)
61−80 (n=15)
31−45 (n=3)
20−30 (n=2)
Age_category

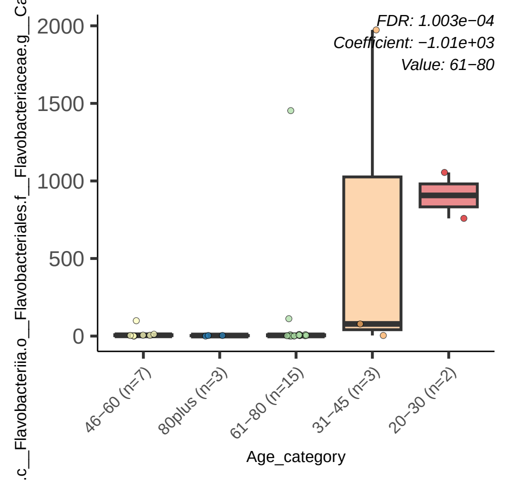

FDR: 1.003e−04
Coefficient: −1.01e+03
Value: 61−80
2000
1500
1000
500
0
46−60 (n=7)
80plus (n=3)
61−80 (n=15)
31−45 (n=3)
20−30 (n=2)
Age_category
.c__Flavobacteriia.o__Flavobacteriales.f__Flavobacteriaceae.g__Ca

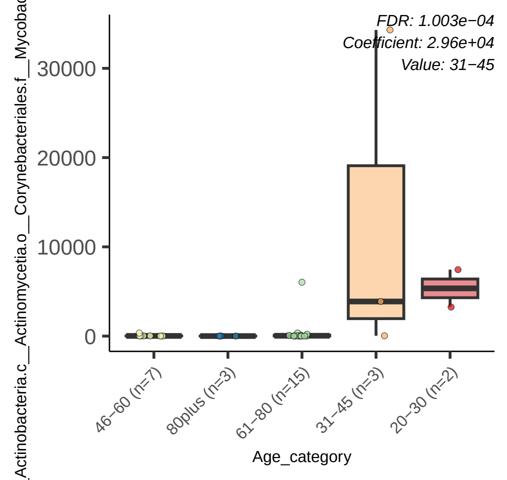

FDR: 1.003e−04
Coefficient: 2.96e+04
Value: 31−45
Actinobacteria.c__Actinomycetia.o__Corynebacteriales.f__Mycobac
30000
20000
10000
0
46−60 (n=7)
80plus (n=3)
61−80 (n=15)
31−45 (n=3)
20−30 (n=2)
Age_category

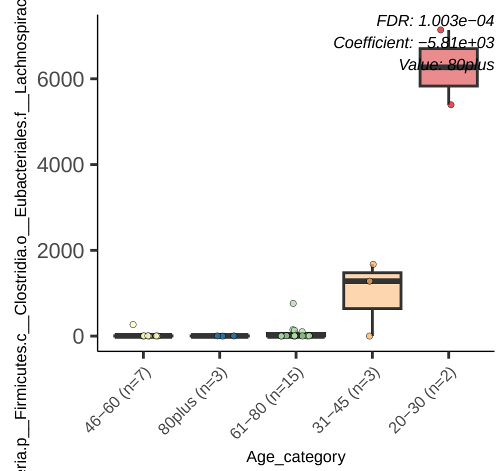
FDR: 1.003e−04
Coefficient: −5.81e+03
Value: 80plus
teria.p__Firmicutes.c__Clostridia.o__Eubacteriales.f__Lachnospirac
6000
4000
2000
0
46−60 (n=7)
80plus (n=3)
61−80 (n=15)
31−45 (n=3)
20−30 (n=2)
Age_category

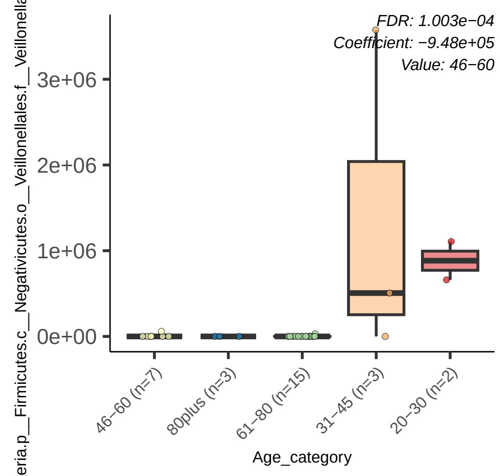

FDR: 1.003e−04
Coefficient: −9.48e+05
Value: 46−60
3e+06
2e+06
1e+06
0e+00
teria.p__Firmicutes.c__Negativicutes.o__Veillonellales.f__Veillonella
46−60 (n=7)
80plus (n=3)
61−80 (n=15)
31−45 (n=3)
20−30 (n=2)
Age_category

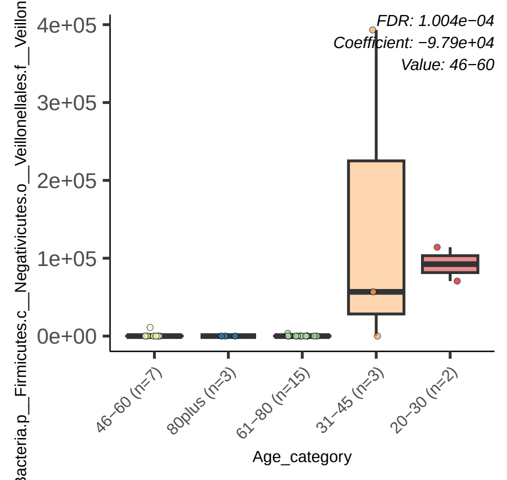
FDR: 1.004e−04
Coefficient: −9.79e+04
Value: 46−60
4e+05
3e+05
2e+05
1e+05
0e+00
Bacteria.p__Firmicutes.c__Negativicutes.o__Veillonellales.f__Veillon
46−60 (n=7)
80plus (n=3)
61−80 (n=15)
31−45 (n=3)
20−30 (n=2)
Age_category

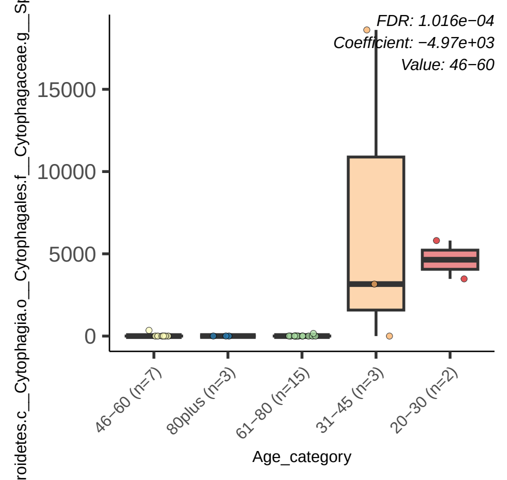

FDR: 1.016e−04
Coefficient: −4.97e+03
Value: 46−60
15000
10000
5000
0
roidetes.c__Cytophagia.o__Cytophagales.f__Cytophagaceae.g__Sp
46−60 (n=7)
80plus (n=3)
61−80 (n=15)
31−45 (n=3)
20−30 (n=2)
Age_category

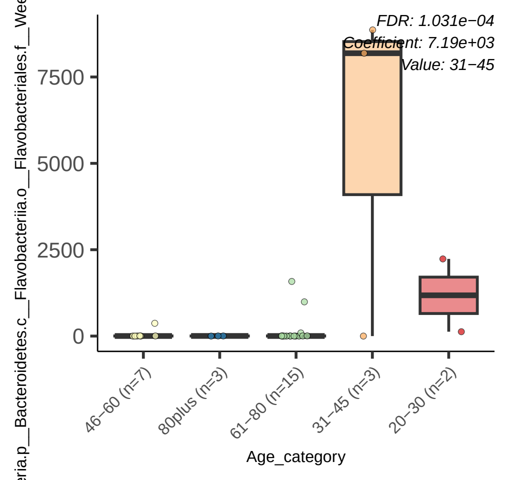

FDR: 1.031e−04
Coefficient: 7.19e+03
Value: 31−45
7500
5000
2500
0
46−60 (n=7)
80plus (n=3)
61−80 (n=15)
31−45 (n=3)
20−30 (n=2)
Age_category
eria.p__Bacteroidetes.c__Flavobacteriia.o__Flavobacteriales.f__Wee

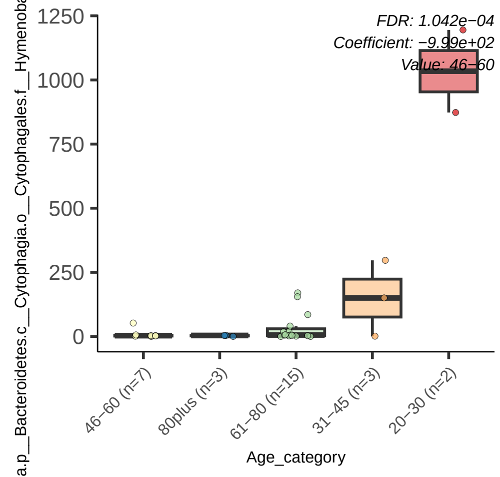

FDR: 1.042e−04
Coefficient: −9.99e+02
Value: 46−60
ia.p__Bacteroidetes.c__Cytophagia.o__Cytophagales.f__Hymenoba
1250
1000
750
500
250
0
46−60 (n=7)
80plus (n=3)
61−80 (n=15)
31−45 (n=3)
20−30 (n=2)
Age_category

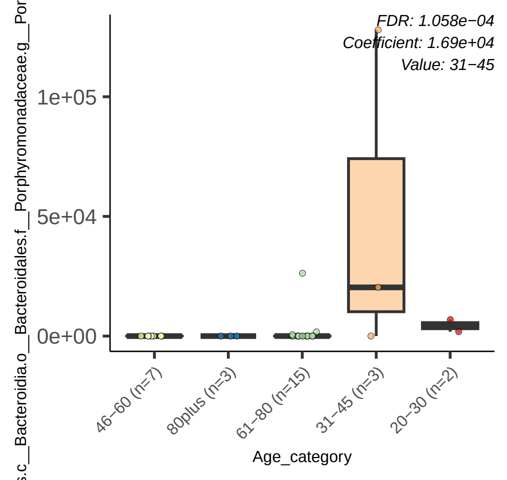

FDR: 1.058e−04
Coefficient: 1.69e+04
Value: 31−45
s.c__Bacteroidia.o__Bacteroidales.f__Porphyromonadaceae.g__Por
1e+05
5e+04
0e+00
46−60 (n=7)
80plus (n=3)
61−80 (n=15)
31−45 (n=3)
20−30 (n=2)
Age_category

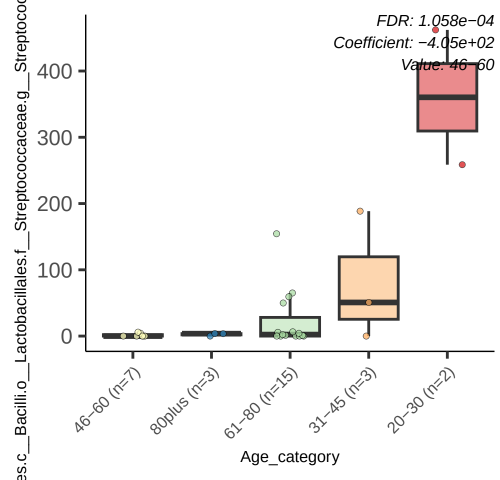

FDR: 1.058e−04
Coefficient: −4.05e+02
Value: 46−60
400
300
200
100
0
46−60 (n=7)
80plus (n=3)
61−80 (n=15)
31−45 (n=3)
20−30 (n=2)
Age_category

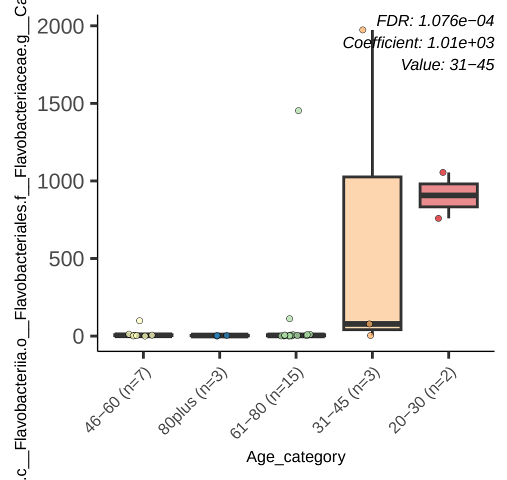

FDR: 1.076e−04
Coefficient: 1.01e+03
Value: 31−45
2000
1500
1000
500
0
.c__Flavobacteriia.o__Flavobacteriales.f__Flavobacteriaceae.g__Ca
46−60 (n=7)
80plus (n=3)
61−80 (n=15)
31−45 (n=3)
20−30 (n=2)
Age_category

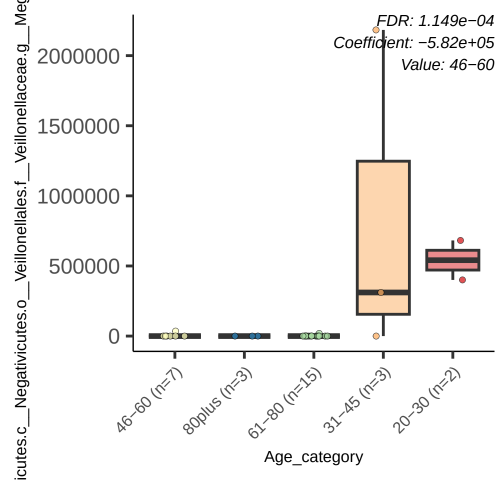

FDR: 1.149e−04
Coefficient: −5.82e+05
Value: 46−60
2000000
1500000
1000000
500000
0
46−60 (n=7)
80plus (n=3)
61−80 (n=15)
31−45 (n=3)
20−30 (n=2)
Age_category
icutes.c__Negativicutes.o__Veillonellales.f__Veillonellaceae.g__Meg

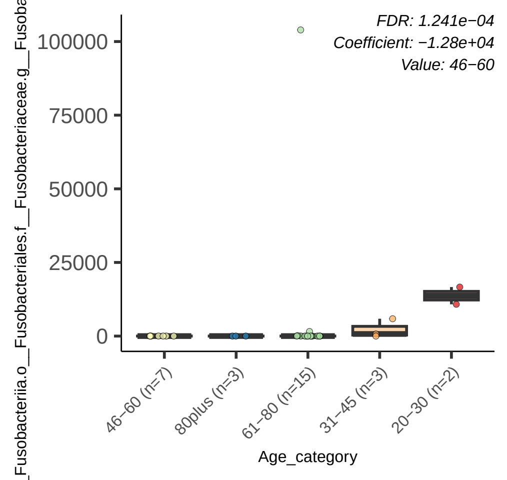
FDR: 1.241e−04
Coefficient: −1.28e+04
Value: 46−60
100000
75000
50000
25000
0
_Fusobacteriia.o__Fusobacteriales.f__Fusobacteriaceae.g__Fusoba
46−60 (n=7)
80plus (n=3)
61−80 (n=15)
31−45 (n=3)
20−30 (n=2)
Age_category

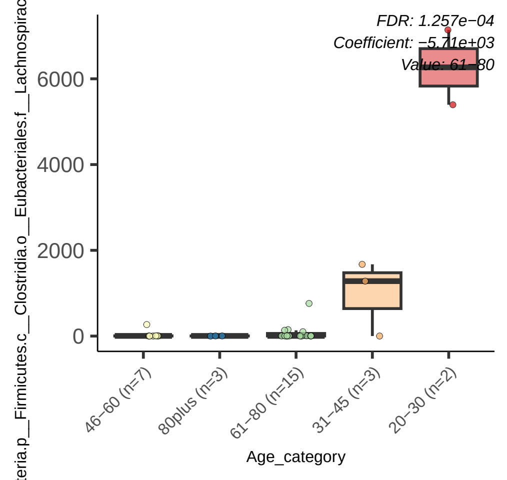

FDR: 1.257e−04
Coefficient: −5.71e+03
Value: 61−80
6000
4000
2000
0
46−60 (n=7)
80plus (n=3)
61−80 (n=15)
31−45 (n=3)
20−30 (n=2)
Age_category
teria.p__Firmicutes.c__Clostridia.o__Eubacteriales.f__Lachnospirac

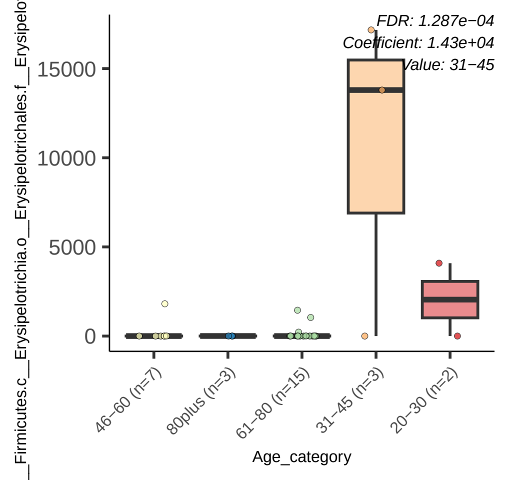

FDR: 1.287e−04
Coefficient: 1.43e+04
Value: 31−45
15000
10000
5000
0
46−60 (n=7)
80plus (n=3)
61−80 (n=15)
31−45 (n=3)
20−30 (n=2)
Age_category
Firmicutes.c__Erysipelotrichia.o__Erysipelotrichales.f__Erysipelo

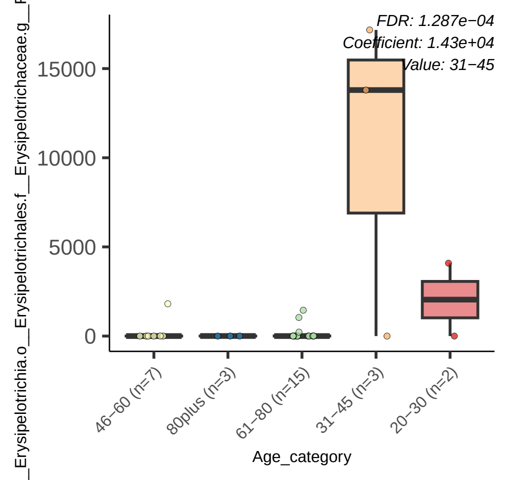

FDR: 1.287e−04
Coefficient: 1.43e+04
Value: 31−45
15000
10000
5000
0
_Erysipelotrichia.o__Erysipelotrichales.f__Erysipelotrichaceae.g__F
46−60 (n=7)
80plus (n=3)
61−80 (n=15)
31−45 (n=3)
20−30 (n=2)
Age_category

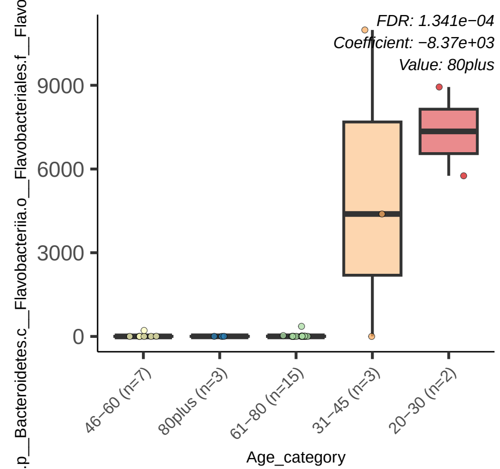

FDR: 1.341e−04
Coefficient: −8.37e+03
Value: 80plus
ia.p__Bacteroidetes.c__Flavobacteriia.o__Flavobacteriales.f__Flavo
9000
6000
3000
0
46−60 (n=7)
80plus (n=3)
61−80 (n=15)
31−45 (n=3)
20−30 (n=2)
Age_category

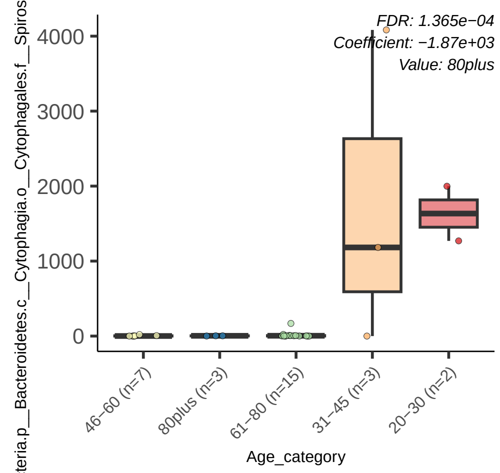

FDR: 1.365e−04
Coefficient: −1.87e+03
Value: 80plus
cteria.p__Bacteroidetes.c__Cytophagia.o__Cytophagales.f__Spiros
4000
3000
2000
1000
0
46−60 (n=7)
80plus (n=3)
61−80 (n=15)
31−45 (n=3)
20−30 (n=2)
Age_category

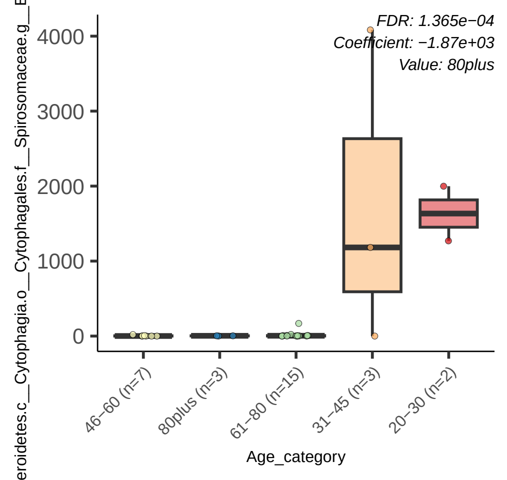

FDR: 1.365e−04
Coefficient: −1.87e+03
Value: 80plus
4000
3000
2000
1000
0
eroidetes.c__Cytophagia.o__Cytophagales.f__Spirosomaceae.g__E
46−60 (n=7)
80plus (n=3)
61−80 (n=15)
31−45 (n=3)
20−30 (n=2)
Age_category

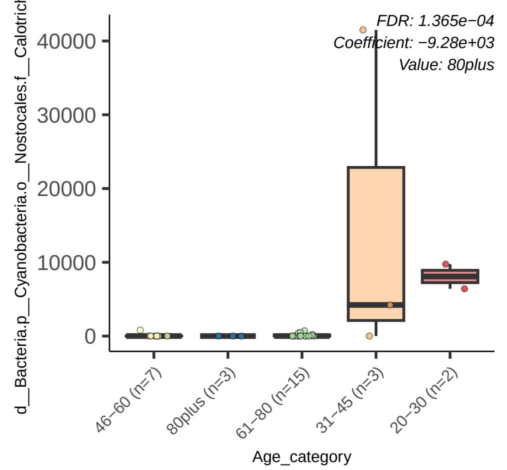

FDR: 1.365e−04
Coefficient: −9.28e+03
Value: 80plus
d__Bacteria.p__Cyanobacteria.o__Nostocales.f__Calotrich
40000
30000
20000
10000
0
46−60 (n=7)
80plus (n=3)
61−80 (n=15)
31−45 (n=3)
20−30 (n=2)
Age_category

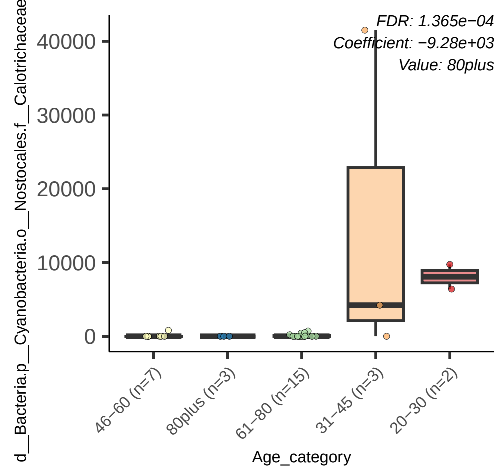
FDR: 1.365e−04
Coefficient: −9.28e+03
Value: 80plus
d__Bacteria.p__Cyanobacteria.o__Nostocales.f__Calotrichaceae
40000
30000
20000
10000
0
46−60 (n=7)
80plus (n=3)
61−80 (n=15)
31−45 (n=3)
20−30 (n=2)
Age_category

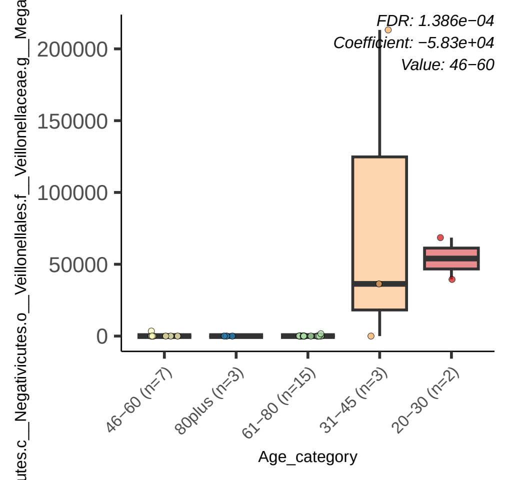
FDR: 1.386e−04
Coefficient: −5.83e+04
Value: 46−60
200000
150000
100000
50000
0
46−60 (n=7)
80plus (n=3)
61−80 (n=15)
31−45 (n=3)
20−30 (n=2)
Age_category
utes.c__Negativicutes.o__Veillonellales.f__Veillonellaceae.g__Mega

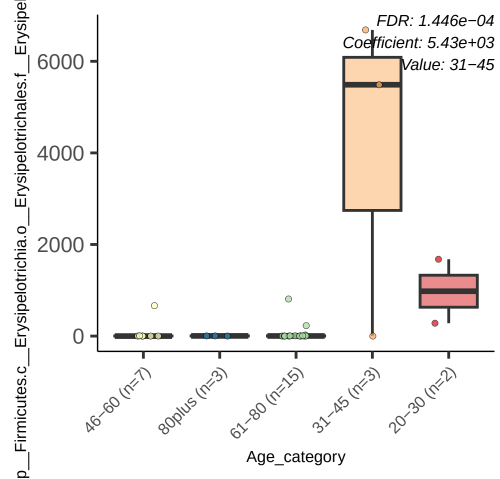

FDR: 1.446e−04
Coefficient: 5.43e+03
Value: 31−45
p__Firmicutes.c__Erysipelotrichia.o__Erysipelotrichales.f__Erysipel
6000
4000
2000
0
46−60 (n=7)
80plus (n=3)
61−80 (n=15)
31−45 (n=3)
20−30 (n=2)
Age_category

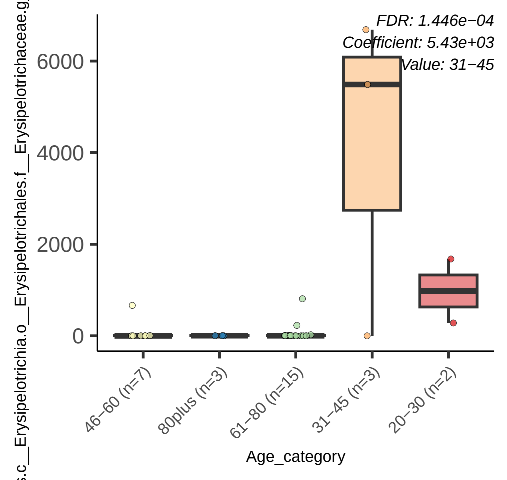

FDR: 1.446e−04
Coefficient: 5.43e+03
Value: 31−45
6000
4000
2000
0
s.c__Erysipelotrichia.o__Erysipelotrichales.f__Erysipelotrichaceae.g
46−60 (n=7)
80plus (n=3)
61−80 (n=15)
31−45 (n=3)
20−30 (n=2)
Age_category

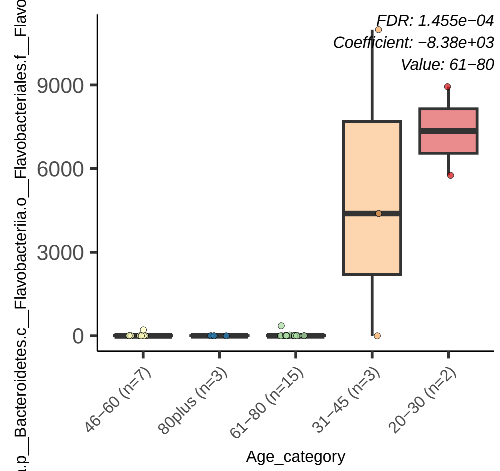

FDR: 1.455e−04
Coefficient: −8.38e+03
Value: 61−80
ia.p__Bacteroidetes.c__Flavobacteriia.o__Flavobacteriales.f__Flavo
9000
6000
3000
0
46−60 (n=7)
80plus (n=3)
61−80 (n=15)
31−45 (n=3)
20−30 (n=2)
Age_category

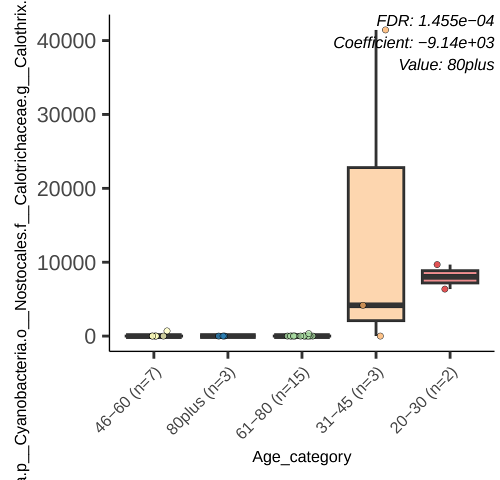

FDR: 1.455e−04
Coefficient: −9.14e+03
Value: 80plus
a.p__Cyanobacteria.o__Nostocales.f__Calotrichaceae.g__Calothrix.
40000
30000
20000
10000
0
46−60 (n=7)
80plus (n=3)
61−80 (n=15)
31−45 (n=3)
20−30 (n=2)
Age_category

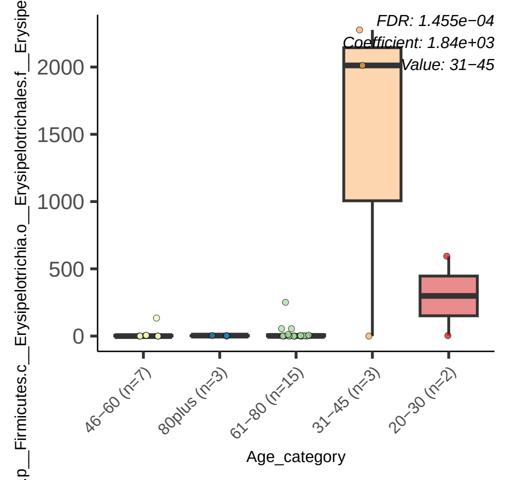

FDR: 1.455e−04
Coefficient: 1.84e+03
Value: 31−45
p__Firmicutes.c__Erysipelotrichia.o__Erysipelotrichales.f__Erysipe
2000
1500
1000
500
0
46−60 (n=7)
80plus (n=3)
61−80 (n=15)
31−45 (n=3)
20−30 (n=2)
Age_category

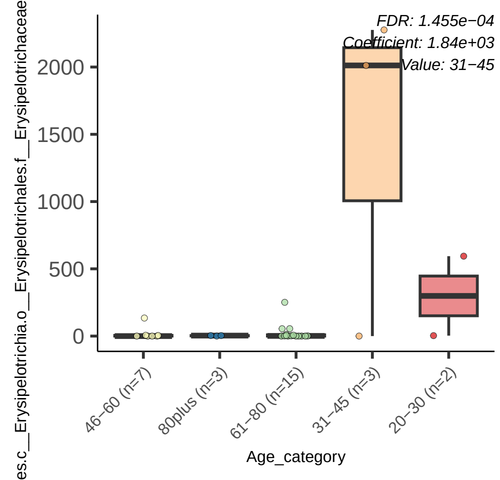

FDR: 1.455e−04
Coefficient: 1.84e+03
Value: 31−45
es.c__Erysipelotrichia.o__Erysipelotrichales.f__Erysipelotrichaceae
2000
1500
1000
500
0
46−60 (n=7)
80plus (n=3)
61−80 (n=15)
31−45 (n=3)
20−30 (n=2)
Age_category

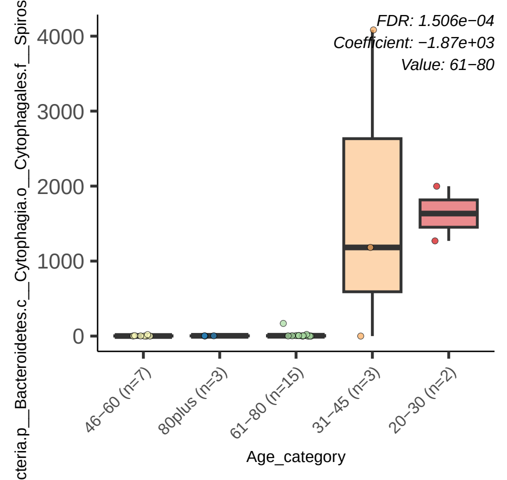

FDR: 1.506e−04
Coefficient: −1.87e+03
Value: 61−80
4000
3000
2000
1000
0
cteria.p__Bacteroidetes.c__Cytophagia.o__Cytophagales.f__Spiros
46−60 (n=7)
80plus (n=3)
61−80 (n=15)
31−45 (n=3)
20−30 (n=2)
Age_category

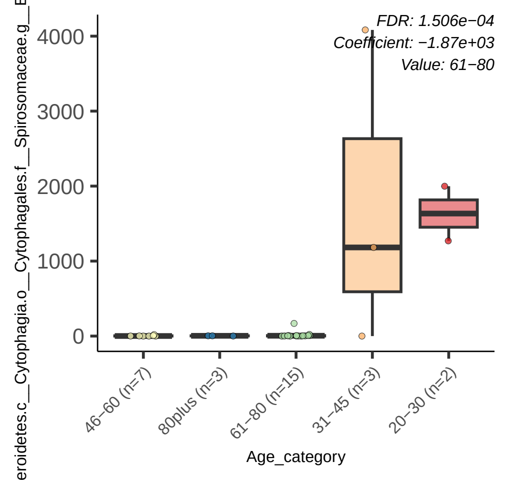

FDR: 1.506e−04
Coefficient: −1.87e+03
Value: 61−80
4000
3000
2000
1000
0
46−60 (n=7)
80plus (n=3)
61−80 (n=15)
31−45 (n=3)
20−30 (n=2)
Age_category
eroidetes.c__Cytophagia.o__Cytophagales.f__Spirosomaceae.g__E

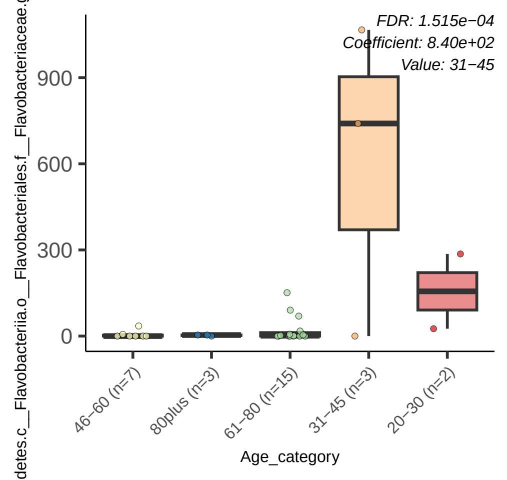

FDR: 1.515e−04
Coefficient: 8.40e+02
Value: 31−45
900
600
300
0
detes.c__Flavobacteriia.o__Flavobacteriales.f__Flavobacteriaceae.g
46−60 (n=7)
80plus (n=3)
61−80 (n=15)
31−45 (n=3)
20−30 (n=2)
Age_category

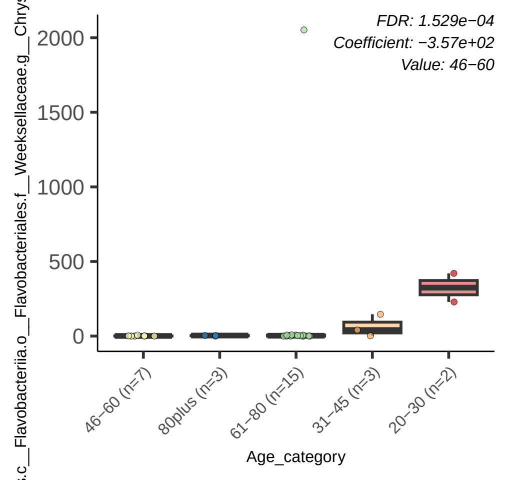
FDR: 1.529e−04
Coefficient: −3.57e+02
Value: 46−60
2000
1500
1000
500
0
46−60 (n=7)
80plus (n=3)
61−80 (n=15)
31−45 (n=3)
20−30 (n=2)
Age_category
s.c__Flavobacteriia.o__Flavobacteriales.f__Weeksellaceae.g__Chrys

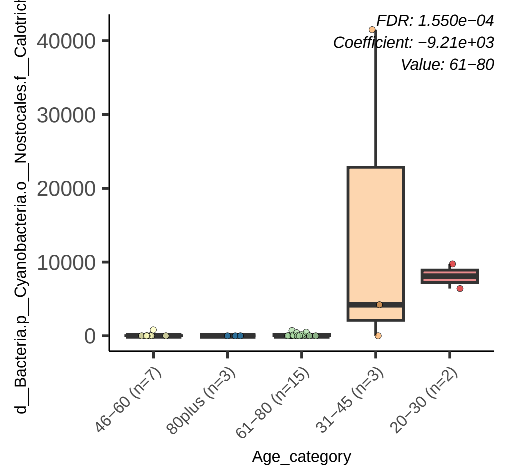

FDR: 1.550e−04
Coefficient: −9.21e+03
Value: 61−80
40000
30000
20000
10000
0
d__Bacteria.p__Cyanobacteria.o__Nostocales.f__Calotrich
46−60 (n=7)
80plus (n=3)
61−80 (n=15)
31−45 (n=3)
20−30 (n=2)
Age_category

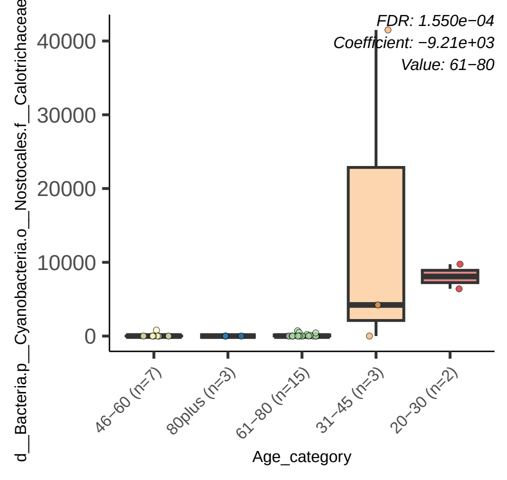

FDR: 1.550e−04
Coefficient: −9.21e+03
Value: 61−80
d__Bacteria.p__Cyanobacteria.o__Nostocales.f__Calotrichaceae
40000
30000
20000
10000
0
46−60 (n=7)
80plus (n=3)
61−80 (n=15)
31−45 (n=3)
20−30 (n=2)
Age_category

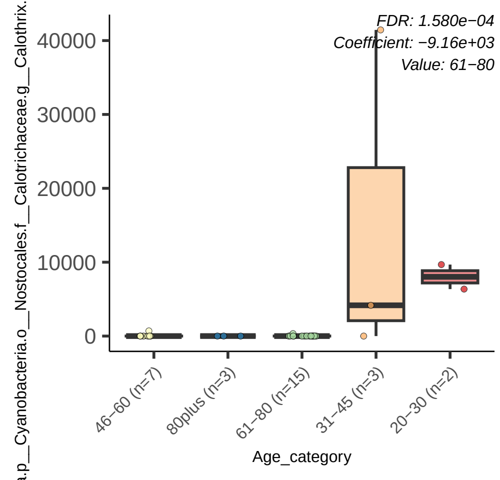

FDR: 1.580e−04
Coefficient: −9.16e+03
Value: 61−80
40000
30000
20000
10000
0
a.p__Cyanobacteria.o__Nostocales.f__Calotrichaceae.g__Calothrix.
46−60 (n=7)
80plus (n=3)
61−80 (n=15)
31−45 (n=3)
20−30 (n=2)
Age_category

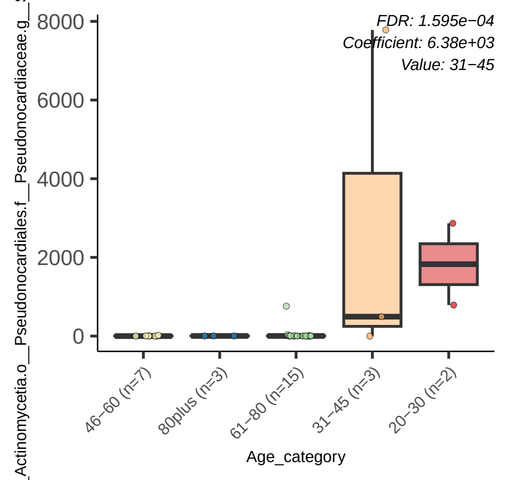

FDR: 1.595e−04
Coefficient: 6.38e+03
Value: 31−45
8000
6000
4000
2000
0
Actinomycetia.o__Pseudonocardiales.f__Pseudonocardiaceae.g__
46−60 (n=7)
80plus (n=3)
61−80 (n=15)
31−45 (n=3)
20−30 (n=2)
Age_category

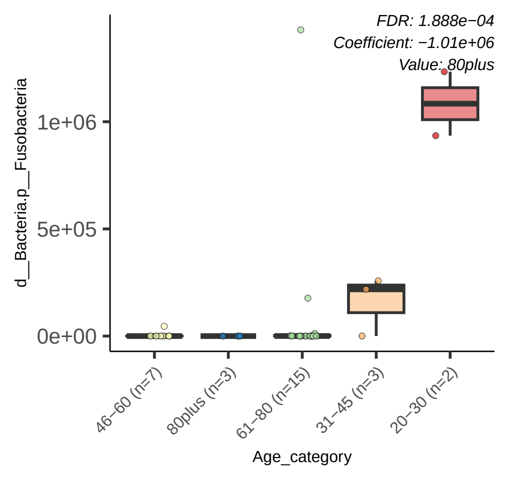

FDR: 1.888e−04
Coefficient: −1.01e+06
Value: 80plus
d__Bacteria.p__Fusobacteria
1e+06
5e+05
0e+00
46−60 (n=7)
80plus (n=3)
61−80 (n=15)
31−45 (n=3)
20−30 (n=2)
Age_category

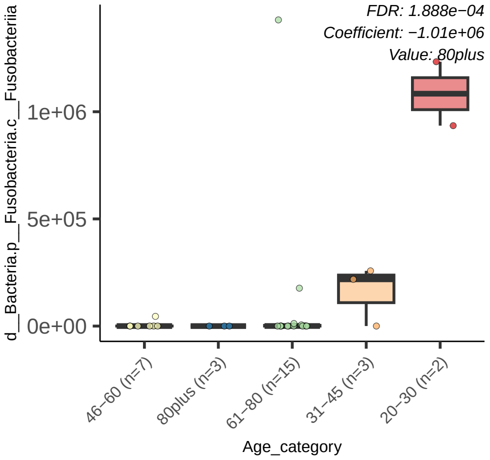
FDR: 1.888e−04
Coefficient: −1.01e+06
Value: 80plus
d__Bacteria.p__Fusobacteria.c__Fusobacteriia
1e+06
5e+05
0e+00
46−60 (n=7)
80plus (n=3)
61−80 (n=15)
31−45 (n=3)
20−30 (n=2)
Age_category

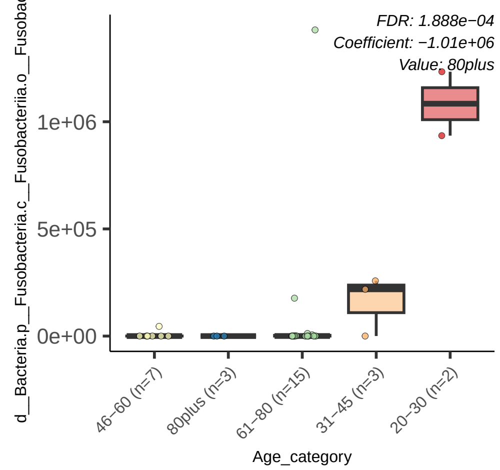

FDR: 1.888e−04
Coefficient: −1.01e+06
Value: 80plus
d__Bacteria.p__Fusobacteria.c__Fusobacteriia.o__Fusobac
1e+06
5e+05
0e+00
46−60 (n=7)
80plus (n=3)
61−80 (n=15)
31−45 (n=3)
20−30 (n=2)
Age_category

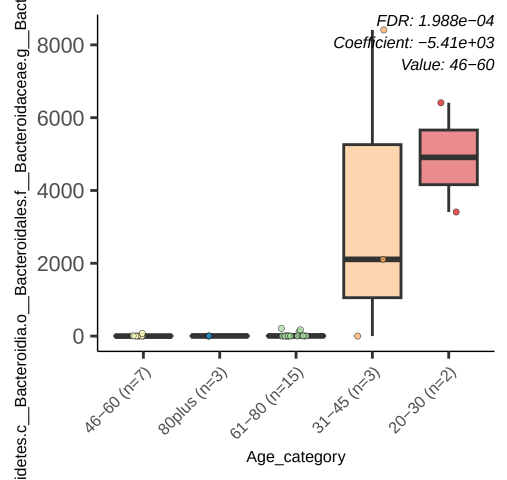

FDR: 1.988e−04
Coefficient: −5.41e+03
Value: 46−60
8000
6000
4000
2000
0
idetes.c__Bacteroidia.o__Bacteroidales.f__Bacteroidaceae.g__Bact
46−60 (n=7)
80plus (n=3)
61−80 (n=15)
31−45 (n=3)
20−30 (n=2)
Age_category

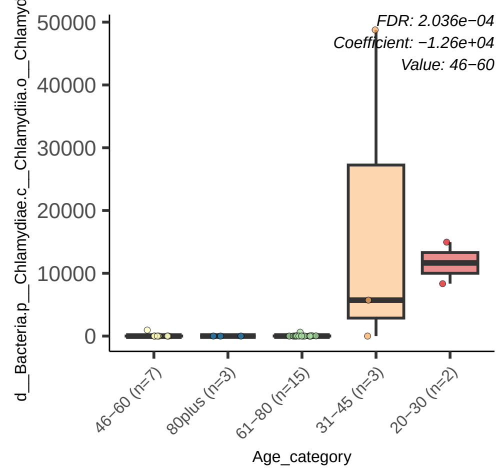

FDR: 2.036e−04
Coefficient: −1.26e+04
Value: 46−60
50000
40000
30000
20000
10000
0
46−60 (n=7)
80plus (n=3)
61−80 (n=15)
31−45 (n=3)
20−30 (n=2)
Age_category
d__Bacteria.p__Chlamydiae.c__Chlamydiia.o__Chlamyd

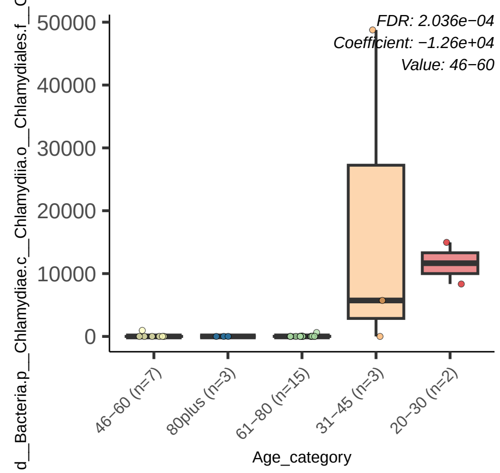

FDR: 2.036e−04
Coefficient: −1.26e+04
Value: 46−60
d__Bacteria.p__Chlamydiae.c__Chlamydiia.o__Chlamydiales.f__C
50000
40000
30000
20000
10000
0
46−60 (n=7)
80plus (n=3)
61−80 (n=15)
31−45 (n=3)
20−30 (n=2)
Age_category

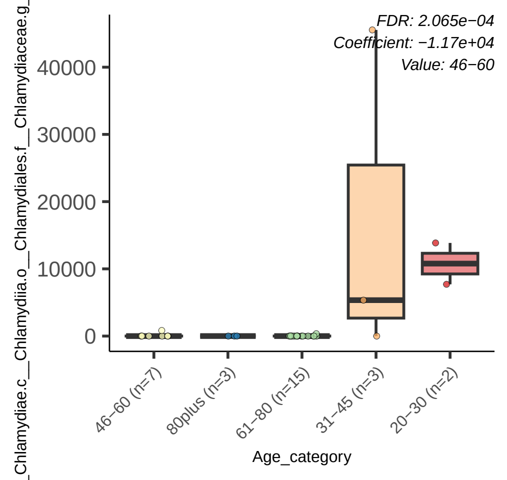

FDR: 2.065e−04
Coefficient: −1.17e+04
Value: 46−60
40000
30000
20000
10000
0
_Chlamydiae.c__Chlamydiia.o__Chlamydiales.f__Chlamydiaceae.g_
46−60 (n=7)
80plus (n=3)
61−80 (n=15)
31−45 (n=3)
20−30 (n=2)
Age_category

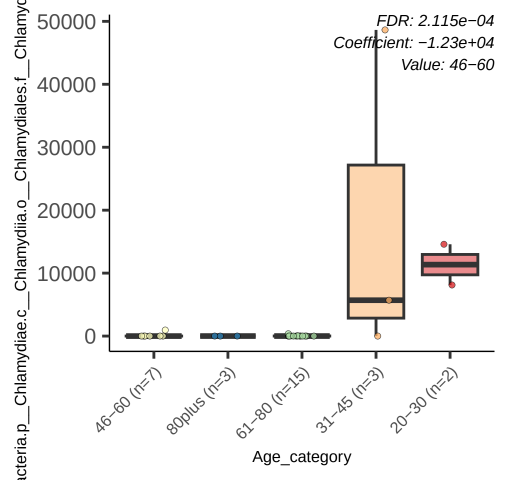

FDR: 2.115e−04
Coefficient: −1.23e+04
Value: 46−60
50000
40000
30000
20000
10000
0
acteria.p__Chlamydiae.c__Chlamydiia.o__Chlamydiales.f__Chlamyd
46−60 (n=7)
80plus (n=3)
61−80 (n=15)
31−45 (n=3)
20−30 (n=2)
Age_category

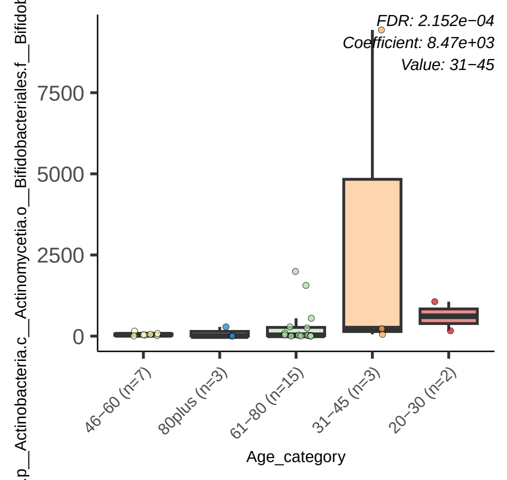

FDR: 2.152e−04
Coefficient: 8.47e+03
Value: 31−45
p__Actinobacteria.c__Actinomycetia.o__Bifidobacteriales.f__Bifidob
7500
5000
2500
0
46−60 (n=7)
80plus (n=3)
61−80 (n=15)
31−45 (n=3)
20−30 (n=2)
Age_category

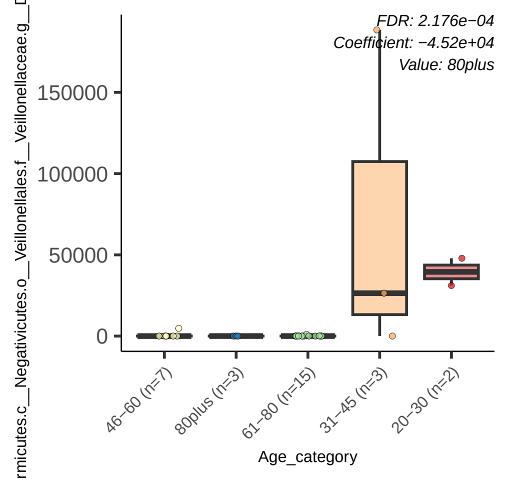
FDR: 2.176e−04
Coefficient: −4.52e+04
Value: 80plus
150000
100000
50000
0
rmicutes.c__Negativicutes.o__Veillonellales.f__Veillonellaceae.g__D
46−60 (n=7)
80plus (n=3)
61−80 (n=15)
31−45 (n=3)
20−30 (n=2)
Age_category

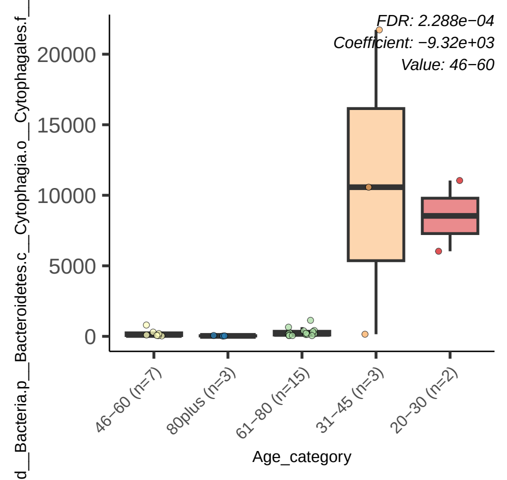
FDR: 2.288e−04
Coefficient: −9.32e+03
Value: 46−60
d__Bacteria.p__Bacteroidetes.c__Cytophagia.o__Cytophagales.f__
20000
15000
10000
5000
0
46−60 (n=7)
80plus (n=3)
61−80 (n=15)
31−45 (n=3)
20−30 (n=2)
Age_category

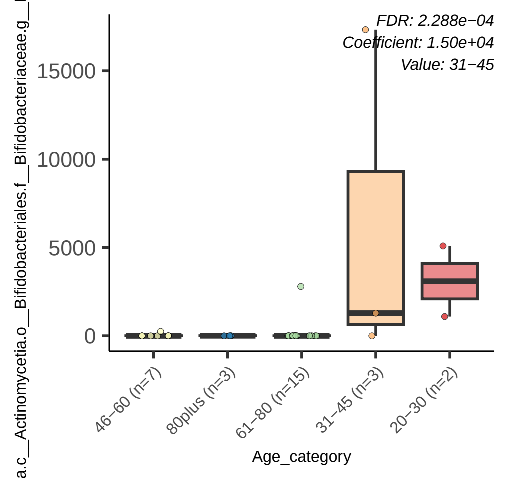

FDR: 2.288e−04
Coefficient: 1.50e+04
Value: 31−45
a.c__Actinomycetia.o__Bifidobacteriales.f__Bifidobacteriaceae.g__
15000
10000
5000
0
46−60 (n=7)
80plus (n=3)
61−80 (n=15)
31−45 (n=3)
20−30 (n=2)
Age_category

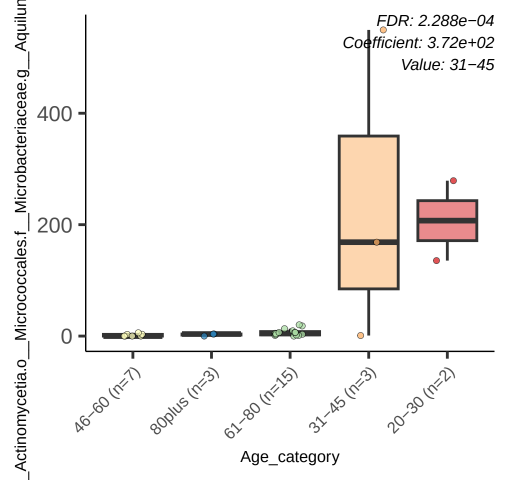

FDR: 2.288e−04
Coefficient: 3.72e+02
Value: 31−45
0
200
400
46−60 (n=7)
80plus (n=3)
61−80 (n=15)
31−45 (n=3)
20−30 (n=2)
Age_category
Actinomycetia.o__Micrococcales.f__Microbacteriaceae.g__Aquilun

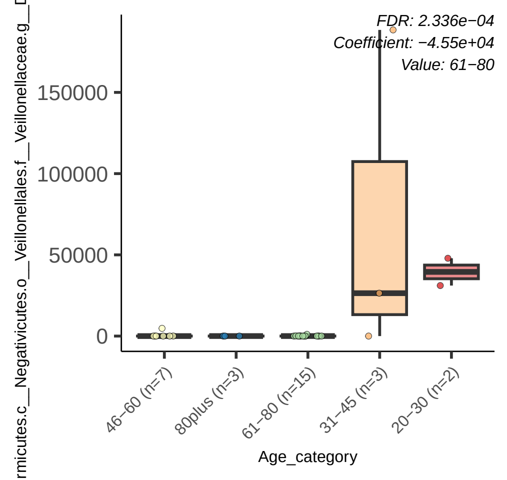

FDR: 2.336e−04
Coefficient: −4.55e+04
Value: 61−80
150000
100000
50000
0
rmicutes.c__Negativicutes.o__Veillonellales.f__Veillonellaceae.g__D
46−60 (n=7)
80plus (n=3)
61−80 (n=15)
31−45 (n=3)
20−30 (n=2)
Age_category

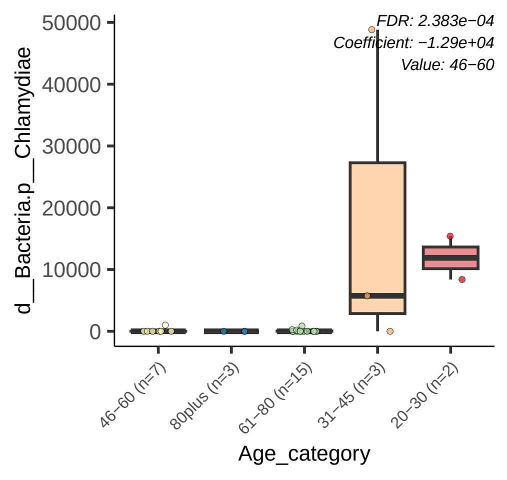

FDR: 2.383e−04
Coefficient: −1.29e+04
Value: 46−60
d__Bacteria.p__Chlamydiae
50000
40000
30000
20000
10000
0
46−60 (n=7)
80plus (n=3)
61−80 (n=15)
31−45 (n=3)
20−30 (n=2)
Age_category

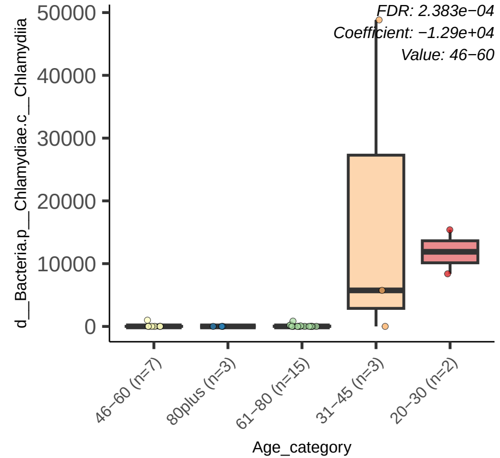
FDR: 2.383e−04
Coefficient: −1.29e+04
Value: 46−60
d__Bacteria.p__Chlamydiae.c__Chlamydiia
50000
40000
30000
20000
10000
0
46−60 (n=7)
80plus (n=3)
61−80 (n=15)
31−45 (n=3)
20−30 (n=2)
Age_category

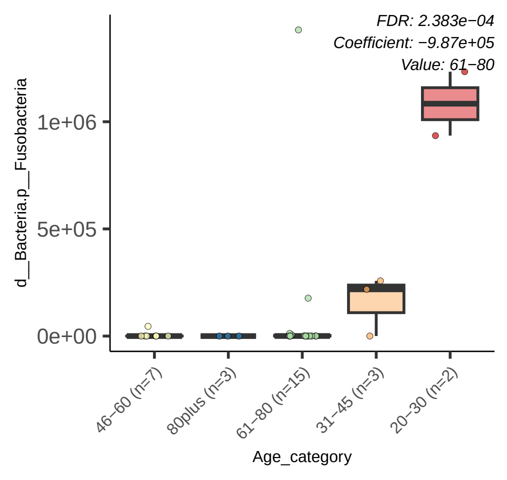
FDR: 2.383e−04
Coefficient: −9.87e+05
Value: 61−80
d__Bacteria.p__Fusobacteria
1e+06
5e+05
0e+00
46−60 (n=7)
80plus (n=3)
61−80 (n=15)
31−45 (n=3)
20−30 (n=2)
Age_category

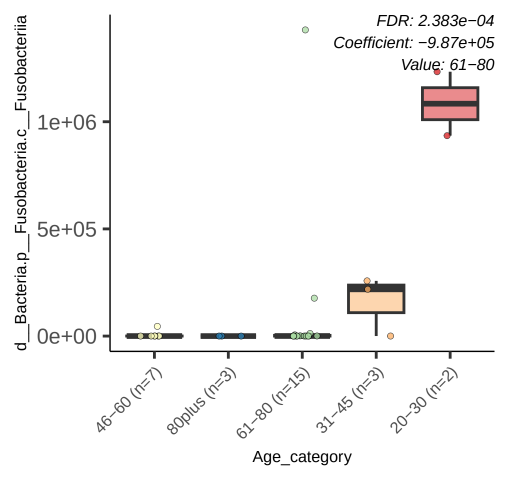

FDR: 2.383e−04
Coefficient: −9.87e+05
Value: 61−80
d__Bacteria.p__Fusobacteria.c__Fusobacteriia
1e+06
5e+05
0e+00
46−60 (n=7)
80plus (n=3)
61−80 (n=15)
31−45 (n=3)
20−30 (n=2)
Age_category

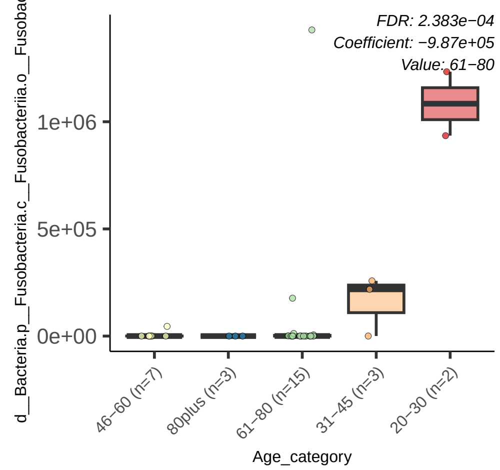

FDR: 2.383e−04
Coefficient: −9.87e+05
Value: 61−80
1e+06
5e+05
0e+00
d__Bacteria.p__Fusobacteria.c__Fusobacteriia.o__Fusobac
46−60 (n=7)
80plus (n=3)
61−80 (n=15)
31−45 (n=3)
20−30 (n=2)
Age_category

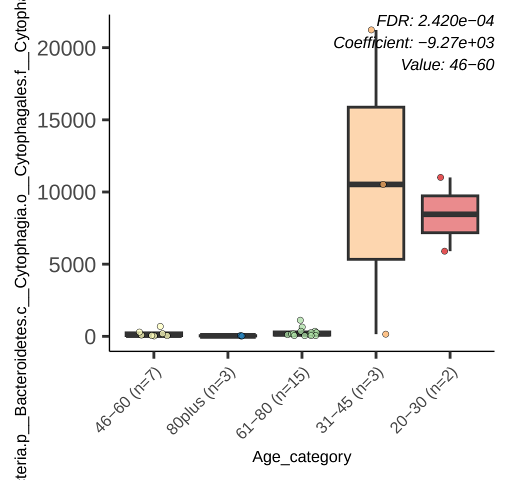

FDR: 2.420e−04
Coefficient: −9.27e+03
Value: 46−60
20000
15000
10000
5000
0
teria.p__Bacteroidetes.c__Cytophagia.o__Cytophagales.f__Cytoph
46−60 (n=7)
80plus (n=3)
61−80 (n=15)
31−45 (n=3)
20−30 (n=2)
Age_category

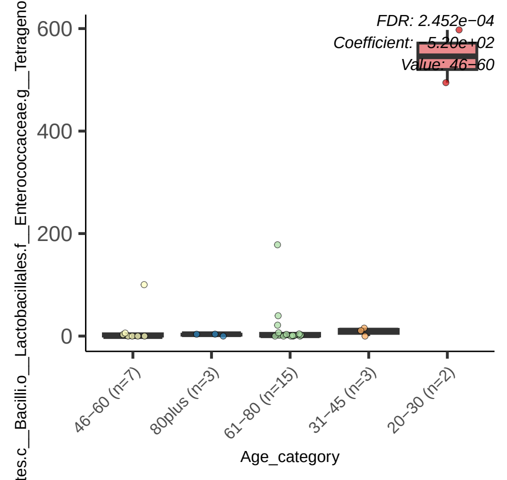
FDR: 2.452e−04
Coefficient: −5.20e+02
Value: 46−60
600
400
200
0
tes.c__Bacilli.o__Lactobacillales.f__Enterococcaceae.g__Tetrageno
46−60 (n=7)
80plus (n=3)
61−80 (n=15)
31−45 (n=3)
20−30 (n=2)
Age_category

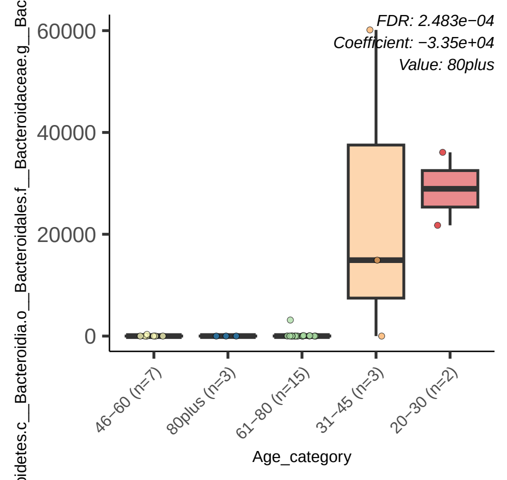
FDR: 2.483e−04
Coefficient: −3.35e+04
Value: 80plus
60000
40000
20000
0
46−60 (n=7)
80plus (n=3)
61−80 (n=15)
31−45 (n=3)
20−30 (n=2)
Age_category
oidetes.c__Bacteroidia.o__Bacteroidales.f__Bacteroidaceae.g__Bac

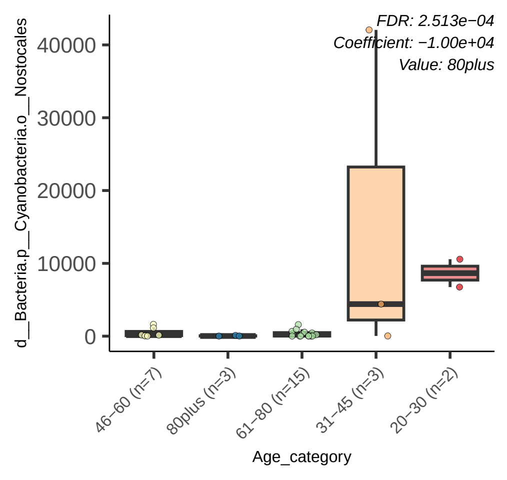

FDR: 2.513e−04
Coefficient: −1.00e+04
Value: 80plus
d__Bacteria.p__Cyanobacteria.o__Nostocales
40000
30000
20000
10000
0
46−60 (n=7)
80plus (n=3)
61−80 (n=15)
31−45 (n=3)
20−30 (n=2)
Age_category

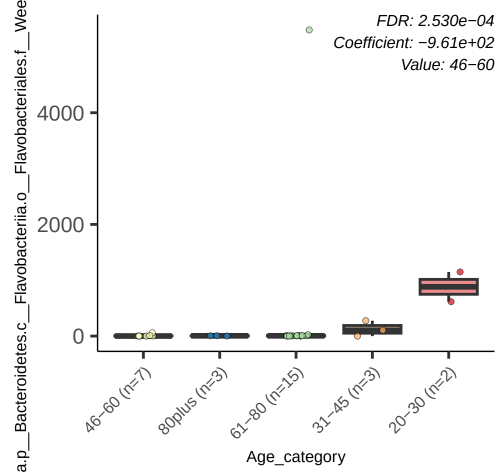

FDR: 2.530e−04
Coefficient: −9.61e+02
Value: 46−60
ria.p__Bacteroidetes.c__Flavobacteriia.o__Flavobacteriales.f__Wee
4000
2000
0
46−60 (n=7)
80plus (n=3)
61−80 (n=15)
31−45 (n=3)
20−30 (n=2)
Age_category

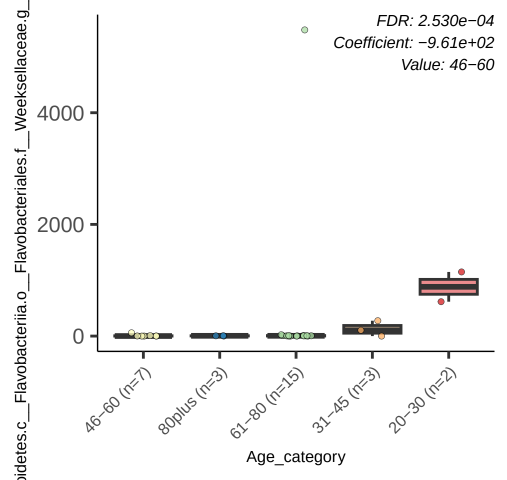

FDR: 2.530e−04
Coefficient: −9.61e+02
Value: 46−60
4000
2000
0
46−60 (n=7)
80plus (n=3)
61−80 (n=15)
31−45 (n=3)
20−30 (n=2)
Age_category
oidetes.c__Flavobacteriia.o__Flavobacteriales.f__Weeksellaceae.g__

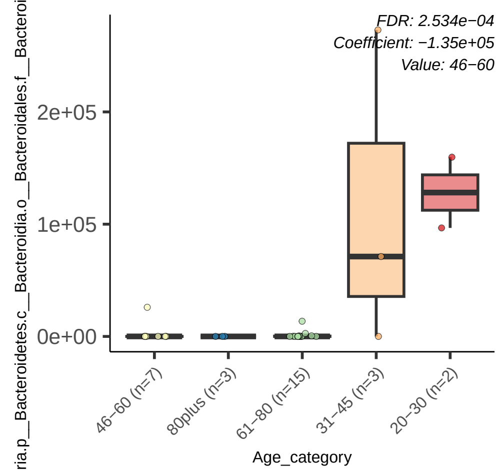

FDR: 2.534e−04
Coefficient: −1.35e+05
Value: 46−60
2e+05
1e+05
0e+00
46−60 (n=7)
80plus (n=3)
61−80 (n=15)
31−45 (n=3)
20−30 (n=2)
Age_category

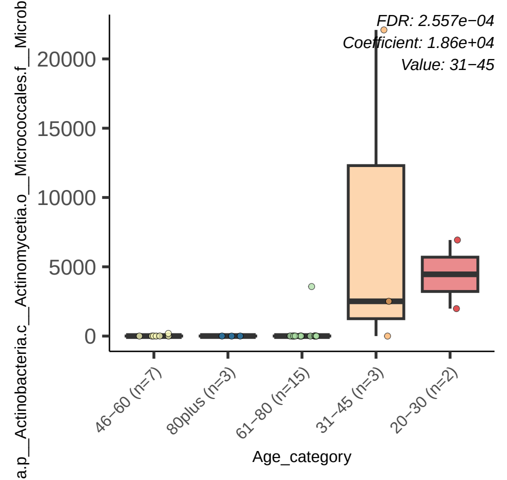

FDR: 2.557e−04
Coefficient: 1.86e+04
Value: 31−45
a.p__Actinobacteria.c__Actinomycetia.o__Micrococcales.f__Microb
20000
15000
10000
5000
0
46−60 (n=7)
80plus (n=3)
61−80 (n=15)
31−45 (n=3)
20−30 (n=2)
Age_category

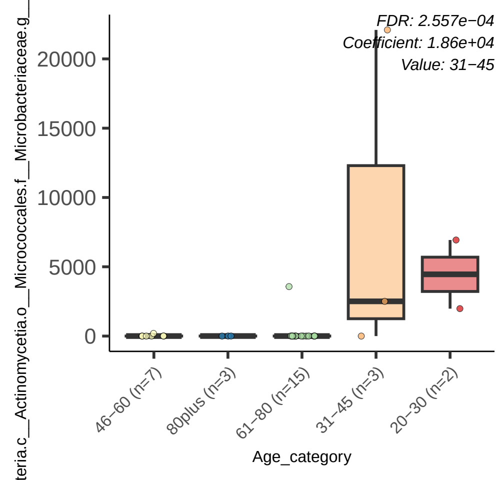

FDR: 2.557e−04
Coefficient: 1.86e+04
Value: 31−45
teria.c__Actinomycetia.o__Micrococcales.f__Microbacteriaceae.g__
20000
15000
10000
5000
0
46−60 (n=7)
80plus (n=3)
61−80 (n=15)
31−45 (n=3)
20−30 (n=2)
Age_category

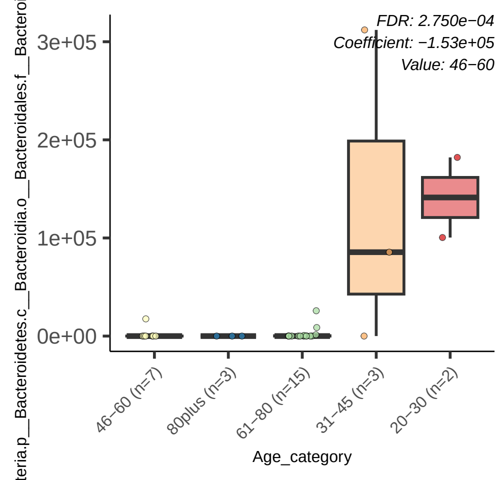

FDR: 2.750e−04
Coefficient: −1.53e+05
Value: 46−60
3e+05
2e+05
1e+05
0e+00
teria.p__Bacteroidetes.c__Bacteroidia.o__Bacteroidales.f__Bacteroi
46−60 (n=7)
80plus (n=3)
61−80 (n=15)
31−45 (n=3)
20−30 (n=2)
Age_category

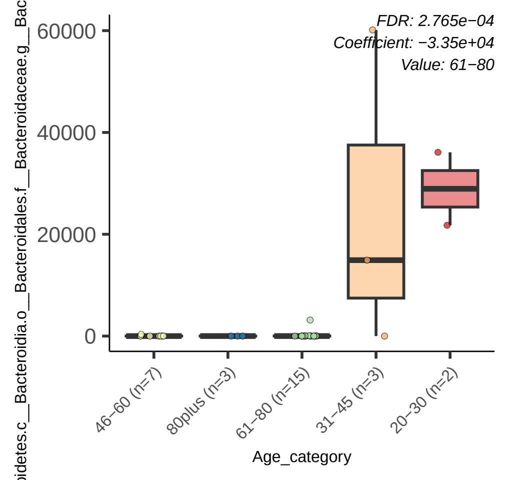

FDR: 2.765e−04
Coefficient: −3.35e+04
Value: 61−80
60000
40000
20000
0
oidetes.c__Bacteroidia.o__Bacteroidales.f__Bacteroidaceae.g__Bac
46−60 (n=7)
80plus (n=3)
61−80 (n=15)
31−45 (n=3)
20−30 (n=2)
Age_category

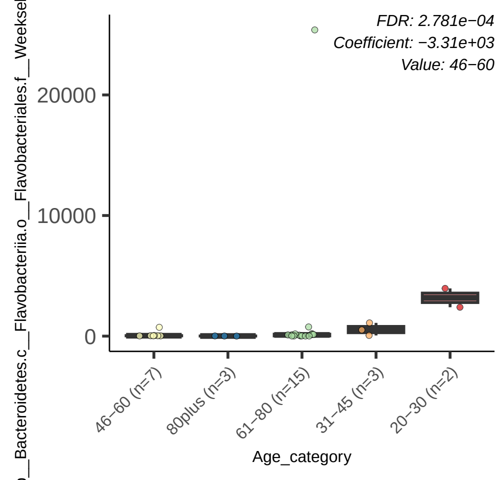
FDR: 2.781e−04
Coefficient: −3.31e+03
Value: 46−60
20000
10000
0
46−60 (n=7)
80plus (n=3)
61−80 (n=15)
31−45 (n=3)
20−30 (n=2)
Age_category
Bacteroidetes.c__Flavobacteriia.o__Flavobacteriales.f__Weeksel

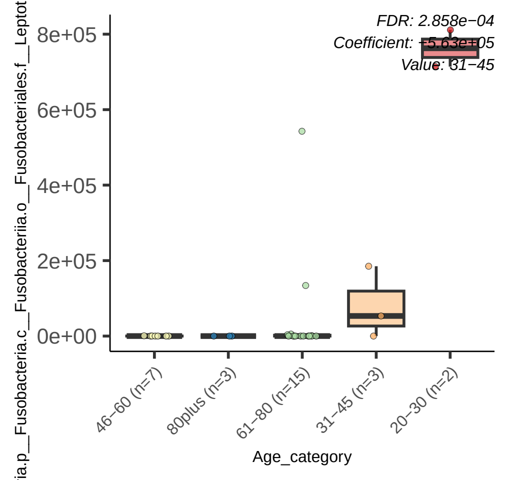

FDR: 2.858e−04
Coefficient: −5.63e+05
Value: 31−45
8e+05
6e+05
4e+05
2e+05
0e+00
46−60 (n=7)
80plus (n=3)
61−80 (n=15)
31−45 (n=3)
20−30 (n=2)
Age_category
ia.p__Fusobacteria.c__Fusobacteriia.o__Fusobacteriales.f__Leptot

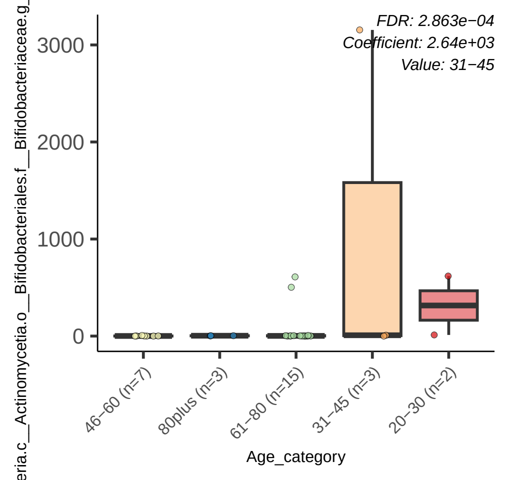

FDR: 2.863e−04
Coefficient: 2.64e+03
Value: 31−45
3000
2000
1000
0
eria.c__Actinomycetia.o__Bifidobacteriales.f__Bifidobacteriaceae.g_
46−60 (n=7)
80plus (n=3)
61−80 (n=15)
31−45 (n=3)
20−30 (n=2)
Age_category

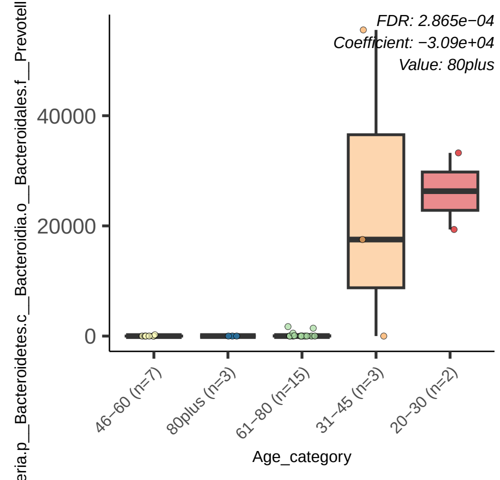

FDR: 2.865e−04
Coefficient: −3.09e+04
Value: 80plus
40000
20000
0
46−60 (n=7)
80plus (n=3)
61−80 (n=15)
31−45 (n=3)
20−30 (n=2)
Age_category
eria.p__Bacteroidetes.c__Bacteroidia.o__Bacteroidales.f__Prevotell

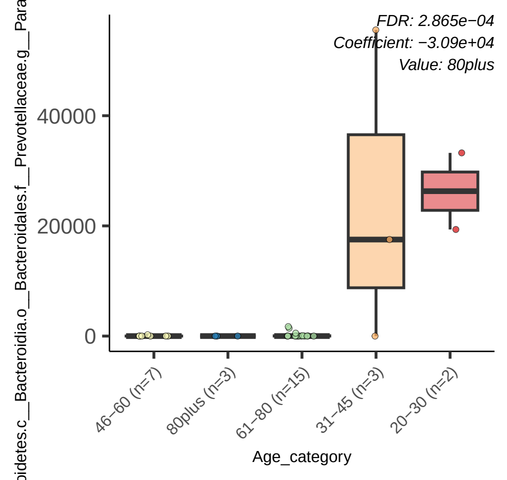

FDR: 2.865e−04
Coefficient: −3.09e+04
Value: 80plus
oidetes.c__Bacteroidia.o__Bacteroidales.f__Prevotellaceae.g__Para
40000
20000
0
46−60 (n=7)
80plus (n=3)
61−80 (n=15)
31−45 (n=3)
20−30 (n=2)
Age_category

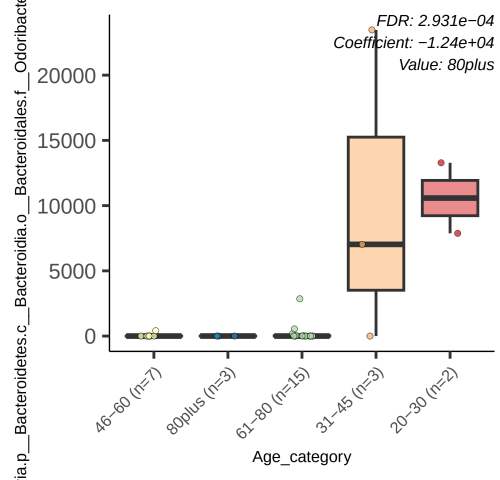

FDR: 2.931e−04
Coefficient: −1.24e+04
Value: 80plus
20000
15000
10000
5000
0
46−60 (n=7)
80plus (n=3)
61−80 (n=15)
31−45 (n=3)
20−30 (n=2)
Age_category
ia.p__Bacteroidetes.c__Bacteroidia.o__Bacteroidales.f__Odoribacte

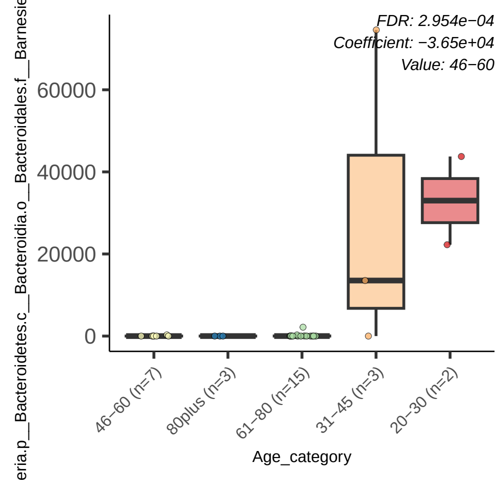
FDR: 2.954e−04
Coefficient: −3.65e+04
Value: 46−60
60000
40000
20000
0
eria.p__Bacteroidetes.c__Bacteroidia.o__Bacteroidales.f__Barnesie
46−60 (n=7)
80plus (n=3)
61−80 (n=15)
31−45 (n=3)
20−30 (n=2)
Age_category

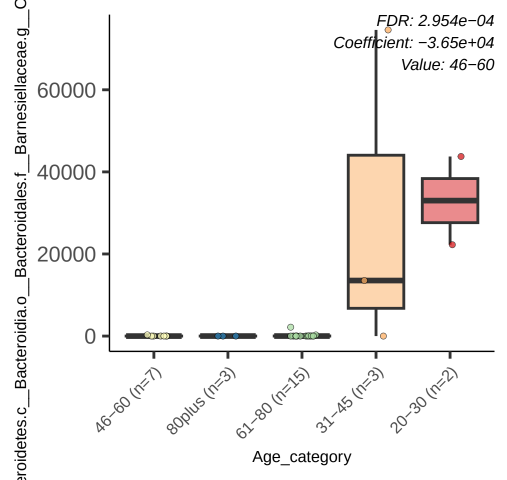

FDR: 2.954e−04
Coefficient: −3.65e+04
Value: 46−60
60000
40000
20000
0
eroidetes.c__Bacteroidia.o__Bacteroidales.f__Barnesiellaceae.g__C
46−60 (n=7)
80plus (n=3)
61−80 (n=15)
31−45 (n=3)
20−30 (n=2)
Age_category

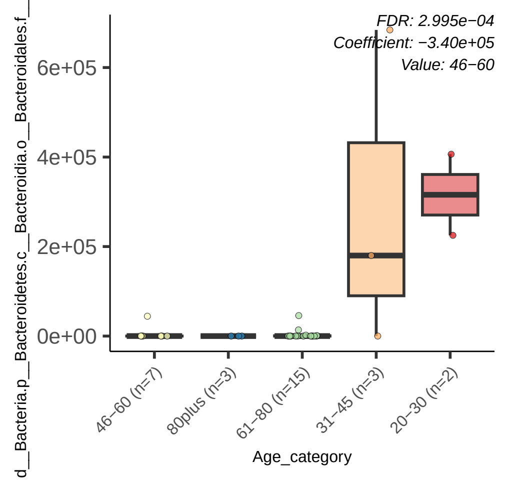

FDR: 2.995e−04
Coefficient: −3.40e+05
Value: 46−60
d__Bacteria.p__Bacteroidetes.c__Bacteroidia.o__Bacteroidales.f__
6e+05
4e+05
2e+05
0e+00
46−60 (n=7)
80plus (n=3)
61−80 (n=15)
31−45 (n=3)
20−30 (n=2)
Age_category

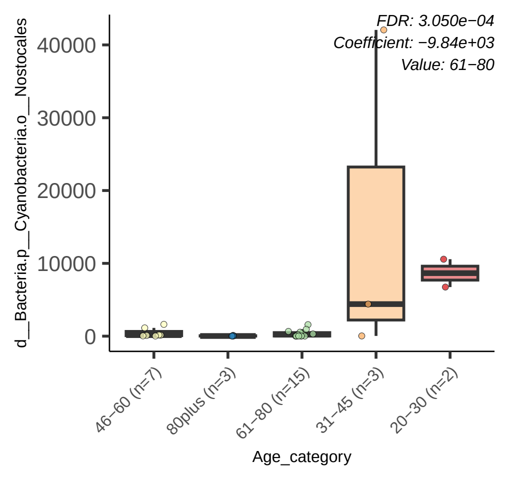

FDR: 3.050e−04
Coefficient: −9.84e+03
Value: 61−80
d__Bacteria.p__Cyanobacteria.o__Nostocales
40000
30000
20000
10000
0
46−60 (n=7)
80plus (n=3)
61−80 (n=15)
31−45 (n=3)
20−30 (n=2)
Age_category

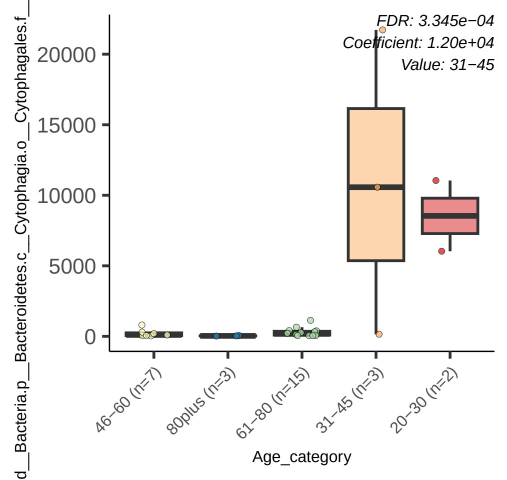

FDR: 3.345e−04
Coefficient: 1.20e+04
Value: 31−45
d__Bacteria.p__Bacteroidetes.c__Cytophagia.o__Cytophagales.f__
20000
15000
10000
5000
0
46−60 (n=7)
80plus (n=3)
61−80 (n=15)
31−45 (n=3)
20−30 (n=2)
Age_category

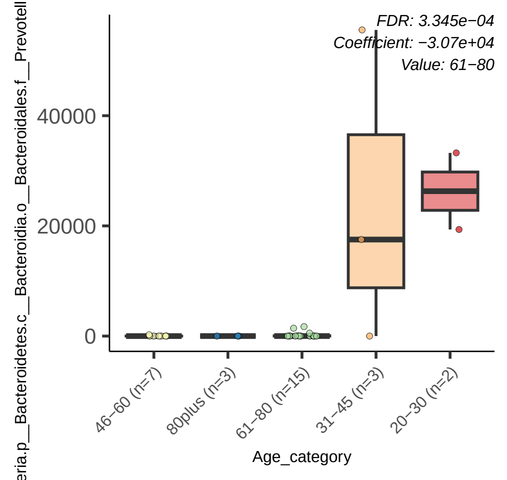

FDR: 3.345e−04
Coefficient: −3.07e+04
Value: 61−80
40000
20000
0
eria.p__Bacteroidetes.c__Bacteroidia.o__Bacteroidales.f__Prevotell
46−60 (n=7)
80plus (n=3)
61−80 (n=15)
31−45 (n=3)
20−30 (n=2)
Age_category

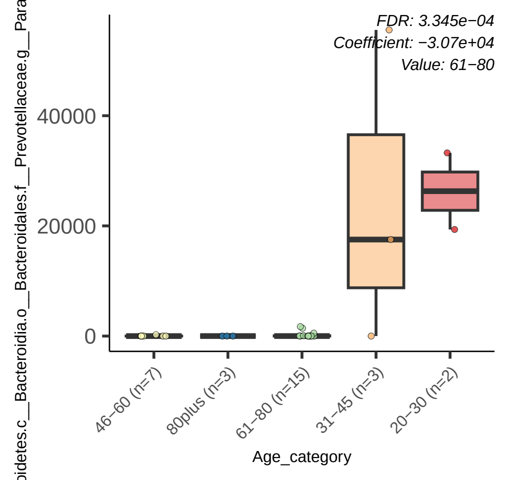

FDR: 3.345e−04
Coefficient: −3.07e+04
Value: 61−80
oidetes.c__Bacteroidia.o__Bacteroidales.f__Prevotellaceae.g__Para
40000
20000
0
46−60 (n=7)
80plus (n=3)
61−80 (n=15)
31−45 (n=3)
20−30 (n=2)
Age_category

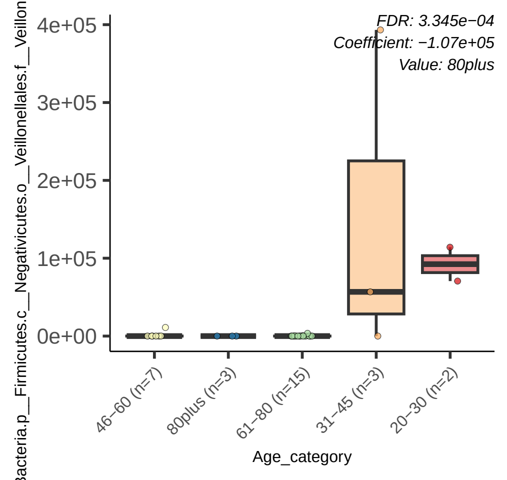

FDR: 3.345e−04
Coefficient: −1.07e+05
Value: 80plus
4e+05
3e+05
2e+05
1e+05
0e+00
Bacteria.p__Firmicutes.c__Negativicutes.o__Veillonellales.f__Veillon
46−60 (n=7)
80plus (n=3)
61−80 (n=15)
31−45 (n=3)
20−30 (n=2)
Age_category

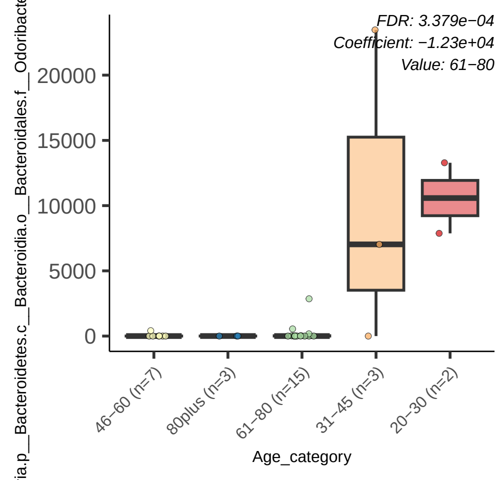

FDR: 3.379e−04
Coefficient: −1.23e+04
Value: 61−80
ia.p__Bacteroidetes.c__Bacteroidia.o__Bacteroidales.f__Odoribacte
20000
15000
10000
5000
0
46−60 (n=7)
80plus (n=3)
61−80 (n=15)
31−45 (n=3)
20−30 (n=2)
Age_category

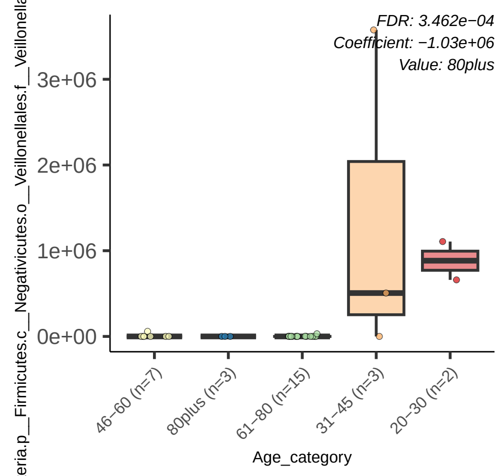
FDR: 3.462e−04
Coefficient: −1.03e+06
Value: 80plus
3e+06
2e+06
1e+06
0e+00
teria.p__Firmicutes.c__Negativicutes.o__Veillonellales.f__Veillonella
46−60 (n=7)
80plus (n=3)
61−80 (n=15)
31−45 (n=3)
20−30 (n=2)
Age_category

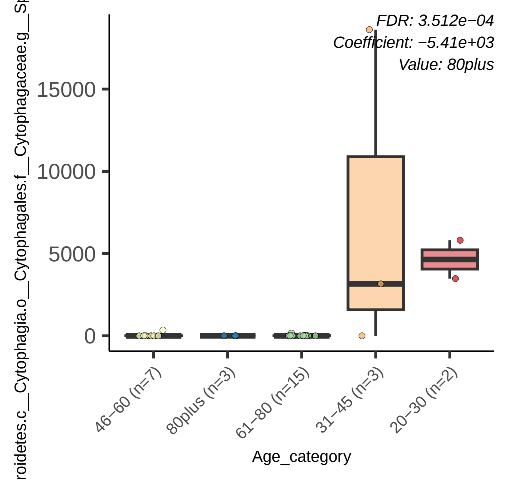

FDR: 3.512e−04
Coefficient: −5.41e+03
Value: 80plus
15000
10000
5000
0
roidetes.c__Cytophagia.o__Cytophagales.f__Cytophagaceae.g__Sp
46−60 (n=7)
80plus (n=3)
61−80 (n=15)
31−45 (n=3)
20−30 (n=2)
Age_category

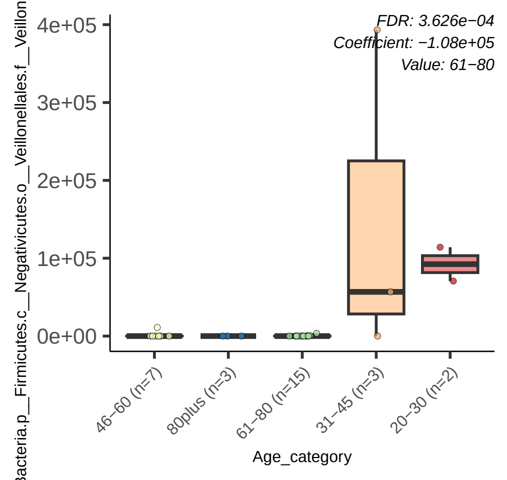

FDR: 3.626e−04
Coefficient: −1.08e+05
Value: 61−80
4e+05
3e+05
2e+05
1e+05
0e+00
Bacteria.p__Firmicutes.c__Negativicutes.o__Veillonellales.f__Veillon
46−60 (n=7)
80plus (n=3)
61−80 (n=15)
31−45 (n=3)
20−30 (n=2)
Age_category

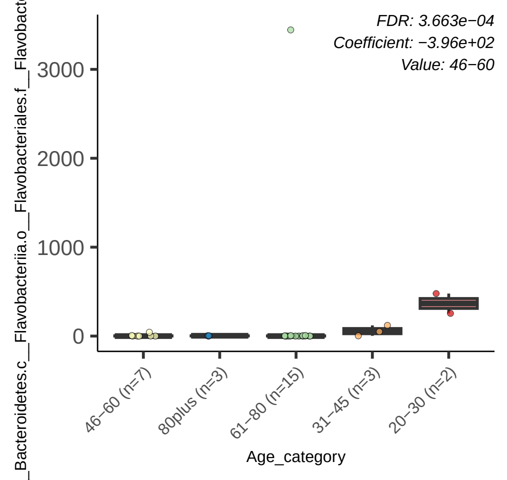

FDR: 3.663e−04
Coefficient: −3.96e+02
Value: 46−60
3000
2000
1000
0
Bacteroidetes.c__Flavobacteriia.o__Flavobacteriales.f__Flavobact
46−60 (n=7)
80plus (n=3)
61−80 (n=15)
31−45 (n=3)
20−30 (n=2)
Age_category

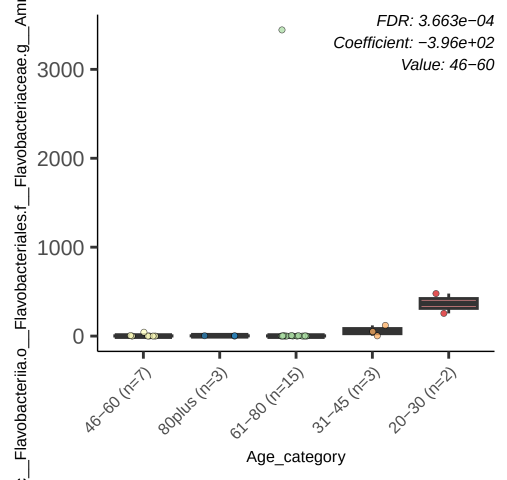

FDR: 3.663e−04
Coefficient: −3.96e+02
Value: 46−60
3000
2000
1000
0
Flavobacteriia.o__Flavobacteriales.f__Flavobacteriaceae.g__Am
46−60 (n=7)
80plus (n=3)
61−80 (n=15)
31−45 (n=3)
20−30 (n=2)
Age_category

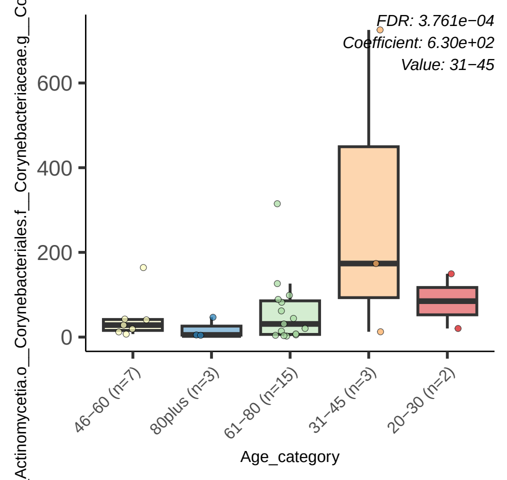
FDR: 3.761e−04
Coefficient: 6.30e+02
Value: 31−45
Actinomycetia.o__Corynebacteriales.f__Corynebacteriaceae.g__Co
600
400
200
0
46−60 (n=7)
80plus (n=3)
61−80 (n=15)
31−45 (n=3)
20−30 (n=2)
Age_category

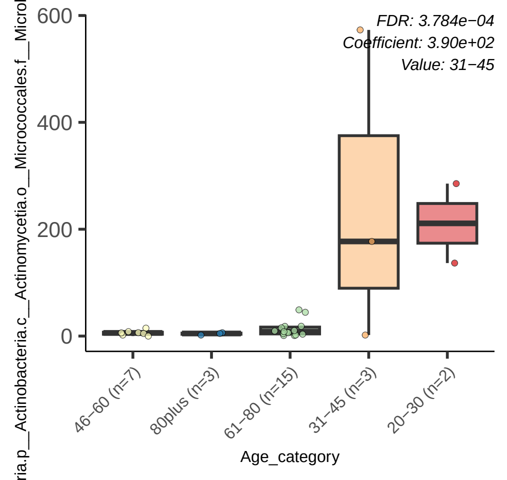

FDR: 3.784e−04
Coefficient: 3.90e+02
Value: 31−45
600
400
200
0
ria.p__Actinobacteria.c__Actinomycetia.o__Micrococcales.f__Microb
46−60 (n=7)
80plus (n=3)
61−80 (n=15)
31−45 (n=3)
20−30 (n=2)
Age_category

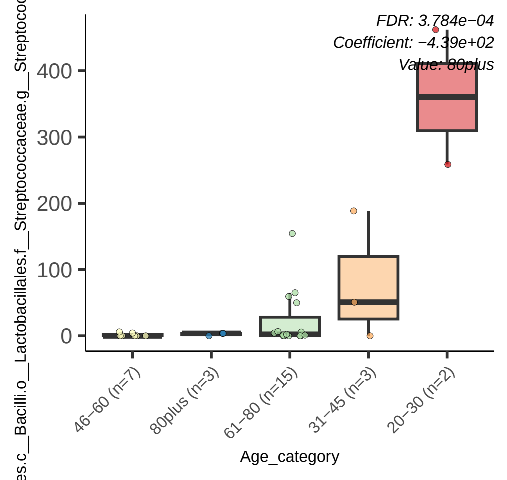

FDR: 3.784e−04
Coefficient: −4.39e+02
Value: 80plus
400
300
200
100
0
46−60 (n=7)
80plus (n=3)
61−80 (n=15)
31−45 (n=3)
20−30 (n=2)
Age_category
es.c__Bacilli.o__Lactobacillales.f__Streptococcaceae.g__Streptococ

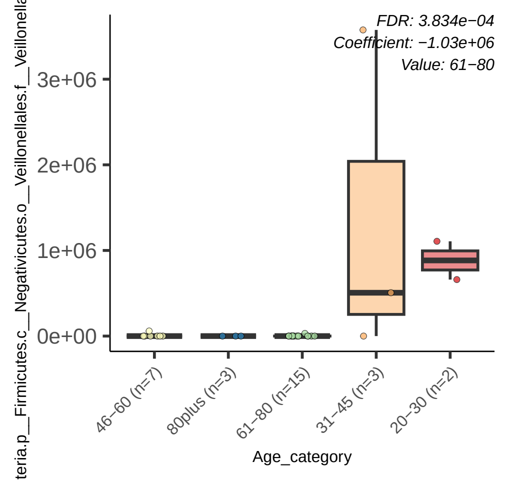

FDR: 3.834e−04
Coefficient: −1.03e+06
Value: 61−80
3e+06
2e+06
1e+06
0e+00
teria.p__Firmicutes.c__Negativicutes.o__Veillonellales.f__Veillonella
46−60 (n=7)
80plus (n=3)
61−80 (n=15)
31−45 (n=3)
20−30 (n=2)
Age_category

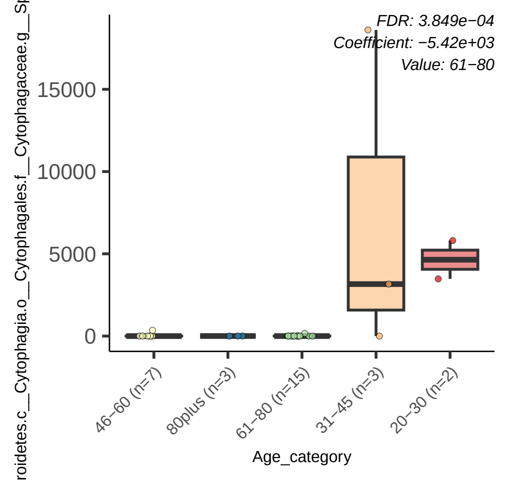

FDR: 3.849e−04
Coefficient: −5.42e+03
Value: 61−80
15000
10000
5000
0
46−60 (n=7)
80plus (n=3)
61−80 (n=15)
31−45 (n=3)
20−30 (n=2)
Age_category
roidetes.c__Cytophagia.o__Cytophagales.f__Cytophagaceae.g__Sp

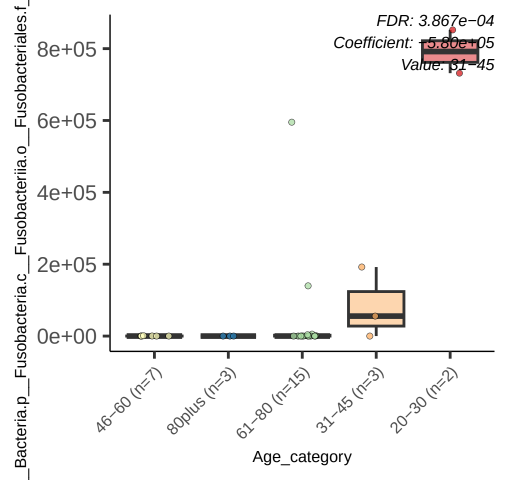

FDR: 3.867e−04
Coefficient: -5.80e+05
Value: 31−45
8e+05
6e+05
4e+05
2e+05
0e+00
_Bacteria.p__Fusobacteria.c__Fusobacteriia.o__Fusobacteriales.f_
46−60 (n=7)
80plus (n=3)
61−80 (n=15)
31−45 (n=3)
20−30 (n=2)
Age_category

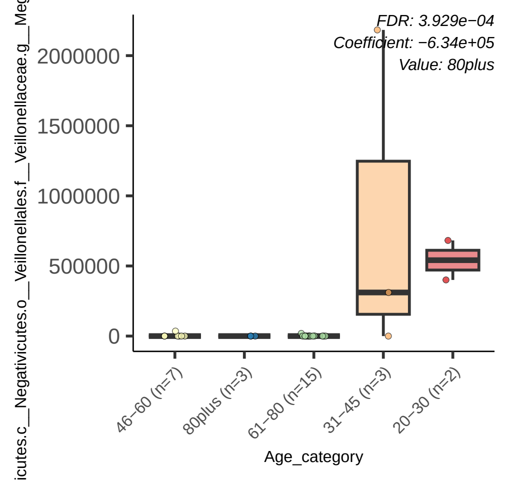

FDR: 3.929e−04
Coefficient: −6.34e+05
Value: 80plus
2000000
1500000
1000000
500000
0
icutes.c__Negativicutes.o__Veillonellales.f__Veillonellaceae.g__Meg
46−60 (n=7)
80plus (n=3)
61−80 (n=15)
31−45 (n=3)
20−30 (n=2)
Age_category

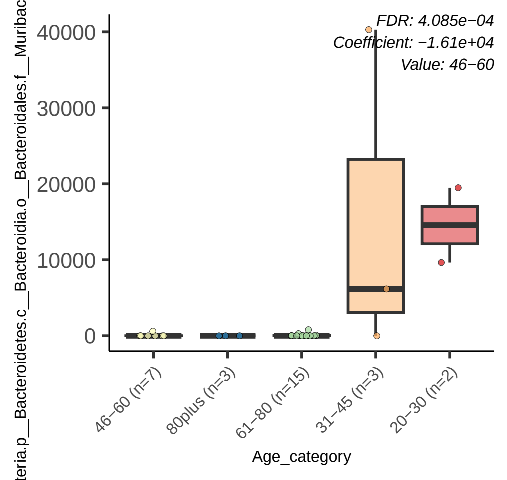

FDR: 4.085e−04
Coefficient: −1.61e+04
Value: 46−60
40000
30000
20000
10000
0
teria.p__Bacteroidetes.c__Bacteroidia.o__Bacteroidales.f__Muribac
46−60 (n=7)
80plus (n=3)
61−80 (n=15)
31−45 (n=3)
20−30 (n=2)
Age_category

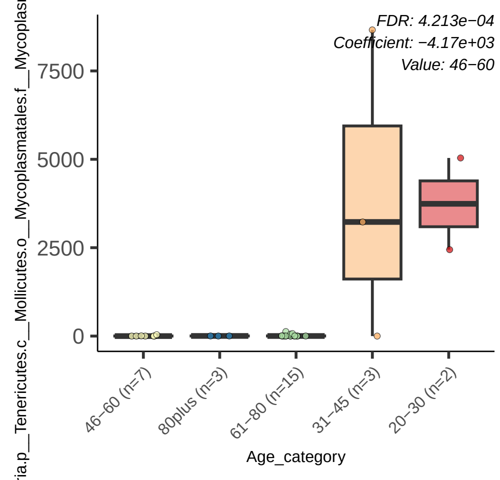
FDR: 4.213e−04
Coefficient: −4.17e+03
Value: 46−60
ria.p__Tenericutes.c__Mollicutes.o__Mycoplasmatales.f__Mycoplas
7500
5000
2500
0
46−60 (n=7)
80plus (n=3)
61−80 (n=15)
31−45 (n=3)
20−30 (n=2)
Age_category

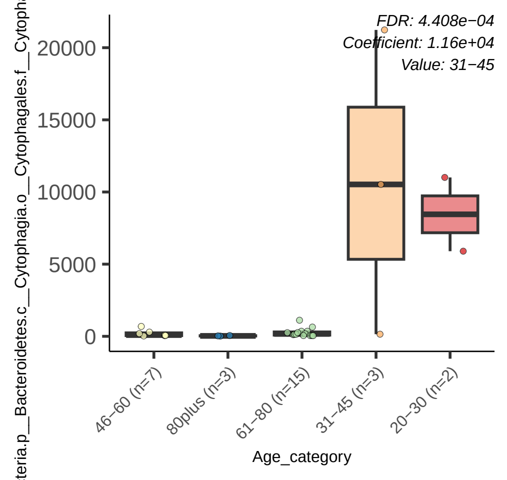

FDR: 4.408e−04
Coefficient: 1.16e+04
Value: 31−45
20000
15000
10000
5000
0
46−60 (n=7)
80plus (n=3)
61−80 (n=15)
31−45 (n=3)
20−30 (n=2)
Age_category
teria.p__Bacteroidetes.c__Cytophagia.o__Cytophagales.f__Cytopha

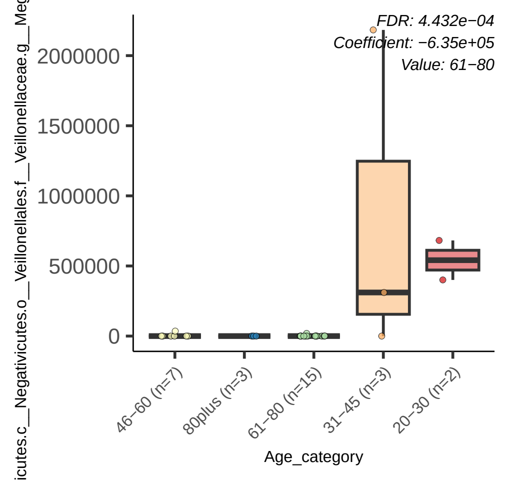
FDR: 4.432e−04
Coefficient: −6.35e+05
Value: 61−80
2000000
1500000
1000000
500000
0
46−60 (n=7)
80plus (n=3)
61−80 (n=15)
31−45 (n=3)
20−30 (n=2)
Age_category
icutes.c__Negativicutes.o__Veillonellales.f__Veillonellaceae.g__Meg

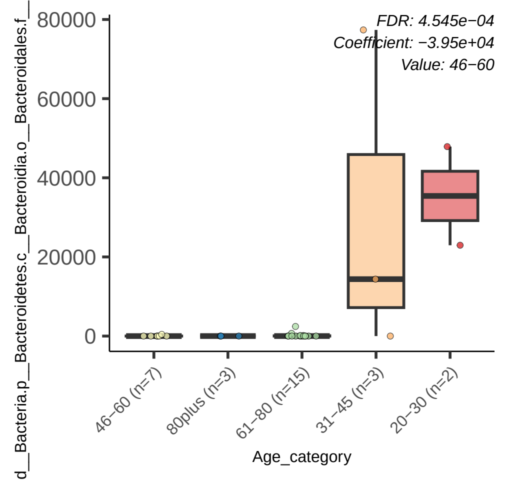
FDR: 4.545e−04
Coefficient: −3.95e+04
Value: 46−60
d__Bacteria.p__Bacteroidetes.c__Bacteroidia.o__Bacteroidales.f__
80000
60000
40000
20000
0
46−60 (n=7)
80plus (n=3)
61−80 (n=15)
31−45 (n=3)
20−30 (n=2)
Age_category

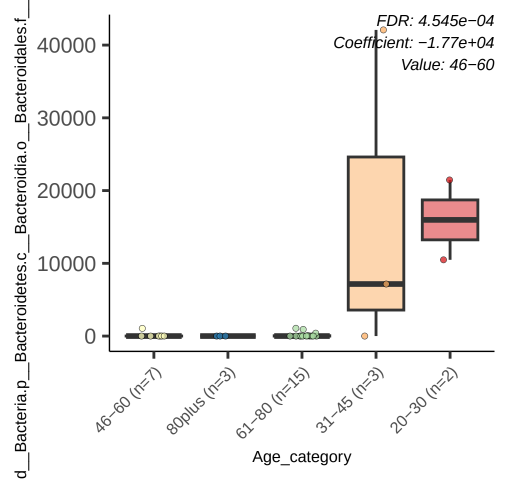

FDR: 4.545e−04
Coefficient: −1.77e+04
Value: 46−60
d__Bacteria.p__Bacteroidetes.c__Bacteroidia.o__Bacteroidales.f__
40000
30000
20000
10000
0
46−60 (n=7)
80plus (n=3)
61−80 (n=15)
31−45 (n=3)
20−30 (n=2)
Age_category

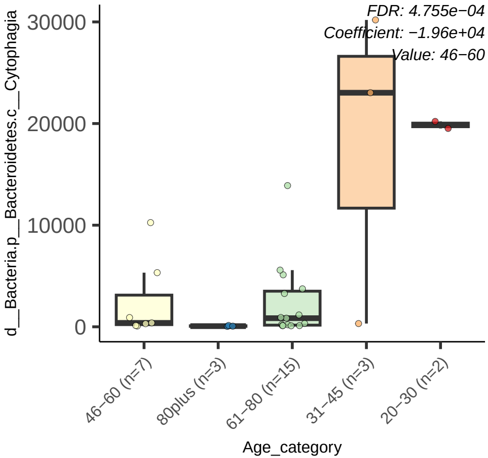

FDR: 4.755e−04
Coefficient: −1.96e+04
Value: 46−60
d__Bacteria.p__Bacteroidetes.c__Cytophagia
30000
20000
10000
0
46−60 (n=7)
80plus (n=3)
61−80 (n=15)
31−45 (n=3)
20−30 (n=2)
Age_category

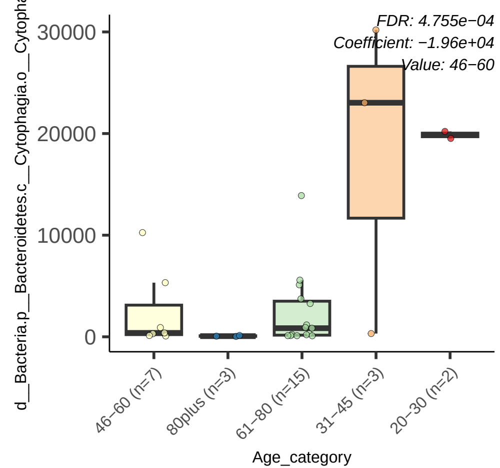

FDR: 4.755e−04
Coefficient: −1.96e+04
Value: 46−60
d__Bacteria.p__Bacteroidetes.c__Cytophagia.o__Cytophaga
30000
20000
10000
0
46−60 (n=7)
80plus (n=3)
61−80 (n=15)
31−45 (n=3)
20−30 (n=2)
Age_category

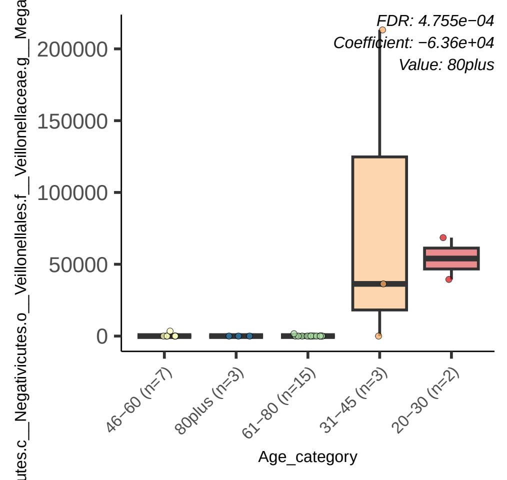

FDR: 4.755e−04
Coefficient: −6.36e+04
Value: 80plus
200000
150000
100000
50000
0
utes.c__Negativicutes.o__Veillonellales.f__Veillonellaceae.g__Mega
46−60 (n=7)
80plus (n=3)
61−80 (n=15)
31−45 (n=3)
20−30 (n=2)
Age_category

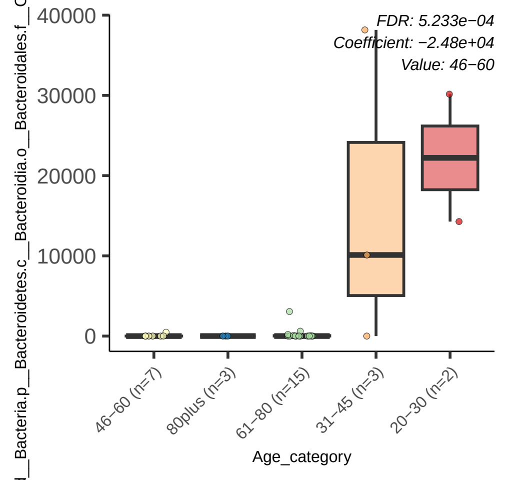
FDR: 5.233e−04
Coefficient: −2.48e+04
Value: 46−60
40000
30000
20000
10000
0
46−60 (n=7)
80plus (n=3)
61−80 (n=15)
31−45 (n=3)
20−30 (n=2)
Age_category
d__Bacteria.p__Bacteroidetes.c__Bacteroidia.o__Bacteroidales.f__

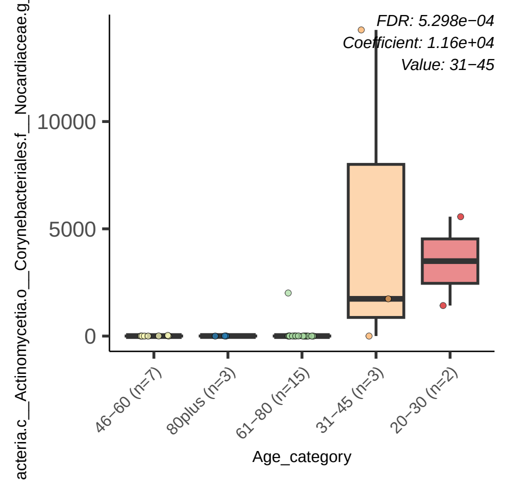

FDR: 5.298e−04
Coefficient: 1.16e+04
Value: 31−45
10000
5000
0
acteria.c__Actinomycetia.o__Corynebacteriales.f__Nocardiaceae.g_
46−60 (n=7)
80plus (n=3)
61−80 (n=15)
31−45 (n=3)
20−30 (n=2)
Age_category

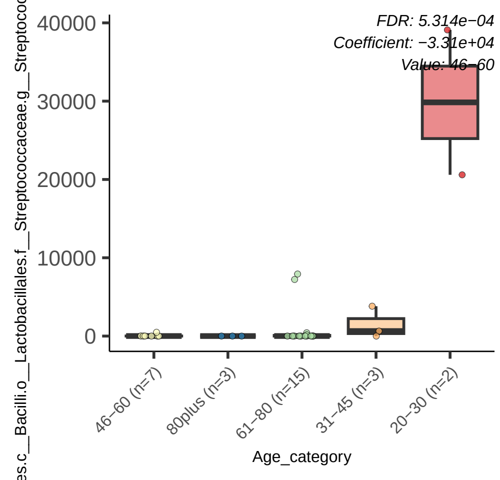

es.c__Bacilli.o__Lactobacillales.f__Streptococcaceae.g__Streptococ
FDR: 5.314e−04
Coefficient: −3.31e+04
Value: 46−60
40000
30000
20000
10000
0
46−60 (n=7)
80plus (n=3)
61−80 (n=15)
31−45 (n=3)
20−30 (n=2)
Age_category

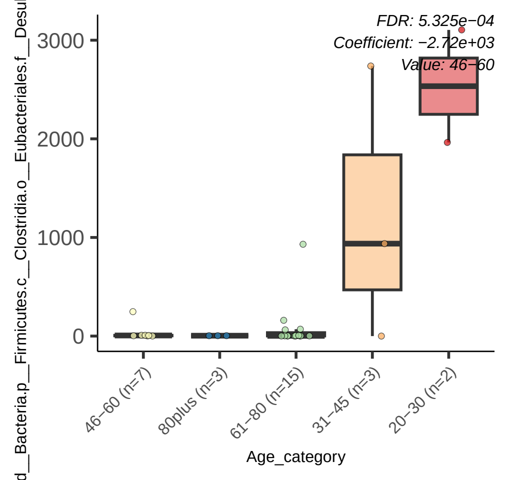

FDR: 5.325e−04
Coefficient: −2.72e+03
Value: 46−60
d__Bacteria.p__Firmicutes.c__Clostridia.o__Eubacteriales.f__Desul
3000
2000
1000
0
46−60 (n=7)
80plus (n=3)
61−80 (n=15)
31−45 (n=3)
20−30 (n=2)
Age_category

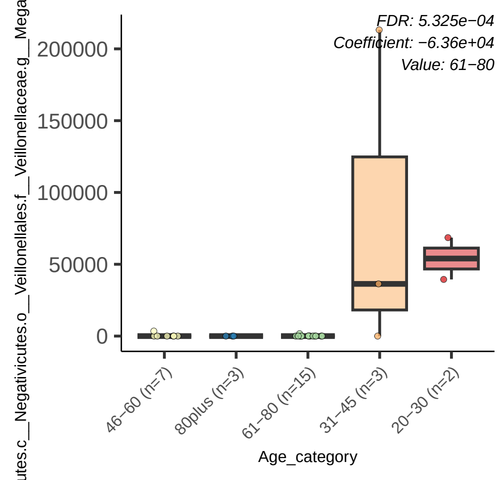
FDR: 5.325e−04
Coefficient: −6.36e+04
Value: 61−80
utes.c__Negativicutes.o__Veillonellales.f__Veillonellaceae.g__Mega
200000
150000
100000
50000
0
46−60 (n=7)
80plus (n=3)
61−80 (n=15)
31−45 (n=3)
20−30 (n=2)
Age_category

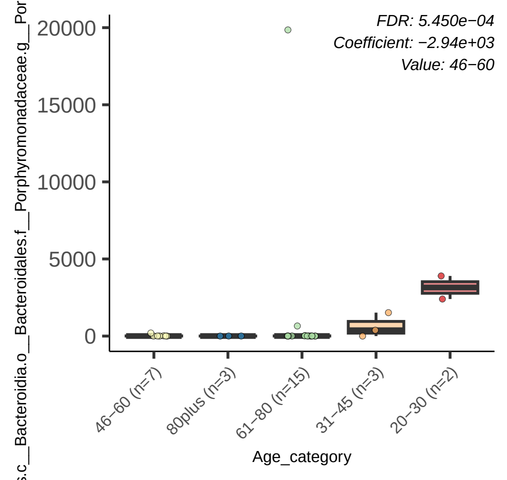

FDR: 5.450e−04
Coefficient: −2.94e+03
Value: 46−60
20000
15000
10000
5000
0
46−60 (n=7)
80plus (n=3)
61−80 (n=15)
31−45 (n=3)
20−30 (n=2)
Age_category
s.c__Bacteroidia.o__Bacteroidales.f__Porphyromonadaceae.g__Por

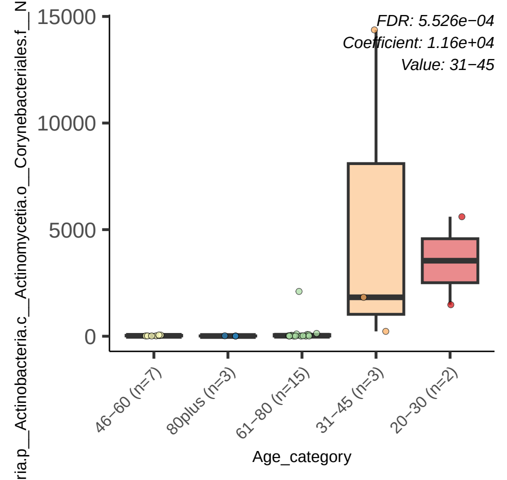
FDR: 5.526e−04
Coefficient: 1.16e+04
Value: 31−45
ria.p__Actinobacteria.c__Actinomycetia.o__Corynebacteriales.f__N
15000
10000
5000
0
46−60 (n=7)
80plus (n=3)
61−80 (n=15)
31−45 (n=3)
20−30 (n=2)
Age_category

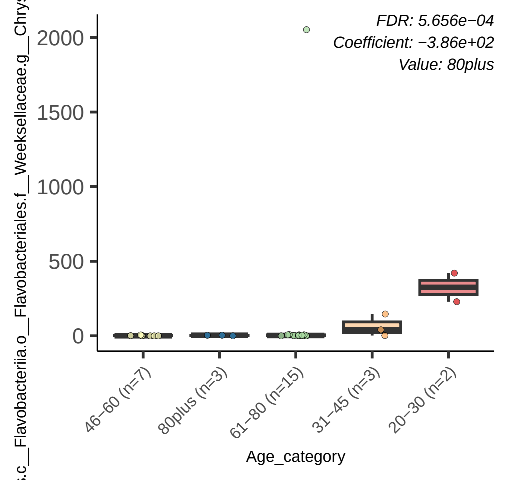

s.c__Flavobacteriia.o__Flavobacteriales.f__Weeksellaceae.g__Chrys
FDR: 5.656e−04
Coefficient: −3.86e+02
Value: 80plus
2000
1500
1000
500
0
46−60 (n=7)
80plus (n=3)
61−80 (n=15)
31−45 (n=3)
20−30 (n=2)
Age_category

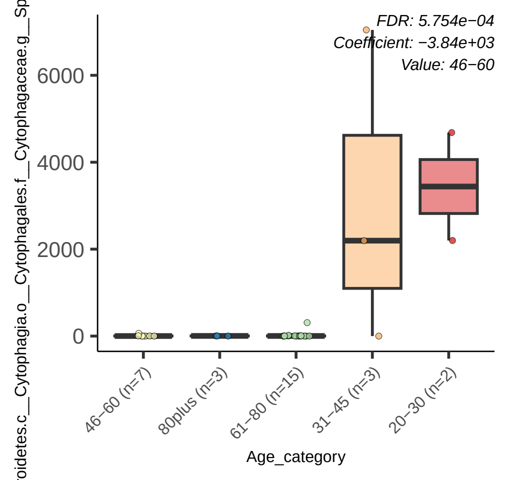

FDR: 5.754e−04
Coefficient: −3.84e+03
Value: 46−60
6000
4000
2000
0
roidetes.c__Cytophagia.o__Cytophagales.f__Cytophagaceae.g__Sp
46−60 (n=7)
80plus (n=3)
61−80 (n=15)
31−45 (n=3)
20−30 (n=2)
Age_category

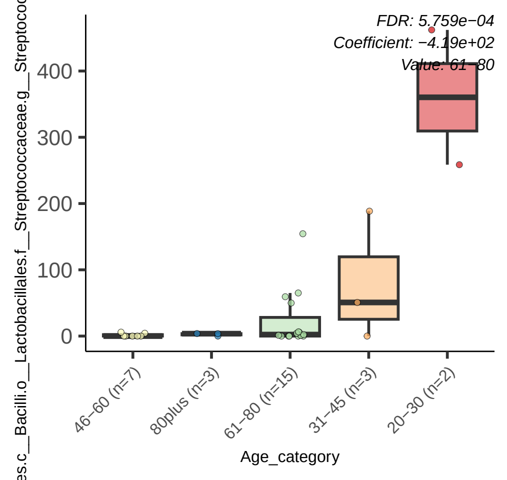

FDR: 5.759e−04
Coefficient: −4.19e+02
Value: 61−80
es.c__Bacilli.o__Lactobacillales.f__Streptococcaceae.g__Streptococ
400
300
200
100
0
46−60 (n=7)
80plus (n=3)
61−80 (n=15)
31−45 (n=3)
20−30 (n=2)
Age_category

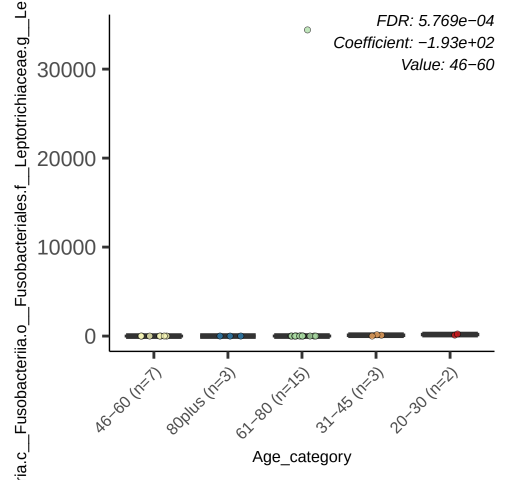

FDR: 5.769e−04
Coefficient: −1.93e+02
Value: 46−60
30000
20000
10000
0
46−60 (n=7)
80plus (n=3)
61−80 (n=15)
31−45 (n=3)
20−30 (n=2)
Age_category
ria.c__Fusobacteriia.o__Fusobacteriales.f__Leptotrichiaceae.g__Le

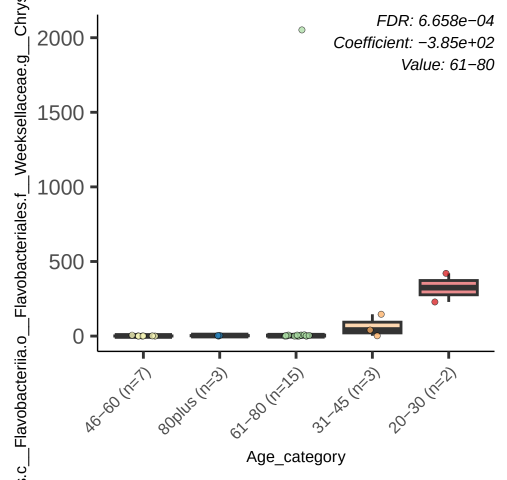

FDR: 6.658e−04
Coefficient: −3.85e+02
Value: 61−80
2000
1500
1000
500
0
46−60 (n=7)
80plus (n=3)
61−80 (n=15)
31−45 (n=3)
20−30 (n=2)
Age_category
Flavobacteriia.o__Flavobacteriales.f__Weeksellaceae.g__Chrys

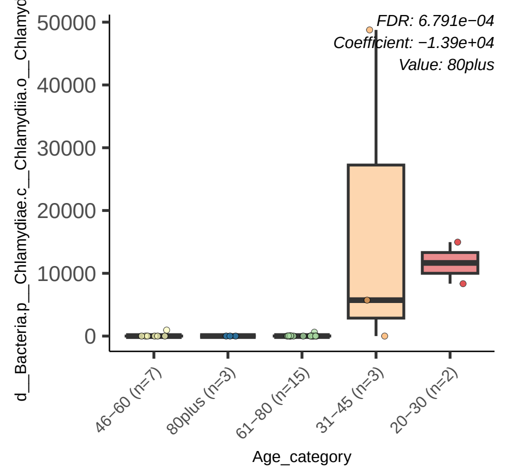
FDR: 6.791e−04
Coefficient: −1.39e+04
Value: 80plus
d__Bacteria.p__Chlamydiae.c__Chlamydiia.o__Chlamyd
50000
40000
30000
20000
10000
0
46−60 (n=7)
80plus (n=3)
61−80 (n=15)
31−45 (n=3)
20−30 (n=2)
Age_category

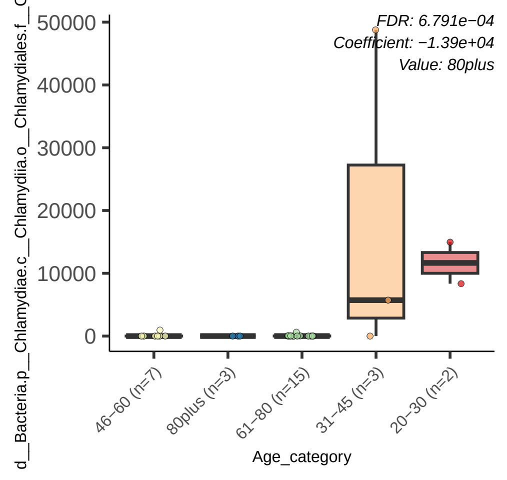

d__Bacteria.p__Chlamydiae.c__Chlamydiia.o__Chlamydiales.f__C
FDR: 6.791e−04
Coefficient: −1.39e+04
Value: 80plus
50000
40000
30000
20000
10000
0
46−60 (n=7)
80plus (n=3)
61−80 (n=15)
31−45 (n=3)
20−30 (n=2)
Age_category

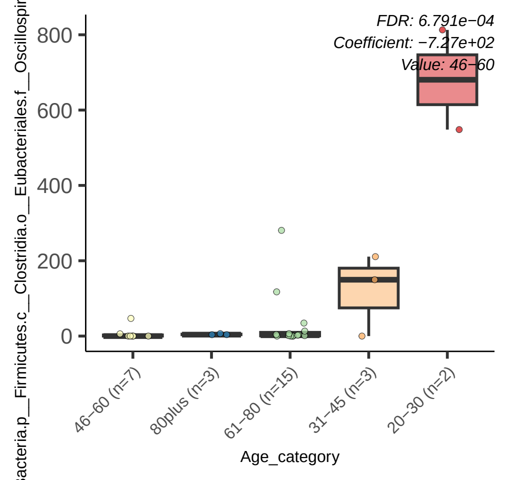

FDR: 6.791e−04
Coefficient: −7.27e+02
Value: 46−60
800
600
400
200
0
acteria.p__Firmicutes.c__Clostridia.o__Eubacteriales.f__Oscillospir
46−60 (n=7)
80plus (n=3)
61−80 (n=15)
31−45 (n=3)
20−30 (n=2)
Age_category

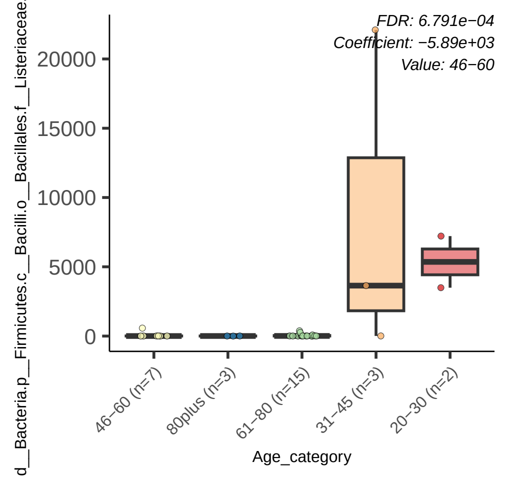

FDR: 6.791e−04
Coefficient: −5.89e+03
Value: 46−60
d__Bacteria.p__Firmicutes.c__Bacilli.o__Bacillales.f__Listeriaceae
20000
15000
10000
5000
0
46−60 (n=7)
80plus (n=3)
61−80 (n=15)
31−45 (n=3)
20−30 (n=2)
Age_category

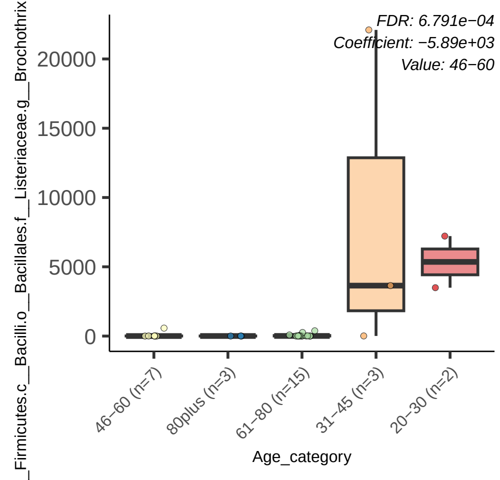

FDR: 6.791e−04
Coefficient: −5.89e+03
Value: 46−60
Firmicutes.c__Bacilli.o__Bacillales.f__Listeriaceae.g__Brochothrix
0
5000
10000
15000
20000
46−60 (n=7)
80plus (n=3)
61−80 (n=15)
31−45 (n=3)
20−30 (n=2)
Age_category

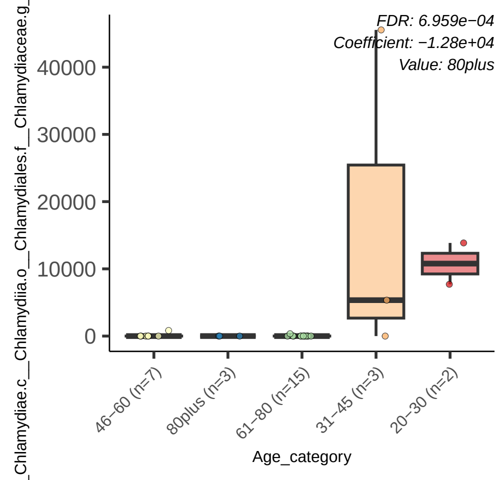

FDR: 6.959e−04
Coefficient: −1.28e+04
Value: 80plus
40000
30000
20000
10000
0
Chlamydiae.c__Chlamydiia.o__Chlamydiales.f__Chlamydiaceae.g__
46−60 (n=7)
80plus (n=3)
61−80 (n=15)
31−45 (n=3)
20−30 (n=2)
Age_category

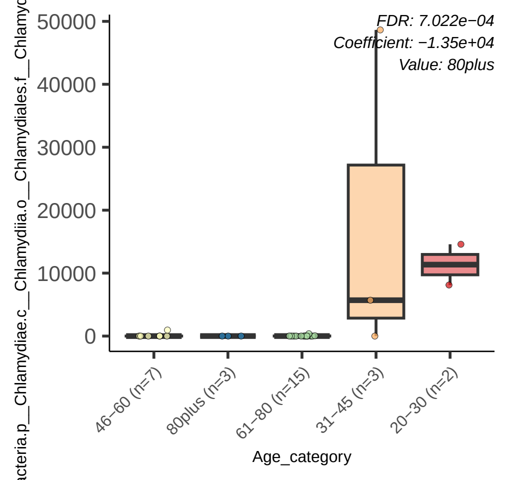

FDR: 7.022e−04
Coefficient: −1.35e+04
Value: 80plus
50000
40000
30000
20000
10000
0
acteria.p__Chlamydiae.c__Chlamydiia.o__Chlamydiales.f__Chlamyd
46−60 (n=7)
80plus (n=3)
61−80 (n=15)
31−45 (n=3)
20−30 (n=2)
Age_category

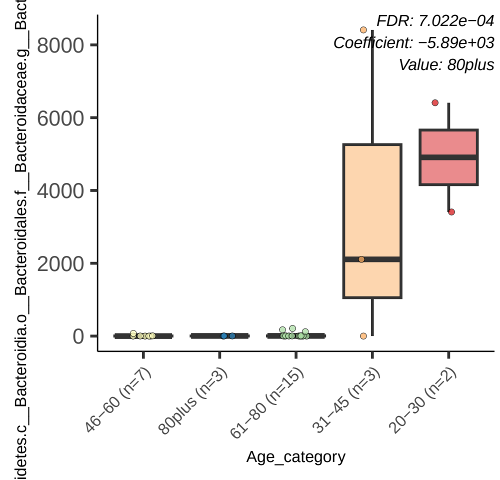

FDR: 7.022e−04
Coefficient: −5.89e+03
Value: 80plus
8000
6000
4000
2000
0
idetes.c__Bacteroidia.o__Bacteroidales.f__Bacteroidaceae.g__Bact
46−60 (n=7)
80plus (n=3)
61−80 (n=15)
31−45 (n=3)
20−30 (n=2)
Age_category

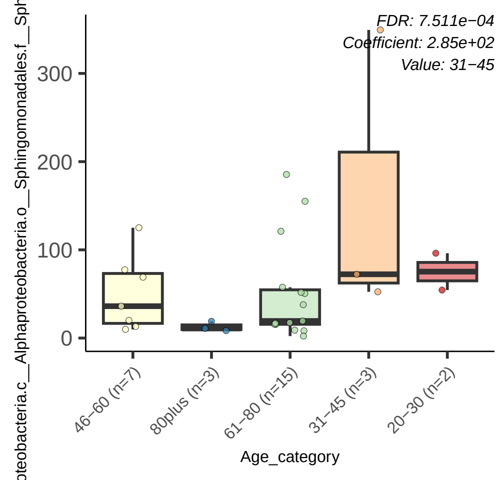

FDR: 7.511e−04
Coefficient: 2.85e+02
Value: 31−45
oteobacteria.c__Alphaproteobacteria.o__Sphingomonadales.f__Sph
300
200
100
0
46−60 (n=7)
80plus (n=3)
61−80 (n=15)
31−45 (n=3)
20−30 (n=2)
Age_category

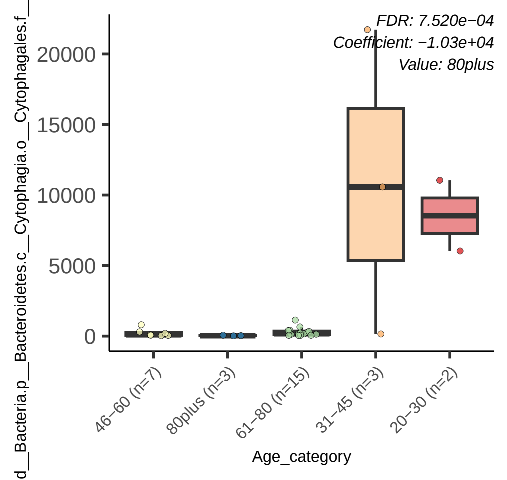
FDR: 7.520e−04
Coefficient: −1.03e+04
Value: 80plus
d__Bacteria.p__Bacteroidetes.c__Cytophagia.o__Cytophagales.f__
20000
15000
10000
5000
0
46−60 (n=7)
80plus (n=3)
61−80 (n=15)
31−45 (n=3)
20−30 (n=2)
Age_category

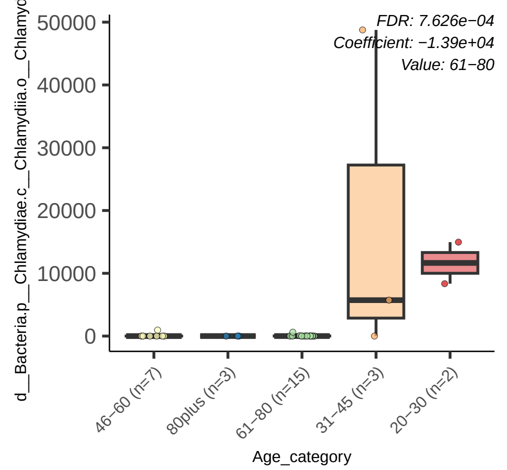
FDR: 7.626e−04
Coefficient: −1.39e+04
Value: 61−80
d__Bacteria.p__Chlamydiae.c__Chlamydiia.o__Chlamyd
50000
40000
30000
20000
10000
0
46−60 (n=7)
80plus (n=3)
61−80 (n=15)
31−45 (n=3)
20−30 (n=2)
Age_category

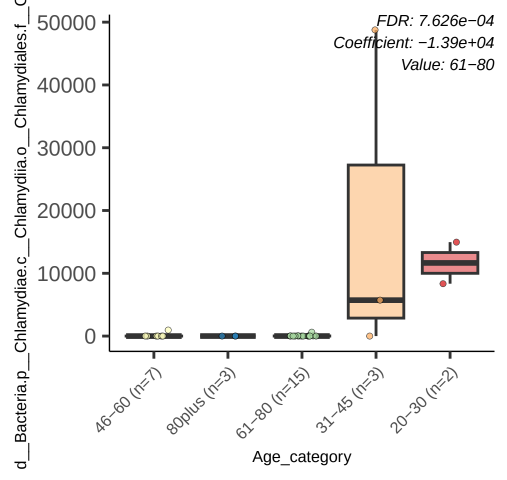

FDR: 7.626e−04
Coefficient: −1.39e+04
Value: 61−80
50000
40000
30000
20000
10000
0
d__Bacteria.p__Chlamydiae.c__Chlamydiia.o__Chlamydiales.f__C
46−60 (n=7)
80plus (n=3)
61−80 (n=15)
31−45 (n=3)
20−30 (n=2)
Age_category

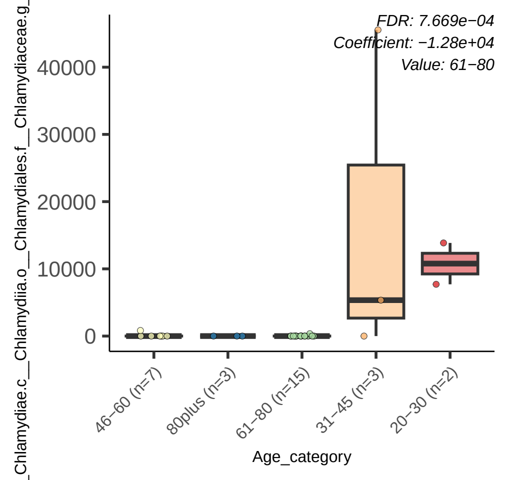
FDR: 7.669e−04
Coefficient: −1.28e+04
Value: 61−80
40000
30000
20000
10000
0
Chlamydiae.c__Chlamydiia.o__Chlamydiales.f__Chlamydiaceae.g_
46−60 (n=7)
80plus (n=3)
61−80 (n=15)
31−45 (n=3)
20−30 (n=2)
Age_category

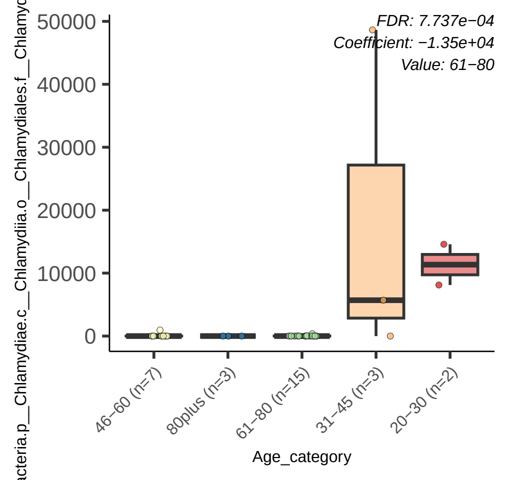

FDR: 7.737e−04
Coefficient: −1.35e+04
Value: 61−80
50000
40000
30000
20000
10000
0
acteria.p__Chlamydiae.c__Chlamydiia.o__Chlamydiales.f__Chlamyd
46−60 (n=7)
80plus (n=3)
61−80 (n=15)
31−45 (n=3)
20−30 (n=2)
Age_category

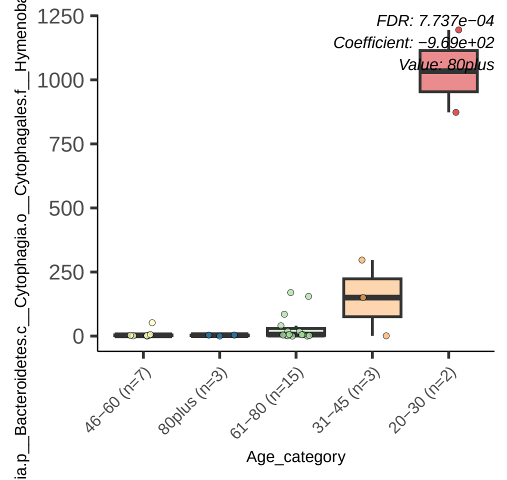

ia.p__Bacteroidetes.c__Cytophagia.o__Cytophagales.f__Hymenoba
1250
1000
750
500
250
0
FDR: 7.737e−04
Coefficient: −9.69e+02
Value: 80plus
46−60 (n=7)
80plus (n=3)
61−80 (n=15)
31−45 (n=3)
20−30 (n=2)
Age_category

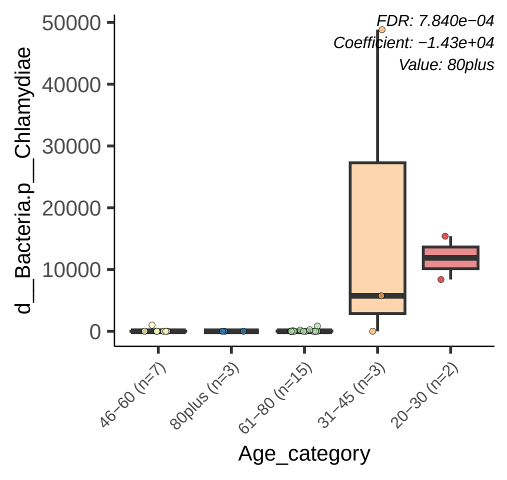

FDR: 7.840e−04
Coefficient: −1.43e+04
Value: 80plus
d__Bacteria.p__Chlamydiae
50000
40000
30000
20000
10000
0
46−60 (n=7)
80plus (n=3)
61−80 (n=15)
31−45 (n=3)
20−30 (n=2)
Age_category

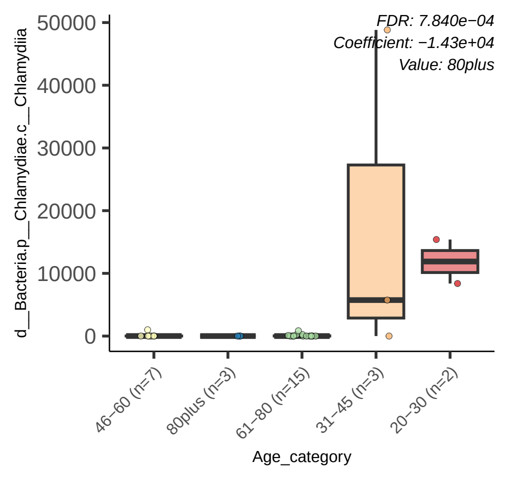

FDR: 7.840e−04
Coefficient: −1.43e+04
Value: 80plus
d__Bacteria.p__Chlamydiae.c__Chlamydiia
50000
40000
30000
20000
10000
0
46−60 (n=7)
80plus (n=3)
61−80 (n=15)
31−45 (n=3)
20−30 (n=2)
Age_category

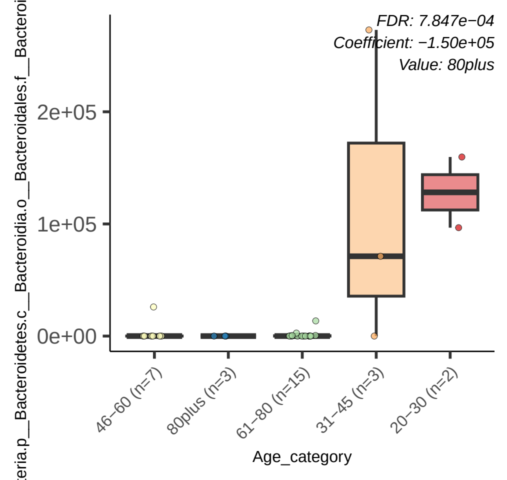
FDR: 7.847e−04
Coefficient: −1.50e+05
Value: 80plus
2e+05
1e+05
0e+00
teria.p__Bacteroidetes.c__Bacteroidia.o__Bacteroidales.f__Bacteroi
46−60 (n=7)
80plus (n=3)
61−80 (n=15)
31−45 (n=3)
20−30 (n=2)
Age_category

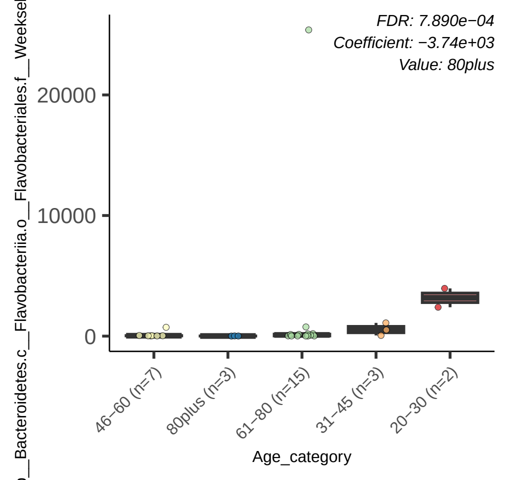
FDR: 7.890e−04
Coefficient: −3.74e+03
Value: 80plus
20000
10000
0
46−60 (n=7)
80plus (n=3)
61−80 (n=15)
31−45 (n=3)
20−30 (n=2)
Age_category
o__Bacteroidetes.c__Flavobacteriia.o__Flavobacteriales.f__Weeksel

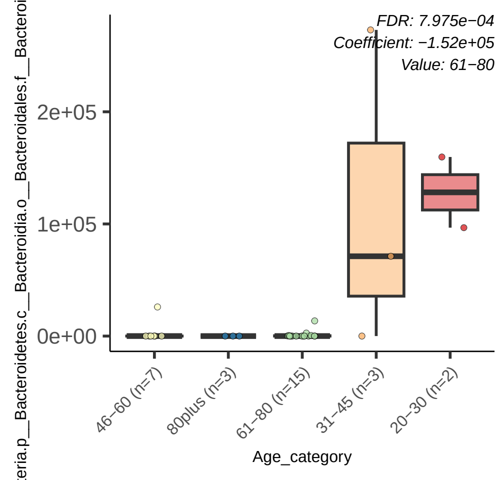

FDR: 7.975e−04
Coefficient: −1.52e+05
Value: 61−80
2e+05
1e+05
0e+00
46−60 (n=7)
80plus (n=3)
61−80 (n=15)
31−45 (n=3)
20−30 (n=2)
Age_category

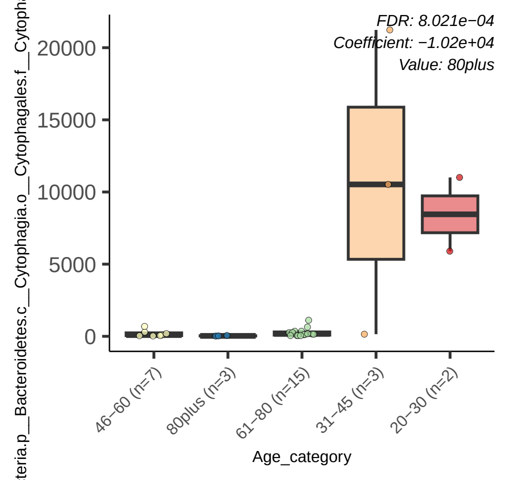

FDR: 8.021e−04
Coefficient: −1.02e+04
Value: 80plus
teria.p__Bacteroidetes.c__Cytophagia.o__Cytophagales.f__Cytopha
20000
15000
10000
5000
0
46−60 (n=7)
80plus (n=3)
61−80 (n=15)
31−45 (n=3)
20−30 (n=2)
Age_category

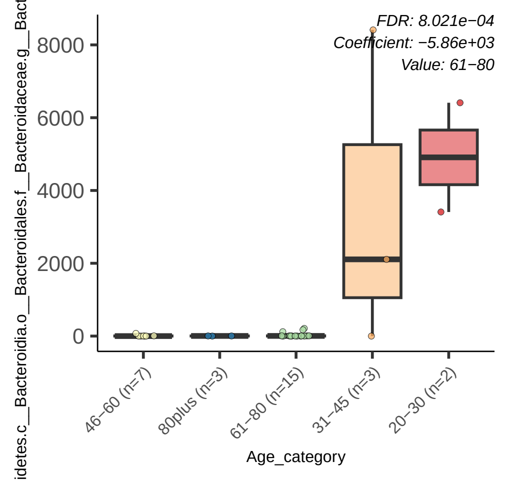

FDR: 8.021e−04
Coefficient: −5.86e+03
Value: 61−80
8000
6000
4000
2000
0
46−60 (n=7)
80plus (n=3)
61−80 (n=15)
31−45 (n=3)
20−30 (n=2)
Age_category
idetes.c__Bacteroidia.o__Bacteroidales.f__Bacteroidaceae.g__Bact

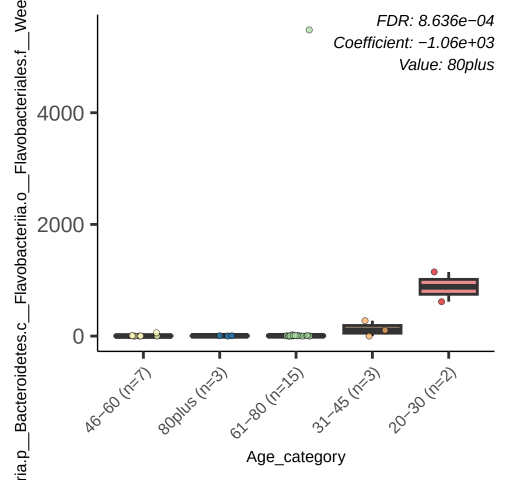

FDR: 8.636e−04
Coefficient: −1.06e+03
Value: 80plus
4000
2000
0
46−60 (n=7)
80plus (n=3)
61−80 (n=15)
31−45 (n=3)
20−30 (n=2)
Age_category
ria.p__Bacteroidetes.c__Flavobacteriia.o__Flavobacteriales.f__Wee

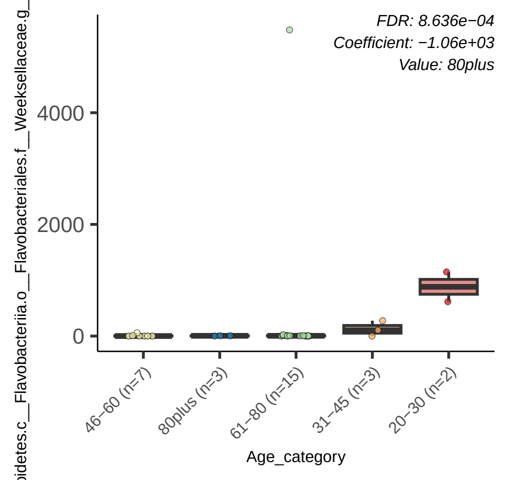

FDR: 8.636e−04
Coefficient: −1.06e+03
Value: 80plus
4000
2000
0
46−60 (n=7)
80plus (n=3)
61−80 (n=15)
31−45 (n=3)
20−30 (n=2)
Age_category
oidetes.c__Flavobacteriia.o__Flavobacteriales.f__Weeksellaceae.g__

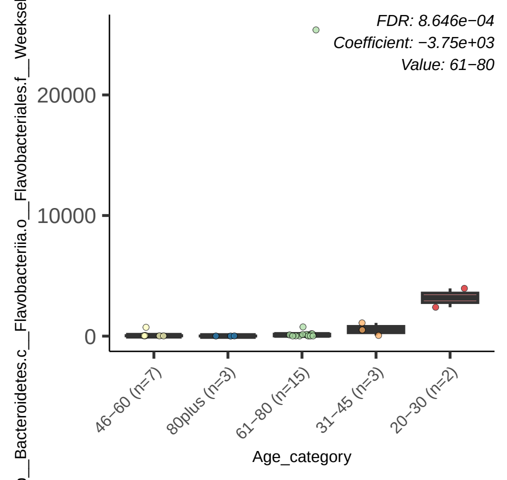

FDR: 8.646e−04
Coefficient: −3.75e+03
Value: 61−80
20000
10000
0
46−60 (n=7)
80plus (n=3)
61−80 (n=15)
31−45 (n=3)
20−30 (n=2)
Age_category
o__Bacteroidetes.c__Flavobacteriia.o__Flavobacteriales.f__Weeksel

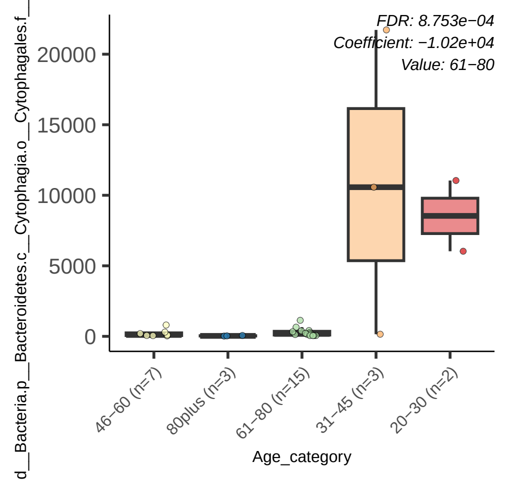

FDR: 8.753e−04
Coefficient: −1.02e+04
Value: 61−80
d__Bacteria.p__Bacteroidetes.c__Cytophagia.o__Cytophagales.f__
20000
15000
10000
5000
0
46−60 (n=7)
80plus (n=3)
61−80 (n=15)
31−45 (n=3)
20−30 (n=2)
Age_category

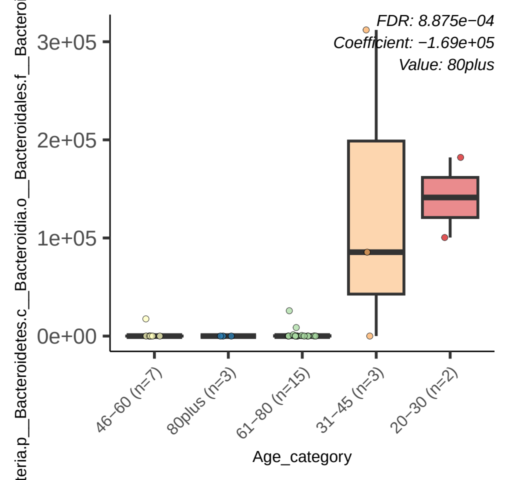

FDR: 8.875e−04
Coefficient: −1.69e+05
Value: 80plus
3e+05
2e+05
1e+05
0e+00
teria.p__Bacteroidetes.c__Bacteroidia.o__Bacteroidales.f__Bacteroi
46−60 (n=7)
80plus (n=3)
61−80 (n=15)
31−45 (n=3)
20−30 (n=2)
Age_category

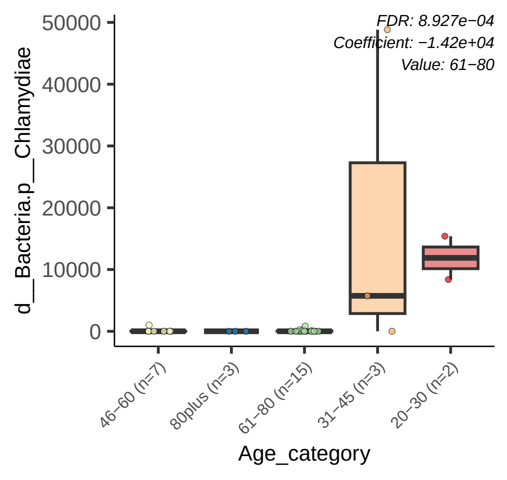
FDR: 8.927e−04
Coefficient: −1.42e+04
Value: 61−80
d__Bacteria.p__Chlamydiae
0
10000
20000
30000
40000
50000
46−60 (n=7)
80plus (n=3)
61−80 (n=15)
31−45 (n=3)
20−30 (n=2)
Age_category

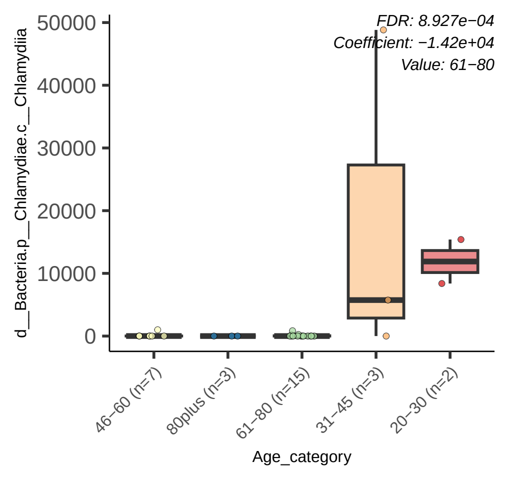

FDR: 8.927e−04
Coefficient: −1.42e+04
Value: 61−80
d__Bacteria.p__Chlamydiae.c__Chlamydiia
50000
40000
30000
20000
10000
0
46−60 (n=7)
80plus (n=3)
61−80 (n=15)
31−45 (n=3)
20−30 (n=2)
Age_category

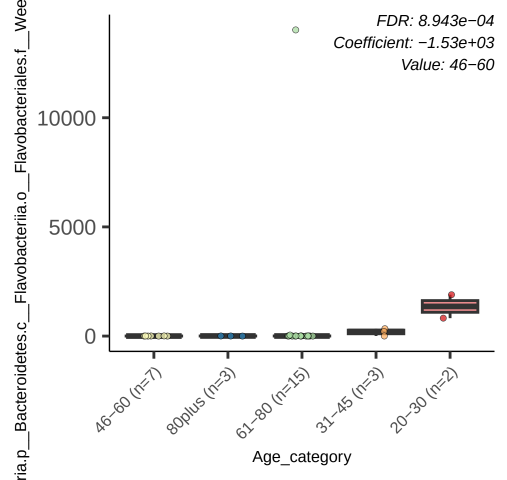
FDR: 8.943e−04
Coefficient: −1.53e+03
Value: 46−60
10000
5000
0
46−60 (n=7)
80plus (n=3)
61−80 (n=15)
31−45 (n=3)
20−30 (n=2)
Age_category
ria.p__Bacteroidetes.c__Flavobacteriia.o__Flavobacteriales.f__Wee

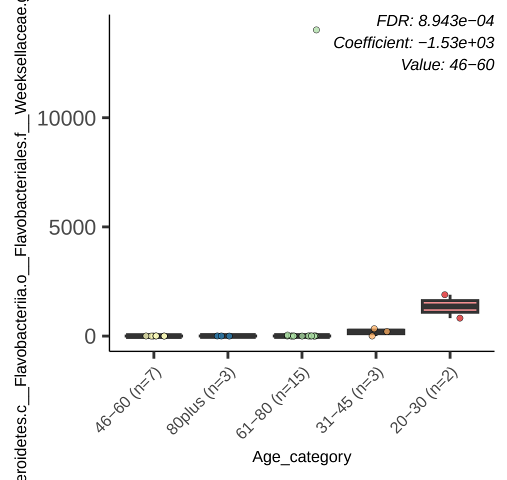

FDR: 8.943e−04
Coefficient: −1.53e+03
Value: 46−60
10000
5000
0
46−60 (n=7)
80plus (n=3)
61−80 (n=15)
31−45 (n=3)
20−30 (n=2)
Age_category

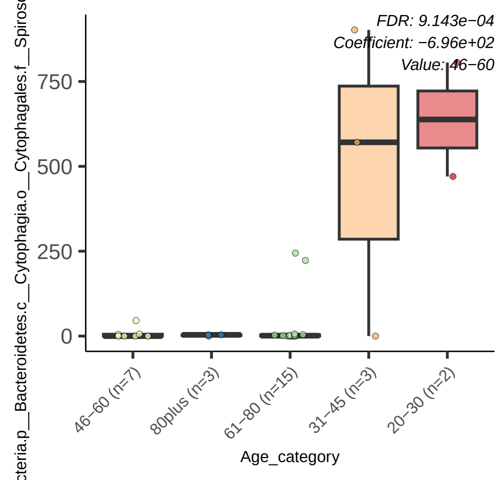
FDR: 9.143e−04
Coefficient: −6.96e+02
Value: 46−60
750
500
250
0
46−60 (n=7)
80plus (n=3)
61−80 (n=15)
31−45 (n=3)
20−30 (n=2)
Age_category
cteria.p__Bacteroidetes.c__Cytophagia.o__Cytophagales.f__Spiroso

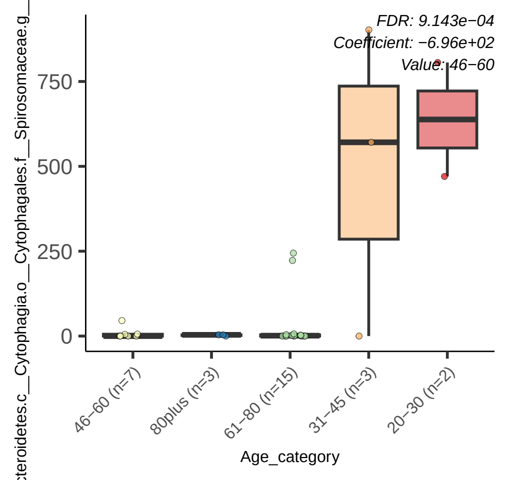
FDR: 9.143e−04
Coefficient: −6.96e+02
Value: 46−60
750
500
250
0
cteroidetes.c__Cytophagia.o__Cytophagales.f__Spirosomaceae.g__
46−60 (n=7)
80plus (n=3)
61−80 (n=15)
31−45 (n=3)
20−30 (n=2)
Age_category

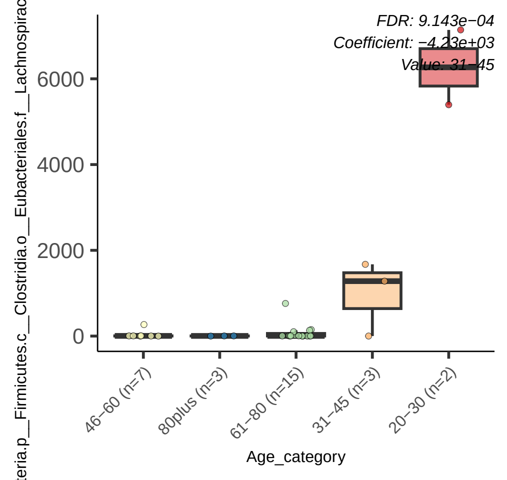

FDR: 9.143e−04
Coefficient: −4.23e+03
Value: 31−45
6000
4000
2000
0
teria.p__Firmicutes.c__Clostridia.o__Eubacteriales.f__Lachnospirac
46−60 (n=7)
80plus (n=3)
61−80 (n=15)
31−45 (n=3)
20−30 (n=2)
Age_category

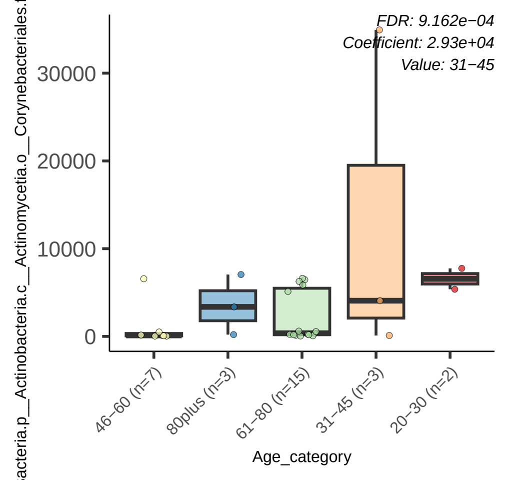

FDR: 9.162e−04
Coefficient: 2.93e+04
Value: 31−45
30000
20000
10000
0
46−60 (n=7)
80plus (n=3)
61−80 (n=15)
31−45 (n=3)
20−30 (n=2)
Age_category

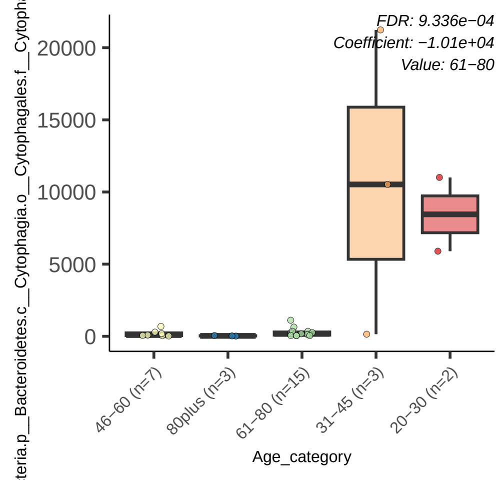
FDR: 9.336e−04
Coefficient: −1.01e+04
Value: 61−80
0
5000
10000
15000
20000
46−60 (n=7)
80plus (n=3)
61−80 (n=15)
31−45 (n=3)
20−30 (n=2)
Age_category

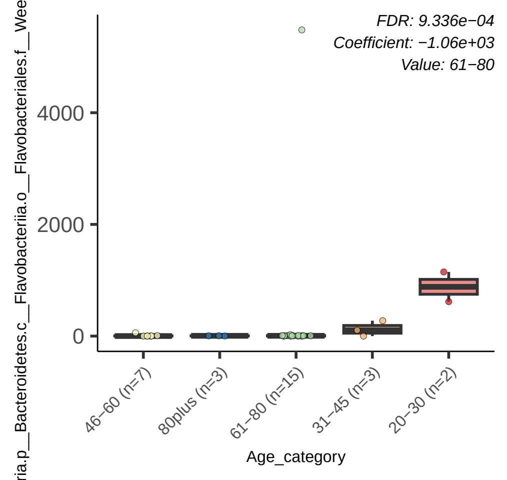

FDR: 9.336e−04
Coefficient: −1.06e+03
Value: 61−80
4000
2000
0
46−60 (n=7)
80plus (n=3)
61−80 (n=15)
31−45 (n=3)
20−30 (n=2)
Age_category
ria.p__Bacteroidetes.c__Flavobacteriia.o__Flavobacteriales.f__Wee

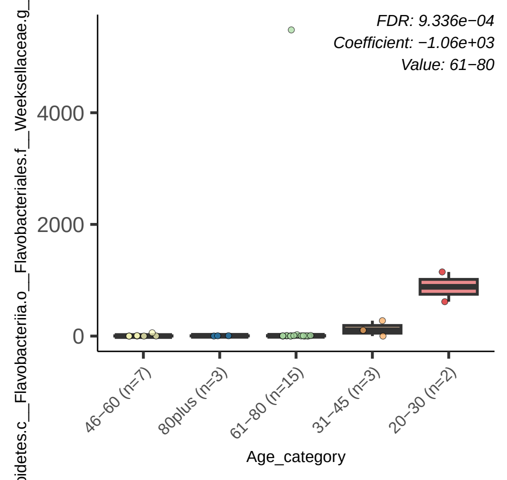

FDR: 9.336e−04
Coefficient: −1.06e+03
Value: 61−80
4000
2000
0
46−60 (n=7)
80plus (n=3)
61−80 (n=15)
31−45 (n=3)
20−30 (n=2)
Age_category
oidetes.c__Flavobacteriia.o__Flavobacteriales.f__Weeksellaceae.g__

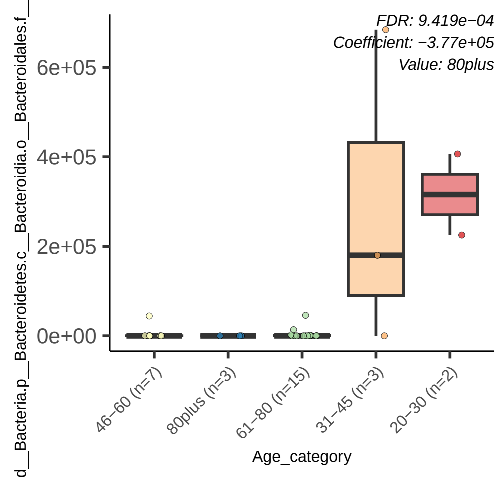

FDR: 9.419e−04
Coefficient: −3.77e+05
Value: 80plus
d__Bacteria.p__Bacteroidetes.c__Bacteroidia.o__Bacteroidales.f__
6e+05
4e+05
2e+05
0e+00
46−60 (n=7)
80plus (n=3)
61−80 (n=15)
31−45 (n=3)
20−30 (n=2)
Age_category

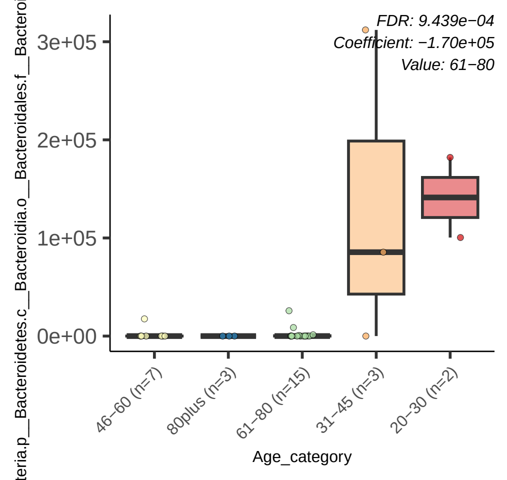

FDR: 9.439e−04
Coefficient: −1.70e+05
Value: 61−80
3e+05
2e+05
1e+05
0e+00
teria.p__Bacteroidetes.c__Bacteroidia.o__Bacteroidales.f__Bacteroi
46−60 (n=7)
80plus (n=3)
61−80 (n=15)
31−45 (n=3)
20−30 (n=2)
Age_category

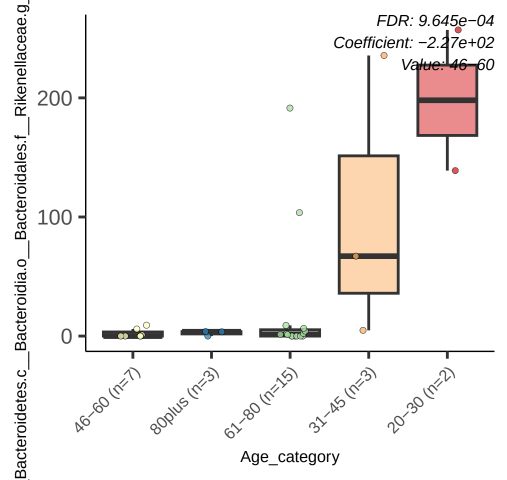
FDR: 9.645e−04
Coefficient: −2.27e+02
Value: 46−60
Bacteroidetes.c__Bacteroidia.o__Bacteroidales.f__Rikenellaceae.g_
200
100
0
46−60 (n=7)
80plus (n=3)
61−80 (n=15)
31−45 (n=3)
20−30 (n=2)
Age_category

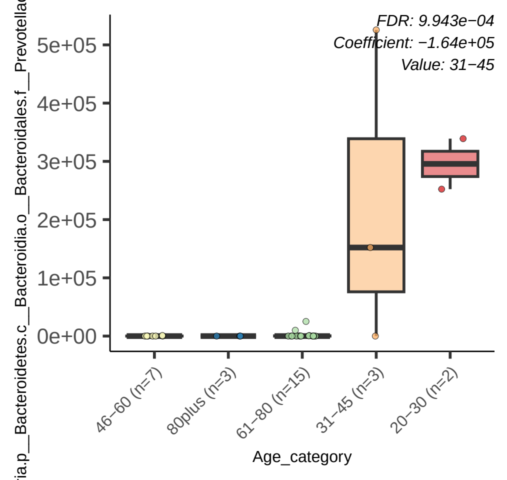

FDR: 9.943e−04
Coefficient: −1.64e+05
Value: 31−45
ria.p__Bacteroidetes.c__Bacteroidia.o__Bacteroidales.f__Prevotellac
5e+05
4e+05
3e+05
2e+05
1e+05
0e+00
46−60 (n=7)
80plus (n=3)
61−80 (n=15)
31−45 (n=3)
20−30 (n=2)
Age_category

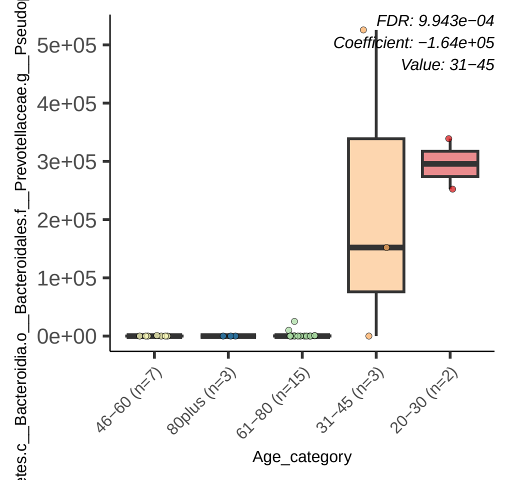

FDR: 9.943e−04
Coefficient: −1.64e+05
Value: 31−45
5e+05
4e+05
3e+05
2e+05
1e+05
0e+00
etes.c__Bacteroidia.o__Bacteroidales.f__Prevotellaceae.g__Pseudo
46−60 (n=7)
80plus (n=3)
61−80 (n=15)
31−45 (n=3)
20−30 (n=2)
Age_category

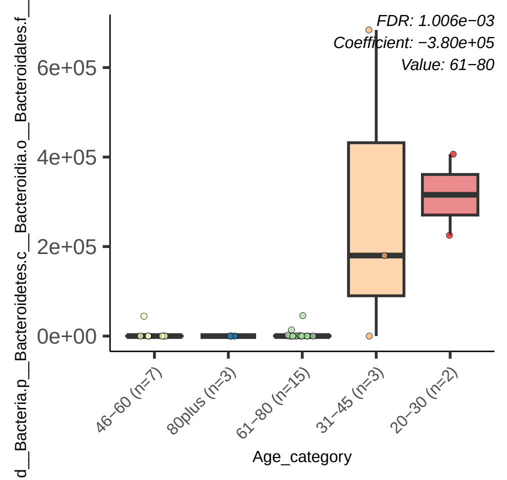

FDR: 1.006e−03
Coefficient: −3.80e+05
Value: 61−80
d__Bacteria.p__Bacteroidetes.c__Bacteroidia.o__Bacteroidales.f__
6e+05
4e+05
2e+05
0e+00
46−60 (n=7)
80plus (n=3)
61−80 (n=15)
31−45 (n=3)
20−30 (n=2)
Age_category

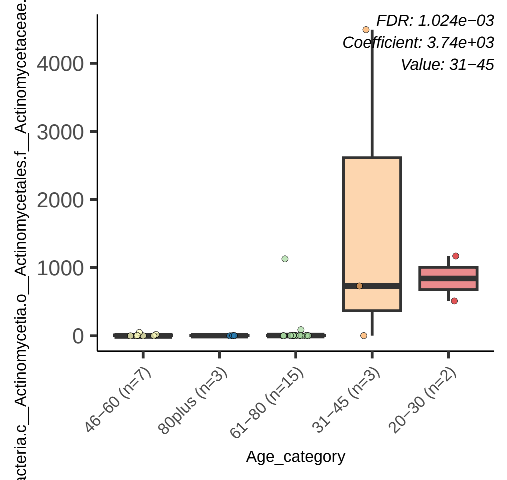

FDR: 1.024e−03
Coefficient: 3.74e+03
Value: 31−45
0
1000
2000
3000
4000
acteria.c__Actinomycetia.o__Actinomycetales.f__Actinomycetaceae.
46−60 (n=7)
80plus (n=3)
61−80 (n=15)
31−45 (n=3)
20−30 (n=2)
Age_category

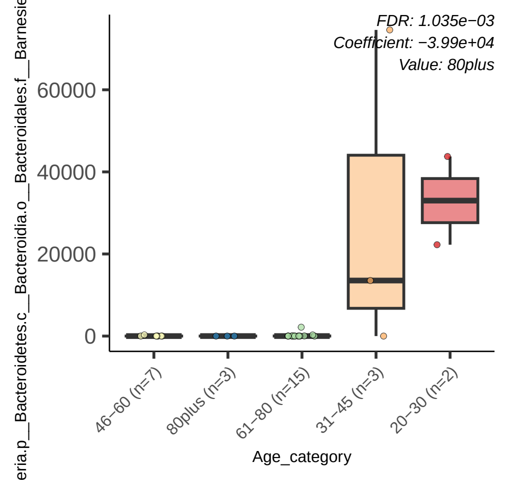

FDR: 1.035e−03
Coefficient: −3.99e+04
Value: 80plus
60000
40000
20000
0
eria.p__Bacteroidetes.c__Bacteroidia.o__Bacteroidales.f__Barnesie
46−60 (n=7)
80plus (n=3)
61−80 (n=15)
31−45 (n=3)
20−30 (n=2)
Age_category

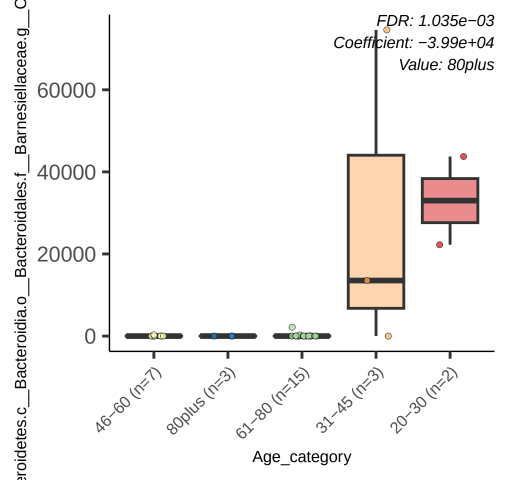

FDR: 1.035e−03
Coefficient: −3.99e+04
Value: 80plus
60000
40000
20000
0
46−60 (n=7)
80plus (n=3)
61−80 (n=15)
31−45 (n=3)
20−30 (n=2)
Age_category
eroidetes.c__Bacteroidia.o__Bacteroidales.f__Barnesiellaceae.g__C

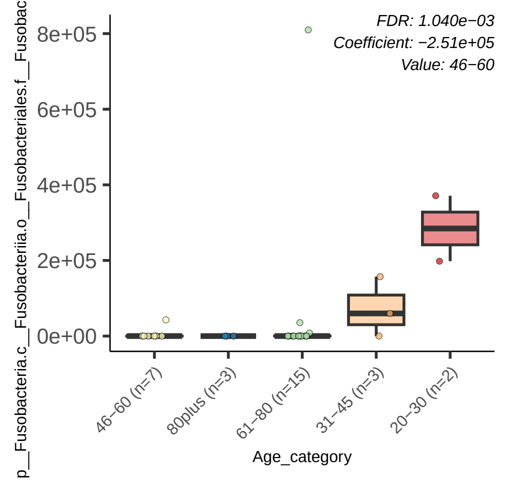
FDR: 1.040e−03
Coefficient: −2.51e+05
Value: 46−60
p__Fusobacteria.c__Fusobacteriia.o__Fusobacteriales.f__Fusobac
8e+05
6e+05
4e+05
2e+05
0e+00
46−60 (n=7)
80plus (n=3)
61−80 (n=15)
31−45 (n=3)
20−30 (n=2)
Age_category

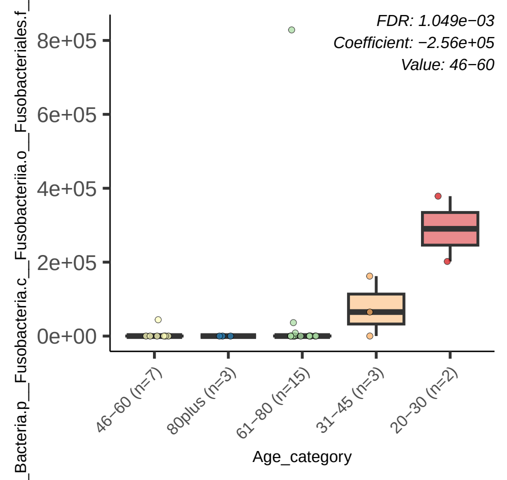

FDR: 1.049e−03
Coefficient: −2.56e+05
Value: 46−60
8e+05
6e+05
4e+05
2e+05
0e+00
_Bacteria.p__Fusobacteria.c__Fusobacteriia.o__Fusobacteriales.f
46−60 (n=7)
80plus (n=3)
61−80 (n=15)
31−45 (n=3)
20−30 (n=2)
Age_category

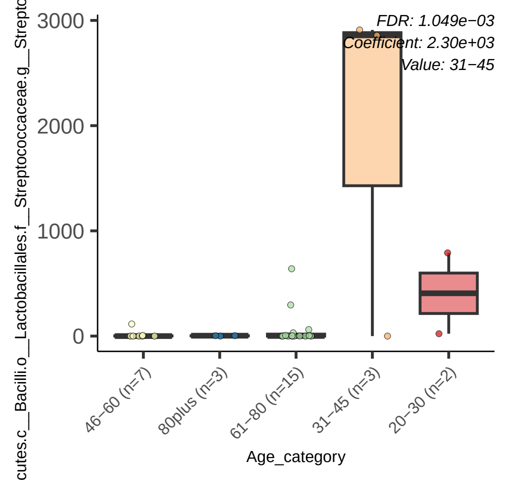
FDR: 1.049e−03
Coefficient: 2.30e+03
Value: 31−45
3000
2000
1000
0
cutes.c__Bacilli.o__Lactobacillales.f__Streptococcaceae.g__Strepto
46−60 (n=7)
80plus (n=3)
61−80 (n=15)
31−45 (n=3)
20−30 (n=2)
Age_category

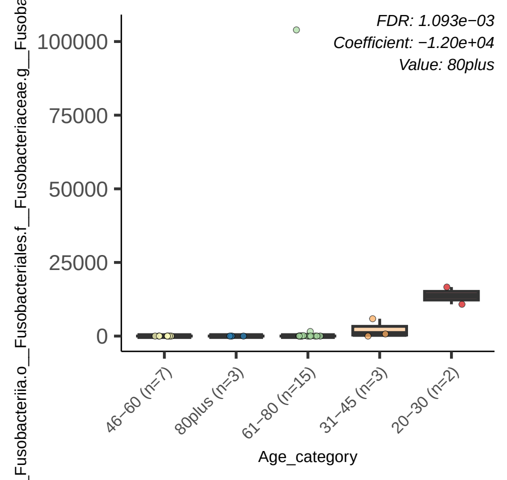

FDR: 1.093e−03
Coefficient: −1.20e+04
Value: 80plus
100000
75000
50000
25000
0
46−60 (n=7)
80plus (n=3)
61−80 (n=15)
31−45 (n=3)
20−30 (n=2)
Age_category
Fusobacteriia.o__Fusobacteriales.f__Fusobacteriaceae.g__Fusoba

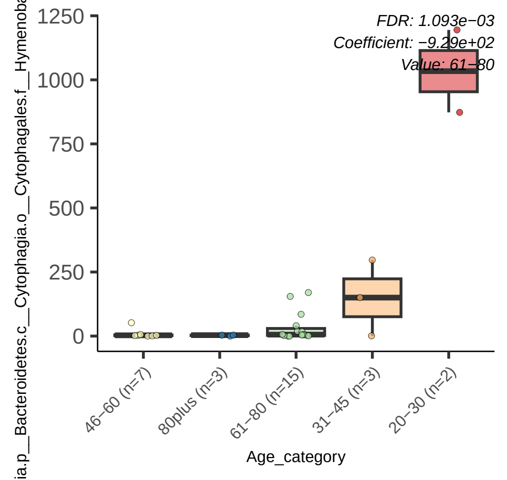

FDR: 1.093e−03
Coefficient: −9.29e+02
Value: 61−80
1250
1000
750
500
250
0
ia.p__Bacteroidetes.c__Cytophagia.o__Cytophagales.f__Hymenoba
46−60 (n=7)
80plus (n=3)
61−80 (n=15)
31−45 (n=3)
20−30 (n=2)
Age_category

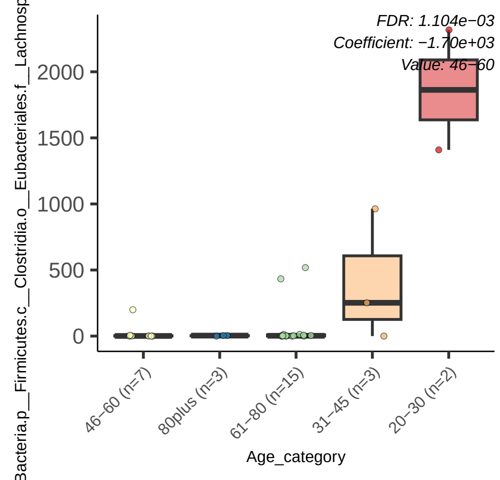

FDR: 1.104e−03
Coefficient: −1.70e+03
Value: 46−60
Bacteria.p__Firmicutes.c__Clostridia.o__Eubacteriales.f__Lachnosp
2000
1500
1000
500
0
46−60 (n=7)
80plus (n=3)
61−80 (n=15)
31−45 (n=3)
20−30 (n=2)
Age_category

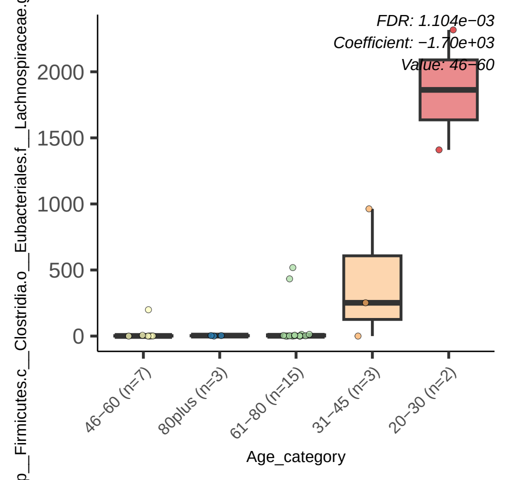
FDR: 1.104e−03
Coefficient: −1.70e+03
Value: 46−60
2000
1500
1000
500
0
Firmicutes.c__Clostridia.o__Eubacteriales.f__Lachnospiraceae.g
46−60 (n=7)
80plus (n=3)
61−80 (n=15)
31−45 (n=3)
20−30 (n=2)
Age_category

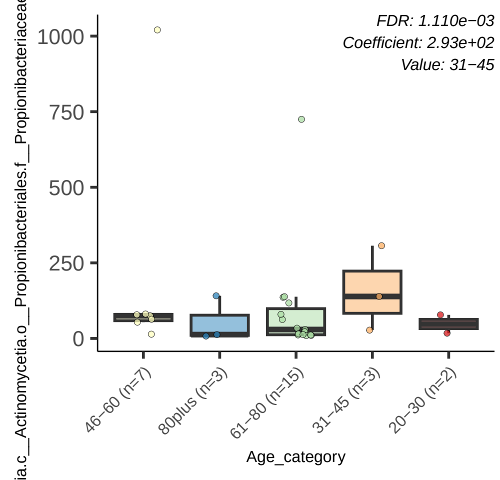

FDR: 1.110e−03
Coefficient: 2.93e+02
Value: 31−45
ia.c__Actinomycetia.o__Propionibacteriales.f__Propionibacteriaceae
1000
750
500
250
0
46−60 (n=7)
80plus (n=3)
61−80 (n=15)
31−45 (n=3)
20−30 (n=2)
Age_category

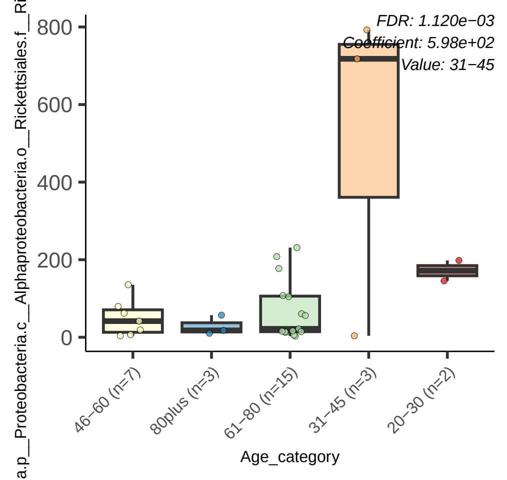

FDR: 1.120e−03
Coefficient: 5.98e+02
Value: 31−45
a.p__Proteobacteria.c__Alphaproteobacteria.o__Rickettsiales.f__Ri
800
600
400
200
0
46−60 (n=7)
80plus (n=3)
61−80 (n=15)
31−45 (n=3)
20−30 (n=2)
Age_category

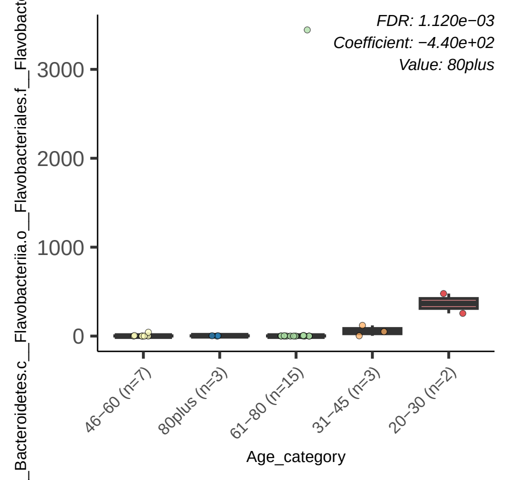

FDR: 1.120e−03
Coefficient: −4.40e+02
Value: 80plus
3000
2000
1000
0
46−60 (n=7)
80plus (n=3)
61−80 (n=15)
31−45 (n=3)
20−30 (n=2)
Age_category
Bacteroidetes.c__Flavobacteriia.o__Flavobacteriales.f__Flavobacte

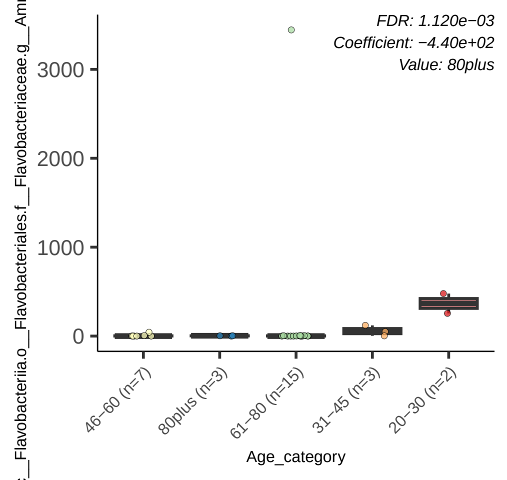

FDR: 1.120e−03
Coefficient: −4.40e+02
Value: 80plus
3000
2000
1000
0
46−60 (n=7)
80plus (n=3)
61−80 (n=15)
31−45 (n=3)
20−30 (n=2)
Age_category
Flavobacteriia.o__Flavobacteriales.f__Flavobacteriaceae.g__Am

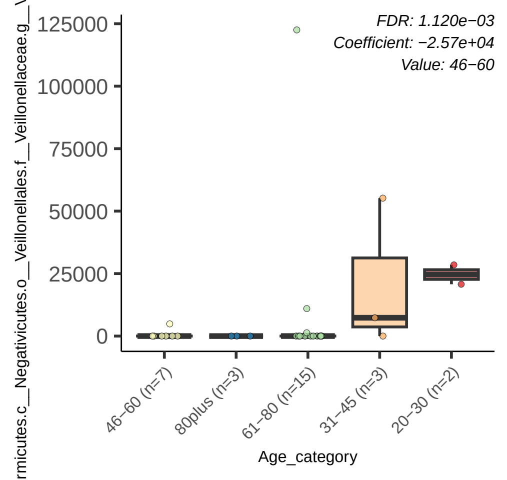

FDR: 1.120e−03
Coefficient: −2.57e+04
Value: 46−60
rmicutes.c__Negativicutes.o__Veillonellales.f__Veillonellaceae.g__V
125000
100000
75000
50000
25000
0
46−60 (n=7)
80plus (n=3)
61−80 (n=15)
31−45 (n=3)
20−30 (n=2)
Age_category

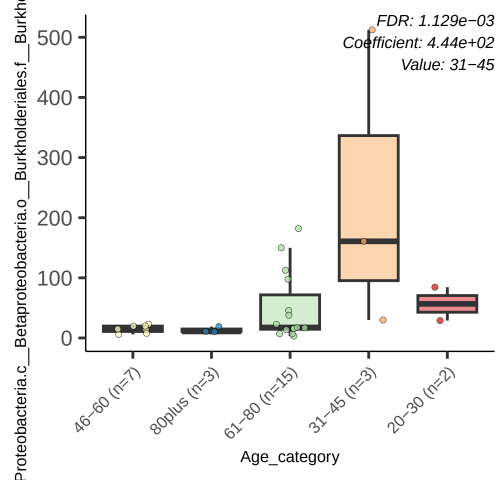
FDR: 1.129e−03
Coefficient: 4.44e+02
Value: 31−45
Proteobacteria.c__Betaproteobacteria.o__Burkholderiales.f__Burkho
500
400
300
200
100
0
46−60 (n=7)
80plus (n=3)
61−80 (n=15)
31−45 (n=3)
20−30 (n=2)
Age_category

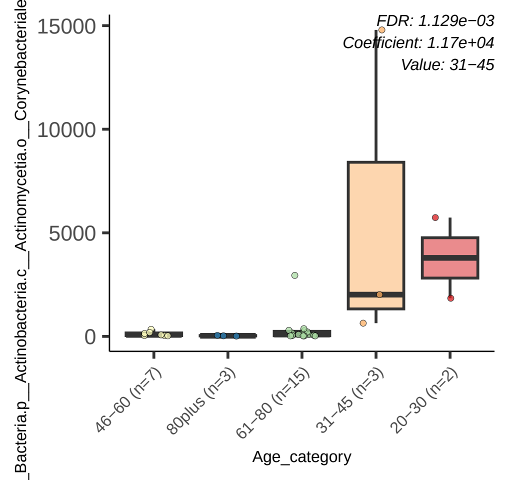
FDR: 1.129e−03
Coefficient: 1.17e+04
Value: 31−45
15000
10000
5000
0
_Bacteria.p__Actinobacteria.c__Actinomycetia.o__Corynebacteriale
46−60 (n=7)
80plus (n=3)
61−80 (n=15)
31−45 (n=3)
20−30 (n=2)
Age_category

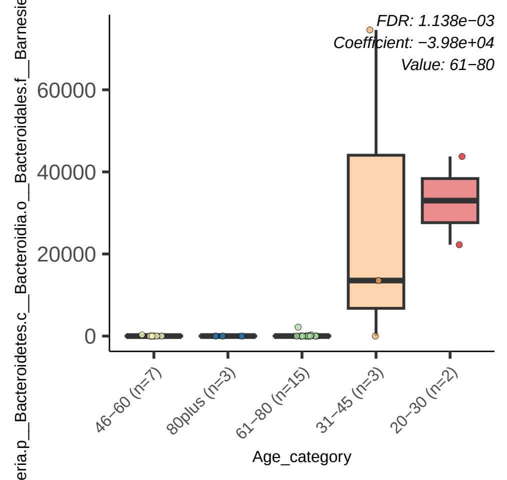
FDR: 1.138e−03
Coefficient: −3.98e+04
Value: 61−80
60000
40000
20000
0
eria.p__Bacteroidetes.c__Bacteroidia.o__Bacteroidales.f__Barnesie
46−60 (n=7)
80plus (n=3)
61−80 (n=15)
31−45 (n=3)
20−30 (n=2)
Age_category

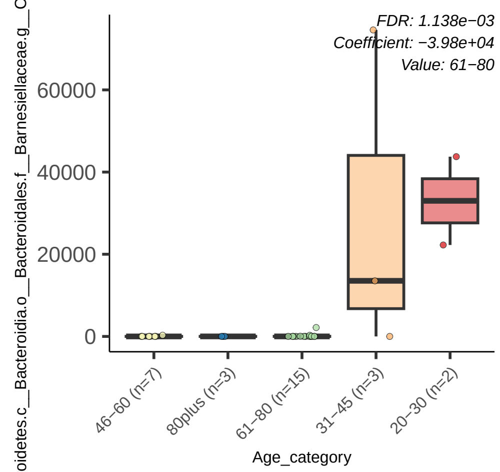

FDR: 1.138e−03
Coefficient: −3.98e+04
Value: 61−80
60000
40000
20000
0
eroidetes.c__Bacteroidia.o__Bacteroidales.f__Barnesiellaceae.g__C
46−60 (n=7)
80plus (n=3)
61−80 (n=15)
31−45 (n=3)
20−30 (n=2)
Age_category

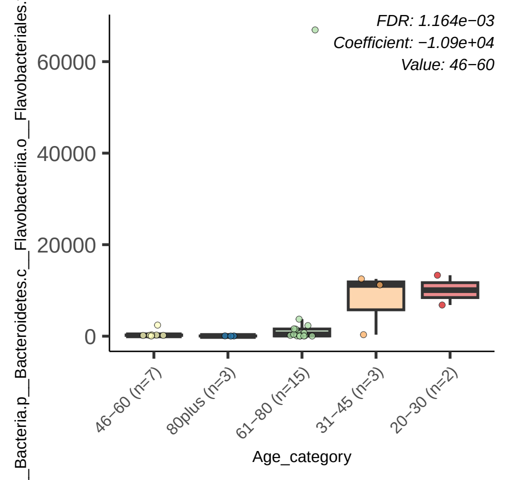

FDR: 1.164e−03
Coefficient: −1.09e+04
Value: 46−60
60000
40000
20000
0
46−60 (n=7)
80plus (n=3)
61−80 (n=15)
31−45 (n=3)
20−30 (n=2)
Age_category
_Bacteria.p__Bacteroidetes.c__Flavobacteriia.o__Flavobacteriales.

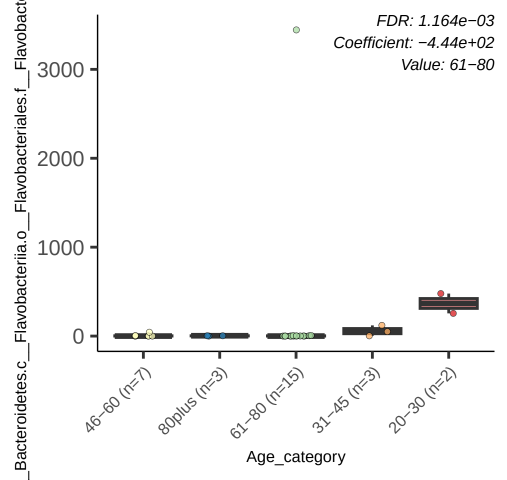

FDR: 1.164e−03
Coefficient: −4.44e+02
Value: 61−80
Bacteroidetes.c__Flavobacteriia.o__Flavobacteriales.f__Flavobact
3000
2000
1000
0
46−60 (n=7)
80plus (n=3)
61−80 (n=15)
31−45 (n=3)
20−30 (n=2)
Age_category

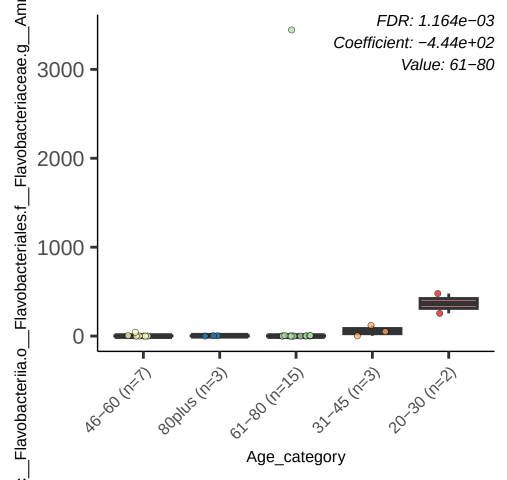

FDR: 1.164e−03
Coefficient: −4.44e+02
Value: 61−80
3000
2000
1000
0
46−60 (n=7)
80plus (n=3)
61−80 (n=15)
31−45 (n=3)
20−30 (n=2)
Age_category
Flavobacteriia.o__Flavobacteriales.f__Flavobacteriaceae.g__

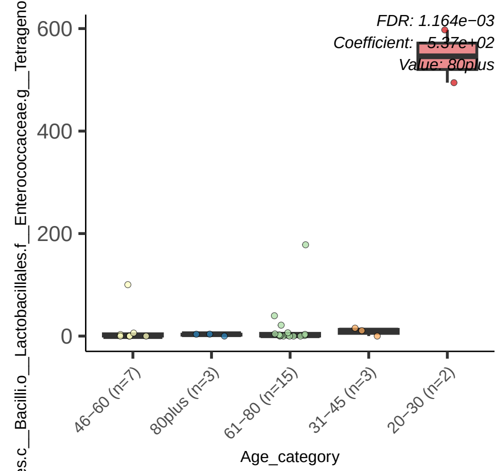

FDR: 1.164e−03
Coefficient: −5.37e+02
Value: 80plus
600
400
200
0
tes.c__Bacilli.o__Lactobacillales.f__Enterococcaceae.g__Tetrageno
46−60 (n=7)
80plus (n=3)
61−80 (n=15)
31−45 (n=3)
20−30 (n=2)
Age_category

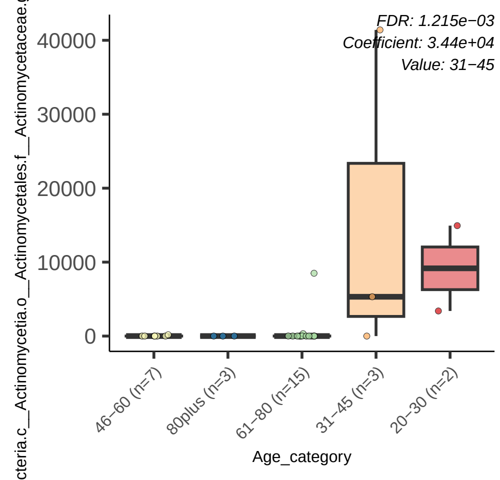

FDR: 1.215e−03
Coefficient: 3.44e+04
Value: 31−45
40000
30000
20000
10000
0
46−60 (n=7)
80plus (n=3)
61−80 (n=15)
31−45 (n=3)
20−30 (n=2)
Age_category
cteria.c__Actinomycetia.o__Actinomycetales.f__Actinomycetaceae.g

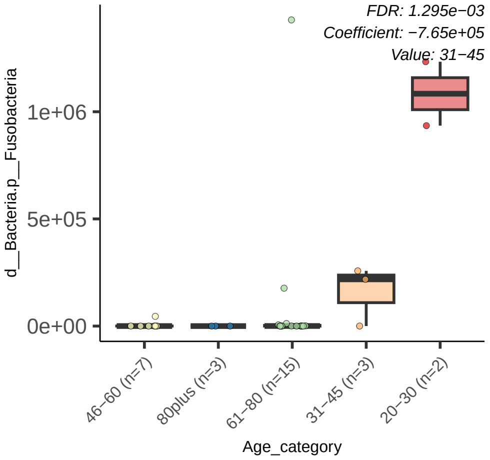
FDR: 1.295e−03
Coefficient: −7.65e+05
Value: 31−45
d__Bacteria.p__Fusobacteria
1e+06
5e+05
0e+00
46−60 (n=7)
80plus (n=3)
61−80 (n=15)
31−45 (n=3)
20−30 (n=2)
Age_category

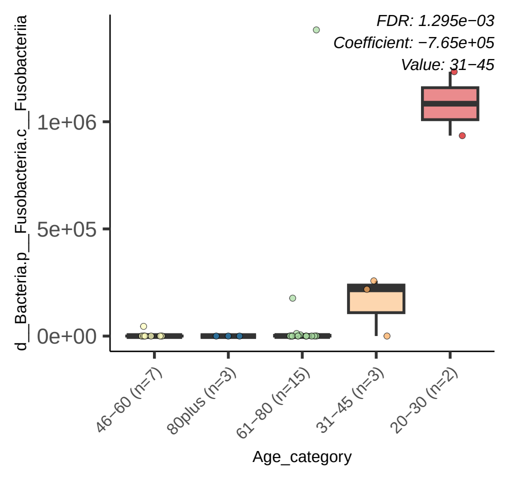

FDR: 1.295e−03
Coefficient: −7.65e+05
Value: 31−45
d__Bacteria.p__Fusobacteria.c__Fusobacteriia
1e+06
5e+05
0e+00
46−60 (n=7)
80plus (n=3)
61−80 (n=15)
31−45 (n=3)
20−30 (n=2)
Age_category

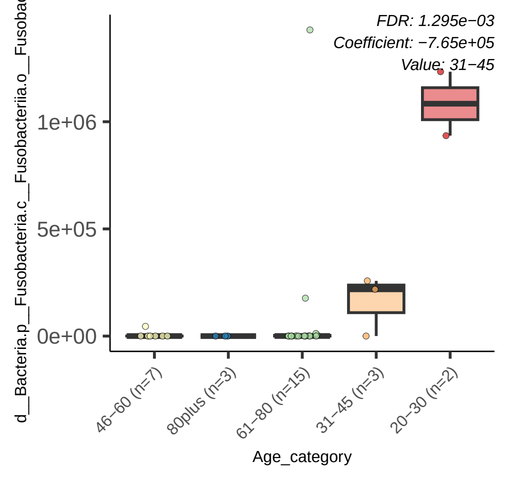

FDR: 1.295e−03
Coefficient: −7.65e+05
Value: 31−45
d__Bacteria.p__Fusobacteria.c__Fusobacteriia.o__Fusobac
1e+06
5e+05
0e+00
46−60 (n=7)
80plus (n=3)
61−80 (n=15)
31−45 (n=3)
20−30 (n=2)
Age_category

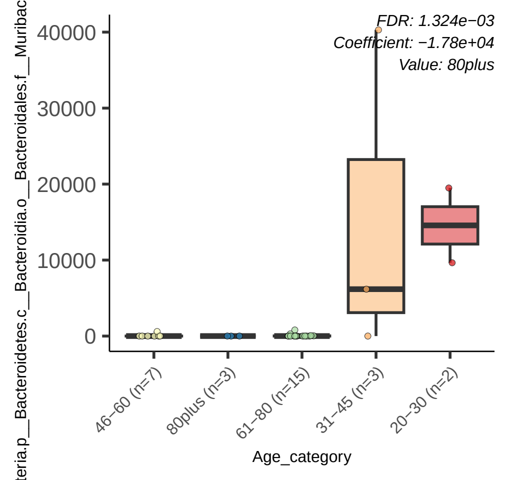

FDR: 1.324e−03
Coefficient: −1.78e+04
Value: 80plus
40000
30000
20000
10000
0
teria.p__Bacteroidetes.c__Bacteroidia.o__Bacteroidales.f__Muribac
46−60 (n=7)
80plus (n=3)
61−80 (n=15)
31−45 (n=3)
20−30 (n=2)
Age_category

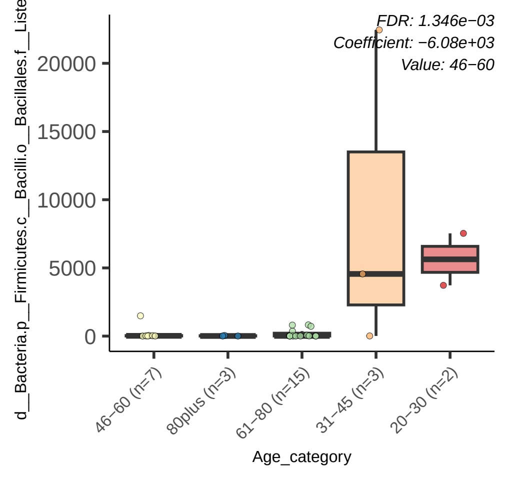

FDR: 1.346e−03
Coefficient: −6.08e+03
Value: 46−60
20000
15000
10000
5000
0
d__Bacteria.p__Firmicutes.c__Bacilli.o__Bacillales.f__Liste
46−60 (n=7)
80plus (n=3)
61−80 (n=15)
31−45 (n=3)
20−30 (n=2)
Age_category

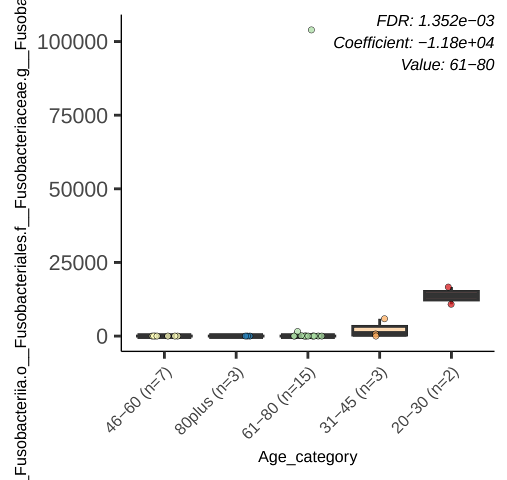

FDR: 1.352e−03
Coefficient: −1.18e+04
Value: 61−80
Fusobacteriia.o__Fusobacteriales.f__Fusobacteriaceae.g__Fusoba
100000
75000
50000
25000
0
46−60 (n=7)
80plus (n=3)
61−80 (n=15)
31−45 (n=3)
20−30 (n=2)
Age_category

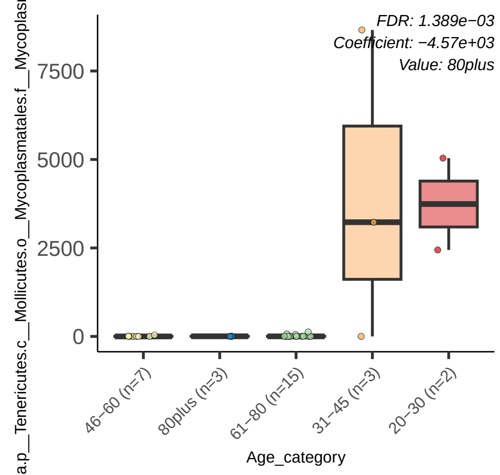

FDR: 1.389e−03
Coefficient: −4.57e+03
Value: 80plus
ria.p__Tenericutes.c__Mollicutes.o__Mycoplasmatales.f__Mycoplasm
7500
5000
2500
0
46−60 (n=7)
80plus (n=3)
61−80 (n=15)
31−45 (n=3)
20−30 (n=2)
Age_category

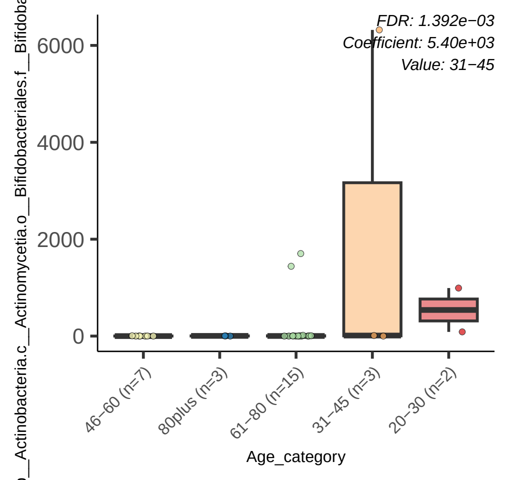

FDR: 1.392e−03
Coefficient: 5.40e+03
Value: 31−45
6000
4000
2000
0
o__Actinobacteria.c__Actinomycetia.o__Bifidobacteriales.f__Bifidoba
46−60 (n=7)
80plus (n=3)
61−80 (n=15)
31−45 (n=3)
20−30 (n=2)
Age_category

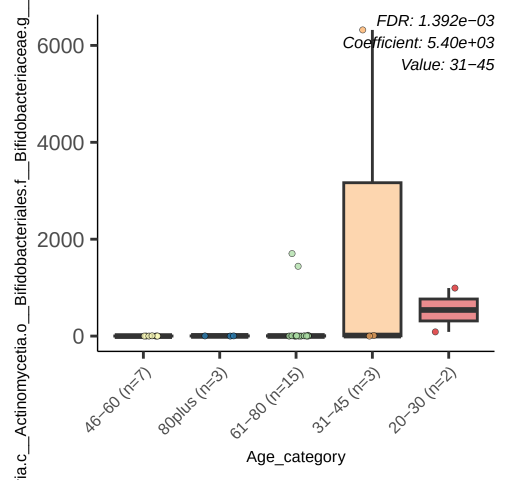

FDR: 1.392e−03
Coefficient: 5.40e+03
Value: 31−45
0
2000
4000
6000
ia.c__Actinomycetia.o__Bifidobacteriales.f__Bifidobacteriaceae.g__
46−60 (n=7)
80plus (n=3)
61−80 (n=15)
31−45 (n=3)
20−30 (n=2)
Age_category

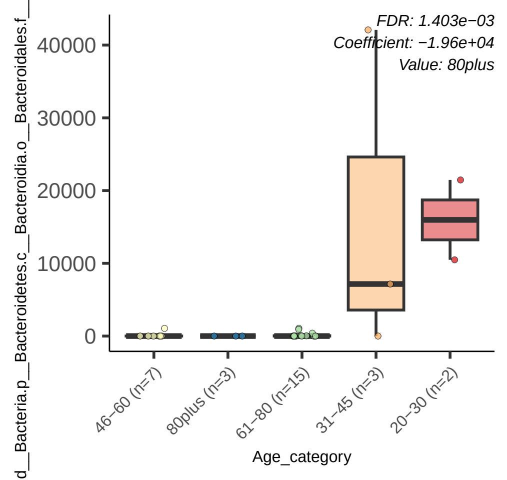

FDR: 1.403e−03
Coefficient: −1.96e+04
Value: 80plus
40000
30000
20000
10000
0
d__Bacteria.p__Bacteroidetes.c__Bacteroidia.o__Bacteroidales.f__
46−60 (n=7)
80plus (n=3)
61−80 (n=15)
31−45 (n=3)
20−30 (n=2)
Age_category

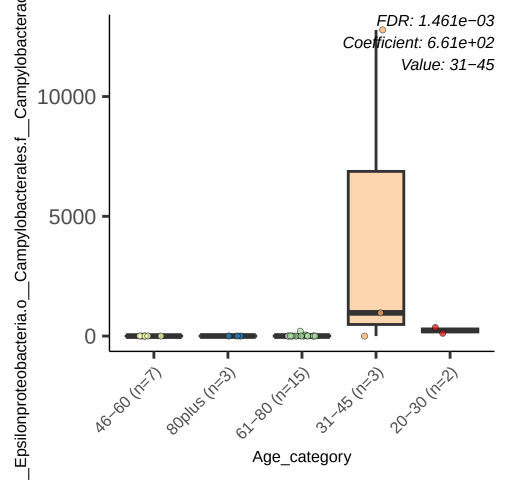

FDR: 1.461e−03
Coefficient: 6.61e+02
Value: 31−45
10000
5000
0
_Epsilonproteobacteria.o__Campylobacterales.f__Campylobacterac
46−60 (n=7)
80plus (n=3)
61−80 (n=15)
31−45 (n=3)
20−30 (n=2)
Age_category

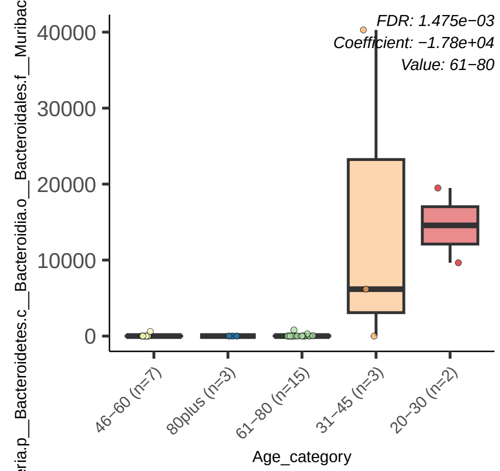

FDR: 1.475e−03
Coefficient: −1.78e+04
Value: 61−80
40000
30000
20000
10000
0
teria.p__Bacteroidetes.c__Bacteroidia.o__Bacteroidales.f__Muribac
46−60 (n=7)
80plus (n=3)
61−80 (n=15)
31−45 (n=3)
20−30 (n=2)
Age_category

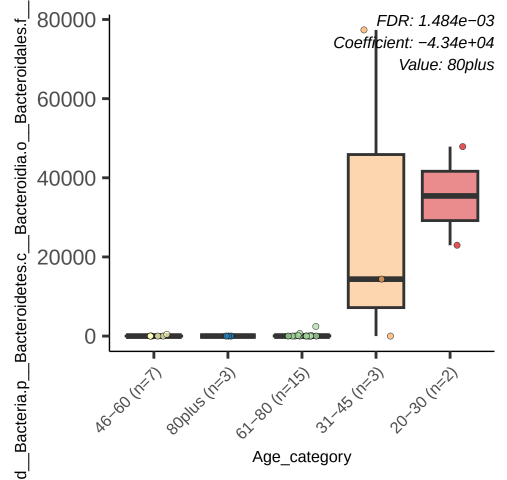

FDR: 1.484e−03
Coefficient: −4.34e+04
Value: 80plus
d__Bacteria.p__Bacteroidetes.c__Bacteroidia.o__Bacteroidales.f__
80000
60000
40000
20000
0
46−60 (n=7)
80plus (n=3)
61−80 (n=15)
31−45 (n=3)
20−30 (n=2)
Age_category

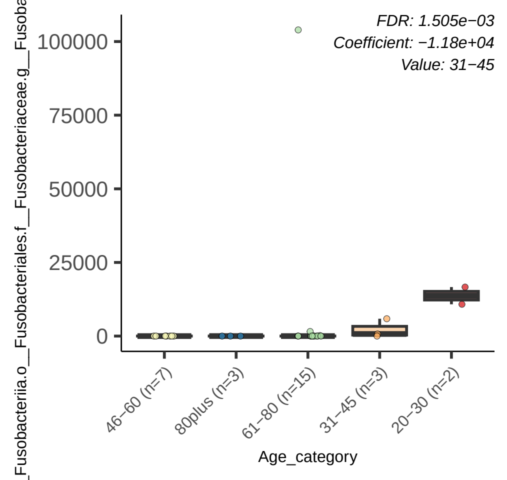

FDR: 1.505e−03
Coefficient: −1.18e+04
Value: 31−45
100000
75000
50000
25000
0
Fusobacteriia.o__Fusobacteriales.f__Fusobacteriaceae.g__Fusoba
46−60 (n=7)
80plus (n=3)
61−80 (n=15)
31−45 (n=3)
20−30 (n=2)
Age_category

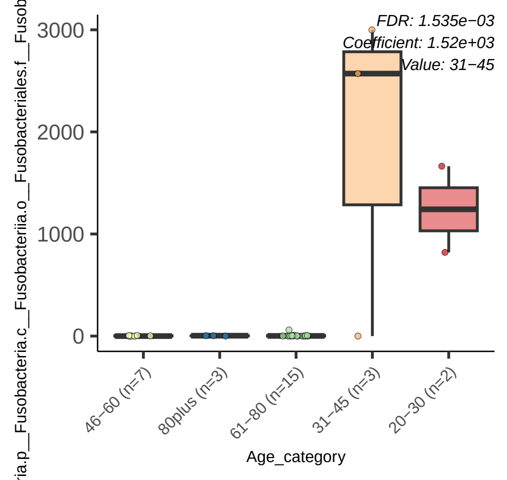

FDR: 1.535e−03
Coefficient: 1.52e+03
Value: 31−45
ria.p__Fusobacteria.c__Fusobacteriia.o__Fusobacteriales.f__Fusob
3000
2000
1000
0
46−60 (n=7)
80plus (n=3)
61−80 (n=15)
31−45 (n=3)
20−30 (n=2)
Age_category

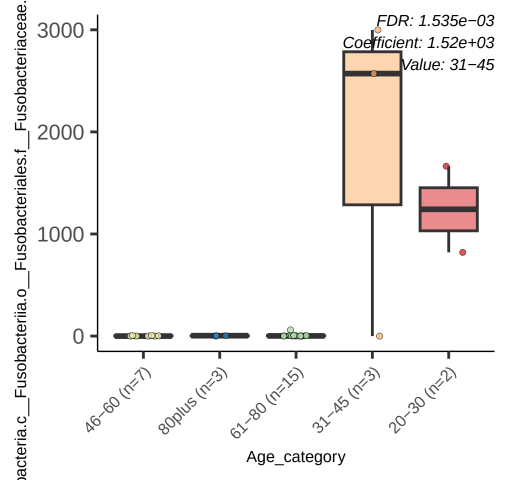
FDR: 1.535e−03
Coefficient: 1.52e+03
Value: 31−45
3000
2000
1000
0
46−60 (n=7)
80plus (n=3)
61−80 (n=15)
31−45 (n=3)
20−30 (n=2)
Age_category
bacteria.c__Fusobacteriia.o__Fusobacteriales.f__Fusobacteriaceae.

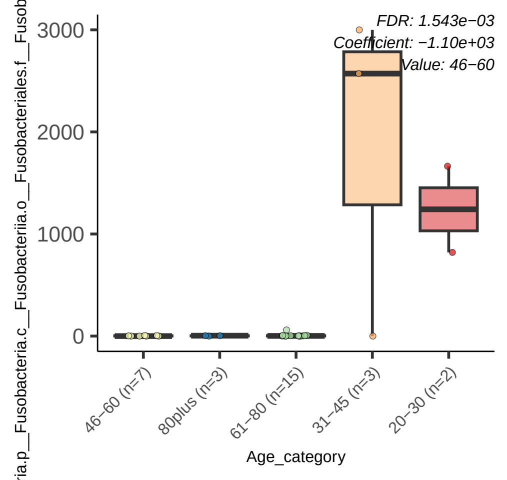

FDR: 1.543e−03
Coefficient: −1.10e+03
Value: 46−60
3000
2000
1000
0
ria.p__Fusobacteria.c__Fusobacteriia.o__Fusobacteriales.f__Fusob
46−60 (n=7)
80plus (n=3)
61−80 (n=15)
31−45 (n=3)
20−30 (n=2)
Age_category

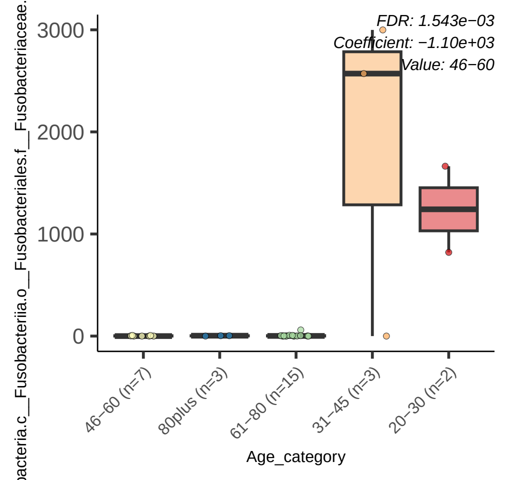

FDR: 1.543e−03
Coefficient: −1.10e+03
Value: 46−60
3000
2000
1000
0
bacteria.c__Fusobacteriia.o__Fusobacteriales.f__Fusobacteriaceae.
46−60 (n=7)
80plus (n=3)
61−80 (n=15)
31−45 (n=3)
20−30 (n=2)
Age_category

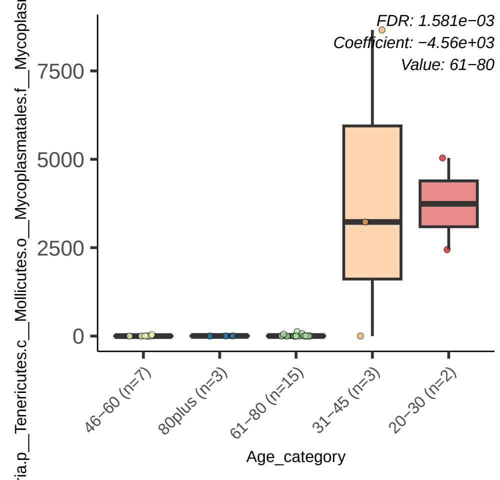

FDR: 1.581e−03
Coefficient: −4.56e+03
Value: 61−80
ria.p__Tenericutes.c__Mollicutes.o__Mycoplasmatales.f__Mycoplasm
7500
5000
2500
0
46−60 (n=7)
80plus (n=3)
61−80 (n=15)
31−45 (n=3)
20−30 (n=2)
Age_category

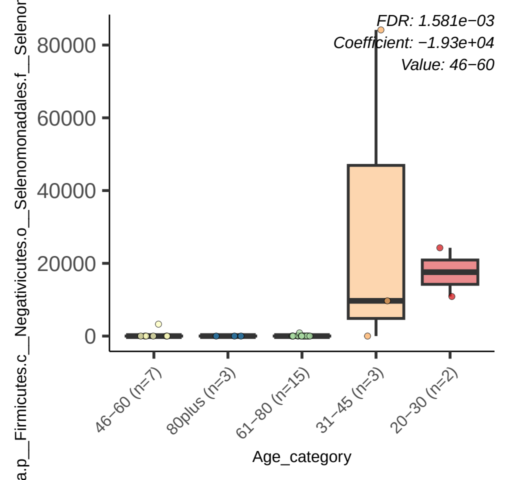

FDR: 1.581e−03
Coefficient: −1.93e+04
Value: 46−60
80000
60000
40000
20000
0
a.p__Firmicutes.c__Negativicutes.o__Selenomonadales.f__Selenor
46−60 (n=7)
80plus (n=3)
61−80 (n=15)
31−45 (n=3)
20−30 (n=2)
Age_category

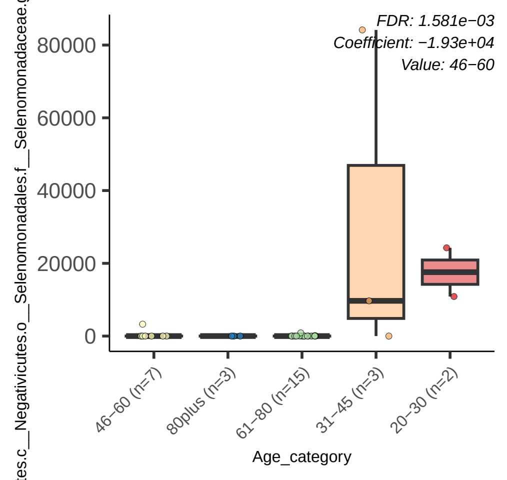

FDR: 1.581e−03
Coefficient: −1.93e+04
Value: 46−60
80000
60000
40000
20000
0
tes.c__Negativicutes.o__Selenomonadales.f__Selenomonadaceae.g
46−60 (n=7)
80plus (n=3)
61−80 (n=15)
31−45 (n=3)
20−30 (n=2)
Age_category

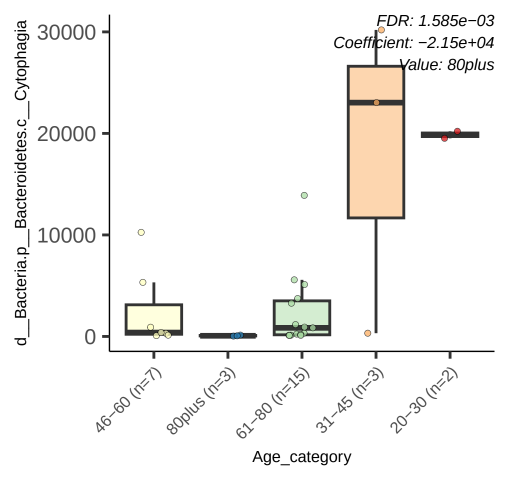

FDR: 1.585e−03
Coefficient: −2.15e+04
Value: 80plus
d__Bacteria.p__Bacteroidetes.c__Cytophagia
30000
20000
10000
0
46−60 (n=7)
80plus (n=3)
61−80 (n=15)
31−45 (n=3)
20−30 (n=2)
Age_category

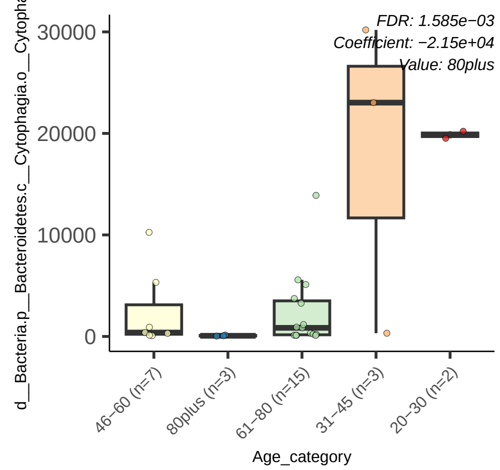

FDR: 1.585e−03
Coefficient: −2.15e+04
Value: 80plus
d__Bacteria.p__Bacteroidetes.c__Cytophagia.o__Cytopha
30000
20000
10000
0
46−60 (n=7)
80plus (n=3)
61−80 (n=15)
31−45 (n=3)
20−30 (n=2)
Age_category

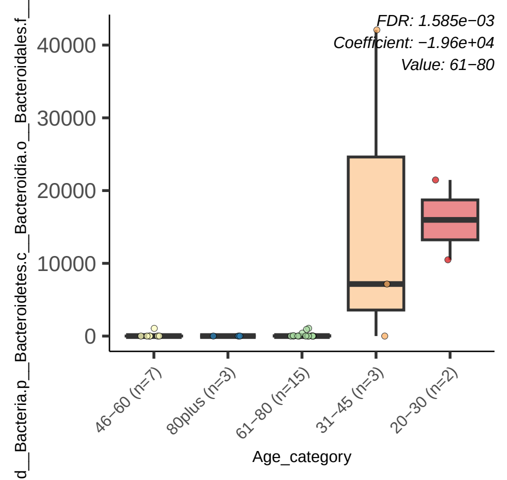
FDR: 1.585e−03
Coefficient: −1.96e+04
Value: 61−80
d__Bacteria.p__Bacteroidetes.c__Bacteroidia.o__Bacteroidales.f__
40000
30000
20000
10000
0
46−60 (n=7)
80plus (n=3)
61−80 (n=15)
31−45 (n=3)
20−30 (n=2)
Age_category

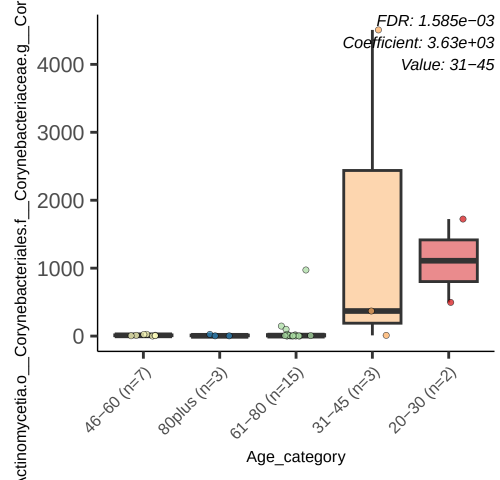

FDR: 1.585e−03
Coefficient: 3.63e+03
Value: 31−45
Actinomycetia.o__Corynebacteriales.f__Corynebacteriaceae.g__Cor
4000
3000
2000
1000
0
46−60 (n=7)
80plus (n=3)
61−80 (n=15)
31−45 (n=3)
20−30 (n=2)
Age_category

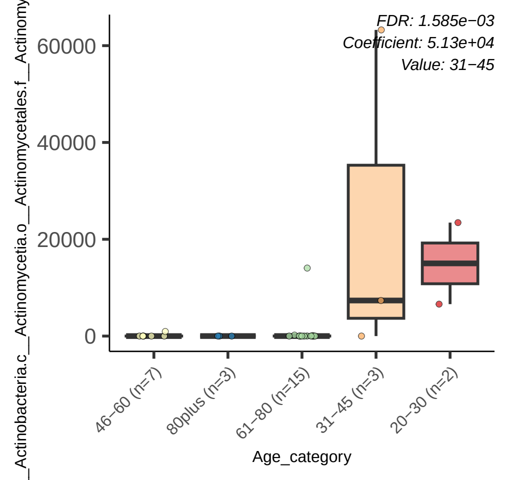

FDR: 1.585e−03
Coefficient: 5.13e+04
Value: 31−45
60000
40000
20000
0
Actinobacteria.c__Actinomycetia.o__Actinomycetales.f__Actinomy
46−60 (n=7)
80plus (n=3)
61−80 (n=15)
31−45 (n=3)
20−30 (n=2)
Age_category

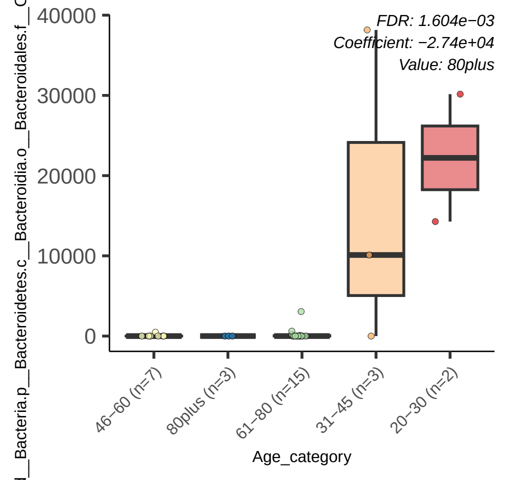

FDR: 1.604e−03
Coefficient: −2.74e+04
Value: 80plus
40000
30000
20000
10000
0
46−60 (n=7)
80plus (n=3)
61−80 (n=15)
31−45 (n=3)
20−30 (n=2)
Age_category
Bacteria.p__Bacteroidetes.c__Bacteroidia.o__Bacteroidales.f__

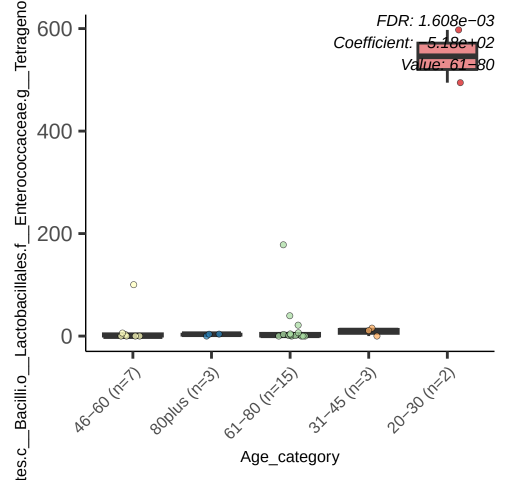
FDR: 1.608e−03
Coefficient: −5.18e+02
Value: 61−80
600
400
200
0
tes.c__Bacilli.o__Lactobacillales.f__Enterococcaceae.g__Tetrageno
46−60 (n=7)
80plus (n=3)
61−80 (n=15)
31−45 (n=3)
20−30 (n=2)
Age_category

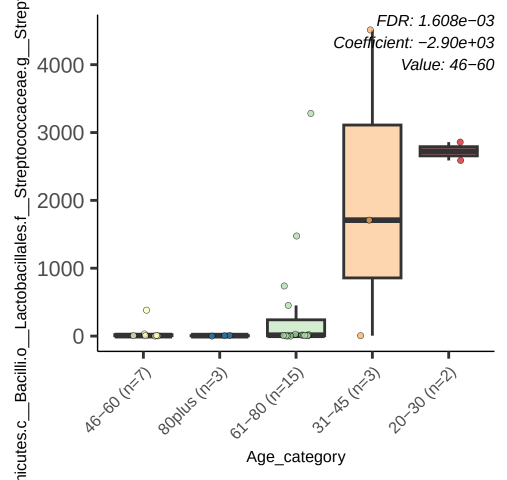
FDR: 1.608e−03
Coefficient: −2.90e+03
Value: 46−60
4000
3000
2000
1000
0
icutes.c__Bacilli.o__Lactobacillales.f__Streptococcaceae.g__Strep
46−60 (n=7)
80plus (n=3)
61−80 (n=15)
31−45 (n=3)
20−30 (n=2)
Age_category

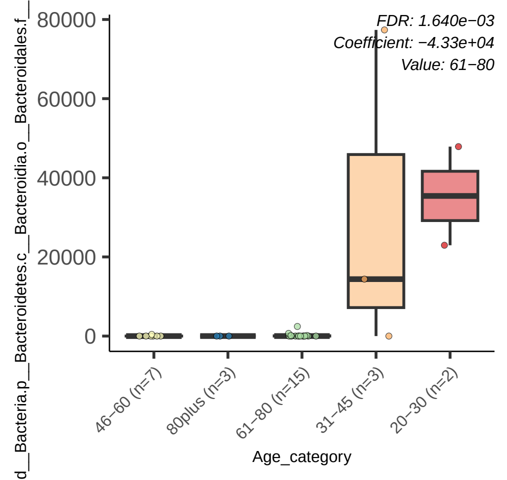

FDR: 1.640e−03
Coefficient: −4.33e+04
Value: 61−80
d__Bacteria.p__Bacteroidetes.c__Bacteroidia.o__Bacteroidales.f__
80000
60000
40000
20000
0
46−60 (n=7)
80plus (n=3)
61−80 (n=15)
31−45 (n=3)
20−30 (n=2)
Age_category

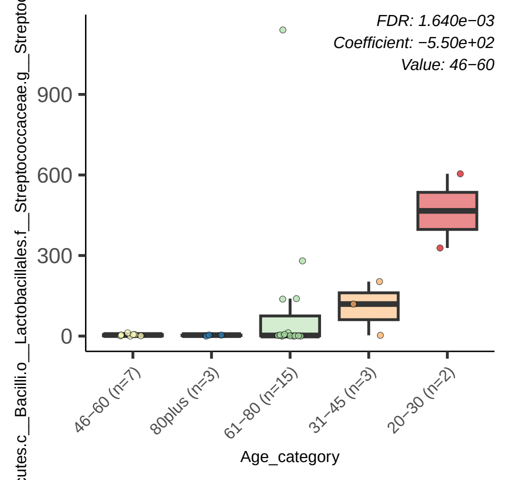
FDR: 1.640e−03
Coefficient: −5.50e+02
Value: 46−60
900
600
300
0
46−60 (n=7)
80plus (n=3)
61−80 (n=15)
31−45 (n=3)
20−30 (n=2)
Age_category

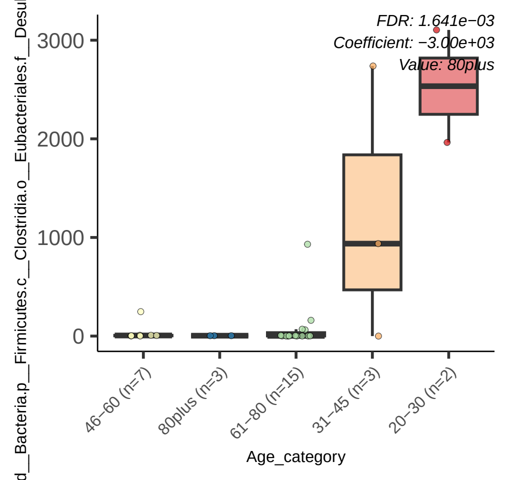

FDR: 1.641e−03
Coefficient: −3.00e+03
Value: 80plus
d__Bacteria.p__Firmicutes.c__Clostridia.o__Eubacteriales.f__Desu
3000
2000
1000
0
46−60 (n=7)
80plus (n=3)
61−80 (n=15)
31−45 (n=3)
20−30 (n=2)
Age_category

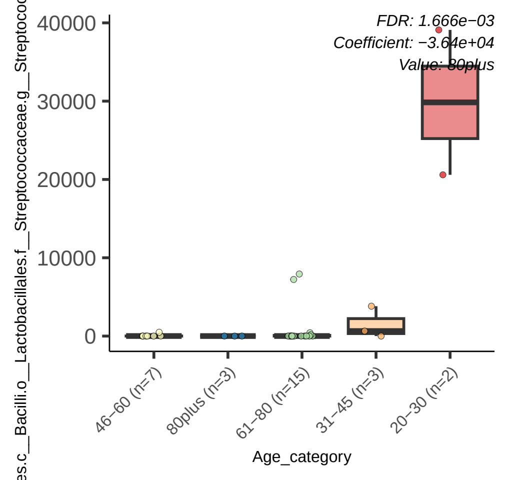

FDR: 1.666e−03
Coefficient: −3.64e+04
Value: 80plus
40000
30000
20000
10000
0
es.c__Bacilli.o__Lactobacillales.f__Streptococcaceae.g__Streptococ
46−60 (n=7)
80plus (n=3)
61−80 (n=15)
31−45 (n=3)
20−30 (n=2)
Age_category

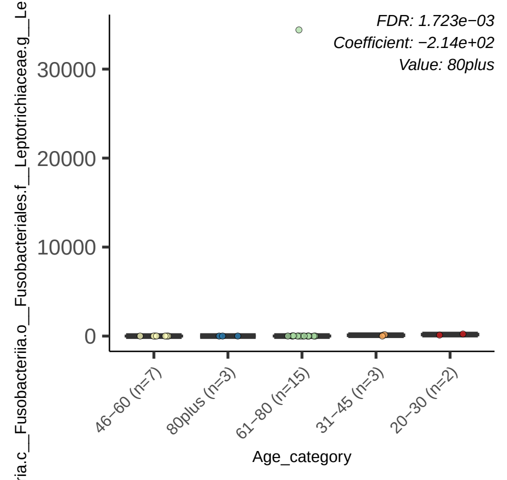

FDR: 1.723e−03
Coefficient: −2.14e+02
Value: 80plus
30000
20000
10000
0
46−60 (n=7)
80plus (n=3)
61−80 (n=15)
31−45 (n=3)
20−30 (n=2)
Age_category
ria.c__Fusobacteriia.o__Fusobacteriales.f__Leptotrichiaceae.g__Le

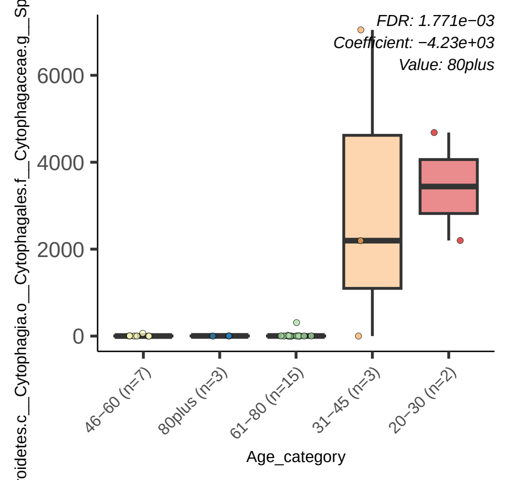

FDR: 1.771e−03
Coefficient: −4.23e+03
Value: 80plus
6000
4000
2000
0
46−60 (n=7)
80plus (n=3)
61−80 (n=15)
31−45 (n=3)
20−30 (n=2)
Age_category
roidetes.c__Cytophagia.o__Cytophagales.f__Cytophagaceae.g__Sp

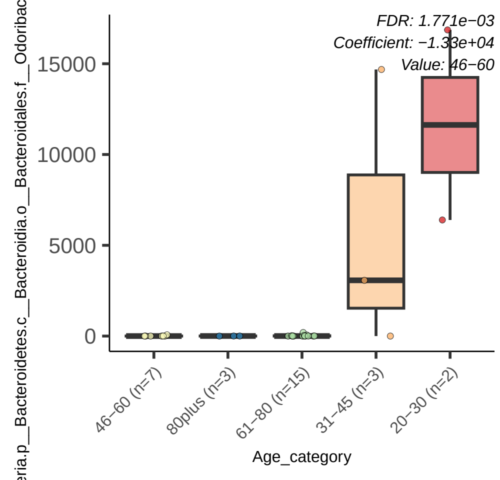

FDR: 1.771e−03
Coefficient: −1.33e+04
Value: 46−60
15000
10000
5000
0
46−60 (n=7)
80plus (n=3)
61−80 (n=15)
31−45 (n=3)
20−30 (n=2)
Age_category
eria.p__Bacteroidetes.c__Bacteroidia.o__Bacteroidales.f__Odoribac

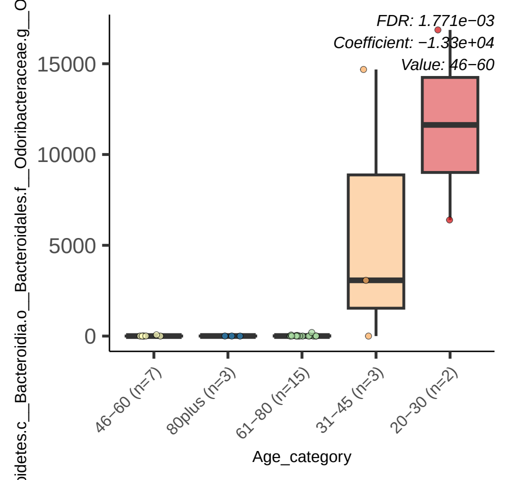

FDR: 1.771e−03
Coefficient: −1.33e+04
Value: 46−60
15000
10000
5000
0
oidetes.c__Bacteroidia.o__Bacteroidales.f__Odoribacteraceae.g__O
46−60 (n=7)
80plus (n=3)
61−80 (n=15)
31−45 (n=3)
20−30 (n=2)
Age_category

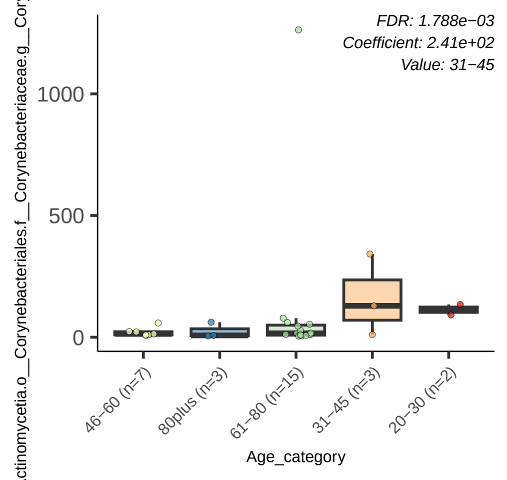

FDR: 1.788e−03
Coefficient: 2.41e+02
Value: 31−45
1000
500
0
ctinomycetia.o__Corynebacteriales.f__Corynebacteriaceae.g__Cor
46−60 (n=7)
80plus (n=3)
61−80 (n=15)
31−45 (n=3)
20−30 (n=2)
Age_category

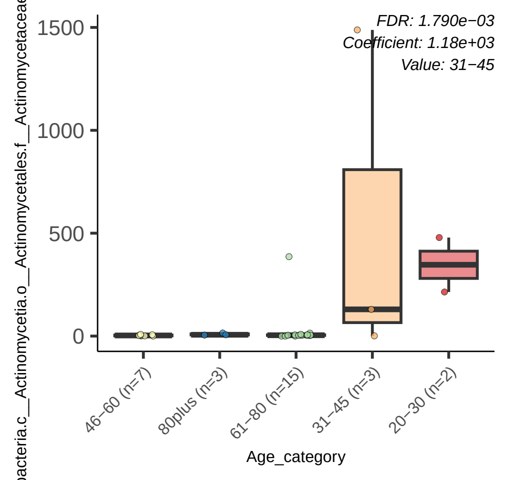

FDR: 1.790e−03
Coefficient: 1.18e+03
Value: 31−45
1500
1000
500
0
46−60 (n=7)
80plus (n=3)
61−80 (n=15)
31−45 (n=3)
20−30 (n=2)
Age_category
bacteria.c__Actinomycetia.o__Actinomycetales.f__Actinomycetaceae

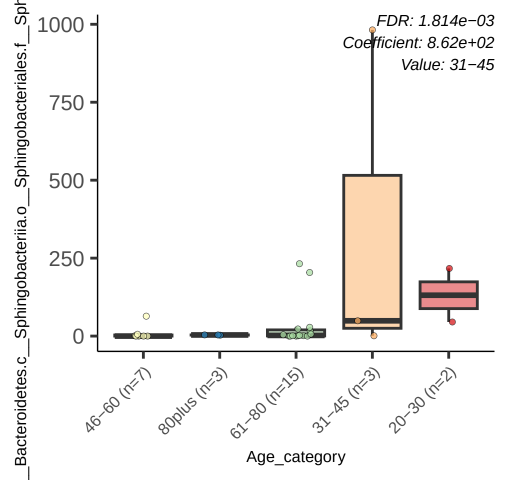

FDR: 1.814e−03
Coefficient: 8.62e+02
Value: 31−45
1000
750
500
250
0
__Bacteroidetes.c__Sphingobacteriia.o__Sphingobacteriales.f__Sph
46−60 (n=7)
80plus (n=3)
61−80 (n=15)
31−45 (n=3)
20−30 (n=2)
Age_category

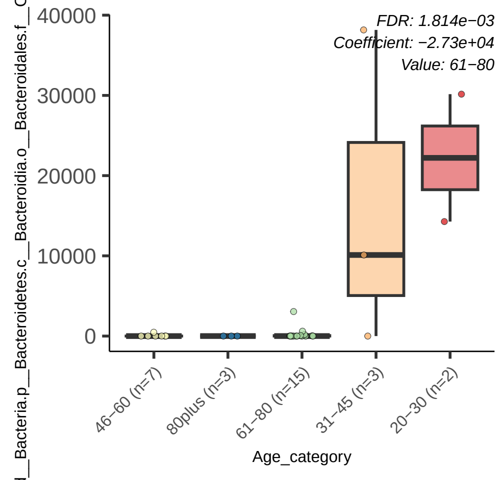
FDR: 1.814e−03
Coefficient: −2.73e+04
Value: 61−80
40000
30000
20000
10000
0
46−60 (n=7)
80plus (n=3)
61−80 (n=15)
31−45 (n=3)
20−30 (n=2)
Age_category

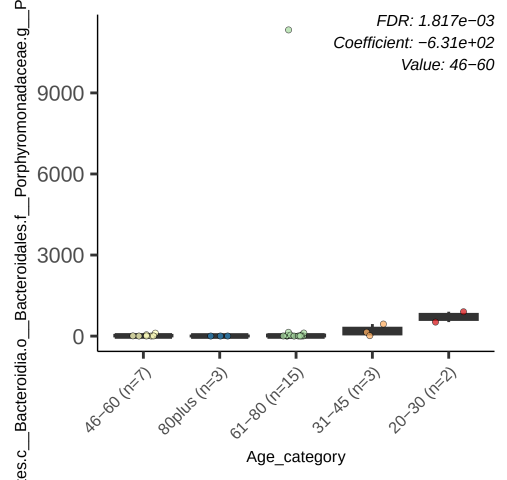
FDR: 1.817e−03
Coefficient: −6.31e+02
Value: 46−60
9000
6000
3000
0
46−60 (n=7)
80plus (n=3)
61−80 (n=15)
31−45 (n=3)
20−30 (n=2)
Age_category
tes.c__Bacteroidia.o__Bacteroidales.f__Porphyromonadaceae.g__P

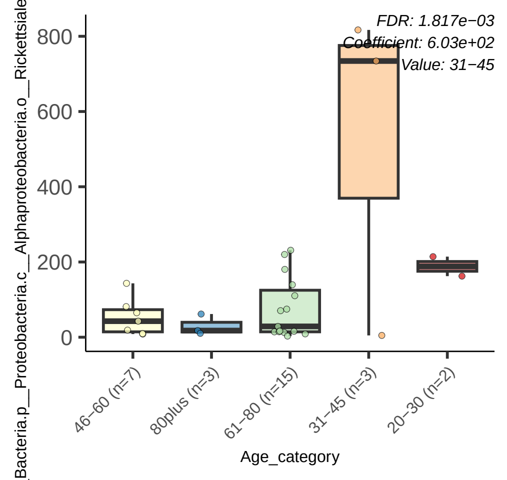

FDR: 1.817e−03
Coefficient: 6.03e+02
Value: 31−45
800
600
400
200
0
Bacteria.p__Proteobacteria.c__Alphaproteobacteria.o__Rickettsiale
46−60 (n=7)
80plus (n=3)
61−80 (n=15)
31−45 (n=3)
20−30 (n=2)
Age_category

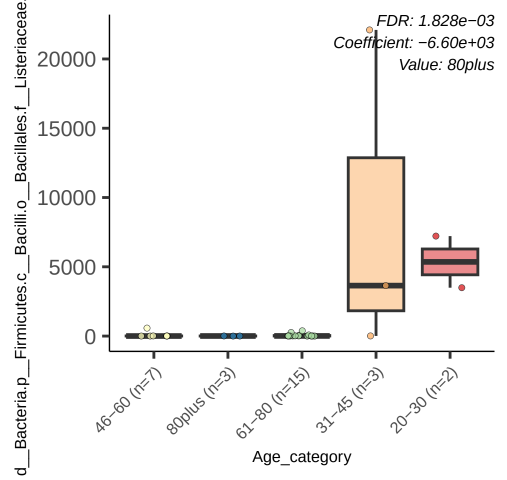

d__Bacteria.p__Firmicutes.c__Bacilli.o__Bacillales.f__Listeriaceae.
FDR: 1.828e−03
Coefficient: −6.60e+03
Value: 80plus
20000
15000
10000
5000
0
46−60 (n=7)
80plus (n=3)
61−80 (n=15)
31−45 (n=3)
20−30 (n=2)
Age_category

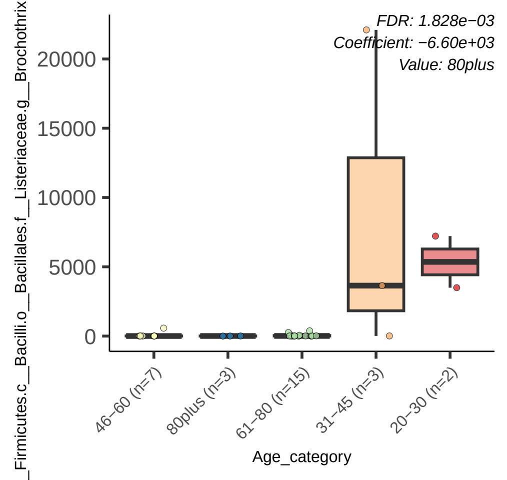
FDR: 1.828e−03
Coefficient: −6.60e+03
Value: 80plus
20000
15000
10000
5000
0
_Firmicutes.c__Bacilli.o__Bacillales.f__Listeriaceae.g__Brochothrix
46−60 (n=7)
80plus (n=3)
61−80 (n=15)
31−45 (n=3)
20−30 (n=2)
Age_category

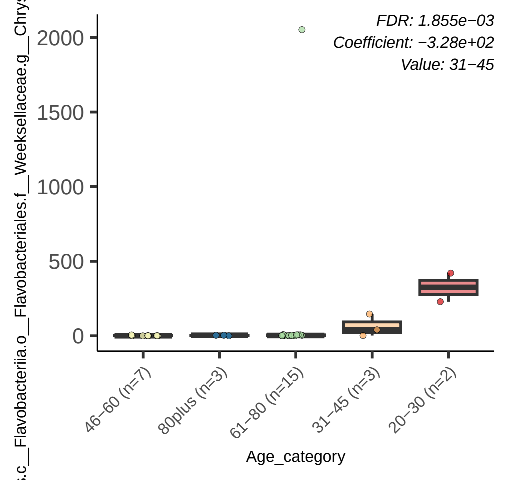

s.c__Flavobacteriia.o__Flavobacteriales.f__Weeksellaceae.g__Chrys
FDR: 1.855e−03
Coefficient: −3.28e+02
Value: 31−45
2000
1500
1000
500
0
46−60 (n=7)
80plus (n=3)
61−80 (n=15)
31−45 (n=3)
20−30 (n=2)
Age_category

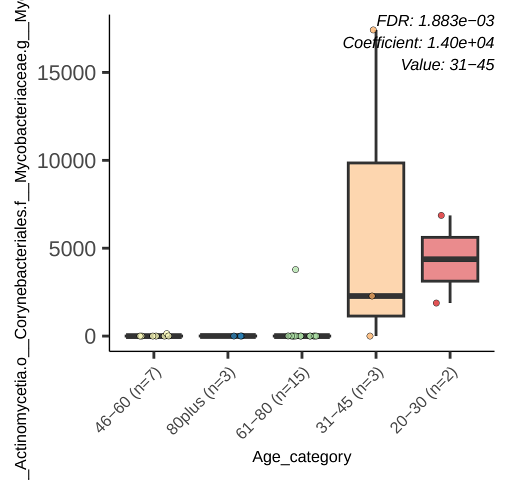

FDR: 1.883e−03
Coefficient: 1.40e+04
Value: 31−45
15000
10000
5000
0
Actinomycetia.o__Corynebacteriales.f__Mycobacteriaceae.g__My
46−60 (n=7)
80plus (n=3)
61−80 (n=15)
31−45 (n=3)
20−30 (n=2)
Age_category

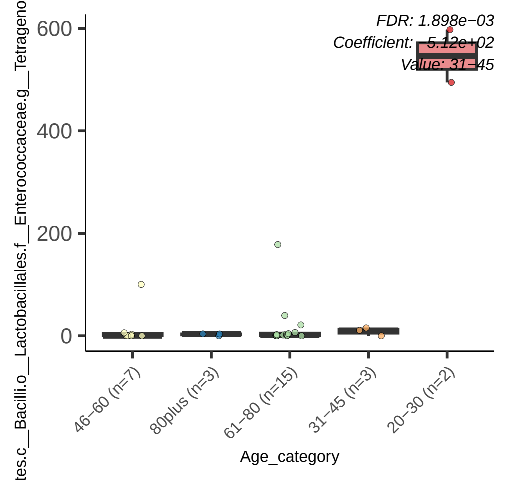

FDR: 1.898e−03
Coefficient: −5.12e+02
Value: 31−45
600
400
200
0
tes.c__Bacilli.o__Lactobacillales.f__Enterococcaceae.g__Tetrageno
46−60 (n=7)
80plus (n=3)
61−80 (n=15)
31−45 (n=3)
20−30 (n=2)
Age_category

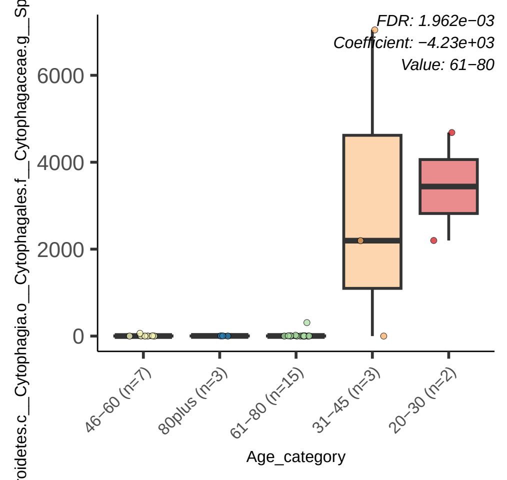

FDR: 1.962e−03
Coefficient: −4.23e+03
Value: 61−80
6000
4000
2000
0
46−60 (n=7)
80plus (n=3)
61−80 (n=15)
31−45 (n=3)
20−30 (n=2)
Age_category

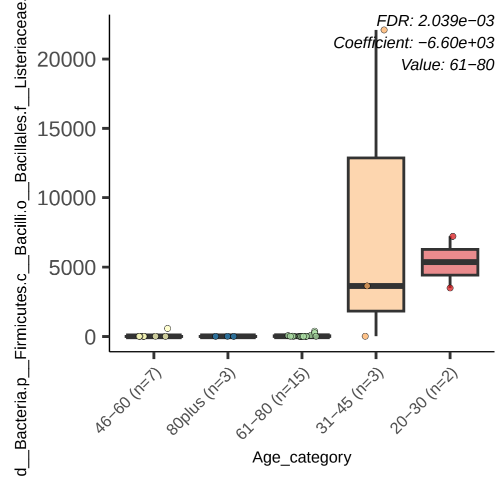

FDR: 2.039e−03
Coefficient: −6.60e+03
Value: 61−80
d__Bacteria.p__Firmicutes.c__Bacilli.o__Bacillales.f__Listeriaceae
20000
15000
10000
5000
0
46−60 (n=7)
80plus (n=3)
61−80 (n=15)
31−45 (n=3)
20−30 (n=2)
Age_category

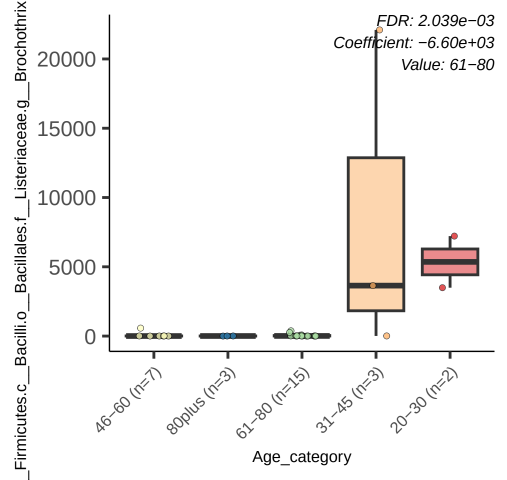
FDR: 2.039e−03
Coefficient: −6.60e+03
Value: 61−80
Firmicutes.c__Bacilli.o__Bacillales.f__Listeriaceae.g__Brochothrix
20000
15000
10000
5000
0
46−60 (n=7)
80plus (n=3)
61−80 (n=15)
31−45 (n=3)
20−30 (n=2)
Age_category

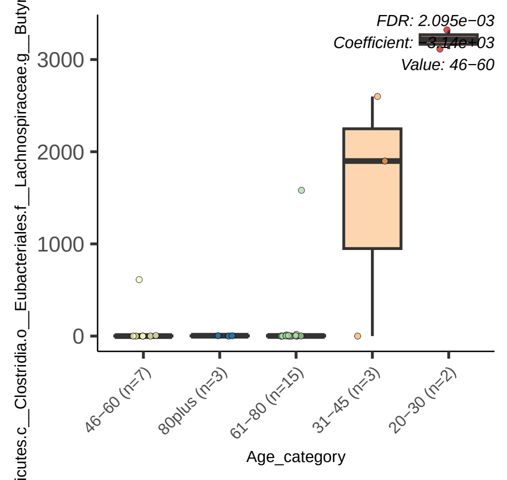

FDR: 2.095e−03
Coefficient: −3.14e+03
Value: 46−60
3000
2000
1000
0
46−60 (n=7)
80plus (n=3)
61−80 (n=15)
31−45 (n=3)
20−30 (n=2)
Age_category
icutes.c__Clostridia.o__Eubacteriales.f__Lachnospiraceae.g__Butyr

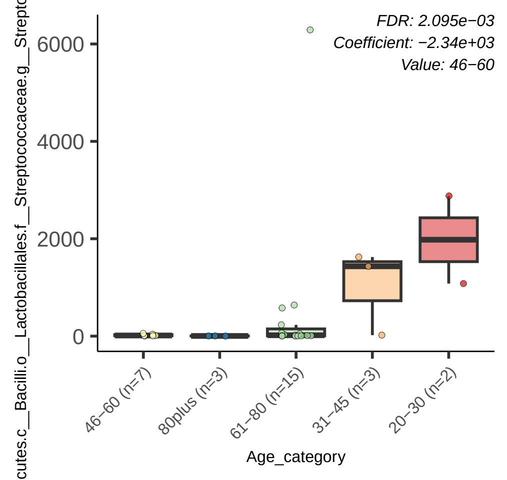

FDR: 2.095e−03
Coefficient: −2.34e+03
Value: 46−60
6000
4000
2000
0
46−60 (n=7)
80plus (n=3)
61−80 (n=15)
31−45 (n=3)
20−30 (n=2)
Age_category
cutes.c__Bacilli.o__Lactobacillales.f__Streptococcaceae.g__Strepto

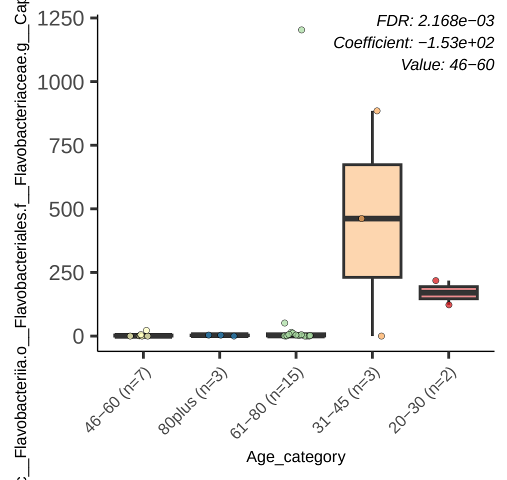

FDR: 2.168e−03
Coefficient: −1.53e+02
Value: 46−60
1250
1000
750
500
250
0
c__Flavobacteriia.o__Flavobacteriales.f__Flavobacteriaceae.g__Cap
46−60 (n=7)
80plus (n=3)
61−80 (n=15)
31−45 (n=3)
20−30 (n=2)
Age_category

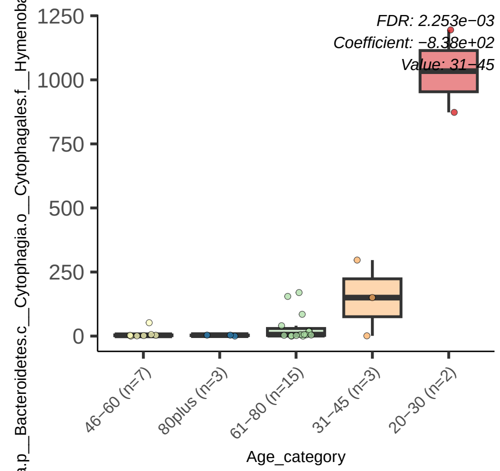

FDR: 2.253e−03
Coefficient: −8.38e+02
Value: 31−45
1250
1000
750
500
250
0
ia.p__Bacteroidetes.c__Cytophagia.o__Cytophagales.f__Hymenoba
46−60 (n=7)
80plus (n=3)
61−80 (n=15)
31−45 (n=3)
20−30 (n=2)
Age_category

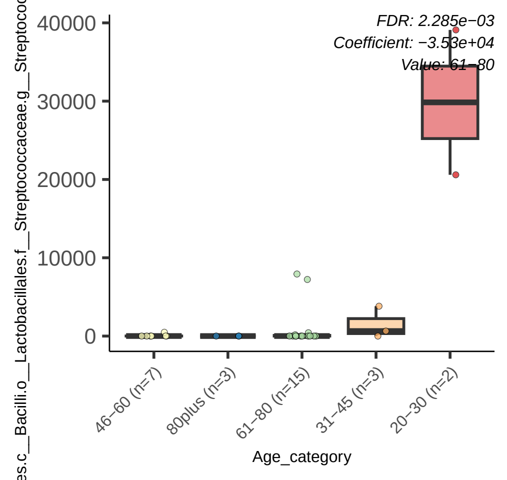

FDR: 2.285e−03
Coefficient: −3.53e+04
Value: 61−80
Lactobacillales.f__Streptococcaceae.g__Streptococ
40000
30000
20000
10000
0
46−60 (n=7)
80plus (n=3)
61−80 (n=15)
31−45 (n=3)
20−30 (n=2)
Age_category

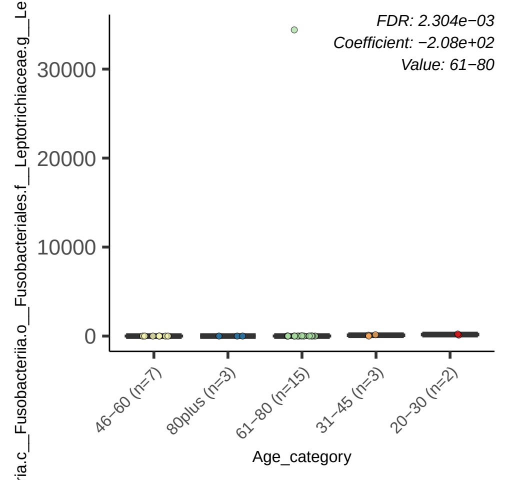

FDR: 2.304e−03
Coefficient: −2.08e+02
Value: 61−80
30000
20000
10000
0
46−60 (n=7)
80plus (n=3)
61−80 (n=15)
31−45 (n=3)
20−30 (n=2)
Age_category
ria.c__Fusobacteriia.o__Fusobacteriales.f__Leptotrichiaceae.g__Le

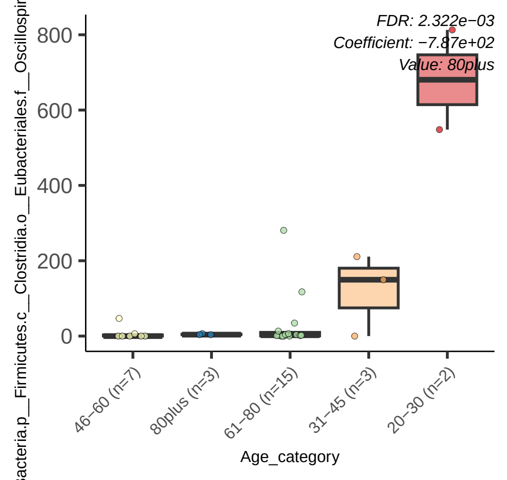
FDR: 2.322e−03
Coefficient: −7.87e+02
Value: 80plus
800
600
400
200
0
acteria.p__Firmicutes.c__Clostridia.o__Eubacteriales.f__Oscillospi
46−60 (n=7)
80plus (n=3)
61−80 (n=15)
31−45 (n=3)
20−30 (n=2)
Age_category

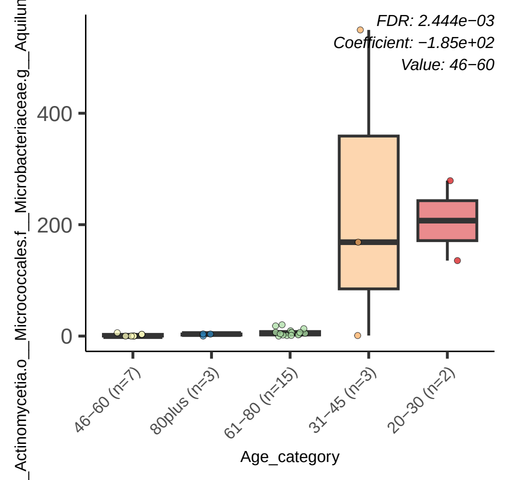
FDR: 2.444e−03
Coefficient: −1.85e+02
Value: 46−60
Actinomycetia.o__Micrococcales.f__Microbacteriaceae.g__Aquilun
400
200
0
46−60 (n=7)
80plus (n=3)
61−80 (n=15)
31−45 (n=3)
20−30 (n=2)
Age_category

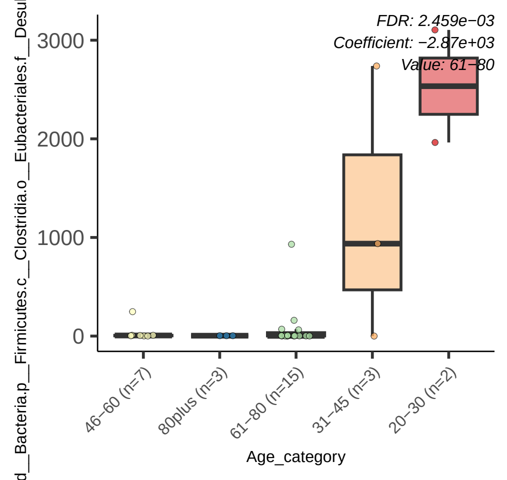

FDR: 2.459e−03
Coefficient: −2.87e+03
Value: 61−80
0
1000
2000
3000
d__Bacteria.p__Firmicutes.c__Clostridia.o__Eubacteriales.f__Desul
46−60 (n=7)
80plus (n=3)
61−80 (n=15)
31−45 (n=3)
20−30 (n=2)
Age_category

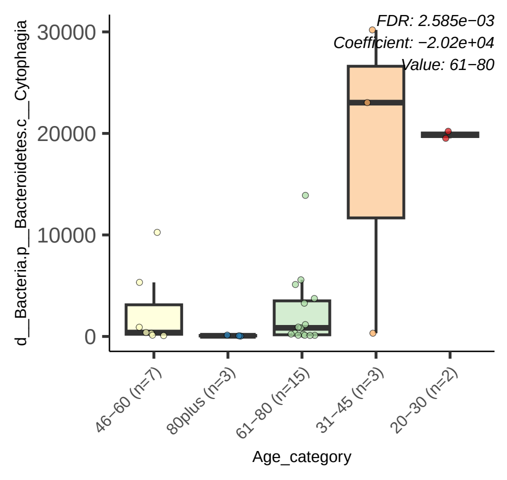

FDR: 2.585e−03
Coefficient: −2.02e+04
Value: 61−80
30000
20000
10000
0
d__Bacteria.p__Bacteroidetes.c__Cytophagia
46−60 (n=7)
80plus (n=3)
61−80 (n=15)
31−45 (n=3)
20−30 (n=2)
Age_category

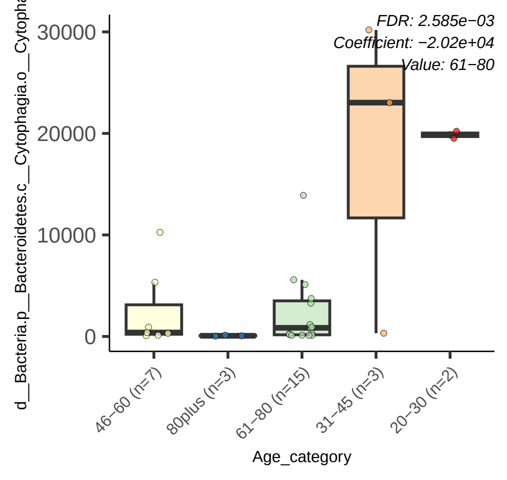

FDR: 2.585e−03
Coefficient: −2.02e+04
Value: 61−80
d__Bacteria.p__Bacteroidetes.c__Cytophagia.o__Cytopha
30000
20000
10000
0
46−60 (n=7)
80plus (n=3)
61−80 (n=15)
31−45 (n=3)
20−30 (n=2)
Age_category

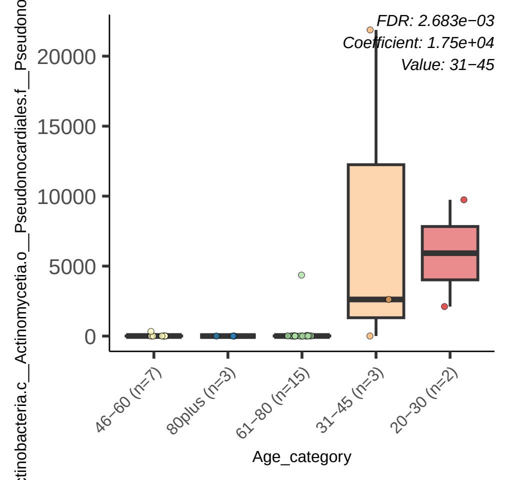

FDR: 2.683e−03
Coefficient: 1.75e+04
Value: 31−45
0
5000
10000
15000
20000
46−60 (n=7)
80plus (n=3)
61−80 (n=15)
31−45 (n=3)
20−30 (n=2)
Age_category
ctinobacteria.c__Actinomycetia.o__Pseudonocardiales.f__Pseudono

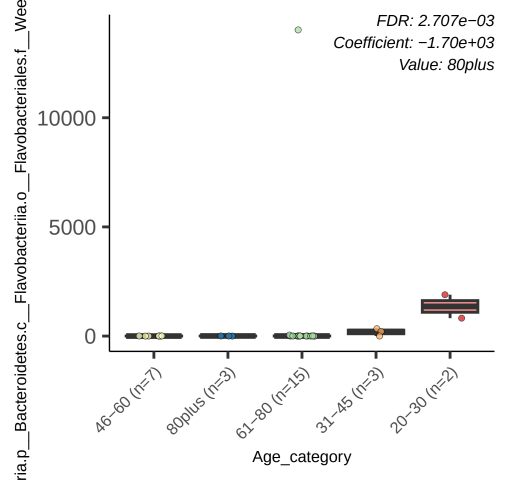

FDR: 2.707e−03
Coefficient: −1.70e+03
Value: 80plus
10000
5000
0
46−60 (n=7)
80plus (n=3)
61−80 (n=15)
31−45 (n=3)
20−30 (n=2)
Age_category
ria.p__Bacteroidetes.c__Flavobacteriia.o__Flavobacteriales.f__Wee

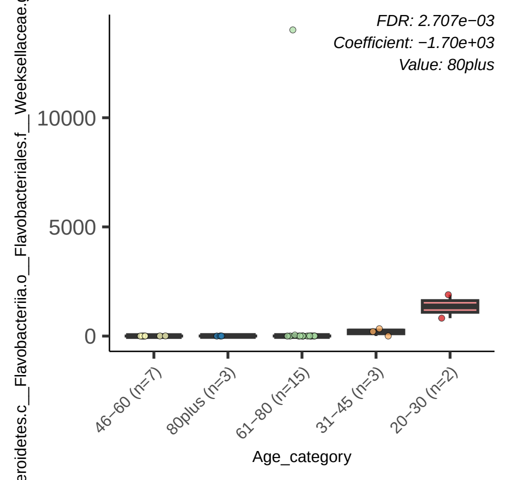

FDR: 2.707e−03
Coefficient: −1.70e+03
Value: 80plus
10000
5000
0
46−60 (n=7)
80plus (n=3)
61−80 (n=15)
31−45 (n=3)
20−30 (n=2)
Age_category
eroidetes.c__Flavobacteriia.o__Flavobacteriales.f__Weeksellaceae.g

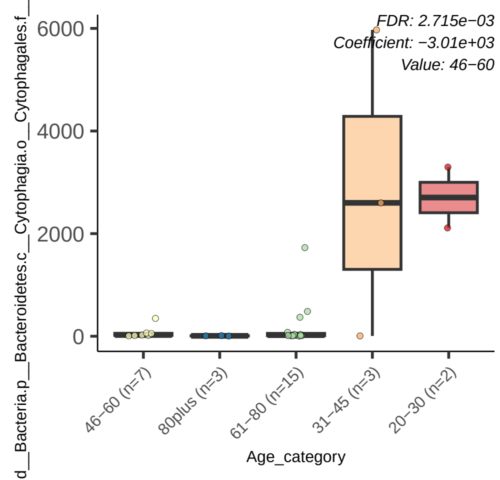

FDR: 2.715e−03
Coefficient: −3.01e+03
Value: 46−60
d__Bacteria.p__Bacteroidetes.c__Cytophagia.o__Cytophagales.f__
6000
4000
2000
0
46−60 (n=7)
80plus (n=3)
61−80 (n=15)
31−45 (n=3)
20−30 (n=2)
Age_category

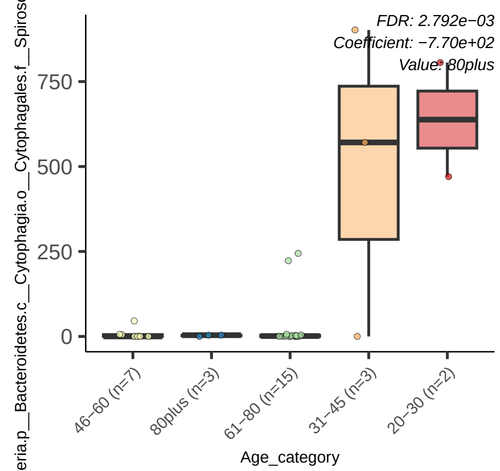

FDR: 2.792e−03
Coefficient: −7.70e+02
Value: 80plus
750
500
250
0
cteria.p__Bacteroidetes.c__Cytophagia.o__Cytophagales.f__Spiros
46−60 (n=7)
80plus (n=3)
61−80 (n=15)
31−45 (n=3)
20−30 (n=2)
Age_category

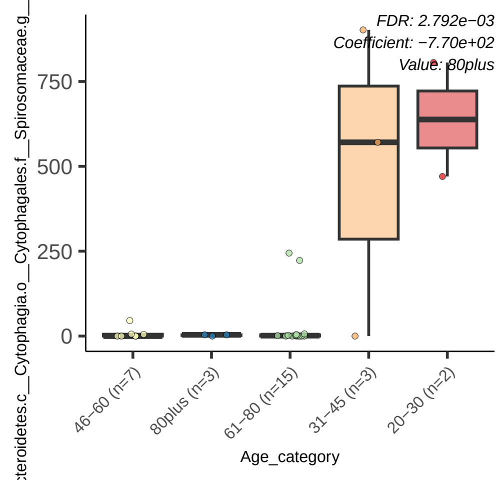

FDR: 2.792e−03
Coefficient: −7.70e+02
Value: 80plus
750
500
250
0
cteroidetes.c__Cytophagia.o__Cytophagales.f__Spirosomaceae.g__
46−60 (n=7)
80plus (n=3)
61−80 (n=15)
31−45 (n=3)
20−30 (n=2)
Age_category

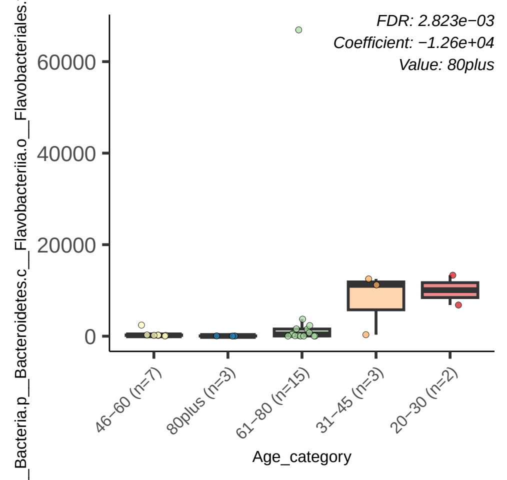

FDR: 2.823e−03
Coefficient: −1.26e+04
Value: 80plus
_Bacteria.p__Bacteroidetes.c__Flavobacteriia.o__Flavobacteriales.
60000
40000
20000
0
46−60 (n=7)
80plus (n=3)
61−80 (n=15)
31−45 (n=3)
20−30 (n=2)
Age_category

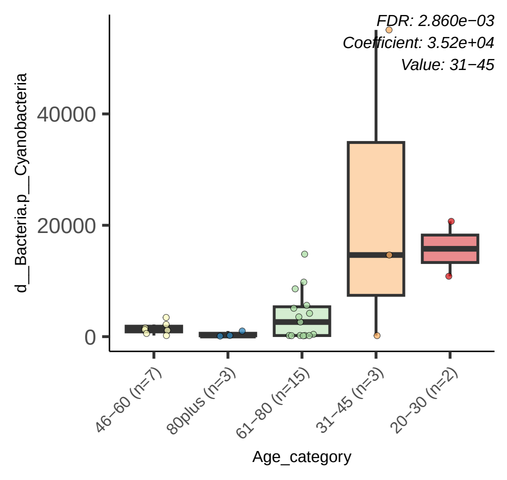

FDR: 2.860e−03
Coefficient: 3.52e+04
Value: 31−45
d__Bacteria.p__Cyanobacteria
40000
20000
0
46−60 (n=7)
80plus (n=3)
61−80 (n=15)
31−45 (n=3)
20−30 (n=2)
Age_category

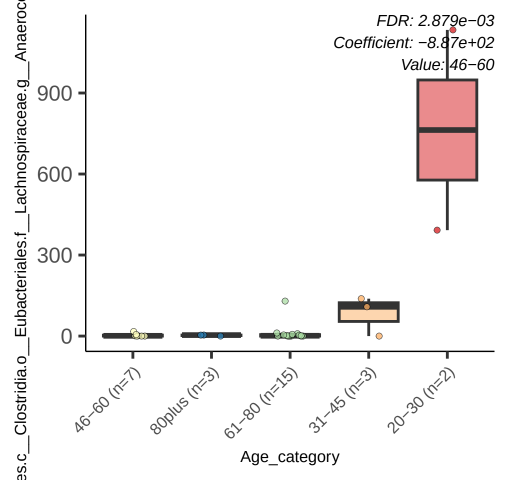
FDR: 2.879e−03
Coefficient: −8.87e+02
Value: 46−60
900
600
300
0
es.c__Clostridia.o__Eubacteriales.f__Lachnospiraceae.g__Anaeroc
46−60 (n=7)
80plus (n=3)
61−80 (n=15)
31−45 (n=3)
20−30 (n=2)
Age_category

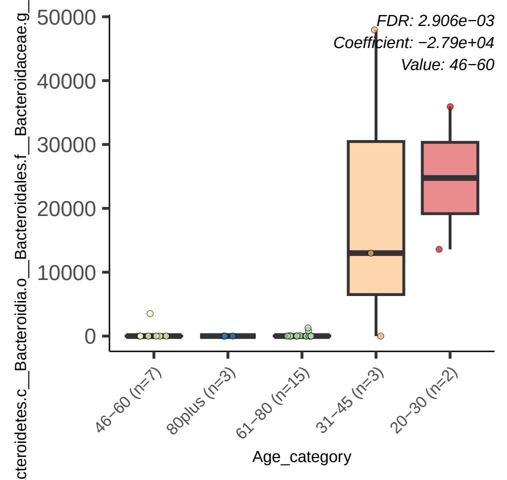

FDR: 2.906e−03
Coefficient: −2.79e+04
Value: 46−60
50000
40000
30000
20000
10000
0
cteroidetes.c__Bacteroidia.o__Bacteroidales.f__Bacteroidaceae.g_
46−60 (n=7)
80plus (n=3)
61−80 (n=15)
31−45 (n=3)
20−30 (n=2)
Age_category

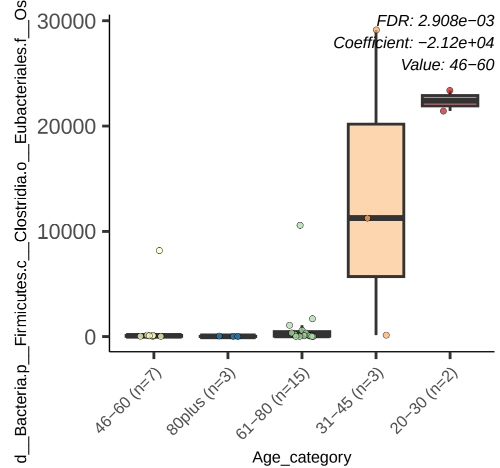

d__Bacteria.p__Firmicutes.c__Clostridia.o__Eubacteriales.f__Os
30000
20000
10000
0
FDR: 2.908e−03
Coefficient: −2.12e+04
Value: 46−60
46−60 (n=7)
80plus (n=3)
61−80 (n=15)
31−45 (n=3)
20−30 (n=2)
Age_category

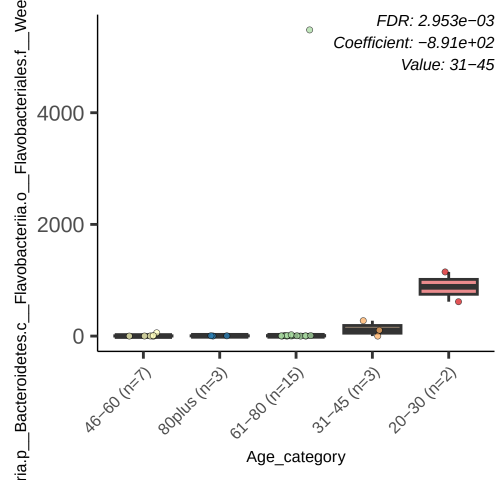

FDR: 2.953e−03
Coefficient: −8.91e+02
Value: 31−45
ria.p__Bacteroidetes.c__Flavobacteriia.o__Flavobacteriales.f__Wee
4000
2000
0
46−60 (n=7)
80plus (n=3)
61−80 (n=15)
31−45 (n=3)
20−30 (n=2)
Age_category

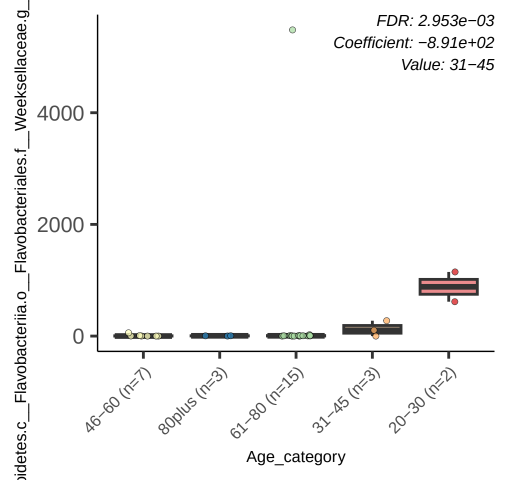

FDR: 2.953e−03
Coefficient: −8.91e+02
Value: 31−45
4000
2000
0
46−60 (n=7)
80plus (n=3)
61−80 (n=15)
31−45 (n=3)
20−30 (n=2)
Age_category
oidetes.c__Flavobacteriia.o__Flavobacteriales.f__Weeksellaceae.g__

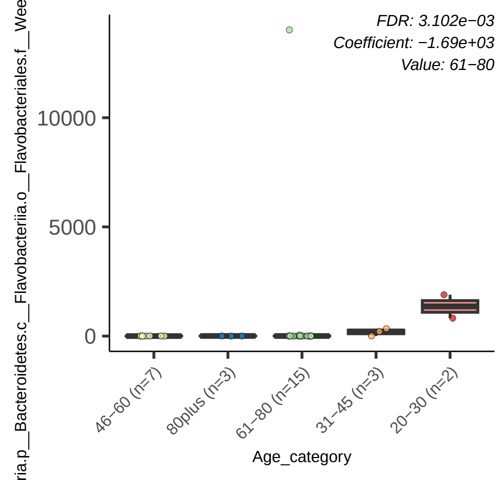

FDR: 3.102e−03
Coefficient: −1.69e+03
Value: 61−80
10000
5000
0
46−60 (n=7)
80plus (n=3)
61−80 (n=15)
31−45 (n=3)
20−30 (n=2)
Age_category
ria.p__Bacteroidetes.c__Flavobacteriia.o__Flavobacteriales.f__Wee

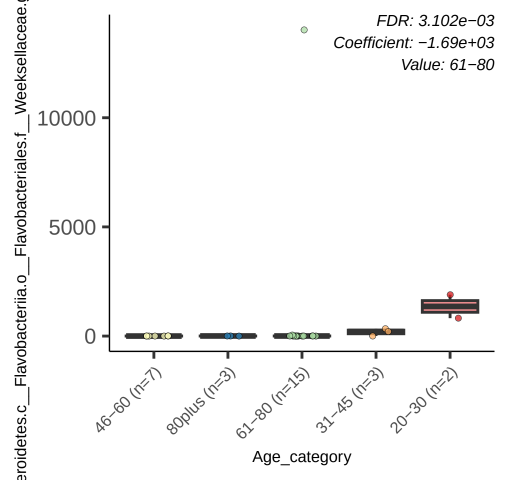
FDR: 3.102e−03
Coefficient: −1.69e+03
Value: 61−80
eroidetes.c__Flavobacteriia.o__Flavobacteriales.f__Weeksellaceae.g
10000
5000
0
46−60 (n=7)
80plus (n=3)
61−80 (n=15)
31−45 (n=3)
20−30 (n=2)
Age_category

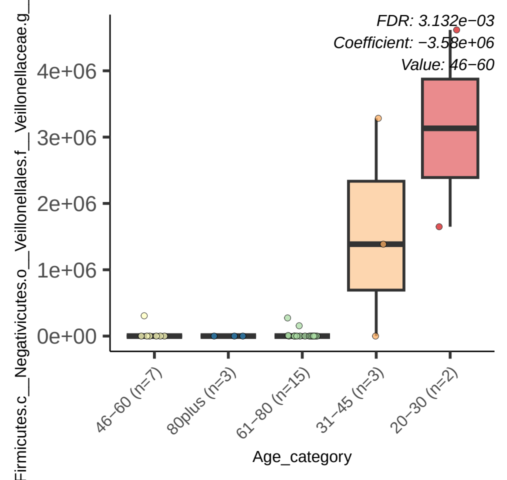
FDR: 3.132e−03
Coefficient: −3.58e+06
Value: 46−60
Firmicutes.c__Negativicutes.o__Veillonellales.f__Veillonellaceae.g__
4e+06
3e+06
2e+06
1e+06
0e+00
46−60 (n=7)
80plus (n=3)
61−80 (n=15)
31−45 (n=3)
20−30 (n=2)
Age_category

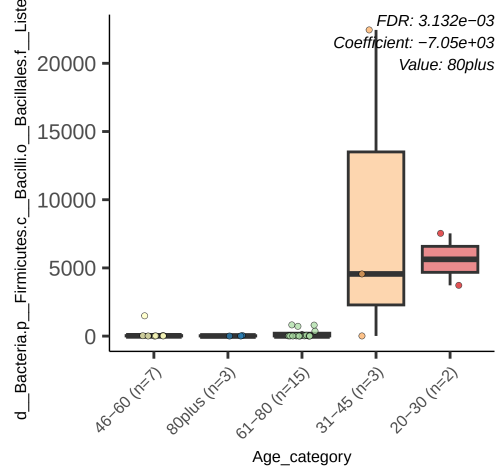

FDR: 3.132e−03
Coefficient: −7.05e+03
Value: 80plus
d__Bacteria.p__Firmicutes.c__Bacilli.o__Bacillales.f__Liste
20000
15000
10000
5000
0
46−60 (n=7)
80plus (n=3)
61−80 (n=15)
31−45 (n=3)
20−30 (n=2)
Age_category

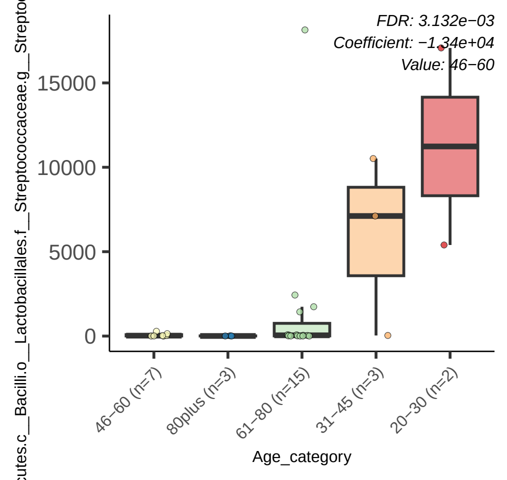

FDR: 3.132e−03
Coefficient: −1.34e+04
Value: 46−60
cutes.c__Bacilli.o__Lactobacillales.f__Streptococcaceae.g__Strepto
15000
10000
5000
0
46−60 (n=7)
80plus (n=3)
61−80 (n=15)
31−45 (n=3)
20−30 (n=2)
Age_category

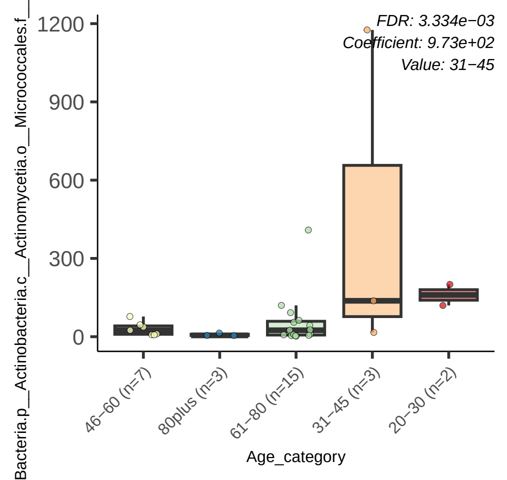
FDR: 3.334e−03
Coefficient: 9.73e+02
Value: 31−45
Bacteria.p__Actinobacteria.c__Actinomycetia.o__Micrococcales.f__
1200
900
600
300
0
46−60 (n=7)
80plus (n=3)
61−80 (n=15)
31−45 (n=3)
20−30 (n=2)
Age_category

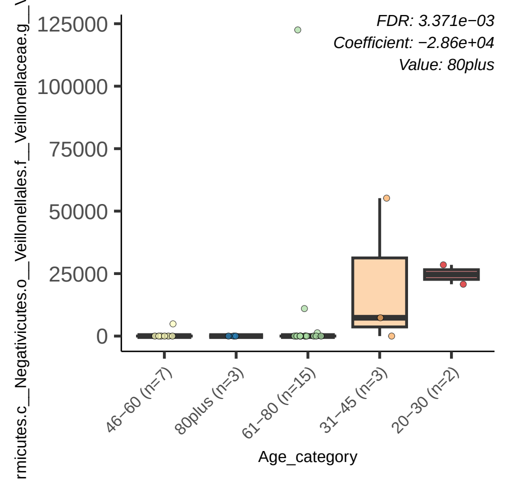

FDR: 3.371e−03
Coefficient: −2.86e+04
Value: 80plus
rmicutes.c__Negativicutes.o__Veillonellales.f__Veillonellaceae.g__V
125000
100000
75000
50000
25000
0
46−60 (n=7)
80plus (n=3)
61−80 (n=15)
31−45 (n=3)
20−30 (n=2)
Age_category

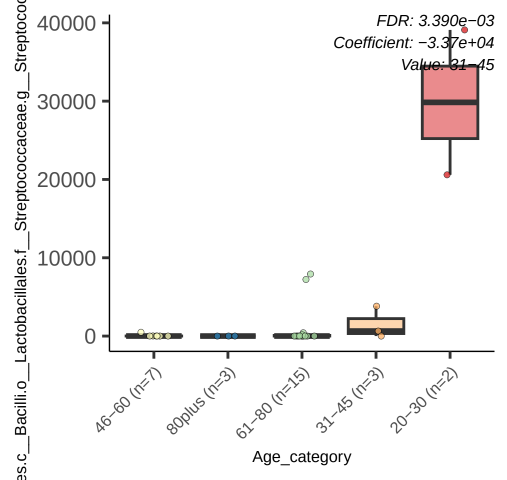

FDR: 3.390e−03
Coefficient: −3.37e+04
Value: 31−45
es.c__Bacilli.o__Lactobacillales.f__Streptococcaceae.g__Streptococ
40000
30000
20000
10000
0
46−60 (n=7)
80plus (n=3)
61−80 (n=15)
31−45 (n=3)
20−30 (n=2)
Age_category

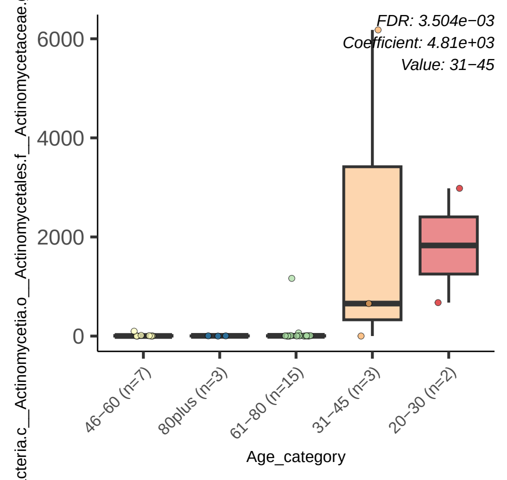

FDR: 3.504e−03
Coefficient: 4.81e+03
Value: 31−45
6000
4000
2000
0
cteria.c__Actinomycetia.o__Actinomycetales.f__Actinomycetaceae.
46−60 (n=7)
80plus (n=3)
61−80 (n=15)
31−45 (n=3)
20−30 (n=2)
Age_category

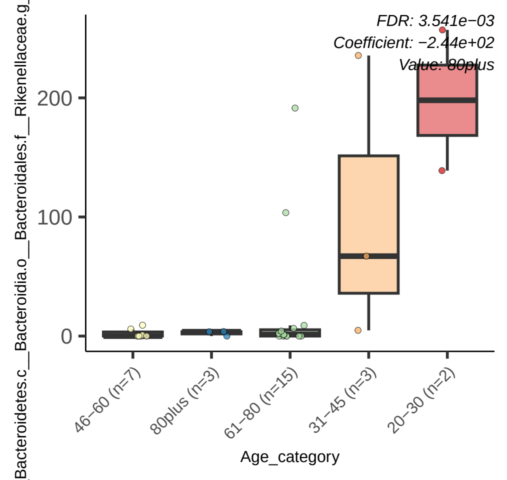
FDR: 3.541e−03
Coefficient: −2.44e+02
Value: 80plus
Bacteroidetes.c__Bacteroidia.o__Bacteroidales.f__Rikenellaceae.g_
200
100
0
46−60 (n=7)
80plus (n=3)
61−80 (n=15)
31−45 (n=3)
20−30 (n=2)
Age_category

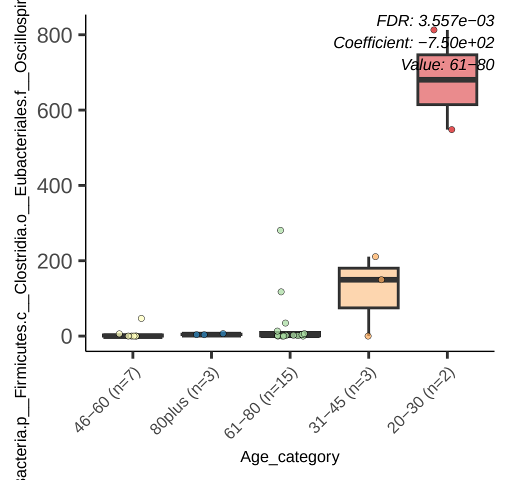

FDR: 3.557e−03
Coefficient: −7.50e+02
Value: 61−80
800
600
400
200
0
acteria.p__Firmicutes.c__Clostridia.o__Eubacteriales.f__Oscillospir
46−60 (n=7)
80plus (n=3)
61−80 (n=15)
31−45 (n=3)
20−30 (n=2)
Age_category

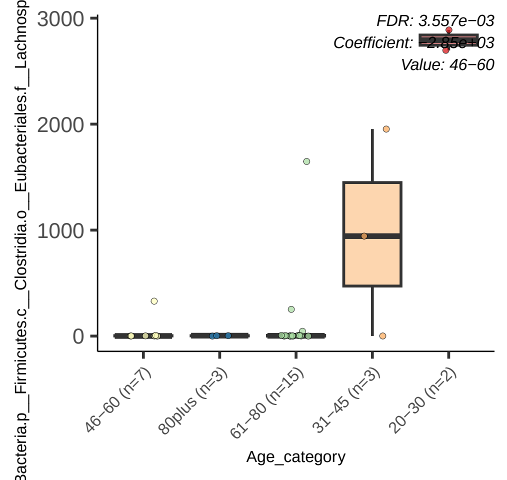

FDR: 3.557e−03
Coefficient: −2.85e+03
Value: 46−60
3000
2000
1000
0
Bacteria.p__Firmicutes.c__Clostridia.o__Eubacteriales.f__Lachnosp
46−60 (n=7)
80plus (n=3)
61−80 (n=15)
31−45 (n=3)
20−30 (n=2)
Age_category

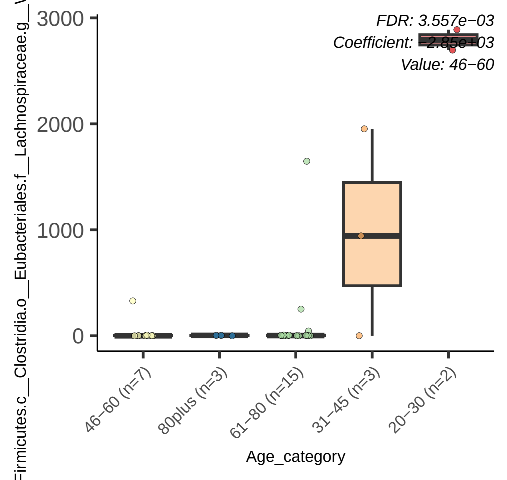

FDR: 3.557e−03
Coefficient: −2.85e+03
Value: 46−60
Firmicutes.c__Clostridia.o__Eubacteriales.f__Lachnospiraceae.g__
3000
2000
1000
0
46−60 (n=7)
80plus (n=3)
61−80 (n=15)
31−45 (n=3)
20−30 (n=2)
Age_category

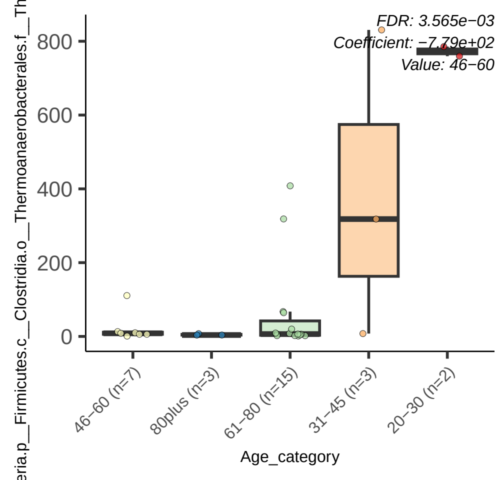

FDR: 3.565e−03
Coefficient: −7.79e+02
Value: 46−60
800
600
400
200
0
eria.p__Firmicutes.c__Clostridia.o__Thermoanaerobacterales.f__Th
46−60 (n=7)
80plus (n=3)
61−80 (n=15)
31−45 (n=3)
20−30 (n=2)
Age_category

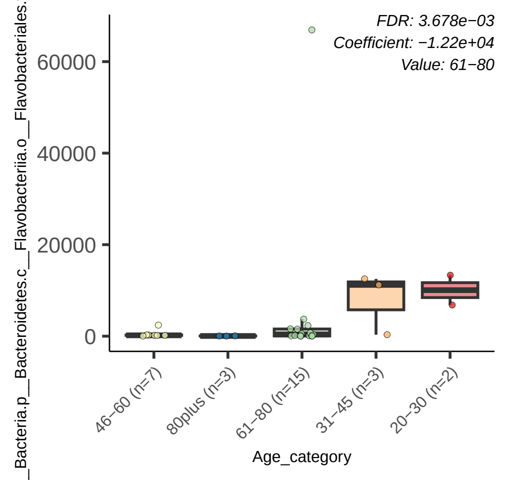

FDR: 3.678e−03
Coefficient: −1.22e+04
Value: 61−80
60000
40000
20000
0
_Bacteria.p__Bacteroidetes.c__Flavobacteriia.o__Flavobacteriales.
46−60 (n=7)
80plus (n=3)
61−80 (n=15)
31−45 (n=3)
20−30 (n=2)
Age_category

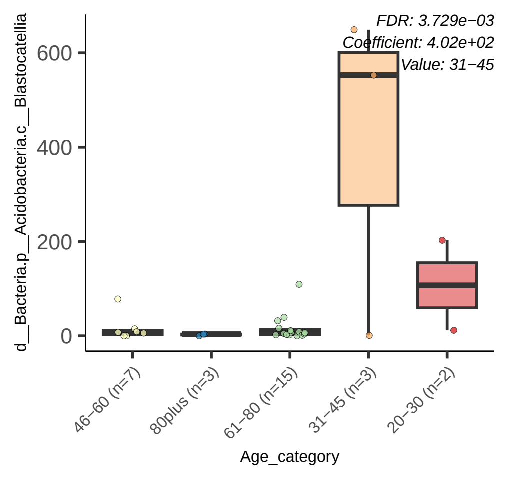
FDR: 3.729e−03
Coefficient: 4.02e+02
Value: 31−45
d__Bacteria.p__Acidobacteria.c__Blastocatellia
600
400
200
0
46−60 (n=7)
80plus (n=3)
61−80 (n=15)
31−45 (n=3)
20−30 (n=2)
Age_category

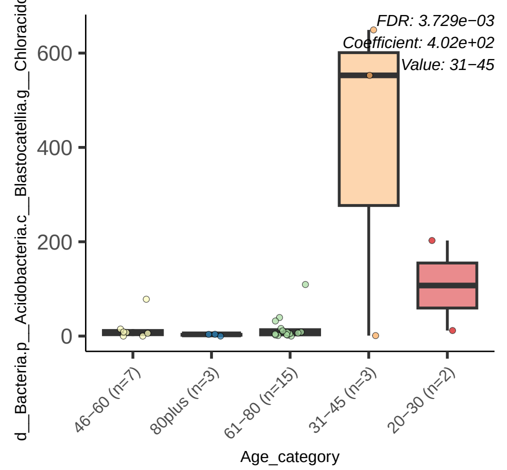
FDR: 3.729e−03
Coefficient: 4.02e+02
Value: 31−45
d__Bacteria.p__Acidobacteria.c__Blastocatellia.g__Chloracido
600
400
200
0
46−60 (n=7)
80plus (n=3)
61−80 (n=15)
31−45 (n=3)
20−30 (n=2)
Age_category

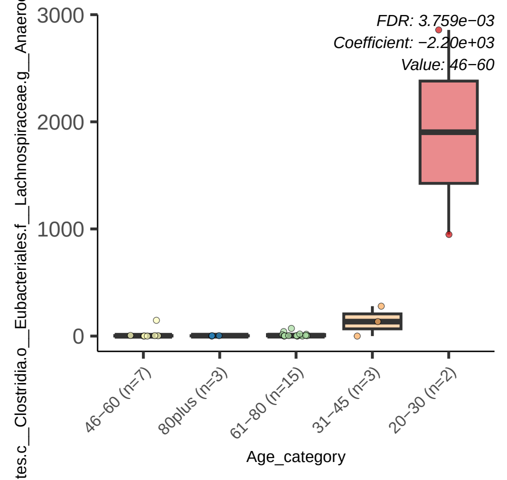

FDR: 3.759e−03
Coefficient: −2.20e+03
Value: 46−60
3000
2000
1000
0
tes.c__Clostridia.o__Eubacteriales.f__Lachnospiraceae.g__Anaero
46−60 (n=7)
80plus (n=3)
61−80 (n=15)
31−45 (n=3)
20−30 (n=2)
Age_category

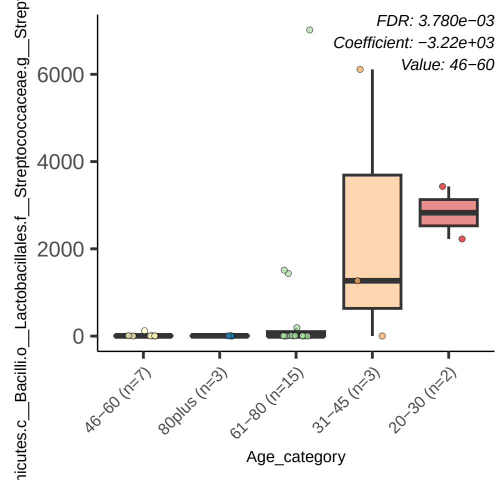
FDR: 3.780e−03
Coefficient: −3.22e+03
Value: 46−60
6000
4000
2000
0
nicutes.c__Bacilli.o__Lactobacillales.f__Streptococcaceae.g__Strep
46−60 (n=7)
80plus (n=3)
61−80 (n=15)
31−45 (n=3)
20−30 (n=2)
Age_category

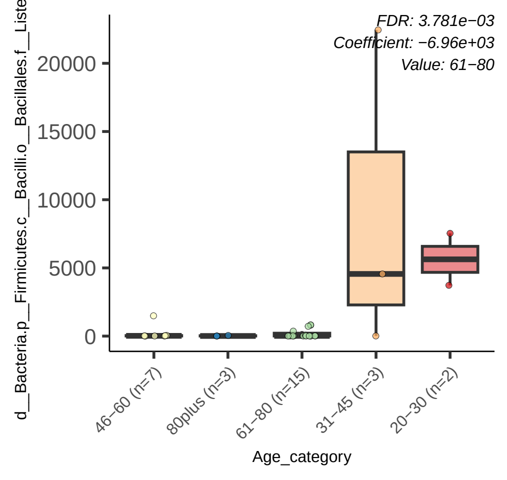

FDR: 3.781e−03
Coefficient: −6.96e+03
Value: 61−80
20000
15000
10000
5000
0
d__Bacteria.p__Firmicutes.c__Bacilli.o__Bacillales.f__Liste
46−60 (n=7)
80plus (n=3)
61−80 (n=15)
31−45 (n=3)
20−30 (n=2)
Age_category

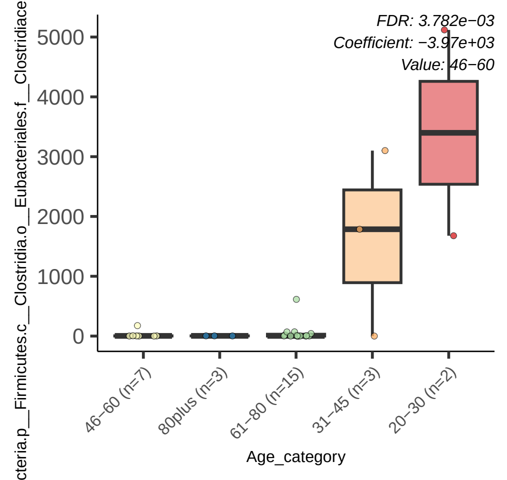
FDR: 3.782e−03
Coefficient: −3.97e+03
Value: 46−60
cteria.p__Firmicutes.c__Clostridia.o__Eubacteriales.f__Clostridiace
5000
4000
3000
2000
1000
0
46−60 (n=7)
80plus (n=3)
61−80 (n=15)
31−45 (n=3)
20−30 (n=2)
Age_category

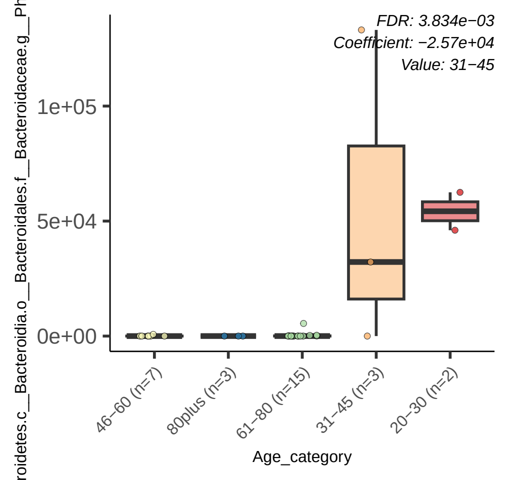
FDR: 3.834e−03
Coefficient: −2.57e+04
Value: 31−45
1e+05
5e+04
0e+00
46−60 (n=7)
80plus (n=3)
61−80 (n=15)
31−45 (n=3)
20−30 (n=2)
Age_category
roidetes.c__Bacteroidia.o__Bacteroidales.f__Bacteroidaceae.g__Ph

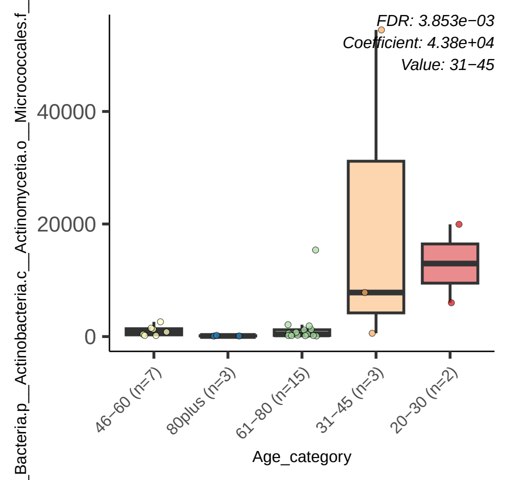

FDR: 3.853e−03
Coefficient: 4.38e+04
Value: 31−45
Bacteria.p__Actinobacteria.c__Actinomycetia.o__Micrococcales.f__
0
20000
40000
46−60 (n=7)
80plus (n=3)
61−80 (n=15)
31−45 (n=3)
20−30 (n=2)
Age_category

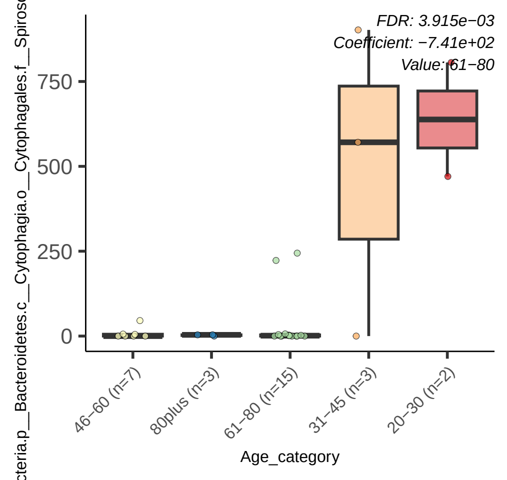

FDR: 3.915e−03
Coefficient: −7.41e+02
Value: 61−80
750
500
250
0
cteria.p__Bacteroidetes.c__Cytophagia.o__Cytophagales.f__Spiros
46−60 (n=7)
80plus (n=3)
61−80 (n=15)
31−45 (n=3)
20−30 (n=2)
Age_category

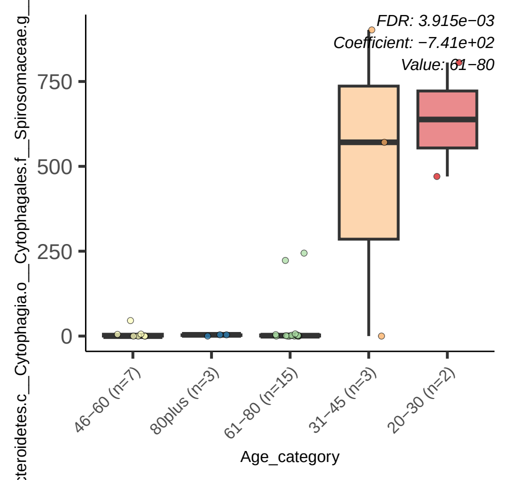

FDR: 3.915e−03
Coefficient: −7.41e+02
Value: 61−80
750
500
250
0
cteroidetes.c__Cytophagia.o__Cytophagales.f__Spirosomaceae.g__
46−60 (n=7)
80plus (n=3)
61−80 (n=15)
31−45 (n=3)
20−30 (n=2)
Age_category

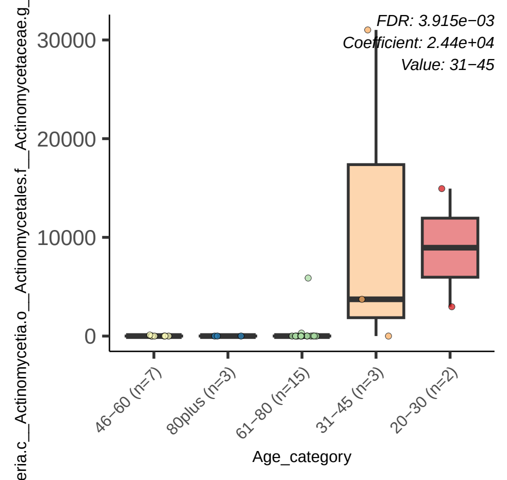

FDR: 3.915e−03
Coefficient: 2.44e+04
Value: 31−45
eria.c__Actinomycetia.o__Actinomycetales.f__Actinomycetaceae.g_
30000
20000
10000
0
46−60 (n=7)
80plus (n=3)
61−80 (n=15)
31−45 (n=3)
20−30 (n=2)
Age_category

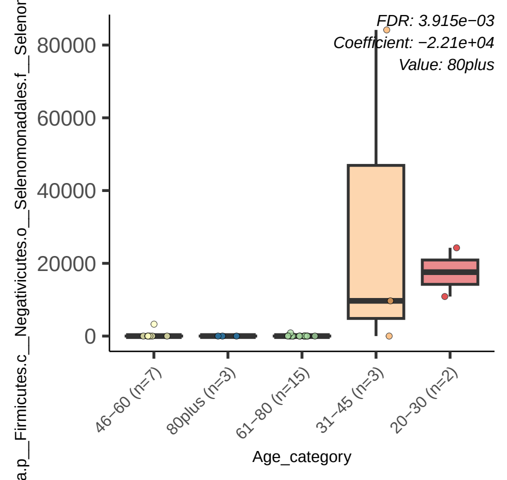

FDR: 3.915e−03
Coefficient: −2.21e+04
Value: 80plus
a.p__Firmicutes.c__Negativicutes.o__Selenomonadales.f__Selenor
80000
60000
40000
20000
0
46−60 (n=7)
80plus (n=3)
61−80 (n=15)
31−45 (n=3)
20−30 (n=2)
Age_category

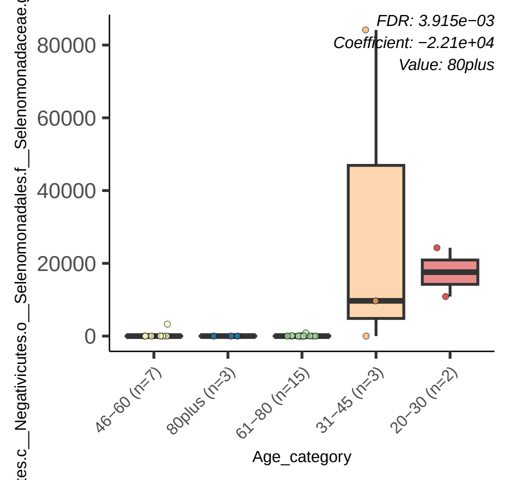

FDR: 3.915e−03
Coefficient: −2.21e+04
Value: 80plus
80000
60000
40000
20000
0
tes.c__Negativicutes.o__Selenomonadales.f__Selenomonadaceae.g
46−60 (n=7)
80plus (n=3)
61−80 (n=15)
31−45 (n=3)
20−30 (n=2)
Age_category

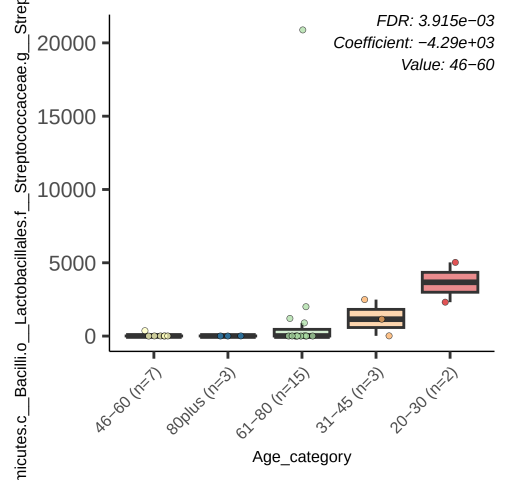

FDR: 3.915e−03
Coefficient: −4.29e+03
Value: 46−60
20000
15000
10000
5000
0
micutes.c__Bacilli.o__Lactobacillales.f__Streptococcaceae.g__Strep
46−60 (n=7)
80plus (n=3)
61−80 (n=15)
31−45 (n=3)
20−30 (n=2)
Age_category

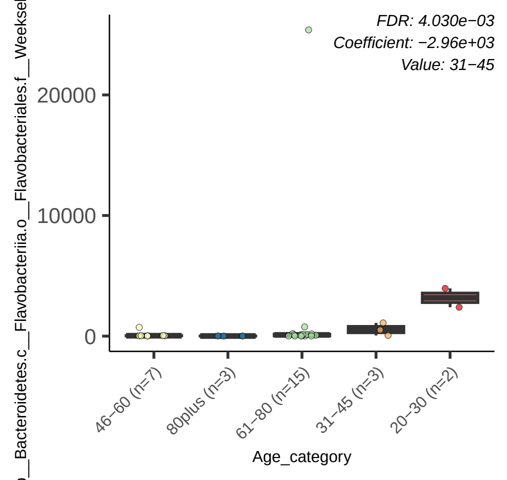

FDR: 4.030e−03
Coefficient: −2.96e+03
Value: 31−45
20000
10000
0
o__Bacteroidetes.c__Flavobacteriia.o__Flavobacteriales.f__Weeksel
46−60 (n=7)
80plus (n=3)
61−80 (n=15)
31−45 (n=3)
20−30 (n=2)
Age_category

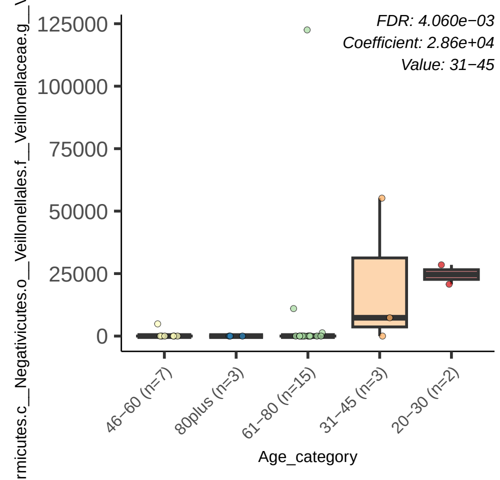

FDR: 4.060e−03
Coefficient: 2.86e+04
Value: 31−45
125000
100000
75000
50000
25000
0
rmicutes.c__Negativicutes.o__Veillonellales.f__Veillonellaceae.g__
46−60 (n=7)
80plus (n=3)
61−80 (n=15)
31−45 (n=3)
20−30 (n=2)
Age_category

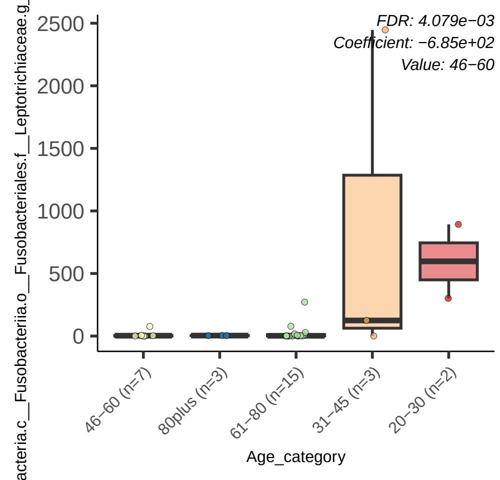

FDR: 4.079e−03
Coefficient: −6.85e+02
Value: 46−60
2500
2000
1500
1000
500
0
46−60 (n=7)
80plus (n=3)
61−80 (n=15)
31−45 (n=3)
20−30 (n=2)
Age_category
acteria.c__Fusobacteriia.o__Fusobacteriales.f__Leptotrichiaceae.g_

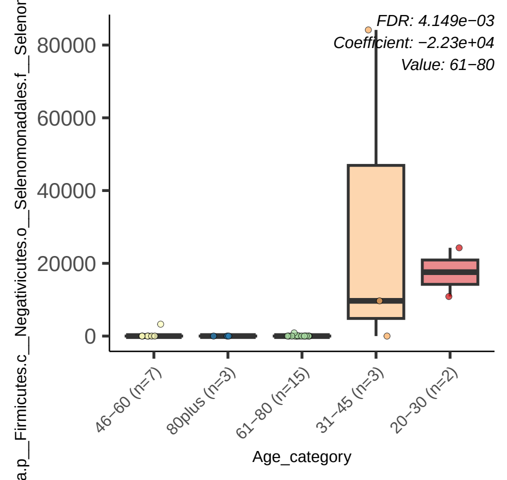
FDR: 4.149e−03
Coefficient: −2.23e+04
Value: 61−80
80000
60000
40000
20000
0
a.p__Firmicutes.c__Negativicutes.o__Selenomonadales.f__Selenor
46−60 (n=7)
80plus (n=3)
61−80 (n=15)
31−45 (n=3)
20−30 (n=2)
Age_category

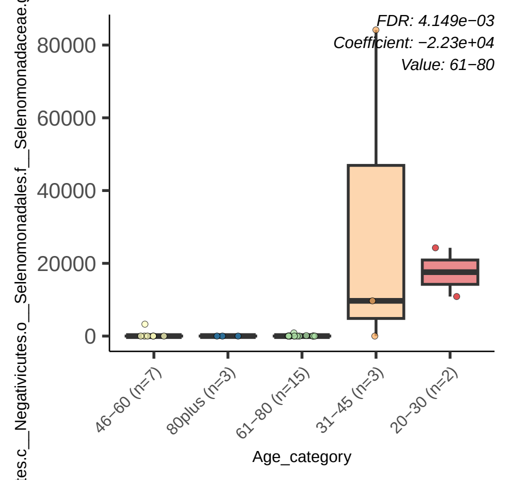

FDR: 4.149e−03
Coefficient: −2.23e+04
Value: 61−80
80000
60000
40000
20000
0
tes.c__Negativicutes.o__Selenomonadales.f__Selenomonadaceae.g
46−60 (n=7)
80plus (n=3)
61−80 (n=15)
31−45 (n=3)
20−30 (n=2)
Age_category

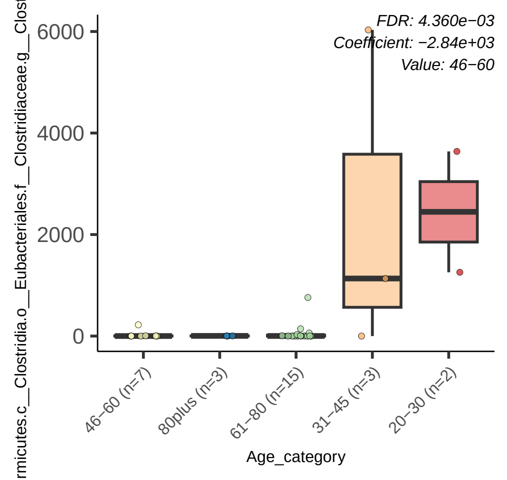
FDR: 4.360e−03
Coefficient: −2.84e+03
Value: 46−60
rmicutes.c__Clostridia.o__Eubacteriales.f__Clostridiaceae.g__Clost
6000
4000
2000
0
46−60 (n=7)
80plus (n=3)
61−80 (n=15)
31−45 (n=3)
20−30 (n=2)
Age_category

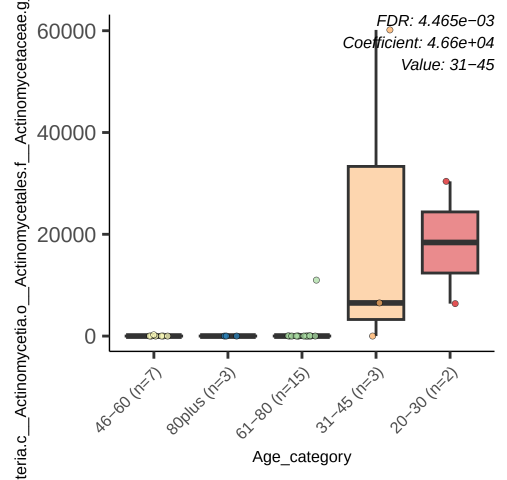
FDR: 4.465e−03
Coefficient: 4.66e+04
Value: 31−45
60000
40000
20000
0
teria.c__Actinomycetia.o__Actinomycetales.f__Actinomycetaceae.g
46−60 (n=7)
80plus (n=3)
61−80 (n=15)
31−45 (n=3)
20−30 (n=2)
Age_category

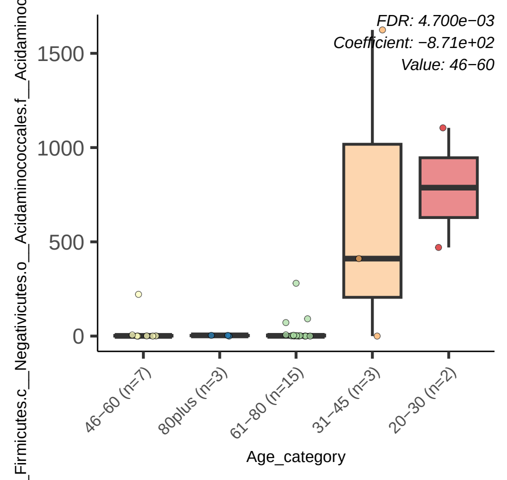

FDR: 4.700e−03
Coefficient: −8.71e+02
Value: 46−60
_Firmicutes.c__Negativicutes.o__Acidaminococcales.f__Acidaminoc
1500
1000
500
0
46−60 (n=7)
80plus (n=3)
61−80 (n=15)
31−45 (n=3)
20−30 (n=2)
Age_category

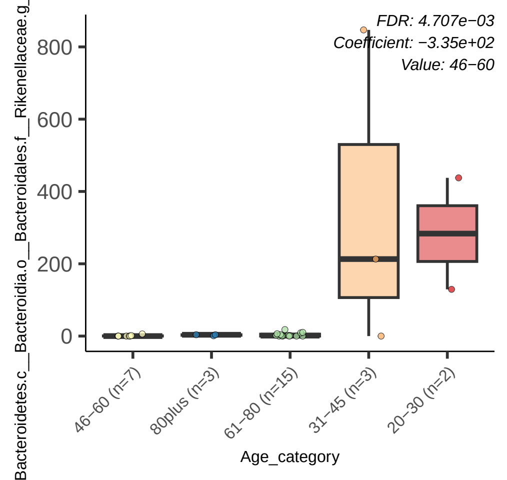

FDR: 4.707e−03
Coefficient: −3.35e+02
Value: 46−60
Bacteroidetes.c__Bacteroidia.o__Bacteroidales.f__Rikenellaceae.g__
800
600
400
200
0
46−60 (n=7)
80plus (n=3)
61−80 (n=15)
31−45 (n=3)
20−30 (n=2)
Age_category

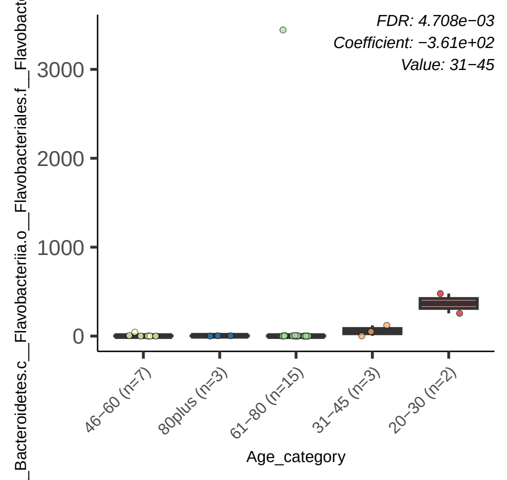

FDR: 4.708e−03
Coefficient: −3.61e+02
Value: 31−45
3000
2000
1000
0
46−60 (n=7)
80plus (n=3)
61−80 (n=15)
31−45 (n=3)
20−30 (n=2)
Age_category
Bacteroidetes.c__Flavobacteriia.o__Flavobacteriales.f__Flavobact

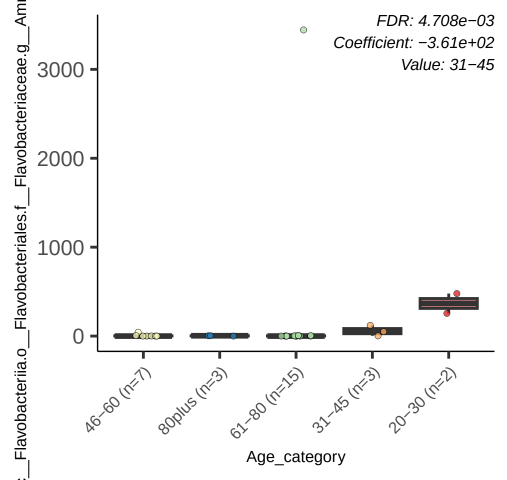

FDR: 4.708e−03
Coefficient: −3.61e+02
Value: 31−45
3000
2000
1000
0
Flavobacteriia.o__Flavobacteriales.f__Flavobacteriaceae.g__Am
46−60 (n=7)
80plus (n=3)
61−80 (n=15)
31−45 (n=3)
20−30 (n=2)
Age_category

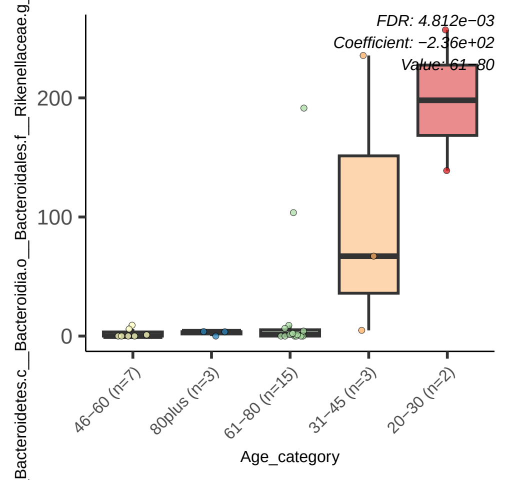

FDR: 4.812e−03
Coefficient: −2.36e+02
Value: 61−80
Bacteroidetes.c__Bacteroidia.o__Bacteroidales.f__Rikenellaceae.g_
200
100
0
46−60 (n=7)
80plus (n=3)
61−80 (n=15)
31−45 (n=3)
20−30 (n=2)
Age_category

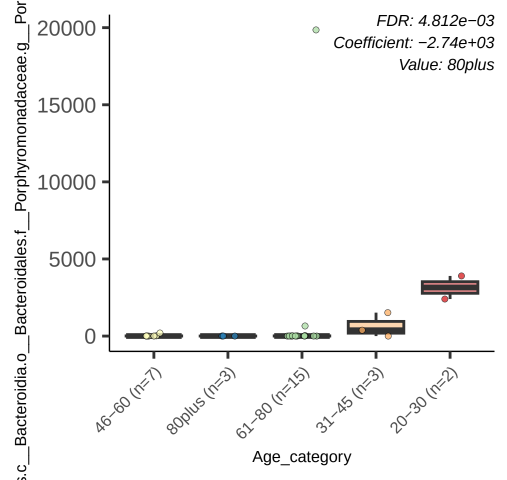

FDR: 4.812e−03
Coefficient: −2.74e+03
Value: 80plus
20000
15000
10000
5000
0
s.c__Bacteroidia.o__Bacteroidales.f__Porphyromonadaceae.g__Por
46−60 (n=7)
80plus (n=3)
61−80 (n=15)
31−45 (n=3)
20−30 (n=2)
Age_category

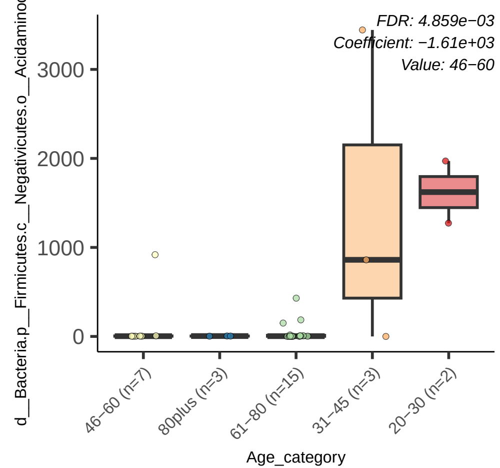

FDR: 4.859e−03
Coefficient: −1.61e+03
Value: 46−60
d__Bacteria.p__Firmicutes.c__Negativicutes.o__Acidaminoc
3000
2000
1000
0
46−60 (n=7)
80plus (n=3)
61−80 (n=15)
31−45 (n=3)
20−30 (n=2)
Age_category

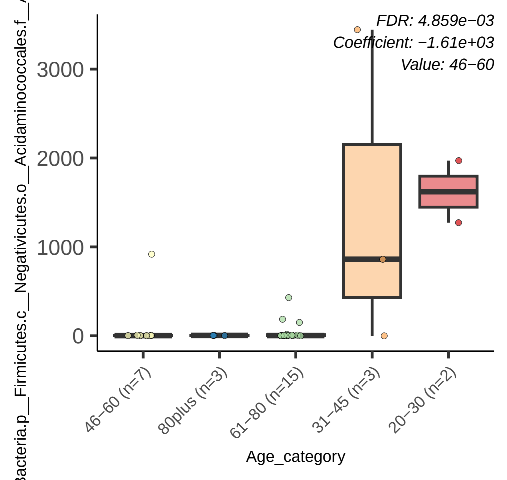

FDR: 4.859e−03
Coefficient: −1.61e+03
Value: 46−60
Bacteria.p__Firmicutes.c__Negativicutes.o__Acidaminococcales.f__
3000
2000
1000
0
46−60 (n=7)
80plus (n=3)
61−80 (n=15)
31−45 (n=3)
20−30 (n=2)
Age_category

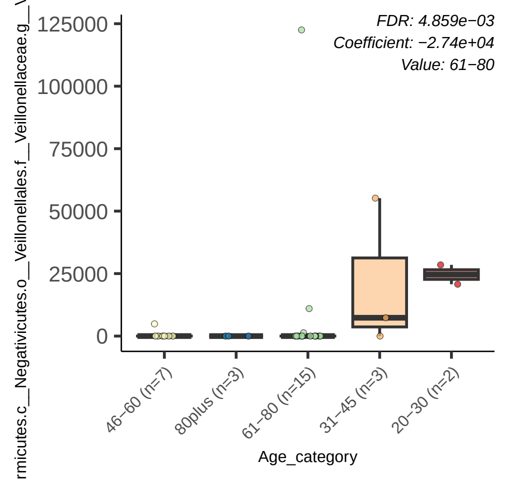

FDR: 4.859e−03
Coefficient: −2.74e+04
Value: 61−80
125000
100000
75000
50000
25000
0
rmicutes.c__Negativicutes.o__Veillonellales.f__Veillonellaceae.g__V
46−60 (n=7)
80plus (n=3)
61−80 (n=15)
31−45 (n=3)
20−30 (n=2)
Age_category

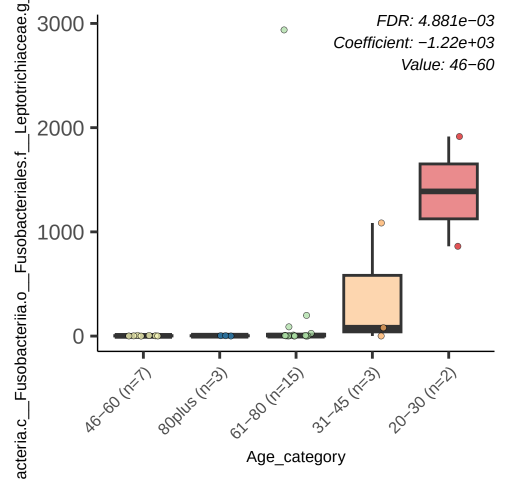

FDR: 4.881e−03
Coefficient: −1.22e+03
Value: 46−60
acteria.c__Fusobacteriia.o__Fusobacteriales.f__Leptotrichiaceae.g_
3000
2000
1000
0
46−60 (n=7)
80plus (n=3)
61−80 (n=15)
31−45 (n=3)
20−30 (n=2)
Age_category

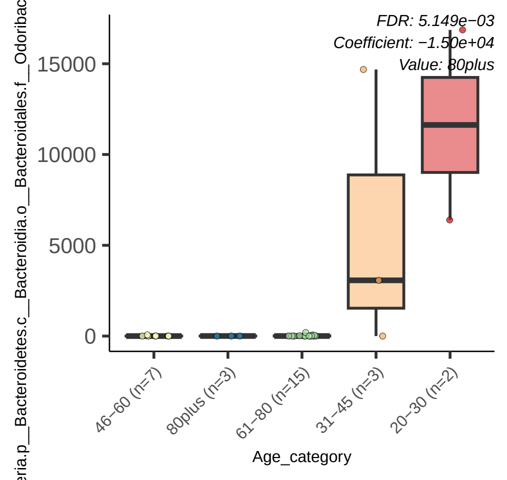

FDR: 5.149e−03
Coefficient: −1.50e+04
Value: 80plus
15000
10000
5000
0
46−60 (n=7)
80plus (n=3)
61−80 (n=15)
31−45 (n=3)
20−30 (n=2)
Age_category
eria.p__Bacteroidetes.c__Bacteroidia.o__Bacteroidales.f__Odoribac

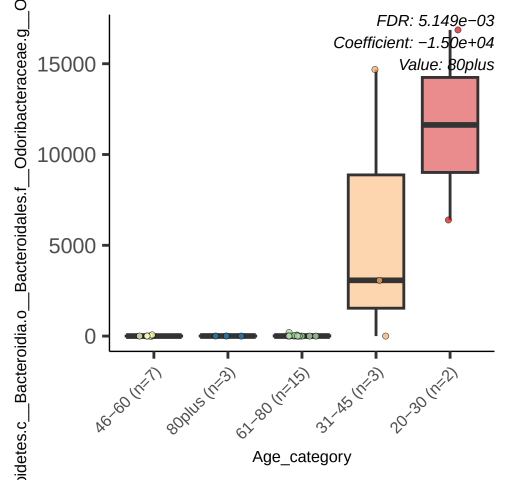

FDR: 5.149e−03
Coefficient: −1.50e+04
Value: 80plus
15000
10000
5000
0
oidetes.c__Bacteroidia.o__Bacteroidales.f__Odoribacteraceae.g__Odo
46−60 (n=7)
80plus (n=3)
61−80 (n=15)
31−45 (n=3)
20−30 (n=2)
Age_category

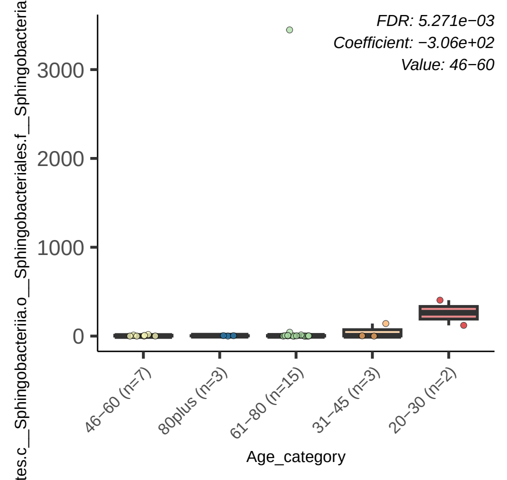

FDR: 5.271e−03
Coefficient: −3.06e+02
Value: 46−60
3000
2000
1000
0
tes.c__Sphingobacteriia.o__Sphingobacteriales.f__Sphingobacteria
46−60 (n=7)
80plus (n=3)
61−80 (n=15)
31−45 (n=3)
20−30 (n=2)
Age_category

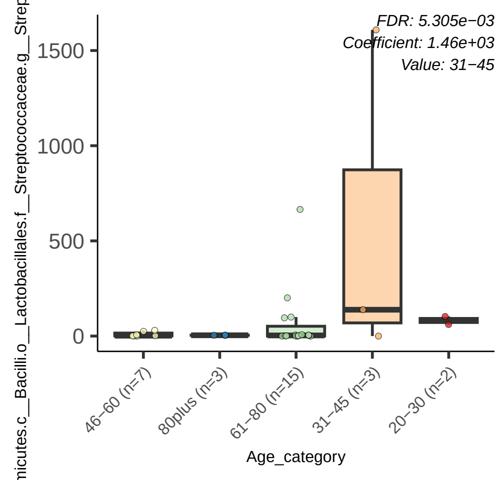

FDR: 5.305e−03
Coefficient: 1.46e+03
Value: 31−45
1500
1000
500
0
micutes.c__Bacilli.o__Lactobacillales.f__Streptococcaceae.g__Strep
46−60 (n=7)
80plus (n=3)
61−80 (n=15)
31−45 (n=3)
20−30 (n=2)
Age_category

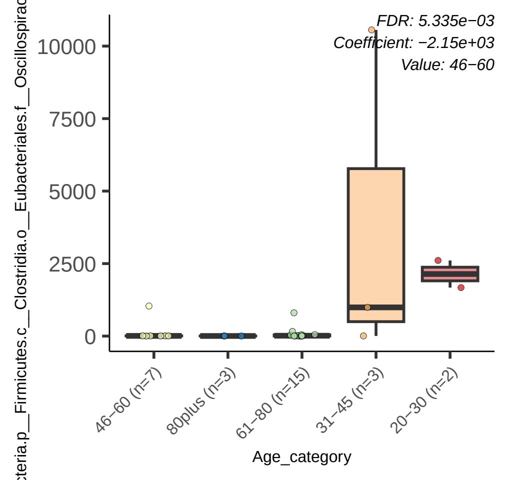

FDR: 5.335e−03
Coefficient: −2.15e+03
Value: 46−60
10000
7500
5000
2500
0
cteria.p__Firmicutes.c__Clostridia.o__Eubacteriales.f__Oscillospirac
46−60 (n=7)
80plus (n=3)
61−80 (n=15)
31−45 (n=3)
20−30 (n=2)
Age_category

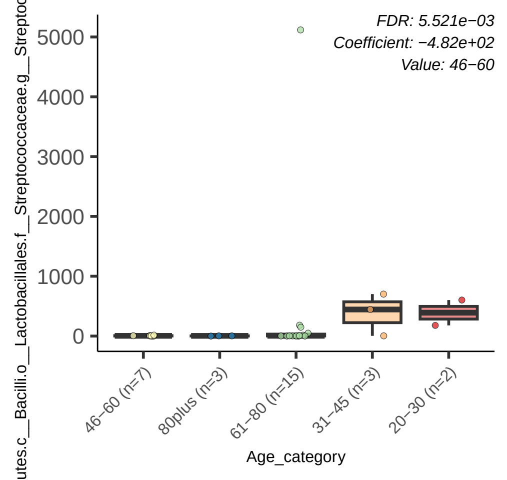

FDR: 5.521e−03
Coefficient: −4.82e+02
Value: 46−60
5000
4000
3000
2000
1000
0
utes.c__Bacilli.o__Lactobacillales.f__Streptococcaceae.g__Streptoc
46−60 (n=7)
80plus (n=3)
61−80 (n=15)
31−45 (n=3)
20−30 (n=2)
Age_category

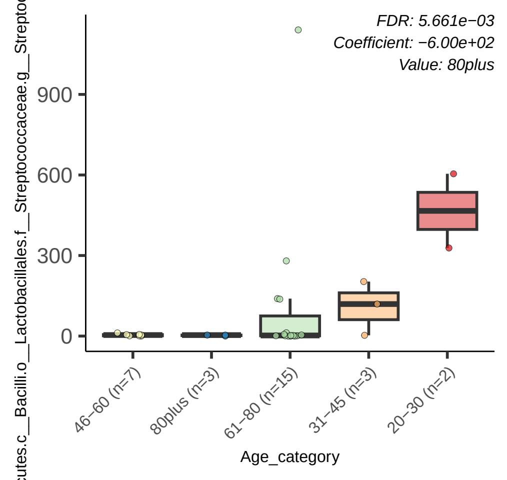

FDR: 5.661e−03
Coefficient: −6.00e+02
Value: 80plus
cutes.c__Bacilli.o__Lactobacillales.f__Streptococcaceae.g__Streptoc
900
600
300
0
46−60 (n=7)
80plus (n=3)
61−80 (n=15)
31−45 (n=3)
20−30 (n=2)
Age_category

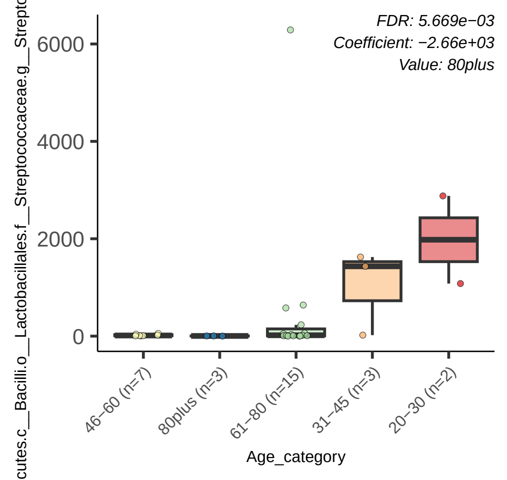

FDR: 5.669e−03
Coefficient: −2.66e+03
Value: 80plus
6000
4000
2000
0
cutes.c__Bacilli.o__Lactobacillales.f__Streptococcaceae.g__Strepto
46−60 (n=7)
80plus (n=3)
61−80 (n=15)
31−45 (n=3)
20−30 (n=2)
Age_category

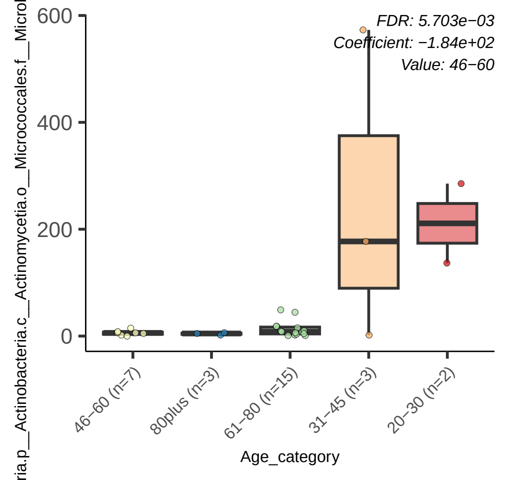
FDR: 5.703e−03
Coefficient: −1.84e+02
Value: 46−60
600
400
200
0
ria.p__Actinobacteria.c__Actinomycetia.o__Micrococcales.f__Microb
46−60 (n=7)
80plus (n=3)
61−80 (n=15)
31−45 (n=3)
20−30 (n=2)
Age_category

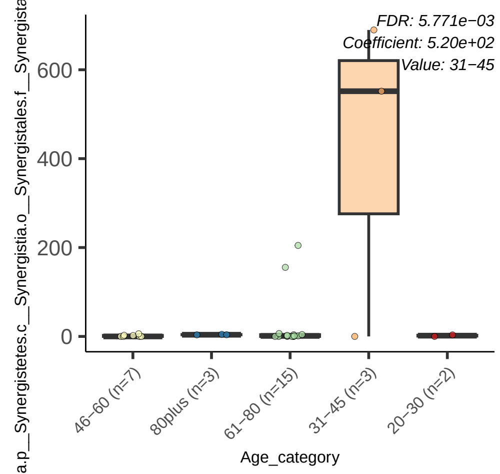

FDR: 5.771e−03
Coefficient: 5.20e+02
Value: 31−45
600
400
200
0
ria.p__Synergistetes.c__Synergistia.o__Synergistales.f__Synergista
46−60 (n=7)
80plus (n=3)
61−80 (n=15)
31−45 (n=3)
20−30 (n=2)
Age_category

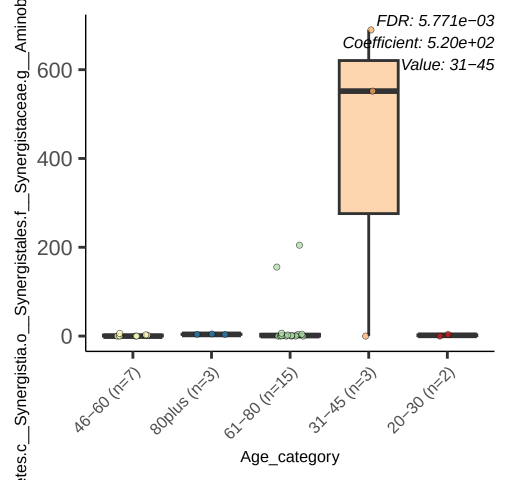

FDR: 5.771e−03
Coefficient: 5.20e+02
Value: 31−45
0
200
400
600
etes.c__Synergistia.o__Synergistales.f__Synergistaceae.g__Aminob
46−60 (n=7)
80plus (n=3)
61−80 (n=15)
31−45 (n=3)
20−30 (n=2)
Age_category

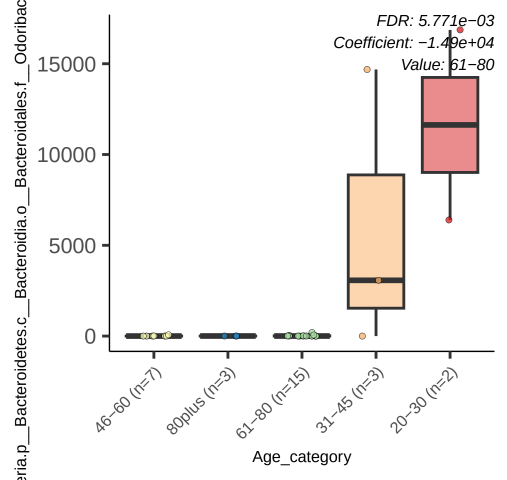

FDR: 5.771e−03
Coefficient: −1.49e+04
Value: 61−80
eria.p__Bacteroidetes.c__Bacteroidia.o__Bacteroidales.f__Odoribac
15000
10000
5000
0
46−60 (n=7)
80plus (n=3)
61−80 (n=15)
31−45 (n=3)
20−30 (n=2)
Age_category

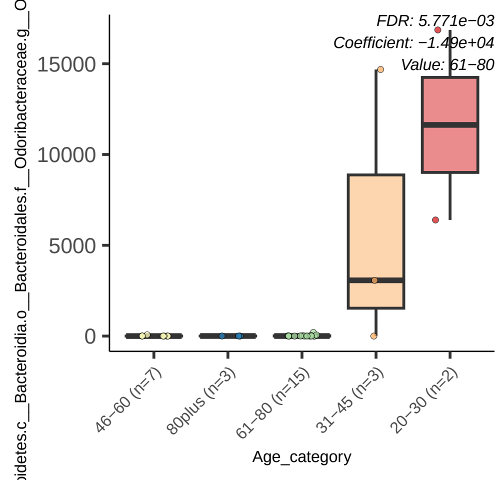

FDR: 5.771e−03
Coefficient: −1.49e+04
Value: 61−80
oidetes.c__Bacteroidia.o__Bacteroidales.f__Odoribacteraceae.g__Odori
0
5000
10000
15000
46−60 (n=7)
80plus (n=3)
61−80 (n=15)
31−45 (n=3)
20−30 (n=2)
Age_category

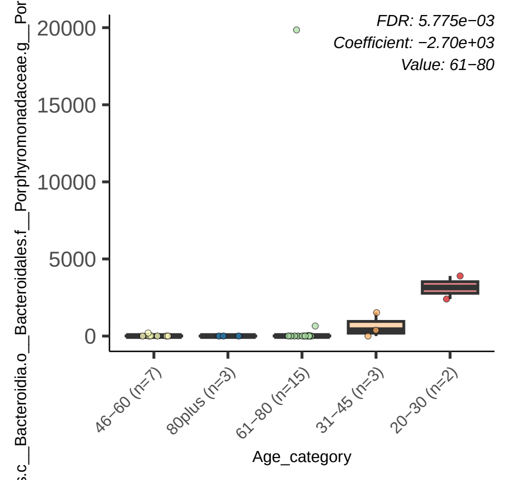

FDR: 5.775e−03
Coefficient: −2.70e+03
Value: 61−80
20000
15000
10000
5000
0
s.c__Bacteroidia.o__Bacteroidales.f__Porphyromonadaceae.g__Por
46−60 (n=7)
80plus (n=3)
61−80 (n=15)
31−45 (n=3)
20−30 (n=2)
Age_category

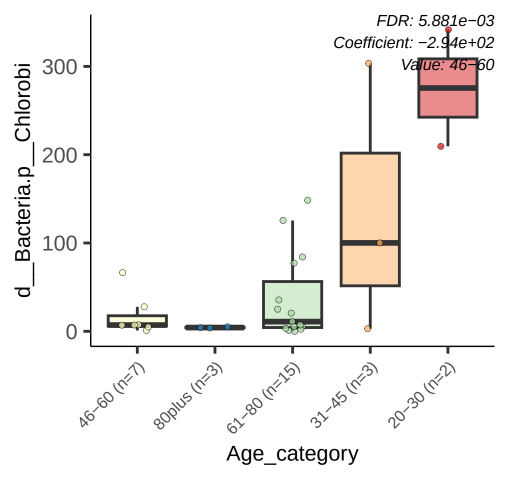

FDR: 5.881e−03
Coefficient: −2.94e+02
Value: 46−60
d__Bacteria.p__Chlorobi
300
200
100
0
46−60 (n=7)
80plus (n=3)
61−80 (n=15)
31−45 (n=3)
20−30 (n=2)
Age_category

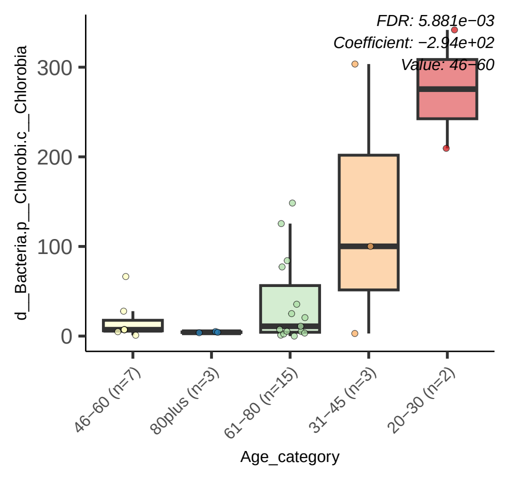
FDR: 5.881e−03
Coefficient: −2.94e+02
Value: 46−60
d__Bacteria.p__Chlorobi.c__Chlorobia
300
200
100
0
46−60 (n=7)
80plus (n=3)
61−80 (n=15)
31−45 (n=3)
20−30 (n=2)
Age_category

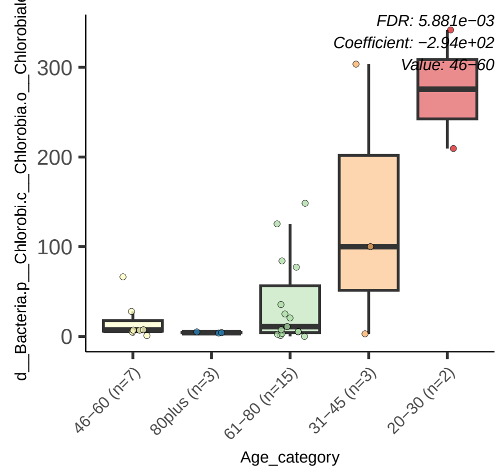

FDR: 5.881e−03
Coefficient: −2.94e+02
Value: 46−60
d__Bacteria.p__Chlorobi.c__Chlorobia.o__Chlorobiale
300
200
100
0
46−60 (n=7)
80plus (n=3)
61−80 (n=15)
31−45 (n=3)
20−30 (n=2)
Age_category

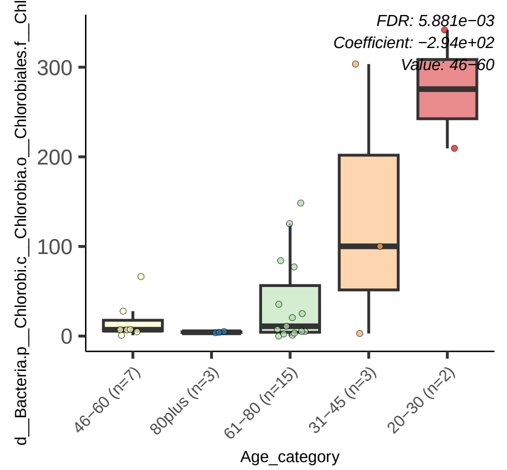

FDR: 5.881e−03
Coefficient: −2.94e+02
Value: 46−60
d__Bacteria.p__Chlorobi.c__Chlorobia.o__Chlorobiales.f__Chl
300
200
100
0
46−60 (n=7)
80plus (n=3)
61−80 (n=15)
31−45 (n=3)
20−30 (n=2)
Age_category

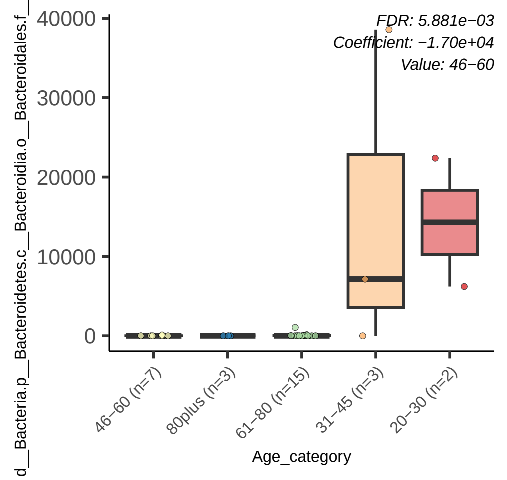
FDR: 5.881e−03
Coefficient: −1.70e+04
Value: 46−60
d__Bacteria.p__Bacteroidetes.c__Bacteroidia.o__Bacteroidales.f
40000
30000
20000
10000
0
46−60 (n=7)
80plus (n=3)
61−80 (n=15)
31−45 (n=3)
20−30 (n=2)
Age_category

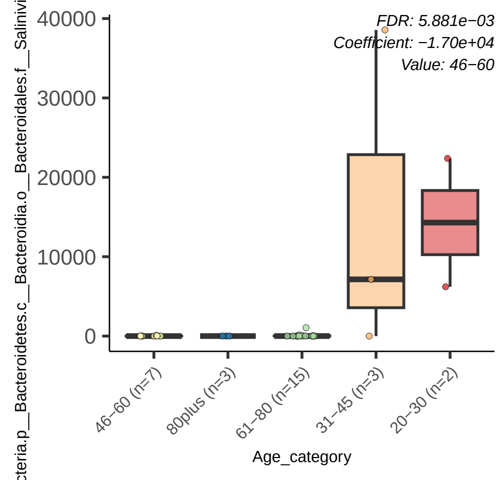

FDR: 5.881e−03
Coefficient: −1.70e+04
Value: 46−60
40000
30000
20000
10000
0
46−60 (n=7)
80plus (n=3)
61−80 (n=15)
31−45 (n=3)
20−30 (n=2)
Age_category
cteria.p__Bacteroidetes.c__Bacteroidia.o__Bacteroidales.f__Salinivi

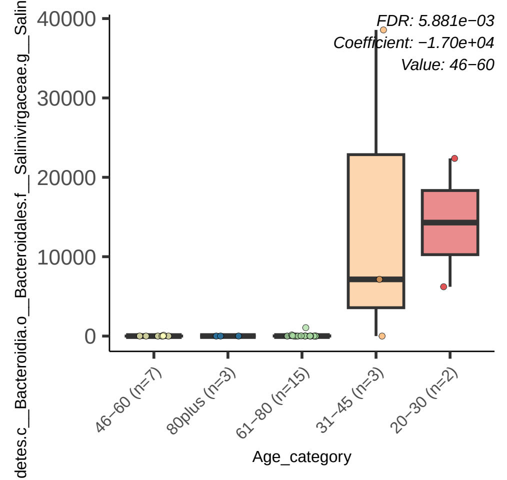

detes.c__Bacteroidia.o__Bacteroidales.f__Salinivirgaceae.g__Salin
40000
30000
20000
10000
0
FDR: 5.881e−03
Coefficient: −1.70e+04
Value: 46−60
46−60 (n=7)
80plus (n=3)
61−80 (n=15)
31−45 (n=3)
20−30 (n=2)
Age_category

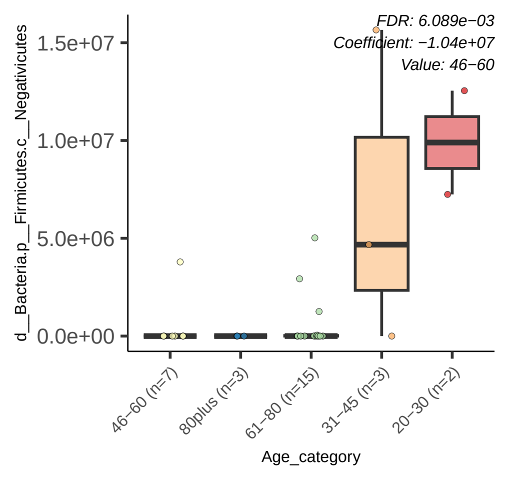

FDR: 6.089e−03
Coefficient: −1.04e+07
Value: 46−60
d__Bacteria.p__Firmicutes.c__Negativicutes
1.5e+07
1.0e+07
5.0e+06
0.0e+00
46−60 (n=7)
80plus (n=3)
61−80 (n=15)
31−45 (n=3)
20−30 (n=2)
Age_category

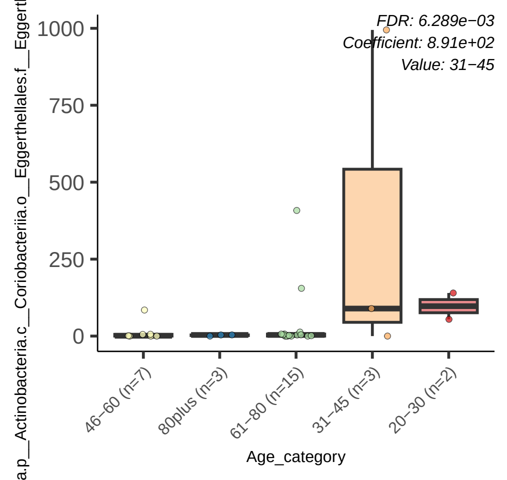

FDR: 6.289e−03
Coefficient: 8.91e+02
Value: 31−45
1000
750
500
250
0
46−60 (n=7)
80plus (n=3)
61−80 (n=15)
31−45 (n=3)
20−30 (n=2)
Age_category
a.p__Actinobacteria.c__Coriobacteriia.o__Eggerthellales.f__Eggerth

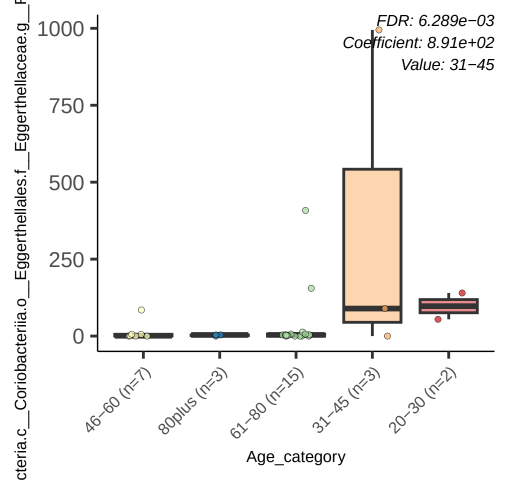
FDR: 6.289e−03
Coefficient: 8.91e+02
Value: 31−45
1000
750
500
250
0
46−60 (n=7)
80plus (n=3)
61−80 (n=15)
31−45 (n=3)
20−30 (n=2)
Age_category
cteria.c__Coriobacteriia.o__Eggerthellales.f__Eggerthellaceae.g__F

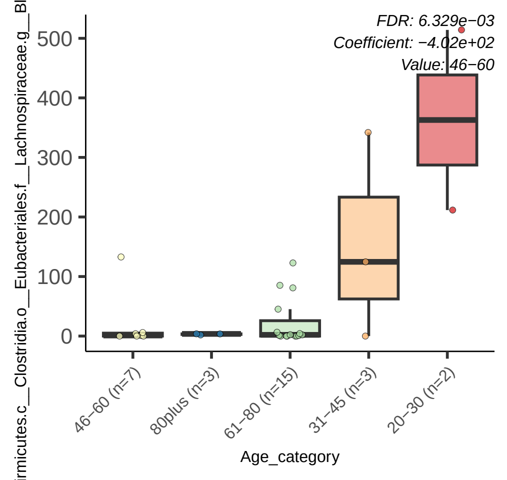

FDR: 6.329e−03
Coefficient: −4.02e+02
Value: 46−60
500
400
300
200
100
0
irmicutes.c__Clostridia.o__Eubacteriales.f__Lachnospiraceae.g__Bl
46−60 (n=7)
80plus (n=3)
61−80 (n=15)
31−45 (n=3)
20−30 (n=2)
Age_category

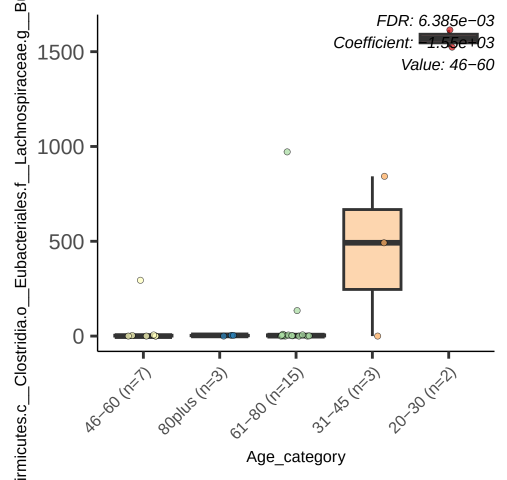

FDR: 6.385e−03
Coefficient: −1.55e+03
Value: 46−60
1500
1000
500
0
irmicutes.c__Clostridia.o__Eubacteriales.f__Lachnospiraceae.g__B
46−60 (n=7)
80plus (n=3)
61−80 (n=15)
31−45 (n=3)
20−30 (n=2)
Age_category

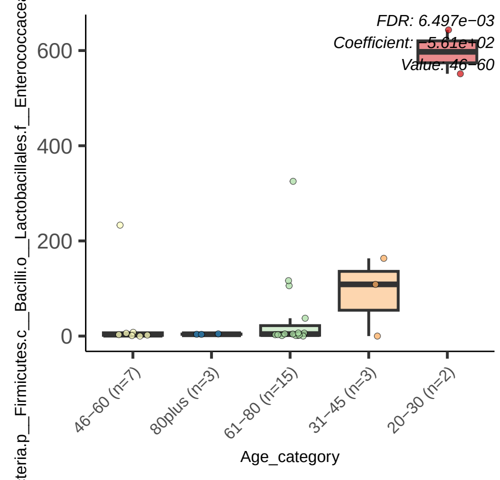

FDR: 6.497e−03
Coefficient: 5.61e+02
Value: 46−60
600
400
200
0
46−60 (n=7)
80plus (n=3)
61−80 (n=15)
31−45 (n=3)
20−30 (n=2)
Age_category
Firmicutes.c__Bacilli.o__Lactobacillales.f__Enterococcace

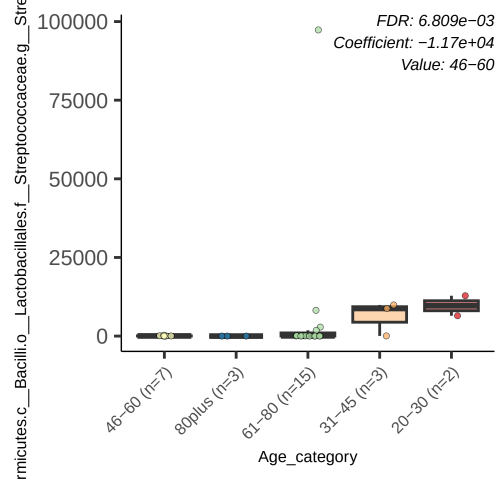

rmicutes.c__Bacilli.o__Lactobacillales.f__Streptococcaceae.g__Stre
100000
75000
50000
25000
0
FDR: 6.809e−03
Coefficient: −1.17e+04
Value: 46−60
46−60 (n=7)
80plus (n=3)
61−80 (n=15)
31−45 (n=3)
20−30 (n=2)
Age_category

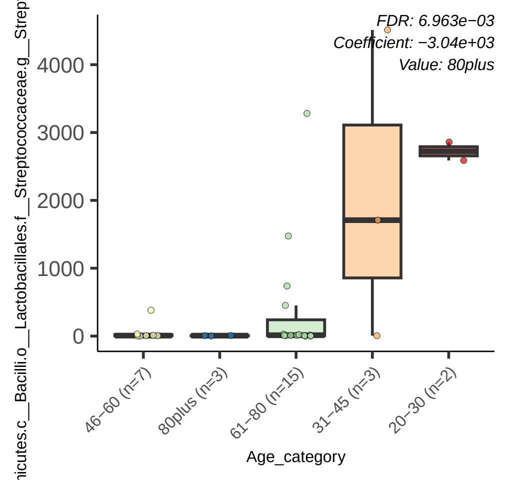
FDR: 6.963e−03
Coefficient: −3.04e+03
Value: 80plus
4000
3000
2000
1000
0
46−60 (n=7)
80plus (n=3)
61−80 (n=15)
31−45 (n=3)
20−30 (n=2)
Age_category
nicutes.c__Bacilli.o__Lactobacillales.f__Streptococcaceae.g__Strep

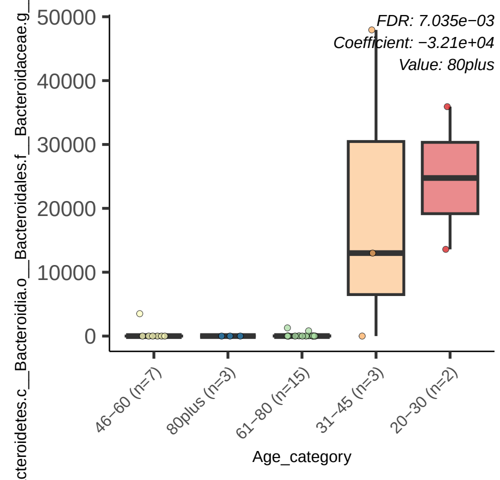
FDR: 7.035e−03
Coefficient: −3.21e+04
Value: 80plus
50000
40000
30000
20000
10000
0
cteroidetes.c__Bacteroidia.o__Bacteroidales.f__Bacteroidaceae.g__
46−60 (n=7)
80plus (n=3)
61−80 (n=15)
31−45 (n=3)
20−30 (n=2)
Age_category

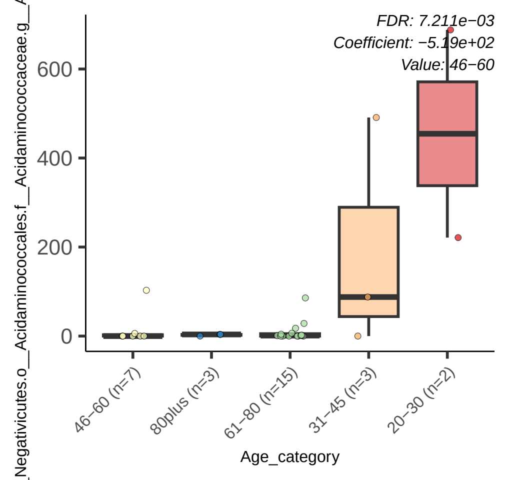

FDR: 7.211e−03
Coefficient: −5.19e+02
Value: 46−60
0
200
400
600
Negativicutes.o__Acidaminococcales.f__Acidaminococcaceae.g__A
46−60 (n=7)
80plus (n=3)
61−80 (n=15)
31−45 (n=3)
20−30 (n=2)
Age_category

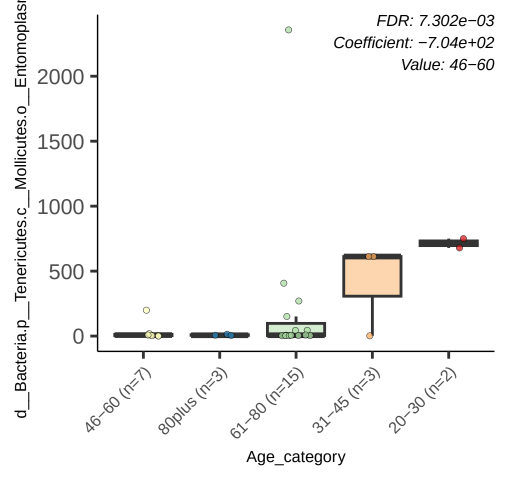

FDR: 7.302e−03
Coefficient: −7.04e+02
Value: 46−60
d__Bacteria.p__Tenericutes.c__Mollicutes.o__Entomoplasm
2000
1500
1000
500
0
46−60 (n=7)
80plus (n=3)
61−80 (n=15)
31−45 (n=3)
20−30 (n=2)
Age_category

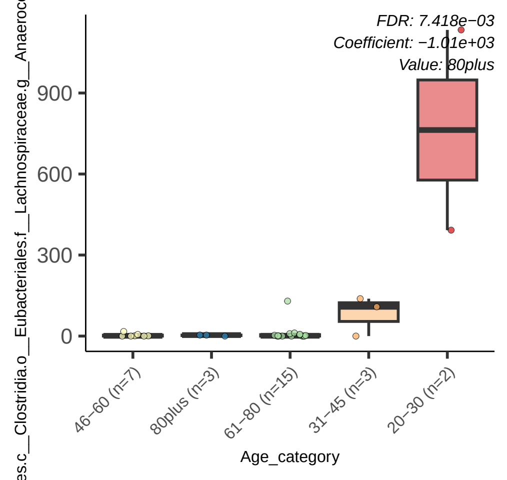

FDR: 7.418e−03
Coefficient: −1.01e+03
Value: 80plus
900
600
300
0
es.c__Clostridia.o__Eubacteriales.f__Lachnospiraceae.g__Anaeroc
46−60 (n=7)
80plus (n=3)
61−80 (n=15)
31−45 (n=3)
20−30 (n=2)
Age_category

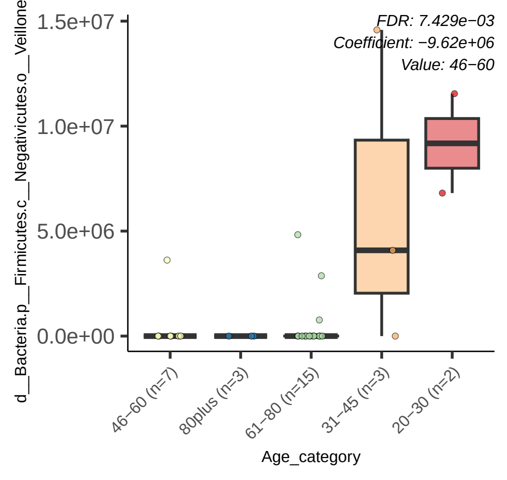

FDR: 7.429e−03
Coefficient: −9.62e+06
Value: 46−60
1.5e+07
1.0e+07
5.0e+06
0.0e+00
d__Bacteria.p__Firmicutes.c__Negativicutes.o__Veillone
46−60 (n=7)
80plus (n=3)
61−80 (n=15)
31−45 (n=3)
20−30 (n=2)
Age_category

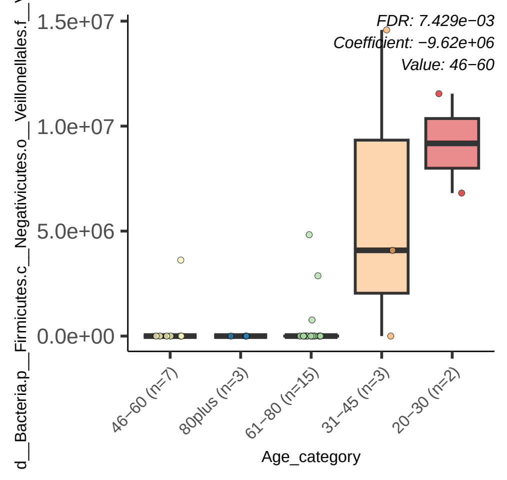

d__Bacteria.p__Firmicutes.c__Negativicutes.o__Veillonellales.f__V
1.5e+07
1.0e+07
5.0e+06
0.0e+00
FDR: 7.429e−03
Coefficient: −9.62e+06
Value: 46−60
46−60 (n=7)
80plus (n=3)
61−80 (n=15)
31−45 (n=3)
20−30 (n=2)
Age_category

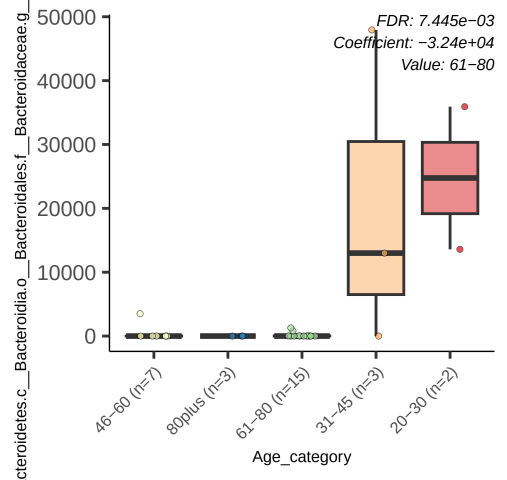
FDR: 7.445e−03
Coefficient: −3.24e+04
Value: 61−80
cteroidetes.c__Bacteroidia.o__Bacteroidales.f__Bacteroidaceae.g__
50000
40000
30000
20000
10000
0
46−60 (n=7)
80plus (n=3)
61−80 (n=15)
31−45 (n=3)
20−30 (n=2)
Age_category

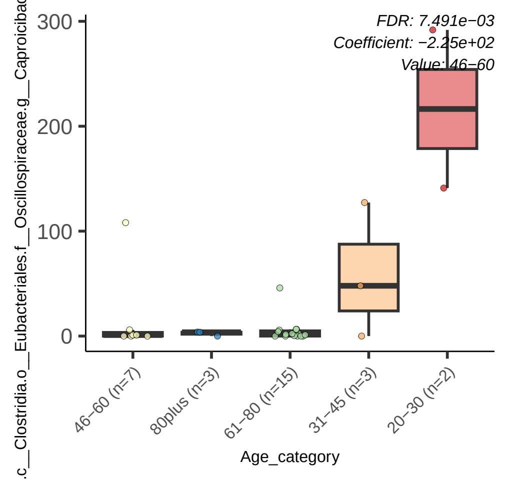

c__Clostridia.o__Eubacteriales.f__Oscillospiraceae.g__Caproicibac
FDR: 7.491e−03
Coefficient: −2.25e+02
Value: 46−60
300
200
100
0
46−60 (n=7)
80plus (n=3)
61−80 (n=15)
31−45 (n=3)
20−30 (n=2)
Age_category

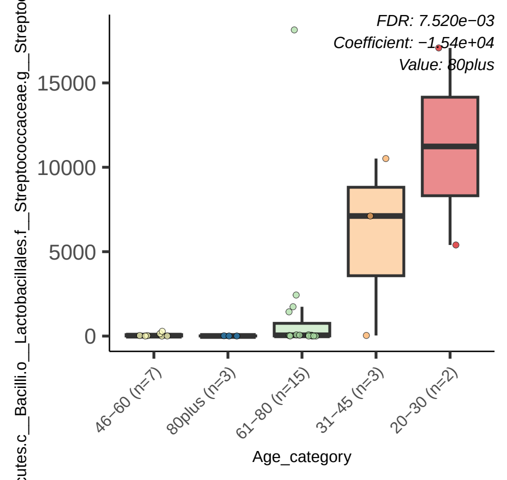

FDR: 7.520e−03
Coefficient: −1.54e+04
Value: 80plus
15000
10000
5000
0
46−60 (n=7)
80plus (n=3)
61−80 (n=15)
31−45 (n=3)
20−30 (n=2)
Age_category

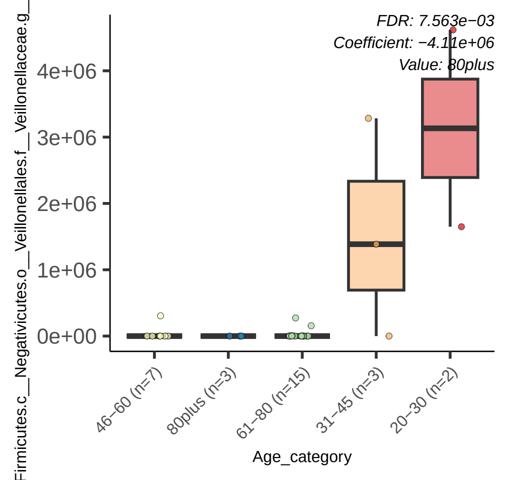
FDR: 7.563e−03
Coefficient: −4.11e+06
Value: 80plus
Firmicutes.c__Negativicutes.o__Veillonellales.f__Veillonellaceae.g__
4e+06
3e+06
2e+06
1e+06
0e+00
46−60 (n=7)
80plus (n=3)
61−80 (n=15)
31−45 (n=3)
20−30 (n=2)
Age_category

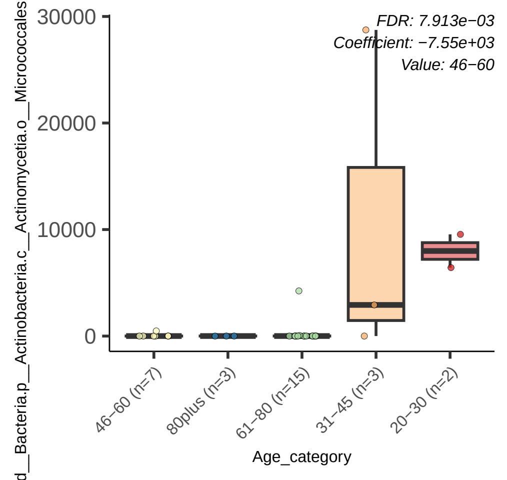

FDR: 7.913e−03
Coefficient: −7.55e+03
Value: 46−60
d__Bacteria.p__Actinobacteria.c__Actinomycetia.o__Micrococcales
30000
20000
10000
0
46−60 (n=7)
80plus (n=3)
61−80 (n=15)
31−45 (n=3)
20−30 (n=2)
Age_category

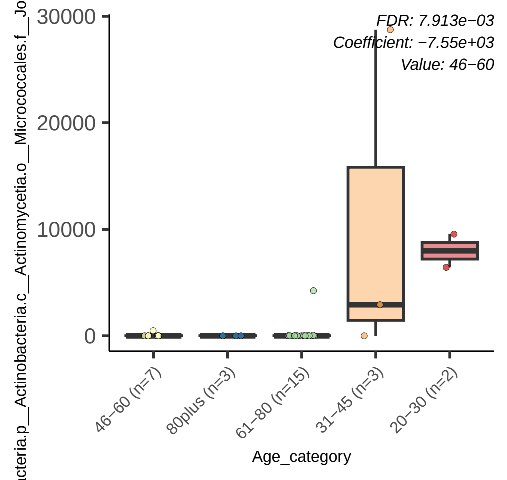

FDR: 7.913e−03
Coefficient: −7.55e+03
Value: 46−60
30000
20000
10000
0
46−60 (n=7)
80plus (n=3)
61−80 (n=15)
31−45 (n=3)
20−30 (n=2)
Age_category

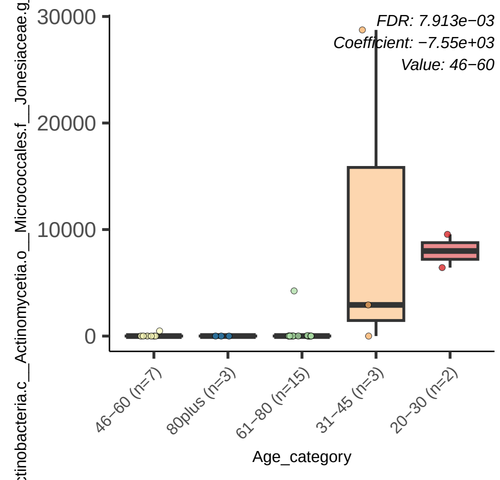

FDR: 7.913e−03
Coefficient: −7.55e+03
Value: 46−60
30000
20000
10000
0
46−60 (n=7)
80plus (n=3)
61−80 (n=15)
31−45 (n=3)
20−30 (n=2)
Age_category

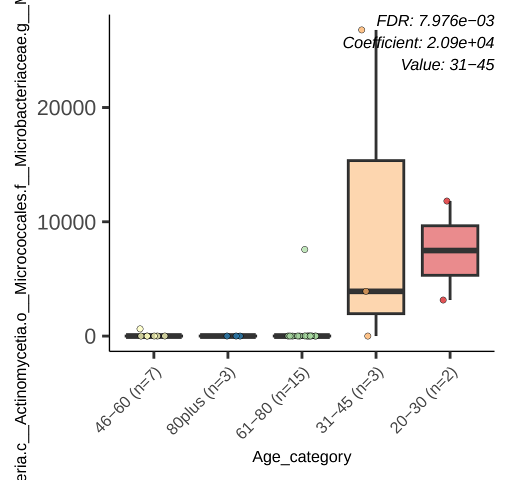

FDR: 7.976e−03
Coefficient: 2.09e+04
Value: 31−45
20000
10000
0
46−60 (n=7)
80plus (n=3)
61−80 (n=15)
31−45 (n=3)
20−30 (n=2)
Age_category
eria.c__Actinomycetia.o__Micrococcales.f__Microbacteriaceae.g__M

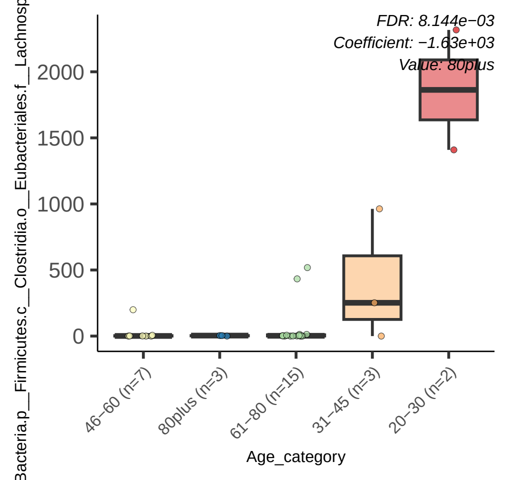

FDR: 8.144e−03
Coefficient: −1.63e+03
Value: 80plus
Bacteria.p__Firmicutes.c__Clostridia.o__Eubacteriales.f__Lachnosp
2000
1500
1000
500
0
46−60 (n=7)
80plus (n=3)
61−80 (n=15)
31−45 (n=3)
20−30 (n=2)
Age_category

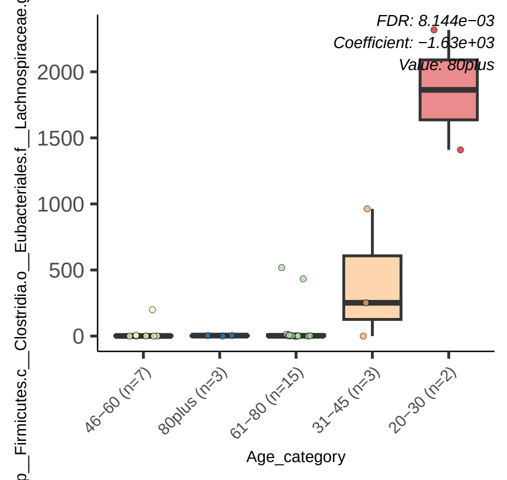

FDR: 8.144e−03
Coefficient: −1.63e+03
Value: 80plus
2000
1500
1000
500
0
Firmicutes.c__Clostridia.o__Eubacteriales.f__Lachnospiraceae.g
46−60 (n=7)
80plus (n=3)
61−80 (n=15)
31−45 (n=3)
20−30 (n=2)
Age_category

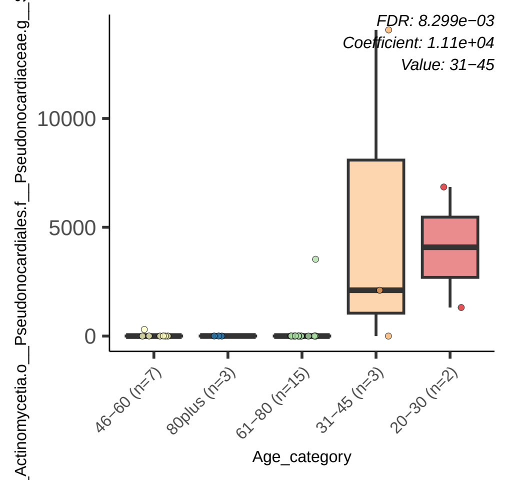

FDR: 8.299e−03
Coefficient: 1.11e+04
Value: 31−45
10000
5000
0
Actinomycetia.o__Pseudonocardiales.f__Pseudonocardiaceae.g__
46−60 (n=7)
80plus (n=3)
61−80 (n=15)
31−45 (n=3)
20−30 (n=2)
Age_category

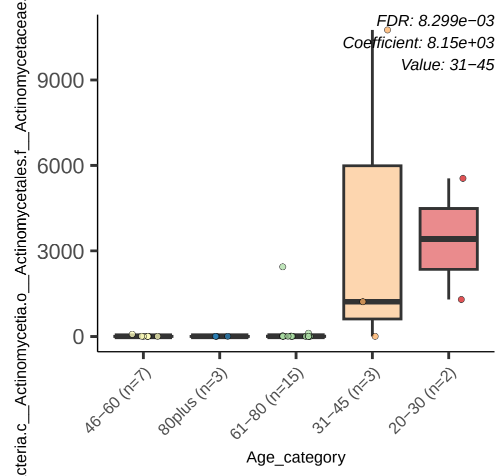

FDR: 8.299e−03
Coefficient: 8.15e+03
Value: 31−45
9000
6000
3000
0
acteria.c__Actinomycetia.o__Actinomycetales.f__Actinomycetaceae.
46−60 (n=7)
80plus (n=3)
61−80 (n=15)
31−45 (n=3)
20−30 (n=2)
Age_category

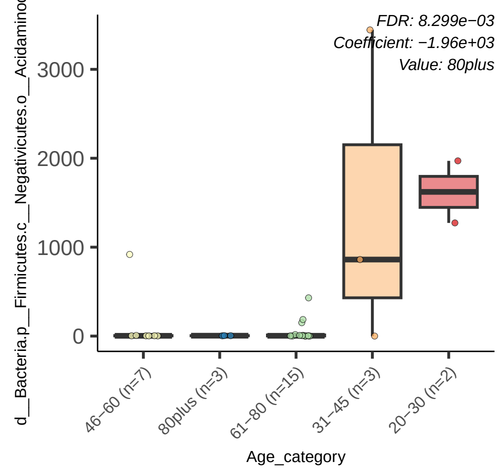

FDR: 8.299e−03
Coefficient: −1.96e+03
Value: 80plus
3000
2000
1000
0
d__Bacteria.p__Firmicutes.c__Negativicutes.o__Acidaminoc
46−60 (n=7)
80plus (n=3)
61−80 (n=15)
31−45 (n=3)
20−30 (n=2)
Age_category

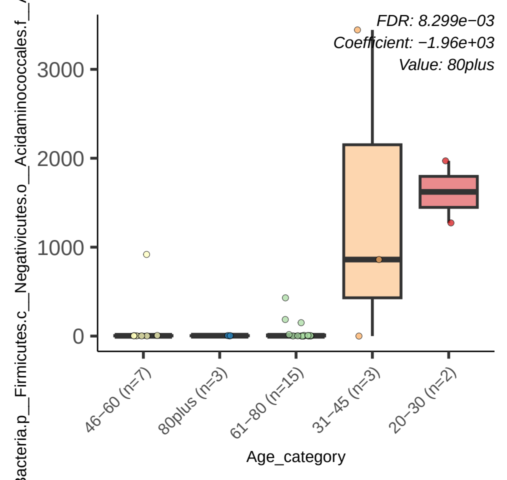

FDR: 8.299e−03
Coefficient: −1.96e+03
Value: 80plus
Bacteria.p__Firmicutes.c__Negativicutes.o__Acidaminococcales.f__
3000
2000
1000
0
46−60 (n=7)
80plus (n=3)
61−80 (n=15)
31−45 (n=3)
20−30 (n=2)
Age_category

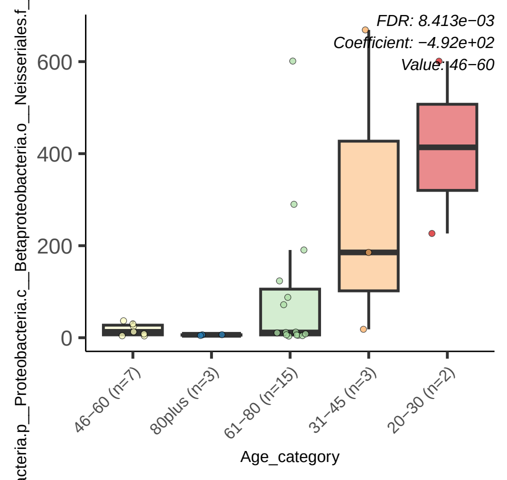

FDR: 8.413e−03
Coefficient: −4.92e+02
Value: 46−60
600
400
200
0
acteria.p__Proteobacteria.c__Betaproteobacteria.o__Neisseriales.f_
46−60 (n=7)
80plus (n=3)
61−80 (n=15)
31−45 (n=3)
20−30 (n=2)
Age_category

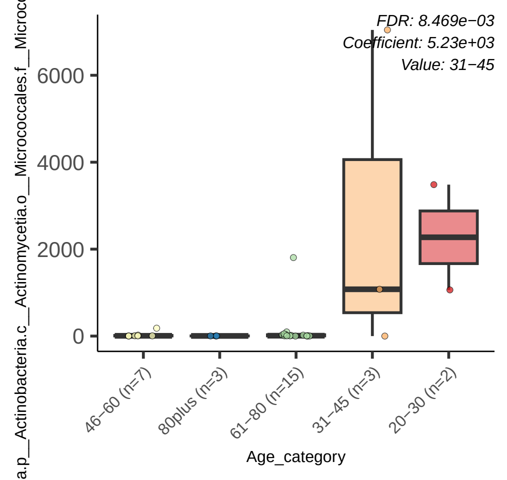

FDR: 8.469e−03
Coefficient: 5.23e+03
Value: 31−45
a.p__Actinobacteria.c__Actinomycetia.o__Micrococcales.f__Microco
6000
4000
2000
0
46−60 (n=7)
80plus (n=3)
61−80 (n=15)
31−45 (n=3)
20−30 (n=2)
Age_category

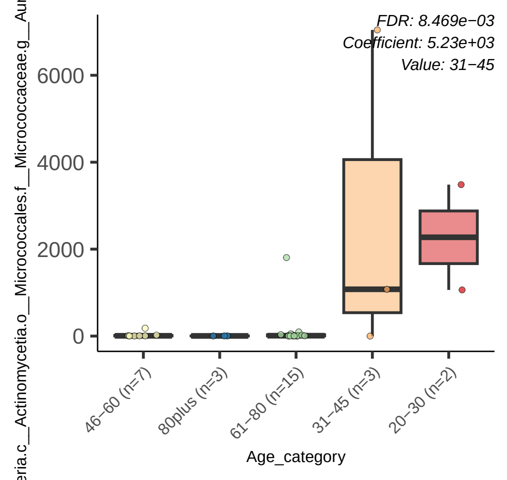

FDR: 8.469e−03
Coefficient: 5.23e+03
Value: 31−45
6000
4000
2000
0
46−60 (n=7)
80plus (n=3)
61−80 (n=15)
31−45 (n=3)
20−30 (n=2)
Age_category
eria.c__Actinomycetia.o__Micrococcales.f__Micrococcaceae.g__Aur

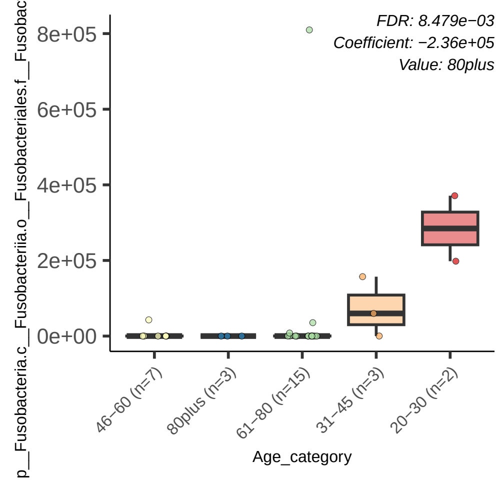
FDR: 8.479e−03
Coefficient: −2.36e+05
Value: 80plus
8e+05
6e+05
4e+05
2e+05
0e+00
p__Fusobacteria.c__Fusobacteriia.o__Fusobacteriales.f__Fusobac
46−60 (n=7)
80plus (n=3)
61−80 (n=15)
31−45 (n=3)
20−30 (n=2)
Age_category

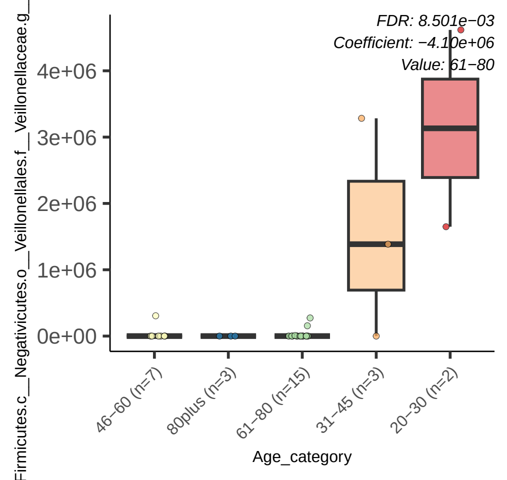

FDR: 8.501e−03
Coefficient: −4.10e+06
Value: 61−80
4e+06
3e+06
2e+06
1e+06
0e+00
Firmicutes.c__Negativicutes.o__Veillonellales.f__Veillonellaceae.g__
46−60 (n=7)
80plus (n=3)
61−80 (n=15)
31−45 (n=3)
20−30 (n=2)
Age_category

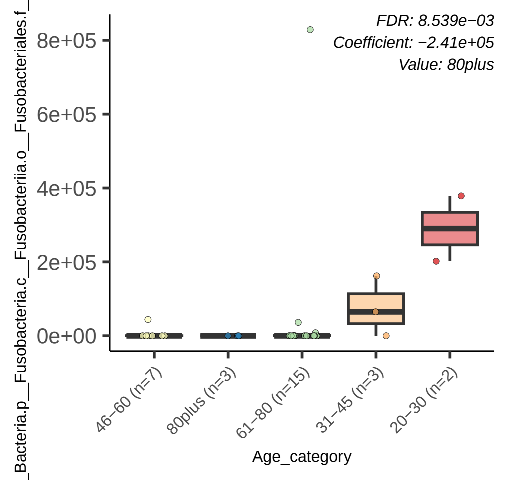

FDR: 8.539e−03
Coefficient: −2.41e+05
Value: 80plus
8e+05
6e+05
4e+05
2e+05
0e+00
Bacteria.p__Fusobacteria.c__Fusobacteriia.o__Fusobacteriales.f
46−60 (n=7)
80plus (n=3)
61−80 (n=15)
31−45 (n=3)
20−30 (n=2)
Age_category

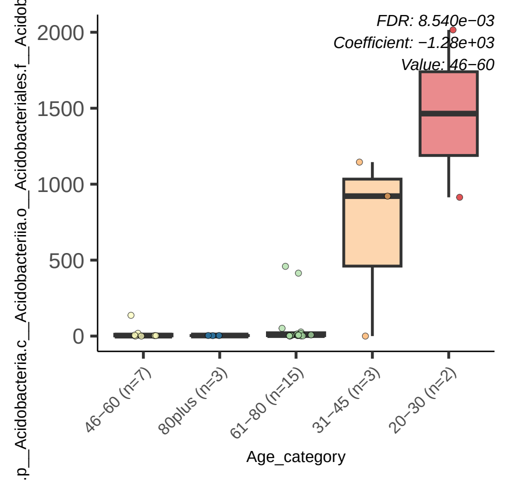

FDR: 8.540e−03
Coefficient: −1.28e+03
Value: 46−60
2000
1500
1000
500
0
p__Acidobacteria.c__Acidobacteriia.o__Acidobacteriales.f__Acidob
46−60 (n=7)
80plus (n=3)
61−80 (n=15)
31−45 (n=3)
20−30 (n=2)
Age_category

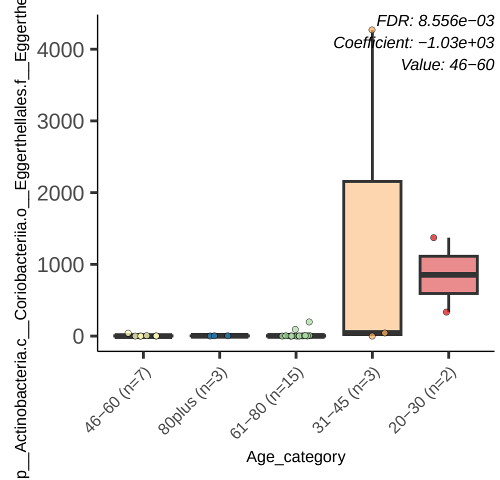

FDR: 8.556e−03
Coefficient: −1.03e+03
Value: 46−60
p__Actinobacteria.c__Coriobacteriia.o__Eggerthellales.f__Eggerthe
4000
3000
2000
1000
0
46−60 (n=7)
80plus (n=3)
61−80 (n=15)
31−45 (n=3)
20−30 (n=2)
Age_category

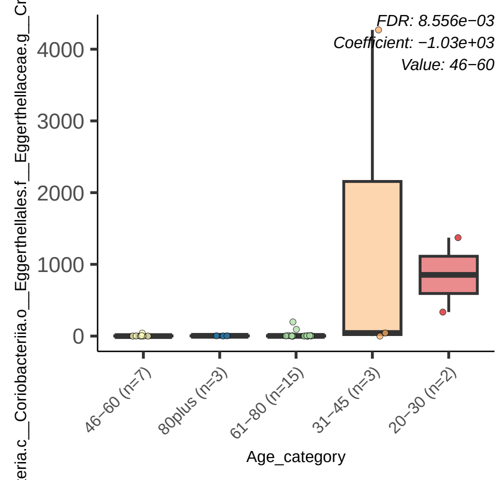

FDR: 8.556e−03
Coefficient: −1.03e+03
Value: 46−60
4000
3000
2000
1000
0
46−60 (n=7)
80plus (n=3)
61−80 (n=15)
31−45 (n=3)
20−30 (n=2)
Age_category
Coriobacteriia.o__Eggerthellales.f__Eggerthellaceae.g__

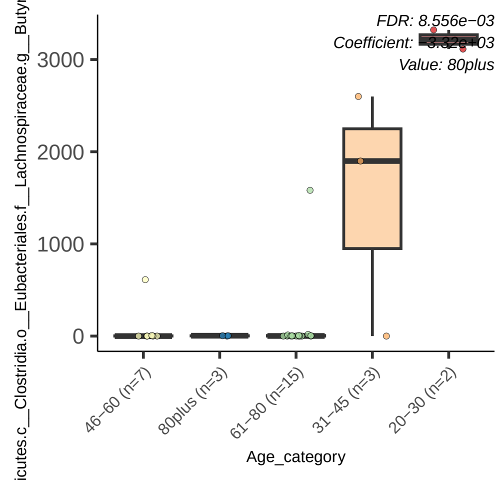

FDR: 8.556e−03
Coefficient: −3.32e+03
Value: 80plus
icutes.c__Clostridia.o__Eubacteriales.f__Lachnospiraceae.g__Butyr
3000
2000
1000
0
46−60 (n=7)
80plus (n=3)
61−80 (n=15)
31−45 (n=3)
20−30 (n=2)
Age_category

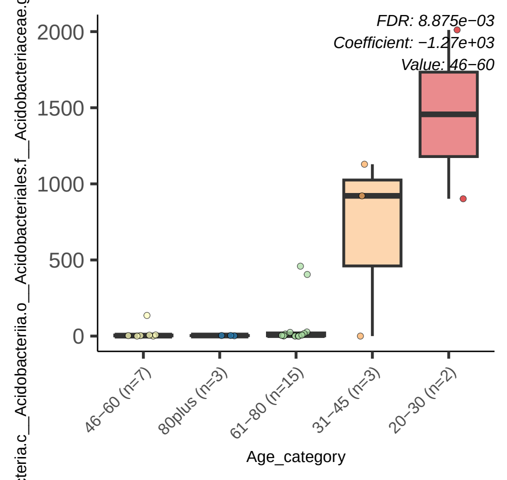
FDR: 8.875e−03
Coefficient: −1.27e+03
Value: 46−60
2000
1500
1000
500
0
46−60 (n=7)
80plus (n=3)
61−80 (n=15)
31−45 (n=3)
20−30 (n=2)
Age_category

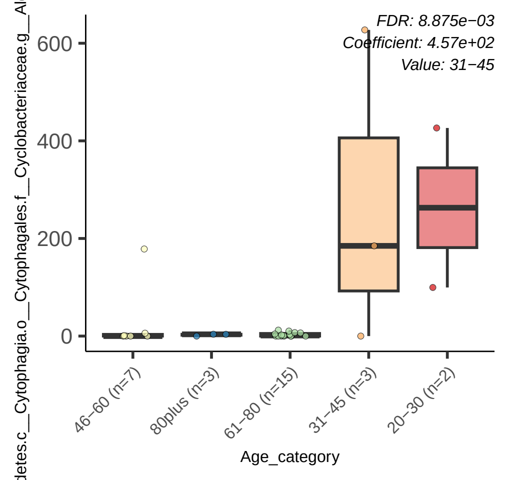

FDR: 8.875e−03
Coefficient: 4.57e+02
Value: 31−45
600
400
200
0
46−60 (n=7)
80plus (n=3)
61−80 (n=15)
31−45 (n=3)
20−30 (n=2)
Age_category
detes.c__Cytophagia.o__Cytophagales.f__Cyclobacteriaceae.g__Al

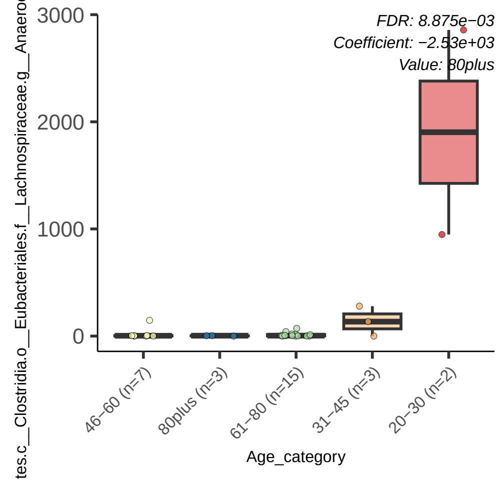

FDR: 8.875e−03
Coefficient: −2.53e+03
Value: 80plus
3000
2000
1000
0
tes.c__Clostridia.o__Eubacteriales.f__Lachnospiraceae.g__Anaero
46−60 (n=7)
80plus (n=3)
61−80 (n=15)
31−45 (n=3)
20−30 (n=2)
Age_category

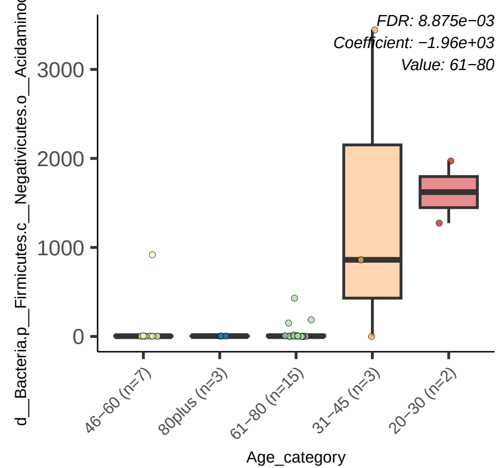

FDR: 8.875e−03
Coefficient: −1.96e+03
Value: 61−80
d__Bacteria.p__Firmicutes.c__Negativicutes.o__Acidaminoc
3000
2000
1000
0
46−60 (n=7)
80plus (n=3)
61−80 (n=15)
31−45 (n=3)
20−30 (n=2)
Age_category

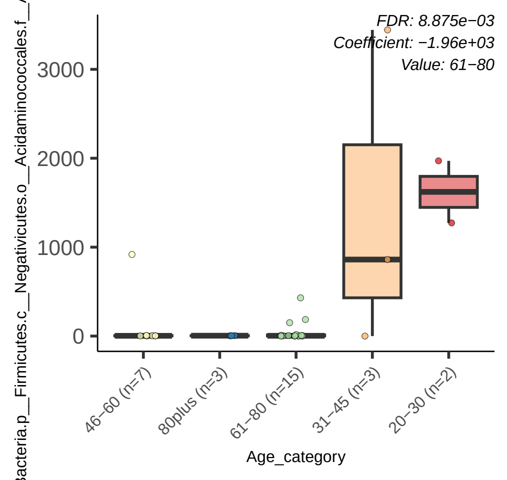

FDR: 8.875e−03
Coefficient: −1.96e+03
Value: 61−80
3000
2000
1000
0
Bacteria.p__Firmicutes.c__Negativicutes.o__Acidaminococcales.f__
46−60 (n=7)
80plus (n=3)
61−80 (n=15)
31−45 (n=3)
20−30 (n=2)
Age_category

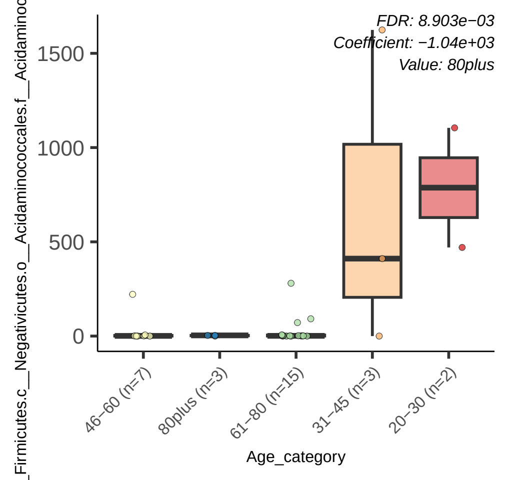

FDR: 8.903e−03
Coefficient: −1.04e+03
Value: 80plus
Firmicutes.c__Negativicutes.o__Acidaminococcales.f__Acidaminoc
1500
1000
500
0
46−60 (n=7)
80plus (n=3)
61−80 (n=15)
31−45 (n=3)
20−30 (n=2)
Age_category

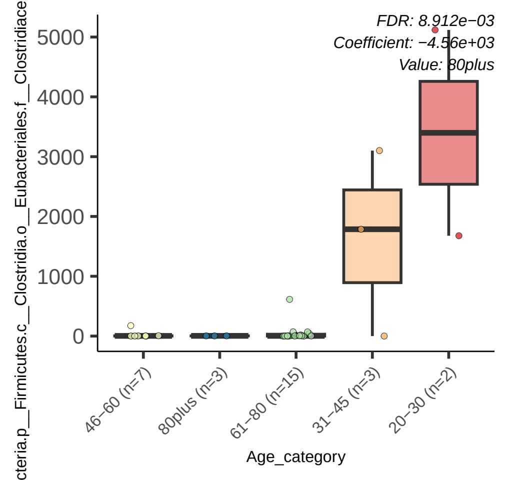
FDR: 8.912e−03
Coefficient: −4.56e+03
Value: 80plus
5000
4000
3000
2000
1000
0
cteria.p__Firmicutes.c__Clostridia.o__Eubacteriales.f__Clostridiace
46−60 (n=7)
80plus (n=3)
61−80 (n=15)
31−45 (n=3)
20−30 (n=2)
Age_category

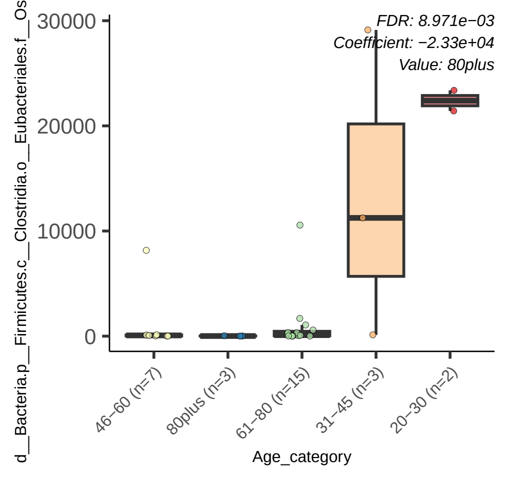

FDR: 8.971e−03
Coefficient: −2.33e+04
Value: 80plus
30000
20000
10000
0
d__Bacteria.p__Firmicutes.c__Clostridia.o__Eubacteriales.f__Os
46−60 (n=7)
80plus (n=3)
61−80 (n=15)
31−45 (n=3)
20−30 (n=2)
Age_category

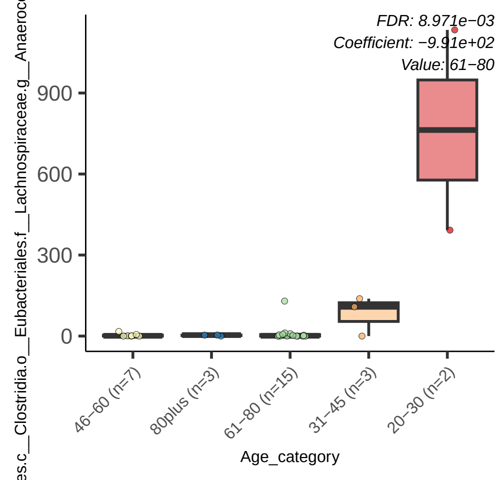

FDR: 8.971e−03
Coefficient: −9.91e+02
Value: 61−80
900
600
300
0
es.c__Clostridia.o__Eubacteriales.f__Lachnospiraceae.g__Anaeroc
46−60 (n=7)
80plus (n=3)
61−80 (n=15)
31−45 (n=3)
20−30 (n=2)
Age_category

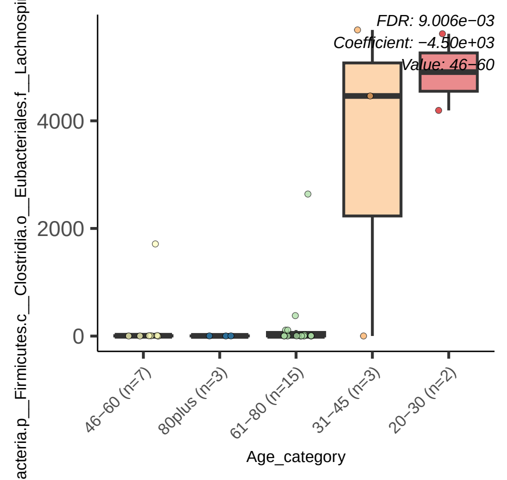

FDR: 9.006e−03
Coefficient: −4.50e+03
Value: 46−60
acteria.p__Firmicutes.c__Clostridia.o__Eubacteriales.f__Lachnospi
4000
2000
0
46−60 (n=7)
80plus (n=3)
61−80 (n=15)
31−45 (n=3)
20−30 (n=2)
Age_category

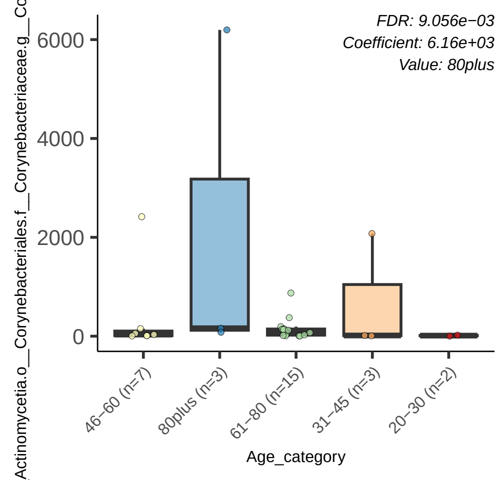

FDR: 9.056e−03
Coefficient: 6.16e+03
Value: 80plus
Actinomycetia.o__Corynebacteriales.f__Corynebacteriaceae.g__Co
6000
4000
2000
0
46−60 (n=7)
80plus (n=3)
61−80 (n=15)
31−45 (n=3)
20−30 (n=2)
Age_category

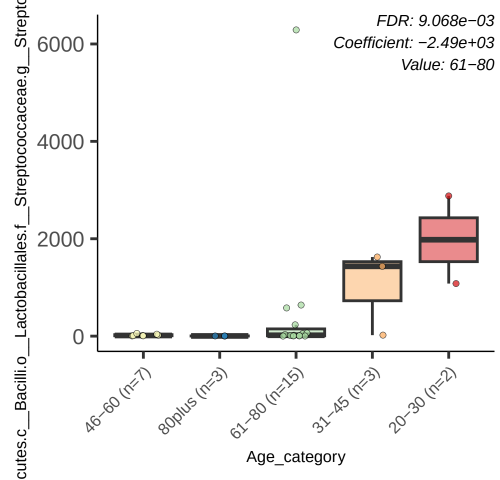

FDR: 9.068e−03
Coefficient: −2.49e+03
Value: 61−80
6000
4000
2000
0
46−60 (n=7)
80plus (n=3)
61−80 (n=15)
31−45 (n=3)
20−30 (n=2)
Age_category
cutes.c__Bacilli.o__Lactobacillales.f__Streptococcaceae.g__Strepto

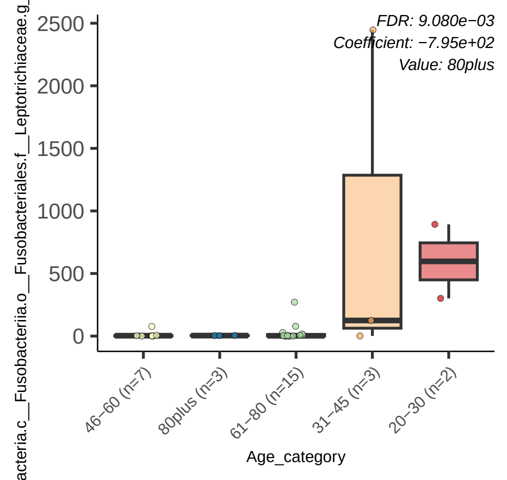

FDR: 9.080e−03
Coefficient: −7.95e+02
Value: 80plus
2500
2000
1500
1000
500
0
acteria.c__Fusobacteriia.o__Fusobacteriales.f__Leptotrichiaceae.g_
46−60 (n=7)
80plus (n=3)
61−80 (n=15)
31−45 (n=3)
20−30 (n=2)
Age_category

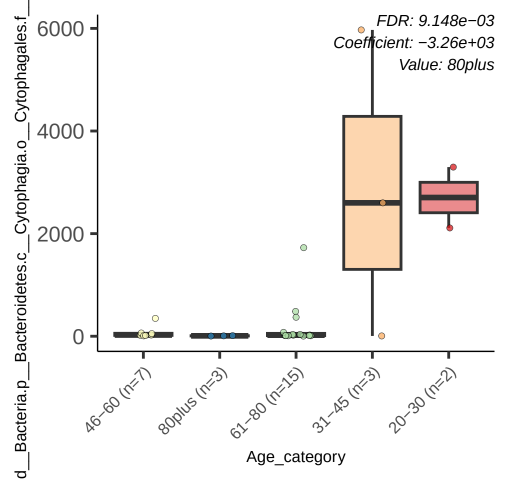

FDR: 9.148e−03
Coefficient: −3.26e+03
Value: 80plus
d__Bacteria.p__Bacteroidetes.c__Cytophagia.o__Cytophagales.f__
6000
4000
2000
0
46−60 (n=7)
80plus (n=3)
61−80 (n=15)
31−45 (n=3)
20−30 (n=2)
Age_category

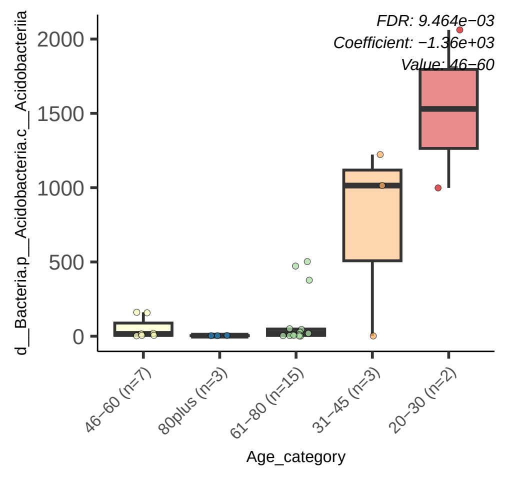

FDR: 9.464e−03
Coefficient: −1.36e+03
Value: 46−60
d__Bacteria.p__Acidobacteria.c__Acidobacteriia
2000
1500
1000
500
0
46−60 (n=7)
80plus (n=3)
61−80 (n=15)
31−45 (n=3)
20−30 (n=2)
Age_category

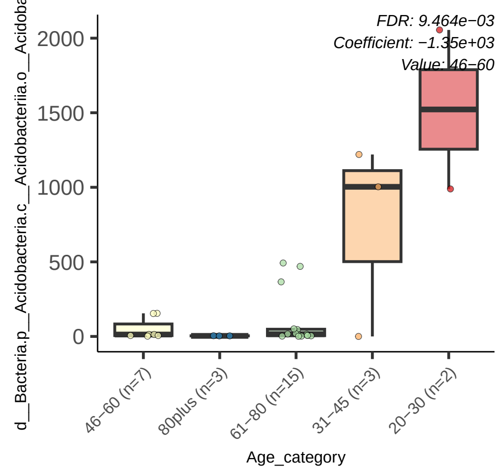

FDR: 9.464e−03
Coefficient: −1.35e+03
Value: 46−60
d__Bacteria.p__Acidobacteria.c__Acidobacteriia.o__Acidoba
2000
1500
1000
500
0
46−60 (n=7)
80plus (n=3)
61−80 (n=15)
31−45 (n=3)
20−30 (n=2)
Age_category

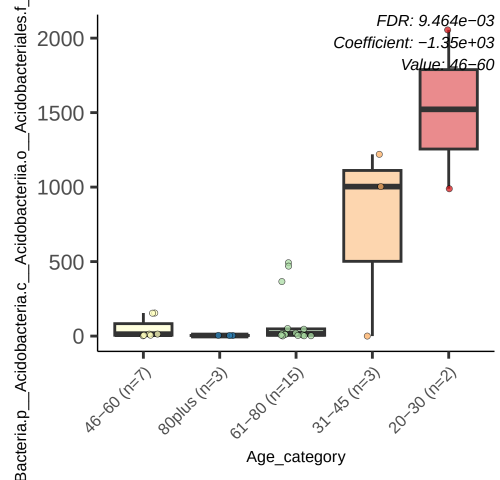

FDR: 9.464e−03
Coefficient: −1.35e+03
Value: 46−60
Bacteria.p__Acidobacteria.c__Acidobacteriia.o__Acidobacteriales.f__
2000
1500
1000
500
0
46−60 (n=7)
80plus (n=3)
61−80 (n=15)
31−45 (n=3)
20−30 (n=2)
Age_category

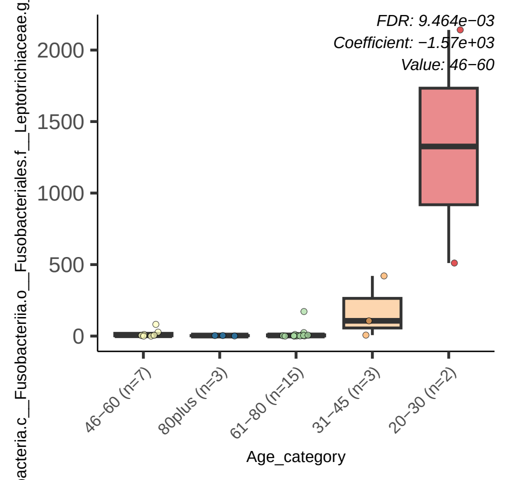

FDR: 9.464e−03
Coefficient: −1.57e+03
Value: 46−60
2000
1500
1000
500
0
46−60 (n=7)
80plus (n=3)
61−80 (n=15)
31−45 (n=3)
20−30 (n=2)
Age_category

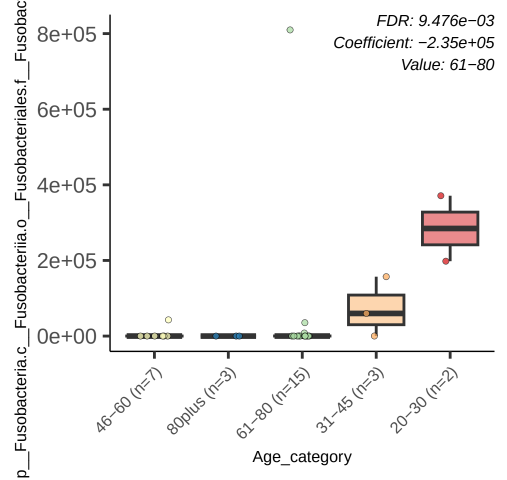

FDR: 9.476e−03
Coefficient: −2.35e+05
Value: 61−80
p__Fusobacteria.c__Fusobacteriia.o__Fusobacteriales.f__Fusobac
8e+05
6e+05
4e+05
2e+05
0e+00
46−60 (n=7)
80plus (n=3)
61−80 (n=15)
31−45 (n=3)
20−30 (n=2)
Age_category

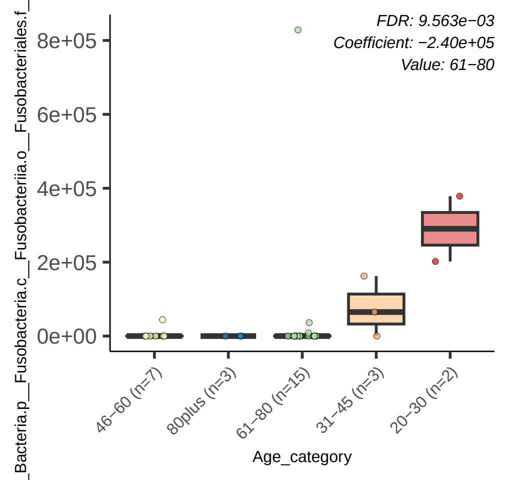
FDR: 9.563e−03
Coefficient: −2.40e+05
Value: 61−80
Bacteria.p__Fusobacteria.c__Fusobacteriia.o__Fusobacteriales.f
8e+05
6e+05
4e+05
2e+05
0e+00
46−60 (n=7)
80plus (n=3)
61−80 (n=15)
31−45 (n=3)
20−30 (n=2)
Age_category

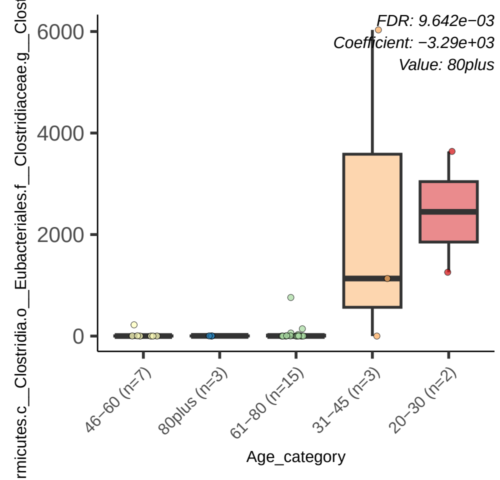

FDR: 9.642e−03
Coefficient: −3.29e+03
Value: 80plus
6000
4000
2000
0
rmicutes.c__Clostridia.o__Eubacteriales.f__Clostridiaceae.g__Clost
46−60 (n=7)
80plus (n=3)
61−80 (n=15)
31−45 (n=3)
20−30 (n=2)
Age_category

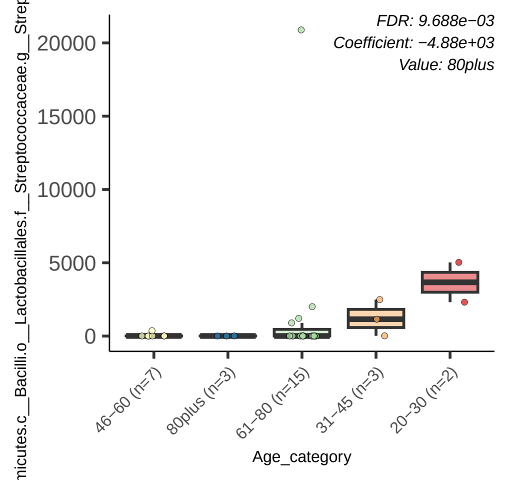

FDR: 9.688e−03
Coefficient: −4.88e+03
Value: 80plus
20000
15000
10000
5000
0
46−60 (n=7)
80plus (n=3)
61−80 (n=15)
31−45 (n=3)
20−30 (n=2)
Age_category
micutes.c__Bacilli.o__Lactobacillales.f__Streptococcaceae.g__Strep

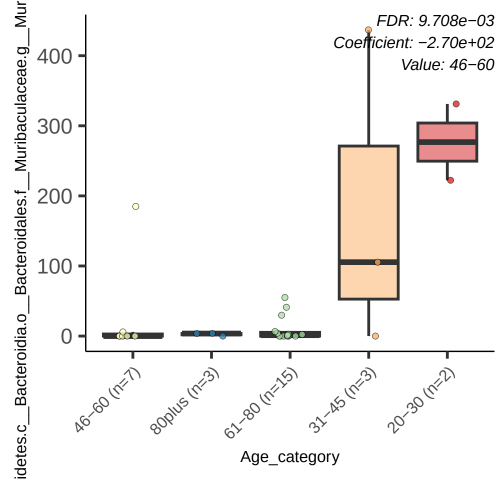

FDR: 9.708e−03
Coefficient: −2.70e+02
Value: 46−60
idetes.c__Bacteroidia.o__Bacteroidales.f__Muribaculaceae.g__Mur
0
100
200
300
400
46−60 (n=7)
80plus (n=3)
61−80 (n=15)
31−45 (n=3)
20−30 (n=2)
Age_category

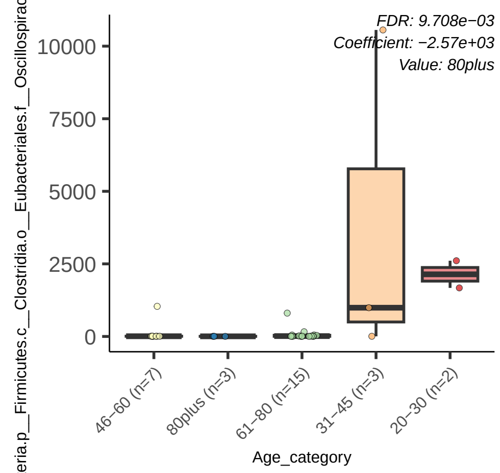

FDR: 9.708e−03
Coefficient: −2.57e+03
Value: 80plus
10000
7500
5000
2500
0
cteria.p__Firmicutes.c__Clostridia.o__Eubacteriales.f__Oscillospirac
46−60 (n=7)
80plus (n=3)
61−80 (n=15)
31−45 (n=3)
20−30 (n=2)
Age_category

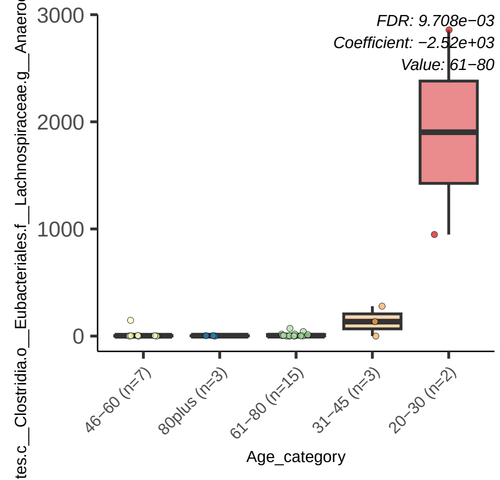

FDR: 9.708e−03
Coefficient: −2.52e+03
Value: 61−80
3000
2000
1000
0
tes.c__Clostridia.o__Eubacteriales.f__Lachnospiraceae.g__Anaero
46−60 (n=7)
80plus (n=3)
61−80 (n=15)
31−45 (n=3)
20−30 (n=2)
Age_category

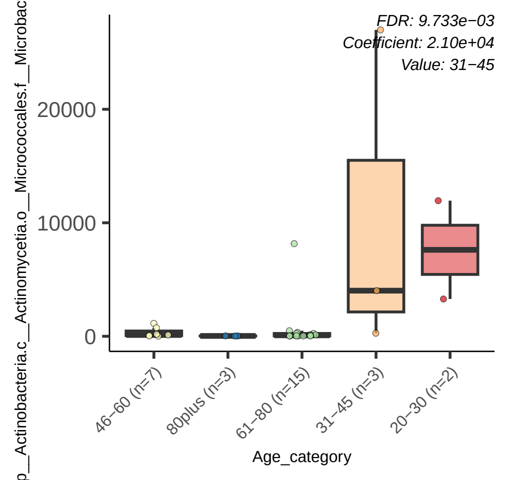

FDR: 9.733e−03
Coefficient: 2.10e+04
Value: 31−45
20000
10000
0
46−60 (n=7)
80plus (n=3)
61−80 (n=15)
31−45 (n=3)
20−30 (n=2)
Age_category
p__Actinobacteria.c__Actinomycetia.o__Micrococcales.f__Microbac

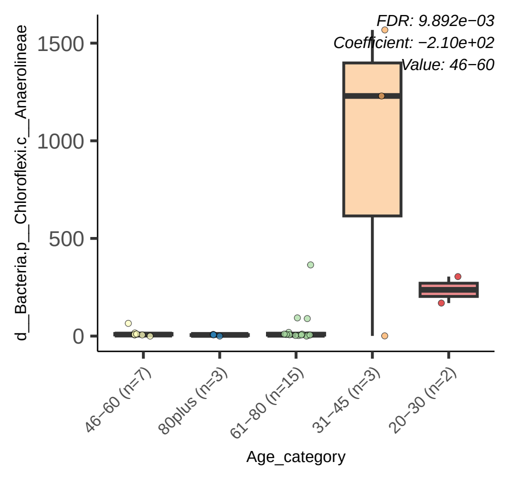

FDR: 9.892e−03
Coefficient: −2.10e+02
Value: 46−60
d__Bacteria.p__Chloroflexi.c__Anaerolineae
1500
1000
500
0
46−60 (n=7)
80plus (n=3)
61−80 (n=15)
31−45 (n=3)
20−30 (n=2)
Age_category

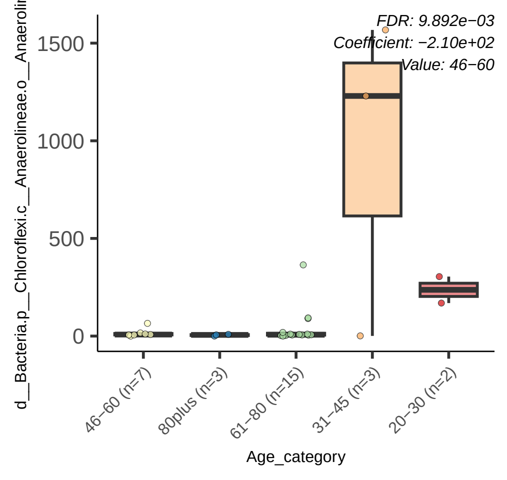

FDR: 9.892e−03
Coefficient: −2.10e+02
Value: 46−60
d__Bacteria.p__Chloroflexi.c__Anaerolineae.o__Anaerolin
1500
1000
500
0
46−60 (n=7)
80plus (n=3)
61−80 (n=15)
31−45 (n=3)
20−30 (n=2)
Age_category

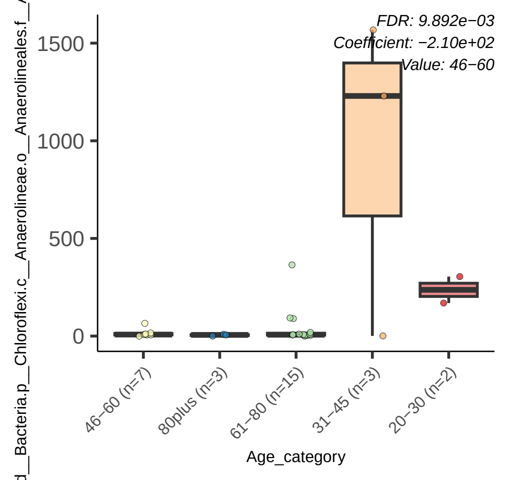

FDR: 9.892e−03
Coefficient: −2.10e+02
Value: 46−60
Bacteria.p__Chloroflexi.c__Anaerolineae.o__Anaerolineales.f__
1500
1000
500
0
46−60 (n=7)
80plus (n=3)
61−80 (n=15)
31−45 (n=3)
20−30 (n=2)
Age_category

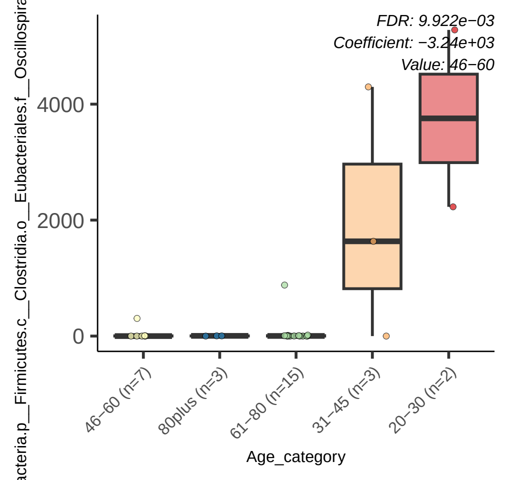

FDR: 9.922e−03
Coefficient: −3.24e+03
Value: 46−60
4000
2000
0
acteria.p__Firmicutes.c__Clostridia.o__Eubacteriales.f__Oscillospira
46−60 (n=7)
80plus (n=3)
61−80 (n=15)
31−45 (n=3)
20−30 (n=2)
Age_category

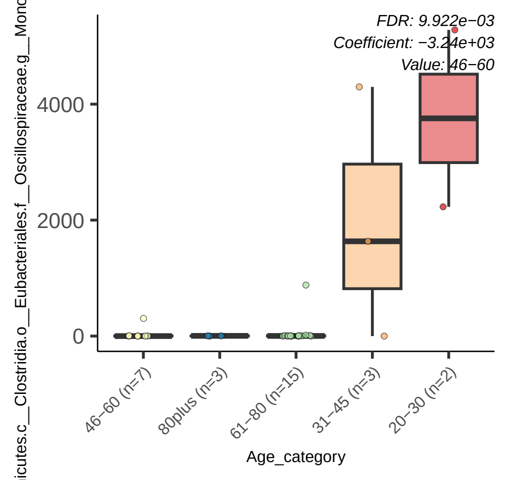

FDR: 9.922e−03
Coefficient: −3.24e+03
Value: 46−60
4000
2000
0
icutes.c__Clostridia.o__Eubacteriales.f__Oscillospiraceae.g__Monc
46−60 (n=7)
80plus (n=3)
61−80 (n=15)
31−45 (n=3)
20−30 (n=2)
Age_category

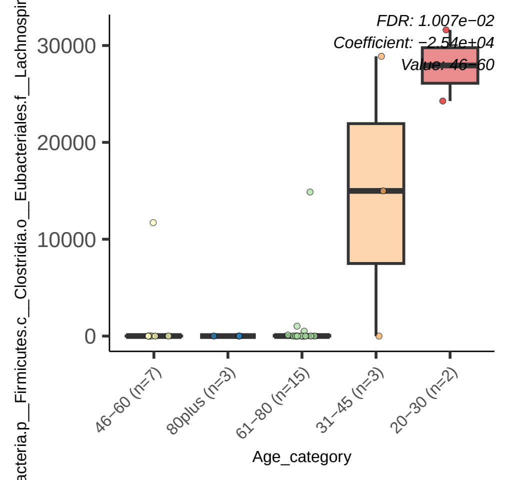

FDR: 1.007e−02
Coefficient: −2.54e+04
Value: 46−60
30000
20000
10000
0
46−60 (n=7)
80plus (n=3)
61−80 (n=15)
31−45 (n=3)
20−30 (n=2)
Age_category
acteria.p__Firmicutes.c__Clostridia.o__Eubacteriales.f__Lachnospir

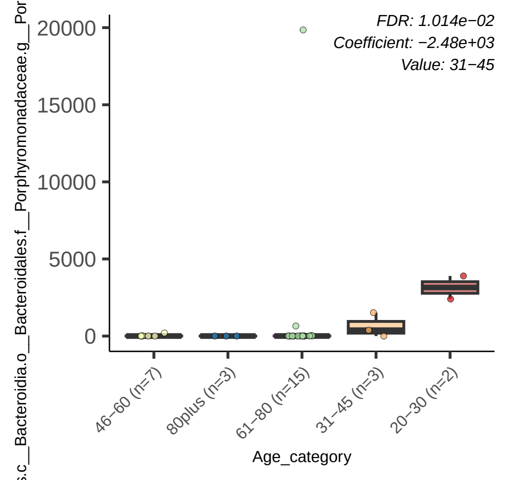
FDR: 1.014e−02
Coefficient: −2.48e+03
Value: 31−45
20000
15000
10000
5000
0
s.c__Bacteroidia.o__Bacteroidales.f__Porphyromonadaceae.g__Por
46−60 (n=7)
80plus (n=3)
61−80 (n=15)
31−45 (n=3)
20−30 (n=2)
Age_category

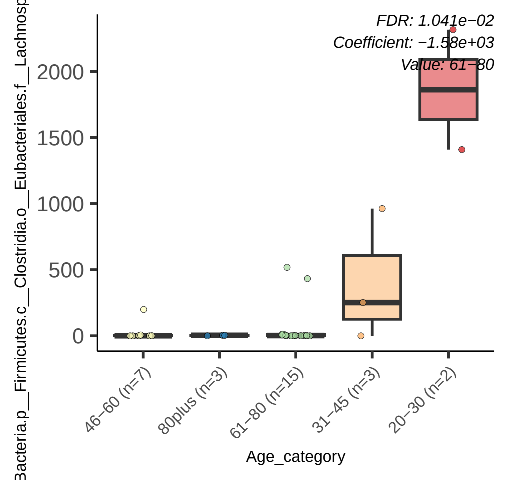

FDR: 1.041e−02
Coefficient: −1.58e+03
Value: 61−80
Bacteria.p__Firmicutes.c__Clostridia.o__Eubacteriales.f__Lachnosp
2000
1500
1000
500
0
46−60 (n=7)
80plus (n=3)
61−80 (n=15)
31−45 (n=3)
20−30 (n=2)
Age_category

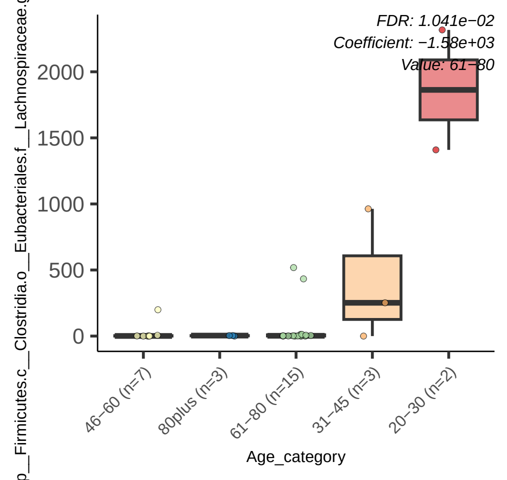

FDR: 1.041e−02
Coefficient: −1.58e+03
Value: 61−80
0
500
1000
1500
2000
p__Firmicutes.c__Clostridia.o__Eubacteriales.f__Lachnospiraceae.g
46−60 (n=7)
80plus (n=3)
61−80 (n=15)
31−45 (n=3)
20−30 (n=2)
Age_category

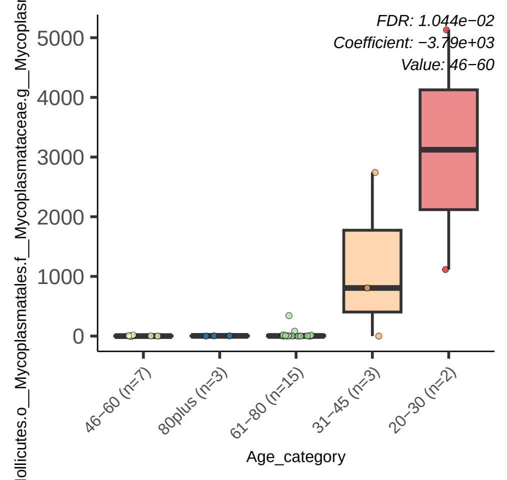
FDR: 1.044e−02
Coefficient: −3.79e+03
Value: 46−60
5000
4000
3000
2000
1000
0
46−60 (n=7)
80plus (n=3)
61−80 (n=15)
31−45 (n=3)
20−30 (n=2)
Age_category
ollicutes.o__Mycoplasmatales.f__Mycoplasmataceae.g__Mycoplasm

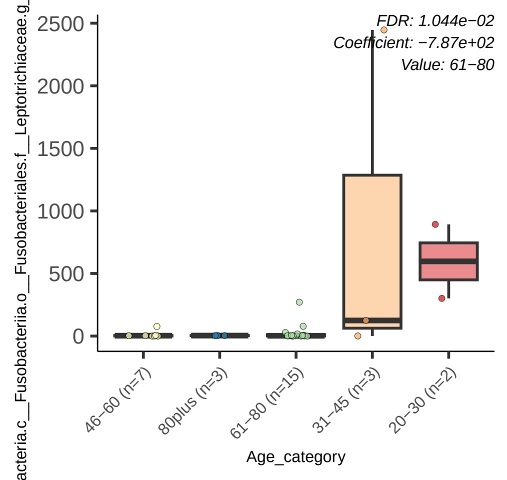

FDR: 1.044e−02
Coefficient: −7.87e+02
Value: 61−80
acteria.c__Fusobacteriia.o__Fusobacteriales.f__Leptotrichiaceae.g_
2500
2000
1500
1000
500
0
46−60 (n=7)
80plus (n=3)
61−80 (n=15)
31−45 (n=3)
20−30 (n=2)
Age_category

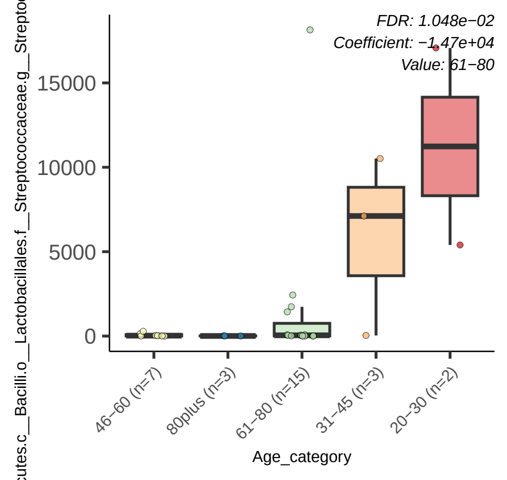

FDR: 1.048e−02
Coefficient: −1.47e+04
Value: 61−80
15000
10000
5000
0
cutes.c__Bacilli.o__Lactobacillales.f__Streptococcaceae.g__Strepto
46−60 (n=7)
80plus (n=3)
61−80 (n=15)
31−45 (n=3)
20−30 (n=2)
Age_category

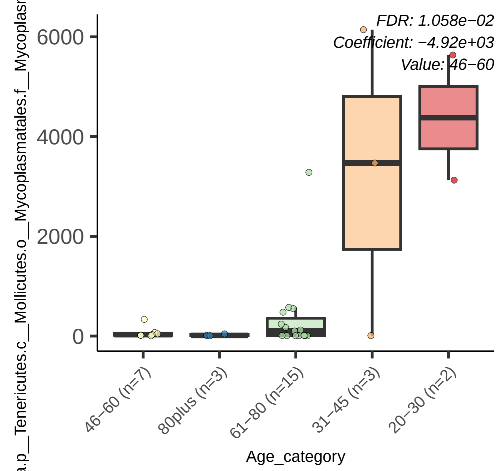

FDR: 1.058e−02
Coefficient: −4.92e+03
Value: 46−60
6000
4000
2000
0
ia.p__Tenericutes.c__Mollicutes.o__Mycoplasmatales.f__Mycoplasr
46−60 (n=7)
80plus (n=3)
61−80 (n=15)
31−45 (n=3)
20−30 (n=2)
Age_category

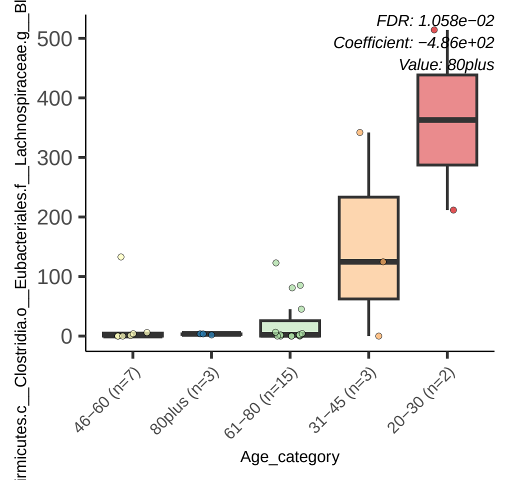
FDR: 1.058e−02
Coefficient: −4.86e+02
Value: 80plus
0
100
200
300
400
500
46−60 (n=7)
80plus (n=3)
61−80 (n=15)
31−45 (n=3)
20−30 (n=2)
Age_category
irmicutes.c__Clostridia.o__Eubacteriales.f__Lachnospiraceae.g__Bl

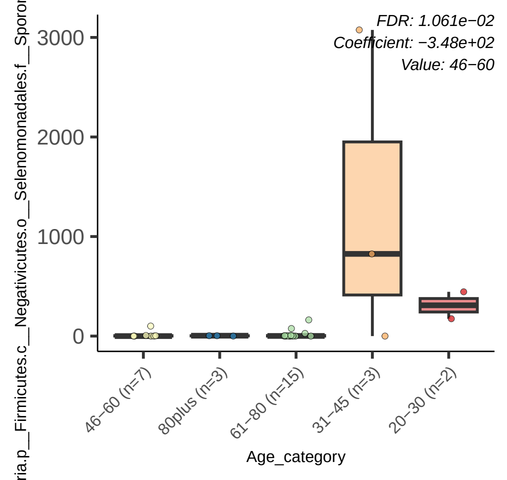

FDR: 1.061e−02
Coefficient: −3.48e+02
Value: 46−60
ria.p__Firmicutes.c__Negativicutes.o__Selenomonadales.f__Sporor
3000
2000
1000
0
46−60 (n=7)
80plus (n=3)
61−80 (n=15)
31−45 (n=3)
20−30 (n=2)
Age_category

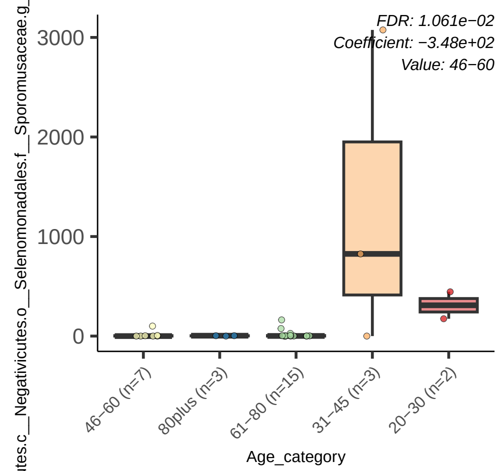
FDR: 1.061e−02
Coefficient: −3.48e+02
Value: 46−60
3000
2000
1000
0
cutes.c__Negativicutes.o__Selenomonadales.f__Sporomusaceae.g_
46−60 (n=7)
80plus (n=3)
61−80 (n=15)
31−45 (n=3)
20−30 (n=2)
Age_category

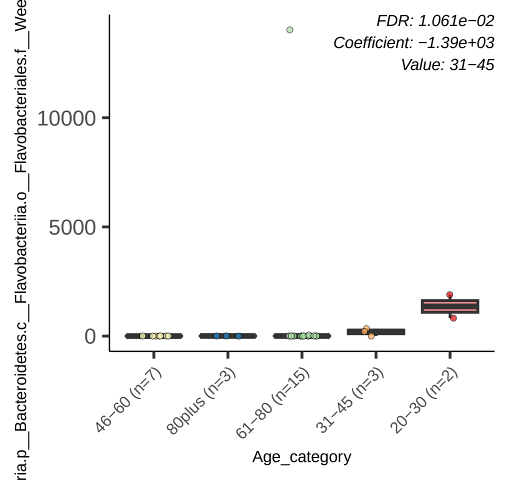

FDR: 1.061e−02
Coefficient: −1.39e+03
Value: 31−45
10000
5000
0
46−60 (n=7)
80plus (n=3)
61−80 (n=15)
31−45 (n=3)
20−30 (n=2)
Age_category
ria.p__Bacteroidetes.c__Flavobacteriia.o__Flavobacteriales.f__Wee

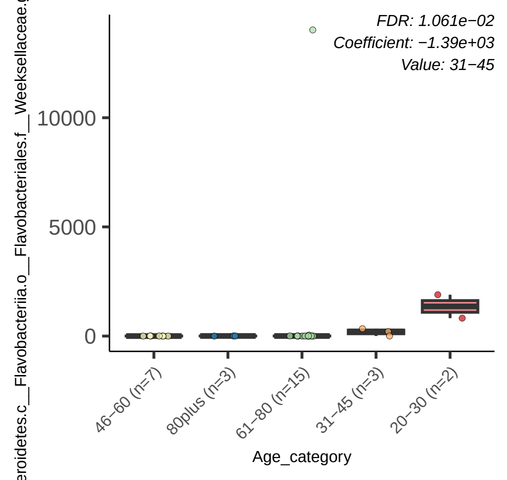

FDR: 1.061e−02
Coefficient: −1.39e+03
Value: 31−45
10000
5000
0
46−60 (n=7)
80plus (n=3)
61−80 (n=15)
31−45 (n=3)
20−30 (n=2)
Age_category
eroidetes.c__Flavobacteriia.o__Flavobacteriales.f__Weeksellaceae.g

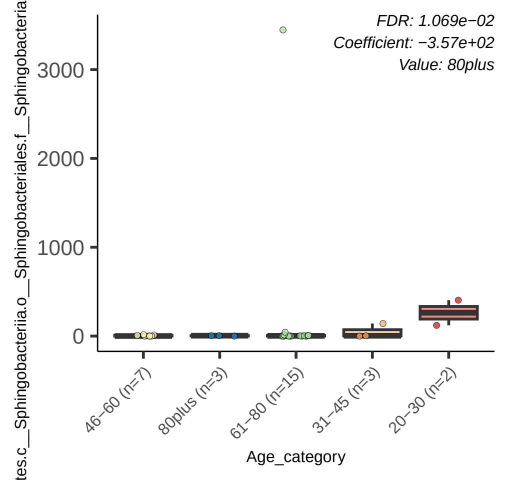

FDR: 1.069e−02
Coefficient: −3.57e+02
Value: 80plus
tes.c__Sphingobacteriia.o__Sphingobacteriales.f__Sphingobacteria
3000
2000
1000
0
46−60 (n=7)
80plus (n=3)
61−80 (n=15)
31−45 (n=3)
20−30 (n=2)
Age_category

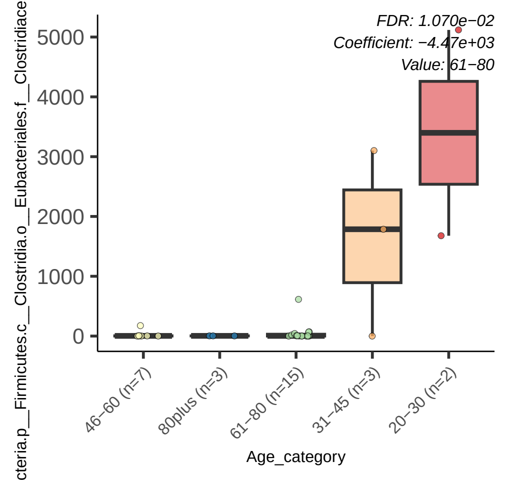
FDR: 1.070e−02
Coefficient: −4.47e+03
Value: 61−80
5000
4000
3000
2000
1000
0
cteria.p__Firmicutes.c__Clostridia.o__Eubacteriales.f__Clostridiace
46−60 (n=7)
80plus (n=3)
61−80 (n=15)
31−45 (n=3)
20−30 (n=2)
Age_category

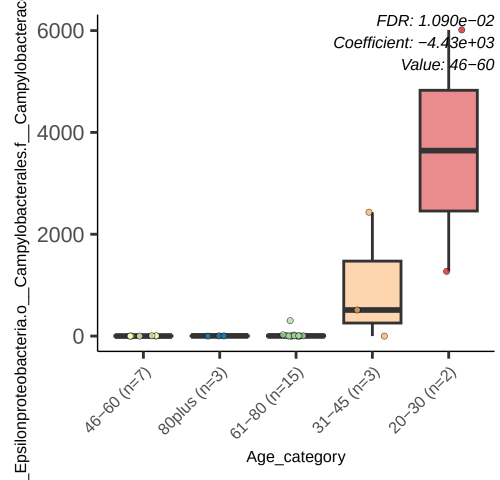

FDR: 1.090e−02
Coefficient: −4.43e+03
Value: 46−60
6000
4000
2000
0
_Epsilonproteobacteria.o__Campylobacterales.f__Campylobacterac
46−60 (n=7)
80plus (n=3)
61−80 (n=15)
31−45 (n=3)
20−30 (n=2)
Age_category

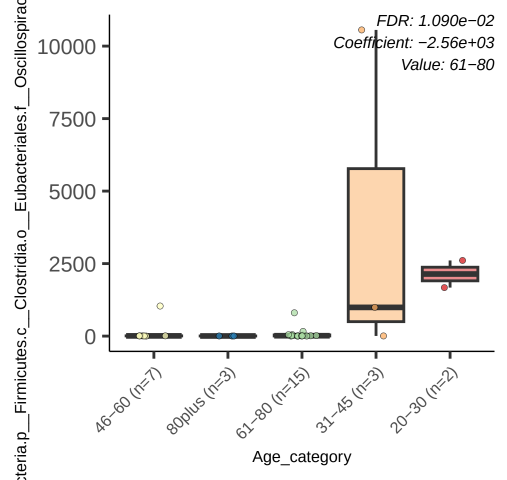

FDR: 1.090e−02
Coefficient: −2.56e+03
Value: 61−80
10000
7500
5000
2500
0
46−60 (n=7)
80plus (n=3)
61−80 (n=15)
31−45 (n=3)
20−30 (n=2)
Age_category

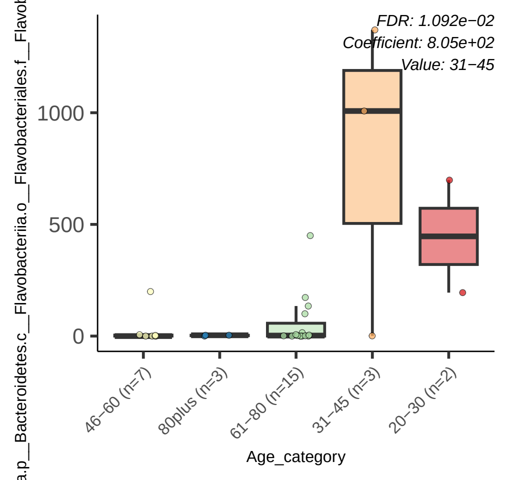

FDR: 1.092e−02
Coefficient: 8.05e+02
Value: 31−45
a.p__Bacteroidetes.c__Flavobacteriia.o__Flavobacteriales.f__Flavob
1000
500
0
46−60 (n=7)
80plus (n=3)
61−80 (n=15)
31−45 (n=3)
20−30 (n=2)
Age_category

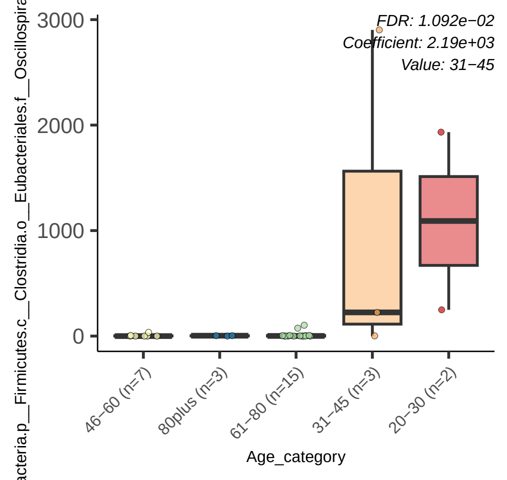

FDR: 1.092e−02
Coefficient: 2.19e+03
Value: 31−45
acteria.p__Firmicutes.c__Clostridia.o__Eubacteriales.f__Oscillospira
3000
2000
1000
0
46−60 (n=7)
80plus (n=3)
61−80 (n=15)
31−45 (n=3)
20−30 (n=2)
Age_category

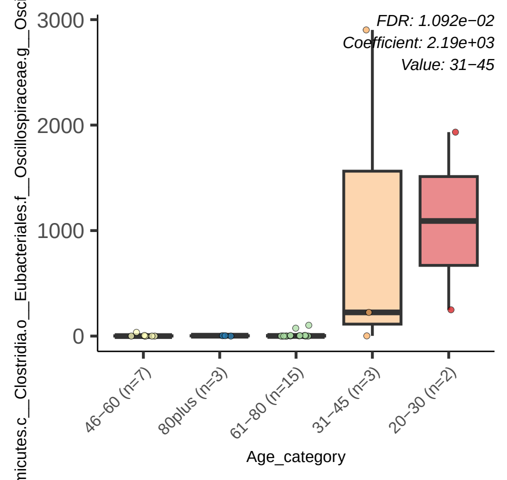

FDR: 1.092e−02
Coefficient: 2.19e+03
Value: 31−45
3000
2000
1000
0
46−60 (n=7)
80plus (n=3)
61−80 (n=15)
31−45 (n=3)
20−30 (n=2)
Age_category
micutes.c__Clostridia.o__Eubacteriales.f__Oscillospiraceae.g__Osci

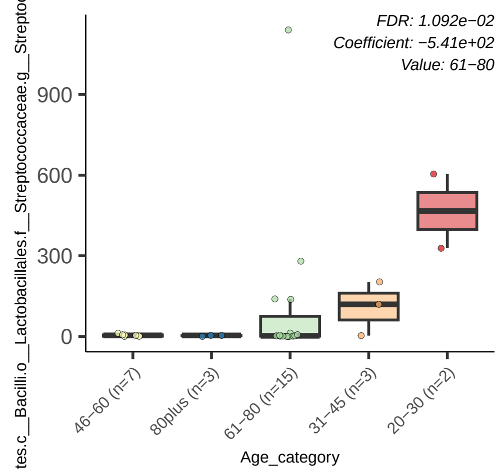

FDR: 1.092e−02
Coefficient: −5.41e+02
Value: 61−80
0
300
600
900
cutes.c__Bacilli.o__Lactobacillales.f__Streptococcaceae.g__Streptoc
46−60 (n=7)
80plus (n=3)
61−80 (n=15)
31−45 (n=3)
20−30 (n=2)
Age_category

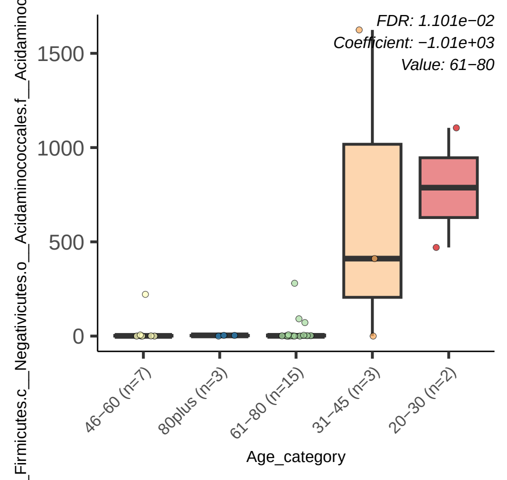

FDR: 1.101e−02
Coefficient: −1.01e+03
Value: 61−80
1500
1000
500
0
_Firmicutes.c__Negativicutes.o__Acidaminococcales.f__Acidaminoc
46−60 (n=7)
80plus (n=3)
61−80 (n=15)
31−45 (n=3)
20−30 (n=2)
Age_category

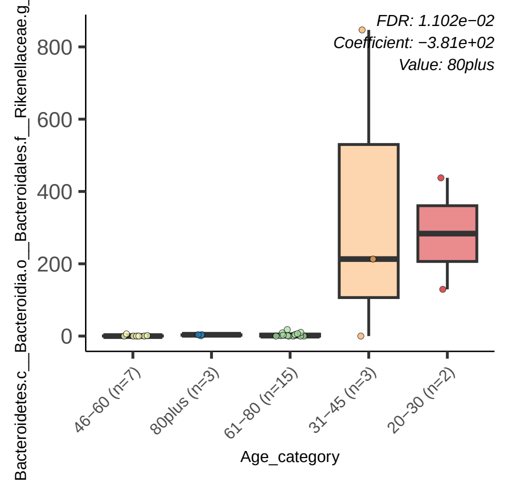

FDR: 1.102e−02
Coefficient: −3.81e+02
Value: 80plus
Bacteroidetes.c__Bacteroidia.o__Bacteroidales.f__Rikenellaceae.g__
800
600
400
200
0
46−60 (n=7)
80plus (n=3)
61−80 (n=15)
31−45 (n=3)
20−30 (n=2)
Age_category

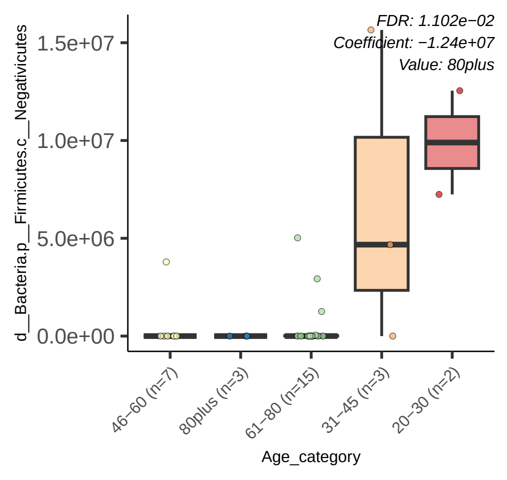
FDR: 1.102e−02
Coefficient: −1.24e+07
Value: 80plus
1.5e+07
1.0e+07
5.0e+06
0.0e+00
d__Bacteria.p__Firmicutes.c__Negativicutes
46−60 (n=7)
80plus (n=3)
61−80 (n=15)
31−45 (n=3)
20−30 (n=2)
Age_category

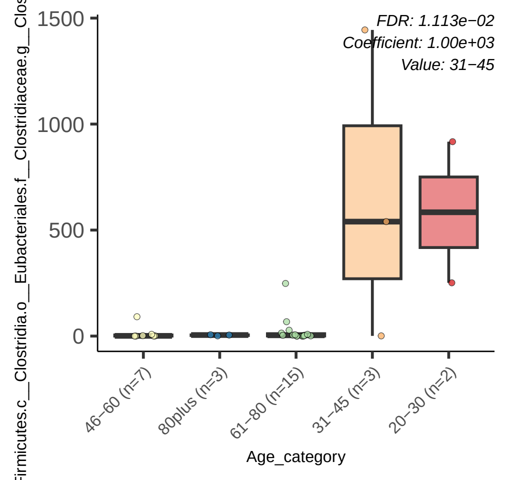

FDR: 1.113e−02
Coefficient: 1.00e+03
Value: 31−45
1500
1000
500
0
46−60 (n=7)
80plus (n=3)
61−80 (n=15)
31−45 (n=3)
20−30 (n=2)
Age_category
Firmicutes.c__Clostridia.o__Eubacteriales.f__Clostridiaceae.g__Clos

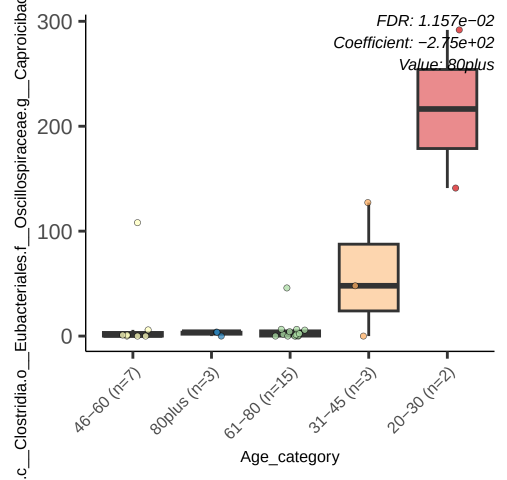

FDR: 1.157e−02
Coefficient: −2.75e+02
Value: 80plus
300
200
100
0
c__Clostridia.o__Eubacteriales.f__Oscillospiraceae.g__Caproicibac
46−60 (n=7)
80plus (n=3)
61−80 (n=15)
31−45 (n=3)
20−30 (n=2)
Age_category

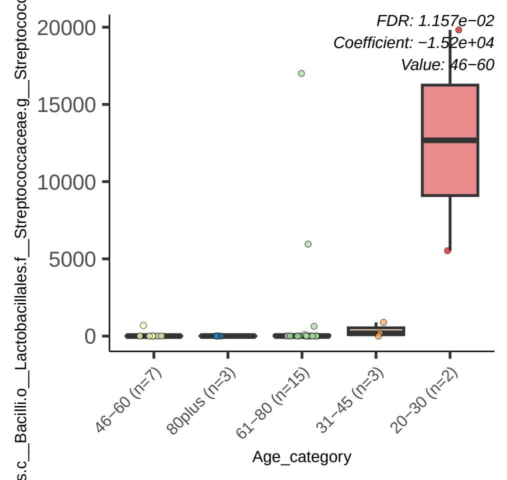
FDR: 1.157e−02
Coefficient: −1.52e+04
Value: 46−60
20000
15000
10000
5000
0
s.c__Bacilli.o__Lactobacillales.f__Streptococcaceae.g__Streptococc
46−60 (n=7)
80plus (n=3)
61−80 (n=15)
31−45 (n=3)
20−30 (n=2)
Age_category

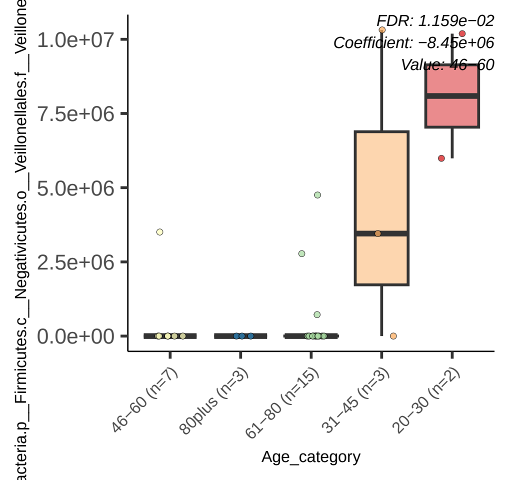

FDR: 1.159e−02
Coefficient: −8.45e+06
Value: 46−60
1.0e+07
7.5e+06
5.0e+06
2.5e+06
0.0e+00
acteria.p__Firmicutes.c__Negativicutes.o__Veillonellales.f__Veillone
46−60 (n=7)
80plus (n=3)
61−80 (n=15)
31−45 (n=3)
20−30 (n=2)
Age_category

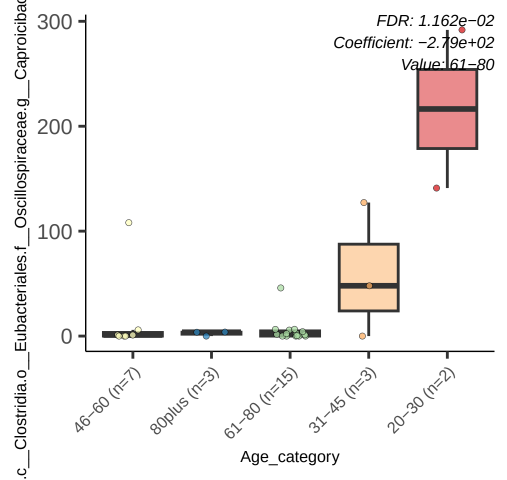
FDR: 1.162e−02
Coefficient: −2.79e+02
Value: 61−80
c__Clostridia.o__Eubacteriales.f__Oscillospiraceae.g__Caproicibac
300
200
100
0
46−60 (n=7)
80plus (n=3)
61−80 (n=15)
31−45 (n=3)
20−30 (n=2)
Age_category

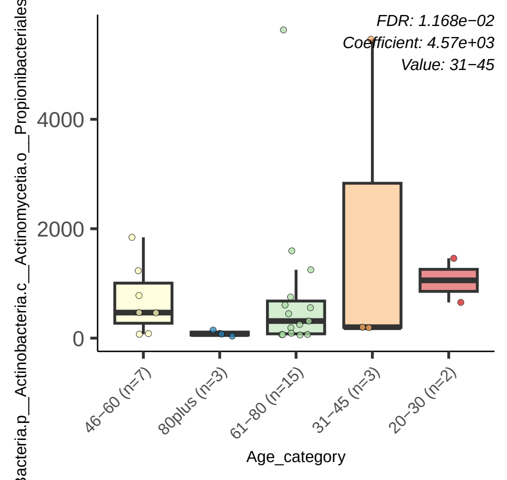
FDR: 1.168e−02
Coefficient: 4.57e+03
Value: 31−45
Bacteria.p__Actinobacteria.c__Actinomycetia.o__Propionibacteriales
4000
2000
0
46−60 (n=7)
80plus (n=3)
61−80 (n=15)
31−45 (n=3)
20−30 (n=2)
Age_category

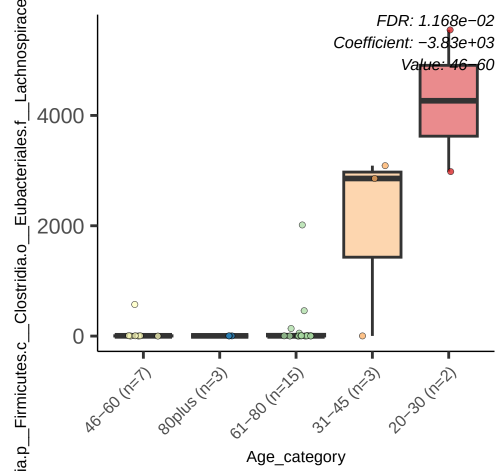

FDR: 1.168e−02
Coefficient: −3.83e+03
Value: 46−60
4000
2000
0
46−60 (n=7)
80plus (n=3)
61−80 (n=15)
31−45 (n=3)
20−30 (n=2)
Age_category
eria.p__Firmicutes.c__Clostridia.o__Eubacteriales.f__Lachnospirace

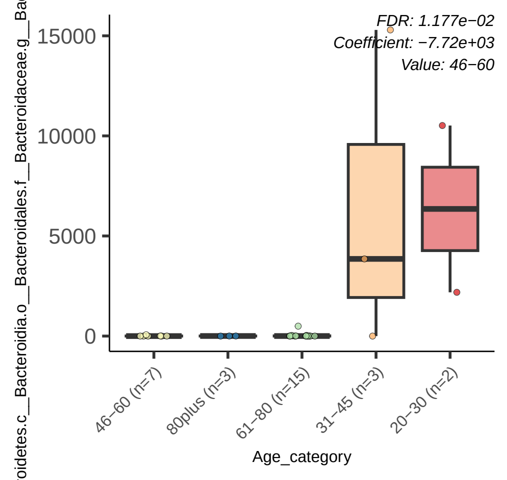

FDR: 1.177e−02
Coefficient: −7.72e+03
Value: 46−60
15000
10000
5000
0
46−60 (n=7)
80plus (n=3)
61−80 (n=15)
31−45 (n=3)
20−30 (n=2)
Age_category
oidetes.c__Bacteroidia.o__Bacteroidales.f__Bacteroidaceae.g__Ba

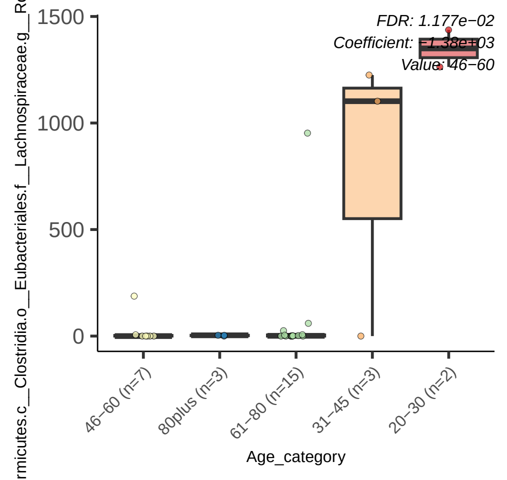

FDR: 1.177e−02
Coefficient: −1.38e+03
Value: 46−60
1500
1000
500
0
rmicutes.c__Clostridia.o__Eubacteriales.f__Lachnospiraceae.g__Ro
46−60 (n=7)
80plus (n=3)
61−80 (n=15)
31−45 (n=3)
20−30 (n=2)
Age_category

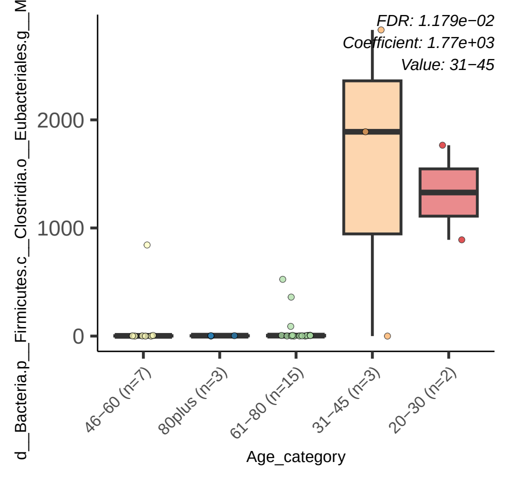

FDR: 1.179e−02
Coefficient: 1.77e+03
Value: 31−45
d__Bacteria.p__Firmicutes.c__Clostridia.o__Eubacteriales.g__M
2000
1000
0
46−60 (n=7)
80plus (n=3)
61−80 (n=15)
31−45 (n=3)
20−30 (n=2)
Age_category

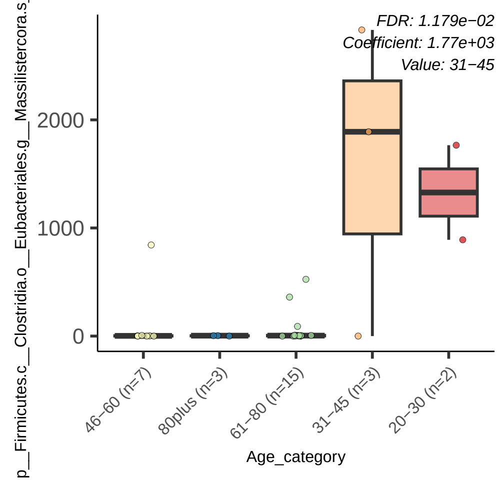

FDR: 1.179e−02
Coefficient: 1.77e+03
Value: 31−45
p__Firmicutes.c__Clostridia.o__Eubacteriales.g__Massilistercora.s_
2000
1000
0
46−60 (n=7)
80plus (n=3)
61−80 (n=15)
31−45 (n=3)
20−30 (n=2)
Age_category

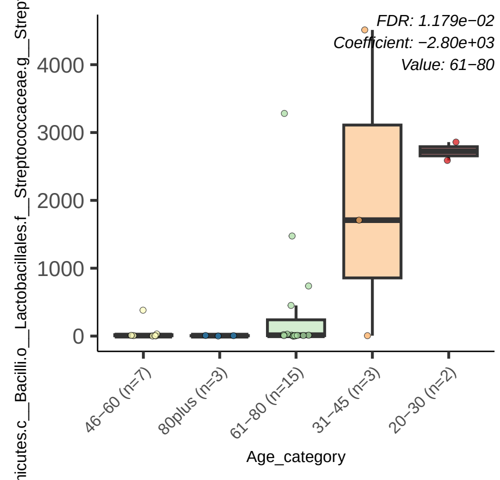

FDR: 1.179e−02
Coefficient: −2.80e+03
Value: 61−80
4000
3000
2000
1000
0
46−60 (n=7)
80plus (n=3)
61−80 (n=15)
31−45 (n=3)
20−30 (n=2)
Age_category
nicutes.c__Bacilli.o__Lactobacillales.f__Streptococcaceae.g__Strep

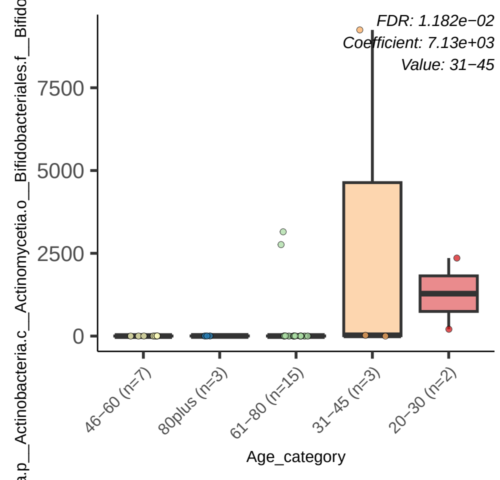

FDR: 1.182e−02
Coefficient: 7.13e+03
Value: 31−45
a.p__Actinobacteria.c__Actinomycetia.o__Bifidobacteriales.f__Bifido
7500
5000
2500
0
46−60 (n=7)
80plus (n=3)
61−80 (n=15)
31−45 (n=3)
20−30 (n=2)
Age_category

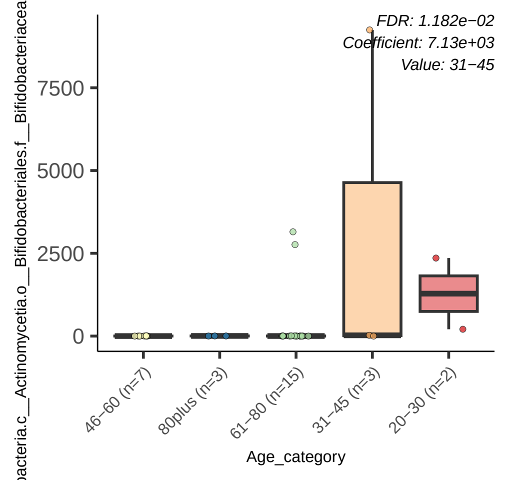

FDR: 1.182e−02
Coefficient: 7.13e+03
Value: 31−45
bacteria.c__Actinomycetia.o__Bifidobacteriales.f__Bifidobacteriacea
7500
5000
2500
0
46−60 (n=7)
80plus (n=3)
61−80 (n=15)
31−45 (n=3)
20−30 (n=2)
Age_category

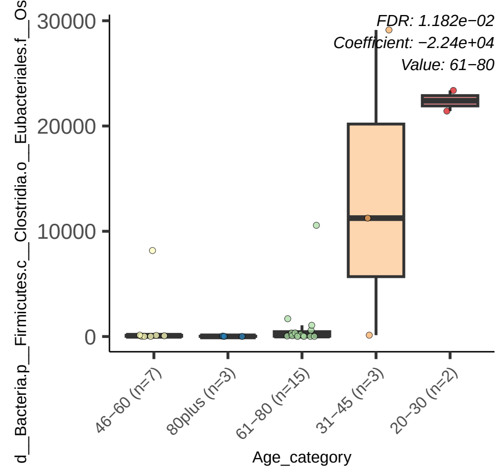

FDR: 1.182e−02
Coefficient: −2.24e+04
Value: 61−80
30000
20000
10000
0
d__Bacteria.p__Firmicutes.c__Clostridia.o__Eubacteriales.f__Os
46−60 (n=7)
80plus (n=3)
61−80 (n=15)
31−45 (n=3)
20−30 (n=2)
Age_category

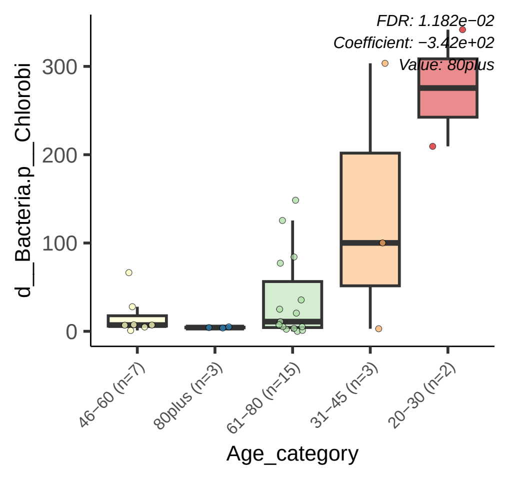
FDR: 1.182e−02
Coefficient: −3.42e+02
Value: 80plus
d__Bacteria.p__Chlorobi
300
200
100
0
46−60 (n=7)
80plus (n=3)
61−80 (n=15)
31−45 (n=3)
20−30 (n=2)
Age_category

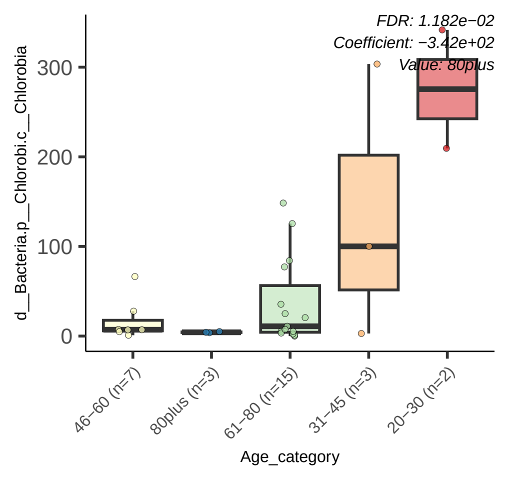

FDR: 1.182e−02
Coefficient: −3.42e+02
Value: 80plus
d__Bacteria.p__Chlorobi.c__Chlorobia
300
200
100
0
46−60 (n=7)
80plus (n=3)
61−80 (n=15)
31−45 (n=3)
20−30 (n=2)
Age_category

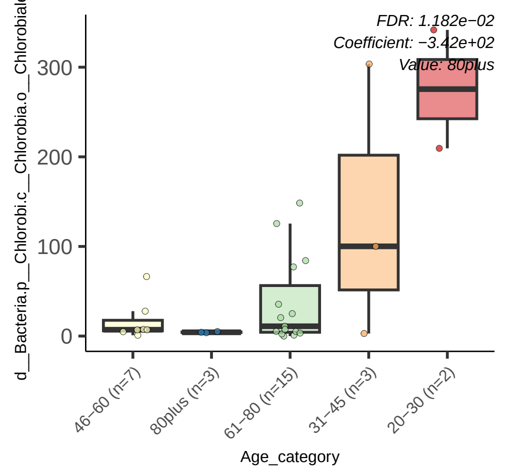
FDR: 1.182e−02
Coefficient: −3.42e+02
Value: 80plus
d__Bacteria.p__Chlorobi.c__Chlorobia.o__Chlorobiale
300
200
100
0
46−60 (n=7)
80plus (n=3)
61−80 (n=15)
31−45 (n=3)
20−30 (n=2)
Age_category

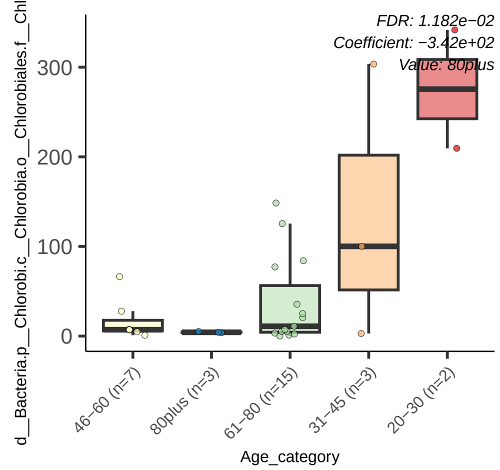

FDR: 1.182e−02
Coefficient: −3.42e+02
Value: 80plus
d__Bacteria.p__Chlorobi.c__Chlorobia.o__Chlorobiales.f__Chl
300
200
100
0
46−60 (n=7)
80plus (n=3)
61−80 (n=15)
31−45 (n=3)
20−30 (n=2)
Age_category

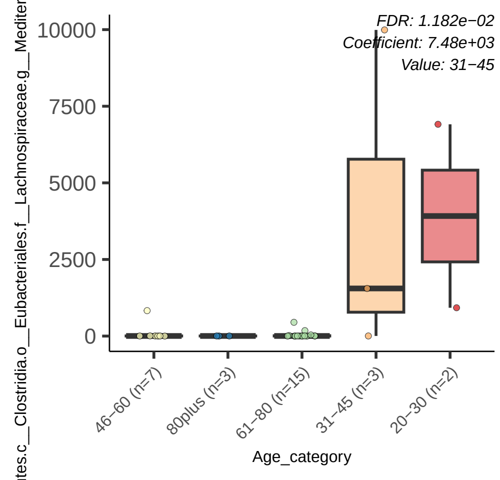

FDR: 1.182e−02
Coefficient: 7.48e+03
Value: 31−45
10000
7500
5000
2500
0
46−60 (n=7)
80plus (n=3)
61−80 (n=15)
31−45 (n=3)
20−30 (n=2)
Age_category
tes.c__Clostridia.o__Eubacteriales.f__Lachnospiraceae.g__Mediter

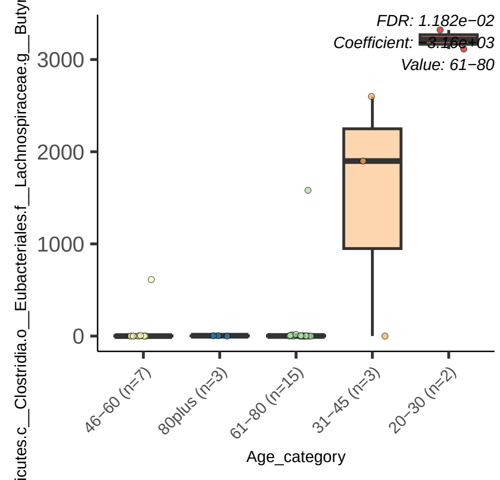

FDR: 1.182e−02
Coefficient: −3.16e+03
Value: 61−80
3000
2000
1000
0
46−60 (n=7)
80plus (n=3)
61−80 (n=15)
31−45 (n=3)
20−30 (n=2)
Age_category
icutes.c__Clostridia.o__Eubacteriales.f__Lachnospiraceae.g__Butyr

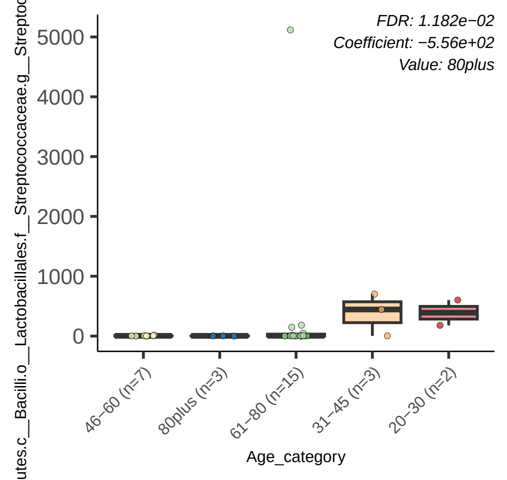

FDR: 1.182e−02
Coefficient: −5.56e+02
Value: 80plus
5000
4000
3000
2000
1000
0
utes.c__Bacilli.o__Lactobacillales.f__Streptococcaceae.g__Streptoc
46−60 (n=7)
80plus (n=3)
61−80 (n=15)
31−45 (n=3)
20−30 (n=2)
Age_category

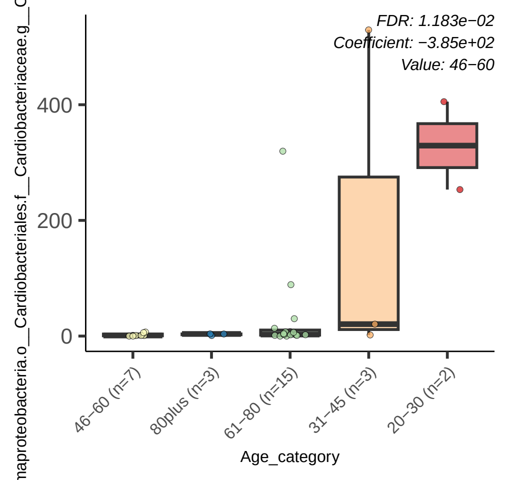

FDR: 1.183e−02
Coefficient: −3.85e+02
Value: 46−60
400
200
0
maproteobacteria.o__Cardiobacteriales.f__Cardiobacteriaceae.g__C
46−60 (n=7)
80plus (n=3)
61−80 (n=15)
31−45 (n=3)
20−30 (n=2)
Age_category

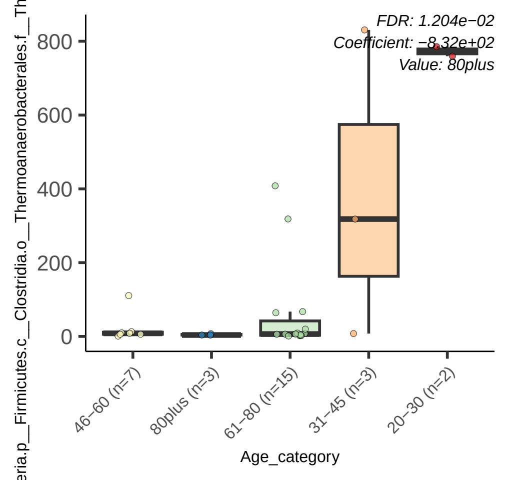

FDR: 1.204e−02
Coefficient: −8.32e+02
Value: 80plus
eria.p__Firmicutes.c__Clostridia.o__Thermoanaerobacterales.f__Th
800
600
400
200
0
46−60 (n=7)
80plus (n=3)
61−80 (n=15)
31−45 (n=3)
20−30 (n=2)
Age_category

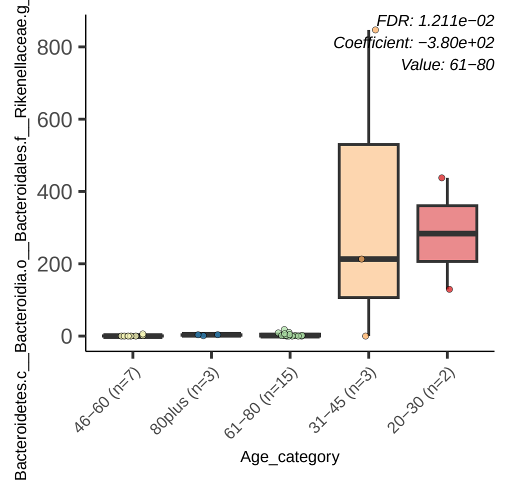

FDR: 1.211e−02
Coefficient: −3.80e+02
Value: 61−80
Bacteroidetes.c__Bacteroidia.o__Bacteroidales.f__Rikenellaceae.g__
800
600
400
200
0
46−60 (n=7)
80plus (n=3)
61−80 (n=15)
31−45 (n=3)
20−30 (n=2)
Age_category

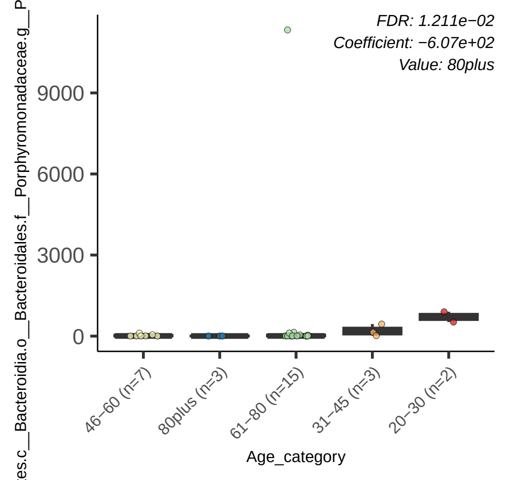

FDR: 1.211e−02
Coefficient: −6.07e+02
Value: 80plus
9000
6000
3000
0
46−60 (n=7)
80plus (n=3)
61−80 (n=15)
31−45 (n=3)
20−30 (n=2)
Age_category
tes.c__Bacteroidia.o__Bacteroidales.f__Porphyromonadaceae.g__P

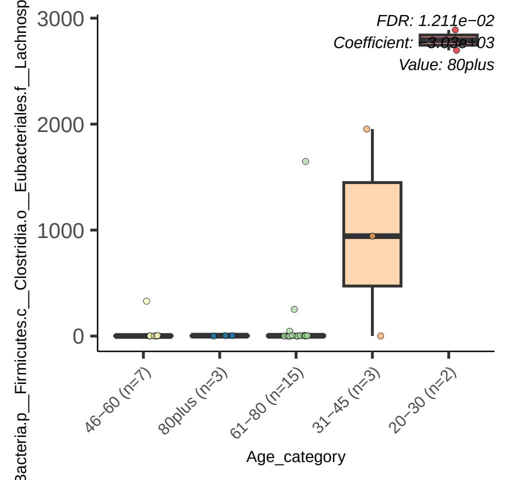

FDR: 1.211e−02
Coefficient: -3.03e+03
Value: 80plus
3000
2000
1000
0
Bacteria.p__Firmicutes.c__Clostridia.o__Eubacteriales.f__Lachnosp
46−60 (n=7)
80plus (n=3)
61−80 (n=15)
31−45 (n=3)
20−30 (n=2)
Age_category

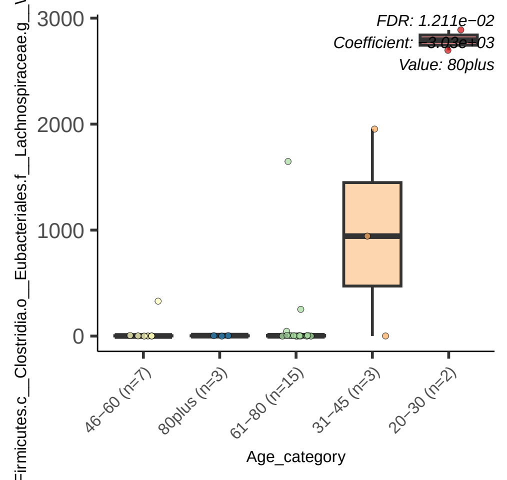
FDR: 1.211e−02
Coefficient: −3.03e+03
Value: 80plus
3000
2000
1000
0
Firmicutes.c__Clostridia.o__Eubacteriales.f__Lachnospiraceae.g__
46−60 (n=7)
80plus (n=3)
61−80 (n=15)
31−45 (n=3)
20−30 (n=2)
Age_category

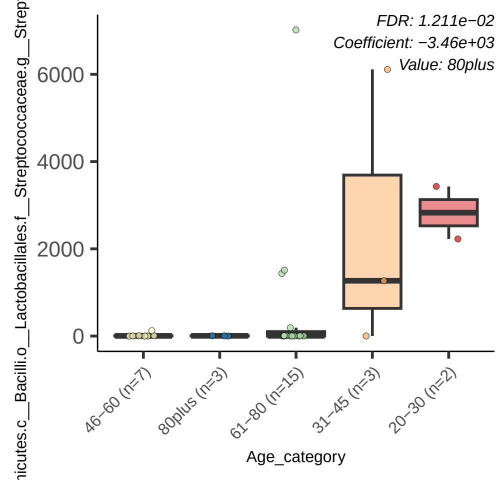

FDR: 1.211e−02
Coefficient: −3.46e+03
Value: 80plus
6000
4000
2000
0
nicutes.c__Bacilli.o__Lactobacillales.f__Streptococcaceae.g__Strep
46−60 (n=7)
80plus (n=3)
61−80 (n=15)
31−45 (n=3)
20−30 (n=2)
Age_category

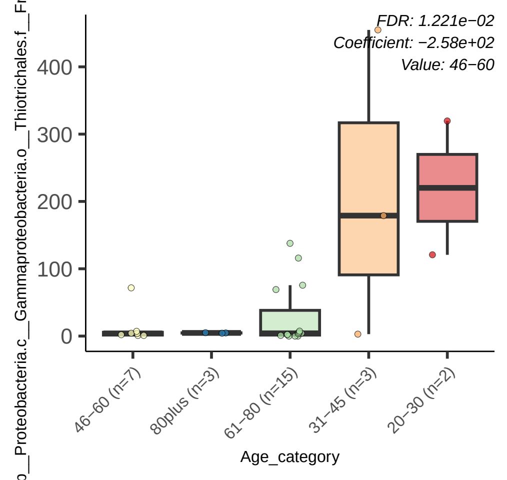

FDR: 1.221e−02
Coefficient: −2.58e+02
Value: 46−60
o__Proteobacteria.c__Gammaproteobacteria.o__Thiotrichales.f__Fr
0
100
200
300
400
46−60 (n=7)
80plus (n=3)
61−80 (n=15)
31−45 (n=3)
20−30 (n=2)
Age_category

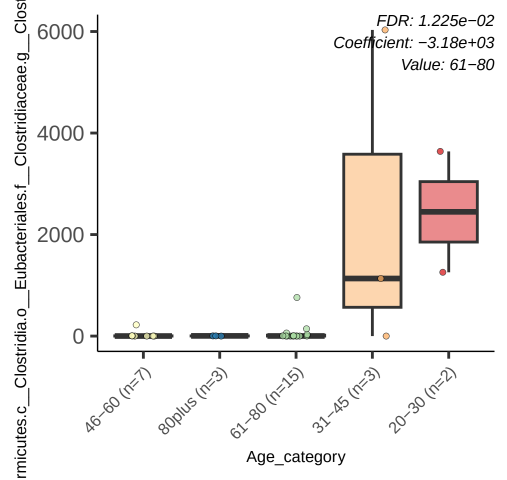

FDR: 1.225e−02
Coefficient: −3.18e+03
Value: 61−80
6000
4000
2000
0
rmicutes.c__Clostridia.o__Eubacteriales.f__Clostridiaceae.g__Clost
46−60 (n=7)
80plus (n=3)
61−80 (n=15)
31−45 (n=3)
20−30 (n=2)
Age_category

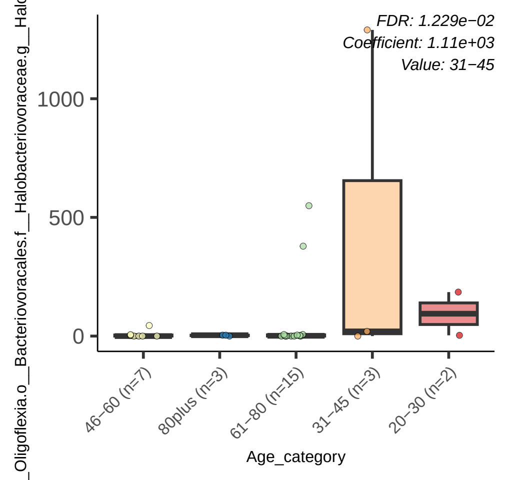
FDR: 1.229e−02
Coefficient: 1.11e+03
Value: 31−45
1000
500
0
_Oligoflexia.o__Bacteriovoracales.f__Halobacteriovoraceae.g__Halo
46−60 (n=7)
80plus (n=3)
61−80 (n=15)
31−45 (n=3)
20−30 (n=2)
Age_category

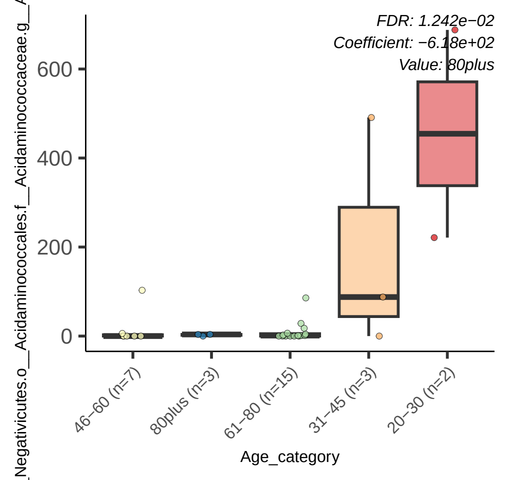

FDR: 1.242e−02
Coefficient: −6.18e+02
Value: 80plus
600
400
200
0
46−60 (n=7)
80plus (n=3)
61−80 (n=15)
31−45 (n=3)
20−30 (n=2)
Age_category
Negativicutes.o__Acidaminococcales.f__Acidaminococcaceae.g__A

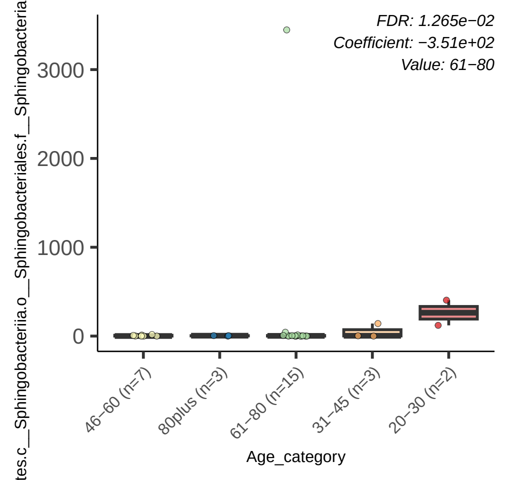

FDR: 1.265e−02
Coefficient: −3.51e+02
Value: 61−80
3000
2000
1000
0
tes.c__Sphingobacteriia.o__Sphingobacteriales.f__Sphingobacteria
46−60 (n=7)
80plus (n=3)
61−80 (n=15)
31−45 (n=3)
20−30 (n=2)
Age_category

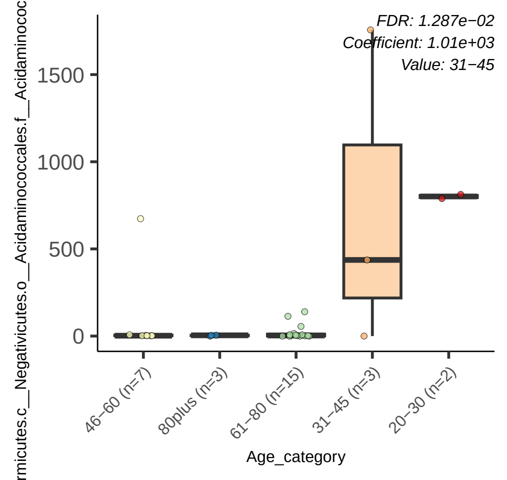

FDR: 1.287e−02
Coefficient: 1.01e+03
Value: 31−45
1500
1000
500
0
rmicutes.c__Negativicutes.o__Acidaminococcales.f__Acidaminococ
46−60 (n=7)
80plus (n=3)
61−80 (n=15)
31−45 (n=3)
20−30 (n=2)
Age_category

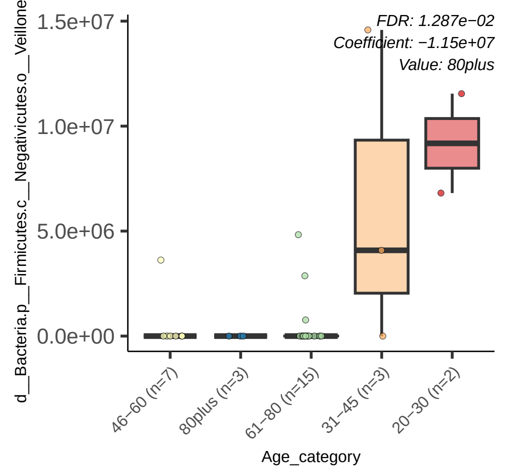

FDR: 1.287e−02
Coefficient: −1.15e+07
Value: 80plus
d__Bacteria.p__Firmicutes.c__Negativicutes.o__Veillone
1.5e+07
1.0e+07
5.0e+06
0.0e+00
46−60 (n=7)
80plus (n=3)
61−80 (n=15)
31−45 (n=3)
20−30 (n=2)
Age_category

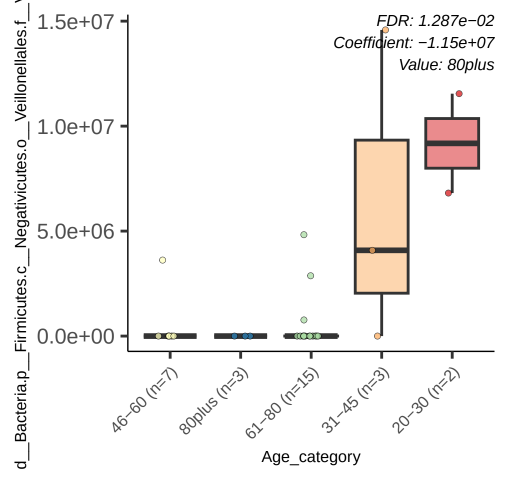

d__Bacteria.p__Firmicutes.c__Negativicutes.o__Veillonellales.f__V
FDR: 1.287e−02
Coefficient: −1.15e+07
Value: 80plus
1.5e+07
1.0e+07
5.0e+06
0.0e+00
46−60 (n=7)
80plus (n=3)
61−80 (n=15)
31−45 (n=3)
20−30 (n=2)
Age_category

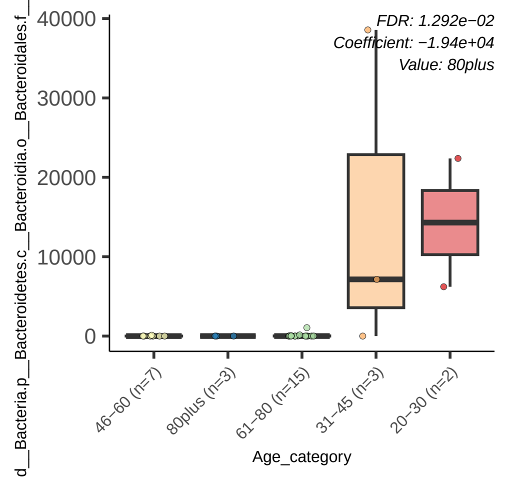

FDR: 1.292e−02
Coefficient: −1.94e+04
Value: 80plus
40000
30000
20000
10000
0
46−60 (n=7)
80plus (n=3)
61−80 (n=15)
31−45 (n=3)
20−30 (n=2)
Age_category
d__Bacteria.p__Bacteroidetes.c__Bacteroidia.o__Bacteroidales.f

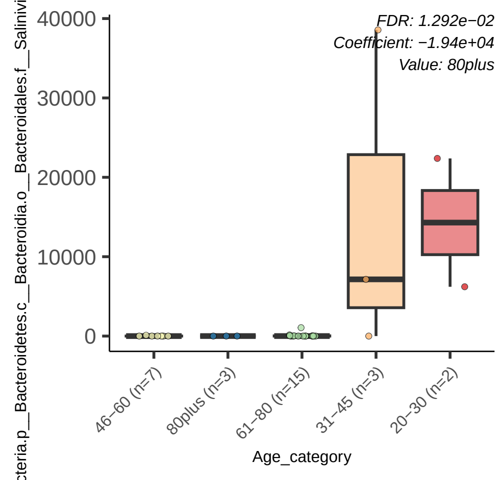

FDR: 1.292e−02
Coefficient: −1.94e+04
Value: 80plus
40000
30000
20000
10000
0
46−60 (n=7)
80plus (n=3)
61−80 (n=15)
31−45 (n=3)
20−30 (n=2)
Age_category
cteria.p__Bacteroidetes.c__Bacteroidia.o__Bacteroidales.f__Salinivi

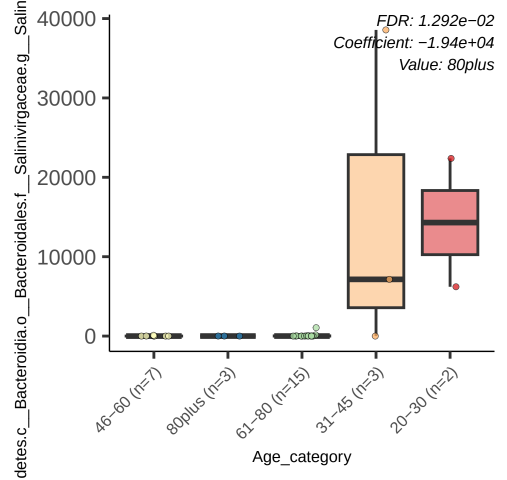
FDR: 1.292e−02
Coefficient: −1.94e+04
Value: 80plus
40000
30000
20000
10000
0
detes.c__Bacteroidia.o__Bacteroidales.f__Salinivirgaceae.g__Salin
46−60 (n=7)
80plus (n=3)
61−80 (n=15)
31−45 (n=3)
20−30 (n=2)
Age_category

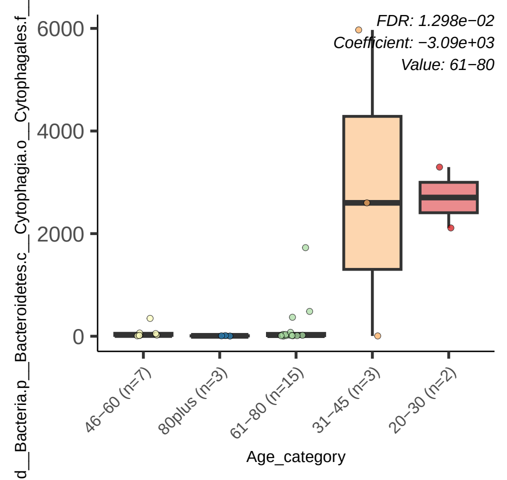

FDR: 1.298e−02
Coefficient: −3.09e+03
Value: 61−80
d__Bacteria.p__Bacteroidetes.c__Cytophagia.o__Cytophagales.f__
6000
4000
2000
0
46−60 (n=7)
80plus (n=3)
61−80 (n=15)
31−45 (n=3)
20−30 (n=2)
Age_category

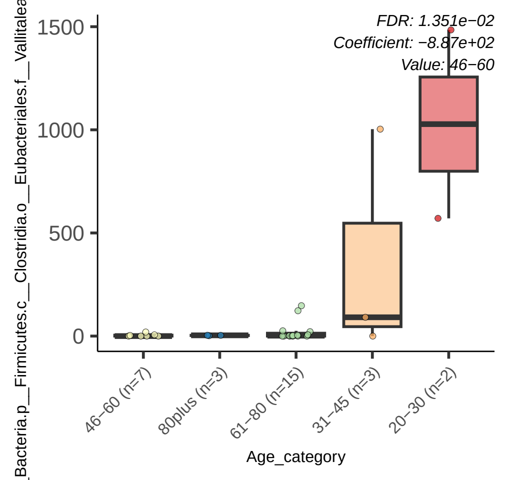
FDR: 1.351e−02
Coefficient: −8.87e+02
Value: 46−60
1500
1000
500
0
Bacteria.p__Firmicutes.c__Clostridia.o__Eubacteriales.f__Vallitalea
46−60 (n=7)
80plus (n=3)
61−80 (n=15)
31−45 (n=3)
20−30 (n=2)
Age_category

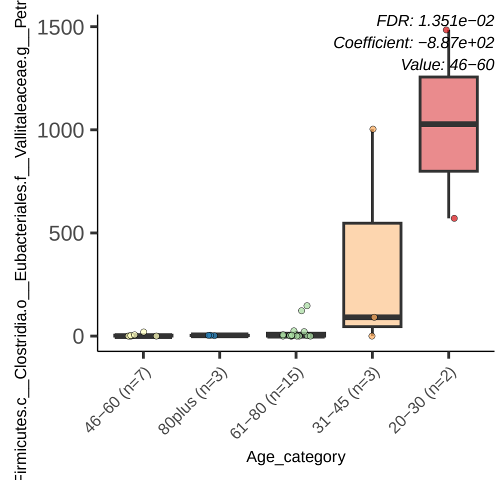
FDR: 1.351e−02
Coefficient: −8.87e+02
Value: 46−60
1500
1000
500
0
46−60 (n=7)
80plus (n=3)
61−80 (n=15)
31−45 (n=3)
20−30 (n=2)
Age_category
Firmicutes.c__Clostridia.o__Eubacteriales.f__Vallitaleaceae.g__Petr

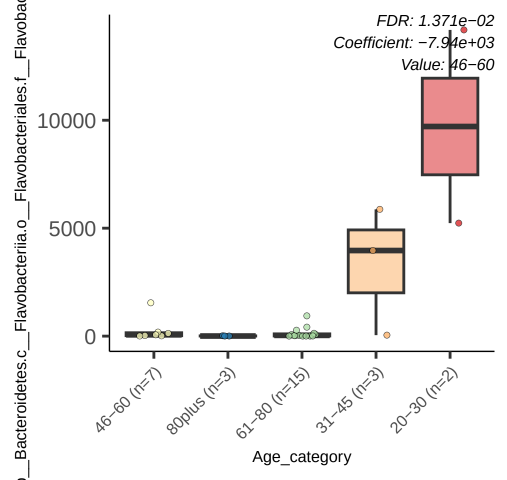

FDR: 1.371e−02
Coefficient: −7.94e+03
Value: 46−60
10000
5000
0
o__Bacteroidetes.c__Flavobacteriia.o__Flavobacteriales.f__Flavobac
46−60 (n=7)
80plus (n=3)
61−80 (n=15)
31−45 (n=3)
20−30 (n=2)
Age_category

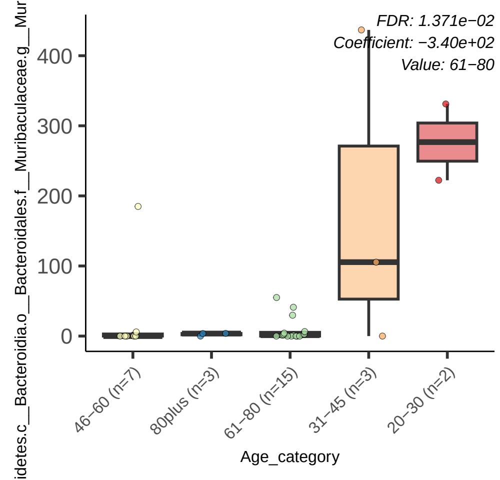

FDR: 1.371e−02
Coefficient: −3.40e+02
Value: 61−80
idetes.c__Bacteroidia.o__Bacteroidales.f__Muribaculaceae.g__Mur
400
300
200
100
0
46−60 (n=7)
80plus (n=3)
61−80 (n=15)
31−45 (n=3)
20−30 (n=2)
Age_category

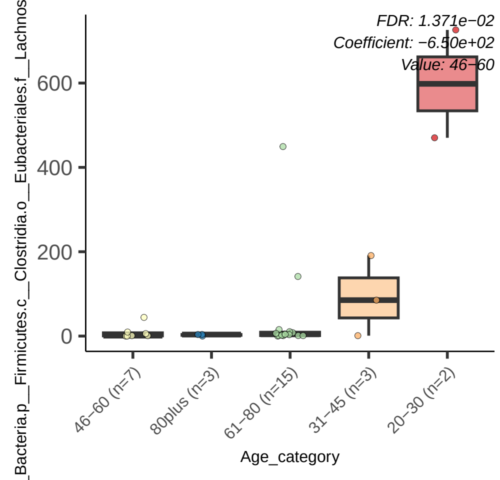
FDR: 1.371e−02
Coefficient: −6.50e+02
Value: 46−60
Bacteria.p__Firmicutes.c__Clostridia.o__Eubacteriales.f__Lachnos
0
200
400
600
46−60 (n=7)
80plus (n=3)
61−80 (n=15)
31−45 (n=3)
20−30 (n=2)
Age_category

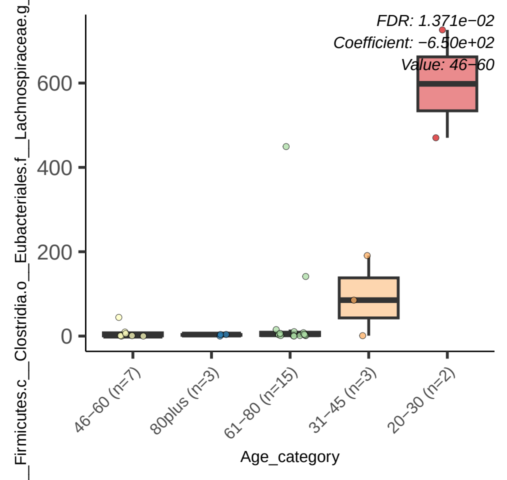
FDR: 1.371e−02
Coefficient: −6.50e+02
Value: 46−60
Firmicutes.c__Clostridia.o__Eubacteriales.f__Lachnospiraceae.g_
0
200
400
600
46−60 (n=7)
80plus (n=3)
61−80 (n=15)
31−45 (n=3)
20−30 (n=2)
Age_category

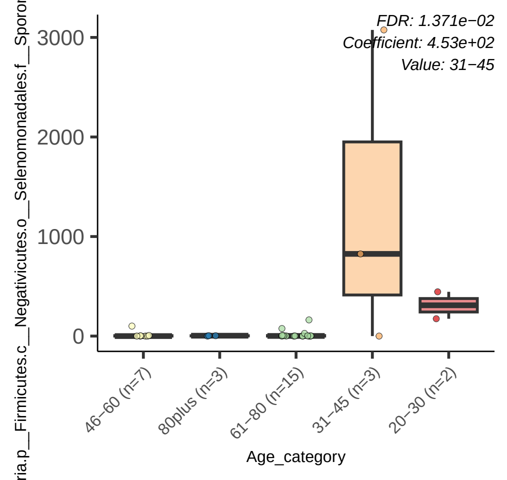

ria.p__Firmicutes.c__Negativicutes.o__Selenomonadales.f__Sporo
FDR: 1.371e−02
Coefficient: 4.53e+02
Value: 31−45
3000
2000
1000
0
46−60 (n=7)
80plus (n=3)
61−80 (n=15)
31−45 (n=3)
20−30 (n=2)
Age_category

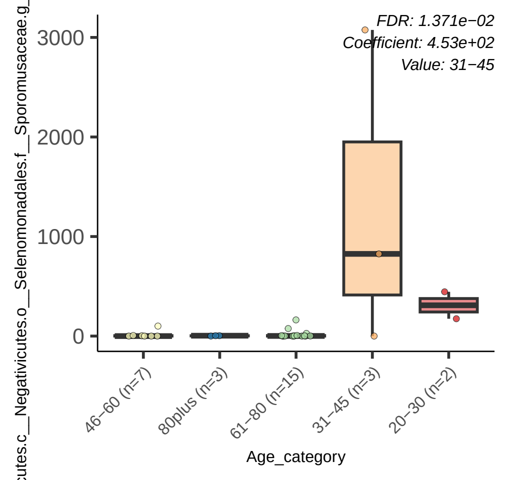

FDR: 1.371e−02
Coefficient: 4.53e+02
Value: 31−45
cutes.c__Negativicutes.o__Selenomonadales.f__Sporomusaceae.g_
3000
2000
1000
0
46−60 (n=7)
80plus (n=3)
61−80 (n=15)
31−45 (n=3)
20−30 (n=2)
Age_category

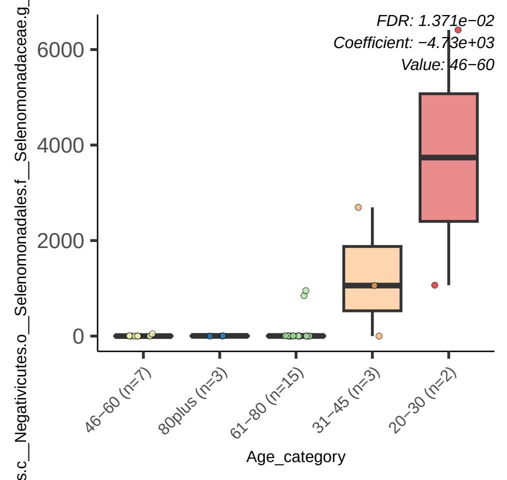

FDR: 1.371e−02
Coefficient: −4.73e+03
Value: 46−60
6000
4000
2000
0
s.c__Negativicutes.o__Selenomonadales.f__Selenomonadaceae.g__
46−60 (n=7)
80plus (n=3)
61−80 (n=15)
31−45 (n=3)
20−30 (n=2)
Age_category

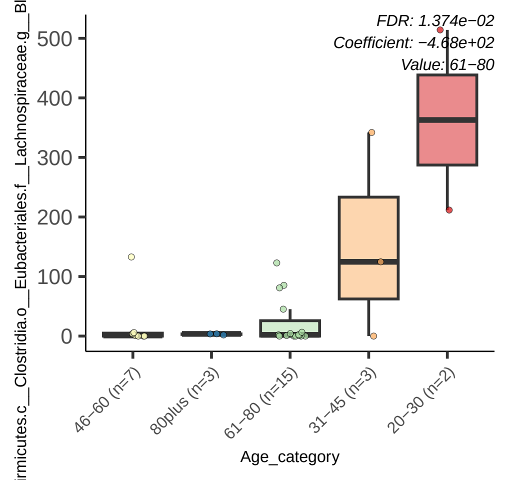

FDR: 1.374e−02
Coefficient: −4.68e+02
Value: 61−80
irmicutes.c__Clostridia.o__Eubacteriales.f__Lachnospiraceae.g__Bl
500
400
300
200
100
0
46−60 (n=7)
80plus (n=3)
61−80 (n=15)
31−45 (n=3)
20−30 (n=2)
Age_category

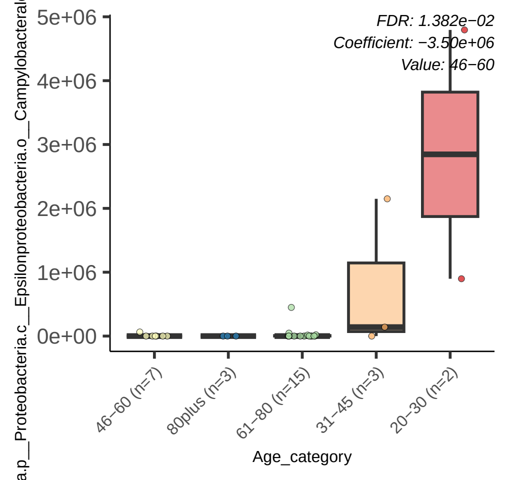
FDR: 1.382e−02
Coefficient: −3.50e+06
Value: 46−60
5e+06
4e+06
3e+06
2e+06
1e+06
0e+00
a.p__Proteobacteria.c__Epsilonproteobacteria.o__Campylobacterale
46−60 (n=7)
80plus (n=3)
61−80 (n=15)
31−45 (n=3)
20−30 (n=2)
Age_category

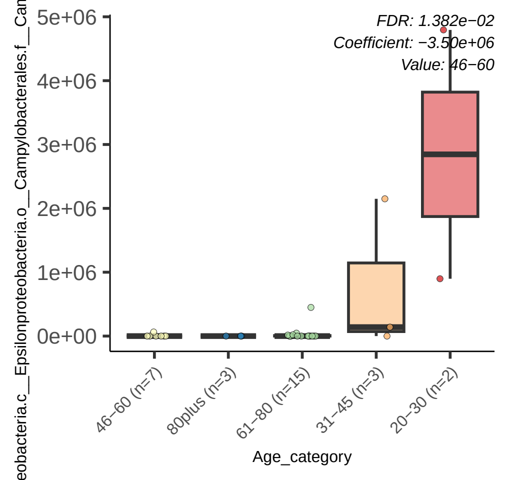

FDR: 1.382e−02
Coefficient: −3.50e+06
Value: 46−60
5e+06
4e+06
3e+06
2e+06
1e+06
0e+00
46−60 (n=7)
80plus (n=3)
61−80 (n=15)
31−45 (n=3)
20−30 (n=2)
Age_category
eobacteria.c__Epsilonproteobacteria.o__Campylobacterales.f__Cam

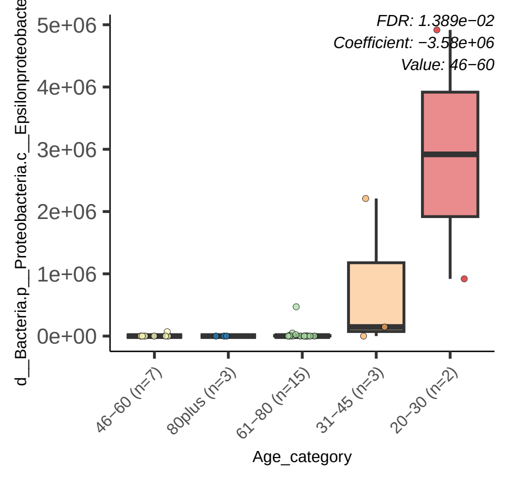

FDR: 1.389e−02
Coefficient: −3.58e+06
Value: 46−60
d__Bacteria.p__Proteobacteria.c__Epsilonproteobacte
5e+06
4e+06
3e+06
2e+06
1e+06
0e+00
46−60 (n=7)
80plus (n=3)
61−80 (n=15)
31−45 (n=3)
20−30 (n=2)
Age_category

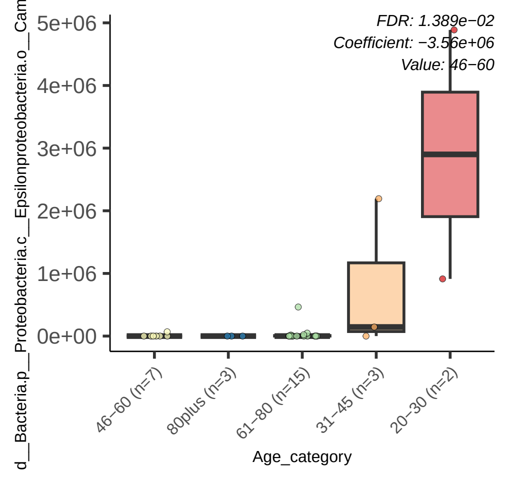

FDR: 1.389e−02
Coefficient: −3.56e+06
Value: 46−60
d__Bacteria.p__Proteobacteria.c__Epsilonproteobacteria.o__Cam
5e+06
4e+06
3e+06
2e+06
1e+06
0e+00
46−60 (n=7)
80plus (n=3)
61−80 (n=15)
31−45 (n=3)
20−30 (n=2)
Age_category

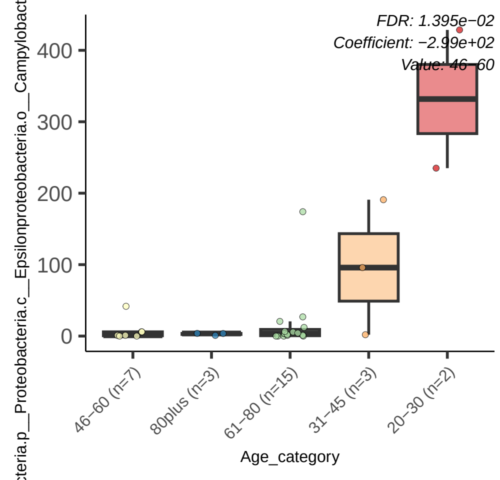

FDR: 1.395e−02
Coefficient: −2.99e+02
Value: 46−60
400
300
200
100
0
cteria.p__Proteobacteria.c__Epsilonproteobacteria.o__Campylobact
46−60 (n=7)
80plus (n=3)
61−80 (n=15)
31−45 (n=3)
20−30 (n=2)
Age_category

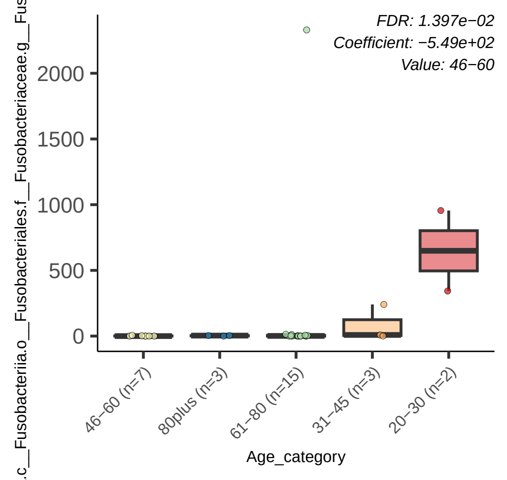

FDR: 1.397e−02
Coefficient: −5.49e+02
Value: 46−60
2000
1500
1000
500
0
46−60 (n=7)
80plus (n=3)
61−80 (n=15)
31−45 (n=3)
20−30 (n=2)
Age_category
.c__Fusobacteriia.o__Fusobacteriales.f__Fusobacteriaceae.g__Fus

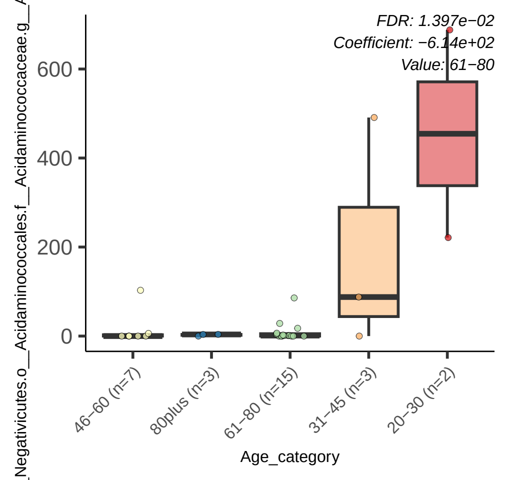

FDR: 1.397e−02
Coefficient: −6.14e+02
Value: 61−80
Negativicutes.o__Acidaminococcales.f__Acidaminococcaceae.g__
0
200
400
600
46−60 (n=7)
80plus (n=3)
61−80 (n=15)
31−45 (n=3)
20−30 (n=2)
Age_category

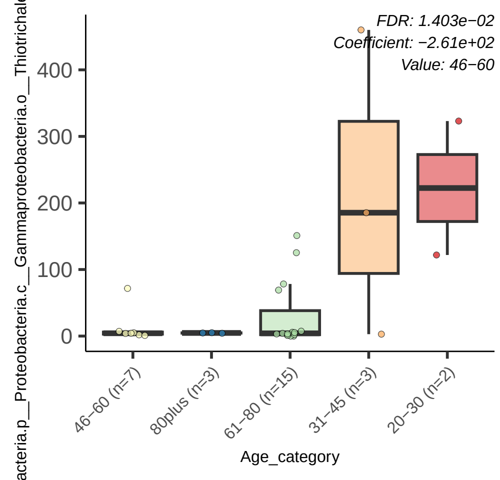

FDR: 1.403e−02
Coefficient: −2.61e+02
Value: 46−60
acteria.p__Proteobacteria.c__Gammaproteobacteria.o__Thiotrichale
400
300
200
100
0
46−60 (n=7)
80plus (n=3)
61−80 (n=15)
31−45 (n=3)
20−30 (n=2)
Age_category

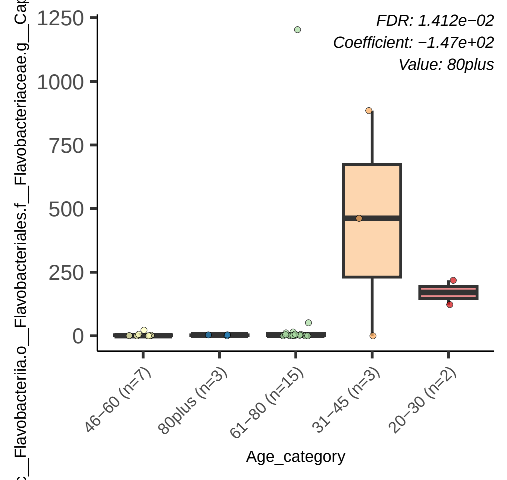
FDR: 1.412e−02
Coefficient: −1.47e+02
Value: 80plus
1250
1000
750
500
250
0
46−60 (n=7)
80plus (n=3)
61−80 (n=15)
31−45 (n=3)
20−30 (n=2)
Age_category
c__Flavobacteriia.o__Flavobacteriales.f__Flavobacteriaceae.g__Cap

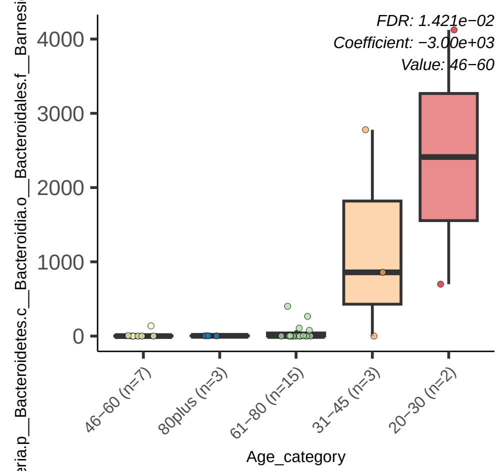
FDR: 1.421e−02
Coefficient: −3.00e+03
Value: 46−60
teria.p__Bacteroidetes.c__Bacteroidia.o__Bacteroidales.f__Barnesi
4000
3000
2000
1000
0
46−60 (n=7)
80plus (n=3)
61−80 (n=15)
31−45 (n=3)
20−30 (n=2)
Age_category

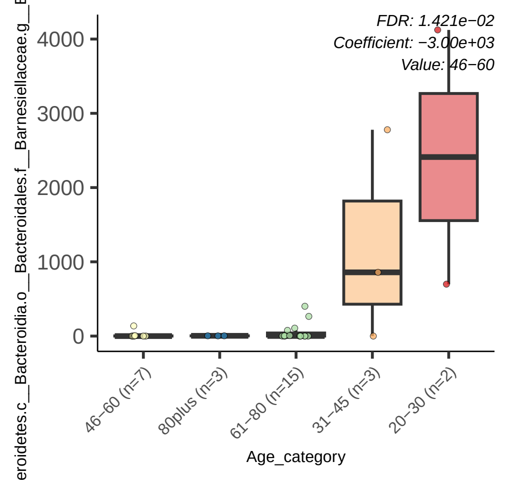

FDR: 1.421e−02
Coefficient: −3.00e+03
Value: 46−60
4000
3000
2000
1000
0
eroidetes.c__Bacteroidia.o__Bacteroidales.f__Barnesiellaceae.g__B
46−60 (n=7)
80plus (n=3)
61−80 (n=15)
31−45 (n=3)
20−30 (n=2)
Age_category

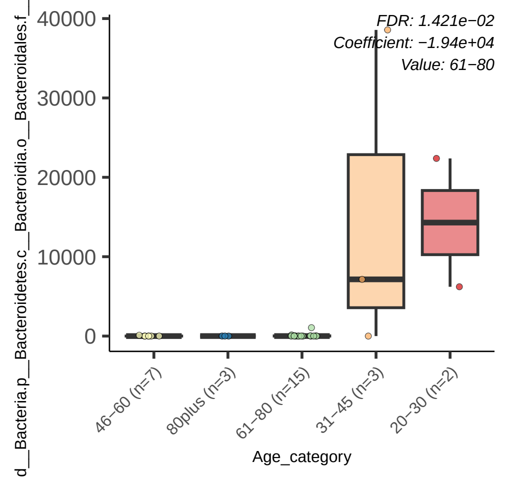

FDR: 1.421e−02
Coefficient: −1.94e+04
Value: 61−80
d__Bacteria.p__Bacteroidetes.c__Bacteroidia.o__Bacteroidales.f__
40000
30000
20000
10000
0
46−60 (n=7)
80plus (n=3)
61−80 (n=15)
31−45 (n=3)
20−30 (n=2)
Age_category

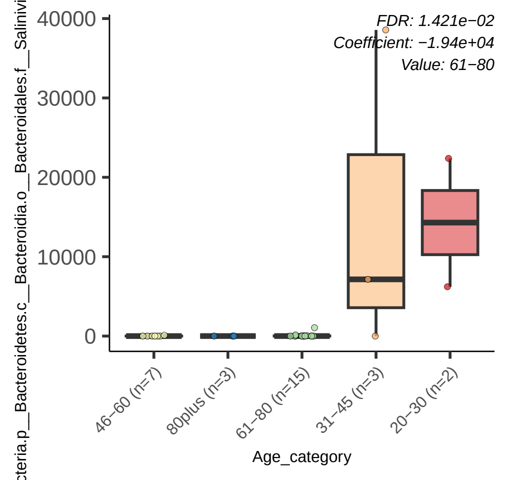

FDR: 1.421e−02
Coefficient: −1.94e+04
Value: 61−80
40000
30000
20000
10000
0
cteria.p__Bacteroidetes.c__Bacteroidia.o__Bacteroidales.f__Salinivi
46−60 (n=7)
80plus (n=3)
61−80 (n=15)
31−45 (n=3)
20−30 (n=2)
Age_category

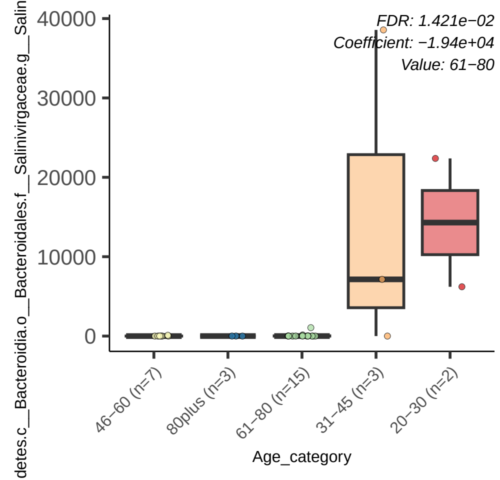

FDR: 1.421e−02
Coefficient: −1.94e+04
Value: 61−80
detes.c__Bacteroidia.o__Bacteroidales.f__Salinivirgaceae.g__Salin
40000
30000
20000
10000
0
46−60 (n=7)
80plus (n=3)
61−80 (n=15)
31−45 (n=3)
20−30 (n=2)
Age_category

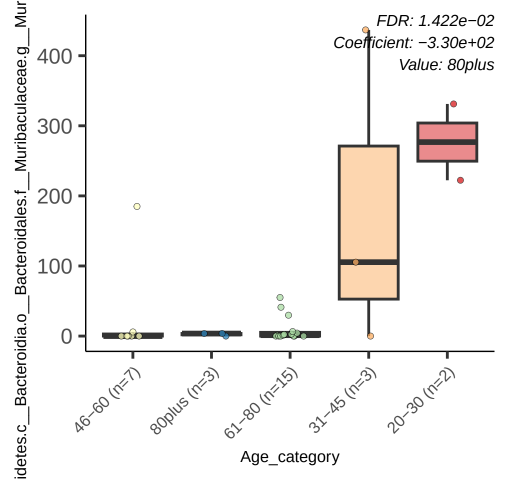

FDR: 1.422e−02
Coefficient: −3.30e+02
Value: 80plus
400
300
200
100
0
46−60 (n=7)
80plus (n=3)
61−80 (n=15)
31−45 (n=3)
20−30 (n=2)
Age_category
idetes.c__Bacteroidia.o__Bacteroidales.f__Muribaculaceae.g__Mur

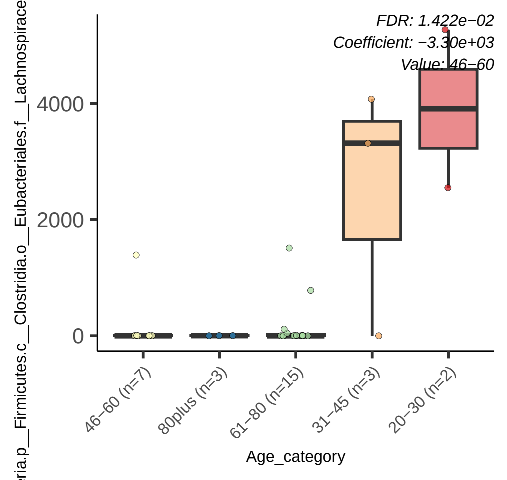

FDR: 1.422e−02
Coefficient: −3.30e+03
Value: 46−60
4000
2000
0
46−60 (n=7)
80plus (n=3)
61−80 (n=15)
31−45 (n=3)
20−30 (n=2)
Age_category
eria.p__Firmicutes.c__Clostridia.o__Eubacteriales.f__Lachnospirace

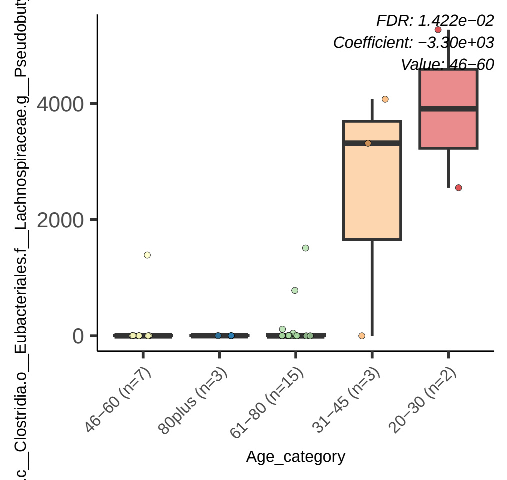

FDR: 1.422e−02
Coefficient: −3.30e+03
Value: 46−60
c__Clostridia.o__Eubacteriales.f__Lachnospiraceae.g__Pseudobuty
4000
2000
0
46−60 (n=7)
80plus (n=3)
61−80 (n=15)
31−45 (n=3)
20−30 (n=2)
Age_category

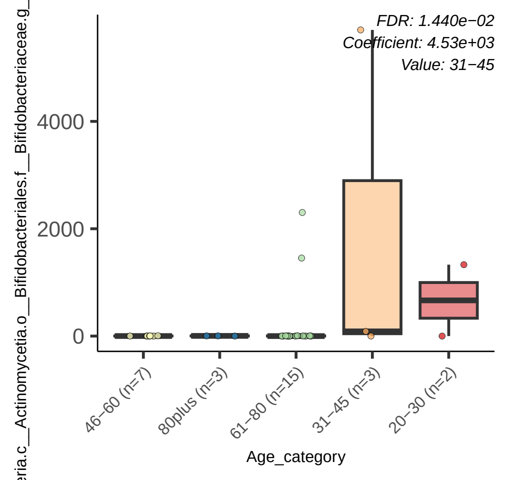

FDR: 1.440e−02
Coefficient: 4.53e+03
Value: 31−45
4000
2000
0
46−60 (n=7)
80plus (n=3)
61−80 (n=15)
31−45 (n=3)
20−30 (n=2)
Age_category
eria.c__Actinomycetia.o__Bifidobacteriales.f__Bifidobacteriaceae.g_

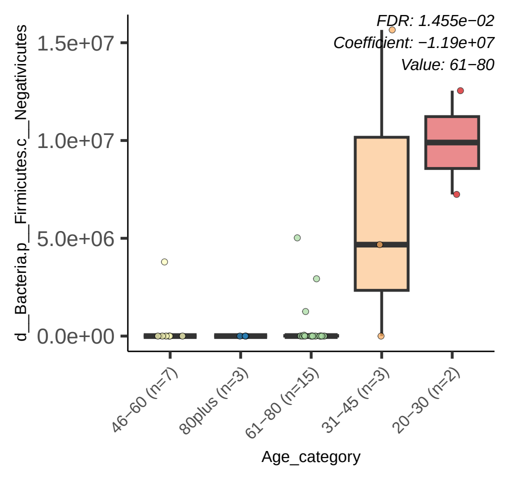
FDR: 1.455e−02
Coefficient: −1.19e+07
Value: 61−80
d__Bacteria.p__Firmicutes.c__Negativicutes
1.5e+07
1.0e+07
5.0e+06
0.0e+00
46−60 (n=7)
80plus (n=3)
61−80 (n=15)
31−45 (n=3)
20−30 (n=2)
Age_category

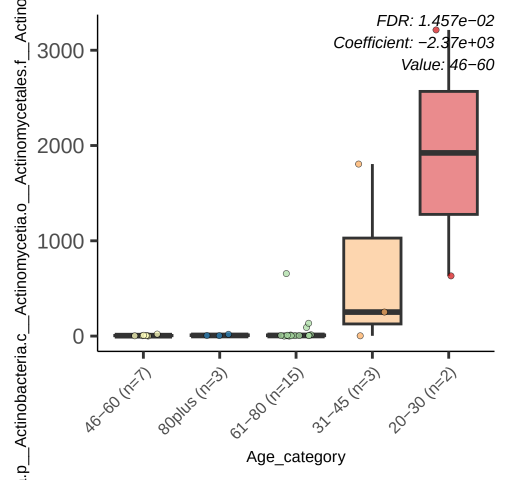

FDR: 1.457e−02
Coefficient: −2.37e+03
Value: 46−60
3000
2000
1000
0
p__Actinobacteria.c__Actinomycetia.o__Actinomycetales.f__Actino
46−60 (n=7)
80plus (n=3)
61−80 (n=15)
31−45 (n=3)
20−30 (n=2)
Age_category

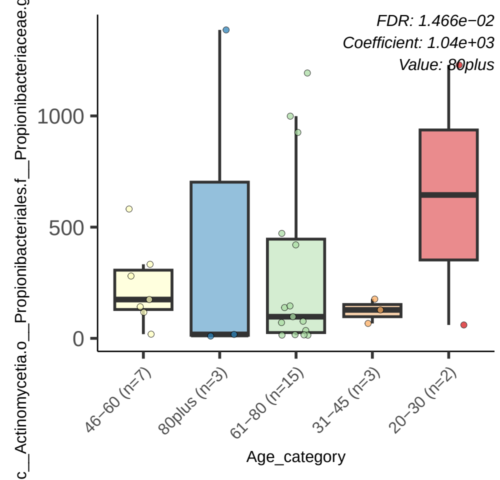

FDR: 1.466e−02
Coefficient: 1.04e+03
Value: 80plus
c__Actinomycetia.o__Propionibacteriales.f__Propionibacteriaceae.g
1000
500
0
46−60 (n=7)
80plus (n=3)
61−80 (n=15)
31−45 (n=3)
20−30 (n=2)
Age_category

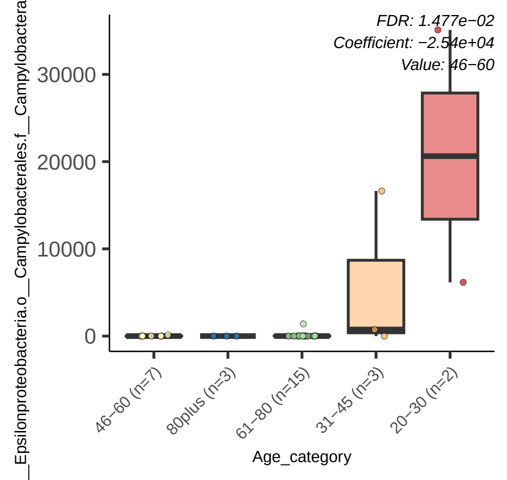

FDR: 1.477e−02
Coefficient: −2.54e+04
Value: 46−60
30000
20000
10000
0
__Epsilonproteobacteria.o__Campylobacterales.f__Campylobactera
46−60 (n=7)
80plus (n=3)
61−80 (n=15)
31−45 (n=3)
20−30 (n=2)
Age_category

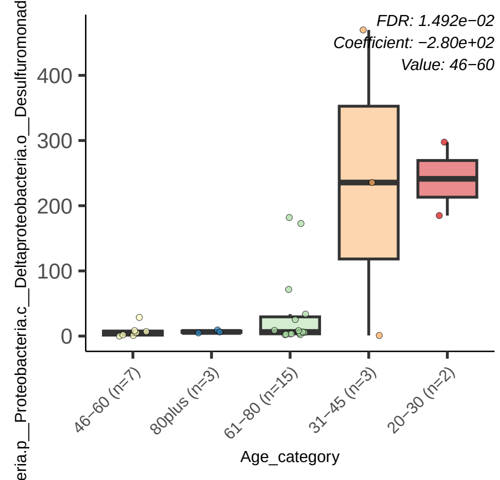

FDR: 1.492e−02
Coefficient: −2.80e+02
Value: 46−60
400
300
200
100
0
eria.p__Proteobacteria.c__Deltaproteobacteria.o__Desulfuromonad
46−60 (n=7)
80plus (n=3)
61−80 (n=15)
31−45 (n=3)
20−30 (n=2)
Age_category

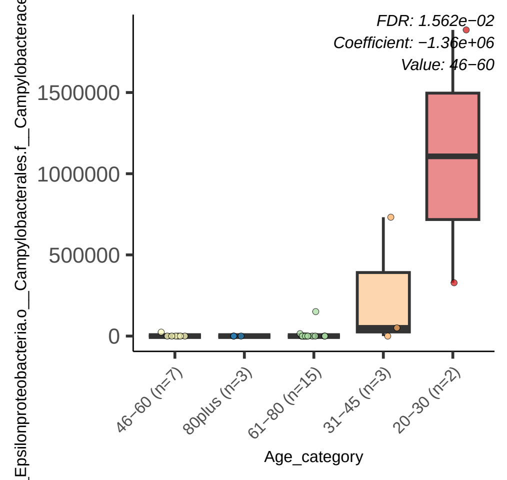

FDR: 1.562e−02
Coefficient: −1.36e+06
Value: 46−60
1500000
1000000
500000
0
_Epsilonproteobacteria.o__Campylobacterales.f__Campylobacterace
46−60 (n=7)
80plus (n=3)
61−80 (n=15)
31−45 (n=3)
20−30 (n=2)
Age_category

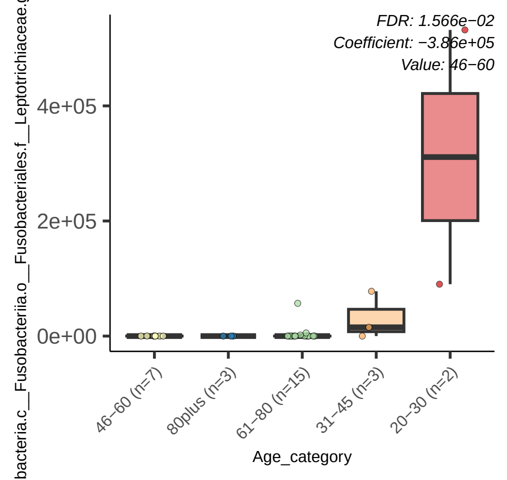

FDR: 1.566e−02
Coefficient: −3.86e+05
Value: 46−60
4e+05
2e+05
0e+00
46−60 (n=7)
80plus (n=3)
61−80 (n=15)
31−45 (n=3)
20−30 (n=2)
Age_category

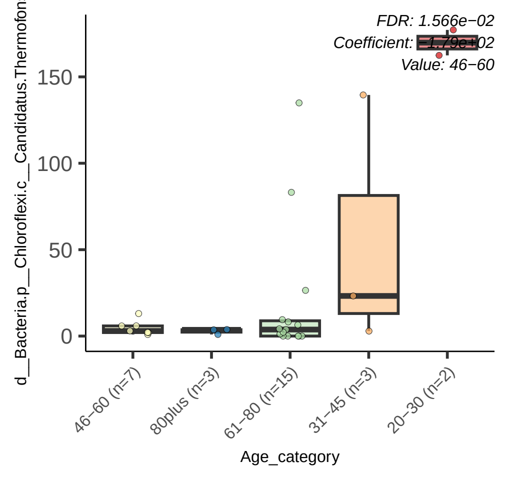

FDR: 1.566e−02
Coefficient: −1.79e+02
Value: 46−60
d__Bacteria.p__Chloroflexi.c__Candidatus.Thermofon
150
100
50
0
46−60 (n=7)
80plus (n=3)
61−80 (n=15)
31−45 (n=3)
20−30 (n=2)
Age_category

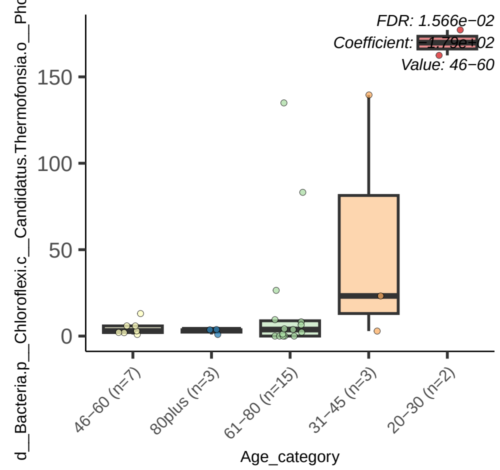

FDR: 1.566e−02
Coefficient: −1.79e+02
Value: 46−60
d__Bacteria.p__Chloroflexi.c__Candidatus.Thermofonsia.o__Pho
150
100
50
0
46−60 (n=7)
80plus (n=3)
61−80 (n=15)
31−45 (n=3)
20−30 (n=2)
Age_category

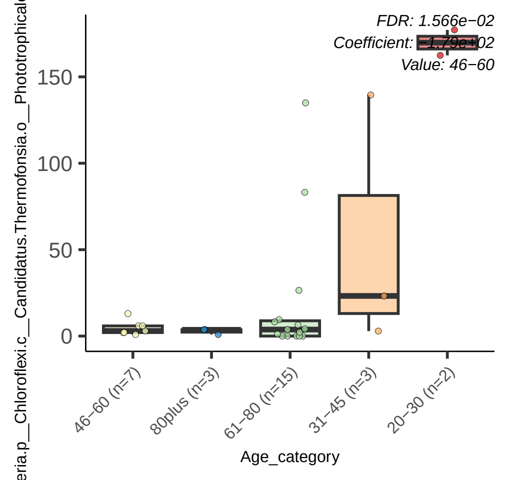

FDR: 1.566e−02
Coefficient: −1.79e+02
Value: 46−60
150
100
50
0
46−60 (n=7)
80plus (n=3)
61−80 (n=15)
31−45 (n=3)
20−30 (n=2)
Age_category
eria.p__Chloroflexi.c__Candidatus.Thermofonsia.o__Phototrophical

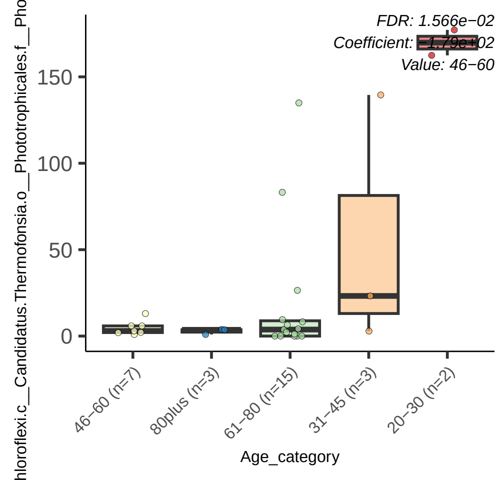

FDR: 1.566e−02
Coefficient: −1.79e+02
Value: 46−60
150
100
50
0
hloroflexi.c__Candidatus.Thermofonsia.o__Phototrophicales.f__Pho
46−60 (n=7)
80plus (n=3)
61−80 (n=15)
31−45 (n=3)
20−30 (n=2)
Age_category

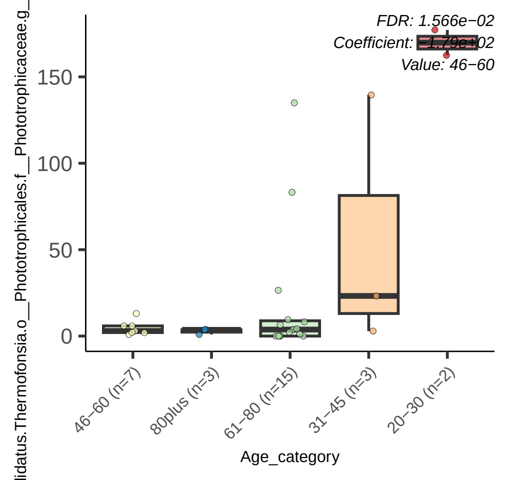

FDR: 1.566e−02
Coefficient: −1.79e+02
Value: 46−60
150
100
50
0
46−60 (n=7)
80plus (n=3)
61−80 (n=15)
31−45 (n=3)
20−30 (n=2)
Age_category
lidatus.Thermofonsia.o__Phototrophicales.f__Phototrophicaceae.g__

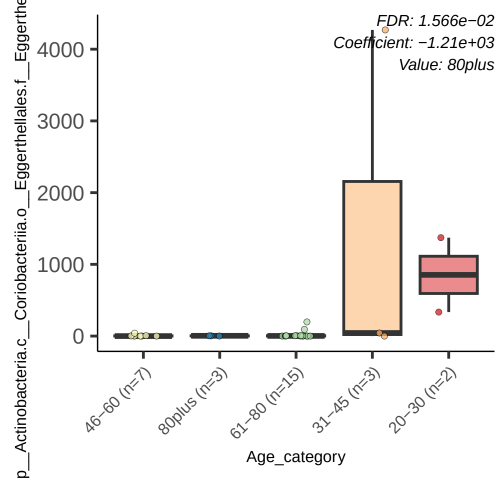
FDR: 1.566e−02
Coefficient: −1.21e+03
Value: 80plus
p__Actinobacteria.c__Coriobacteriia.o__Eggerthellales.f__Eggerthe
4000
3000
2000
1000
0
46−60 (n=7)
80plus (n=3)
61−80 (n=15)
31−45 (n=3)
20−30 (n=2)
Age_category

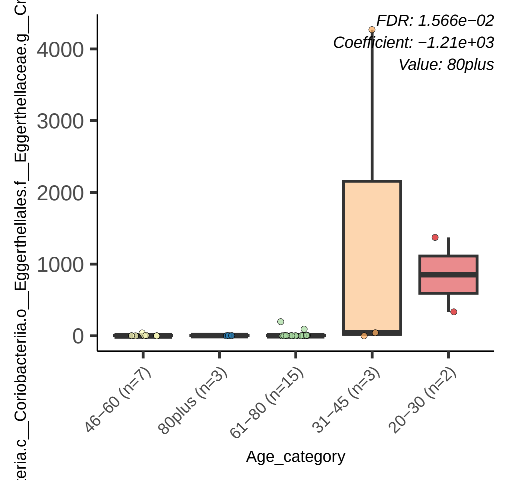
FDR: 1.566e−02
Coefficient: −1.21e+03
Value: 80plus
4000
3000
2000
1000
0
46−60 (n=7)
80plus (n=3)
61−80 (n=15)
31−45 (n=3)
20−30 (n=2)
Age_category
teria.c__Coriobacteriia.o__Eggerthellales.f__Eggerthellaceae.g__Cr

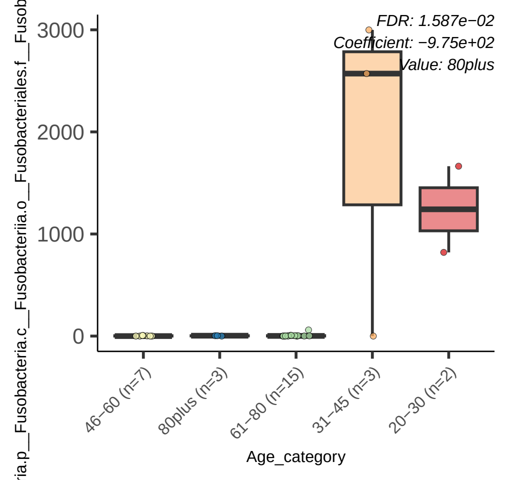

FDR: 1.587e−02
Coefficient: −9.75e+02
Value: 80plus
3000
2000
1000
0
46−60 (n=7)
80plus (n=3)
61−80 (n=15)
31−45 (n=3)
20−30 (n=2)
Age_category
ria.p__Fusobacteria.c__Fusobacteriia.o__Fusobacteriales.f__Fusob

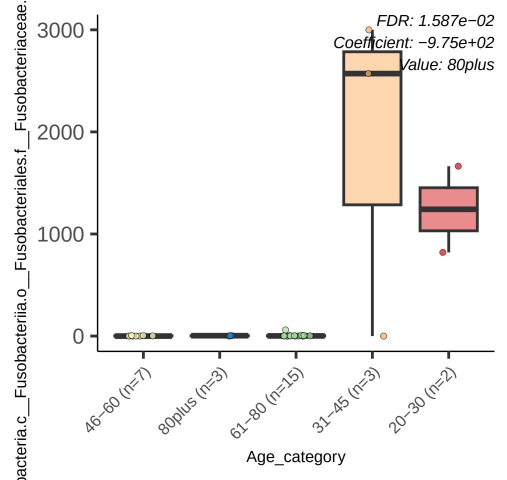

FDR: 1.587e−02
Coefficient: −9.75e+02
Value: 80plus
3000
2000
1000
0
bacteria.c__Fusobacteriia.o__Fusobacteriales.f__Fusobacteriaceae.
46−60 (n=7)
80plus (n=3)
61−80 (n=15)
31−45 (n=3)
20−30 (n=2)
Age_category

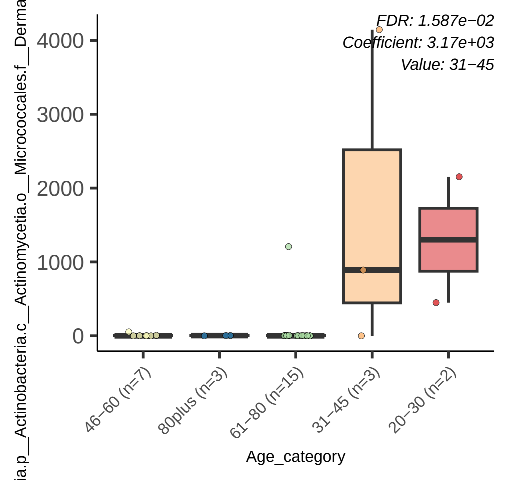

FDR: 1.587e−02
Coefficient: 3.17e+03
Value: 31−45
4000
3000
2000
1000
0
ia.p__Actinobacteria.c__Actinomycetia.o__Micrococcales.f__Derma
46−60 (n=7)
80plus (n=3)
61−80 (n=15)
31−45 (n=3)
20−30 (n=2)
Age_category

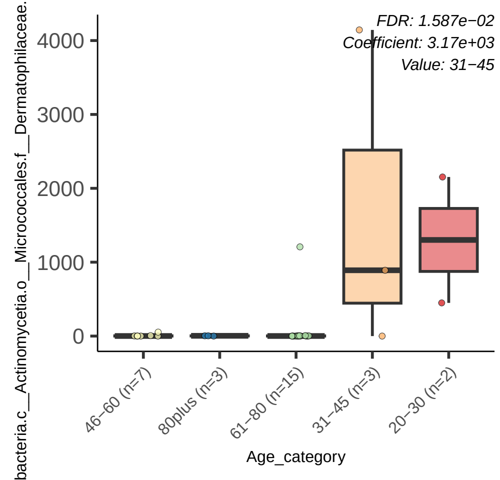

FDR: 1.587e−02
Coefficient: 3.17e+03
Value: 31−45
bacteria.c__Actinomycetia.o__Micrococcales.f__Dermatophilaceae.
4000
3000
2000
1000
0
46−60 (n=7)
80plus (n=3)
61−80 (n=15)
31−45 (n=3)
20−30 (n=2)
Age_category

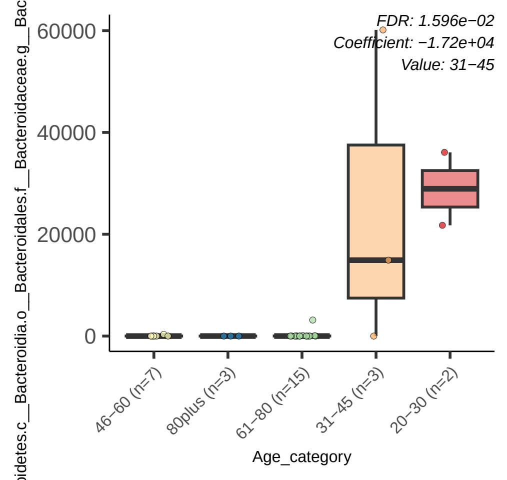

FDR: 1.596e−02
Coefficient: −1.72e+04
Value: 31−45
60000
40000
20000
0
46−60 (n=7)
80plus (n=3)
61−80 (n=15)
31−45 (n=3)
20−30 (n=2)
Age_category
oidetes.c__Bacteroidia.o__Bacteroidales.f__Bacteroidaceae.g__Bac

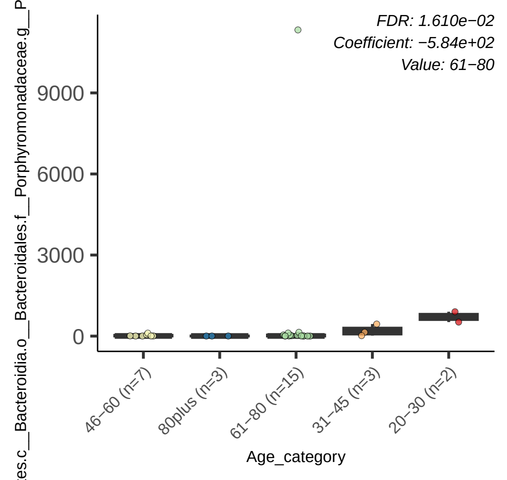
FDR: 1.610e−02
Coefficient: −5.84e+02
Value: 61−80
9000
6000
3000
0
46−60 (n=7)
80plus (n=3)
61−80 (n=15)
31−45 (n=3)
20−30 (n=2)
Age_category
es.c__Bacteroidia.o__Bacteroidales.f__Porphyromonadaceae.g__P

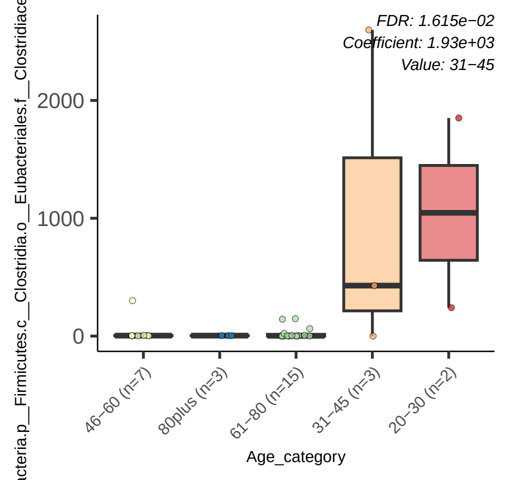
FDR: 1.615e−02
Coefficient: 1.93e+03
Value: 31−45
2000
1000
0
46−60 (n=7)
80plus (n=3)
61−80 (n=15)
31−45 (n=3)
20−30 (n=2)
Age_category
acteria.p__Firmicutes.c__Clostridia.o__Eubacteriales.f__Clostridiace

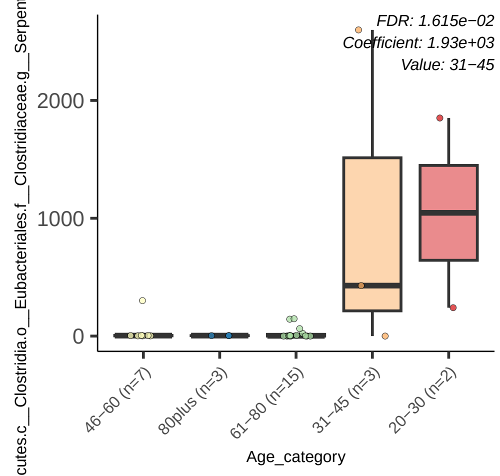

FDR: 1.615e−02
Coefficient: 1.93e+03
Value: 31−45
2000
1000
0
icutes.c__Clostridia.o__Eubacteriales.f__Clostridiaceae.g__Serpent
46−60 (n=7)
80plus (n=3)
61−80 (n=15)
31−45 (n=3)
20−30 (n=2)
Age_category

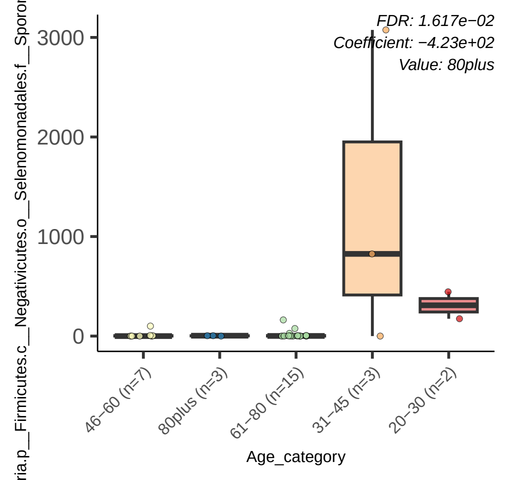

FDR: 1.617e−02
Coefficient: −4.23e+02
Value: 80plus
3000
2000
1000
0
ria.p__Firmicutes.c__Negativicutes.o__Selenomonadales.f__Sporo
46−60 (n=7)
80plus (n=3)
61−80 (n=15)
31−45 (n=3)
20−30 (n=2)
Age_category

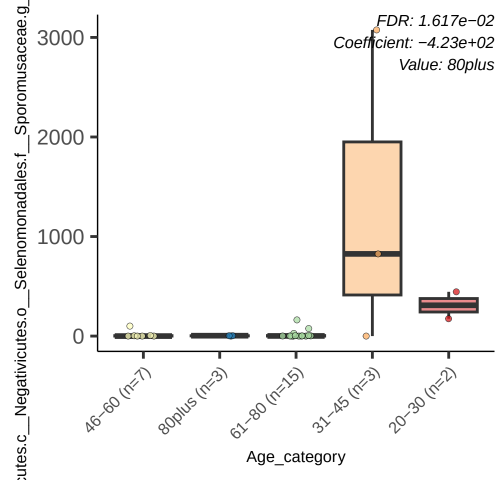

FDR: 1.617e−02
Coefficient: −4.23e+02
Value: 80plus
3000
2000
1000
0
cutes.c__Negativicutes.o__Selenomonadales.f__Sporomusaceae.g_
46−60 (n=7)
80plus (n=3)
61−80 (n=15)
31−45 (n=3)
20−30 (n=2)
Age_category

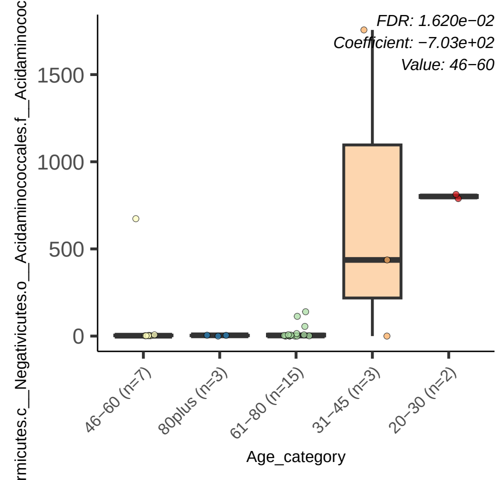
FDR: 1.620e−02
Coefficient: −7.03e+02
Value: 46−60
rmicutes.c__Negativicutes.o__Acidaminococcales.f__Acidaminococ
1500
1000
500
0
46−60 (n=7)
80plus (n=3)
61−80 (n=15)
31−45 (n=3)
20−30 (n=2)
Age_category

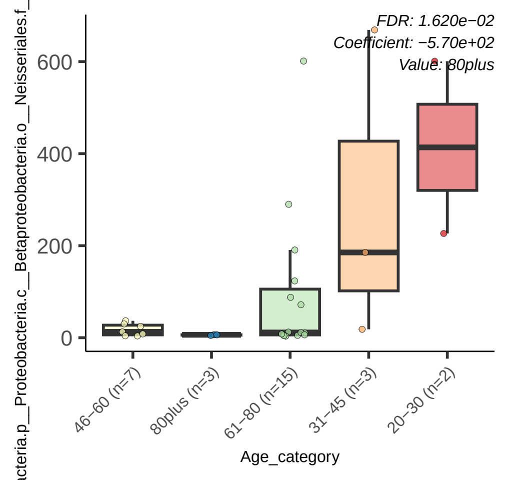

FDR: 1.620e−02
Coefficient: −5.70e+02
Value: 80plus
acteria.p__Proteobacteria.c__Betaproteobacteria.o__Neisseriales.f__
600
400
200
0
46−60 (n=7)
80plus (n=3)
61−80 (n=15)
31−45 (n=3)
20−30 (n=2)
Age_category

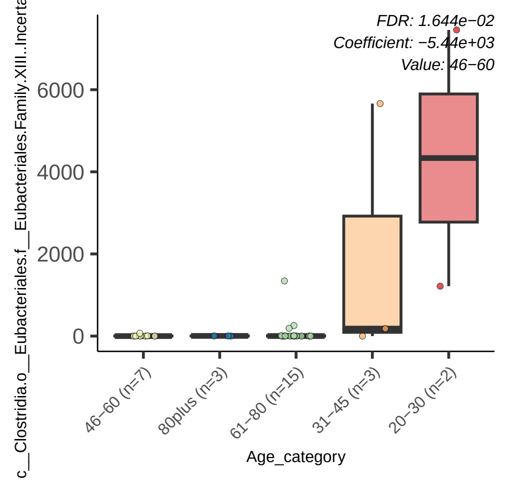
FDR: 1.644e−02
Coefficient: −5.44e+03
Value: 46−60
c__Clostridia.o__Eubacteriales.f__Eubacteriales.Family.XIII..Incerta
6000
4000
2000
0
46−60 (n=7)
80plus (n=3)
61−80 (n=15)
31−45 (n=3)
20−30 (n=2)
Age_category

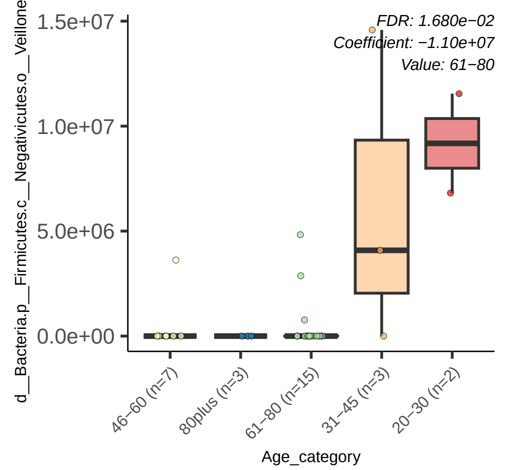

FDR: 1.680e−02
Coefficient: −1.10e+07
Value: 61−80
1.5e+07
1.0e+07
5.0e+06
0.0e+00
d__Bacteria.p__Firmicutes.c__Negativicutes.o__Veillone
46−60 (n=7)
80plus (n=3)
61−80 (n=15)
31−45 (n=3)
20−30 (n=2)
Age_category

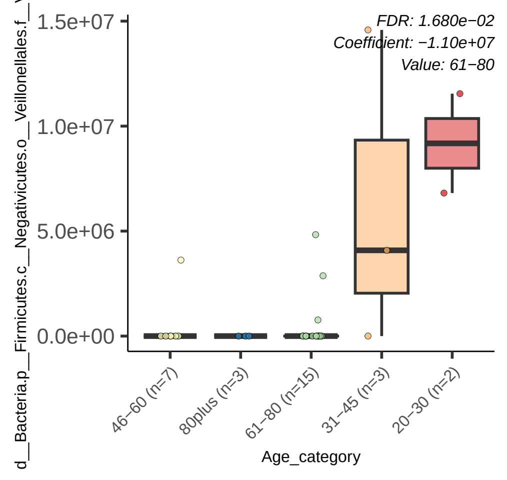

d__Bacteria.p__Firmicutes.c__Negativicutes.o__Veillonellales.f__
1.5e+07
1.0e+07
5.0e+06
0.0e+00
FDR: 1.680e−02
Coefficient: −1.10e+07
Value: 61−80
46−60 (n=7)
80plus (n=3)
61−80 (n=15)
31−45 (n=3)
20−30 (n=2)
Age_category

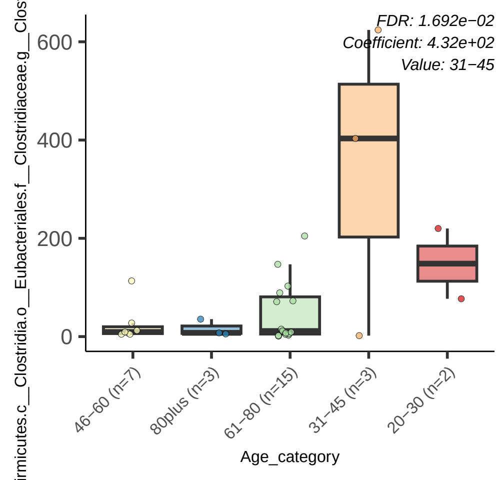
FDR: 1.692e−02
Coefficient: 4.32e+02
Value: 31−45
0
200
400
600
46−60 (n=7)
80plus (n=3)
61−80 (n=15)
31−45 (n=3)
20−30 (n=2)
Age_category

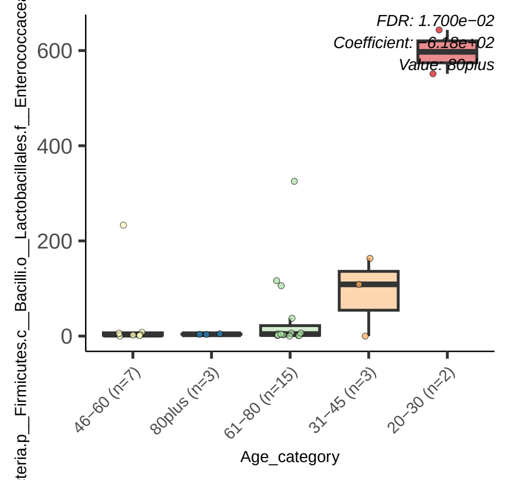

FDR: 1.700e−02
Coefficient: 6.18e+02
Value: 80plus
600
400
200
0
Firmicutes.c__Bacilli.o__Lactobacillales.f__Enterococcace
46−60 (n=7)
80plus (n=3)
61−80 (n=15)
31−45 (n=3)
20−30 (n=2)
Age_category

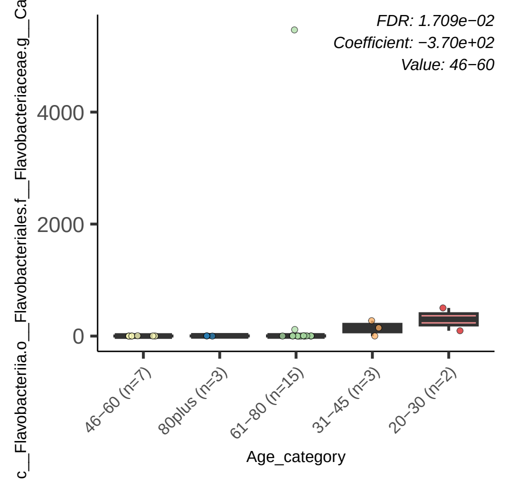

FDR: 1.709e−02
Coefficient: −3.70e+02
Value: 46−60
4000
2000
0
46−60 (n=7)
80plus (n=3)
61−80 (n=15)
31−45 (n=3)
20−30 (n=2)
Age_category
c__Flavobacteriia.o__Flavobacteriales.f__Flavobacteriaceae.g__Ca

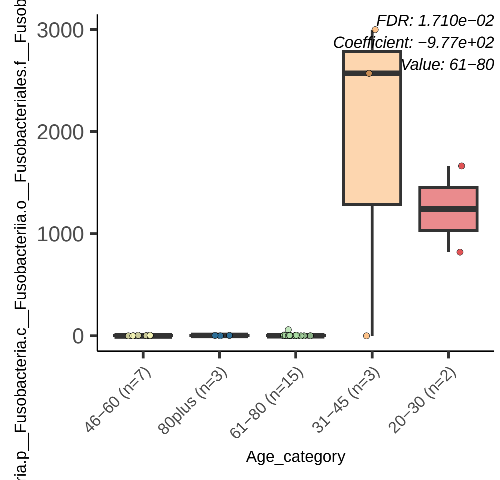

FDR: 1.710e−02
Coefficient: −9.77e+02
Value: 61−80
3000
2000
1000
0
46−60 (n=7)
80plus (n=3)
61−80 (n=15)
31−45 (n=3)
20−30 (n=2)
Age_category
ria.p__Fusobacteria.c__Fusobacteriia.o__Fusobacteriales.f__Fusob

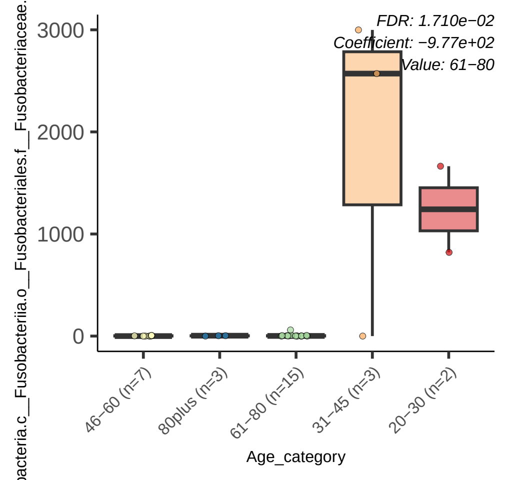

FDR: 1.710e−02
Coefficient: −9.77e+02
Value: 61−80
3000
2000
1000
0
46−60 (n=7)
80plus (n=3)
61−80 (n=15)
31−45 (n=3)
20−30 (n=2)
Age_category
bacteria.c__Fusobacteriia.o__Fusobacteriales.f__Fusobacteriaceae.

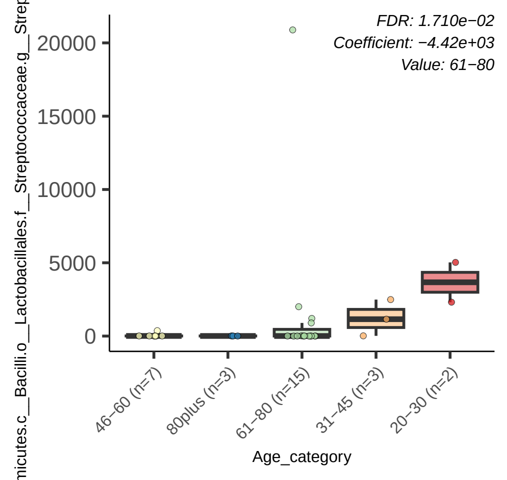
FDR: 1.710e−02
Coefficient: −4.42e+03
Value: 61−80
micutes.c__Bacilli.o__Lactobacillales.f__Streptococcaceae.g__Strep
20000
15000
10000
5000
0
46−60 (n=7)
80plus (n=3)
61−80 (n=15)
31−45 (n=3)
20−30 (n=2)
Age_category

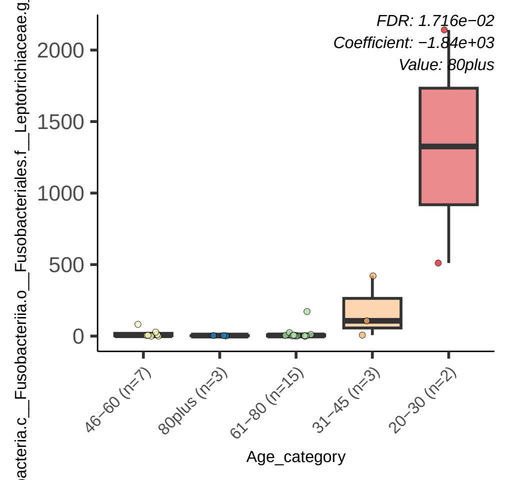

FDR: 1.716e−02
Coefficient: −1.84e+03
Value: 80plus
2000
1500
1000
500
0
bacteria.c__Fusobacteriia.o__Fusobacteriales.f__Leptotrichiaceae.g
46−60 (n=7)
80plus (n=3)
61−80 (n=15)
31−45 (n=3)
20−30 (n=2)
Age_category

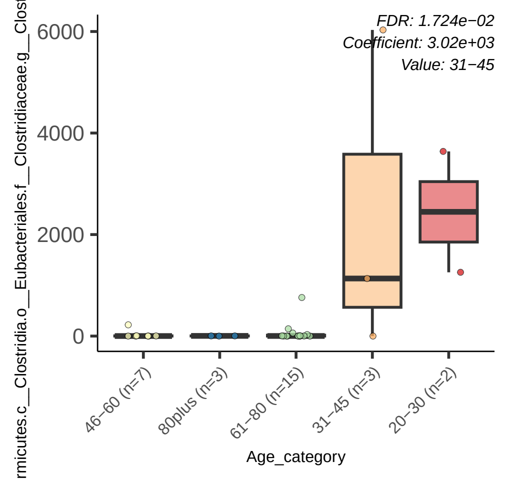

FDR: 1.724e−02
Coefficient: 3.02e+03
Value: 31−45
6000
4000
2000
0
rmicutes.c__Clostridia.o__Eubacteriales.f__Clostridiaceae.g__Clost
46−60 (n=7)
80plus (n=3)
61−80 (n=15)
31−45 (n=3)
20−30 (n=2)
Age_category

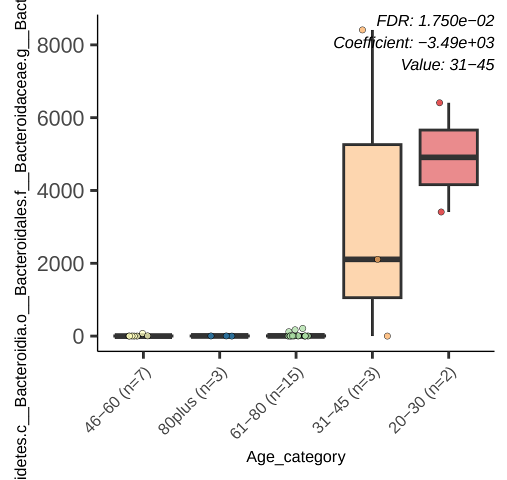

FDR: 1.750e−02
Coefficient: −3.49e+03
Value: 31−45
8000
6000
4000
2000
0
idetes.c__Bacteroidia.o__Bacteroidales.f__Bacteroidaceae.g__Bact
46−60 (n=7)
80plus (n=3)
61−80 (n=15)
31−45 (n=3)
20−30 (n=2)
Age_category

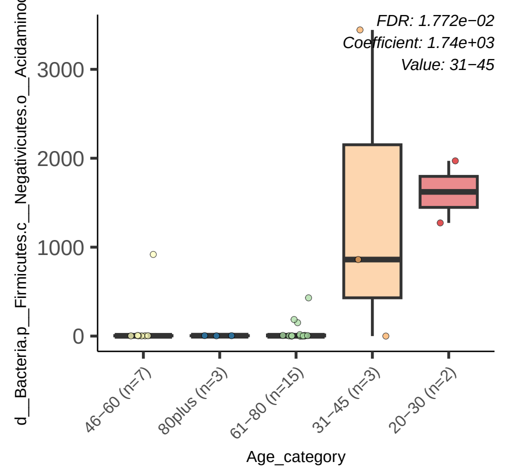

FDR: 1.772e−02
Coefficient: 1.74e+03
Value: 31−45
d__Bacteria.p__Firmicutes.c__Negativicutes.o__Acidaminoc
3000
2000
1000
0
46−60 (n=7)
80plus (n=3)
61−80 (n=15)
31−45 (n=3)
20−30 (n=2)
Age_category

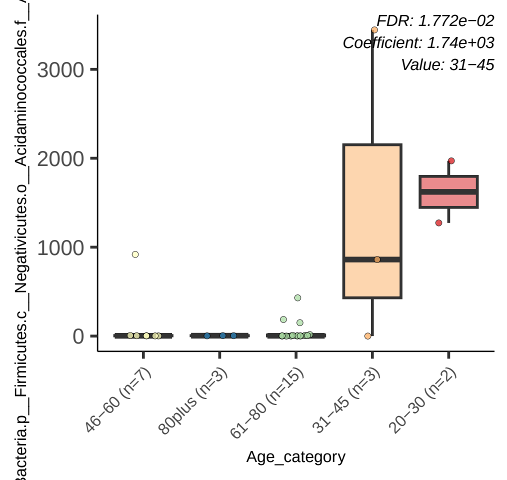

FDR: 1.772e−02
Coefficient: 1.74e+03
Value: 31−45
3000
2000
1000
0
Bacteria.p__Firmicutes.c__Negativicutes.o__Acidaminococcales.f__
46−60 (n=7)
80plus (n=3)
61−80 (n=15)
31−45 (n=3)
20−30 (n=2)
Age_category

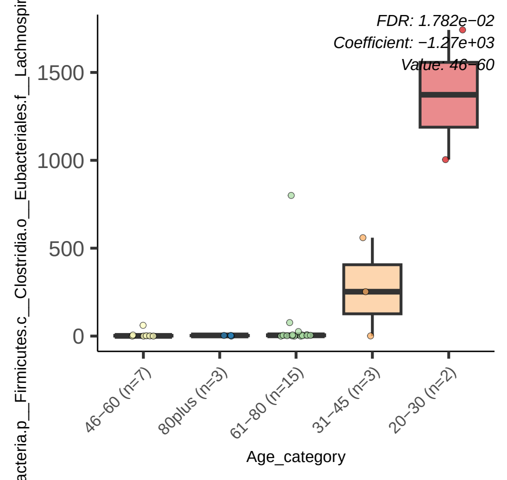

FDR: 1.782e−02
Coefficient: −1.27e+03
Value: 46−60
acteria.p__Firmicutes.c__Clostridia.o__Eubacteriales.f__Lachnospi
1500
1000
500
0
46−60 (n=7)
80plus (n=3)
61−80 (n=15)
31−45 (n=3)
20−30 (n=2)
Age_category

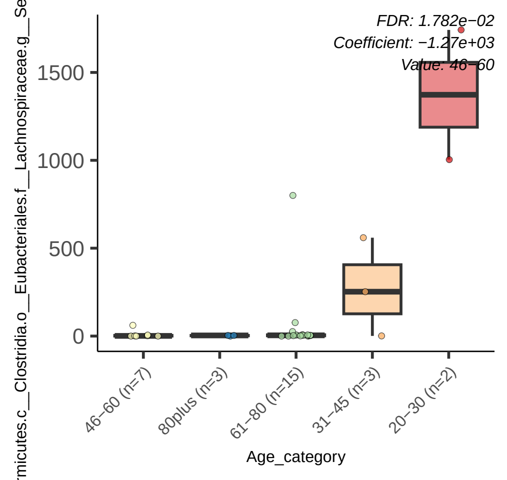

FDR: 1.782e−02
Coefficient: −1.27e+03
Value: 46−60
rmicutes.c__Clostridia.o__Eubacteriales.f__Lachnospiraceae.g__Se
0
500
1000
1500
46−60 (n=7)
80plus (n=3)
61−80 (n=15)
31−45 (n=3)
20−30 (n=2)
Age_category

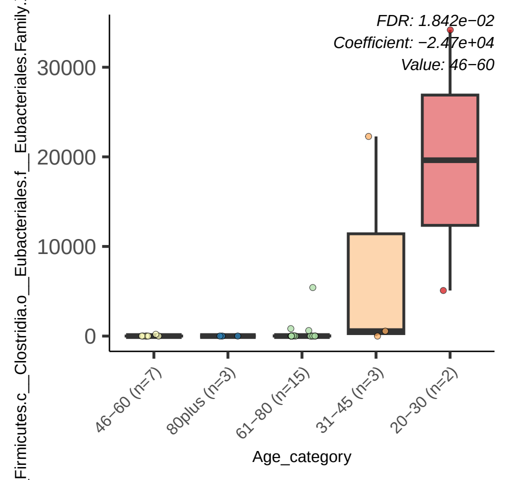

FDR: 1.842e−02
Coefficient: −2.47e+04
Value: 46−60
Firmicutes.c__Clostridia.o__Eubacteriales.f__Eubacteriales.Family.
30000
20000
10000
0
46−60 (n=7)
80plus (n=3)
61−80 (n=15)
31−45 (n=3)
20−30 (n=2)
Age_category

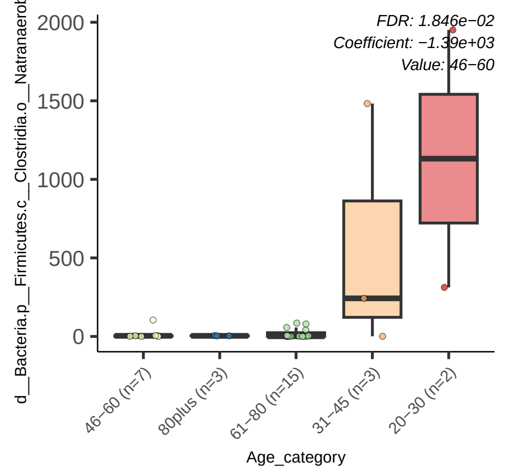

FDR: 1.846e−02
Coefficient: −1.39e+03
Value: 46−60
d__Bacteria.p__Firmicutes.c__Clostridia.o__Natranaerob
2000
1500
1000
500
0
46−60 (n=7)
80plus (n=3)
61−80 (n=15)
31−45 (n=3)
20−30 (n=2)
Age_category

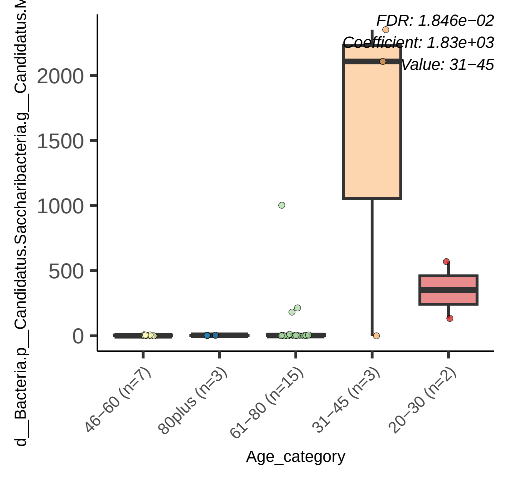

FDR: 1.846e−02
Coefficient: 1.83e+03
Value: 31−45
d__Bacteria.p__Candidatus.Saccharibacteria.g__Candidatus.M
0
500
1000
1500
2000
46−60 (n=7)
80plus (n=3)
61−80 (n=15)
31−45 (n=3)
20−30 (n=2)
Age_category

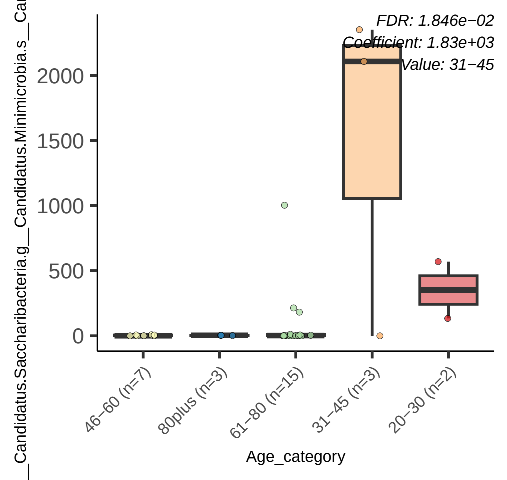

FDR: 1.846e−02
Coefficient: 1.83e+03
Value: 31−45
2000
1500
1000
500
0
Candidatus.Saccharibacteria.g__Candidatus.Minimicrobia.s__Ca
46−60 (n=7)
80plus (n=3)
61−80 (n=15)
31−45 (n=3)
20−30 (n=2)
Age_category

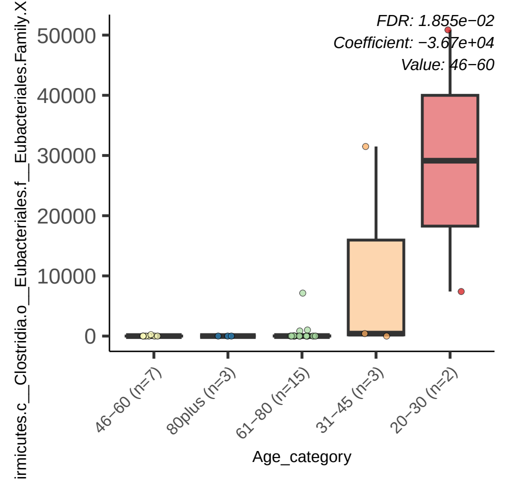

FDR: 1.855e−02
Coefficient: −3.67e+04
Value: 46−60
50000
40000
30000
20000
10000
0
irmicutes.c__Clostridia.o__Eubacteriales.f__Eubacteriales.Family.X
46−60 (n=7)
80plus (n=3)
61−80 (n=15)
31−45 (n=3)
20−30 (n=2)
Age_category

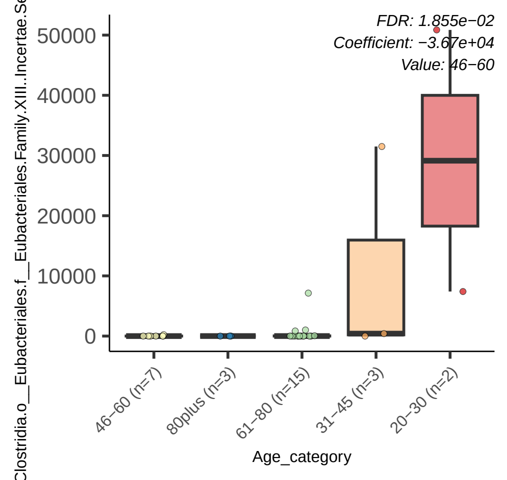

FDR: 1.855e−02
Coefficient: −3.67e+04
Value: 46−60
Clostridia.o__Eubacteriales.f__Eubacteriales.Family.XIII..Incertae.Se
50000
40000
30000
20000
10000
0
46−60 (n=7)
80plus (n=3)
61−80 (n=15)
31−45 (n=3)
20−30 (n=2)
Age_category

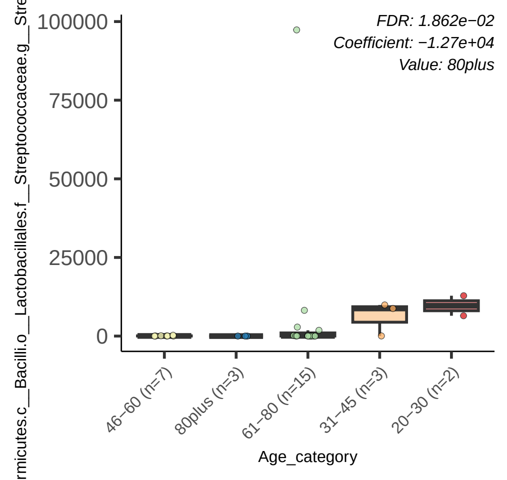

FDR: 1.862e−02
Coefficient: −1.27e+04
Value: 80plus
100000
75000
50000
25000
0
46−60 (n=7)
80plus (n=3)
61−80 (n=15)
31−45 (n=3)
20−30 (n=2)
Age_category
rmicutes.c__Bacilli.o__Lactobacillales.f__Streptococcaceae.g__Stre

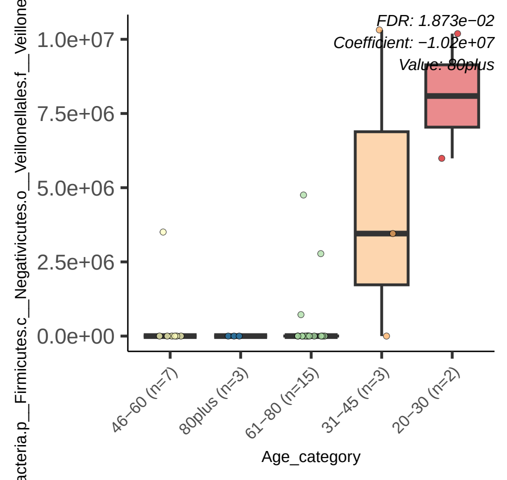

FDR: 1.873e−02
Coefficient: −1.02e+07
Value: 80plus
1.0e+07
7.5e+06
5.0e+06
2.5e+06
0.0e+00
46−60 (n=7)
80plus (n=3)
61−80 (n=15)
31−45 (n=3)
20−30 (n=2)
Age_category
acteria.p__Firmicutes.c__Negativicutes.o__Veillonellales.f__Veillone

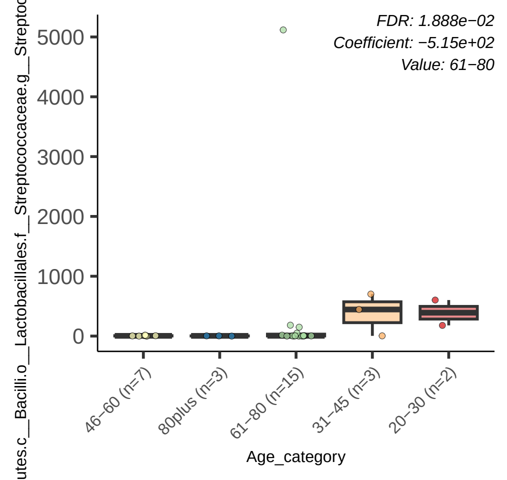
FDR: 1.888e−02
Coefficient: −5.15e+02
Value: 61−80
5000
4000
3000
2000
1000
0
utes.c__Bacilli.o__Lactobacillales.f__Streptococcaceae.g__Streptoc
46−60 (n=7)
80plus (n=3)
61−80 (n=15)
31−45 (n=3)
20−30 (n=2)
Age_category

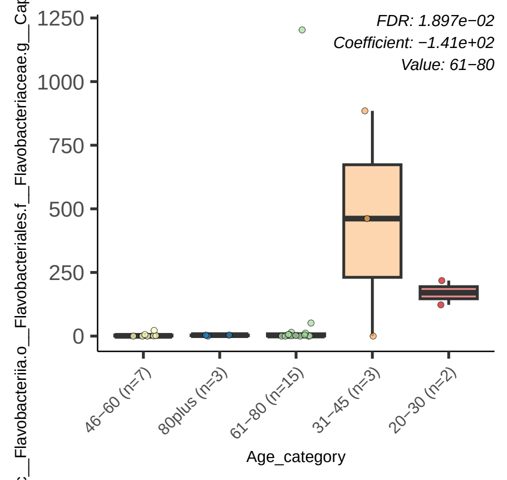

FDR: 1.897e−02
Coefficient: −1.41e+02
Value: 61−80
c__Flavobacteriia.o__Flavobacteriales.f__Flavobacteriaceae.g__Cap
1250
1000
750
500
250
0
46−60 (n=7)
80plus (n=3)
61−80 (n=15)
31−45 (n=3)
20−30 (n=2)
Age_category

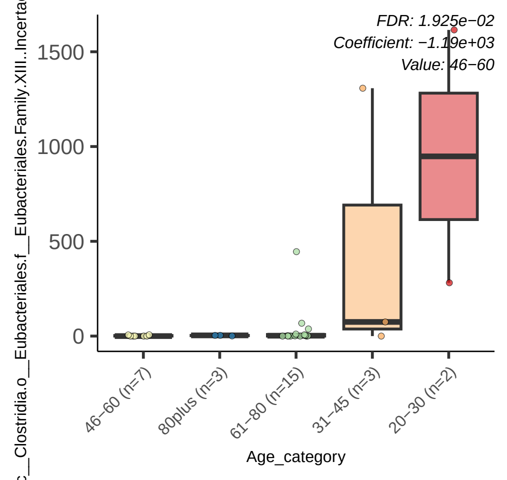

FDR: 1.925e−02
Coefficient: −1.19e+03
Value: 46−60
1500
1000
500
0
46−60 (n=7)
80plus (n=3)
61−80 (n=15)
31−45 (n=3)
20−30 (n=2)
Age_category
c__Clostridia.o__Eubacteriales.f__Eubacteriales.Family.XIII..Incertae

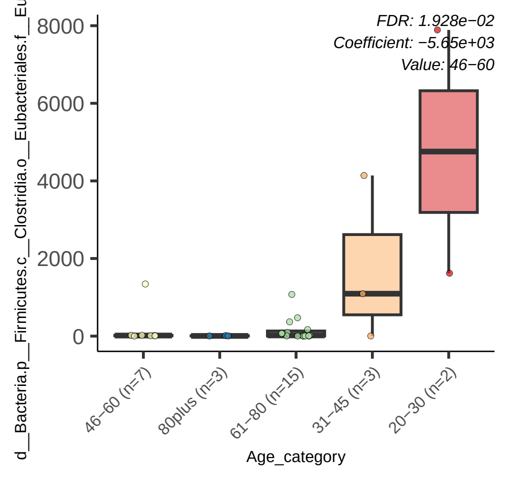

FDR: 1.928e−02
Coefficient: −5.65e+03
Value: 46−60
8000
6000
4000
2000
0
d__Bacteria.p__Firmicutes.c__Clostridia.o__Eubacteriales.f__Eu
46−60 (n=7)
80plus (n=3)
61−80 (n=15)
31−45 (n=3)
20−30 (n=2)
Age_category

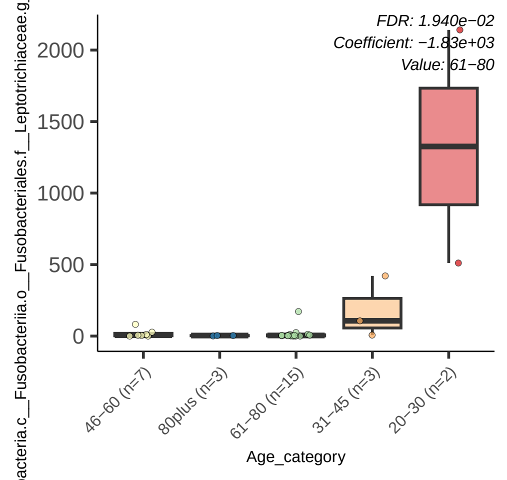
FDR: 1.940e−02
Coefficient: −1.83e+03
Value: 61−80
bacteria.c__Fusobacteriia.o__Fusobacteriales.f__Leptotrichiaceae.g
2000
1500
1000
500
0
46−60 (n=7)
80plus (n=3)
61−80 (n=15)
31−45 (n=3)
20−30 (n=2)
Age_category

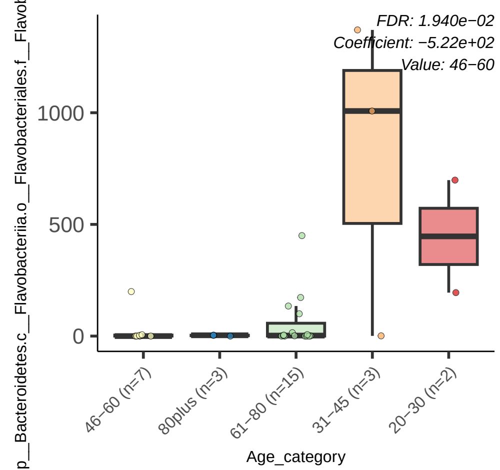

FDR: 1.940e−02
Coefficient: −5.22e+02
Value: 46−60
a.p__Bacteroidetes.c__Flavobacteriia.o__Flavobacteriales.f__Flavob
1000
500
0
46−60 (n=7)
80plus (n=3)
61−80 (n=15)
31−45 (n=3)
20−30 (n=2)
Age_category

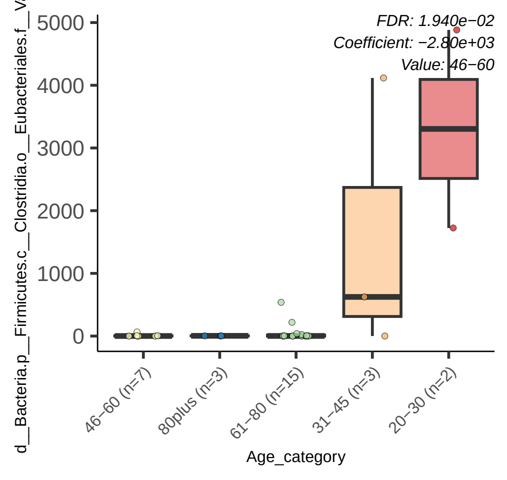

FDR: 1.940e−02
Coefficient: −2.80e+03
Value: 46−60
d__Bacteria.p__Firmicutes.c__Clostridia.o__Eubacteriales.f__
5000
4000
3000
2000
1000
0
46−60 (n=7)
80plus (n=3)
61−80 (n=15)
31−45 (n=3)
20−30 (n=2)
Age_category

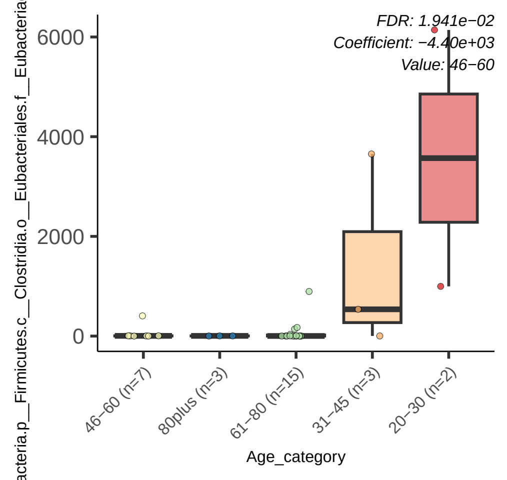
FDR: 1.941e−02
Coefficient: −4.40e+03
Value: 46−60
6000
4000
2000
0
acteria.p__Firmicutes.c__Clostridia.o__Eubacteriales.f__Eubacteria
46−60 (n=7)
80plus (n=3)
61−80 (n=15)
31−45 (n=3)
20−30 (n=2)
Age_category

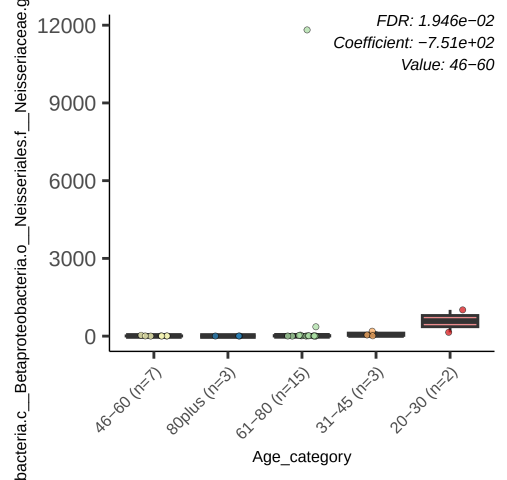

FDR: 1.946e−02
Coefficient: −7.51e+02
Value: 46−60
12000
9000
6000
3000
0
46−60 (n=7)
80plus (n=3)
61−80 (n=15)
31−45 (n=3)
20−30 (n=2)
Age_category
bacteria.c__Betaproteobacteria.o__Neisseriales.f__Neisseriaceae.g

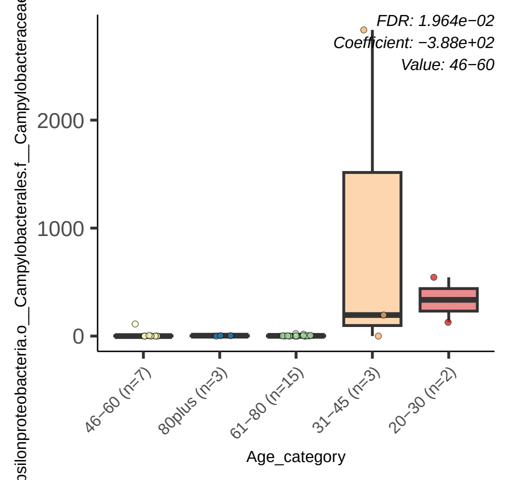
FDR: 1.964e−02
Coefficient: −3.88e+02
Value: 46−60
2000
1000
0
osilonproteobacteria.o__Campylobacterales.f__Campylobacteraceae
46−60 (n=7)
80plus (n=3)
61−80 (n=15)
31−45 (n=3)
20−30 (n=2)
Age_category

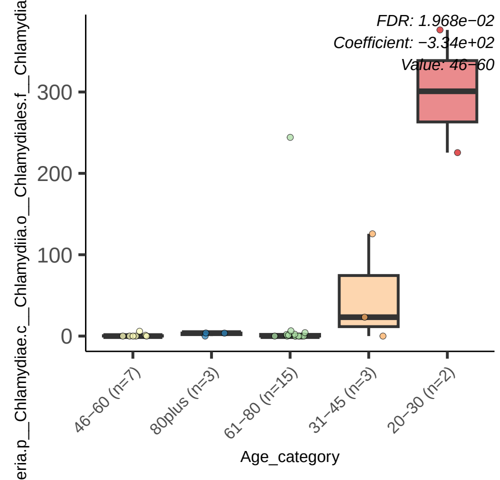

FDR: 1.968e−02
Coefficient: −3.34e+02
Value: 46−60
300
200
100
0
eria.p__Chlamydiae.c__Chlamydiia.o__Chlamydiales.f__Chlamydia
46−60 (n=7)
80plus (n=3)
61−80 (n=15)
31−45 (n=3)
20−30 (n=2)
Age_category

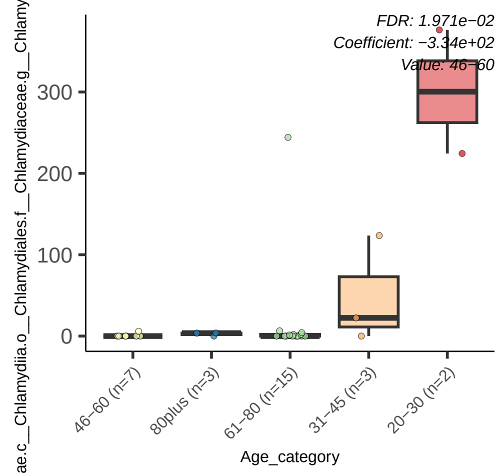

FDR: 1.971e−02
Coefficient: −3.34e+02
Value: 46−60
300
200
100
0
iae.c__Chlamydiia.o__Chlamydiales.f__Chlamydiaceae.g__Chlamy
46−60 (n=7)
80plus (n=3)
61−80 (n=15)
31−45 (n=3)
20−30 (n=2)
Age_category

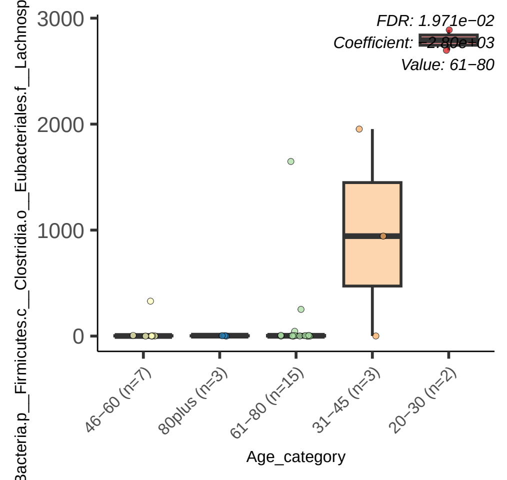

FDR: 1.971e−02
Coefficient: −2.80e+03
Value: 61−80
3000
2000
1000
0
Bacteria.p__Firmicutes.c__Clostridia.o__Eubacteriales.f__Lachnosp
46−60 (n=7)
80plus (n=3)
61−80 (n=15)
31−45 (n=3)
20−30 (n=2)
Age_category

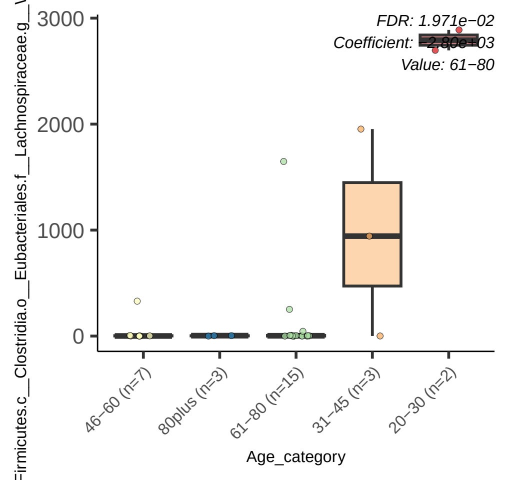

FDR: 1.971e−02
Coefficient: −2.80e+03
Value: 61−80
3000
2000
1000
0
Firmicutes.c__Clostridia.o__Eubacteriales.f__Lachnospiraceae.g__
46−60 (n=7)
80plus (n=3)
61−80 (n=15)
31−45 (n=3)
20−30 (n=2)
Age_category

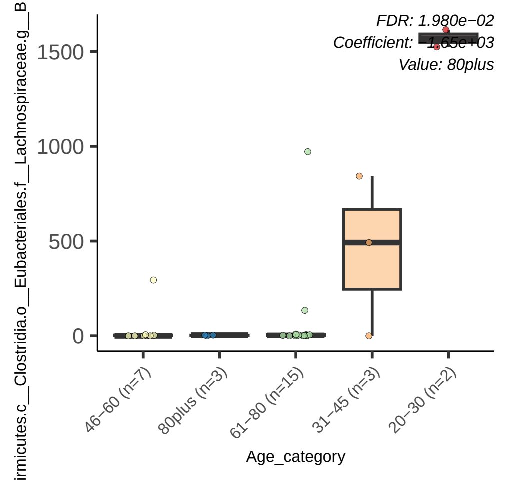

FDR: 1.980e−02
Coefficient: −1.65e+03
Value: 80plus
1500
1000
500
0
irmicutes.c__Clostridia.o__Eubacteriales.f__Lachnospiraceae.g__B
46−60 (n=7)
80plus (n=3)
61−80 (n=15)
31−45 (n=3)
20−30 (n=2)
Age_category

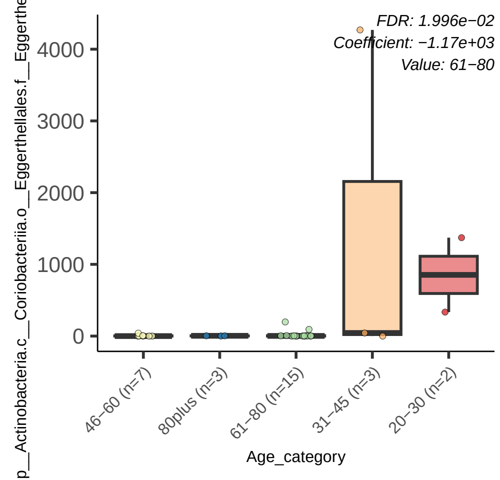
FDR: 1.996e−02
Coefficient: −1.17e+03
Value: 61−80
p__Actinobacteria.c__Coriobacteriia.o__Eggerthellales.f__Eggerthe
4000
3000
2000
1000
0
46−60 (n=7)
80plus (n=3)
61−80 (n=15)
31−45 (n=3)
20−30 (n=2)
Age_category

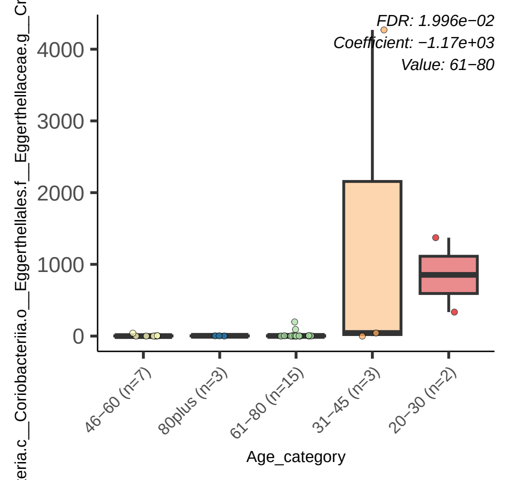

FDR: 1.996e−02
Coefficient: −1.17e+03
Value: 61−80
4000
3000
2000
1000
0
teria.c__Coriobacteriia.o__Eggerthellales.f__Eggerthellaceae.g__Cr
46−60 (n=7)
80plus (n=3)
61−80 (n=15)
31−45 (n=3)
20−30 (n=2)
Age_category

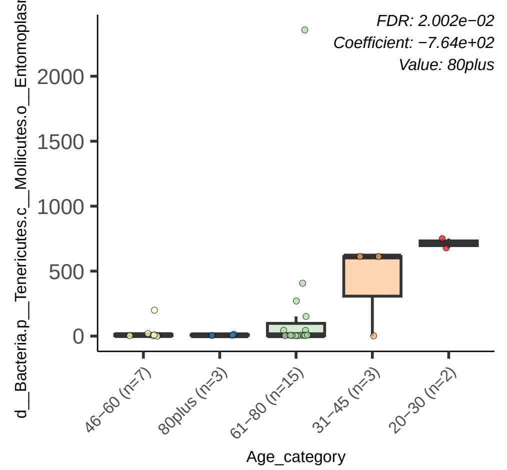

FDR: 2.002e−02
Coefficient: −7.64e+02
Value: 80plus
d__Bacteria.p__Tenericutes.c__Mollicutes.o__Entomoplasm
2000
1500
1000
500
0
46−60 (n=7)
80plus (n=3)
61−80 (n=15)
31−45 (n=3)
20−30 (n=2)
Age_category

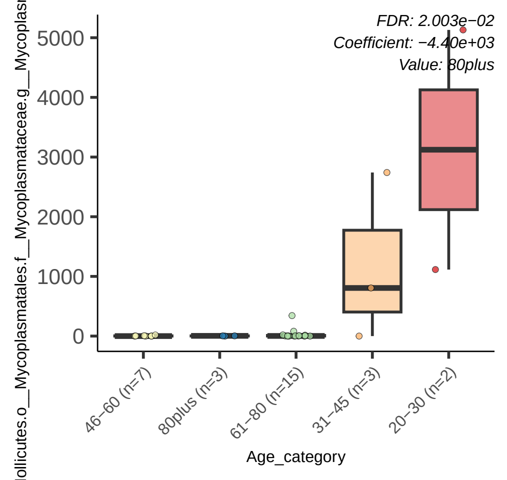

FDR: 2.003e−02
Coefficient: −4.40e+03
Value: 80plus
5000
4000
3000
2000
1000
0
ollicutes.o__Mycoplasmatales.f__Mycoplasmataceae.g__Mycoplasn
46−60 (n=7)
80plus (n=3)
61−80 (n=15)
31−45 (n=3)
20−30 (n=2)
Age_category

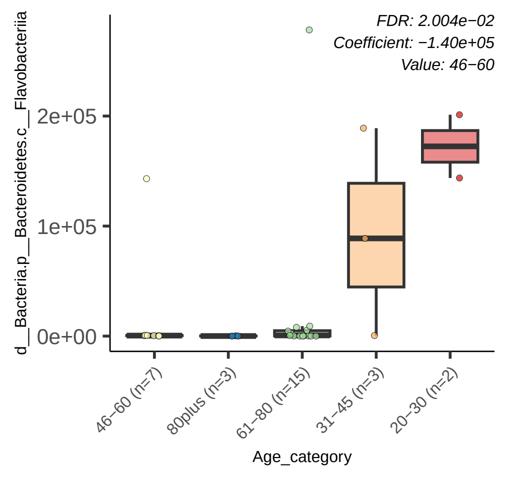
FDR: 2.004e−02
Coefficient: −1.40e+05
Value: 46−60
d__Bacteria.p__Bacteroidetes.c__Flavobacteriia
2e+05
1e+05
0e+00
46−60 (n=7)
80plus (n=3)
61−80 (n=15)
31−45 (n=3)
20−30 (n=2)
Age_category

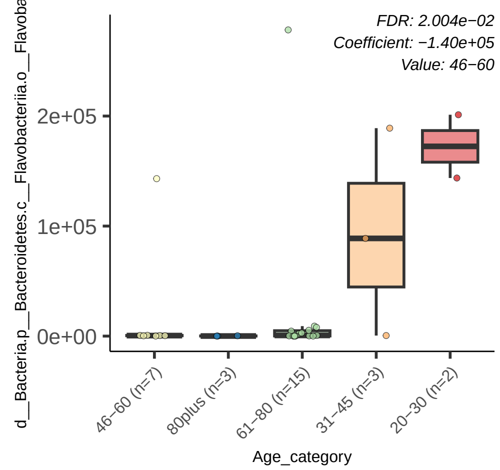

FDR: 2.004e−02
Coefficient: −1.40e+05
Value: 46−60
d__Bacteria.p__Bacteroidetes.c__Flavobacteriia.o__Flavoba
2e+05
1e+05
0e+00
46−60 (n=7)
80plus (n=3)
61−80 (n=15)
31−45 (n=3)
20−30 (n=2)
Age_category

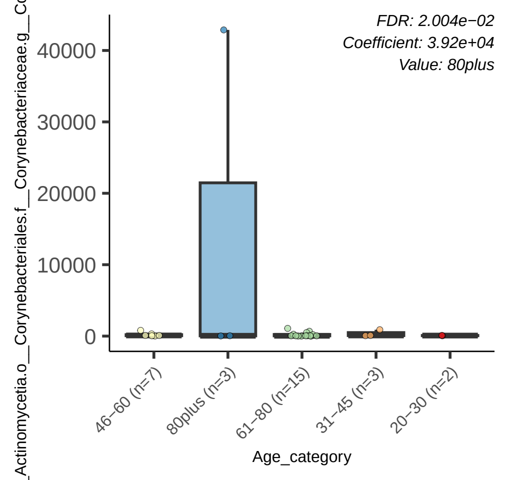

FDR: 2.004e−02
Coefficient: 3.92e+04
Value: 80plus
40000
30000
20000
10000
0
46−60 (n=7)
80plus (n=3)
61−80 (n=15)
31−45 (n=3)
20−30 (n=2)
Age_category
Actinomycetia.o__Corynebacteriales.f__Corynebacteriaceae.g__Co

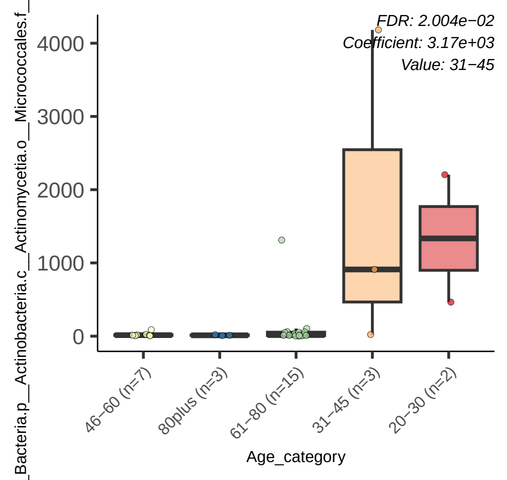

FDR: 2.004e−02
Coefficient: 3.17e+03
Value: 31−45
Bacteria.p__Actinobacteria.c__Actinomycetia.o__Micrococcales.f
4000
3000
2000
1000
0
46−60 (n=7)
80plus (n=3)
61−80 (n=15)
31−45 (n=3)
20−30 (n=2)
Age_category

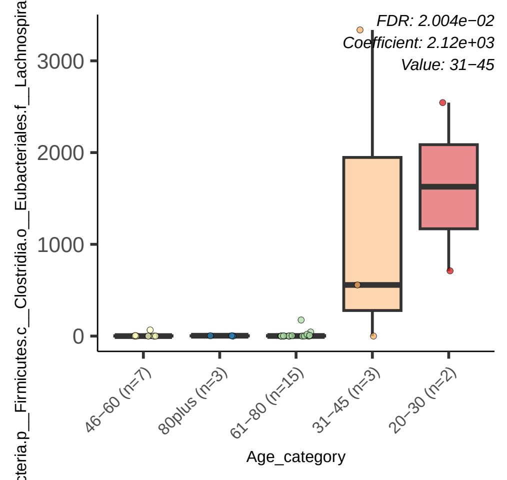

FDR: 2.004e−02
Coefficient: 2.12e+03
Value: 31−45
3000
2000
1000
0
cteria.p__Firmicutes.c__Clostridia.o__Eubacteriales.f__Lachnospira
46−60 (n=7)
80plus (n=3)
61−80 (n=15)
31−45 (n=3)
20−30 (n=2)
Age_category

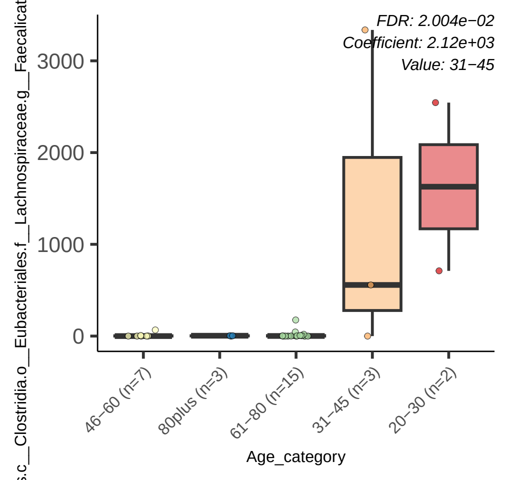

FDR: 2.004e−02
Coefficient: 2.12e+03
Value: 31−45
3000
2000
1000
0
s.c__Clostridia.o__Eubacteriales.f__Lachnospiraceae.g__Faecalicat
46−60 (n=7)
80plus (n=3)
61−80 (n=15)
31−45 (n=3)
20−30 (n=2)
Age_category

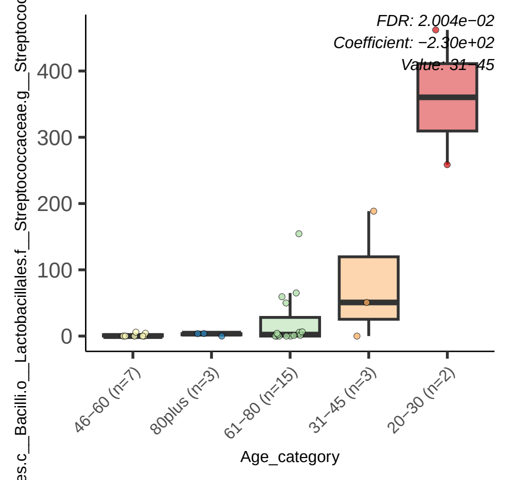

FDR: 2.004e−02
Coefficient: −2.30e+02
Value: 31−45
es.c__Bacilli.o__Lactobacillales.f__Streptococcaceae.g__Streptococ
400
300
200
100
0
46−60 (n=7)
80plus (n=3)
61−80 (n=15)
31−45 (n=3)
20−30 (n=2)
Age_category

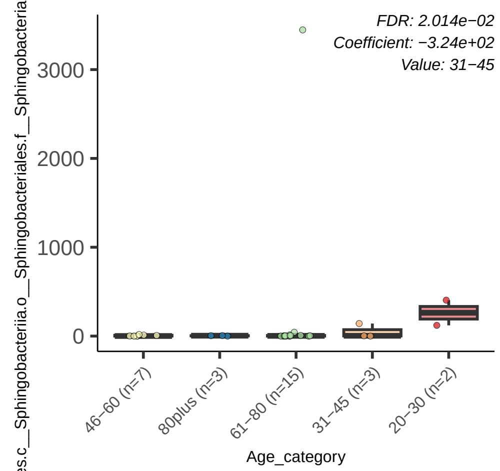

FDR: 2.014e−02
Coefficient: −3.24e+02
Value: 31−45
tes.c__Sphingobacteriia.o__Sphingobacteriales.f__Sphingobacteria
3000
2000
1000
0
46−60 (n=7)
80plus (n=3)
61−80 (n=15)
31−45 (n=3)
20−30 (n=2)
Age_category

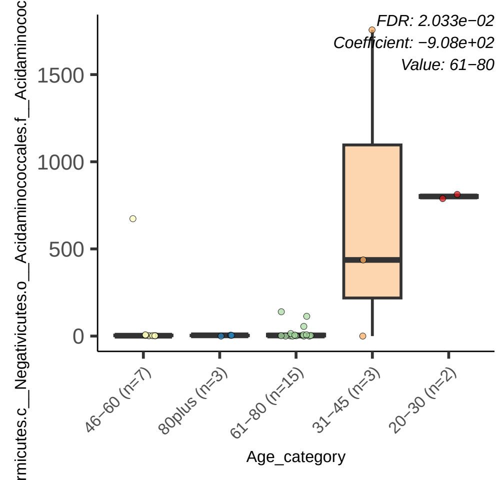

FDR: 2.033e−02
Coefficient: −9.08e+02
Value: 61−80
1500
1000
500
0
46−60 (n=7)
80plus (n=3)
61−80 (n=15)
31−45 (n=3)
20−30 (n=2)
Age_category
rmicutes.c__Negativicutes.o__Acidaminococcales.f__Acidaminococ

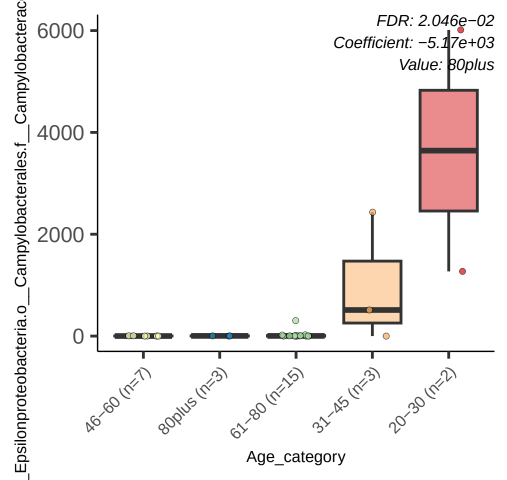

FDR: 2.046e−02
Coefficient: −5.17e+03
Value: 80plus
6000
4000
2000
0
_Epsilonproteobacteria.o__Campylobacterales.f__Campylobacterac
46−60 (n=7)
80plus (n=3)
61−80 (n=15)
31−45 (n=3)
20−30 (n=2)
Age_category

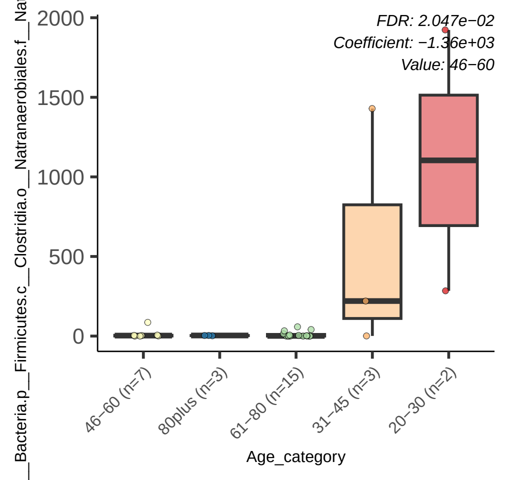

FDR: 2.047e−02
Coefficient: −1.36e+03
Value: 46−60
2000
1500
1000
500
0
46−60 (n=7)
80plus (n=3)
61−80 (n=15)
31−45 (n=3)
20−30 (n=2)
Age_category
Bacteria.p__Firmicutes.c__Clostridia.o__Natranaerobiales.f__Nat

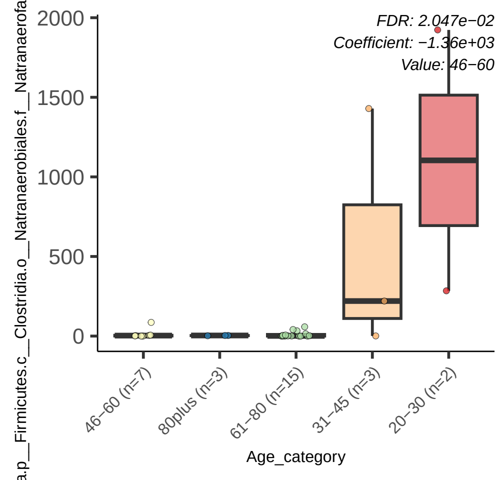
FDR: 2.047e−02
Coefficient: −1.36e+03
Value: 46−60
2000
1500
1000
500
0
a.p__Firmicutes.c__Clostridia.o__Natranaerobiales.f__Natranaerofa
46−60 (n=7)
80plus (n=3)
61−80 (n=15)
31−45 (n=3)
20−30 (n=2)
Age_category

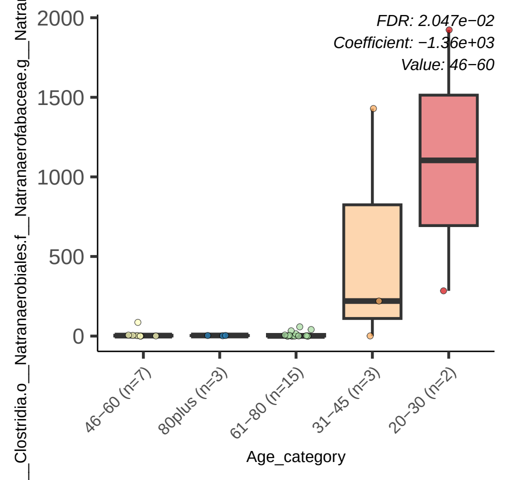

FDR: 2.047e−02
Coefficient: −1.36e+03
Value: 46−60
2000
1500
1000
500
0
Clostridia.o__Natranaerobiales.f__Natranaerofabaceae.g__Natran
46−60 (n=7)
80plus (n=3)
61−80 (n=15)
31−45 (n=3)
20−30 (n=2)
Age_category

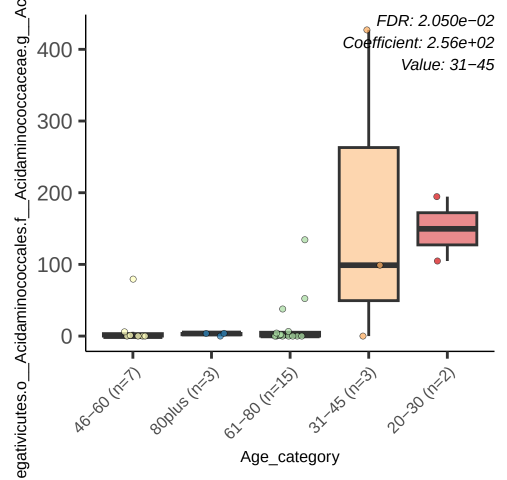

FDR: 2.050e−02
Coefficient: 2.56e+02
Value: 31−45
egativicutes.o__Acidaminococcales.f__Acidaminococcaceae.g__Ac
0
100
200
300
400
46−60 (n=7)
80plus (n=3)
61−80 (n=15)
31−45 (n=3)
20−30 (n=2)
Age_category

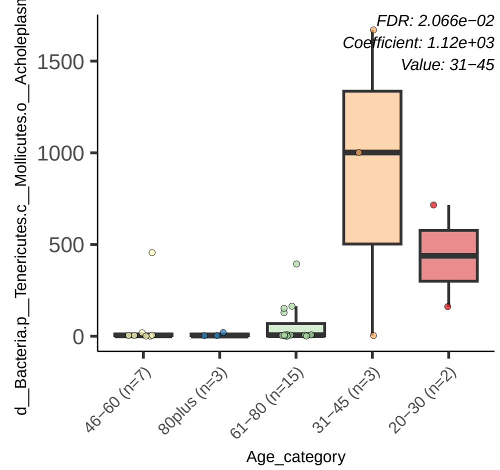

FDR: 2.066e−02
Coefficient: 1.12e+03
Value: 31−45
1500
1000
500
0
d__Bacteria.p__Tenericutes.c__Mollicutes.o__Acholeplasm
46−60 (n=7)
80plus (n=3)
61−80 (n=15)
31−45 (n=3)
20−30 (n=2)
Age_category

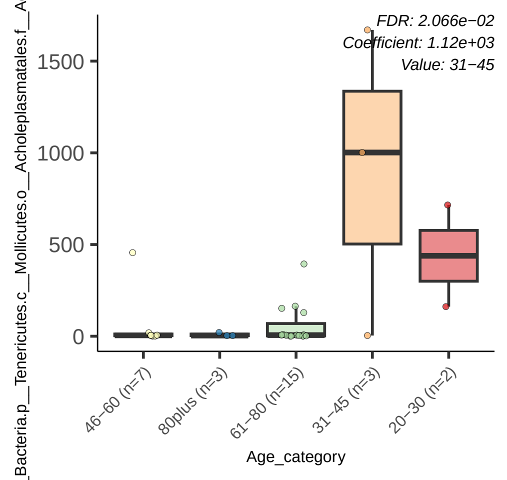
FDR: 2.066e−02
Coefficient: 1.12e+03
Value: 31−45
1500
1000
500
0
46−60 (n=7)
80plus (n=3)
61−80 (n=15)
31−45 (n=3)
20−30 (n=2)
Age_category
Bacteria.p__Tenericutes.c__Mollicutes.o__Acholeplasmatales.f__A

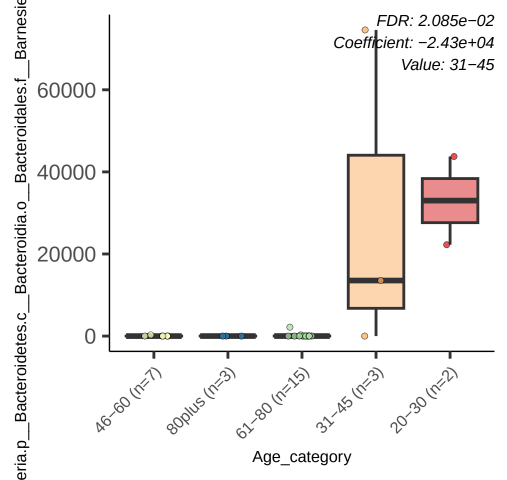

FDR: 2.085e−02
Coefficient: −2.43e+04
Value: 31−45
60000
40000
20000
0
46−60 (n=7)
80plus (n=3)
61−80 (n=15)
31−45 (n=3)
20−30 (n=2)
Age_category
eria.p__Bacteroidetes.c__Bacteroidia.o__Bacteroidales.f__Barnesie

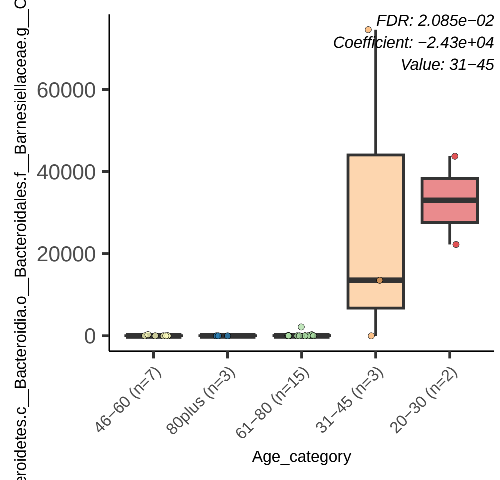

FDR: 2.085e−02
Coefficient: −2.43e+04
Value: 31−45
60000
40000
20000
0
eroidetes.c__Bacteroidia.o__Bacteroidales.f__Barnesiellaceae.g__C
46−60 (n=7)
80plus (n=3)
61−80 (n=15)
31−45 (n=3)
20−30 (n=2)
Age_category

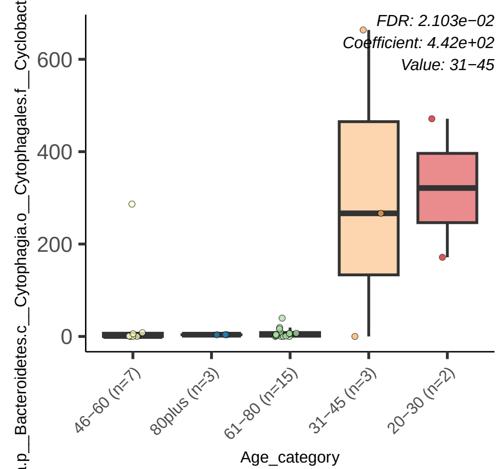

FDR: 2.103e−02
Coefficient: 4.42e+02
Value: 31−45
ria.p__Bacteroidetes.c__Cytophagia.o__Cytophagales.f__Cyclobact
600
400
200
0
46−60 (n=7)
80plus (n=3)
61−80 (n=15)
31−45 (n=3)
20−30 (n=2)
Age_category

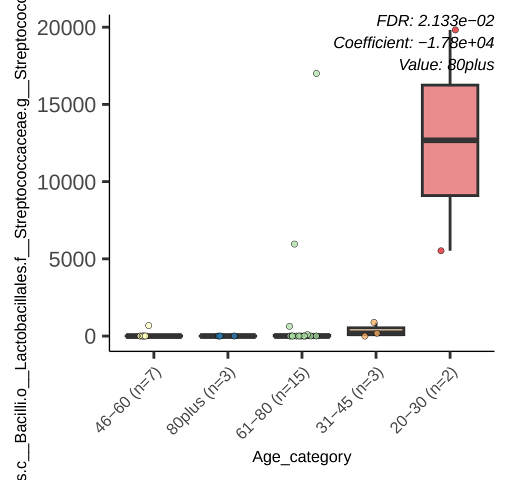

FDR: 2.133e−02
Coefficient: −1.78e+04
Value: 80plus
s.c__Bacilli.o__Lactobacillales.f__Streptococcaceae.g__Streptococc
20000
15000
10000
5000
0
46−60 (n=7)
80plus (n=3)
61−80 (n=15)
31−45 (n=3)
20−30 (n=2)
Age_category

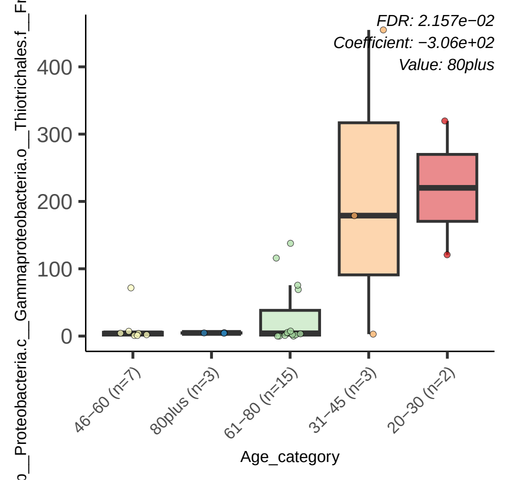

FDR: 2.157e−02
Coefficient: −3.06e+02
Value: 80plus
400
300
200
100
0
o__Proteobacteria.c__Gammaproteobacteria.o__Thiotrichales.f__Fr
46−60 (n=7)
80plus (n=3)
61−80 (n=15)
31−45 (n=3)
20−30 (n=2)
Age_category

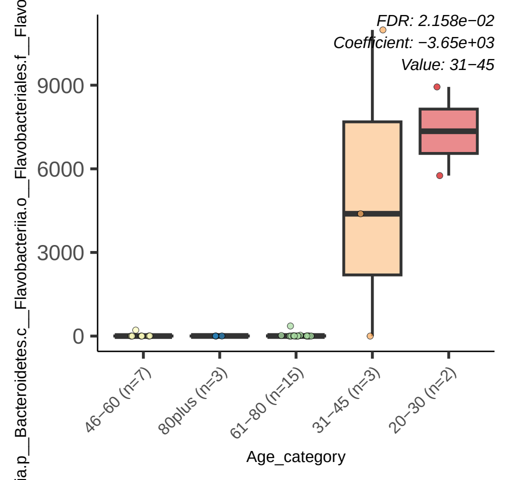
FDR: 2.158e−02
Coefficient: −3.65e+03
Value: 31−45
9000
6000
3000
0
ia.p__Bacteroidetes.c__Flavobacteriia.o__Flavobacteriales.f__Flavo
46−60 (n=7)
80plus (n=3)
61−80 (n=15)
31−45 (n=3)
20−30 (n=2)
Age_category

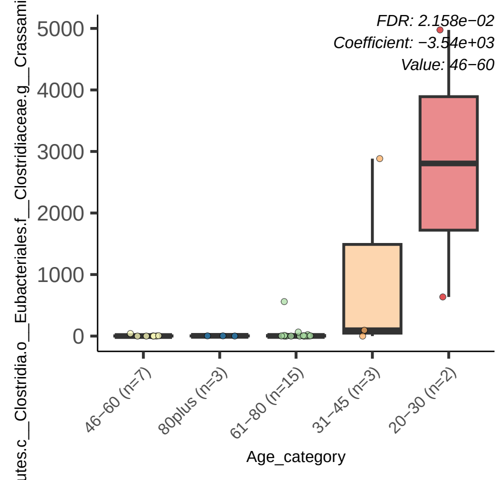
FDR: 2.158e−02
Coefficient: −3.54e+03
Value: 46−60
5000
4000
3000
2000
1000
0
utes.c__Clostridia.o__Eubacteriales.f__Clostridiaceae.g__Crassami
46−60 (n=7)
80plus (n=3)
61−80 (n=15)
31−45 (n=3)
20−30 (n=2)
Age_category

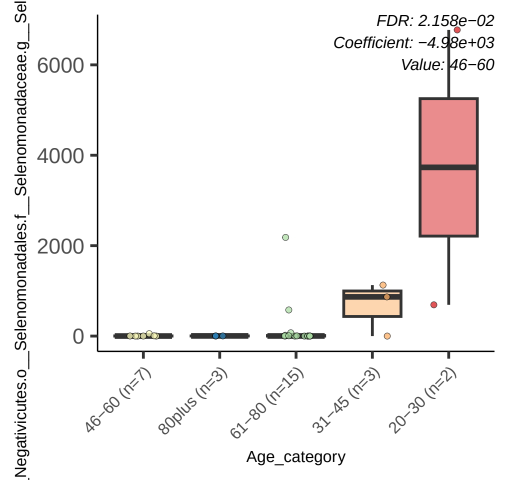

FDR: 2.158e−02
Coefficient: −4.98e+03
Value: 46−60
0
2000
4000
6000
Negativicutes.o__Selenomonadales.f__Selenomonadaceae.g__Sel
46−60 (n=7)
80plus (n=3)
61−80 (n=15)
31−45 (n=3)
20−30 (n=2)
Age_category

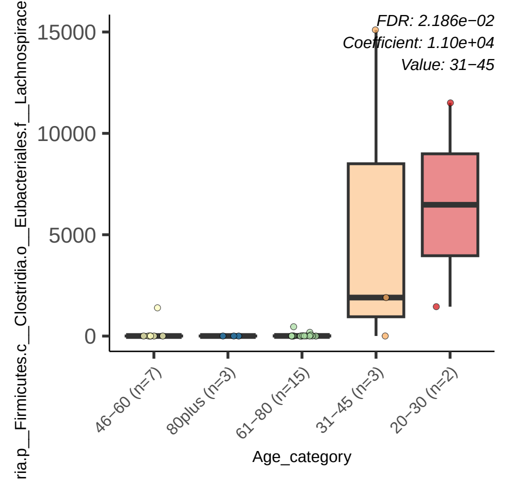

FDR: 2.186e−02
Coefficient: 1.10e+04
Value: 31−45
15000
10000
5000
0
ria.p__Firmicutes.c__Clostridia.o__Eubacteriales.f__Lachnospirace
46−60 (n=7)
80plus (n=3)
61−80 (n=15)
31−45 (n=3)
20−30 (n=2)
Age_category

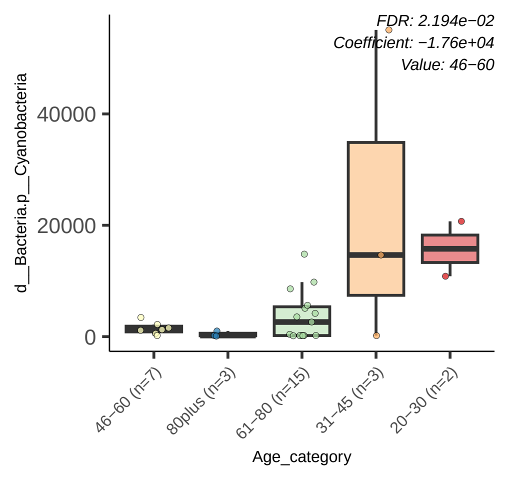

FDR: 2.194e−02
Coefficient: −1.76e+04
Value: 46−60
d__Bacteria.p__Cyanobacteria
40000
20000
0
46−60 (n=7)
80plus (n=3)
61−80 (n=15)
31−45 (n=3)
20−30 (n=2)
Age_category

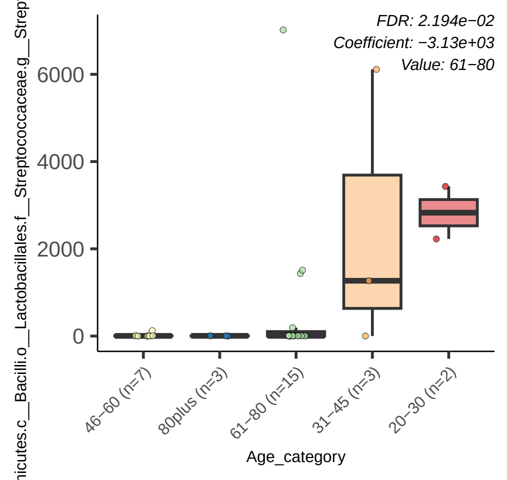

FDR: 2.194e−02
Coefficient: −3.13e+03
Value: 61−80
6000
4000
2000
0
46−60 (n=7)
80plus (n=3)
61−80 (n=15)
31−45 (n=3)
20−30 (n=2)
Age_category

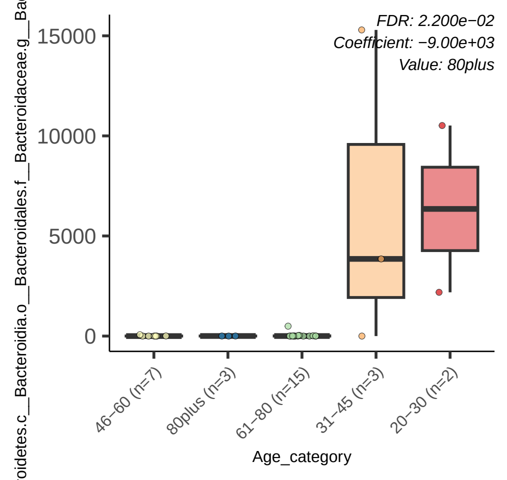

FDR: 2.200e−02
Coefficient: −9.00e+03
Value: 80plus
15000
10000
5000
0
46−60 (n=7)
80plus (n=3)
61−80 (n=15)
31−45 (n=3)
20−30 (n=2)
Age_category
oidetes.c__Bacteroidia.o__Bacteroidales.f__Bacteroidaceae.g__Bac

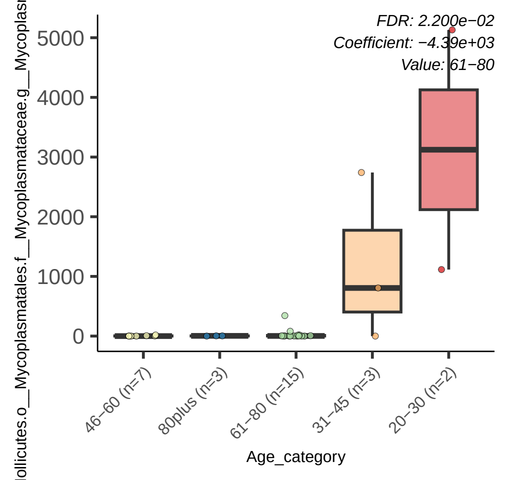

FDR: 2.200e−02
Coefficient: −4.39e+03
Value: 61−80
5000
4000
3000
2000
1000
0
46−60 (n=7)
80plus (n=3)
61−80 (n=15)
31−45 (n=3)
20−30 (n=2)
Age_category
Mollicutes.o__Mycoplasmatales.f__Mycoplasmataceae.g__Mycoplasm

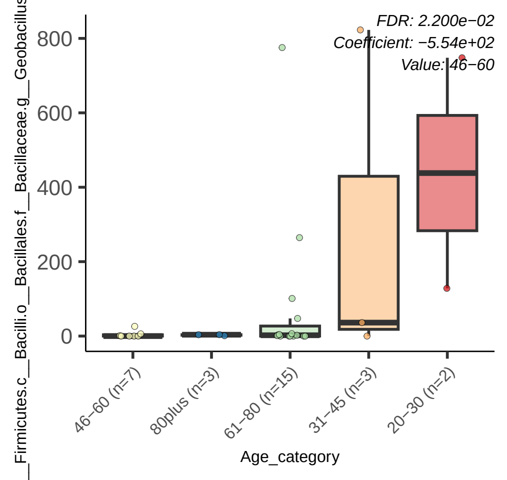
FDR: 2.200e−02
Coefficient: −5.54e+02
Value: 46−60
Firmicutes.c__Bacilli.o__Bacillales.f__Bacillaceae.g__Geobacillus
800
600
400
200
0
46−60 (n=7)
80plus (n=3)
61−80 (n=15)
31−45 (n=3)
20−30 (n=2)
Age_category

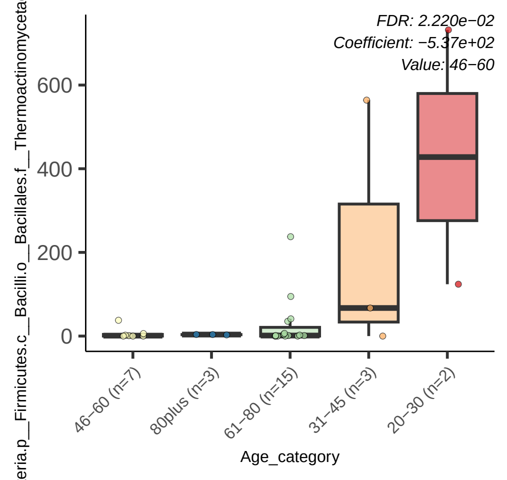

FDR: 2.220e−02
Coefficient: −5.37e+02
Value: 46−60
600
400
200
0
46−60 (n=7)
80plus (n=3)
61−80 (n=15)
31−45 (n=3)
20−30 (n=2)
Age_category

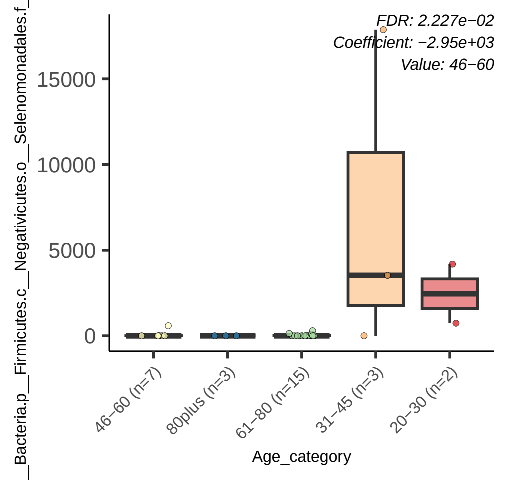

FDR: 2.227e−02
Coefficient: −2.95e+03
Value: 46−60
Bacteria.p__Firmicutes.c__Negativicutes.o__Selenomonadales.f
15000
10000
5000
0
46−60 (n=7)
80plus (n=3)
61−80 (n=15)
31−45 (n=3)
20−30 (n=2)
Age_category

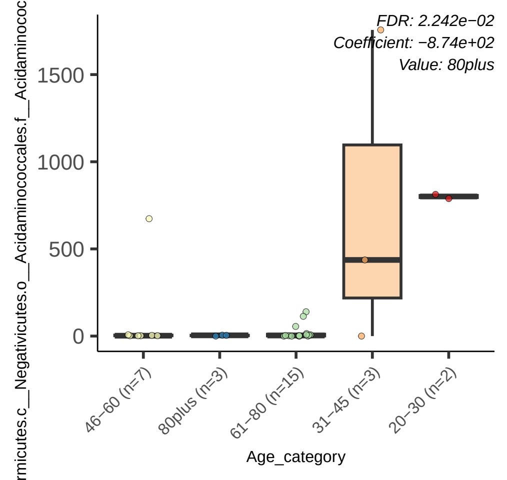
FDR: 2.242e−02
Coefficient: −8.74e+02
Value: 80plus
rmicutes.c__Negativicutes.o__Acidaminococcales.f__Acidaminococ
1500
1000
500
0
46−60 (n=7)
80plus (n=3)
61−80 (n=15)
31−45 (n=3)
20−30 (n=2)
Age_category

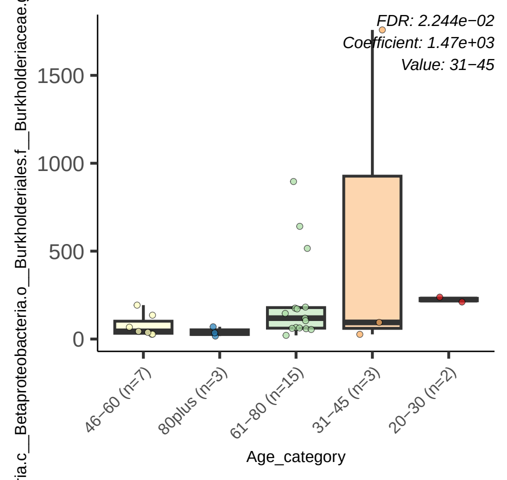
FDR: 2.244e−02
Coefficient: 1.47e+03
Value: 31−45
1500
1000
500
0
46−60 (n=7)
80plus (n=3)
61−80 (n=15)
31−45 (n=3)
20−30 (n=2)
Age_category
ria.c__Betaproteobacteria.o__Burkholderiales.f__Burkholderiaceae.g

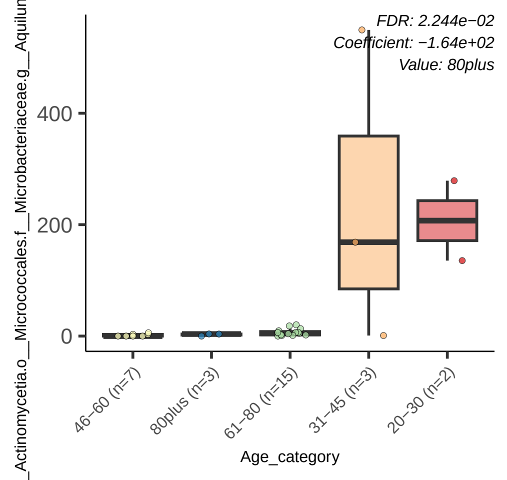

FDR: 2.244e−02
Coefficient: −1.64e+02
Value: 80plus
400
200
0
46−60 (n=7)
80plus (n=3)
61−80 (n=15)
31−45 (n=3)
20−30 (n=2)
Age_category
Actinomycetia.o__Micrococcales.f__Microbacteriaceae.g__Aquilun

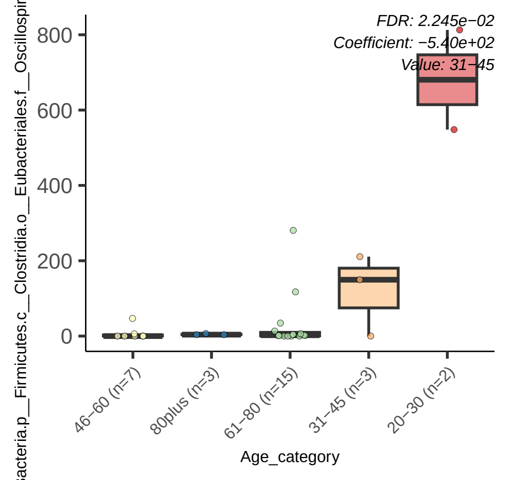

FDR: 2.245e−02
Coefficient: −5.40e+02
Value: 31−45
0
200
400
600
800
Firmicutes.c__Clostridia.o__Eubacteriales.f__Oscillospir
46−60 (n=7)
80plus (n=3)
61−80 (n=15)
31−45 (n=3)
20−30 (n=2)
Age_category

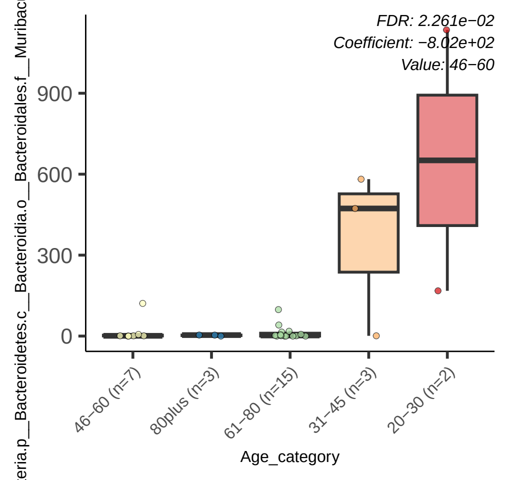

FDR: 2.261e−02
Coefficient: −8.02e+02
Value: 46−60
900
600
300
0
46−60 (n=7)
80plus (n=3)
61−80 (n=15)
31−45 (n=3)
20−30 (n=2)
Age_category
teria.p__Bacteroidetes.c__Bacteroidia.o__Bacteroidales.f__Muribac

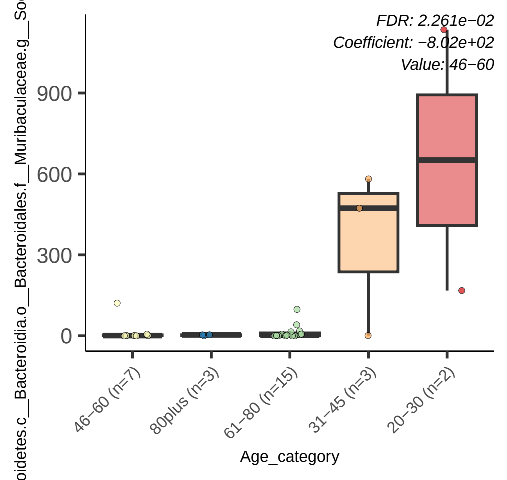

FDR: 2.261e−02
Coefficient: −8.02e+02
Value: 46−60
oidetes.c__Bacteroidia.o__Bacteroidales.f__Muribaculaceae.g__So
900
600
300
0
46−60 (n=7)
80plus (n=3)
61−80 (n=15)
31−45 (n=3)
20−30 (n=2)
Age_category

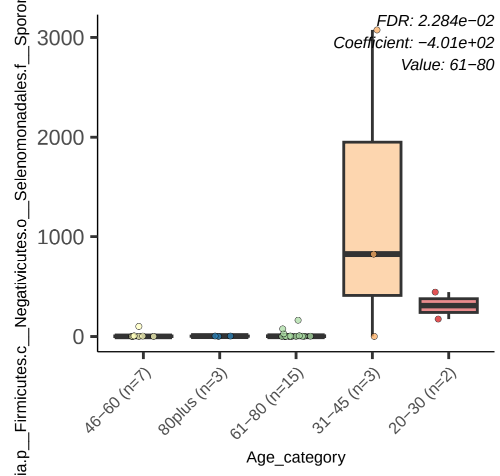

FDR: 2.284e−02
Coefficient: −4.01e+02
Value: 61−80
3000
2000
1000
0
ria.p__Firmicutes.c__Negativicutes.o__Selenomonadales.f__Sporo
46−60 (n=7)
80plus (n=3)
61−80 (n=15)
31−45 (n=3)
20−30 (n=2)
Age_category

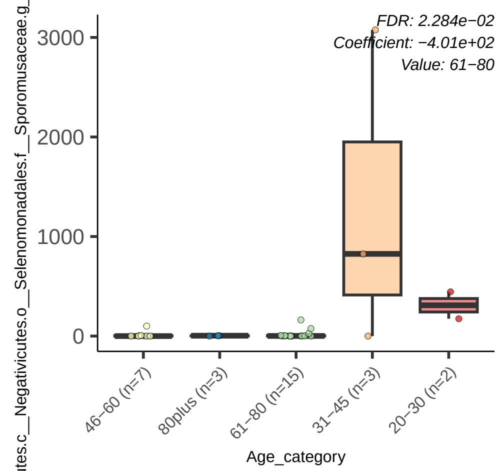

FDR: 2.284e−02
Coefficient: −4.01e+02
Value: 61−80
cutes.c__Negativicutes.o__Selenomonadales.f__Sporomusaceae.g_
3000
2000
1000
0
46−60 (n=7)
80plus (n=3)
61−80 (n=15)
31−45 (n=3)
20−30 (n=2)
Age_category

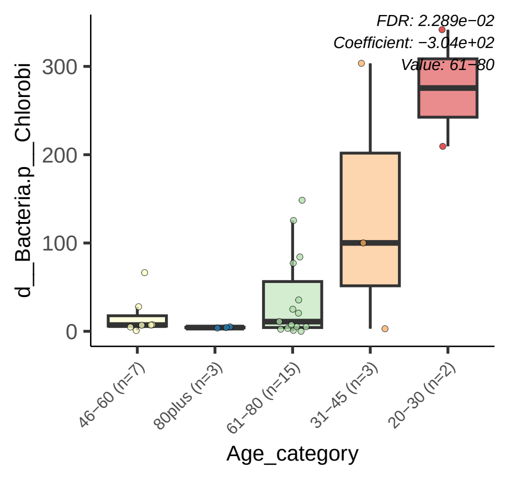

FDR: 2.289e−02
Coefficient: −3.04e+02
Value: 61−80
d__Bacteria.p__Chlorobi
300
200
100
0
46−60 (n=7)
80plus (n=3)
61−80 (n=15)
31−45 (n=3)
20−30 (n=2)
Age_category

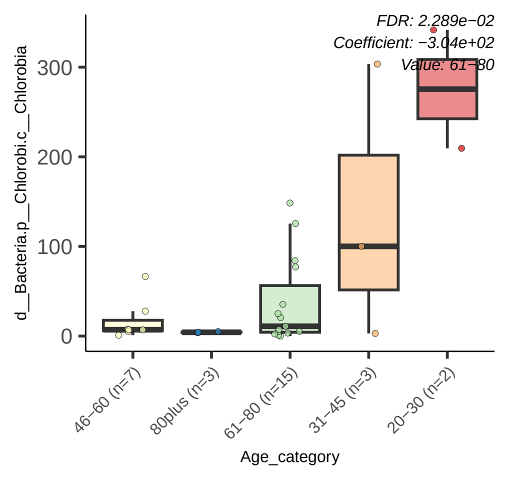

FDR: 2.289e−02
Coefficient: −3.04e+02
Value: 61−80
d__Bacteria.p__Chlorobi.c__Chlorobia
300
200
100
0
46−60 (n=7)
80plus (n=3)
61−80 (n=15)
31−45 (n=3)
20−30 (n=2)
Age_category

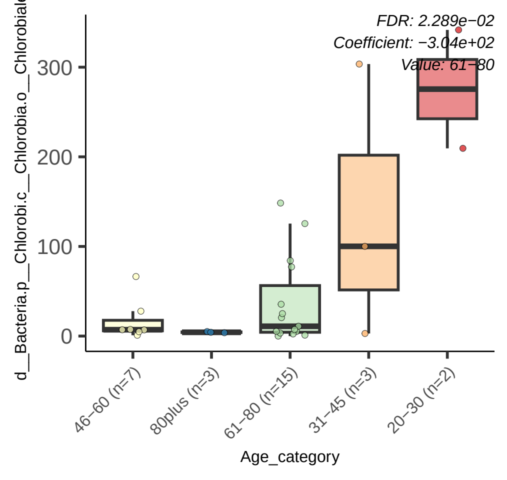

FDR: 2.289e−02
Coefficient: −3.04e+02
Value: 61−80
d__Bacteria.p__Chlorobi.c__Chlorobia.o__Chlorobiale
300
200
100
0
46−60 (n=7)
80plus (n=3)
61−80 (n=15)
31−45 (n=3)
20−30 (n=2)
Age_category

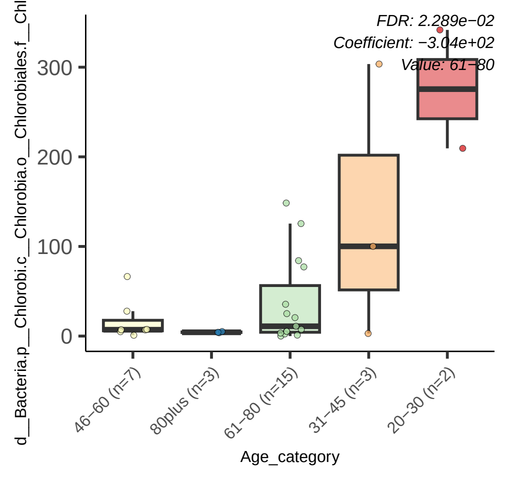

FDR: 2.289e−02
Coefficient: −3.04e+02
Value: 61−80
d__Bacteria.p__Chlorobi.c__Chlorobia.o__Chlorobiales.f__Chl
0
100
200
300
46−60 (n=7)
80plus (n=3)
61−80 (n=15)
31−45 (n=3)
20−30 (n=2)
Age_category

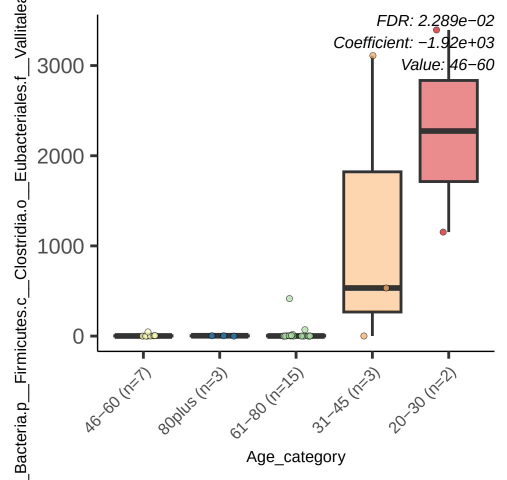

FDR: 2.289e−02
Coefficient: −1.92e+03
Value: 46−60
_Bacteria.p__Firmicutes.c__Clostridia.o__Eubacteriales.f__Vallitalea
3000
2000
1000
0
46−60 (n=7)
80plus (n=3)
61−80 (n=15)
31−45 (n=3)
20−30 (n=2)
Age_category

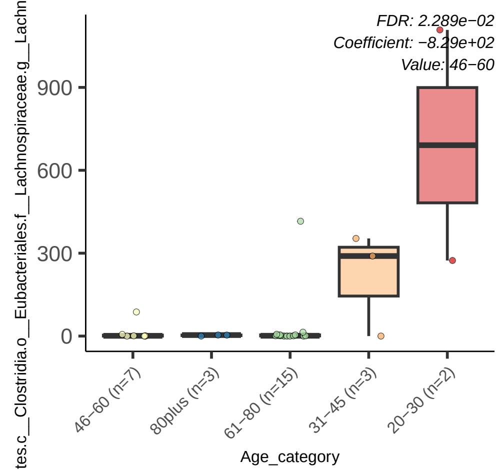

FDR: 2.289e−02
Coefficient: −8.29e+02
Value: 46−60
900
600
300
0
cutes.c__Clostridia.o__Eubacteriales.f__Lachnospiraceae.g__Lachn
46−60 (n=7)
80plus (n=3)
61−80 (n=15)
31−45 (n=3)
20−30 (n=2)
Age_category

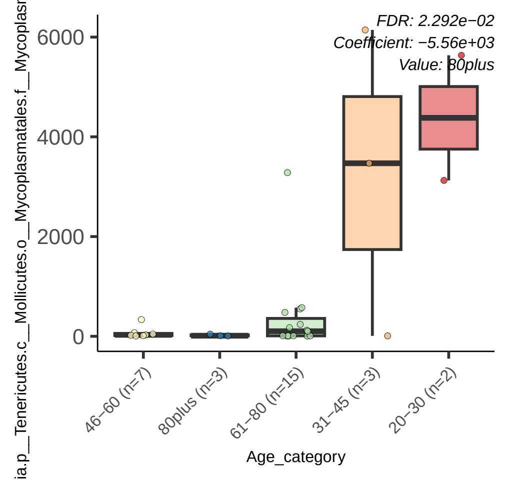
FDR: 2.292e−02
Coefficient: −5.56e+03
Value: 80plus
6000
4000
2000
0
46−60 (n=7)
80plus (n=3)
61−80 (n=15)
31−45 (n=3)
20−30 (n=2)
Age_category
ia.p__Tenericutes.c__Mollicutes.o__Mycoplasmatales.f__Mycoplasm

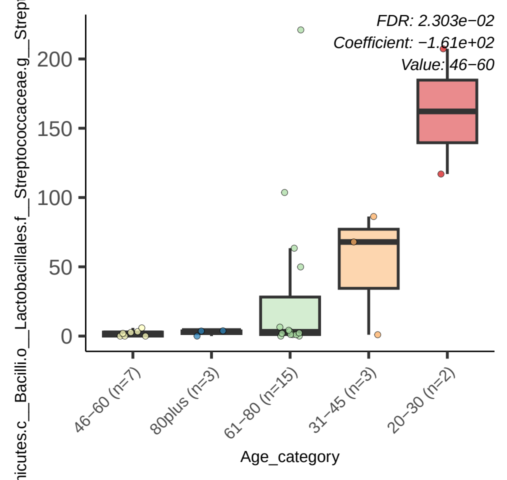

FDR: 2.303e−02
Coefficient: −1.61e+02
Value: 46−60
nicutes.c__Bacilli.o__Lactobacillales.f__Streptococcaceae.g__Strep
0
50
100
150
200
46−60 (n=7)
80plus (n=3)
61−80 (n=15)
31−45 (n=3)
20−30 (n=2)
Age_category

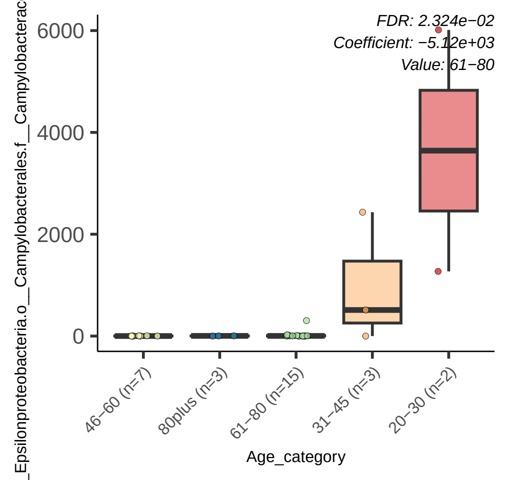

FDR: 2.324e−02
Coefficient: −5.12e+03
Value: 61−80
6000
4000
2000
0
_Epsilonproteobacteria.o__Campylobacterales.f__Campylobacterac
46−60 (n=7)
80plus (n=3)
61−80 (n=15)
31−45 (n=3)
20−30 (n=2)
Age_category

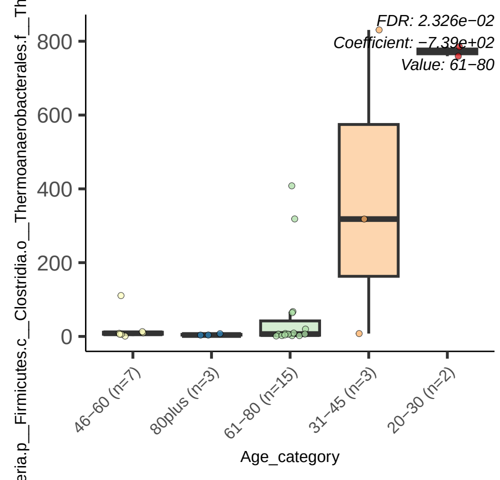

FDR: 2.326e−02
Coefficient: −7.39e+02
Value: 61−80
800
600
400
200
0
eria.p__Firmicutes.c__Clostridia.o__Thermoanaerobacterales.f__Th
46−60 (n=7)
80plus (n=3)
61−80 (n=15)
31−45 (n=3)
20−30 (n=2)
Age_category

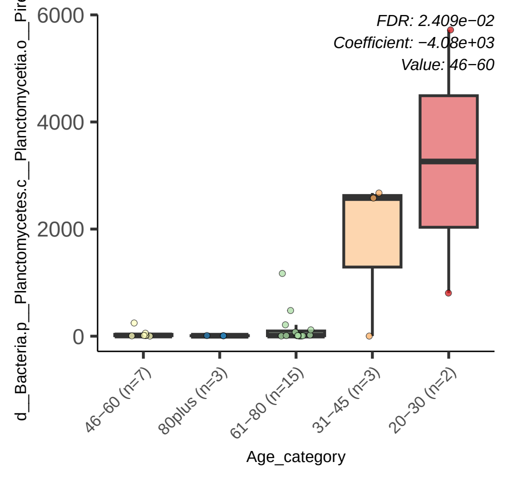

FDR: 2.409e−02
Coefficient: −4.08e+03
Value: 46−60
d__Bacteria.p__Planctomycetes.c__Planctomycetia.o__Pir
6000
4000
2000
0
46−60 (n=7)
80plus (n=3)
61−80 (n=15)
31−45 (n=3)
20−30 (n=2)
Age_category

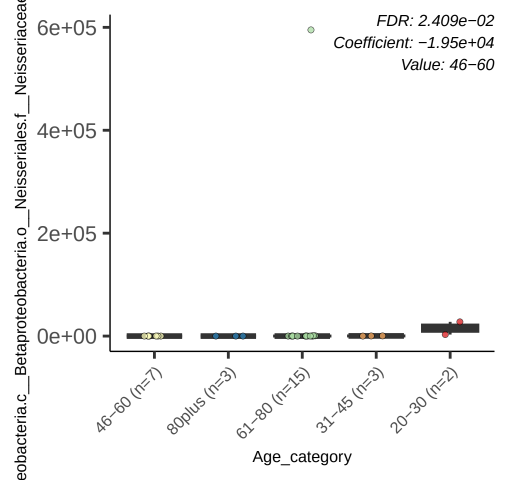
FDR: 2.409e−02
Coefficient: −1.95e+04
Value: 46−60
6e+05
4e+05
2e+05
0e+00
eobacteria.c__Betaproteobacteria.o__Neisseriales.f__Neisseriacea
46−60 (n=7)
80plus (n=3)
61−80 (n=15)
31−45 (n=3)
20−30 (n=2)
Age_category

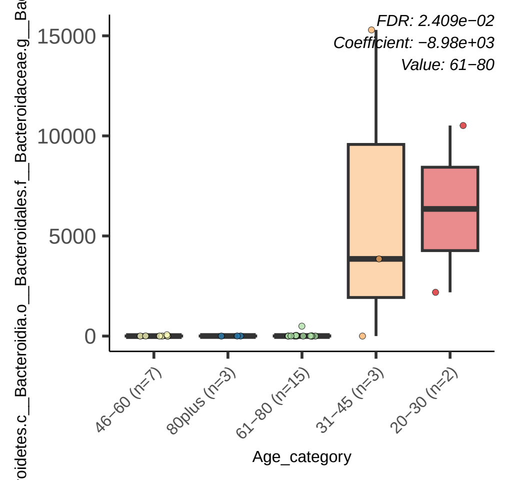

FDR: 2.409e−02
Coefficient: −8.98e+03
Value: 61−80
15000
10000
5000
0
46−60 (n=7)
80plus (n=3)
61−80 (n=15)
31−45 (n=3)
20−30 (n=2)
Age_category
oidetes.c__Bacteroidia.o__Bacteroidales.f__Bacteroidaceae.g__Bac

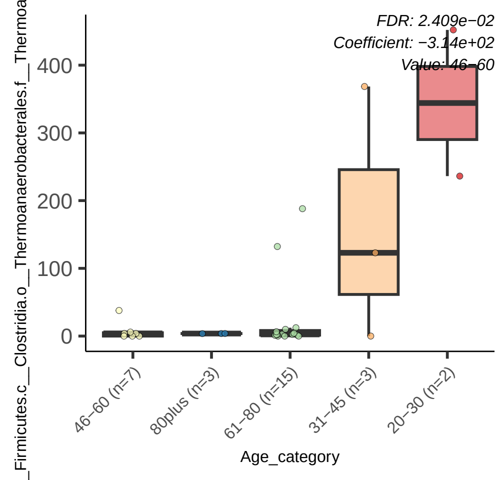

FDR: 2.409e−02
Coefficient: −3.14e+02
Value: 46−60
Firmicutes.c__Clostridia.o__Thermoanaerobacterales.f__Thermoa
400
300
200
100
0
46−60 (n=7)
80plus (n=3)
61−80 (n=15)
31−45 (n=3)
20−30 (n=2)
Age_category

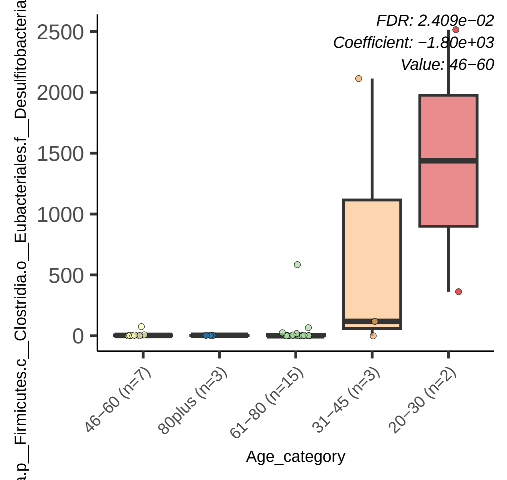

FDR: 2.409e−02
Coefficient: −1.80e+03
Value: 46−60
Firmicutes.c__Clostridia.o__Eubacteriales.f__Desulfitobacteria
2500
2000
1500
1000
500
0
46−60 (n=7)
80plus (n=3)
61−80 (n=15)
31−45 (n=3)
20−30 (n=2)
Age_category

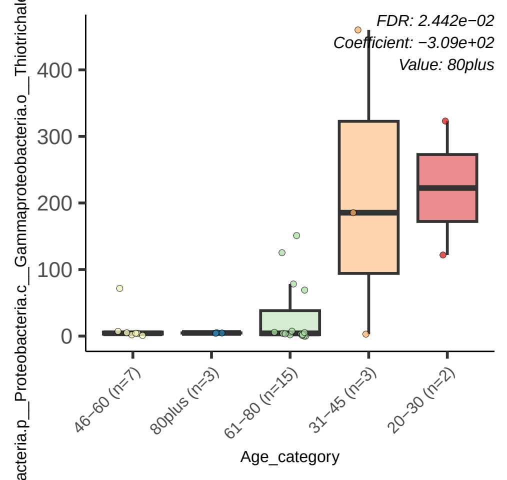
FDR: 2.442e−02
Coefficient: −3.09e+02
Value: 80plus
0
100
200
300
400
46−60 (n=7)
80plus (n=3)
61−80 (n=15)
31−45 (n=3)
20−30 (n=2)
Age_category
acteria.p__Proteobacteria.c__Gammaproteobacteria.o__Thiotrichale

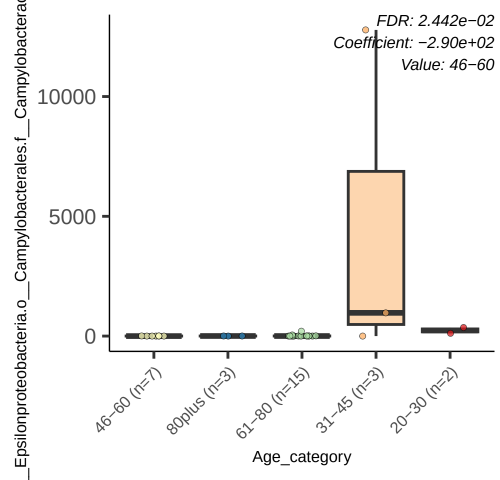

FDR: 2.442e−02
Coefficient: −2.90e+02
Value: 46−60
10000
5000
0
_Epsilonproteobacteria.o__Campylobacterales.f__Campylobacterac
46−60 (n=7)
80plus (n=3)
61−80 (n=15)
31−45 (n=3)
20−30 (n=2)
Age_category

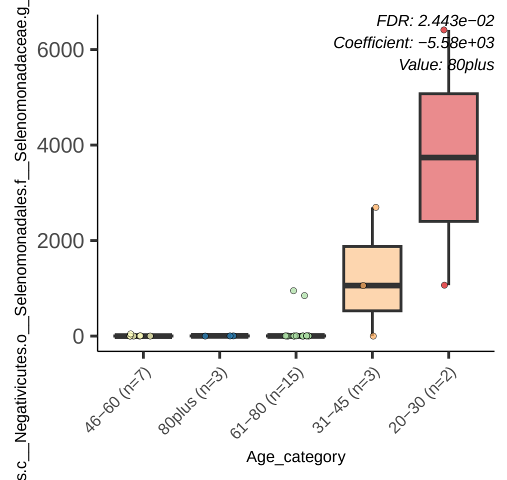

FDR: 2.443e−02
Coefficient: −5.58e+03
Value: 80plus
6000
4000
2000
0
s.c__Negativicutes.o__Selenomonadales.f__Selenomonadaceae.g_
46−60 (n=7)
80plus (n=3)
61−80 (n=15)
31−45 (n=3)
20−30 (n=2)
Age_category

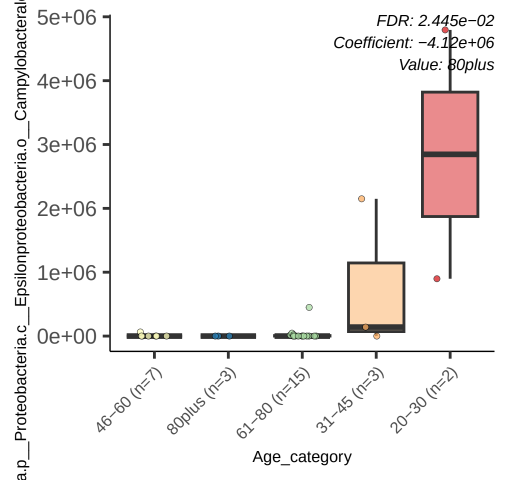
FDR: 2.445e−02
Coefficient: −4.12e+06
Value: 80plus
5e+06
4e+06
3e+06
2e+06
1e+06
0e+00
a.p__Proteobacteria.c__Epsilonproteobacteria.o__Campylobacteral
46−60 (n=7)
80plus (n=3)
61−80 (n=15)
31−45 (n=3)
20−30 (n=2)
Age_category

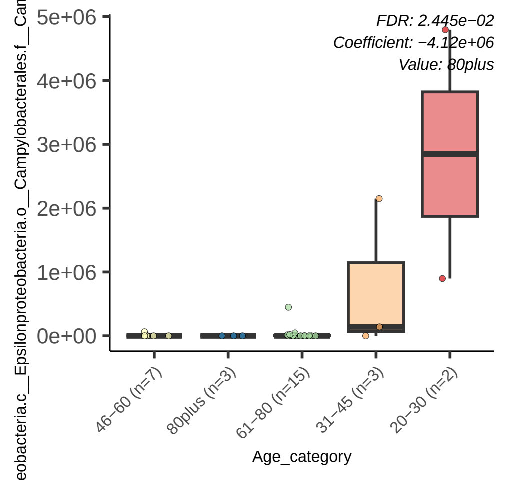

FDR: 2.445e−02
Coefficient: −4.12e+06
Value: 80plus
eobacteria.c__Epsilonproteobacteria.o__Campylobacterales.f__Cam
5e+06
4e+06
3e+06
2e+06
1e+06
0e+00
46−60 (n=7)
80plus (n=3)
61−80 (n=15)
31−45 (n=3)
20−30 (n=2)
Age_category

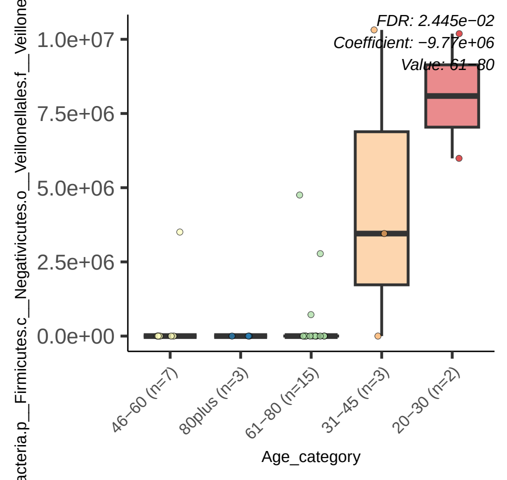

FDR: 2.445e−02
Coefficient: −9.77e+06
Value: 61−80
1.0e+07
7.5e+06
5.0e+06
2.5e+06
0.0e+00
acteria.p__Firmicutes.c__Negativicutes.o__Veillonellales.f__Veillone
46−60 (n=7)
80plus (n=3)
61−80 (n=15)
31−45 (n=3)
20−30 (n=2)
Age_category

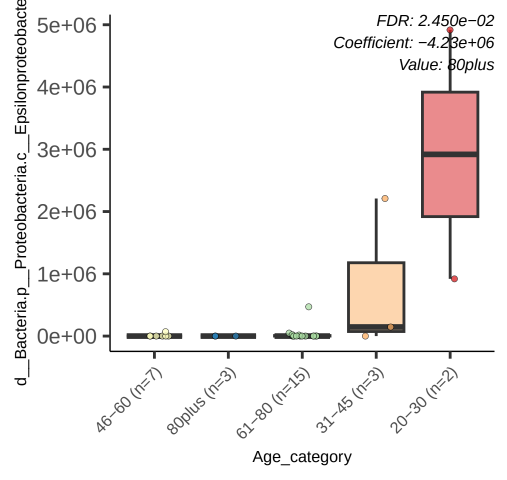

FDR: 2.450e−02
Coefficient: −4.23e+06
Value: 80plus
d__Bacteria.p__Proteobacteria.c__Epsilonproteobacte
5e+06
4e+06
3e+06
2e+06
1e+06
0e+00
46−60 (n=7)
80plus (n=3)
61−80 (n=15)
31−45 (n=3)
20−30 (n=2)
Age_category

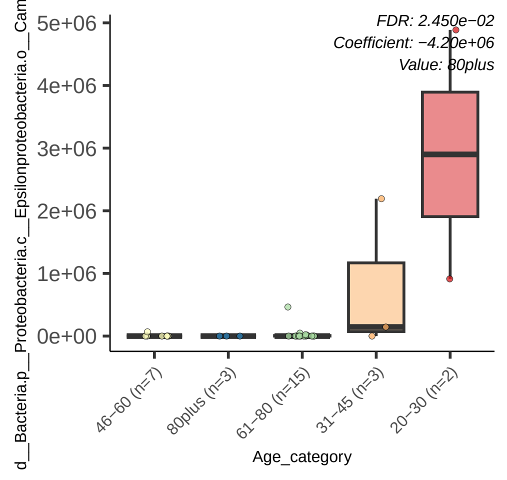

d__Bacteria.p__Proteobacteria.c__Epsilonproteobacteria.o__Cam
FDR: 2.450e−02
Coefficient: −4.20e+06
Value: 80plus
5e+06
4e+06
3e+06
2e+06
1e+06
0e+00
46−60 (n=7)
80plus (n=3)
61−80 (n=15)
31−45 (n=3)
20−30 (n=2)
Age_category

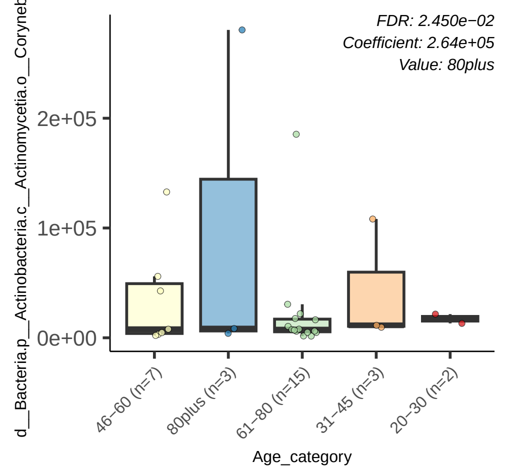

FDR: 2.450e−02
Coefficient: 2.64e+05
Value: 80plus
d__Bacteria.p__Actinobacteria.c__Actinomycetia.o__Coryneb
2e+05
1e+05
0e+00
46−60 (n=7)
80plus (n=3)
61−80 (n=15)
31−45 (n=3)
20−30 (n=2)
Age_category

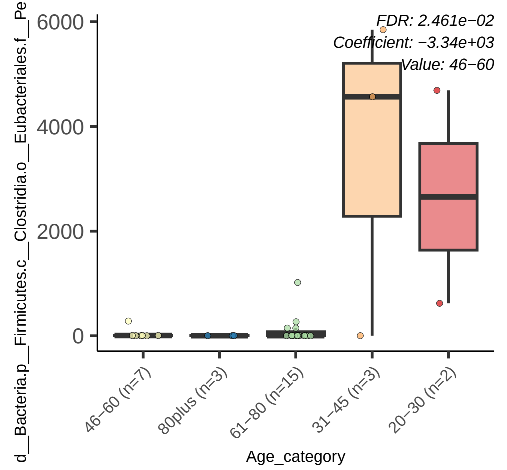

FDR: 2.461e−02
Coefficient: −3.34e+03
Value: 46−60
d__Bacteria.p__Firmicutes.c__Clostridia.o__Eubacteriales.f__Pe
6000
4000
2000
0
46−60 (n=7)
80plus (n=3)
61−80 (n=15)
31−45 (n=3)
20−30 (n=2)
Age_category

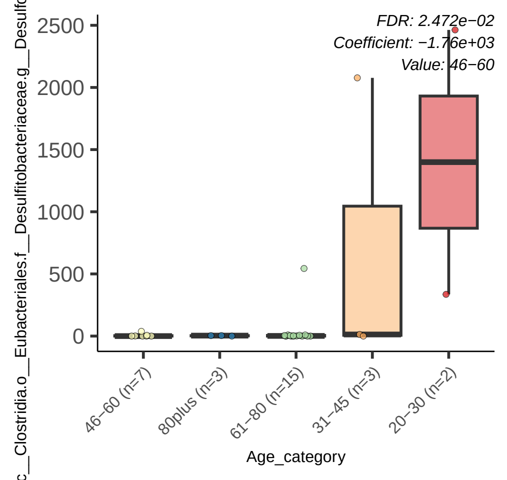

FDR: 2.472e−02
Coefficient: −1.76e+03
Value: 46−60
c__Clostridia.o__Eubacteriales.f__Desulfitobacteriaceae.g__Desulfo
2500
2000
1500
1000
500
0
46−60 (n=7)
80plus (n=3)
61−80 (n=15)
31−45 (n=3)
20−30 (n=2)
Age_category

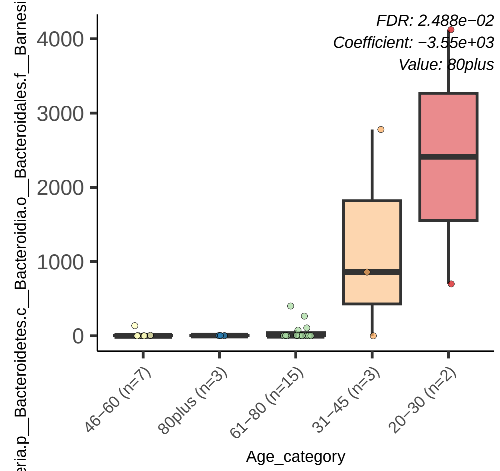

FDR: 2.488e−02
Coefficient: −3.55e+03
Value: 80plus
4000
3000
2000
1000
0
teria.p__Bacteroidetes.c__Bacteroidia.o__Bacteroidales.f__Barnesi
46−60 (n=7)
80plus (n=3)
61−80 (n=15)
31−45 (n=3)
20−30 (n=2)
Age_category

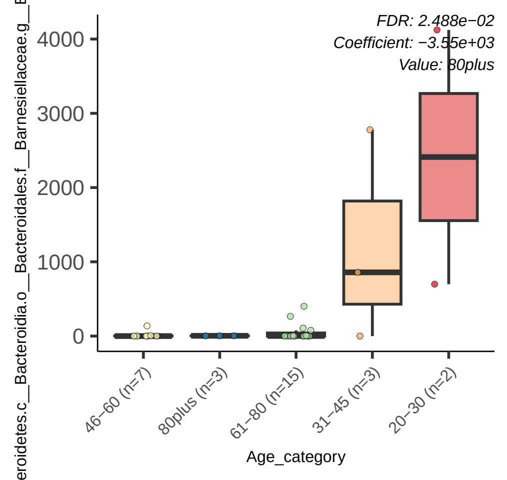
FDR: 2.488e−02
Coefficient: −3.55e+03
Value: 80plus
4000
3000
2000
1000
0
46−60 (n=7)
80plus (n=3)
61−80 (n=15)
31−45 (n=3)
20−30 (n=2)
Age_category
eroidetes.c__Bacteroidia.o__Bacteroidales.f__Barnesiellaceae.g__B

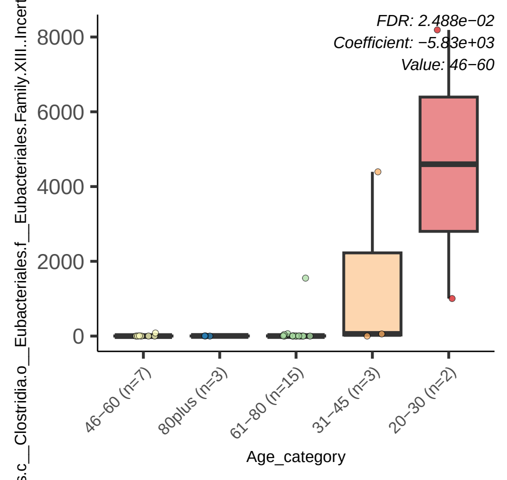
FDR: 2.488e−02
Coefficient: −5.83e+03
Value: 46−60
8000
6000
4000
2000
0
s.c__Clostridia.o__Eubacteriales.f__Eubacteriales.Family.XIII..Incert
46−60 (n=7)
80plus (n=3)
61−80 (n=15)
31−45 (n=3)
20−30 (n=2)
Age_category

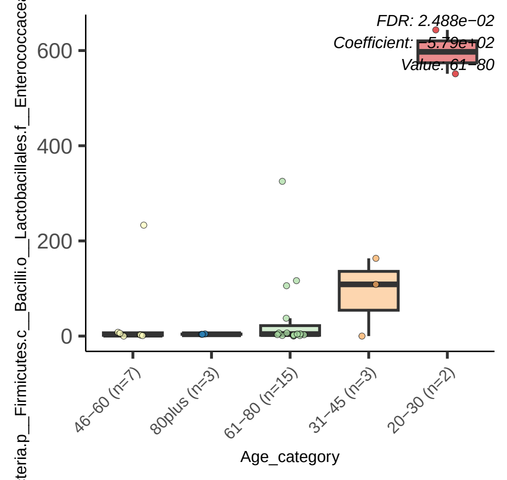

FDR: 2.488e−02
Coefficient: 5.79e+02
Value: 61−80
600
400
200
0
teria.p__Firmicutes.c__Bacilli.o__Lactobacillales.f__Enterococcace
46−60 (n=7)
80plus (n=3)
61−80 (n=15)
31−45 (n=3)
20−30 (n=2)
Age_category

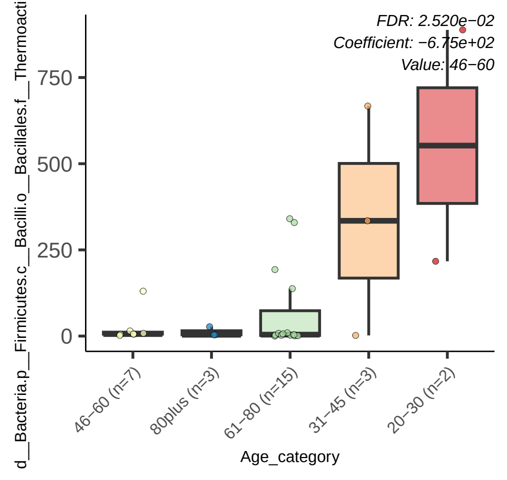
FDR: 2.520e−02
Coefficient: −6.75e+02
Value: 46−60
d__Bacteria.p__Firmicutes.c__Bacilli.o__Bacillales.f__Thermoacti
750
500
250
0
46−60 (n=7)
80plus (n=3)
61−80 (n=15)
31−45 (n=3)
20−30 (n=2)
Age_category

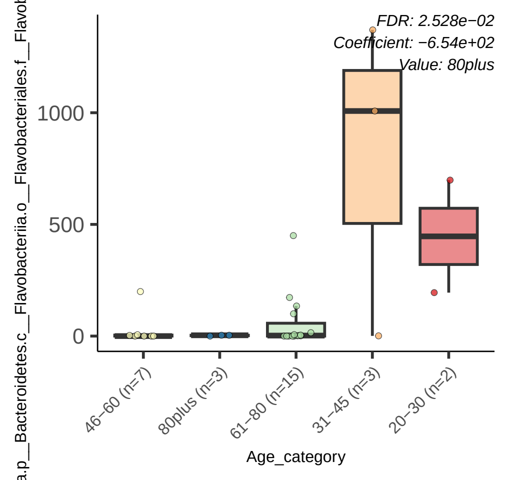

FDR: 2.528e−02
Coefficient: −6.54e+02
Value: 80plus
a.p__Bacteroidetes.c__Flavobacteriia.o__Flavobacteriales.f__Flavob
1000
500
0
46−60 (n=7)
80plus (n=3)
61−80 (n=15)
31−45 (n=3)
20−30 (n=2)
Age_category

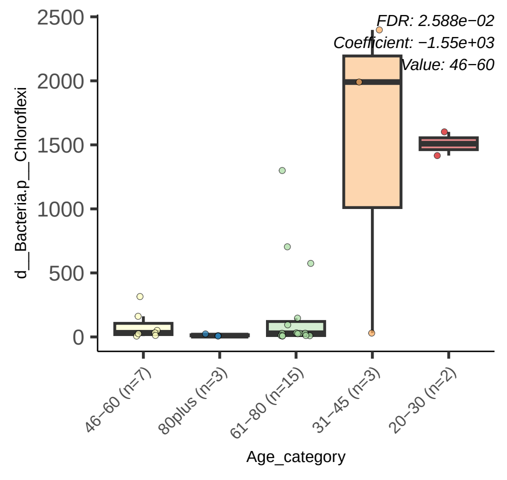
FDR: 2.588e−02
Coefficient: −1.55e+03
Value: 46−60
d__Bacteria.p__Chloroflexi
2500
2000
1500
1000
500
0
46−60 (n=7)
80plus (n=3)
61−80 (n=15)
31−45 (n=3)
20−30 (n=2)
Age_category

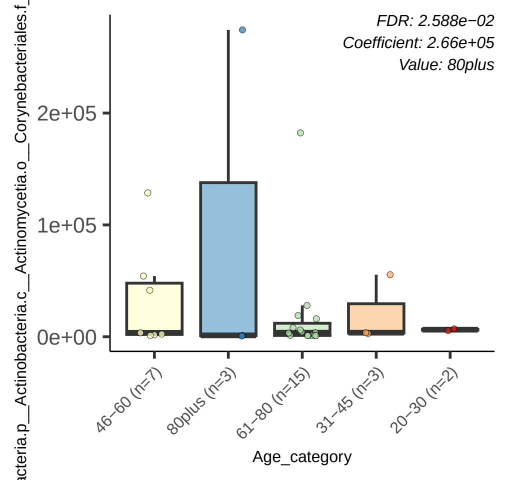

FDR: 2.588e−02
Coefficient: 2.66e+05
Value: 80plus
2e+05
1e+05
0e+00
acteria.p__Actinobacteria.c__Actinomycetia.o__Corynebacteriales.f_
46−60 (n=7)
80plus (n=3)
61−80 (n=15)
31−45 (n=3)
20−30 (n=2)
Age_category

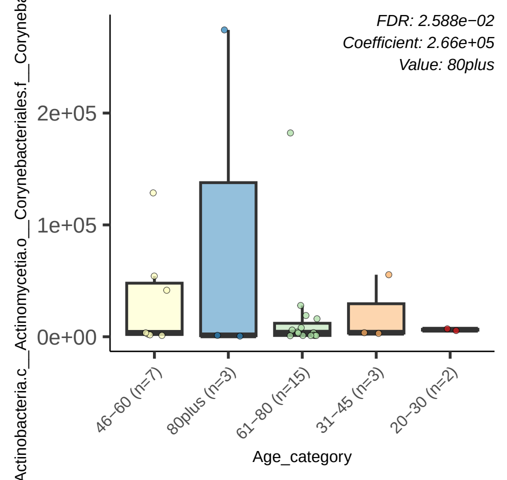

FDR: 2.588e−02
Coefficient: 2.66e+05
Value: 80plus
Actinobacteria.c__Actinomycetia.o__Corynebacteriales.f__Coryneba
2e+05
1e+05
0e+00
46−60 (n=7)
80plus (n=3)
61−80 (n=15)
31−45 (n=3)
20−30 (n=2)
Age_category

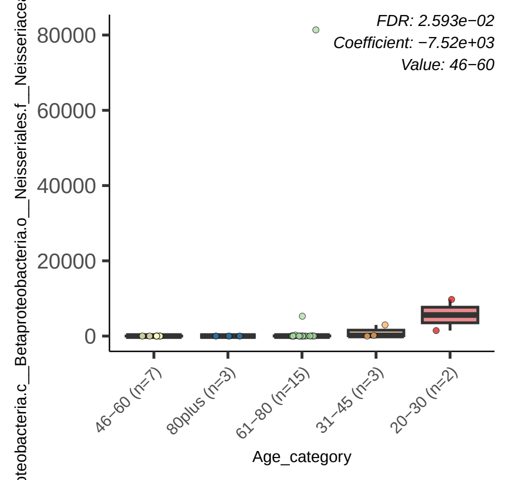

FDR: 2.593e−02
Coefficient: −7.52e+03
Value: 46−60
80000
60000
40000
20000
0
46−60 (n=7)
80plus (n=3)
61−80 (n=15)
31−45 (n=3)
20−30 (n=2)
Age_category
oteobacteria.c__Betaproteobacteria.o__Neisseriales.f__Neisseriace

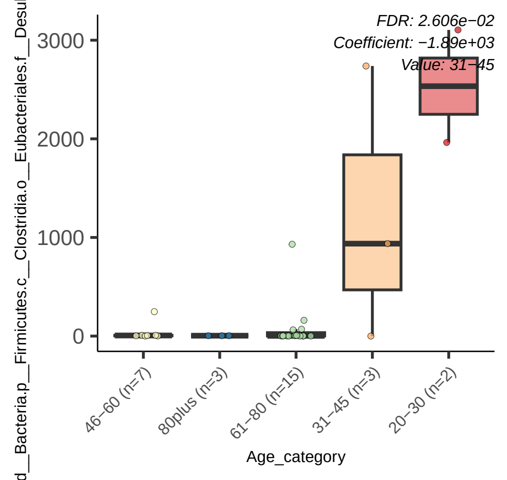

FDR: 2.606e−02
Coefficient: −1.89e+03
Value: 31−45
3000
2000
1000
0
46−60 (n=7)
80plus (n=3)
61−80 (n=15)
31−45 (n=3)
20−30 (n=2)
Age_category
d__Bacteria.p__Firmicutes.c__Clostridia.o__Eubacteriales.f__Desul

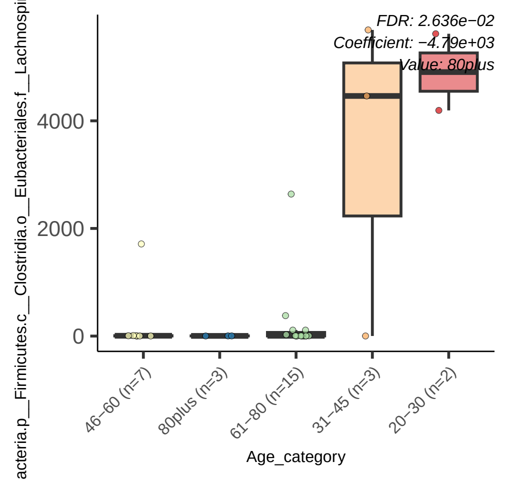

FDR: 2.636e−02
Coefficient: −4.79e+03
Value: 80plus
acteria.p__Firmicutes.c__Clostridia.o__Eubacteriales.f__Lachnospi
4000
2000
0
46−60 (n=7)
80plus (n=3)
61−80 (n=15)
31−45 (n=3)
20−30 (n=2)
Age_category

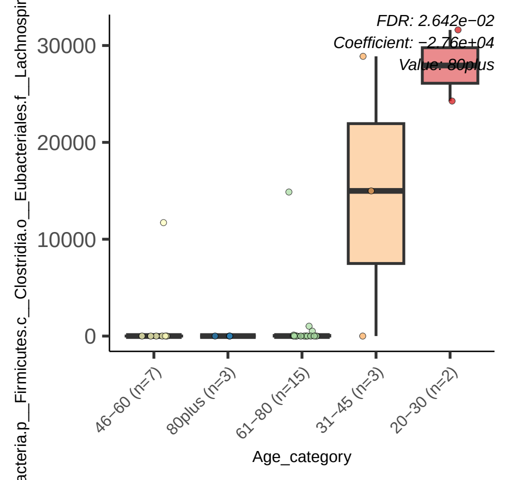

FDR: 2.642e−02
Coefficient: −2.76e+04
Value: 80plus
30000
20000
10000
0
acteria.p__Firmicutes.c__Clostridia.o__Eubacteriales.f__Lachnospir
46−60 (n=7)
80plus (n=3)
61−80 (n=15)
31−45 (n=3)
20−30 (n=2)
Age_category

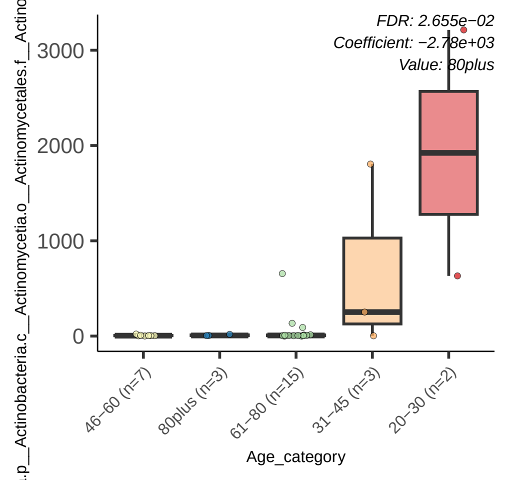
FDR: 2.655e−02
Coefficient: −2.78e+03
Value: 80plus
3000
2000
1000
0
46−60 (n=7)
80plus (n=3)
61−80 (n=15)
31−45 (n=3)
20−30 (n=2)
Age_category
p__Actinobacteria.c__Actinomycetia.o__Actinomycetales.f__Actino

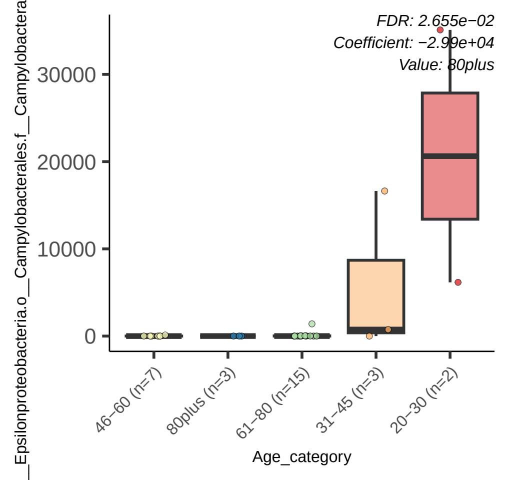

FDR: 2.655e−02
Coefficient: −2.99e+04
Value: 80plus
Epsilonproteobacteria.o__Campylobacterales.f__Campylobactera
30000
20000
10000
0
46−60 (n=7)
80plus (n=3)
61−80 (n=15)
31−45 (n=3)
20−30 (n=2)
Age_category

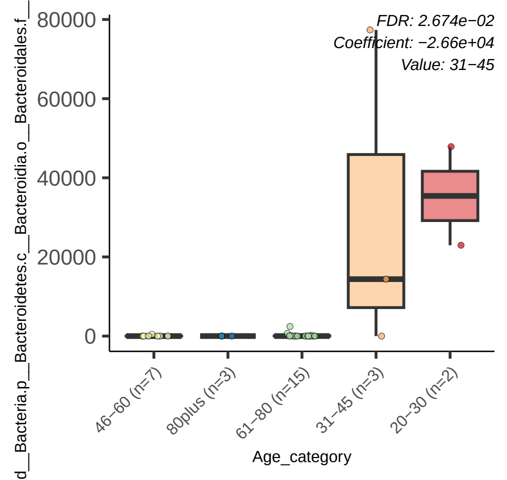
FDR: 2.674e−02
Coefficient: −2.66e+04
Value: 31−45
d__Bacteria.p__Bacteroidetes.c__Bacteroidia.o__Bacteroidales.f__
80000
60000
40000
20000
0
46−60 (n=7)
80plus (n=3)
61−80 (n=15)
31−45 (n=3)
20−30 (n=2)
Age_category

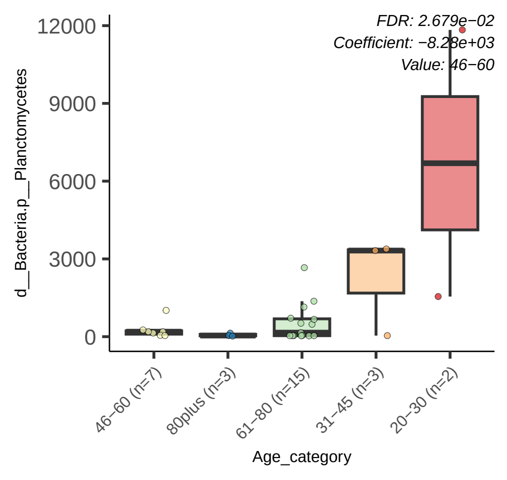

FDR: 2.679e−02
Coefficient: −8.28e+03
Value: 46−60
d__Bacteria.p__Planctomycetes
12000
9000
6000
3000
0
46−60 (n=7)
80plus (n=3)
61−80 (n=15)
31−45 (n=3)
20−30 (n=2)
Age_category

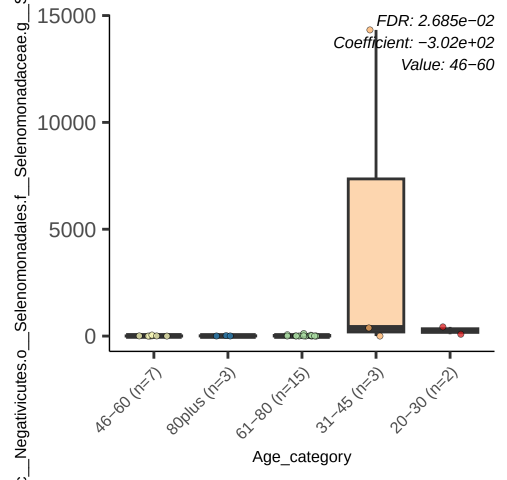

FDR: 2.685e−02
Coefficient: −3.02e+02
Value: 46−60
15000
10000
5000
0
Negativicutes.o__Selenomonadales.f__Selenomonadaceae.g__
46−60 (n=7)
80plus (n=3)
61−80 (n=15)
31−45 (n=3)
20−30 (n=2)
Age_category

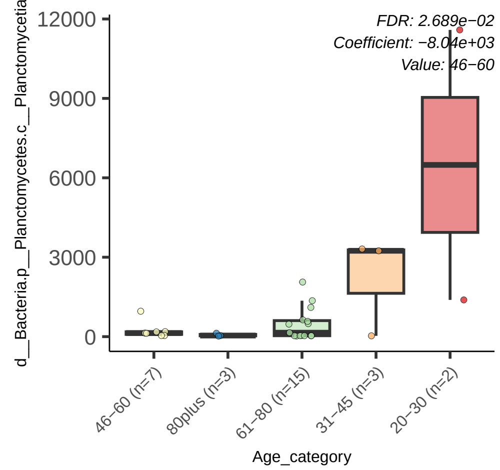

FDR: 2.689e−02
Coefficient: −8.04e+03
Value: 46−60
12000
9000
6000
3000
0
d__Bacteria.p__Planctomycetes.c__Planctomycetia
46−60 (n=7)
80plus (n=3)
61−80 (n=15)
31−45 (n=3)
20−30 (n=2)
Age_category

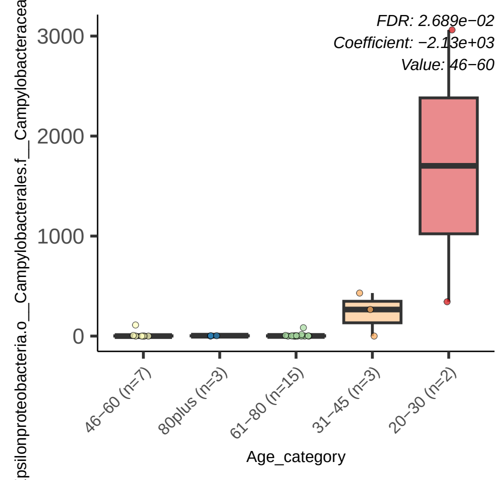

FDR: 2.689e−02
Coefficient: −2.13e+03
Value: 46−60
3000
2000
1000
0
psilonproteobacteria.o__Campylobacterales.f__Campylobacteracea
46−60 (n=7)
80plus (n=3)
61−80 (n=15)
31−45 (n=3)
20−30 (n=2)
Age_category

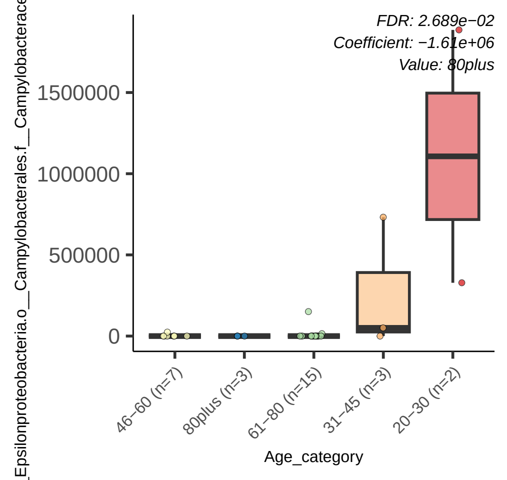

FDR: 2.689e−02
Coefficient: −1.61e+06
Value: 80plus
1500000
1000000
500000
0
46−60 (n=7)
80plus (n=3)
61−80 (n=15)
31−45 (n=3)
20−30 (n=2)
Age_category
Epsilonproteobacteria.o__Campylobacterales.f__Campylobacterace

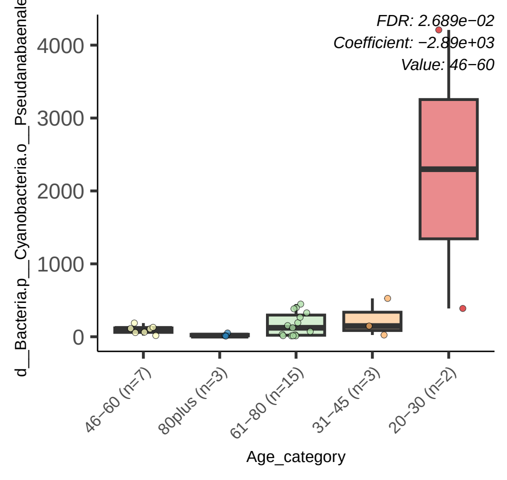
FDR: 2.689e−02
Coefficient: −2.89e+03
Value: 46−60
d__Bacteria.p__Cyanobacteria.o__Pseudanabaenale
4000
3000
2000
1000
0
46−60 (n=7)
80plus (n=3)
61−80 (n=15)
31−45 (n=3)
20−30 (n=2)
Age_category

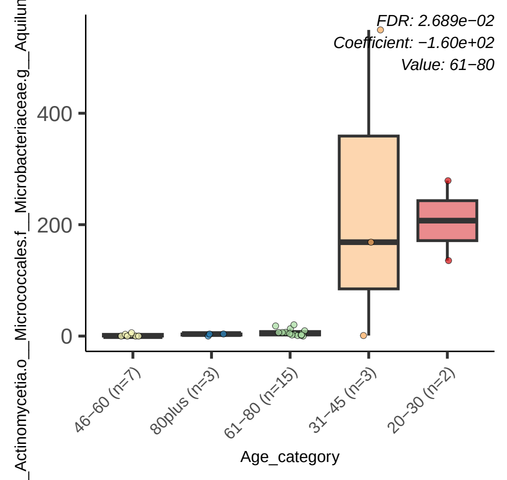
FDR: 2.689e−02
Coefficient: −1.60e+02
Value: 61−80
400
200
0
46−60 (n=7)
80plus (n=3)
61−80 (n=15)
31−45 (n=3)
20−30 (n=2)
Age_category
_Actinomycetia.o__Micrococcales.f__Microbacteriaceae.g__Aquilun

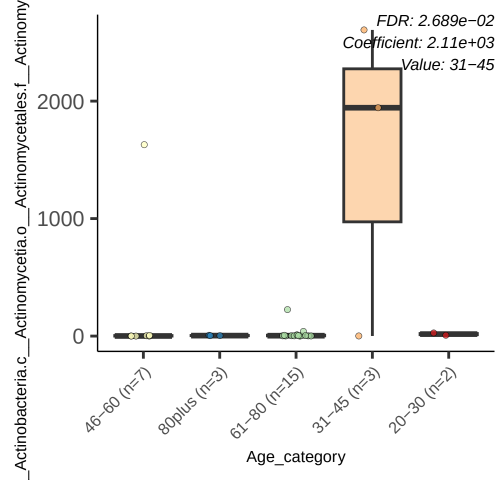

FDR: 2.689e−02
Coefficient: 2.11e+03
Value: 31−45
_Actinobacteria.c__Actinomycetia.o__Actinomycetales.f__Actinomy
2000
1000
0
46−60 (n=7)
80plus (n=3)
61−80 (n=15)
31−45 (n=3)
20−30 (n=2)
Age_category

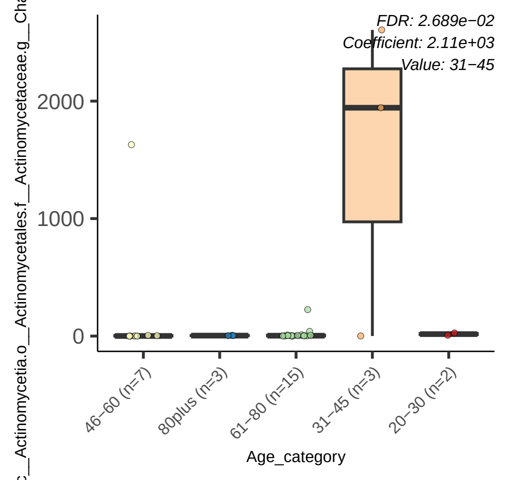

FDR: 2.689e−02
Coefficient: 2.11e+03
Value: 31−45
2000
1000
0
c__Actinomycetia.o__Actinomycetales.f__Actinomycetaceae.g__Cha
46−60 (n=7)
80plus (n=3)
61−80 (n=15)
31−45 (n=3)
20−30 (n=2)
Age_category

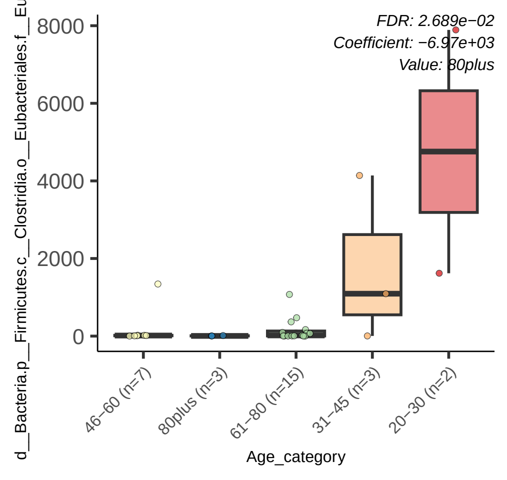

FDR: 2.689e−02
Coefficient: −6.97e+03
Value: 80plus
8000
6000
4000
2000
0
d__Bacteria.p__Firmicutes.c__Clostridia.o__Eubacteriales.f__Eu
46−60 (n=7)
80plus (n=3)
61−80 (n=15)
31−45 (n=3)
20−30 (n=2)
Age_category

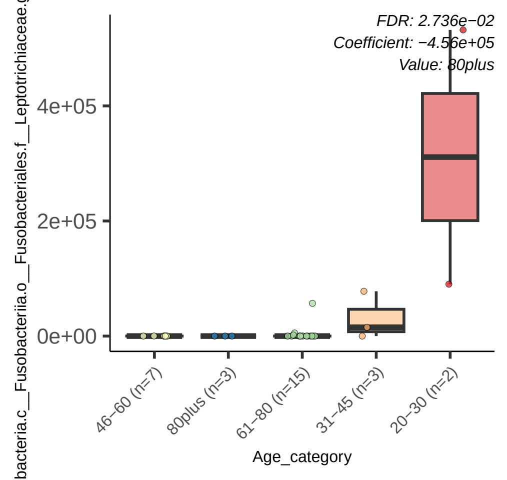

FDR: 2.736e−02
Coefficient: −4.56e+05
Value: 80plus
bacteria.c__Fusobacteriia.o__Fusobacteriales.f__Leptotrichiaceae.g
4e+05
2e+05
0e+00
46−60 (n=7)
80plus (n=3)
61−80 (n=15)
31−45 (n=3)
20−30 (n=2)
Age_category

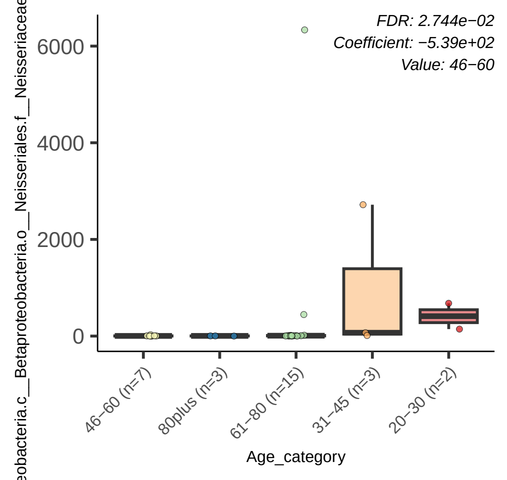

FDR: 2.744e−02
Coefficient: −5.39e+02
Value: 46−60
6000
4000
2000
0
eobacteria.c__Betaproteobacteria.o__Neisseriales.f__Neisseriaceae
46−60 (n=7)
80plus (n=3)
61−80 (n=15)
31−45 (n=3)
20−30 (n=2)
Age_category

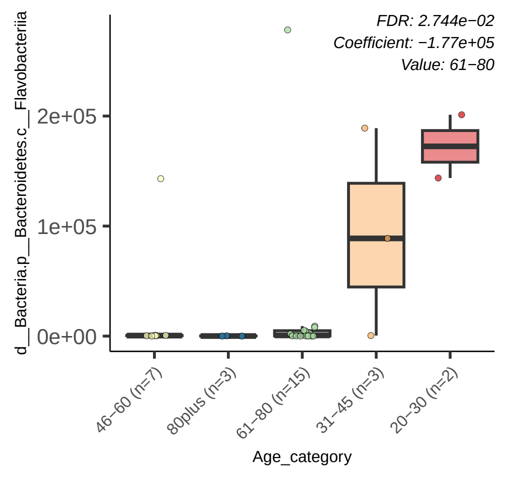

FDR: 2.744e−02
Coefficient: −1.77e+05
Value: 61−80
d__Bacteria.p__Bacteroidetes.c__Flavobacteriia
2e+05
1e+05
0e+00
46−60 (n=7)
80plus (n=3)
61−80 (n=15)
31−45 (n=3)
20−30 (n=2)
Age_category

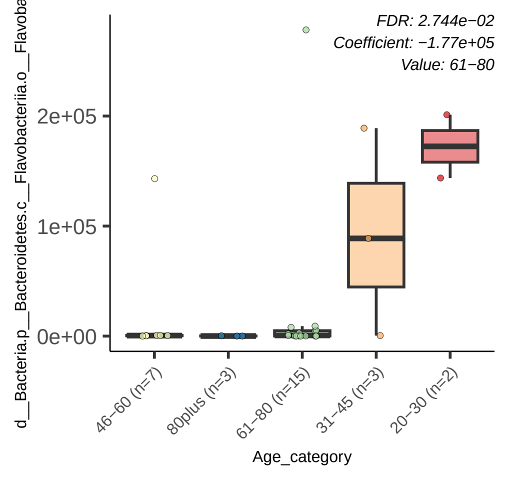

FDR: 2.744e−02
Coefficient: −1.77e+05
Value: 61−80
2e+05
1e+05
0e+00
d__Bacteria.p__Bacteroidetes.c__Flavobacteriia.o__Flavoba
46−60 (n=7)
80plus (n=3)
61−80 (n=15)
31−45 (n=3)
20−30 (n=2)
Age_category

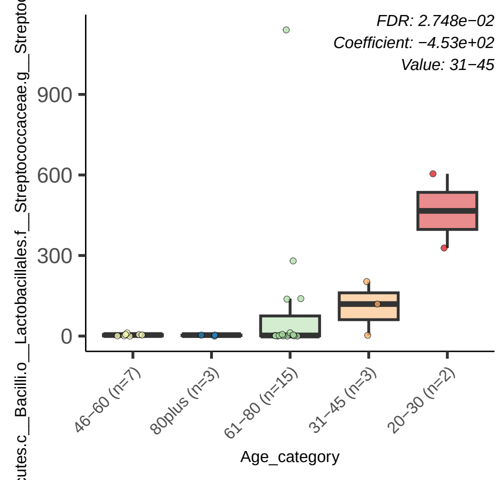

FDR: 2.748e−02
Coefficient: −4.53e+02
Value: 31−45
900
600
300
0
cutes.c__Bacilli.o__Lactobacillales.f__Streptococcaceae.g__Streptoc
46−60 (n=7)
80plus (n=3)
61−80 (n=15)
31−45 (n=3)
20−30 (n=2)
Age_category

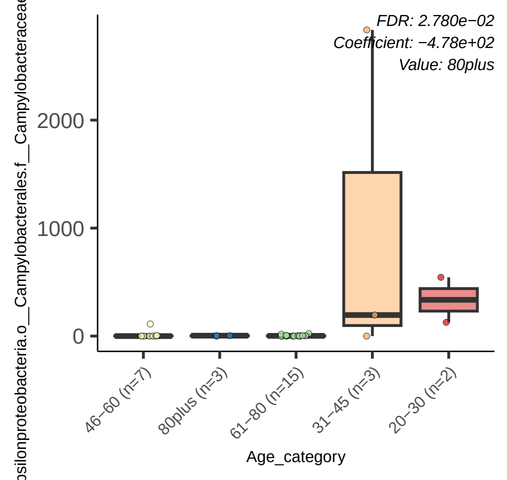

FDR: 2.780e−02
Coefficient: −4.78e+02
Value: 80plus
2000
1000
0
46−60 (n=7)
80plus (n=3)
61−80 (n=15)
31−45 (n=3)
20−30 (n=2)
Age_category
psilonproteobacteria.o__Campylobacterales.f__Campylobacterace

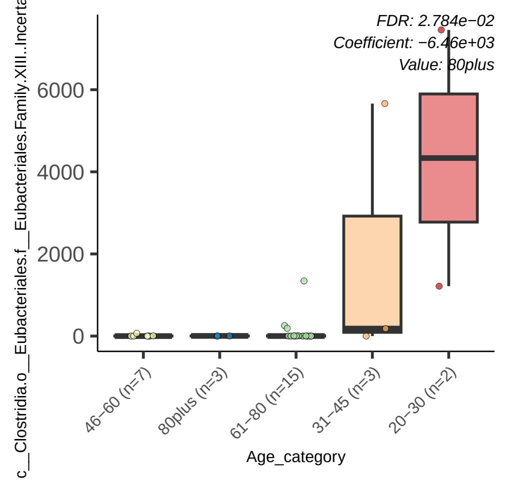
FDR: 2.784e−02
Coefficient: −6.46e+03
Value: 80plus
6000
4000
2000
0
c__Clostridia.o__Eubacteriales.f__Eubacteriales.Family.XIII..Incerta
46−60 (n=7)
80plus (n=3)
61−80 (n=15)
31−45 (n=3)
20−30 (n=2)
Age_category

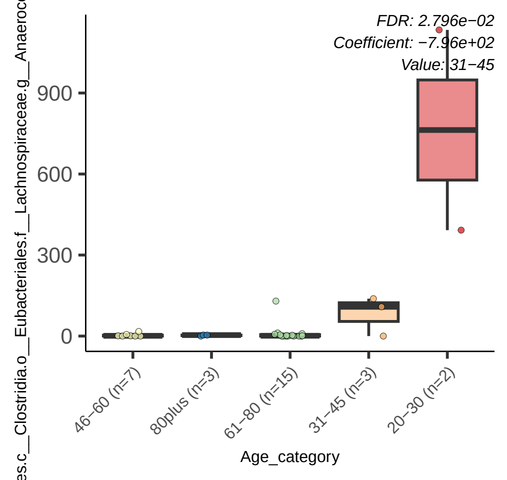
FDR: 2.796e−02
Coefficient: −7.96e+02
Value: 31−45
es.c__Clostridia.o__Eubacteriales.f__Lachnospiraceae.g__Anaeroc
900
600
300
0
46−60 (n=7)
80plus (n=3)
61−80 (n=15)
31−45 (n=3)
20−30 (n=2)
Age_category

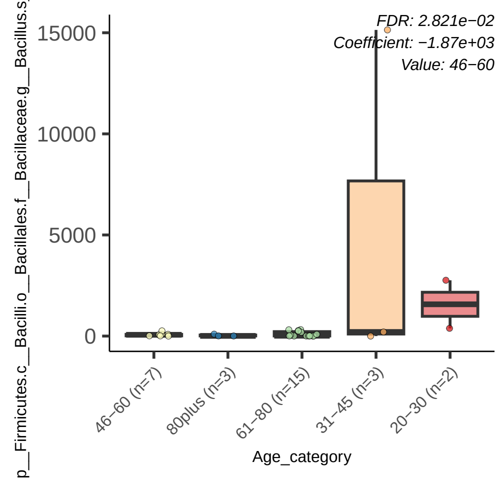

FDR: 2.821e−02
Coefficient: −1.87e+03
Value: 46−60
15000
10000
5000
0
p__Firmicutes.c__Bacilli.o__Bacillales.f__Bacillaceae.g__Bacillus.s_
46−60 (n=7)
80plus (n=3)
61−80 (n=15)
31−45 (n=3)
20−30 (n=2)
Age_category

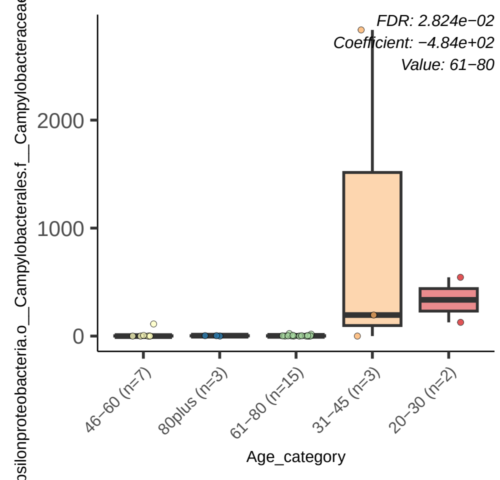

FDR: 2.824e−02
Coefficient: −4.84e+02
Value: 61−80
2000
1000
0
psilonproteobacteria.o__Campylobacterales.f__Campylobacterace
46−60 (n=7)
80plus (n=3)
61−80 (n=15)
31−45 (n=3)
20−30 (n=2)
Age_category

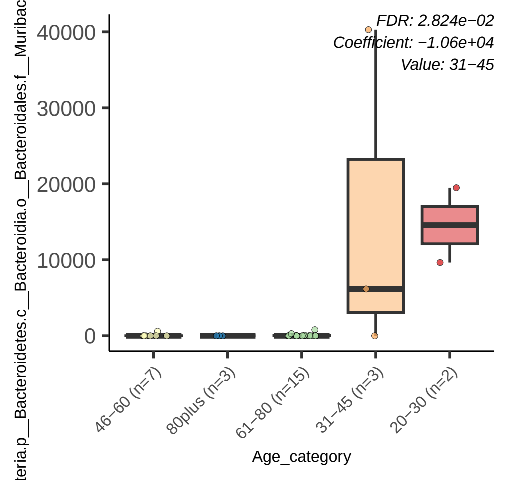

FDR: 2.824e−02
Coefficient: −1.06e+04
Value: 31−45
40000
30000
20000
10000
0
46−60 (n=7)
80plus (n=3)
61−80 (n=15)
31−45 (n=3)
20−30 (n=2)
Age_category
teria.p__Bacteroidetes.c__Bacteroidia.o__Bacteroidales.f__Muribac

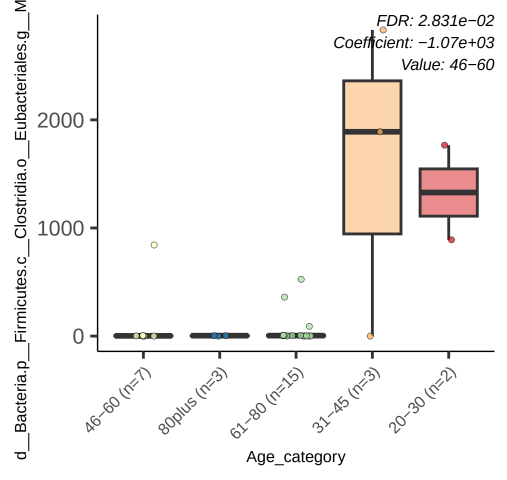

FDR: 2.831e−02
Coefficient: −1.07e+03
Value: 46−60
d__Bacteria.p__Firmicutes.c__Clostridia.o__Eubacteriales.g__M
2000
1000
0
46−60 (n=7)
80plus (n=3)
61−80 (n=15)
31−45 (n=3)
20−30 (n=2)
Age_category

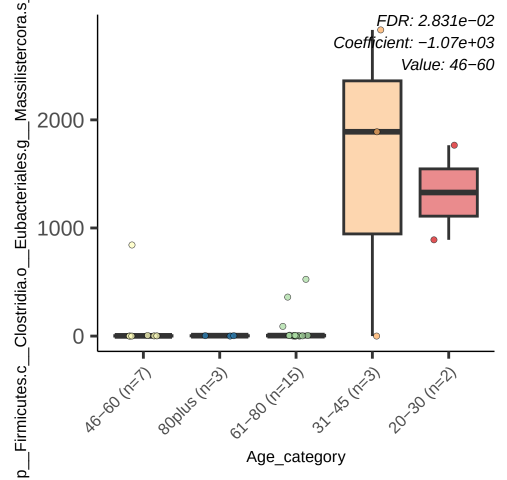

p__Firmicutes.c__Clostridia.o__Eubacteriales.g__Massilistercora.s_
FDR: 2.831e−02
Coefficient: −1.07e+03
Value: 46−60
2000
1000
0
46−60 (n=7)
80plus (n=3)
61−80 (n=15)
31−45 (n=3)
20−30 (n=2)
Age_category

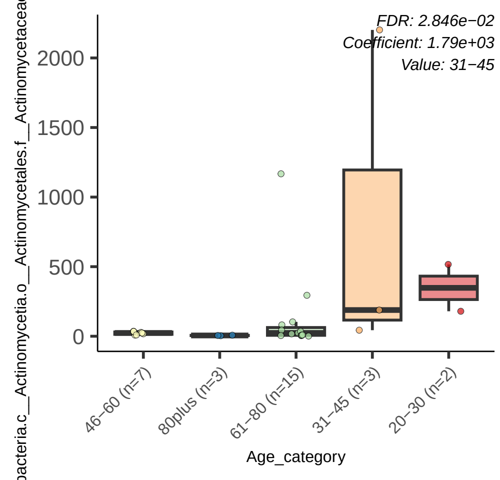

FDR: 2.846e−02
Coefficient: 1.79e+03
Value: 31−45
2000
1500
1000
500
0
bacteria.c__Actinomycetia.o__Actinomycetales.f__Actinomycetaceae
46−60 (n=7)
80plus (n=3)
61−80 (n=15)
31−45 (n=3)
20−30 (n=2)
Age_category

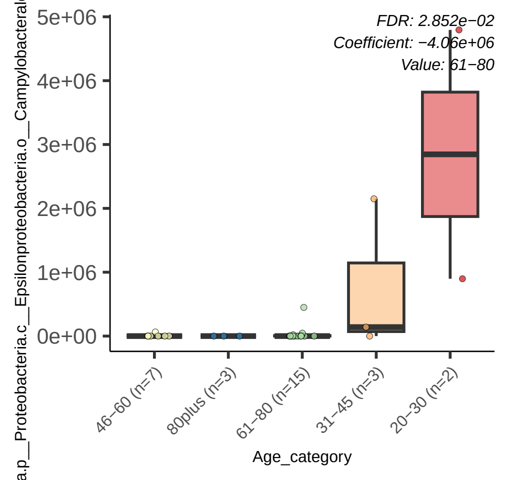

FDR: 2.852e−02
Coefficient: −4.06e+06
Value: 61−80
5e+06
4e+06
3e+06
2e+06
1e+06
0e+00
a.p__Proteobacteria.c__Epsilonproteobacteria.o__Campylobacterale
46−60 (n=7)
80plus (n=3)
61−80 (n=15)
31−45 (n=3)
20−30 (n=2)
Age_category

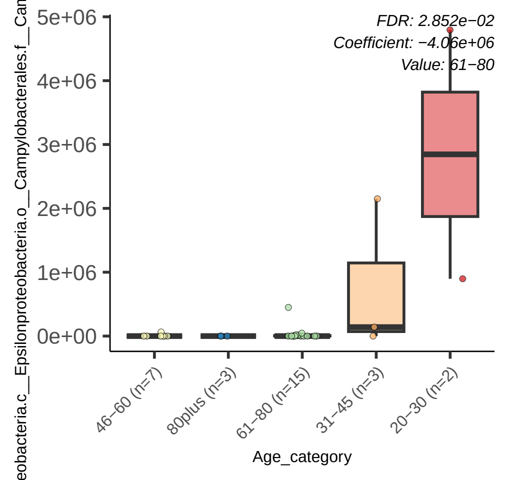

FDR: 2.852e−02
Coefficient: −4.06e+06
Value: 61−80
5e+06
4e+06
3e+06
2e+06
1e+06
0e+00
eobacteria.c__Epsilonproteobacteria.o__Campylobacterales.f__Cam
46−60 (n=7)
80plus (n=3)
61−80 (n=15)
31−45 (n=3)
20−30 (n=2)
Age_category

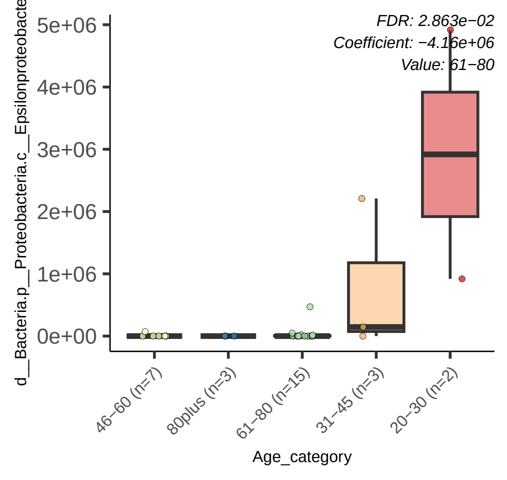

FDR: 2.863e−02
Coefficient: −4.16e+06
Value: 61−80
d__Bacteria.p__Proteobacteria.c__Epsilonproteobacte
5e+06
4e+06
3e+06
2e+06
1e+06
0e+00
46−60 (n=7)
80plus (n=3)
61−80 (n=15)
31−45 (n=3)
20−30 (n=2)
Age_category

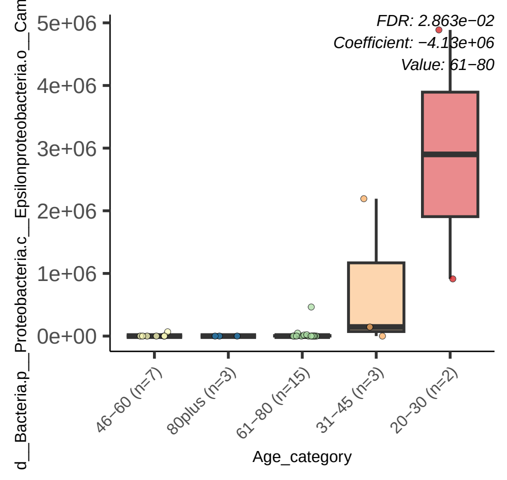

FDR: 2.863e−02
Coefficient: −4.13e+06
Value: 61−80
5e+06
4e+06
3e+06
2e+06
1e+06
0e+00
d__Bacteria.p__Proteobacteria.c__Epsilonproteobacteria.o__Cam
46−60 (n=7)
80plus (n=3)
61−80 (n=15)
31−45 (n=3)
20−30 (n=2)
Age_category

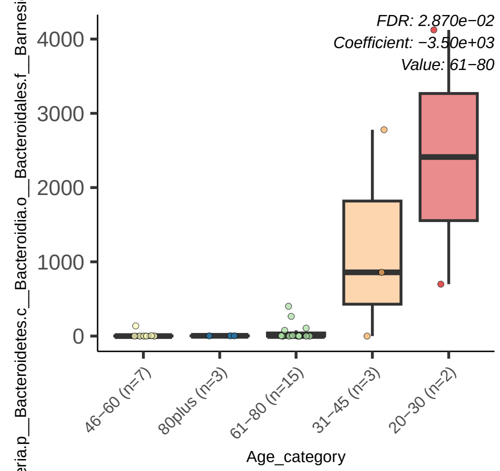

FDR: 2.870e−02
Coefficient: −3.50e+03
Value: 61−80
4000
3000
2000
1000
0
teria.p__Bacteroidetes.c__Bacteroidia.o__Bacteroidales.f__Barnesi
46−60 (n=7)
80plus (n=3)
61−80 (n=15)
31−45 (n=3)
20−30 (n=2)
Age_category

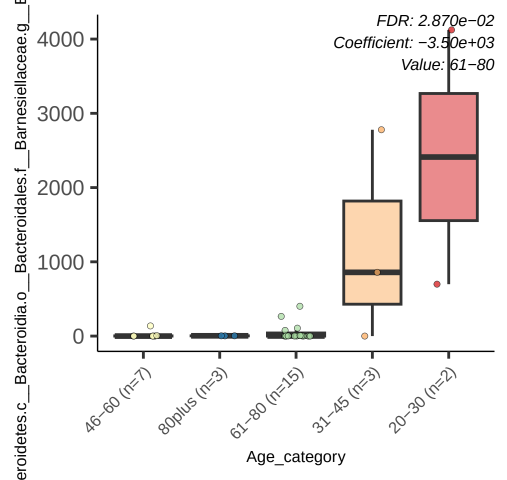

FDR: 2.870e−02
Coefficient: −3.50e+03
Value: 61−80
4000
3000
2000
1000
0
46−60 (n=7)
80plus (n=3)
61−80 (n=15)
31−45 (n=3)
20−30 (n=2)
Age_category
eroidetes.c__Bacteroidia.o__Bacteroidales.f__Barnesiellaceae.g__B

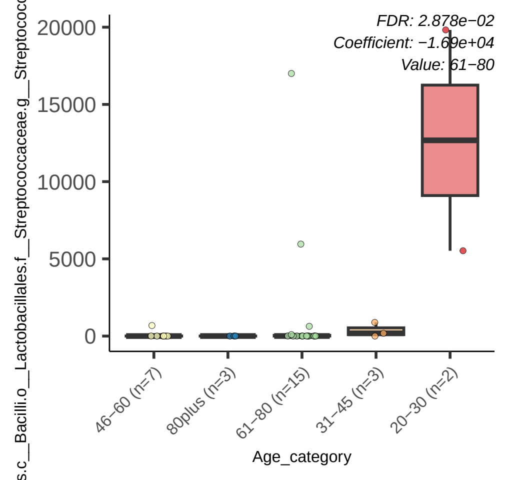

FDR: 2.878e−02
Coefficient: −1.69e+04
Value: 61−80
20000
15000
10000
5000
0
s.c__Bacilli.o__Lactobacillales.f__Streptococcaceae.g__Streptococc
46−60 (n=7)
80plus (n=3)
61−80 (n=15)
31−45 (n=3)
20−30 (n=2)
Age_category

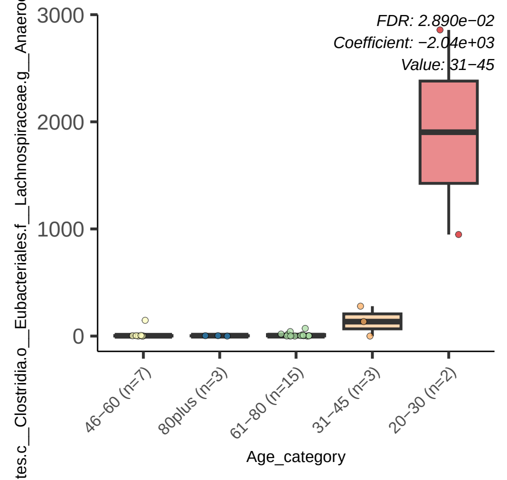

FDR: 2.890e−02
Coefficient: −2.04e+03
Value: 31−45
3000
2000
1000
0
tes.c__Clostridia.o__Eubacteriales.f__Lachnospiraceae.g__Anaero
46−60 (n=7)
80plus (n=3)
61−80 (n=15)
31−45 (n=3)
20−30 (n=2)
Age_category

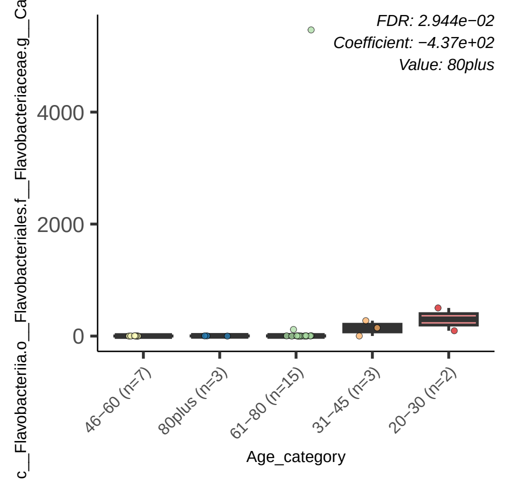

FDR: 2.944e−02
Coefficient: −4.37e+02
Value: 80plus
4000
2000
0
c__Flavobacteriia.o__Flavobacteriales.f__Flavobacteriaceae.g__Ca
46−60 (n=7)
80plus (n=3)
61−80 (n=15)
31−45 (n=3)
20−30 (n=2)
Age_category

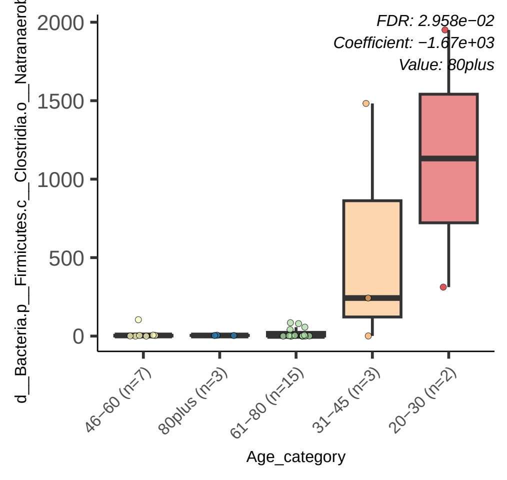

FDR: 2.958e−02
Coefficient: −1.67e+03
Value: 80plus
d__Bacteria.p__Firmicutes.c__Clostridia.o__Natranaerob
2000
1500
1000
500
0
46−60 (n=7)
80plus (n=3)
61−80 (n=15)
31−45 (n=3)
20−30 (n=2)
Age_category

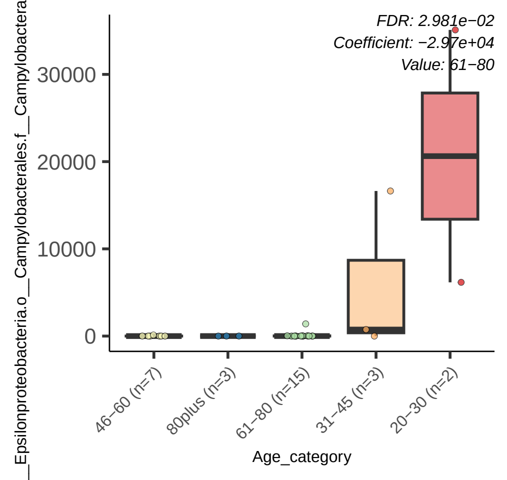

FDR: 2.981e−02
Coefficient: −2.97e+04
Value: 61−80
30000
20000
10000
0
__Epsilonproteobacteria.o__Campylobacterales.f__Campylobactera
46−60 (n=7)
80plus (n=3)
61−80 (n=15)
31−45 (n=3)
20−30 (n=2)
Age_category

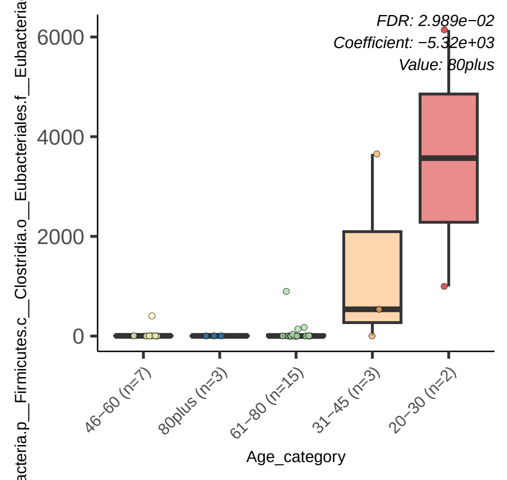

FDR: 2.989e−02
Coefficient: −5.32e+03
Value: 80plus
6000
4000
2000
0
46−60 (n=7)
80plus (n=3)
61−80 (n=15)
31−45 (n=3)
20−30 (n=2)
Age_category
acteria.p__Firmicutes.c__Clostridia.o__Eubacteriales.f__Eubacteria

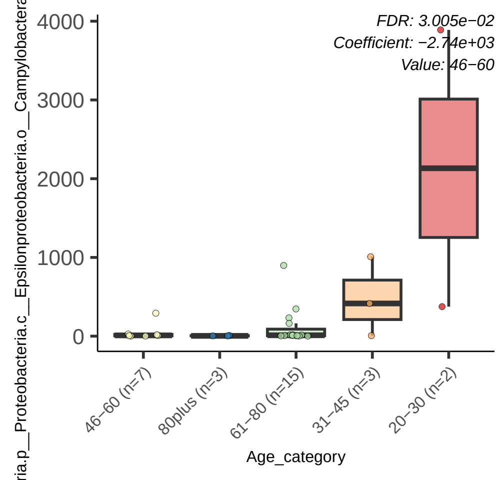

FDR: 3.005e−02
Coefficient: −2.74e+03
Value: 46−60
ria.p__Proteobacteria.c__Epsilonproteobacteria.o__Campylobactera
4000
3000
2000
1000
0
46−60 (n=7)
80plus (n=3)
61−80 (n=15)
31−45 (n=3)
20−30 (n=2)
Age_category

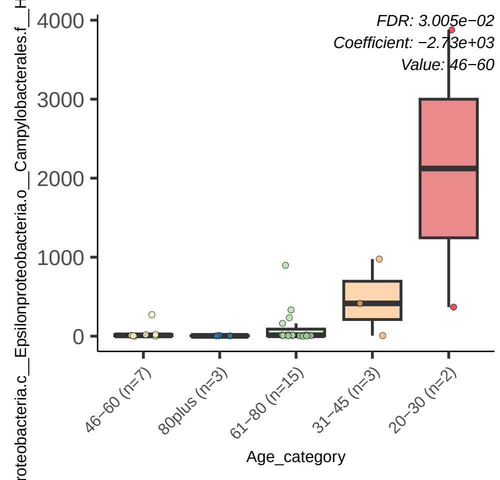
FDR: 3.005e−02
Coefficient: −2.73e+03
Value: 46−60
4000
3000
2000
1000
0
46−60 (n=7)
80plus (n=3)
61−80 (n=15)
31−45 (n=3)
20−30 (n=2)
Age_category
roteobacteria.c__Epsilonproteobacteria.o__Campylobacterales.f__H

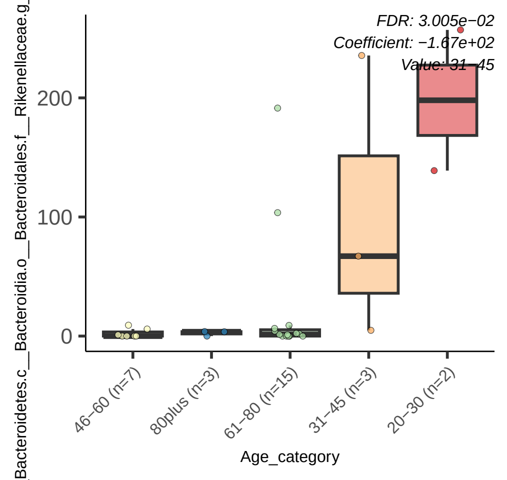

FDR: 3.005e−02
Coefficient: −1.67e+02
Value: 31−45
Bacteroidetes.c__Bacteroidia.o__Bacteroidales.f__Rikenellaceae.g_
200
100
0
46−60 (n=7)
80plus (n=3)
61−80 (n=15)
31−45 (n=3)
20−30 (n=2)
Age_category

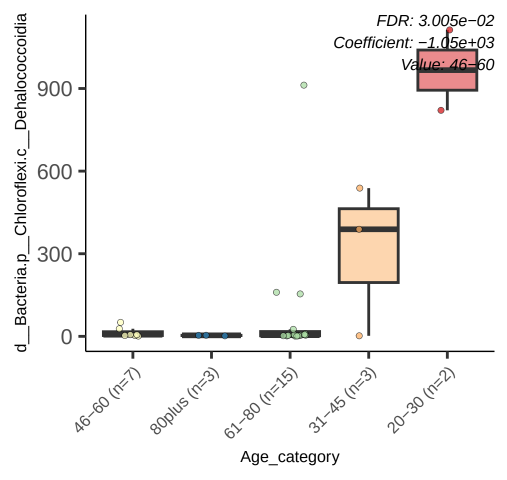
FDR: 3.005e−02
Coefficient: −1.05e+03
Value: 46−60
d__Bacteria.p__Chloroflexi.c__Dehalococcoidia
900
600
300
0
46−60 (n=7)
80plus (n=3)
61−80 (n=15)
31−45 (n=3)
20−30 (n=2)
Age_category

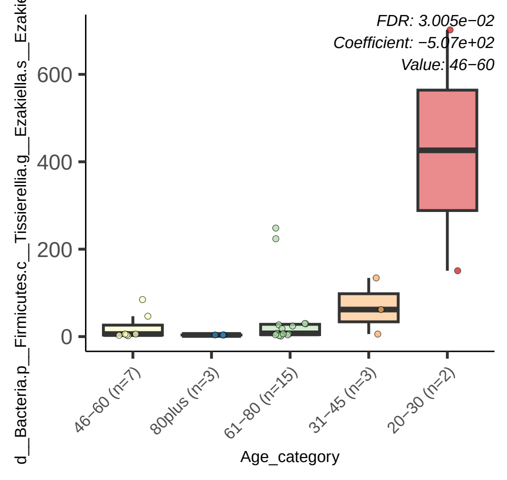

FDR: 3.005e−02
Coefficient: −5.07e+02
Value: 46−60
d__Bacteria.p__Firmicutes.c__Tissierellia.g__Ezakiella.s__Ezakie
0
200
400
600
46−60 (n=7)
80plus (n=3)
61−80 (n=15)
31−45 (n=3)
20−30 (n=2)
Age_category

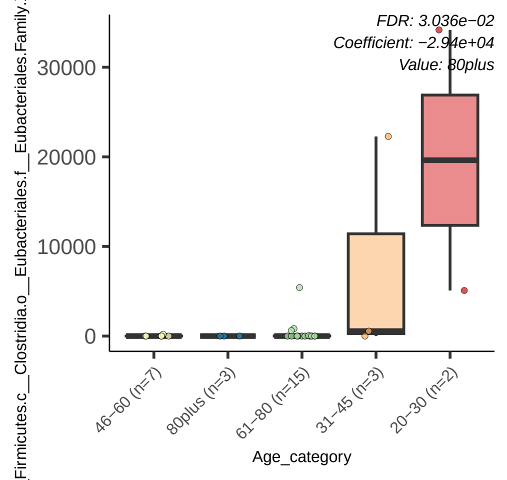
FDR: 3.036e−02
Coefficient: −2.94e+04
Value: 80plus
30000
20000
10000
0
Firmicutes.c__Clostridia.o__Eubacteriales.f__Eubacteriales.Family.
46−60 (n=7)
80plus (n=3)
61−80 (n=15)
31−45 (n=3)
20−30 (n=2)
Age_category

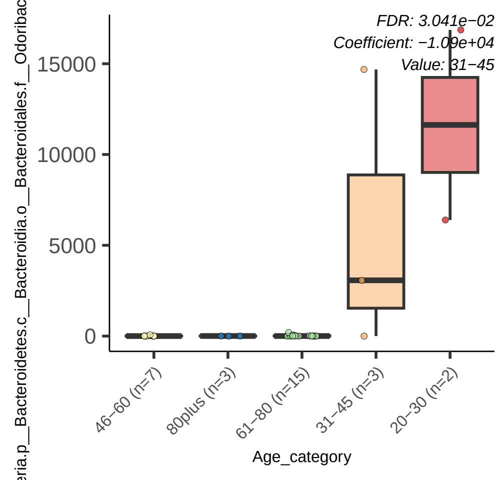
FDR: 3.041e−02
Coefficient: −1.09e+04
Value: 31−45
15000
10000
5000
0
46−60 (n=7)
80plus (n=3)
61−80 (n=15)
31−45 (n=3)
20−30 (n=2)
Age_category
eria.p__Bacteroidetes.c__Bacteroidia.o__Bacteroidales.f__Odoribac

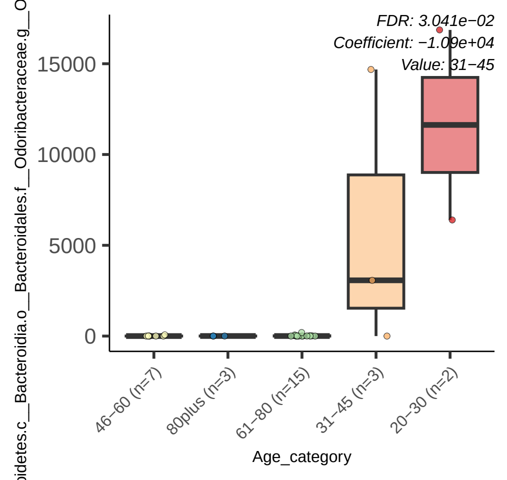

FDR: 3.041e−02
Coefficient: −1.09e+04
Value: 31−45
15000
10000
5000
0
46−60 (n=7)
80plus (n=3)
61−80 (n=15)
31−45 (n=3)
20−30 (n=2)
Age_category
oidetes.c__Bacteroidia.o__Bacteroidales.f__Odoribacteraceae.g__O

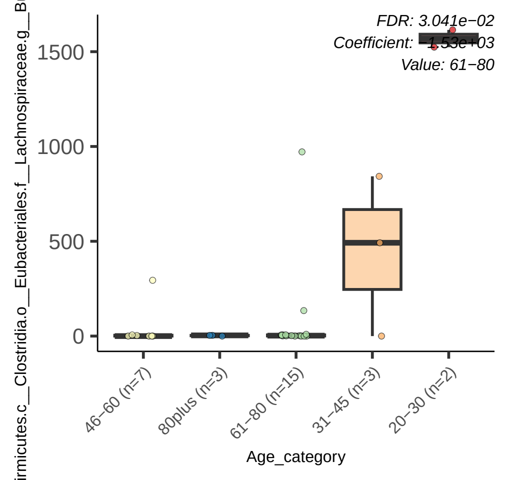

FDR: 3.041e−02
Coefficient: −1.53e+03
Value: 61−80
1500
1000
500
0
irmicutes.c__Clostridia.o__Eubacteriales.f__Lachnospiraceae.g__B
46−60 (n=7)
80plus (n=3)
61−80 (n=15)
31−45 (n=3)
20−30 (n=2)
Age_category

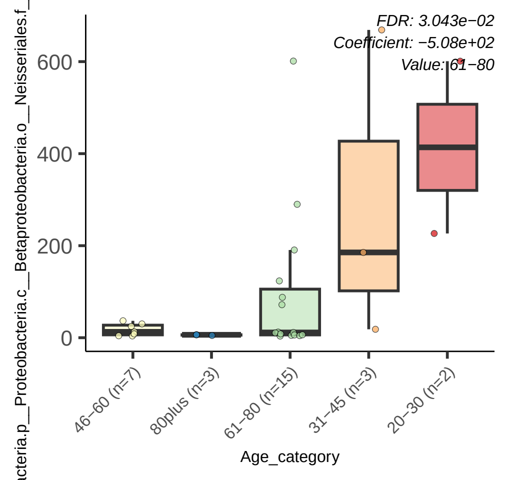

FDR: 3.043e−02
Coefficient: −5.08e+02
Value: 61−80
600
400
200
0
acteria.p__Proteobacteria.c__Betaproteobacteria.o__Neisseriales.f_
46−60 (n=7)
80plus (n=3)
61−80 (n=15)
31−45 (n=3)
20−30 (n=2)
Age_category

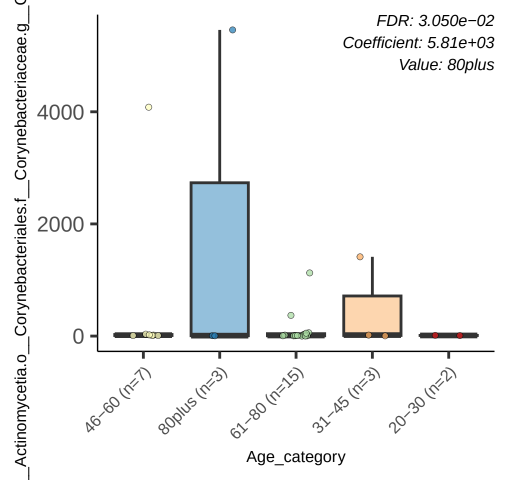

FDR: 3.050e−02
Coefficient: 5.81e+03
Value: 80plus
4000
2000
0
46−60 (n=7)
80plus (n=3)
61−80 (n=15)
31−45 (n=3)
20−30 (n=2)
Age_category
Actinomycetia.o__Corynebacteriales.f__Corynebacteriaceae.g__C

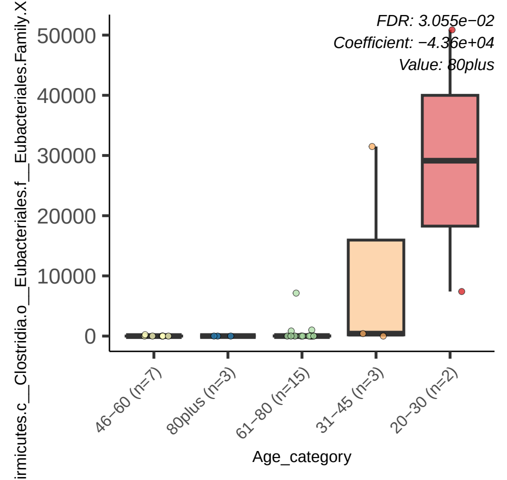
FDR: 3.055e−02
Coefficient: −4.36e+04
Value: 80plus
irmicutes.c__Clostridia.o__Eubacteriales.f__Eubacteriales.Family.X
50000
40000
30000
20000
10000
0
46−60 (n=7)
80plus (n=3)
61−80 (n=15)
31−45 (n=3)
20−30 (n=2)
Age_category

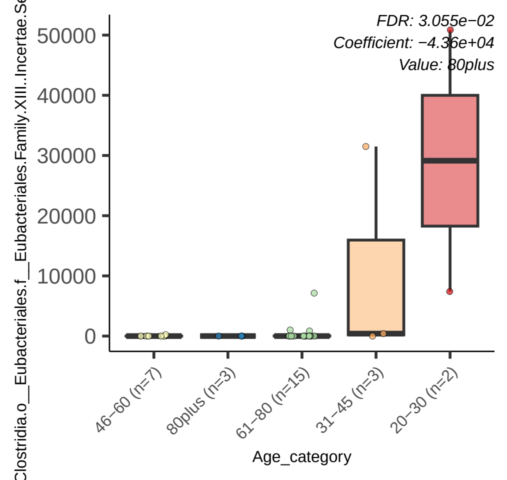
FDR: 3.055e−02
Coefficient: −4.36e+04
Value: 80plus
Clostridia.o__Eubacteriales.f__Eubacteriales.Family.XIII..Incertae.Se
50000
40000
30000
20000
10000
0
46−60 (n=7)
80plus (n=3)
61−80 (n=15)
31−45 (n=3)
20−30 (n=2)
Age_category

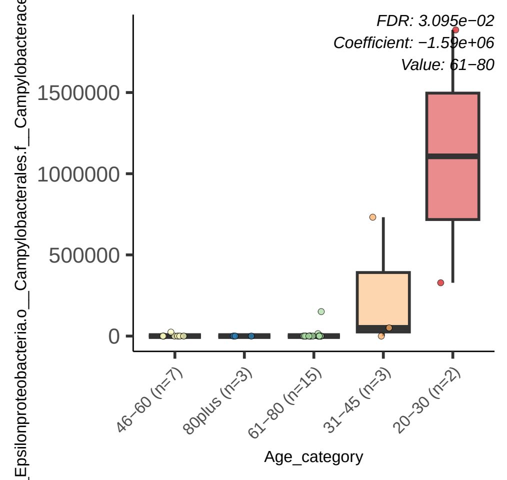

FDR: 3.095e−02
Coefficient: −1.59e+06
Value: 61−80
1500000
1000000
500000
0
_Epsilonproteobacteria.o__Campylobacterales.f__Campylobacterace
46−60 (n=7)
80plus (n=3)
61−80 (n=15)
31−45 (n=3)
20−30 (n=2)
Age_category

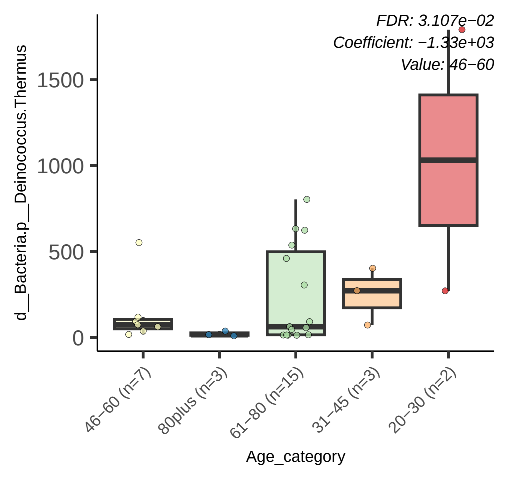

FDR: 3.107e−02
Coefficient: −1.33e+03
Value: 46−60
d__Bacteria.p__Deinococcus.Thermus
1500
1000
500
0
46−60 (n=7)
80plus (n=3)
61−80 (n=15)
31−45 (n=3)
20−30 (n=2)
Age_category

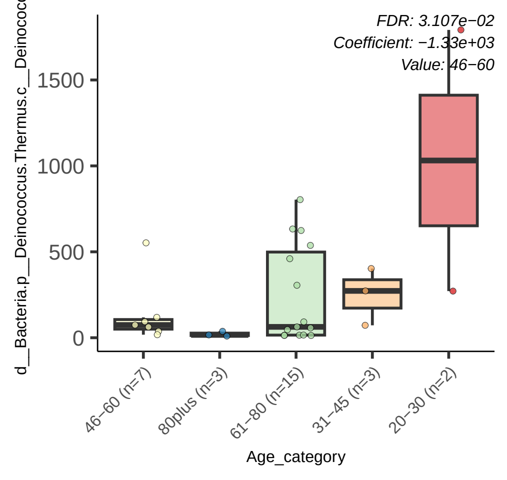
FDR: 3.107e−02
Coefficient: −1.33e+03
Value: 46−60
d__Bacteria.p__Deinococcus.Thermus.c__Deinococ
1500
1000
500
0
46−60 (n=7)
80plus (n=3)
61−80 (n=15)
31−45 (n=3)
20−30 (n=2)
Age_category

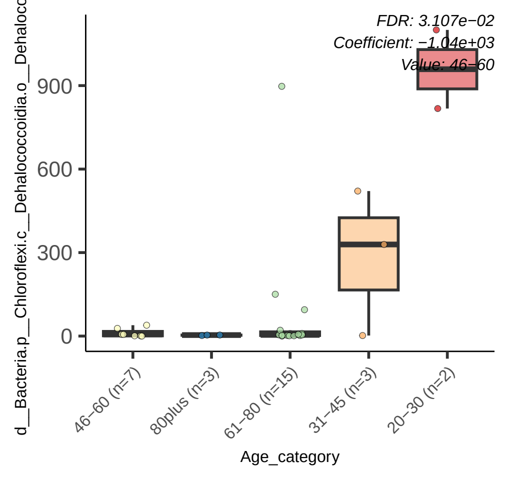

FDR: 3.107e−02
Coefficient: −1.04e+03
Value: 46−60
d__Bacteria.p__Chloroflexi.c__Dehalococcoidia.o__Dehalocc
900
600
300
0
46−60 (n=7)
80plus (n=3)
61−80 (n=15)
31−45 (n=3)
20−30 (n=2)
Age_category

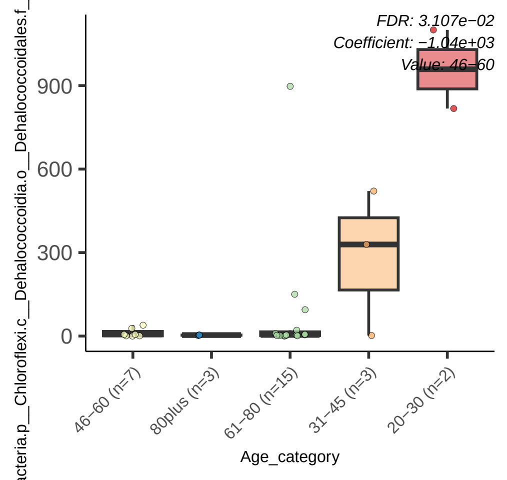
FDR: 3.107e−02
Coefficient: −1.04e+03
Value: 46−60
0
300
600
900
acteria.p__Chloroflexi.c__Dehalococcoidia.o__Dehalococcoidales.f_
46−60 (n=7)
80plus (n=3)
61−80 (n=15)
31−45 (n=3)
20−30 (n=2)
Age_category

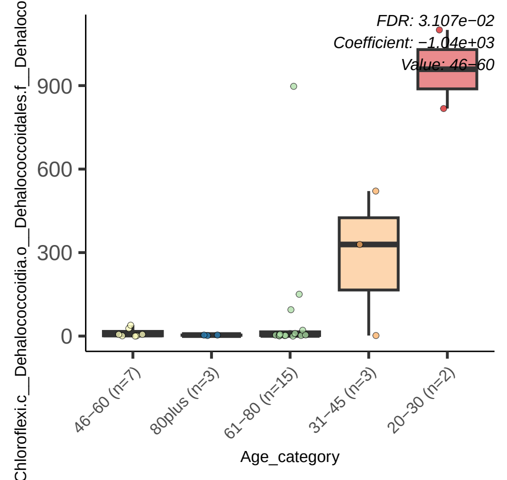

FDR: 3.107e−02
Coefficient: −1.04e+03
Value: 46−60
900
600
300
0
Chloroflexi.c__Dehalococcoidia.o__Dehalococcoidales.f__Dehaloco
46−60 (n=7)
80plus (n=3)
61−80 (n=15)
31−45 (n=3)
20−30 (n=2)
Age_category

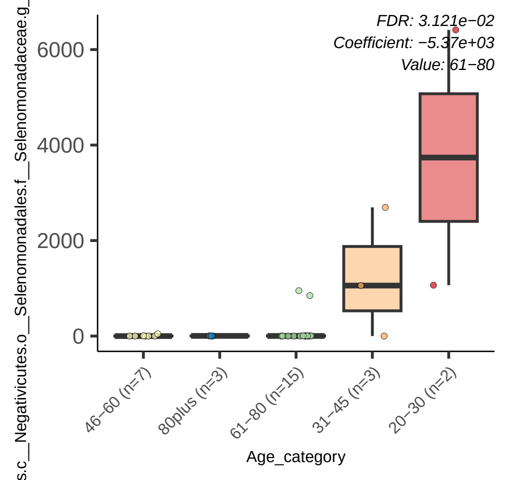

FDR: 3.121e−02
Coefficient: −5.37e+03
Value: 61−80
s.c__Negativicutes.o__Selenomonadales.f__Selenomonadaceae.g_
6000
4000
2000
0
46−60 (n=7)
80plus (n=3)
61−80 (n=15)
31−45 (n=3)
20−30 (n=2)
Age_category

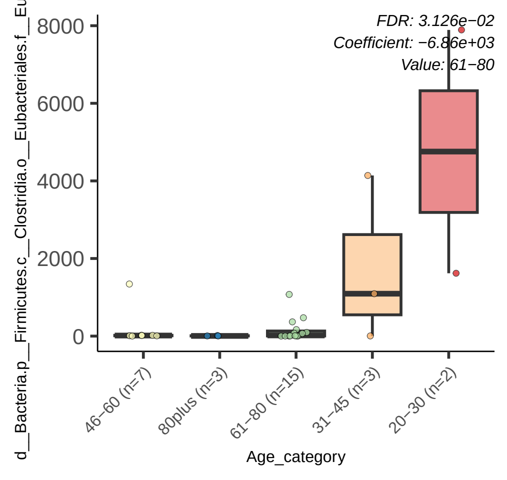

FDR: 3.126e−02
Coefficient: −6.86e+03
Value: 61−80
d__Bacteria.p__Firmicutes.c__Clostridia.o__Eubacteriales.f__Eu
8000
6000
4000
2000
0
46−60 (n=7)
80plus (n=3)
61−80 (n=15)
31−45 (n=3)
20−30 (n=2)
Age_category

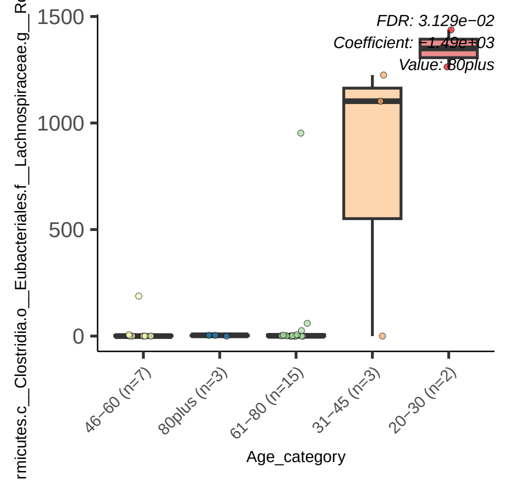

FDR: 3.129e−02
Coefficient: -1.49e+03
Value: 80plus
1500
1000
500
0
46−60 (n=7)
80plus (n=3)
61−80 (n=15)
31−45 (n=3)
20−30 (n=2)
Age_category
rmicutes.c__Clostridia.o__Eubacteriales.f__Lachnospiraceae.g__Ro

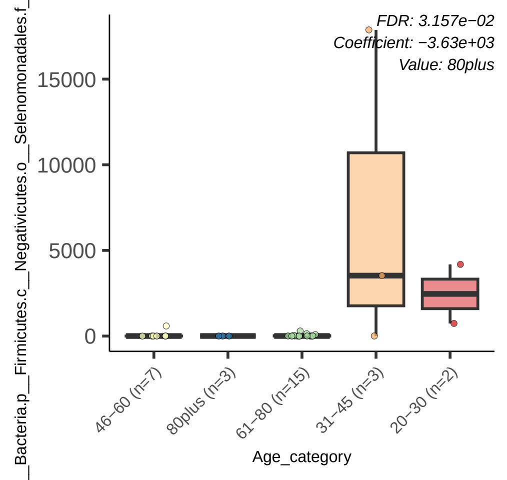

FDR: 3.157e−02
Coefficient: −3.63e+03
Value: 80plus
Bacteria.p__Firmicutes.c__Negativicutes.o__Selenomonadales.f
15000
10000
5000
0
46−60 (n=7)
80plus (n=3)
61−80 (n=15)
31−45 (n=3)
20−30 (n=2)
Age_category

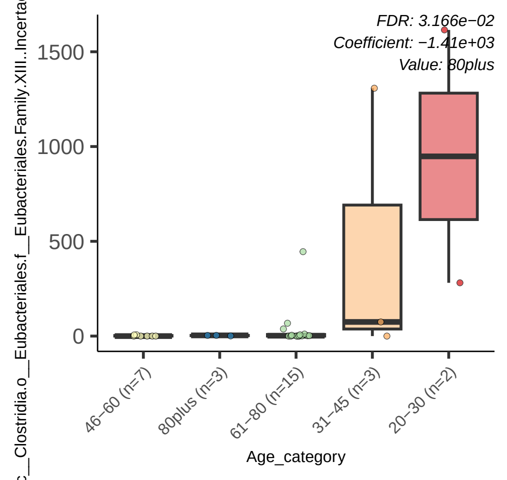

FDR: 3.166e−02
Coefficient: −1.41e+03
Value: 80plus
1500
1000
500
0
c__Clostridia.o__Eubacteriales.f__Eubacteriales.Family.XIII..Incertae
46−60 (n=7)
80plus (n=3)
61−80 (n=15)
31−45 (n=3)
20−30 (n=2)
Age_category

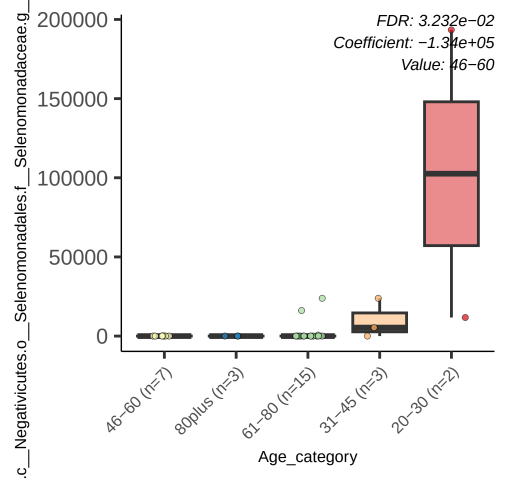

FDR: 3.232e−02
Coefficient: −1.34e+05
Value: 46−60
200000
150000
100000
50000
0
c__Negativicutes.o__Selenomonadales.f__Selenomonadaceae.g__
46−60 (n=7)
80plus (n=3)
61−80 (n=15)
31−45 (n=3)
20−30 (n=2)
Age_category

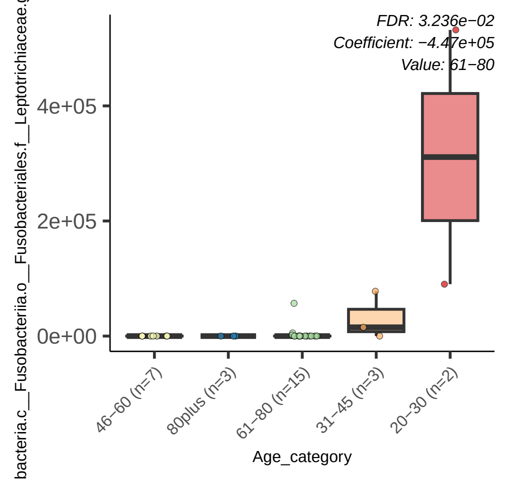
FDR: 3.236e−02
Coefficient: −4.47e+05
Value: 61−80
bacteria.c__Fusobacteriia.o__Fusobacteriales.f__Leptotrichiaceae.g
4e+05
2e+05
0e+00
46−60 (n=7)
80plus (n=3)
61−80 (n=15)
31−45 (n=3)
20−30 (n=2)
Age_category

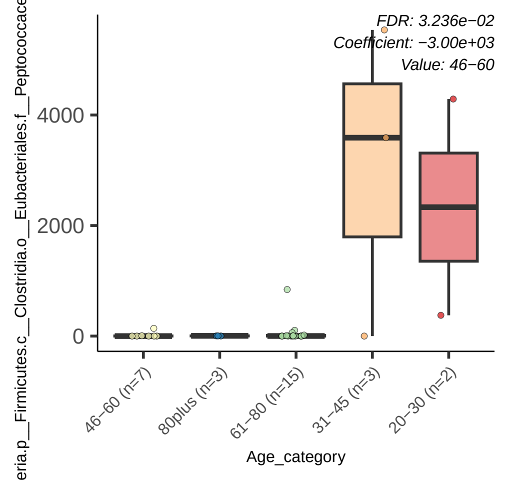

FDR: 3.236e−02
Coefficient: −3.00e+03
Value: 46−60
eria.p__Firmicutes.c__Clostridia.o__Eubacteriales.f__Peptococcace
4000
2000
0
46−60 (n=7)
80plus (n=3)
61−80 (n=15)
31−45 (n=3)
20−30 (n=2)
Age_category

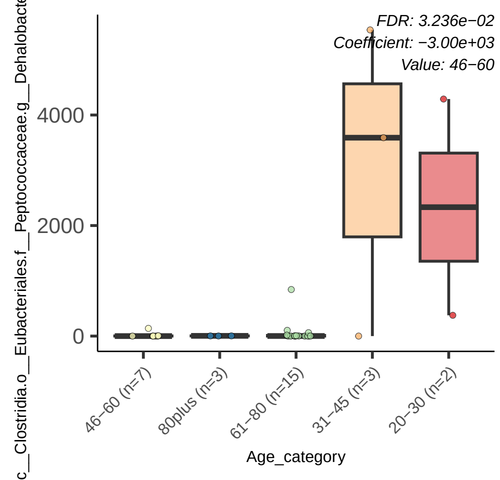

FDR: 3.236e−02
Coefficient: −3.00e+03
Value: 46−60
c__Clostridia.o__Eubacteriales.f__Peptococcaceae.g__Dehalobacte
4000
2000
0
46−60 (n=7)
80plus (n=3)
61−80 (n=15)
31−45 (n=3)
20−30 (n=2)
Age_category

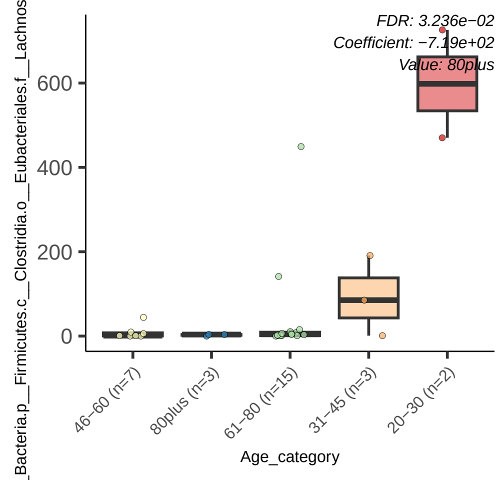

FDR: 3.236e−02
Coefficient: −7.19e+02
Value: 80plus
0
200
400
600
Bacteria.p__Firmicutes.c__Clostridia.o__Eubacteriales.f__Lachnos
46−60 (n=7)
80plus (n=3)
61−80 (n=15)
31−45 (n=3)
20−30 (n=2)
Age_category

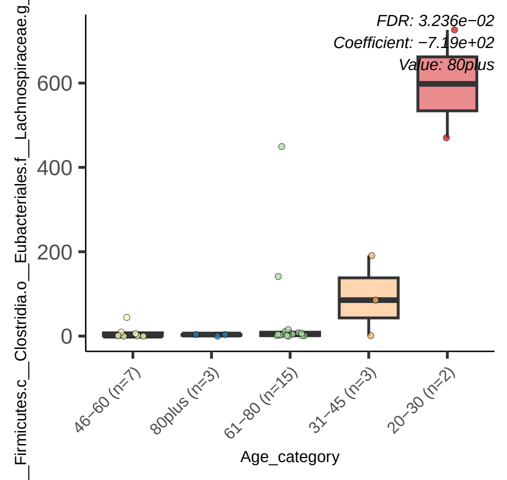

FDR: 3.236e−02
Coefficient: −7.19e+02
Value: 80plus
Firmicutes.c__Clostridia.o__Eubacteriales.f__Lachnospiraceae.g_
0
200
400
600
46−60 (n=7)
80plus (n=3)
61−80 (n=15)
31−45 (n=3)
20−30 (n=2)
Age_category

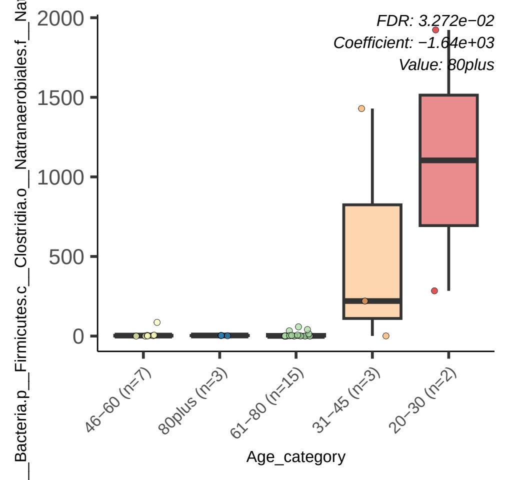

FDR: 3.272e−02
Coefficient: −1.64e+03
Value: 80plus
2000
1500
1000
500
0
46−60 (n=7)
80plus (n=3)
61−80 (n=15)
31−45 (n=3)
20−30 (n=2)
Age_category
Bacteria.p__Firmicutes.c__Clostridia.o__Natranaerobiales.f__Na

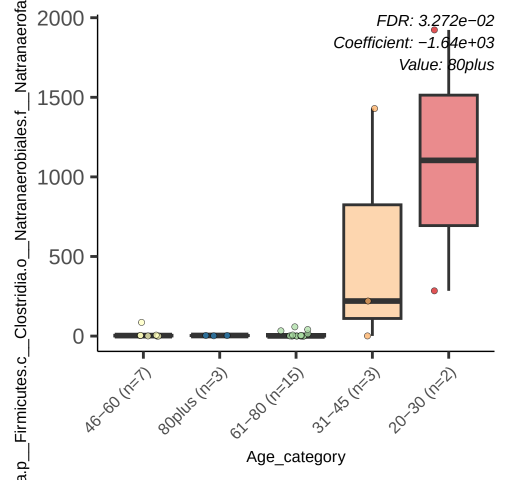

FDR: 3.272e−02
Coefficient: −1.64e+03
Value: 80plus
2000
1500
1000
500
0
a.p__Firmicutes.c__Clostridia.o__Natranaerobiales.f__Natranaerofa
46−60 (n=7)
80plus (n=3)
61−80 (n=15)
31−45 (n=3)
20−30 (n=2)
Age_category

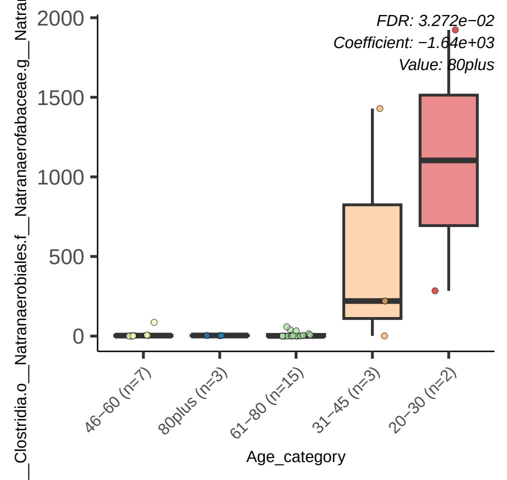

FDR: 3.272e−02
Coefficient: −1.64e+03
Value: 80plus
2000
1500
1000
500
0
46−60 (n=7)
80plus (n=3)
61−80 (n=15)
31−45 (n=3)
20−30 (n=2)
Age_category

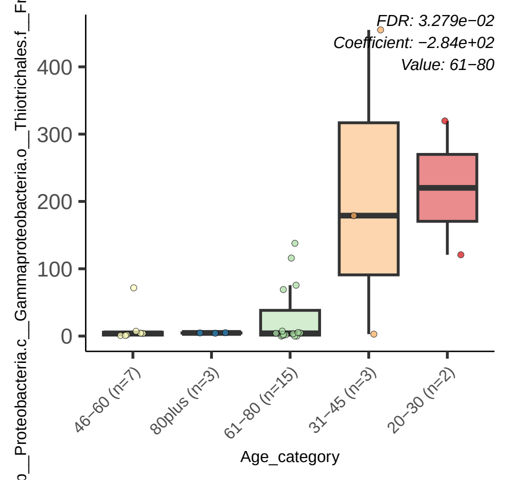

FDR: 3.279e−02
Coefficient: −2.84e+02
Value: 61−80
o__Proteobacteria.c__Gammaproteobacteria.o__Thiotrichales.f__Fr
400
300
200
100
0
46−60 (n=7)
80plus (n=3)
61−80 (n=15)
31−45 (n=3)
20−30 (n=2)
Age_category

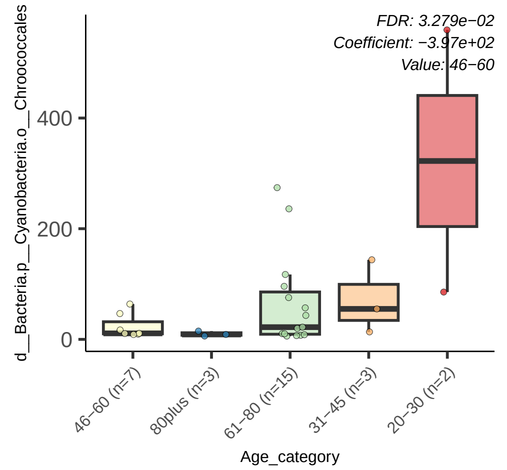

FDR: 3.279e−02
Coefficient: −3.97e+02
Value: 46−60
d__Bacteria.p__Cyanobacteria.o__Chroococcales
400
200
0
46−60 (n=7)
80plus (n=3)
61−80 (n=15)
31−45 (n=3)
20−30 (n=2)
Age_category

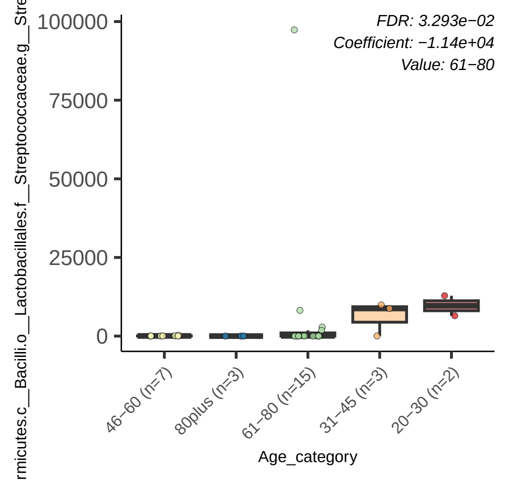
FDR: 3.293e−02
Coefficient: −1.14e+04
Value: 61−80
100000
75000
50000
25000
0
46−60 (n=7)
80plus (n=3)
61−80 (n=15)
31−45 (n=3)
20−30 (n=2)
Age_category
rmicutes.c__Bacilli.o__Lactobacillales.f__Streptococcaceae.g__Stre

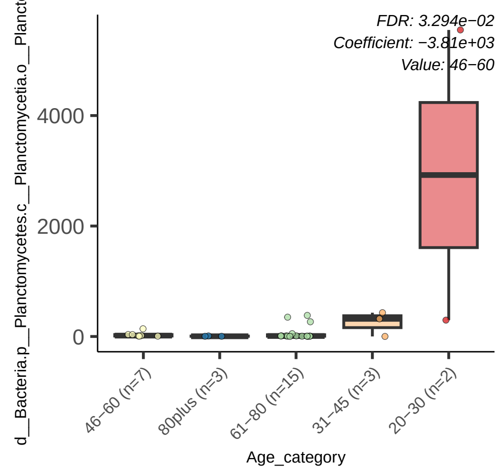

FDR: 3.294e−02
Coefficient: −3.81e+03
Value: 46−60
d__Bacteria.p__Planctomycetes.c__Planctomycetia.o__Planct
4000
2000
0
46−60 (n=7)
80plus (n=3)
61−80 (n=15)
31−45 (n=3)
20−30 (n=2)
Age_category

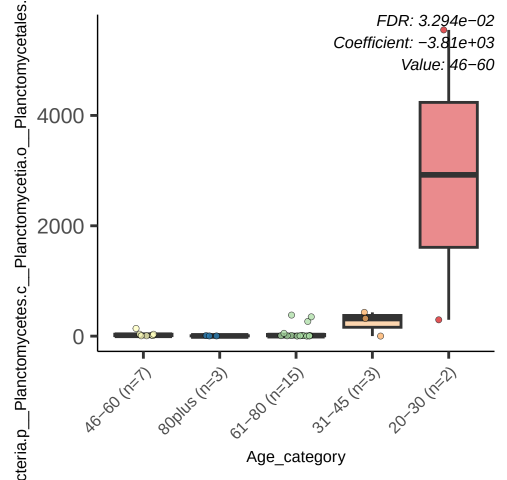
FDR: 3.294e−02
Coefficient: −3.81e+03
Value: 46−60
4000
2000
0
cteria.p__Planctomycetes.c__Planctomycetia.o__Planctomycetales.
46−60 (n=7)
80plus (n=3)
61−80 (n=15)
31−45 (n=3)
20−30 (n=2)
Age_category

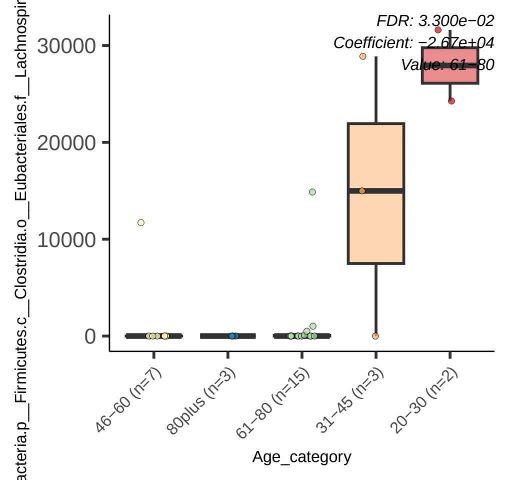

FDR: 3.300e−02
Coefficient: −2.67e+04
Value: 61−80
30000
20000
10000
0
acteria.p__Firmicutes.c__Clostridia.o__Eubacteriales.f__Lachnospir
46−60 (n=7)
80plus (n=3)
61−80 (n=15)
31−45 (n=3)
20−30 (n=2)
Age_category

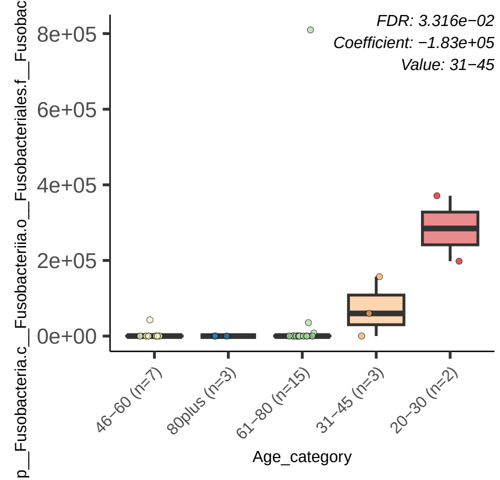

FDR: 3.316e−02
Coefficient: −1.83e+05
Value: 31−45
8e+05
6e+05
4e+05
2e+05
0e+00
p__Fusobacteria.c__Fusobacteriia.o__Fusobacteriales.f__Fusobac
46−60 (n=7)
80plus (n=3)
61−80 (n=15)
31−45 (n=3)
20−30 (n=2)
Age_category

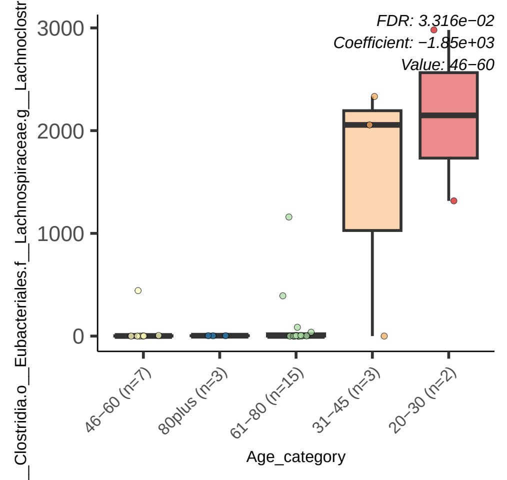
FDR: 3.316e−02
Coefficient: −1.85e+03
Value: 46−60
3000
2000
1000
0
Clostridia.o__Eubacteriales.f__Lachnospiraceae.g__Lachnoclostr
46−60 (n=7)
80plus (n=3)
61−80 (n=15)
31−45 (n=3)
20−30 (n=2)
Age_category

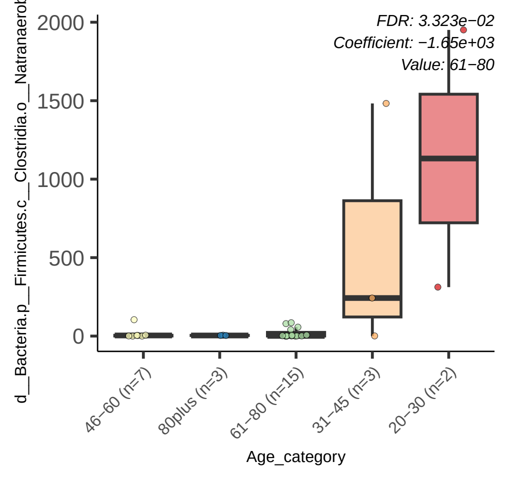

FDR: 3.323e−02
Coefficient: −1.65e+03
Value: 61−80
d__Bacteria.p__Firmicutes.c__Clostridia.o__Natranaerob
2000
1500
1000
500
0
46−60 (n=7)
80plus (n=3)
61−80 (n=15)
31−45 (n=3)
20−30 (n=2)
Age_category

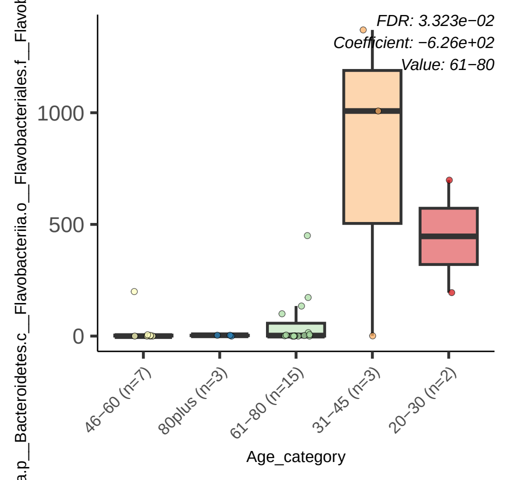
FDR: 3.323e−02
Coefficient: −6.26e+02
Value: 61−80
1000
500
0
a.p__Bacteroidetes.c__Flavobacteriia.o__Flavobacteriales.f__Flavob
46−60 (n=7)
80plus (n=3)
61−80 (n=15)
31−45 (n=3)
20−30 (n=2)
Age_category

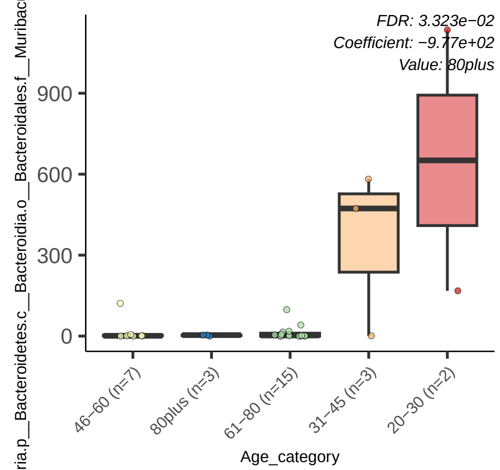

FDR: 3.323e−02
Coefficient: −9.77e+02
Value: 80plus
900
600
300
0
46−60 (n=7)
80plus (n=3)
61−80 (n=15)
31−45 (n=3)
20−30 (n=2)
Age_category
teria.p__Bacteroidetes.c__Bacteroidia.o__Bacteroidales.f__Muribac

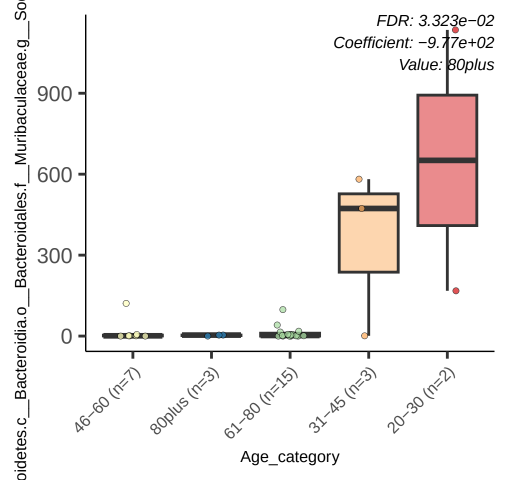

FDR: 3.323e−02
Coefficient: −9.77e+02
Value: 80plus
900
600
300
0
oidetes.c__Bacteroidia.o__Bacteroidales.f__Muribaculaceae.g__So
46−60 (n=7)
80plus (n=3)
61−80 (n=15)
31−45 (n=3)
20−30 (n=2)
Age_category

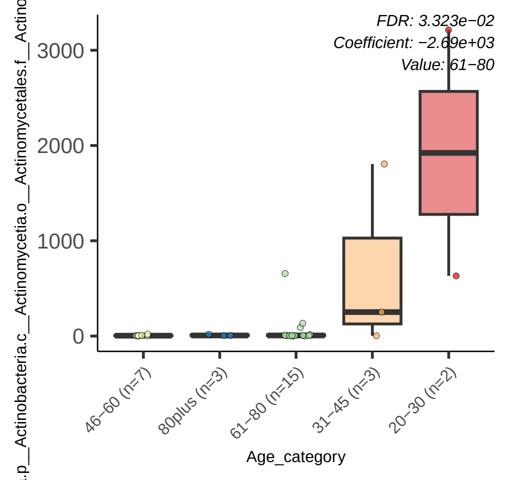

FDR: 3.323e−02
Coefficient: −2.69e+03
Value: 61−80
3000
2000
1000
0
46−60 (n=7)
80plus (n=3)
61−80 (n=15)
31−45 (n=3)
20−30 (n=2)
Age_category
p__Actinobacteria.c__Actinomycetia.o__Actinomycetales.f__Actino

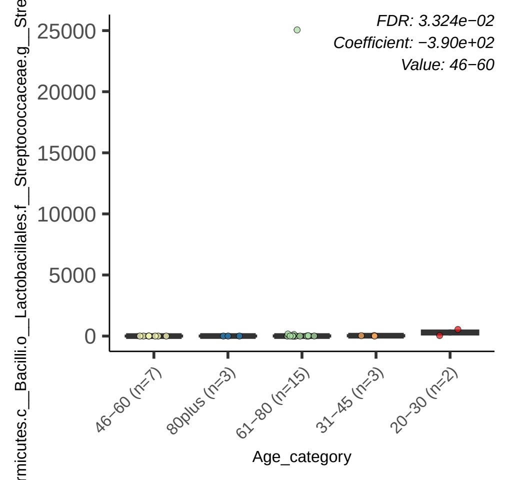

FDR: 3.324e−02
Coefficient: −3.90e+02
Value: 46−60
25000
20000
15000
10000
5000
0
46−60 (n=7)
80plus (n=3)
61−80 (n=15)
31−45 (n=3)
20−30 (n=2)
Age_category
rmicutes.c__Bacilli.o__Lactobacillales.f__Streptococcaceae.g__Stre

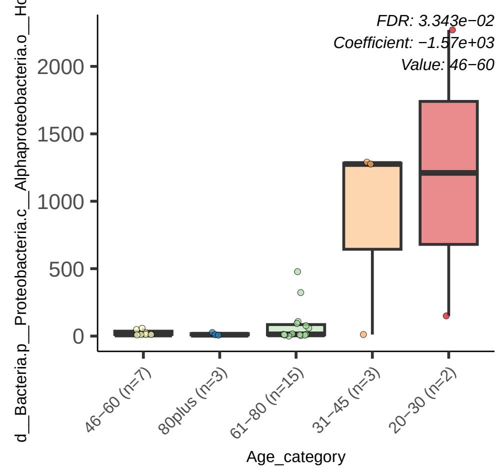

FDR: 3.343e−02
Coefficient: −1.57e+03
Value: 46−60
d__Bacteria.p__Proteobacteria.c__Alphaproteobacteria.o__Ho
0
500
1000
1500
2000
46−60 (n=7)
80plus (n=3)
61−80 (n=15)
31−45 (n=3)
20−30 (n=2)
Age_category

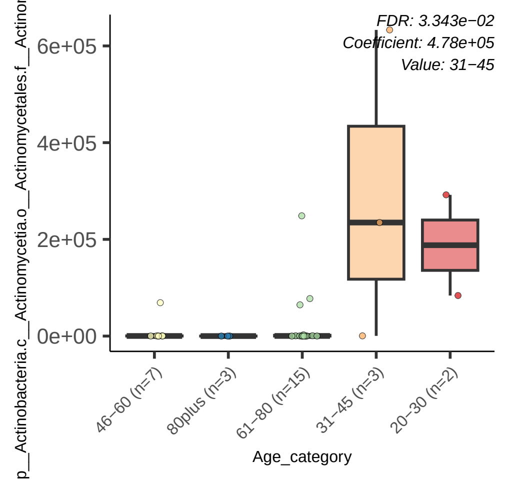

FDR: 3.343e−02
Coefficient: 4.78e+05
Value: 31−45
p__Actinobacteria.c__Actinomycetia.o__Actinomycetales.f__Actinor
6e+05
4e+05
2e+05
0e+00
46−60 (n=7)
80plus (n=3)
61−80 (n=15)
31−45 (n=3)
20−30 (n=2)
Age_category

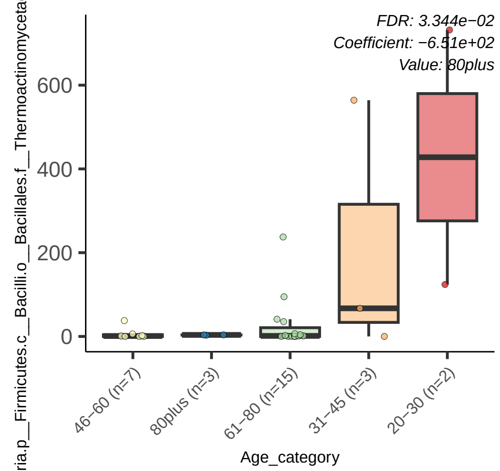

FDR: 3.344e−02
Coefficient: −6.51e+02
Value: 80plus
600
400
200
0
46−60 (n=7)
80plus (n=3)
61−80 (n=15)
31−45 (n=3)
20−30 (n=2)
Age_category

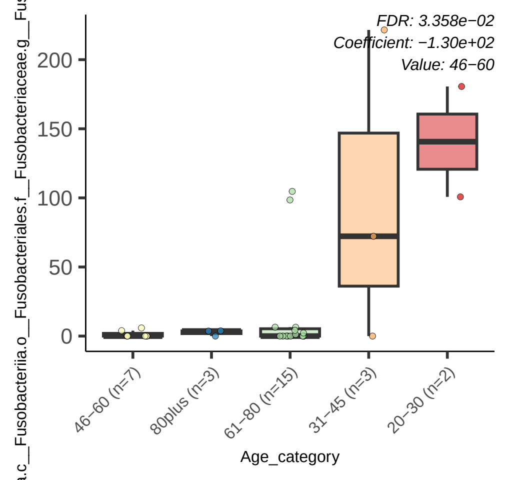

FDR: 3.358e−02
Coefficient: −1.30e+02
Value: 46−60
200
150
100
50
0
Fusobacteria.c__Fusobacteriia.o__Fusobacteriales.f__Fusobacteriaceae.g__Fus
46−60 (n=7)
80plus (n=3)
61−80 (n=15)
31−45 (n=3)
20−30 (n=2)
Age_category

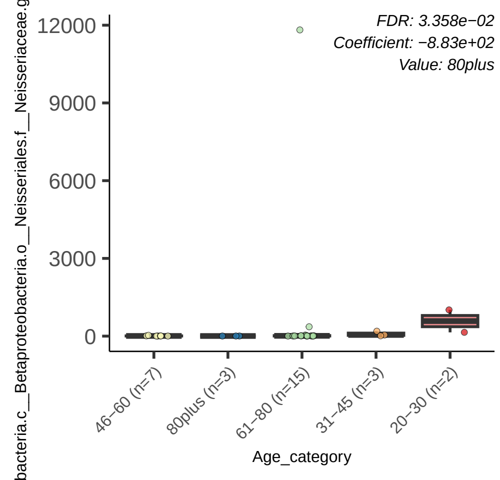

FDR: 3.358e−02
Coefficient: −8.83e+02
Value: 80plus
12000
9000
6000
3000
0
46−60 (n=7)
80plus (n=3)
61−80 (n=15)
31−45 (n=3)
20−30 (n=2)
Age_category
bacteria.c__Betaproteobacteria.o__Neisseriales.f__Neisseriaceae.g

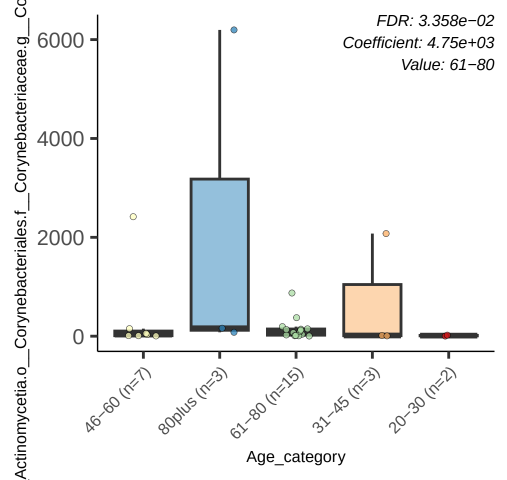

FDR: 3.358e−02
Coefficient: 4.75e+03
Value: 61−80
6000
4000
2000
0
Actinomycetia.o__Corynebacteriales.f__Corynebacteriaceae.g__Co
46−60 (n=7)
80plus (n=3)
61−80 (n=15)
31−45 (n=3)
20−30 (n=2)
Age_category

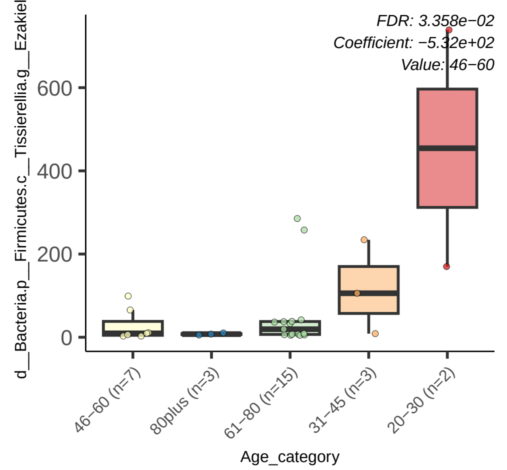

FDR: 3.358e−02
Coefficient: −5.32e+02
Value: 46−60
d__Bacteria.p__Firmicutes.c__Tissierellia.g__Ezakiel
0
200
400
600
46−60 (n=7)
80plus (n=3)
61−80 (n=15)
31−45 (n=3)
20−30 (n=2)
Age_category

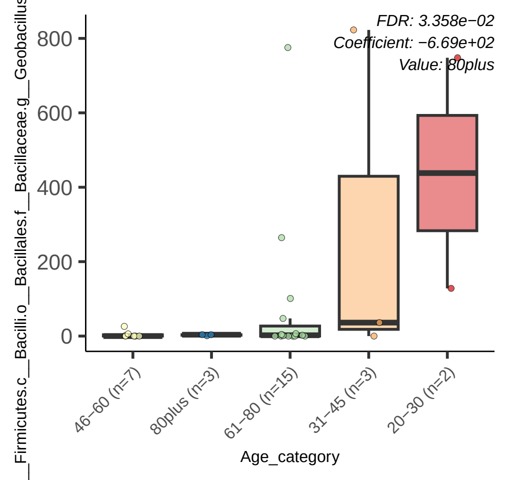

FDR: 3.358e−02
Coefficient: −6.69e+02
Value: 80plus
Firmicutes.c__Bacilli.o__Bacillales.f__Bacillaceae.g__Geobacillus
800
600
400
200
0
46−60 (n=7)
80plus (n=3)
61−80 (n=15)
31−45 (n=3)
20−30 (n=2)
Age_category

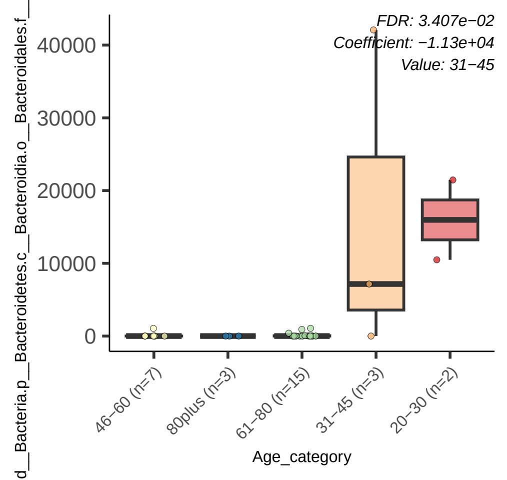
FDR: 3.407e−02
Coefficient: −1.13e+04
Value: 31−45
d__Bacteria.p__Bacteroidetes.c__Bacteroidia.o__Bacteroidales.f__
40000
30000
20000
10000
0
46−60 (n=7)
80plus (n=3)
61−80 (n=15)
31−45 (n=3)
20−30 (n=2)
Age_category

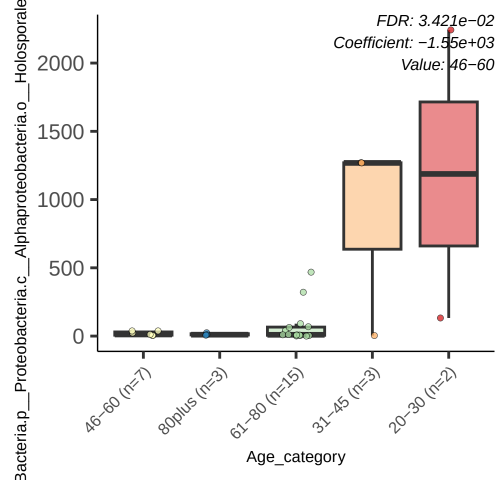

FDR: 3.421e−02
Coefficient: −1.55e+03
Value: 46−60
2000
1500
1000
500
0
Bacteria.p__Proteobacteria.c__Alphaproteobacteria.o__Holosporale
46−60 (n=7)
80plus (n=3)
61−80 (n=15)
31−45 (n=3)
20−30 (n=2)
Age_category

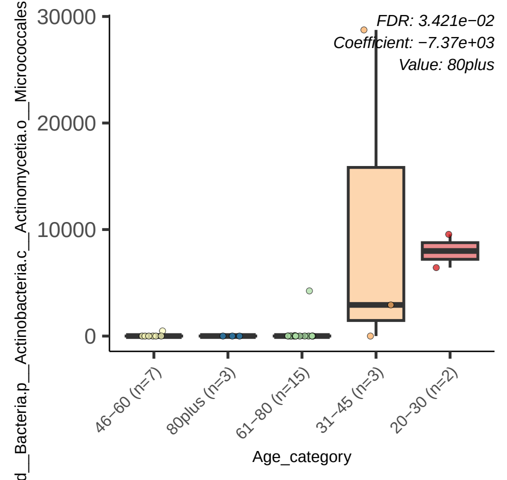
FDR: 3.421e−02
Coefficient: −7.37e+03
Value: 80plus
30000
20000
10000
0
d__Bacteria.p__Actinobacteria.c__Actinomycetia.o__Micrococcales
46−60 (n=7)
80plus (n=3)
61−80 (n=15)
31−45 (n=3)
20−30 (n=2)
Age_category

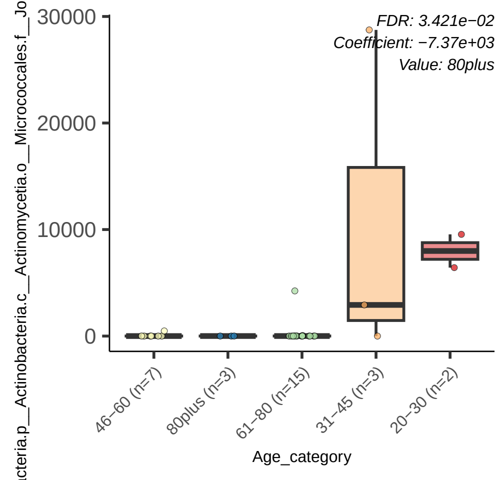

FDR: 3.421e−02
Coefficient: −7.37e+03
Value: 80plus
30000
20000
10000
0
46−60 (n=7)
80plus (n=3)
61−80 (n=15)
31−45 (n=3)
20−30 (n=2)
Age_category
acteria.p__Actinobacteria.c__Actinomycetia.o__Micrococcales.f__Jo

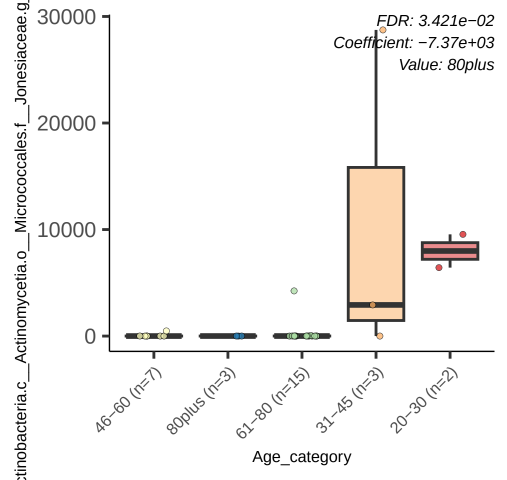
FDR: 3.421e−02
Coefficient: −7.37e+03
Value: 80plus
30000
20000
10000
0
46−60 (n=7)
80plus (n=3)
61−80 (n=15)
31−45 (n=3)
20−30 (n=2)
Age_category
ctinobacteria.c__Actinomycetia.o__Micrococcales.f__Jonesiaceae.g_

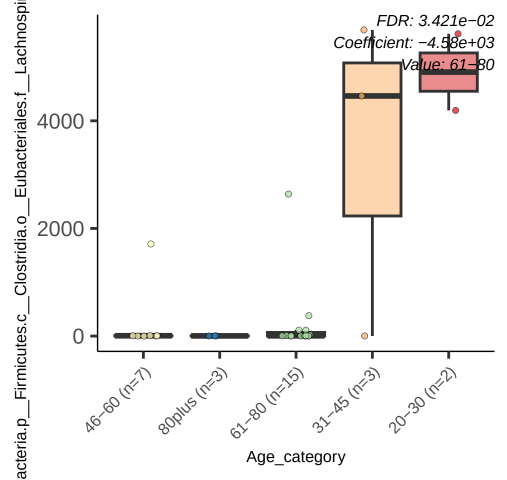

FDR: 3.421e−02
Coefficient: −4.58e+03
Value: 61−80
4000
2000
0
46−60 (n=7)
80plus (n=3)
61−80 (n=15)
31−45 (n=3)
20−30 (n=2)
Age_category
acteria.p__Firmicutes.c__Clostridia.o__Eubacteriales.f__Lachnospi

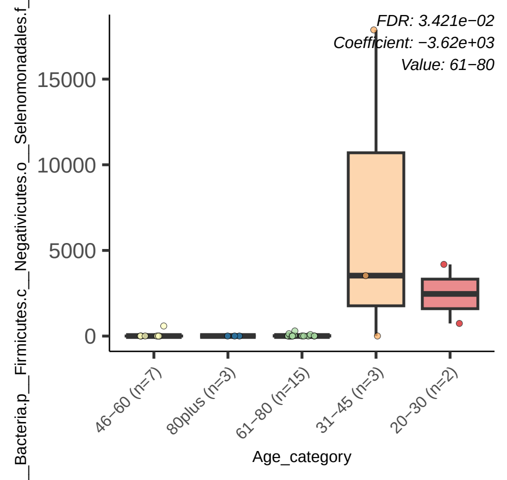

FDR: 3.421e−02
Coefficient: −3.62e+03
Value: 61−80
Bacteria.p__Firmicutes.c__Negativicutes.o__Selenomonadales.f
15000
10000
5000
0
46−60 (n=7)
80plus (n=3)
61−80 (n=15)
31−45 (n=3)
20−30 (n=2)
Age_category

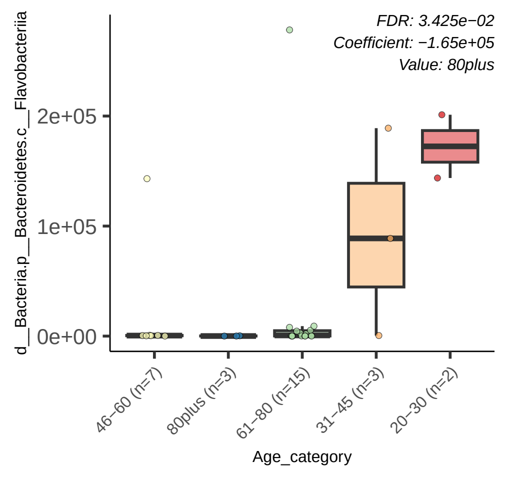
FDR: 3.425e−02
Coefficient: −1.65e+05
Value: 80plus
d__Bacteria.p__Bacteroidetes.c__Flavobacteriia
2e+05
1e+05
0e+00
46−60 (n=7)
80plus (n=3)
61−80 (n=15)
31−45 (n=3)
20−30 (n=2)
Age_category

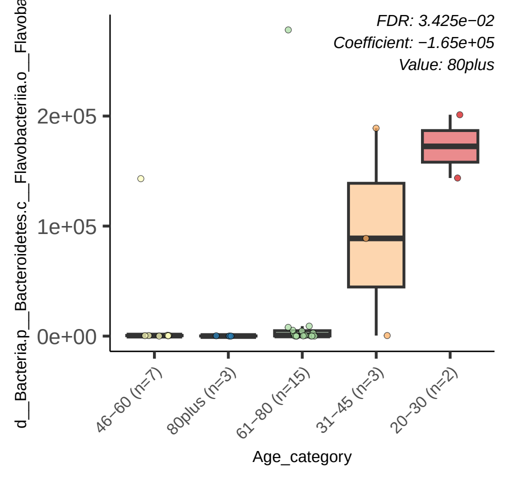

FDR: 3.425e−02
Coefficient: −1.65e+05
Value: 80plus
d__Bacteria.p__Bacteroidetes.c__Flavobacteriia.o__Flavoba
2e+05
1e+05
0e+00
46−60 (n=7)
80plus (n=3)
61−80 (n=15)
31−45 (n=3)
20−30 (n=2)
Age_category

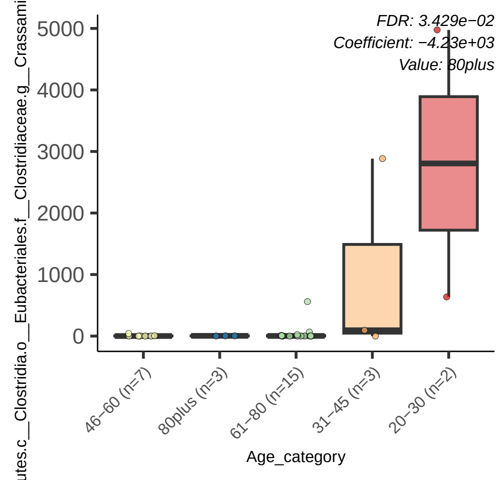

FDR: 3.429e−02
Coefficient: −4.23e+03
Value: 80plus
5000
4000
3000
2000
1000
0
utes.c__Clostridia.o__Eubacteriales.f__Clostridiaceae.g__Crassami
46−60 (n=7)
80plus (n=3)
61−80 (n=15)
31−45 (n=3)
20−30 (n=2)
Age_category

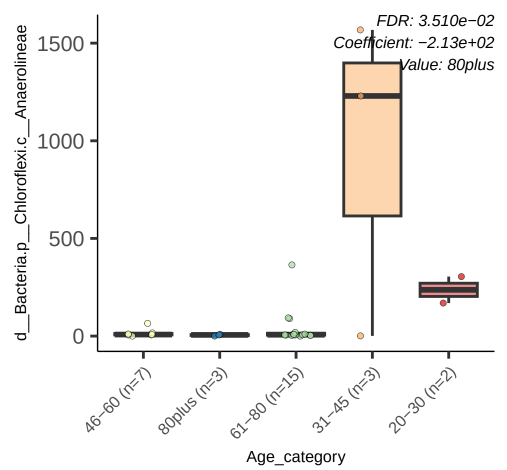

FDR: 3.510e−02
Coefficient: −2.13e+02
Value: 80plus
d__Bacteria.p__Chloroflexi.c__Anaerolineae
1500
1000
500
0
46−60 (n=7)
80plus (n=3)
61−80 (n=15)
31−45 (n=3)
20−30 (n=2)
Age_category

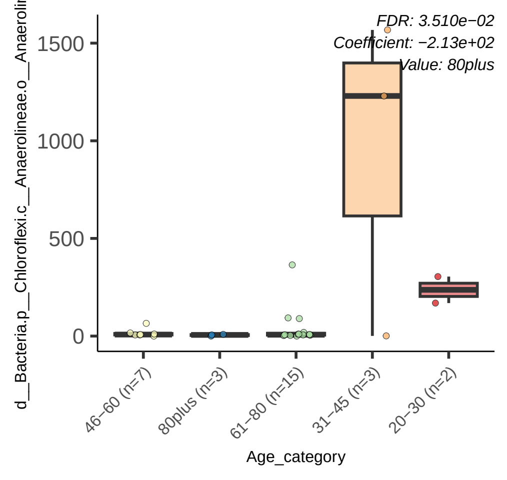

FDR: 3.510e−02
Coefficient: −2.13e+02
Value: 80plus
d__Bacteria.p__Chloroflexi.c__Anaerolineae.o__Anaeroli
1500
1000
500
0
46−60 (n=7)
80plus (n=3)
61−80 (n=15)
31−45 (n=3)
20−30 (n=2)
Age_category

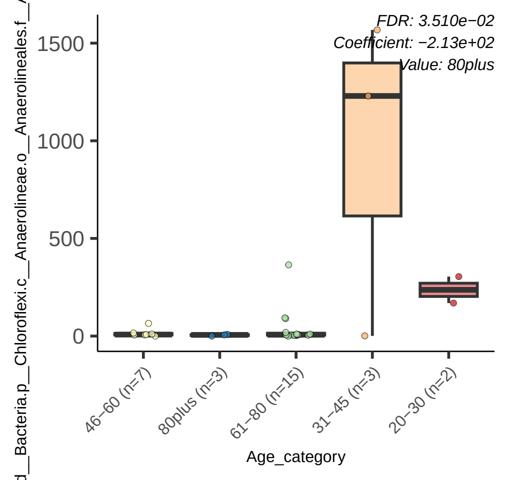

FDR: 3.510e−02
Coefficient: −2.13e+02
Value: 80plus
1500
1000
500
0
46−60 (n=7)
80plus (n=3)
61−80 (n=15)
31−45 (n=3)
20−30 (n=2)
Age_category
d__Bacteria.p__Chloroflexi.c__Anaerolineae.o__Anaerolineales.f__

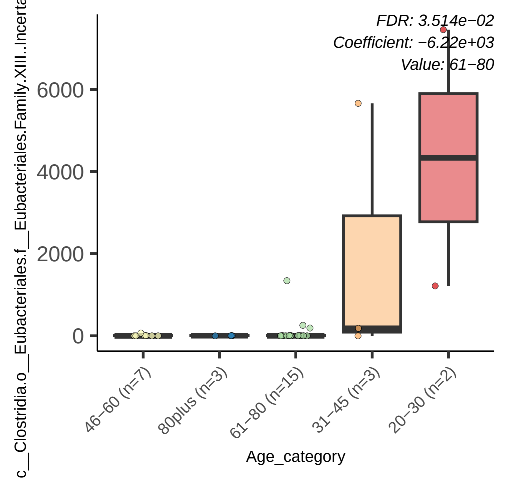

FDR: 3.514e−02
Coefficient: −6.22e+03
Value: 61−80
c__Clostridia.o__Eubacteriales.f__Eubacteriales.Family.XIII..Incerta
6000
4000
2000
0
46−60 (n=7)
80plus (n=3)
61−80 (n=15)
31−45 (n=3)
20−30 (n=2)
Age_category

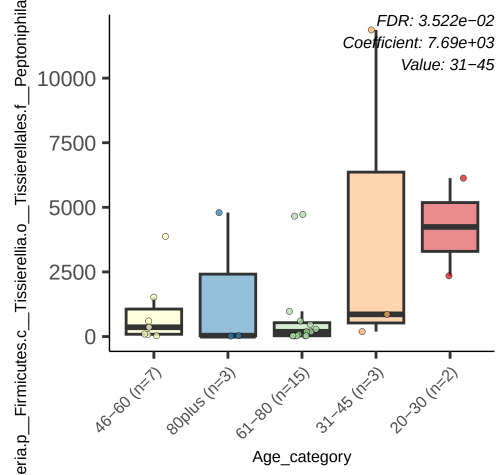

FDR: 3.522e−02
Coefficient: 7.69e+03
Value: 31−45
10000
7500
5000
2500
0
46−60 (n=7)
80plus (n=3)
61−80 (n=15)
31−45 (n=3)
20−30 (n=2)
Age_category

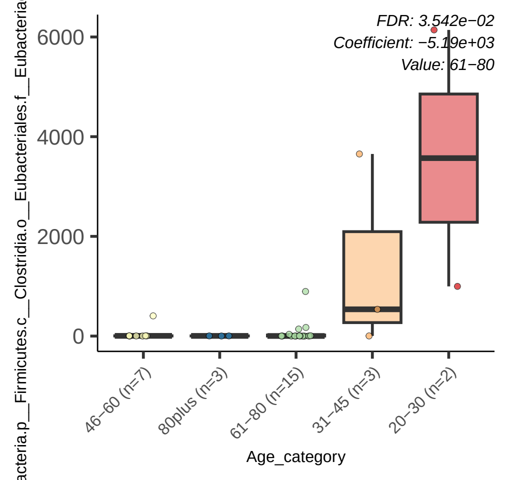

FDR: 3.542e−02
Coefficient: −5.19e+03
Value: 61−80
6000
4000
2000
0
46−60 (n=7)
80plus (n=3)
61−80 (n=15)
31−45 (n=3)
20−30 (n=2)
Age_category
acteria.p__Firmicutes.c__Clostridia.o__Eubacteriales.f__Eubacteria

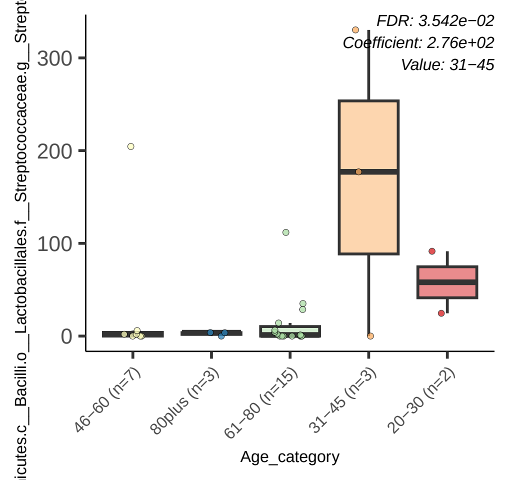

FDR: 3.542e−02
Coefficient: 2.76e+02
Value: 31−45
300
200
100
0
46−60 (n=7)
80plus (n=3)
61−80 (n=15)
31−45 (n=3)
20−30 (n=2)
Age_category

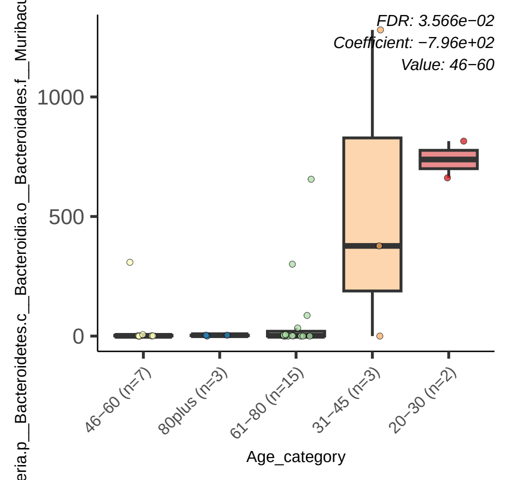

FDR: 3.566e−02
Coefficient: −7.96e+02
Value: 46−60
1000
500
0
eria.p__Bacteroidetes.c__Bacteroidia.o__Bacteroidales.f__Muribacu
46−60 (n=7)
80plus (n=3)
61−80 (n=15)
31−45 (n=3)
20−30 (n=2)
Age_category

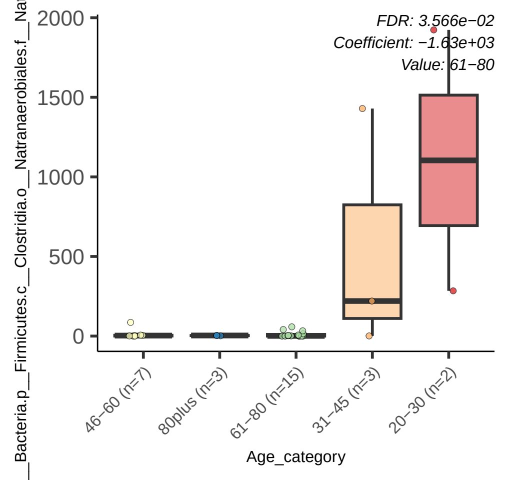

FDR: 3.566e−02
Coefficient: −1.63e+03
Value: 61−80
2000
1500
1000
500
0
Bacteria.p__Firmicutes.c__Clostridia.o__Natranaerobiales.f__Na
46−60 (n=7)
80plus (n=3)
61−80 (n=15)
31−45 (n=3)
20−30 (n=2)
Age_category

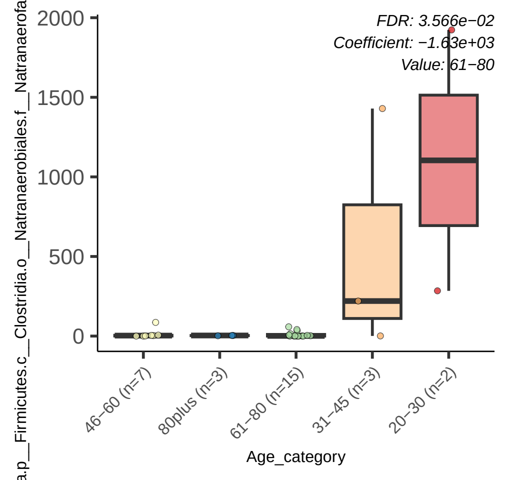
FDR: 3.566e−02
Coefficient: −1.63e+03
Value: 61−80
2000
1500
1000
500
0
a.p__Firmicutes.c__Clostridia.o__Natranaerobiales.f__Natranaerofa
46−60 (n=7)
80plus (n=3)
61−80 (n=15)
31−45 (n=3)
20−30 (n=2)
Age_category

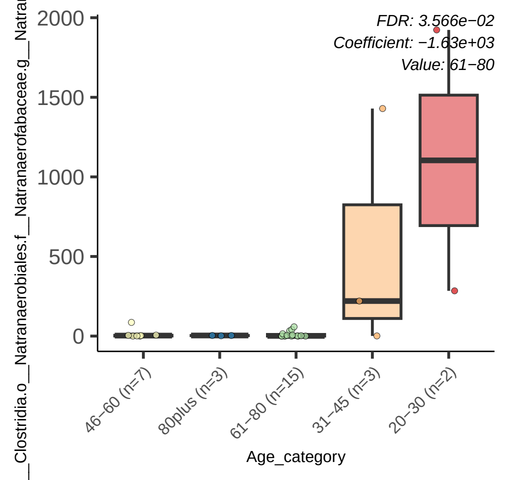

FDR: 3.566e−02
Coefficient: −1.63e+03
Value: 61−80
2000
1500
1000
500
0
__Clostridia.o__Natranaerobiales.f__Natranaerofabaceae.g__Natran
46−60 (n=7)
80plus (n=3)
61−80 (n=15)
31−45 (n=3)
20−30 (n=2)
Age_category

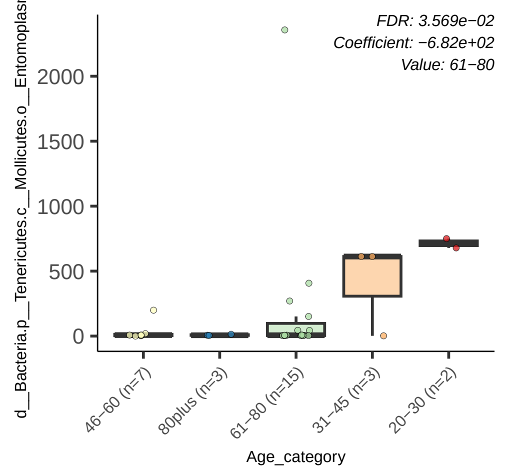

FDR: 3.569e−02
Coefficient: −6.82e+02
Value: 61−80
d__Bacteria.p__Tenericutes.c__Mollicutes.o__Entomoplasm
2000
1500
1000
500
0
46−60 (n=7)
80plus (n=3)
61−80 (n=15)
31−45 (n=3)
20−30 (n=2)
Age_category

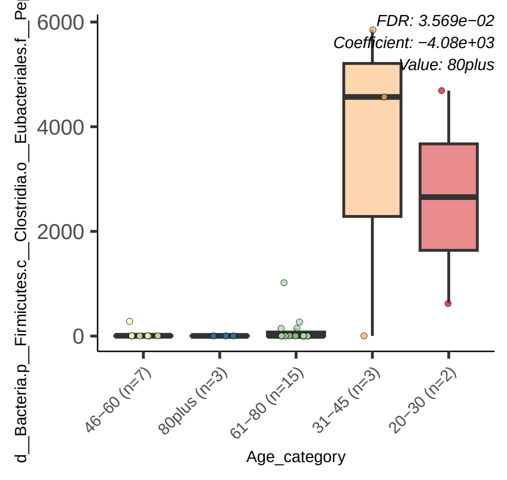

FDR: 3.569e−02
Coefficient: −4.08e+03
Value: 80plus
6000
4000
2000
0
d__Bacteria.p__Firmicutes.c__Clostridia.o__Eubacteriales.f__Pe
46−60 (n=7)
80plus (n=3)
61−80 (n=15)
31−45 (n=3)
20−30 (n=2)
Age_category

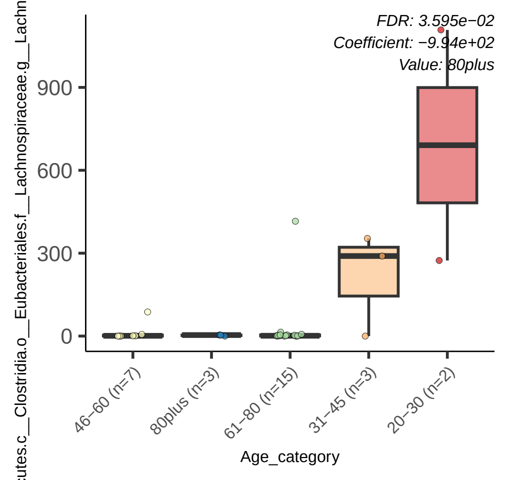
FDR: 3.595e−02
Coefficient: −9.94e+02
Value: 80plus
900
600
300
0
46−60 (n=7)
80plus (n=3)
61−80 (n=15)
31−45 (n=3)
20−30 (n=2)
Age_category
cutes.c__Clostridia.o__Eubacteriales.f__Lachnospiraceae.g__Lachn

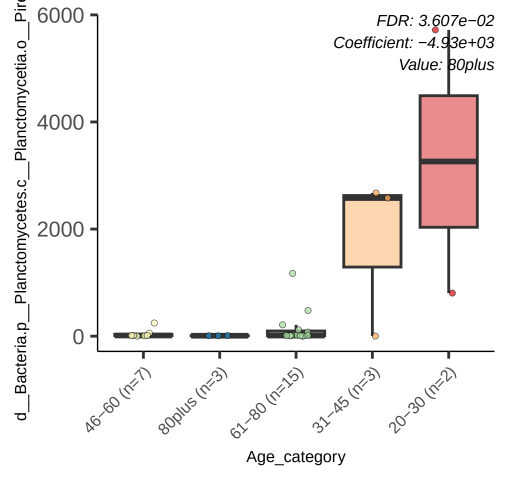

d__Bacteria.p__Planctomycetes.c__Planctomycetia.o__Pir
6000
4000
2000
0
FDR: 3.607e−02
Coefficient: −4.93e+03
Value: 80plus
46−60 (n=7)
80plus (n=3)
61−80 (n=15)
31−45 (n=3)
20−30 (n=2)
Age_category

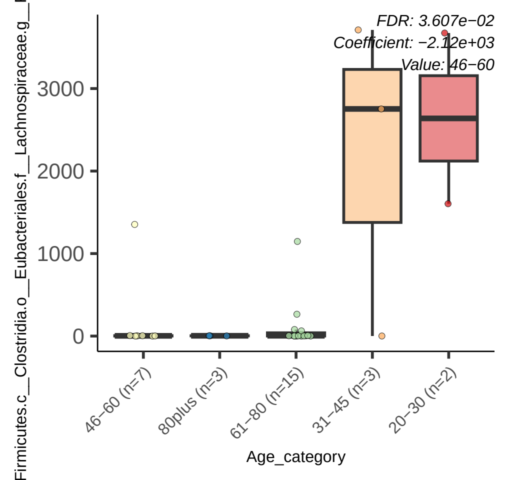

FDR: 3.607e−02
Coefficient: −2.12e+03
Value: 46−60
Firmicutes.c__Clostridia.o__Eubacteriales.f__Lachnospiraceae.g__
3000
2000
1000
0
46−60 (n=7)
80plus (n=3)
61−80 (n=15)
31−45 (n=3)
20−30 (n=2)
Age_category

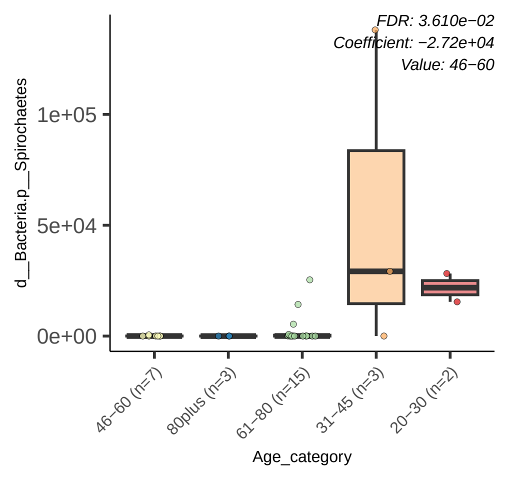
FDR: 3.610e−02
Coefficient: −2.72e+04
Value: 46−60
d__Bacteria.p__Spirochaetes
1e+05
5e+04
0e+00
46−60 (n=7)
80plus (n=3)
61−80 (n=15)
31−45 (n=3)
20−30 (n=2)
Age_category

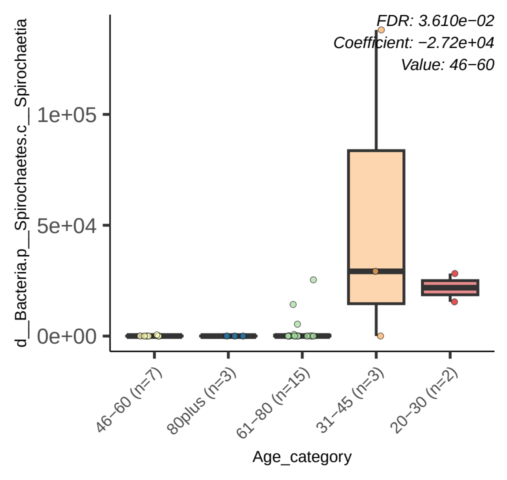

FDR: 3.610e−02
Coefficient: −2.72e+04
Value: 46−60
d__Bacteria.p__Spirochaetes.c__Spirochaetia
1e+05
5e+04
0e+00
46−60 (n=7)
80plus (n=3)
61−80 (n=15)
31−45 (n=3)
20−30 (n=2)
Age_category

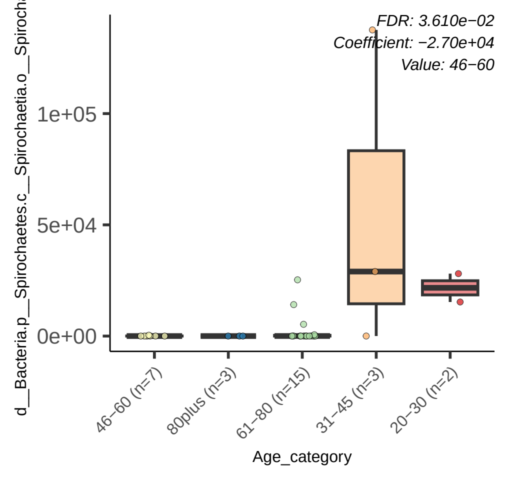

FDR: 3.610e−02
Coefficient: −2.70e+04
Value: 46−60
d__Bacteria.p__Spirochaetes.c__Spirochaetia.o__Spiroch
1e+05
5e+04
0e+00
46−60 (n=7)
80plus (n=3)
61−80 (n=15)
31−45 (n=3)
20−30 (n=2)
Age_category

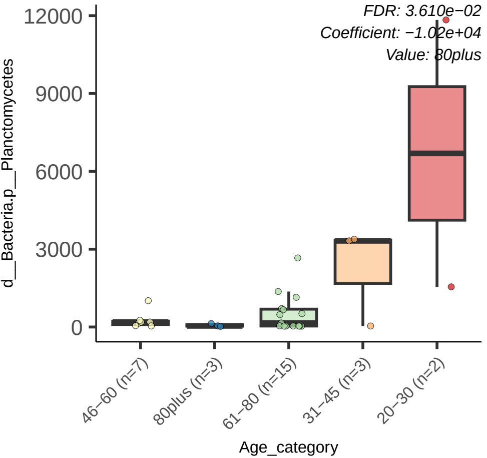

FDR: 3.610e−02
Coefficient: −1.02e+04
Value: 80plus
12000
9000
6000
3000
0
d__Bacteria.p__Planctomycetes
46−60 (n=7)
80plus (n=3)
61−80 (n=15)
31−45 (n=3)
20−30 (n=2)
Age_category

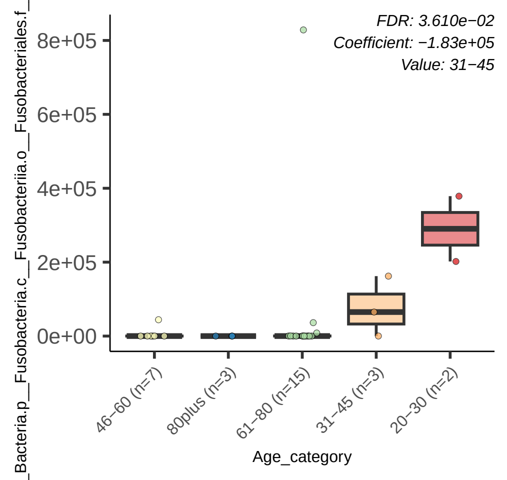
FDR: 3.610e−02
Coefficient: −1.83e+05
Value: 31−45
8e+05
6e+05
4e+05
2e+05
0e+00
Bacteria.p__Fusobacteria.c__Fusobacteriia.o__Fusobacteriales.f
46−60 (n=7)
80plus (n=3)
61−80 (n=15)
31−45 (n=3)
20−30 (n=2)
Age_category

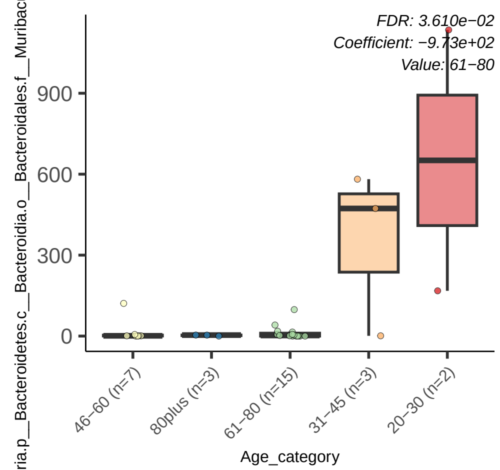

FDR: 3.610e−02
Coefficient: −9.73e+02
Value: 61−80
900
600
300
0
Bacteria.p__Bacteroidetes.c__Bacteroidia.o__Bacteroidales.f__Muribac
46−60 (n=7)
80plus (n=3)
61−80 (n=15)
31−45 (n=3)
20−30 (n=2)
Age_category

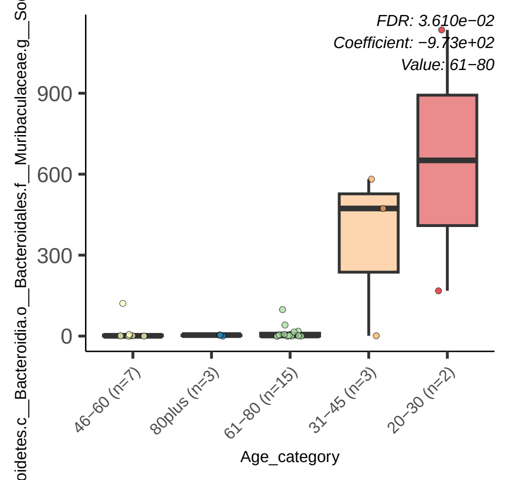

FDR: 3.610e−02
Coefficient: −9.73e+02
Value: 61−80
900
600
300
0
oidetes.c__Bacteroidia.o__Bacteroidales.f__Muribaculaceae.g__So
46−60 (n=7)
80plus (n=3)
61−80 (n=15)
31−45 (n=3)
20−30 (n=2)
Age_category

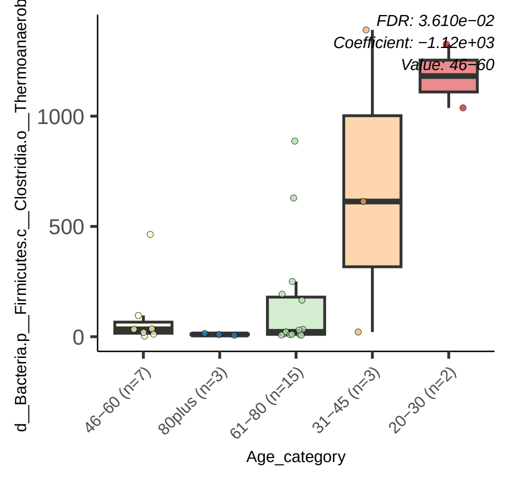

FDR: 3.610e−02
Coefficient: −1.12e+03
Value: 46−60
d__Bacteria.p__Firmicutes.c__Clostridia.o__Thermoanaerob
1000
500
0
46−60 (n=7)
80plus (n=3)
61−80 (n=15)
31−45 (n=3)
20−30 (n=2)
Age_category

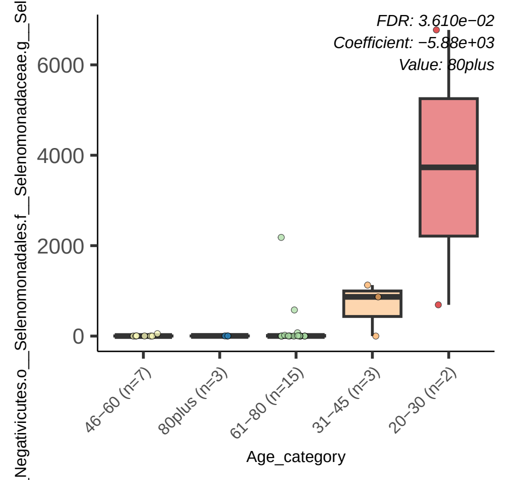

FDR: 3.610e−02
Coefficient: −5.88e+03
Value: 80plus
Negativicutes.o__Selenomonadales.f__Selenomonadaceae.g__Sel
6000
4000
2000
0
46−60 (n=7)
80plus (n=3)
61−80 (n=15)
31−45 (n=3)
20−30 (n=2)
Age_category

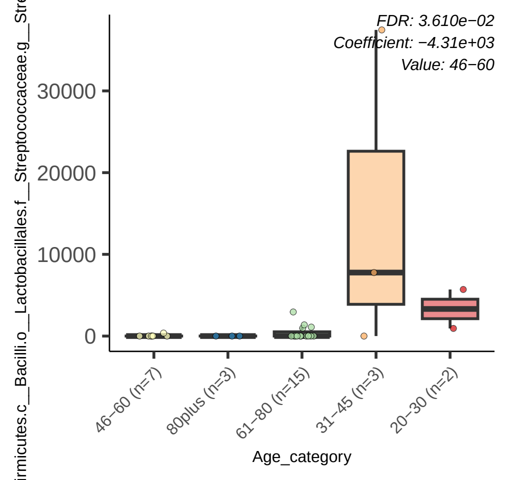

FDR: 3.610e−02
Coefficient: −4.31e+03
Value: 46−60
30000
20000
10000
0
46−60 (n=7)
80plus (n=3)
61−80 (n=15)
31−45 (n=3)
20−30 (n=2)
Age_category
irmicutes.c__Bacilli.o__Lactobacillales.f__Streptococcaceae.g__Stre

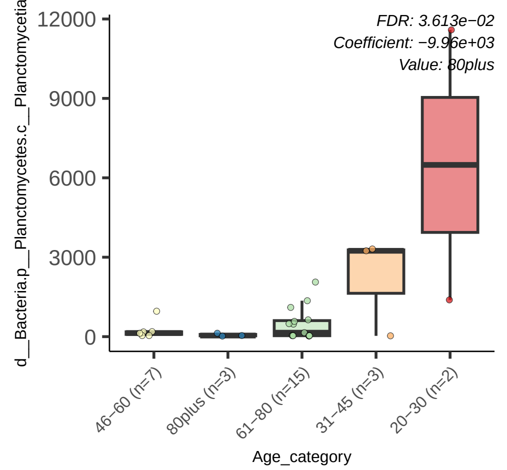

FDR: 3.613e−02
Coefficient: −9.96e+03
Value: 80plus
12000
9000
6000
3000
0
d__Bacteria.p__Planctomycetes.c__Planctomycetia
46−60 (n=7)
80plus (n=3)
61−80 (n=15)
31−45 (n=3)
20−30 (n=2)
Age_category

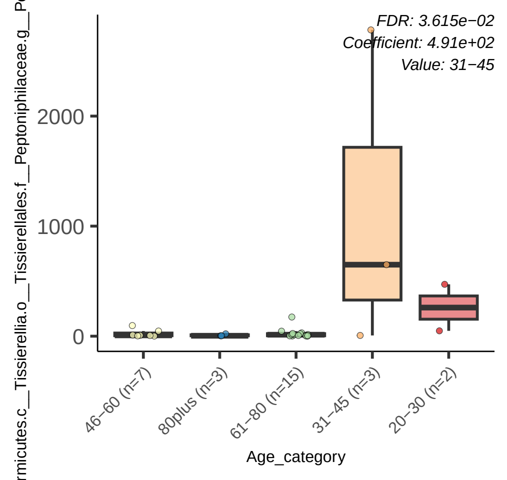
FDR: 3.615e−02
Coefficient: 4.91e+02
Value: 31−45
2000
1000
0
46−60 (n=7)
80plus (n=3)
61−80 (n=15)
31−45 (n=3)
20−30 (n=2)
Age_category
rmicutes.c__Tissierellia.o__Tissierellales.f__Peptoniphilaceae.g__Pe

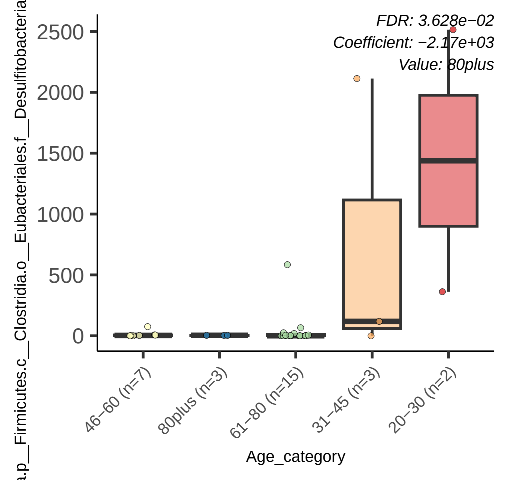

FDR: 3.628e−02
Coefficient: −2.17e+03
Value: 80plus
0
500
1000
1500
2000
2500
Firmicutes.c__Clostridia.o__Eubacteriales.f__Desulfitobacteria
46−60 (n=7)
80plus (n=3)
61−80 (n=15)
31−45 (n=3)
20−30 (n=2)
Age_category

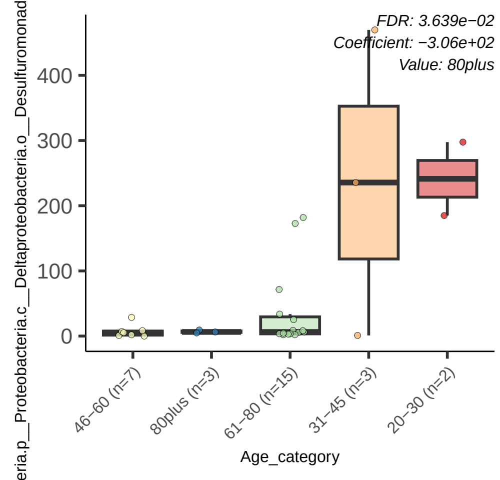

FDR: 3.639e−02
Coefficient: −3.06e+02
Value: 80plus
400
300
200
100
0
46−60 (n=7)
80plus (n=3)
61−80 (n=15)
31−45 (n=3)
20−30 (n=2)
Age_category
eria.p__Proteobacteria.c__Deltaproteobacteria.o__Desulfuromonad

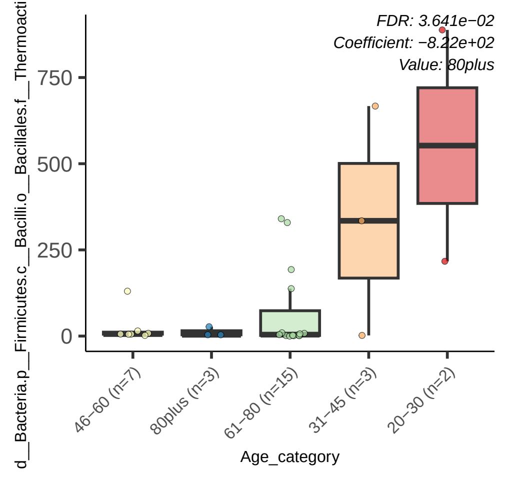
FDR: 3.641e−02
Coefficient: −8.22e+02
Value: 80plus
d__Bacteria.p__Firmicutes.c__Bacilli.o__Bacillales.f__Thermoacti
750
500
250
0
46−60 (n=7)
80plus (n=3)
61−80 (n=15)
31−45 (n=3)
20−30 (n=2)
Age_category

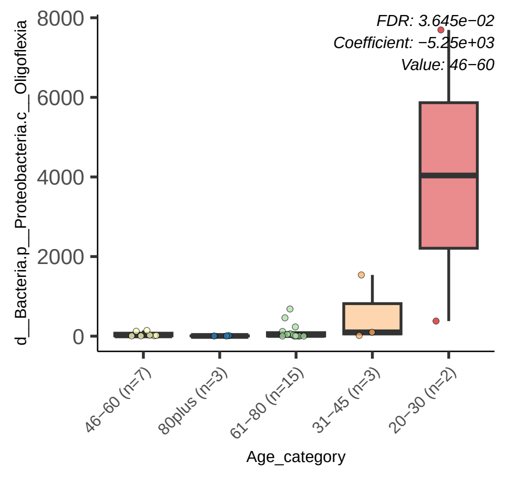

FDR: 3.645e−02
Coefficient: −5.25e+03
Value: 46−60
d__Bacteria.p__Proteobacteria.c__Oligoflexia
8000
6000
4000
2000
0
46−60 (n=7)
80plus (n=3)
61−80 (n=15)
31−45 (n=3)
20−30 (n=2)
Age_category

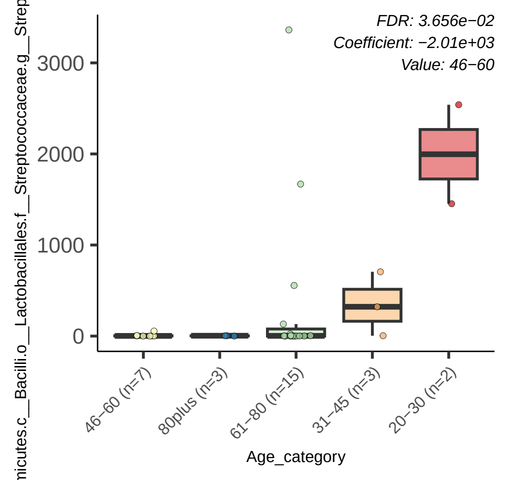

FDR: 3.656e−02
Coefficient: −2.01e+03
Value: 46−60
micutes.c__Bacilli.o__Lactobacillales.f__Streptococcaceae.g__Strep
3000
2000
1000
0
46−60 (n=7)
80plus (n=3)
61−80 (n=15)
31−45 (n=3)
20−30 (n=2)
Age_category

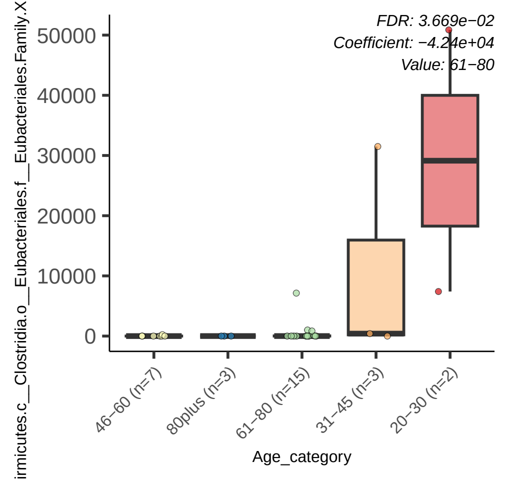

FDR: 3.669e−02
Coefficient: −4.24e+04
Value: 61−80
irmicutes.c__Clostridia.o__Eubacteriales.f__Eubacteriales.Family.X
50000
40000
30000
20000
10000
0
46−60 (n=7)
80plus (n=3)
61−80 (n=15)
31−45 (n=3)
20−30 (n=2)
Age_category

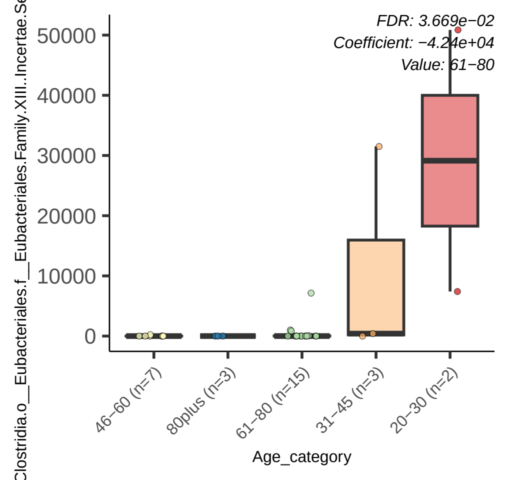

FDR: 3.669e−02
Coefficient: −4.24e+04
Value: 61−80
Clostridia.o__Eubacteriales.f__Eubacteriales.Family.XIII..Incertae.Se
50000
40000
30000
20000
10000
0
46−60 (n=7)
80plus (n=3)
61−80 (n=15)
31−45 (n=3)
20−30 (n=2)
Age_category

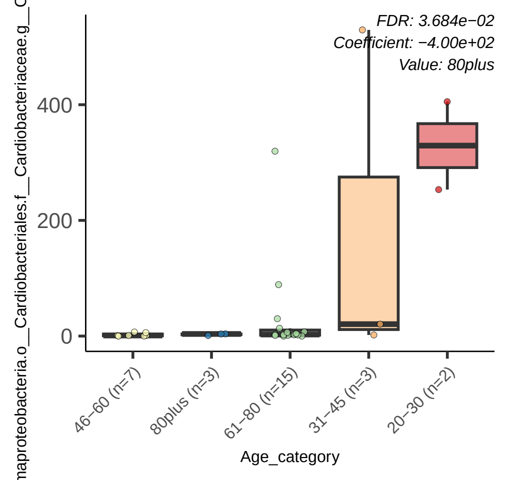

FDR: 3.684e−02
Coefficient: −4.00e+02
Value: 80plus
400
200
0
maproteobacteria.o__Cardiobacteriales.f__Cardiobacteriaceae.g__C
46−60 (n=7)
80plus (n=3)
61−80 (n=15)
31−45 (n=3)
20−30 (n=2)
Age_category

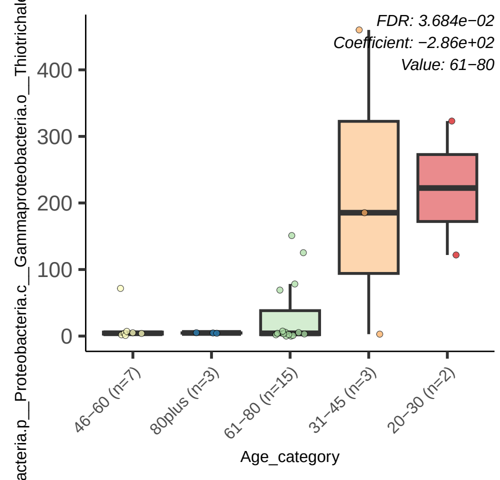

FDR: 3.684e−02
Coefficient: −2.86e+02
Value: 61−80
acteria.p__Proteobacteria.c__Gammaproteobacteria.o__Thiotrichale
0
100
200
300
400
46−60 (n=7)
80plus (n=3)
61−80 (n=15)
31−45 (n=3)
20−30 (n=2)
Age_category

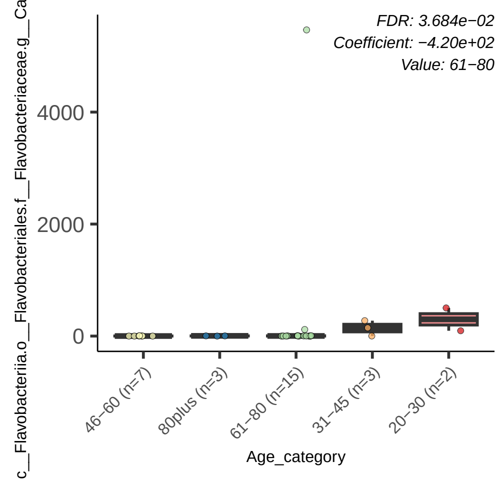

FDR: 3.684e−02
Coefficient: −4.20e+02
Value: 61−80
4000
2000
0
c__Flavobacteriia.o__Flavobacteriales.f__Flavobacteriaceae.g__Ca
46−60 (n=7)
80plus (n=3)
61−80 (n=15)
31−45 (n=3)
20−30 (n=2)
Age_category

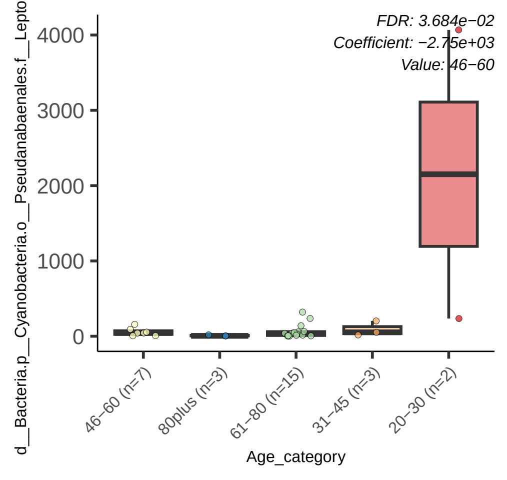

FDR: 3.684e−02
Coefficient: −2.75e+03
Value: 46−60
d__Bacteria.p__Cyanobacteria.o__Pseudanabaenales.f__Lepto
4000
3000
2000
1000
0
46−60 (n=7)
80plus (n=3)
61−80 (n=15)
31−45 (n=3)
20−30 (n=2)
Age_category

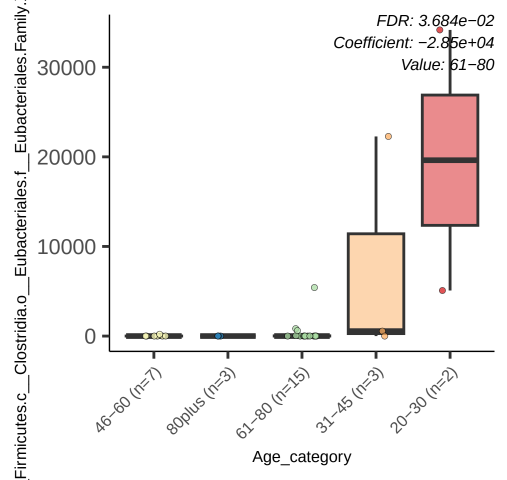

FDR: 3.684e−02
Coefficient: −2.85e+04
Value: 61−80
Firmicutes.c__Clostridia.o__Eubacteriales.f__Eubacteriales.Family.
30000
20000
10000
0
46−60 (n=7)
80plus (n=3)
61−80 (n=15)
31−45 (n=3)
20−30 (n=2)
Age_category

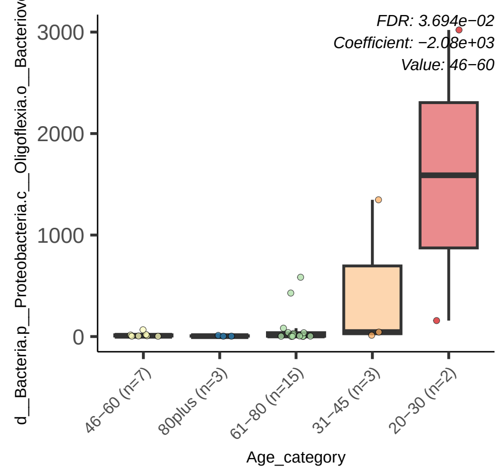

FDR: 3.694e−02
Coefficient: −2.08e+03
Value: 46−60
d__Bacteria.p__Proteobacteria.c__Oligoflexia.o__Bacteriov
3000
2000
1000
0
46−60 (n=7)
80plus (n=3)
61−80 (n=15)
31−45 (n=3)
20−30 (n=2)
Age_category

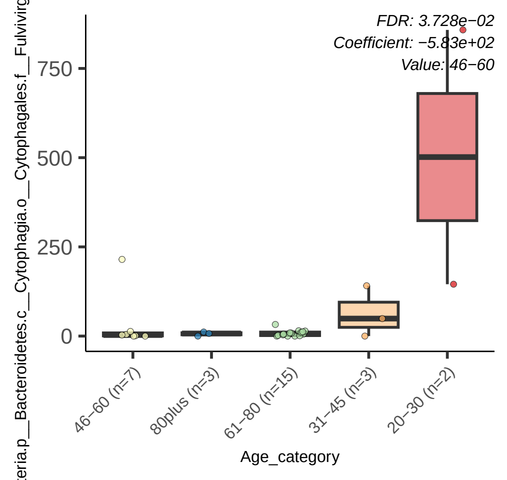

FDR: 3.728e−02
Coefficient: −5.83e+02
Value: 46−60
750
500
250
0
eria.p__Bacteroidetes.c__Cytophagia.o__Cytophagales.f__Fulvivirg
46−60 (n=7)
80plus (n=3)
61−80 (n=15)
31−45 (n=3)
20−30 (n=2)
Age_category

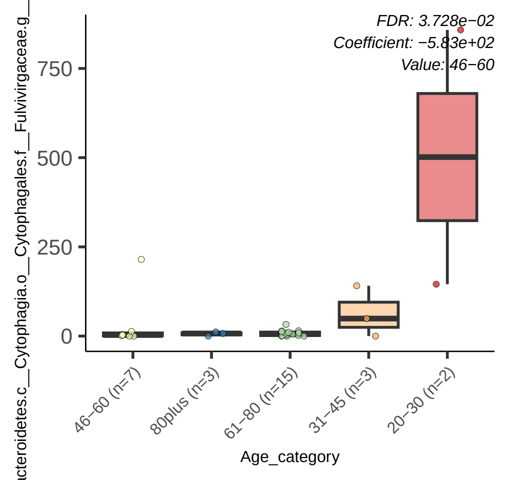

FDR: 3.728e−02
Coefficient: −5.83e+02
Value: 46−60
750
500
250
0
acteroidetes.c__Cytophagia.o__Cytophagales.f__Fulvivirgaceae.g__
46−60 (n=7)
80plus (n=3)
61−80 (n=15)
31−45 (n=3)
20−30 (n=2)
Age_category

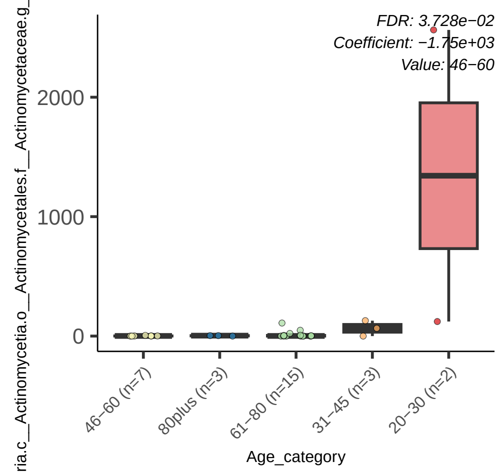
FDR: 3.728e−02
Coefficient: −1.75e+03
Value: 46−60
2000
1000
0
46−60 (n=7)
80plus (n=3)
61−80 (n=15)
31−45 (n=3)
20−30 (n=2)
Age_category
eria.c__Actinomycetia.o__Actinomycetales.f__Actinomycetaceae.g_

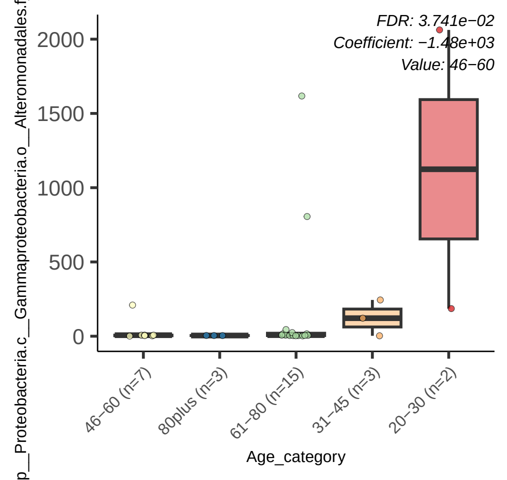

FDR: 3.741e−02
Coefficient: −1.48e+03
Value: 46−60
p__Proteobacteria.c__Gammaproteobacteria.o__Alteromonadales.f
2000
1500
1000
500
0
46−60 (n=7)
80plus (n=3)
61−80 (n=15)
31−45 (n=3)
20−30 (n=2)
Age_category

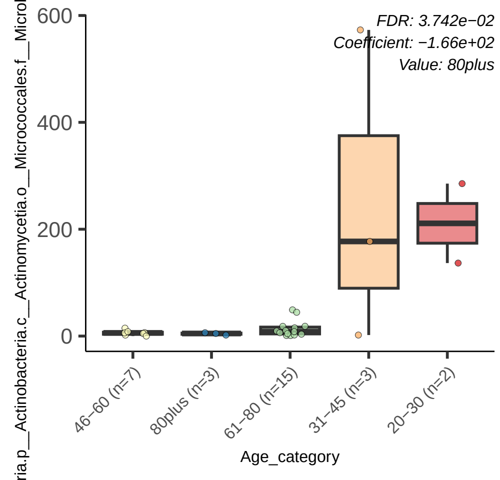

FDR: 3.742e−02
Coefficient: −1.66e+02
Value: 80plus
600
400
200
0
ria.p__Actinobacteria.c__Actinomycetia.o__Micrococcales.f__Microl
46−60 (n=7)
80plus (n=3)
61−80 (n=15)
31−45 (n=3)
20−30 (n=2)
Age_category

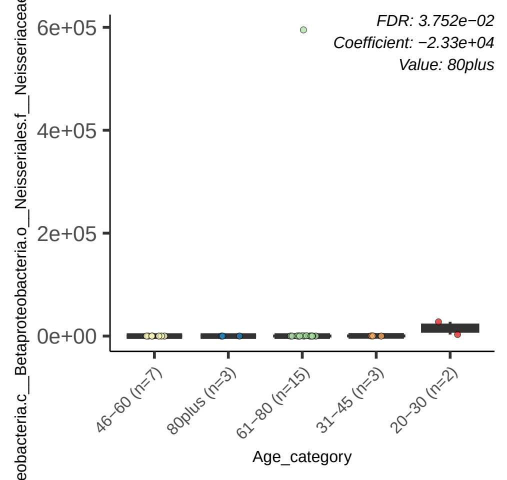

FDR: 3.752e−02
Coefficient: −2.33e+04
Value: 80plus
6e+05
4e+05
2e+05
0e+00
eobacteria.c__Betaproteobacteria.o__Neisseriales.f__Neisseriacea
46−60 (n=7)
80plus (n=3)
61−80 (n=15)
31−45 (n=3)
20−30 (n=2)
Age_category

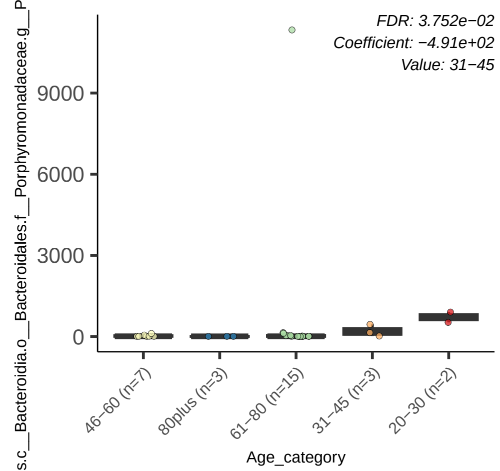
FDR: 3.752e−02
Coefficient: −4.91e+02
Value: 31−45
9000
6000
3000
0
46−60 (n=7)
80plus (n=3)
61−80 (n=15)
31−45 (n=3)
20−30 (n=2)
Age_category
es.c__Bacteroidia.o__Bacteroidales.f__Porphyromonadaceae.g__P

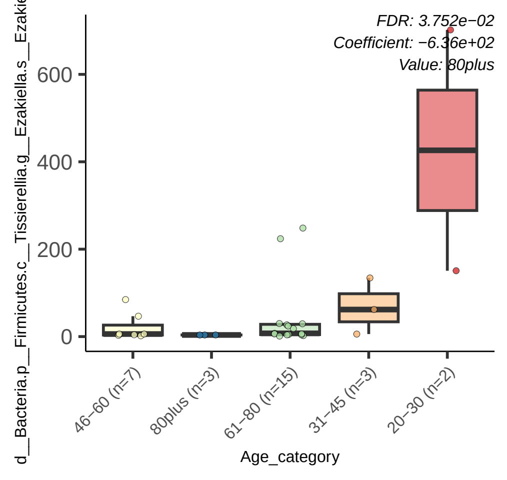
FDR: 3.752e−02
Coefficient: −6.36e+02
Value: 80plus
d__Bacteria.p__Firmicutes.c__Tissierellia.g__Ezakiella.s__Ezakie
600
400
200
0
46−60 (n=7)
80plus (n=3)
61−80 (n=15)
31−45 (n=3)
20−30 (n=2)
Age_category

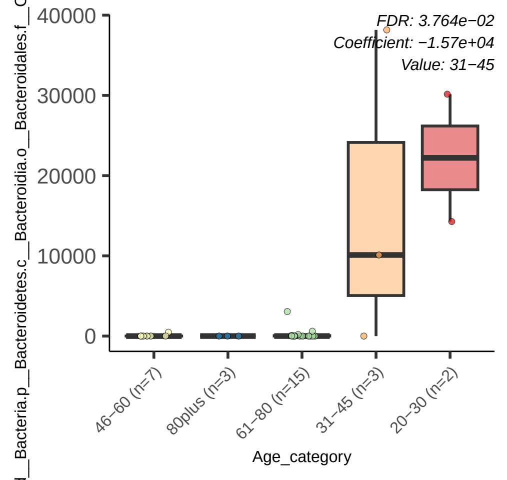

FDR: 3.764e−02
Coefficient: −1.57e+04
Value: 31−45
40000
30000
20000
10000
0
d__Bacteria.p__Bacteroidetes.c__Bacteroidia.o__Bacteroidales.f__
46−60 (n=7)
80plus (n=3)
61−80 (n=15)
31−45 (n=3)
20−30 (n=2)
Age_category

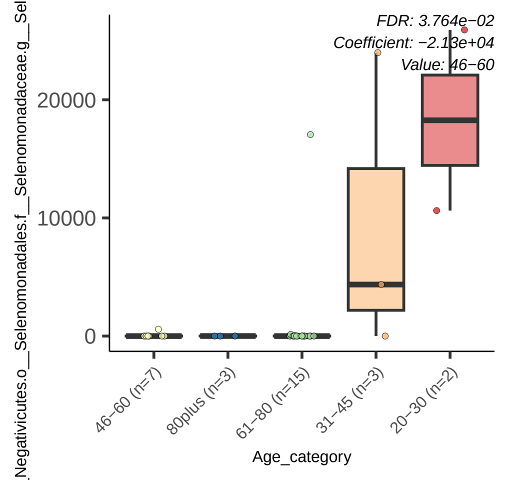

FDR: 3.764e−02
Coefficient: −2.13e+04
Value: 46−60
20000
10000
0
Negativicutes.o__Selenomonadales.f__Selenomonadaceae.g__Sel
46−60 (n=7)
80plus (n=3)
61−80 (n=15)
31−45 (n=3)
20−30 (n=2)
Age_category

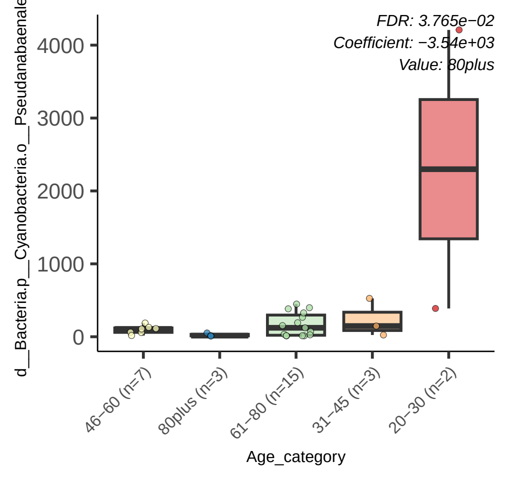
FDR: 3.765e−02
Coefficient: −3.54e+03
Value: 80plus
d__Bacteria.p__Cyanobacteria.o__Pseudanabaenale
4000
3000
2000
1000
0
46−60 (n=7)
80plus (n=3)
61−80 (n=15)
31−45 (n=3)
20−30 (n=2)
Age_category

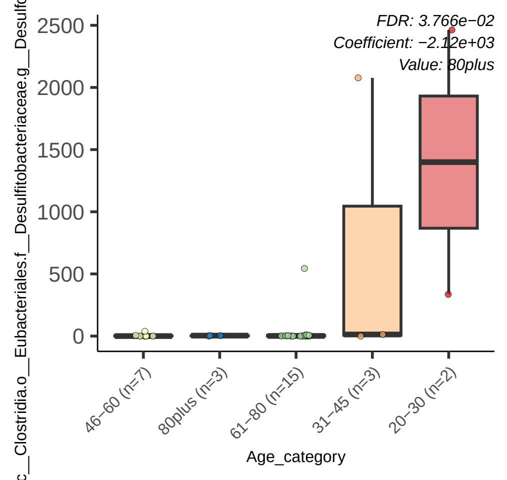
FDR: 3.766e−02
Coefficient: −2.12e+03
Value: 80plus
c__Clostridia.o__Eubacteriales.f__Desulfitobacteriaceae.g__Desulfo
2500
2000
1500
1000
500
0
46−60 (n=7)
80plus (n=3)
61−80 (n=15)
31−45 (n=3)
20−30 (n=2)
Age_category

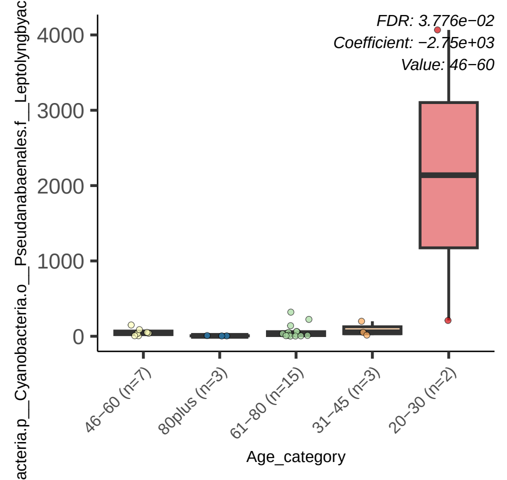

FDR: 3.776e−02
Coefficient: −2.75e+03
Value: 46−60
acteria.p__Cyanobacteria.o__Pseudanabaenales.f__Leptolyngbyac
4000
3000
2000
1000
0
46−60 (n=7)
80plus (n=3)
61−80 (n=15)
31−45 (n=3)
20−30 (n=2)
Age_category

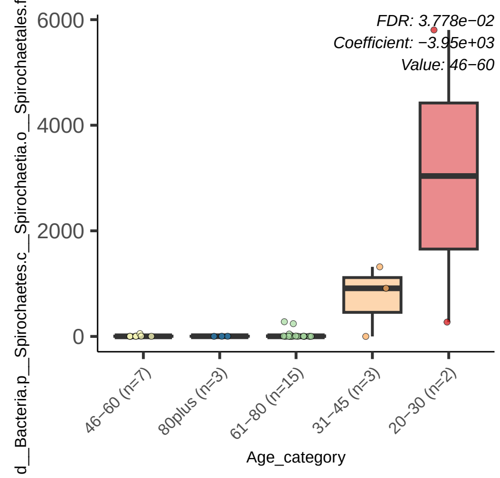
FDR: 3.778e−02
Coefficient: −3.95e+03
Value: 46−60
d__Bacteria.p__Spirochaetes.c__Spirochaetia.o__Spirochaetales.f
6000
4000
2000
0
46−60 (n=7)
80plus (n=3)
61−80 (n=15)
31−45 (n=3)
20−30 (n=2)
Age_category

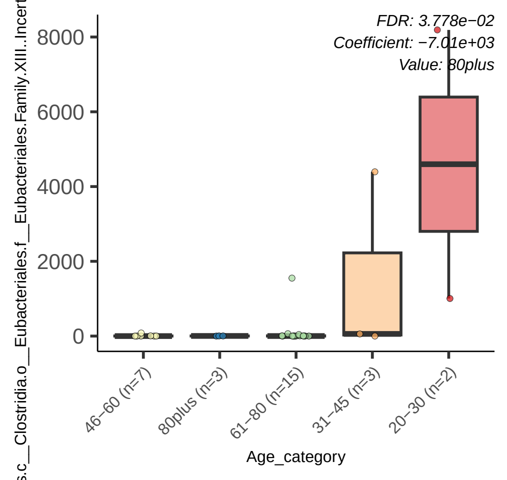
FDR: 3.778e−02
Coefficient: −7.01e+03
Value: 80plus
8000
6000
4000
2000
0
s.c__Clostridia.o__Eubacteriales.f__Eubacteriales.Family.XIII..Incert
46−60 (n=7)
80plus (n=3)
61−80 (n=15)
31−45 (n=3)
20−30 (n=2)
Age_category

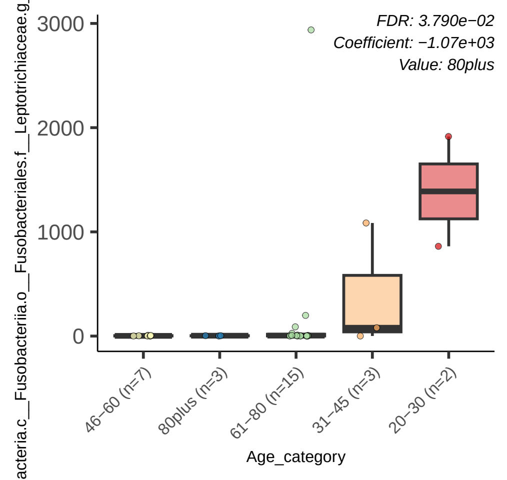

FDR: 3.790e−02
Coefficient: −1.07e+03
Value: 80plus
acteria.c__Fusobacteriia.o__Fusobacteriales.f__Leptotrichiaceae.g_
3000
2000
1000
0
46−60 (n=7)
80plus (n=3)
61−80 (n=15)
31−45 (n=3)
20−30 (n=2)
Age_category

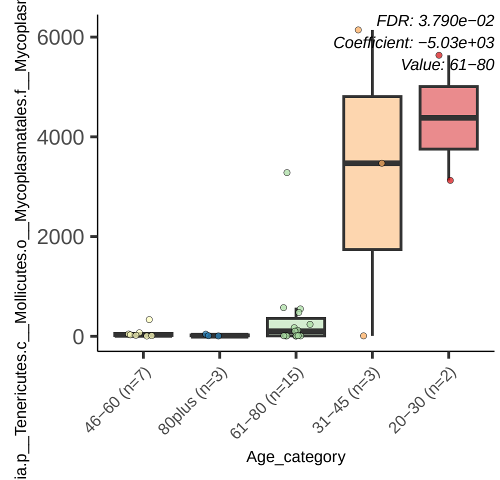
FDR: 3.790e−02
Coefficient: −5.03e+03
Value: 61−80
6000
4000
2000
0
ia.p__Tenericutes.c__Mollicutes.o__Mycoplasmatales.f__Mycoplasm
46−60 (n=7)
80plus (n=3)
61−80 (n=15)
31−45 (n=3)
20−30 (n=2)
Age_category

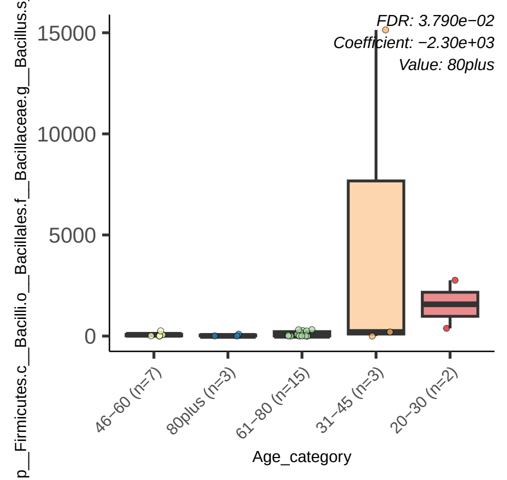
FDR: 3.790e−02
Coefficient: −2.30e+03
Value: 80plus
15000
10000
5000
0
p__Firmicutes.c__Bacilli.o__Bacillales.f__Bacillaceae.g__Bacillus.s_
46−60 (n=7)
80plus (n=3)
61−80 (n=15)
31−45 (n=3)
20−30 (n=2)
Age_category

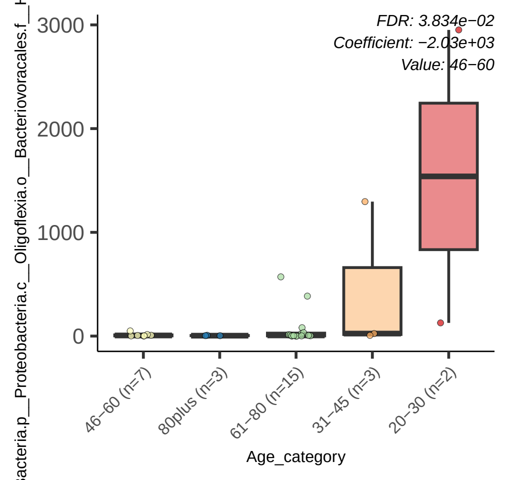

FDR: 3.834e−02
Coefficient: −2.03e+03
Value: 46−60
3000
2000
1000
0
46−60 (n=7)
80plus (n=3)
61−80 (n=15)
31−45 (n=3)
20−30 (n=2)
Age_category
acteria.p__Proteobacteria.c__Oligoflexia.o__Bacteriovoracales.f__H

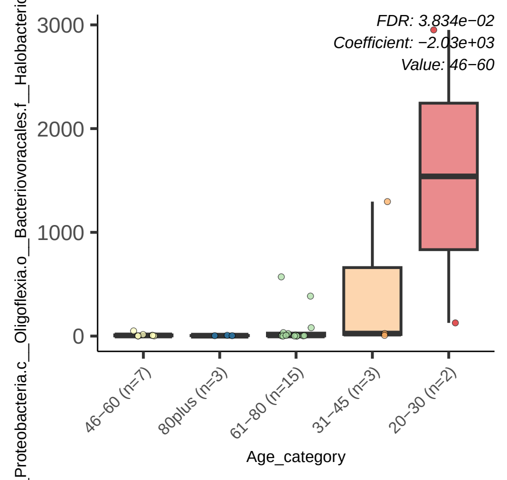

FDR: 3.834e−02
Coefficient: −2.03e+03
Value: 46−60
Proteobacteria.c__Oligoflexia.o__Bacteriovoracales.f__Halobacterio
3000
2000
1000
0
46−60 (n=7)
80plus (n=3)
61−80 (n=15)
31−45 (n=3)
20−30 (n=2)
Age_category

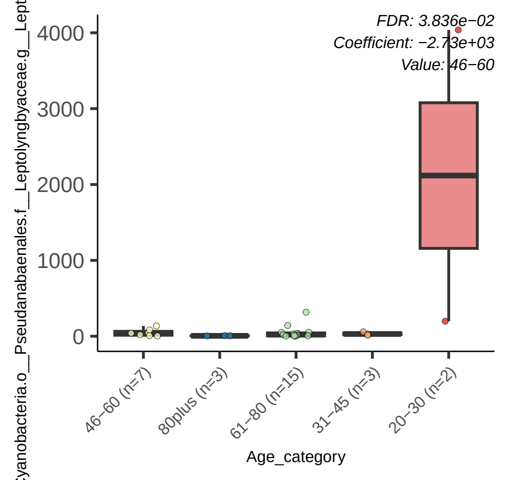

FDR: 3.836e−02
Coefficient: −2.73e+03
Value: 46−60
4000
3000
2000
1000
0
Cyanobacteria.o__Pseudanabaenales.f__Leptolyngbyaceae.g__Lept
46−60 (n=7)
80plus (n=3)
61−80 (n=15)
31−45 (n=3)
20−30 (n=2)
Age_category

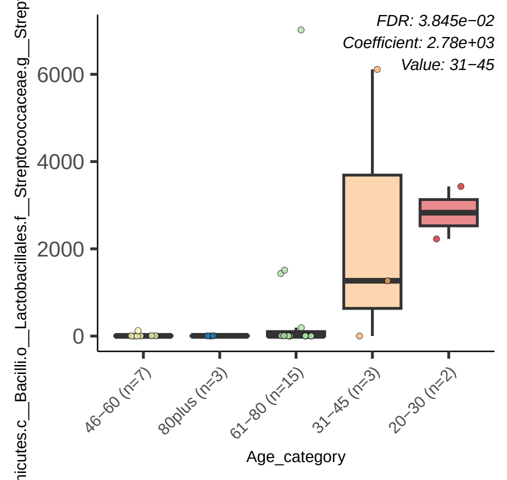
FDR: 3.845e−02
Coefficient: 2.78e+03
Value: 31−45
6000
4000
2000
0
46−60 (n=7)
80plus (n=3)
61−80 (n=15)
31−45 (n=3)
20−30 (n=2)
Age_category
nicutes.c__Bacilli.o__Lactobacillales.f__Streptococcaceae.g__Strep

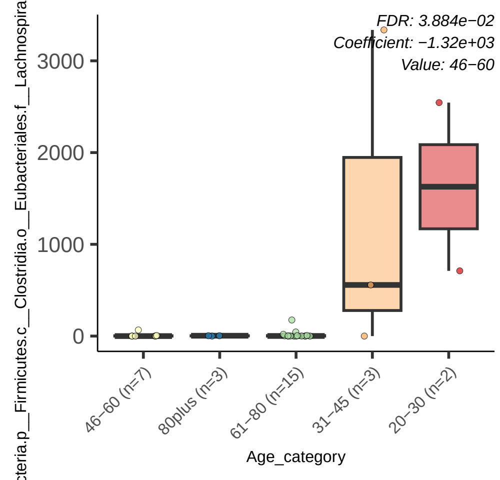

FDR: 3.884e−02
Coefficient: −1.32e+03
Value: 46−60
3000
2000
1000
0
cteria.p__Firmicutes.c__Clostridia.o__Eubacteriales.f__Lachnospira
46−60 (n=7)
80plus (n=3)
61−80 (n=15)
31−45 (n=3)
20−30 (n=2)
Age_category

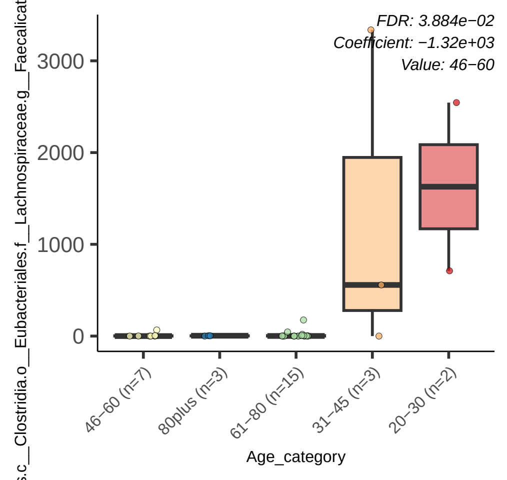

FDR: 3.884e−02
Coefficient: −1.32e+03
Value: 46−60
3000
2000
1000
0
46−60 (n=7)
80plus (n=3)
61−80 (n=15)
31−45 (n=3)
20−30 (n=2)
Age_category
Clostridia.o__Eubacteriales.f__Lachnospiraceae.g__Faecalicat

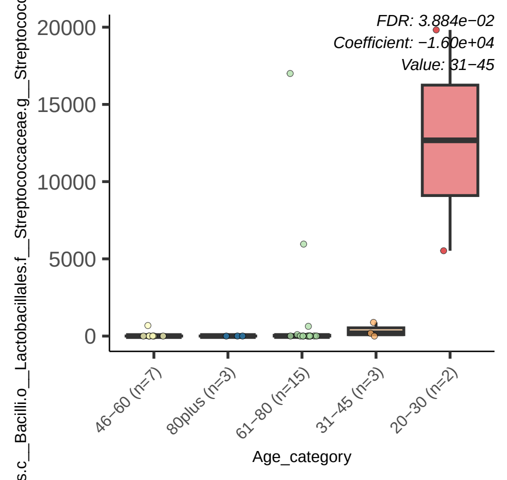
FDR: 3.884e−02
Coefficient: −1.60e+04
Value: 31−45
20000
15000
10000
5000
0
s.c__Bacilli.o__Lactobacillales.f__Streptococcaceae.g__Streptococc
46−60 (n=7)
80plus (n=3)
61−80 (n=15)
31−45 (n=3)
20−30 (n=2)
Age_category

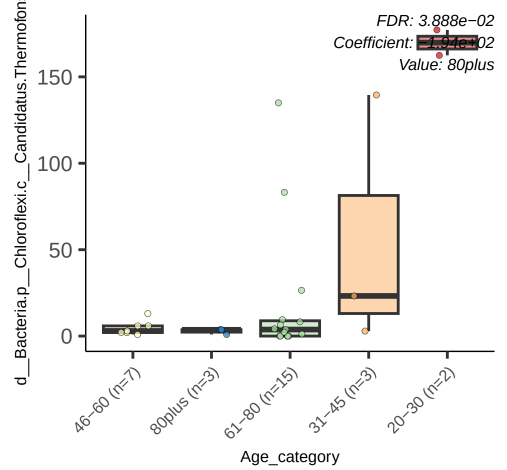

FDR: 3.888e−02
Coefficient: −1.94e+02
Value: 80plus
d__Bacteria.p__Chloroflexi.c__Candidatus.Thermofon
150
100
50
0
46−60 (n=7)
80plus (n=3)
61−80 (n=15)
31−45 (n=3)
20−30 (n=2)
Age_category

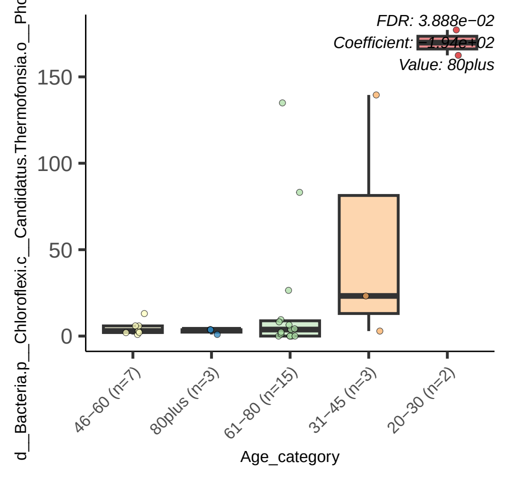

FDR: 3.888e−02
Coefficient: −1.94e+02
Value: 80plus
d__Bacteria.p__Chloroflexi.c__Candidatus.Thermofonsia.o__Pho
150
100
50
0
46−60 (n=7)
80plus (n=3)
61−80 (n=15)
31−45 (n=3)
20−30 (n=2)
Age_category

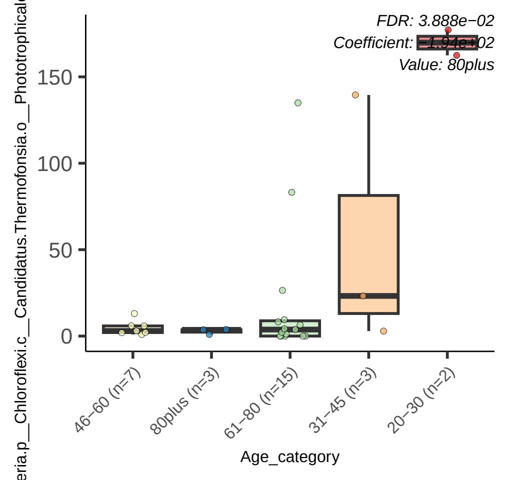
FDR: 3.888e−02
Coefficient: −1.94e+02
Value: 80plus
150
100
50
0
46−60 (n=7)
80plus (n=3)
61−80 (n=15)
31−45 (n=3)
20−30 (n=2)
Age_category
eria.p__Chloroflexi.c__Candidatus.Thermofonsia.o__Phototrophicale

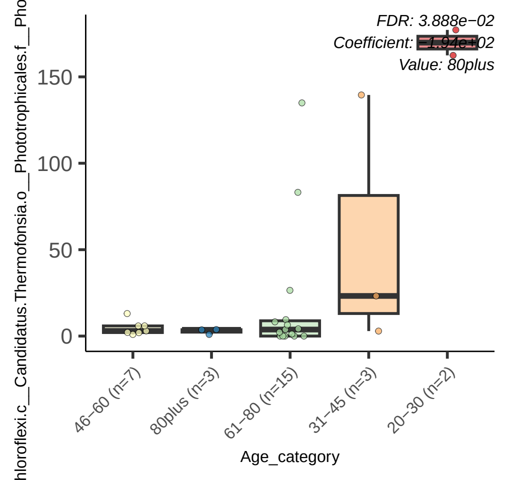

FDR: 3.888e−02
Coefficient: −1.94e+02
Value: 80plus
150
100
50
0
hloroflexi.c__Candidatus.Thermofonsia.o__Phototrophicales.f__Pho
46−60 (n=7)
80plus (n=3)
61−80 (n=15)
31−45 (n=3)
20−30 (n=2)
Age_category

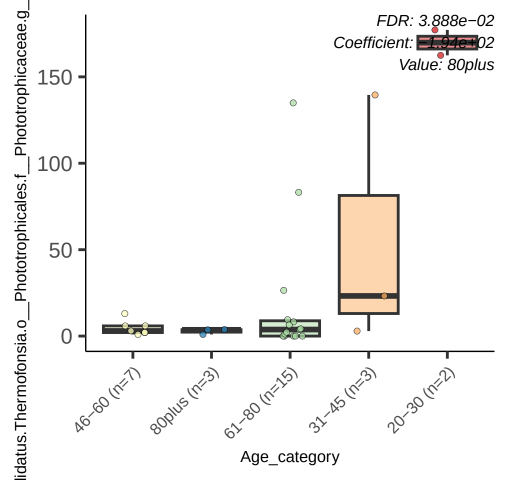

FDR: 3.888e−02
Coefficient: −1.94e+02
Value: 80plus
lidatus.Thermofonsia.o__Photrophicales.f__Photrophicaceae.g__
150
100
50
0
46−60 (n=7)
80plus (n=3)
61−80 (n=15)
31−45 (n=3)
20−30 (n=2)
Age_category

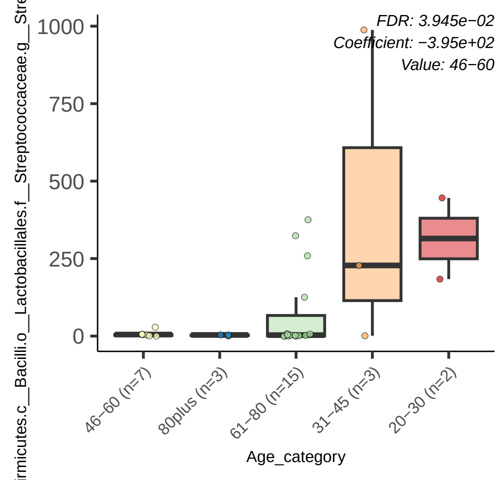
FDR: 3.945e−02
Coefficient: −3.95e+02
Value: 46−60
1000
750
500
250
0
irmicutes.c__Bacilli.o__Lactobacillales.f__Streptococcaceae.g__Stre
46−60 (n=7)
80plus (n=3)
61−80 (n=15)
31−45 (n=3)
20−30 (n=2)
Age_category

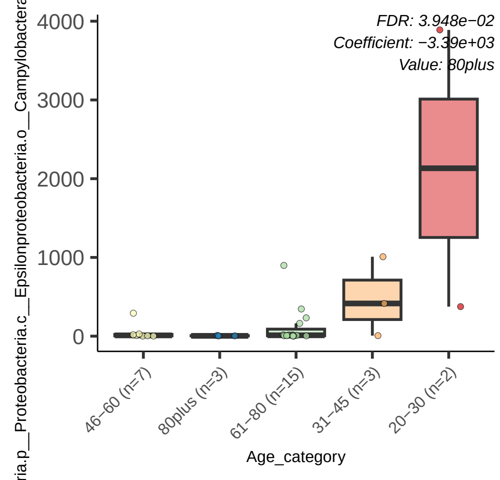
FDR: 3.948e−02
Coefficient: −3.39e+03
Value: 80plus
ria.p__Proteobacteria.c__Epsilonproteobacteria.o__Campylobactera
4000
3000
2000
1000
0
46−60 (n=7)
80plus (n=3)
61−80 (n=15)
31−45 (n=3)
20−30 (n=2)
Age_category

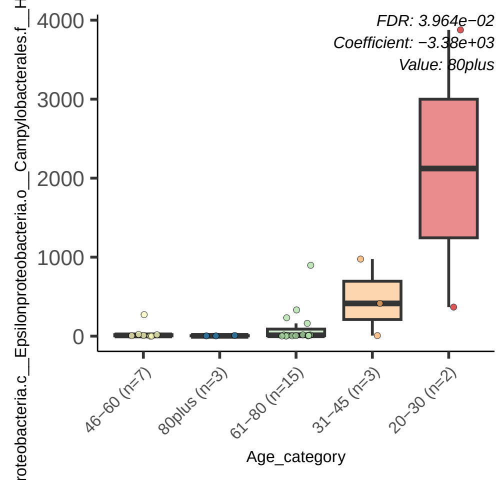

FDR: 3.964e−02
Coefficient: −3.38e+03
Value: 80plus
4000
3000
2000
1000
0
46−60 (n=7)
80plus (n=3)
61−80 (n=15)
31−45 (n=3)
20−30 (n=2)
Age_category
roteobacteria.c__Epsilonproteobacteria.o__Campylobacterales.f__H

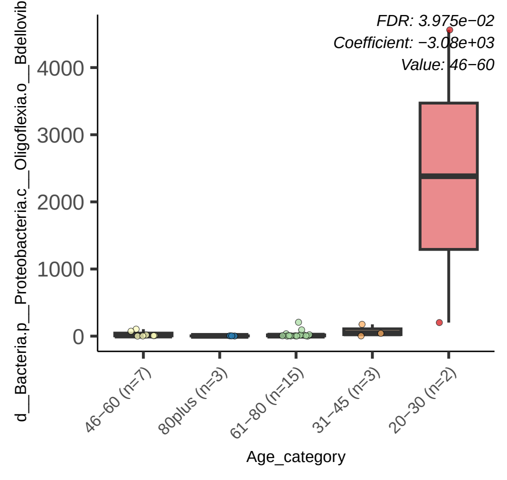

FDR: 3.975e−02
Coefficient: −3.08e+03
Value: 46−60
d__Bacteria.p__Proteobacteria.c__Oligoflexia.o__Bdellovib
4000
3000
2000
1000
0
46−60 (n=7)
80plus (n=3)
61−80 (n=15)
31−45 (n=3)
20−30 (n=2)
Age_category

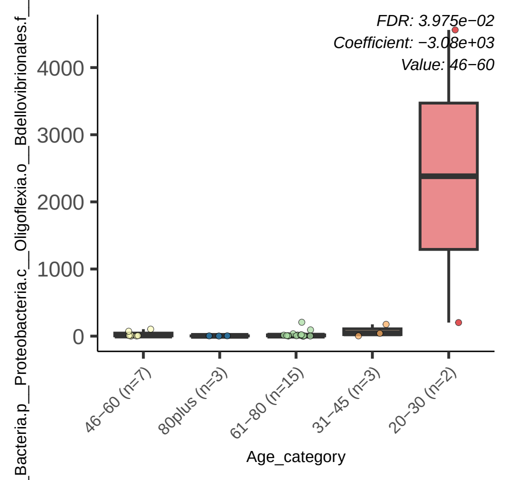
FDR: 3.975e−02
Coefficient: −3.08e+03
Value: 46−60
Bacteria.p__Proteobacteria.c__Oligoflexia.o__Bdellovibrionales.f__
4000
3000
2000
1000
0
46−60 (n=7)
80plus (n=3)
61−80 (n=15)
31−45 (n=3)
20−30 (n=2)
Age_category

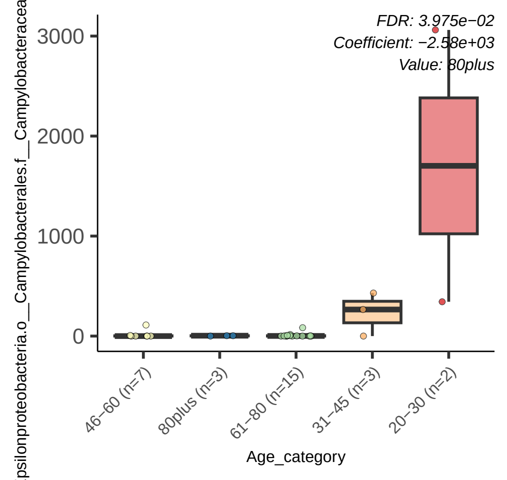

FDR: 3.975e−02
Coefficient: −2.58e+03
Value: 80plus
psilonproteobacteria.o__Campylobacterales.f__Campylobacteracea
3000
2000
1000
0
46−60 (n=7)
80plus (n=3)
61−80 (n=15)
31−45 (n=3)
20−30 (n=2)
Age_category

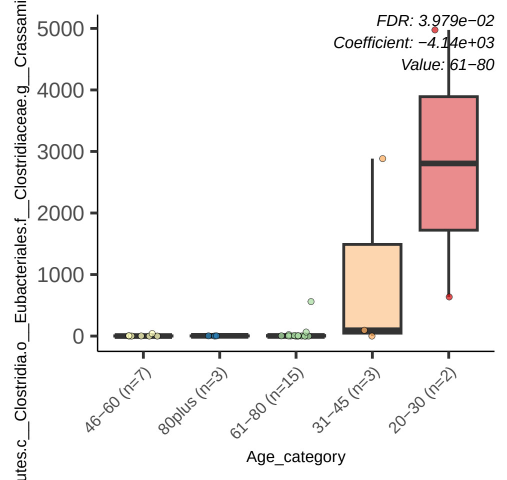

FDR: 3.979e−02
Coefficient: −4.14e+03
Value: 61−80
5000
4000
3000
2000
1000
0
46−60 (n=7)
80plus (n=3)
61−80 (n=15)
31−45 (n=3)
20−30 (n=2)
Age_category

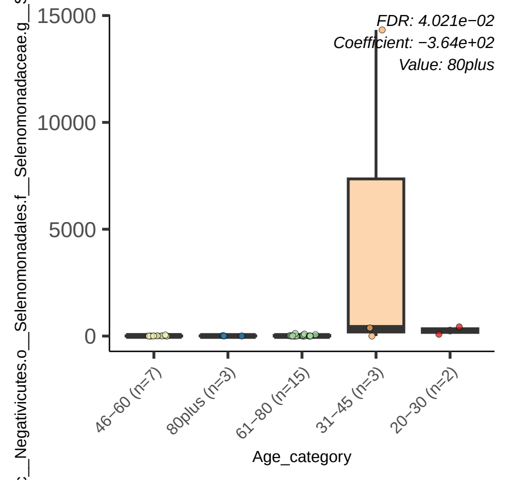

FDR: 4.021e−02
Coefficient: −3.64e+02
Value: 80plus
15000
10000
5000
0
Negativicutes.o__Selenomonadales.f__Selenomonadaceae.g__
46−60 (n=7)
80plus (n=3)
61−80 (n=15)
31−45 (n=3)
20−30 (n=2)
Age_category

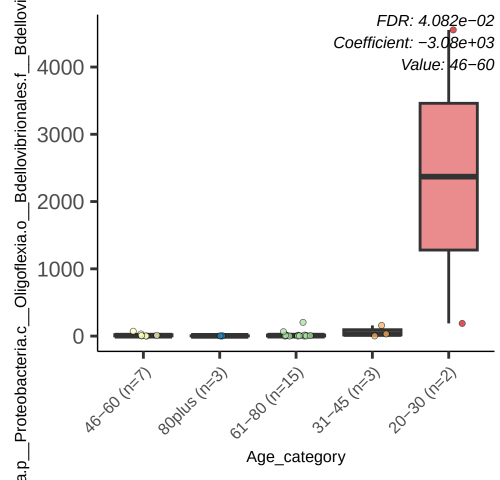

FDR: 4.082e−02
Coefficient: −3.08e+03
Value: 46−60
a.p__Proteobacteria.c__Oligoflexia.o__Bdellovibrionales.f__Bdellovi
4000
3000
2000
1000
0
46−60 (n=7)
80plus (n=3)
61−80 (n=15)
31−45 (n=3)
20−30 (n=2)
Age_category

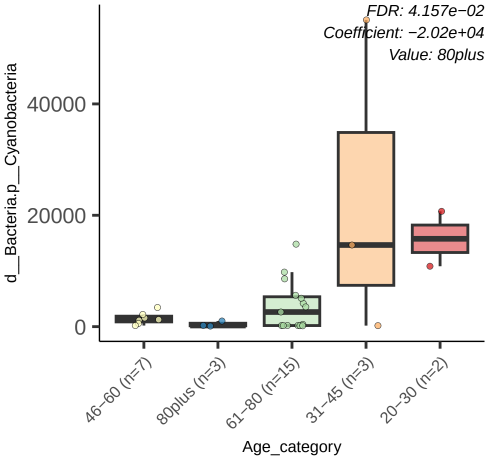

FDR: 4.157e−02
Coefficient: −2.02e+04
Value: 80plus
d__Bacteria.p__Cyanobacteria
40000
20000
0
46−60 (n=7)
80plus (n=3)
61−80 (n=15)
31−45 (n=3)
20−30 (n=2)
Age_category

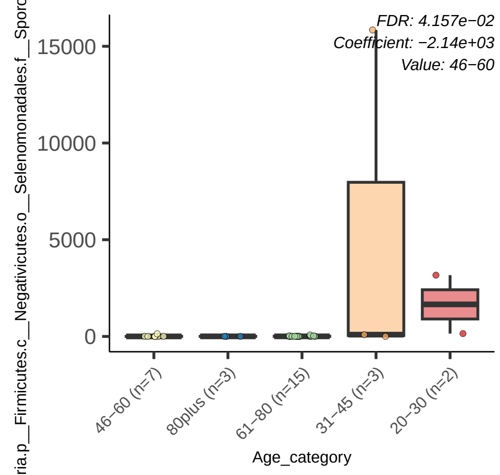

FDR: 4.157e−02
Coefficient: −2.14e+03
Value: 46−60
15000
10000
5000
0
eria.p__Firmicutes.c__Negativicutes.o__Selenomonadales.f__Sporo
46−60 (n=7)
80plus (n=3)
61−80 (n=15)
31−45 (n=3)
20−30 (n=2)
Age_category

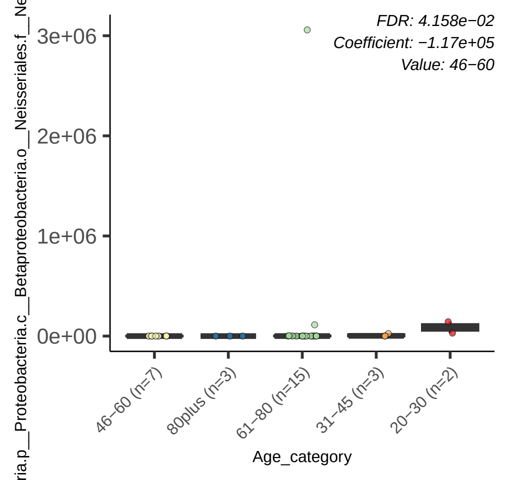

FDR: 4.158e−02
Coefficient: −1.17e+05
Value: 46−60
3e+06
2e+06
1e+06
0e+00
ria.p__Proteobacteria.c__Betaproteobacteria.o__Neisseriales.f__Ne
46−60 (n=7)
80plus (n=3)
61−80 (n=15)
31−45 (n=3)
20−30 (n=2)
Age_category

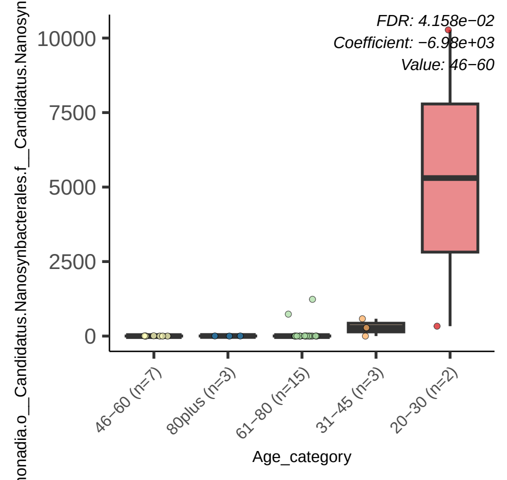
FDR: 4.158e−02
Coefficient: −6.98e+03
Value: 46−60
10000
7500
5000
2500
0
nonadia.o__Candidatus.Nanosynbacterales.f__Candidatus.Nanosyn
46−60 (n=7)
80plus (n=3)
61−80 (n=15)
31−45 (n=3)
20−30 (n=2)
Age_category

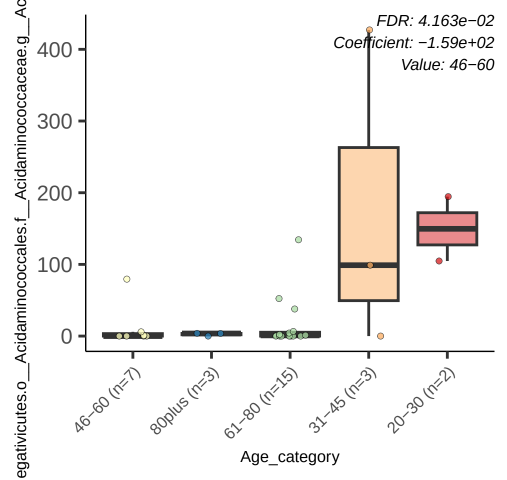
FDR: 4.163e−02
Coefficient: −1.59e+02
Value: 46−60
egativicutes.o__Acidaminococcales.f__Acidaminococcaceae.g__Ac
0
100
200
300
400
46−60 (n=7)
80plus (n=3)
61−80 (n=15)
31−45 (n=3)
20−30 (n=2)
Age_category

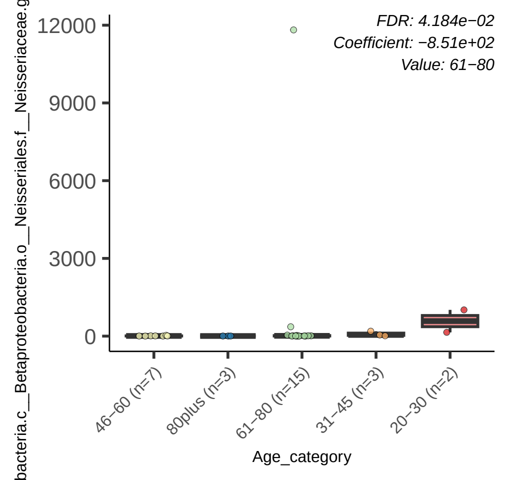
FDR: 4.184e−02
Coefficient: −8.51e+02
Value: 61−80
12000
9000
6000
3000
0
bacteria.c__Betaproteobacteria.o__Neisseriales.f__Neisseriaceae.g
46−60 (n=7)
80plus (n=3)
61−80 (n=15)
31−45 (n=3)
20−30 (n=2)
Age_category

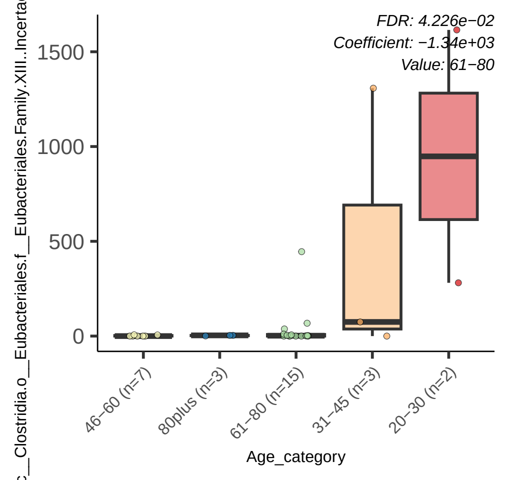
FDR: 4.226e−02
Coefficient: −1.34e+03
Value: 61−80
1500
1000
500
0
46−60 (n=7)
80plus (n=3)
61−80 (n=15)
31−45 (n=3)
20−30 (n=2)
Age_category
Clostridia.o__Eubacteriales.f__Eubacteriales.Family.XIII..Incerta

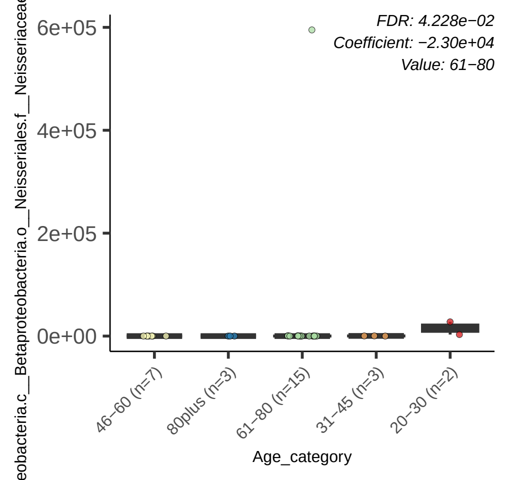

FDR: 4.228e−02
Coefficient: −2.30e+04
Value: 61−80
6e+05
4e+05
2e+05
0e+00
eobacteria.c__Betaproteobacteria.o__Neisseriales.f__Neisseriaceae
46−60 (n=7)
80plus (n=3)
61−80 (n=15)
31−45 (n=3)
20−30 (n=2)
Age_category

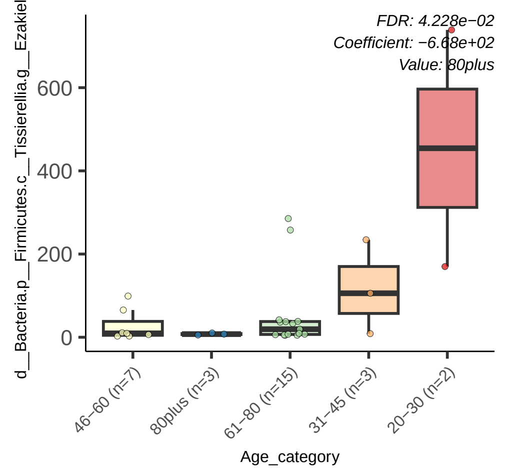
FDR: 4.228e−02
Coefficient: −6.68e+02
Value: 80plus
d__Bacteria.p__Firmicutes.c__Tissierellia.g__Ezakiel
600
400
200
0
46−60 (n=7)
80plus (n=3)
61−80 (n=15)
31−45 (n=3)
20−30 (n=2)
Age_category

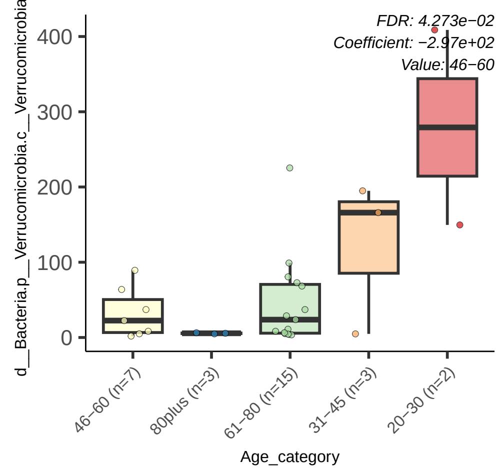

FDR: 4.273e−02
Coefficient: −2.97e+02
Value: 46−60
d__Bacteria.p__Verrucomicrobia.c__Verrucomicrobia
400
300
200
100
0
46−60 (n=7)
80plus (n=3)
61−80 (n=15)
31−45 (n=3)
20−30 (n=2)
Age_category

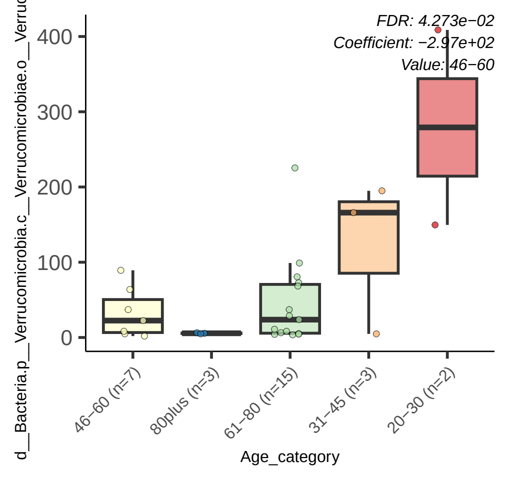

FDR: 4.273e−02
Coefficient: −2.97e+02
Value: 46−60
d__Bacteria.p__Verrucomicrobia.c__Verrucomicrobiae.o__Verruc
0
100
200
300
400
46−60 (n=7)
80plus (n=3)
61−80 (n=15)
31−45 (n=3)
20−30 (n=2)
Age_category

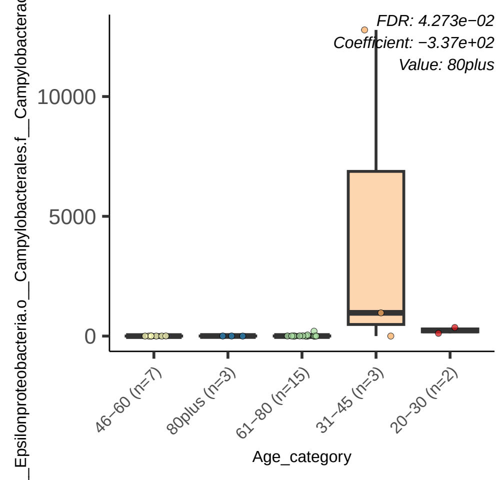

FDR: 4.273e−02
Coefficient: −3.37e+02
Value: 80plus
10000
5000
0
_Epsilonproteobacteria.o__Campylobacterales.f__Campylobacterac
46−60 (n=7)
80plus (n=3)
61−80 (n=15)
31−45 (n=3)
20−30 (n=2)
Age_category

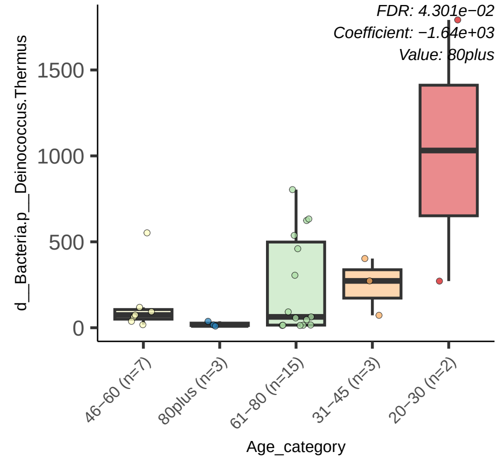

FDR: 4.301e−02
Coefficient: −1.64e+03
Value: 80plus
d__Bacteria.p__Deinococcus.Thermus
1500
1000
500
0
46−60 (n=7)
80plus (n=3)
61−80 (n=15)
31−45 (n=3)
20−30 (n=2)
Age_category

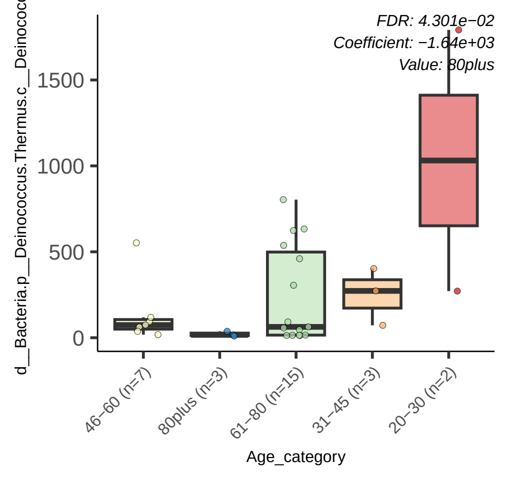

FDR: 4.301e−02
Coefficient: −1.64e+03
Value: 80plus
d__Bacteria.p__Deinococcus.Thermus.c__Deinococ
1500
1000
500
0
46−60 (n=7)
80plus (n=3)
61−80 (n=15)
31−45 (n=3)
20−30 (n=2)
Age_category

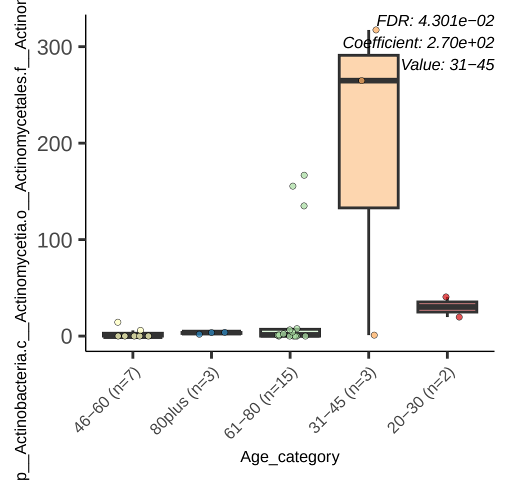

FDR: 4.301e−02
Coefficient: 2.70e+02
Value: 31−45
p__Actinobacteria.c__Actinomycetia.o__Actinomycetales.f__Actinor
300
200
100
0
46−60 (n=7)
80plus (n=3)
61−80 (n=15)
31−45 (n=3)
20−30 (n=2)
Age_category

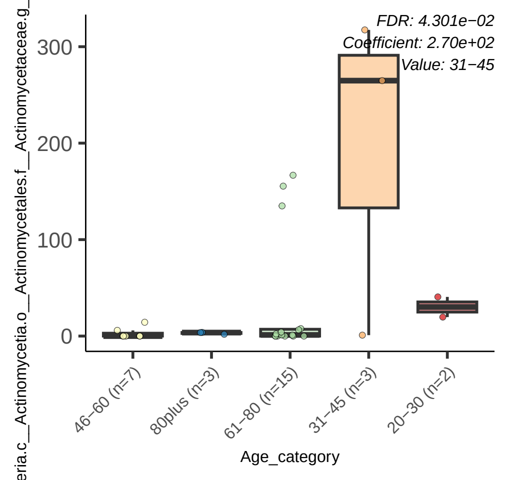

FDR: 4.301e−02
Coefficient: 2.70e+02
Value: 31−45
300
200
100
0
eria.c__Actinomycetia.o__Actinomycetales.f__Actinomycetaceae.g_
46−60 (n=7)
80plus (n=3)
61−80 (n=15)
31−45 (n=3)
20−30 (n=2)
Age_category

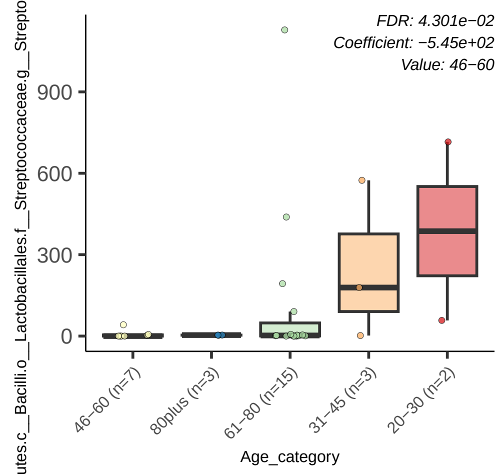

FDR: 4.301e−02
Coefficient: −5.45e+02
Value: 46−60
900
600
300
0
cutes.c__Bacilli.o__Lactobacillales.f__Streptococcaceae.g__Strepto
46−60 (n=7)
80plus (n=3)
61−80 (n=15)
31−45 (n=3)
20−30 (n=2)
Age_category

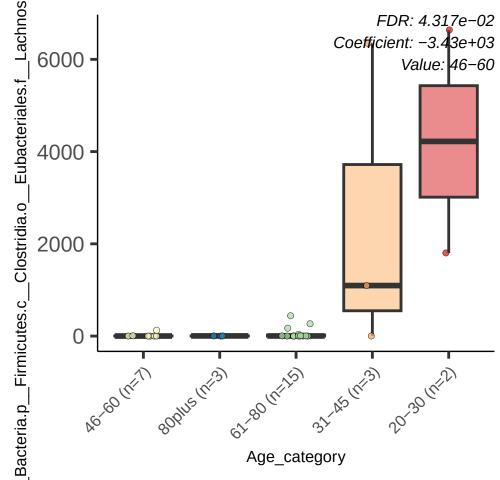
FDR: 4.317e−02
Coefficient: −3.43e+03
Value: 46−60
_Bacteria.p__Firmicutes.c__Clostridia.o__Eubacteriales.f__Lachnos
0
2000
4000
6000
46−60 (n=7)
80plus (n=3)
61−80 (n=15)
31−45 (n=3)
20−30 (n=2)
Age_category

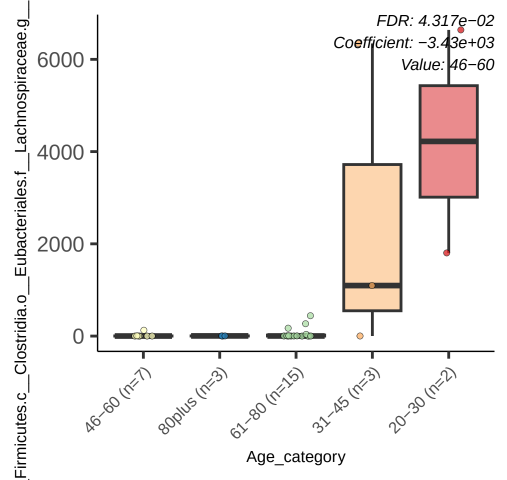

FDR: 4.317e−02
Coefficient: −3.43e+03
Value: 46−60
Firmicutes.c__Clostridia.o__Eubacteriales.f__Lachnospiraceae.g__
6000
4000
2000
0
46−60 (n=7)
80plus (n=3)
61−80 (n=15)
31−45 (n=3)
20−30 (n=2)
Age_category

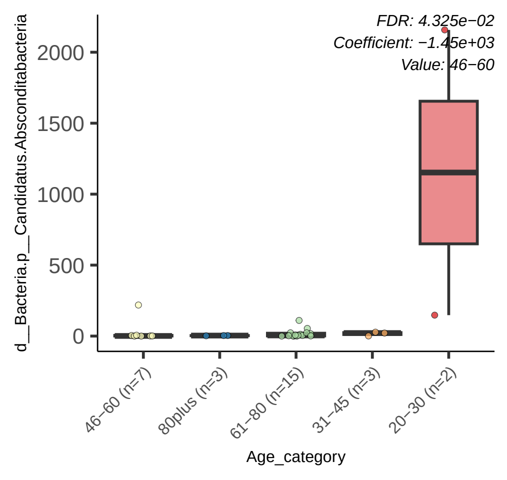

FDR: 4.325e−02
Coefficient: −1.45e+03
Value: 46−60
d__Bacteria.p__Candidatus.Absconditabacteria
2000
1500
1000
500
0
46−60 (n=7)
80plus (n=3)
61−80 (n=15)
31−45 (n=3)
20−30 (n=2)
Age_category

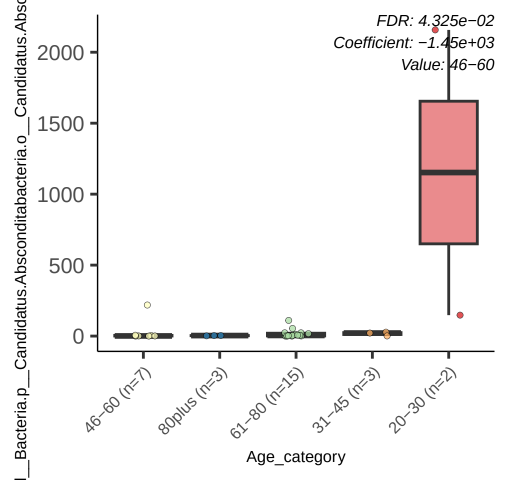
FDR: 4.325e−02
Coefficient: −1.45e+03
Value: 46−60
2000
1500
1000
500
0
l__Bacteria.p__Candidatus.Absconditabacteria.o__Candidatus.Absco
46−60 (n=7)
80plus (n=3)
61−80 (n=15)
31−45 (n=3)
20−30 (n=2)
Age_category

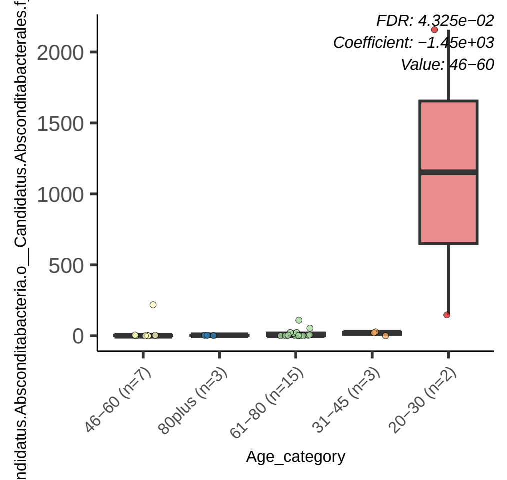

FDR: 4.325e−02
Coefficient: −1.45e+03
Value: 46−60
ndidatus.Absconditabacteria.o__Candidatus.Absconditabacterales.f_
2000
1500
1000
500
0
46−60 (n=7)
80plus (n=3)
61−80 (n=15)
31−45 (n=3)
20−30 (n=2)
Age_category

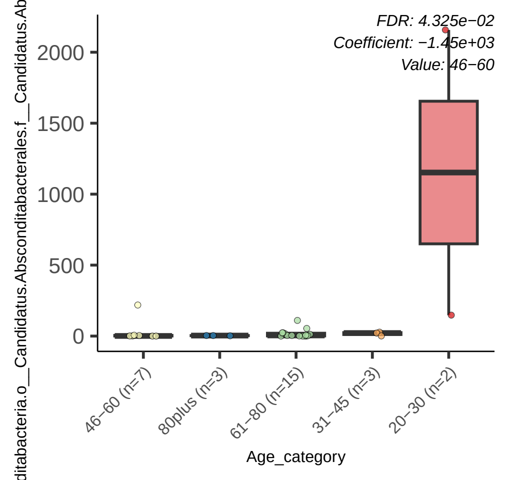
FDR: 4.325e−02
Coefficient: −1.45e+03
Value: 46−60
ditabacteria.o__Candidatus.Absconditabacterales.f__Candidatus.Ab
2000
1500
1000
500
0
46−60 (n=7)
80plus (n=3)
61−80 (n=15)
31−45 (n=3)
20−30 (n=2)
Age_category

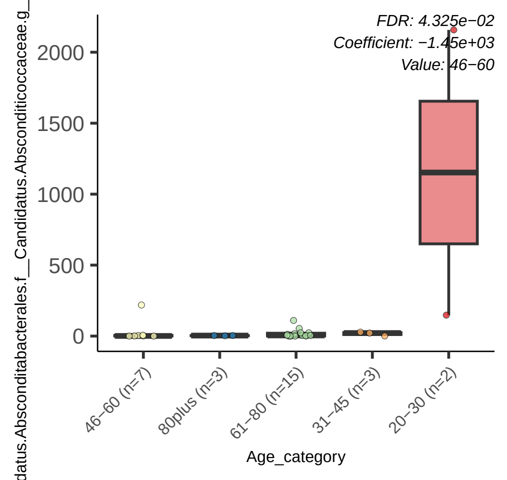

FDR: 4.325e−02
Coefficient: −1.45e+03
Value: 46−60
2000
1500
1000
500
0
46−60 (n=7)
80plus (n=3)
61−80 (n=15)
31−45 (n=3)
20−30 (n=2)
Age_category
datus.Absconditabacterales.f__Candidatus.Absconditicoccaceae.g__

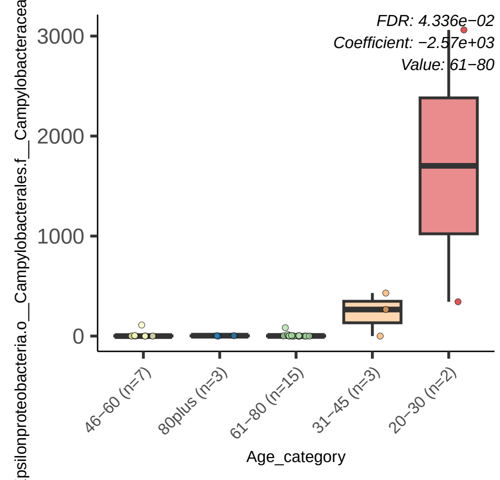

FDR: 4.336e−02
Coefficient: −2.57e+03
Value: 61−80
3000
2000
1000
0
psilonproteobacteria.o__Campylobacterales.f__Campylobacteracea
46−60 (n=7)
80plus (n=3)
61−80 (n=15)
31−45 (n=3)
20−30 (n=2)
Age_category

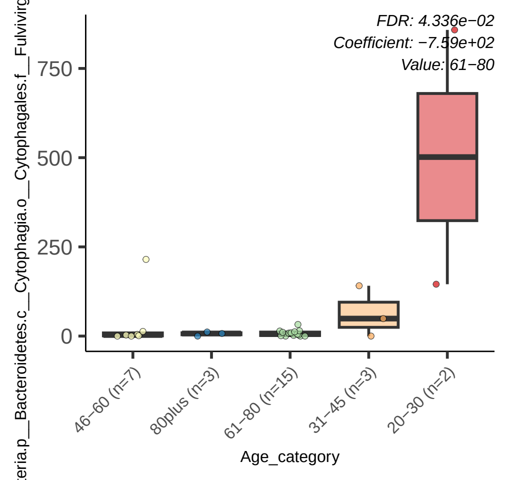
FDR: 4.336e−02
Coefficient: −7.59e+02
Value: 61−80
0
250
500
750
46−60 (n=7)
80plus (n=3)
61−80 (n=15)
31−45 (n=3)
20−30 (n=2)
Age_category

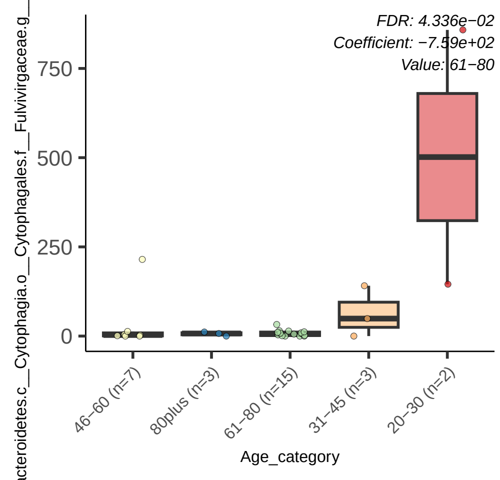

FDR: 4.336e−02
Coefficient: −7.59e+02
Value: 61−80
750
500
250
0
acteroidetes.c__Cytophagia.o__Cytophagales.f__Fulvivirgaceae.g__
46−60 (n=7)
80plus (n=3)
61−80 (n=15)
31−45 (n=3)
20−30 (n=2)
Age_category

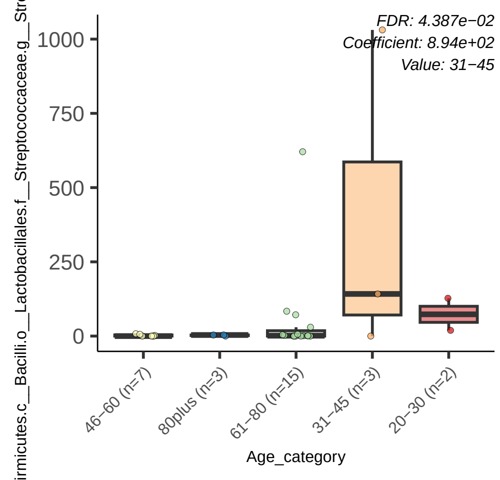
FDR: 4.387e−02
Coefficient: 8.94e+02
Value: 31−45
irmicutes.c__Bacilli.o__Lactobacillales.f__Streptococcaceae.g__Stre
1000
750
500
250
0
46−60 (n=7)
80plus (n=3)
61−80 (n=15)
31−45 (n=3)
20−30 (n=2)
Age_category

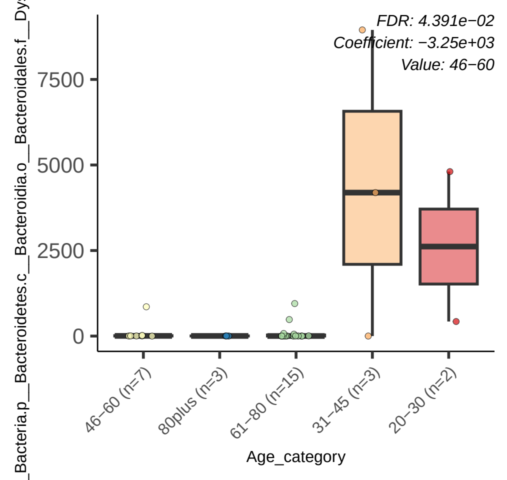
FDR: 4.391e−02
Coefficient: −3.25e+03
Value: 46−60
7500
5000
2500
0
_Bacteria.p__Bacteroidetes.c__Bacteroidia.o__Bacteroidales.f__Dys
46−60 (n=7)
80plus (n=3)
61−80 (n=15)
31−45 (n=3)
20−30 (n=2)
Age_category

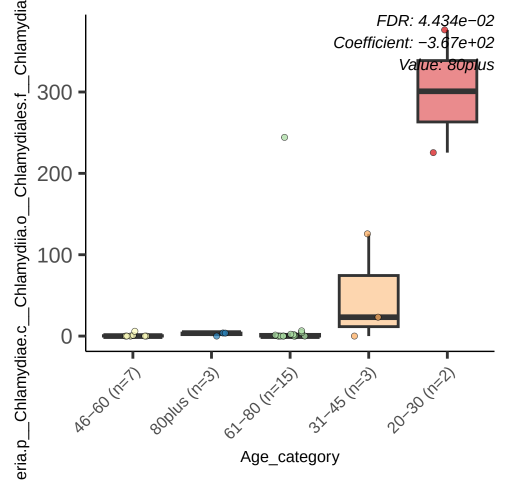

FDR: 4.434e−02
Coefficient: −3.67e+02
Value: 80plus
eria.p__Chlamydiae.c__Chlamydiia.o__Chlamydiales.f__Chlamydia
300
200
100
0
46−60 (n=7)
80plus (n=3)
61−80 (n=15)
31−45 (n=3)
20−30 (n=2)
Age_category

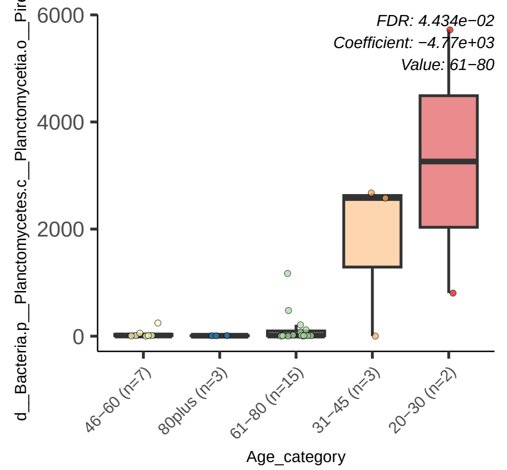
FDR: 4.434e−02
Coefficient: −4.77e+03
Value: 61−80
d__Bacteria.p__Planctomycetes.c__Planctomycetia.o__Pir
6000
4000
2000
0
46−60 (n=7)
80plus (n=3)
61−80 (n=15)
31−45 (n=3)
20−30 (n=2)
Age_category

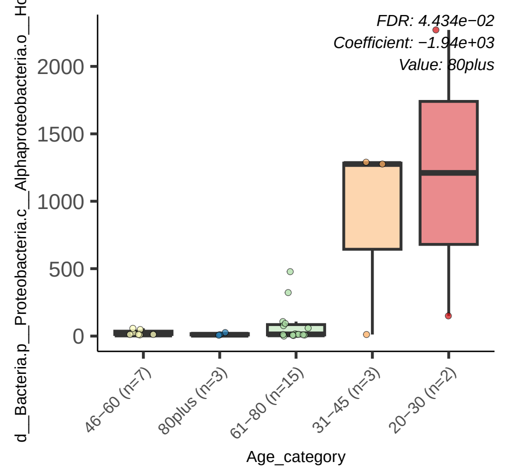

FDR: 4.434e−02
Coefficient: −1.94e+03
Value: 80plus
d__Bacteria.p__Proteobacteria.c__Alphaproteobacteria.o__Ho
2000
1500
1000
500
0
46−60 (n=7)
80plus (n=3)
61−80 (n=15)
31−45 (n=3)
20−30 (n=2)
Age_category

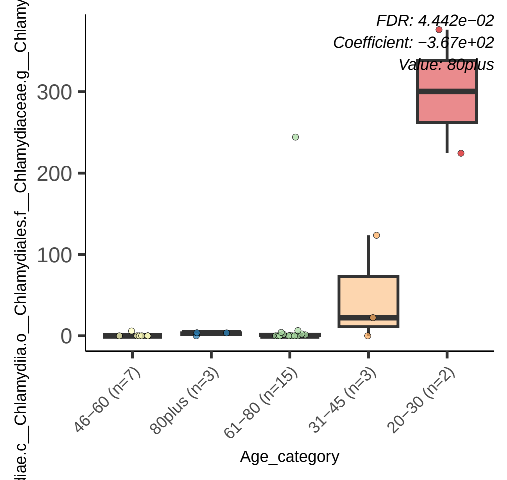

FDR: 4.442e−02
Coefficient: −3.67e+02
Value: 80plus
300
200
100
0
46−60 (n=7)
80plus (n=3)
61−80 (n=15)
31−45 (n=3)
20−30 (n=2)
Age_category
diae.c__Chlamydiia.o__Chlamydiales.f__Chlamydiaceae.g__Chlamy

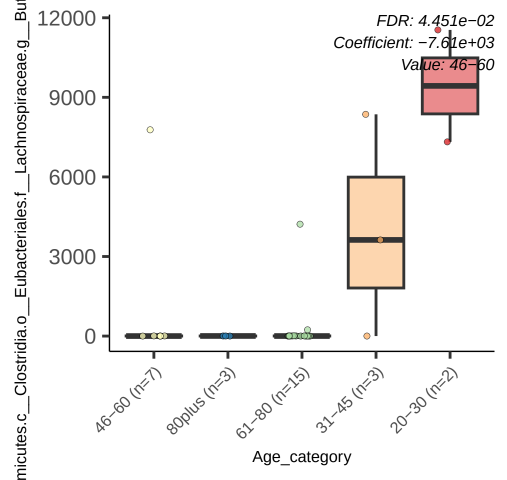
FDR: 4.451e−02
Coefficient: −7.61e+03
Value: 46−60
12000
9000
6000
3000
0
46−60 (n=7)
80plus (n=3)
61−80 (n=15)
31−45 (n=3)
20−30 (n=2)
Age_category
micutes.c__Clostridia.o__Eubacteriales.f__Lachnospiraceae.g__Bu

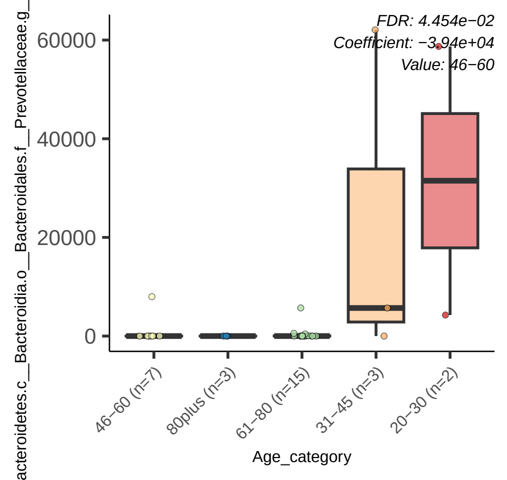

FDR: 4.454e−02
Coefficient: −3.94e+04
Value: 46−60
acteroidetes.c__Bacteroidia.o__Bacteroidales.f__Prevotellaceae.g__
60000
40000
20000
0
46−60 (n=7)
80plus (n=3)
61−80 (n=15)
31−45 (n=3)
20−30 (n=2)
Age_category

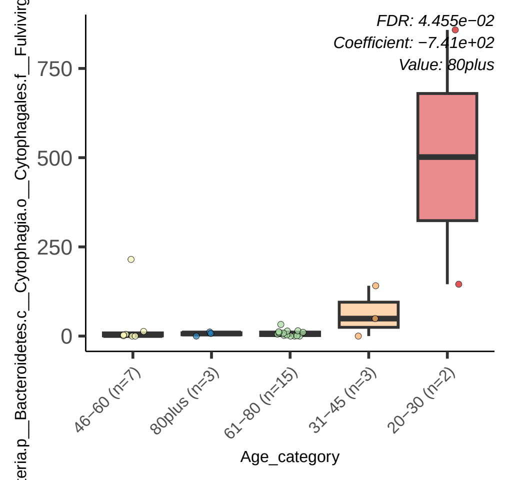

FDR: 4.455e−02
Coefficient: −7.41e+02
Value: 80plus
750
500
250
0
teria.p__Bacteroidetes.c__Cytophagia.o__Cytophagales.f__Fulvivirg
46−60 (n=7)
80plus (n=3)
61−80 (n=15)
31−45 (n=3)
20−30 (n=2)
Age_category

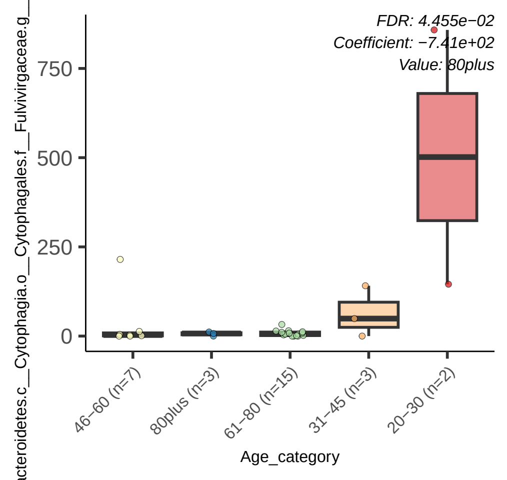
FDR: 4.455e−02
Coefficient: −7.41e+02
Value: 80plus
acteroidetes.c__Cytophagia.o__Cytophagales.f__Fulvivirgaceae.g__
750
500
250
0
46−60 (n=7)
80plus (n=3)
61−80 (n=15)
31−45 (n=3)
20−30 (n=2)
Age_category

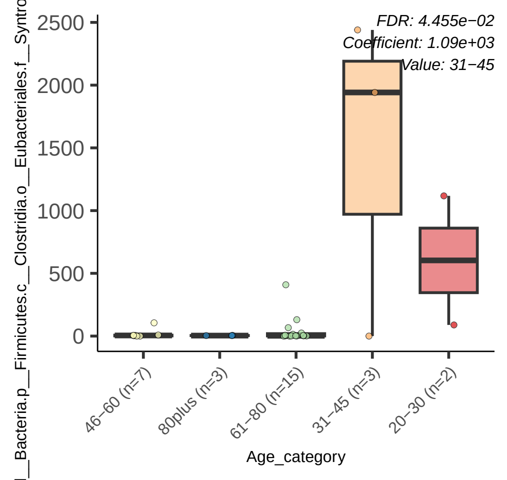

FDR: 4.455e−02
Coefficient: 1.09e+03
Value: 31−45
2500
2000
1500
1000
500
0
Bacteria.p__Firmicutes.c__Clostridia.o__Eubacteriales.f__Syntro
46−60 (n=7)
80plus (n=3)
61−80 (n=15)
31−45 (n=3)
20−30 (n=2)
Age_category

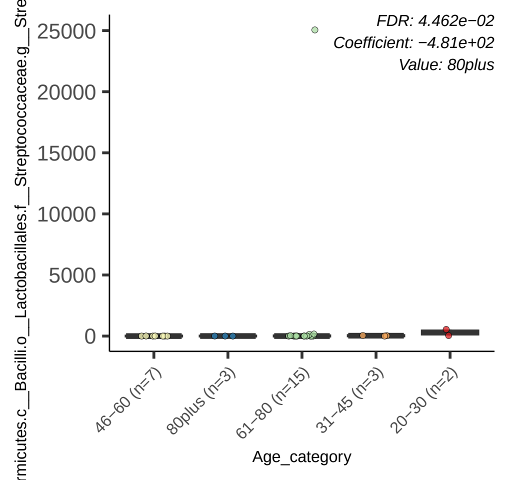

FDR: 4.462e−02
Coefficient: −4.81e+02
Value: 80plus
25000
20000
15000
10000
5000
0
46−60 (n=7)
80plus (n=3)
61−80 (n=15)
31−45 (n=3)
20−30 (n=2)
Age_category
rmicutes.c__Bacilli.o__Lactobacillales.f__Streptococcaceae.g__Stre

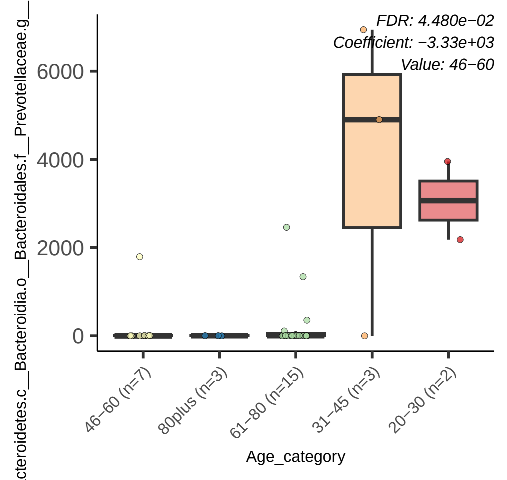

FDR: 4.480e−02
Coefficient: −3.33e+03
Value: 46−60
6000
4000
2000
0
cteroidetes.c__Bacteroidia.o__Bacteroidales.f__Prevotellaceae.g__
46−60 (n=7)
80plus (n=3)
61−80 (n=15)
31−45 (n=3)
20−30 (n=2)
Age_category

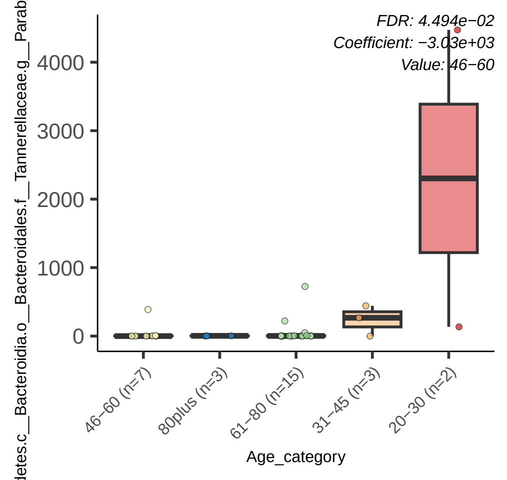

FDR: 4.494e−02
Coefficient: −3.03e+03
Value: 46−60
detes.c__Bacteroidia.o__Bacteroidales.f__Tannerellaceae.g__Parab
4000
3000
2000
1000
0
46−60 (n=7)
80plus (n=3)
61−80 (n=15)
31−45 (n=3)
20−30 (n=2)
Age_category

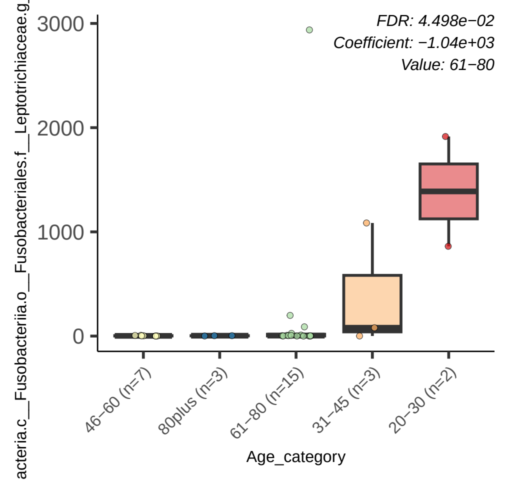

FDR: 4.498e−02
Coefficient: −1.04e+03
Value: 61−80
3000
2000
1000
0
acteria.c__Fusobacteriia.o__Fusobacteriales.f__Leptotrichiaceae.g_
46−60 (n=7)
80plus (n=3)
61−80 (n=15)
31−45 (n=3)
20−30 (n=2)
Age_category

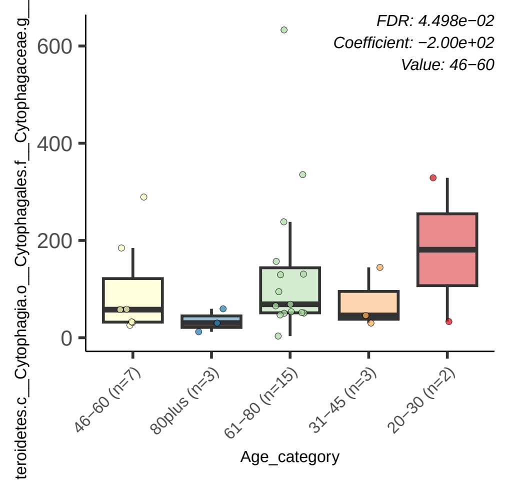
FDR: 4.498e−02
Coefficient: −2.00e+02
Value: 46−60
0
200
400
600
teroidetes.c__Cytophagia.o__Cytophagales.f__Cytophagaceae.g__
46−60 (n=7)
80plus (n=3)
61−80 (n=15)
31−45 (n=3)
20−30 (n=2)
Age_category

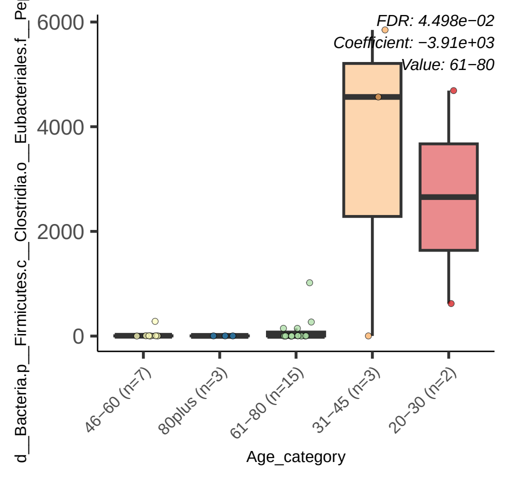

FDR: 4.498e−02
Coefficient: −3.91e+03
Value: 61−80
d__Bacteria.p__Firmicutes.c__Clostridia.o__Eubacteriales.f__Pe
6000
4000
2000
0
46−60 (n=7)
80plus (n=3)
61−80 (n=15)
31−45 (n=3)
20−30 (n=2)
Age_category

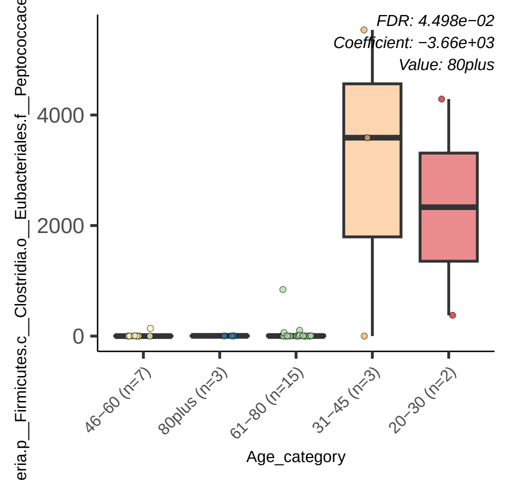

FDR: 4.498e−02
Coefficient: −3.66e+03
Value: 80plus
0
2000
4000
eria.p__Firmicutes.c__Clostridia.o__Eubacteriales.f__Peptococcace
46−60 (n=7)
80plus (n=3)
61−80 (n=15)
31−45 (n=3)
20−30 (n=2)
Age_category

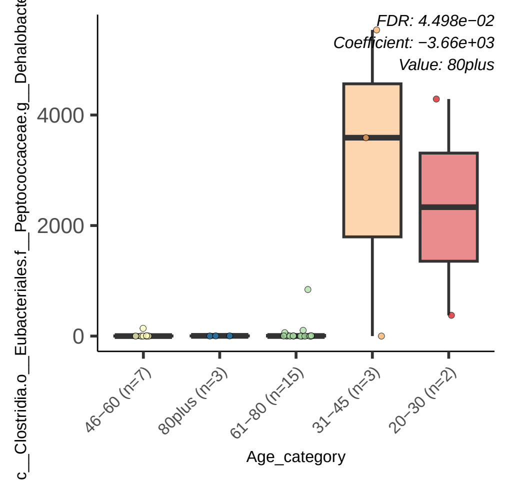
FDR: 4.498e−02
Coefficient: −3.66e+03
Value: 80plus
c__Clostridia.o__Eubacteriales.f__Peptococcaceae.g__Dehalobacte
4000
2000
0
46−60 (n=7)
80plus (n=3)
61−80 (n=15)
31−45 (n=3)
20−30 (n=2)
Age_category

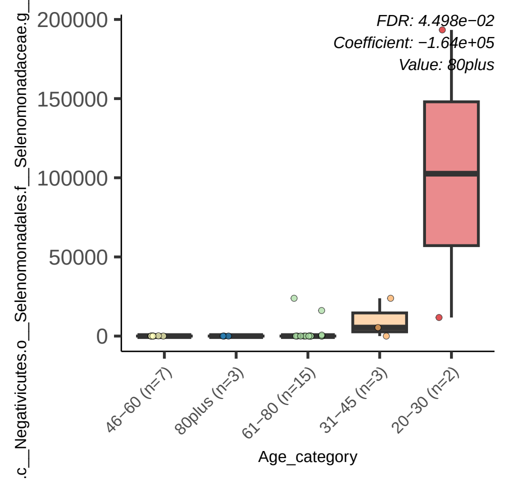
FDR: 4.498e−02
Coefficient: −1.64e+05
Value: 80plus
c__Negativicutes.o__Selenomonadales.f__Selenomonadaceae.g__
200000
150000
100000
50000
0
46−60 (n=7)
80plus (n=3)
61−80 (n=15)
31−45 (n=3)
20−30 (n=2)
Age_category

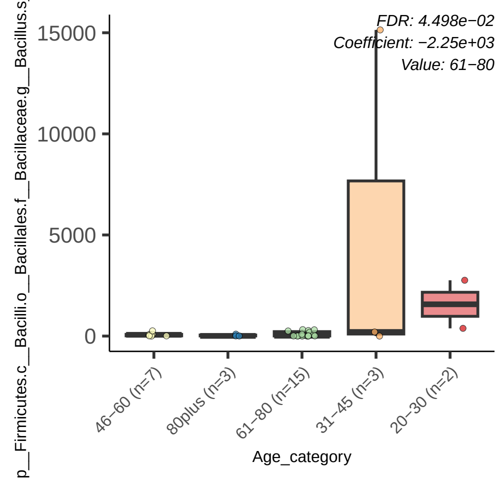

FDR: 4.498e−02
Coefficient: −2.25e+03
Value: 61−80
15000
10000
5000
0
p__Firmicutes.c__Bacilli.o__Bacillales.f__Bacillaceae.g__Bacillus.s_
46−60 (n=7)
80plus (n=3)
61−80 (n=15)
31−45 (n=3)
20−30 (n=2)
Age_category

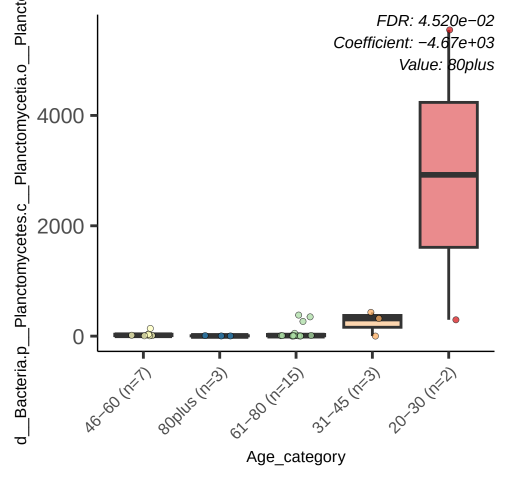

FDR: 4.520e−02
Coefficient: −4.67e+03
Value: 80plus
d__Bacteria.p__Planctomycetes.c__Planctomycetia.o__Plancto
4000
2000
0
46−60 (n=7)
80plus (n=3)
61−80 (n=15)
31−45 (n=3)
20−30 (n=2)
Age_category

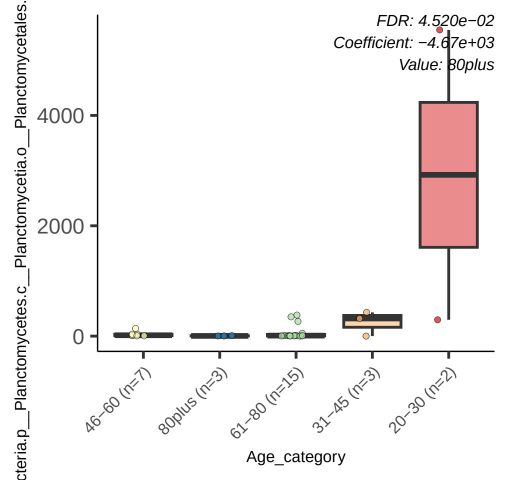

FDR: 4.520e−02
Coefficient: −4.67e+03
Value: 80plus
4000
2000
0
cteria.p__Planctomycetes.c__Planctomycetia.o__Planctomycetales.
46−60 (n=7)
80plus (n=3)
61−80 (n=15)
31−45 (n=3)
20−30 (n=2)
Age_category

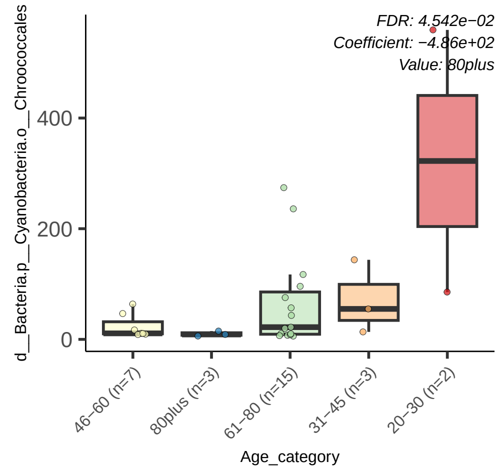

FDR: 4.542e−02
Coefficient: −4.86e+02
Value: 80plus
d__Bacteria.p__Cyanobacteria.o__Chroococcales
0
200
400
46−60 (n=7)
80plus (n=3)
61−80 (n=15)
31−45 (n=3)
20−30 (n=2)
Age_category

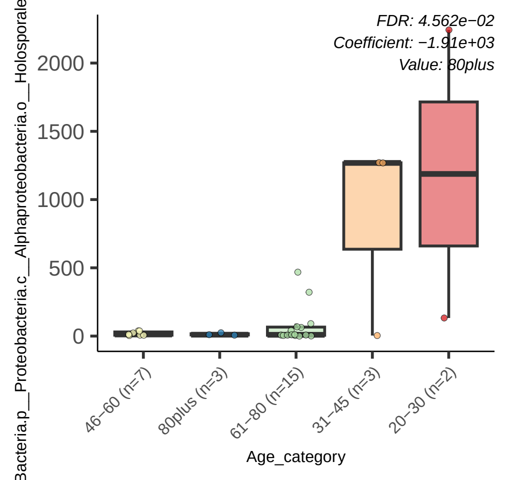

FDR: 4.562e−02
Coefficient: −1.91e+03
Value: 80plus
Bacteria.p__Proteobacteria.c__Alphaproteobacteria.o__Holosporale
2000
1500
1000
500
0
46−60 (n=7)
80plus (n=3)
61−80 (n=15)
31−45 (n=3)
20−30 (n=2)
Age_category

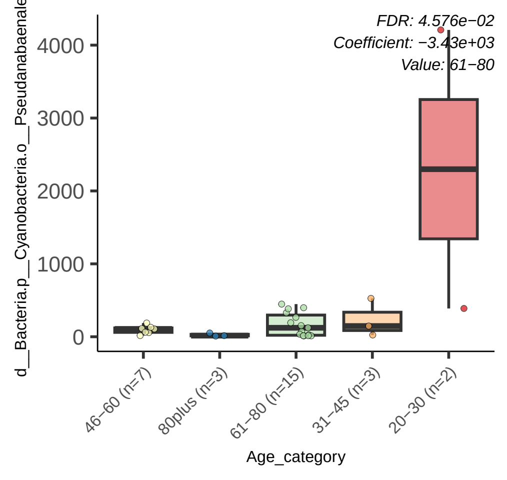

FDR: 4.576e−02
Coefficient: −3.43e+03
Value: 61−80
d__Bacteria.p__Cyanobacteria.o__Pseudanabaenale
4000
3000
2000
1000
0
46−60 (n=7)
80plus (n=3)
61−80 (n=15)
31−45 (n=3)
20−30 (n=2)
Age_category

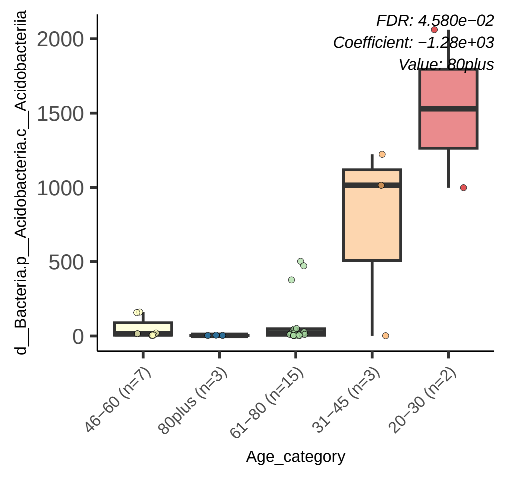

FDR: 4.580e−02
Coefficient: −1.28e+03
Value: 80plus
d__Bacteria.p__Acidobacteria.c__Acidobacteriia
2000
1500
1000
500
0
46−60 (n=7)
80plus (n=3)
61−80 (n=15)
31−45 (n=3)
20−30 (n=2)
Age_category

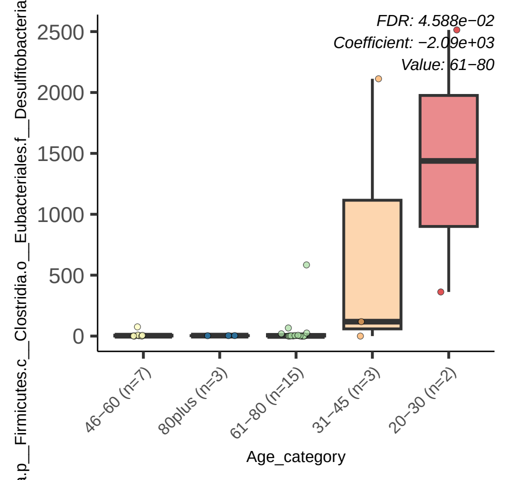
FDR: 4.588e−02
Coefficient: −2.09e+03
Value: 61−80
2500
2000
1500
1000
500
0
Firmicutes.c__Clostridia.o__Eubacteriales.f__Desulfitobacteria
46−60 (n=7)
80plus (n=3)
61−80 (n=15)
31−45 (n=3)
20−30 (n=2)
Age_category

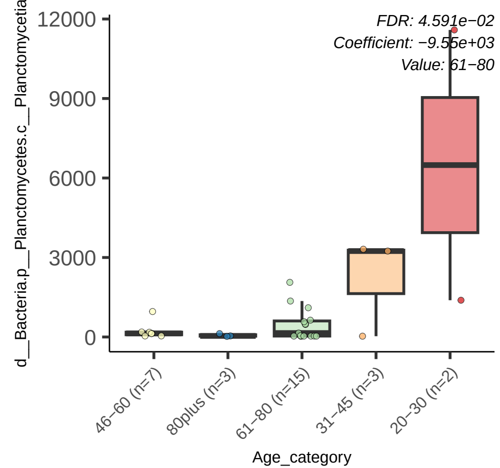

FDR: 4.591e−02
Coefficient: −9.55e+03
Value: 61−80
d__Bacteria.p__Planctomycetes.c__Planctomycetia
12000
9000
6000
3000
0
46−60 (n=7)
80plus (n=3)
61−80 (n=15)
31−45 (n=3)
20−30 (n=2)
Age_category

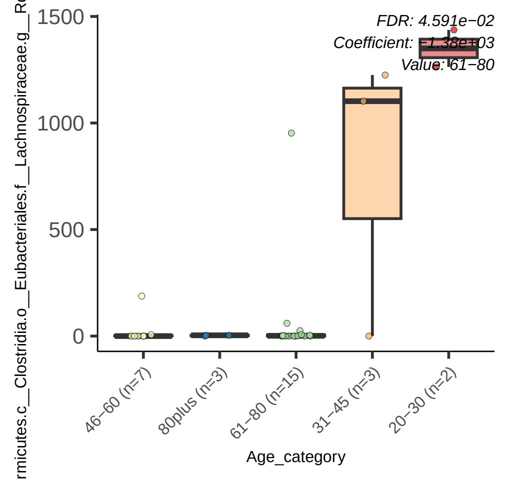
FDR: 4.591e−02
Coefficient: -1.38e+03
Value: 61−80
1500
1000
500
0
rmicutes.c__Clostridia.o__Eubacteriales.f__Lachnospiraceae.g__Ro
46−60 (n=7)
80plus (n=3)
61−80 (n=15)
31−45 (n=3)
20−30 (n=2)
Age_category

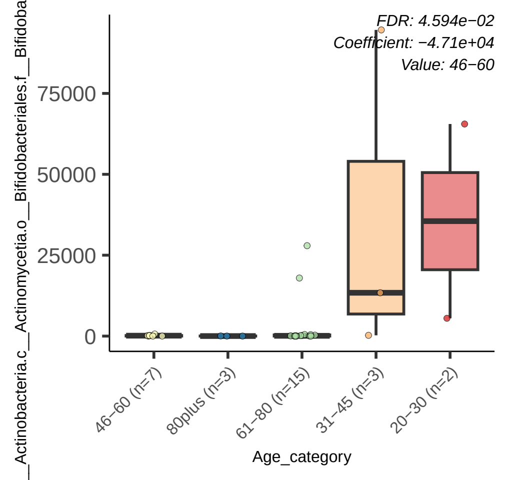
FDR: 4.594e−02
Coefficient: −4.71e+04
Value: 46−60
Actinobacteria.c__Actinomycetia.o__Bifidobacteriales.f__Bifidoba
75000
50000
25000
0
46−60 (n=7)
80plus (n=3)
61−80 (n=15)
31−45 (n=3)
20−30 (n=2)
Age_category

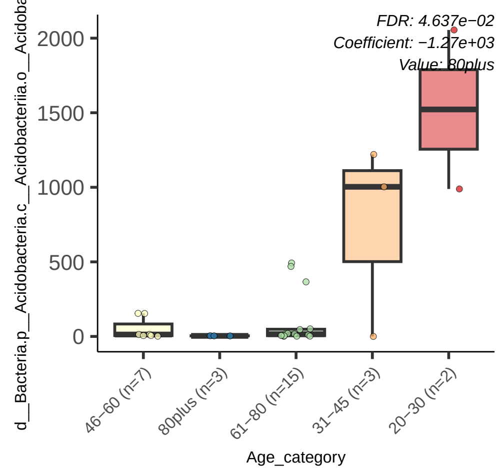

FDR: 4.637e−02
Coefficient: −1.27e+03
Value: 80plus
d__Bacteria.p__Acidobacteria.c__Acidobacteriia.o__Acidoba
2000
1500
1000
500
0
46−60 (n=7)
80plus (n=3)
61−80 (n=15)
31−45 (n=3)
20−30 (n=2)
Age_category

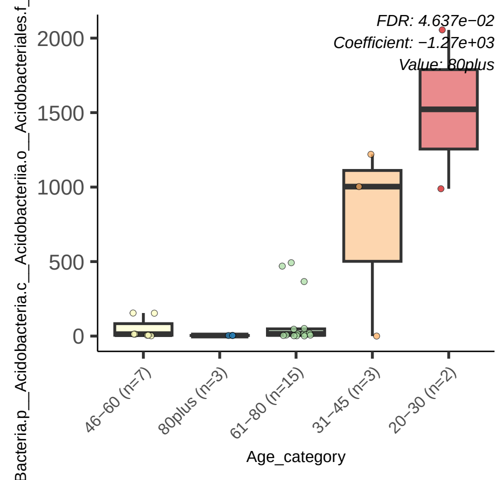

FDR: 4.637e−02
Coefficient: −1.27e+03
Value: 80plus
Bacteria.p__Acidobacteria.c__Acidobacteriia.o__Acidobacteriales.f__
2000
1500
1000
500
0
46−60 (n=7)
80plus (n=3)
61−80 (n=15)
31−45 (n=3)
20−30 (n=2)
Age_category

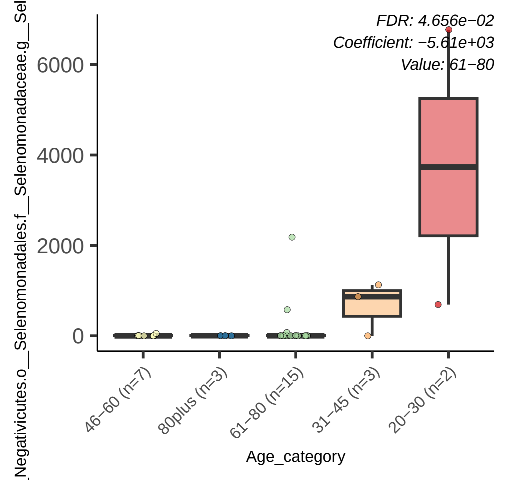

FDR: 4.656e−02
Coefficient: −5.61e+03
Value: 61−80
Negativicutes.o__Selenomonadales.f__Selenomonadaceae.g__Sel
6000
4000
2000
0
46−60 (n=7)
80plus (n=3)
61−80 (n=15)
31−45 (n=3)
20−30 (n=2)
Age_category

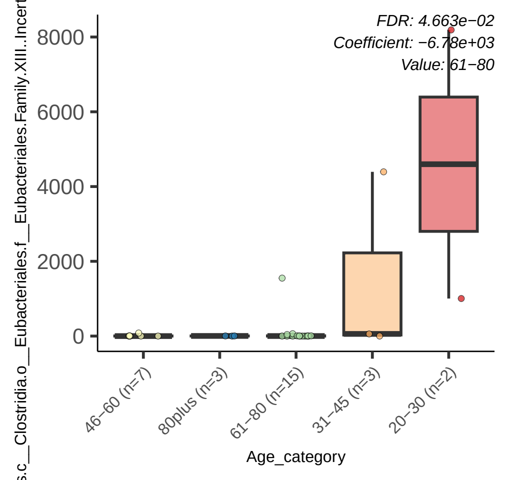

FDR: 4.663e−02
Coefficient: −6.78e+03
Value: 61−80
s.c__Clostridia.o__Eubacteriales.f__Eubacteriales.Family.XIII..Incert
8000
6000
4000
2000
0
46−60 (n=7)
80plus (n=3)
61−80 (n=15)
31−45 (n=3)
20−30 (n=2)
Age_category

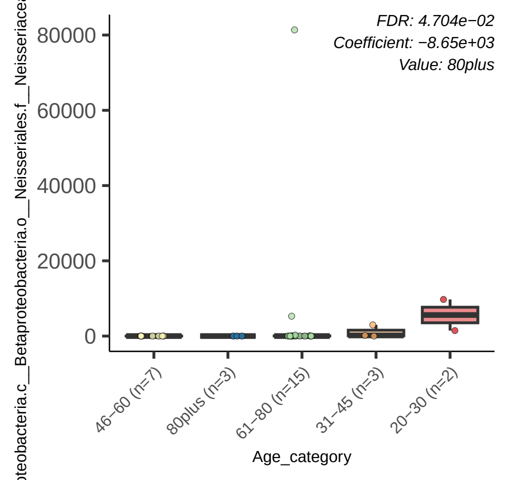

FDR: 4.704e−02
Coefficient: −8.65e+03
Value: 80plus
80000
60000
40000
20000
0
oteobacteria.c__Betaproteobacteria.o__Neisseriales.f__Neisseriace
46−60 (n=7)
80plus (n=3)
61−80 (n=15)
31−45 (n=3)
20−30 (n=2)
Age_category

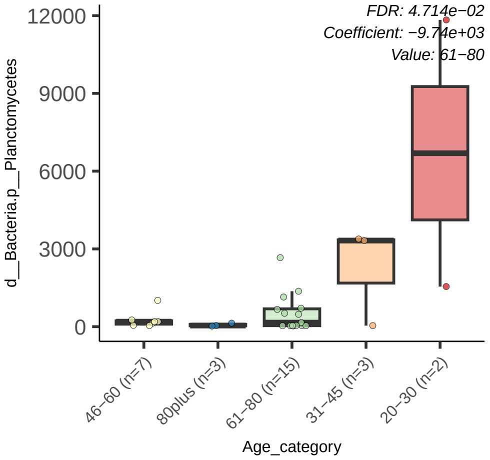

FDR: 4.714e−02
Coefficient: −9.74e+03
Value: 61−80
d__Bacteria.p__Planctomycetes
12000
9000
6000
3000
0
46−60 (n=7)
80plus (n=3)
61−80 (n=15)
31−45 (n=3)
20−30 (n=2)
Age_category

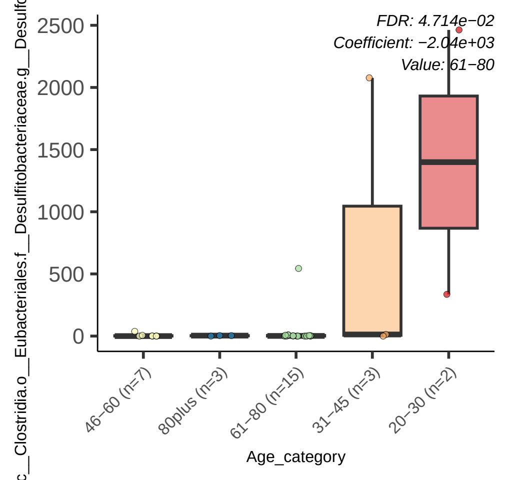

FDR: 4.714e−02
Coefficient: −2.04e+03
Value: 61−80
c__Clostridia.o__Eubacteriales.f__Desulfitobacteriaceae.g__Desulfo
2500
2000
1500
1000
500
0
46−60 (n=7)
80plus (n=3)
61−80 (n=15)
31−45 (n=3)
20−30 (n=2)
Age_category

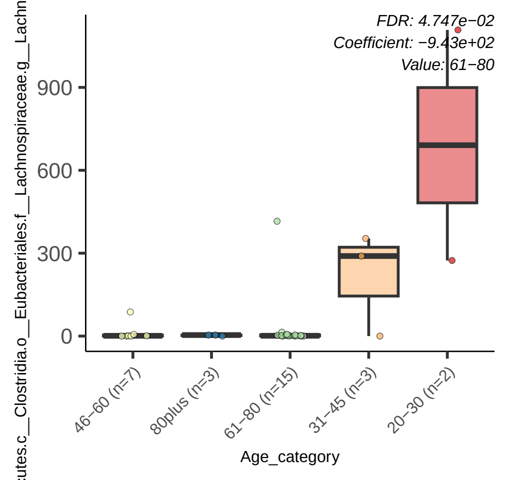
FDR: 4.747e−02
Coefficient: −9.43e+02
Value: 61−80
900
600
300
0
cutes.c__Clostridia.o__Eubacteriales.f__Lachnospiraceae.g__Lachn
46−60 (n=7)
80plus (n=3)
61−80 (n=15)
31−45 (n=3)
20−30 (n=2)
Age_category

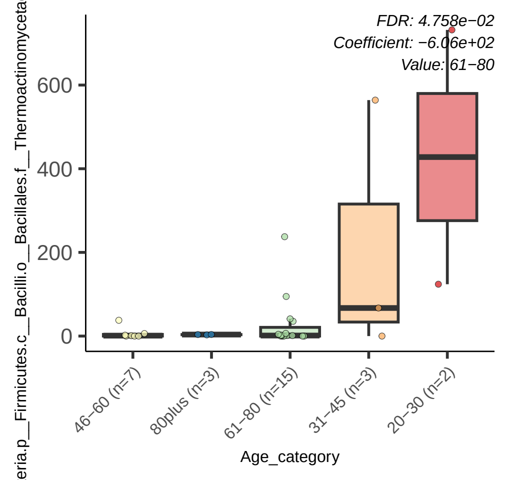

FDR: 4.758e−02
Coefficient: −6.06e+02
Value: 61−80
0
200
400
600
46−60 (n=7)
80plus (n=3)
61−80 (n=15)
31−45 (n=3)
20−30 (n=2)
Age_category
eria.p__Firmicutes.c__Bacilli.o__Bacillales.f__Thermoactinomyceta

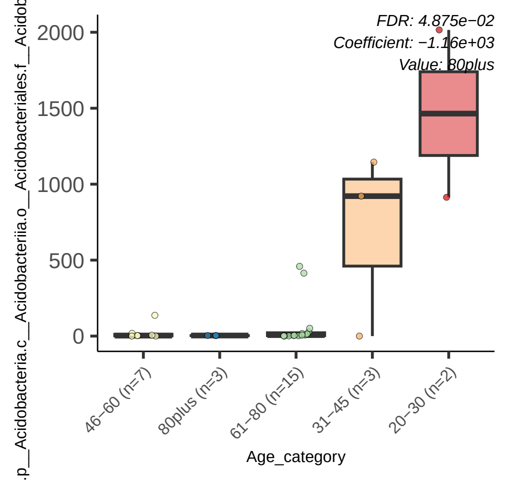

p__Acidobacteria.c__Acidobacteriia.o__Acidobacteriales.f__Acidob
FDR: 4.875e−02
Coefficient: −1.16e+03
Value: 80plus
2000
1500
1000
500
0
46−60 (n=7)
80plus (n=3)
61−80 (n=15)
31−45 (n=3)
20−30 (n=2)
Age_category

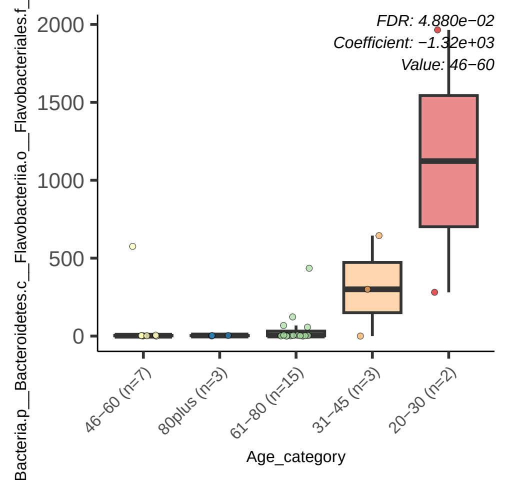

FDR: 4.880e−02
Coefficient: −1.32e+03
Value: 46−60
Bacteria.p__Bacteroidetes.c__Flavobacteriia.o__Flavobacteriales.f__
2000
1500
1000
500
0
46−60 (n=7)
80plus (n=3)
61−80 (n=15)
31−45 (n=3)
20−30 (n=2)
Age_category

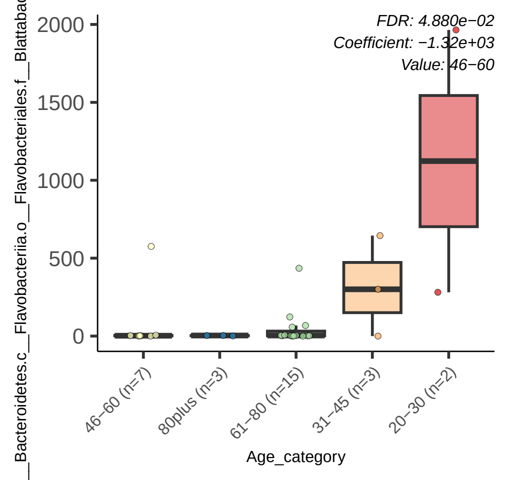

FDR: 4.880e−02
Coefficient: −1.32e+03
Value: 46−60
2000
1500
1000
500
0
Bacteroidetes.c__Flavobacteriia.o__Flavobacteriales.f__Blattabac
46−60 (n=7)
80plus (n=3)
61−80 (n=15)
31−45 (n=3)
20−30 (n=2)
Age_category

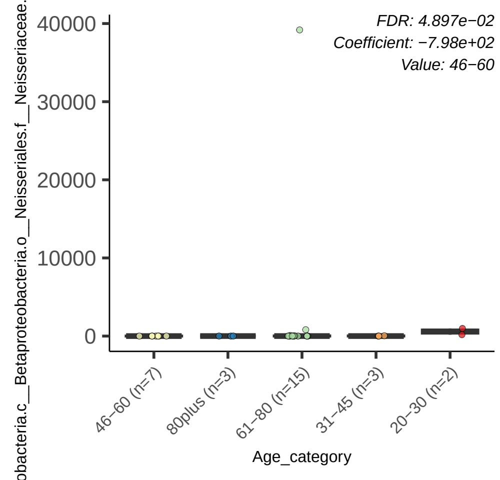

FDR: 4.897e−02
Coefficient: −7.98e+02
Value: 46−60
40000
30000
20000
10000
0
obacteria.c__Betaproteobacteria.o__Neisseriales.f__Neisseriaceae.
46−60 (n=7)
80plus (n=3)
61−80 (n=15)
31−45 (n=3)
20−30 (n=2)
Age_category

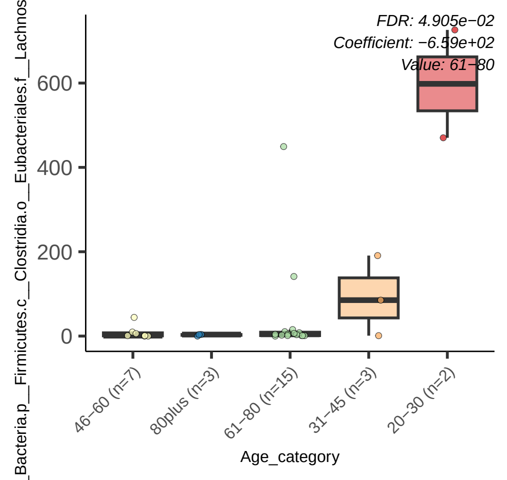

FDR: 4.905e−02
Coefficient: −6.59e+02
Value: 61−80
Bacteria.p__Firmicutes.c__Clostridia.o__Eubacteriales.f__Lachnos
600
400
200
0
46−60 (n=7)
80plus (n=3)
61−80 (n=15)
31−45 (n=3)
20−30 (n=2)
Age_category

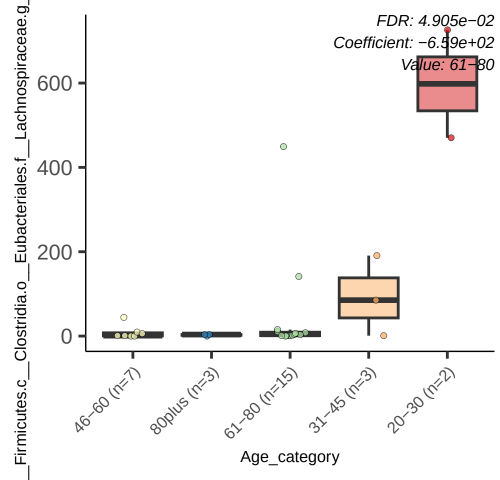
FDR: 4.905e−02
Coefficient: −6.59e+02
Value: 61−80
Firmicutes.c__Clostridia.o__Eubacteriales.f__Lachnospiraceae.g_
0
200
400
600
46−60 (n=7)
80plus (n=3)
61−80 (n=15)
31−45 (n=3)
20−30 (n=2)
Age_category

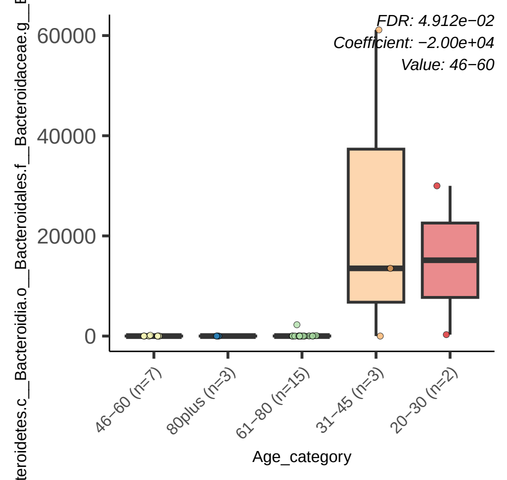
FDR: 4.912e−02
Coefficient: −2.00e+04
Value: 46−60
60000
40000
20000
0
teroidetes.c__Bacteroidia.o__Bacteroidales.f__Bacteroidaceae.g__B
46−60 (n=7)
80plus (n=3)
61−80 (n=15)
31−45 (n=3)
20−30 (n=2)
Age_category

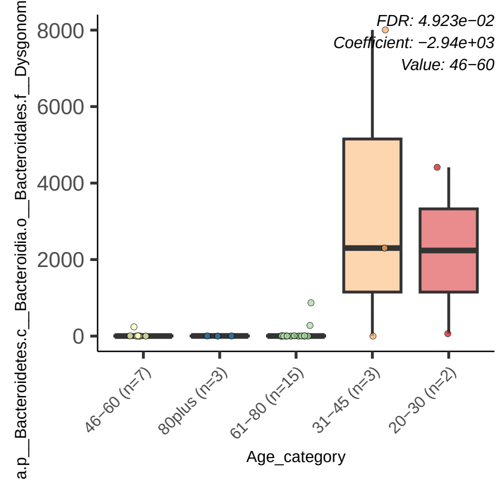
FDR: 4.923e−02
Coefficient: −2.94e+03
Value: 46−60
8000
6000
4000
2000
0
a.p__Bacteroidetes.c__Bacteroidia.o__Bacteroidales.f__Dysgonom
46−60 (n=7)
80plus (n=3)
61−80 (n=15)
31−45 (n=3)
20−30 (n=2)
Age_category

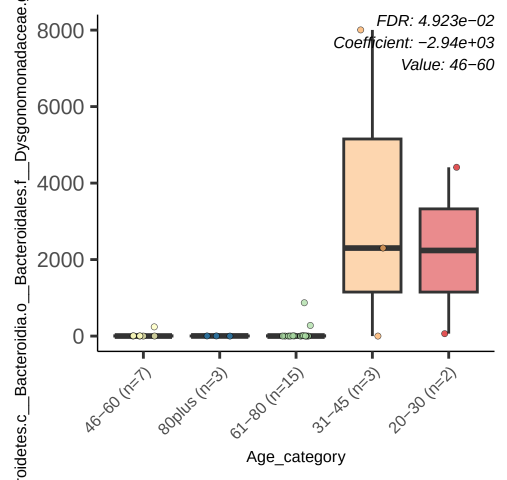

FDR: 4.923e−02
Coefficient: −2.94e+03
Value: 46−60
8000
6000
4000
2000
0
roidetes.c__Bacteroidia.o__Bacteroidales.f__Dysgonomonadaceae.g
46−60 (n=7)
80plus (n=3)
61−80 (n=15)
31−45 (n=3)
20−30 (n=2)
Age_category

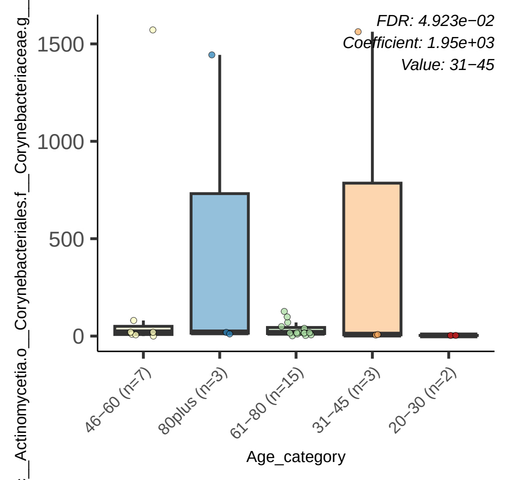

FDR: 4.923e−02
Coefficient: 1.95e+03
Value: 31−45
Actinomycetia.o__Corynebacteriales.f__Corynebacteriaceae.g__
1500
1000
500
0
46−60 (n=7)
80plus (n=3)
61−80 (n=15)
31−45 (n=3)
20−30 (n=2)
Age_category

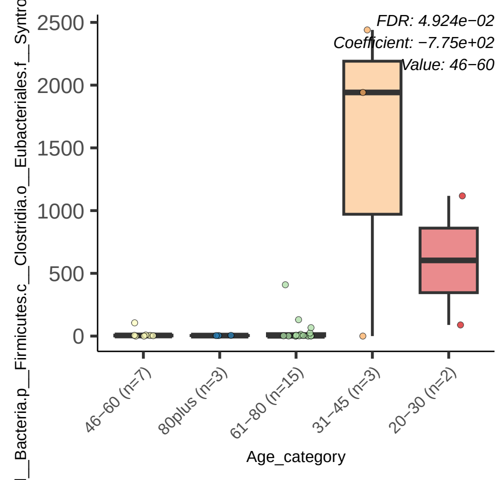

FDR: 4.924e−02
Coefficient: −7.75e+02
Value: 46−60
2500
2000
1500
1000
500
0
46−60 (n=7)
80plus (n=3)
61−80 (n=15)
31−45 (n=3)
20−30 (n=2)
Age_category
_Bacteria.p__Firmicutes.c__Clostridia.o__Eubacteriales.f__Syntro

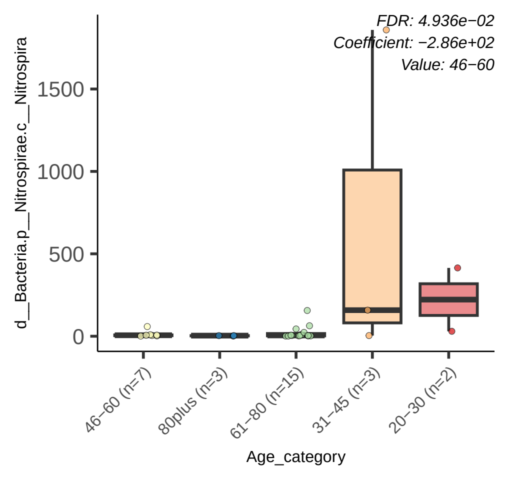
FDR: 4.936e−02
Coefficient: −2.86e+02
Value: 46−60
d__Bacteria.p__Nitrospirae.c__Nitrospira
1500
1000
500
0
46−60 (n=7)
80plus (n=3)
61−80 (n=15)
31−45 (n=3)
20−30 (n=2)
Age_category

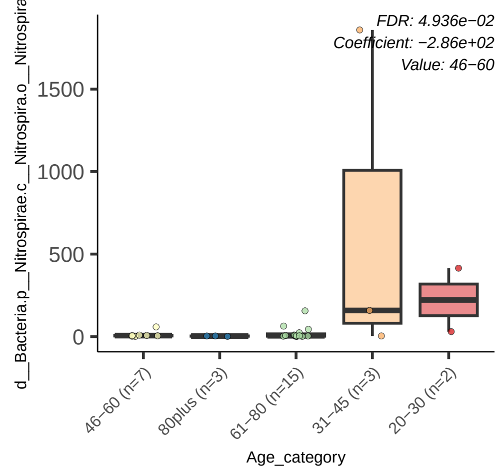

FDR: 4.936e−02
Coefficient: −2.86e+02
Value: 46−60
d__Bacteria.p__Nitrospirae.c__Nitrospira.o__Nitrospira
0
500
1000
1500
46−60 (n=7)
80plus (n=3)
61−80 (n=15)
31−45 (n=3)
20−30 (n=2)
Age_category

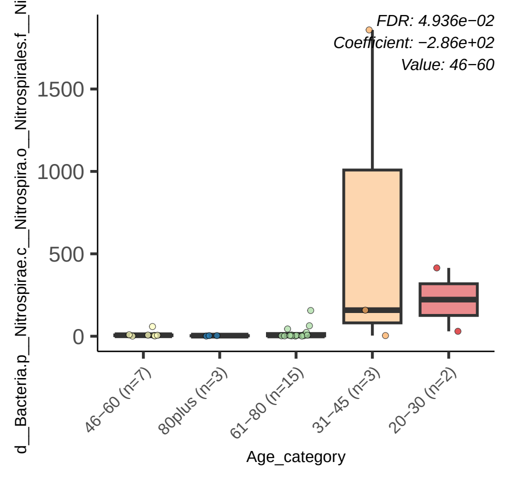
FDR: 4.936e−02
Coefficient: −2.86e+02
Value: 46−60
1500
1000
500
0
d__Bacteria.p__Nitrospirae.c__Nitrospira.o__Nitrospirales.f__Ni
46−60 (n=7)
80plus (n=3)
61−80 (n=15)
31−45 (n=3)
20−30 (n=2)
Age_category

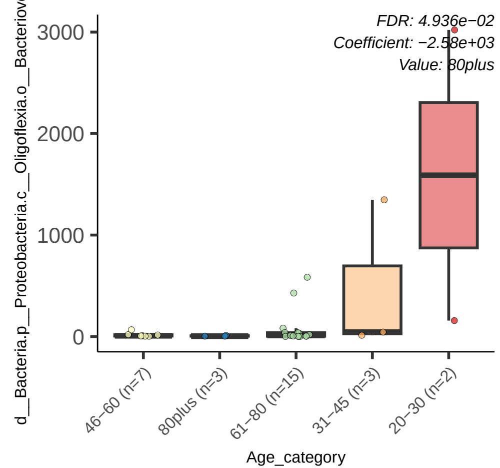

FDR: 4.936e−02
Coefficient: −2.58e+03
Value: 80plus
3000
2000
1000
0
d__Bacteria.p__Proteobacteria.c__Oligoflexia.o__Bacteriov
46−60 (n=7)
80plus (n=3)
61−80 (n=15)
31−45 (n=3)
20−30 (n=2)
Age_category

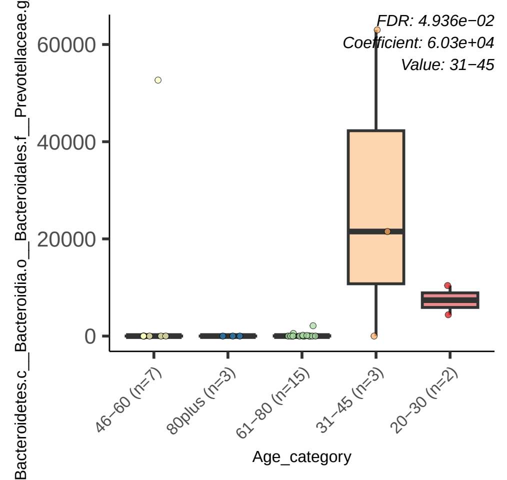

FDR: 4.936e−02
Coefficient: 6.03e+04
Value: 31−45
60000
40000
20000
0
Bacteroidetes.c__Bacteroidia.o__Bacteroidales.f__Prevotellaceae.g
46−60 (n=7)
80plus (n=3)
61−80 (n=15)
31−45 (n=3)
20−30 (n=2)
Age_category

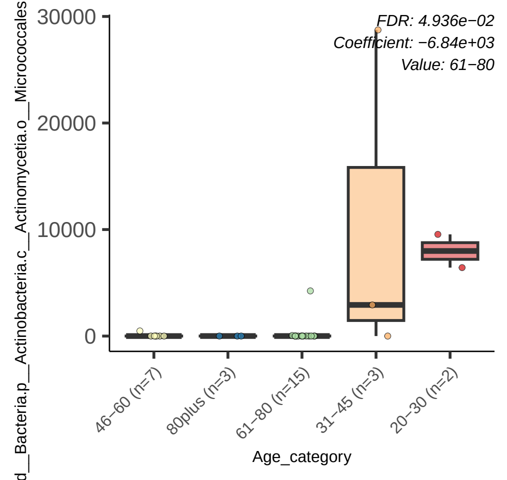

FDR: 4.936e−02
Coefficient: −6.84e+03
Value: 61−80
30000
20000
10000
0
d__Bacteria.p__Actinobacteria.c__Actinomycetia.o__Micrococcales
46−60 (n=7)
80plus (n=3)
61−80 (n=15)
31−45 (n=3)
20−30 (n=2)
Age_category

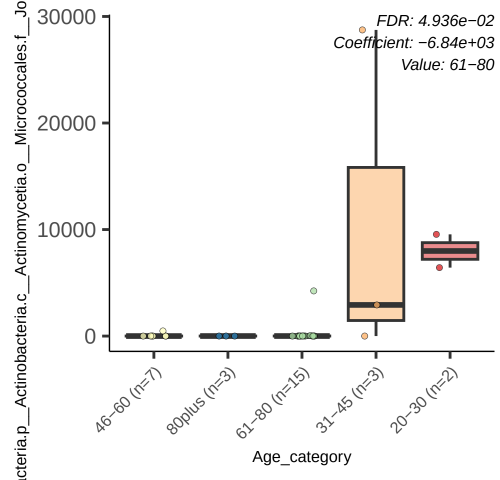

FDR: 4.936e−02
Coefficient: −6.84e+03
Value: 61−80
30000
20000
10000
0
46−60 (n=7)
80plus (n=3)
61−80 (n=15)
31−45 (n=3)
20−30 (n=2)
Age_category
cteria.p__Actinobacteria.c__Actinomycetia.o__Micrococcales.f__Jo

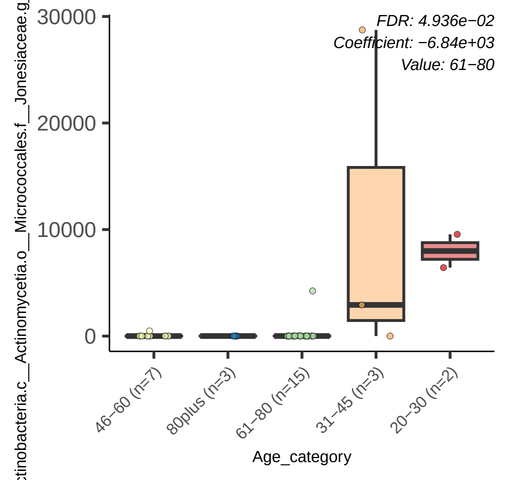

FDR: 4.936e−02
Coefficient: −6.84e+03
Value: 61−80
30000
20000
10000
0
Actinobacteria.c__Actinomycetia.o__Micrococcales.f__Jonesiaceae.g
46−60 (n=7)
80plus (n=3)
61−80 (n=15)
31−45 (n=3)
20−30 (n=2)
Age_category

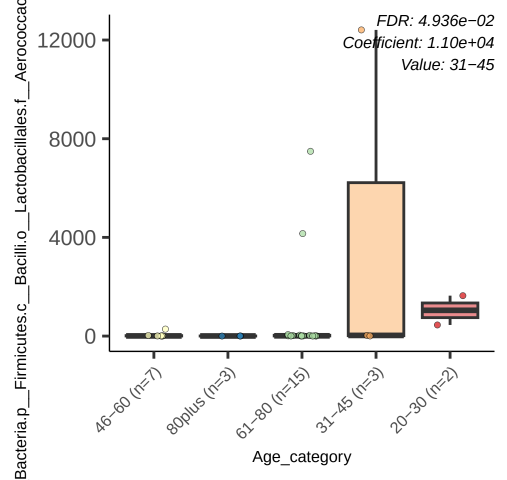

FDR: 4.936e−02
Coefficient: 1.10e+04
Value: 31−45
Bacteria.p__Firmicutes.c__Bacilli.o__Lactobacillales.f__Aerococcac
12000
8000
4000
0
46−60 (n=7)
80plus (n=3)
61−80 (n=15)
31−45 (n=3)
20−30 (n=2)
Age_category

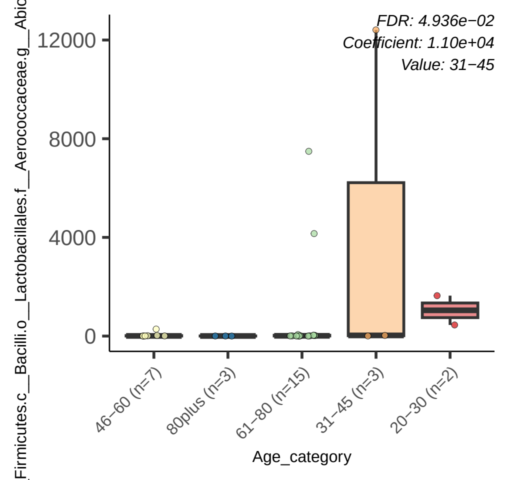

FDR: 4.936e−02
Coefficient: 1.10e+04
Value: 31−45
12000
8000
4000
0
Firmicutes.c__Bacilli.o__Lactobacillales.f__Aerococcaceae.g__Abio
46−60 (n=7)
80plus (n=3)
61−80 (n=15)
31−45 (n=3)
20−30 (n=2)
Age_category

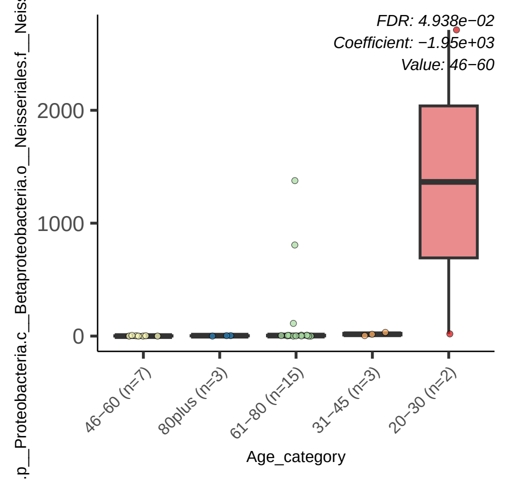

FDR: 4.938e−02
Coefficient: −1.95e+03
Value: 46−60
p__Proteobacteria.c__Betaproteobacteria.o__Neisseriales.f__Neis
2000
1000
0
46−60 (n=7)
80plus (n=3)
61−80 (n=15)
31−45 (n=3)
20−30 (n=2)
Age_category

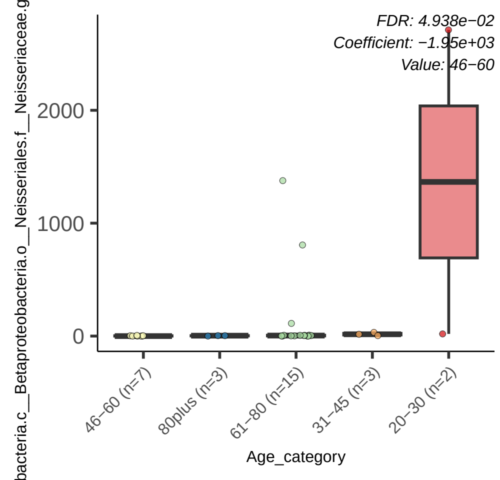

FDR: 4.938e−02
Coefficient: −1.95e+03
Value: 46−60
bacteria.c__Betaproteobacteria.o__Neisseriales.f__Neisseriaceae.g
2000
1000
0
46−60 (n=7)
80plus (n=3)
61−80 (n=15)
31−45 (n=3)
20−30 (n=2)
Age_category

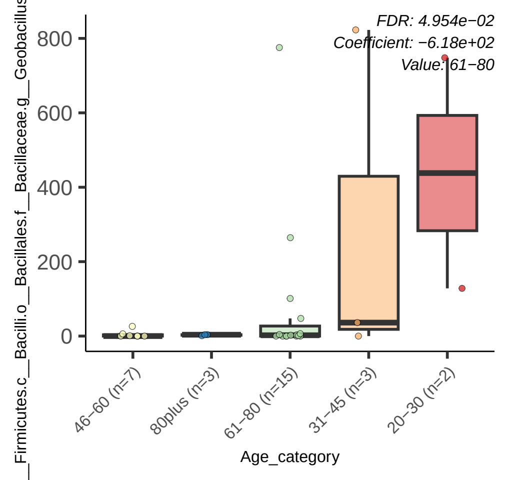

FDR: 4.954e−02
Coefficient: −6.18e+02
Value: 61−80
Firmicutes.c__Bacilli.o__Bacillales.f__Bacillaceae.g__Geobacillus
800
600
400
200
0
46−60 (n=7)
80plus (n=3)
61−80 (n=15)
31−45 (n=3)
20−30 (n=2)
Age_category

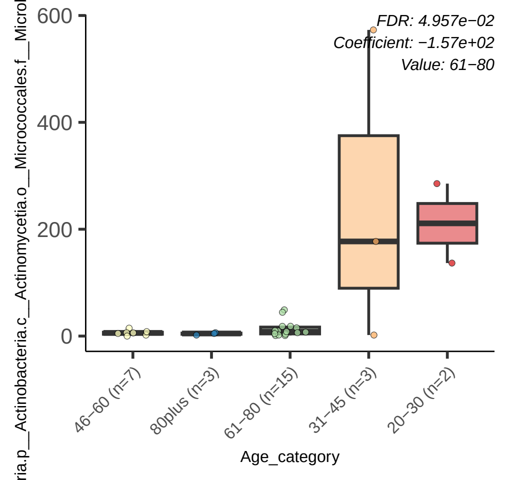
FDR: 4.957e−02
Coefficient: −1.57e+02
Value: 61−80
600
400
200
0
ria.p__Actinobacteria.c__Actinomycetia.o__Micrococcales.f__Microl
46−60 (n=7)
80plus (n=3)
61−80 (n=15)
31−45 (n=3)
20−30 (n=2)
Age_category

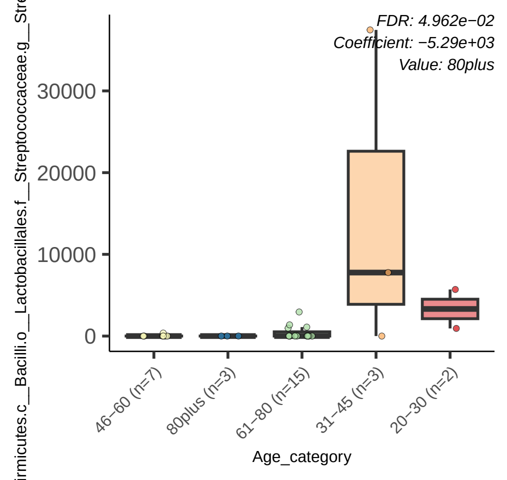

FDR: 4.962e−02
Coefficient: −5.29e+03
Value: 80plus
30000
20000
10000
0
46−60 (n=7)
80plus (n=3)
61−80 (n=15)
31−45 (n=3)
20−30 (n=2)
Age_category
irmicutes.c__Bacilli.o__Lactobacillales.f__Streptococcaceae.g__Stre

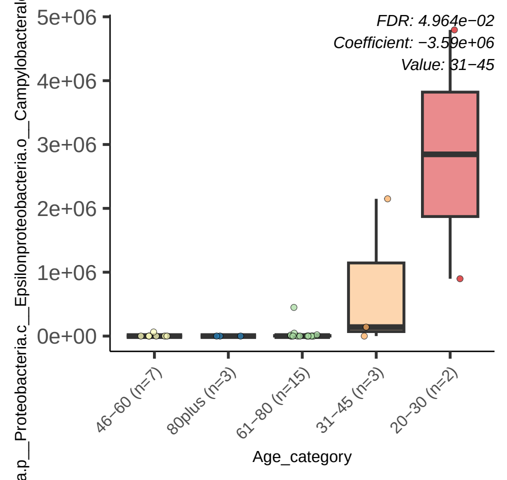

FDR: 4.964e−02
Coefficient: −3.59e+06
Value: 31−45
5e+06
4e+06
3e+06
2e+06
1e+06
0e+00
a.p__Proteobacteria.c__Epsilonproteobacteria.o__Campylobacterale
46−60 (n=7)
80plus (n=3)
61−80 (n=15)
31−45 (n=3)
20−30 (n=2)
Age_category

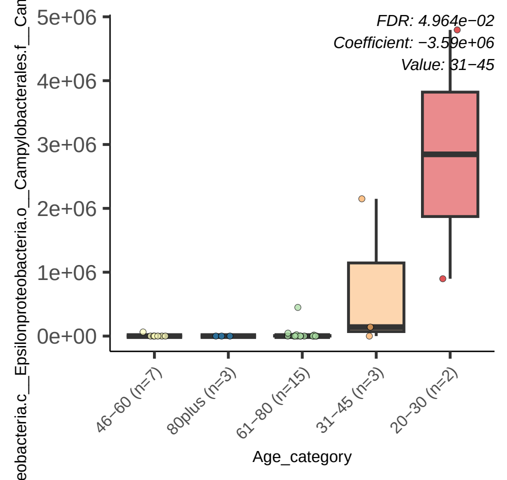

FDR: 4.964e−02
Coefficient: −3.59e+06
Value: 31−45
5e+06
4e+06
3e+06
2e+06
1e+06
0e+00
eobacteria.c__Epsilonproteobacteria.o__Campylobacterales.f__Cam
46−60 (n=7)
80plus (n=3)
61−80 (n=15)
31−45 (n=3)
20−30 (n=2)
Age_category

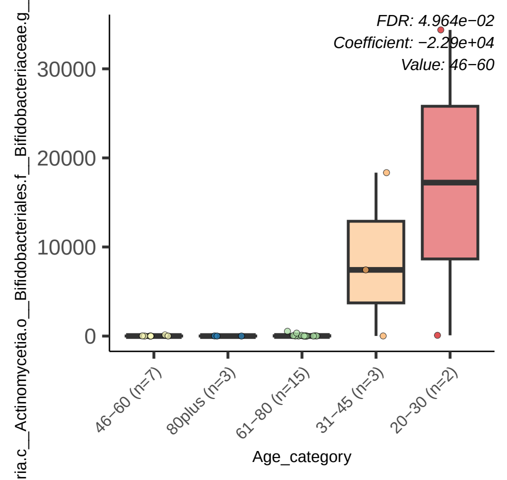

FDR: 4.964e−02
Coefficient: −2.29e+04
Value: 46−60
ria.c__Actinomycetia.o__Bifidobacteriales.f__Bifidobacteriaceae.g__
30000
20000
10000
0
46−60 (n=7)
80plus (n=3)
61−80 (n=15)
31−45 (n=3)
20−30 (n=2)
Age_category

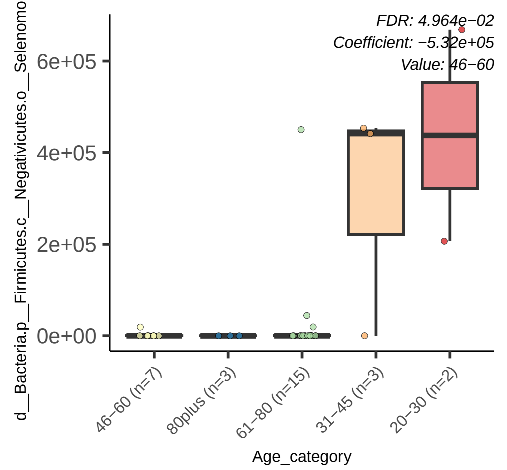

FDR: 4.964e−02
Coefficient: −5.32e+05
Value: 46−60
d__Bacteria.p__Firmicutes.c__Negativicutes.o__Selenomo
6e+05
4e+05
2e+05
0e+00
46−60 (n=7)
80plus (n=3)
61−80 (n=15)
31−45 (n=3)
20−30 (n=2)
Age_category

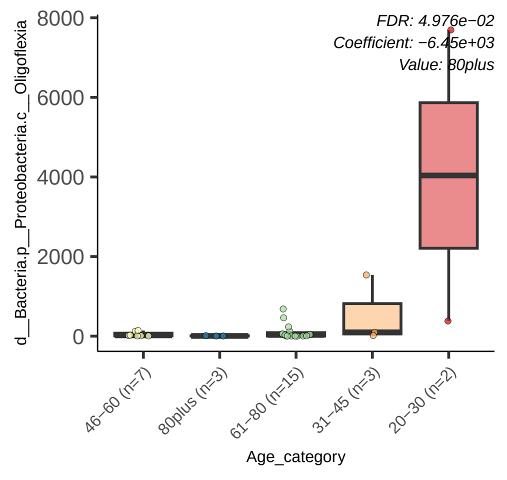

FDR: 4.976e−02
Coefficient: −6.45e+03
Value: 80plus
d__Bacteria.p__Proteobacteria.c__Oligoflexia
8000
6000
4000
2000
0
46−60 (n=7)
80plus (n=3)
61−80 (n=15)
31−45 (n=3)
20−30 (n=2)
Age_category

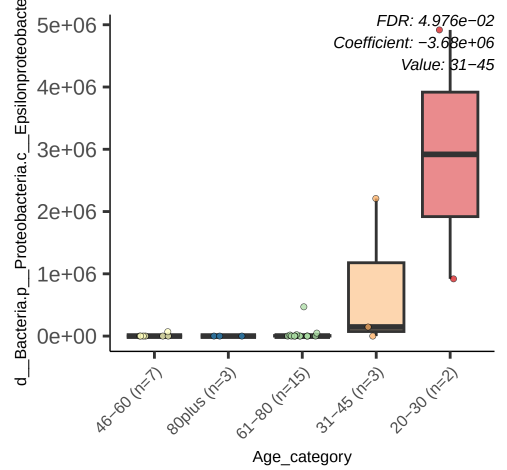

FDR: 4.976e−02
Coefficient: −3.68e+06
Value: 31−45
d__Bacteria.p__Proteobacteria.c__Epsilonproteobacte
5e+06
4e+06
3e+06
2e+06
1e+06
0e+00
46−60 (n=7)
80plus (n=3)
61−80 (n=15)
31−45 (n=3)
20−30 (n=2)
Age_category

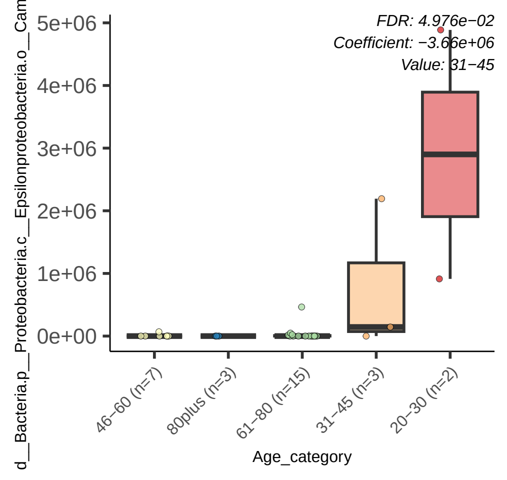

FDR: 4.976e−02
Coefficient: −3.66e+06
Value: 31−45
d__Bacteria.p__Proteobacteria.c__Epsilonproteobacteria.o__Cam
5e+06
4e+06
3e+06
2e+06
1e+06
0e+00
46−60 (n=7)
80plus (n=3)
61−80 (n=15)
31−45 (n=3)
20−30 (n=2)
Age_category

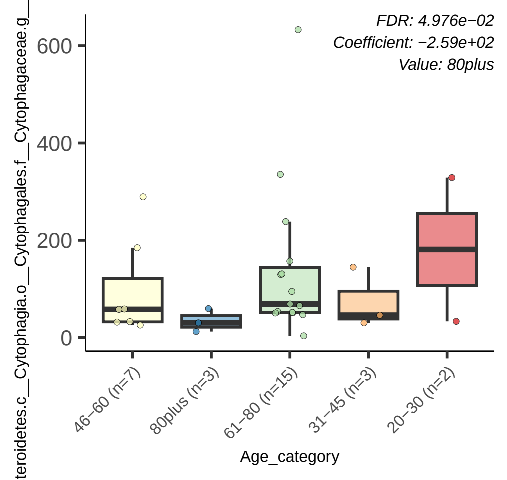
FDR: 4.976e−02
Coefficient: −2.59e+02
Value: 80plus
0
200
400
600
teroidetes.c__Cytophagia.o__Cytophagales.f__Cytophagaceae.g__
46−60 (n=7)
80plus (n=3)
61−80 (n=15)
31−45 (n=3)
20−30 (n=2)
Age_category

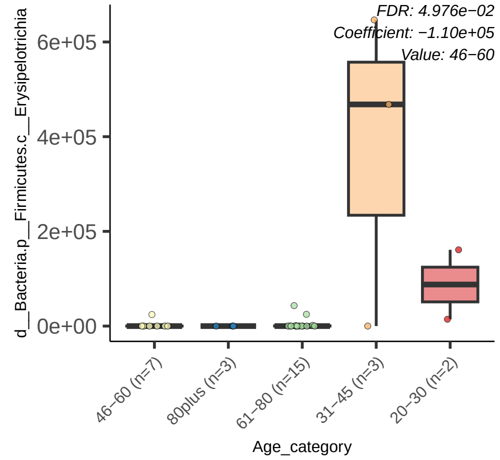

FDR: 4.976e−02
Coefficient: −1.10e+05
Value: 46−60
d__Bacteria.p__Firmicutes.c__Erysipelotrichia
6e+05
4e+05
2e+05
0e+00
46−60 (n=7)
80plus (n=3)
61−80 (n=15)
31−45 (n=3)
20−30 (n=2)
Age_category

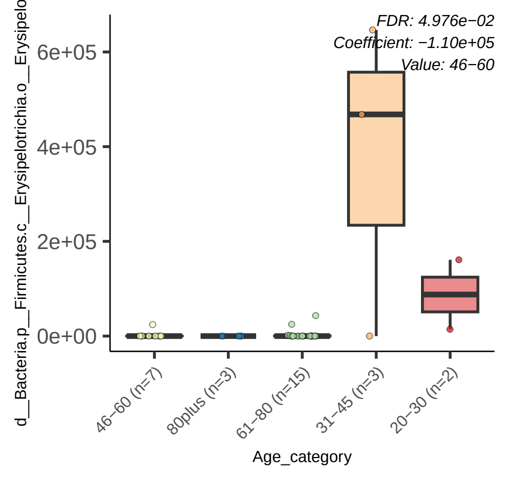

FDR: 4.976e−02
Coefficient: −1.10e+05
Value: 46−60
d__Bacteria.p__Firmicutes.c__Erysipelotrichia.o__Erysipelo
6e+05
4e+05
2e+05
0e+00
46−60 (n=7)
80plus (n=3)
61−80 (n=15)
31−45 (n=3)
20−30 (n=2)
Age_category

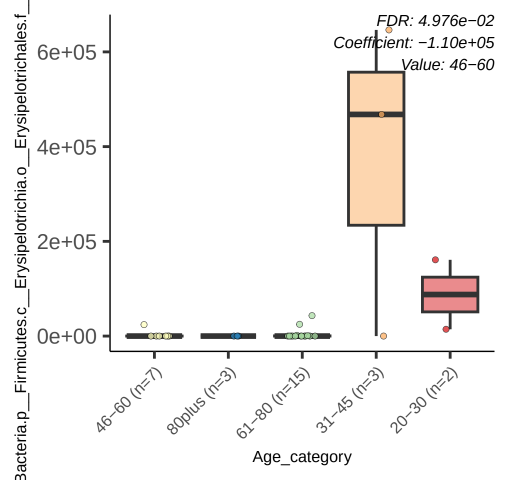
FDR: 4.976e−02
Coefficient: −1.10e+05
Value: 46−60
Bacteria.p__Firmicutes.c__Erysipelotrichia.o__Erysipelotrichales.f__
6e+05
4e+05
2e+05
0e+00
46−60 (n=7)
80plus (n=3)
61−80 (n=15)
31−45 (n=3)
20−30 (n=2)
Age_category

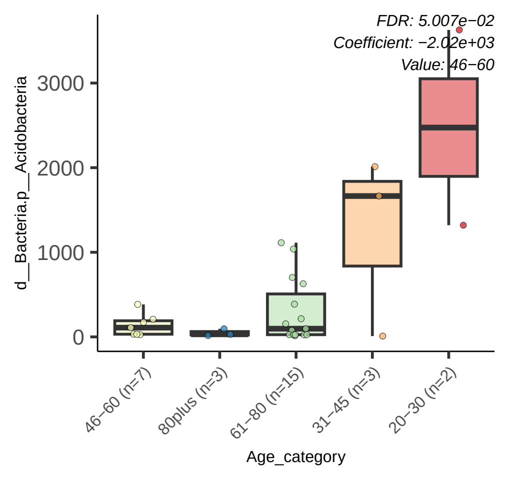

FDR: 5.007e−02
Coefficient: −2.02e+03
Value: 46−60
d__Bacteria.p__Acidobacteria
3000
2000
1000
0
46−60 (n=7)
80plus (n=3)
61−80 (n=15)
31−45 (n=3)
20−30 (n=2)
Age_category

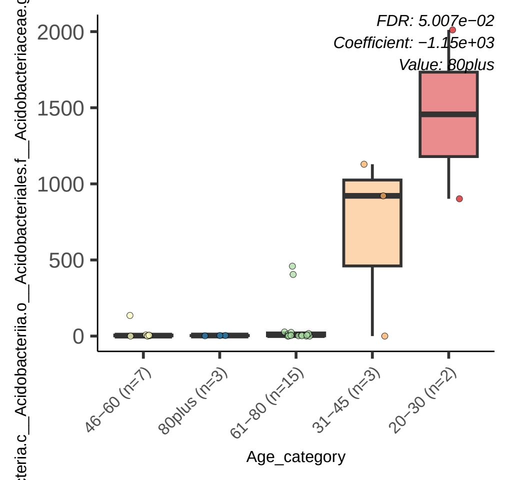
FDR: 5.007e−02
Coefficient: −1.15e+03
Value: 80plus
teria.c__Acidobacteriia.o__Acidobacteriales.f__Acidobacteriaceae.g
2000
1500
1000
500
0
46−60 (n=7)
80plus (n=3)
61−80 (n=15)
31−45 (n=3)
20−30 (n=2)
Age_category

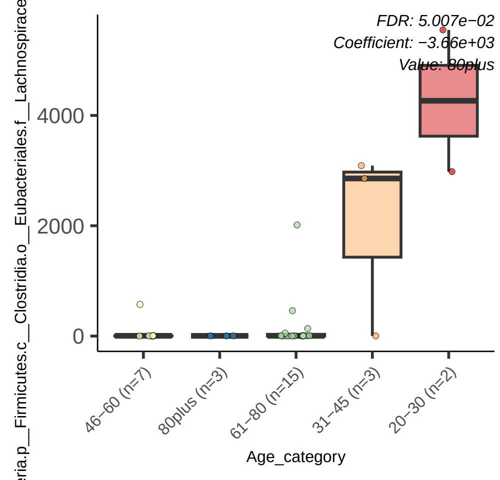

FDR: 5.007e−02
Coefficient: −3.66e+03
Value: 80plus
4000
2000
0
46−60 (n=7)
80plus (n=3)
61−80 (n=15)
31−45 (n=3)
20−30 (n=2)
Age_category
eria.p__Firmicutes.c__Clostridia.o__Eubacteriales.f__Lachnospirace

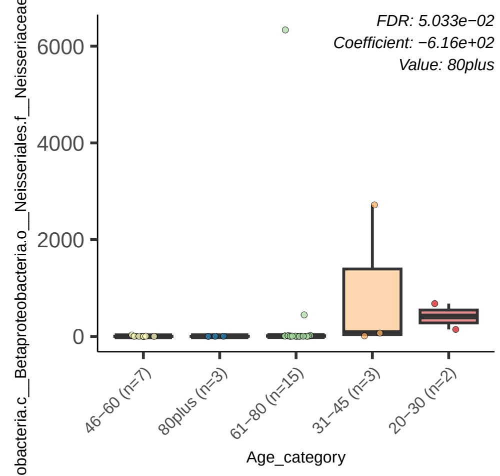

FDR: 5.033e−02
Coefficient: −6.16e+02
Value: 80plus
6000
4000
2000
0
eobacteria.c__Betaproteobacteria.o__Neisseriales.f__Neisseriaceae
46−60 (n=7)
80plus (n=3)
61−80 (n=15)
31−45 (n=3)
20−30 (n=2)
Age_category

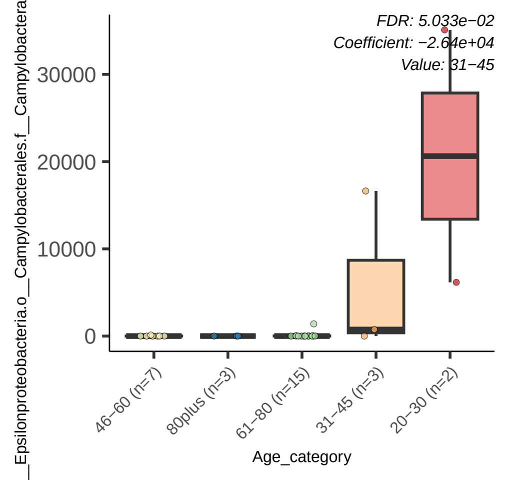

FDR: 5.033e−02
Coefficient: −2.64e+04
Value: 31−45
30000
20000
10000
0
_Epsilonproteobacteria.o__Campylobacterales.f__Campylobactera
46−60 (n=7)
80plus (n=3)
61−80 (n=15)
31−45 (n=3)
20−30 (n=2)
Age_category

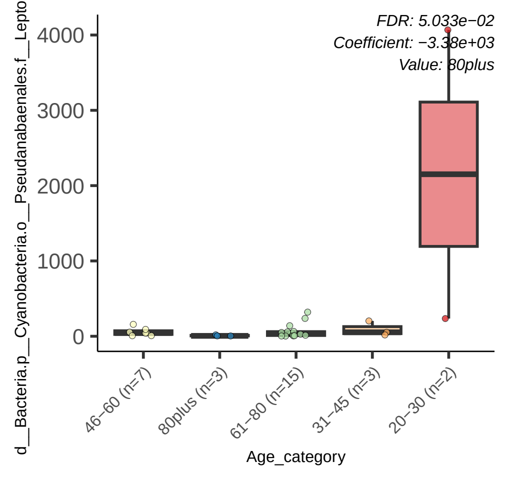

FDR: 5.033e−02
Coefficient: −3.38e+03
Value: 80plus
d__Bacteria.p__Cyanobacteria.o__Pseudanabaenales.f__Lepto
4000
3000
2000
1000
0
46−60 (n=7)
80plus (n=3)
61−80 (n=15)
31−45 (n=3)
20−30 (n=2)
Age_category

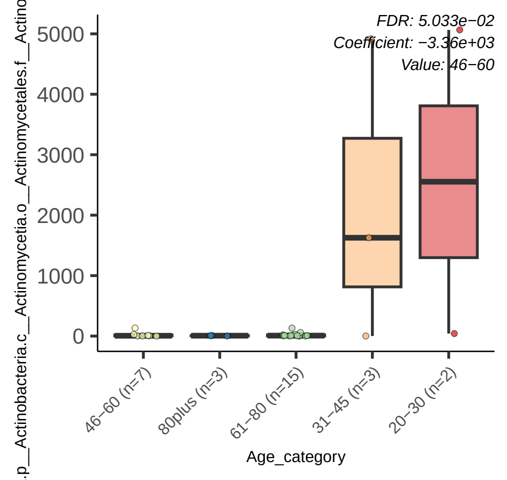

FDR: 5.033e−02
Coefficient: −3.36e+03
Value: 46−60
.p__Actinobacteria.c__Actinomycetia.o__Actinomycetales.f__Actino
5000
4000
3000
2000
1000
0
46−60 (n=7)
80plus (n=3)
61−80 (n=15)
31−45 (n=3)
20−30 (n=2)
Age_category

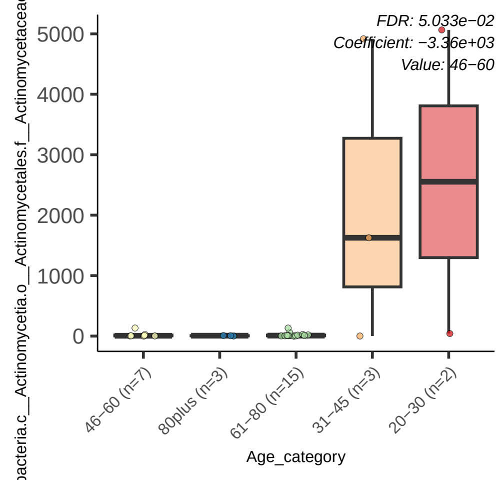

FDR: 5.033e−02
Coefficient: −3.36e+03
Value: 46−60
5000
4000
3000
2000
1000
0
bacteria.c__Actinomycetia.o__Actinomycetales.f__Actinomycetaceae
46−60 (n=7)
80plus (n=3)
61−80 (n=15)
31−45 (n=3)
20−30 (n=2)
Age_category

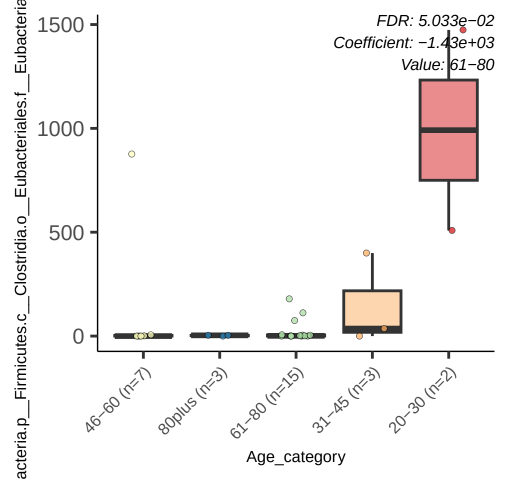

FDR: 5.033e−02
Coefficient: −1.43e+03
Value: 61−80
1500
1000
500
0
acteria.p__Firmicutes.c__Clostridia.o__Eubacteriales.f__Eubacteria
46−60 (n=7)
80plus (n=3)
61−80 (n=15)
31−45 (n=3)
20−30 (n=2)
Age_category

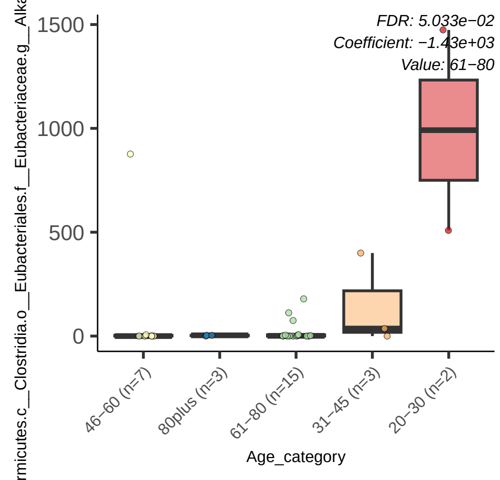

FDR: 5.033e−02
Coefficient: −1.43e+03
Value: 61−80
rmicutes.c__Clostridia.o__Eubacteriales.f__Eubacteriaceae.g__Alka
1500
1000
500
0
46−60 (n=7)
80plus (n=3)
61−80 (n=15)
31−45 (n=3)
20−30 (n=2)
Age_category

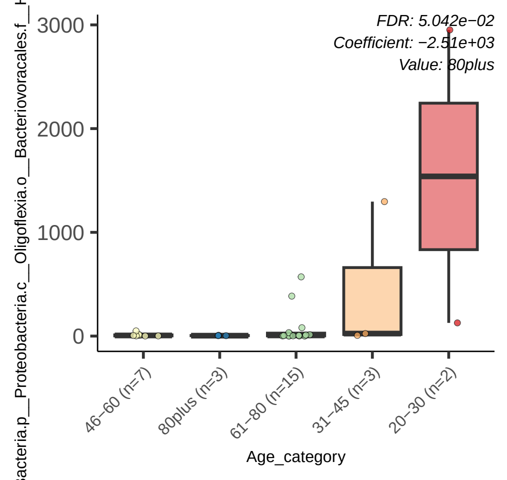

FDR: 5.042e−02
Coefficient: −2.51e+03
Value: 80plus
acteria.p__Proteobacteria.c__Oligoflexia.o__Bacteriovoracales.f__H
3000
2000
1000
0
46−60 (n=7)
80plus (n=3)
61−80 (n=15)
31−45 (n=3)
20−30 (n=2)
Age_category

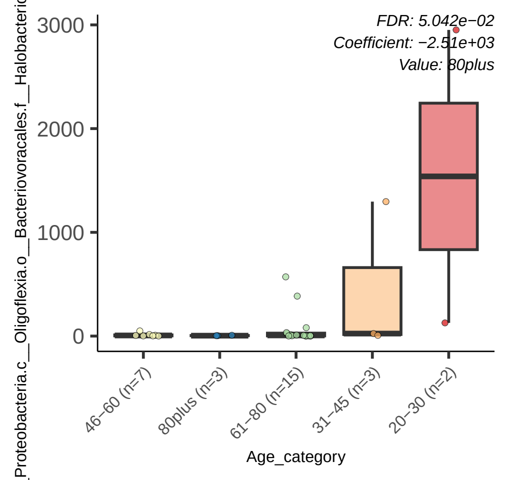

FDR: 5.042e−02
Coefficient: −2.51e+03
Value: 80plus
Proteobacteria.c__Oligoflexia.o__Bacteriovoracales.f__Halobacterio
3000
2000
1000
0
46−60 (n=7)
80plus (n=3)
61−80 (n=15)
31−45 (n=3)
20−30 (n=2)
Age_category

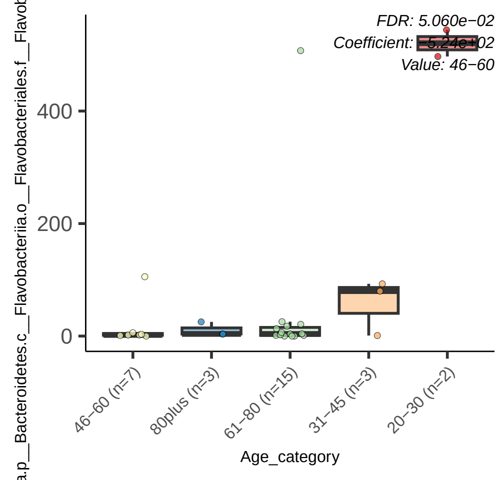

FDR: 5.060e−02
Coefficient: −5.24e+02
Value: 46−60
400
200
0
a.p__Bacteroidetes.c__Flavobacteriia.o__Flavobacteriales.f__Flavob
46−60 (n=7)
80plus (n=3)
61−80 (n=15)
31−45 (n=3)
20−30 (n=2)
Age_category

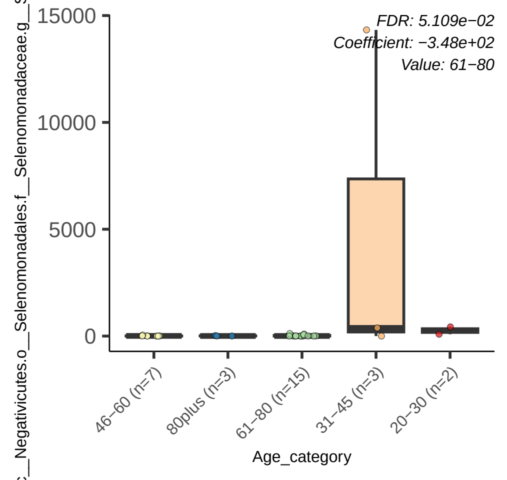

FDR: 5.109e−02
Coefficient: −3.48e+02
Value: 61−80
15000
10000
5000
0
c__Negativicutes.o__Selenomonadales.f__Selenomonadaceae.g__
46−60 (n=7)
80plus (n=3)
61−80 (n=15)
31−45 (n=3)
20−30 (n=2)
Age_category

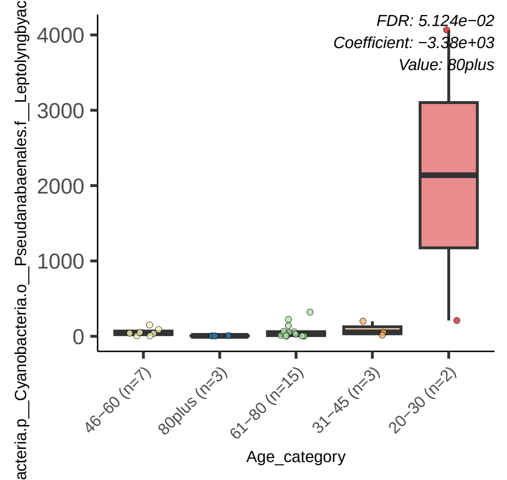
FDR: 5.124e−02
Coefficient: −3.38e+03
Value: 80plus
acteria.p__Cyanobacteria.o__Pseudanabaenales.f__Leptolyngbyac
4000
3000
2000
1000
0
46−60 (n=7)
80plus (n=3)
61−80 (n=15)
31−45 (n=3)
20−30 (n=2)
Age_category

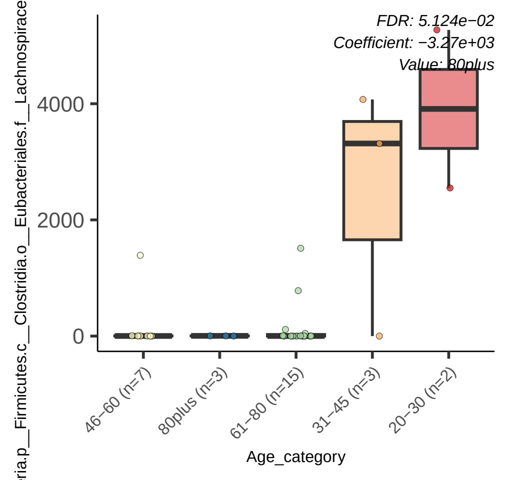

FDR: 5.124e−02
Coefficient: −3.27e+03
Value: 80plus
ria.p__Firmicutes.c__Clostridia.o__Eubacteriales.f__Lachnospirace
4000
2000
0
46−60 (n=7)
80plus (n=3)
61−80 (n=15)
31−45 (n=3)
20−30 (n=2)
Age_category

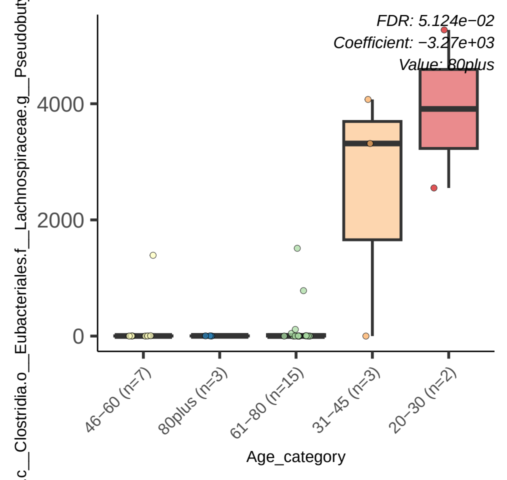
FDR: 5.124e−02
Coefficient: −3.27e+03
Value: 80plus
c__Clostridia.o__Eubacteriales.f__Lachnospiraceae.g__Pseudobuty
4000
2000
0
46−60 (n=7)
80plus (n=3)
61−80 (n=15)
31−45 (n=3)
20−30 (n=2)
Age_category

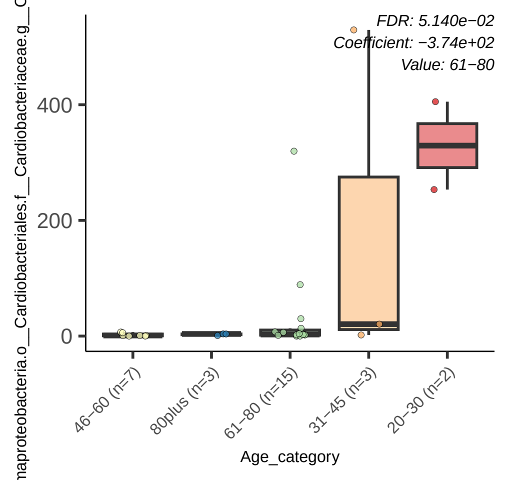

FDR: 5.140e−02
Coefficient: −3.74e+02
Value: 61−80
400
200
0
maproteobacteria.o__Cardiobacteriales.f__Cardiobacteriaceae.g__C
46−60 (n=7)
80plus (n=3)
61−80 (n=15)
31−45 (n=3)
20−30 (n=2)
Age_category

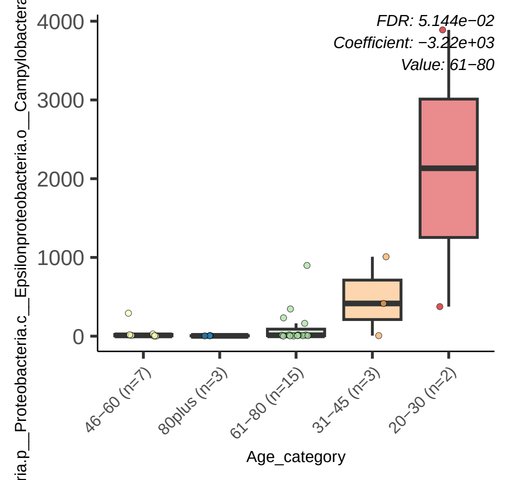

FDR: 5.144e−02
Coefficient: −3.22e+03
Value: 61−80
ria.p__Proteobacteria.c__Epsilonproteobacteria.o__Campylobactera
4000
3000
2000
1000
0
46−60 (n=7)
80plus (n=3)
61−80 (n=15)
31−45 (n=3)
20−30 (n=2)
Age_category

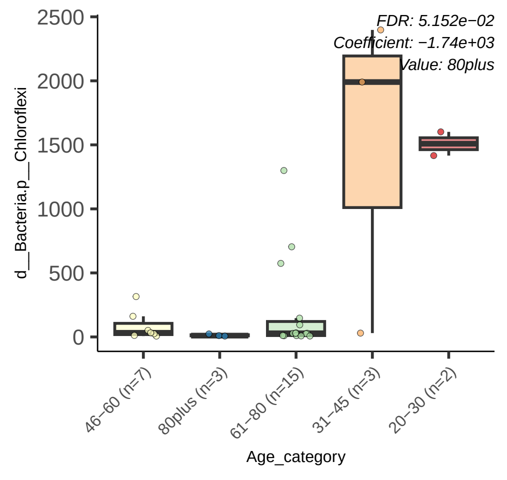

FDR: 5.152e−02
Coefficient: −1.74e+03
Value: 80plus
2500
2000
1500
1000
500
0
d__Bacteria.p__Chloroflexi
46−60 (n=7)
80plus (n=3)
61−80 (n=15)
31−45 (n=3)
20−30 (n=2)
Age_category

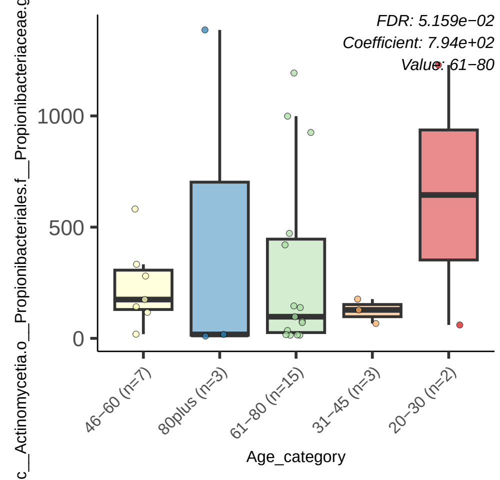
FDR: 5.159e−02
Coefficient: 7.94e+02
Value: 61−80
c__Actinomycetia.o__Propionibacteriales.f__Propionibacteriaceae.g
1000
500
0
46−60 (n=7)
80plus (n=3)
61−80 (n=15)
31−45 (n=3)
20−30 (n=2)
Age_category

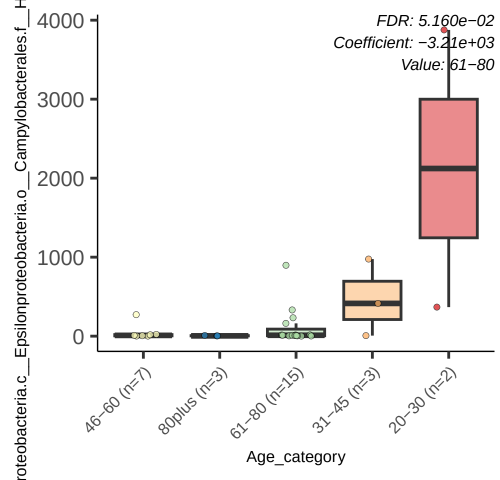

FDR: 5.160e−02
Coefficient: −3.21e+03
Value: 61−80
4000
3000
2000
1000
0
roteobacteria.c__Epsilonproteobacteria.o__Campylobacterales.f__H
46−60 (n=7)
80plus (n=3)
61−80 (n=15)
31−45 (n=3)
20−30 (n=2)
Age_category

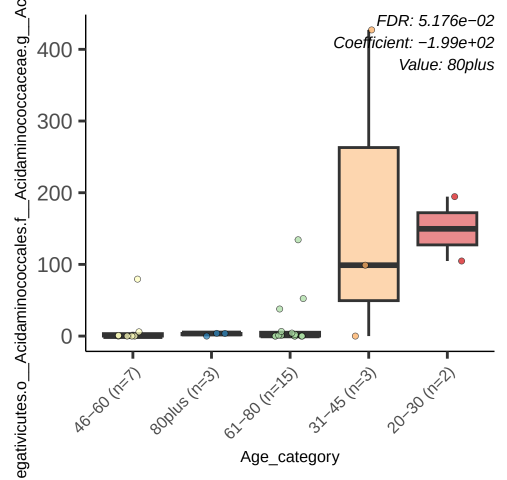

FDR: 5.176e−02
Coefficient: −1.99e+02
Value: 80plus
400
300
200
100
0
egativicutes.o__Acidaminococcales.f__Acidaminococcaceae.g__Ac
46−60 (n=7)
80plus (n=3)
61−80 (n=15)
31−45 (n=3)
20−30 (n=2)
Age_category

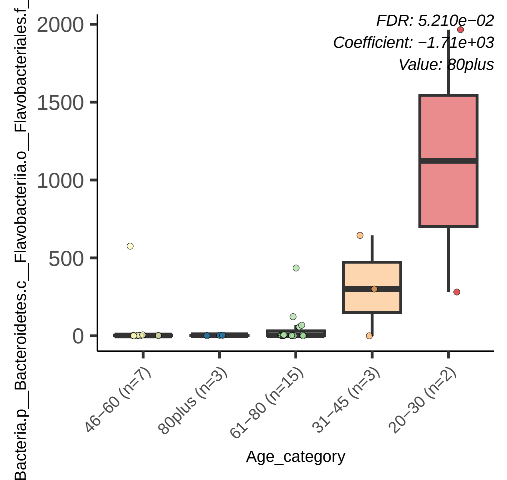

FDR: 5.210e−02
Coefficient: −1.71e+03
Value: 80plus
Bacteria.p__Bacteroidetes.c__Flavobacteriia.o__Flavobacteriales.f__
2000
1500
1000
500
0
46−60 (n=7)
80plus (n=3)
61−80 (n=15)
31−45 (n=3)
20−30 (n=2)
Age_category

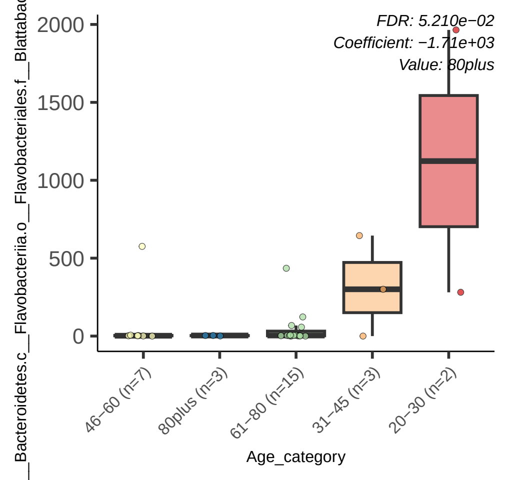

FDR: 5.210e−02
Coefficient: −1.71e+03
Value: 80plus
Bacteroidetes.c__Flavobacteriia.o__Flavobacteriales.f__Blattabac
2000
1500
1000
500
0
46−60 (n=7)
80plus (n=3)
61−80 (n=15)
31−45 (n=3)
20−30 (n=2)
Age_category

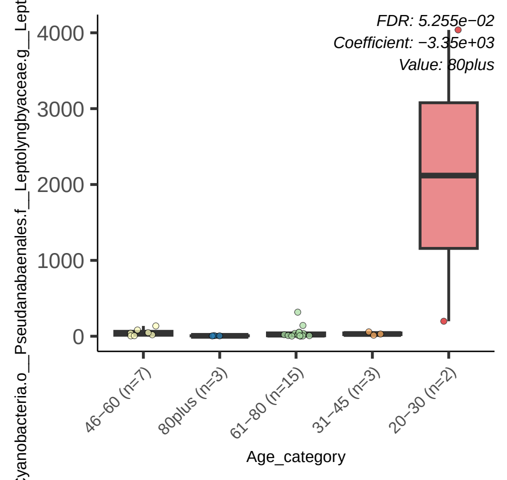

FDR: 5.255e−02
Coefficient: −3.35e+03
Value: 80plus
4000
3000
2000
1000
0
Cyanobacteria.o__Pseudanabaenales.f__Leptolyngbyaceae.g__Lept
46−60 (n=7)
80plus (n=3)
61−80 (n=15)
31−45 (n=3)
20−30 (n=2)
Age_category

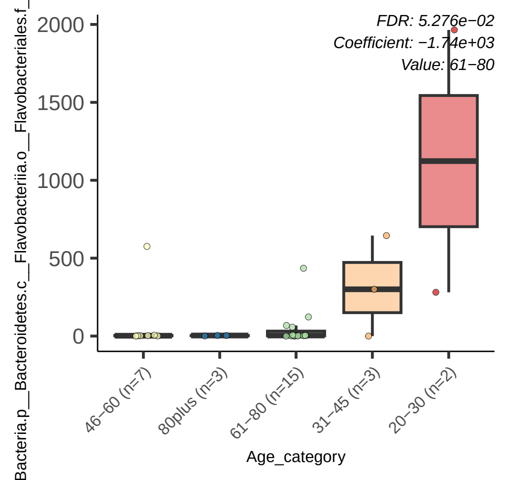

FDR: 5.276e−02
Coefficient: −1.74e+03
Value: 61−80
2000
1500
1000
500
0
Bacteria.p__Bacteroidetes.c__Flavobacteriia.o__Flavobacteriales.f_
46−60 (n=7)
80plus (n=3)
61−80 (n=15)
31−45 (n=3)
20−30 (n=2)
Age_category

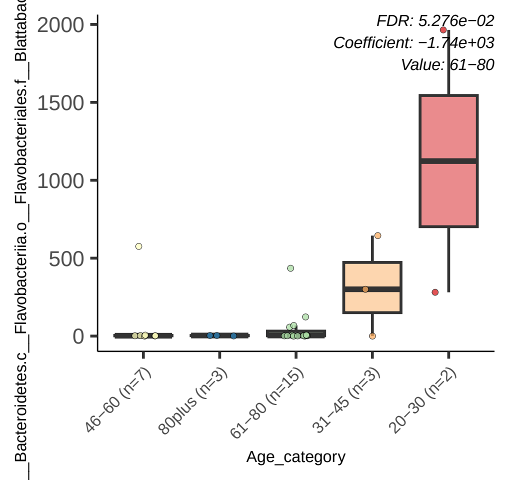

FDR: 5.276e−02
Coefficient: −1.74e+03
Value: 61−80
2000
1500
1000
500
0
Bacteroidetes.c__Flavobacteriia.o__Flavobacteriales.f__Blattabac
46−60 (n=7)
80plus (n=3)
61−80 (n=15)
31−45 (n=3)
20−30 (n=2)
Age_category

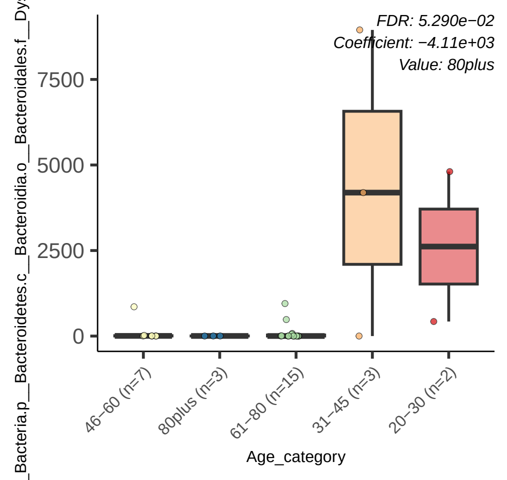

FDR: 5.290e−02
Coefficient: −4.11e+03
Value: 80plus
7500
5000
2500
0
46−60 (n=7)
80plus (n=3)
61−80 (n=15)
31−45 (n=3)
20−30 (n=2)
Age_category
_Bacteria.p__Bacteroidetes.c__Bacteroidia.o__Bacteroidales.f__Dy

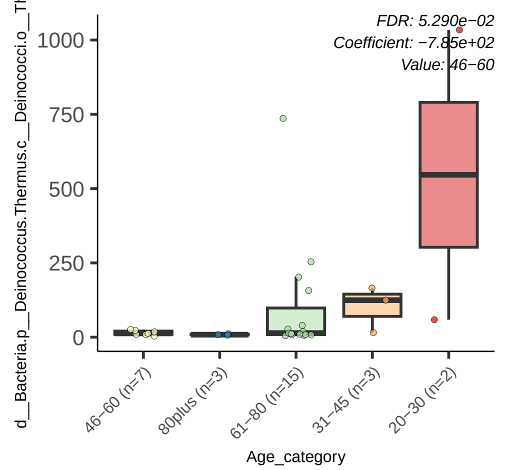

FDR: 5.290e−02
Coefficient: −7.85e+02
Value: 46−60
1000
750
500
250
0
d__Bacteria.p__Deinococcus.Thermus.c__Deinococci.o__Th
46−60 (n=7)
80plus (n=3)
61−80 (n=15)
31−45 (n=3)
20−30 (n=2)
Age_category

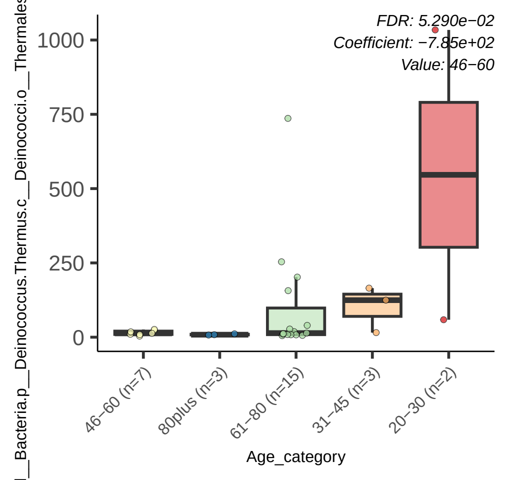
FDR: 5.290e−02
Coefficient: −7.85e+02
Value: 46−60
1000
750
500
250
0
_Bacteria.p__Deinococcus.Thermus.c__Deinococci.o__Thermales
46−60 (n=7)
80plus (n=3)
61−80 (n=15)
31−45 (n=3)
20−30 (n=2)
Age_category

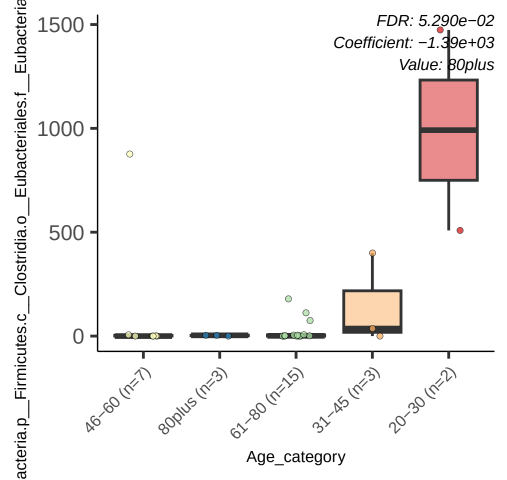

FDR: 5.290e−02
Coefficient: −1.39e+03
Value: 80plus
1500
1000
500
0
acteria.p__Firmicutes.c__Clostridia.o__Eubacteriales.f__Eubacteria
46−60 (n=7)
80plus (n=3)
61−80 (n=15)
31−45 (n=3)
20−30 (n=2)
Age_category

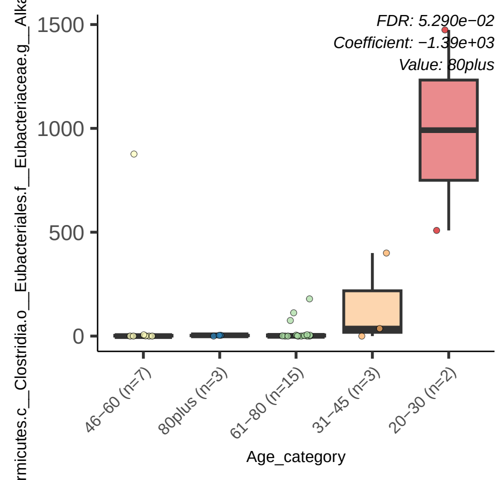

FDR: 5.290e−02
Coefficient: −1.39e+03
Value: 80plus
1500
1000
500
0
46−60 (n=7)
80plus (n=3)
61−80 (n=15)
31−45 (n=3)
20−30 (n=2)
Age_category
rmicutes.c__Clostridia.o__Eubacteriales.f__Eubacteriaceae.g__Alka

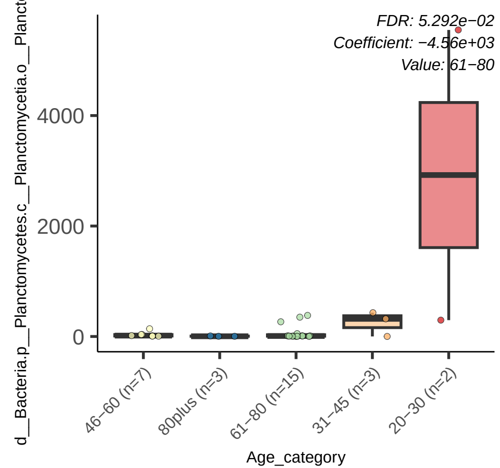

FDR: 5.292e−02
Coefficient: −4.56e+03
Value: 61−80
d__Bacteria.p__Planctomycetes.c__Planctomycetia.o__Plancto
4000
2000
0
46−60 (n=7)
80plus (n=3)
61−80 (n=15)
31−45 (n=3)
20−30 (n=2)
Age_category

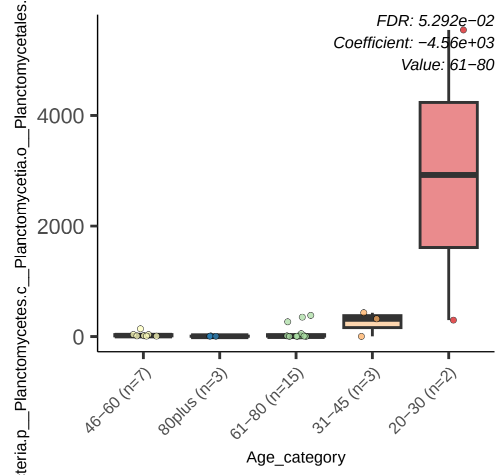

FDR: 5.292e−02
Coefficient: −4.56e+03
Value: 61−80
4000
2000
0
46−60 (n=7)
80plus (n=3)
61−80 (n=15)
31−45 (n=3)
20−30 (n=2)
Age_category
cteria.p__Planctomycetes.c__Planctomycetia.o__Planctomycetales.

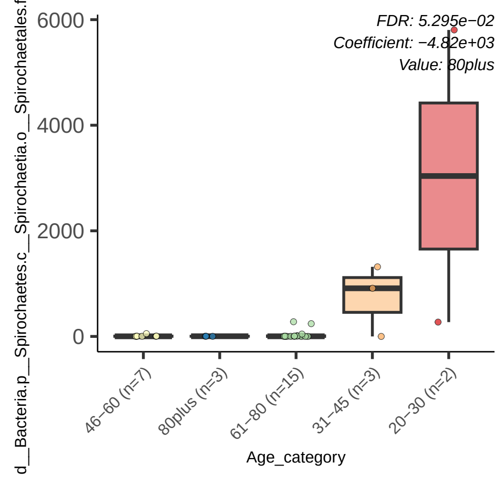

FDR: 5.295e−02
Coefficient: −4.82e+03
Value: 80plus
6000
4000
2000
0
d__Bacteria.p__Spirochaetes.c__Spirochaetia.o__Spirochaetales.f
46−60 (n=7)
80plus (n=3)
61−80 (n=15)
31−45 (n=3)
20−30 (n=2)
Age_category

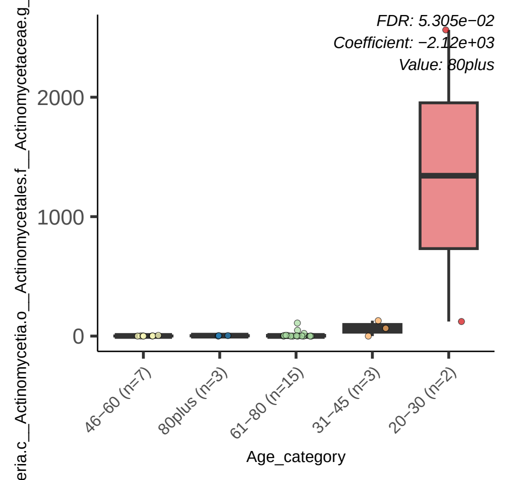

FDR: 5.305e−02
Coefficient: −2.12e+03
Value: 80plus
2000
1000
0
eria.c__Actinomycetia.o__Actinomycetales.f__Actinomycetaceae.g_
46−60 (n=7)
80plus (n=3)
61−80 (n=15)
31−45 (n=3)
20−30 (n=2)
Age_category

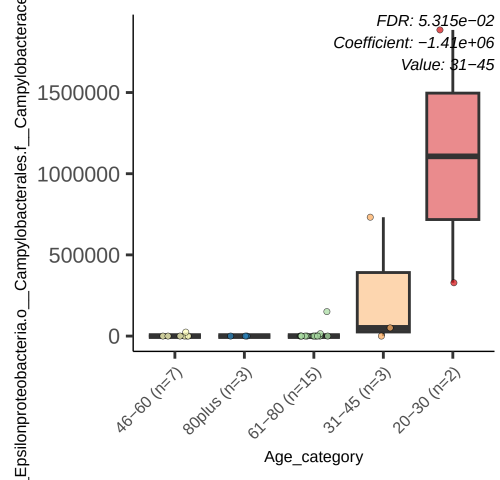

FDR: 5.315e−02
Coefficient: −1.41e+06
Value: 31−45
1500000
1000000
500000
0
_Epsilonproteobacteria.o__Campylobacterales.f__Campylobacterace
46−60 (n=7)
80plus (n=3)
61−80 (n=15)
31−45 (n=3)
20−30 (n=2)
Age_category

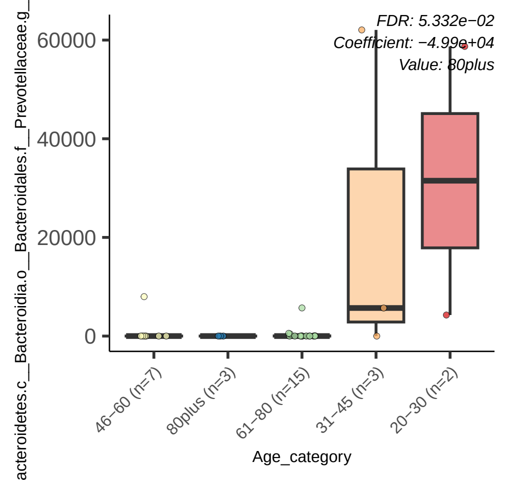
FDR: 5.332e−02
Coefficient: −4.99e+04
Value: 80plus
acteroidetes.c__Bacteroidia.o__Bacteroidales.f__Prevotellaceae.g__
60000
40000
20000
0
46−60 (n=7)
80plus (n=3)
61−80 (n=15)
31−45 (n=3)
20−30 (n=2)
Age_category

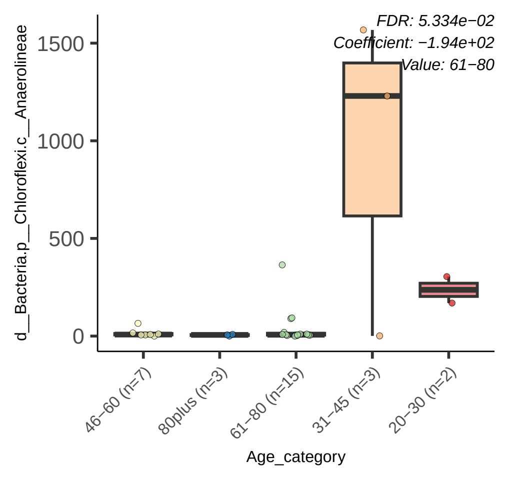

FDR: 5.334e−02
Coefficient: −1.94e+02
Value: 61−80
d__Bacteria.p__Chloroflexi.c__Anaerolineae
1500
1000
500
0
46−60 (n=7)
80plus (n=3)
61−80 (n=15)
31−45 (n=3)
20−30 (n=2)
Age_category

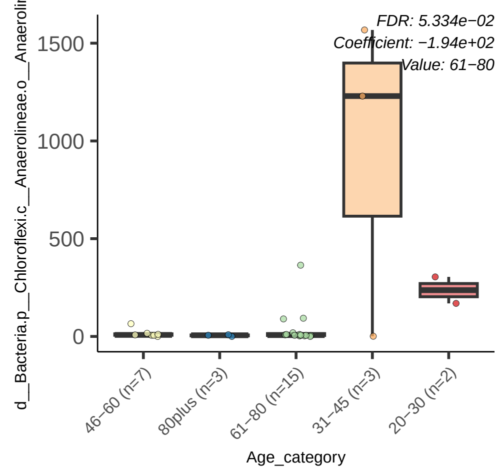

FDR: 5.334e−02
Coefficient: −1.94e+02
Value: 61−80
d__Bacteria.p__Chloroflexi.c__Anaerolineae.o__Anaeroli
1500
1000
500
0
46−60 (n=7)
80plus (n=3)
61−80 (n=15)
31−45 (n=3)
20−30 (n=2)
Age_category

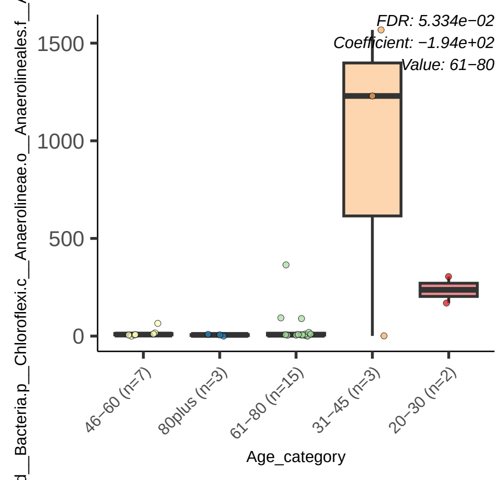

FDR: 5.334e−02
Coefficient: −1.94e+02
Value: 61−80
Bacteria.p__Chloroflexi.c__Anaerolineae.o__Anaerolineales.f__
1500
1000
500
0
46−60 (n=7)
80plus (n=3)
61−80 (n=15)
31−45 (n=3)
20−30 (n=2)
Age_category

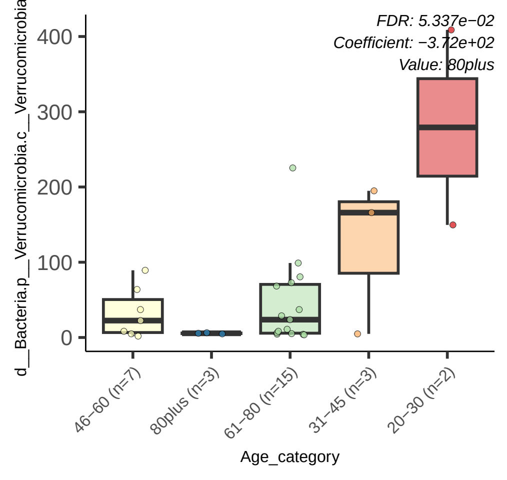

FDR: 5.337e−02
Coefficient: −3.72e+02
Value: 80plus
d__Bacteria.p__Verrucomicrobia.c__Verrucomicrobia
400
300
200
100
0
46−60 (n=7)
80plus (n=3)
61−80 (n=15)
31−45 (n=3)
20−30 (n=2)
Age_category

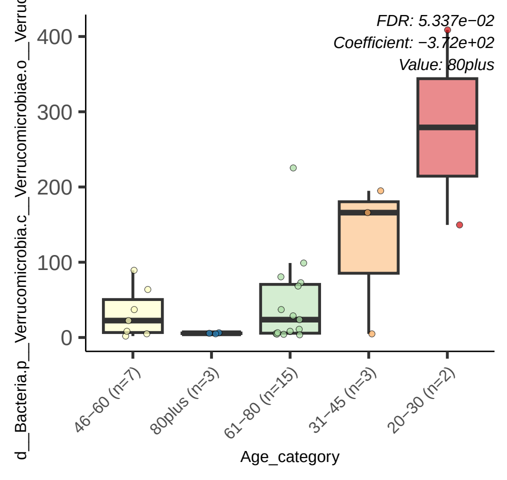

FDR: 5.337e−02
Coefficient: −3.72e+02
Value: 80plus
d__Bacteria.p__Verrucomicrobia.c__Verrucomicrobiae.o__Verruc
400
300
200
100
0
46−60 (n=7)
80plus (n=3)
61−80 (n=15)
31−45 (n=3)
20−30 (n=2)
Age_category

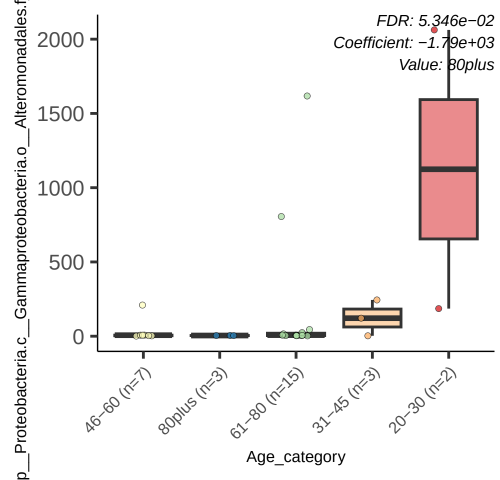

FDR: 5.346e−02
Coefficient: −1.79e+03
Value: 80plus
p__Proteobacteria.c__Gammaproteobacteria.o__Alteromonadales.f
2000
1500
1000
500
0
46−60 (n=7)
80plus (n=3)
61−80 (n=15)
31−45 (n=3)
20−30 (n=2)
Age_category

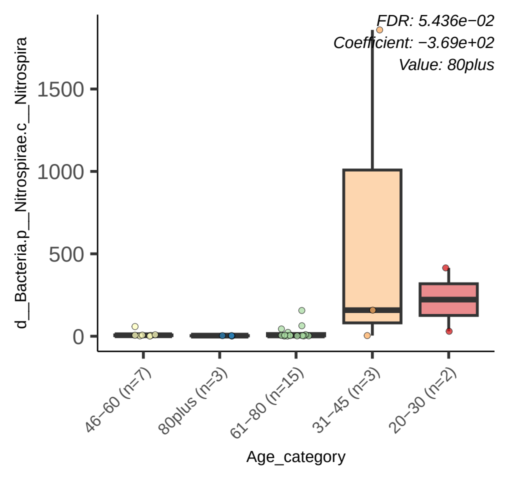
FDR: 5.436e−02
Coefficient: −3.69e+02
Value: 80plus
d__Bacteria.p__Nitrospirae.c__Nitrospira
1500
1000
500
0
46−60 (n=7)
80plus (n=3)
61−80 (n=15)
31−45 (n=3)
20−30 (n=2)
Age_category

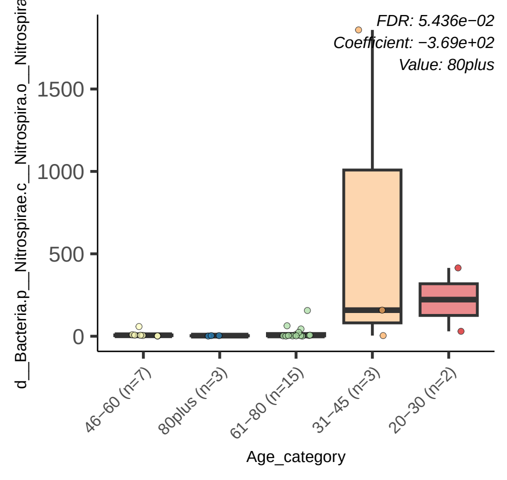

FDR: 5.436e−02
Coefficient: −3.69e+02
Value: 80plus
d__Bacteria.p__Nitrospirae.c__Nitrospira.o__Nitrospira
1500
1000
500
0
46−60 (n=7)
80plus (n=3)
61−80 (n=15)
31−45 (n=3)
20−30 (n=2)
Age_category

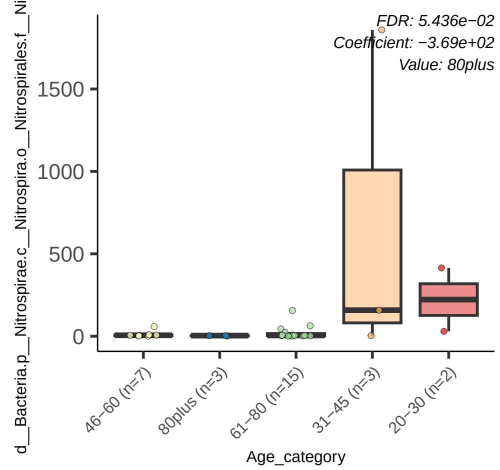
FDR: 5.436e−02
Coefficient: −3.69e+02
Value: 80plus
1500
1000
500
0
d__Bacteria.p__Nitrospirae.c__Nitrospira.o__Nitrospirales.f__Ni
46−60 (n=7)
80plus (n=3)
61−80 (n=15)
31−45 (n=3)
20−30 (n=2)
Age_category

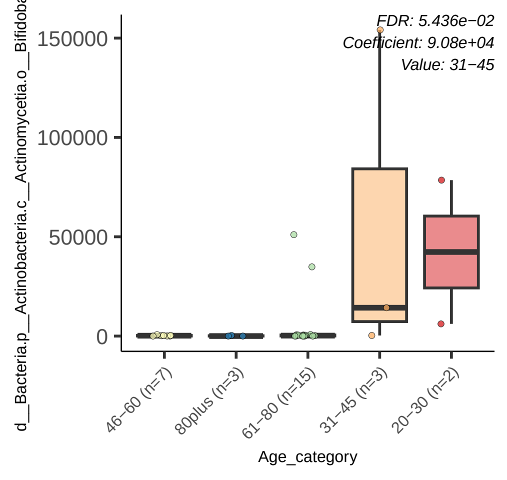

FDR: 5.436e−02
Coefficient: 9.08e+04
Value: 31−45
d__Bacteria.p__Actinobacteria.c__Actinomycetia.o__Bifidoba
150000
100000
50000
0
46−60 (n=7)
80plus (n=3)
61−80 (n=15)
31−45 (n=3)
20−30 (n=2)
Age_category

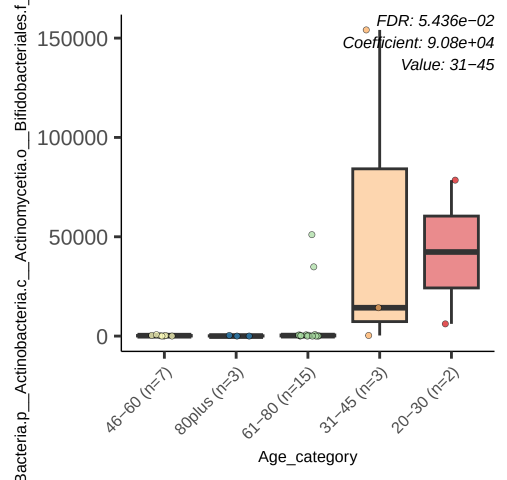

FDR: 5.436e−02
Coefficient: 9.08e+04
Value: 31−45
Bacteria.p__Actinobacteria.c__Actinomycetia.o__Bifidobacteriales.f_
150000
100000
50000
0
46−60 (n=7)
80plus (n=3)
61−80 (n=15)
31−45 (n=3)
20−30 (n=2)
Age_category

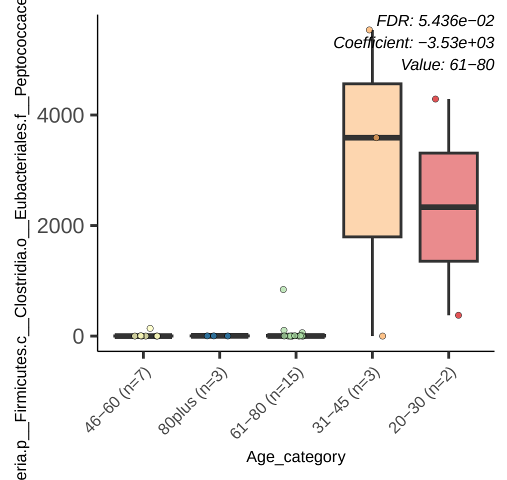

FDR: 5.436e−02
Coefficient: −3.53e+03
Value: 61−80
eria.p__Firmicutes.c__Clostridia.o__Eubacteriales.f__Peptococcace
4000
2000
0
46−60 (n=7)
80plus (n=3)
61−80 (n=15)
31−45 (n=3)
20−30 (n=2)
Age_category

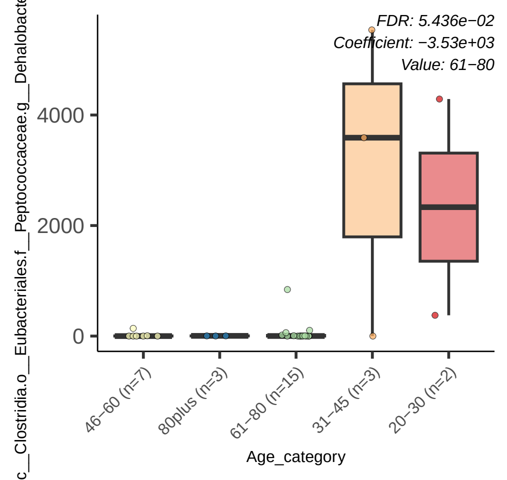

FDR: 5.436e−02
Coefficient: −3.53e+03
Value: 61−80
c__Clostridia.o__Eubacteriales.f__Peptococcaceae.g__Dehalobacte
4000
2000
0
46−60 (n=7)
80plus (n=3)
61−80 (n=15)
31−45 (n=3)
20−30 (n=2)
Age_category

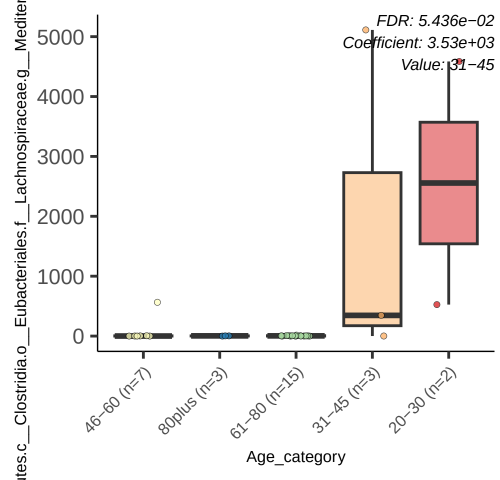

FDR: 5.436e−02
Coefficient: 3.53e+03
Value: 31−45
5000
4000
3000
2000
1000
0
ites.c__Clostridia.o__Eubacteriales.f__Lachnospiraceae.g__Mediter
46−60 (n=7)
80plus (n=3)
61−80 (n=15)
31−45 (n=3)
20−30 (n=2)
Age_category

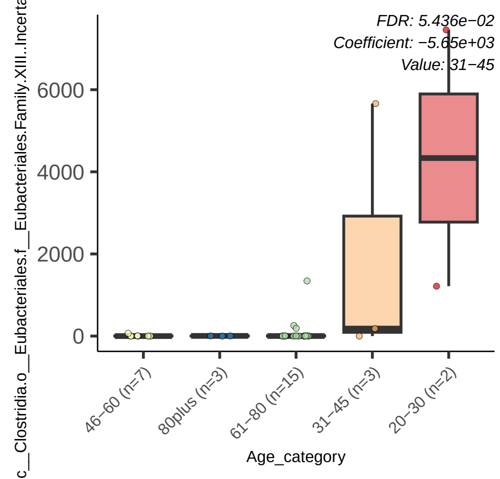
FDR: 5.436e−02
Coefficient: −5.65e+03
Value: 31−45
6000
4000
2000
0
c__Clostridia.o__Eubacteriales.f__Eubacteriales.Family.XIII..Incerta
46−60 (n=7)
80plus (n=3)
61−80 (n=15)
31−45 (n=3)
20−30 (n=2)
Age_category

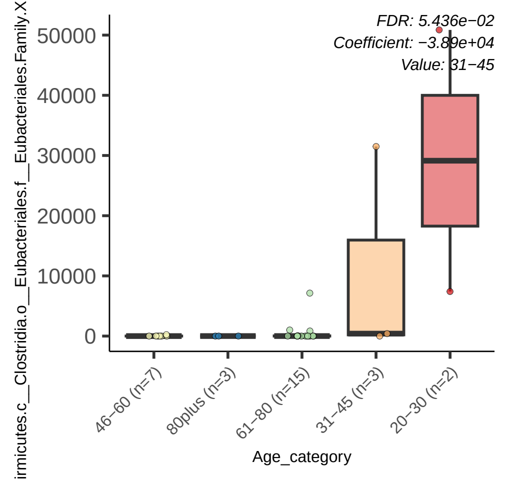

FDR: 5.436e−02
Coefficient: −3.89e+04
Value: 31−45
irmicutes.c__Clostridia.o__Eubacteriales.f__Eubacteriales.Family.X
50000
40000
30000
20000
10000
0
46−60 (n=7)
80plus (n=3)
61−80 (n=15)
31−45 (n=3)
20−30 (n=2)
Age_category

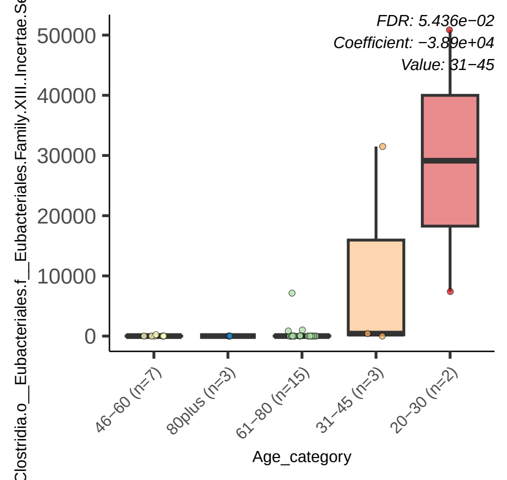

FDR: 5.436e−02
Coefficient: −3.89e+04
Value: 31−45
50000
40000
30000
20000
10000
0
Clostridia.o__Eubacteriales.f__Eubacteriales.Family.XIII..Incertae.Se
46−60 (n=7)
80plus (n=3)
61−80 (n=15)
31−45 (n=3)
20−30 (n=2)
Age_category

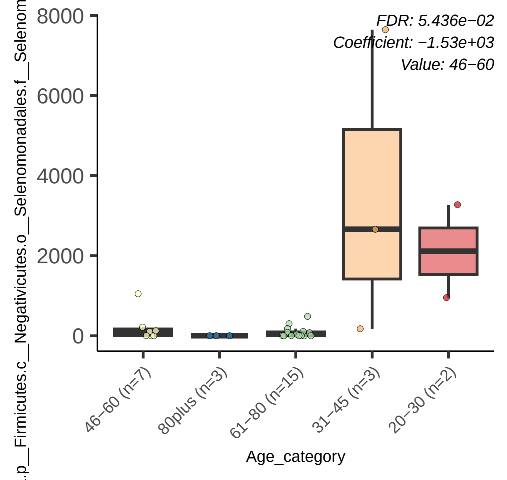

FDR: 5.436e−02
Coefficient: −1.53e+03
Value: 46−60
8000
6000
4000
2000
0
.p__Firmicutes.c__Negativicutes.o__Selenomonadales.f__Selenom
46−60 (n=7)
80plus (n=3)
61−80 (n=15)
31−45 (n=3)
20−30 (n=2)
Age_category

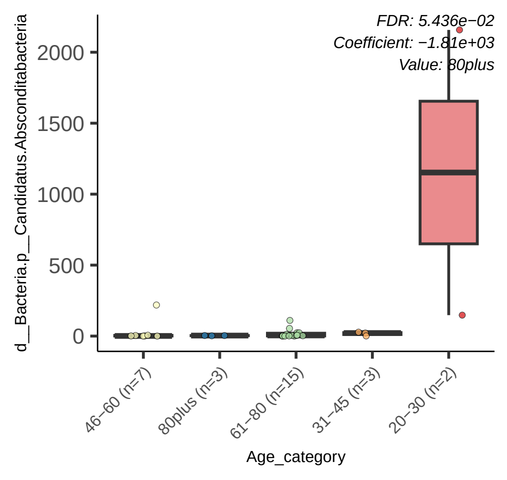

FDR: 5.436e−02
Coefficient: −1.81e+03
Value: 80plus
d__Bacteria.p__Candidatus.Absconditabacteria
2000
1500
1000
500
0
46−60 (n=7)
80plus (n=3)
61−80 (n=15)
31−45 (n=3)
20−30 (n=2)
Age_category

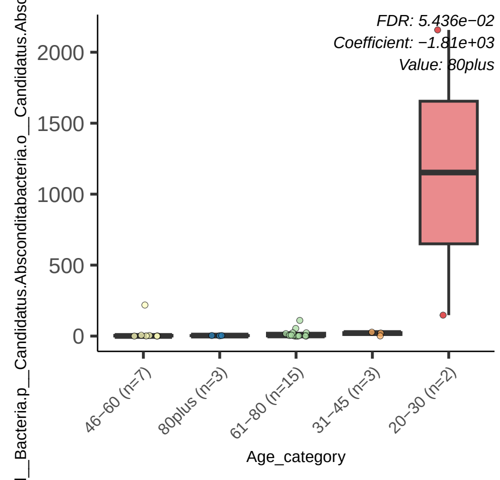

FDR: 5.436e−02
Coefficient: −1.81e+03
Value: 80plus
2000
1500
1000
500
0
46−60 (n=7)
80plus (n=3)
61−80 (n=15)
31−45 (n=3)
20−30 (n=2)
Age_category
l__Bacteria.p__Candidatus.Absconditabacteria.o__Candidatus.Absc

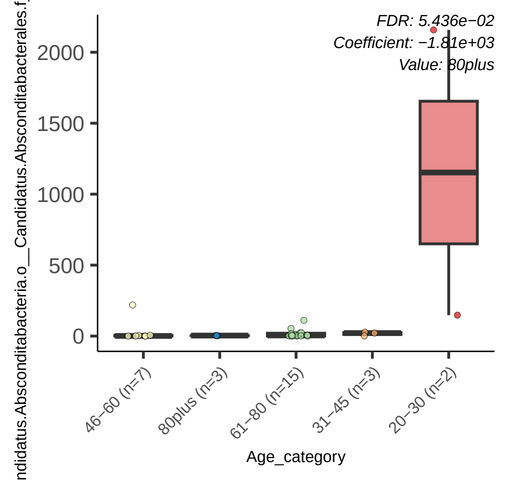

FDR: 5.436e−02
Coefficient: −1.81e+03
Value: 80plus
ndidatus.Absconditabacteria.o__Candidatus.Absconditabacterales.f_
2000
1500
1000
500
0
46−60 (n=7)
80plus (n=3)
61−80 (n=15)
31−45 (n=3)
20−30 (n=2)
Age_category

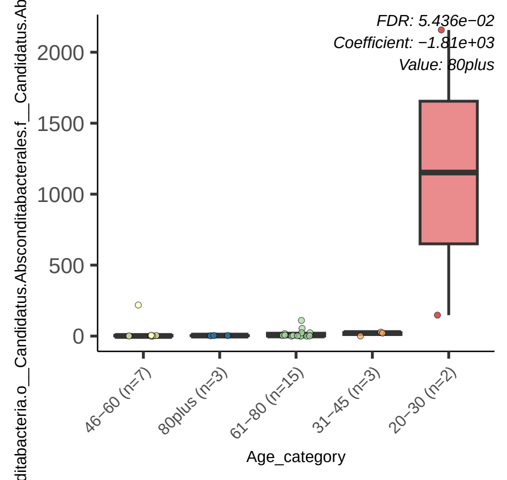

FDR: 5.436e−02
Coefficient: −1.81e+03
Value: 80plus
2000
1500
1000
500
0
ditabacteria.o__Candidatus.Absconditabacterales.f__Candidatus.Ab
46−60 (n=7)
80plus (n=3)
61−80 (n=15)
31−45 (n=3)
20−30 (n=2)
Age_category

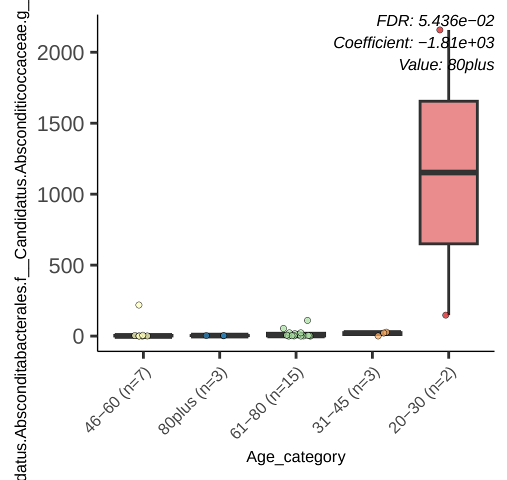

FDR: 5.436e−02
Coefficient: −1.81e+03
Value: 80plus
2000
1500
1000
500
0
datus.Abscönditabacterales.f__Candidatus.Absconditicoccaceae.g__
46−60 (n=7)
80plus (n=3)
61−80 (n=15)
31−45 (n=3)
20−30 (n=2)
Age_category

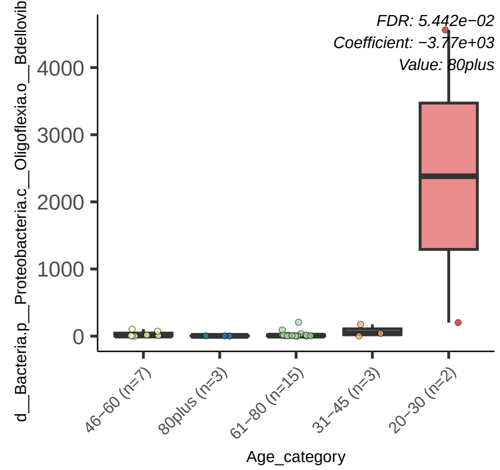

FDR: 5.442e−02
Coefficient: −3.77e+03
Value: 80plus
d__Bacteria.p__Proteobacteria.c__Oligoflexia.o__Bdellovib
4000
3000
2000
1000
0
46−60 (n=7)
80plus (n=3)
61−80 (n=15)
31−45 (n=3)
20−30 (n=2)
Age_category

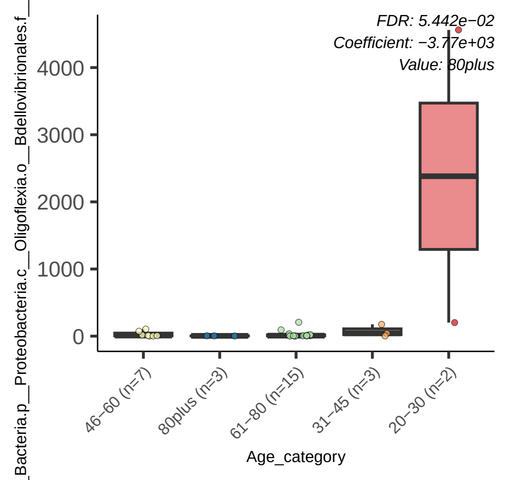
FDR: 5.442e−02
Coefficient: −3.77e+03
Value: 80plus
Bacteria.p__Proteobacteria.c__Oligoflexia.o__Bdellovibrionales.f__
4000
3000
2000
1000
0
46−60 (n=7)
80plus (n=3)
61−80 (n=15)
31−45 (n=3)
20−30 (n=2)
Age_category

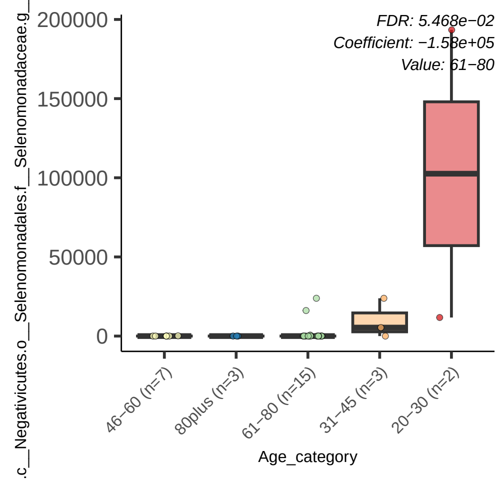

FDR: 5.468e−02
Coefficient: −1.58e+05
Value: 61−80
200000
150000
100000
50000
0
c__Negativicutes.o__Selenomonadales.f__Selenomonadaceae.g__
46−60 (n=7)
80plus (n=3)
61−80 (n=15)
31−45 (n=3)
20−30 (n=2)
Age_category

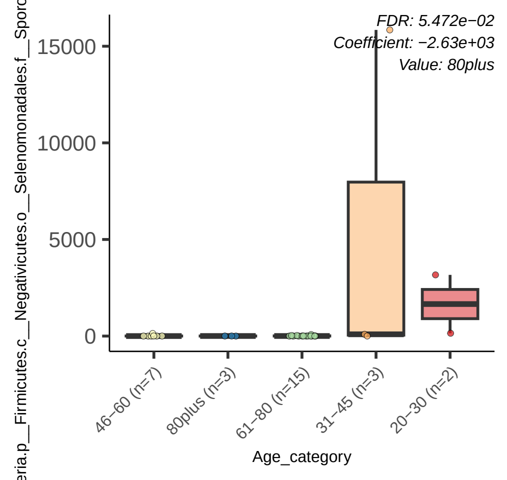

FDR: 5.472e−02
Coefficient: −2.63e+03
Value: 80plus
15000
10000
5000
0
eria.p__Firmicutes.c__Negativicutes.o__Selenomonadales.f__Sporo
46−60 (n=7)
80plus (n=3)
61−80 (n=15)
31−45 (n=3)
20−30 (n=2)
Age_category

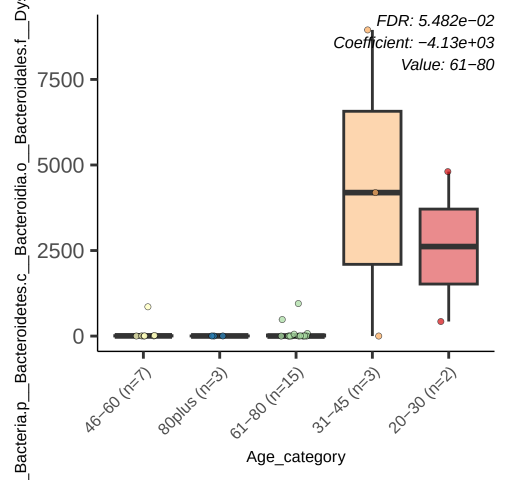

FDR: 5.482e−02
Coefficient: −4.13e+03
Value: 61−80
_Bacteria.p__Bacteroidetes.c__Bacteroidia.o__Bacteroidales.f__Dys
7500
5000
2500
0
46−60 (n=7)
80plus (n=3)
61−80 (n=15)
31−45 (n=3)
20−30 (n=2)
Age_category

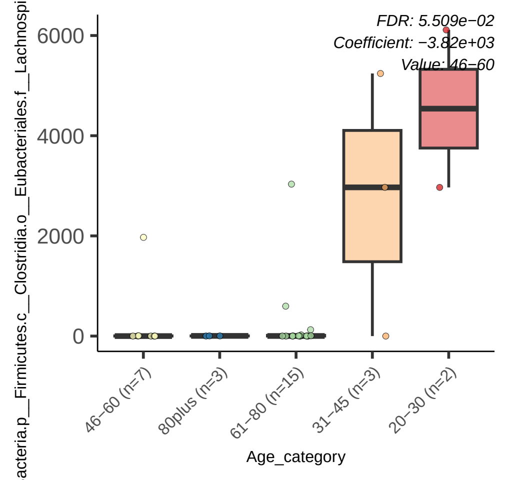

FDR: 5.509e−02
Coefficient: −3.82e+03
Value: 46−60
6000
4000
2000
0
acteria.p__Firmicutes.c__Clostridia.o__Eubacteriales.f__Lachnospi
46−60 (n=7)
80plus (n=3)
61−80 (n=15)
31−45 (n=3)
20−30 (n=2)
Age_category

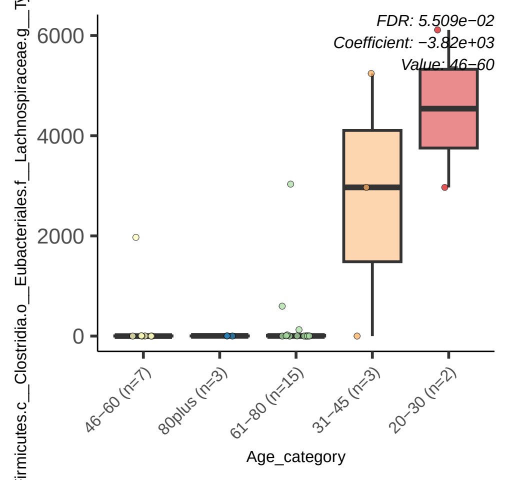

FDR: 5.509e−02
Coefficient: −3.82e+03
Value: 46−60
6000
4000
2000
0
irmicutes.c__Clostridia.o__Eubacteriales.f__Lachnospiraceae.g__Ty
46−60 (n=7)
80plus (n=3)
61−80 (n=15)
31−45 (n=3)
20−30 (n=2)
Age_category

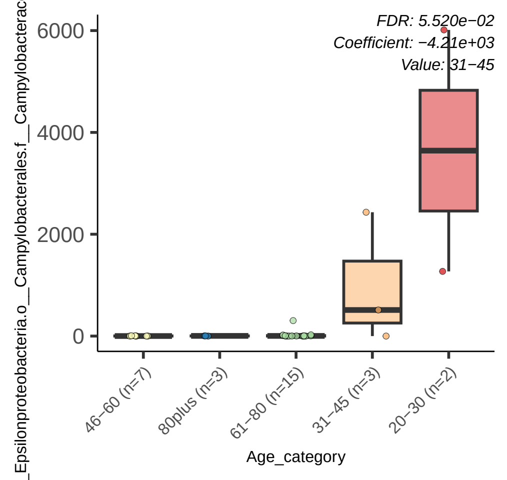

FDR: 5.520e−02
Coefficient: −4.21e+03
Value: 31−45
6000
4000
2000
0
_Epsilonproteobacteria.o__Campylobacterales.f__Campylobacterac
46−60 (n=7)
80plus (n=3)
61−80 (n=15)
31−45 (n=3)
20−30 (n=2)
Age_category

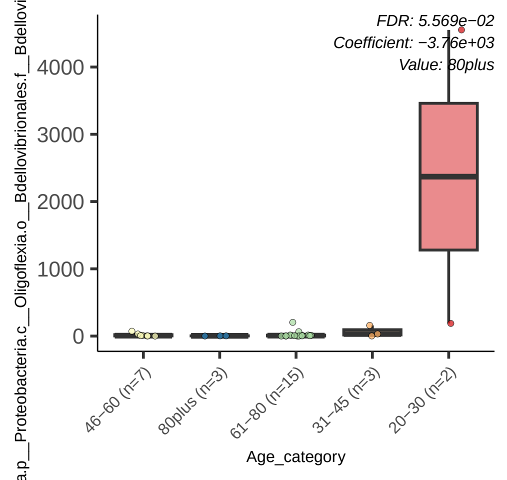

FDR: 5.569e−02
Coefficient: −3.76e+03
Value: 80plus
a.p__Proteobacteria.c__Oligoflexia.o__Bdellovibrionales.f__Bdellovi
4000
3000
2000
1000
0
46−60 (n=7)
80plus (n=3)
61−80 (n=15)
31−45 (n=3)
20−30 (n=2)
Age_category

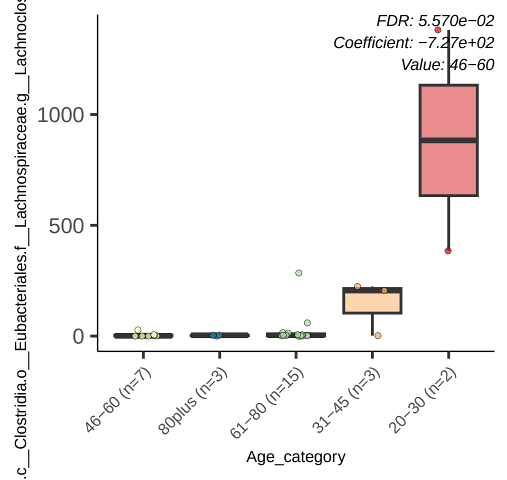

FDR: 5.570e−02
Coefficient: −7.27e+02
Value: 46−60
.c__Clostridia.o__Eubacteriales.f__Lachnospiraceae.g__Lachnoclos
1000
500
0
46−60 (n=7)
80plus (n=3)
61−80 (n=15)
31−45 (n=3)
20−30 (n=2)
Age_category

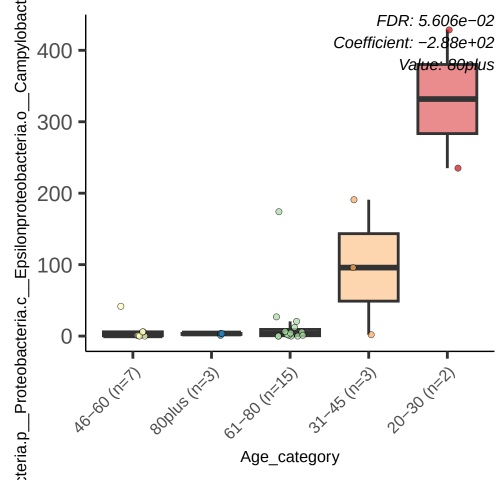

FDR: 5.606e−02
Coefficient: −2.88e+02
Value: 80plus
400
300
200
100
0
cteria.p__Proteobacteria.c__Epsilonproteobacteria.o__Campylobact
46−60 (n=7)
80plus (n=3)
61−80 (n=15)
31−45 (n=3)
20−30 (n=2)
Age_category

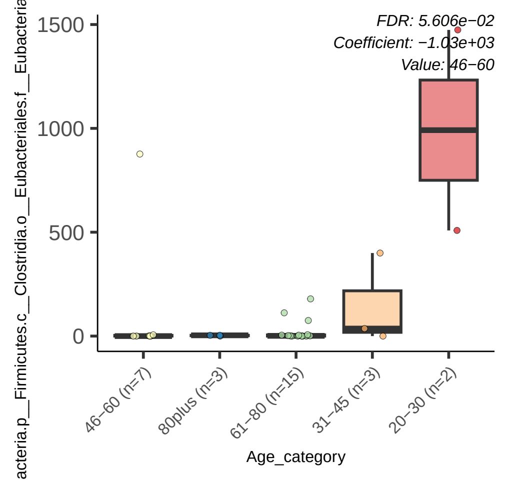

FDR: 5.606e−02
Coefficient: −1.03e+03
Value: 46−60
1500
1000
500
0
acteria.p__Firmicutes.c__Clostridia.o__Eubacteriales.f__Eubacteria
46−60 (n=7)
80plus (n=3)
61−80 (n=15)
31−45 (n=3)
20−30 (n=2)
Age_category

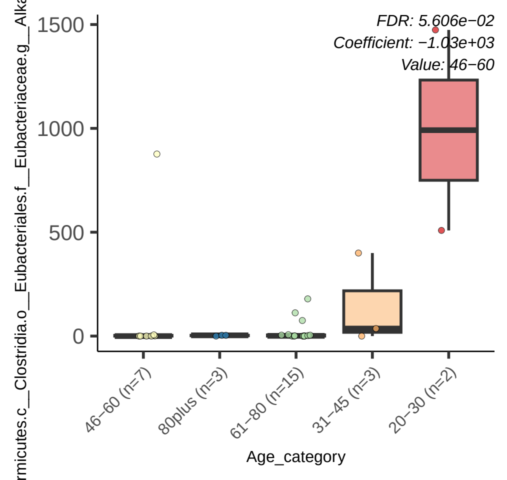

FDR: 5.606e−02
Coefficient: −1.03e+03
Value: 46−60
0
500
1000
1500
46−60 (n=7)
80plus (n=3)
61−80 (n=15)
31−45 (n=3)
20−30 (n=2)
Age_category
rmicutes.c__Clostridia.o__Eubacteriales.f__Eubacteriaceae.g__Alka

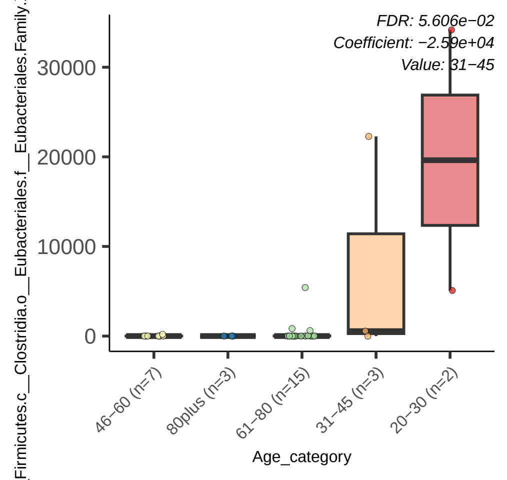

FDR: 5.606e−02
Coefficient: −2.59e+04
Value: 31−45
30000
20000
10000
0
Firmicutes.c__Clostridia.o__Eubacteriales.f__Eubacteriales.Family.
46−60 (n=7)
80plus (n=3)
61−80 (n=15)
31−45 (n=3)
20−30 (n=2)
Age_category

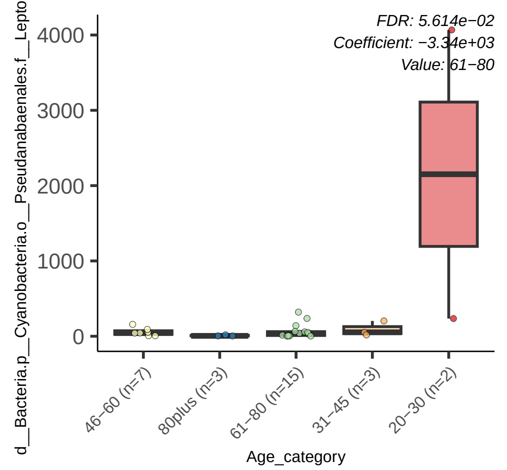
FDR: 5.614e−02
Coefficient: −3.34e+03
Value: 61−80
d__Bacteria.p__Cyanobacteria.o__Pseudanabaenales.f__Lepto
4000
3000
2000
1000
0
46−60 (n=7)
80plus (n=3)
61−80 (n=15)
31−45 (n=3)
20−30 (n=2)
Age_category

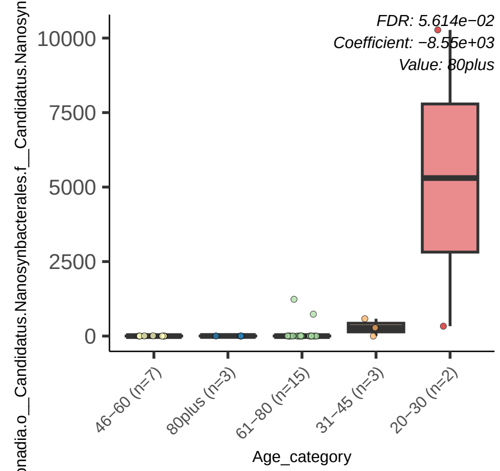

FDR: 5.614e−02
Coefficient: −8.55e+03
Value: 80plus
10000
7500
5000
2500
0
nonadia.o__Candidatus.Nanosynbacterales.f__Candidatus.Nanosyn
46−60 (n=7)
80plus (n=3)
61−80 (n=15)
31−45 (n=3)
20−30 (n=2)
Age_category

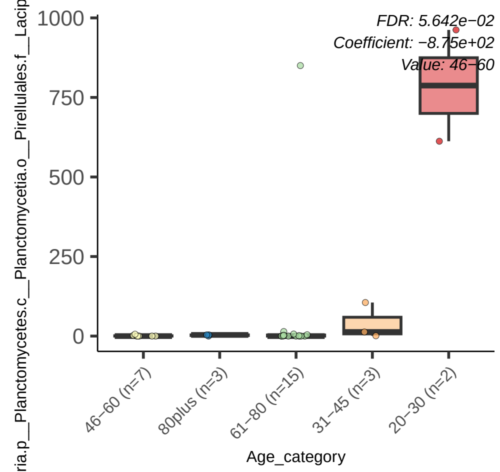

FDR: 5.642e−02
Coefficient: −8.75e+02
Value: 46−60
1000
750
500
250
0
eria.p__Planctomycetes.c__Planctomycetia.o__Pirellulales.f__Lacip
46−60 (n=7)
80plus (n=3)
61−80 (n=15)
31−45 (n=3)
20−30 (n=2)
Age_category

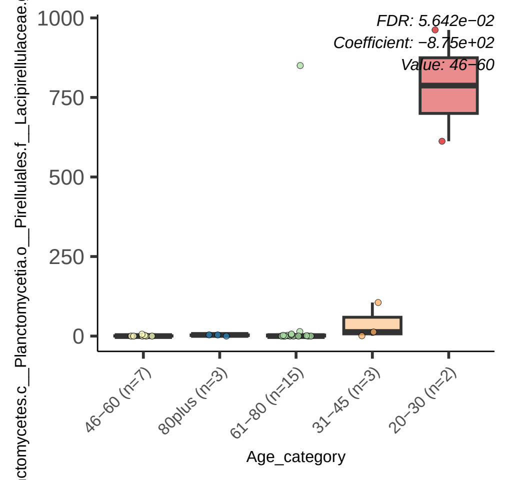

FDR: 5.642e−02
Coefficient: −8.75e+02
Value: 46−60
1000
750
500
250
0
ctomycetes.c__Planctomycetia.o__Pirellulales.f__Lacipirellulaceae.
46−60 (n=7)
80plus (n=3)
61−80 (n=15)
31−45 (n=3)
20−30 (n=2)
Age_category

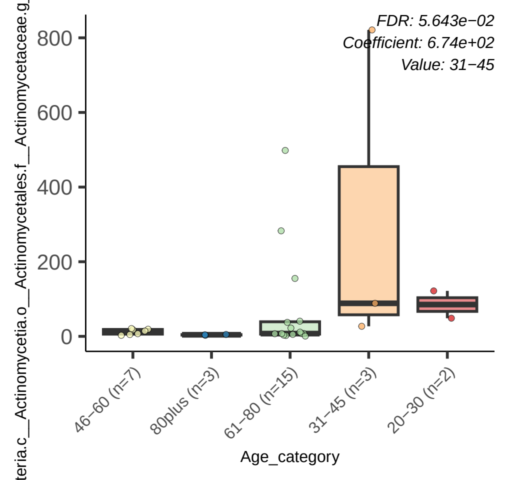
FDR: 5.643e−02
Coefficient: 6.74e+02
Value: 31−45
800
600
400
200
0
teria.c__Actinomycetia.o__Actinomycetales.f__Actinomycetaceae.g_
46−60 (n=7)
80plus (n=3)
61−80 (n=15)
31−45 (n=3)
20−30 (n=2)
Age_category

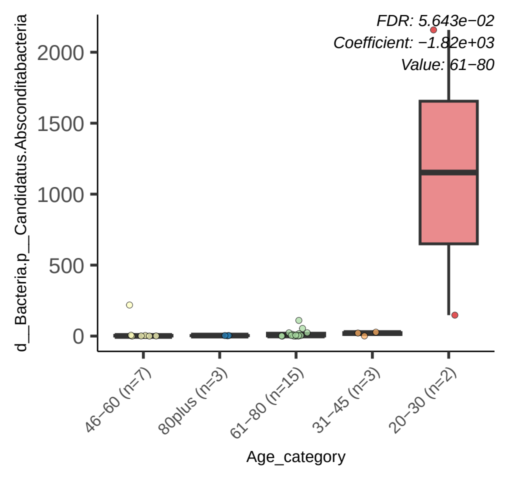

FDR: 5.643e−02
Coefficient: −1.82e+03
Value: 61−80
d__Bacteria.p__Candidatus.Absconditabacteria
2000
1500
1000
500
0
46−60 (n=7)
80plus (n=3)
61−80 (n=15)
31−45 (n=3)
20−30 (n=2)
Age_category

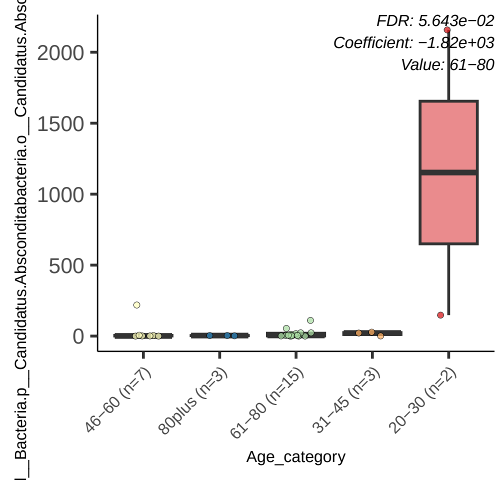
FDR: 5.643e−02
Coefficient: −1.82e+03
Value: 61−80
2000
1500
1000
500
0
Bacteria.p__Candidatus.Absconditabacteria.o__Candidatus.Absc
46−60 (n=7)
80plus (n=3)
61−80 (n=15)
31−45 (n=3)
20−30 (n=2)
Age_category

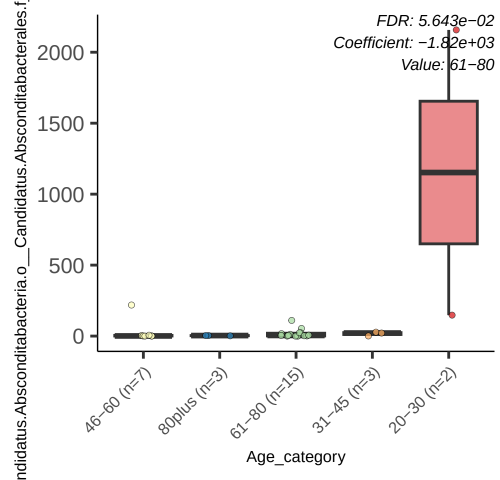

FDR: 5.643e−02
Coefficient: −1.82e+03
Value: 61−80
ndidatus.Absconditabacteria.o__Candidatus.Absconditabacterales.f_
2000
1500
1000
500
0
46−60 (n=7)
80plus (n=3)
61−80 (n=15)
31−45 (n=3)
20−30 (n=2)
Age_category

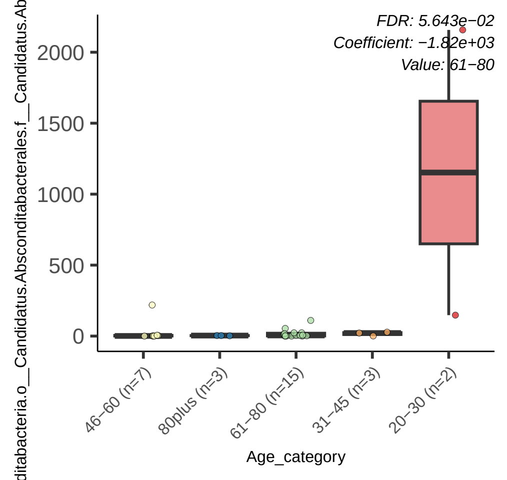

FDR: 5.643e−02
Coefficient: −1.82e+03
Value: 61−80
2000
1500
1000
500
0
ditabacteria.o__Candidatus.Absconditabacterales.f__Candidatus.Ab
46−60 (n=7)
80plus (n=3)
61−80 (n=15)
31−45 (n=3)
20−30 (n=2)
Age_category

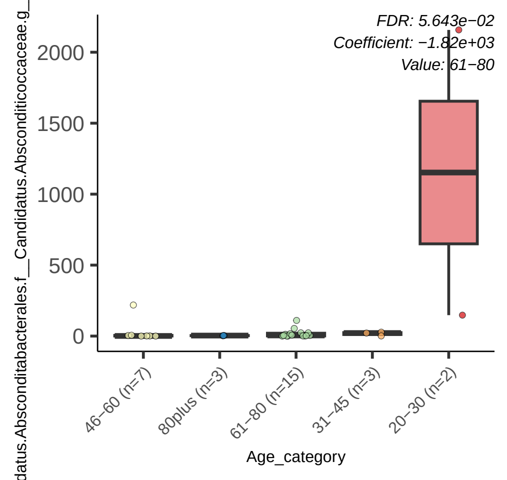
FDR: 5.643e−02
Coefficient: −1.82e+03
Value: 61−80
2000
1500
1000
500
0
46−60 (n=7)
80plus (n=3)
61−80 (n=15)
31−45 (n=3)
20−30 (n=2)
Age_category
datus.Absconditabacterales.f__Candidatus.Absconditicoccaceae.g_

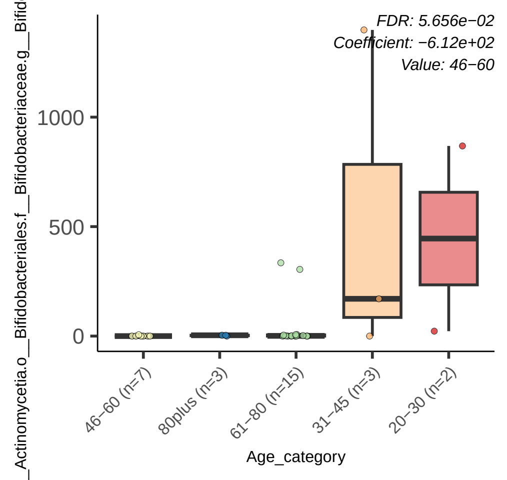

FDR: 5.656e−02
Coefficient: −6.12e+02
Value: 46−60
1000
500
0
46−60 (n=7)
80plus (n=3)
61−80 (n=15)
31−45 (n=3)
20−30 (n=2)
Age_category
Actinomycetia.o__Bifidobacteriales.f__Bifidobacteriaceae.g__Bifid

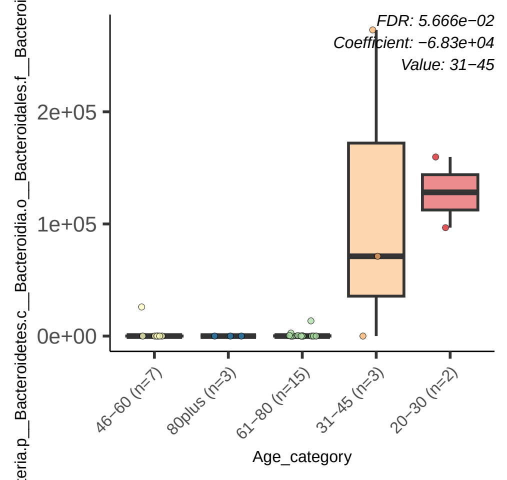
FDR: 5.666e−02
Coefficient: −6.83e+04
Value: 31−45
2e+05
1e+05
0e+00
46−60 (n=7)
80plus (n=3)
61−80 (n=15)
31−45 (n=3)
20−30 (n=2)
Age_category
teria.p__Bacteroidetes.c__Bacteroidia.o__Bacteroidales.f__Bacteroi

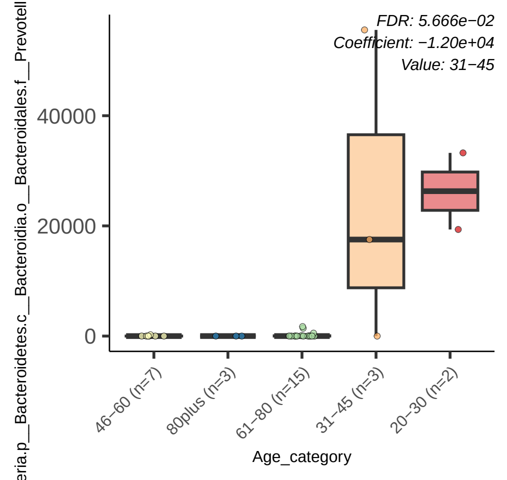
FDR: 5.666e−02
Coefficient: −1.20e+04
Value: 31−45
40000
20000
0
eria.p__Bacteroidetes.c__Bacteroidia.o__Bacteroidales.f__Prevotell
46−60 (n=7)
80plus (n=3)
61−80 (n=15)
31−45 (n=3)
20−30 (n=2)
Age_category

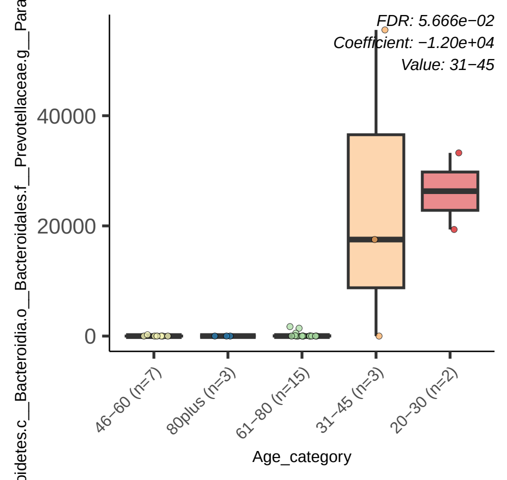

FDR: 5.666e−02
Coefficient: −1.20e+04
Value: 31−45
oidetes.c__Bacteroidia.o__Bacteroidales.f__Prevotellaceae.g__Para
40000
20000
0
46−60 (n=7)
80plus (n=3)
61−80 (n=15)
31−45 (n=3)
20−30 (n=2)
Age_category

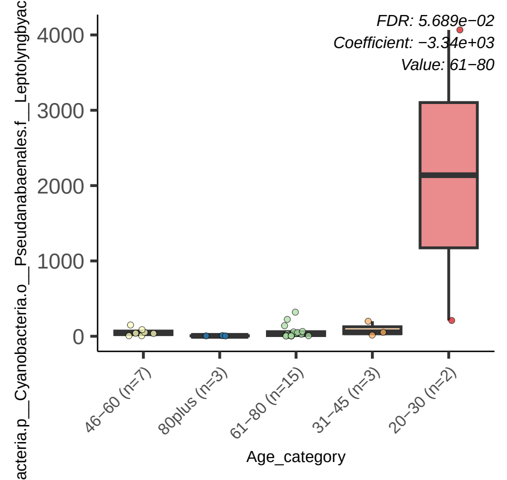

FDR: 5.689e−02
Coefficient: −3.34e+03
Value: 61−80
acteria.p__Cyanobacteria.o__Pseudanabaenales.f__Leptolyngbyac
4000
3000
2000
1000
0
46−60 (n=7)
80plus (n=3)
61−80 (n=15)
31−45 (n=3)
20−30 (n=2)
Age_category

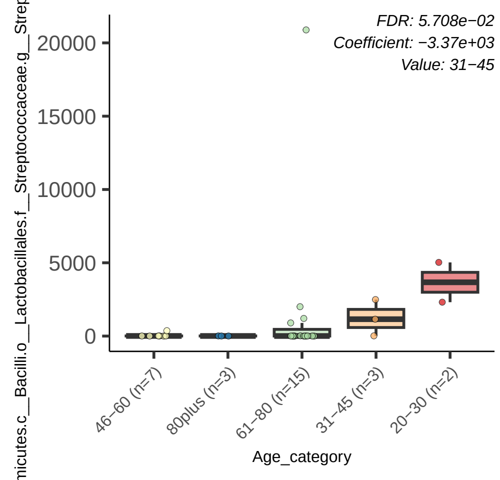

FDR: 5.708e−02
Coefficient: −3.37e+03
Value: 31−45
20000
15000
10000
5000
0
micutes.c__Bacilli.o__Lactobacillales.f__Streptococcaceae.g__Strep
46−60 (n=7)
80plus (n=3)
61−80 (n=15)
31−45 (n=3)
20−30 (n=2)
Age_category

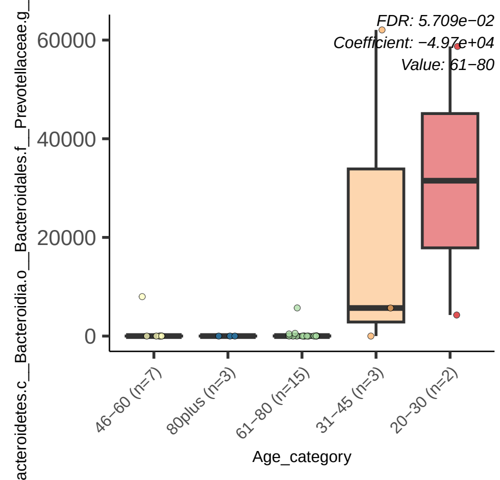

FDR: 5.709e−02
Coefficient: −4.97e+04
Value: 61−80
acteroidetes.c__Bacteroidia.o__Bacteroidales.f__Prevotellaceae.g__
60000
40000
20000
0
46−60 (n=7)
80plus (n=3)
61−80 (n=15)
31−45 (n=3)
20−30 (n=2)
Age_category

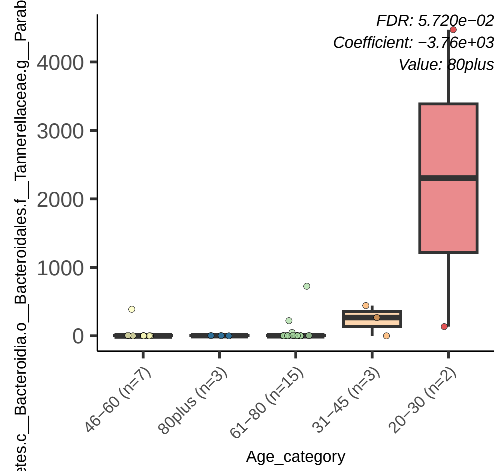

FDR: 5.720e−02
Coefficient: −3.76e+03
Value: 80plus
4000
3000
2000
1000
0
detes.c__Bacteroidia.o__Bacteroidales.f__Tannerellaceae.g__Parab
46−60 (n=7)
80plus (n=3)
61−80 (n=15)
31−45 (n=3)
20−30 (n=2)
Age_category

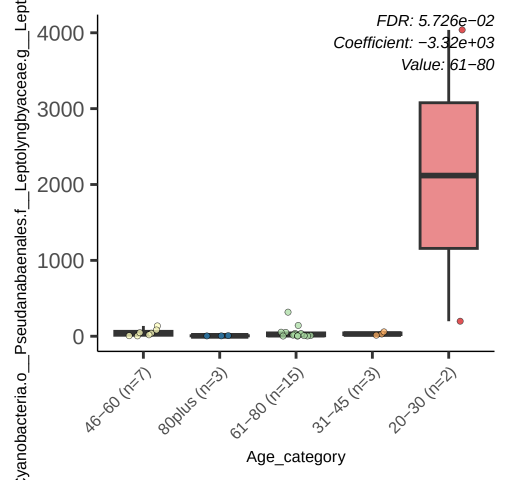

FDR: 5.726e−02
Coefficient: −3.32e+03
Value: 61−80
Cyanobacteria.o__Pseudanabaenales.f__Leptolyngbyaceae.g__Lept
4000
3000
2000
1000
0
46−60 (n=7)
80plus (n=3)
61−80 (n=15)
31−45 (n=3)
20−30 (n=2)
Age_category

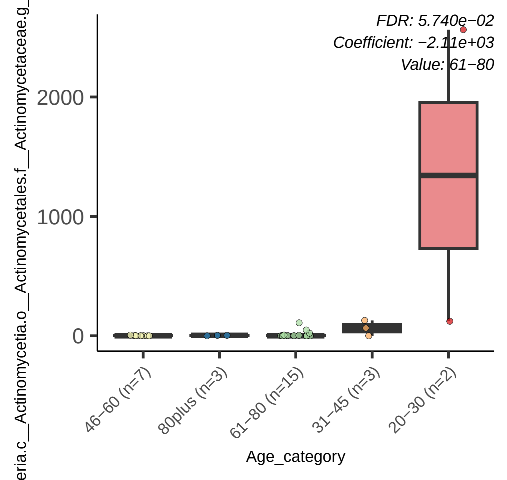

FDR: 5.740e−02
Coefficient: −2.11e+03
Value: 61−80
2000
1000
0
eria.c__Actinomycetia.o__Actinomycetales.f__Actinomycetaceae.g_
46−60 (n=7)
80plus (n=3)
61−80 (n=15)
31−45 (n=3)
20−30 (n=2)
Age_category

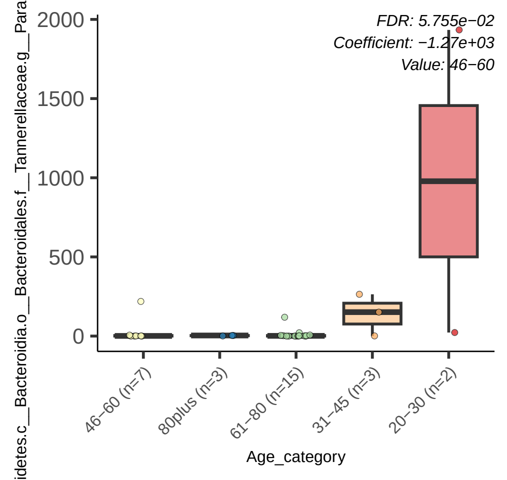

FDR: 5.755e−02
Coefficient: −1.27e+03
Value: 46−60
idetes.c__Bacteroidia.o__Bacteroidales.f__Tannerellaceae.g__Para
2000
1500
1000
500
0
46−60 (n=7)
80plus (n=3)
61−80 (n=15)
31−45 (n=3)
20−30 (n=2)
Age_category

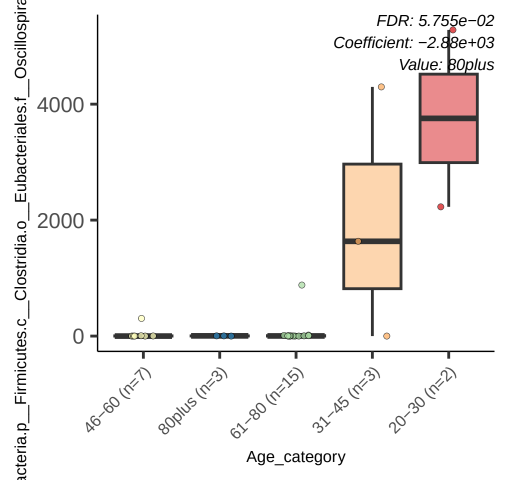
FDR: 5.755e−02
Coefficient: −2.88e+03
Value: 80plus
4000
2000
0
46−60 (n=7)
80plus (n=3)
61−80 (n=15)
31−45 (n=3)
20−30 (n=2)
Age_category
acteria.p__Firmicutes.c__Clostridia.o__Eubacteriales.f__Oscillospira

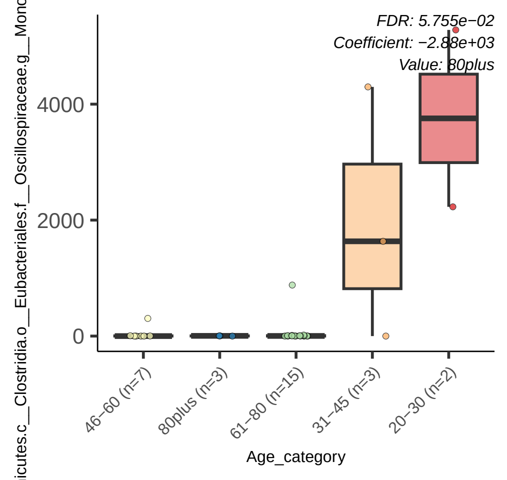

FDR: 5.755e−02
Coefficient: −2.88e+03
Value: 80plus
4000
2000
0
46−60 (n=7)
80plus (n=3)
61−80 (n=15)
31−45 (n=3)
20−30 (n=2)
Age_category
icutes.c__Clostridia.o__Eubacteriales.f__Oscillospiraceae.g__Mono

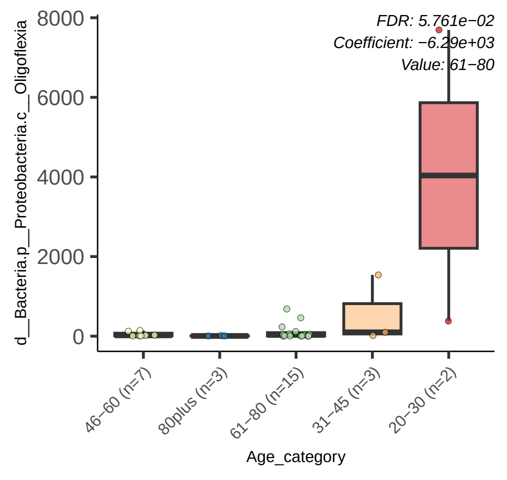
FDR: 5.761e−02
Coefficient: −6.29e+03
Value: 61−80
d__Bacteria.p__Proteobacteria.c__Oligoflexia
8000
6000
4000
2000
0
46−60 (n=7)
80plus (n=3)
61−80 (n=15)
31−45 (n=3)
20−30 (n=2)
Age_category

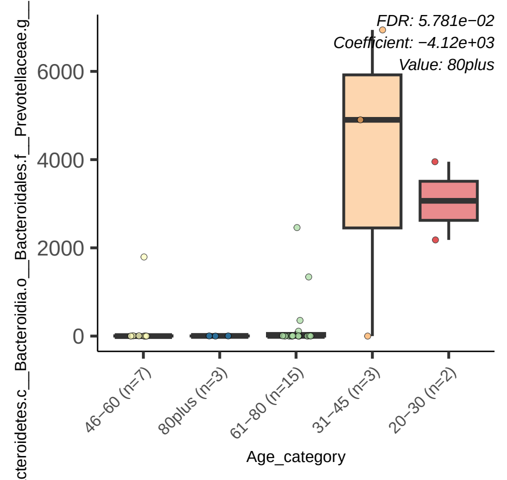

FDR: 5.781e−02
Coefficient: −4.12e+03
Value: 80plus
6000
4000
2000
0
cteroidetes.c__Bacteroidia.o__Bacteroidales.f__Prevotellaceae.g__
46−60 (n=7)
80plus (n=3)
61−80 (n=15)
31−45 (n=3)
20−30 (n=2)
Age_category

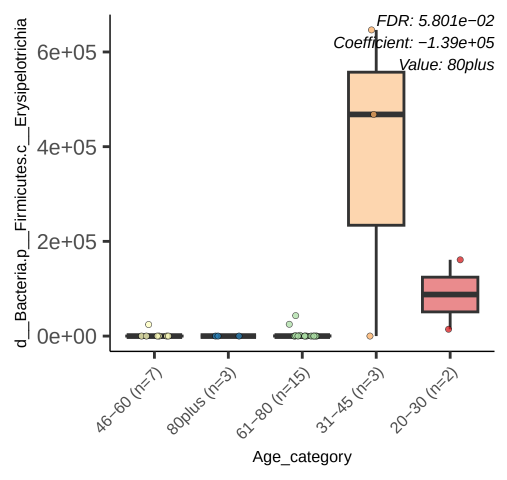

FDR: 5.801e−02
Coefficient: −1.39e+05
Value: 80plus
d__Bacteria.p__Firmicutes.c__Erysipelotrichia
6e+05
4e+05
2e+05
0e+00
46−60 (n=7)
80plus (n=3)
61−80 (n=15)
31−45 (n=3)
20−30 (n=2)
Age_category

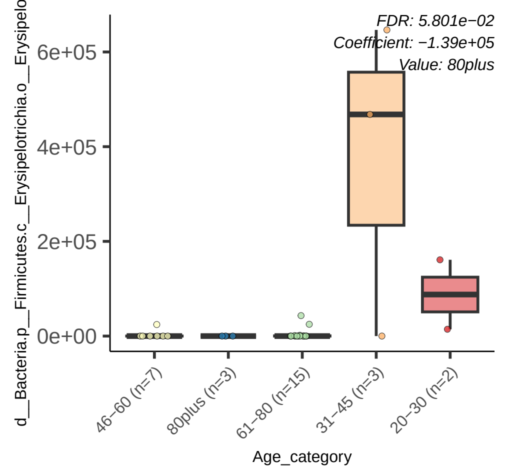

FDR: 5.801e−02
Coefficient: −1.39e+05
Value: 80plus
d__Bacteria.p__Firmicutes.c__Erysipelotrichia.o__Erysipelo
6e+05
4e+05
2e+05
0e+00
46−60 (n=7)
80plus (n=3)
61−80 (n=15)
31−45 (n=3)
20−30 (n=2)
Age_category

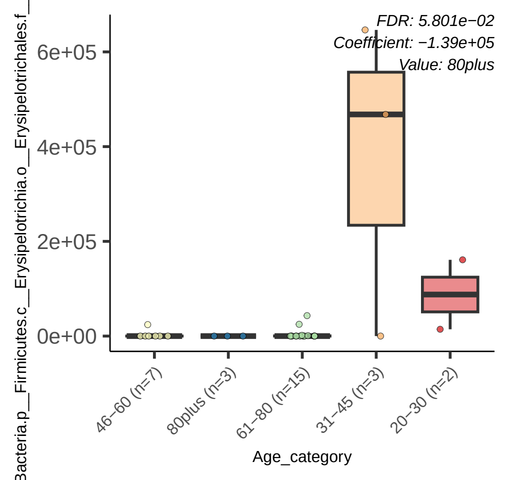
FDR: 5.801e−02
Coefficient: −1.39e+05
Value: 80plus
Bacteria.p__Firmicutes.c__Erysipelotrichia.o__Erysipelotrichales.f__
6e+05
4e+05
2e+05
0e+00
46−60 (n=7)
80plus (n=3)
61−80 (n=15)
31−45 (n=3)
20−30 (n=2)
Age_category

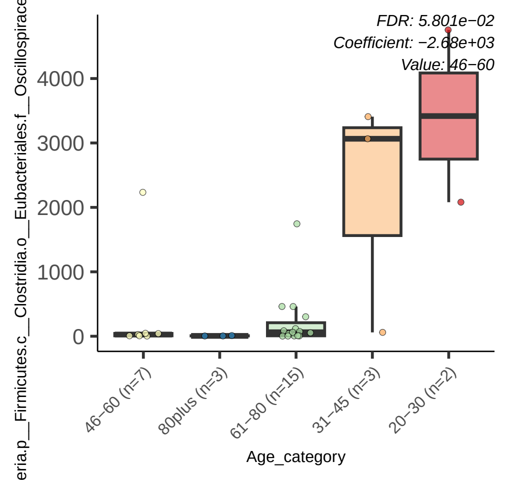

FDR: 5.801e−02
Coefficient: −2.68e+03
Value: 46−60
eria.p__Firmicutes.c__Clostridia.o__Eubacteriales.f__Oscillospirace
0
1000
2000
3000
4000
46−60 (n=7)
80plus (n=3)
61−80 (n=15)
31−45 (n=3)
20−30 (n=2)
Age_category

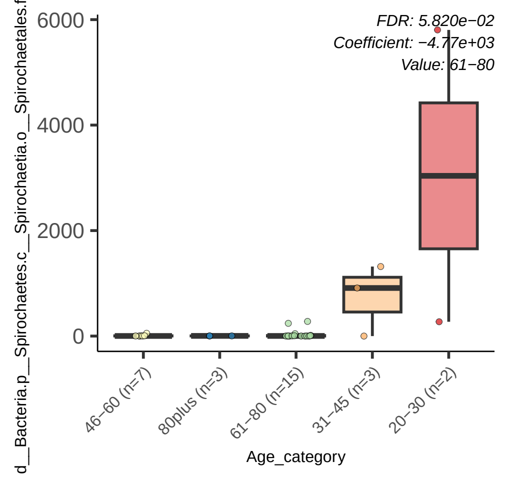

FDR: 5.820e−02
Coefficient: −4.77e+03
Value: 61−80
6000
4000
2000
0
d__Bacteria.p__Spirochaetes.c__Spirochaetia.o__Spirochaetales.f
46−60 (n=7)
80plus (n=3)
61−80 (n=15)
31−45 (n=3)
20−30 (n=2)
Age_category

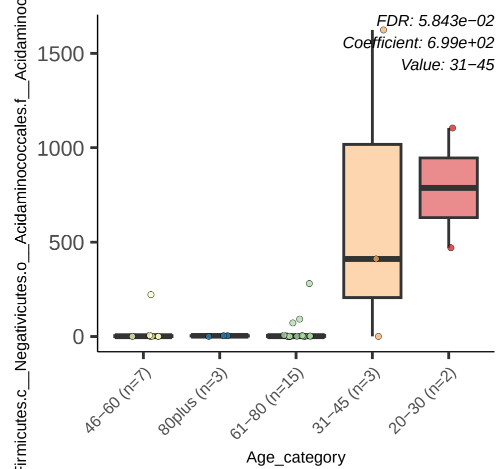

FDR: 5.843e−02
Coefficient: 6.99e+02
Value: 31−45
_Firmicutes.c__Negativicutes.o__Acidaminococcales.f__Acidaminoc
1500
1000
500
0
46−60 (n=7)
80plus (n=3)
61−80 (n=15)
31−45 (n=3)
20−30 (n=2)
Age_category

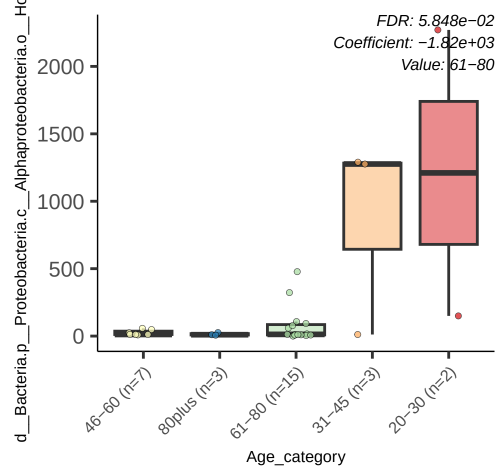

FDR: 5.848e−02
Coefficient: −1.82e+03
Value: 61−80
d__Bacteria.p__Proteobacteria.c__Alphaproteobacteria.o__Ho
2000
1500
1000
500
0
46−60 (n=7)
80plus (n=3)
61−80 (n=15)
31−45 (n=3)
20−30 (n=2)
Age_category

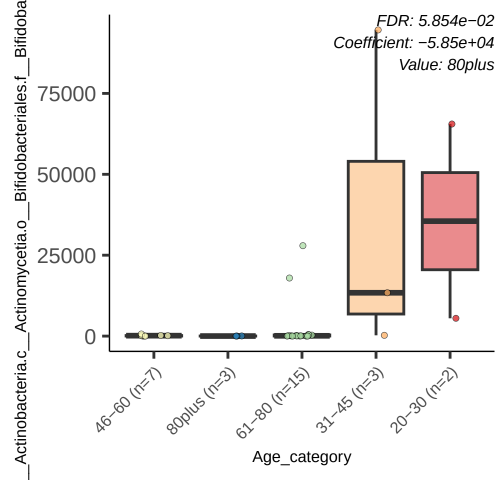

FDR: 5.854e−02
Coefficient: −5.85e+04
Value: 80plus
Actinobacteria.c__Actinomycetia.o__Bifidobacteriales.f__Bifidoba
75000
50000
25000
0
46−60 (n=7)
80plus (n=3)
61−80 (n=15)
31−45 (n=3)
20−30 (n=2)
Age_category

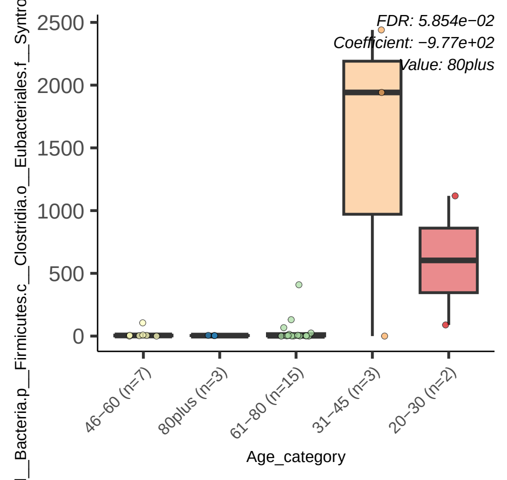

FDR: 5.854e−02
Coefficient: −9.77e+02
Value: 80plus
Bacteria.p__Firmicutes.c__Clostridia.o__Eubacteriales.f__Syntro
2500
2000
1500
1000
500
0
46−60 (n=7)
80plus (n=3)
61−80 (n=15)
31−45 (n=3)
20−30 (n=2)
Age_category

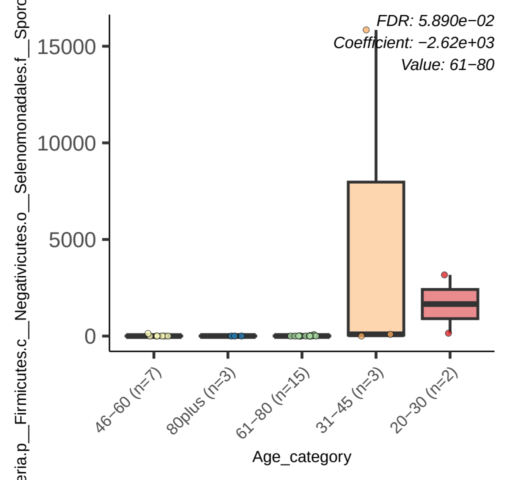

FDR: 5.890e−02
Coefficient: −2.62e+03
Value: 61−80
15000
10000
5000
0
eria.p__Firmicutes.c__Negativicutes.o__Selenomonadales.f__Sporo
46−60 (n=7)
80plus (n=3)
61−80 (n=15)
31−45 (n=3)
20−30 (n=2)
Age_category

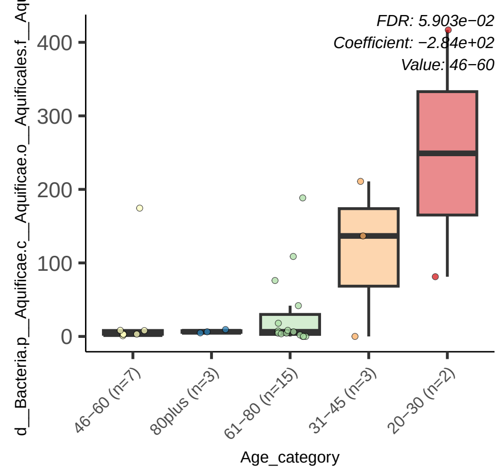

FDR: 5.903e−02
Coefficient: −2.84e+02
Value: 46−60
0
100
200
300
400
d__Bacteria.p__Aquificae.c__Aquificae.o__Aquificales.f__Aqu
46−60 (n=7)
80plus (n=3)
61−80 (n=15)
31−45 (n=3)
20−30 (n=2)
Age_category

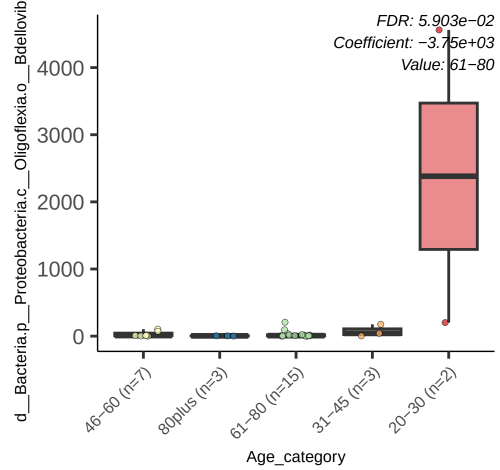

FDR: 5.903e−02
Coefficient: −3.75e+03
Value: 61−80
d__Bacteria.p__Proteobacteria.c__Oligoflexia.o__Bdellovib
4000
3000
2000
1000
0
46−60 (n=7)
80plus (n=3)
61−80 (n=15)
31−45 (n=3)
20−30 (n=2)
Age_category

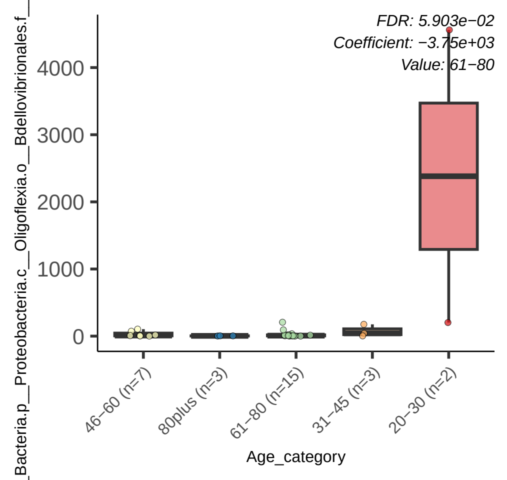

FDR: 5.903e−02
Coefficient: −3.75e+03
Value: 61−80
Bacteria.p__Proteobacteria.c__Oligoflexia.o__Bdellovibrionales.f
4000
3000
2000
1000
0
46−60 (n=7)
80plus (n=3)
61−80 (n=15)
31−45 (n=3)
20−30 (n=2)
Age_category

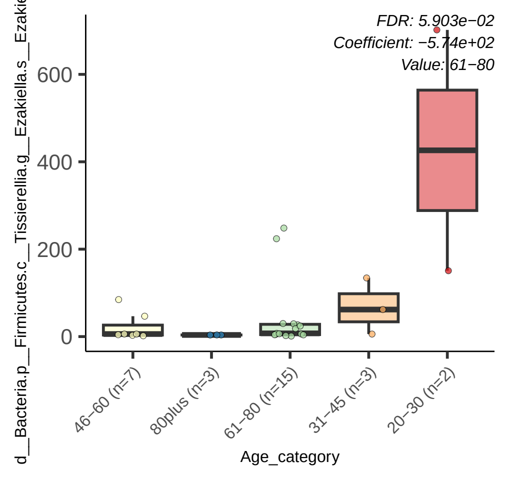

FDR: 5.903e−02
Coefficient: −5.74e+02
Value: 61−80
d__Bacteria.p__Firmicutes.c__Tissierellia.g__Ezakiella.s__Ezakie
0
200
400
600
46−60 (n=7)
80plus (n=3)
61−80 (n=15)
31−45 (n=3)
20−30 (n=2)
Age_category

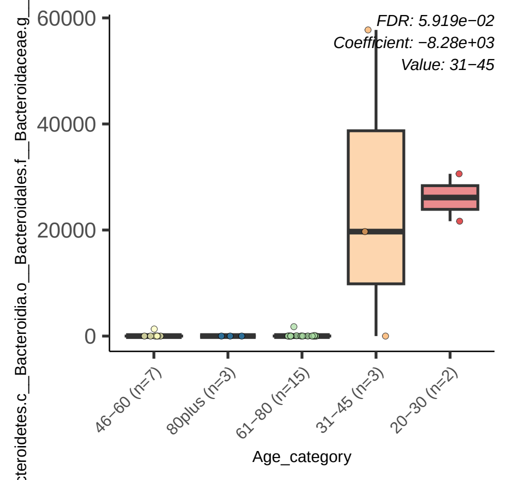
FDR: 5.919e−02
Coefficient: −8.28e+03
Value: 31−45
60000
40000
20000
0
cteroidetes.c__Bacteroidia.o__Bacteroidales.f__Bacteroidaceae.g__
46−60 (n=7)
80plus (n=3)
61−80 (n=15)
31−45 (n=3)
20−30 (n=2)
Age_category

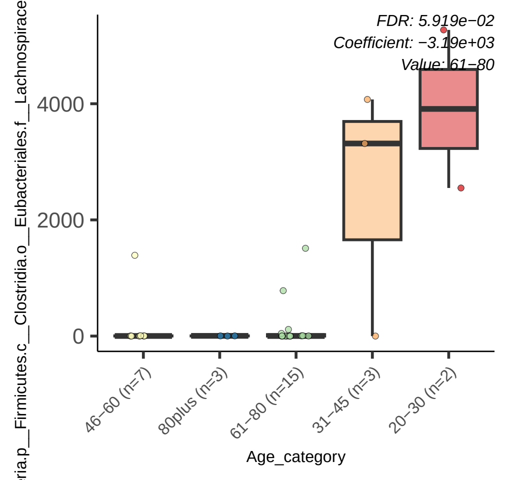

FDR: 5.919e−02
Coefficient: −3.19e+03
Value: 61−80
4000
2000
0
46−60 (n=7)
80plus (n=3)
61−80 (n=15)
31−45 (n=3)
20−30 (n=2)
Age_category
ria.p__Firmicutes.c__Clostridia.o__Eubacteriales.f__Lachnospirace

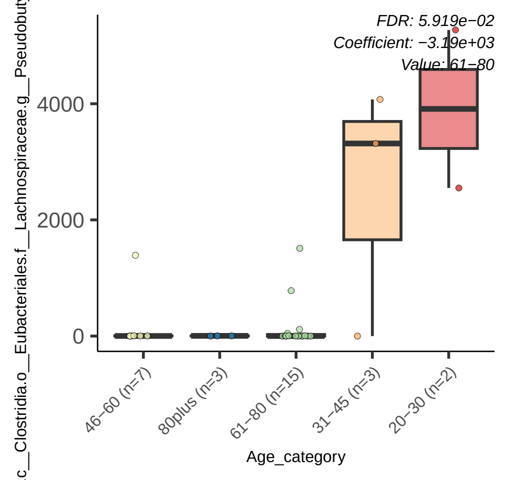

FDR: 5.919e−02
Coefficient: −3.19e+03
Value: 61−80
c__Clostridia.o__Eubacteriales.f__Lachnospiraceae.g__Pseudobuty
4000
2000
0
46−60 (n=7)
80plus (n=3)
61−80 (n=15)
31−45 (n=3)
20−30 (n=2)
Age_category

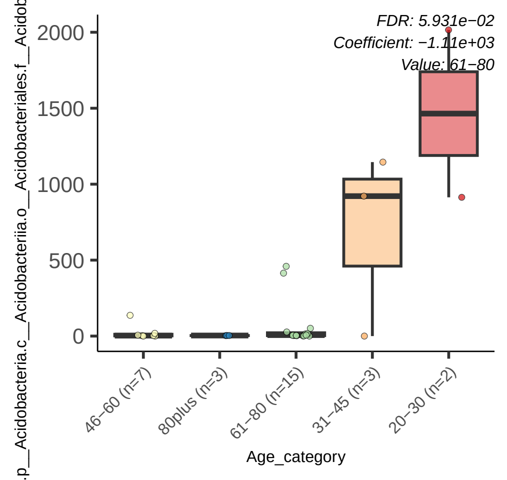

FDR: 5.931e−02
Coefficient: −1.11e+03
Value: 61−80
p__Acidobacteria.c__Acidobacteriia.o__Acidobacteriales.f__Acidob
2000
1500
1000
500
0
46−60 (n=7)
80plus (n=3)
61−80 (n=15)
31−45 (n=3)
20−30 (n=2)
Age_category

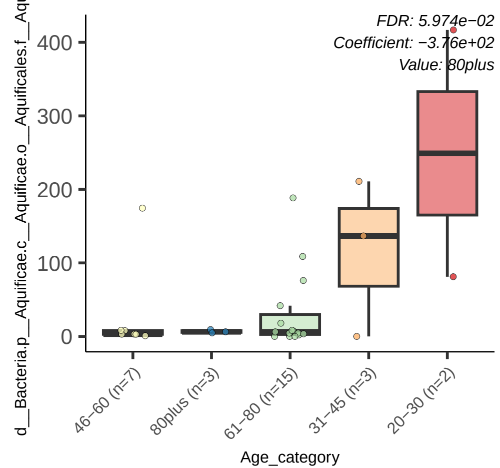

FDR: 5.974e−02
Coefficient: −3.76e+02
Value: 80plus
d__Bacteria.p__Aquificae.c__Aquificae.o__Aquificales.f__Aqu
400
300
200
100
0
46−60 (n=7)
80plus (n=3)
61−80 (n=15)
31−45 (n=3)
20−30 (n=2)
Age_category

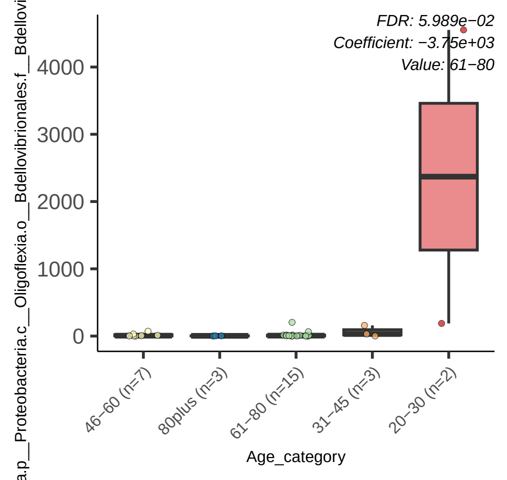

FDR: 5.989e−02
Coefficient: −3.75e+03
Value: 61−80
a.p__Proteobacteria.c__Oligoflexia.o__Bdellovibrionales.f__Bdellovi
4000
3000
2000
1000
0
46−60 (n=7)
80plus (n=3)
61−80 (n=15)
31−45 (n=3)
20−30 (n=2)
Age_category

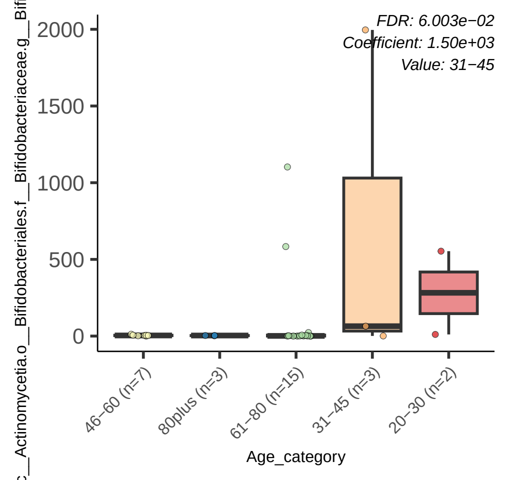
FDR: 6.003e−02
Coefficient: 1.50e+03
Value: 31−45
2000
1500
1000
500
0
46−60 (n=7)
80plus (n=3)
61−80 (n=15)
31−45 (n=3)
20−30 (n=2)
Age_category
Actinomycetia.o__Bifidobacteriales.f__Bifidobacteriaceae.g__Bifi

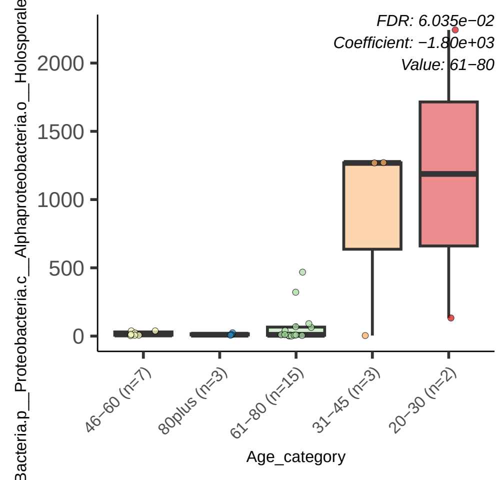

FDR: 6.035e−02
Coefficient: −1.80e+03
Value: 61−80
Bacteria.p__Proteobacteria.c__Alphaproteobacteria.o__Holosporale
2000
1500
1000
500
0
46−60 (n=7)
80plus (n=3)
61−80 (n=15)
31−45 (n=3)
20−30 (n=2)
Age_category

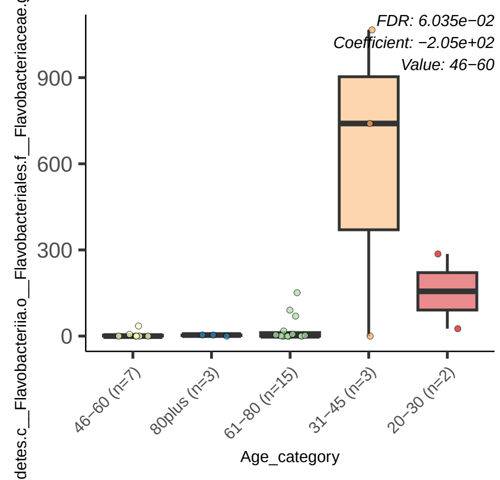

FDR: 6.035e−02
Coefficient: −2.05e+02
Value: 46−60
900
600
300
0
46−60 (n=7)
80plus (n=3)
61−80 (n=15)
31−45 (n=3)
20−30 (n=2)
Age_category
detes.c__Flavobacteriia.o__Flavobacteriales.f__Flavobacteriaceae.g

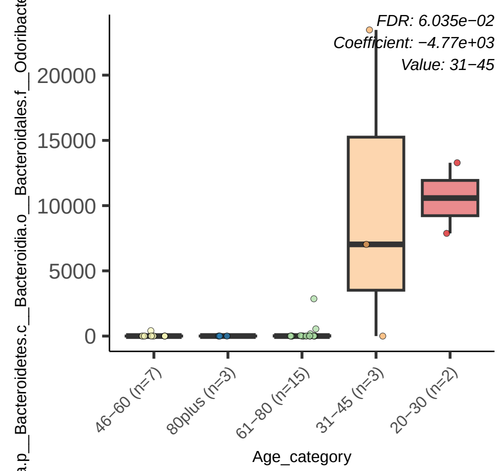
FDR: 6.035e−02
Coefficient: −4.77e+03
Value: 31−45
ia.p__Bacteroidetes.c__Bacteroidia.o__Bacteroidales.f__Odoribacte
20000
15000
10000
5000
0
46−60 (n=7)
80plus (n=3)
61−80 (n=15)
31−45 (n=3)
20−30 (n=2)
Age_category

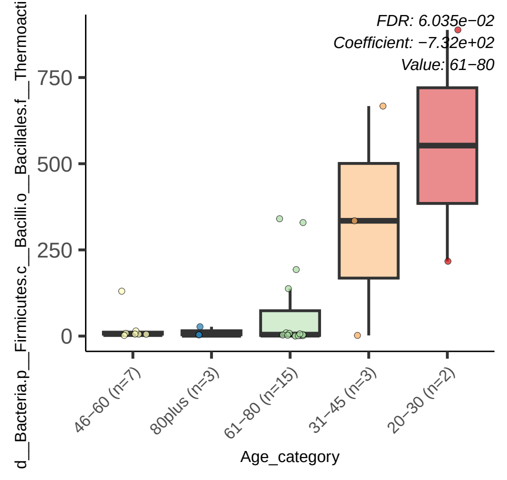
FDR: 6.035e−02
Coefficient: −7.32e+02
Value: 61−80
d__Bacteria.p__Firmicutes.c__Bacilli.o__Bacillales.f__Thermoacti
750
500
250
0
46−60 (n=7)
80plus (n=3)
61−80 (n=15)
31−45 (n=3)
20−30 (n=2)
Age_category

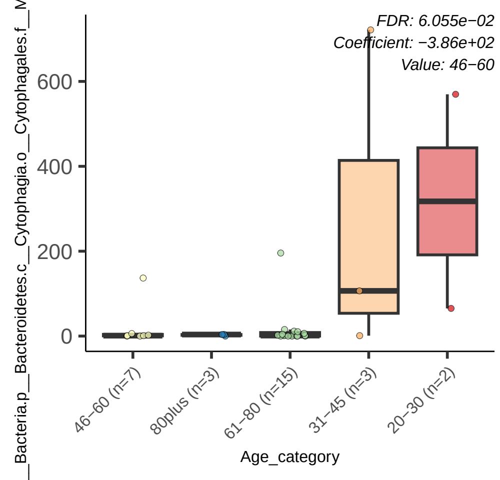
FDR: 6.055e−02
Coefficient: −3.86e+02
Value: 46−60
Bacteria.p__Bacteroidetes.c__Cytophagia.o__Cytophagales.f__M
0
200
400
600
46−60 (n=7)
80plus (n=3)
61−80 (n=15)
31−45 (n=3)
20−30 (n=2)
Age_category

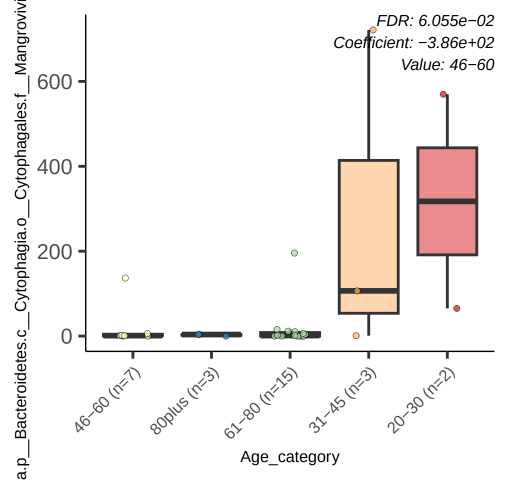
FDR: 6.055e−02
Coefficient: −3.86e+02
Value: 46−60
a.p__Bacteroidetes.c__Cytophagia.o__Cytophagales.f__Mangrovivi
600
400
200
0
46−60 (n=7)
80plus (n=3)
61−80 (n=15)
31−45 (n=3)
20−30 (n=2)
Age_category

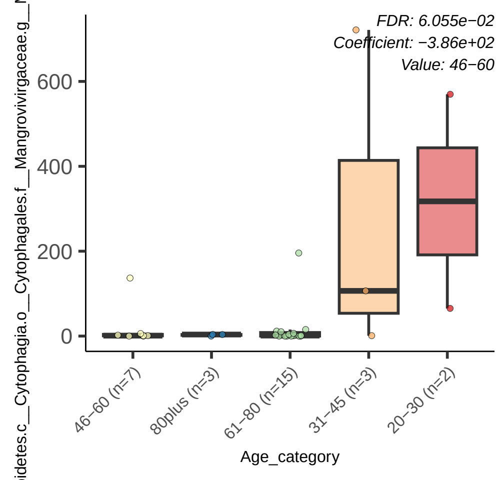

FDR: 6.055e−02
Coefficient: −3.86e+02
Value: 46−60
600
400
200
0
46−60 (n=7)
80plus (n=3)
61−80 (n=15)
31−45 (n=3)
20−30 (n=2)
Age_category
idetes.c__Cytophagia.o__Cytophagales.f__Mangrovivirgaceae.g__

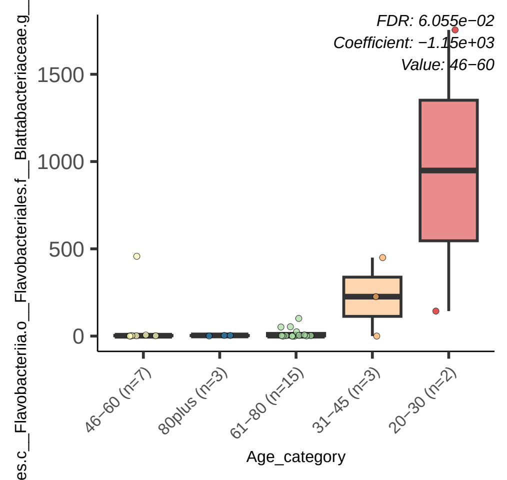
FDR: 6.055e−02
Coefficient: −1.15e+03
Value: 46−60
1500
1000
500
0
es.c__Flavobacteriia.o__Flavobacteriales.f__Blattabacteriaceae.g__
46−60 (n=7)
80plus (n=3)
61−80 (n=15)
31−45 (n=3)
20−30 (n=2)
Age_category

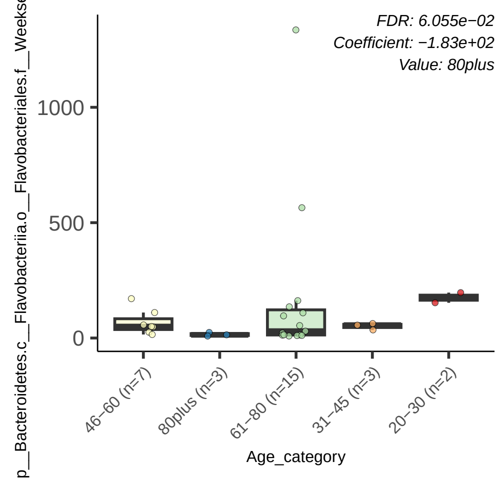

FDR: 6.055e−02
Coefficient: −1.83e+02
Value: 80plus
1000
500
0
p__Bacteroidetes.c__Flavobacteriia.o__Flavobacteriales.f__Weekse
46−60 (n=7)
80plus (n=3)
61−80 (n=15)
31−45 (n=3)
20−30 (n=2)
Age_category

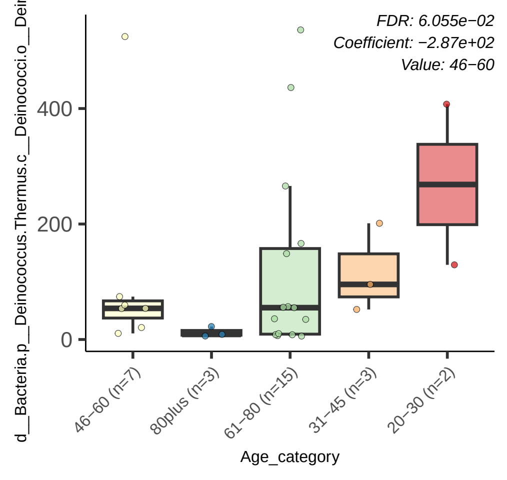

FDR: 6.055e−02
Coefficient: −2.87e+02
Value: 46−60
d__Bacteria.p__Deinococcus.Thermus.c__Deinococci.o__Dei
400
200
0
46−60 (n=7)
80plus (n=3)
61−80 (n=15)
31−45 (n=3)
20−30 (n=2)
Age_category

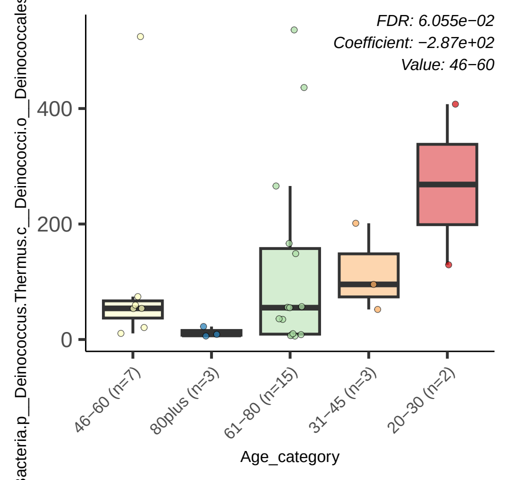

FDR: 6.055e−02
Coefficient: −2.87e+02
Value: 46−60
Bacteria.p__Deinococcus.Thermus.c__Deinococci.o__Deinococcales
0
200
400
46−60 (n=7)
80plus (n=3)
61−80 (n=15)
31−45 (n=3)
20−30 (n=2)
Age_category

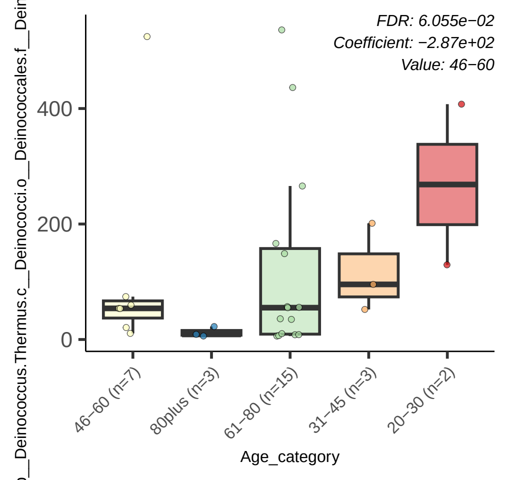
FDR: 6.055e−02
Coefficient: −2.87e+02
Value: 46−60
400
200
0
46−60 (n=7)
80plus (n=3)
61−80 (n=15)
31−45 (n=3)
20−30 (n=2)
Age_category
o__Deinococcus.Thermus.c__Deinococci.o__Deinococcales.f__Dein

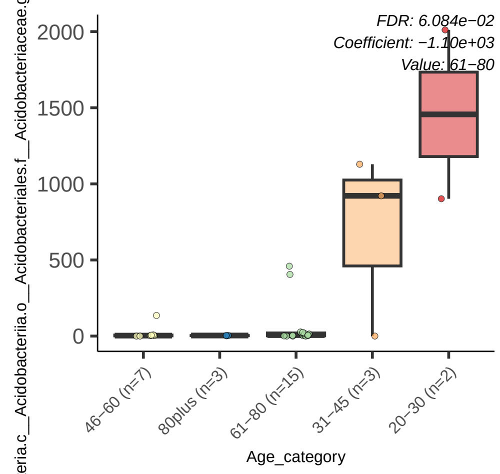

FDR: 6.084e−02
Coefficient: −1.10e+03
Value: 61−80
2000
1500
1000
500
0
46−60 (n=7)
80plus (n=3)
61−80 (n=15)
31−45 (n=3)
20−30 (n=2)
Age_category
cteria.c__Acidobacteriia.o__Acidobacteriales.f__Acidobacteriaceae.g

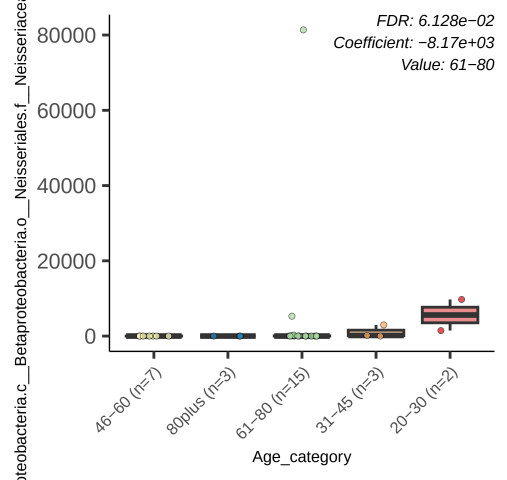
FDR: 6.128e−02
Coefficient: −8.17e+03
Value: 61−80
80000
60000
40000
20000
0
46−60 (n=7)
80plus (n=3)
61−80 (n=15)
31−45 (n=3)
20−30 (n=2)
Age_category
oteobacteria.c__Betaproteobacteria.o__Neisseriales.f__Neisseriace

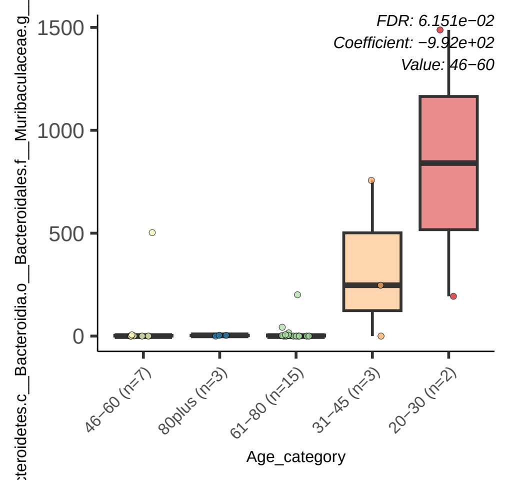
FDR: 6.151e−02
Coefficient: −9.92e+02
Value: 46−60
Bacteroidetes.c__Bacteroidia.o__Bacteroidales.f__Muribaculaceae.g__
1500
1000
500
0
46−60 (n=7)
80plus (n=3)
61−80 (n=15)
31−45 (n=3)
20−30 (n=2)
Age_category

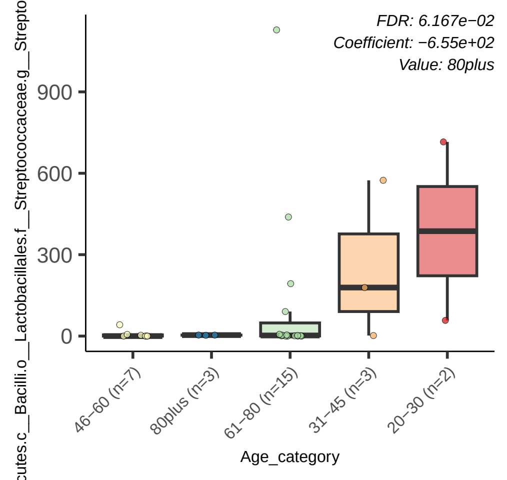
FDR: 6.167e−02
Coefficient: −6.55e+02
Value: 80plus
cutes.c__Bacilli.o__Lactobacillales.f__Streptococcaceae.g__Strepto
900
600
300
0
46−60 (n=7)
80plus (n=3)
61−80 (n=15)
31−45 (n=3)
20−30 (n=2)
Age_category

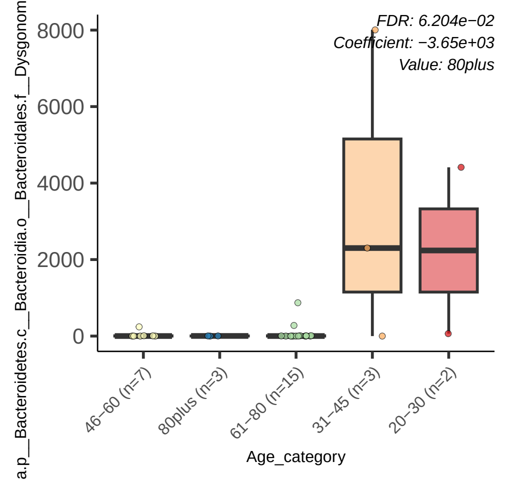

FDR: 6.204e−02
Coefficient: −3.65e+03
Value: 80plus
a.p__Bacteroidetes.c__Bacteroidia.o__Bacteroidales.f__Dysgonom
8000
6000
4000
2000
0
46−60 (n=7)
80plus (n=3)
61−80 (n=15)
31−45 (n=3)
20−30 (n=2)
Age_category

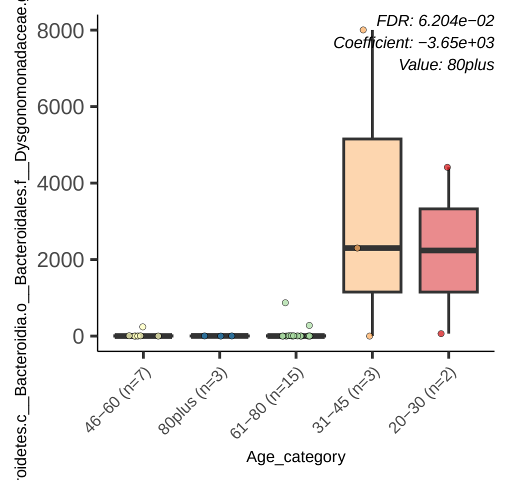

FDR: 6.204e−02
Coefficient: −3.65e+03
Value: 80plus
8000
6000
4000
2000
0
oidetes.c__Bacteroidia.o__Bacteroidales.f__Dysgonomonadaceae.g
46−60 (n=7)
80plus (n=3)
61−80 (n=15)
31−45 (n=3)
20−30 (n=2)
Age_category

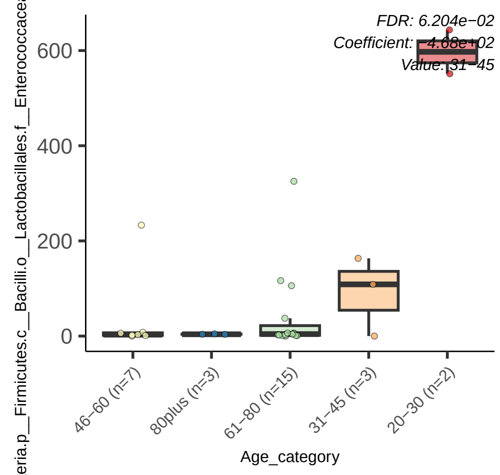

FDR: 6.204e−02
Coefficient: 4.68e+02
Value: 31−45
600
400
200
0
teria.p__Firmicutes.c__Bacilli.o__Lactobacillales.f__Enterococcace
46−60 (n=7)
80plus (n=3)
61−80 (n=15)
31−45 (n=3)
20−30 (n=2)
Age_category

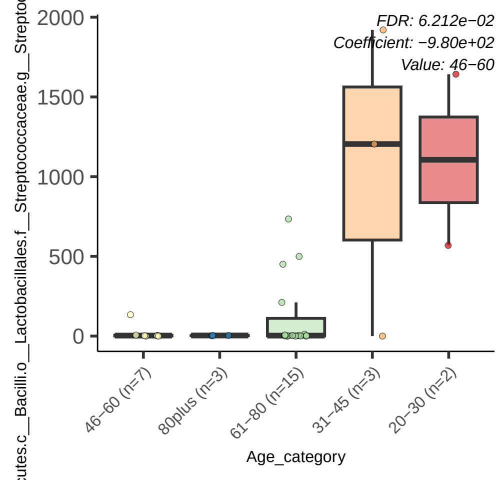

FDR: 6.212e−02
Coefficient: −9.80e+02
Value: 46−60
2000
1500
1000
500
0
46−60 (n=7)
80plus (n=3)
61−80 (n=15)
31−45 (n=3)
20−30 (n=2)
Age_category
cutes.c__Bacilli.o__Lactobacillales.f__Streptococcaceae.g__Streptoc

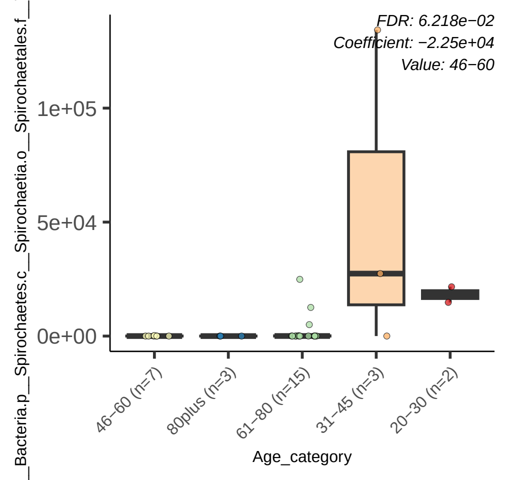

FDR: 6.218e−02
Coefficient: −2.25e+04
Value: 46−60
1e+05
5e+04
0e+00
Bacteria.p__Spirochaetes.c__Spirochaetia.o__Spirochaetales.f__
46−60 (n=7)
80plus (n=3)
61−80 (n=15)
31−45 (n=3)
20−30 (n=2)
Age_category

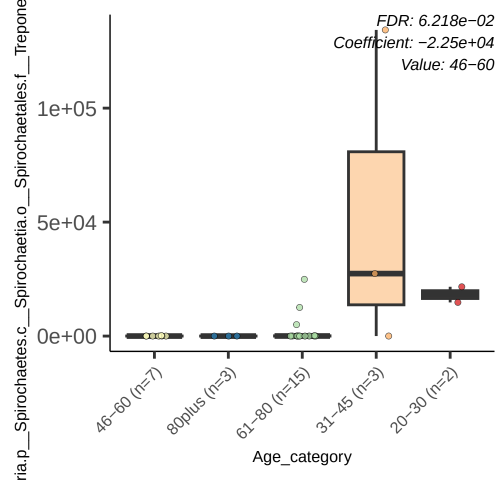
FDR: 6.218e−02
Coefficient: −2.25e+04
Value: 46−60
1e+05
5e+04
0e+00
ria.p__Spirochaetes.c__Spirochaetia.o__Spirochaetales.f__Trepone
46−60 (n=7)
80plus (n=3)
61−80 (n=15)
31−45 (n=3)
20−30 (n=2)
Age_category

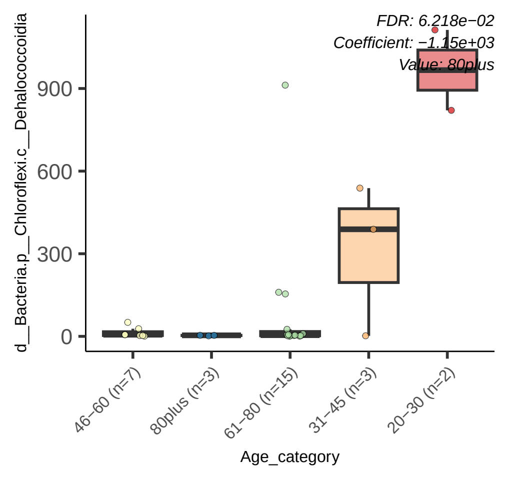
FDR: 6.218e−02
Coefficient: −1.15e+03
Value: 80plus
d__Bacteria.p__Chloroflexi.c__Dehalococcoidia
900
600
300
0
46−60 (n=7)
80plus (n=3)
61−80 (n=15)
31−45 (n=3)
20−30 (n=2)
Age_category

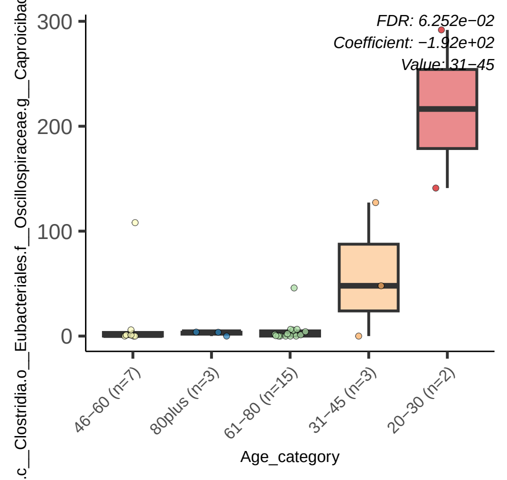

FDR: 6.252e−02
Coefficient: −1.92e+02
Value: 31−45
300
200
100
0
c__Clostridia.o__Eubacteriales.f__Oscillospiraceae.g__Caproicibac
46−60 (n=7)
80plus (n=3)
61−80 (n=15)
31−45 (n=3)
20−30 (n=2)
Age_category

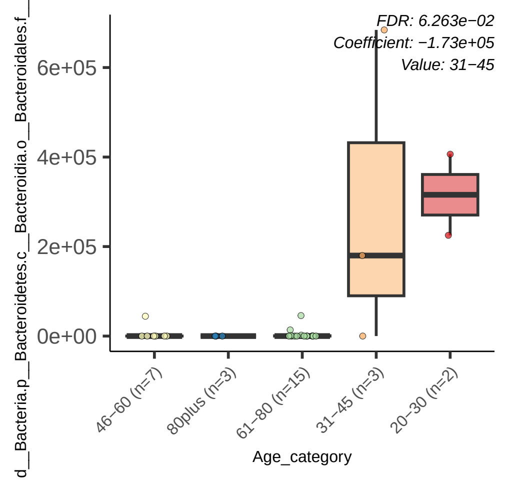

FDR: 6.263e−02
Coefficient: −1.73e+05
Value: 31−45
6e+05
4e+05
2e+05
0e+00
d__Bacteria.p__Bacteroidetes.c__Bacteroidia.o__Bacteroidales.f__
46−60 (n=7)
80plus (n=3)
61−80 (n=15)
31−45 (n=3)
20−30 (n=2)
Age_category

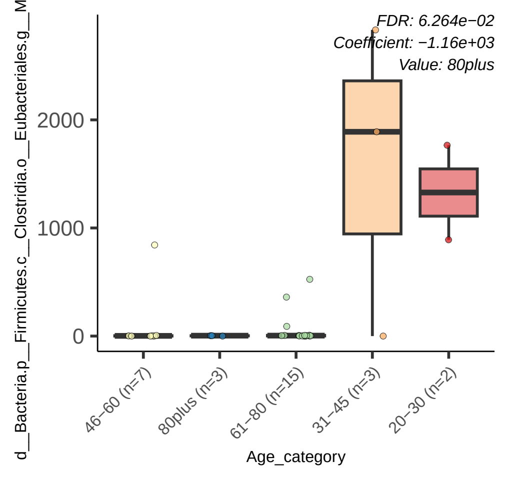

FDR: 6.264e−02
Coefficient: −1.16e+03
Value: 80plus
2000
1000
0
46−60 (n=7)
80plus (n=3)
61−80 (n=15)
31−45 (n=3)
20−30 (n=2)
Age_category
d__Bacteria.p__Firmicutes.c__Clostridia.o__Eubacteriales.g__M

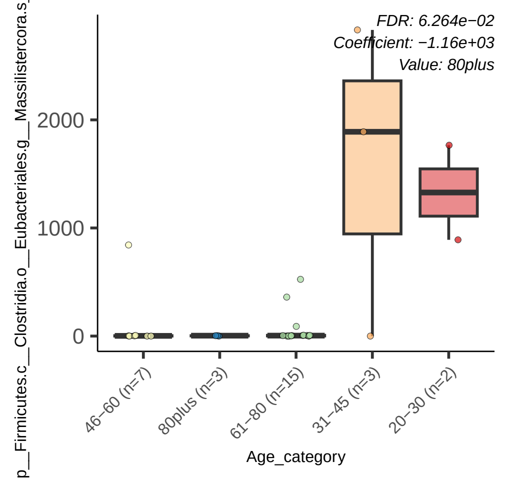
FDR: 6.264e−02
Coefficient: −1.16e+03
Value: 80plus
p__Firmicutes.c__Clostridia.o__Eubacteriales.g__Massilistercora.s_
2000
1000
0
46−60 (n=7)
80plus (n=3)
61−80 (n=15)
31−45 (n=3)
20−30 (n=2)
Age_category

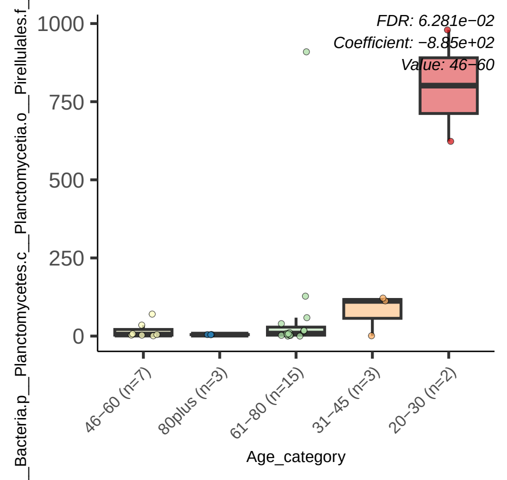

FDR: 6.281e−02
Coefficient: −8.85e+02
Value: 46−60
Bacteria.p__Planctomycetes.c__Planctomycetia.o__Pirellulales.f
1000
750
500
250
0
46−60 (n=7)
80plus (n=3)
61−80 (n=15)
31−45 (n=3)
20−30 (n=2)
Age_category

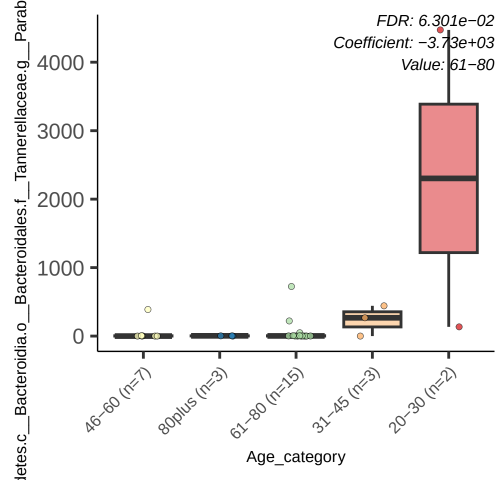

FDR: 6.301e−02
Coefficient: −3.73e+03
Value: 61−80
4000
3000
2000
1000
0
46−60 (n=7)
80plus (n=3)
61−80 (n=15)
31−45 (n=3)
20−30 (n=2)
Age_category
detes.c__Bacteroidia.o__Bacteroidales.f__Tannerellaceae.g__Parab

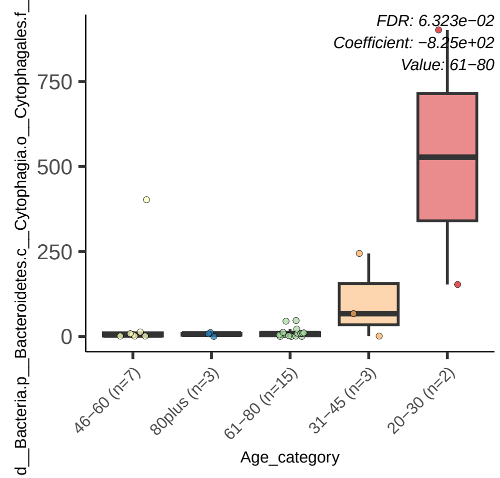

FDR: 6.323e−02
Coefficient: −8.25e+02
Value: 61−80
d__Bacteria.p__Bacteroidetes.c__Cytophagia.o__Cytophagales.f__
0
250
500
750
46−60 (n=7)
80plus (n=3)
61−80 (n=15)
31−45 (n=3)
20−30 (n=2)
Age_category

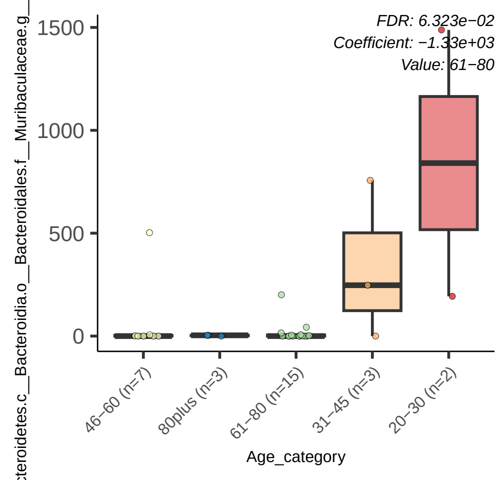
FDR: 6.323e−02
Coefficient: −1.33e+03
Value: 61−80
1500
1000
500
0
cteroidetes.c__Bacteroidia.o__Bacteroidales.f__Muribaculaceae.g__
46−60 (n=7)
80plus (n=3)
61−80 (n=15)
31−45 (n=3)
20−30 (n=2)
Age_category

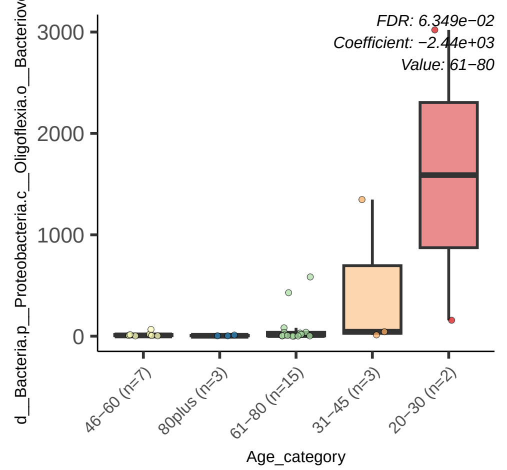

FDR: 6.349e−02
Coefficient: −2.44e+03
Value: 61−80
d__Bacteria.p__Proteobacteria.c__Oligoflexia.o__Bacteriov
3000
2000
1000
0
46−60 (n=7)
80plus (n=3)
61−80 (n=15)
31−45 (n=3)
20−30 (n=2)
Age_category

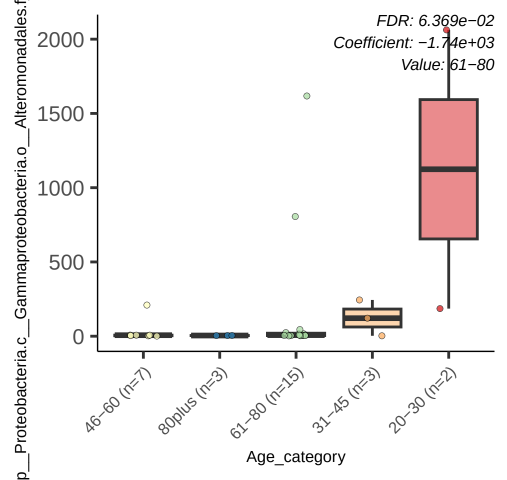

FDR: 6.369e−02
Coefficient: −1.74e+03
Value: 61−80
2000
1500
1000
500
0
p__Proteobacteria.c__Gammaproteobacteria.o__Alteromonadales.f
46−60 (n=7)
80plus (n=3)
61−80 (n=15)
31−45 (n=3)
20−30 (n=2)
Age_category

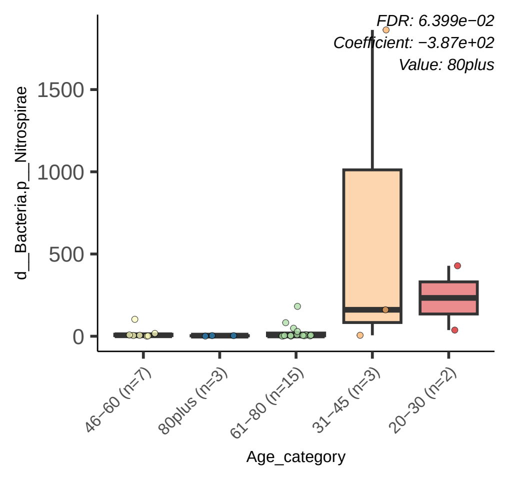

FDR: 6.399e−02
Coefficient: −3.87e+02
Value: 80plus
d__Bacteria.p__Nitrospirae
1500
1000
500
0
46−60 (n=7)
80plus (n=3)
61−80 (n=15)
31−45 (n=3)
20−30 (n=2)
Age_category

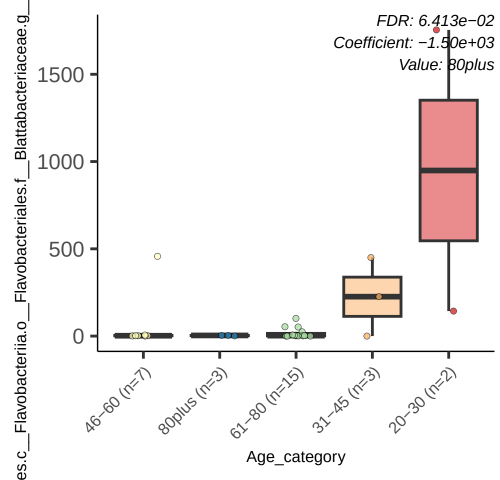

FDR: 6.413e−02
Coefficient: −1.50e+03
Value: 80plus
es.c__Flavobacteriia.o__Flavobacteriales.f__Blattabacteriaceae.g__
1500
1000
500
0
46−60 (n=7)
80plus (n=3)
61−80 (n=15)
31−45 (n=3)
20−30 (n=2)
Age_category

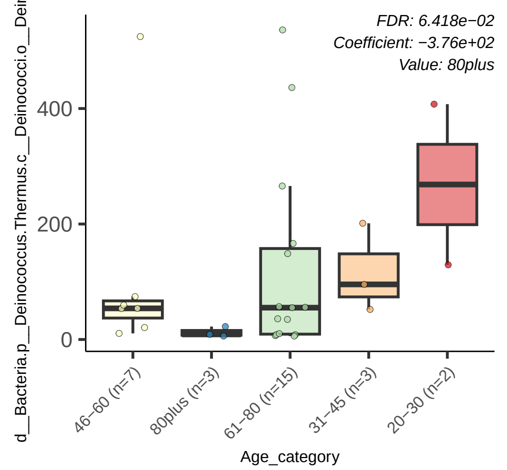

FDR: 6.418e−02
Coefficient: −3.76e+02
Value: 80plus
0
200
400
d__Bacteria.p__Deinococcus.Thermus.c__Deinococci.o__Dei
46−60 (n=7)
80plus (n=3)
61−80 (n=15)
31−45 (n=3)
20−30 (n=2)
Age_category

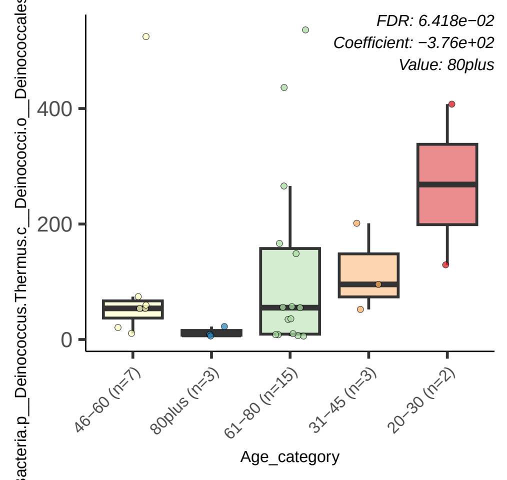

FDR: 6.418e−02
Coefficient: −3.76e+02
Value: 80plus
0
200
400
Bacteria.p__Deinococcus.Thermus.c__Deinococci.o__Deinococcales
46−60 (n=7)
80plus (n=3)
61−80 (n=15)
31−45 (n=3)
20−30 (n=2)
Age_category

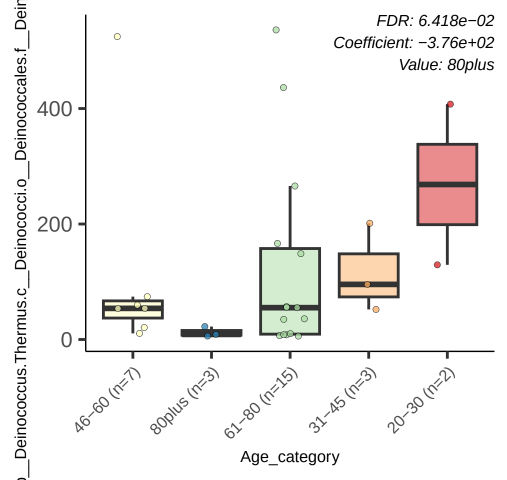

FDR: 6.418e−02
Coefficient: −3.76e+02
Value: 80plus
0
200
400
o__Deinococcus.Thermus.c__Deinococci.o__Deinococcales.f__Dein
46−60 (n=7)
80plus (n=3)
61−80 (n=15)
31−45 (n=3)
20−30 (n=2)
Age_category

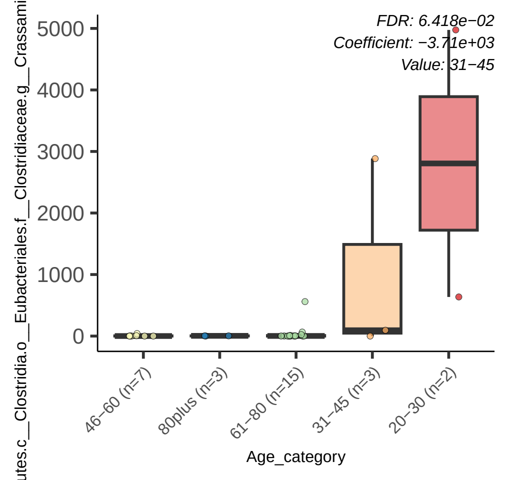
FDR: 6.418e−02
Coefficient: −3.71e+03
Value: 31−45
utes.c__Clostridia.o__Eubacteriales.f__Clostridiaceae.g__Crassami
5000
4000
3000
2000
1000
0
46−60 (n=7)
80plus (n=3)
61−80 (n=15)
31−45 (n=3)
20−30 (n=2)
Age_category

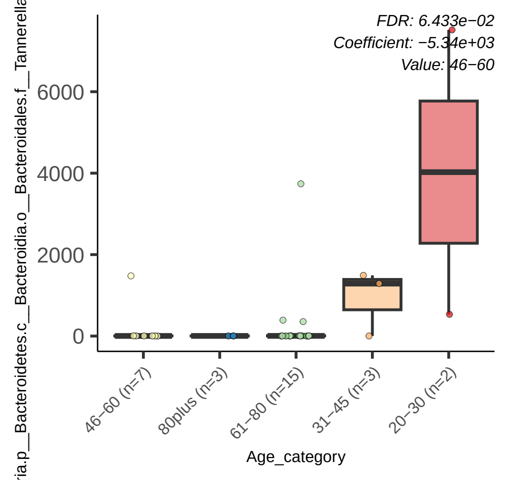

FDR: 6.433e−02
Coefficient: −5.34e+03
Value: 46−60
ria.p__Bacteroidetes.c__Bacteroidia.o__Bacteroidales.f__Tannerella
6000
4000
2000
0
46−60 (n=7)
80plus (n=3)
61−80 (n=15)
31−45 (n=3)
20−30 (n=2)
Age_category

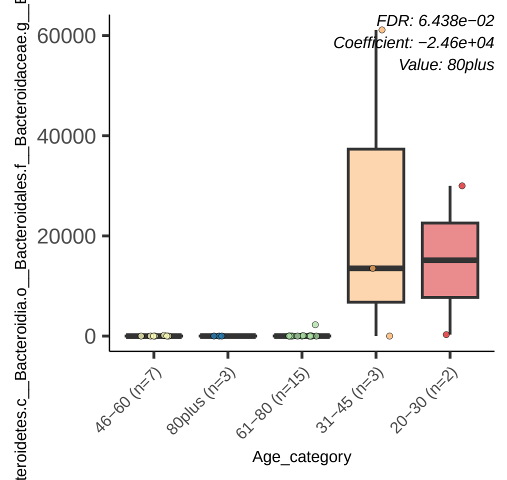

FDR: 6.438e−02
Coefficient: −2.46e+04
Value: 80plus
60000
40000
20000
0
teroidetes.c__Bacteroidia.o__Bacteroidales.f__Bacteroidaceae.g__B
46−60 (n=7)
80plus (n=3)
61−80 (n=15)
31−45 (n=3)
20−30 (n=2)
Age_category

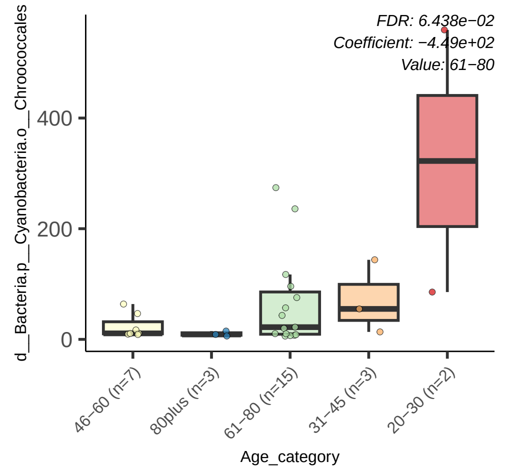

FDR: 6.438e−02
Coefficient: −4.49e+02
Value: 61−80
d__Bacteria.p__Cyanobacteria.o__Chroococcales
0
200
400
46−60 (n=7)
80plus (n=3)
61−80 (n=15)
31−45 (n=3)
20−30 (n=2)
Age_category

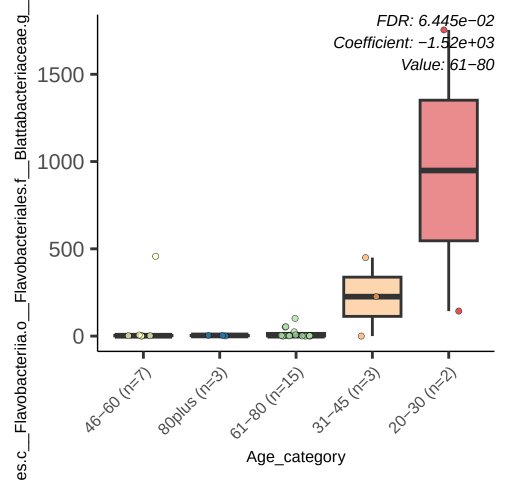

FDR: 6.445e−02
Coefficient: −1.52e+03
Value: 61−80
1500
1000
500
0
46−60 (n=7)
80plus (n=3)
61−80 (n=15)
31−45 (n=3)
20−30 (n=2)
Age_category
es.c__Flavobacteriia.o__Flavobacteriales.f__Blattabacteriaceae.g__

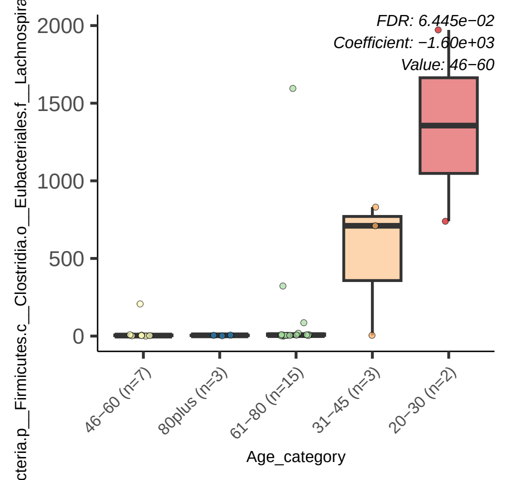

FDR: 6.445e−02
Coefficient: −1.60e+03
Value: 46−60
2000
1500
1000
500
0
cteria.p__Firmicutes.c__Clostridia.o__Eubacteriales.f__Lachnospira
46−60 (n=7)
80plus (n=3)
61−80 (n=15)
31−45 (n=3)
20−30 (n=2)
Age_category

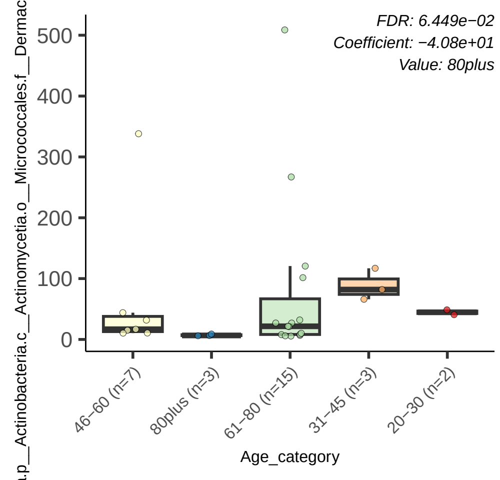
FDR: 6.449e−02
Coefficient: −4.08e+01
Value: 80plus
a.p__Actinobacteria.c__Actinomycetia.o__Micrococcales.f__Dermac
0
100
200
300
400
500
46−60 (n=7)
80plus (n=3)
61−80 (n=15)
31−45 (n=3)
20−30 (n=2)
Age_category

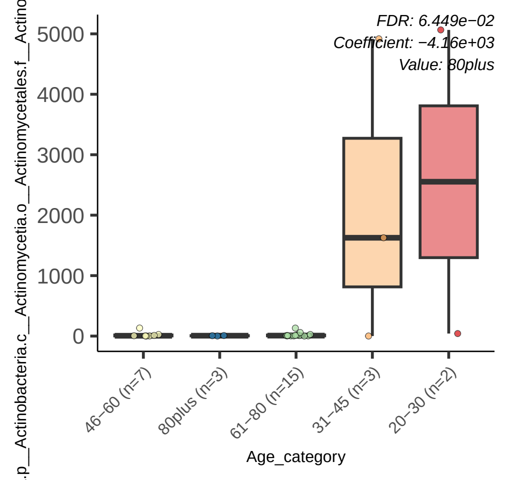

p__Actinobacteria.c__Actinomycetia.o__Actinomycetales.f__Actino
FDR: 6.449e−02
Coefficient: −4.16e+03
Value: 80plus
5000
4000
3000
2000
1000
0
46−60 (n=7)
80plus (n=3)
61−80 (n=15)
31−45 (n=3)
20−30 (n=2)
Age_category

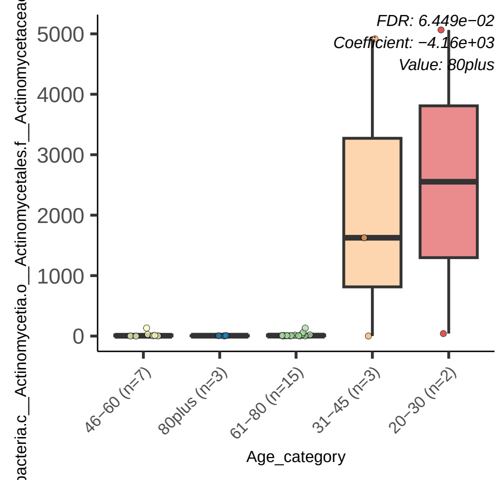

FDR: 6.449e−02
Coefficient: −4.16e+03
Value: 80plus
5000
4000
3000
2000
1000
0
bacteria.c__Actinomycetia.o__Actinomycetales.f__Actinomycetaceae
46−60 (n=7)
80plus (n=3)
61−80 (n=15)
31−45 (n=3)
20−30 (n=2)
Age_category

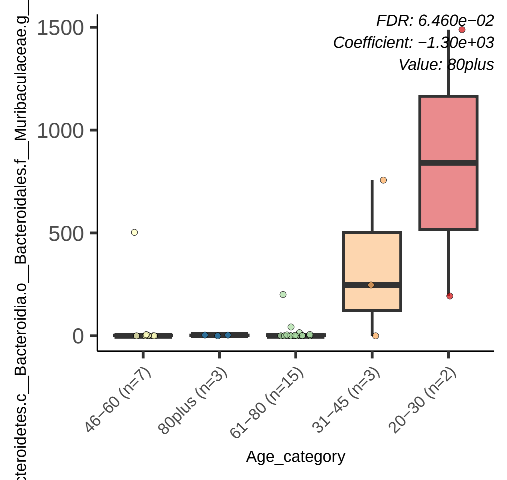

FDR: 6.460e−02
Coefficient: −1.30e+03
Value: 80plus
1500
1000
500
0
46−60 (n=7)
80plus (n=3)
61−80 (n=15)
31−45 (n=3)
20−30 (n=2)
Age_category
cteroidetes.c__Bacteroidia.o__Bacteroidales.f__Muribaculaceae.g__

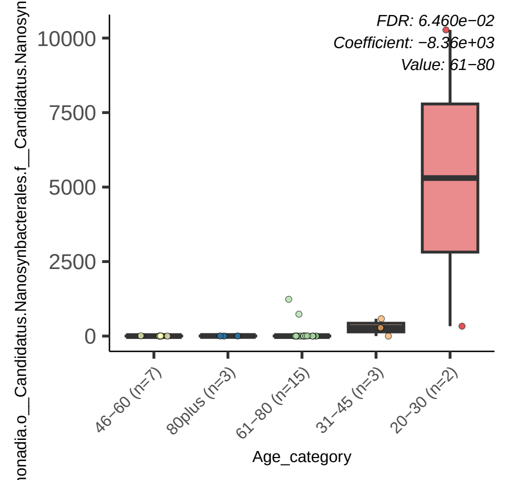

FDR: 6.460e−02
Coefficient: −8.36e+03
Value: 61−80
10000
7500
5000
2500
0
46−60 (n=7)
80plus (n=3)
61−80 (n=15)
31−45 (n=3)
20−30 (n=2)
Age_category
nonadia.o__Candidatus.Nanosynbacterales.f__Candidatus.Nanosyn

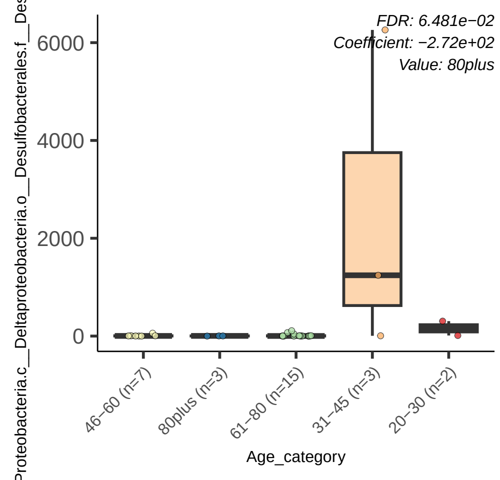

FDR: 6.481e−02
Coefficient: −2.72e+02
Value: 80plus
Proteobacteria.c__Deltaproteobacteria.o__Desulfobacterales.f__Des
6000
4000
2000
0
46−60 (n=7)
80plus (n=3)
61−80 (n=15)
31−45 (n=3)
20−30 (n=2)
Age_category

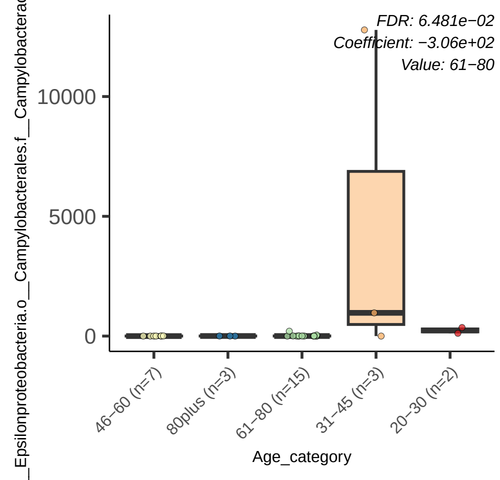

FDR: 6.481e−02
Coefficient: −3.06e+02
Value: 61−80
10000
5000
0
_Epsilonproteobacteria.o__Campylobacterales.f__Campylobacterac
46−60 (n=7)
80plus (n=3)
61−80 (n=15)
31−45 (n=3)
20−30 (n=2)
Age_category

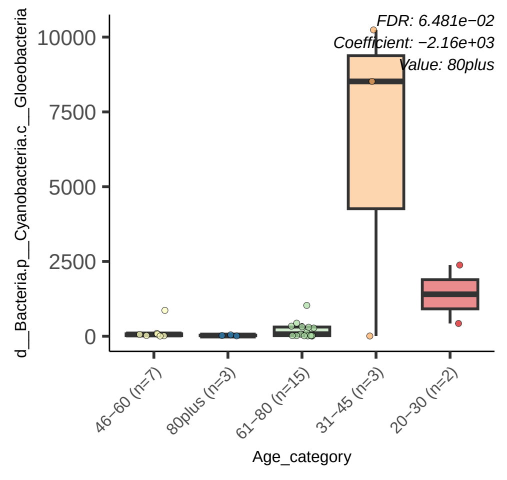
FDR: 6.481e−02
Coefficient: −2.16e+03
Value: 80plus
d__Bacteria.p__Cyanobacteria.c__Gloeobacteria
10000
7500
5000
2500
0
46−60 (n=7)
80plus (n=3)
61−80 (n=15)
31−45 (n=3)
20−30 (n=2)
Age_category

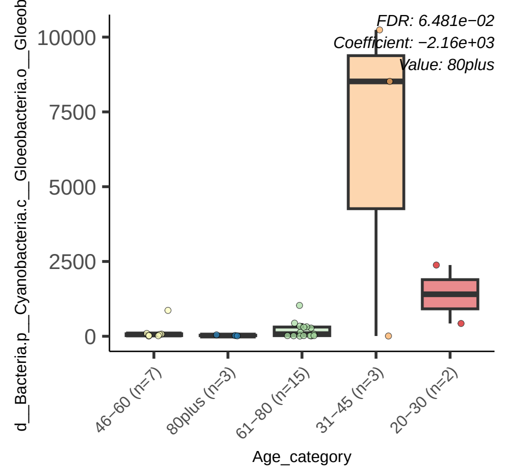

FDR: 6.481e−02
Coefficient: −2.16e+03
Value: 80plus
10000
7500
5000
2500
0
d__Bacteria.p__Cyanobacteria.c__Gloeobacteria.o__Gloeob
46−60 (n=7)
80plus (n=3)
61−80 (n=15)
31−45 (n=3)
20−30 (n=2)
Age_category

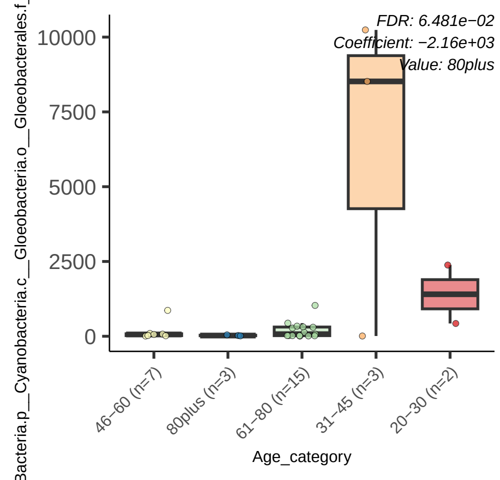

FDR: 6.481e−02
Coefficient: −2.16e+03
Value: 80plus
Bacteria.p__Cyanobacteria.c__Gloeobacteria.o__Gloeobacterales.f_
10000
7500
5000
2500
0
46−60 (n=7)
80plus (n=3)
61−80 (n=15)
31−45 (n=3)
20−30 (n=2)
Age_category

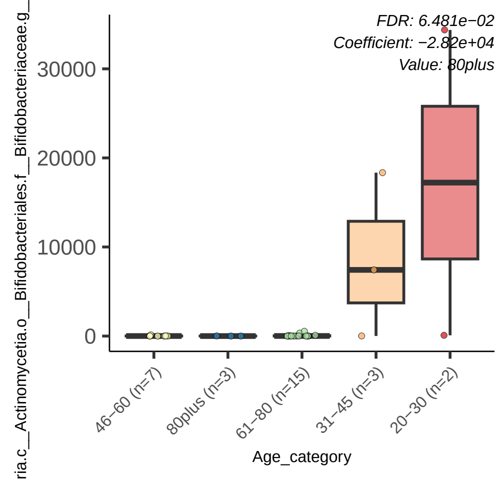

FDR: 6.481e−02
Coefficient: −2.82e+04
Value: 80plus
ria.c__Actinomycetia.o__Bifidobacteriales.f__Bifidobacteriaceae.g__
30000
20000
10000
0
46−60 (n=7)
80plus (n=3)
61−80 (n=15)
31−45 (n=3)
20−30 (n=2)
Age_category

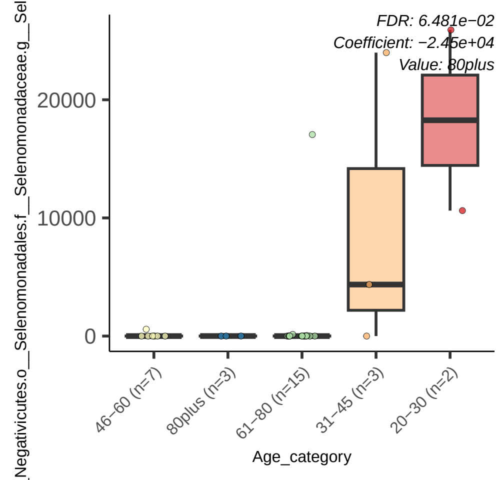

FDR: 6.481e−02
Coefficient: −2.45e+04
Value: 80plus
20000
10000
0
46−60 (n=7)
80plus (n=3)
61−80 (n=15)
31−45 (n=3)
20−30 (n=2)
Age_category
Negativicutes.o__Selenomonadales.f__Selenomonadaceae.g__Sel

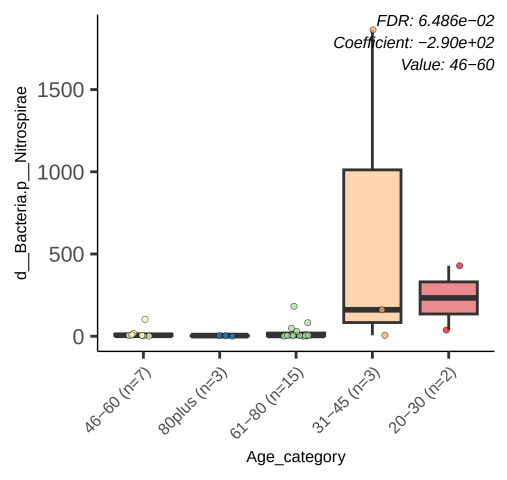
FDR: 6.486e−02
Coefficient: −2.90e+02
Value: 46−60
d__Bacteria.p__Nitrospirae
1500
1000
500
0
46−60 (n=7)
80plus (n=3)
61−80 (n=15)
31−45 (n=3)
20−30 (n=2)
Age_category

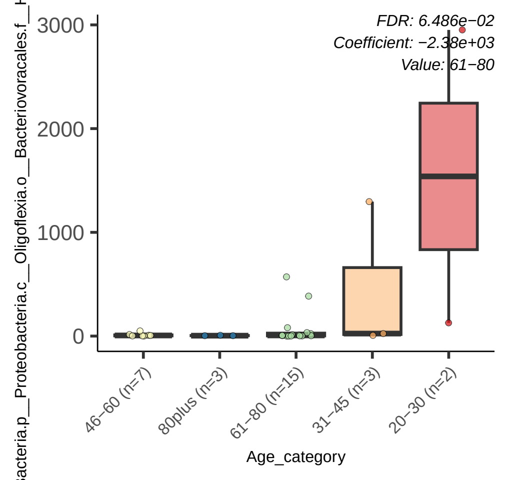

FDR: 6.486e−02
Coefficient: −2.38e+03
Value: 61−80
3000
2000
1000
0
46−60 (n=7)
80plus (n=3)
61−80 (n=15)
31−45 (n=3)
20−30 (n=2)
Age_category
acteria.p__Proteobacteria.c__Oligoflexia.o__Bacteriovoracales.f__H

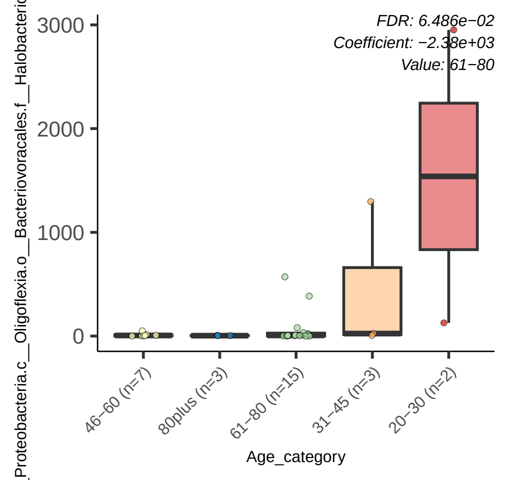

FDR: 6.486e−02
Coefficient: −2.38e+03
Value: 61−80
3000
2000
1000
0
Proteobacteria.c__Oligoflexia.o__Bacteriovoracales.f__Halobacterio
46−60 (n=7)
80plus (n=3)
61−80 (n=15)
31−45 (n=3)
20−30 (n=2)
Age_category

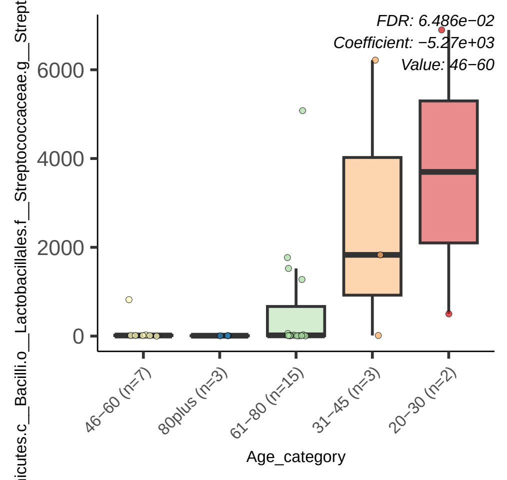

FDR: 6.486e−02
Coefficient: −5.27e+03
Value: 46−60
6000
4000
2000
0
icutes.c__Bacilli.o__Lactobacillales.f__Streptococcaceae.g__Strept
46−60 (n=7)
80plus (n=3)
61−80 (n=15)
31−45 (n=3)
20−30 (n=2)
Age_category

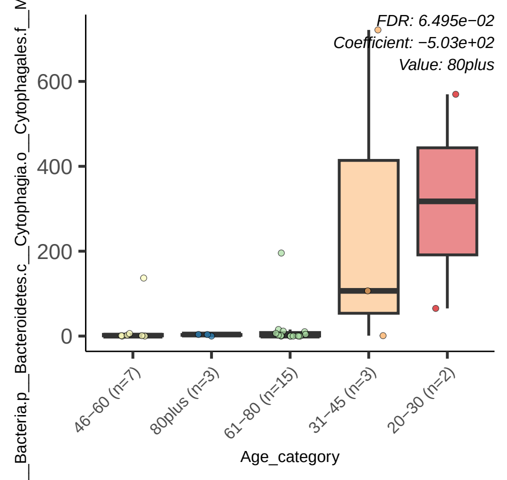
FDR: 6.495e−02
Coefficient: −5.03e+02
Value: 80plus
0
200
400
600
46−60 (n=7)
80plus (n=3)
61−80 (n=15)
31−45 (n=3)
20−30 (n=2)
Age_category
Bacteria.p__Bacteroidetes.c__Cytophagia.o__Cytophagales.f__M

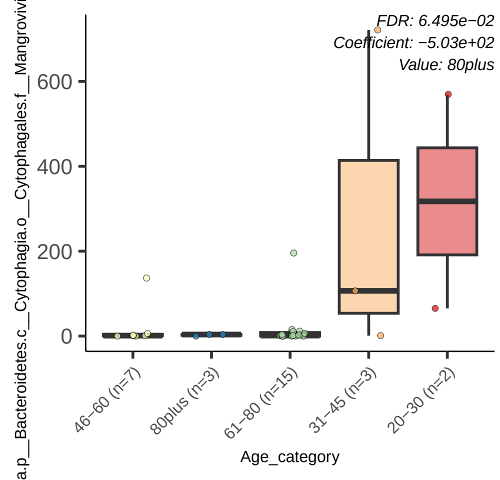
FDR: 6.495e−02
Coefficient: −5.03e+02
Value: 80plus
a.p__Bacteroidetes.c__Cytophagia.o__Cytophagales.f__Mangrovivi
600
400
200
0
46−60 (n=7)
80plus (n=3)
61−80 (n=15)
31−45 (n=3)
20−30 (n=2)
Age_category

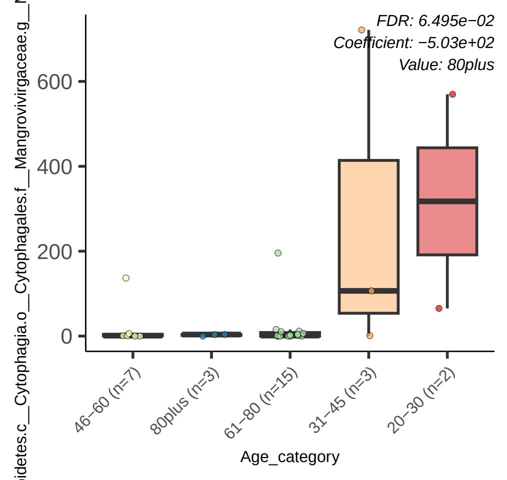

FDR: 6.495e−02
Coefficient: −5.03e+02
Value: 80plus
600
400
200
0
46−60 (n=7)
80plus (n=3)
61−80 (n=15)
31−45 (n=3)
20−30 (n=2)
Age_category
Cytophagia.o__Cytophagales.f__Mangrovivirgaceae.g__

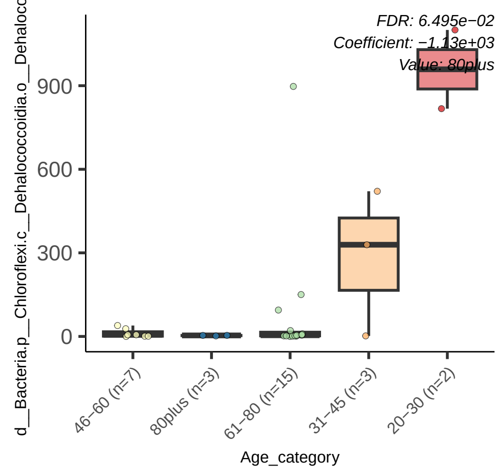

FDR: 6.495e−02
Coefficient: −1.13e+03
Value: 80plus
d__Bacteria.p__Chloroflexi.c__Dehalococcoidia.o__Dehalocc
900
600
300
0
46−60 (n=7)
80plus (n=3)
61−80 (n=15)
31−45 (n=3)
20−30 (n=2)
Age_category

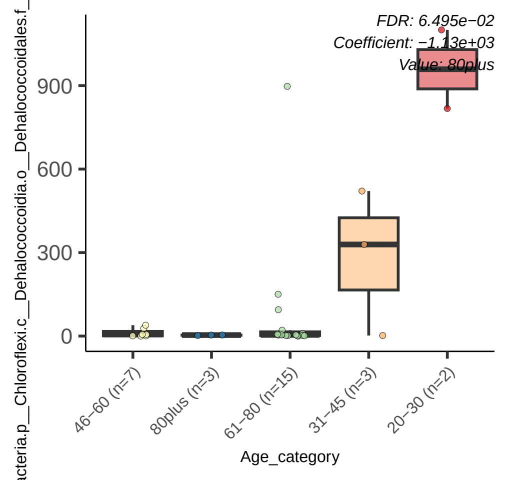
FDR: 6.495e−02
Coefficient: −1.13e+03
Value: 80plus
acteria.p__Chloroflexi.c__Dehalococcoidia.o__Dehalococcoidales.f__
900
600
300
0
46−60 (n=7)
80plus (n=3)
61−80 (n=15)
31−45 (n=3)
20−30 (n=2)
Age_category

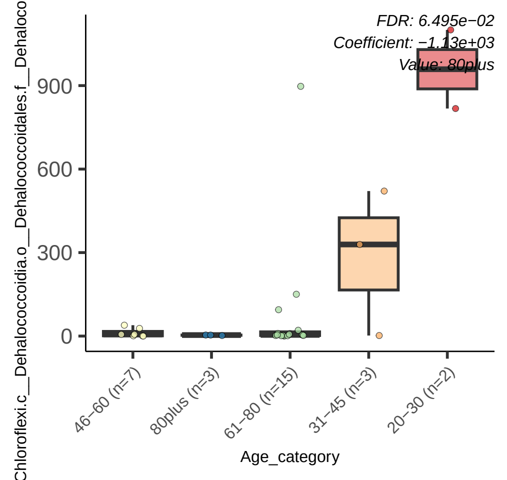
FDR: 6.495e−02
Coefficient: −1.13e+03
Value: 80plus
900
600
300
0
Chloroflexi.c__Dehalococcoidia.o__Dehalococcoidales.f__Dehaloco
46−60 (n=7)
80plus (n=3)
61−80 (n=15)
31−45 (n=3)
20−30 (n=2)
Age_category

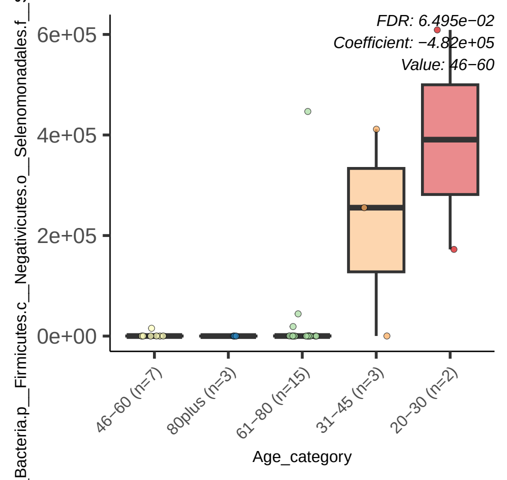

FDR: 6.495e−02
Coefficient: −4.82e+05
Value: 46−60
Bacteria.p__Firmicutes.c__Negativicutes.o__Selenomonadales.f__
6e+05
4e+05
2e+05
0e+00
46−60 (n=7)
80plus (n=3)
61−80 (n=15)
31−45 (n=3)
20−30 (n=2)
Age_category

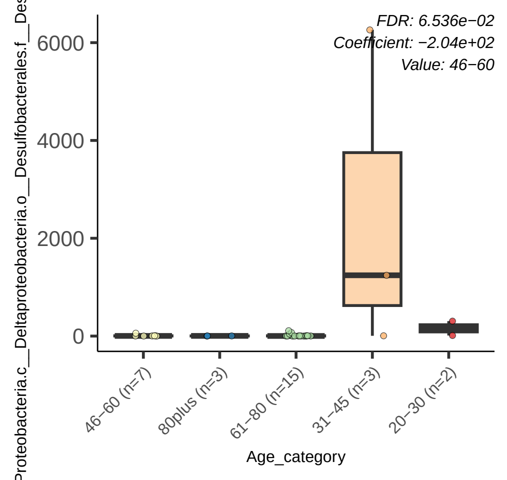

FDR: 6.536e−02
Coefficient: −2.04e+02
Value: 46−60
6000
4000
2000
0
Proteobacteria.c__Deltaproteobacteria.o__Desulfobacterales.f__Des
46−60 (n=7)
80plus (n=3)
61−80 (n=15)
31−45 (n=3)
20−30 (n=2)
Age_category

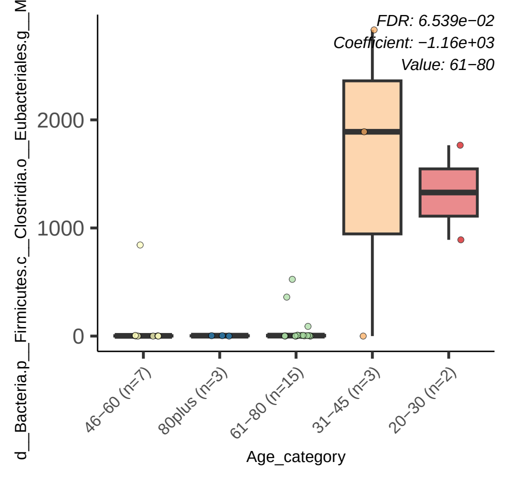

FDR: 6.539e−02
Coefficient: −1.16e+03
Value: 61−80
d__Bacteria.p__Firmicutes.c__Clostridia.o__Eubacteriales.g__M
2000
1000
0
46−60 (n=7)
80plus (n=3)
61−80 (n=15)
31−45 (n=3)
20−30 (n=2)
Age_category

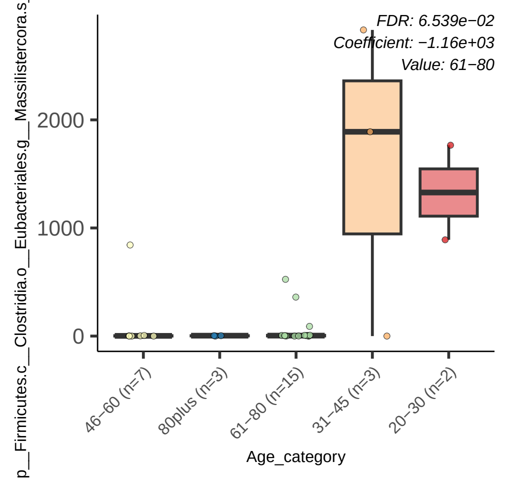

FDR: 6.539e−02
Coefficient: −1.16e+03
Value: 61−80
p__Firmicutes.c__Clostridia.o__Eubacteriales.g__Massilistercora.s_
2000
1000
0
46−60 (n=7)
80plus (n=3)
61−80 (n=15)
31−45 (n=3)
20−30 (n=2)
Age_category

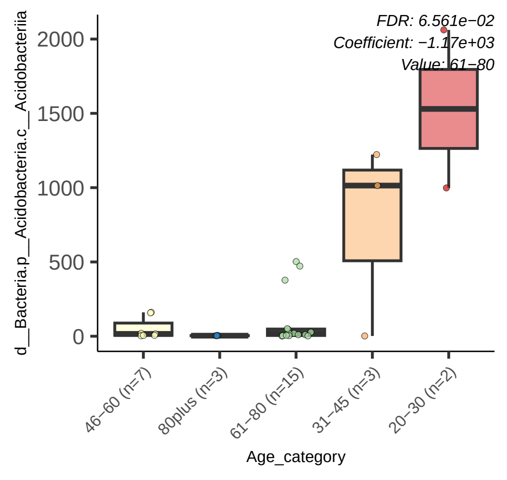

FDR: 6.561e−02
Coefficient: −1.17e+03
Value: 61−80
d__Bacteria.p__Acidobacteria.c__Acidobacteriia
2000
1500
1000
500
0
46−60 (n=7)
80plus (n=3)
61−80 (n=15)
31−45 (n=3)
20−30 (n=2)
Age_category

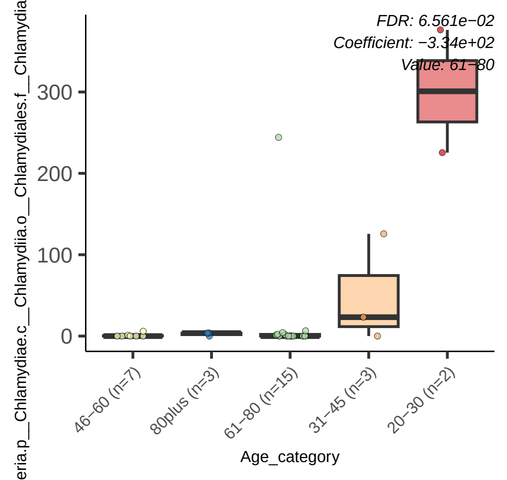

FDR: 6.561e−02
Coefficient: −3.34e+02
Value: 61−80
eria.p__Chlamydiae.c__Chlamydiia.o__Chlamydiales.f__Chlamydia
300
200
100
0
46−60 (n=7)
80plus (n=3)
61−80 (n=15)
31−45 (n=3)
20−30 (n=2)
Age_category

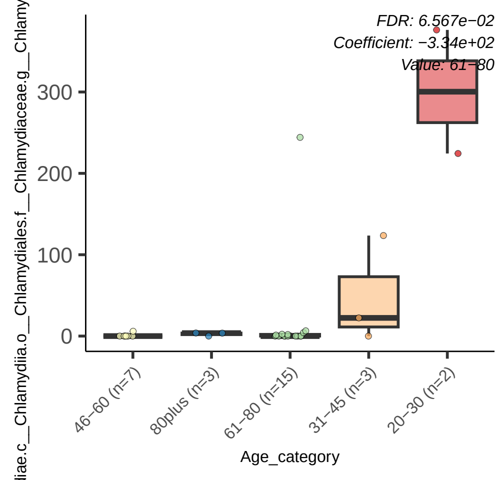

FDR: 6.567e−02
Coefficient: −3.34e+02
Value: 61−80
300
200
100
0
46−60 (n=7)
80plus (n=3)
61−80 (n=15)
31−45 (n=3)
20−30 (n=2)
Age_category
diae.c__Chlamydiia.o__Chlamydiales.f__Chlamydiaceae.g__Chlamy

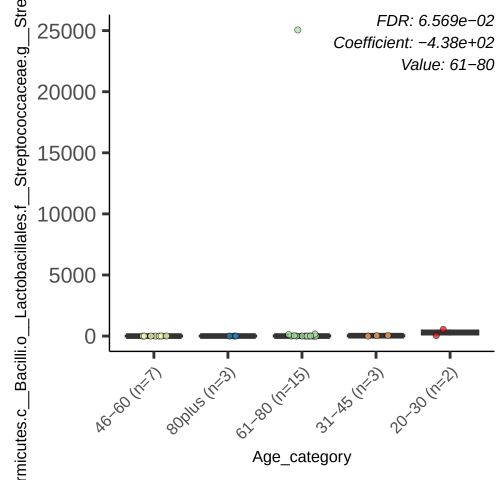
FDR: 6.569e−02
Coefficient: −4.38e+02
Value: 61−80
rmicutes.c__Bacilli.o__Lactobacillales.f__Streptococcaceae.g__Stre
25000
20000
15000
10000
5000
0
46−60 (n=7)
80plus (n=3)
61−80 (n=15)
31−45 (n=3)
20−30 (n=2)
Age_category

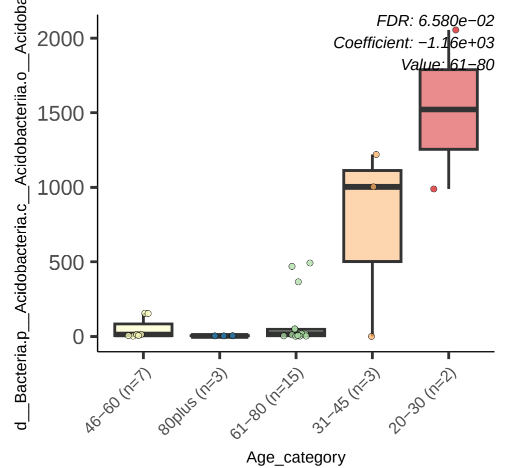

FDR: 6.580e−02
Coefficient: −1.16e+03
Value: 61−80
d__Bacteria.p__Acidobacteria.c__Acidobacteriia.o__Acidoba
2000
1500
1000
500
0
46−60 (n=7)
80plus (n=3)
61−80 (n=15)
31−45 (n=3)
20−30 (n=2)
Age_category

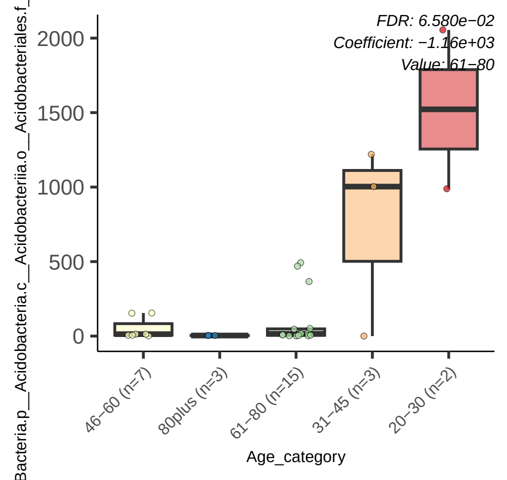

FDR: 6.580e−02
Coefficient: −1.16e+03
Value: 61−80
Bacteria.p__Acidobacteria.c__Acidobacteriia.o__Acidobacteriales.f__
2000
1500
1000
500
0
46−60 (n=7)
80plus (n=3)
61−80 (n=15)
31−45 (n=3)
20−30 (n=2)
Age_category

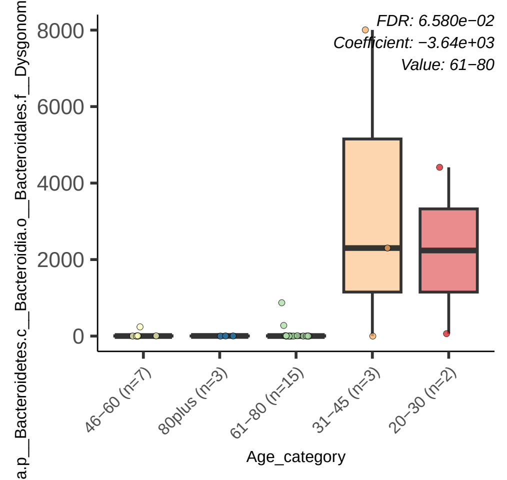
FDR: 6.580e−02
Coefficient: −3.64e+03
Value: 61−80
8000
6000
4000
2000
0
a.p__Bacteroidetes.c__Bacteroidia.o__Bacteroidales.f__Dysgonom
46−60 (n=7)
80plus (n=3)
61−80 (n=15)
31−45 (n=3)
20−30 (n=2)
Age_category

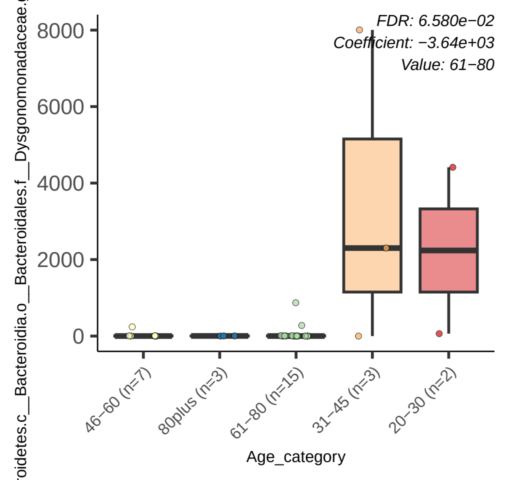
FDR: 6.580e−02
Coefficient: −3.64e+03
Value: 61−80
8000
6000
4000
2000
0
oidetes.c__Bacteroidia.o__Bacteroidales.f__Dysgonomonadaceae.g
46−60 (n=7)
80plus (n=3)
61−80 (n=15)
31−45 (n=3)
20−30 (n=2)
Age_category

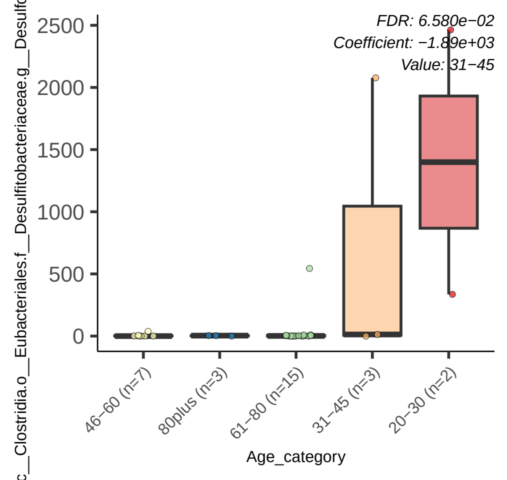
FDR: 6.580e−02
Coefficient: −1.89e+03
Value: 31−45
c__Clostridia.o__Eubacteriales.f__Desulfitobacteriaceae.g__Desulfo
2500
2000
1500
1000
500
0
46−60 (n=7)
80plus (n=3)
61−80 (n=15)
31−45 (n=3)
20−30 (n=2)
Age_category

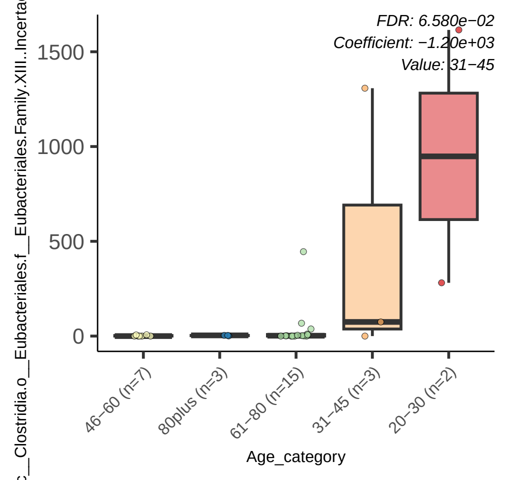

FDR: 6.580e−02
Coefficient: −1.20e+03
Value: 31−45
1500
1000
500
0
c__Clostridia.o__Eubacteriales.f__Eubacteriales.Family.XIII..Incertae
46−60 (n=7)
80plus (n=3)
61−80 (n=15)
31−45 (n=3)
20−30 (n=2)
Age_category

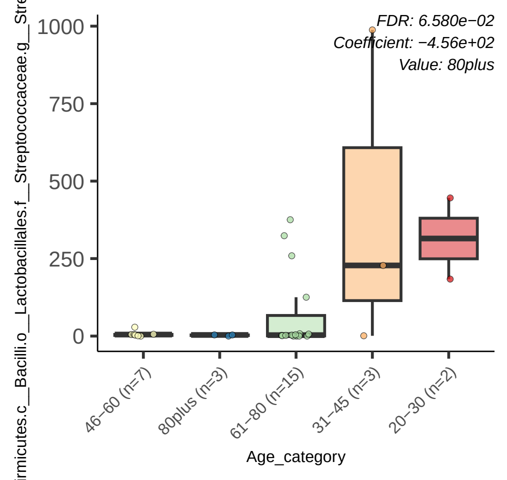

FDR: 6.580e−02
Coefficient: −4.56e+02
Value: 80plus
1000
750
500
250
0
46−60 (n=7)
80plus (n=3)
61−80 (n=15)
31−45 (n=3)
20−30 (n=2)
Age_category
irmicutes.c__Bacilli.o__Lactobacillales.f__Streptococcaceae.g__Stre

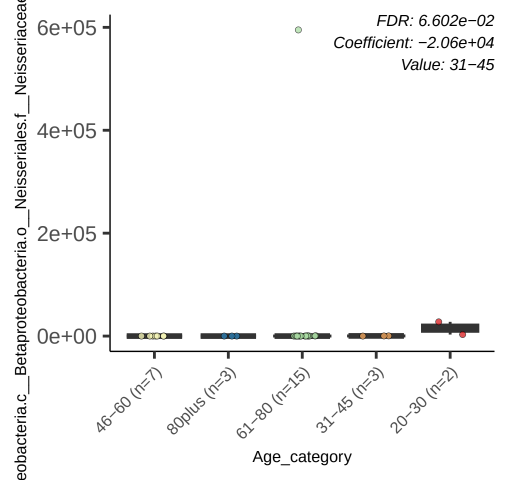

FDR: 6.602e−02
Coefficient: −2.06e+04
Value: 31−45
6e+05
4e+05
2e+05
0e+00
eobacteria.c__Betaproteobacteria.o__Neisseriales.f__Neisseriacea
46−60 (n=7)
80plus (n=3)
61−80 (n=15)
31−45 (n=3)
20−30 (n=2)
Age_category

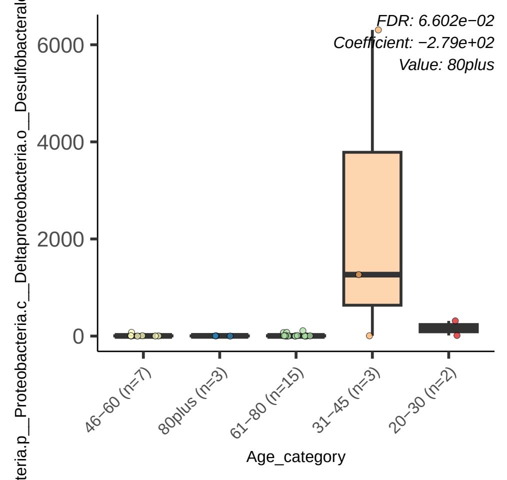
FDR: 6.602e−02
Coefficient: −2.79e+02
Value: 80plus
6000
4000
2000
0
teria.p__Proteobacteria.c__Deltaproteobacteria.o__Desulfobacteral
46−60 (n=7)
80plus (n=3)
61−80 (n=15)
31−45 (n=3)
20−30 (n=2)
Age_category

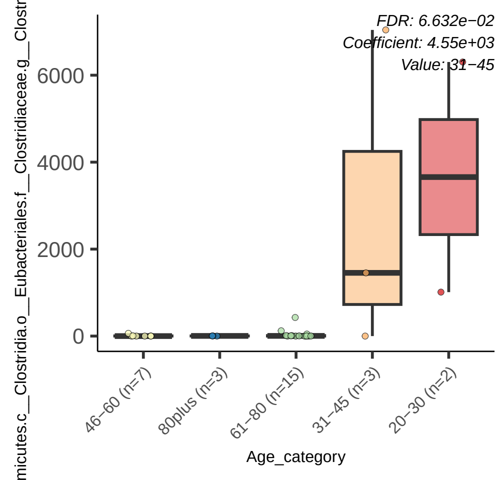

FDR: 6.632e−02
Coefficient: 4.55e+03
Value: 31−45
6000
4000
2000
0
micutes.c__Clostridia.o__Eubacteriales.f__Clostridiaceae.g__Clostr
46−60 (n=7)
80plus (n=3)
61−80 (n=15)
31−45 (n=3)
20−30 (n=2)
Age_category

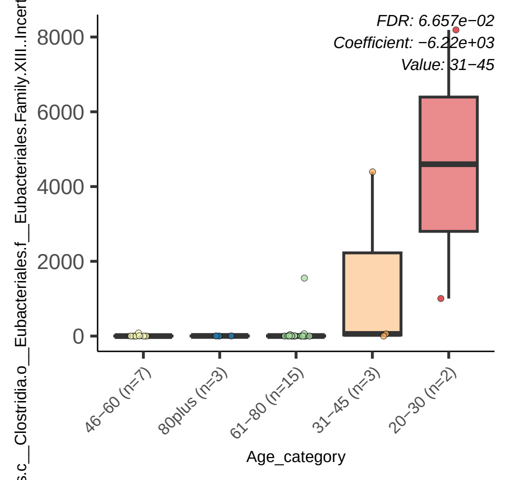

FDR: 6.657e−02
Coefficient: −6.22e+03
Value: 31−45
8000
6000
4000
2000
0
s.c__Clostridia.o__Eubacteriales.f__Eubacteriales.Family.XIII..Incert
46−60 (n=7)
80plus (n=3)
61−80 (n=15)
31−45 (n=3)
20−30 (n=2)
Age_category

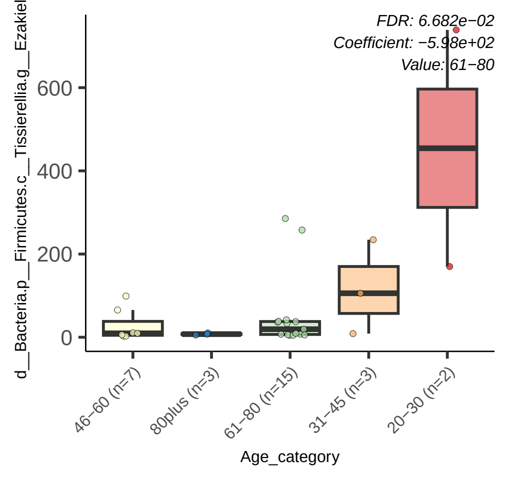
FDR: 6.682e−02
Coefficient: −5.98e+02
Value: 61−80
d__Bacteria.p__Firmicutes.c__Tissierellia.g__Ezakiel
600
400
200
0
46−60 (n=7)
80plus (n=3)
61−80 (n=15)
31−45 (n=3)
20−30 (n=2)
Age_category

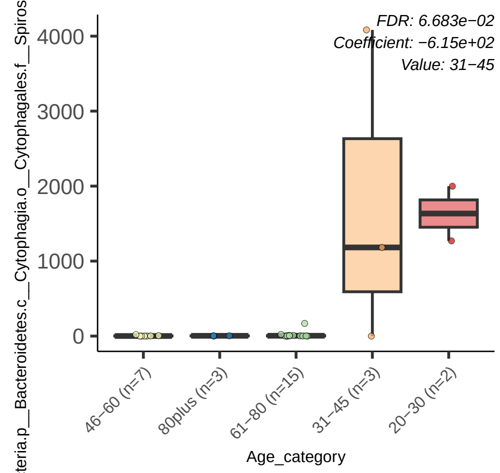
FDR: 6.683e−02
Coefficient: −6.15e+02
Value: 31−45
4000
3000
2000
1000
0
cteria.p__Bacteroidetes.c__Cytophagia.o__Cytophagales.f__Spiros
46−60 (n=7)
80plus (n=3)
61−80 (n=15)
31−45 (n=3)
20−30 (n=2)
Age_category

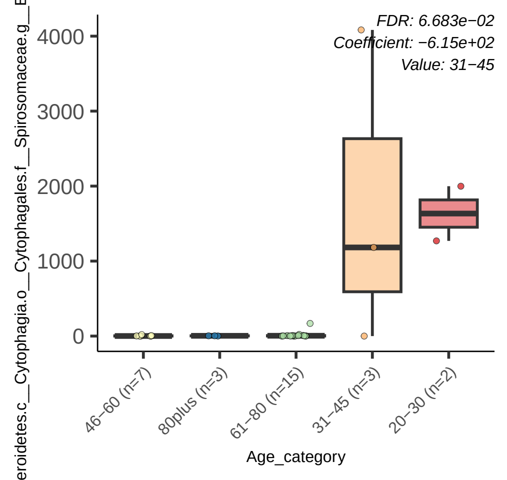

FDR: 6.683e−02
Coefficient: −6.15e+02
Value: 31−45
4000
3000
2000
1000
0
eroidetes.c__Cytophagia.o__Cytophagales.f__Spirosomaceae.g__E
46−60 (n=7)
80plus (n=3)
61−80 (n=15)
31−45 (n=3)
20−30 (n=2)
Age_category

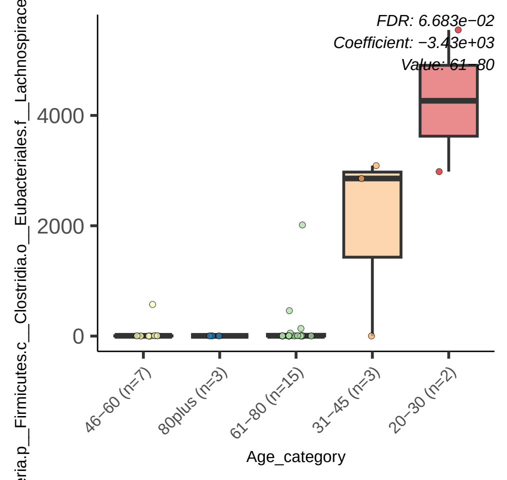
FDR: 6.683e−02
Coefficient: −3.43e+03
Value: 61−80
0
2000
4000
46−60 (n=7)
80plus (n=3)
61−80 (n=15)
31−45 (n=3)
20−30 (n=2)
Age_category

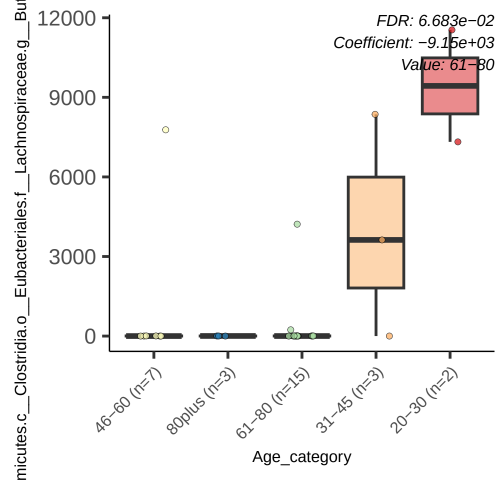

micutes.c__Clostridia.o__Eubacteriales.f__Lachnospiraceae.g__Bu
12000
9000
6000
3000
0
FDR: 6.683e−02
Coefficient: −9.15e+03
Value: 61−80
46−60 (n=7)
80plus (n=3)
61−80 (n=15)
31−45 (n=3)
20−30 (n=2)
Age_category

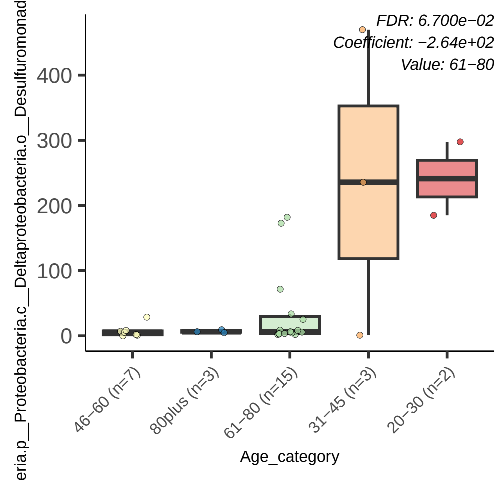
FDR: 6.700e−02
Coefficient: −2.64e+02
Value: 61−80
400
300
200
100
0
eria.p__Proteobacteria.c__Deltaproteobacteria.o__Desulfuromonad
46−60 (n=7)
80plus (n=3)
61−80 (n=15)
31−45 (n=3)
20−30 (n=2)
Age_category

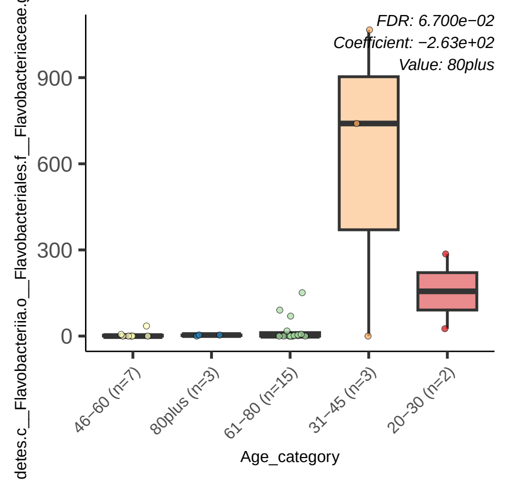

FDR: 6.700e−02
Coefficient: −2.63e+02
Value: 80plus
900
600
300
0
detes.c__Flavobacteriia.o__Flavobacteriales.f__Flavobacteriaceae.g
46−60 (n=7)
80plus (n=3)
61−80 (n=15)
31−45 (n=3)
20−30 (n=2)
Age_category

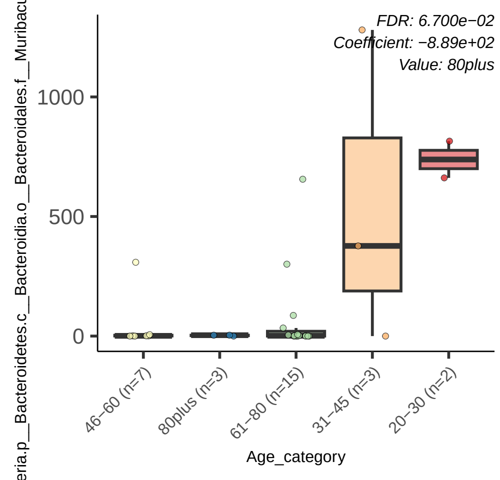

FDR: 6.700e−02
Coefficient: −8.89e+02
Value: 80plus
1000
500
0
eria.p__Bacteroidetes.c__Bacteroidia.o__Bacteroidales.f__Muribacu
46−60 (n=7)
80plus (n=3)
61−80 (n=15)
31−45 (n=3)
20−30 (n=2)
Age_category

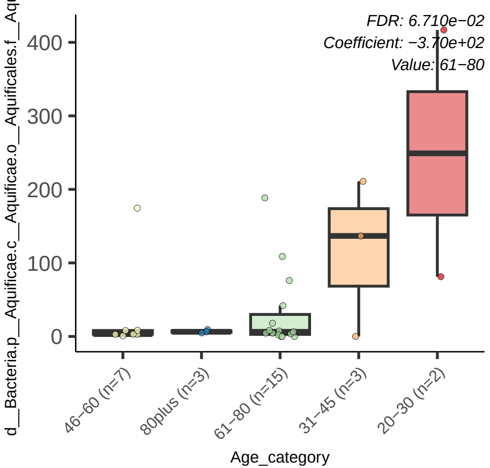

FDR: 6.710e−02
Coefficient: −3.70e+02
Value: 61−80
d__Bacteria.p__Aquificae.c__Aquificae.o__Aquificales.f__Aqu
0
100
200
300
400
46−60 (n=7)
80plus (n=3)
61−80 (n=15)
31−45 (n=3)
20−30 (n=2)
Age_category

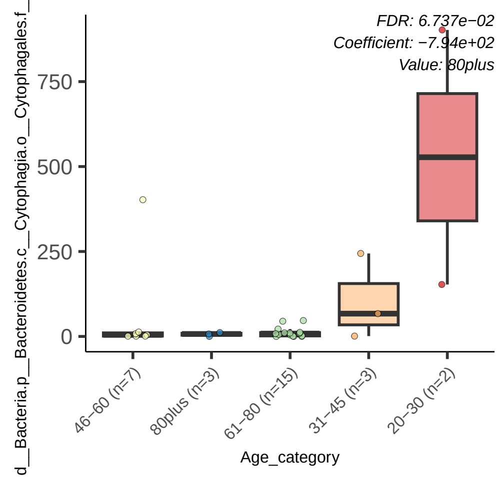
FDR: 6.737e−02
Coefficient: −7.94e+02
Value: 80plus
d__Bacteria.p__Bacteroidetes.c__Cytophagia.o__Cytophagales.f__
750
500
250
0
46−60 (n=7)
80plus (n=3)
61−80 (n=15)
31−45 (n=3)
20−30 (n=2)
Age_category

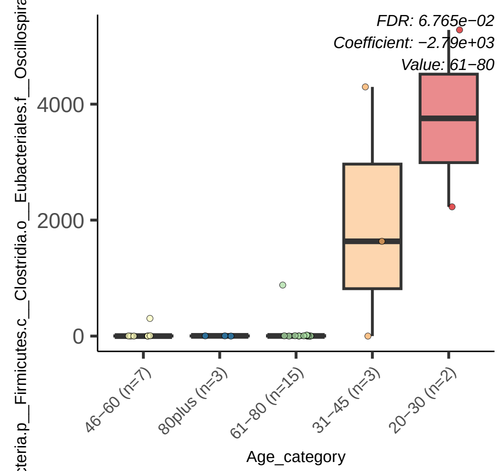

FDR: 6.765e−02
Coefficient: −2.79e+03
Value: 61−80
acteria.p__Firmicutes.c__Clostridia.o__Eubacteriales.f__Oscillospira
4000
2000
0
46−60 (n=7)
80plus (n=3)
61−80 (n=15)
31−45 (n=3)
20−30 (n=2)
Age_category

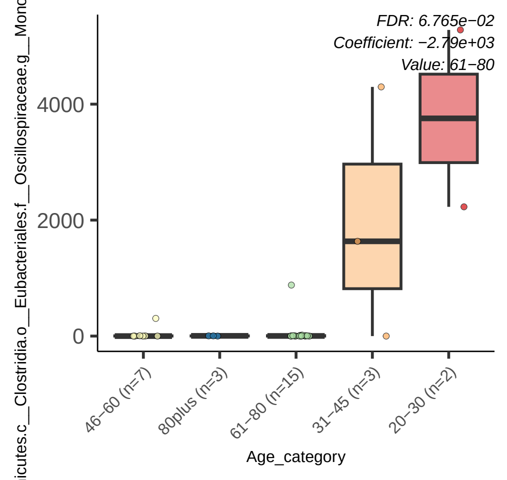

FDR: 6.765e−02
Coefficient: −2.79e+03
Value: 61−80
4000
2000
0
46−60 (n=7)
80plus (n=3)
61−80 (n=15)
31−45 (n=3)
20−30 (n=2)
Age_category
icutes.c__Clostridia.o__Eubacteriales.f__Oscillospiraceae.g__Monc

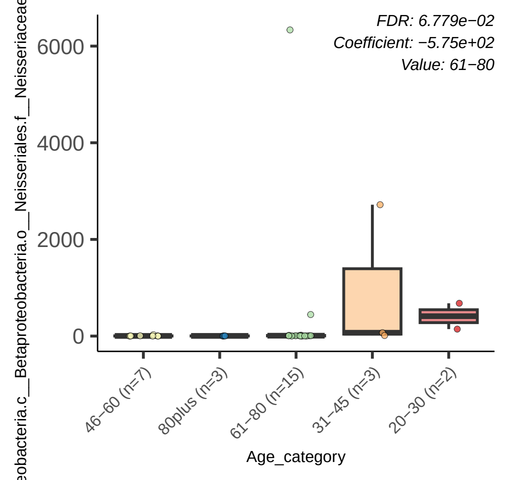

FDR: 6.779e−02
Coefficient: −5.75e+02
Value: 61−80
6000
4000
2000
0
eobacteria.c__Betaproteobacteria.o__Neisseriales.f__Neisseriaceae
46−60 (n=7)
80plus (n=3)
61−80 (n=15)
31−45 (n=3)
20−30 (n=2)
Age_category

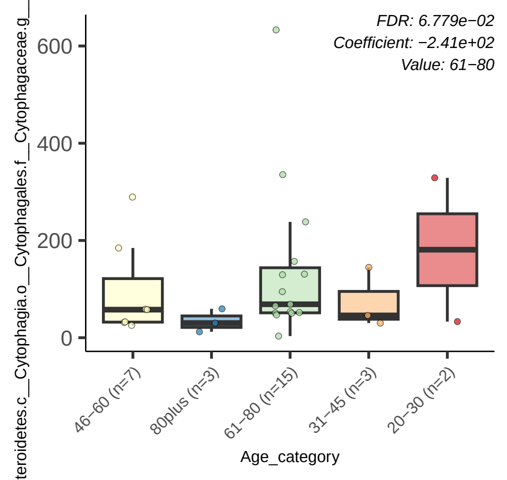

FDR: 6.779e−02
Coefficient: −2.41e+02
Value: 61−80
teroidetes.c__Cytophagia.o__Cytophagales.f__Cytophagaceae.g__
0
200
400
600
46−60 (n=7)
80plus (n=3)
61−80 (n=15)
31−45 (n=3)
20−30 (n=2)
Age_category

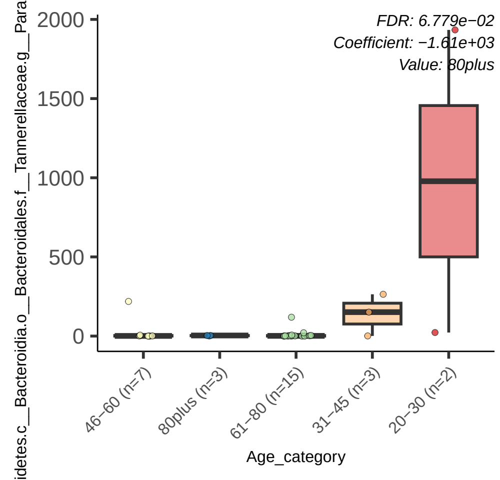

FDR: 6.779e−02
Coefficient: −1.61e+03
Value: 80plus
idetes.c__Bacteroidia.o__Bacteroidales.f__Tannerellaceae.g__Para
2000
1500
1000
500
0
46−60 (n=7)
80plus (n=3)
61−80 (n=15)
31−45 (n=3)
20−30 (n=2)
Age_category

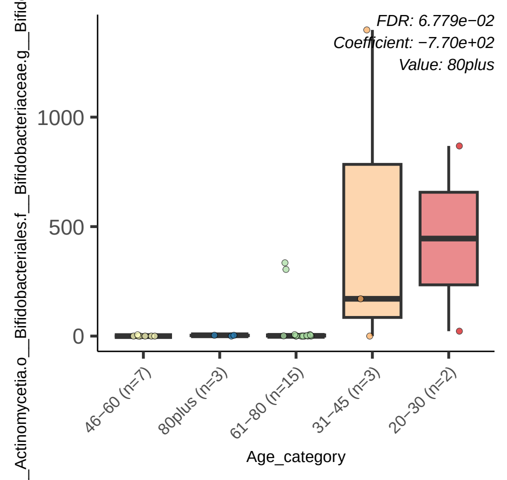

FDR: 6.779e−02
Coefficient: −7.70e+02
Value: 80plus
0
500
1000
Actinomycetia.o__Bifidobacteriales.f__Bifidobacteriaceae.g__Bifid
46−60 (n=7)
80plus (n=3)
61−80 (n=15)
31−45 (n=3)
20−30 (n=2)
Age_category

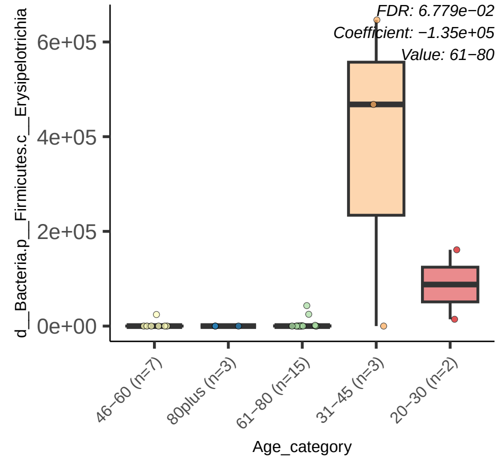

FDR: 6.779e−02
Coefficient: −1.35e+05
Value: 61−80
d__Bacteria.p__Firmicutes.c__Erysipelotrichia
6e+05
4e+05
2e+05
0e+00
46−60 (n=7)
80plus (n=3)
61−80 (n=15)
31−45 (n=3)
20−30 (n=2)
Age_category

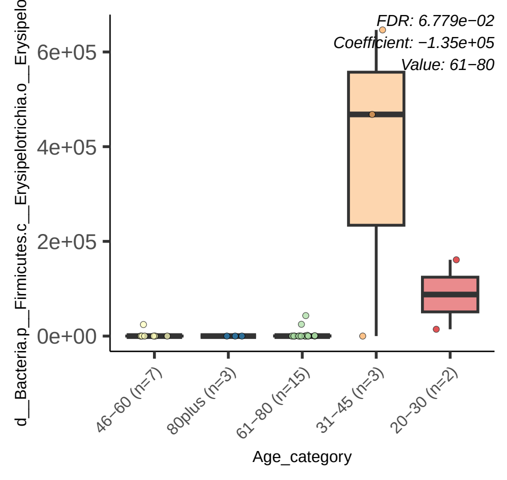

FDR: 6.779e−02
Coefficient: −1.35e+05
Value: 61−80
6e+05
4e+05
2e+05
0e+00
d__Bacteria.p__Firmicutes.c__Erysipelotrichia.o__Erysipelo
46−60 (n=7)
80plus (n=3)
61−80 (n=15)
31−45 (n=3)
20−30 (n=2)
Age_category

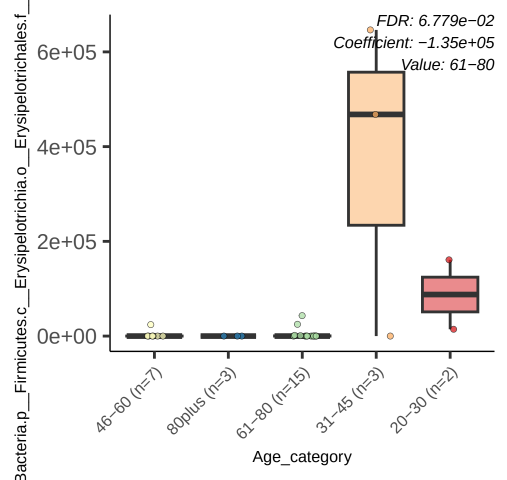

FDR: 6.779e−02
Coefficient: −1.35e+05
Value: 61−80
Bacteria.p__Firmicutes.c__Erysipelotrichia.o__Erysipelotrichales.f__
6e+05
4e+05
2e+05
0e+00
46−60 (n=7)
80plus (n=3)
61−80 (n=15)
31−45 (n=3)
20−30 (n=2)
Age_category

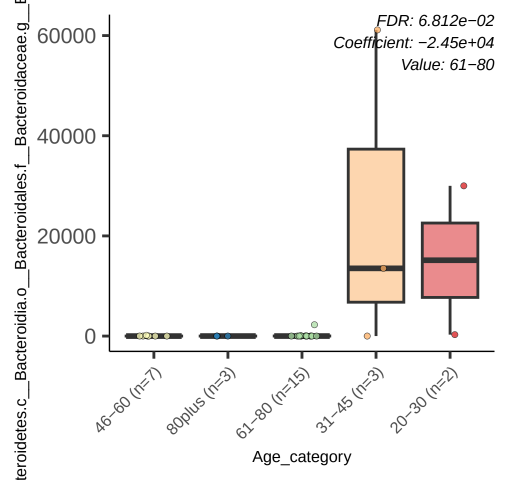

FDR: 6.812e−02
Coefficient: −2.45e+04
Value: 61−80
60000
40000
20000
0
teroidetes.c__Bacteroidia.o__Bacteroidales.f__Bacteroidaceae.g__B
46−60 (n=7)
80plus (n=3)
61−80 (n=15)
31−45 (n=3)
20−30 (n=2)
Age_category

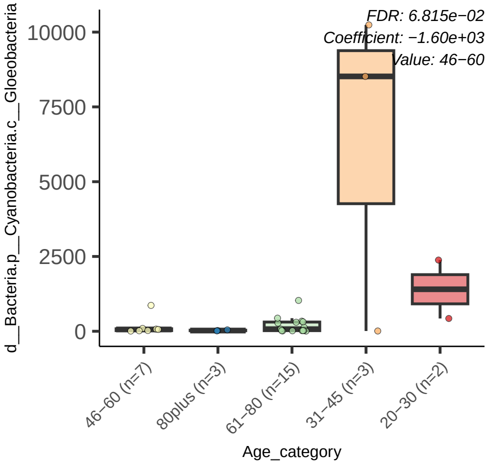
FDR: 6.815e−02
Coefficient: −1.60e+03
Value: 46−60
d__Bacteria.p__Cyanobacteria.c__Gloeobacteria
10000
7500
5000
2500
0
46−60 (n=7)
80plus (n=3)
61−80 (n=15)
31−45 (n=3)
20−30 (n=2)
Age_category

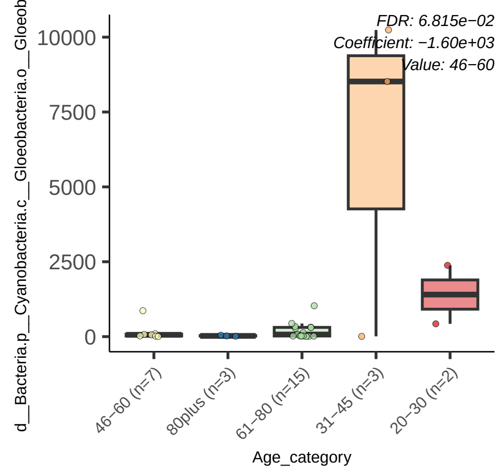

FDR: 6.815e−02
Coefficient: −1.60e+03
Value: 46−60
d__Bacteria.p__Cyanobacteria.c__Gloeobacteria.o__Gloeob
10000
7500
5000
2500
0
46−60 (n=7)
80plus (n=3)
61−80 (n=15)
31−45 (n=3)
20−30 (n=2)
Age_category

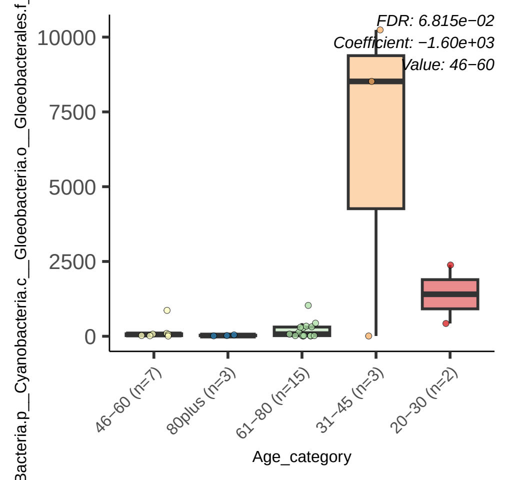

FDR: 6.815e−02
Coefficient: −1.60e+03
Value: 46−60
10000
7500
5000
2500
0
Bacteria.p__Cyanobacteria.c__Gloeobacteria.o__Gloeobacterales.f_
46−60 (n=7)
80plus (n=3)
61−80 (n=15)
31−45 (n=3)
20−30 (n=2)
Age_category

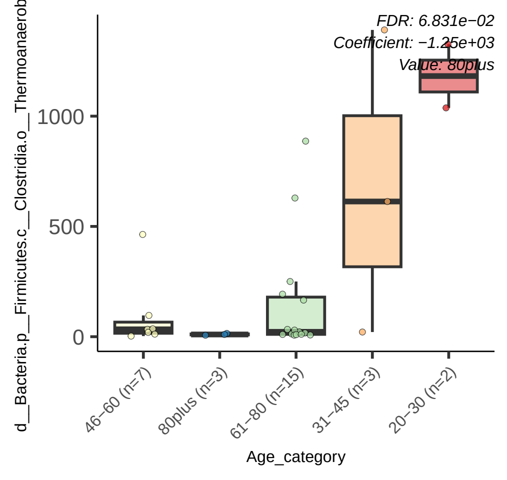

FDR: 6.831e−02
Coefficient: −1.25e+03
Value: 80plus
d__Bacteria.p__Firmicutes.c__Clostridia.o__Thermoanaerob
1000
500
0
46−60 (n=7)
80plus (n=3)
61−80 (n=15)
31−45 (n=3)
20−30 (n=2)
Age_category

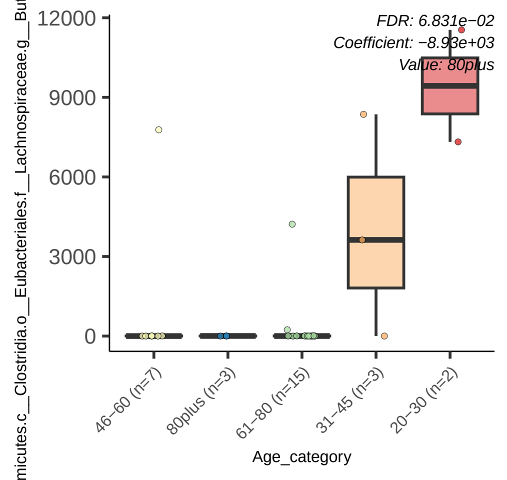

FDR: 6.831e−02
Coefficient: −8.93e+03
Value: 80plus
micutes.c__Clostridia.o__Eubacteriales.f__Lachnospiraceae.g__Bu
12000
9000
6000
3000
0
46−60 (n=7)
80plus (n=3)
61−80 (n=15)
31−45 (n=3)
20−30 (n=2)
Age_category

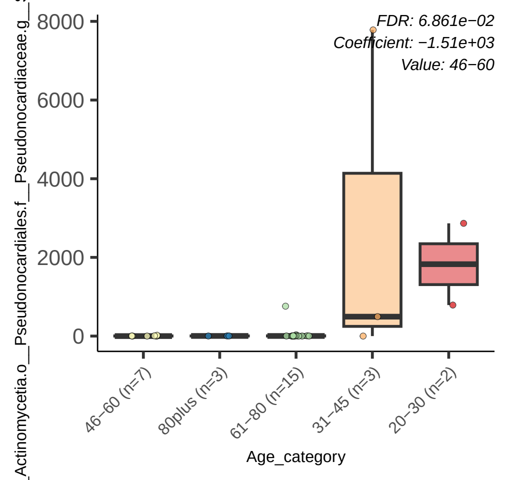

FDR: 6.861e−02
Coefficient: −1.51e+03
Value: 46−60
Actinomycetia.o__Pseudonocardiales.f__Pseudonocardiaceae.g__
8000
6000
4000
2000
0
46−60 (n=7)
80plus (n=3)
61−80 (n=15)
31−45 (n=3)
20−30 (n=2)
Age_category

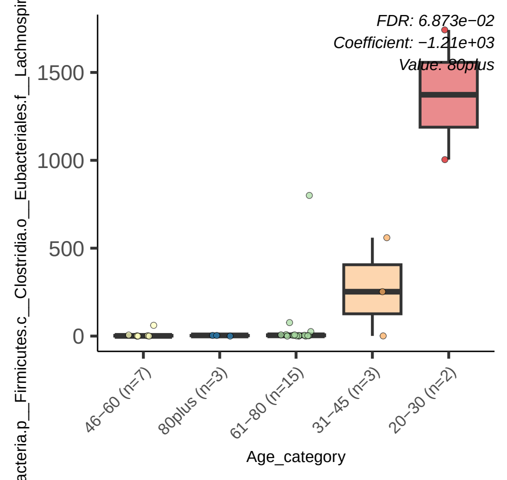

FDR: 6.873e−02
Coefficient: −1.21e+03
Value: 80plus
acteria.p__Firmicutes.c__Clostridia.o__Eubacteriales.f__Lachnospi
1500
1000
500
0
46−60 (n=7)
80plus (n=3)
61−80 (n=15)
31−45 (n=3)
20−30 (n=2)
Age_category

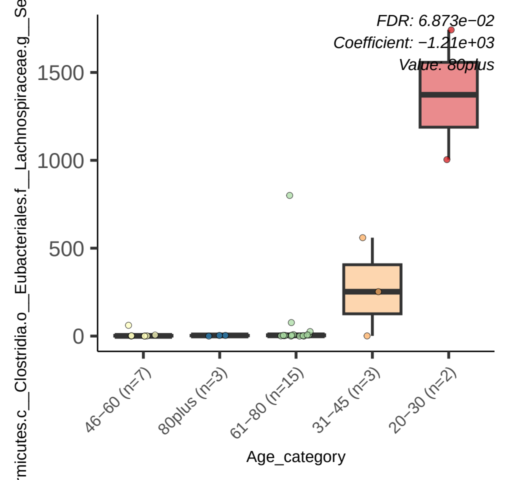
FDR: 6.873e−02
Coefficient: −1.21e+03
Value: 80plus
1500
1000
500
0
46−60 (n=7)
80plus (n=3)
61−80 (n=15)
31−45 (n=3)
20−30 (n=2)
Age_category
rmicutes.c__Clostridia.o__Eubacteriales.f__Lachnospiraceae.g__Se

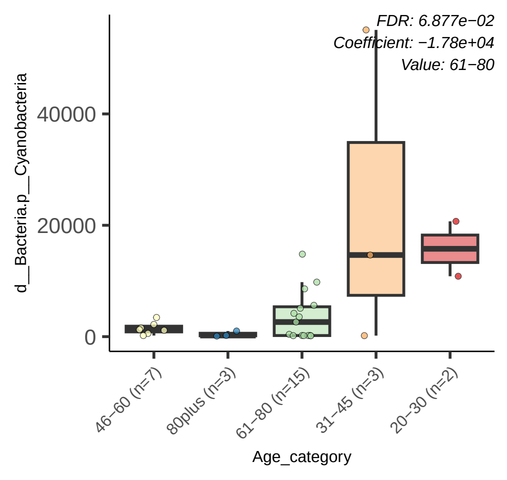
FDR: 6.877e−02
Coefficient: −1.78e+04
Value: 61−80
d__Bacteria.p__Cyanobacteria
40000
20000
0
46−60 (n=7)
80plus (n=3)
61−80 (n=15)
31−45 (n=3)
20−30 (n=2)
Age_category

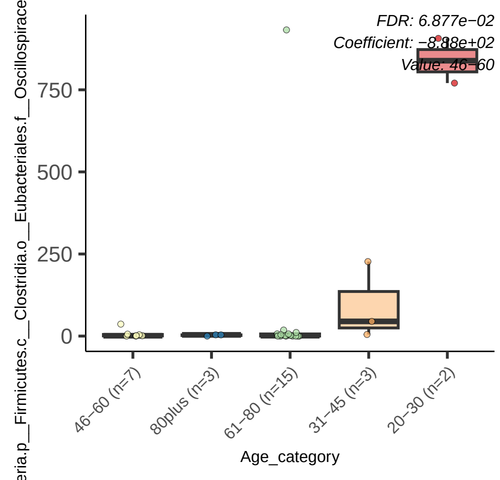

FDR: 6.877e−02
Coefficient: −8.88e+02
Value: 46−60
0
250
500
750
eria.p__Firmicutes.c__Clostridia.o__Eubacteriales.f__Oscillospirace
46−60 (n=7)
80plus (n=3)
61−80 (n=15)
31−45 (n=3)
20−30 (n=2)
Age_category

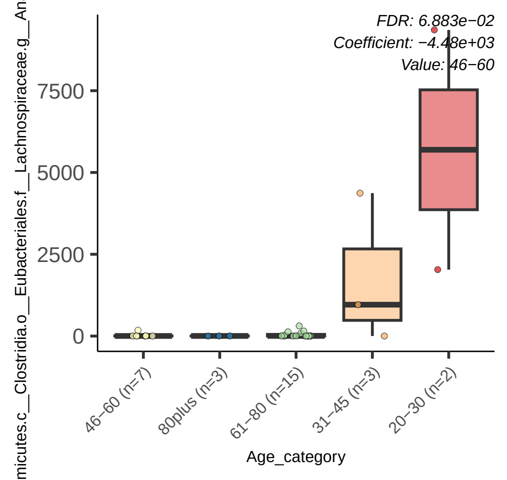

FDR: 6.883e−02
Coefficient: −4.48e+03
Value: 46−60
micutes.c__Clostridia.o__Eubacteriales.f__Lachnospiraceae.g__An
7500
5000
2500
0
46−60 (n=7)
80plus (n=3)
61−80 (n=15)
31−45 (n=3)
20−30 (n=2)
Age_category

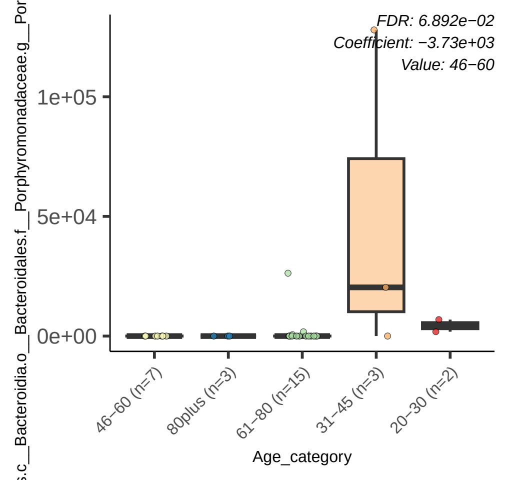

FDR: 6.892e−02
Coefficient: −3.73e+03
Value: 46−60
1e+05
5e+04
0e+00
s.c__Bacteroidia.o__Bacteroidales.f__Porphyromonadaceae.g__Por
46−60 (n=7)
80plus (n=3)
61−80 (n=15)
31−45 (n=3)
20−30 (n=2)
Age_category

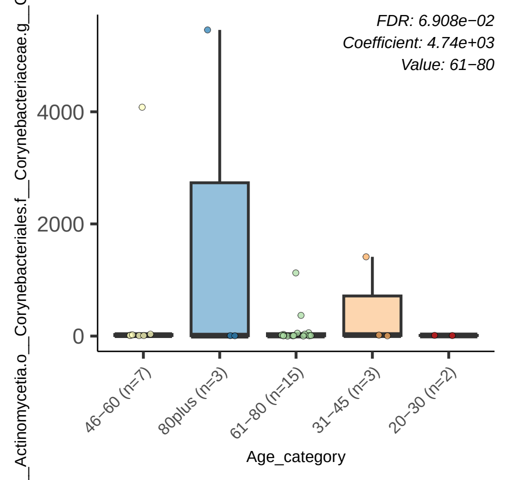

FDR: 6.908e−02
Coefficient: 4.74e+03
Value: 61−80
__Actinomycetia.o__Corynebacteriales.f__Corynebacteriaceae.g__C
4000
2000
0
46−60 (n=7)
80plus (n=3)
61−80 (n=15)
31−45 (n=3)
20−30 (n=2)
Age_category

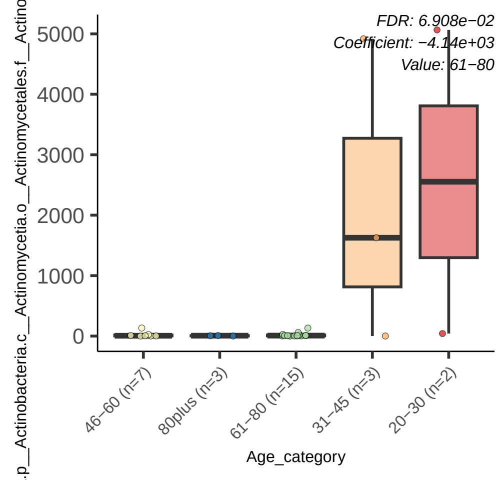

FDR: 6.908e−02
Coefficient: −4.14e+03
Value: 61−80
p__Actinobacteria.c__Actinomycetia.o__Actinomycetales.f__Actino
5000
4000
3000
2000
1000
0
46−60 (n=7)
80plus (n=3)
61−80 (n=15)
31−45 (n=3)
20−30 (n=2)
Age_category

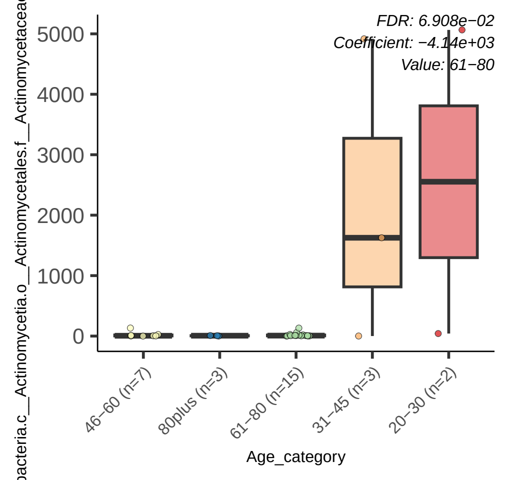

FDR: 6.908e−02
Coefficient: −4.14e+03
Value: 61−80
5000
4000
3000
2000
1000
0
bacteria.c__Actinomycetia.o__Actinomycetales.f__Actinomycetacea
46−60 (n=7)
80plus (n=3)
61−80 (n=15)
31−45 (n=3)
20−30 (n=2)
Age_category

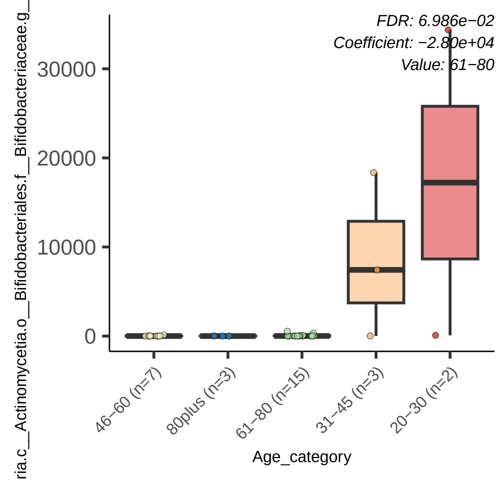
FDR: 6.986e−02
Coefficient: −2.80e+04
Value: 61−80
ria.c__Actinomycetia.o__Bifidobacteriales.f__Bifidobacteriaceae.g__
30000
20000
10000
0
46−60 (n=7)
80plus (n=3)
61−80 (n=15)
31−45 (n=3)
20−30 (n=2)
Age_category

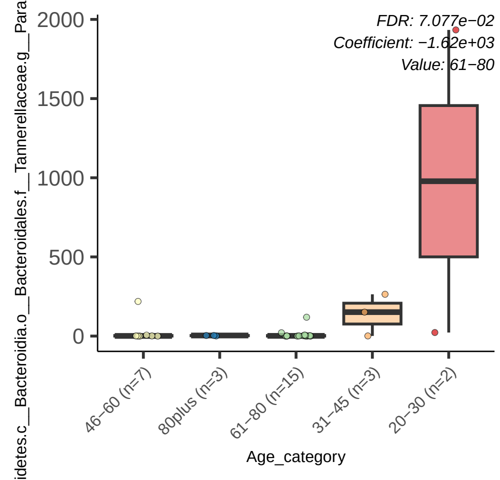

FDR: 7.077e−02
Coefficient: −1.62e+03
Value: 61−80
2000
1500
1000
500
0
idetes.c__Bacteroidia.o__Bacteroidales.f__Tannerellaceae.g__Para
46−60 (n=7)
80plus (n=3)
61−80 (n=15)
31−45 (n=3)
20−30 (n=2)
Age_category

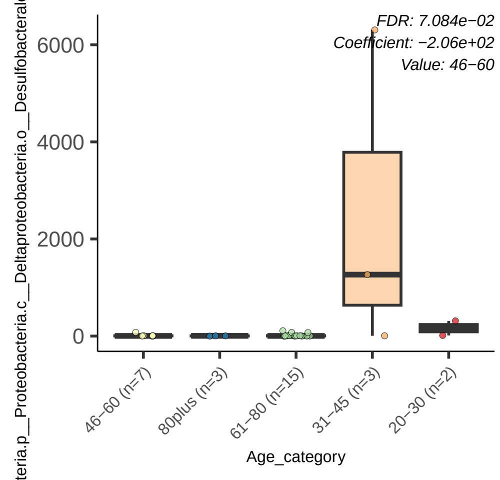

FDR: 7.084e−02
Coefficient: −2.06e+02
Value: 46−60
6000
4000
2000
0
teria.p__Proteobacteria.c__Deltaproteobacteria.o__Desulfobacteral
46−60 (n=7)
80plus (n=3)
61−80 (n=15)
31−45 (n=3)
20−30 (n=2)
Age_category

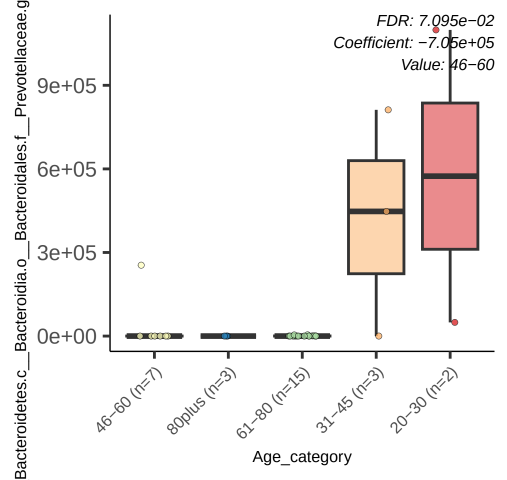

FDR: 7.095e−02
Coefficient: −7.05e+05
Value: 46−60
9e+05
6e+05
3e+05
0e+00
Bacteroidetes.c__Bacteroidia.o__Bacteroidales.f__Prevotellaceae.g
46−60 (n=7)
80plus (n=3)
61−80 (n=15)
31−45 (n=3)
20−30 (n=2)
Age_category

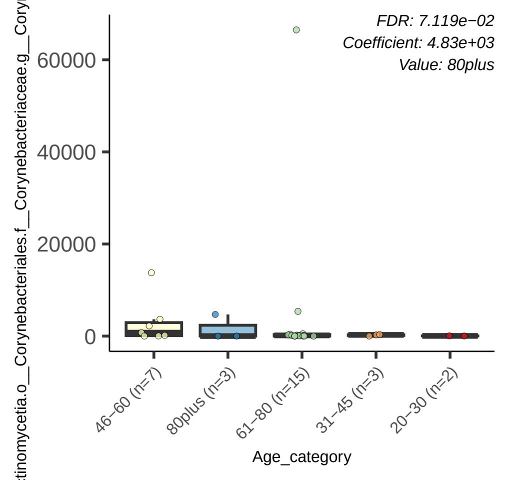

FDR: 7.119e−02
Coefficient: 4.83e+03
Value: 80plus
60000
40000
20000
0
ctinomycetia.o__Corynebacteriales.f__Corynebacteriaceae.g__Cory
46−60 (n=7)
80plus (n=3)
61−80 (n=15)
31−45 (n=3)
20−30 (n=2)
Age_category

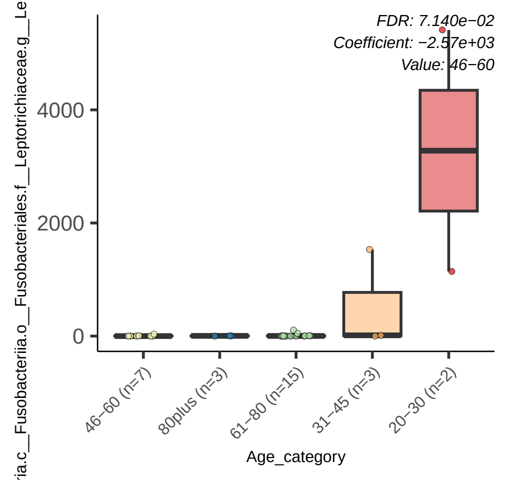

FDR: 7.140e−02
Coefficient: −2.57e+03
Value: 46−60
ria.c__Fusobacteriia.o__Fusobacteriales.f__Leptotrichiaceae.g__Le
4000
2000
0
46−60 (n=7)
80plus (n=3)
61−80 (n=15)
31−45 (n=3)
20−30 (n=2)
Age_category

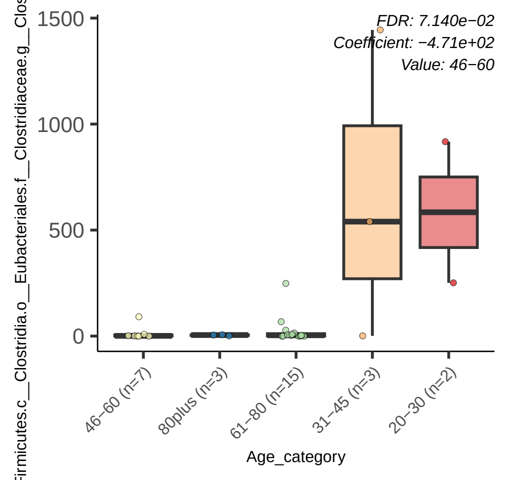
FDR: 7.140e−02
Coefficient: −4.71e+02
Value: 46−60
1500
1000
500
0
Firmicutes.c__Clostridia.o__Eubacteriales.f__Clostridiaceae.g__Clos
46−60 (n=7)
80plus (n=3)
61−80 (n=15)
31−45 (n=3)
20−30 (n=2)
Age_category

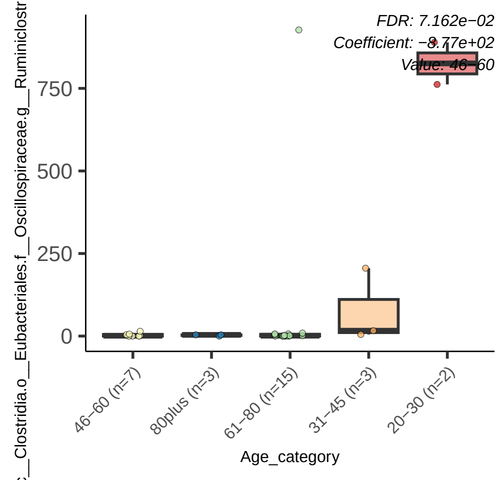

FDR: 7.162e−02
Coefficient: −8.77e+02
Value: 46−60
c__Clostridia.o__Eubacteriales.f__Oscillospiraceae.g__Ruminiclostr
750
500
250
0
46−60 (n=7)
80plus (n=3)
61−80 (n=15)
31−45 (n=3)
20−30 (n=2)
Age_category

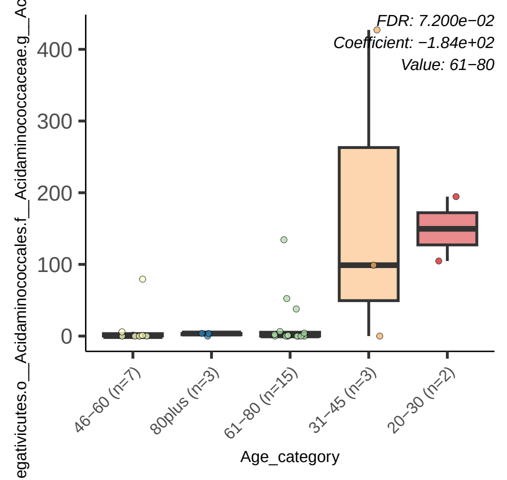

FDR: 7.200e−02
Coefficient: −1.84e+02
Value: 61−80
400
300
200
100
0
egativicutes.o__Acidaminococcales.f__Acidaminococcaceae.g__Ac
46−60 (n=7)
80plus (n=3)
61−80 (n=15)
31−45 (n=3)
20−30 (n=2)
Age_category

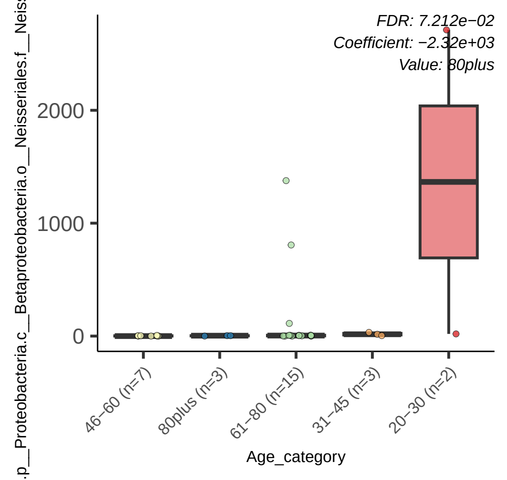

FDR: 7.212e−02
Coefficient: −2.32e+03
Value: 80plus
2000
1000
0
p__Proteobacteria.c__Betaproteobacteria.o__Neisseriales.f__Neis
46−60 (n=7)
80plus (n=3)
61−80 (n=15)
31−45 (n=3)
20−30 (n=2)
Age_category

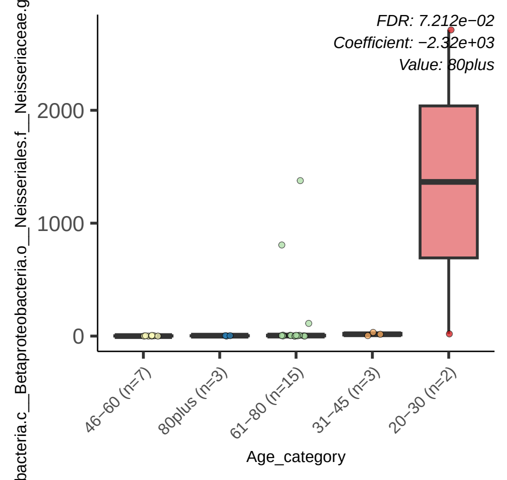
FDR: 7.212e−02
Coefficient: −2.32e+03
Value: 80plus
bacteria.c__Betaproteobacteria.o__Neisseriales.f__Neisseriaceae.g
2000
1000
0
46−60 (n=7)
80plus (n=3)
61−80 (n=15)
31−45 (n=3)
20−30 (n=2)
Age_category

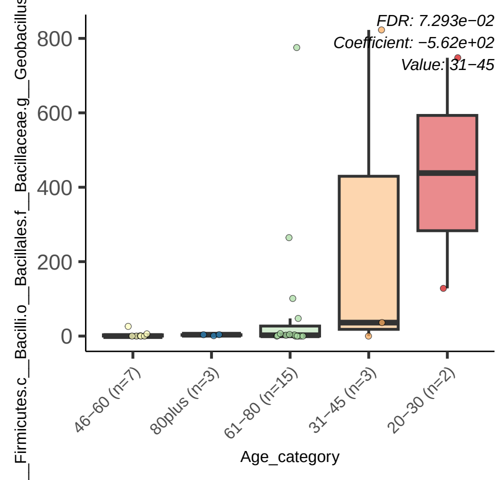

FDR: 7.293e−02
Coefficient: −5.62e+02
Value: 31−45
Firmicutes.c__Bacilli.o__Bacillales.f__Bacillaceae.g__Geobacillus
800
600
400
200
0
46−60 (n=7)
80plus (n=3)
61−80 (n=15)
31−45 (n=3)
20−30 (n=2)
Age_category

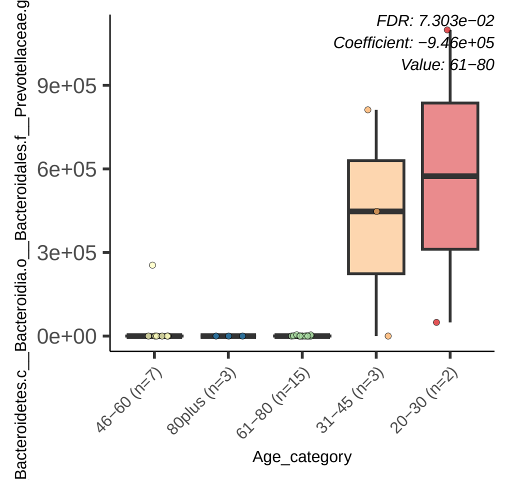

FDR: 7.303e−02
Coefficient: −9.46e+05
Value: 61−80
Bacteroidetes.c__Bacteroidia.o__Bacteroidales.f__Prevotellaceae.g
9e+05
6e+05
3e+05
0e+00
46−60 (n=7)
80plus (n=3)
61−80 (n=15)
31−45 (n=3)
20−30 (n=2)
Age_category

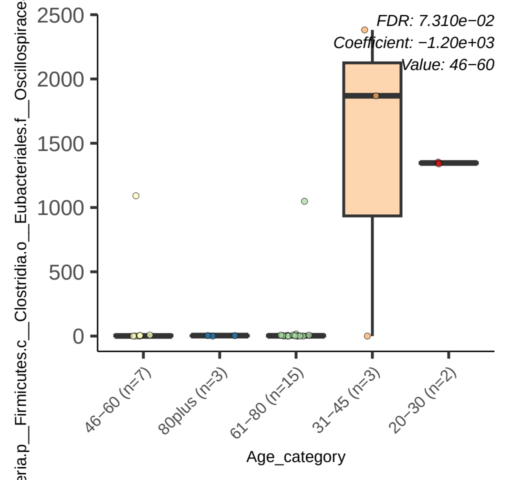

FDR: 7.310e−02
Coefficient: −1.20e+03
Value: 46−60
2500
2000
1500
1000
500
0
46−60 (n=7)
80plus (n=3)
61−80 (n=15)
31−45 (n=3)
20−30 (n=2)
Age_category
eria.p__Firmicutes.c__Clostridia.o__Eubacteriales.f__Oscillospirace

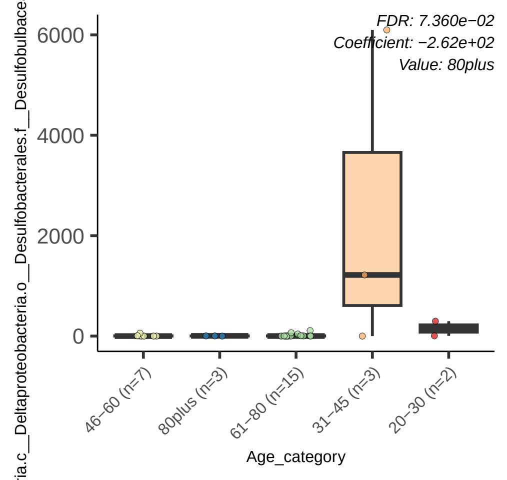

FDR: 7.360e−02
Coefficient: −2.62e+02
Value: 80plus
6000
4000
2000
0
ria.c__Deltaproteobacteria.o__Desulfobacterales.f__Desulfobulbace
46−60 (n=7)
80plus (n=3)
61−80 (n=15)
31−45 (n=3)
20−30 (n=2)
Age_category

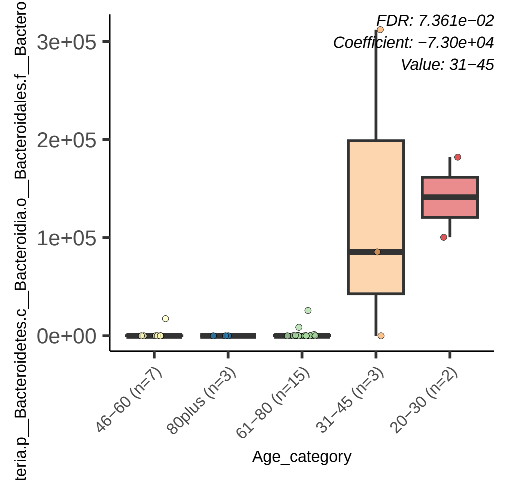

FDR: 7.361e−02
Coefficient: −7.30e+04
Value: 31−45
3e+05
2e+05
1e+05
0e+00
teria.p__Bacteroidetes.c__Bacteroidia.o__Bacteroidales.f__Bacteroi
46−60 (n=7)
80plus (n=3)
61−80 (n=15)
31−45 (n=3)
20−30 (n=2)
Age_category

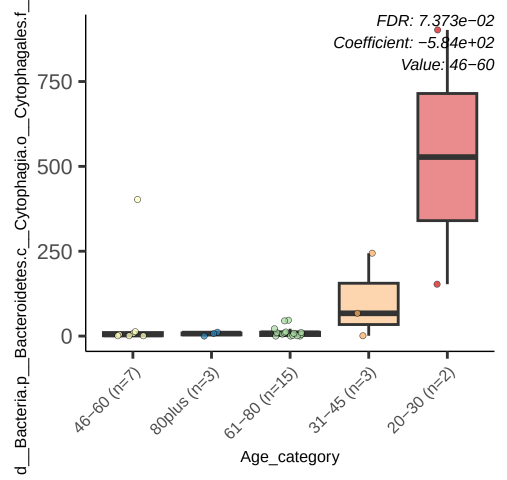
FDR: 7.373e−02
Coefficient: −5.84e+02
Value: 46−60
d__Bacteria.p__Bacteroidetes.c__Cytophagia.o__Cytophagales.f__
750
500
250
0
46−60 (n=7)
80plus (n=3)
61−80 (n=15)
31−45 (n=3)
20−30 (n=2)
Age_category

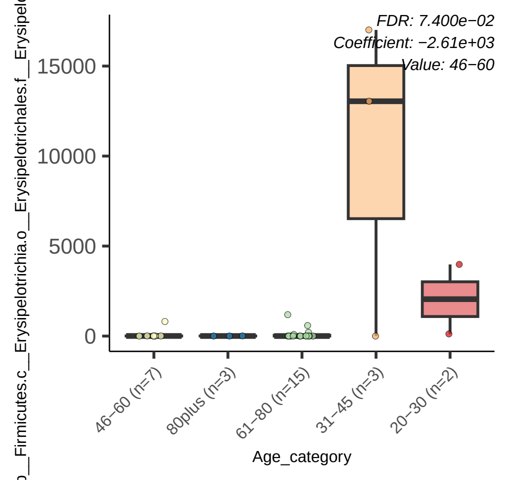

FDR: 7.400e−02
Coefficient: −2.61e+03
Value: 46−60
15000
10000
5000
0
o__Firmicutes.c__Erysipelotrichia.o__Erysipelotrichales.f__Erysipelo
46−60 (n=7)
80plus (n=3)
61−80 (n=15)
31−45 (n=3)
20−30 (n=2)
Age_category

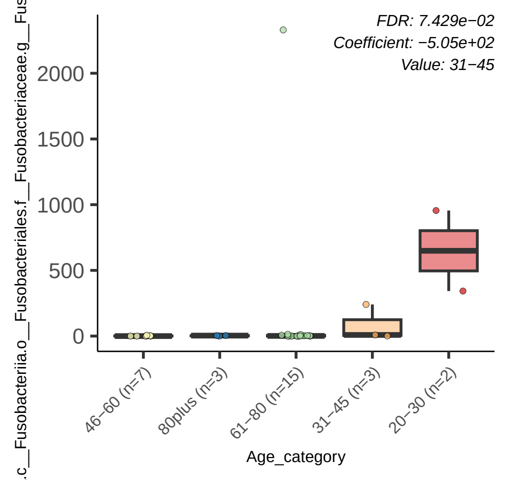

FDR: 7.429e−02
Coefficient: −5.05e+02
Value: 31−45
2000
1500
1000
500
0
c__Fusobacteriia.o__Fusobacteriales.f__Fusobacteriaceae.g__Fus
46−60 (n=7)
80plus (n=3)
61−80 (n=15)
31−45 (n=3)
20−30 (n=2)
Age_category

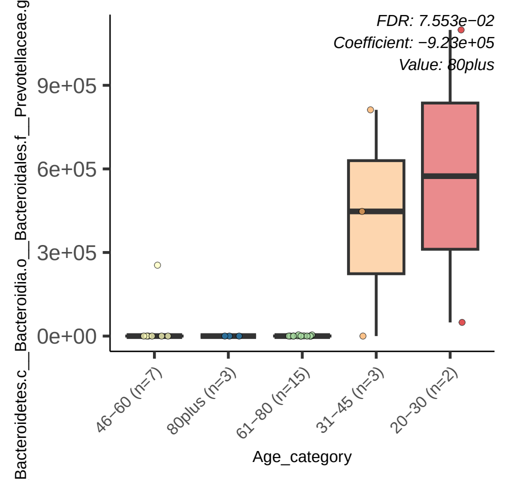

FDR: 7.553e−02
Coefficient: −9.23e+05
Value: 80plus
Bacteroidetes.c__Bacteroidia.o__Bacteroidales.f__Prevotellaceae.g
9e+05
6e+05
3e+05
0e+00
46−60 (n=7)
80plus (n=3)
61−80 (n=15)
31−45 (n=3)
20−30 (n=2)
Age_category

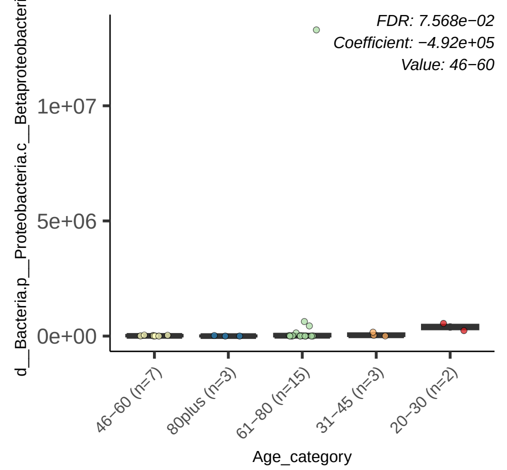

FDR: 7.568e−02
Coefficient: −4.92e+05
Value: 46−60
d__Bacteria.p__Proteobacteria.c__Betaproteobacteri
1e+07
5e+06
0e+00
46−60 (n=7)
80plus (n=3)
61−80 (n=15)
31−45 (n=3)
20−30 (n=2)
Age_category

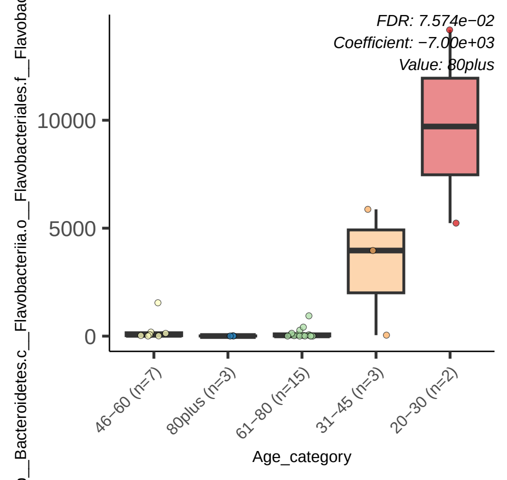

FDR: 7.574e−02
Coefficient: −7.00e+03
Value: 80plus
10000
5000
0
46−60 (n=7)
80plus (n=3)
61−80 (n=15)
31−45 (n=3)
20−30 (n=2)
Age_category
o__Bacteroidetes.c__Flavobacteriia.o__Flavobacteriales.f__Flavobac

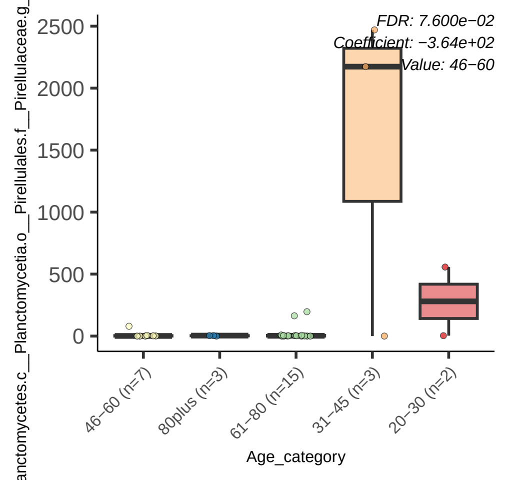

FDR: 7.600e−02
Coefficient: −3.64e+02
Value: 46−60
2500
2000
1500
1000
500
0
Planctomycetes.c__Planctomycetia.o__Pirellulales.f__Pirellulaceae.g_
46−60 (n=7)
80plus (n=3)
61−80 (n=15)
31−45 (n=3)
20−30 (n=2)
Age_category

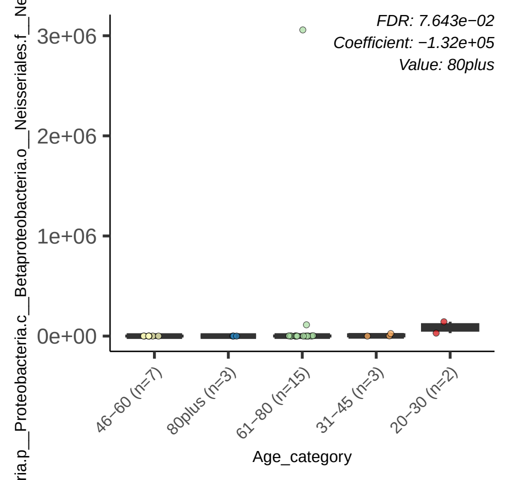

FDR: 7.643e−02
Coefficient: −1.32e+05
Value: 80plus
3e+06
2e+06
1e+06
0e+00
ria.p__Proteobacteria.c__Betaproteobacteria.o__Neisseriales.f__Ne
46−60 (n=7)
80plus (n=3)
61−80 (n=15)
31−45 (n=3)
20−30 (n=2)
Age_category

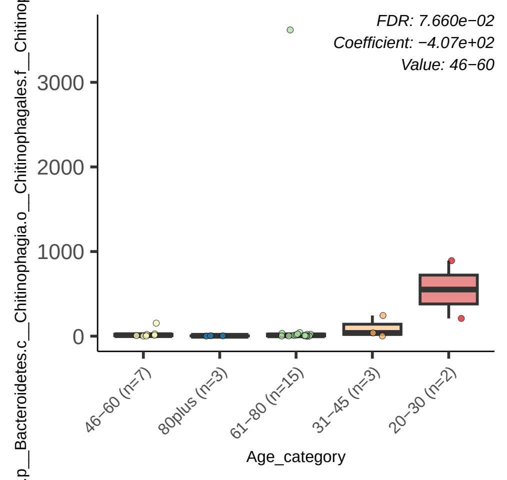

FDR: 7.660e−02
Coefficient: −4.07e+02
Value: 46−60
3000
2000
1000
0
p__Bacteroidetes.c__Chitinophagia.o__Chitinophagales.f__Chitinop
46−60 (n=7)
80plus (n=3)
61−80 (n=15)
31−45 (n=3)
20−30 (n=2)
Age_category

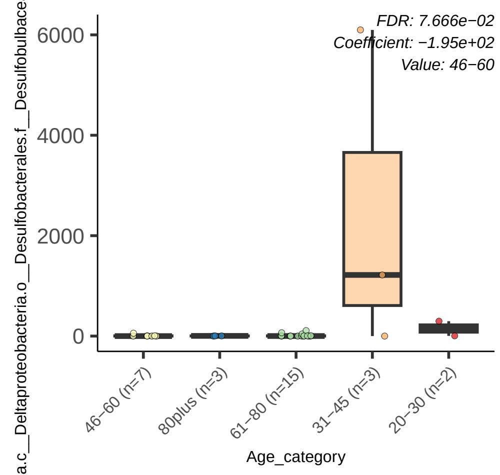

FDR: 7.666e−02
Coefficient: −1.95e+02
Value: 46−60
6000
4000
2000
0
ria.c__Deltaproteobacteria.o__Desulfobacterales.f__Desulfobulbace
46−60 (n=7)
80plus (n=3)
61−80 (n=15)
31−45 (n=3)
20−30 (n=2)
Age_category

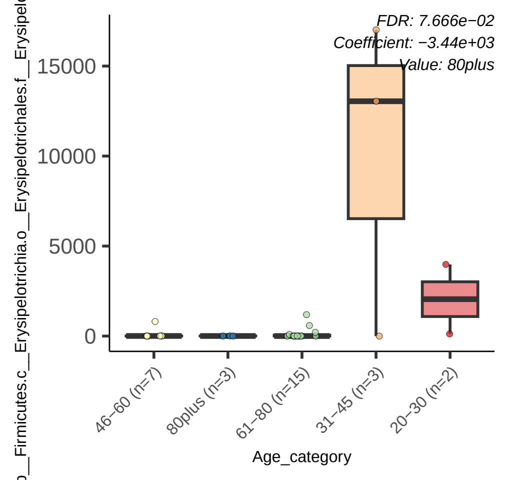

FDR: 7.666e−02
Coefficient: −3.44e+03
Value: 80plus
15000
10000
5000
0
o__Firmicutes.c__Erysipelotrichia.o__Erysipelotrichales.f__Erysipelo
46−60 (n=7)
80plus (n=3)
61−80 (n=15)
31−45 (n=3)
20−30 (n=2)
Age_category

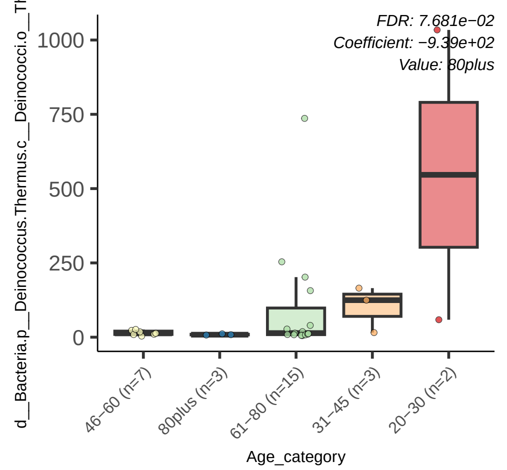

FDR: 7.681e−02
Coefficient: −9.39e+02
Value: 80plus
d__Bacteria.p__Deinococcus.Thermus.c__Deinococci.o__Th
1000
750
500
250
0
46−60 (n=7)
80plus (n=3)
61−80 (n=15)
31−45 (n=3)
20−30 (n=2)
Age_category

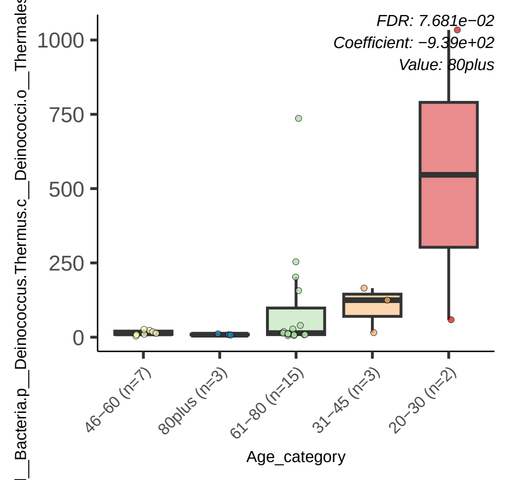

FDR: 7.681e−02
Coefficient: −9.39e+02
Value: 80plus
1000
750
500
250
0
Bacteria.p__Deinococcus.Thermus.c__Deinococci.o__Thermales
46−60 (n=7)
80plus (n=3)
61−80 (n=15)
31−45 (n=3)
20−30 (n=2)
Age_category

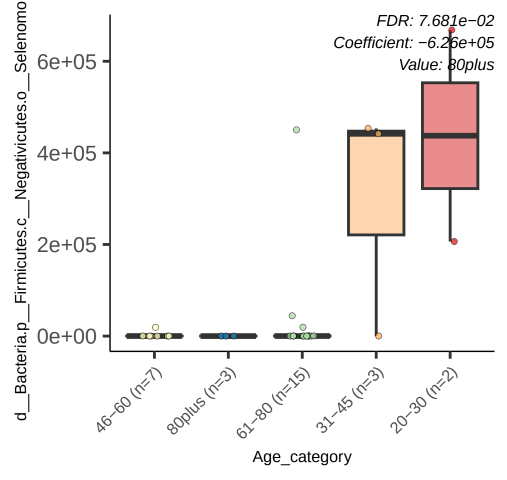

FDR: 7.681e−02
Coefficient: −6.26e+05
Value: 80plus
d__Bacteria.p__Firmicutes.c__Negativicutes.o__Selenomo
6e+05
4e+05
2e+05
0e+00
46−60 (n=7)
80plus (n=3)
61−80 (n=15)
31−45 (n=3)
20−30 (n=2)
Age_category

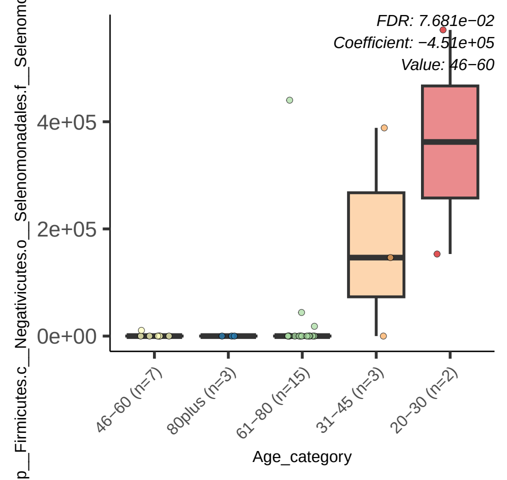

FDR: 7.681e−02
Coefficient: −4.51e+05
Value: 46−60
p__Firmicutes.c__Negativicutes.o__Selenomonadales.f__Selenomo
4e+05
2e+05
0e+00
46−60 (n=7)
80plus (n=3)
61−80 (n=15)
31−45 (n=3)
20−30 (n=2)
Age_category

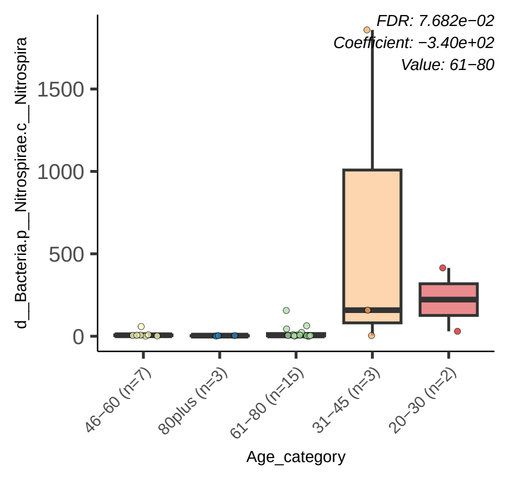

FDR: 7.682e−02
Coefficient: −3.40e+02
Value: 61−80
d__Bacteria.p__Nitrospirae.c__Nitrospira
1500
1000
500
0
46−60 (n=7)
80plus (n=3)
61−80 (n=15)
31−45 (n=3)
20−30 (n=2)
Age_category

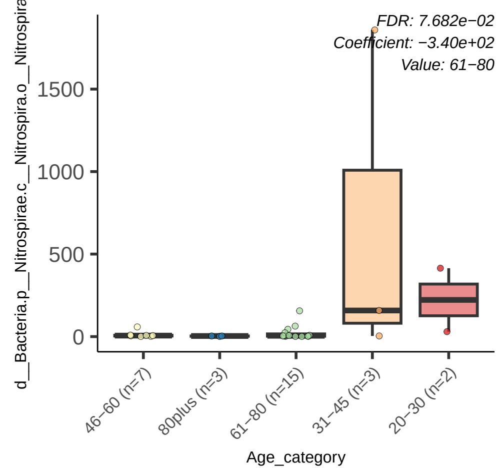

FDR: 7.682e−02
Coefficient: −3.40e+02
Value: 61−80
d__Bacteria.p__Nitrospirae.c__Nitrospira.o__Nitrospira
1500
1000
500
0
46−60 (n=7)
80plus (n=3)
61−80 (n=15)
31−45 (n=3)
20−30 (n=2)
Age_category

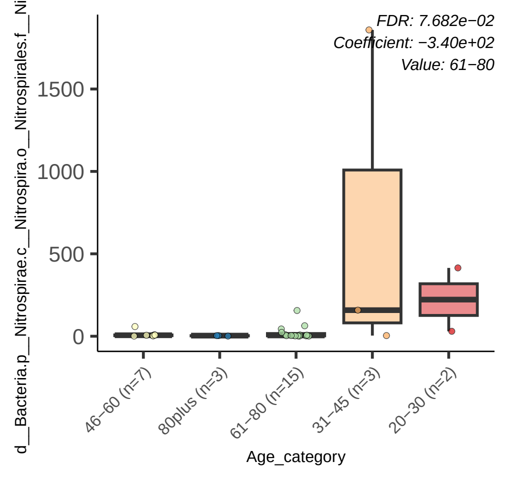

FDR: 7.682e−02
Coefficient: −3.40e+02
Value: 61−80
d__Bacteria.p__Nitrospirae.c__Nitrospira.o__Nitrospirales.f__Ni
1500
1000
500
0
46−60 (n=7)
80plus (n=3)
61−80 (n=15)
31−45 (n=3)
20−30 (n=2)
Age_category

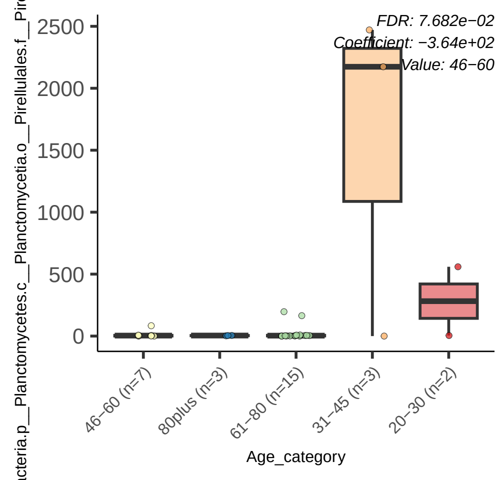
FDR: 7.682e−02
Coefficient: −3.64e+02
Value: 46−60
2500
2000
1500
1000
500
0
acteria.p__Planctomycetes.c__Planctomycetia.o__Pirellulales.f__Pir
46−60 (n=7)
80plus (n=3)
61−80 (n=15)
31−45 (n=3)
20−30 (n=2)
Age_category

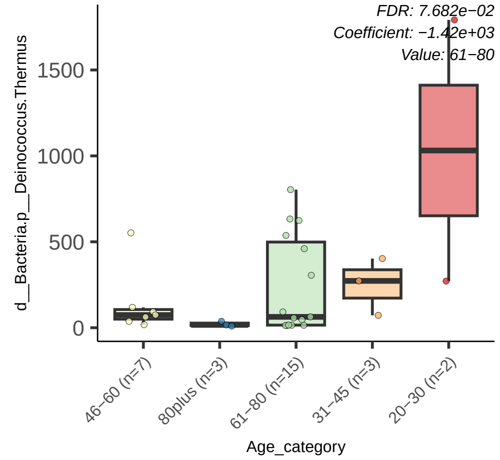
FDR: 7.682e−02
Coefficient: −1.42e+03
Value: 61−80
d__Bacteria.p__Deinococcus.Thermus
1500
1000
500
0
46−60 (n=7)
80plus (n=3)
61−80 (n=15)
31−45 (n=3)
20−30 (n=2)
Age_category

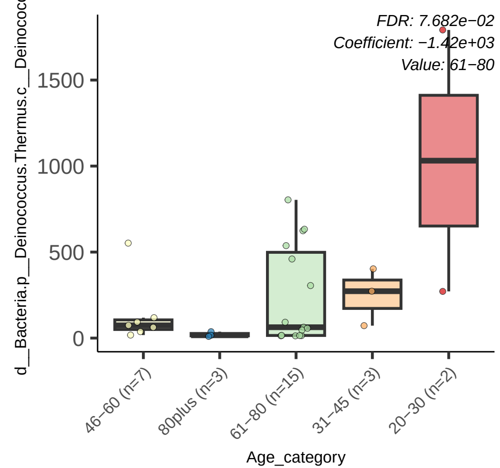

FDR: 7.682e−02
Coefficient: −1.42e+03
Value: 61−80
d__Bacteria.p__Deinococcus.Thermus.c__Deinococ
1500
1000
500
0
46−60 (n=7)
80plus (n=3)
61−80 (n=15)
31−45 (n=3)
20−30 (n=2)
Age_category

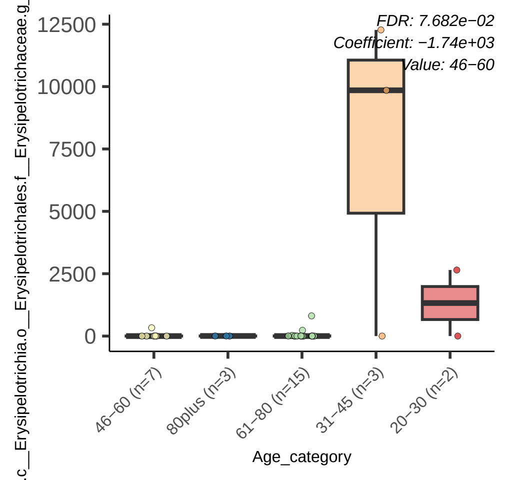

FDR: 7.682e−02
Coefficient: −1.74e+03
Value: 46−60
12500
10000
7500
5000
2500
0
c__Erysipelotrichia.o__Erysipelotrichales.f__Erysipelotrichaceae.g_
46−60 (n=7)
80plus (n=3)
61−80 (n=15)
31−45 (n=3)
20−30 (n=2)
Age_category

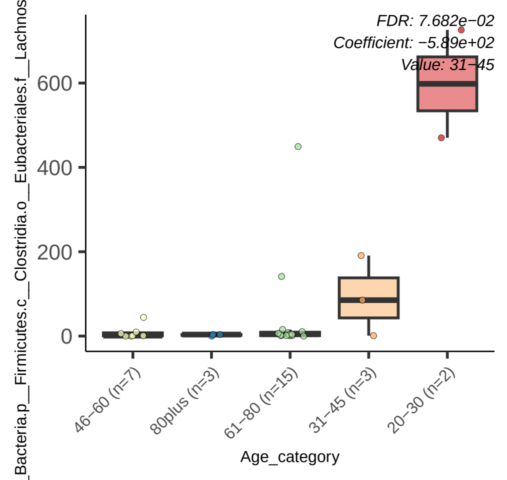

FDR: 7.682e−02
Coefficient: −5.89e+02
Value: 31−45
Bacteria.p__Firmicutes.c__Clostridia.o__Eubacteriales.f__Lachnos
0
200
400
600
46−60 (n=7)
80plus (n=3)
61−80 (n=15)
31−45 (n=3)
20−30 (n=2)
Age_category

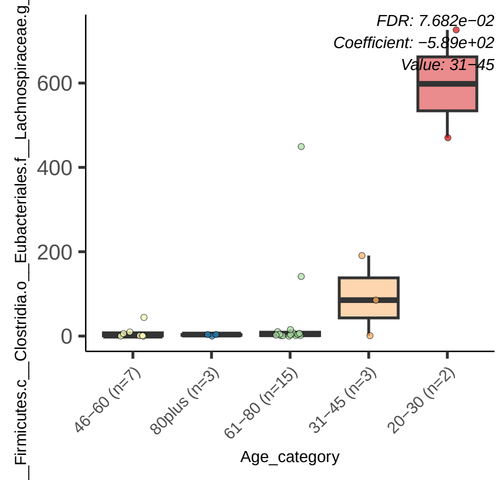

FDR: 7.682e−02
Coefficient: −5.89e+02
Value: 31−45
Firmicutes.c__Clostridia.o__Eubacteriales.f__Lachnospiraceae.g__
0
200
400
600
46−60 (n=7)
80plus (n=3)
61−80 (n=15)
31−45 (n=3)
20−30 (n=2)
Age_category

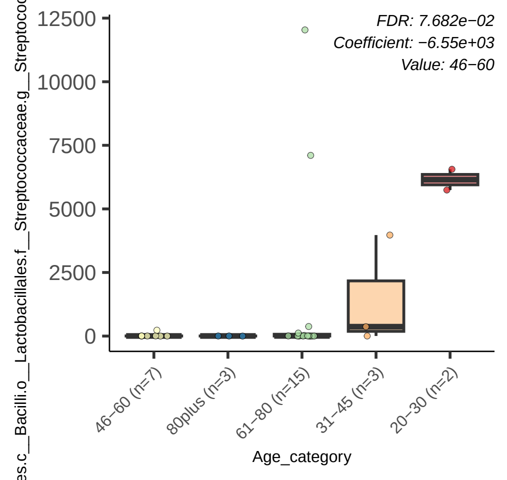

FDR: 7.682e−02
Coefficient: −6.55e+03
Value: 46−60
12500
10000
7500
5000
2500
0
es.c__Bacilli.o__Lactobacillales.f__Streptococcaceae.g__Streptococ
46−60 (n=7)
80plus (n=3)
61−80 (n=15)
31−45 (n=3)
20−30 (n=2)
Age_category

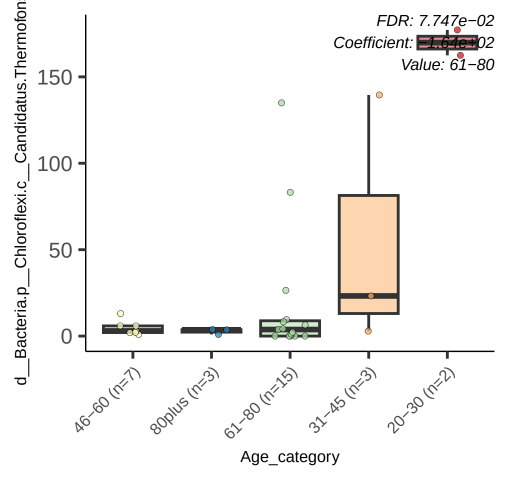
FDR: 7.747e−02
Coefficient: −1.64e+02
Value: 61−80
d__Bacteria.p__Chloroflexi.c__Candidatus.Thermofon
150
100
50
0
46−60 (n=7)
80plus (n=3)
61−80 (n=15)
31−45 (n=3)
20−30 (n=2)
Age_category

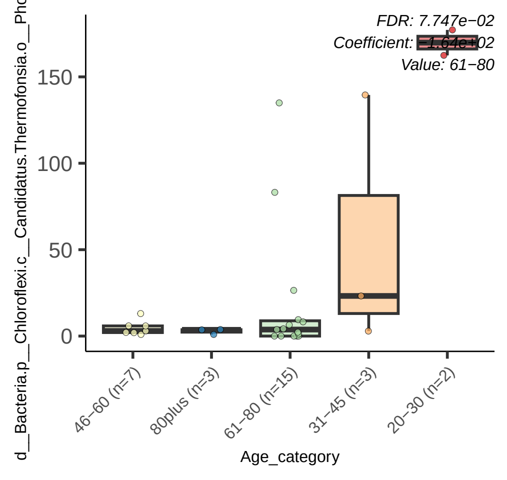

FDR: 7.747e−02
Coefficient: −1.64e+02
Value: 61−80
d__Bacteria.p__Chloroflexi.c__Candidatus.Thermofonsia.o__Pho
150
100
50
0
46−60 (n=7)
80plus (n=3)
61−80 (n=15)
31−45 (n=3)
20−30 (n=2)
Age_category

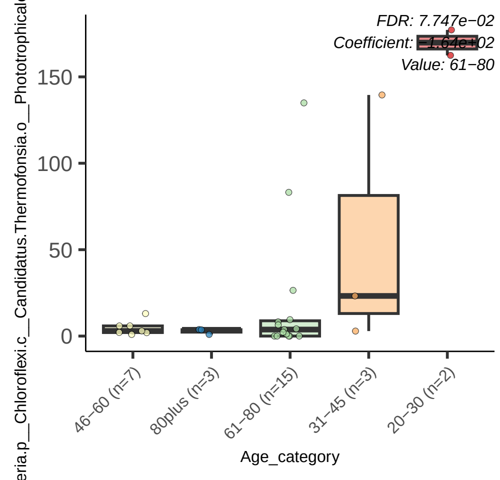

FDR: 7.747e−02
Coefficient: −1.64e+02
Value: 61−80
150
100
50
0
46−60 (n=7)
80plus (n=3)
61−80 (n=15)
31−45 (n=3)
20−30 (n=2)
Age_category
eria.p__Chloroflexi.c__Candidatus.Thermofonsia.o__Phototrophicalo

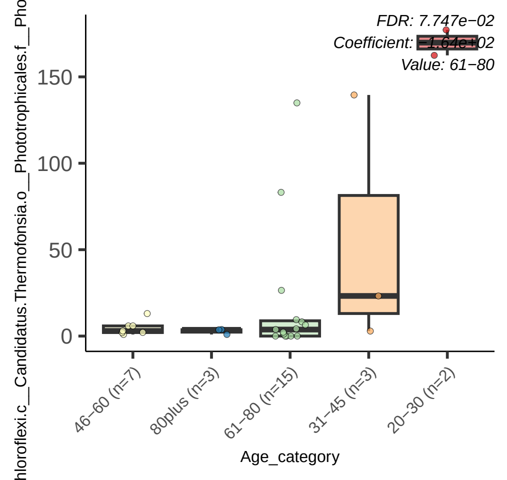

FDR: 7.747e−02
Coefficient: −1.64e+02
Value: 61−80
150
100
50
0
46−60 (n=7)
80plus (n=3)
61−80 (n=15)
31−45 (n=3)
20−30 (n=2)
Age_category
hloroflexi.c__Candidatus.Thermofonsia.o__Phototrophicales.f__Pho

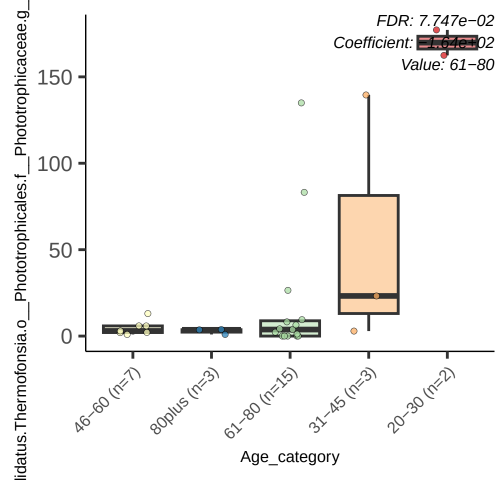

FDR: 7.747e−02
Coefficient: −1.64e+02
Value: 61−80
150
100
50
0
lidatus.Thermofonsia.o__Photrophicales.f__Phototrophicaceae.g__
46−60 (n=7)
80plus (n=3)
61−80 (n=15)
31−45 (n=3)
20−30 (n=2)
Age_category

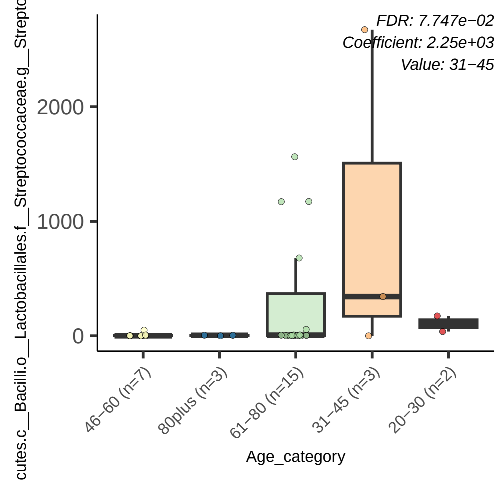

FDR: 7.747e−02
Coefficient: 2.25e+03
Value: 31−45
cutes.c__Bacilli.o__Lactobacillales.f__Streptococcaceae.g__Strepto
0
1000
2000
46−60 (n=7)
80plus (n=3)
61−80 (n=15)
31−45 (n=3)
20−30 (n=2)
Age_category

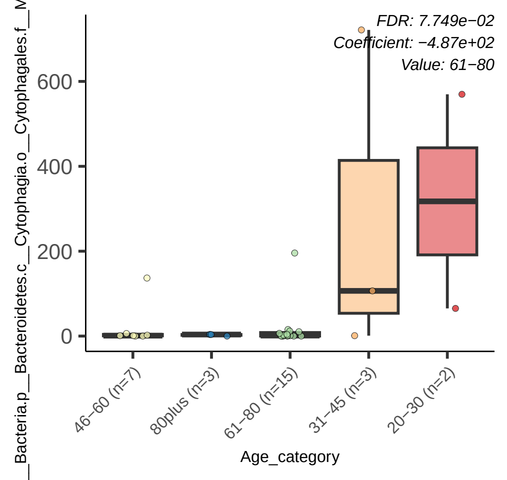

Bacteria.p__Bacteroidetes.c__Cytophagia.o__Cytophagales.f__M
FDR: 7.749e−02
Coefficient: −4.87e+02
Value: 61−80
600
400
200
0
46−60 (n=7)
80plus (n=3)
61−80 (n=15)
31−45 (n=3)
20−30 (n=2)
Age_category

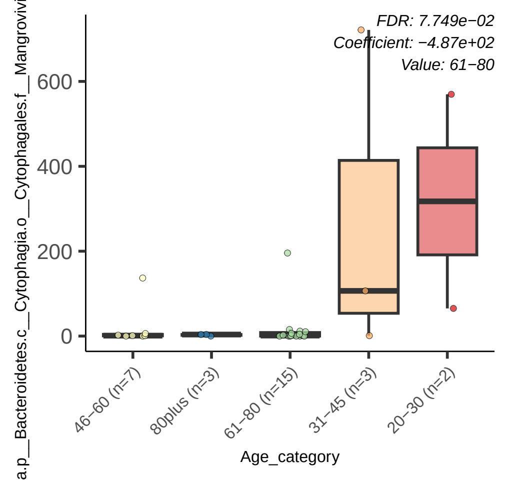

FDR: 7.749e−02
Coefficient: −4.87e+02
Value: 61−80
a.p__Bacteroidetes.c__Cytophagia.o__Cytophagales.f__Mangrovivi
0
200
400
600
46−60 (n=7)
80plus (n=3)
61−80 (n=15)
31−45 (n=3)
20−30 (n=2)
Age_category

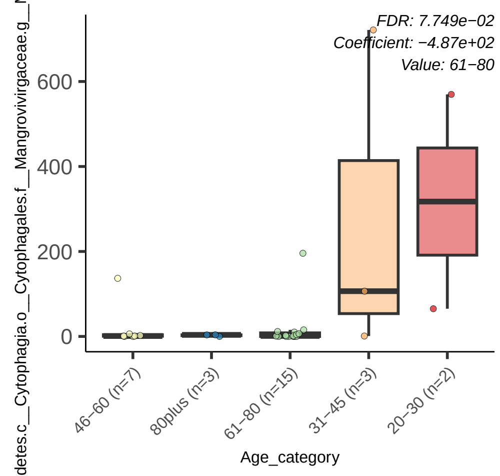

FDR: 7.749e−02
Coefficient: −4.87e+02
Value: 61−80
600
400
200
0
46−60 (n=7)
80plus (n=3)
61−80 (n=15)
31−45 (n=3)
20−30 (n=2)
Age_category
idetes.c__Cytophagia.o__Cytophagales.f__Mangrovivirgaceae.g__

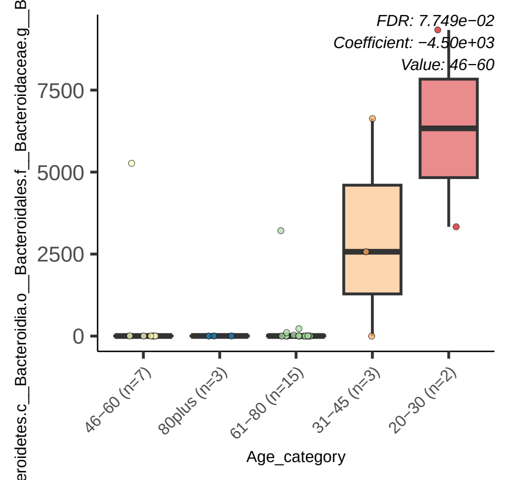
FDR: 7.749e−02
Coefficient: −4.50e+03
Value: 46−60
7500
5000
2500
0
46−60 (n=7)
80plus (n=3)
61−80 (n=15)
31−45 (n=3)
20−30 (n=2)
Age_category
eroidetes.c__Bacteroidia.o__Bacteroidales.f__Bacteroidaceae.g__B

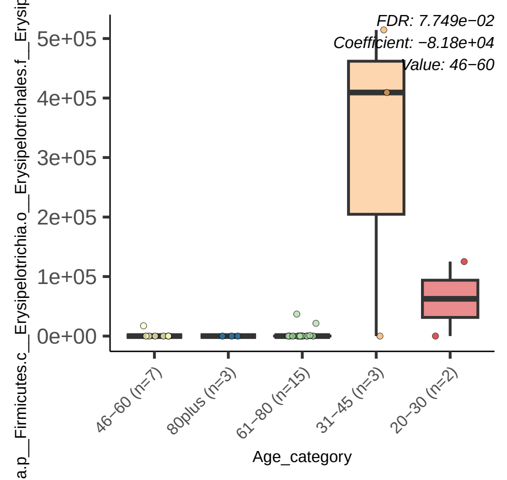

FDR: 7.749e−02
Coefficient: −8.18e+04
Value: 46−60
a.p__Firmicutes.c__Erysipelotrichia.o__Erysipelotrichales.f__Erysip
5e+05
4e+05
3e+05
2e+05
1e+05
0e+00
46−60 (n=7)
80plus (n=3)
61−80 (n=15)
31−45 (n=3)
20−30 (n=2)
Age_category

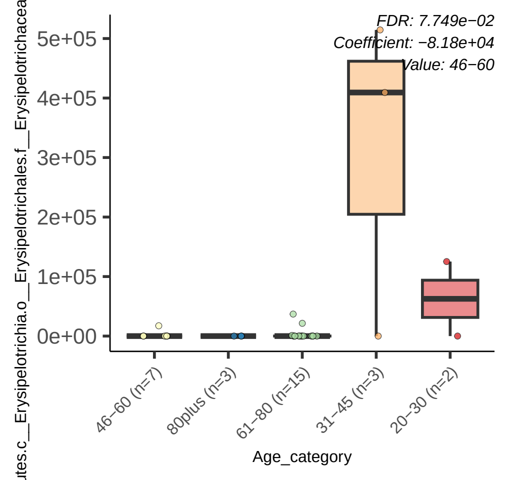

FDR: 7.749e−02
Coefficient: −8.18e+04
Value: 46−60
5e+05
4e+05
3e+05
2e+05
1e+05
0e+00
utes.c__Erysipelotrichia.o__Erysipelotrichales.f__Erysipelotrichacea
46−60 (n=7)
80plus (n=3)
61−80 (n=15)
31−45 (n=3)
20−30 (n=2)
Age_category

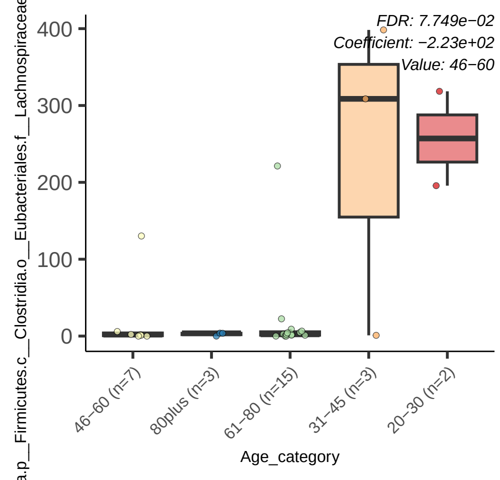

FDR: 7.749e−02
Coefficient: −2.23e+02
Value: 46−60
a.p__Firmicutes.c__Clostridia.o__Eubacteriales.f__Lachnospiraceae
400
300
200
100
0
46−60 (n=7)
80plus (n=3)
61−80 (n=15)
31−45 (n=3)
20−30 (n=2)
Age_category

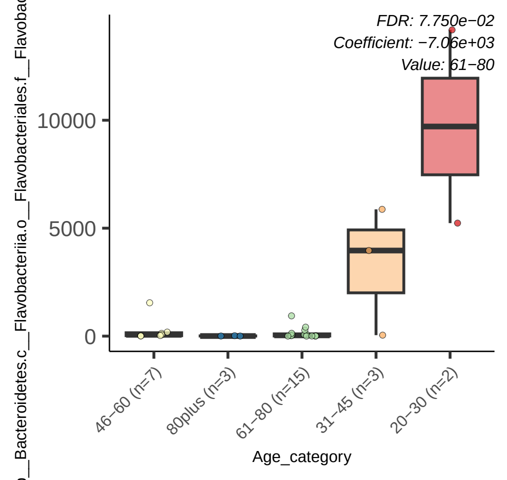

FDR: 7.750e−02
Coefficient: −7.06e+03
Value: 61−80
10000
5000
0
o__Bacteroidetes.c__Flavobacteriia.o__Flavobacteriales.f__Flavobac
46−60 (n=7)
80plus (n=3)
61−80 (n=15)
31−45 (n=3)
20−30 (n=2)
Age_category

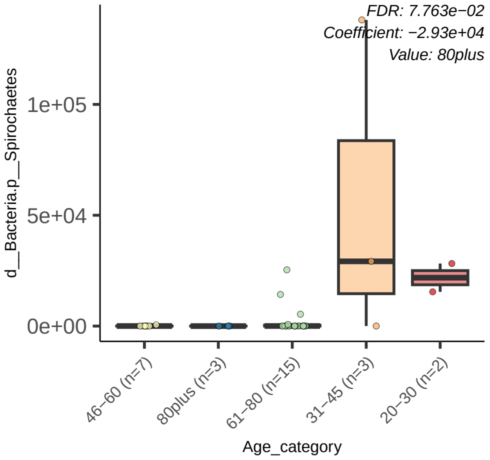

FDR: 7.763e−02
Coefficient: −2.93e+04
Value: 80plus
d__Bacteria.p__Spirochaetes
1e+05
5e+04
0e+00
46−60 (n=7)
80plus (n=3)
61−80 (n=15)
31−45 (n=3)
20−30 (n=2)
Age_category

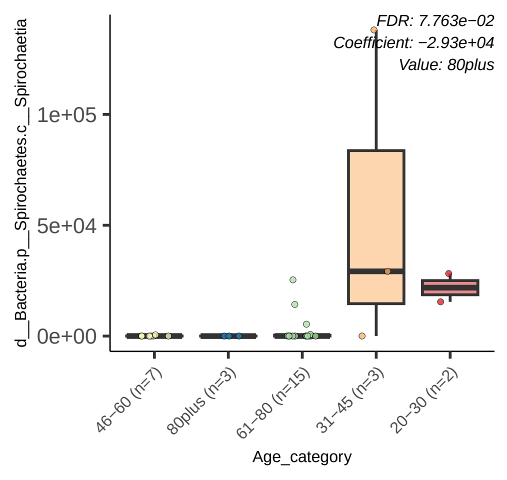

FDR: 7.763e−02
Coefficient: −2.93e+04
Value: 80plus
d__Bacteria.p__Spirochaetes.c__Spirochaetia
1e+05
5e+04
0e+00
46−60 (n=7)
80plus (n=3)
61−80 (n=15)
31−45 (n=3)
20−30 (n=2)
Age_category

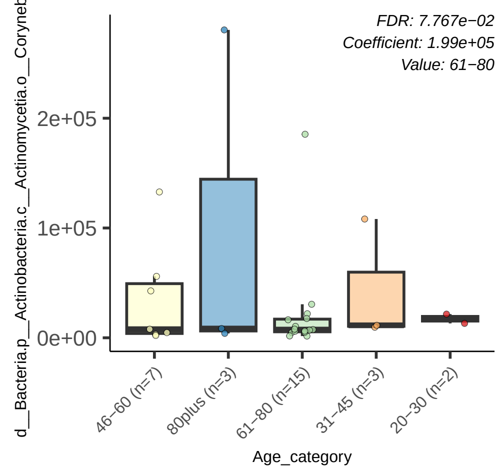

FDR: 7.767e−02
Coefficient: 1.99e+05
Value: 61−80
d__Bacteria.p__Actinobacteria.c__Actinomycetia.o__Coryneb
2e+05
1e+05
0e+00
46−60 (n=7)
80plus (n=3)
61−80 (n=15)
31−45 (n=3)
20−30 (n=2)
Age_category

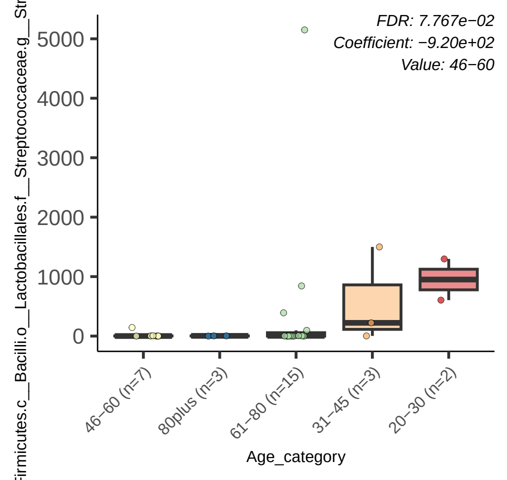

FDR: 7.767e−02
Coefficient: −9.20e+02
Value: 46−60
Firmicutes.c__Bacilli.o__Lactobacillales.f__Streptococcaceae.g__Str
5000
4000
3000
2000
1000
0
46−60 (n=7)
80plus (n=3)
61−80 (n=15)
31−45 (n=3)
20−30 (n=2)
Age_category

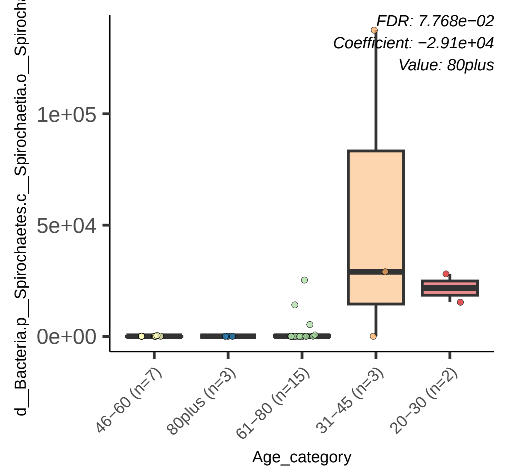

FDR: 7.768e−02
Coefficient: −2.91e+04
Value: 80plus
d__Bacteria.p__Spirochaetes.c__Spirochaetia.o__Spiroch
1e+05
5e+04
0e+00
46−60 (n=7)
80plus (n=3)
61−80 (n=15)
31−45 (n=3)
20−30 (n=2)
Age_category

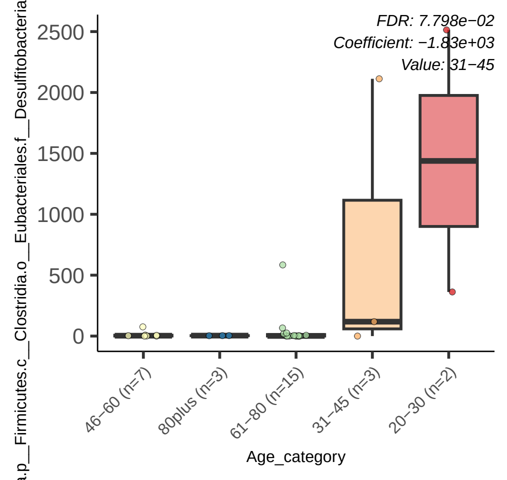
FDR: 7.798e−02
Coefficient: −1.83e+03
Value: 31−45
2500
2000
1500
1000
500
0
a.p__Firmicutes.c__Clostridia.o__Eubacteriales.f__Desulfitobacteria
46−60 (n=7)
80plus (n=3)
61−80 (n=15)
31−45 (n=3)
20−30 (n=2)
Age_category

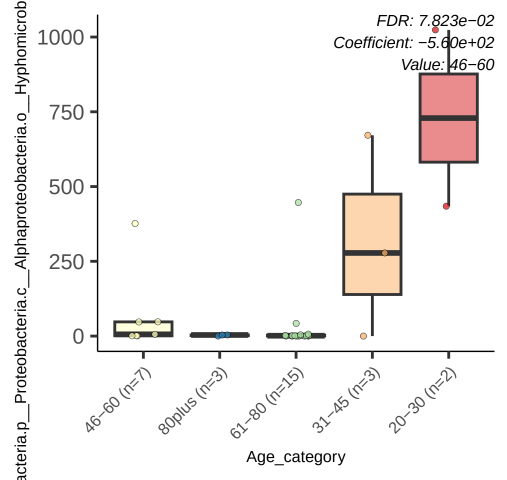

FDR: 7.823e−02
Coefficient: −5.60e+02
Value: 46−60
1000
750
500
250
0
46−60 (n=7)
80plus (n=3)
61−80 (n=15)
31−45 (n=3)
20−30 (n=2)
Age_category
acteria.p__Proteobacteria.c__Alphaproteobacteria.o__Hyphomicrob

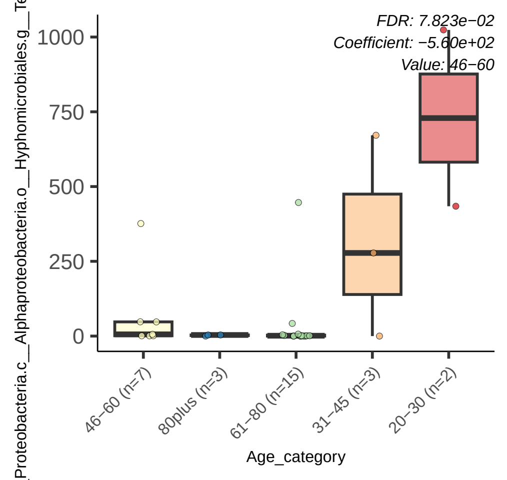

FDR: 7.823e−02
Coefficient: −5.60e+02
Value: 46−60
Proteobacteria.c__Alphaproteobacteria.o__Hyphomicrobiales.g__Te
1000
750
500
250
0
46−60 (n=7)
80plus (n=3)
61−80 (n=15)
31−45 (n=3)
20−30 (n=2)
Age_category

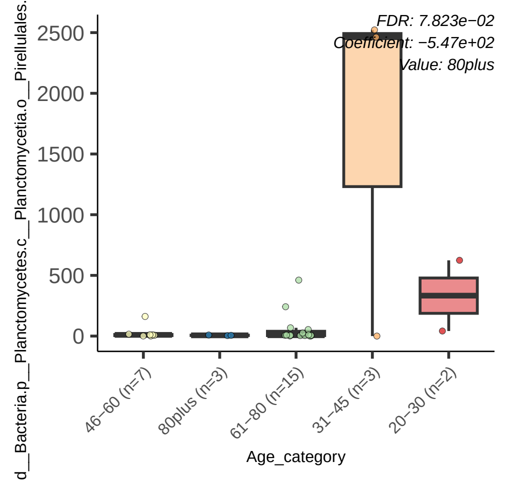

FDR: 7.823e−02
Coefficient: −5.47e+02
Value: 80plus
d__Bacteria.p__Planctomycetes.c__Planctomycetia.o__Pirellulales.
2500
2000
1500
1000
500
0
46−60 (n=7)
80plus (n=3)
61−80 (n=15)
31−45 (n=3)
20−30 (n=2)
Age_category

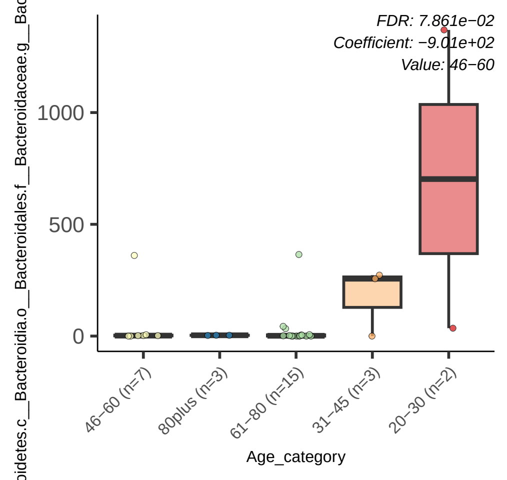

FDR: 7.861e−02
Coefficient: −9.01e+02
Value: 46−60
1000
500
0
46−60 (n=7)
80plus (n=3)
61−80 (n=15)
31−45 (n=3)
20−30 (n=2)
Age_category
oidetes.c__Bacteroidia.o__Bacteroidales.f__Bacteroidaceae.g__Bac

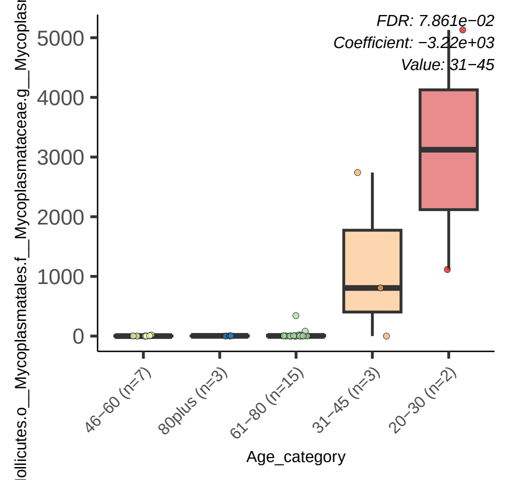

FDR: 7.861e−02
Coefficient: −3.22e+03
Value: 31−45
5000
4000
3000
2000
1000
0
46−60 (n=7)
80plus (n=3)
61−80 (n=15)
31−45 (n=3)
20−30 (n=2)
Age_category

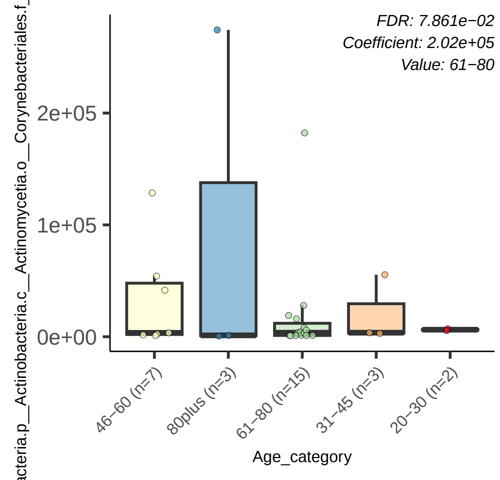

FDR: 7.861e−02
Coefficient: 2.02e+05
Value: 61−80
acteria.p__Actinobacteria.c__Actinomycetia.o__Corynebacteriales.f_
2e+05
1e+05
0e+00
46−60 (n=7)
80plus (n=3)
61−80 (n=15)
31−45 (n=3)
20−30 (n=2)
Age_category

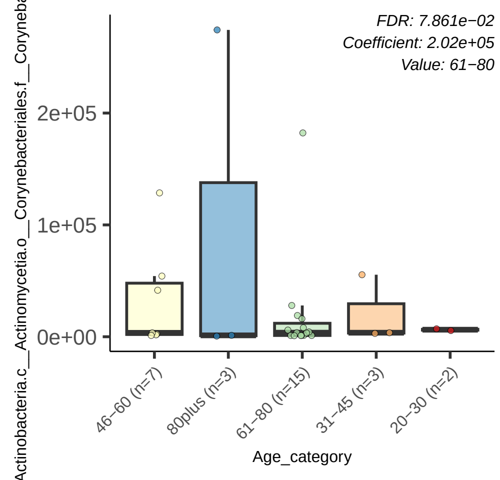

FDR: 7.861e−02
Coefficient: 2.02e+05
Value: 61−80
Actinobacteria.c__Actinomycetia.o__Corynebacteriales.f__Corynebacteriales.f__Coryneba
2e+05
1e+05
0e+00
46−60 (n=7)
80plus (n=3)
61−80 (n=15)
31−45 (n=3)
20−30 (n=2)
Age_category

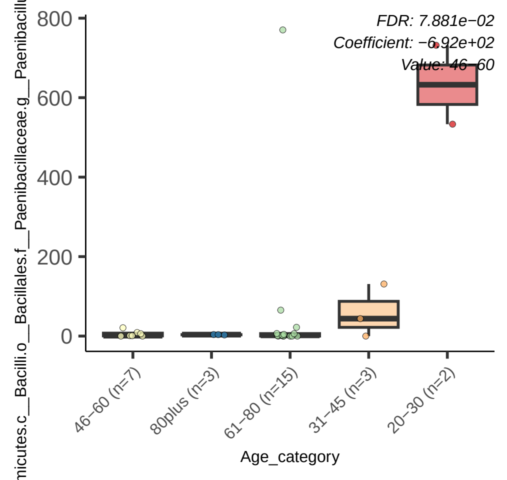
FDR: 7.881e−02
Coefficient: −6.92e+02
Value: 46−60
800
600
400
200
0
micutes.c__Bacilli.o__Bacillales.f__Paenibacillaceae.g__Paenibacillu
46−60 (n=7)
80plus (n=3)
61−80 (n=15)
31−45 (n=3)
20−30 (n=2)
Age_category

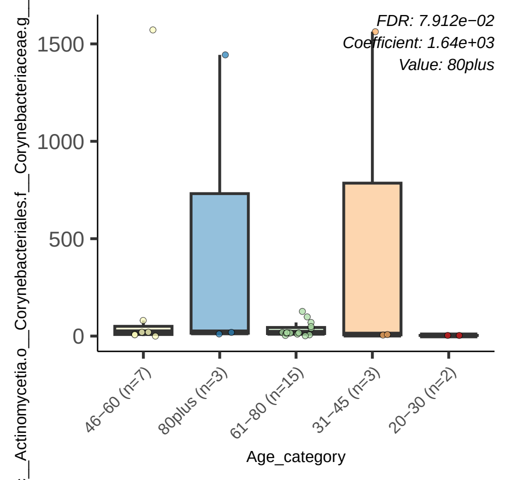

FDR: 7.912e−02
Coefficient: 1.64e+03
Value: 80plus
Actinomycetia.o__Corynebacteriales.f__Corynebacteriaceae.g__
1500
1000
500
0
46−60 (n=7)
80plus (n=3)
61−80 (n=15)
31−45 (n=3)
20−30 (n=2)
Age_category

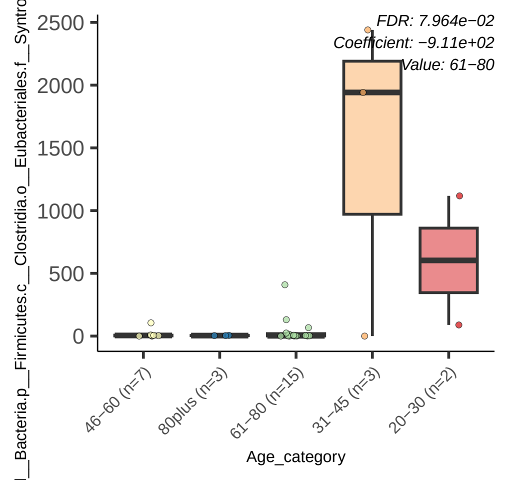
FDR: 7.964e−02
Coefficient: −9.11e+02
Value: 61−80
_Bacteria.p__Firmicutes.c__Clostridia.o__Eubacteriales.f__Syntro
2500
2000
1500
1000
500
0
46−60 (n=7)
80plus (n=3)
61−80 (n=15)
31−45 (n=3)
20−30 (n=2)
Age_category

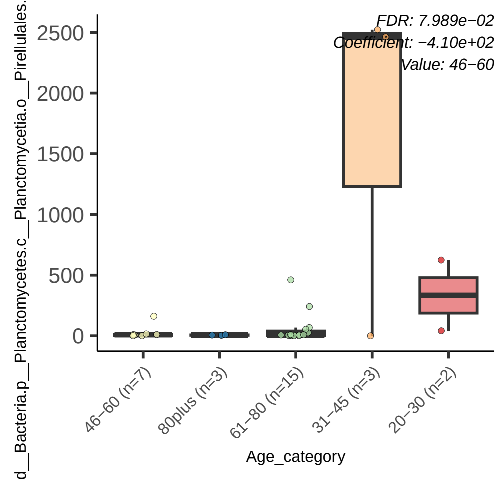

FDR: 7.989e−02
Coefficient: −4.10e+02
Value: 46−60
d__Bacteria.p__Planctomycetes.c__Planctomycetia.o__Pirellulales.
2500
2000
1500
1000
500
0
46−60 (n=7)
80plus (n=3)
61−80 (n=15)
31−45 (n=3)
20−30 (n=2)
Age_category

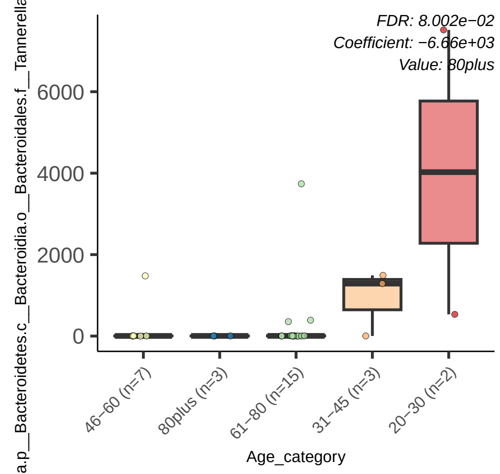

FDR: 8.002e−02
Coefficient: −6.66e+03
Value: 80plus
6000
4000
2000
0
ria.p__Bacteroidetes.c__Bacteroidia.o__Bacteroidales.f__Tannerella
46−60 (n=7)
80plus (n=3)
61−80 (n=15)
31−45 (n=3)
20−30 (n=2)
Age_category

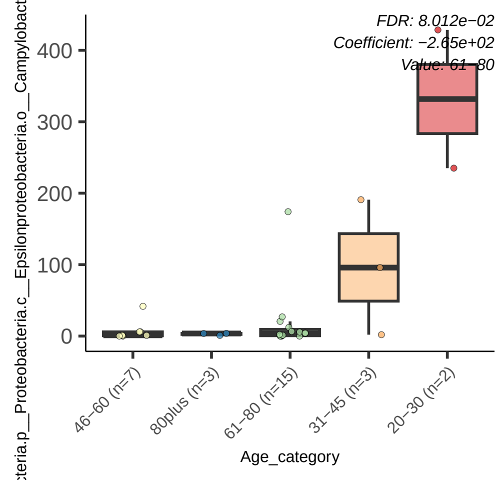

FDR: 8.012e−02
Coefficient: −2.65e+02
Value: 61−80
cteria.p__Proteobacteria.c__Epsilonproteobacteria.o__Campylobact
0
100
200
300
400
46−60 (n=7)
80plus (n=3)
61−80 (n=15)
31−45 (n=3)
20−30 (n=2)
Age_category

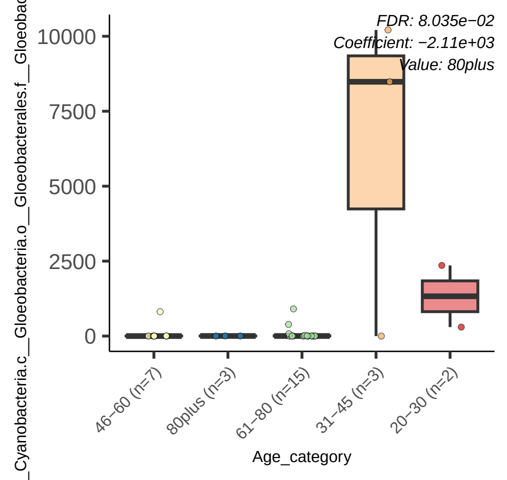
FDR: 8.035e−02
Coefficient: −2.11e+03
Value: 80plus
10000
7500
5000
2500
0
Cyanobacteria.c__Gloeobacteria.o__Gloeobacterales.f__Gloeobac
46−60 (n=7)
80plus (n=3)
61−80 (n=15)
31−45 (n=3)
20−30 (n=2)
Age_category

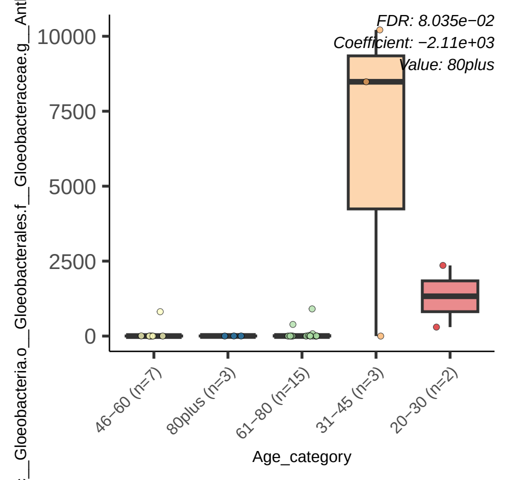

FDR: 8.035e−02
Coefficient: −2.11e+03
Value: 80plus
10000
7500
5000
2500
0
Gloeobacteria.o__Gloeobacterales.f__Gloeobacteraceae.g__Ant
46−60 (n=7)
80plus (n=3)
61−80 (n=15)
31−45 (n=3)
20−30 (n=2)
Age_category

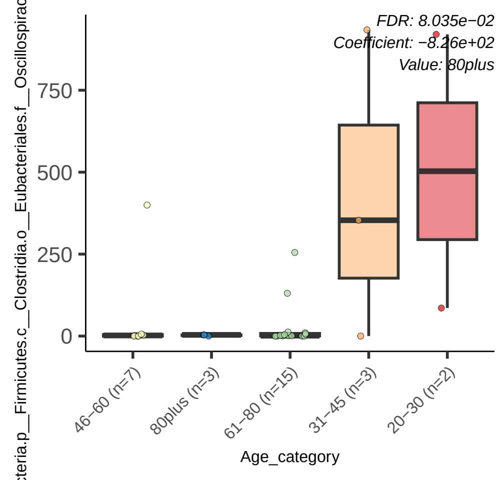

FDR: 8.035e−02
Coefficient: −8.26e+02
Value: 80plus
750
500
250
0
46−60 (n=7)
80plus (n=3)
61−80 (n=15)
31−45 (n=3)
20−30 (n=2)
Age_category
cteria.p__Firmicutes.c__Clostridia.o__Eubacteriales.f__Oscillospirac

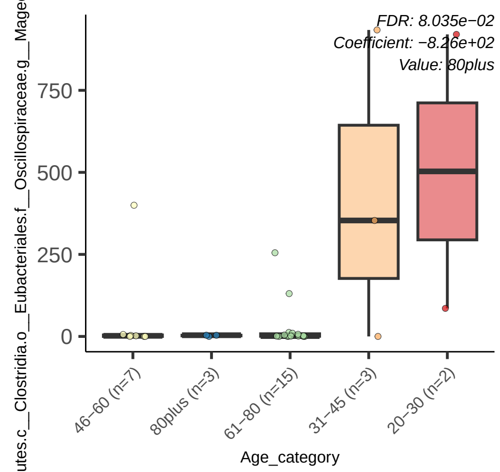

FDR: 8.035e−02
Coefficient: −8.26e+02
Value: 80plus
750
500
250
0
cutes.c__Clostridia.o__Eubacteriales.f__Oscillospiraceae.g__Mage
46−60 (n=7)
80plus (n=3)
61−80 (n=15)
31−45 (n=3)
20−30 (n=2)
Age_category

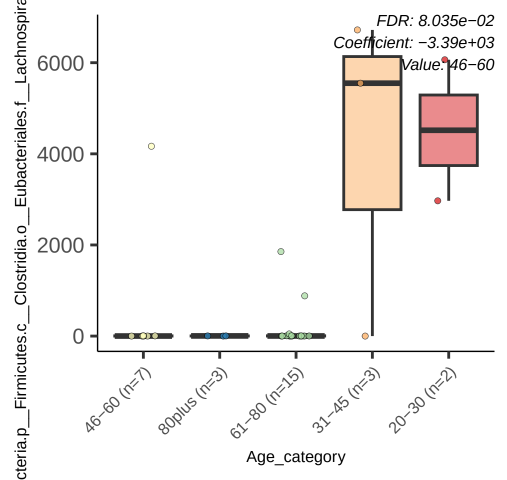

FDR: 8.035e−02
Coefficient: −3.39e+03
Value: 46−60
cteria.p__Firmicutes.c__Clostridia.o__Eubacteriales.f__Lachnospira
6000
4000
2000
0
46−60 (n=7)
80plus (n=3)
61−80 (n=15)
31−45 (n=3)
20−30 (n=2)
Age_category

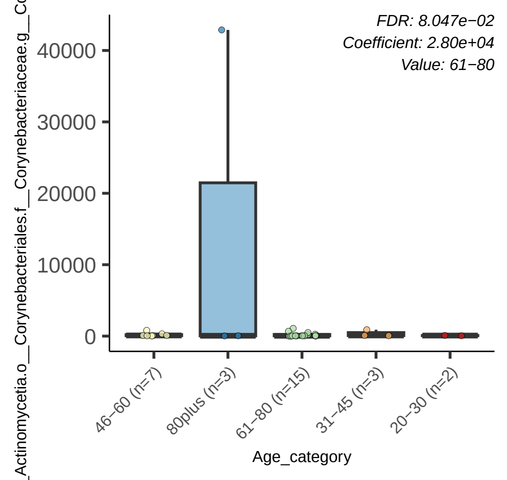
FDR: 8.047e−02
Coefficient: 2.80e+04
Value: 61−80
Actinomycetia.o__Corynebacteriales.f__Corynebacteriaceae.g__Co
40000
30000
20000
10000
0
46−60 (n=7)
80plus (n=3)
61−80 (n=15)
31−45 (n=3)
20−30 (n=2)
Age_category

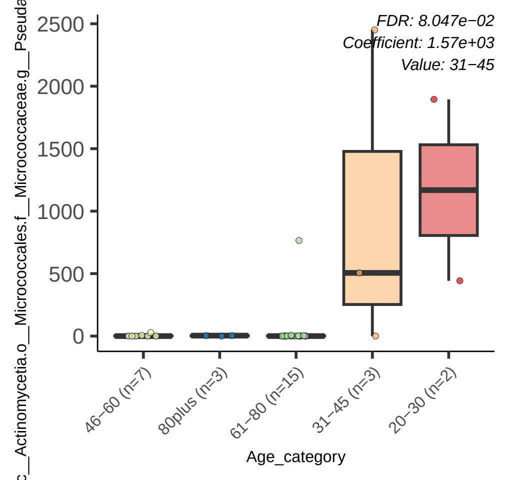

FDR: 8.047e−02
Coefficient: 1.57e+03
Value: 31−45
2500
2000
1500
1000
500
0
c__Actinomycetia.o__Micrococcales.f__Micrococcaceae.g__Pseuda
46−60 (n=7)
80plus (n=3)
61−80 (n=15)
31−45 (n=3)
20−30 (n=2)
Age_category

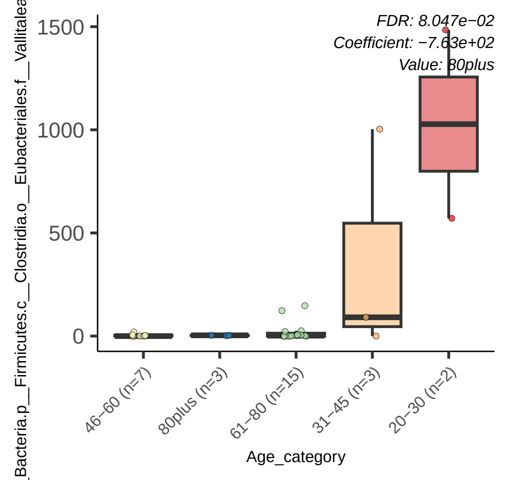

FDR: 8.047e−02
Coefficient: −7.63e+02
Value: 80plus
1500
1000
500
0
Bacteria.p__Firmicutes.c__Clostridia.o__Eubacteriales.f__Vallitalea
46−60 (n=7)
80plus (n=3)
61−80 (n=15)
31−45 (n=3)
20−30 (n=2)
Age_category

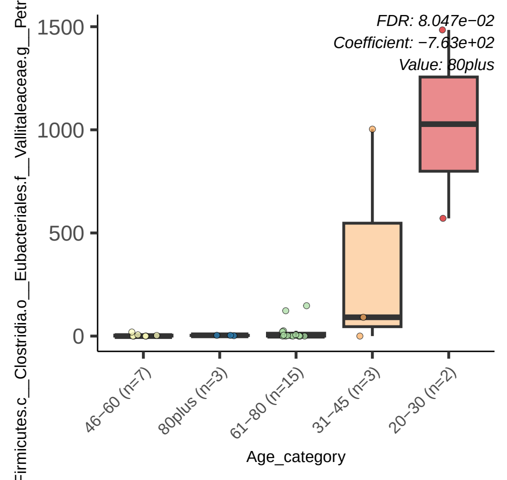

FDR: 8.047e−02
Coefficient: −7.63e+02
Value: 80plus
1500
1000
500
0
46−60 (n=7)
80plus (n=3)
61−80 (n=15)
31−45 (n=3)
20−30 (n=2)
Age_category
Firmicutes.c__Clostridia.o__Eubacteriales.f__Vallitaleaceae.g__Petr

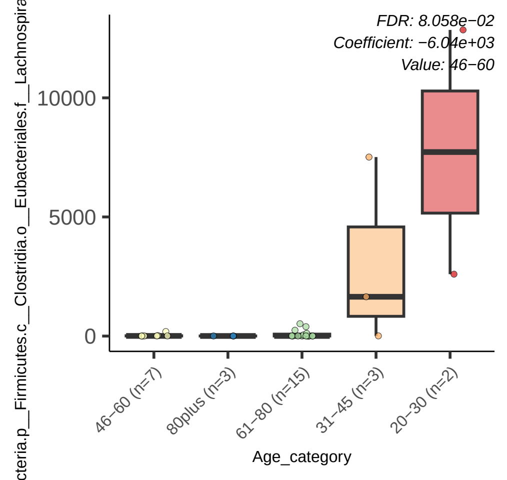

FDR: 8.058e−02
Coefficient: −6.04e+03
Value: 46−60
10000
5000
0
46−60 (n=7)
80plus (n=3)
61−80 (n=15)
31−45 (n=3)
20−30 (n=2)
Age_category
cteria.p__Firmicutes.c__Clostridia.o__Eubacteriales.f__Lachnospira

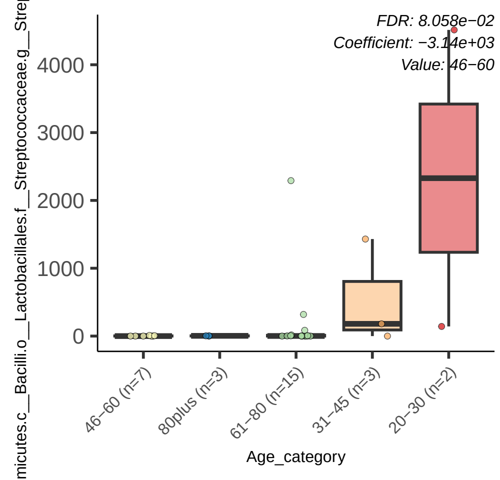

FDR: 8.058e−02
Coefficient: −3.14e+03
Value: 46−60
micutes.c__Bacilli.o__Lactobacillales.f__Streptococcaceae.g__Strep
4000
3000
2000
1000
0
46−60 (n=7)
80plus (n=3)
61−80 (n=15)
31−45 (n=3)
20−30 (n=2)
Age_category

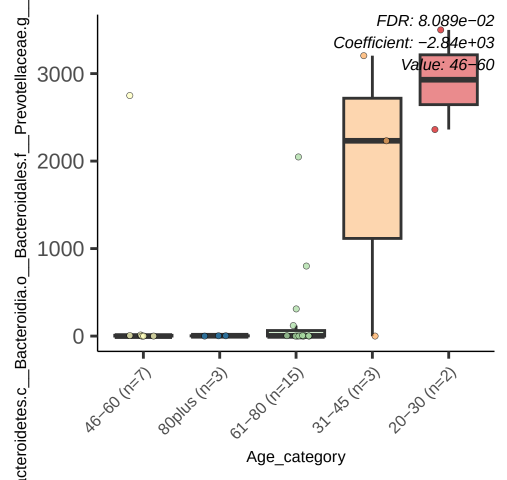
FDR: 8.089e−02
Coefficient: −2.84e+03
Value: 46−60
3000
2000
1000
0
acteroidetes.c__Bacteroidia.o__Bacteroidales.f__Prevotellaceae.g__
46−60 (n=7)
80plus (n=3)
61−80 (n=15)
31−45 (n=3)
20−30 (n=2)
Age_category

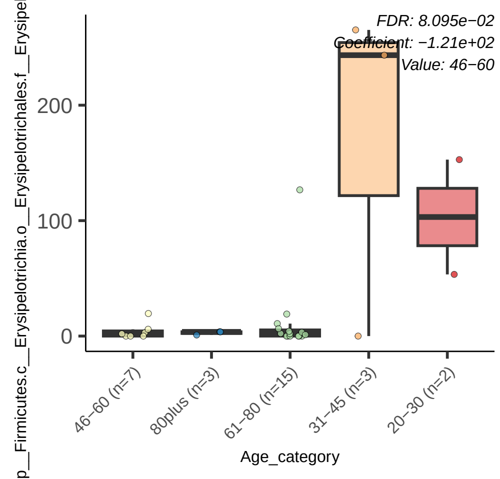

FDR: 8.095e−02
Coefficient: −1.21e+02
Value: 46−60
p__Firmicutes.c__Erysipelotrichia.o__Erysipelotrichales.f__Erysipel
200
100
0
46−60 (n=7)
80plus (n=3)
61−80 (n=15)
31−45 (n=3)
20−30 (n=2)
Age_category

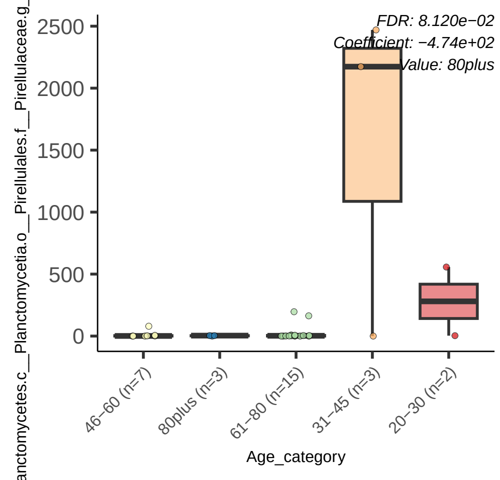

FDR: 8.120e−02
Coefficient: −4.74e+02
Value: 80plus
Planctomycetes.c__Planctomycetia.o__Pirellulales.f__Pirellulaceae.g_
2500
2000
1500
1000
500
0
46−60 (n=7)
80plus (n=3)
61−80 (n=15)
31−45 (n=3)
20−30 (n=2)
Age_category

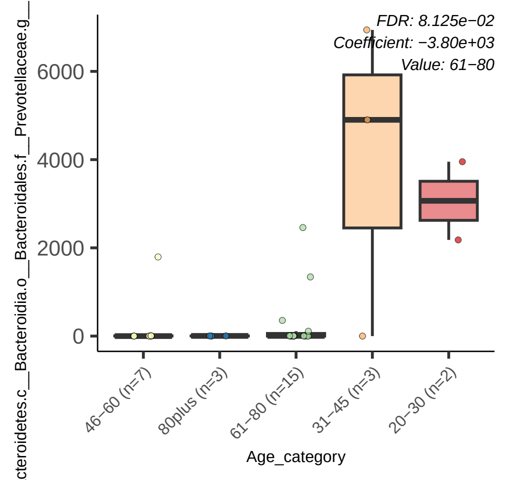
FDR: 8.125e−02
Coefficient: −3.80e+03
Value: 61−80
6000
4000
2000
0
cteroidetes.c__Bacteroidia.o__Bacteroidales.f__Prevotellaceae.g__
46−60 (n=7)
80plus (n=3)
61−80 (n=15)
31−45 (n=3)
20−30 (n=2)
Age_category

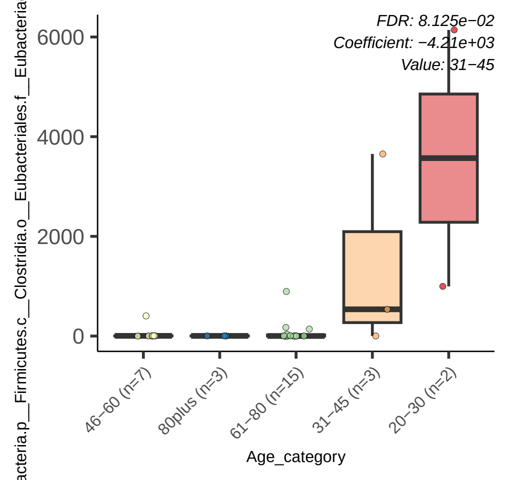

FDR: 8.125e−02
Coefficient: −4.21e+03
Value: 31−45
6000
4000
2000
0
acteria.p__Firmicutes.c__Clostridia.o__Eubacteriales.f__Eubacteria
46−60 (n=7)
80plus (n=3)
61−80 (n=15)
31−45 (n=3)
20−30 (n=2)
Age_category

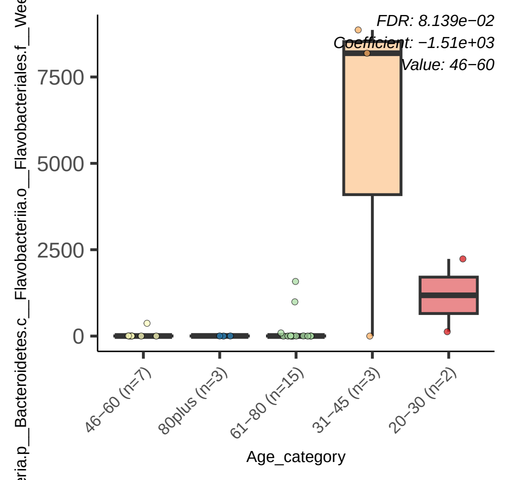

FDR: 8.139e−02
Coefficient: −1.51e+03
Value: 46−60
7500
5000
2500
0
Bacteroidetes.c__Flavobacteriia.o__Flavobacteriales.f__Wee
46−60 (n=7)
80plus (n=3)
61−80 (n=15)
31−45 (n=3)
20−30 (n=2)
Age_category

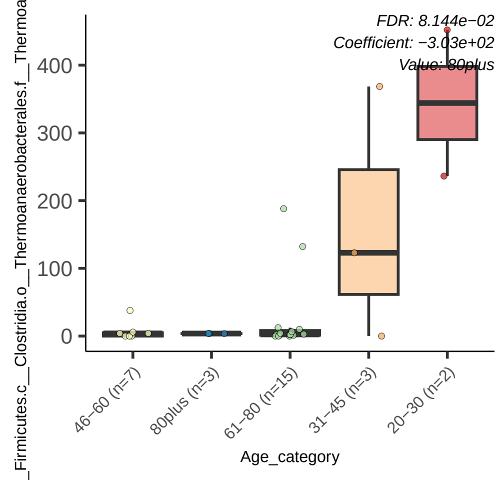

FDR: 8.144e−02
Coefficient: −3.03e+02
Value: 80plus
Firmicutes.c__Clostridia.o__Thermoanaerobacterales.f__Thermoa
400
300
200
100
0
46−60 (n=7)
80plus (n=3)
61−80 (n=15)
31−45 (n=3)
20−30 (n=2)
Age_category

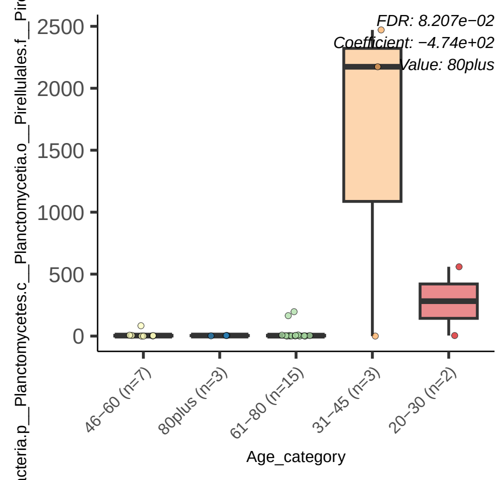

FDR: 8.207e−02
Coefficient: −4.74e+02
Value: 80plus
2500
2000
1500
1000
500
0
46−60 (n=7)
80plus (n=3)
61−80 (n=15)
31−45 (n=3)
20−30 (n=2)
Age_category

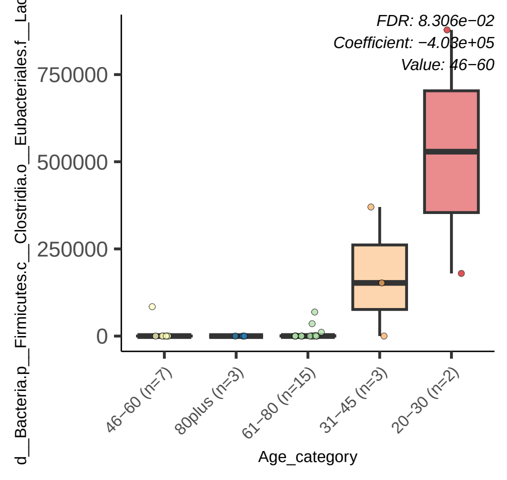

FDR: 8.306e−02
Coefficient: −4.03e+05
Value: 46−60
d__Bacteria.p__Firmicutes.c__Clostridia.o__Eubacteriales.f__Lac
750000
500000
250000
0
46−60 (n=7)
80plus (n=3)
61−80 (n=15)
31−45 (n=3)
20−30 (n=2)
Age_category

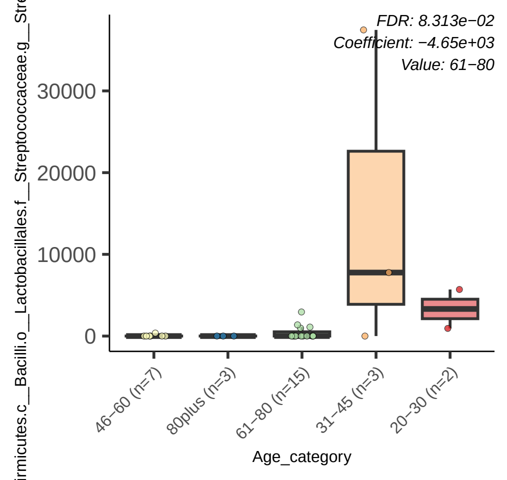

FDR: 8.313e−02
Coefficient: −4.65e+03
Value: 61−80
30000
20000
10000
0
irmicutes.c__Bacilli.o__Lactobacillales.f__Streptococcaceae.g__Stre
46−60 (n=7)
80plus (n=3)
61−80 (n=15)
31−45 (n=3)
20−30 (n=2)
Age_category

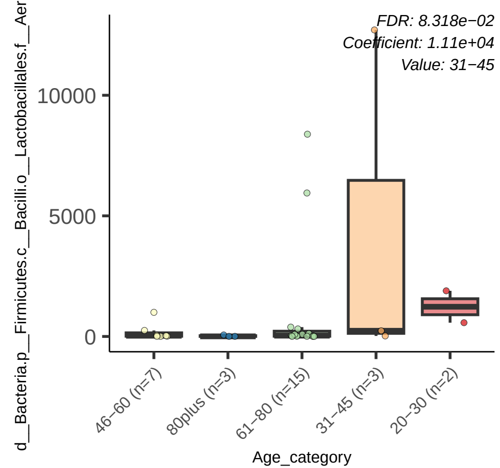

FDR: 8.318e−02
Coefficient: 1.11e+04
Value: 31−45
10000
5000
0
d__Bacteria.p__Firmicutes.c__Bacilli.o__Lactobacillales.f__Aer
46−60 (n=7)
80plus (n=3)
61−80 (n=15)
31−45 (n=3)
20−30 (n=2)
Age_category

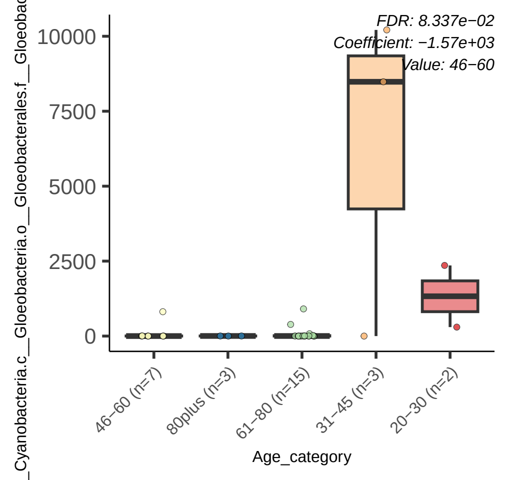

FDR: 8.337e−02
Coefficient: −1.57e+03
Value: 46−60
Cyanobacteria.c__Gloeobacteria.o__Gloeobacterales.f__Gloeobac
10000
7500
5000
2500
0
46−60 (n=7)
80plus (n=3)
61−80 (n=15)
31−45 (n=3)
20−30 (n=2)
Age_category

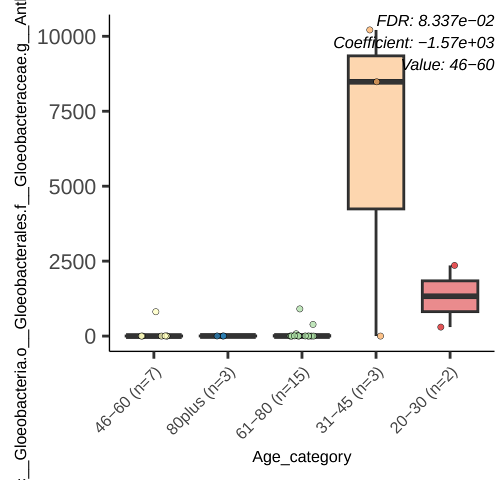

FDR: 8.337e−02
Coefficient: −1.57e+03
Value: 46−60
10000
7500
5000
2500
0
Gloeobacteria.o__Gloeobacterales.f__Gloeobacteraceae.g__Ant
46−60 (n=7)
80plus (n=3)
61−80 (n=15)
31−45 (n=3)
20−30 (n=2)
Age_category

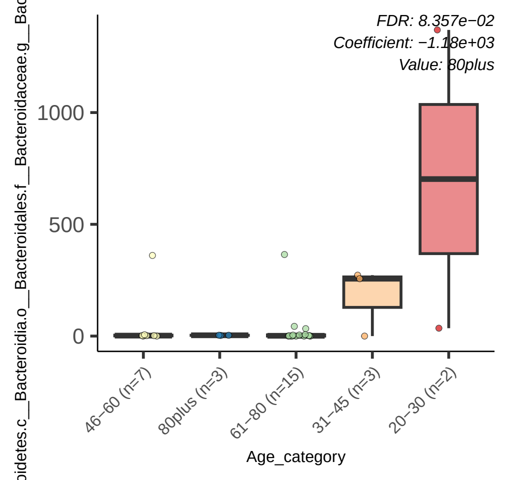

FDR: 8.357e−02
Coefficient: −1.18e+03
Value: 80plus
1000
500
0
46−60 (n=7)
80plus (n=3)
61−80 (n=15)
31−45 (n=3)
20−30 (n=2)
Age_category
oidetes.c__Bacteroidia.o__Bacteroidales.f__Bacteroidaceae.g__Bac

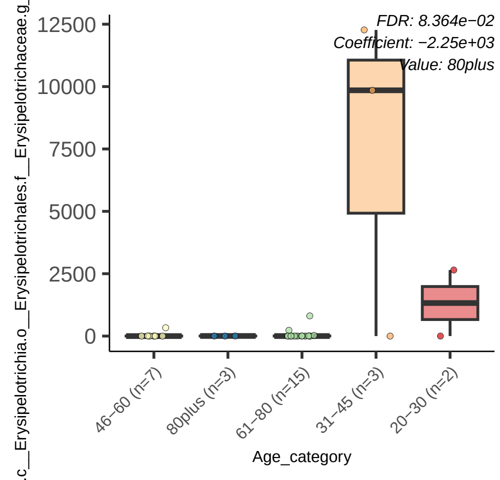

FDR: 8.364e−02
Coefficient: −2.25e+03
Value: 80plus
12500
10000
7500
5000
2500
0
c__Erysipelotrichia.o__Erysipelotrichales.f__Erysipelotrichaceae.g__
46−60 (n=7)
80plus (n=3)
61−80 (n=15)
31−45 (n=3)
20−30 (n=2)
Age_category

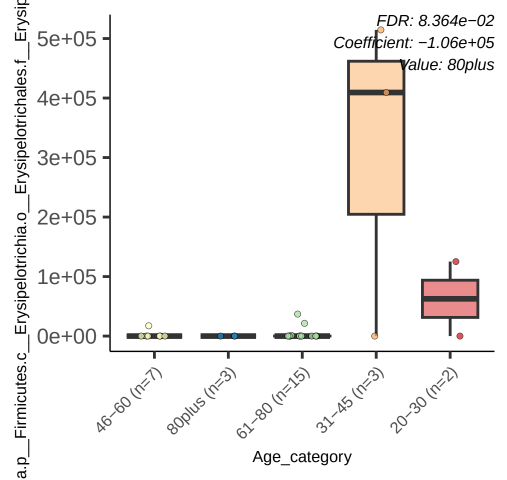
FDR: 8.364e−02
Coefficient: −1.06e+05
Value: 80plus
5e+05
4e+05
3e+05
2e+05
1e+05
0e+00
a.p__Firmicutes.c__Erysipelotrichia.o__Erysipelotrichales.f__Erysip
46−60 (n=7)
80plus (n=3)
61−80 (n=15)
31−45 (n=3)
20−30 (n=2)
Age_category

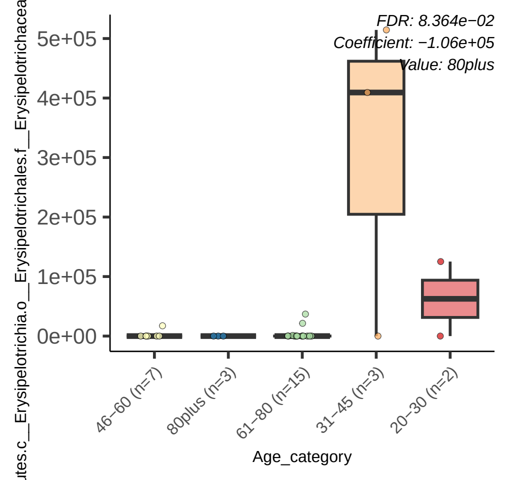

FDR: 8.364e−02
Coefficient: −1.06e+05
Value: 80plus
utes.c__Erysipelotrichia.o__Erysipelotrichales.f__Erysipelotrichacea
5e+05
4e+05
3e+05
2e+05
1e+05
0e+00
46−60 (n=7)
80plus (n=3)
61−80 (n=15)
31−45 (n=3)
20−30 (n=2)
Age_category

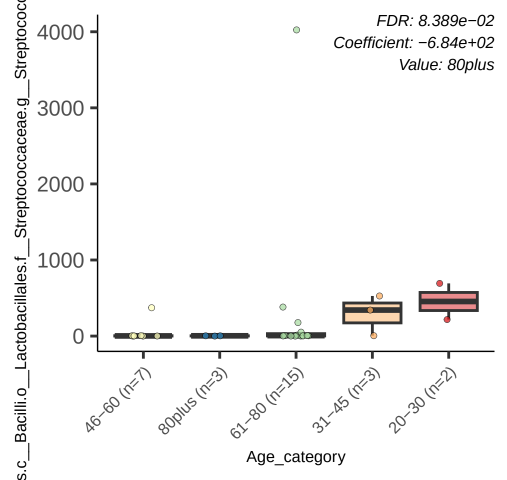

FDR: 8.389e−02
Coefficient: −6.84e+02
Value: 80plus
s.c__Bacilli.o__Lactobacillales.f__Streptococcaceae.g__Streptococc
4000
3000
2000
1000
0
46−60 (n=7)
80plus (n=3)
61−80 (n=15)
31−45 (n=3)
20−30 (n=2)
Age_category

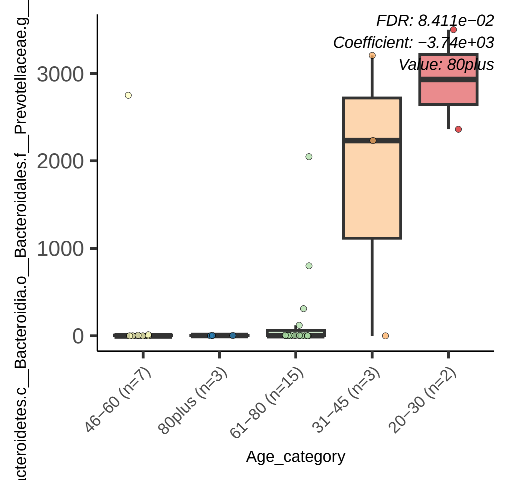

FDR: 8.411e−02
Coefficient: −3.74e+03
Value: 80plus
3000
2000
1000
0
acteroidetes.c__Bacteroidia.o__Bacteroidales.f__Prevotellaceae.g__
46−60 (n=7)
80plus (n=3)
61−80 (n=15)
31−45 (n=3)
20−30 (n=2)
Age_category

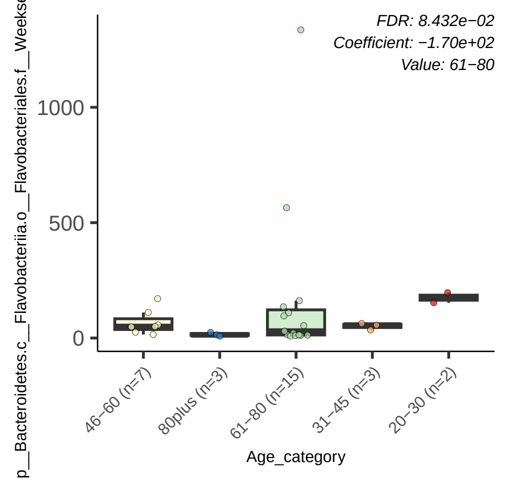
FDR: 8.432e−02
Coefficient: −1.70e+02
Value: 61−80
1000
500
0
46−60 (n=7)
80plus (n=3)
61−80 (n=15)
31−45 (n=3)
20−30 (n=2)
Age_category
p__Bacteroidetes.c__Flavobacteriia.o__Flavobacteriales.f__Weekse

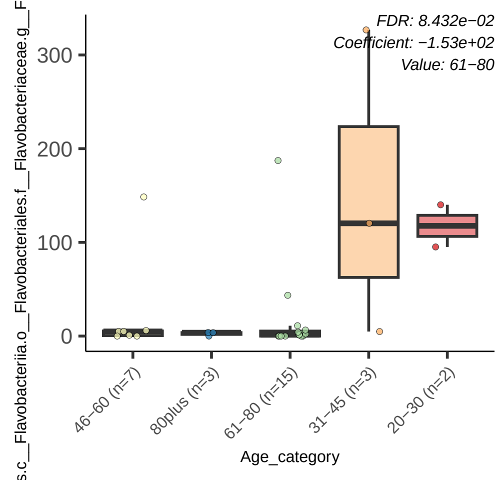

FDR: 8.432e−02
Coefficient: −1.53e+02
Value: 61−80
300
200
100
0
s.c__Flavobacteriia.o__Flavobacteriales.f__Flavobacteriaceae.g__F
46−60 (n=7)
80plus (n=3)
61−80 (n=15)
31−45 (n=3)
20−30 (n=2)
Age_category

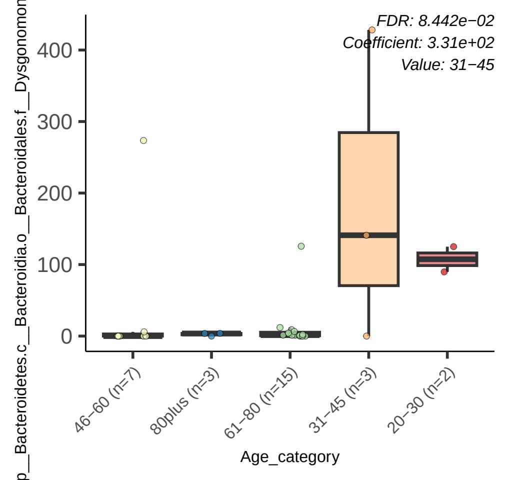

FDR: 8.442e−02
Coefficient: 3.31e+02
Value: 31−45
p__Bacteroidetes.c__Bacteroidia.o__Bacteroidales.f__Dysgonomon
400
300
200
100
0
46−60 (n=7)
80plus (n=3)
61−80 (n=15)
31−45 (n=3)
20−30 (n=2)
Age_category

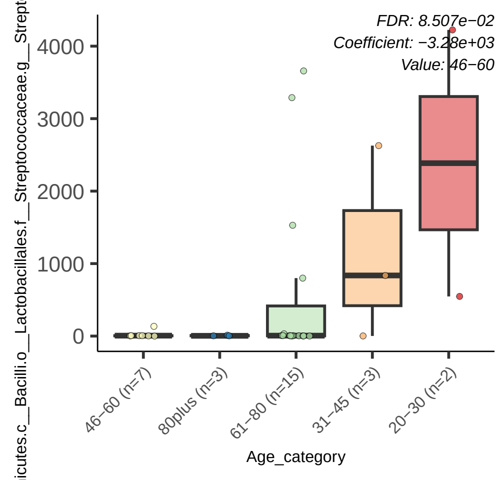

FDR: 8.507e−02
Coefficient: −3.28e+03
Value: 46−60
0
1000
2000
3000
4000
46−60 (n=7)
80plus (n=3)
61−80 (n=15)
31−45 (n=3)
20−30 (n=2)
Age_category
icutes.c__Bacilli.o__Lactobacillales.f__Streptococcaceae.g__Strept

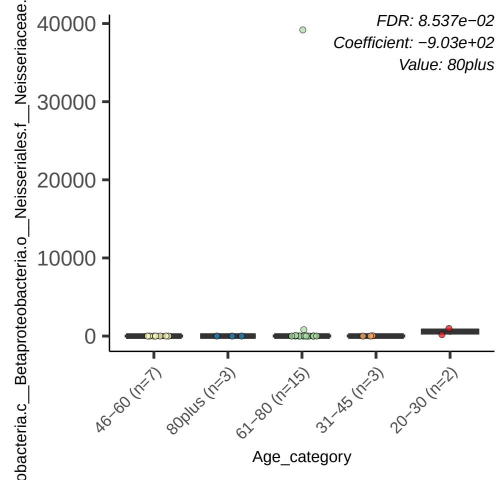
FDR: 8.537e−02
Coefficient: −9.03e+02
Value: 80plus
obacteria.c__Betaproteobacteria.o__Neisseriales.f__Neisseriaceae.
40000
30000
20000
10000
0
46−60 (n=7)
80plus (n=3)
61−80 (n=15)
31−45 (n=3)
20−30 (n=2)
Age_category

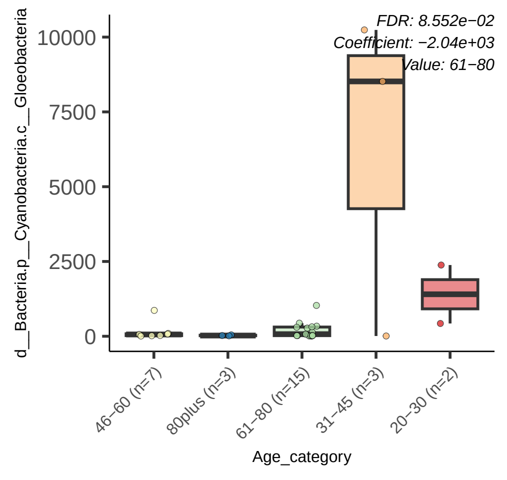

FDR: 8.552e−02
Coefficient: −2.04e+03
Value: 61−80
d__Bacteria.p__Cyanobacteria.c__Gloeobacteria
10000
7500
5000
2500
0
46−60 (n=7)
80plus (n=3)
61−80 (n=15)
31−45 (n=3)
20−30 (n=2)
Age_category

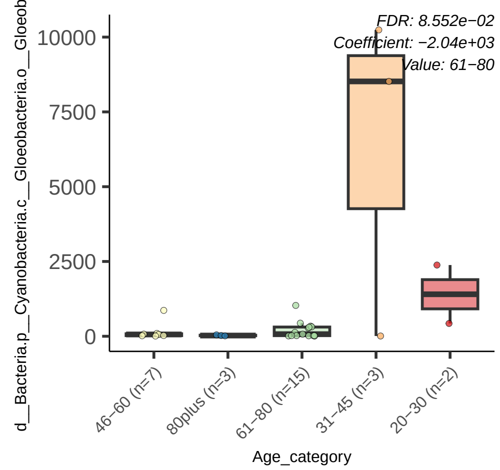

FDR: 8.552e−02
Coefficient: −2.04e+03
Value: 61−80
10000
7500
5000
2500
0
d__Bacteria.p__Cyanobacteria.c__Gloeobacteria.o__Gloeob
46−60 (n=7)
80plus (n=3)
61−80 (n=15)
31−45 (n=3)
20−30 (n=2)
Age_category

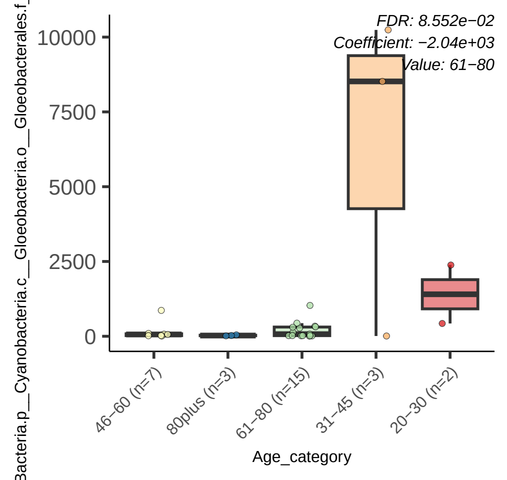

FDR: 8.552e−02
Coefficient: −2.04e+03
Value: 61−80
10000
7500
5000
2500
0
Bacteria.p__Cyanobacteria.c__Gloeobacteria.o__Gloeobacterales.f_
46−60 (n=7)
80plus (n=3)
61−80 (n=15)
31−45 (n=3)
20−30 (n=2)
Age_category

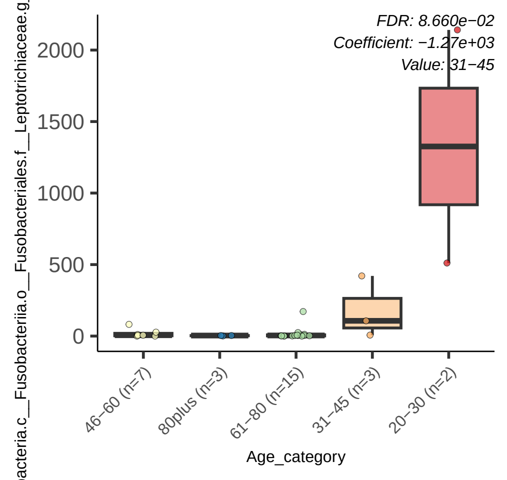

FDR: 8.660e−02
Coefficient: −1.27e+03
Value: 31−45
2000
1500
1000
500
0
bacteria.c__Fusobacteriia.o__Fusobacteriales.f__Leptotrichiaceae.g
46−60 (n=7)
80plus (n=3)
61−80 (n=15)
31−45 (n=3)
20−30 (n=2)
Age_category

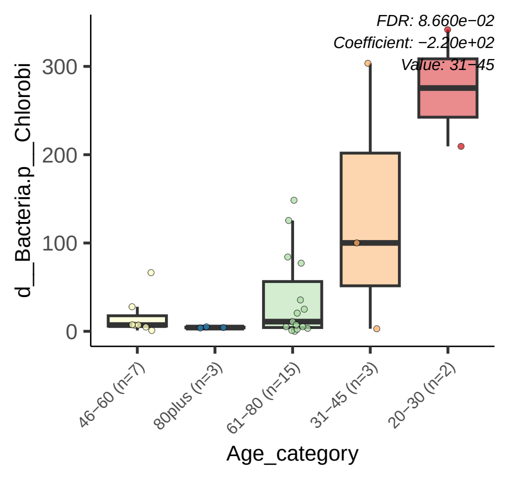
FDR: 8.660e−02
Coefficient: −2.20e+02
Value: 31−45
d__Bacteria.p__Chlorobi
300
200
100
0
46−60 (n=7)
80plus (n=3)
61−80 (n=15)
31−45 (n=3)
20−30 (n=2)
Age_category

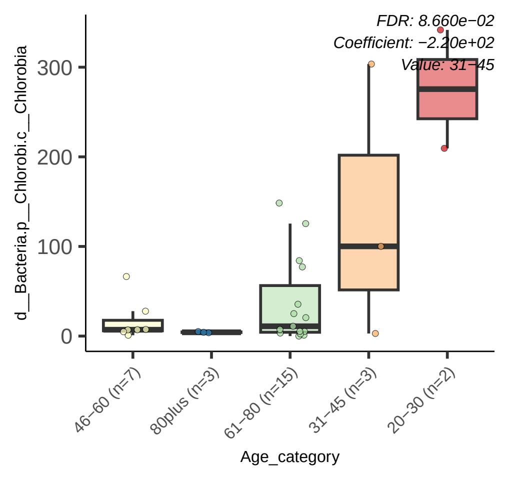

FDR: 8.660e−02
Coefficient: −2.20e+02
Value: 31−45
d__Bacteria.p__Chlorobi.c__Chlorobia
300
200
100
0
46−60 (n=7)
80plus (n=3)
61−80 (n=15)
31−45 (n=3)
20−30 (n=2)
Age_category

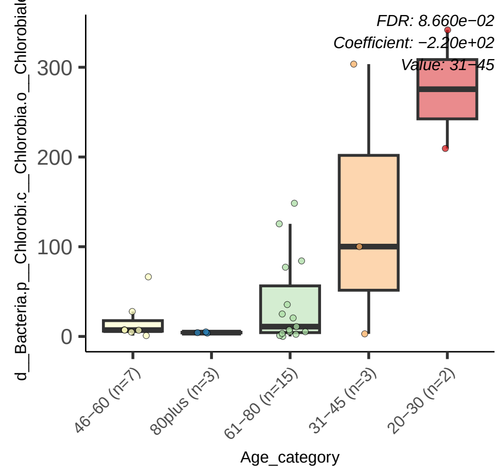

FDR: 8.660e−02
Coefficient: −2.20e+02
Value: 31−45
d__Bacteria.p__Chlorobi.c__Chlorobia.o__Chlorobiale
0
100
200
300
46−60 (n=7)
80plus (n=3)
61−80 (n=15)
31−45 (n=3)
20−30 (n=2)
Age_category

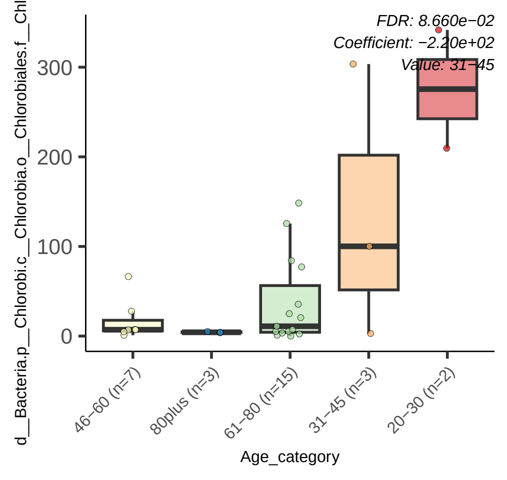

FDR: 8.660e−02
Coefficient: −2.20e+02
Value: 31−45
d__Bacteria.p__Chlorobi.c__Chlorobia.o__Chlorobiales.f__Chl
300
200
100
0
46−60 (n=7)
80plus (n=3)
61−80 (n=15)
31−45 (n=3)
20−30 (n=2)
Age_category

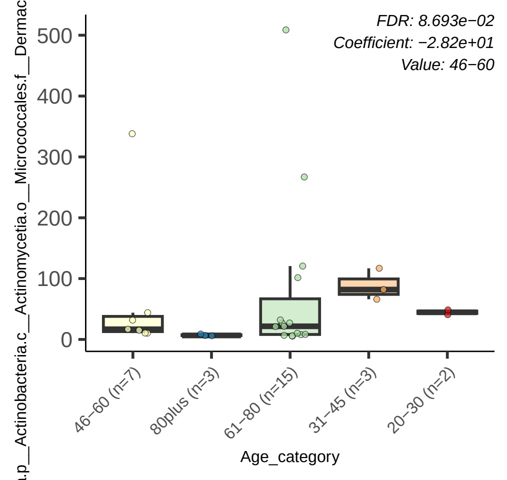

FDR: 8.693e−02
Coefficient: −2.82e+01
Value: 46−60
a.p__Actinobacteria.c__Actinomycetia.o__Micrococcales.f__Dermac
0
100
200
300
400
500
46−60 (n=7)
80plus (n=3)
61−80 (n=15)
31−45 (n=3)
20−30 (n=2)
Age_category

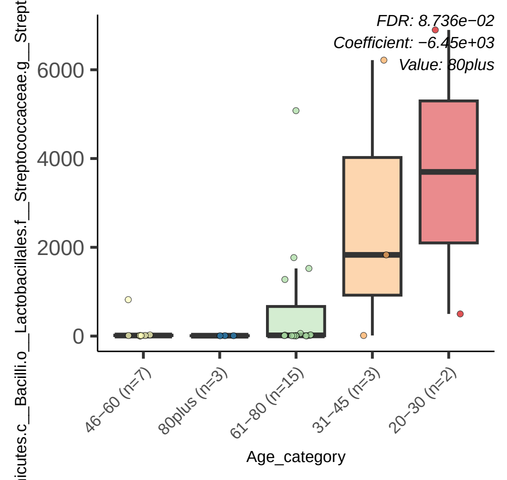

FDR: 8.736e−02
Coefficient: −6.45e+03
Value: 80plus
6000
4000
2000
0
icutes.c__Bacilli.o__Lactobacillales.f__Streptococcaceae.g__Strept
46−60 (n=7)
80plus (n=3)
61−80 (n=15)
31−45 (n=3)
20−30 (n=2)
Age_category

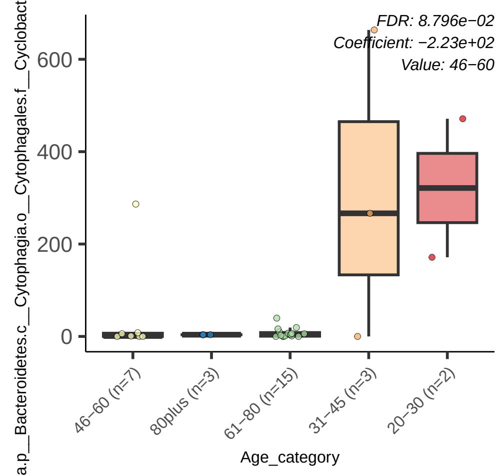

FDR: 8.796e−02
Coefficient: −2.23e+02
Value: 46−60
0
200
400
600
ria.p__Bacteroidetes.c__Cytophagia.o__Cytophagales.f__Cyclobact
46−60 (n=7)
80plus (n=3)
61−80 (n=15)
31−45 (n=3)
20−30 (n=2)
Age_category

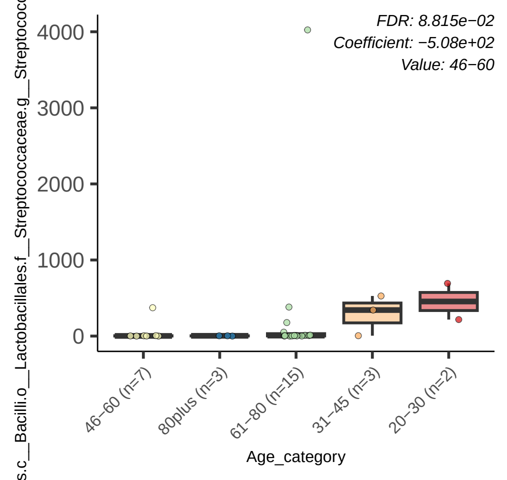

FDR: 8.815e−02
Coefficient: −5.08e+02
Value: 46−60
4000
3000
2000
1000
0
s.c__Bacilli.o__Lactobacillales.f__Streptococcaceae.g__Streptococ
46−60 (n=7)
80plus (n=3)
61−80 (n=15)
31−45 (n=3)
20−30 (n=2)
Age_category

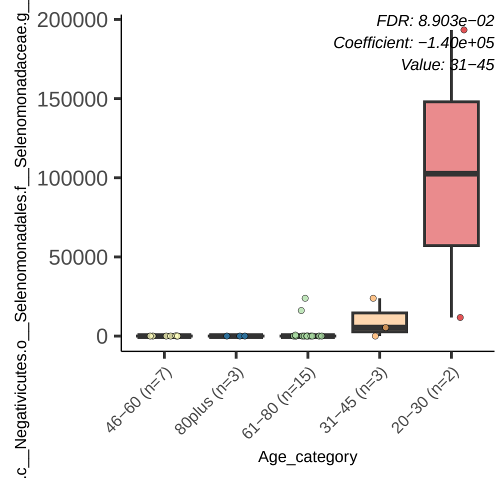
FDR: 8.903e−02
Coefficient: −1.40e+05
Value: 31−45
c__Negativicutes.o__Selenomonadales.f__Selenomonadaceae.g__
200000
150000
100000
50000
0
46−60 (n=7)
80plus (n=3)
61−80 (n=15)
31−45 (n=3)
20−30 (n=2)
Age_category

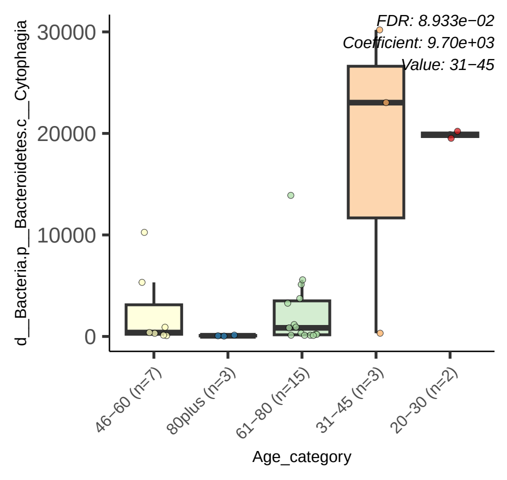

FDR: 8.933e−02
Coefficient: 9.70e+03
Value: 31−45
d__Bacteria.p__Bacteroidetes.c__Cytophagia
30000
20000
10000
0
46−60 (n=7)
80plus (n=3)
61−80 (n=15)
31−45 (n=3)
20−30 (n=2)
Age_category

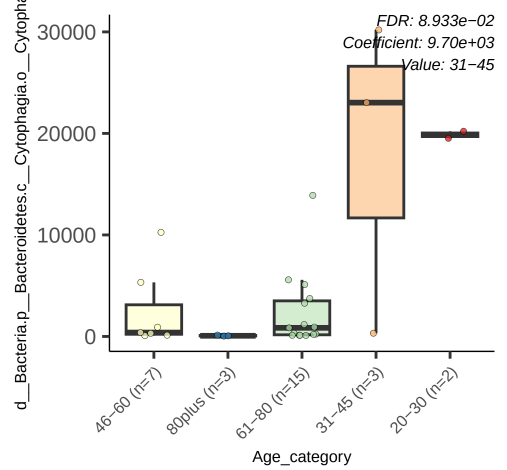
FDR: 8.933e−02
Coefficient: 9.70e+03
Value: 31−45
d__Bacteria.p__Bacteroidetes.c__Cytophagia.o__Cytopha
30000
20000
10000
0
46−60 (n=7)
80plus (n=3)
61−80 (n=15)
31−45 (n=3)
20−30 (n=2)
Age_category

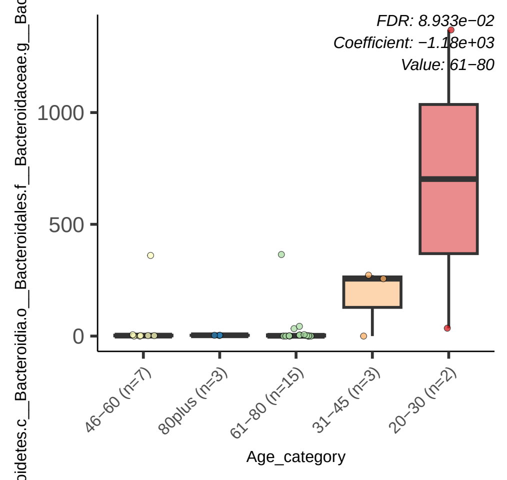

FDR: 8.933e−02
Coefficient: −1.18e+03
Value: 61−80
oidetes.c__Bacteroidia.o__Bacteroidales.f__Bacteroidaceae.g__Bac
1000
500
0
46−60 (n=7)
80plus (n=3)
61−80 (n=15)
31−45 (n=3)
20−30 (n=2)
Age_category

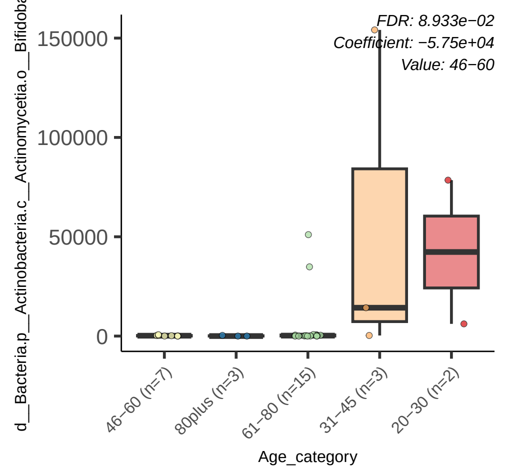

FDR: 8.933e−02
Coefficient: −5.75e+04
Value: 46−60
d__Bacteria.p__Actinobacteria.c__Actinomycetia.o__Bifidoba
150000
100000
50000
0
46−60 (n=7)
80plus (n=3)
61−80 (n=15)
31−45 (n=3)
20−30 (n=2)
Age_category

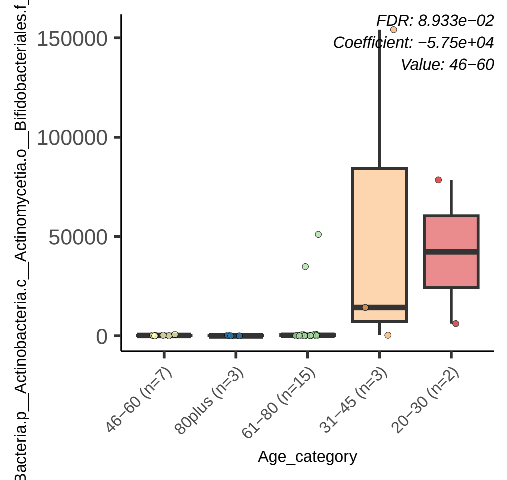

FDR: 8.933e−02
Coefficient: −5.75e+04
Value: 46−60
150000
100000
50000
0
46−60 (n=7)
80plus (n=3)
61−80 (n=15)
31−45 (n=3)
20−30 (n=2)
Age_category
Bacteria.p__Actinobacteria.c__Actinomycetia.o__Bifidobacteriales.f_

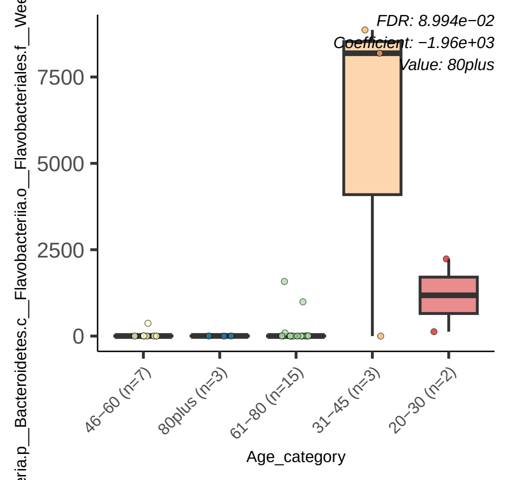

FDR: 8.994e−02
Coefficient: −1.96e+03
Value: 80plus
7500
5000
2500
0
46−60 (n=7)
80plus (n=3)
61−80 (n=15)
31−45 (n=3)
20−30 (n=2)
Age_category
eria.p__Bacteroidetes.c__Flavobacteriia.o__Flavobacteriales.f__Wee

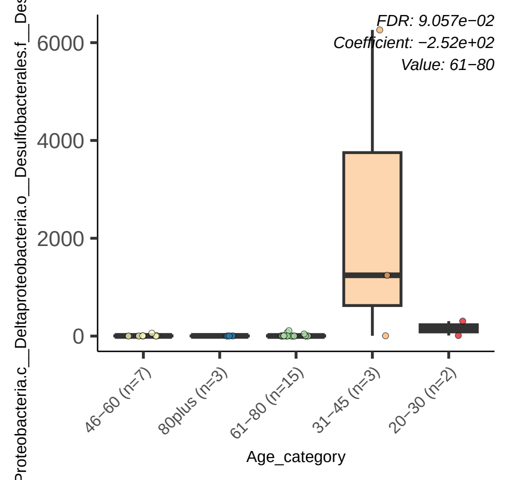

FDR: 9.057e−02
Coefficient: −2.52e+02
Value: 61−80
6000
4000
2000
0
Proteobacteria.c__Deltaproteobacteria.o__Desulfobacterales.f__Des
46−60 (n=7)
80plus (n=3)
61−80 (n=15)
31−45 (n=3)
20−30 (n=2)
Age_category

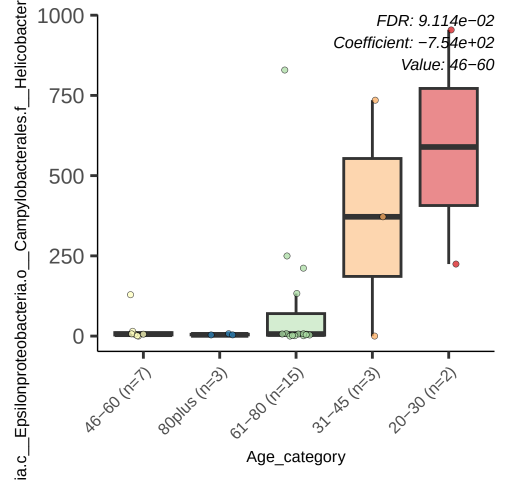

FDR: 9.114e−02
Coefficient: −7.54e+02
Value: 46−60
ia.c__Epsilonproteobacteria.o__Campylobacterales.f__Helicobacter
1000
750
500
250
0
46−60 (n=7)
80plus (n=3)
61−80 (n=15)
31−45 (n=3)
20−30 (n=2)
Age_category

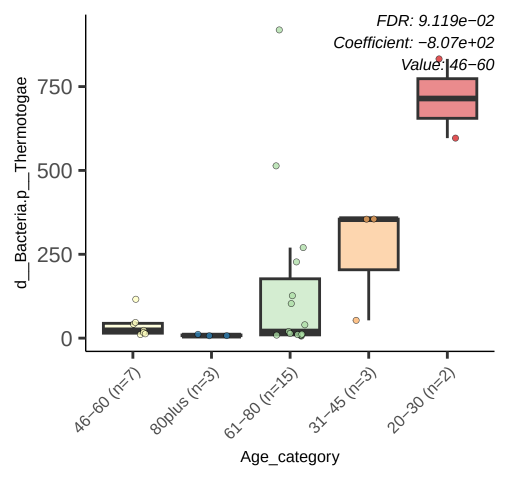

FDR: 9.119e−02
Coefficient: −8.07e+02
Value: 46−60
d__Bacteria.p__Thermotogae
0
250
500
750
46−60 (n=7)
80plus (n=3)
61−80 (n=15)
31−45 (n=3)
20−30 (n=2)
Age_category

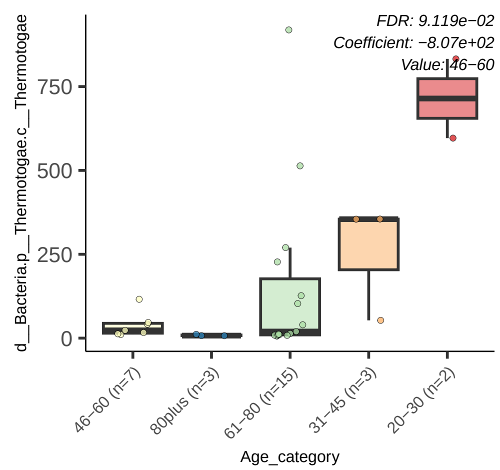
FDR: 9.119e−02
Coefficient: −8.07e+02
Value: 46−60
d__Bacteria.p__Thermotogae.c__Thermotogae
750
500
250
0
46−60 (n=7)
80plus (n=3)
61−80 (n=15)
31−45 (n=3)
20−30 (n=2)
Age_category

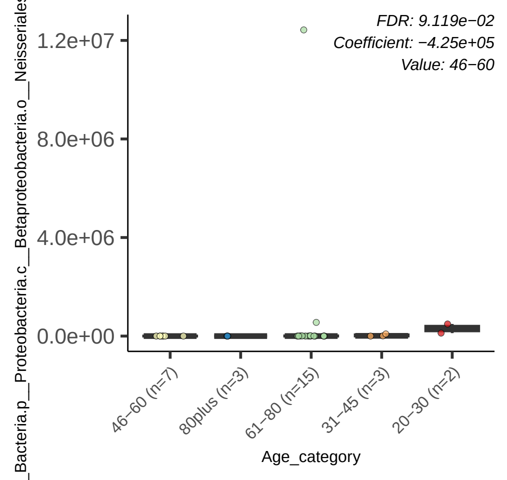

FDR: 9.119e−02
Coefficient: −4.25e+05
Value: 46−60
Bacteria.p__Proteobacteria.c__Betaproteobacteria.o__Neisseriales
1.2e+07
8.0e+06
4.0e+06
0.0e+00
46−60 (n=7)
80plus (n=3)
61−80 (n=15)
31−45 (n=3)
20−30 (n=2)
Age_category

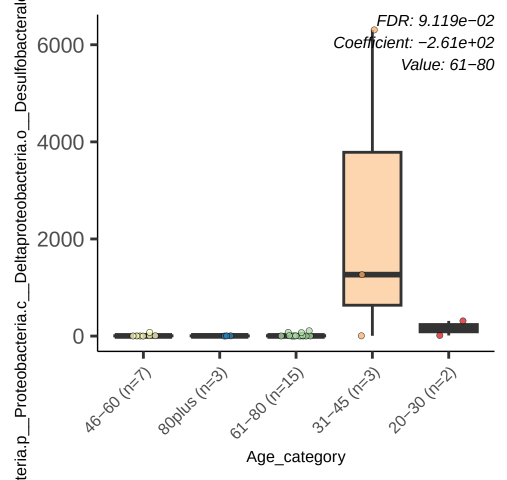

FDR: 9.119e−02
Coefficient: −2.61e+02
Value: 61−80
6000
4000
2000
0
teria.p__Proteobacteria.c__Deltaproteobacteria.o__Desulfobacteral
46−60 (n=7)
80plus (n=3)
61−80 (n=15)
31−45 (n=3)
20−30 (n=2)
Age_category

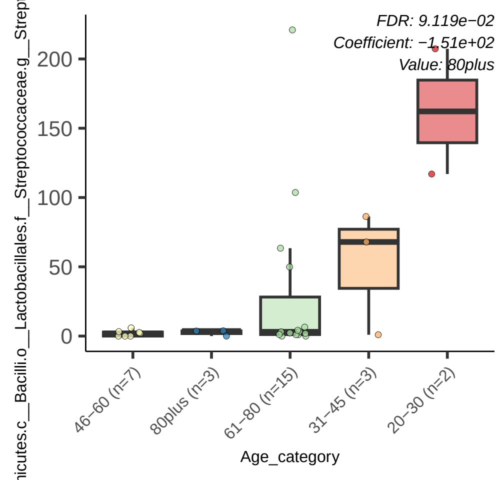

FDR: 9.119e−02
Coefficient: −1.51e+02
Value: 80plus
200
150
100
50
0
46−60 (n=7)
80plus (n=3)
61−80 (n=15)
31−45 (n=3)
20−30 (n=2)
Age_category

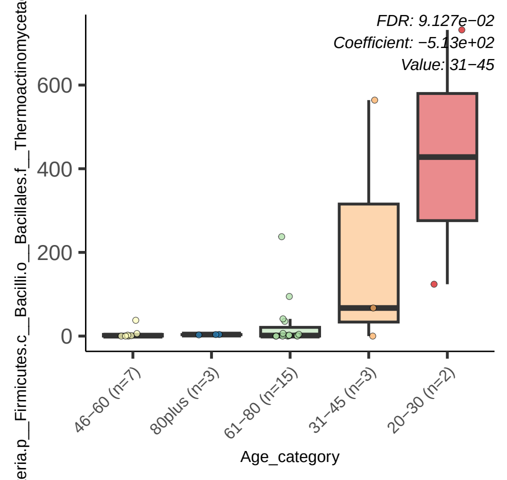

FDR: 9.127e−02
Coefficient: −5.13e+02
Value: 31−45
0
200
400
600
46−60 (n=7)
80plus (n=3)
61−80 (n=15)
31−45 (n=3)
20−30 (n=2)
Age_category
eria.p__Firmicutes.c__Bacilli.o__Bacillales.f__Thermoactinomyceta

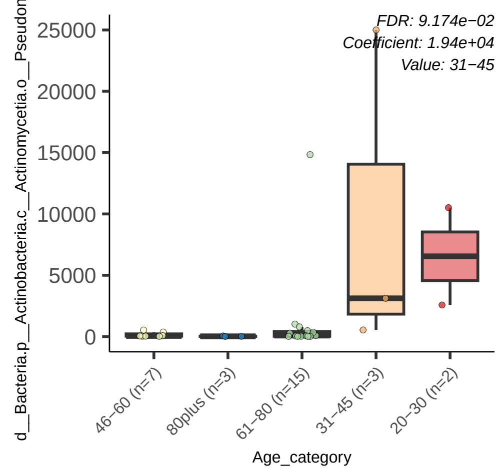

FDR: 9.174e−02
Coefficient: 1.94e+04
Value: 31−45
d__Bacteria.p__Actinobacteria.c__Actinomycetia.o__Pseudon
25000
20000
15000
10000
5000
0
46−60 (n=7)
80plus (n=3)
61−80 (n=15)
31−45 (n=3)
20−30 (n=2)
Age_category

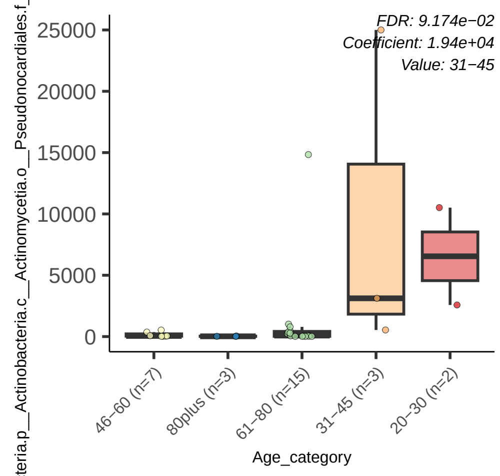

FDR: 9.174e−02
Coefficient: 1.94e+04
Value: 31−45
25000
20000
15000
10000
5000
0
cteria.p__Actinobacteria.c__Actinomycetia.o__Pseudonocardiales.f_
46−60 (n=7)
80plus (n=3)
61−80 (n=15)
31−45 (n=3)
20−30 (n=2)
Age_category

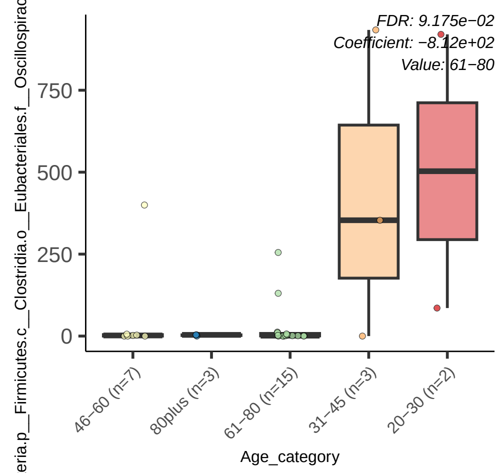

FDR: 9.175e−02
Coefficient: −8.12e+02
Value: 61−80
0
250
500
750
cteria.p__Firmicutes.c__Clostridia.o__Eubacteriales.f__Oscillospirac
46−60 (n=7)
80plus (n=3)
61−80 (n=15)
31−45 (n=3)
20−30 (n=2)
Age_category

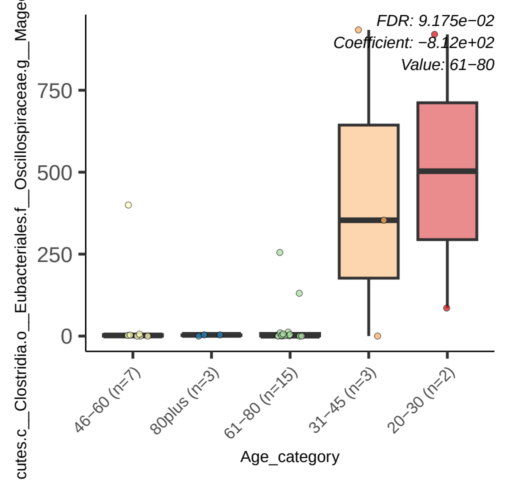

FDR: 9.175e−02
Coefficient: −8.12e+02
Value: 61−80
750
500
250
0
cutes.c__Clostridia.o__Eubacteriales.f__Oscillospiraceae.g__Mageo
46−60 (n=7)
80plus (n=3)
61−80 (n=15)
31−45 (n=3)
20−30 (n=2)
Age_category

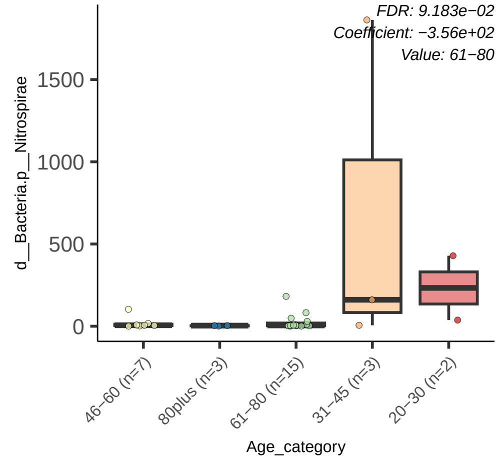
FDR: 9.183e−02
Coefficient: −3.56e+02
Value: 61−80
d__Bacteria.p__Nitrospirae
1500
1000
500
0
46−60 (n=7)
80plus (n=3)
61−80 (n=15)
31−45 (n=3)
20−30 (n=2)
Age_category

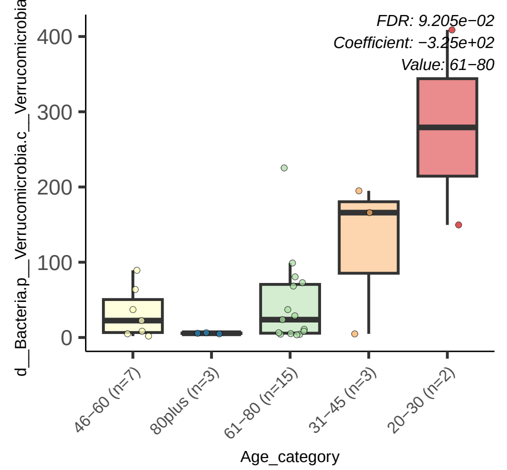

FDR: 9.205e−02
Coefficient: −3.25e+02
Value: 61−80
d__Bacteria.p__Verrucomicrobia.c__Verrucomicrobia
400
300
200
100
0
46−60 (n=7)
80plus (n=3)
61−80 (n=15)
31−45 (n=3)
20−30 (n=2)
Age_category

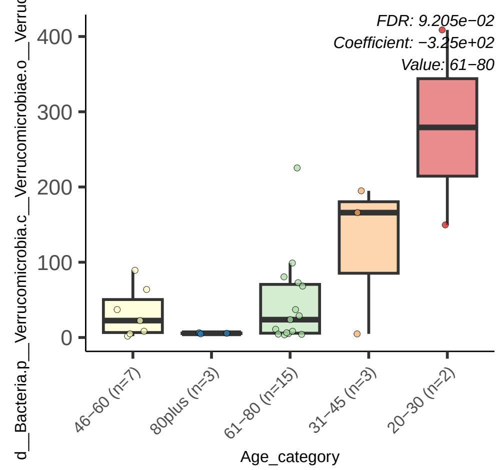

FDR: 9.205e−02
Coefficient: −3.25e+02
Value: 61−80
d__Bacteria.p__Verrucomicrobia.c__Verrucomicrobiae.o__Verruc
400
300
200
100
0
46−60 (n=7)
80plus (n=3)
61−80 (n=15)
31−45 (n=3)
20−30 (n=2)
Age_category

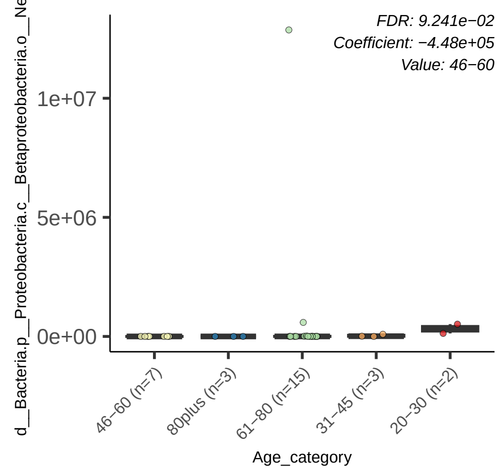

FDR: 9.241e−02
Coefficient: −4.48e+05
Value: 46−60
1e+07
5e+06
0e+00
d__Bacteria.p__Proteobacteria.c__Betaproteobacteria.o__Ne
46−60 (n=7)
80plus (n=3)
61−80 (n=15)
31−45 (n=3)
20−30 (n=2)
Age_category

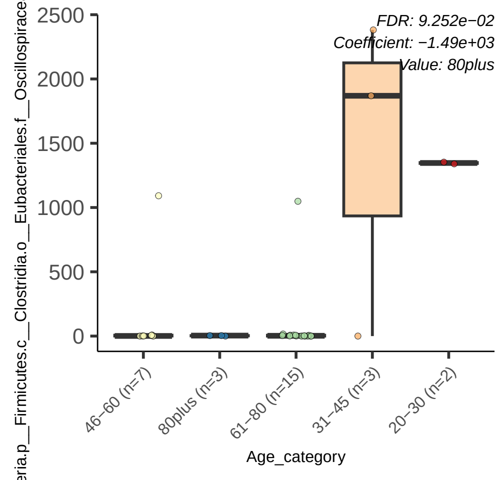

FDR: 9.252e−02
Coefficient: −1.49e+03
Value: 80plus
2500
2000
1500
1000
500
0
eria.p__Firmicutes.c__Clostridia.o__Eubacteriales.f__Oscillospirace
46−60 (n=7)
80plus (n=3)
61−80 (n=15)
31−45 (n=3)
20−30 (n=2)
Age_category

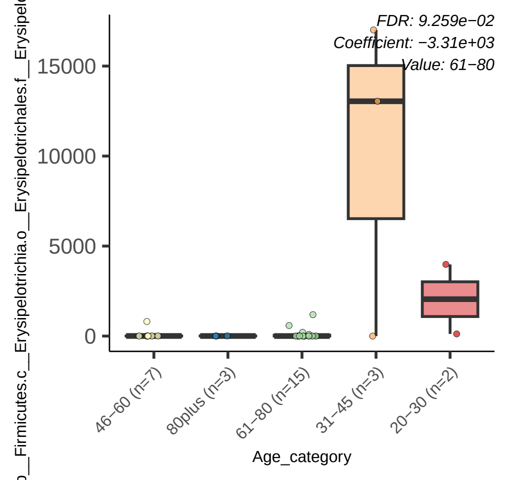

FDR: 9.259e−02
Coefficient: −3.31e+03
Value: 61−80
15000
10000
5000
0
46−60 (n=7)
80plus (n=3)
61−80 (n=15)
31−45 (n=3)
20−30 (n=2)
Age_category
o__Firmicutes.c__Erysipelotrichia.o__Erysipelotrichales.f__Erysipel

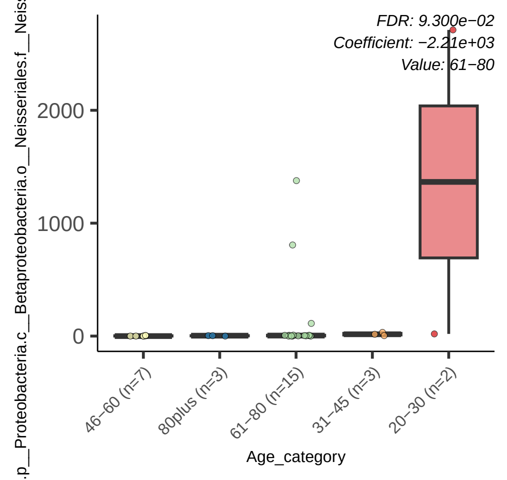

FDR: 9.300e−02
Coefficient: −2.21e+03
Value: 61−80
2000
1000
0
46−60 (n=7)
80plus (n=3)
61−80 (n=15)
31−45 (n=3)
20−30 (n=2)
Age_category
p__Proteobacteria.c__Betaproteobacteria.o__Neisseriales.f__Neiss

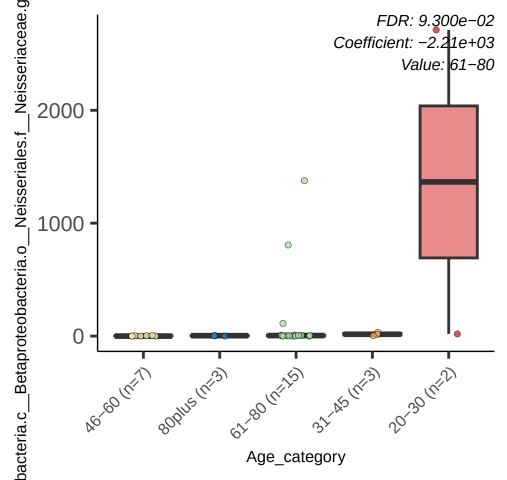

FDR: 9.300e−02
Coefficient: −2.21e+03
Value: 61−80
bacteria.c__Betaproteobacteria.o__Neisseriales.f__Neisseriaceae.g
2000
1000
0
46−60 (n=7)
80plus (n=3)
61−80 (n=15)
31−45 (n=3)
20−30 (n=2)
Age_category

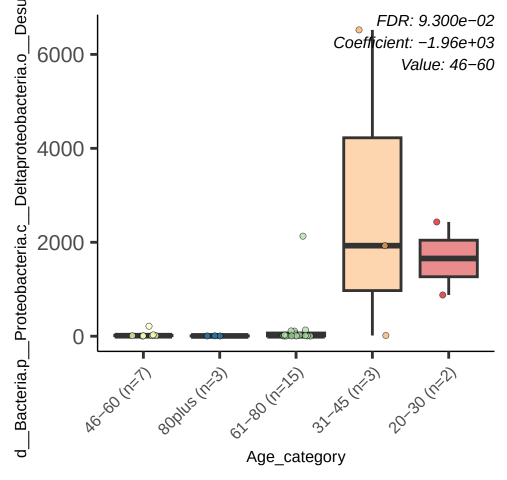

FDR: 9.300e−02
Coefficient: −1.96e+03
Value: 46−60
d__Bacteria.p__Proteobacteria.c__Deltaproteobacteria.o__Desu
6000
4000
2000
0
46−60 (n=7)
80plus (n=3)
61−80 (n=15)
31−45 (n=3)
20−30 (n=2)
Age_category

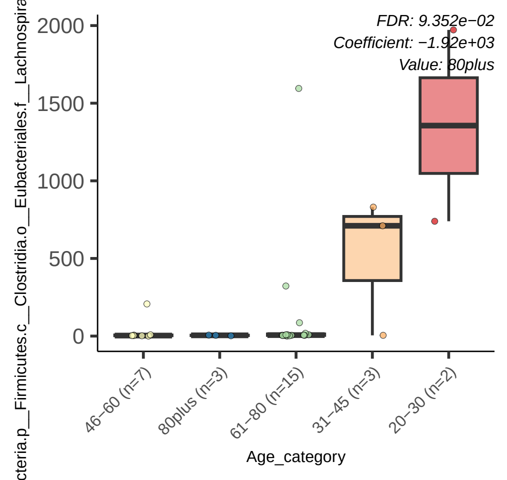

cteria.p__Firmicutes.c__Clostridia.o__Eubacteriales.f__Lachnospira
2000
1500
1000
500
0
FDR: 9.352e−02
Coefficient: −1.92e+03
Value: 80plus
46−60 (n=7)
80plus (n=3)
61−80 (n=15)
31−45 (n=3)
20−30 (n=2)
Age_category

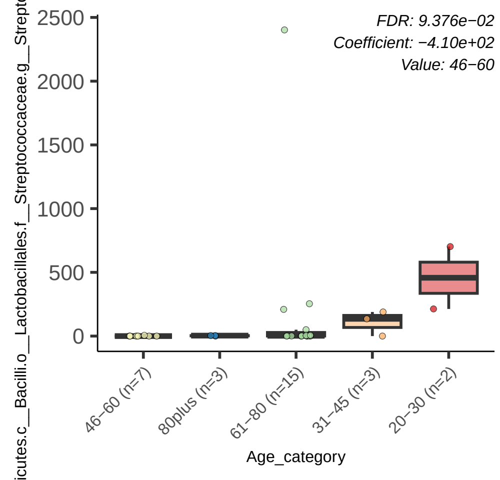
FDR: 9.376e−02
Coefficient: −4.10e+02
Value: 46−60
2500
2000
1500
1000
500
0
46−60 (n=7)
80plus (n=3)
61−80 (n=15)
31−45 (n=3)
20−30 (n=2)
Age_category
Bacilli.o__Lactobacillales.f__Streptococcaceae.g__Strept

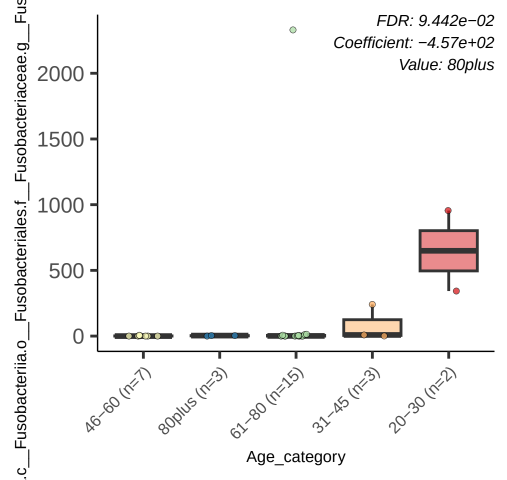

FDR: 9.442e−02
Coefficient: −4.57e+02
Value: 80plus
2000
1500
1000
500
0
c__Fusobacteriia.o__Fusobacteriales.f__Fusobacteriaceae.g__Fus
46−60 (n=7)
80plus (n=3)
61−80 (n=15)
31−45 (n=3)
20−30 (n=2)
Age_category

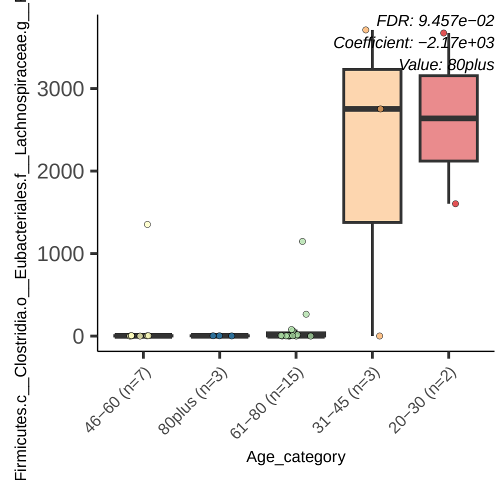
FDR: 9.457e−02
Coefficient: −2.17e+03
Value: 80plus
Firmicutes.c__Clostridia.o__Eubacteriales.f__Lachnospiraceae.g__
3000
2000
1000
0
46−60 (n=7)
80plus (n=3)
61−80 (n=15)
31−45 (n=3)
20−30 (n=2)
Age_category

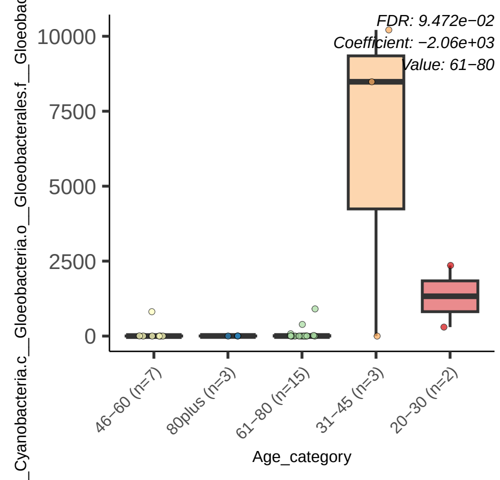

FDR: 9.472e−02
Coefficient: −2.06e+03
Value: 61−80
10000
7500
5000
2500
0
46−60 (n=7)
80plus (n=3)
61−80 (n=15)
31−45 (n=3)
20−30 (n=2)
Age_category

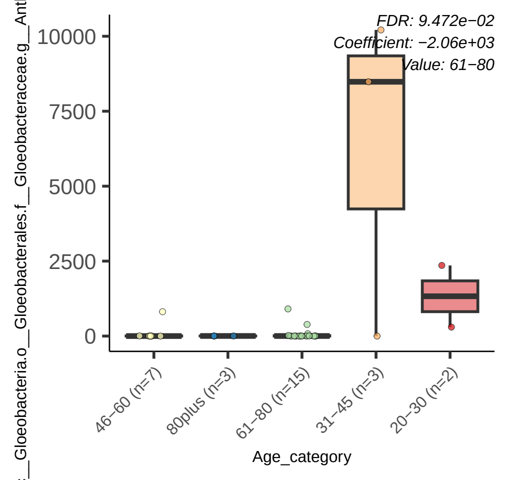
FDR: 9.472e−02
Coefficient: −2.06e+03
Value: 61−80
10000
7500
5000
2500
0
Gloeobacteria.o__Gloeobacterales.f__Gloeobacteraceae.g__Ant
46−60 (n=7)
80plus (n=3)
61−80 (n=15)
31−45 (n=3)
20−30 (n=2)
Age_category

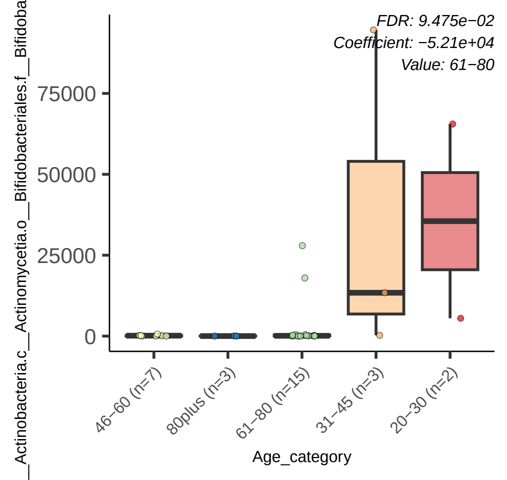

FDR: 9.475e−02
Coefficient: −5.21e+04
Value: 61−80
75000
50000
25000
0
46−60 (n=7)
80plus (n=3)
61−80 (n=15)
31−45 (n=3)
20−30 (n=2)
Age_category
_Actinobacteria.c__Actinomycetia.o__Bifidobacteriales.f__Bifidoba

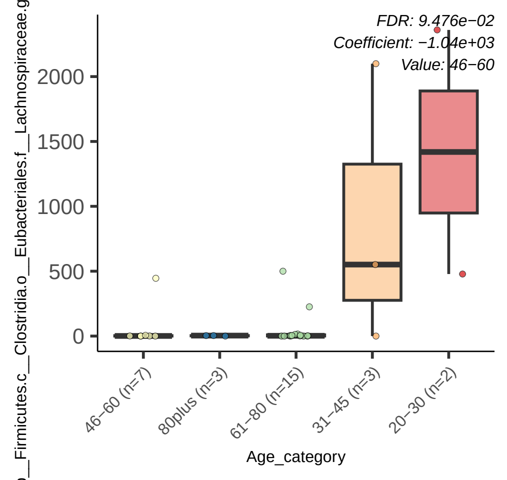

FDR: 9.476e−02
Coefficient: −1.04e+03
Value: 46−60
0
500
1000
1500
2000
o__Firmicutes.c__Clostridia.o__Eubacteriales.f__Lachnospiraceae.g
46−60 (n=7)
80plus (n=3)
61−80 (n=15)
31−45 (n=3)
20−30 (n=2)
Age_category

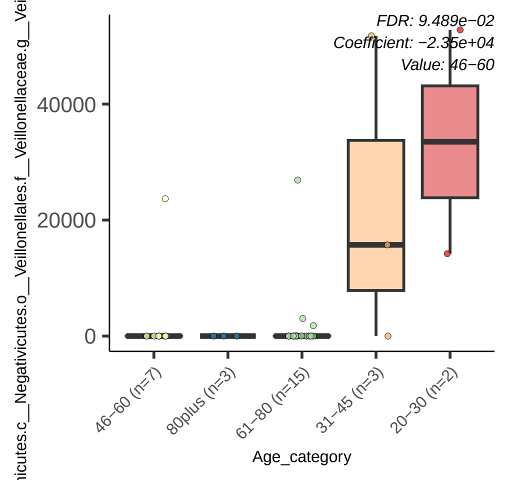

FDR: 9.489e−02
Coefficient: −2.35e+04
Value: 46−60
40000
20000
0
46−60 (n=7)
80plus (n=3)
61−80 (n=15)
31−45 (n=3)
20−30 (n=2)
Age_category
icutes.c__Negativicutes.o__Veillonellales.f__Veillonellaceae.g__Vei

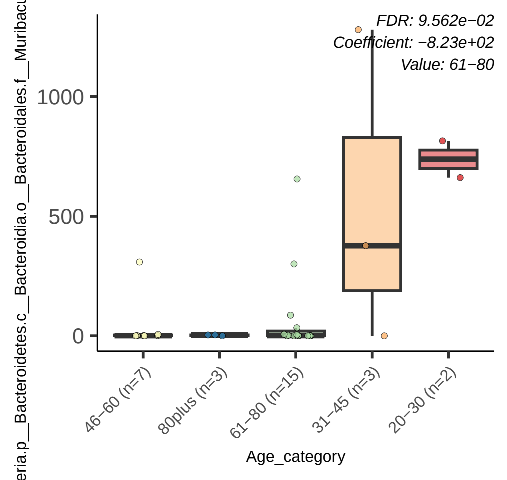

FDR: 9.562e−02
Coefficient: −8.23e+02
Value: 61−80
1000
500
0
46−60 (n=7)
80plus (n=3)
61−80 (n=15)
31−45 (n=3)
20−30 (n=2)
Age_category
eria.p__Bacteroidetes.c__Bacteroidia.o__Bacteroidales.f__Muribacu

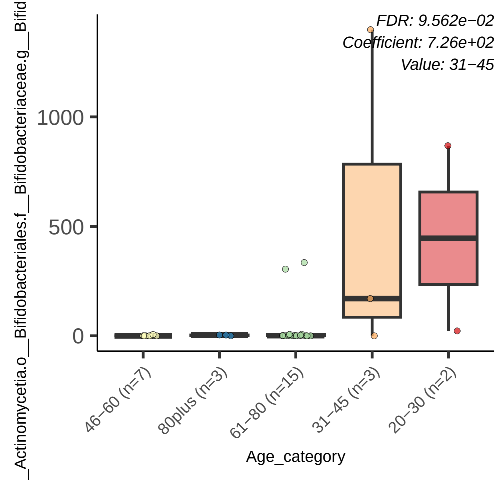

FDR: 9.562e−02
Coefficient: 7.26e+02
Value: 31−45
1000
500
0
46−60 (n=7)
80plus (n=3)
61−80 (n=15)
31−45 (n=3)
20−30 (n=2)
Age_category
_Actinomycetia.o__Bifidobacteriales.f__Bifidobacteriaceae.g__Bifid

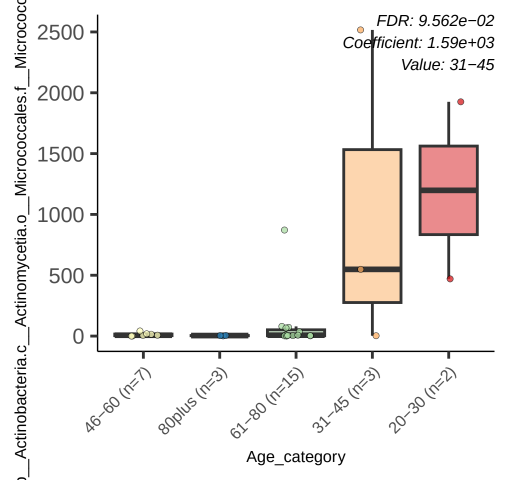

FDR: 9.562e−02
Coefficient: 1.59e+03
Value: 31−45
o__Actinobacteria.c__Actinomycetia.o__Micrococcales.f__Micrococc
2500
2000
1500
1000
500
0
46−60 (n=7)
80plus (n=3)
61−80 (n=15)
31−45 (n=3)
20−30 (n=2)
Age_category

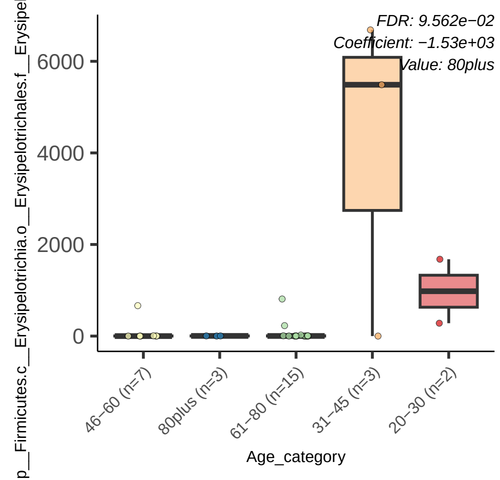

FDR: 9.562e−02
Coefficient: −1.53e+03
Value: 80plus
6000
4000
2000
0
p__Firmicutes.c__Erysipelotrichia.o__Erysipelotrichales.f__Erysipel
46−60 (n=7)
80plus (n=3)
61−80 (n=15)
31−45 (n=3)
20−30 (n=2)
Age_category

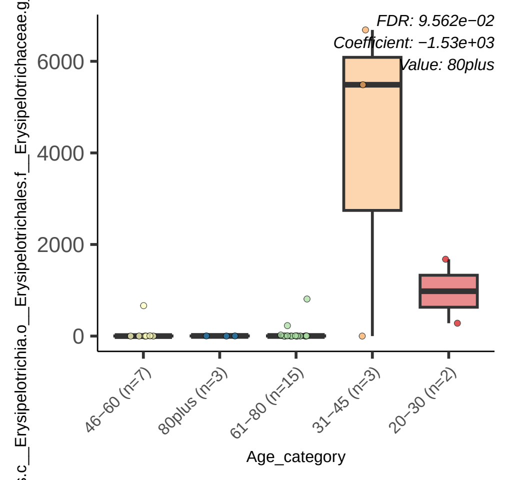
FDR: 9.562e−02
Coefficient: −1.53e+03
Value: 80plus
6000
4000
2000
0
46−60 (n=7)
80plus (n=3)
61−80 (n=15)
31−45 (n=3)
20−30 (n=2)
Age_category
Erysipelotrichia.o__Erysipelotrichales.f__Erysipelotrichaceae.g

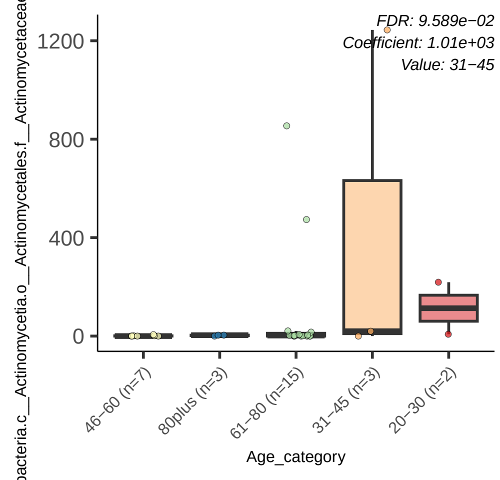

FDR: 9.589e−02
Coefficient: 1.01e+03
Value: 31−45
1200
800
400
0
46−60 (n=7)
80plus (n=3)
61−80 (n=15)
31−45 (n=3)
20−30 (n=2)
Age_category
bacteria.c__Actinomycetia.o__Actinomycetales.f__Actinomycetaceae

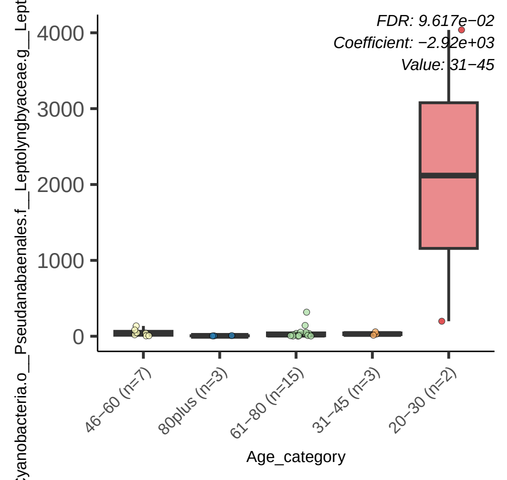

FDR: 9.617e−02
Coefficient: −2.92e+03
Value: 31−45
4000
3000
2000
1000
0
Cyanobacteria.o__Pseudanabaenales.f__Leptolyngbyaceae.g__Lept
46−60 (n=7)
80plus (n=3)
61−80 (n=15)
31−45 (n=3)
20−30 (n=2)
Age_category

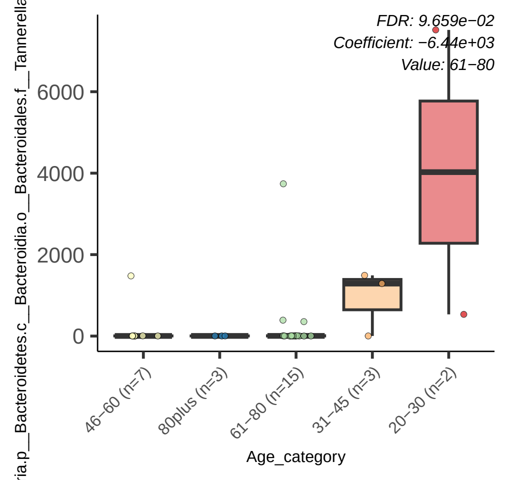
FDR: 9.659e−02
Coefficient: −6.44e+03
Value: 61−80
6000
4000
2000
0
ria.p__Bacteroidetes.c__Bacteroidia.o__Bacteroidales.f__Tannerella
46−60 (n=7)
80plus (n=3)
61−80 (n=15)
31−45 (n=3)
20−30 (n=2)
Age_category

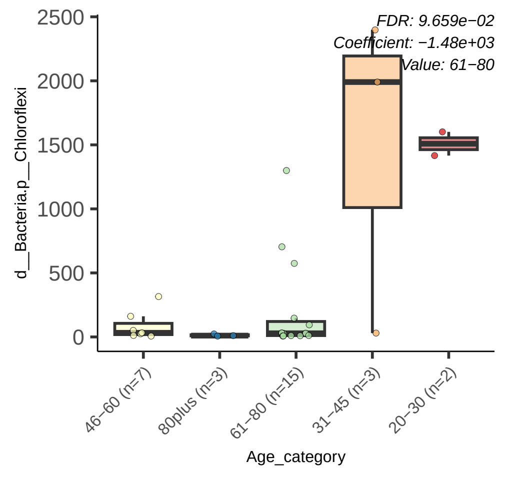

FDR: 9.659e−02
Coefficient: −1.48e+03
Value: 61−80
d__Bacteria.p__Chloroflexi
2500
2000
1500
1000
500
0
46−60 (n=7)
80plus (n=3)
61−80 (n=15)
31−45 (n=3)
20−30 (n=2)
Age_category

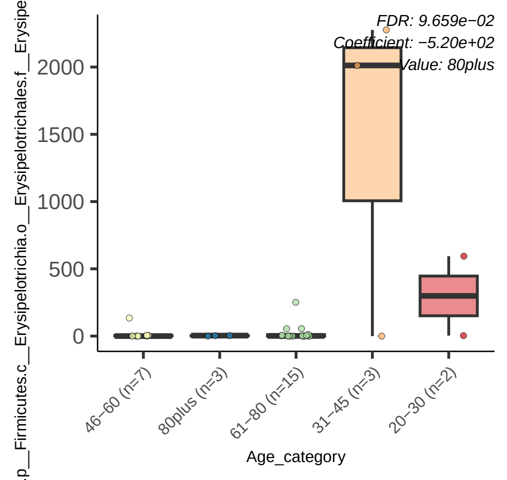

FDR: 9.659e−02
Coefficient: −5.20e+02
Value: 80plus
p__Firmicutes.c__Erysipelotrichia.o__Erysipelotrichales.f__Erysipe
2000
1500
1000
500
0
46−60 (n=7)
80plus (n=3)
61−80 (n=15)
31−45 (n=3)
20−30 (n=2)
Age_category

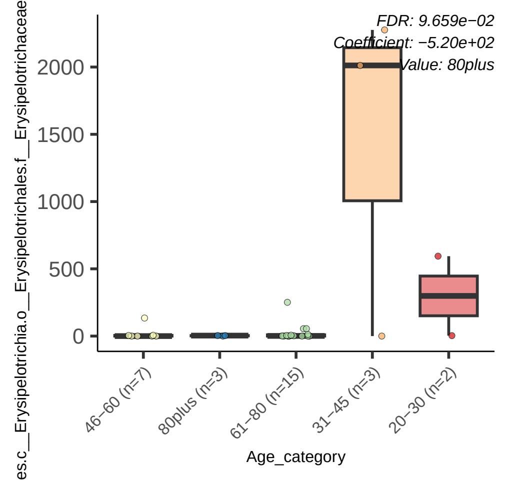

FDR: 9.659e−02
Coefficient: −5.20e+02
Value: 80plus
es.c__Erysipelotrichia.o__Erysipelotrichales.f__Erysipelotrichaceae
2000
1500
1000
500
0
46−60 (n=7)
80plus (n=3)
61−80 (n=15)
31−45 (n=3)
20−30 (n=2)
Age_category

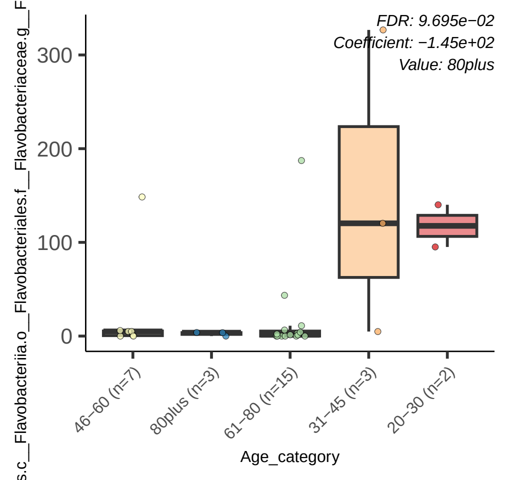

FDR: 9.695e−02
Coefficient: −1.45e+02
Value: 80plus
300
200
100
0
s.c__Flavobacteriia.o__Flavobacteriales.f__Flavobacteriaceae.g__F
46−60 (n=7)
80plus (n=3)
61−80 (n=15)
31−45 (n=3)
20−30 (n=2)
Age_category

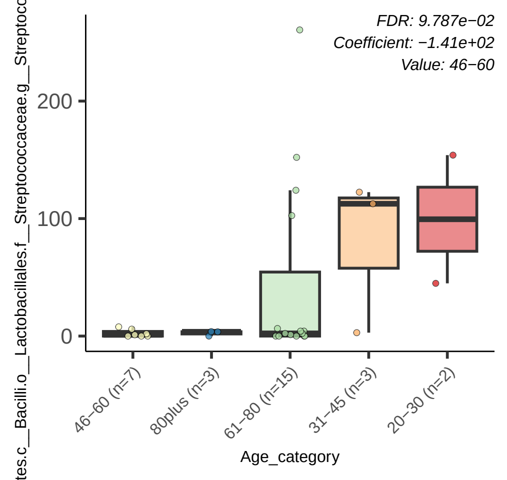

FDR: 9.787e−02
Coefficient: −1.41e+02
Value: 46−60
0
100
200
tes.c__Bacilli.o__Lactobacillales.f__Streptococcaceae.g__Streptoco
46−60 (n=7)
80plus (n=3)
61−80 (n=15)
31−45 (n=3)
20−30 (n=2)
Age_category

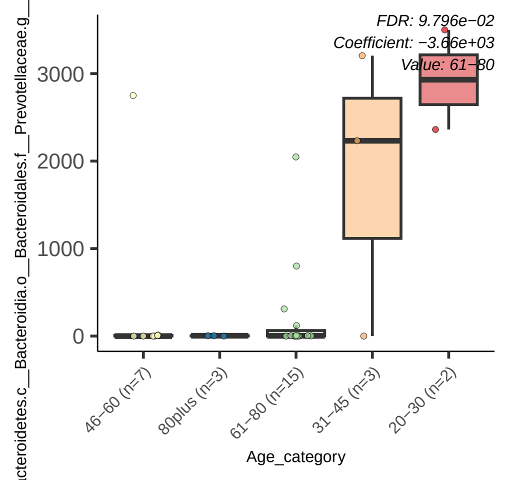

FDR: 9.796e−02
Coefficient: −3.66e+03
Value: 61−80
3000
2000
1000
0
acteroidetes.c__Bacteroidia.o__Bacteroidales.f__Prevotellaceae.g__
46−60 (n=7)
80plus (n=3)
61−80 (n=15)
31−45 (n=3)
20−30 (n=2)
Age_category

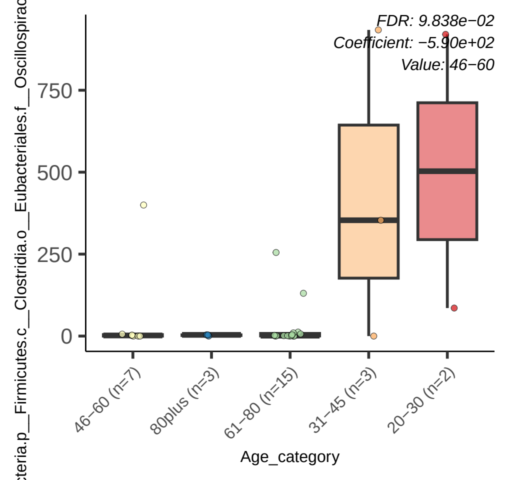

FDR: 9.838e−02
Coefficient: −5.90e+02
Value: 46−60
750
500
250
0
46−60 (n=7)
80plus (n=3)
61−80 (n=15)
31−45 (n=3)
20−30 (n=2)
Age_category
cteria.p__Firmicutes.c__Clostridia.o__Eubacteriales.f__Oscillospirac

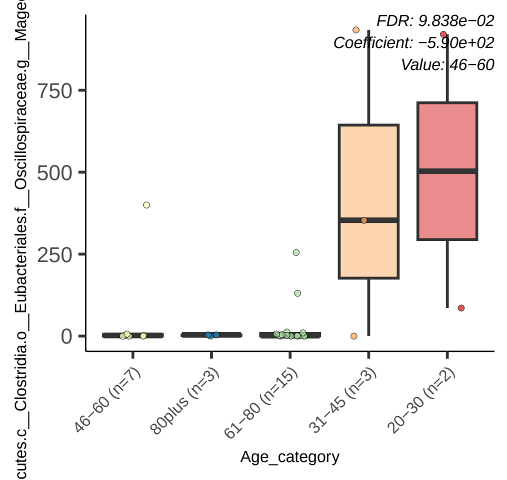

FDR: 9.838e−02
Coefficient: −5.90e+02
Value: 46−60
cutes.c__Clostridia.o__Eubacteriales.f__Oscillospiraceae.g__Mage
750
500
250
0
46−60 (n=7)
80plus (n=3)
61−80 (n=15)
31−45 (n=3)
20−30 (n=2)
Age_category

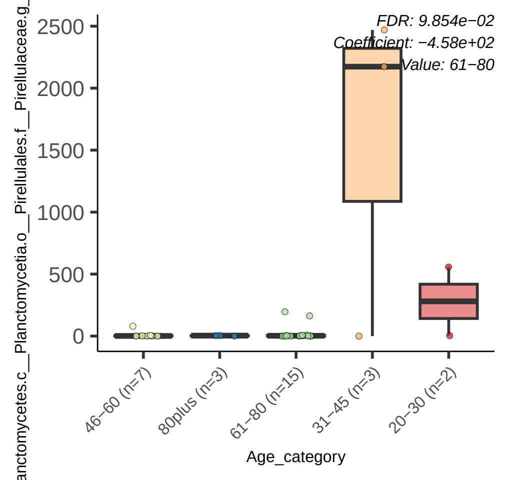

FDR: 9.854e−02
Coefficient: −4.58e+02
Value: 61−80
2500
2000
1500
1000
500
0
Planctomycetes.c__Planctomycetia.o__Pirellulales.f__Pirellulaceae.g__
46−60 (n=7)
80plus (n=3)
61−80 (n=15)
31−45 (n=3)
20−30 (n=2)
Age_category

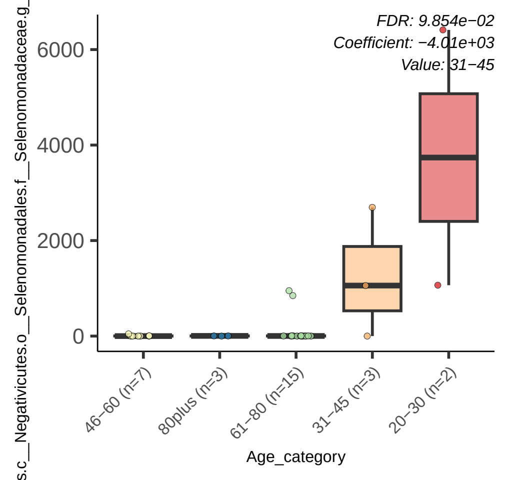

FDR: 9.854e−02
Coefficient: −4.01e+03
Value: 31−45
6000
4000
2000
0
s.c__Negativicutes.o__Selenomonadales.f__Selenomonadaceae.g__
46−60 (n=7)
80plus (n=3)
61−80 (n=15)
31−45 (n=3)
20−30 (n=2)
Age_category

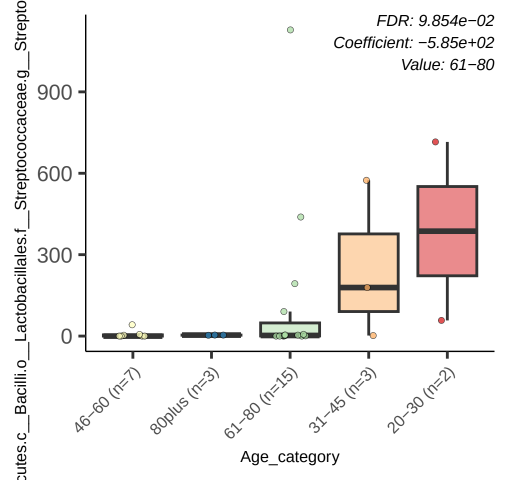

FDR: 9.854e−02
Coefficient: −5.85e+02
Value: 61−80
900
600
300
0
cutes.c__Bacilli.o__Lactobacillales.f__Streptococcaceae.g__Strepto
46−60 (n=7)
80plus (n=3)
61−80 (n=15)
31−45 (n=3)
20−30 (n=2)
Age_category

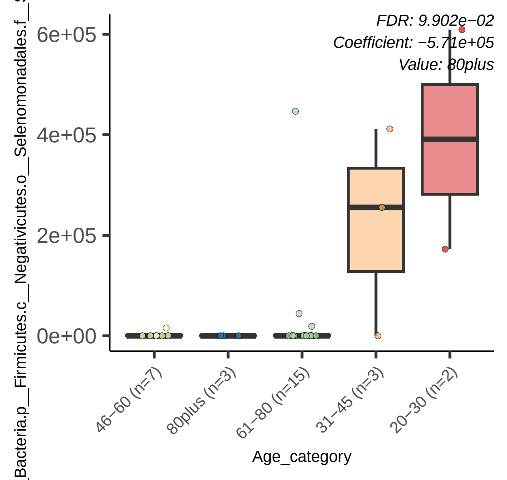

FDR: 9.902e−02
Coefficient: −5.71e+05
Value: 80plus
6e+05
4e+05
2e+05
0e+00
Bacteria.p__Firmicutes.c__Negativicutes.o__Selenomonadales.f__
46−60 (n=7)
80plus (n=3)
61−80 (n=15)
31−45 (n=3)
20−30 (n=2)
Age_category

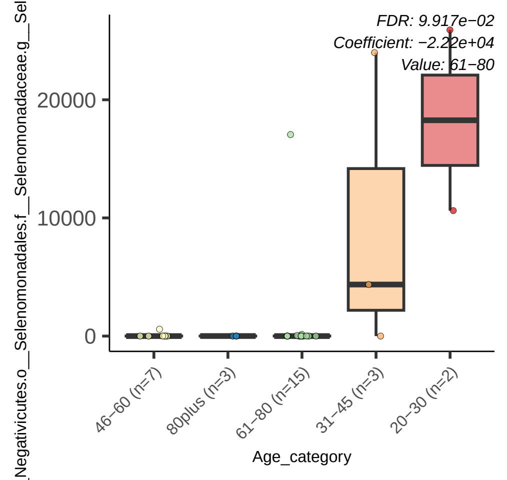
FDR: 9.917e−02
Coefficient: −2.22e+04
Value: 61−80
20000
10000
0
Negativicutes.o__Selenomonadales.f__Selenomonadaceae.g__Sel
46−60 (n=7)
80plus (n=3)
61−80 (n=15)
31−45 (n=3)
20−30 (n=2)
Age_category

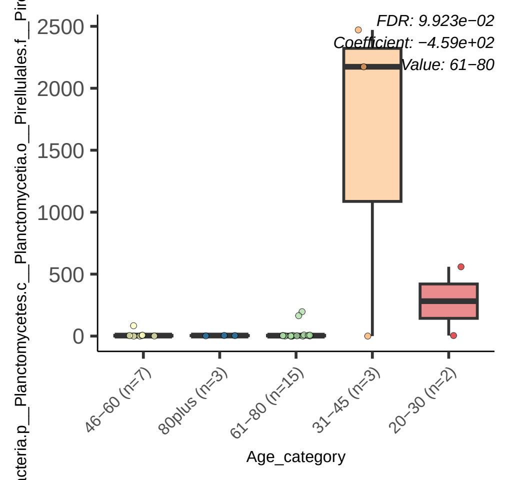

FDR: 9.923e−02
Coefficient: −4.59e+02
Value: 61−80
2500
2000
1500
1000
500
0
46−60 (n=7)
80plus (n=3)
61−80 (n=15)
31−45 (n=3)
20−30 (n=2)
Age_category

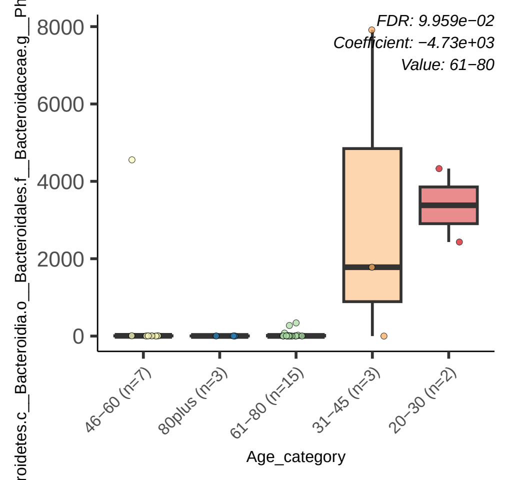

FDR: 9.959e−02
Coefficient: −4.73e+03
Value: 61−80
8000
6000
4000
2000
0
roidetes.c__Bacteroidia.o__Bacteroidales.f__Bacteroidaceae.g__Ph
46−60 (n=7)
80plus (n=3)
61−80 (n=15)
31−45 (n=3)
20−30 (n=2)
Age_category

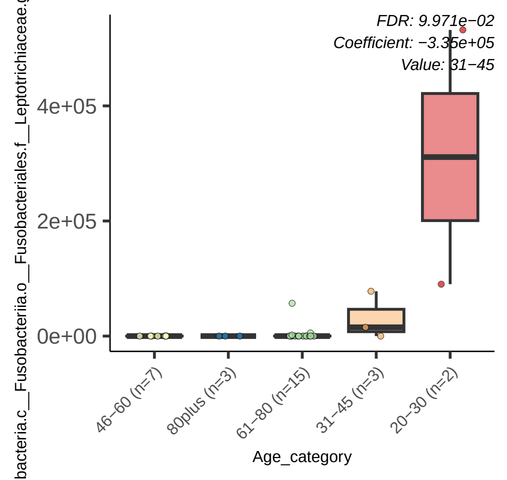
FDR: 9.971e−02
Coefficient: −3.35e+05
Value: 31−45
bacteria.c__Fusobacteriia.o__Fusobacteriales.f__Leptotrichiaceae.g
4e+05
2e+05
0e+00
46−60 (n=7)
80plus (n=3)
61−80 (n=15)
31−45 (n=3)
20−30 (n=2)
Age_category

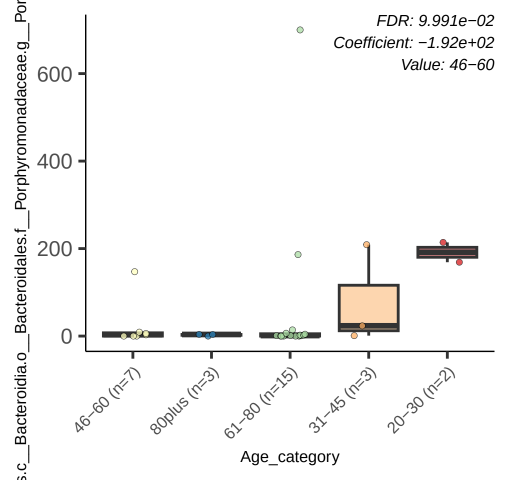

FDR: 9.991e−02
Coefficient: −1.92e+02
Value: 46−60
600
400
200
0
s.c__Bacteroidia.o__Bacteroidales.f__Porphyromonadaceae.g__Por
46−60 (n=7)
80plus (n=3)
61−80 (n=15)
31−45 (n=3)
20−30 (n=2)
Age_category

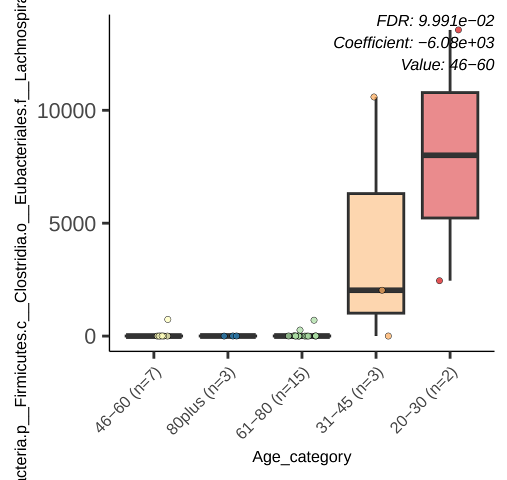

FDR: 9.991e−02
Coefficient: −6.08e+03
Value: 46−60
10000
5000
0
cteria.p__Firmicutes.c__Clostridia.o__Eubacteriales.f__Lachnospira
46−60 (n=7)
80plus (n=3)
61−80 (n=15)
31−45 (n=3)
20−30 (n=2)
Age_category

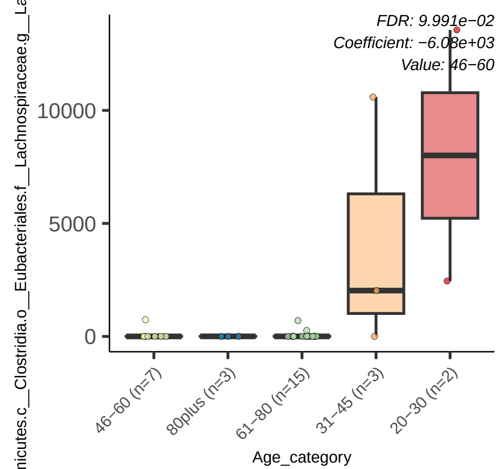

FDR: 9.991e−02
Coefficient: −6.08e+03
Value: 46−60
rmicutes.c__Clostridia.o__Eubacteriales.f__Lachnospiraceae.g__La
10000
5000
0
46−60 (n=7)
80plus (n=3)
61−80 (n=15)
31−45 (n=3)
20−30 (n=2)
Age_category

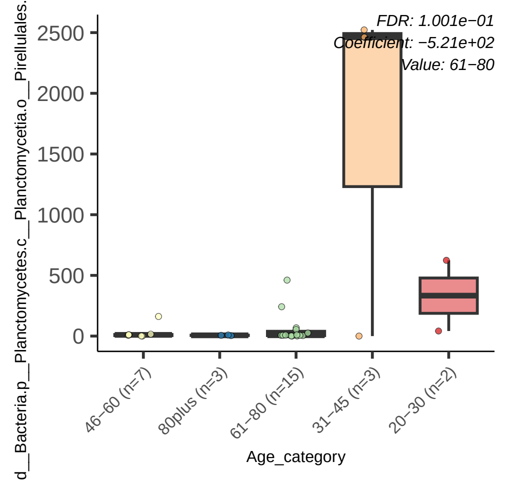

FDR: 1.001e−01
Coefficient: −5.21e+02
Value: 61−80
d__Bacteria.p__Planctomycetes.c__Planctomycetia.o__Pirellulales.
2500
2000
1500
1000
500
0
46−60 (n=7)
80plus (n=3)
61−80 (n=15)
31−45 (n=3)
20−30 (n=2)
Age_category

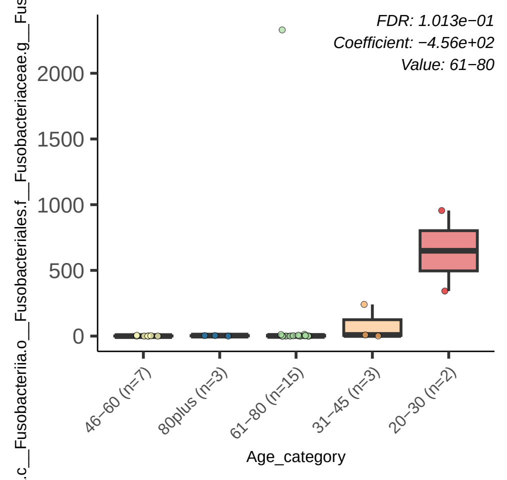
FDR: 1.013e−01
Coefficient: −4.56e+02
Value: 61−80
2000
1500
1000
500
0
46−60 (n=7)
80plus (n=3)
61−80 (n=15)
31−45 (n=3)
20−30 (n=2)
Age_category
c__Fusobacteriia.o__Fusobacteriales.f__Fusobacteriaceae.g__Fus

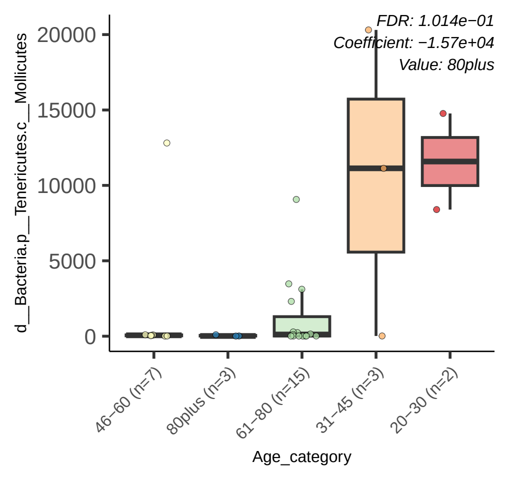

FDR: 1.014e−01
Coefficient: −1.57e+04
Value: 80plus
d__Bacteria.p__Tenericutes.c__Mollicutes
20000
15000
10000
5000
0
46−60 (n=7)
80plus (n=3)
61−80 (n=15)
31−45 (n=3)
20−30 (n=2)
Age_category

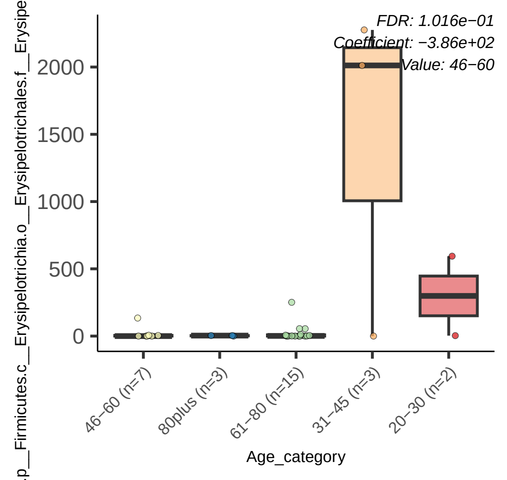

FDR: 1.016e−01
Coefficient: −3.86e+02
Value: 46−60
p__Firmicutes.c__Erysipelotrichia.o__Erysipelotrichales.f__Erysipe
2000
1500
1000
500
0
46−60 (n=7)
80plus (n=3)
61−80 (n=15)
31−45 (n=3)
20−30 (n=2)
Age_category

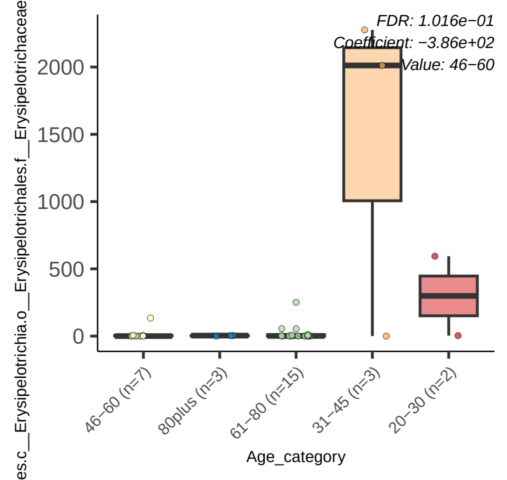

FDR: 1.016e−01
Coefficient: −3.86e+02
Value: 46−60
es.c__Erysipelotrichia.o__Erysipelotrichales.f__Erysipelotrichaceae
2000
1500
1000
500
0
46−60 (n=7)
80plus (n=3)
61−80 (n=15)
31−45 (n=3)
20−30 (n=2)
Age_category

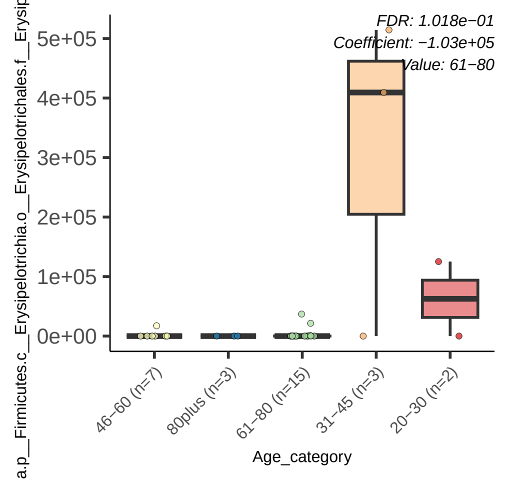
FDR: 1.018e−01
Coefficient: −1.03e+05
Value: 61−80
a.p__Firmicutes.c__Erysipelotrichia.o__Erysipelotrichales.f__Erysip
5e+05
4e+05
3e+05
2e+05
1e+05
0e+00
46−60 (n=7)
80plus (n=3)
61−80 (n=15)
31−45 (n=3)
20−30 (n=2)
Age_category

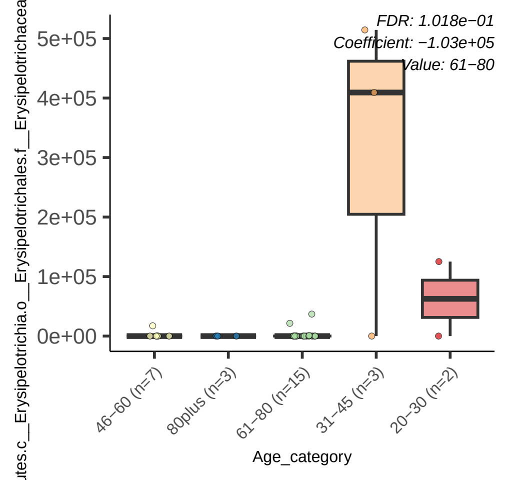

FDR: 1.018e−01
Coefficient: −1.03e+05
Value: 61−80
5e+05
4e+05
3e+05
2e+05
1e+05
0e+00
utes.c__Erysipelotrichia.o__Erysipelotrichales.f__Erysipelotrichacea
46−60 (n=7)
80plus (n=3)
61−80 (n=15)
31−45 (n=3)
20−30 (n=2)
Age_category

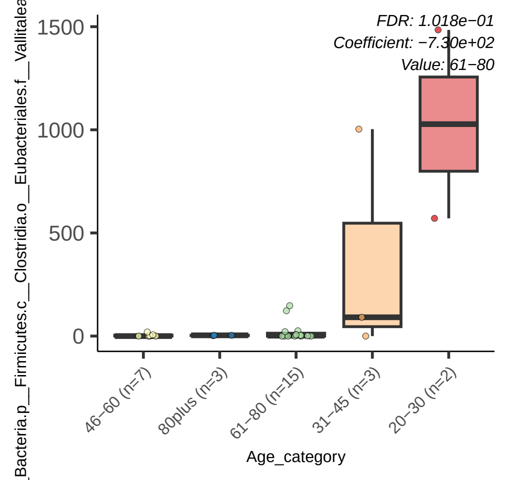
FDR: 1.018e−01
Coefficient: −7.30e+02
Value: 61−80
_Bacteria.p__Firmicutes.c__Clostridia.o__Eubacteriales.f__Vallitalea
1500
1000
500
0
46−60 (n=7)
80plus (n=3)
61−80 (n=15)
31−45 (n=3)
20−30 (n=2)
Age_category

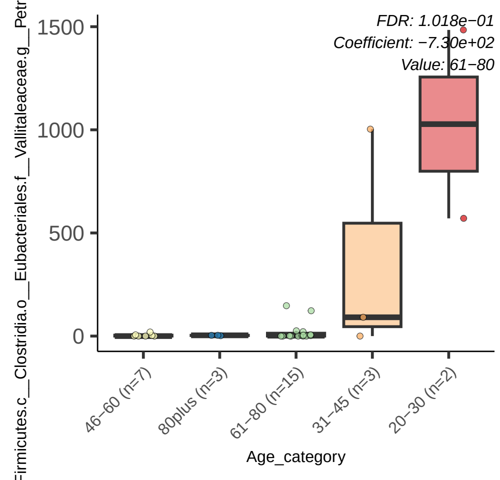

FDR: 1.018e−01
Coefficient: −7.30e+02
Value: 61−80
Firmicutes.c__Clostridia.o__Eubacteriales.f__Vallitaleaceae.g__Petr
1500
1000
500
0
46−60 (n=7)
80plus (n=3)
61−80 (n=15)
31−45 (n=3)
20−30 (n=2)
Age_category

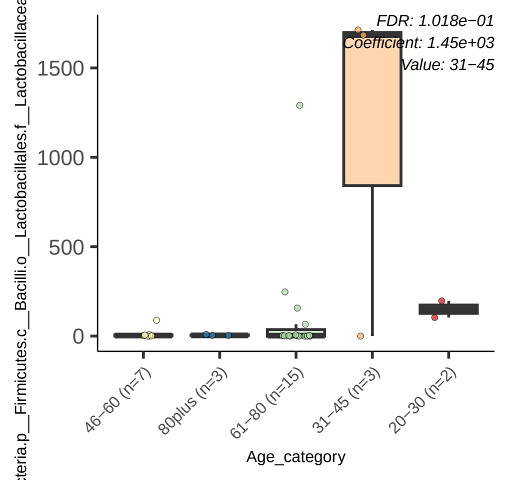

FDR: 1.018e−01
Coefficient: 1.45e+03
Value: 31−45
cteria.p__Firmicutes.c__Bacilli.o__Lactobacillales.f__Lactobacillacea
0
500
1000
1500
46−60 (n=7)
80plus (n=3)
61−80 (n=15)
31−45 (n=3)
20−30 (n=2)
Age_category

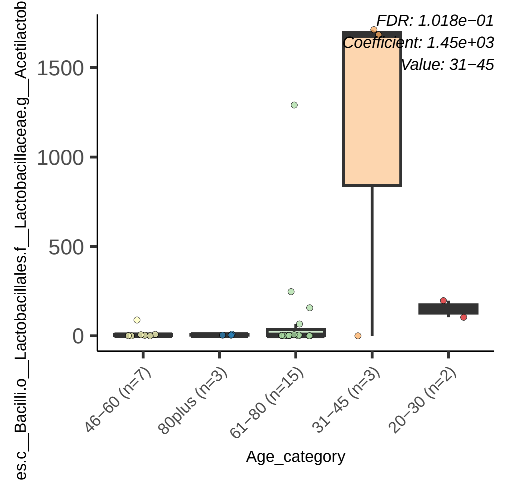

FDR: 1.018e−01
Coefficient: 1.45e+03
Value: 31−45
es.c__Bacilli.o__Lactobacillales.f__Lactobacillaceae.g__Acetilactob
1500
1000
500
0
46−60 (n=7)
80plus (n=3)
61−80 (n=15)
31−45 (n=3)
20−30 (n=2)
Age_category

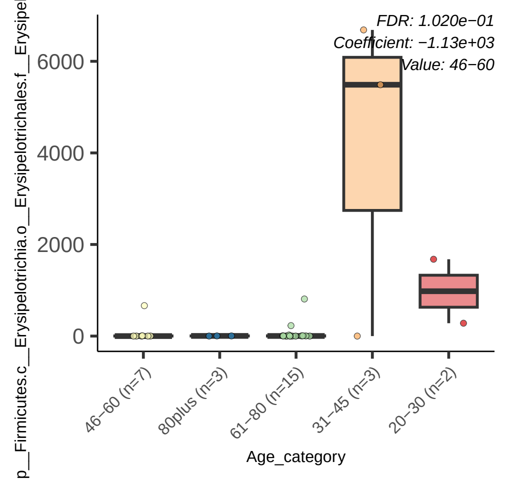

FDR: 1.020e−01
Coefficient: −1.13e+03
Value: 46−60
p__Firmicutes.c__Erysipelotrichia.o__Erysipelotrichales.f__Erysipel
6000
4000
2000
0
46−60 (n=7)
80plus (n=3)
61−80 (n=15)
31−45 (n=3)
20−30 (n=2)
Age_category

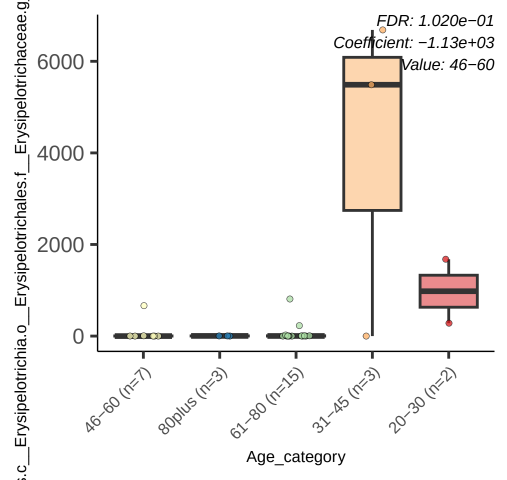

FDR: 1.020e−01
Coefficient: −1.13e+03
Value: 46−60
6000
4000
2000
0
s.c__Erysipelotrichia.o__Erysipelotrichales.f__Erysipelotrichaceae.g
46−60 (n=7)
80plus (n=3)
61−80 (n=15)
31−45 (n=3)
20−30 (n=2)
Age_category

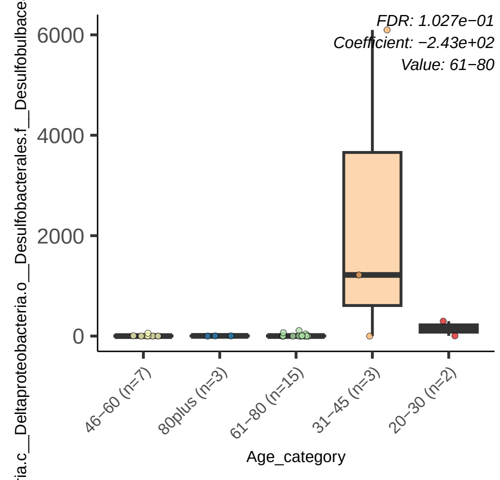

FDR: 1.027e−01
Coefficient: −2.43e+02
Value: 61−80
6000
4000
2000
0
ria.c__Deltaproteobacteria.o__Desulfobacterales.f__Desulfobulbace
46−60 (n=7)
80plus (n=3)
61−80 (n=15)
31−45 (n=3)
20−30 (n=2)
Age_category

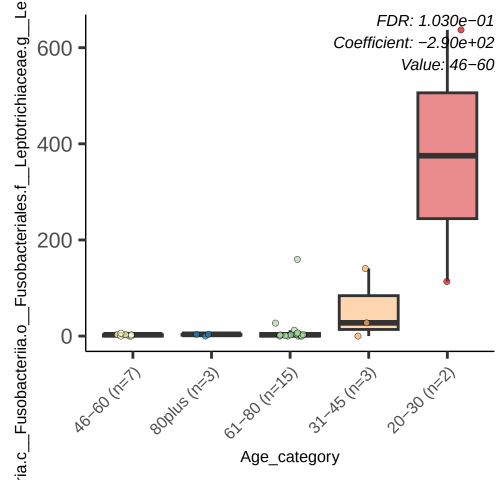
FDR: 1.030e−01
Coefficient: −2.90e+02
Value: 46−60
ria.c__Fusobacteriia.o__Fusobacteriales.f__Leptotrichiaceae.g__Le
600
400
200
0
46−60 (n=7)
80plus (n=3)
61−80 (n=15)
31−45 (n=3)
20−30 (n=2)
Age_category

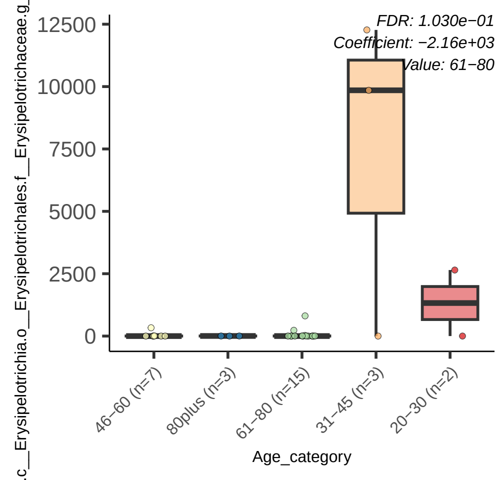

FDR: 1.030e−01
Coefficient: −2.16e+03
Value: 61−80
c__Erysipelotrichia.o__Erysipelotrichales.f__Erysipelotrichaceae.g_
12500
10000
7500
5000
2500
0
46−60 (n=7)
80plus (n=3)
61−80 (n=15)
31−45 (n=3)
20−30 (n=2)
Age_category

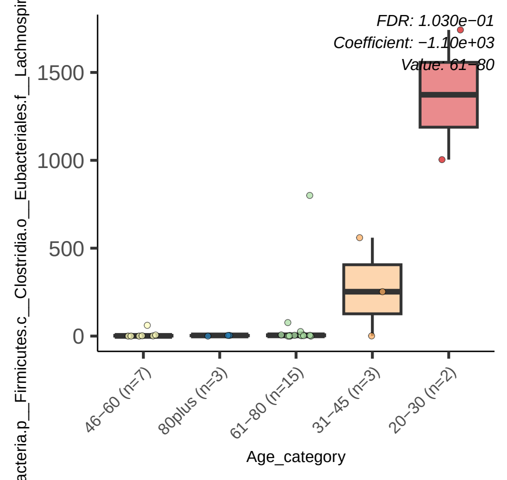

FDR: 1.030e−01
Coefficient: −1.10e+03
Value: 61−80
1500
1000
500
0
46−60 (n=7)
80plus (n=3)
61−80 (n=15)
31−45 (n=3)
20−30 (n=2)
Age_category
acteria.p__Firmicutes.c__Clostridia.o__Eubacteriales.f__Lachnospi

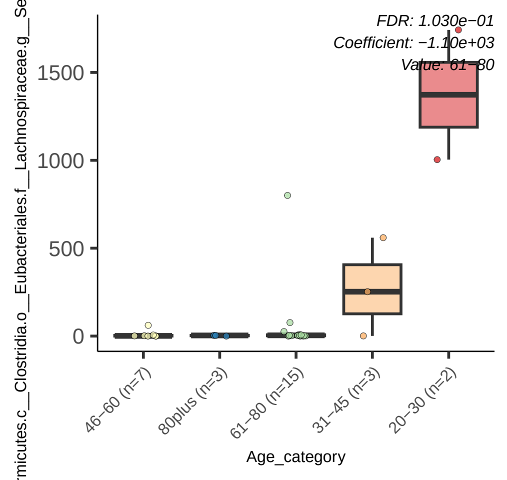

FDR: 1.030e−01
Coefficient: −1.10e+03
Value: 61−80
rmicutes.c__Clostridia.o__Eubacteriales.f__Lachnospiraceae.g__Se
1500
1000
500
0
46−60 (n=7)
80plus (n=3)
61−80 (n=15)
31−45 (n=3)
20−30 (n=2)
Age_category

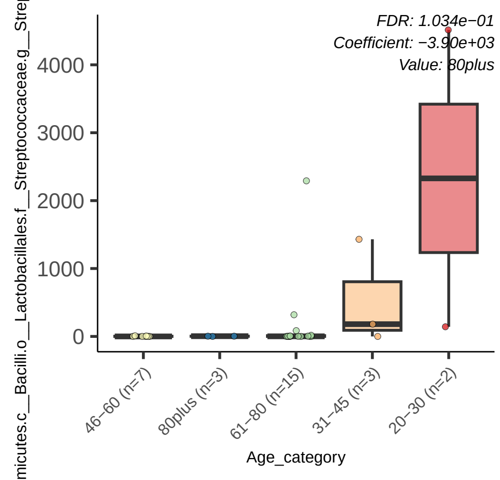

FDR: 1.034e−01
Coefficient: −3.90e+03
Value: 80plus
4000
3000
2000
1000
0
micutes.c__Bacilli.o__Lactobacillales.f__Streptococcaceae.g__Strep
46−60 (n=7)
80plus (n=3)
61−80 (n=15)
31−45 (n=3)
20−30 (n=2)
Age_category

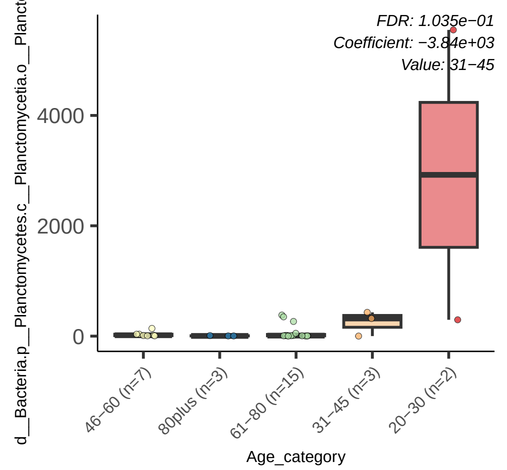
FDR: 1.035e−01
Coefficient: −3.84e+03
Value: 31−45
d__Bacteria.p__Planctomycetes.c__Planctomycetia.o__Planct
4000
2000
0
46−60 (n=7)
80plus (n=3)
61−80 (n=15)
31−45 (n=3)
20−30 (n=2)
Age_category

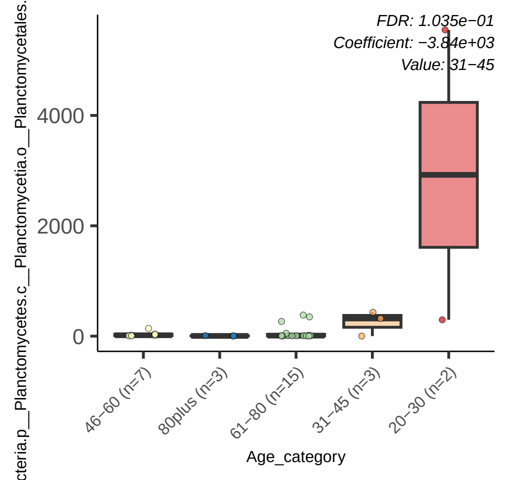
FDR: 1.035e−01
Coefficient: −3.84e+03
Value: 31−45
4000
2000
0
46−60 (n=7)
80plus (n=3)
61−80 (n=15)
31−45 (n=3)
20−30 (n=2)
Age_category
cteria.p__Planctomycetes.c__Planctomycetia.o__Planctomycetales.

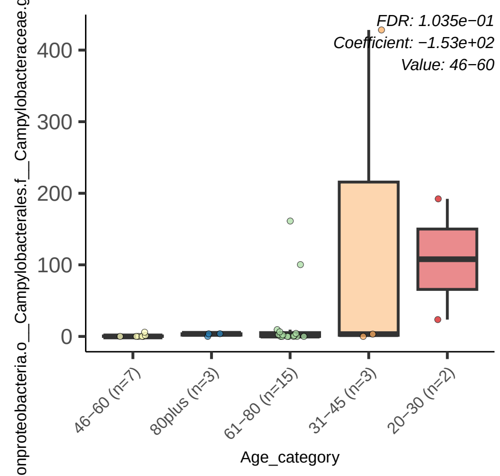

FDR: 1.035e−01
Coefficient: −1.53e+02
Value: 46−60
0
100
200
300
400
lonproteobacteria.o__Campylobacterales.f__Campylobacteraceae.g
46−60 (n=7)
80plus (n=3)
61−80 (n=15)
31−45 (n=3)
20−30 (n=2)
Age_category

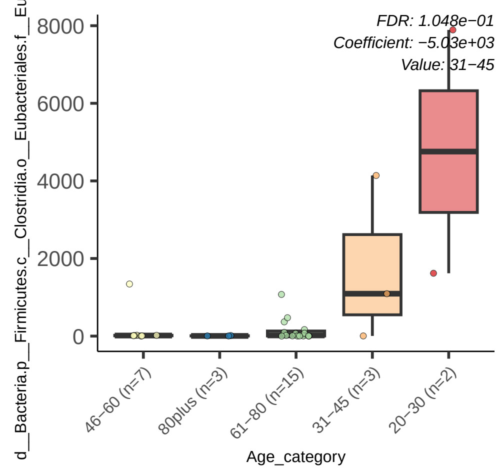

d__Bacteria.p__Firmicutes.c__Clostridia.o__Eubacteriales.f__Eu
8000
6000
4000
2000
0
FDR: 1.048e−01
Coefficient: −5.03e+03
Value: 31−45
46−60 (n=7)
80plus (n=3)
61−80 (n=15)
31−45 (n=3)
20−30 (n=2)
Age_category

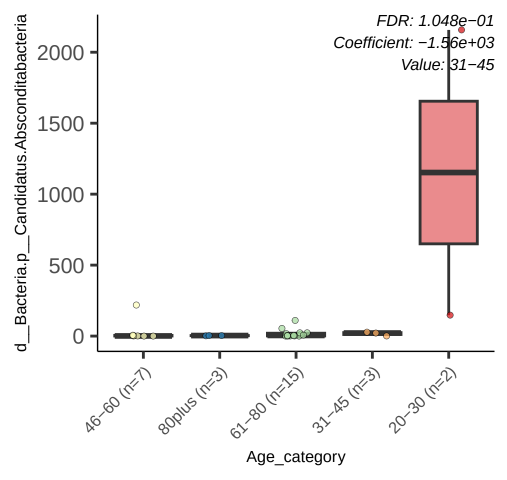

FDR: 1.048e−01
Coefficient: −1.56e+03
Value: 31−45
2000
1500
1000
500
0
d__Bacteria.p__Candidatus.Absconditabacteria
46−60 (n=7)
80plus (n=3)
61−80 (n=15)
31−45 (n=3)
20−30 (n=2)
Age_category

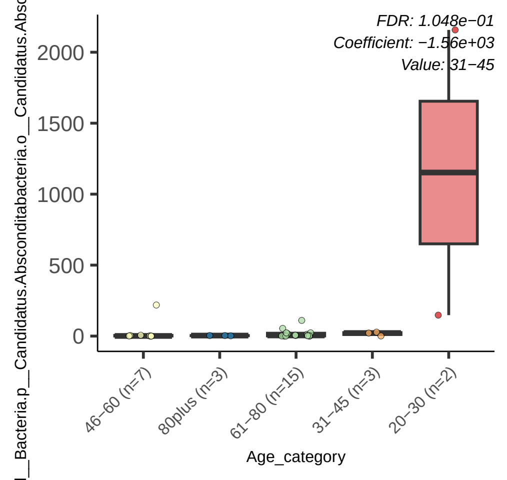

FDR: 1.048e−01
Coefficient: −1.56e+03
Value: 31−45
2000
1500
1000
500
0
46−60 (n=7)
80plus (n=3)
61−80 (n=15)
31−45 (n=3)
20−30 (n=2)
Age_category
l__Bacteria.p__Candidatus.Absconditabacteria.o__Candidatus.Absc

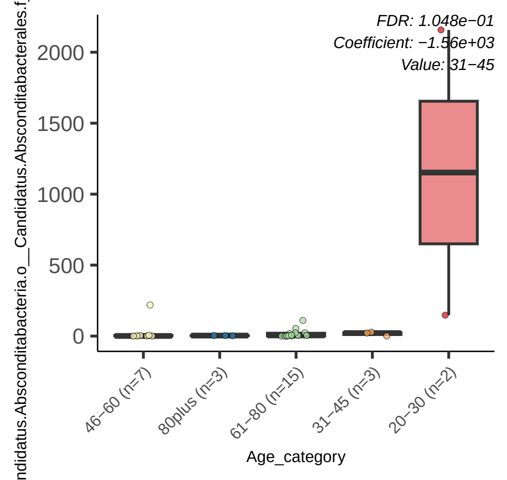

FDR: 1.048e−01
Coefficient: −1.56e+03
Value: 31−45
2000
1500
1000
500
0
ndidatus.Absconditabacteria.o__Candidatus.Absconditabacterales.f_
46−60 (n=7)
80plus (n=3)
61−80 (n=15)
31−45 (n=3)
20−30 (n=2)
Age_category

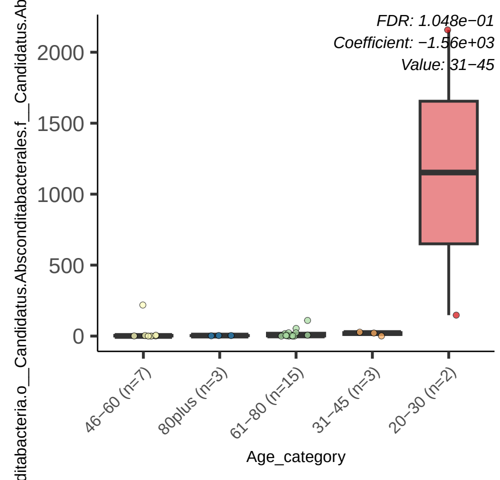

FDR: 1.048e−01
Coefficient: −1.56e+03
Value: 31−45
2000
1500
1000
500
0
ditabacteria.o__Candidatus.Absconditabacterales.f__Candidatus.Ab
46−60 (n=7)
80plus (n=3)
61−80 (n=15)
31−45 (n=3)
20−30 (n=2)
Age_category

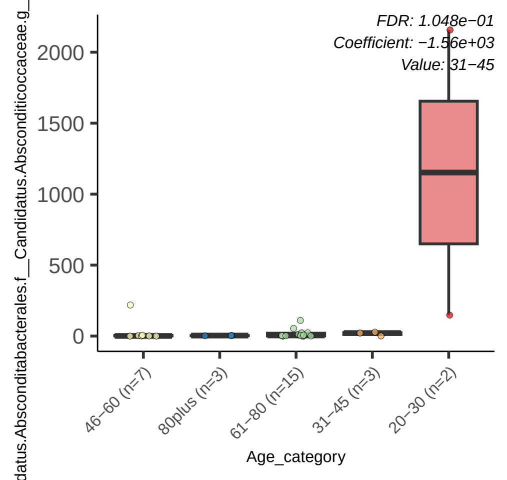

FDR: 1.048e−01
Coefficient: −1.56e+03
Value: 31−45
2000
1500
1000
500
0
datus.Absconditabacterales.f__Candidatus.Absconditicoccaceae.g__
46−60 (n=7)
80plus (n=3)
61−80 (n=15)
31−45 (n=3)
20−30 (n=2)
Age_category

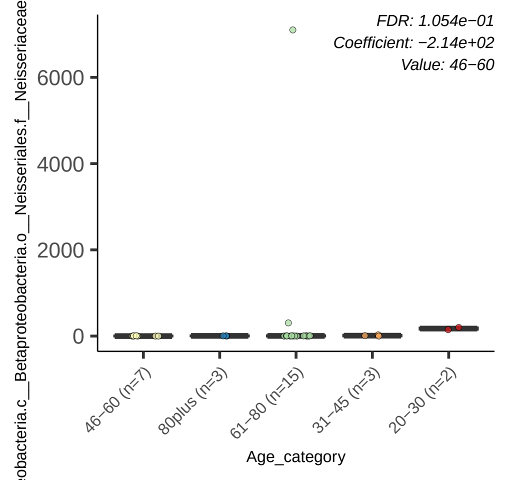

FDR: 1.054e−01
Coefficient: −2.14e+02
Value: 46−60
6000
4000
2000
0
46−60 (n=7)
80plus (n=3)
61−80 (n=15)
31−45 (n=3)
20−30 (n=2)
Age_category
eobacteria.c__Betaproteobacteria.o__Neisseriales.f__Neisseriaceae

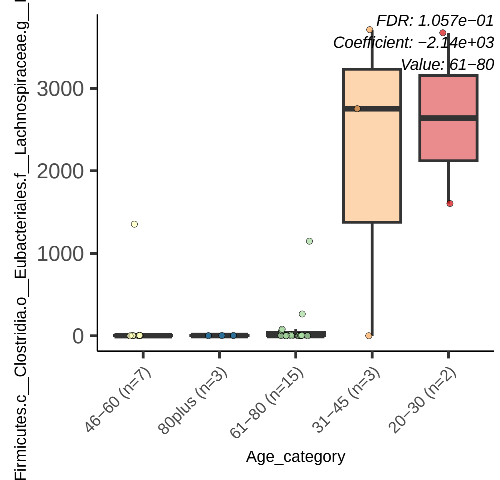

FDR: 1.057e−01
Coefficient: −2.14e+03
Value: 61−80
Firmicutes.c__Clostridia.o__Eubacteriales.f__Lachnospiraceae.g__
3000
2000
1000
0
46−60 (n=7)
80plus (n=3)
61−80 (n=15)
31−45 (n=3)
20−30 (n=2)
Age_category

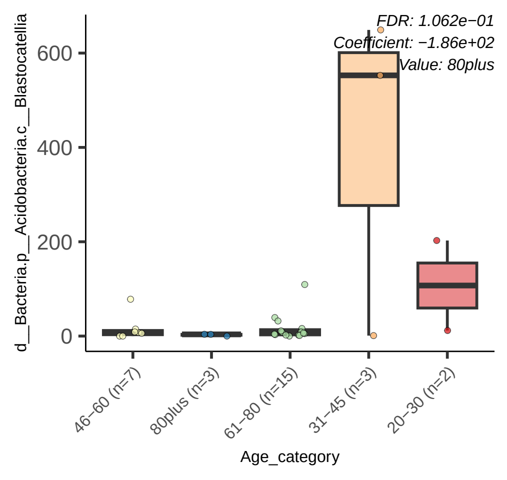

FDR: 1.062e−01
Coefficient: −1.86e+02
Value: 80plus
d__Bacteria.p__Acidobacteria.c__Blastocatellia
600
400
200
0
46−60 (n=7)
80plus (n=3)
61−80 (n=15)
31−45 (n=3)
20−30 (n=2)
Age_category

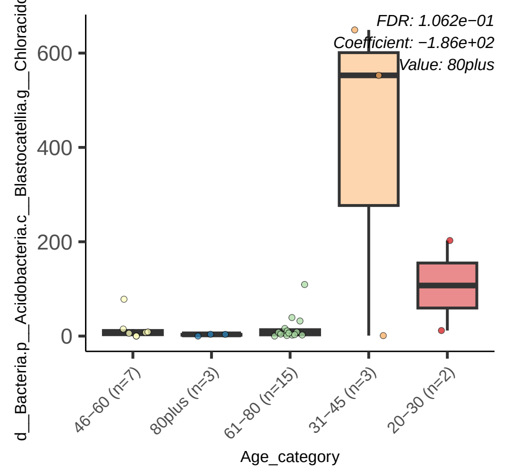

FDR: 1.062e−01
Coefficient: −1.86e+02
Value: 80plus
d__Bacteria.p__Acidobacteria.c__Blastocatellia.g__Chloracido
600
400
200
0
46−60 (n=7)
80plus (n=3)
61−80 (n=15)
31−45 (n=3)
20−30 (n=2)
Age_category

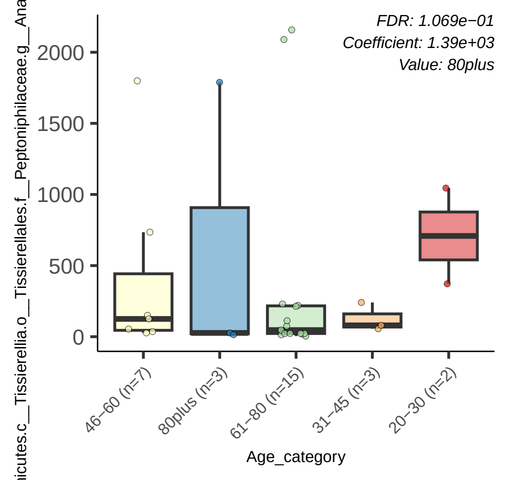

FDR: 1.069e−01
Coefficient: 1.39e+03
Value: 80plus
2000
1500
1000
500
0
46−60 (n=7)
80plus (n=3)
61−80 (n=15)
31−45 (n=3)
20−30 (n=2)
Age_category
icutes.c__Tissierellia.o__Tissierellales.f__Peptoniphilaceae.g__Ana

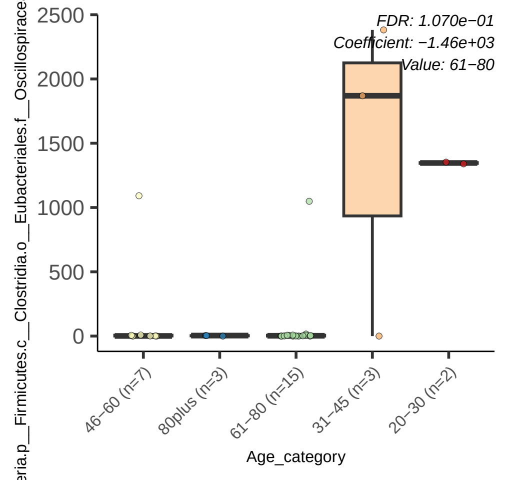

FDR: 1.070e−01
Coefficient: −1.46e+03
Value: 61−80
eria.p__Firmicutes.c__Clostridia.o__Eubacteriales.f__Oscillospirace
2500
2000
1500
1000
500
0
46−60 (n=7)
80plus (n=3)
61−80 (n=15)
31−45 (n=3)
20−30 (n=2)
Age_category

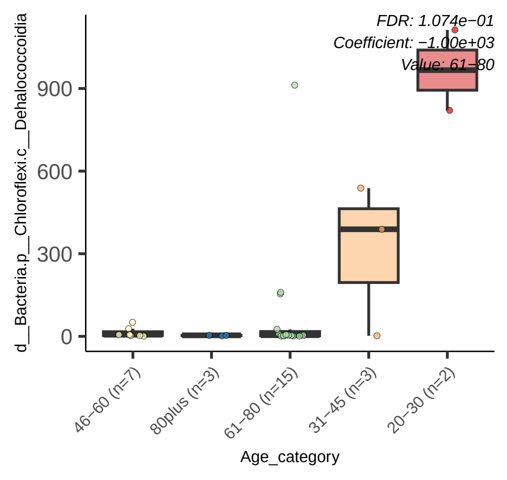
FDR: 1.074e−01
Coefficient: −1.00e+03
Value: 61−80
d__Bacteria.p__Chloroflexi.c__Dehalococcoidia
900
600
300
0
46−60 (n=7)
80plus (n=3)
61−80 (n=15)
31−45 (n=3)
20−30 (n=2)
Age_category

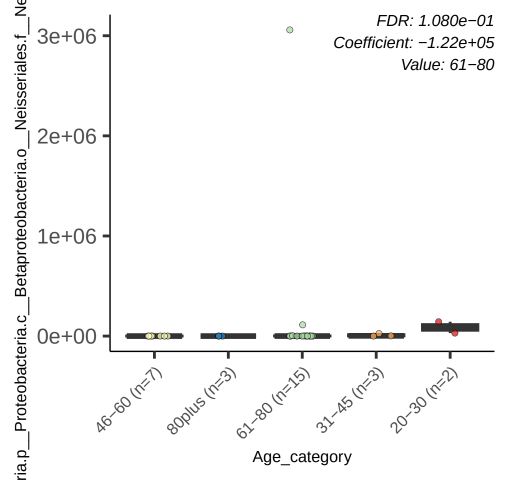
FDR: 1.080e−01
Coefficient: −1.22e+05
Value: 61−80
ria.p__Proteobacteria.c__Betaproteobacteria.o__Neisseriales.f__Ne
3e+06
2e+06
1e+06
0e+00
46−60 (n=7)
80plus (n=3)
61−80 (n=15)
31−45 (n=3)
20−30 (n=2)
Age_category

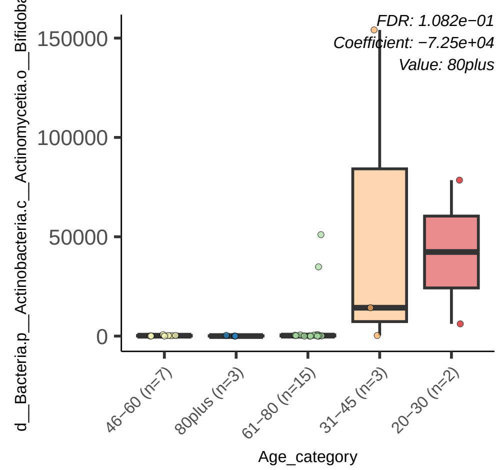
FDR: 1.082e−01
Coefficient: −7.25e+04
Value: 80plus
d__Bacteria.p__Actinobacteria.c__Actinomycetia.o__Bifidoba
150000
100000
50000
0
46−60 (n=7)
80plus (n=3)
61−80 (n=15)
31−45 (n=3)
20−30 (n=2)
Age_category

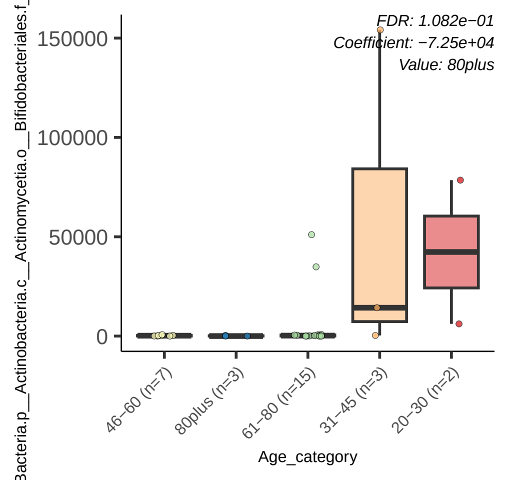

FDR: 1.082e−01
Coefficient: −7.25e+04
Value: 80plus
150000
100000
50000
0
Bacteria.p__Actinobacteria.c__Actinomycetia.o__Bifidobacteriales.f_
46−60 (n=7)
80plus (n=3)
61−80 (n=15)
31−45 (n=3)
20−30 (n=2)
Age_category

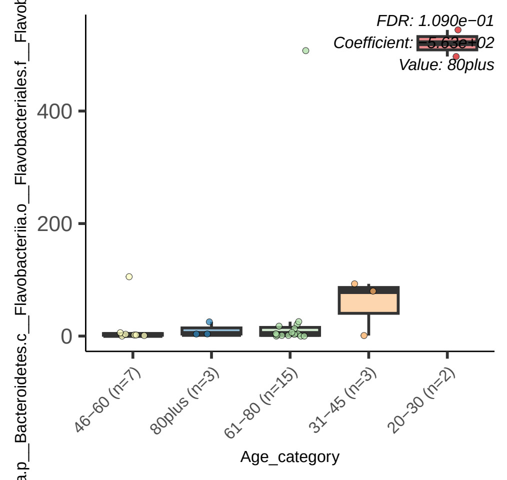

FDR: 1.090e−01
Coefficient: −5.63e+02
Value: 80plus
400
200
0
46−60 (n=7)
80plus (n=3)
61−80 (n=15)
31−45 (n=3)
20−30 (n=2)
Age_category
a.p__Bacteroidetes.c__Flavobacteriia.o__Flavobacteriales.f__Flavob

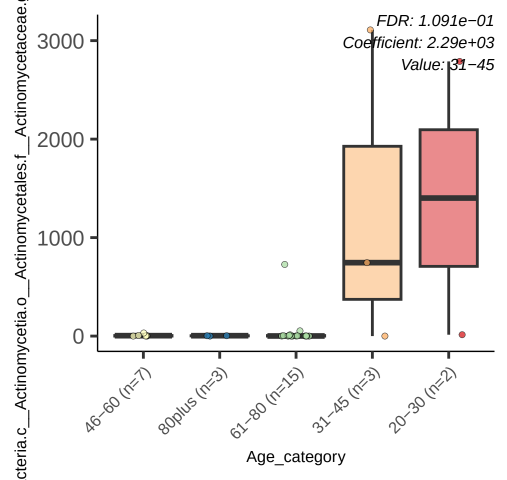

FDR: 1.091e−01
Coefficient: 2.29e+03
Value: 31−45
3000
2000
1000
0
46−60 (n=7)
80plus (n=3)
61−80 (n=15)
31−45 (n=3)
20−30 (n=2)
Age_category
cteria.c__Actinomycetia.o__Actinomycetales.f__Actinomycetaceae.g

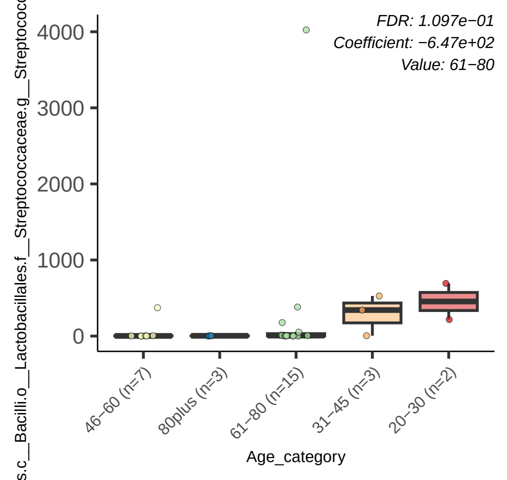

FDR: 1.097e−01
Coefficient: −6.47e+02
Value: 61−80
s.c__Bacilli.o__Lactobacillales.f__Streptococcaceae.g__Streptococc
4000
3000
2000
1000
0
46−60 (n=7)
80plus (n=3)
61−80 (n=15)
31−45 (n=3)
20−30 (n=2)
Age_category

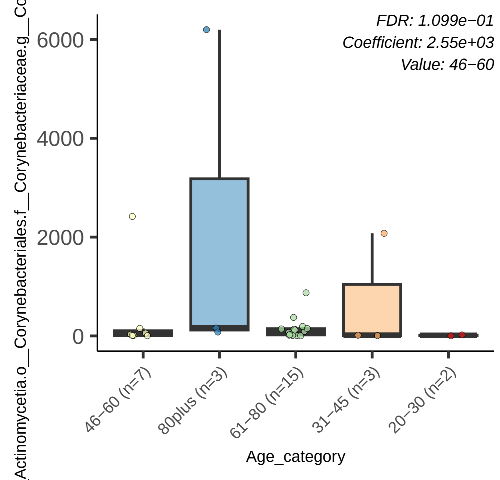

FDR: 1.099e−01
Coefficient: 2.55e+03
Value: 46−60
6000
4000
2000
0
Actinomycetia.o__Corynebacteriales.f__Corynebacteriaceae.g__Co
46−60 (n=7)
80plus (n=3)
61−80 (n=15)
31−45 (n=3)
20−30 (n=2)
Age_category

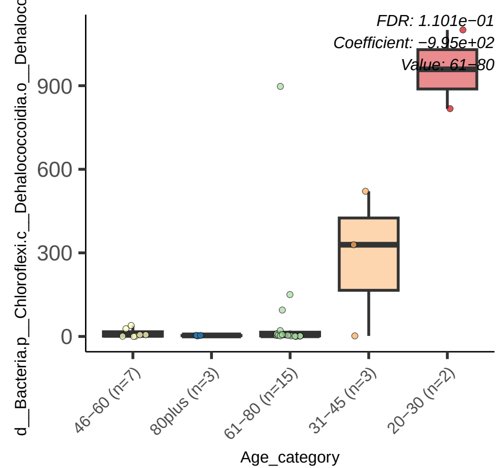

FDR: 1.101e−01
Coefficient: −9.95e+02
Value: 61−80
d__Bacteria.p__Chloroflexi.c__Dehalococcoidia.o__Dehaloco
0
300
600
900
46−60 (n=7)
80plus (n=3)
61−80 (n=15)
31−45 (n=3)
20−30 (n=2)
Age_category

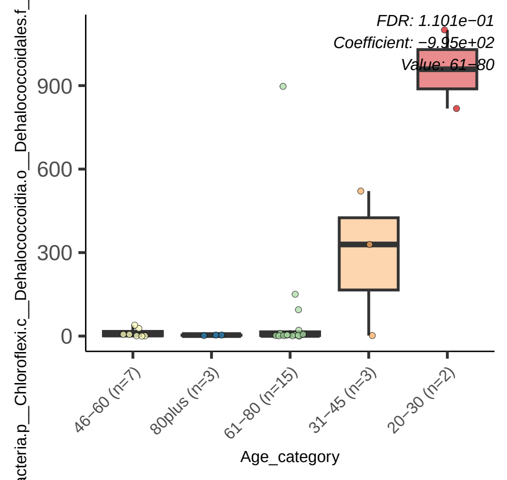

FDR: 1.101e−01
Coefficient: −9.95e+02
Value: 61−80
acteria.p__Chloroflexi.c__Dehalococcoidia.o__Dehalococcoidales.f__
900
600
300
0
46−60 (n=7)
80plus (n=3)
61−80 (n=15)
31−45 (n=3)
20−30 (n=2)
Age_category

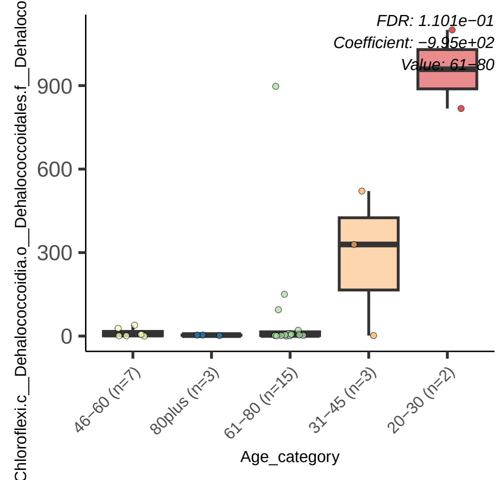
FDR: 1.101e−01
Coefficient: −9.95e+02
Value: 61−80
900
600
300
0
46−60 (n=7)
80plus (n=3)
61−80 (n=15)
31−45 (n=3)
20−30 (n=2)
Age_category
Chloroflexi.c__Dehalococcoidia.o__Dehalococcoidales.f__Dehaloco

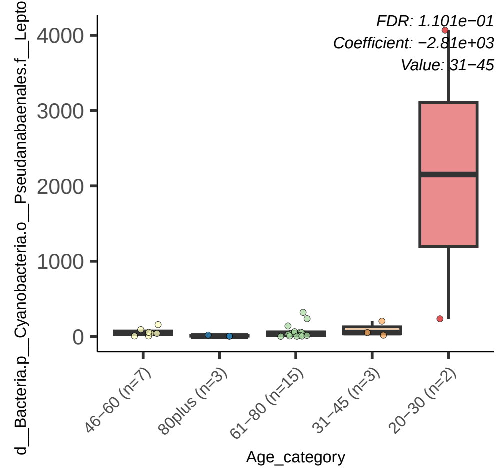

FDR: 1.101e−01
Coefficient: −2.81e+03
Value: 31−45
d__Bacteria.p__Cyanobacteria.o__Pseudanabaenales.f__Lepto
4000
3000
2000
1000
0
46−60 (n=7)
80plus (n=3)
61−80 (n=15)
31−45 (n=3)
20−30 (n=2)
Age_category

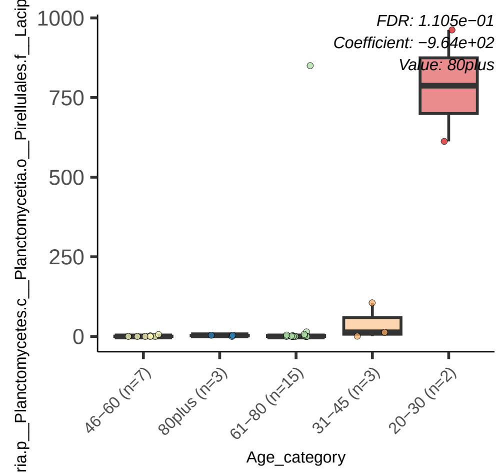
FDR: 1.105e−01
Coefficient: −9.64e+02
Value: 80plus
1000
750
500
250
0
eria.p__Planctomycetes.c__Planctomycetia.o__Pirellulales.f__Lacip
46−60 (n=7)
80plus (n=3)
61−80 (n=15)
31−45 (n=3)
20−30 (n=2)
Age_category

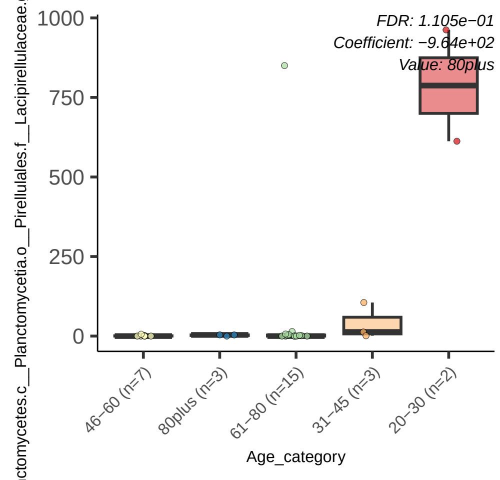

FDR: 1.105e−01
Coefficient: −9.64e+02
Value: 80plus
ctomycetes.c__Planctomycetia.o__Pirellulales.f__Lacipirellulaceae.
1000
750
500
250
0
46−60 (n=7)
80plus (n=3)
61−80 (n=15)
31−45 (n=3)
20−30 (n=2)
Age_category

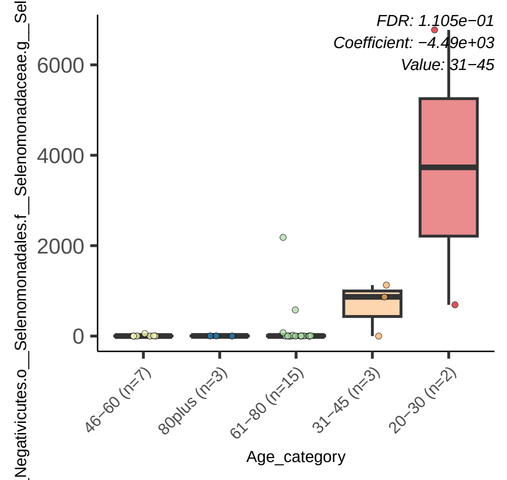

FDR: 1.105e−01
Coefficient: −4.49e+03
Value: 31−45
6000
4000
2000
0
Negativicutes.o__Selenomonadales.f__Selenomonadaceae.g__Sel
46−60 (n=7)
80plus (n=3)
61−80 (n=15)
31−45 (n=3)
20−30 (n=2)
Age_category

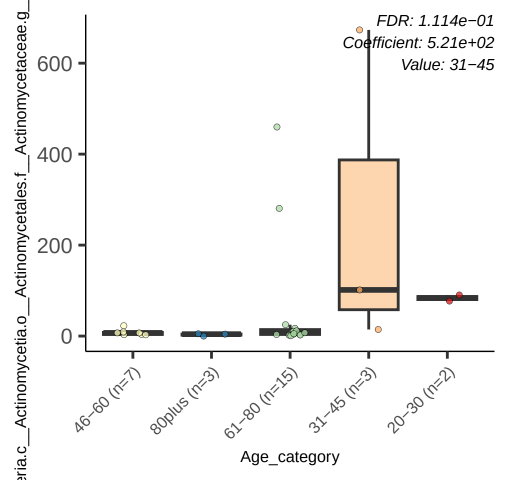

FDR: 1.114e−01
Coefficient: 5.21e+02
Value: 31−45
0
200
400
600
eria.c__Actinomycetia.o__Actinomycetales.f__Actinomycetaceae.g__
46−60 (n=7)
80plus (n=3)
61−80 (n=15)
31−45 (n=3)
20−30 (n=2)
Age_category

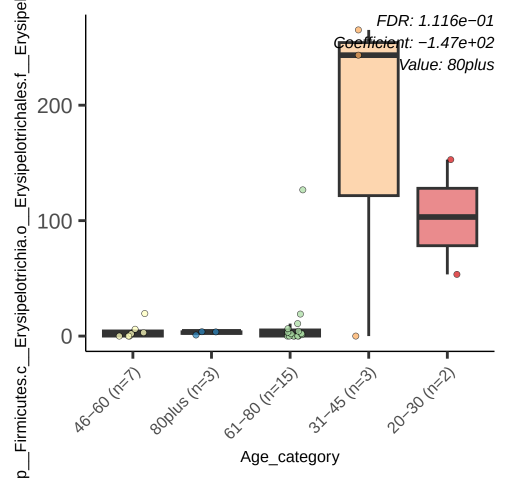
FDR: 1.116e−01
Coefficient: −1.47e+02
Value: 80plus
p__Firmicutes.c__Erysipelotrichia.o__Erysipelotrichales.f__Erysipel
200
100
0
46−60 (n=7)
80plus (n=3)
61−80 (n=15)
31−45 (n=3)
20−30 (n=2)
Age_category

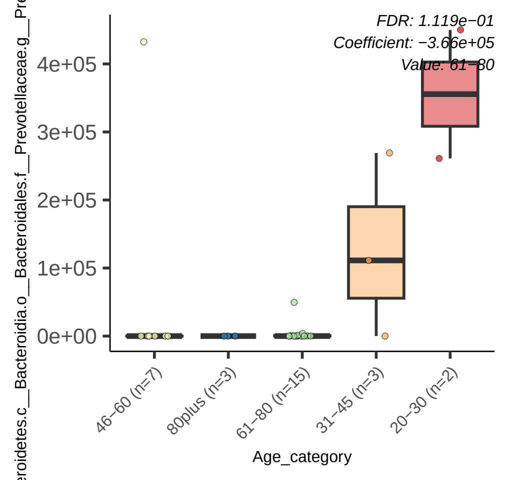

FDR: 1.119e−01
Coefficient: −3.66e+05
Value: 61−80
eroidetes.c__Bacteroidia.o__Bacteroidales.f__Prevotellaceae.g__Pre
4e+05
3e+05
2e+05
1e+05
0e+00
46−60 (n=7)
80plus (n=3)
61−80 (n=15)
31−45 (n=3)
20−30 (n=2)
Age_category

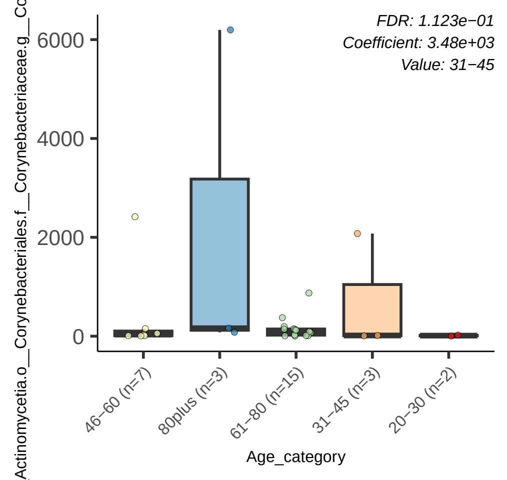

FDR: 1.123e−01
Coefficient: 3.48e+03
Value: 31−45
Actinomycetia.o__Corynebacteriales.f__Corynebacteriaceae.g__Co
6000
4000
2000
0
46−60 (n=7)
80plus (n=3)
61−80 (n=15)
31−45 (n=3)
20−30 (n=2)
Age_category

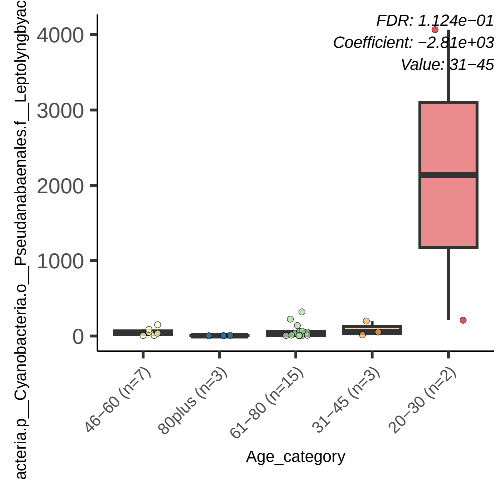

FDR: 1.124e−01
Coefficient: −2.81e+03
Value: 31−45
acteria.p__Cyanobacteria.o__Pseudanabaenales.f__Leptolyngbyac
4000
3000
2000
1000
0
46−60 (n=7)
80plus (n=3)
61−80 (n=15)
31−45 (n=3)
20−30 (n=2)
Age_category

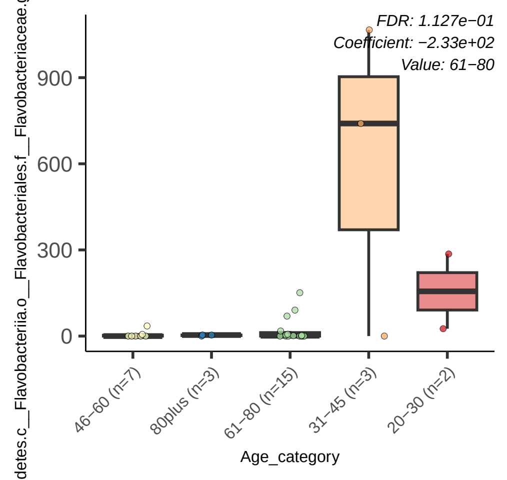

FDR: 1.127e−01
Coefficient: −2.33e+02
Value: 61−80
900
600
300
0
detes.c__Flavobacteriia.o__Flavobacteriales.f__Flavobacteriaceae.g
46−60 (n=7)
80plus (n=3)
61−80 (n=15)
31−45 (n=3)
20−30 (n=2)
Age_category

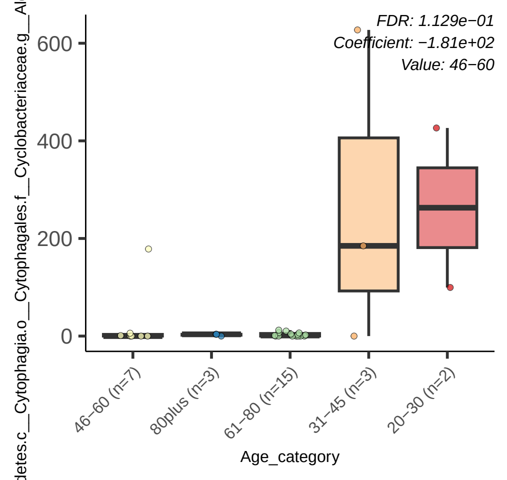

FDR: 1.129e−01
Coefficient: −1.81e+02
Value: 46−60
detes.c__Cytophagia.o__Cytophagales.f__Cyclobacteriaceae.g__Al
600
400
200
0
46−60 (n=7)
80plus (n=3)
61−80 (n=15)
31−45 (n=3)
20−30 (n=2)
Age_category

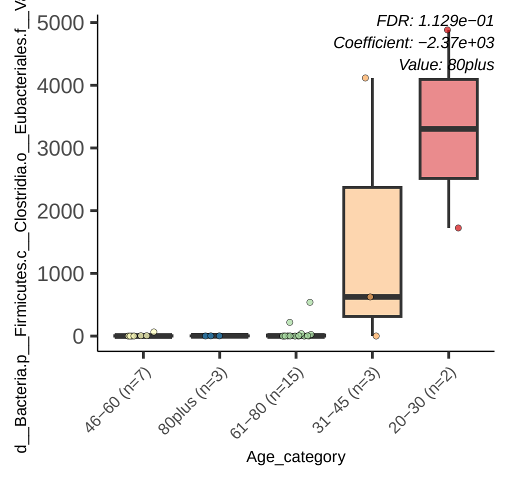
FDR: 1.129e−01
Coefficient: −2.37e+03
Value: 80plus
d__Bacteria.p__Firmicutes.c__Clostridia.o__Eubacteriales.f__
5000
4000
3000
2000
1000
0
46−60 (n=7)
80plus (n=3)
61−80 (n=15)
31−45 (n=3)
20−30 (n=2)
Age_category

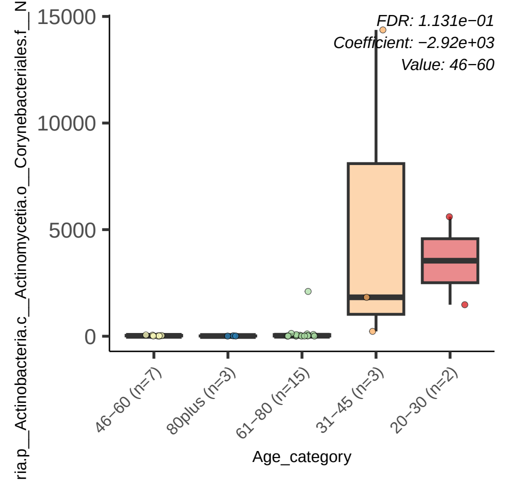

FDR: 1.131e−01
Coefficient: −2.92e+03
Value: 46−60
ria.p__Actinobacteria.c__Actinomycetia.o__Corynebacteriales.f__N
15000
10000
5000
0
46−60 (n=7)
80plus (n=3)
61−80 (n=15)
31−45 (n=3)
20−30 (n=2)
Age_category

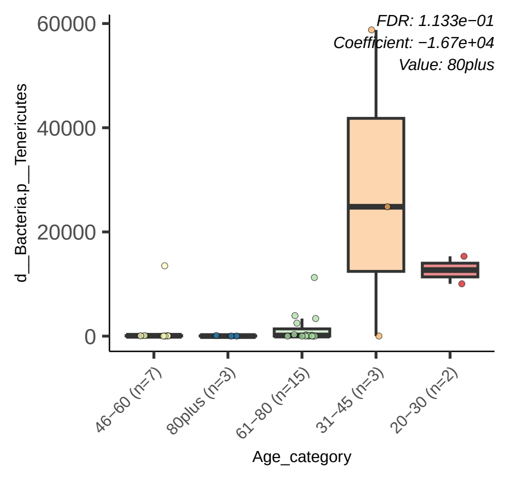

FDR: 1.133e−01
Coefficient: −1.67e+04
Value: 80plus
60000
40000
20000
0
d__Bacteria.p__Tenericutes
46−60 (n=7)
80plus (n=3)
61−80 (n=15)
31−45 (n=3)
20−30 (n=2)
Age_category

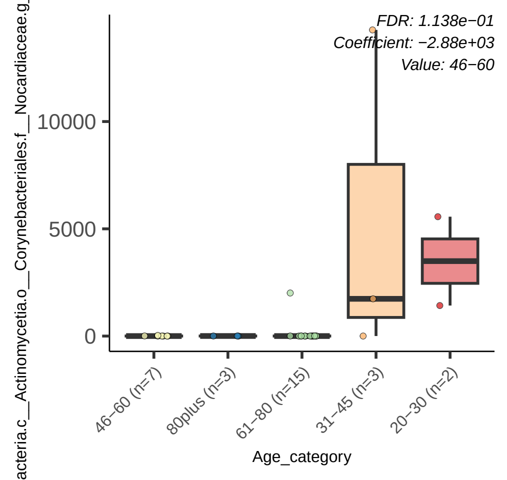
FDR: 1.138e−01
Coefficient: −2.88e+03
Value: 46−60
acteria.c__Actinomycetia.o__Corynebacteriales.f__Nocardiaceae.g_
10000
5000
0
46−60 (n=7)
80plus (n=3)
61−80 (n=15)
31−45 (n=3)
20−30 (n=2)
Age_category

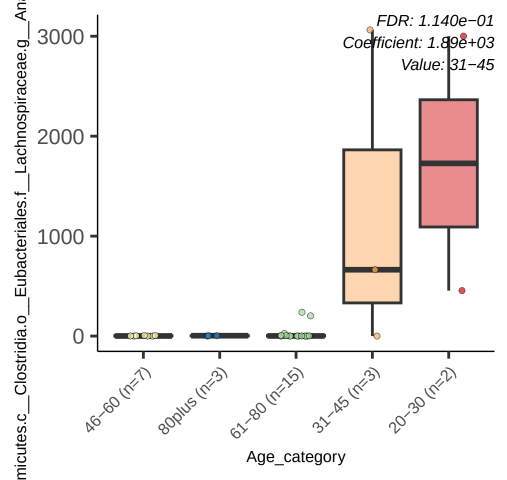
FDR: 1.140e−01
Coefficient: 1.89e+03
Value: 31−45
3000
2000
1000
0
46−60 (n=7)
80plus (n=3)
61−80 (n=15)
31−45 (n=3)
20−30 (n=2)
Age_category
micutes.c__Clostridia.o__Eubacteriales.f__Lachnospiraceae.g__An

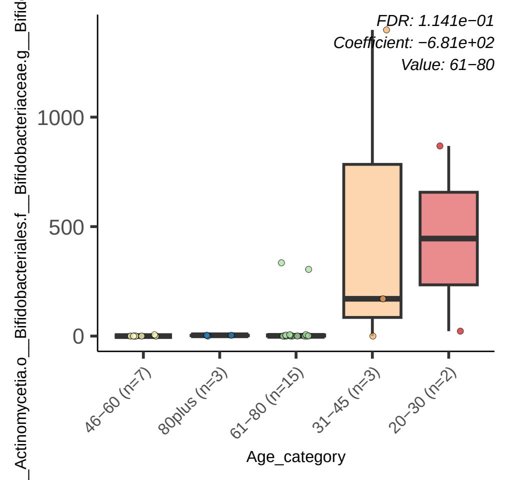

FDR: 1.141e−01
Coefficient: −6.81e+02
Value: 61−80
1000
500
0
Actinomycetia.o__Bifidobacteriales.f__Bifidobacteriaceae.g__Bifid
46−60 (n=7)
80plus (n=3)
61−80 (n=15)
31−45 (n=3)
20−30 (n=2)
Age_category

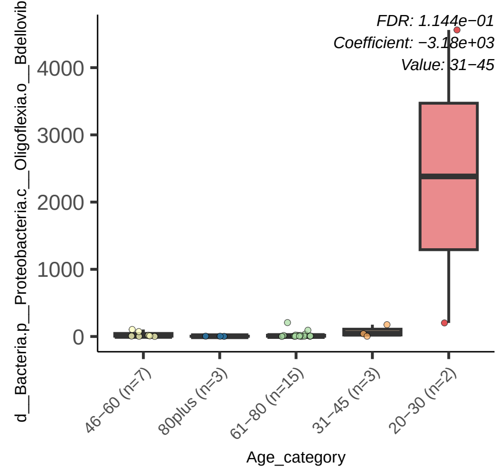

FDR: 1.144e−01
Coefficient: −3.18e+03
Value: 31−45
d__Bacteria.p__Proteobacteria.c__Oligoflexia.o__Bdellovib
4000
3000
2000
1000
0
46−60 (n=7)
80plus (n=3)
61−80 (n=15)
31−45 (n=3)
20−30 (n=2)
Age_category

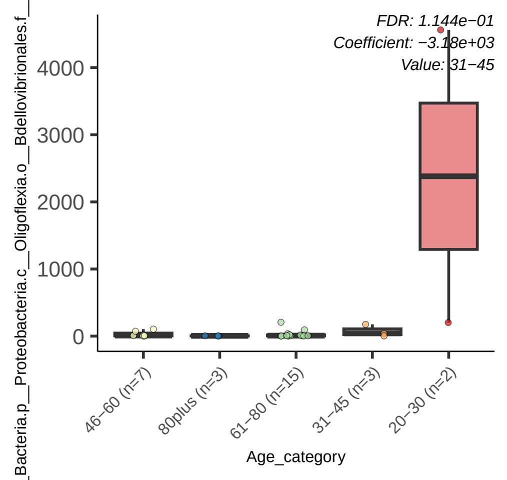

FDR: 1.144e−01
Coefficient: −3.18e+03
Value: 31−45
Bacteria.p__Proteobacteria.c__Oligoflexia.o__Bdellovibrionales.f
4000
3000
2000
1000
0
46−60 (n=7)
80plus (n=3)
61−80 (n=15)
31−45 (n=3)
20−30 (n=2)
Age_category

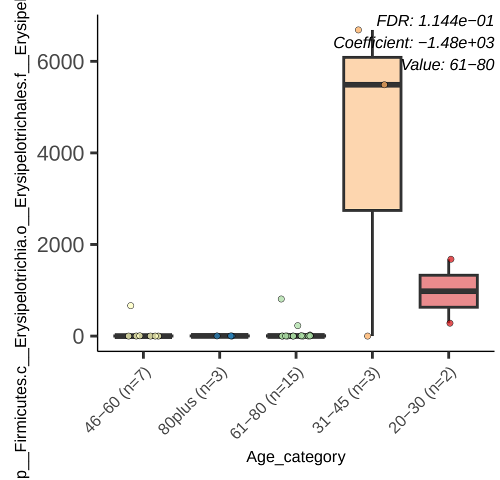

FDR: 1.144e−01
Coefficient: −1.48e+03
Value: 61−80
6000
4000
2000
0
p__Firmicutes.c__Erysipelotrichia.o__Erysipelotrichales.f__Erysipel
46−60 (n=7)
80plus (n=3)
61−80 (n=15)
31−45 (n=3)
20−30 (n=2)
Age_category

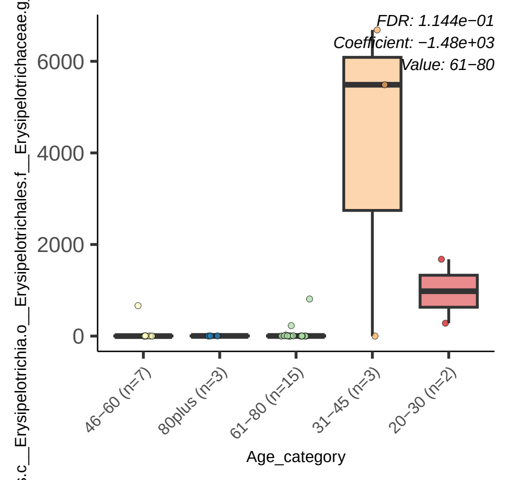

FDR: 1.144e−01
Coefficient: −1.48e+03
Value: 61−80
6000
4000
2000
0
s.c__Erysipelotrichia.o__Erysipelotrichales.f__Erysipelotrichaceae.g
46−60 (n=7)
80plus (n=3)
61−80 (n=15)
31−45 (n=3)
20−30 (n=2)
Age_category

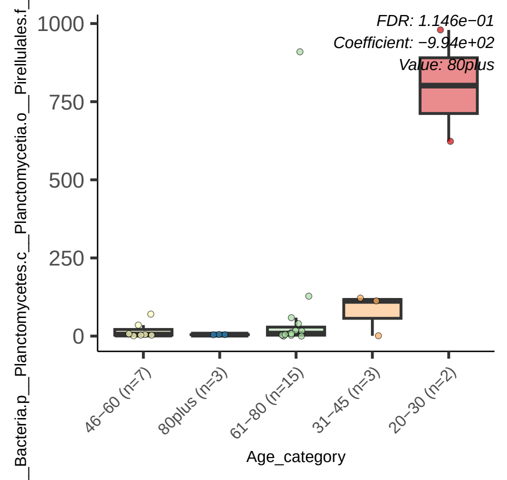

FDR: 1.146e−01
Coefficient: −9.94e+02
Value: 80plus
Bacteria.p__Planctomycetes.c__Planctomycetia.o__Pirellulales.f__
1000
750
500
250
0
46−60 (n=7)
80plus (n=3)
61−80 (n=15)
31−45 (n=3)
20−30 (n=2)
Age_category

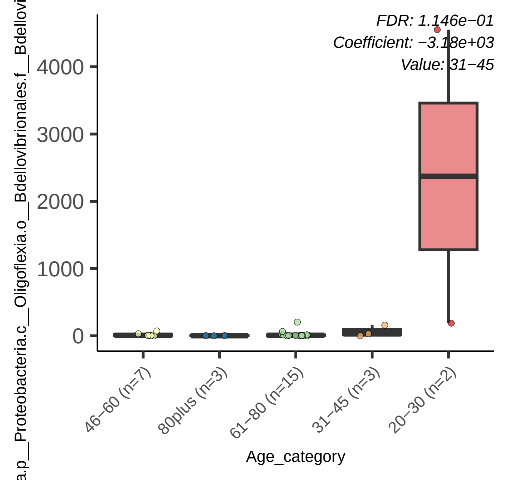

FDR: 1.146e−01
Coefficient: −3.18e+03
Value: 31−45
a.p__Proteobacteria.c__Oligoflexia.o__Bdellovibrionales.f__Bdellovi
4000
3000
2000
1000
0
46−60 (n=7)
80plus (n=3)
61−80 (n=15)
31−45 (n=3)
20−30 (n=2)
Age_category

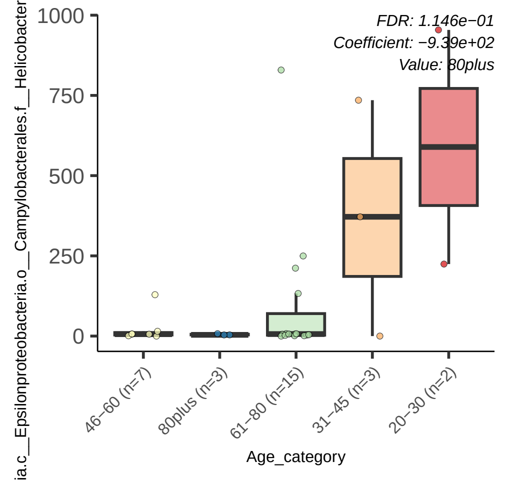

FDR: 1.146e−01
Coefficient: −9.39e+02
Value: 80plus
1000
750
500
250
0
ia.c__Epsilonproteobacteria.o__Campylobacterales.f__Helicobacter
46−60 (n=7)
80plus (n=3)
61−80 (n=15)
31−45 (n=3)
20−30 (n=2)
Age_category

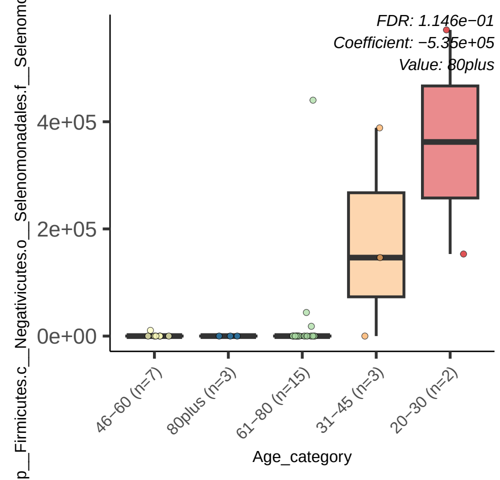
FDR: 1.146e−01
Coefficient: −5.35e+05
Value: 80plus
p__Firmicutes.c__Negativicutes.o__Selenomonadales.f__Selenomo
4e+05
2e+05
0e+00
46−60 (n=7)
80plus (n=3)
61−80 (n=15)
31−45 (n=3)
20−30 (n=2)
Age_category

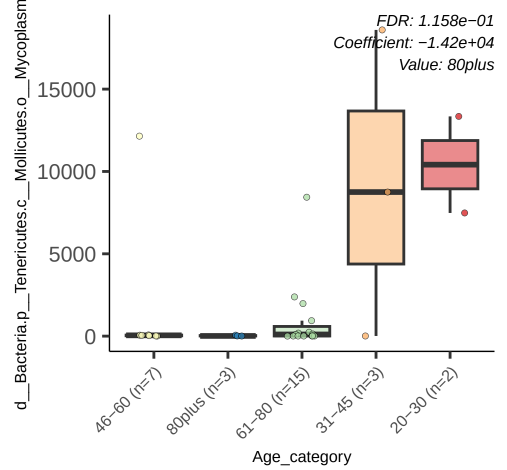
FDR: 1.158e−01
Coefficient: −1.42e+04
Value: 80plus
d__Bacteria.p__Tenericutes.c__Mollicutes.o__Mycoplasm
15000
10000
5000
0
46−60 (n=7)
80plus (n=3)
61−80 (n=15)
31−45 (n=3)
20−30 (n=2)
Age_category

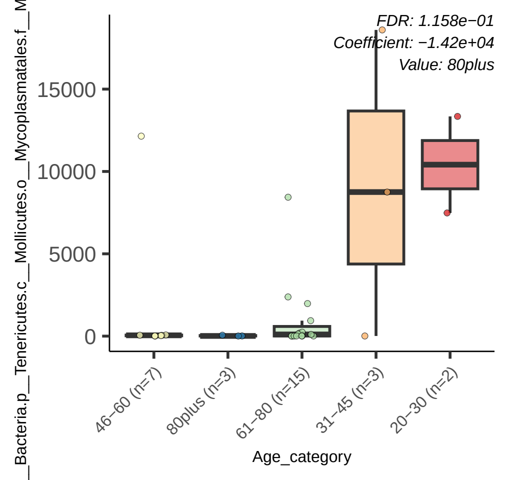
FDR: 1.158e−01
Coefficient: −1.42e+04
Value: 80plus
15000
10000
5000
0
Bacteria.p__Tenericutes.c__Mollicutes.o__Mycoplasmatales.f__M
46−60 (n=7)
80plus (n=3)
61−80 (n=15)
31−45 (n=3)
20−30 (n=2)
Age_category

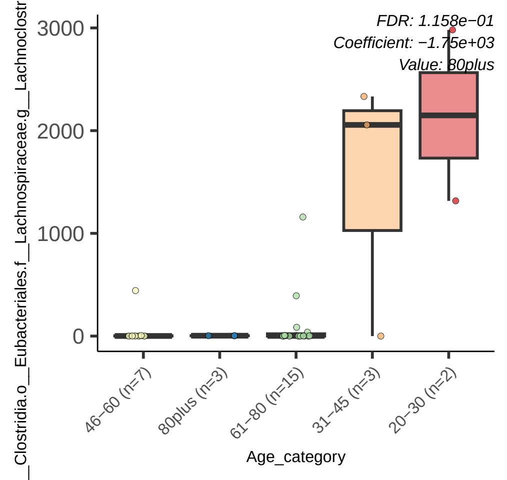

FDR: 1.158e−01
Coefficient: −1.75e+03
Value: 80plus
3000
2000
1000
0
46−60 (n=7)
80plus (n=3)
61−80 (n=15)
31−45 (n=3)
20−30 (n=2)
Age_category
Clostridia.o__Eubacteriales.f__Lachnospiraceae.g__Lachnoclostr

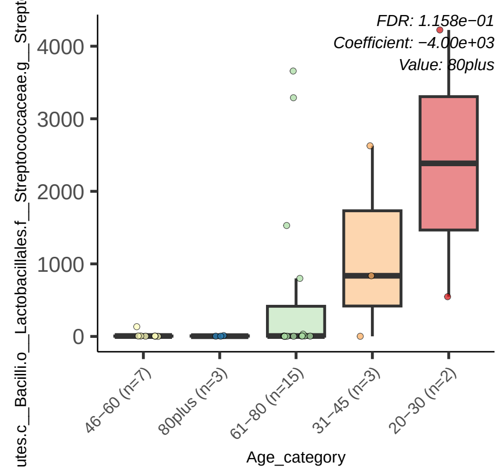

FDR: 1.158e−01
Coefficient: −4.00e+03
Value: 80plus
icutes.c__Bacilli.o__Lactobacillales.f__Streptococcaceae.g__Strept
0
1000
2000
3000
4000
46−60 (n=7)
80plus (n=3)
61−80 (n=15)
31−45 (n=3)
20−30 (n=2)
Age_category

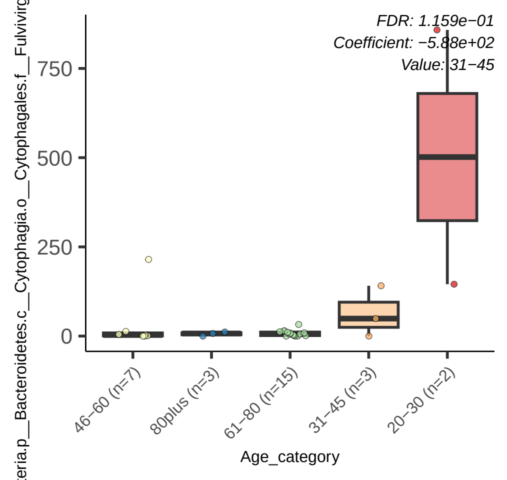
FDR: 1.159e−01
Coefficient: −5.88e+02
Value: 31−45
750
500
250
0
46−60 (n=7)
80plus (n=3)
61−80 (n=15)
31−45 (n=3)
20−30 (n=2)
Age_category
teria.p__Bacteroidetes.c__Cytophagia.o__Cytophagales.f__Fulvivirg

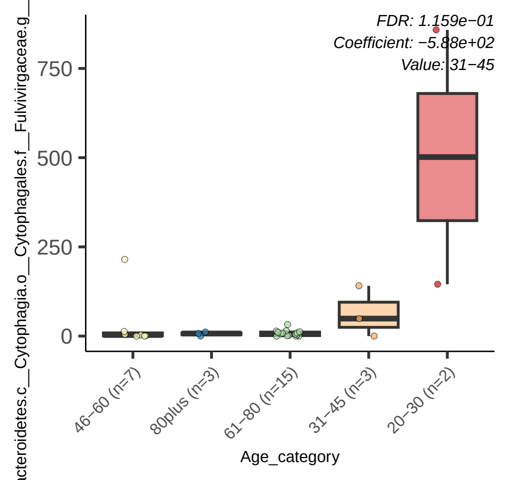
FDR: 1.159e−01
Coefficient: −5.88e+02
Value: 31−45
acteroidetes.c__Cytophagia.o__Cytophagales.f__Fulvivirgaceae.g__
750
500
250
0
46−60 (n=7)
80plus (n=3)
61−80 (n=15)
31−45 (n=3)
20−30 (n=2)
Age_category

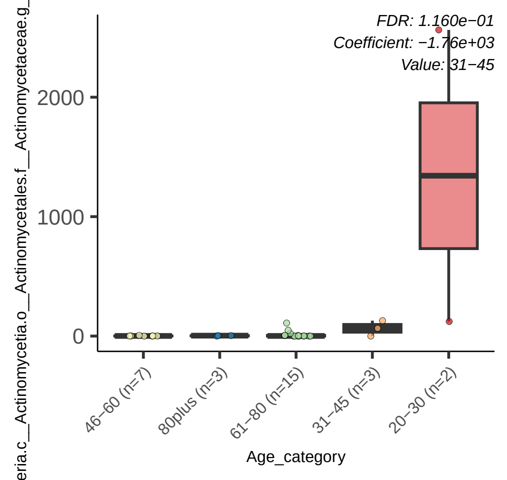

FDR: 1.160e−01
Coefficient: −1.76e+03
Value: 31−45
2000
1000
0
eria.c__Actinomycetia.o__Actinomycetales.f__Actinomycetaceae.g_
46−60 (n=7)
80plus (n=3)
61−80 (n=15)
31−45 (n=3)
20−30 (n=2)
Age_category

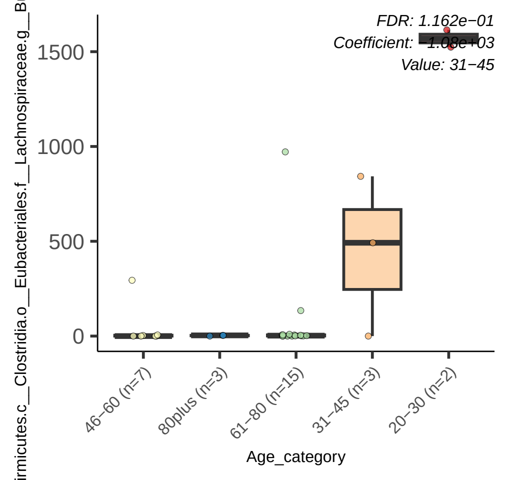

FDR: 1.162e−01
Coefficient: −1.08e+03
Value: 31−45
1500
1000
500
0
46−60 (n=7)
80plus (n=3)
61−80 (n=15)
31−45 (n=3)
20−30 (n=2)
Age_category
irmicutes.c__Clostridia.o__Eubacteriales.f__Lachnospiraceae.g__B

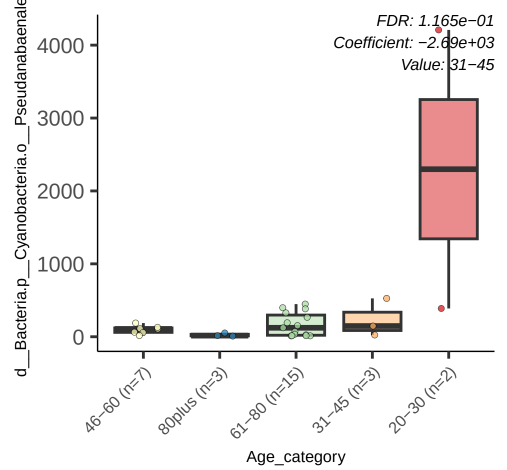
FDR: 1.165e−01
Coefficient: −2.69e+03
Value: 31−45
d__Bacteria.p__Cyanobacteria.o__Pseudanabaenale
4000
3000
2000
1000
0
46−60 (n=7)
80plus (n=3)
61−80 (n=15)
31−45 (n=3)
20−30 (n=2)
Age_category

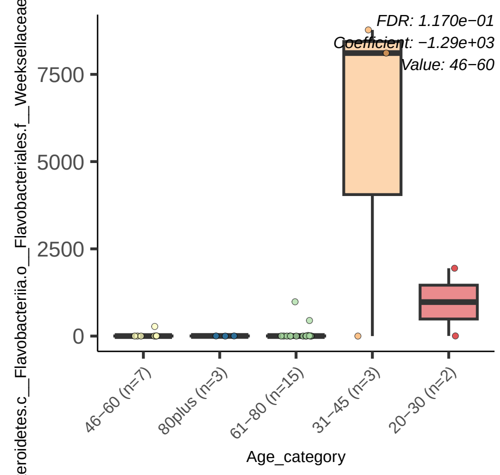

FDR: 1.170e−01
Coefficient: −1.29e+03
Value: 46−60
7500
5000
2500
0
46−60 (n=7)
80plus (n=3)
61−80 (n=15)
31−45 (n=3)
20−30 (n=2)
Age_category
teroidetes.c__Flavobacteriia.o__Flavobacteriales.f__Weeksellaceae

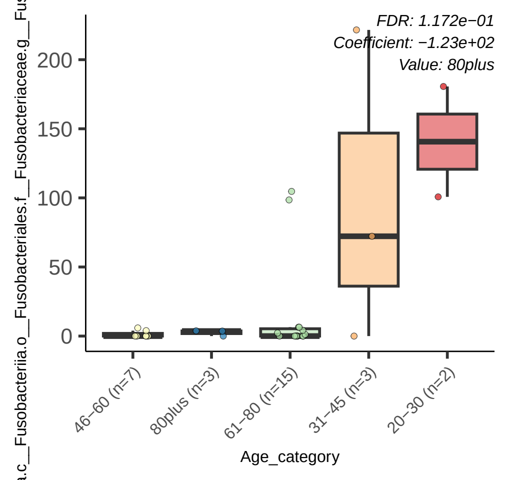

FDR: 1.172e−01
Coefficient: −1.23e+02
Value: 80plus
a.c__Fusobacteriia.o__Fusobacteriales.f__Fusobacteriaceae.g__Fus
200
150
100
50
0
46−60 (n=7)
80plus (n=3)
61−80 (n=15)
31−45 (n=3)
20−30 (n=2)
Age_category

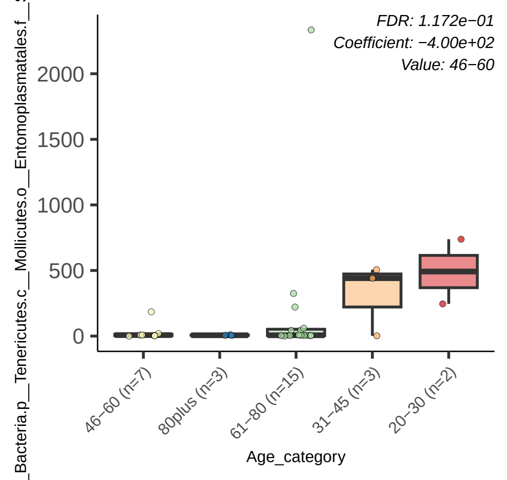

FDR: 1.172e−01
Coefficient: −4.00e+02
Value: 46−60
2000
1500
1000
500
0
Bacteria.p__Tenericutes.c__Mollicutes.o__Entomoplasmatales.f__
46−60 (n=7)
80plus (n=3)
61−80 (n=15)
31−45 (n=3)
20−30 (n=2)
Age_category

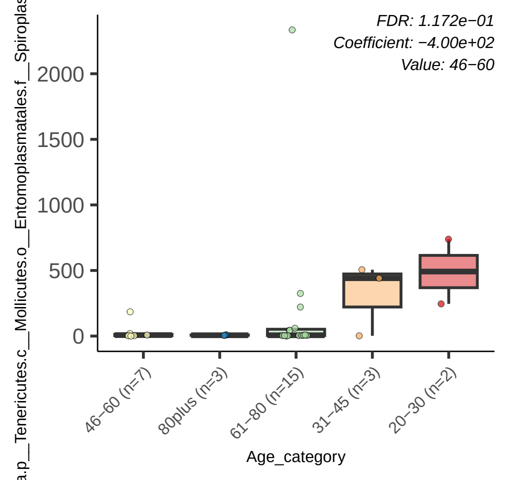

FDR: 1.172e−01
Coefficient: −4.00e+02
Value: 46−60
a.p__Tenericutes.c__Mollicutes.o__Entomoplasmatales.f__Spiroplas
2000
1500
1000
500
0
46−60 (n=7)
80plus (n=3)
61−80 (n=15)
31−45 (n=3)
20−30 (n=2)
Age_category

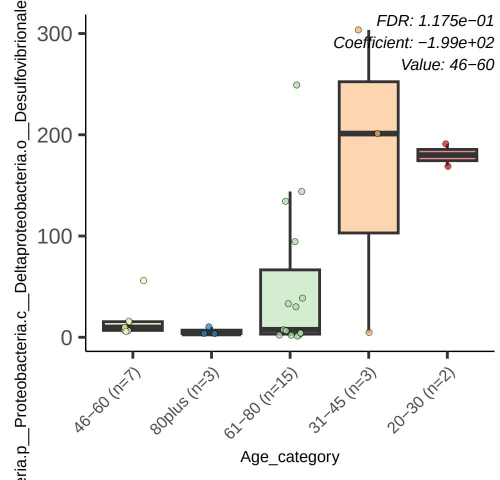

FDR: 1.175e−01
Coefficient: −1.99e+02
Value: 46−60
300
200
100
0
eria.p__Proteobacteria.c__Deltaproteobacteria.o__Desulfovibrionale
46−60 (n=7)
80plus (n=3)
61−80 (n=15)
31−45 (n=3)
20−30 (n=2)
Age_category

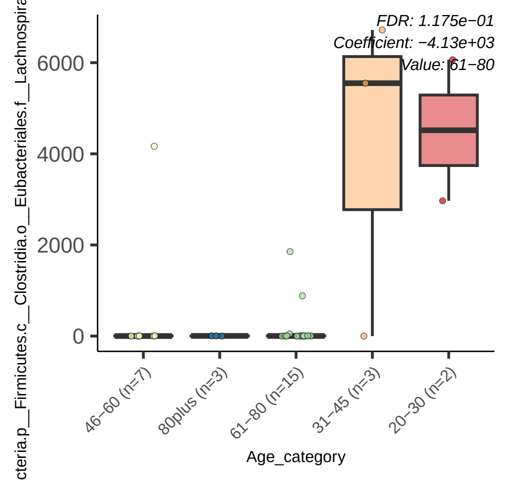

FDR: 1.175e−01
Coefficient: −4.13e+03
Value: 61−80
6000
4000
2000
0
cteria.p__Firmicutes.c__Clostridia.o__Eubacteriales.f__Lachnospira
46−60 (n=7)
80plus (n=3)
61−80 (n=15)
31−45 (n=3)
20−30 (n=2)
Age_category

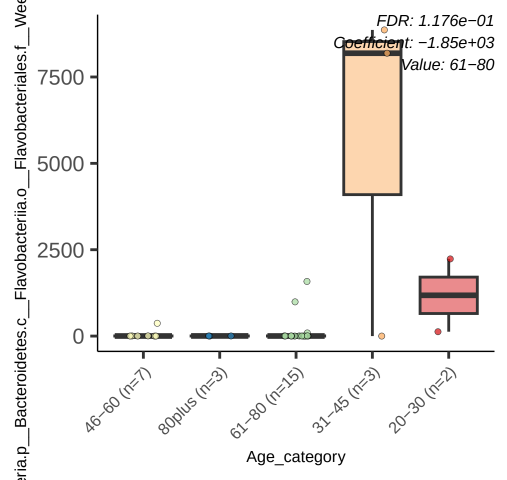

FDR: 1.176e−01
Coefficient: −1.85e+03
Value: 61−80
7500
5000
2500
0
eria.p__Bacteroidetes.c__Flavobacteriia.o__Flavobacteriales.f__Wee
46−60 (n=7)
80plus (n=3)
61−80 (n=15)
31−45 (n=3)
20−30 (n=2)
Age_category

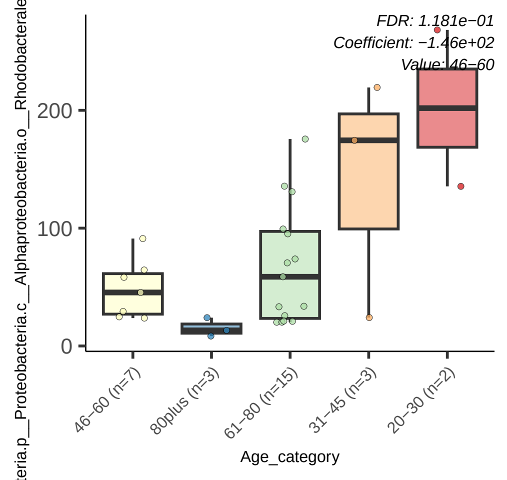
FDR: 1.181e−01
Coefficient: −1.46e+02
Value: 46−60
teria.p__Proteobacteria.c__Alphaproteobacteria.o__Rhodobacterale
200
100
0
46−60 (n=7)
80plus (n=3)
61−80 (n=15)
31−45 (n=3)
20−30 (n=2)
Age_category

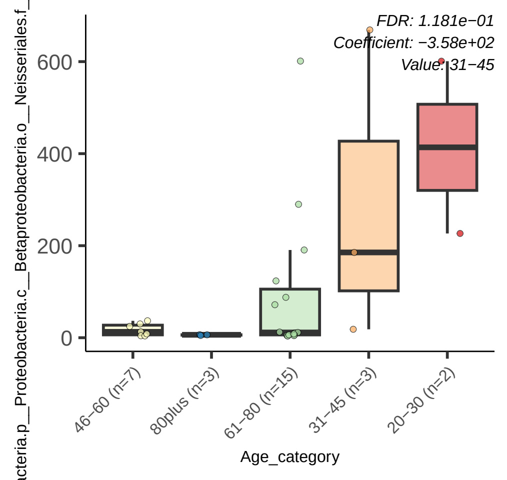

FDR: 1.181e−01
Coefficient: −3.58e+02
Value: 31−45
acteria.p__Proteobacteria.c__Betaproteobacteria.o__Neisseriales.f__
600
400
200
0
46−60 (n=7)
80plus (n=3)
61−80 (n=15)
31−45 (n=3)
20−30 (n=2)
Age_category

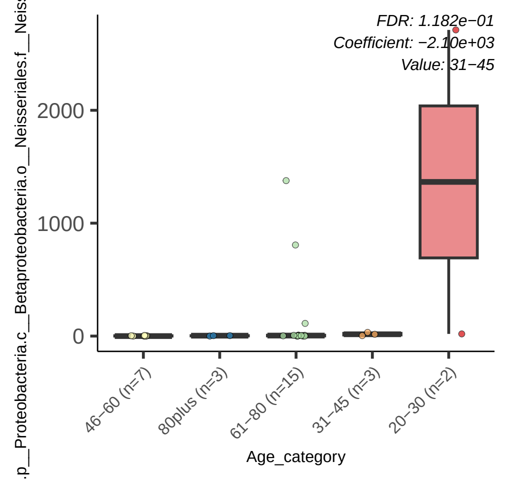

FDR: 1.182e−01
Coefficient: −2.10e+03
Value: 31−45
2000
1000
0
46−60 (n=7)
80plus (n=3)
61−80 (n=15)
31−45 (n=3)
20−30 (n=2)
Age_category
.p__Proteobacteria.c__Betaproteobacteria.o__Neisseriales.f__Neiss

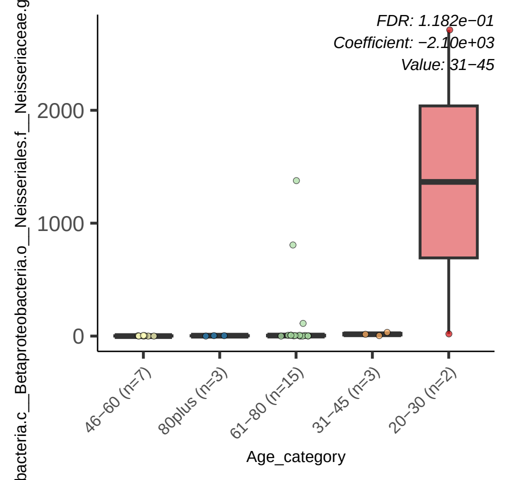

FDR: 1.182e−01
Coefficient: −2.10e+03
Value: 31−45
2000
1000
0
46−60 (n=7)
80plus (n=3)
61−80 (n=15)
31−45 (n=3)
20−30 (n=2)
Age_category
bacteria.c__Betaproteobacteria.o__Neisseriales.f__Neisseriaceae.g

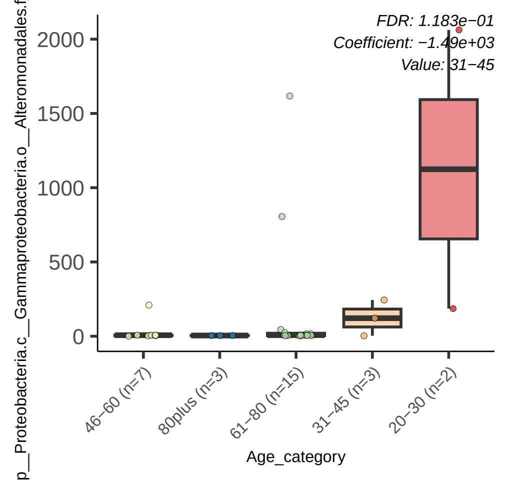

FDR: 1.183e−01
Coefficient: −1.49e+03
Value: 31−45
p__Proteobacteria.c__Gammaproteobacteria.o__Alteromonadales.f
2000
1500
1000
500
0
46−60 (n=7)
80plus (n=3)
61−80 (n=15)
31−45 (n=3)
20−30 (n=2)
Age_category

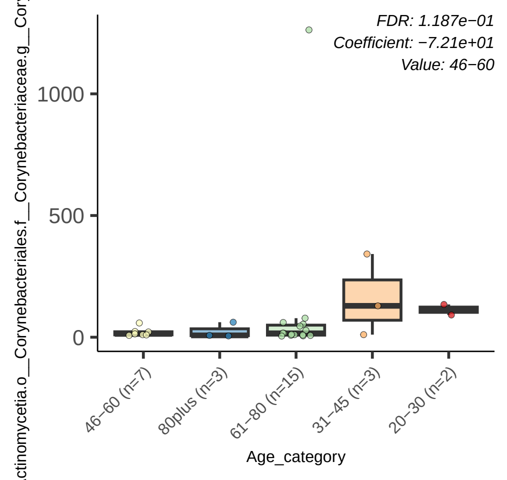

FDR: 1.187e−01
Coefficient: −7.21e+01
Value: 46−60
ctinomycetia.o__Corynebacteriales.f__Corynebacteriaceae.g__Cory
1000
500
0
46−60 (n=7)
80plus (n=3)
61−80 (n=15)
31−45 (n=3)
20−30 (n=2)
Age_category

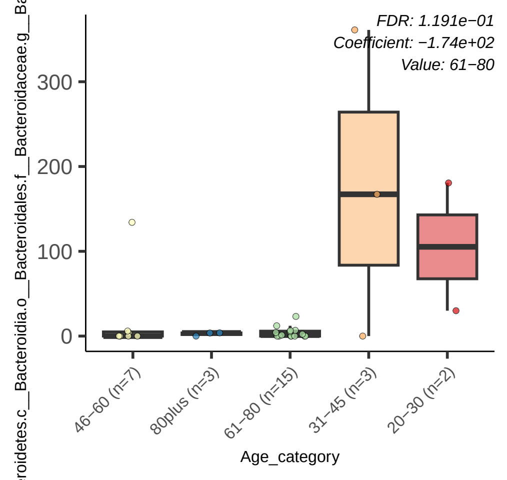

FDR: 1.191e−01
Coefficient: −1.74e+02
Value: 61−80
300
200
100
0
roidetes.c__Bacteroidia.o__Bacteroidales.f__Bacteroidaceae.g__Ba
46−60 (n=7)
80plus (n=3)
61−80 (n=15)
31−45 (n=3)
20−30 (n=2)
Age_category

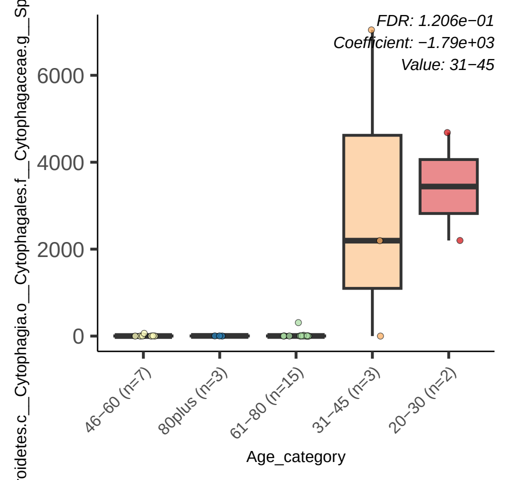

FDR: 1.206e−01
Coefficient: −1.79e+03
Value: 31−45
6000
4000
2000
0
roidetes.c__Cytophagia.o__Cytophagales.f__Cytophagaceae.g__Sp
46−60 (n=7)
80plus (n=3)
61−80 (n=15)
31−45 (n=3)
20−30 (n=2)
Age_category

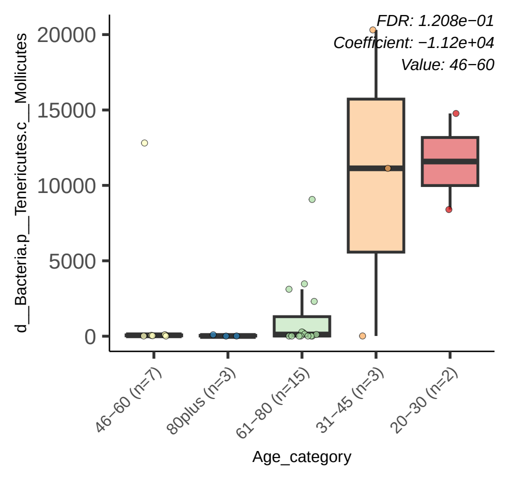

FDR: 1.208e−01
Coefficient: −1.12e+04
Value: 46−60
d__Bacteria.p__Tenericutes.c__Mollicutes
20000
15000
10000
5000
0
46−60 (n=7)
80plus (n=3)
61−80 (n=15)
31−45 (n=3)
20−30 (n=2)
Age_category

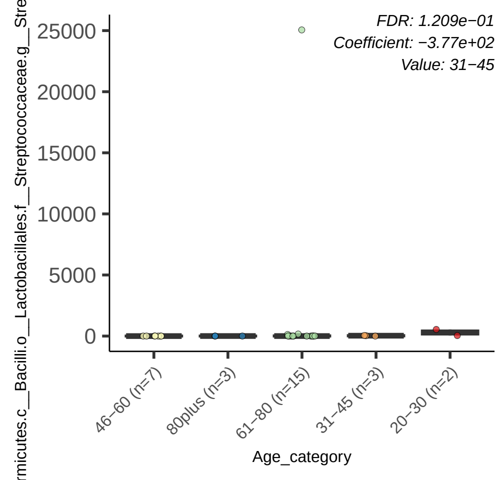

FDR: 1.209e−01
Coefficient: −3.77e+02
Value: 31−45
25000
20000
15000
10000
5000
0
46−60 (n=7)
80plus (n=3)
61−80 (n=15)
31−45 (n=3)
20−30 (n=2)
Age_category
rmicutes.c__Bacilli.o__Lactobacillales.f__Streptococcaceae.g__Stre

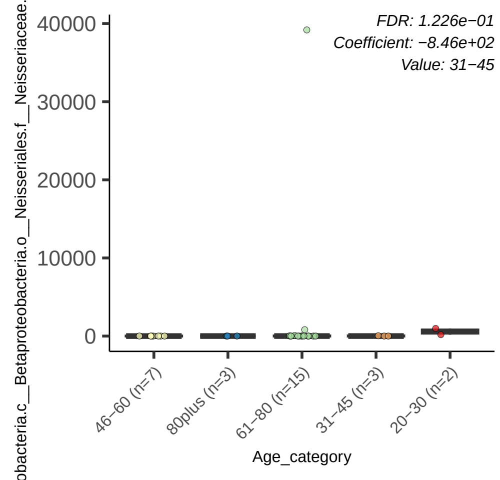

FDR: 1.226e−01
Coefficient: −8.46e+02
Value: 31−45
obacteria.c__Betaproteobacteria.o__Neisseriales.f__Neisseriaceae.
40000
30000
20000
10000
0
46−60 (n=7)
80plus (n=3)
61−80 (n=15)
31−45 (n=3)
20−30 (n=2)
Age_category

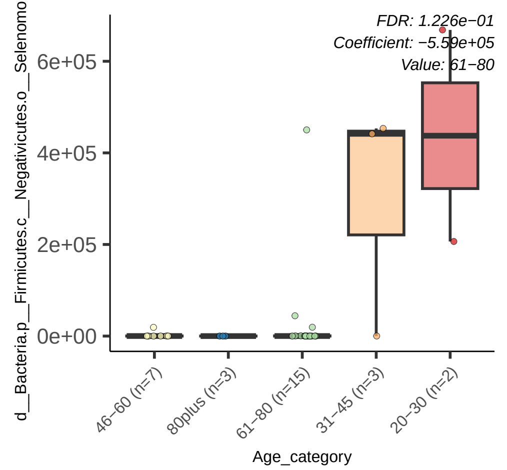
FDR: 1.226e−01
Coefficient: −5.59e+05
Value: 61−80
d__Bacteria.p__Firmicutes.c__Negativicutes.o__Selenomo
6e+05
4e+05
2e+05
0e+00
46−60 (n=7)
80plus (n=3)
61−80 (n=15)
31−45 (n=3)
20−30 (n=2)
Age_category

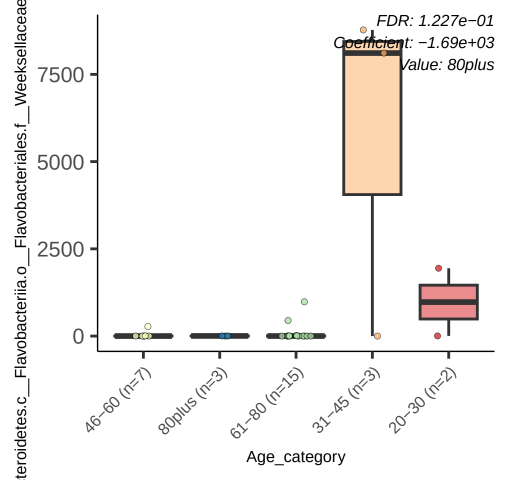

FDR: 1.227e−01
Coefficient: −1.69e+03
Value: 80plus
7500
5000
2500
0
46−60 (n=7)
80plus (n=3)
61−80 (n=15)
31−45 (n=3)
20−30 (n=2)
Age_category

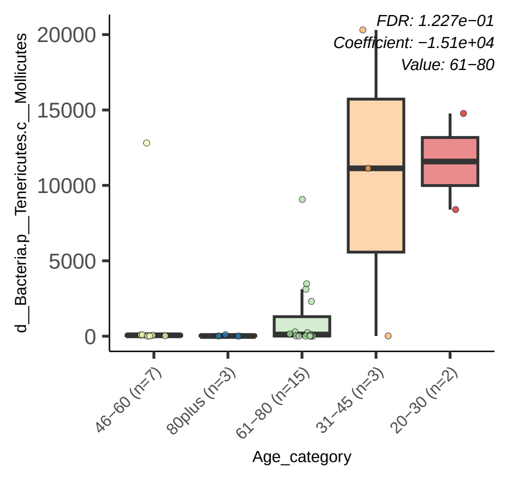

FDR: 1.227e−01
Coefficient: −1.51e+04
Value: 61−80
d__Bacteria.p__Tenericutes.c__Mollicutes
20000
15000
10000
5000
0
46−60 (n=7)
80plus (n=3)
61−80 (n=15)
31−45 (n=3)
20−30 (n=2)
Age_category

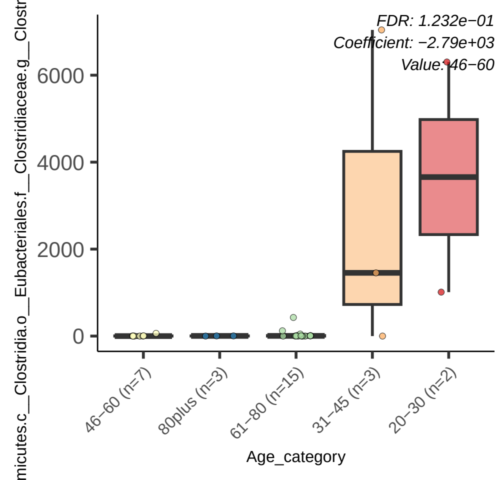

FDR: 1.232e−01
Coefficient: −2.79e+03
Value: 46−60
6000
4000
2000
0
micutes.c__Clostridia.o__Eubacteriales.f__Clostridiaceae.g__Clostr
46−60 (n=7)
80plus (n=3)
61−80 (n=15)
31−45 (n=3)
20−30 (n=2)
Age_category

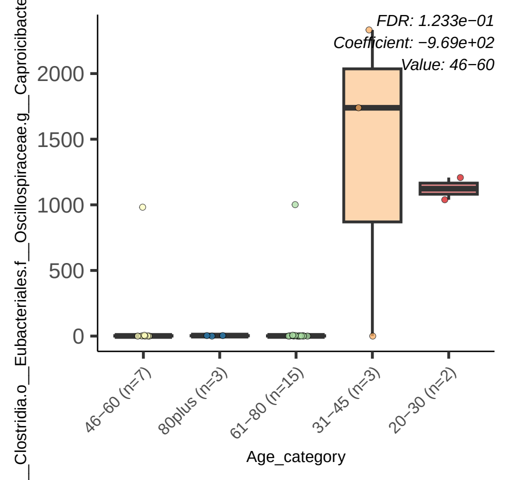

FDR: 1.233e−01
Coefficient: −9.69e+02
Value: 46−60
Clostridia.o__Eubacteriales.f__Oscillospiraceae.g__Caproicibacte
2000
1500
1000
500
0
46−60 (n=7)
80plus (n=3)
61−80 (n=15)
31−45 (n=3)
20−30 (n=2)
Age_category

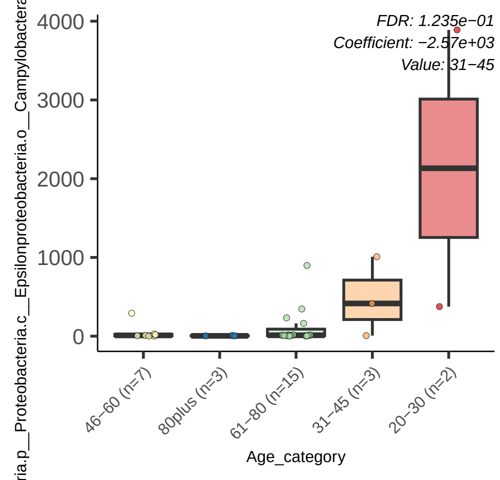

FDR: 1.235e−01
Coefficient: −2.57e+03
Value: 31−45
4000
3000
2000
1000
0
ria.p__Proteobacteria.c__Epsilonproteobacteria.o__Campylobacteral
46−60 (n=7)
80plus (n=3)
61−80 (n=15)
31−45 (n=3)
20−30 (n=2)
Age_category

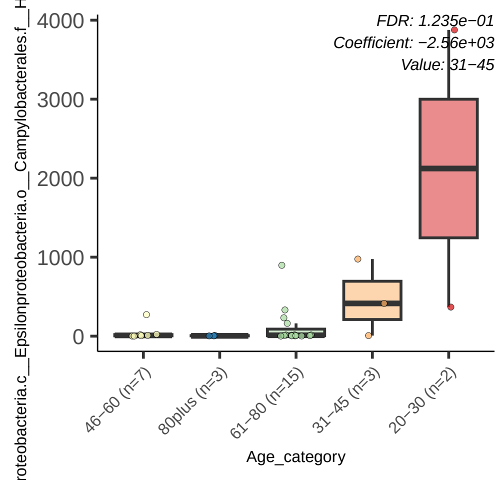

FDR: 1.235e−01
Coefficient: −2.56e+03
Value: 31−45
4000
3000
2000
1000
0
proteobacteria.c__Epsilonproteobacteria.o__Campylobacterales.f__H
46−60 (n=7)
80plus (n=3)
61−80 (n=15)
31−45 (n=3)
20−30 (n=2)
Age_category

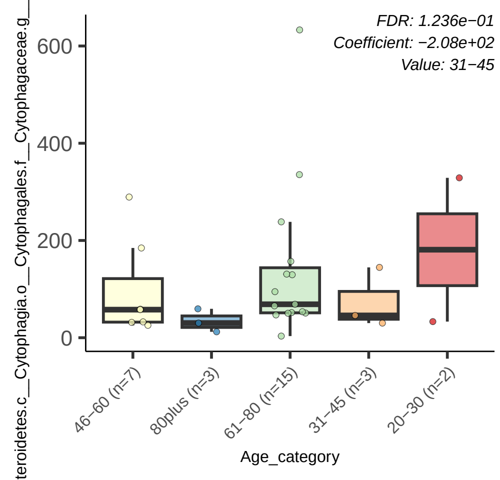

FDR: 1.236e−01
Coefficient: −2.08e+02
Value: 31−45
600
400
200
0
teroidetes.c__Cytophagia.o__Cytophagales.f__Cytophagaceae.g__
46−60 (n=7)
80plus (n=3)
61−80 (n=15)
31−45 (n=3)
20−30 (n=2)
Age_category

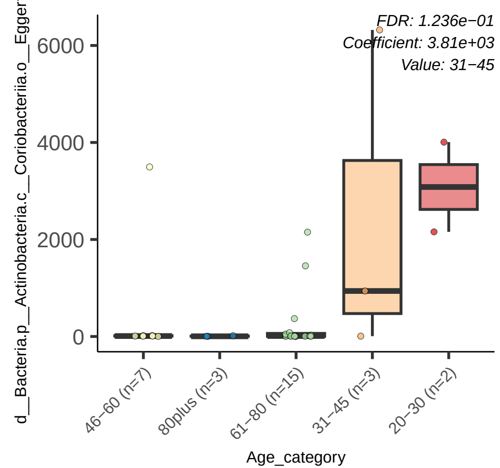
FDR: 1.236e−01
Coefficient: 3.81e+03
Value: 31−45
d__Bacteria.p__Actinobacteria.c__Coriobacteriia.o__Egger
6000
4000
2000
0
46−60 (n=7)
80plus (n=3)
61−80 (n=15)
31−45 (n=3)
20−30 (n=2)
Age_category

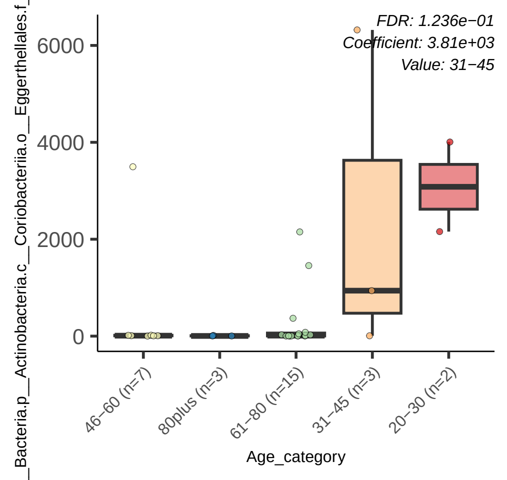

FDR: 1.236e−01
Coefficient: 3.81e+03
Value: 31−45
_Bacteria.p__Actinobacteria.c__Coriobacteriia.o__Eggerthellales.f_
6000
4000
2000
0
46−60 (n=7)
80plus (n=3)
61−80 (n=15)
31−45 (n=3)
20−30 (n=2)
Age_category

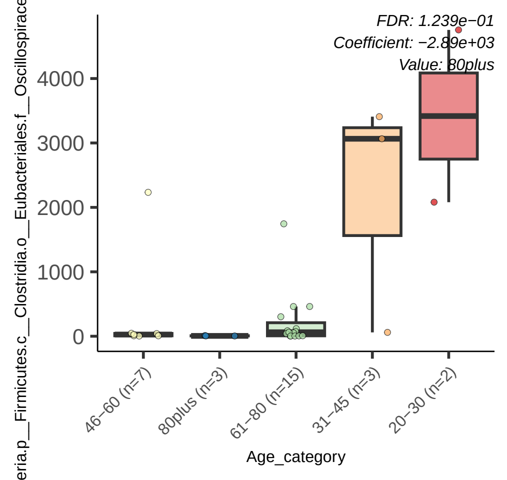
FDR: 1.239e−01
Coefficient: −2.89e+03
Value: 80plus
eria.p__Firmicutes.c__Clostridia.o__Eubacteriales.f__Oscillospirace
4000
3000
2000
1000
0
46−60 (n=7)
80plus (n=3)
61−80 (n=15)
31−45 (n=3)
20−30 (n=2)
Age_category

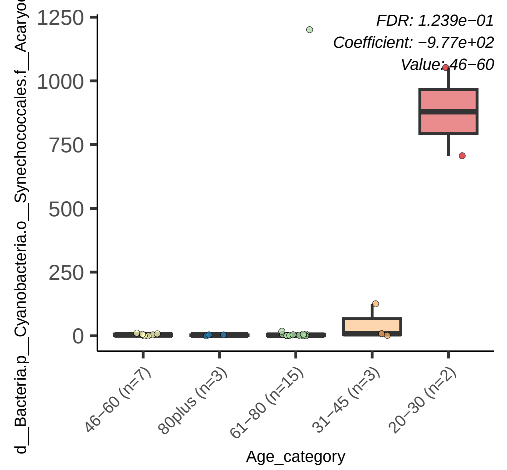

FDR: 1.239e−01
Coefficient: −9.77e+02
Value: 46−60
d__Bacteria.p__Cyanobacteria.o__Synechococcales.f__Acaryoc
1250
1000
750
500
250
0
46−60 (n=7)
80plus (n=3)
61−80 (n=15)
31−45 (n=3)
20−30 (n=2)
Age_category

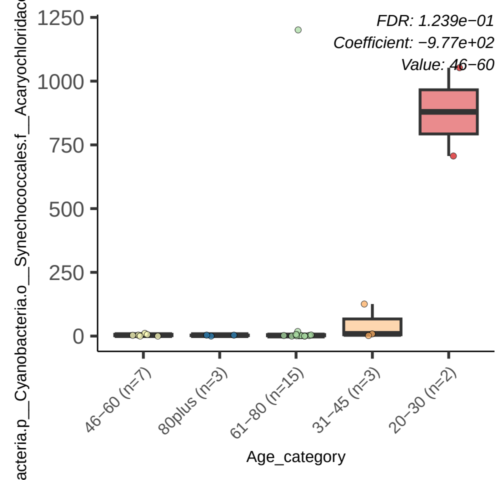

FDR: 1.239e−01
Coefficient: −9.77e+02
Value: 46−60
acteria.p__Cyanobacteria.o__Synechococcales.f__Acaryochloridac
1250
1000
750
500
250
0
46−60 (n=7)
80plus (n=3)
61−80 (n=15)
31−45 (n=3)
20−30 (n=2)
Age_category

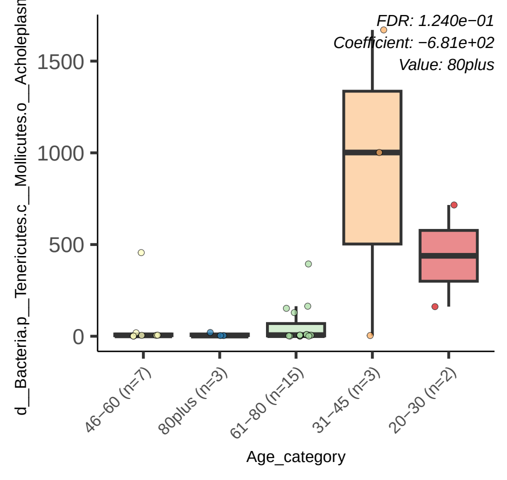

FDR: 1.240e−01
Coefficient: −6.81e+02
Value: 80plus
d__Bacteria.p__Tenericutes.c__Mollicutes.o__Acholeplasm
1500
1000
500
0
46−60 (n=7)
80plus (n=3)
61−80 (n=15)
31−45 (n=3)
20−30 (n=2)
Age_category

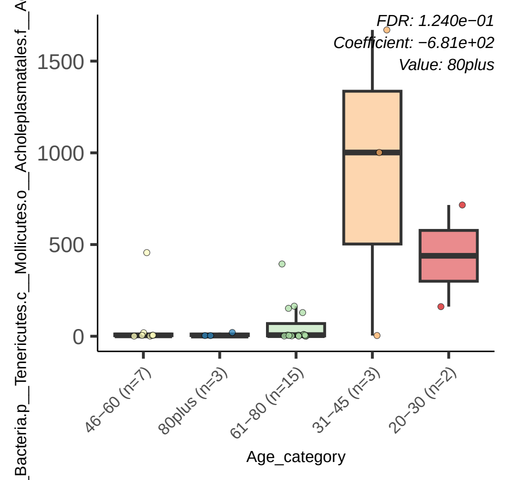
FDR: 1.240e−01
Coefficient: −6.81e+02
Value: 80plus
Bacteria.p__Tenericutes.c__Mollicutes.o__Acholeplasmatales.f__A
1500
1000
500
0
46−60 (n=7)
80plus (n=3)
61−80 (n=15)
31−45 (n=3)
20−30 (n=2)
Age_category

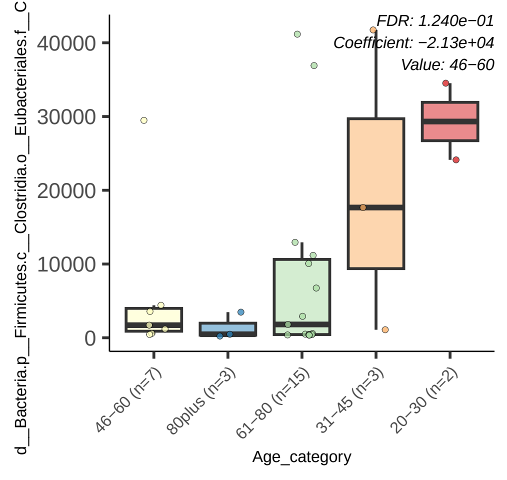

FDR: 1.240e−01
Coefficient: −2.13e+04
Value: 46−60
d__Bacteria.p__Firmicutes.c__Clostridia.o__Eubacteriales.f__C
40000
30000
20000
10000
0
46−60 (n=7)
80plus (n=3)
61−80 (n=15)
31−45 (n=3)
20−30 (n=2)
Age_category

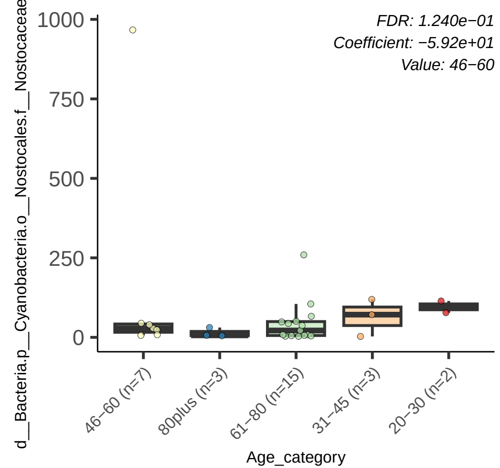

FDR: 1.240e−01
Coefficient: −5.92e+01
Value: 46−60
d__Bacteria.p__Cyanobacteria.o__Nostocales.f__Nostocaceae
1000
750
500
250
0
46−60 (n=7)
80plus (n=3)
61−80 (n=15)
31−45 (n=3)
20−30 (n=2)
Age_category

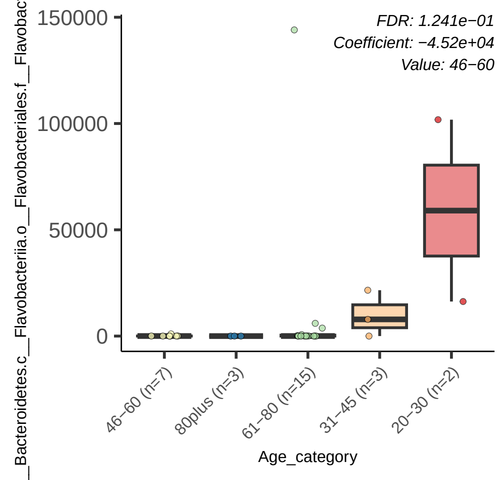

FDR: 1.241e−01
Coefficient: −4.52e+04
Value: 46−60
150000
100000
50000
0
46−60 (n=7)
80plus (n=3)
61−80 (n=15)
31−45 (n=3)
20−30 (n=2)
Age_category
_Bacteroidetes.c__Flavobacteriia.o__Flavobacteriales.f__Flavobac

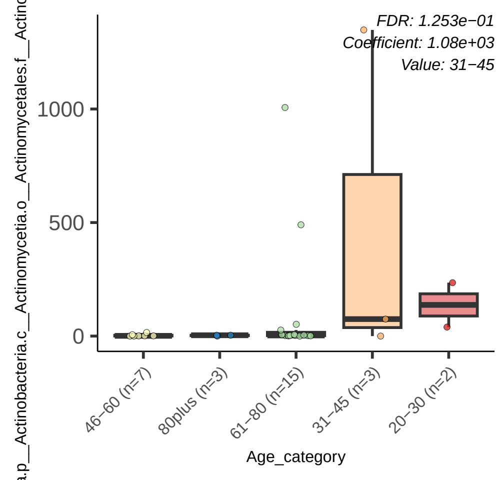

FDR: 1.253e−01
Coefficient: 1.08e+03
Value: 31−45
1000
500
0
a.p__Actinobacteria.c__Actinomycetia.o__Actinomycetales.f__Actino
46−60 (n=7)
80plus (n=3)
61−80 (n=15)
31−45 (n=3)
20−30 (n=2)
Age_category

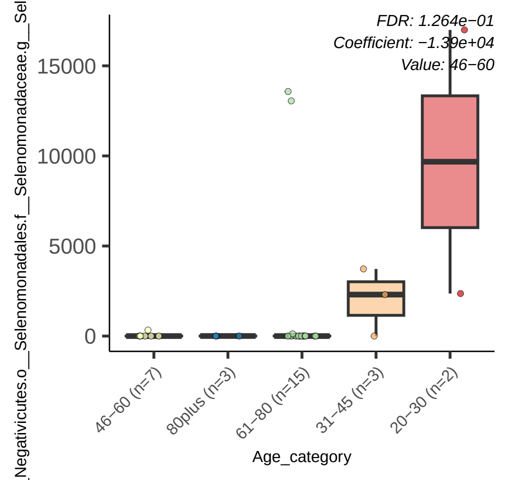

FDR: 1.264e−01
Coefficient: −1.39e+04
Value: 46−60
15000
10000
5000
0
Negativicutes.o__Selenomonadales.f__Selenomonadaceae.g__Sel
46−60 (n=7)
80plus (n=3)
61−80 (n=15)
31−45 (n=3)
20−30 (n=2)
Age_category

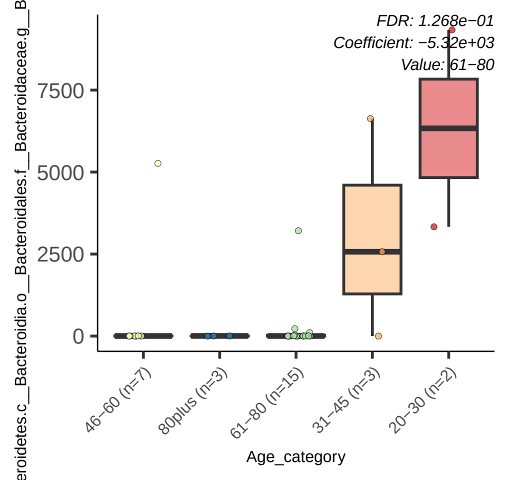

FDR: 1.268e−01
Coefficient: −5.32e+03
Value: 61−80
7500
5000
2500
0
46−60 (n=7)
80plus (n=3)
61−80 (n=15)
31−45 (n=3)
20−30 (n=2)
Age_category

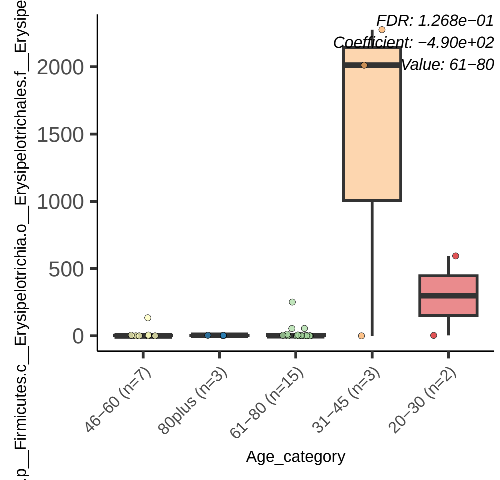
FDR: 1.268e−01
Coefficient: −4.90e+02
Value: 61−80
2000
1500
1000
500
0
46−60 (n=7)
80plus (n=3)
61−80 (n=15)
31−45 (n=3)
20−30 (n=2)
Age_category

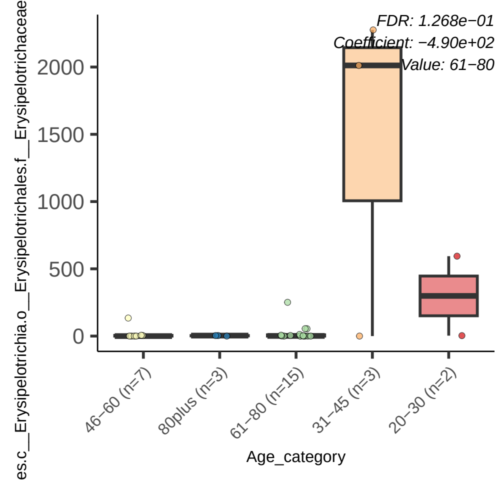

FDR: 1.268e−01
Coefficient: −4.90e+02
Value: 61−80
es.c__Erysipelotrichia.o__Erysipelotrichales.f__Erysipelotrichaceae
2000
1500
1000
500
0
46−60 (n=7)
80plus (n=3)
61−80 (n=15)
31−45 (n=3)
20−30 (n=2)
Age_category

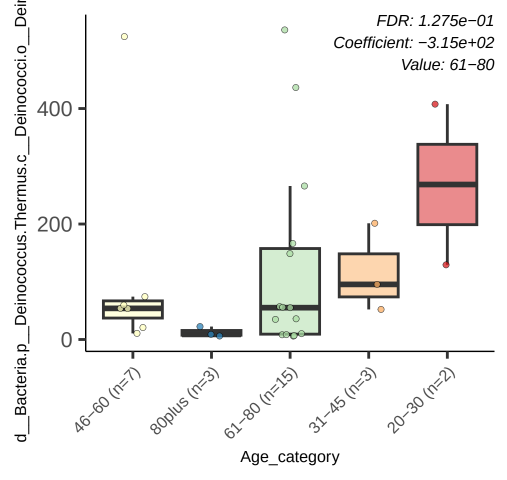
FDR: 1.275e−01
Coefficient: −3.15e+02
Value: 61−80
d__Bacteria.p__Deinococcus.Thermus.c__Deinococci.o__Dei
400
200
0
46−60 (n=7)
80plus (n=3)
61−80 (n=15)
31−45 (n=3)
20−30 (n=2)
Age_category

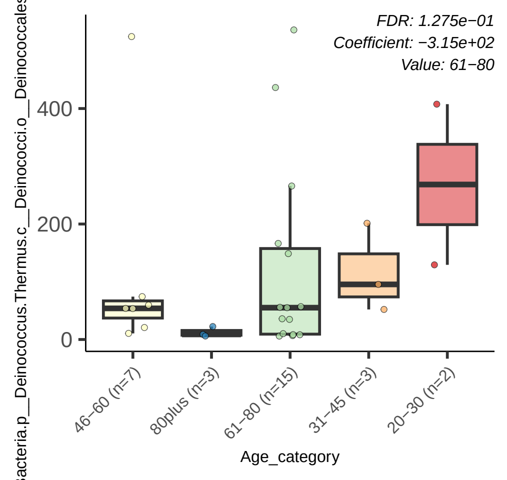
FDR: 1.275e−01
Coefficient: −3.15e+02
Value: 61−80
Bacteria.p__Deinococcus.Thermus.c__Deinococci.o__Deinococcales
400
200
0
46−60 (n=7)
80plus (n=3)
61−80 (n=15)
31−45 (n=3)
20−30 (n=2)
Age_category

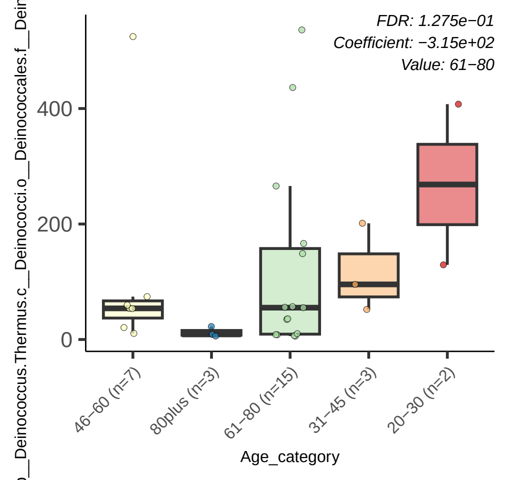
FDR: 1.275e−01
Coefficient: −3.15e+02
Value: 61−80
o__Deinococcus.Thermus.c__Deinococci.o__Deinococcales.f__Dei
400
200
0
46−60 (n=7)
80plus (n=3)
61−80 (n=15)
31−45 (n=3)
20−30 (n=2)
Age_category

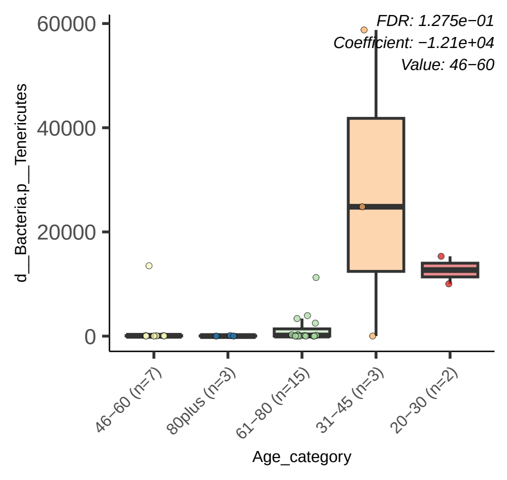

FDR: 1.275e−01
Coefficient: −1.21e+04
Value: 46−60
60000
40000
20000
0
d__Bacteria.p__Tenericutes
46−60 (n=7)
80plus (n=3)
61−80 (n=15)
31−45 (n=3)
20−30 (n=2)
Age_category

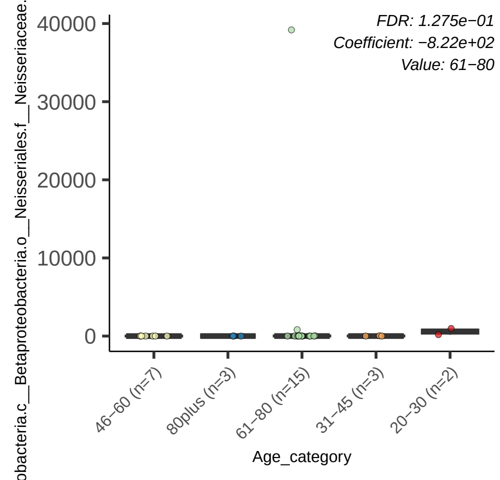

FDR: 1.275e−01
Coefficient: −8.22e+02
Value: 61−80
40000
30000
20000
10000
0
obacteria.c__Betaproteobacteria.o__Neisseriales.f__Neisseriaceae.
46−60 (n=7)
80plus (n=3)
61−80 (n=15)
31−45 (n=3)
20−30 (n=2)
Age_category

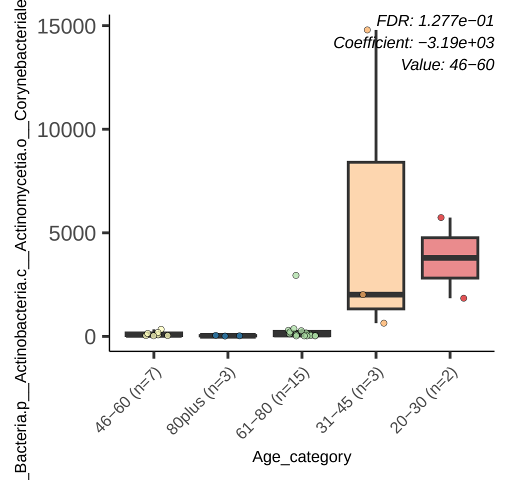

FDR: 1.277e−01
Coefficient: −3.19e+03
Value: 46−60
15000
10000
5000
0
Bacteria.p__Actinobacteria.c__Actinomycetia.o__Corynebacteriale
46−60 (n=7)
80plus (n=3)
61−80 (n=15)
31−45 (n=3)
20−30 (n=2)
Age_category

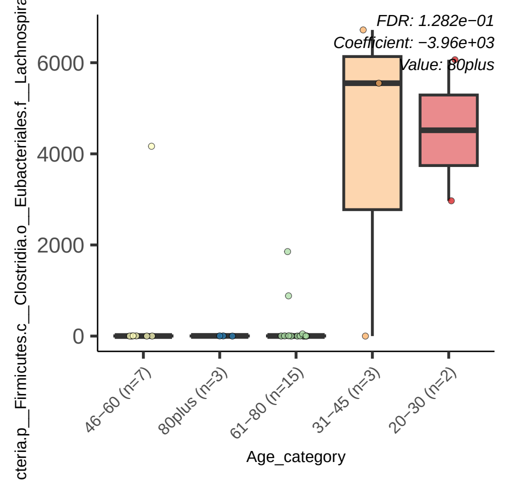
FDR: 1.282e−01
Coefficient: −3.96e+03
Value: 80plus
cteria.p__Firmicutes.c__Clostridia.o__Eubacteriales.f__Lachnospira
0
2000
4000
6000
46−60 (n=7)
80plus (n=3)
61−80 (n=15)
31−45 (n=3)
20−30 (n=2)
Age_category

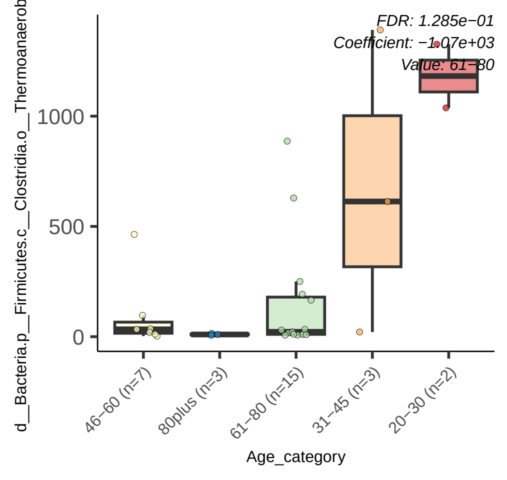

FDR: 1.285e−01
Coefficient: −1.07e+03
Value: 61−80
d__Bacteria.p__Firmicutes.c__Clostridia.o__Thermoanaerob
1000
500
0
46−60 (n=7)
80plus (n=3)
61−80 (n=15)
31−45 (n=3)
20−30 (n=2)
Age_category

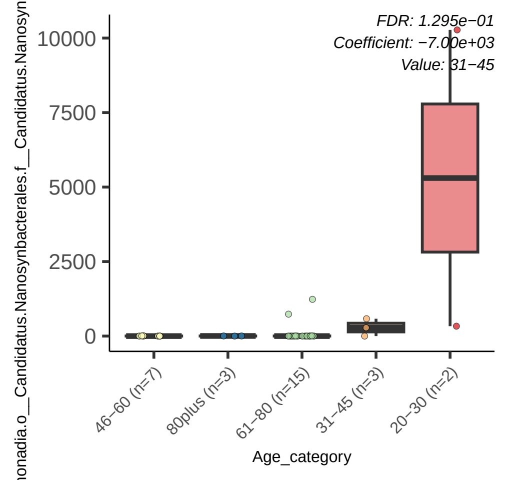

FDR: 1.295e−01
Coefficient: −7.00e+03
Value: 31−45
10000
7500
5000
2500
0
onadia.o__Candidatus.Nanosynbacterales.f__Candidatus.Nanosyn
46−60 (n=7)
80plus (n=3)
61−80 (n=15)
31−45 (n=3)
20−30 (n=2)
Age_category

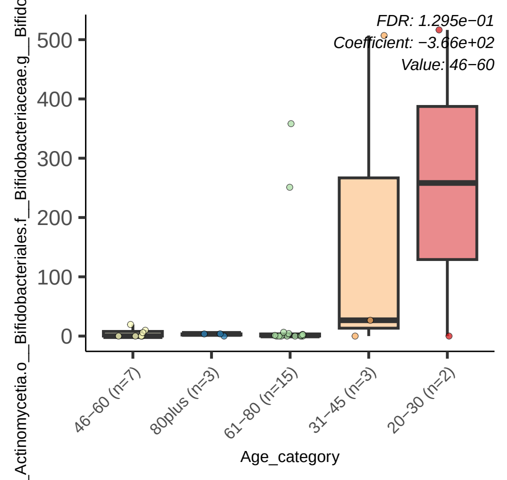

FDR: 1.295e−01
Coefficient: −3.66e+02
Value: 46−60
_Actinomycetia.o__Bifidobacteriales.f__Bifidobacteriaceae.g__Bifido
0
100
200
300
400
500
46−60 (n=7)
80plus (n=3)
61−80 (n=15)
31−45 (n=3)
20−30 (n=2)
Age_category

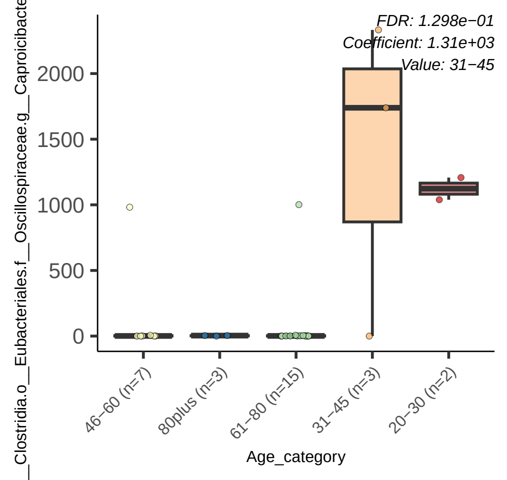

FDR: 1.298e−01
Coefficient: 1.31e+03
Value: 31−45
Clostridia.o__Eubacteriales.f__Oscillospiraceae.g__Caproicibacte
2000
1500
1000
500
0
46−60 (n=7)
80plus (n=3)
61−80 (n=15)
31−45 (n=3)
20−30 (n=2)
Age_category

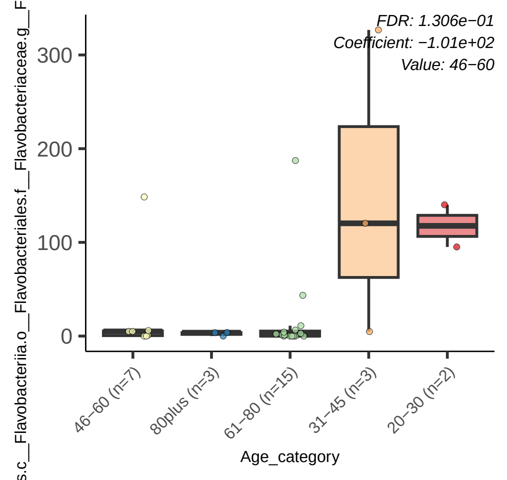

FDR: 1.306e−01
Coefficient: −1.01e+02
Value: 46−60
300
200
100
0
s.c__Flavobacteriia.o__Flavobacteriales.f__Flavobacteriaceae.g__F
46−60 (n=7)
80plus (n=3)
61−80 (n=15)
31−45 (n=3)
20−30 (n=2)
Age_category

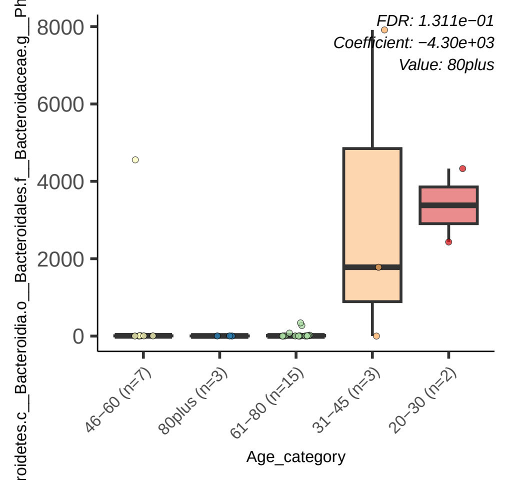
FDR: 1.311e−01
Coefficient: −4.30e+03
Value: 80plus
roidetes.c__Bacteroidia.o__Bacteroidales.f__Bacteroidaceae.g__Ph
8000
6000
4000
2000
0
46−60 (n=7)
80plus (n=3)
61−80 (n=15)
31−45 (n=3)
20−30 (n=2)
Age_category

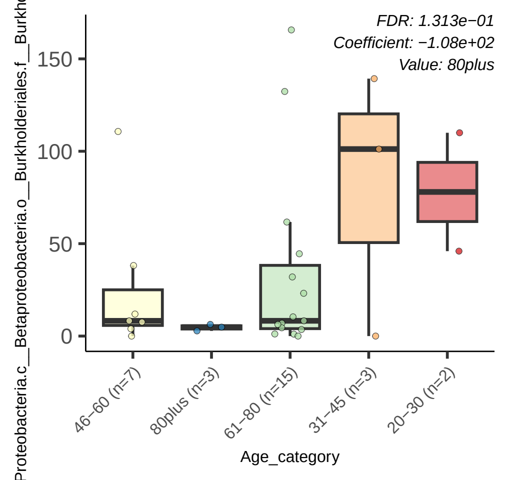
FDR: 1.313e−01
Coefficient: −1.08e+02
Value: 80plus
Proteobacteria.c__Betaproteobacteria.o__Burkholderiales.f__Burkho
150
100
50
0
46−60 (n=7)
80plus (n=3)
61−80 (n=15)
31−45 (n=3)
20−30 (n=2)
Age_category

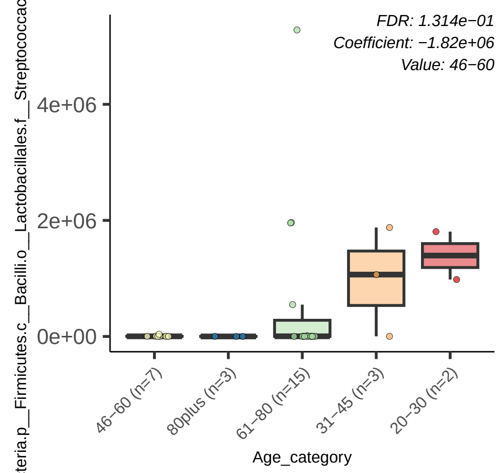

FDR: 1.314e−01
Coefficient: −1.82e+06
Value: 46−60
cteria.p__Firmicutes.c__Bacilli.o__Lactobacillales.f__Streptococcac
4e+06
2e+06
0e+00
46−60 (n=7)
80plus (n=3)
61−80 (n=15)
31−45 (n=3)
20−30 (n=2)
Age_category

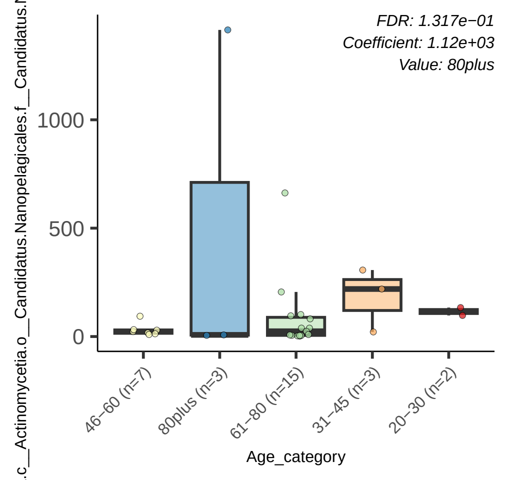

FDR: 1.317e−01
Coefficient: 1.12e+03
Value: 80plus
1000
500
0
.c__Actinomycetia.o__Candidatus.Nanopelagicales.f__Candidatus.N
46−60 (n=7)
80plus (n=3)
61−80 (n=15)
31−45 (n=3)
20−30 (n=2)
Age_category

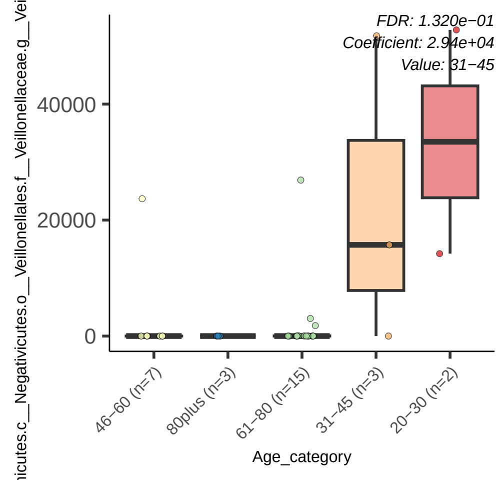

FDR: 1.320e−01
Coefficient: 2.94e+04
Value: 31−45
40000
20000
0
46−60 (n=7)
80plus (n=3)
61−80 (n=15)
31−45 (n=3)
20−30 (n=2)
Age_category
icutes.c__Negativicutes.o__Veillonellales.f__Veillonellaceae.g__Vei

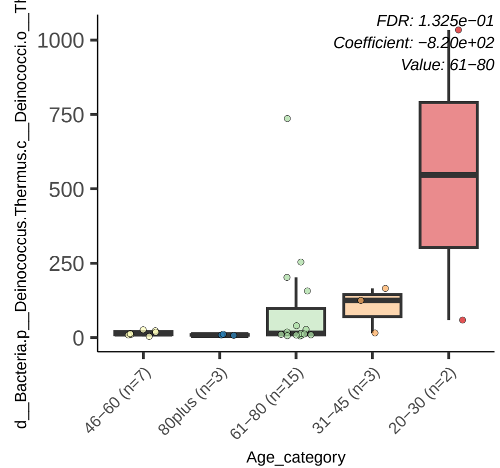

FDR: 1.325e−01
Coefficient: −8.20e+02
Value: 61−80
d__Bacteria.p__Deinococcus.Thermus.c__Deinococci.o__Th
1000
750
500
250
0
46−60 (n=7)
80plus (n=3)
61−80 (n=15)
31−45 (n=3)
20−30 (n=2)
Age_category

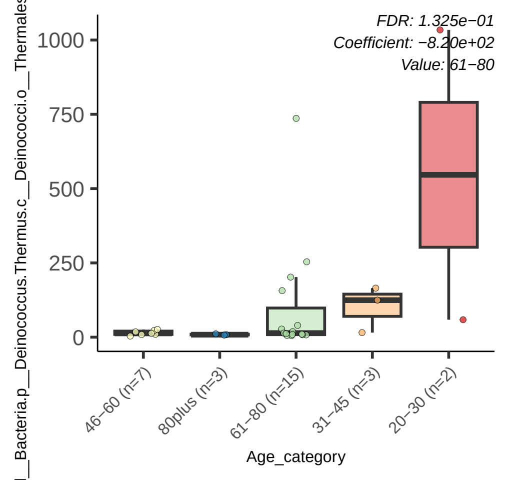

FDR: 1.325e−01
Coefficient: −8.20e+02
Value: 61−80
1000
750
500
250
0
_Bacteria.p__Deinococcus.Thermus.c__Deinococci.o__Thermales
46−60 (n=7)
80plus (n=3)
61−80 (n=15)
31−45 (n=3)
20−30 (n=2)
Age_category

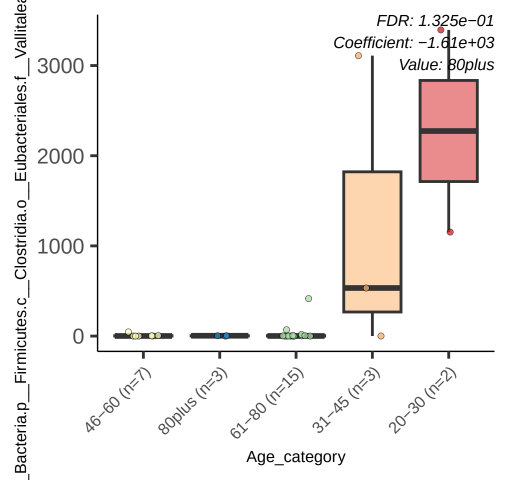

FDR: 1.325e−01
Coefficient: −1.61e+03
Value: 80plus
3000
2000
1000
0
46−60 (n=7)
80plus (n=3)
61−80 (n=15)
31−45 (n=3)
20−30 (n=2)
Age_category
Bacteria.p__Firmicutes.c__Clostridia.o__Eubacteriales.f__Vallitalea

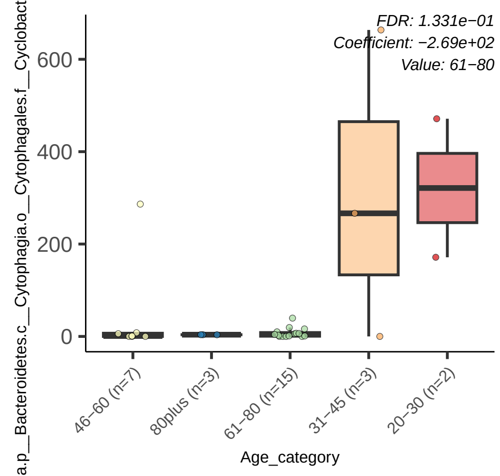

FDR: 1.331e−01
Coefficient: −2.69e+02
Value: 61−80
ria.p__Bacteroidetes.c__Cytophagia.o__Cytophagales.f__Cyclobact
600
400
200
0
46−60 (n=7)
80plus (n=3)
61−80 (n=15)
31−45 (n=3)
20−30 (n=2)
Age_category

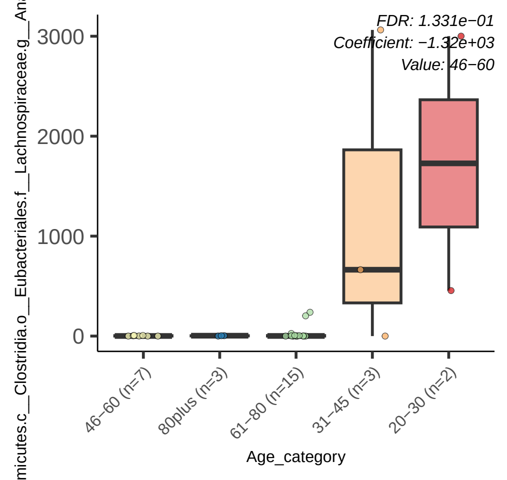

FDR: 1.331e−01
Coefficient: −1.32e+03
Value: 46−60
micutes.c__Clostridia.o__Eubacteriales.f__Lachnospiraceae.g__An
3000
2000
1000
0
46−60 (n=7)
80plus (n=3)
61−80 (n=15)
31−45 (n=3)
20−30 (n=2)
Age_category

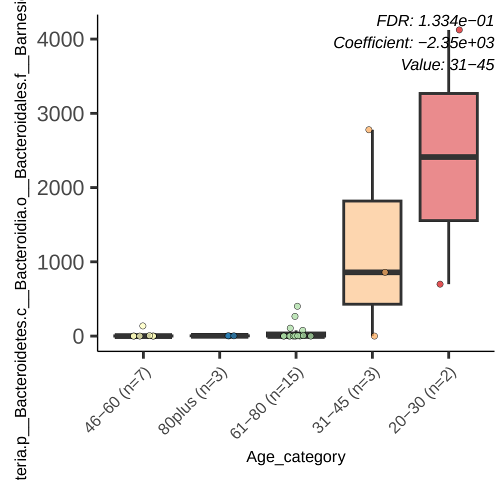

FDR: 1.334e−01
Coefficient: −2.35e+03
Value: 31−45
4000
3000
2000
1000
0
46−60 (n=7)
80plus (n=3)
61−80 (n=15)
31−45 (n=3)
20−30 (n=2)
Age_category
teria.p__Bacteroidetes.c__Bacteroidia.o__Bacteroidales.f__Barnesi

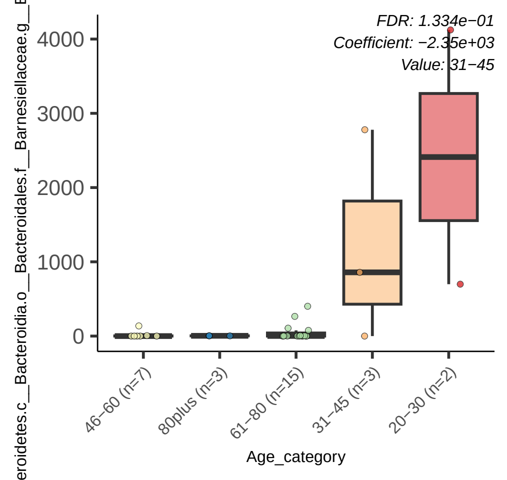

FDR: 1.334e−01
Coefficient: −2.35e+03
Value: 31−45
4000
3000
2000
1000
0
46−60 (n=7)
80plus (n=3)
61−80 (n=15)
31−45 (n=3)
20−30 (n=2)
Age_category
eroidetes.c__Bacteroidia.o__Bacteroidales.f__Barnesiellaceae.g__B

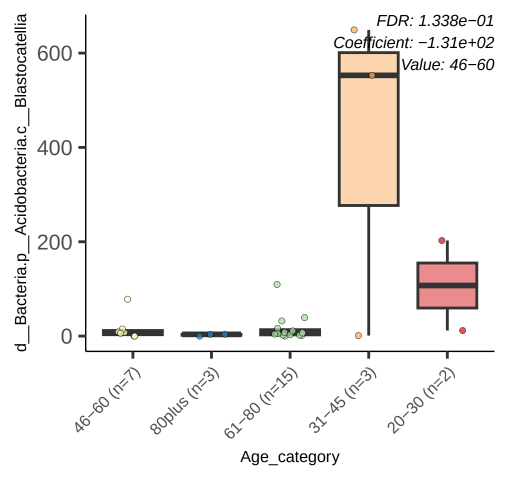

FDR: 1.338e−01
Coefficient: −1.31e+02
Value: 46−60
d__Bacteria.p__Acidobacteria.c__Blastocatellia
600
400
200
0
46−60 (n=7)
80plus (n=3)
61−80 (n=15)
31−45 (n=3)
20−30 (n=2)
Age_category

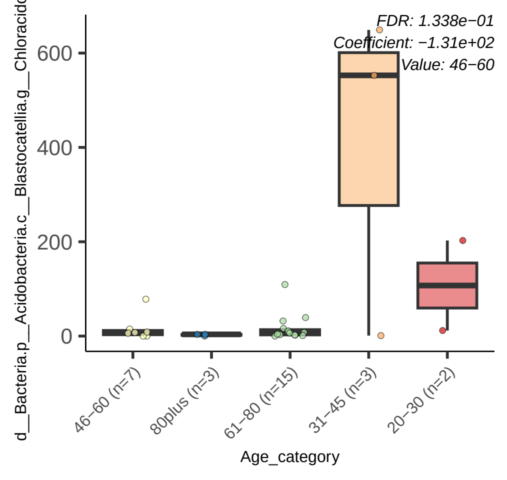
FDR: 1.338e−01
Coefficient: −1.31e+02
Value: 46−60
d__Bacteria.p__Acidobacteria.c__Blastocatellia.g__Chloracido
0
200
400
600
46−60 (n=7)
80plus (n=3)
61−80 (n=15)
31−45 (n=3)
20−30 (n=2)
Age_category

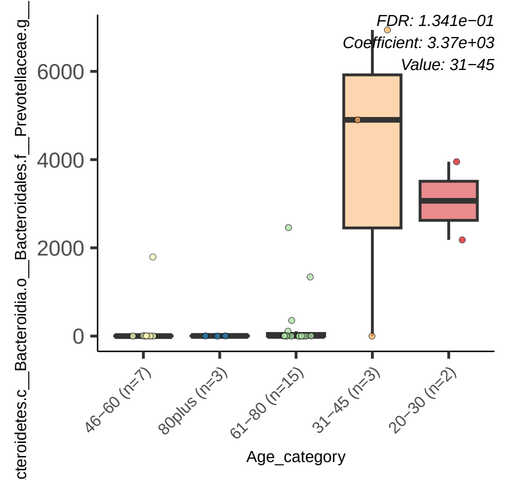

FDR: 1.341e−01
Coefficient: 3.37e+03
Value: 31−45
6000
4000
2000
0
46−60 (n=7)
80plus (n=3)
61−80 (n=15)
31−45 (n=3)
20−30 (n=2)
Age_category
cteroidetes.c__Bacteroidia.o__Bacteroidales.f__Prevotellaceae.g__

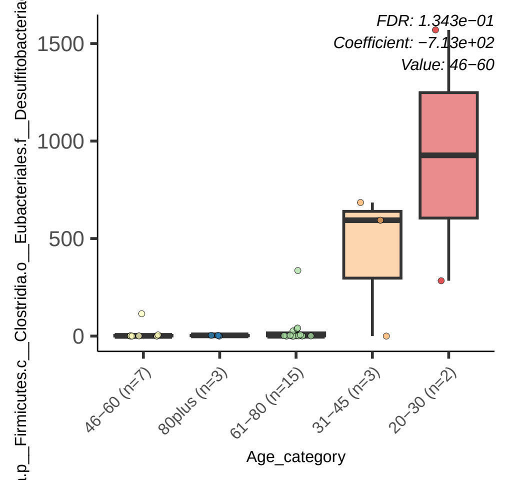

FDR: 1.343e−01
Coefficient: −7.13e+02
Value: 46−60
Firmicutes.c__Clostridia.o__Eubacteriales.f__Desulfitobacteria
1500
1000
500
0
46−60 (n=7)
80plus (n=3)
61−80 (n=15)
31−45 (n=3)
20−30 (n=2)
Age_category

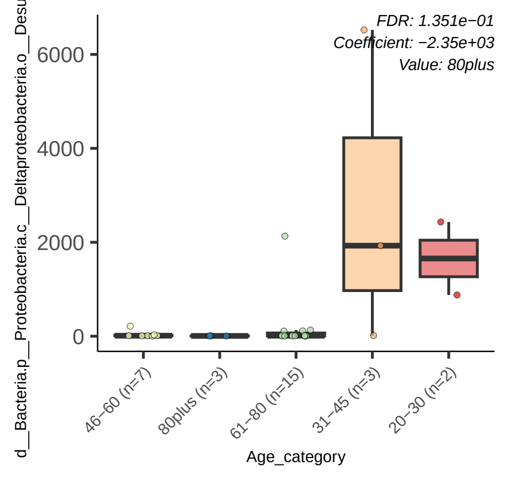

FDR: 1.351e−01
Coefficient: −2.35e+03
Value: 80plus
6000
4000
2000
0
d__Bacteria.p__Proteobacteria.c__Deltaproteobacteria.o__Desu
46−60 (n=7)
80plus (n=3)
61−80 (n=15)
31−45 (n=3)
20−30 (n=2)
Age_category

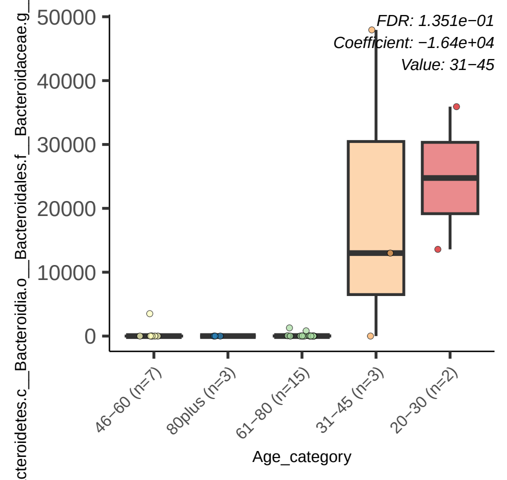
FDR: 1.351e−01
Coefficient: −1.64e+04
Value: 31−45
50000
40000
30000
20000
10000
0
cteroidetes.c__Bacteroidia.o__Bacteroidales.f__Bacteroidaceae.g__
46−60 (n=7)
80plus (n=3)
61−80 (n=15)
31−45 (n=3)
20−30 (n=2)
Age_category

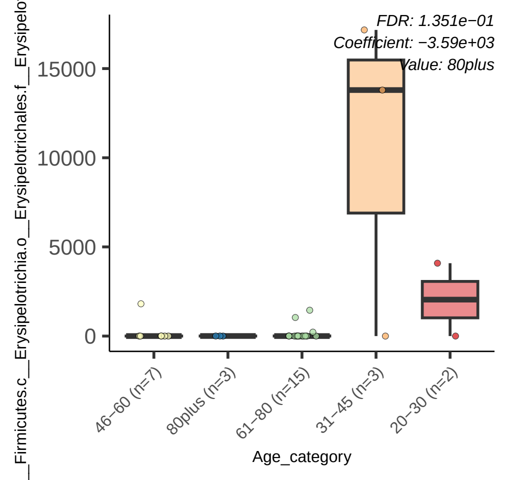

FDR: 1.351e−01
Coefficient: −3.59e+03
Value: 80plus
15000
10000
5000
0
Firmicutes.c__Erysipelotrichia.o__Erysipelotrichales.f__Erysipelo
46−60 (n=7)
80plus (n=3)
61−80 (n=15)
31−45 (n=3)
20−30 (n=2)
Age_category

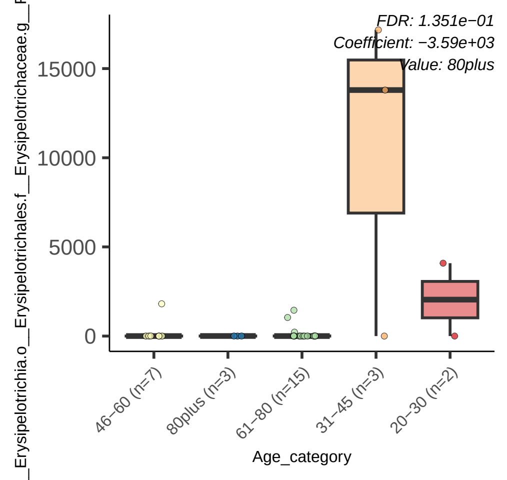

FDR: 1.351e−01
Coefficient: −3.59e+03
Value: 80plus
15000
10000
5000
0
Erysipelotrichia.o__Erysipelotrichales.f__Erysipelotrichaceae.g__F
46−60 (n=7)
80plus (n=3)
61−80 (n=15)
31−45 (n=3)
20−30 (n=2)
Age_category

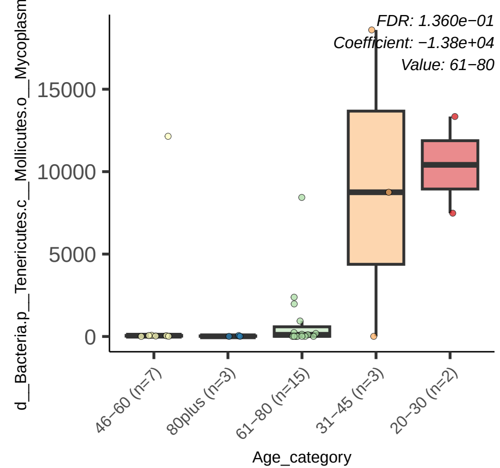

FDR: 1.360e−01
Coefficient: −1.38e+04
Value: 61−80
d__Bacteria.p__Tenericutes.c__Mollicutes.o__Mycoplasm
15000
10000
5000
0
46−60 (n=7)
80plus (n=3)
61−80 (n=15)
31−45 (n=3)
20−30 (n=2)
Age_category

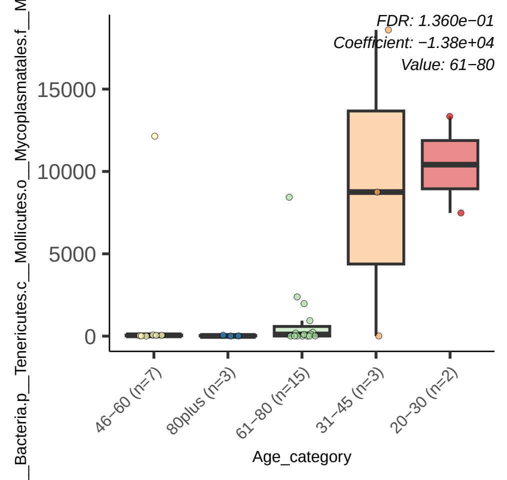

FDR: 1.360e−01
Coefficient: −1.38e+04
Value: 61−80
Bacteria.p__Tenericutes.c__Mollicutes.o__Mycoplasmatales.f__M
15000
10000
5000
0
46−60 (n=7)
80plus (n=3)
61−80 (n=15)
31−45 (n=3)
20−30 (n=2)
Age_category

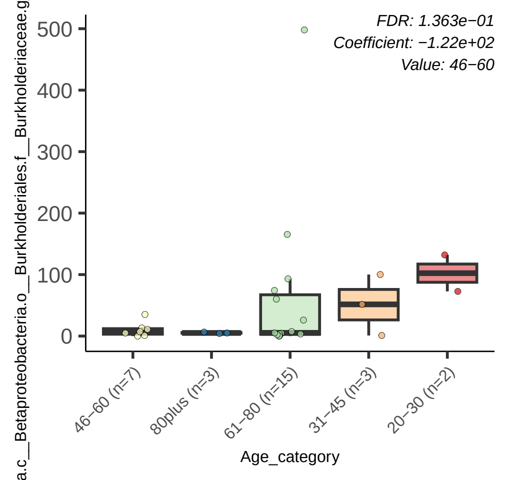

FDR: 1.363e−01
Coefficient: −1.22e+02
Value: 46−60
a.c__Betaproteobacteria.o__Burkholderiales.f__Burkholderiaceae.g
500
400
300
200
100
0
46−60 (n=7)
80plus (n=3)
61−80 (n=15)
31−45 (n=3)
20−30 (n=2)
Age_category

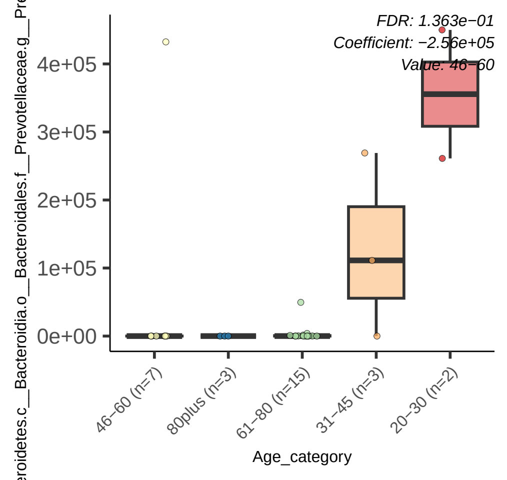
FDR: 1.363e−01
Coefficient: −2.56e+05
Value: 46−60
4e+05
3e+05
2e+05
1e+05
0e+00
eroidetes.c__Bacteroidia.o__Bacteroidales.f__Prevotellaceae.g__Pre
46−60 (n=7)
80plus (n=3)
61−80 (n=15)
31−45 (n=3)
20−30 (n=2)
Age_category

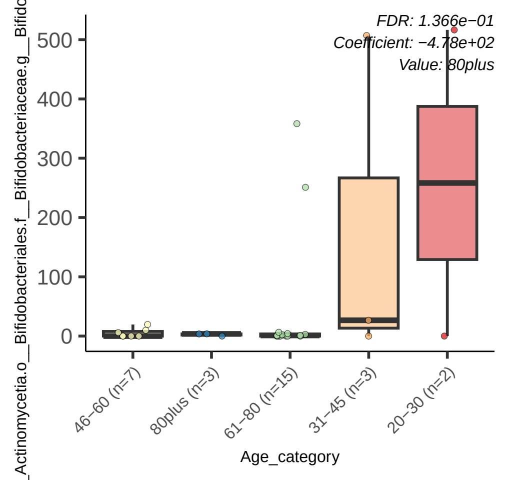

FDR: 1.366e−01
Coefficient: −4.78e+02
Value: 80plus
0
100
200
300
400
500
Actinomycetia.o__Bifidobacteriales.f__Bifidobacteriaceae.g__Bifido
46−60 (n=7)
80plus (n=3)
61−80 (n=15)
31−45 (n=3)
20−30 (n=2)
Age_category

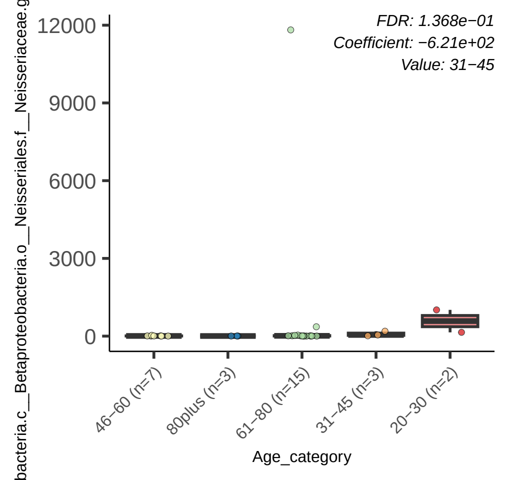
FDR: 1.368e−01
Coefficient: −6.21e+02
Value: 31−45
12000
9000
6000
3000
0
bacteria.c__Betaproteobacteria.o__Neisseriales.f__Neisseriaceae.g
46−60 (n=7)
80plus (n=3)
61−80 (n=15)
31−45 (n=3)
20−30 (n=2)
Age_category

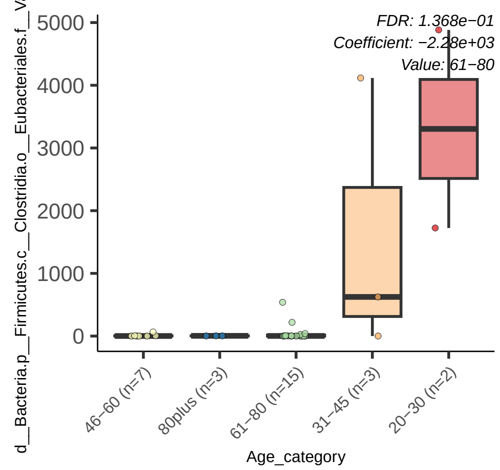
FDR: 1.368e−01
Coefficient: −2.28e+03
Value: 61−80
d__Bacteria.p__Firmicutes.c__Clostridia.o__Eubacteriales.f__Va
5000
4000
3000
2000
1000
0
46−60 (n=7)
80plus (n=3)
61−80 (n=15)
31−45 (n=3)
20−30 (n=2)
Age_category

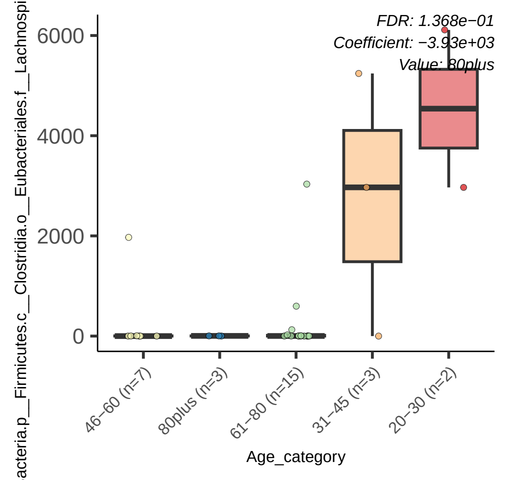

FDR: 1.368e−01
Coefficient: −3.93e+03
Value: 80plus
6000
4000
2000
0
acteria.p__Firmicutes.c__Clostridia.o__Eubacteriales.f__Lachnospi
46−60 (n=7)
80plus (n=3)
61−80 (n=15)
31−45 (n=3)
20−30 (n=2)
Age_category

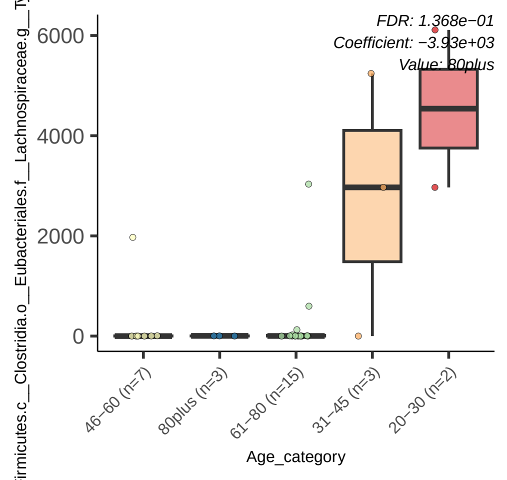

FDR: 1.368e−01
Coefficient: −3.93e+03
Value: 80plus
6000
4000
2000
0
irmicutes.c__Clostridia.o__Eubacteriales.f__Lachnospiraceae.g__Ty
46−60 (n=7)
80plus (n=3)
61−80 (n=15)
31−45 (n=3)
20−30 (n=2)
Age_category

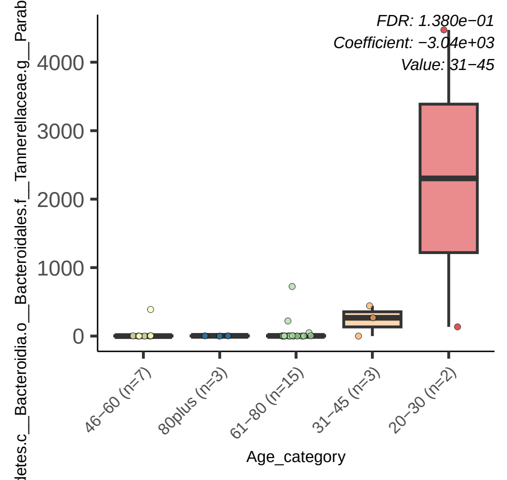

FDR: 1.380e−01
Coefficient: −3.04e+03
Value: 31−45
4000
3000
2000
1000
0
dетes.c__Bacteroidia.o__Bacteroidales.f__Tannerellaceae.g__Parab
46−60 (n=7)
80plus (n=3)
61−80 (n=15)
31−45 (n=3)
20−30 (n=2)
Age_category

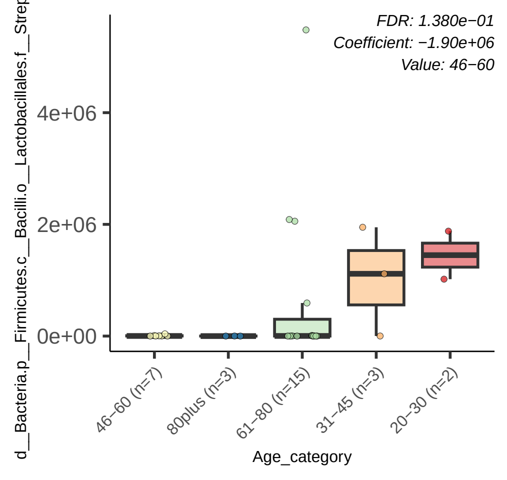

FDR: 1.380e−01
Coefficient: −1.90e+06
Value: 46−60
d__Bacteria.p__Firmicutes.c__Bacilli.o__Lactobacillales.f__Strep
4e+06
2e+06
0e+00
46−60 (n=7)
80plus (n=3)
61−80 (n=15)
31−45 (n=3)
20−30 (n=2)
Age_category

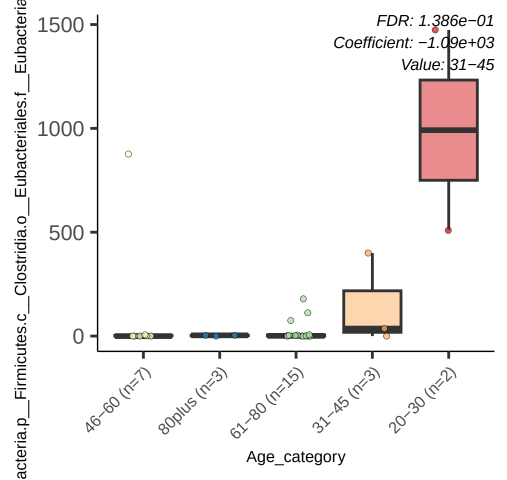

FDR: 1.386e−01
Coefficient: −1.09e+03
Value: 31−45
1500
1000
500
0
acteria.p__Firmicutes.c__Clostridia.o__Eubacteriales.f__Eubacteria
46−60 (n=7)
80plus (n=3)
61−80 (n=15)
31−45 (n=3)
20−30 (n=2)
Age_category

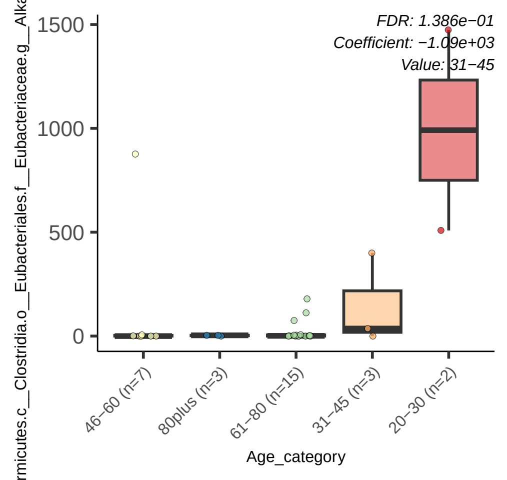

FDR: 1.386e−01
Coefficient: −1.09e+03
Value: 31−45
rmicutes.c__Clostridia.o__Eubacteriales.f__Eubacteriaceae.g__Alka
1500
1000
500
0
46−60 (n=7)
80plus (n=3)
61−80 (n=15)
31−45 (n=3)
20−30 (n=2)
Age_category

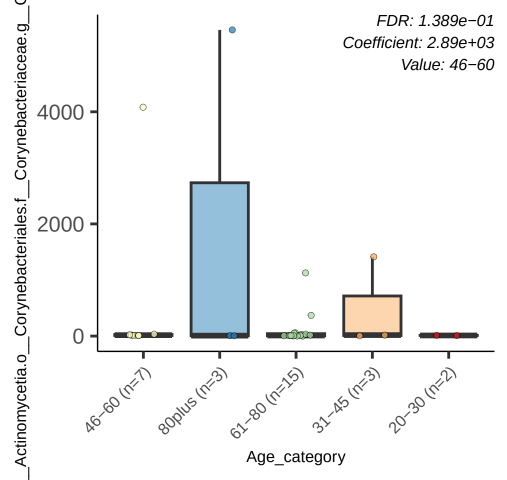

FDR: 1.389e−01
Coefficient: 2.89e+03
Value: 46−60
4000
2000
0
46−60 (n=7)
80plus (n=3)
61−80 (n=15)
31−45 (n=3)
20−30 (n=2)
Age_category
Actinomycetia.o__Corynebacteriales.f__Corynebacteriaceae.g__C

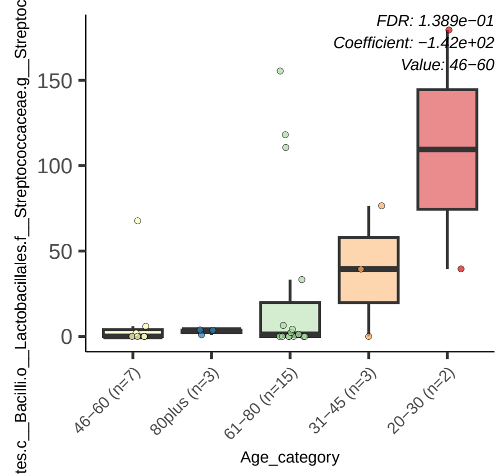

FDR: 1.389e−01
Coefficient: −1.42e+02
Value: 46−60
0
50
100
150
utes.c__Bacilli.o__Lactobacillales.f__Streptococcaceae.g__Streptoc
46−60 (n=7)
80plus (n=3)
61−80 (n=15)
31−45 (n=3)
20−30 (n=2)
Age_category

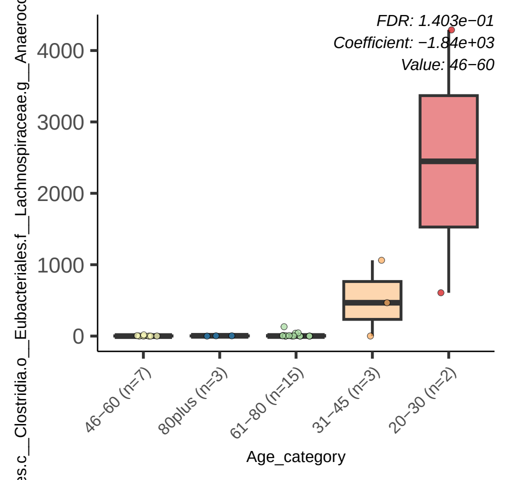

FDR: 1.403e−01
Coefficient: −1.84e+03
Value: 46−60
4000
3000
2000
1000
0
46−60 (n=7)
80plus (n=3)
61−80 (n=15)
31−45 (n=3)
20−30 (n=2)
Age_category
es.c__Clostridia.o__Eubacteriales.f__Lachnospiraceae.g__Anaeroco

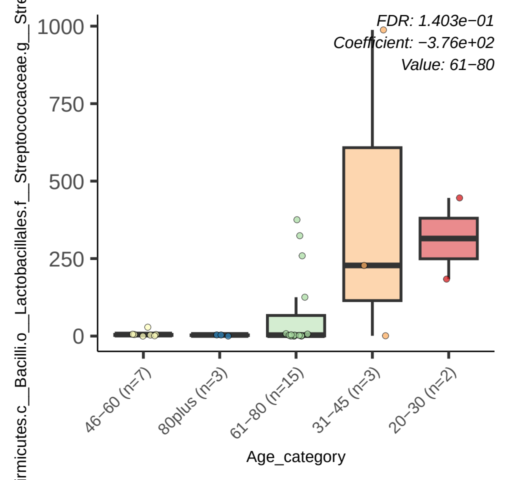

FDR: 1.403e−01
Coefficient: −3.76e+02
Value: 61−80
irmicutes.c__Bacilli.o__Lactobacillales.f__Streptococcaceae.g__Stre
1000
750
500
250
0
46−60 (n=7)
80plus (n=3)
61−80 (n=15)
31−45 (n=3)
20−30 (n=2)
Age_category

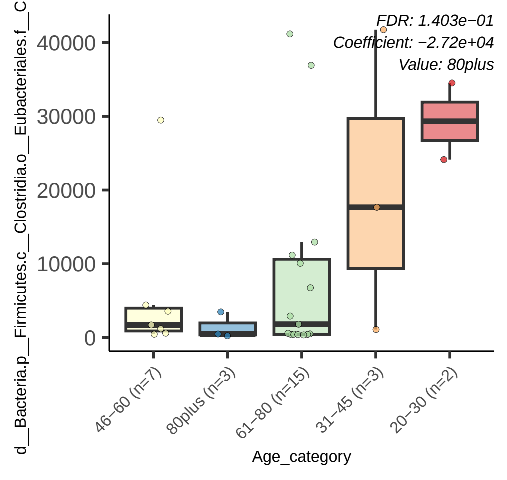

FDR: 1.403e−01
Coefficient: −2.72e+04
Value: 80plus
d__Bacteria.p__Firmicutes.c__Clostridia.o__Eubacteriales.f__C
0
10000
20000
30000
40000
46−60 (n=7)
80plus (n=3)
61−80 (n=15)
31−45 (n=3)
20−30 (n=2)
Age_category

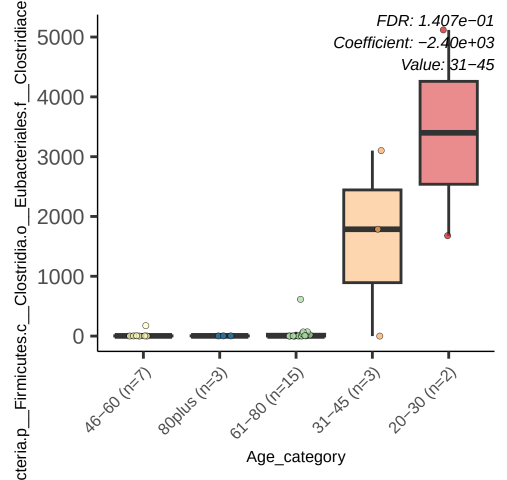

FDR: 1.407e−01
Coefficient: −2.40e+03
Value: 31−45
cteria.p__Firmicutes.c__Clostridia.o__Eubacteriales.f__Clostridiace
5000
4000
3000
2000
1000
0
46−60 (n=7)
80plus (n=3)
61−80 (n=15)
31−45 (n=3)
20−30 (n=2)
Age_category

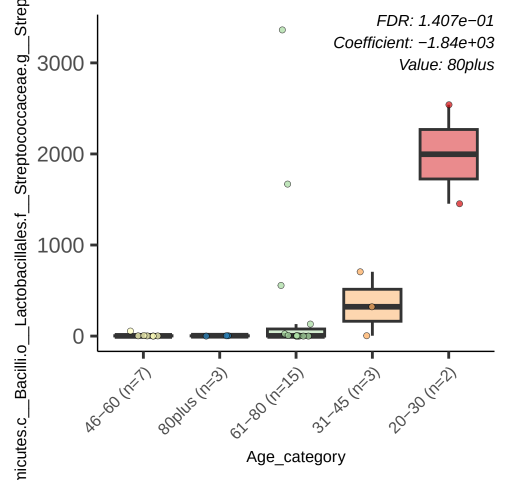
FDR: 1.407e−01
Coefficient: −1.84e+03
Value: 80plus
3000
2000
1000
0
micutes.c__Bacilli.o__Lactobacillales.f__Streptococcaceae.g__Strep
46−60 (n=7)
80plus (n=3)
61−80 (n=15)
31−45 (n=3)
20−30 (n=2)
Age_category

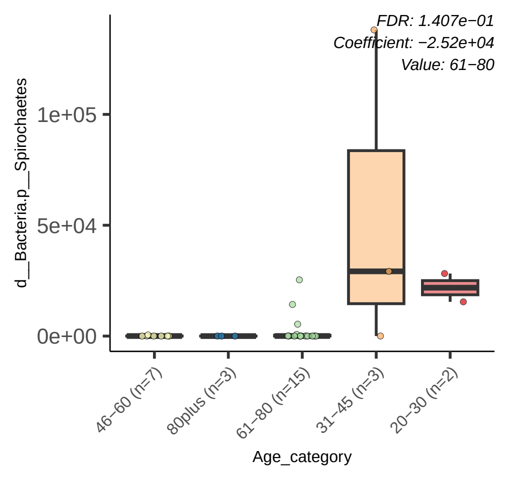

FDR: 1.407e−01
Coefficient: −2.52e+04
Value: 61−80
d__Bacteria.p__Spirochaetes
1e+05
5e+04
0e+00
46−60 (n=7)
80plus (n=3)
61−80 (n=15)
31−45 (n=3)
20−30 (n=2)
Age_category

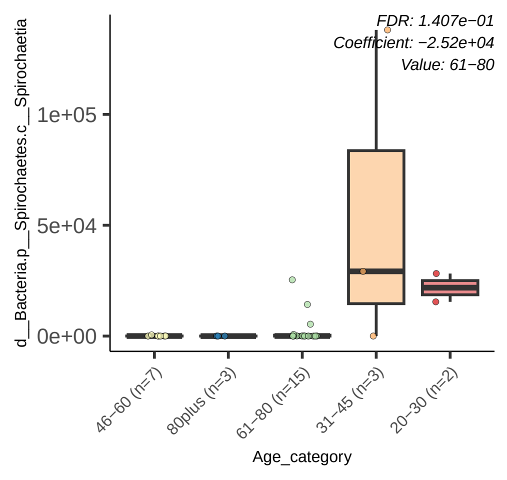

FDR: 1.407e−01
Coefficient: −2.52e+04
Value: 61−80
d__Bacteria.p__Spirochaetes.c__Spirochaetia
1e+05
5e+04
0e+00
46−60 (n=7)
80plus (n=3)
61−80 (n=15)
31−45 (n=3)
20−30 (n=2)
Age_category

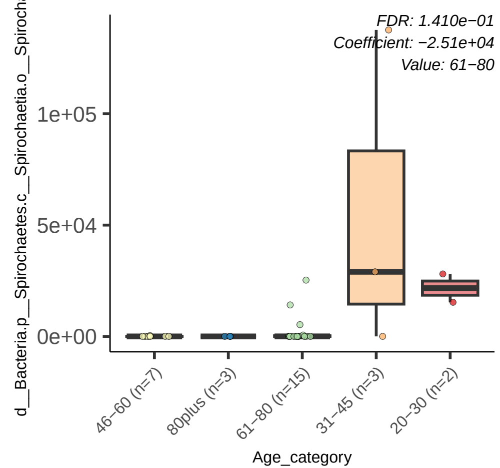

FDR: 1.410e−01
Coefficient: −2.51e+04
Value: 61−80
d__Bacteria.p__Spirochaetes.c__Spirochaetia.o__Spiroch
1e+05
5e+04
0e+00
46−60 (n=7)
80plus (n=3)
61−80 (n=15)
31−45 (n=3)
20−30 (n=2)
Age_category

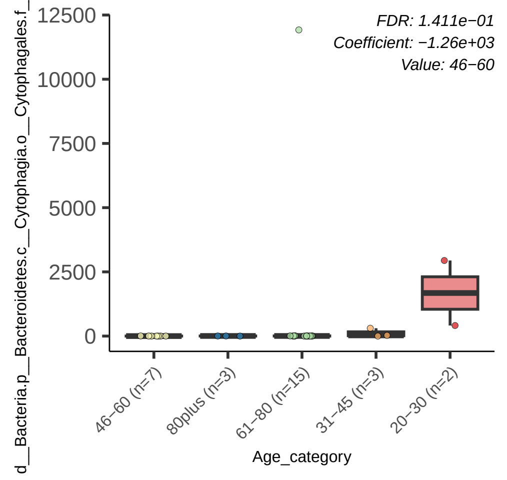

FDR: 1.411e−01
Coefficient: −1.26e+03
Value: 46−60
d__Bacteria.p__Bacteroidetes.c__Cytophagia.o__Cytophagales.f__
12500
10000
7500
5000
2500
0
46−60 (n=7)
80plus (n=3)
61−80 (n=15)
31−45 (n=3)
20−30 (n=2)
Age_category

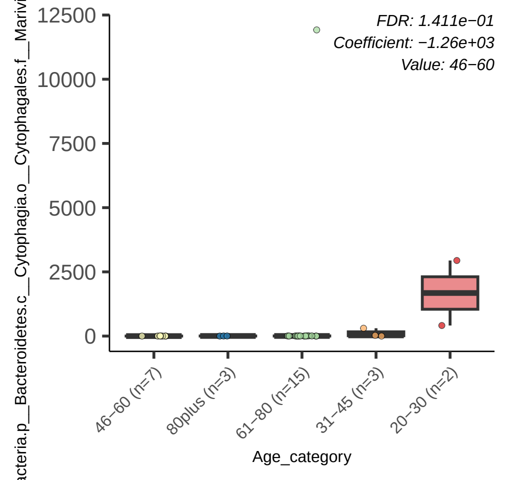
FDR: 1.411e−01
Coefficient: −1.26e+03
Value: 46−60
12500
10000
7500
5000
2500
0
acteria.p__Bacteroidetes.c__Cytophagia.o__Cytophagales.f__Marivi
46−60 (n=7)
80plus (n=3)
61−80 (n=15)
31−45 (n=3)
20−30 (n=2)
Age_category

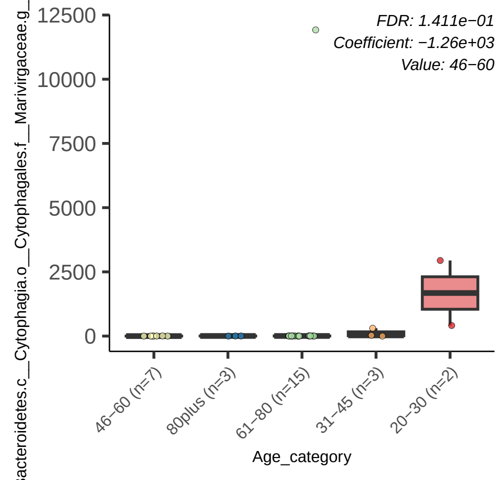
FDR: 1.411e−01
Coefficient: −1.26e+03
Value: 46−60
Bacteroidetes.c__Cytophagia.o__Cytophagales.f__Marivirgaceae.g__
12500
10000
7500
5000
2500
0
46−60 (n=7)
80plus (n=3)
61−80 (n=15)
31−45 (n=3)
20−30 (n=2)
Age_category

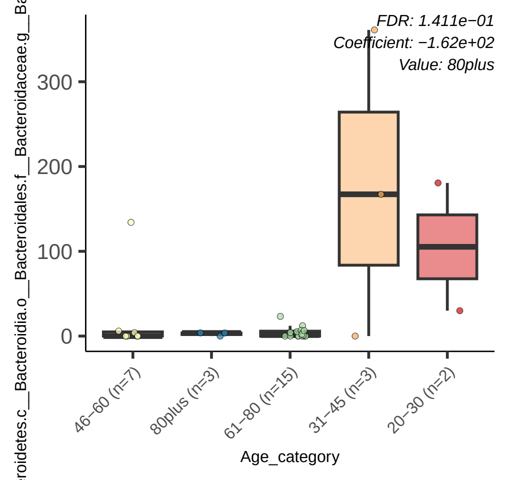

FDR: 1.411e−01
Coefficient: −1.62e+02
Value: 80plus
0
100
200
300
46−60 (n=7)
80plus (n=3)
61−80 (n=15)
31−45 (n=3)
20−30 (n=2)
Age_category
roidetes.c__Bacteroidia.o__Bacteroidales.f__Bacteroidaceae.g__Ba

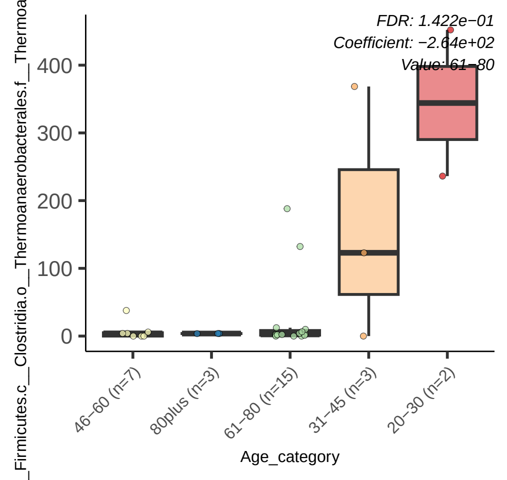

FDR: 1.422e−01
Coefficient: −2.64e+02
Value: 61−80
Firmicutes.c__Clostridia.o__Thermoanaerobacterales.f__Thermoa
0
100
200
300
400
46−60 (n=7)
80plus (n=3)
61−80 (n=15)
31−45 (n=3)
20−30 (n=2)
Age_category

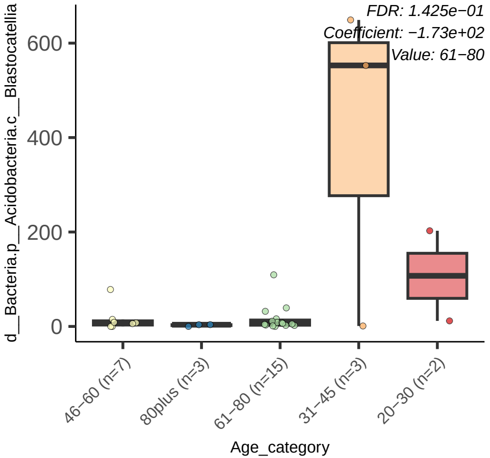

FDR: 1.425e−01
Coefficient: −1.73e+02
Value: 61−80
d__Bacteria.p__Acidobacteria.c__Blastocatellia
600
400
200
0
46−60 (n=7)
80plus (n=3)
61−80 (n=15)
31−45 (n=3)
20−30 (n=2)
Age_category

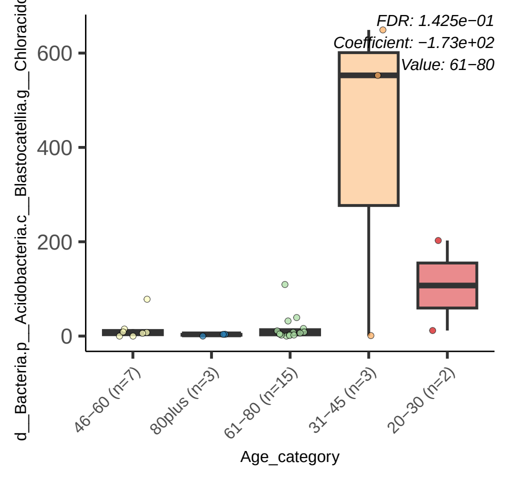

FDR: 1.425e−01
Coefficient: −1.73e+02
Value: 61−80
d__Bacteria.p__Acidobacteria.c__Blastocatellia.g__Chloracido
0
200
400
600
46−60 (n=7)
80plus (n=3)
61−80 (n=15)
31−45 (n=3)
20−30 (n=2)
Age_category

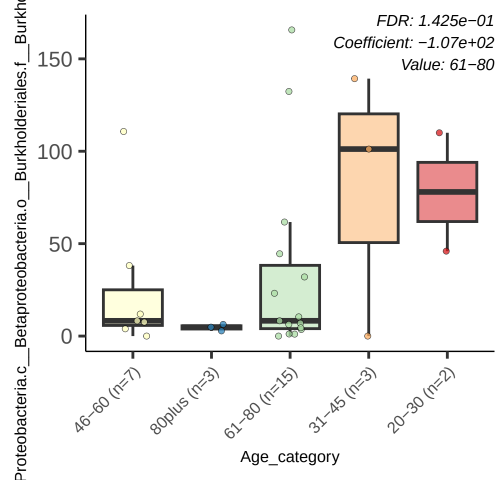
FDR: 1.425e−01
Coefficient: −1.07e+02
Value: 61−80
Proteobacteria.c__Betaproteobacteria.o__Burkholderiales.f__Burkho
150
100
50
0
46−60 (n=7)
80plus (n=3)
61−80 (n=15)
31−45 (n=3)
20−30 (n=2)
Age_category

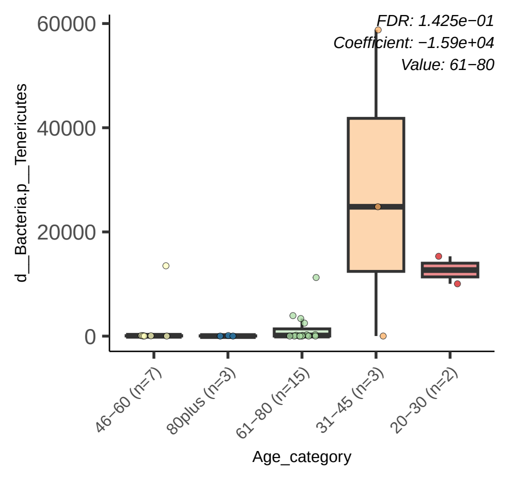
FDR: 1.425e−01
Coefficient: −1.59e+04
Value: 61−80
60000
40000
20000
0
d__Bacteria.p__Tenericutes
46−60 (n=7)
80plus (n=3)
61−80 (n=15)
31−45 (n=3)
20−30 (n=2)
Age_category

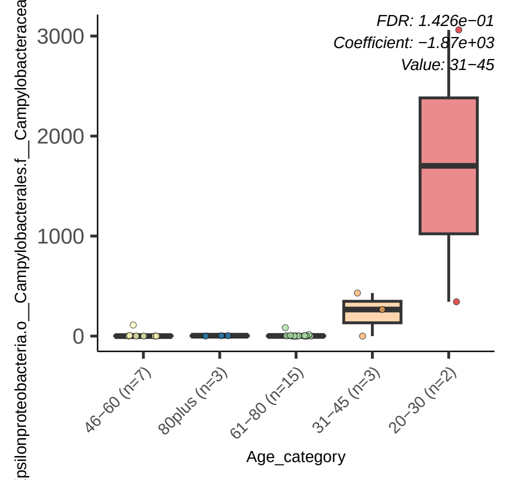

FDR: 1.426e−01
Coefficient: −1.87e+03
Value: 31−45
3000
2000
1000
0
psilonproteobacteria.o__Campylobacterales.f__Campylobacteracea
46−60 (n=7)
80plus (n=3)
61−80 (n=15)
31−45 (n=3)
20−30 (n=2)
Age_category

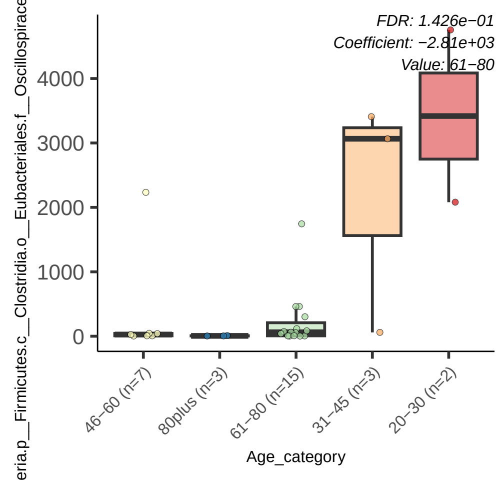

FDR: 1.426e−01
Coefficient: −2.81e+03
Value: 61−80
eria.p__Firmicutes.c__Clostridia.o__Eubacteriales.f__Oscillospirace
4000
3000
2000
1000
0
46−60 (n=7)
80plus (n=3)
61−80 (n=15)
31−45 (n=3)
20−30 (n=2)
Age_category

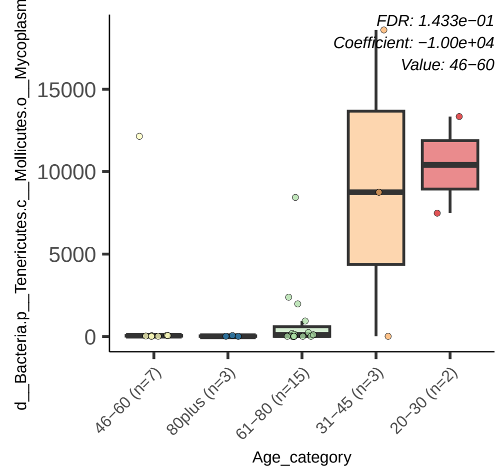

FDR: 1.433e−01
Coefficient: −1.00e+04
Value: 46−60
d__Bacteria.p__Tenericutes.c__Mollicutes.o__Mycoplasm
15000
10000
5000
0
46−60 (n=7)
80plus (n=3)
61−80 (n=15)
31−45 (n=3)
20−30 (n=2)
Age_category

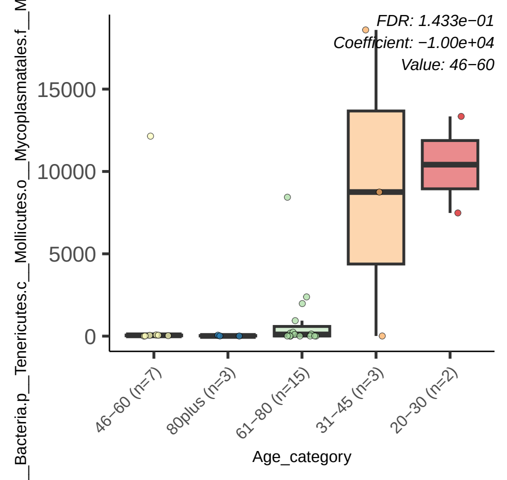

FDR: 1.433e−01
Coefficient: −1.00e+04
Value: 46−60
Bacteria.p__Tenericutes.c__Mollicutes.o__Mycoplasmatales.f__M
15000
10000
5000
0
46−60 (n=7)
80plus (n=3)
61−80 (n=15)
31−45 (n=3)
20−30 (n=2)
Age_category

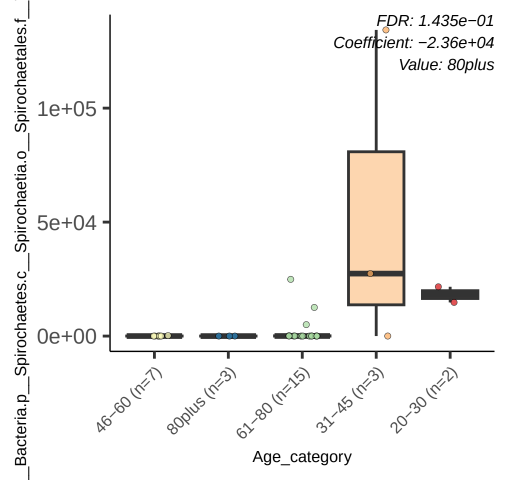

FDR: 1.435e−01
Coefficient: −2.36e+04
Value: 80plus
Bacteria.p__Spirochaetes.c__Spirochaetia.o__Spirochaetales.f__
1e+05
5e+04
0e+00
46−60 (n=7)
80plus (n=3)
61−80 (n=15)
31−45 (n=3)
20−30 (n=2)
Age_category

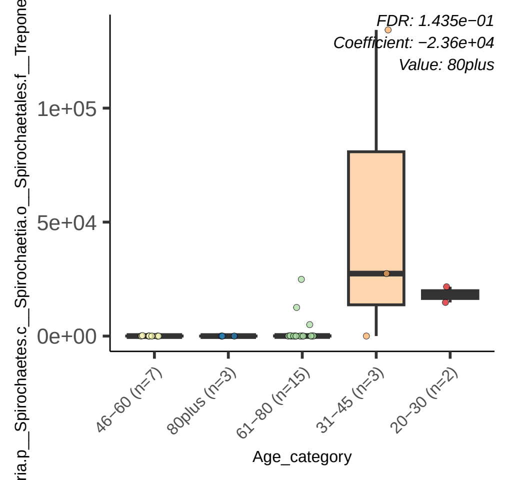

FDR: 1.435e−01
Coefficient: −2.36e+04
Value: 80plus
1e+05
5e+04
0e+00
ria.p__Spirochaetes.c__Spirochaetia.o__Spirochaetales.f__Trepone
46−60 (n=7)
80plus (n=3)
61−80 (n=15)
31−45 (n=3)
20−30 (n=2)
Age_category

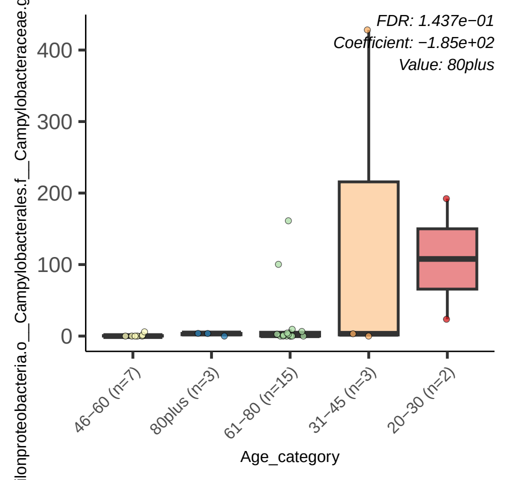

FDR: 1.437e−01
Coefficient: −1.85e+02
Value: 80plus
400
300
200
100
0
46−60 (n=7)
80plus (n=3)
61−80 (n=15)
31−45 (n=3)
20−30 (n=2)
Age_category
ilonproteobacteria.o__Campylobacterales.f__Campylobacteraceae.g

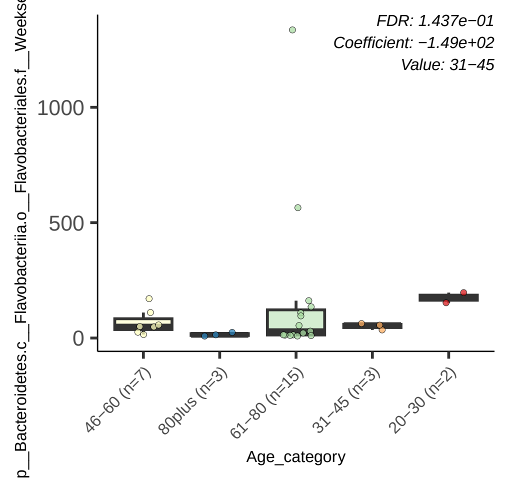
FDR: 1.437e−01
Coefficient: −1.49e+02
Value: 31−45
1000
500
0
46−60 (n=7)
80plus (n=3)
61−80 (n=15)
31−45 (n=3)
20−30 (n=2)
Age_category
p__Bacteroidetes.c__Flavobacteriia.o__Flavobacteriales.f__Weekse

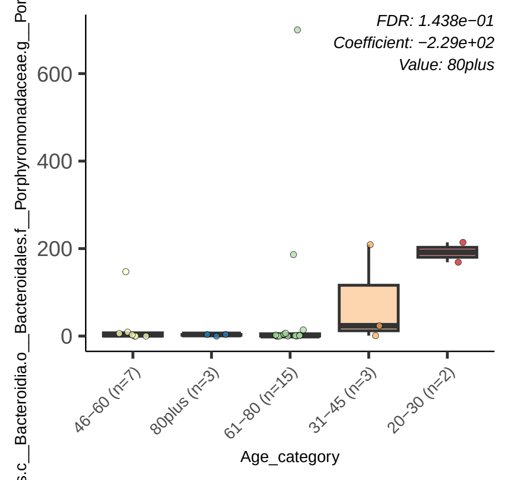

FDR: 1.438e−01
Coefficient: −2.29e+02
Value: 80plus
0
200
400
600
46−60 (n=7)
80plus (n=3)
61−80 (n=15)
31−45 (n=3)
20−30 (n=2)
Age_category
s.c__Bacteroidia.o__Bacteroidales.f__Porphyromonadaceae.g__Por

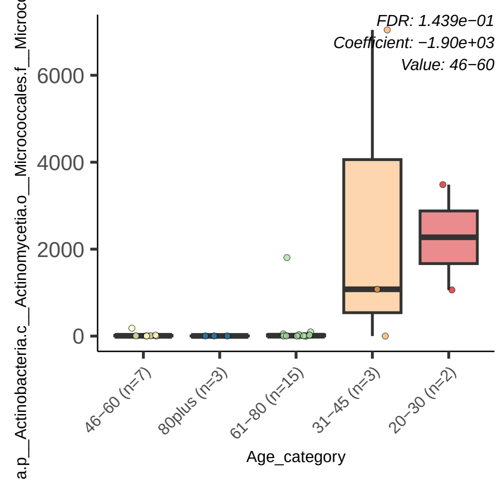

FDR: 1.439e−01
Coefficient: −1.90e+03
Value: 46−60
6000
4000
2000
0
a.p__Actinobacteria.c__Actinomycetia.o__Micrococcales.f__Microco
46−60 (n=7)
80plus (n=3)
61−80 (n=15)
31−45 (n=3)
20−30 (n=2)
Age_category

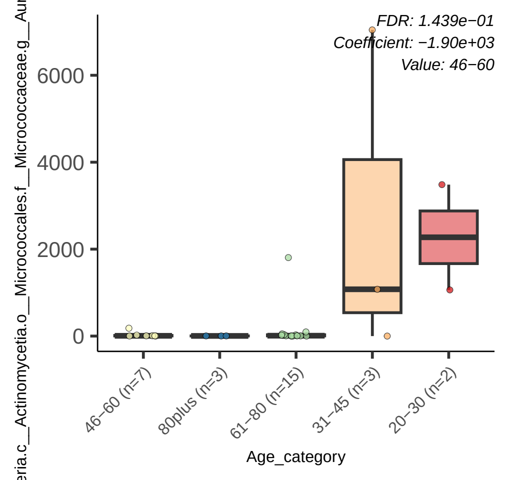

FDR: 1.439e−01
Coefficient: −1.90e+03
Value: 46−60
6000
4000
2000
0
eria.c__Actinomycetia.o__Micrococcales.f__Micrococcaceae.g__Aur
46−60 (n=7)
80plus (n=3)
61−80 (n=15)
31−45 (n=3)
20−30 (n=2)
Age_category

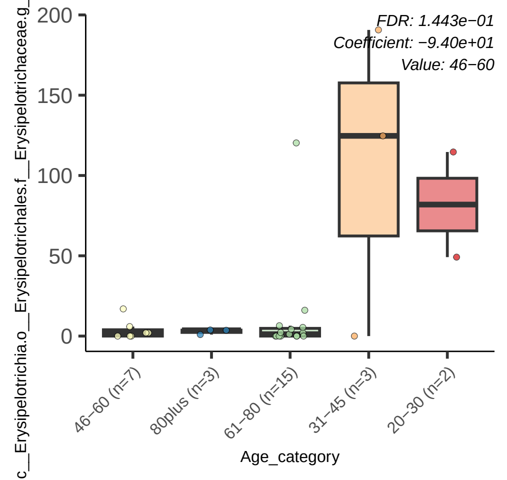

FDR: 1.443e−01
Coefficient: −9.40e+01
Value: 46−60
200
150
100
50
0
c__Erysipelotrichia.o__Erysipelotrichales.f__Erysipelotrichaceae.g__
46−60 (n=7)
80plus (n=3)
61−80 (n=15)
31−45 (n=3)
20−30 (n=2)
Age_category

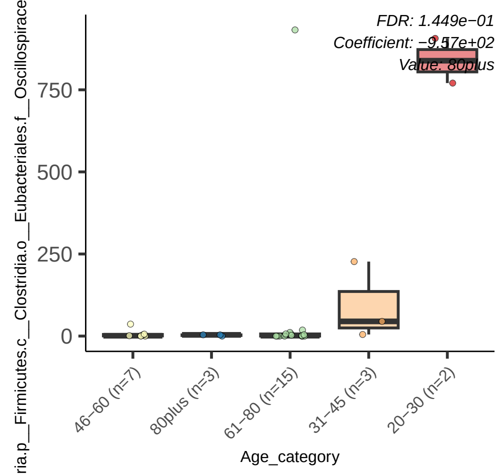

FDR: 1.449e-01
Coefficient: −9.57e+02
Value: 80plus
0
250
500
750
eria.p__Firmicutes.c__Clostridia.o__Eubacteriales.f__Oscillospirace
46−60 (n=7)
80plus (n=3)
61−80 (n=15)
31−45 (n=3)
20−30 (n=2)
Age_category

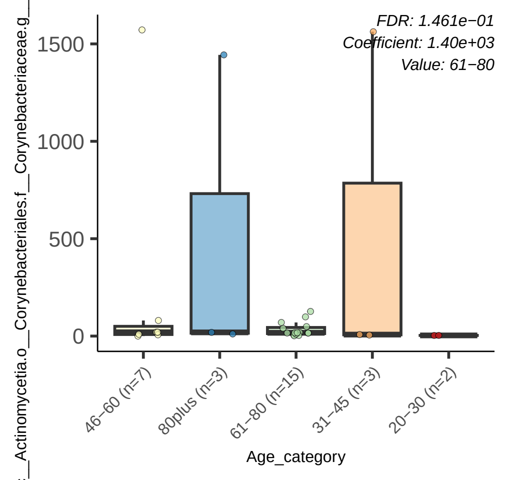

FDR: 1.461e−01
Coefficient: 1.40e+03
Value: 61−80
Actinomycetia.o__Corynebacteriales.f__Corynebacteriaceae.g__
1500
1000
500
0
46−60 (n=7)
80plus (n=3)
61−80 (n=15)
31−45 (n=3)
20−30 (n=2)
Age_category

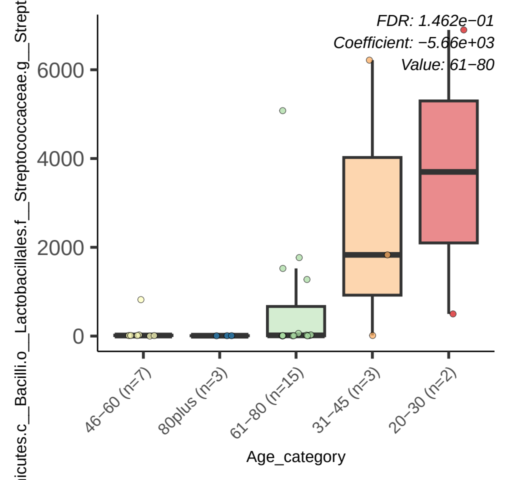

FDR: 1.462e−01
Coefficient: −5.66e+03
Value: 61−80
icutes.c__Bacilli.o__Lactobacillales.f__Streptococcaceae.g__Strept
6000
4000
2000
0
46−60 (n=7)
80plus (n=3)
61−80 (n=15)
31−45 (n=3)
20−30 (n=2)
Age_category

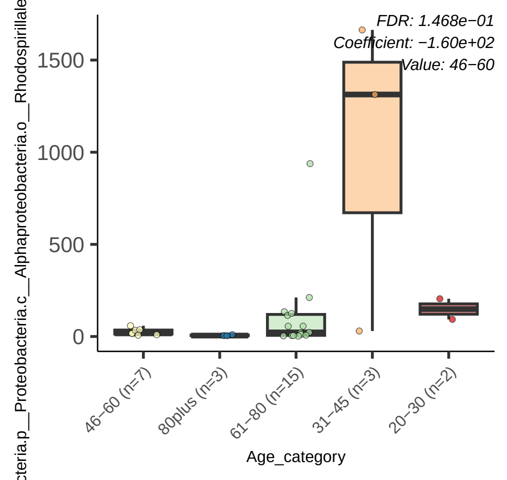
FDR: 1.468e−01
Coefficient: −1.60e+02
Value: 46−60
1500
1000
500
0
cteria.p__Proteobacteria.c__Alphaproteobacteria.o__Rhodospirillale
46−60 (n=7)
80plus (n=3)
61−80 (n=15)
31−45 (n=3)
20−30 (n=2)
Age_category

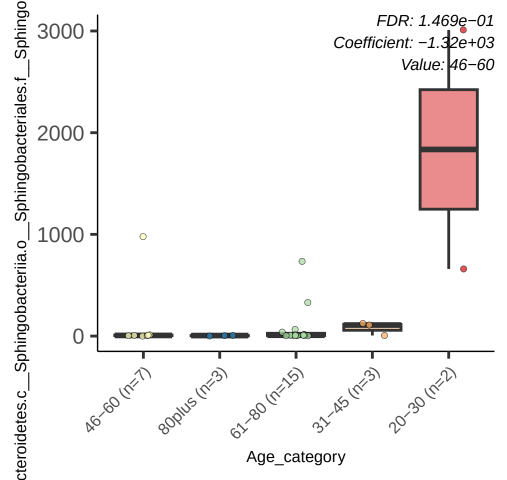

FDR: 1.469e−01
Coefficient: −1.32e+03
Value: 46−60
3000
2000
1000
0
46−60 (n=7)
80plus (n=3)
61−80 (n=15)
31−45 (n=3)
20−30 (n=2)
Age_category
cteroidetes.c__Sphingobacteriia.o__Sphingobacteriales.f__Sphingo

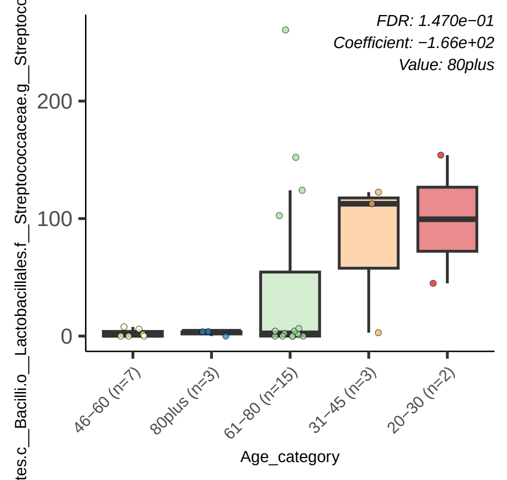

FDR: 1.470e−01
Coefficient: −1.66e+02
Value: 80plus
200
100
0
tes.c__Bacilli.o__Lactobacillales.f__Streptococcaceae.g__Streptoco
46−60 (n=7)
80plus (n=3)
61−80 (n=15)
31−45 (n=3)
20−30 (n=2)
Age_category

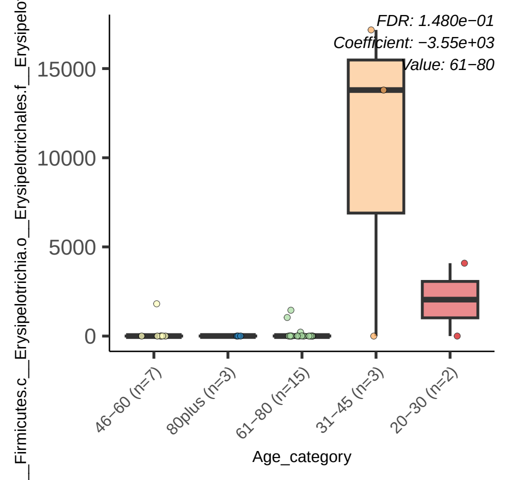

FDR: 1.480e−01
Coefficient: −3.55e+03
Value: 61−80
15000
10000
5000
0
46−60 (n=7)
80plus (n=3)
61−80 (n=15)
31−45 (n=3)
20−30 (n=2)
Age_category
Firmicutes.c__Erysipelotrichia.o__Erysipelotrichales.f__Erysipelo

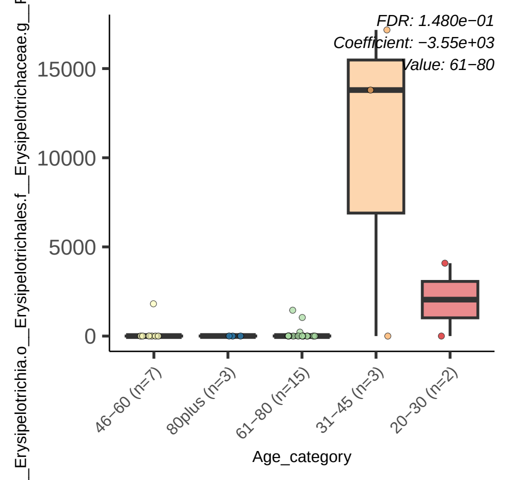
FDR: 1.480e−01
Coefficient: −3.55e+03
Value: 61−80
15000
10000
5000
0
_Erysipelotrichia.o__Erysipelotrichales.f__Erysipelotrichaceae.g__F
46−60 (n=7)
80plus (n=3)
61−80 (n=15)
31−45 (n=3)
20−30 (n=2)
Age_category

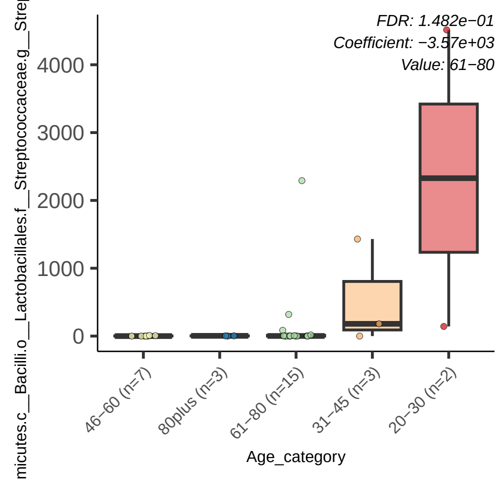

FDR: 1.482e−01
Coefficient: −3.57e+03
Value: 61−80
4000
3000
2000
1000
0
micutes.c__Bacilli.o__Lactobacillales.f__Streptococcaceae.g__Stre
46−60 (n=7)
80plus (n=3)
61−80 (n=15)
31−45 (n=3)
20−30 (n=2)
Age_category

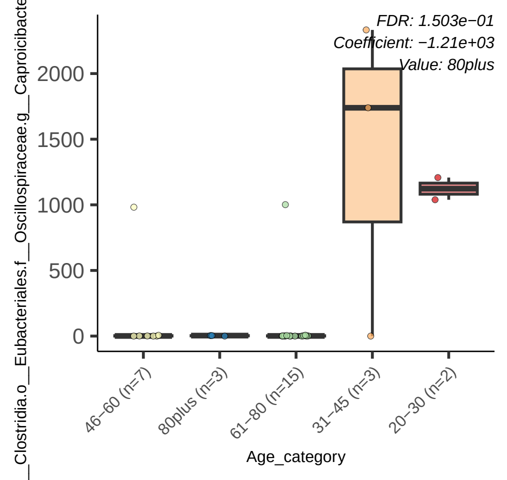
FDR: 1.503e−01
Coefficient: −1.21e+03
Value: 80plus
Clostridia.o__Eubacteriales.f__Oscillospiraceae.g__Caproicibacte
0
500
1000
1500
2000
46−60 (n=7)
80plus (n=3)
61−80 (n=15)
31−45 (n=3)
20−30 (n=2)
Age_category

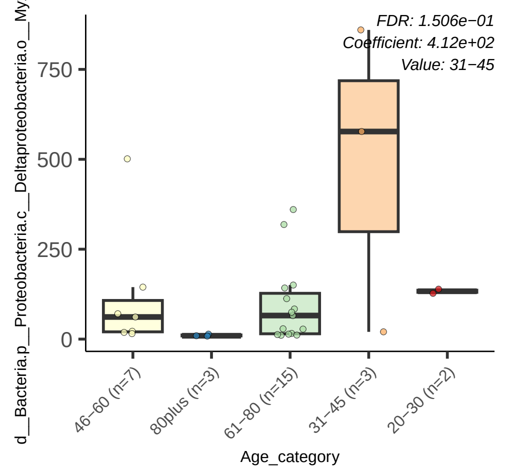

FDR: 1.506e−01
Coefficient: 4.12e+02
Value: 31−45
d__Bacteria.p__Proteobacteria.c__Deltaproteobacteria.o__My
750
500
250
0
46−60 (n=7)
80plus (n=3)
61−80 (n=15)
31−45 (n=3)
20−30 (n=2)
Age_category

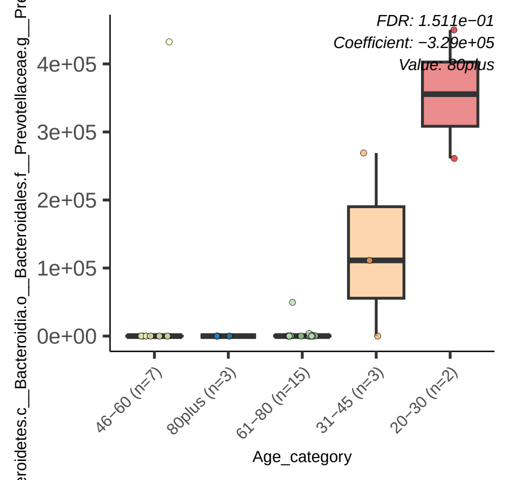

FDR: 1.511e−01
Coefficient: −3.29e+05
Value: 80plus
eroidetes.c__Bacteroidia.o__Bacteroidales.f__Prevotellaceae.g__Pre
4e+05
3e+05
2e+05
1e+05
0e+00
46−60 (n=7)
80plus (n=3)
61−80 (n=15)
31−45 (n=3)
20−30 (n=2)
Age_category

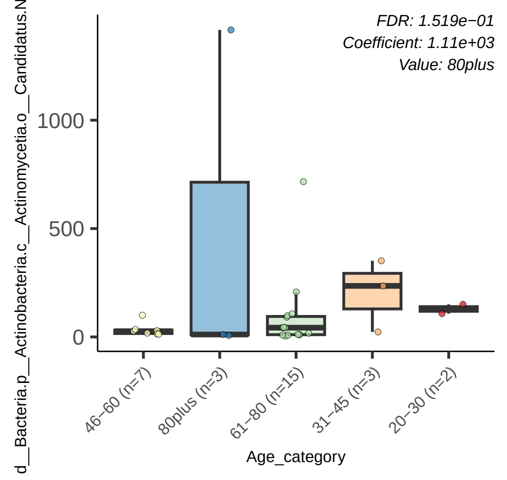

FDR: 1.519e−01
Coefficient: 1.11e+03
Value: 80plus
d__Bacteria.p__Actinobacteria.c__Actinomycetia.o__Candidatus.N
1000
500
0
46−60 (n=7)
80plus (n=3)
61−80 (n=15)
31−45 (n=3)
20−30 (n=2)
Age_category

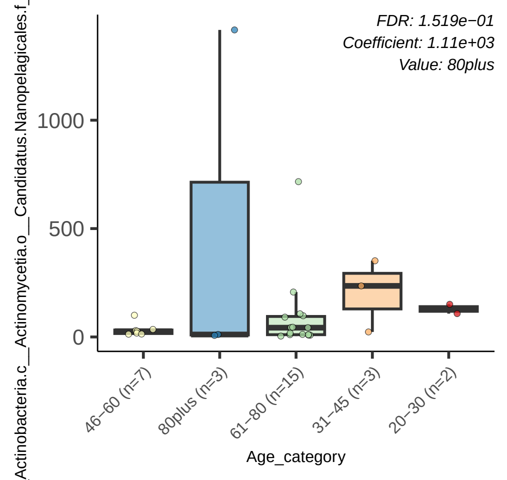
FDR: 1.519e−01
Coefficient: 1.11e+03
Value: 80plus
Actinobacteria.c__Actinomycetia.o__Candidatus.Nanopelagicales.f_
1000
500
0
46−60 (n=7)
80plus (n=3)
61−80 (n=15)
31−45 (n=3)
20−30 (n=2)
Age_category

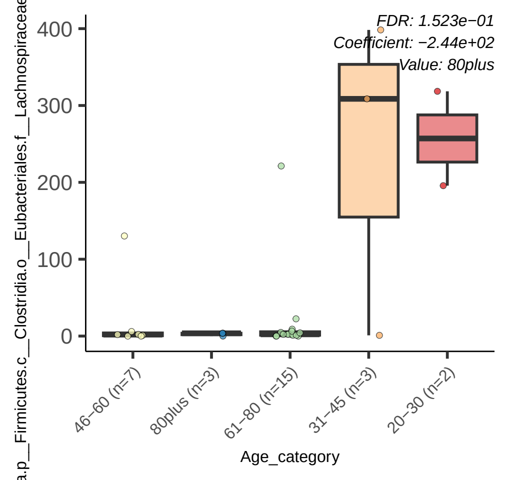

FDR: 1.523e−01
Coefficient: −2.44e+02
Value: 80plus
a.p__Firmicutes.c__Clostridia.o__Eubacteriales.f__Lachnospiraceae
400
300
200
100
0
46−60 (n=7)
80plus (n=3)
61−80 (n=15)
31−45 (n=3)
20−30 (n=2)
Age_category

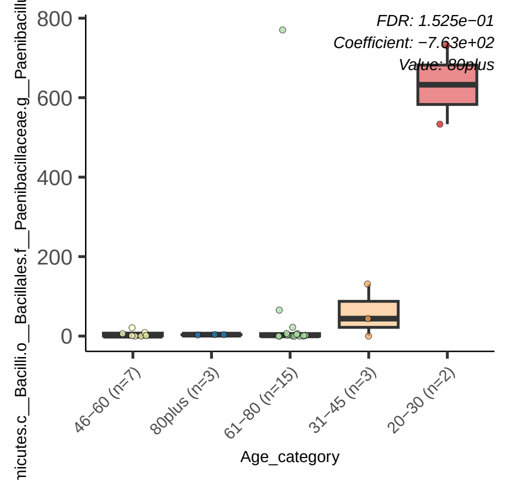

FDR: 1.525e−01
Coefficient: −7.63e+02
Value: 80plus
800
600
400
200
0
micutes.c__Bacilli.o__Bacillales.f__Paenibacillaceae.g__Paenibacillu
46−60 (n=7)
80plus (n=3)
61−80 (n=15)
31−45 (n=3)
20−30 (n=2)
Age_category

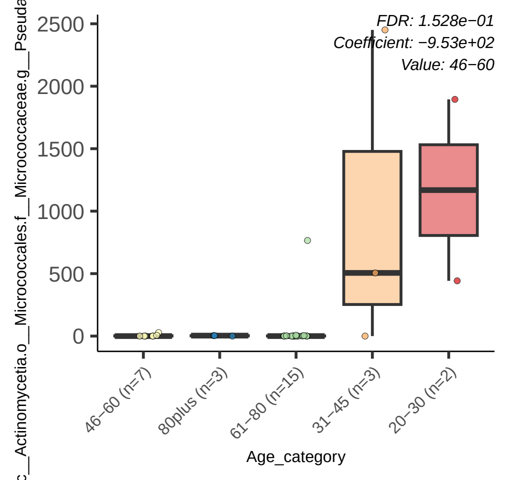

FDR: 1.528e−01
Coefficient: −9.53e+02
Value: 46−60
0
500
1000
1500
2000
2500
c__Actinomycetia.o__Micrococcales.f__Micrococcaceae.g__Pseuda
46−60 (n=7)
80plus (n=3)
61−80 (n=15)
31−45 (n=3)
20−30 (n=2)
Age_category

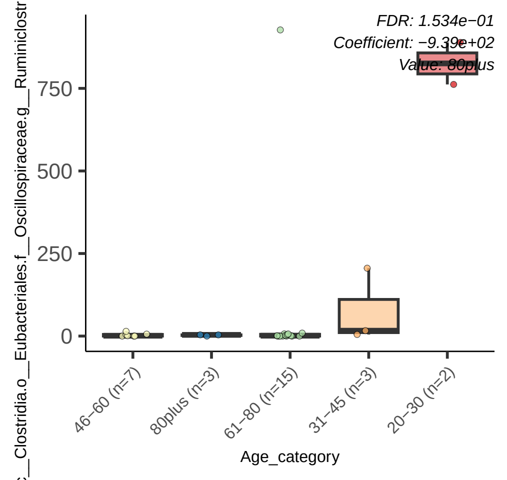

FDR: 1.534e−01
Coefficient: −9.39e+02
Value: 80plus
Clostridia.o__Eubacteriales.f__Oscillospiraceae.g__Ruminiclostr
750
500
250
0
46−60 (n=7)
80plus (n=3)
61−80 (n=15)
31−45 (n=3)
20−30 (n=2)
Age_category

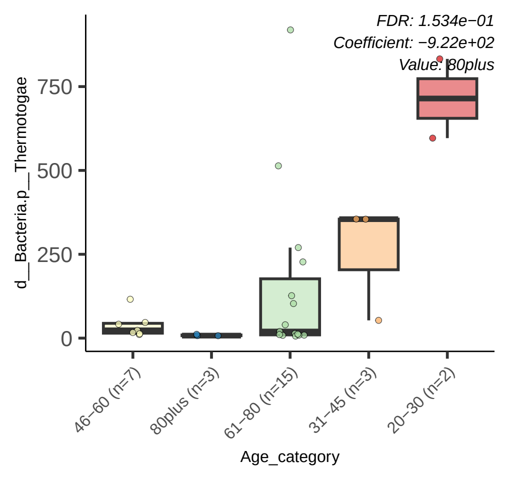

FDR: 1.534e−01
Coefficient: −9.22e+02
Value: 80plus
d__Bacteria.p__Thermotogae
750
500
250
0
46−60 (n=7)
80plus (n=3)
61−80 (n=15)
31−45 (n=3)
20−30 (n=2)
Age_category

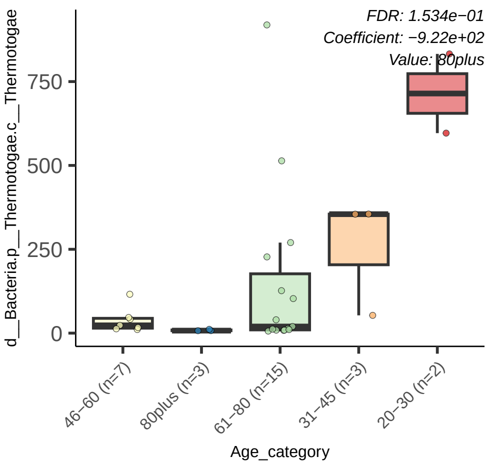

FDR: 1.534e−01
Coefficient: −9.22e+02
Value: 80plus
d__Bacteria.p__Thermotogae.c__Thermotogae
750
500
250
0
46−60 (n=7)
80plus (n=3)
61−80 (n=15)
31−45 (n=3)
20−30 (n=2)
Age_category

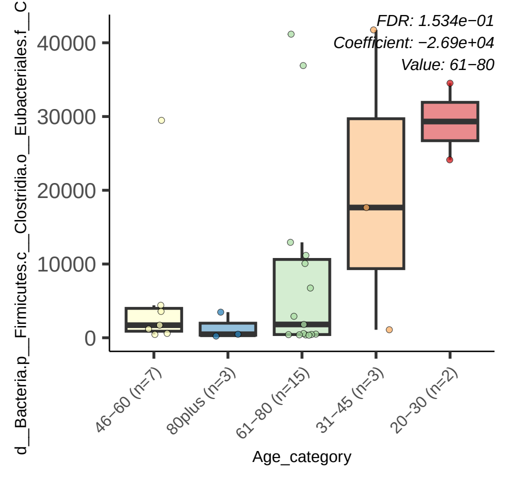

FDR: 1.534e−01
Coefficient: −2.69e+04
Value: 61−80
d__Bacteria.p__Firmicutes.c__Clostridia.o__Eubacteriales.f__C
40000
30000
20000
10000
0
46−60 (n=7)
80plus (n=3)
61−80 (n=15)
31−45 (n=3)
20−30 (n=2)
Age_category

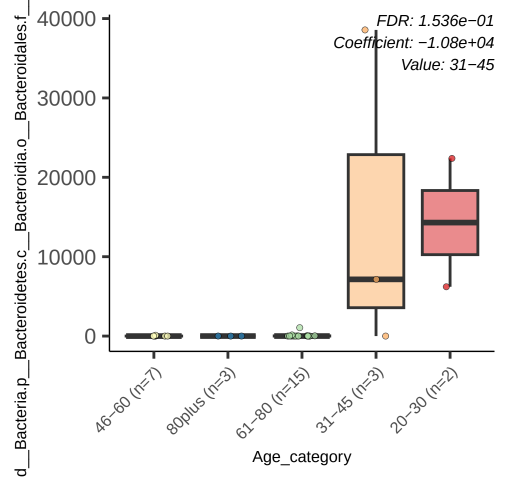

FDR: 1.536e−01
Coefficient: −1.08e+04
Value: 31−45
d__Bacteria.p__Bacteroidetes.c__Bacteroidia.o__Bacteroidales.f
40000
30000
20000
10000
0
46−60 (n=7)
80plus (n=3)
61−80 (n=15)
31−45 (n=3)
20−30 (n=2)
Age_category

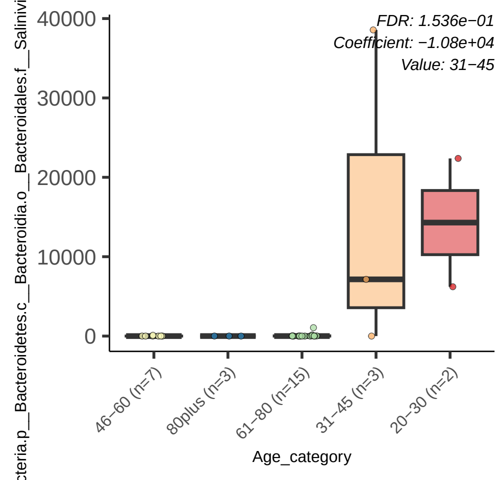

FDR: 1.536e−01
Coefficient: −1.08e+04
Value: 31−45
40000
30000
20000
10000
0
46−60 (n=7)
80plus (n=3)
61−80 (n=15)
31−45 (n=3)
20−30 (n=2)
Age_category
cteria.p__Bacteroidetes.c__Bacteroidia.o__Bacteroidales.f__Salinivi

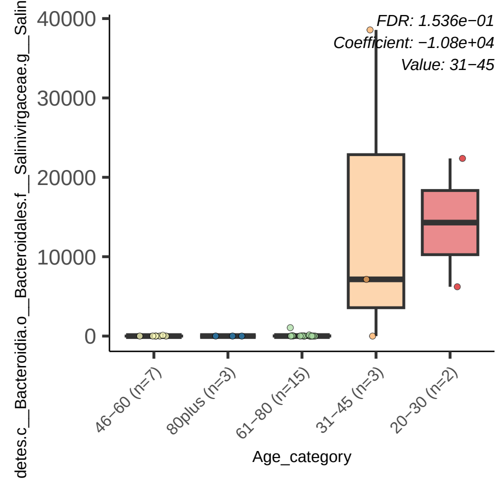

FDR: 1.536e−01
Coefficient: −1.08e+04
Value: 31−45
40000
30000
20000
10000
0
46−60 (n=7)
80plus (n=3)
61−80 (n=15)
31−45 (n=3)
20−30 (n=2)
Age_category
detes.c__Bacteroidia.o__Bacteroidales.f__Salinivirgaceae.g__Salin

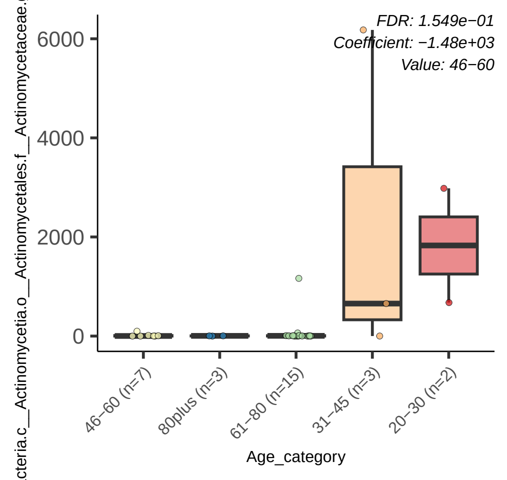

FDR: 1.549e−01
Coefficient: −1.48e+03
Value: 46−60
6000
4000
2000
0
cteria.c__Actinomycetia.o__Actinomycetales.f__Actinomycetaceae.
46−60 (n=7)
80plus (n=3)
61−80 (n=15)
31−45 (n=3)
20−30 (n=2)
Age_category

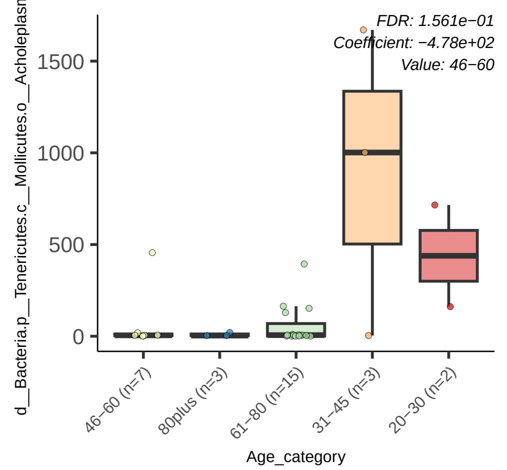

FDR: 1.561e−01
Coefficient: −4.78e+02
Value: 46−60
d__Bacteria.p__Tenericutes.c__Mollicutes.o__Acholeplasm
1500
1000
500
0
46−60 (n=7)
80plus (n=3)
61−80 (n=15)
31−45 (n=3)
20−30 (n=2)
Age_category

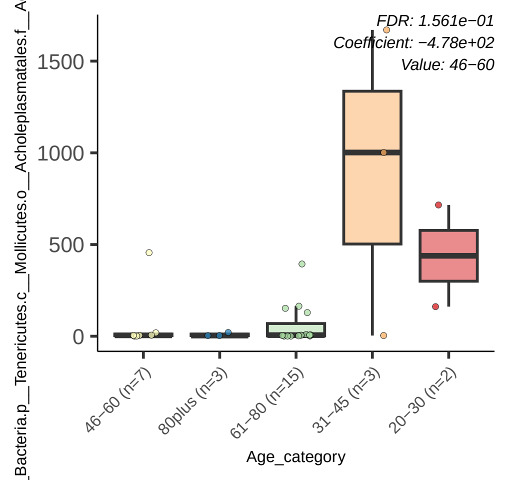
FDR: 1.561e−01
Coefficient: −4.78e+02
Value: 46−60
1500
1000
500
0
_Bacteria.p__Tenericutes.c__Mollicutes.o__Acholeplasmatales.f__A
46−60 (n=7)
80plus (n=3)
61−80 (n=15)
31−45 (n=3)
20−30 (n=2)
Age_category

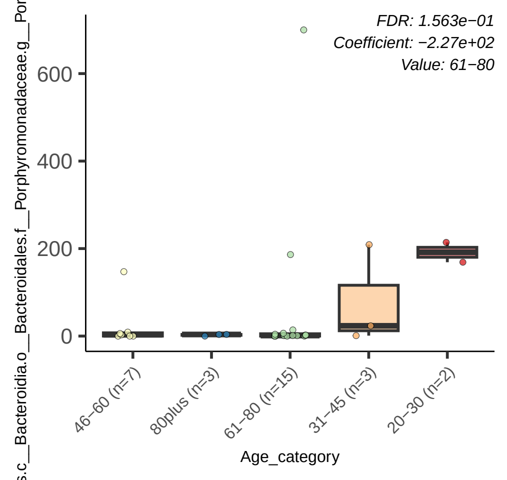

FDR: 1.563e−01
Coefficient: −2.27e+02
Value: 61−80
600
400
200
0
s.c__Bacteroidia.o__Bacteroidales.f__Porphyromonadaceae.g__Por
46−60 (n=7)
80plus (n=3)
61−80 (n=15)
31−45 (n=3)
20−30 (n=2)
Age_category

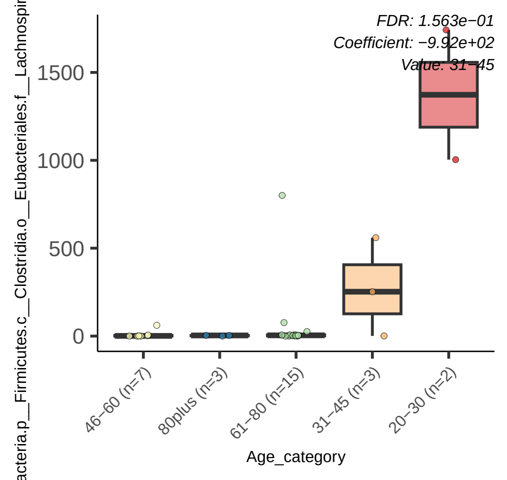

FDR: 1.563e−01
Coefficient: −9.92e+02
Value: 31−45
acteria.p__Firmicutes.c__Clostridia.o__Eubacteriales.f__Lachnospi
1500
1000
500
0
46−60 (n=7)
80plus (n=3)
61−80 (n=15)
31−45 (n=3)
20−30 (n=2)
Age_category

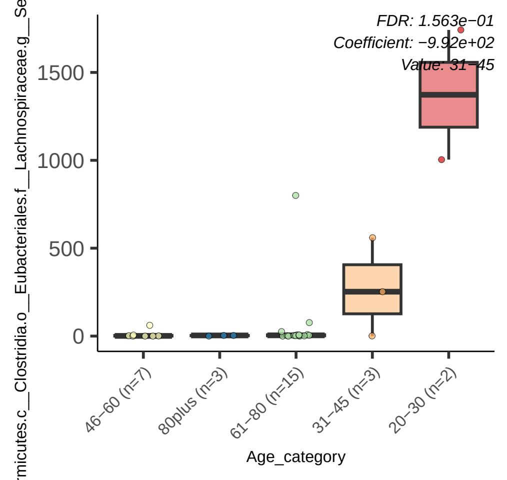

FDR: 1.563e−01
Coefficient: −9.92e+02
Value: 31−45
0
500
1000
1500
46−60 (n=7)
80plus (n=3)
61−80 (n=15)
31−45 (n=3)
20−30 (n=2)
Age_category
rmicutes.c__Clostridia.o__Eubacteriales.f__Lachnospiraceae.g__Se

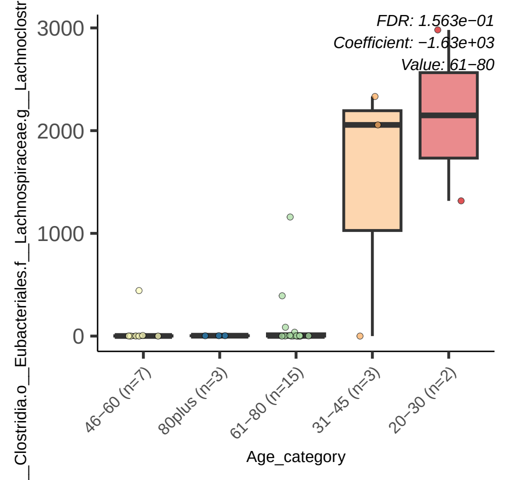

FDR: 1.563e−01
Coefficient: −1.63e+03
Value: 61−80
3000
2000
1000
0
__Clostridia.o__Eubacteriales.f__Lachnospiraceae.g__Lachnoclostr
46−60 (n=7)
80plus (n=3)
61−80 (n=15)
31−45 (n=3)
20−30 (n=2)
Age_category

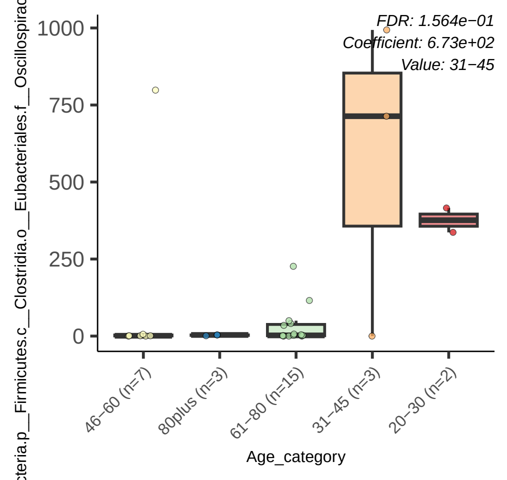

FDR: 1.564e−01
Coefficient: 6.73e+02
Value: 31−45
1000
750
500
250
0
46−60 (n=7)
80plus (n=3)
61−80 (n=15)
31−45 (n=3)
20−30 (n=2)
Age_category
cteria.p__Firmicutes.c__Clostridia.o__Eubacteriales.f__Oscillospirac

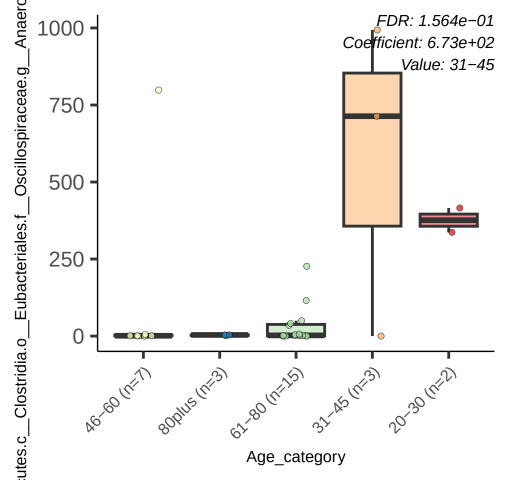

FDR: 1.564e−01
Coefficient: 6.73e+02
Value: 31−45
1000
750
500
250
0
46−60 (n=7)
80plus (n=3)
61−80 (n=15)
31−45 (n=3)
20−30 (n=2)
Age_category
cutes.c__Clostridia.o__Eubacteriales.f__Oscillospiraceae.g__Anaero

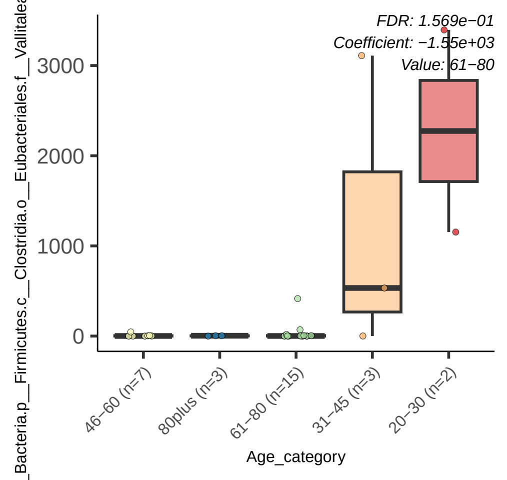

FDR: 1.569e−01
Coefficient: −1.55e+03
Value: 61−80
3000
2000
1000
0
Bacteria.p__Firmicutes.c__Clostridia.o__Eubacteriales.f__Vallitalea
46−60 (n=7)
80plus (n=3)
61−80 (n=15)
31−45 (n=3)
20−30 (n=2)
Age_category

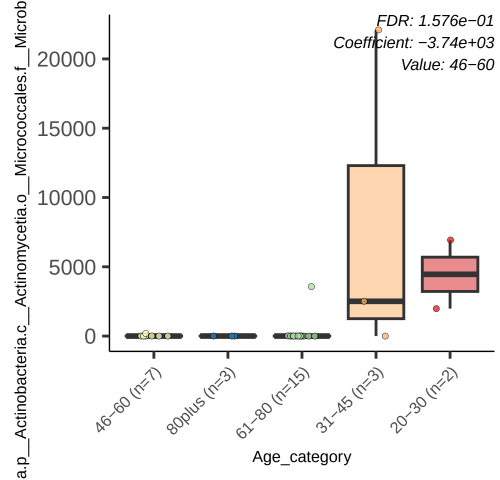

FDR: 1.576e−01
Coefficient: −3.74e+03
Value: 46−60
a.p__Actinobacteria.c__Actinomycetia.o__Micrococcales.f__Microb
20000
15000
10000
5000
0
46−60 (n=7)
80plus (n=3)
61−80 (n=15)
31−45 (n=3)
20−30 (n=2)
Age_category

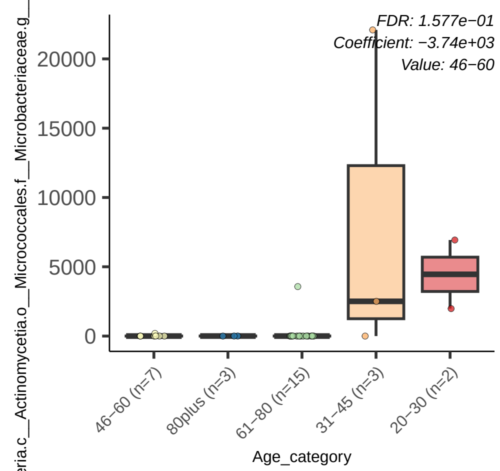
FDR: 1.577e−01
Coefficient: −3.74e+03
Value: 46−60
20000
15000
10000
5000
0
teria.c__Actinomycetia.o__Micrococcales.f__Microbacteriaceae.g__
46−60 (n=7)
80plus (n=3)
61−80 (n=15)
31−45 (n=3)
20−30 (n=2)
Age_category

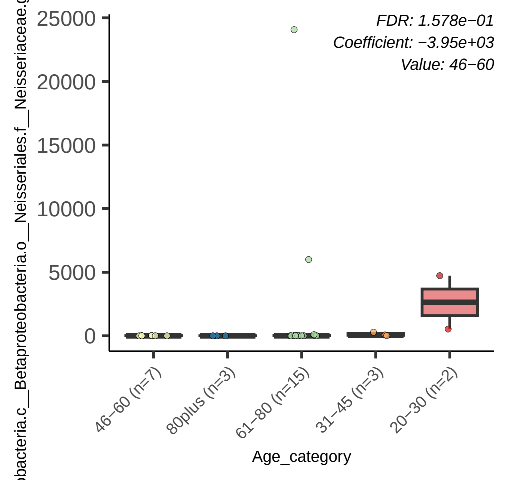

FDR: 1.578e−01
Coefficient: −3.95e+03
Value: 46−60
25000
20000
15000
10000
5000
0
obacteria.c__Betaproteobacteria.o__Neisseriales.f__Neisseriaceae.g
46−60 (n=7)
80plus (n=3)
61−80 (n=15)
31−45 (n=3)
20−30 (n=2)
Age_category

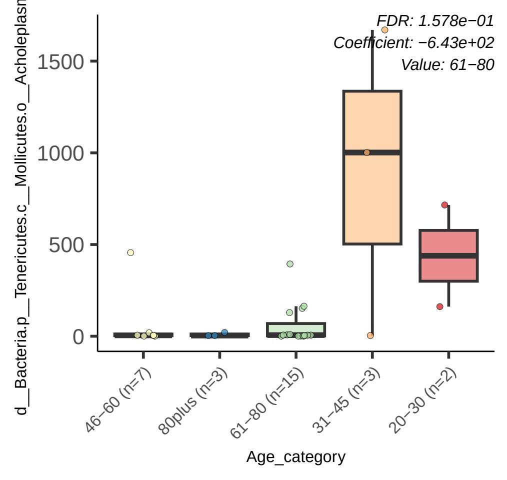

FDR: 1.578e−01
Coefficient: −6.43e+02
Value: 61−80
d__Bacteria.p__Tenericutes.c__Mollicutes.o__Acholeplasm
1500
1000
500
0
46−60 (n=7)
80plus (n=3)
61−80 (n=15)
31−45 (n=3)
20−30 (n=2)
Age_category

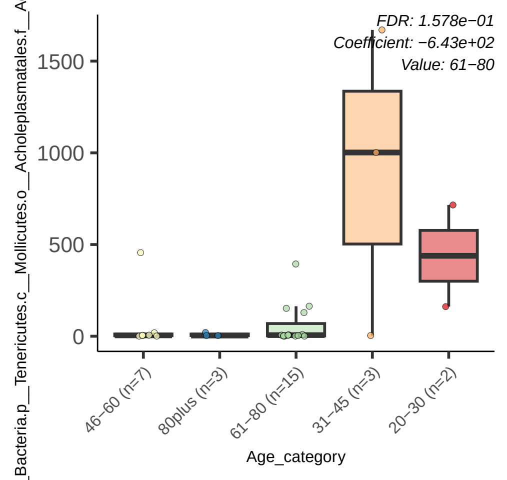

FDR: 1.578e−01
Coefficient: −6.43e+02
Value: 61−80
1500
1000
500
0
Bacteria.p__Tenericutes.c__Mollicutes.o__Acholeplasmatales.f__A
46−60 (n=7)
80plus (n=3)
61−80 (n=15)
31−45 (n=3)
20−30 (n=2)
Age_category

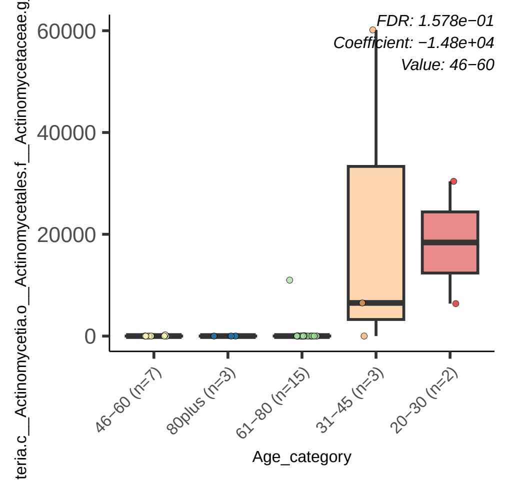

FDR: 1.578e−01
Coefficient: −1.48e+04
Value: 46−60
60000
40000
20000
0
teria.c__Actinomycetia.o__Actinomycetales.f__Actinomycetaceae.g
46−60 (n=7)
80plus (n=3)
61−80 (n=15)
31−45 (n=3)
20−30 (n=2)
Age_category

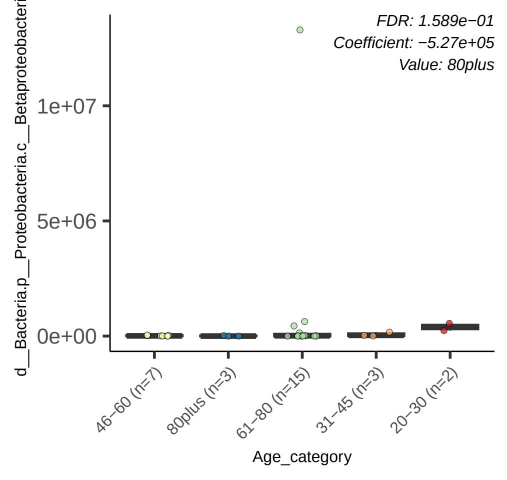
FDR: 1.589e−01
Coefficient: −5.27e+05
Value: 80plus
d__Bacteria.p__Proteobacteria.c__Betaproteobacteri
1e+07
5e+06
0e+00
46−60 (n=7)
80plus (n=3)
61−80 (n=15)
31−45 (n=3)
20−30 (n=2)
Age_category

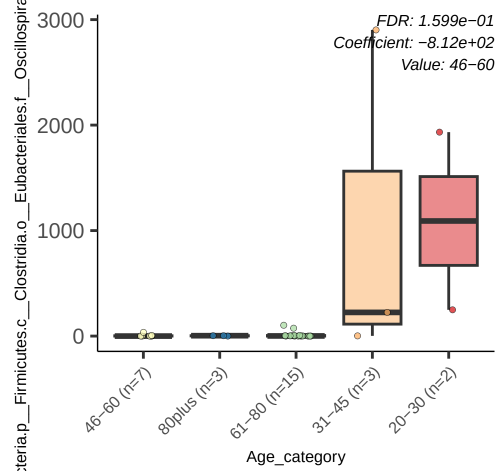

FDR: 1.599e−01
Coefficient: −8.12e+02
Value: 46−60
3000
2000
1000
0
acteria.p__Firmicutes.c__Clostridia.o__Eubacteriales.f__Oscillospira
46−60 (n=7)
80plus (n=3)
61−80 (n=15)
31−45 (n=3)
20−30 (n=2)
Age_category

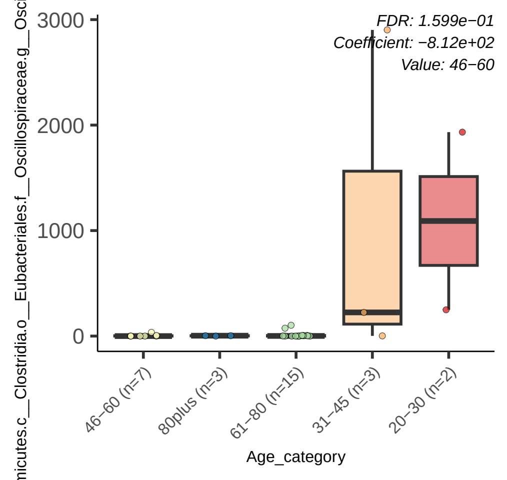
FDR: 1.599e−01
Coefficient: −8.12e+02
Value: 46−60
3000
2000
1000
0
micutes.c__Clostridia.o__Eubacteriales.f__Oscillospiraceae.g__Osc
46−60 (n=7)
80plus (n=3)
61−80 (n=15)
31−45 (n=3)
20−30 (n=2)
Age_category

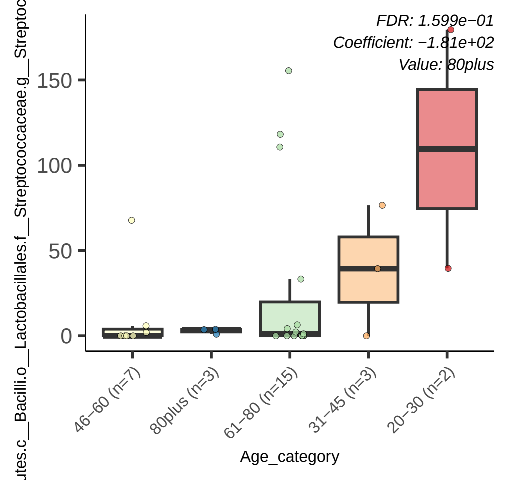

FDR: 1.599e−01
Coefficient: −1.81e+02
Value: 80plus
utes.c__Bacilli.o__Lactobacillales.f__Streptococcaceae.g__Streptoc
150
100
50
0
46−60 (n=7)
80plus (n=3)
61−80 (n=15)
31−45 (n=3)
20−30 (n=2)
Age_category

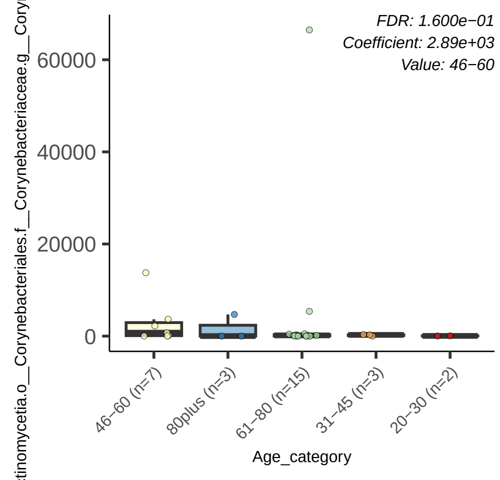

FDR: 1.600e−01
Coefficient: 2.89e+03
Value: 46−60
60000
40000
20000
0
ctinomycetia.o__Corynebacteriales.f__Corynebacteriaceae.g__Cory
46−60 (n=7)
80plus (n=3)
61−80 (n=15)
31−45 (n=3)
20−30 (n=2)
Age_category

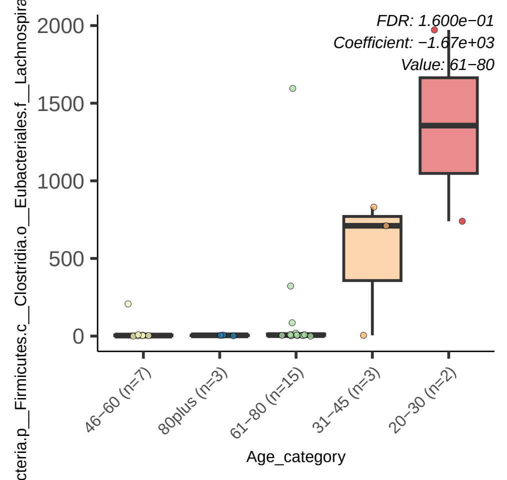

FDR: 1.600e−01
Coefficient: −1.67e+03
Value: 61−80
cteria.p__Firmicutes.c__Clostridia.o__Eubacteriales.f__Lachnospira
2000
1500
1000
500
0
46−60 (n=7)
80plus (n=3)
61−80 (n=15)
31−45 (n=3)
20−30 (n=2)
Age_category

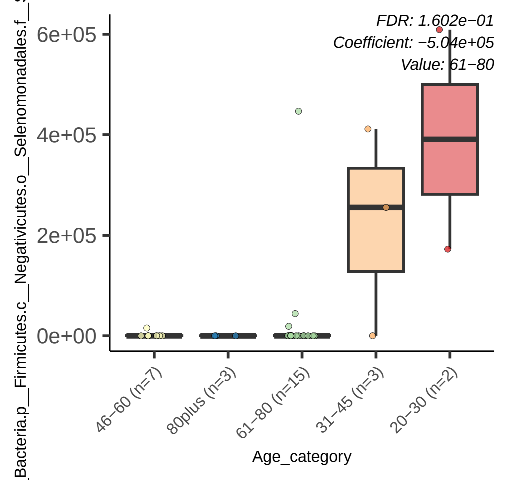

FDR: 1.602e−01
Coefficient: −5.04e+05
Value: 61−80
6e+05
4e+05
2e+05
0e+00
Bacteria.p__Firmicutes.c__Negativicutes.o__Selenomonadales.f__
46−60 (n=7)
80plus (n=3)
61−80 (n=15)
31−45 (n=3)
20−30 (n=2)
Age_category

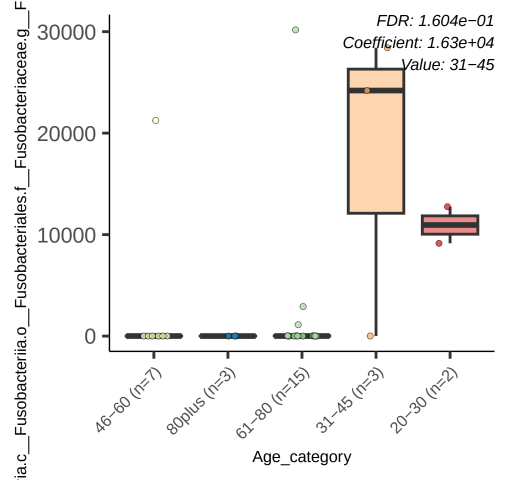

FDR: 1.604e−01
Coefficient: 1.63e+04
Value: 31−45
30000
20000
10000
0
46−60 (n=7)
80plus (n=3)
61−80 (n=15)
31−45 (n=3)
20−30 (n=2)
Age_category
ia.c__Fusobacteriia.o__Fusobacteriales.f__Fusobacteriaceae.g__F

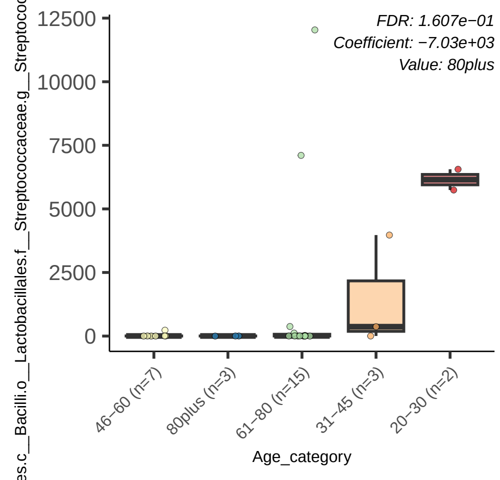

FDR: 1.607e−01
Coefficient: −7.03e+03
Value: 80plus
12500
10000
7500
5000
2500
0
es.c__Bacilli.o__Lactobacillales.f__Streptococcaceae.g__Streptococ
46−60 (n=7)
80plus (n=3)
61−80 (n=15)
31−45 (n=3)
20−30 (n=2)
Age_category

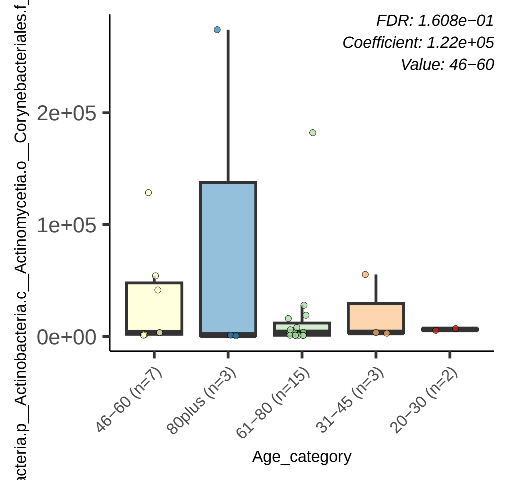

FDR: 1.608e−01
Coefficient: 1.22e+05
Value: 46−60
2e+05
1e+05
0e+00
acteria.p__Actinobacteria.c__Actinomycetia.o__Corynebacteriales.f_
46−60 (n=7)
80plus (n=3)
61−80 (n=15)
31−45 (n=3)
20−30 (n=2)
Age_category

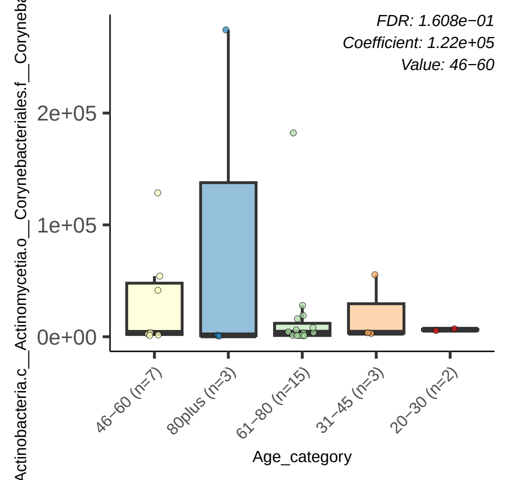

FDR: 1.608e−01
Coefficient: 1.22e+05
Value: 46−60
Actinobacteria.c__Actinomycetia.o__Corynebacteriales.f__Corynebacteria
2e+05
1e+05
0e+00
46−60 (n=7)
80plus (n=3)
61−80 (n=15)
31−45 (n=3)
20−30 (n=2)
Age_category

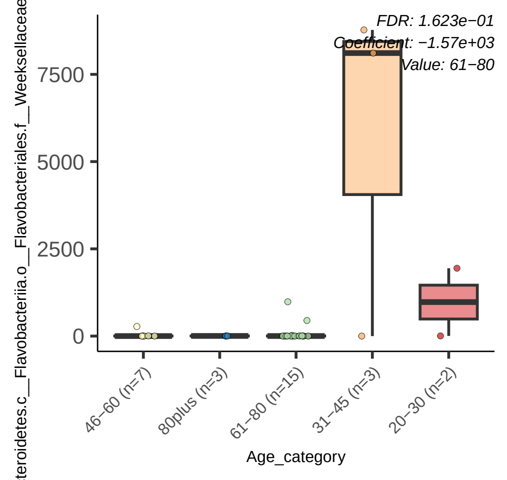
FDR: 1.623e−01
Coefficient: −1.57e+03
Value: 61−80
7500
5000
2500
0
teroidetes.c__Flavobacteriia.o__Flavobacteriales.f__Weeksellaceae
46−60 (n=7)
80plus (n=3)
61−80 (n=15)
31−45 (n=3)
20−30 (n=2)
Age_category

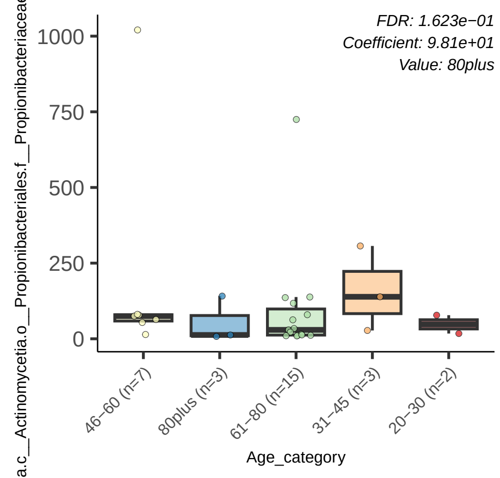
FDR: 1.623e−01
Coefficient: 9.81e+01
Value: 80plus
ia.c__Actinomycetia.o__Propionibacteriales.f__Propionibacteriaceae
1000
750
500
250
0
46−60 (n=7)
80plus (n=3)
61−80 (n=15)
31−45 (n=3)
20−30 (n=2)
Age_category

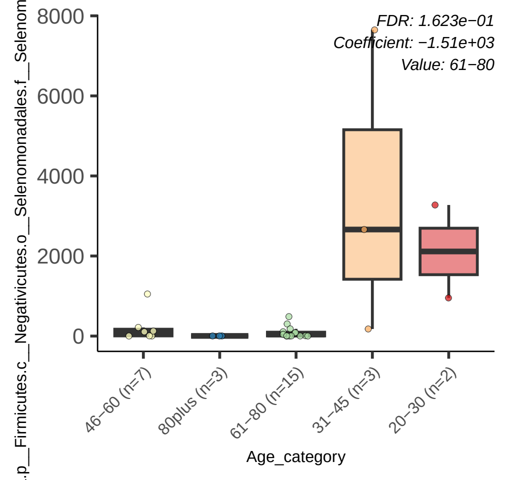

FDR: 1.623e−01
Coefficient: −1.51e+03
Value: 61−80
p__Firmicutes.c__Negativicutes.o__Selenomonadales.f__Selenom
8000
6000
4000
2000
0
46−60 (n=7)
80plus (n=3)
61−80 (n=15)
31−45 (n=3)
20−30 (n=2)
Age_category

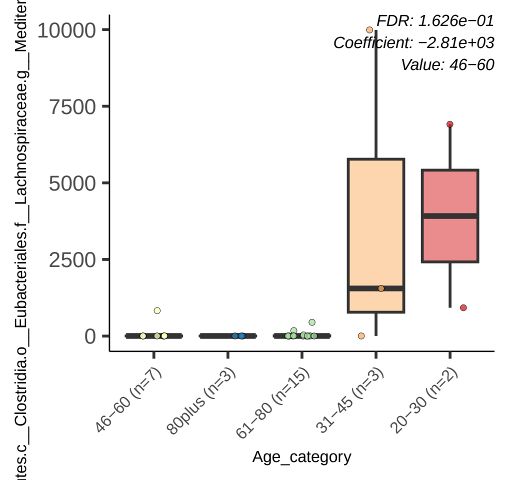

FDR: 1.626e−01
Coefficient: −2.81e+03
Value: 46−60
10000
7500
5000
2500
0
46−60 (n=7)
80plus (n=3)
61−80 (n=15)
31−45 (n=3)
20−30 (n=2)
Age_category
ites.c__Clostridia.o__Eubacteriales.f__Lachnospiraceae.g__Mediter

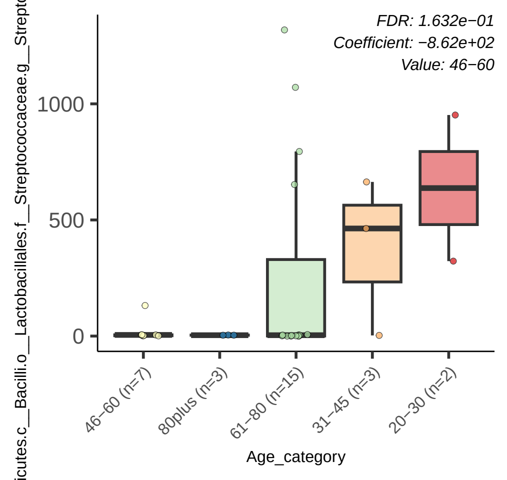

FDR: 1.632e−01
Coefficient: −8.62e+02
Value: 46−60
1000
500
0
46−60 (n=7)
80plus (n=3)
61−80 (n=15)
31−45 (n=3)
20−30 (n=2)
Age_category
icutes.c__Bacilli.o__Lactobacillales.f__Streptococcaceae.g__Strept

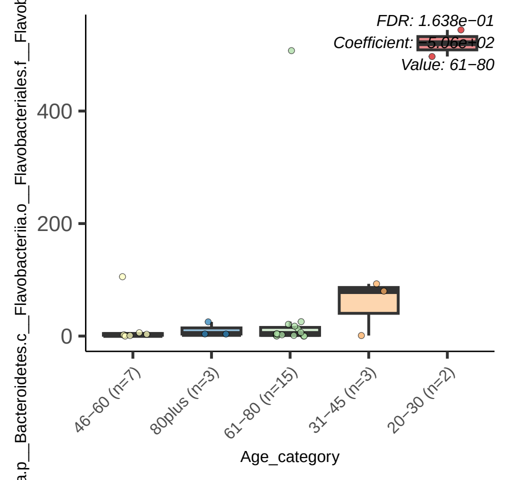

FDR: 1.638e−01
Coefficient: −5.06e+02
Value: 61−80
400
200
0
46−60 (n=7)
80plus (n=3)
61−80 (n=15)
31−45 (n=3)
20−30 (n=2)
Age_category
a.p__Bacteroidetes.c__Flavobacteriia.o__Flavobacteriales.f__Flavob

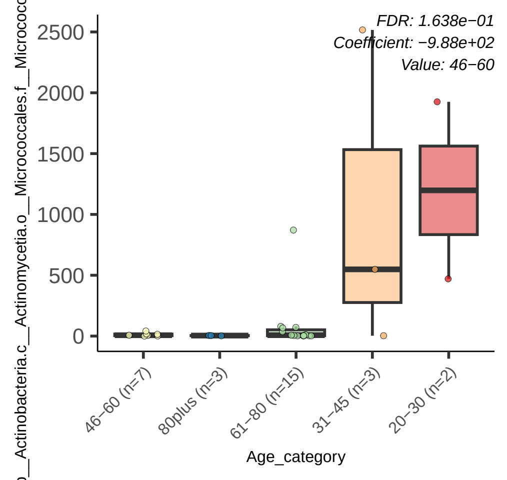

FDR: 1.638e−01
Coefficient: −9.88e+02
Value: 46−60
0
500
1000
1500
2000
2500
o__Actinobacteria.c__Actinomycetia.o__Micrococcales.f__Micrococc
46−60 (n=7)
80plus (n=3)
61−80 (n=15)
31−45 (n=3)
20−30 (n=2)
Age_category

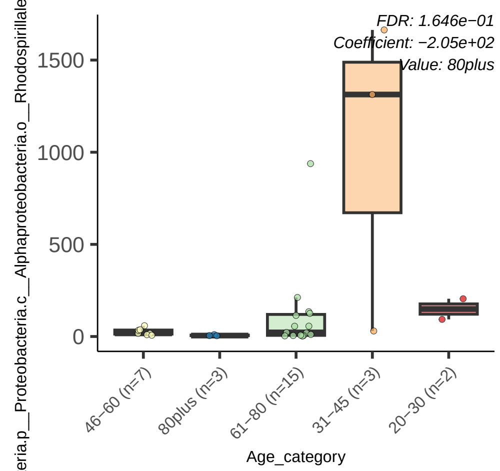

FDR: 1.646e−01
Coefficient: −2.05e+02
Value: 80plus
cteria.p__Proteobacteria.c__Alphaproteobacteria.o__Rhodospirillale
1500
1000
500
0
46−60 (n=7)
80plus (n=3)
61−80 (n=15)
31−45 (n=3)
20−30 (n=2)
Age_category

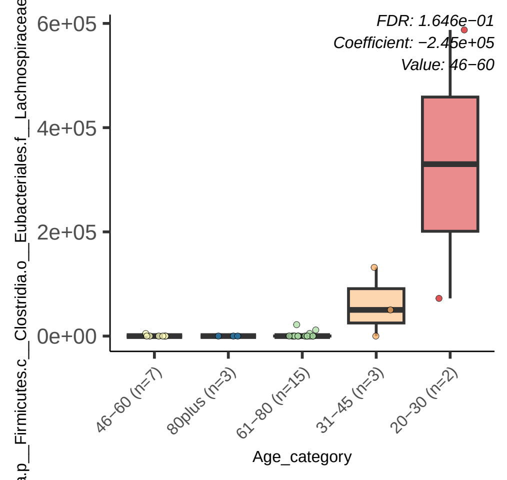

FDR: 1.646e−01
Coefficient: −2.45e+05
Value: 46−60
6e+05
4e+05
2e+05
0e+00
a.p__Firmicutes.c__Clostridia.o__Eubacteriales.f__Lachnospiraceae
46−60 (n=7)
80plus (n=3)
61−80 (n=15)
31−45 (n=3)
20−30 (n=2)
Age_category

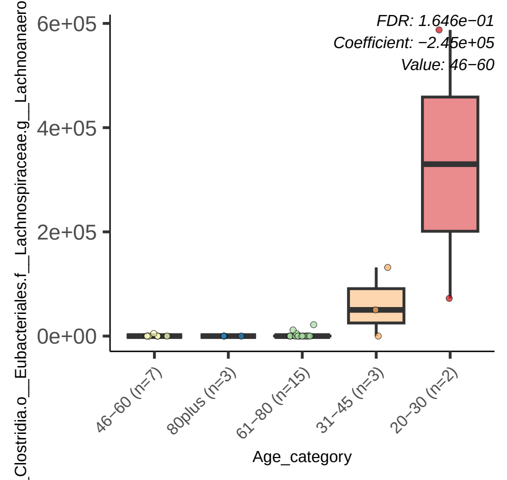

FDR: 1.646e−01
Coefficient: −2.45e+05
Value: 46−60
6e+05
4e+05
2e+05
0e+00
Clostridia.o__Eubacteriales.f__Lachnospiraceae.g__Lachnoanaero
46−60 (n=7)
80plus (n=3)
61−80 (n=15)
31−45 (n=3)
20−30 (n=2)
Age_category

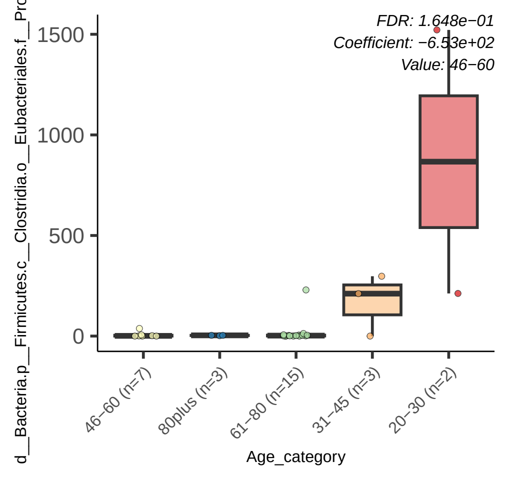

FDR: 1.648e−01
Coefficient: −6.53e+02
Value: 46−60
d__Bacteria.p__Firmicutes.c__Clostridia.o__Eubacteriales.f__Pro
1500
1000
500
0
46−60 (n=7)
80plus (n=3)
61−80 (n=15)
31−45 (n=3)
20−30 (n=2)
Age_category

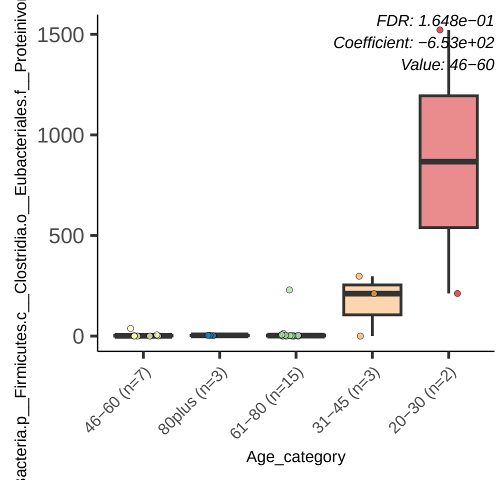

FDR: 1.648e−01
Coefficient: −6.53e+02
Value: 46−60
1500
1000
500
0
46−60 (n=7)
80plus (n=3)
61−80 (n=15)
31−45 (n=3)
20−30 (n=2)
Age_category
Bacteria.p__Firmicutes.c__Clostridia.o__Eubacteriales.f__Proteinivor

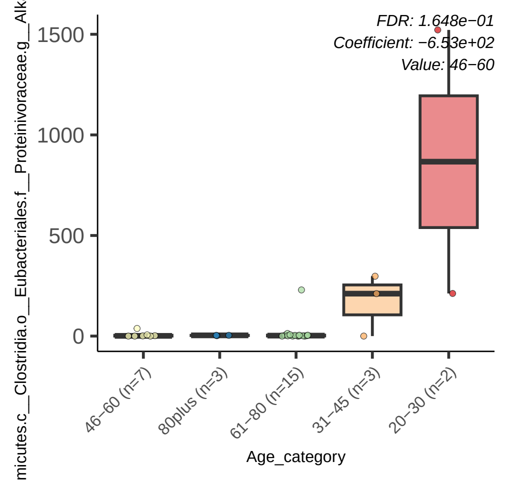

FDR: 1.648e−01
Coefficient: −6.53e+02
Value: 46−60
1500
1000
500
0
micutes.c__Clostridia.o__Eubacteriales.f__Proteinivoraceae.g__Alk
46−60 (n=7)
80plus (n=3)
61−80 (n=15)
31−45 (n=3)
20−30 (n=2)
Age_category

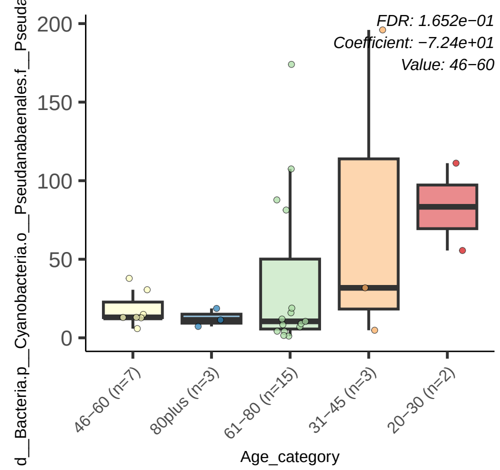

FDR: 1.652e−01
Coefficient: −7.24e+01
Value: 46−60
d__Bacteria.p__Cyanobacteria.o__Pseudanabaenales.f__Pseuda
200
150
100
50
0
46−60 (n=7)
80plus (n=3)
61−80 (n=15)
31−45 (n=3)
20−30 (n=2)
Age_category

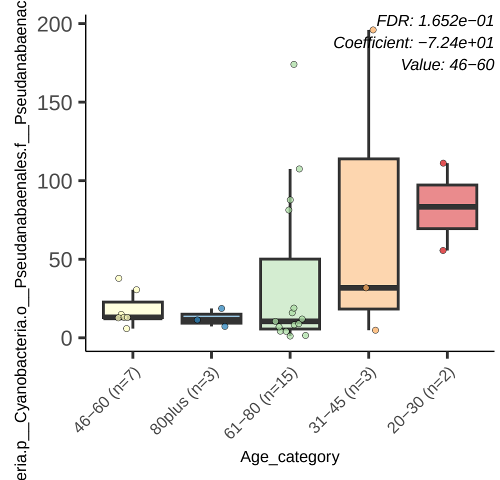

FDR: 1.652e−01
Coefficient: −7.24e+01
Value: 46−60
eria.p__Cyanobacteria.o__Pseudanabaenales.f__Pseudanabaenac
200
150
100
50
0
46−60 (n=7)
80plus (n=3)
61−80 (n=15)
31−45 (n=3)
20−30 (n=2)
Age_category

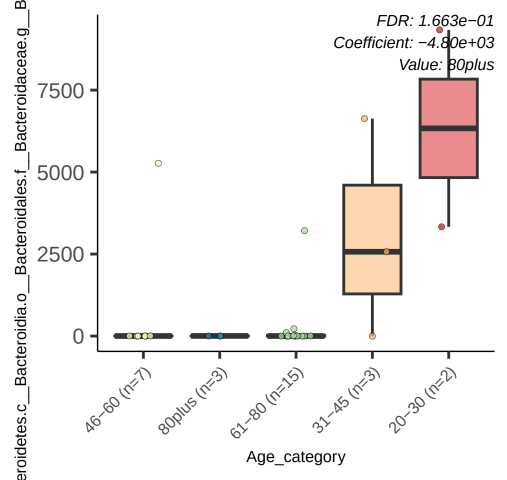

FDR: 1.663e−01
Coefficient: −4.80e+03
Value: 80plus
7500
5000
2500
0
46−60 (n=7)
80plus (n=3)
61−80 (n=15)
31−45 (n=3)
20−30 (n=2)
Age_category
eroidetes.c__Bacteroidia.o__Bacteroidales.f__Bacteroidaceae.g__B

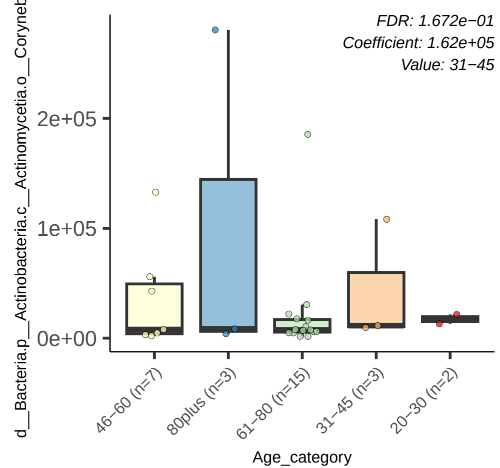

FDR: 1.672e−01
Coefficient: 1.62e+05
Value: 31−45
2e+05
1e+05
0e+00
d__Bacteria.p__Actinobacteria.c__Actinomycetia.o__Coryneb
46−60 (n=7)
80plus (n=3)
61−80 (n=15)
31−45 (n=3)
20−30 (n=2)
Age_category

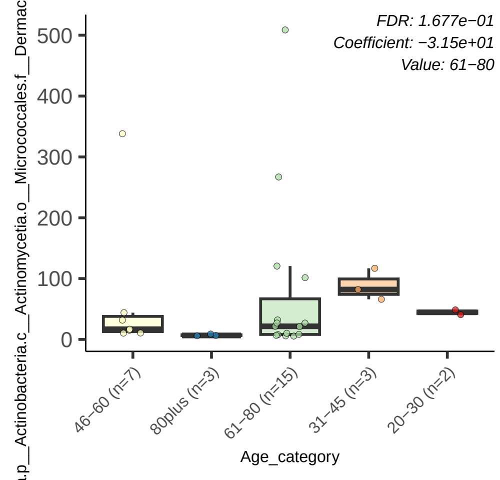

FDR: 1.677e−01
Coefficient: −3.15e+01
Value: 61−80
a.p__Actinobacteria.c__Actinomycetia.o__Micrococcales.f__Dermac
500
400
300
200
100
0
46−60 (n=7)
80plus (n=3)
61−80 (n=15)
31−45 (n=3)
20−30 (n=2)
Age_category

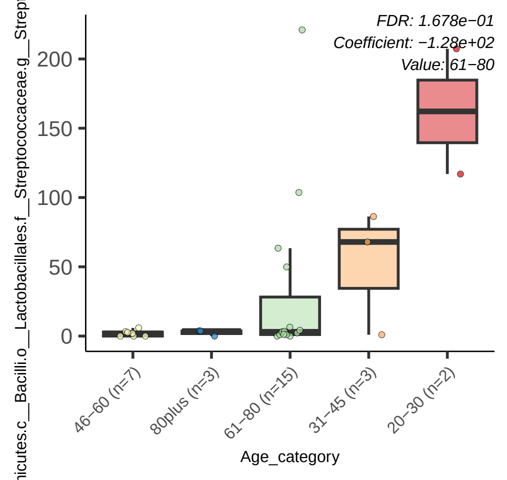

FDR: 1.678e−01
Coefficient: −1.28e+02
Value: 61−80
200
150
100
50
0
nicutes.c__Bacilli.o__Lactobacillales.f__Streptococcaceae.g__Strep
46−60 (n=7)
80plus (n=3)
61−80 (n=15)
31−45 (n=3)
20−30 (n=2)
Age_category

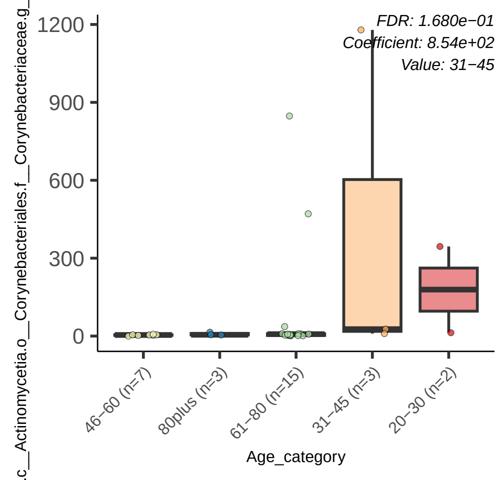

FDR: 1.680e−01
Coefficient: 8.54e+02
Value: 31−45
c__Actinomycetia.o__Corynebacteriales.f__Corynebacteriaceae.g__
1200
900
600
300
0
46−60 (n=7)
80plus (n=3)
61−80 (n=15)
31−45 (n=3)
20−30 (n=2)
Age_category

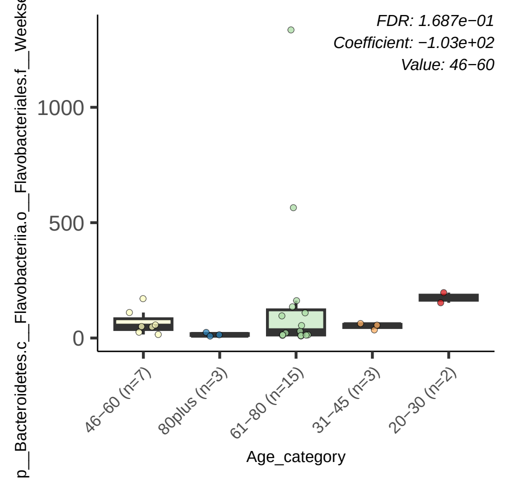

FDR: 1.687e−01
Coefficient: −1.03e+02
Value: 46−60
p__Bacteroidetes.c__Flavobacteriia.o__Flavobacteriales.f__Weekse
1000
500
0
46−60 (n=7)
80plus (n=3)
61−80 (n=15)
31−45 (n=3)
20−30 (n=2)
Age_category

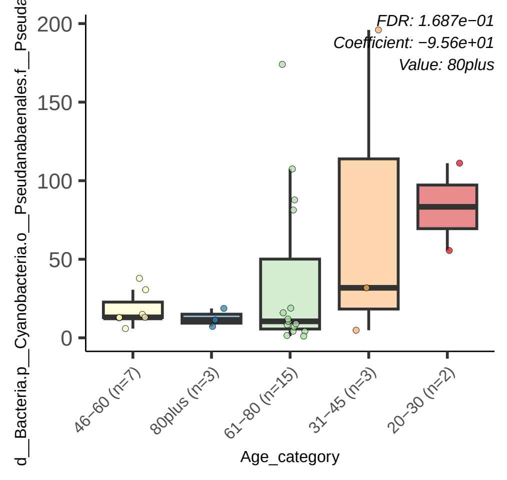

FDR: 1.687e−01
Coefficient: −9.56e+01
Value: 80plus
d__Bacteria.p__Cyanobacteria.o__Pseudanabaenales.f__Pseuda
200
150
100
50
0
46−60 (n=7)
80plus (n=3)
61−80 (n=15)
31−45 (n=3)
20−30 (n=2)
Age_category

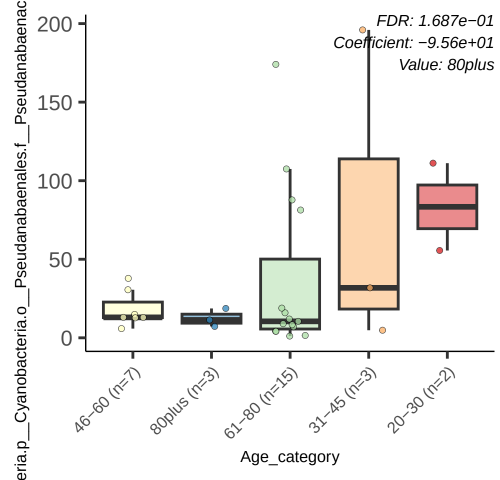
FDR: 1.687e−01
Coefficient: −9.56e+01
Value: 80plus
200
150
100
50
0
eria.p__Cyanobacteria.o__Pseudanabaenales.f__Pseudanabaenac
46−60 (n=7)
80plus (n=3)
61−80 (n=15)
31−45 (n=3)
20−30 (n=2)
Age_category

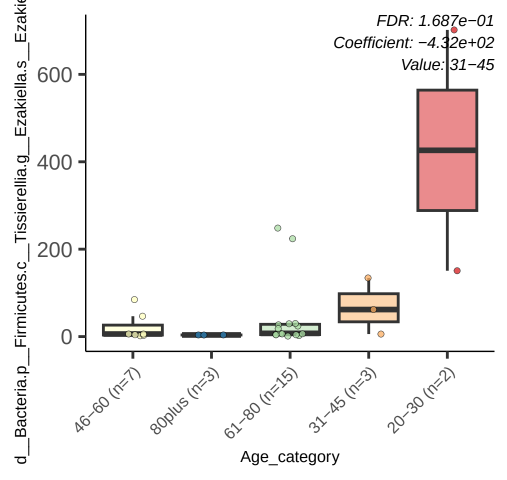

FDR: 1.687e−01
Coefficient: −4.32e+02
Value: 31−45
d__Bacteria.p__Firmicutes.c__Tissierellia.g__Ezakiella.s__Ezakie
0
200
400
600
46−60 (n=7)
80plus (n=3)
61−80 (n=15)
31−45 (n=3)
20−30 (n=2)
Age_category

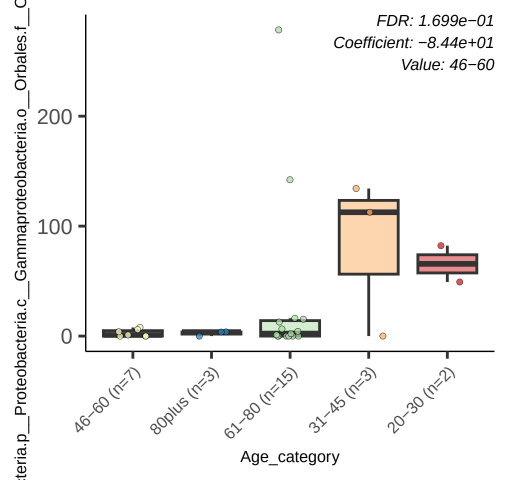

cteria.p__Proteobacteria.c__Gammaproteobacteria.o__Orbales.f__O
FDR: 1.699e−01
Coefficient: −8.44e+01
Value: 46−60
200
100
0
46−60 (n=7)
80plus (n=3)
61−80 (n=15)
31−45 (n=3)
20−30 (n=2)
Age_category

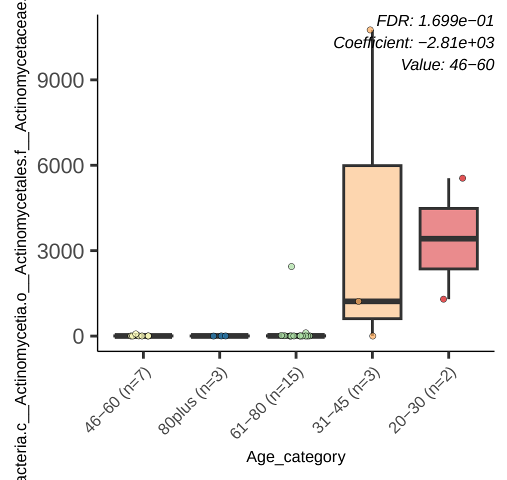

FDR: 1.699e−01
Coefficient: −2.81e+03
Value: 46−60
9000
6000
3000
0
acteria.c__Actinomycetia.o__Actinomycetales.f__Actinomycetaceae.
46−60 (n=7)
80plus (n=3)
61−80 (n=15)
31−45 (n=3)
20−30 (n=2)
Age_category

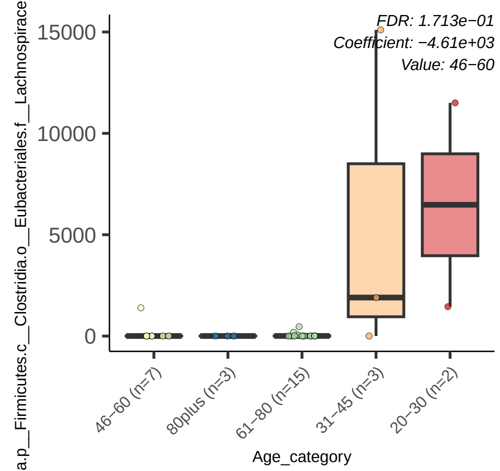

FDR: 1.713e−01
Coefficient: −4.61e+03
Value: 46−60
15000
10000
5000
0
ria.p__Firmicutes.c__Clostridia.o__Eubacteriales.f__Lachnospirace
46−60 (n=7)
80plus (n=3)
61−80 (n=15)
31−45 (n=3)
20−30 (n=2)
Age_category

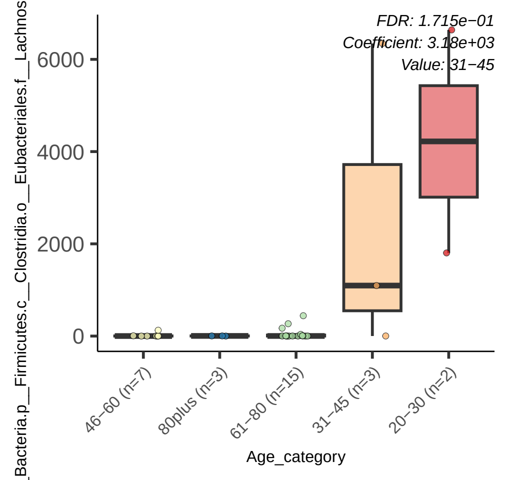

FDR: 1.715e−01
Coefficient: 3.18e+03
Value: 31−45
Bacteria.p__Firmicutes.c__Clostridia.o__Eubacteriales.f__Lachnos
6000
4000
2000
0
46−60 (n=7)
80plus (n=3)
61−80 (n=15)
31−45 (n=3)
20−30 (n=2)
Age_category

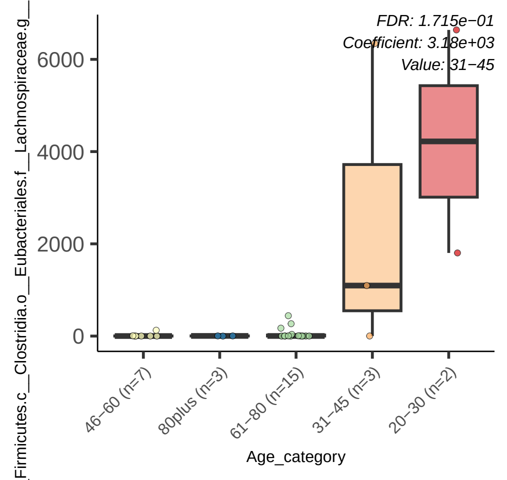
FDR: 1.715e−01
Coefficient: 3.18e+03
Value: 31−45
Firmicutes.c__Clostridia.o__Eubacteriales.f__Lachnospiraceae.g__
6000
4000
2000
0
46−60 (n=7)
80plus (n=3)
61−80 (n=15)
31−45 (n=3)
20−30 (n=2)
Age_category

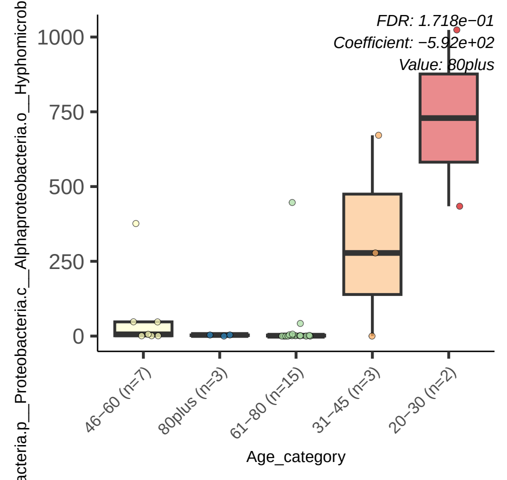

FDR: 1.718e−01
Coefficient: −5.92e+02
Value: 80plus
acteria.p__Proteobacteria.c__Alphaproteobacteria.o__Hyphomicrob
1000
750
500
250
0
46−60 (n=7)
80plus (n=3)
61−80 (n=15)
31−45 (n=3)
20−30 (n=2)
Age_category

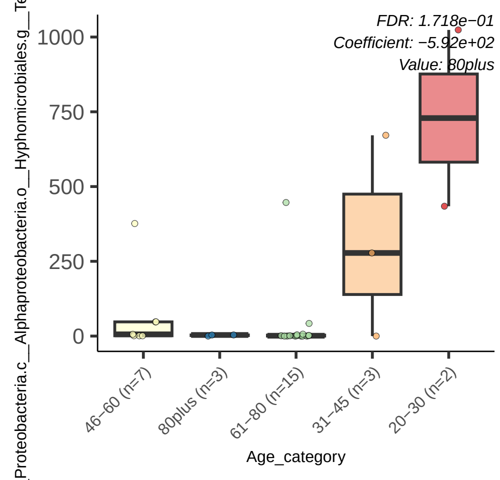

FDR: 1.718e−01
Coefficient: −5.92e+02
Value: 80plus
Proteobacteria.c__Alphaproteobacteria.o__Hyphomicrobiales.g__Te
1000
750
500
250
0
46−60 (n=7)
80plus (n=3)
61−80 (n=15)
31−45 (n=3)
20−30 (n=2)
Age_category

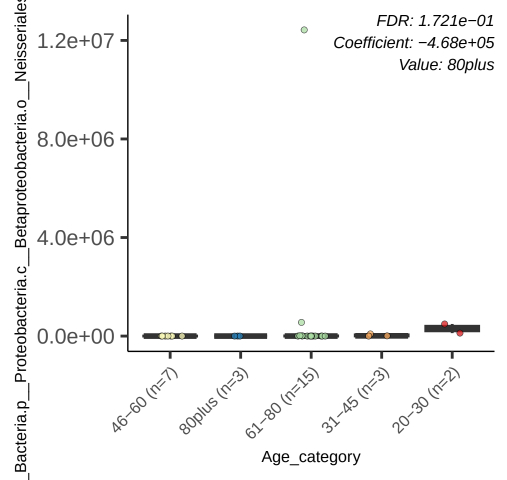

FDR: 1.721e−01
Coefficient: −4.68e+05
Value: 80plus
1.2e+07
8.0e+06
4.0e+06
0.0e+00
Bacteria.p__Proteobacteria.c__Betaproteobacteria.o__Neisseriales
46−60 (n=7)
80plus (n=3)
61−80 (n=15)
31−45 (n=3)
20−30 (n=2)
Age_category

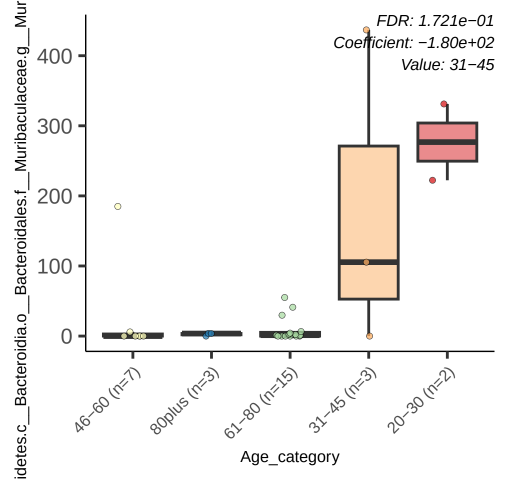
FDR: 1.721e−01
Coefficient: −1.80e+02
Value: 31−45
400
300
200
100
0
46−60 (n=7)
80plus (n=3)
61−80 (n=15)
31−45 (n=3)
20−30 (n=2)
Age_category
idetes.c__Bacteroidia.o__Bacteroidales.f__Muribaculaceae.g__Mur

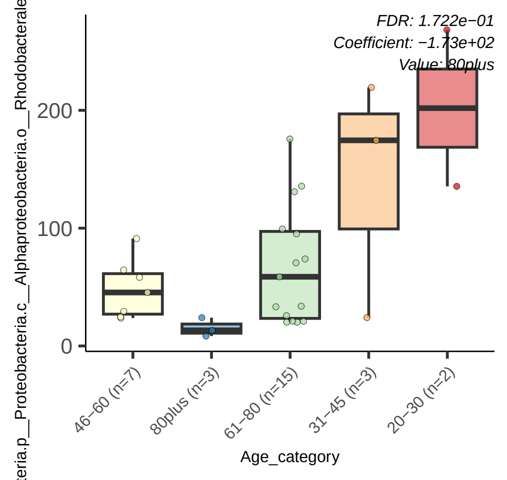
FDR: 1.722e−01
Coefficient: −1.73e+02
Value: 80plus
200
100
0
46−60 (n=7)
80plus (n=3)
61−80 (n=15)
31−45 (n=3)
20−30 (n=2)
Age_category
teria.p__Proteobacteria.c__Alphaproteobacteria.o__Rhodobacterale

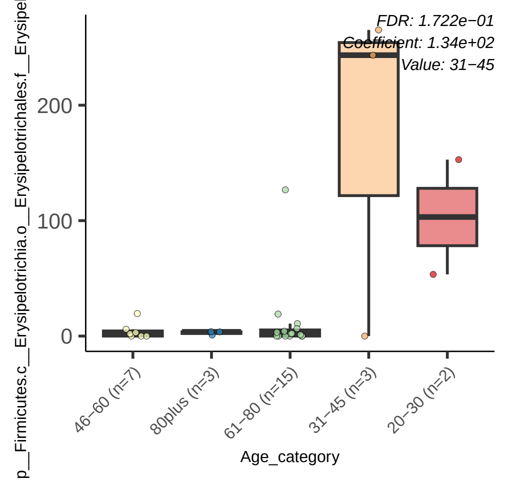
FDR: 1.722e−01
Coefficient: 1.34e+02
Value: 31−45
p__Firmicutes.c__Erysipelotrichia.o__Erysipelotrichales.f__Erysipel
0
100
200
46−60 (n=7)
80plus (n=3)
61−80 (n=15)
31−45 (n=3)
20−30 (n=2)
Age_category

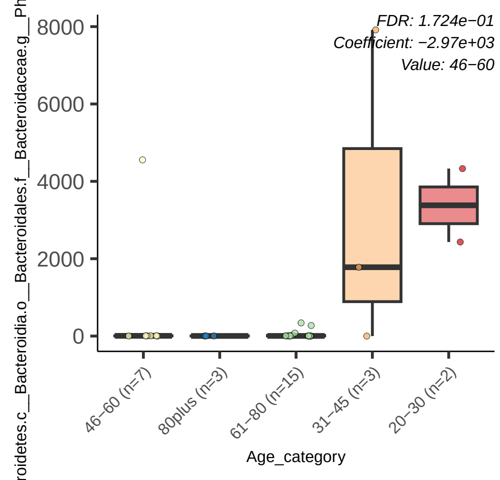

FDR: 1.724e−01
Coefficient: −2.97e+03
Value: 46−60
8000
6000
4000
2000
0
roidetes.c__Bacteroidia.o__Bacteroidales.f__Bacteroidaceae.g__Ph
46−60 (n=7)
80plus (n=3)
61−80 (n=15)
31−45 (n=3)
20−30 (n=2)
Age_category

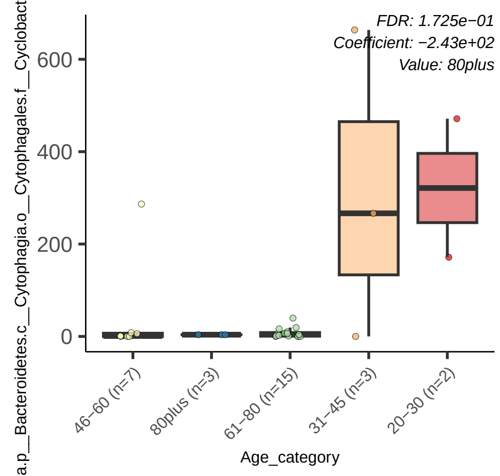

FDR: 1.725e−01
Coefficient: −2.43e+02
Value: 80plus
ia.p__Bacteroidetes.c__Cytophagia.o__Cytophagales.f__Cyclobact
0
200
400
600
46−60 (n=7)
80plus (n=3)
61−80 (n=15)
31−45 (n=3)
20−30 (n=2)
Age_category

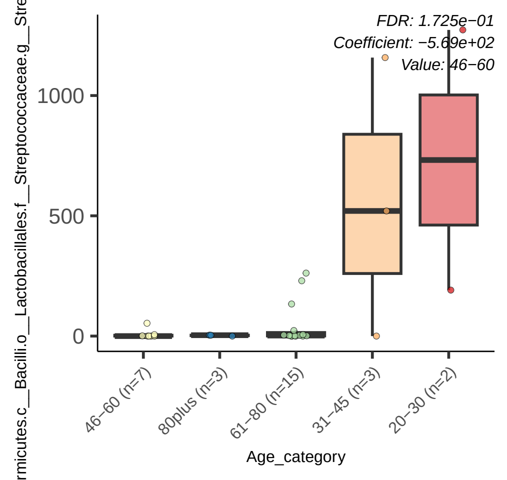
FDR: 1.725e−01
Coefficient: −5.69e+02
Value: 46−60
1000
500
0
46−60 (n=7)
80plus (n=3)
61−80 (n=15)
31−45 (n=3)
20−30 (n=2)
Age_category
rmicutes.c__Bacilli.o__Lactobacillales.f__Streptococcaceae.g__Stre

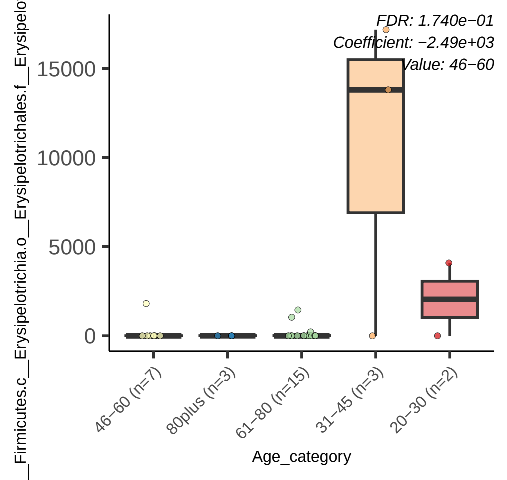

FDR: 1.740e−01
Coefficient: −2.49e+03
Value: 46−60
15000
10000
5000
0
46−60 (n=7)
80plus (n=3)
61−80 (n=15)
31−45 (n=3)
20−30 (n=2)
Age_category
Firmicutes.c__Erysipelotrichia.o__Erysipelotrichales.f__Erysipelo

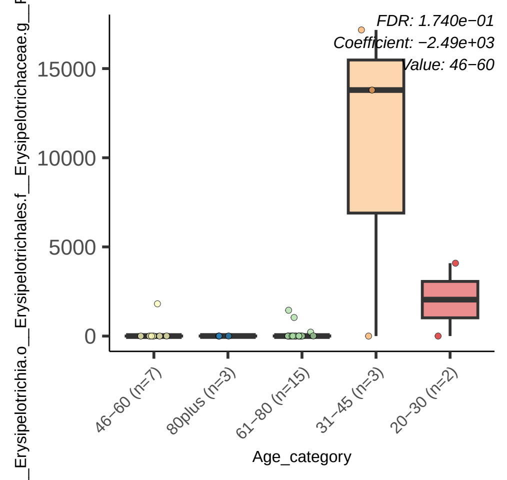

FDR: 1.740e−01
Coefficient: −2.49e+03
Value: 46−60
15000
10000
5000
0
Erysipelotrichia.o__Erysipelotrichales.f__Erysipelotrichaceae.g__F
46−60 (n=7)
80plus (n=3)
61−80 (n=15)
31−45 (n=3)
20−30 (n=2)
Age_category

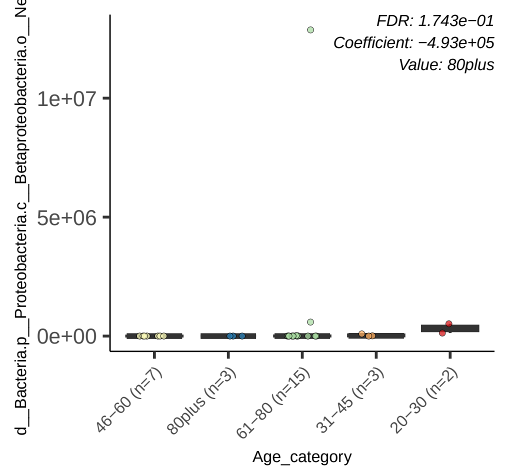

FDR: 1.743e−01
Coefficient: −4.93e+05
Value: 80plus
d__Bacteria.p__Proteobacteria.c__Betaproteobacteria.o__Ne
1e+07
5e+06
0e+00
46−60 (n=7)
80plus (n=3)
61−80 (n=15)
31−45 (n=3)
20−30 (n=2)
Age_category

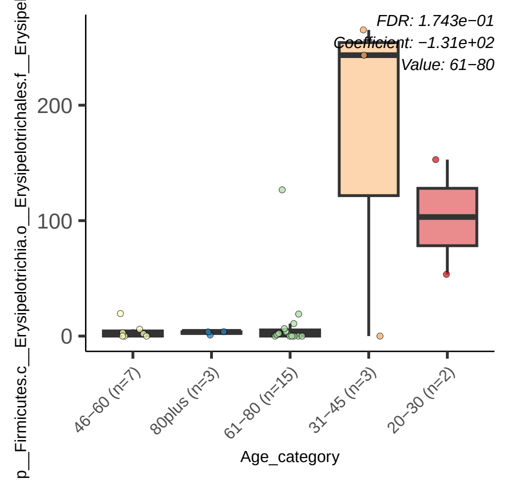

FDR: 1.743e−01
Coefficient: −1.31e+02
Value: 61−80
p__Firmicutes.c__Erysipelotrichia.o__Erysipelotrichales.f__Erysipel
200
100
0
46−60 (n=7)
80plus (n=3)
61−80 (n=15)
31−45 (n=3)
20−30 (n=2)
Age_category

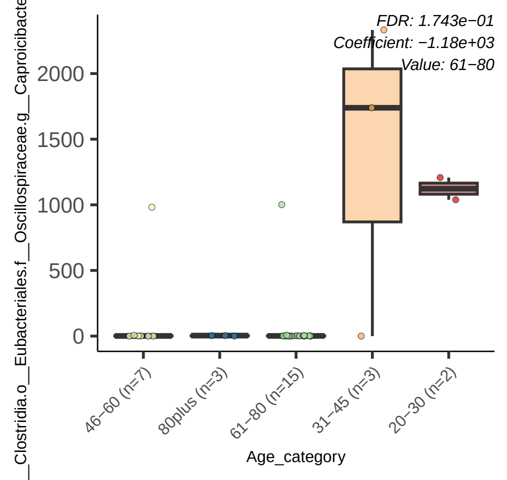

FDR: 1.743e−01
Coefficient: −1.18e+03
Value: 61−80
Clostridia.o__Eubacteriales.f__Oscillospiraceae.g__Caproicibacte
0
500
1000
1500
2000
46−60 (n=7)
80plus (n=3)
61−80 (n=15)
31−45 (n=3)
20−30 (n=2)
Age_category

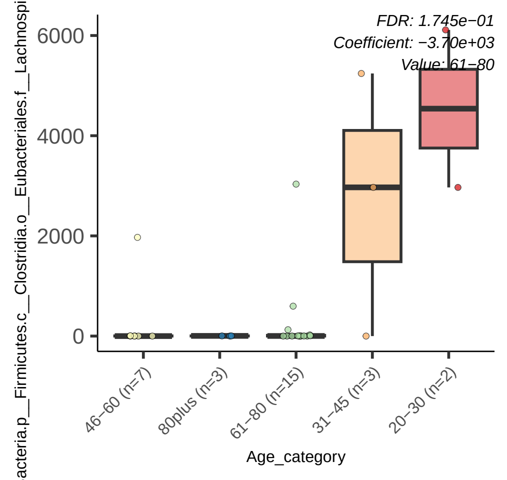

FDR: 1.745e−01
Coefficient: −3.70e+03
Value: 61−80
6000
4000
2000
0
acteria.p__Firmicutes.c__Clostridia.o__Eubacteriales.f__Lachnospi
46−60 (n=7)
80plus (n=3)
61−80 (n=15)
31−45 (n=3)
20−30 (n=2)
Age_category

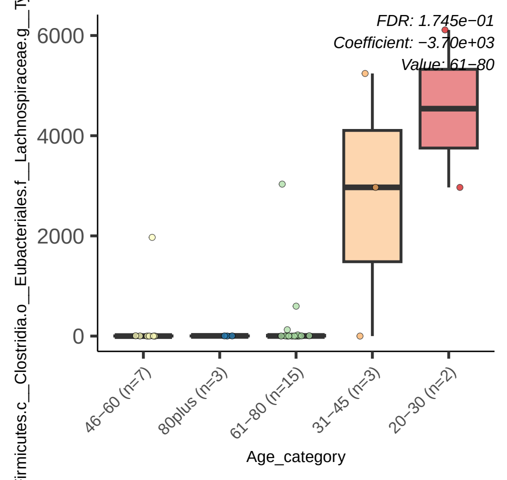

FDR: 1.745e−01
Coefficient: −3.70e+03
Value: 61−80
6000
4000
2000
0
irmicutes.c__Clostridia.o__Eubacteriales.f__Lachnospiraceae.g__Ty
46−60 (n=7)
80plus (n=3)
61−80 (n=15)
31−45 (n=3)
20−30 (n=2)
Age_category

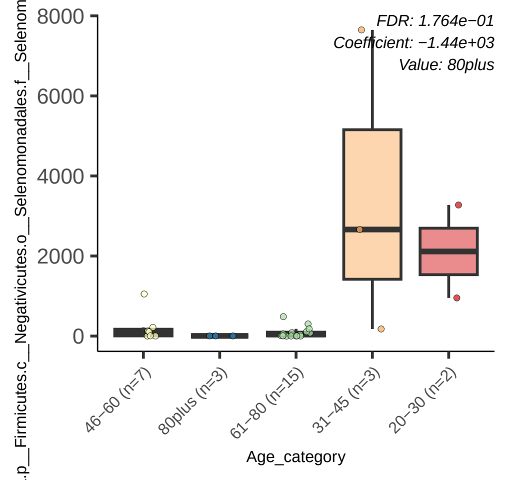

FDR: 1.764e−01
Coefficient: −1.44e+03
Value: 80plus
8000
6000
4000
2000
0
p__Firmicutes.c__Negativicutes.o__Selenomonadales.f__Selenom
46−60 (n=7)
80plus (n=3)
61−80 (n=15)
31−45 (n=3)
20−30 (n=2)
Age_category

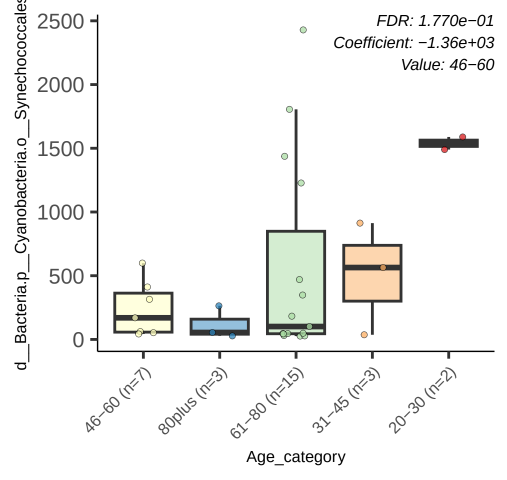
FDR: 1.770e−01
Coefficient: −1.36e+03
Value: 46−60
d__Bacteria.p__Cyanobacteria.o__Synechococcales
2500
2000
1500
1000
500
0
46−60 (n=7)
80plus (n=3)
61−80 (n=15)
31−45 (n=3)
20−30 (n=2)
Age_category

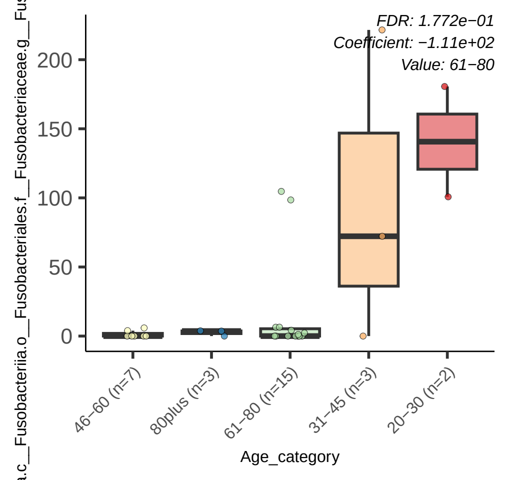

FDR: 1.772e−01
Coefficient: −1.11e+02
Value: 61−80
0
50
100
150
200
a.c__Fusobacteriia.o__Fusobacteriales.f__Fusobacteriaceae.g__Fus
46−60 (n=7)
80plus (n=3)
61−80 (n=15)
31−45 (n=3)
20−30 (n=2)
Age_category

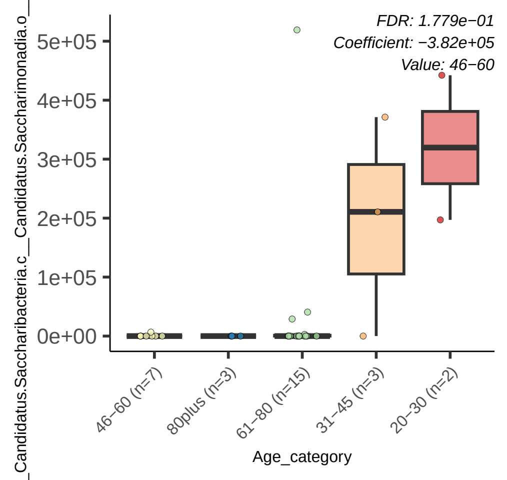

FDR: 1.779e−01
Coefficient: −3.82e+05
Value: 46−60
5e+05
4e+05
3e+05
2e+05
1e+05
0e+00
Candidatus.Saccharibacteria.c__Candidatus.Saccharimonadia.o__
46−60 (n=7)
80plus (n=3)
61−80 (n=15)
31−45 (n=3)
20−30 (n=2)
Age_category

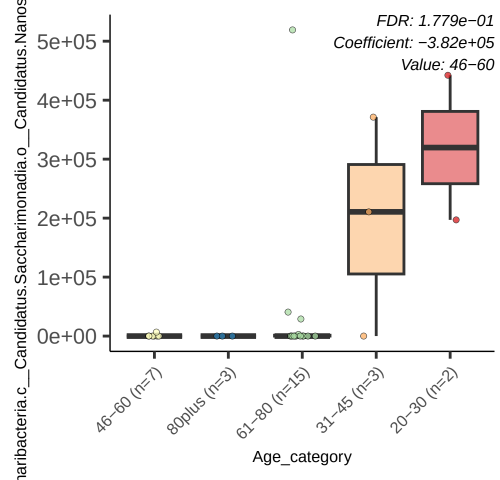

FDR: 1.779e−01
Coefficient: −3.82e+05
Value: 46−60
5e+05
4e+05
3e+05
2e+05
1e+05
0e+00
haribacteria.c__Candidatus.Saccharimonadia.o__Candidatus.Nanos
46−60 (n=7)
80plus (n=3)
61−80 (n=15)
31−45 (n=3)
20−30 (n=2)
Age_category

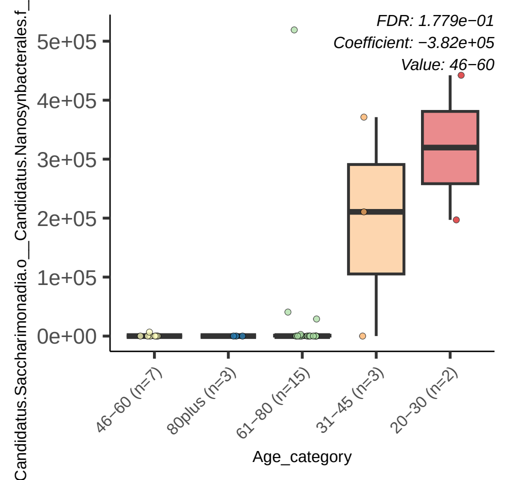
FDR: 1.779e−01
Coefficient: −3.82e+05
Value: 46−60
Candidatus.Saccharimonadia.o__Candidatus.Nanosynbacterales.f__
5e+05
4e+05
3e+05
2e+05
1e+05
0e+00
46−60 (n=7)
80plus (n=3)
61−80 (n=15)
31−45 (n=3)
20−30 (n=2)
Age_category

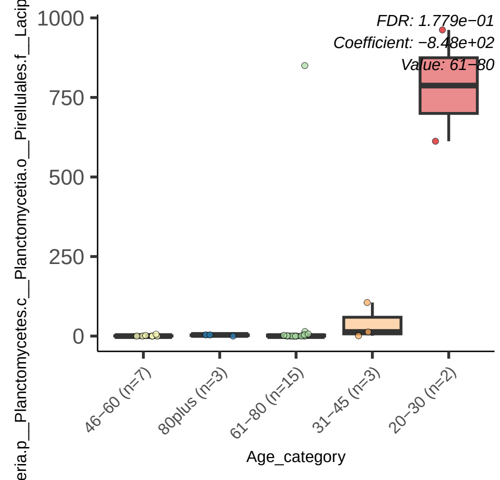

FDR: 1.779e−01
Coefficient: −8.48e+02
Value: 61−80
1000
750
500
250
0
46−60 (n=7)
80plus (n=3)
61−80 (n=15)
31−45 (n=3)
20−30 (n=2)
Age_category
eria.p__Planctomycetes.c__Planctomycetia.o__Pirellulales.f__Lacip

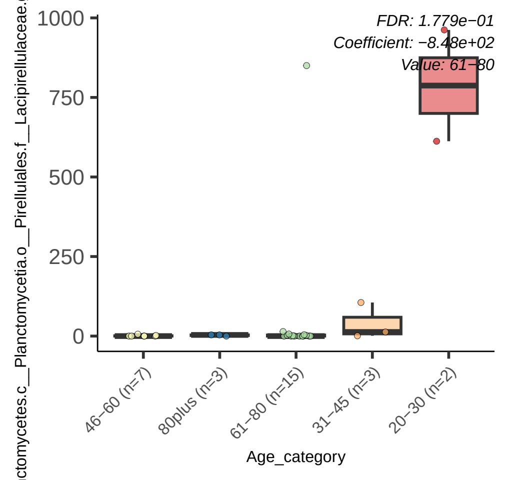

FDR: 1.779e−01
Coefficient: −8.48e+02
Value: 61−80
1000
750
500
250
0
ctomycetes.c__Planctomycetia.o__Pirellulales.f__Lacipirellulaceae.
46−60 (n=7)
80plus (n=3)
61−80 (n=15)
31−45 (n=3)
20−30 (n=2)
Age_category

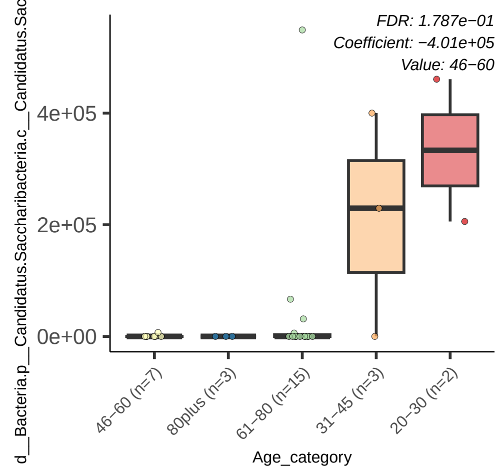

FDR: 1.787e−01
Coefficient: −4.01e+05
Value: 46−60
d__Bacteria.p__Candidatus.Saccharibacteria.c__Candidatus.Sac
4e+05
2e+05
0e+00
46−60 (n=7)
80plus (n=3)
61−80 (n=15)
31−45 (n=3)
20−30 (n=2)
Age_category

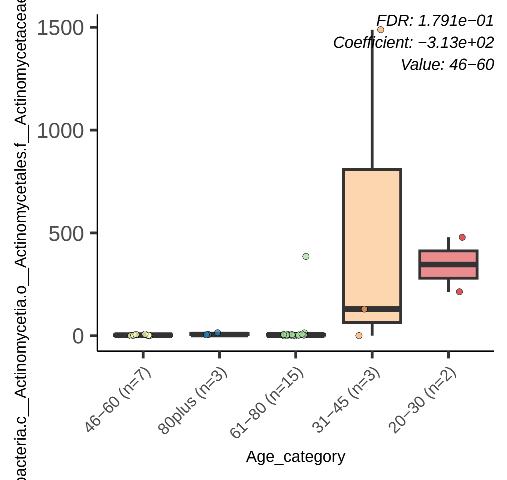
FDR: 1.791e−01
Coefficient: −3.13e+02
Value: 46−60
1500
1000
500
0
bacteria.c__Actinomycetia.o__Actinomycetales.f__Actinomycetaceae
46−60 (n=7)
80plus (n=3)
61−80 (n=15)
31−45 (n=3)
20−30 (n=2)
Age_category

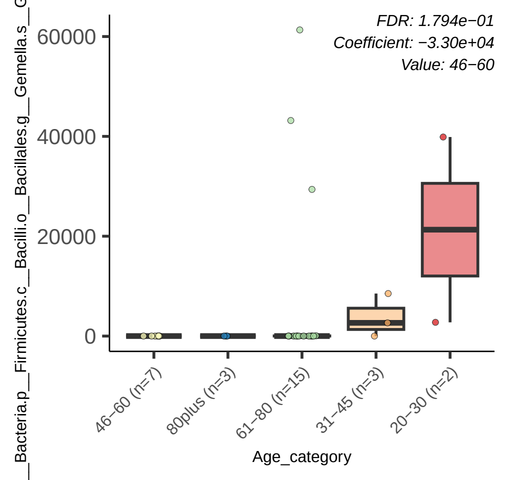
FDR: 1.794e−01
Coefficient: −3.30e+04
Value: 46−60
60000
40000
20000
0
Bacteria.p__Firmicutes.c__Bacilli.o__Bacillales.g__Gemella.s__G
46−60 (n=7)
80plus (n=3)
61−80 (n=15)
31−45 (n=3)
20−30 (n=2)
Age_category

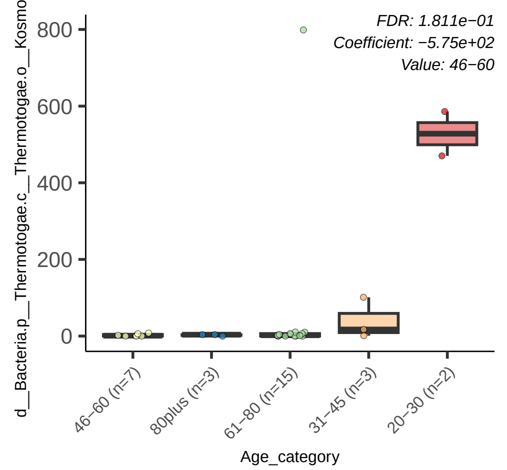
FDR: 1.811e−01
Coefficient: −5.75e+02
Value: 46−60
d__Bacteria.p__Thermotogae.c__Thermotogae.o__Kosmo
800
600
400
200
0
46−60 (n=7)
80plus (n=3)
61−80 (n=15)
31−45 (n=3)
20−30 (n=2)
Age_category

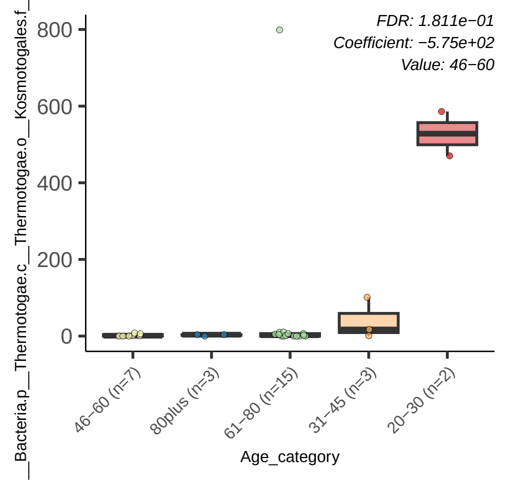

FDR: 1.811e−01
Coefficient: −5.75e+02
Value: 46−60
Bacteria.p__Thermotogae.c__Thermotogae.o__Kosmotogales.f__
800
600
400
200
0
46−60 (n=7)
80plus (n=3)
61−80 (n=15)
31−45 (n=3)
20−30 (n=2)
Age_category

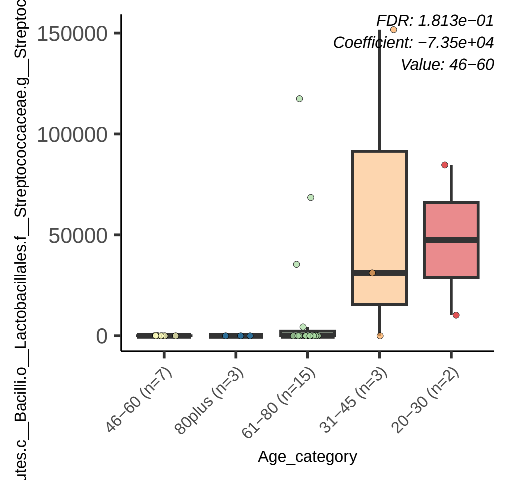

FDR: 1.813e−01
Coefficient: −7.35e+04
Value: 46−60
150000
100000
50000
0
46−60 (n=7)
80plus (n=3)
61−80 (n=15)
31−45 (n=3)
20−30 (n=2)
Age_category
utes.c__Bacilli.o__Lactobacillales.f__Streptococcaceae.g__Streptoc

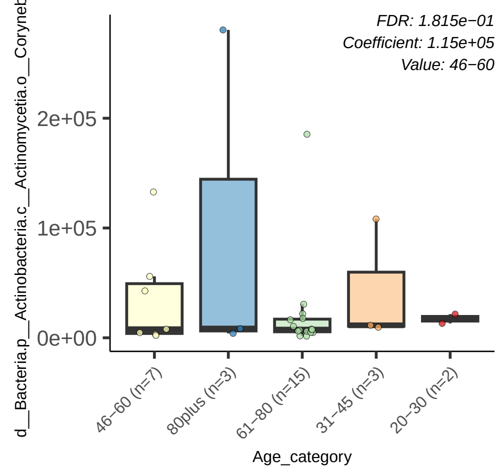

FDR: 1.815e−01
Coefficient: 1.15e+05
Value: 46−60
d__Bacteria.p__Actinobacteria.c__Actinomycetia.o__Coryneb
2e+05
1e+05
0e+00
46−60 (n=7)
80plus (n=3)
61−80 (n=15)
31−45 (n=3)
20−30 (n=2)
Age_category

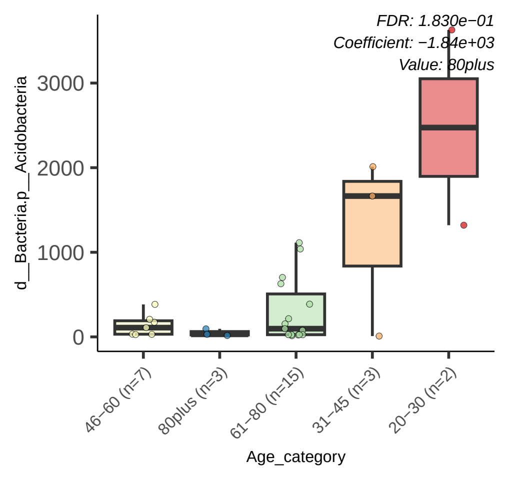

FDR: 1.830e−01
Coefficient: −1.84e+03
Value: 80plus
d__Bacteria.p__Acidobacteria
3000
2000
1000
0
46−60 (n=7)
80plus (n=3)
61−80 (n=15)
31−45 (n=3)
20−30 (n=2)
Age_category

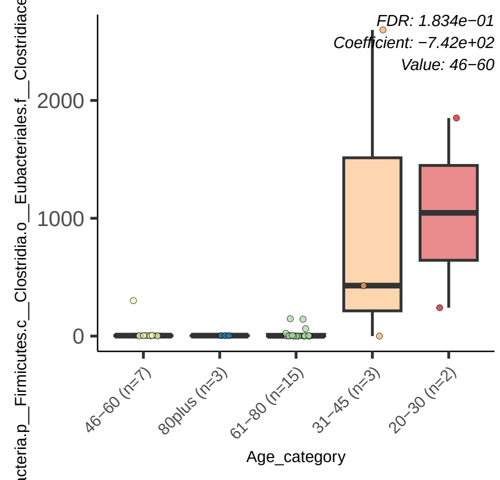

FDR: 1.834e−01
Coefficient: −7.42e+02
Value: 46−60
acteria.p__Firmicutes.c__Clostridia.o__Eubacteriales.f__Clostridiace
2000
1000
0
46−60 (n=7)
80plus (n=3)
61−80 (n=15)
31−45 (n=3)
20−30 (n=2)
Age_category

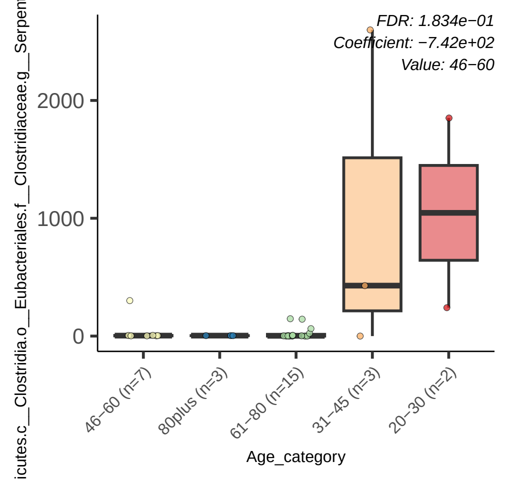

FDR: 1.834e−01
Coefficient: −7.42e+02
Value: 46−60
2000
1000
0
46−60 (n=7)
80plus (n=3)
61−80 (n=15)
31−45 (n=3)
20−30 (n=2)
Age_category
icutes.c__Clostridia.o__Eubacteriales.f__Clostridiaceae.g__Serpent

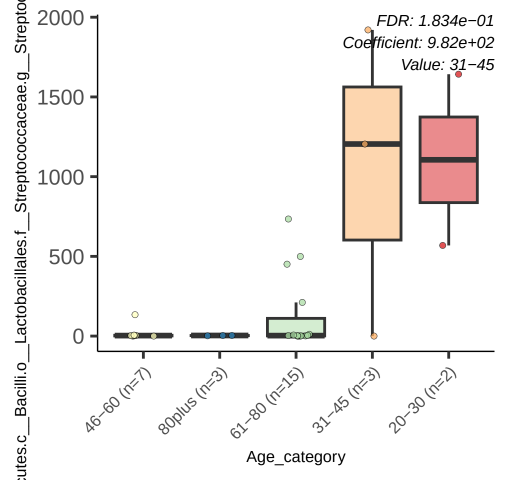

FDR: 1.834e−01
Coefficient: 9.82e+02
Value: 31−45
2000
1500
1000
500
0
cutes.c__Bacilli.o__Lactobacillales.f__Streptococcaceae.g__Streptoc
46−60 (n=7)
80plus (n=3)
61−80 (n=15)
31−45 (n=3)
20−30 (n=2)
Age_category

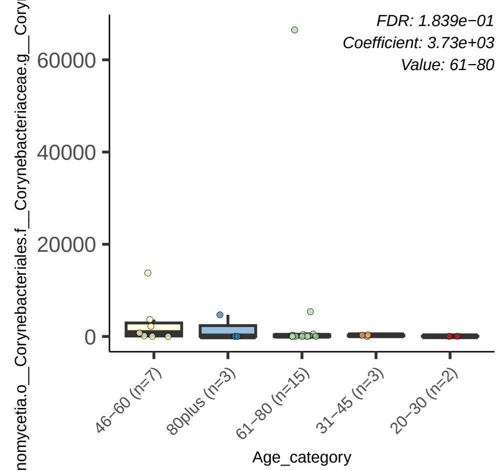

FDR: 1.839e−01
Coefficient: 3.73e+03
Value: 61−80
60000
40000
20000
0
46−60 (n=7)
80plus (n=3)
61−80 (n=15)
31−45 (n=3)
20−30 (n=2)
Age_category

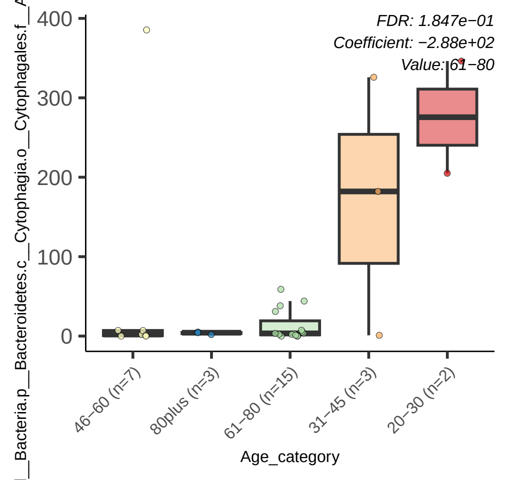

FDR: 1.847e−01
Coefficient: −2.88e+02
Value: 61−80
400
300
200
100
0
__Bacteria.p__Bacteroidetes.c__Cytophagia.o__Cytophagales.f__A
46−60 (n=7)
80plus (n=3)
61−80 (n=15)
31−45 (n=3)
20−30 (n=2)
Age_category

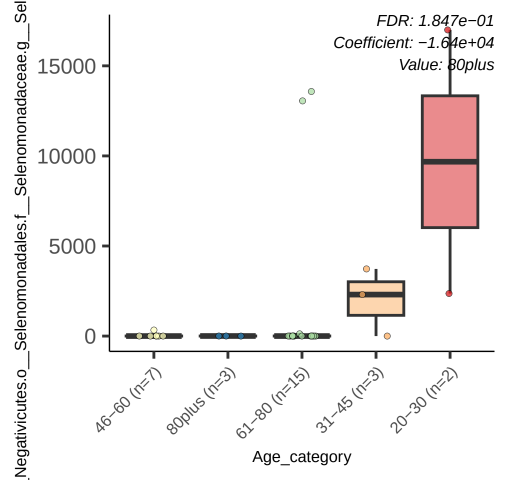
FDR: 1.847e−01
Coefficient: −1.64e+04
Value: 80plus
15000
10000
5000
0
Negativicutes.o__Selenomonadales.f__Selenomonadaceae.g__Sel
46−60 (n=7)
80plus (n=3)
61−80 (n=15)
31−45 (n=3)
20−30 (n=2)
Age_category

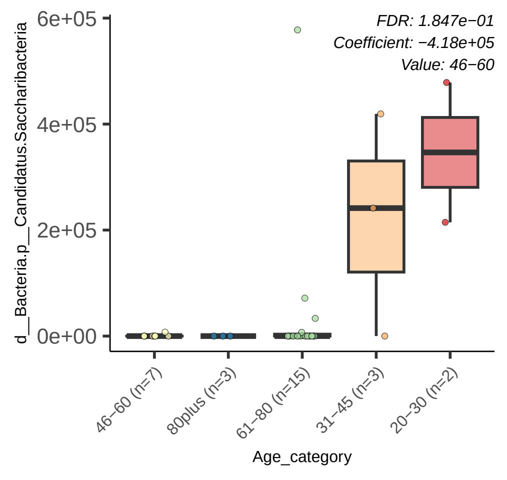
FDR: 1.847e−01
Coefficient: −4.18e+05
Value: 46−60
6e+05
4e+05
2e+05
0e+00
d__Bacteria.p__Candidatus.Saccharibacteria
46−60 (n=7)
80plus (n=3)
61−80 (n=15)
31−45 (n=3)
20−30 (n=2)
Age_category

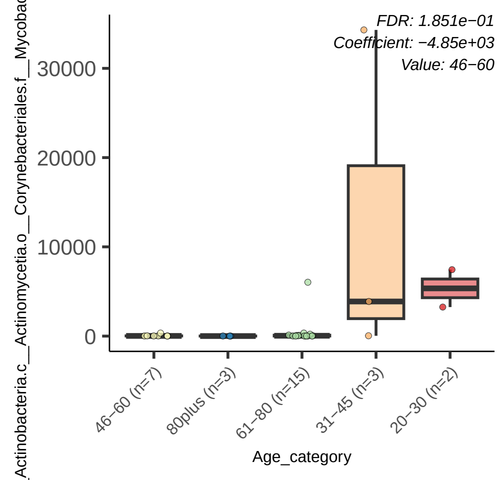

FDR: 1.851e−01
Coefficient: −4.85e+03
Value: 46−60
Actinobacteria.c__Actinomycetia.o__Corynebacteriales.f__Mycobac
30000
20000
10000
0
46−60 (n=7)
80plus (n=3)
61−80 (n=15)
31−45 (n=3)
20−30 (n=2)
Age_category

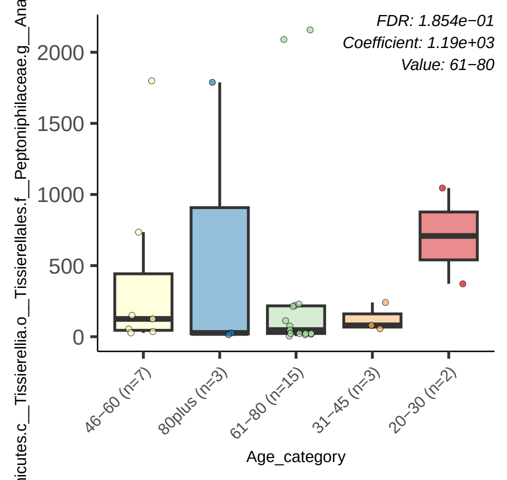

FDR: 1.854e−01
Coefficient: 1.19e+03
Value: 61−80
2000
1500
1000
500
0
46−60 (n=7)
80plus (n=3)
61−80 (n=15)
31−45 (n=3)
20−30 (n=2)
Age_category

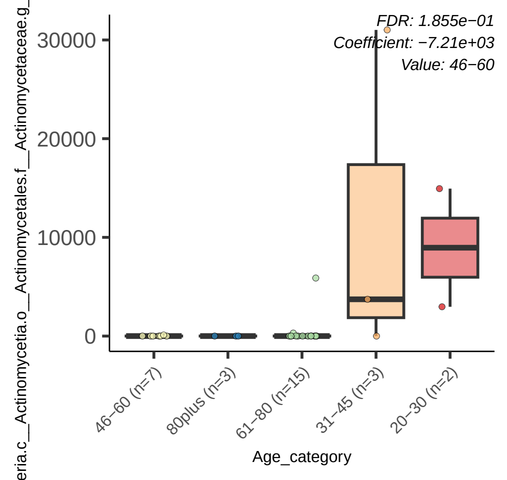

FDR: 1.855e−01
Coefficient: −7.21e+03
Value: 46−60
30000
20000
10000
0
eria.c__Actinomycetia.o__Actinomycetales.f__Actinomycetaceae.g_
46−60 (n=7)
80plus (n=3)
61−80 (n=15)
31−45 (n=3)
20−30 (n=2)
Age_category

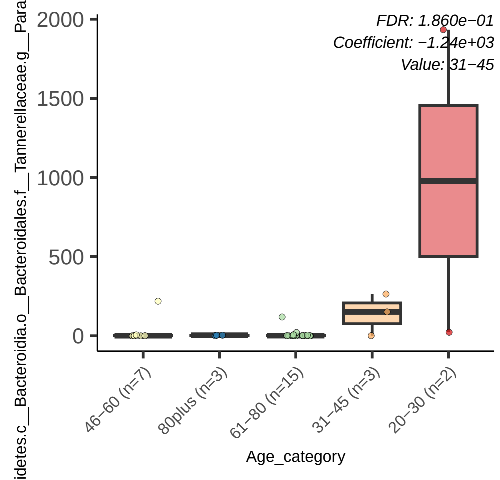

FDR: 1.860e−01
Coefficient: −1.24e+03
Value: 31−45
idetes.c__Bacteroidia.o__Bacteroidales.f__Tannerellaceae.g__Para
2000
1500
1000
500
0
46−60 (n=7)
80plus (n=3)
61−80 (n=15)
31−45 (n=3)
20−30 (n=2)
Age_category

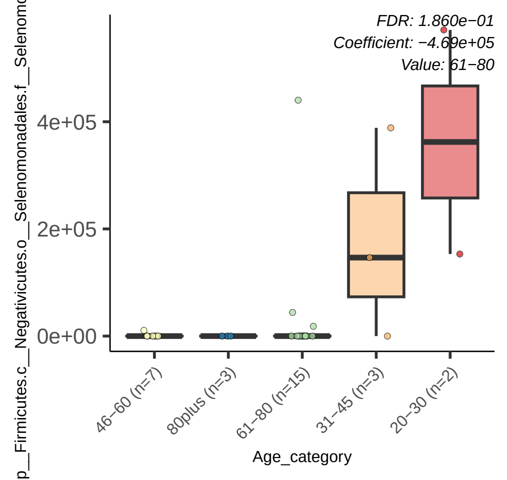

FDR: 1.860e−01
Coefficient: −4.69e+05
Value: 61−80
p__Firmicutes.c__Negativicutes.o__Selenomonadales.f__Selenomo
4e+05
2e+05
0e+00
46−60 (n=7)
80plus (n=3)
61−80 (n=15)
31−45 (n=3)
20−30 (n=2)
Age_category

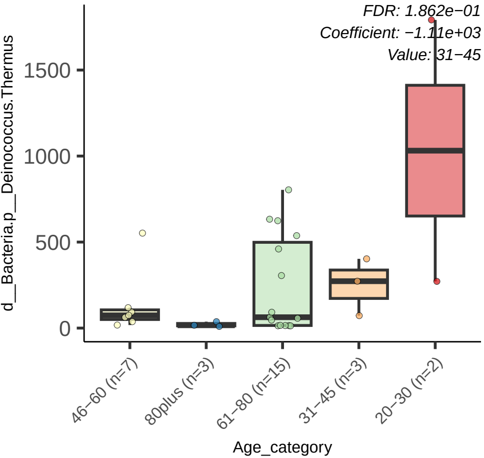
FDR: 1.862e−01
Coefficient: −1.11e+03
Value: 31−45
d__Bacteria.p__Deinococcus.Thermus
1500
1000
500
0
46−60 (n=7)
80plus (n=3)
61−80 (n=15)
31−45 (n=3)
20−30 (n=2)
Age_category

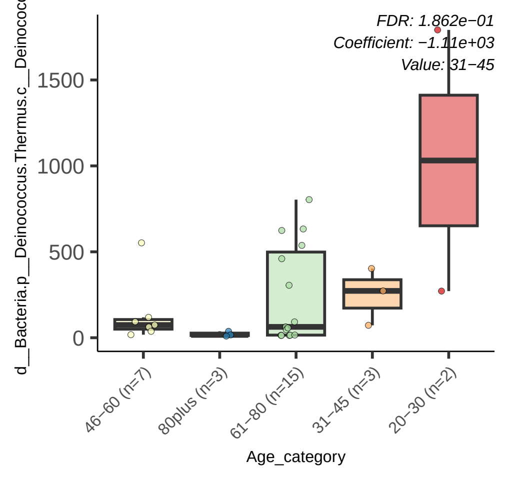

FDR: 1.862e−01
Coefficient: −1.11e+03
Value: 31−45
d__Bacteria.p__Deinococcus.Thermus.c__Deinococ
1500
1000
500
0
46−60 (n=7)
80plus (n=3)
61−80 (n=15)
31−45 (n=3)
20−30 (n=2)
Age_category

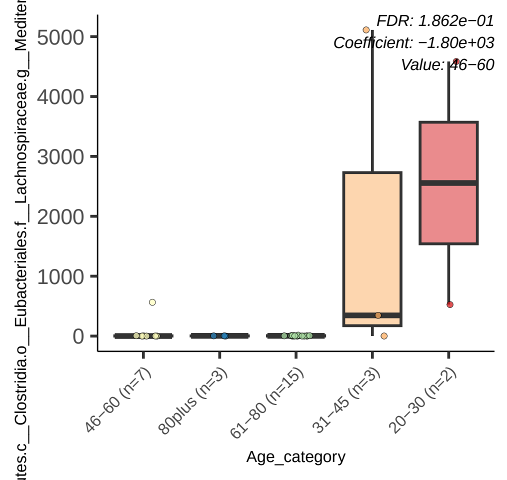

FDR: 1.862e−01
Coefficient: −1.80e+03
Value: 46−60
5000
4000
3000
2000
1000
0
ites.c__Clostridia.o__Eubacteriales.f__Lachnospiraceae.g__Mediter
46−60 (n=7)
80plus (n=3)
61−80 (n=15)
31−45 (n=3)
20−30 (n=2)
Age_category

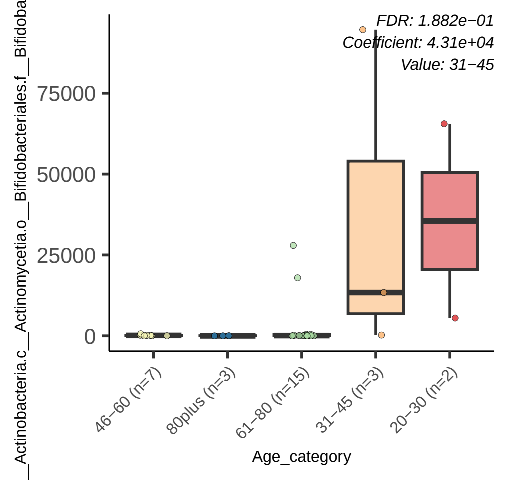

FDR: 1.882e−01
Coefficient: 4.31e+04
Value: 31−45
Actinobacteria.c__Actinomycetia.o__Bifidobacteriales.f__Bifidoba
75000
50000
25000
0
46−60 (n=7)
80plus (n=3)
61−80 (n=15)
31−45 (n=3)
20−30 (n=2)
Age_category

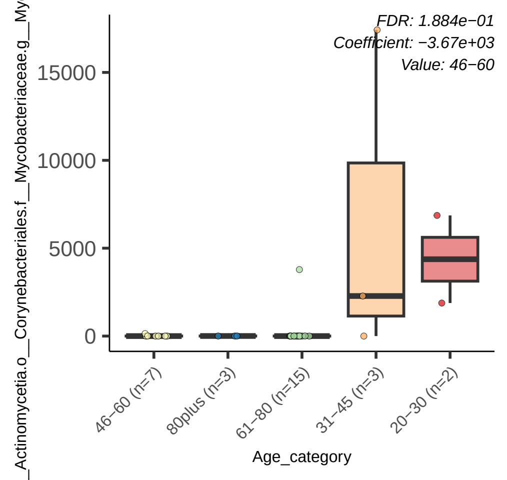

FDR: 1.884e−01
Coefficient: −3.67e+03
Value: 46−60
15000
10000
5000
0
_Actinomycetia.o__Corynebacteriales.f__Mycobacteriaceae.g__My
46−60 (n=7)
80plus (n=3)
61−80 (n=15)
31−45 (n=3)
20−30 (n=2)
Age_category

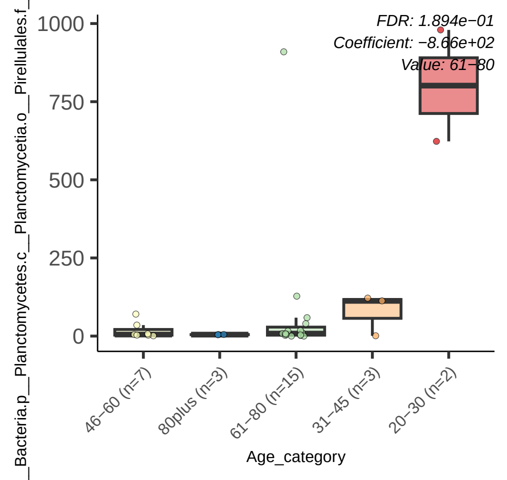

FDR: 1.894e−01
Coefficient: −8.66e+02
Value: 61−80
Bacteria.p__Planctomycetes.c__Planctomycetia.o__Pirellulales.f__
1000
750
500
250
0
46−60 (n=7)
80plus (n=3)
61−80 (n=15)
31−45 (n=3)
20−30 (n=2)
Age_category

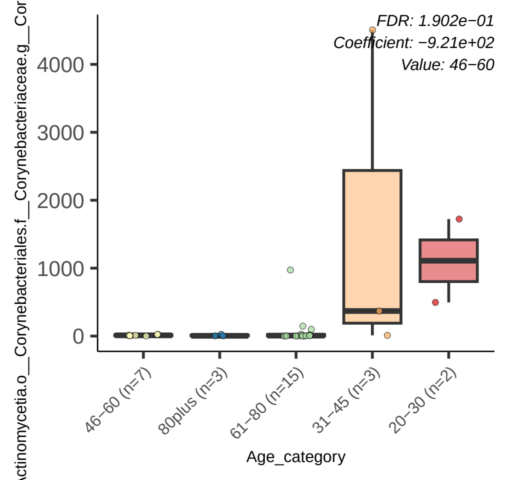

FDR: 1.902e−01
Coefficient: −9.21e+02
Value: 46−60
Actinomycetia.o__Corynebacteriales.f__Corynebacteriaceae.g__Cor
4000
3000
2000
1000
0
46−60 (n=7)
80plus (n=3)
61−80 (n=15)
31−45 (n=3)
20−30 (n=2)
Age_category

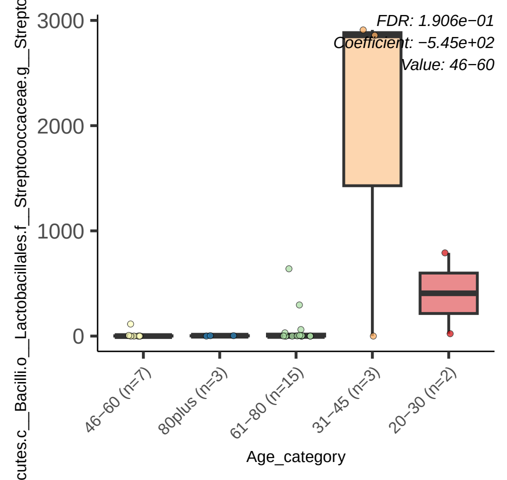

FDR: 1.906e−01
Coefficient: −5.45e+02
Value: 46−60
3000
2000
1000
0
cutes.c__Bacilli.o__Lactobacillales.f__Streptococcaceae.g__Strepto
46−60 (n=7)
80plus (n=3)
61−80 (n=15)
31−45 (n=3)
20−30 (n=2)
Age_category

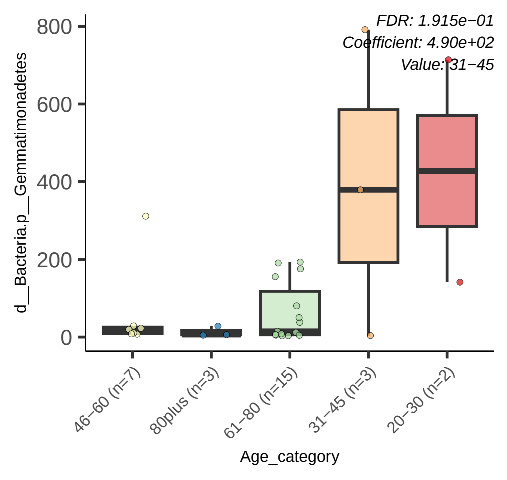
FDR: 1.915e−01
Coefficient: 4.90e+02
Value: 31−45
d__Bacteria.p__Gemmatimonadetes
800
600
400
200
0
46−60 (n=7)
80plus (n=3)
61−80 (n=15)
31−45 (n=3)
20−30 (n=2)
Age_category

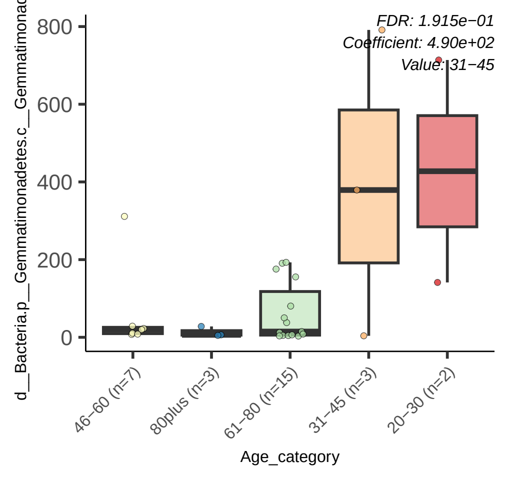
FDR: 1.915e−01
Coefficient: 4.90e+02
Value: 31−45
d__Bacteria.p__Gemmatimonadetes.c__Gemmatimonad
800
600
400
200
0
46−60 (n=7)
80plus (n=3)
61−80 (n=15)
31−45 (n=3)
20−30 (n=2)
Age_category

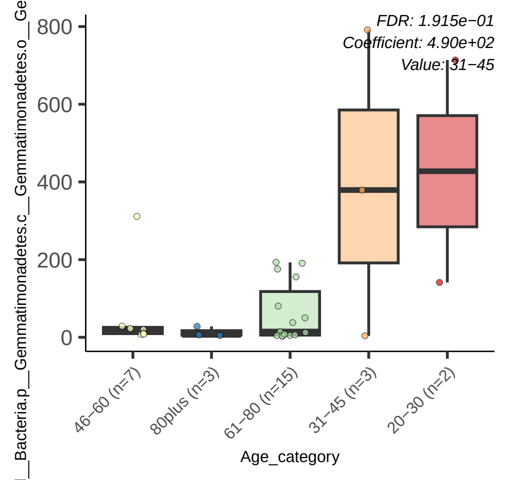

FDR: 1.915e−01
Coefficient: 4.90e+02
Value: 31−45
800
600
400
200
0
__Bacteria.p__Gemmatimonadetes.c__Gemmatimonadetes.o__Ge
46−60 (n=7)
80plus (n=3)
61−80 (n=15)
31−45 (n=3)
20−30 (n=2)
Age_category

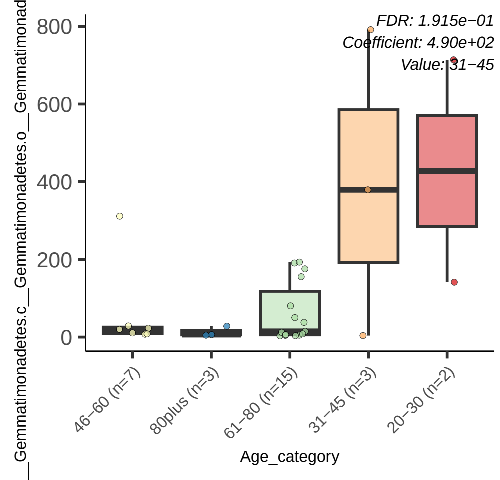

FDR: 1.915e−01
Coefficient: 4.90e+02
Value: 31−45
_Gemmatimonadetes.c__Gemmatimonadetes.o__Gemmatimonad
800
600
400
200
0
46−60 (n=7)
80plus (n=3)
61−80 (n=15)
31−45 (n=3)
20−30 (n=2)
Age_category

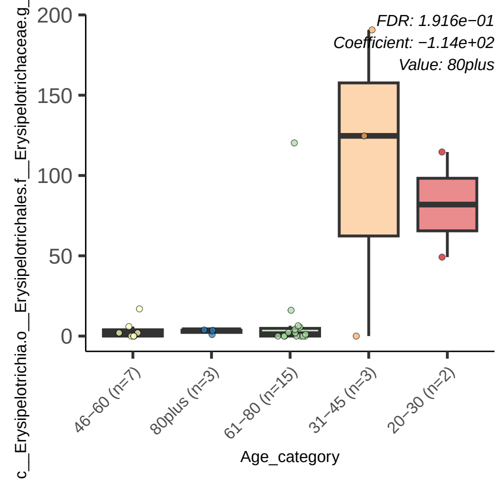

FDR: 1.916e−01
Coefficient: −1.14e+02
Value: 80plus
c__Erysipelotrichia.o__Erysipelotrichales.f__Erysipelotrichaceae.g__
200
150
100
50
0
46−60 (n=7)
80plus (n=3)
61−80 (n=15)
31−45 (n=3)
20−30 (n=2)
Age_category

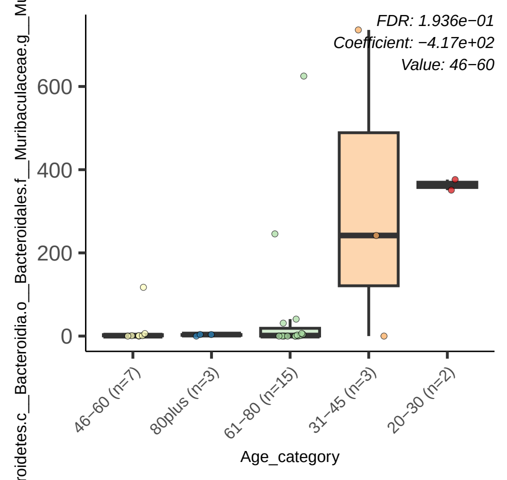

FDR: 1.936e−01
Coefficient: −4.17e+02
Value: 46−60
0
200
400
600
46−60 (n=7)
80plus (n=3)
61−80 (n=15)
31−45 (n=3)
20−30 (n=2)
Age_category

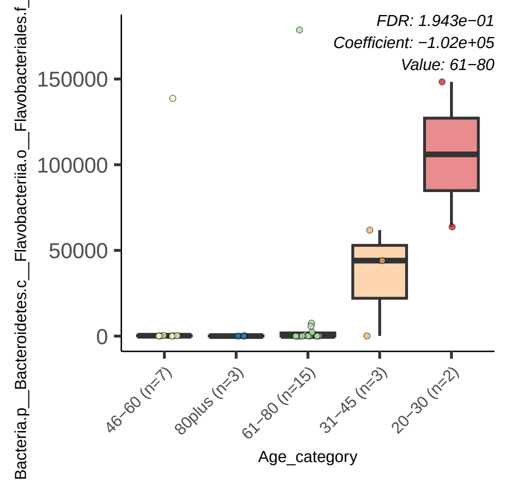

FDR: 1.943e−01
Coefficient: −1.02e+05
Value: 61−80
Bacteria.p__Bacteroidetes.c__Flavobacteriia.o__Flavobacteriales.f_
150000
100000
50000
0
46−60 (n=7)
80plus (n=3)
61−80 (n=15)
31−45 (n=3)
20−30 (n=2)
Age_category

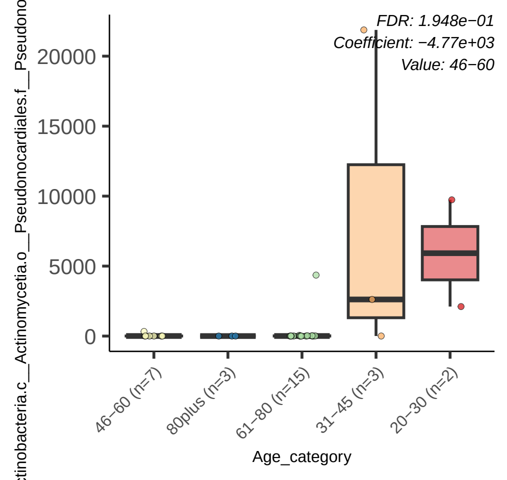

FDR: 1.948e−01
Coefficient: −4.77e+03
Value: 46−60
20000
15000
10000
5000
0
ctinobacteria.c__Actinomycetia.o__Pseudonocardiales.f__Pseudono
46−60 (n=7)
80plus (n=3)
61−80 (n=15)
31−45 (n=3)
20−30 (n=2)
Age_category

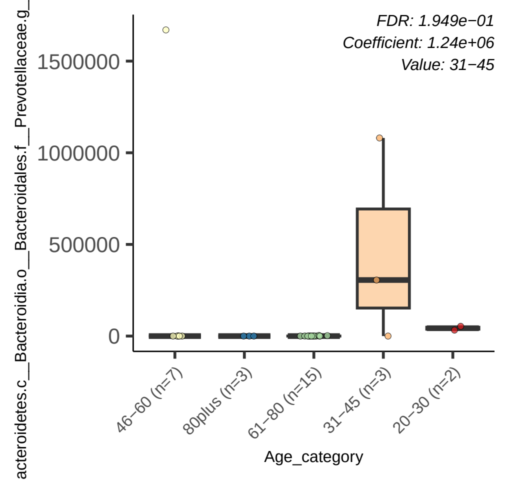

FDR: 1.949e−01
Coefficient: 1.24e+06
Value: 31−45
1500000
1000000
500000
0
acteroidetes.c__Bacteroidia.o__Bacteroidales.f__Prevotellaceae.g_
46−60 (n=7)
80plus (n=3)
61−80 (n=15)
31−45 (n=3)
20−30 (n=2)
Age_category

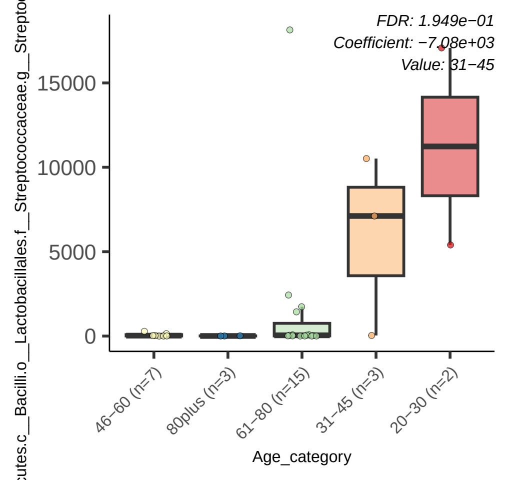

FDR: 1.949e−01
Coefficient: −7.08e+03
Value: 31−45
15000
10000
5000
0
cutes.c__Bacilli.o__Lactobacillales.f__Streptococcaceae.g__Strepto
46−60 (n=7)
80plus (n=3)
61−80 (n=15)
31−45 (n=3)
20−30 (n=2)
Age_category

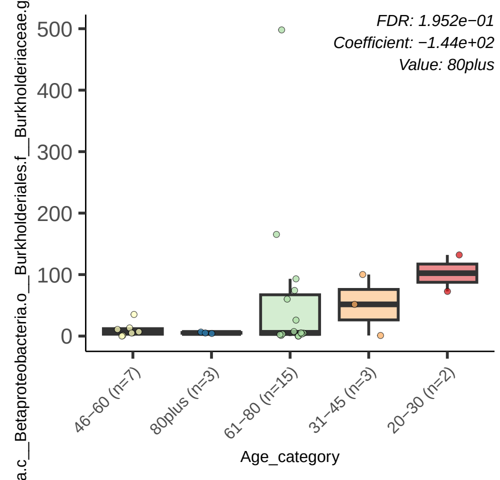

FDR: 1.952e−01
Coefficient: −1.44e+02
Value: 80plus
a.c__Betaproteobacteria.o__Burkholderiales.f__Burkholderiaceae.g
500
400
300
200
100
0
46−60 (n=7)
80plus (n=3)
61−80 (n=15)
31−45 (n=3)
20−30 (n=2)
Age_category

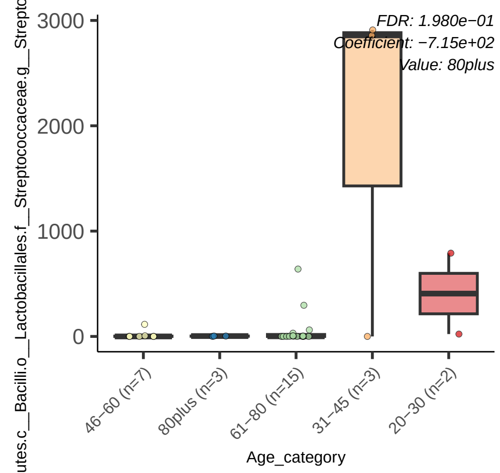

FDR: 1.980e−01
Coefficient: −7.15e+02
Value: 80plus
3000
2000
1000
0
cutes.c__Bacilli.o__Lactobacillales.f__Streptococcaceae.g__Strepto
46−60 (n=7)
80plus (n=3)
61−80 (n=15)
31−45 (n=3)
20−30 (n=2)
Age_category

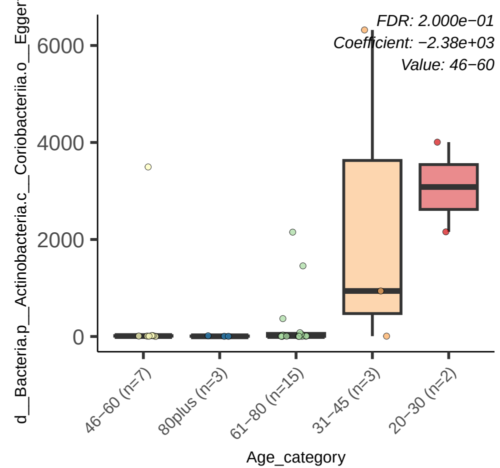

FDR: 2.000e−01
Coefficient: −2.38e+03
Value: 46−60
d__Bacteria.p__Actinobacteria.c__Coriobacteriia.o__Egger
6000
4000
2000
0
46−60 (n=7)
80plus (n=3)
61−80 (n=15)
31−45 (n=3)
20−30 (n=2)
Age_category

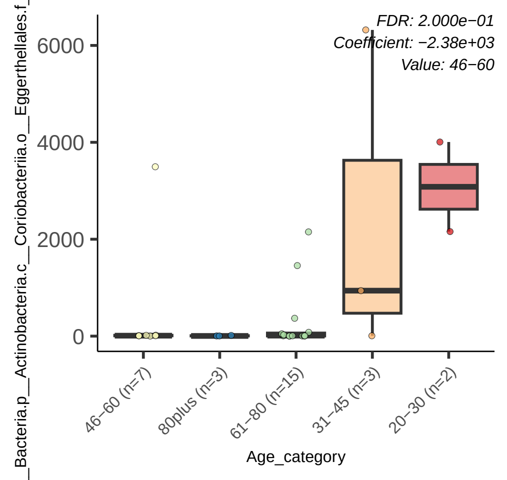
FDR: 2.000e−01
Coefficient: −2.38e+03
Value: 46−60
_Bacteria.p__Actinobacteria.c__Coriobacteria.o__Eggerthellales.f_
6000
4000
2000
0
46−60 (n=7)
80plus (n=3)
61−80 (n=15)
31−45 (n=3)
20−30 (n=2)
Age_category

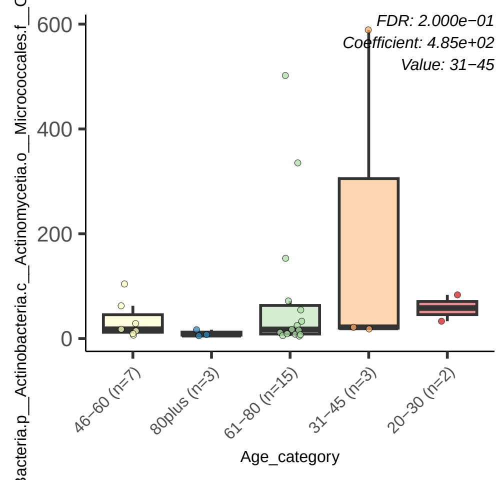

FDR: 2.000e−01
Coefficient: 4.85e+02
Value: 31−45
Bacteria.p__Actinobacteria.c__Actinomycetia.o__Micrococcales.f__C
600
400
200
0
46−60 (n=7)
80plus (n=3)
61−80 (n=15)
31−45 (n=3)
20−30 (n=2)
Age_category

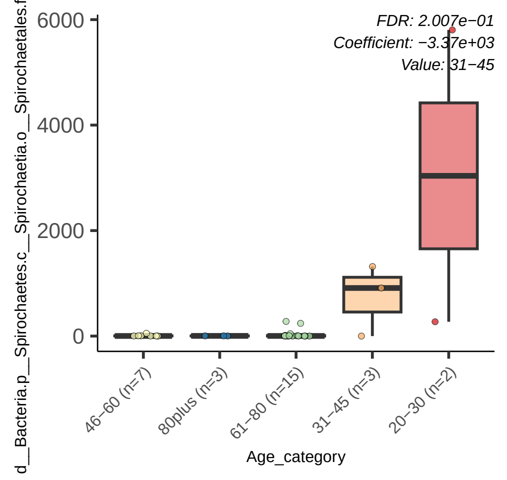

FDR: 2.007e−01
Coefficient: −3.37e+03
Value: 31−45
d__Bacteria.p__Spirochaetes.c__Spirochaetia.o__Spirochaetales.f
6000
4000
2000
0
46−60 (n=7)
80plus (n=3)
61−80 (n=15)
31−45 (n=3)
20−30 (n=2)
Age_category

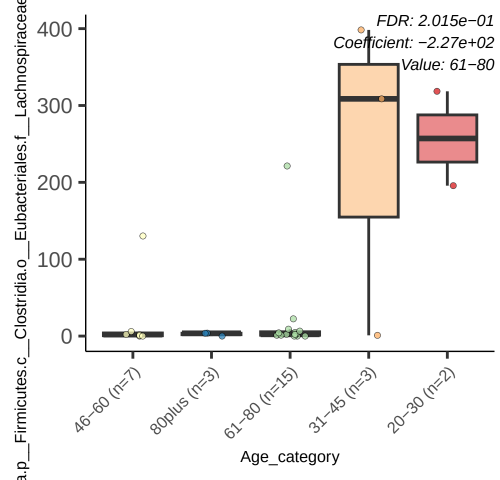

FDR: 2.015e−01
Coefficient: −2.27e+02
Value: 61−80
a.p__Firmicutes.c__Clostridia.o__Eubacteriales.f__Lachnospiraceae
400
300
200
100
0
46−60 (n=7)
80plus (n=3)
61−80 (n=15)
31−45 (n=3)
20−30 (n=2)
Age_category

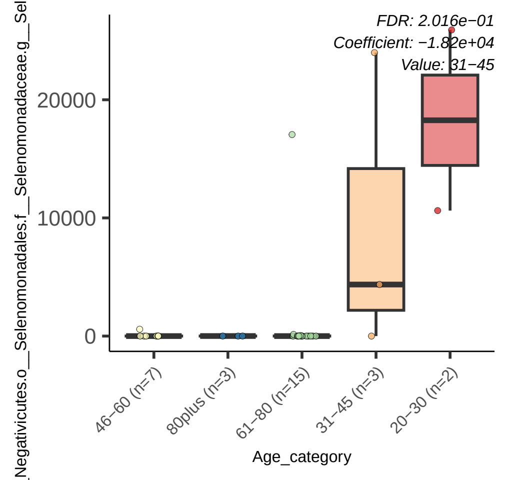

FDR: 2.016e−01
Coefficient: −1.82e+04
Value: 31−45
Negativicutes.o__Selenomonadales.f__Selenomonadaceae.g__Sel
20000
10000
0
46−60 (n=7)
80plus (n=3)
61−80 (n=15)
31−45 (n=3)
20−30 (n=2)
Age_category

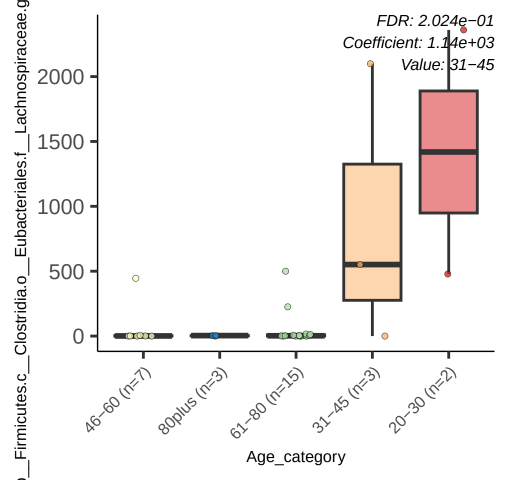

FDR: 2.024e−01
Coefficient: 1.14e+03
Value: 31−45
2000
1500
1000
500
0
Firmicutes.c__Clostridia.o__Eubacteriales.f__Lachnospiraceae.g
46−60 (n=7)
80plus (n=3)
61−80 (n=15)
31−45 (n=3)
20−30 (n=2)
Age_category

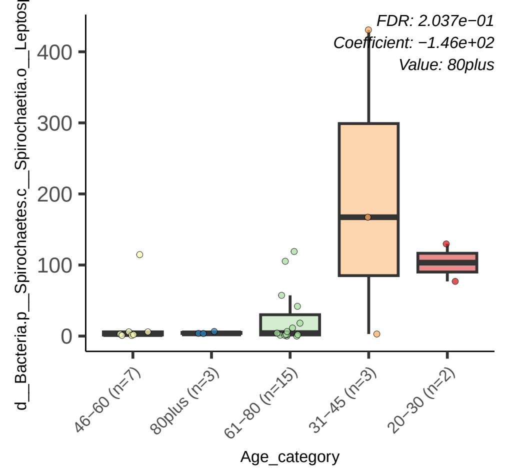

FDR: 2.037e−01
Coefficient: −1.46e+02
Value: 80plus
d__Bacteria.p__Spirochaetes.c__Spirochaetia.o__Leptosp
400
300
200
100
0
46−60 (n=7)
80plus (n=3)
61−80 (n=15)
31−45 (n=3)
20−30 (n=2)
Age_category

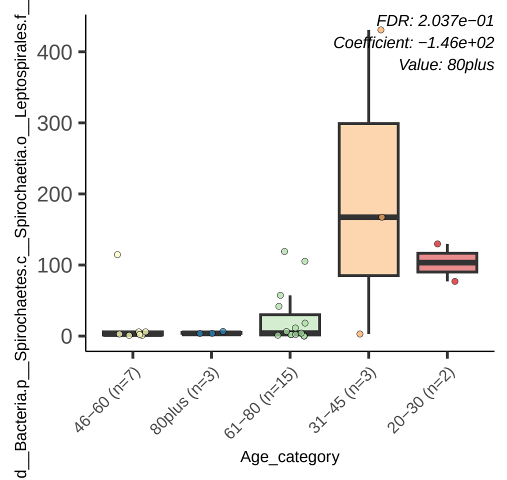

FDR: 2.037e−01
Coefficient: −1.46e+02
Value: 80plus
d__Bacteria.p__Spirochaetes.c__Spirochaetia.o__Leptospirales.f__
0
100
200
300
400
46−60 (n=7)
80plus (n=3)
61−80 (n=15)
31−45 (n=3)
20−30 (n=2)
Age_category

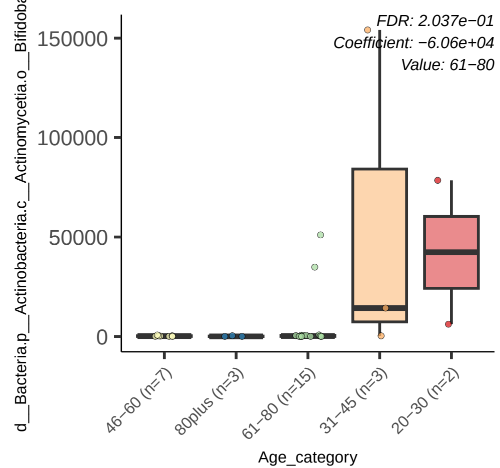

FDR: 2.037e−01
Coefficient: −6.06e+04
Value: 61−80
d__Bacteria.p__Actinobacteria.c__Actinomycetia.o__Bifidoba
150000
100000
50000
0
46−60 (n=7)
80plus (n=3)
61−80 (n=15)
31−45 (n=3)
20−30 (n=2)
Age_category

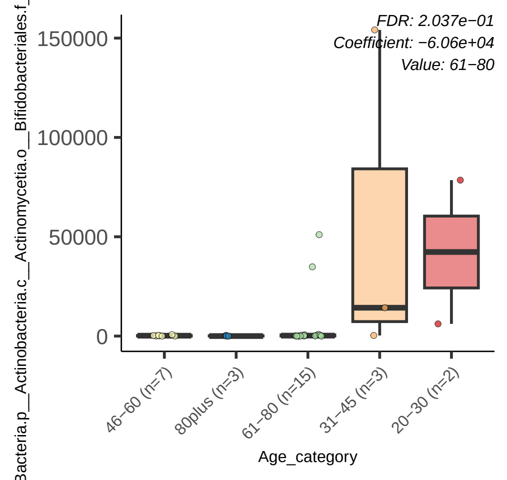

FDR: 2.037e−01
Coefficient: −6.06e+04
Value: 61−80
Bacteria.p__Actinobacteria.c__Actinomycetia.o__Bifidobacteriales.f_
150000
100000
50000
0
46−60 (n=7)
80plus (n=3)
61−80 (n=15)
31−45 (n=3)
20−30 (n=2)
Age_category

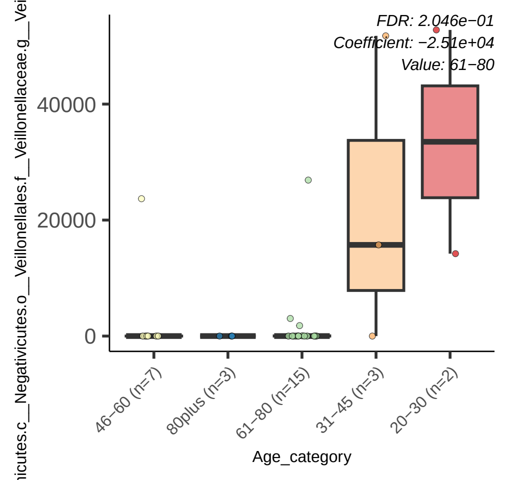

FDR: 2.046e−01
Coefficient: −2.51e+04
Value: 61−80
icutes.c__Negativicutes.o__Veillonellales.f__Veillonellaceae.g__Vei
40000
20000
0
46−60 (n=7)
80plus (n=3)
61−80 (n=15)
31−45 (n=3)
20−30 (n=2)
Age_category

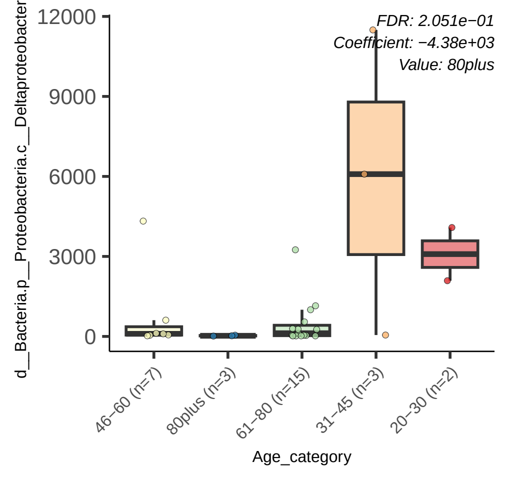
FDR: 2.051e−01
Coefficient: −4.38e+03
Value: 80plus
d__Bacteria.p__Proteobacteria.c__Deltaproteobacter
12000
9000
6000
3000
0
46−60 (n=7)
80plus (n=3)
61−80 (n=15)
31−45 (n=3)
20−30 (n=2)
Age_category

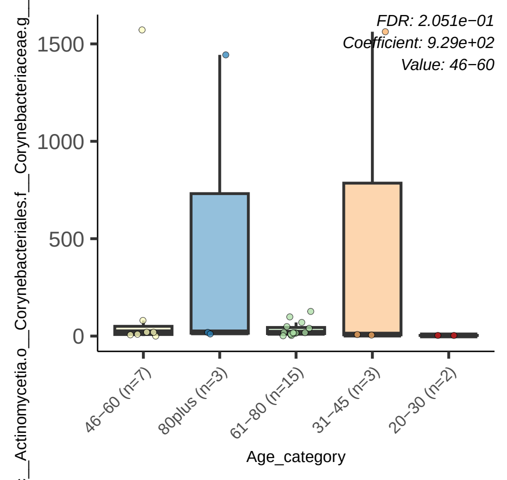
FDR: 2.051e−01
Coefficient: 9.29e+02
Value: 46−60
Actinomycetia.o__Corynebacteriales.f__Corynebacteriaceae.g__
1500
1000
500
0
46−60 (n=7)
80plus (n=3)
61−80 (n=15)
31−45 (n=3)
20−30 (n=2)
Age_category

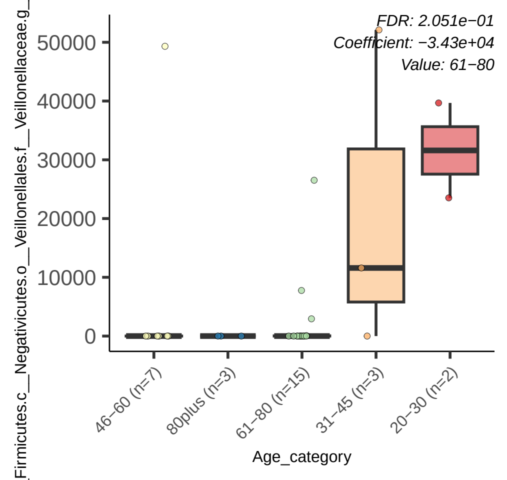

FDR: 2.051e−01
Coefficient: −3.43e+04
Value: 61−80
Firmicutes.c__Negativicutes.o__Veillonellales.f__Veillonellaceae.g__
50000
40000
30000
20000
10000
0
46−60 (n=7)
80plus (n=3)
61−80 (n=15)
31−45 (n=3)
20−30 (n=2)
Age_category

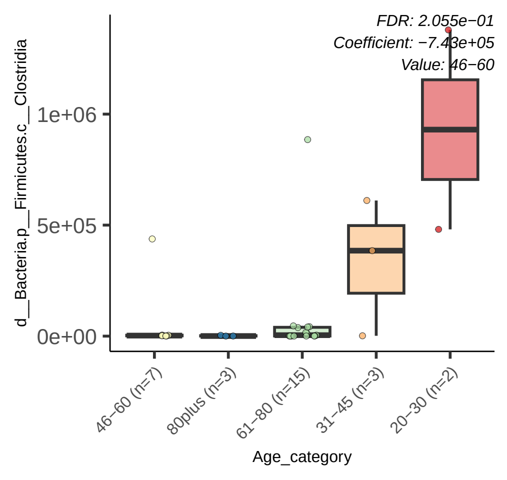
FDR: 2.055e−01
Coefficient: −7.43e+05
Value: 46−60
d__Bacteria.p__Firmicutes.c__Clostridia
1e+06
5e+05
0e+00
46−60 (n=7)
80plus (n=3)
61−80 (n=15)
31−45 (n=3)
20−30 (n=2)
Age_category

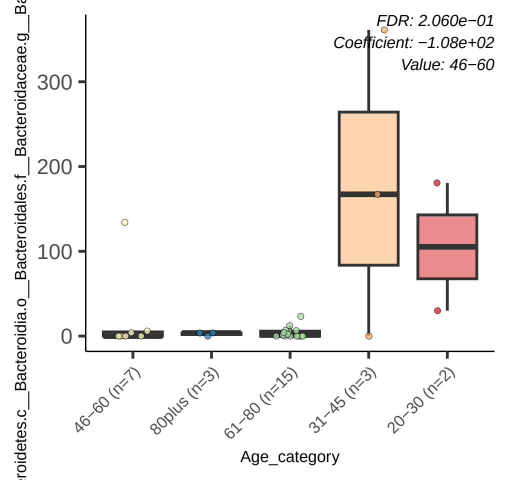

FDR: 2.060e−01
Coefficient: −1.08e+02
Value: 46−60
0
100
200
300
46−60 (n=7)
80plus (n=3)
61−80 (n=15)
31−45 (n=3)
20−30 (n=2)
Age_category
roidetes.c__Bacteroidia.o__Bacteroidales.f__Bacteroidaceae.g__Ba

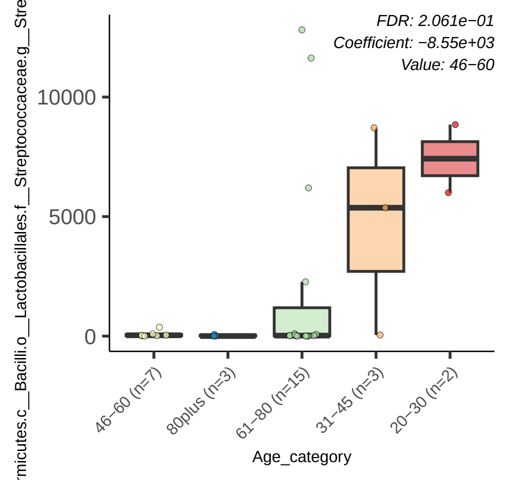

FDR: 2.061e−01
Coefficient: −8.55e+03
Value: 46−60
10000
5000
0
micutes.c__Bacilli.o__Lactobacillales.f__Streptococcaceae.g__Stre
46−60 (n=7)
80plus (n=3)
61−80 (n=15)
31−45 (n=3)
20−30 (n=2)
Age_category

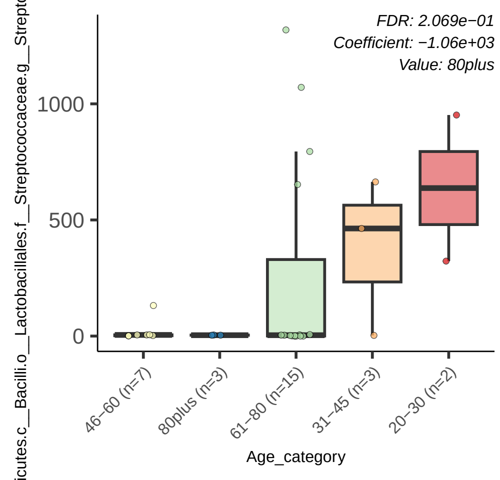

FDR: 2.069e−01
Coefficient: −1.06e+03
Value: 80plus
1000
500
0
46−60 (n=7)
80plus (n=3)
61−80 (n=15)
31−45 (n=3)
20−30 (n=2)
Age_category
icutes.c__Bacilli.o__Lactobacillales.f__Streptococcaceae.g__Strept

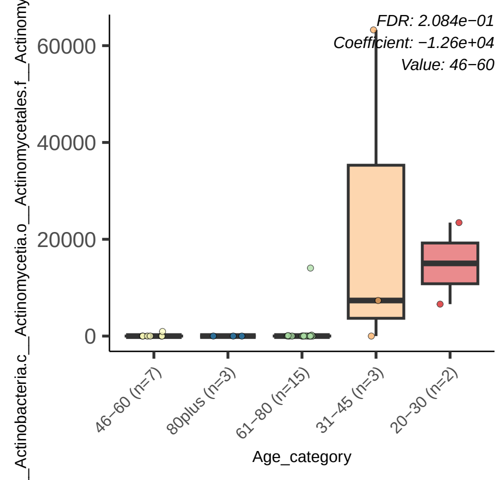

FDR: 2.084e−01
Coefficient: −1.26e+04
Value: 46−60
60000
40000
20000
0
_Actinobacteria.c__Actinomycetia.o__Actinomycetales.f__Actinomy
46−60 (n=7)
80plus (n=3)
61−80 (n=15)
31−45 (n=3)
20−30 (n=2)
Age_category

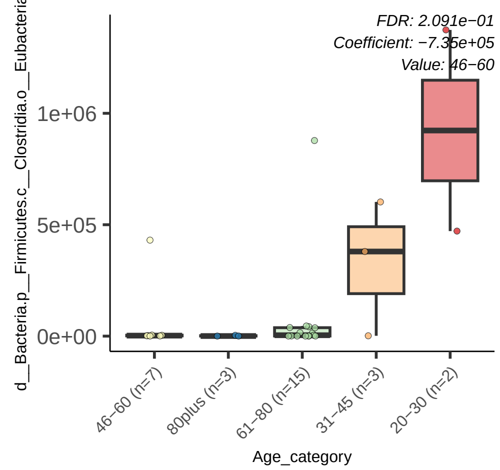

FDR: 2.091e−01
Coefficient: −7.35e+05
Value: 46−60
d__Bacteria.p__Firmicutes.c__Clostridia.o__Eubacteria
1e+06
5e+05
0e+00
46−60 (n=7)
80plus (n=3)
61−80 (n=15)
31−45 (n=3)
20−30 (n=2)
Age_category

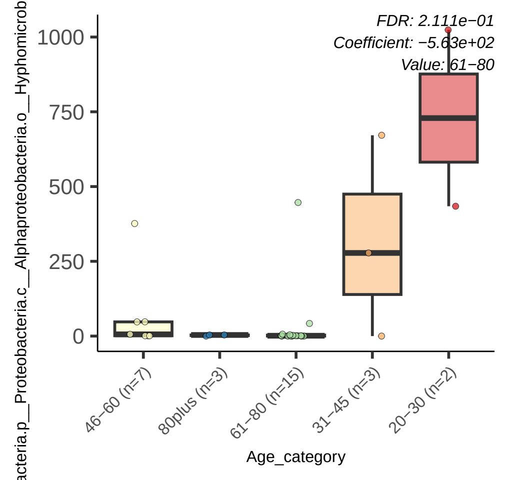

FDR: 2.111e−01
Coefficient: −5.63e+02
Value: 61−80
acteria.p__Proteobacteria.c__Alphaproteobacteria.o__Hyphomicrob
1000
750
500
250
0
46−60 (n=7)
80plus (n=3)
61−80 (n=15)
31−45 (n=3)
20−30 (n=2)
Age_category

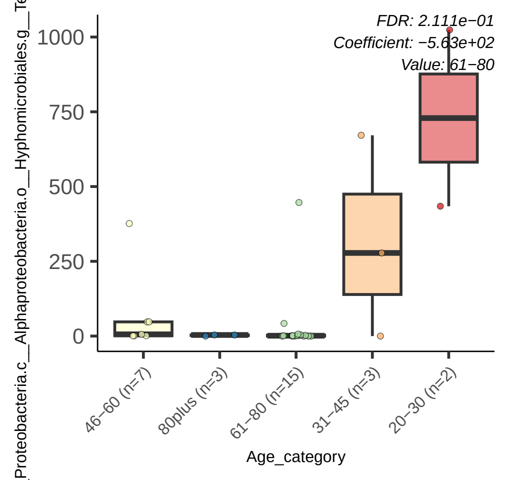
FDR: 2.111e−01
Coefficient: −5.63e+02
Value: 61−80
Proteobacteria.c__Alphaproteobacteria.o__Hyphomicrobiales.g__Te
1000
750
500
250
0
46−60 (n=7)
80plus (n=3)
61−80 (n=15)
31−45 (n=3)
20−30 (n=2)
Age_category

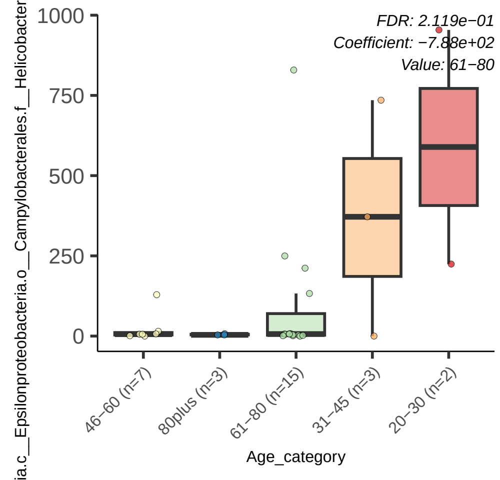

FDR: 2.119e−01
Coefficient: −7.88e+02
Value: 61−80
1000
750
500
250
0
ia.c__Epsilonproteobacteria.o__Campylobacterales.f__Helicobacter
46−60 (n=7)
80plus (n=3)
61−80 (n=15)
31−45 (n=3)
20−30 (n=2)
Age_category

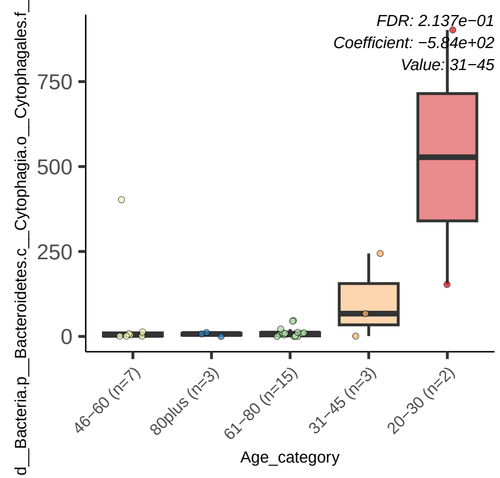

FDR: 2.137e−01
Coefficient: −5.84e+02
Value: 31−45
d__Bacteria.p__Bacteroidetes.c__Cytophagia.o__Cytophagales.f__
750
500
250
0
46−60 (n=7)
80plus (n=3)
61−80 (n=15)
31−45 (n=3)
20−30 (n=2)
Age_category

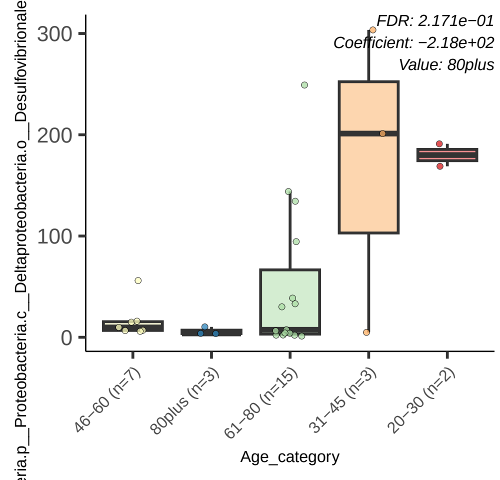

FDR: 2.171e−01
Coefficient: −2.18e+02
Value: 80plus
300
200
100
0
eria.p__Proteobacteria.c__Deltaproteobacteria.o__Desulfovibrionale
46−60 (n=7)
80plus (n=3)
61−80 (n=15)
31−45 (n=3)
20−30 (n=2)
Age_category

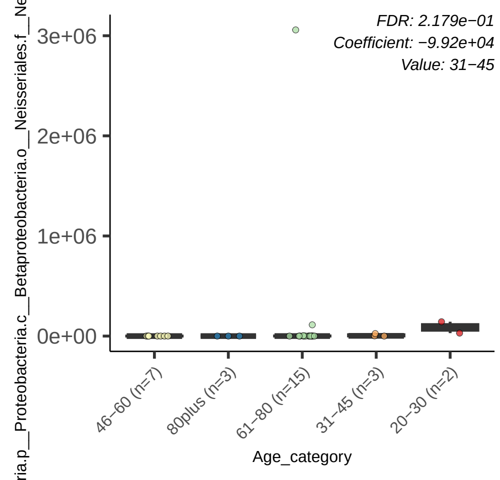

ria.p__Proteobacteria.c__Betaproteobacteria.o__Neisseriales.f__Ne
FDR: 2.179e−01
Coefficient: −9.92e+04
Value: 31−45
3e+06
2e+06
1e+06
0e+00
46−60 (n=7)
80plus (n=3)
61−80 (n=15)
31−45 (n=3)
20−30 (n=2)
Age_category

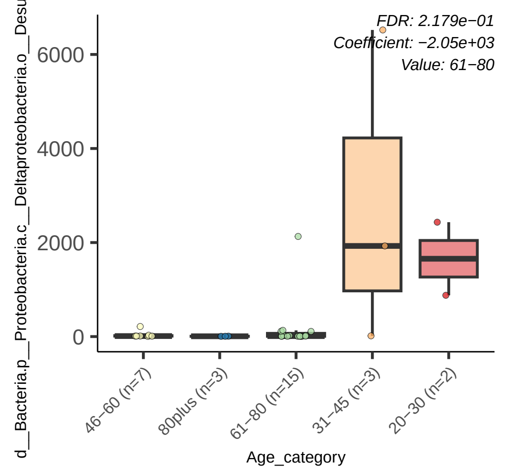

FDR: 2.179e−01
Coefficient: −2.05e+03
Value: 61−80
d__Bacteria.p__Proteobacteria.c__Deltaproteobacteria.o__Desu
0
2000
4000
6000
46−60 (n=7)
80plus (n=3)
61−80 (n=15)
31−45 (n=3)
20−30 (n=2)
Age_category

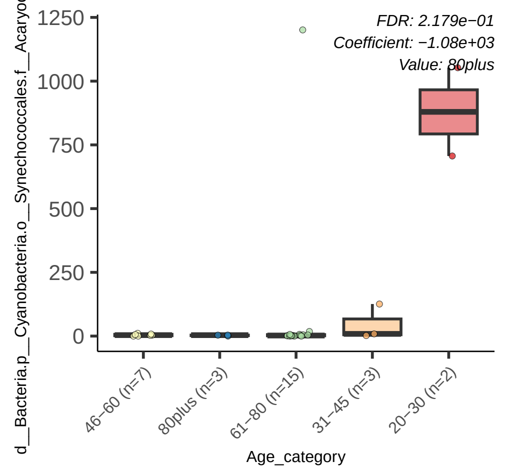
FDR: 2.179e−01
Coefficient: −1.08e+03
Value: 80plus
d__Bacteria.p__Cyanobacteria.o__Synechococcales.f__Acaryoc
1250
1000
750
500
250
0
46−60 (n=7)
80plus (n=3)
61−80 (n=15)
31−45 (n=3)
20−30 (n=2)
Age_category

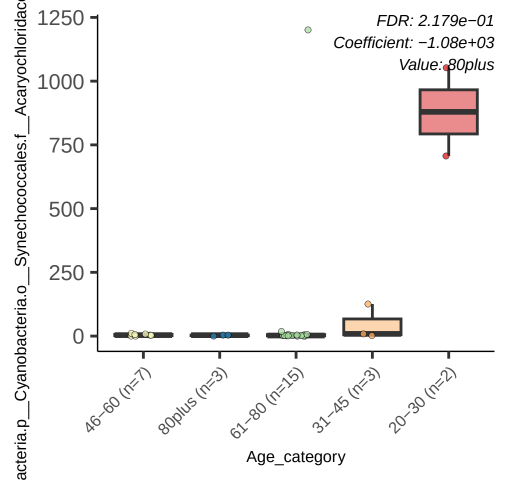

FDR: 2.179e−01
Coefficient: −1.08e+03
Value: 80plus
acteria.p__Cyanobacteria.o__Synechococcales.f__Acaryochloridac
1250
1000
750
500
250
0
46−60 (n=7)
80plus (n=3)
61−80 (n=15)
31−45 (n=3)
20−30 (n=2)
Age_category

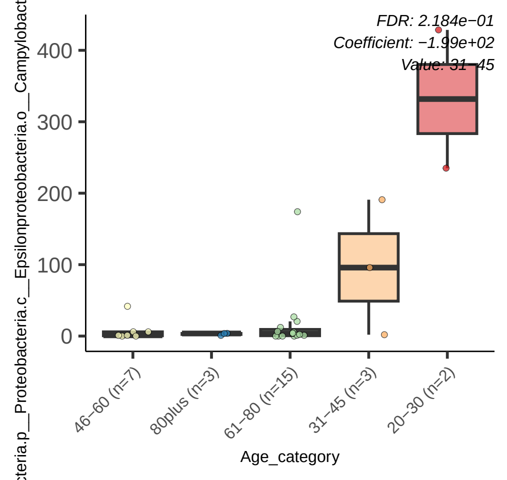

FDR: 2.184e−01
Coefficient: −1.99e+02
Value: 31−45
cteria.p__Proteobacteria.c__Epsilonproteobacteria.o__Campylobact
400
300
200
100
0
46−60 (n=7)
80plus (n=3)
61−80 (n=15)
31−45 (n=3)
20−30 (n=2)
Age_category

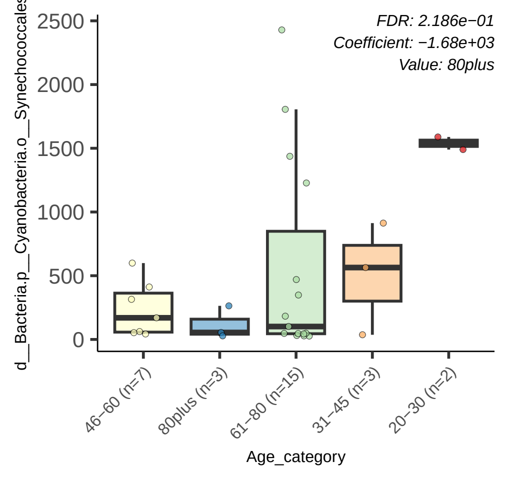

FDR: 2.186e−01
Coefficient: −1.68e+03
Value: 80plus
d__Bacteria.p__Cyanobacteria.o__Synechococcales
2500
2000
1500
1000
500
0
46−60 (n=7)
80plus (n=3)
61−80 (n=15)
31−45 (n=3)
20−30 (n=2)
Age_category

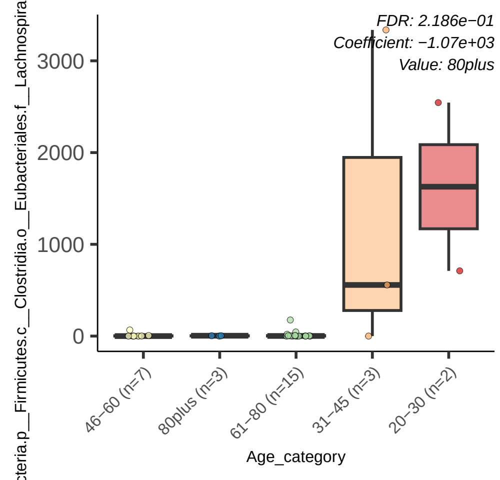

FDR: 2.186e−01
Coefficient: −1.07e+03
Value: 80plus
3000
2000
1000
0
cteria.p__Firmicutes.c__Clostridia.o__Eubacteriales.f__Lachnospira
46−60 (n=7)
80plus (n=3)
61−80 (n=15)
31−45 (n=3)
20−30 (n=2)
Age_category

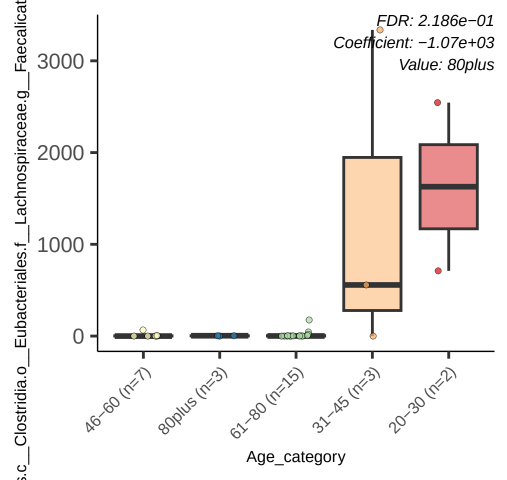

FDR: 2.186e−01
Coefficient: −1.07e+03
Value: 80plus
3000
2000
1000
0
s.c__Clostridia.o__Eubacteriales.f__Lachnospiraceae.g__Faecalicat
46−60 (n=7)
80plus (n=3)
61−80 (n=15)
31−45 (n=3)
20−30 (n=2)
Age_category

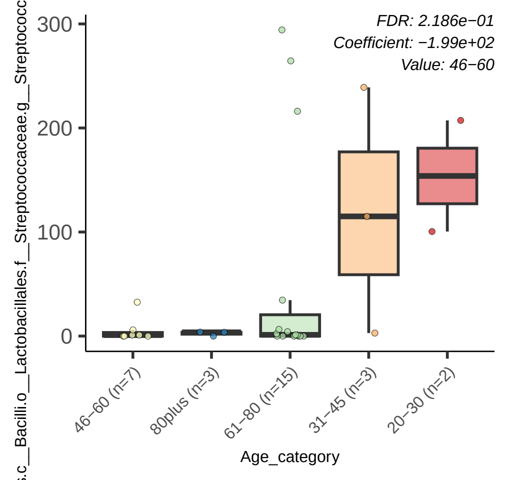

FDR: 2.186e−01
Coefficient: −1.99e+02
Value: 46−60
300
200
100
0
Bacilli.o__Lactobacillales.f__Streptococcaceae.g__Streptococc
46−60 (n=7)
80plus (n=3)
61−80 (n=15)
31−45 (n=3)
20−30 (n=2)
Age_category

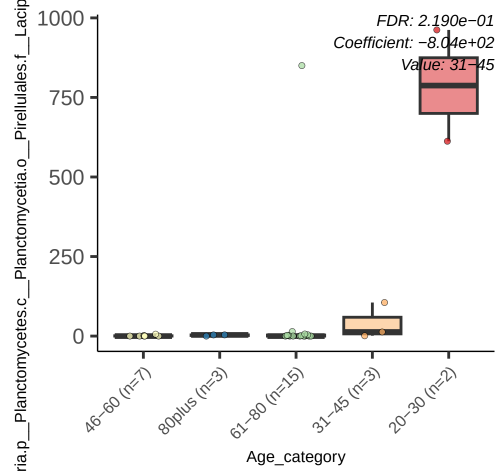

FDR: 2.190e−01
Coefficient: −8.04e+02
Value: 31−45
1000
750
500
250
0
eria.p__Planctomycetes.c__Planctomycetia.o__Pirellulales.f__Lacip
46−60 (n=7)
80plus (n=3)
61−80 (n=15)
31−45 (n=3)
20−30 (n=2)
Age_category

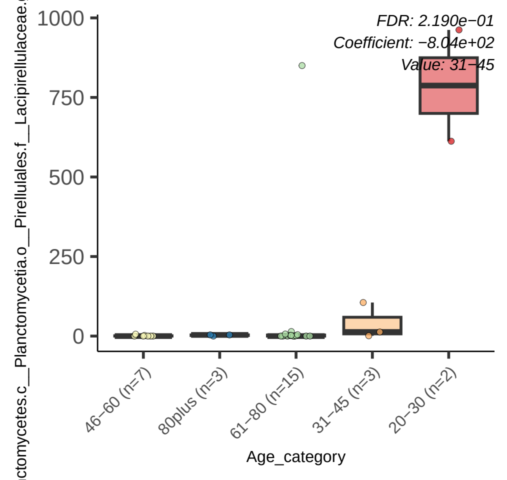
FDR: 2.190e−01
Coefficient: −8.04e+02
Value: 31−45
1000
750
500
250
0
ctomycetes.c__Planctomycetia.o__Pirellulales.f__Lacipirellulaceae.
46−60 (n=7)
80plus (n=3)
61−80 (n=15)
31−45 (n=3)
20−30 (n=2)
Age_category

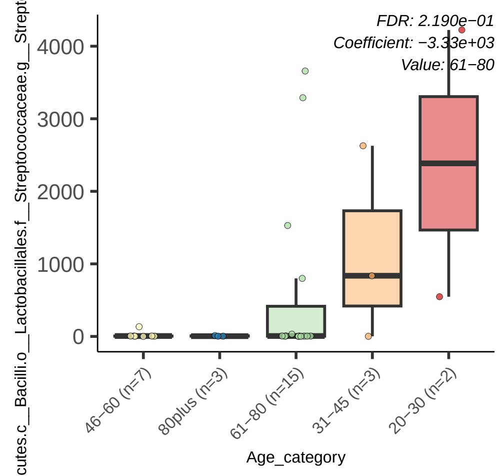
FDR: 2.190e−01
Coefficient: −3.33e+03
Value: 61−80
4000
3000
2000
1000
0
46−60 (n=7)
80plus (n=3)
61−80 (n=15)
31−45 (n=3)
20−30 (n=2)
Age_category
icutes.c__Bacilli.o__Lactobacillales.f__Streptococcaceae.g__Strept

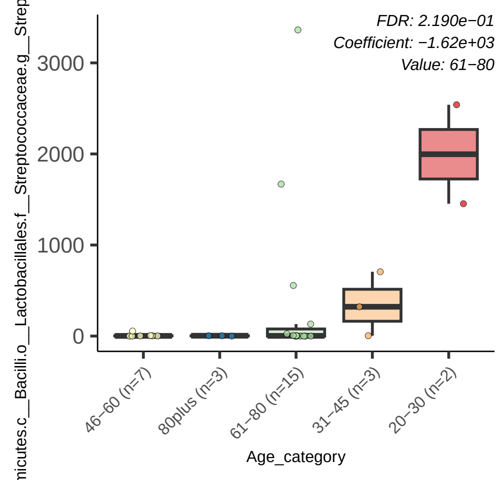

FDR: 2.190e−01
Coefficient: −1.62e+03
Value: 61−80
3000
2000
1000
0
micutes.c__Bacilli.o__Lactobacillales.f__Streptococcaceae.g__Strep
46−60 (n=7)
80plus (n=3)
61−80 (n=15)
31−45 (n=3)
20−30 (n=2)
Age_category

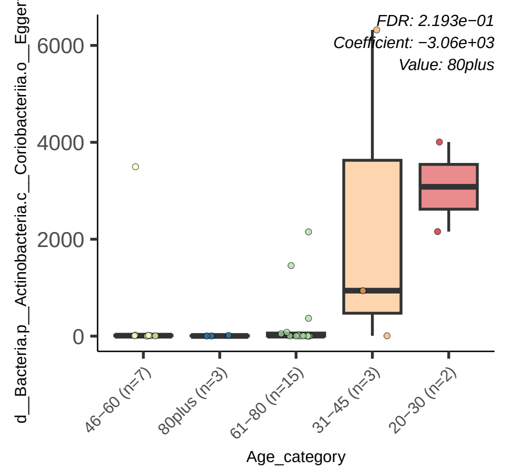
FDR: 2.193e−01
Coefficient: −3.06e+03
Value: 80plus
d__Bacteria.p__Actinobacteria.c__Coriobacteriia.o__Egger
6000
4000
2000
0
46−60 (n=7)
80plus (n=3)
61−80 (n=15)
31−45 (n=3)
20−30 (n=2)
Age_category

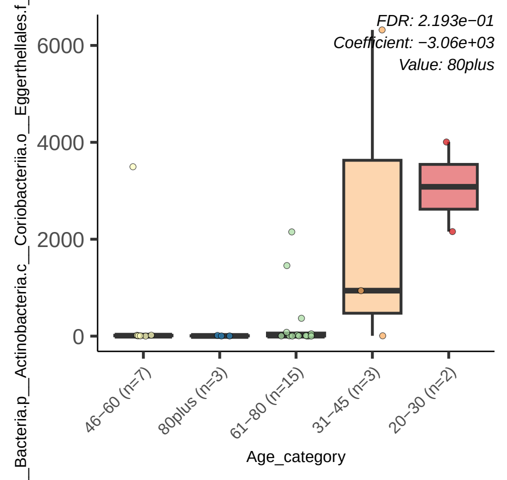
FDR: 2.193e−01
Coefficient: −3.06e+03
Value: 80plus
Bacteria.p__Actinobacteria.c__Coriobacteriia.o__Eggerthellales.f__
6000
4000
2000
0
46−60 (n=7)
80plus (n=3)
61−80 (n=15)
31−45 (n=3)
20−30 (n=2)
Age_category

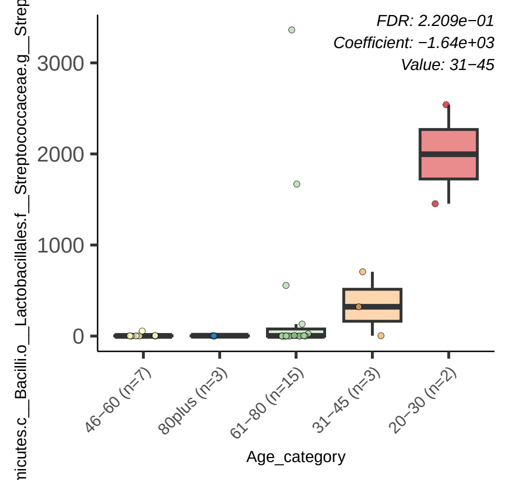

FDR: 2.209e−01
Coefficient: −1.64e+03
Value: 31−45
3000
2000
1000
0
micutes.c__Bacilli.o__Lactobacillales.f__Streptococcaceae.g__Strep
46−60 (n=7)
80plus (n=3)
61−80 (n=15)
31−45 (n=3)
20−30 (n=2)
Age_category

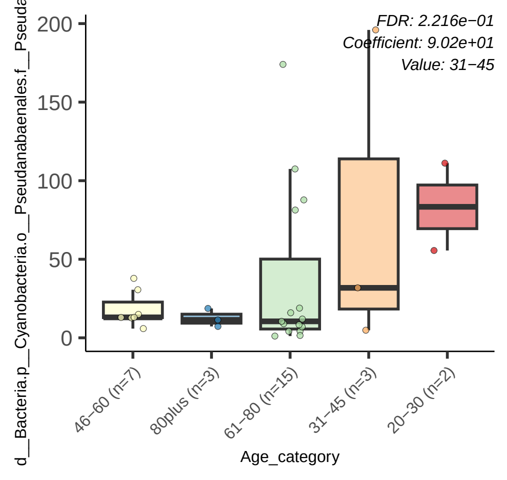
FDR: 2.216e−01
Coefficient: 9.02e+01
Value: 31−45
d__Bacteria.p__Cyanobacteria.o__Pseudanabaenales.f__Pseuda
200
150
100
50
0
46−60 (n=7)
80plus (n=3)
61−80 (n=15)
31−45 (n=3)
20−30 (n=2)
Age_category

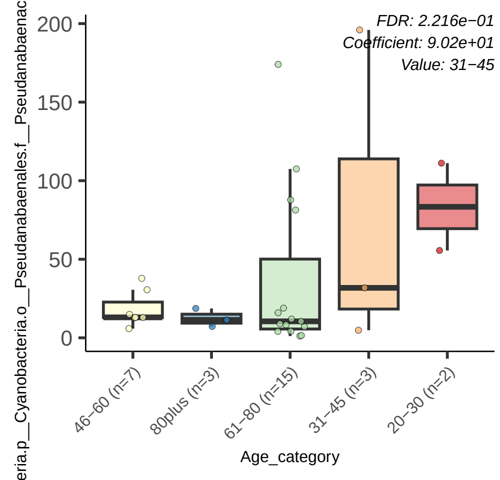

FDR: 2.216e−01
Coefficient: 9.02e+01
Value: 31−45
200
150
100
50
0
eria.p__Cyanobacteria.o__Pseudanabaenales.f__Pseudanabaenac
46−60 (n=7)
80plus (n=3)
61−80 (n=15)
31−45 (n=3)
20−30 (n=2)
Age_category

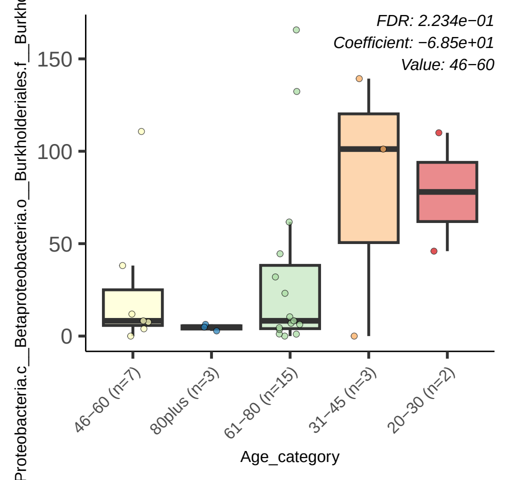

FDR: 2.234e−01
Coefficient: −6.85e+01
Value: 46−60
Proteobacteria.c__Betaproteobacteria.o__Burkholderiales.f__Burkho
150
100
50
0
46−60 (n=7)
80plus (n=3)
61−80 (n=15)
31−45 (n=3)
20−30 (n=2)
Age_category

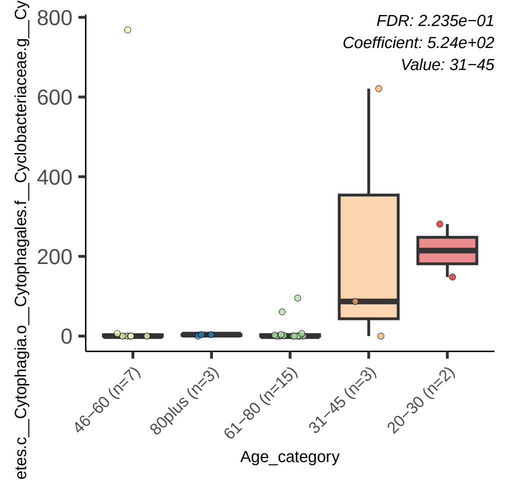

FDR: 2.235e−01
Coefficient: 5.24e+02
Value: 31−45
etes.c__Cytophagia.o__Cytophagales.f__Cyclobacteriaceae.g__Cy
800
600
400
200
0
46−60 (n=7)
80plus (n=3)
61−80 (n=15)
31−45 (n=3)
20−30 (n=2)
Age_category

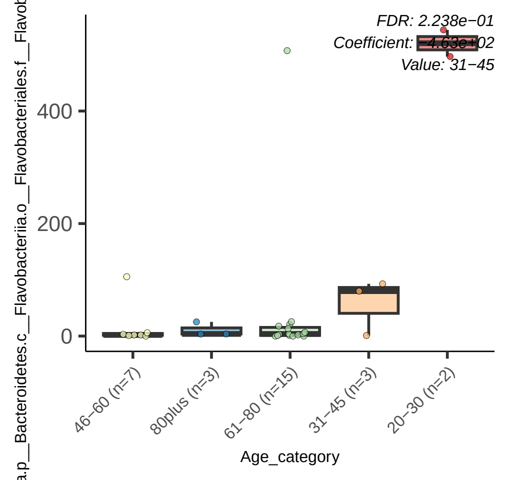

FDR: 2.238e−01
Coefficient: −4.63e+02
Value: 31−45
400
200
0
a.p__Bacteroidetes.c__Flavobacteriia.o__Flavobacteriales.f__Flavob
46−60 (n=7)
80plus (n=3)
61−80 (n=15)
31−45 (n=3)
20−30 (n=2)
Age_category

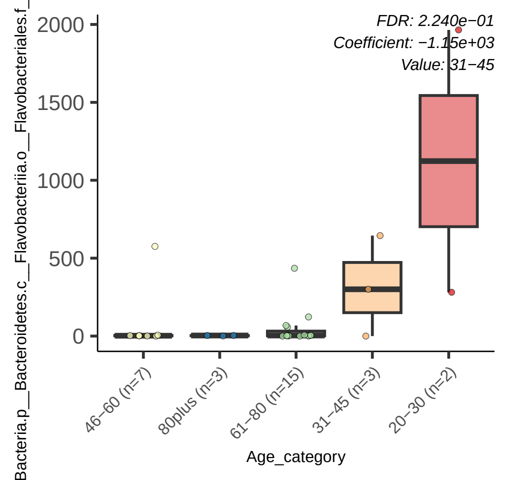

FDR: 2.240e−01
Coefficient: −1.15e+03
Value: 31−45
Bacteria.p__Bacteroidetes.c__Flavobacteriia.o__Flavobacteriales.f__
2000
1500
1000
500
0
46−60 (n=7)
80plus (n=3)
61−80 (n=15)
31−45 (n=3)
20−30 (n=2)
Age_category

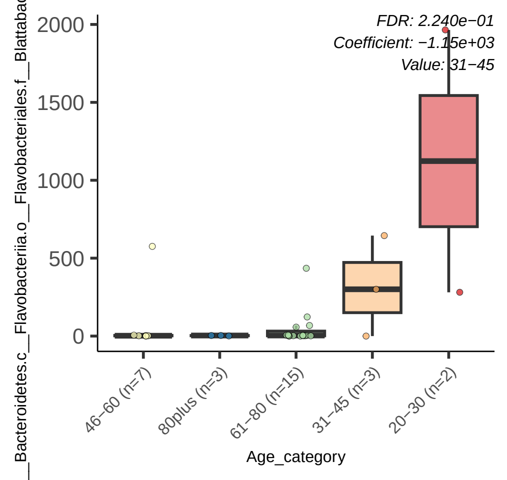

FDR: 2.240e−01
Coefficient: −1.15e+03
Value: 31−45
2000
1500
1000
500
0
Bacteroidetes.c__Flavobacteriia.o__Flavobacteriales.f__Blattabac
46−60 (n=7)
80plus (n=3)
61−80 (n=15)
31−45 (n=3)
20−30 (n=2)
Age_category

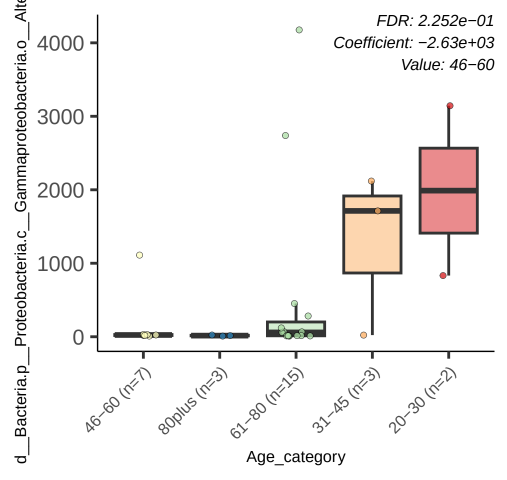

FDR: 2.252e−01
Coefficient: −2.63e+03
Value: 46−60
d__Bacteria.p__Proteobacteria.c__Gammaproteobacteria.o__Alte
4000
3000
2000
1000
0
46−60 (n=7)
80plus (n=3)
61−80 (n=15)
31−45 (n=3)
20−30 (n=2)
Age_category

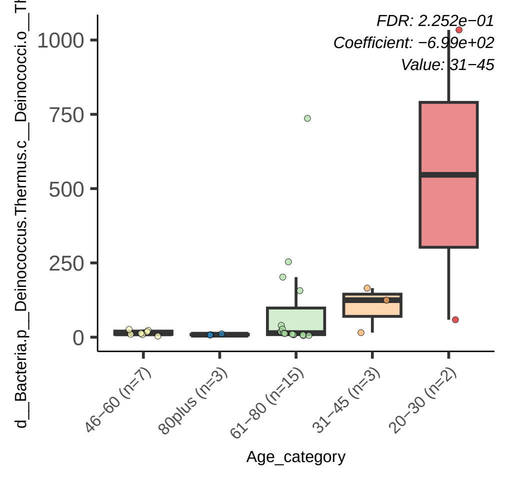

FDR: 2.252e−01
Coefficient: −6.99e+02
Value: 31−45
1000
750
500
250
0
46−60 (n=7)
80plus (n=3)
61−80 (n=15)
31−45 (n=3)
20−30 (n=2)
Age_category
d__Bacteria.p__Deinococcus.Thermus.c__Deinococci.o__Th

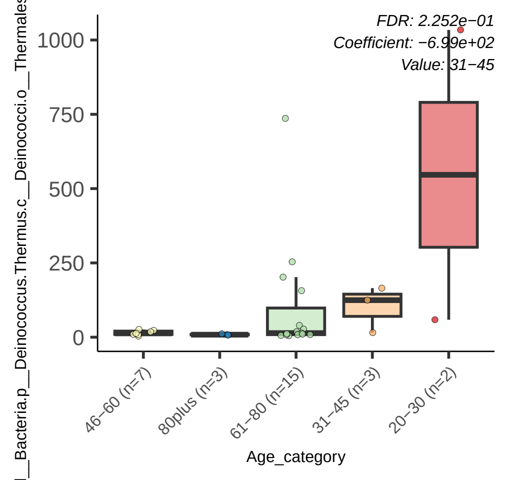

FDR: 2.252e−01
Coefficient: −6.99e+02
Value: 31−45
Bacteria.p__Deinococcus.Thermus.c__Deinococci.o__Thermales
1000
750
500
250
0
46−60 (n=7)
80plus (n=3)
61−80 (n=15)
31−45 (n=3)
20−30 (n=2)
Age_category

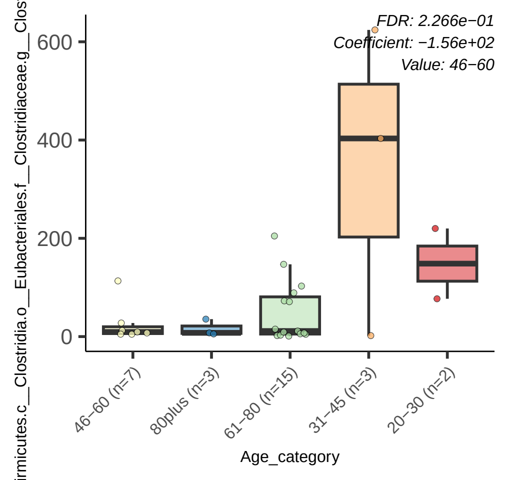

FDR: 2.266e−01
Coefficient: −1.56e+02
Value: 46−60
irmicutes.c__Clostridia.o__Eubacteriales.f__Clostridiaceae.g__Clos
0
200
400
600
46−60 (n=7)
80plus (n=3)
61−80 (n=15)
31−45 (n=3)
20−30 (n=2)
Age_category

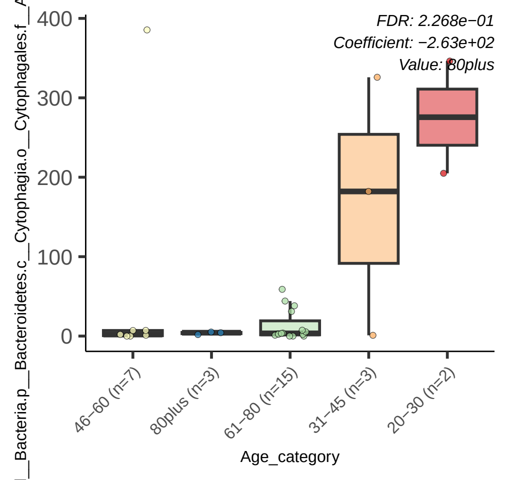
FDR: 2.268e−01
Coefficient: −2.63e+02
Value: 80plus
400
300
200
100
0
__Bacteria.p__Bacteroidetes.c__Cytophagia.o__Cytophagales.f__A
46−60 (n=7)
80plus (n=3)
61−80 (n=15)
31−45 (n=3)
20−30 (n=2)
Age_category

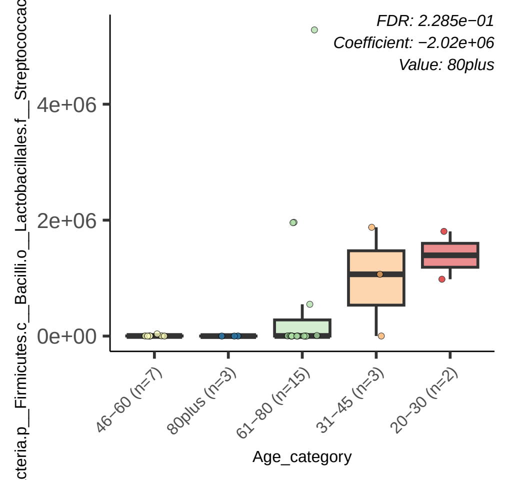

FDR: 2.285e−01
Coefficient: −2.02e+06
Value: 80plus
4e+06
2e+06
0e+00
46−60 (n=7)
80plus (n=3)
61−80 (n=15)
31−45 (n=3)
20−30 (n=2)
Age_category
cteria.p__Firmicutes.c__Bacilli.o__Lactobacillales.f__Streptococcac

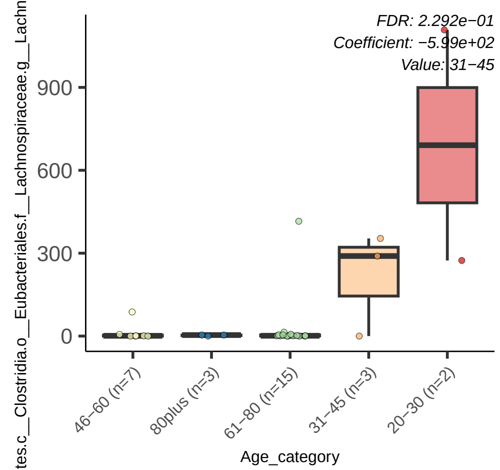

FDR: 2.292e−01
Coefficient: −5.99e+02
Value: 31−45
900
600
300
0
cutes.c__Clostridia.o__Eubacteriales.f__Lachnospiraceae.g__Lachn
46−60 (n=7)
80plus (n=3)
61−80 (n=15)
31−45 (n=3)
20−30 (n=2)
Age_category

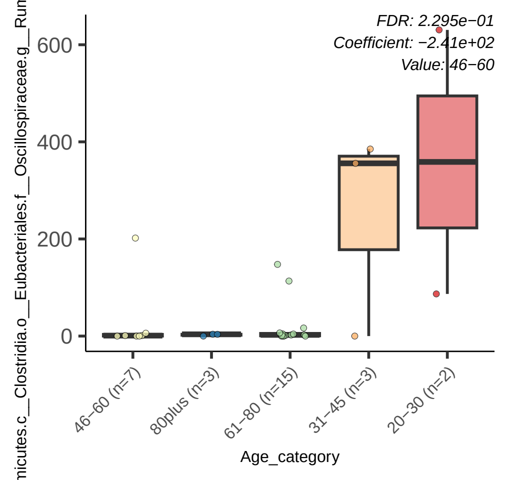

FDR: 2.295e−01
Coefficient: −2.41e+02
Value: 46−60
600
400
200
0
micutes.c__Clostridia.o__Eubacteriales.f__Oscillospiraceae.g__Rum
46−60 (n=7)
80plus (n=3)
61−80 (n=15)
31−45 (n=3)
20−30 (n=2)
Age_category

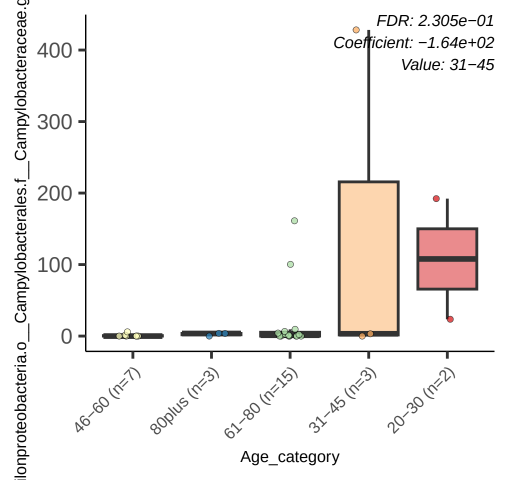
FDR: 2.305e−01
Coefficient: −1.64e+02
Value: 31−45
0
100
200
300
400
ilonproteobacteria.o__Campylobacterales.f__Campylobacteraceae.g
46−60 (n=7)
80plus (n=3)
61−80 (n=15)
31−45 (n=3)
20−30 (n=2)
Age_category

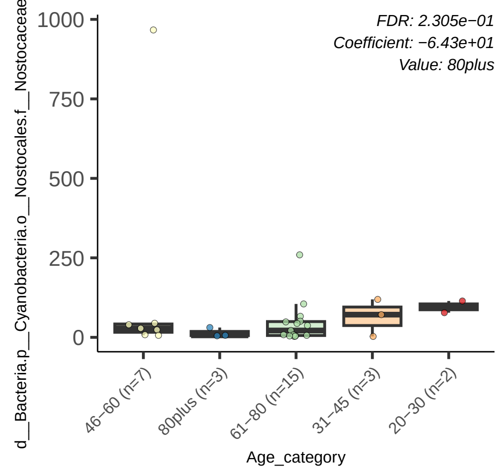

FDR: 2.305e−01
Coefficient: −6.43e+01
Value: 80plus
d__Bacteria.p__Cyanobacteria.o__Nostocales.f__Nostocaceae
1000
750
500
250
0
46−60 (n=7)
80plus (n=3)
61−80 (n=15)
31−45 (n=3)
20−30 (n=2)
Age_category

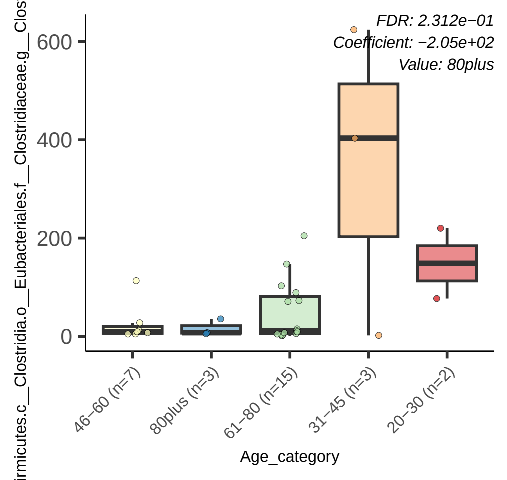

FDR: 2.312e−01
Coefficient: −2.05e+02
Value: 80plus
0
200
400
600
irmicutes.c__Clostridia.o__Eubacteriales.f__Clostridiaceae.g__Clos
46−60 (n=7)
80plus (n=3)
61−80 (n=15)
31−45 (n=3)
20−30 (n=2)
Age_category

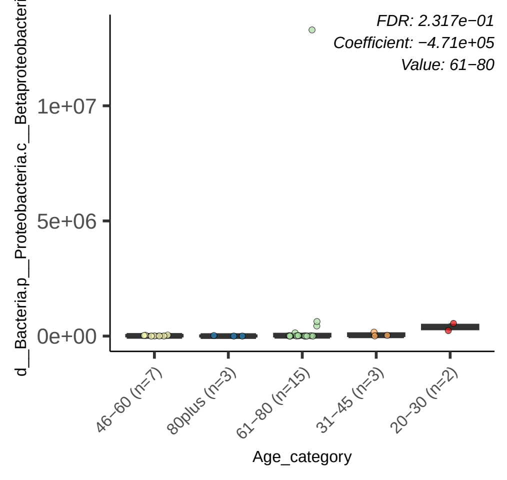
FDR: 2.317e−01
Coefficient: −4.71e+05
Value: 61−80
d__Bacteria.p__Proteobacteria.c__Betaproteobacteri
1e+07
5e+06
0e+00
46−60 (n=7)
80plus (n=3)
61−80 (n=15)
31−45 (n=3)
20−30 (n=2)
Age_category

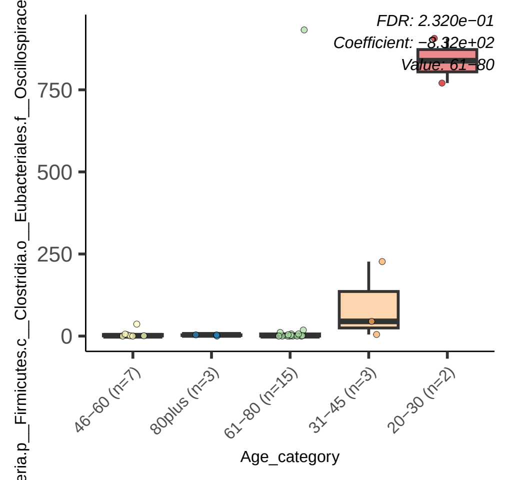
FDR: 2.320e−01
Coefficient: −8.32e+02
Value: 61−80
eria.p__Firmicutes.c__Clostridia.o__Eubacteriales.f__Oscillospirace
750
500
250
0
46−60 (n=7)
80plus (n=3)
61−80 (n=15)
31−45 (n=3)
20−30 (n=2)
Age_category

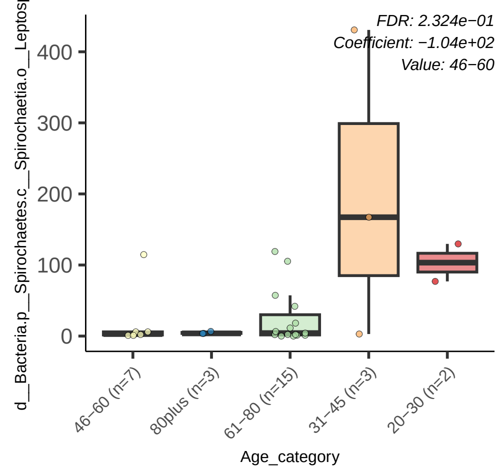

FDR: 2.324e−01
Coefficient: −1.04e+02
Value: 46−60
d__Bacteria.p__Spirochaetes.c__Spirochaetia.o__Leptos
0
100
200
300
400
46−60 (n=7)
80plus (n=3)
61−80 (n=15)
31−45 (n=3)
20−30 (n=2)
Age_category

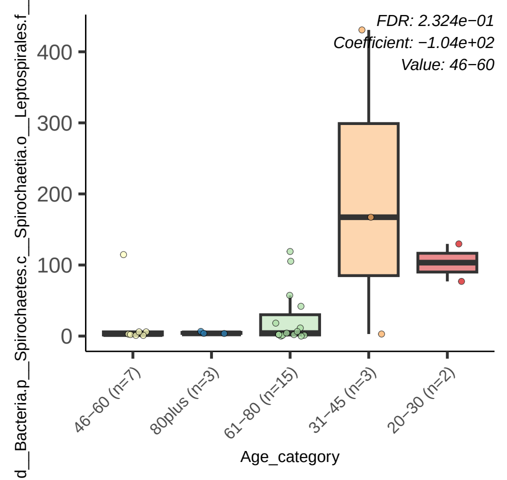

FDR: 2.324e−01
Coefficient: −1.04e+02
Value: 46−60
d__Bacteria.p__Spirochaetes.c__Spirochaetia.o__Leptospirales.f__
0
100
200
300
400
46−60 (n=7)
80plus (n=3)
61−80 (n=15)
31−45 (n=3)
20−30 (n=2)
Age_category

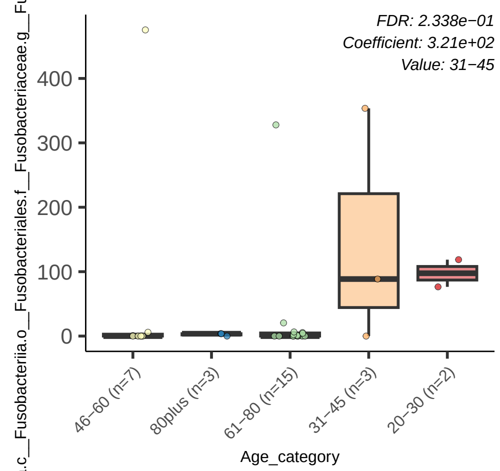

FDR: 2.338e−01
Coefficient: 3.21e+02
Value: 31−45
ia.c__Fusobacteriia.o__Fusobacteriales.f__Fusobacteriaceae.g__Fu
400
300
200
100
0
46−60 (n=7)
80plus (n=3)
61−80 (n=15)
31−45 (n=3)
20−30 (n=2)
Age_category

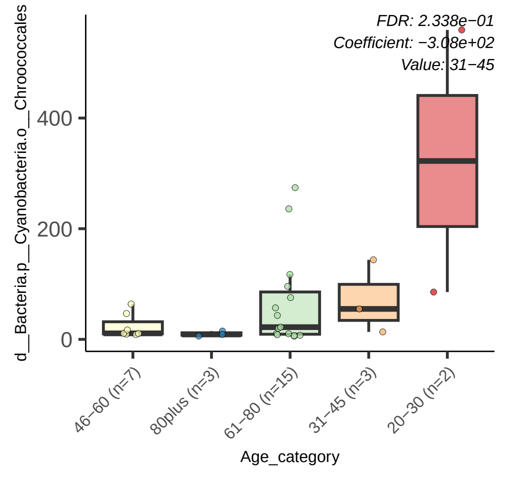

FDR: 2.338e−01
Coefficient: −3.08e+02
Value: 31−45
d__Bacteria.p__Cyanobacteria.o__Chroococcales
400
200
0
46−60 (n=7)
80plus (n=3)
61−80 (n=15)
31−45 (n=3)
20−30 (n=2)
Age_category

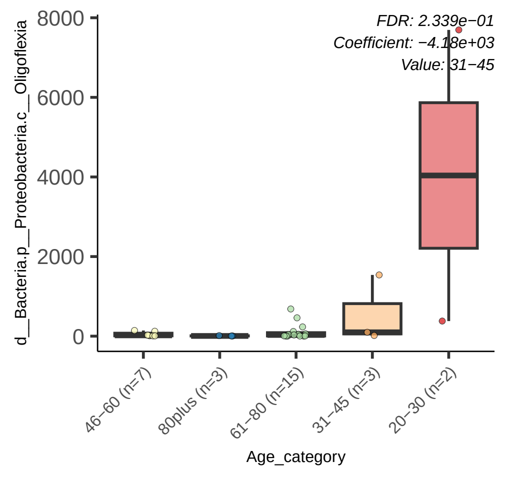

FDR: 2.339e−01
Coefficient: −4.18e+03
Value: 31−45
d__Bacteria.p__Proteobacteria.c__Oligoflexia
8000
6000
4000
2000
0
46−60 (n=7)
80plus (n=3)
61−80 (n=15)
31−45 (n=3)
20−30 (n=2)
Age_category

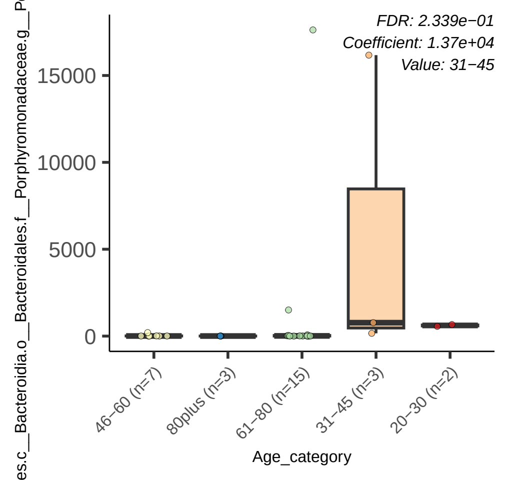

FDR: 2.339e−01
Coefficient: 1.37e+04
Value: 31−45
15000
10000
5000
0
46−60 (n=7)
80plus (n=3)
61−80 (n=15)
31−45 (n=3)
20−30 (n=2)
Age_category
es.c__Bacteroidia.o__Bacteroidales.f__Porphyromonadaceae.g__Po

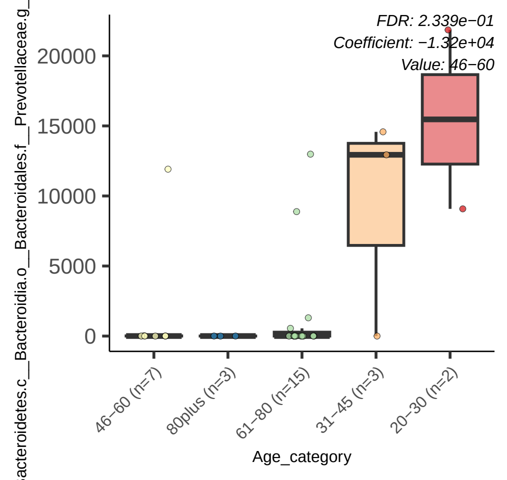

FDR: 2.339e−01
Coefficient: −1.32e+04
Value: 46−60
Bacteroidetes.c__Bacteroidia.o__Bacteroidales.f__Prevotellaceae.g__
20000
15000
10000
5000
0
46−60 (n=7)
80plus (n=3)
61−80 (n=15)
31−45 (n=3)
20−30 (n=2)
Age_category

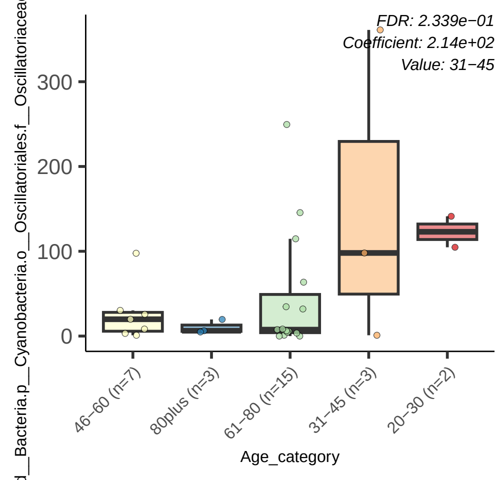

FDR: 2.339e−01
Coefficient: 2.14e+02
Value: 31−45
d__Bacteria.p__Cyanobacteria.o__Oscillatoriales.f__Oscillatoriaceae
300
200
100
0
46−60 (n=7)
80plus (n=3)
61−80 (n=15)
31−45 (n=3)
20−30 (n=2)
Age_category

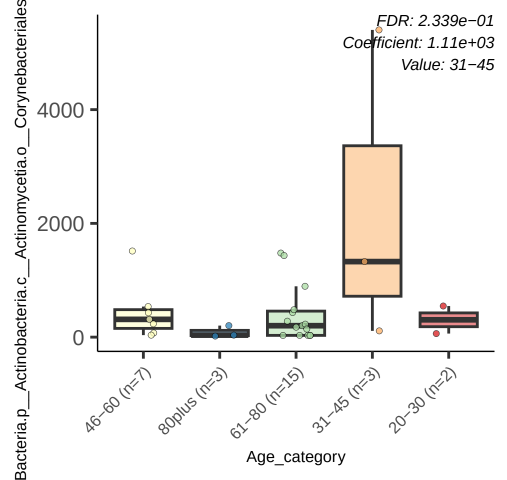

FDR: 2.339e−01
Coefficient: 1.11e+03
Value: 31−45
Bacteria.p__Actinobacteria.c__Actinomycetia.o__Corynebacteriales
4000
2000
0
46−60 (n=7)
80plus (n=3)
61−80 (n=15)
31−45 (n=3)
20−30 (n=2)
Age_category

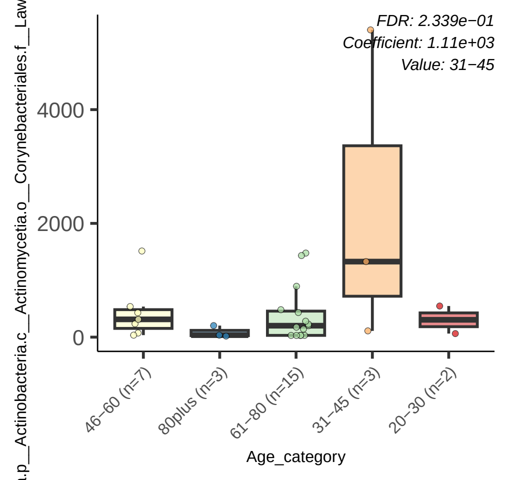

FDR: 2.339e−01
Coefficient: 1.11e+03
Value: 31−45
a.p__Actinobacteria.c__Actinomycetia.o__Corynebacteriales.f__Law
4000
2000
0
46−60 (n=7)
80plus (n=3)
61−80 (n=15)
31−45 (n=3)
20−30 (n=2)
Age_category

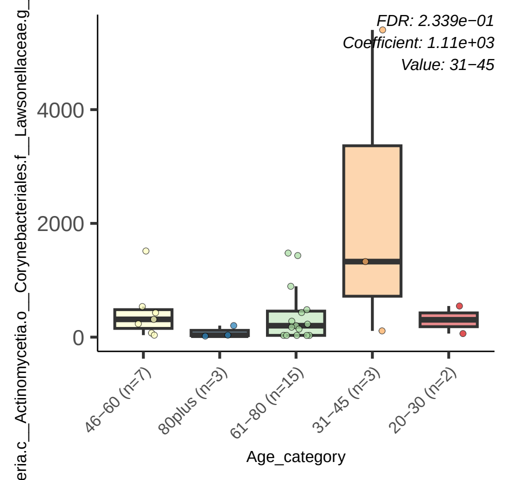

FDR: 2.339e−01
Coefficient: 1.11e+03
Value: 31−45
4000
2000
0
eria.c__Actinomycetia.o__Corynebacteriales.f__Lawsonellaceae.g__
46−60 (n=7)
80plus (n=3)
61−80 (n=15)
31−45 (n=3)
20−30 (n=2)
Age_category

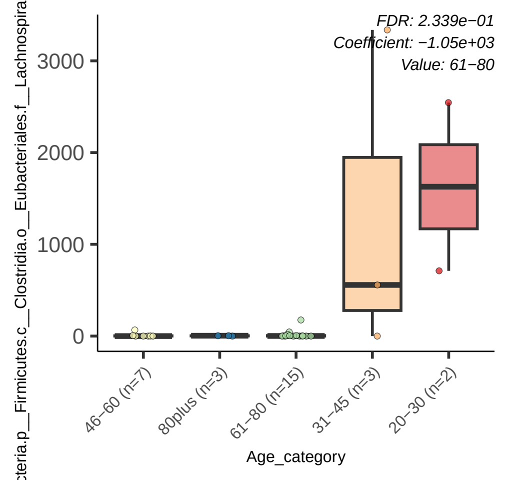

FDR: 2.339e−01
Coefficient: −1.05e+03
Value: 61−80
3000
2000
1000
0
cteria.p__Firmicutes.c__Clostridia.o__Eubacteriales.f__Lachnospira
46−60 (n=7)
80plus (n=3)
61−80 (n=15)
31−45 (n=3)
20−30 (n=2)
Age_category

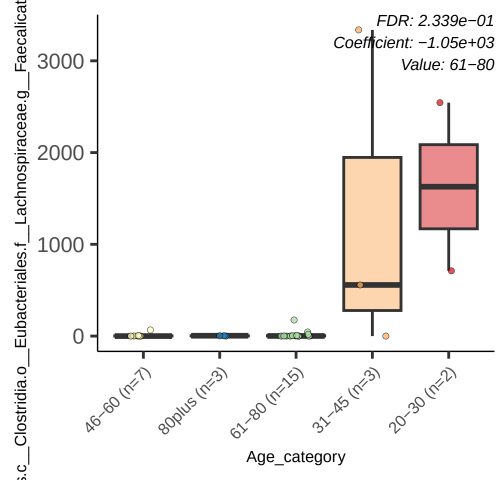

FDR: 2.339e−01
Coefficient: −1.05e+03
Value: 61−80
s.c__Clostridia.o__Eubacteriales.f__Lachnospiraceae.g__Faecalicat
3000
2000
1000
0
46−60 (n=7)
80plus (n=3)
61−80 (n=15)
31−45 (n=3)
20−30 (n=2)
Age_category

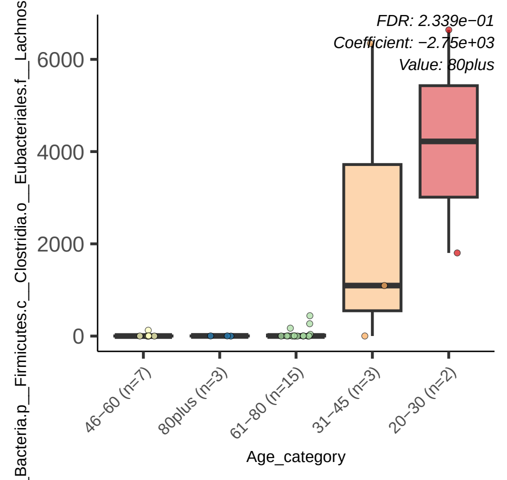

FDR: 2.339e−01
Coefficient: −2.75e+03
Value: 80plus
6000
4000
2000
0
_Bacteria.p__Firmicutes.c__Clostridia.o__Eubacteriales.f__Lachnos
46−60 (n=7)
80plus (n=3)
61−80 (n=15)
31−45 (n=3)
20−30 (n=2)
Age_category

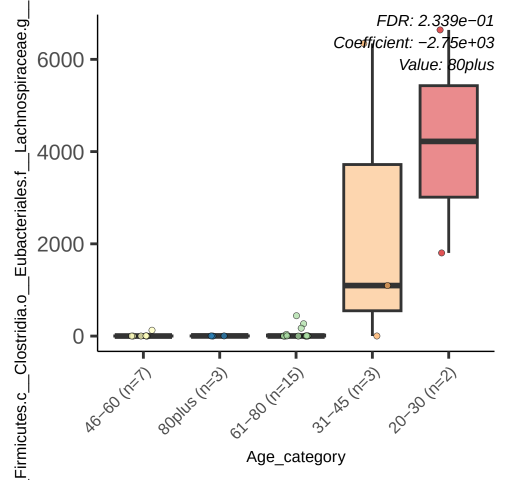

FDR: 2.339e−01
Coefficient: −2.75e+03
Value: 80plus
Firmicutes.c__Clostridia.o__Eubacteriales.f__Lachnospiraceae.g__
6000
4000
2000
0
46−60 (n=7)
80plus (n=3)
61−80 (n=15)
31−45 (n=3)
20−30 (n=2)
Age_category

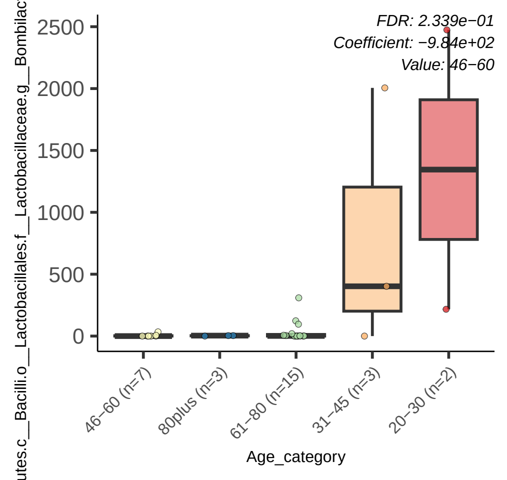

FDR: 2.339e−01
Coefficient: −9.84e+02
Value: 46−60
2500
2000
1500
1000
500
0
46−60 (n=7)
80plus (n=3)
61−80 (n=15)
31−45 (n=3)
20−30 (n=2)
Age_category
utes.c__Bacilli.o__Lactobacillales.f__Lactobacillaceae.g__Bombilac

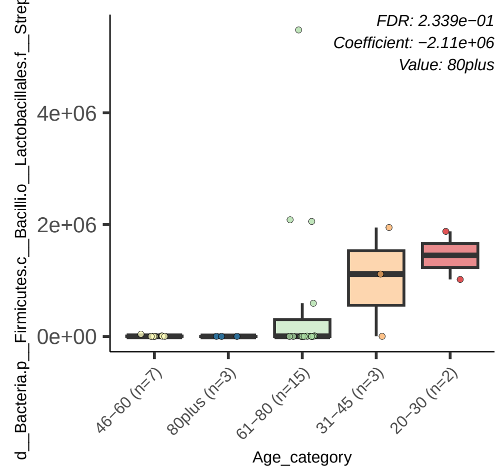

FDR: 2.339e−01
Coefficient: −2.11e+06
Value: 80plus
d__Bacteria.p__Firmicutes.c__Bacilli.o__Lactobacillales.f__Strep
4e+06
2e+06
0e+00
46−60 (n=7)
80plus (n=3)
61−80 (n=15)
31−45 (n=3)
20−30 (n=2)
Age_category

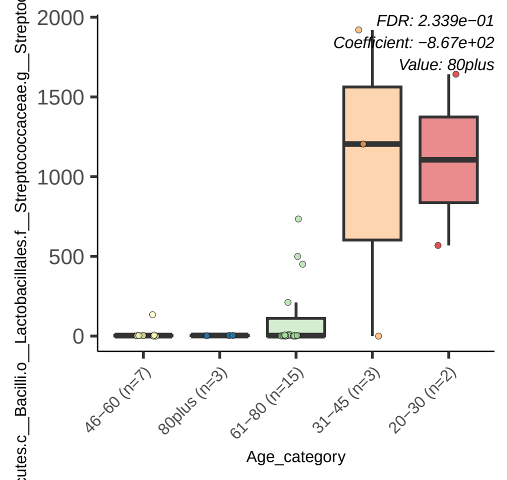

FDR: 2.339e−01
Coefficient: −8.67e+02
Value: 80plus
2000
1500
1000
500
0
cutes.c__Bacilli.o__Lactobacillales.f__Streptococcaceae.g__Streptoc
46−60 (n=7)
80plus (n=3)
61−80 (n=15)
31−45 (n=3)
20−30 (n=2)
Age_category

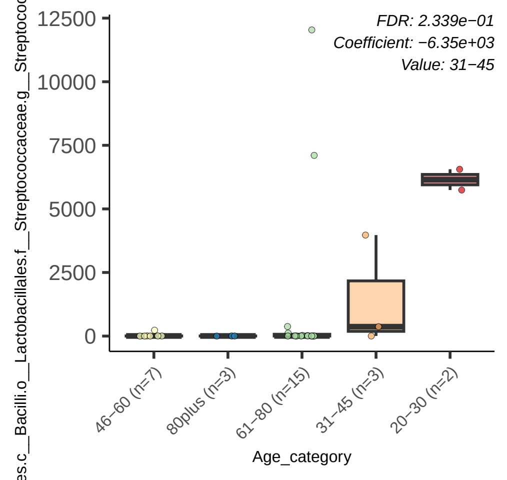

FDR: 2.339e−01
Coefficient: −6.35e+03
Value: 31−45
12500
10000
7500
5000
2500
0
46−60 (n=7)
80plus (n=3)
61−80 (n=15)
31−45 (n=3)
20−30 (n=2)
Age_category
es.c__Bacilli.o__Lactobacillales.f__Streptococcaceae.g__Streptococ

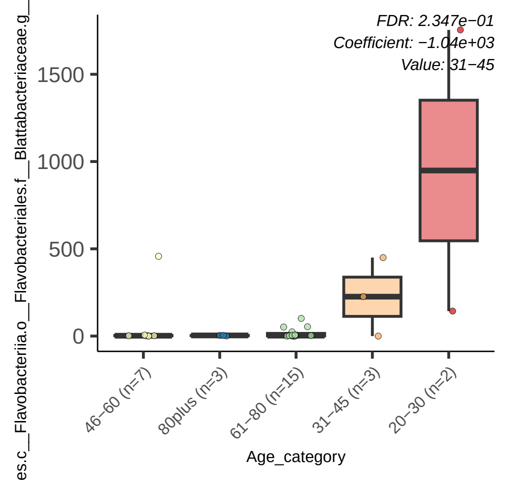
FDR: 2.347e−01
Coefficient: −1.04e+03
Value: 31−45
es.c__Flavobacteriia.o__Flavobacteriales.f__Blattabacteriaceae.g__
1500
1000
500
0
46−60 (n=7)
80plus (n=3)
61−80 (n=15)
31−45 (n=3)
20−30 (n=2)
Age_category

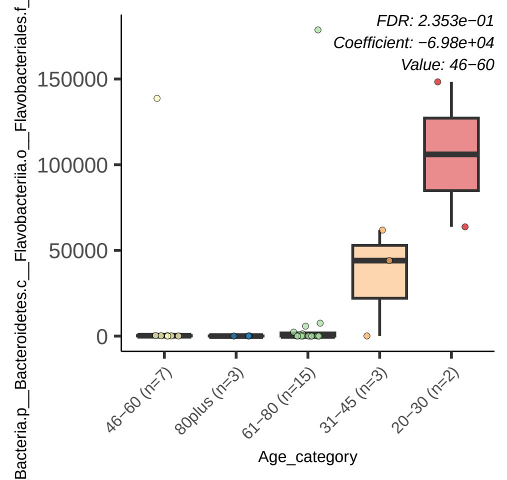
FDR: 2.353e−01
Coefficient: −6.98e+04
Value: 46−60
Bacteria.p__Bacteroidetes.c__Flavobacteriia.o__Flavobacteriales.f_
150000
100000
50000
0
46−60 (n=7)
80plus (n=3)
61−80 (n=15)
31−45 (n=3)
20−30 (n=2)
Age_category

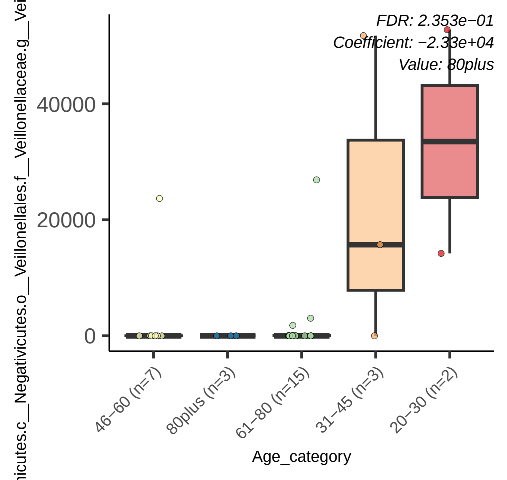
FDR: 2.353e−01
Coefficient: −2.33e+04
Value: 80plus
icutes.c__Negativicutes.o__Veillonellales.f__Veillonellaceae.g__Vei
40000
20000
0
46−60 (n=7)
80plus (n=3)
61−80 (n=15)
31−45 (n=3)
20−30 (n=2)
Age_category

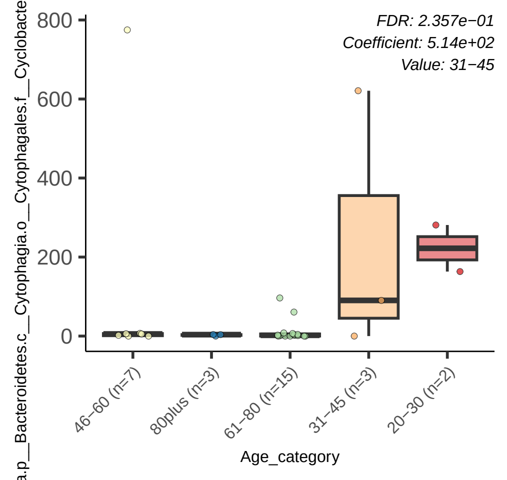

FDR: 2.357e−01
Coefficient: 5.14e+02
Value: 31−45
800
600
400
200
0
a.p__Bacteroidetes.c__Cytophagia.o__Cytophagales.f__Cyclobacte
46−60 (n=7)
80plus (n=3)
61−80 (n=15)
31−45 (n=3)
20−30 (n=2)
Age_category

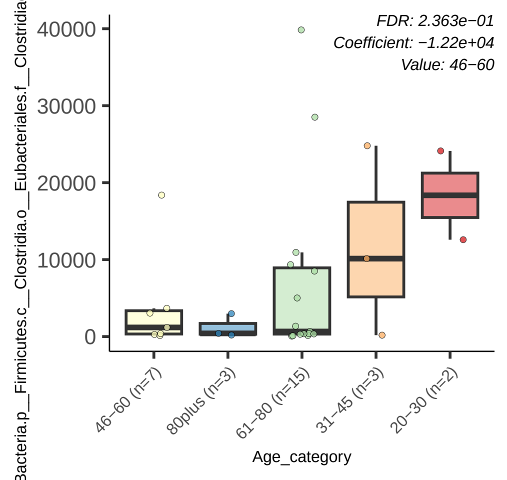

FDR: 2.363e−01
Coefficient: −1.22e+04
Value: 46−60
Bacteria.p__Firmicutes.c__Clostridia.o__Eubacteriales.f__Clostridiac
40000
30000
20000
10000
0
46−60 (n=7)
80plus (n=3)
61−80 (n=15)
31−45 (n=3)
20−30 (n=2)
Age_category

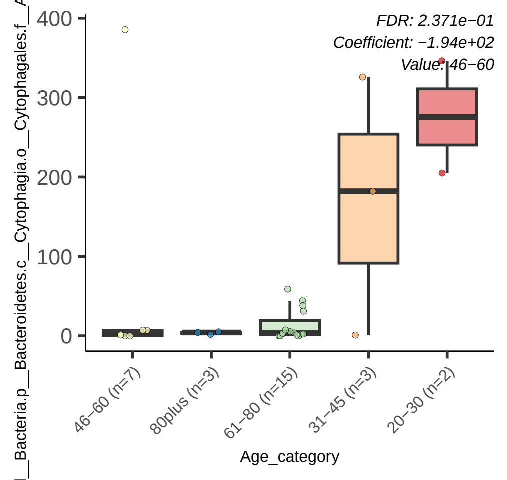

__Bacteria.p__Bacteroidetes.c__Cytophagia.o__Cytophagales.f__A
400
300
200
100
0
FDR: 2.371e−01
Coefficient: −1.94e+02
Value: 46−60
46−60 (n=7)
80plus (n=3)
61−80 (n=15)
31−45 (n=3)
20−30 (n=2)
Age_category

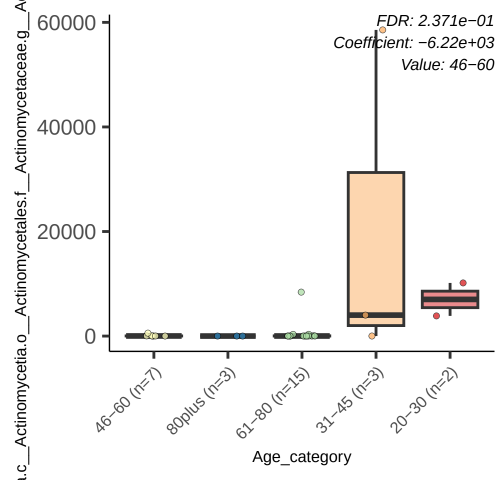

a.c__Actinomycetia.o__Actinomycetales.f__Actinomycetaceae.g__Ac
60000
40000
20000
0
FDR: 2.371e−01
Coefficient: −6.22e+03
Value: 46−60
46−60 (n=7)
80plus (n=3)
61−80 (n=15)
31−45 (n=3)
20−30 (n=2)
Age_category

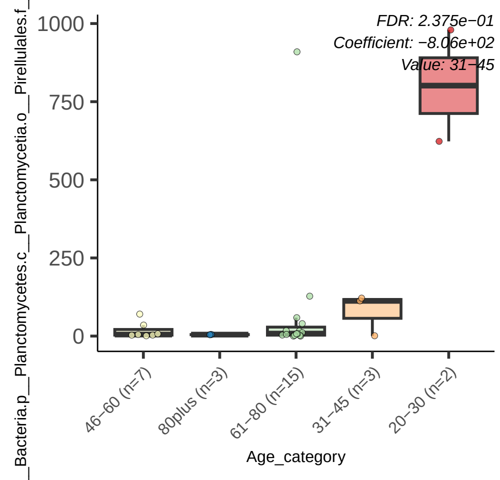

FDR: 2.375e−01
Coefficient: −8.06e+02
Value: 31−45
Bacteria.p__Planctomycetes.c__Planctomycetia.o__Pirellulales.f__
1000
750
500
250
0
46−60 (n=7)
80plus (n=3)
61−80 (n=15)
31−45 (n=3)
20−30 (n=2)
Age_category

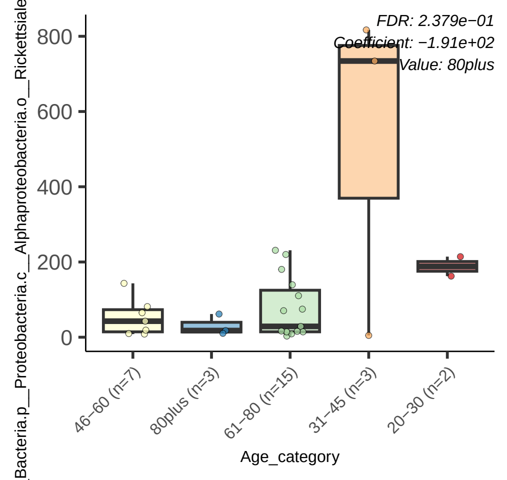

FDR: 2.379e−01
Coefficient: −1.91e+02
Value: 80plus
Bacteria.p__Proteobacteria.c__Alphaproteobacteria.o__Rickettsiale
800
600
400
200
0
46−60 (n=7)
80plus (n=3)
61−80 (n=15)
31−45 (n=3)
20−30 (n=2)
Age_category

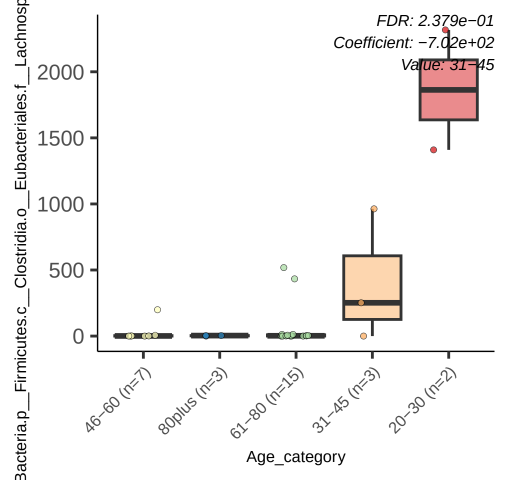

FDR: 2.379e−01
Coefficient: −7.02e+02
Value: 31−45
2000
1500
1000
500
0
Bacteria.p__Firmicutes.c__Clostridia.o__Eubacteriales.f__Lachnosp
46−60 (n=7)
80plus (n=3)
61−80 (n=15)
31−45 (n=3)
20−30 (n=2)
Age_category

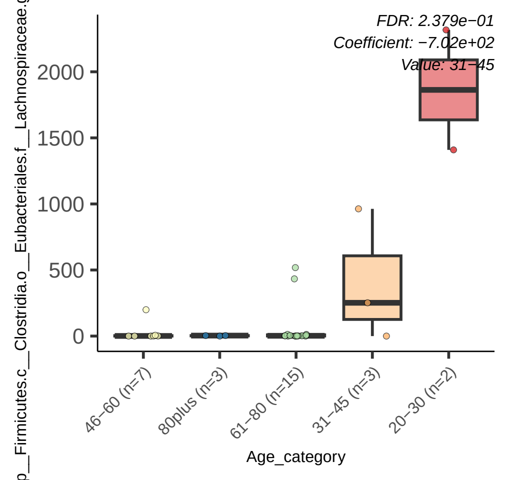

FDR: 2.379e−01
Coefficient: −7.02e+02
Value: 31−45
Firmicutes.c__Clostridia.o__Eubacteriales.f__Lachnospiraceae.g
0
500
1000
1500
2000
46−60 (n=7)
80plus (n=3)
61−80 (n=15)
31−45 (n=3)
20−30 (n=2)
Age_category

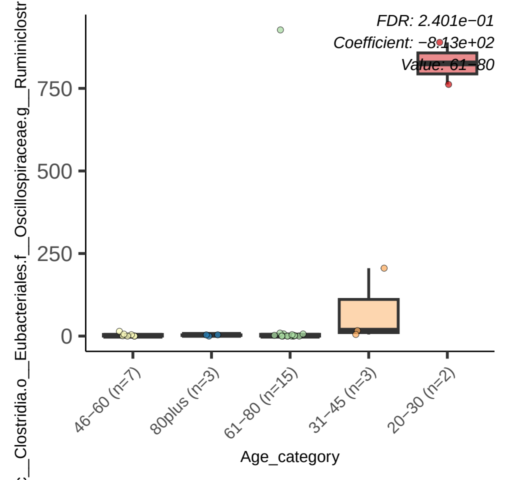

FDR: 2.401e−01
Coefficient: −8.13e+02
Value: 61−80
Clostridia.o__Eubacteriales.f__Oscillospiraceae.g__Ruminiclostr
750
500
250
0
46−60 (n=7)
80plus (n=3)
61−80 (n=15)
31−45 (n=3)
20−30 (n=2)
Age_category

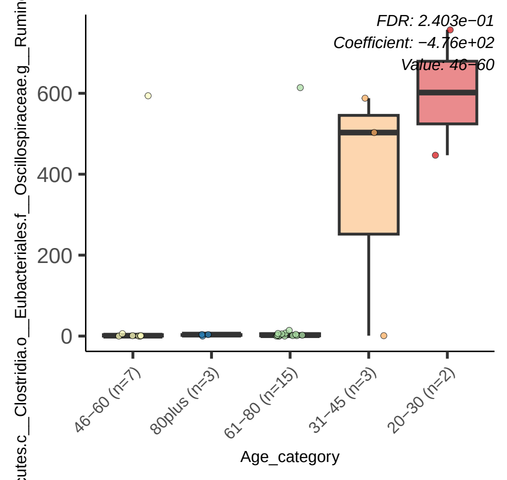

FDR: 2.403e−01
Coefficient: −4.76e+02
Value: 46−60
0
200
400
600
cutes.c__Clostridia.o__Eubacteriales.f__Oscillospiraceae.g__Rumin
46−60 (n=7)
80plus (n=3)
61−80 (n=15)
31−45 (n=3)
20−30 (n=2)
Age_category

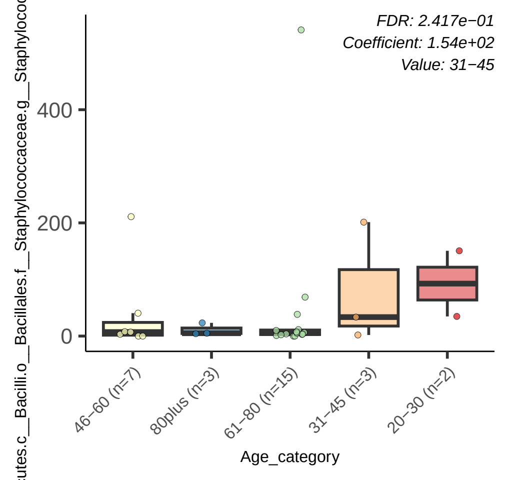

FDR: 2.417e−01
Coefficient: 1.54e+02
Value: 31−45
cutes.c__Bacilli.o__Bacillales.f__Staphylococcaceae.g__Staphylococ
400
200
0
46−60 (n=7)
80plus (n=3)
61−80 (n=15)
31−45 (n=3)
20−30 (n=2)
Age_category

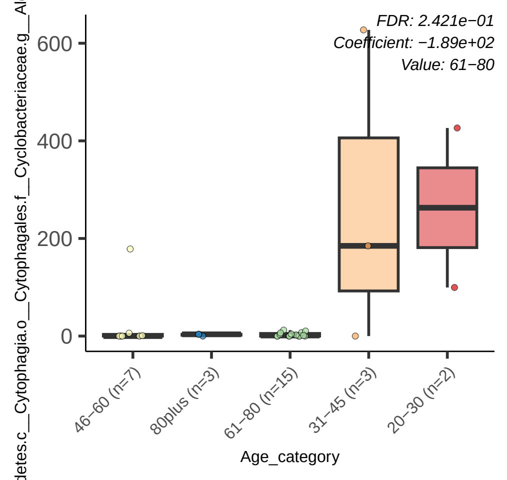

FDR: 2.421e−01
Coefficient: −1.89e+02
Value: 61−80
600
400
200
0
detes.c__Cytophagia.o__Cytophagales.f__Cyclobacteriaceae.g__Al
46−60 (n=7)
80plus (n=3)
61−80 (n=15)
31−45 (n=3)
20−30 (n=2)
Age_category

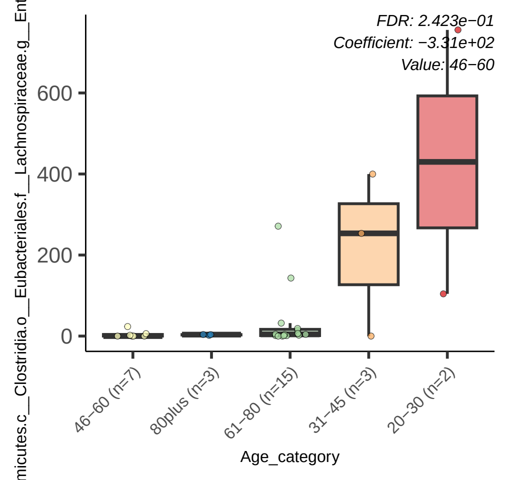

FDR: 2.423e−01
Coefficient: −3.31e+02
Value: 46−60
micutes.c__Clostridia.o__Eubacteriales.f__Lachnospiraceae.g__Ent
600
400
200
0
46−60 (n=7)
80plus (n=3)
61−80 (n=15)
31−45 (n=3)
20−30 (n=2)
Age_category

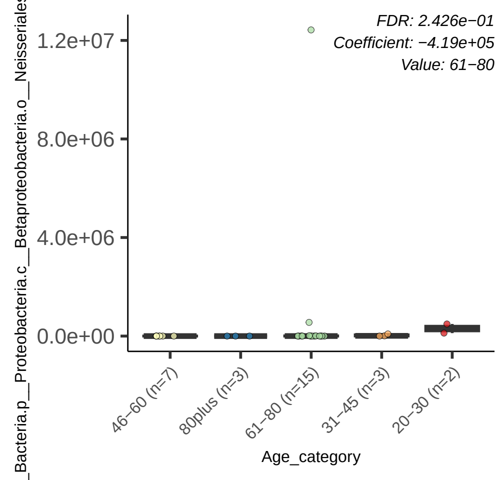
FDR: 2.426e−01
Coefficient: −4.19e+05
Value: 61−80
1.2e+07
8.0e+06
4.0e+06
0.0e+00
_Bacteria.p__Proteobacteria.c__Betaproteobacteria.o__Neisseriales
46−60 (n=7)
80plus (n=3)
61−80 (n=15)
31−45 (n=3)
20−30 (n=2)
Age_category

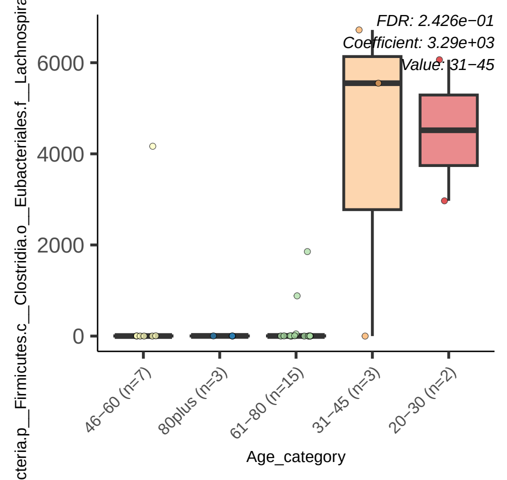

FDR: 2.426e−01
Coefficient: 3.29e+03
Value: 31−45
6000
4000
2000
0
cteria.p__Firmicutes.c__Clostridia.o__Eubacteriales.f__Lachnospira
46−60 (n=7)
80plus (n=3)
61−80 (n=15)
31−45 (n=3)
20−30 (n=2)
Age_category

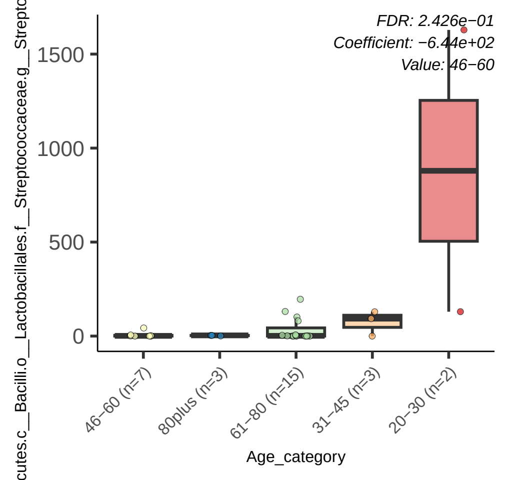
FDR: 2.426e−01
Coefficient: −6.44e+02
Value: 46−60
1500
1000
500
0
cutes.c__Bacilli.o__Lactobacillales.f__Streptococcaceae.g__Strepto
46−60 (n=7)
80plus (n=3)
61−80 (n=15)
31−45 (n=3)
20−30 (n=2)
Age_category

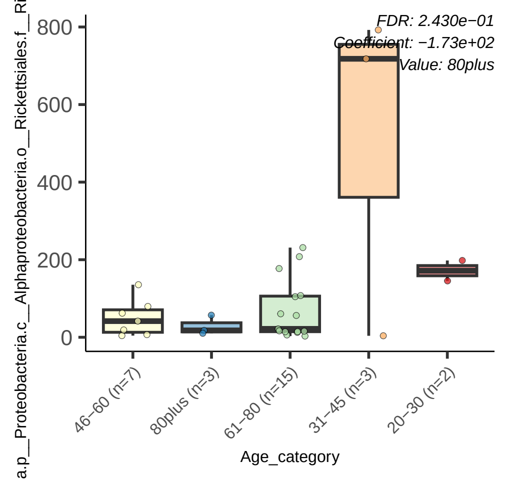

FDR: 2.430e−01
Coefficient: −1.73e+02
Value: 80plus
0
200
400
600
800
a.p__Proteobacteria.c__Alphaproteobacteria.o__Rickettsiales.f__Ri
46−60 (n=7)
80plus (n=3)
61−80 (n=15)
31−45 (n=3)
20−30 (n=2)
Age_category

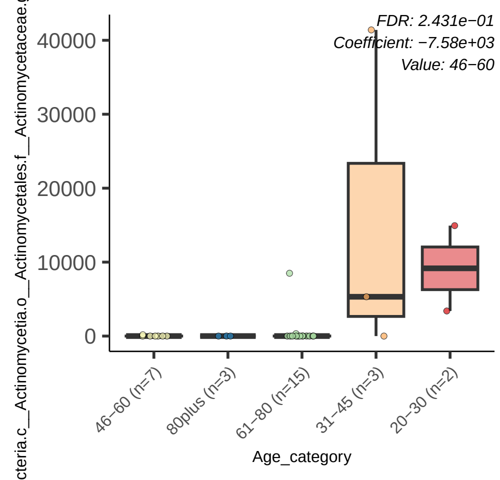

FDR: 2.431e−01
Coefficient: −7.58e+03
Value: 46−60
40000
30000
20000
10000
0
cteria.c__Actinomycetia.o__Actinomycetales.f__Actinomycetaceae.g
46−60 (n=7)
80plus (n=3)
61−80 (n=15)
31−45 (n=3)
20−30 (n=2)
Age_category

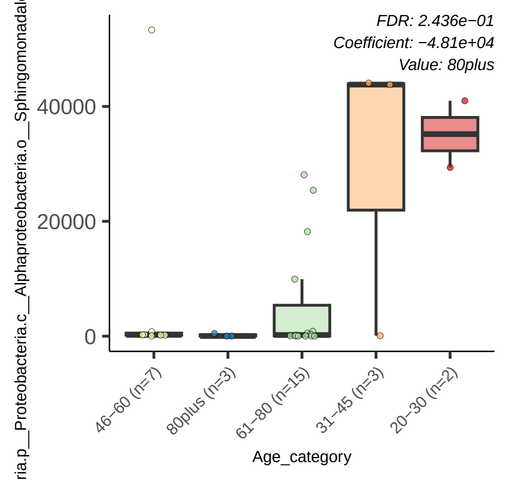

FDR: 2.436e−01
Coefficient: −4.81e+04
Value: 80plus
ria.p__Proteobacteria.c__Alphaproteobacteria.o__Sphingomonadal
40000
20000
0
46−60 (n=7)
80plus (n=3)
61−80 (n=15)
31−45 (n=3)
20−30 (n=2)
Age_category

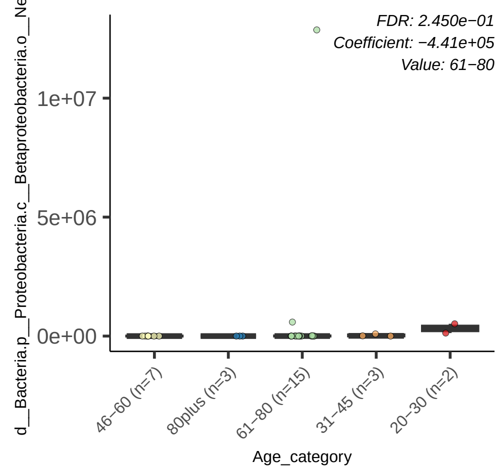

FDR: 2.450e−01
Coefficient: −4.41e+05
Value: 61−80
1e+07
5e+06
0e+00
d__Bacteria.p__Proteobacteria.c__Betaproteobacteria.o__Ne
46−60 (n=7)
80plus (n=3)
61−80 (n=15)
31−45 (n=3)
20−30 (n=2)
Age_category

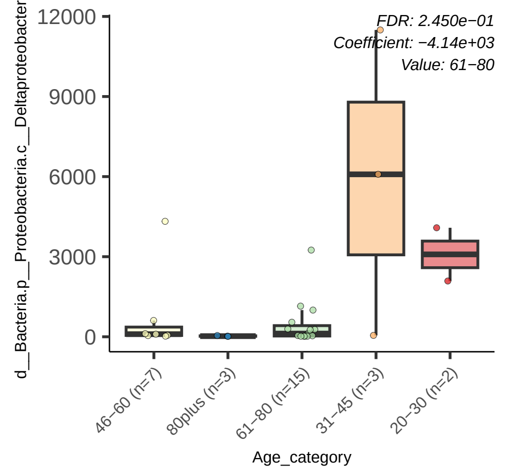

FDR: 2.450e−01
Coefficient: −4.14e+03
Value: 61−80
d__Bacteria.p__Proteobacteria.c__Deltaproteobacter
12000
9000
6000
3000
0
46−60 (n=7)
80plus (n=3)
61−80 (n=15)
31−45 (n=3)
20−30 (n=2)
Age_category

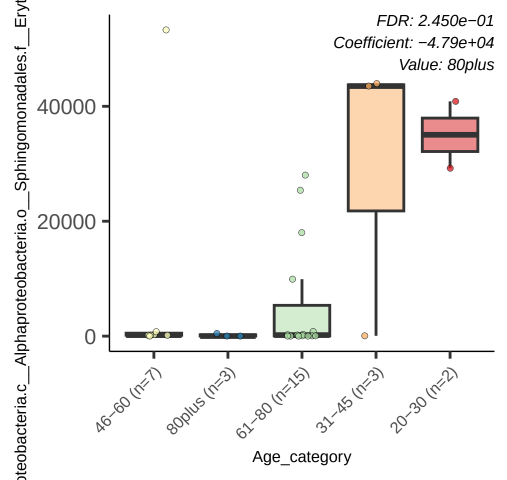

FDR: 2.450e−01
Coefficient: −4.79e+04
Value: 80plus
40000
20000
0
46−60 (n=7)
80plus (n=3)
61−80 (n=15)
31−45 (n=3)
20−30 (n=2)
Age_category
oteobacteria.c__Alphaproteobacteria.o__Sphingomonadales.f__Eryt

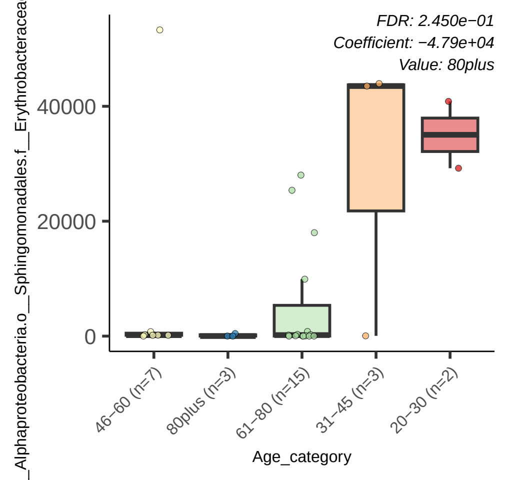

FDR: 2.450e−01
Coefficient: −4.79e+04
Value: 80plus
_Alphaproteobacteria.o__Sphingomonadales.f__Erythrobacteraceae
0
20000
40000
46−60 (n=7)
80plus (n=3)
61−80 (n=15)
31−45 (n=3)
20−30 (n=2)
Age_category

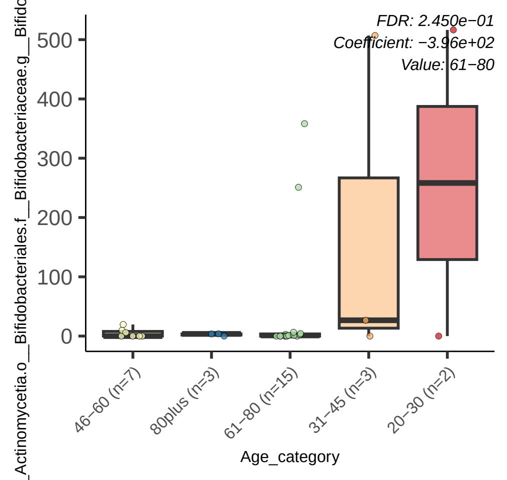

FDR: 2.450e−01
Coefficient: −3.96e+02
Value: 61−80
500
400
300
200
100
0
46−60 (n=7)
80plus (n=3)
61−80 (n=15)
31−45 (n=3)
20−30 (n=2)
Age_category
Actinomycetia.o__Bifidobacteriales.f__Bifidobacteriaceae.g__Bifido

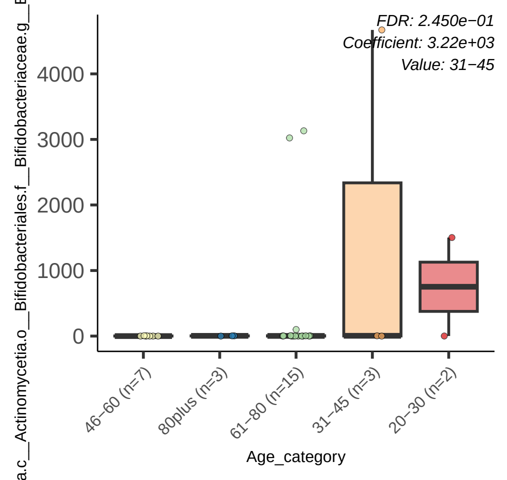

FDR: 2.450e−01
Coefficient: 3.22e+03
Value: 31−45
4000
3000
2000
1000
0
46−60 (n=7)
80plus (n=3)
61−80 (n=15)
31−45 (n=3)
20−30 (n=2)
Age_category
a.c__Actinomycetia.o__Bifidobacteriales.f__Bifidobacteriaceae.g__B

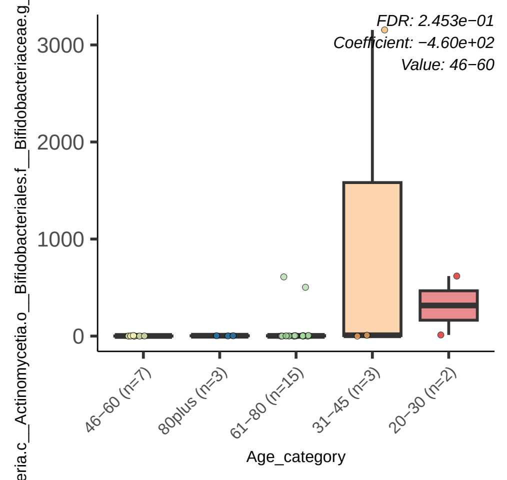

FDR: 2.453e−01
Coefficient: −4.60e+02
Value: 46−60
3000
2000
1000
0
eria.c__Actinomycetia.o__Bifidobacteriales.f__Bifidobacteriaceae.g_
46−60 (n=7)
80plus (n=3)
61−80 (n=15)
31−45 (n=3)
20−30 (n=2)
Age_category

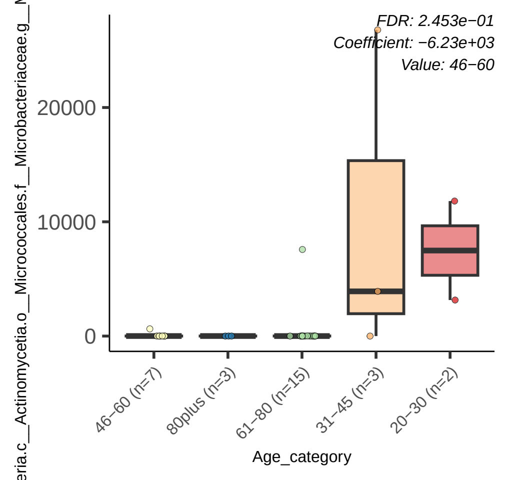

FDR: 2.453e−01
Coefficient: −6.23e+03
Value: 46−60
20000
10000
0
46−60 (n=7)
80plus (n=3)
61−80 (n=15)
31−45 (n=3)
20−30 (n=2)
Age_category

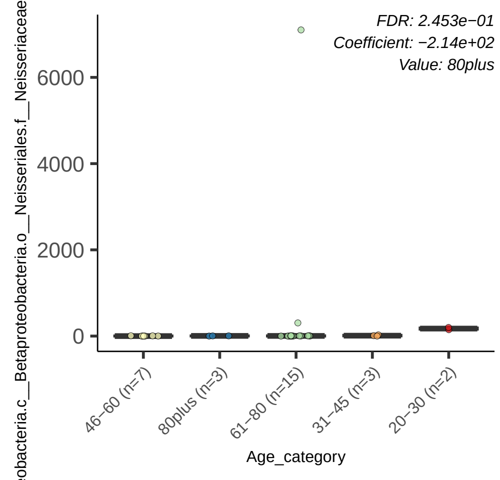

FDR: 2.453e−01
Coefficient: −2.14e+02
Value: 80plus
6000
4000
2000
0
eobacteria.c__Betaproteobacteria.o__Neisseriales.f__Neisseriaceae
46−60 (n=7)
80plus (n=3)
61−80 (n=15)
31−45 (n=3)
20−30 (n=2)
Age_category

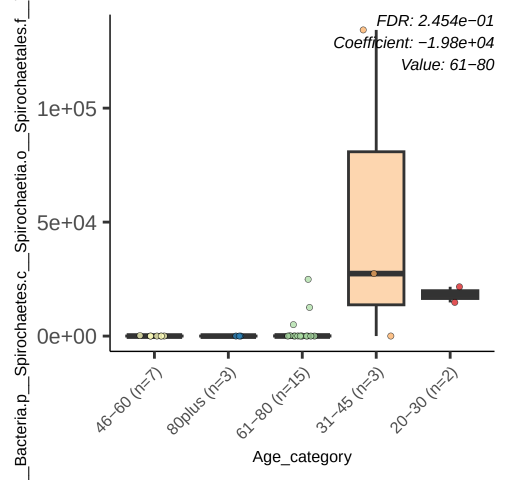

FDR: 2.454e−01
Coefficient: −1.98e+04
Value: 61−80
1e+05
5e+04
0e+00
Bacteria.p__Spirochaetes.c__Spirochaetia.o__Spirochaetales.f__
46−60 (n=7)
80plus (n=3)
61−80 (n=15)
31−45 (n=3)
20−30 (n=2)
Age_category

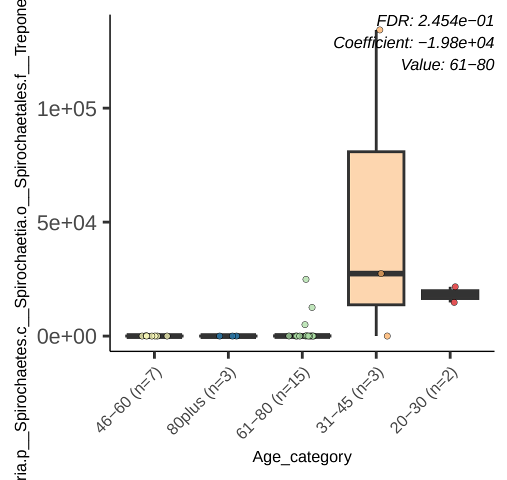
FDR: 2.454e−01
Coefficient: −1.98e+04
Value: 61−80
1e+05
5e+04
0e+00
ria.p__Spirochaetes.c__Spirochaetia.o__Spirochaetales.f__Trepone
46−60 (n=7)
80plus (n=3)
61−80 (n=15)
31−45 (n=3)
20−30 (n=2)
Age_category

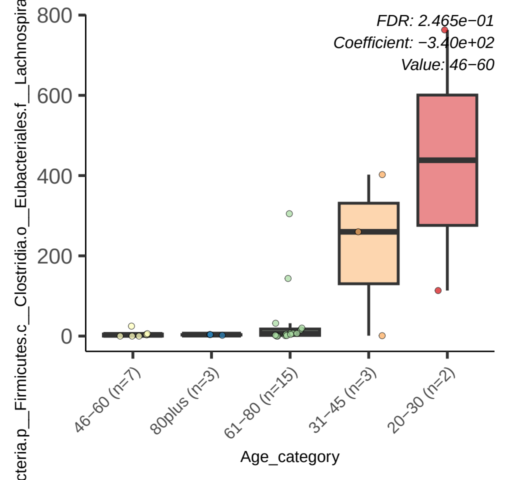

FDR: 2.465e−01
Coefficient: −3.40e+02
Value: 46−60
cteria.p__Firmicutes.c__Clostridia.o__Eubacteriales.f__Lachnospira
800
600
400
200
0
46−60 (n=7)
80plus (n=3)
61−80 (n=15)
31−45 (n=3)
20−30 (n=2)
Age_category

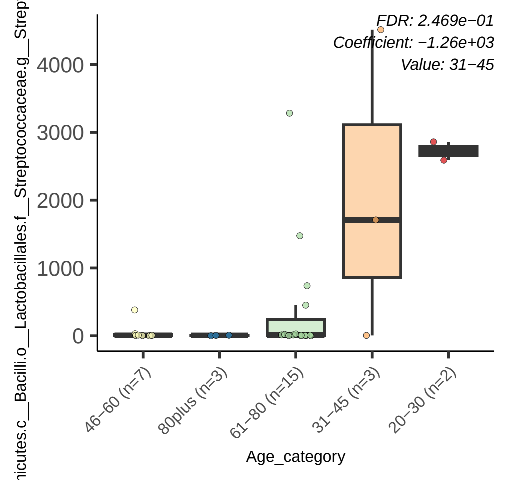

FDR: 2.469e−01
Coefficient: −1.26e+03
Value: 31−45
4000
3000
2000
1000
0
46−60 (n=7)
80plus (n=3)
61−80 (n=15)
31−45 (n=3)
20−30 (n=2)
Age_category
nicutes.c__Bacilli.o__Lactobacillales.f__Streptococcaceae.g__Strep

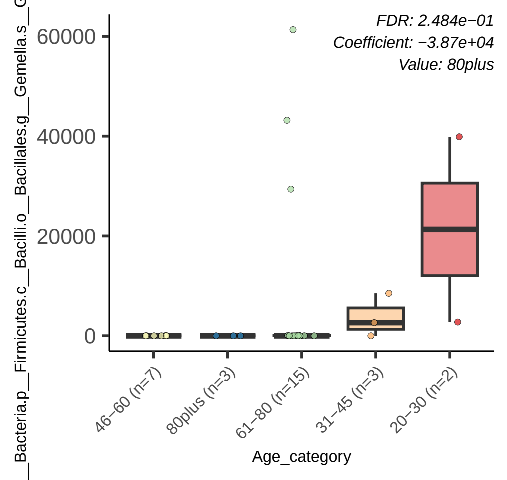

FDR: 2.484e−01
Coefficient: −3.87e+04
Value: 80plus
60000
40000
20000
0
46−60 (n=7)
80plus (n=3)
61−80 (n=15)
31−45 (n=3)
20−30 (n=2)
Age_category
Bacteria.p__Firmicutes.c__Bacilli.o__Bacillales.g__Gemella.s__G

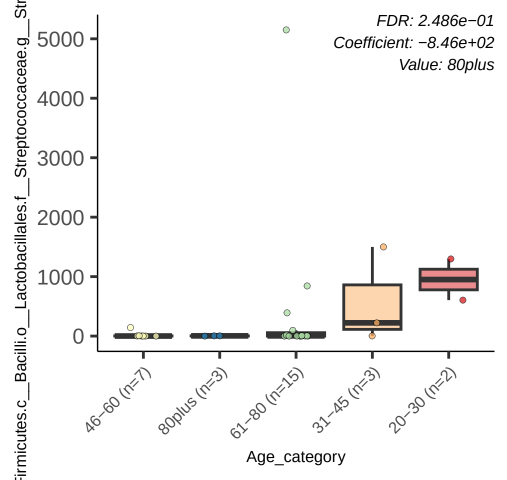

FDR: 2.486e−01
Coefficient: −8.46e+02
Value: 80plus
Firmicutes.c__Bacilli.o__Lactobacillales.f__Streptococcaceae.g__Str
5000
4000
3000
2000
1000
0
46−60 (n=7)
80plus (n=3)
61−80 (n=15)
31−45 (n=3)
20−30 (n=2)
Age_category

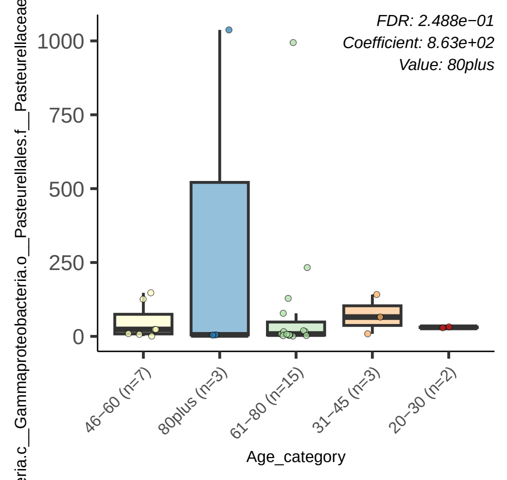
FDR: 2.488e−01
Coefficient: 8.63e+02
Value: 80plus
1000
750
500
250
0
46−60 (n=7)
80plus (n=3)
61−80 (n=15)
31−45 (n=3)
20−30 (n=2)
Age_category
eria.c__Gammaproteobacteria.o__Pasteurellales.f__Pasteurellaceae

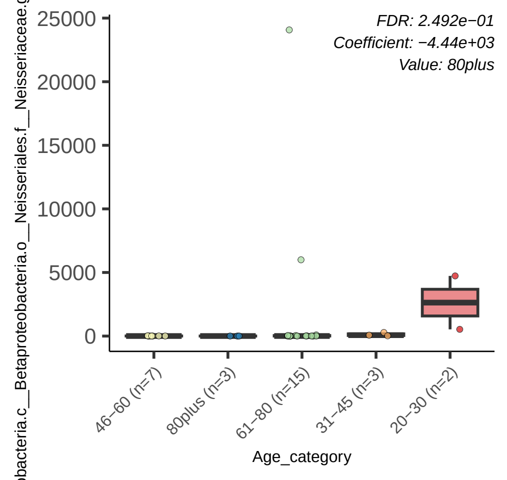

FDR: 2.492e−01
Coefficient: −4.44e+03
Value: 80plus
25000
20000
15000
10000
5000
0
obacteria.c__Betaproteobacteria.o__Neisseriales.f__Neisseriaceae.g
46−60 (n=7)
80plus (n=3)
61−80 (n=15)
31−45 (n=3)
20−30 (n=2)
Age_category

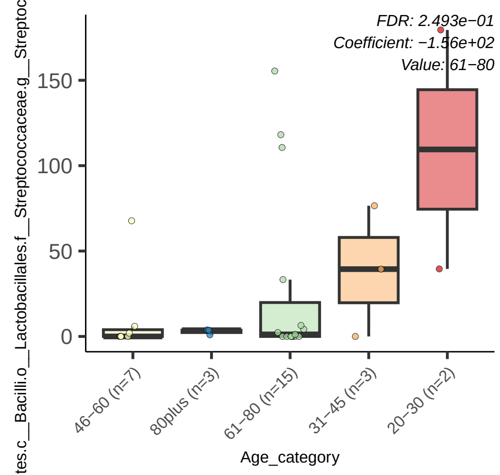

FDR: 2.493e−01
Coefficient: −1.56e+02
Value: 61−80
utes.c__Bacilli.o__Lactobacillales.f__Streptococcaceae.g__Streptoc
150
100
50
0
46−60 (n=7)
80plus (n=3)
61−80 (n=15)
31−45 (n=3)
20−30 (n=2)
Age_category

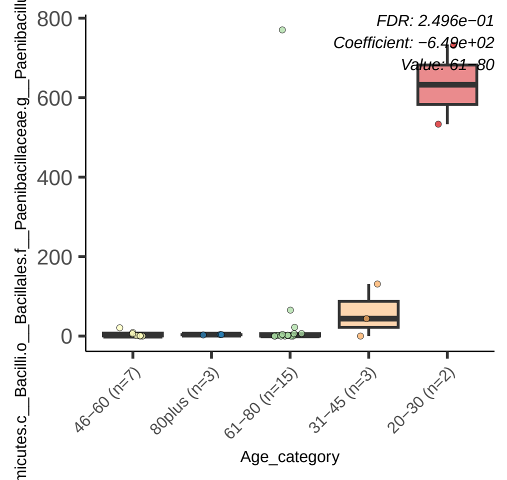

FDR: 2.496e−01
Coefficient: −6.49e+02
Value: 61−80
800
600
400
200
0
micutes.c__Bacilli.o__Bacillales.f__Paenibacillaceae.g__Paenibacill
46−60 (n=7)
80plus (n=3)
61−80 (n=15)
31−45 (n=3)
20−30 (n=2)
Age_category

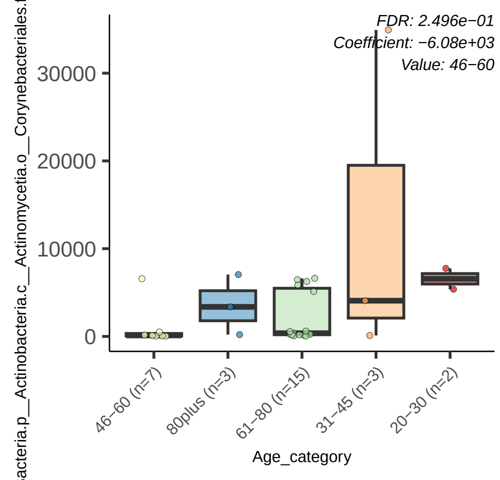
FDR: 2.496e−01
Coefficient: −6.08e+03
Value: 46−60
30000
20000
10000
0
46−60 (n=7)
80plus (n=3)
61−80 (n=15)
31−45 (n=3)
20−30 (n=2)
Age_category
Bacteria.p__Actinobacteria.c__Actinomycetia.o__Corynebacteriales.f
