# Supplementary material for: Metatranscriptome Analysis of Nasopharyngeal Swabs across the Varying Severity of COVID-19 Disease Demonstrated Unprecedented Species Diversity
Source: Microorganisms. 2023 Jul 14;11(7):1804. doi: 10.3390/microorganisms11071804 (PMC10384460; doi:10.3390/microorganisms11071804)
Supplement: Supplementary file 1 [file microorganisms-11-01804-s001.zip › Maaslin_results_ONLYsevere.zip!/Maaslin_results_ONLYsevere/ATBTreatment.pdf]

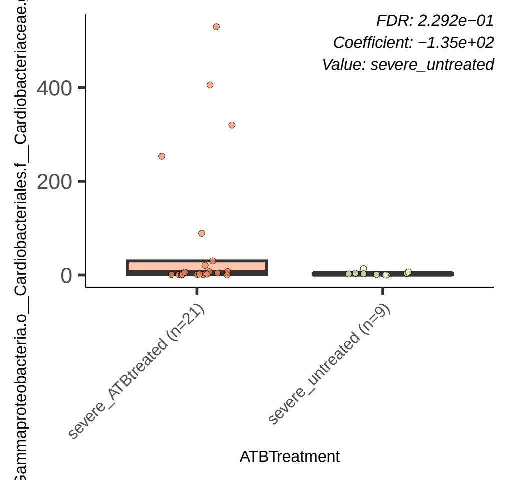

FDR: 2.292e−01
Coefficient: −1.35e+02
Value: severe_untreated
Gammaproteobacteria.o__Cardiobacteriales.f__Cardiobacteriaceae.g
400
200
0
severe_ATBtreated (n=21)
severe_untreated (n=9)
ATBTreatment

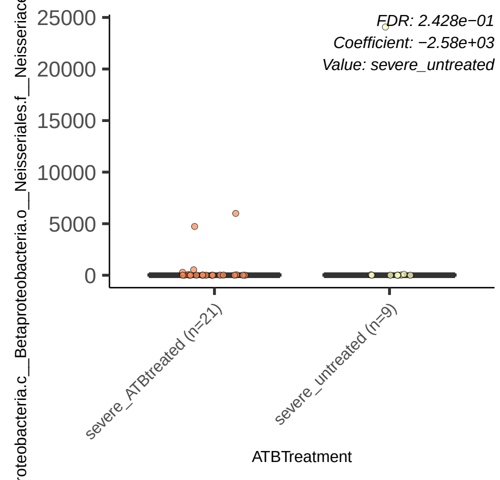

roteobacteria.c__Betaproteobacteria.o__Neisseriales.f__Neisseriace
25000
20000
15000
10000
5000
0
FDR: 2.428e−01
Coefficient: −2.58e+03
Value: severe_untreated
severe_ATBtreated (n=21)
severe_untreated (n=9)
ATBTreatment

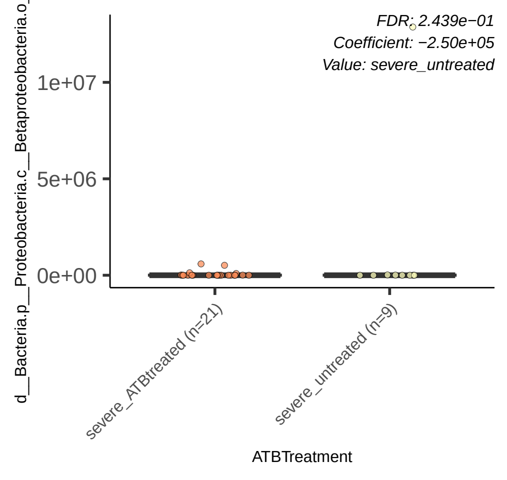
FDR: 2.439e−01
Coefficient: −2.50e+05
Value: severe_untreated
1e+07
5e+06
0e+00
d__Bacteria.p__Proteobacteria.c__Betaproteobacteria.o_
severe_ATBtreated (n=21)
severe_untreated (n=9)
ATBTreatment

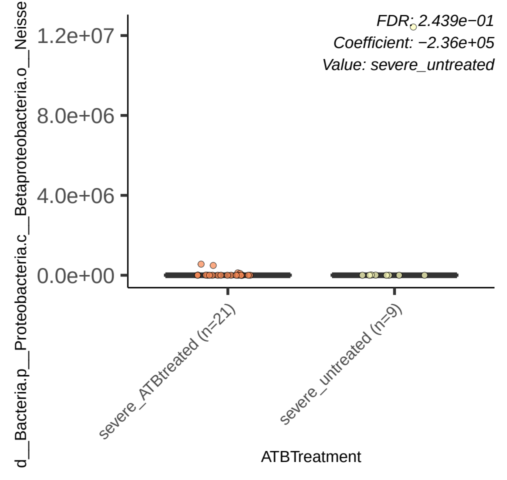

FDR: 2.439e−01
Coefficient: −2.36e+05
Value: severe_untreated
1.2e+07
8.0e+06
4.0e+06
0.0e+00
d__Bacteria.p__Proteobacteria.c__Betaproteobacteria.o__Neisse
severe_ATBtreated (n=21)
severe_untreated (n=9)
ATBTreatment

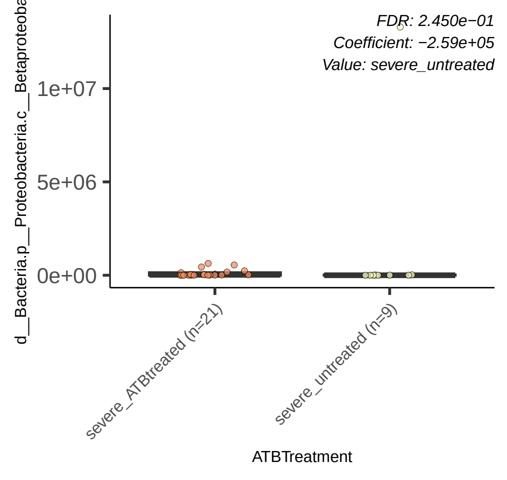

FDR: 2.450e−01
Coefficient: −2.59e+05
Value: severe_untreated
d__Bacteria.p__Proteobacteria.c__Betaproteoba
1e+07
5e+06
0e+00
severe_ATBtreated (n=21)
severe_untreated (n=9)
ATBTreatment
