# Supplementary material for: Metatranscriptome Analysis of Nasopharyngeal Swabs across the Varying Severity of COVID-19 Disease Demonstrated Unprecedented Species Diversity
Source: Microorganisms. 2023 Jul 14;11(7):1804. doi: 10.3390/microorganisms11071804 (PMC10384460; doi:10.3390/microorganisms11071804)

icant associations (−log(qval)*sign(coeff))

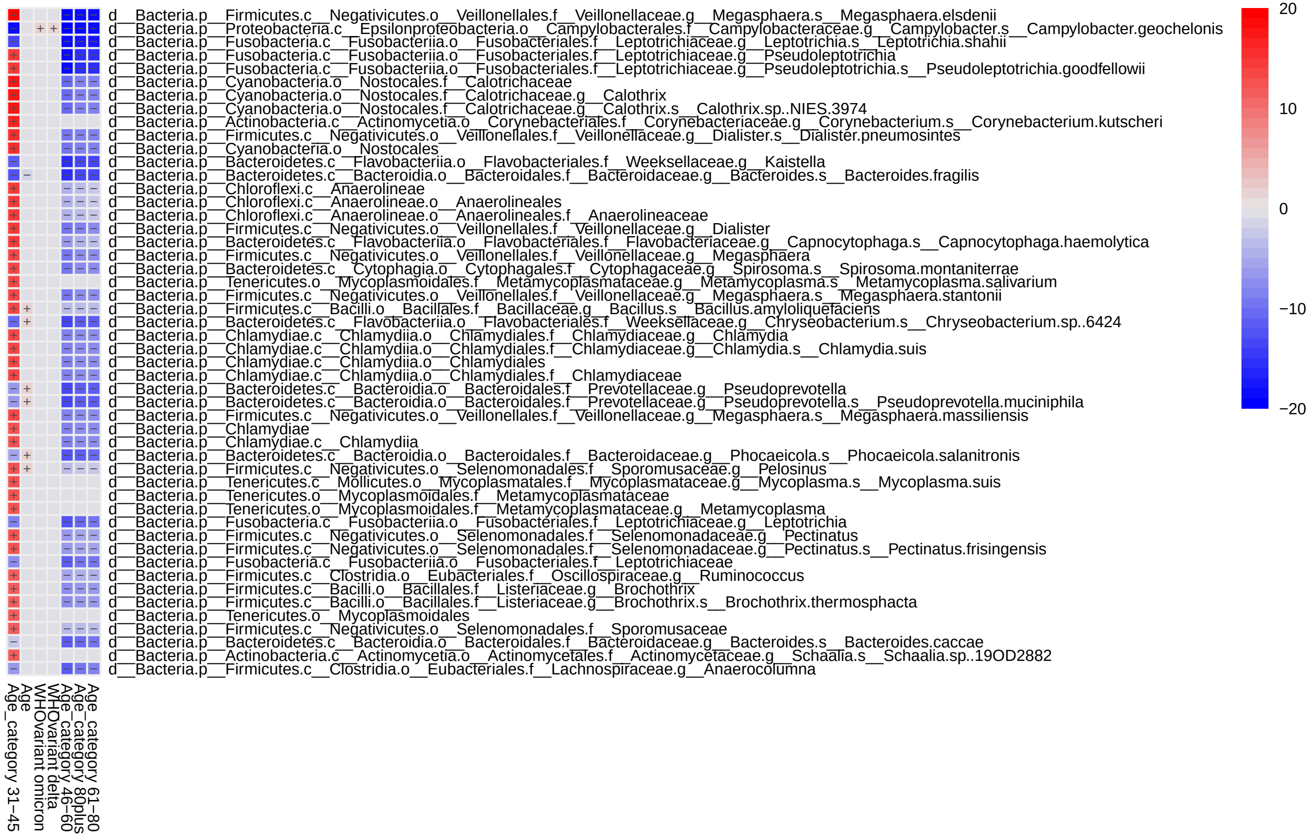

Supplement: Supplementary file 1 [file microorganisms-11-01804-s001.zip › Maaslin_results_ONLYsevere.zip!/Maaslin_results_ONLYsevere/heatmap.pdf]
