# Supplementary material for: Metatranscriptome Analysis of Nasopharyngeal Swabs across the Varying Severity of COVID-19 Disease Demonstrated Unprecedented Species Diversity
Source: Microorganisms. 2023 Jul 14;11(7):1804. doi: 10.3390/microorganisms11071804 (PMC10384460; doi:10.3390/microorganisms11071804)
Supplement: Supplementary file 1 [file microorganisms-11-01804-s001.zip › Maaslin_results_ONLYsevere.zip!/Maaslin_results_ONLYsevere/WHOvariant.pdf]

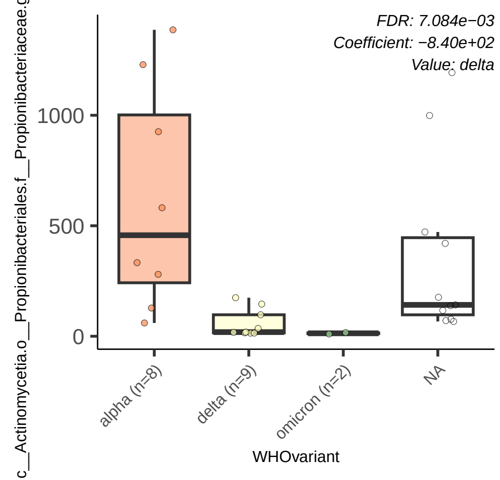

FDR: 7.084e−03
Coefficient: −8.40e+02
Value: delta
c__Actinomycetia.o__Propionibacteriales.f__Propionibacteriaceae.g
1000
500
0
alpha (n=8)
delta (n=9)
omicron (n=2)
NA
WHOvariant

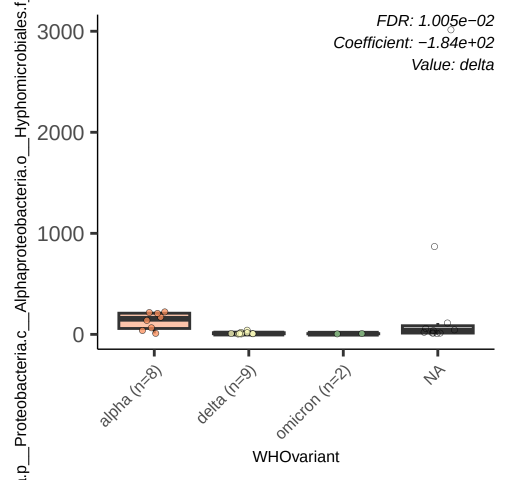
FDR: 1.005e−02
Coefficient: −1.84e+02
Value: delta
3000
2000
1000
0
a.p__Proteobacteria.c__Alphaproteobacteria.o__Hyphomicrobiales.f_
alpha (n=8)
delta (n=9)
omicron (n=2)
NA
WHOvariant

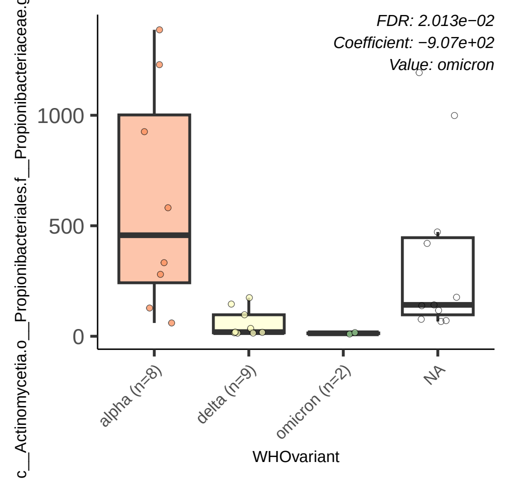
FDR: 2.013e−02
Coefficient: −9.07e+02
Value: omicron
1000
500
0
c__Actinomycetia.o__Propionibacteriales.f__Propionibacteriaceae.g
alpha (n=8)
delta (n=9)
omicron (n=2)
NA
WHOvariant

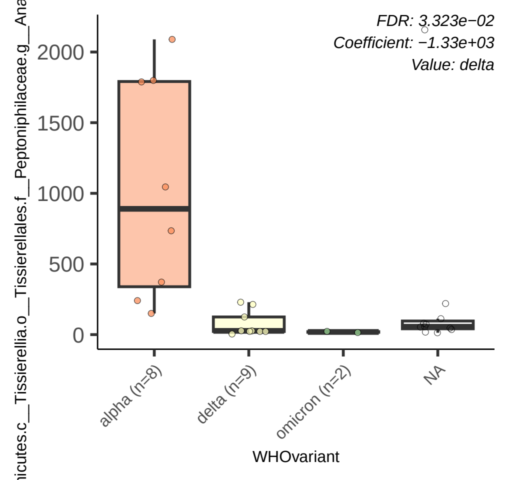

FDR: 3.323e−02
Coefficient: −1.33e+03
Value: delta
Firmicutes.c__Tissierellia.o__Tissierellales.f__Peptoniphilaceae.g__Ana
2000
1500
1000
500
0
alpha (n=8)
delta (n=9)
omicron (n=2)
NA
WHOvariant

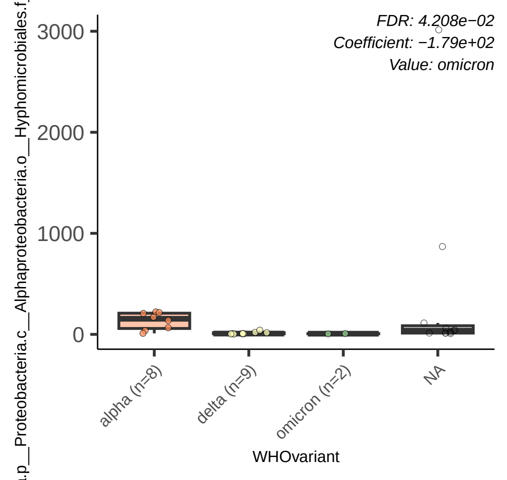
FDR: 4.208e−02
Coefficient: −1.79e+02
Value: omicron
3000
2000
1000
0
alpha (n=8)
delta (n=9)
omicron (n=2)
NA
WHOvariant
a.p__Proteobacteria.c__Alphaproteobacteria.o__Hyphomicrobiales.f_

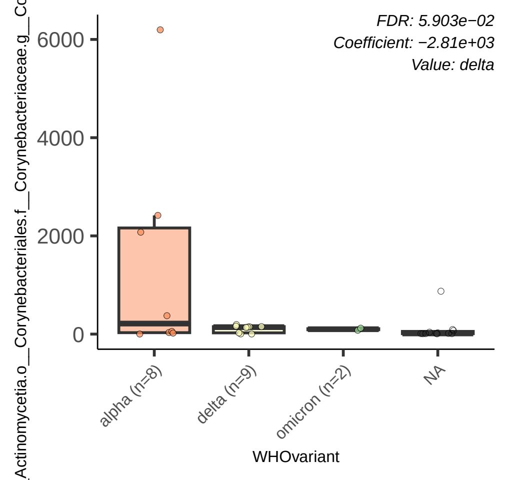
FDR: 5.903e−02
Coefficient: −2.81e+03
Value: delta
6000
4000
2000
0
Actinomycetia.o__Corynebacteriales.f__Corynebacteriaceae.g__Co
alpha (n=8)
delta (n=9)
omicron (n=2)
NA
WHOvariant

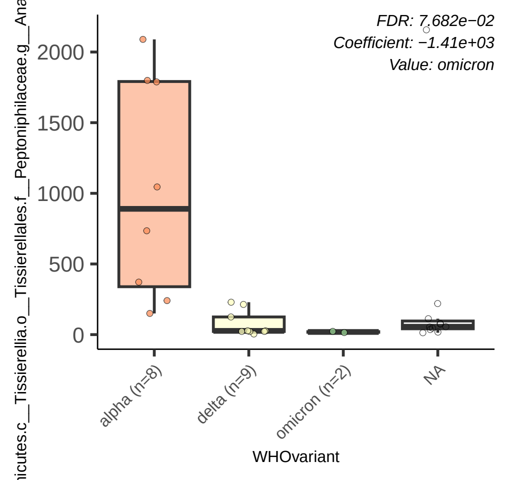

FDR: 7.682e−02
Coefficient: −1.41e+03
Value: omicron
nicutes.c__Tissierellia.o__Tissierellales.f__Peptoniphilaceae.g__Ana
2000
1500
1000
500
0
alpha (n=8)
delta (n=9)
omicron (n=2)
NA
WHOvariant

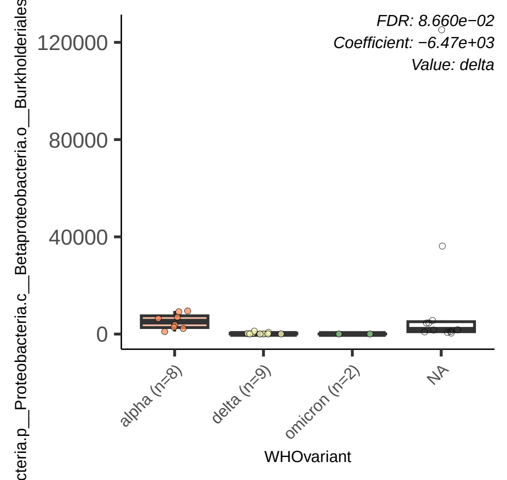

FDR: 8.660e−02
Coefficient: −6.47e+03
Value: delta
120000
80000
40000
0
alpha (n=8)
delta (n=9)
omicron (n=2)
NA
WHOvariant
cteria.p__Proteobacteria.c__Betaproteobacteria.o__Burkholderiales

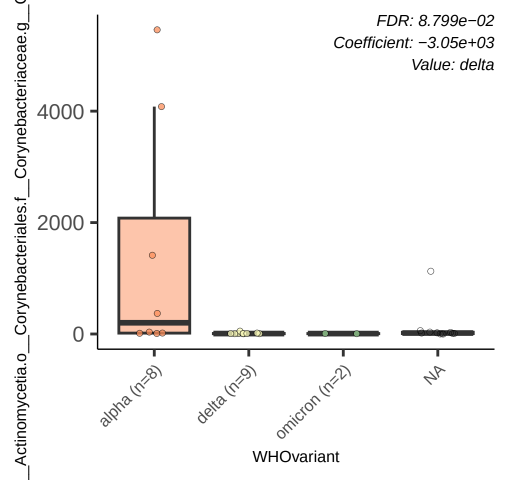

FDR: 8.799e−02
Coefficient: −3.05e+03
Value: delta
Actinomycetia.o__Corynebacteriales.f__Corynebacteriaceae.g__C
4000
2000
0
alpha (n=8)
delta (n=9)
omicron (n=2)
NA
WHOvariant

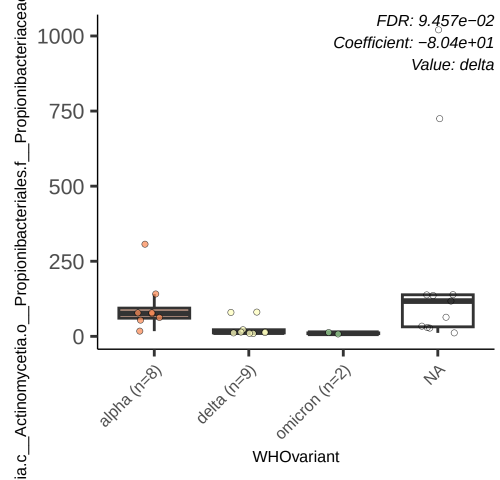
FDR: 9.457e−02
Coefficient: −8.04e+01
Value: delta
1000
750
500
250
0
ia.c__Actinomycetia.o__Propionibacteriales.f__Propionibacteriaceae
alpha (n=8)
delta (n=9)
omicron (n=2)
NA
WHOvariant

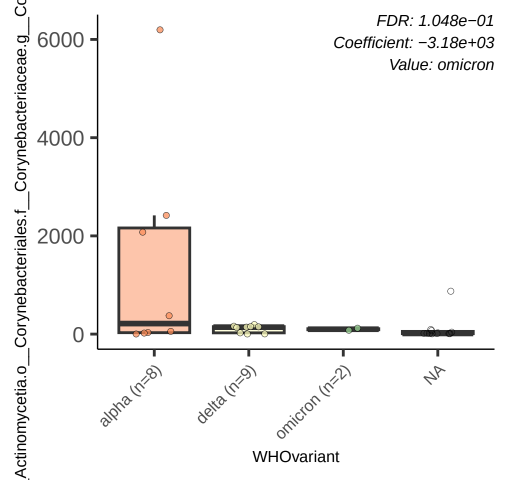

FDR: 1.048e−01
Coefficient: −3.18e+03
Value: omicron
6000
4000
2000
0
Actinomycetia.o__Corynebacteriales.f__Corynebacteriaceae.g__Co
alpha (n=8)
delta (n=9)
omicron (n=2)
NA
WHOvariant

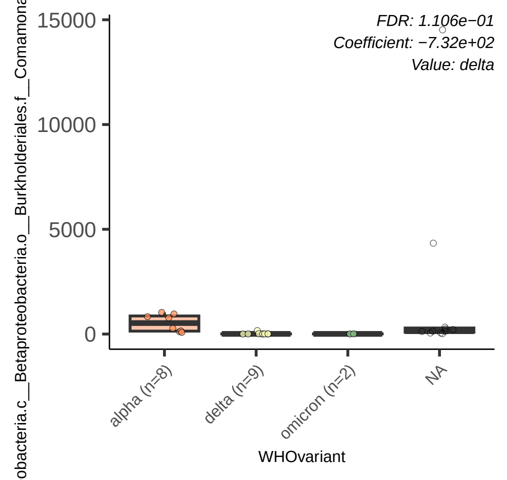
FDR: 1.106e−01
Coefficient: −7.32e+02
Value: delta
15000
10000
5000
0
alpha (n=8)
delta (n=9)
omicron (n=2)
NA
WHOvariant
obacteria.c__Betaproteobacteria.o__Burkholderiales.f__Comamona

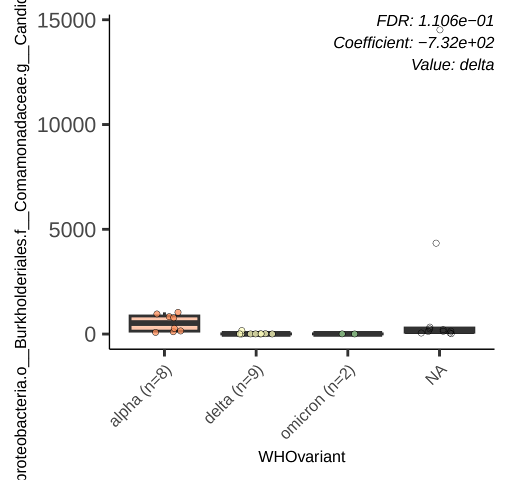
FDR: 1.106e−01
Coefficient: −7.32e+02
Value: delta
15000
10000
5000
0
alpha (n=8)
delta (n=9)
omicron (n=2)
NA
WHOvariant
roteobacteria.o__Burkholderiales.f__Comamonadaceae.g__Candid

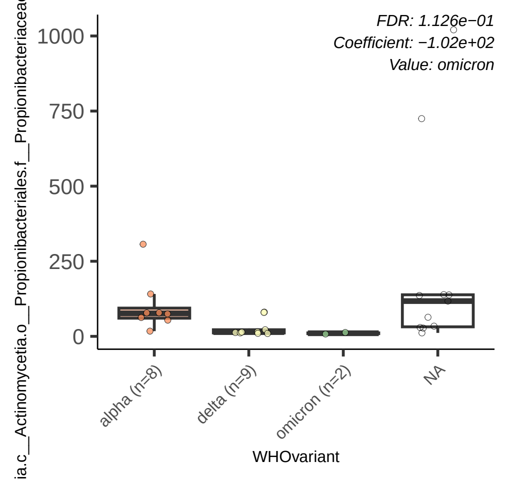

FDR: 1.126e−01
Coefficient: −1.02e+02
Value: omicron
1000
750
500
250
0
alpha (n=8)
delta (n=9)
omicron (n=2)
NA
WHOvariant
ia.c__Actinomycetia.o__Propionibacteriales.f__Propionibacteriaceae

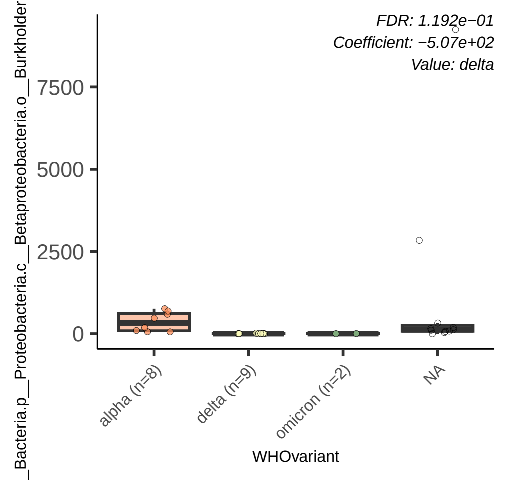

FDR: 1.192e−01
Coefficient: −5.07e+02
Value: delta
7500
5000
2500
0
Bacteria.p__Proteobacteria.c__Betaproteobacteria.o__Burkholder
alpha (n=8)
delta (n=9)
omicron (n=2)
NA
WHOvariant

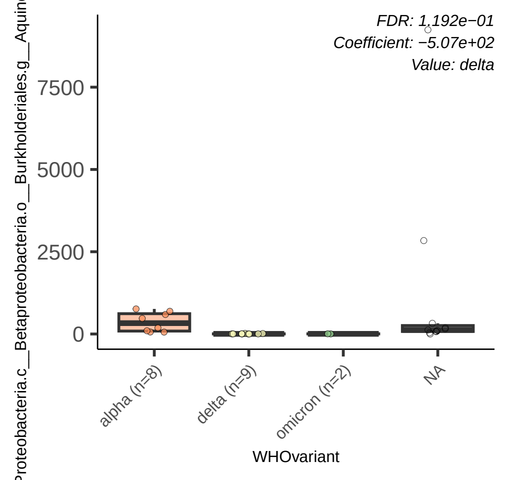

FDR: 1.192e−01
Coefficient: −5.07e+02
Value: delta
7500
5000
2500
0
alpha (n=8)
delta (n=9)
omicron (n=2)
NA
WHOvariant
Proteobacteria.c__Betaproteobacteria.o__Burkholderiales.g__Aquin

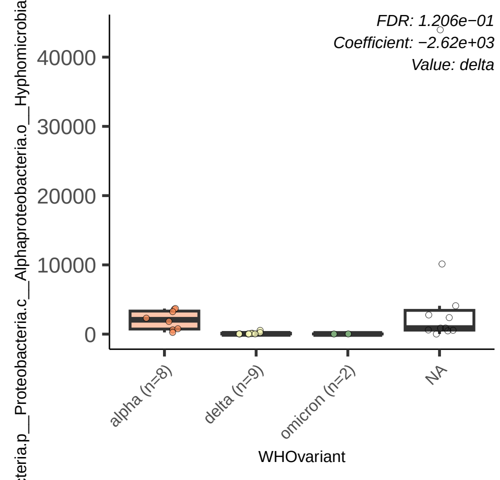
FDR: 1.206e−01
Coefficient: −2.62e+03
Value: delta
40000
30000
20000
10000
0
alpha (n=8)
delta (n=9)
omicron (n=2)
NA
WHOvariant
cteria.p__Proteobacteria.c__Alphaproteobacteria.o__Hyphomicrobia

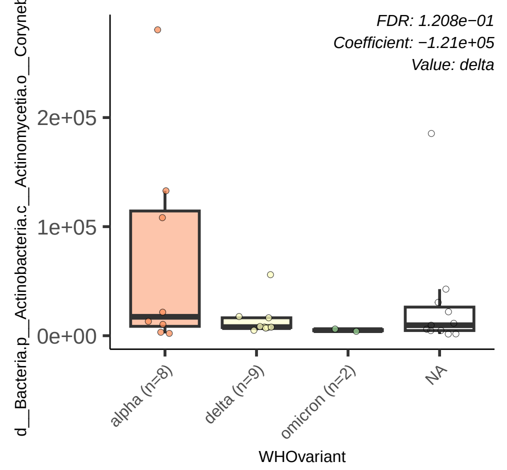

FDR: 1.208e−01
Coefficient: −1.21e+05
Value: delta
d__Bacteria.p__Actinobacteria.c__Actinomycetia.o__Coryneb
2e+05
1e+05
0e+00
alpha (n=8)
delta (n=9)
omicron (n=2)
NA
WHOvariant

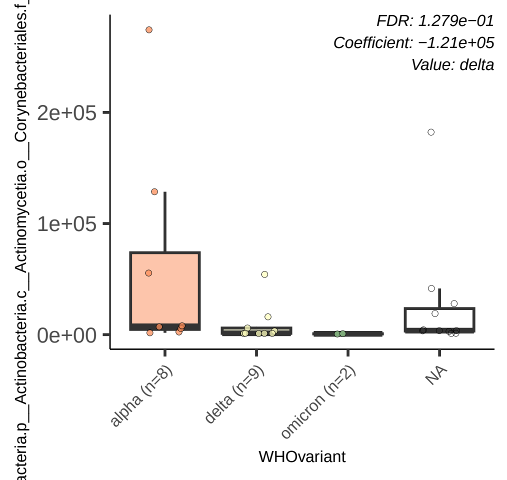

FDR: 1.279e−01
Coefficient: −1.21e+05
Value: delta
acteria.p__Actinobacteria.c__Actinomycetia.o__Corynebacteriales.f_
2e+05
1e+05
0e+00
alpha (n=8)
delta (n=9)
omicron (n=2)
NA
WHOvariant

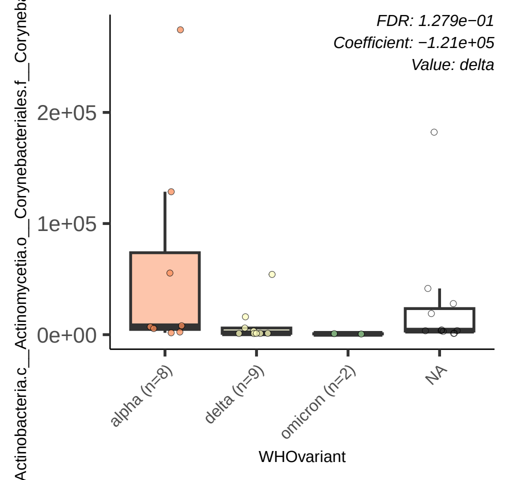
FDR: 1.279e−01
Coefficient: −1.21e+05
Value: delta
Actinobacteria.c__Actinomycetia.o__Corynebacteriales.f__Corynebacteriaceae
2e+05
1e+05
0e+00
alpha (n=8)
delta (n=9)
omicron (n=2)
NA
WHOvariant

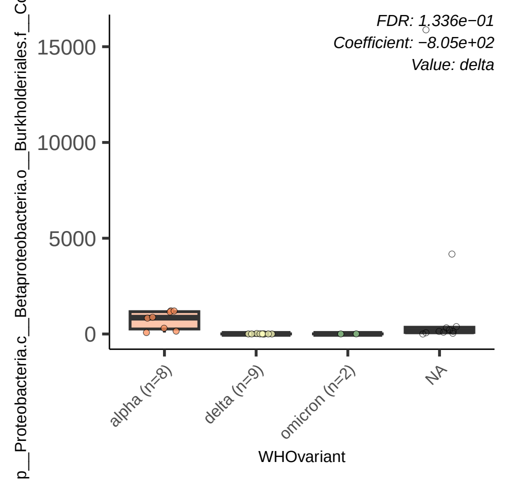
FDR: 1.336e−01
Coefficient: −8.05e+02
Value: delta
15000
10000
5000
0
p__Proteobacteria.c__Betaproteobacteria.o__Burkholderiales.f__Co
alpha (n=8)
delta (n=9)
omicron (n=2)
NA
WHOvariant

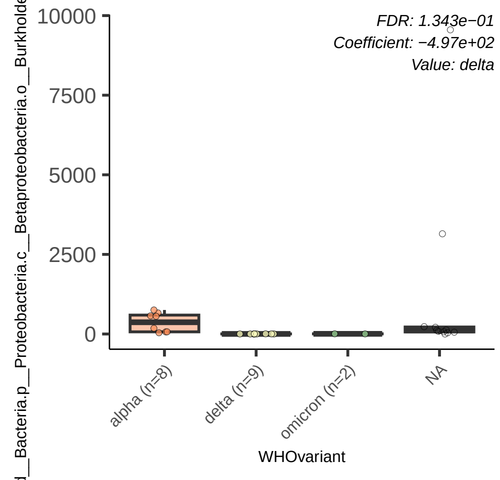

FDR: 1.343e−01
Coefficient: −4.97e+02
Value: delta
10000
7500
5000
2500
0
d__Bacteria.p__Proteobacteria.c__Betaproteobacteria.o__Burkholde
alpha (n=8)
delta (n=9)
omicron (n=2)
NA
WHOvariant

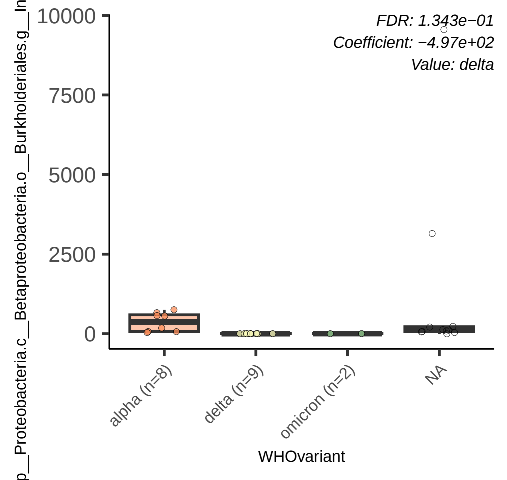
FDR: 1.343e−01
Coefficient: −4.97e+02
Value: delta
10000
7500
5000
2500
0
alpha (n=8)
delta (n=9)
omicron (n=2)
NA
WHOvariant
p__Proteobacteria.c__Betaproteobacteria.o__Burkholderiales.g__In

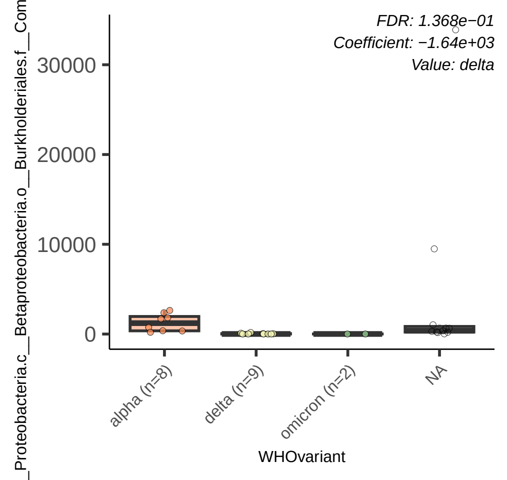

FDR: 1.368e−01
Coefficient: −1.64e+03
Value: delta
30000
20000
10000
0
_Proteobacteria.c__Betaproteobacteria.o__Burkholderiales.f__Com
alpha (n=8)
delta (n=9)
omicron (n=2)
NA
WHOvariant

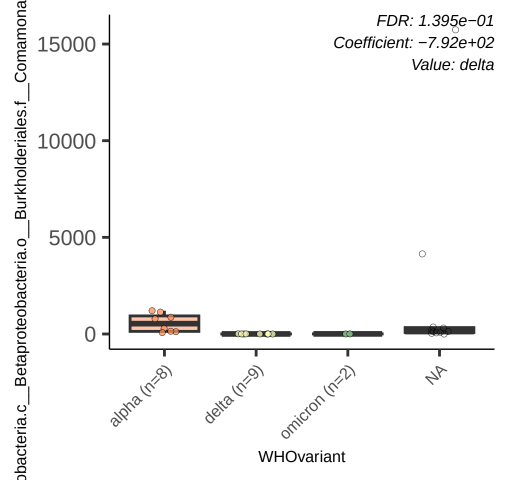

FDR: 1.395e−01
Coefficient: −7.92e+02
Value: delta
15000
10000
5000
0
obacteria.c__Betaproteobacteria.o__Burkholderiales.f__Comamona
alpha (n=8)
delta (n=9)
omicron (n=2)
NA
WHOvariant

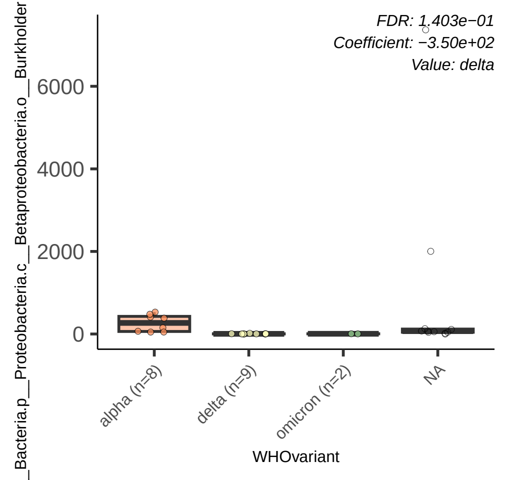
FDR: 1.403e−01
Coefficient: −3.50e+02
Value: delta
_Bacteria.p__Proteobacteria.c__Betaproteobacteria.o__Burkholder
6000
4000
2000
0
alpha (n=8)
delta (n=9)
omicron (n=2)
NA
WHOvariant

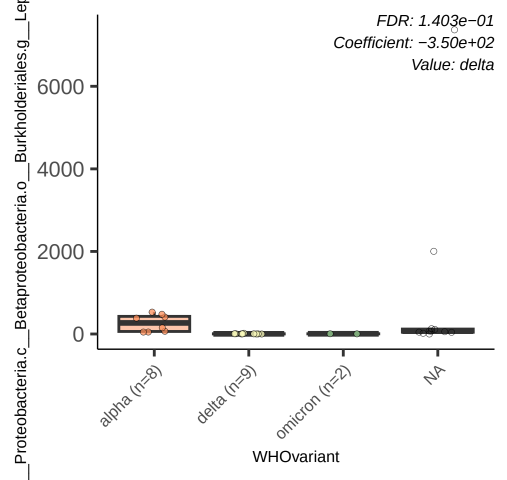

FDR: 1.403e−01
Coefficient: −3.50e+02
Value: delta
6000
4000
2000
0
alpha (n=8)
delta (n=9)
omicron (n=2)
NA
WHOvariant
__Proteobacteria.c__Betaproteobacteria.o__Burkholderiales.g__Lep

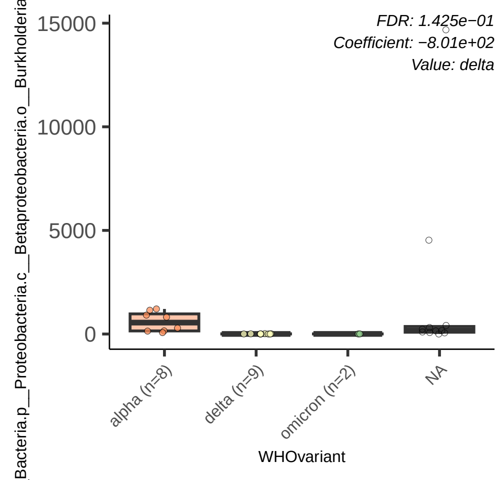
FDR: 1.425e−01
Coefficient: −8.01e+02
Value: delta
15000
10000
5000
0
Bacteria.p__Proteobacteria.c__Betaproteobacteria.o__Burkholderia
alpha (n=8)
delta (n=9)
omicron (n=2)
NA
WHOvariant

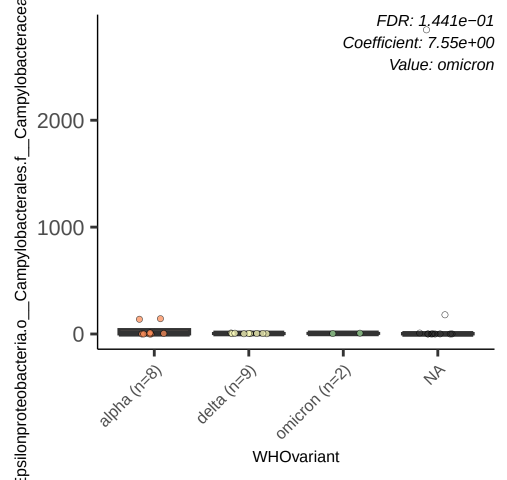

FDR: 1.441e−01
Coefficient: 7.55e+00
Value: omicron
2000
1000
0
alpha (n=8)
delta (n=9)
omicron (n=2)
NA
WHOvariant
Epsilonproteobacteria.o__Campylobacterales.f__Campylobacteraceae

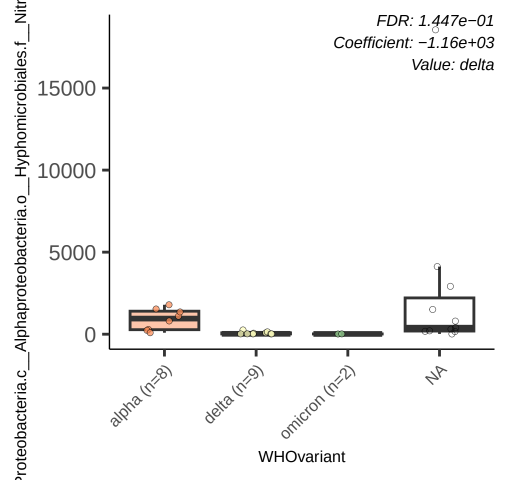
FDR: 1.447e−01
Coefficient: −1.16e+03
Value: delta
Proteobacteria.c__Alphaproteobacteria.o__Hyphomicrobiales.f__Nitr
15000
10000
5000
0
alpha (n=8)
delta (n=9)
omicron (n=2)
NA
WHOvariant

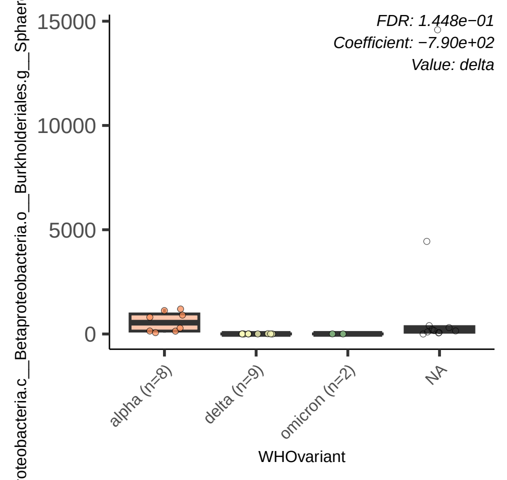
FDR: 1.448e−01
Coefficient: −7.90e+02
Value: delta
15000
10000
5000
0
roteobacteria.c__Betaproteobacteria.o__Burkholderiales.g__Sphaer
alpha (n=8)
delta (n=9)
omicron (n=2)
NA
WHOvariant

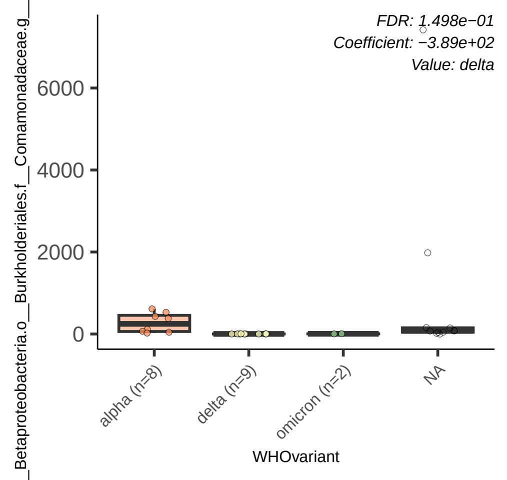

FDR: 1.498e−01
Coefficient: −3.89e+02
Value: delta
Betaproteobacteria.o__Burkholderiales.f__Comamonadaceae.g__
6000
4000
2000
0
alpha (n=8)
delta (n=9)
omicron (n=2)
NA
WHOvariant

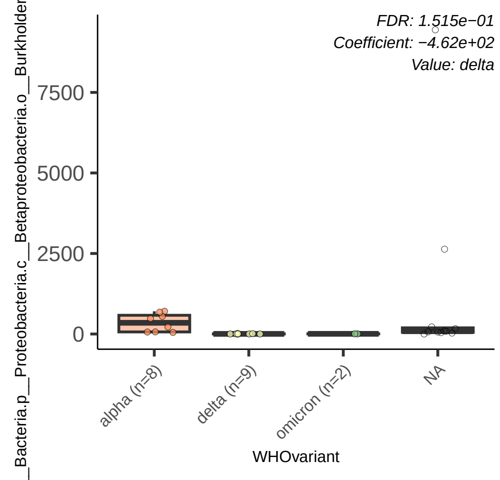
FDR: 1.515e−01
Coefficient: −4.62e+02
Value: delta
7500
5000
2500
0
alpha (n=8)
delta (n=9)
omicron (n=2)
NA
WHOvariant
Bacteria.p__Proteobacteria.c__Betaproteobacteria.o__Burkholder

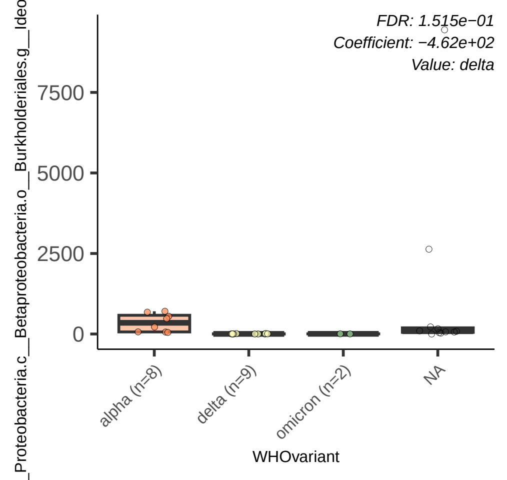

FDR: 1.515e−01
Coefficient: −4.62e+02
Value: delta
7500
5000
2500
0
_Proteobacteria.c__Betaproteobacteria.o__Burkholderiales.g__Ideo
alpha (n=8)
delta (n=9)
omicron (n=2)
NA
WHOvariant

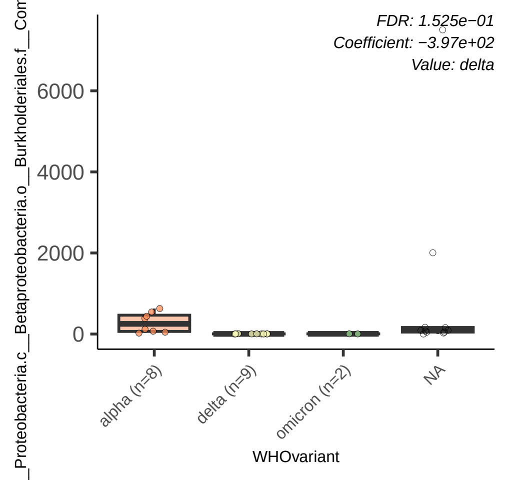

FDR: 1.525e−01
Coefficient: −3.97e+02
Value: delta
6000
4000
2000
0
_Proteobacteria.c__Betaproteobacteria.o__Burkholderiales.f__Com
alpha (n=8)
delta (n=9)
omicron (n=2)
NA
WHOvariant

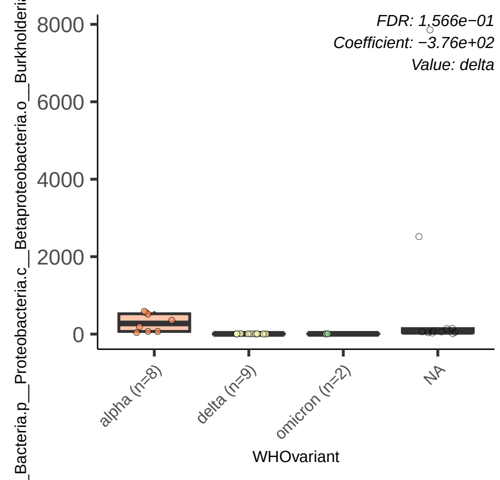

FDR: 1.566e−01
Coefficient: −3.76e+02
Value: delta
8000
6000
4000
2000
0
alpha (n=8)
delta (n=9)
omicron (n=2)
NA
WHOvariant
Bacteria.p__Proteobacteria.c__Betaproteobacteria.o__Burkholderia

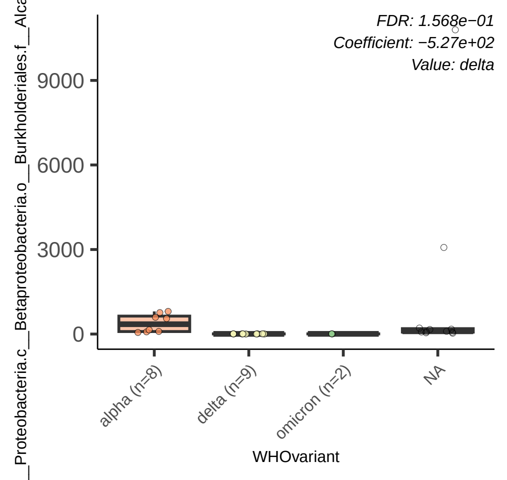

FDR: 1.568e−01
Coefficient: −5.27e+02
Value: delta
9000
6000
3000
0
Proteobacteria.c__Betaproteobacteria.o__Burkholderiales.f__Alca
alpha (n=8)
delta (n=9)
omicron (n=2)
NA
WHOvariant

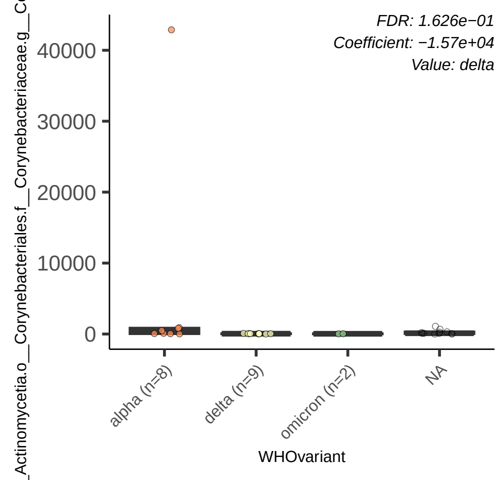

FDR: 1.626e−01
Coefficient: −1.57e+04
Value: delta
Actinomycetia.o__Corynebacteriales.f__Corynebacteriaceae.g__Co
40000
30000
20000
10000
0
alpha (n=8)
delta (n=9)
omicron (n=2)
NA
WHOvariant

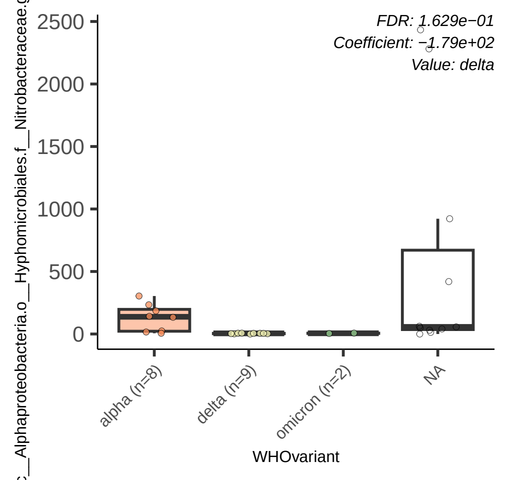

FDR: 1.629e−01
Coefficient: −1.79e+02
Value: delta
2500
2000
1500
1000
500
0
alpha (n=8)
delta (n=9)
omicron (n=2)
NA
WHOvariant
c__Alphaproteobacteria.o__Hyphomicrobiales.f__Nitrobacteraceae.g

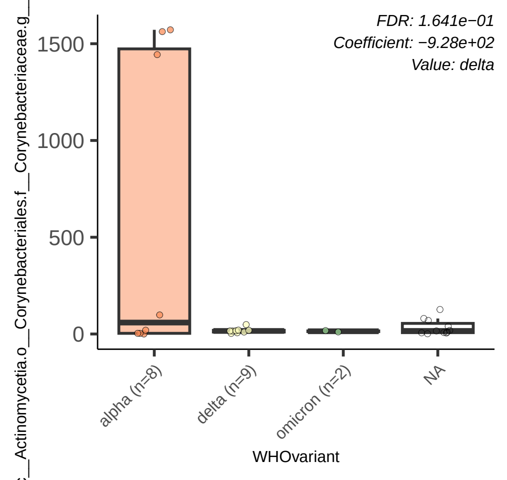

FDR: 1.641e−01
Coefficient: −9.28e+02
Value: delta
c__Actinomycetia.o__Corynebacteriales.f__Corynebacteriaceae.g__
1500
1000
500
0
alpha (n=8)
delta (n=9)
omicron (n=2)
NA
WHOvariant

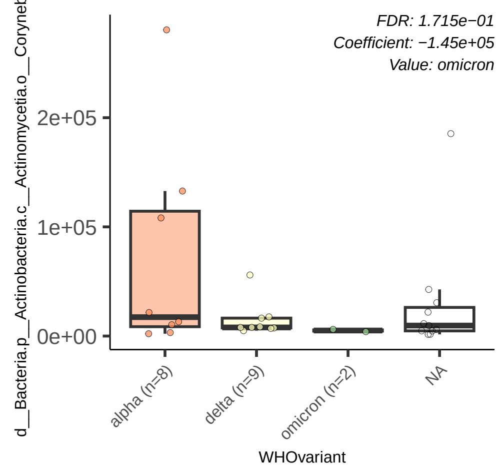

FDR: 1.715e−01
Coefficient: −1.45e+05
Value: omicron
2e+05
1e+05
0e+00
d__Bacteria.p__Actinobacteria.c__Actinomycetia.o__Coryneb
alpha (n=8)
delta (n=9)
omicron (n=2)
NA
WHOvariant

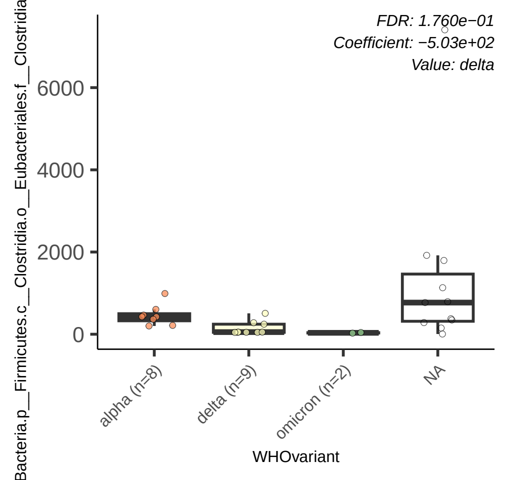

FDR: 1.760e−01
Coefficient: −5.03e+02
Value: delta
6000
4000
2000
0
Bacteria.p__Firmicutes.c__Clostridia.o__Eubacteriales.f__Clostridia
alpha (n=8)
delta (n=9)
omicron (n=2)
NA
WHOvariant

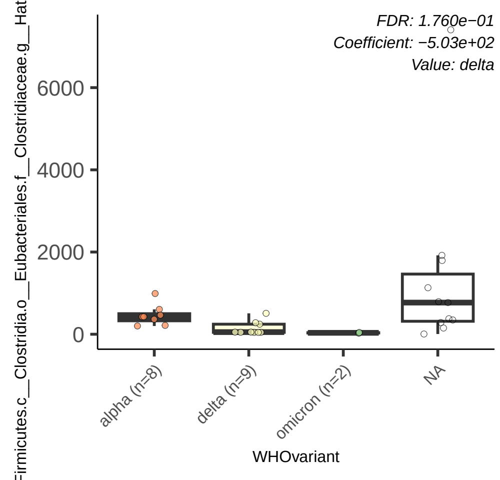

FDR: 1.760e−01
Coefficient: −5.03e+02
Value: delta
6000
4000
2000
0
Firmicutes.c__Clostridia.o__Eubacteriales.f__Clostridiaceae.g__Hat
alpha (n=8)
delta (n=9)
omicron (n=2)
NA
WHOvariant

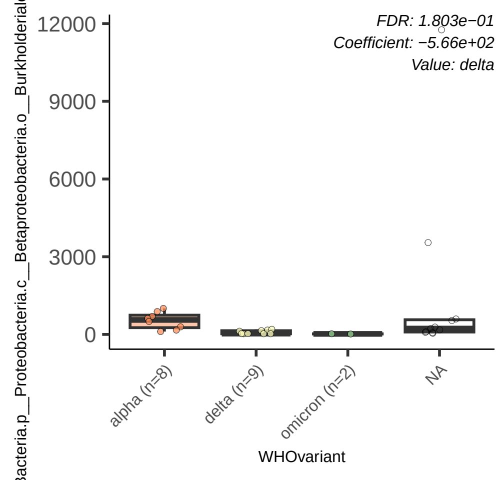

FDR: 1.803e−01
Coefficient: −5.66e+02
Value: delta
Bacteria.p__Proteobacteria.c__Betaproteobacteria.o__Burkholderiale
12000
9000
6000
3000
0
alpha (n=8)
delta (n=9)
omicron (n=2)
NA
WHOvariant

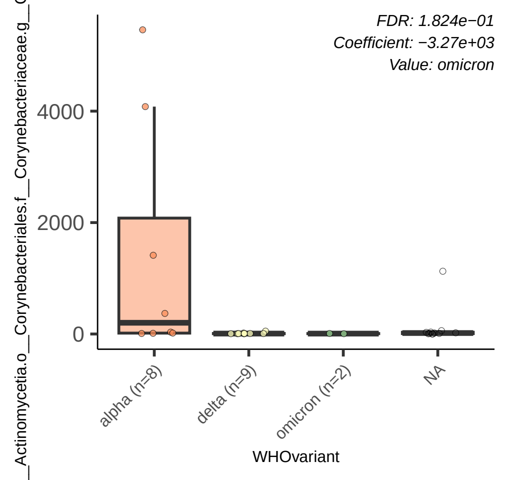
FDR: 1.824e−01
Coefficient: −3.27e+03
Value: omicron
4000
2000
0
Actinomycetia.o__Corynebacteriales.f__Corynebacteriaceae.g__C
alpha (n=8)
delta (n=9)
omicron (n=2)
NA
WHOvariant

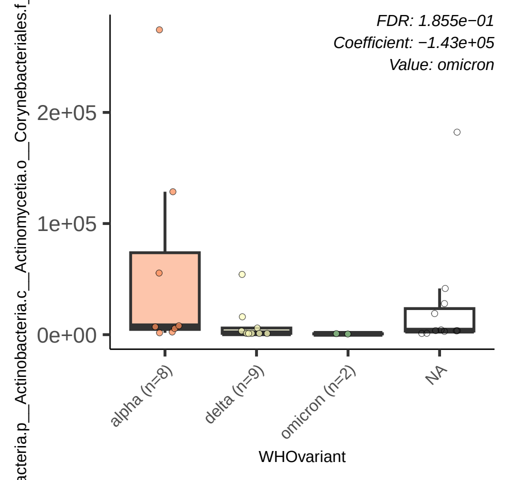
FDR: 1.855e−01
Coefficient: −1.43e+05
Value: omicron
acteria.p__Actinobacteria.c__Actinomycetia.o__Corynebacteriales.f_
2e+05
1e+05
0e+00
alpha (n=8)
delta (n=9)
omicron (n=2)
NA
WHOvariant

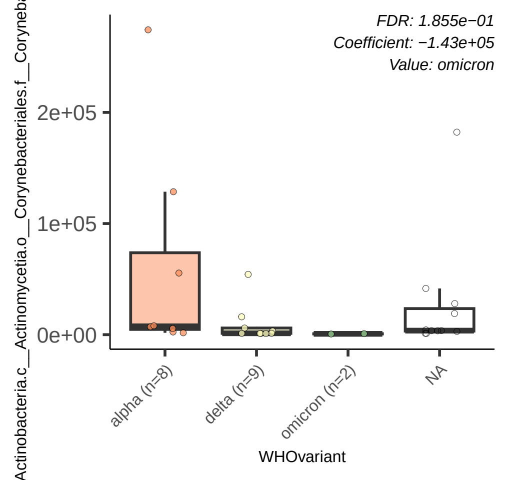
FDR: 1.855e−01
Coefficient: −1.43e+05
Value: omicron
Actinobacteria.c__Actinomycetia.o__Corynebacteriales.f__Corynebac
2e+05
1e+05
0e+00
alpha (n=8)
delta (n=9)
omicron (n=2)
NA
WHOvariant

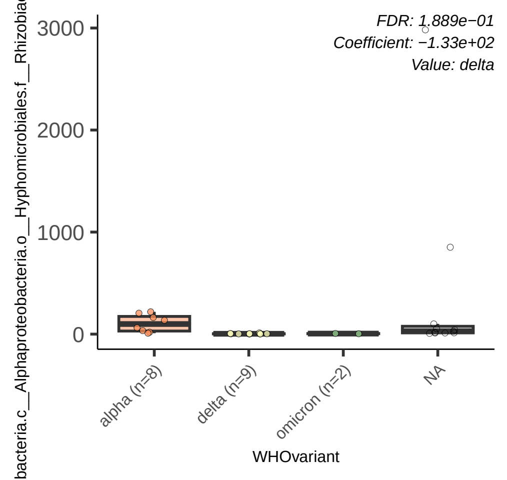

FDR: 1.889e−01
Coefficient: −1.33e+02
Value: delta
3000
2000
1000
0
bacteria.c__Alphaproteobacteria.o__Hyphomicrobiales.f__Rhizobia
alpha (n=8)
delta (n=9)
omicron (n=2)
NA
WHOvariant

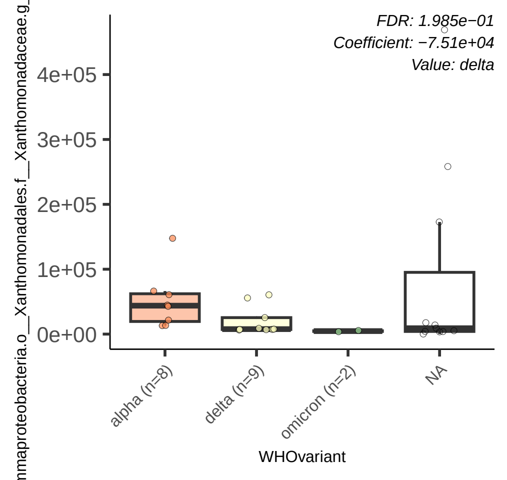
FDR: 1.985e−01
Coefficient: −7.51e+04
Value: delta
4e+05
3e+05
2e+05
1e+05
0e+00
nmaproteobacteria.o__Xanthomonadales.f__Xanthomonadaceae.g_
alpha (n=8)
delta (n=9)
omicron (n=2)
NA
WHOvariant

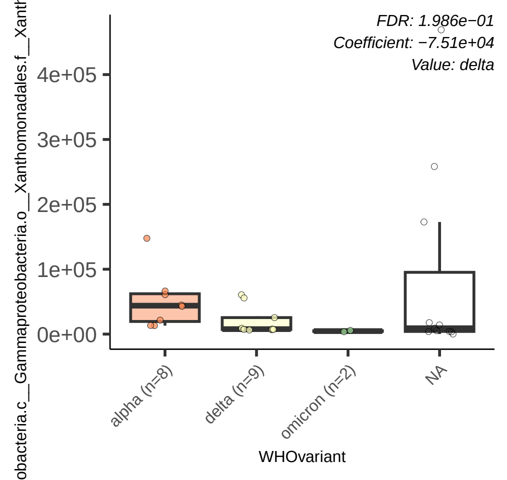

FDR: 1.986e−01
Coefficient: −7.51e+04
Value: delta
4e+05
3e+05
2e+05
1e+05
0e+00
obacteria.c__Gammaproteobacteria.o__Xanthomonadales.f__Xanth
alpha (n=8)
delta (n=9)
omicron (n=2)
NA
WHOvariant

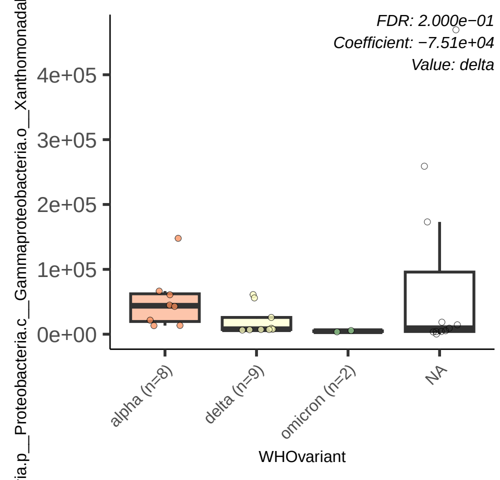
FDR: 2.000e−01
Coefficient: −7.51e+04
Value: delta
4e+05
3e+05
2e+05
1e+05
0e+00
ia.p__Proteobacteria.c__Gammaproteobacteria.o__Xanthomonadal
alpha (n=8)
delta (n=9)
omicron (n=2)
NA
WHOvariant

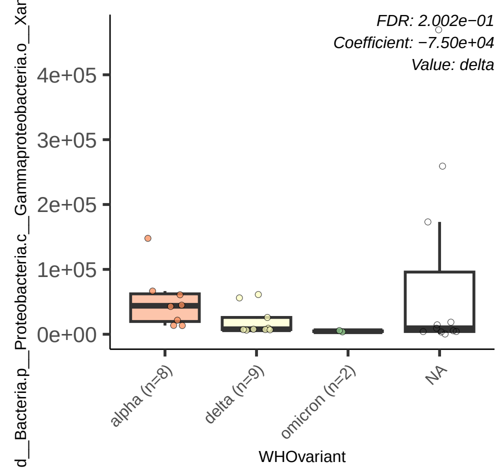

FDR: 2.002e−01
Coefficient: −7.50e+04
Value: delta
4e+05
3e+05
2e+05
1e+05
0e+00
d__Bacteria.p__Proteobacteria.c__Gammaproteobacteria.o__Xan
alpha (n=8)
delta (n=9)
omicron (n=2)
NA
WHOvariant

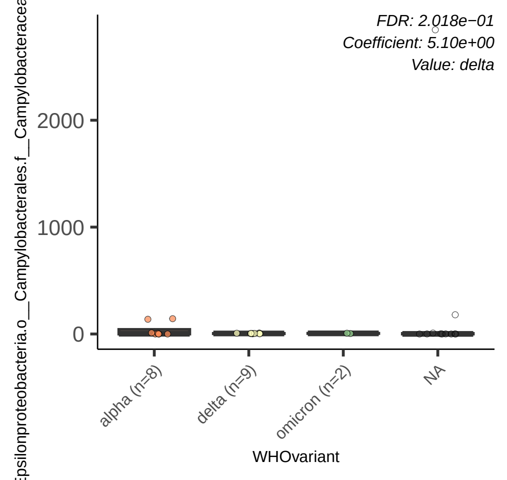

FDR: 2.018e−01
Coefficient: 5.10e+00
Value: delta
Epsilonproteobacteria.o__Campylobacterales.f__Campylobacterace
2000
1000
0
alpha (n=8)
delta (n=9)
omicron (n=2)
NA
WHOvariant

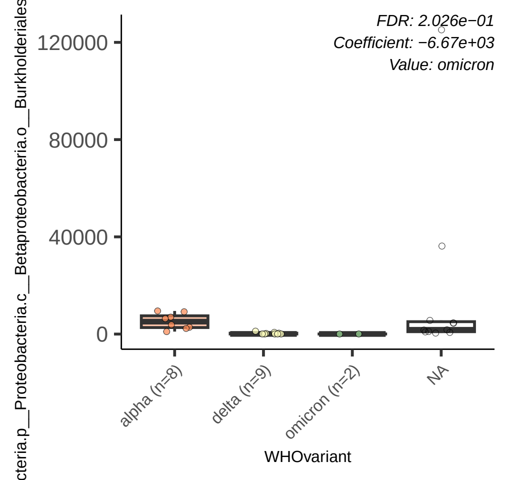

FDR: 2.026e−01
Coefficient: −6.67e+03
Value: omicron
120000
80000
40000
0
cteria.p__Proteobacteria.c__Betaproteobacteria.o__Burkholderiales
alpha (n=8)
delta (n=9)
omicron (n=2)
NA
WHOvariant

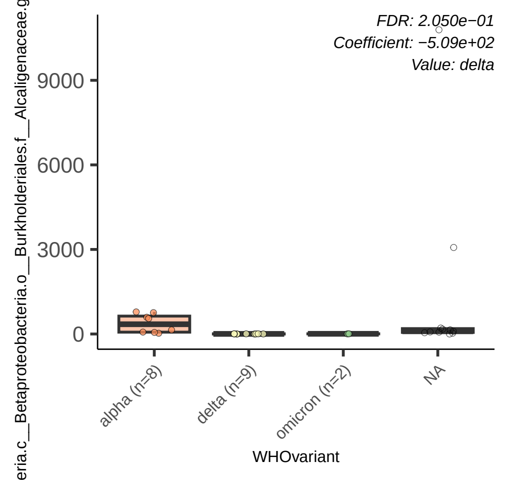
FDR: 2.050e−01
Coefficient: −5.09e+02
Value: delta
9000
6000
3000
0
eria.c__Betaproteobacteria.o__Burkholderiales.f__Alcaligenaceae.g
alpha (n=8)
delta (n=9)
omicron (n=2)
NA
WHOvariant

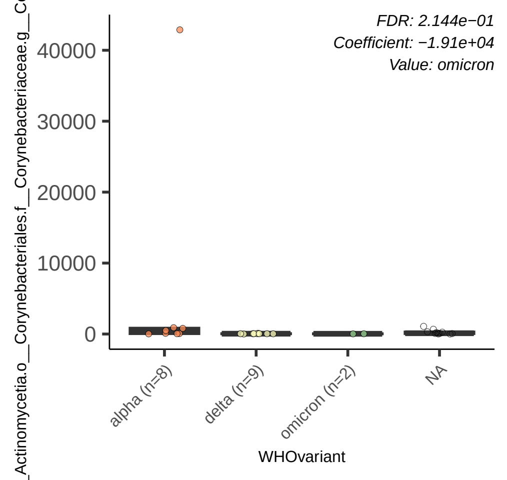

FDR: 2.144e−01
Coefficient: −1.91e+04
Value: omicron
40000
30000
20000
10000
0
alpha (n=8)
delta (n=9)
omicron (n=2)
NA
WHOvariant
_Actinomycetia.o__Corynebacteriales.f__Corynebacteriaceae.g__Co

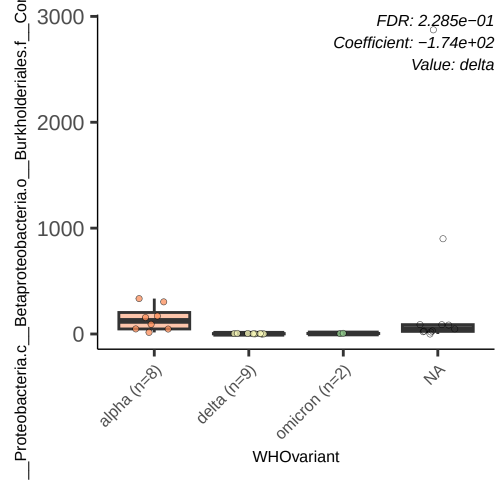

FDR: 2.285e−01
Coefficient: −1.74e+02
Value: delta
3000
2000
1000
0
Proteobacteria.c__Betaproteobacteria.o__Burkholderiales.f__Co
alpha (n=8)
delta (n=9)
omicron (n=2)
NA
WHOvariant

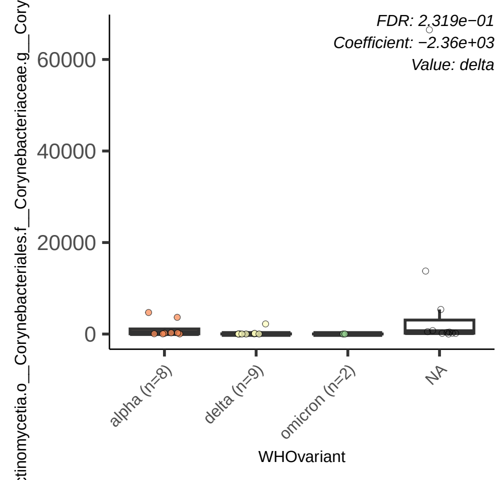
FDR: 2.319e−01
Coefficient: −2.36e+03
Value: delta
60000
40000
20000
0
alpha (n=8)
delta (n=9)
omicron (n=2)
NA
WHOvariant
ctinomycetia.o__Corynebacteriales.f__Corynebacteriaceae.g__Cory

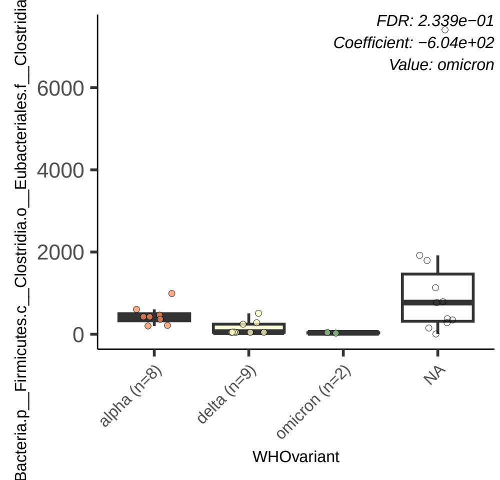

FDR: 2.339e−01
Coefficient: −6.04e+02
Value: omicron
6000
4000
2000
0
Bacteria.p__Firmicutes.c__Clostridia.o__Eubacteriales.f__Clostridia
alpha (n=8)
delta (n=9)
omicron (n=2)
NA
WHOvariant

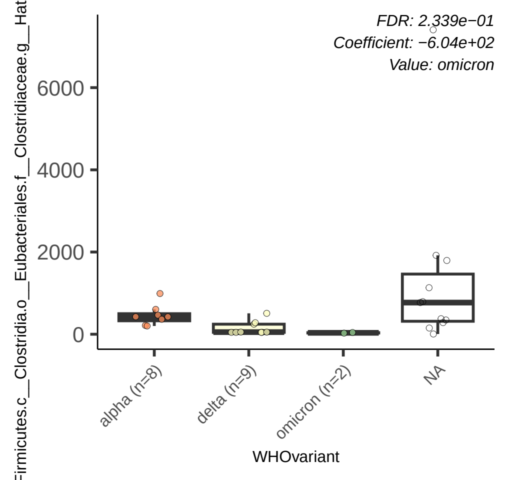

FDR: 2.339e−01
Coefficient: −6.04e+02
Value: omicron
6000
4000
2000
0
Firmicutes.c__Clostridia.o__Eubacteriales.f__Clostridiaceae.g__Hat
alpha (n=8)
delta (n=9)
omicron (n=2)
NA
WHOvariant

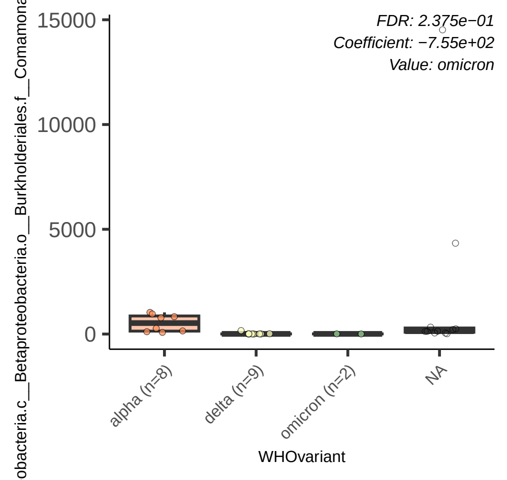

FDR: 2.375e−01
Coefficient: −7.55e+02
Value: omicron
15000
10000
5000
0
obacteria.c__Betaproteobacteria.o__Burkholderiales.f__Comamona
alpha (n=8)
delta (n=9)
omicron (n=2)
NA
WHOvariant

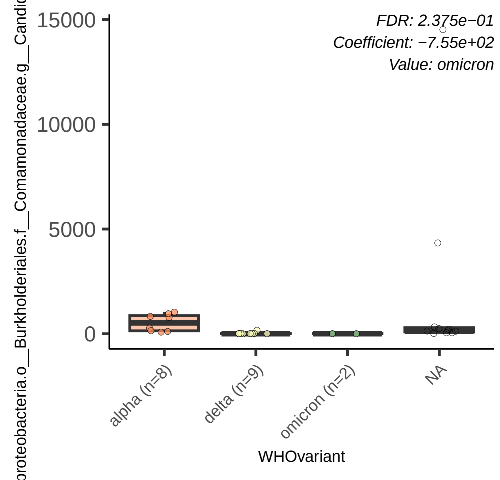
FDR: 2.375e−01
Coefficient: −7.55e+02
Value: omicron
15000
10000
5000
0
alpha (n=8)
delta (n=9)
omicron (n=2)
NA
WHOvariant
Burkholderiales.f__Comamonadaceae.g__Candid
oteobacteria.o__

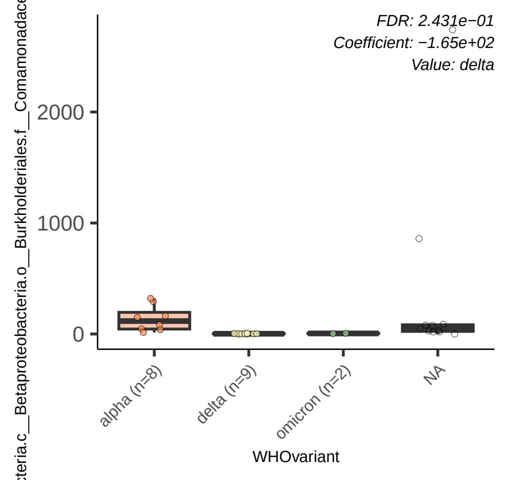
FDR: 2.431e−01
Coefficient: −1.65e+02
Value: delta
2000
1000
0
alpha (n=8)
delta (n=9)
omicron (n=2)
NA
WHOvariant
cteria.c__Betaproteobacteria.o__Burkholderiales.f__Comamonadace

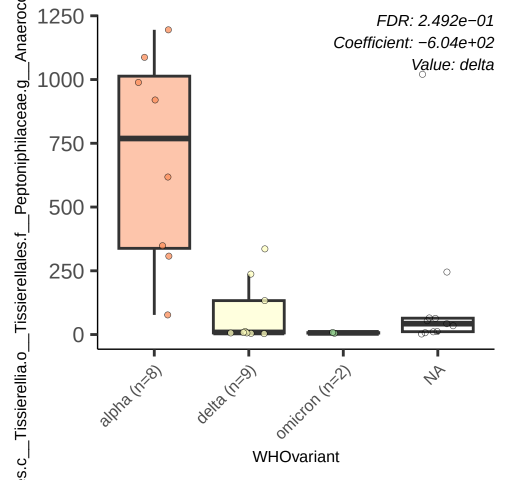

FDR: 2.492e−01
Coefficient: −6.04e+02
Value: delta
1250
1000
750
500
250
0
s.c__Tissierellia.o__Tissierellales.f__Peptoniphilaceae.g__Anaeroco
alpha (n=8)
delta (n=9)
omicron (n=2)
NA
WHOvariant
